# Temporal Analysis of Protein Ubiquitylation and Phosphorylation During Parkin-Dependent Mitophagy

**DOI:** 10.1016/j.mcpro.2021.100191

**Published:** 2021-12-30

**Authors:** Katharina I. Zittlau, Anna Lechado-Terradas, Nicolas Nalpas, Sven Geisler, Philipp J. Kahle, Boris Macek

**Affiliations:** 1 Department of Biology, Quantitative Proteomics Group, Interfaculty Institute of Cell Biology, University of Tübingen, Tübingen, Germany; 2 Functional Neurogenetics, Laboratory of Neurodegeneration, Faculty of Medicine, Hertie Institute for Clinical Brain Research and German Center for Neurodegenerative Diseases, University of Tübingen, Tübingen, Germany; 3 Department of Biochemistry, Faculty of Science, University of Tübingen, Tübingen, Germany

**Keywords:** parkin, mitochondria, mitophagy, ubiquitin, quantitative proteomics, ACN, acetonitrile, CCCP, carbonyl cyanide *m*-chlorophenyl hydrazine, COX4, Cytochrome c oxidase polypeptide IV, CS, citrate synthase, FA, formic acid, FBS, fetal bovine serum, FDR, false discovery rate, IAP, immunoaffinity purification, MFN, mitofusin, MIM, mitochondrial inner membrane, MOM, mitochondrial outer membrane, MS, mass spectrometry, NEB, nuclear extraction buffer, Ni–NTA, nickel–nitrilotriacetic acid, PINK1, phosphatase and tensin homolog–induced kinase 1, RHOT1/2, Ras homolog family member T1/2, RT, room temperature, TMT, tandem mass tag, TOM70, translocase of outer mitochondrial membrane protein 70, VDAC1/2, voltage-dependent anion channel 1/2

## Abstract

Mitophagy, the selective degradation of mitochondria by autophagy, affects defective mitochondria following damage or stress. At the onset of mitophagy, parkin ubiquitylates proteins on the mitochondrial outer membrane. While the role of parkin at the onset of mitophagy is well understood, less is known about its activity during later stages in the process. Here, we used HeLa cells expressing catalytically active or inactive parkin to perform temporal analysis of the proteome, ubiquitylome, and phosphoproteome during 18 h after induction of mitophagy by mitochondrial uncoupler carbonyl cyanide *m*-chlorophenyl hydrazine. Abundance profiles of proteins downregulated in parkin-dependent manner revealed a stepwise and “outside–in” directed degradation of mitochondrial subcompartments. While ubiquitylation of mitochondrial outer membrane proteins was enriched among early parkin-dependent targets, numerous mitochondrial inner membrane, matrix, and cytosolic proteins were also found ubiquitylated at later stages of mitophagy. Phosphoproteome analysis revealed a possible crosstalk between phosphorylation and ubiquitylation during mitophagy on key parkin targets, such as voltage-dependent anion channel 2.

## Highlights

• We report dynamics of the mitochondrial proteome during parkin-dependent mitophagy.
• Our data point to outside–in degradation of mitochondria.
• Inner membrane and matrix proteins are ubiquitylated after 12 and 18 h of mitophagy.
• Interplay of phosphorylation and ubiquitylation leads to degradation of VDAC2.

## Graphical Abstract

**Figure:**
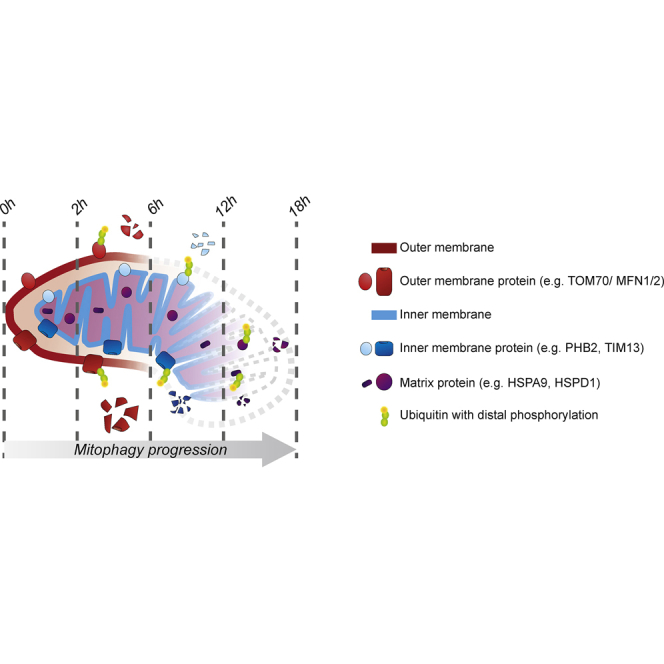

## In Brief

We used a quantitative proteomics approach to study dynamics of the mitochondrial proteome, ubiquitylome, and phosphoproteome during early (2–6 h) and late stages (12–18 h) of mitophagy. We focused on parkin-initiated ubiquitylation and the impact on the proteome level, which pointed to an outside–in progression of ubiquitylation and protein degradation of mitochondrial subcompartments. In addition, we validated the interplay of phosphorylation and ubiquitylation on VDAC2, which directly influenced its degradation over the course of mitophagy.

Mitochondria are involved in many cellular processes, such as apoptosis, lipid transfer, and cellular energy production. Under stress conditions that compromise their multifaceted functions (1, 2), selective autophagic elimination of mitochondria (mitophagy) is activated. One of the most studied mitophagy pathways is regulated by the serine/threonine-protein kinase phosphatase and tensin homolog–induced kinase 1 (PINK1) and the E3-ubiquitin ligase parkin. Mutations in these proteins have been directly linked with autosomal–recessive familial forms of Parkinson's disease (3, 4). Under basal conditions, PINK1 is constantly imported and degraded within mitochondria (5, 6, 7). Once the mitochondrial membrane potential is compromised, PINK1 accumulates at the mitochondrial outer membrane (MOM), where it becomes fully active upon autophosphorylation (8, 9). PINK1 phosphorylates parkin within its highly conserved ubiquitin-like domain at S65, inducing a conformational change on the E3-ligase enzyme that allows parkin to reach its complete active state (9, 10). Upon translocation to the MOM, parkin starts ubiquitylating several MOM proteins. The ubiquitin molecules added by parkin serve as a phosphorylation substrate to PINK1 and vice versa. This promotes a feed-forward signaling loop that amplifies the mitochondrial ubiquitylation signal (11).

Parkin is known to build different polyubiquitin chains but has different preferences for chain-linkage types, K63 and K48 being the most abundant chain types on MOM proteins (11). Other linkage chains, such as K27, K29, and K33, are known to be parkin dependent but are found to be less abundant on MOM proteins (11, 12). During the course of mitophagy, some MOM proteins are known to be eliminated at early stages (*i.e.*, mitofusins 1/2 [MFN1/2], mitochondrial GTPase miro1/2 [Ras homolog family member T1/2 [RHOT1/2], and translocase of outer mitochondrial membrane protein 70 [TOM70]), whereas some mitochondrial proteins remain ubiquitylated after intermediate depolarization times on damaged mitochondria (*i.e.*, voltage-dependent anion channel 1/2 [VDAC1/2], TOM20) (12, 13, 14). After extensive parkin-dependent ubiquitylation events, ubiquitin-binding autophagy receptor proteins are recruited, which trigger the engulfment of damaged mitochondria by the autophagosome. This eventually promotes mitochondrial degradation after the autophagosome fused with lysosomes (1, 15, 16, 17, 18). Even though it is well accepted that damaged mitochondria are engulfed as a single whole entity (19, 20), an alternative hypothesis suggests a sequential subcompartment degradation. In this context, Yoshii *et al.* (21) demonstrated that autophagosomes engulf complete mitochondria after 6 h of depolarization, but MOM-ruptured and mitochondrial inner membrane (MIM)-ruptured mitochondria are engulfed after extensive depolarization times (12 h carbonyl cyanide *m*-chlorophenyl hydrazine [CCCP]). Recent advances in the field of quantitative proteomics and investigation of low-abundant ubiquitylation have allowed an in-depth analysis of parkin-dependent mitophagy (11). However, our understanding of molecular processes during later stages of mitophagy as well as the interplay between parkin-dependent ubiquitylation and phosphorylation is limited.

Here, we performed an in-depth temporal analysis of the proteome, ubiquitylome, and phosphoproteome during early and late stages of mitophagy. We identify novel parkin and downstream-acting substrates under mitophagy conditions; suggest that parkin-dependent mitochondrial degradation is tightly regulated in space and time; and indicate a potential crosstalk between ubiquitylation and phosphorylation able to influence specific protein degradation during PINK1/parkin-dependent mitophagy. Furthermore, we suggest an outside–in degradation of mitochondria, initiated by parkin activity during mitophagy.

## Experimental Procedures

### Experimental Design and Statistical Rationale

In this study, we used HeLa cells in all experiments. This cell line was chosen because of the absence of endogenous parkin and the possibility to study late mitochondria-depleted stages of mitophagy (22) (supplemental Fig. S1*A*). To increase the level of mitophagy-related low-abundant proteins from different organelles, cells were first fractionated using a subcellular protein fractionation kit and pooled to remove highly abundant nuclear and cytoskeletal proteins. All Western blot analyses were performed at least twice for endogenous proteins and up to five times for transient overexpressed proteins (*i.e.*, VDAC2). All microscopy analyses were performed in duplicates. For in-depth proteome and phosphoproteome analysis, we chose a triple dimethyl labeling approach that covers all five treatment conditions in two sets sharing the untreated control. Both proteomic analyses were performed in two biological replicates. To study dynamics in the ubiquitylome, we adapted the recently presented Tandem Mass Tag 10-plex (TMT10-plex) approach by Udeshi *et al.* (23). In total, three replicates were performed to stabilize the quality of the quantified ubiquitylation sites. Only proteins identified in both replicates were further analyzed to investigate parkin-related dynamics on the proteome level. For phosphoproteome and ubiquitylome analysis, sites identified in at least one replicate were further processed. Mitochondrial proteins were annotated based on MitoCarta3.0 (24). Significantly regulated proteins (ANOVA *p* < 0.1 followed by post hoc test false discovery rate [FDR] <0.1) were subjected to hierarchical clustering. To identify parkin-dependent ubiquitylation sites, only strongly regulated ubiquitylation sites (z-scored fold change outside −2 and 2) were used for hierarchical clustering. This ratiometric approach was chosen based on the work from Hung *et al.* (25) because of varying identification rates of replicates that would have reduced the number of significant hits. To investigate dynamics on the phosphoproteome level, significantly regulated sites were determined (ANOVA *p* < 0.1 followed by post hoc test FDR < 0.1).

### Cell Culture, Transfection, and Treatments

HeLa cells expressing stable 3xFlag-parkin WT or ligase-dead C431A parkin were generated as mentioned by Geisler *et al.* (13). Cells were cultured in Dulbecco's modified Eagle's medium supplemented with 10% (v/v) fetal bovine serum (FBS) at 37 °C with 5% CO_2_. Cells were transfected with Fugene (Promega) with the indicated plasmids for at least 24 h. Mitochondrial depolarization was achieved by adding 10 μM of CCCP in the cell media. Upon harvest, cells were washed once with Dulbecco's PBS and stored until fractionation at −80 °C.

### Expression Constructs

Full-length VDAC2 complementary DNA was amplified from HeLa 3xFlag-parkin stable cells. VDAC2 was cloned in pCMV-6xHIS vector using SalI and EcoRI fast digestion enzymes (Thermo Fisher Scientific), yielding an N-terminal tagged pCMV-6xHIS-VDAC2 construct. Phosphomimic and phosphodead point mutations were introduced in 6xHIS-VDAC2 using a two-step point-directed mutagenesis protocol. All complementary DNA sequences were verified by Sanger sequencing.

### Western Blotting and Nickel–Nitrilotriacetic Acid Purification

Cells were washed with PBS and further pelleted at 972 g for 1 min at 4 °C. Cell pellets were resuspended in urea lysis buffer (10 mM Tris, pH 8.0, 100 mM NaH_2_PO_4_, and 8 M urea), passed five times through a 26-gauge needle, and pelleted at 3151 g for 15 min at 4 °C to obtain whole cell lysate fractions. Total protein measurement was performed using Bradford Protein Assay Kit (Bio-Rad). Samples were separated on 4 to 20% polyacrylamide gradient gels (TruPAGE precast gels; Merck) and run for 1 h at 100 V in Mops buffer. Proteins were transferred to poly(vinylidene fluoride) membranes (Immobilon-P; Merck) and blocked with 5% bovine serum albumin or nonfat dried milk in Tris-buffered saline with 0.1% Tween-20. Membranes were incubated overnight at 4 °C with the following primary antibodies: TOM70, 1:1000, ProteinTech (#14528-1-AP); TOM20, 1:6000, Santa Cruz (#sc-11415); VDAC1, 1:30000, Millipore (#AB10527); VDAC2, 1:50000, ProteinTech (#1663-1-AP); cytochrome c oxidase polypeptide IV (COX4), 1:1000, Cell Signaling (#4850); citrate synthase (CS), 1:6000, GeneTex (#GTX110624); SSBP1, 1:1000, R&D Systems (#AF6588); Vinculin, 1:6000, Sigma Aldrich (#V9131); Mfn1, 1:10000, Abnova (#H00055669-M04); 6HIS, 1:10000, Invitrogen (#HIS.H8); K27-ubiquitin, 1:50000, Abcam (#ab181537); Total Ubiquitin, 1:30000, Millipore (#MAB1510); pS65-ubiquitin, 1:1000, Millipore (#ABS1513); Parkin, 1:50000, Cell Signaling (#4211S); Flag, 1:10000, Sigma Aldrich (#F1804); pS757-ULK1, 1:1000, Cell signaling (#6888); PINK1, 1:1000, Cell Signaling (#6946S); and B-Actin, 1:5000, Sigma Aldrich (#A5441). Secondary antibody incubation were performed at room temperature (RT) for 1 h in 5% nonfat dried milk with the following antibodies: Goat anti-mouse HRP, Amersham Pharmacia (#115-035-003); Goat anti-rabbit HRP, Amersham Pharmacia (#111-035-003); and Goat anti-sheep HRP, Amersham Pharmacia (#718-035-147). Horseradish peroxidase chemiluminescent reaction was achieved using Western chemiluminescent-horseradish peroxidase substrate (Millipore) on Ultracruz autoradiography films (Santacruz). For nickel–nitrilotriacetic acid (Ni–NTA) pulldown, cell pellets were resuspended in urea lysis buffer at pH 8.0. At least 350 μg of total protein supplemented with 10 mM imidazole was incubated with 30 μl of Ni–NTA beads for 21 h at 4 °C. Incubated lysates were shortly pelleted at 972 g for 1 min at 4 °C and washed three times with urea lysis buffer at pH 6.0 containing 20 mM of imidazole. Proteins were eluted with 50 μl of 2× Laemmli buffer for 15 min at 95 °C. One-fifth of the total eluate was subjected to Western blot analysis.

### Immunostaining and Microscopy

Cells were seeded on precoated coverslips and treated with CCCP for the indicated time points. Cells were fixed with 4% paraformaldehyde in PBS for 20 min at RT and further permeabilized with 1% Triton X-100 for 5 min at RT. After three additional washing steps with PBS, cells were blocked with 10% of FBS in PBS for 1 h at RT. Cells were then incubated with the primary antibodies diluted in 5% FBS for 2 h at RT, and after three times washing with PBS, secondary antibodies diluted with 10% FBS were incubated for 1 h at RT in the dark. After two washing steps, cell nuclei were stained with 2 μg/ml Hoechst 33342 (Molecular Probes) in PBS for 5 min at RT. Cells were mounted onto coverslips using Fluorescent Mounting Medium (Dako). Imaging was performed using an AxioImager microscope equipped with an ApoTome imaging system using 63× objective (Carl Zeiss). The images were processed with AxioVision 4.9.1 software (Carl Zeiss). Primary and secondary antibodies used were the following: SSBP1, 1:1000, R&D Systems (#AF6588); K63-Ubiquitin, 1:1000, Millipore (#05-1308); Parkin, 1:1000, Cell Signaling (#4211S); TOM20, 1:1000, Santa Cruz (#sc-17764); Total Ubiquitin, 1:1000, Millipore (#MAB1510); COX4, 1:1000, Cell Signaling (#4850); p62/SQSTM, 1:1000, BD Biosciences (#610832); 6HIS, 1:1000, Invitrogen (#HIS.H8); CS, 1:1000, GeneTex (#GTX110624); Alexa Fluor 647 donkey anti-sheep, Invitrogen (A-21448); Alexa Fluor 488 donkey anti-mouse, Invitrogen (A-21202); Alexa Fluor 488 donkey anti-rabbit, Invitrogen (A-21206); Alexa Fluor donkey anti-mouse, Invitrogen (A-10037); and Alexa Fluor donkey anti-rabbit, Invitrogen (A-10042).

### Subcellular Protein Fractionation

Mitochondrial proteins were enriched using subcellular protein fractionation kit for cultured cells (kit no.: 78840; Thermo Fisher Scientific) (supplemental Fig. S1*B* and supplemental Table S7). Cells were washed once with PBS, scraped in PBS, and further pelleted at 2188 g for 5 min at 4 °C. All steps were performed according to the vendor with around 1 × 10^7^ cells per sample. Cytoplasmic extraction buffer, membrane extraction buffer, nuclear extraction buffer (NEB), and pellet extraction buffer were supplemented with Halt Protease Inhibitor Cocktail (1:100) immediately before use. Frozen cell pellets were resuspended in cytoplasmic extraction buffer and incubated for 10 min at 4 °C. After centrifugation at 500*g* for 10 min at 4 °C, the supernatants were stored as “cytoplasmic extract.” The pellet was resuspended in membrane extraction buffer and incubated for 10 min at 4 °C. The supernatant was taken as “membrane extract” after centrifugation at 3000*g* for 10 min at 4 °C. “Soluble nuclear extract” was collected after incubation with NEB for 30 min at 4 °C and centrifugation at 5000*g* at 4 °C. “Chromatin-bound nuclear extract” was obtained after incubation with NEB supplemented with CaCl_2_ and micrococcal nuclease for 15 min at RT and centrifugation at 16,000*g* for 5 min. For preparation of “cytoskeletal extract,” pellets were incubated with phosphoenolpyruvate for 10 min at RT followed by centrifugation at 16,000*g* for 5 min. All namings are according to the vendor.

In a pilot experiment, the extracted cytoplasmic, membrane, soluble nuclear, chromatin-bound nuclear, and cytoskeletal fractions of the 3xFLAG-parkin clones and control cells were subjected to proteome analysis (supplemental Fig. S1, *C* and *D*). The principal component analysis revealed high similarity between fractions, independent of the expression of parkin WT or C431A and replicates (supplemental Fig. S1*C*). The first component explains 82% of the variance and allows discrimination of cytoplasmic, membrane, and soluble nuclear fractions (F1–F3) *versus* chromatin-bound and cytoskeletal fractions (F4–F5), whereas the second dimension (explaining 6% of the variance) differentiates fraction 4 from 5. Gene Ontology analysis of subcellular localization of detected proteins agreed with the vendor-defined naming of fractions, albeit with minor contamination in adjacent fractions (supplemental Fig. S1*D*). Therefore, all further analyses were performed on the pooled “cytoplasmic,” “membrane,” and “soluble nuclear” fractions that enabled the optimal recovery of mitochondrial and cytosolic proteins, while depleting the heavily post-translationally–modified cytoskeletal and chromatin-bound nuclear proteins.

### Sample Preparation for Mass Spectrometry Analysis

Potentially present detergents in protein extracts were removed by acetone–methanol (acetone: eight volumes and methanol: one volume) precipitation overnight at −20 °C. Proteins were pelleted by centrifugation at 2500*g* for 20 min, and the remaining methanol was washed out with 80% of ice-cold acetone. Pellets were resuspended in denaturation buffer (6 M urea, 2 M thiourea, 10 mM Tris, pH 8), and the protein concentration was determined by Bradford assay. Within each experimental condition, the cytoplasmic, membrane, and nuclear extracts were mixed 1:1:1. Disulfide bonds were reduced with 10 mM DTT for 1 h, before alkylation for 1 h with either 55 mM iodoacetamide (phosphoproteome) or 55 mM chloracetamide (ubiquitylome). Predigestion was performed with Lys-C (Lysyl endopeptidase; Wako Chemicals) in a peptidase–protein ratio of 1:100 for 3 h at RT. For full overnight digestion, the fourfold volume of 50 mM ammonium bicarbonate was added before trypsin (Promega Corporation) in a peptidase–protein ratio of 1:100. Digestion was stopped by adding 1% TFA. Peptides were purified on Sep-Pak C18 Cartridges (Waters). For ubiquitylome analysis, peptides were eluted with 80% acetonitrile (ACN) and lyophilized for 24 h. Peptides were stored at −80 °C until GlyGly-peptide enrichment.

### Sample Preparation for Quantitative Phosphorylation and Ubiquitylation Analysis

Peptides of the phosphoproteome samples were dimethyl-labeled on Sep-Pak C18 Cartridges. Except for 0 h CCCP-treated samples (2 mg), 1 mg of peptide per sample were labeled as previously described (26). Early and late triple dimethyl sets were mixed in a peptide ratio of 1:1:1 per labeling channel with 0 h light as a common time point. Successful label efficiency and label channel mixing were checked in pilot LC–MS/MS runs.

Each sample from GlyGly proteome (15 μg) was loaded on C18 StageTips and flushed once with Hepes buffer at pH 8. TMT10-plex labeling reagent was adjusted to RT and constituted in 40 μl ACN. Six microliters of TMT reagent was added to each sample and pushed through the C18 material to fully cover loaded peptides. After 1 h, labeled peptides were eluted with 80% ACN, and labeling reaction was quenched for 15 min with 5% hydroxylamine in 80% ACN. Peptides were concentrated prior to label efficiency check in pilot LC–MS/MS run.

### High pH Reverse-Phase Chromatography

An off-line Ultimate 3000 HPLC System (Dionex, Thermo Fisher Scientific) equipped with xBridge BEH130 C_18_ 130 A, 3.5 μm, 4.6 × 250 mm column (Waters) was used to fractionate 3 mg per triple dimethyl (phosphoproteome set under basic conditions, as previously described (27)). In total, 66 fractions were collected every minute in an 80 min gradient at a flow rate of 1 ml/min by compositing buffer A (5 mM NH_4_OH) and B (5 mM NH_4_OH in 90% ACN). From minute 0 to 45, buffer B composition was increased from 5 to 25%. For 10 min, the gradient was maintained at 40% B followed by 70% B for 5 min. For column re-equilibration, the gradient was reduced to 5% B over 5 min and held for further 10 min. Fractions were concentrated into 33 pools and dried by vacuum centrifugation overnight. After reconstitution in 80% ACN, 10 μg per fraction were concentrated and used for proteome analysis by LC–MS/MS measurement.

The Pierce High pH Reversed-Phase Peptide Fractionation Kit (kit no.: 84868; Thermo Fisher Scientific) was used to fractionate 50 μg of TMT10-plex–labeled and mixed proteome. Spin columns were conditioned twice with ACN and 0.1% TFA according to the vendor. Acidified peptides were loaded on columns and washed once with water. Peptides were eluted stepwise with 5%, 7.5%, 10%, 12.5%, 13.3%, 15%, 17.5%, 20%, and final 50% ACN/ammonia. Fractions were acidified to pH 2 to 3 and desalted on C18 StageTips prior to LC–MS/MS measurement.

### Phosphopeptide Enrichment

Phosphopeptides were enriched using MagReSyn Ti-IMAC (titanium-immobilized metal affinity chromatography; ReSyn Bioscience) in two consecutive rounds of enrichment. About 15 μl of magnetic bead suspension per fraction and enrichment round was equilibrated first for 5 min with 70% ethanol, followed by washing twice for 10 min with 1% NH_4_OH. Beads were washed further three times in 1-min intervals with loading buffer (1 M glycolic acid and 5% TFA in 80% ACN). Reconstituted peptides were mixed 1:1 with loading buffer and transferred to the equilibrated beads. The mixture was incubated for 20 min, and the flow-through was applied for a second round of enrichment. Nonspecifically bound peptides were removed by washing twice for 2 min with 1% TFA in 80% ACN and twice with 0.2% TFA in 10% ACN. Phosphopeptides were eluted three times with 1% NH_4_OH for 20 min, and the pH was immediately adjusted with formic acid (FA) to 2 to 3. Both eluates of the same fraction were pooled and desalted on C18 StageTips prior to LC–MS/MS measurement.

### GlyGly-Peptide Enrichment and On-Bead TMT Labeling

GlyGly-modified peptides were enriched using PTM-Scan ubiquitin remnant motif (K-ϵ-GG) kit (kit no.: 5562; Cell Signaling Technology) as described previously (23). Dimethyl pimelimidate crosslinked antibodies were washed twice with ice-cold immunoaffinity purification (IAP) buffer (50 mM Mops, pH 7.2, 10 mM sodium phosphate, and 50 mM NaCl). Lyophilized peptides were reconditioned in IAP buffer for 5 min, and insoluble material was removed by centrifugation at 5000*g* for 5 min at 4 °C. Supernatants (pH ∼7) were transferred to the beads and incubated at 4 °C for 2 to 3 h with end-over-end rotation. Unbound peptides were removed by washing twice with ice-cold IAP buffer and once with PBS. For TMT labeling, beads were resuspended in Hepes buffer (pH 8.0–8.5) and incubated with 10 μl TMT for 15 min. The labeling reaction was quenched with 0.05% hydroxylamine for 5 min. Before elution, beads were washed twice with IAP and once with PBS. About 0.15% TFA was used for elution in two consecutive rounds for 5 min. TMT-labeled peptides were desalted on C18 StageTips prior to LC–MS/MS measurement.

### LC–MS Analysis

All phosphoproteome and TMT-labeled samples were analyzed on an Q Exactive HF mass spectrometer (Thermo Fisher Scientific), and all proteome samples were analyzed on an Q Exactive HF-X mass spectrometer (Thermo Fisher Scientific). An online-coupled Easy-nLC 1200 UHPLC (Thermo Fisher Scientific) was used to separate peptides on a 20 cm analytical column (75 μm ID PicoTip fused silica emitter [New Objective]) in-house packed with ReproSil-Pur C18-AQ 1.9 μm resin (Dr Maisch GmbH Ltd). Gradient was generated by solvent A (0.1% FA) and solvent B (0.1% FA in 80% ACN), at 40 °C and a 200 nl/min flow rate. Phosphopeptides were eluted using 90 min, GlyGly peptides in a 130 min gradient. Dimethyl-labeled proteome samples were eluted in a 36 min gradient and TMT-labeled proteome fractions in a fraction-specific segmented linear 90 min gradient. Eluted peptides were ionized on an electrospray ionization source. Both mass spectrometers were operated in a positive ion and data-dependent acquisition mode. All full mass spectrometry (MS) were acquired in a scan range of 300 to 1750 *m/z* at a resolution of 60,000. For proteome samples, the 20 most intense multiple-charged ions were selected for higher-energy collisional dissociation fragmentation at a resolution of 15,000. For phosphoproteome and all TMT samples, top seven most intense peptides were picked with maximum injection time set to 220 ms for phospho and 110 ms set for TMT samples at MS2 resolution of 60,000. In addition, all TMT samples were measured with isolation window set to 0.7 *m/z* and normalized collision energy set to 35.

### MS Data Analysis and Statistical Analysis

Raw data files were processed with the MaxQuant software suite (version 1.6.14.0) (28). MS/MS data were searched against UniProt *Homo sapiens* database (released December 11, 2019; 96,818 entries) containing PARK2 C431A mutant sequence and commonly observed contaminants. The mass tolerance for precursor ions was set to 4.5 ppm and for fragment ions to 20 pm. All search parameters were kept to default values except for the following. Dimethylation for light (28.03 Da), intermediate (32.06 Da), and heavy (36.08 Da) labels was allowed on lysine residues and peptide N termini for phosphoproteome data. Isobaric labeling and quantification on MS2 was enabled on lysine residues and peptide N termini with the TMT lot specific correction factors for ubiquitylome analysis. For all phospho raw files, phosphorylation on STY was defined as variable modification. GlyGly modification on K was configured to not create a new terminus and allowed for all GlyGly samples. Furthermore, oxidation of methionine and protein N-terminal acetylation were set as variable modifications. Carbamidomethylation of cysteine residues was allowed as fixed modification. All searches were performed in trypsin/P-specific digestion mode. Except for the analysis of GlyGly samples, maximum two missed cleavages were allowed. For GlyGly samples, the number of maximum missed cleavages was set to 4. Requantify and match-between runs and intensity-based absolute quantification options were enabled.

For the data analysis, only noncontaminant protein groups with at least two unique peptides were included. For subsequent proteome analysis, only protein groups were kept if quantified in two out of two replicates. For phosphoproteome and ubiquitylome analysis, identification in one replicate out of two or three was accepted after validation of high correlation between replicates. All data analyses were performed using Perseus software (version 1.6.7.0) (29). The density of data points with mitochondrial annotation or total between replicates was estimated by using default settings. For phosphoproteome and ubiquitylome analysis, the identified sites were normalized to the protein group prior to log2 transformation. Proteins were functionally annotated with GO cellular compartments as well as MitoCarta3.0. To investigate significantly changing protein abundances, two-way ANOVA test was performed to examine the influence of the cell line and treatment durations. Significantly regulated proteins (−log10 *p* > 1) were further analyzed by post hoc test (FDR: 0.1). Only proteins were kept that showed significant change between WT-parkin 0 h and longer treatment durations. The protein groups of WT-parkin samples were then hierarchically clustered (Euclidean distance followed by clustering on average). K-means was used to obtain 14 cluster groups for proteome. Similar significant testing was performed for phosphoproteome analysis.

For ubiquitylome analysis, we used the average of all replicates and filtered for ubiquitylation events present in at least 50% in all conditions. Highly regulated ubiquitylation sites (z-score outside −2 and 2) of at least one condition of WT- or C431A-parkin samples across all treatment durations were used for hierarchical clustering. We identified 13 row clusters, based on k-means. For temporal treatment dynamics analysis, the profiles of each clusters were averaged. For overrepresentation analysis within clusters, Fisher exact test (Benjamini–Hochberg FDR ≤0.1) for relative enrichment to majority protein groups was applied. For graphical display, R environment was applied. For phosphoproteome analysis, only sites identified under all experimental conditions in at least one replicate were considered for subsequent analysis, whereas 75% identification in at least one replicate was required for ubiquitylome analysis. For generation of Venn diagrams, the online tool https://www.stefanjol.nl/venny was used.

Western-blot based statistical analysis for degradation of mitochondrial subcompartment proteins and the analysis of the site-directed mutagenesis of VDAC2 effect were performed using one-way ANOVA and Fisher's least significant difference analysis as a post hoc test. All data were from at least three independent experiments. Significance is indicated by asterisks: ∗*p* ≤ 0.05, ∗∗*p* ≤ 0.01, and ∗∗∗*p* < 0.001.

## Results

We applied a quantitative proteomics approach to perform temporal analysis of protein abundance, ubiquitylation, and phosphorylation during early (0–6 h) and late stages (12–18 h) of parkin-dependent mitophagy. As a cellular system, we generated HeLa cell lines stably expressing WT- or ligase-dead (C431A) 3xFLAG-parkin at comparable levels. We chose HeLa cells as they are devoid of endogenous parkin activity (supplemental Fig. S1*A*). We treated these cells with the mitochondrial uncoupler CCCP to induce mitophagy and harvested them after 0, 2, 6, 12, and 18 h. After harvesting, we performed rough subcellular protein fractionation to reduce the levels of nuclear and cytoskeletal proteins (supplemental Fig. S1, *B*–*D*). The pooled “cytoplasmic,” “membrane,” and “soluble nuclear” extracts that enabled the optimal recovery of mitochondrial and cytosolic proteins were further investigated (see the Experimental Procedures section). We integrated the datasets on the temporal analysis of protein abundances, ubiquitylation, and phosphorylation during parkin-dependent mitophagy. In total, we identified 8359 proteins (supplemental Table S1), 1717 ubiquitylation sites (supplemental Table S2), and 20,464 phosphorylation sites (supplemental Table S3) with an FDR of 1% at the peptide and protein level.

### Parkin-Dependent Proteome Dynamics During Mitophagy

**Fig. 1:**
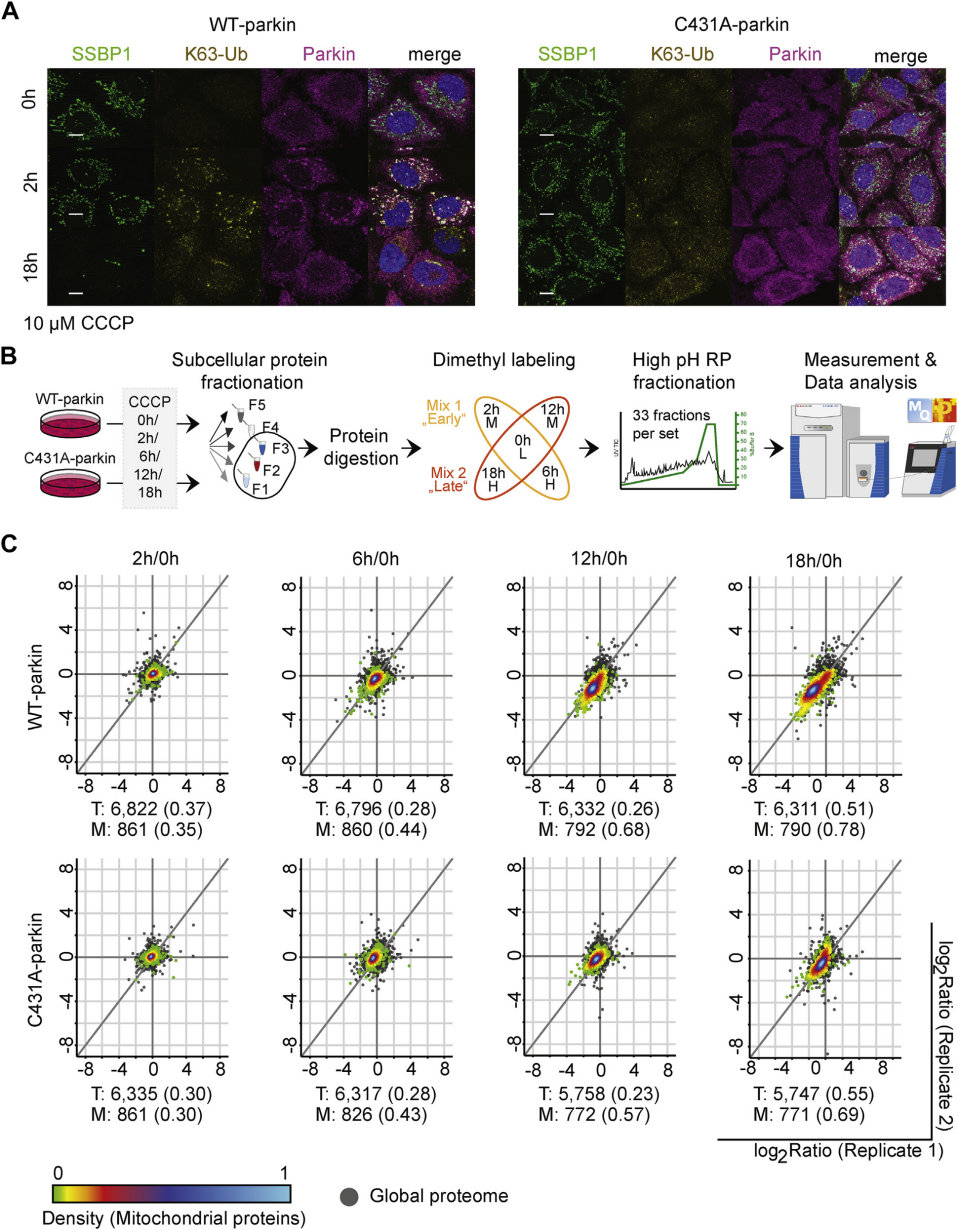
**Parkin activity is essential for the removal of depolarized mitochondria.***A*, immunofluorescence staining of WT-parkin and C431A-parkin cells after early (2 h) and late (18 h) depolarization times. Scale bars represent 5 μm. *B*, experimental setup combining subcellular protein fractionation with peptide dimethyl labeling and high pH reversed-phase fractionation during “early” and “late” mitophagy stages in WT-parkin and C431A-parkin cells. Basic workflow applied for proteome and phosphoproteome analysis. *C*, correlation plot between replicates after log_2_ transformation of protein ratios. Common light dimethyl labeling channel (0 h postdepolarization) allows comparison between “early” and “late” triple dimethyl-labeling sets per cell line. Parkin-dependent degradation of mitochondrial proteins reflected by shifting data points (density colored points) from “unregulated” toward “downregulated” in WT-parkin expressing cells in comparison to total proteome (*gray points*). Number of valid pairs per condition and Spearman rank correlation (*in brackets*) indicated for total (T) and mitochondrial annotated proteins (M).

Fluorescence microscopy confirmed FLAG-parkin recruitment to depolarized mitochondria within 2 h of CCCP treatment and the subsequent elimination of mitochondria in cells expressing WT- but not C431A-parkin (Fig. 1*A*). For temporal profiling of mitophagy-related changes in the proteome and phosphoproteome analyses, we used dimethyl labeling of tryptic peptides. To cover all five time points, we integrated two triple-dimethyl measurements using the 0 h CCCP treatment as the common time point of early (0, 2, and 6 h) and late (0, 12, and 18 h) stages of mitophagy for each cell line (Fig. 1*B*). In total, we identified 8359 proteins, of which 903 are annotated as mitochondrial. Furthermore, 83 of those are annotated as localized to the MOM, validating that our experimental design enriched for mitochondrial proteins. The reproducibility between biological replicates was high (supplemental Table S4). Next, we validated overall mitochondrial protein degradation by observing the density shift of data points related to mitochondrial proteins between cells expressing WT-parkin or C431A-parkin during prolonged CCCP treatment (Fig. 1*C*). In the presence of WT-parkin, we observed a downregulation of mitochondrial proteins after 6 h of mitophagy induction, and this trend continued until 18 h postdepolarization. In contrast, cells expressing C431A-parkin showed a mild shift of highly dense mitochondrial data points after 12 h of CCCP treatment. In addition, we validated mitochondrial protein degradation by Western blot analysis of established marker proteins (supplemental Fig. S1*E*). As expected, MOM proteins were degraded within 2 to 4 h of mitophagy induction only in cells expressing WT-parkin. To further validate that the observed mitochondrial degradation is a result of the activated PINK1/-parkin-dependent mitophagy pathway, we investigated additional hallmarks of mitophagy. First, we validated PINK1 accumulation after 2 h of CCCP treatment in WT-parkin–expressing cells. As PINK1 accumulated, the depolarized mitochondria were not eliminated in C431A-parkin cells (supplemental Fig. S1*E*). At later stages (8–18 h of mitochondrial depolarization), the dephosphorylation of S757 on ULK1 was observed in both WT-parkin and C431A-parkin cell lines, which revealed activation of general autophagy (supplemental Fig. S1*E*). Overall, we could validate successful execution of mitophagy in WT-parkin–expressing cells and also correct initiation in C431A-parkin–expressing cells. Late protein degradation in C431A-parkin cells may be associated with parkin-independent mitophagy and/or general autophagy induction.

### “Outside–In” Degradation of Mitochondrial Subcompartments

**Fig. 2:**
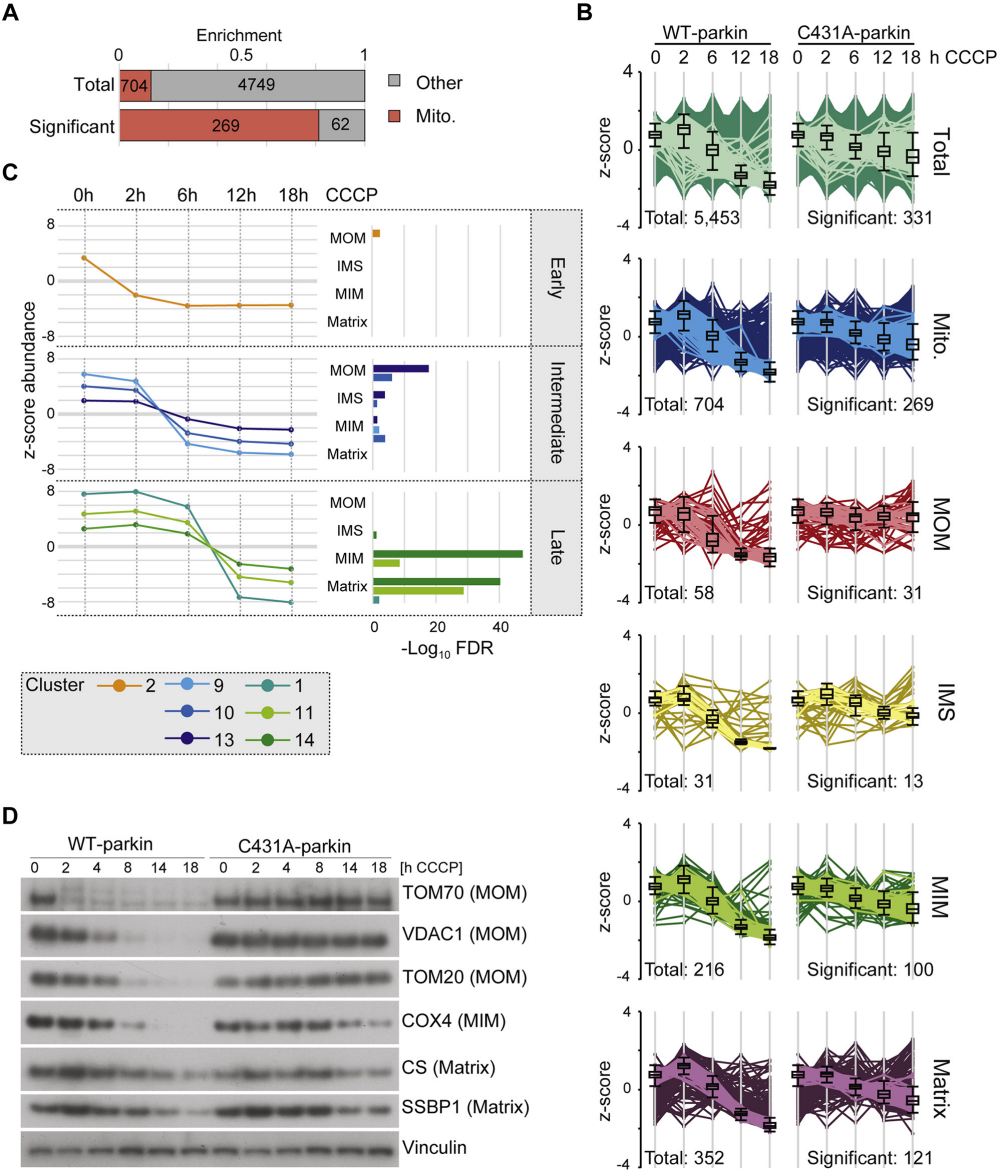
**Outside–in degradation of mitochondrial proteins.***A*, mitochondrial proteins were enriched among significantly regulated proteins. *B*, abundance profiles of significantly regulated proteins from various mitochondrial subcompartments show trends dependent on parkin activity. Indicated are size of total annotated proteins (*left*; *dark profiles*) and those after filtering (*right*; *bright profiles*); four mitochondrial proteins are of unknown or dual sublocalization (data not shown). *C*, average profile of each cluster sorted by half maximum in combination with overrepresentation analysis of mitochondrial subcompartments allows for the discrimination of “early” degraded MOM, “intermediate” degraded MOM/IMS/MIM, and “late” degraded MIM/matrix proteins. *D*, Western blot detection of mitochondrial subcompartment proteins during mitophagy validates stepwise degradation of mitochondrial subcompartments. IMS, inter membrane space; Mito, mitochondrion; MIM, mitochondrial inner membrane; MOM, mitochondrial outer membrane.

During prolonged mitophagy induction, the temporal profiles of 5453 proteins identified in both replicates were obtained in both WT-parkin and C431A-parkin–expressing cells. Of these, 704 were mitochondrial, including 58 MOM annotated proteins (Fig. 2, *A* and *B*). Among proteins significantly downregulated upon CCCP treatment in WT-parkin–expressing cells (ANOVA FDR <0.1), more than 80% (269 proteins) were annotated as mitochondrial (Fig. 2*A*). For all mitochondrial subcompartments, we detected a clear downregulation of protein levels during prolonged CCCP treatment in WT-parkin cells (Fig. 2*B*). Only a minor decrease on the level of mitochondrial proteins was seen in C431A-parkin cells, which are likely because of parkin-independent mitophagy pathways or due to the impaired mitochondrial import machinery.

To assess the dynamics of parkin-dependent mitochondrial protein degradation of early and late substrates, we performed unsupervised hierarchical clustering of significantly downregulated proteins in WT-parkin–expressing cells after mitophagy induction (supplemental Fig. S2*A*). Of the 14 identified protein profile clusters, six did not contain mitochondrial proteins and were not considered for downstream analyses (supplemental Fig. S2*B*). To identify a time line of mitochondrial protein degradation, we averaged the protein profiles within each cluster. By overrepresentation analysis of mitochondrial subcompartments, we could connect cluster profiles of early (0–2 h), intermediate (2–6 h), and late (12–18 h) downregulated proteins to specific mitochondrial subcompartments (Fig. 2*C* and supplemental Fig. S2*C*). Interestingly, proteins annotated for MOM localization were downregulated already after 2 h upon depolarization. Downregulated proteins at intermediate time points (2–6 h) were predominantly annotated for MOM, inner mitochondrial space, and MIM localization. During late stages of CCCP treatment (6–12 h), downregulated proteins were annotated based on MIM or matrix localization. These findings were further supported by analyzing protein levels of representative proteins of each mitochondrial compartment *via* Western blot as well as the relative quantification of the corresponding Western blot (Fig. 2*D* and supplemental Fig. S2*D*). MOM proteins such as TOM70 or VDAC1 were degraded after 8 h of CCCP, whereas MIM and matrix proteins represented by COX4 and CS remained present even after prolonged mitochondrial depolarization times. In addition, we observed differential time-dependent degradation between early and late MOM substrates: TOM70 degradation was obvious after 2 h of CCCP induction, whereas VDAC1 or TOM20 was not completely degraded after 8 h of mitochondrial depolarization (Fig. 2*D*). These results point to an “outside–in” directed sequential degradation of mitochondria subcompartments during parkin-dependent mitophagy. MOM protein degradation took place mainly within 2 to 6 h of CCCP treatment, whereas MIM and matrix protein degradation was delayed until 12 to 18 h of mitophagy induction.

### Protein Ubiquitylation Analysis Supports “Outside–In” Degradation Of Mitochondrial Subcompartments

To link the observed mitochondrial subcompartment protein degradation to parkin-dependent ubiquitylation, we further investigated the effect of parkin activity on site-specific ubiquitylome changes during early and late stages of mitophagy. Here, we applied the workflow of Udeshi *et al.* (23), which is based on the TMT labeling of GlyGly-modified peptides before elution from the ubiquitin remnant motif (K-ε-GG) antibody beads (supplemental Fig. S3*A*). In total, we identified 1717 ubiquitylation sites on 920 proteins. Of these, 245 sites were identified on 108 mitochondrial proteins (supplemental Fig. S3*B*). Although less ubiquitylated peptides were detected in two replicates, correlation of quantified GlyGly sites and proteins was high, and thus, all identified sites were considered in subsequent analyses (supplemental Table S5). Next, we compared levels of GlyGly-modified ubiquitin across time points and parkin cell lines (supplemental Fig. S3*C*). In both cell lines, we found high levels of GlyGly-modified ubiquitin on K48 during late stages of mitophagy (12–18 h). We also found a trend of increased levels of K6 and K63 GlyGly-modified ubiquitin after 2 h of depolarization in WT-parkin, whereas in C431A-parkin cells, these sites did not appear regulated.

**Fig. 3:**
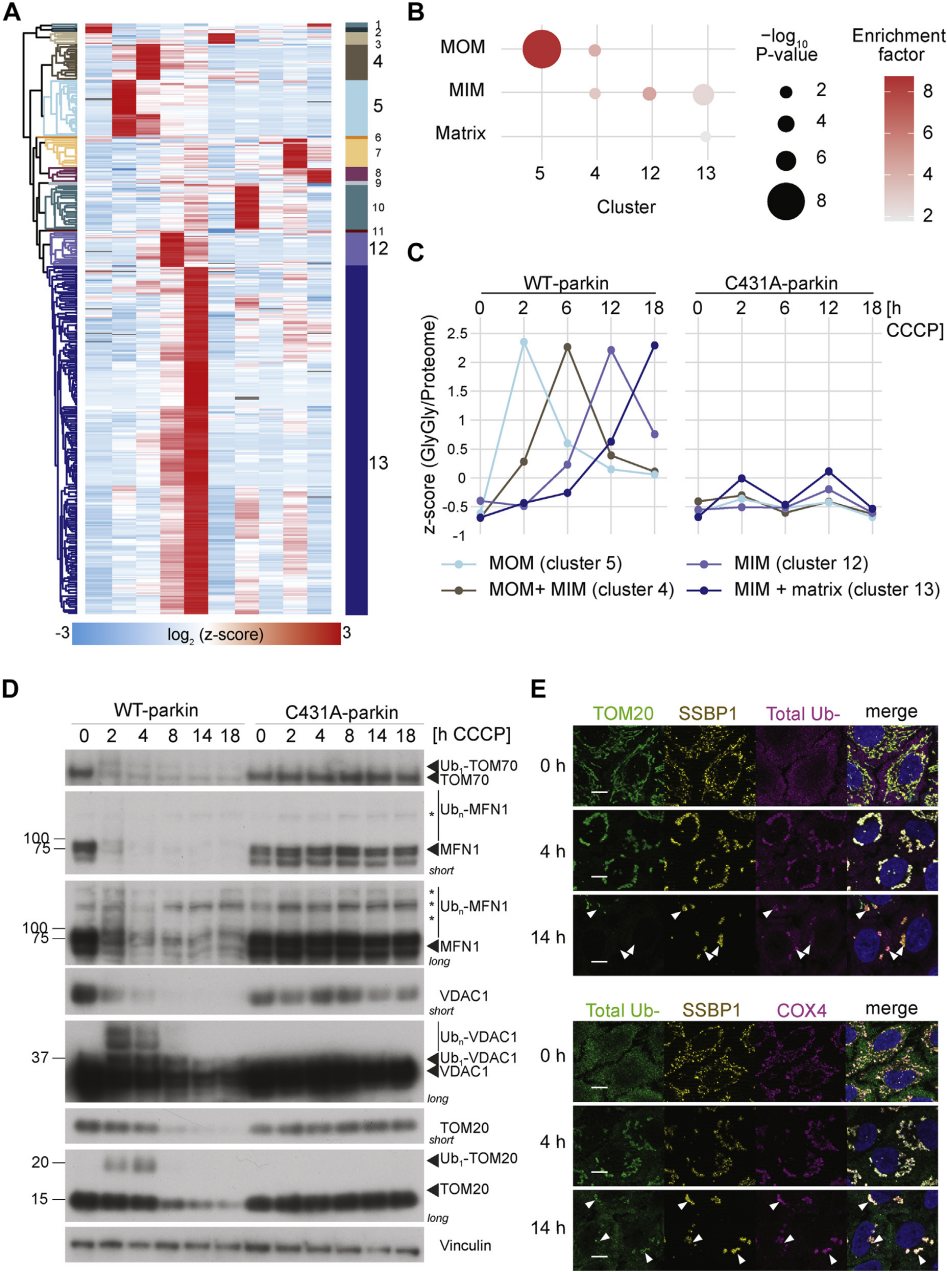
**Ubiquitylation dynamics during mitophagy.***A*, heat map after unsupervised hierarchical clustering into 13 clusters after filtering for significantly changing GlyGly sites (outside −2/2). *B*, average of profiles across CCCP treatment and parkin cell lines showing parkin-dependent and treatment-dependent response. *C*, overrepresentation of mitochondrial subcompartments across treatment duration changing clusters. *D*, Western blot detection of mitochondrial MOM parkin substrates over the course of mitophagy in WT-parkin and C431A-parkin cells. *Asterisks* indicate unspecific bands. “Short” and “long” indicate corresponding developing exposure times. *E*, immunofluorescence staining of ubiquitylated mitochondria (4 h) and ubiquitylated mitochondria subcompartments (14 h). *Arrowheads* indicate inner mitochondrial clumps. The scale bar represents 5 μm. CCCP, carbonyl cyanide *m*-chlorophenyl hydrazine; MOM, mitochondrial outer membrane; Ub, ubiquitylated.

Unsupervised hierarchical clustering identified 13 profile clusters containing regulated ubiquitylation sites (z-scored fold change outside −2 and 2) (Fig. 3*A* and supplemental Fig. S3*D*). We used overrepresentation analysis to connect specific ubiquitylation profile clusters with mitochondrial subcompartments (Fig. 3*B*). We further averaged four profile clusters with a parkin-dependent upregulation of ubiquitylation sites, either after 2, 6, 12, or 18 h of mitophagy induction (Fig. 3*C*). This revealed that MOM-annotated proteins showed an increase in ubiquitylation starting from 2 h of CCCP treatment and continued until 6 h. We found that MIM-annotated proteins predominantly ubiquitylated after 12 h of CCCP treatment, in addition to increased ubiquitylation after 6 and 18 h. In contrast, matrix-annotated proteins peaked after 18 h of mitochondrial depolarization. Interestingly, we identified ubiquitylation on parkin K48 among the late regulated ubiquitylation sites of cluster 13 (data not shown). This sequential pattern of ubiquitylation was almost exclusively found in WT-parkin but not C431A-parkin expressing cells.

We integrated the temporal dynamics of ubiquitylation events with significantly changing protein levels, based on their mitochondrial subcompartment localization (supplemental Fig. S3*E*). We identified 10 MOM annotated proteins with an early upregulated ubiquitylation site (2 h), which were degraded within 6 h of mitophagy induction. Among these, there were several validated parkin targets (*e.g.*, MFN1/2, RHOT2, or TOM70A). In contrast, 12 MIM (*e.g.*, PHB) and 3 matrix (*e.g.*, HSPD1) proteins showed protein degradation after 6 to 18 h with additional ubiquitylation during late stages (12–18 h). Ubiquitylation of MOM proteins was validated by Western blot analysis, which also revealed a time-dependent difference between early and late MOM parkin substrates (Fig. 3*D*). Proteins like MFN1 or TOM70 were rapidly ubiquitylated and degraded within 2 h of CCCP treatment. In contrast, other MOM proteins like TOM20 or VDAC2 became ubiquitylated shortly after incubation with CCCP and remained longer modified for up to 4 h. As expected, ubiquitylation changes were only detected in WT-parkin but not in C431A-parkin expressing cells (Fig. 3, *C* and *D*). However, immunofluorescence microscopy revealed that after 14 h of mitophagy induction—when MOM proteins (represented by TOM20) were mostly degraded—the inner mitochondrial proteins (represented by COX4 and SSBP1) appeared in clumpy profiles together with ubiquitin and the ubiquitin-binding p62/SQSTM autophagy receptor (Fig. 3*E* and supplemental Fig. S3*F*). When analyzing parkin localization during mitophagy, we observed that after intermediate (4–6 h) and late (>12 h) mitophagy induction time points, parkin is not detected on inner mitochondrial (or mitoplast) clumps but in the cytosol (Fig. 1*A* and supplemental Fig. S3*G*).

Taken together, the ubiquitylation data support the notion that parkin-dependent mitochondrial degradation takes place in a sequential “outside–in” manner, where extensive MOM ubiquitylation and further MOM elimination precede ubiquitylation and autophagy degradation of inner mitochondrial subcompartments (*i.e.*, MIM and matrix).

### Protein Phosphorylation Dynamics During Mitophagy

**Fig. 4:**
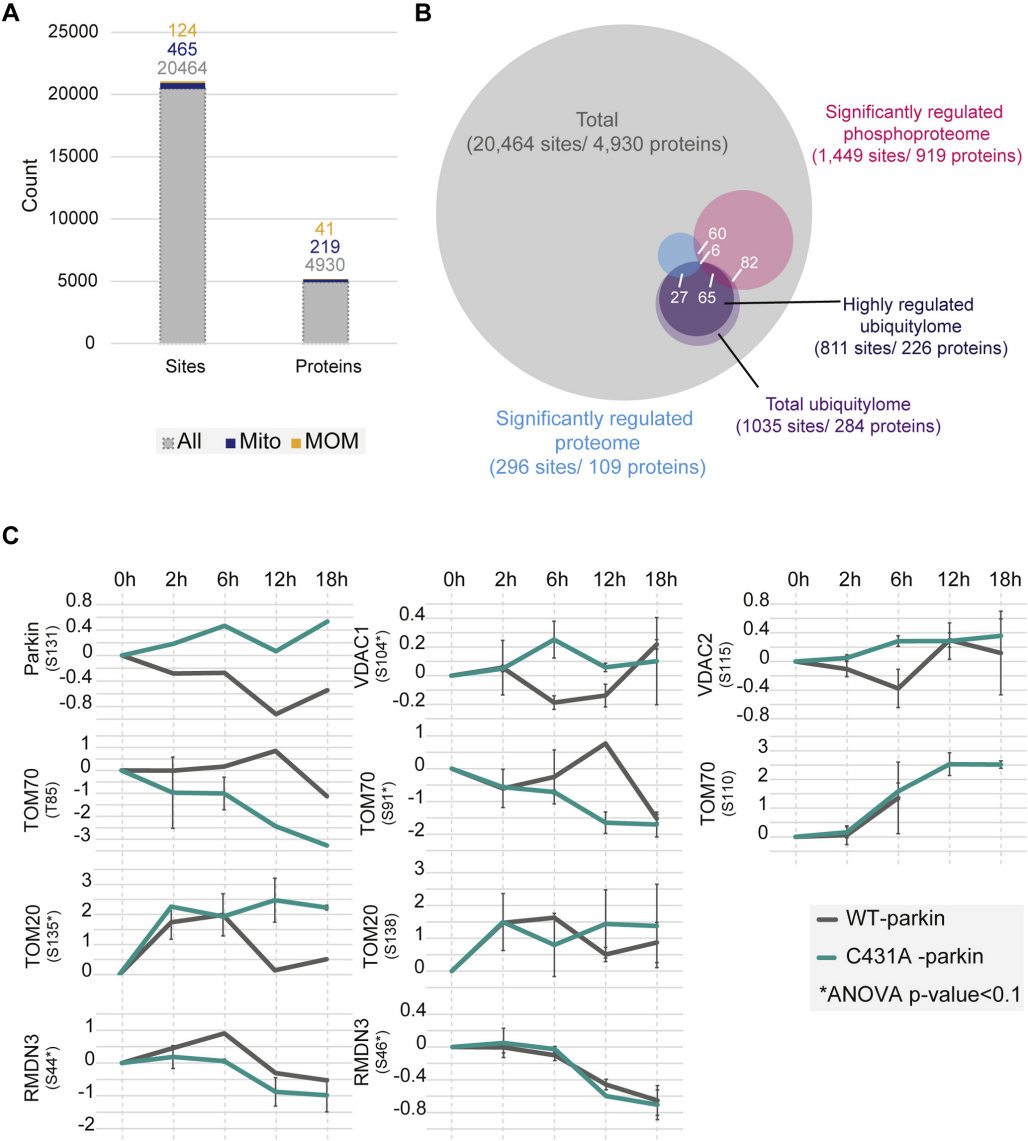
**Phosphorylation dynamics during mitophagy.***A*, identified phosphorylation sites of total, mitochondrial-annotated, and MOM-annotated proteins. *B*, overlap between proteome, ubiquitylome, and phosphoproteome datasets. Bulk-phosphorylation events are not simultaneously regulated on the protein and ubiquitylation levels. *C*, phosphorylation dynamics of MOM-annotated proteins with additional highly regulated ubiquitylation events. MOM, mitochondrial outer membrane.

Since ubiquitylation and phosphorylation play an important role during parkin-dependent mitophagy, we investigated a potential interplay of these two modifications during early and late stages of mitophagy. We extended our workflow to investigate proteome dynamics and integrated a phosphopeptide enrichment step after high pH reverse-phase fractionation. In total, we quantified 20,464 nonredundant phosphorylation events on 4930 proteins with high correlation between replicates (Fig. 4*A*, supplemental Fig. S4*A*, and supplemental Table S6). As shown earlier, we validated the accumulation of PINK1 on the MOM as a well-known initial step during PINK1/parkin-dependent mitophagy *via* Western blot analysis for both cell lines (supplemental Fig. S1*E*). Although we could not quantify PINK1 in our proteomics dataset, we validated the kinase activity by analyzing the phosphorylation dynamics of ubiquitin as it is established as a well characterized PINK1 target. After 2 h of postmitophagy induction, we detected strong upregulation of ubiquitin S65 phosphorylation, independent of parkin activity (supplemental Fig. S4*B*). This phosphorylation event showed a downward trend from 12 h of CCCP treatment in mitophagy-competent WT-parkin expressing cells. In contrast, pS65-ubiquitin continued to accumulate in mutant C431A-parkin expressing cells (supplemental Fig. S4*C*), consistent with the accumulation of PINK1 in the mitophagy-impaired C431A-parkin mutant cells (supplemental Fig. S1*E*). The higher molecular weight phospho-ubi adducts (supplemental Fig. S4*C*) must reflect ubiquitylations by ligases other than parkin, which evidently do not promote mitophagy as efficiently as those formed by parkin. Of all the identified phosphorylation events, 1449 were significantly regulated; however, no clear assignments to cellular compartments, signaling process, or specific regulatory differences between both cell lines or prolonged mitophagy induction were identified (supplemental Fig. S4*D*). We next addressed a potential interplay between phosphorylation and ubiquitylation events on identified significantly regulated proteins. Overall, we detected 284 doubly modified (phosphorylated and ubiquitylated) proteins indicating a modest crosstalk between these modifications during mitophagy (Fig. 4*B*). Six phosphorylation sites (on five proteins) showed significantly reduced protein abundance and highly regulated ubiquitylation and phosphorylation. Importantly, all these are either localized to the MOM (VDAC1, RMDN3, and TOM70) or to the MIM (AIFM1 and SLC25A11). All phosphorylation sites except for VDAC1 were found annotated in UniProt (30), but their functions remain unclear. Furthermore, we detected five phosphorylation sites on four MOM-annotated proteins, which showed a strongly regulated ubiquitylation site together with a nonsignificantly changing phosphorylation event (Fig. 4*C*). Both TOM70 phosphorylation events on T85 and S91 followed a similar trend of upregulation after 12 h in WT-parkin cells and downregulation in C431A-parkin cells. Similar regulation of phosphorylation dynamics between WT-parkin and C431A-parkin cells was found for RMDN3 S44 and S46 phosphorylation, following a downregulation trend after 6 h of CCCP treatment. Interestingly, we found VDAC1 and VDAC2 phosphorylation to be downregulated in WT-parkin expressing cells during intermediate and late stages of mitophagy but not in mutant C431A expressing cells. Taken together, these results point to a potential crosstalk between ubiquitylation and phosphorylation on MOM proteins degraded during parkin-dependent mitophagy.

### Interplay of Phosphorylation and Ubiquitylation on VDAC2 During Mitophagy

In addition to parkin-dependent ubiquitylation events guiding the course of mitophagy, we hypothesized that prior phosphorylation or dephosphorylation events might influence subsequent ubiquitylation. For this purpose, we were interested in parkin substrates that also showed potential phosphorylation or dephosphorylation regulations.

VDAC proteins are parkin ubiquitylation targets that remain in the mitochondria after intermediate times of depolarization (Figs. 2*D* and 3*D*; supplemental Fig. S1*E*). In our screen, VDAC2 was modified by ubiquitin as well as phosphorylation moieties over the course of mitophagy (supplemental Tables S2 and S3). More specifically, MS data indicated that in WT-parkin expressing cells, VDAC2 was ubiquitylated at K120, whereas dephosphorylation of S115 was commencing after 2 h of CCCP treatment. In C431A-parkin cells, phosphorylation of S115 was increasing over time, whereas no ubiquitylation changes were found in K120 (supplemental Fig. S5*A*).

**Fig. 5:**
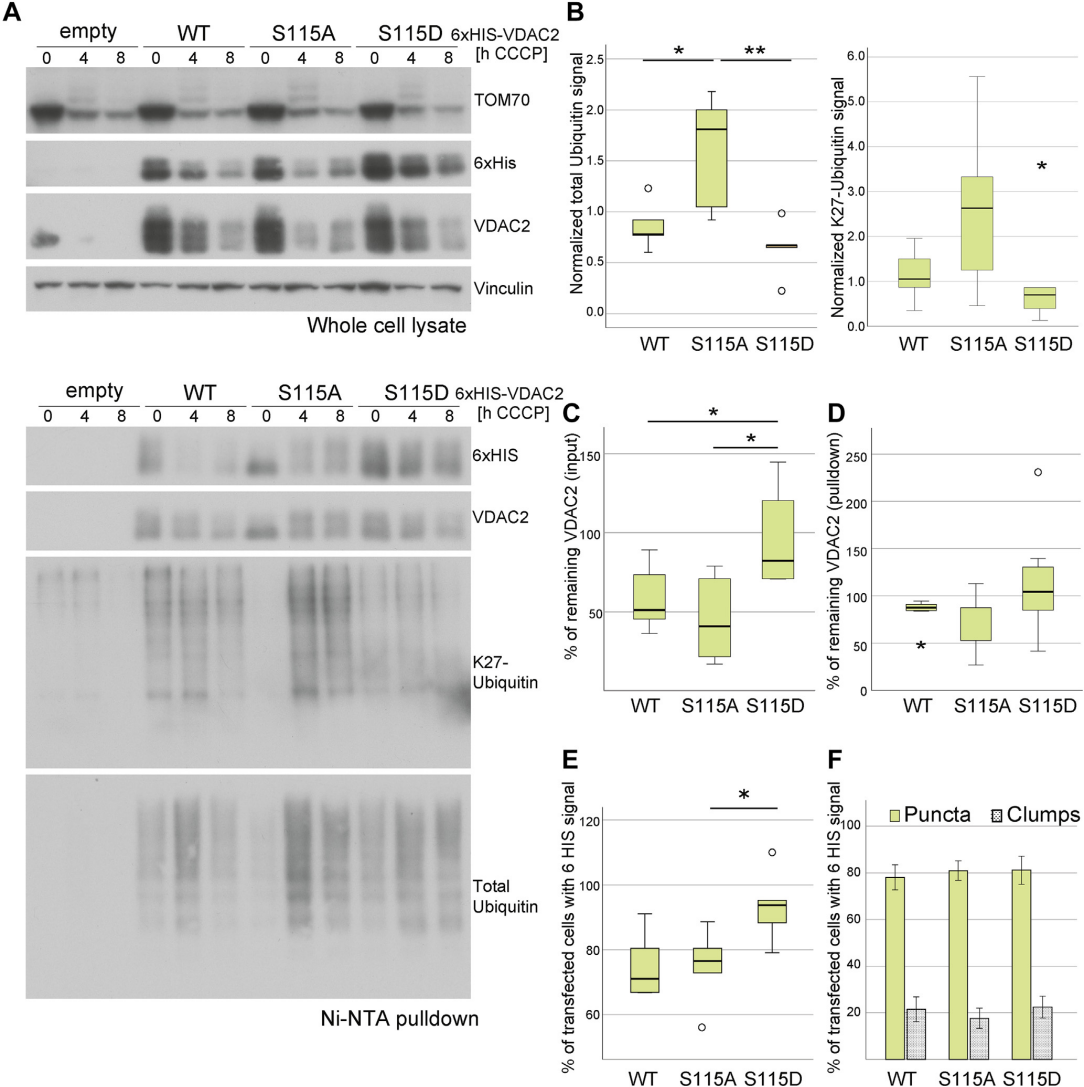
**Effect of phosphomimic and phosphodead mutations on VDAC2 mitophagy-dependent degradation.***A*, whole cell lysate and pulldown of transfected WT-parkin cells with 6xHis-WT and VDAC2 serine mutants. *B*, total ubiquitin and specific K27-linked ubiquitin chain levels of pulldown samples after 4 h of CCCP. *C* and *D*, remaining total VDAC2 levels after 4 h of CCCP in input and pulldown samples, respectively. *E*, percentage of transfected cells still showing VDAC2 signal after 14 h CCCP. *F*, mitochondria morphology of cells showing residual VDAC2 signal after 14 h of CCCP. Data of at least N = 3 independent experiments. Mean ± SEM. ∗*p* ≤ 0.05, ∗∗*p* ≤ 0.01. CCCP, carbonyl cyanide *m*-chlorophenyl hydrazine; VDAC2, voltage-dependent anion channel 2.

In order to decipher any possible crosstalk between dephosphorylation and ubiquitylation at this specific cytosolic loop of VDAC2, we generated 6xHIS-tagged VDAC2 constructs with phosphodead (S115A) and phosphomimic (S115D) serine substitutions. Total transfected levels of VDAC2 in whole cell lysate samples showed degradation of VDAC2 matching with mitophagy-dependent degradation of other MOM proteins (Fig. 5*A*). Moreover, there was an increase in VDAC2-S115D levels after 4 h, indicating a potential slower degradation of the phosphomimic mutant in comparison to the phosphodead form of VDAC2 (Fig. 5, *A*, *C*, and *D*). In order to understand if the slower degradation of the phosphomimic form of VDAC2 could be explained by the total ubiquitylation levels or specific ubiquitin-linkage chain levels, we performed Ni–NTA pull-down analysis. Total ubiquitin levels showed to be significantly increased in VDAC2-S115A in comparison to WT or VDAC2-S115D after mitochondrial depolarization (Fig. 5, *A* and *B*). In addition, there was a mild reduction of K27-ubiquitin–linked chain formation in VDAC2-S115D (Fig. 5, *A* and *B*).

We also analyzed the behavior of all VDAC2 transfected constructs over the course of mitophagy using immunofluorescence-based imaging techniques. Transfection of all 6xHIS-tagged VDAC2 constructs did not have an impact on overall mitophagy time course since we could observe mitochondrial fragmentation and complete elimination of mitochondria after 4 and 14 h of CCCP treatment, respectively (supplemental Fig. S5*B*). In addition, when performing single-cell quantification analysis of the number of cells with residual VDAC2 signal after 14 h CCCP, we observed a significant increase of positive cells, which were previously transfected with VDAC2-S115D (Fig. 5*E*), indicating that mimicking phosphorylation at S115D may delay VDAC2 degradation. We also assessed the impact of the phospho-VDAC2 mutations on the overall speed of mitophagy completion. For this purpose, we analyzed the mitochondria morphology of 6xHIS positive cells with the idea that, if delayed mitophagy was to be found, an increased number of cells would show mitochondrial clumps instead of mitochondria puncta after 14 h CCCP. Overall, around 80% of 6xHIS positive cells showed puncta mitochondrial morphology for all 6xHIS-VDAC2 transfected constructs, suggesting that bulk mitophagy completion was not affected when overexpressing VDAC2 point mutations (Fig. 5*F* and supplemental Fig. S5*B*).

Together, these data suggest that there is an interplay between ubiquitylation and phosphorylation at the fourth cytosolic loop (115–120 amino acids) of VDAC2, which seems to be dephosphorylated in order to be later ubiquitylated under parkin-dependent mitophagy conditions (supplemental Fig. S5*C*). Indeed, modifying phosphorylation status of S115 seems to have an impact on VDAC2 ubiquitylation and further protein degradation. When S115-phosphorylation is abolished, total ubiquitylation of VDAC2 is enhanced, and this leads to a faster VDAC2 degradation. Conversely, when S115-phosphorylation is mimicked, total VDAC2 ubiquitylations seem to mildly decrease, delaying VDAC2 degradation (Fig. 5, *A*, *B*, and *E*). In addition, it seems that mimicking S115-phosphorylation might have an impact on the topology of polyubiquitin chains that are assembled on this particular cytosolic loop of VDAC2, as K27-linked ubiquitin chains were slightly reduced when VDAC2-S115D was expressed (Fig. 5*D*).

## Discussion

Here, we provide a multilevel proteomic dataset comprising (phospho)proteome and ubiquitylome dynamics during early (2–6 h) and late (12–18 h) stages of parkin-dependent mitophagy. To link parkin activity with newly identified post-translational events, we compared conditions of successful and defective mitophagy in HeLa cells overexpressing similar levels of WT-parkin or C431A-parkin, respectively. As expected, we observed general mitochondrial elimination as well as ubiquitylation of known MOM proteins in WT-parkin cells only. GlyGly protein modification patterns of known MOM parkin substrates not only largely matched with the literature (11, 14, 31) but also yielded potential novel substrates, such as FIS1 or MARC1/2 (supplemental Table S2). Because of low protein levels, we could not validate PINK1 accumulation in all conditions *via* MS. Nevertheless, phosphorylation modification dynamics observed in our screen were in agreement with already published phosphoproteomic studies under parkin-dependent mitophagy conditions (32). For example, PINK1-dependent pS65-ubiquitin phosphorylation, together with autophagy adaptor phosphorylation, was observed in our dataset (supplemental Table S3). Taken together, our in-depth proteomic temporal analysis approach defined substrates at the protein, ubiquitylation, and phosphorylation levels, which are affected in early (0–2 h) intermediate (4–8 h), and late (12–18 h) stages of parkin-dependent mitophagy.

### Outside–In Mitochondrial Subcompartment Degradation

Previous studies demonstrated that MOM ubiquitylation promotes recruitment of autophagy adaptors and engulfment of damaged mitochondria by the autophagosome complex (15, 16, 33, 34). However, our data support the sequential subcompartment mitochondrial degradation hypothesis. While we observed extensive MOM ubiquitylation and protein degradation from 2 to 6 h after mitochondrial depolarization, we could also identify an increasing ubiquitylation of inner mitochondrial subcompartments at longer depolarization times (12 and 18 h CCCP) (Fig. 3, *B*, *C*, and *E*). Moreover, ubiquitylation of inner mitochondrial proteins matched with a delayed inner mitochondrial protein degradation (Fig. 2). Parkin is known to preferentially build K63-linked and K48-linked ubiquitin chains (12, 14, 19). Increase of K6 and K63 polyubiquitin chains after 2 h of depolarization in WT-parkin cells was in agreement with the reported increase of ubiquitin ligase activity between 2 and 6 h after mitochondrial depolarization (supplemental Fig. S3*C*). Conversely, K48-linked polyubiquitin chains were increased at late time points of mitochondrial depolarization but not exclusively in WT-parkin expressing cells. These findings suggest that parkin targets the MOM proteins within the initial stages but not the inner mitochondrial proteins during later stages of mitophagy.

Inner mitochondrial protein ubiquitylation has already been reported under parkin-dependent mitophagy conditions but only after relatively early depolarization time points. Sarraf *et al.* identified inner mitochondrial ubiquitylation after 1 h of depolarization under endogenous parkin conditions (31). Similarly, Ordureau *et al.* found such ubiquitylations to be enriched under WT-parkin and inactive parkin (S65A) endogenous expressing conditions, after 6 h of depolarization (11). Remaining mitochondria clumps were found ubiquitylated as well as recognized by p62/SQSTM at later stages of the pathway (Fig. 3*E* and supplemental Fig. S3*E*); this might indicate that inner mitochondrial ubiquitylations may trigger a later autophagy elimination of mitochondria inner compartments. This is in accordance to what we observe after 4 h of mitochondrial depolarization where complete MOM ubiquitylation triggers p62/SQSTM recognition and further MOM elimination (Fig. 3*E* and supplemental Fig. S3*E*). In addition, we identified parkin K48 ubiquitylation that is localized within the ubiquitin-like domain. This site was upregulated especially during late stages of mitophagy (12–18 h) and was not regulated in C431A-parkin expressing cells. Polyubiquitylation in general and specifically K48-linked ubiquitin chains are well-known markers for protein degradation (19, 35). Because we did not find CCCP treatment–dependent changes in the protein level of parkin, we hypothesize that parkin K48 ubiquitylation may affect parkin activity rather than its degradation.

These data, together with the fact that inner mitochondrial ubiquitylations have also been reported in conditions of inactive (11) or nonendogenous parkin (36, 37), point out that inner mitochondrial protein ubiquitylation may happen in a parkin-independent manner. Further experiments are needed to decipher which E3-ubiquitin ligases are responsible for inner mitochondrial subcompartment protein ubiquitylation under WT-parkin expressing conditions. A potential candidate might be the E3-ligase HUEW1 on which K1107 ubiquitylation was upregulated after 2 h of depolarization. Based on the temporal profile of parkin-preferred polyubiquitin chain linkages, with the highest level after 2 h depolarization (supplemental Fig. S3*C*), we hypothesize that HUWE1 ubiquitylation might be regulated by parkin.

### Crosstalk Between Ubiquitylation and Phosphorylation

The initiation of mitophagy relies on PINK1-dependent phosphorylation leading to parkin activation and translocation to the MOM (38, 39, 40, 41, 42). In addition, parkin-dependent ubiquitylation of mitochondrial proteins is known to promote mitochondrial degradation (19, 43). Here, we hypothesized that, in addition to parkin-dependent regulation of mitophagy by ubiquitylation, phosphorylation or dephosphorylation events could also play a role in specific MOM protein degradation or mitophagy progression. Ten phosphorylation sites on six significantly downregulated MOM proteins were detected in vicinity of regulated ubiquitylation sites. Among these were VDAC1 and VDAC2, which showed to be ubiquitylated and dephosphorylated at one of their cytosolic loops: 104 to 109 amino acids and 115 to 120 amino acids, respectively (supplemental Tables S2 and S3). Both are known parkin and non-PINK1 target sites. Site-directed mutagenesis of VDAC2 combined with mitochondrial depolarization assays indicated that mimicking dephosphorylation of S115 (S115A-VDAC2) promoted faster protein degradation because of an increased total ubiquitylation. On the other hand, mimicking phosphorylation of S115 (S115D-VDAC2) had the opposite effect; a delay in protein degradation because of a decreased total ubiquitylation.

Even though the role of K27-linked ubiquitin chains in cellular processes remains unknown, they have been suggested to be strongly increased in VDAC1 upon parkin-dependent ubiquitylation and have been linked with lysosomal targeting (12, 44). Considering that VDAC1 and VDAC2 show similarities in sequence and protein structure, we hypothesized that we might see changes in K27-linked ubiquitin chains when mutating S115. Indeed, even though data were statistically not significant, there seemed to be a decreased trend of K27-linked ubiquitin chain formation when dephosphorylation of S115 was abolished (Fig. 5*C*). This may indicate that K27-linked ubiquitin chains play a role in VDAC2 degradation as well but are not exclusive for the cytosolic loop of 115 to 120 amino acids. Taken together, our data provide evidence that protein degradation under parkin-dependent mitophagy conditions is likely to not only be regulated *via* parkin ubiquitylation but may implicate the role of phosphatases in the regulation of mitochondrial protein elimination.

In summary, our data indicate that degradation of complete mitochondrial or organelle subcompartment might happen in a sequential time-dependent manner and suggest that parkin may not be responsible for inner mitochondrial ubiquitylation events. In addition, we provide evidence that phosphorylation likely influences protein ubiquitylation and further degradation of some parkin substrates and thus, phosphatase and kinases are likely to play a role in the regulation of PINK1/parkin-dependent mitophagy.

## Data Availability

The MS proteomics data have been deposited to the ProteomeXchange Consortium *via* the PRIDE (45) partner repository with the dataset identifier PXD027586. Visualization of MS/MS spectra is possible at https://msviewer.ucsf.edu/prospector/cgi-bin/msform.cgi?form=msviewer *via* the search keys: nrajlpjg0x (subcellular protein fractionation), km5ojtsfoc (proteome), c0geshw3ie (phosphoproteome), and suxnuntnwt (ubiquitylome).

## Supplemental data

This article contains supplemental data.

## Conflict of interest

The authors declare no competing interests.

## References

[1] Dikic I., Elazar Z. (2018). Mechanism and medical implications of mammalian autophagy. Nat. Rev. Mol. Cell Biol..

[2] Giacomello M., Pyakurel A., Glytsou C., Scorrano L. (2020). The cell biology of mitochondrial membrane dynamics. Nat. Rev. Mol. Cell Biol..

[3] Kitada T., Asakawa S., Hattori N., Matsumine H., Yamamura Y., Minoshima S., Yokochi M., Mizuno Y., Shimizu N. (1998). Mutations in the parkin gene cause autosomal recessive juvenile parkinsonism. Nature.

[4] Valente E.M., Abou-Sleiman P.M., Caputo V., Muqit M.M., Harvey K., Gispert S., Ali Z., Del Turco D., Bentivoglio A.R., Healy D.G., Albanese A., Nussbaum R., Gonzalez-Maldonado R., Deller T., Salvi S. (2004). Hereditary early-onset Parkinson's disease caused by mutations in PINK1. Science.

[5] Greene A.W., Grenier K., Aguileta M.A., Muise S., Farazifard R., Haque M.E., McBride H.M., Park D.S., Fon E.A. (2012). Mitochondrial processing peptidase regulates PINK1 processing, import and Parkin recruitment. EMBO Rep..

[6] Jin S.M., Lazarou M., Wang C., Kane L.A., Narendra D.P., Youle R.J. (2010). Mitochondrial membrane potential regulates PINK1 import and proteolytic destabilization by PARL. J. Cell Biol..

[7] Meissner C., Lorenz H., Weihofen A., Selkoe D.J., Lemberg M.K. (2011). The mitochondrial intramembrane protease PARL cleaves human Pink1 to regulate Pink1 trafficking. J. Neurochem..

[8] Aerts L., Craessaerts K., De Strooper B., Morais V.A. (2015). PINK1 kinase catalytic activity is regulated by phosphorylation on serines 228 and 402. J. Biol. Chem..

[9] Okatsu K., Oka T., Iguchi M., Imamura K., Kosako H., Tani N., Kimura M., Go E., Koyano F., Funayama M., Shiba-Fukushima K., Sato S., Shimizu H., Fukunaga Y., Taniguchi H. (2012). PINK1 autophosphorylation upon membrane potential dissipation is essential for Parkin recruitment to damaged mitochondria. Nat. Commun..

[10] Trempe J.F., Sauve V., Grenier K., Seirafi M., Tang M.Y., Menade M., Al-Abdul-Wahid S., Krett J., Wong K., Kozlov G., Nagar B., Fon E.A., Gehring K. (2013). Structure of parkin reveals mechanisms for ubiquitin ligase activation. Science.

[11] Ordureau A., Paulo J.A., Zhang J., An H., Swatek K.N., Cannon J.R., Wan Q., Komander D., Harper J.W. (2020). Global landscape and dynamics of parkin and USP30-dependent ubiquitylomes in iNeurons during mitophagic signaling. Mol. Cell.

[12] Geisler S., Holmstrom K.M., Skujat D., Fiesel F.C., Rothfuss O.C., Kahle P.J., Springer W. (2010). PINK1/Parkin-mediated mitophagy is dependent on VDAC1 and p62/SQSTM1. Nat. Cell Biol..

[13] Geisler S., Jager L., Golombek S., Nakanishi E., Hans F., Casadei N., Terradas A.L., Linnemann C., Kahle P.J. (2019). Ubiquitin-specific protease USP36 knockdown impairs Parkin-dependent mitophagy *via* downregulation of Beclin-1-associated autophagy-related ATG14L. Exp. Cell Res..

[14] Ordureau A., Paulo J.A., Zhang W., Ahfeldt T., Zhang J., Cohn E.F., Hou Z., Heo J.M., Rubin L.L., Sidhu S.S., Gygi S.P., Harper J.W. (2018). Dynamics of PARKIN-dependent mitochondrial ubiquitylation in induced neurons and model systems revealed by digital Snapshot proteomics. Mol. Cell.

[15] Heo J.M., Ordureau A., Paulo J.A., Rinehart J., Harper J.W. (2015). The PINK1-PARKIN mitochondrial ubiquitylation pathway drives a program of OPTN/NDP52 recruitment and TBK1 activation to promote mitophagy. Mol. Cell.

[16] Lazarou M., Sliter D.A., Kane L.A., Sarraf S.A., Wang C., Burman J.L., Sideris D.P., Fogel A.I., Youle R.J. (2015). The ubiquitin kinase PINK1 recruits autophagy receptors to induce mitophagy. Nature.

[17] Padman B.S., Nguyen T.N., Uoselis L., Skulsuppaisarn M., Nguyen L.K., Lazarou M. (2019). LC3/GABARAPs drive ubiquitin-independent recruitment of Optineurin and NDP52 to amplify mitophagy. Nat. Commun..

[18] Yamano K., Matsuda N., Tanaka K. (2016). The ubiquitin signal and autophagy: An orchestrated dance leading to mitochondrial degradation. EMBO Rep..

[19] Harper J.W., Ordureau A., Heo J.M. (2018). Building and decoding ubiquitin chains for mitophagy. Nat. Rev. Mol. Cell Biol..

[20] Pickrell A.M., Youle R.J. (2015). The roles of PINK1, parkin, and mitochondrial fidelity in Parkinson's disease. Neuron.

[21] Yoshii S.R., Kishi C., Ishihara N., Mizushima N. (2011). Parkin mediates proteasome-dependent protein degradation and rupture of the outer mitochondrial membrane. J. Biol. Chem..

[22] Denison S.R., Wang F., Becker N.A., Schule B., Kock N., Phillips L.A., Klein C., Smith D.I. (2003). Alterations in the common fragile site gene Parkin in ovarian and other cancers. Oncogene.

[23] Udeshi N.D., Mani D.C., Satpathy S., Fereshetian S., Gasser J.A., Svinkina T., Olive M.E., Ebert B.L., Mertins P., Carr S.A. (2020). Rapid and deep-scale ubiquitylation profiling for biology and translational research. Nat. Commun..

[24] Rath S., Sharma R., Gupta R., Ast T., Chan C., Durham T.J., Goodman R.P., Grabarek Z., Haas M.E., Hung W.H.W., Joshi P.R., Jourdain A.A., Kim S.H., Kotrys A.V., Lam S.S. (2021). MitoCarta3.0: an updated mitochondrial proteome now with sub-organelle localization and pathway annotations. Nucl. Acids Res..

[25] Hung V., Zou P., Rhee H.W., Udeshi N.D., Cracan V., Svinkina T., Carr S.A., Mootha V.K., Ting A.Y. (2014). Proteomic mapping of the human mitochondrial intermembrane space in live cells *via* ratiometric APEX tagging. Mol. Cell.

[26] Boersema P.J., Raijmakers R., Lemeer S., Mohammed S., Heck A.J. (2009). Multiplex peptide stable isotope dimethyl labeling for quantitative proteomics. Nat. Protoc..

[27] Batth T.S., Olsen J.V. (2016). Offline high pH reversed-phase peptide fractionation for deep phosphoproteome coverage. Methods Mol. Biol..

[28] Cox J., Mann M. (2008). MaxQuant enables high peptide identification rates, individualized p.p.b.-range mass accuracies and proteome-wide protein quantification. Nat. Biotechnol..

[29] Tyanova S., Temu T., Sinitcyn P., Carlson A., Hein M.Y., Geiger T., Mann M., Cox J. (2016). The Perseus computational platform for comprehensive analysis of (prote)omics data. Nat. Methods.

[30] UniProt C. (2021). UniProt: The universal protein knowledgebase in 2021. Nucleic Acids Res..

[31] Sarraf S.A., Raman M., Guarani-Pereira V., Sowa M.E., Huttlin E.L., Gygi S.P., Harper J.W. (2013). Landscape of the PARKIN-dependent ubiquitylome in response to mitochondrial depolarization. Nature.

[32] Lai Y.C., Kondapalli C., Lehneck R., Procter J.B., Dill B.D., Woodroof H.I., Gourlay R., Peggie M., Macartney T.J., Corti O., Corvol J.C., Campbell D.G., Itzen A., Trost M., Muqit M.M. (2015). Phosphoproteomic screening identifies Rab GTPases as novel downstream targets of PINK1. EMBO J..

[33] Nguyen T.N., Padman B.S., Lazarou M. (2016). Deciphering the molecular signals of PINK1/parkin mitophagy. Trends Cell Biol..

[34] Richter B., Sliter D.A., Herhaus L., Stolz A., Wang C., Beli P., Zaffagnini G., Wild P., Martens S., Wagner S.A., Youle R.J., Dikic I. (2016). Phosphorylation of OPTN by TBK1 enhances its binding to Ub chains and promotes selective autophagy of damaged mitochondria. Proc. Natl. Acad. Sci. U. S. A..

[35] Chan N.C., Salazar A.M., Pham A.H., Sweredoski M.J., Kolawa N.J., Graham R.L., Hess S., Chan D.C. (2011). Broad activation of the ubiquitin-proteasome system by Parkin is critical for mitophagy. Hum. Mol. Genet..

[36] Oshima Y., Cartier E., Boyman L., Verhoeven N., Polster B.M., Huang W., Kane M., Lederer W.J., Karbowski M. (2021). Parkin-independent mitophagy *via* Drp1-mediated outer membrane severing and inner membrane ubiquitination. J. Cell Biol..

[37] Sulkshane P., Duek I., Ram J., Thakur A., Reis N., Ziv T., Glickman M.H. (2020). Inhibition of proteasome reveals basal mitochondrial ubiquitination. J. Proteomics.

[38] Kazlauskaite A., Kelly V., Johnson C., Baillie C., Hastie C.J., Peggie M., Macartney T., Woodroof H.I., Alessi D.R., Pedrioli P.G., Muqit M.M. (2014). Phosphorylation of Parkin at Serine65 is essential for activation: Elaboration of a Miro1 substrate-based assay of parkin E3 ligase activity. Open Biol..

[39] Kazlauskaite A., Kondapalli C., Gourlay R., Campbell D.G., Ritorto M.S., Hofmann K., Alessi D.R., Knebel A., Trost M., Muqit M.M. (2014). Parkin is activated by PINK1-dependent phosphorylation of ubiquitin at Ser65. Biochem. J..

[40] Kondapalli C., Kazlauskaite A., Zhang N., Woodroof H.I., Campbell D.G., Gourlay R., Burchell L., Walden H., Macartney T.J., Deak M., Knebel A., Alessi D.R., Muqit M.M. (2012). PINK1 is activated by mitochondrial membrane potential depolarization and stimulates Parkin E3 ligase activity by phosphorylating Serine 65. Open Biol..

[41] McWilliams T.G., Barini E., Pohjolan-Pirhonen R., Brooks S.P., Singh F., Burel S., Balk K., Kumar A., Montava-Garriga L., Prescott A.R., Hassoun S.M., Mouton-Liger F., Ball G., Hills R., Knebel A. (2018). Phosphorylation of Parkin at serine 65 is essential for its activation *in vivo*. Open Biol..

[42] Zhuang N., Li L., Chen S., Wang T. (2016). PINK1-dependent phosphorylation of PINK1 and Parkin is essential for mitochondrial quality control. Cell Death Dis..

[43] Nguyen T.N., Padman B.S., Usher J., Oorschot V., Ramm G., Lazarou M. (2016). Atg8 family LC3/GABARAP proteins are crucial for autophagosome-lysosome fusion but not autophagosome formation during PINK1/Parkin mitophagy and starvation. J. Cell Biol..

[44] Ikeda H., Kerppola T.K. (2008). Lysosomal localization of ubiquitinated Jun requires multiple determinants in a lysine-27–linked polyubiquitin conjugate. Mol. Biol. Cell.

[45] Perez-Riverol Y., Csordas A., Bai J., Bernal-Llinares M., Hewapathirana S., Kundu D.J., Inuganti A., Griss J., Mayer G., Eisenacher M., Perez E., Uszkoreit J., Pfeuffer J., Sachsenberg T., Yilmaz S. (2019). The PRIDE database and related tools and resources in 2019: Improving support for quantification data. Nucleic Acids Res..

