# Supplementary material for: Temporal Analysis of Protein Ubiquitylation and Phosphorylation During Parkin-Dependent Mitophagy
Source: Mol Cell Proteomics. 2021 Dec 30;21(2):100191. doi: 10.1016/j.mcpro.2021.100191 (PMC8808264; doi:10.1016/j.mcpro.2021.100191)
Supplement: Supplemental Figures S1–S5 [file mmc1.docx]

Temporal Analysis of Protein Ubiquitylation and Phosphorylation During Parkin-dependent Mitophagy

Katharina I. Zittlau^1*^, Anna Lechado-Terradas^2,3*^, Nicolas Nalpas^1^, Sven Geisler^2†^, Philipp J. Kahle^2,3#^, Boris Macek^1#^

# **Supplementary Materials**

Supplementary Figure 1: Subcellular protein fractionation enriches mitochondrial and associated proteins

Supplementary Figure 2: Stepwise degradation of mitochondrial sub-compartments

Supplementary Figure 3: Parkin-dependent ubiquitination of mitochondrial proteins

Supplementary Figure 4: Phosphorylation dynamics during PINK1/Parkin-dependent mitophagy.

Supplementary Figure 5: Behavior of VDAC2 upon mitophagy induction in WT-parkin expressing cells.

Supplementary Table 1 (S1): Outside-in degradation of mitochondrial sub-compartments (Figure 2)

Supplementary Table 2 (S2): Protein ubiquitylation analysis supports outside-in directed degradation of mitochondrial subcompartments (Figure 3)

Supplementary Table 3 (S3): Protein phosphorylation dynamics during mitophagy (Figure 4)

Supplementary Table 4 (S4): Correlation between replicates – proteome (Figure 1B)

Supplementary Table 5 (S5): Correlation between replicates - ubiquitylome (Figure 3)

Supplementary Table 6 (61): Correlation between replicates - phosphoproteome (Supplementary Figure 4C)

Supplementary Table 7 (S7): Subcellular protein fractionation (Supplementary Figure 1)


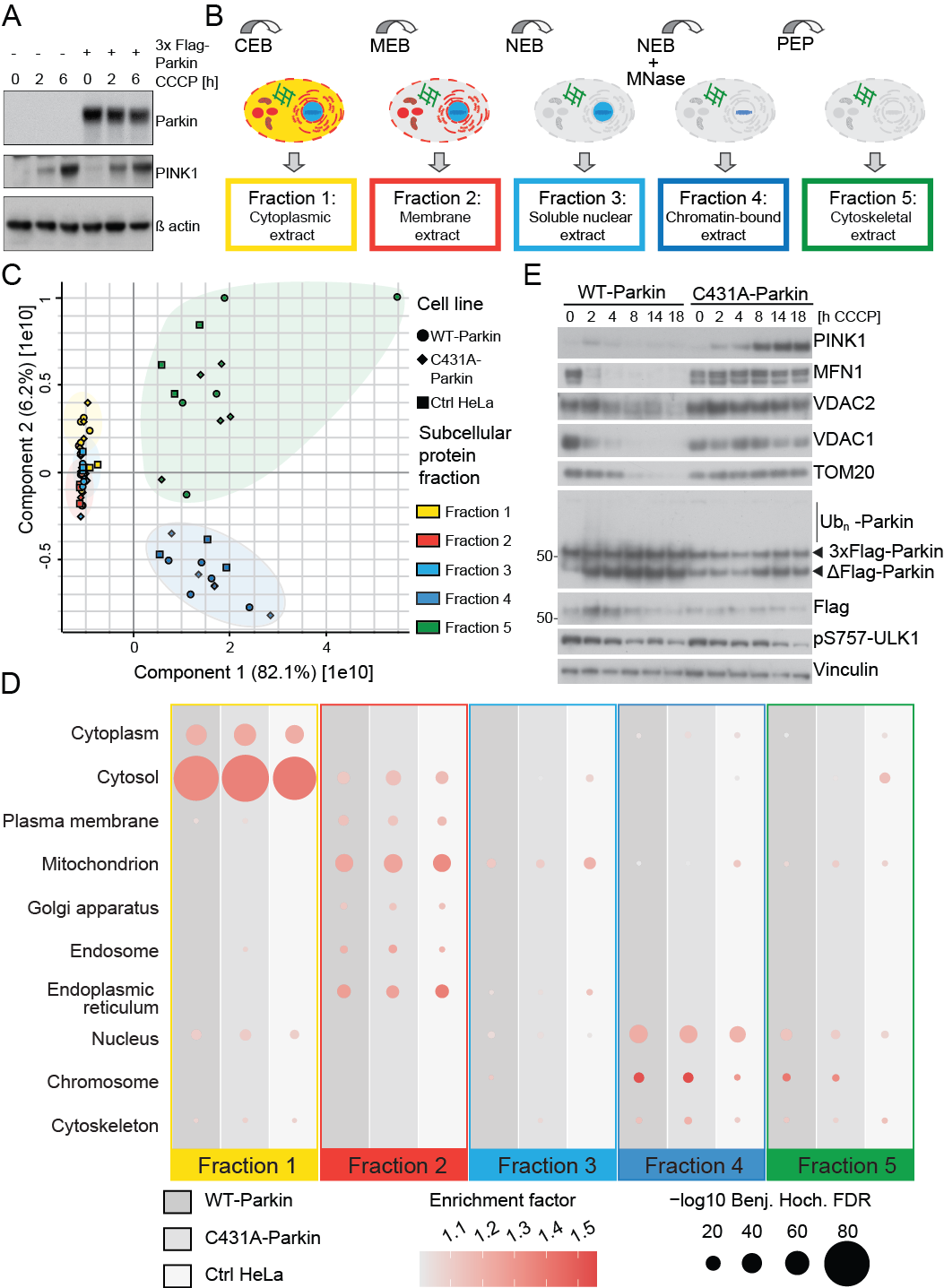


**Supplementary Figure 1: Subcellular protein fractionation enriches mitochondrial and associated proteins (A) Validation of Parkin expression levels in parental and transfected HeLa cells. (B)** General workflow of the subcellular protein fractionation kit for cultured cells provided by Thermo Fisher Scientific. Rough cellular fractionation (naming according to vendor): Fraction 1 “Cytoplasmic extract”, Fraction 2 “Membrane extract”, Fraction 3 “Soluble nuclear extract”, Fraction 4 “Chromatin-bound nuclear extract”, Fraction 5 “Cytoskeletal extract”. **(C)** Principal component analysis shows high similarity between fractions, independent of cell-line and replicate. Analysis based on 0h CCCP treatment. The first component explains 82% of the variance and allows discrimination of fractions 1-3 versus 4-5, while the second dimension (explaining 6% of the variance) differentiates fraction 4 from 5. WT-Parkin: n = 5, C431A-Parkin: n = 5, Ctrl. HeLa: n =3. **(D)** Overrepresentation of selected cellular sub- compartments in WT-Parkin, C431A-Parkin and parental HeLa cells (no endogenous Parkin) after subcellular fractionation. **(E)** Western blot validation of mitophagy induction and mitochondria elimination achieved only in WT-Parkin cells. CEB: Cytoplasmic extraction buffer, MEB: Membrane extraction buffer, NEB: Nuclear extraction buffer, PEP: Pellet Extraction buffer, Ctrl: control; Ub-: Ubiquitinated


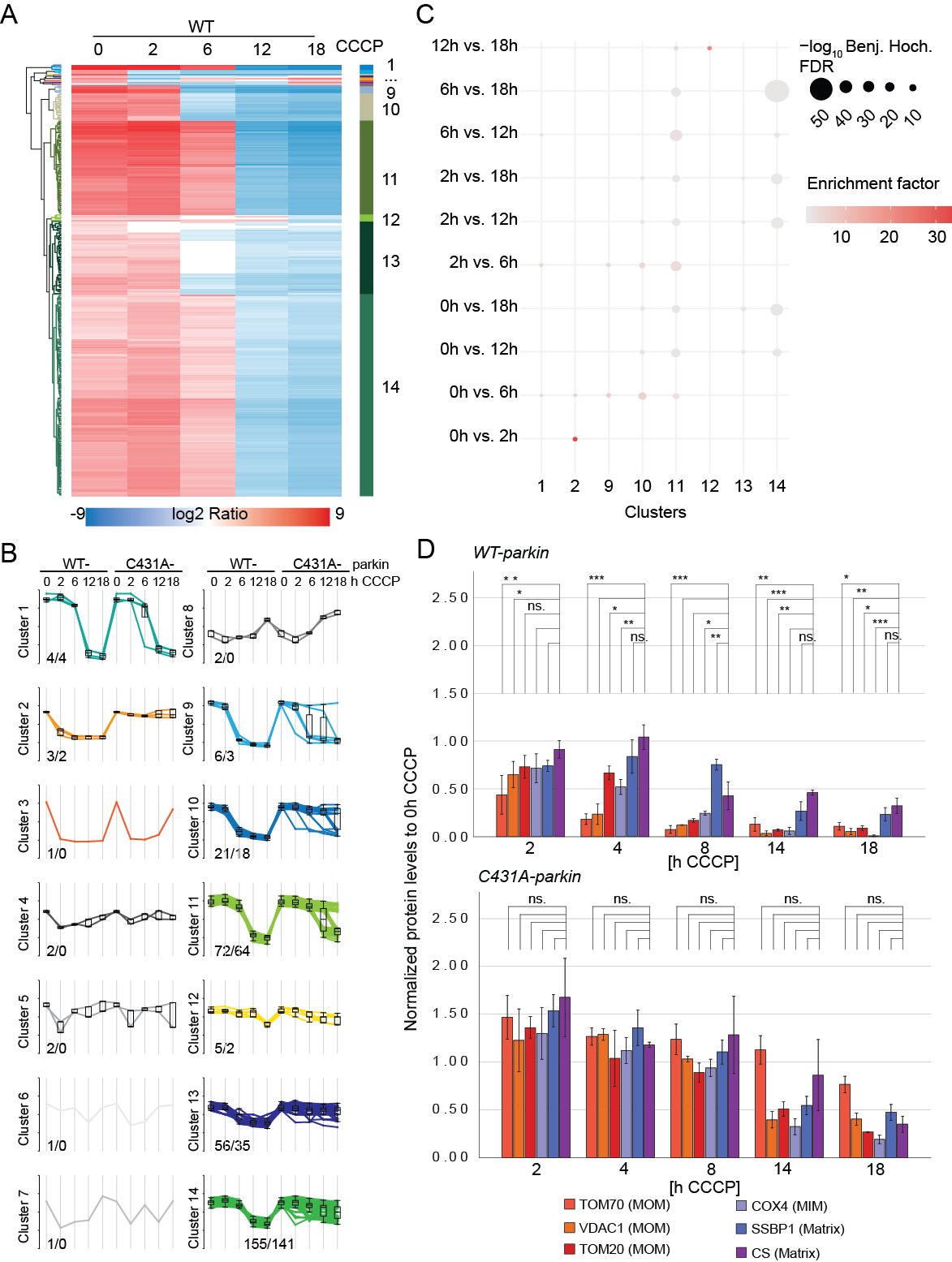


**Supplementary figure 2: Stepwise degradation of mitochondrial sub-compartments**. **(A)** Heatmap after unsupervised hierarchical clustering of significantly different proteins between 0h and prolonged depolarization in WT-Parkin expressing cells. 14 clusters were identified with **(B)** Profile plots after hierarchical clustering of significantly regulated proteins and filtering for significantly changed between WT 0h and prolonged CCCP treatment. Indicated are the cluster size and number of mitochondrial proteins identified. **(C)** Overrepresentation of significantly different pairs of clusters. **(D)** Normalized protein degradation levels of mitochondrial sub- compartments for WT- and C431A-Parkin expressing cells at the indicated depolarization time-points, based on Western blot (Figure 2D) quantification. Data from at least N=4. No significant Mean ± SEM. ns.: p-value>0.05; *: p-value≤0.05, **; p-value≤0.01.


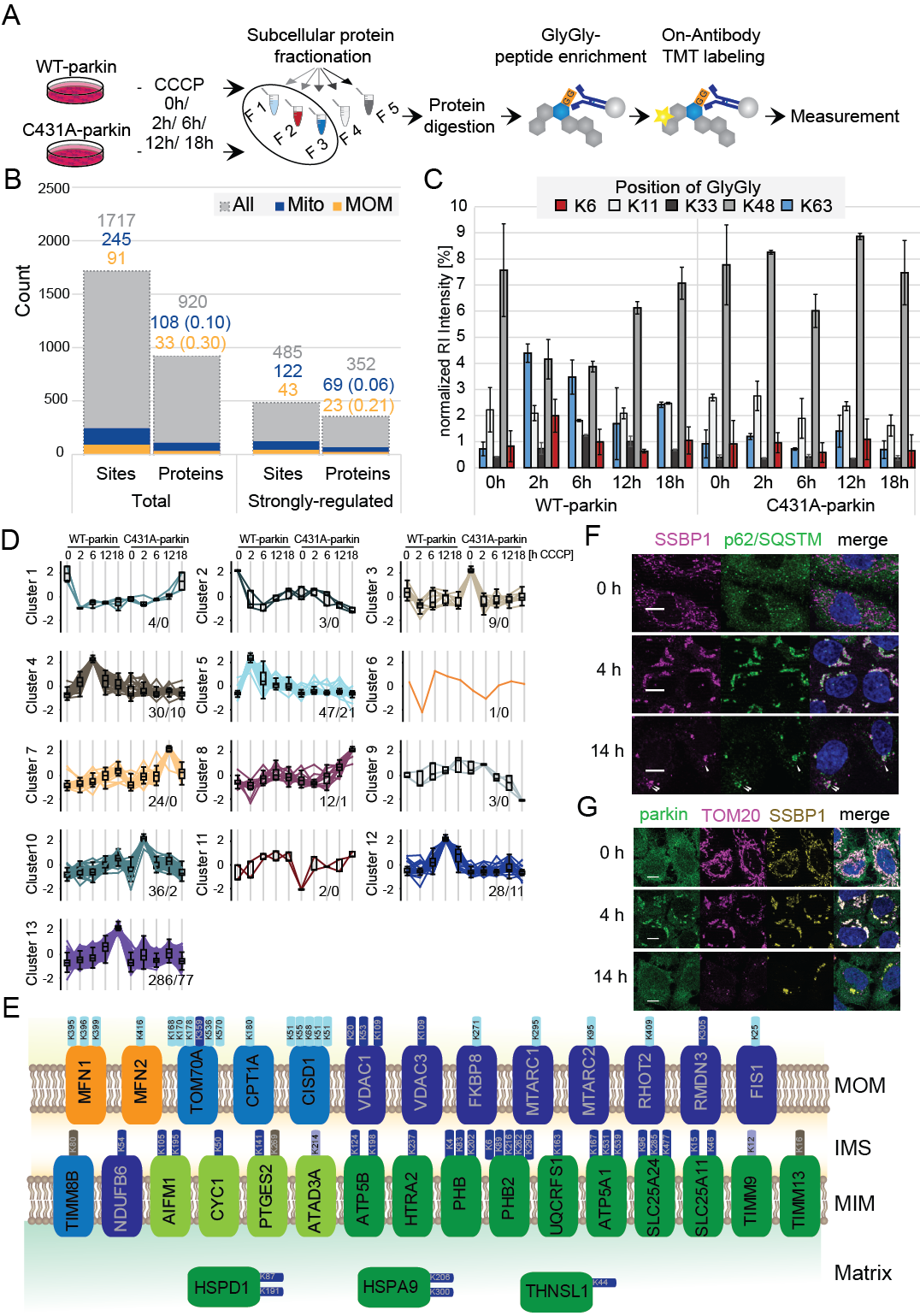


**Supplementary figure 3: Parkin-dependent ubiquitination of mitochondrial proteins**. **(A)** Experimental design to investigate dynamics of the ubiquitylome during Parkin-dependent mitophagy. Depolarization of the mitochondrial membrane potential by CCCP treatment for 0h, 2h, 6h, 12h, and 18h in Parkin-WT- or -C431A expressing cells. Antibody based enrichment of GlyGly-modified peptides after tryptic digestion followed by isobaric on-bead labeling (TMT10-plex).**(B)** Identification of mitochondrial (Mito) and Mitochondrial outer membrane (MOM) ubiquitinated proteins in total and strongly-regulated (criteria for log2 ratio -2<x<2). **(C)** Parkin activity dynamics interpreted by dynamics of GlyGly modified ubiquitin. **(D)** Profile plots after hierarchical clustering of “significantly” regulated GlyGly sites. Indicated are the cluster assignments and portion of mitochondrial proteins. **(E)** Mitochondria regulated clusters and annotations as mitochondria and Proteome. Colour coding corresponds to each proteomic (protein names) or ubiquitin cluster (GlyGly residues). **(F)** Immunofluorescence staining of SSBP1 clumps recognized by p62/SQSTM. Arrow heads indicate inner-mitochondrial clumps recognized by p62/SQSTM. Scale bar = 3 µm **(G)** Parkin cellular localization during mitophagy time-course (4-14h CCCP). Scale bar = 5 µm. MOM: mitochondrial outer membrane, IMS: mitochondrial intermembrane space, MIM: mitochondrial inner membrane.


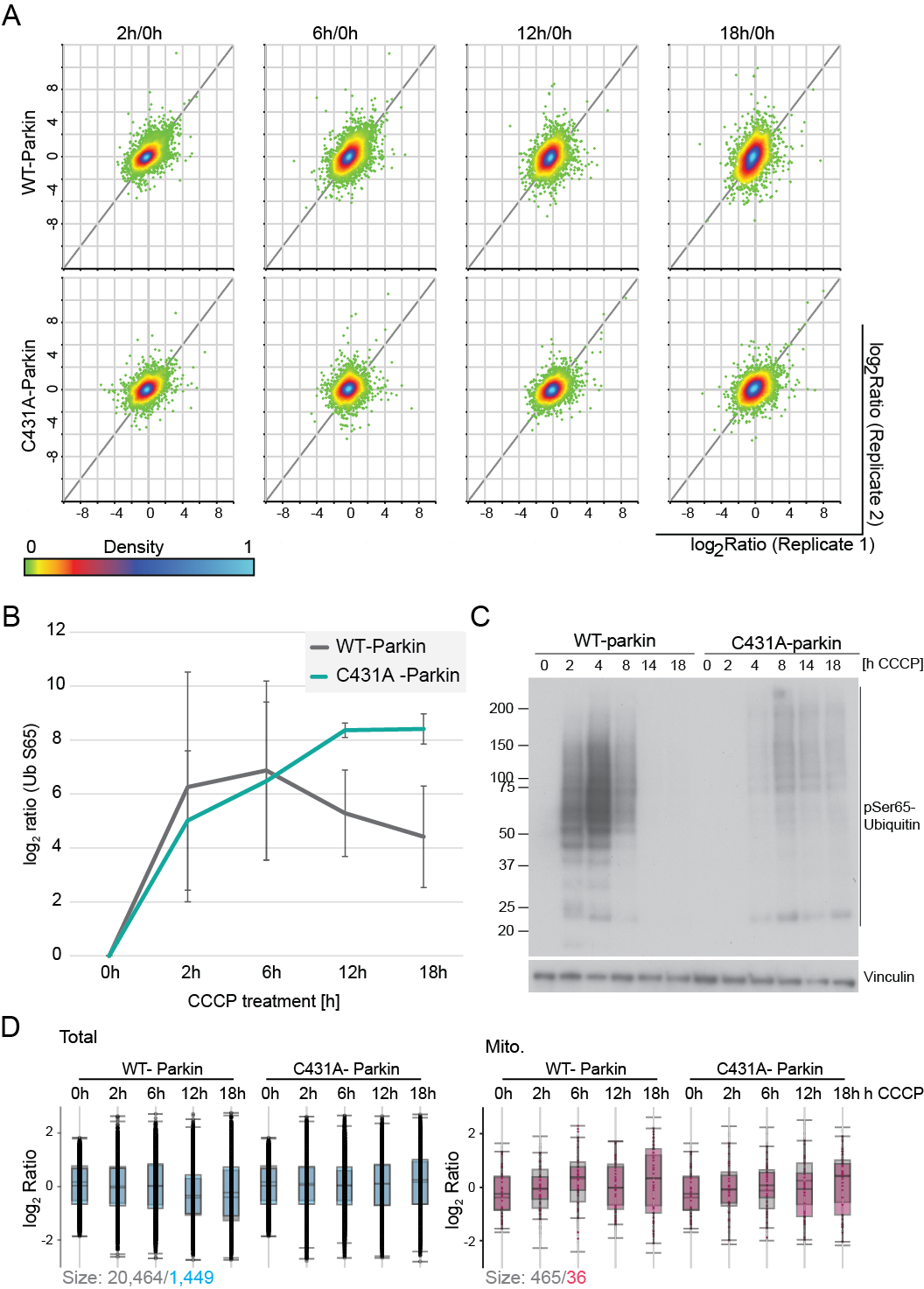


**Supplementary Figure 4: Phosphorylation dynamics during PINK1/Parkin-dependent mitophagy. (A)** Correlation between replicates across CCCP treatments and Parkin expressing cell lines used (**B-C**) Validation of PINK1 kinase activity by upregulation of Ubiquitin S65 phosphorylation after 2h post-mitophagy induction, independent of Parkin-activity. **(C)** Significantly regulated total and mitochondrial phosphorylation events have similar median regulation as bulk (total: grey, colored: significantly regulated phosphorylation events)


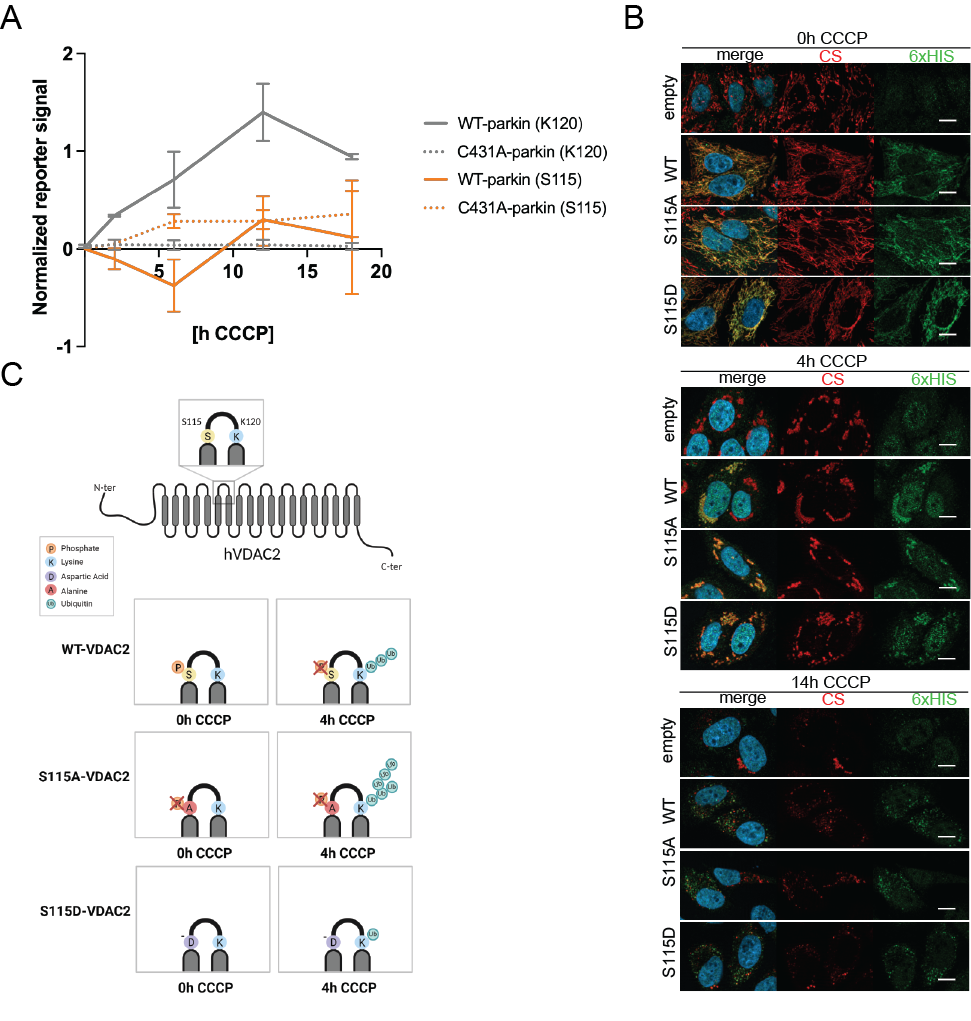


**Supplementary Figure 5: Behavior of VDAC2 upon mitophagy induction in WT-parkin expressing cells.** **(A)** Post-translational modifications observed in VDAC2 for specific S115 and K120 upon mitophagy induction. **(B)** Fragmentation and elimination of mitochondria is not impaired upon overexpression of 6xHIS-VDAC2 constructs. Scale bar = 3 µm **(C)** Schematic representation of phosphorylation and ubiquitination cross-talk in VDAC2 upon mitochondrial depolarization. Created with Biorender®
