# Supplementary material for: Temporal Analysis of Protein Ubiquitylation and Phosphorylation During Parkin-Dependent Mitophagy
Source: Mol Cell Proteomics. 2021 Dec 30;21(2):100191. doi: 10.1016/j.mcpro.2021.100191 (PMC8808264; doi:10.1016/j.mcpro.2021.100191)

20200921_KIZ_MUb4_GlyGly_10plex − Scan number: 15647

Gene Names	Charge	m/z	Mass	Retention time	PEP	Score	Precursor Intensity
PCNX	2	517.7851	1033.556	55.955	0.088642	56.51	5969287

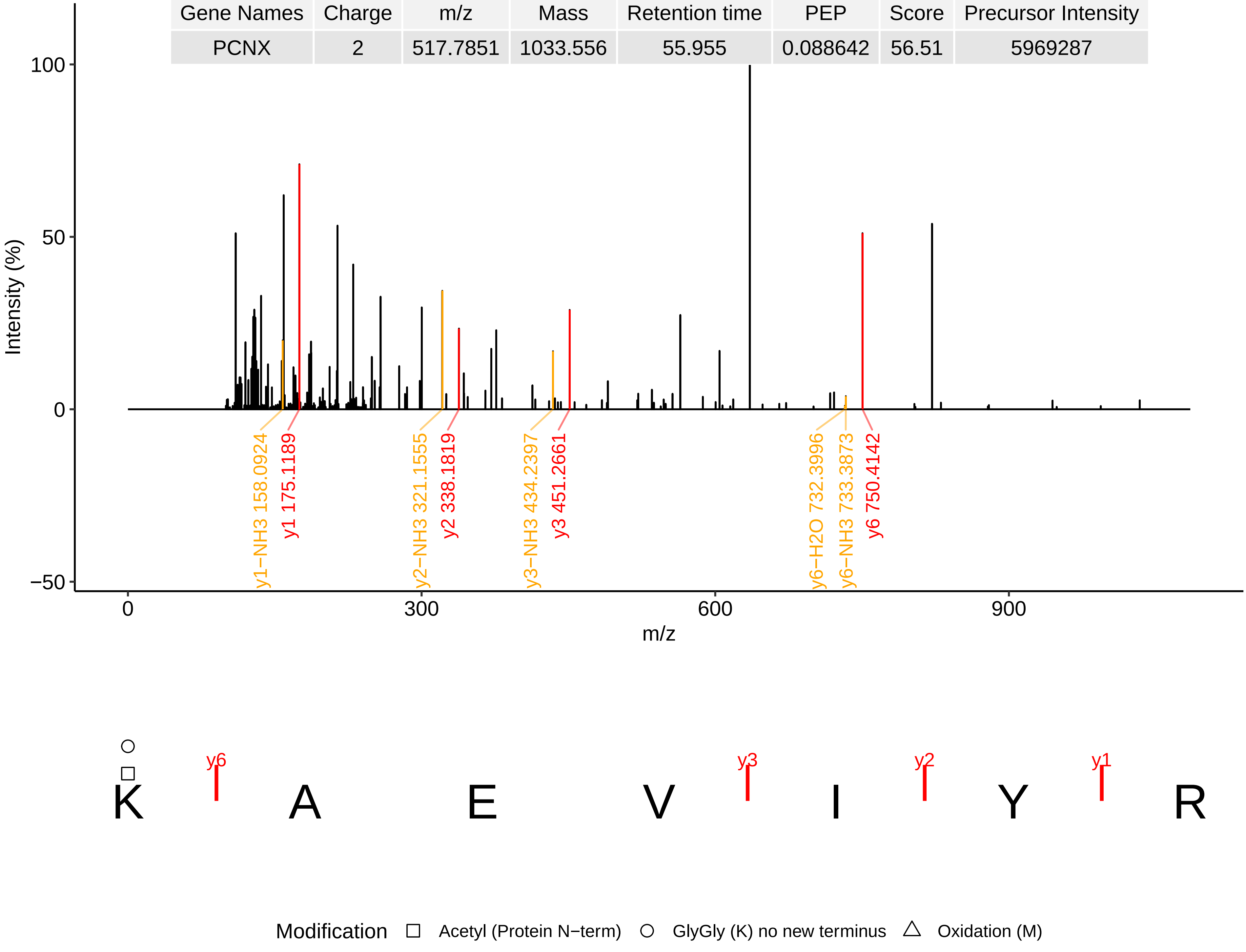

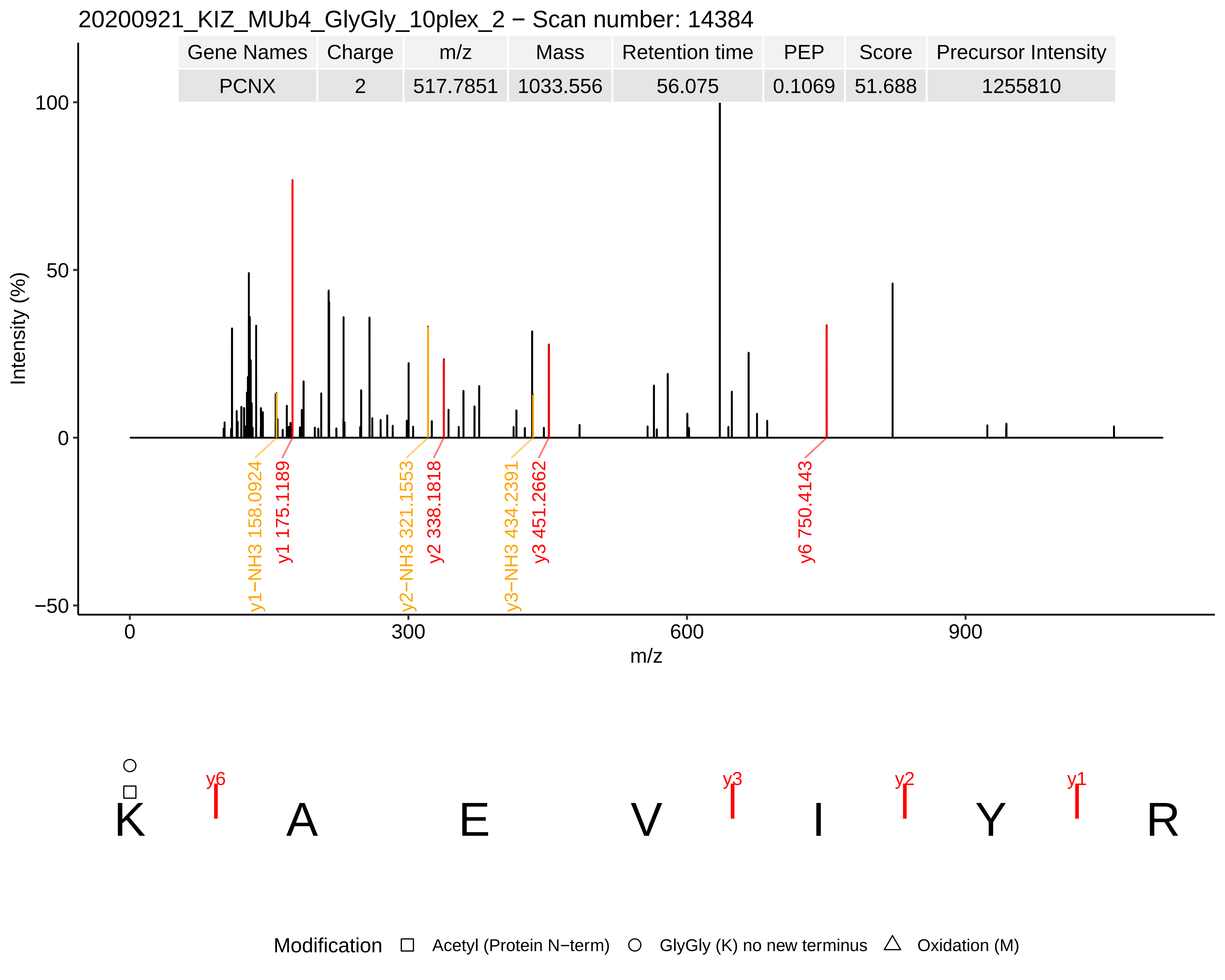
20200921_KIZ_MUb4_GlyGly_10plex_2 − Scan number: 14384
Gene Names | Charge | m/z | Mass | Retention time | PEP | Score | Precursor Intensity
PCNX | 2 | 517.7851 | 1033.556 | 56.075 | 0.1069 | 51.688 | 1255810
Intensity (%)
100
50
0
−50
m/z
0
300
600
900
y1−NH3 158.0924
y1 175.1189
y2−NH3 321.1553
y2 338.1818
y3−NH3 434.2391
y3 451.2662
y6 750.4143
K A E V I Y R
y6
y3
y2
y1
Modification
Acetyl (Protein N−term)
GlyGly (K) no new terminus
Oxidation (M)

20200924_KIZ_MUb5_GlyGly_1 − Scan number: 14843

Gene Names	Charge	m/z	Mass	Retention time	PEP	Score	Precursor Intensity
PCNX	2	517.7851	1033.556	48.479	0.088642	56.51	21135578

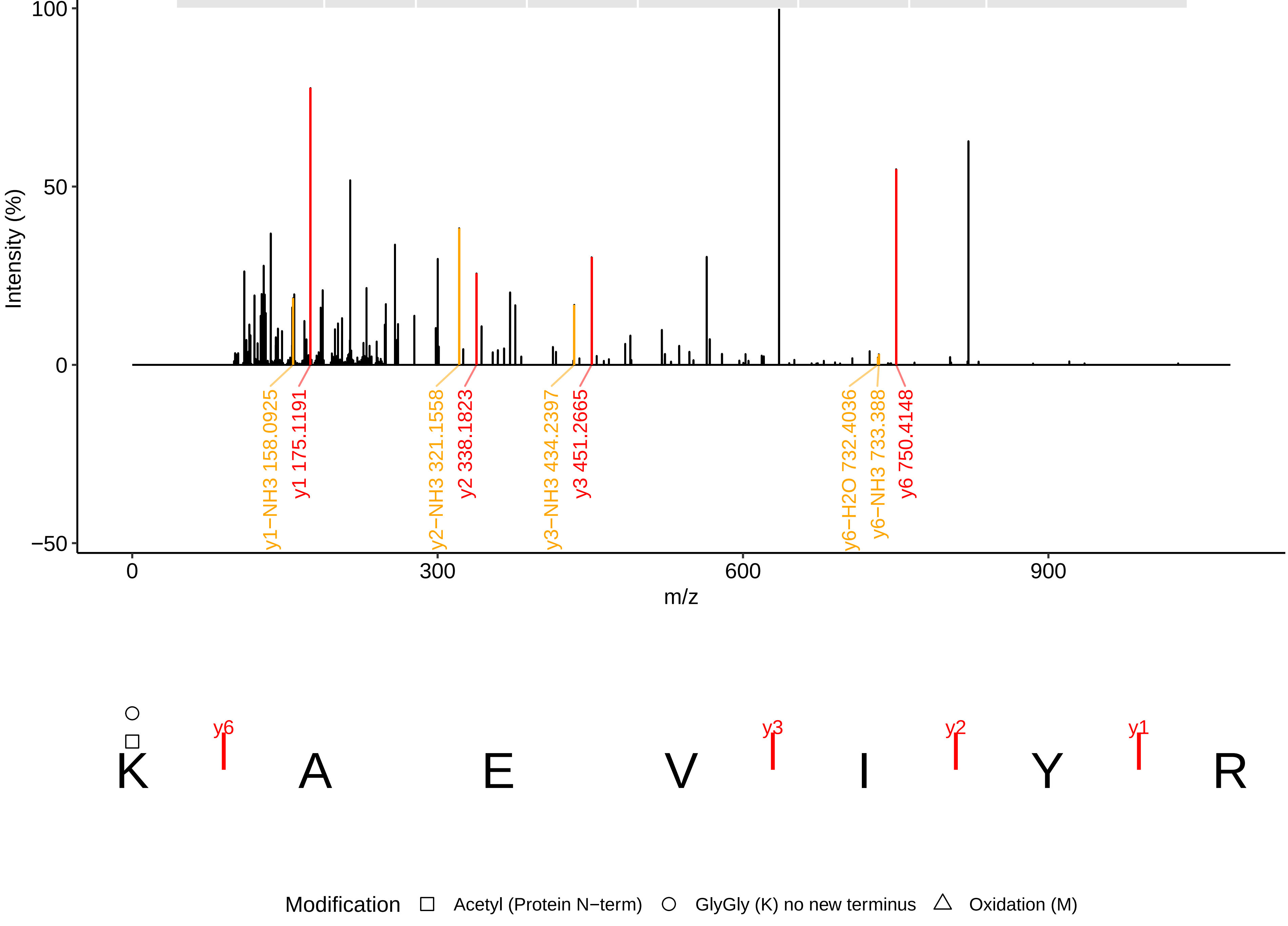

20200924_KIZ_MUb5_GlyGly_2 − Scan number: 13861

Gene Names	Charge	m/z	Mass	Retention time	PEP	Score	Precursor Intensity
PCNX	2	517.7851	1033.556	51.84	0.046138	64.866	5077339

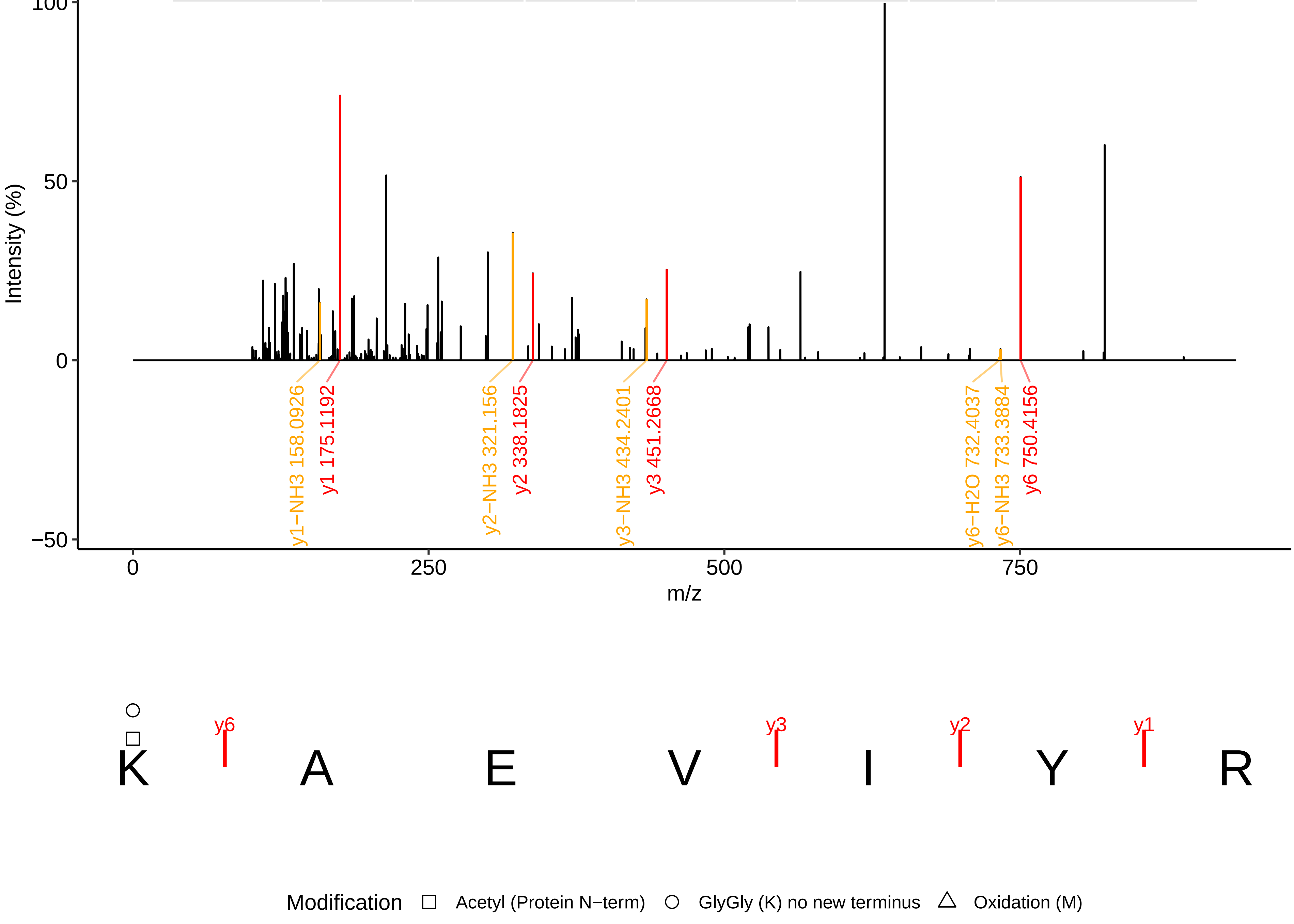

20200921_KIZ_MUb4_GlyGly_10plex − Scan number: 4071

Gene Names	Charge	m/z	Mass	Retention time	PEP	Score	Precursor Intensity
RAB5C	2	437.7407	873.4668	13.24	0.10177	99.799	6267445

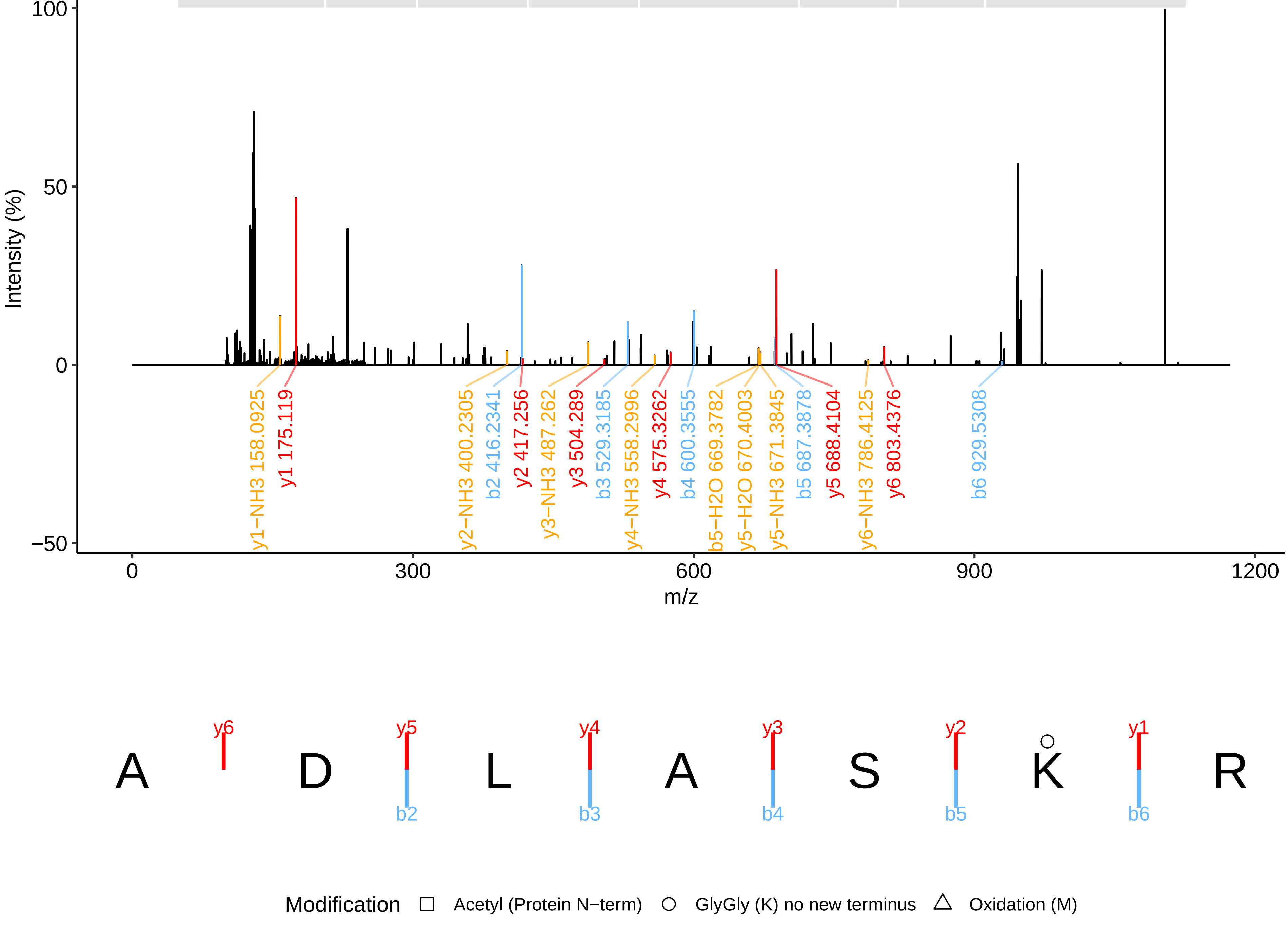

20200924_KIZ_MUb5_GlyGly_1 − Scan number: 3559

Gene Names	Charge	m/z	Mass	Retention time	PEP	Score	Precursor Intensity
RAB5C	2	437.7407	873.4668	10.892	0.12026	87.284	4202041

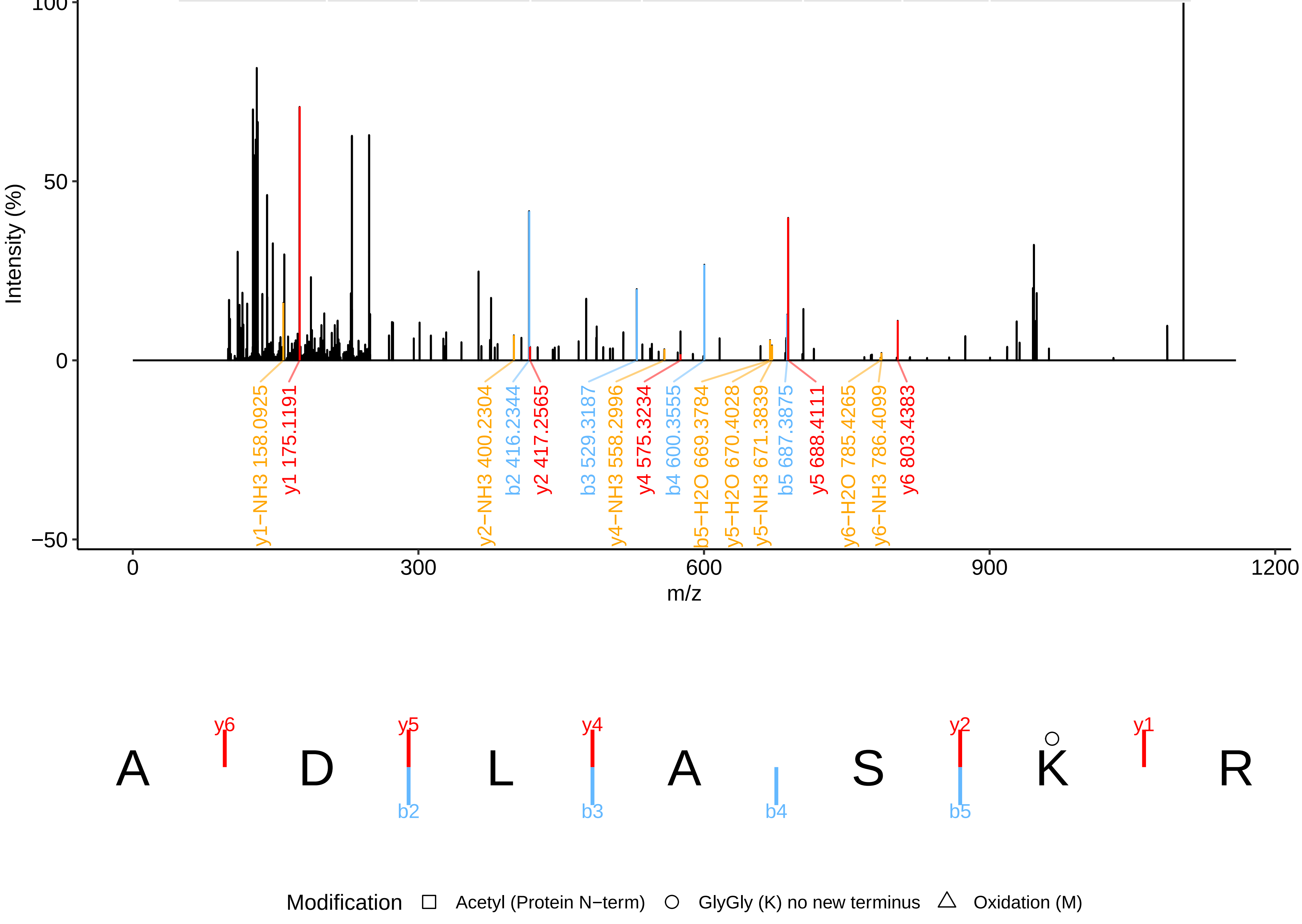

20200924_KIZ_MUb5_GlyGly_1 − Scan number: 3605

Gene Names	Charge	m/z	Mass	Retention time	PEP	Score	Precursor Intensity
CCT6A	2	440.7133	879.412	11.023	0.10614	53.71	4500894

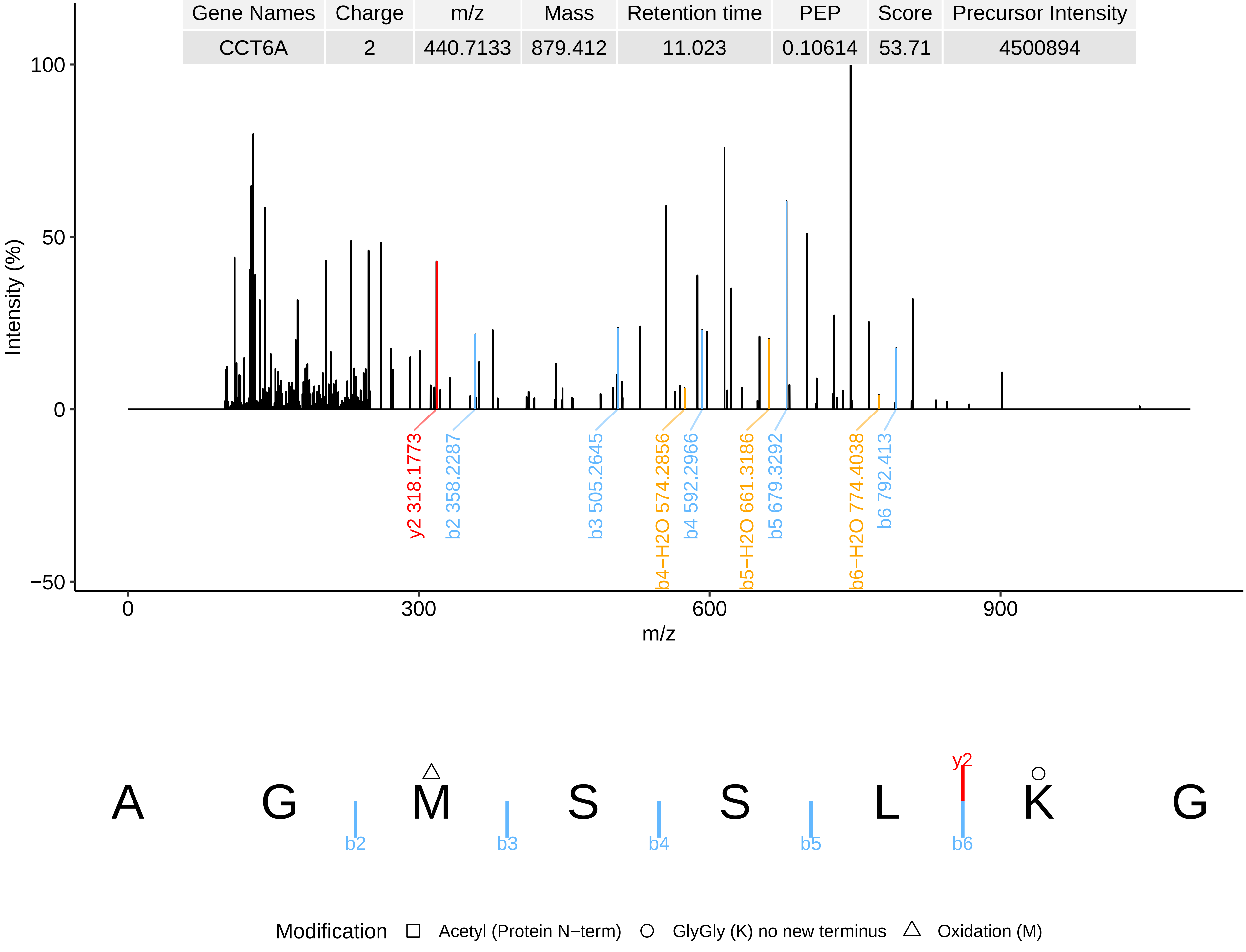

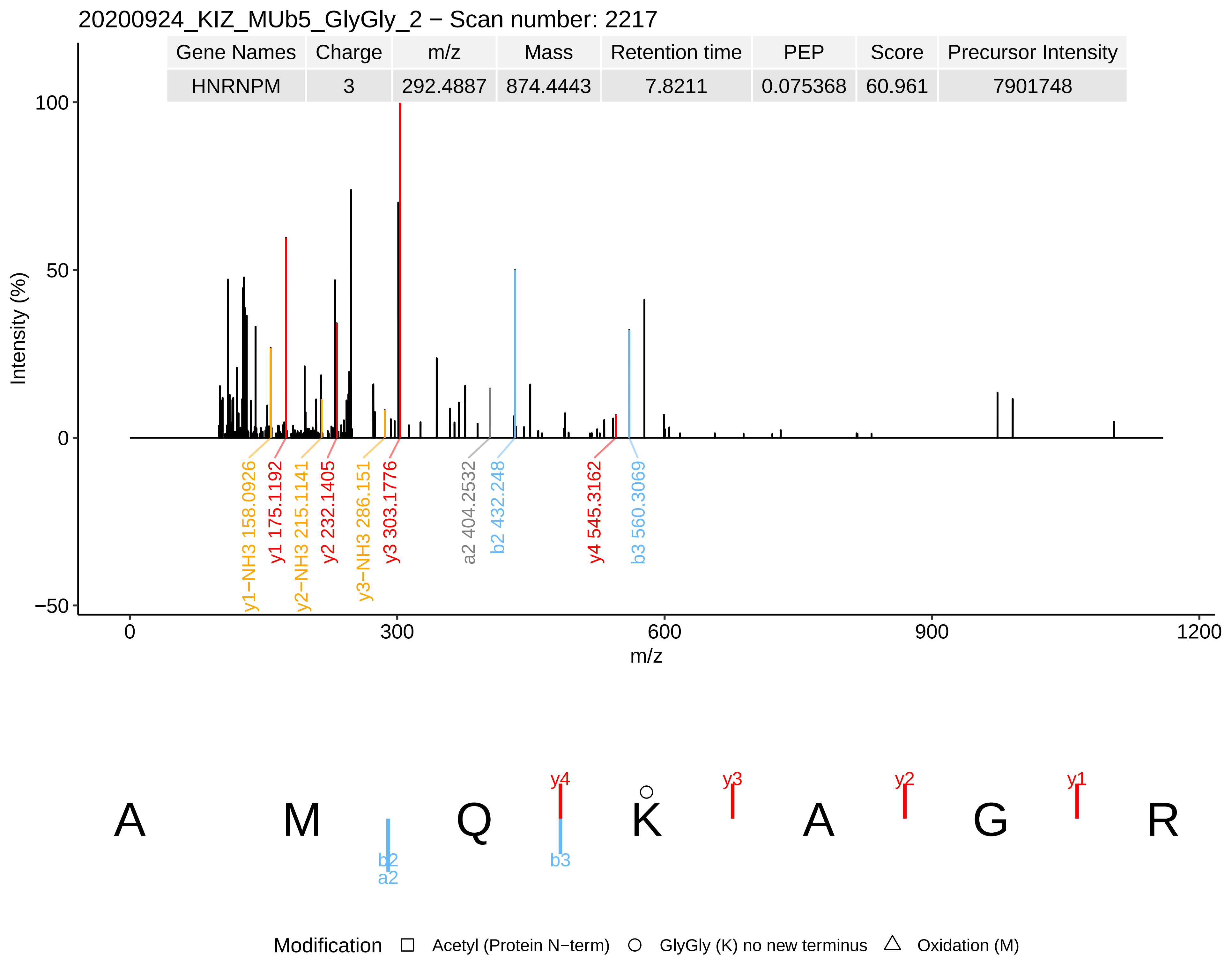
20200924_KIZ_MUb5_GlyGly_2 − Scan number: 2217
Gene Names | Charge | m/z | Mass | Retention time | PEP | Score | Precursor Intensity
HNRNPM | 3 | 292.4887 | 874.4443 | 7.8211 | 0.075368 | 60.961 | 7901748
Intensity (%)
100
50
0
−50
y1−NH3 158.0926
y1 175.1192
y2−NH3 215.1141
y2 232.1405
y3−NH3 286.151
y3 303.1776
a2 404.2532
b2 432.248
y4 545.3162
b3 560.3069
0
300
600
900
1200
m/z
A M Q K A G R
y4 y3 y2 y1
b2 a2 b3
Modification
Acetyl (Protein N−term)
GlyGly (K) no new terminus
Oxidation (M)

20201118_KIZ_MUb6_GlyGly_1 − Scan number: 2639

Gene Names	Charge	m/z	Mass	Retention time	PEP	Score	Precursor Intensity
HNRNPM	3	292.4887	874.4443	8.0755	0.022724	79.454	14639546

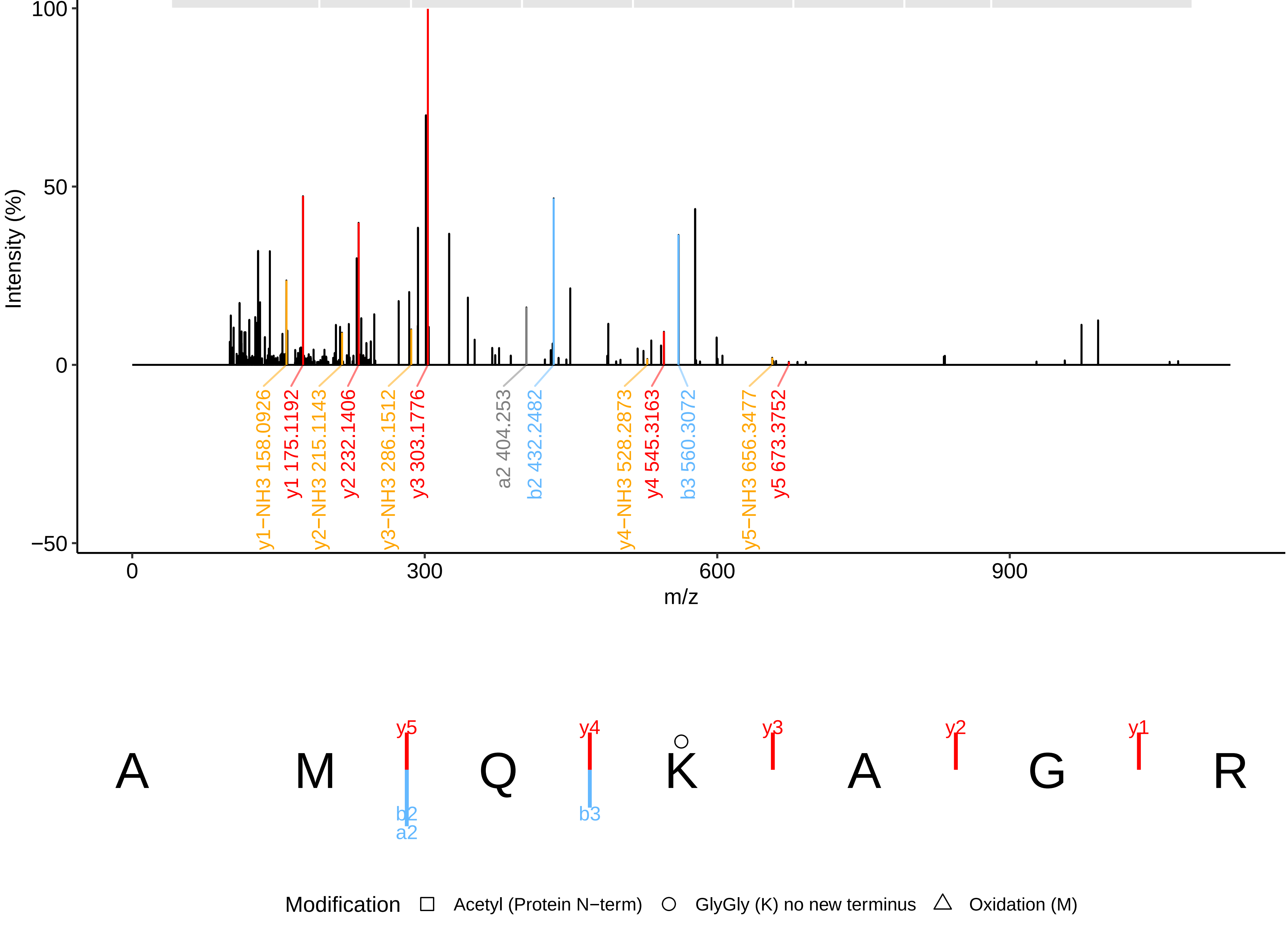

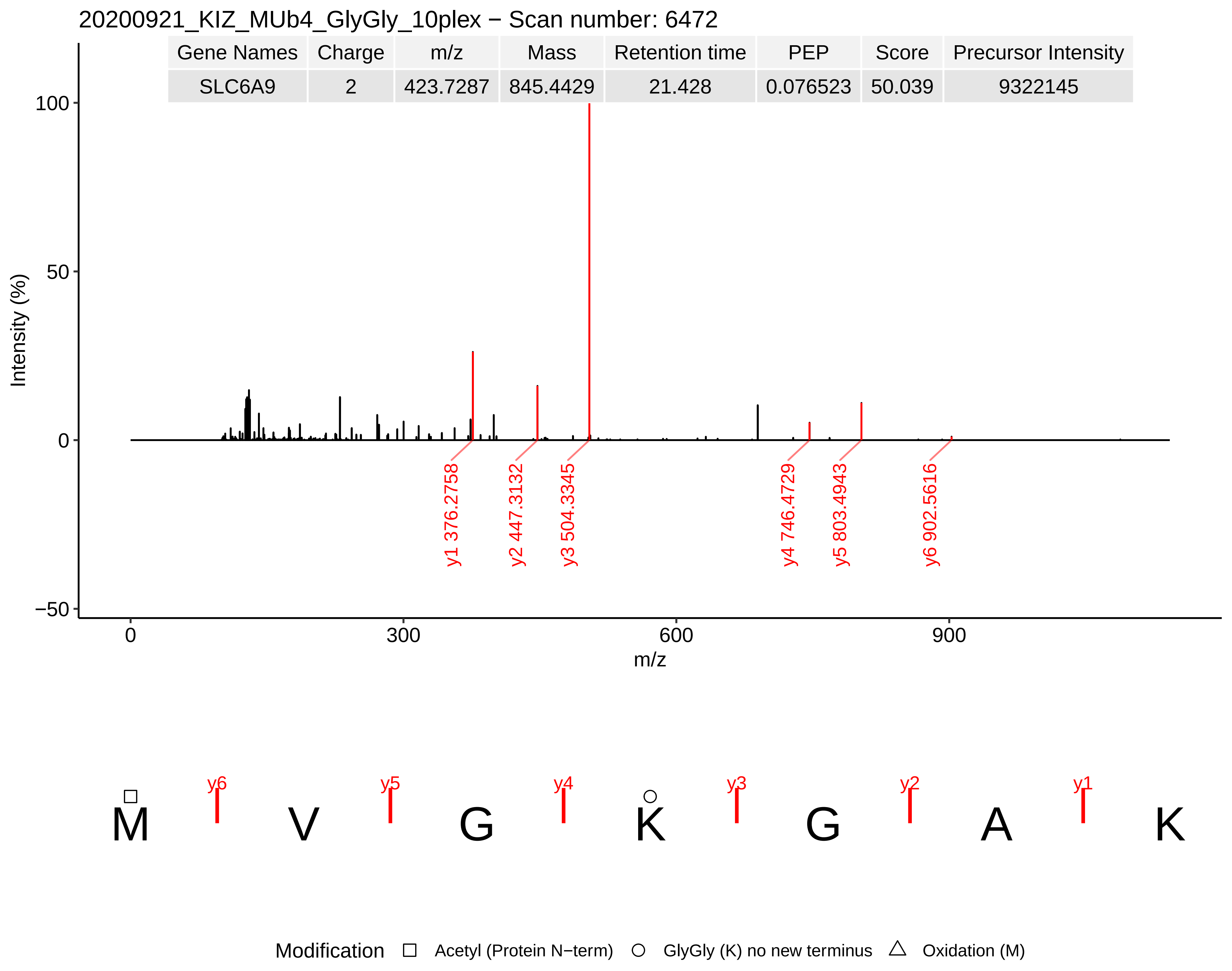

20200921_KIZ_MUb4_GlyGly_10plex − Scan number: 6472

Gene Names	Charge	m/z	Mass	Retention time	PEP	Score	Precursor Intensity
SLC6A9	2	423.7287	845.4429	21.428	0.076523	50.039	9322145

Intensity (%)
100
50
0
−50
0
300
600
900
m/z
y1 376.2758
y2 447.3132
y3 504.3345
y4 746.4729
y5 803.4943
y6 902.5616
M
y6
V
y5
G
y4
K
y3
G
y2
A
y1
K
Modification
Acetyl (Protein N−term)
GlyGly (K) no new terminus
Oxidation (M)

20200921_KIZ_MUb4_GlyGly_10plex − Scan number: 9688

Gene Names	Charge	m/z	Mass	Retention time	PEP	Score	Precursor Intensity
ENO1	3	365.2207	1092.64	33.261	0.010706	77.674	5284676

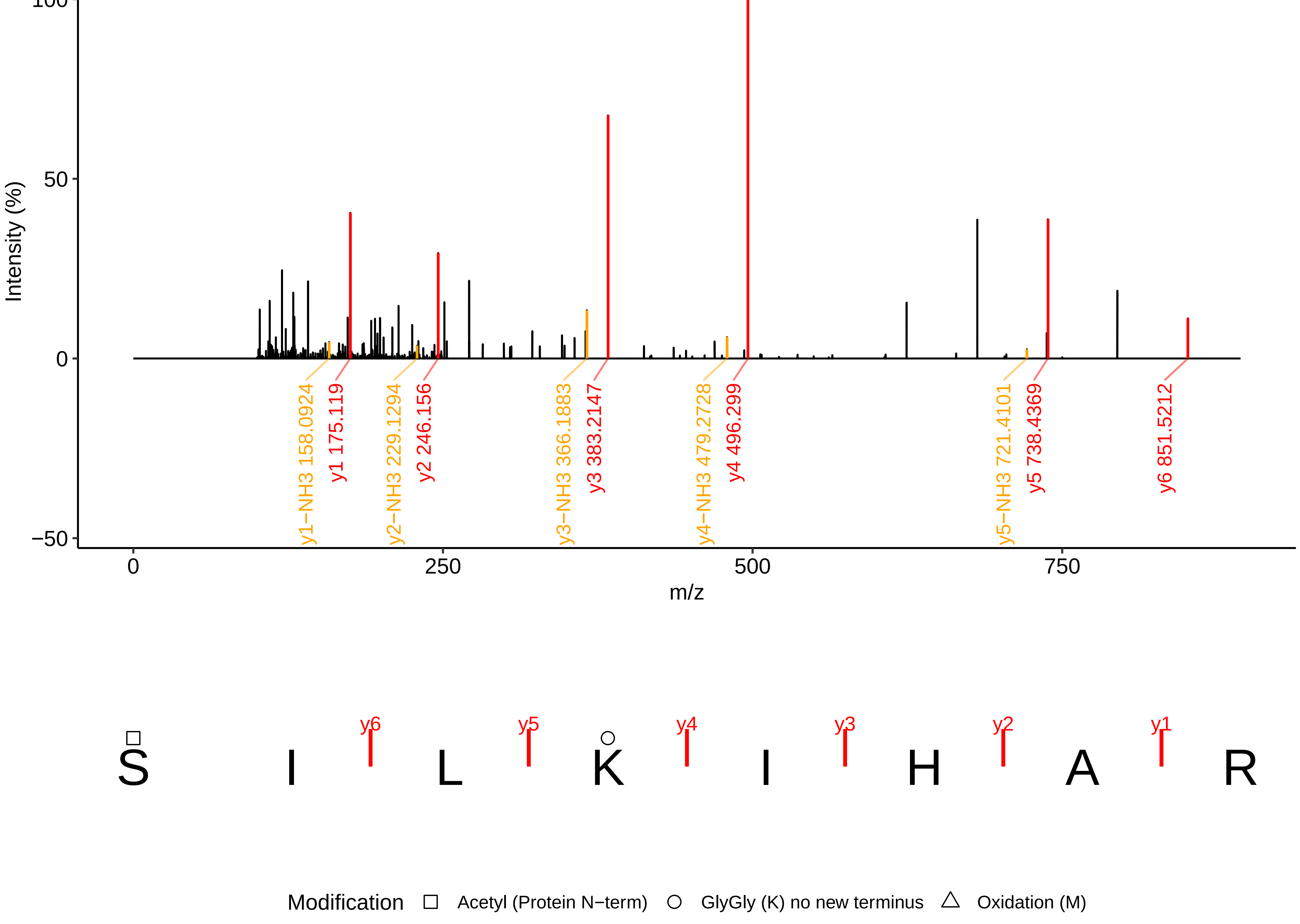

20200921_KIZ_MUb4_GlyGly_10plex − Scan number: 9707

Gene Names	Charge	m/z	Mass	Retention time	PEP	Score	Precursor Intensity
ENO1	2	547.3275	1092.64	33.33	0.00014906	113.25	6248761

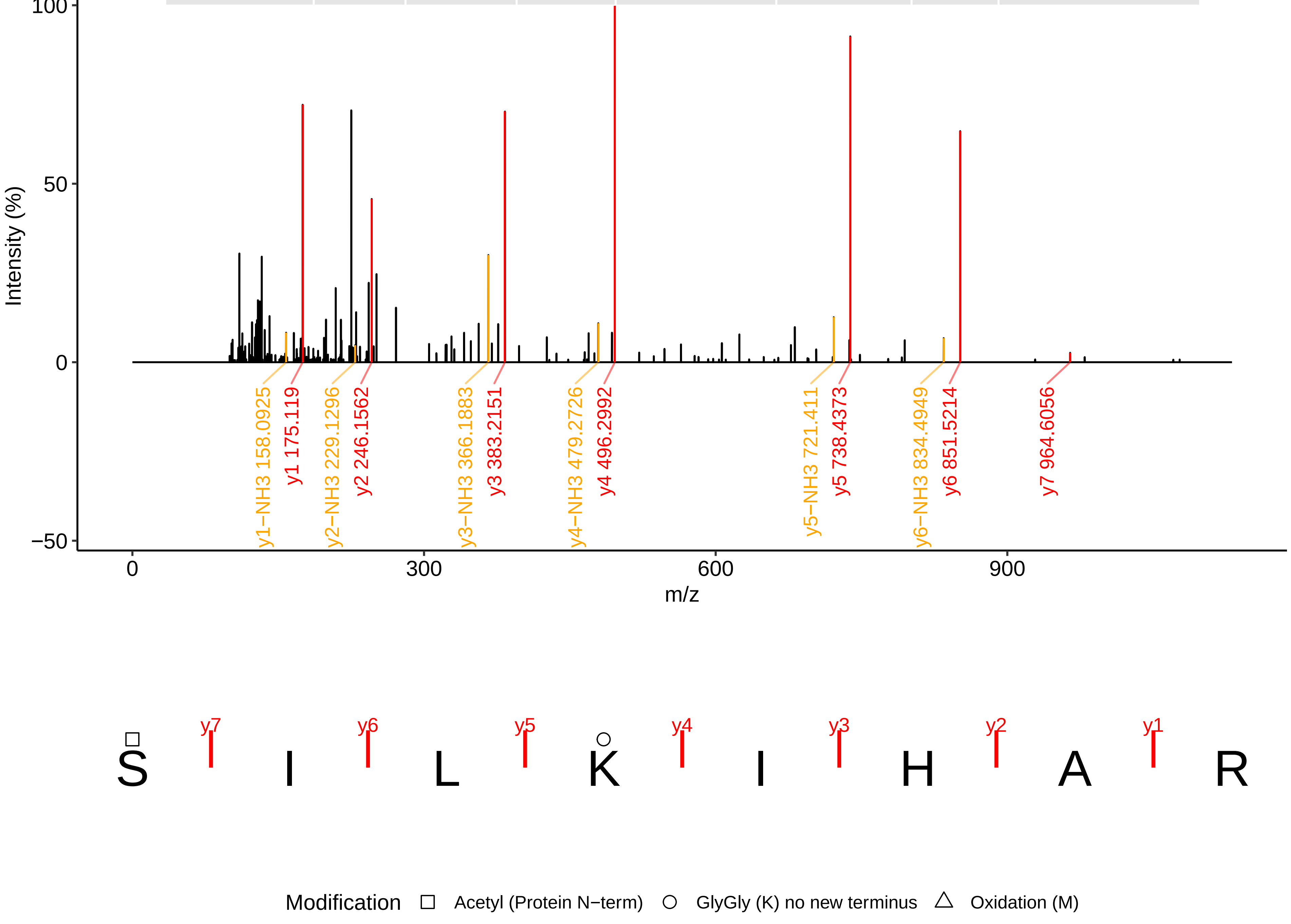

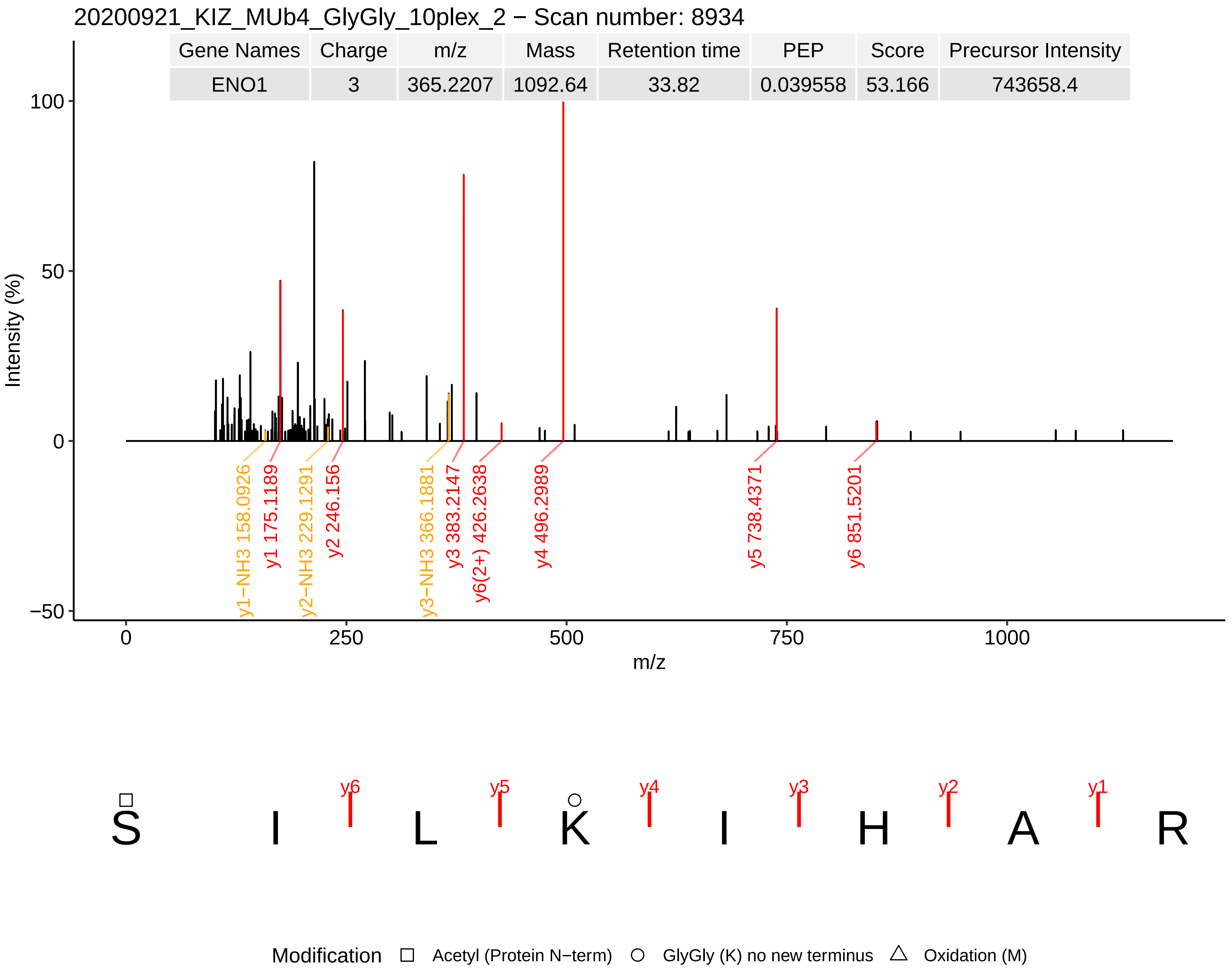
20200921_KIZ_MUb4_GlyGly_10plex_2 − Scan number: 8934
Gene Names | Charge | m/z | Mass | Retention time | PEP | Score | Precursor Intensity
ENO1 | 3 | 365.2207 | 1092.64 | 33.82 | 0.039558 | 53.166 | 743658.4
Intensity (%)
m/z
y1−NH3 158.0926
y1 175.1189
y2−NH3 229.1291
y2 246.156
y3−NH3 366.1881
y3 383.2147
y6(2+) 426.2638
y4 496.2989
y5 738.4371
y6 851.5201
S I L K I H A R
y6 y5 y4 y3 y2 y1
Modification
Acetyl (Protein N−term)
GlyGly (K) no new terminus
Oxidation (M)

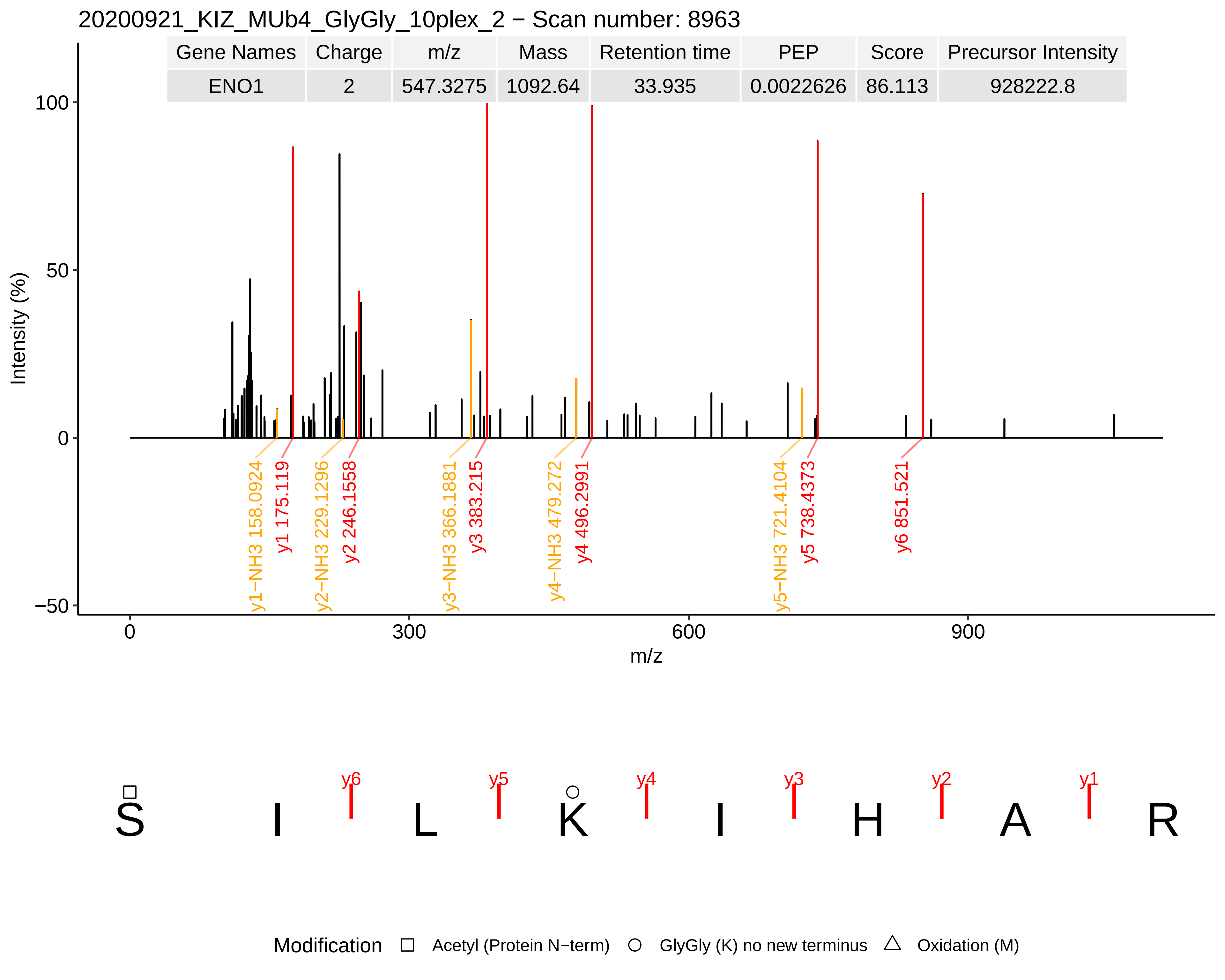

20200921_KIZ_MUb4_GlyGly_10plex_2 − Scan number: 8963
Gene Names | Charge | m/z | Mass | Retention time | PEP | Score | Precursor Intensity
ENO1 | 2 | 547.3275 | 1092.64 | 33.935 | 0.0022626 | 86.113 | 928222.8
Intensity (%)
100
50
0
−50
0
300
600
900
m/z
y1−NH3 158.0924
y1 175.119
y2−NH3 229.1296
y2 246.1558
y3−NH3 366.1881
y3 383.215
y4−NH3 479.272
y4 496.2991
y5−NH3 721.4104
y5 738.4373
y6 851.521
S I L K I H A R
y6 y5 y4 y3 y2 y1
Modification
Acetyl (Protein N−term)
GlyGly (K) no new terminus
Oxidation (M)

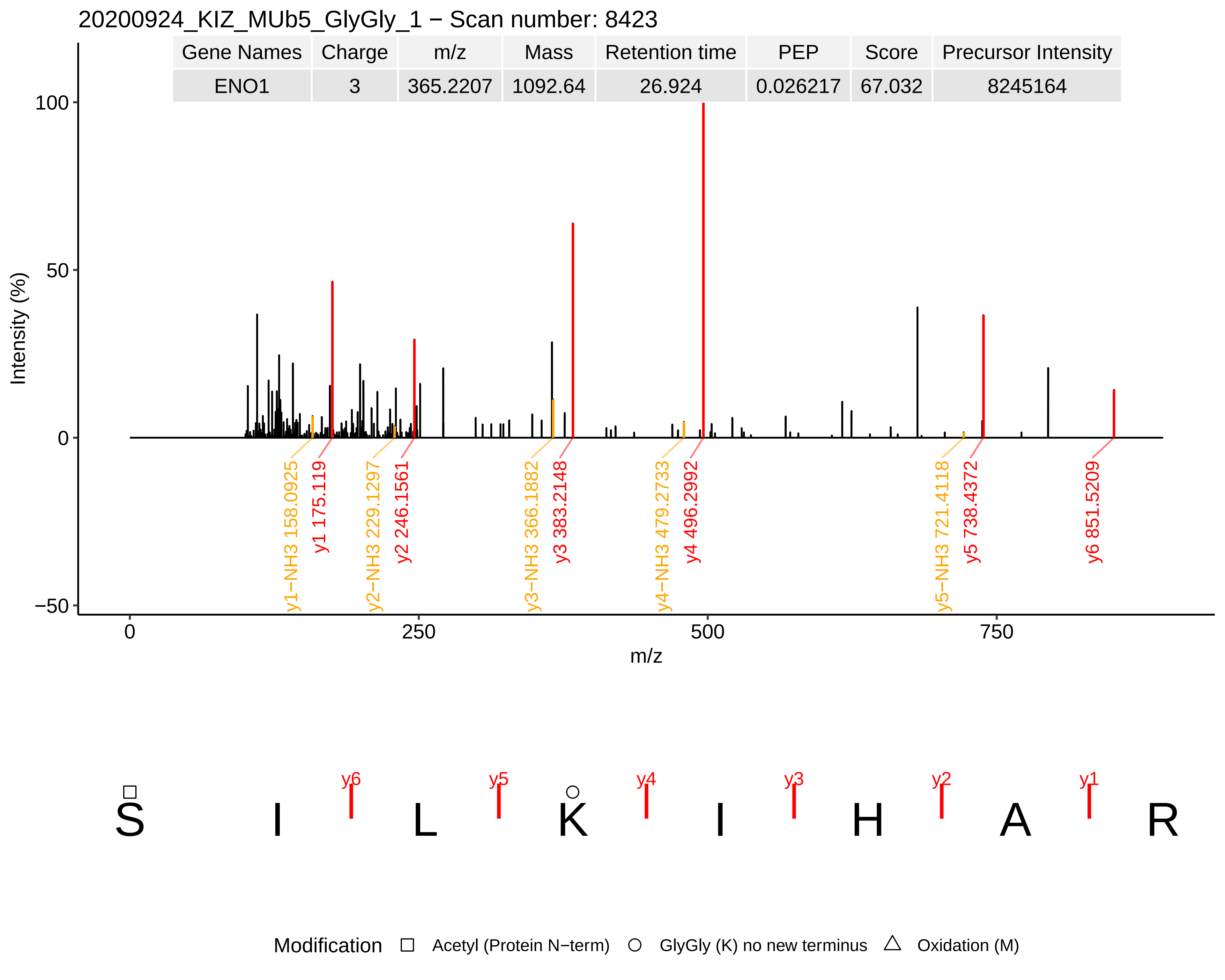
20200924_KIZ_MUb5_GlyGly_1 − Scan number: 8423
Gene Names | Charge | m/z | Mass | Retention time | PEP | Score | Precursor Intensity
ENO1 | 3 | 365.2207 | 1092.64 | 26.924 | 0.026217 | 67.032 | 8245164
Intensity (%)
100
50
0
−50
y1−NH3 158.0925
y1 175.119
y2−NH3 229.1297
y2 246.1561
y3−NH3 366.1882
y3 383.2148
y4−NH3 479.2733
y4 496.2992
y5−NH3 721.4118
y5 738.4372
y6 851.5209
0
250
500
750
m/z
S I L K I H A R
y6 y5 y4 y3 y2 y1
Modification
Acetyl (Protein N−term)
GlyGly (K) no new terminus
Oxidation (M)

20200924_KIZ_MUb5_GlyGly_2 − Scan number: 7919

Gene Names	Charge	m/z	Mass	Retention time	PEP	Score	Precursor Intensity
ENO1	3	365.2207	1092.64	29.591	0.028316	65.038	3228366

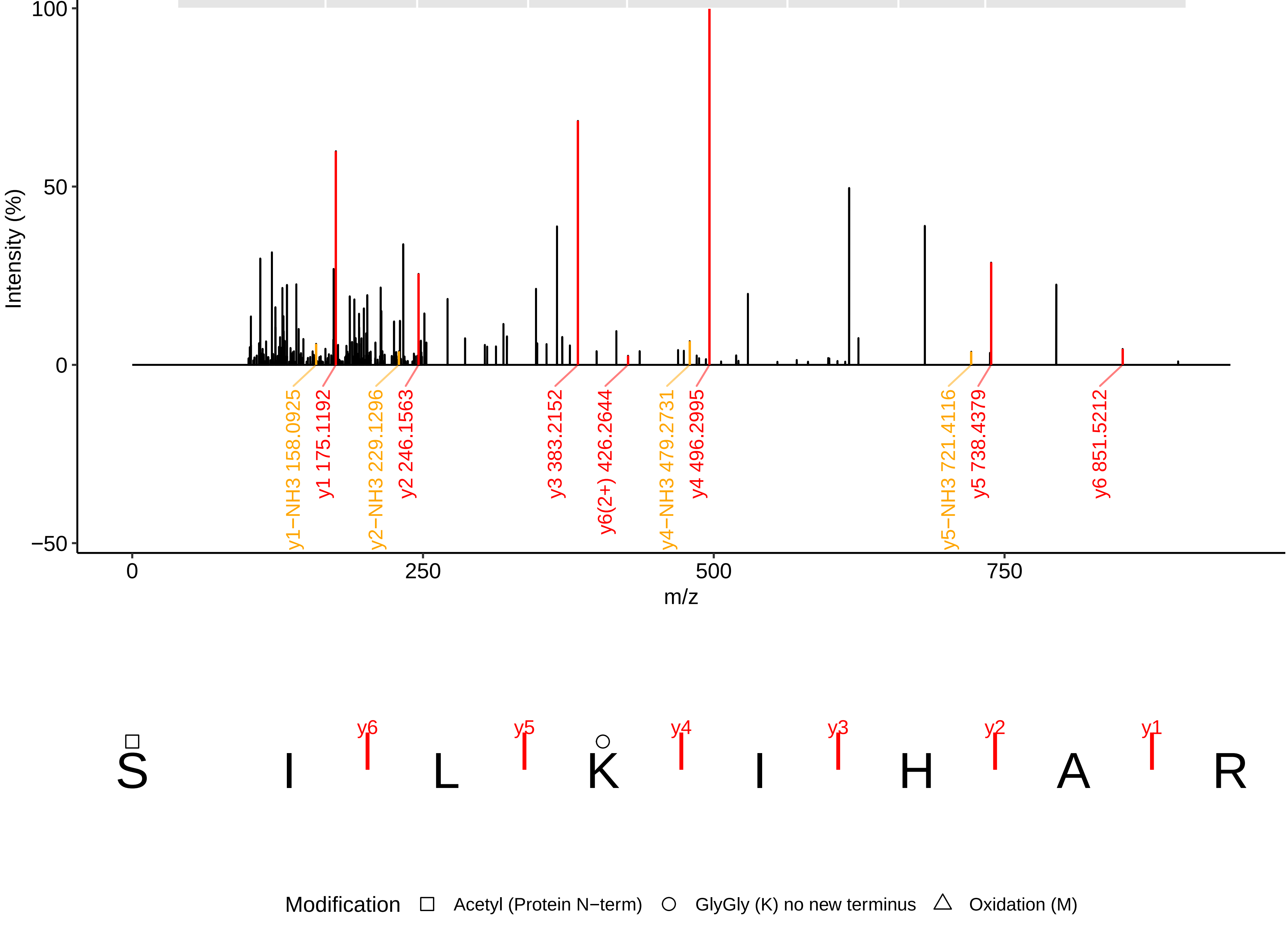

20200921_KIZ_MUb4_GlyGly_10plex_2 − Scan number: 2837

Gene Names	Charge	m/z	Mass	Retention time	PEP	Score	Precursor Intensity
NHP2L1	3	291.8349	872.4828	9.1742	0.11627	52.683	1141371

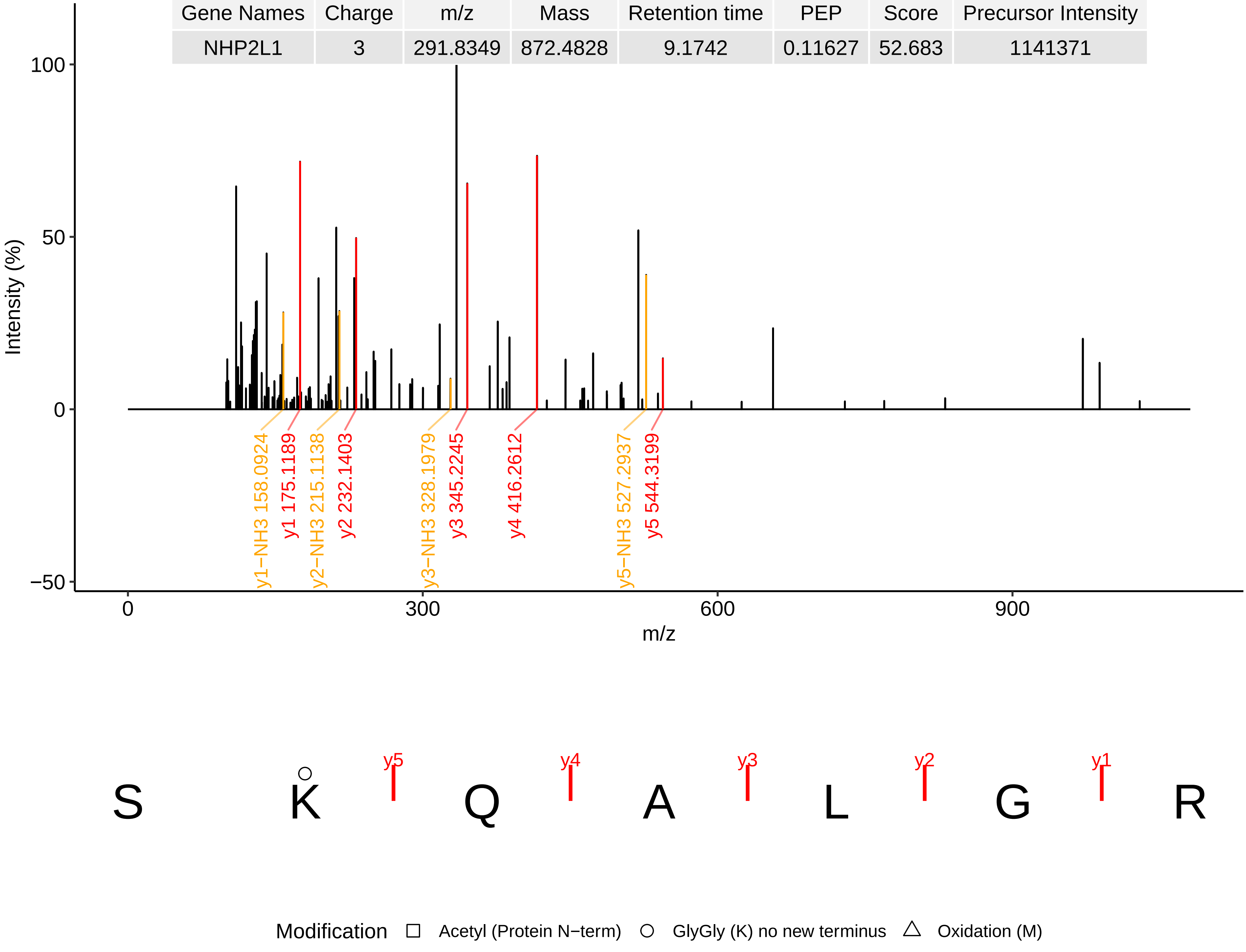

20200924_KIZ_MUb5_GlyGly_1 − Scan number: 3130

Gene Names	Charge	m/z	Mass	Retention time	PEP	Score	Precursor Intensity
USP5	2	428.7354	855.4563	9.6599	0.057466	85.272	10042730

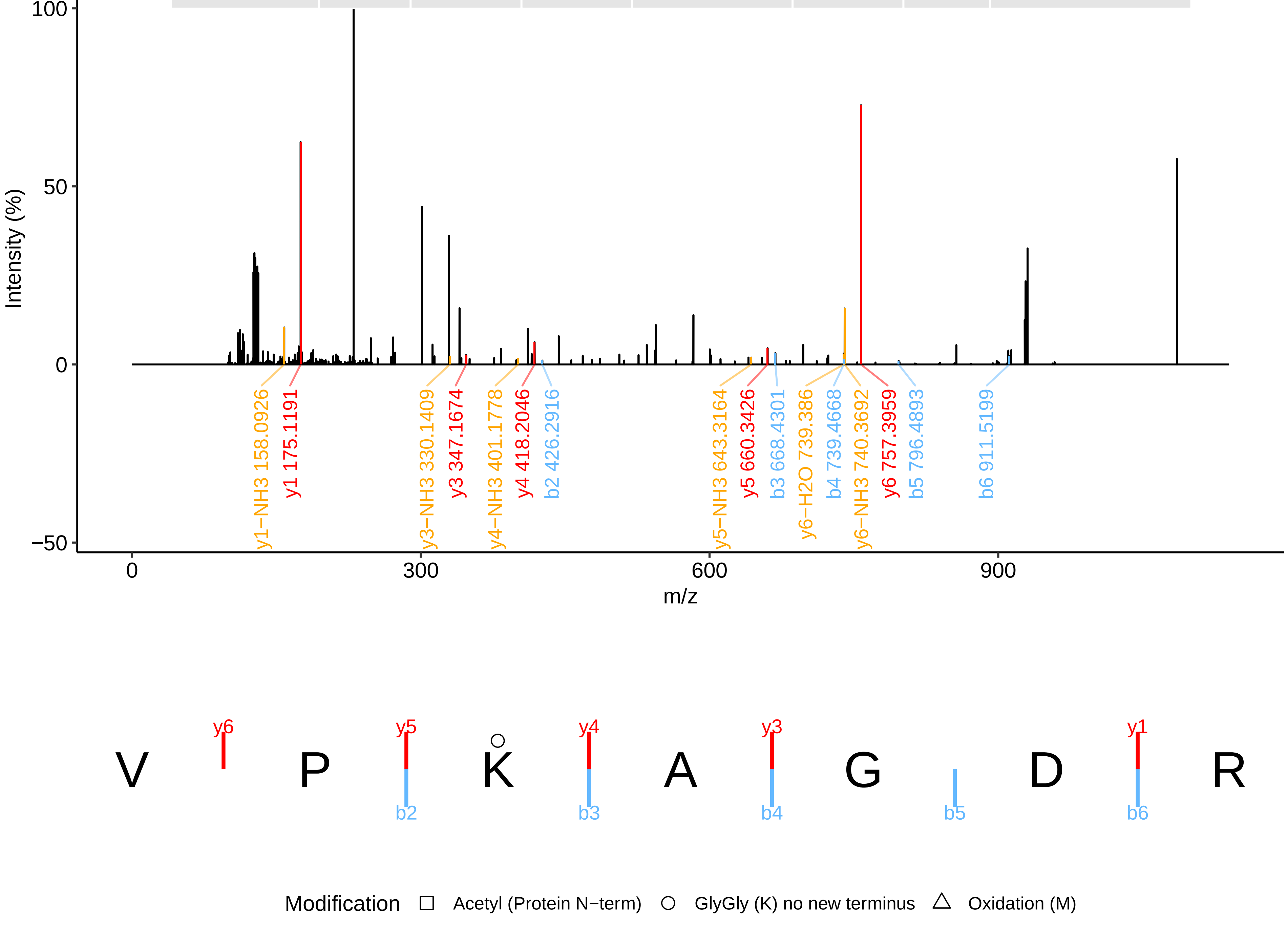

20200921_KIZ_MUb4_GlyGly_10plex − Scan number: 7926

Gene Names	Charge	m/z	Mass	Retention time	PEP	Score	Precursor Intensity
RPS20	2	461.7351	921.4556	26.769	0.033033	72.234	10250500

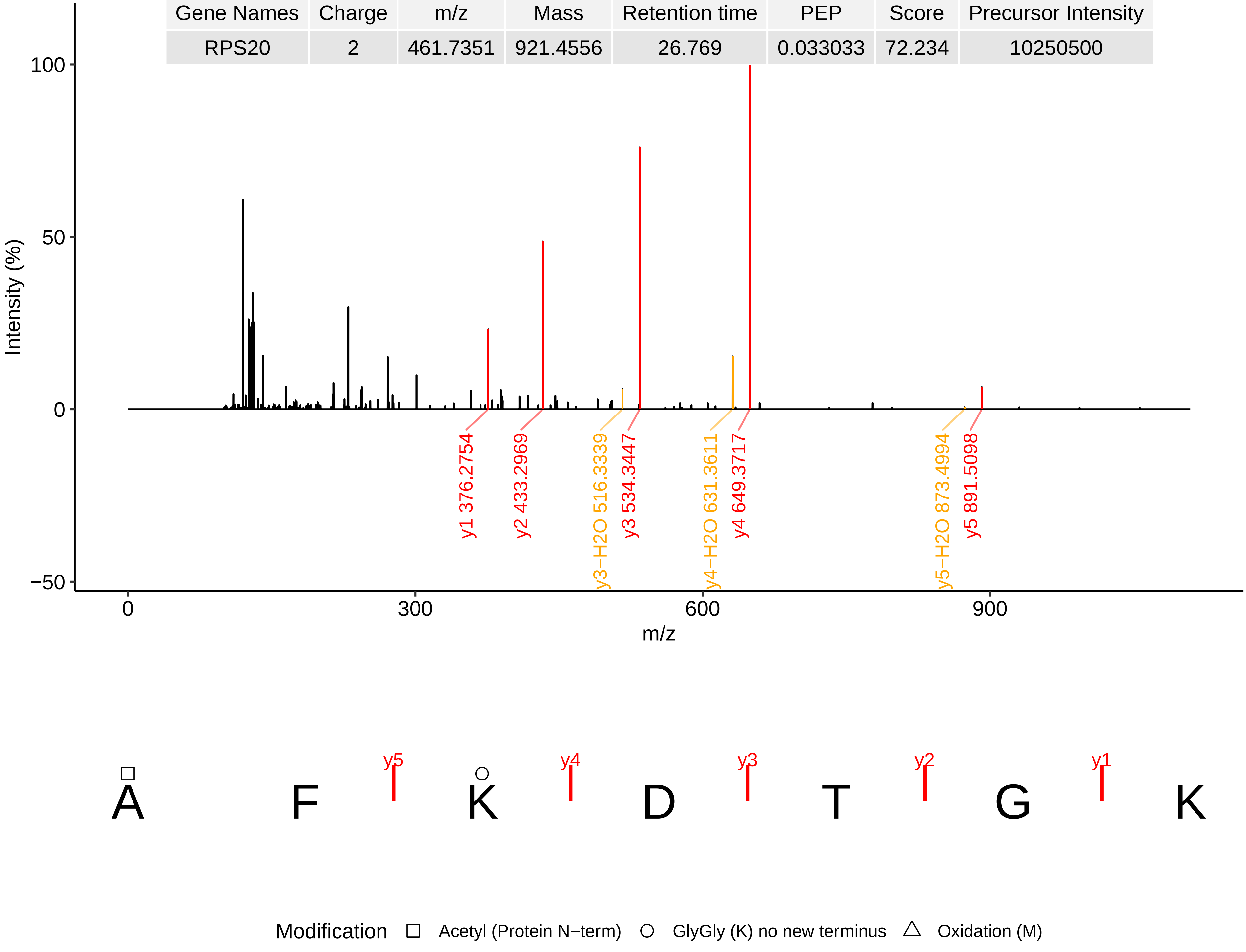

20200921_KIZ_MUb4_GlyGly_10plex_2 − Scan number: 7242

Gene Names	Charge	m/z	Mass	Retention time	PEP	Score	Precursor Intensity
RPS20	2	461.7351	921.4556	26.951	0.060011	59.205	537703.8

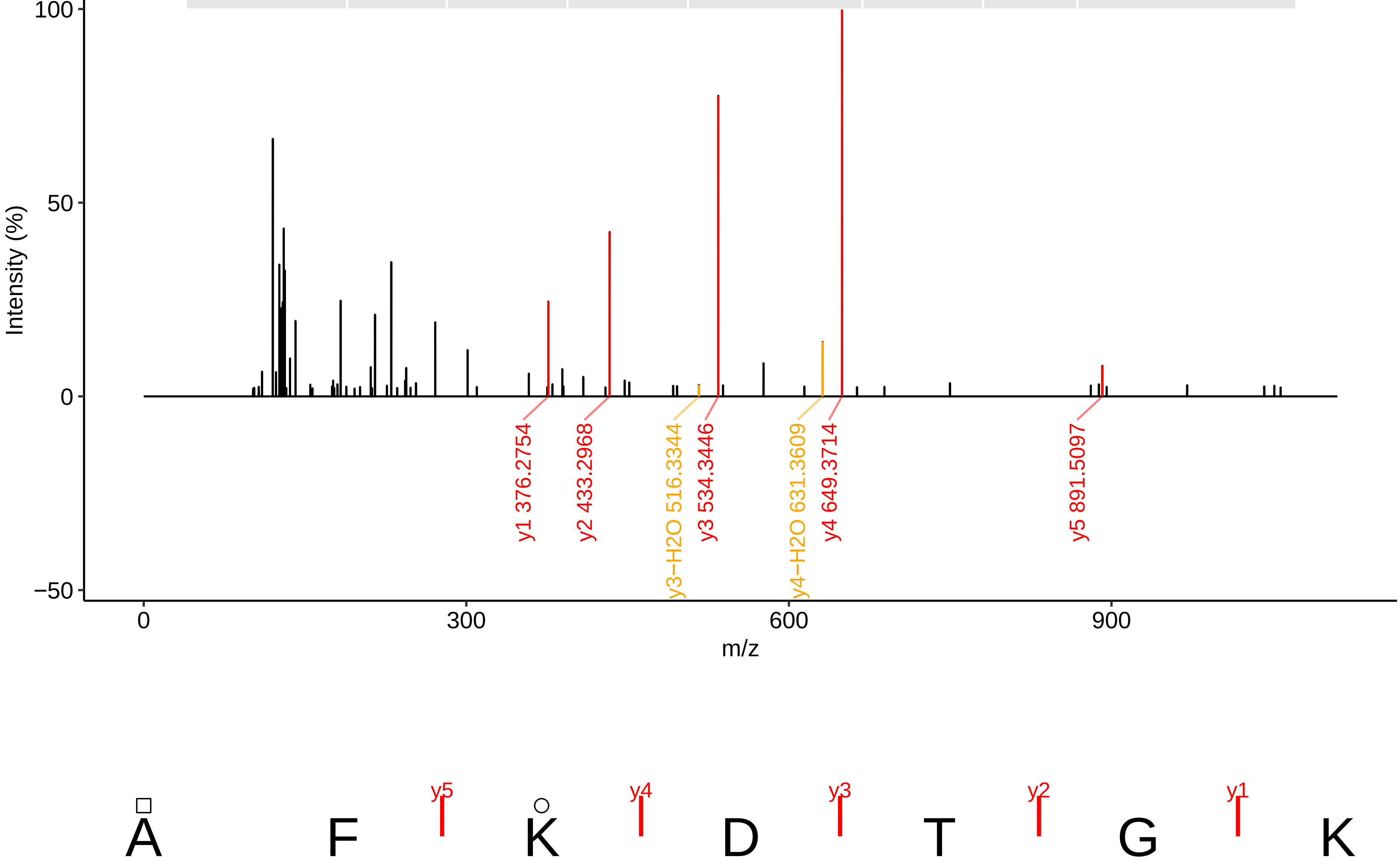

Modification □ Acetyl (Protein N−term) ○ GlyGly (K) no new terminus △ Oxidation (M)

20200921_KIZ_MUb4_GlyGly_10plex_2 − Scan number: 7367

Gene Names	Charge	m/z	Mass	Retention time	PEP	Score	Precursor Intensity
RPS20	2	461.7351	921.4556	27.463	0.060011	59.205	278379.3

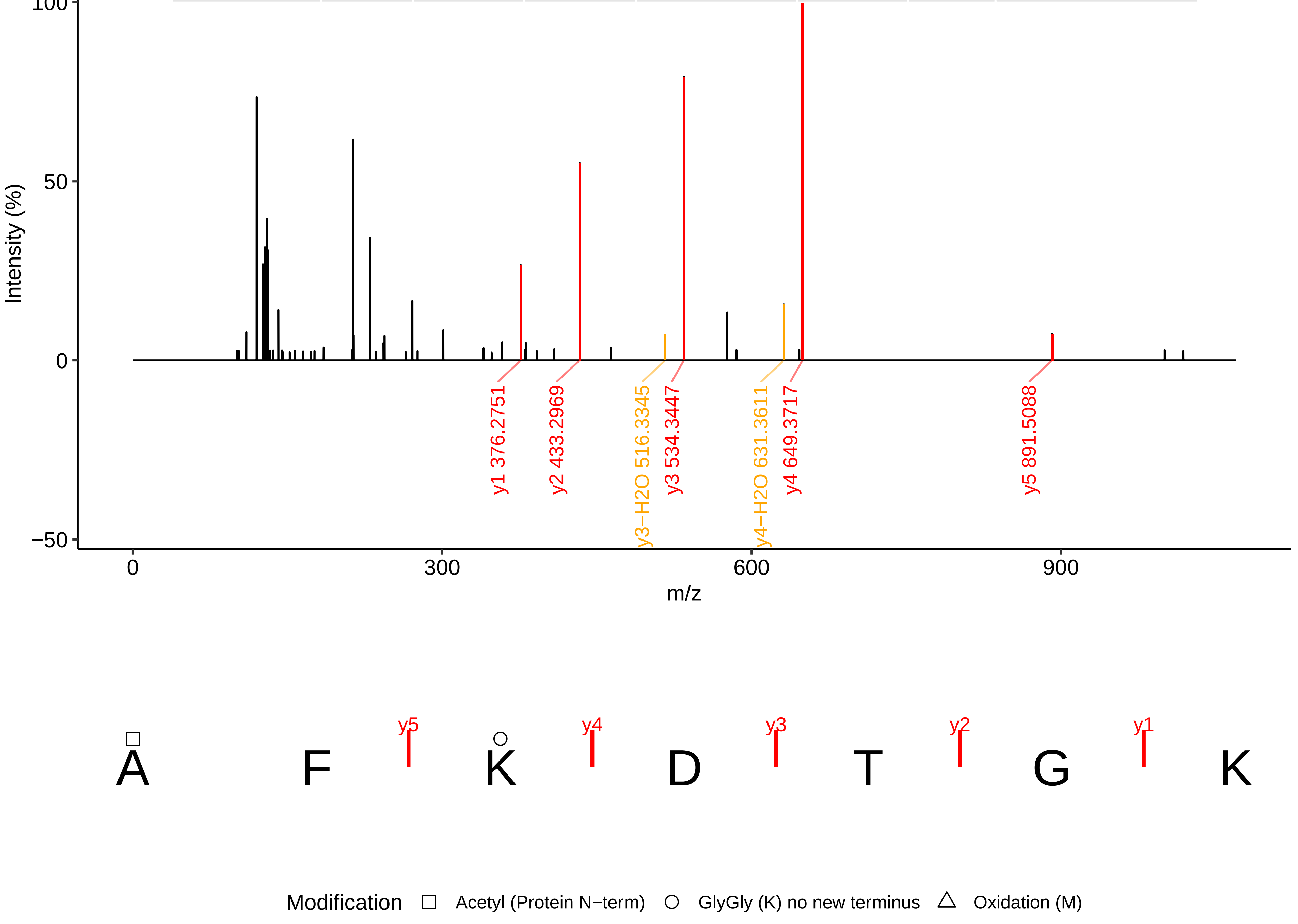

20200924_KIZ_MUb5_GlyGly_1 − Scan number: 7007

Gene Names	Charge	m/z	Mass	Retention time	PEP	Score	Precursor Intensity
RPS20	2	461.7351	921.4556	22.168	0.012488	86.113	5565280

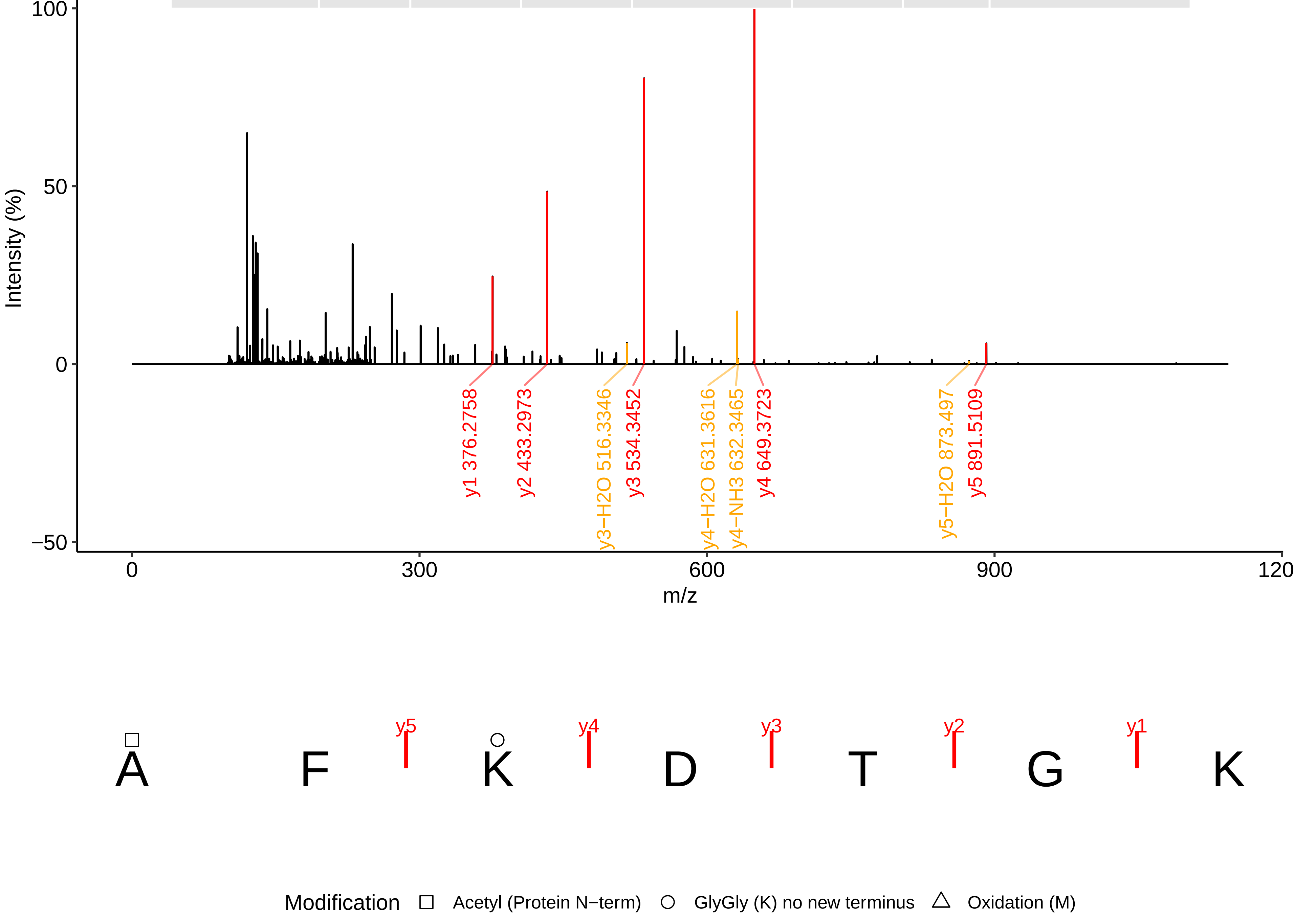

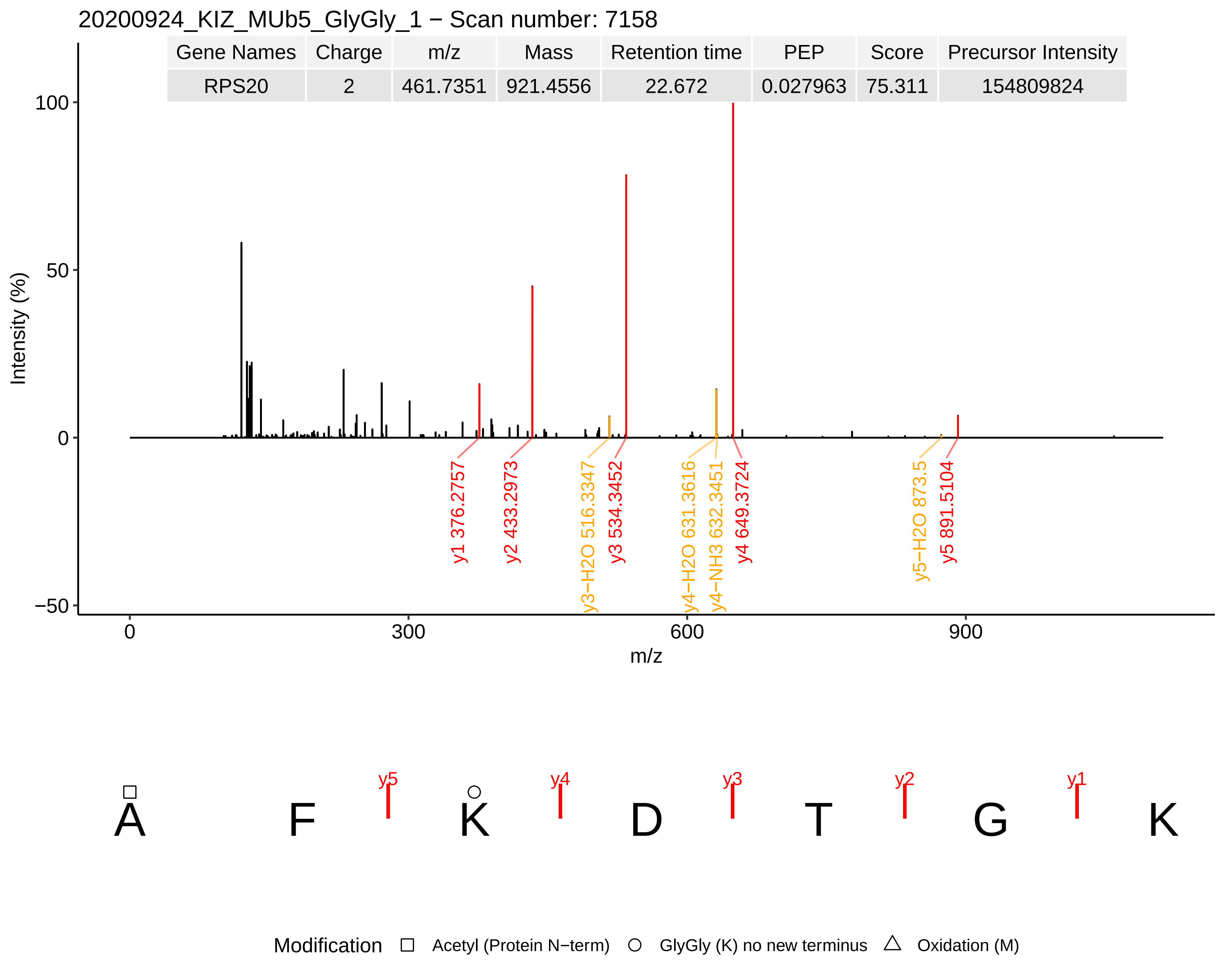
20200924_KIZ_MUb5_GlyGly_1 − Scan number: 7158
Gene Names | Charge | m/z | Mass | Retention time | PEP | Score | Precursor Intensity
RPS20 | 2 | 461.7351 | 921.4556 | 22.672 | 0.027963 | 75.311 | 154809824
Intensity (%)
100
50
0
−50
0
300
600
900
m/z
y1 376.2757
y2 433.2973
y3−H2O 516.3347
y3 534.3452
y4−H2O 631.3616
y4−NH3 632.3451
y4 649.3724
y5−H2O 873.5
y5 891.5104
A F K D T G K
y5 y4 y3 y2 y1
Modification
Acetyl (Protein N−term)
GlyGly (K) no new terminus
Oxidation (M)

20200924_KIZ_MUb5_GlyGly_2 − Scan number: 6469

Gene Names	Charge	m/z	Mass	Retention time	PEP	Score	Precursor Intensity
RPS20	2	461.7351	921.4556	24.125	0.029436	72.234	2672858

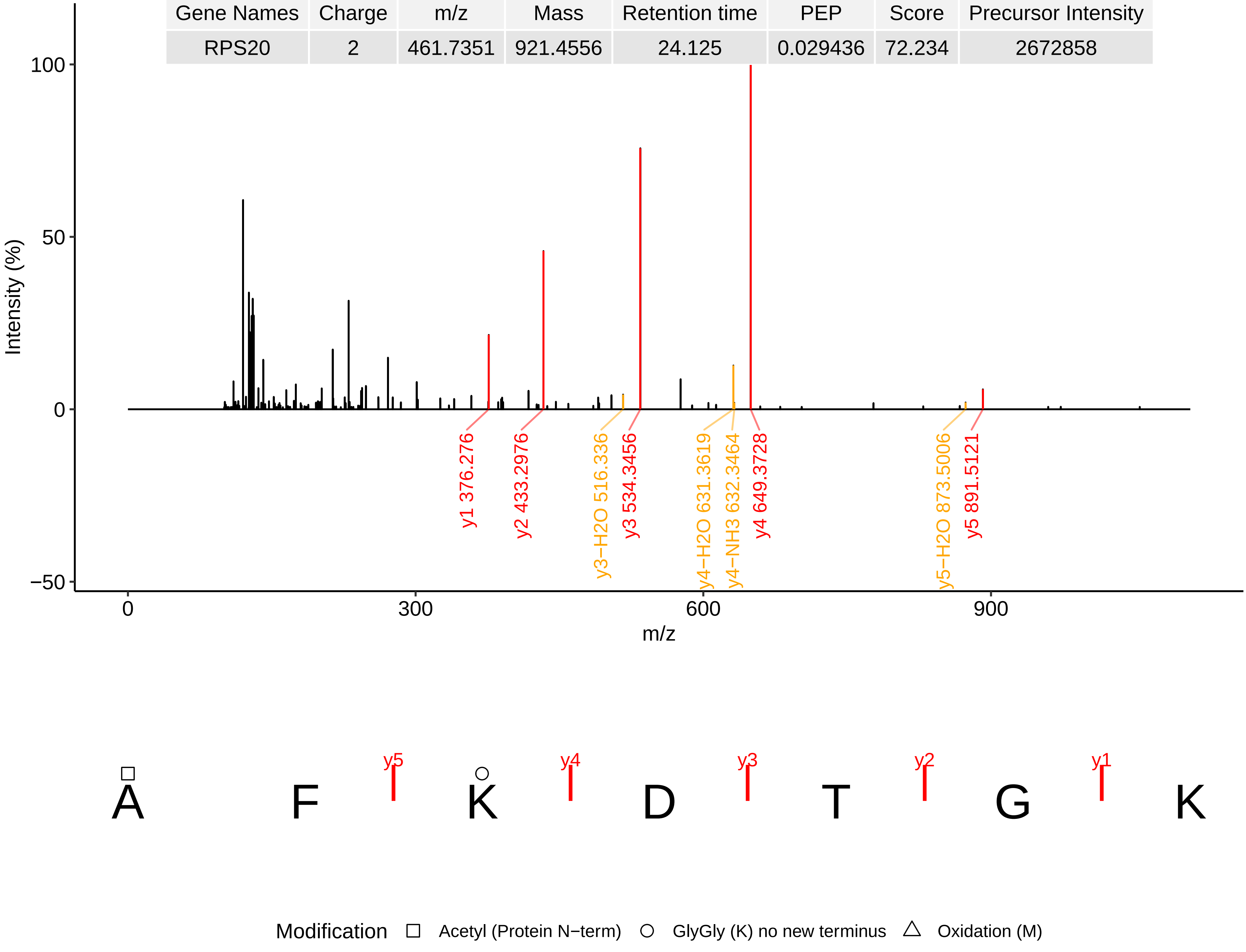

20200924_KIZ_MUb5_GlyGly_2 − Scan number: 6600

Gene Names	Charge	m/z	Mass	Retention time	PEP	Score	Precursor Intensity
RPS20	2	461.7351	921.4556	24.634	0.007581	95.62	67131472

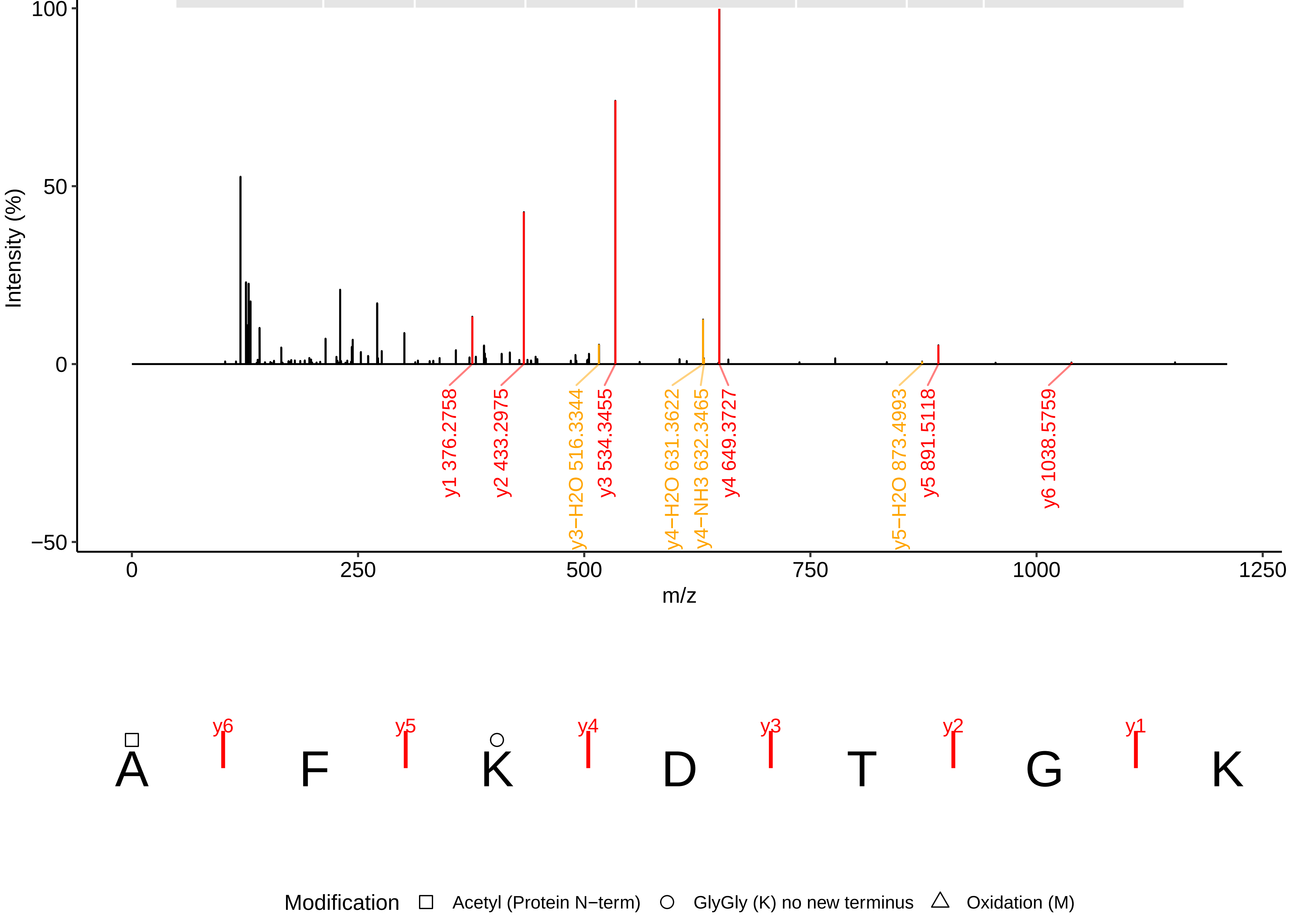

20201118_KIZ_MUb6_GlyGly_1 − Scan number: 6526

Gene Names	Charge	m/z	Mass	Retention time	PEP	Score	Precursor Intensity
RPS20	2	461.7351	921.4556	20.665	0.027963	75.311	18247150

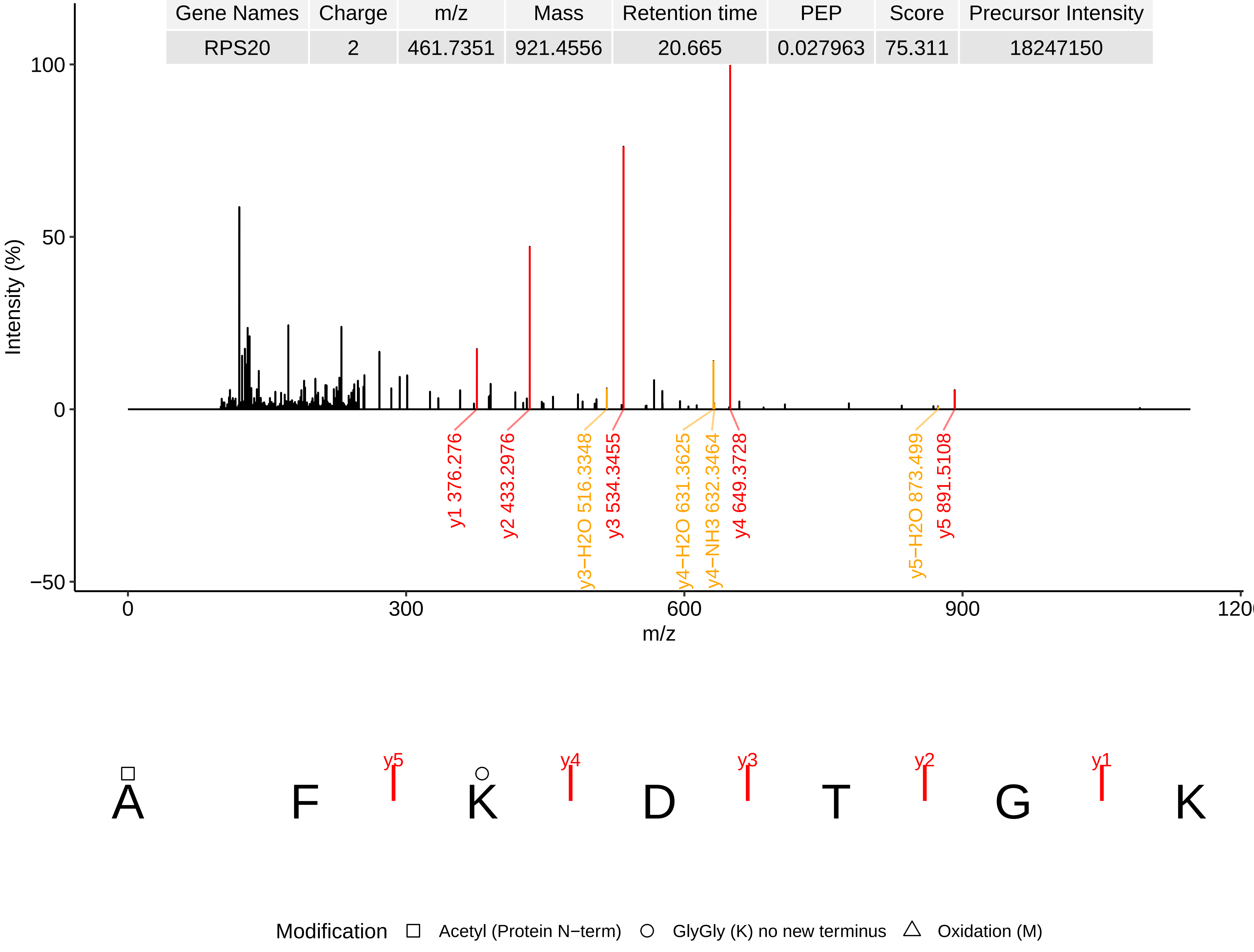

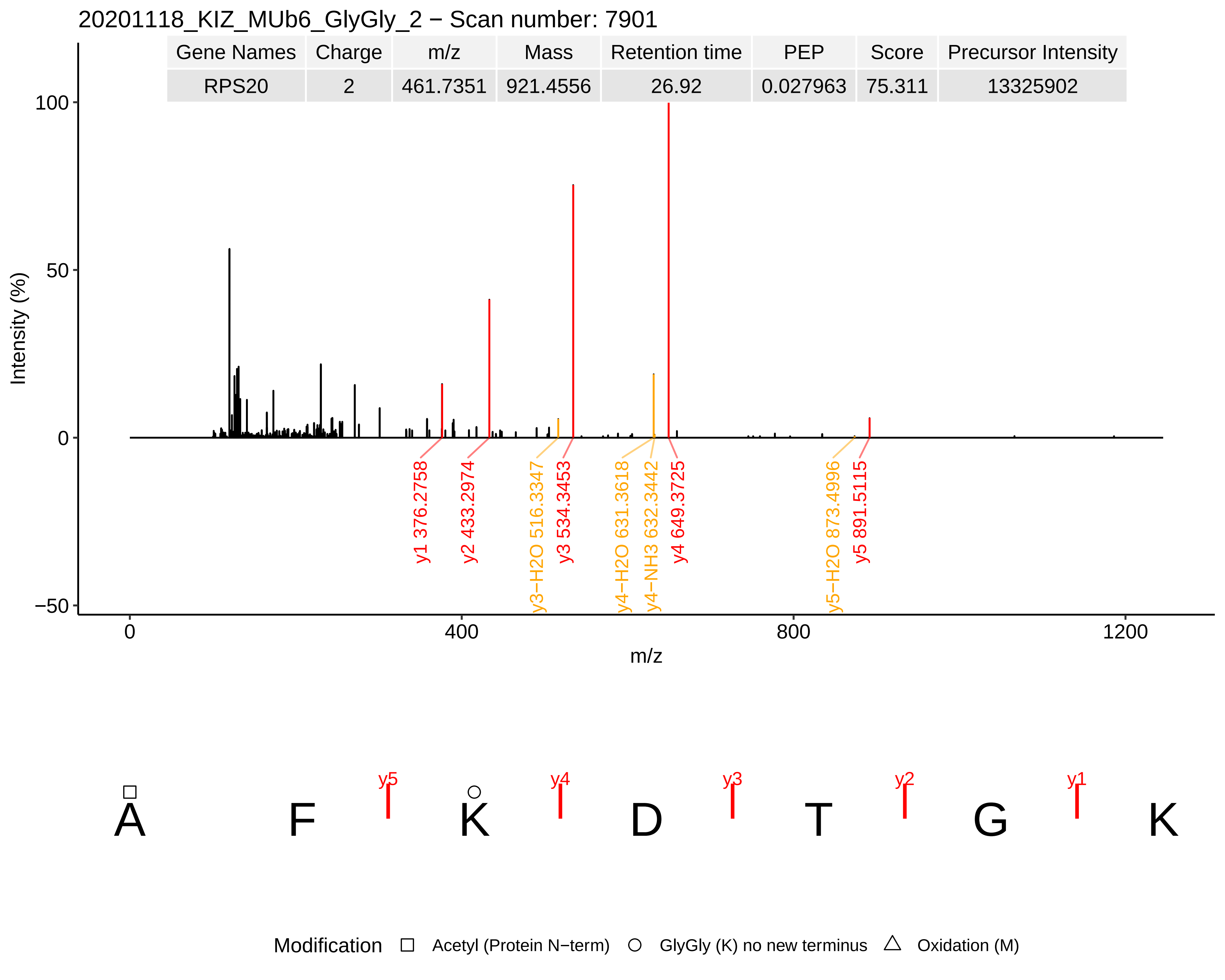
20201118_KIZ_MUb6_GlyGly_2 − Scan number: 7901
Gene Names | Charge | m/z | Mass | Retention time | PEP | Score | Precursor Intensity
RPS20 | 2 | 461.7351 | 921.4556 | 26.92 | 0.027963 | 75.311 | 13325902
Intensity (%)
100
50
0
−50
0
400
800
1200
m/z
y1 376.2758
y2 433.2974
y3−H2O 516.3347
y3 534.3453
y4−H2O 631.3618
y4−NH3 632.3442
y4 649.3725
y5−H2O 873.4996
y5 891.5115
A
F
y5
K
y4
D
y3
T
y2
G
y1
K
Modification
Acetyl (Protein N−term)
GlyGly (K) no new terminus
Oxidation (M)

20200921_KIZ_MUb4_GlyGly_10plex − Scan number: 4702

Gene Names	Charge	m/z	Mass	Retention time	PEP	Score	Precursor Intensity
IMPA2	3	300.5189	898.5349	15.328	0.053123	96.048	7481321

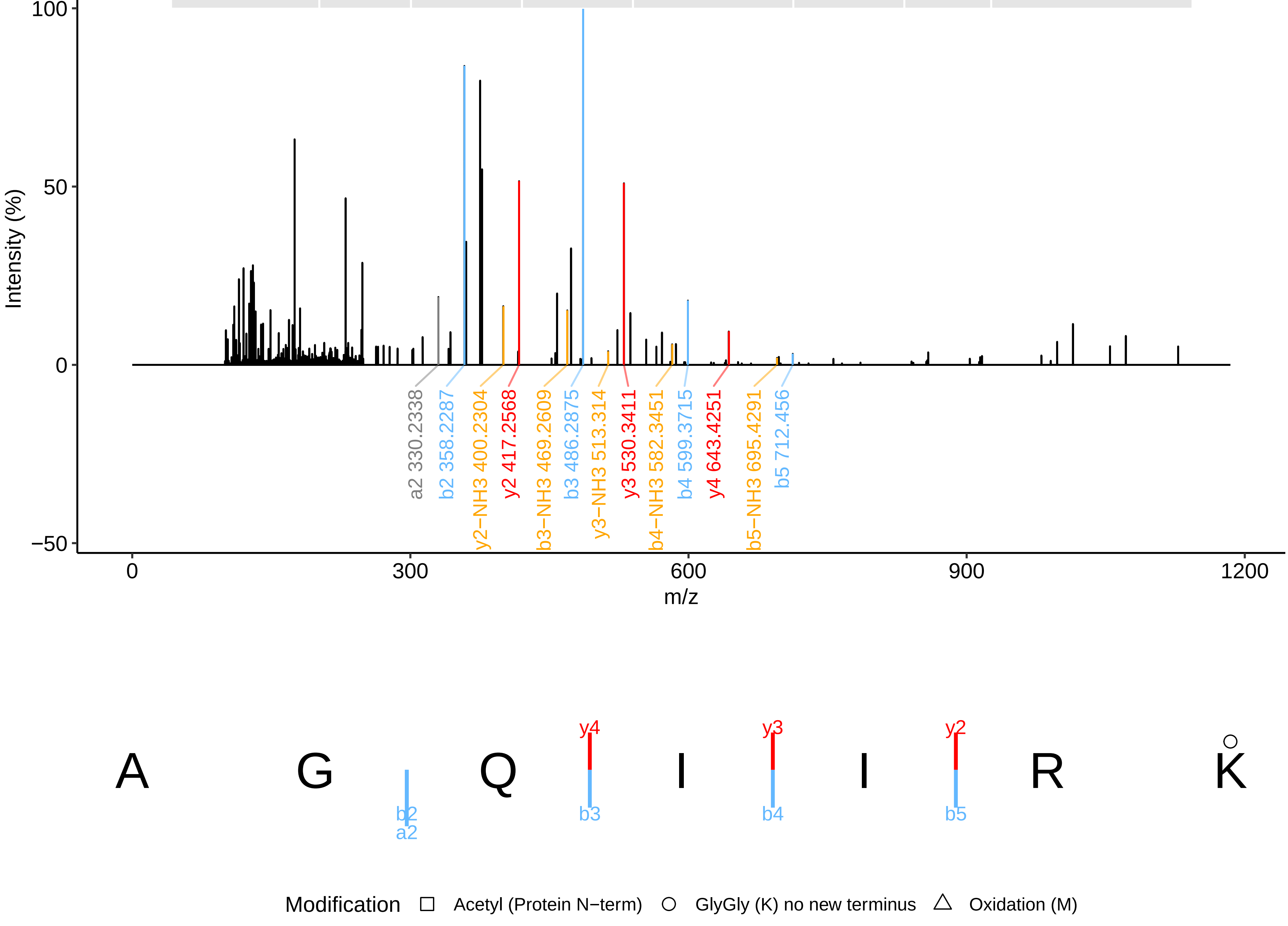

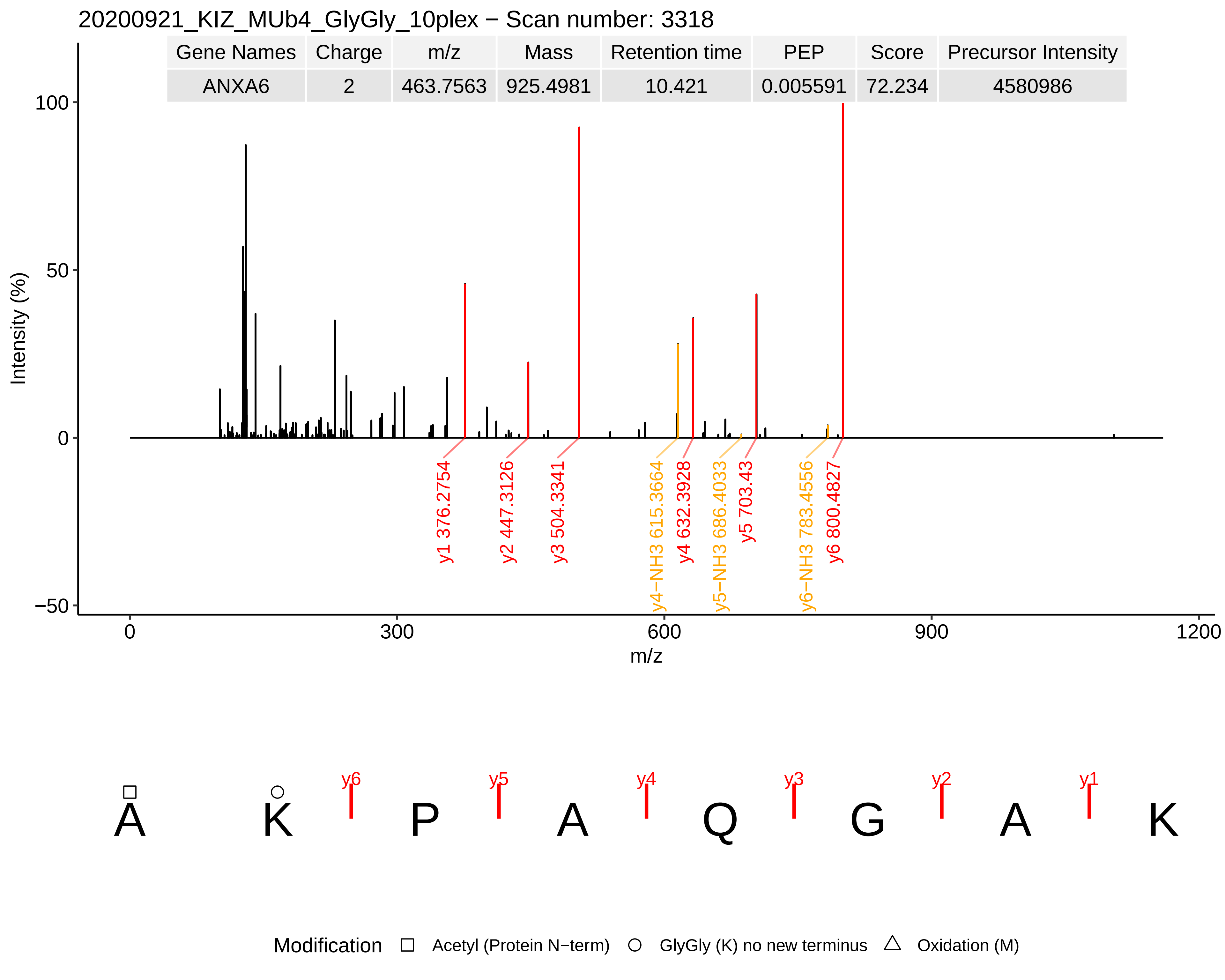
20200921_KIZ_MUb4_GlyGly_10plex − Scan number: 3318
Gene Names
Charge
m/z
Mass
Retention time
PEP
Score
Precursor Intensity
ANXA6
2
463.7563
925.4981
10.421
0.005591
72.234
4580986
Intensity (%)
100
50
0
−50
0
300
600
900
1200
m/z
y1 376.2754
y2 447.3126
y3 504.3341
y4−NH3 615.3664
y4 632.3928
y5−NH3 686.4033
y5 703.43
y6−NH3 783.4556
y6 800.4827
A
K
y6
P
y5
A
y4
Q
y3
G
y2
A
y1
K
Modification
Acetyl (Protein N−term)
GlyGly (K) no new terminus
Oxidation (M)

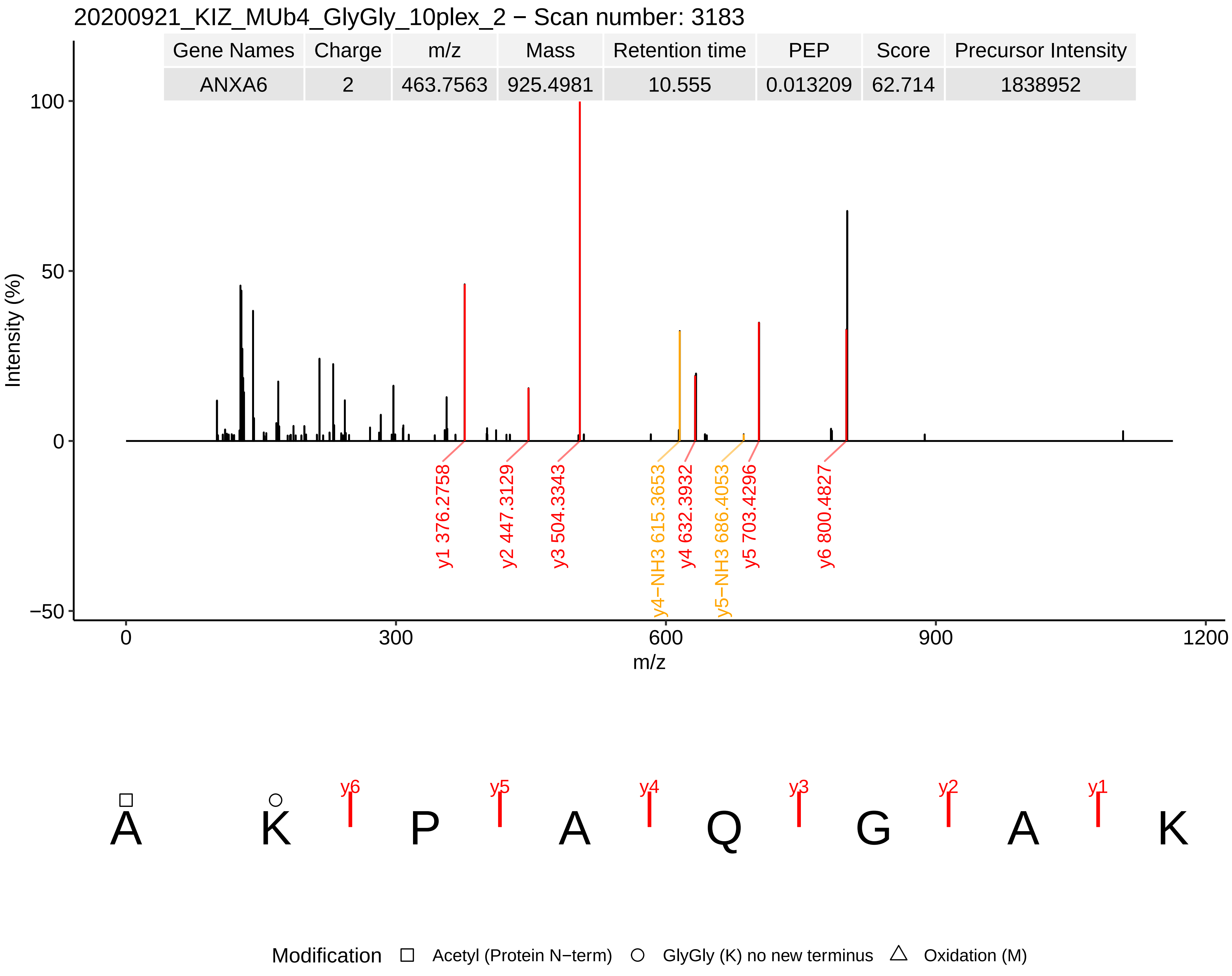
20200921_KIZ_MUb4_GlyGly_10plex_2 − Scan number: 3183
Gene Names | Charge | m/z | Mass | Retention time | PEP | Score | Precursor Intensity
ANXA6 | 2 | 463.7563 | 925.4981 | 10.555 | 0.013209 | 62.714 | 1838952
Intensity (%)
100
50
0
−50
0
300
600
900
1200
m/z
y1 376.2758
y2 447.3129
y3 504.3343
y4−NH3 615.3653
y4 632.3932
y5−NH3 686.4053
y5 703.4296
y6 800.4827
A K P A Q G A K
y6 y5 y4 y3 y2 y1
Modification
Acetyl (Protein N−term)
GlyGly (K) no new terminus
Oxidation (M)

20200924_KIZ_MUb5_GlyGly_1 − Scan number: 3148

Gene Names	Charge	m/z	Mass	Retention time	PEP	Score	Precursor Intensity
ANXA6	2	463.7563	925.4981	9.7164	0.018874	72.234	6193120

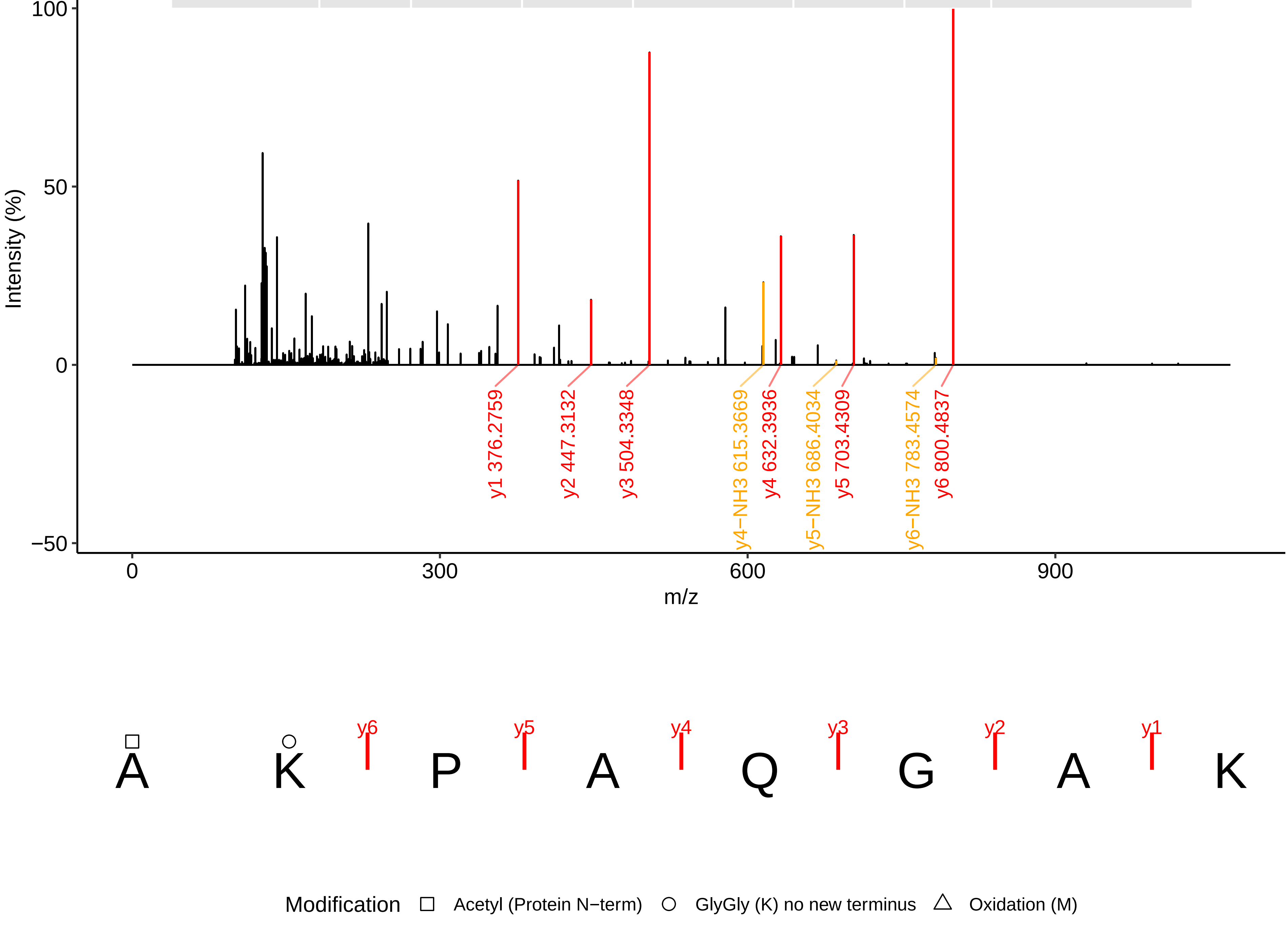

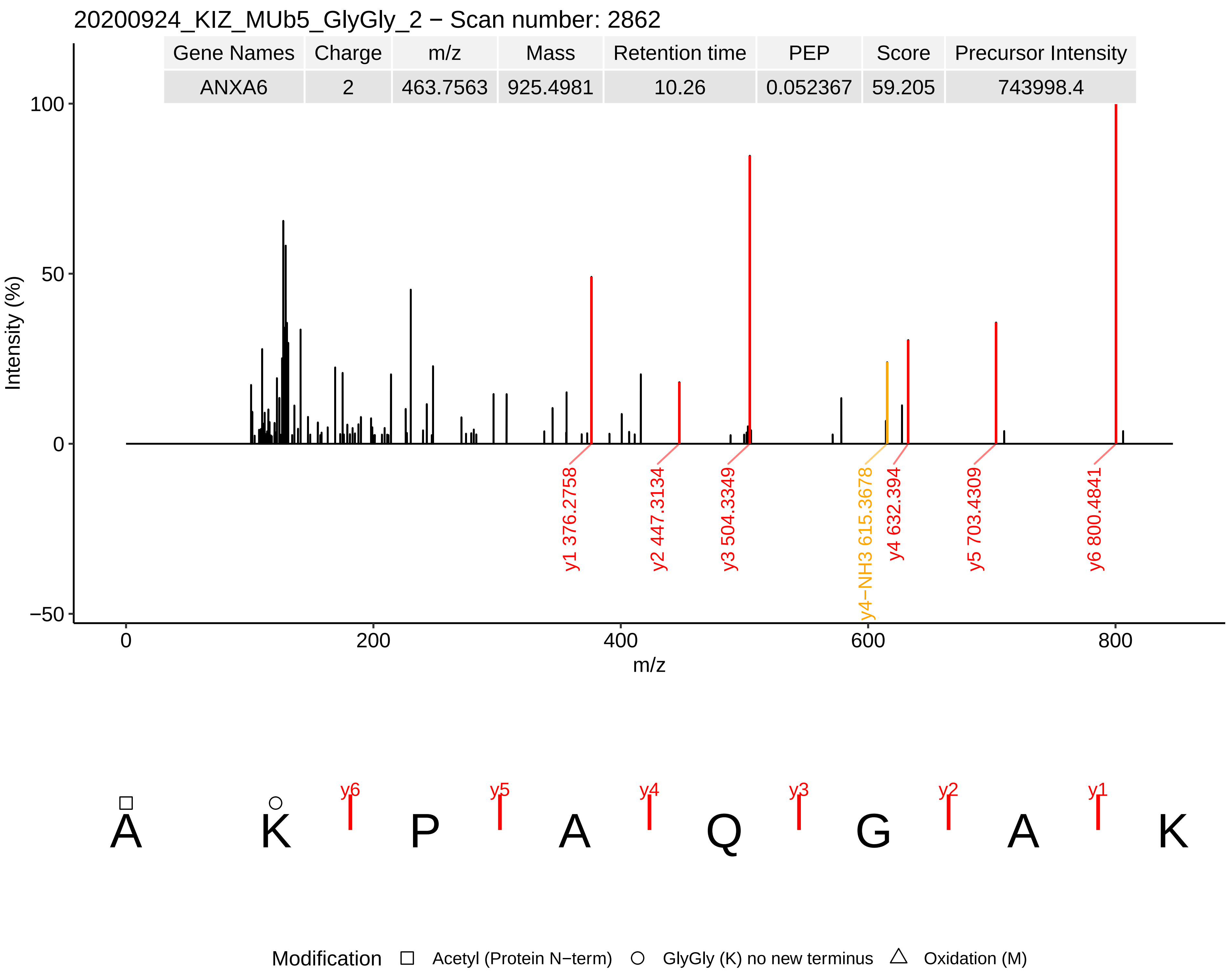

Gene Names	Charge	m/z	Mass	Retention time	PEP	Score	Precursor Intensity
ANXA6	2	463.7563	925.4981	10.26	0.052367	59.205	743998.4

20200921_KIZ_MUb4_GlyGly_10plex_2 − Scan number: 2922

Gene Names	Charge	m/z	Mass	Retention time	PEP	Score	Precursor Intensity
EPS15L1	3	311.17	930.4883	9.5198	0.019308	81.297	1522425

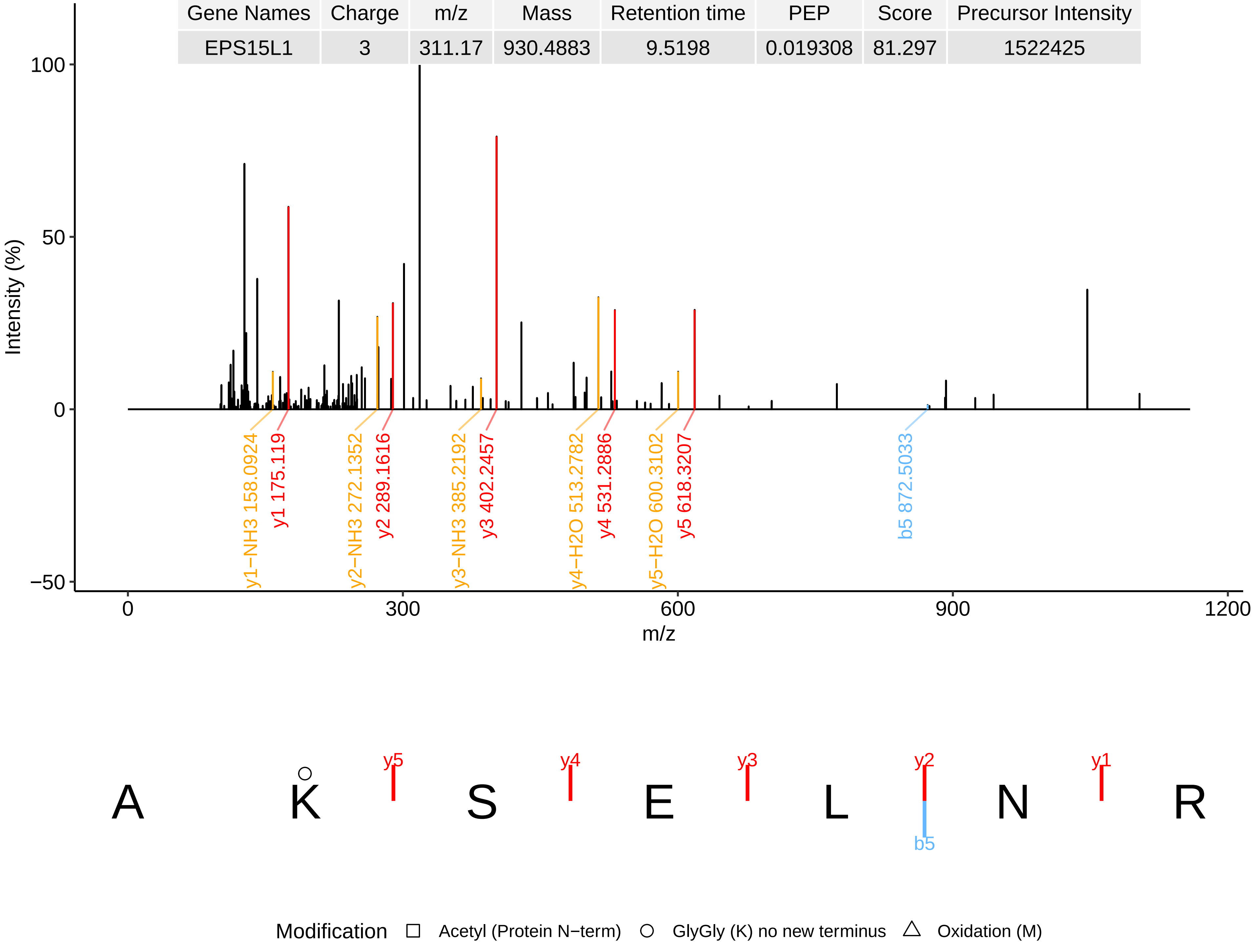

20200924_KIZ_MUb5_GlyGly_1 − Scan number: 2911

Gene Names	Charge	m/z	Mass	Retention time	PEP	Score	Precursor Intensity
EPS15L1	3	311.17	930.4883	9.0034	0.089173	58.167	14297980

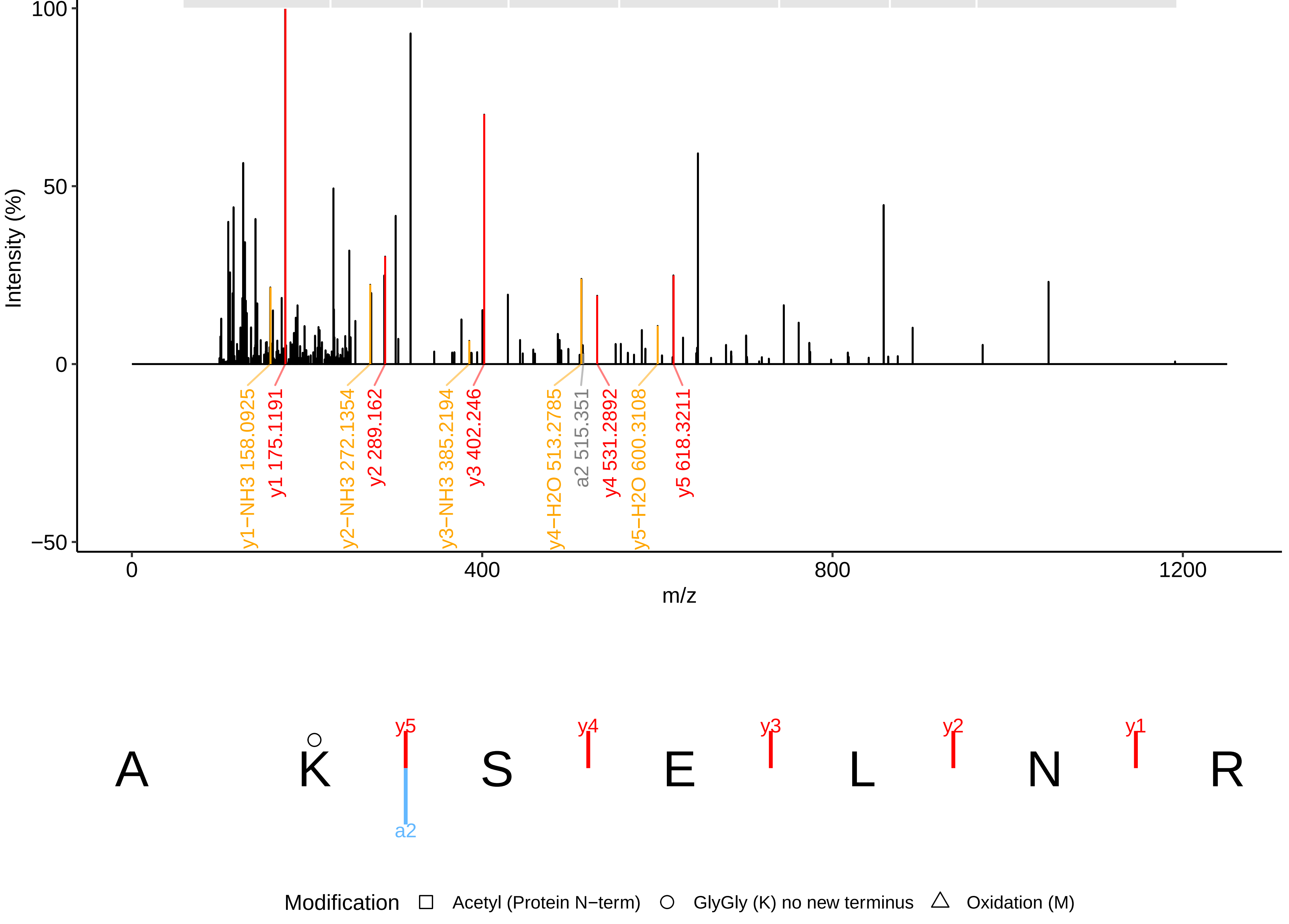

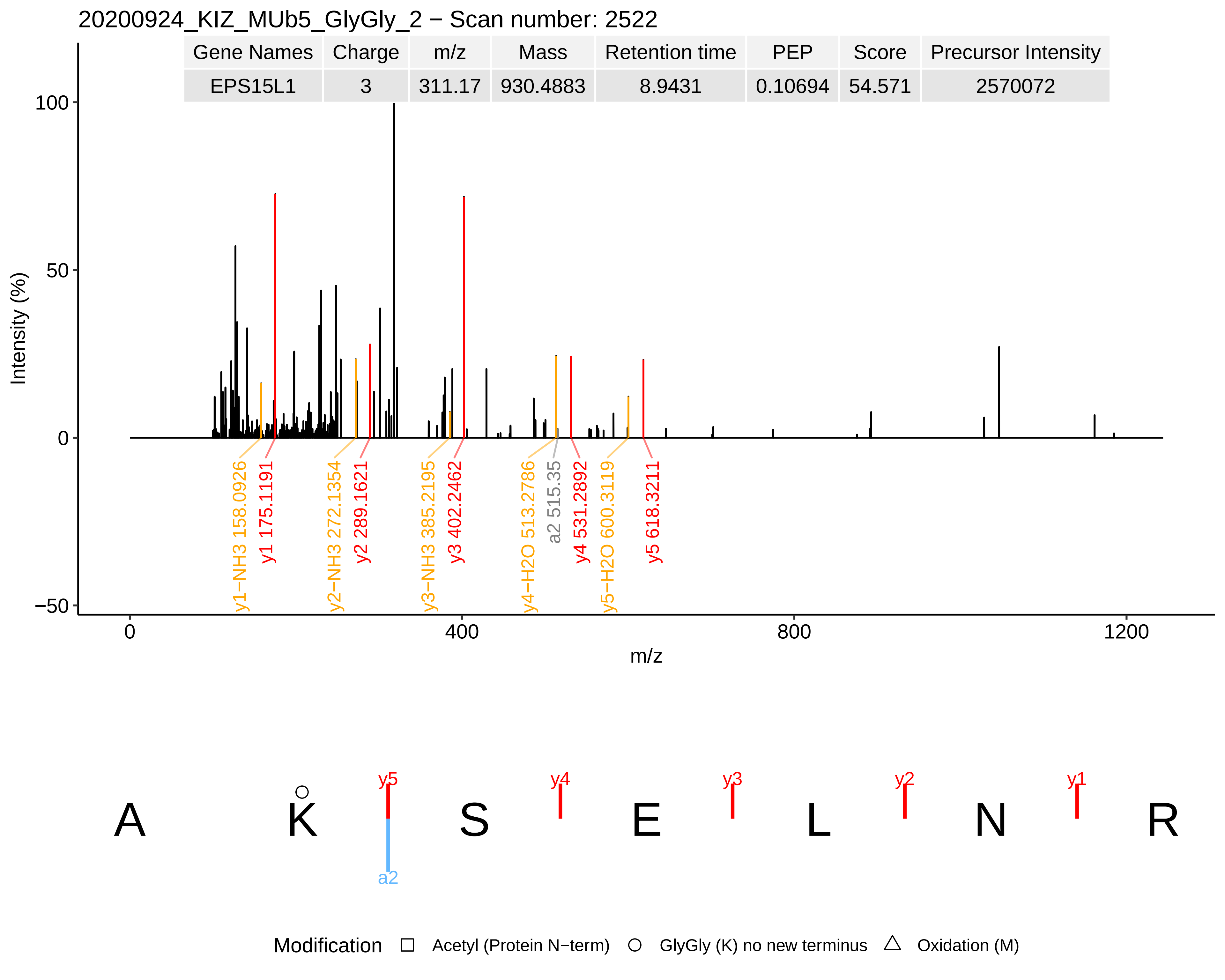

20200924_KIZ_MUb5_GlyGly_2 − Scan number: 2522
Gene Names | Charge | m/z | Mass | Retention time | PEP | Score | Precursor Intensity
EPS15L1 | 3 | 311.17 | 930.4883 | 8.9431 | 0.10694 | 54.571 | 2570072
Intensity (%)
100
50
0
−50
y1−NH3 158.0926
y1 175.1191
y2−NH3 272.1354
y2 289.1621
y3−NH3 385.2195
y3 402.2462
y4−H2O 513.2786
a2 515.35
y4 531.2892
y5−H2O 600.3119
y5 618.3211
0
400
800
1200
m/z
A K S E L N R
y5 y4 y3 y2 y1
a2
Modification
Acetyl (Protein N−term)
GlyGly (K) no new terminus
Oxidation (M)

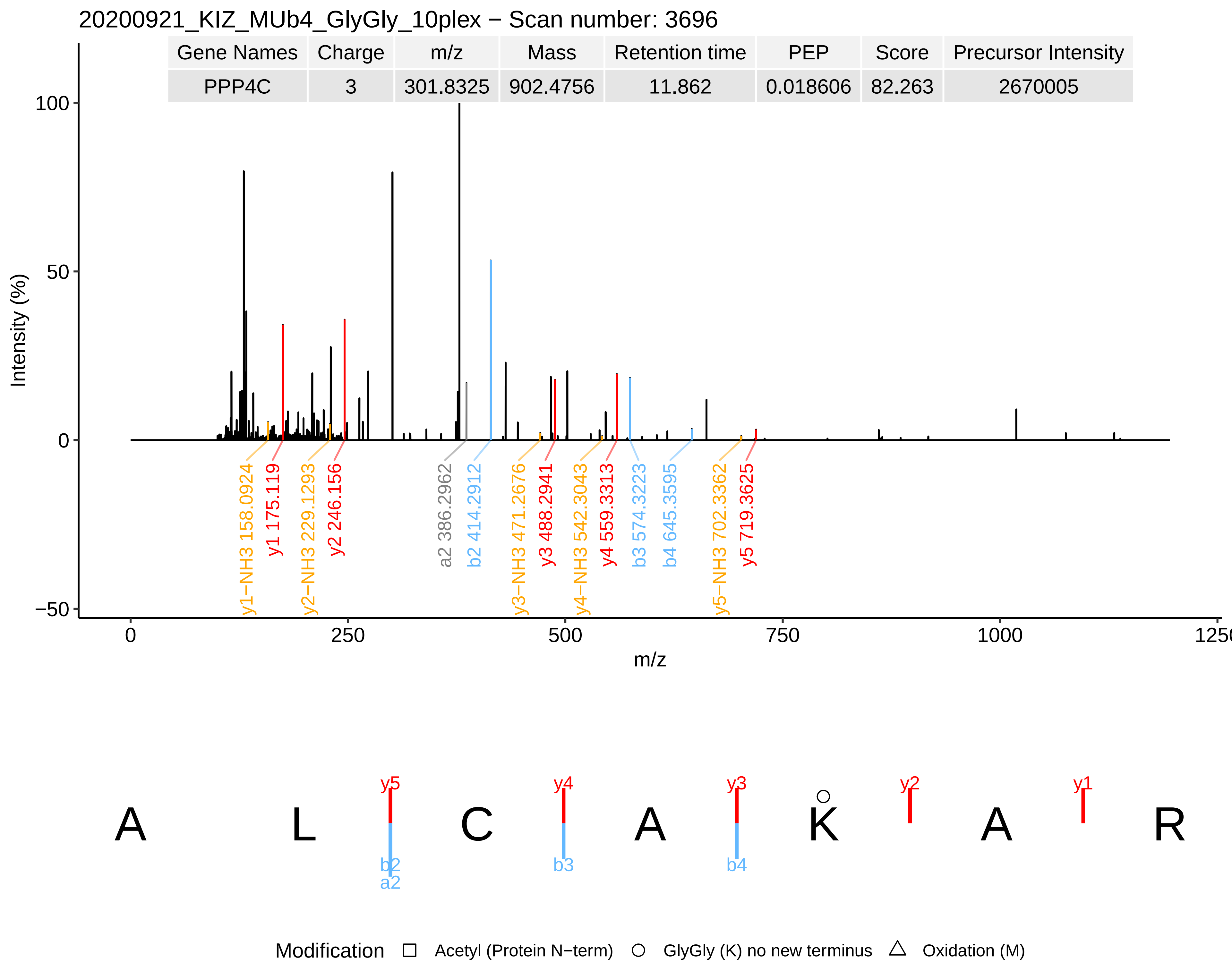
20200921_KIZ_MUb4_GlyGly_10plex − Scan number: 3696
Gene Names | Charge | m/z | Mass | Retention time | PEP | Score | Precursor Intensity
PPP4C | 3 | 301.8325 | 902.4756 | 11.862 | 0.018606 | 82.263 | 2670005
Intensity (%)
m/z
y1−NH3 158.0924
y1 175.119
y2−NH3 229.1293
y2 246.156
a2 386.2962
b2 414.2912
y3−NH3 471.2676
y3 488.2941
y4−NH3 542.3043
y4 559.3313
b3 574.3223
b4 645.3595
y5−NH3 702.3362
y5 719.3625
A L C A K A R
y5 y4 y3 y2 y1
b2 a2 b3 b4
Modification
Acetyl (Protein N−term)
GlyGly (K) no new terminus
Oxidation (M)

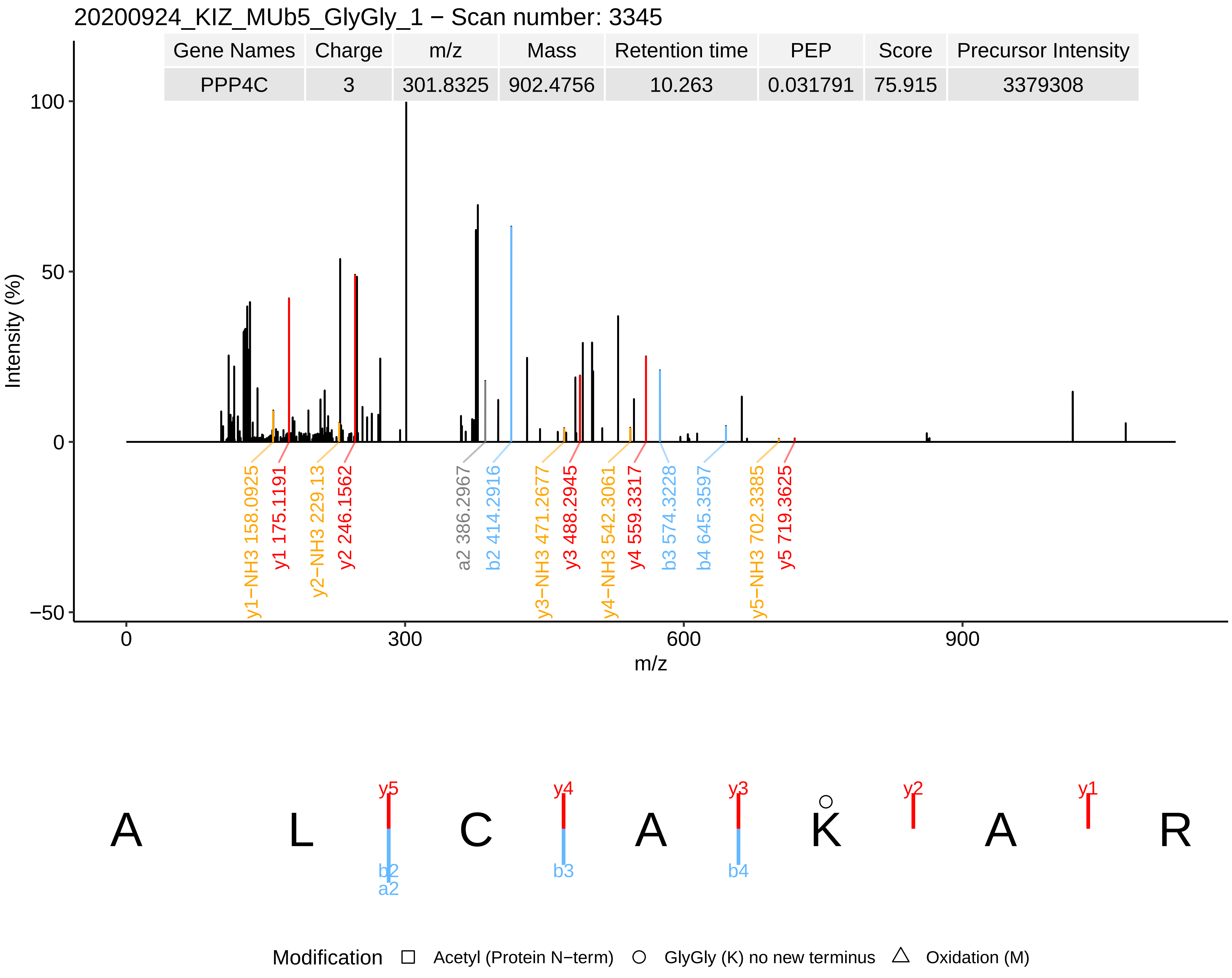

20200924_KIZ_MUb5_GlyGly_1 − Scan number: 3345
Gene Names | Charge | m/z | Mass | Retention time | PEP | Score | Precursor Intensity
PPP4C | 3 | 301.8325 | 902.4756 | 10.263 | 0.031791 | 75.915 | 3379308
Intensity (%)
100
50
0
−50
0
300
600
900
m/z
y1−NH3 158.0925
y1 175.1191
y2−NH3 229.13
y2 246.1562
a2 386.2967
b2 414.2916
y3−NH3 471.2677
y3 488.2945
y4−NH3 542.3061
y4 559.3317
b3 574.3228
b4 645.3597
y5−NH3 702.3385
y5 719.3625
A L C A K A R
y5 y4 y3 y2 y1
b2 a2 b3 b4
Modification
Acetyl (Protein N−term)
GlyGly (K) no new terminus
Oxidation (M)

20200924_KIZ_MUb5_GlyGly_2 − Scan number: 3026

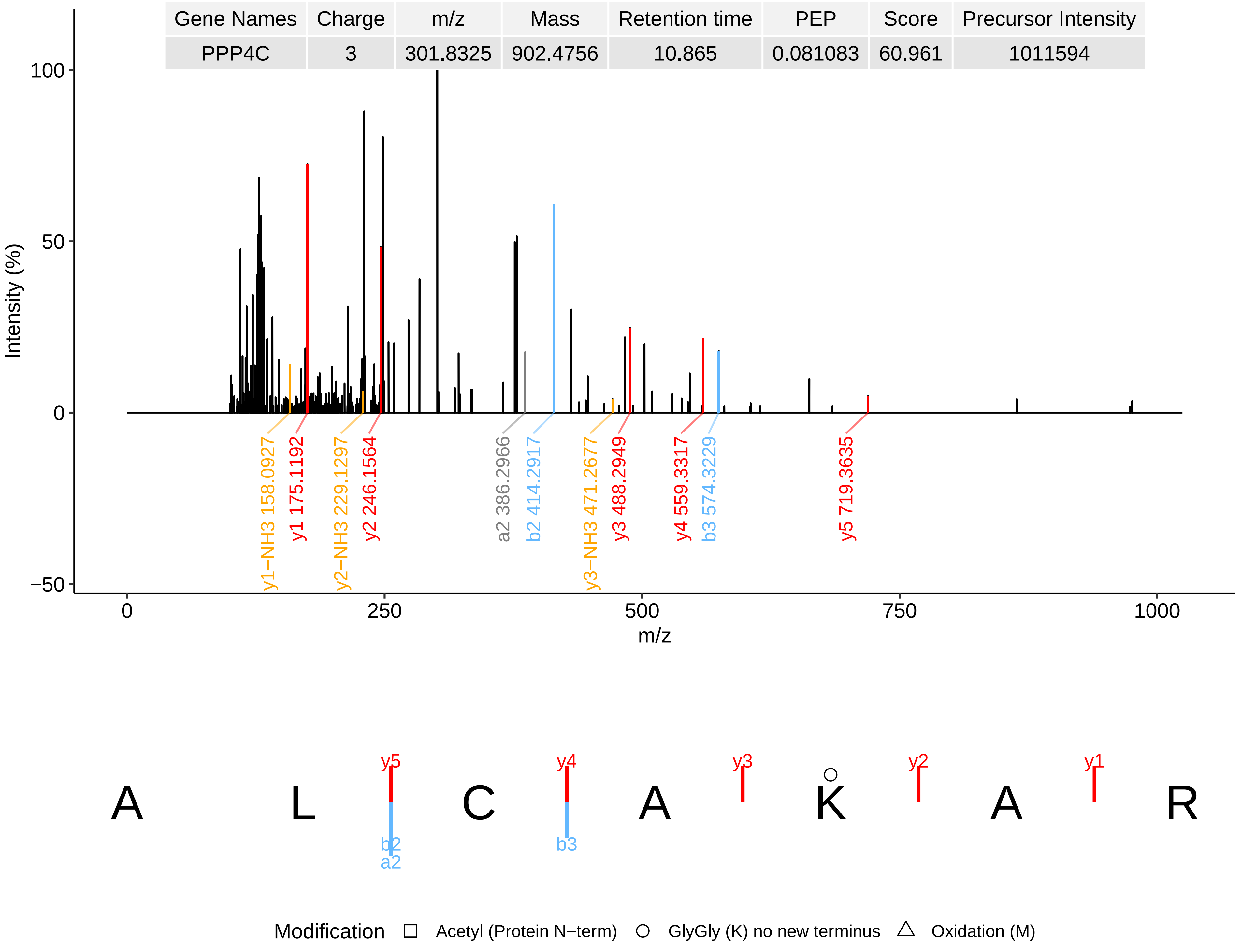

20201118_KIZ_MUb6_GlyGly_1 − Scan number: 3239

Gene Names	Charge	m/z	Mass	Retention time	PEP	Score	Precursor Intensity
PPP4C	3	301.8325	902.4756	9.965	0.027094	77.748	34101364

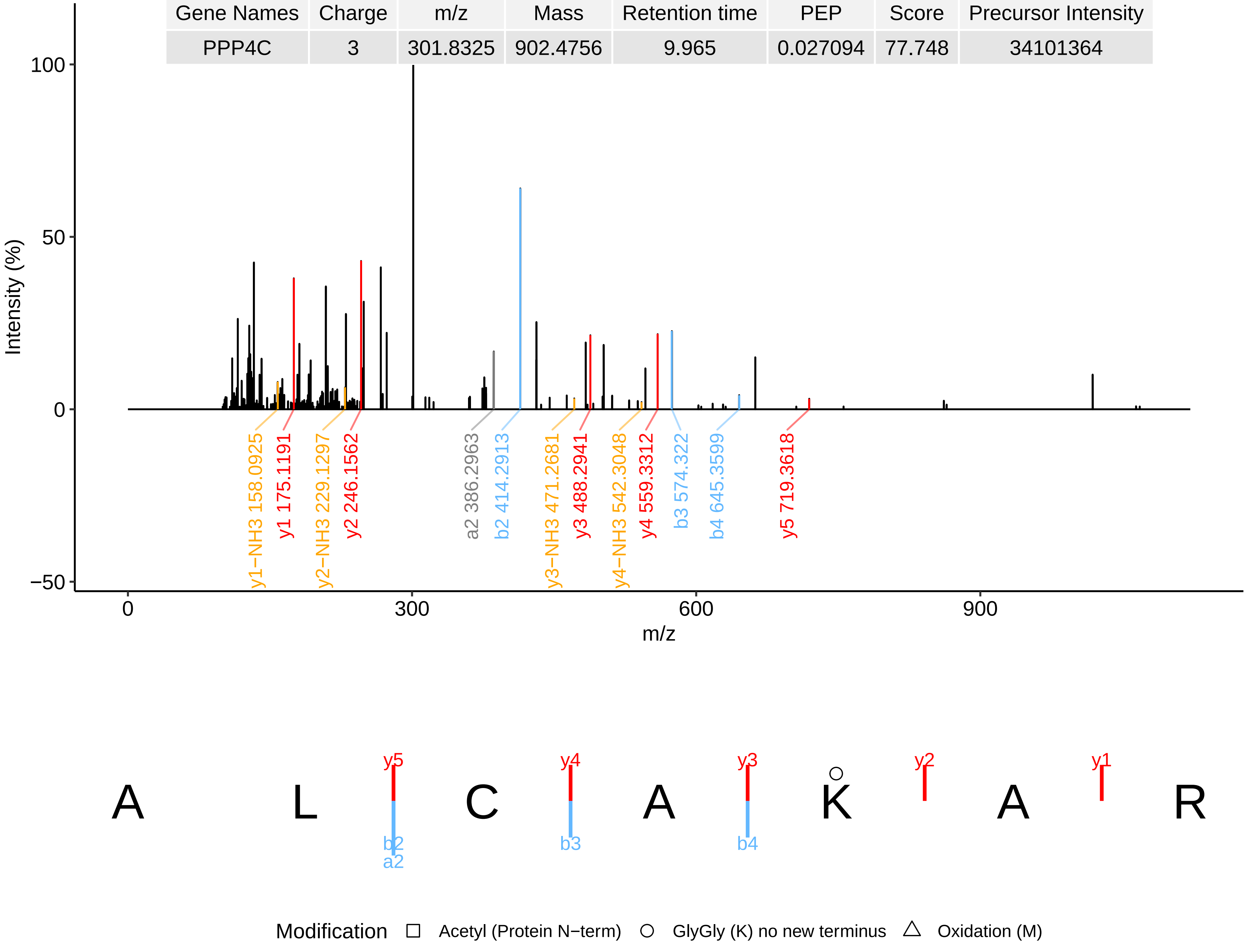

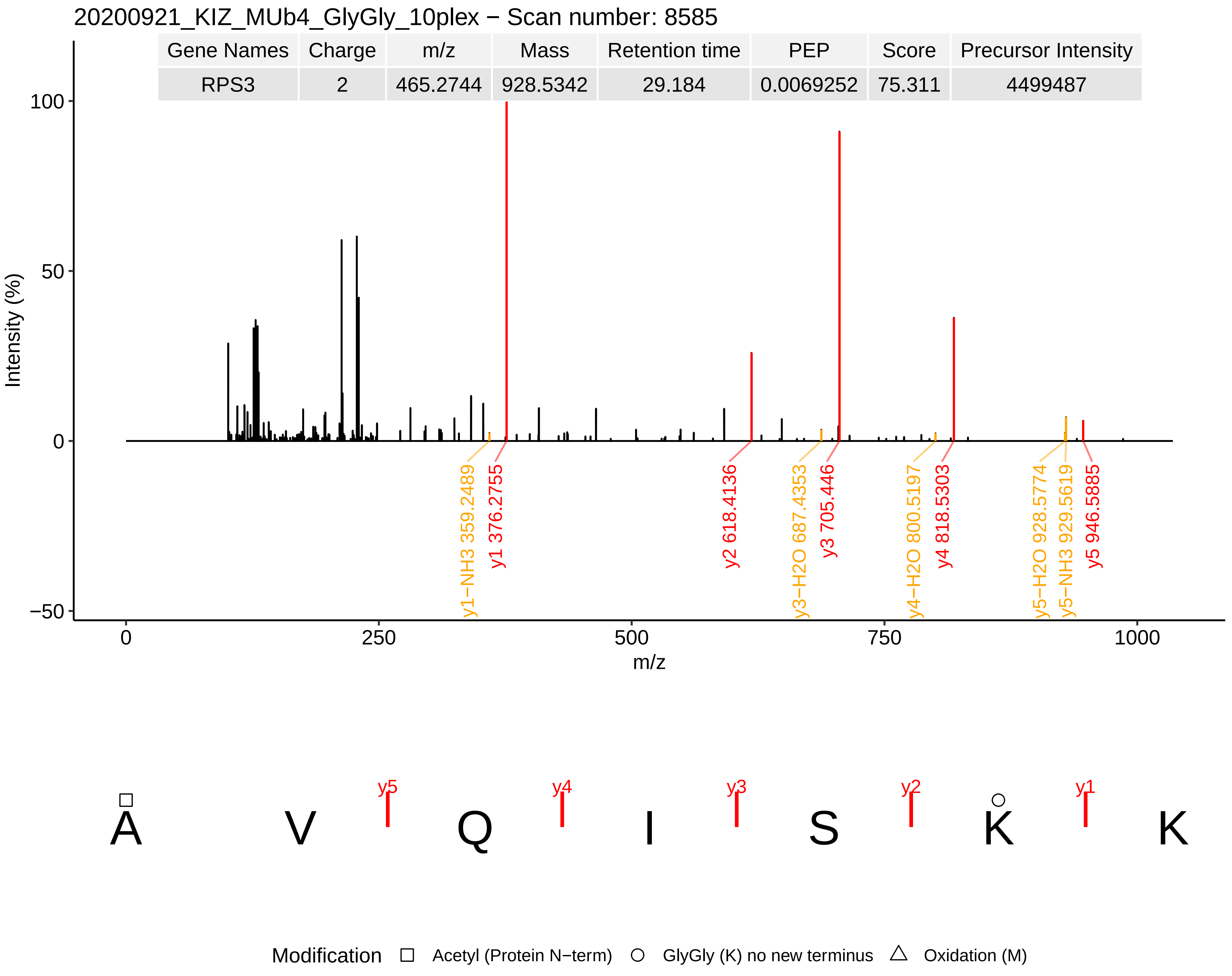
20200921_KIZ_MUb4_GlyGly_10plex − Scan number: 8585
Gene Names | Charge | m/z | Mass | Retention time | PEP | Score | Precursor Intensity
RPS3 | 2 | 465.2744 | 928.5342 | 29.184 | 0.0069252 | 75.311 | 4499487
Intensity (%)
100
50
0
−50
0
250
500
750
1000
m/z
y1−NH3 359.2489
y1 376.2755
y2 618.4136
y3−H2O 687.4353
y3 705.446
y4−H2O 800.5197
y4 818.5303
y5−H2O 928.5774
y5−NH3 929.5619
y5 946.5885
A V Q I S K K
y5
y4
y3
y2
y1
Modification
Acetyl (Protein N−term)
GlyGly (K) no new terminus
Oxidation (M)

20200921_KIZ_MUb4_GlyGly_10plex_2 − Scan number: 7846

Gene Names	Charge	m/z	Mass	Retention time	PEP	Score	Precursor Intensity
RPS3	2	465.2744	928.5342	29.408	0.0079934	72.234	255209.6

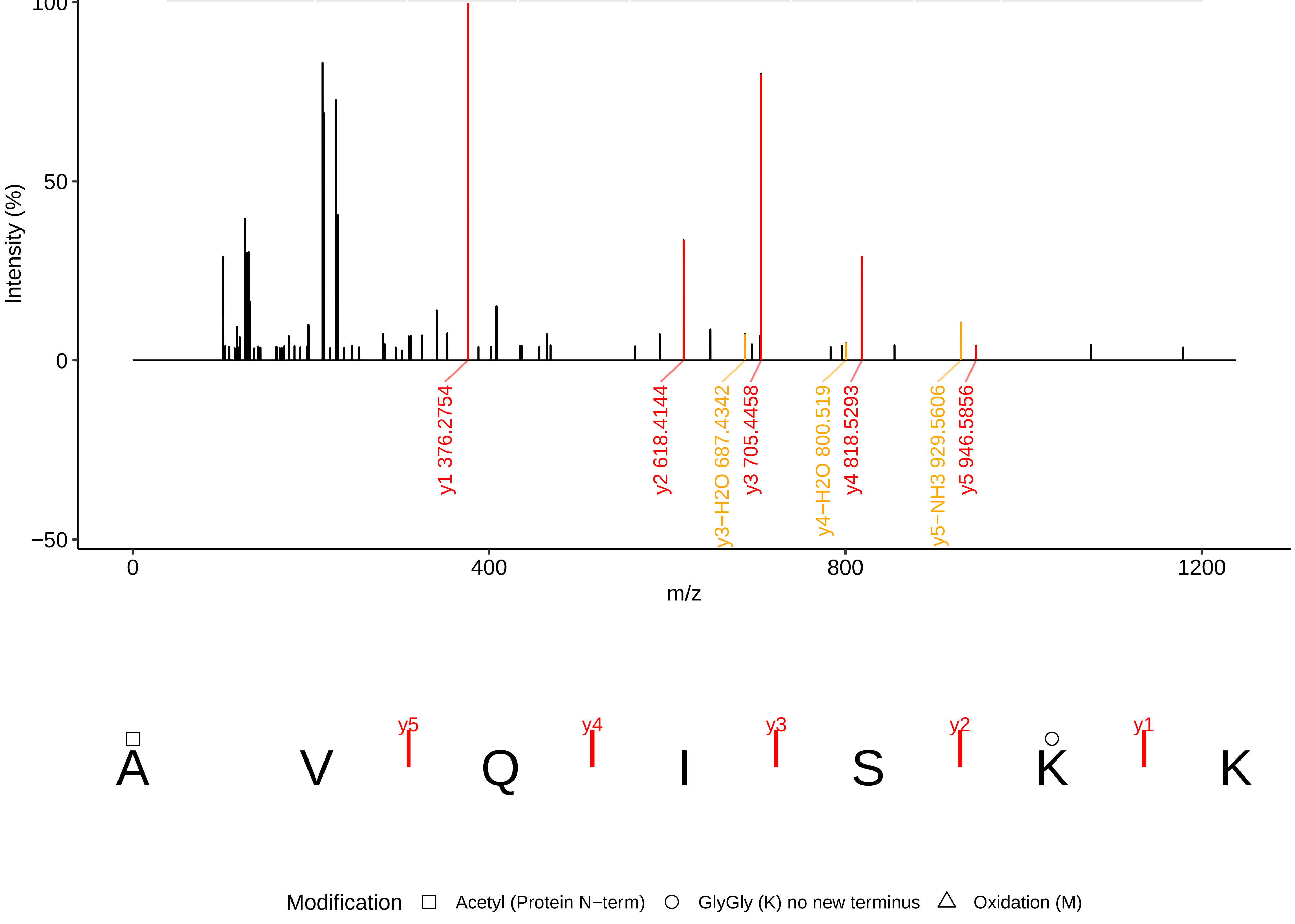

20200924_KIZ_MUb5_GlyGly_1 − Scan number: 7674

Gene Names	Charge	m/z	Mass	Retention time	PEP	Score	Precursor Intensity
RPS3	2	465.2744	928.5342	24.414	0.0083569	75.311	NA

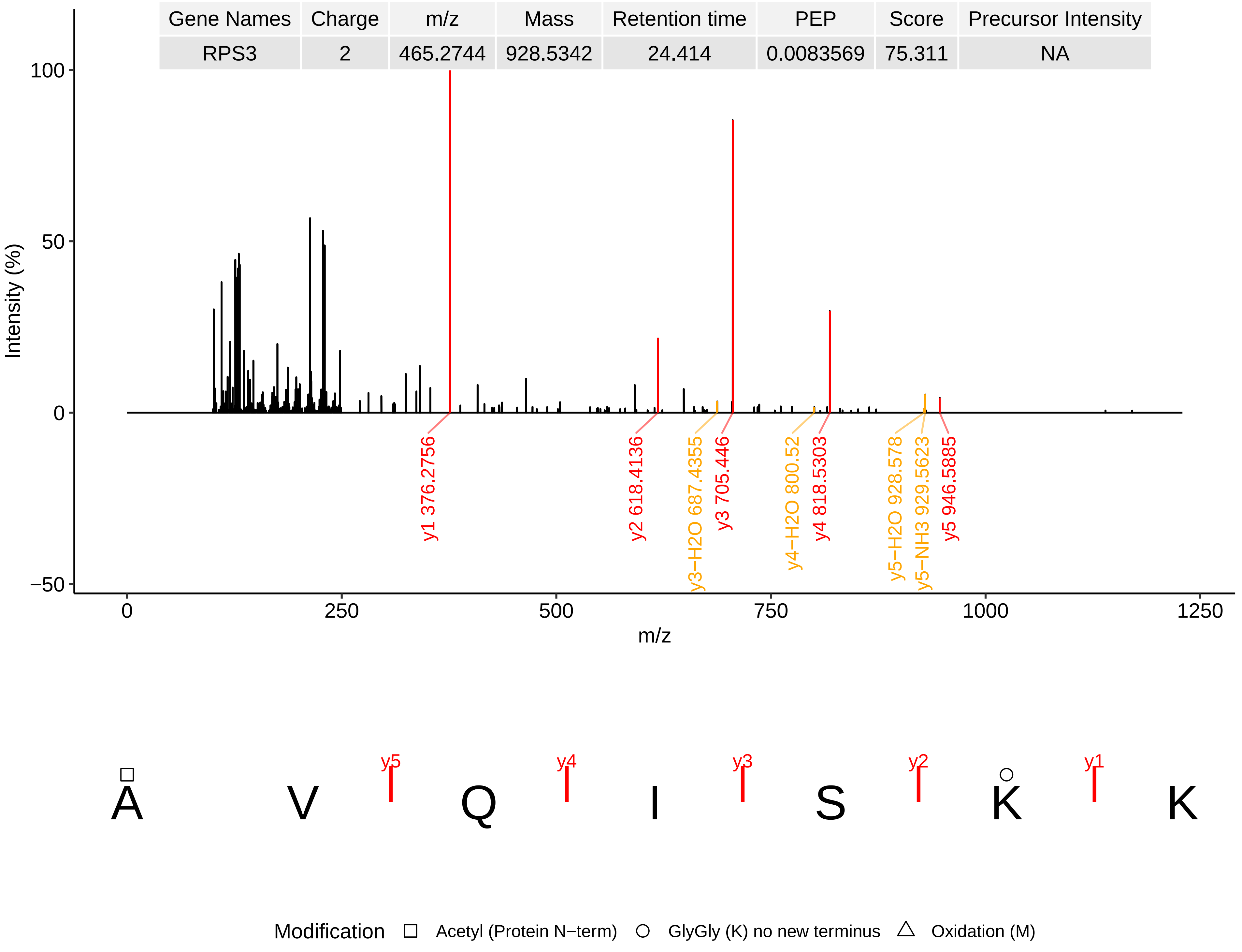

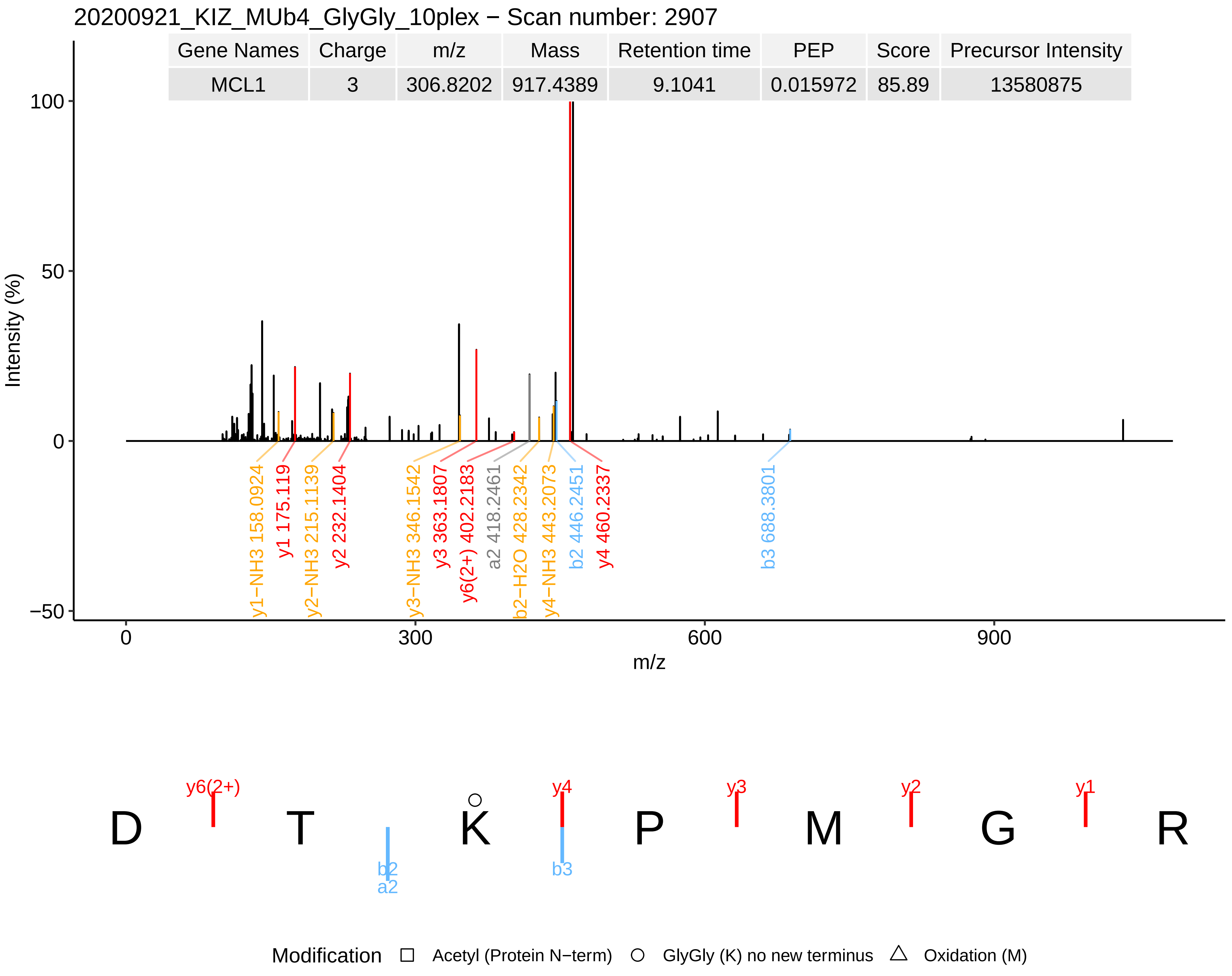
20200921_KIZ_MUb4_GlyGly_10plex − Scan number: 2907
Gene Names | Charge | m/z | Mass | Retention time | PEP | Score | Precursor Intensity
MCL1 | 3 | 306.8202 | 917.4389 | 9.1041 | 0.015972 | 85.89 | 13580875
Intensity (%)
100
50
0
−50
0
300
600
900
m/z
y1−NH3 158.0924
y1 175.119
y2−NH3 215.1139
y2 232.1404
y3−NH3 346.1542
y3 363.1807
y6(2+) 402.2183
a2 418.2461
b2−H2O 428.2342
y4−NH3 443.2073
b2 446.2451
y4 460.2337
b3 688.3801
D
T
K
P
M
G
R
y6(2+)
y4
y3
y2
y1
b2
a2
b3
Modification
Acetyl (Protein N−term)
GlyGly (K) no new terminus
Oxidation (M)

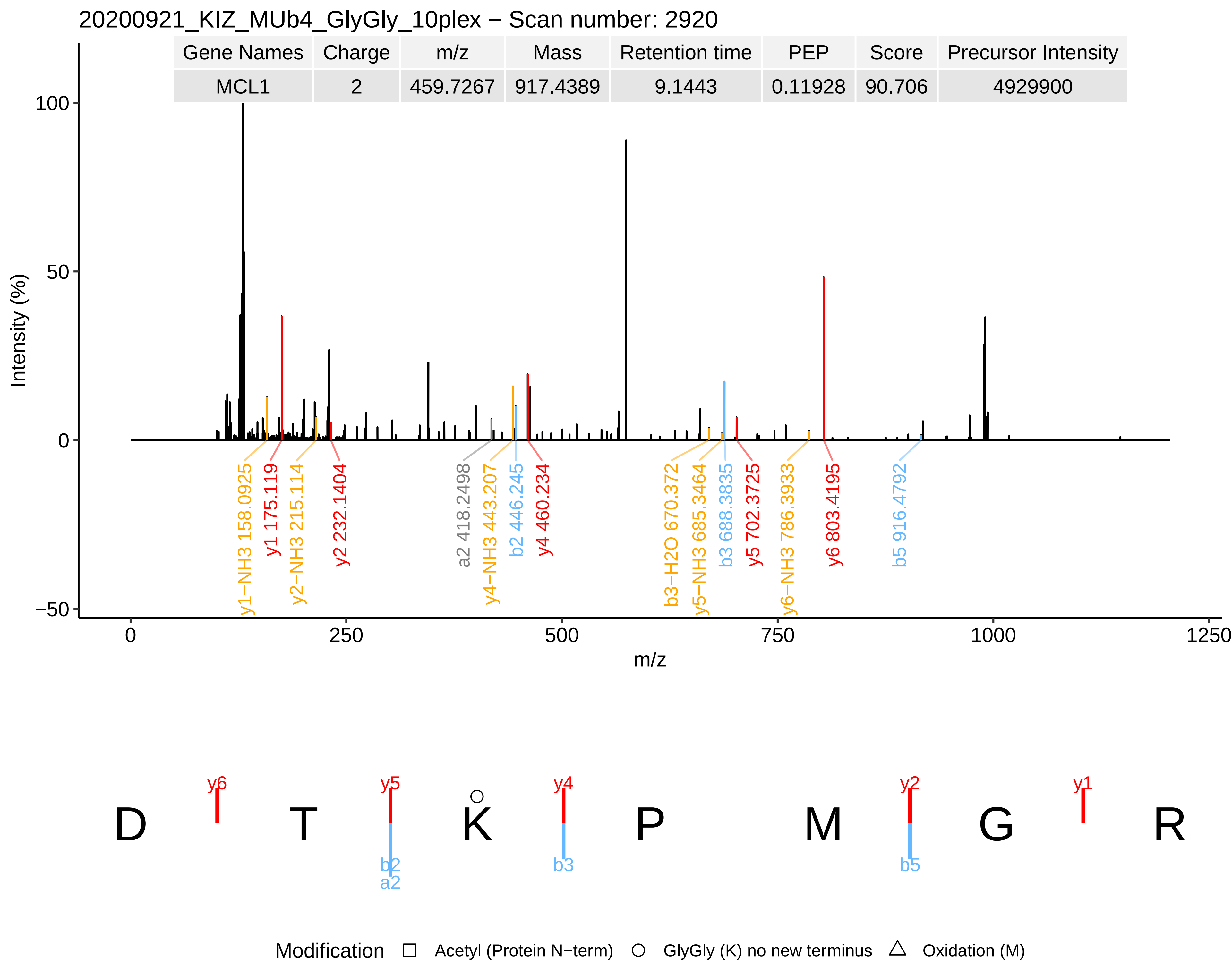
20200921_KIZ_MUb4_GlyGly_10plex − Scan number: 2920
Gene Names | Charge | m/z | Mass | Retention time | PEP | Score | Precursor Intensity
MCL1 | 2 | 459.7267 | 917.4389 | 9.1443 | 0.11928 | 90.706 | 4929900
Intensity (%)
m/z
y1−NH3 158.0925
y1 175.119
y2−NH3 215.114
y2 232.1404
a2 418.2498
y4−NH3 443.207
b2 446.245
y4 460.234
b3−H2O 670.372
y5−NH3 685.3464
b3 688.3835
y5 702.3725
y6−NH3 786.3933
y6 803.4195
b5 916.4792
D T K P M G R
y6 y5 y4 y2 y1
b2 a2 b3 b5
Modification
Acetyl (Protein N−term)
GlyGly (K) no new terminus
Oxidation (M)

20200921_KIZ_MUb4_GlyGly_10plex_2 − Scan number: 2856

Gene Names	Charge	m/z	Mass	Retention time	PEP	Score	Precursor Intensity
MCL1	3	306.8202	917.4389	9.2504	0.01552	86.512	2866538

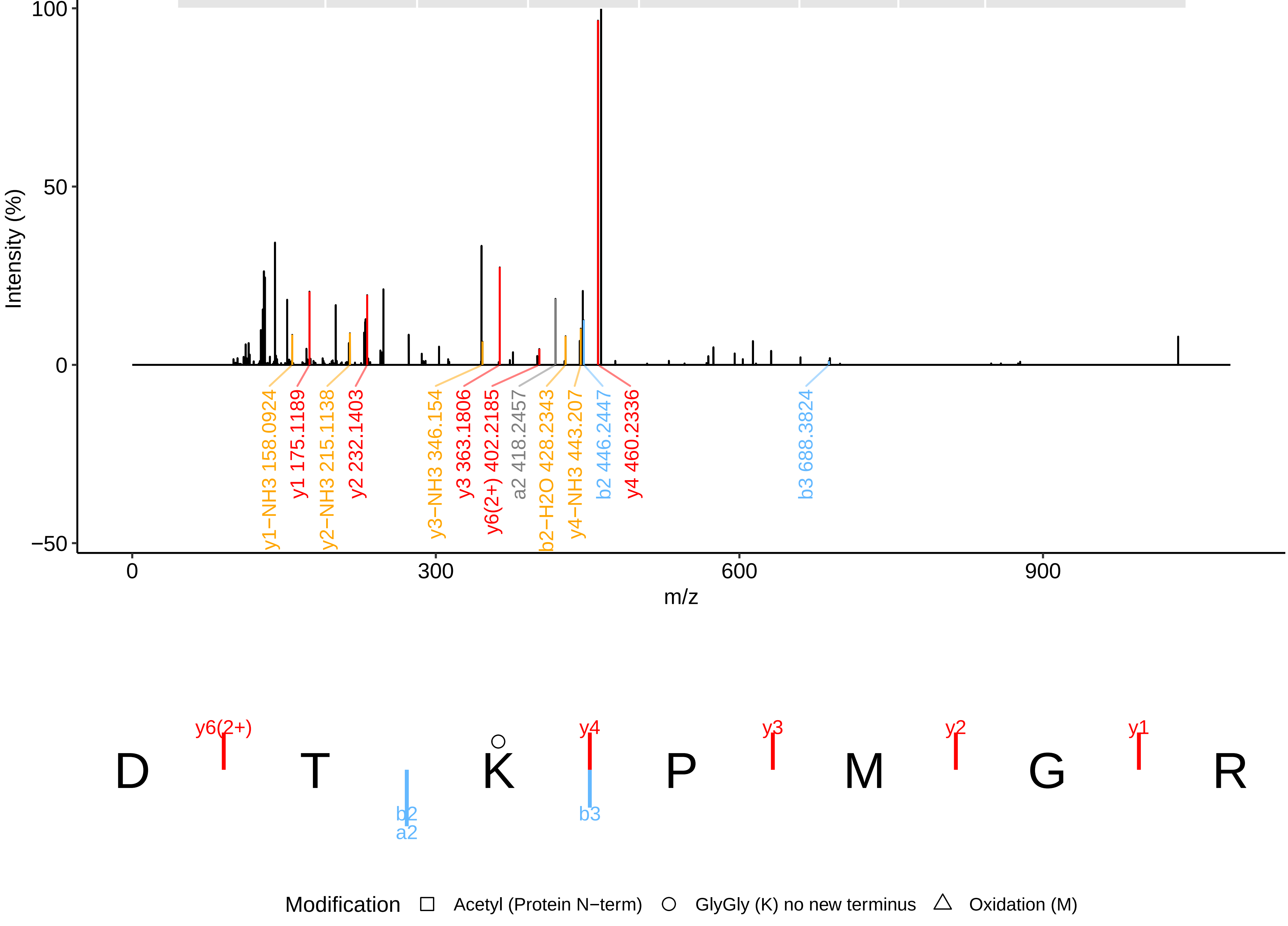

20200924_KIZ_MUb5_GlyGly_1 − Scan number: 2814

Gene Names	Charge	m/z	Mass	Retention time	PEP	Score	Precursor Intensity
MCL1	3	306.8202	917.4389	8.6918	0.02148	79.939	12767144

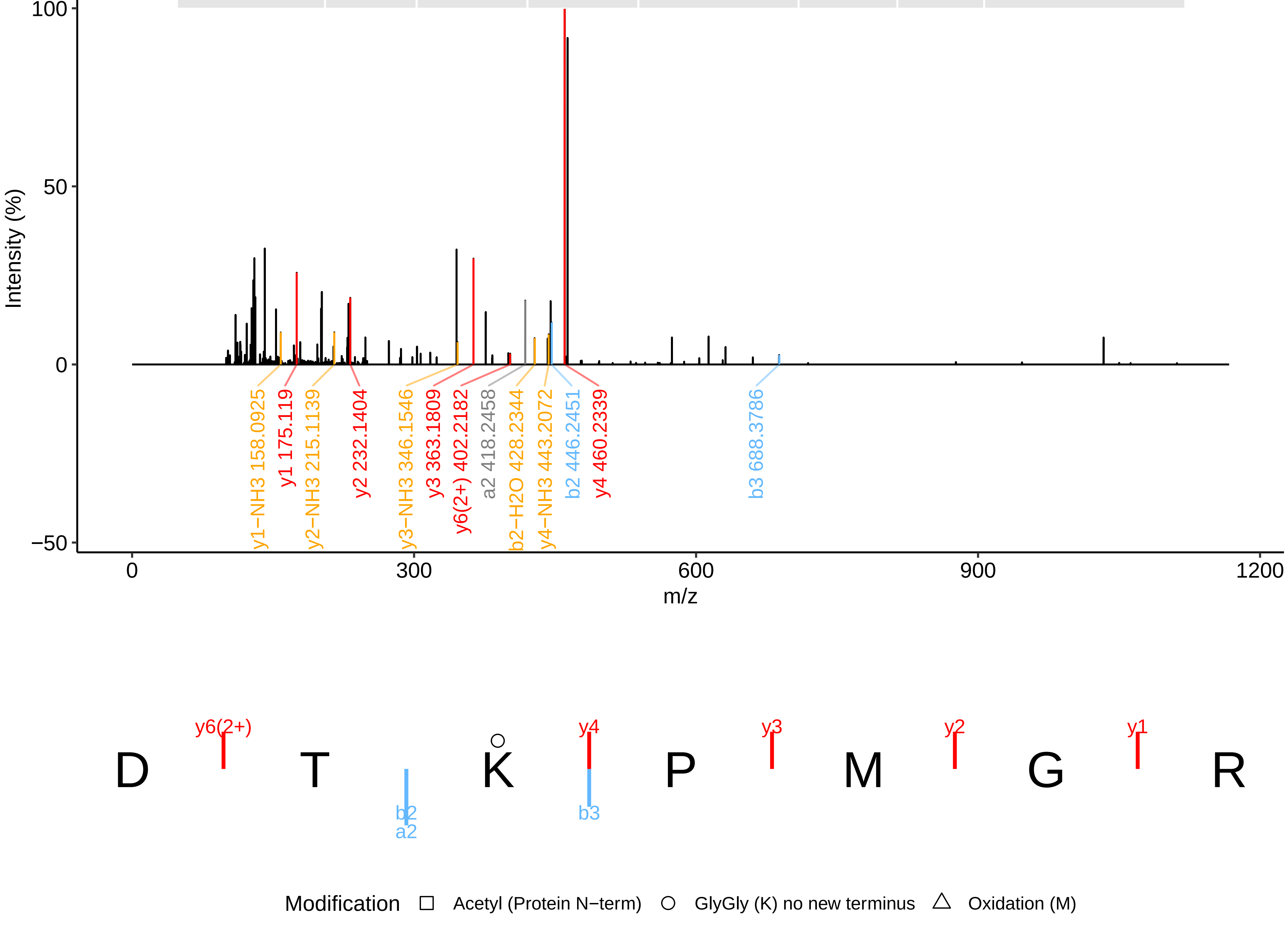

20200924_KIZ_MUb5_GlyGly_2 − Scan number: 2432

Gene Names	Charge	m/z	Mass	Retention time	PEP	Score	Precursor Intensity
MCL1	3	306.8202	917.4389	8.5863	0.049272	69.093	2176674

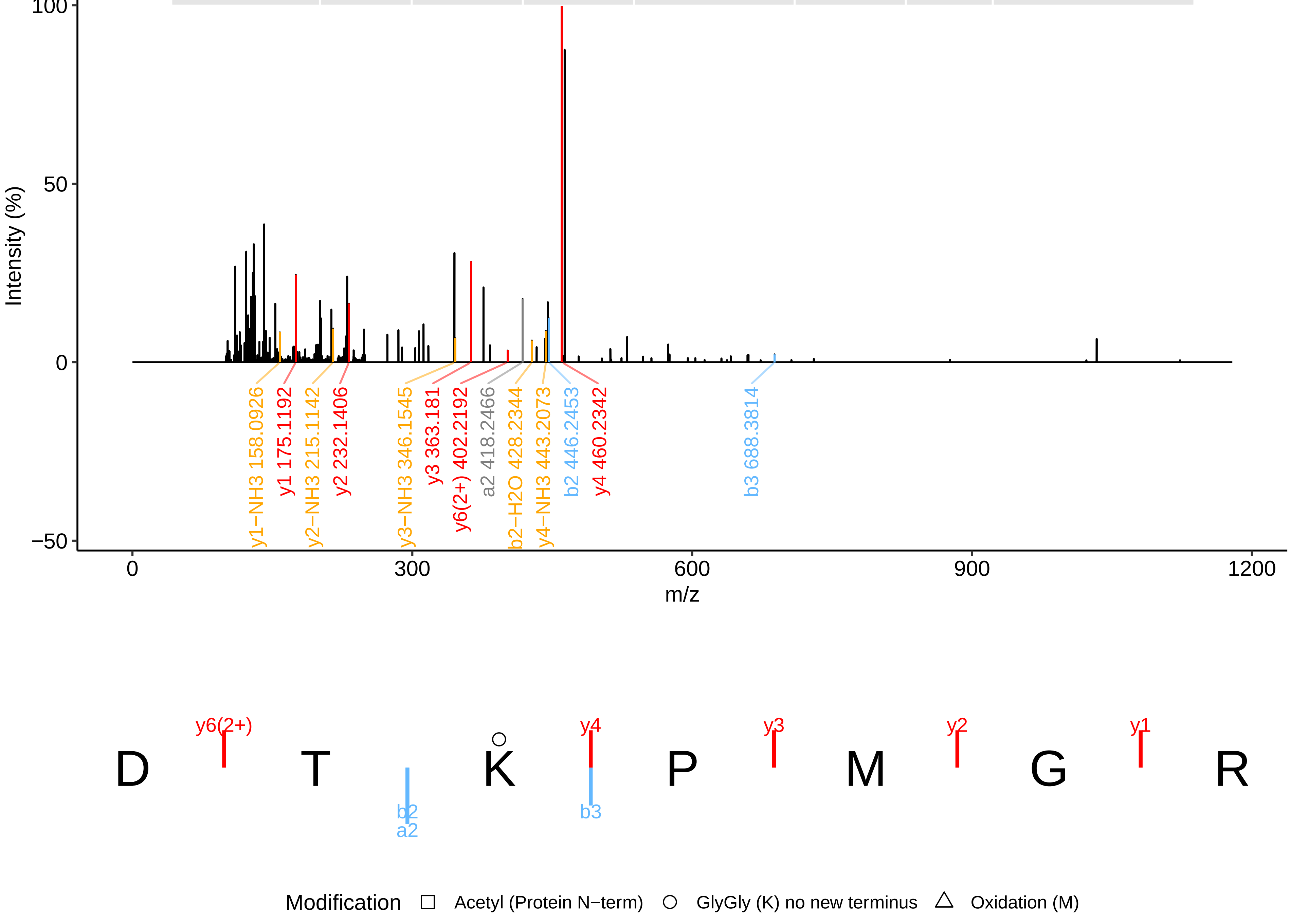

20201118_KIZ_MUb6_GlyGly_1 − Scan number: 2832

Gene Names	Charge	m/z	Mass	Retention time	PEP	Score	Precursor Intensity
MCL1	3	306.8202	917.4389	8.6671	0.033448	75.269	10315756

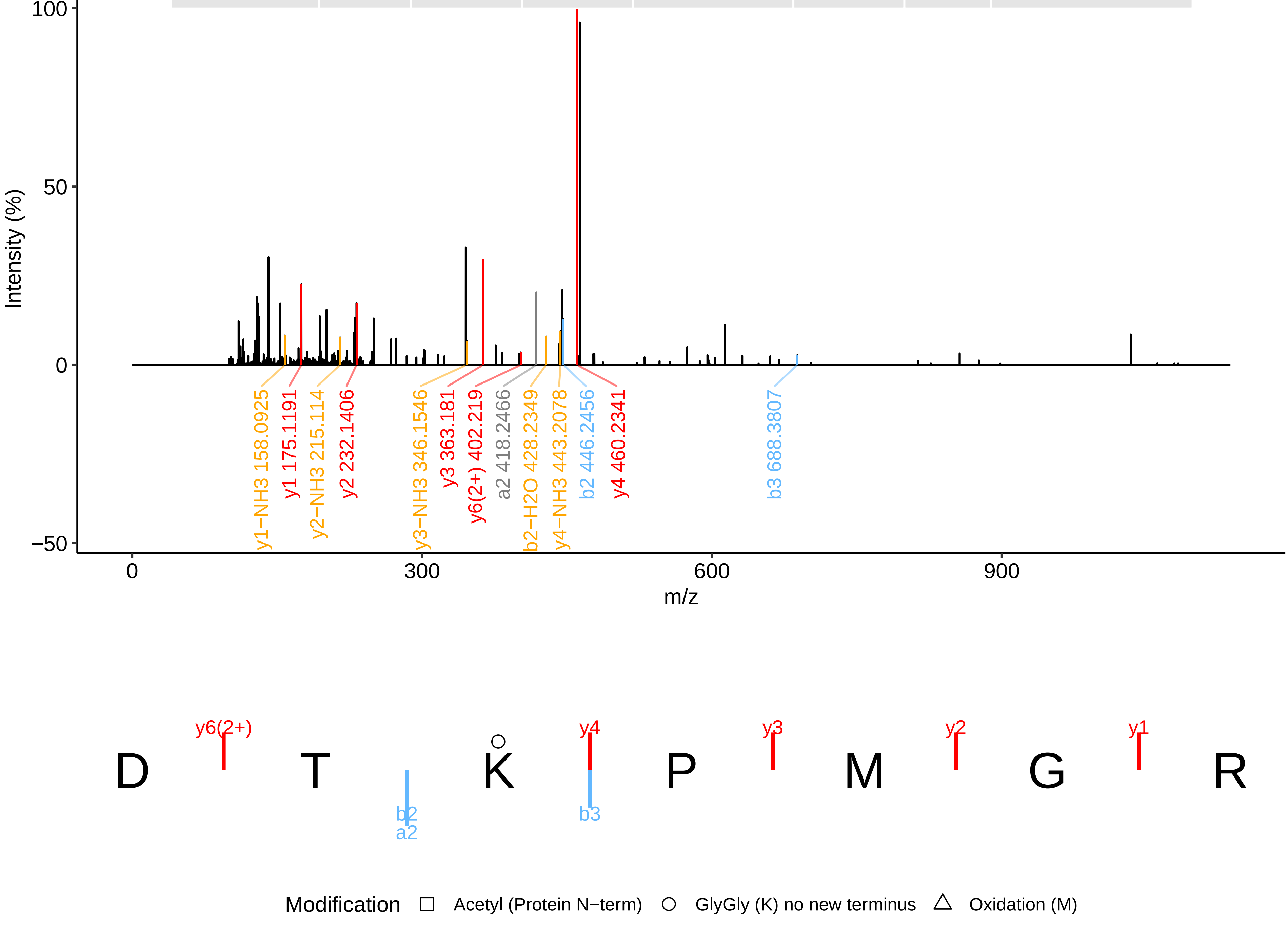

20201118_KIZ_MUb6_GlyGly_2 − Scan number: 3062

Gene Names	Charge	m/z	Mass	Retention time	PEP	Score	Precursor Intensity
MCL1	3	306.8202	917.4389	9.9015	0.048912	69.234	17929420

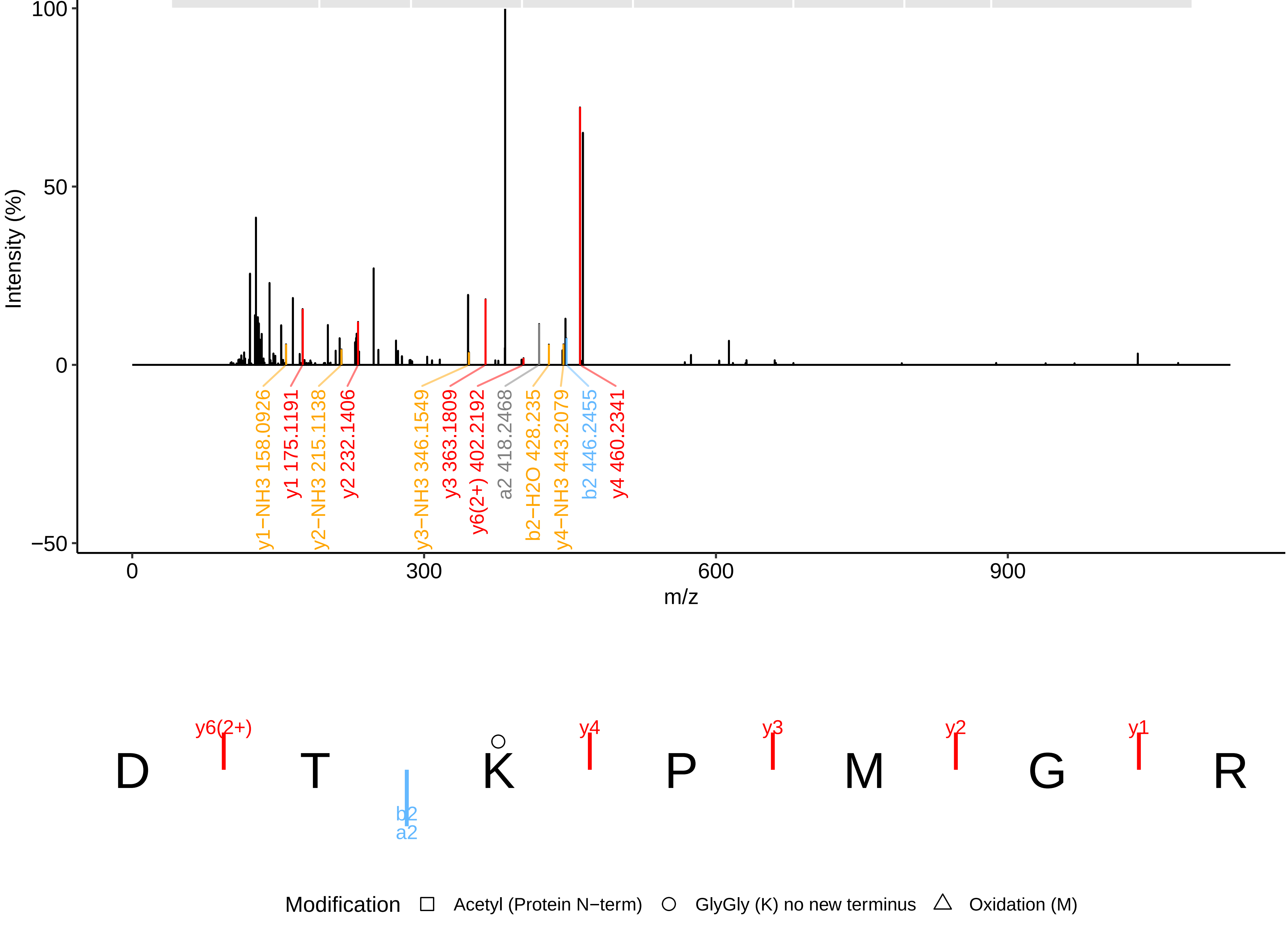

20200921_KIZ_MUb4_GlyGly_10plex_2 − Scan number: 2781

Gene Names	Charge	m/z	Mass	Retention time	PEP	Score	Precursor Intensity
	3	305.8384	914.4934	8.9515	0.078665	60.294	2988433

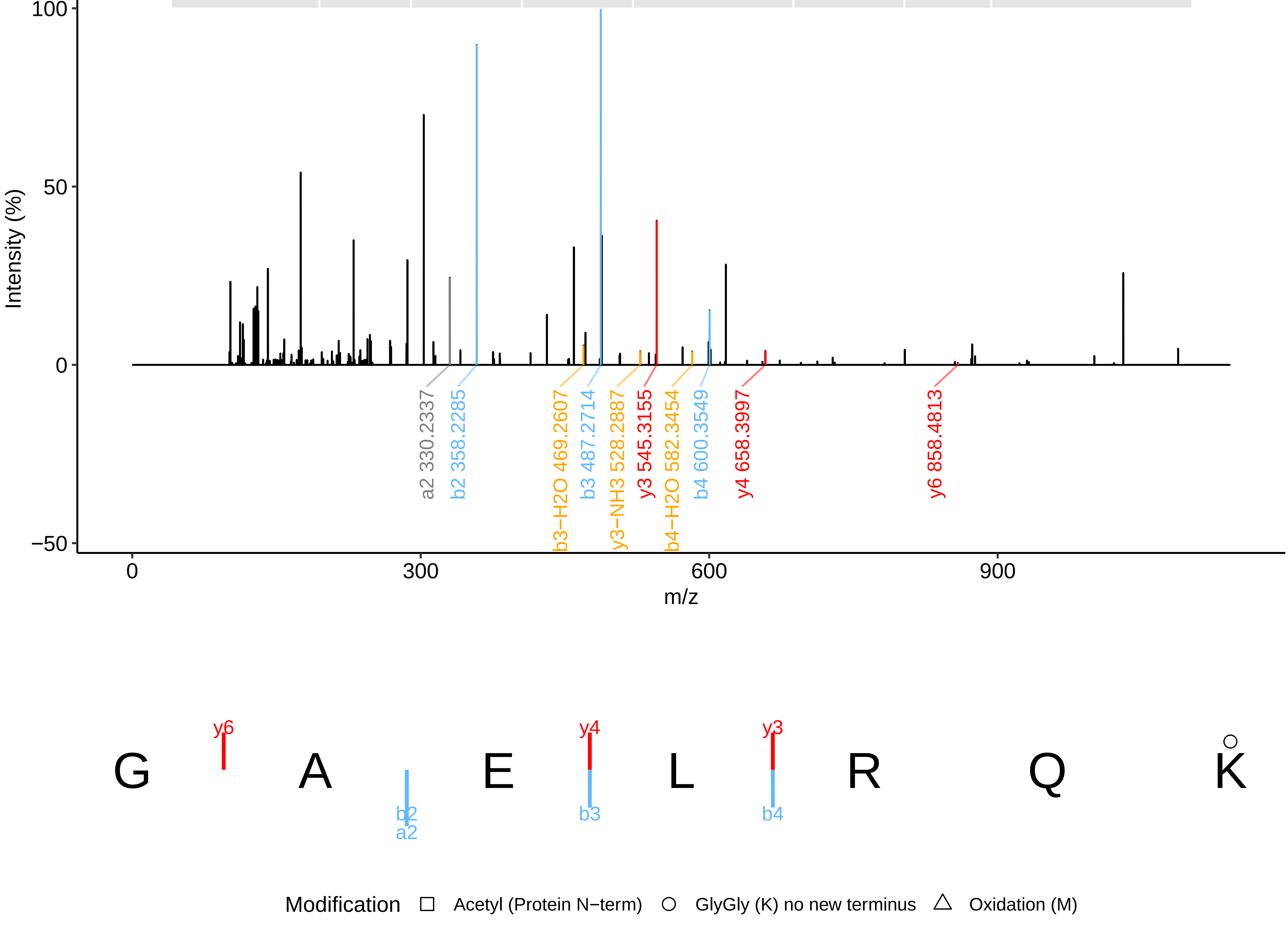

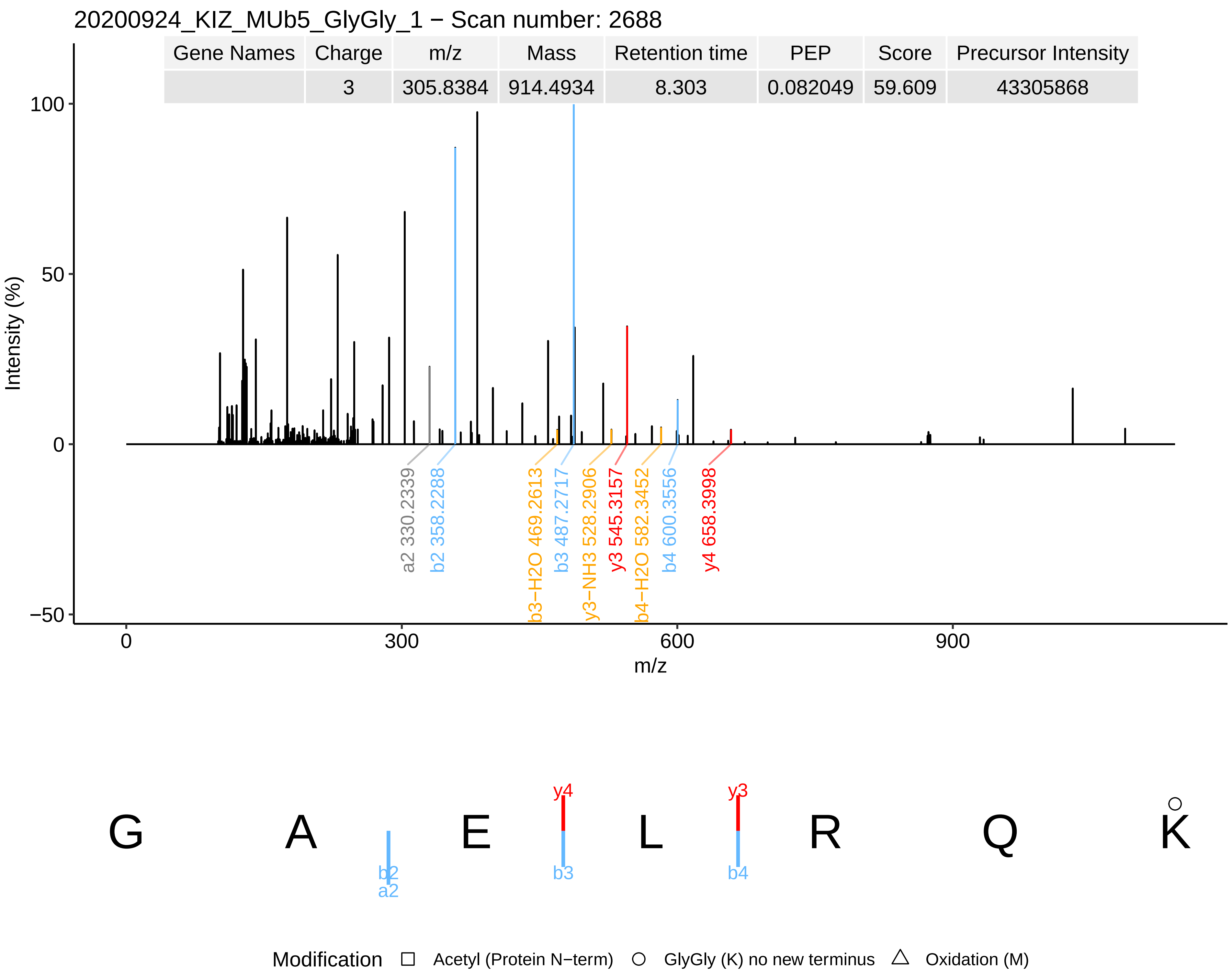

20200924_KIZ_MUb5_GlyGly_1 − Scan number: 2688
Gene Names | Charge | m/z | Mass | Retention time | PEP | Score | Precursor Intensity
3 | 305.8384 | 914.4934 | 8.303 | 0.082049 | 59.609 | 43305868
Intensity (%)
m/z
a2 330.2339
b2 358.2288
b3−H2O 469.2613
b3 487.2717
y3−NH3 528.2906
y3 545.3157
b4−H2O 582.3452
b4 600.3556
y4 658.3998
G A E L R Q K
Modification
Acetyl (Protein N−term)
GlyGly (K) no new terminus
Oxidation (M)

20200924_KIZ_MUb5_GlyGly_2 − Scan number: 2337

Gene Names	Charge	m/z	Mass	Retention time	PEP	Score	Precursor Intensity
	3	305.8384	914.4934	8.2563	0.10599	54.765	1889701

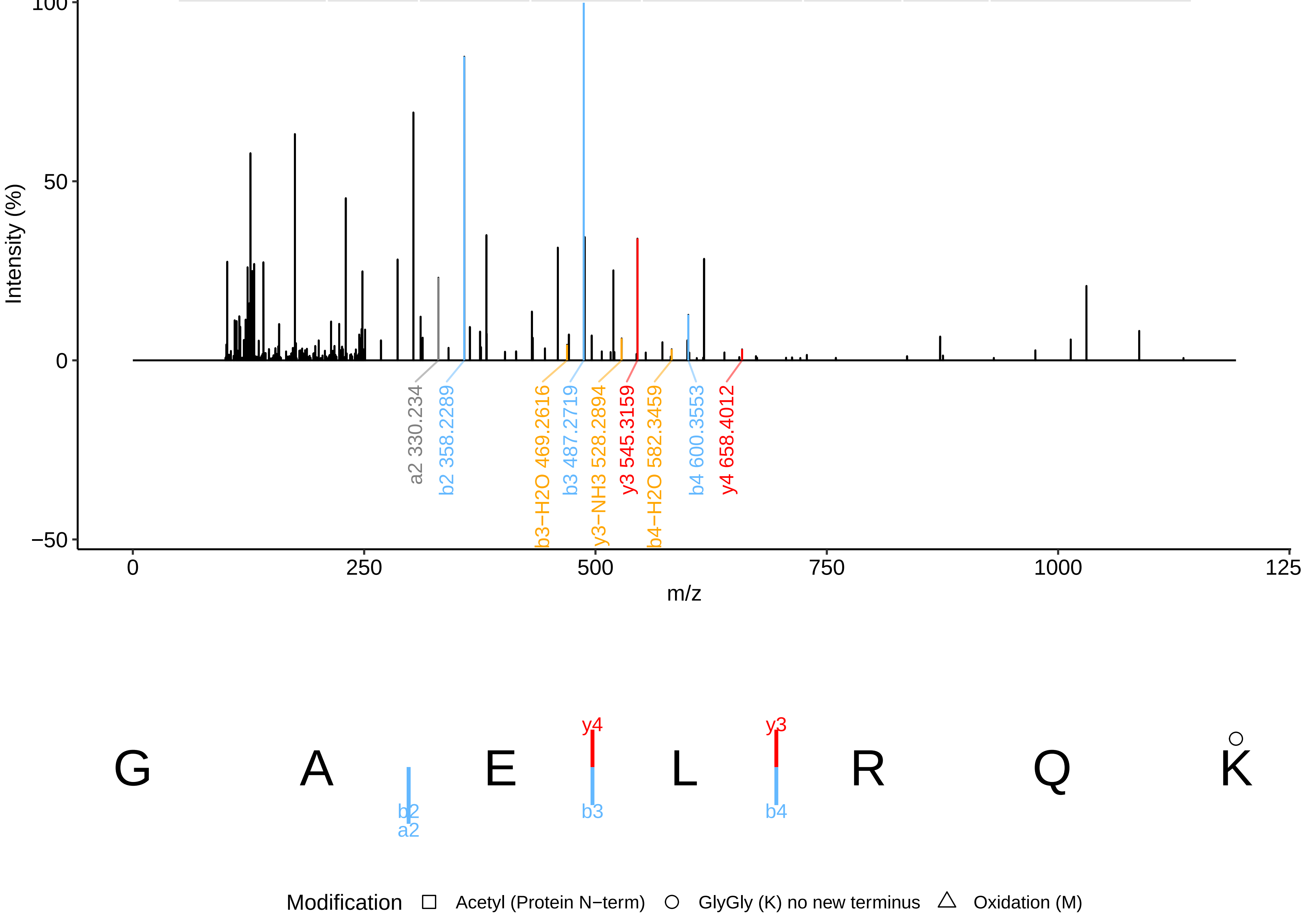

20200921_KIZ_MUb4_GlyGly_10plex − Scan number: 14248

Gene Names	Charge	m/z	Mass	Retention time	PEP	Score	Precursor Intensity
	2	344.6905	687.3664	50.562	0.095078	77.72	2766342

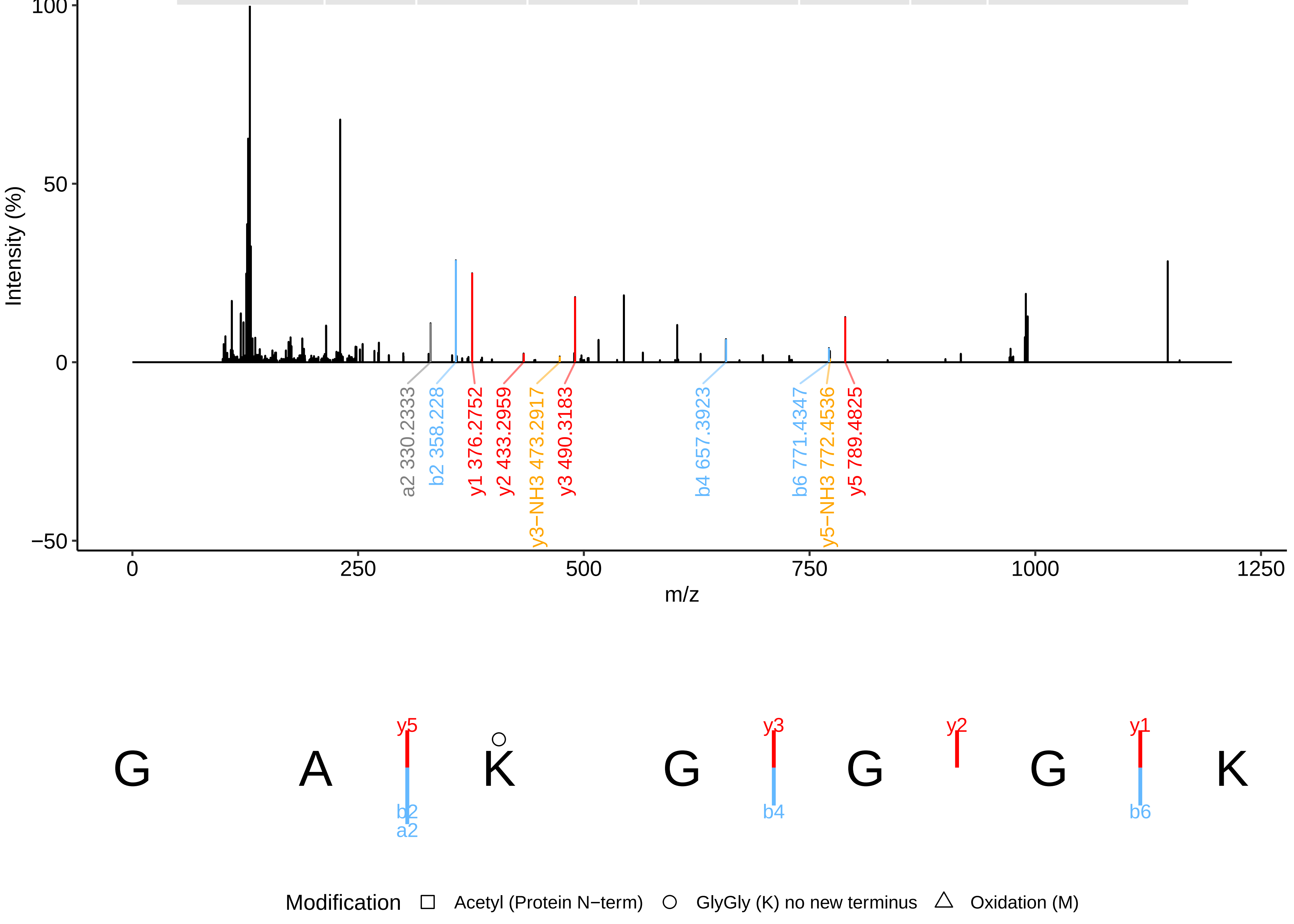

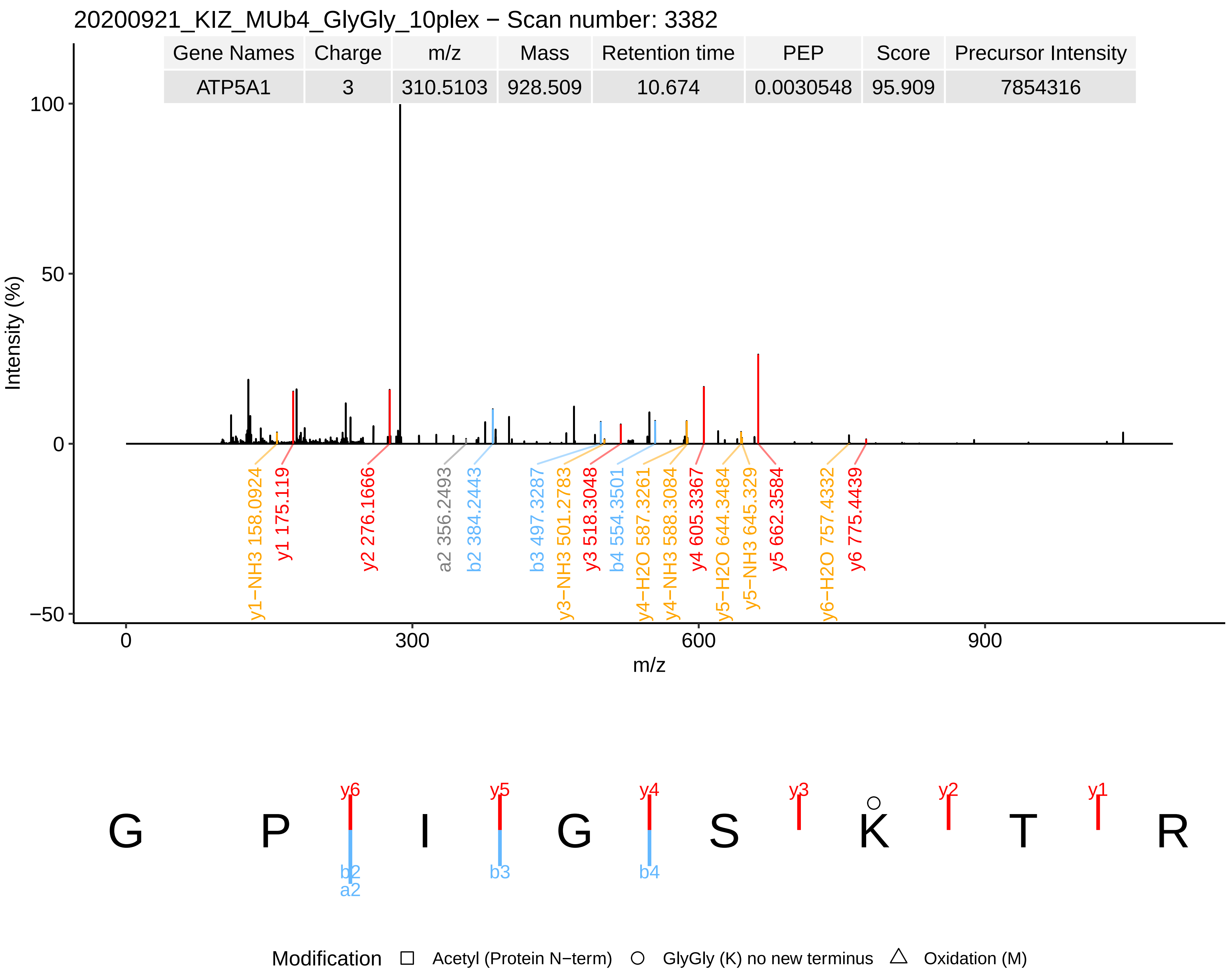

20200921_KIZ_MUb4_GlyGly_10plex − Scan number: 3382
Gene Names | Charge | m/z | Mass | Retention time | PEP | Score | Precursor Intensity
ATP5A1 | 3 | 310.5103 | 928.509 | 10.674 | 0.0030548 | 95.909 | 7854316
Intensity (%)
m/z
y1−NH3 158.0924
y1 175.119
y2 276.1666
a2 356.2493
b2 384.2443
b3 497.3287
y3−NH3 501.2783
y3 518.3048
b4 554.3501
y4−H2O 587.3261
y4−NH3 588.3084
y4 605.3367
y5−H2O 644.3484
y5−NH3 645.329
y5 662.3584
y6−H2O 757.4332
y6 775.4439
G P I G S K T R
Modification
Acetyl (Protein N−term)
GlyGly (K) no new terminus
Oxidation (M)

20200921_KIZ_MUb4_GlyGly_10plex_2 − Scan number: 3276

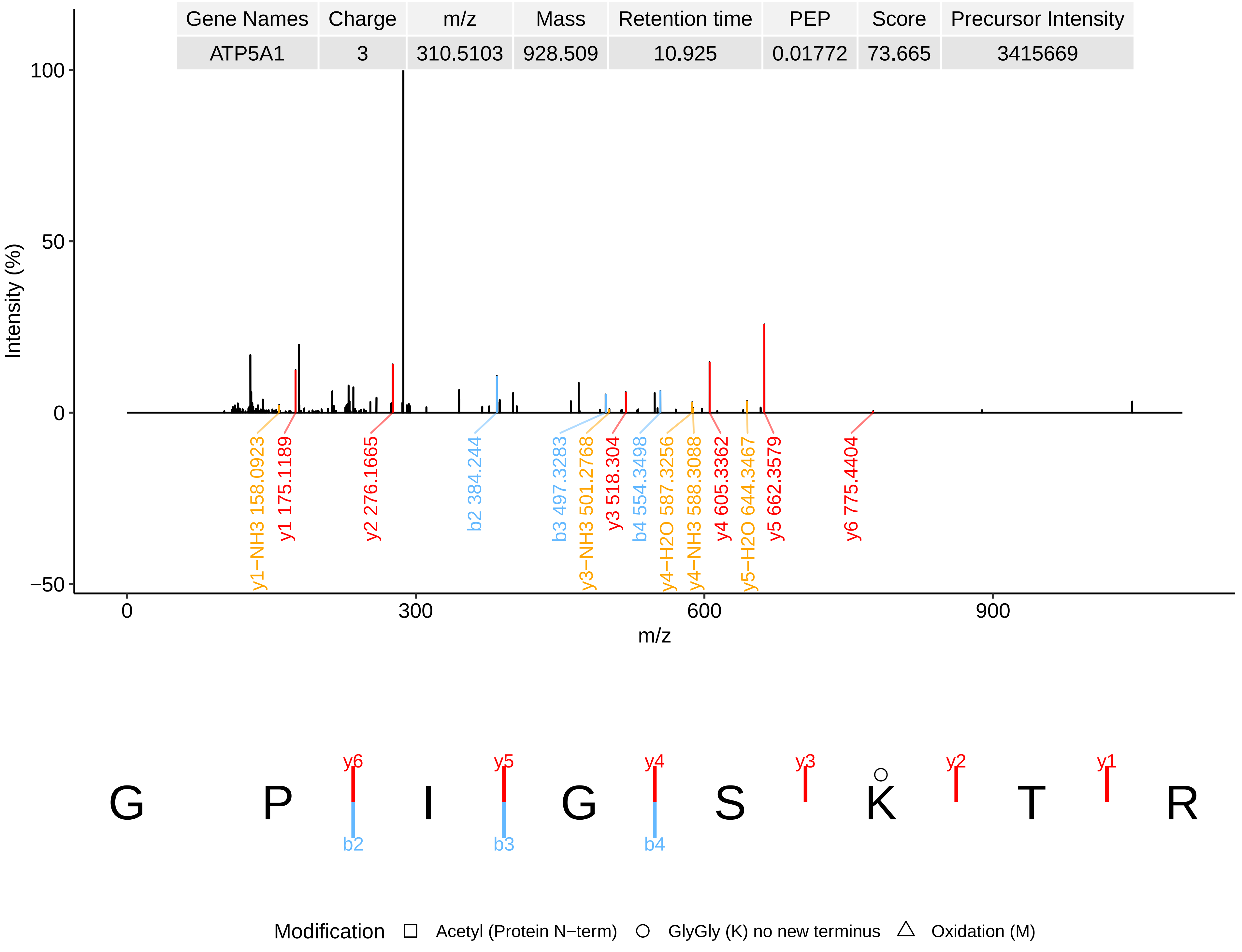

20200924_KIZ_MUb5_GlyGly_1 − Scan number: 3181

Gene Names	Charge	m/z	Mass	Retention time	PEP	Score	Precursor Intensity
ATP5A1	3	310.5103	928.509	9.8111	0.013475	77.072	9457101

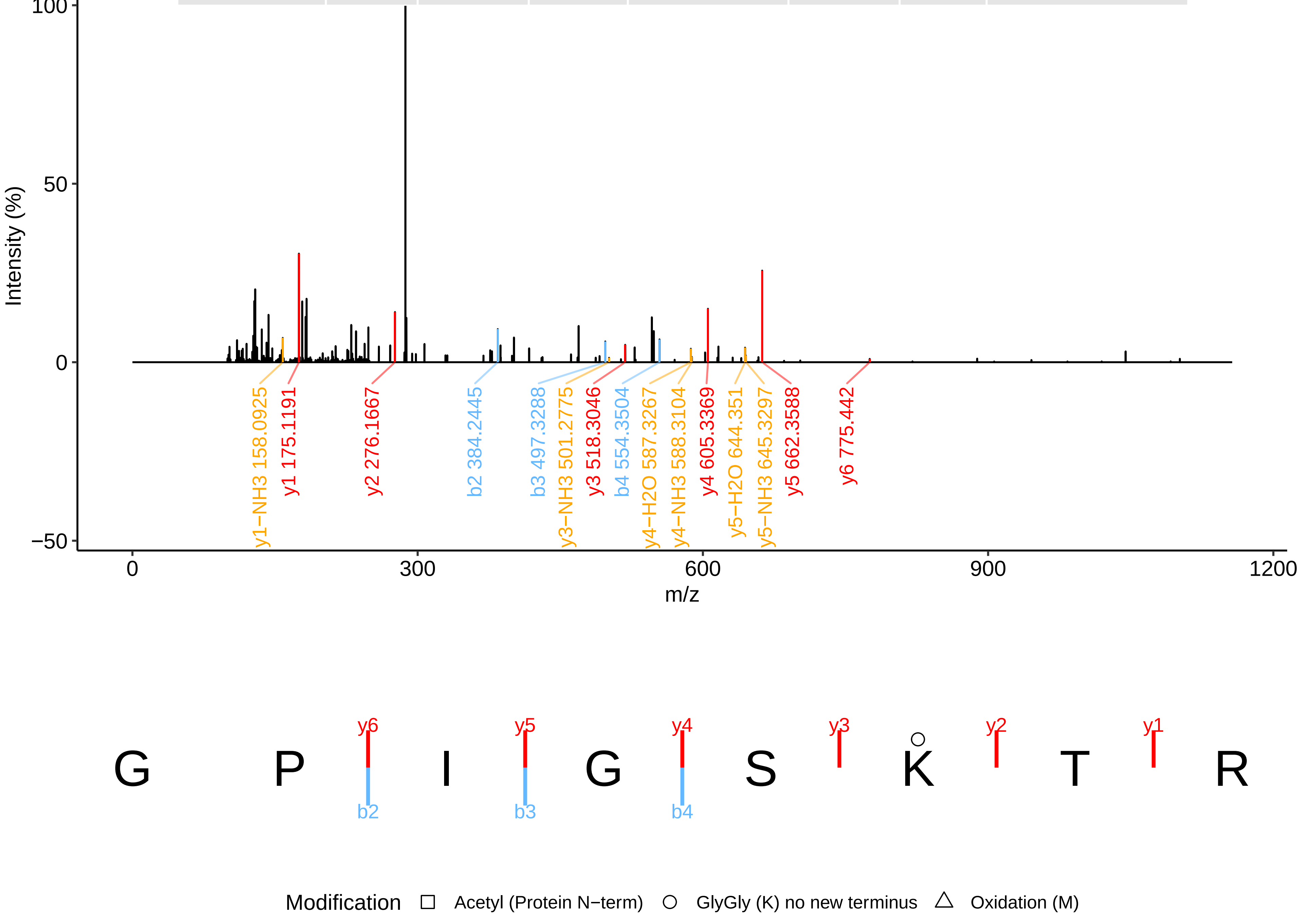

20200924_KIZ_MUb5_GlyGly_2 − Scan number: 2889

Gene Names	Charge	m/z	Mass	Retention time	PEP	Score	Precursor Intensity
ATP5A1	3	310.5103	928.509	10.359	0.0082247	87.181	3891858

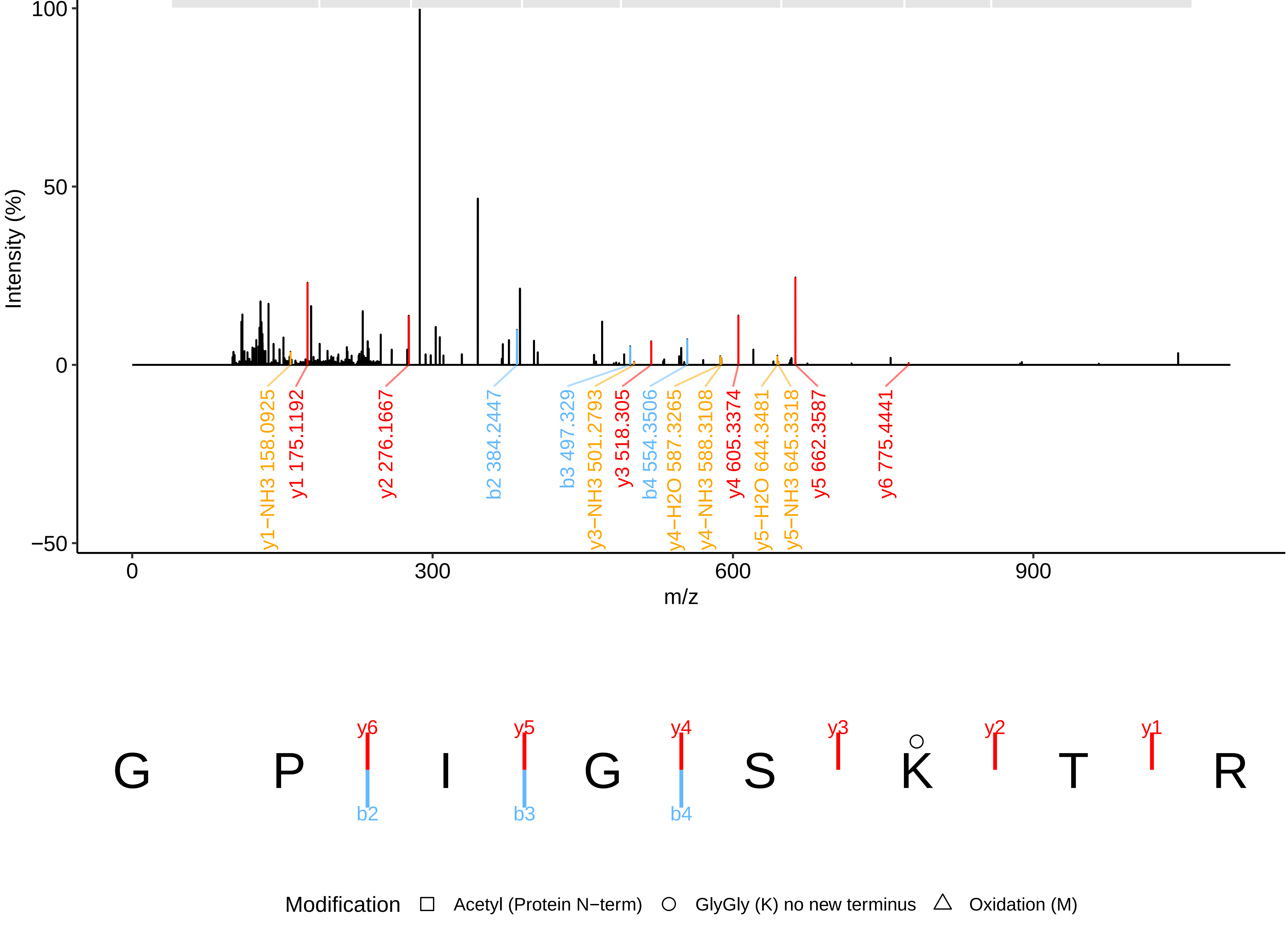

20201118_KIZ_MUb6_GlyGly_1 − Scan number: 3109

Gene Names	Charge	m/z	Mass	Retention time	PEP	Score	Precursor Intensity
ATP5A1	3	310.5103	928.509	9.587	0.0082247	87.181	30447430

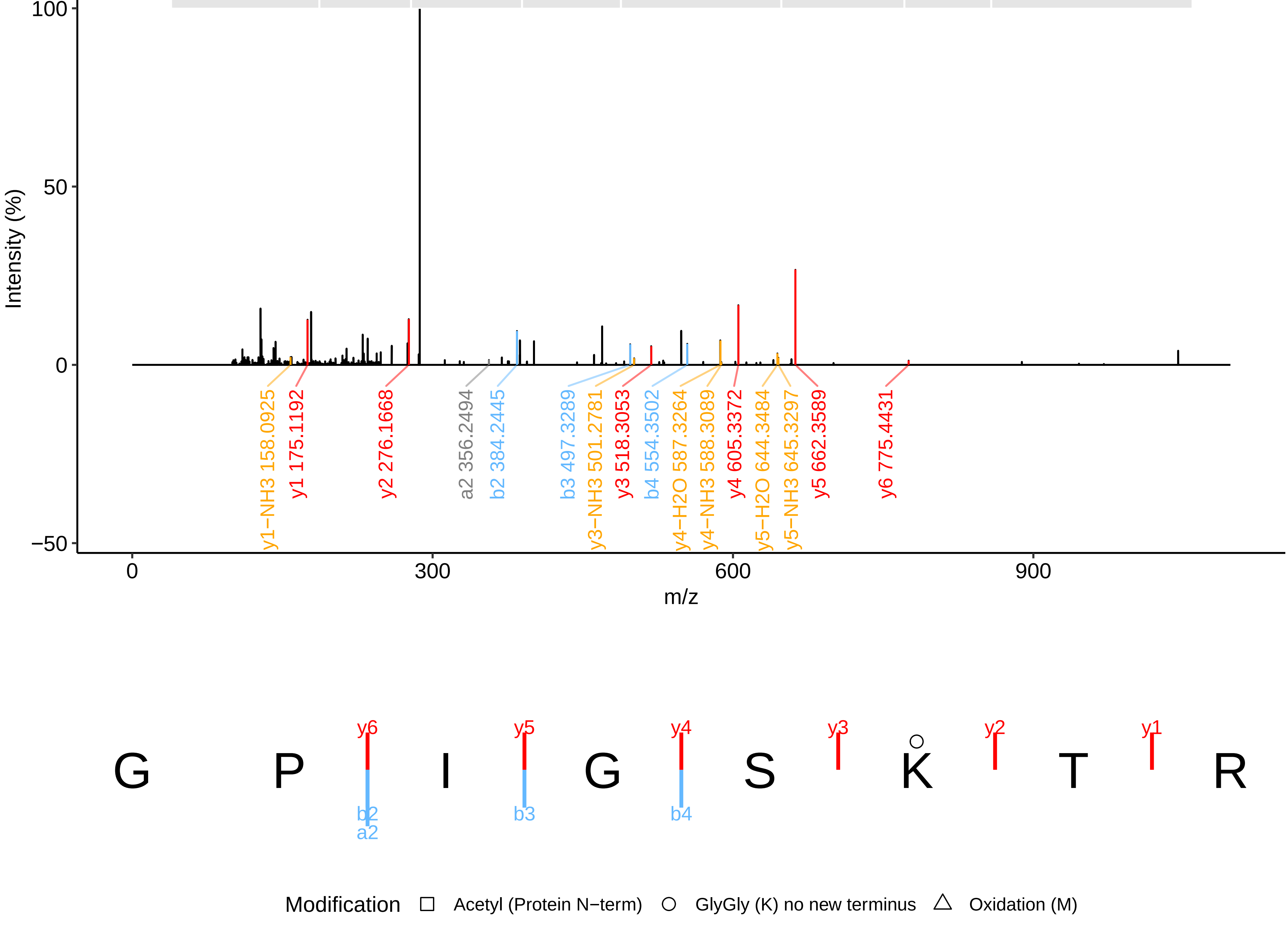

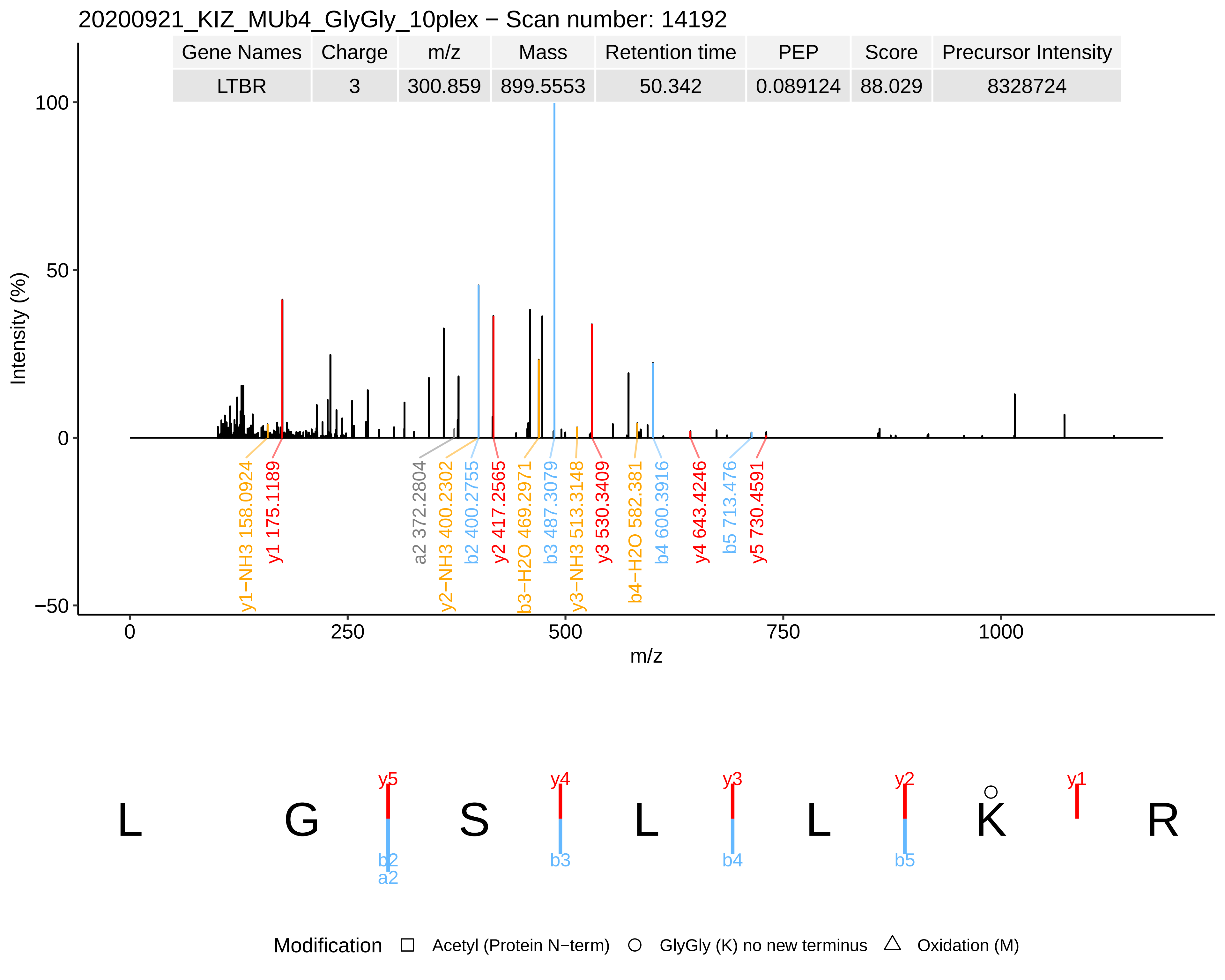
20200921_KIZ_MUb4_GlyGly_10plex − Scan number: 14192
Gene Names | Charge | m/z | Mass | Retention time | PEP | Score | Precursor Intensity
LTBR | 3 | 300.859 | 899.5553 | 50.342 | 0.089124 | 88.029 | 8328724
Intensity (%)
100
50
0
−50
y1−NH3 158.0924
y1 175.1189
a2 372.2804
y2−NH3 400.2302
b2 400.2755
y2 417.2565
b3−H2O 469.2971
b3 487.3079
y3−NH3 513.3148
y3 530.3409
b4−H2O 582.381
b4 600.3916
y4 643.4246
b5 713.476
y5 730.4591
0
250
500
750
1000
m/z
L G S L L K R
y5 y4 y3 y2 y1
b2 a2 b3 b4 b5
Modification
Acetyl (Protein N−term)
GlyGly (K) no new terminus
Oxidation (M)

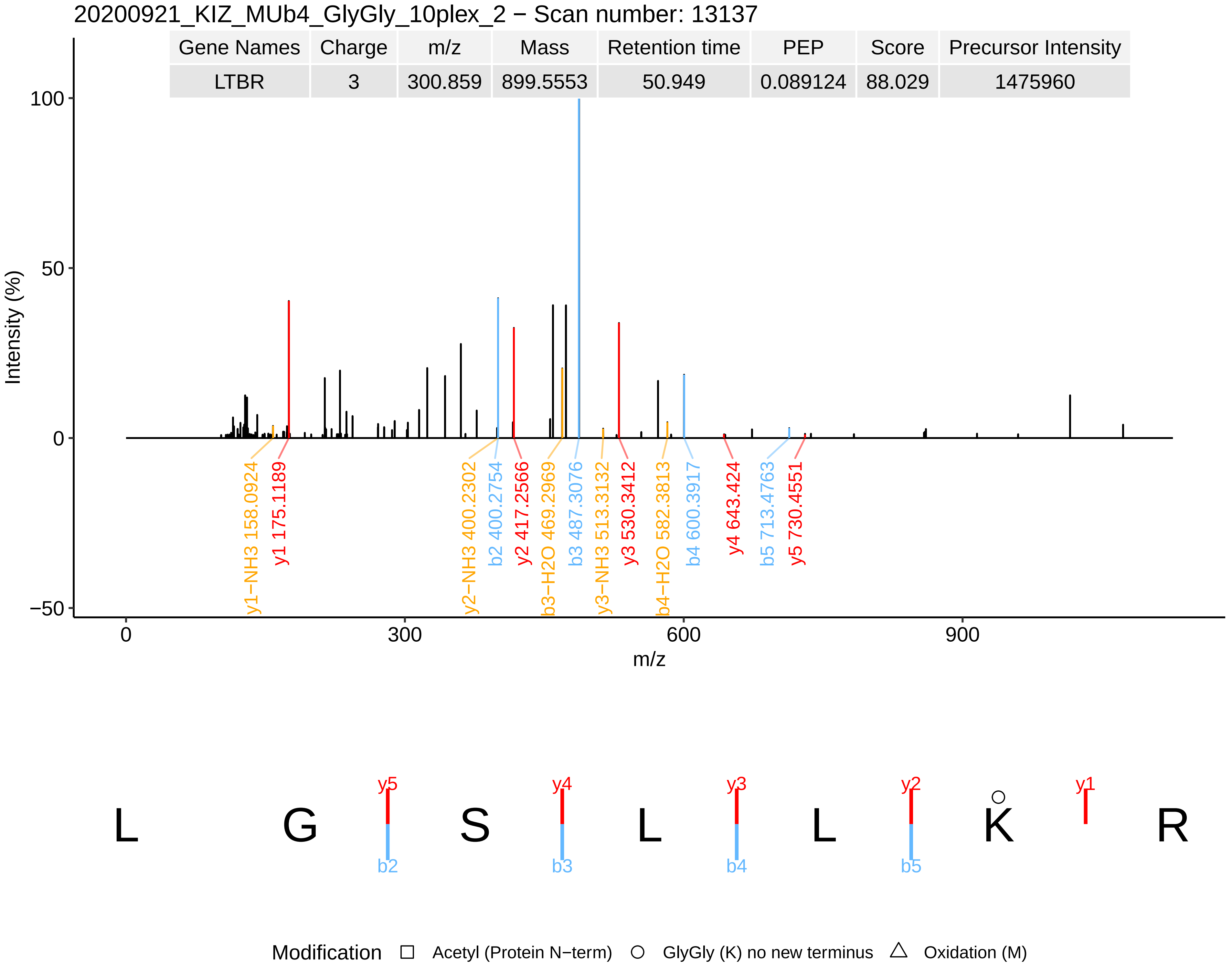

20200921_KIZ_MUb4_GlyGly_10plex_2 − Scan number: 13137
Gene Names | Charge | m/z | Mass | Retention time | PEP | Score | Precursor Intensity
LTBR | 3 | 300.859 | 899.5553 | 50.949 | 0.089124 | 88.029 | 1475960
Intensity (%)
m/z
y1−NH3 158.0924
y1 175.1189
y2−NH3 400.2302
b2 400.2754
y2 417.2566
b3−H2O 469.2969
b3 487.3076
y3−NH3 513.3132
y3 530.3412
b4−H2O 582.3813
b4 600.3917
y4 643.424
b5 713.4763
y5 730.4551
L G S L L K R
y5 y4 y3 y2 y1
b2 b3 b4 b5
Modification
Acetyl (Protein N−term)
GlyGly (K) no new terminus
Oxidation (M)

20201118_KIZ_MUb6_GlyGly_1 − Scan number: 12744

Gene Names	Charge	m/z	Mass	Retention time	PEP	Score	Precursor Intensity
LTBR	3	300.859	899.5553	40.284	0.070594	84.097	18099808

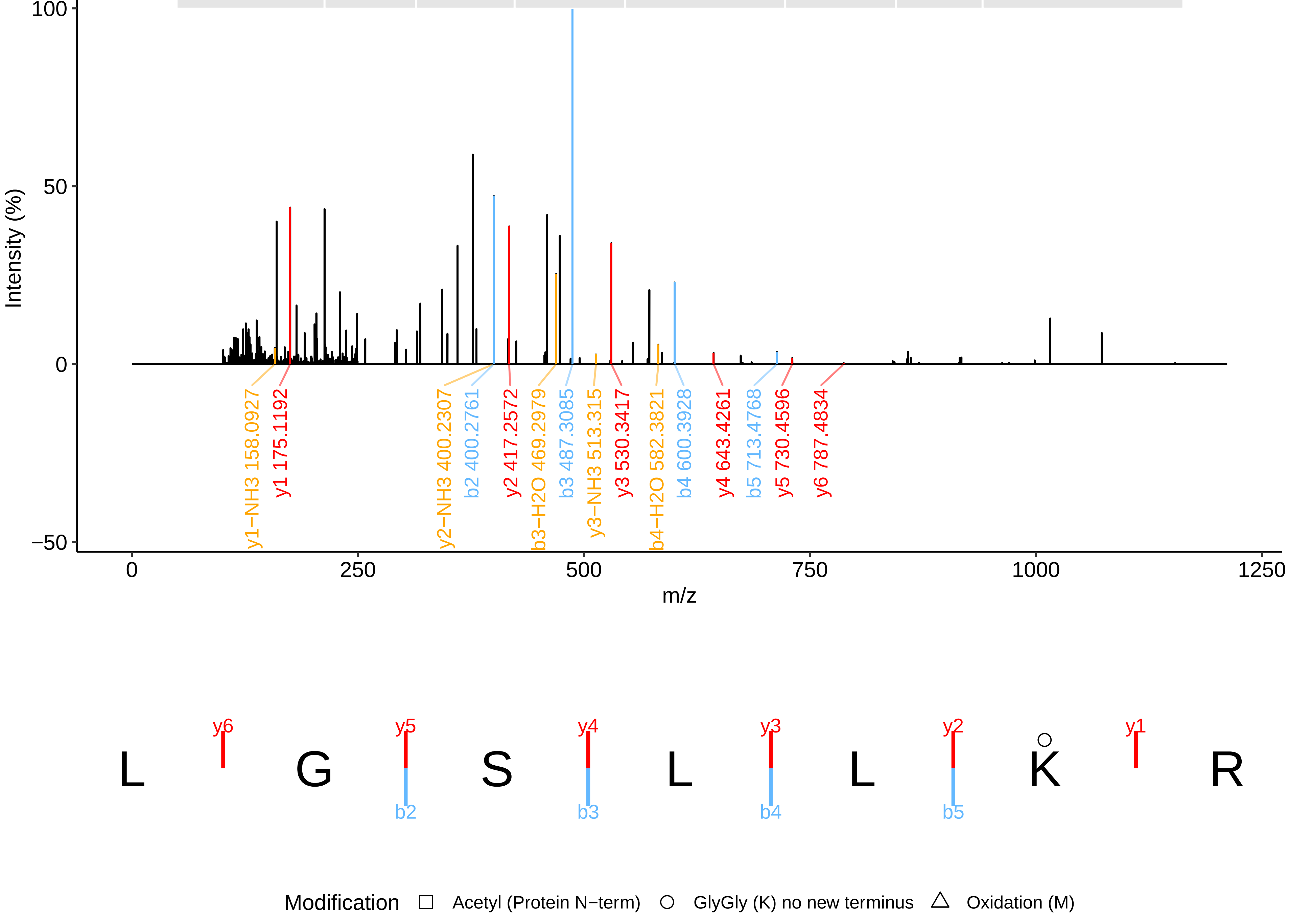

20200921_KIZ_MUb4_GlyGly_10plex − Scan number: 7130

Gene Names	Charge	m/z	Mass	Retention time	PEP	Score	Precursor Intensity
DDR1	3	310.8504	929.5294	23.835	0.058028	65.676	9820001

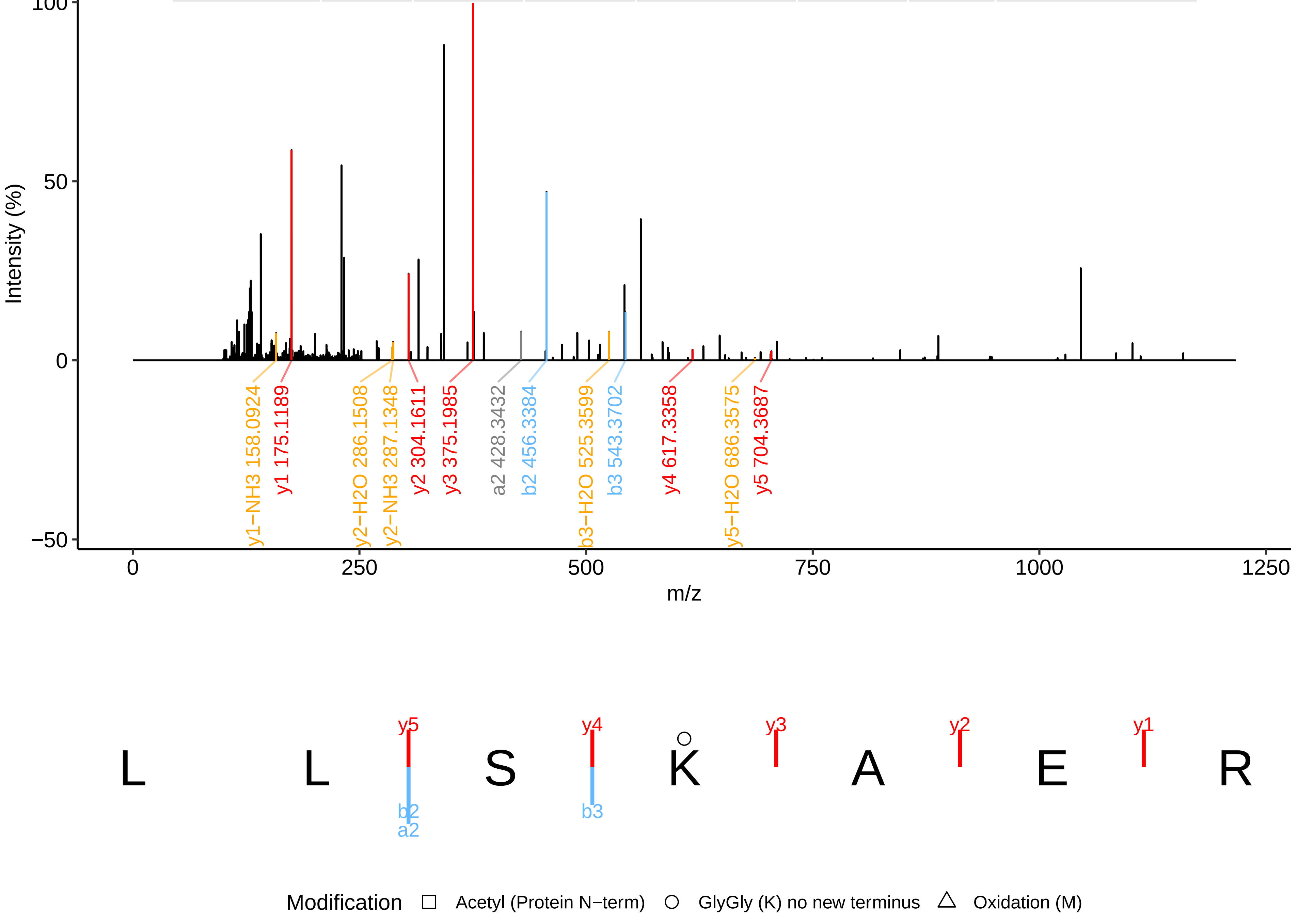

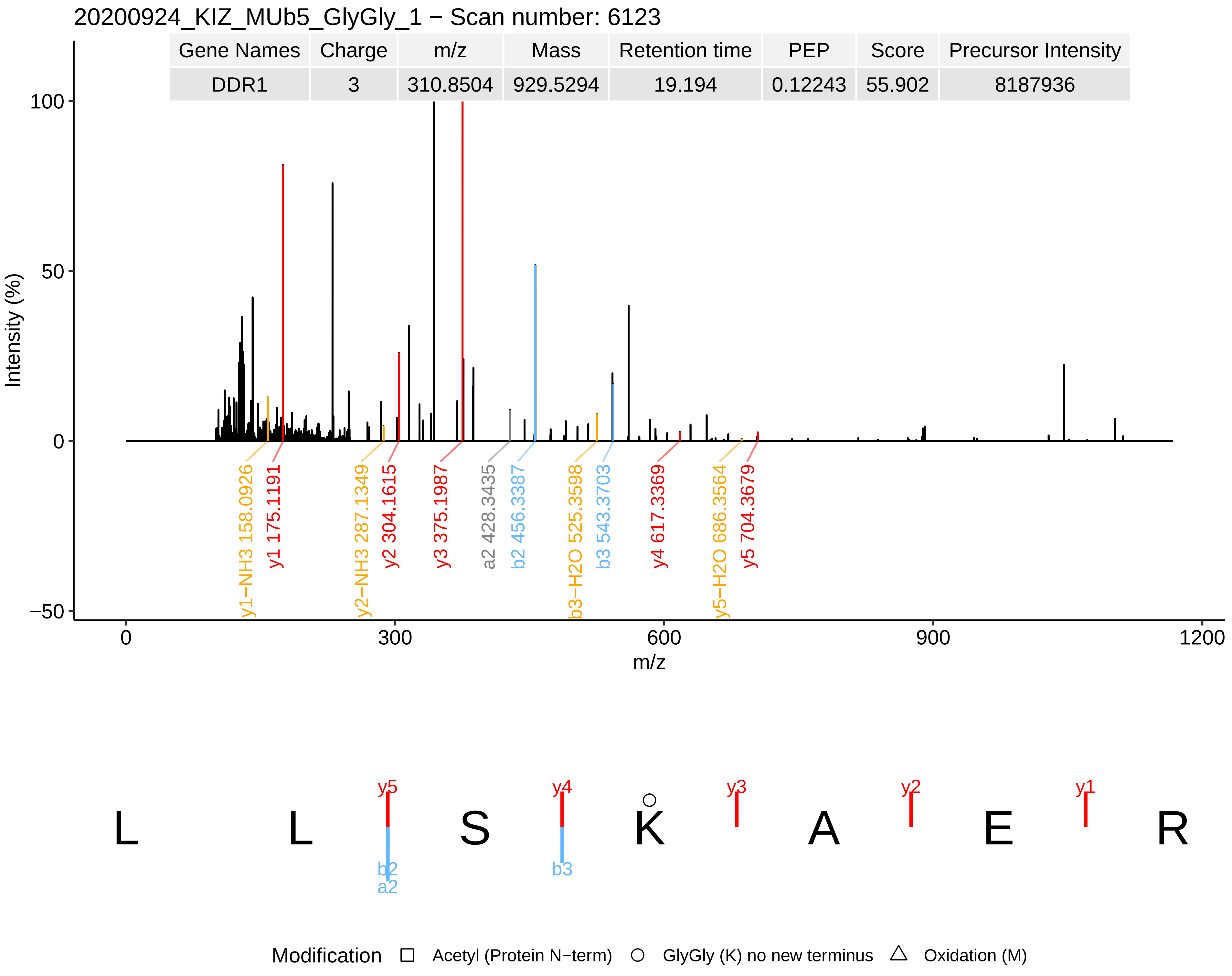

20200924_KIZ_MUb5_GlyGly_1 − Scan number: 6123
Gene Names | Charge | m/z | Mass | Retention time | PEP | Score | Precursor Intensity
DDR1 | 3 | 310.8504 | 929.5294 | 19.194 | 0.12243 | 55.902 | 8187936
Intensity (%)
m/z
y1−NH3 158.0926
y1 175.1191
y2−NH3 287.1349
y2 304.1615
y3 375.1987
a2 428.3435
b2 456.3387
b3−H2O 525.3598
b3 543.3703
y4 617.3369
y5−H2O 686.3564
y5 704.3679
L L S K A E R
y5 y4 y3 y2 y1
b2 a2 b3
Modification
Acetyl (Protein N−term)
GlyGly (K) no new terminus
Oxidation (M)

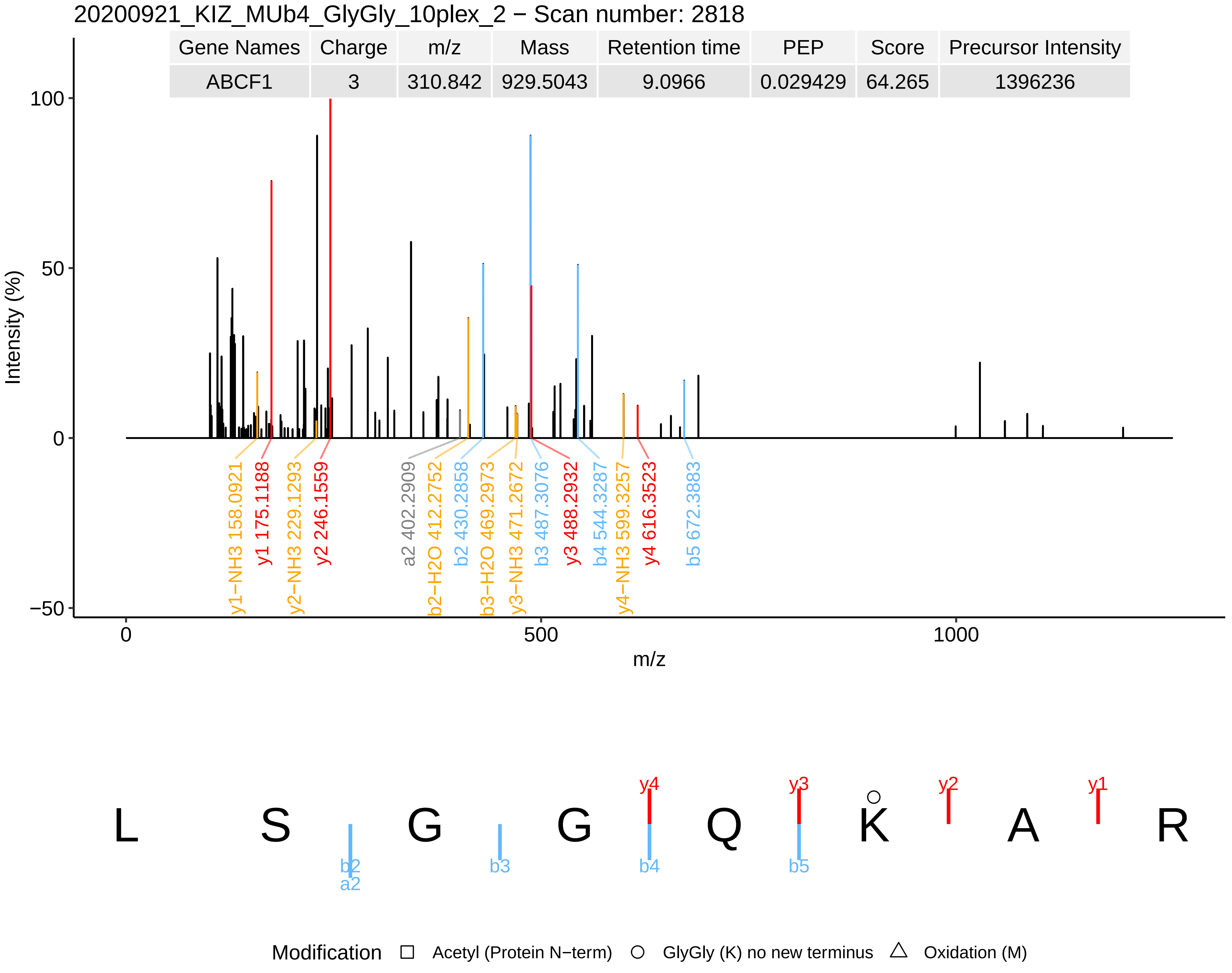
20200921_KIZ_MUb4_GlyGly_10plex_2 − Scan number: 2818
Gene Names | Charge | m/z | Mass | Retention time | PEP | Score | Precursor Intensity
ABCF1 | 3 | 310.842 | 929.5043 | 9.0966 | 0.029429 | 64.265 | 1396236
Intensity (%)
100
50
0
−50
0
500
1000
m/z
y1−NH3 158.0921
y1 175.1188
y2−NH3 229.1293
y2 246.1559
a2 402.2909
b2−H2O 412.2752
b2 430.2858
b3−H2O 469.2973
y3−NH3 471.2672
b3 487.3076
y3 488.2932
b4 544.3287
y4−NH3 599.3257
y4 616.3523
b5 672.3883
L S G G Q K A R
y4 y3 y2 y1
b2 a2 b3 b4 b5
Modification
Acetyl (Protein N−term)
GlyGly (K) no new terminus
Oxidation (M)

20200921_KIZ_MUb4_GlyGly_10plex − Scan number: 10582

Gene Names	Charge	m/z	Mass	Retention time	PEP	Score	Precursor Intensity
LAPTM4A	2	579.2757	1156.537	36.661	3.7884e−05	120.65	5285184

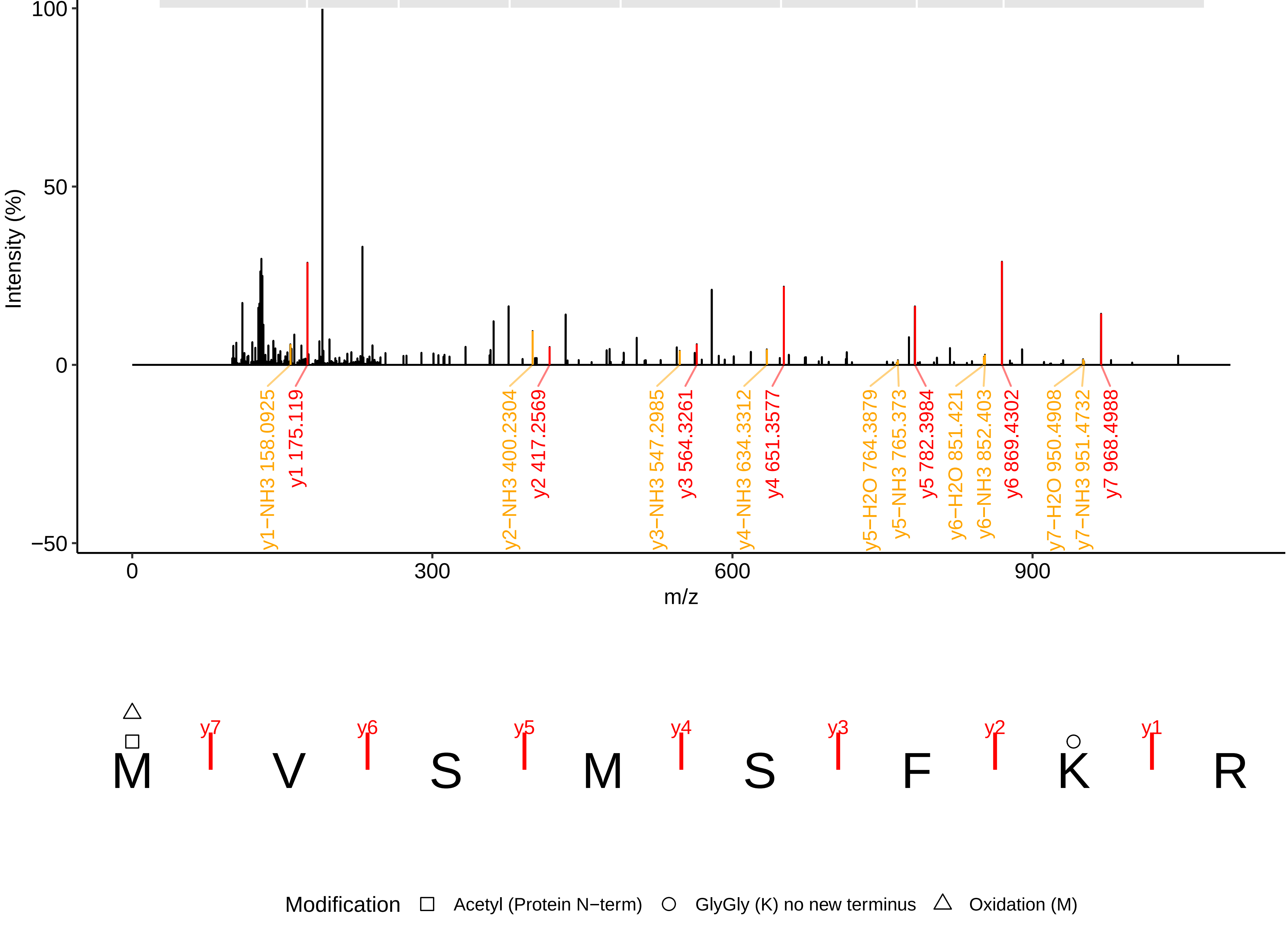

20200921_KIZ_MUb4_GlyGly_10plex − Scan number: 10595

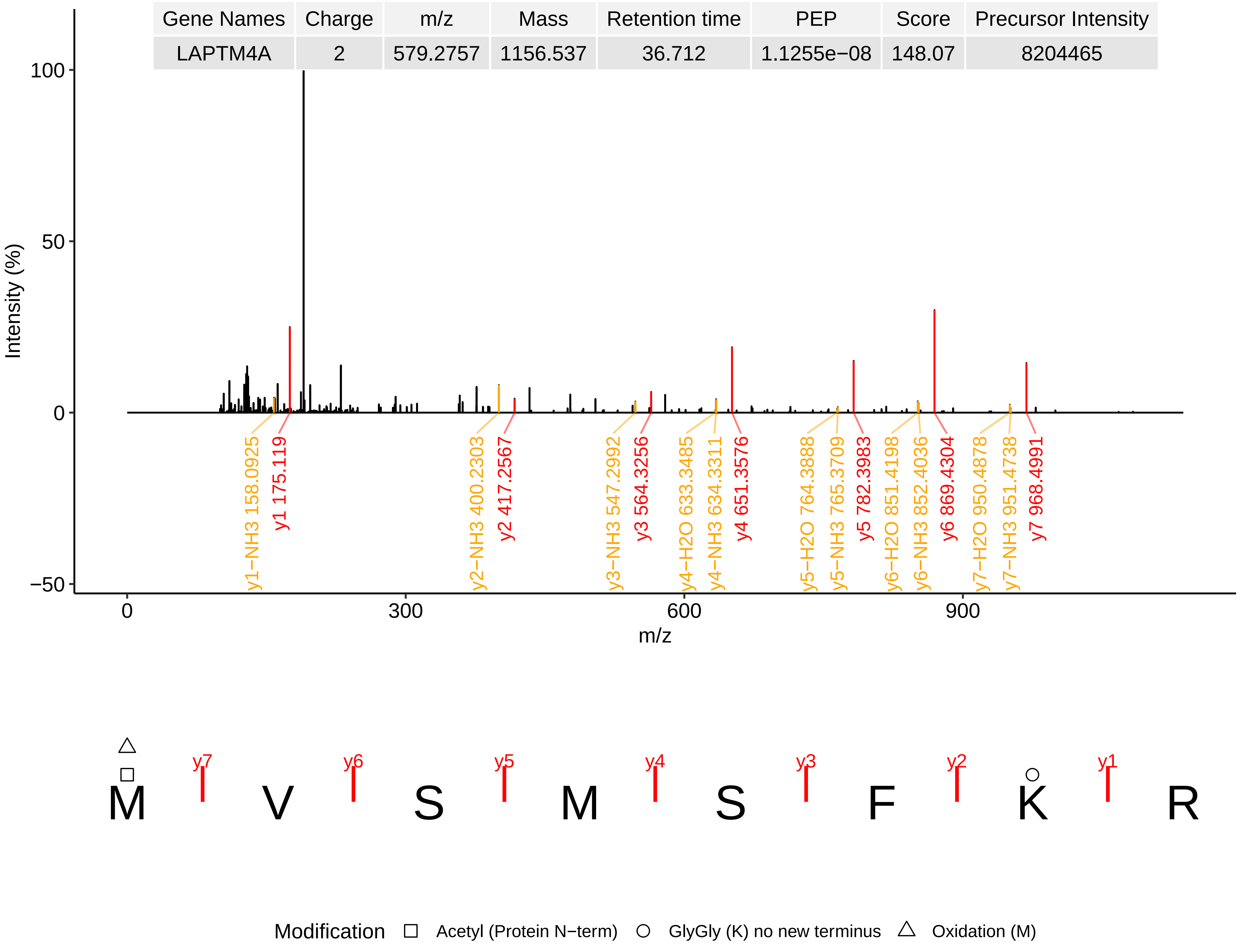

20200921_KIZ_MUb4_GlyGly_10plex_2 − Scan number: 8488

Gene Names	Charge	m/z	Mass	Retention time	PEP	Score	Precursor Intensity
LAPTM4A	2	579.2757	1156.537	31.992	0.0057236	70.268	2172251

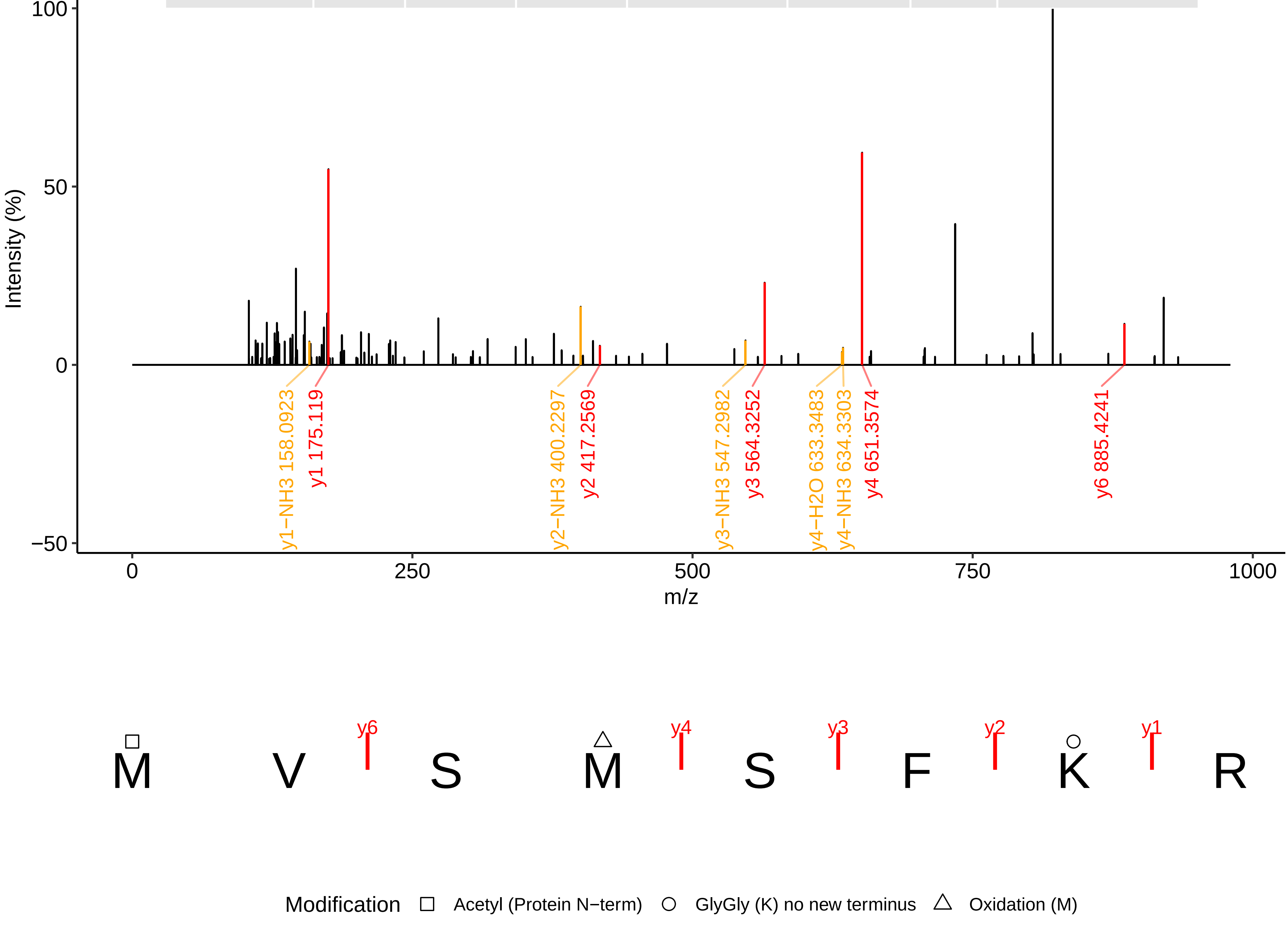

20200924_KIZ_MUb5_GlyGly_1 − Scan number: 8370

Gene Names	Charge	m/z	Mass	Retention time	PEP	Score	Precursor Intensity
LAPTM4A	2	579.2757	1156.537	26.748	0.011314	60.76	NA

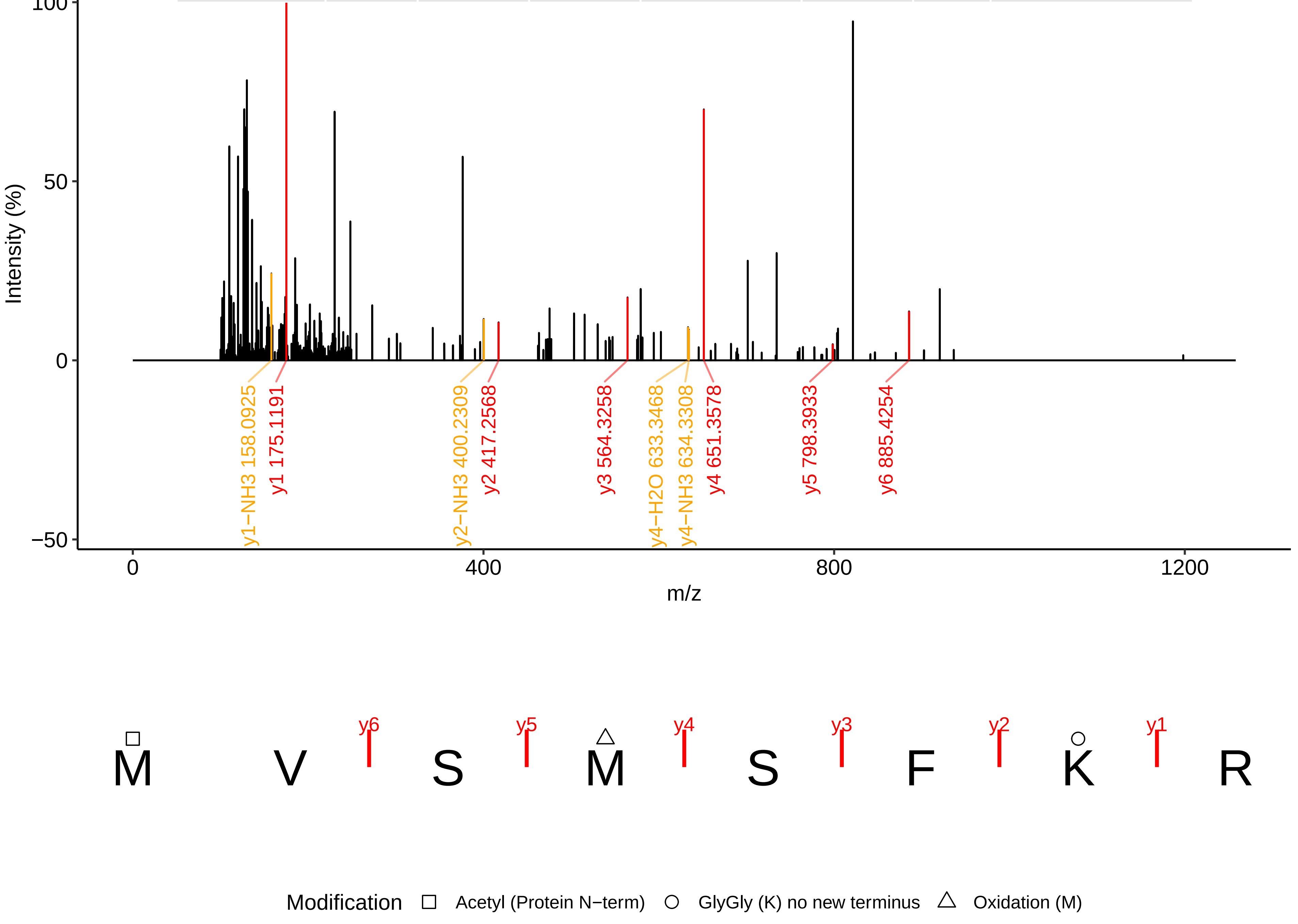

20200924_KIZ_MUb5_GlyGly_2 − Scan number: 9043

Gene Names	Charge	m/z	Mass	Retention time	PEP	Score	Precursor Intensity
LAPTM4A	2	579.2757	1156.537	33.799	0.0060492	71.342	2415414

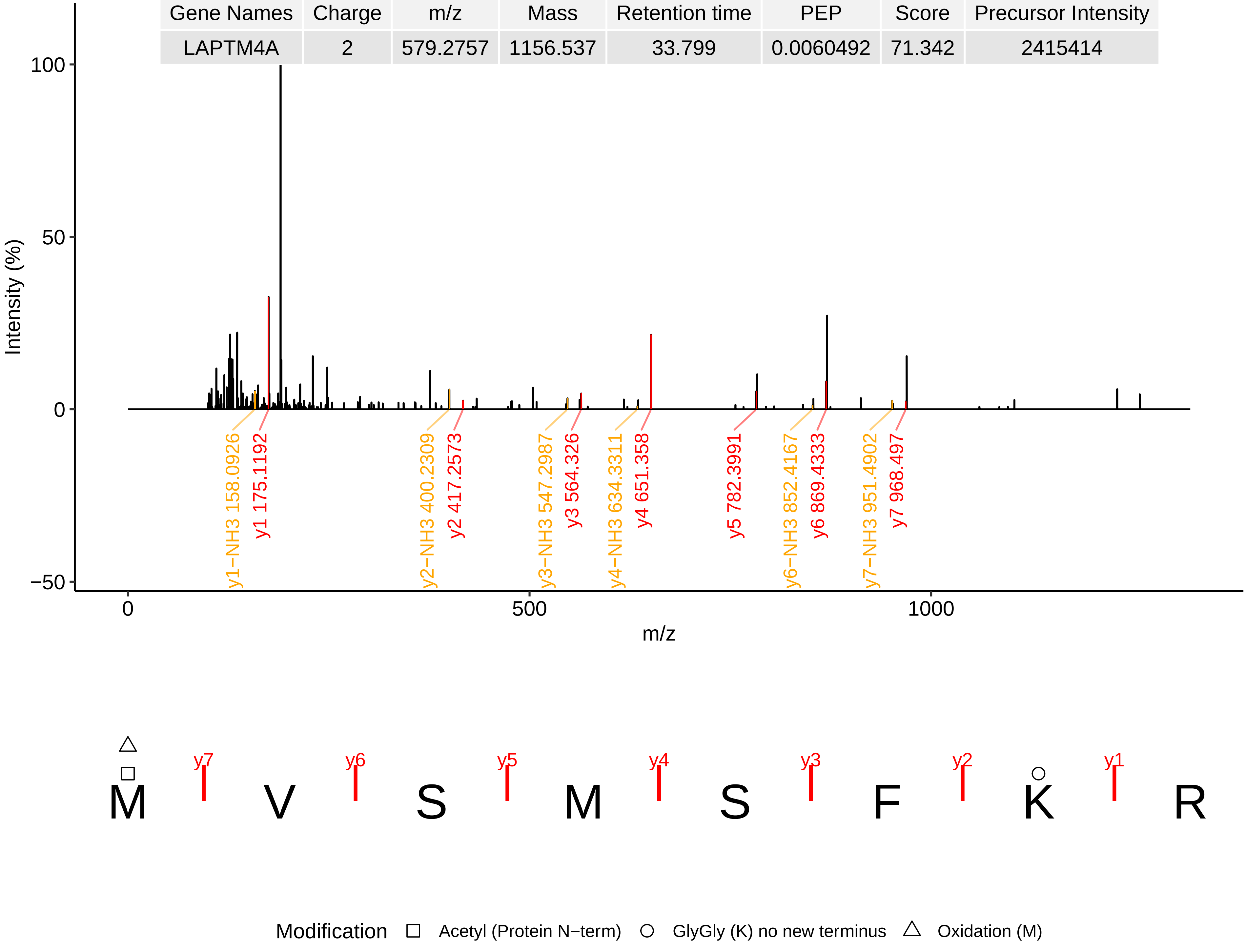

20200921_KIZ_MUb4_GlyGly_10plex − Scan number: 15195

Gene Names	Charge	m/z	Mass	Retention time	PEP	Score	Precursor Intensity
LAPTM4A	2	571.2783	1140.542	54.22	9.3506e−13	164.53	51482528

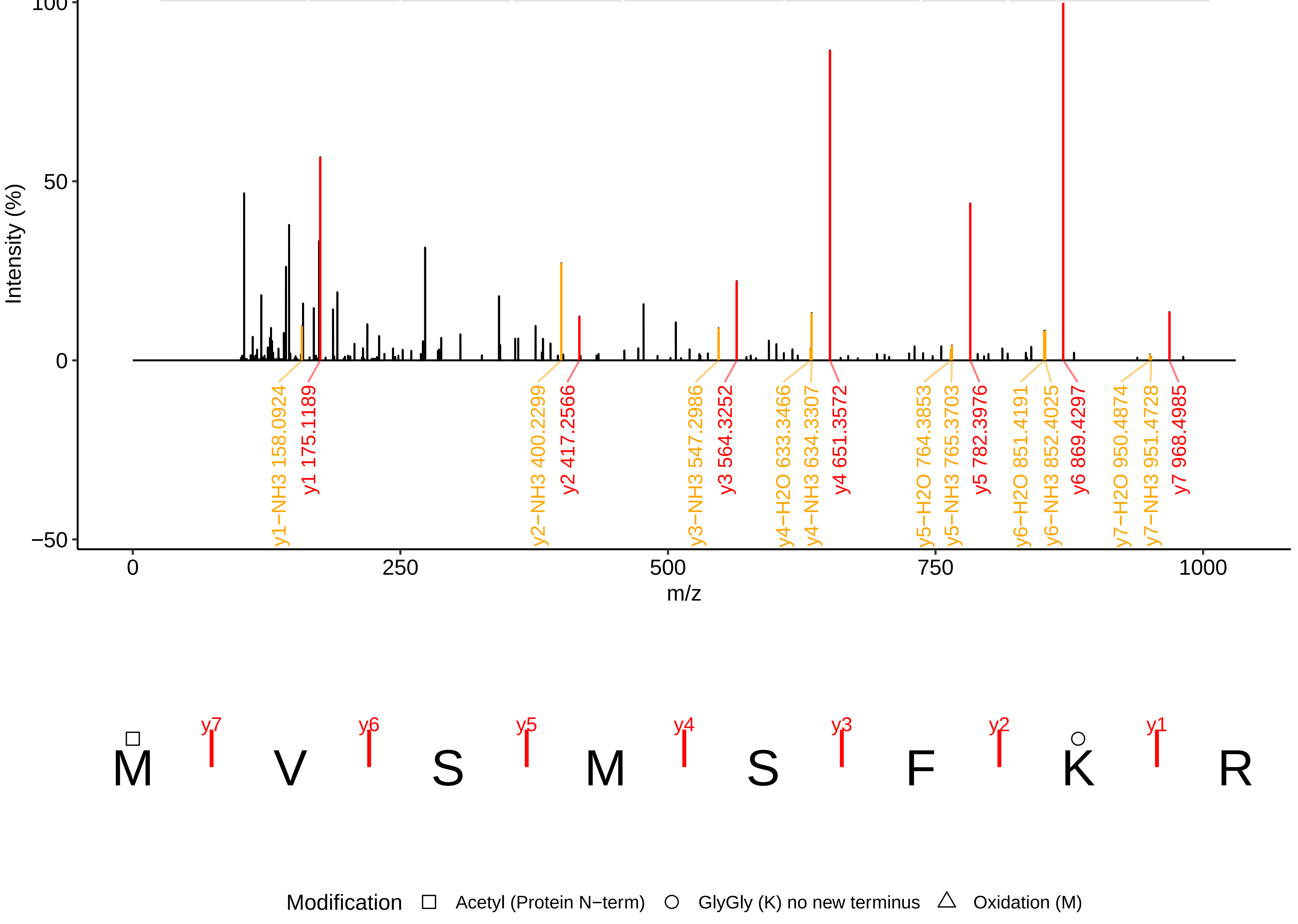

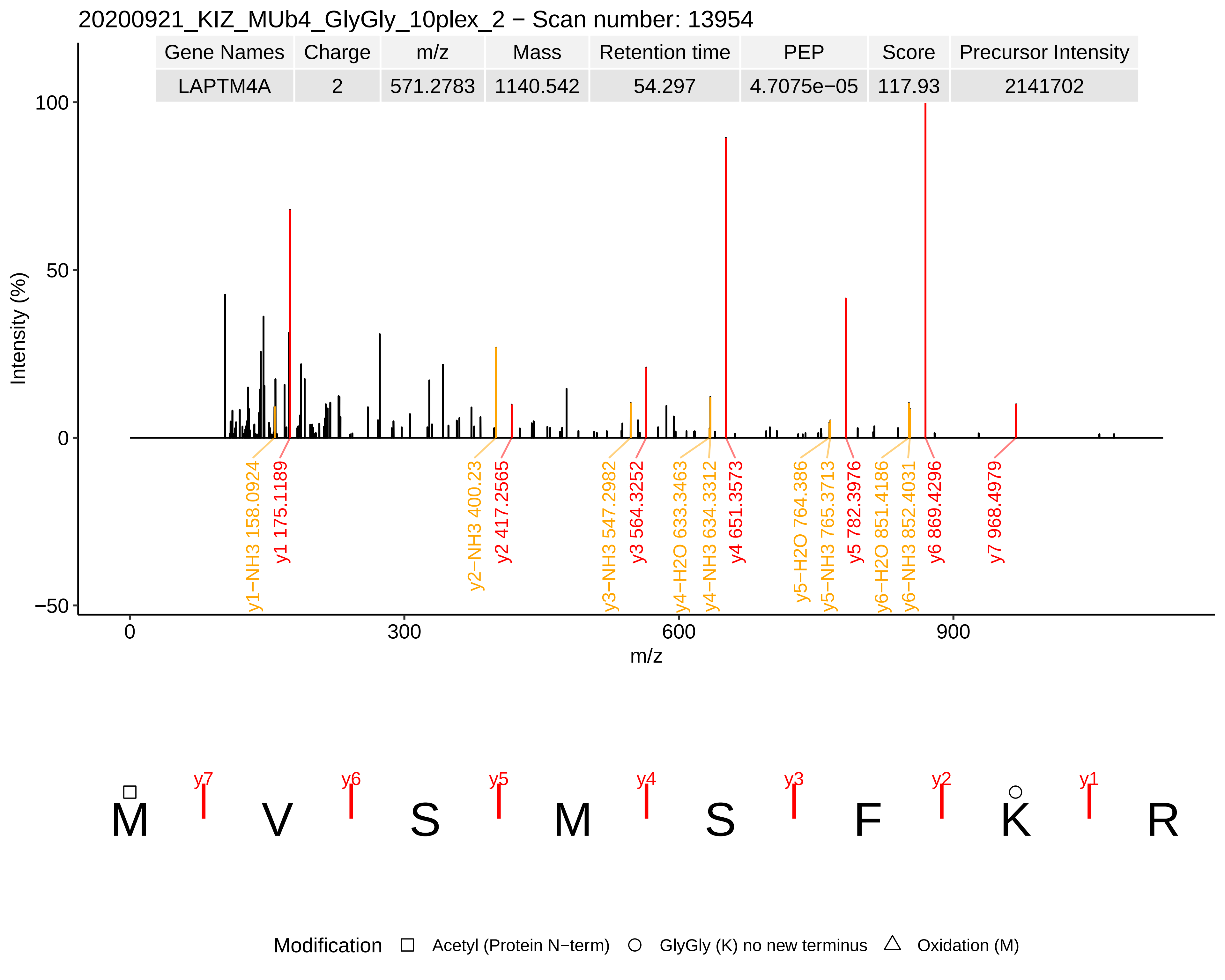

20200921_KIZ_MUb4_GlyGly_10plex_2 − Scan number: 13954
Gene Names
Charge
m/z
Mass
Retention time
PEP
Score
Precursor Intensity
LAPTM4A
2
571.2783
1140.542
54.297
4.7075e−05
117.93
2141702
Intensity (%)
m/z
y1−NH3 158.0924
y1 175.1189
y2−NH3 400.23
y2 417.2565
y3−NH3 547.2982
y3 564.3252
y4−H2O 633.3463
y4−NH3 634.3312
y4 651.3573
y5−H2O 764.386
y5−NH3 765.3713
y5 782.3976
y6−H2O 851.4186
y6−NH3 852.4031
y6 869.4296
y7 968.4979
M V S M S F K R
y7 y6 y5 y4 y3 y2 y1
Modification
Acetyl (Protein N−term)
GlyGly (K) no new terminus
Oxidation (M)

20200924_KIZ_MUb5_GlyGly_1 − Scan number: 14265

Gene Names	Charge	m/z	Mass	Retention time	PEP	Score	Precursor Intensity
LAPTM4A	2	571.2783	1140.542	46.566	0.00022795	103.91	20400198

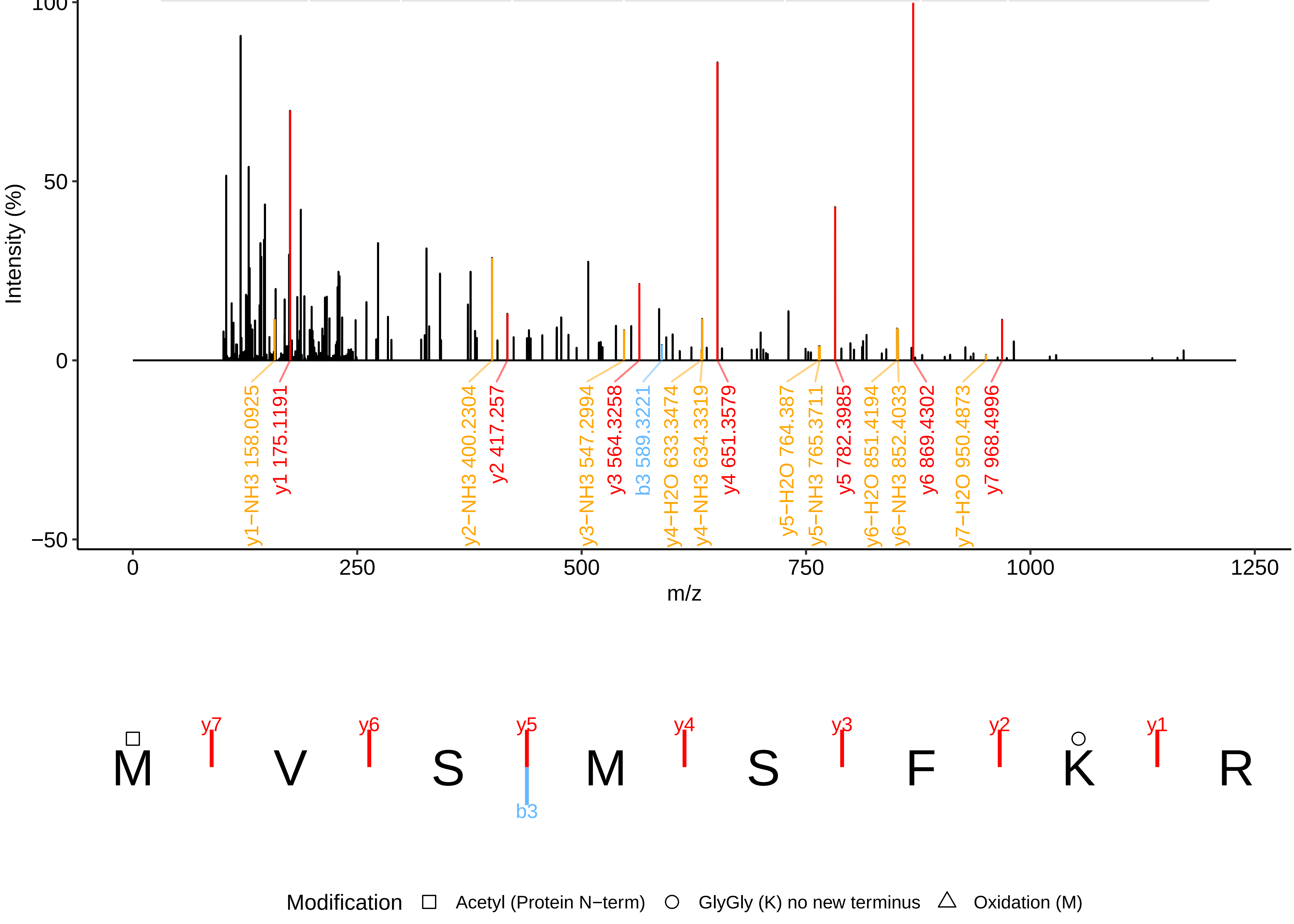

20200924_KIZ_MUb5_GlyGly_1 − Scan number: 14415

Gene Names	Charge	m/z	Mass	Retention time	PEP	Score	Precursor Intensity
LAPTM4A	2	571.2783	1140.542	47.073	1.4436e−12	174.21	27578610

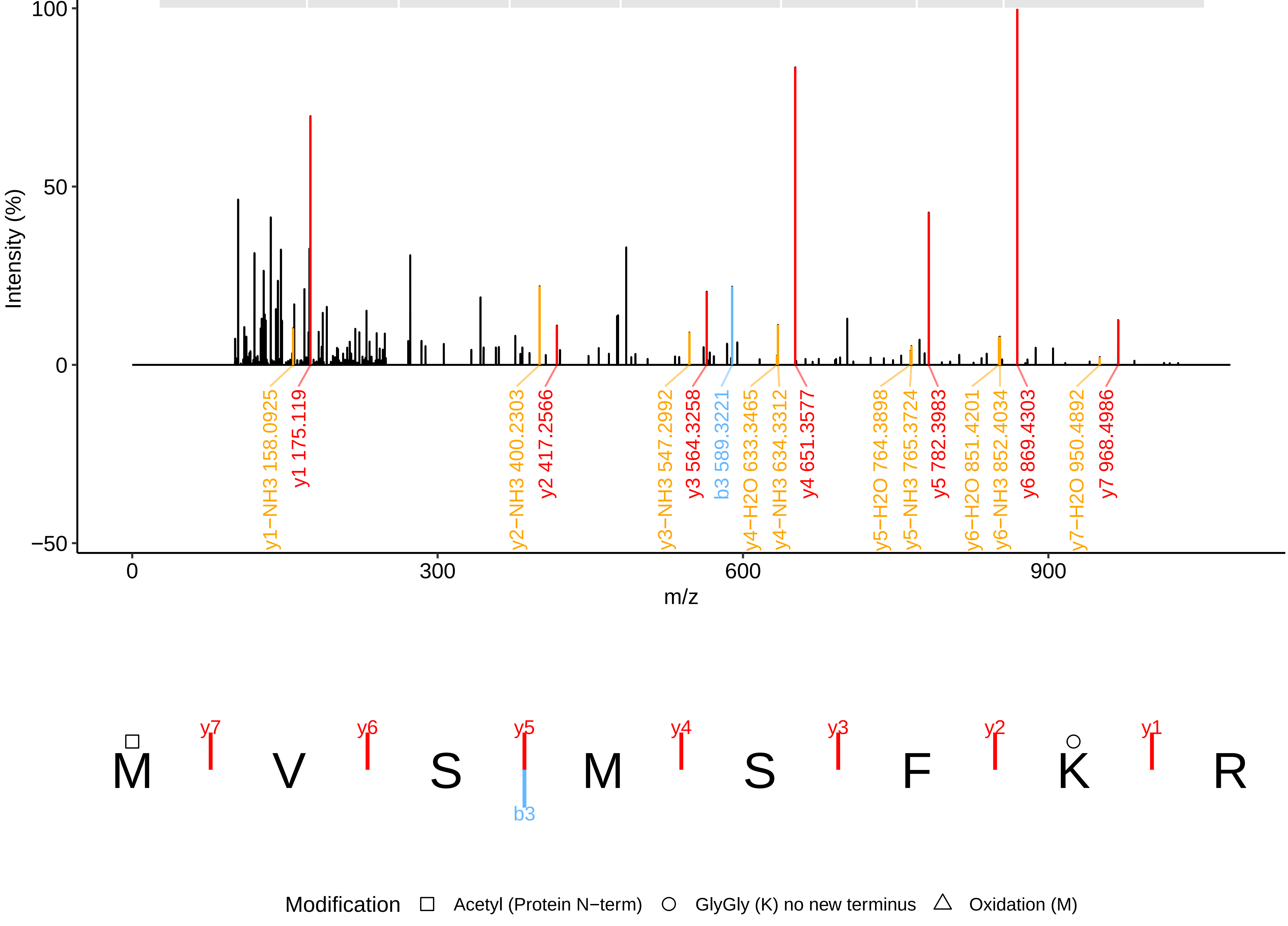

20200924_KIZ_MUb5_GlyGly_2 − Scan number: 13403

Gene Names	Charge	m/z	Mass	Retention time	PEP	Score	Precursor Intensity
LAPTM4A	2	571.2783	1140.542	50.138	0.00013485	114.78	5274851

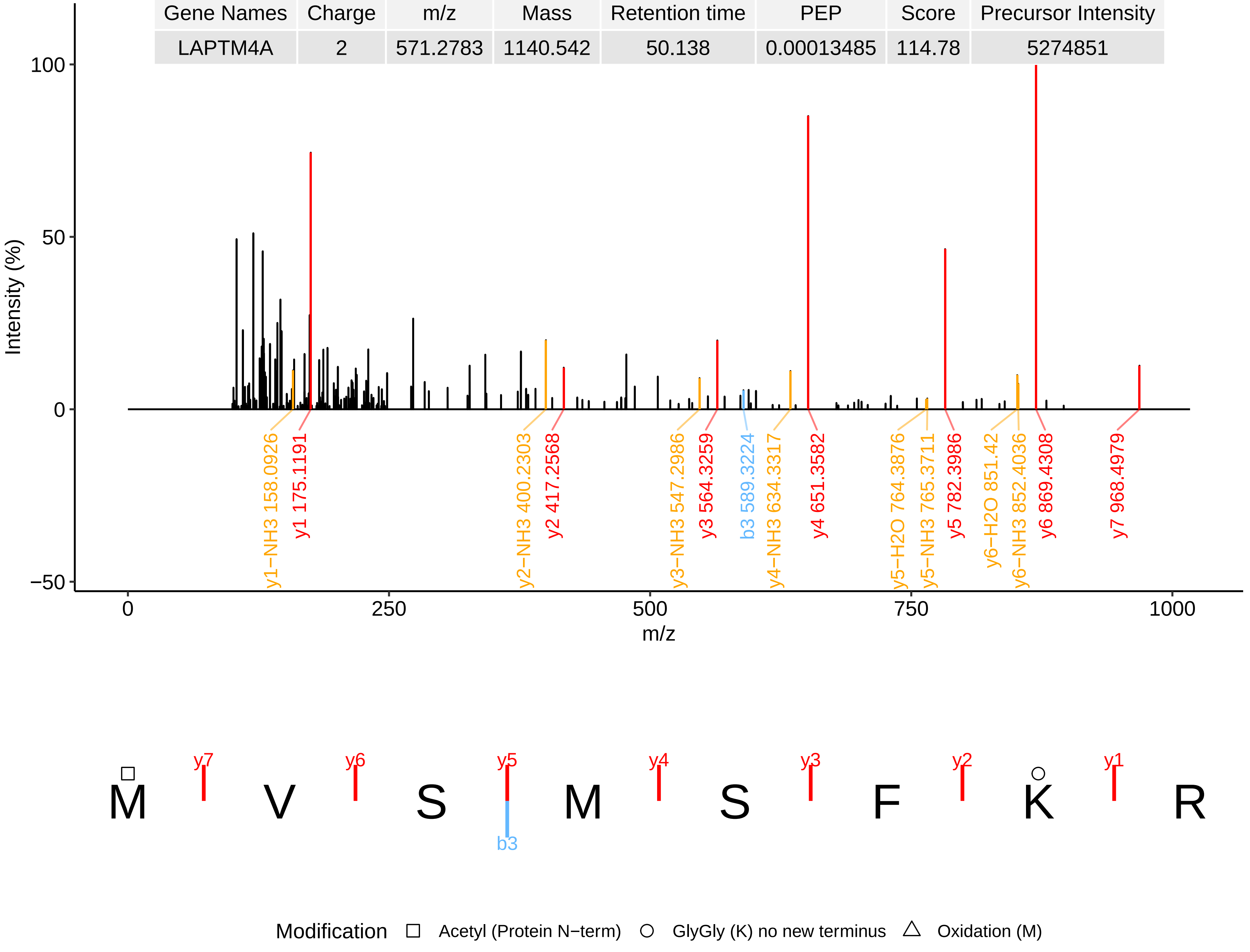

20200924_KIZ_MUb5_GlyGly_2 − Scan number: 13549

Gene Names	Charge	m/z	Mass	Retention time	PEP	Score	Precursor Intensity
LAPTM4A	2	571.2783	1140.542	50.67	0.080062	47.082	4146231

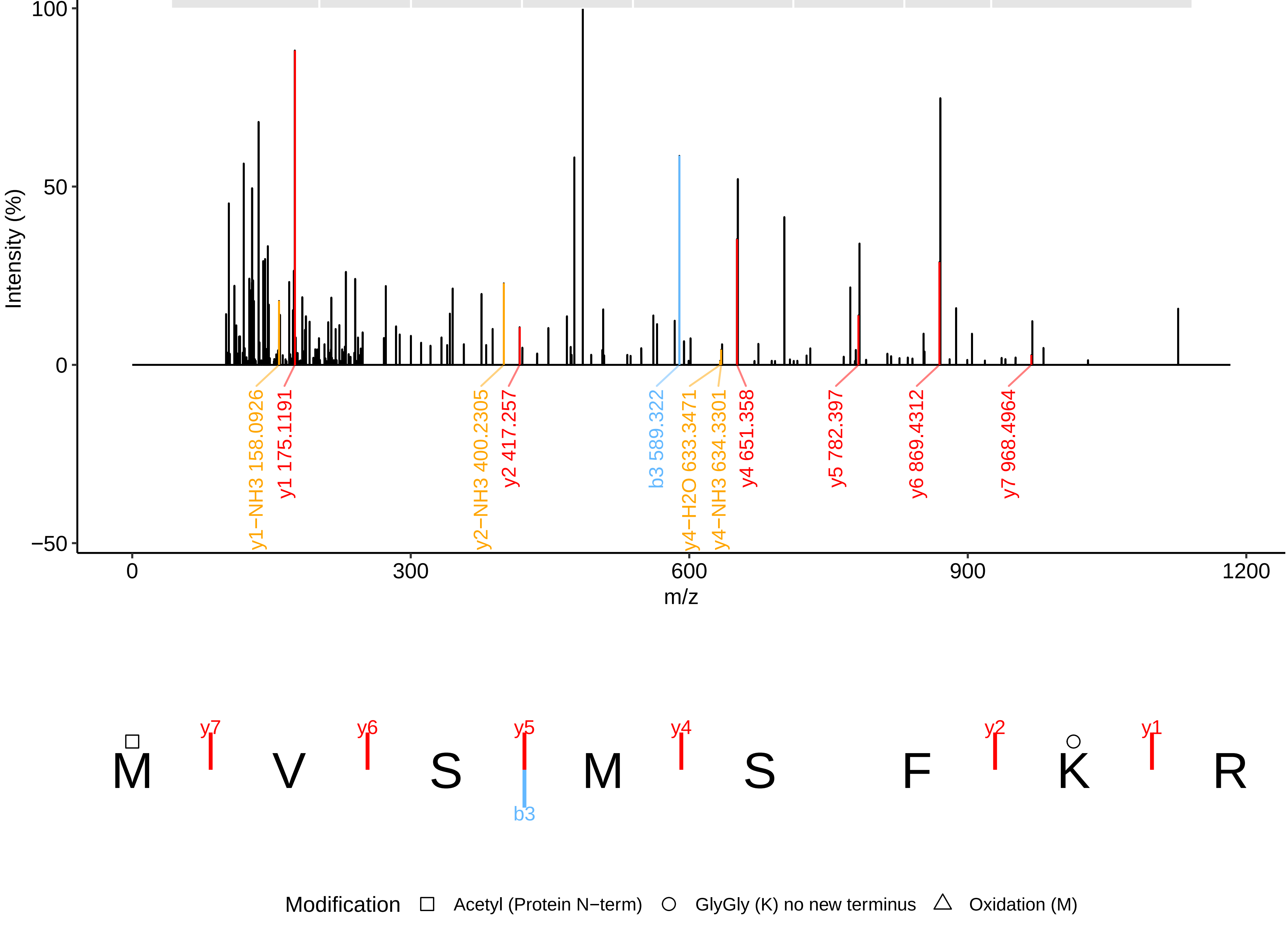

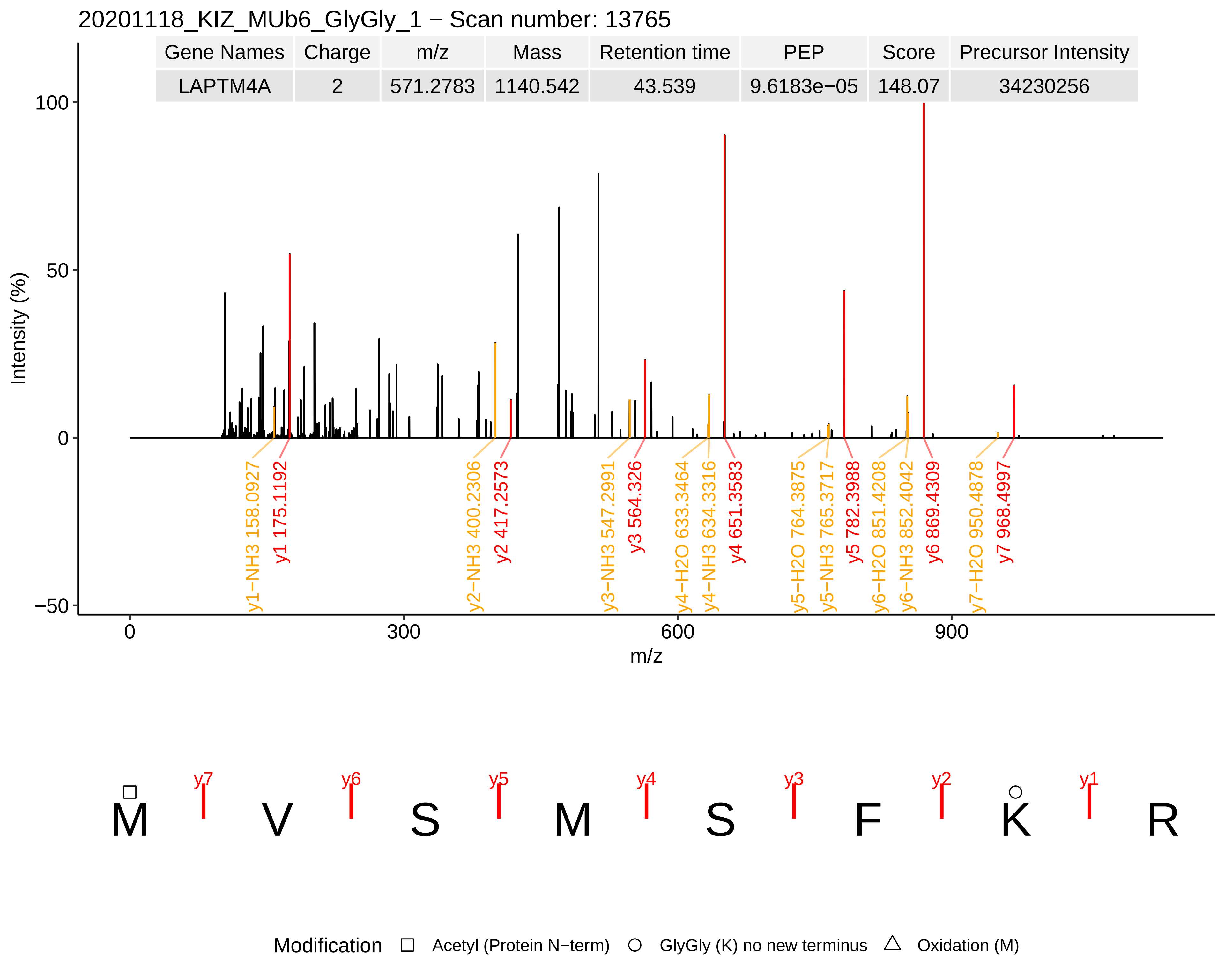

20201118_KIZ_MUb6_GlyGly_1 − Scan number: 13765
Gene Names | Charge | m/z | Mass | Retention time | PEP | Score | Precursor Intensity
LAPTM4A | 2 | 571.2783 | 1140.542 | 43.539 | 9.6183e−05 | 148.07 | 34230256
Intensity (%)
m/z
y1−NH3 158.0927
y1 175.1192
y2−NH3 400.2306
y2 417.2573
y3−NH3 547.2991
y3 564.326
y4−H2O 633.3464
y4−NH3 634.3316
y4 651.3583
y5−H2O 764.3875
y5−NH3 765.3717
y5 782.3988
y6−H2O 851.4208
y6−NH3 852.4042
y6 869.4309
y7−H2O 950.4878
y7 968.4997
M V S M S F K R
y7 y6 y5 y4 y3 y2 y1
Modification □ Acetyl (Protein N−term) ○ GlyGly (K) no new terminus △ Oxidation (M)

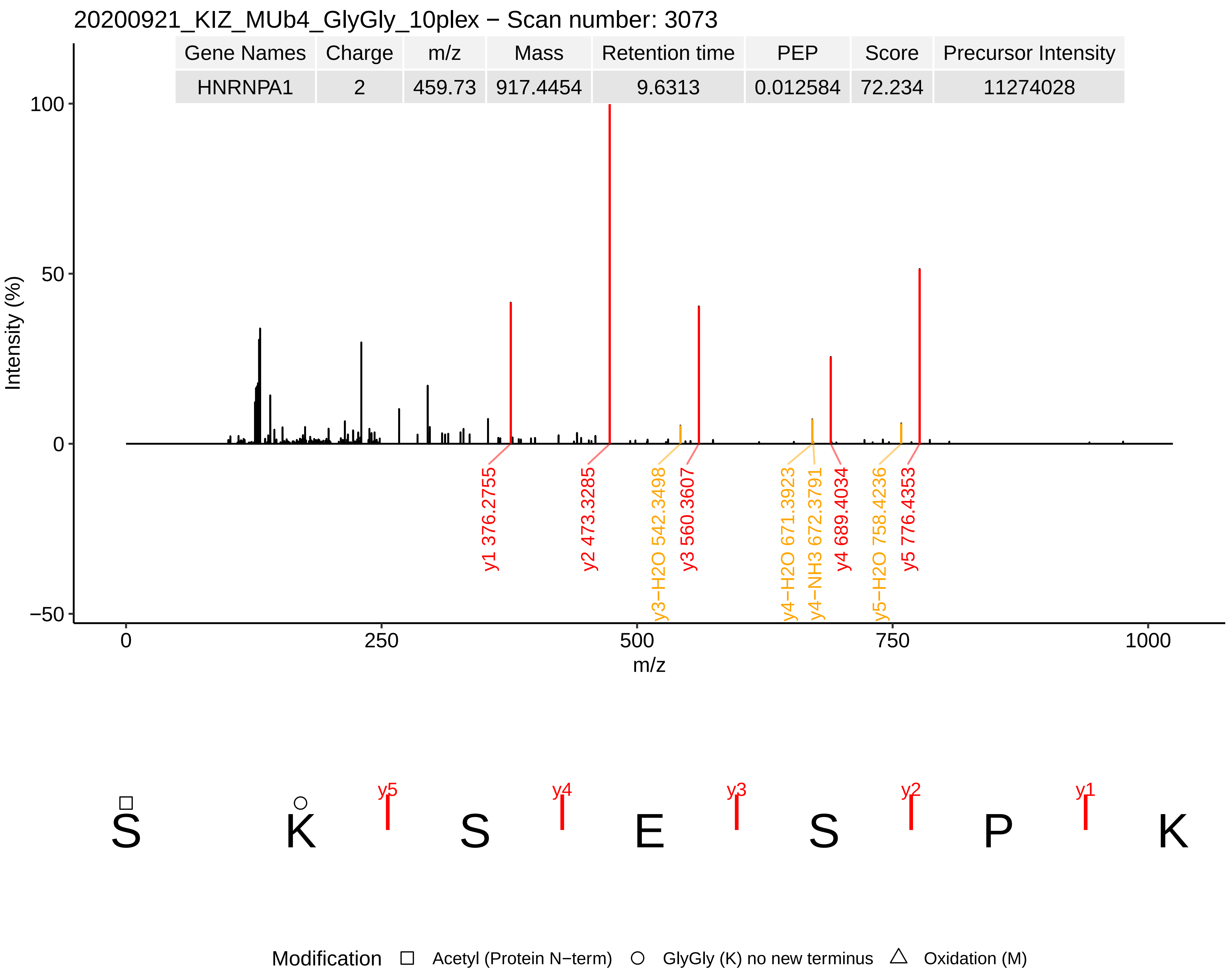

20200921_KIZ_MUb4_GlyGly_10plex − Scan number: 3073
Gene Names | Charge | m/z | Mass | Retention time | PEP | Score | Precursor Intensity
HNRNPA1 | 2 | 459.73 | 917.4454 | 9.6313 | 0.012584 | 72.234 | 11274028
Intensity (%)
100
50
0
−50
y1 376.2755
y2 473.3285
y3−H2O 542.3498
y3 560.3607
y4−H2O 671.3923
y4−NH3 672.3791
y4 689.4034
y5−H2O 758.4236
y5 776.4353
0
250
500
750
1000
m/z
S K S E S P K
y5 y4 y3 y2 y1
Modification
Acetyl (Protein N−term)
GlyGly (K) no new terminus
Oxidation (M)

20200921_KIZ_MUb4_GlyGly_10plex_2 − Scan number: 2967

Gene Names	Charge	m/z	Mass	Retention time	PEP	Score	Precursor Intensity
HNRNPA1	2	459.73	917.4454	9.6917	0.0035974	86.113	1248205

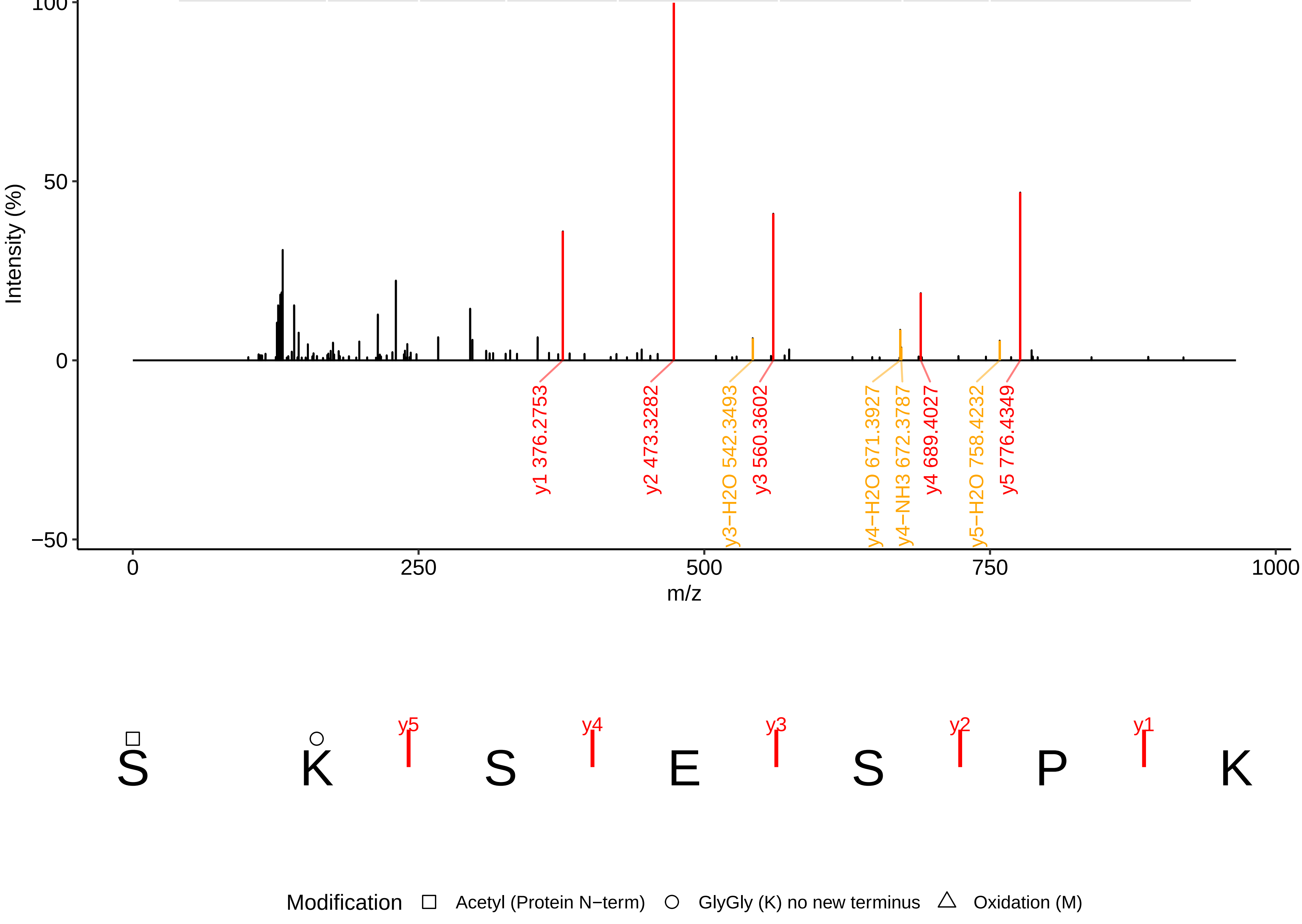

20200924_KIZ_MUb5_GlyGly_1 − Scan number: 2934

Gene Names	Charge	m/z	Mass	Retention time	PEP	Score	Precursor Intensity
HNRNPA1	2	459.73	917.4454	9.0672	0.028514	75.311	11597277

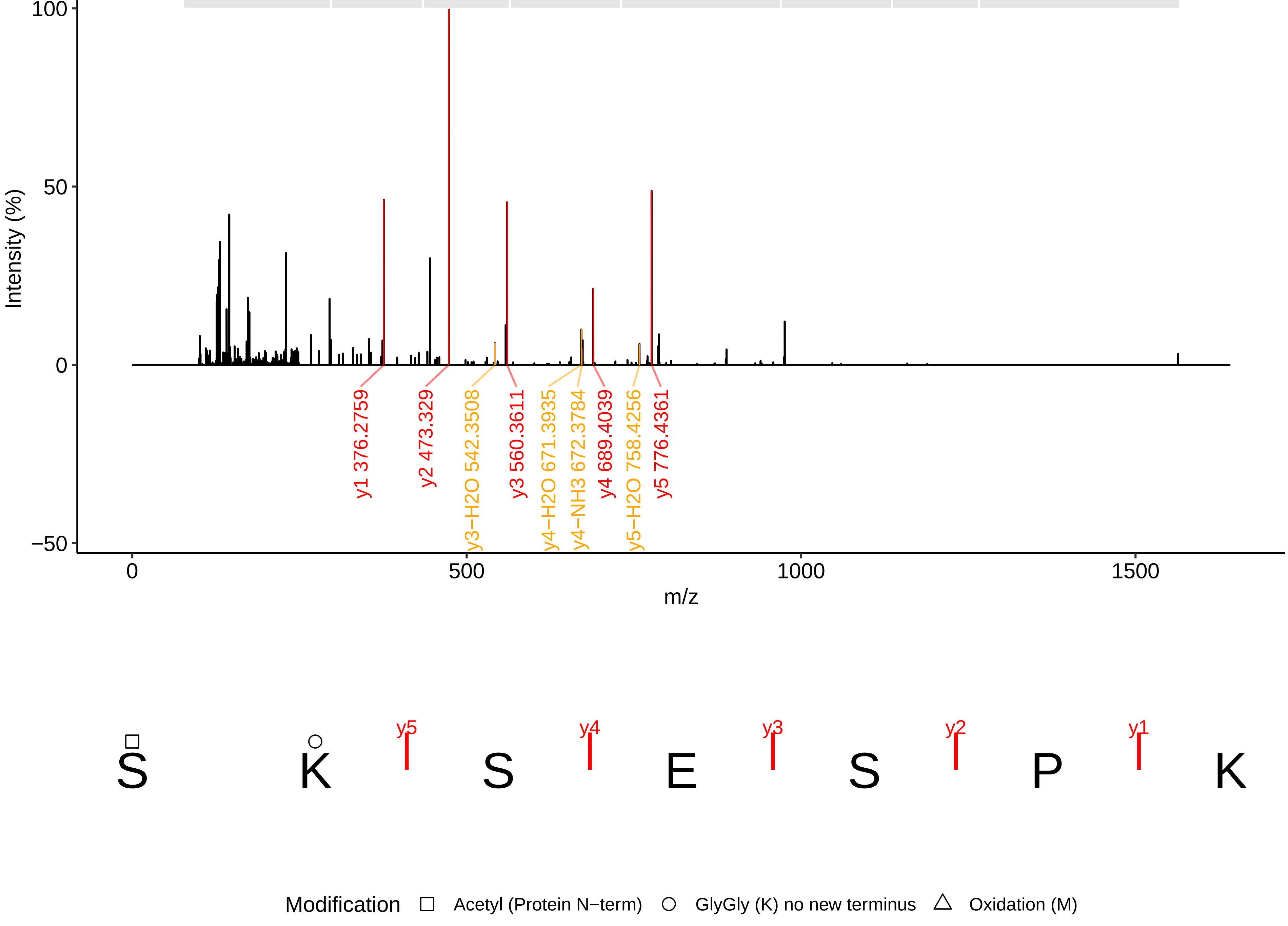

20200924_KIZ_MUb5_GlyGly_2 − Scan number: 2589

Gene Names	Charge	m/z	Mass	Retention time	PEP	Score	Precursor Intensity
HNRNPA1	2	459.73	917.4454	9.1946	0.0035974	86.113	1362295

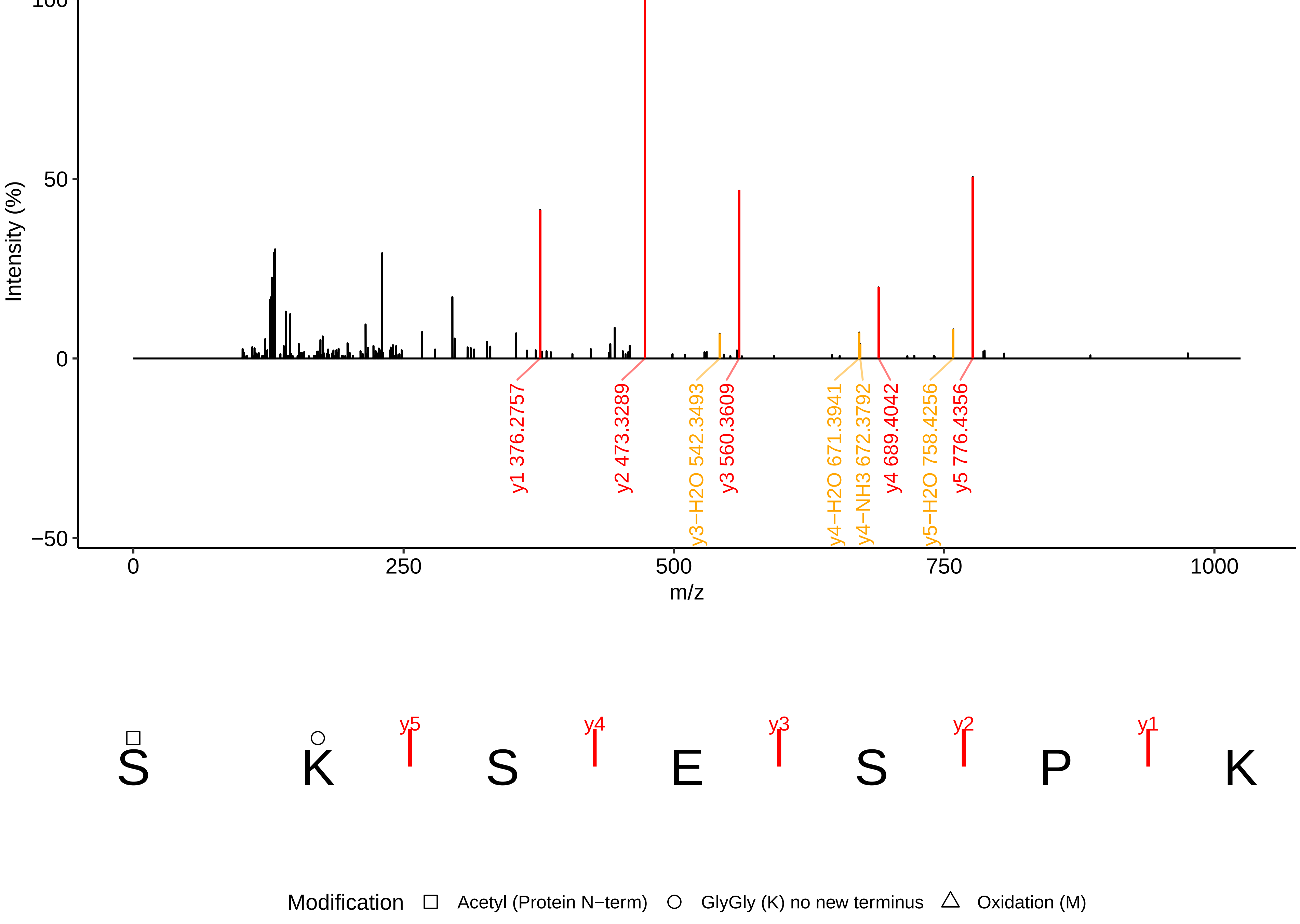

20201118_KIZ_MUb6_GlyGly_1 − Scan number: 2952

Gene Names	Charge	m/z	Mass	Retention time	PEP	Score	Precursor Intensity
HNRNPA1	2	459.73	917.4454	9.0498	0.0082843	75.311	15459488

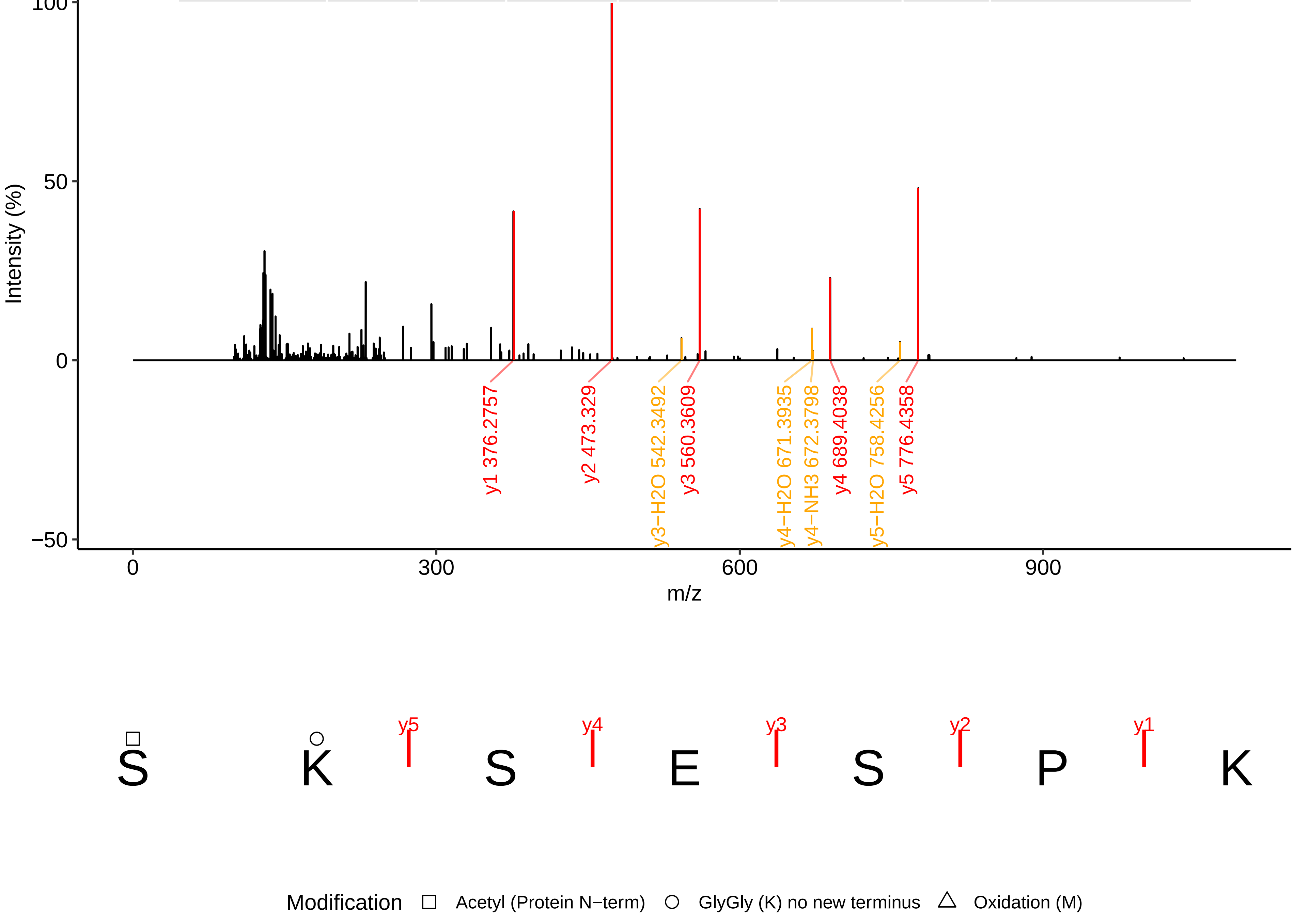

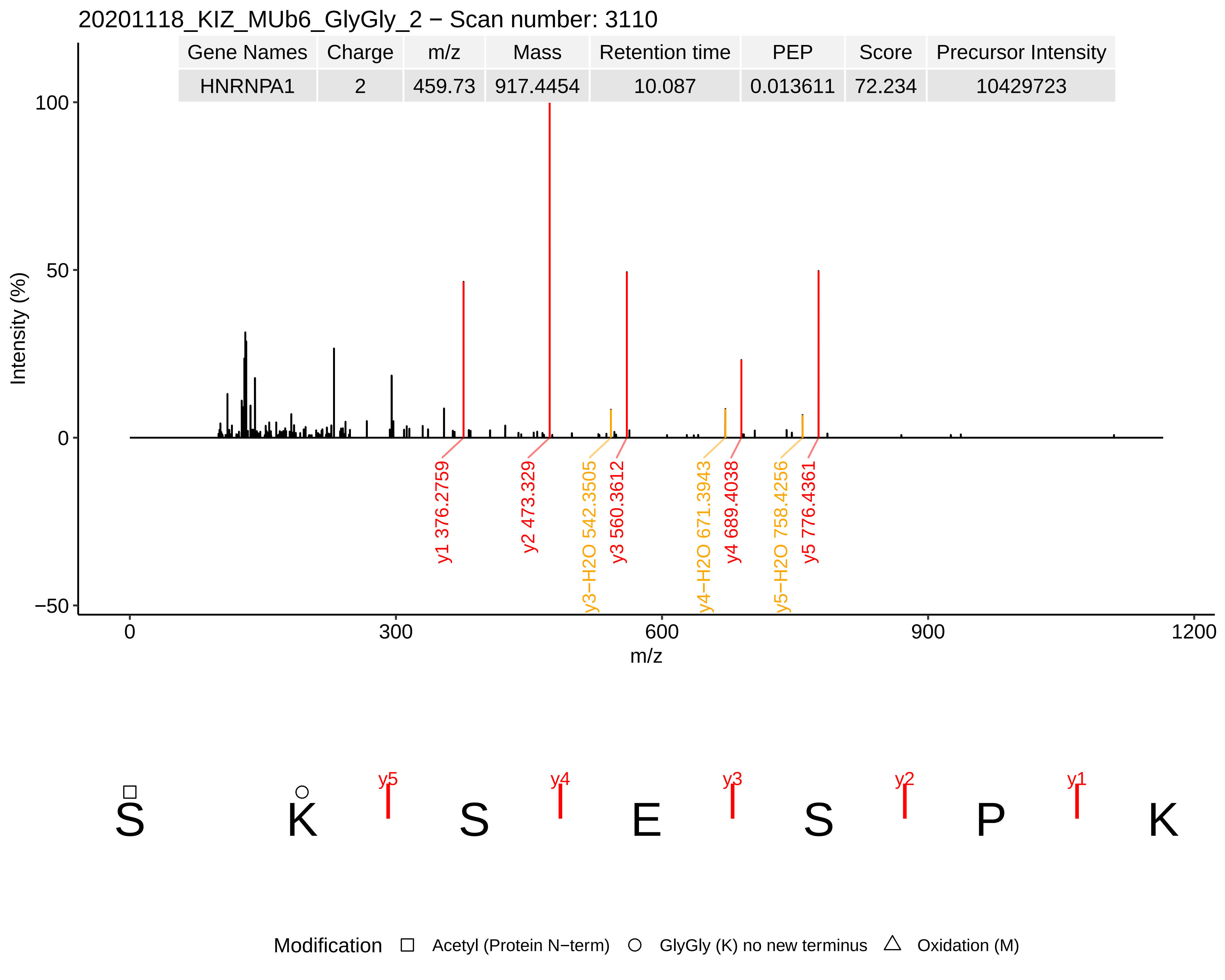

20201118_KIZ_MUb6_GlyGly_2 − Scan number: 3110
Gene Names | Charge | m/z | Mass | Retention time | PEP | Score | Precursor Intensity
HNRNPA1 | 2 | 459.73 | 917.4454 | 10.087 | 0.013611 | 72.234 | 10429723
Intensity (%)
m/z
y1 376.2759
y2 473.329
y3−H2O 542.3505
y3 560.3612
y4−H2O 671.3943
y4 689.4038
y5−H2O 758.4256
y5 776.4361
S K S E S P K
y5 y4 y3 y2 y1
Modification
Acetyl (Protein N−term)
GlyGly (K) no new terminus
Oxidation (M)

20200921_KIZ_MUb4_GlyGly_10plex − Scan number: 2642

Gene Names	Charge	m/z	Mass	Retention time	PEP	Score	Precursor Intensity
SEC31A	3	311.8419	932.5039	8.3266	0.016983	84.498	6724412

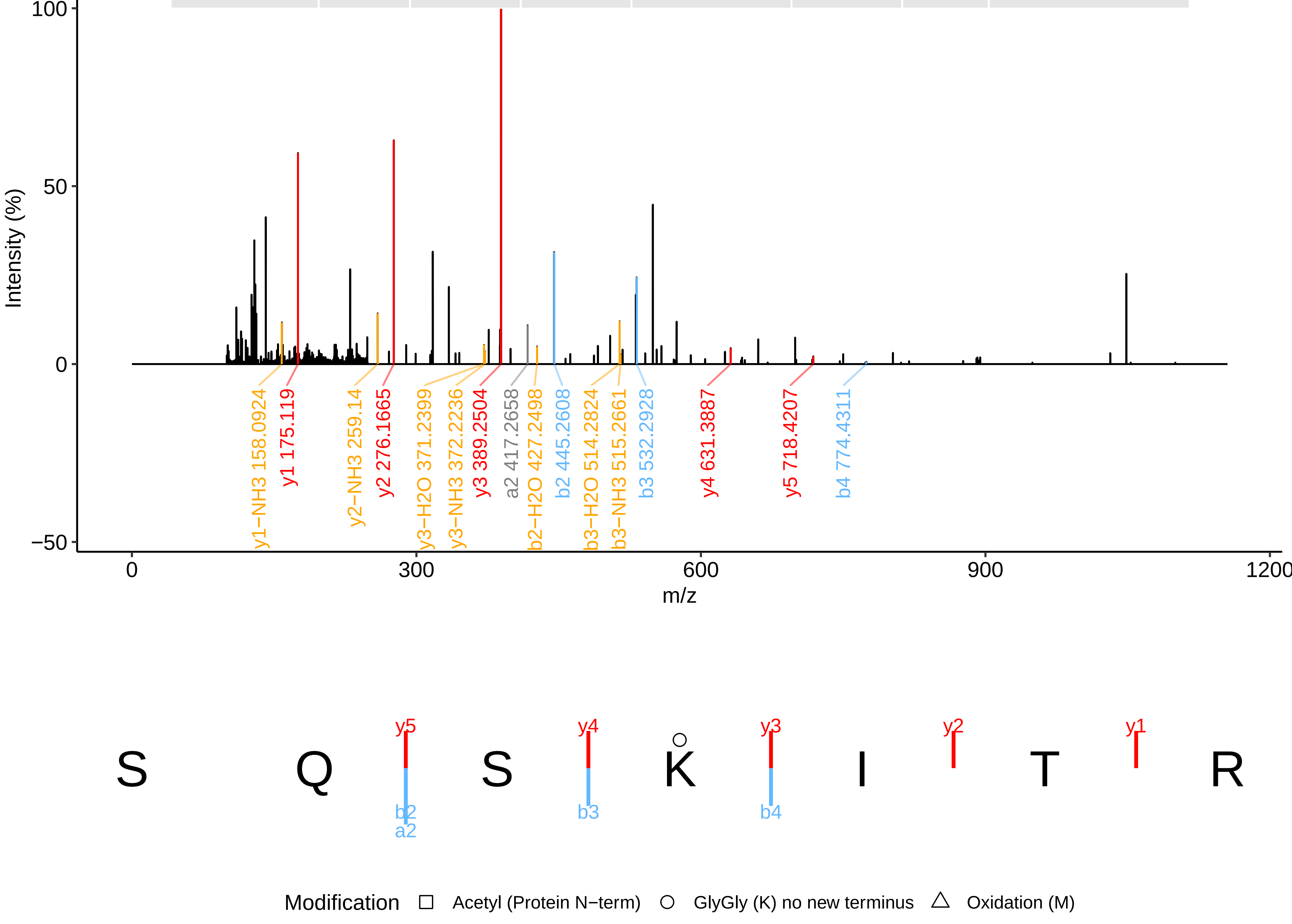

20200921_KIZ_MUb4_GlyGly_10plex_2 − Scan number: 2605

Gene Names	Charge	m/z	Mass	Retention time	PEP	Score	Precursor Intensity
SEC31A	3	311.8419	932.5039	8.3055	0.059114	65.252	1492063

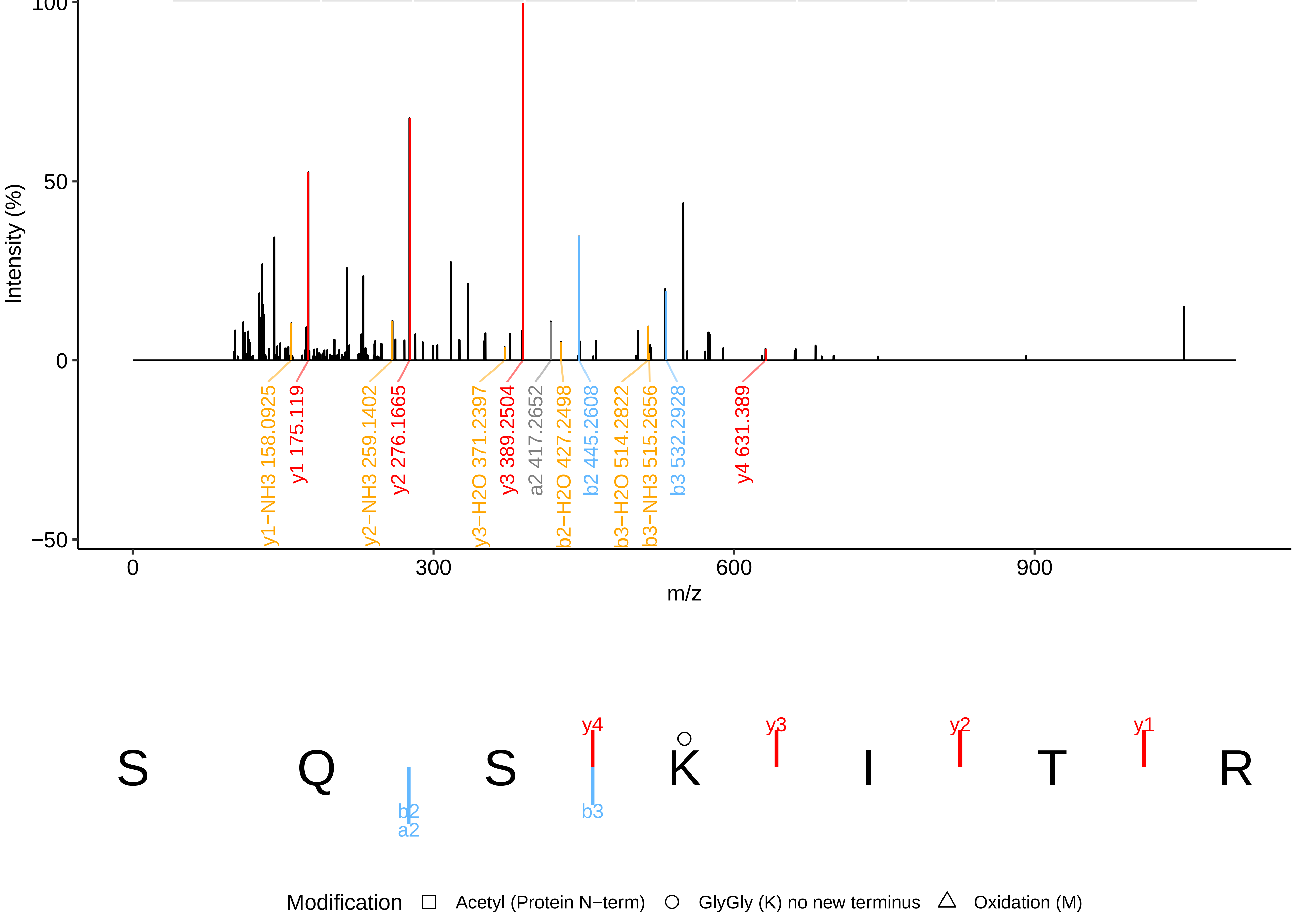

20201118_KIZ_MUb6_GlyGly_1 − Scan number: 2633

Gene Names	Charge	m/z	Mass	Retention time	PEP	Score	Precursor Intensity
SEC31A	3	311.8419	932.5039	8.0562	0.019755	84.916	8007898

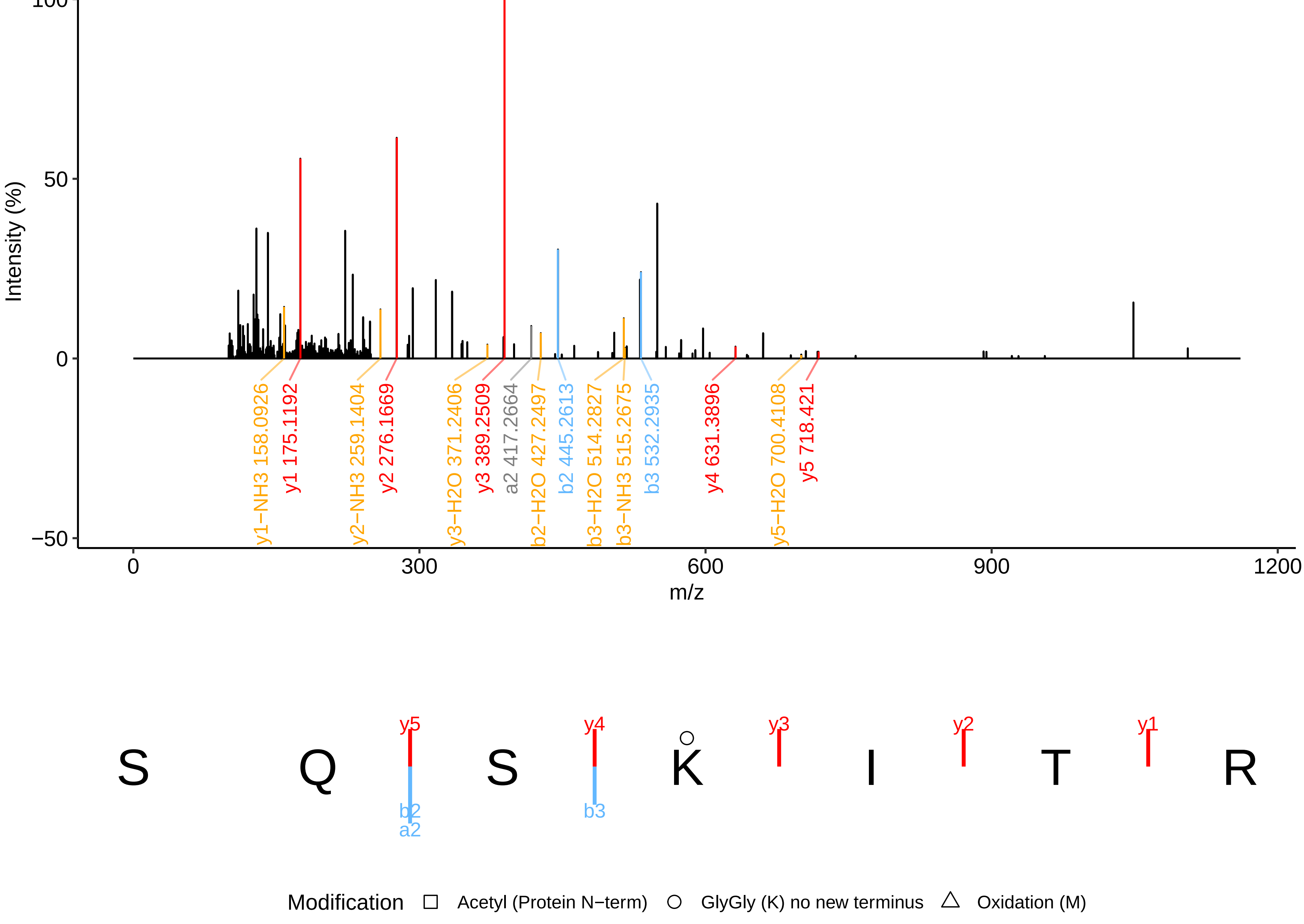

20201118_KIZ_MUb6_GlyGly_2 − Scan number: 2823

Gene Names	Charge	m/z	Mass	Retention time	PEP	Score	Precursor Intensity
SEC31A	3	311.8419	932.5039	9.0043	0.017273	84.097	2237636

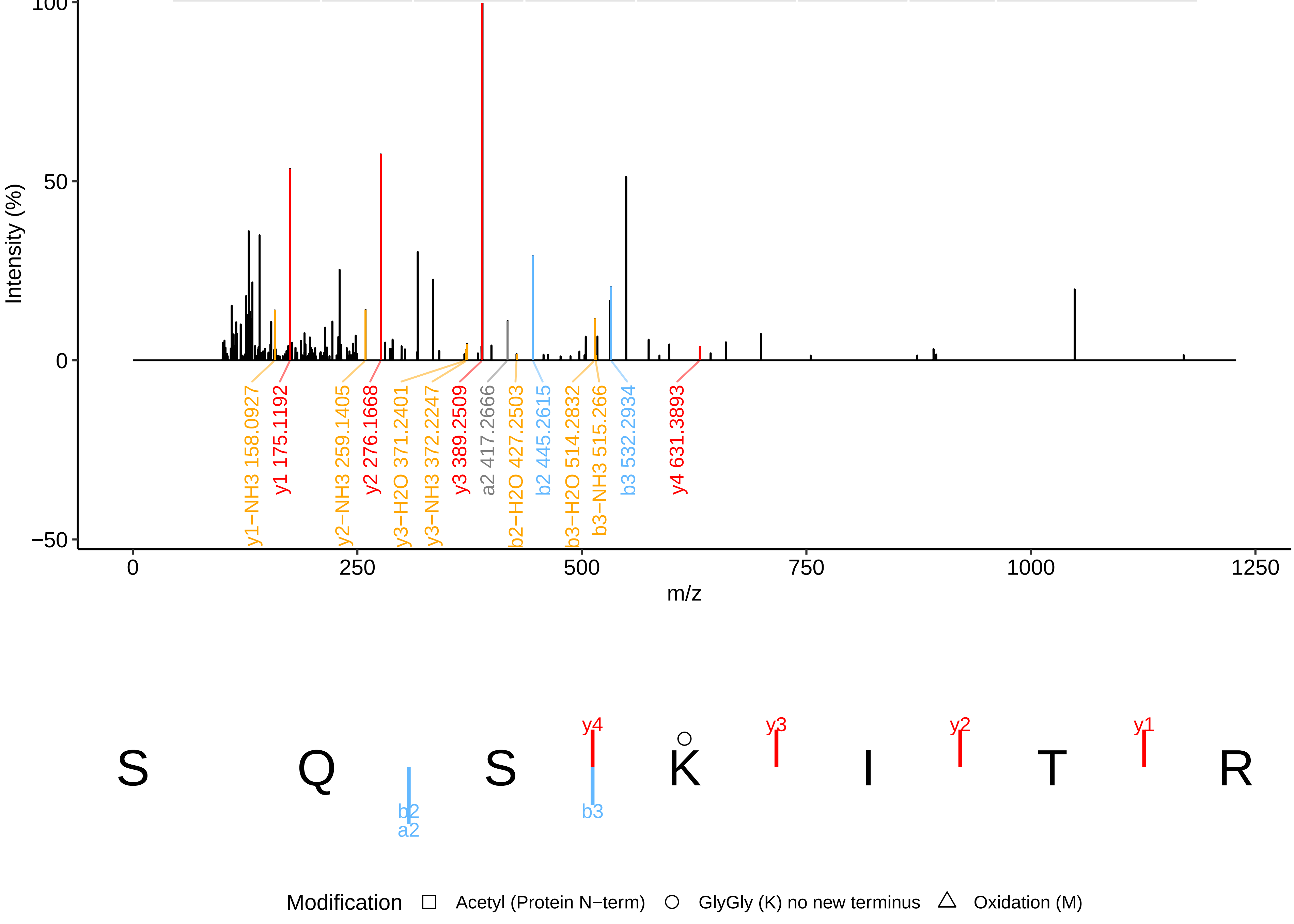

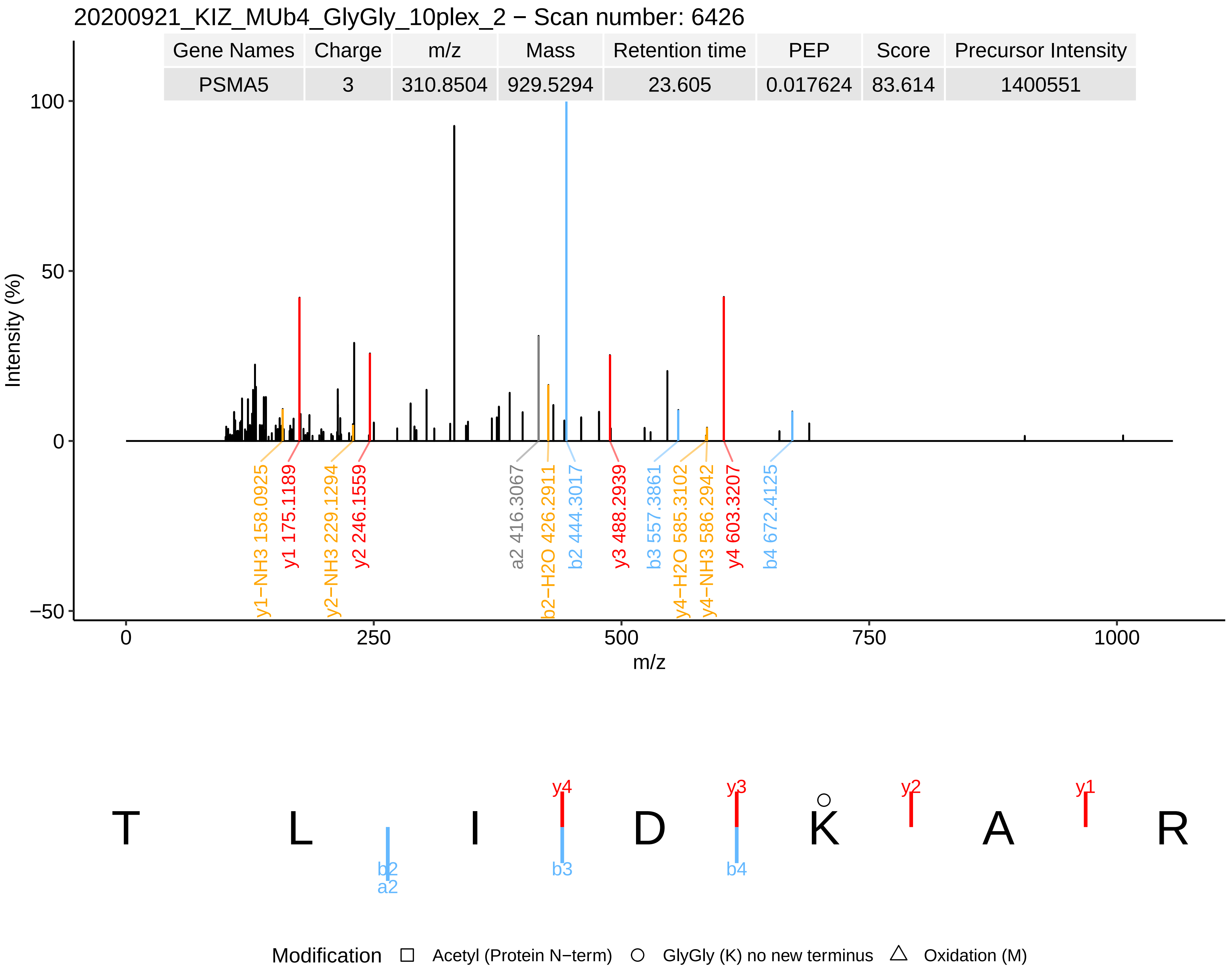
20200921_KIZ_MUb4_GlyGly_10plex_2 − Scan number: 6426
Gene Names
Charge
m/z
Mass
Retention time
PEP
Score
Precursor Intensity
PSMA5
3
310.8504
929.5294
23.605
0.017624
83.614
1400551
Intensity (%)
100
50
0
−50
y1−NH3 158.0925
y1 175.1189
y2−NH3 229.1294
y2 246.1559
a2 416.3067
b2−H2O 426.2911
b2 444.3017
y3 488.2939
b3 557.3861
y4−H2O 585.3102
y4−NH3 586.2942
y4 603.3207
b4 672.4125
0
250
500
750
1000
m/z
T L I D K A R
y4
y3
y2
y1
b2
a2
b3
b4
Modification
Acetyl (Protein N−term)
GlyGly (K) no new terminus
Oxidation (M)

20200924_KIZ_MUb5_GlyGly_2 − Scan number: 5650

Gene Names	Charge	m/z	Mass	Retention time	PEP	Score	Precursor Intensity
PSMA5	3	310.8504	929.5294	20.999	0.13629	54.205	2906253

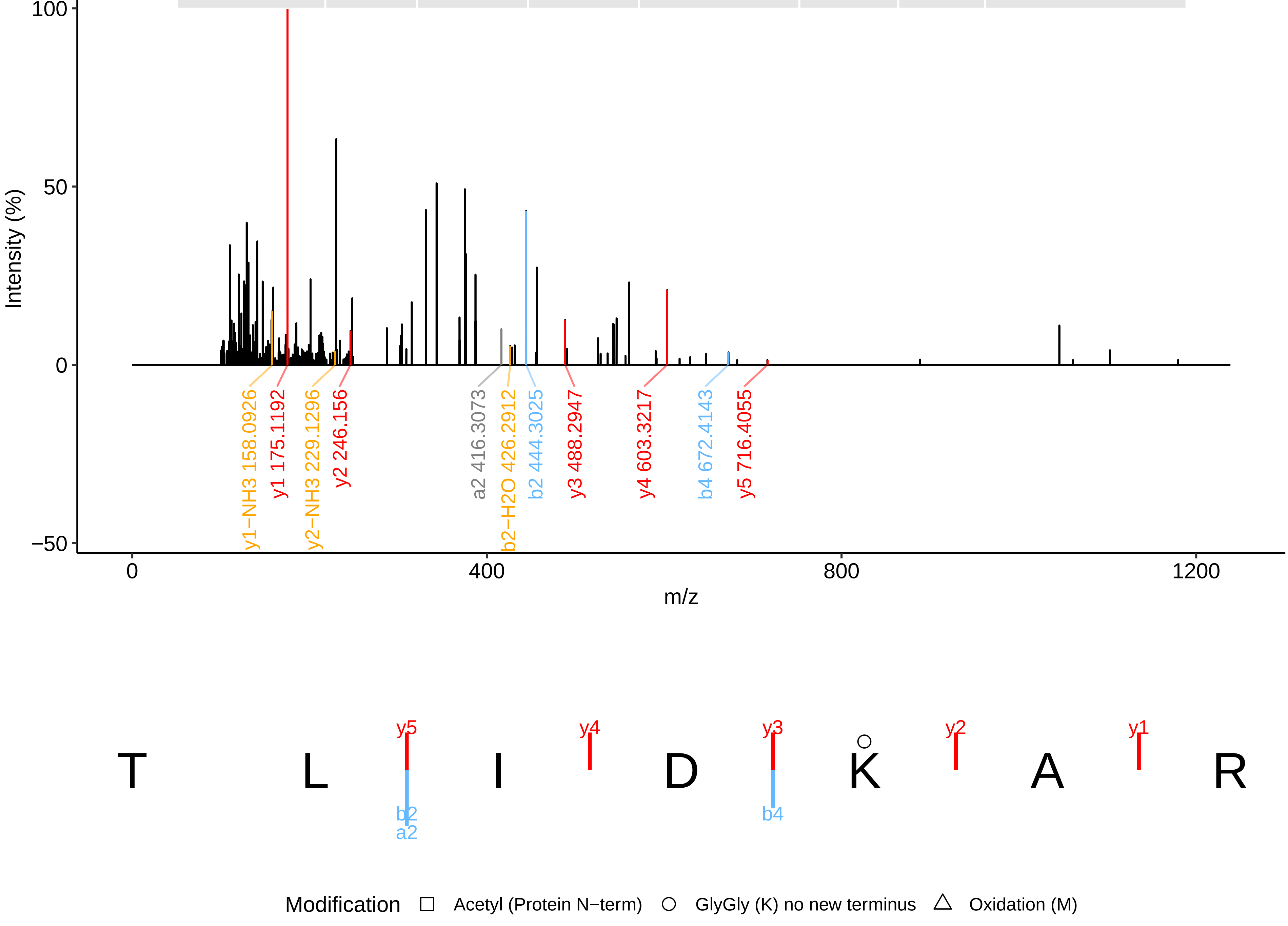

20200921_KIZ_MUb4_GlyGly_10plex_2 − Scan number: 4298

Gene Names	Charge	m/z	Mass	Retention time	PEP	Score	Precursor Intensity
ACE2	3	304.5189	910.5349	15.007	0.075368	60.961	618795.6

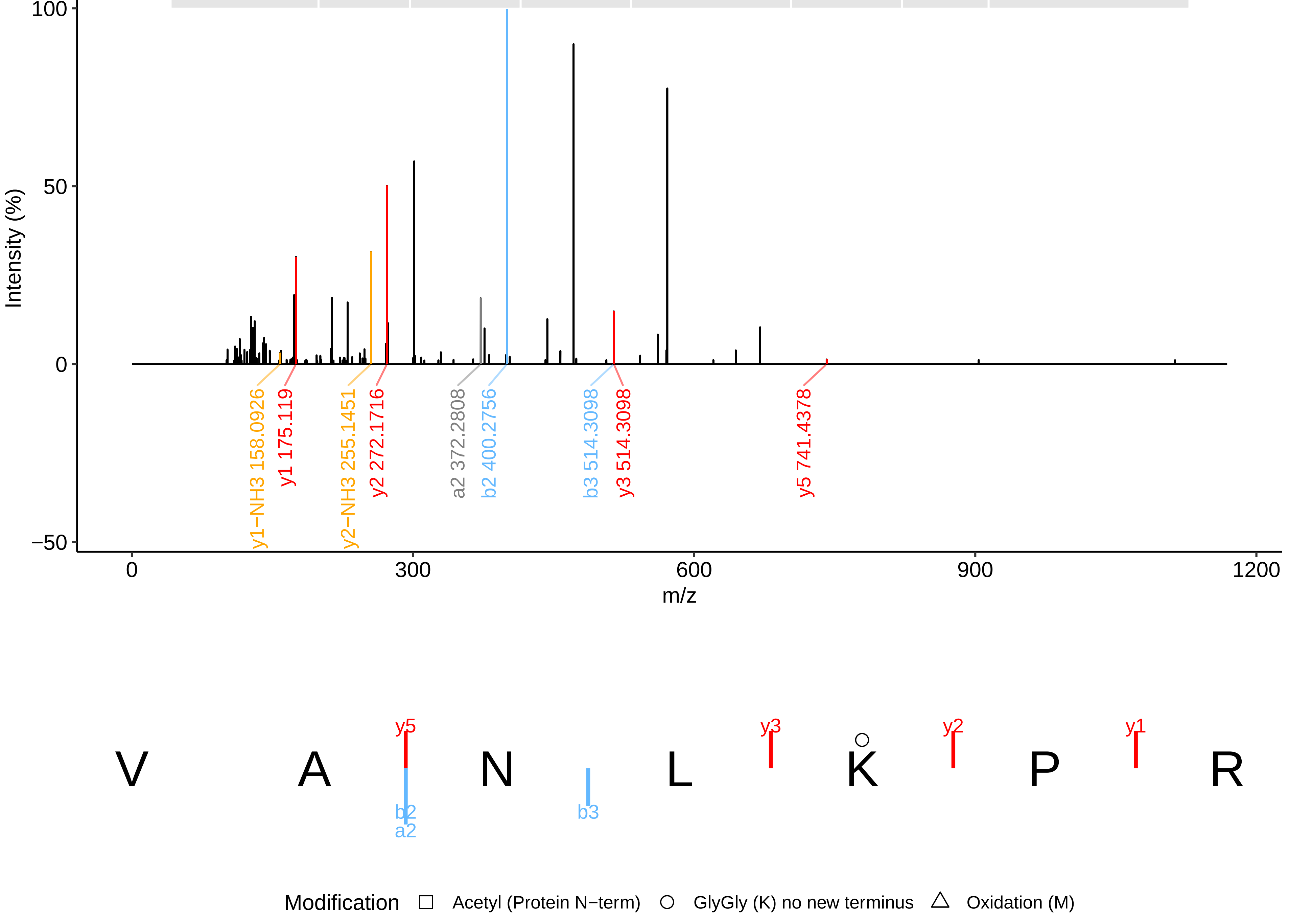

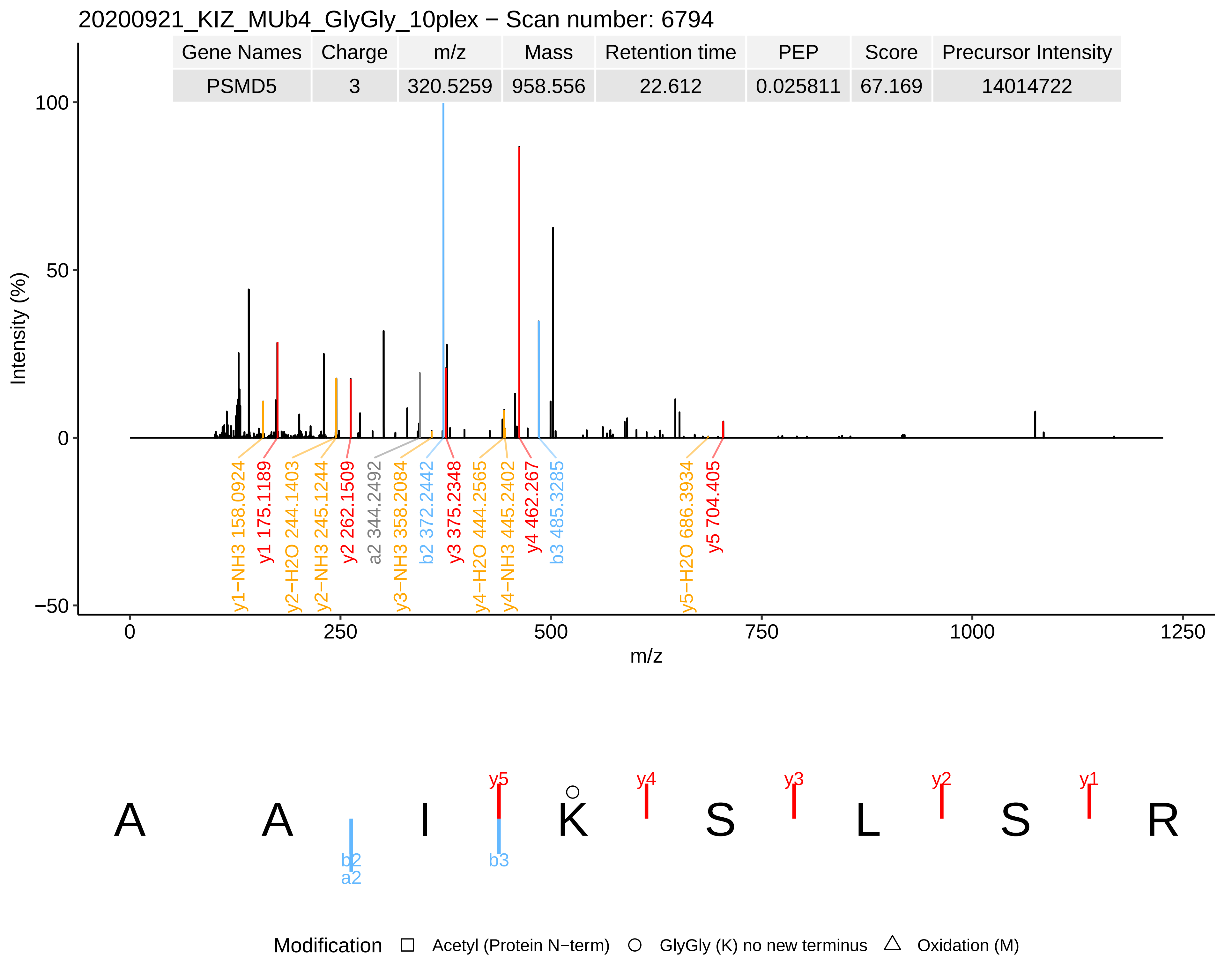

20200921_KIZ_MUb4_GlyGly_10plex − Scan number: 6794

Gene Names	Charge	m/z	Mass	Retention time	PEP	Score	Precursor Intensity
PSMD5	3	320.5259	958.556	22.612	0.025811	67.169	14014722

Intensity (%)
m/z
y1−NH3 158.0924
y1 175.1189
y2−H2O 244.1403
y2−NH3 245.1244
y2 262.1509
a2 344.2492
y3−NH3 358.2084
b2 372.2442
y3 375.2348
y4−H2O 444.2565
y4−NH3 445.2402
y4 462.267
b3 485.3285
y5−H2O 686.3934
y5 704.405
A A I K S L S R
y5 y4 y3 y2 y1
b2 a2 b3
Modification
Acetyl (Protein N−term)
GlyGly (K) no new terminus
Oxidation (M)

20200921_KIZ_MUb4_GlyGly_10plex_2 − Scan number: 6305

Gene Names	Charge	m/z	Mass	Retention time	PEP	Score	Precursor Intensity
PSMD5	3	320.5259	958.556	23.114	0.012415	77.923	2346709

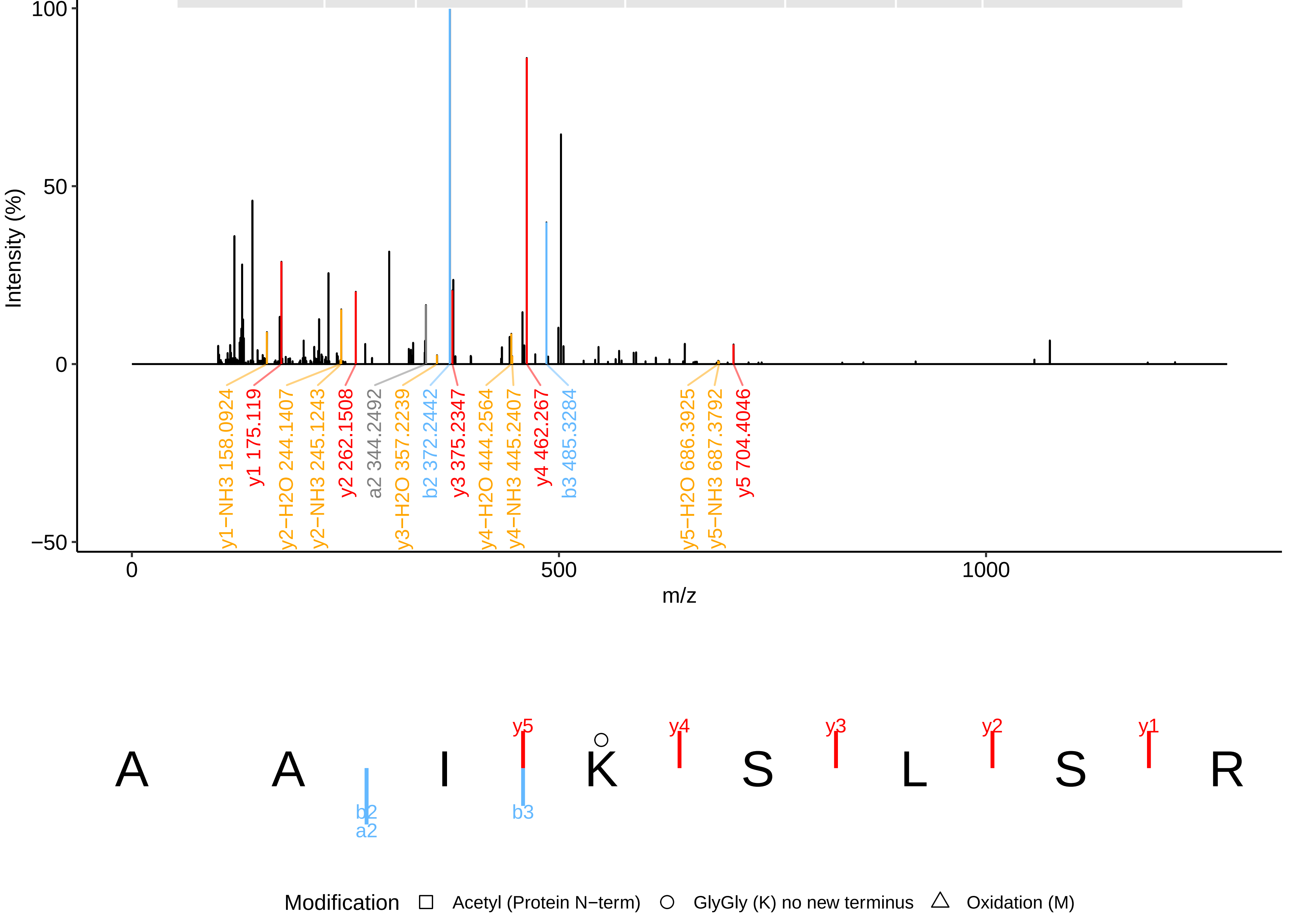

20200924_KIZ_MUb5_GlyGly_1 − Scan number: 5836

Gene Names	Charge	m/z	Mass	Retention time	PEP	Score	Precursor Intensity
PSMD5	3	320.5259	958.556	18.232	0.10782	44.349	36640872

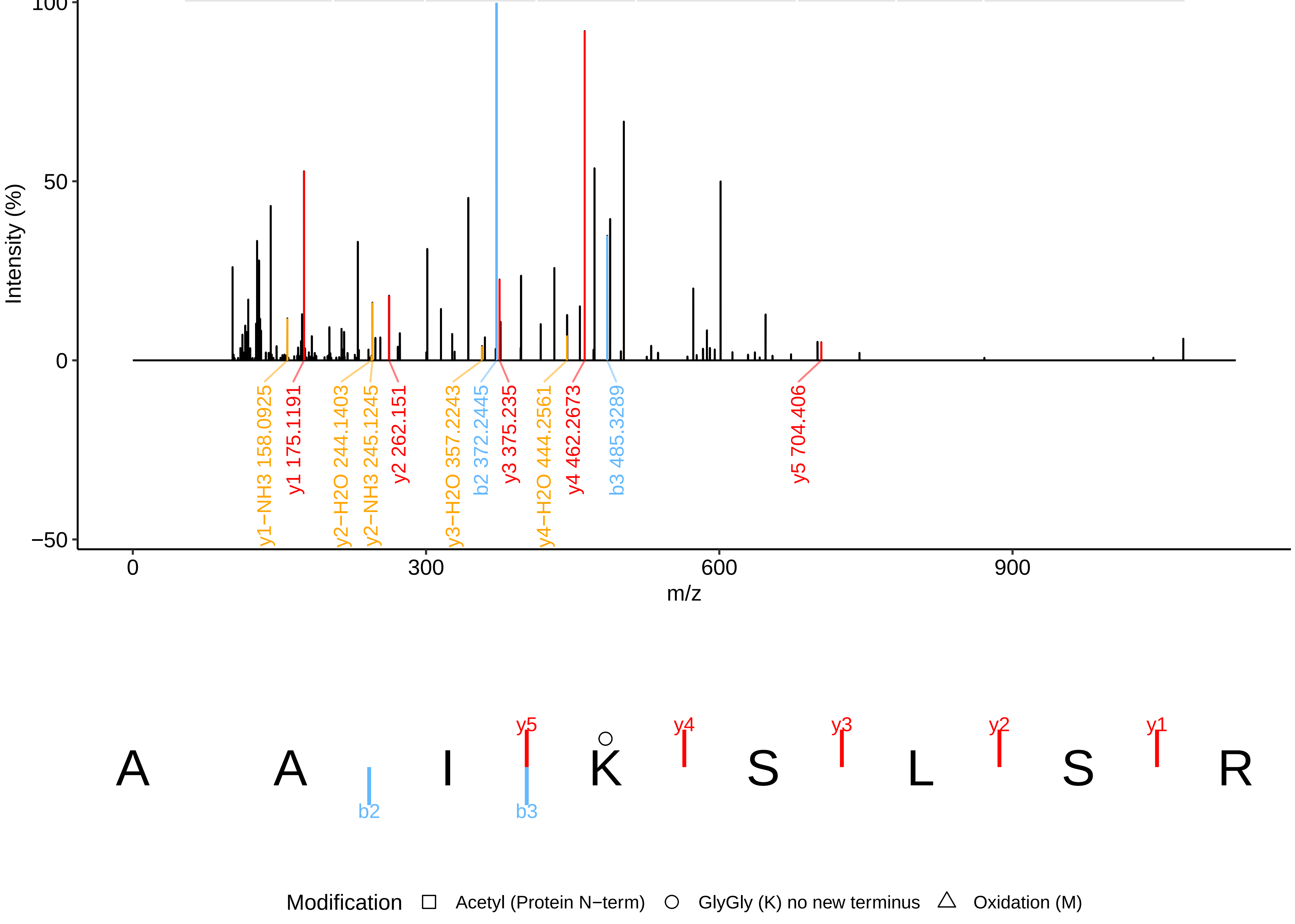

20200924_KIZ_MUb5_GlyGly_2 − Scan number: 5463

Gene Names	Charge	m/z	Mass	Retention time	PEP	Score	Precursor Intensity
PSMD5	3	320.5259	958.556	20.279	0.1025	45.7	1786100

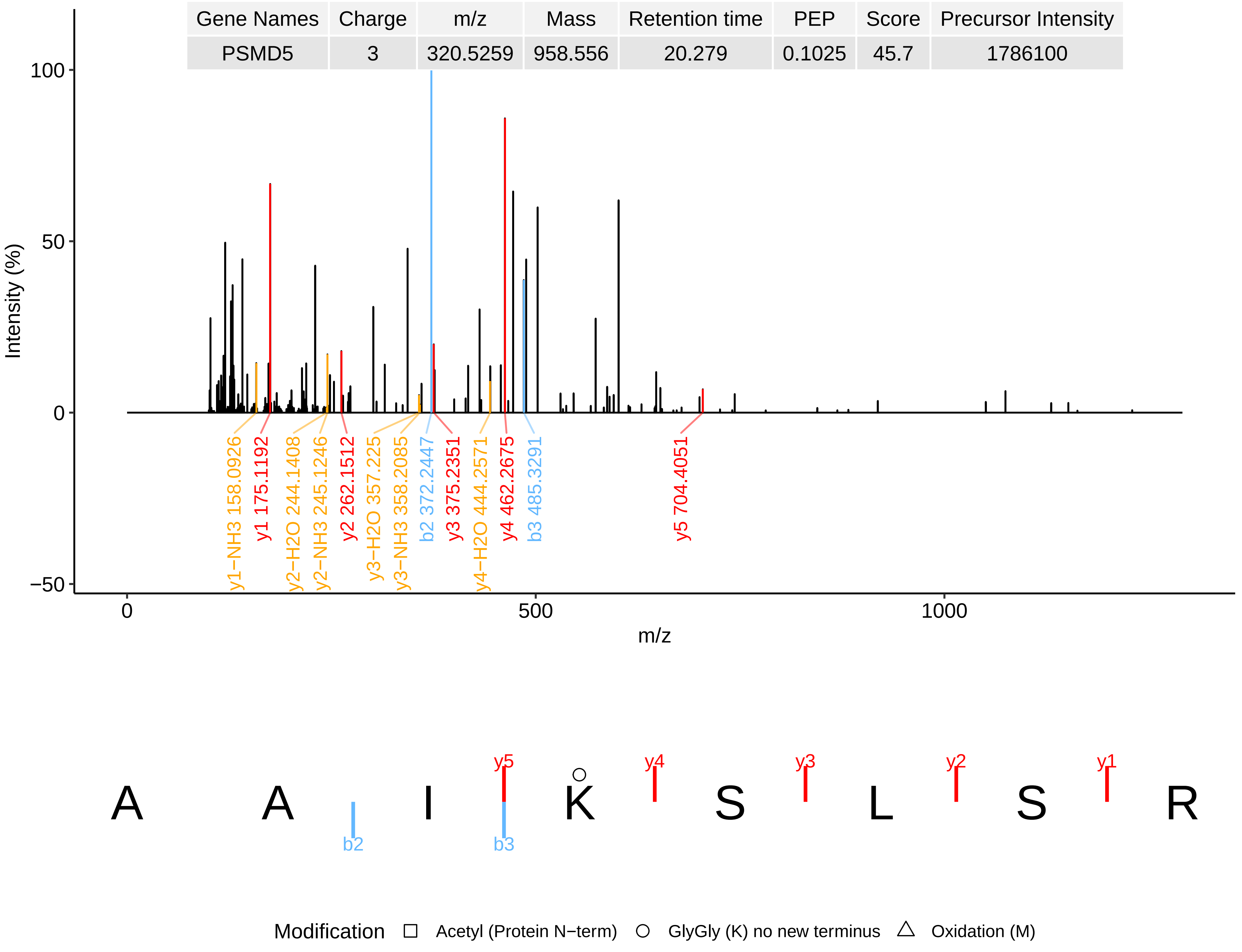

20200924_KIZ_MUb5_GlyGly_2 − Scan number: 5544

Gene Names	Charge	m/z	Mass	Retention time	PEP	Score	Precursor Intensity
PSMD5	3	320.5259	958.556	20.588	0.068571	53.968	3018364

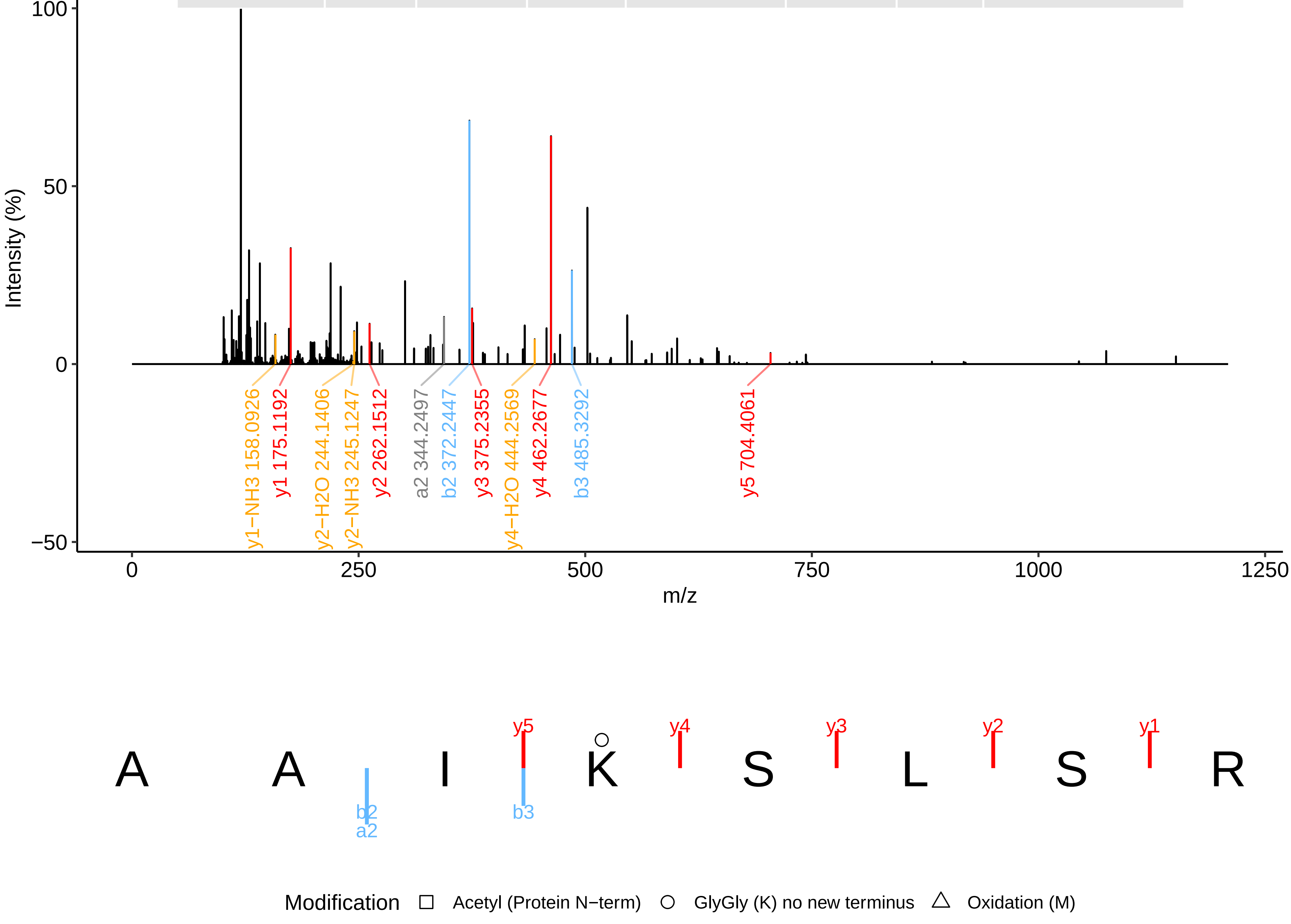

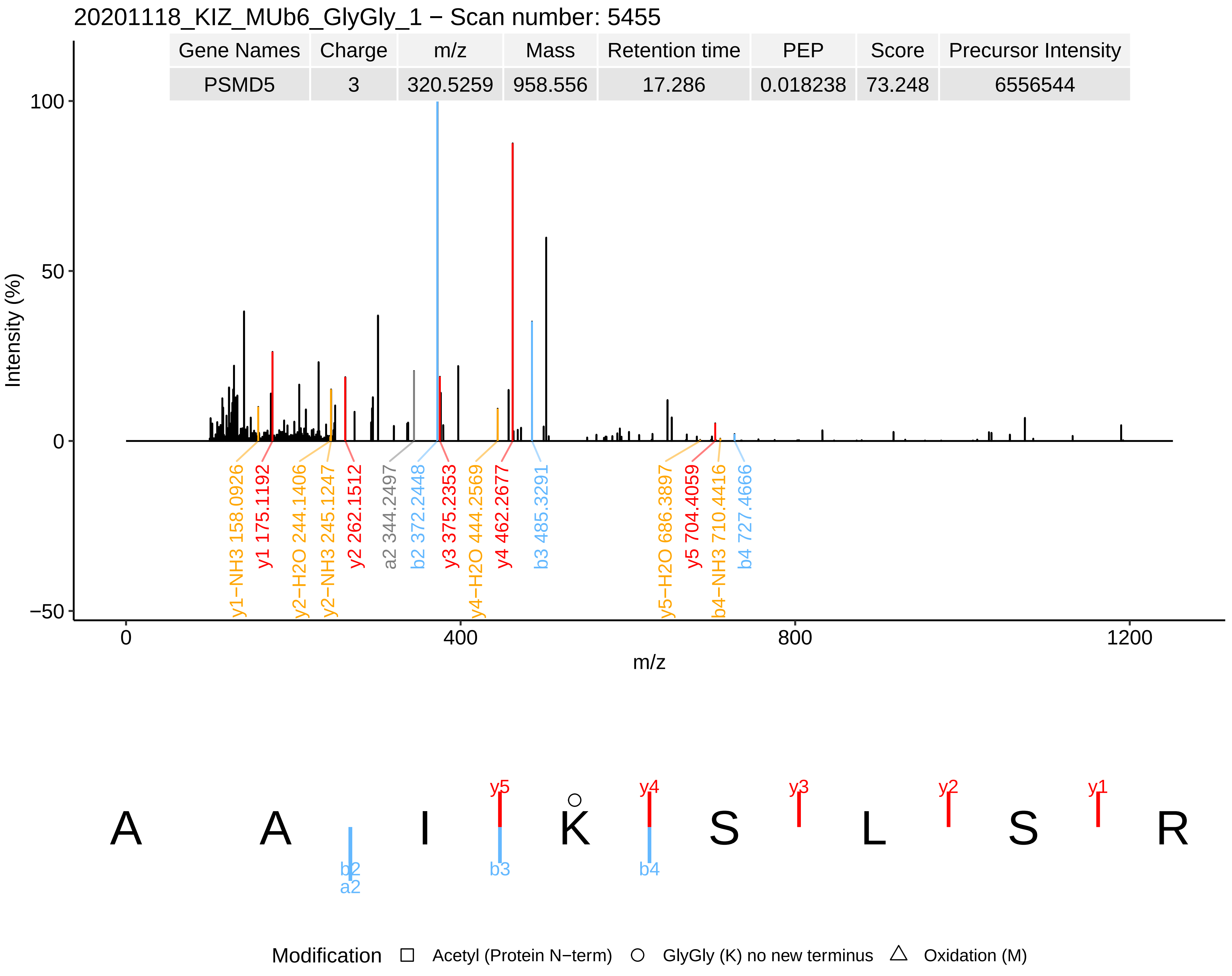

20201118_KIZ_MUb6_GlyGly_1 − Scan number: 5455

Gene Names	Charge	m/z	Mass	Retention time	PEP	Score	Precursor Intensity
PSMD5	3	320.5259	958.556	17.286	0.018238	73.248	6556544

Intensity (%)
m/z
y1−NH3 158.0926
y1 175.1192
y2−H2O 244.1406
y2−NH3 245.1247
y2 262.1512
a2 344.2497
b2 372.2448
y3 375.2353
y4−H2O 444.2569
y4 462.2677
b3 485.3291
y5−H2O 686.3897
y5 704.4059
b4−NH3 710.4416
b4 727.4666
A A I K S L S R
y5 y4 y3 y2 y1
b2 a2 b3 b4
Modification
Acetyl (Protein N−term)
GlyGly (K) no new terminus
Oxidation (M)

20201118_KIZ_MUb6_GlyGly_2 − Scan number: 7124

Gene Names	Charge	m/z	Mass	Retention time	PEP	Score	Precursor Intensity
PSMD5	3	320.5259	958.556	24.322	0.033251	62.573	7164212

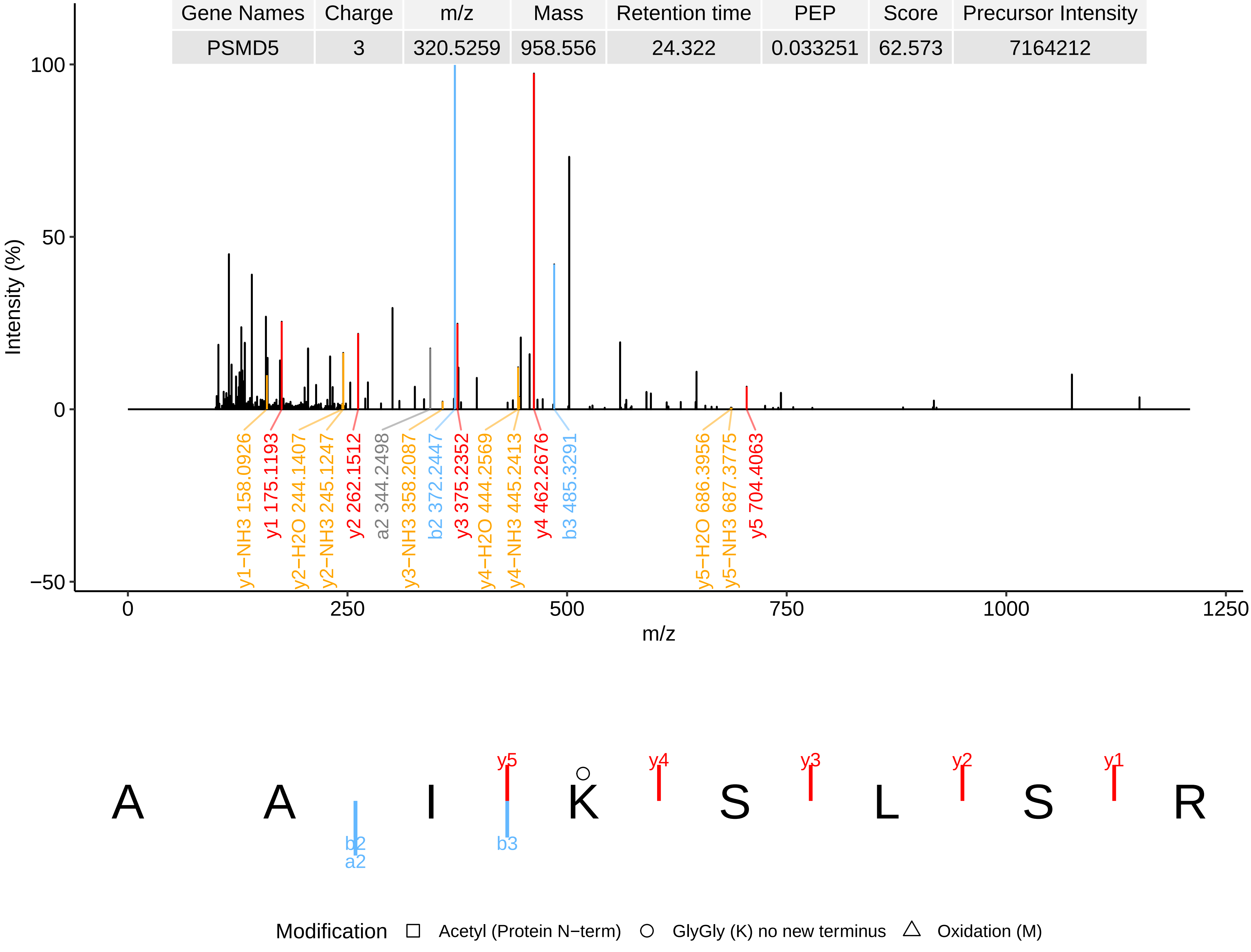

20200921_KIZ_MUb4_GlyGly_10plex − Scan number: 21706

Gene Names	Charge	m/z	Mass	Retention time	PEP	Score	Precursor Intensity
PGAM1;PGAM4	2	601.8664	1201.718	78.652	4.2485e−05	100.74	80800880

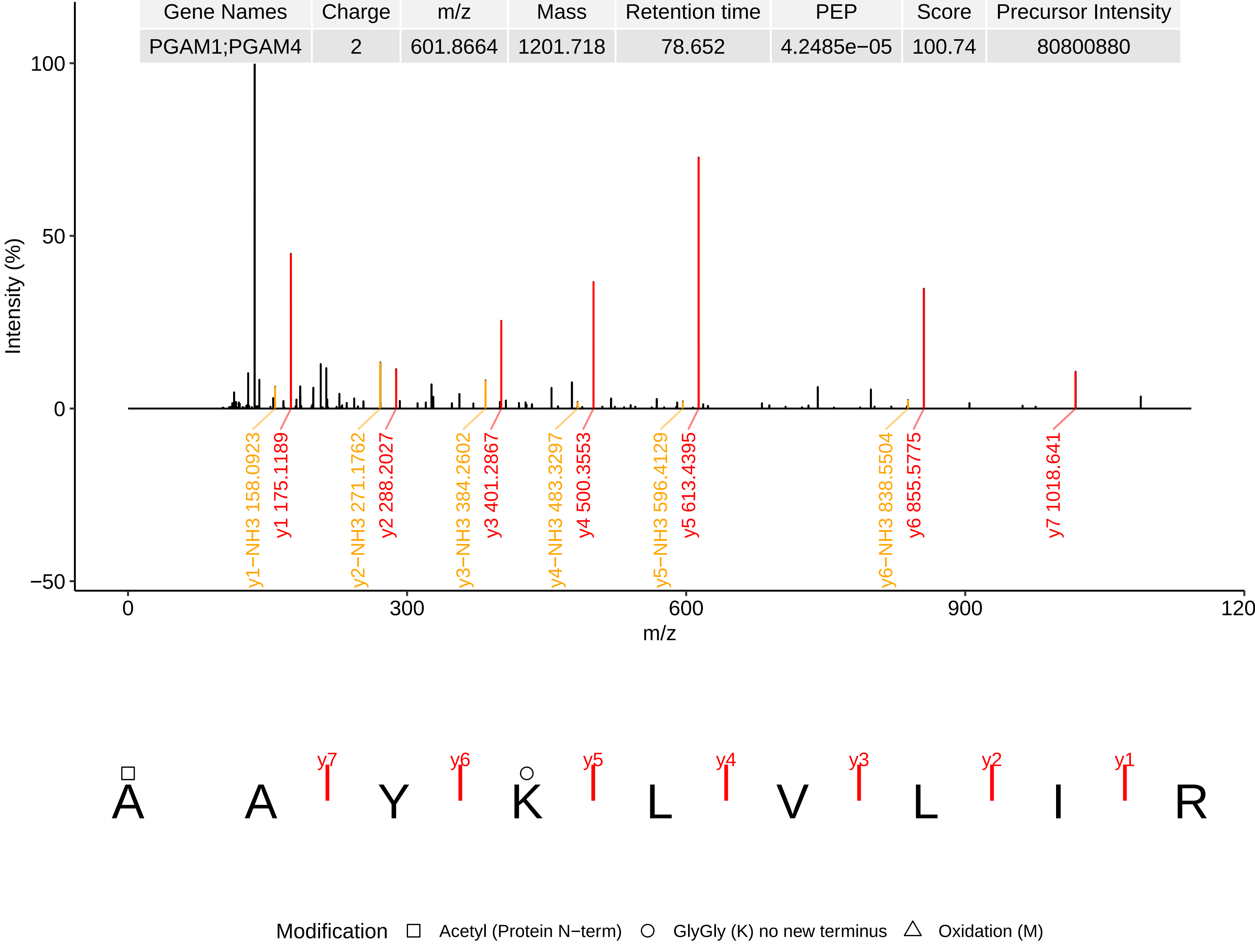

20200921_KIZ_MUb4_GlyGly_10plex − Scan number: 21850

Gene Names	Charge	m/z	Mass	Retention time	PEP	Score	Precursor Intensity
PGAM1;PGAM4	2	601.8664	1201.718	79.199	0.0050827	94.262	1938859

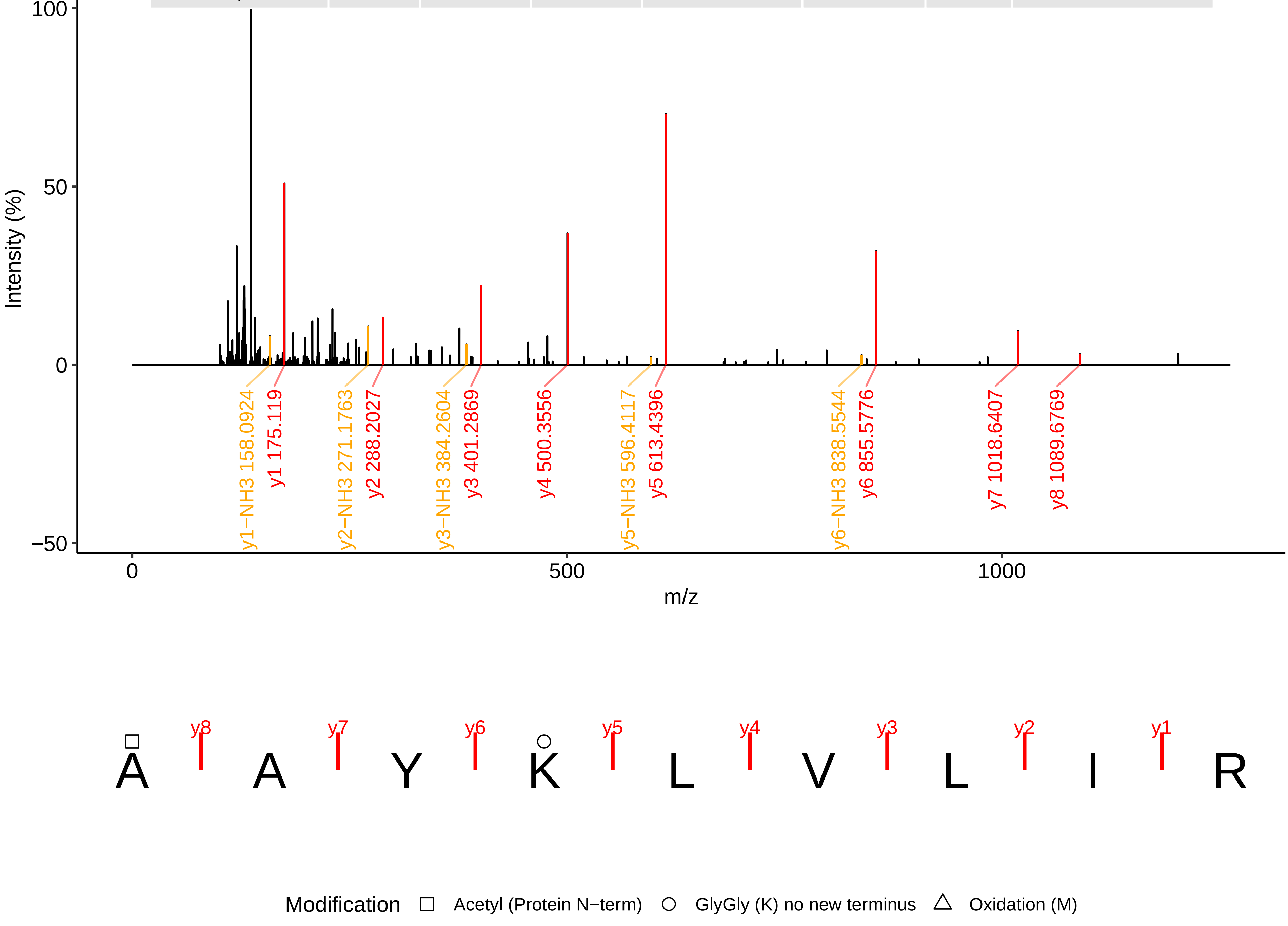

20200921_KIZ_MUb4_GlyGly_10plex_2 − Scan number: 20014

Gene Names	Charge	m/z	Mass	Retention time	PEP	Score	Precursor Intensity
PGAM1;PGAM4	2	601.8664	1201.718	78.622	0.00024091	105.39	1698877

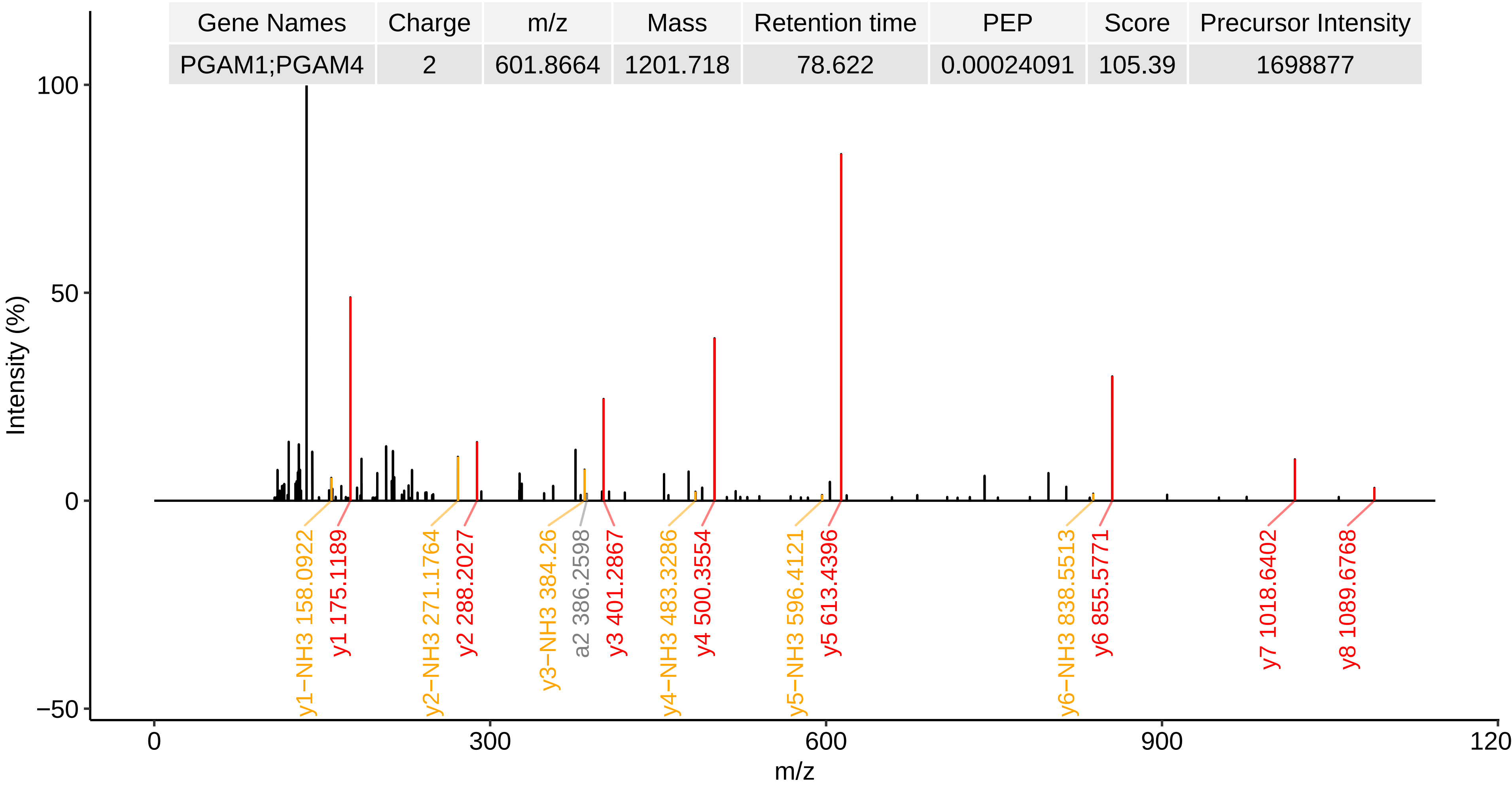

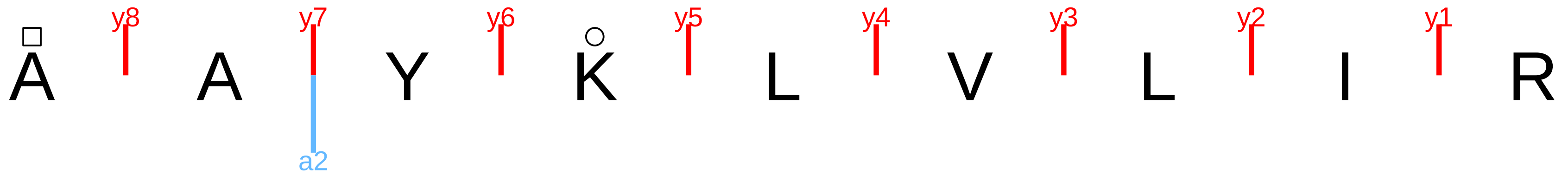

Modification □ Acetyl (Protein N−term) ○ GlyGly (K) no new terminus △ Oxidation (M)

20200924_KIZ_MUb5_GlyGly_1 − Scan number: 21878

Gene Names	Charge	m/z	Mass	Retention time	PEP	Score	Precursor Intensity
PGAM1;PGAM4	2	601.8664	1201.718	70.452	0.013094	66.682	5238437

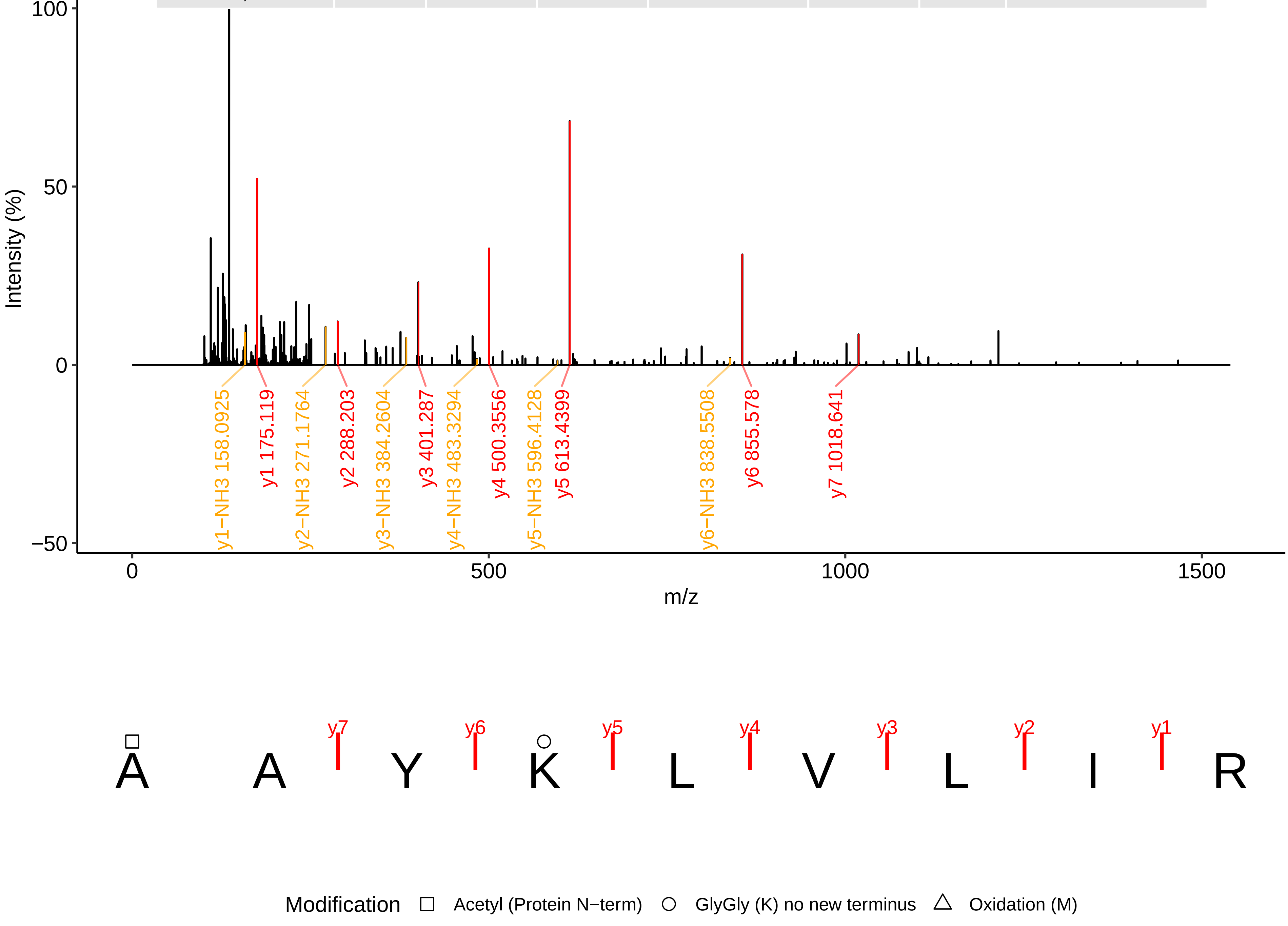

20200924_KIZ_MUb5_GlyGly_1 − Scan number: 22037

Gene Names	Charge	m/z	Mass	Retention time	PEP	Score	Precursor Intensity
PGAM1;PGAM4	2	601.8664	1201.718	70.952	1.9914e−07	121.29	115330608

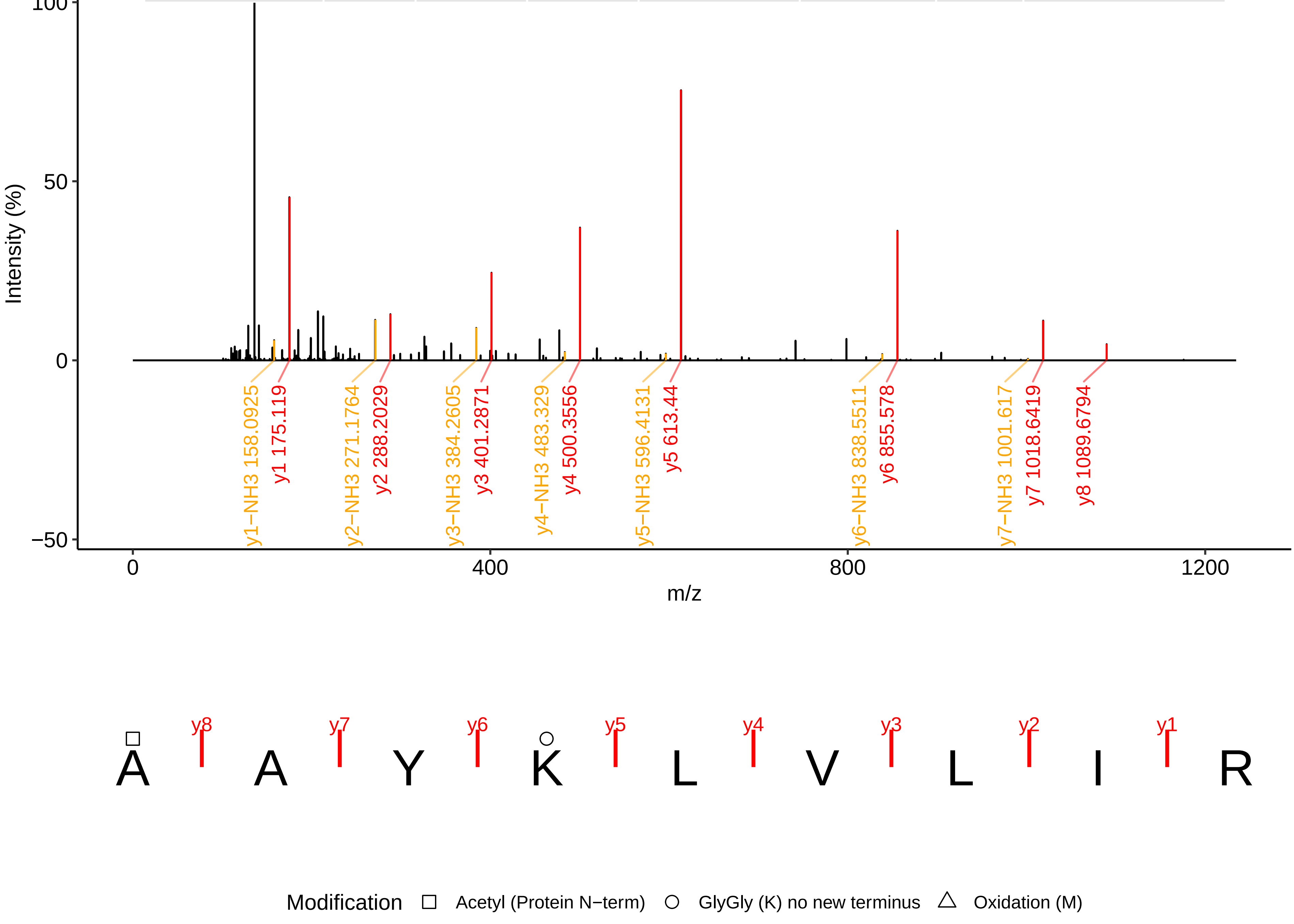

20200924_KIZ_MUb5_GlyGly_1 − Scan number: 22201

Gene Names	Charge	m/z	Mass	Retention time	PEP	Score	Precursor Intensity
PGAM1;PGAM4	2	601.8664	1201.718	71.453	9.8959e−06	121.29	44034056

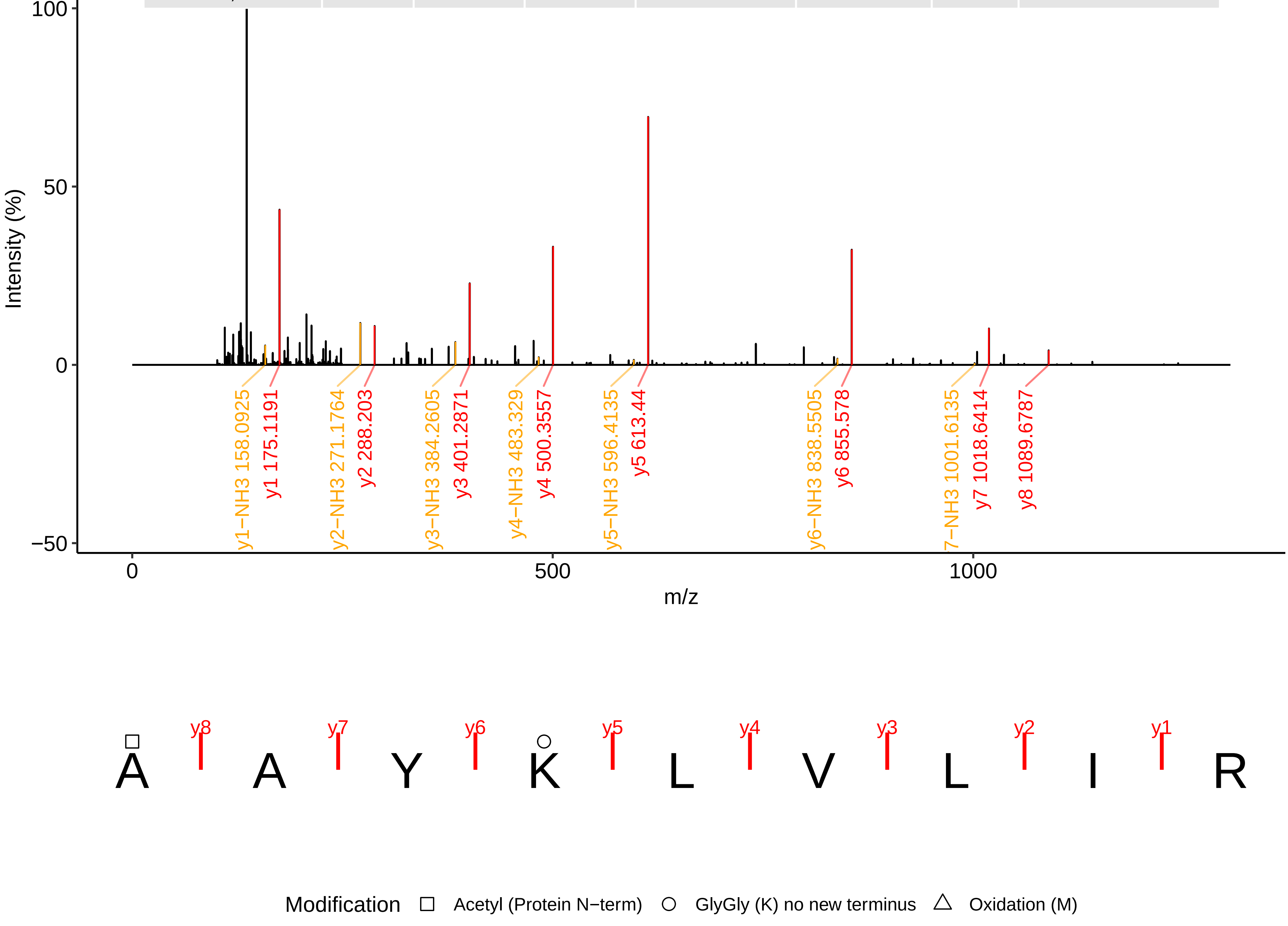

20200924_KIZ_MUb5_GlyGly_2 − Scan number: 19968

Gene Names	Charge	m/z	Mass	Retention time	PEP	Score	Precursor Intensity
PGAM1;PGAM4	2	601.8664	1201.718	74.01	1.172e−07	119.89	70114008

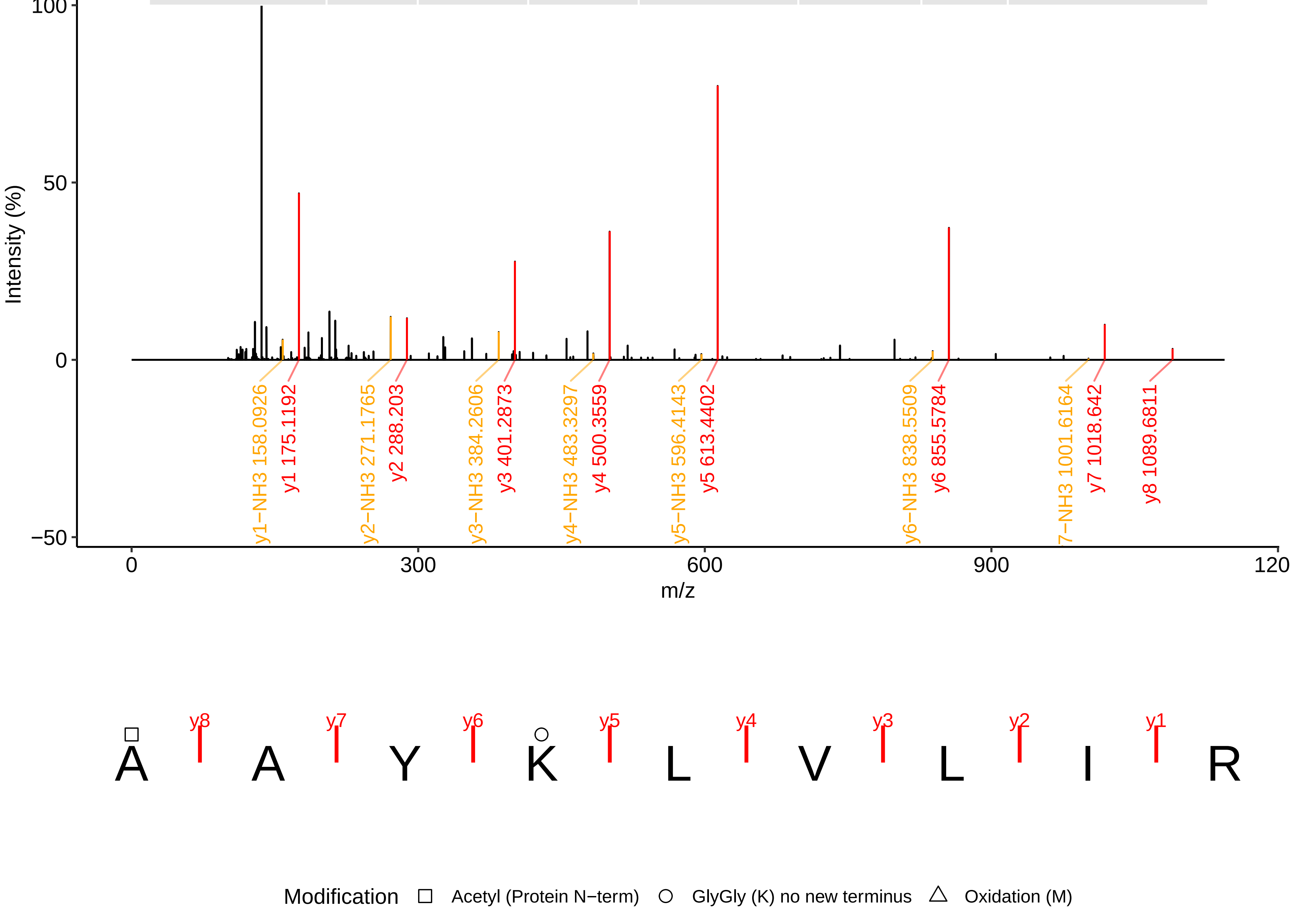

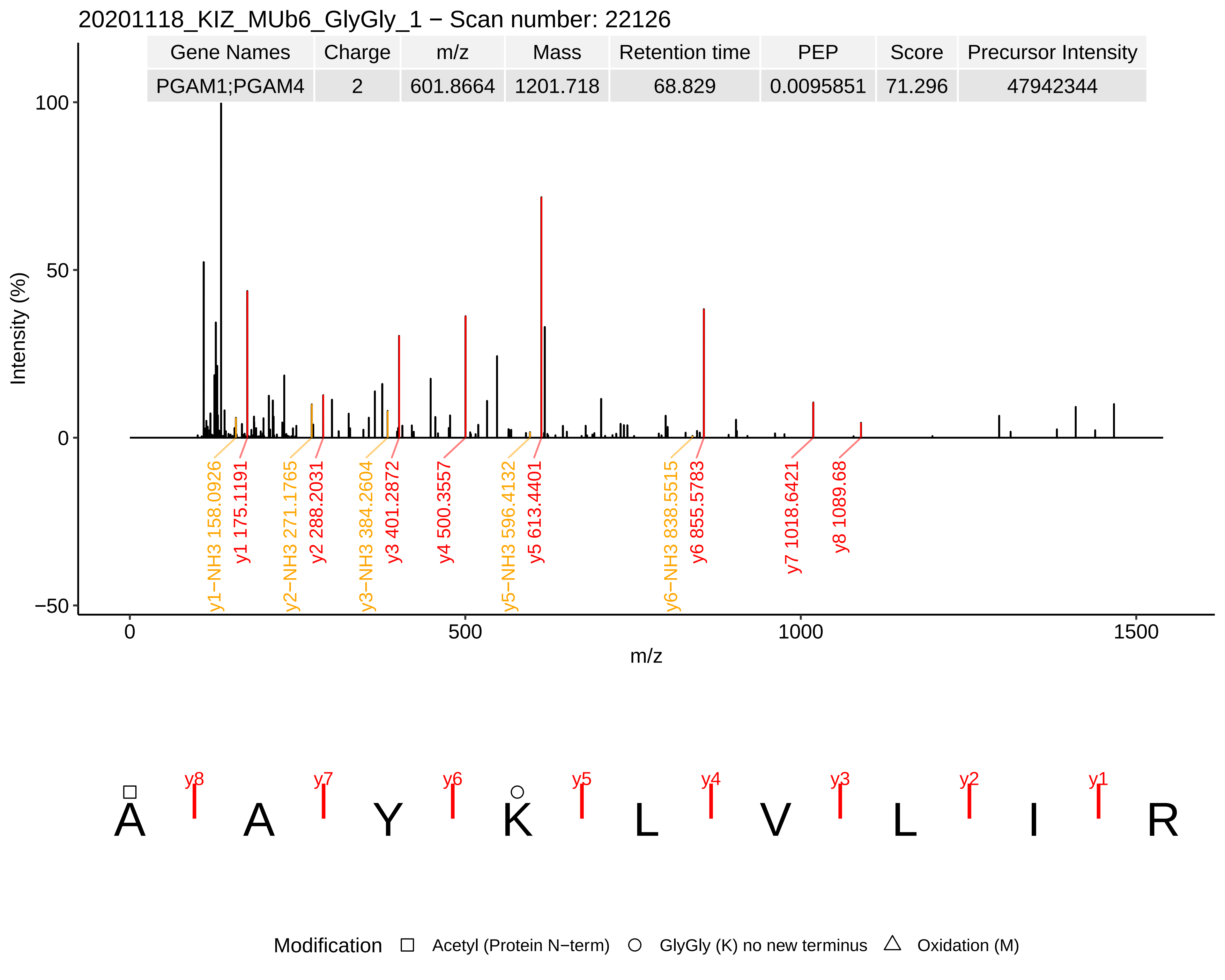
20201118_KIZ_MUb6_GlyGly_1 − Scan number: 22126
Gene Names | Charge | m/z | Mass | Retention time | PEP | Score | Precursor Intensity
PGAM1;PGAM4 | 2 | 601.8664 | 1201.718 | 68.829 | 0.0095851 | 71.296 | 47942344
Intensity (%)
100
50
0
−50
0
500
1000
1500
m/z
y1−NH3 158.0926
y1 175.1191
y2−NH3 271.1765
y2 288.2031
y3−NH3 384.2604
y3 401.2872
y4 500.3557
y5−NH3 596.4132
y5 613.4401
y6−NH3 838.5515
y6 855.5783
y7 1018.6421
y8 1089.68
A y8 A y7 Y y6 K y5 L y4 V y3 L y2 I y1 R
Modification □ Acetyl (Protein N−term) ○ GlyGly (K) no new terminus △ Oxidation (M)

20201118_KIZ_MUb6_GlyGly_2 − Scan number: 23728

Gene Names	Charge	m/z	Mass	Retention time	PEP	Score	Precursor Intensity
PGAM1;PGAM4	2	601.8664	1201.718	79.557	0.0002178	92.925	7383020

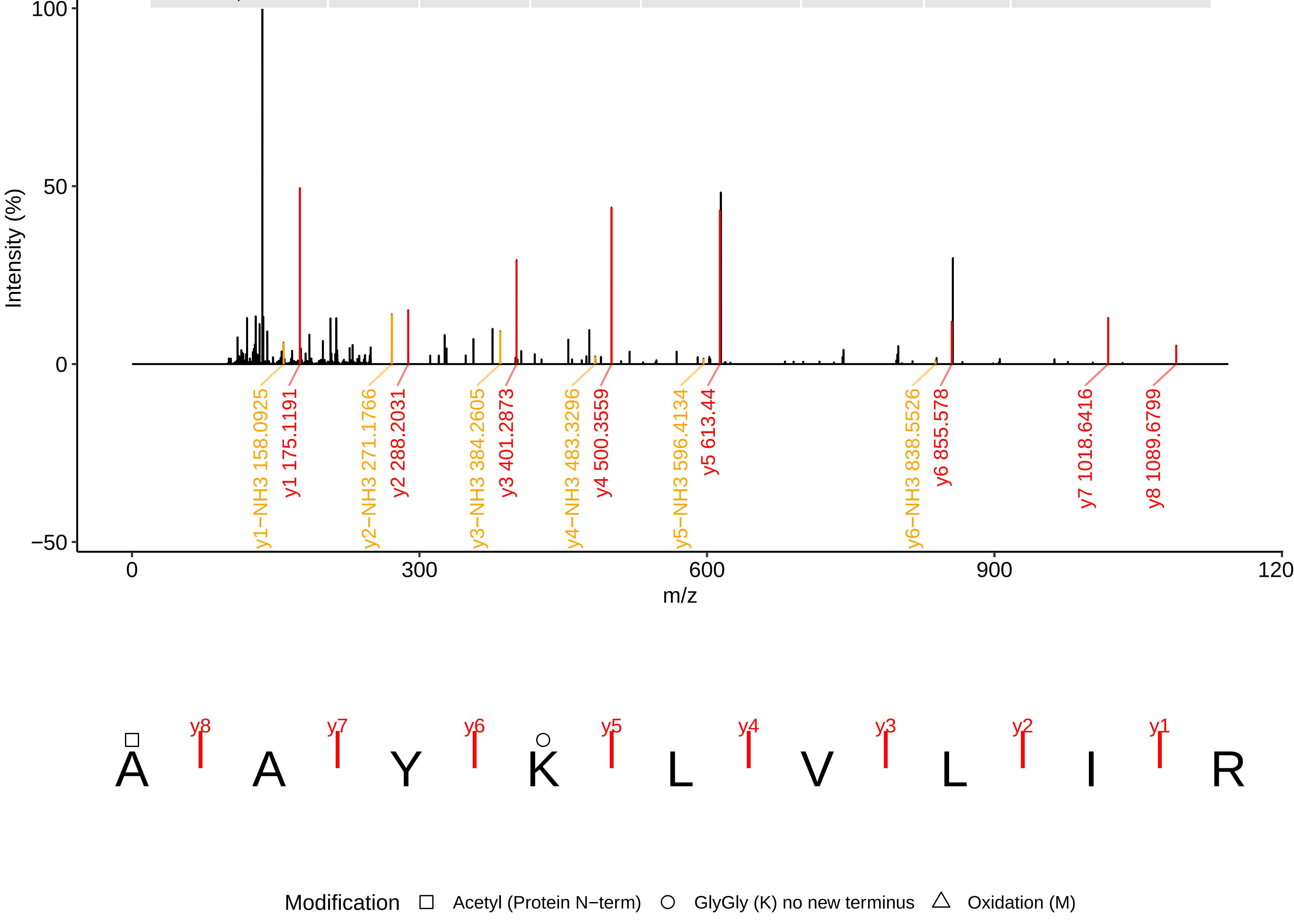

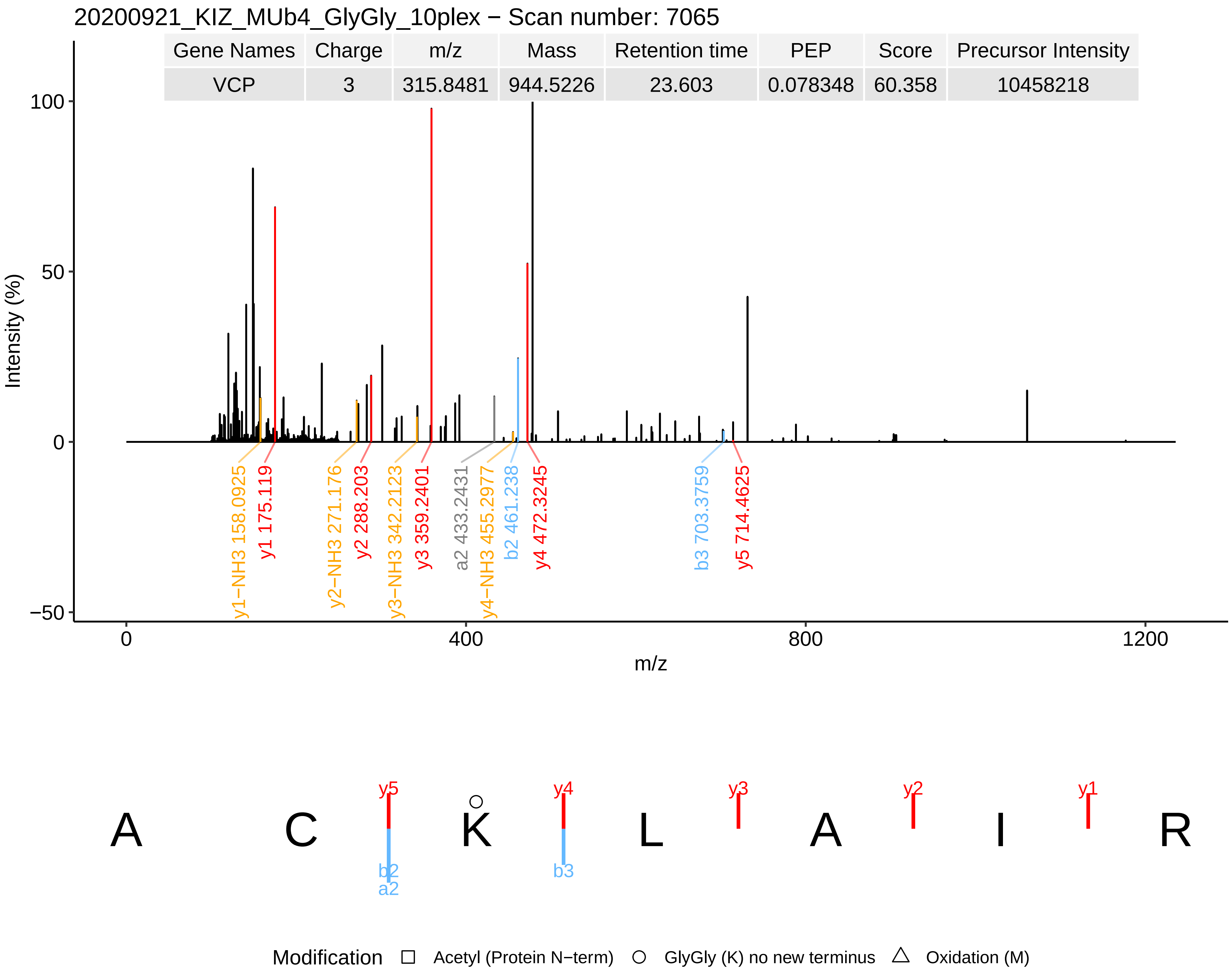
20200921_KIZ_MUb4_GlyGly_10plex − Scan number: 7065
Gene Names | Charge | m/z | Mass | Retention time | PEP | Score | Precursor Intensity
VCP | 3 | 315.8481 | 944.5226 | 23.603 | 0.078348 | 60.358 | 10458218
Intensity (%)
100
50
0
−50
0
400
800
1200
m/z
y1−NH3 158.0925
y1 175.119
y2−NH3 271.176
y2 288.203
y3−NH3 342.2123
y3 359.2401
a2 433.2431
y4−NH3 455.2977
b2 461.238
y4 472.3245
b3 703.3759
y5 714.4625
A C K L A I R
y5
y4
y3
y2
y1
b2
a2
b3
Modification
Acetyl (Protein N−term)
GlyGly (K) no new terminus
Oxidation (M)

20201118_KIZ_MUb6_GlyGly_1 − Scan number: 5624

Gene Names	Charge	m/z	Mass	Retention time	PEP	Score	Precursor Intensity
VCP	3	315.8481	944.5226	17.818	0.042552	72.355	11550014

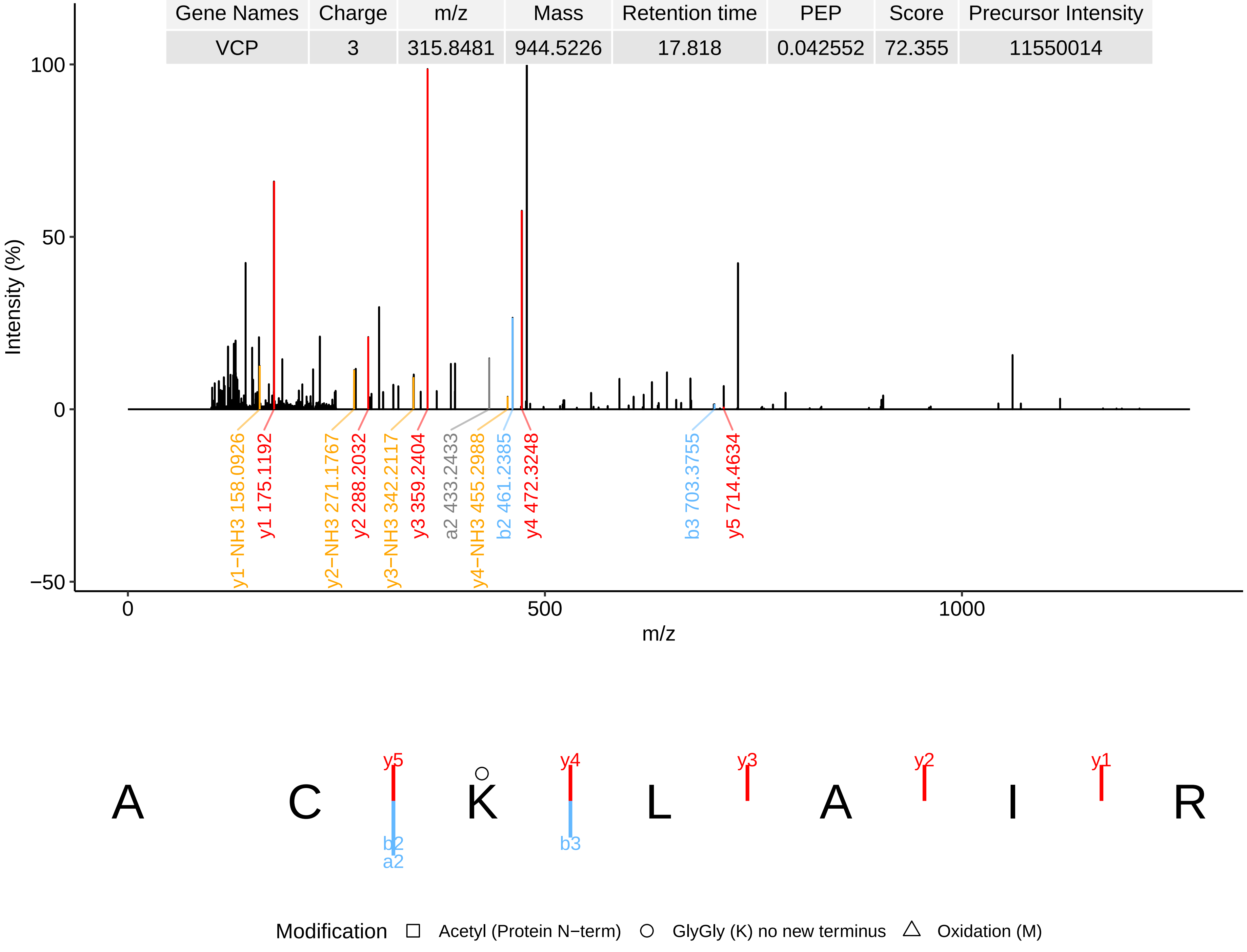

20201118_KIZ_MUb6_GlyGly_2 − Scan number: 7477

Gene Names	Charge	m/z	Mass	Retention time	PEP	Score	Precursor Intensity
VCP	3	315.8481	944.5226	25.505	0.036689	74.717	8776514

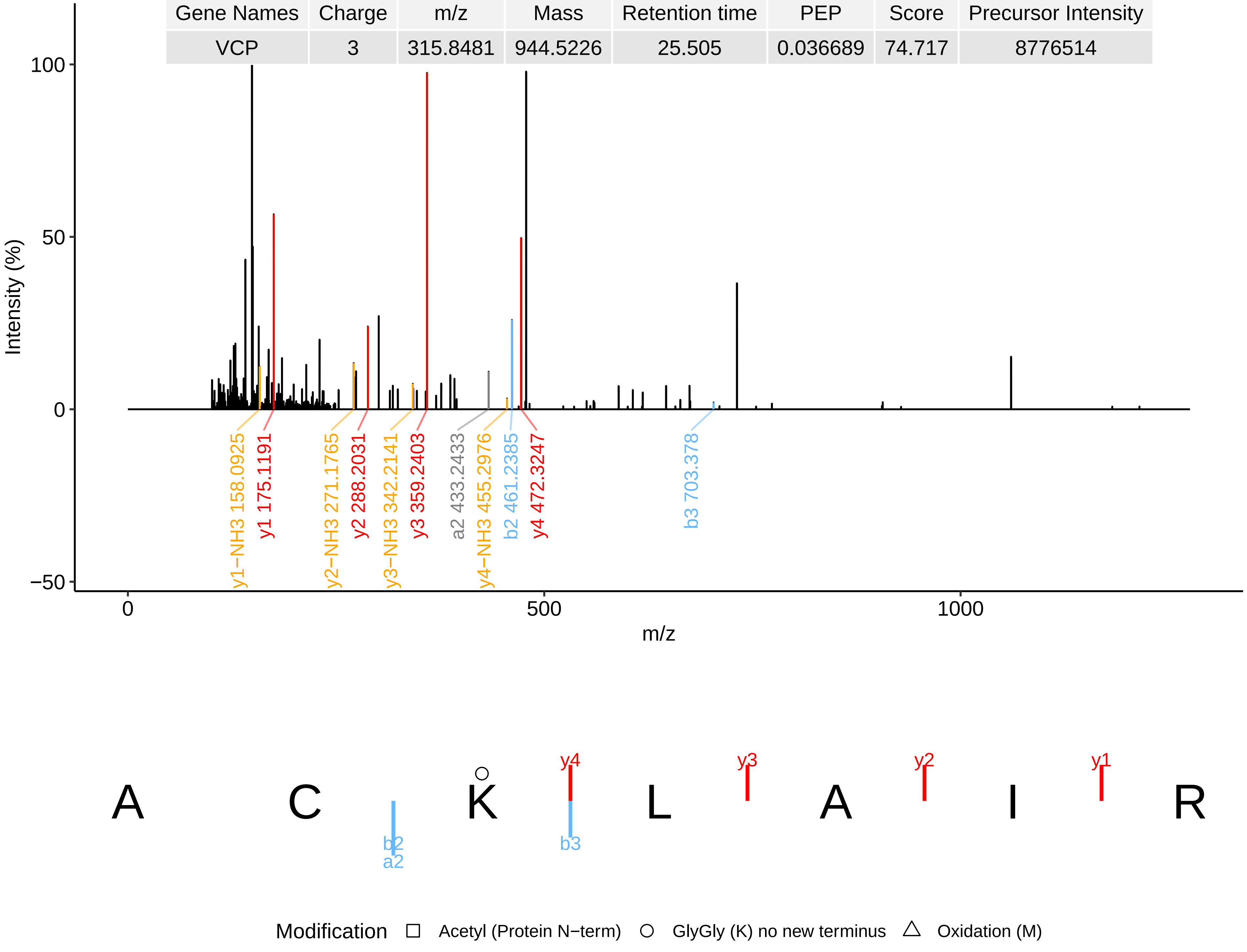

20200921_KIZ_MUb4_GlyGly_10plex − Scan number: 9187

Gene Names	Charge	m/z	Mass	Retention time	PEP	Score	Precursor Intensity
MFAP3L;MFAP3	3	316.8469	947.5189	31.432	0.035388	103.02	10905255

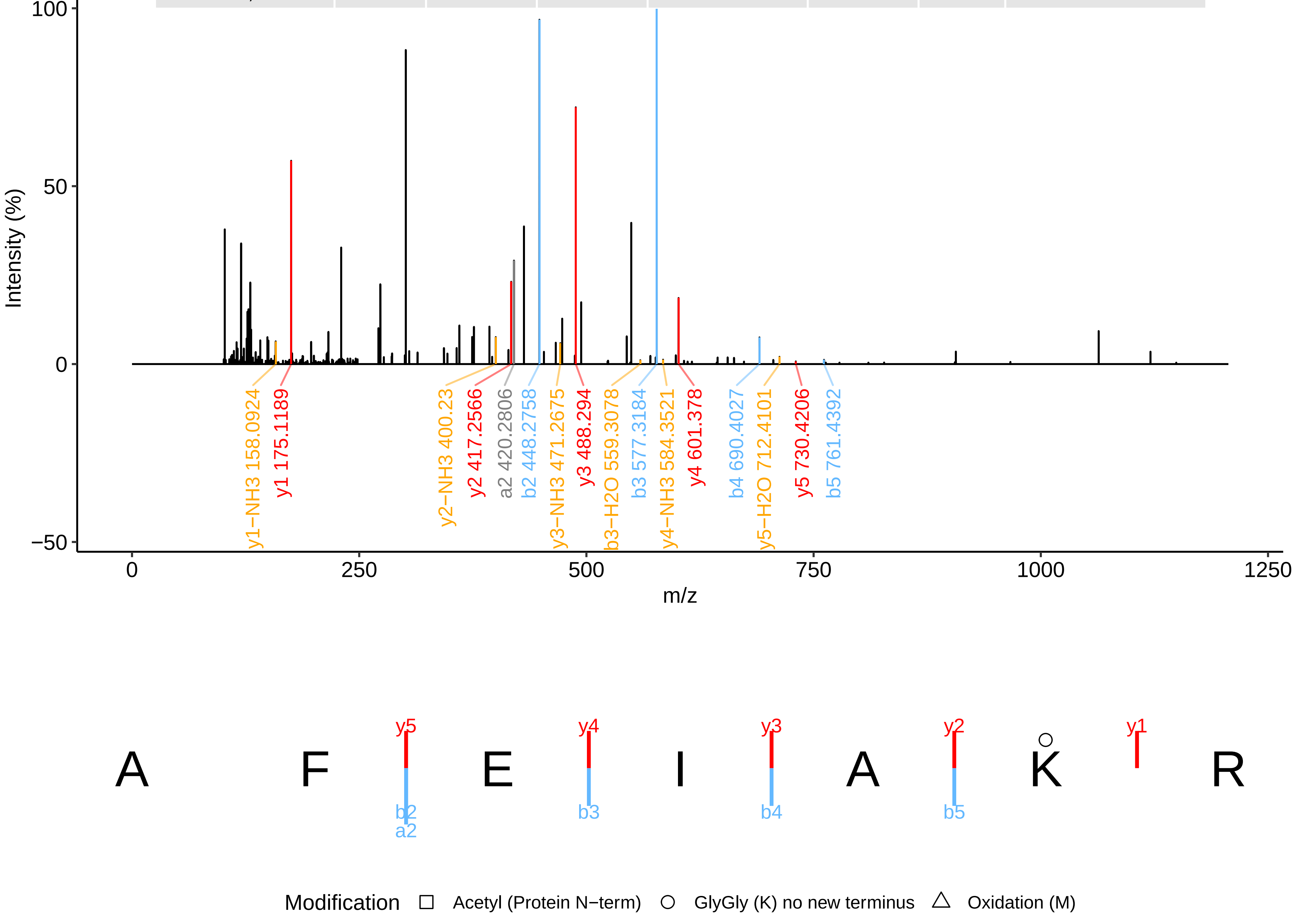

20200924_KIZ_MUb5_GlyGly_2 − Scan number: 13319

Gene Names	Charge	m/z	Mass	Retention time	PEP	Score	Precursor Intensity
COX6C	2	482.2665	962.5185	49.836	0.044425	96.51	12850024

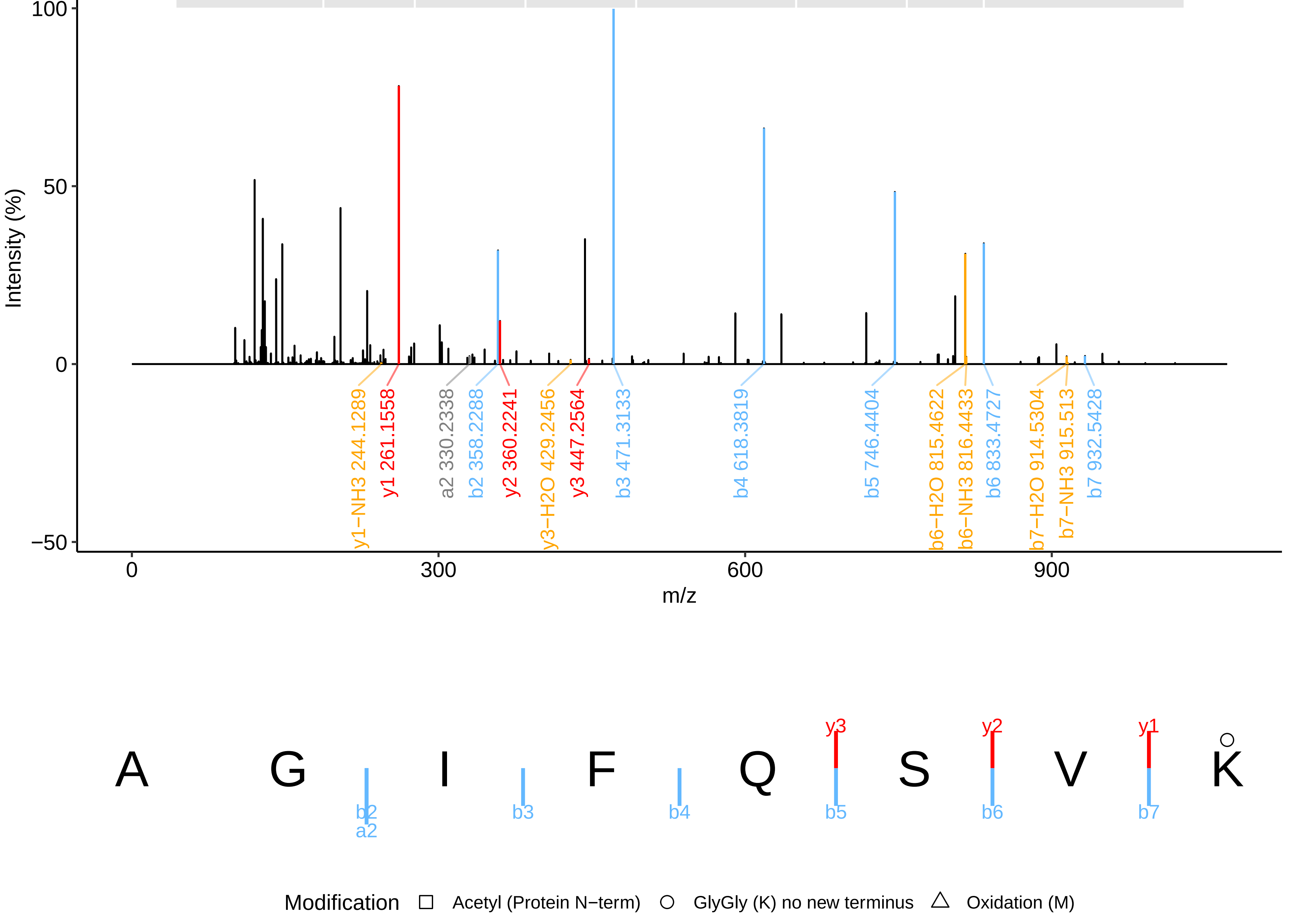

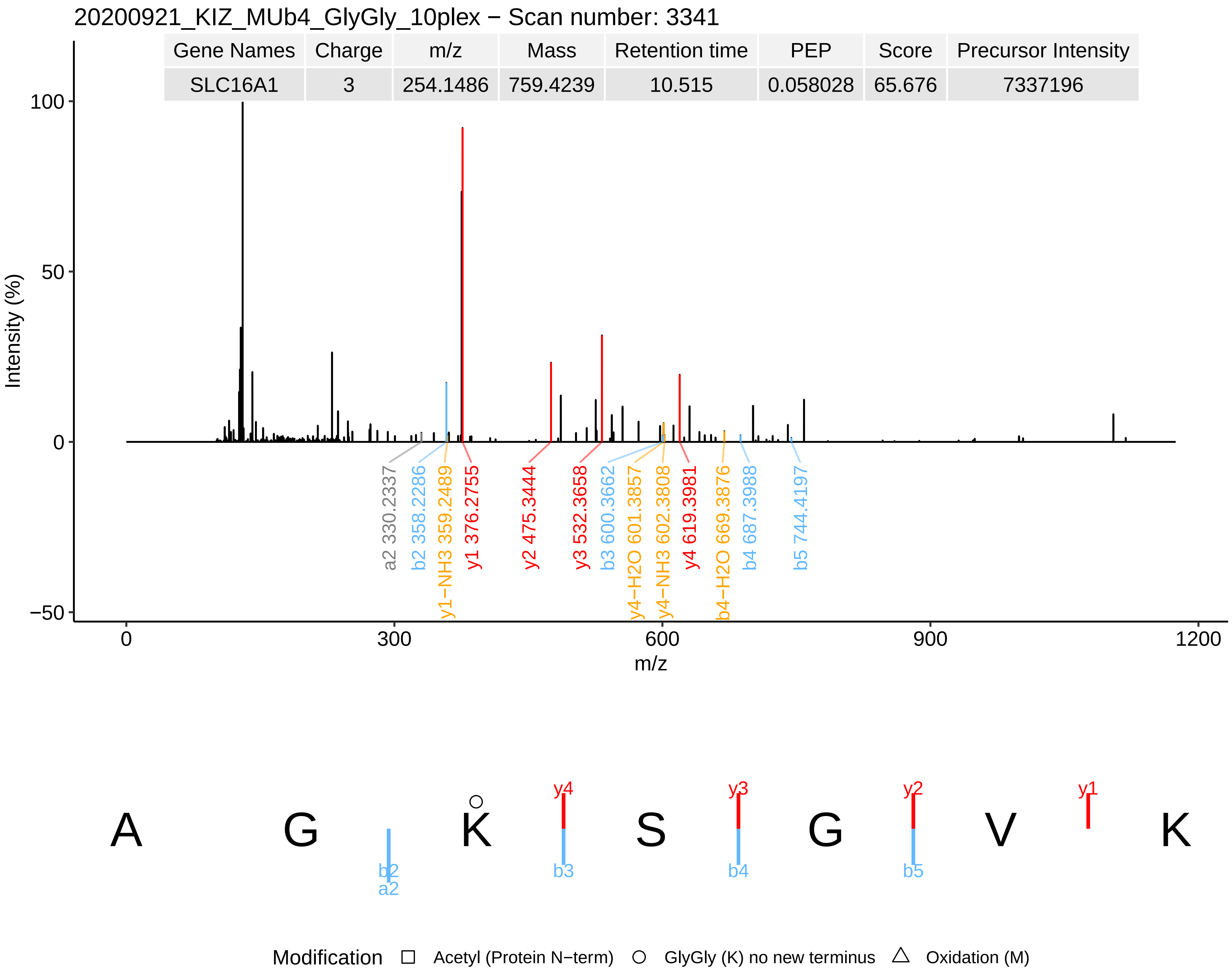

20200921_KIZ_MUb4_GlyGly_10plex − Scan number: 3341
Gene Names | Charge | m/z | Mass | Retention time | PEP | Score | Precursor Intensity
SLC16A1 | 3 | 254.1486 | 759.4239 | 10.515 | 0.058028 | 65.676 | 7337196
Intensity (%)
100
50
0
−50
a2 330.2337
b2 358.2286
y1−NH3 359.2489
y1 376.2755
y2 475.3444
y3 532.3658
b3 600.3662
y4−H2O 601.3857
y4−NH3 602.3808
y4 619.3981
b4−H2O 669.3876
b4 687.3988
b5 744.4197
0
300
600
900
1200
m/z
A G K S G V K
y4 y3 y2 y1
b2 a2 b3 b4 b5
Modification
Acetyl (Protein N−term)
GlyGly (K) no new terminus
Oxidation (M)

20200921_KIZ_MUb4_GlyGly_10plex_2 − Scan number: 3224

Gene Names	Charge	m/z	Mass	Retention time	PEP	Score	Precursor Intensity
SLC16A1	3	254.1486	759.4239	10.717	0.098548	56.27	2654505

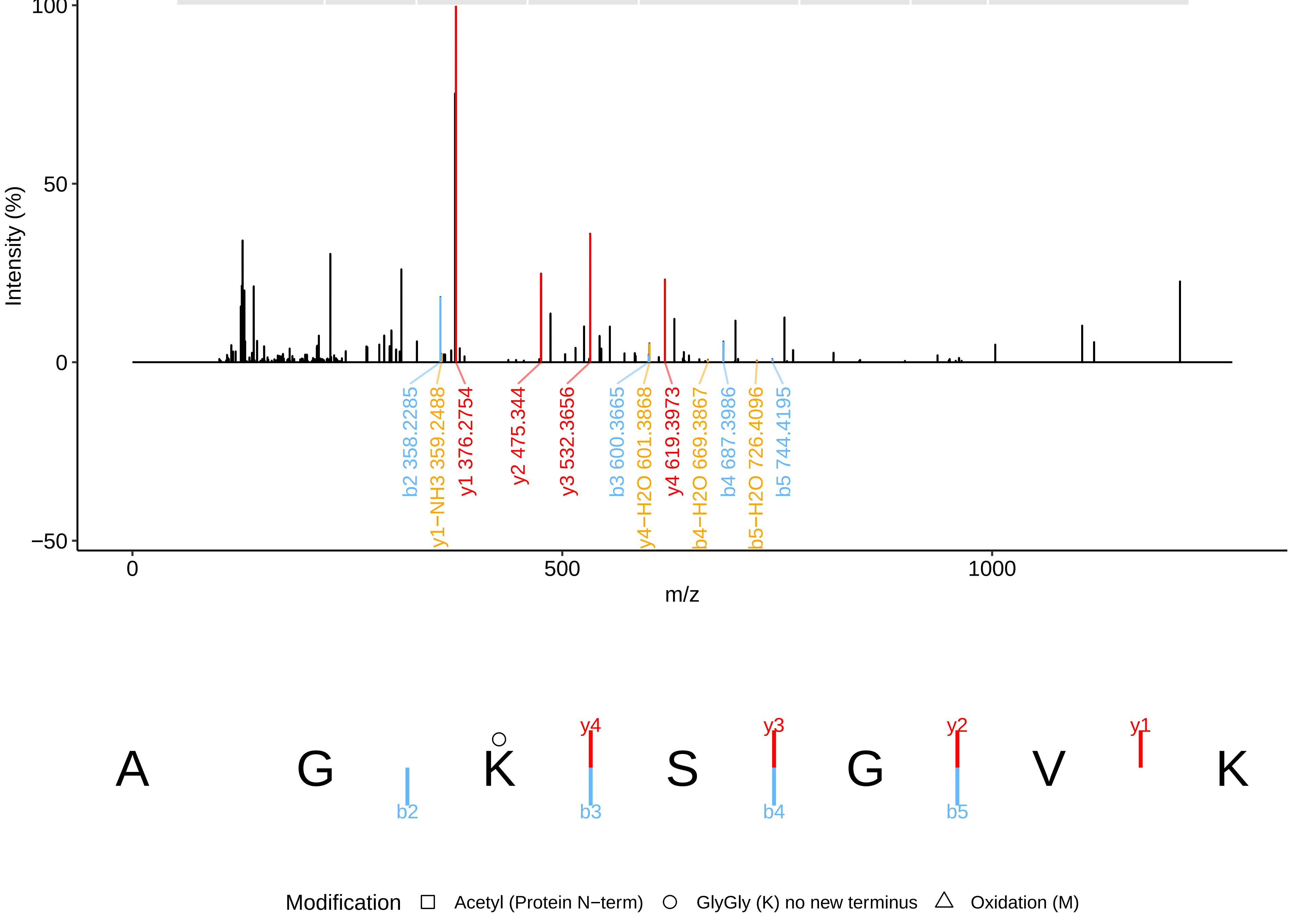

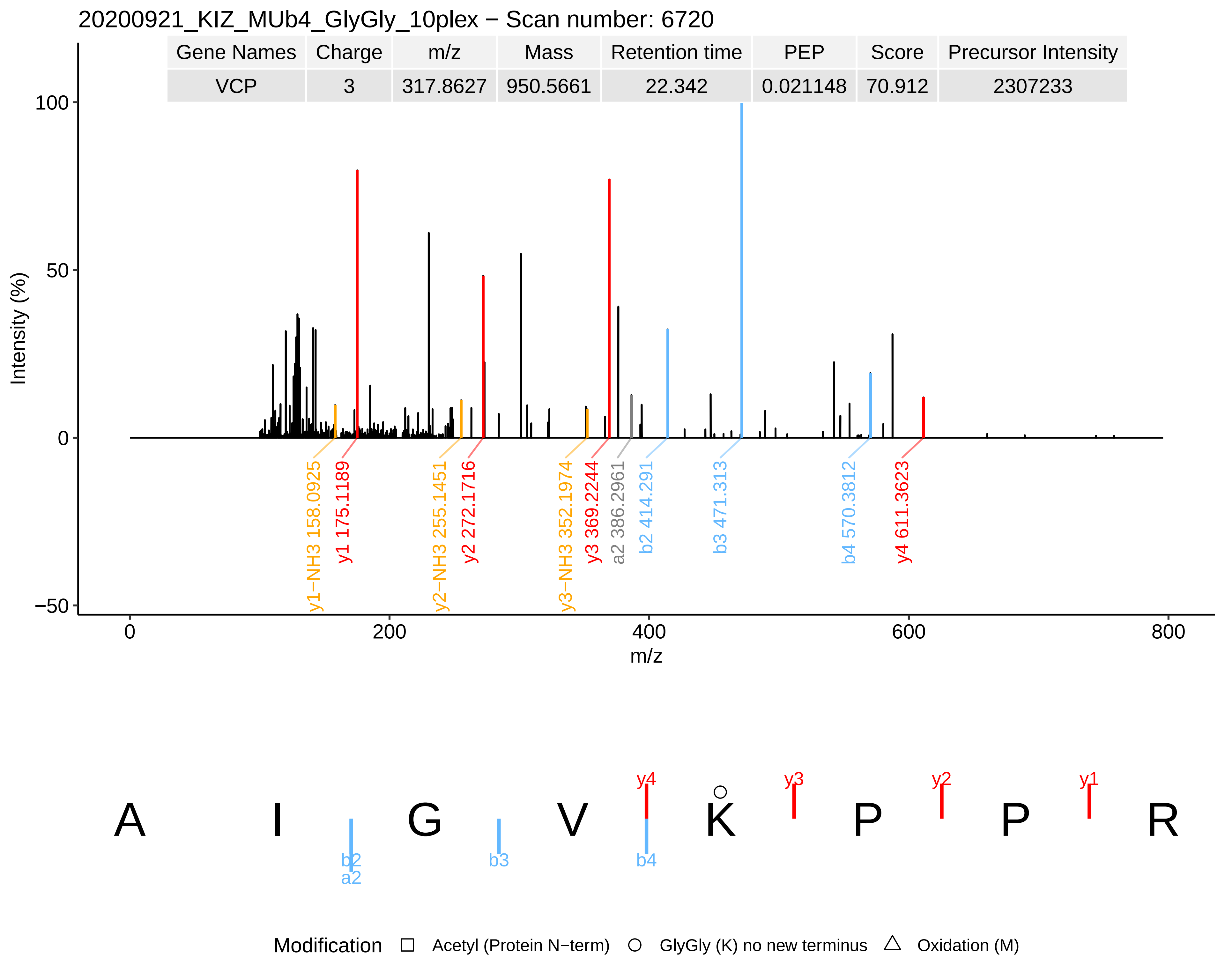

20200921_KIZ_MUb4_GlyGly_10plex − Scan number: 6720
Gene Names | Charge | m/z | Mass | Retention time | PEP | Score | Precursor Intensity
VCP | 3 | 317.8627 | 950.5661 | 22.342 | 0.021148 | 70.912 | 2307233
Intensity (%)
100
50
0
−50
0
200
400
600
800
m/z
y1−NH3 158.0925
y1 175.1189
y2−NH3 255.1451
y2 272.1716
y3−NH3 352.1974
y3 369.2244
a2 386.2961
b2 414.291
b3 471.313
b4 570.3812
y4 611.3623
A I G V K P P R
y4 y3 y2 y1
b2 a2 b3 b4
Modification
Acetyl (Protein N−term)
GlyGly (K) no new terminus
Oxidation (M)

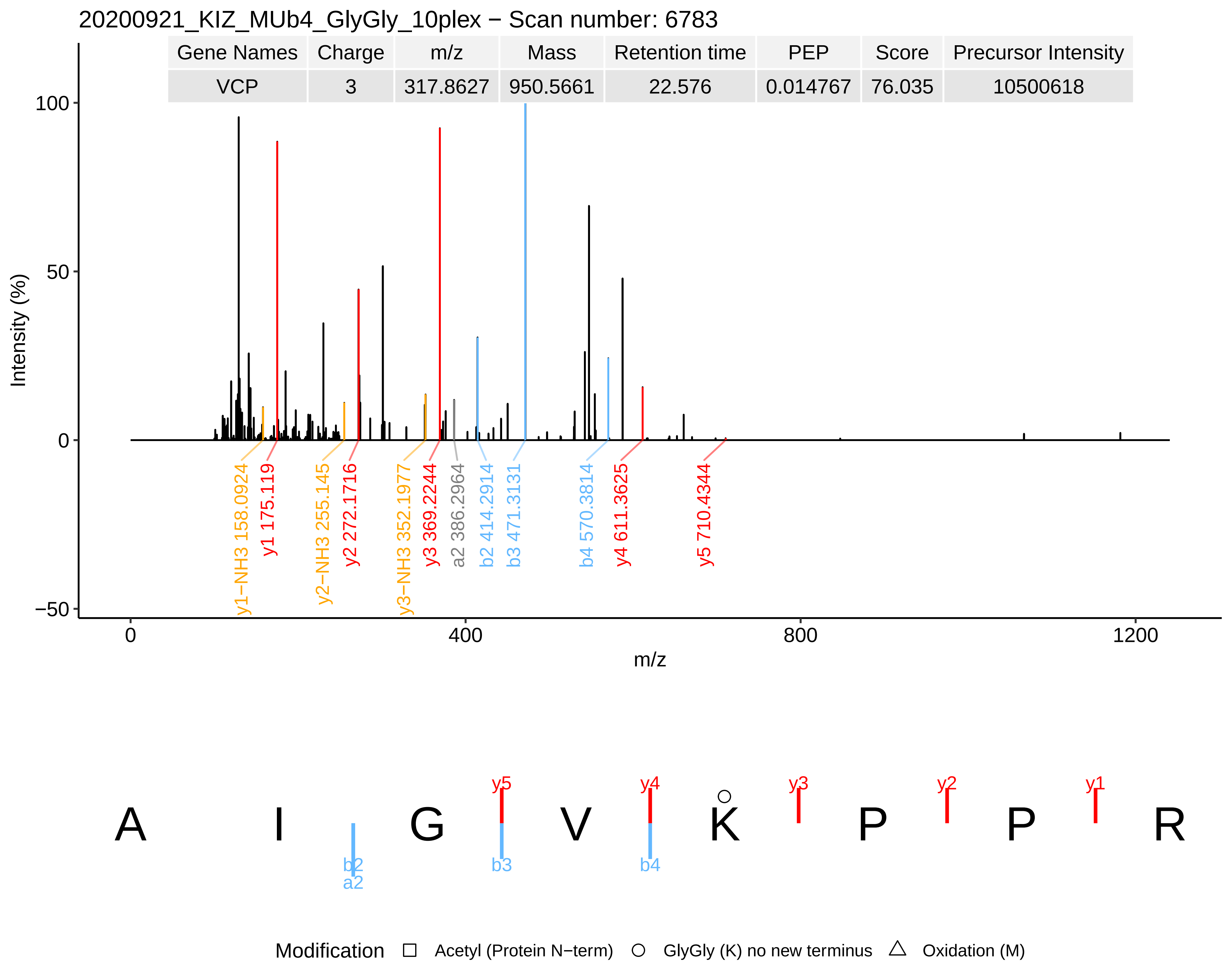

20200921_KIZ_MUb4_GlyGly_10plex − Scan number: 6783
Gene Names | Charge | m/z | Mass | Retention time | PEP | Score | Precursor Intensity
VCP | 3 | 317.8627 | 950.5661 | 22.576 | 0.014767 | 76.035 | 10500618
Intensity (%)
m/z
y1−NH3 158.0924
y1 175.119
y2−NH3 255.145
y2 272.1716
y3−NH3 352.1977
y3 369.2244
a2 386.2964
b2 414.2914
b3 471.3131
b4 570.3814
y4 611.3625
y5 710.4344
A I G V K P P R
y5 y4 y3 y2 y1
b2 a2 b3 b4
Modification
Acetyl (Protein N−term)
GlyGly (K) no new terminus
Oxidation (M)

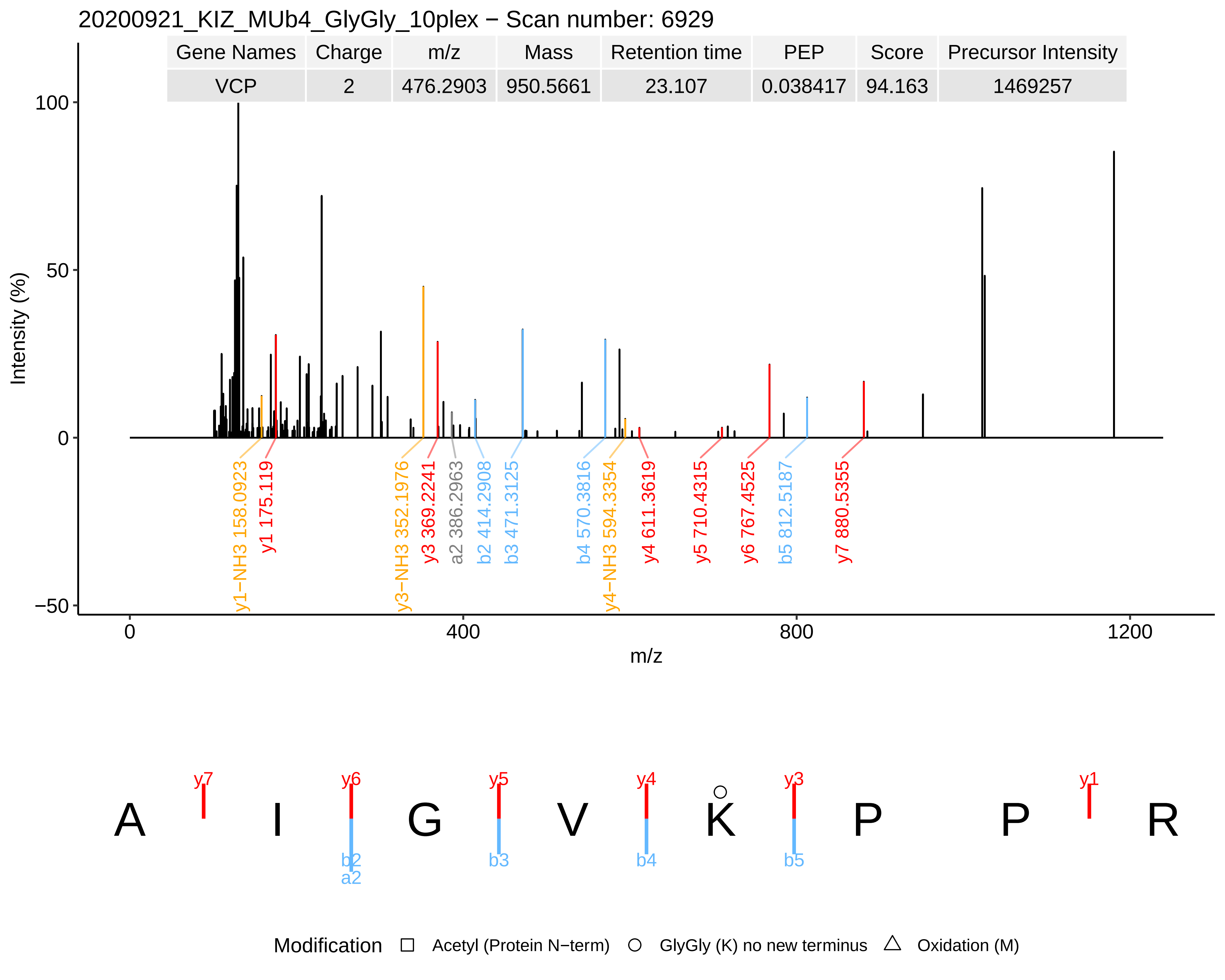
20200921_KIZ_MUb4_GlyGly_10plex − Scan number: 6929
Gene Names
Charge
m/z
Mass
Retention time
PEP
Score
Precursor Intensity
VCP
2
476.2903
950.5661
23.107
0.038417
94.163
1469257
Intensity (%)
100
50
0
−50
0
400
800
1200
m/z
y1−NH3 158.0923
y1 175.119
y3−NH3 352.1976
y3 369.2241
a2 386.2963
b2 414.2908
b3 471.3125
b4 570.3816
y4−NH3 594.3354
y4 611.3619
y5 710.4315
y6 767.4525
b5 812.5187
y7 880.5355
y7
y6
y5
y4
y3
y1
A
I
G
V
K
P
P
R
b2
a2
b3
b4
b5
Modification
Acetyl (Protein N−term)
GlyGly (K) no new terminus
Oxidation (M)

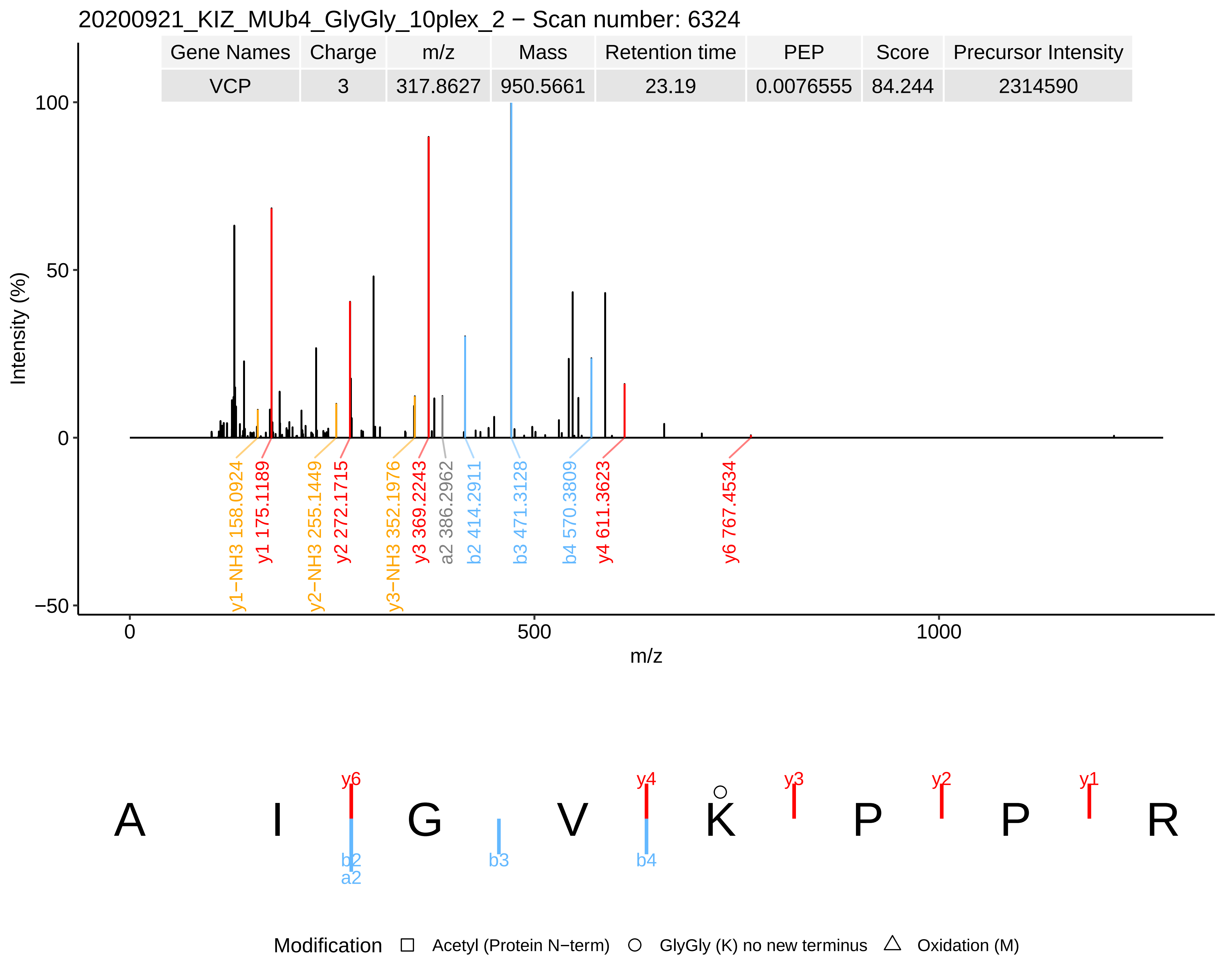
20200921_KIZ_MUb4_GlyGly_10plex_2 − Scan number: 6324
Gene Names | Charge | m/z | Mass | Retention time | PEP | Score | Precursor Intensity
VCP | 3 | 317.8627 | 950.5661 | 23.19 | 0.0076555 | 84.244 | 2314590
Intensity (%)
100
50
0
−50
y1−NH3 158.0924
y1 175.1189
y2−NH3 255.1449
y2 272.1715
y3−NH3 352.1976
y3 369.2243
a2 386.2962
b2 414.2911
b3 471.3128
b4 570.3809
y4 611.3623
y6 767.4534
0
500
1000
m/z
A I G V K P P R
y6 y4 y3 y2 y1
b2 a2 b3 b4
Modification
Acetyl (Protein N−term)
GlyGly (K) no new terminus
Oxidation (M)

20200921_KIZ_MUb4_GlyGly_10plex_2 − Scan number: 6455

Gene Names	Charge	m/z	Mass	Retention time	PEP	Score	Precursor Intensity
VCP	3	317.8627	950.5661	23.728	0.040532	60.799	1083506

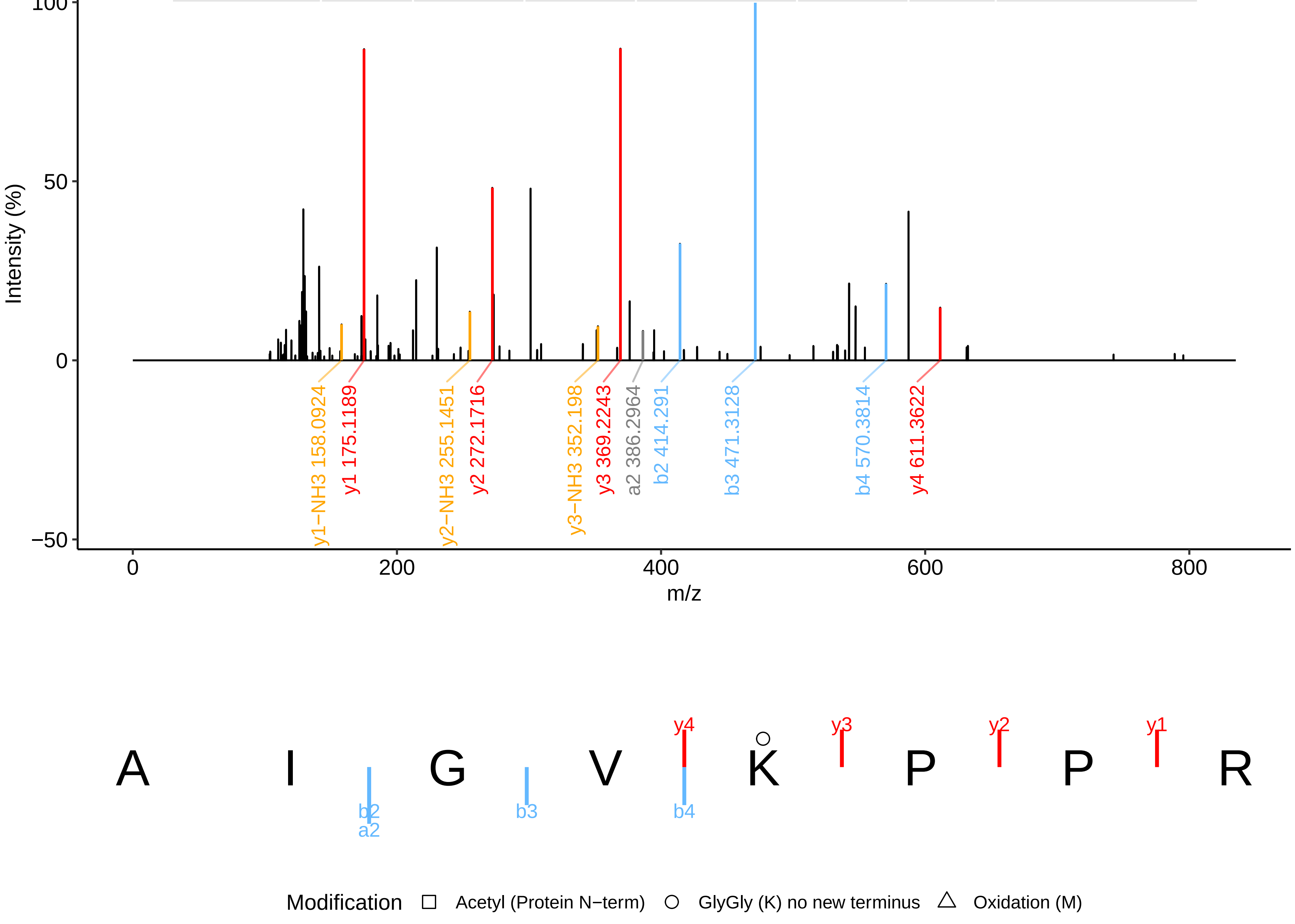

20200924_KIZ_MUb5_GlyGly_1 − Scan number: 5778

Gene Names	Charge	m/z	Mass	Retention time	PEP	Score	Precursor Intensity
VCP	3	317.8627	950.5661	18.047	0.12257	50.898	11918044

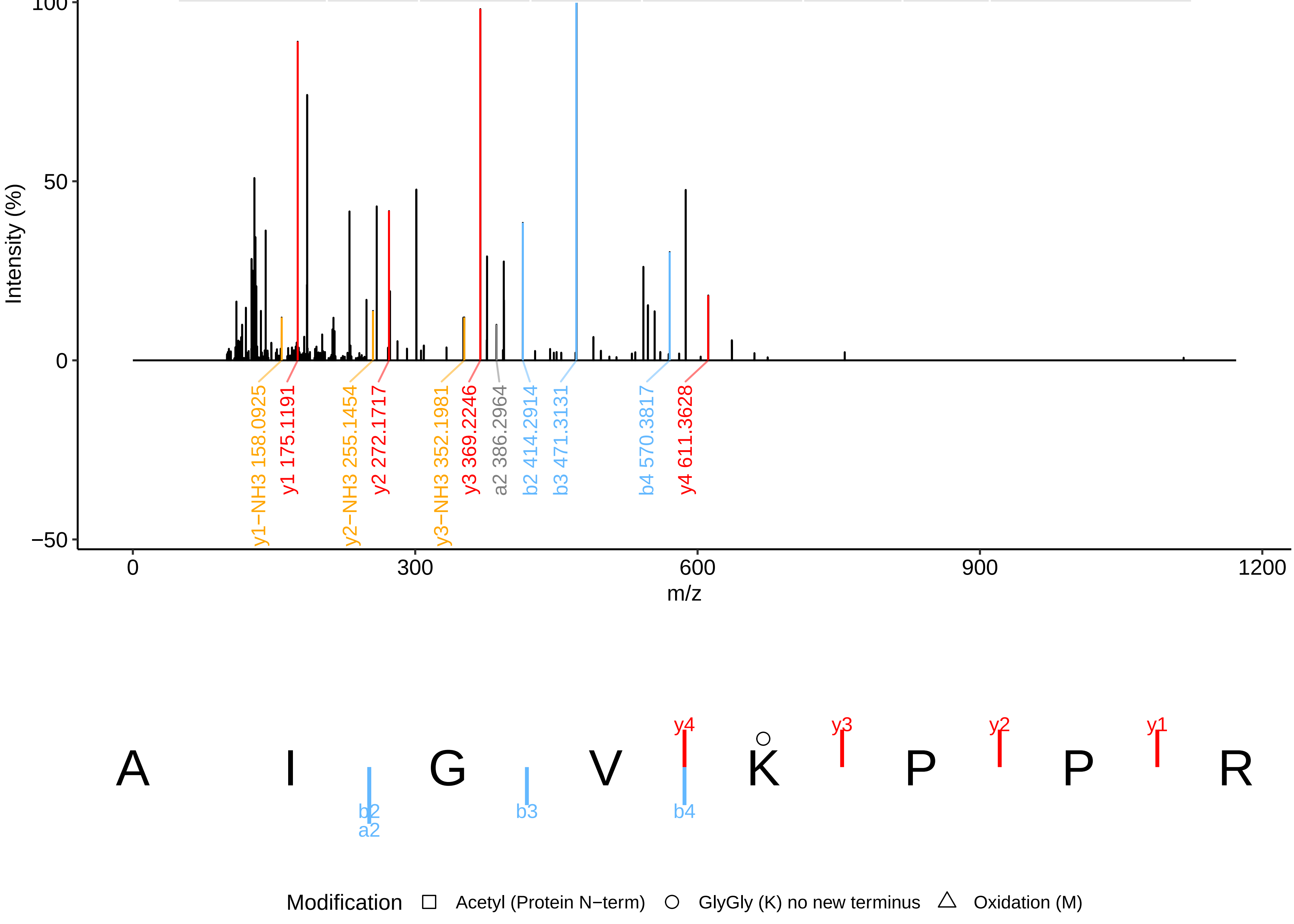

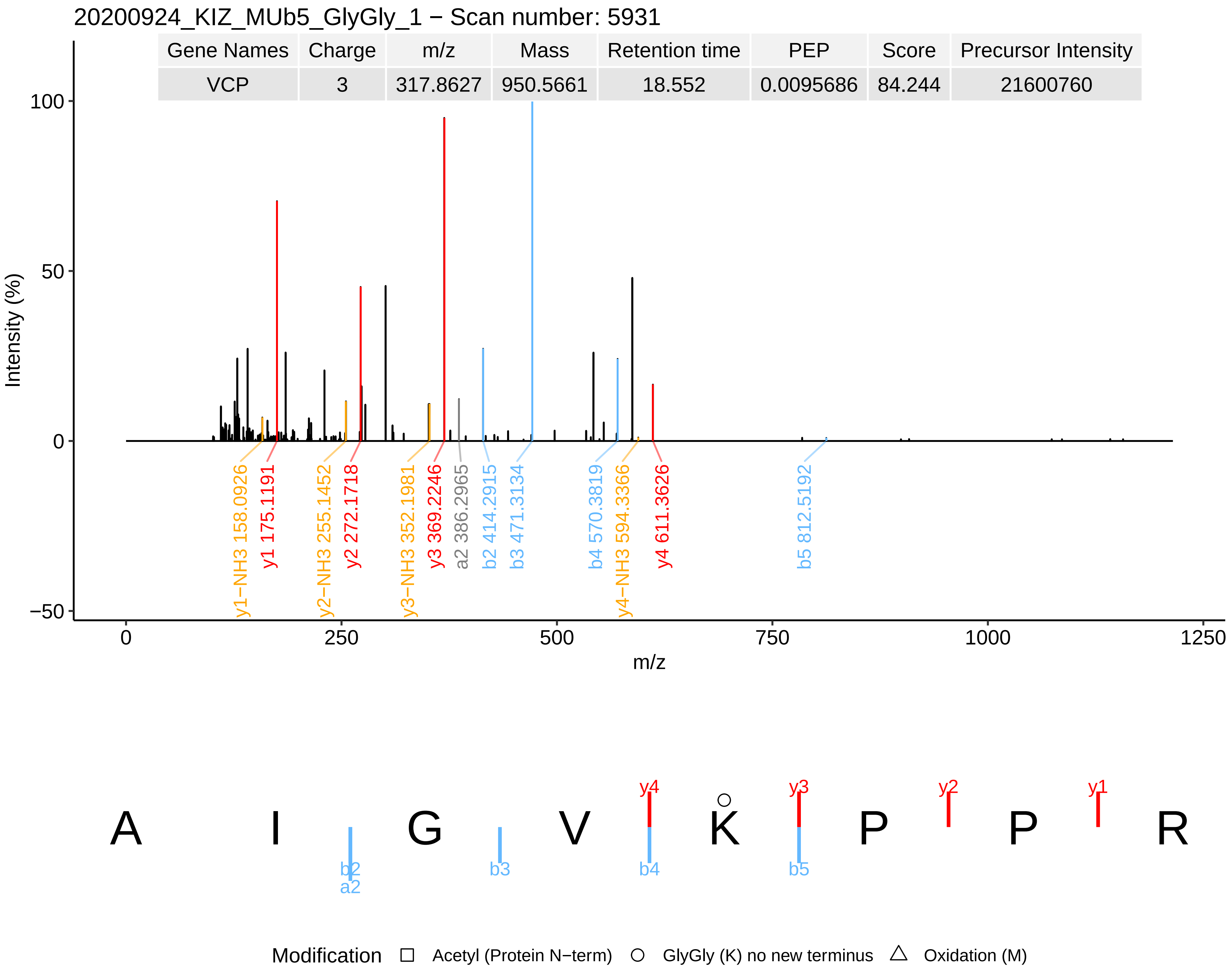
20200924_KIZ_MUb5_GlyGly_1 − Scan number: 5931
Gene Names
Charge
m/z
Mass
Retention time
PEP
Score
Precursor Intensity
VCP
3
317.8627
950.5661
18.552
0.0095686
84.244
21600760
Intensity (%)
100
50
0
−50
0
250
500
750
1000
1250
m/z
y1−NH3 158.0926
y1 175.1191
y2−NH3 255.1452
y2 272.1718
y3−NH3 352.1981
y3 369.2246
a2 386.2965
b2 414.2915
b3 471.3134
b4 570.3819
y4−NH3 594.3366
y4 611.3626
b5 812.5192
A
I
G
V
K
P
P
R
y4
y3
y2
y1
b2
a2
b3
b4
b5
Modification
Acetyl (Protein N−term)
GlyGly (K) no new terminus
Oxidation (M)

20200924_KIZ_MUb5_GlyGly_2 − Scan number: 5353

Gene Names	Charge	m/z	Mass	Retention time	PEP	Score	Precursor Intensity
VCP	3	317.8627	950.5661	19.857	0.014767	76.035	1388330

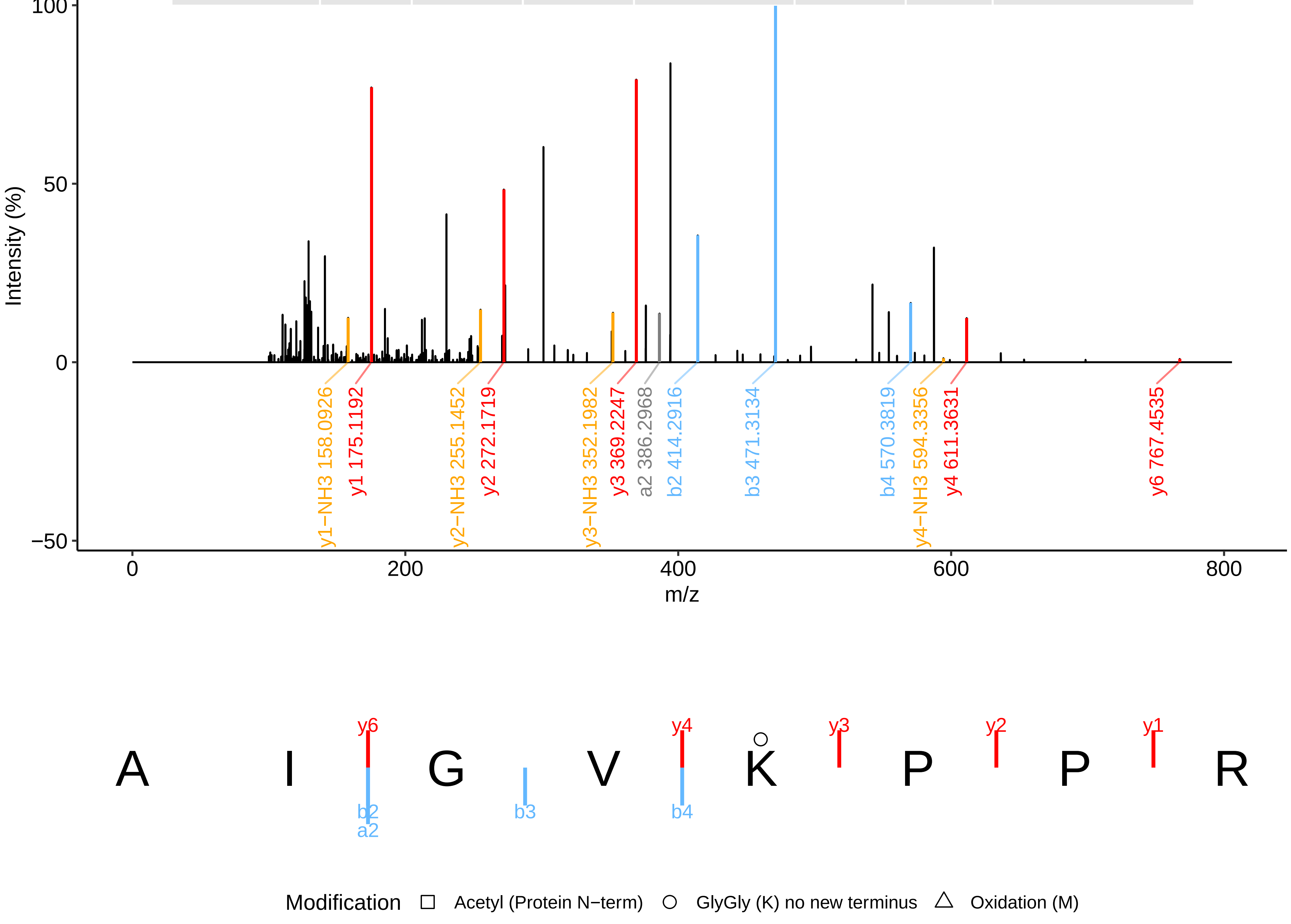

20200924_KIZ_MUb5_GlyGly_2 − Scan number: 5486

Gene Names	Charge	m/z	Mass	Retention time	PEP	Score	Precursor Intensity
VCP	3	317.8627	950.5661	20.366	0.0088177	81.297	11406589

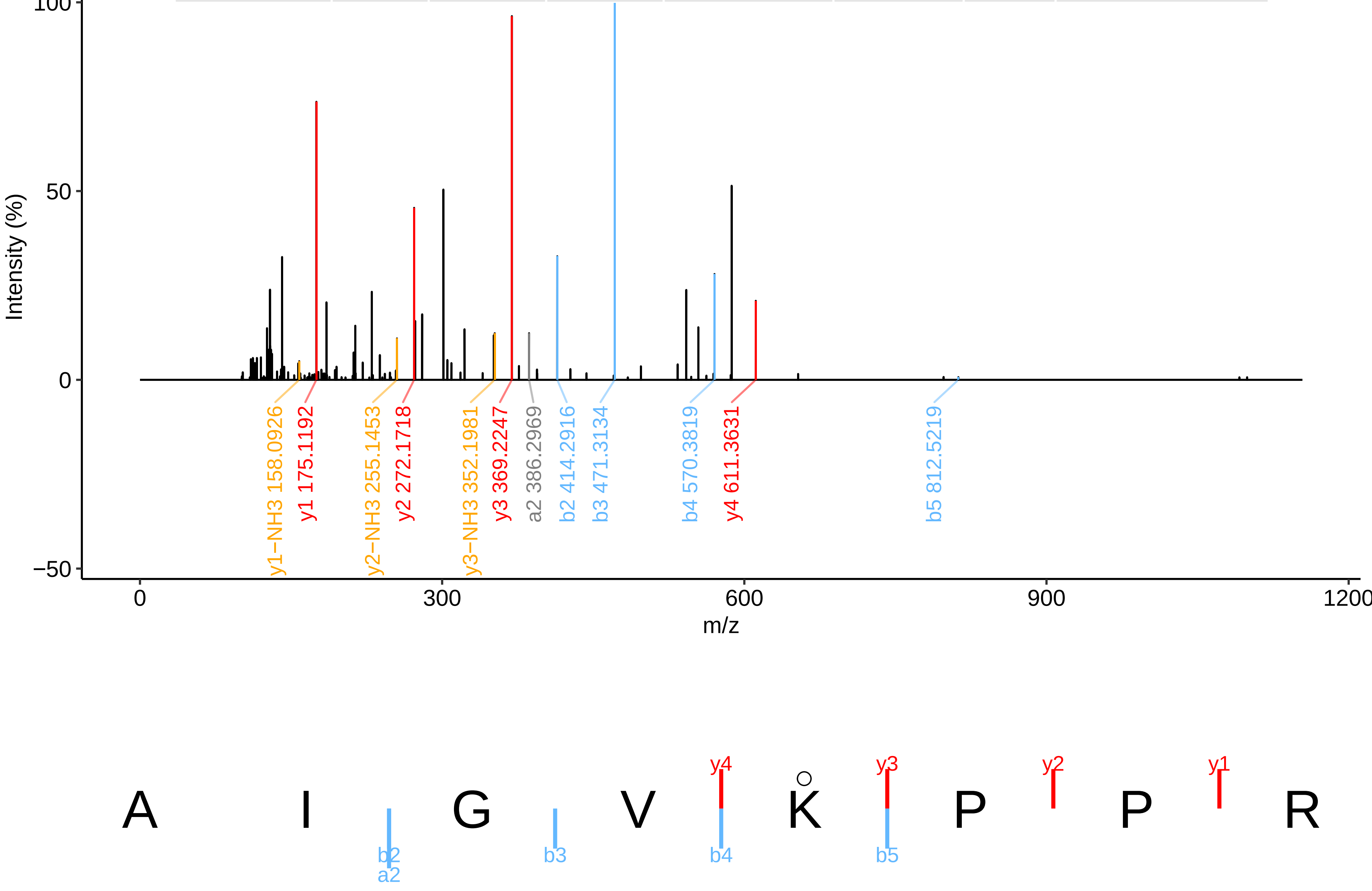

Modification □ Acetyl (Protein N−term) ○ GlyGly (K) no new terminus △ Oxidation (M)

20200924_KIZ_MUb5_GlyGly_2 − Scan number: 5643

Gene Names	Charge	m/z	Mass	Retention time	PEP	Score	Precursor Intensity
VCP	3	317.8627	950.5661	20.972	0.0086256	81.784	2564999

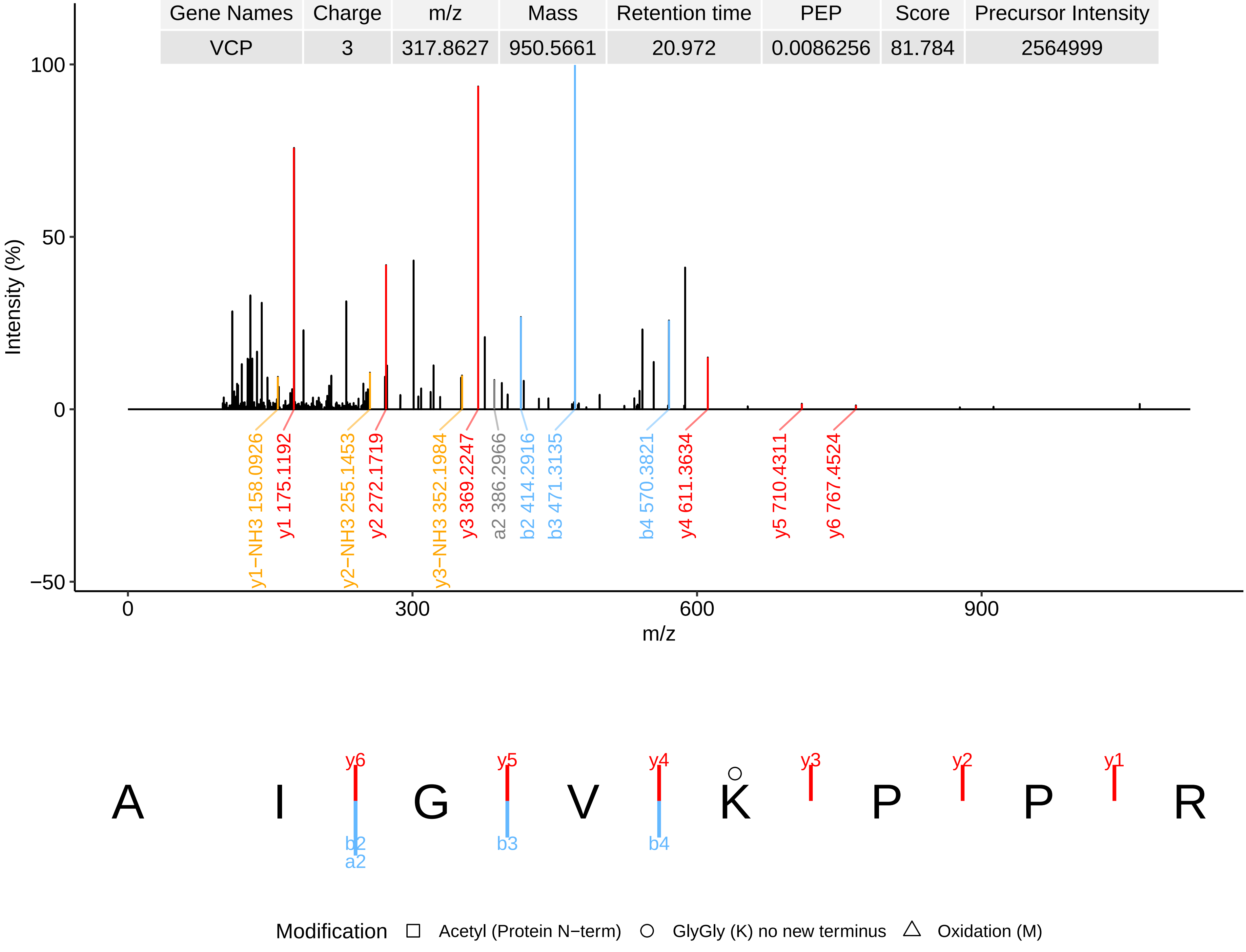

20201118_KIZ_MUb6_GlyGly_2 − Scan number: 7157

Gene Names	Charge	m/z	Mass	Retention time	PEP	Score	Precursor Intensity
VCP	3	317.8627	950.5661	24.443	0.0072527	85.265	2107242

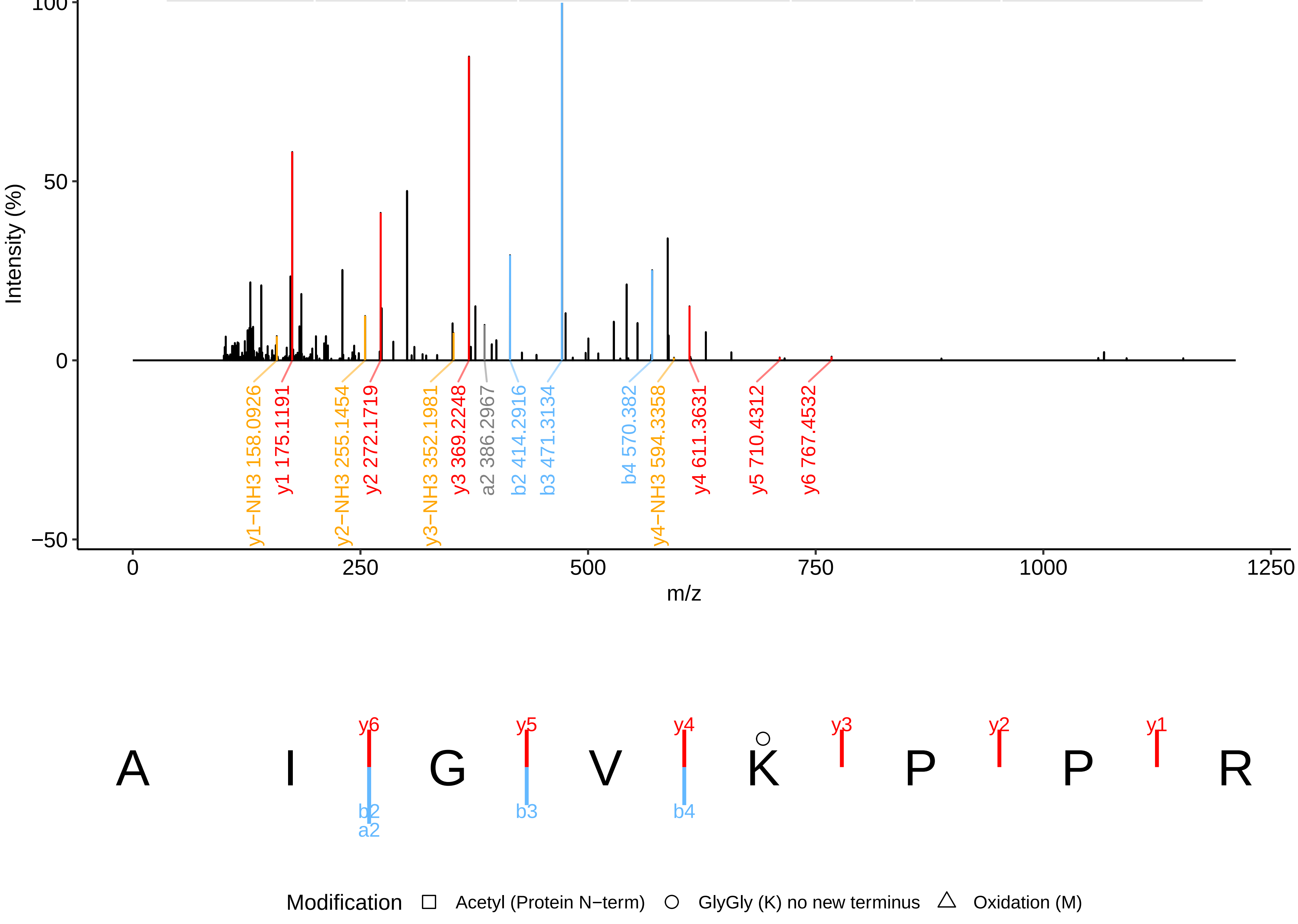

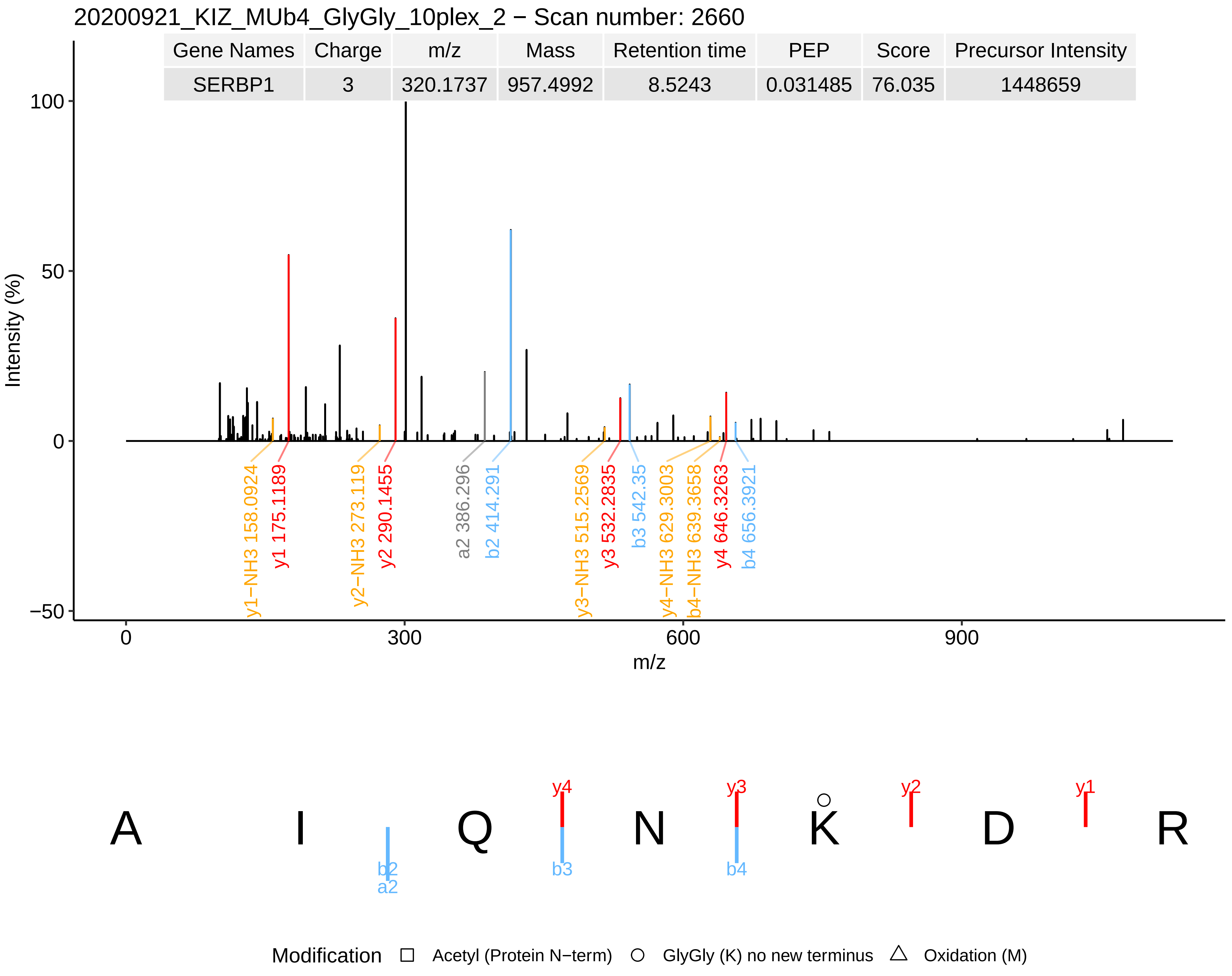

20200921_KIZ_MUb4_GlyGly_10plex_2 − Scan number: 2660
Gene Names | Charge | m/z | Mass | Retention time | PEP | Score | Precursor Intensity
SERBP1 | 3 | 320.1737 | 957.4992 | 8.5243 | 0.031485 | 76.035 | 1448659
Intensity (%)
m/z
y1−NH3 158.0924
y1 175.1189
y2−NH3 273.119
y2 290.1455
a2 386.296
b2 414.291
y3−NH3 515.2569
y3 532.2835
b3 542.35
y4−NH3 629.3003
b4−NH3 639.3658
y4 646.3263
b4 656.3921
A I Q N K D R
y4 y3 y2 y1
b2 a2 b3 b4
Modification
Acetyl (Protein N−term)
GlyGly (K) no new terminus
Oxidation (M)

20200924_KIZ_MUb5_GlyGly_1 − Scan number: 2593

Gene Names	Charge	m/z	Mass	Retention time	PEP	Score	Precursor Intensity
SERBP1	3	320.1737	957.4992	8.0469	0.0096002	94.662	11970684

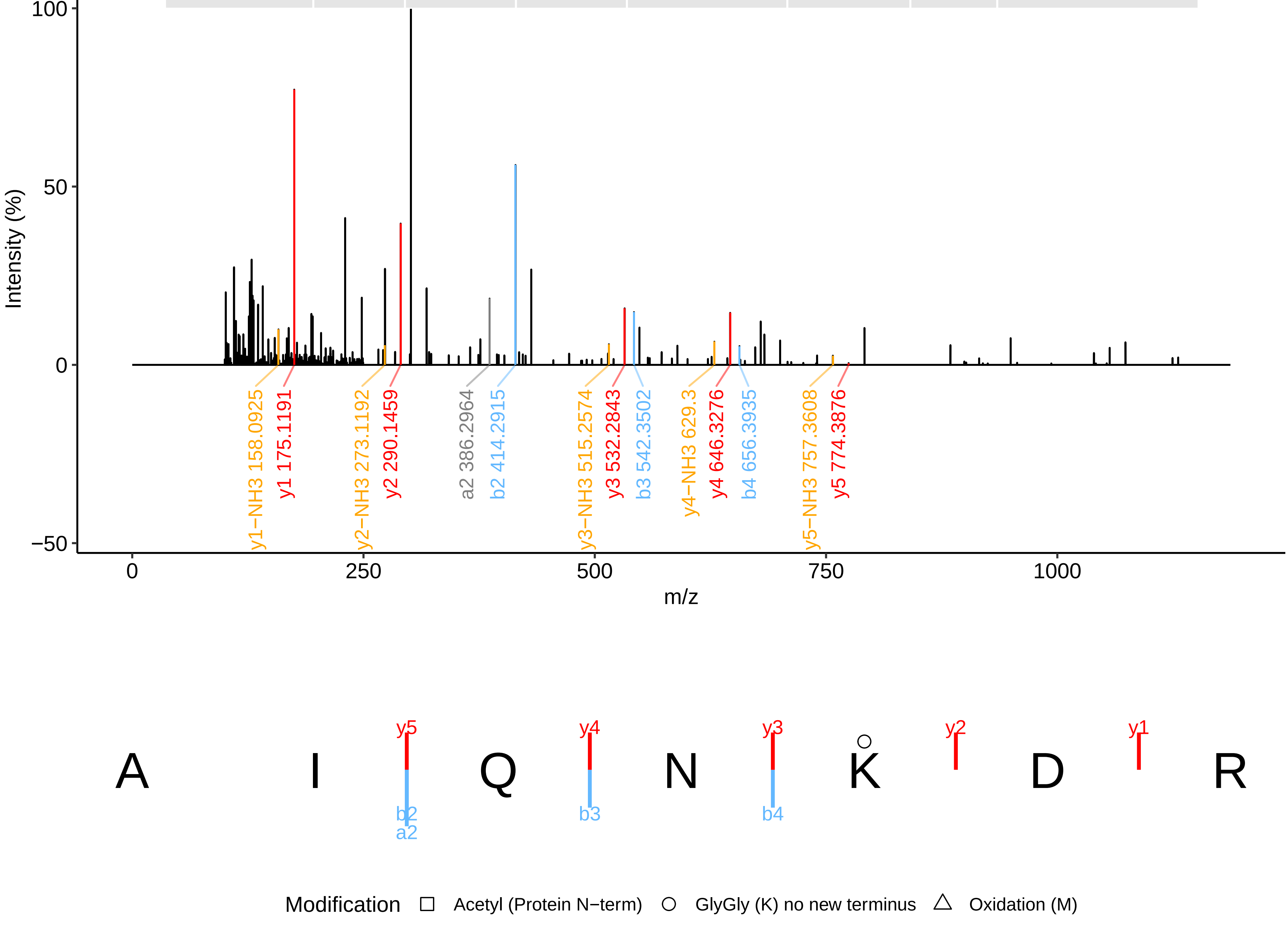

20200921_KIZ_MUb4_GlyGly_10plex − Scan number: 10142

Gene Names	Charge	m/z	Mass	Retention time	PEP	Score	Precursor Intensity
XRCC6	3	314.8625	941.5658	34.995	0.017624	83.614	6201886

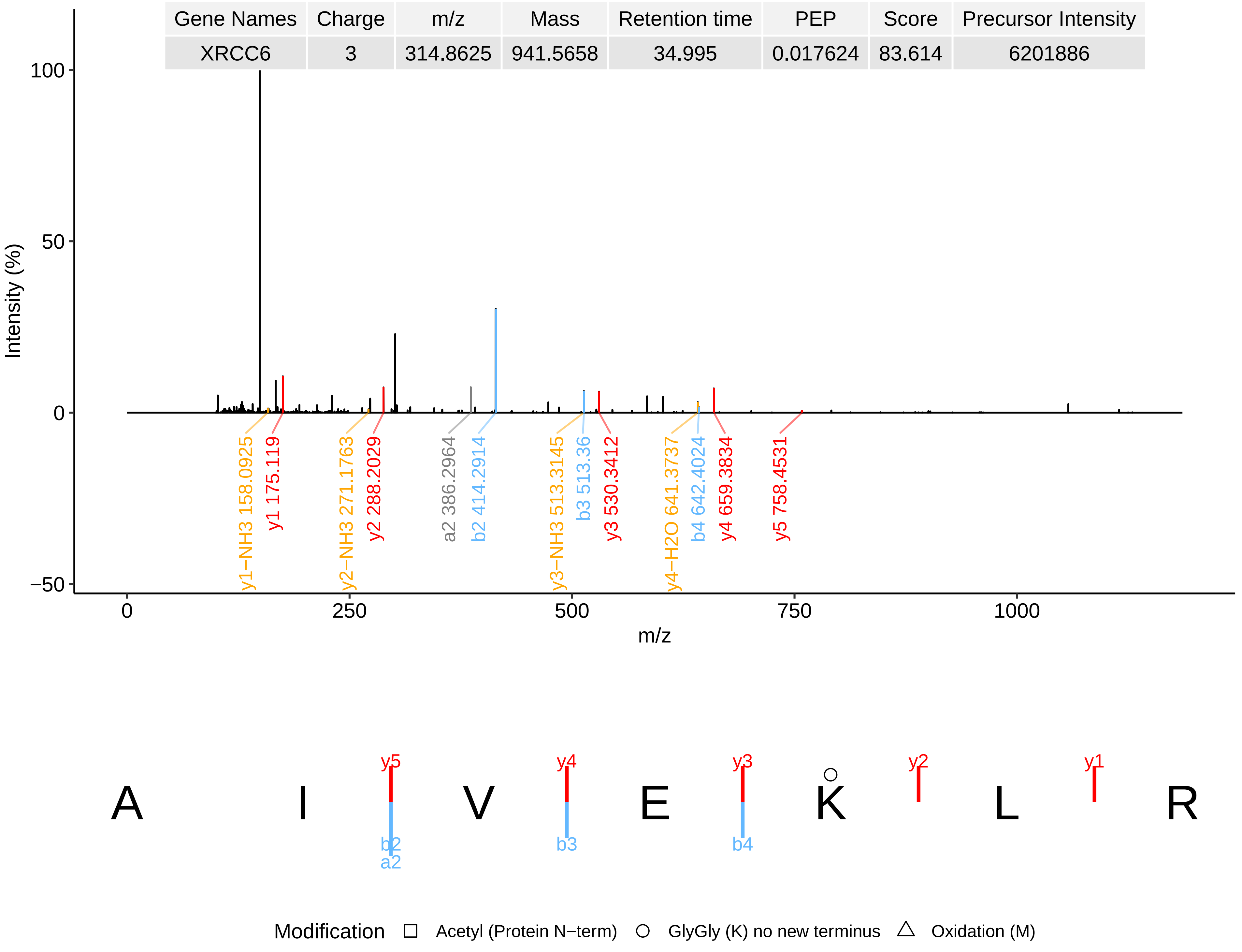

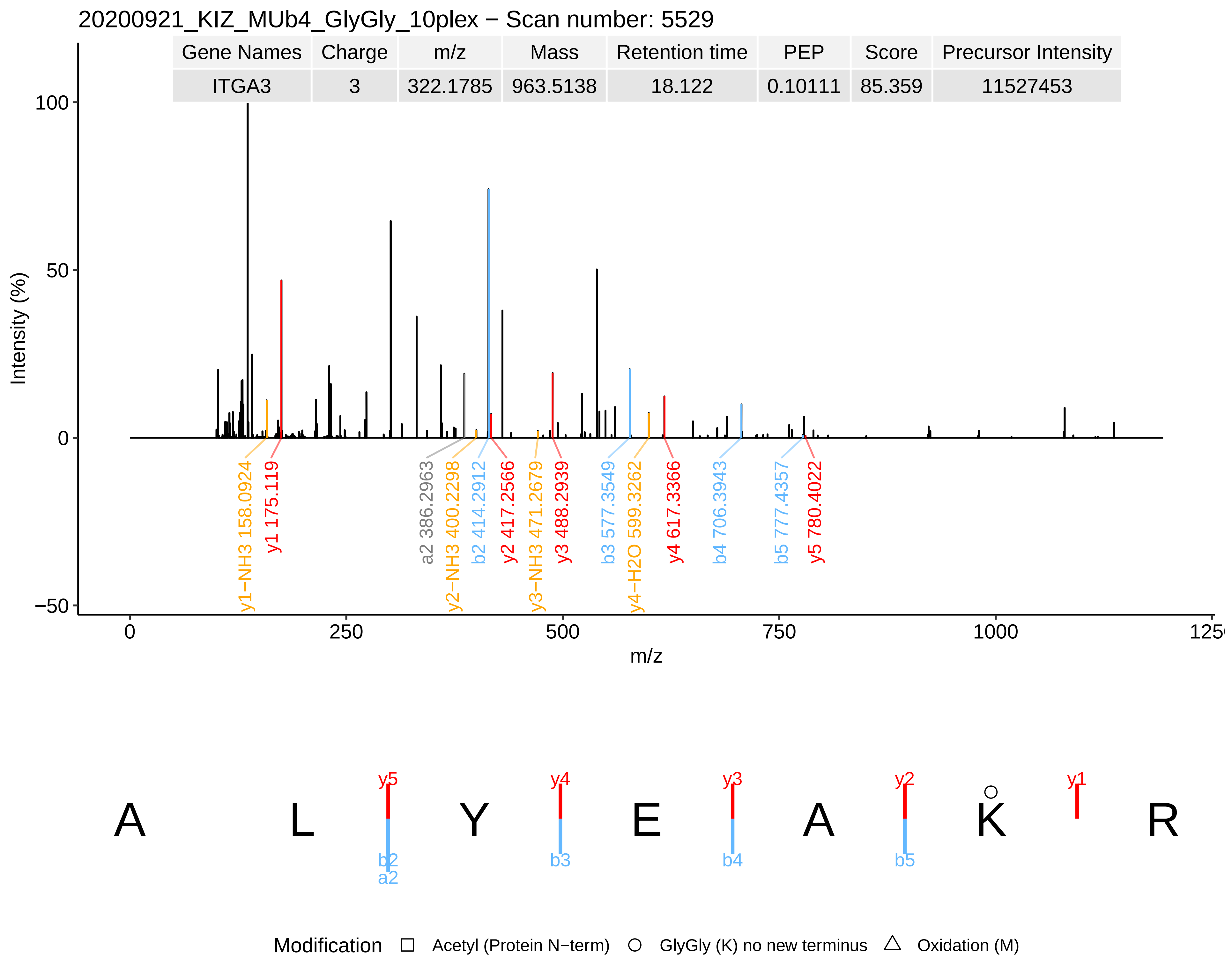

20200921_KIZ_MUb4_GlyGly_10plex − Scan number: 5529
Gene Names | Charge | m/z | Mass | Retention time | PEP | Score | Precursor Intensity
ITGA3 | 3 | 322.1785 | 963.5138 | 18.122 | 0.10111 | 85.359 | 11527453
Intensity (%)
100
50
0
−50
y1−NH3 158.0924
y1 175.119
a2 386.2963
y2−NH3 400.2298
b2 414.2912
y2 417.2566
y3−NH3 471.2679
y3 488.2939
b3 577.3549
y4−H2O 599.3262
y4 617.3366
b4 706.3943
b5 777.4357
y5 780.4022
0
250
500
750
1000
125
m/z
A L Y E A K R
y5 y4 y3 y2 y1
b2 a2 b3 b4 b5
Modification
Acetyl (Protein N−term)
GlyGly (K) no new terminus
Oxidation (M)

20201118_KIZ_MUb6_GlyGly_1 − Scan number: 4585

Gene Names	Charge	m/z	Mass	Retention time	PEP	Score	Precursor Intensity
ITGA3	3	322.1785	963.5138	14.394	0.10407	84.699	16967776

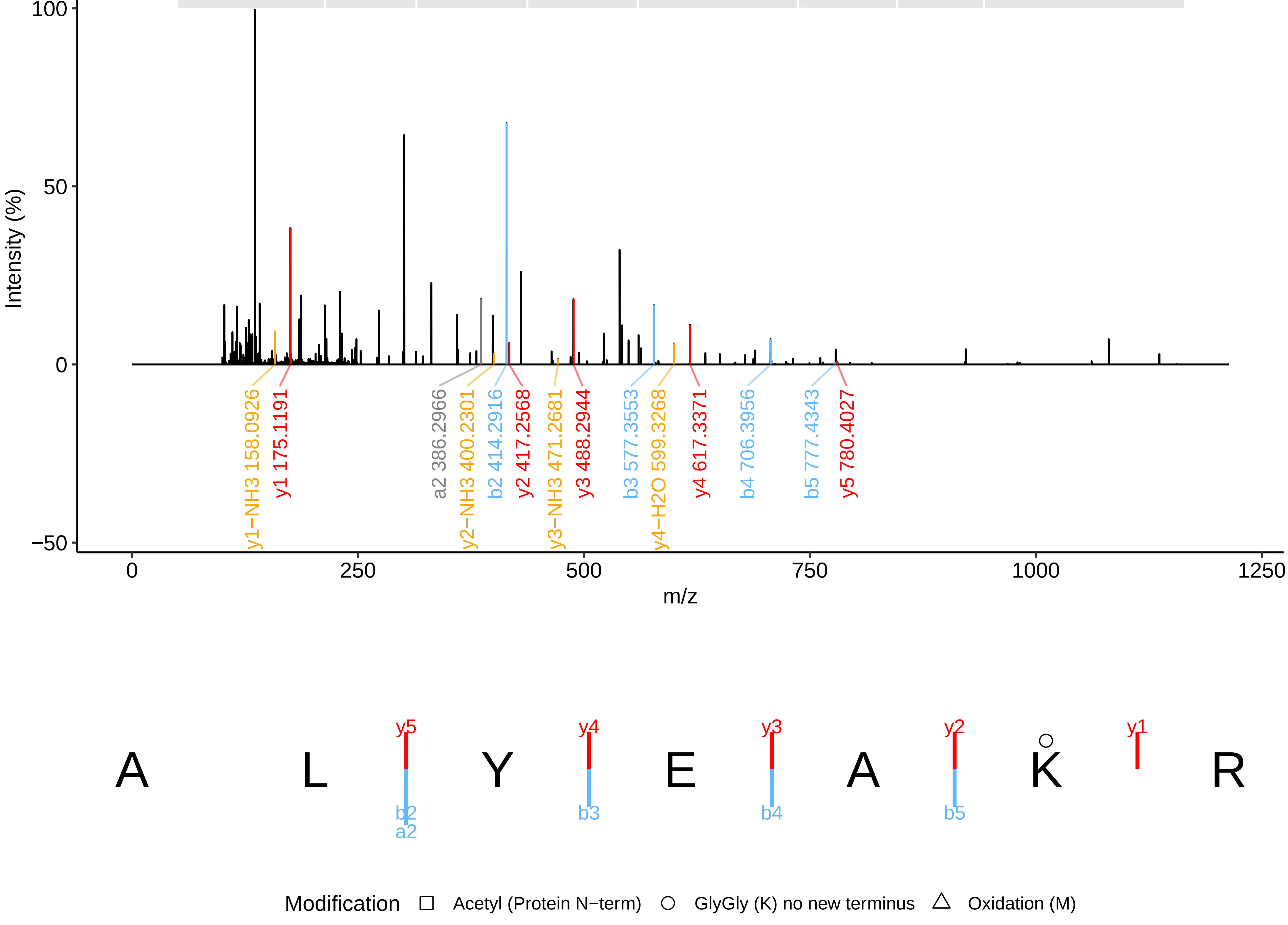

20200921_KIZ_MUb4_GlyGly_10plex − Scan number: 10579

Gene Names	Charge	m/z	Mass	Retention time	PEP	Score	Precursor Intensity
NACA;NACAD	3	330.5235	988.5488	36.65	0.0025879	97.093	8454386

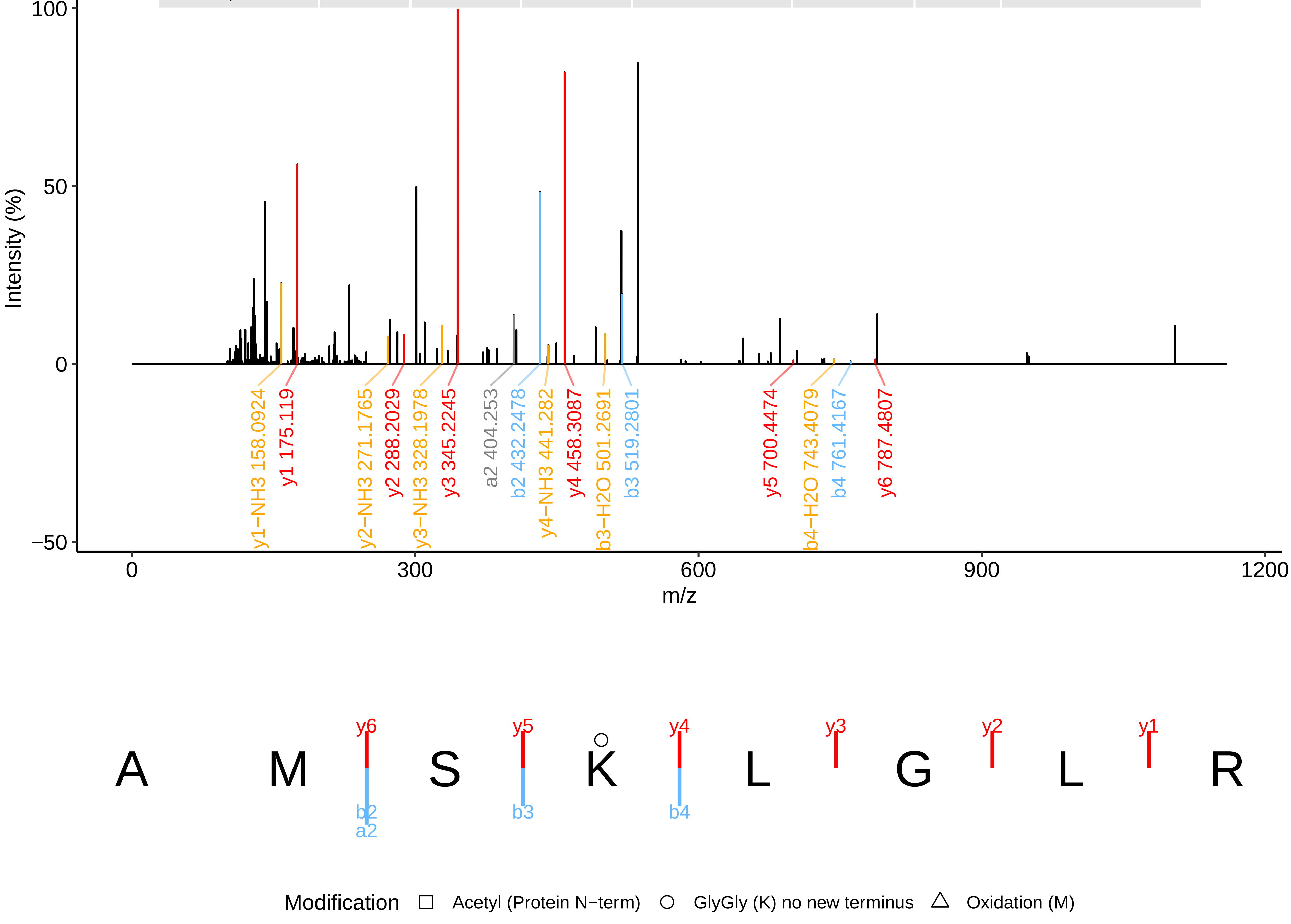

20200921_KIZ_MUb4_GlyGly_10plex_2 − Scan number: 9789

Gene Names	Charge	m/z	Mass	Retention time	PEP	Score	Precursor Intensity
NACA;NACAD	3	330.5235	988.5488	37.323	0.051225	58.194	807312.2

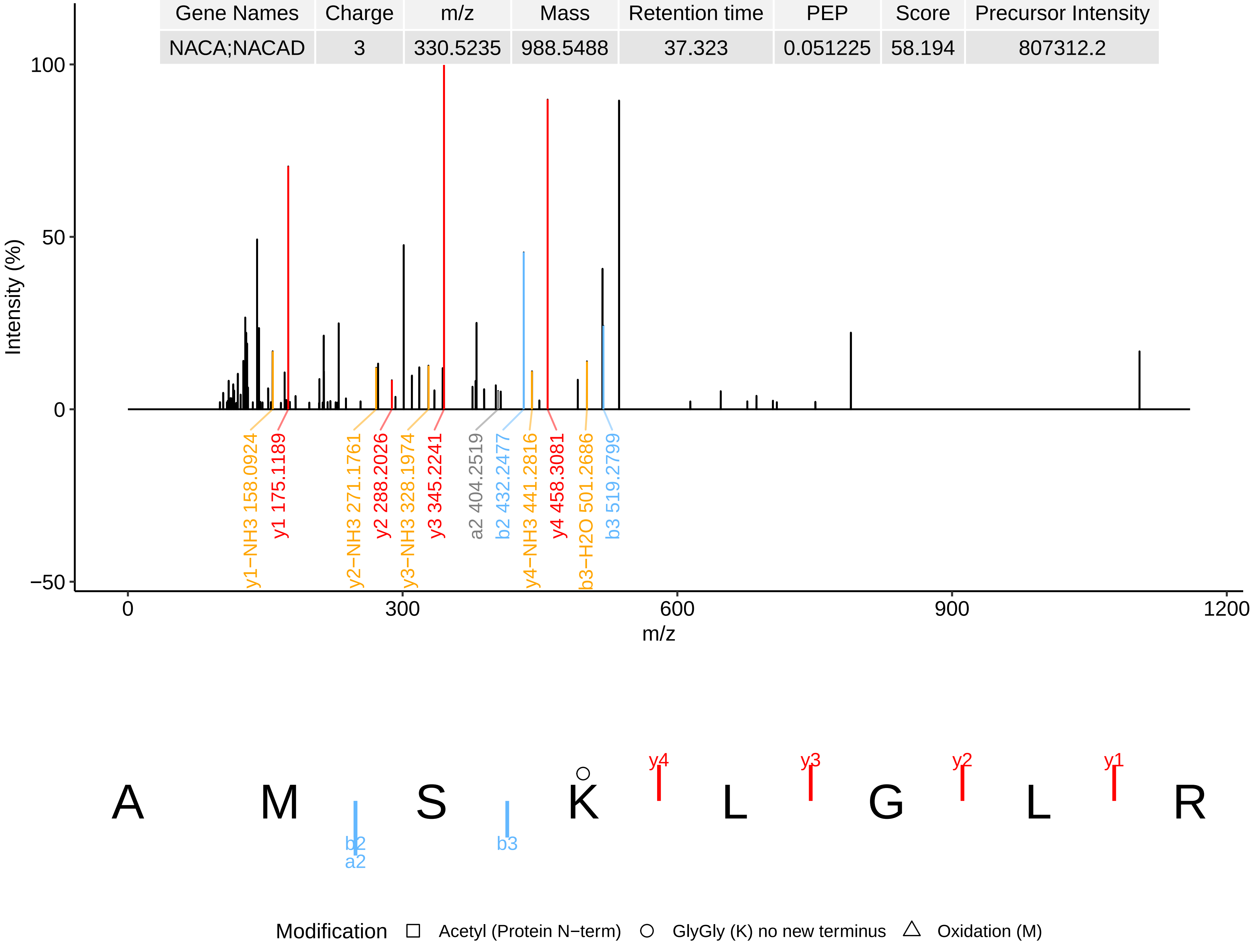

20200921_KIZ_MUb4_GlyGly_10plex − Scan number: 2721

Gene Names	Charge	m/z	Mass	Retention time	PEP	Score	Precursor Inte
HIST1H1E;HIST1H1D;HIST1H1B;HIST1H1C	3	315.8419	944.5039	8.5627	0.001178	107.29	14613001

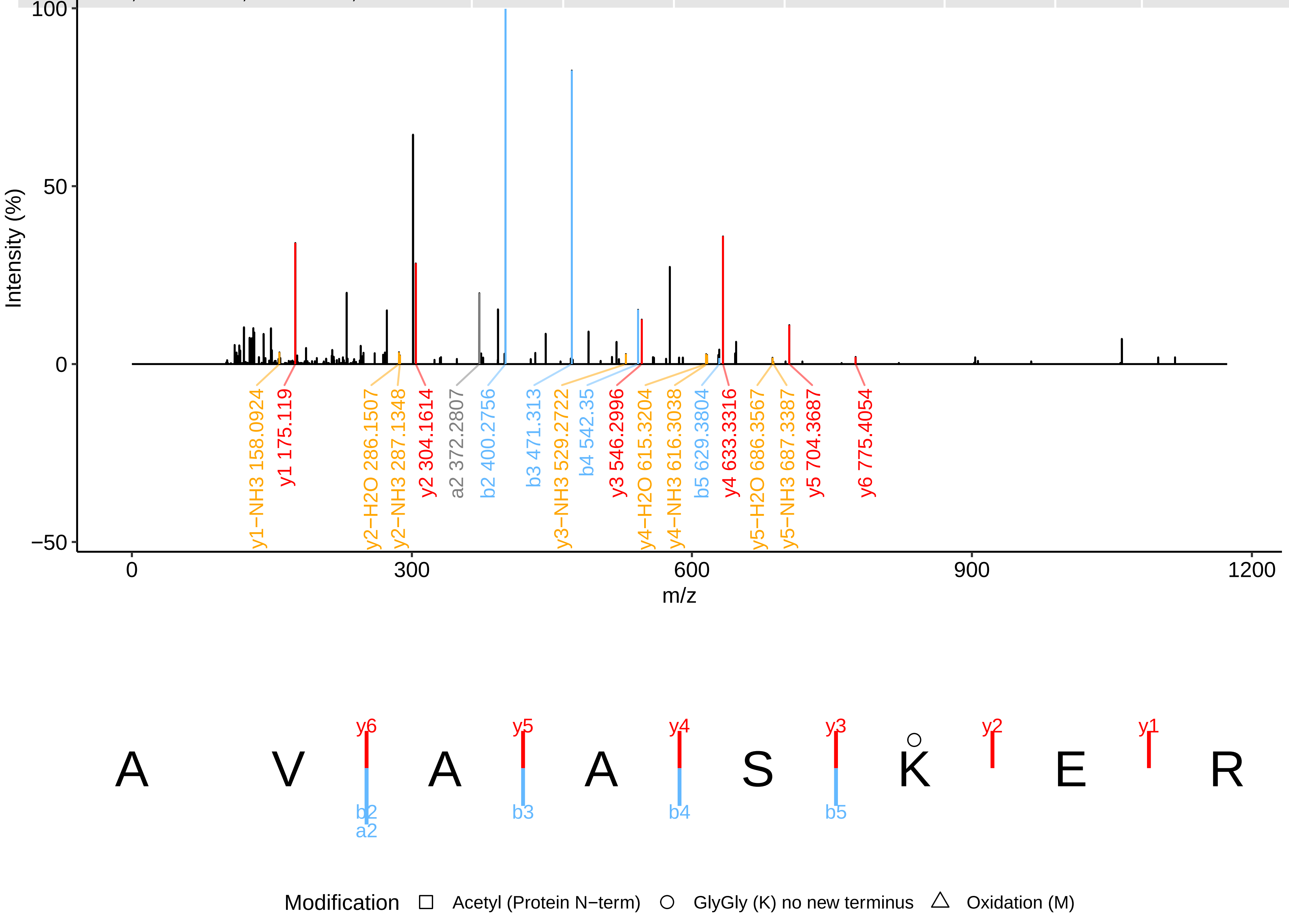

20200921_KIZ_MUb4_GlyGly_10plex_2 − Scan number: 2687

Gene Names	Charge	m/z	Mass	Retention time	PEP	Score	Precursor Inter
HIST1H1E;HIST1H1D;HIST1H1B;HIST1H1C	2	473.2593	944.5039	8.6309	0.062224	73.616	1671159

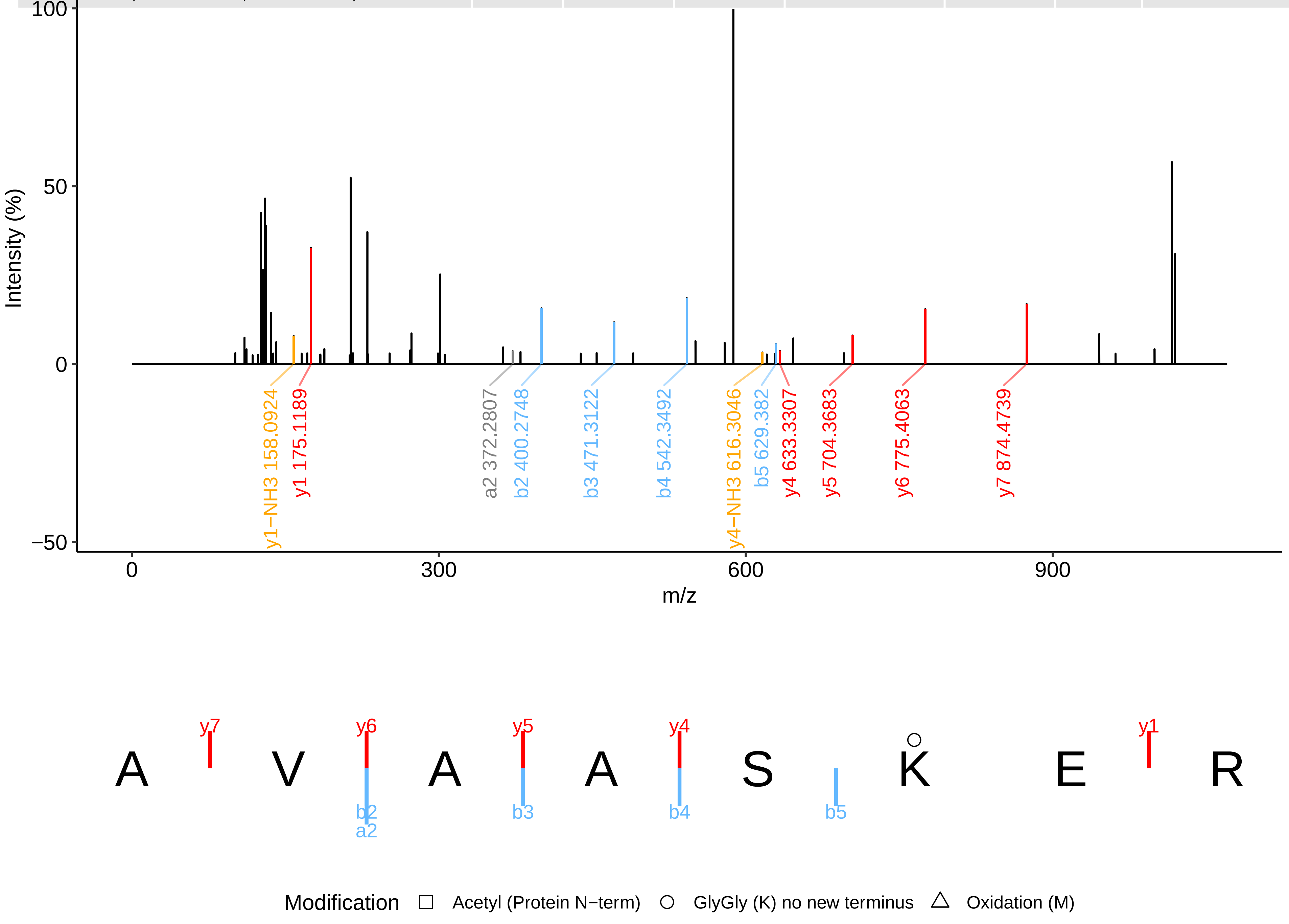

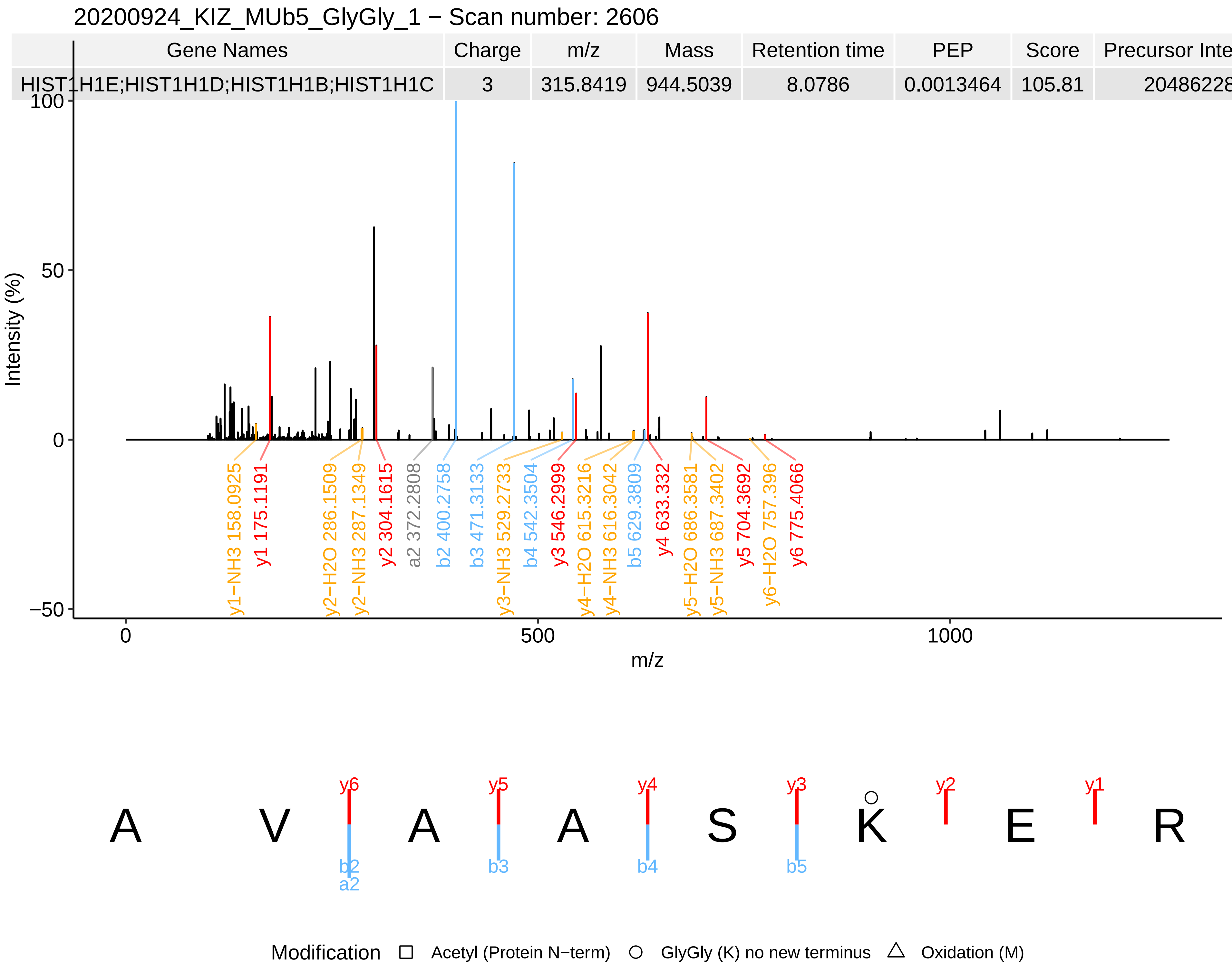
20200924_KIZ_MUb5_GlyGly_1 − Scan number: 2606
Gene Names | Charge | m/z | Mass | Retention time | PEP | Score | Precursor Inte
HIST1H1E;HIST1H1D;HIST1H1B;HIST1H1C | 3 | 315.8419 | 944.5039 | 8.0786 | 0.0013464 | 105.81 | 20486228
Intensity (%)
100
50
0
−50
y1−NH3 158.0925
y1 175.1191
y2−H2O 286.1509
y2−NH3 287.1349
y2 304.1615
a2 372.2808
b2 400.2758
b3 471.3133
y3−NH3 529.2733
b4 542.3504
y3 546.2999
y4−H2O 615.3216
y4−NH3 616.3042
b5 629.3809
y4 633.332
y5−H2O 686.3581
y5−NH3 687.3402
y5 704.3692
y6−H2O 757.396
y6 775.4066
0
500
1000
m/z
A V A A S K E R
y6 y5 y4 y3 y2 y1
b2 a2 b3 b4 b5
Modification
Acetyl (Protein N−term)
GlyGly (K) no new terminus
Oxidation (M)

20200924_KIZ_MUb5_GlyGly_2 − Scan number: 2265

Gene Names	Charge	m/z	Mass	Retention time	PEP	Score	Precursor Inter
HIST1H1E;HIST1H1D;HIST1H1B;HIST1H1C	3	315.8419	944.5039	7.9878	0.010186	79.713	8053397

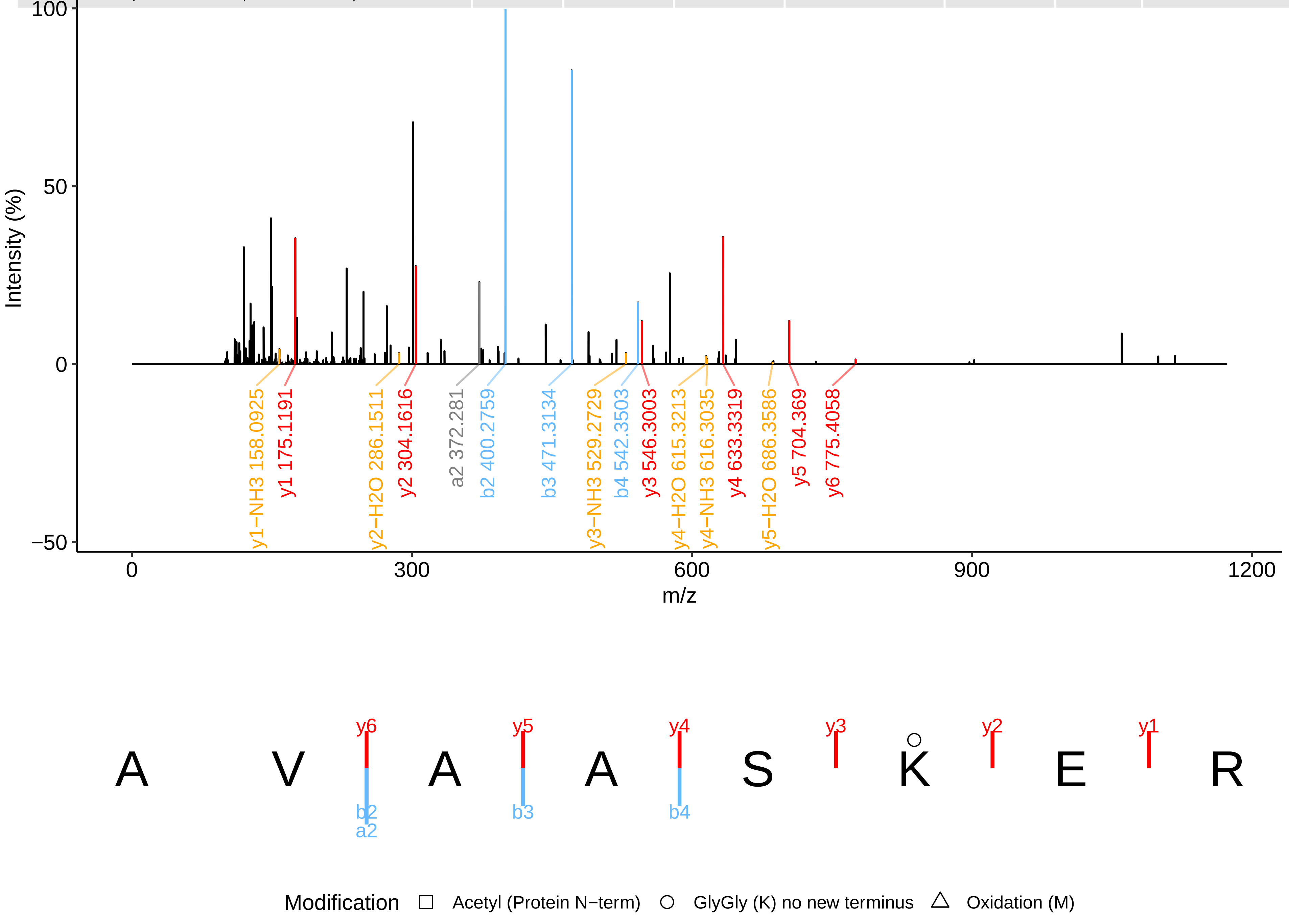

20201118_KIZ_MUb6_GlyGly_1 − Scan number: 2693

Gene Names	Charge	m/z	Mass	Retention time	PEP	Score	Precursor Inte
HIST1H1E;HIST1H1D;HIST1H1B;HIST1H1C	3	315.8419	944.5039	8.2401	0.0046544	91.853	9945485

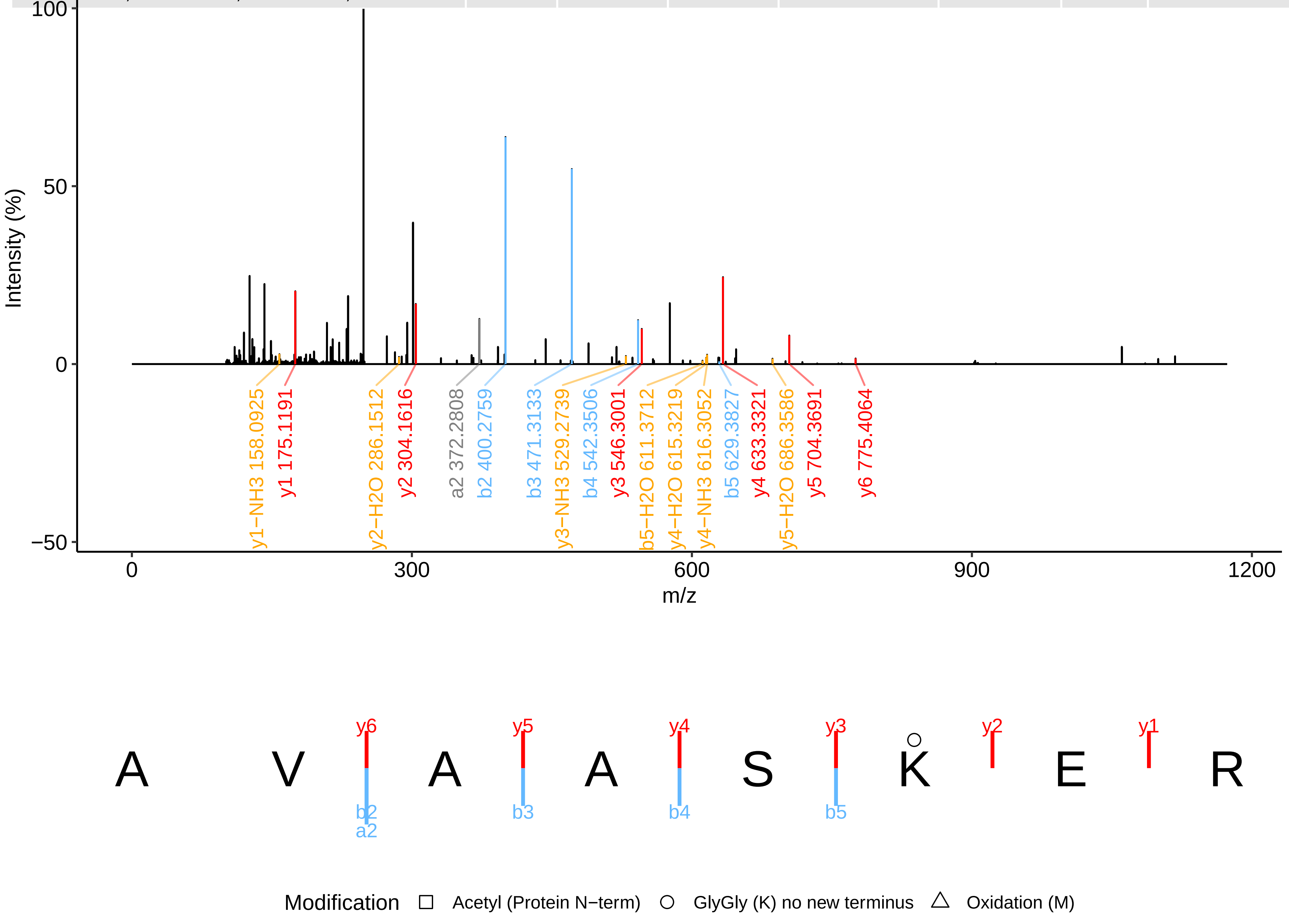

20200921_KIZ_MUb4_GlyGly_10plex_2 − Scan number: 6168

Gene Names	Charge	m/z	Mass	Retention time	PEP	Score	Precursor Intensity
GARS	3	324.5259	970.556	22.546	0.0029193	96.253	2122787

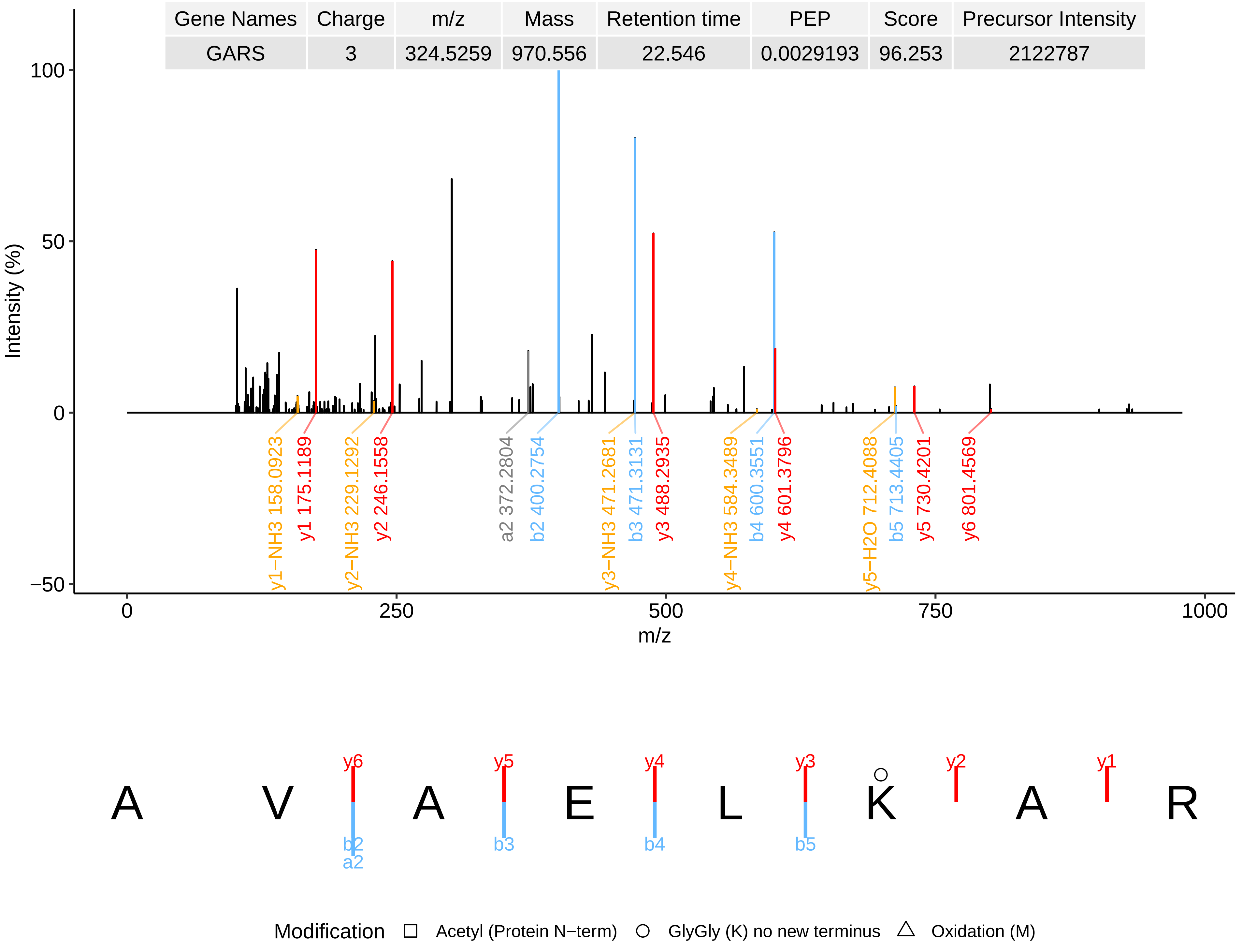

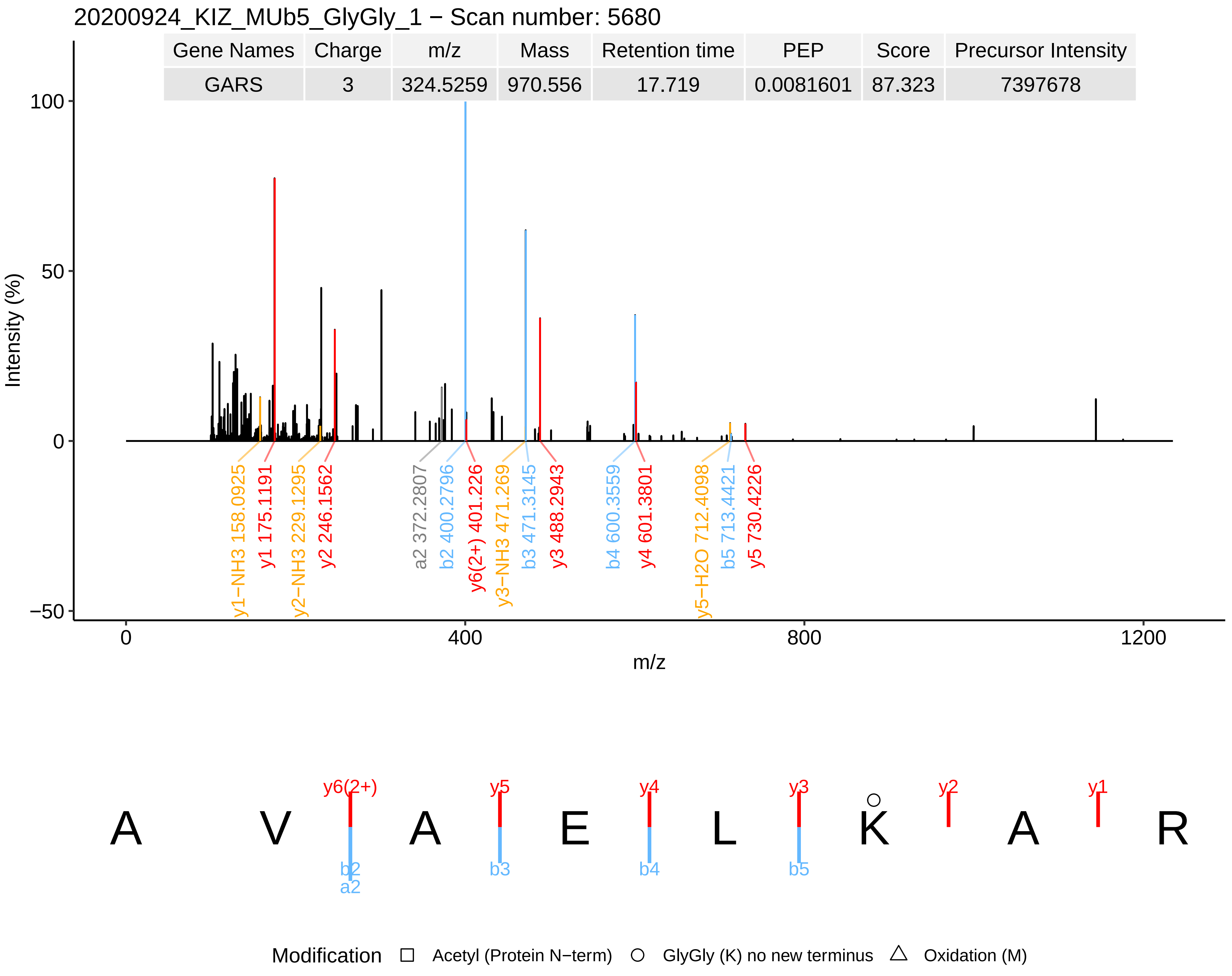
20200924_KIZ_MUb5_GlyGly_1 − Scan number: 5680
Gene Names
Charge
m/z
Mass
Retention time
PEP
Score
Precursor Intensity
GARS
3
324.5259
970.556
17.719
0.0081601
87.323
7397678
Intensity (%)
100
50
0
−50
m/z
0
400
800
1200
y1−NH3 158.0925
y1 175.1191
y2−NH3 229.1295
y2 246.1562
a2 372.2807
b2 400.2796
y6(2+) 401.226
y3−NH3 471.269
b3 471.3145
y3 488.2943
b4 600.3559
y4 601.3801
y5−H2O 712.4098
b5 713.4421
y5 730.4226
A V A E L K A R
y6(2+)
y5
y4
y3
y2
y1
b2
a2
b3
b4
b5
Modification
Acetyl (Protein N−term)
GlyGly (K) no new terminus
Oxidation (M)

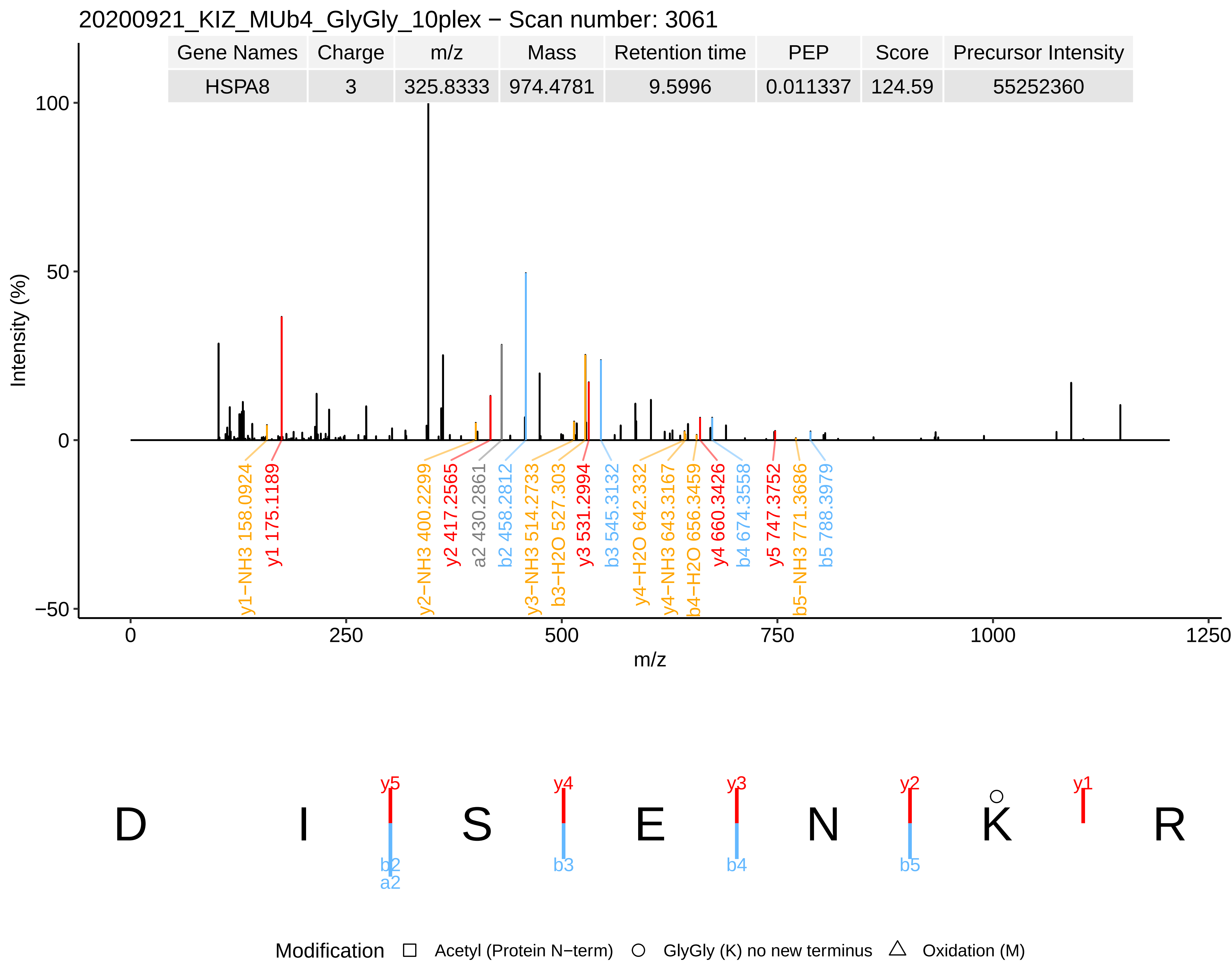

Gene Names	Charge	m/z	Mass	Retention time	PEP	Score	Precursor Intensity
HSPA8	3	325.8333	974.4781	9.5996	0.011337	124.59	55252360

20200921_KIZ_MUb4_GlyGly_10plex − Scan number: 3071

Gene Names	Charge	m/z	Mass	Retention time	PEP	Score	Precursor Intensity
HSPA8	2	488.2463	974.4781	9.6264	0.00031745	164.52	15478977

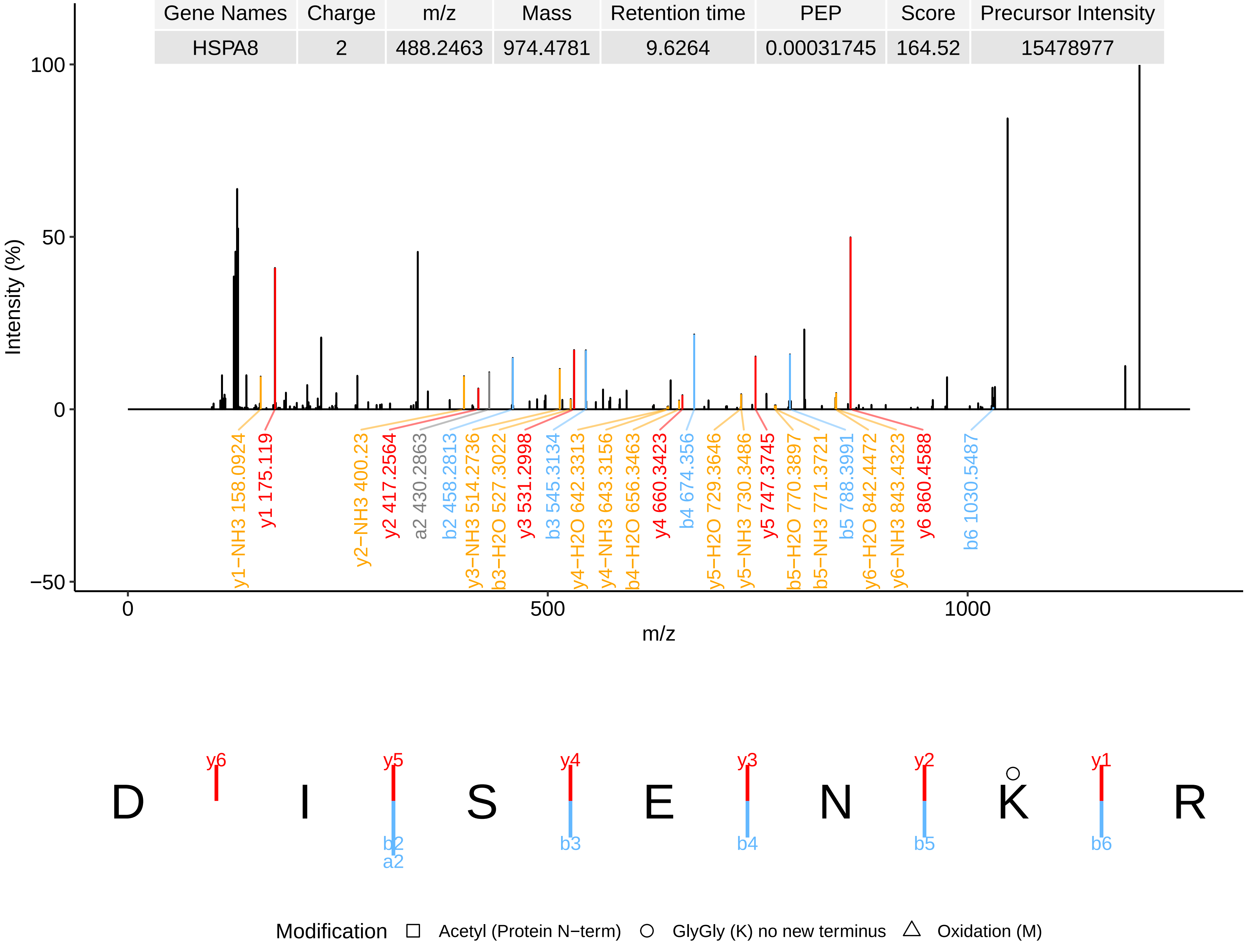

20200921_KIZ_MUb4_GlyGly_10plex_2 − Scan number: 2965

Gene Names	Charge	m/z	Mass	Retention time	PEP	Score	Precursor Intensity
HSPA8	3	325.8333	974.4781	9.6834	0.011967	121.52	2123937

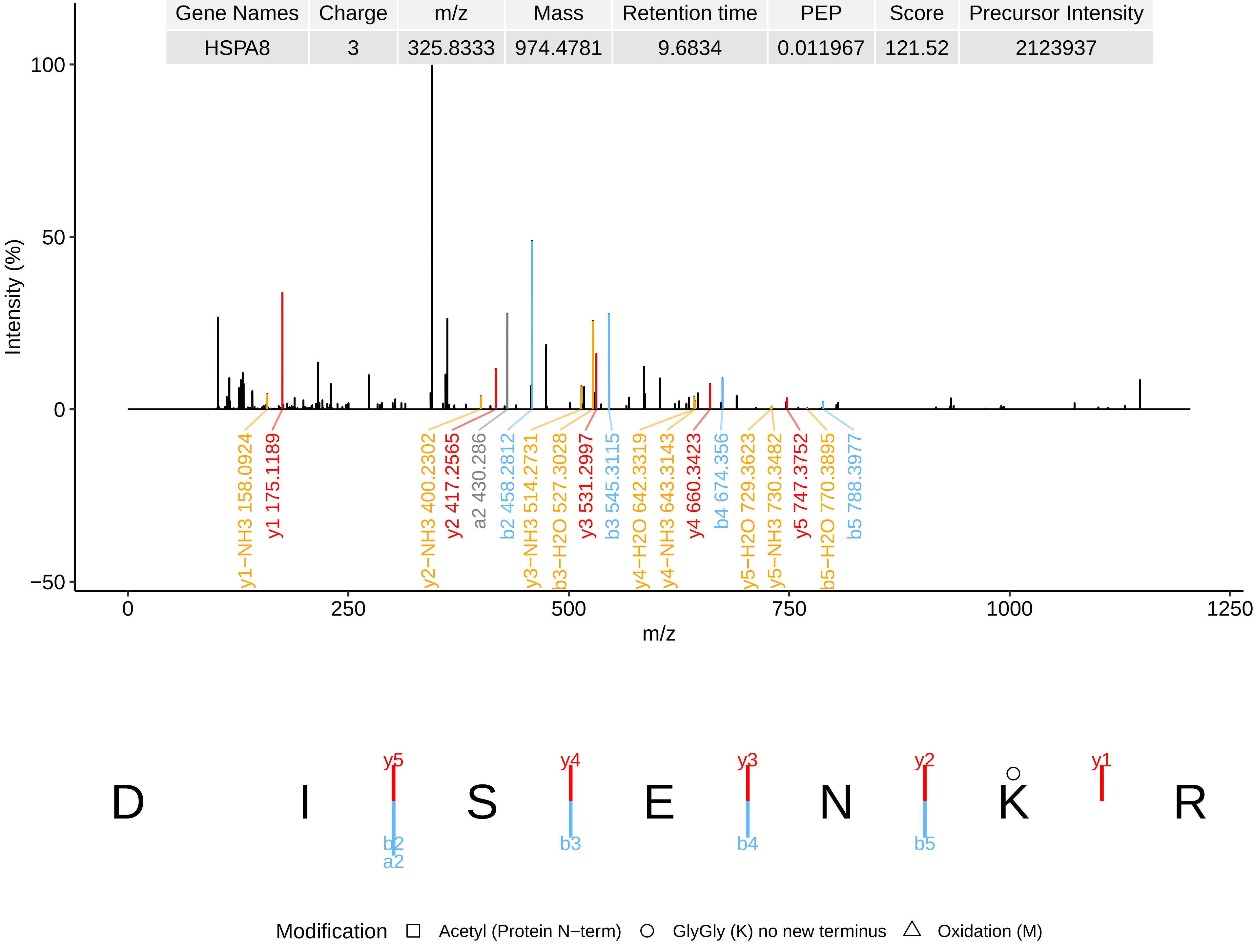

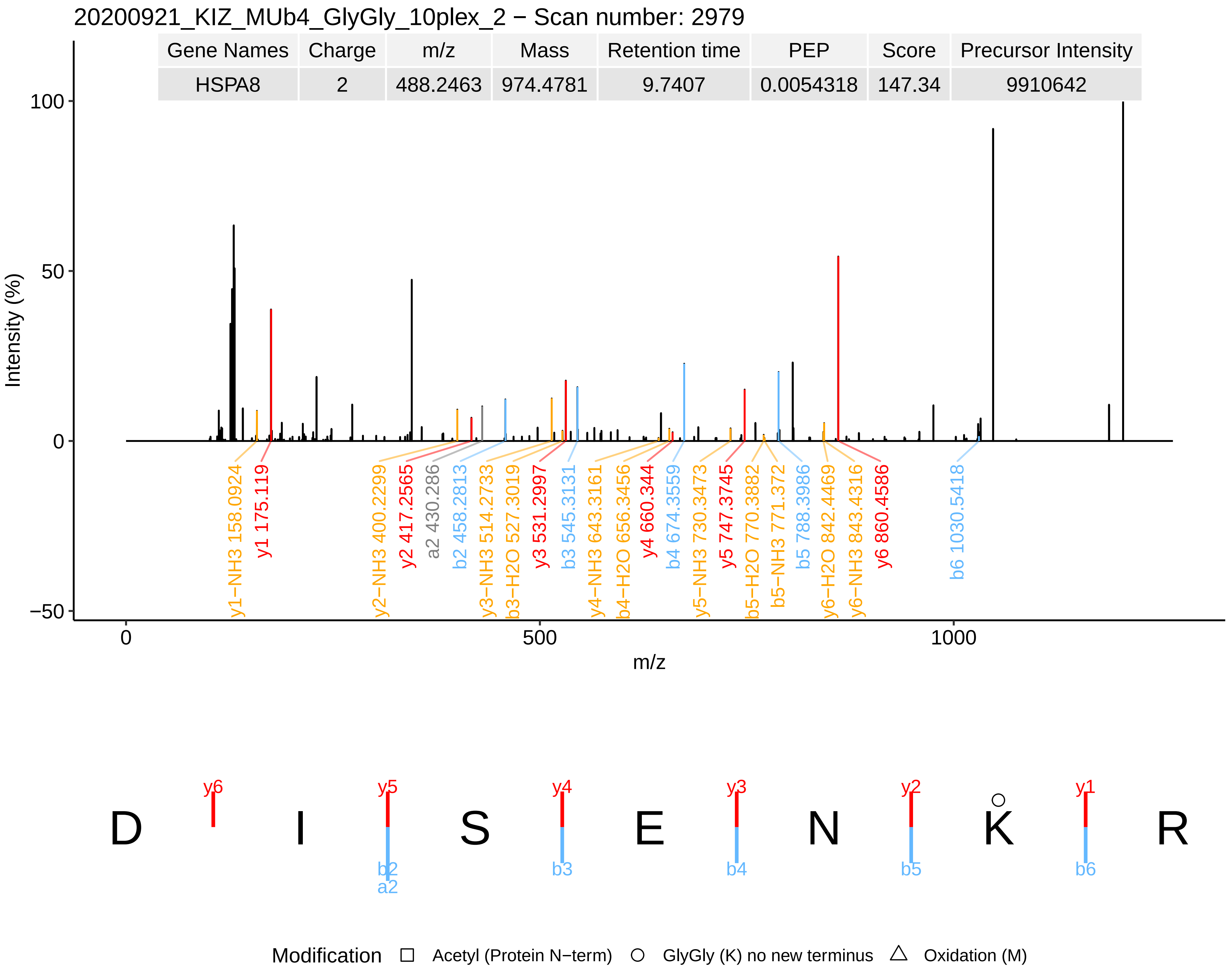

20200921_KIZ_MUb4_GlyGly_10plex_2 − Scan number: 2979
Gene Names | Charge | m/z | Mass | Retention time | PEP | Score | Precursor Intensity
HSPA8 | 2 | 488.2463 | 974.4781 | 9.7407 | 0.0054318 | 147.34 | 9910642
Intensity (%)
m/z
y1−NH3 158.0924
y1 175.119
y2−NH3 400.2299
y2 417.2565
a2 430.286
b2 458.2813
y3−NH3 514.2733
b3−H2O 527.3019
y3 531.2997
b3 545.3131
y4−NH3 643.3161
b4−H2O 656.3456
y4 660.344
b4 674.3559
y5−NH3 730.3473
y5 747.3745
b5−H2O 770.3882
b5−NH3 771.372
b5 788.3986
y6−H2O 842.4469
y6−NH3 843.4316
y6 860.4586
b6 1030.5418
D I S E N K R
Modification
Acetyl (Protein N−term)
GlyGly (K) no new terminus
Oxidation (M)

20200924_KIZ_MUb5_GlyGly_1 − Scan number: 2893

Gene Names	Charge	m/z	Mass	Retention time	PEP	Score	Precursor Intensity
HSPA8	3	325.8333	974.4781	8.9506	0.012186	120.45	10374616

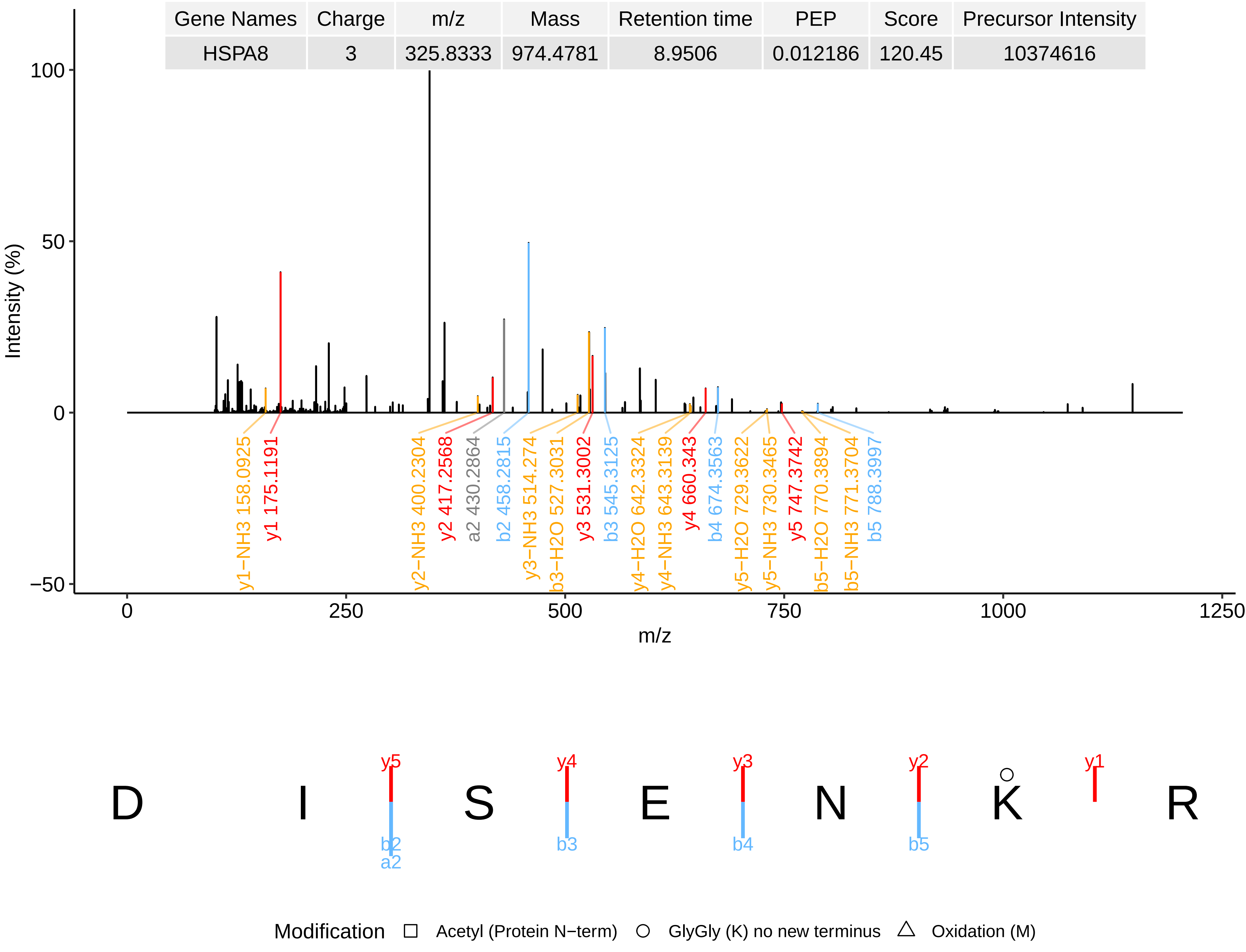

20200924_KIZ_MUb5_GlyGly_1 − Scan number: 2907

Gene Names	Charge	m/z	Mass	Retention time	PEP	Score	Precursor Intensity
HSPA8	2	488.2463	974.4781	8.9944	7.0992e−07	181.49	10554570

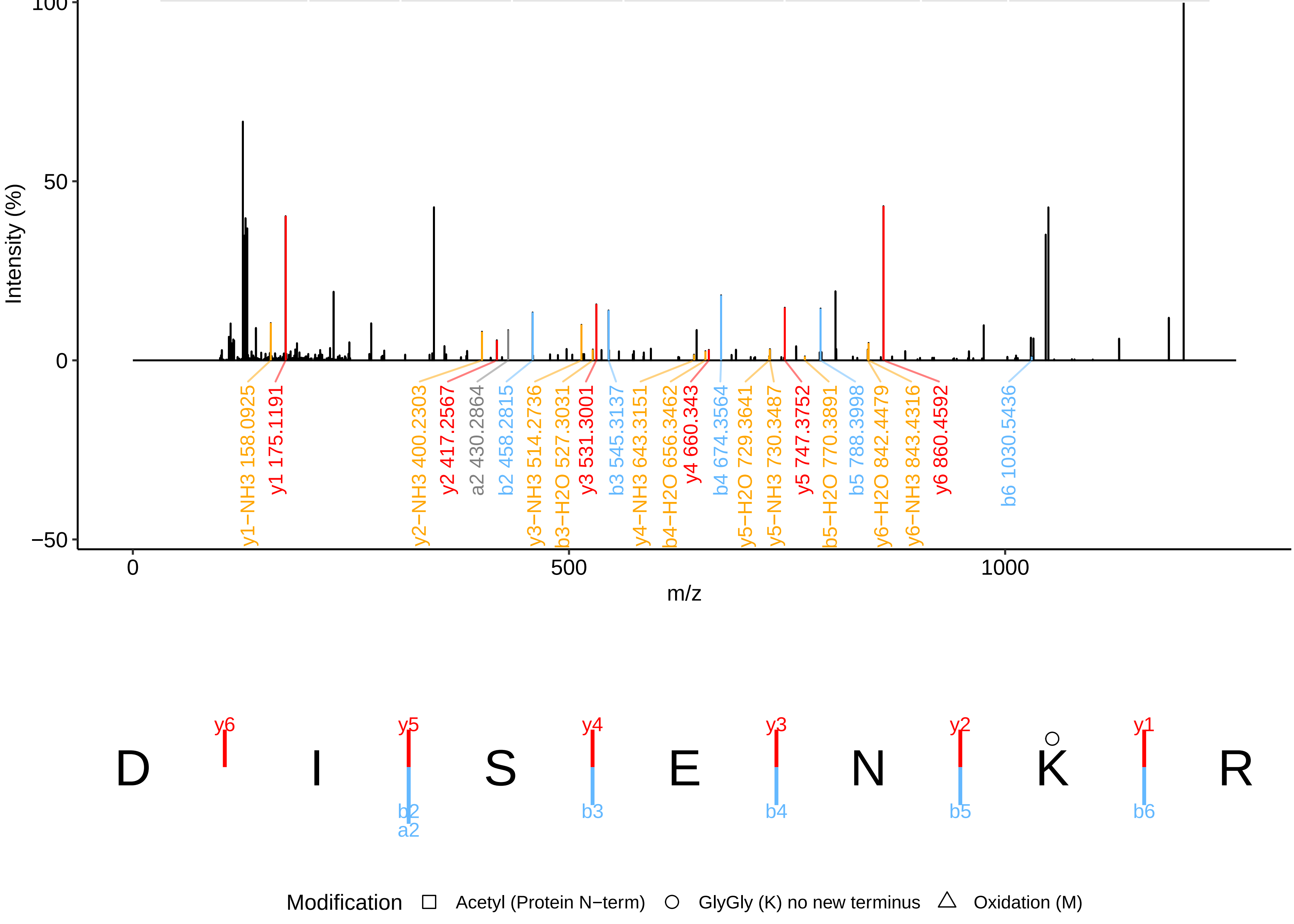

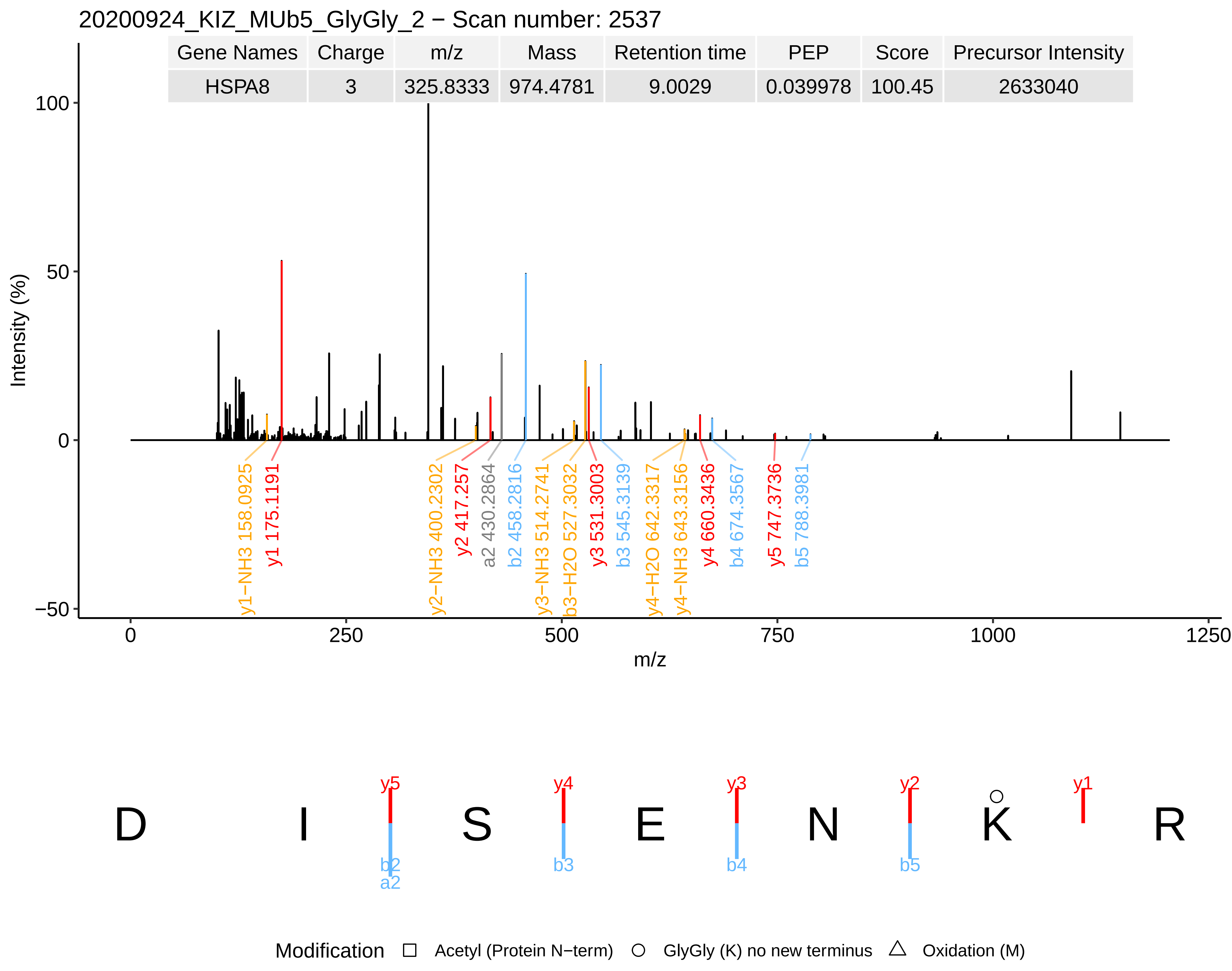

20200924_KIZ_MUb5_GlyGly_2 − Scan number: 2537
Gene Names | Charge | m/z | Mass | Retention time | PEP | Score | Precursor Intensity
HSPA8 | 3 | 325.8333 | 974.4781 | 9.0029 | 0.039978 | 100.45 | 2633040
Intensity (%)
m/z
y1−NH3 158.0925
y1 175.1191
y2−NH3 400.2302
y2 417.257
a2 430.2864
b2 458.2816
y3−NH3 514.2741
b3−H2O 527.3032
y3 531.3003
b3 545.3139
y4−H2O 642.3317
y4−NH3 643.3156
y4 660.3436
b4 674.3567
y5 747.3736
b5 788.3981
D I S E N K R
y5 y4 y3 y2 y1
b2 a2 b3 b4 b5
Modification
Acetyl (Protein N−term)
GlyGly (K) no new terminus
Oxidation (M)

20200924_KIZ_MUb5_GlyGly_2 − Scan number: 2673

Gene Names	Charge	m/z	Mass	Retention time	PEP	Score	Precursor Intensity
HSPA8	3	325.8333	974.4781	9.504	0.076788	90.777	6748492

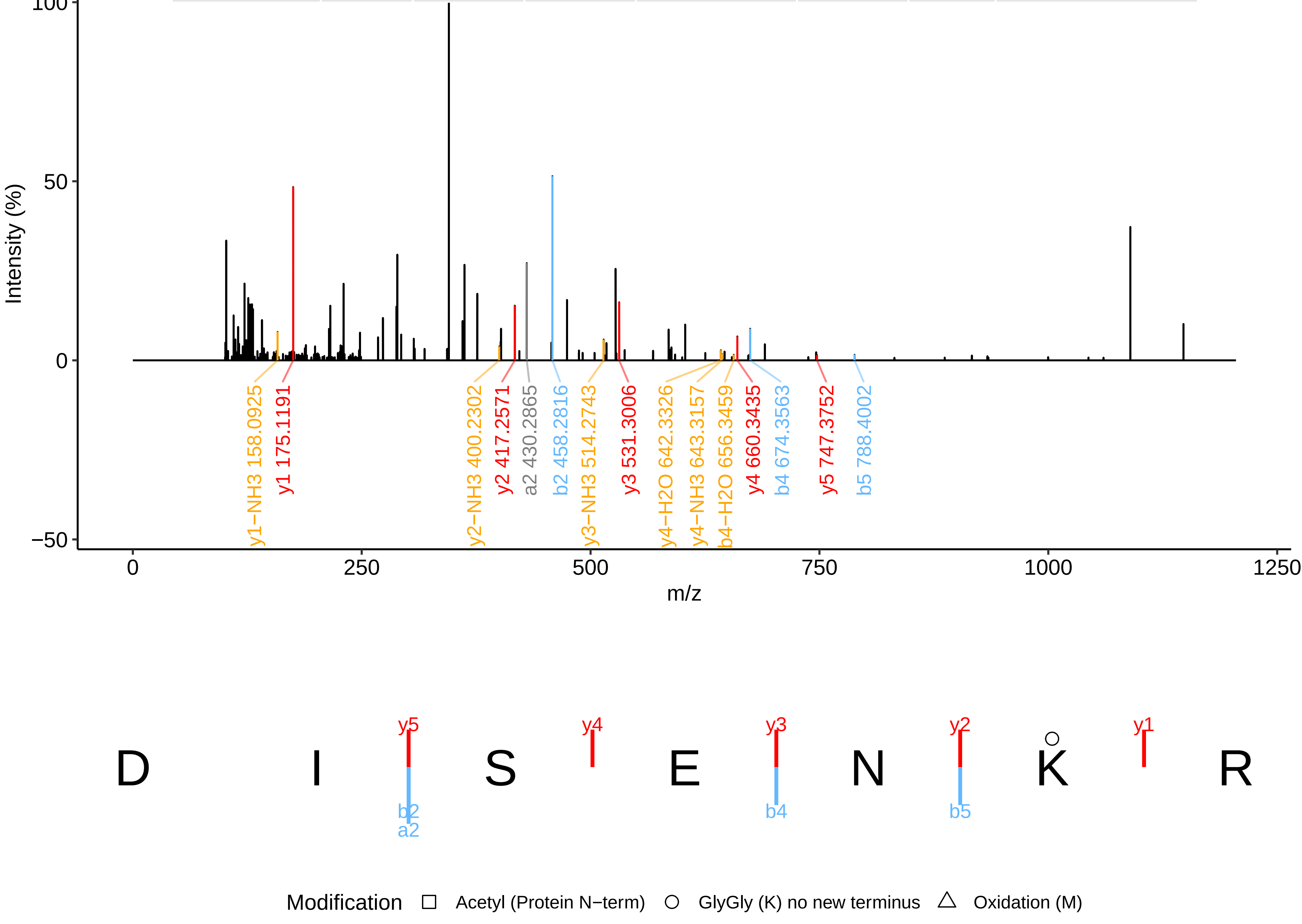

20201118_KIZ_MUb6_GlyGly_1 − Scan number: 2917

Gene Names	Charge	m/z	Mass	Retention time	PEP	Score	Precursor Intensity
HSPA8	3	325.8333	974.4781	8.9462	0.11318	82.671	52062960

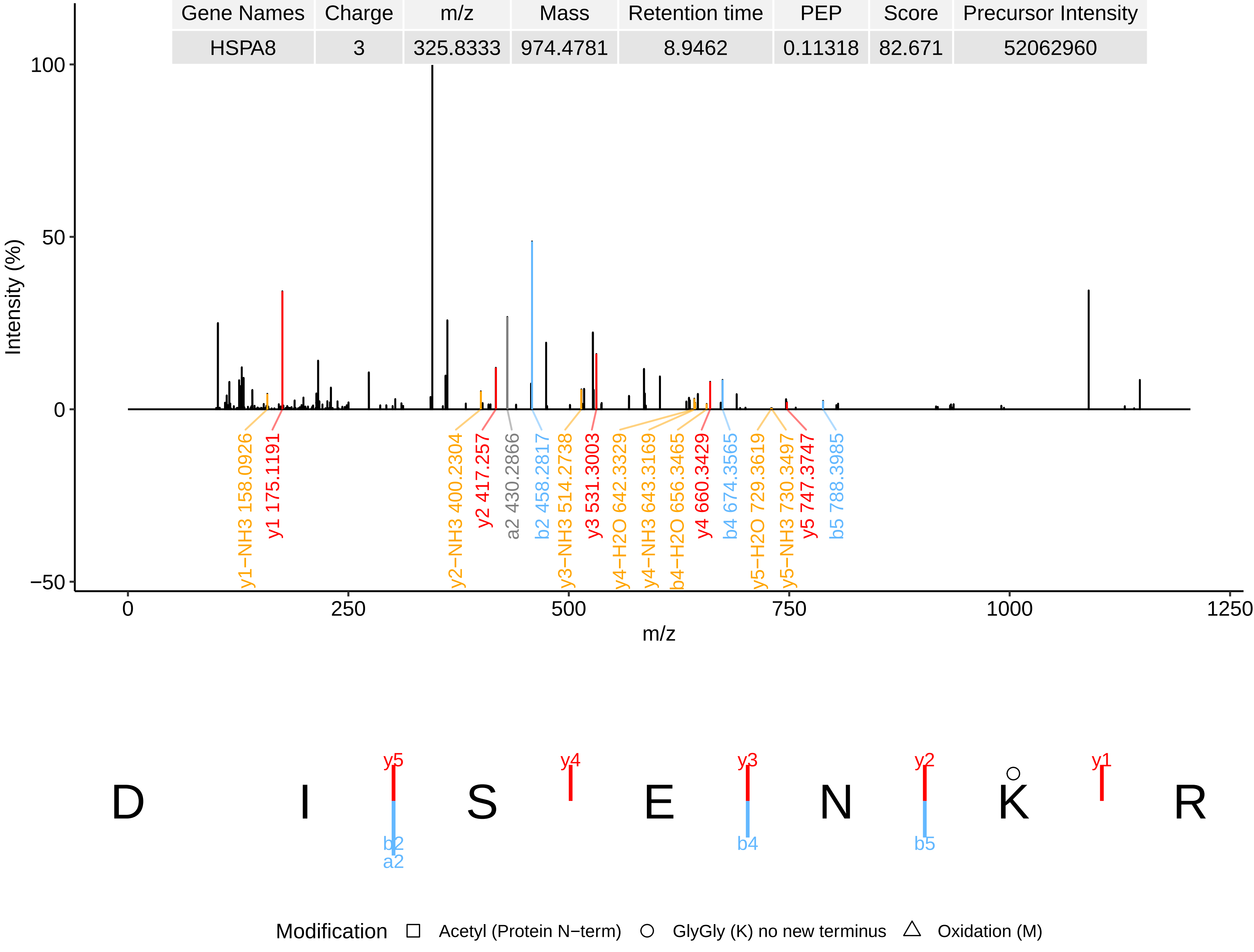

20201118_KIZ_MUb6_GlyGly_1 − Scan number: 2922

Gene Names	Charge	m/z	Mass	Retention time	PEP	Score	Precursor Intensity
HSPA8	2	488.2463	974.4781	8.9593	0.00076684	160.03	11975573

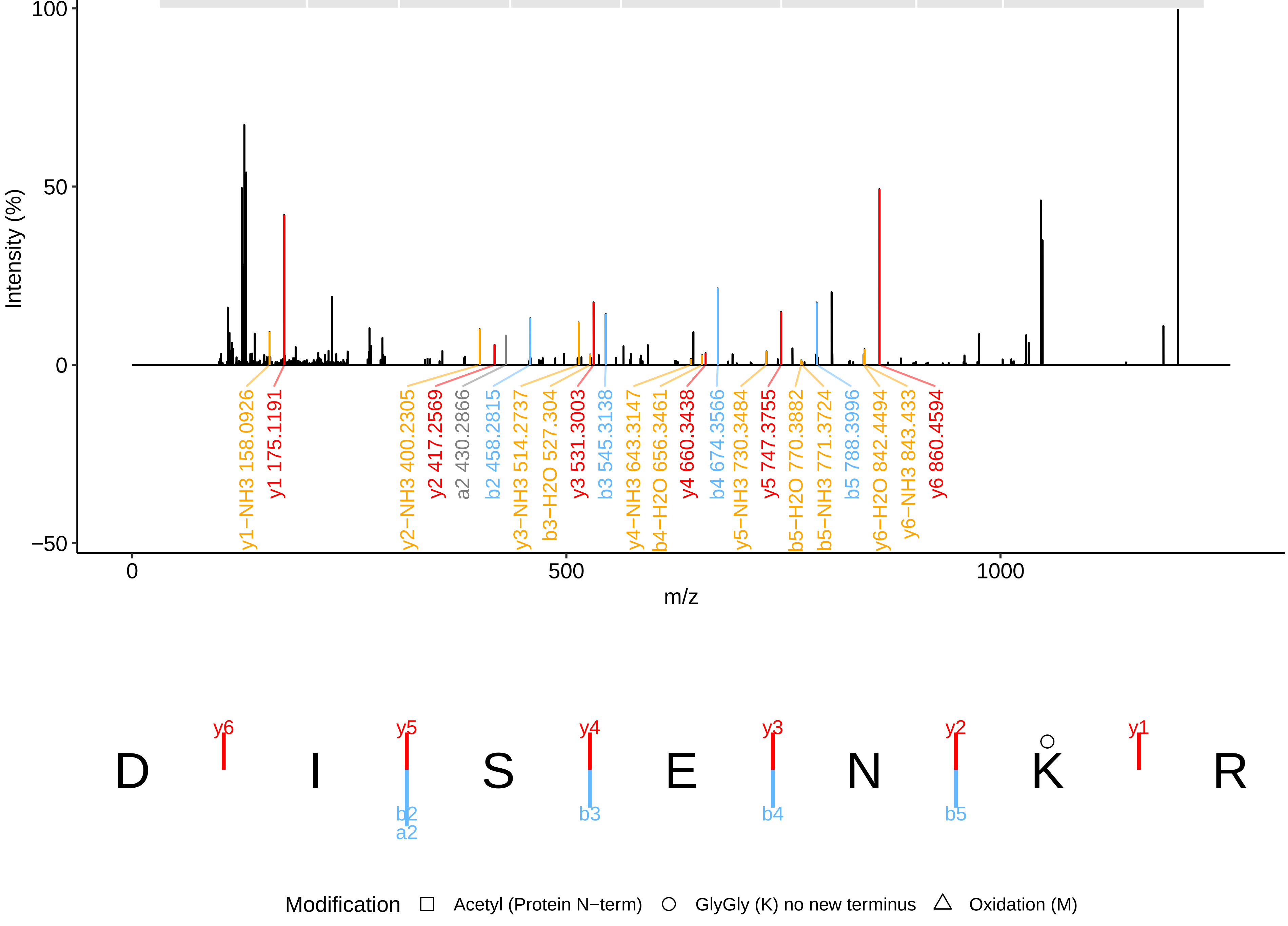

20201118_KIZ_MUb6_GlyGly_2 − Scan number: 3126

Gene Names	Charge	m/z	Mass	Retention time	PEP	Score	Precursor Intensity
HSPA8	3	325.8333	974.4781	10.145	0.0068677	138.24	42991824

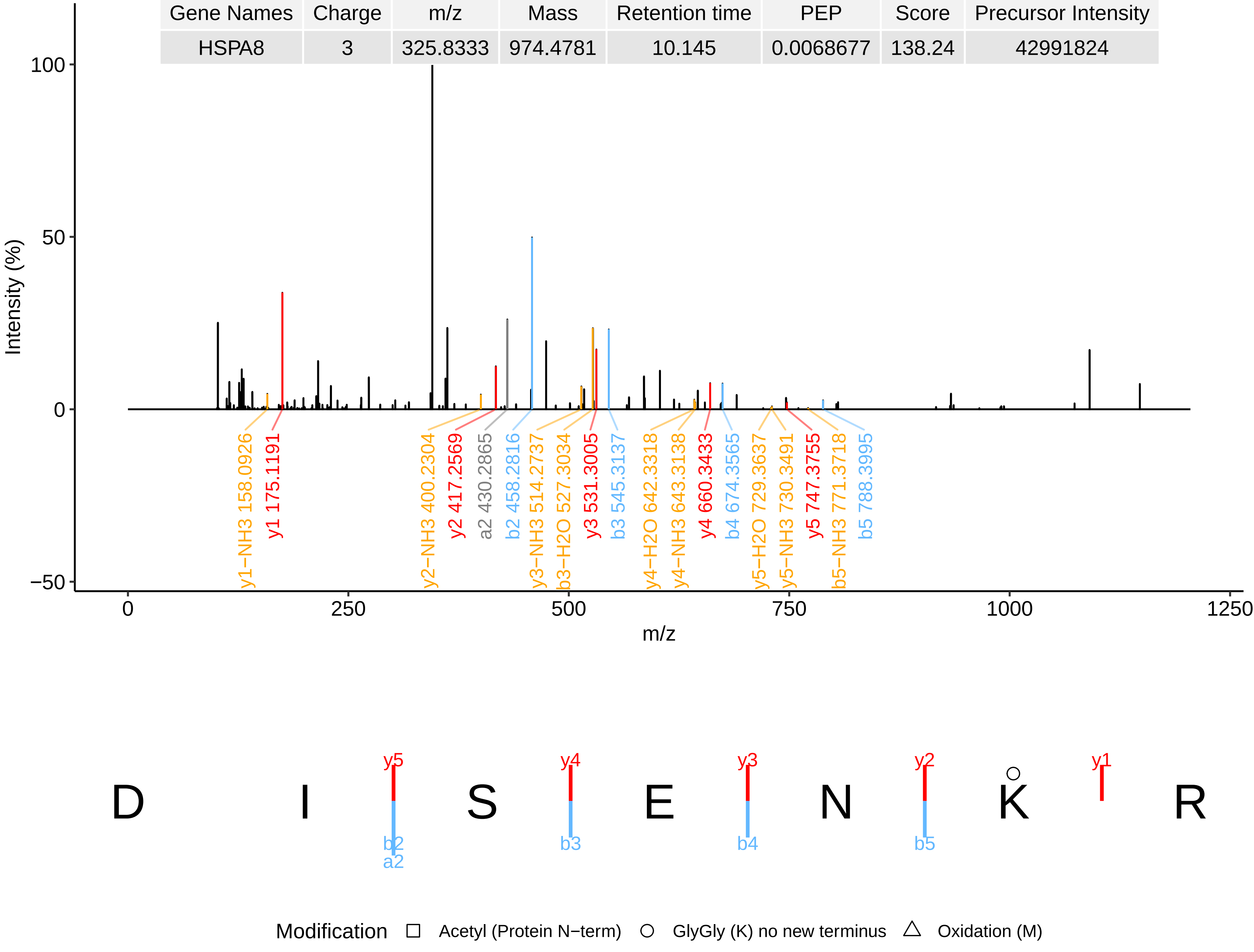

20200924_KIZ_MUb5_GlyGly_1 − Scan number: 2674

Gene Names	Charge	m/z	Mass	Retention time	PEP	Score	Precursor Intensity
HSPA1B;HSPA1A;HSPA1L	2	487.7543	973.4941	8.2633	0.029355	133.7	6979657

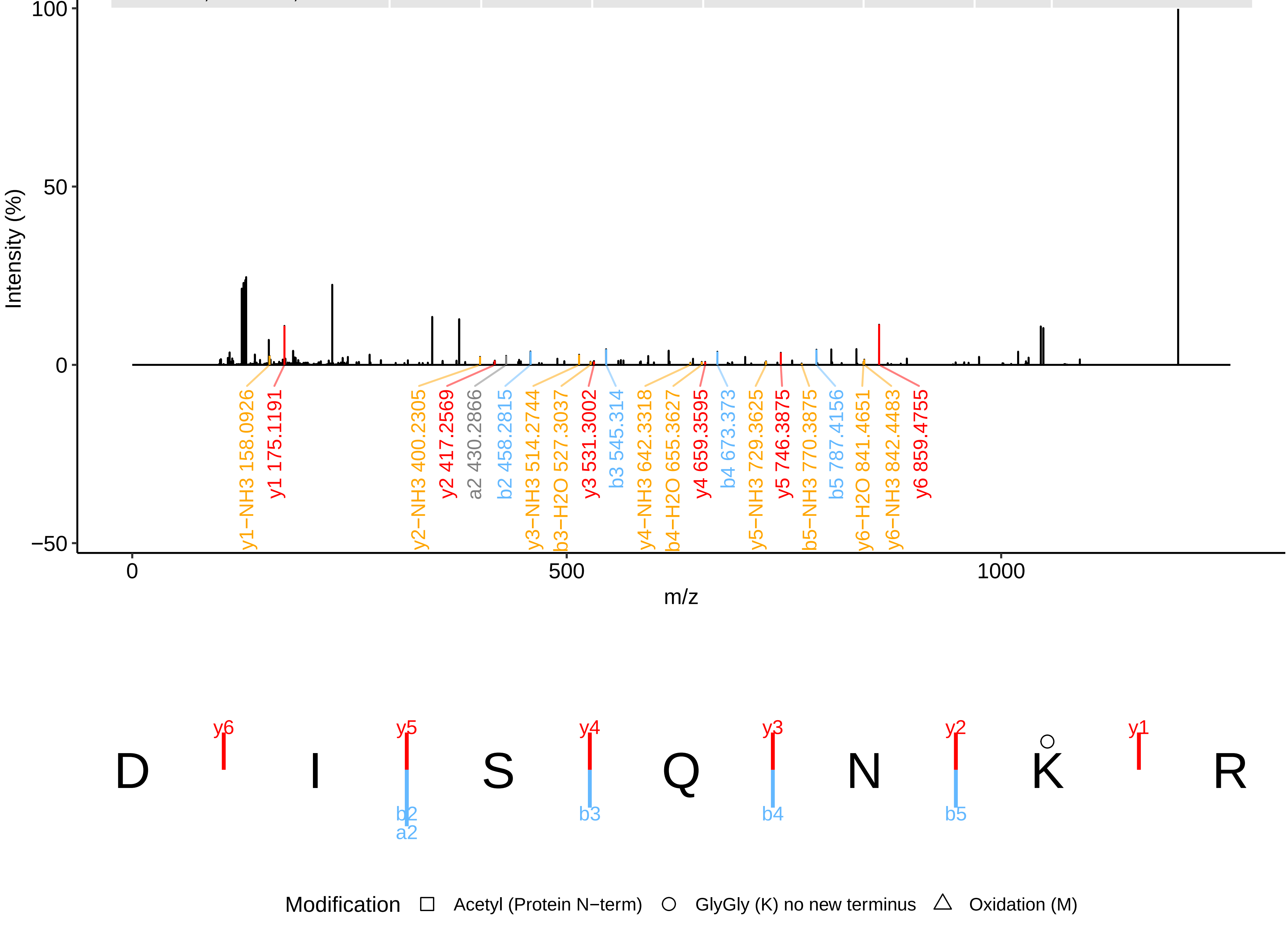

20200924_KIZ_MUb5_GlyGly_2 − Scan number: 2328

Gene Names	Charge	m/z	Mass	Retention time	PEP	Score	Precursor Intensity
HSPA1B;HSPA1A;HSPA1L	2	487.7543	973.4941	8.2195	0.055861	108.92	2929232

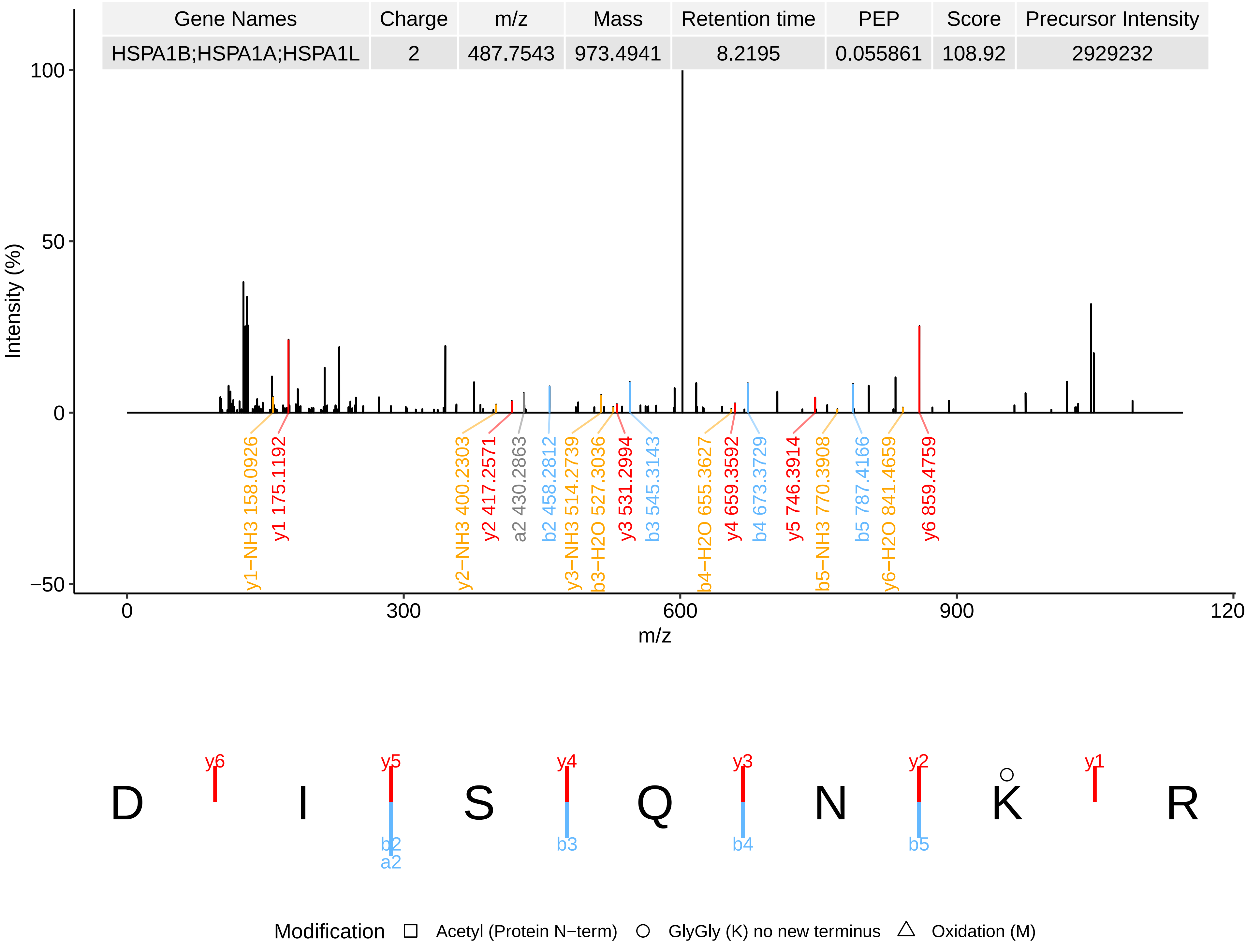

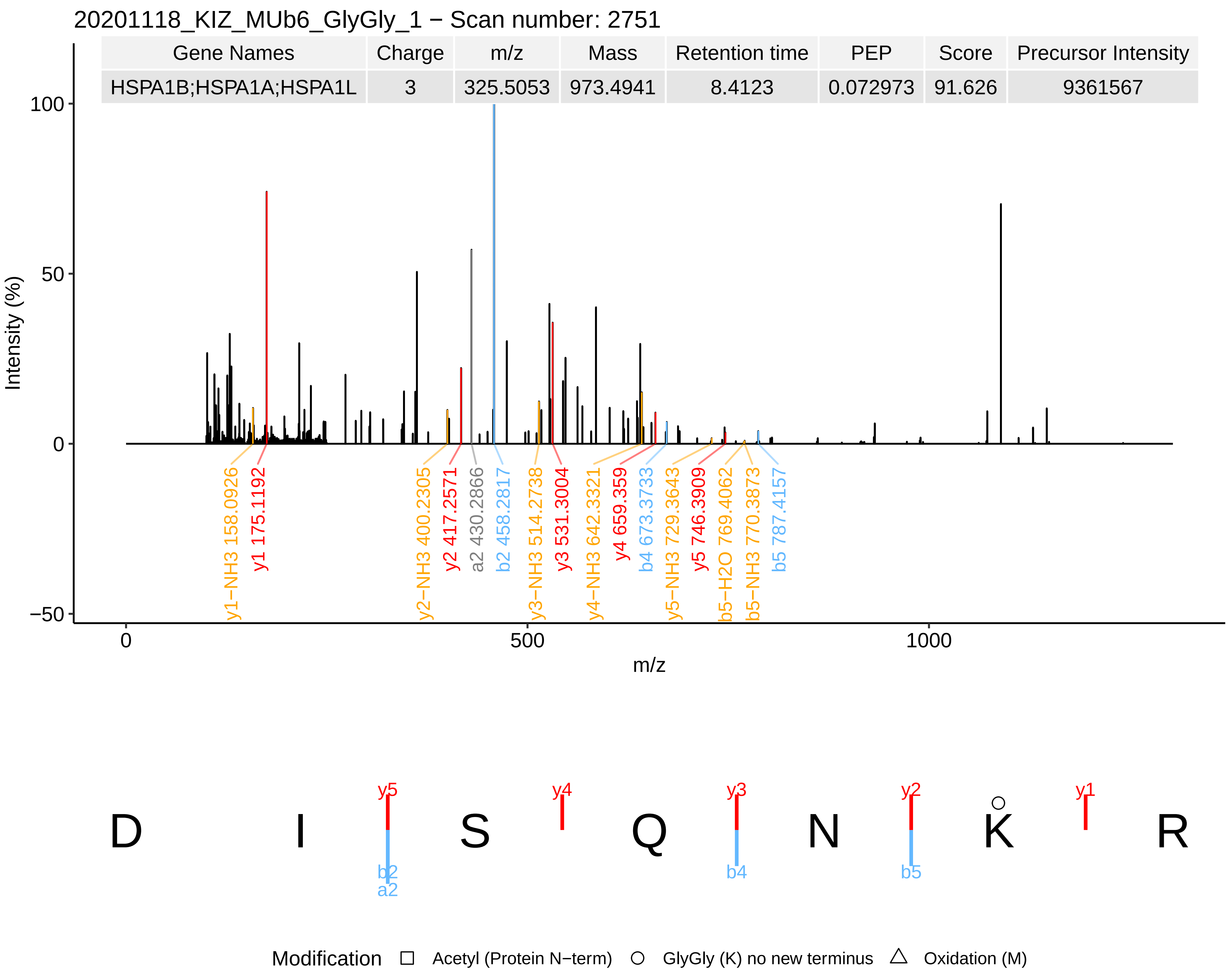

20201118_KIZ_MUb6_GlyGly_1 − Scan number: 2751
Gene Names | Charge | m/z | Mass | Retention time | PEP | Score | Precursor Intensity
HSPA1B;HSPA1A;HSPA1L | 3 | 325.5053 | 973.4941 | 8.4123 | 0.072973 | 91.626 | 9361567
Intensity (%)
m/z
y1−NH3 158.0926
y1 175.1192
y2−NH3 400.2305
y2 417.2571
a2 430.2866
b2 458.2817
y3−NH3 514.2738
y3 531.3004
y4−NH3 642.3321
y4 659.359
b4 673.3733
y5−NH3 729.3643
y5 746.3909
b5−H2O 769.4062
b5−NH3 770.3873
b5 787.4157
D I S Q N K R
Modification
Acetyl (Protein N−term)
GlyGly (K) no new terminus
Oxidation (M)

20201118_KIZ_MUb6_GlyGly_1 − Scan number: 2765

Gene Names	Charge	m/z	Mass	Retention time	PEP	Score	Precursor Intensity
HSPA1B;HSPA1A;HSPA1L	2	487.7543	973.4941	8.4548	0.046412	116.3	NA

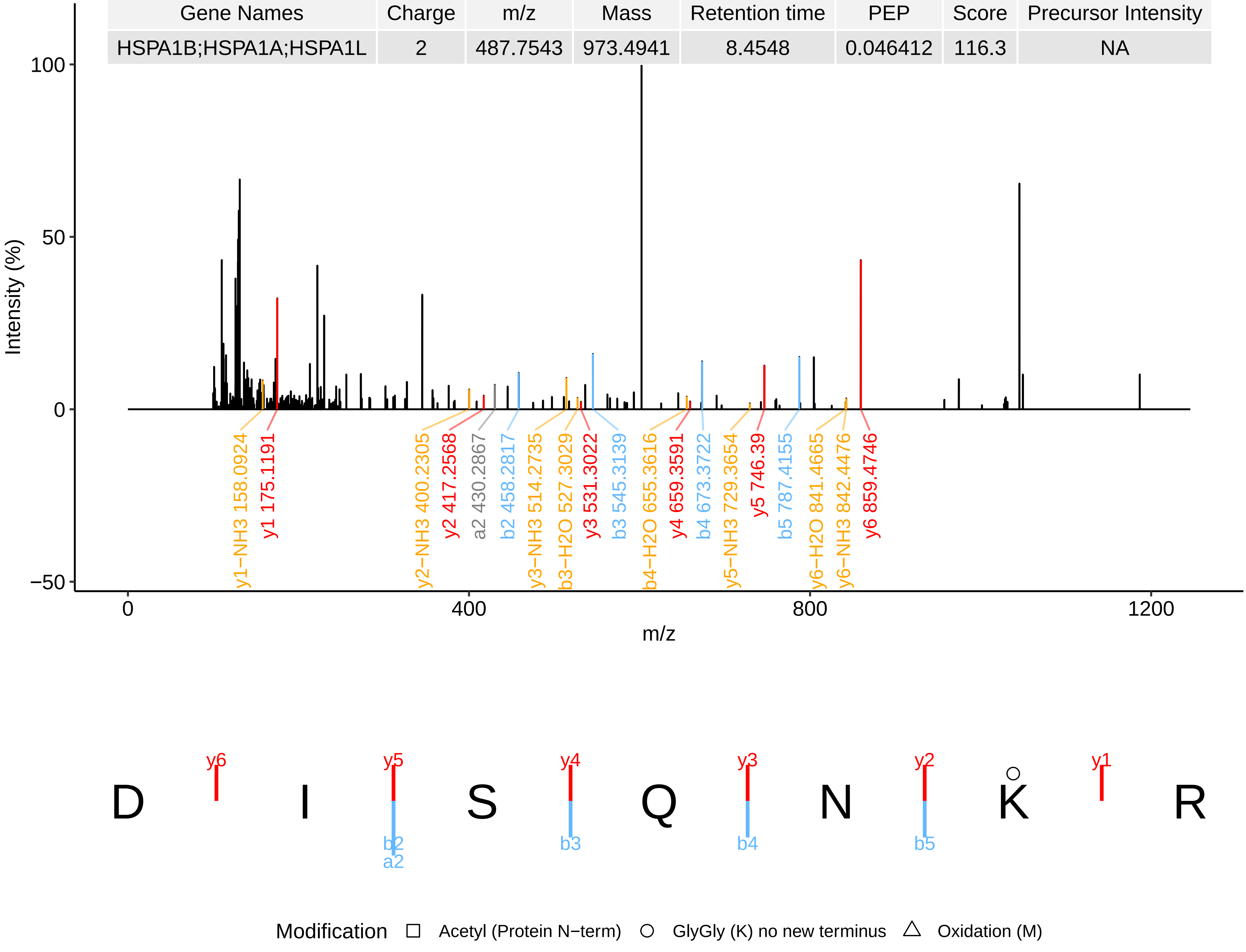

20201118_KIZ_MUb6_GlyGly_2 − Scan number: 2965

Gene Names	Charge	m/z	Mass	Retention time	PEP	Score	Precursor Intensity
HSPA1B;HSPA1A;HSPA1L	3	325.5053	973.4941	9.5326	0.059343	94.662	15786048

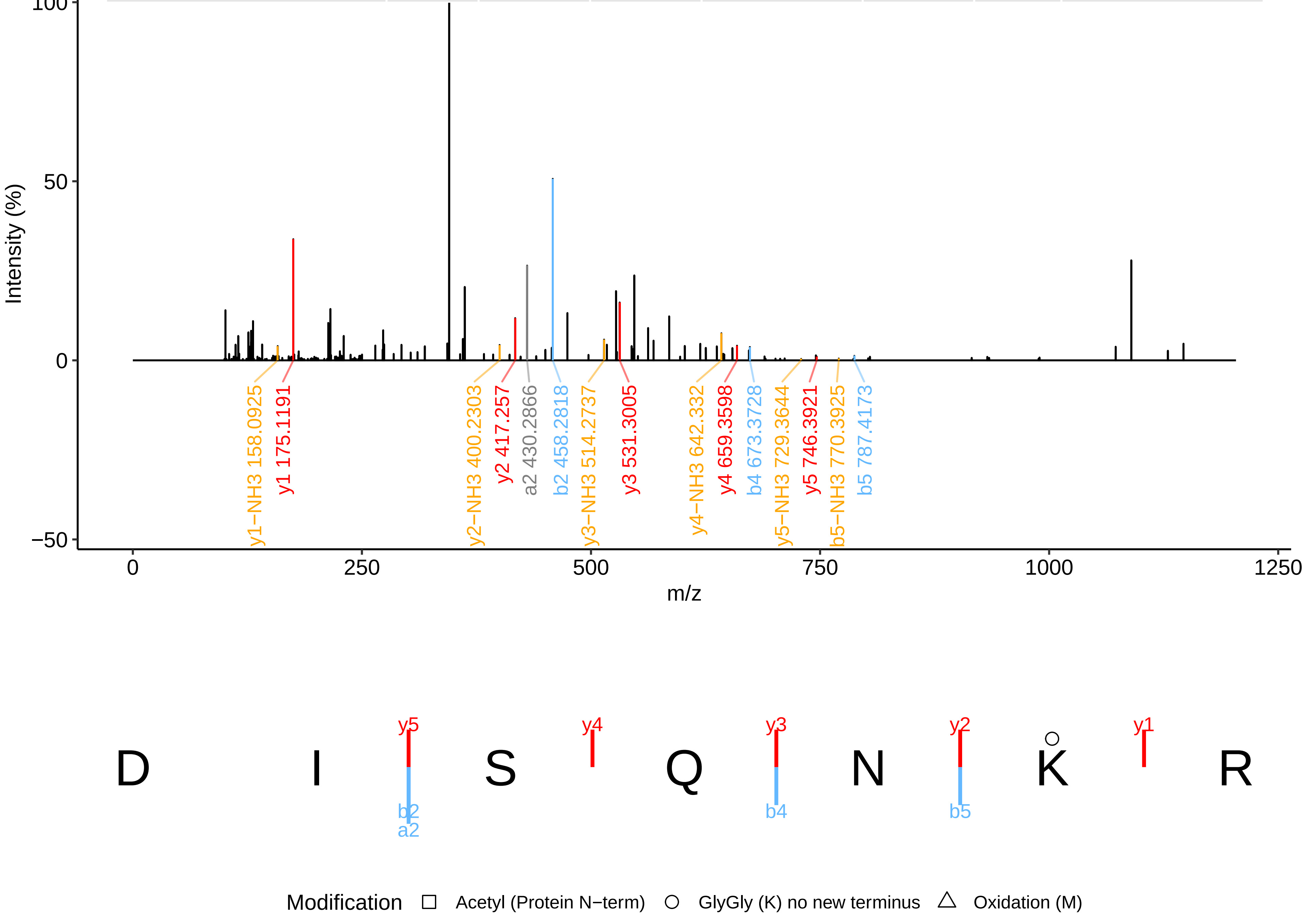

20200921_KIZ_MUb4_GlyGly_10plex − Scan number: 6721

Gene Names	Charge	m/z	Mass	Retention time	PEP	Score	Precursor Intensity
TRABD	3	315.5223	943.5451	22.344	0.027284	77.674	13368706

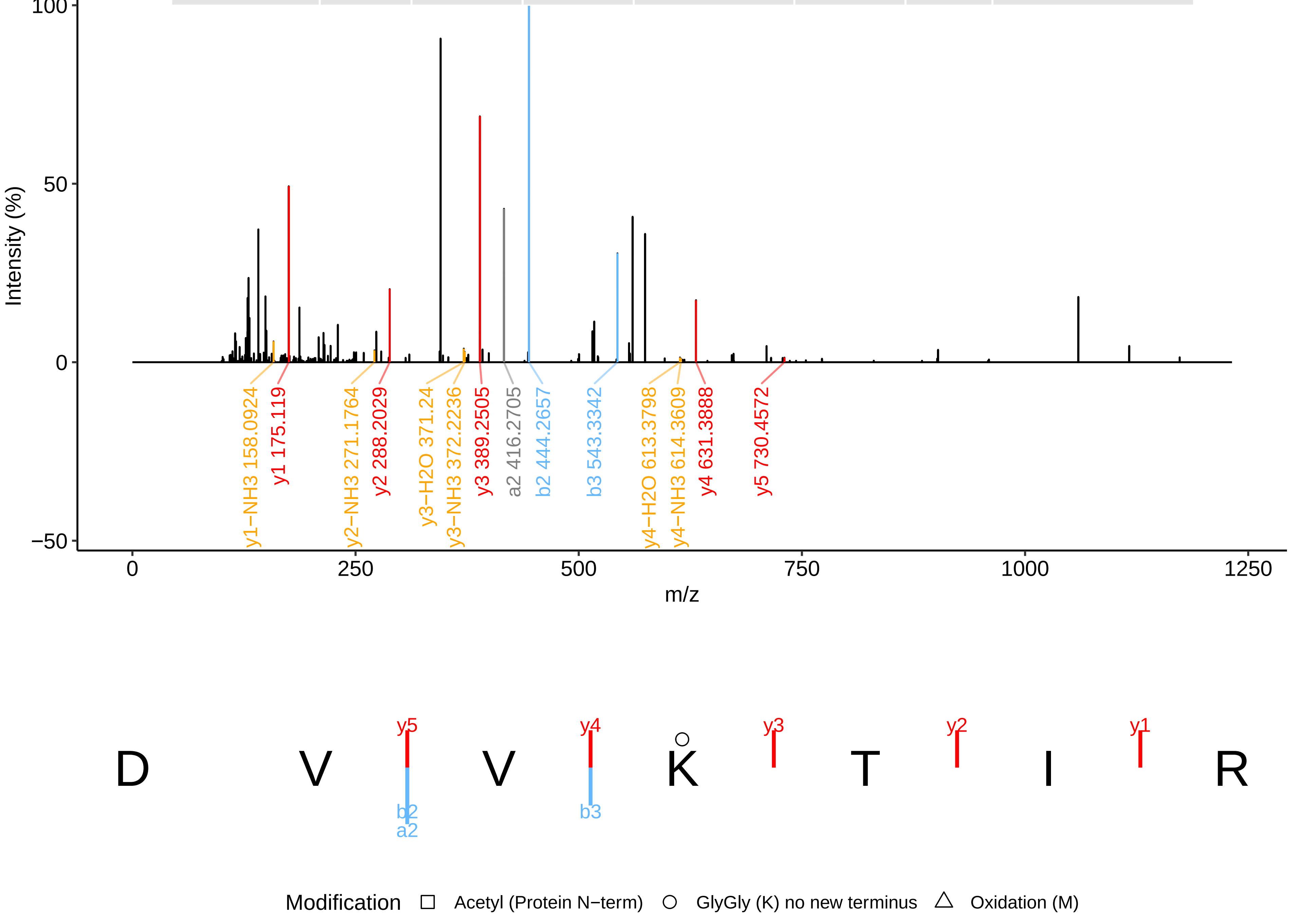

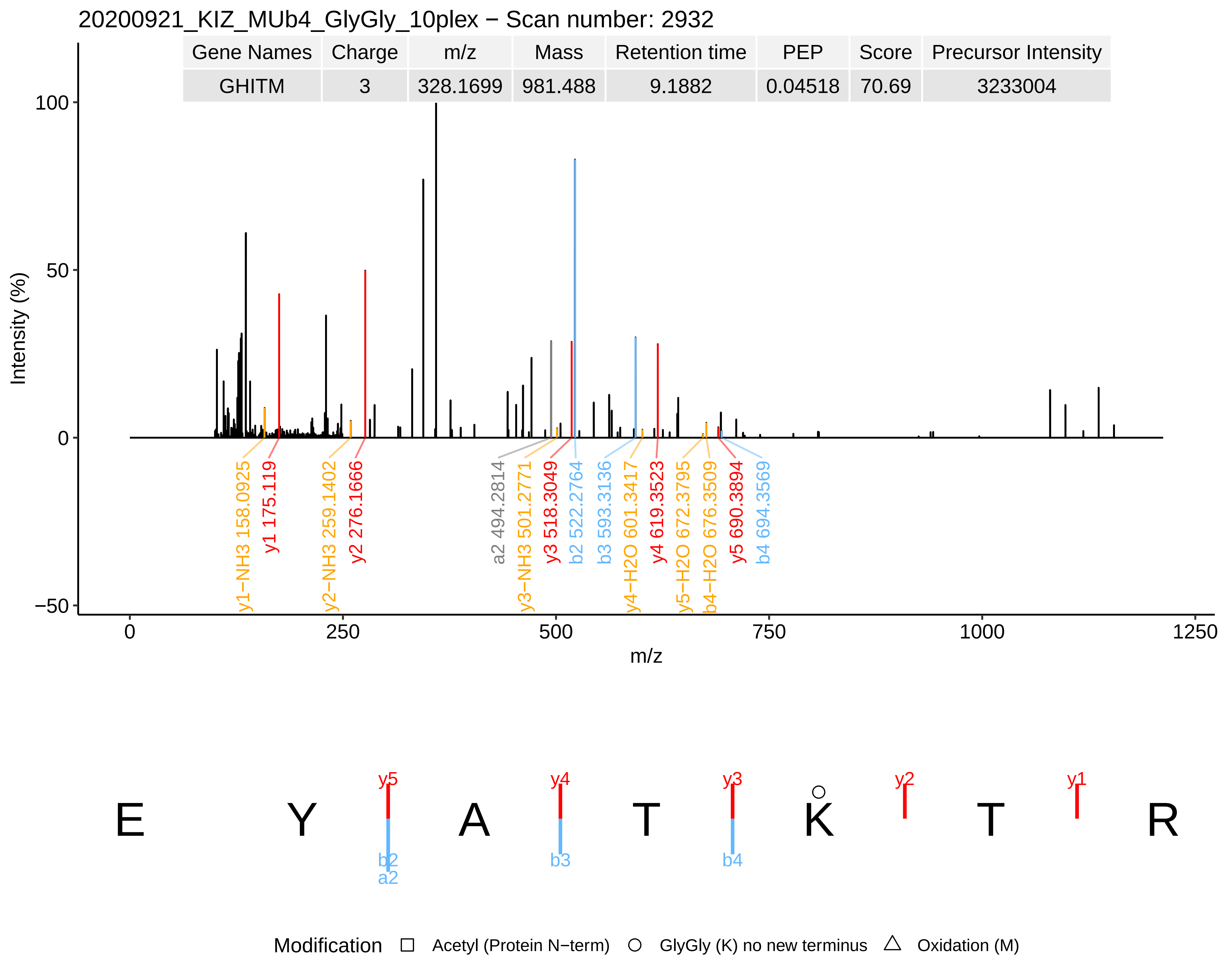

20200921_KIZ_MUb4_GlyGly_10plex − Scan number: 2932
Gene Names | Charge | m/z | Mass | Retention time | PEP | Score | Precursor Intensity
GHITM | 3 | 328.1699 | 981.488 | 9.1882 | 0.04518 | 70.69 | 3233004
Intensity (%)
100
50
0
−50
0
250
500
750
1000
1250
m/z
y1−NH3 158.0925
y1 175.119
y2−NH3 259.1402
y2 276.1666
a2 494.2814
y3−NH3 501.2771
y3 518.3049
b2 522.2764
b3 593.3136
y4−H2O 601.3417
y4 619.3523
y5−H2O 672.3795
b4−H2O 676.3509
y5 690.3894
b4 694.3569
E Y A T K T R
y5 y4 y3 y2 y1
b2 a2 b3 b4
Modification
Acetyl (Protein N−term)
GlyGly (K) no new terminus
Oxidation (M)

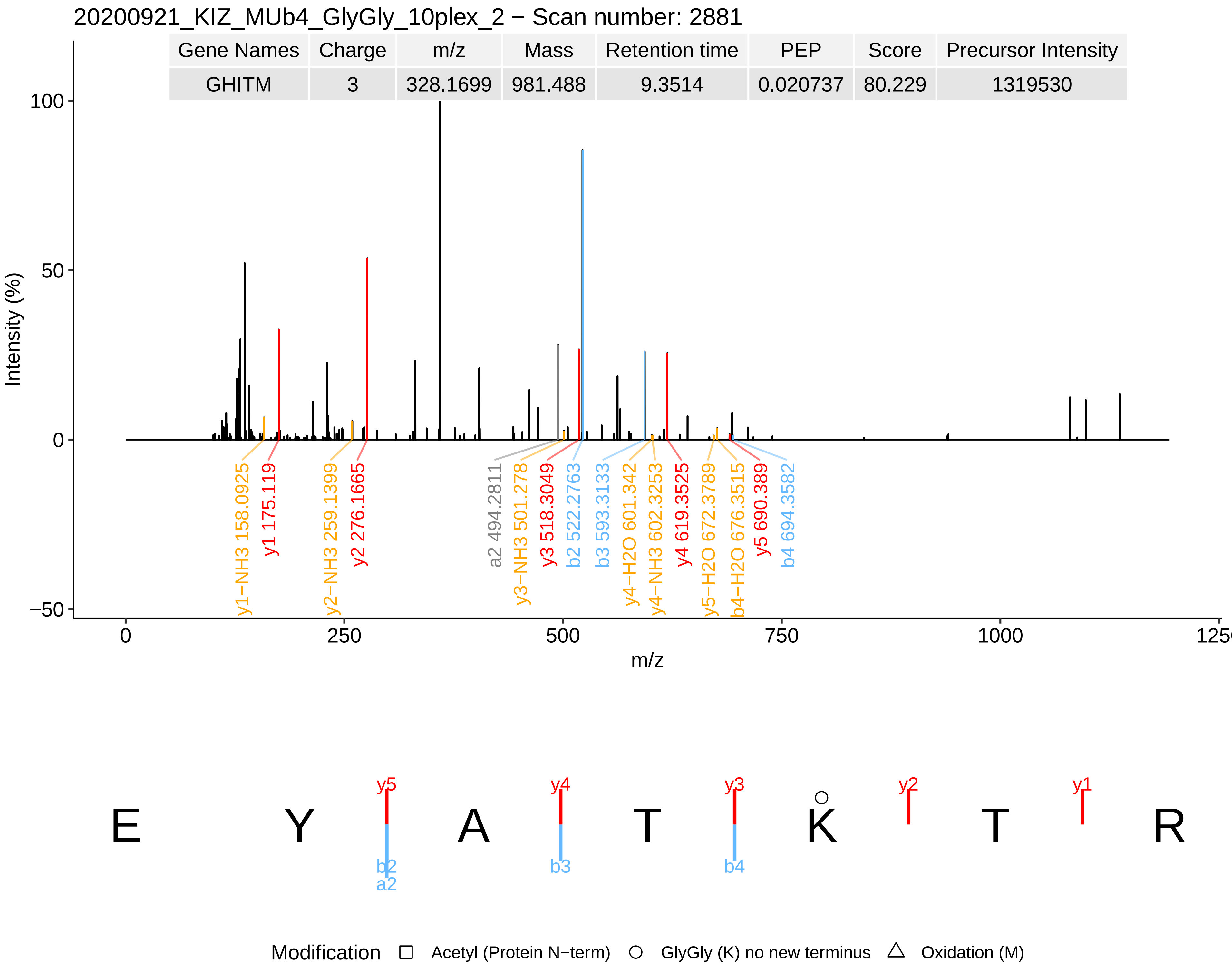

20200921_KIZ_MUb4_GlyGly_10plex_2 − Scan number: 2881
Gene Names | Charge | m/z | Mass | Retention time | PEP | Score | Precursor Intensity
GHITM | 3 | 328.1699 | 981.488 | 9.3514 | 0.020737 | 80.229 | 1319530
Intensity (%)
m/z
y1−NH3 158.0925
y1 175.119
y2−NH3 259.1399
y2 276.1665
a2 494.2811
y3−NH3 501.278
y3 518.3049
b2 522.2763
b3 593.3133
y4−H2O 601.342
y4−NH3 602.3253
y4 619.3525
y5−H2O 672.3789
b4−H2O 676.3515
y5 690.389
b4 694.3582
E Y A T K T R
y5 y4 y3 y2 y1
b2 a2 b3 b4
Modification
Acetyl (Protein N−term)
GlyGly (K) no new terminus
Oxidation (M)

20200924_KIZ_MUb5_GlyGly_2 − Scan number: 2467

Gene Names	Charge	m/z	Mass	Retention time	PEP	Score	Precursor Intensity
GHITM	3	328.1699	981.488	8.7259	0.10899	54.157	1458520

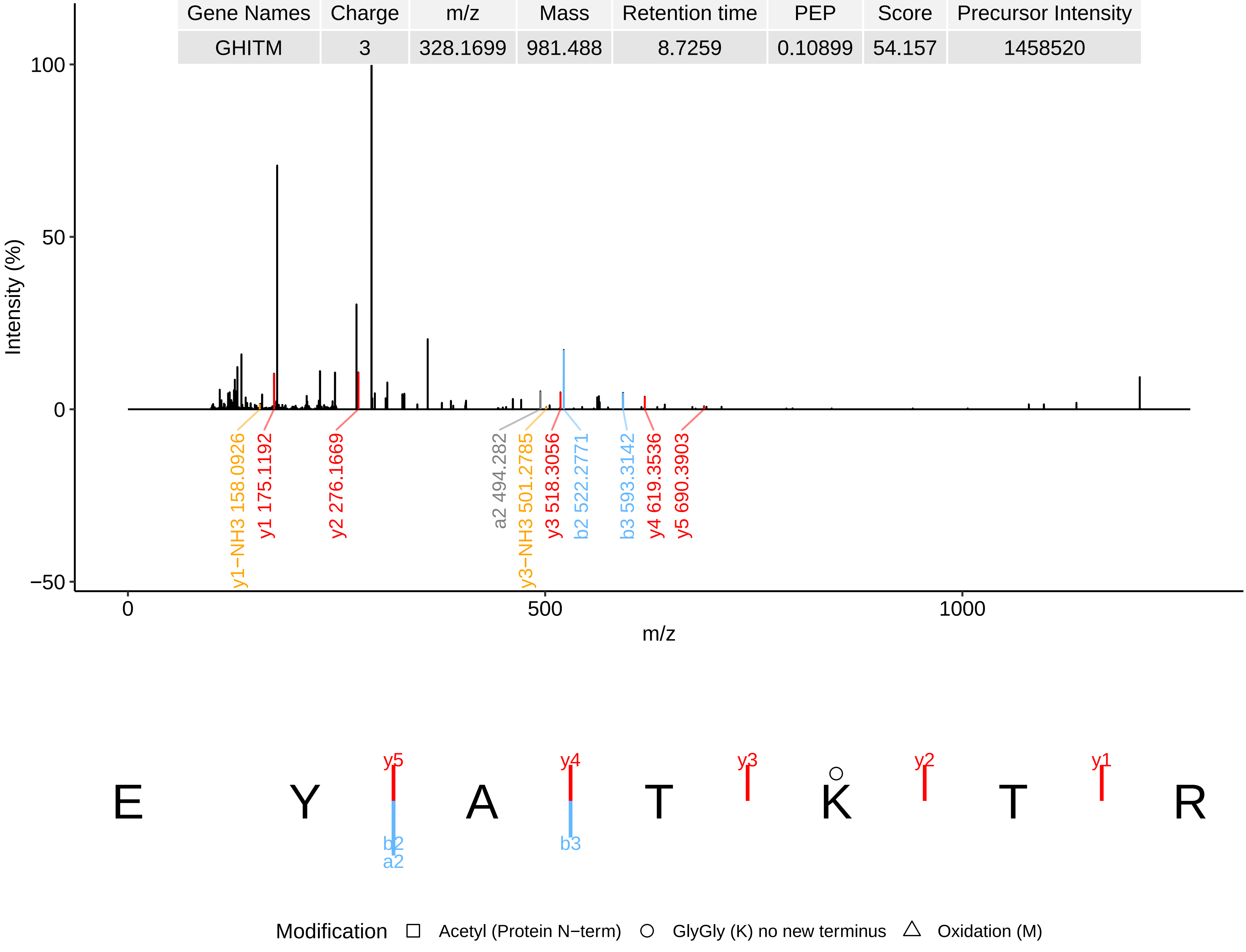

20201118_KIZ_MUb6_GlyGly_1 − Scan number: 2847

Gene Names	Charge	m/z	Mass	Retention time	PEP	Score	Precursor Intensity
GHITM	3	328.1699	981.488	8.7213	0.017179	84.227	10159611

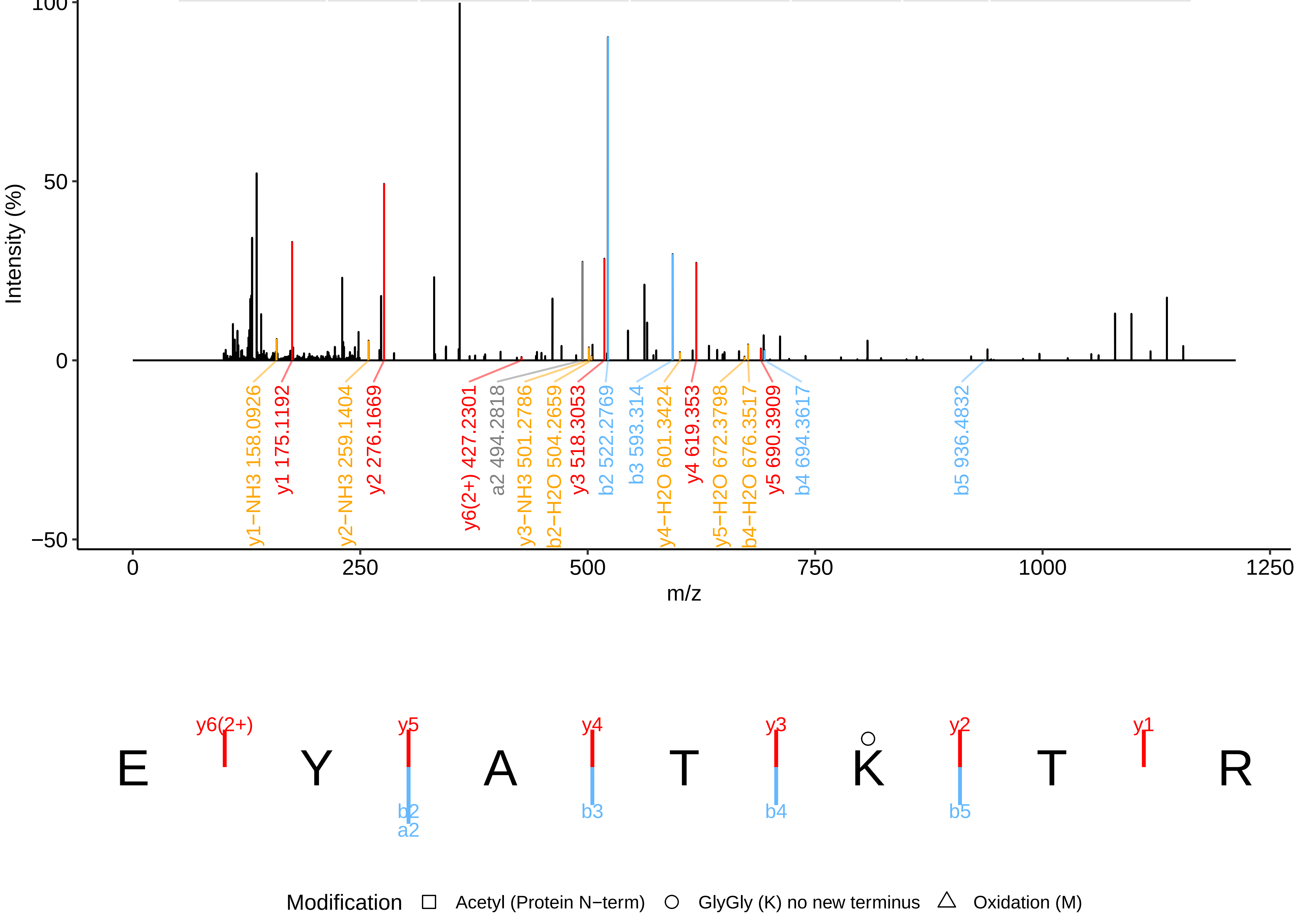

20201118_KIZ_MUb6_GlyGly_2 − Scan number: 3083

Gene Names	Charge	m/z	Mass	Retention time	PEP	Score	Precursor Intensity
GHITM	3	328.1699	981.488	9.9873	0.052322	67.903	7755784

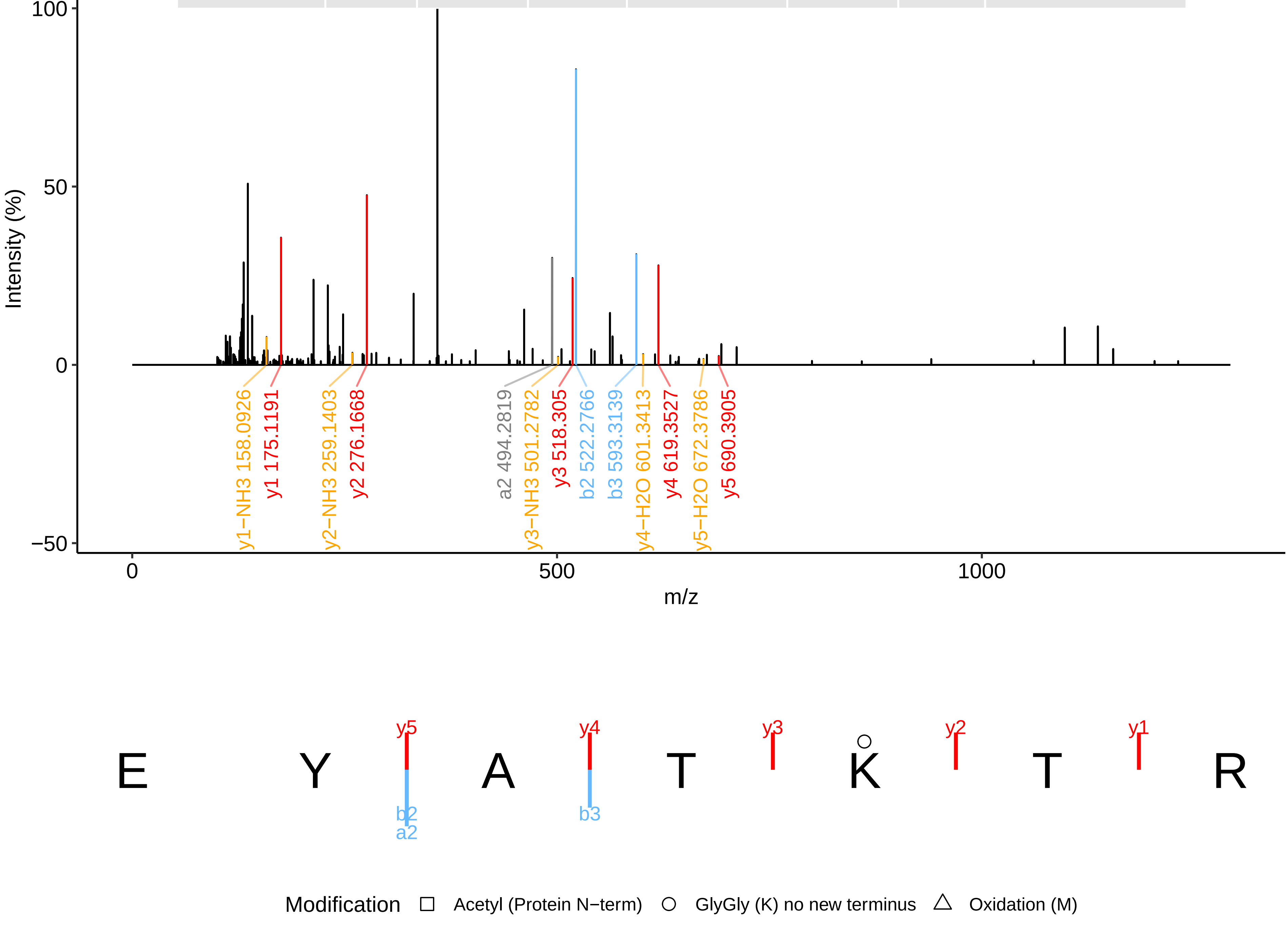

20200921_KIZ_MUb4_GlyGly_10plex − Scan number: 5543

Gene Names	Charge	m/z	Mass	Retention time	PEP	Score	Precursor Intensity
RPL18A	2	482.7642	963.5138	18.171	0.019295	103.75	4489558

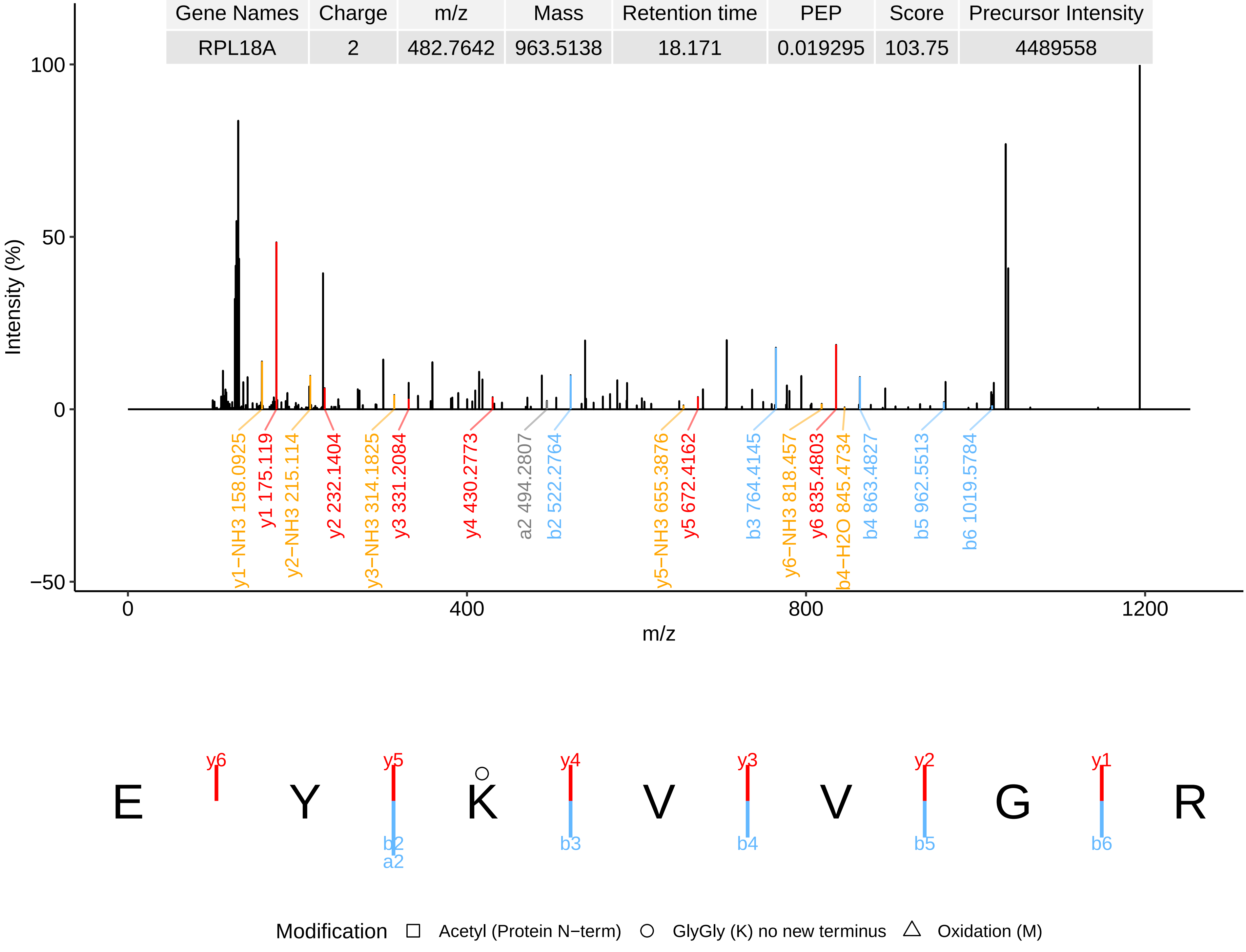

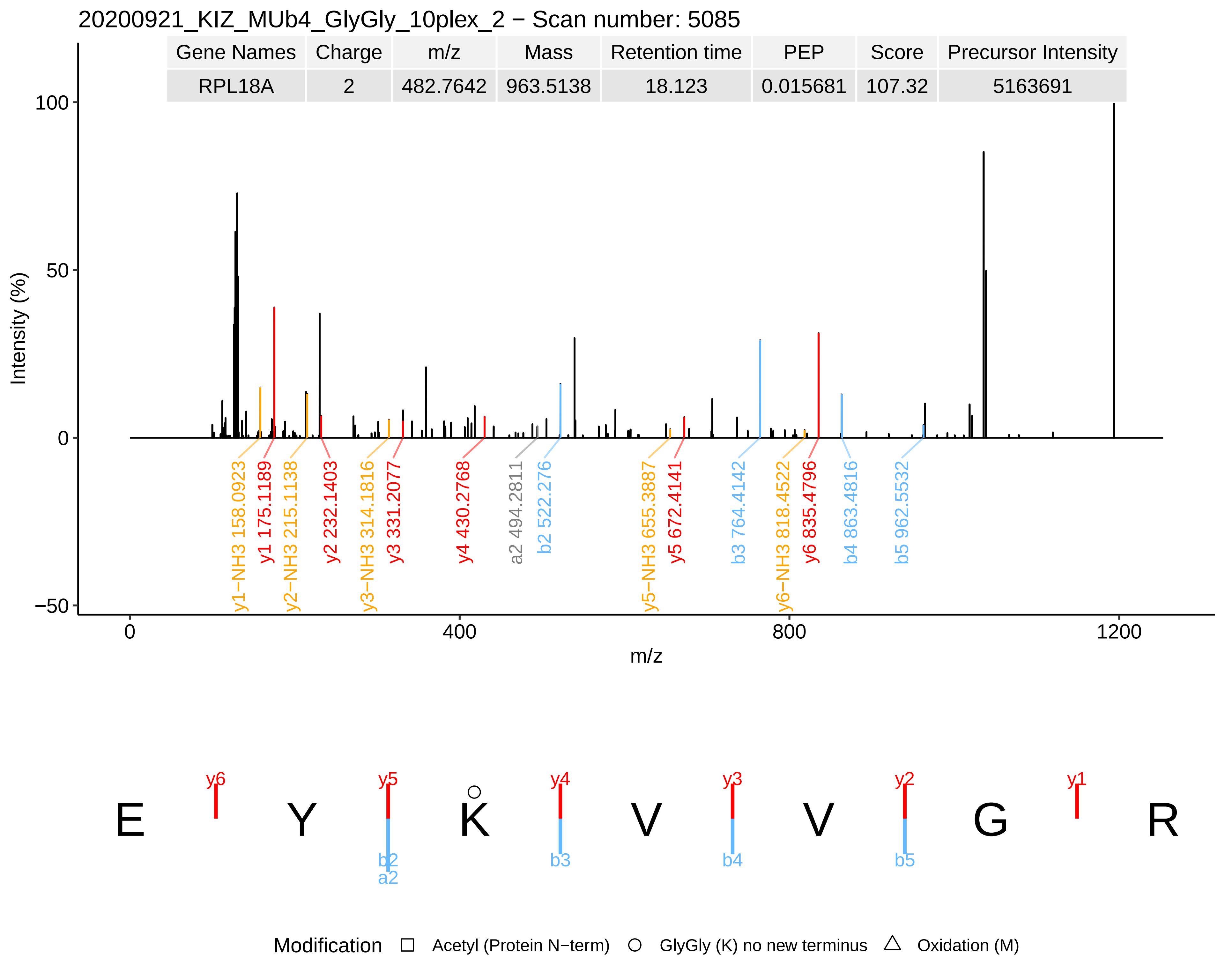
20200921_KIZ_MUb4_GlyGly_10plex_2 − Scan number: 5085
Gene Names | Charge | m/z | Mass | Retention time | PEP | Score | Precursor Intensity
RPL18A | 2 | 482.7642 | 963.5138 | 18.123 | 0.015681 | 107.32 | 5163691
Intensity (%)
m/z
y1−NH3 158.0923
y1 175.1189
y2−NH3 215.1138
y2 232.1403
y3−NH3 314.1816
y3 331.2077
y4 430.2768
a2 494.2811
b2 522.276
y5−NH3 655.3887
y5 672.4141
b3 764.4142
y6−NH3 818.4522
y6 835.4796
b4 863.4816
b5 962.5532
E Y K V V G R
y6 y5 y4 y3 y2 y1
b2 a2 b3 b4 b5
Modification
Acetyl (Protein N−term)
GlyGly (K) no new terminus
Oxidation (M)

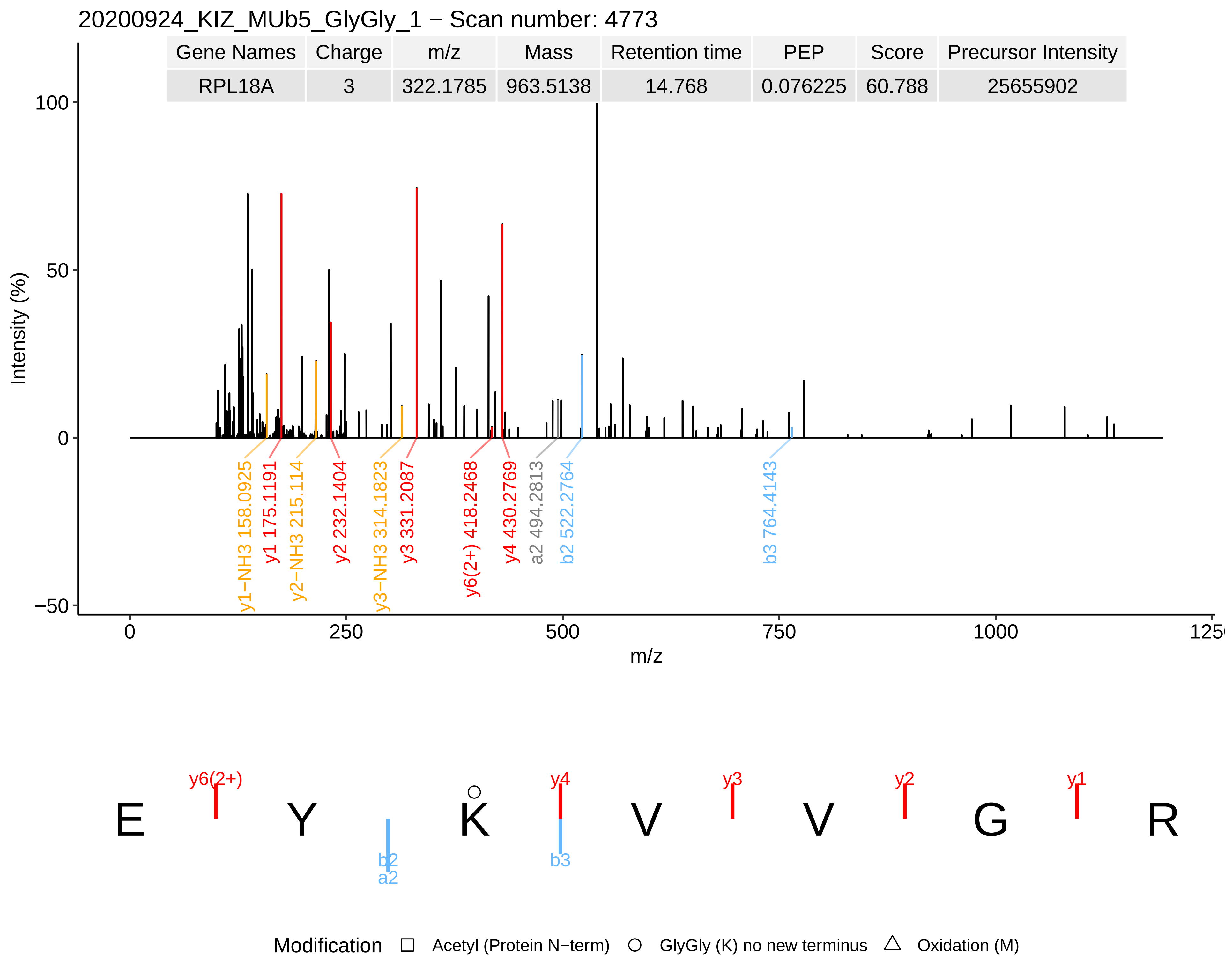
20200924_KIZ_MUb5_GlyGly_1 − Scan number: 4773
Gene Names | Charge | m/z | Mass | Retention time | PEP | Score | Precursor Intensity
RPL18A | 3 | 322.1785 | 963.5138 | 14.768 | 0.076225 | 60.788 | 25655902
Intensity (%)
m/z
y1−NH3 158.0925
y1 175.1191
y2−NH3 215.114
y2 232.1404
y3−NH3 314.1823
y3 331.2087
y6(2+) 418.2468
y4 430.2769
a2 494.2813
b2 522.2764
b3 764.4143
E Y K V V G R
y6(2+) y4 y3 y2 y1
b2 a2 b3
Modification
Acetyl (Protein N−term)
GlyGly (K) no new terminus
Oxidation (M)

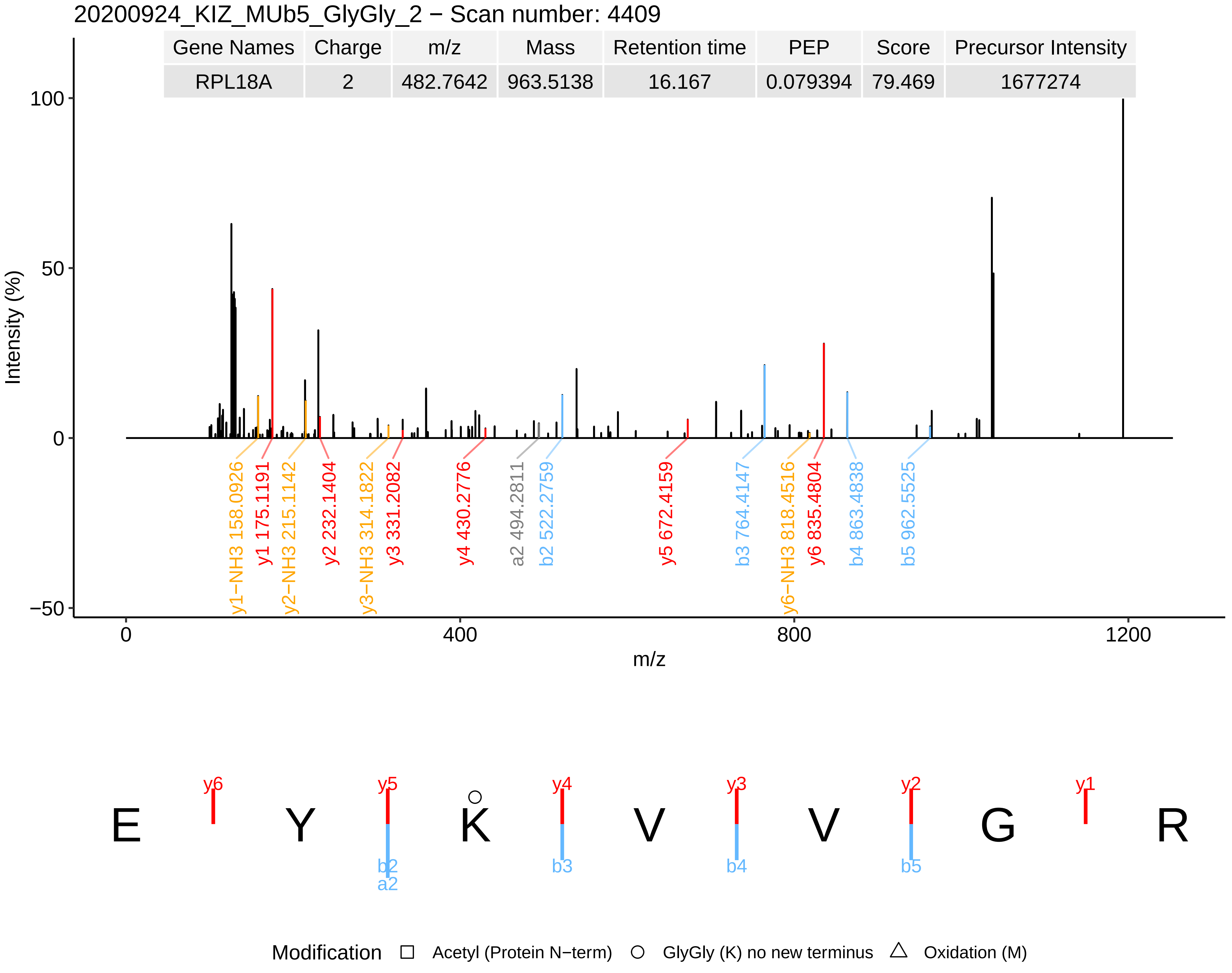

20200924_KIZ_MUb5_GlyGly_2 − Scan number: 4409
Gene Names | Charge | m/z | Mass | Retention time | PEP | Score | Precursor Intensity
RPL18A | 2 | 482.7642 | 963.5138 | 16.167 | 0.079394 | 79.469 | 1677274
Intensity (%)
100
50
0
−50
0
400
800
1200
m/z
y1−NH3 158.0926
y1 175.1191
y2−NH3 215.1142
y2 232.1404
y3−NH3 314.1822
y3 331.2082
y4 430.2776
a2 494.2811
b2 522.2759
y5 672.4159
b3 764.4147
y6−NH3 818.4516
y6 835.4804
b4 863.4838
b5 962.5525
E Y K V V G R
y6 y5 y4 y3 y2 y1
b2 a2 b3 b4 b5
Modification
Acetyl (Protein N−term)
GlyGly (K) no new terminus
Oxidation (M)

20200924_KIZ_MUb5_GlyGly_2 − Scan number: 4498

Gene Names	Charge	m/z	Mass	Retention time	PEP	Score	Precursor Intensity
RPL18A	3	322.1785	963.5138	16.513	0.024872	78.616	8492634

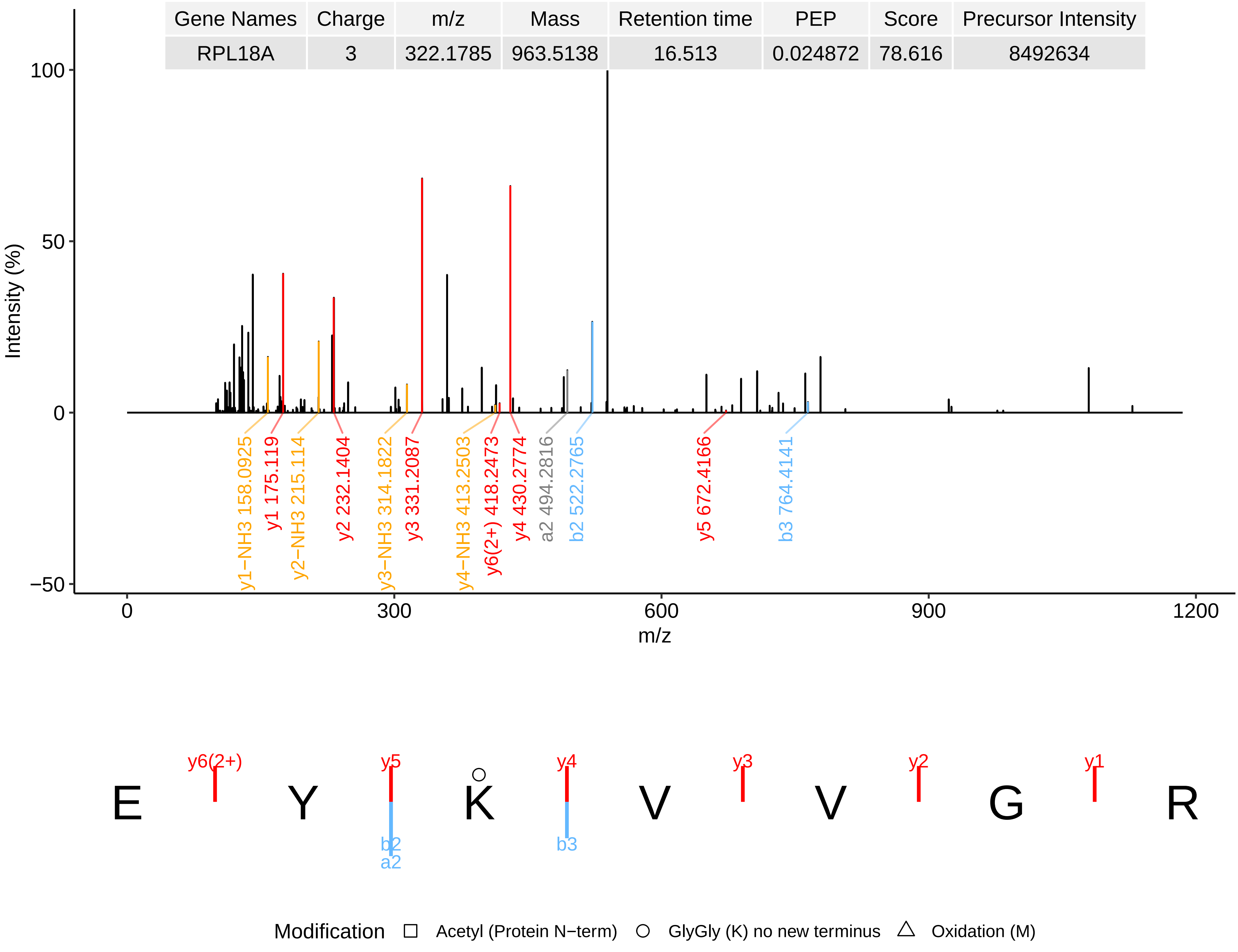

20201118_KIZ_MUb6_GlyGly_2 − Scan number: 5493

Gene Names	Charge	m/z	Mass	Retention time	PEP	Score	Precursor Intensity
RPL18A	3	322.1785	963.5138	18.908	0.029405	76.847	15447725

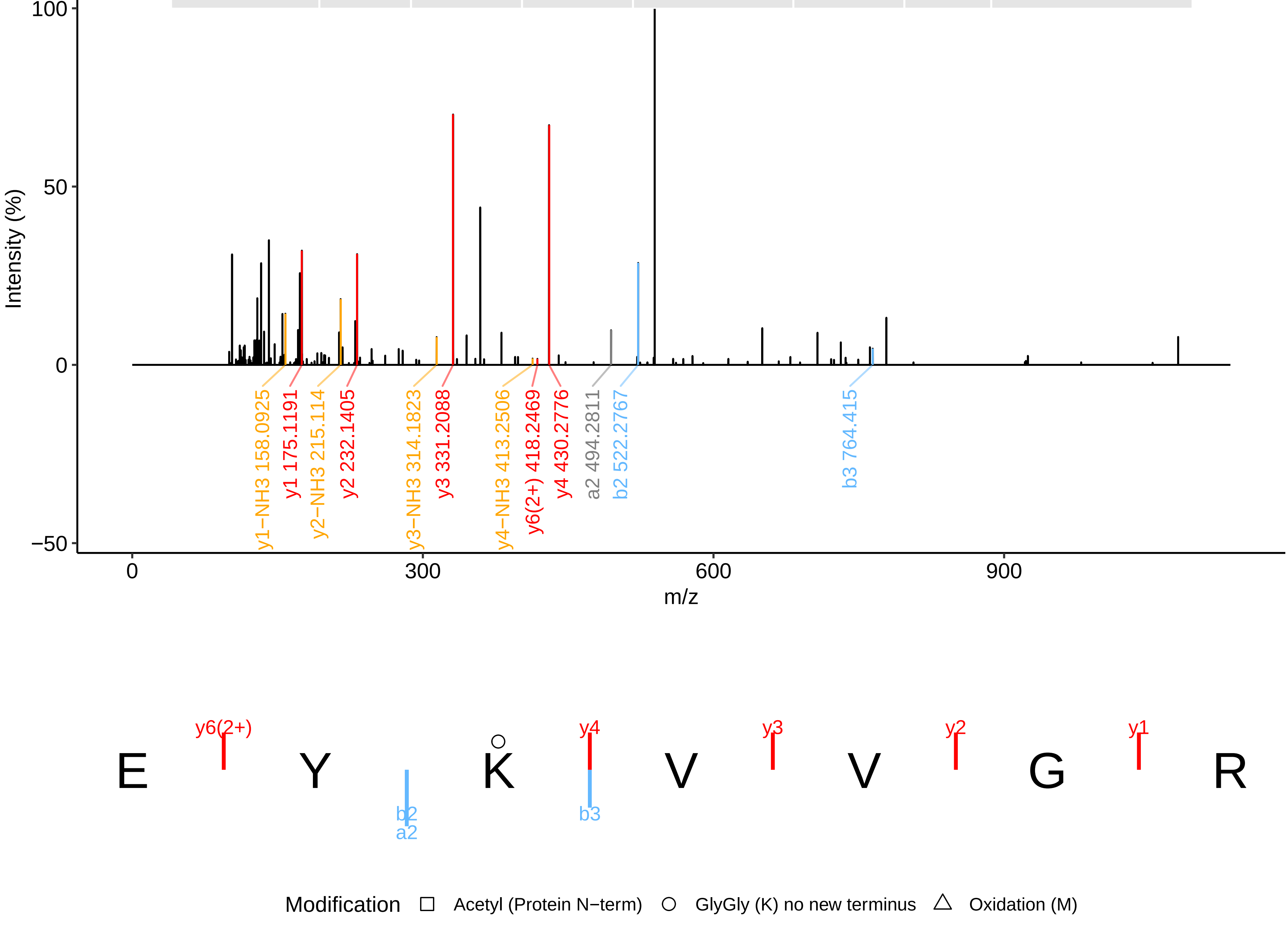

20200921_KIZ_MUb4_GlyGly_10plex − Scan number: 4765

Gene Names	Charge	m/z	Mass	Retention time	PEP	Score	Precursor Intensity
DNAJC11	3	327.17	978.4883	15.536	0.099007	56.177	12955959

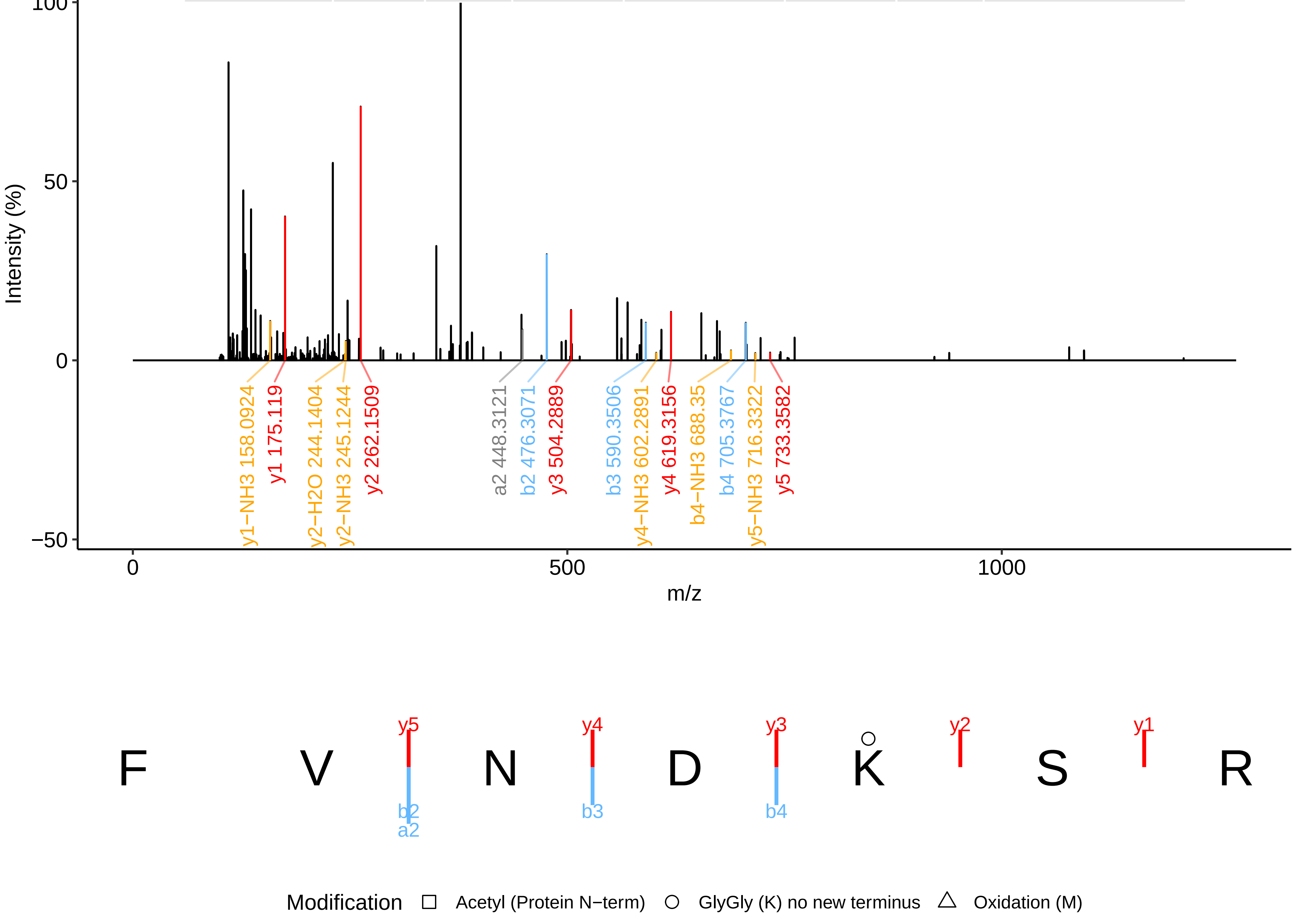

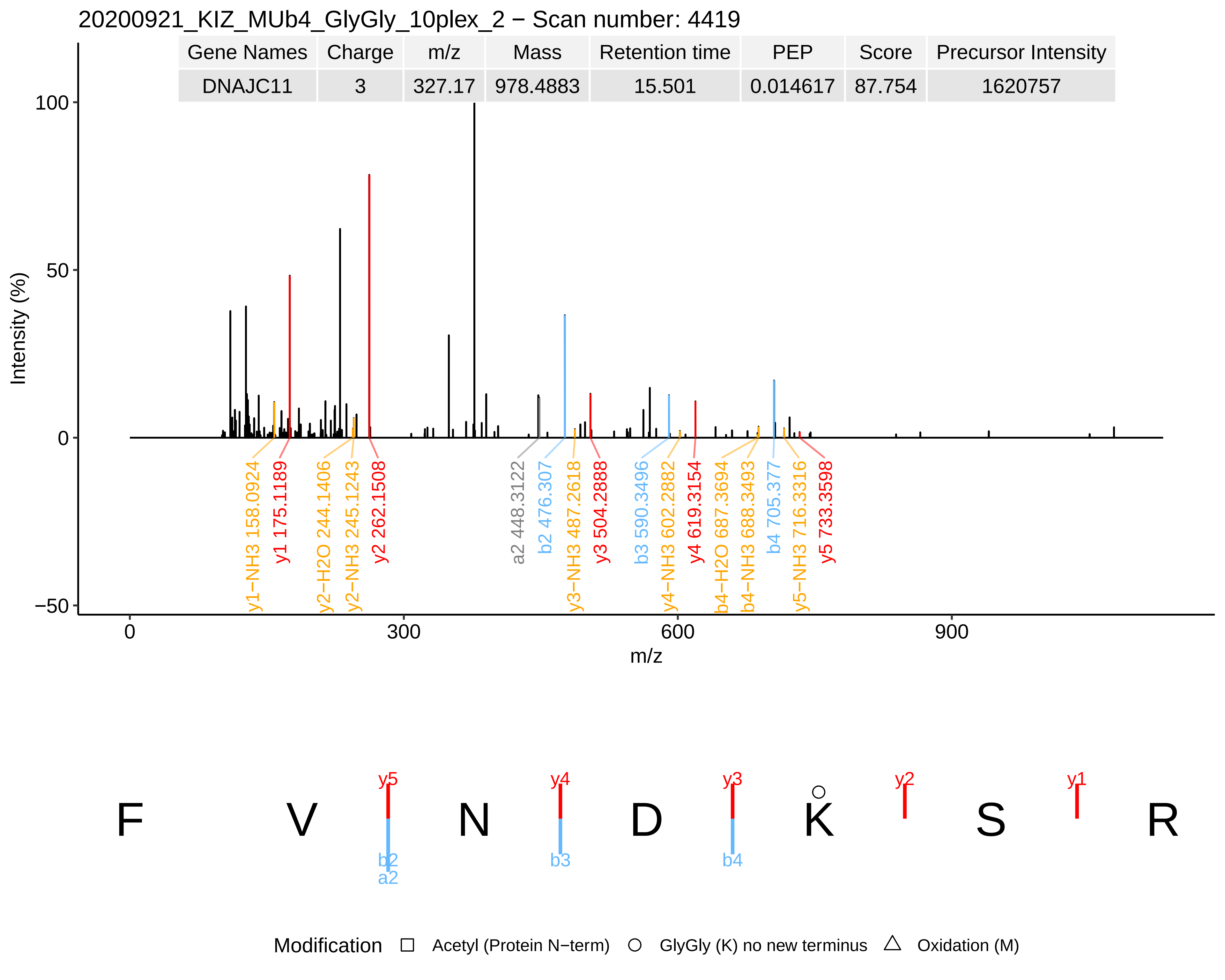

20200921_KIZ_MUb4_GlyGly_10plex_2 − Scan number: 4419
Gene Names | Charge | m/z | Mass | Retention time | PEP | Score | Precursor Intensity
DNAJC11 | 3 | 327.17 | 978.4883 | 15.501 | 0.014617 | 87.754 | 1620757
Intensity (%)
100
50
0
−50
0
300
600
900
m/z
y1−NH3 158.0924
y1 175.1189
y2−H2O 244.1406
y2−NH3 245.1243
y2 262.1508
a2 448.3122
b2 476.307
y3−NH3 487.2618
y3 504.2888
b3 590.3496
y4−NH3 602.2882
y4 619.3154
b4−H2O 687.3694
b4−NH3 688.3493
b4 705.377
y5−NH3 716.3316
y5 733.3598
F V N D K S R
y5 y4 y3 y2 y1
b2 a2 b3 b4
Modification
Acetyl (Protein N−term)
GlyGly (K) no new terminus
Oxidation (M)

20200921_KIZ_MUb4_GlyGly_10plex − Scan number: 13689

Gene Names	Charge	m/z	Mass	Retention time	PEP	Score	Precursor Intensity
TRIM32	3	326.1907	975.5502	48.381	0.016193	89.903	9476896

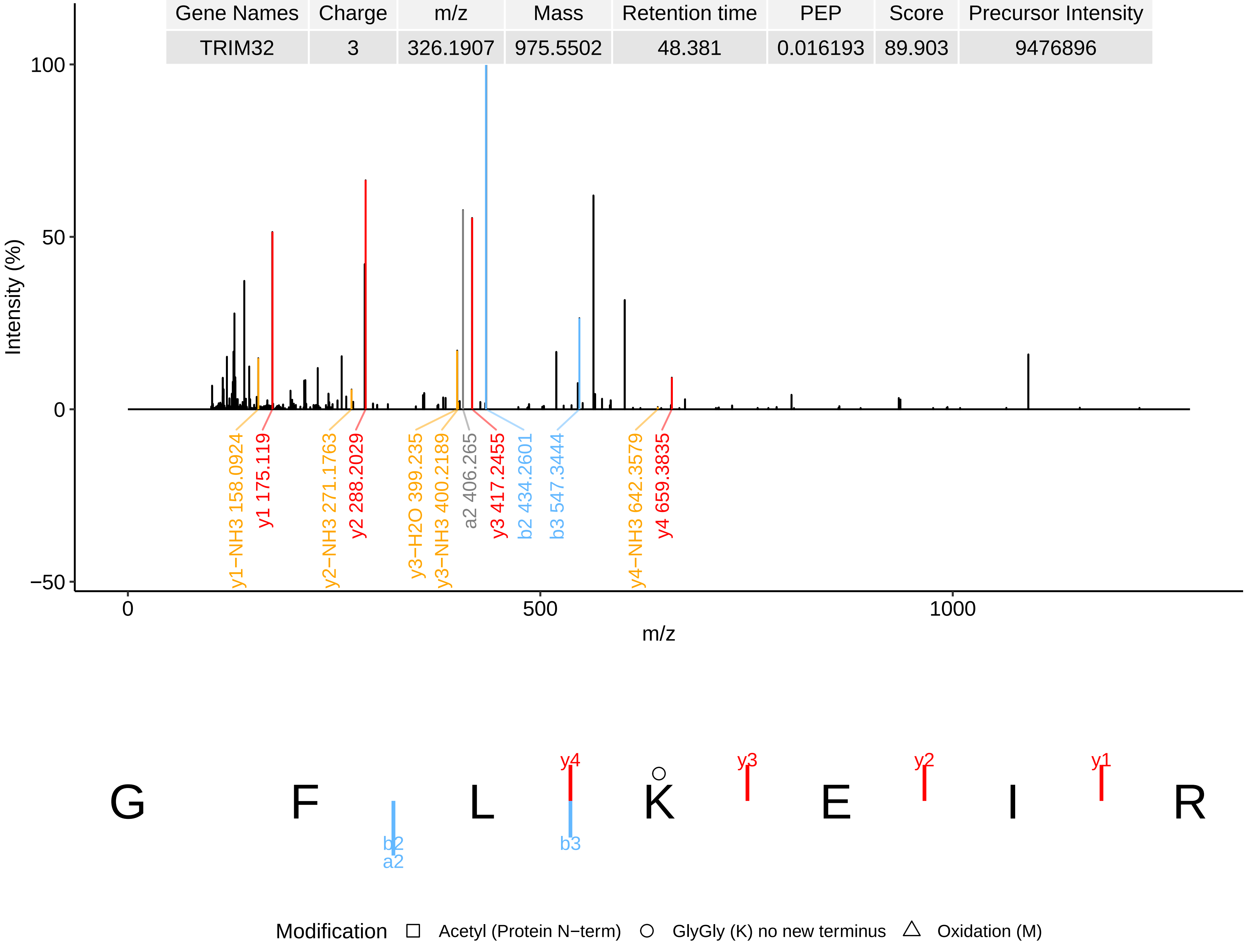

20200921_KIZ_MUb4_GlyGly_10plex − Scan number: 2848

Gene Names	Charge	m/z	Mass	Retention time	PEP	Score	Precursor Intensity
RAB10	3	324.8456	971.5148	8.9162	0.013169	77.318	27758442

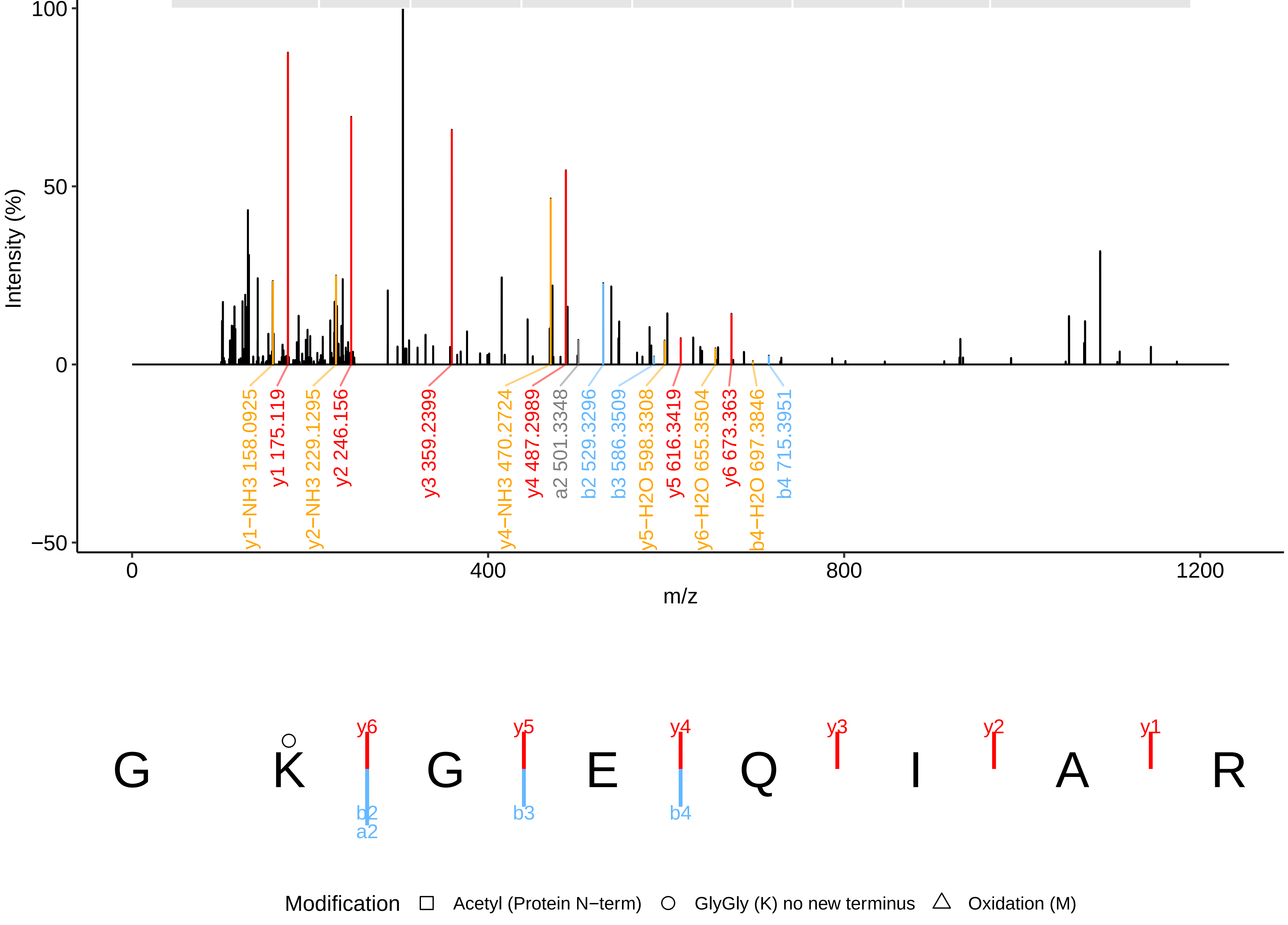

20200921_KIZ_MUb4_GlyGly_10plex_2 − Scan number: 2816

Gene Names	Charge	m/z	Mass	Retention time	PEP	Score	Precursor Intensity
RAB10	2	486.7647	971.5148	9.0882	0.099623	65.038	779728.9

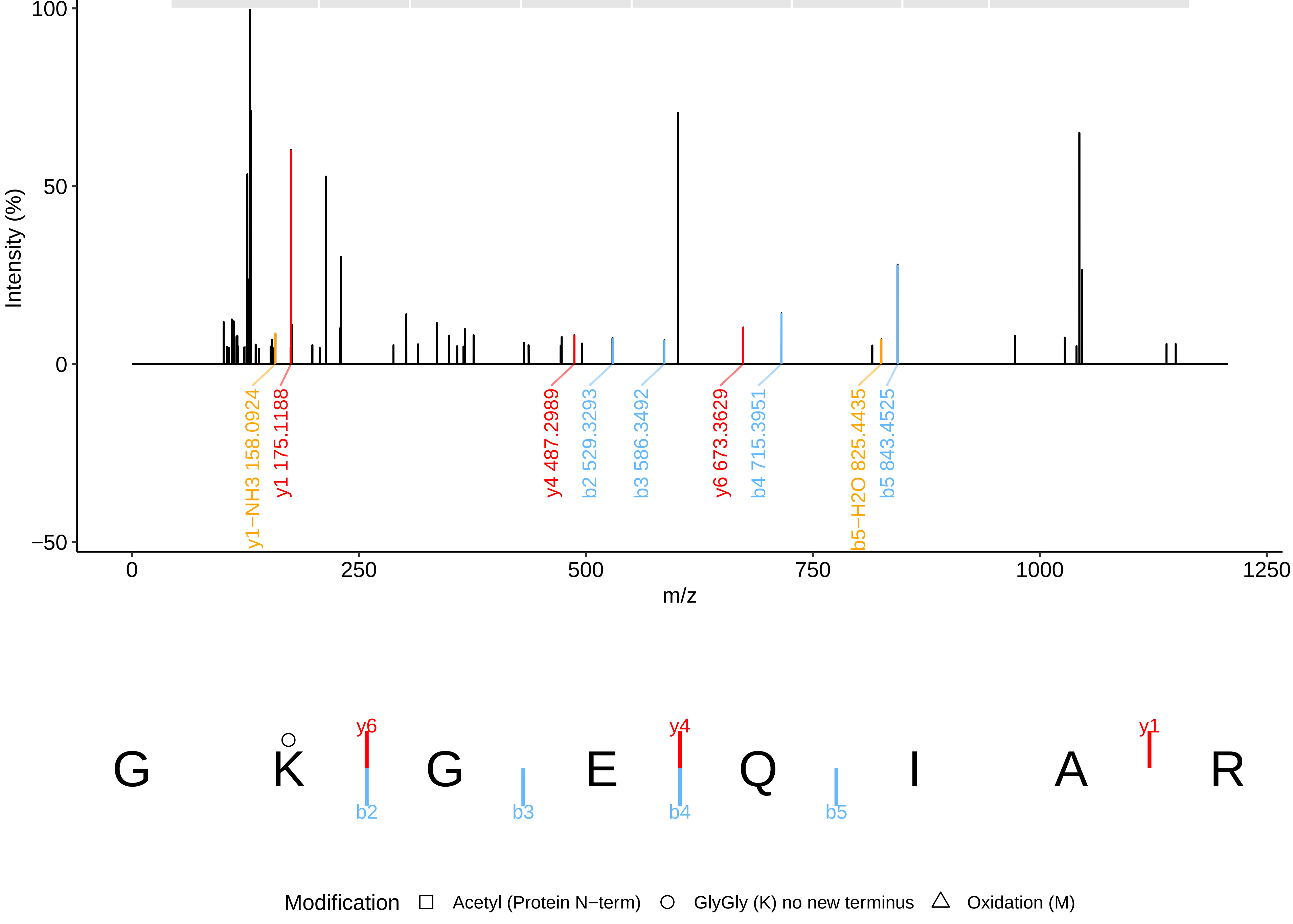

20200924_KIZ_MUb5_GlyGly_1 − Scan number: 2782

Gene Names	Charge	m/z	Mass	Retention time	PEP	Score	Precursor Intensity
RAB10	3	324.8456	971.5148	8.5874	0.0040332	93.429	16880024

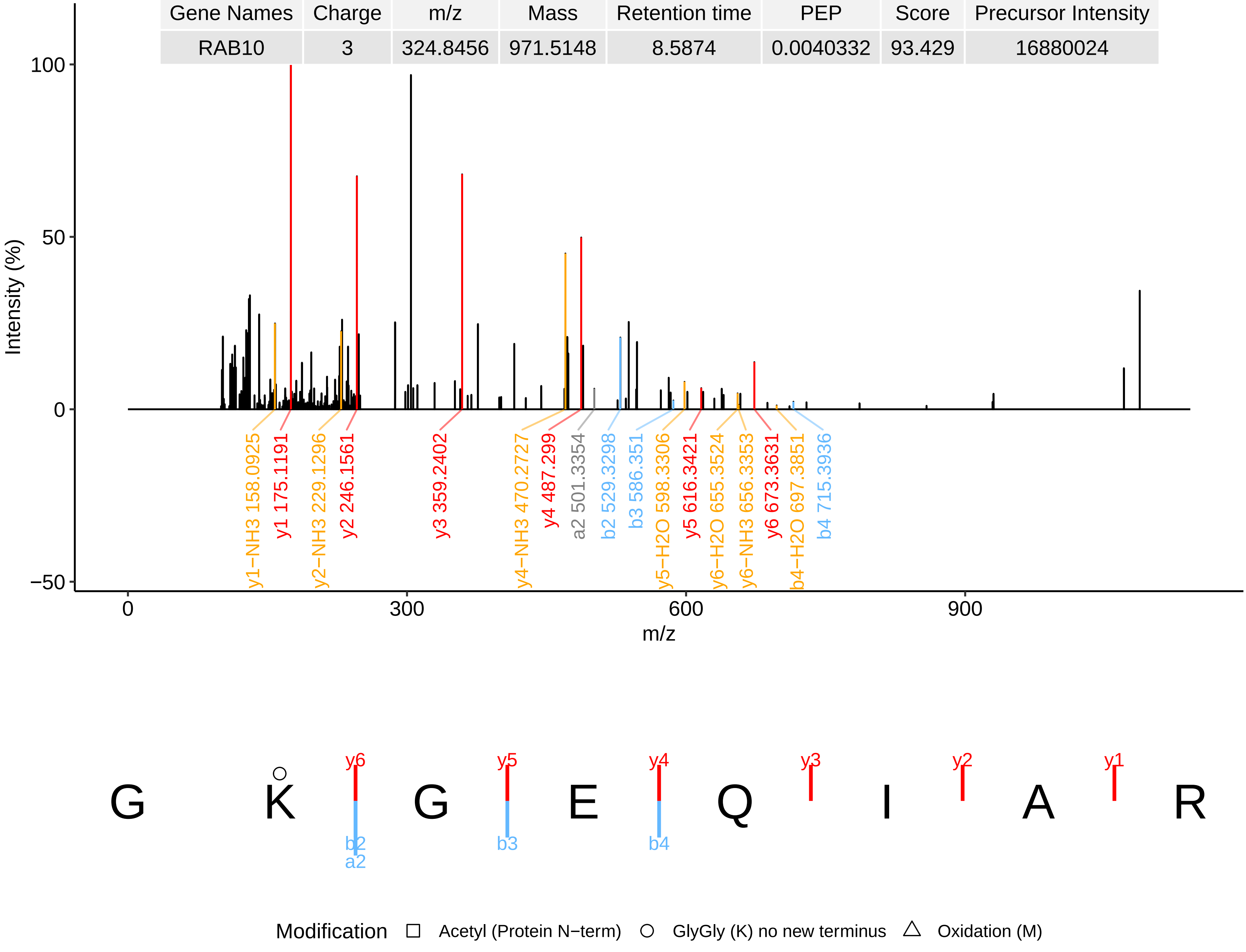

20201118_KIZ_MUb6_GlyGly_1 − Scan number: 2790

Gene Names	Charge	m/z	Mass	Retention time	PEP	Score	Precursor Intensity
RAB10	3	324.8456	971.5148	8.5271	0.004403	92.491	20722596

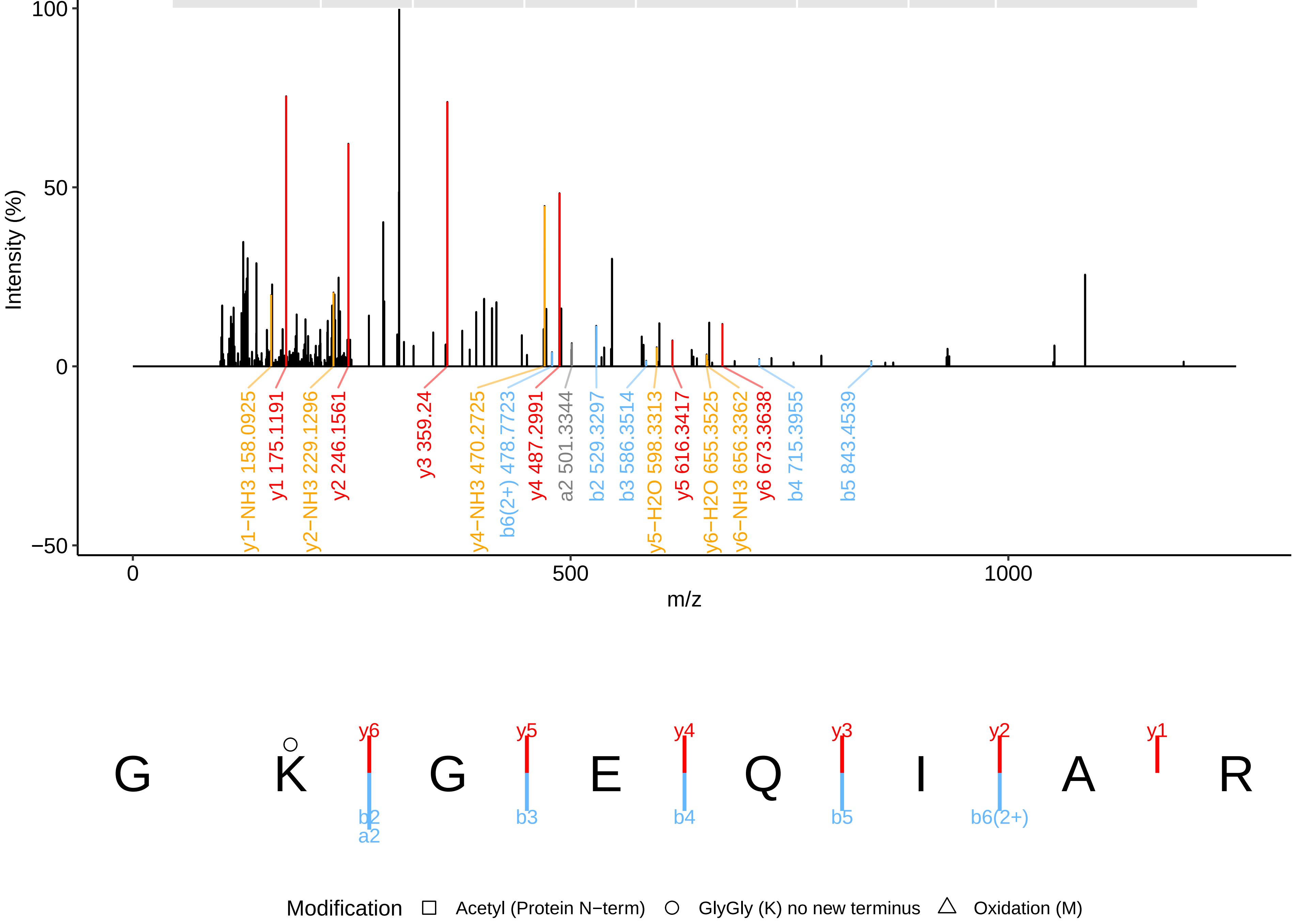

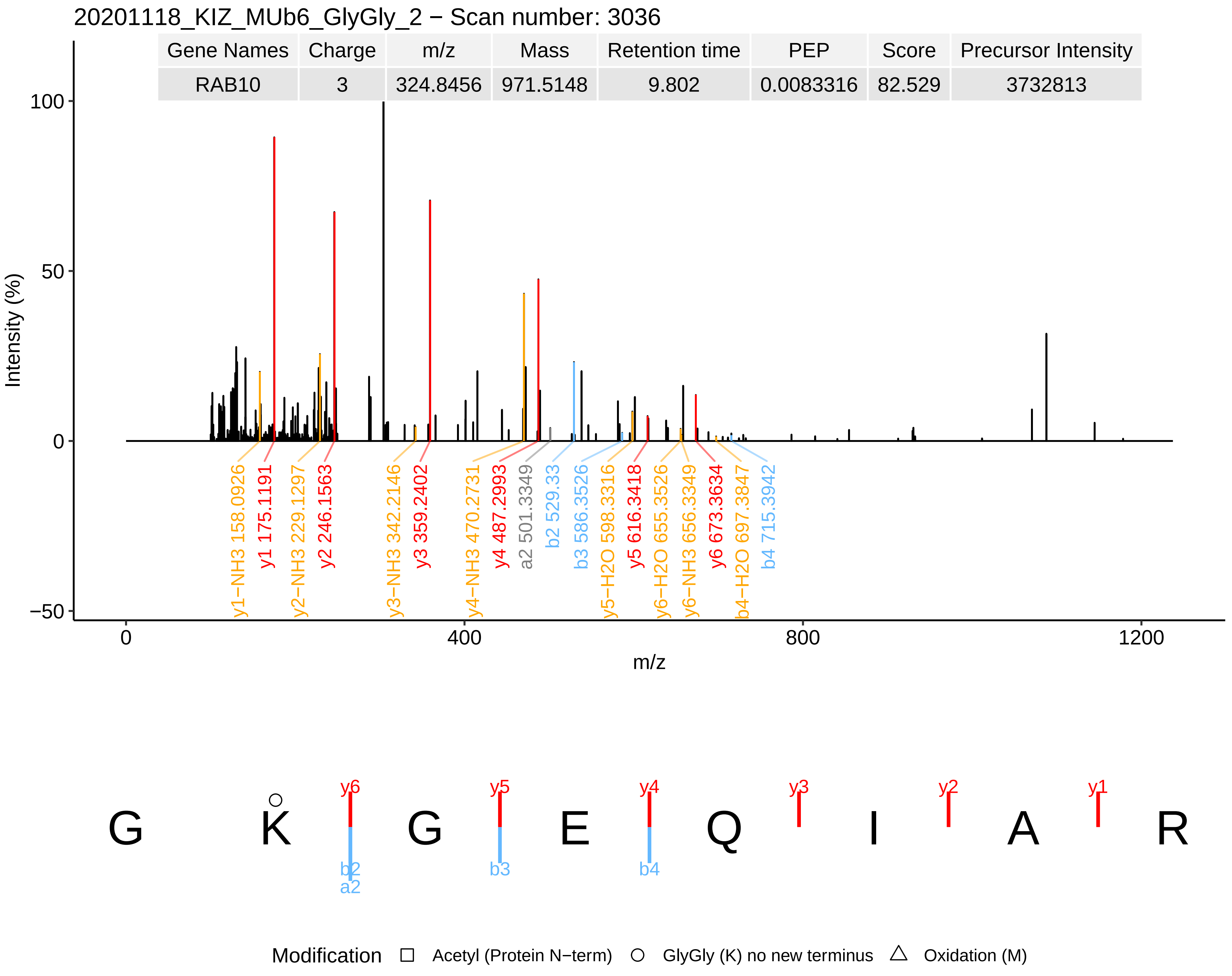

20201118_KIZ_MUb6_GlyGly_2 − Scan number: 3036
Gene Names | Charge | m/z | Mass | Retention time | PEP | Score | Precursor Intensity
RAB10 | 3 | 324.8456 | 971.5148 | 9.802 | 0.0083316 | 82.529 | 3732813
Intensity (%)
100
50
0
−50
0
400
800
1200
m/z
y1−NH3 158.0926
y1 175.1191
y2−NH3 229.1297
y2 246.1563
y3−NH3 342.2146
y3 359.2402
y4−NH3 470.2731
y4 487.2993
a2 501.3349
b2 529.33
b3 586.3526
y5−H2O 598.3316
y5 616.3418
y6−H2O 655.3526
y6−NH3 656.3349
y6 673.3634
b4−H2O 697.3847
b4 715.3942
G K G E Q I A R
y6 y5 y4 y3 y2 y1
b2 a2 b3 b4
Modification
Acetyl (Protein N−term)
GlyGly (K) no new terminus
Oxidation (M)

20200921_KIZ_MUb4_GlyGly_10plex − Scan number: 6149

Gene Names	Charge	m/z	Mass	Retention time	PEP	Score	Precursor Intensity
TRAPPC5	3	329.8576	986.5509	20.242	0.094167	47.732	7567846

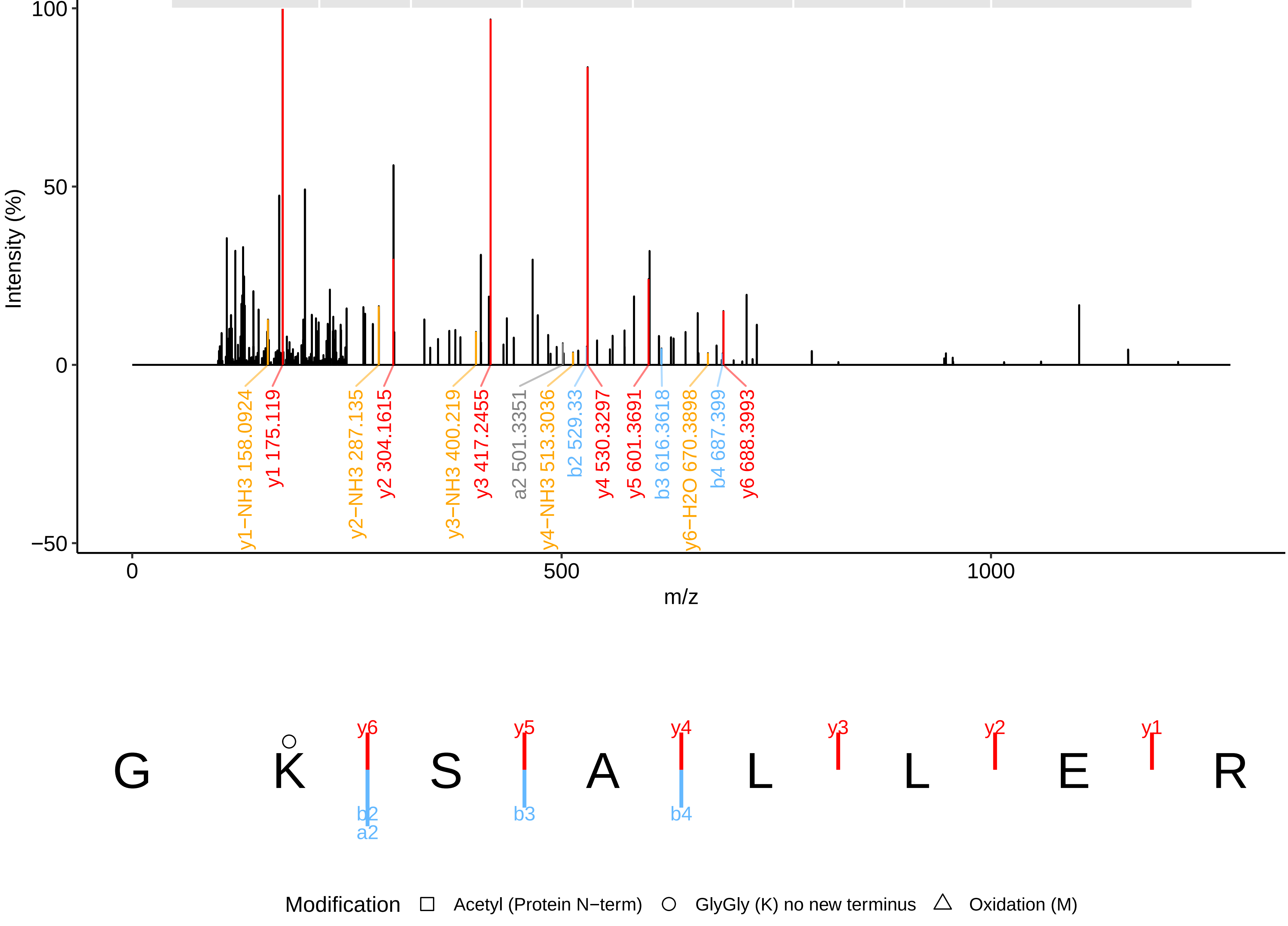

20200921_KIZ_MUb4_GlyGly_10plex_2 − Scan number: 5641

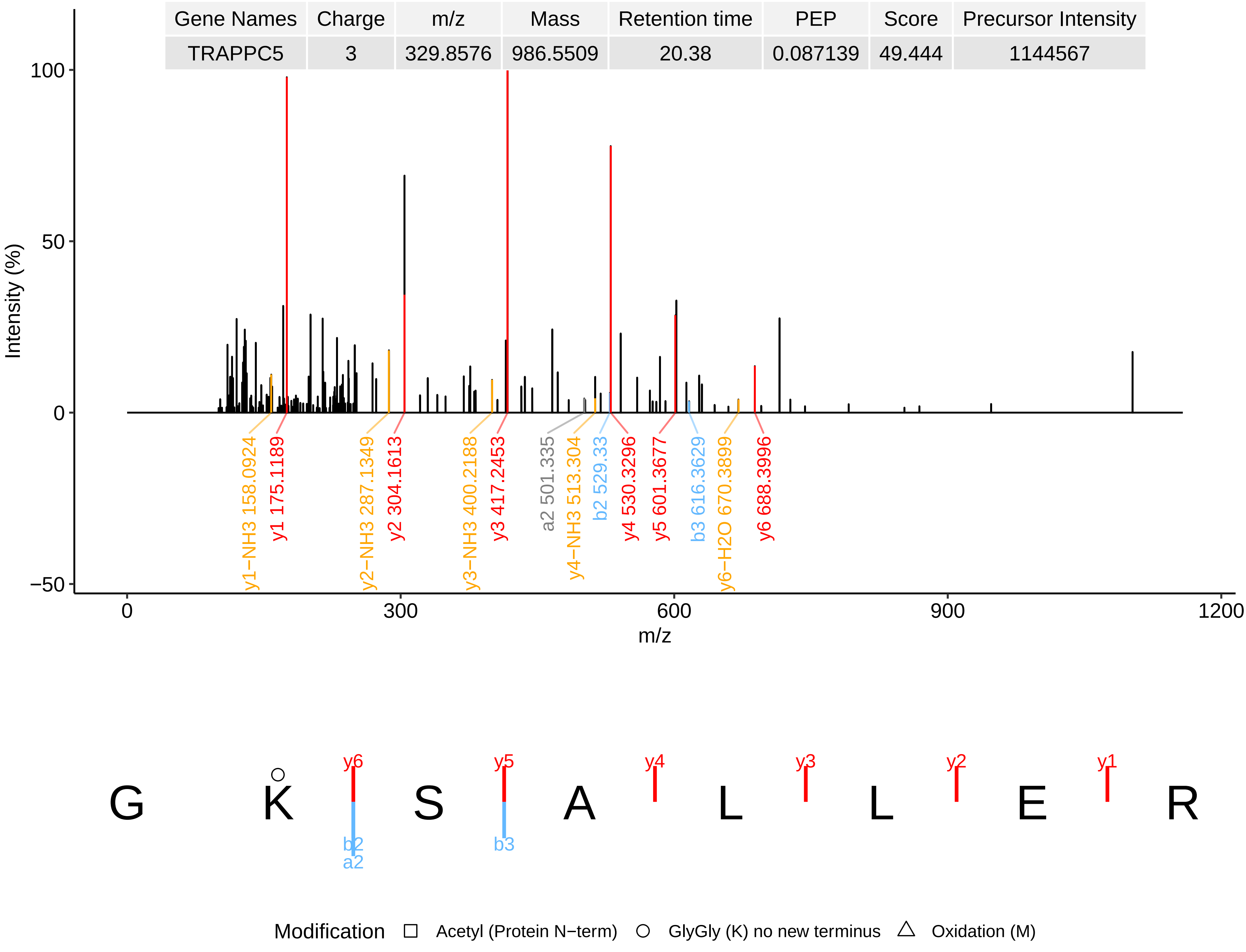

20200921_KIZ_MUb4_GlyGly_10plex − Scan number: 9454

Gene Names	Charge	m/z	Mass	Retention time	PEP	Score	Precursor Intensity
EIF2S3	3	316.5189	946.5349	32.371	0.0095172	94.777	7601372

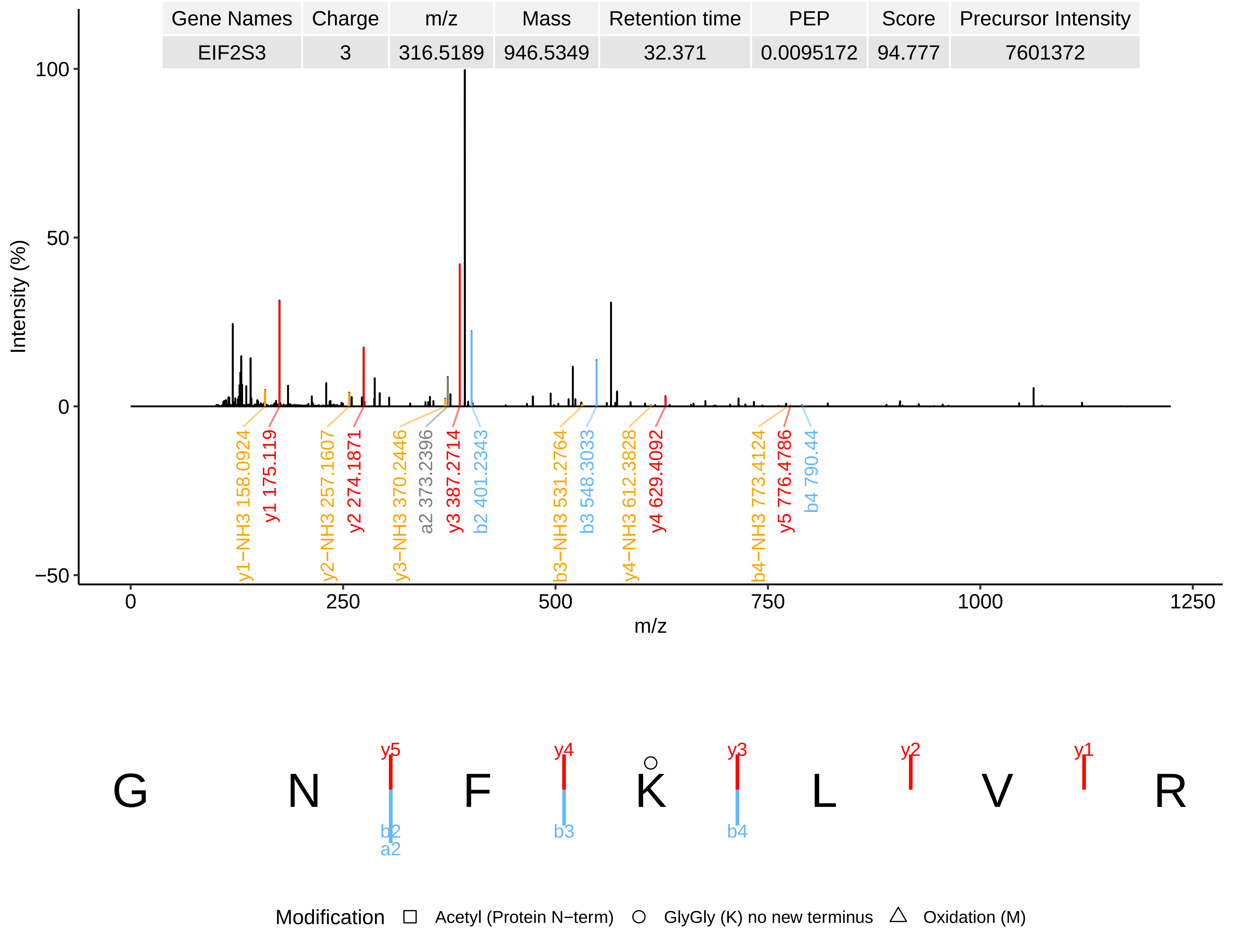

20200921_KIZ_MUb4_GlyGly_10plex − Scan number: 2467

Gene Names	Charge	m/z	Mass	Retention time	PEP	Score	Precursor Intensity
SERP1	3	316.1737	945.4992	7.7657	0.0021648	98.582	10799149

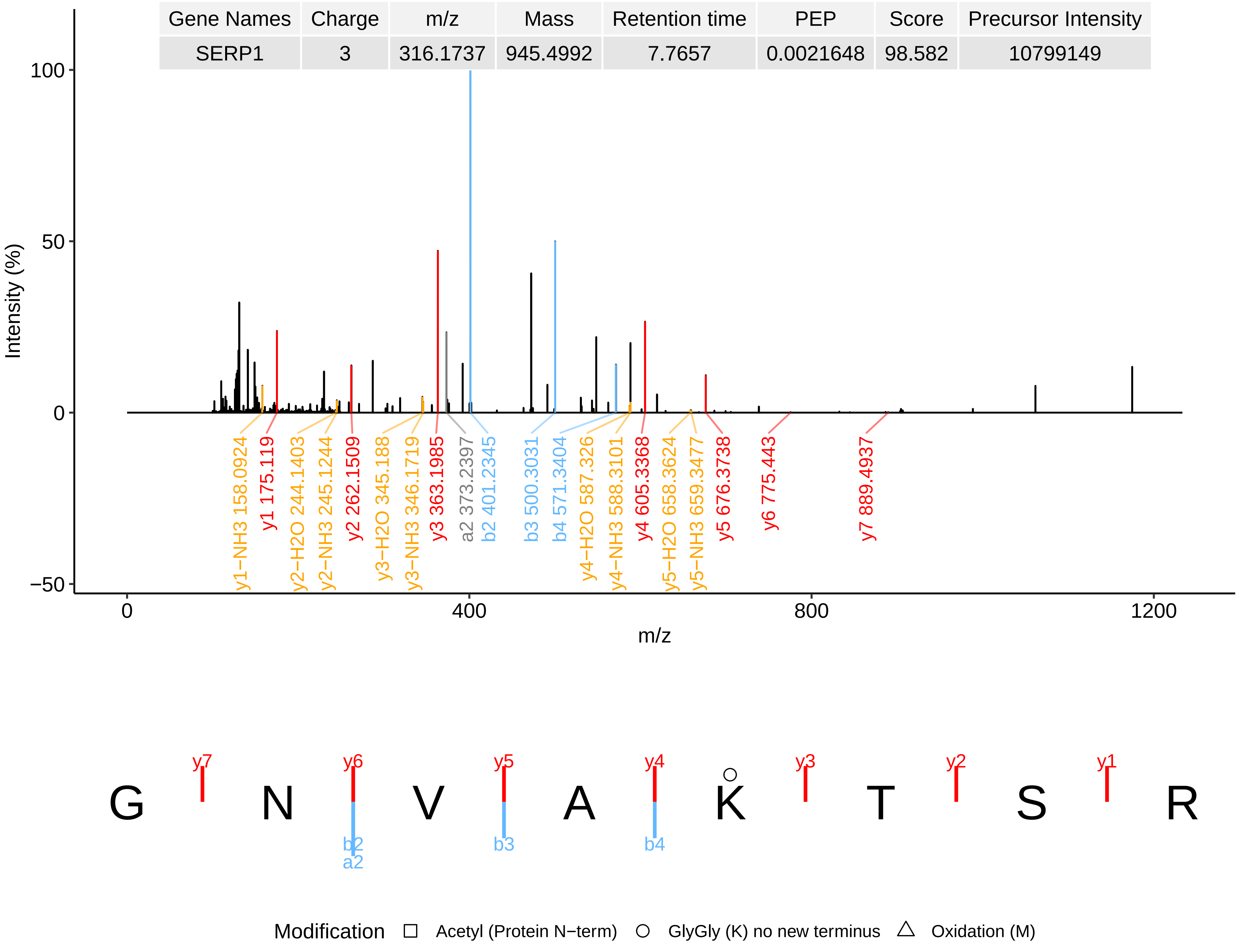

20200921_KIZ_MUb4_GlyGly_10plex − Scan number: 3495

Gene Names	Charge	m/z	Mass	Retention time	PEP	Score	Precursor Intensity
RPL37	3	318.4982	952.4726	11.11	0.046702	84.498	5428176

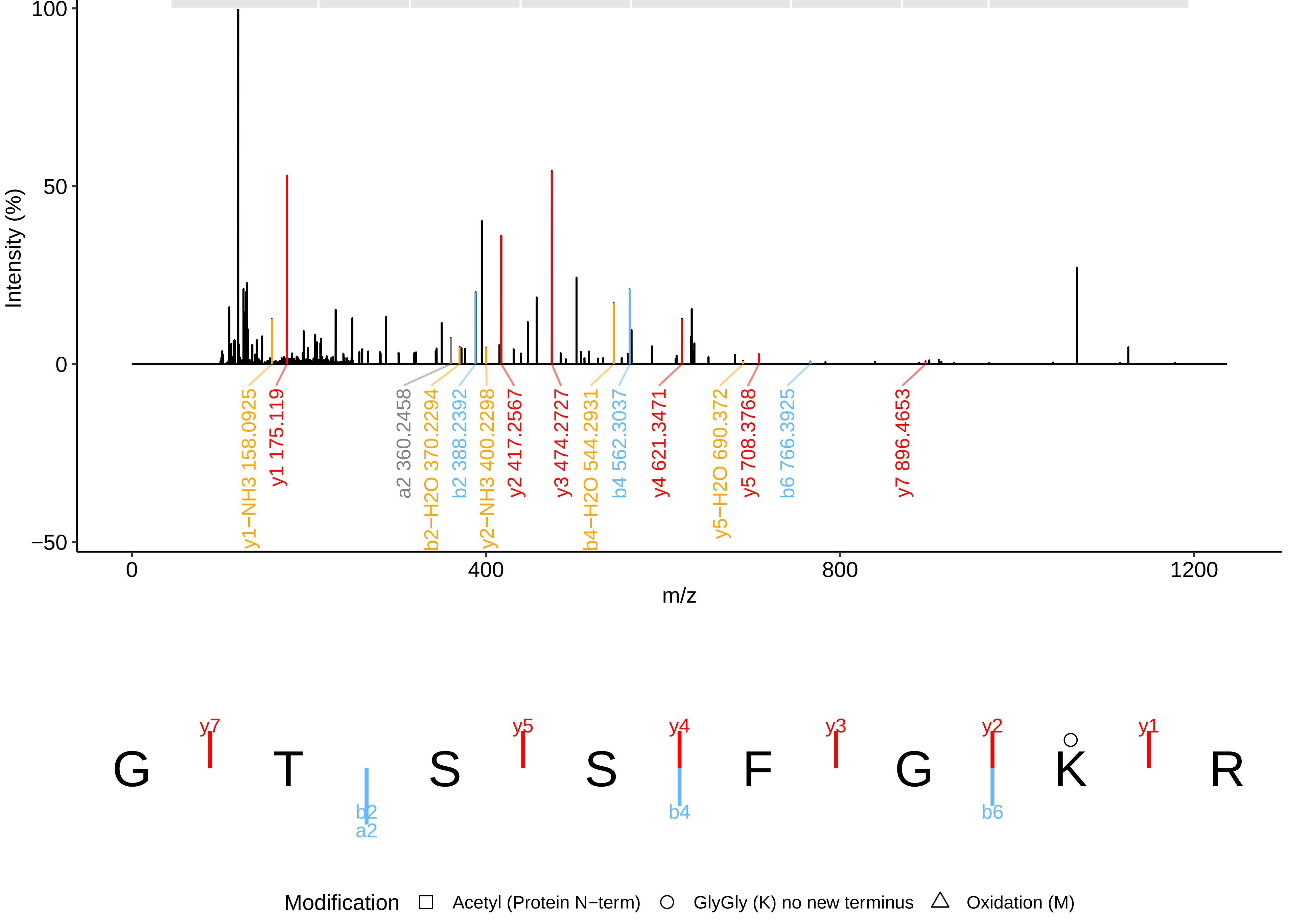

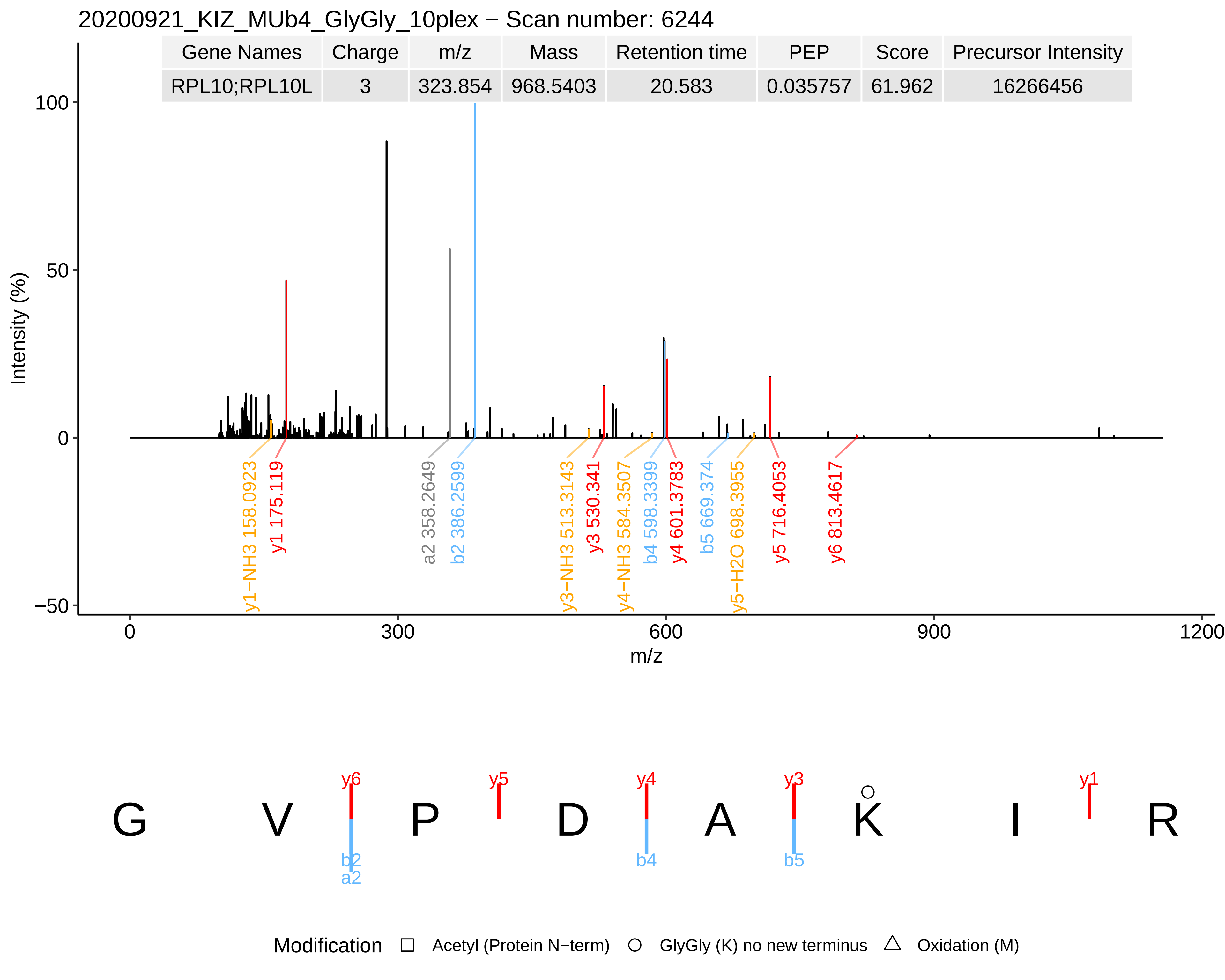

20200921_KIZ_MUb4_GlyGly_10plex − Scan number: 6244
Gene Names | Charge | m/z | Mass | Retention time | PEP | Score | Precursor Intensity
RPL10;RPL10L | 3 | 323.854 | 968.5403 | 20.583 | 0.035757 | 61.962 | 16266456
Intensity (%)
100
50
0
−50
0
300
600
900
1200
m/z
y1−NH3 158.0923
y1 175.119
a2 358.2649
b2 386.2599
y3−NH3 513.3143
y3 530.341
y4−NH3 584.3507
b4 598.3399
y4 601.3783
b5 669.374
y5−H2O 698.3955
y5 716.4053
y6 813.4617
G V P D A K I R
y6 y5 y4 y3 y1
b2 a2 b4 b5
Modification
Acetyl (Protein N−term)
GlyGly (K) no new terminus
Oxidation (M)

20200921_KIZ_MUb4_GlyGly_10plex_2 − Scan number: 5672

Gene Names	Charge	m/z	Mass	Retention time	PEP	Score	Precursor Intensity
RPL10;RPL10L	3	323.854	968.5403	20.504	0.071861	53.166	1233435

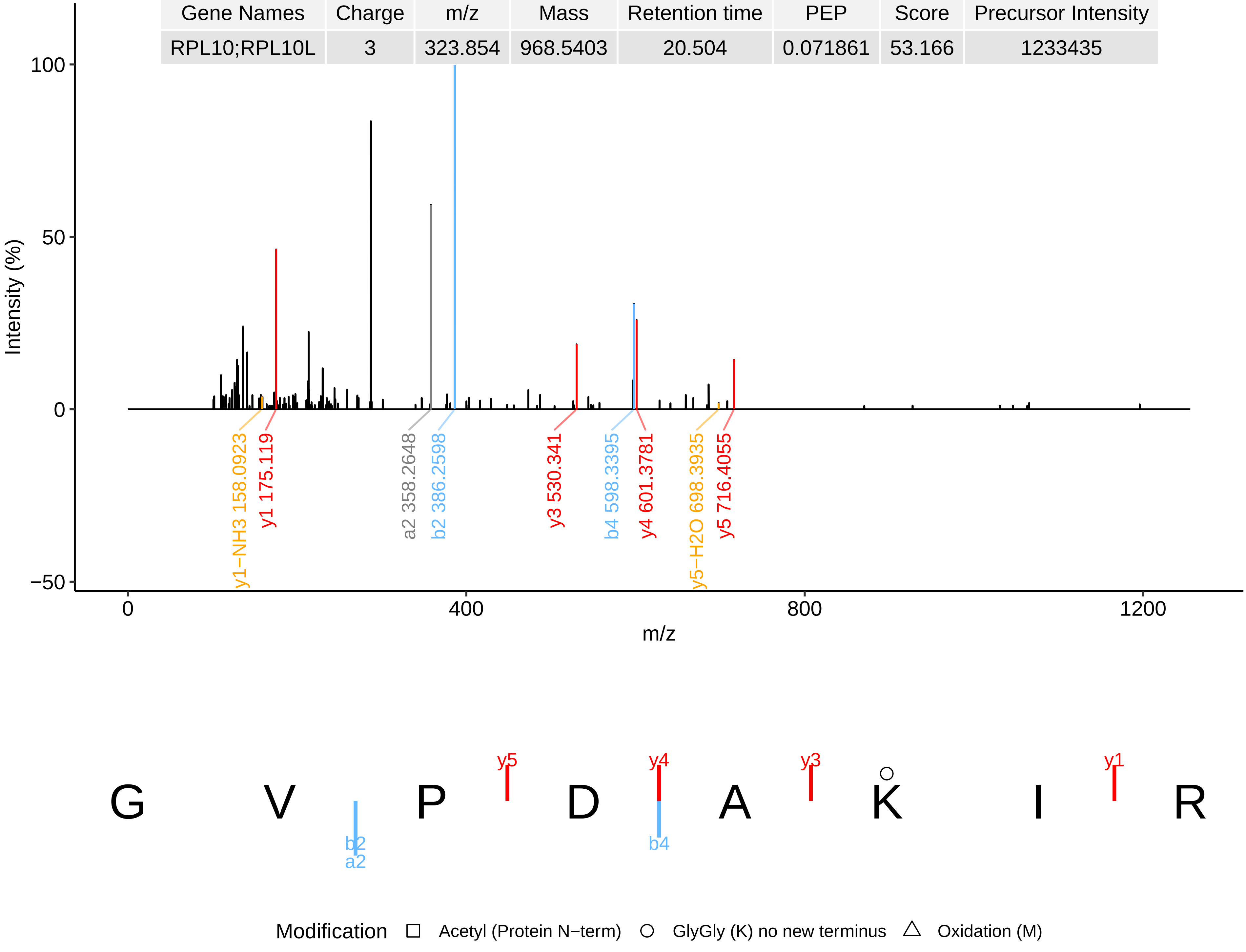

20200924_KIZ_MUb5_GlyGly_1 − Scan number: 5342

Gene Names	Charge	m/z	Mass	Retention time	PEP	Score	Precursor Intensity
RPL10;RPL10L	3	323.854	968.5403	16.591	0.068939	53.878	10050621

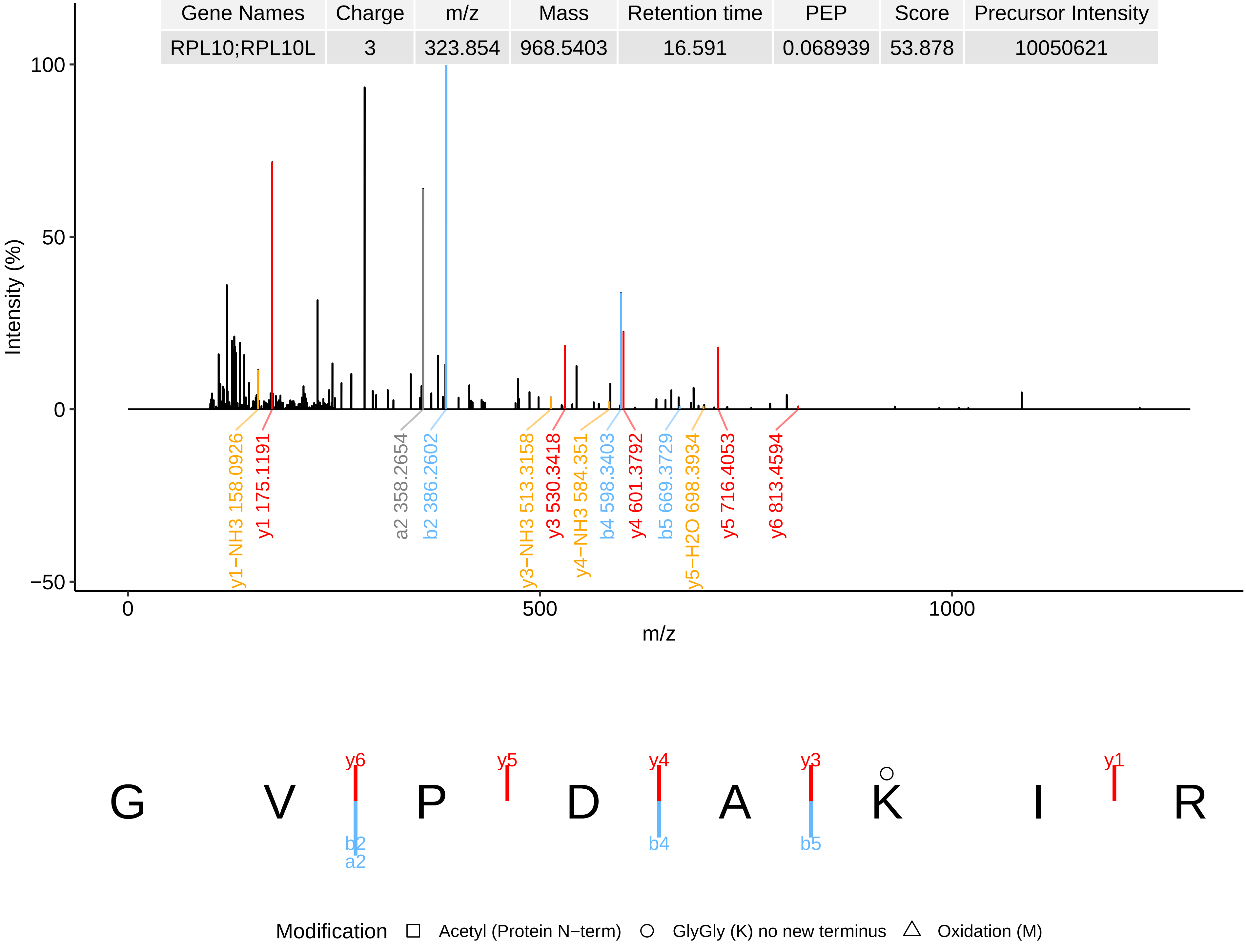

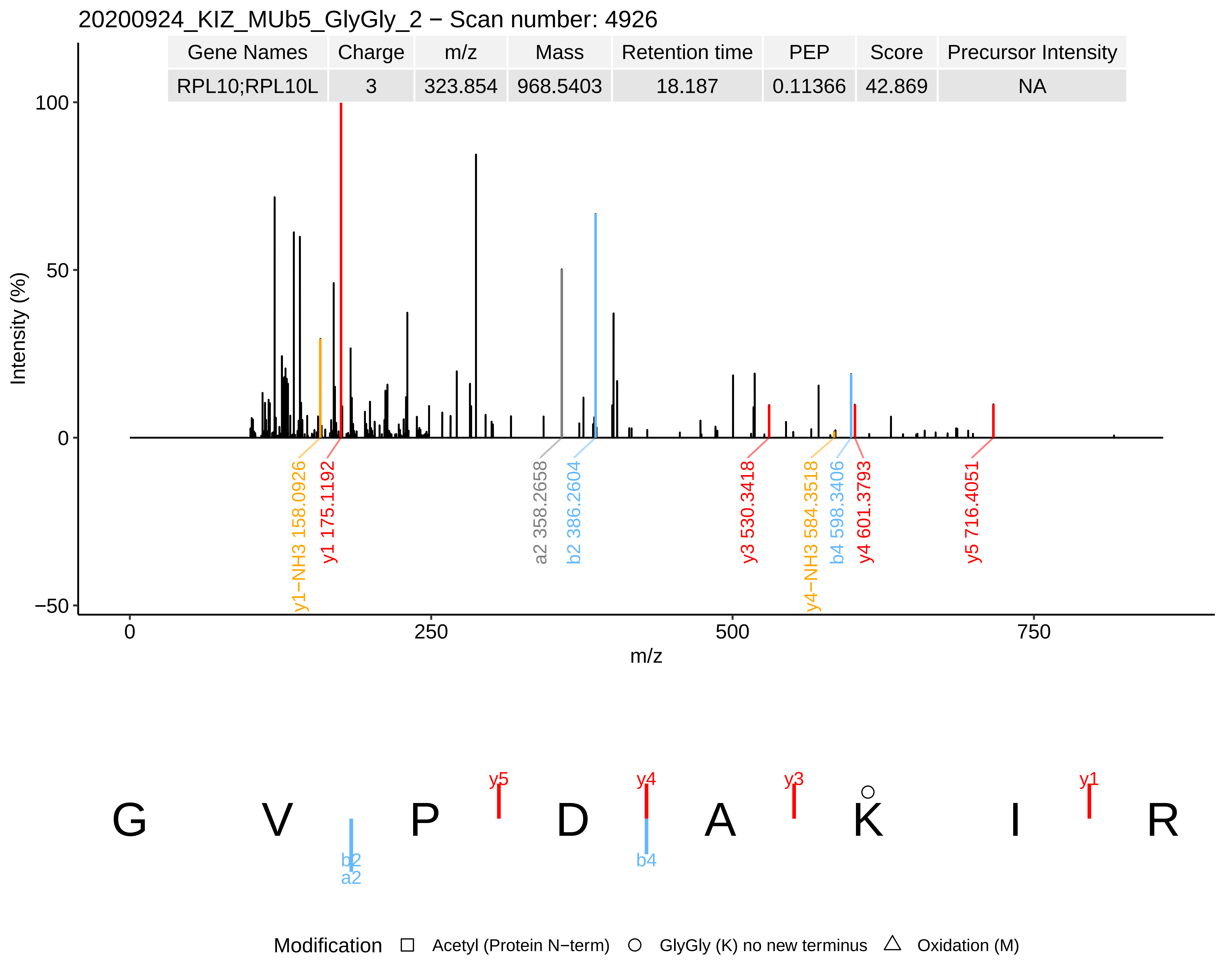

20200924_KIZ_MUb5_GlyGly_2 − Scan number: 4926
Gene Names | Charge | m/z | Mass | Retention time | PEP | Score | Precursor Intensity
RPL10;RPL10L | 3 | 323.854 | 968.5403 | 18.187 | 0.11366 | 42.869 | NA
Intensity (%)
100
50
0
−50
0
250
500
750
m/z
y1−NH3 158.0926
y1 175.1192
a2 358.2658
b2 386.2604
y3 530.3418
y4−NH3 584.3518
b4 598.3406
y4 601.3793
y5 716.4051
G V P D A K I R
y5
y4
y3
y1
b2
a2
b4
Modification
Acetyl (Protein N−term)
GlyGly (K) no new terminus
Oxidation (M)

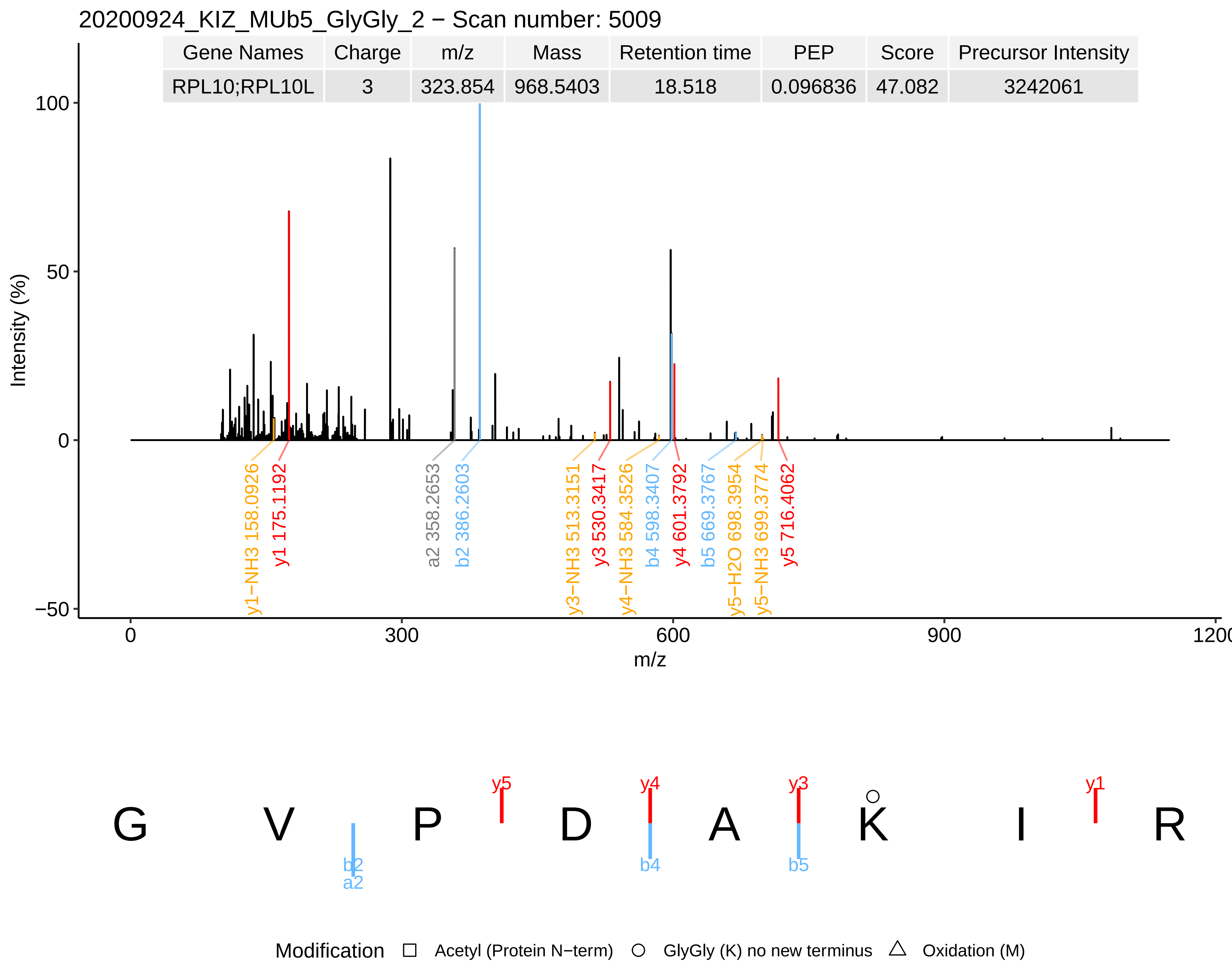
20200924_KIZ_MUb5_GlyGly_2 − Scan number: 5009
Gene Names | Charge | m/z | Mass | Retention time | PEP | Score | Precursor Intensity
RPL10;RPL10L | 3 | 323.854 | 968.5403 | 18.518 | 0.096836 | 47.082 | 3242061
Intensity (%)
m/z
y1−NH3 158.0926
y1 175.1192
a2 358.2653
b2 386.2603
y3−NH3 513.3151
y3 530.3417
y4−NH3 584.3526
b4 598.3407
y4 601.3792
b5 669.3767
y5−H2O 698.3954
y5−NH3 699.3774
y5 716.4062
G V P D A K I R
y5 y4 y3 y1
b2 a2 b4 b5
Modification
Acetyl (Protein N−term)
GlyGly (K) no new terminus
Oxidation (M)

20200921_KIZ_MUb4_GlyGly_10plex − Scan number: 13867

Gene Names	Charge	m/z	Mass	Retention time	PEP	Score	Precursor Intensity
SGTA	3	319.5344	955.5815	49.067	0.095432	59.205	1951172

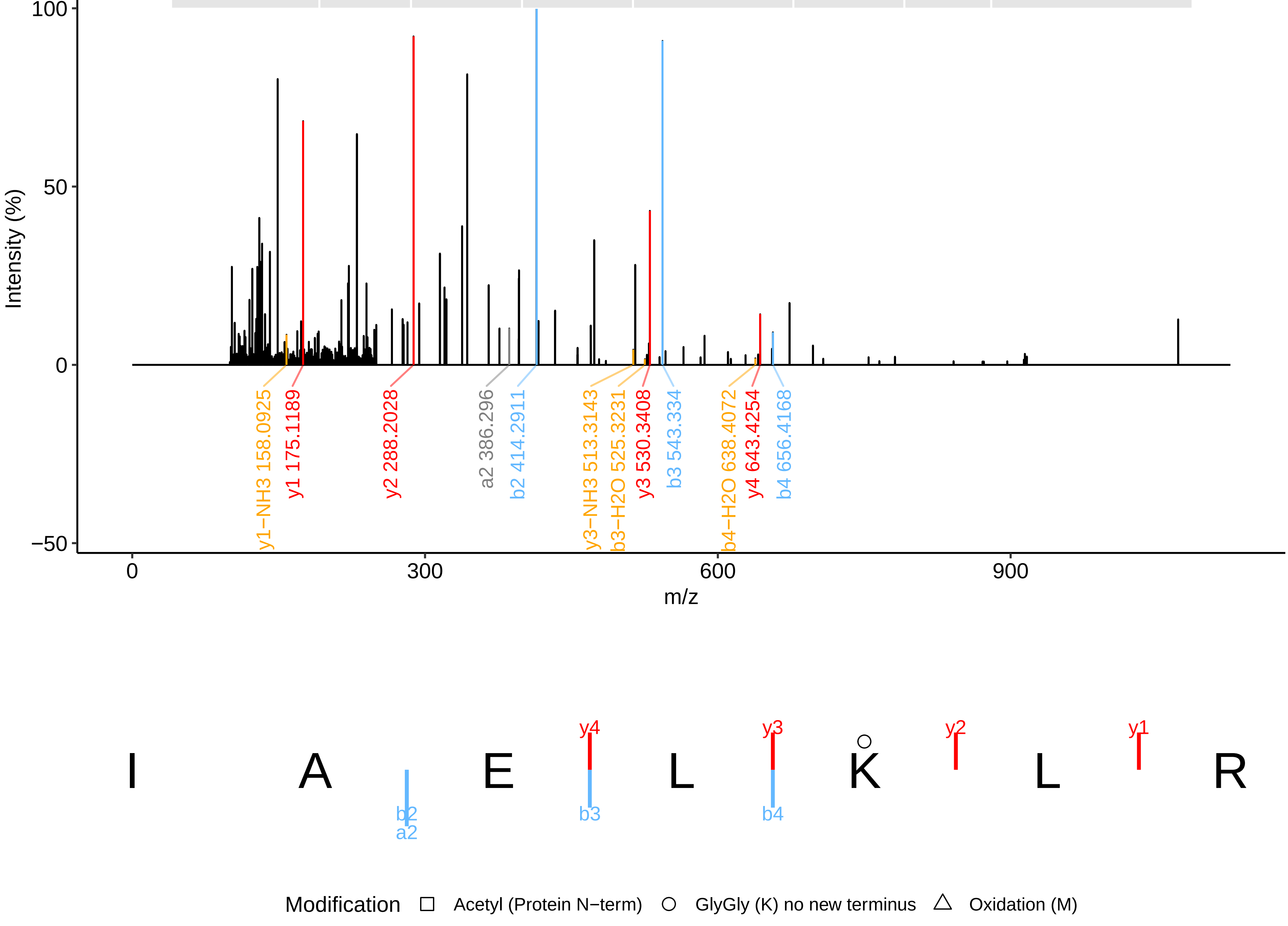

20200921_KIZ_MUb4_GlyGly_10plex − Scan number: 3595

Gene Names	Charge	m/z	Mass	Retention time	PEP	Score	Precursor Intensity
SCAMP3	2	487.7487	973.4829	11.48	0.10398	90.709	NA

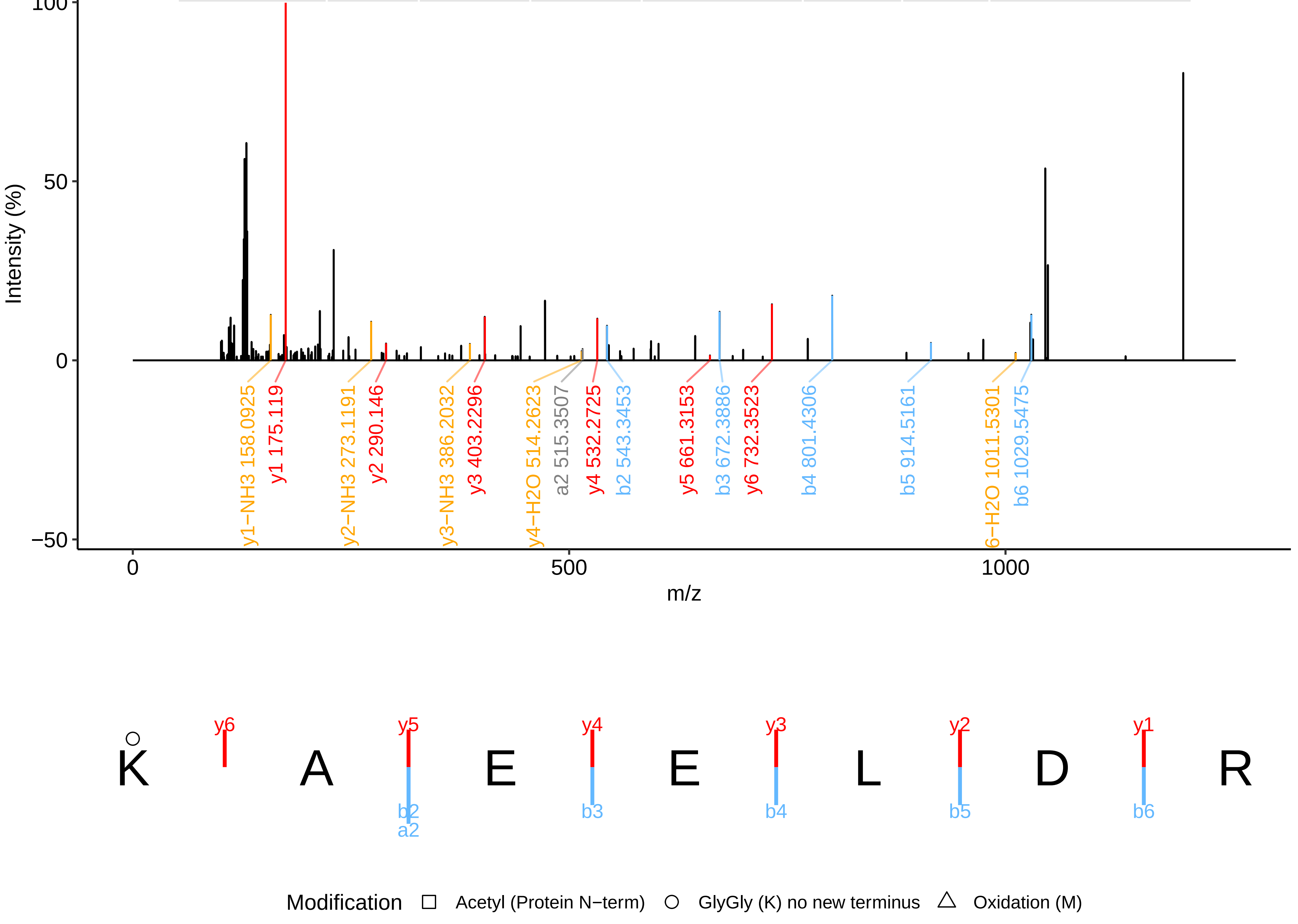

20201118_KIZ_MUb6_GlyGly_1 − Scan number: 3162

Gene Names	Charge	m/z	Mass	Retention time	PEP	Score	Precursor Intensity
SCAMP3	3	325.5016	973.4829	9.7479	0.027202	107.59	29844296

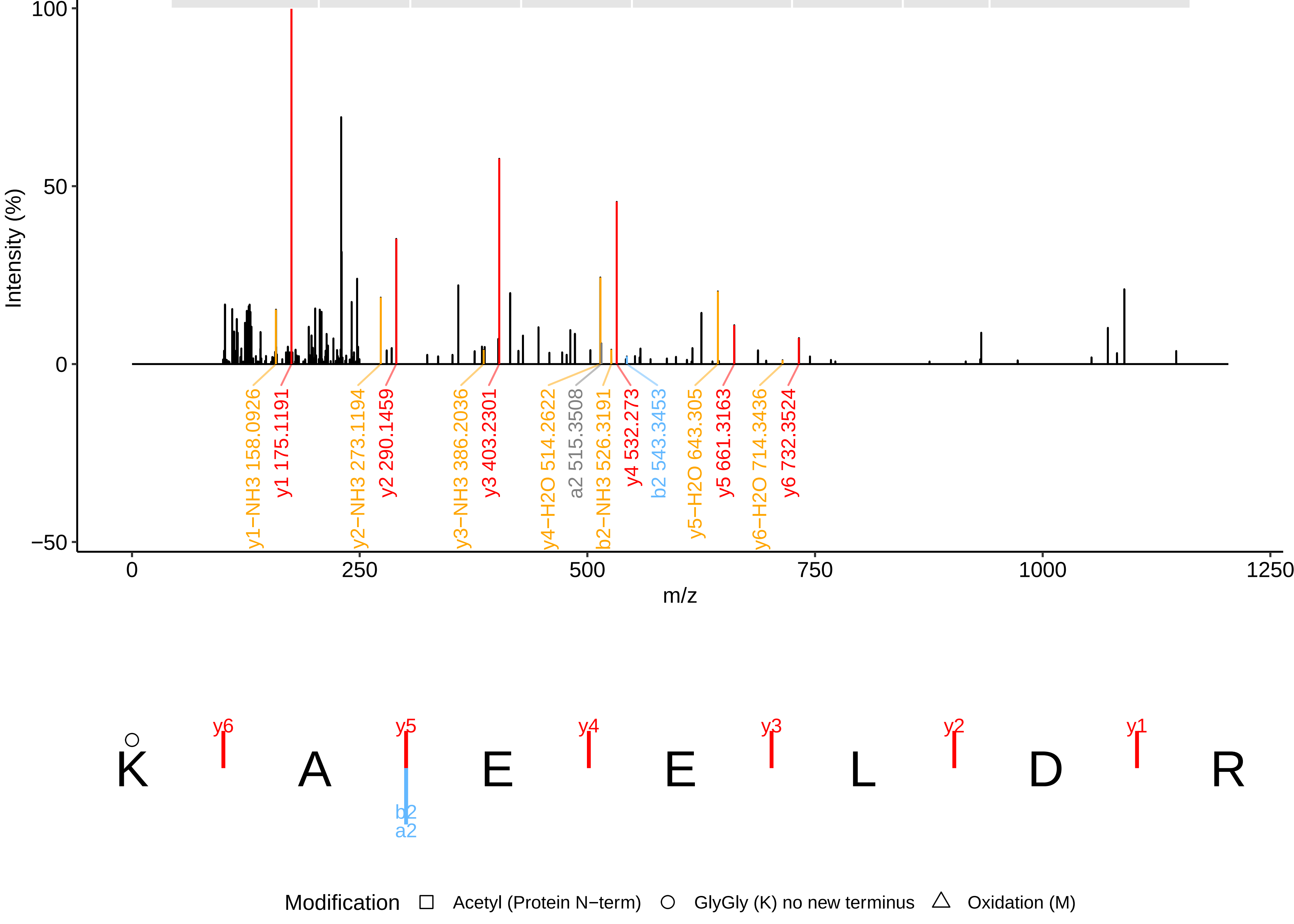

20200921_KIZ_MUb4_GlyGly_10plex − Scan number: 2636

Gene Names	Charge	m/z	Mass	Retention time	PEP	Score	Precursor Intensity
EIF3E	3	325.1773	972.5101	8.3076	0.031769	105.04	11887554

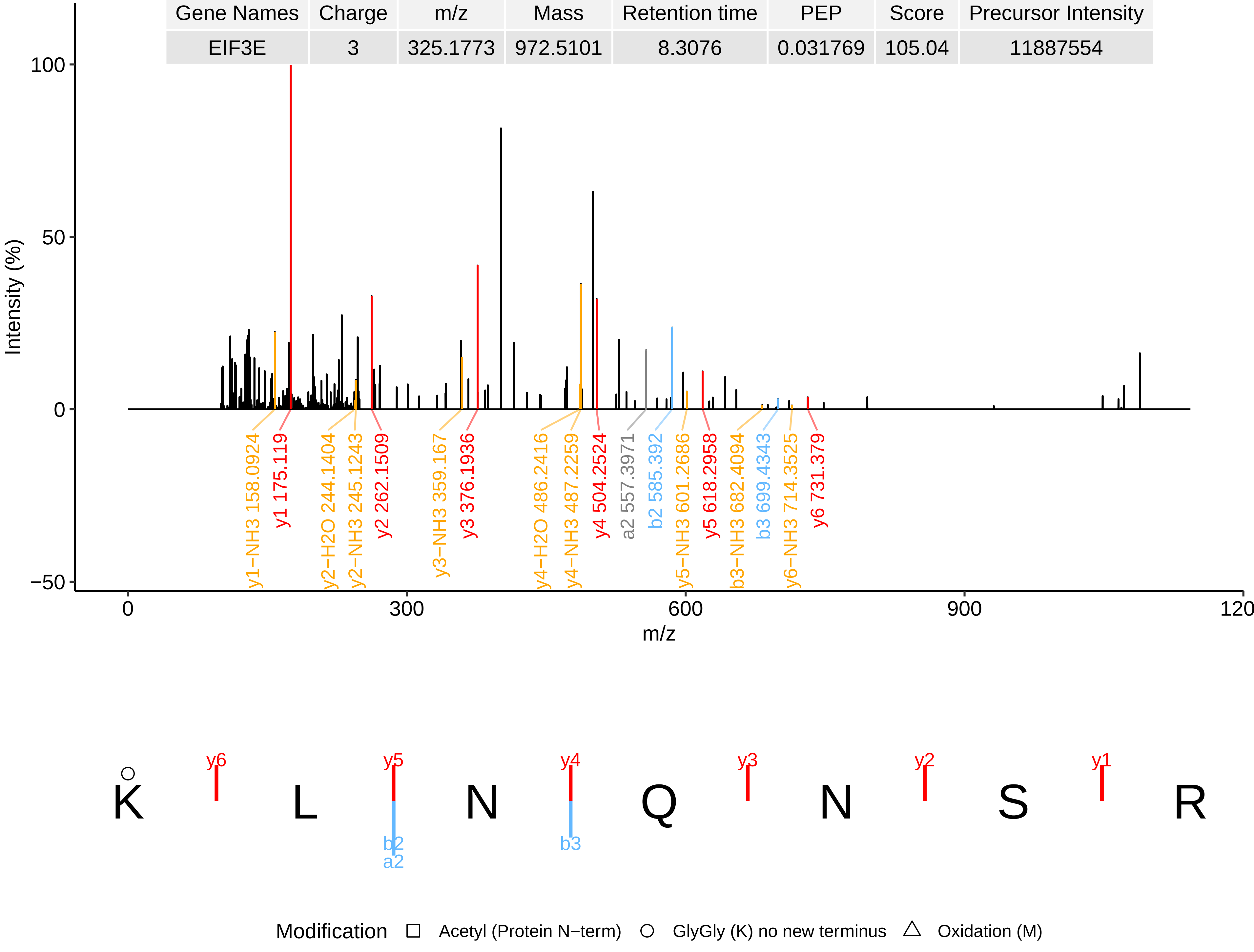

20200921_KIZ_MUb4_GlyGly_10plex_2 − Scan number: 2600

Gene Names	Charge	m/z	Mass	Retention time	PEP	Score	Precursor Intensity
EIF3E	3	325.1773	972.5101	8.2866	0.045265	97.798	835779.7

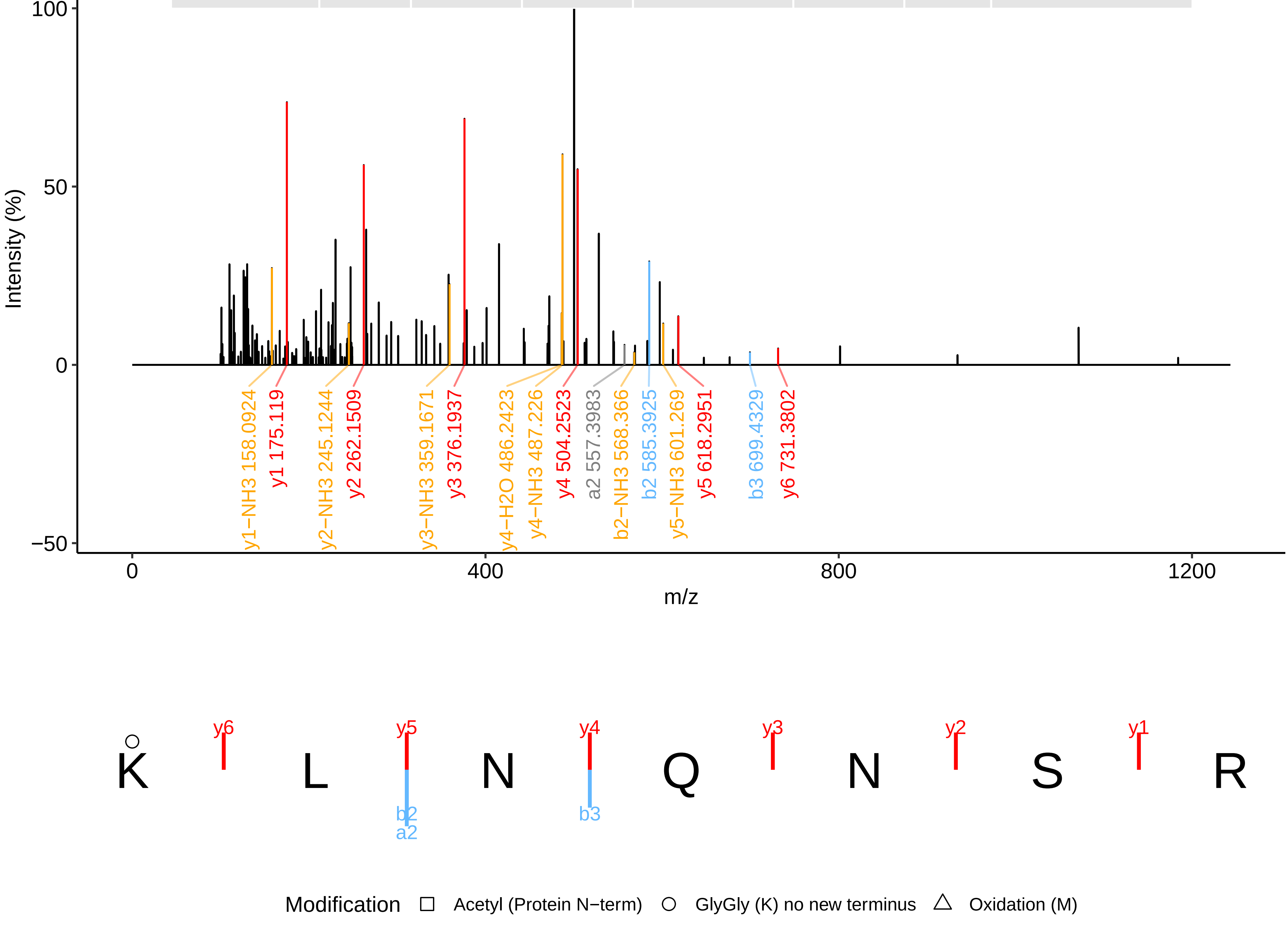

20200924_KIZ_MUb5_GlyGly_1 − Scan number: 2531

Gene Names	Charge	m/z	Mass	Retention time	PEP	Score	Precursor Intensity
EIF3E	3	325.1773	972.5101	7.8717	0.032295	104.75	7919383

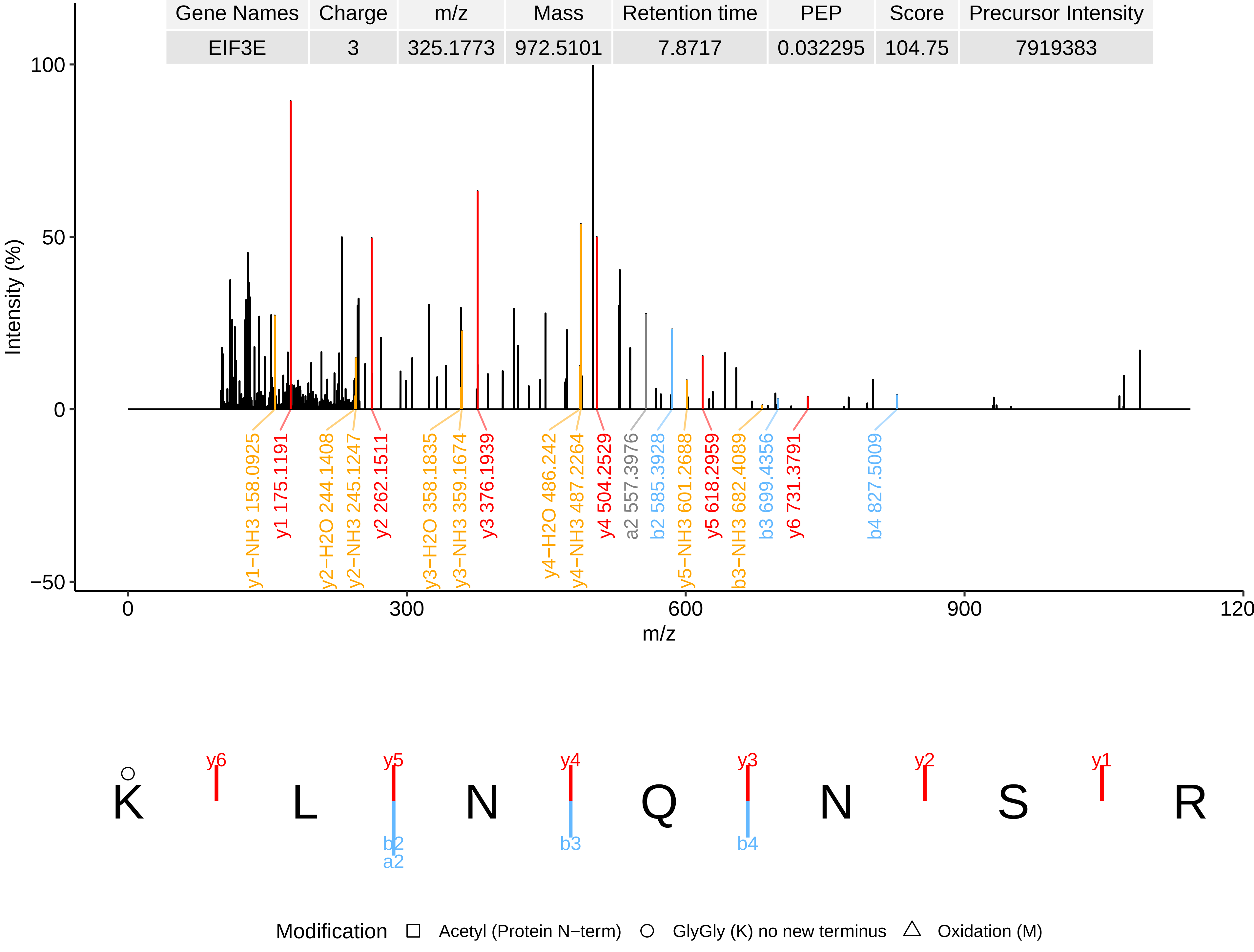

20201118_KIZ_MUb6_GlyGly_1 − Scan number: 2623

Gene Names	Charge	m/z	Mass	Retention time	PEP	Score	Precursor Intensity
EIF3E	3	325.1773	972.5101	8.0269	0.018659	112.37	32243492

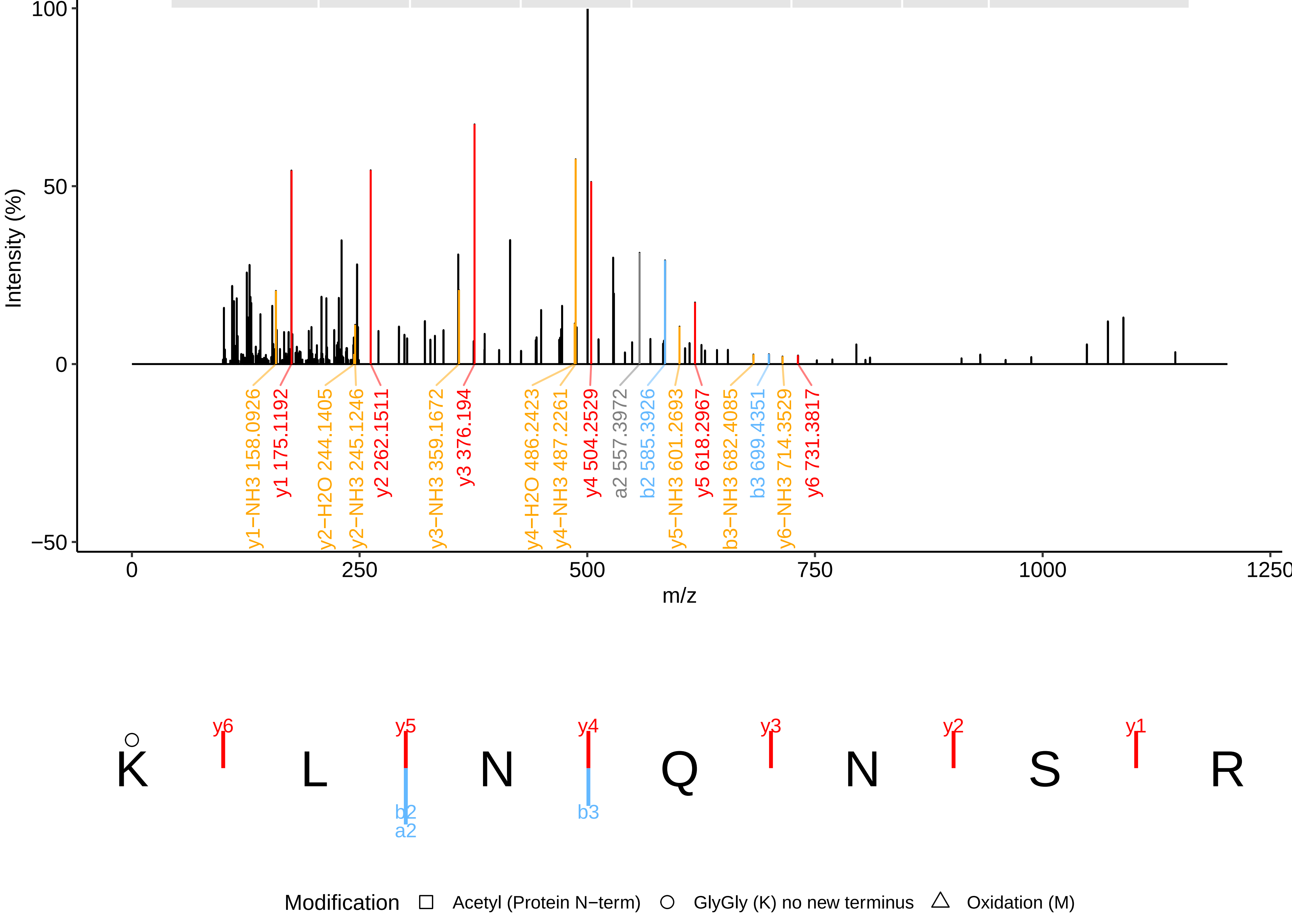

20200921_KIZ_MUb4_GlyGly_10plex − Scan number: 6698

Gene Names	Charge	m/z	Mass	Retention time	PEP	Score	Precursor Intensity
CYB5B	3	320.1821	957.5243	22.261	0.043463	98.503	27206426

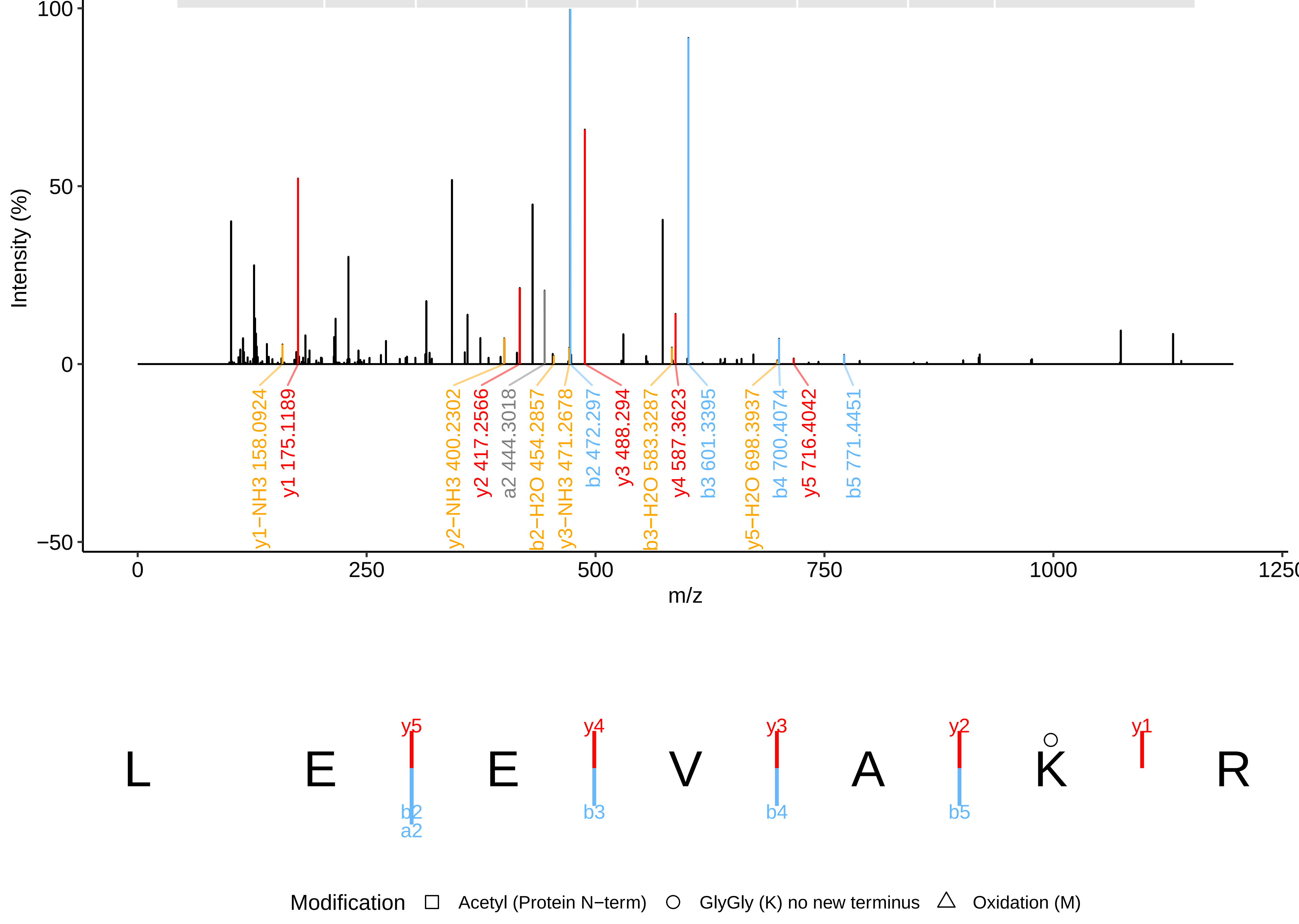

20200921_KIZ_MUb4_GlyGly_10plex − Scan number: 6704

Gene Names	Charge	m/z	Mass	Retention time	PEP	Score	Precursor Intensity
CYB5B	2	479.7695	957.5243	22.283	1.1729e−06	179.16	3935616

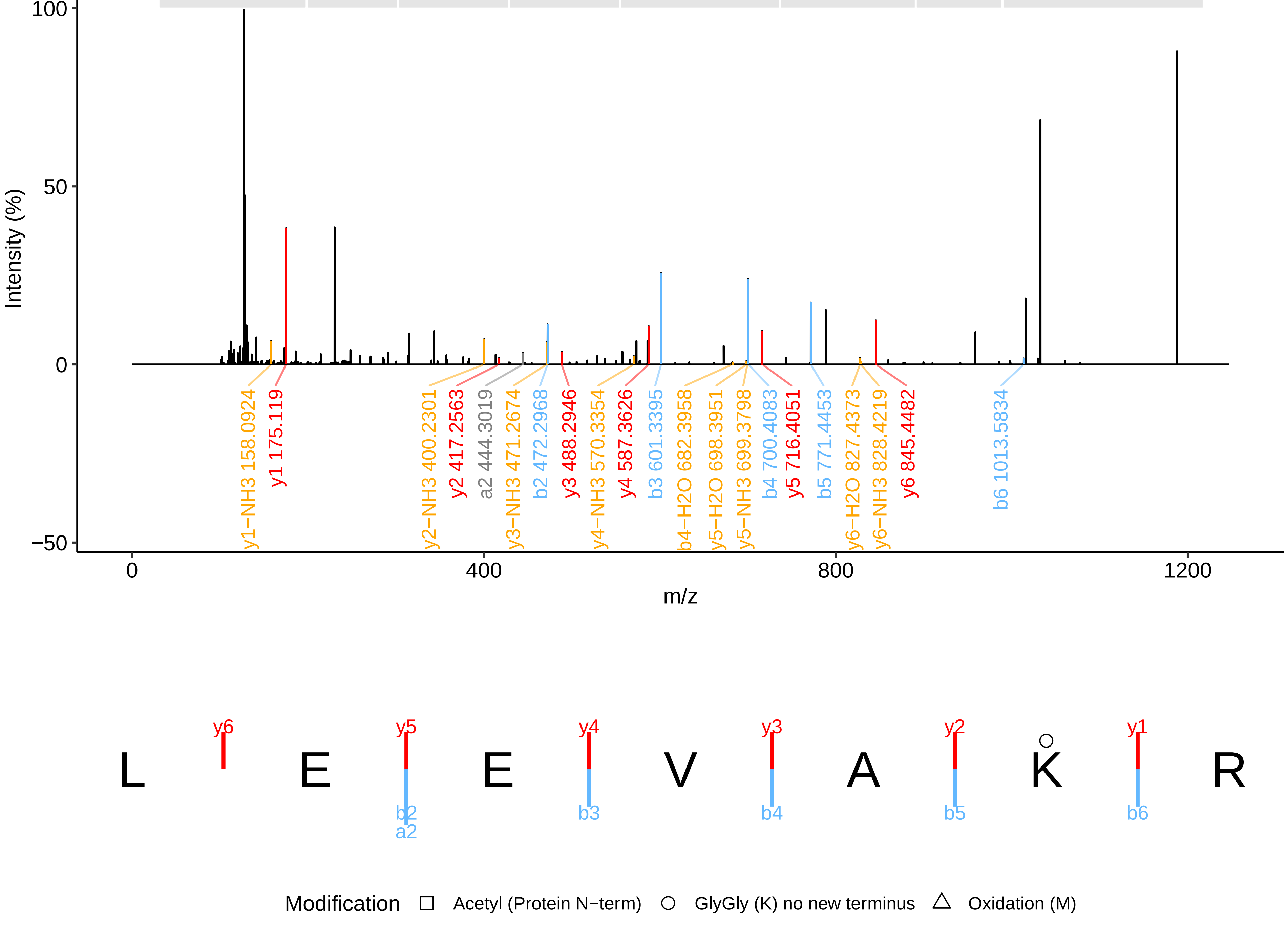

20200921_KIZ_MUb4_GlyGly_10plex_2 − Scan number: 6186

Gene Names	Charge	m/z	Mass	Retention time	PEP	Score	Precursor Intensity
CYB5B	3	320.1821	957.5243	22.62	0.07585	90.986	1731929

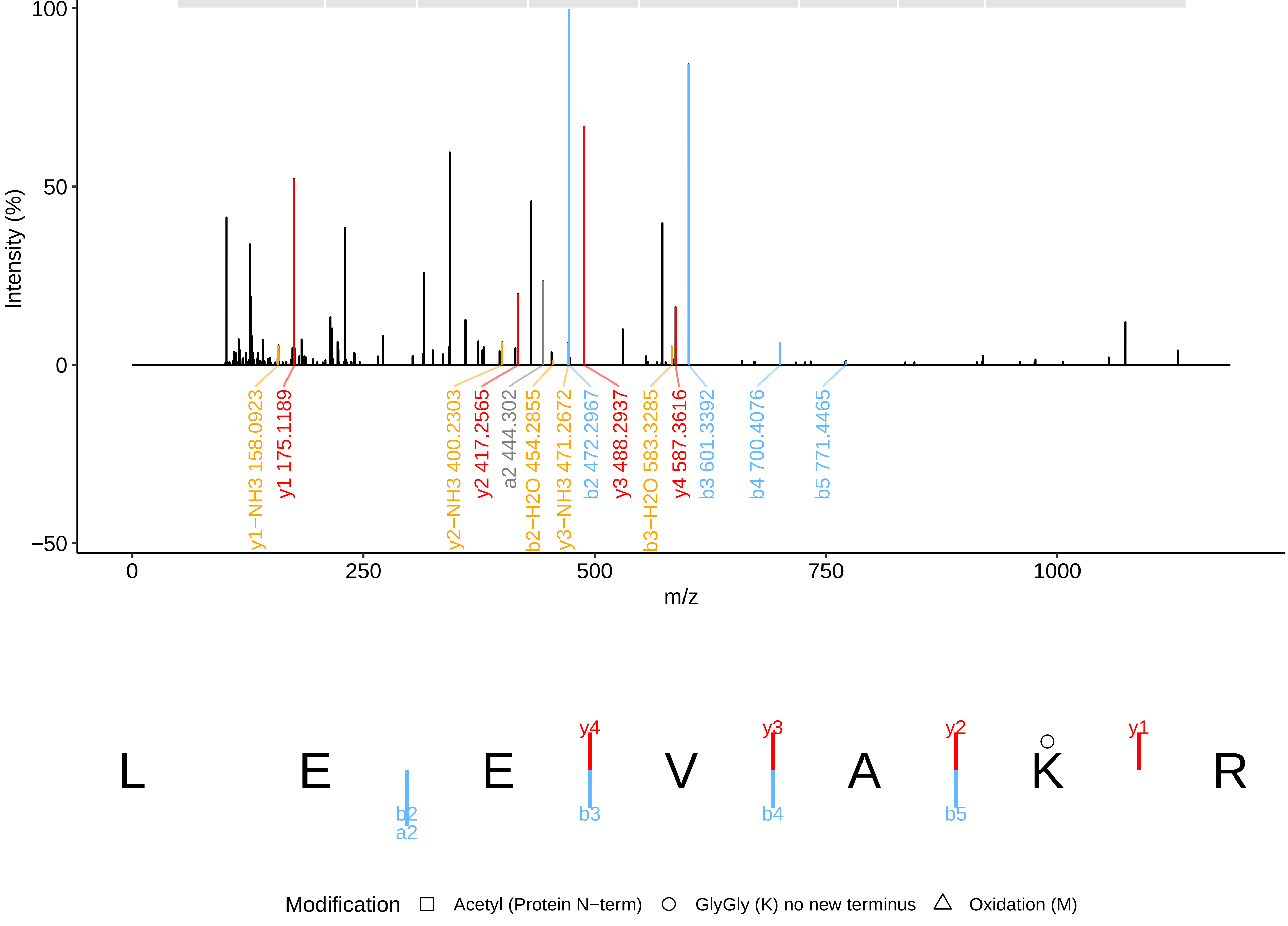

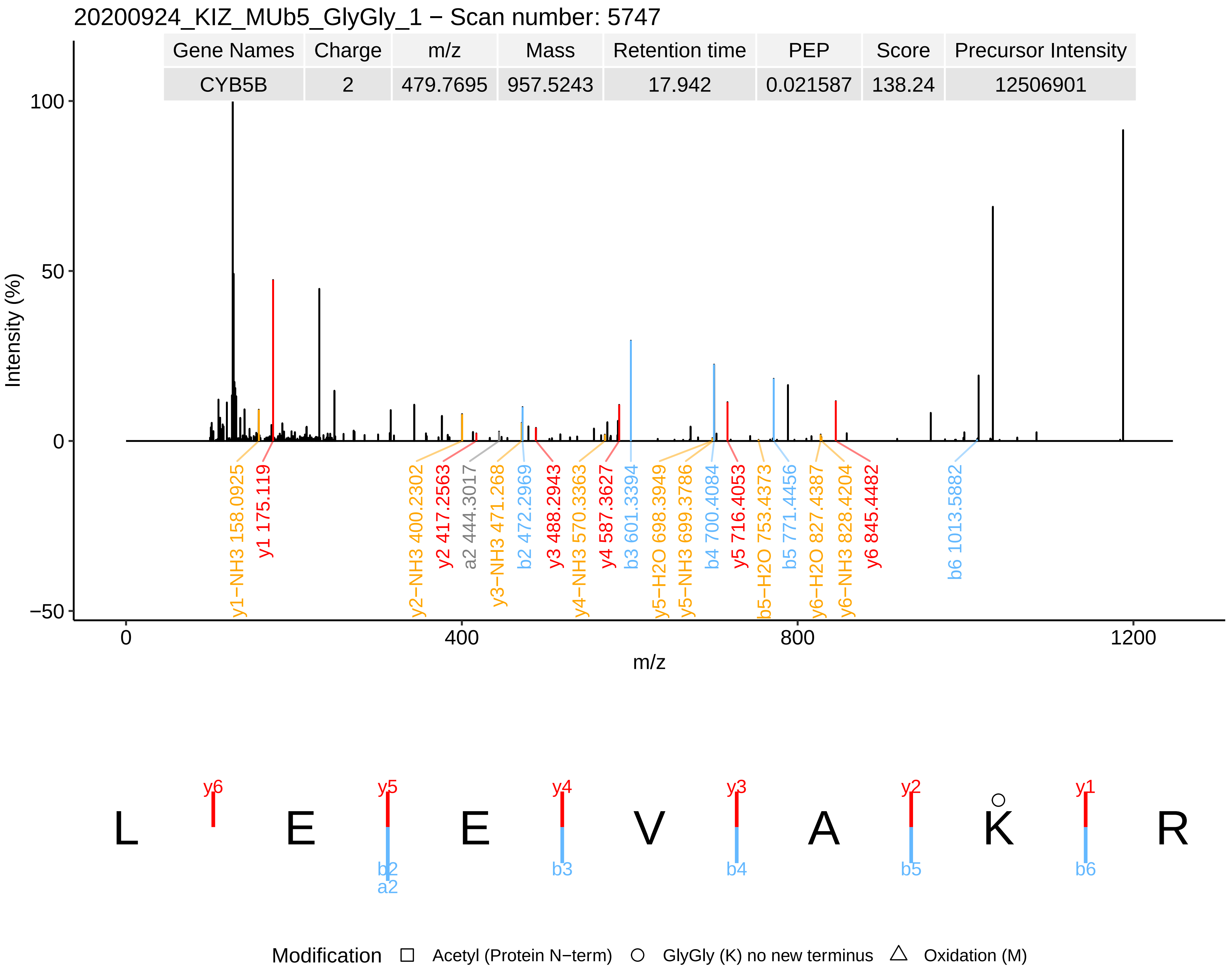

20200924_KIZ_MUb5_GlyGly_1 − Scan number: 5747
Gene Names | Charge | m/z | Mass | Retention time | PEP | Score | Precursor Intensity
CYB5B | 2 | 479.7695 | 957.5243 | 17.942 | 0.021587 | 138.24 | 12506901
Intensity (%)
m/z
y1−NH3 158.0925
y1 175.119
y2−NH3 400.2302
y2 417.2563
a2 444.3017
y3−NH3 471.268
b2 472.2969
y3 488.2943
y4−NH3 570.3363
y4 587.3627
b3 601.3394
y5−H2O 698.3949
y5−NH3 699.3786
b4 700.4084
y5 716.4053
b5−H2O 753.4373
b5 771.4456
y6−H2O 827.4387
y6−NH3 828.4204
y6 845.4482
b6 1013.5882
L E E V A K R
y6 y5 y4 y3 y2 y1
b2 a2 b3 b4 b5 b6
Modification
Acetyl (Protein N−term)
GlyGly (K) no new terminus
Oxidation (M)

20201118_KIZ_MUb6_GlyGly_1 − Scan number: 5421

Gene Names	Charge	m/z	Mass	Retention time	PEP	Score	Precursor Intensity
CYB5B	3	320.1821	957.5243	17.164	0.01277	117.6	26039912

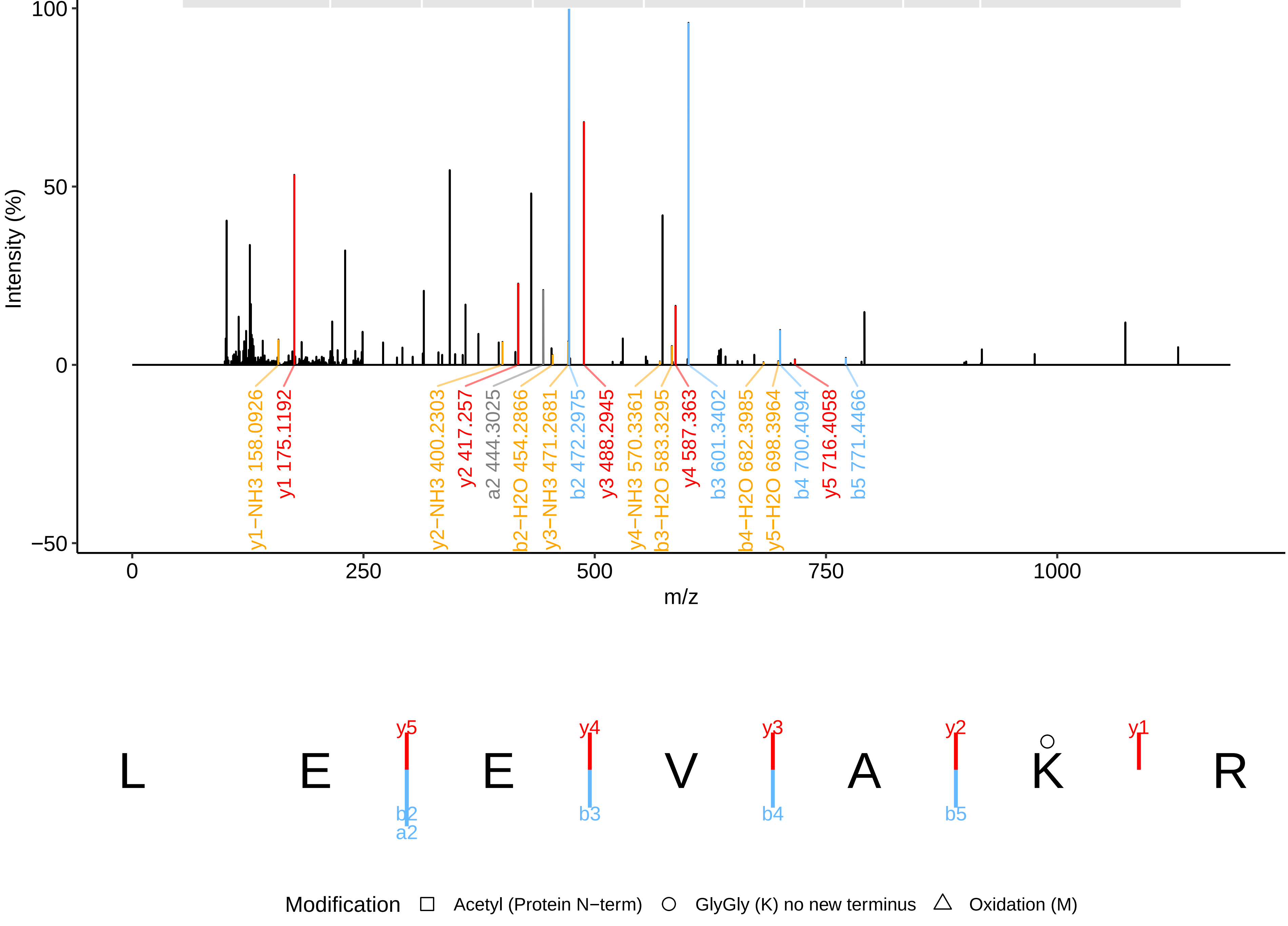

20201118_KIZ_MUb6_GlyGly_2 − Scan number: 6939

Gene Names	Charge	m/z	Mass	Retention time	PEP	Score	Precursor Intensity
CYB5B	3	320.1821	957.5243	23.677	0.046146	97.602	14248670

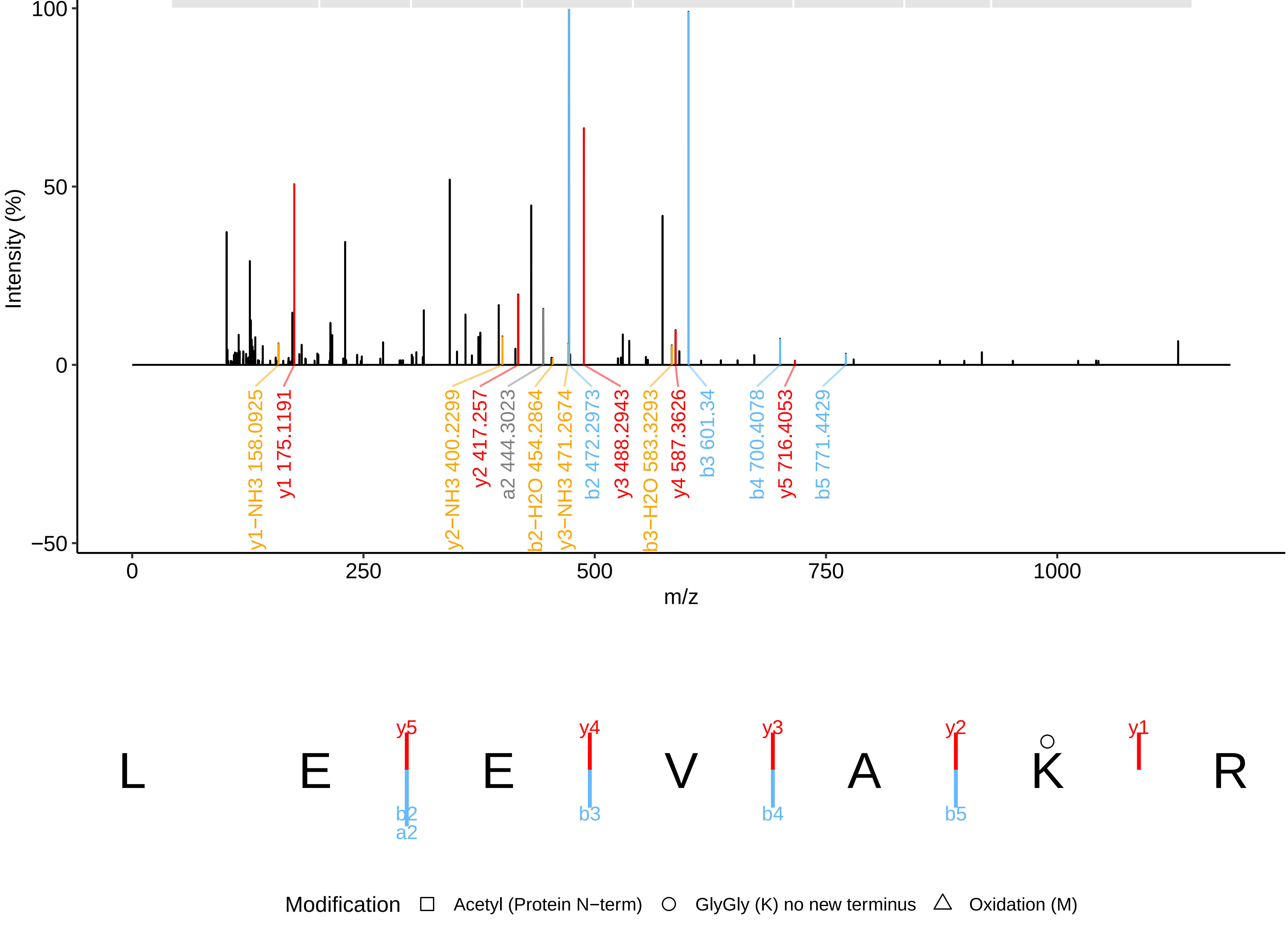

20200924_KIZ_MUb5_GlyGly_1 − Scan number: 2684

Gene Names	Charge	m/z	Mass	Retention time	PEP	Score	Precursor Intensity
ATAD3A	3	313.5104	937.5094	8.2928	0.026066	78.149	39306516

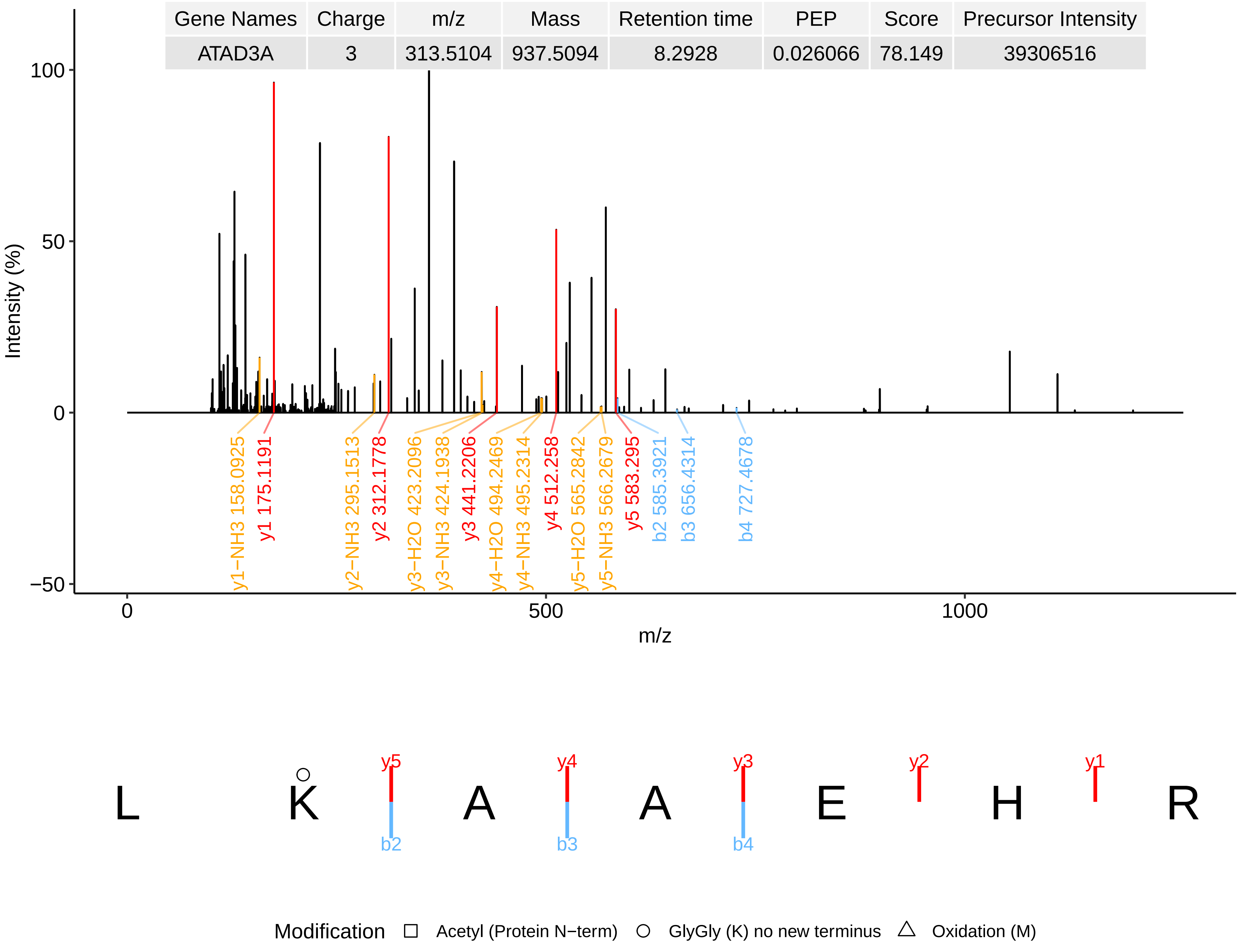

20201118_KIZ_MUb6_GlyGly_1 − Scan number: 2738

Gene Names	Charge	m/z	Mass	Retention time	PEP	Score	Precursor Intensity
ATAD3A	3	313.5104	937.5094	8.3707	0.0281	78.178	20388018

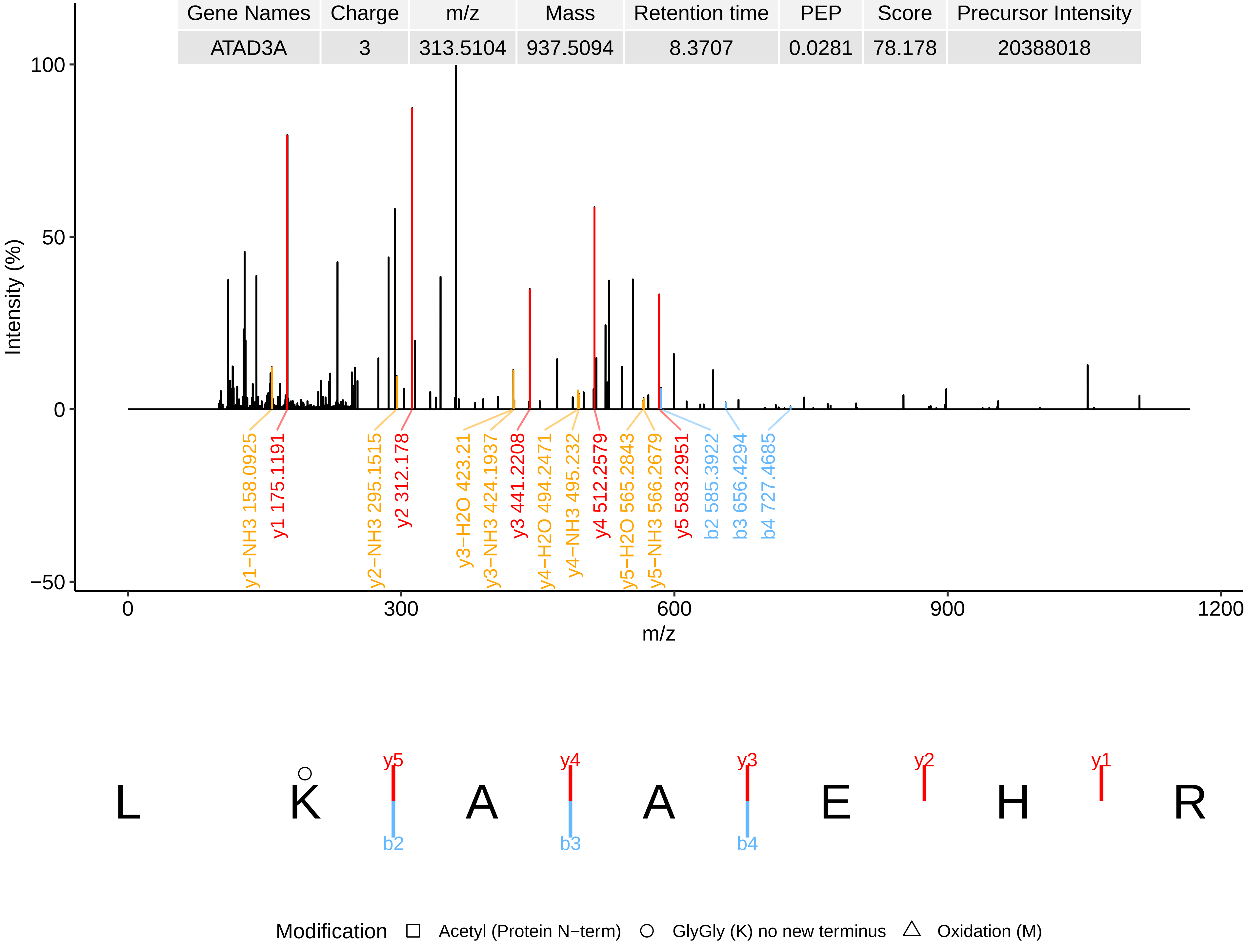

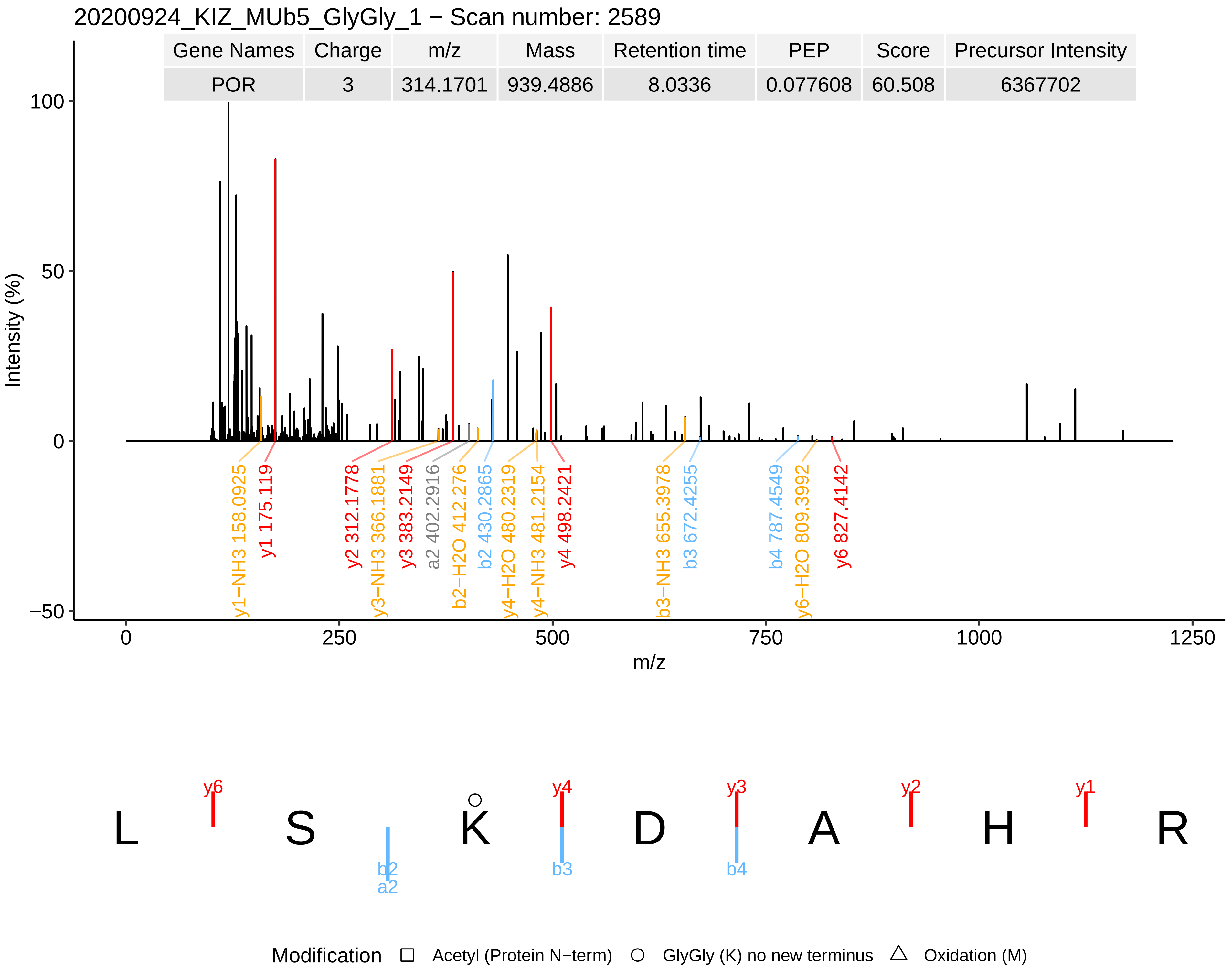
20200924_KIZ_MUb5_GlyGly_1 − Scan number: 2589
Gene Names | Charge | m/z | Mass | Retention time | PEP | Score | Precursor Intensity
POR | 3 | 314.1701 | 939.4886 | 8.0336 | 0.077608 | 60.508 | 6367702
Intensity (%)
100
50
0
−50
y1−NH3 158.0925
y1 175.119
y2 312.1778
y3−NH3 366.1881
y3 383.2149
a2 402.2916
b2−H2O 412.276
b2 430.2865
y4−H2O 480.2319
y4−NH3 481.2154
y4 498.2421
b3−NH3 655.3978
b3 672.4255
b4 787.4549
y6−H2O 809.3992
y6 827.4142
0
250
500
750
1000
1250
m/z
L S K D A H R
y6 y4 y3 y2 y1
b2 a2 b3 b4
Modification
Acetyl (Protein N−term)
GlyGly (K) no new terminus
Oxidation (M)

20200921_KIZ_MUb4_GlyGly_10plex_2 − Scan number: 4864

Gene Names	Charge	m/z	Mass	Retention time	PEP	Score	Precursor Intensity
DDR2	3	316.8359	947.4859	17.243	0.076171	60.799	1171832

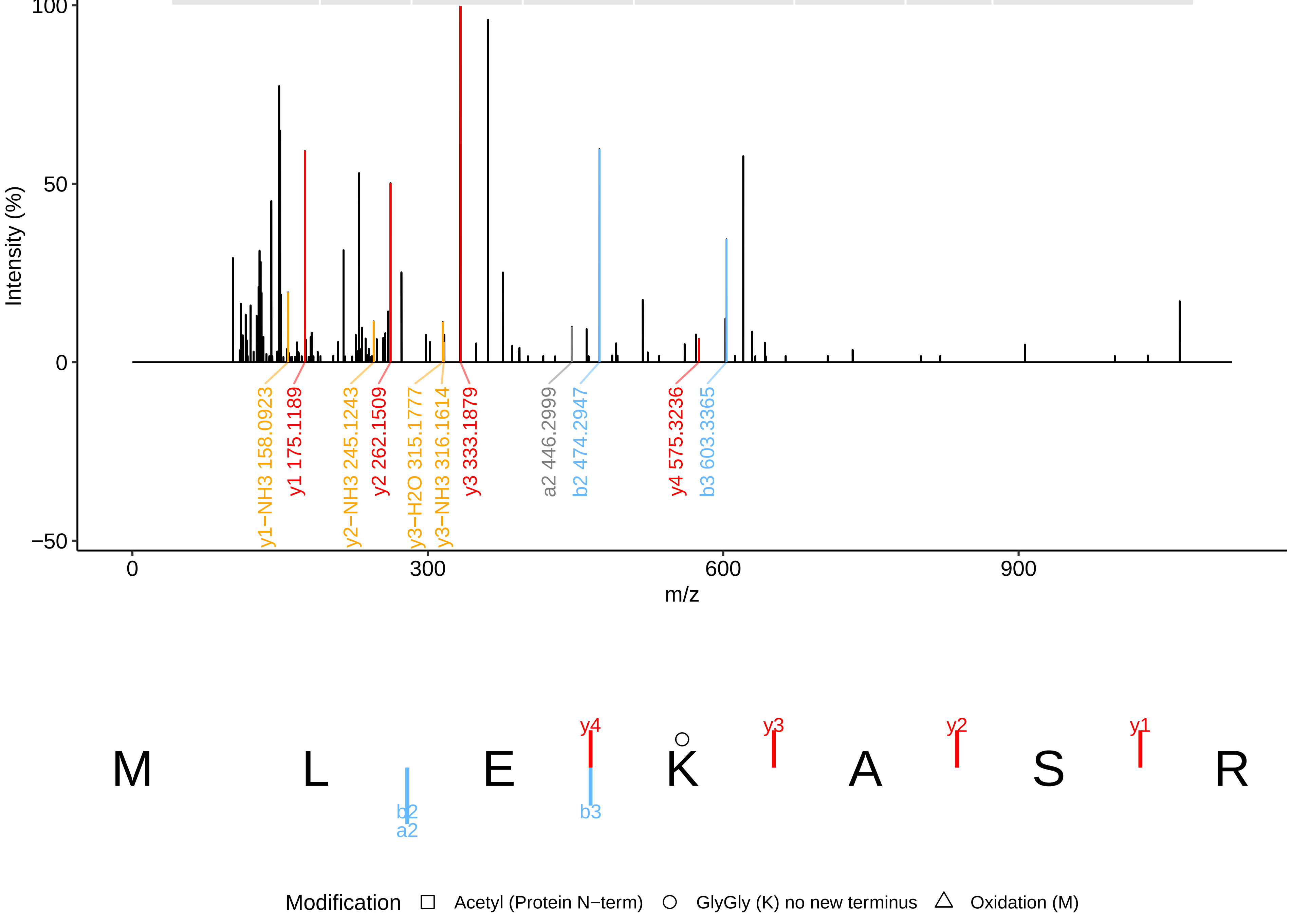

20200924_KIZ_MUb5_GlyGly_2 − Scan number: 4222

Gene Names	Charge	m/z	Mass	Retention time	PEP	Score	Precursor Intensity
DDR2	3	316.8359	947.4859	15.468	0.060708	65.038	3249956

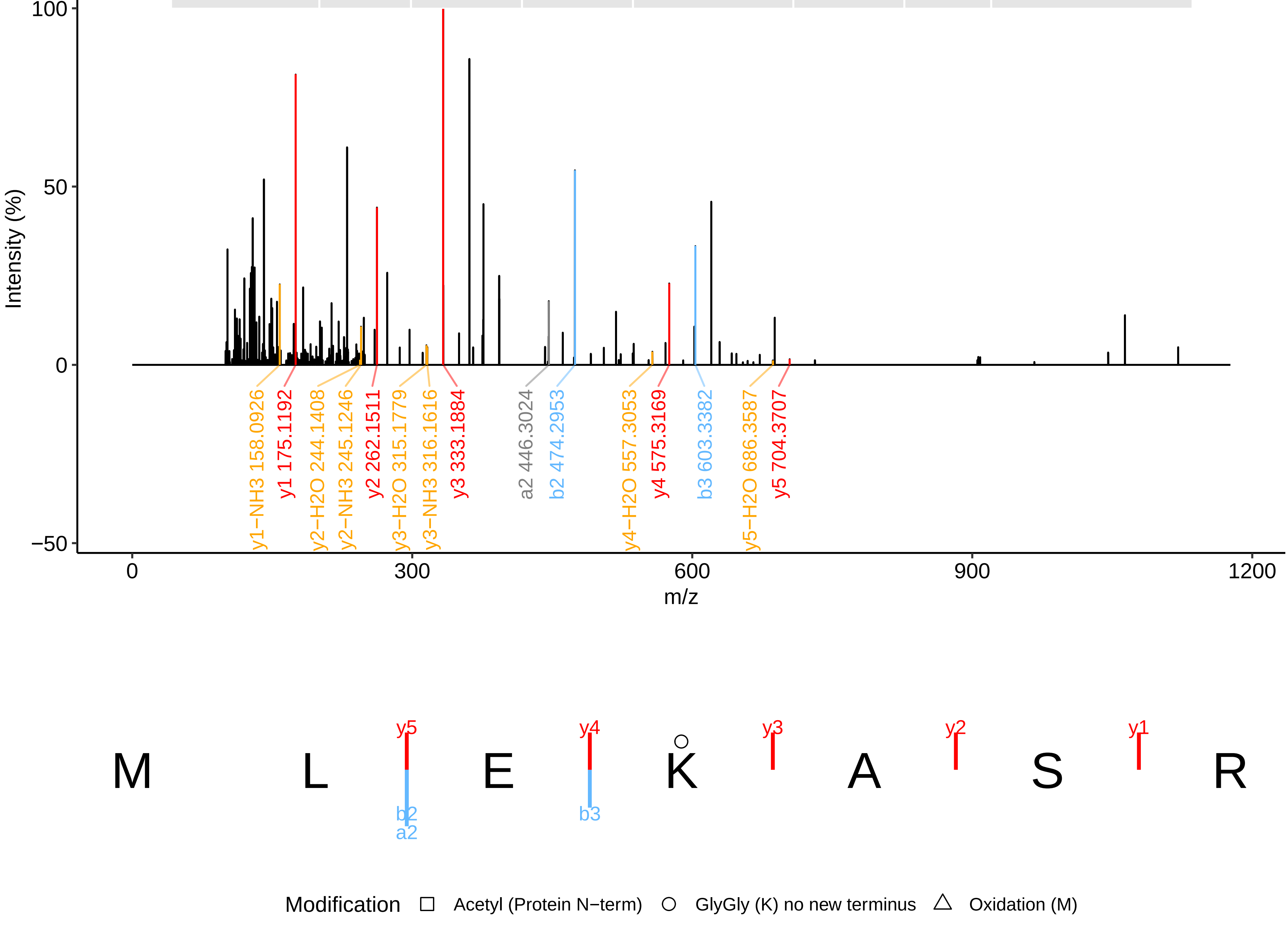

20200921_KIZ_MUb4_GlyGly_10plex − Scan number: 5783

Gene Names	Charge	m/z	Mass	Retention time	PEP	Score	Precursor Intensity
LAPTM4A	2	587.2732	1172.532	18.972	0.095588	49.442	1406073

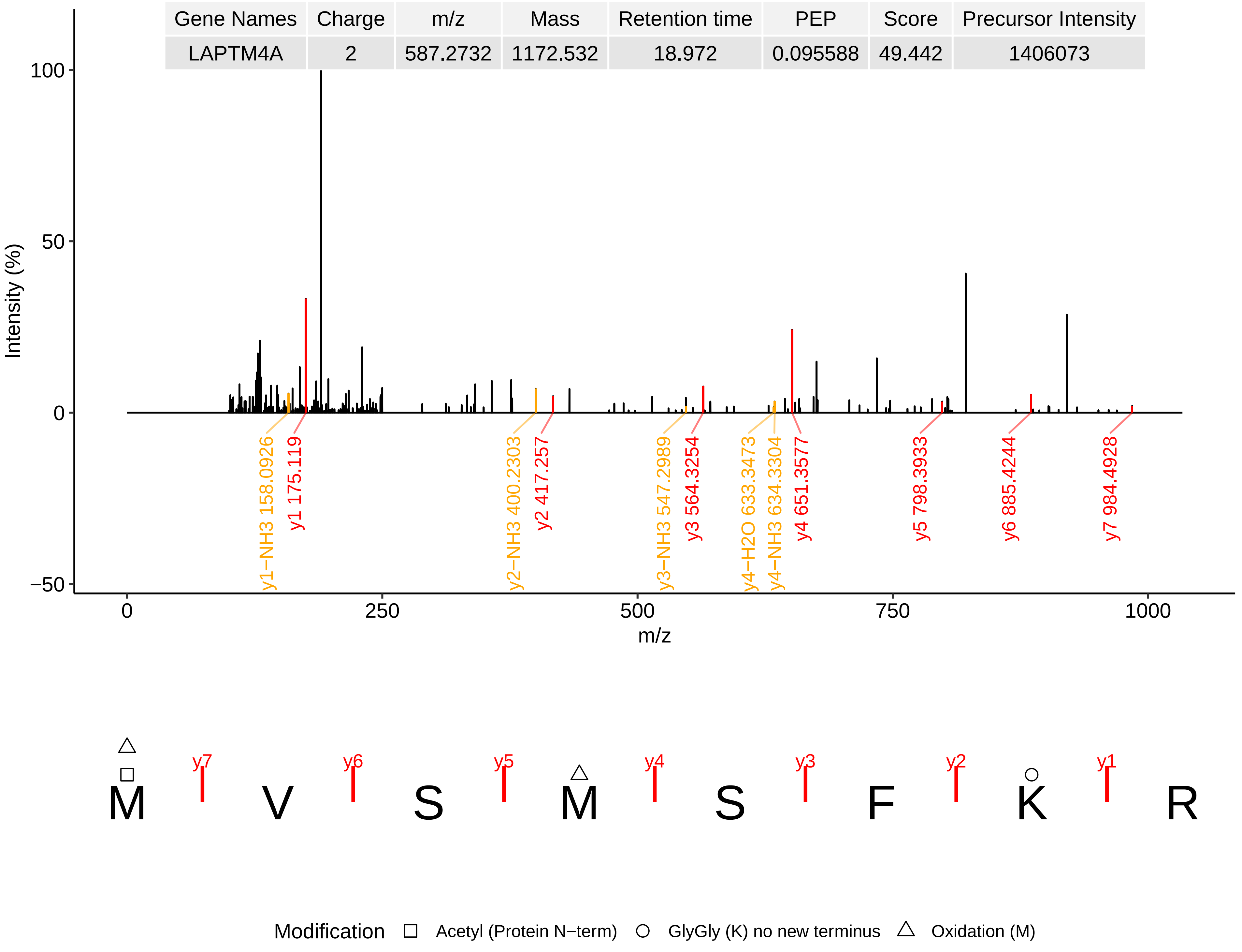

20200921_KIZ_MUb4_GlyGly_10plex − Scan number: 15182

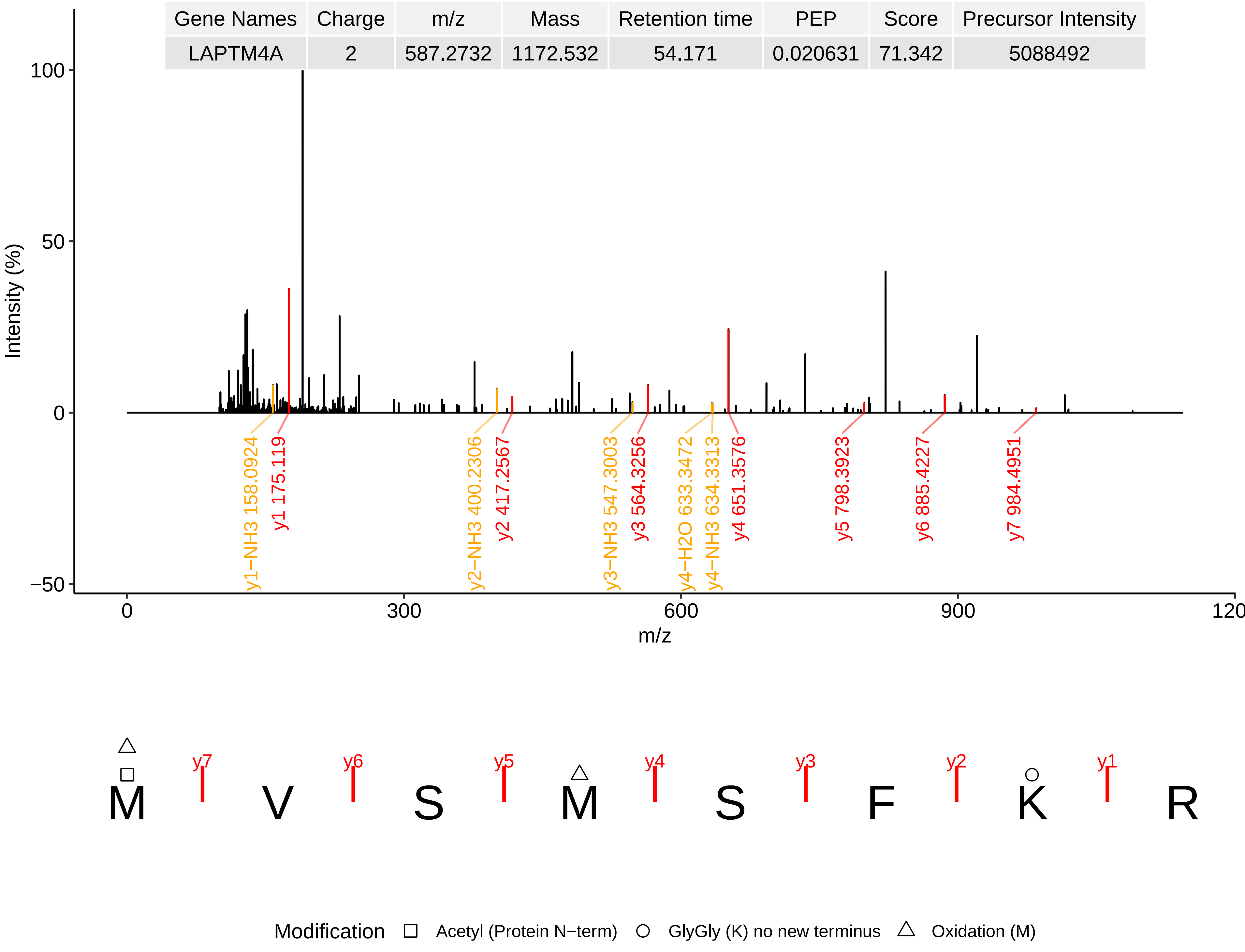

20200921_KIZ_MUb4_GlyGly_10plex_2 − Scan number: 14012

Gene Names	Charge	m/z	Mass	Retention time	PEP	Score	Precursor Intensity
LAPTM4A	2	587.2732	1172.532	54.534	0.021521	72.138	876966.6

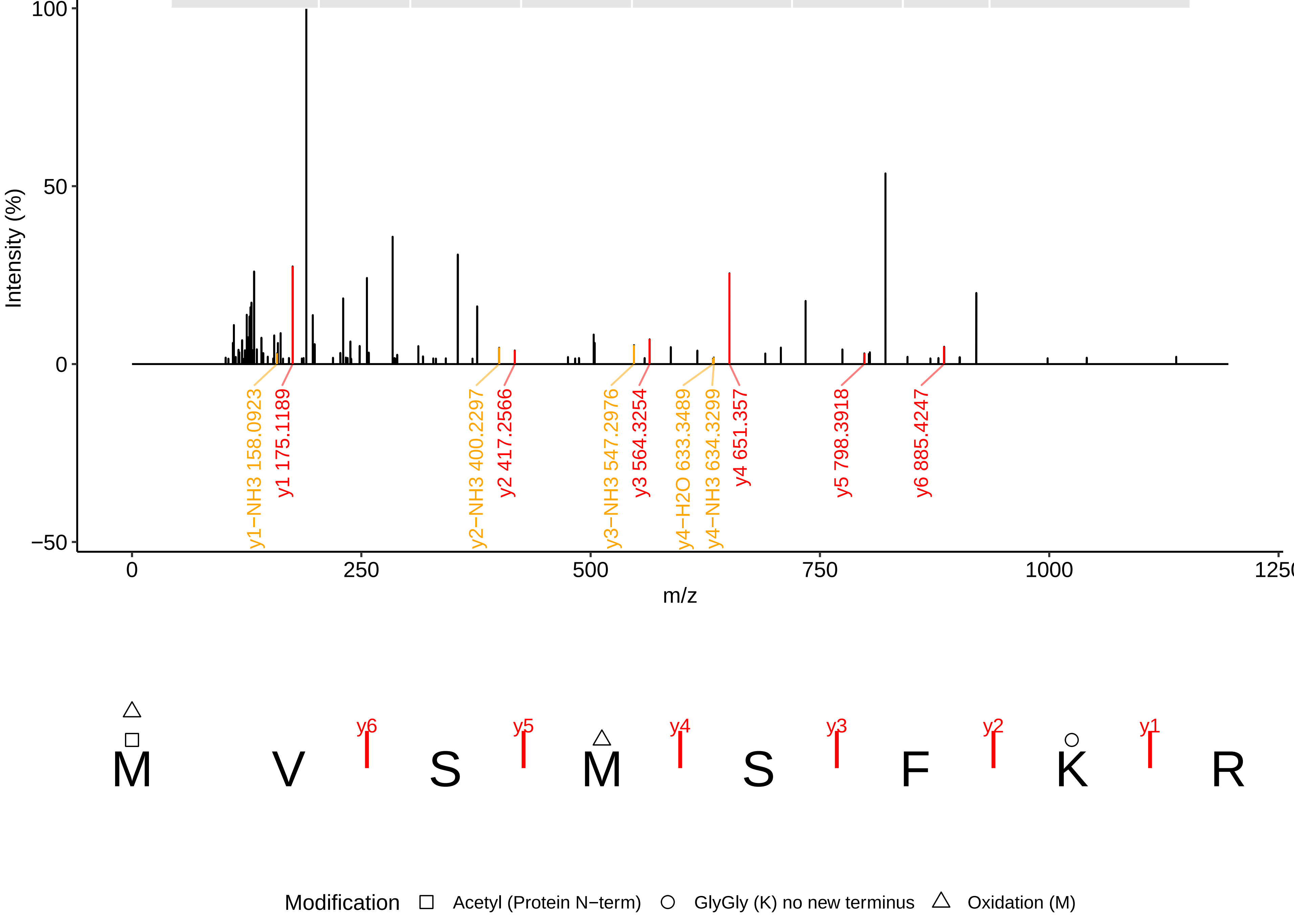

20200921_KIZ_MUb4_GlyGly_10plex_2 − Scan number: 2381

Gene Names	Charge	m/z	Mass	Retention time	PEP	Score	Precursor Intensity
MKRN1	3	326.1772	975.5098	7.4467	0.058677	56.378	861944.8

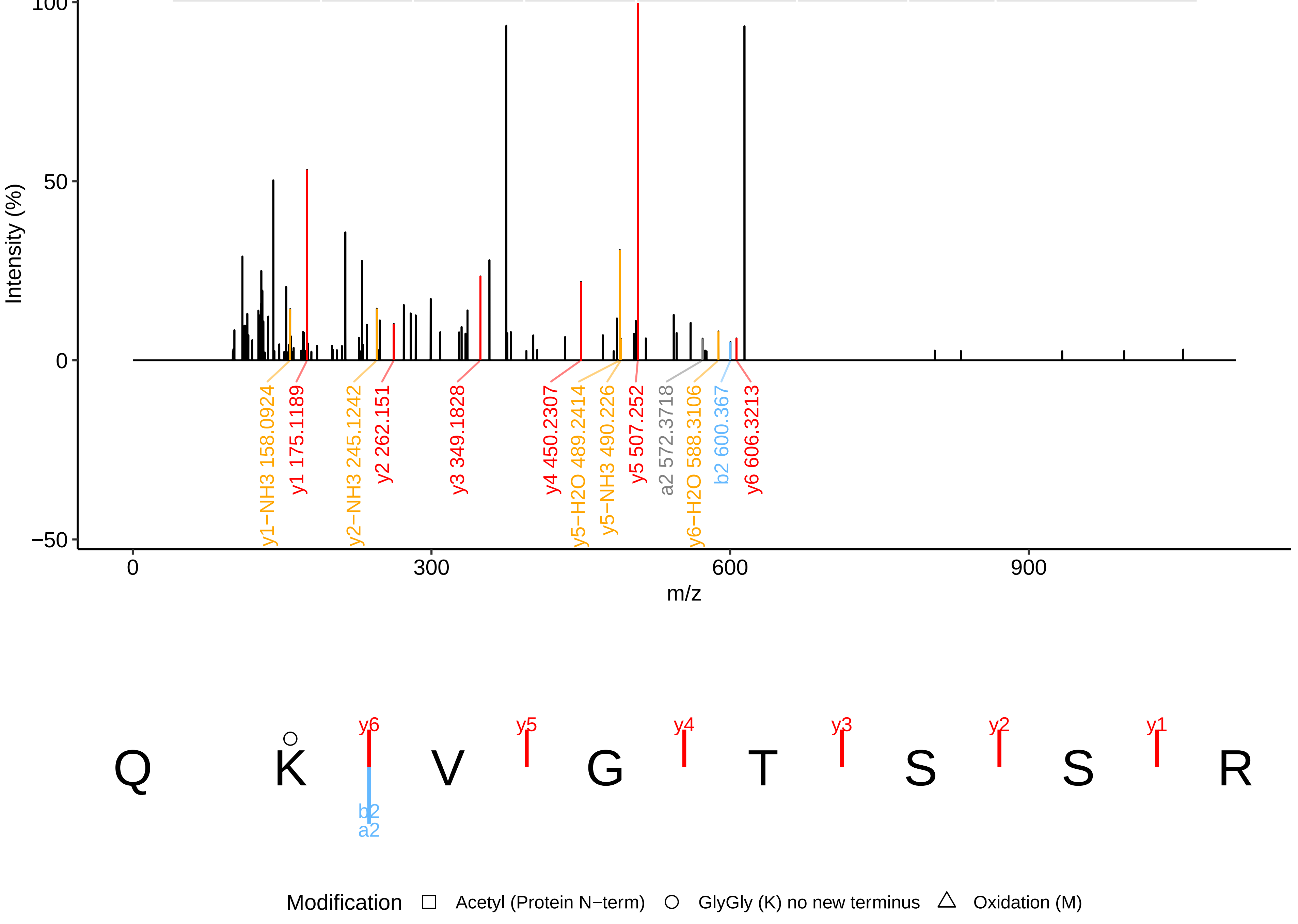

20200924_KIZ_MUb5_GlyGly_1 − Scan number: 2389

Gene Names	Charge	m/z	Mass	Retention time	PEP	Score	Precursor Intensity
MKRN1	3	326.1772	975.5098	7.443	0.026494	66.621	17338680

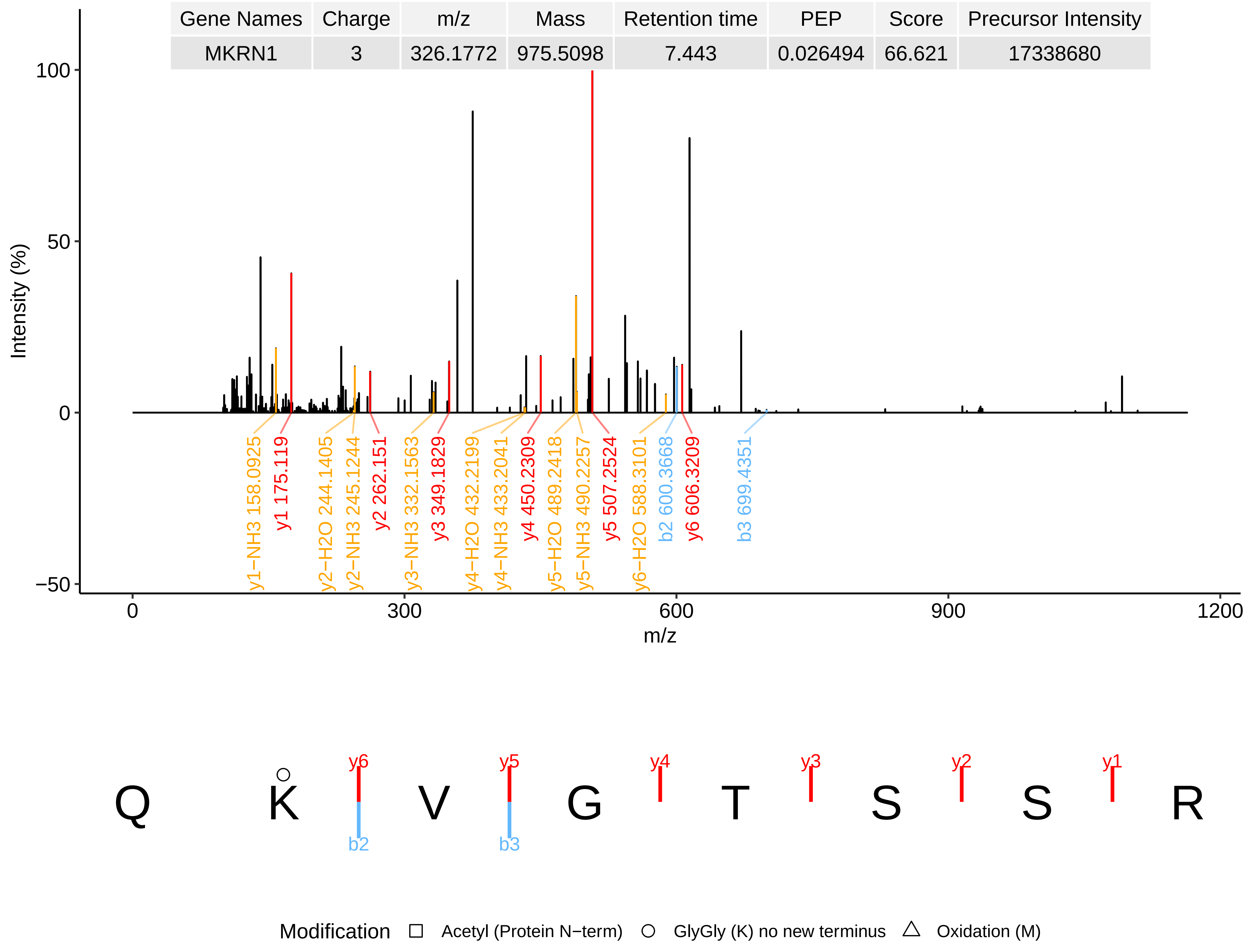

20200921_KIZ_MUb4_GlyGly_10plex − Scan number: 3609

Gene Names	Charge	m/z	Mass	Retention time	PEP	Score	Precurs
3B;H3F3A;H3F3C;HIST1H3A;HIST2H3A;HIST2H3PS2	3	324.8577	971.5512	11.531	0.0040332	93.429	94

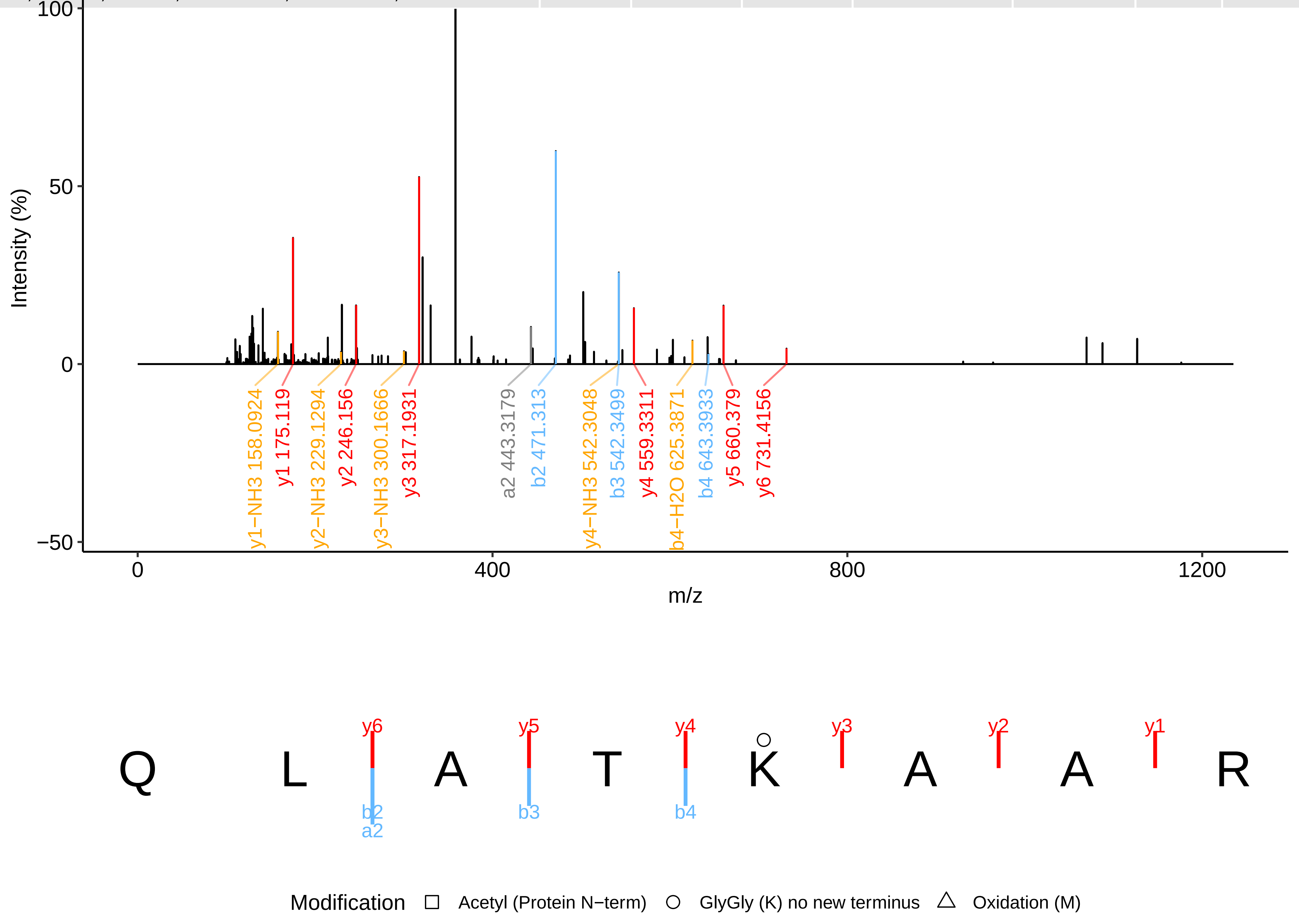

20200921_KIZ_MUb4_GlyGly_10plex_2 − Scan number: 3435

Gene Names	Charge	m/z	Mass	Retention time	PEP	Score	Precurs
3B;H3F3A;H3F3C;HIST1H3A;HIST2H3A;HIST2H3PS2	3	324.8577	971.5512	11.548	0.0021595	98.629	84

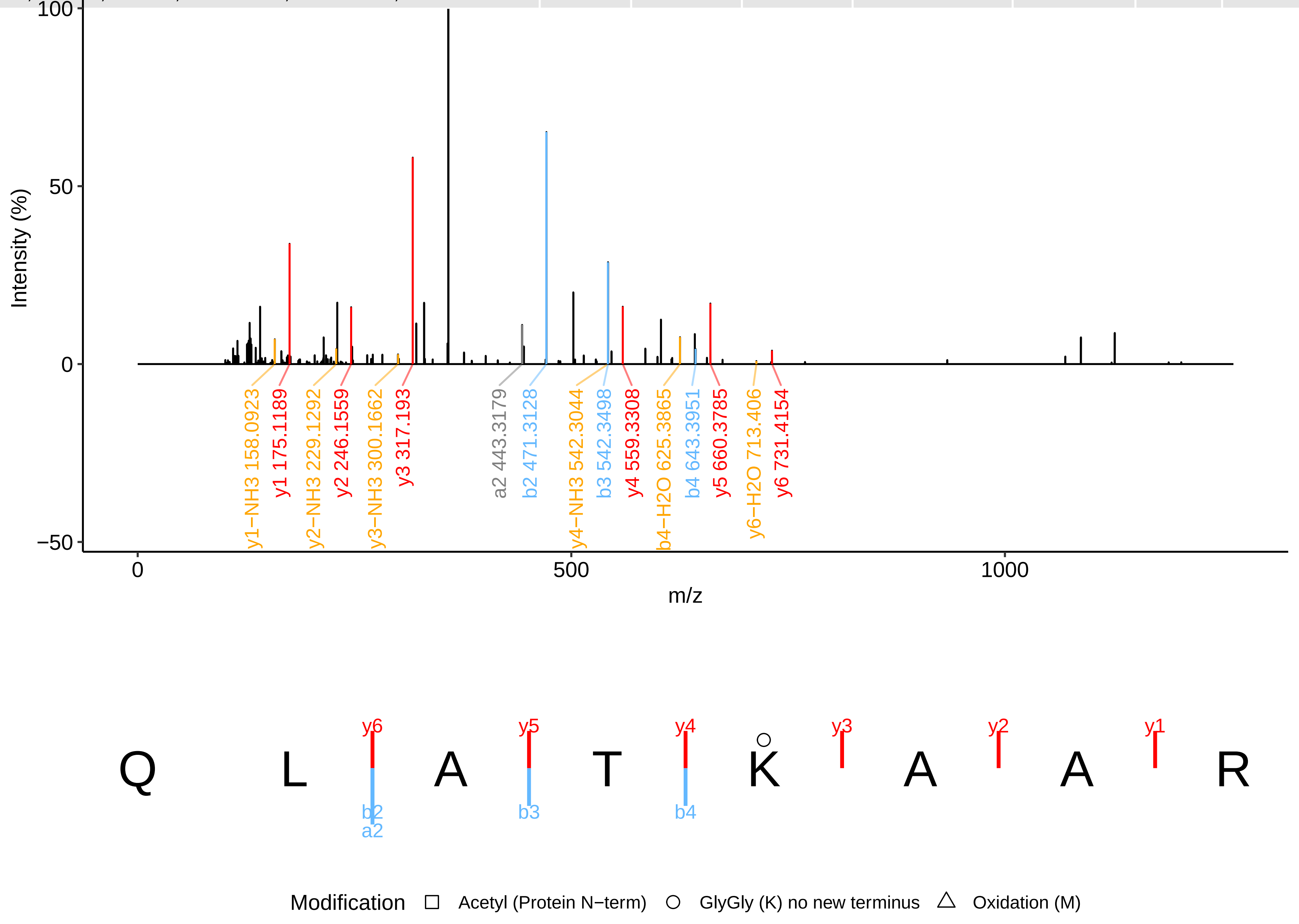

20200924_KIZ_MUb5_GlyGly_1 − Scan number: 3295

Gene Names	Charge	m/z	Mass	Retention time	PEP	Score	Precurs
3B;H3F3A;H3F3C;HIST1H3A;HIST2H3A;HIST2H3PS2	3	324.8577	971.5512	10.127	0.0015701	103.83	35

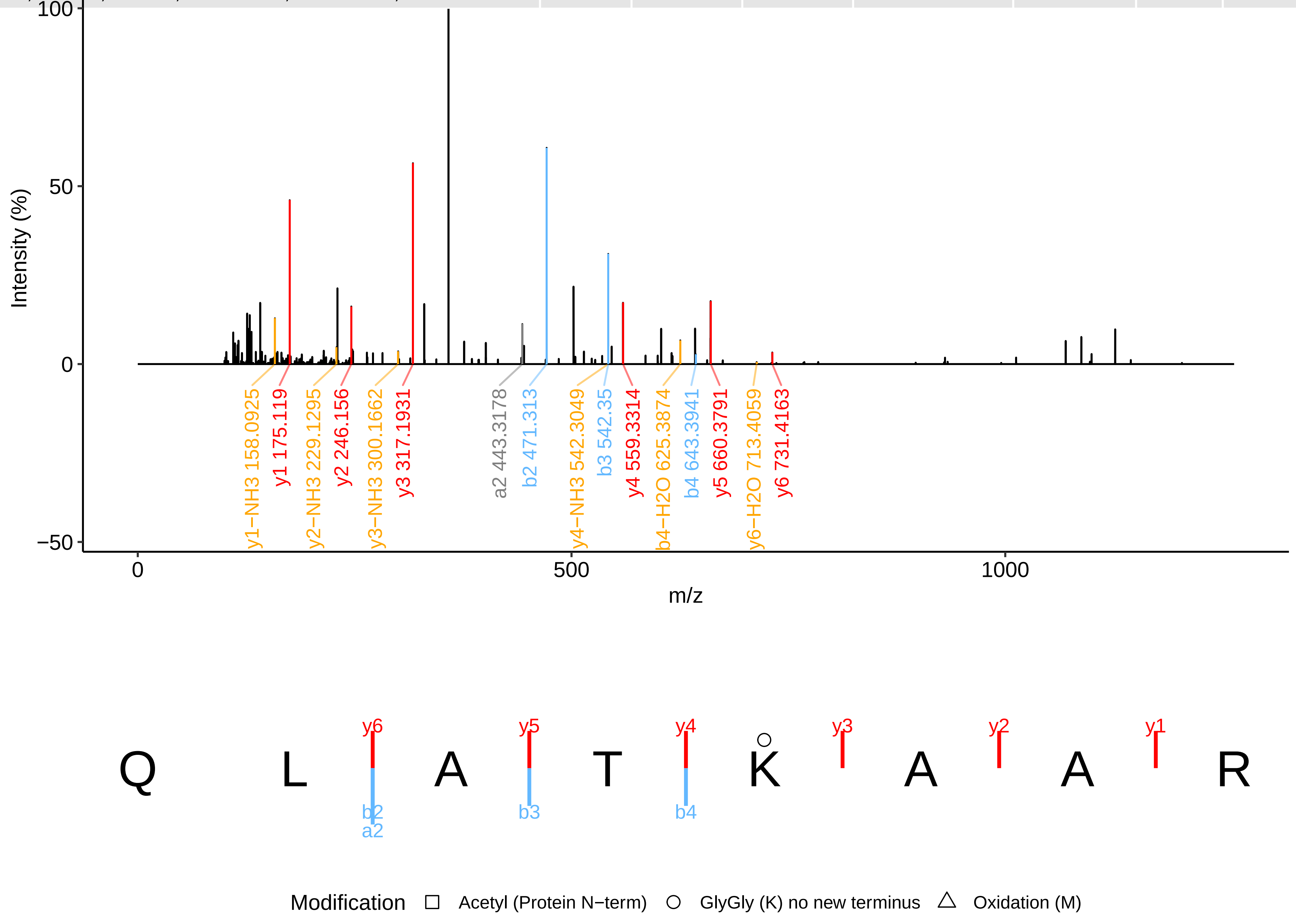

20200924_KIZ_MUb5_GlyGly_2 − Scan number: 2980

Gene Names	Charge	m/z	Mass	Retention time	PEP	Score	Precurso
F3B;H3F3A;H3F3C;HIST1H3A;HIST2H3A;HIST2H3PS2	3	324.8577	971.5512	10.693	0.03336	62.546	178

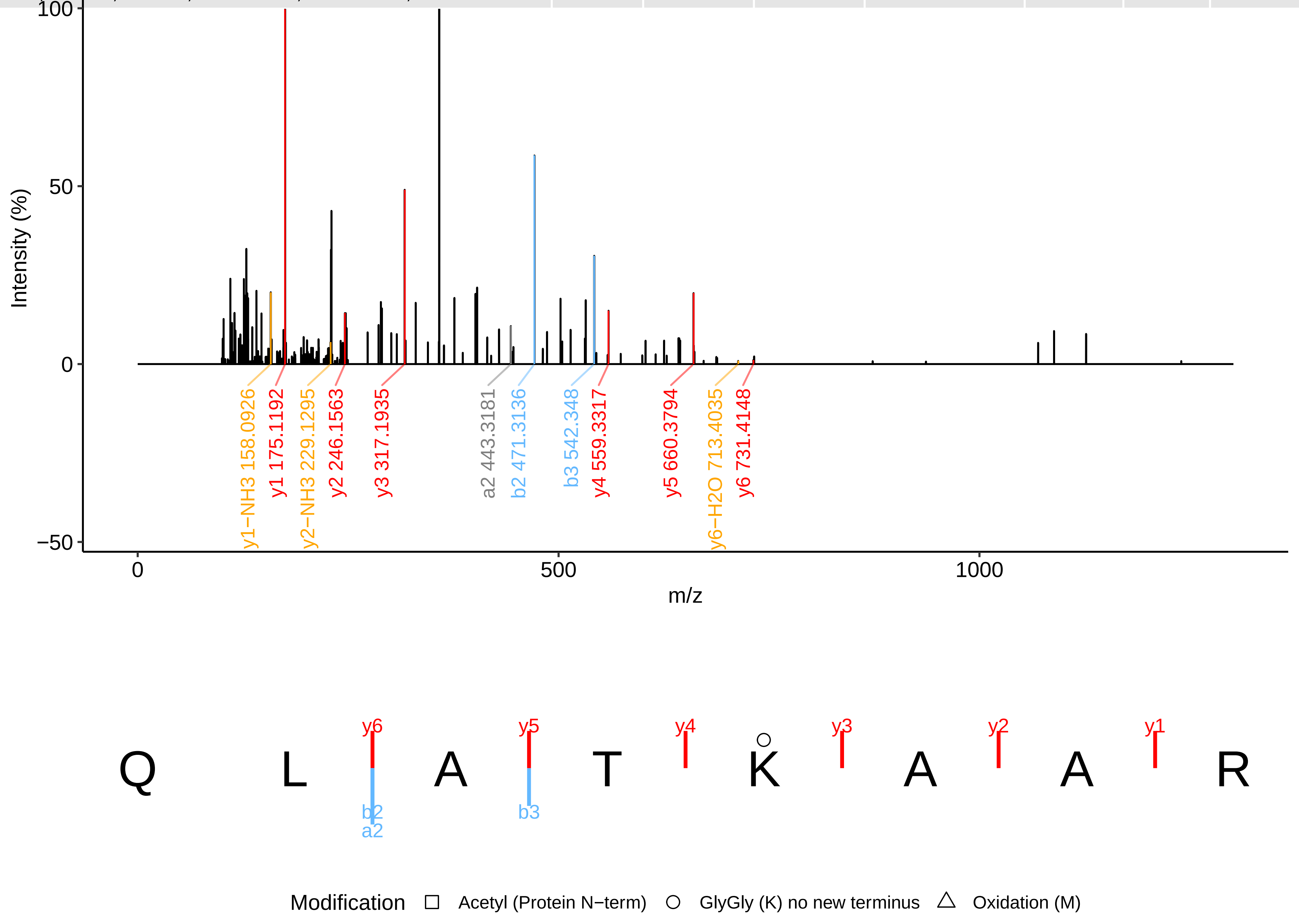

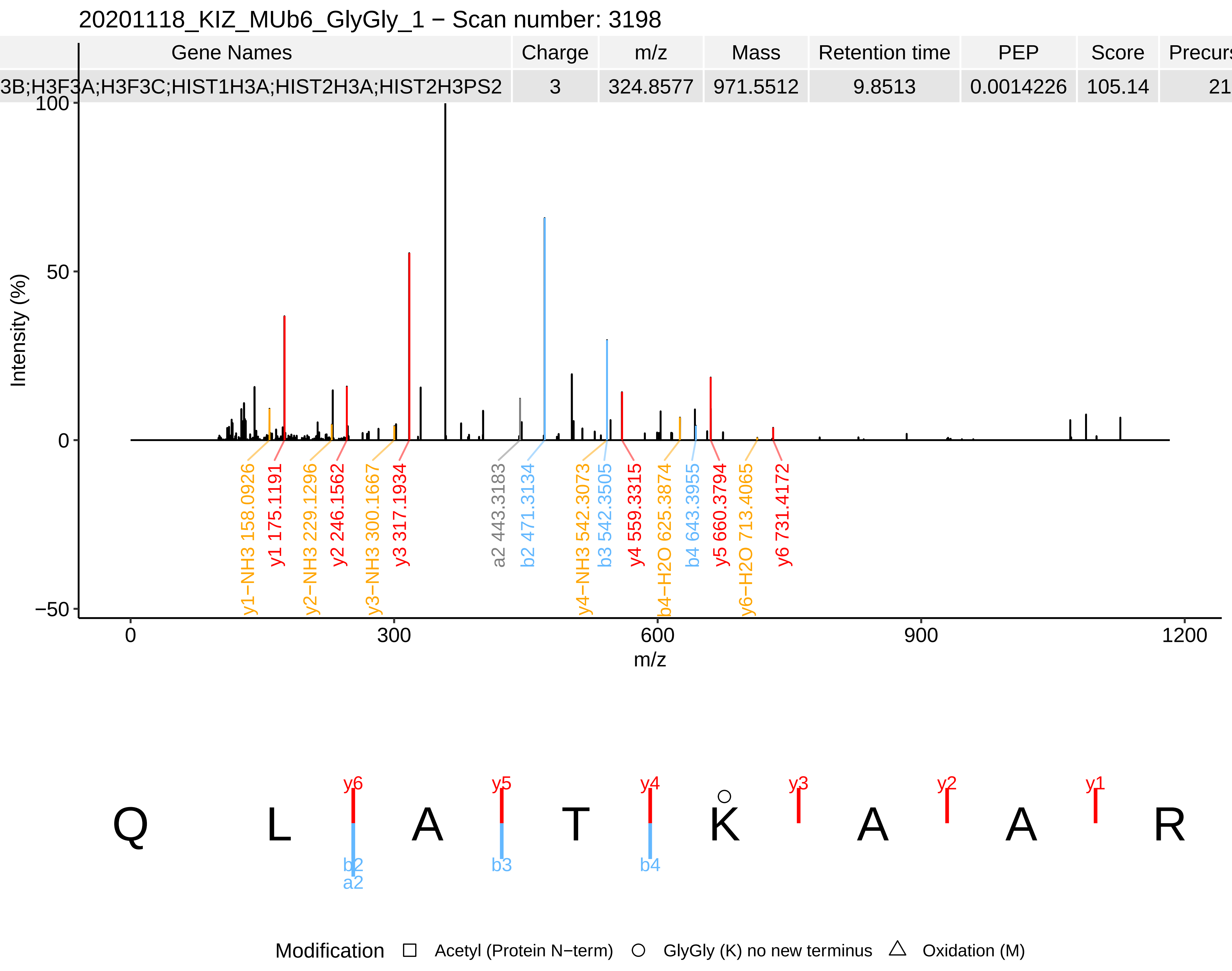

20201118_KIZ_MUb6_GlyGly_1 − Scan number: 3198
Gene Names
Charge
m/z
Mass
Retention time
PEP
Score
Precurs
3B;H3F3A;H3F3C;HIST1H3A;HIST2H3A;HIST2H3PS2
3
324.8577
971.5512
9.8513
0.0014226
105.14
21
Intensity (%)
100
50
0
−50
0
300
600
900
1200
m/z
y1−NH3 158.0926
y1 175.1191
y2−NH3 229.1296
y2 246.1562
y3−NH3 300.1667
y3 317.1934
a2 443.3183
b2 471.3134
y4−NH3 542.3073
b3 542.3505
y4 559.3315
b4−H2O 625.3874
b4 643.3955
y5 660.3794
y6−H2O 713.4065
y6 731.4172
Q L A T K A A R
y6 y5 y4 y3 y2 y1
b2 a2 b3 b4
Modification
Acetyl (Protein N−term)
GlyGly (K) no new terminus
Oxidation (M)

20201118_KIZ_MUb6_GlyGly_2 − Scan number: 3659

Gene Names	Charge	m/z	Mass	Retention time	PEP	Score	Precurs
3B;H3F3A;H3F3C;HIST1H3A;HIST2H3A;HIST2H3PS2	3	324.8577	971.5512	12.222	0.0021595	98.629	52

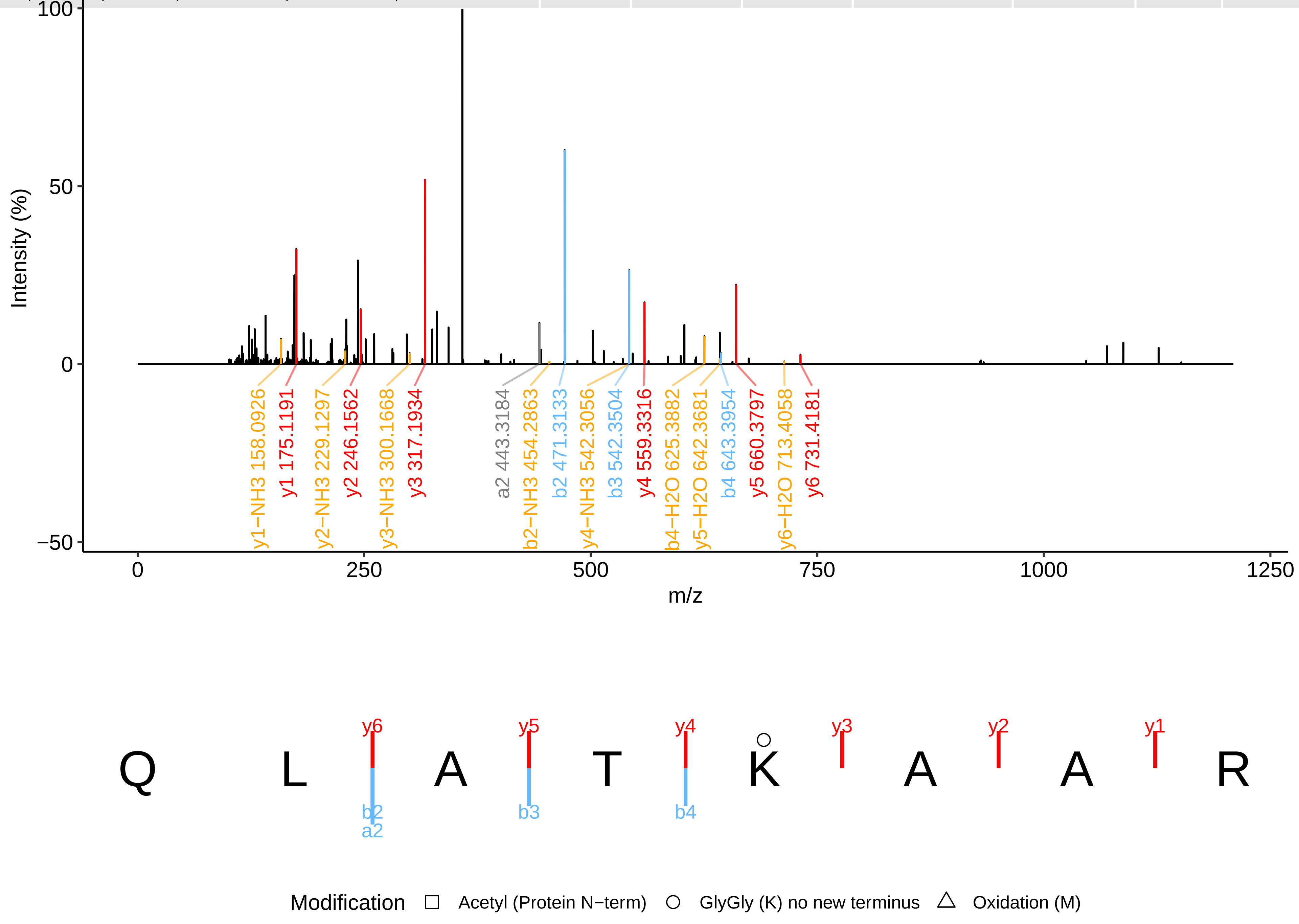

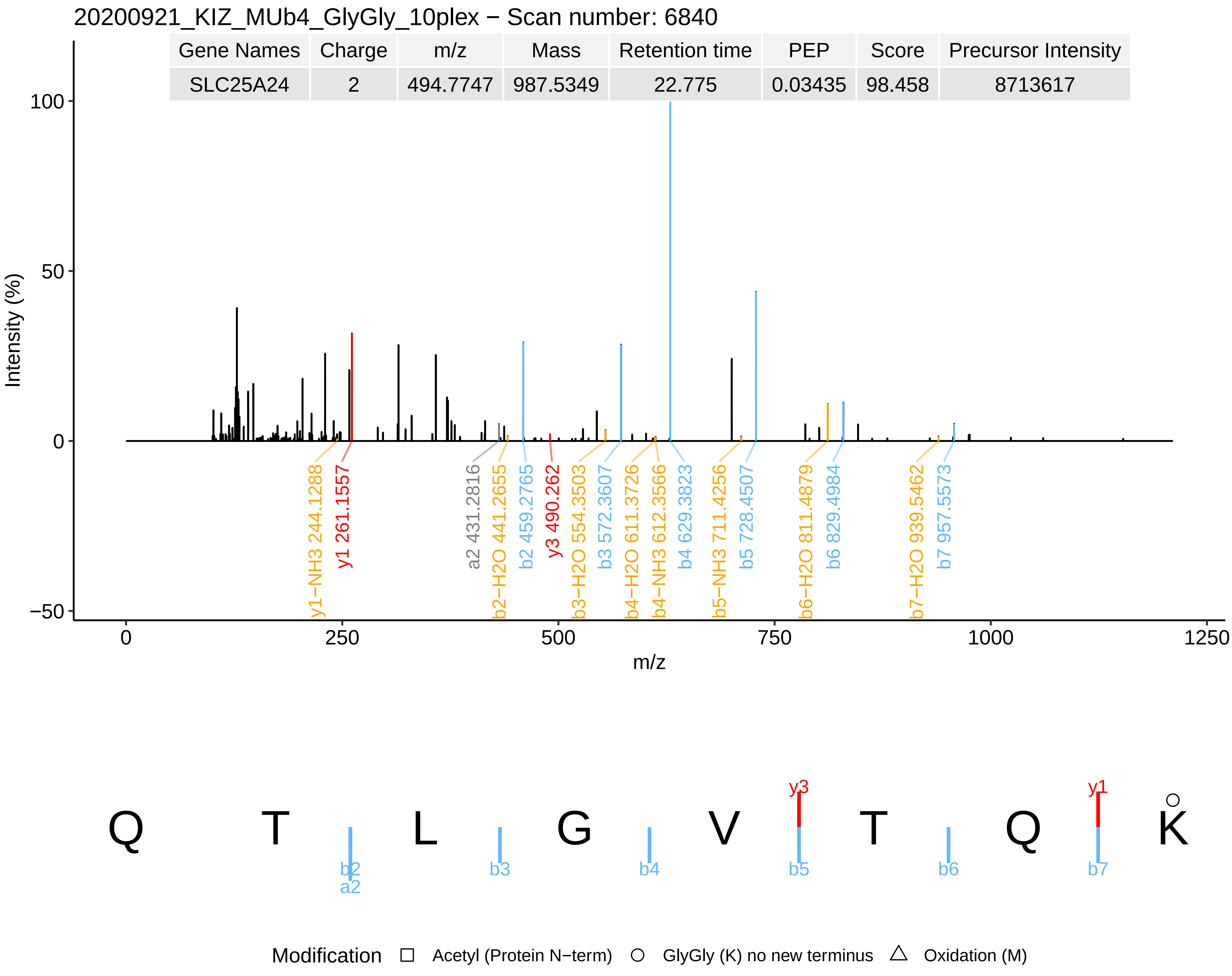
20200921_KIZ_MUb4_GlyGly_10plex − Scan number: 6840
Gene Names: SLC25A24
Charge: 2
m/z: 494.7747
Mass: 987.5349
Retention time: 22.775
PEP: 0.03435
Score: 98.458
Precursor Intensity: 8713617
Intensity (%)
m/z
y1−NH3 244.1288
y1 261.1557
a2 431.2816
b2−H2O 441.2655
b2 459.2765
y3 490.262
b3−H2O 554.3503
b3 572.3607
b4−H2O 611.3726
b4−NH3 612.3566
b4 629.3823
b5−NH3 711.4256
b5 728.4507
b6−H2O 811.4879
b6 829.4984
b7−H2O 939.5462
b7 957.5573
Q T L G V T Q K
y3 y1
b2 a2 b3 b4 b5 b6 b7
Modification
Acetyl (Protein N−term)
GlyGly (K) no new terminus
Oxidation (M)

20201118_KIZ_MUb6_GlyGly_2 − Scan number: 2469

Gene Names	Charge	m/z	Mass	Retention time	PEP	Score	Precursor Intensity
ABCG2	3	326.175	975.5032	7.6738	0.010154	120.49	NA

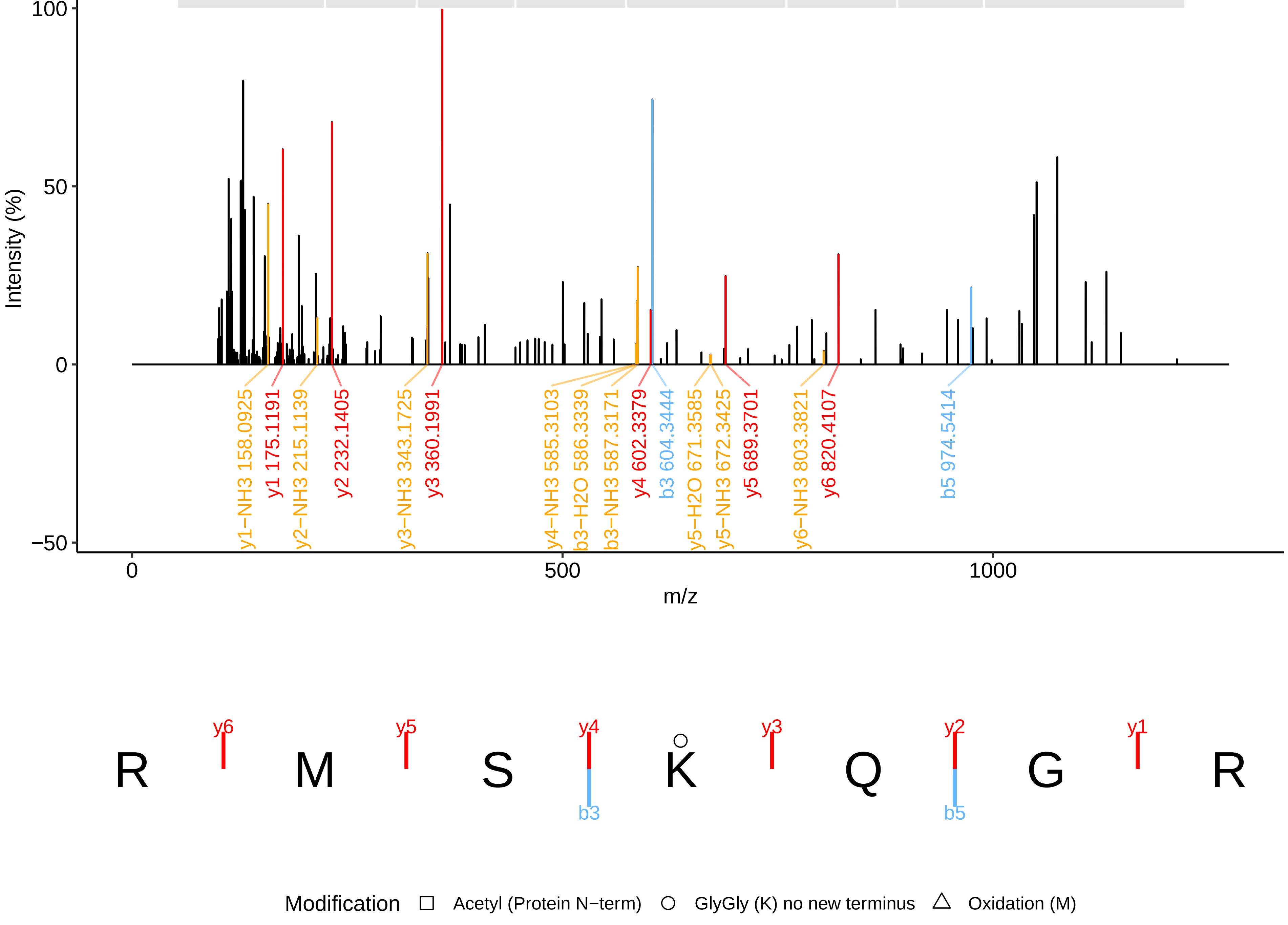

20200921_KIZ_MUb4_GlyGly_10plex − Scan number: 8430

Gene Names	Charge	m/z	Mass	Retention time	PEP	Score	Precursor Intensity
ZFYVE16	3	329.8517	986.5331	28.609	0.018309	82.671	74383464

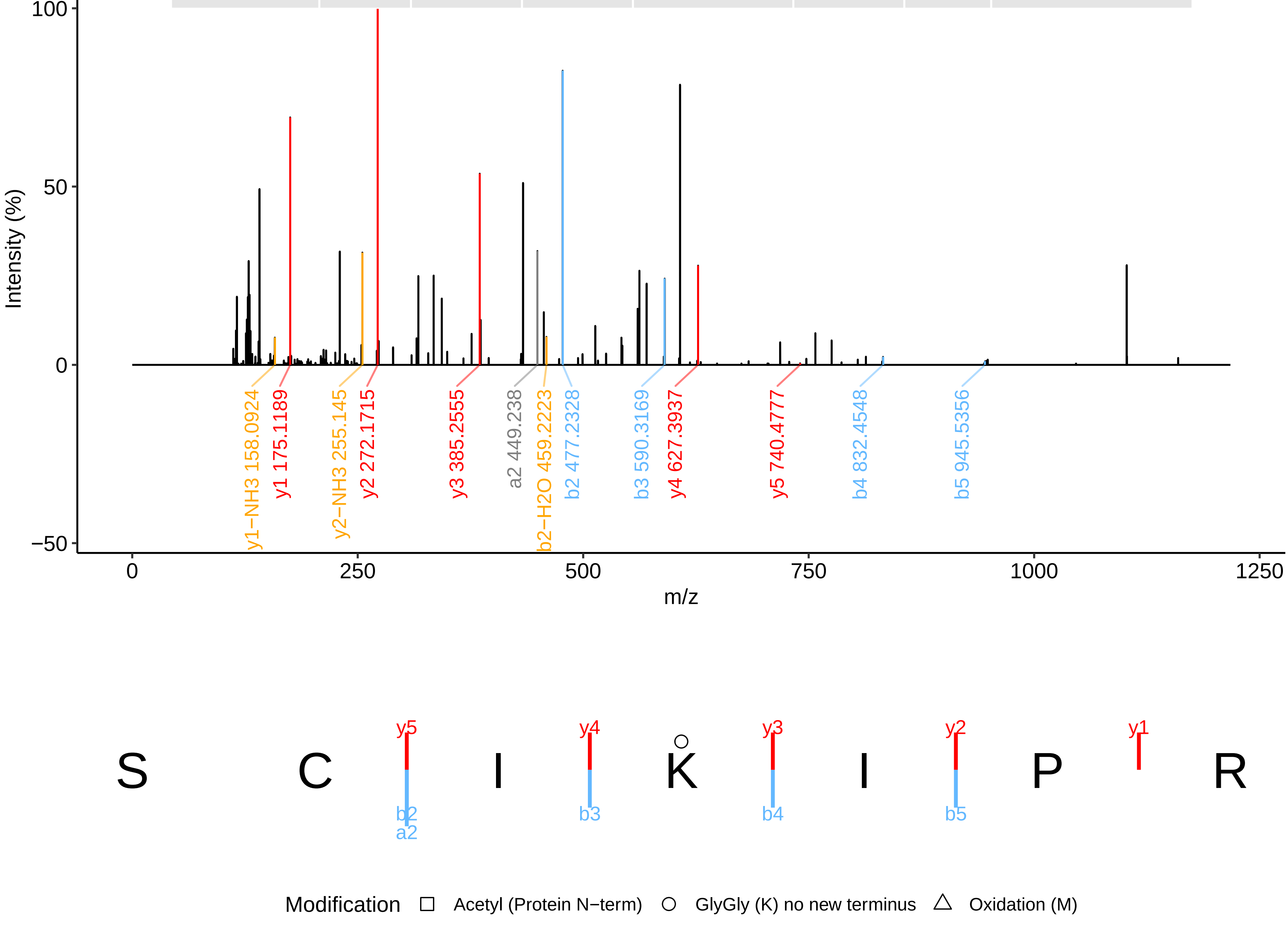

20200921_KIZ_MUb4_GlyGly_10plex − Scan number: 8432

Gene Names	Charge	m/z	Mass	Retention time	PEP	Score	Precursor Intensity
ZFYVE16	2	494.2738	986.5331	28.615	0.033307	94.777	7278211

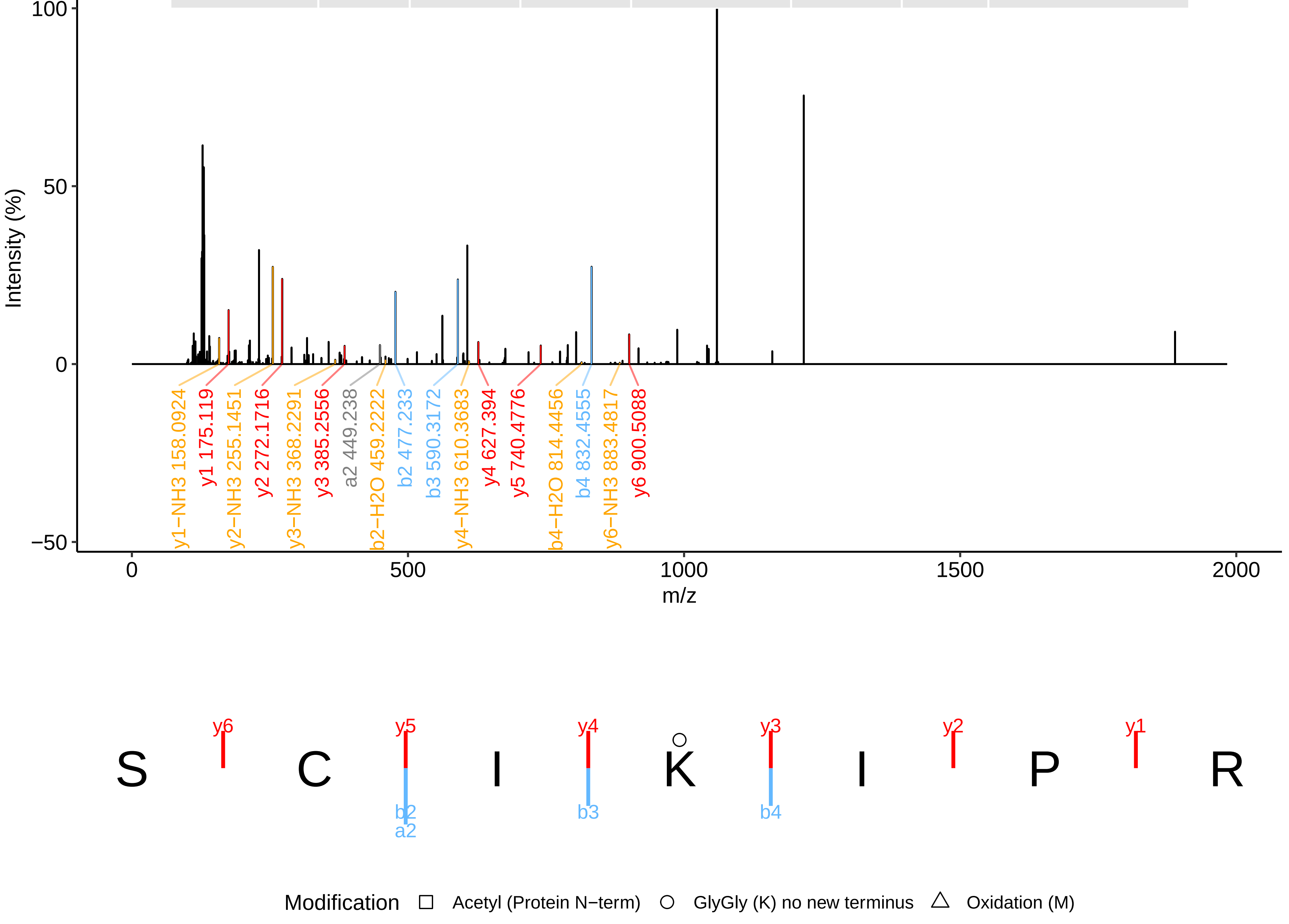

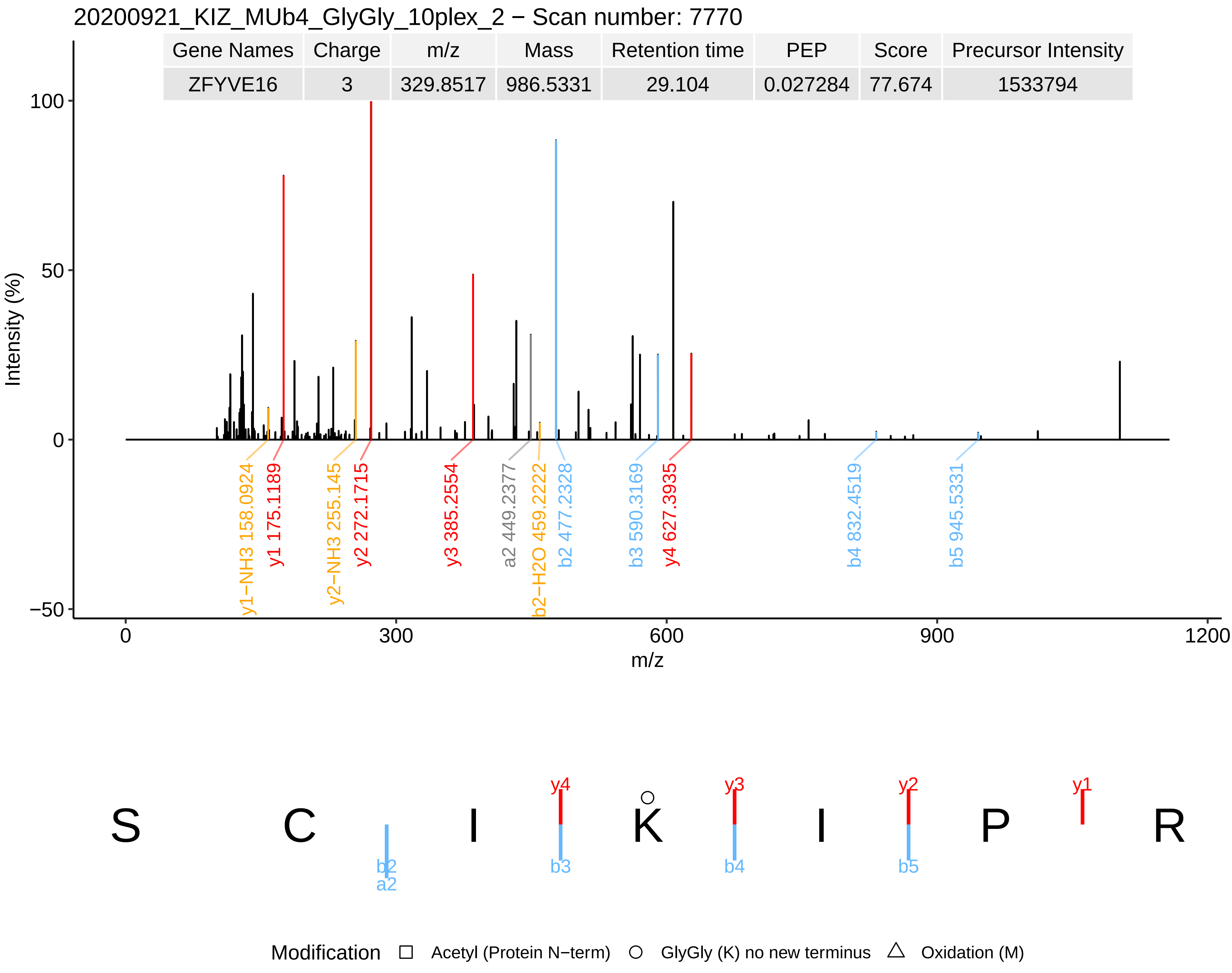

20200921_KIZ_MUb4_GlyGly_10plex_2 − Scan number: 7770
Gene Names | Charge | m/z | Mass | Retention time | PEP | Score | Precursor Intensity
ZFYVE16 | 3 | 329.8517 | 986.5331 | 29.104 | 0.027284 | 77.674 | 1533794
Intensity (%)
m/z
y1−NH3 158.0924
y1 175.1189
y2−NH3 255.145
y2 272.1715
y3 385.2554
a2 449.2377
b2−H2O 459.2222
b2 477.2328
b3 590.3169
y4 627.3935
b4 832.4519
b5 945.5331
S C I K I P R
Modification
Acetyl (Protein N−term)
GlyGly (K) no new terminus
Oxidation (M)

20201118_KIZ_MUb6_GlyGly_1 − Scan number: 6945

Gene Names	Charge	m/z	Mass	Retention time	PEP	Score	Precursor Intensity
ZFYVE16	3	329.8517	986.5331	22.002	0.018309	82.671	37509684

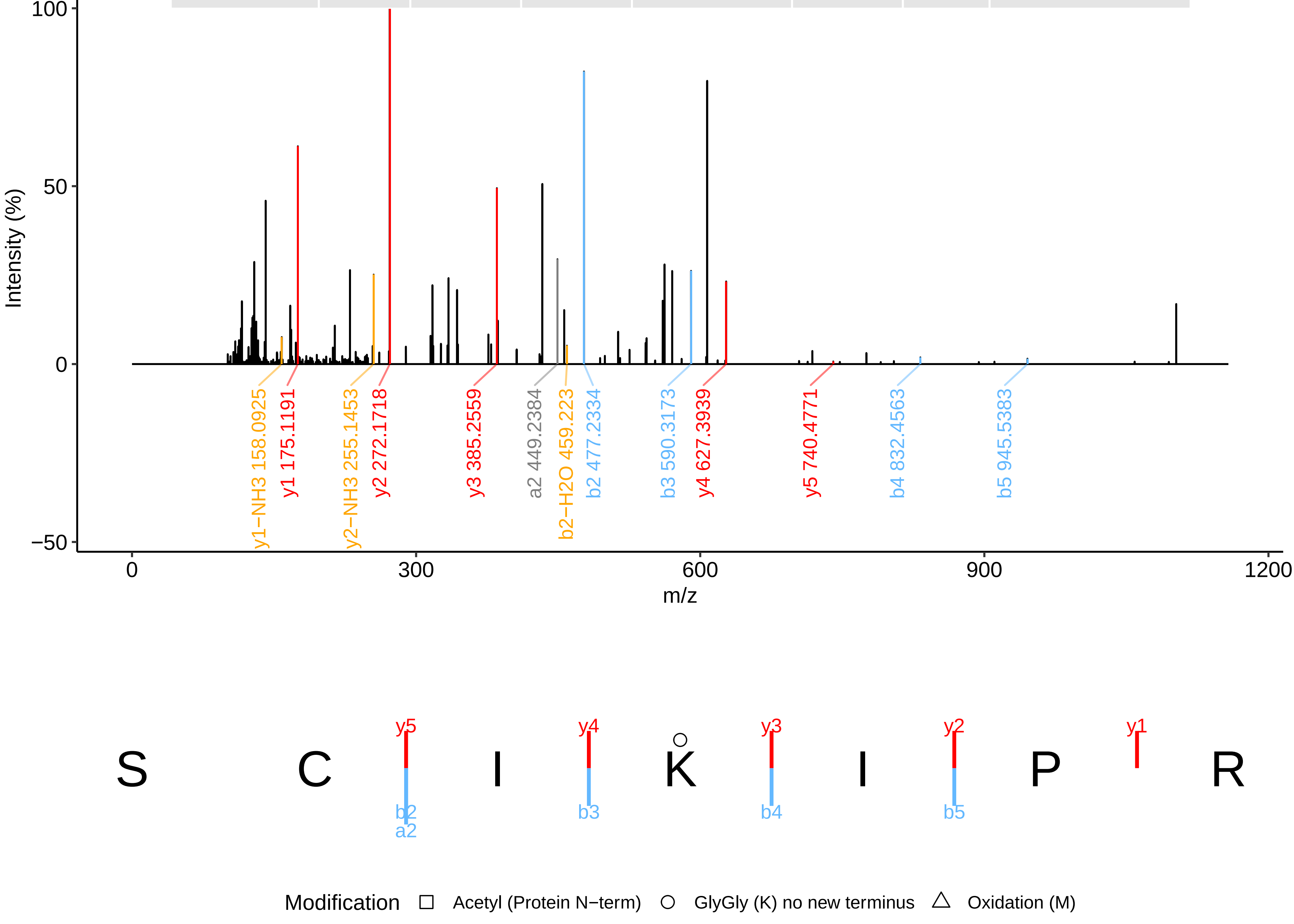

20201118_KIZ_MUb6_GlyGly_2 − Scan number: 8893

Gene Names	Charge	m/z	Mass	Retention time	PEP	Score	Precursor Intensity
ZFYVE16	3	329.8517	986.5331	30.143	0.015263	86.866	10902477

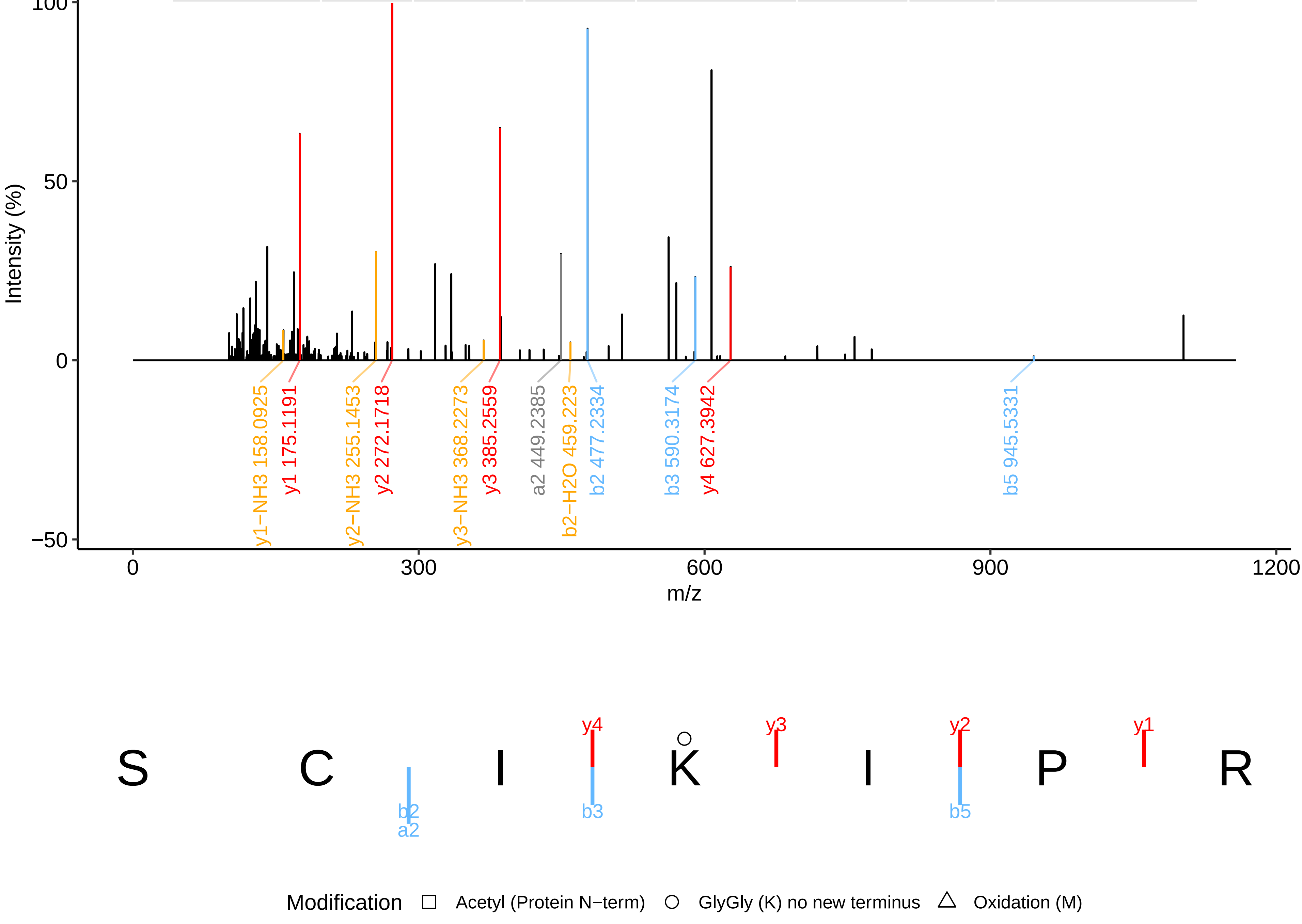

20201118_KIZ_MUb6_GlyGly_2 − Scan number: 8922

Gene Names	Charge	m/z	Mass	Retention time	PEP	Score	Precursor Intensity
ZFYVE16	2	494.2738	986.5331	30.24	0.070006	105.12	3550557

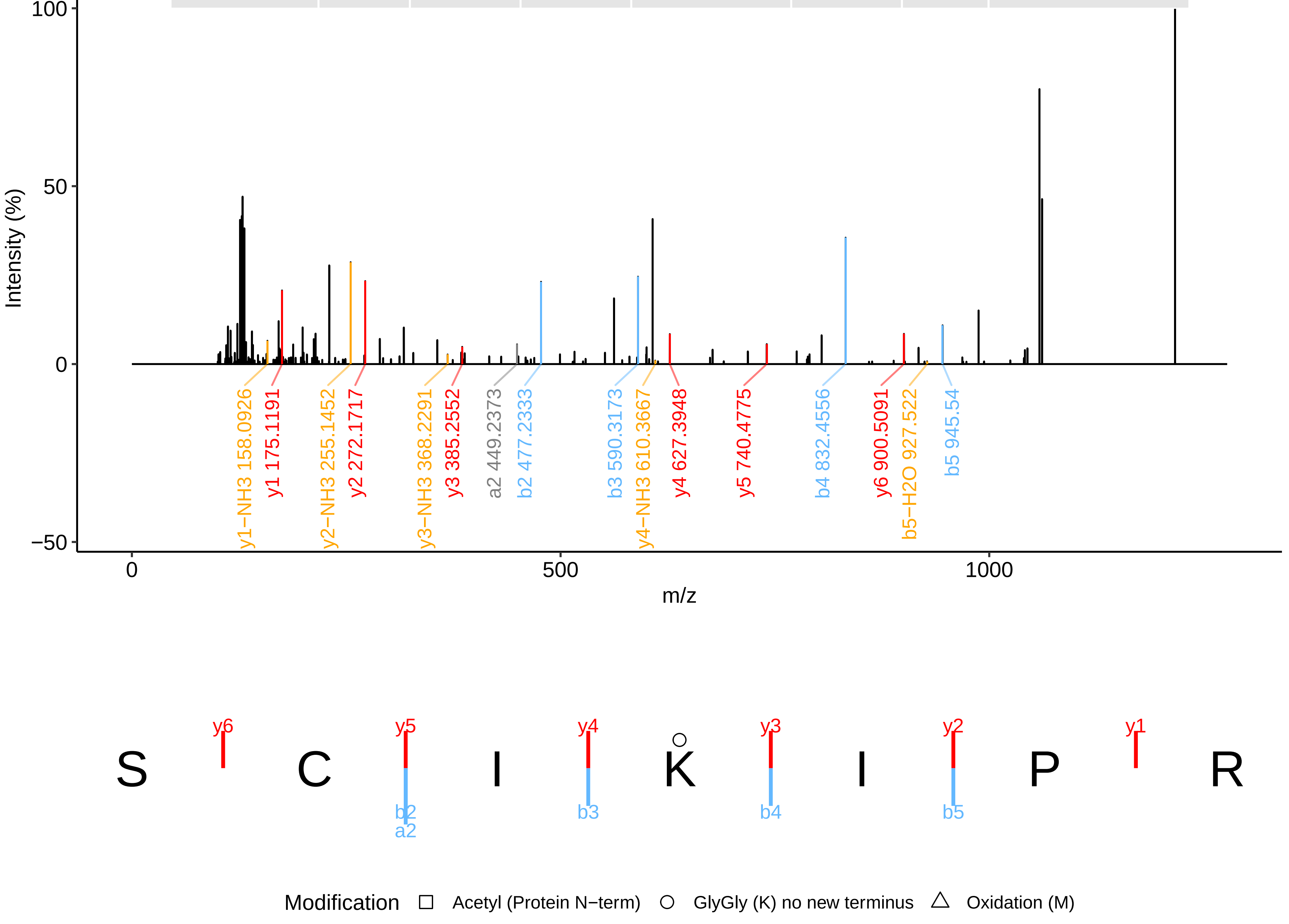

20200921_KIZ_MUb4_GlyGly_10plex − Scan number: 2744

Gene Names	Charge	m/z	Mass	Retention time	PEP	Score	Precursor Intensity
PTTG1IP	3	322.1554	963.4444	8.6212	0.029439	76.833	40365568

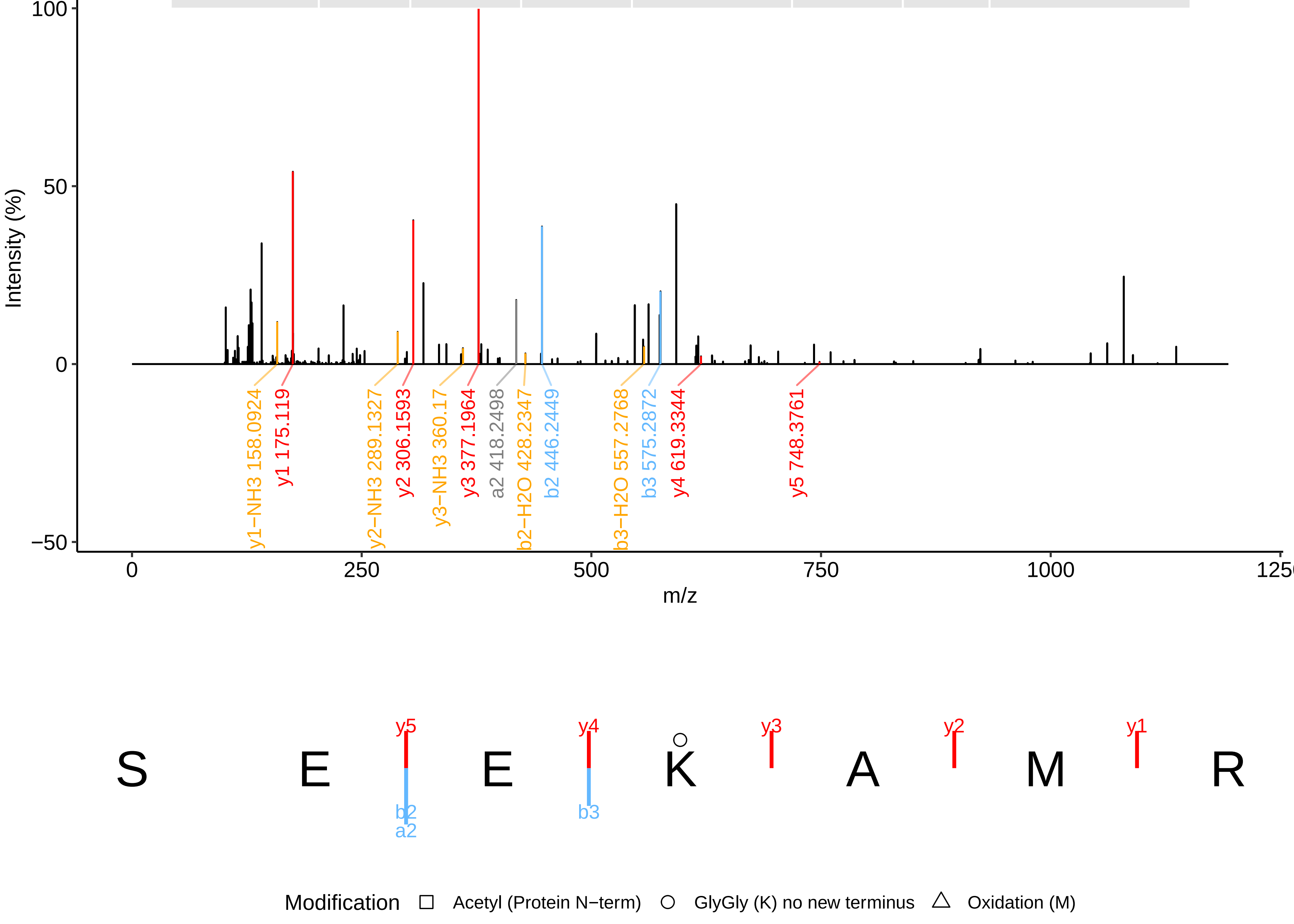

20200921_KIZ_MUb4_GlyGly_10plex − Scan number: 2759

Gene Names	Charge	m/z	Mass	Retention time	PEP	Score	Precursor Intensity
PTTG1IP	2	482.7295	963.4444	8.6589	0.014976	143.23	NA

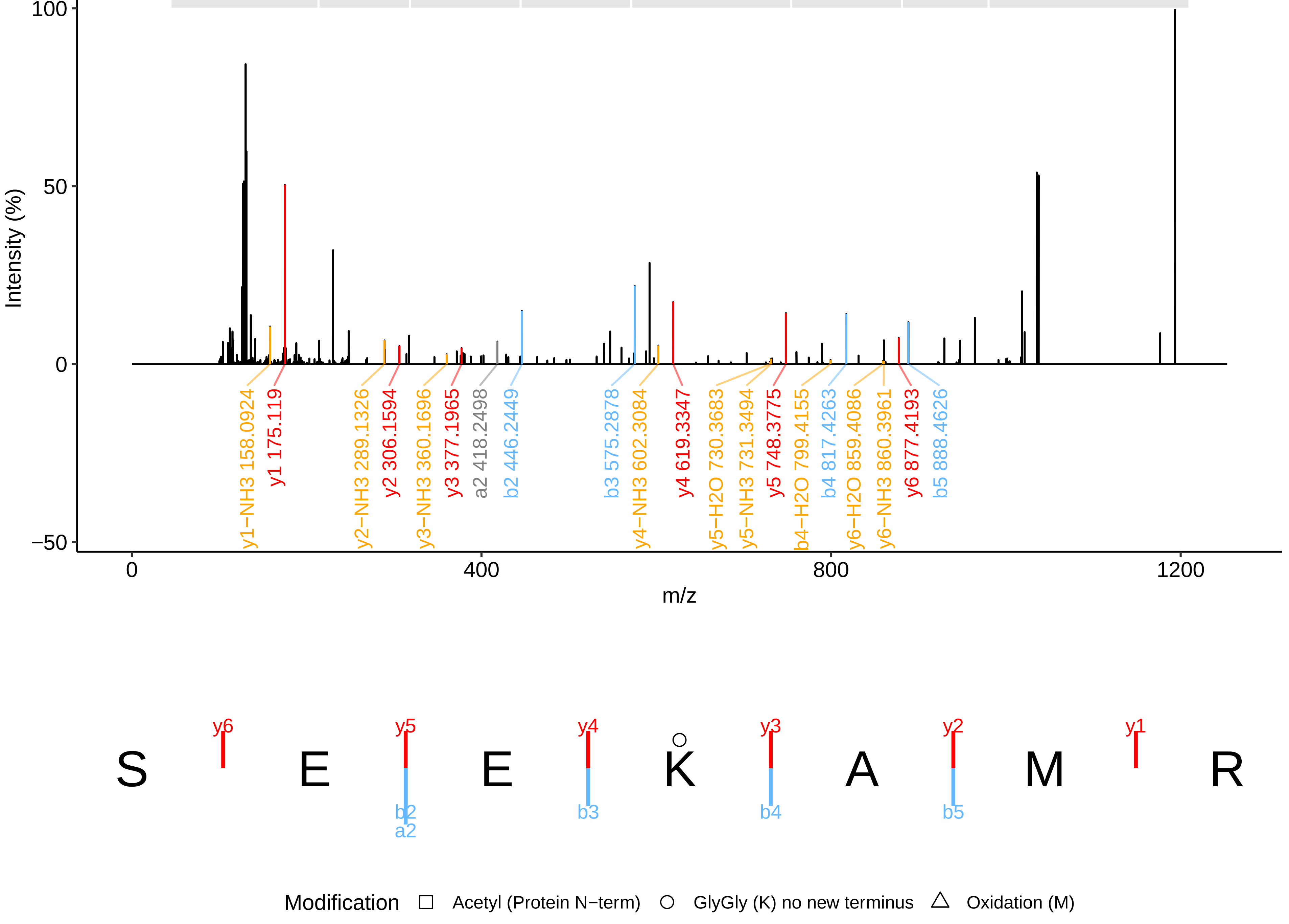

20200921_KIZ_MUb4_GlyGly_10plex_2 − Scan number: 2704

Gene Names	Charge	m/z	Mass	Retention time	PEP	Score	Precursor Intensity
PTTG1IP	3	322.1554	963.4444	8.6953	0.040915	72.355	19610272

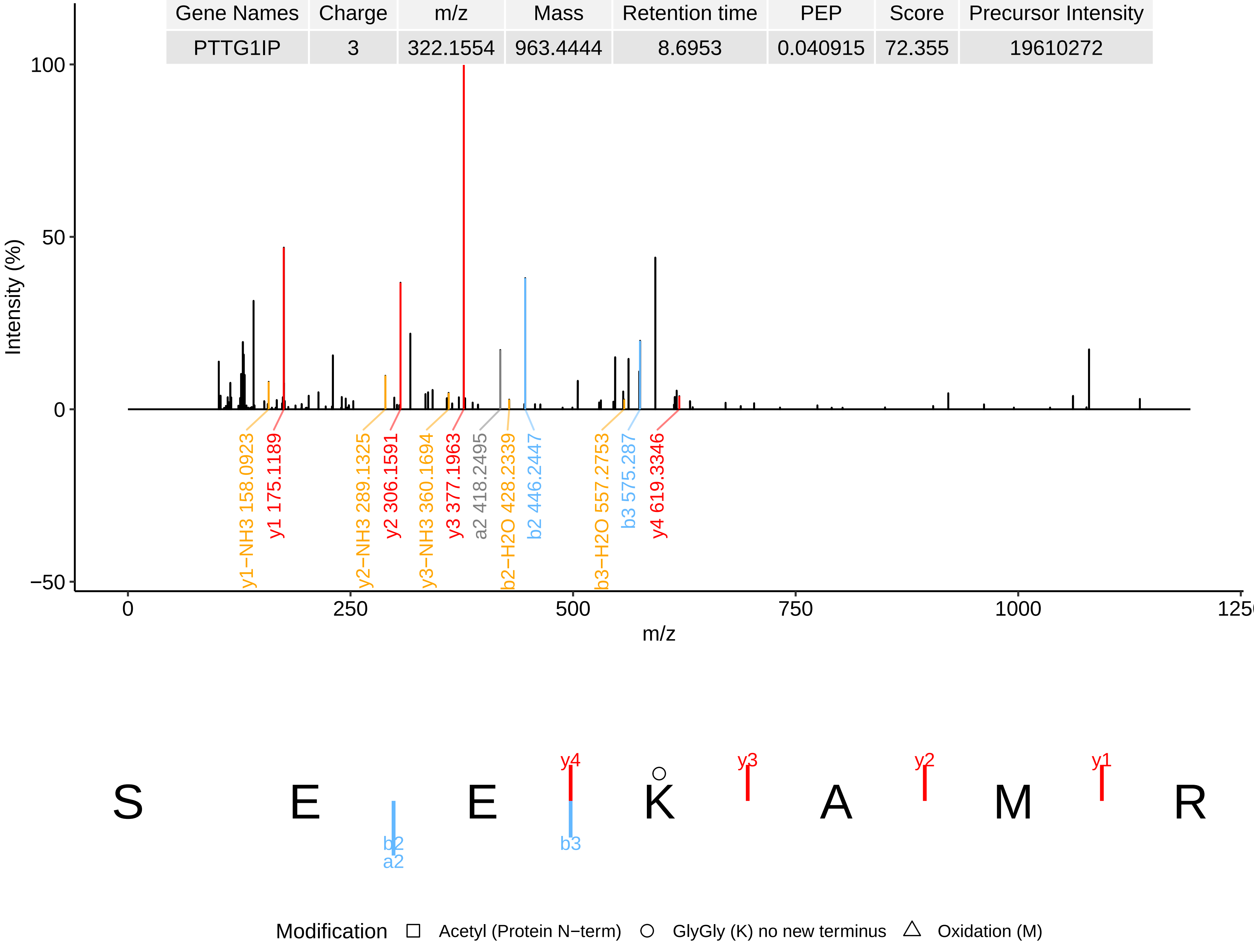

20200924_KIZ_MUb5_GlyGly_1 − Scan number: 2620

Gene Names	Charge	m/z	Mass	Retention time	PEP	Score	Precursor Intensity
PTTG1IP	3	322.1554	963.4444	8.1147	0.040915	72.355	18759470

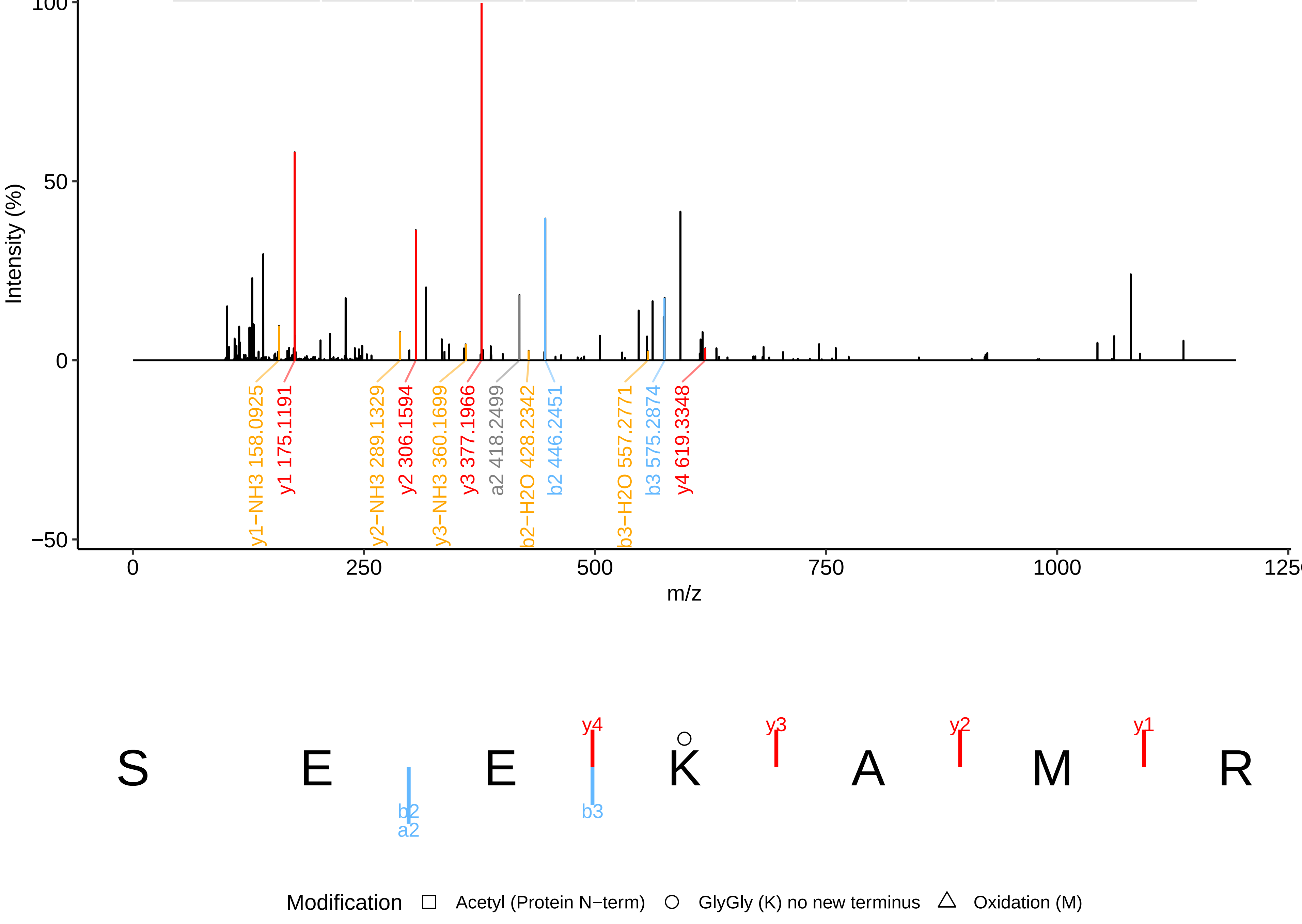

20200924_KIZ_MUb5_GlyGly_1 − Scan number: 2635

Gene Names	Charge	m/z	Mass	Retention time	PEP	Score	Precursor Intensity
PTTG1IP	2	482.7295	963.4444	8.1618	0.032975	134.06	6738576

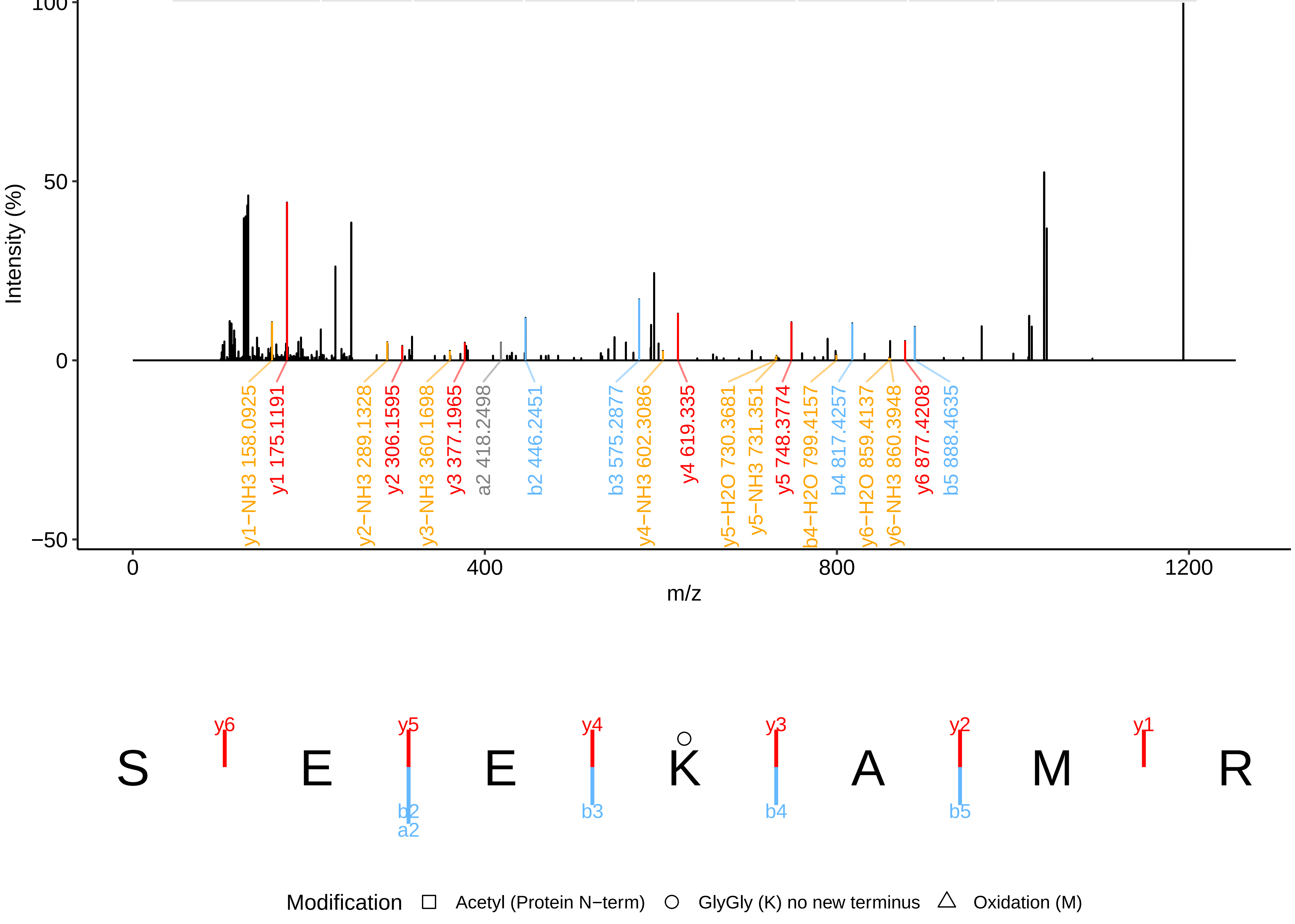

20200924_KIZ_MUb5_GlyGly_2 − Scan number: 2281

Gene Names	Charge	m/z	Mass	Retention time	PEP	Score	Precursor Intensity
PTTG1IP	3	322.1554	963.4444	8.0417	0.084045	59.205	9303047

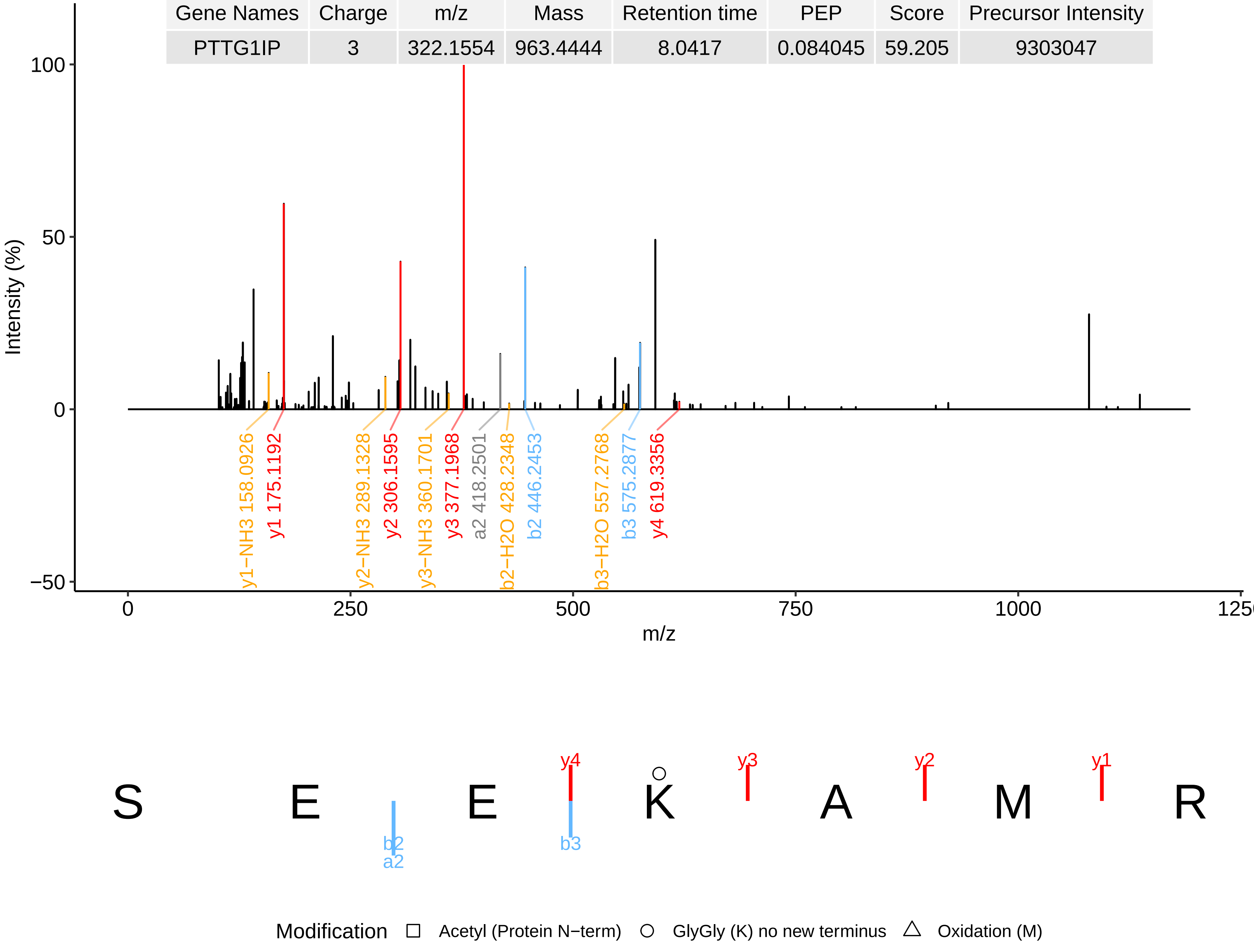

20201118_KIZ_MUb6_GlyGly_2 − Scan number: 2929

Gene Names	Charge	m/z	Mass	Retention time	PEP	Score	Precursor Intensity
PTTG1IP	3	322.1554	963.4444	9.4001	0.040915	72.355	6447468

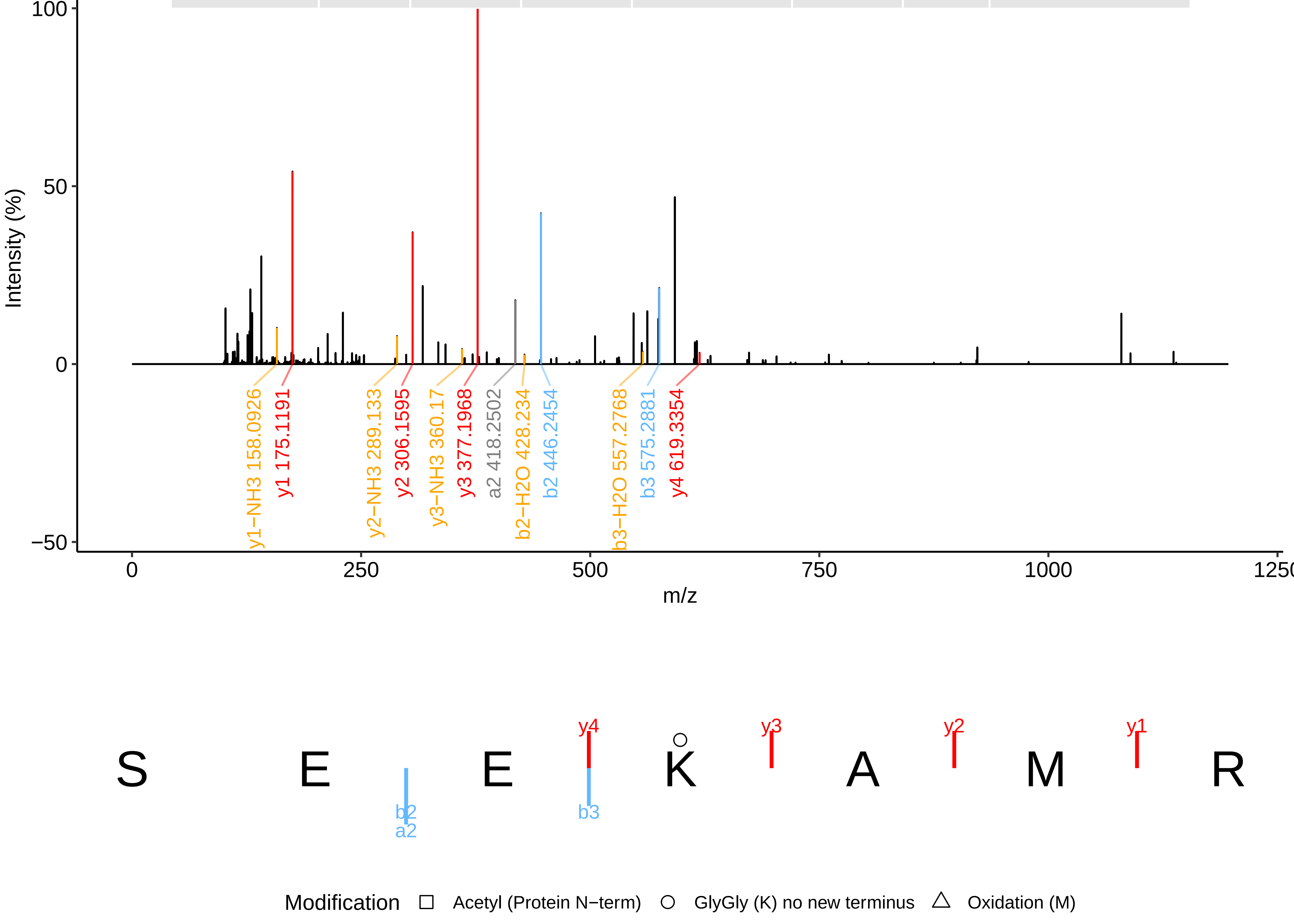

20200921_KIZ_MUb4_GlyGly_10plex − Scan number: 4785

Gene Names	Charge	m/z	Mass	Retention time	PEP	Score	Precursor Intensity
PSMC1	2	473.7513	945.488	15.602	0.016063	106.94	8448121

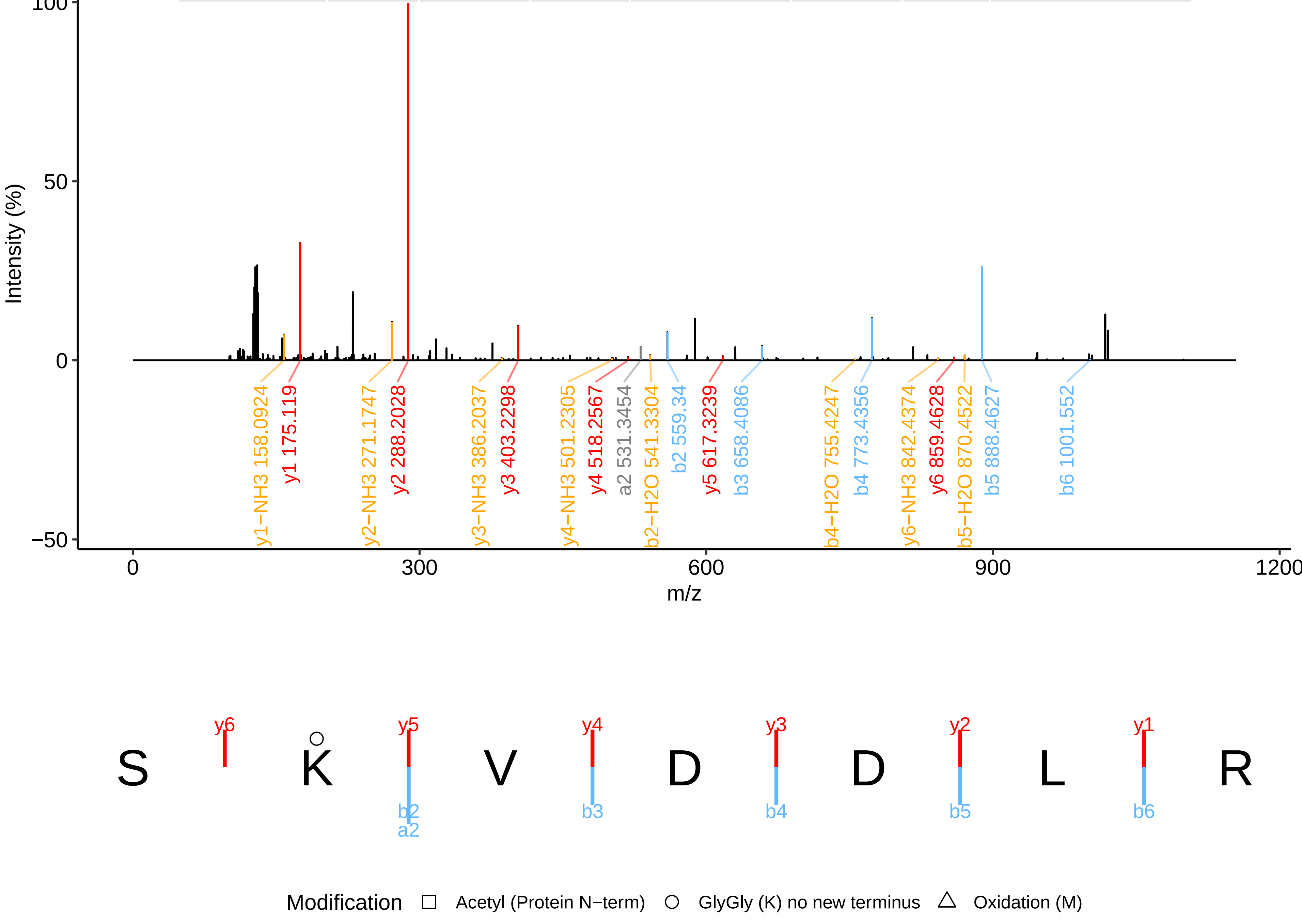

20200924_KIZ_MUb5_GlyGly_1 − Scan number: 4228

Gene Names	Charge	m/z	Mass	Retention time	PEP	Score	Precursor Intensity
PSMC1	2	473.7513	945.488	13.015	0.12145	90.308	10575354

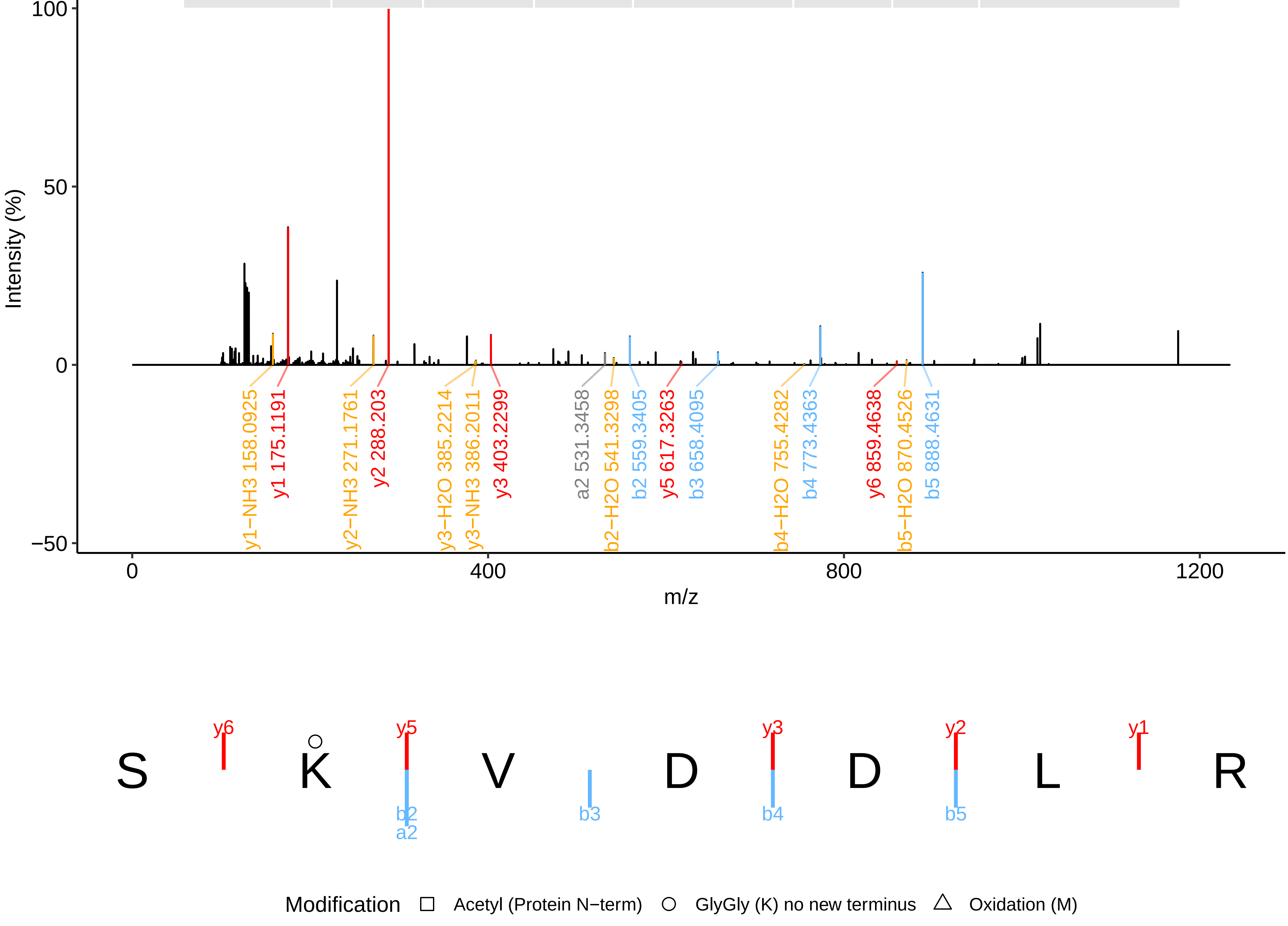

20200924_KIZ_MUb5_GlyGly_2 − Scan number: 2243

Gene Names	Charge	m/z	Mass	Retention time	PEP	Score	Precursor Intensity
IL1RAP	3	317.1665	948.4777	7.9094	0.038531	73.975	3097277

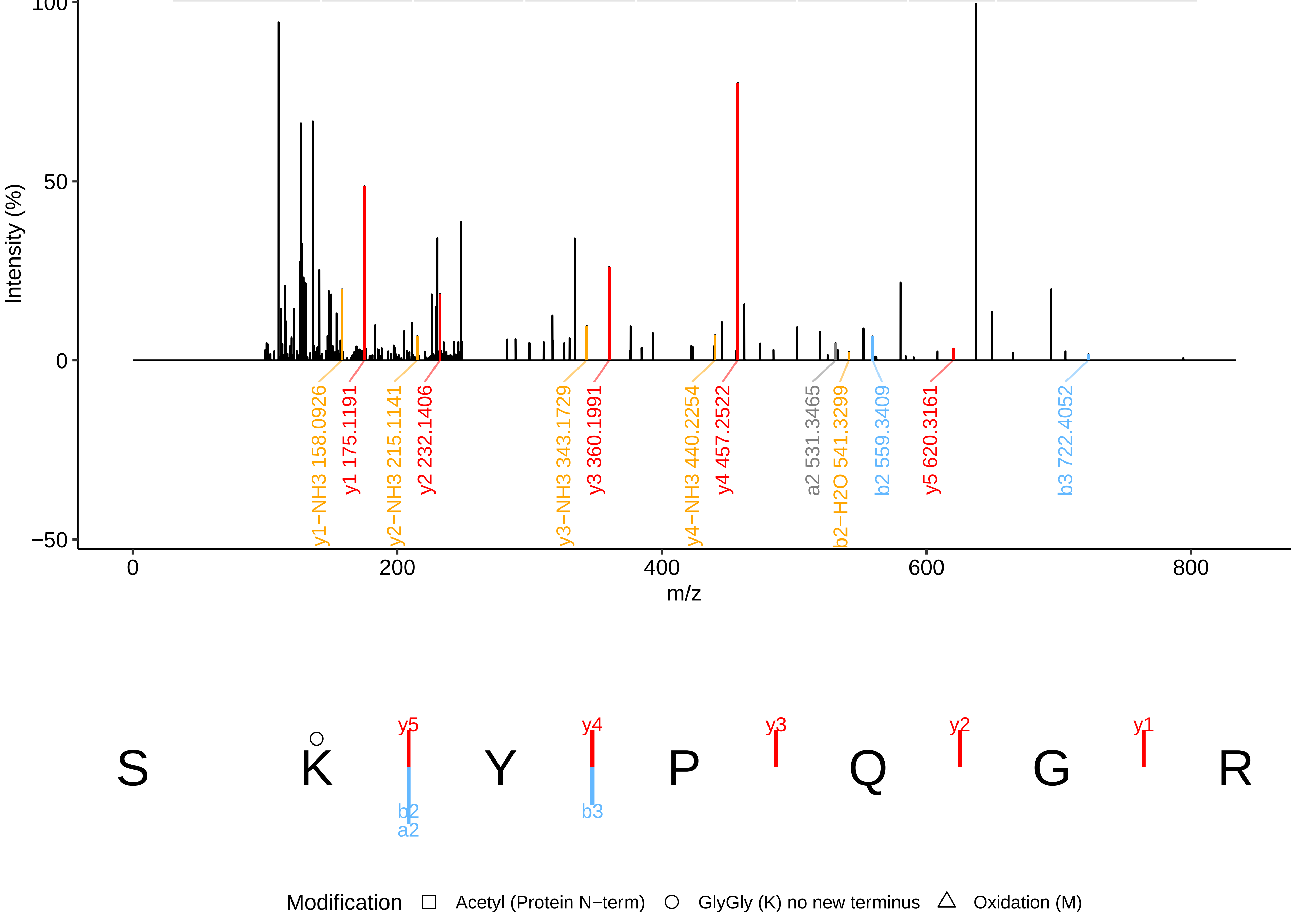

20200921_KIZ_MUb4_GlyGly_10plex − Scan number: 3802

Gene Names	Charge	m/z	Mass	Retention time	PEP	Score	Precursor Intensity
USP5	3	325.8576	974.5509	12.257	0.005811	88.921	11501729

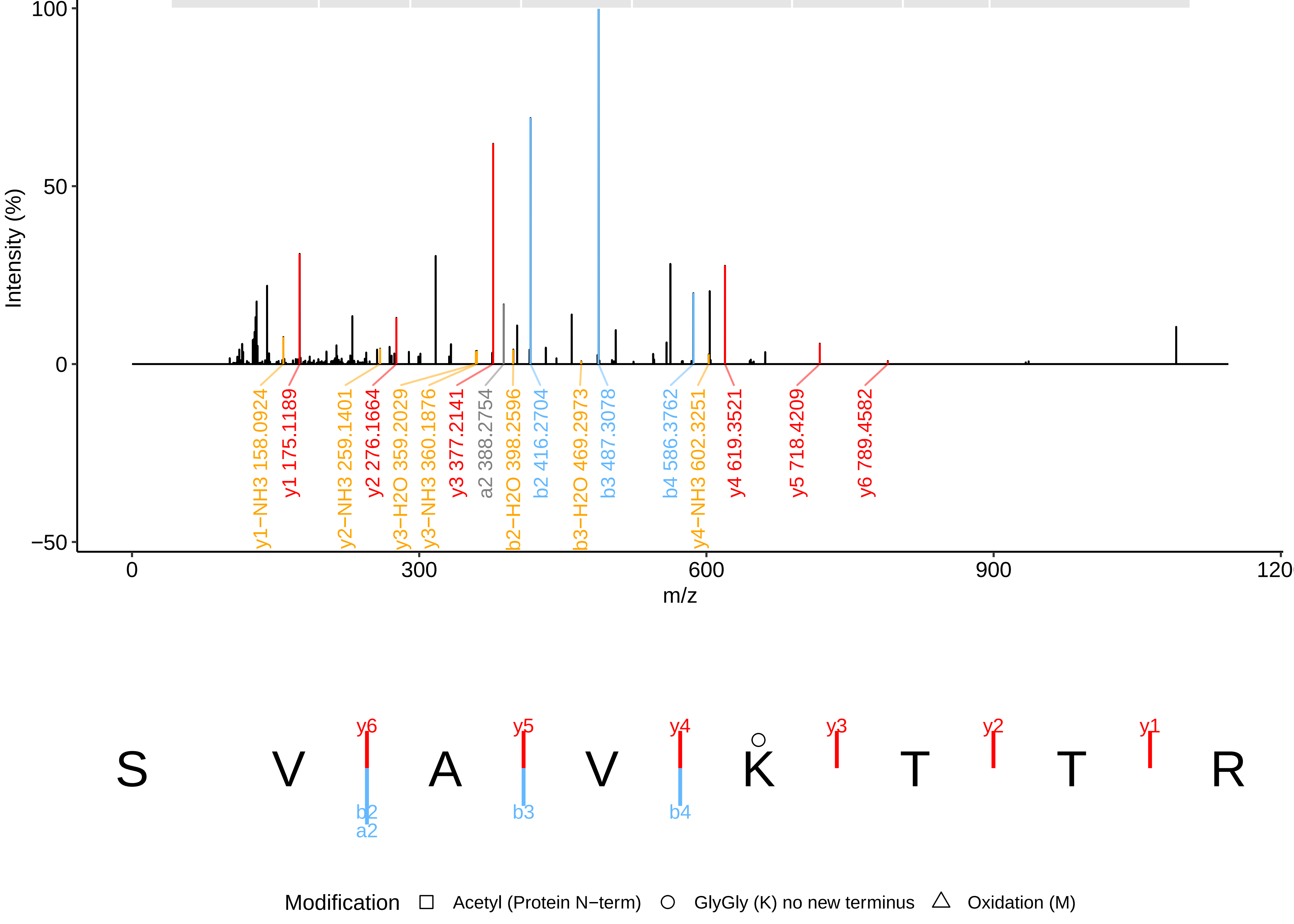

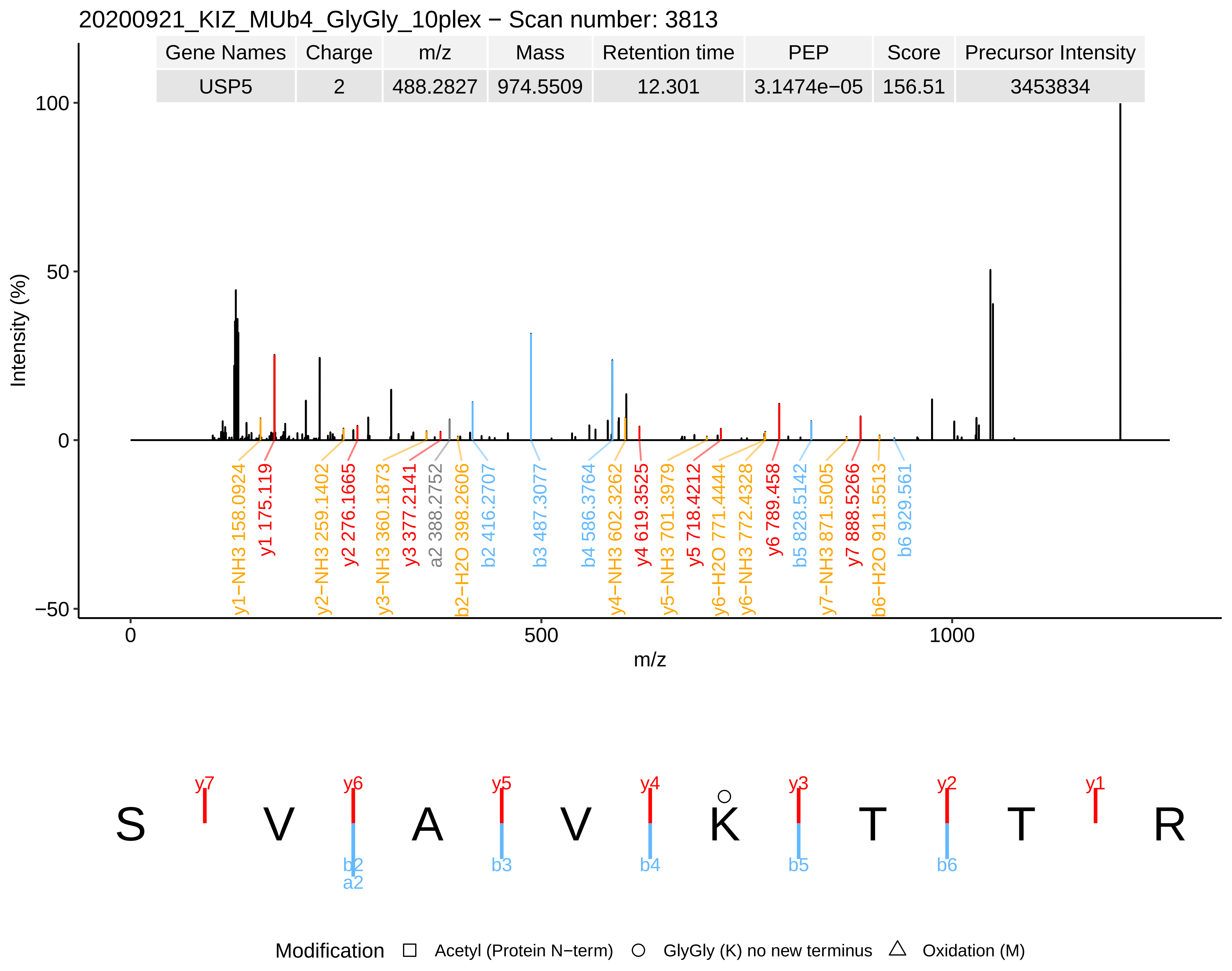

20200921_KIZ_MUb4_GlyGly_10plex − Scan number: 3813
Gene Names | Charge | m/z | Mass | Retention time | PEP | Score | Precursor Intensity
USP5 | 2 | 488.2827 | 974.5509 | 12.301 | 3.1474e−05 | 156.51 | 3453834
Intensity (%)
m/z
y1−NH3 158.0924
y1 175.119
y2−NH3 259.1402
y2 276.1665
y3−NH3 360.1873
y3 377.2141
a2 388.2752
b2−H2O 398.2606
b2 416.2707
b3 487.3077
b4 586.3764
y4−NH3 602.3262
y4 619.3525
y5−NH3 701.3979
y5 718.4212
y6−H2O 771.4444
y6−NH3 772.4328
y6 789.458
b5 828.5142
y7−NH3 871.5005
y7 888.5266
b6−H2O 911.5513
b6 929.561
S V A V K T T R
Modification □ Acetyl (Protein N−term) ○ GlyGly (K) no new terminus △ Oxidation (M)

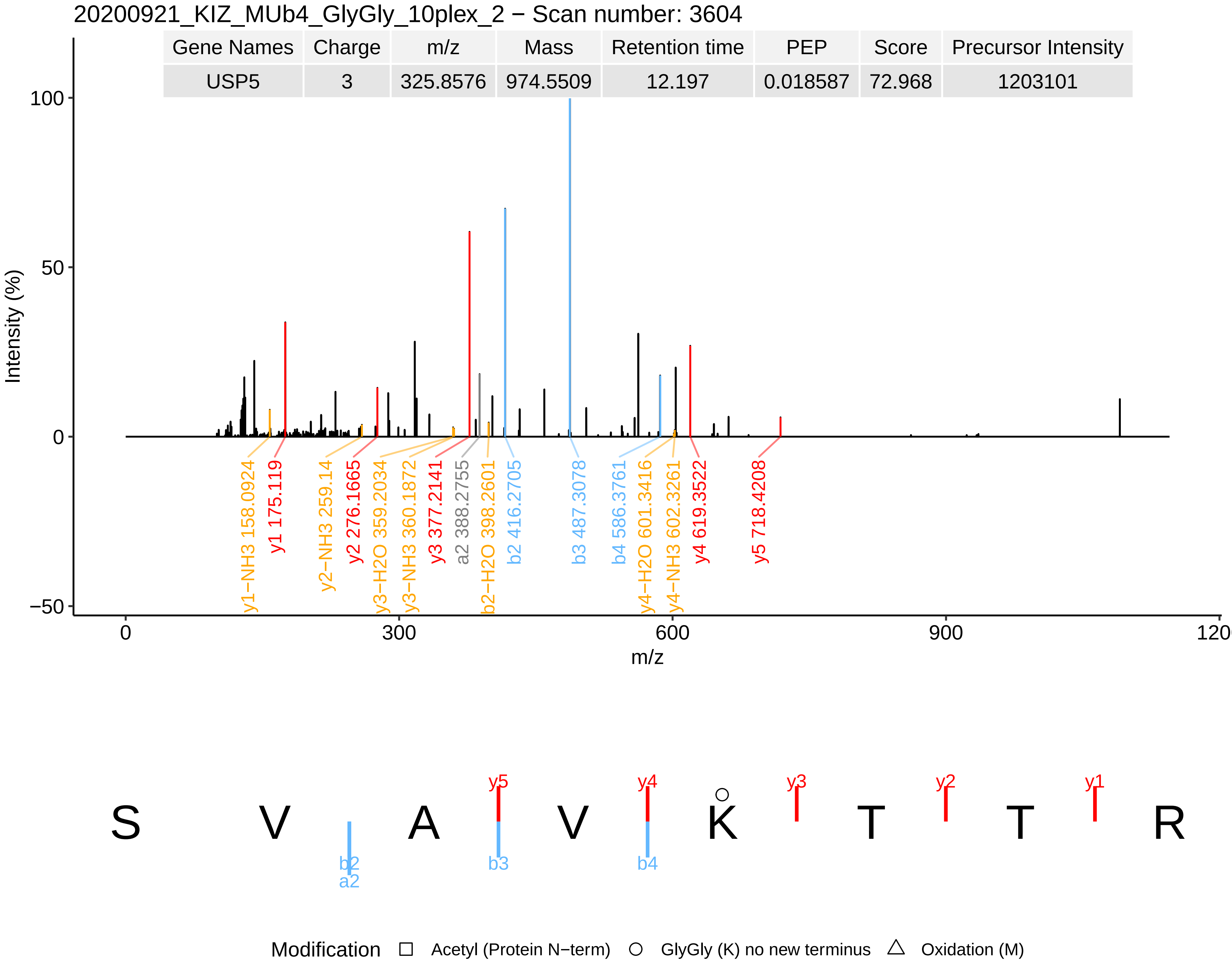
20200921_KIZ_MUb4_GlyGly_10plex_2 − Scan number: 3604
Gene Names | Charge | m/z | Mass | Retention time | PEP | Score | Precursor Intensity
USP5 | 3 | 325.8576 | 974.5509 | 12.197 | 0.018587 | 72.968 | 1203101
Intensity (%)
100
50
0
−50
y1−NH3 158.0924
y1 175.119
y2−NH3 259.14
y2 276.1665
y3−H2O 359.2034
y3−NH3 360.1872
y3 377.2141
a2 388.2755
b2−H2O 398.2601
b2 416.2705
b3 487.3078
b4 586.3761
y4−H2O 601.3416
y4−NH3 602.3261
y4 619.3522
y5 718.4208
0
300
600
900
1200
m/z
S V A V K T T R
y5 y4 y3 y2 y1
b2 a2 b3 b4
Modification
Acetyl (Protein N−term)
GlyGly (K) no new terminus
Oxidation (M)

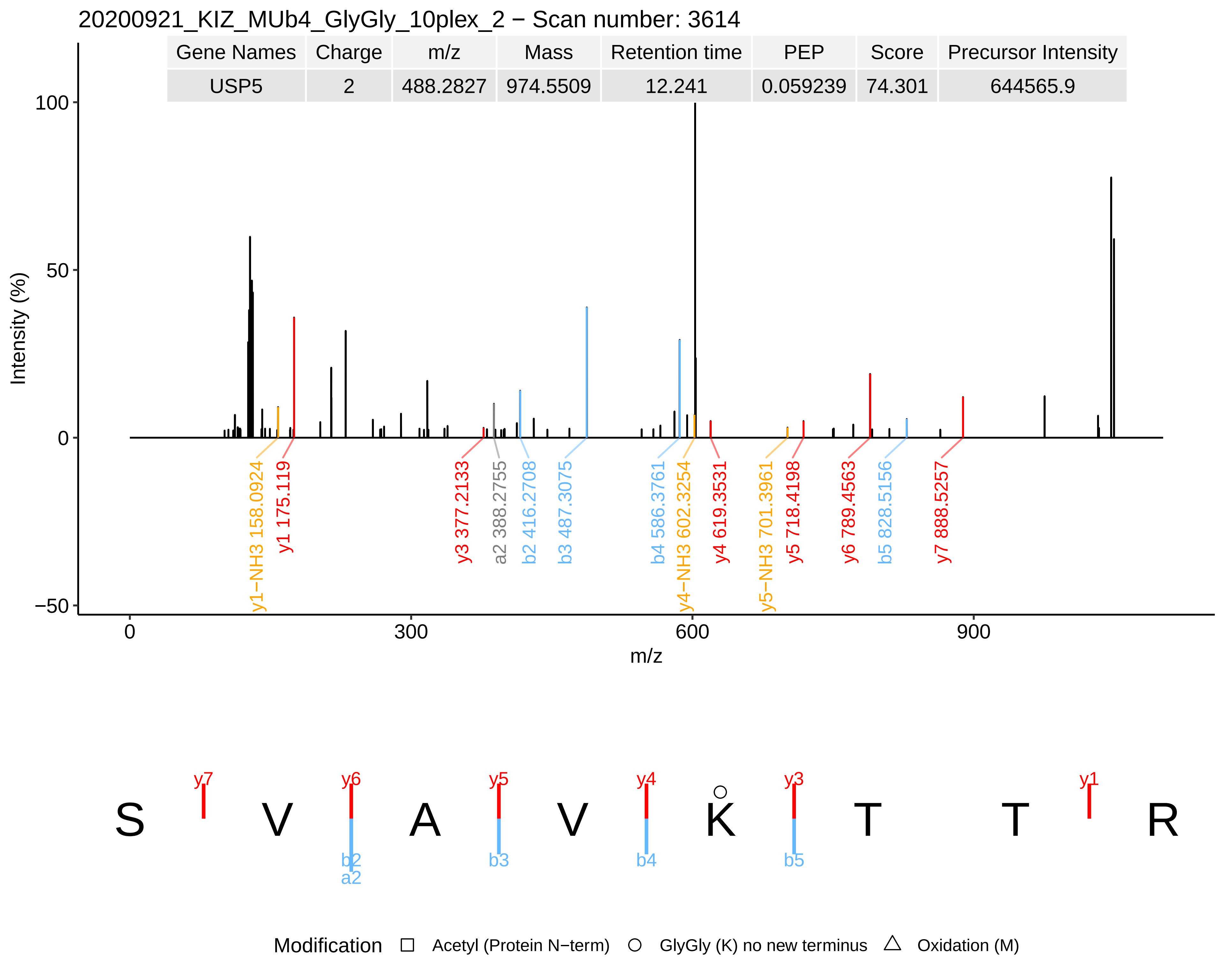
20200921_KIZ_MUb4_GlyGly_10plex_2 − Scan number: 3614
Gene Names
Charge
m/z
Mass
Retention time
PEP
Score
Precursor Intensity
USP5
2
488.2827
974.5509
12.241
0.059239
74.301
644565.9
Intensity (%)
100
50
0
−50
0
300
600
900
m/z
y1−NH3 158.0924
y1 175.119
y3 377.2133
a2 388.2755
b2 416.2708
b3 487.3075
b4 586.3761
y4−NH3 602.3254
y4 619.3531
y5−NH3 701.3961
y5 718.4198
y6 789.4563
b5 828.5156
y7 888.5257
S V A V K T T R
y7
y6
y5
y4
y3
y1
b2
a2
b3
b4
b5
Modification
Acetyl (Protein N−term)
GlyGly (K) no new terminus
Oxidation (M)

20200924_KIZ_MUb5_GlyGly_1 − Scan number: 3371

Gene Names	Charge	m/z	Mass	Retention time	PEP	Score	Precursor Intensity
USP5	3	325.8576	974.5509	10.331	0.0016705	102.95	22195276

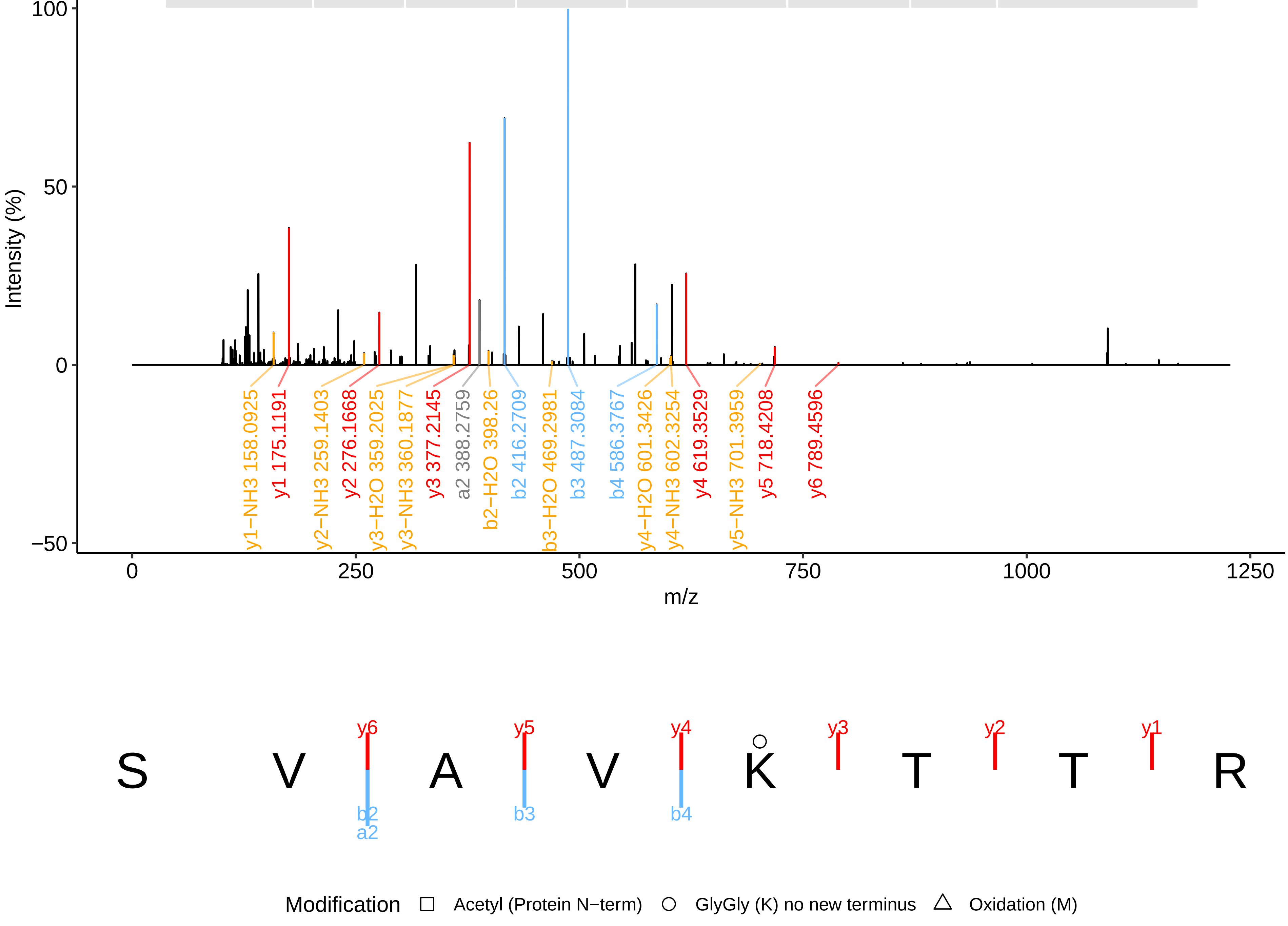

20200924_KIZ_MUb5_GlyGly_1 − Scan number: 3393

Gene Names	Charge	m/z	Mass	Retention time	PEP	Score	Precursor Intensity
USP5	2	488.2827	974.5509	10.398	0.079229	69.716	9430788

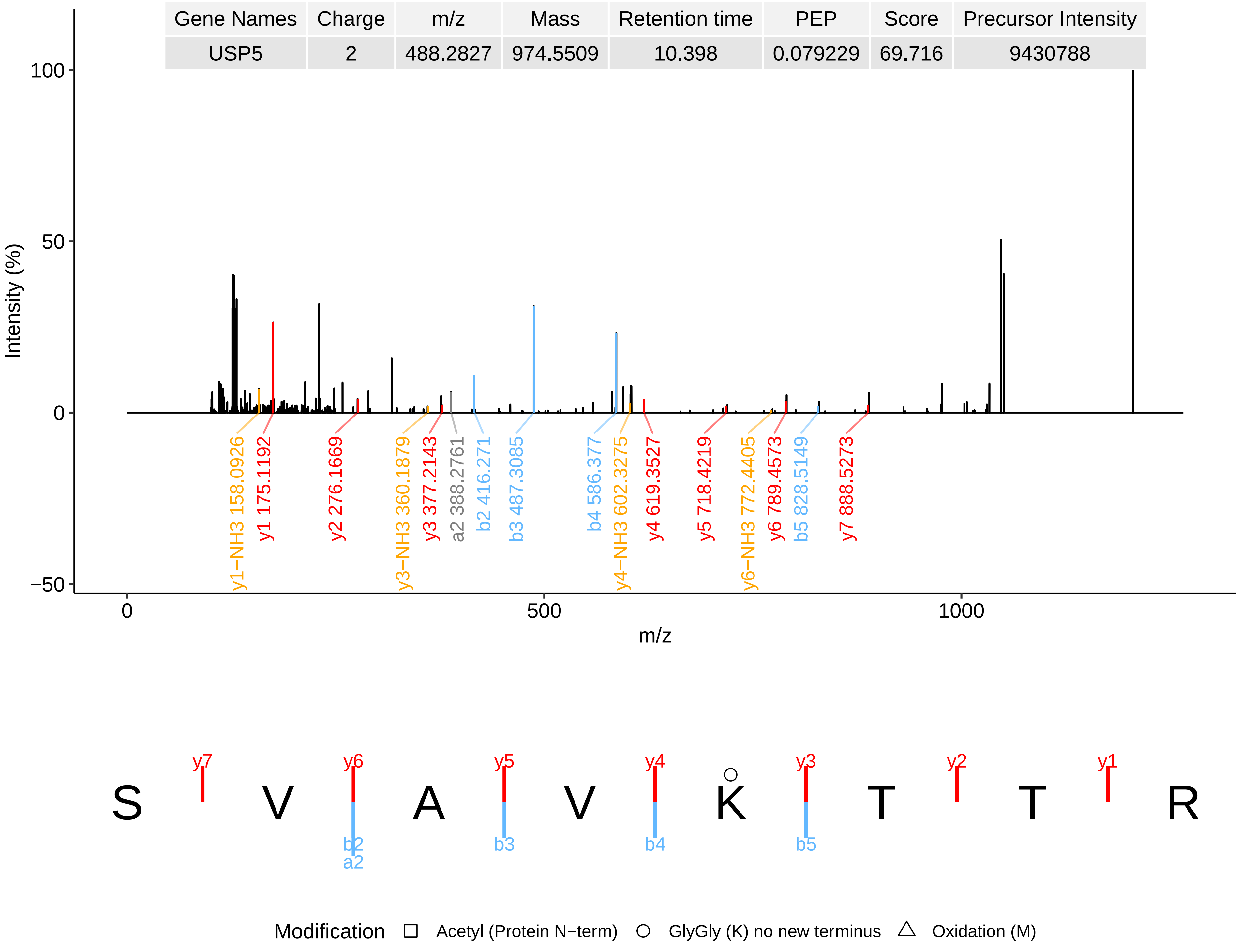

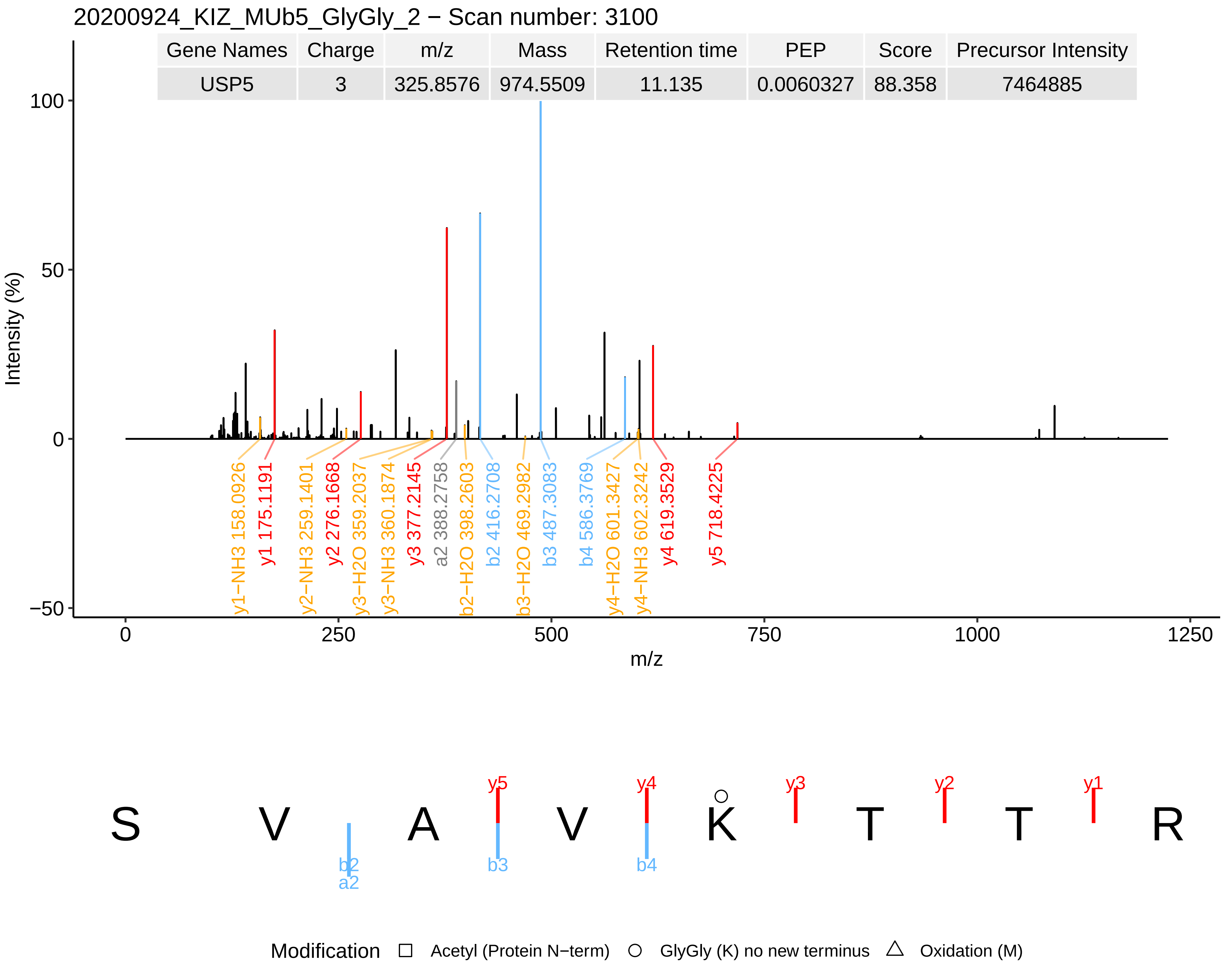
20200924_KIZ_MUb5_GlyGly_2 − Scan number: 3100
Gene Names | Charge | m/z | Mass | Retention time | PEP | Score | Precursor Intensity
USP5 | 3 | 325.8576 | 974.5509 | 11.135 | 0.0060327 | 88.358 | 7464885
Intensity (%)
100
50
0
−50
y1−NH3 158.0926
y1 175.1191
y2−NH3 259.1401
y2 276.1668
y3−H2O 359.2037
y3−NH3 360.1874
y3 377.2145
a2 388.2758
b2−H2O 398.2603
b2 416.2708
b3−H2O 469.2982
b3 487.3083
b4 586.3769
y4−H2O 601.3427
y4−NH3 602.3242
y4 619.3529
y5 718.4225
0
250
500
750
1000
1250
m/z
S V A V K T T R
y5 y4 y3 y2 y1
b2 a2 b3 b4
Modification
Acetyl (Protein N−term)
GlyGly (K) no new terminus
Oxidation (M)

20201118_KIZ_MUb6_GlyGly_1 − Scan number: 3282

Gene Names	Charge	m/z	Mass	Retention time	PEP	Score	Precursor Intensity
USP5	3	325.8576	974.5509	10.087	0.00050292	113.26	86862568

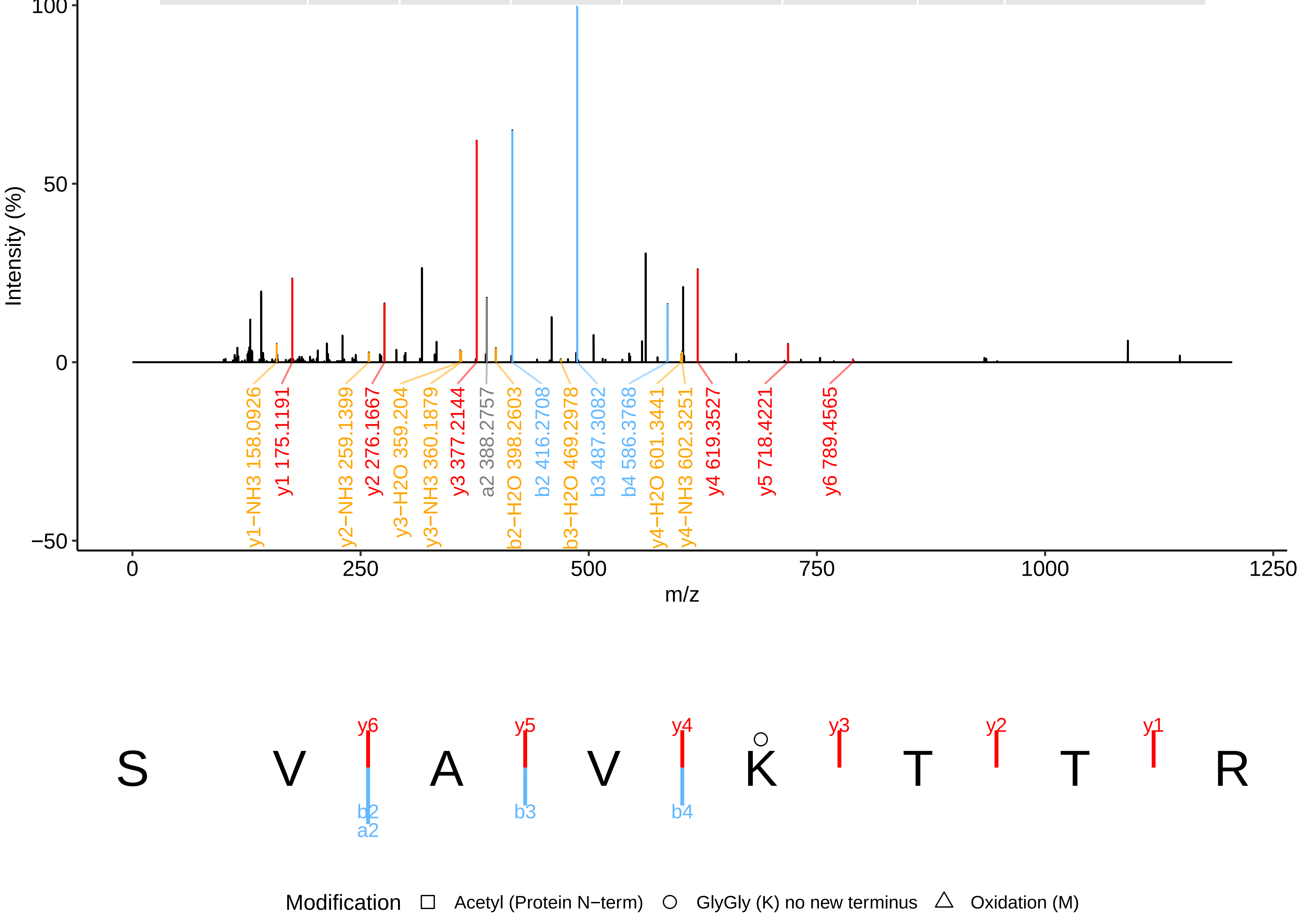

20201118_KIZ_MUb6_GlyGly_1 − Scan number: 3294

Gene Names	Charge	m/z	Mass	Retention time	PEP	Score	Precursor Intensity
USP5	2	488.2827	974.5509	10.12	0.0072876	132.76	13544188

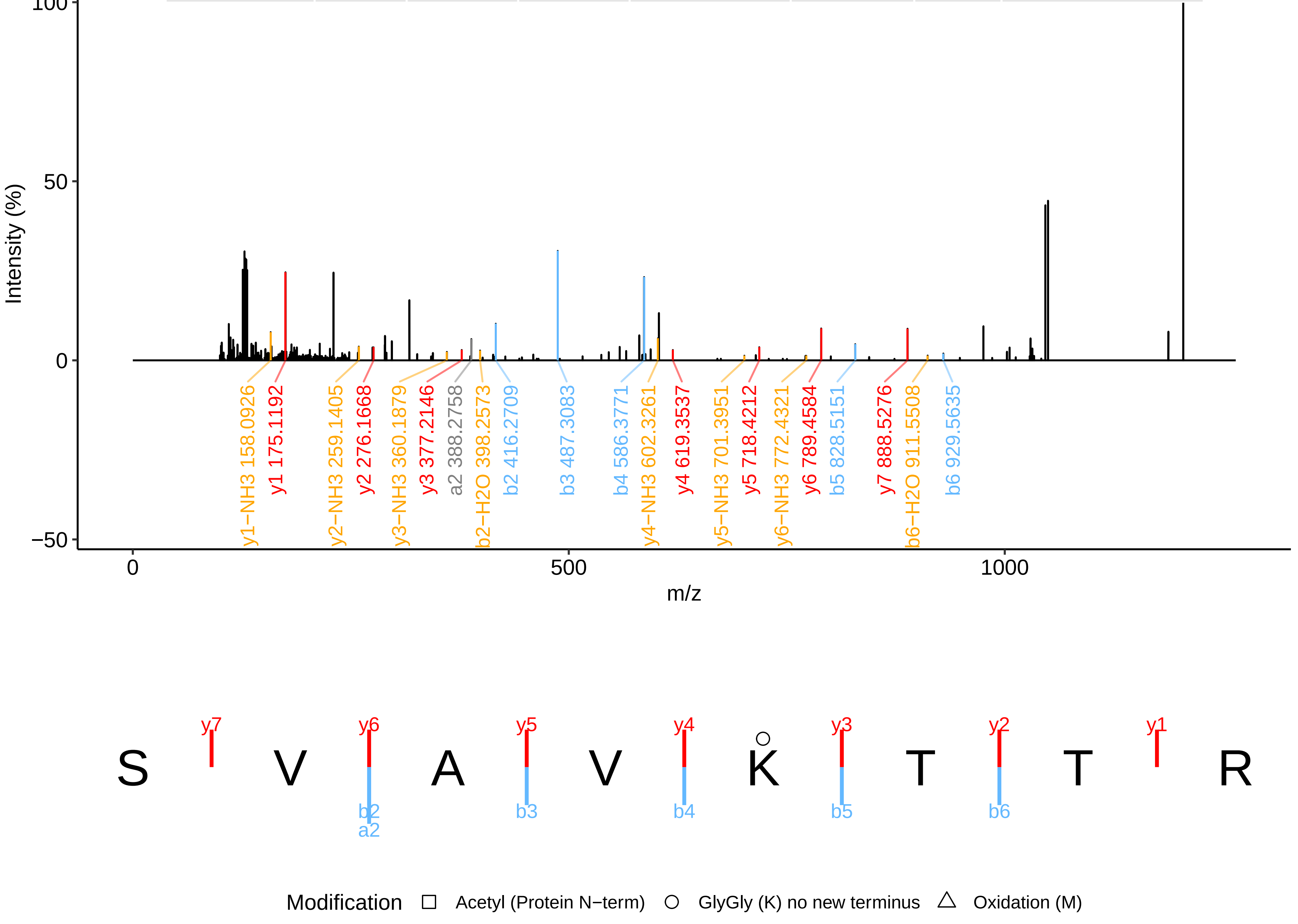

20201118_KIZ_MUb6_GlyGly_2 − Scan number: 3778

Gene Names	Charge	m/z	Mass	Retention time	PEP	Score	Precursor Intensity
USP5	3	325.8576	974.5509	12.642	0.053669	57.598	7257094

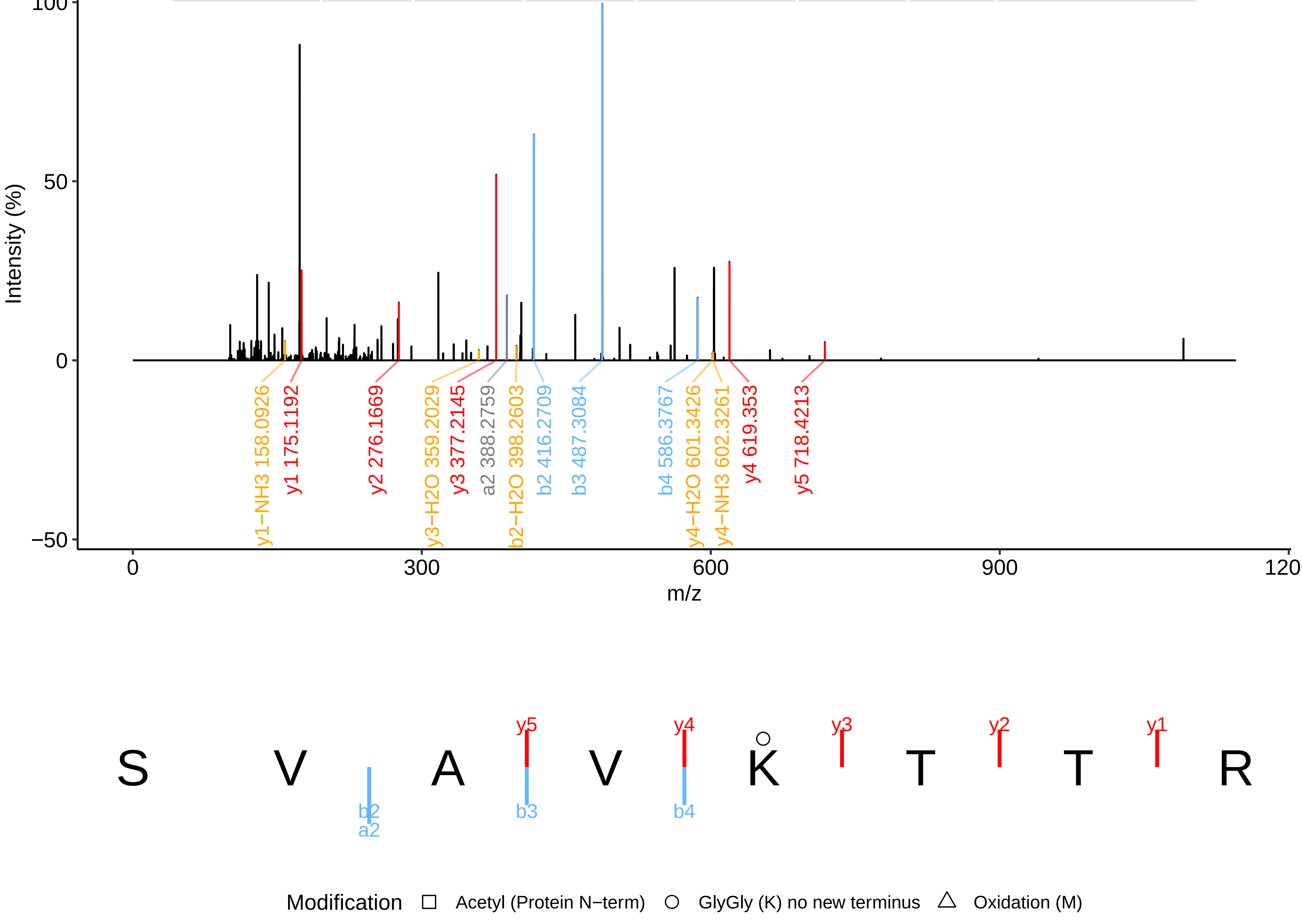

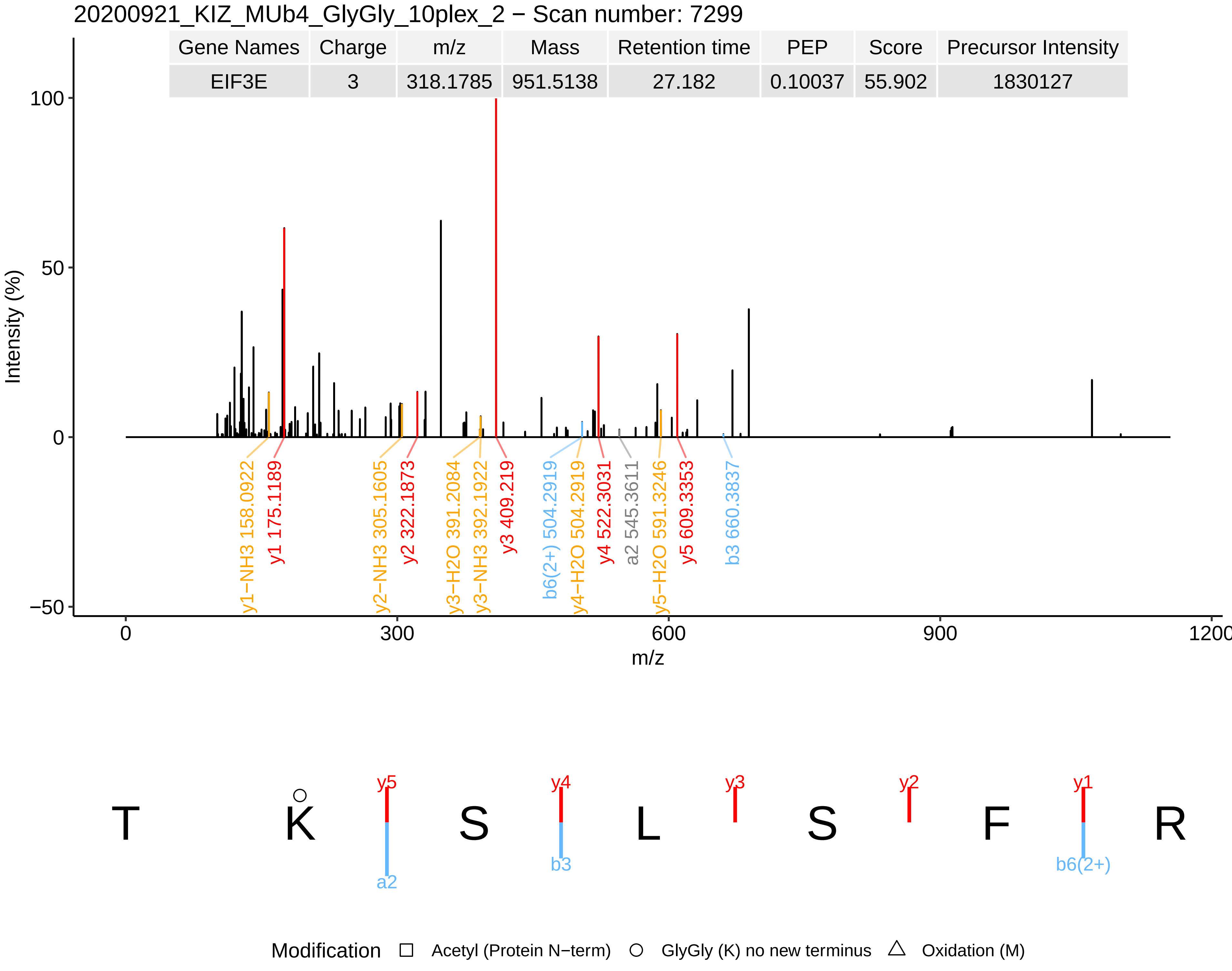
20200921_KIZ_MUb4_GlyGly_10plex_2 − Scan number: 7299
Gene Names | Charge | m/z | Mass | Retention time | PEP | Score | Precursor Intensity
EIF3E | 3 | 318.1785 | 951.5138 | 27.182 | 0.10037 | 55.902 | 1830127
Intensity (%)
100
50
0
−50
y1−NH3 158.0922
y1 175.1189
y2−NH3 305.1605
y2 322.1873
y3−H2O 391.2084
y3−NH3 392.1922
y3 409.219
b6(2+) 504.2919
y4−H2O 504.2919
y4 522.3031
a2 545.3611
y5−H2O 591.3246
y5 609.3353
b3 660.3837
0
300
600
900
1200
m/z
T K S L S F R
y5 y4 y3 y2 y1
a2 b3 b6(2+)
Modification
Acetyl (Protein N−term)
GlyGly (K) no new terminus
Oxidation (M)

20200921_KIZ_MUb4_GlyGly_10plex_2 − Scan number: 6452

Gene Names	Charge	m/z	Mass	Retention time	PEP	Score	Precursor Intensity
NIFK	3	325.1978	972.5716	23.715	0.017532	88.029	1426276

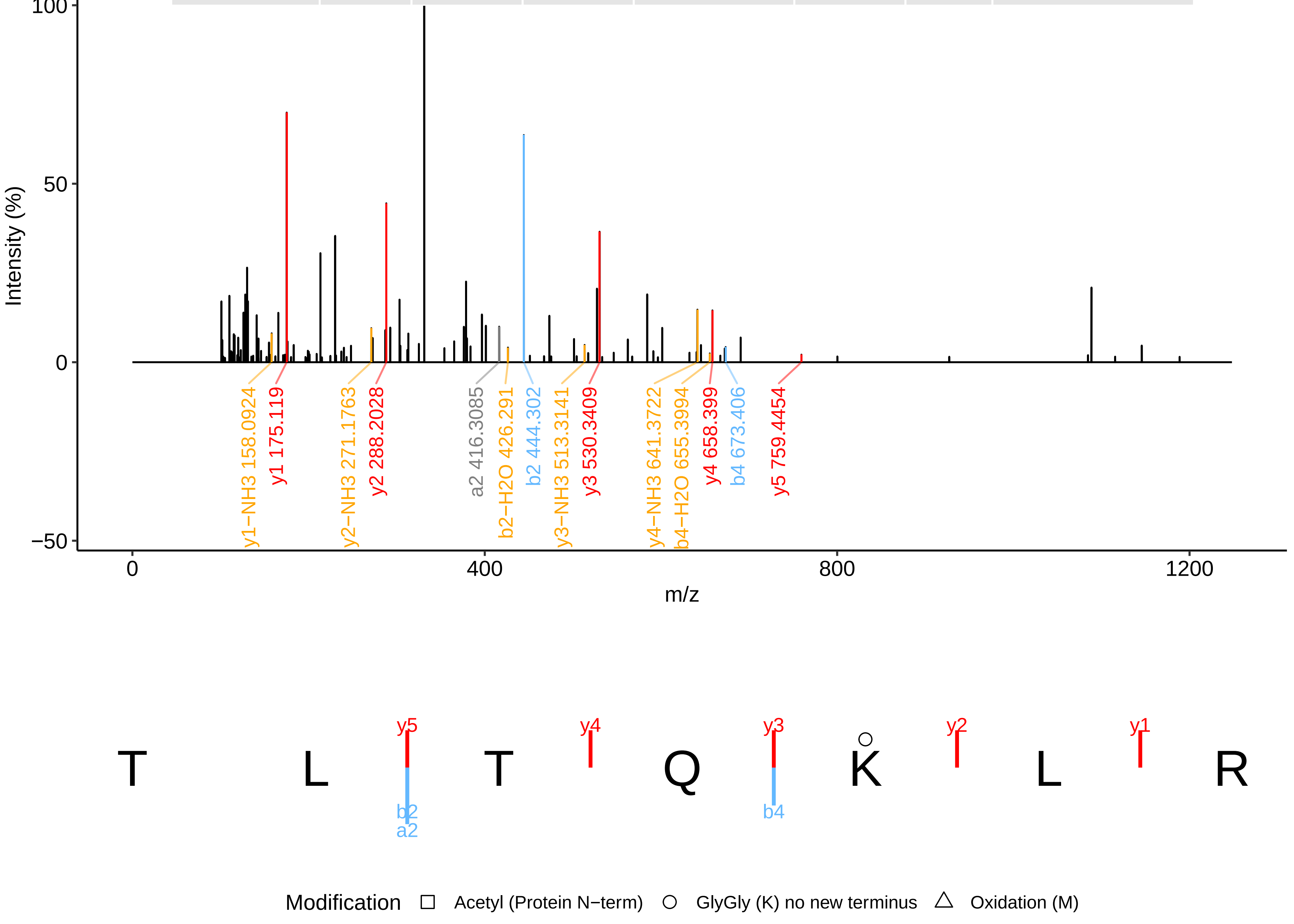

20200921_KIZ_MUb4_GlyGly_10plex − Scan number: 6536

Gene Names	Charge	m/z	Mass	Retention time	PEP	Score	Precursor Intensity
RPL9	3	328.5381	982.5924	21.665	0.0078492	83.753	4849071

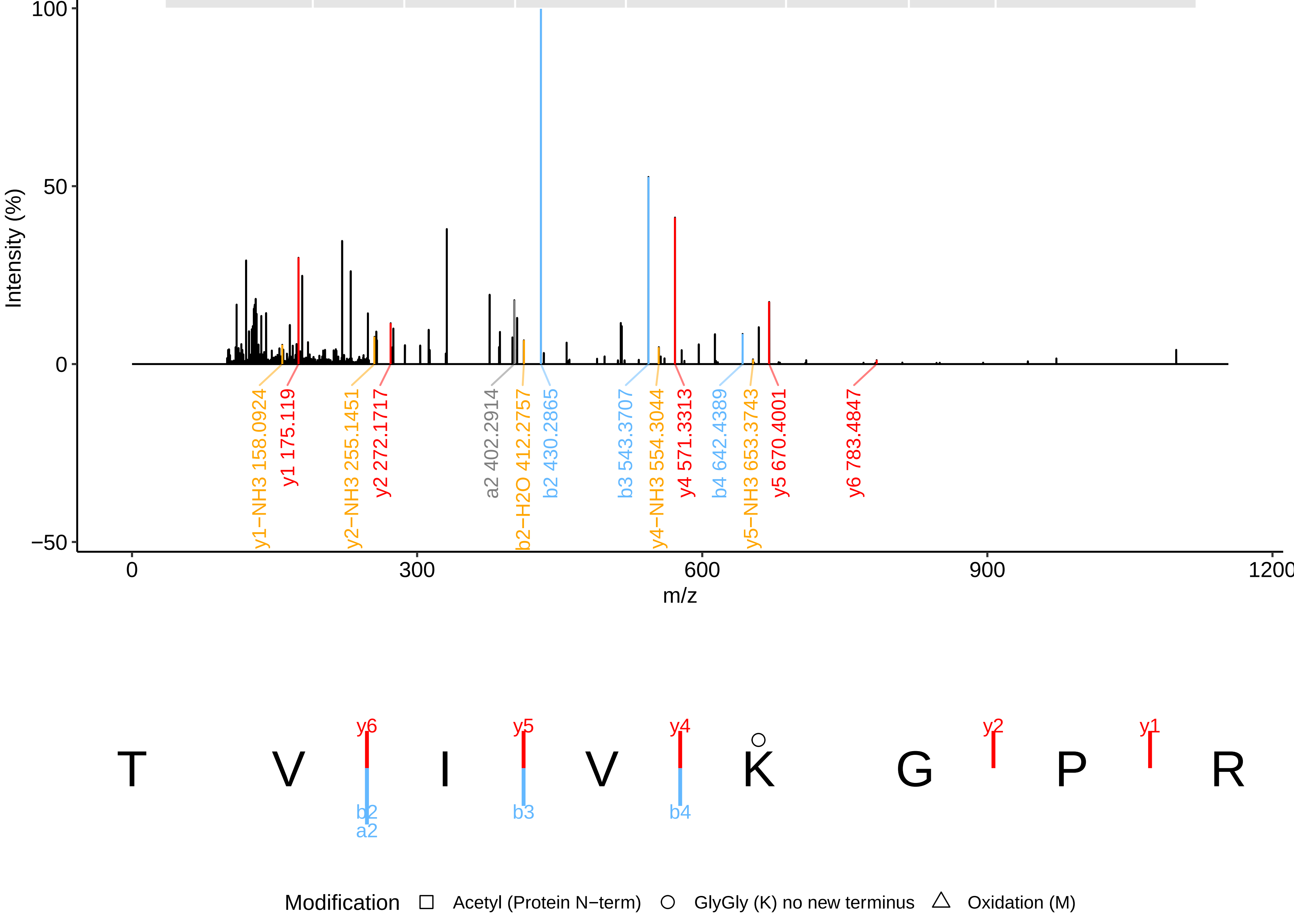

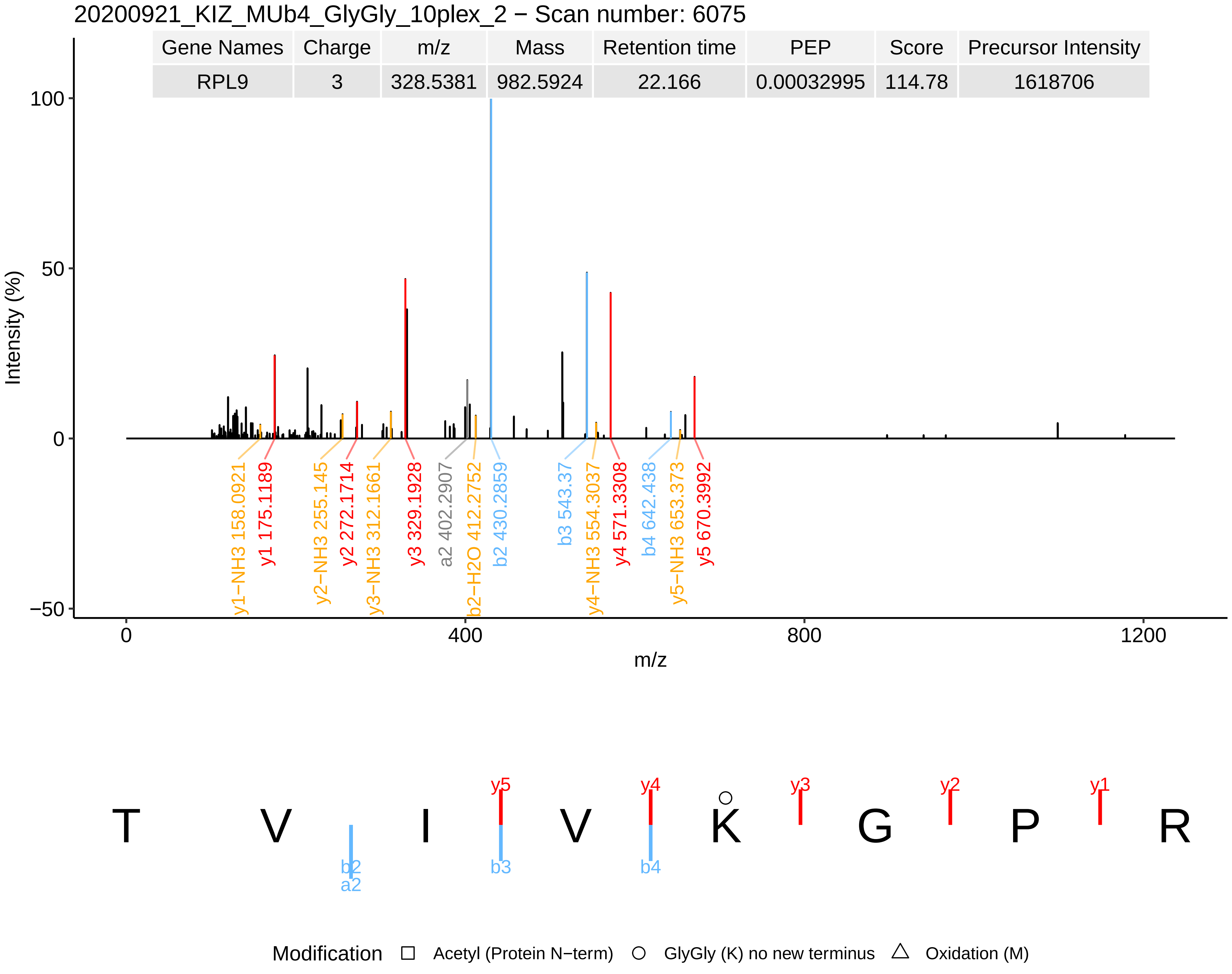

20200921_KIZ_MUb4_GlyGly_10plex_2 − Scan number: 6075
Gene Names | Charge | m/z | Mass | Retention time | PEP | Score | Precursor Intensity
RPL9 | 3 | 328.5381 | 982.5924 | 22.166 | 0.00032995 | 114.78 | 1618706
Intensity (%)
m/z
y1−NH3 158.0921
y1 175.1189
y2−NH3 255.145
y2 272.1714
y3−NH3 312.1661
y3 329.1928
a2 402.2907
b2−H2O 412.2752
b2 430.2859
b3 543.37
y4−NH3 554.3037
y4 571.3308
b4 642.438
y5−NH3 653.373
y5 670.3992
T V I V K G P R
Modification
Acetyl (Protein N−term)
GlyGly (K) no new terminus
Oxidation (M)

20200924_KIZ_MUb5_GlyGly_1 − Scan number: 5688

Gene Names	Charge	m/z	Mass	Retention time	PEP	Score	Precursor Intensity
RPL9	3	328.5381	982.5924	17.747	0.0021715	98.523	10970751

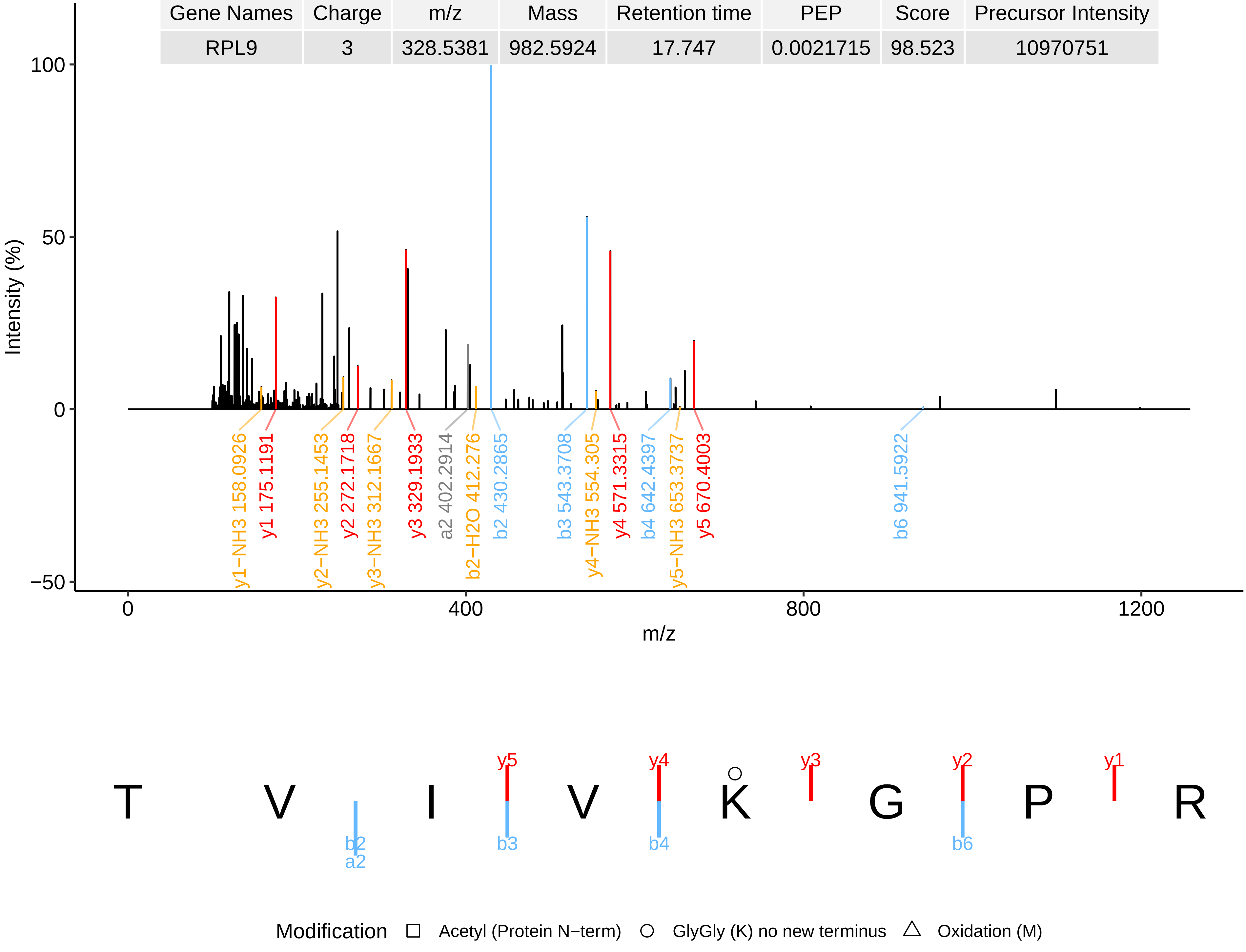

20200924_KIZ_MUb5_GlyGly_2 − Scan number: 11551

Gene Names	Charge	m/z	Mass	Retention time	PEP	Score	Precursor Intensity
	2	480.2671	958.5196	43.191	0.11793	84.097	3913576

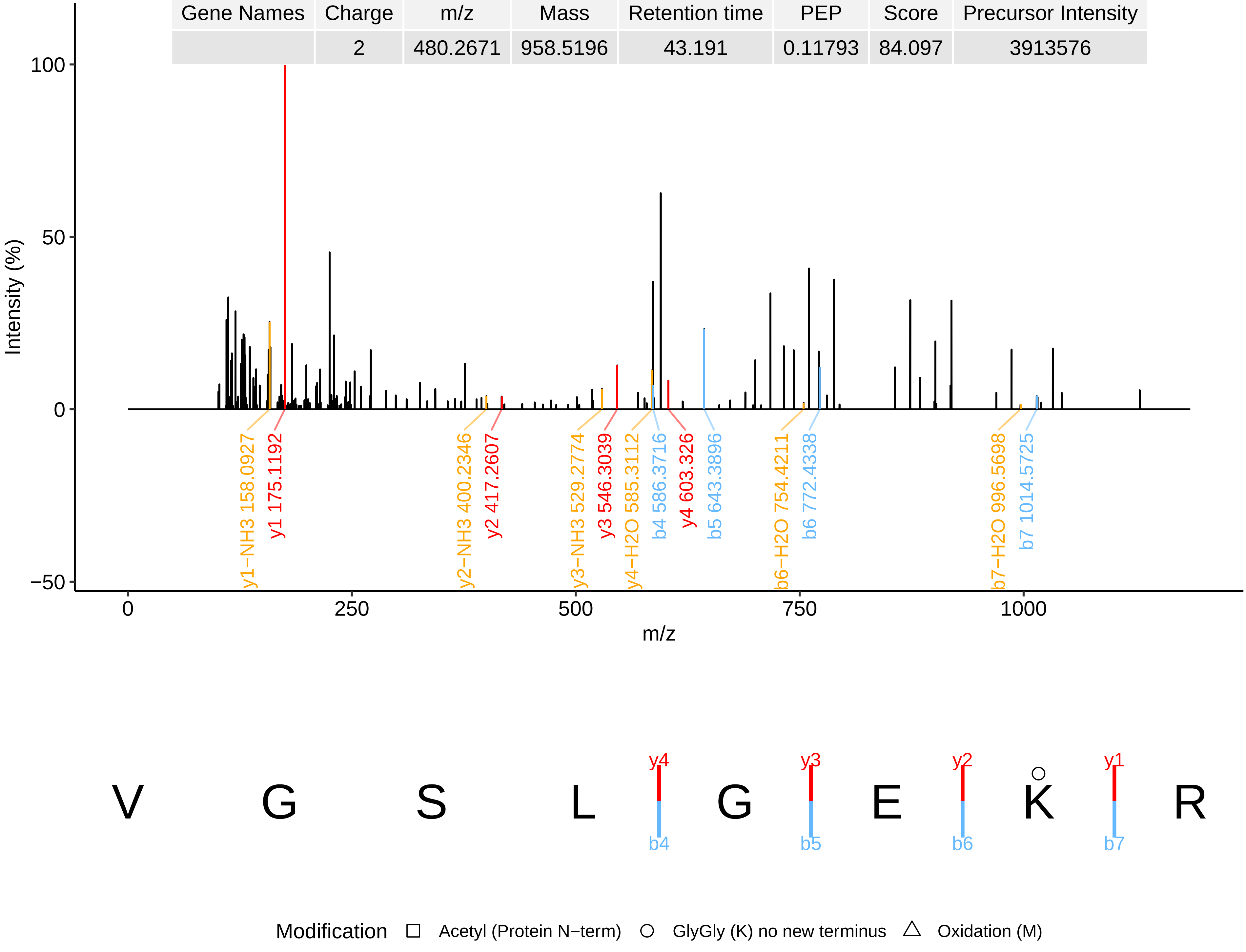

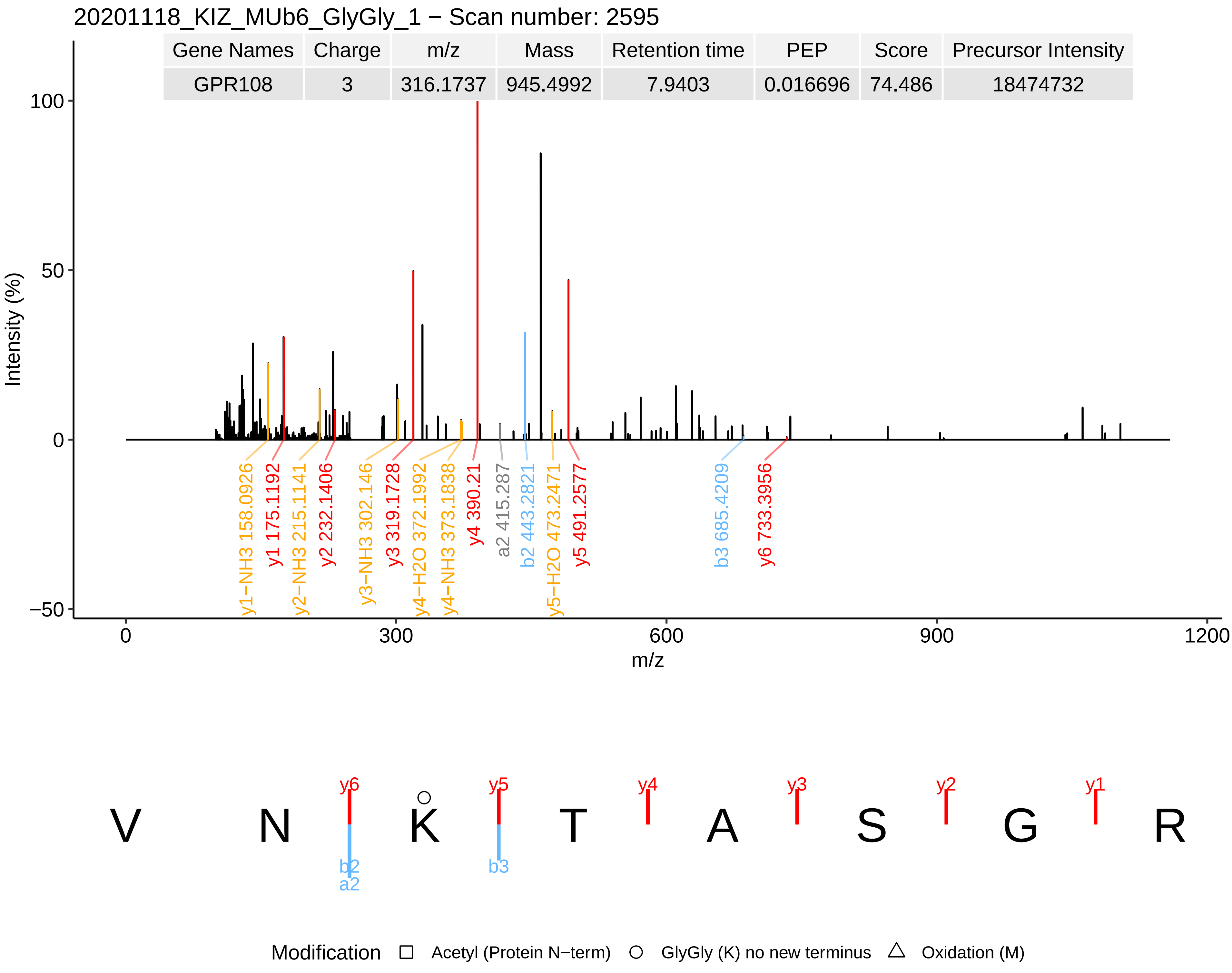
20201118_KIZ_MUb6_GlyGly_1 − Scan number: 2595
Gene Names | Charge | m/z | Mass | Retention time | PEP | Score | Precursor Intensity
GPR108 | 3 | 316.1737 | 945.4992 | 7.9403 | 0.016696 | 74.486 | 18474732
Intensity (%)
m/z
y1−NH3 158.0926
y1 175.1192
y2−NH3 215.1141
y2 232.1406
y3−NH3 302.146
y3 319.1728
y4−H2O 372.1992
y4−NH3 373.1838
y4 390.21
a2 415.287
b2 443.2821
y5−H2O 473.2471
y5 491.2577
b3 685.4209
y6 733.3956
V N K T A S G R
y6 y5 y4 y3 y2 y1
b2 a2 b3
Modification
Acetyl (Protein N−term)
GlyGly (K) no new terminus
Oxidation (M)

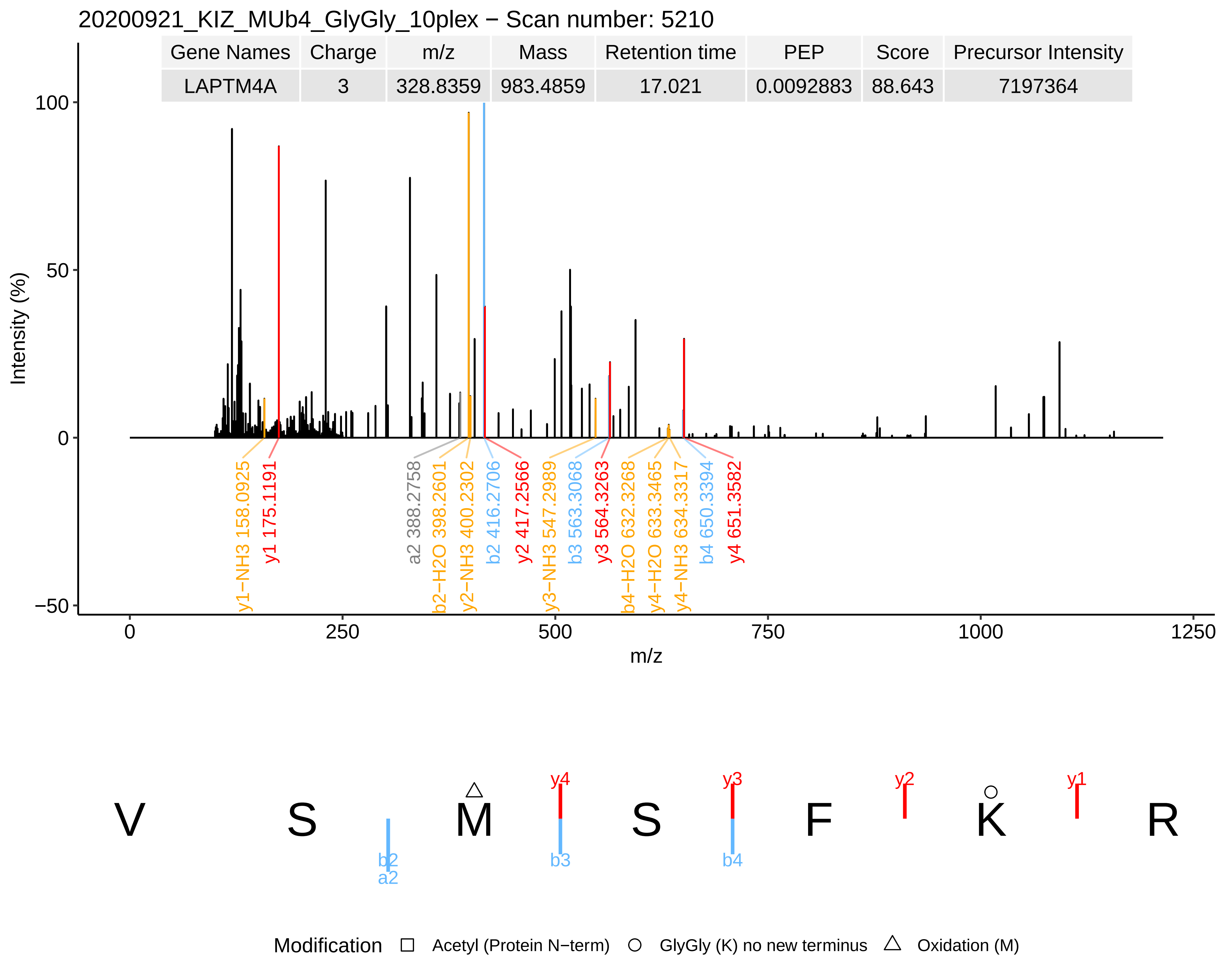
20200921_KIZ_MUb4_GlyGly_10plex − Scan number: 5210
Gene Names | Charge | m/z | Mass | Retention time | PEP | Score | Precursor Intensity
LAPTM4A | 3 | 328.8359 | 983.4859 | 17.021 | 0.0092883 | 88.643 | 7197364
Intensity (%)
100
50
0
−50
y1−NH3 158.0925
y1 175.1191
a2 388.2758
b2−H2O 398.2601
y2−NH3 400.2302
b2 416.2706
y2 417.2566
y3−NH3 547.2989
b3 563.3068
y3 564.3263
b4−H2O 632.3268
y4−H2O 633.3465
y4−NH3 634.3317
b4 650.3394
y4 651.3582
0
250
500
750
1000
1250
m/z
V S M S F K R
y4 y3 y2 y1
b2 a2 b3 b4
Modification
Acetyl (Protein N−term)
GlyGly (K) no new terminus
Oxidation (M)

20200921_KIZ_MUb4_GlyGly_10plex − Scan number: 9763

Gene Names	Charge	m/z	Mass	Retention time	PEP	Score	Precursor Intensity
LAPTM4A	3	328.8359	983.4859	33.54	0.029266	71.141	4050583

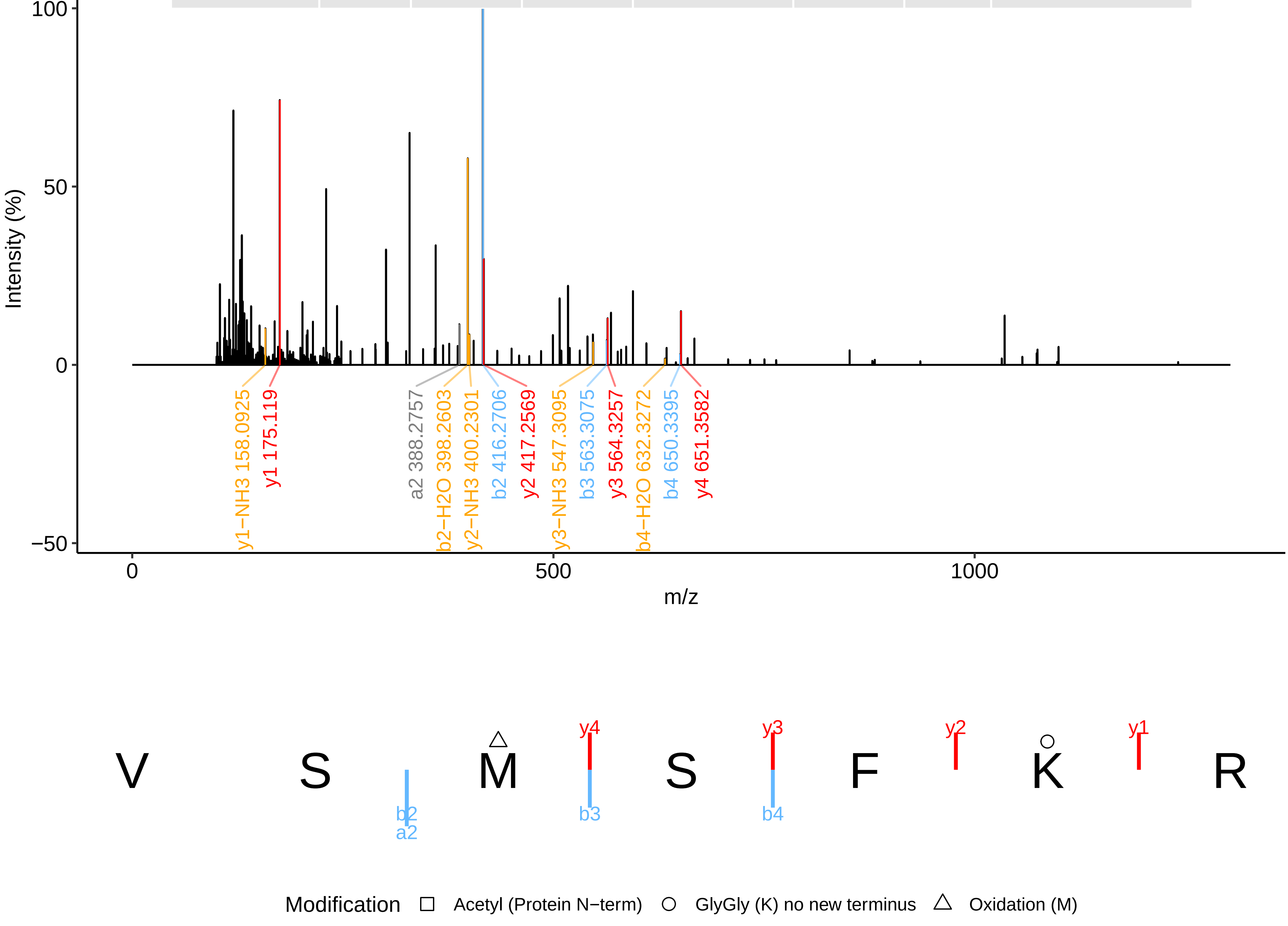

20200921_KIZ_MUb4_GlyGly_10plex_2 − Scan number: 4805

Gene Names	Charge	m/z	Mass	Retention time	PEP	Score	Precursor Intensity
LAPTM4A	3	328.8359	983.4859	17.015	0.011831	83.377	1632023

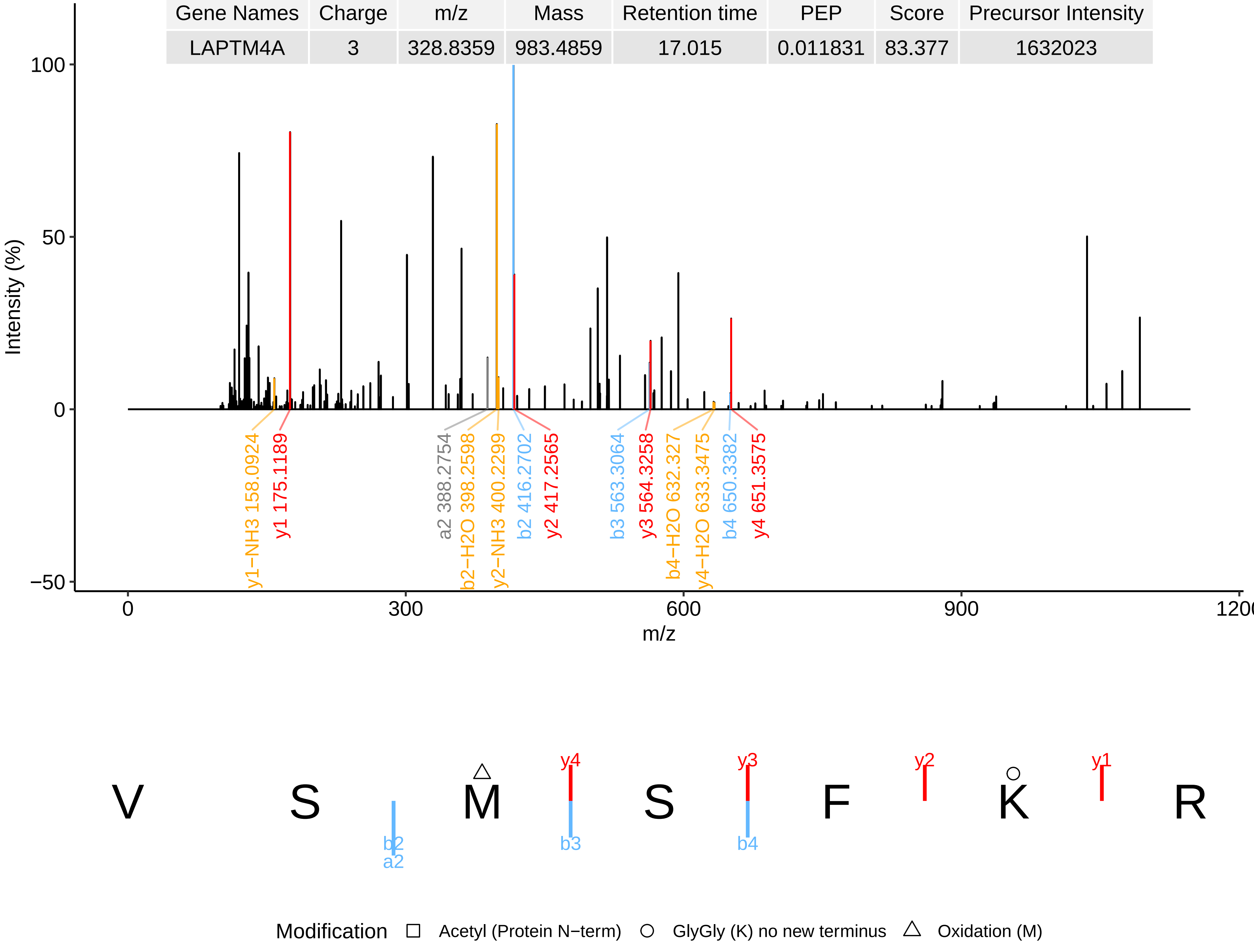

20201118_KIZ_MUb6_GlyGly_1 − Scan number: 4399

Gene Names	Charge	m/z	Mass	Retention time	PEP	Score	Precursor Intensity
LAPTM4A	3	328.8359	983.4859	13.752	0.028289	71.715	10253342

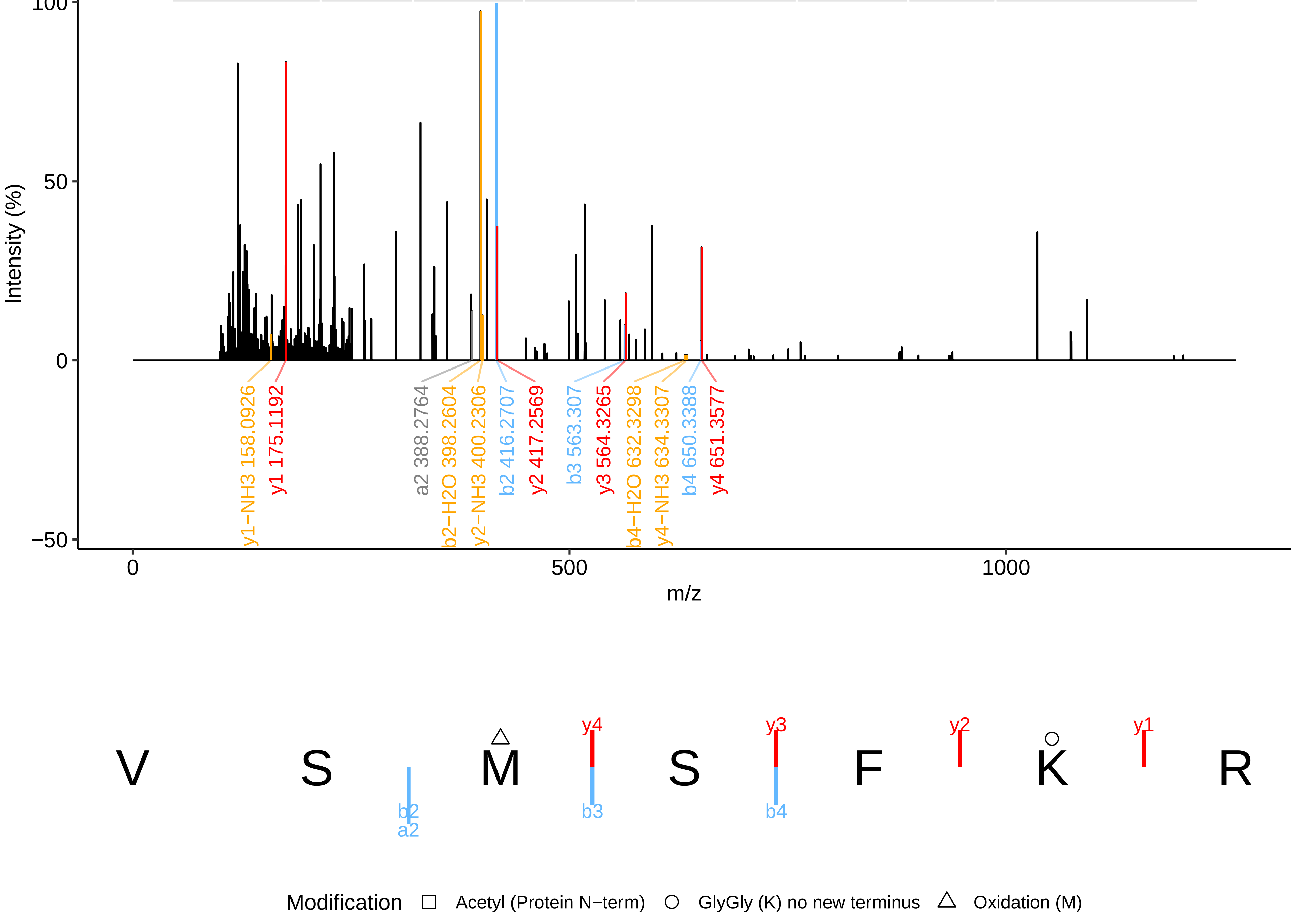

20200921_KIZ_MUb4_GlyGly_10plex_2 − Scan number: 9018

Gene Names	Charge	m/z	Mass	Retention time	PEP	Score	Precursor Intensity
LAPTM4A	3	323.5043	967.4909	34.158	0.021046	111.04	5201472

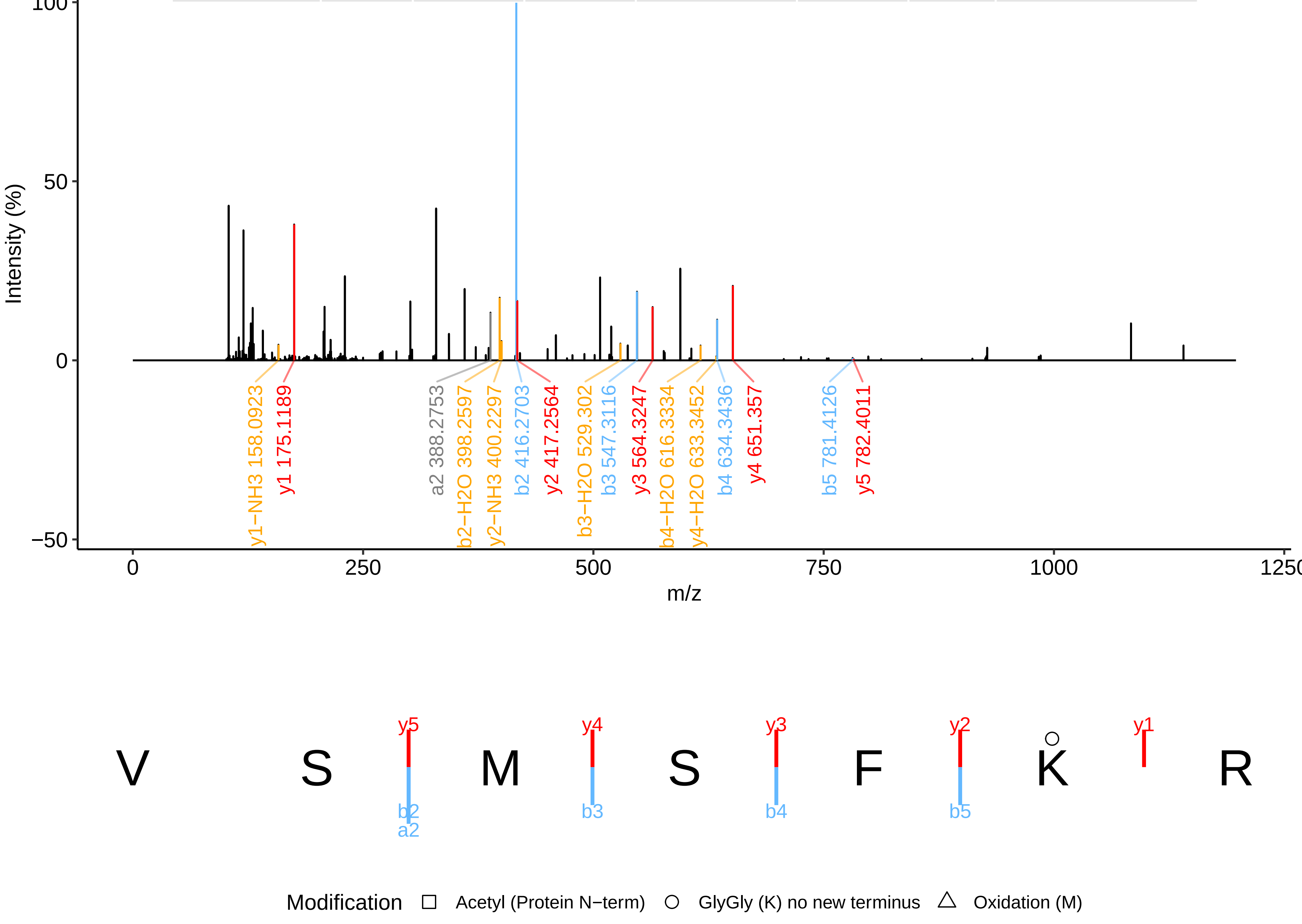

20200924_KIZ_MUb5_GlyGly_1 − Scan number: 8457

Gene Names	Charge	m/z	Mass	Retention time	PEP	Score	Precursor Intensity
LAPTM4A	3	323.5043	967.4909	27.039	0.015411	114.19	21050658

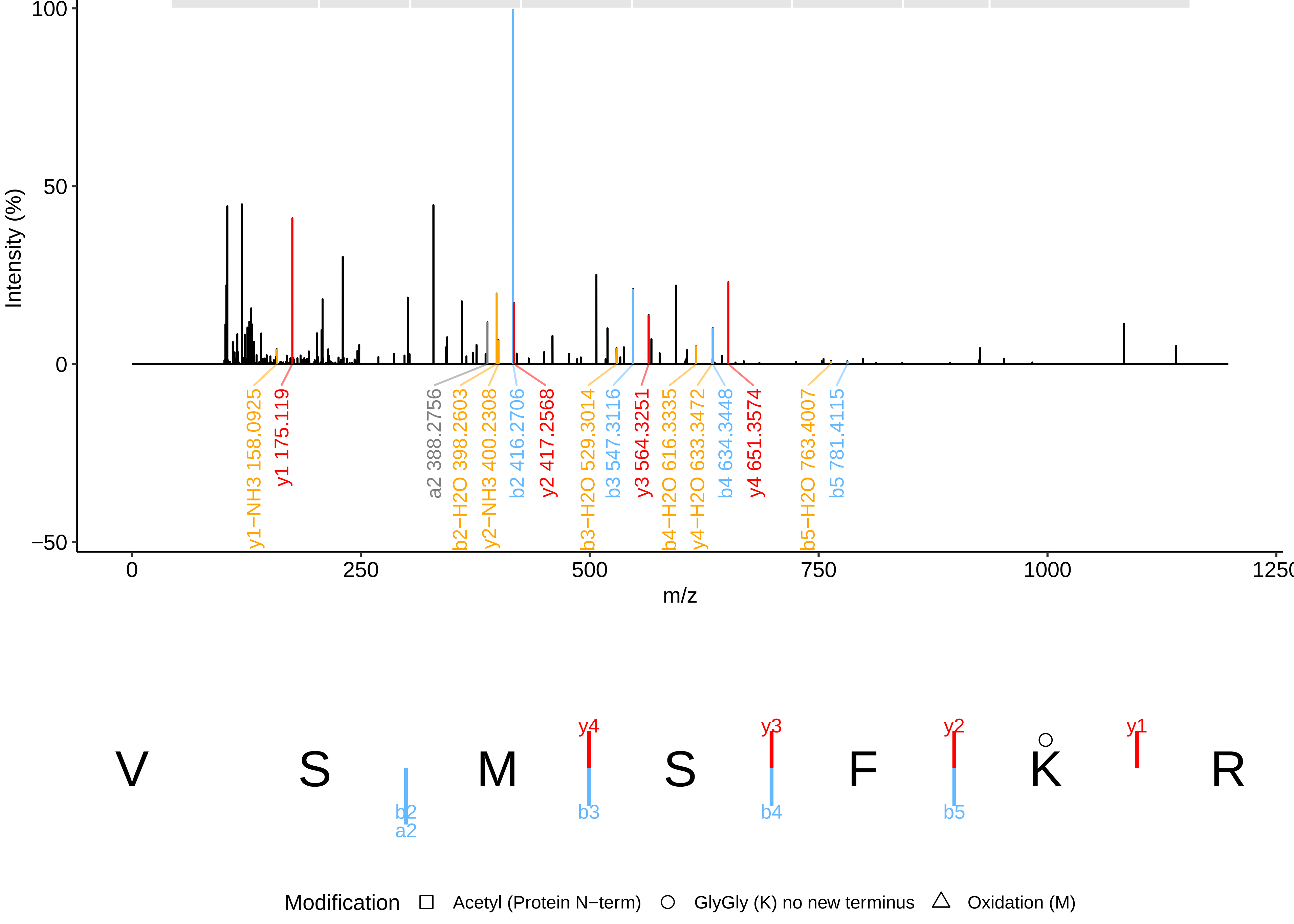

20200924_KIZ_MUb5_GlyGly_1 − Scan number: 8606

Gene Names	Charge	m/z	Mass	Retention time	PEP	Score	Precursor Intensity
LAPTM4A	3	323.5043	967.4909	27.542	0.021046	111.04	34979384

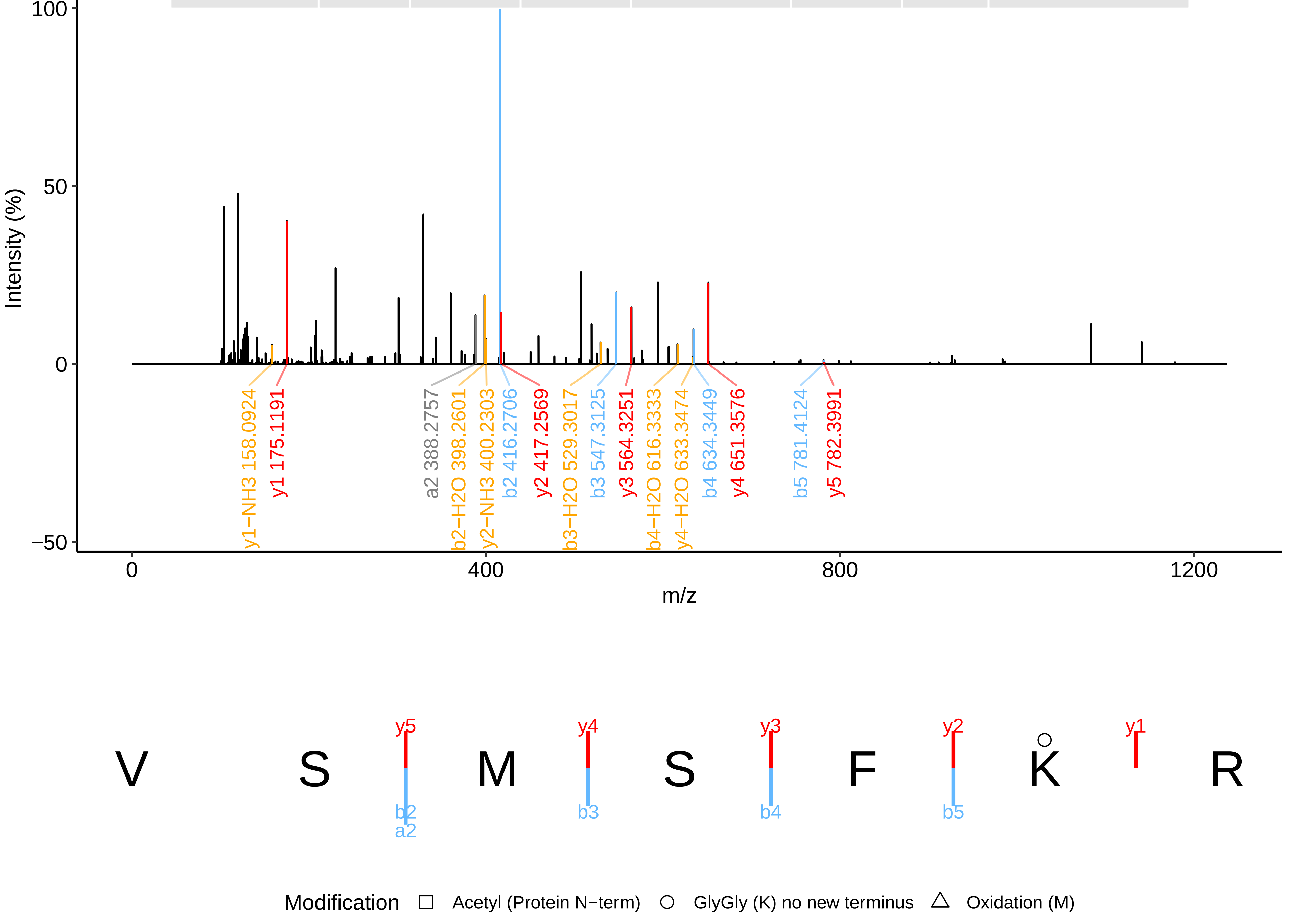

20200924_KIZ_MUb5_GlyGly_1 − Scan number: 8758

Gene Names	Charge	m/z	Mass	Retention time	PEP	Score	Precursor Intensity
LAPTM4A	3	323.5043	967.4909	28.057	0.051047	96.51	18497614

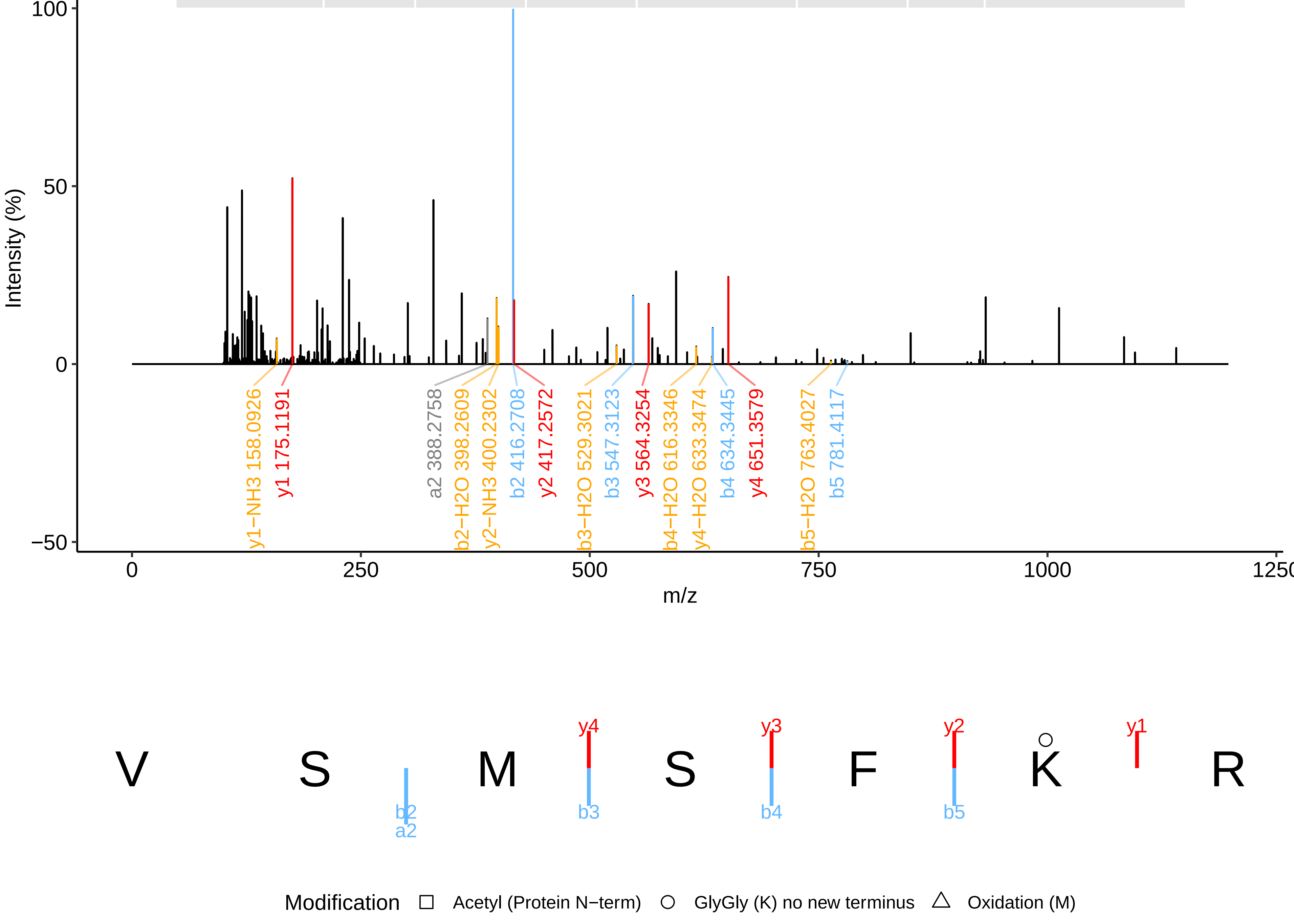

20200924_KIZ_MUb5_GlyGly_1 − Scan number: 8651

Gene Names	Charge	m/z	Mass	Retention time	PEP	Score	Precursor Intensity
LAPTM4A	2	484.7527	967.4909	27.692	0.050879	112.59	8770655

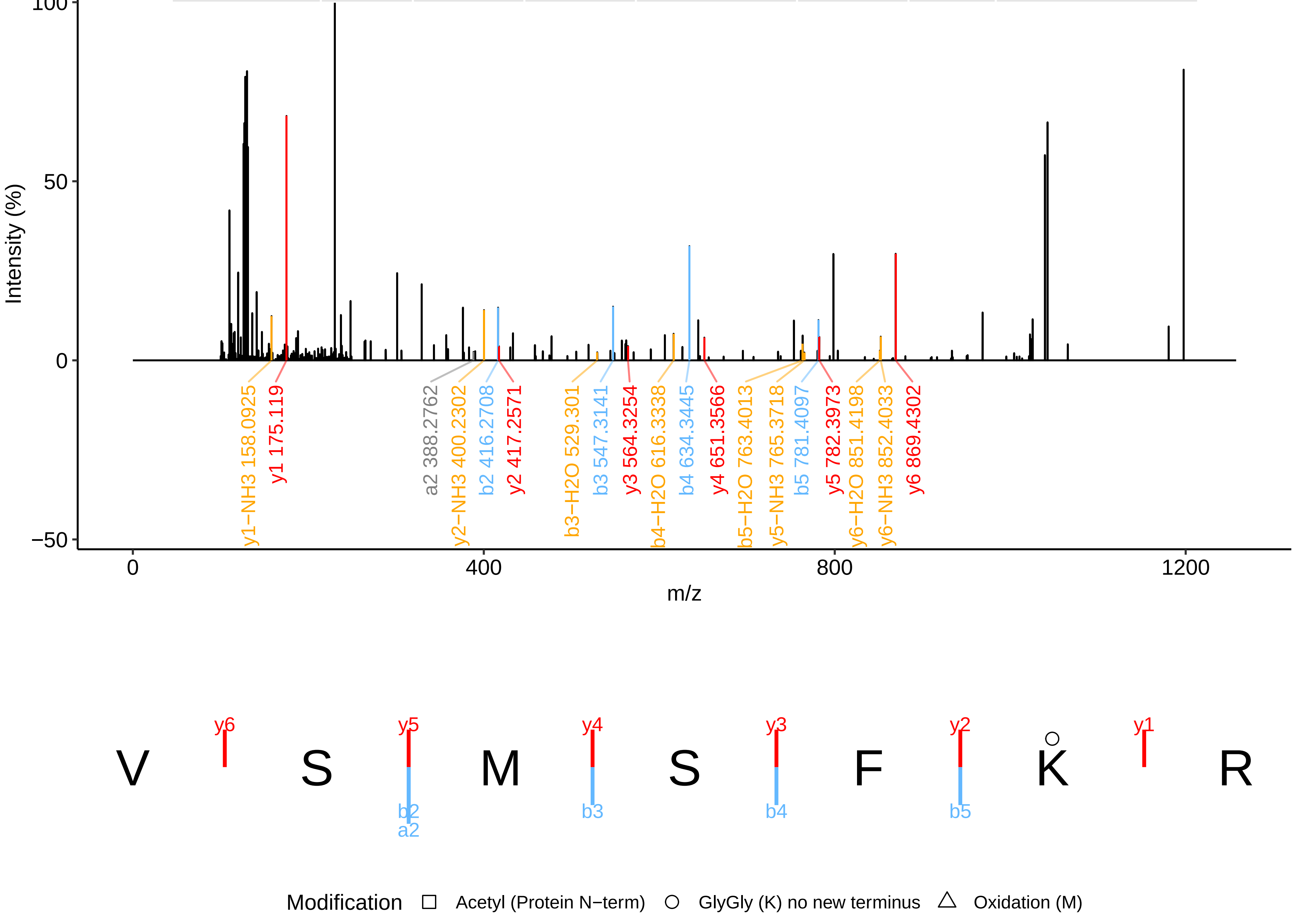

20200924_KIZ_MUb5_GlyGly_2 − Scan number: 8186

Gene Names	Charge	m/z	Mass	Retention time	PEP	Score	Precursor Intensity
LAPTM4A	3	323.5043	967.4909	30.56	0.046726	97.473	7428141

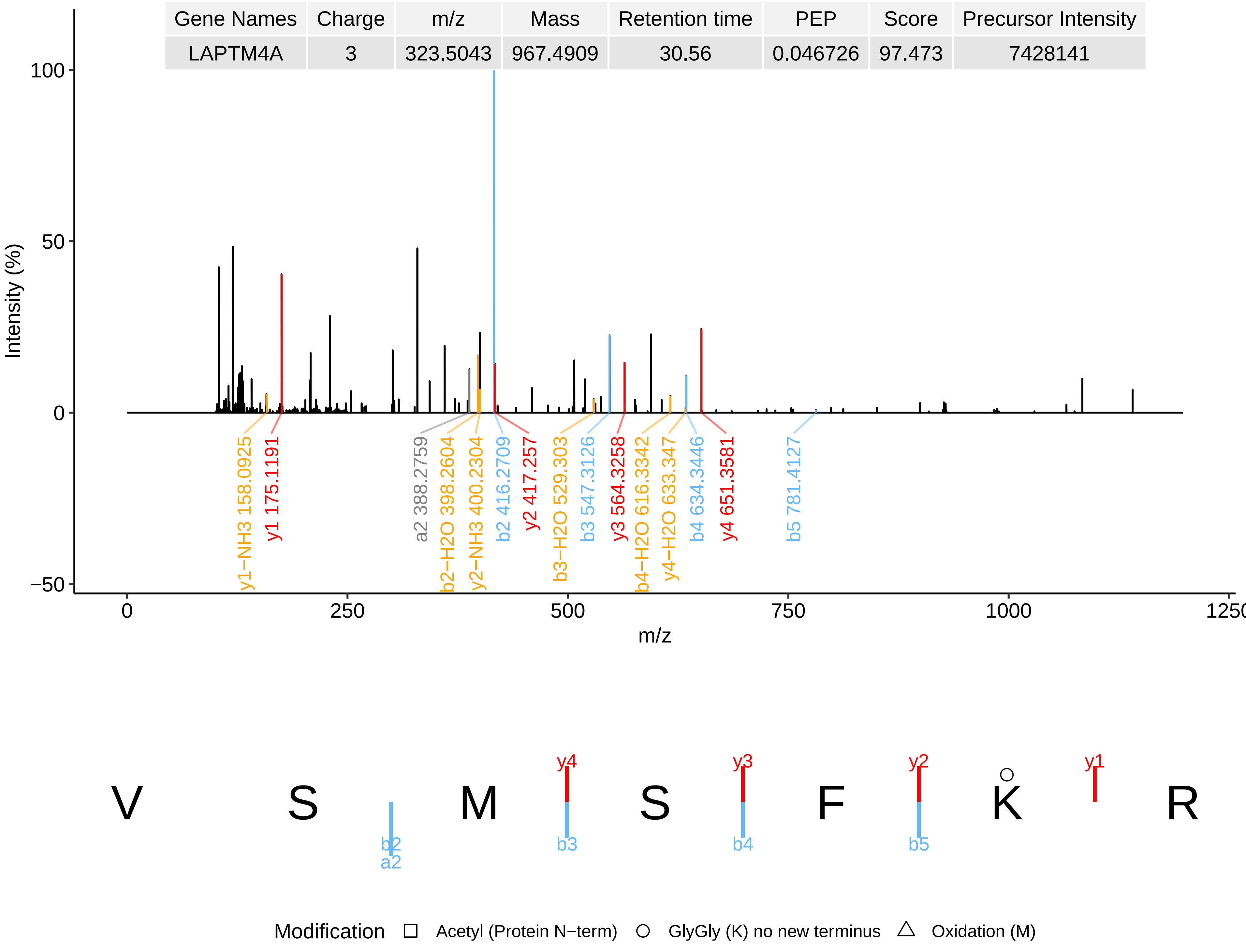

20200924_KIZ_MUb5_GlyGly_2 − Scan number: 8318

Gene Names	Charge	m/z	Mass	Retention time	PEP	Score	Precursor Intensity
LAPTM4A	3	323.5043	967.4909	31.078	0.021046	111.04	12556298

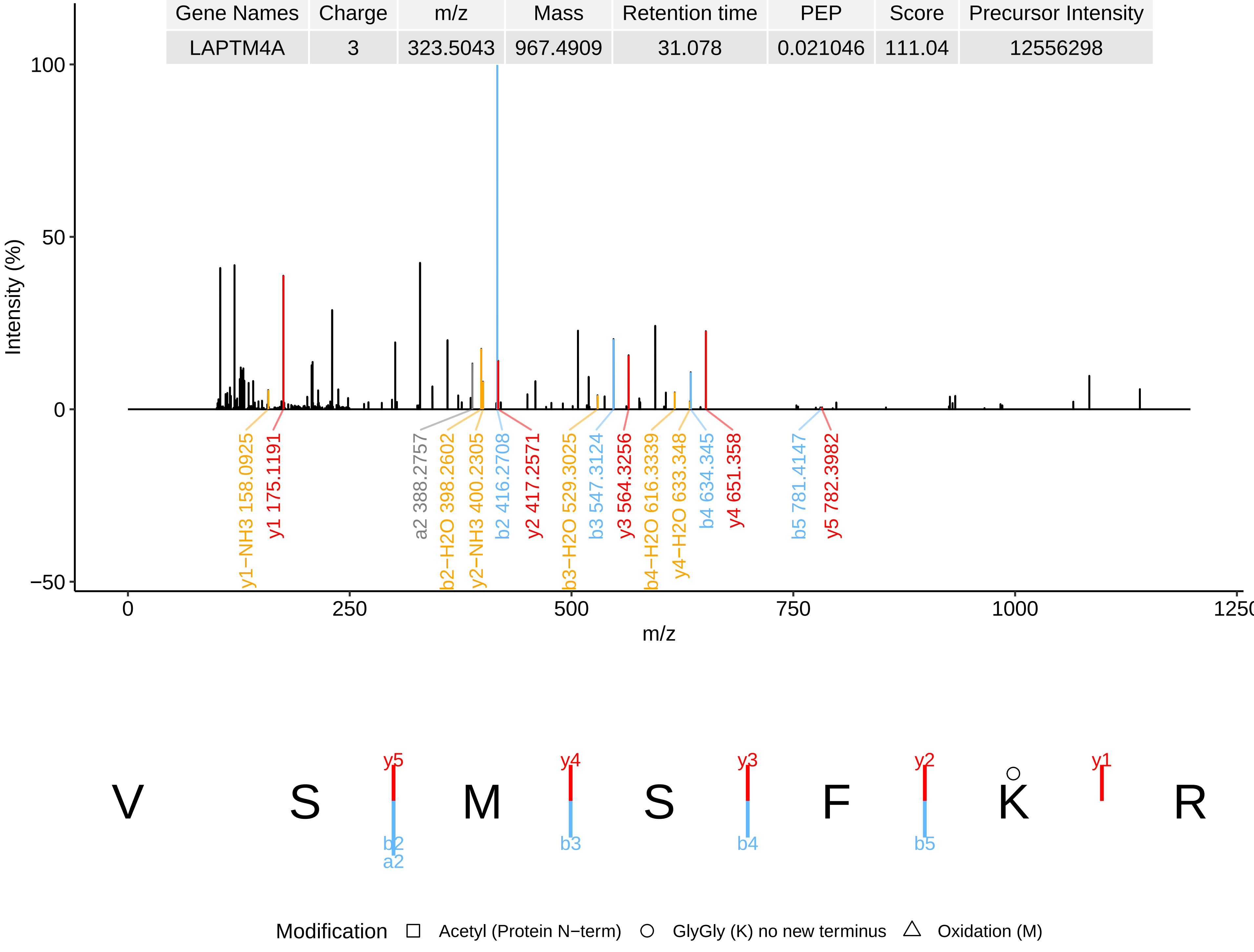

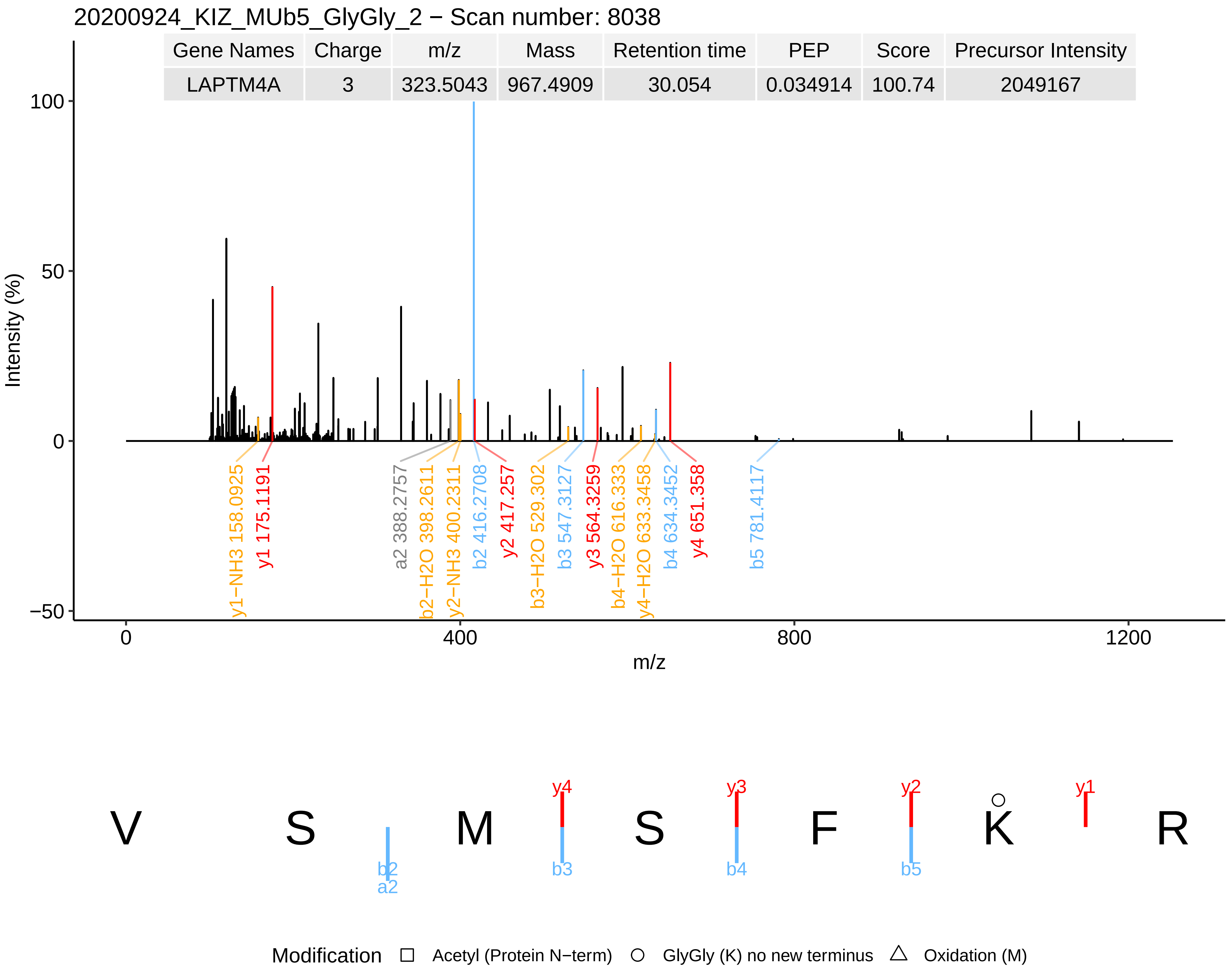

20200924_KIZ_MUb5_GlyGly_2 − Scan number: 8038
Gene Names | Charge | m/z | Mass | Retention time | PEP | Score | Precursor Intensity
LAPTM4A | 3 | 323.5043 | 967.4909 | 30.054 | 0.034914 | 100.74 | 2049167
Intensity (%)
m/z
y1−NH3 158.0925
y1 175.1191
a2 388.2757
b2−H2O 398.2611
y2−NH3 400.2311
b2 416.2708
y2 417.257
b3−H2O 529.302
b3 547.3127
y3 564.3259
b4−H2O 616.333
y4−H2O 633.3458
b4 634.3452
y4 651.358
b5 781.4117
V S M S F K R
y4 y3 y2 y1
b2 a2 b3 b4 b5
Modification
Acetyl (Protein N−term)
GlyGly (K) no new terminus
Oxidation (M)

20201118_KIZ_MUb6_GlyGly_2 − Scan number: 10266

Gene Names	Charge	m/z	Mass	Retention time	PEP	Score	Precursor Intensity
LAPTM4A	3	323.5043	967.4909	34.64	0.069201	92.467	39492780

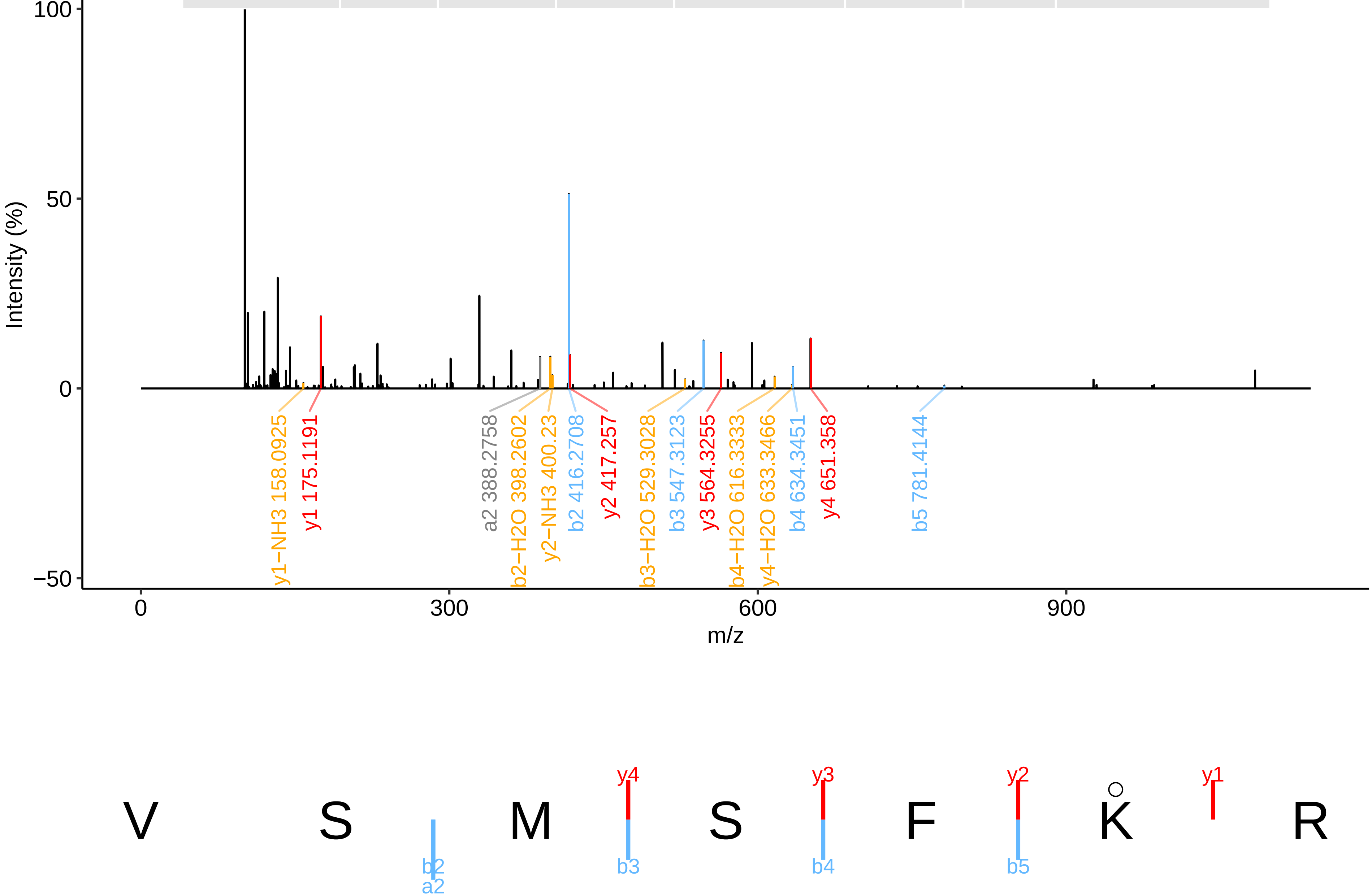

Modification □ Acetyl (Protein N−term) ○ GlyGly (K) no new terminus △ Oxidation (M)

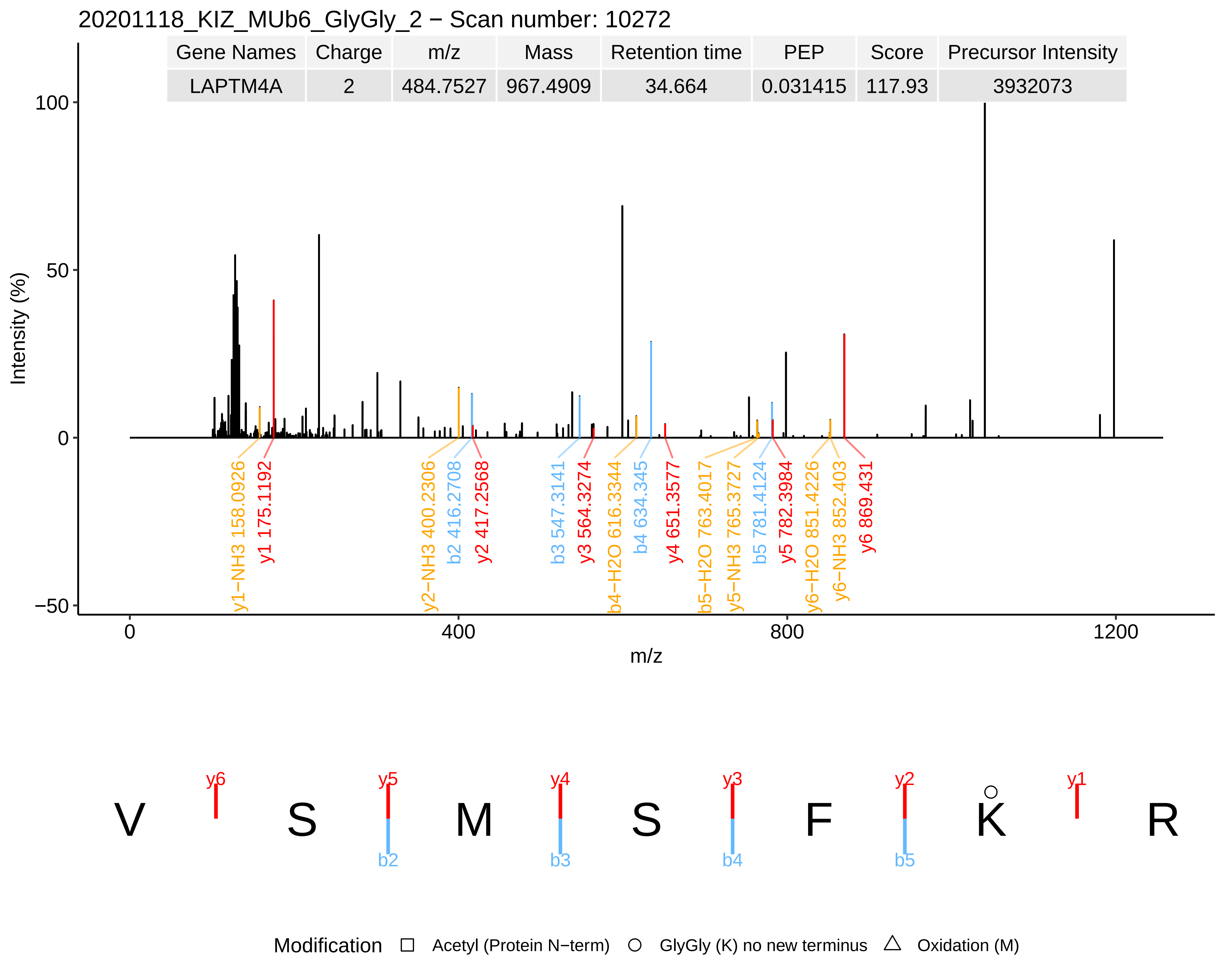

20201118_KIZ_MUb6_GlyGly_2 − Scan number: 10272

Gene Names	Charge	m/z	Mass	Retention time	PEP	Score	Precursor Intensity
LAPTM4A	2	484.7527	967.4909	34.664	0.031415	117.93	3932073

Intensity (%)
m/z
y1−NH3 158.0926
y1 175.1192
y2−NH3 400.2306
b2 416.2708
y2 417.2568
b3 547.3141
y3 564.3274
b4−H2O 616.3344
b4 634.345
y4 651.3577
b5−H2O 763.4017
y5−NH3 765.3727
b5 781.4124
y5 782.3984
y6−H2O 851.4226
y6−NH3 852.403
y6 869.431
V S M S F K R
y6 y5 y4 y3 y2 y1
b2 b3 b4 b5
Modification
Acetyl (Protein N−term)
GlyGly (K) no new terminus
Oxidation (M)

20200921_KIZ_MUb4_GlyGly_10plex − Scan number: 3797

Gene Names	Charge	m/z	Mass	Retention time	PEP	Score	Precursor Intensity
NDUFB10	2	502.754	1003.493	12.238	0.1471	66.621	6306021

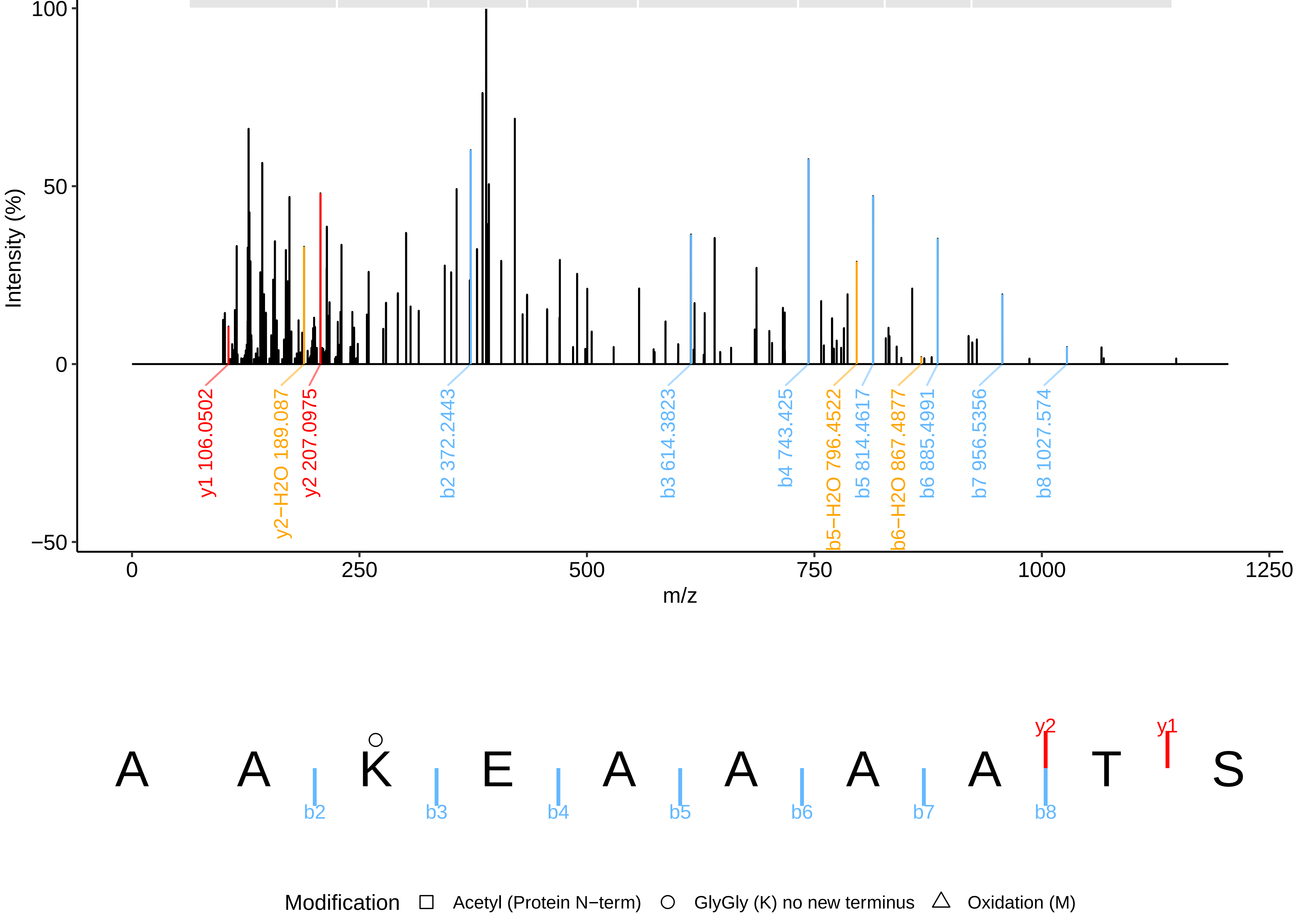

20200921_KIZ_MUb4_GlyGly_10plex − Scan number: 5715

Gene Names	Charge	m/z	Mass	Retention time	PEP	Score	Precursor Intensity
SDCBP	3	343.5331	1027.577	18.743	0.050847	58.286	8260092

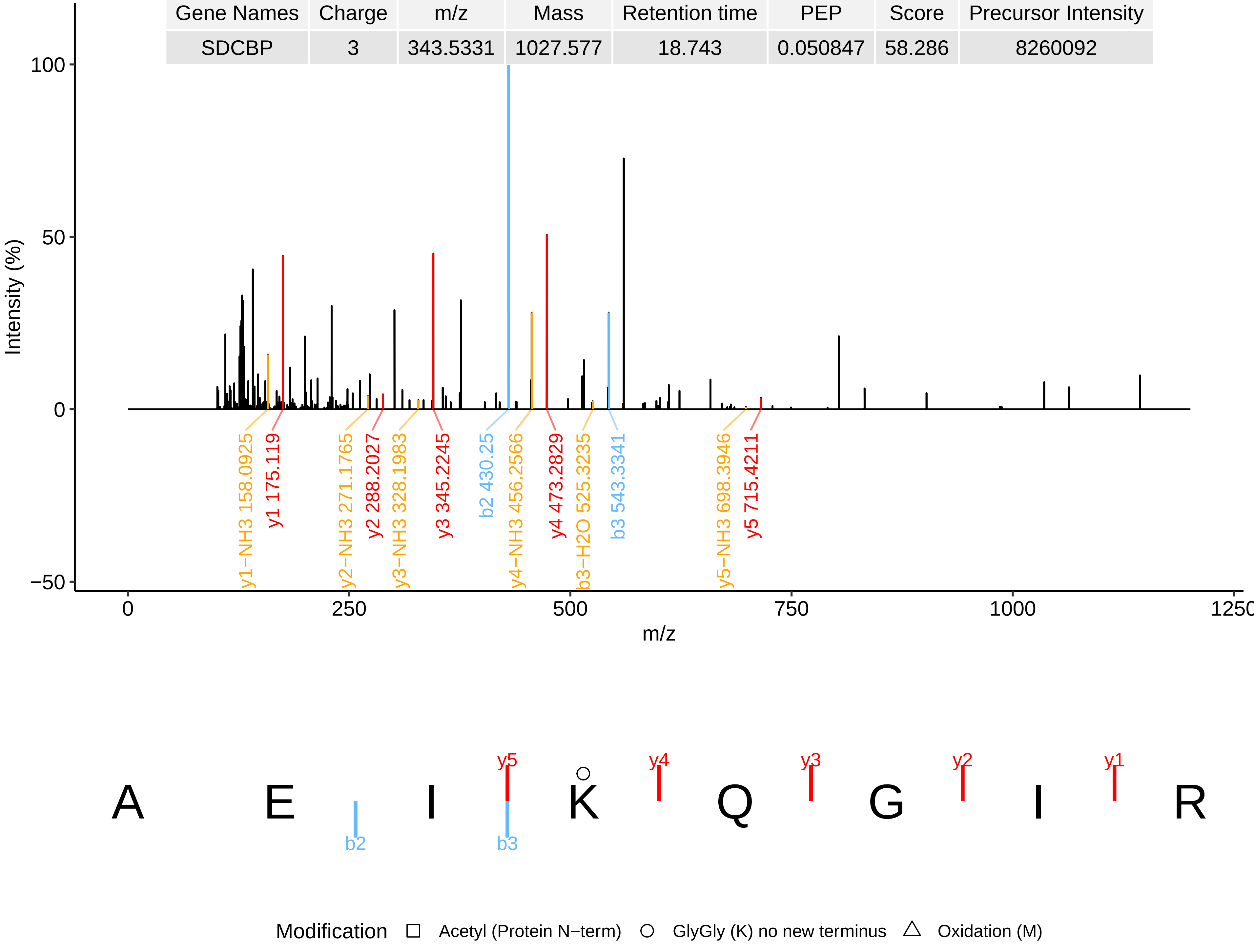

20200921_KIZ_MUb4_GlyGly_10plex − Scan number: 14372

Gene Names	Charge	m/z	Mass	Retention time	PEP	Score	Precursor Intensity
HTATIP2	2	637.341	1272.667	51.064	2.3245e−06	113.71	NA

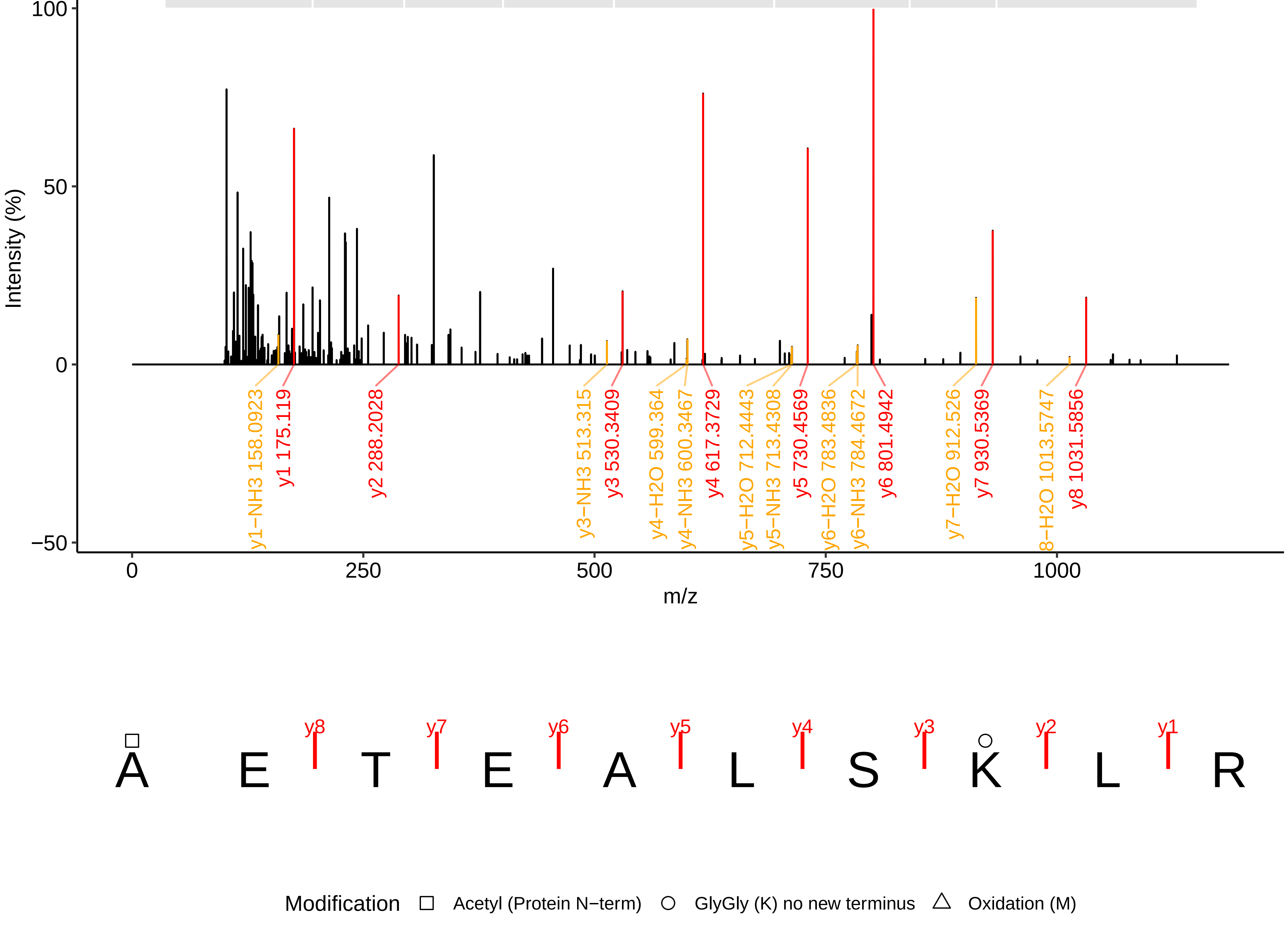

20200921_KIZ_MUb4_GlyGly_10plex_2 − Scan number: 13211

Gene Names	Charge	m/z	Mass	Retention time	PEP	Score	Precursor Intensity
HTATIP2	2	637.341	1272.667	51.249	0.14695	56.563	NA

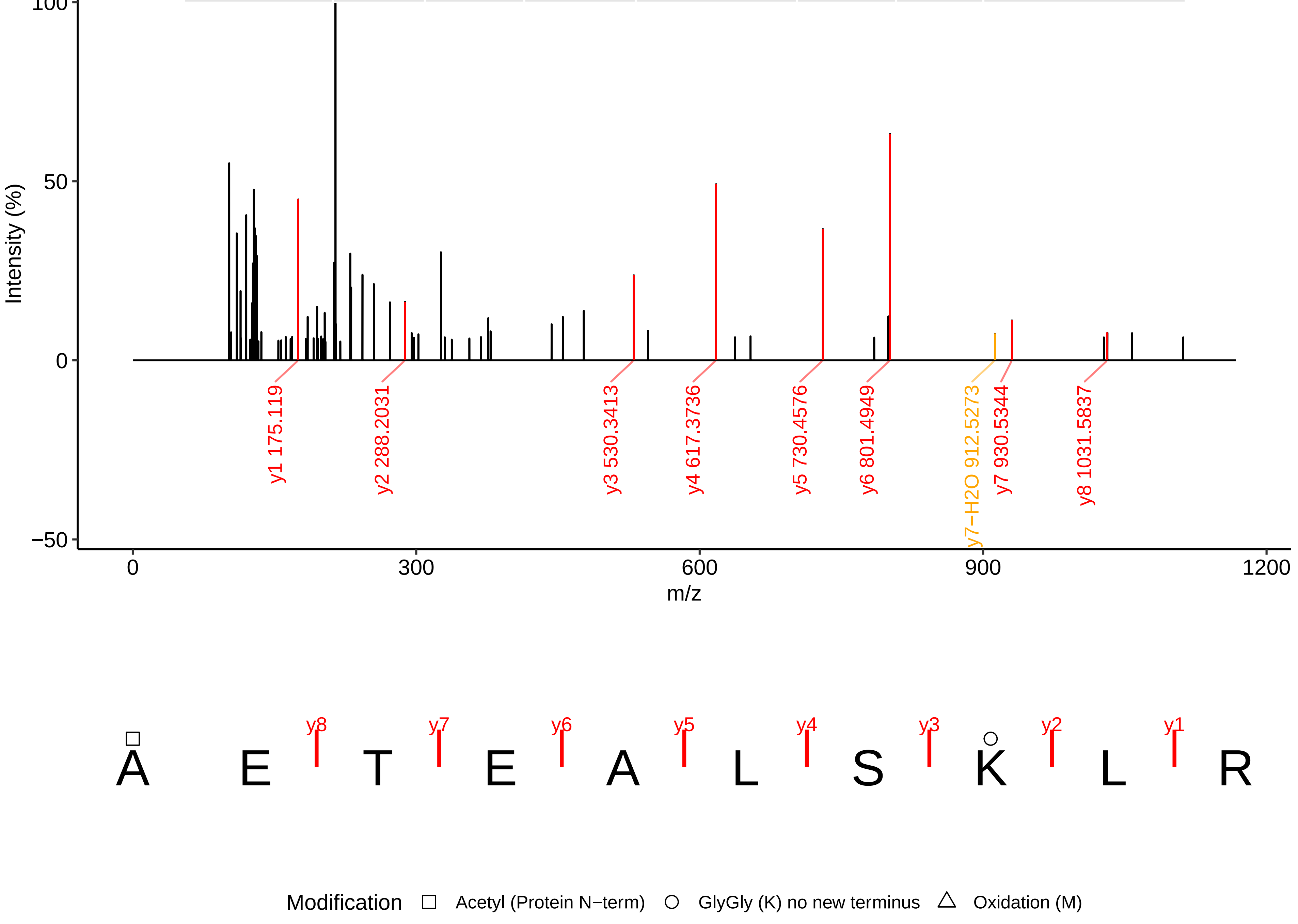

20200921_KIZ_MUb4_GlyGly_10plex − Scan number: 12815

Gene Names	Charge	m/z	Mass	Retention time	PEP	Score	Precursor Intensity
RAB9A	2	497.2718	992.5291	45.073	0.034269	49.358	8869669

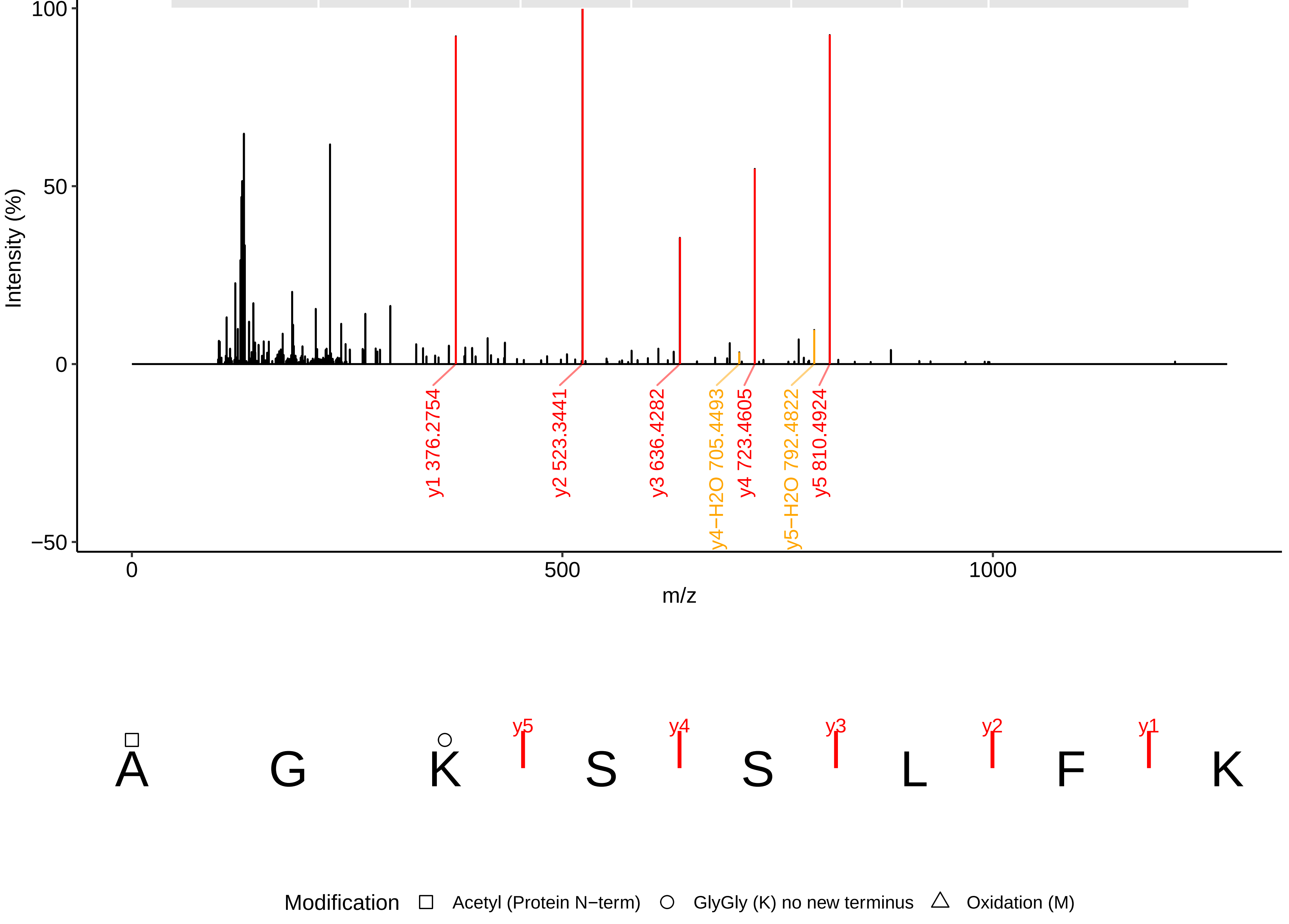

20200921_KIZ_MUb4_GlyGly_10plex_2 − Scan number: 11777

Gene Names	Charge	m/z	Mass	Retention time	PEP	Score	Precursor Intensity
RAB9A	2	497.2718	992.5291	45.413	0.083662	43.027	851353.7

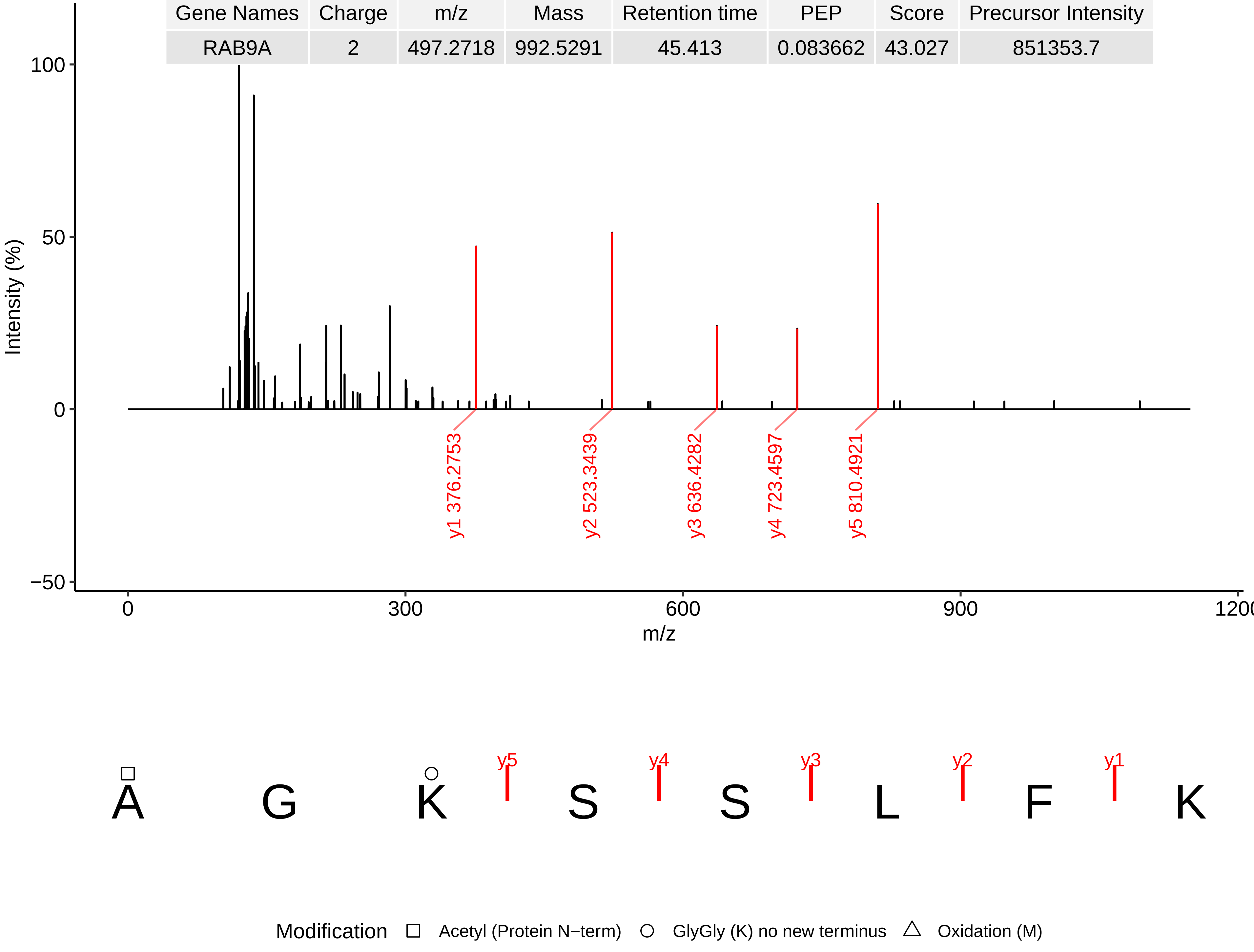

20200924_KIZ_MUb5_GlyGly_1 − Scan number: 11868

Gene Names	Charge	m/z	Mass	Retention time	PEP	Score	Precursor Intensity
RAB9A	2	497.2718	992.5291	38.518	0.05801	57.559	5586492

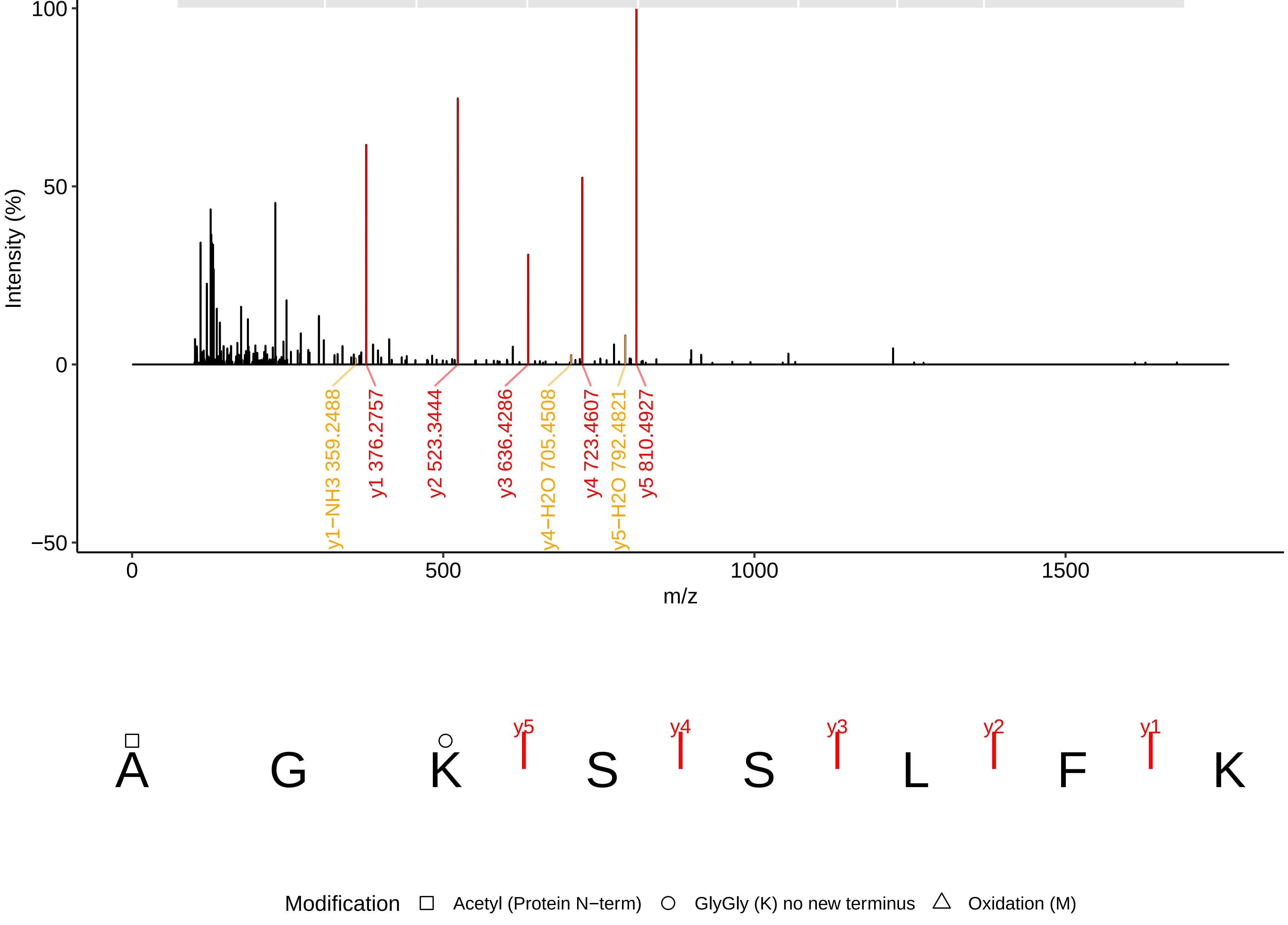

20200921_KIZ_MUb4_GlyGly_10plex − Scan number: 9927

Gene Names	Charge	m/z	Mass	Retention time	PEP	Score	Precursor Intensity
LITAF	3	345.8697	1034.587	34.177	0.011239	101.64	10457883

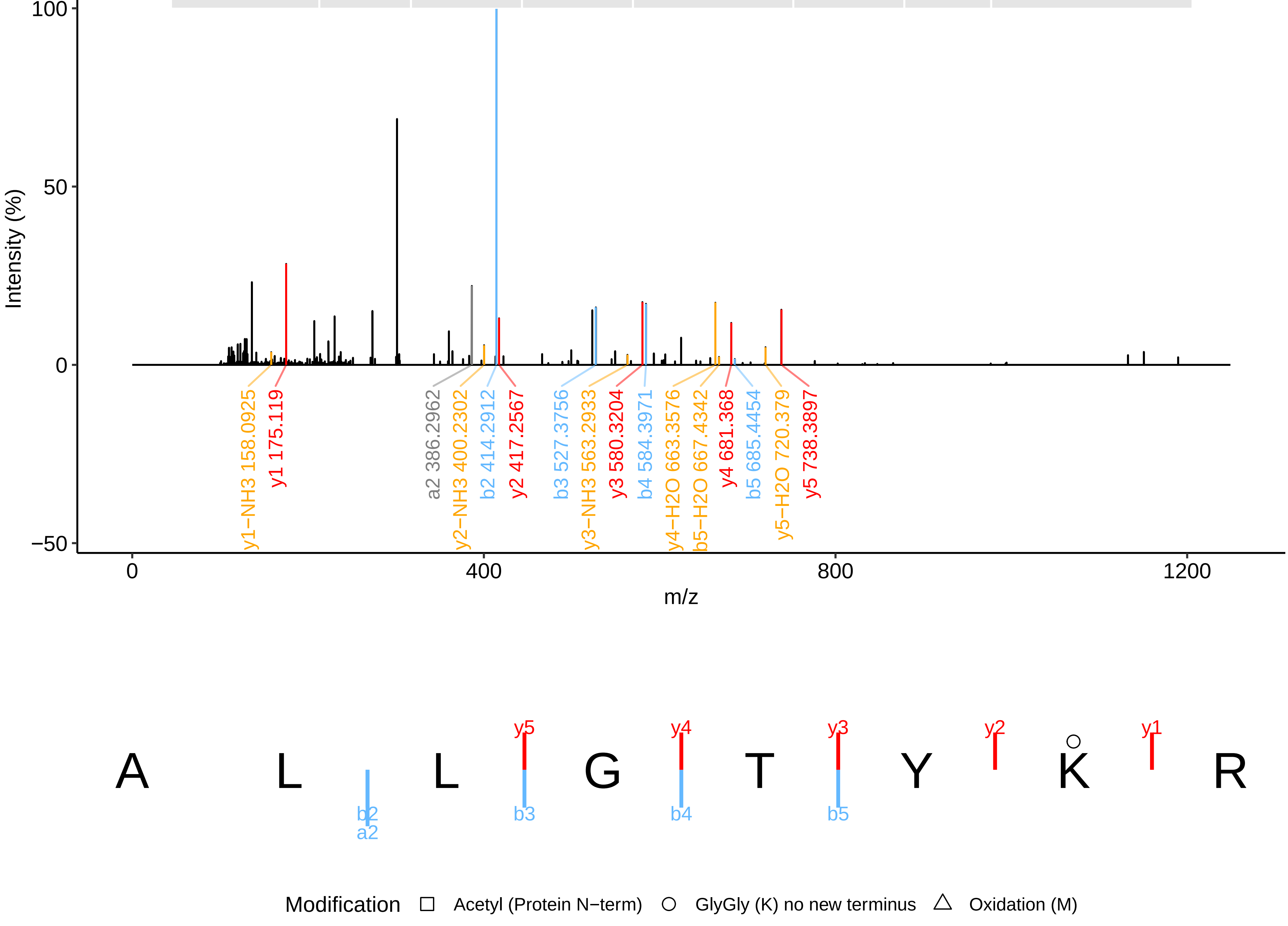

20200921_KIZ_MUb4_GlyGly_10plex_2 − Scan number: 9217

Gene Names	Charge	m/z	Mass	Retention time	PEP	Score	Precursor Intensity
LITAF	3	345.8697	1034.587	34.979	0.0021661	114.6	4099072

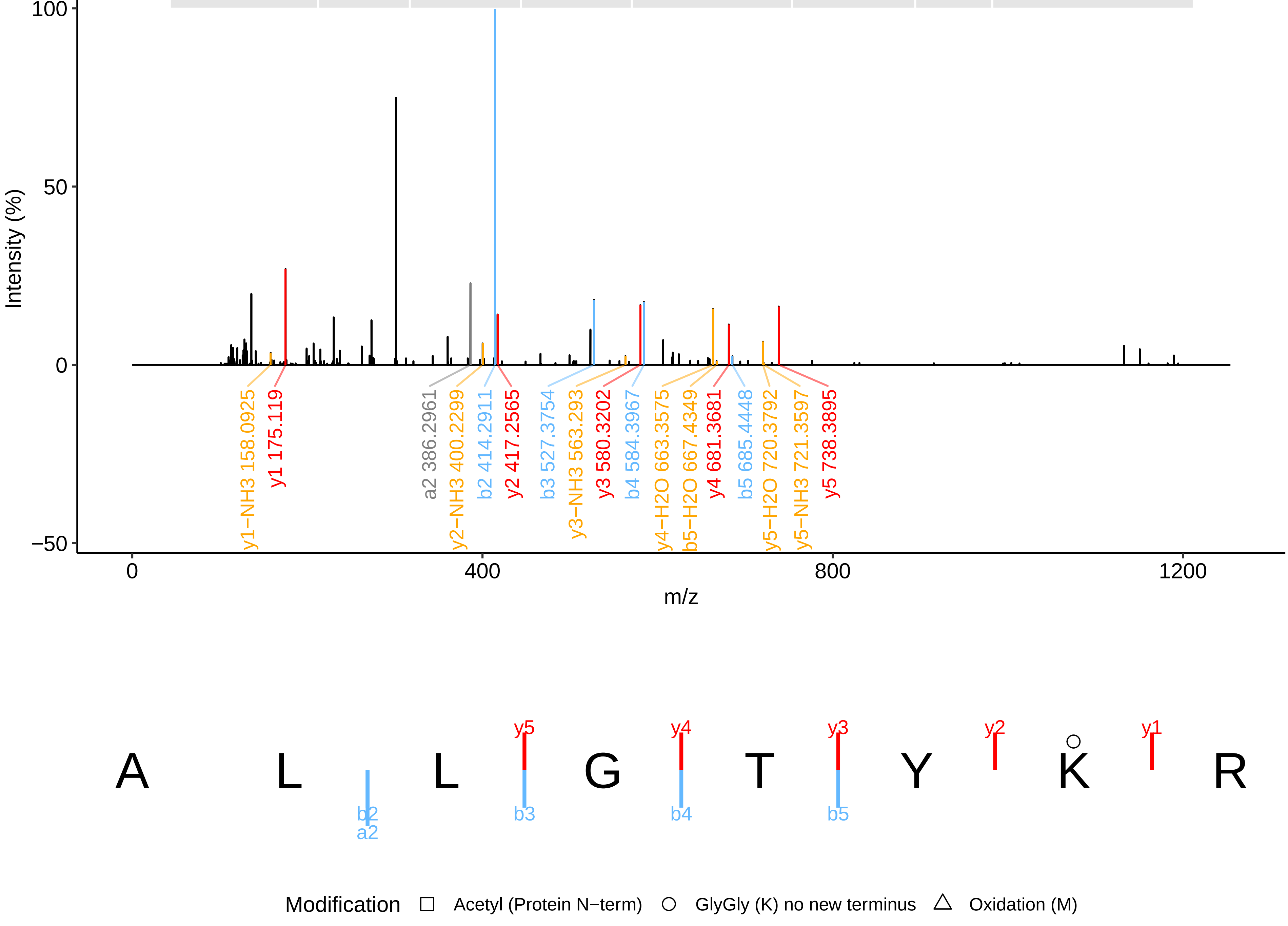

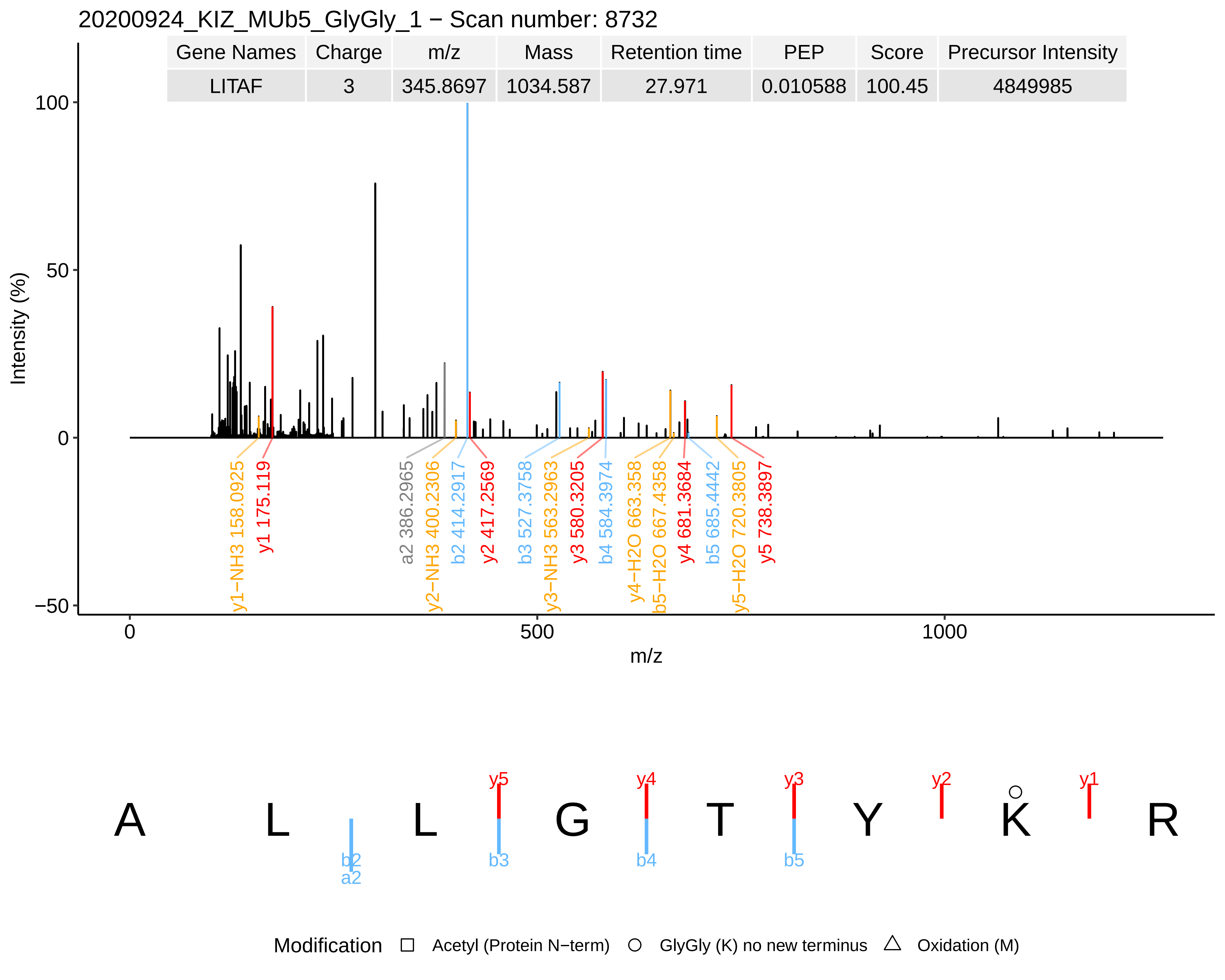

20200924_KIZ_MUb5_GlyGly_1 − Scan number: 8732
Gene Names | Charge | m/z | Mass | Retention time | PEP | Score | Precursor Intensity
LITAF | 3 | 345.8697 | 1034.587 | 27.971 | 0.010588 | 100.45 | 4849985
Intensity (%)
100
50
0
−50
0
500
1000
m/z
y1−NH3 158.0925
y1 175.119
a2 386.2965
y2−NH3 400.2306
b2 414.2917
y2 417.2569
b3 527.3758
y3−NH3 563.2963
y3 580.3205
b4 584.3974
y4−H2O 663.358
b5−H2O 667.4358
y4 681.3684
b5 685.4442
y5−H2O 720.3805
y5 738.3897
A L L G T Y K R
y5 y4 y3 y2 y1
b2 a2 b3 b4 b5
Modification
Acetyl (Protein N−term)
GlyGly (K) no new terminus
Oxidation (M)

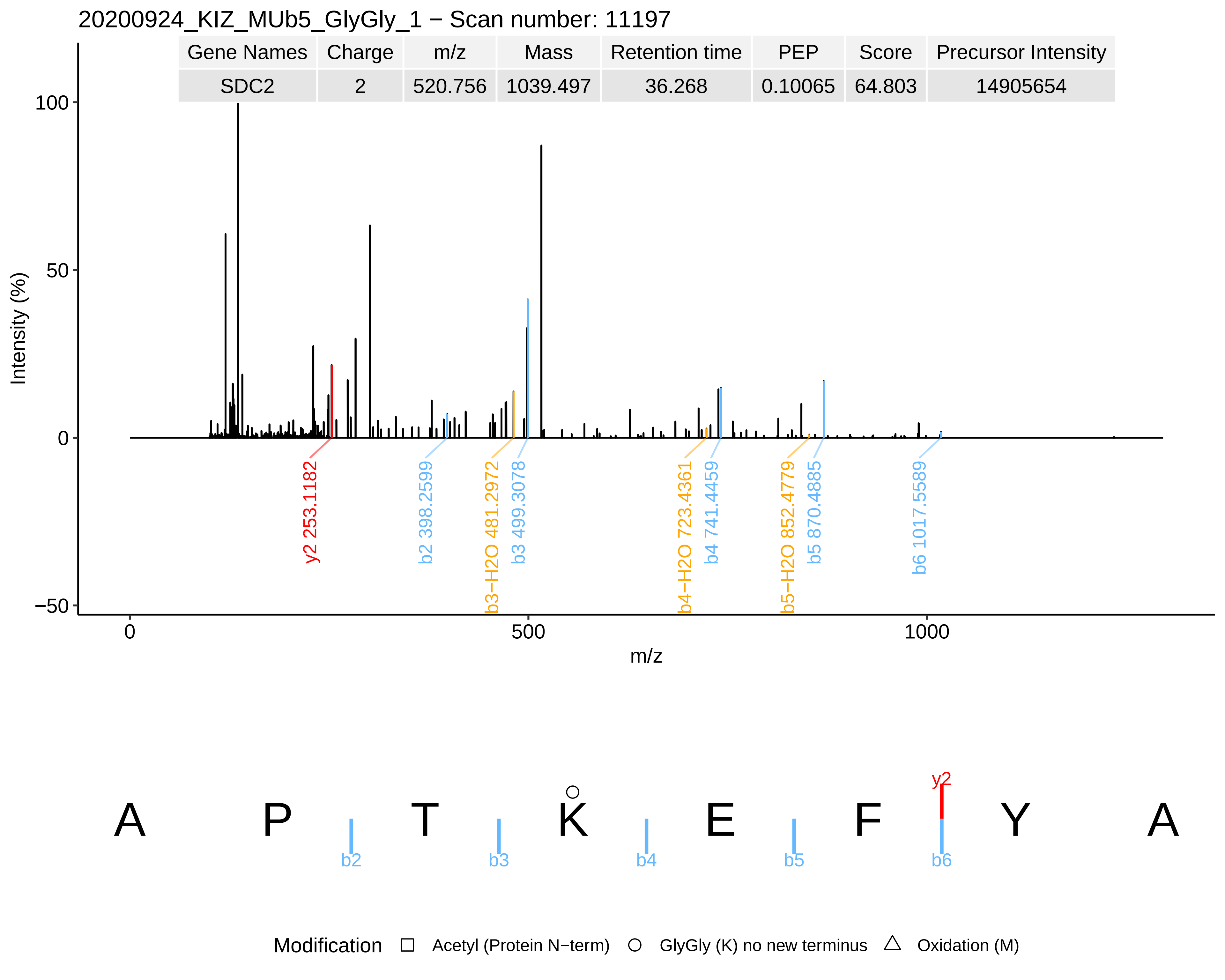

20200924_KIZ_MUb5_GlyGly_1 − Scan number: 11197
Gene Names | Charge | m/z | Mass | Retention time | PEP | Score | Precursor Intensity
SDC2 | 2 | 520.756 | 1039.497 | 36.268 | 0.10065 | 64.803 | 14905654
Intensity (%)
m/z
y2 253.1182
b2 398.2599
b3−H2O 481.2972
b3 499.3078
b4−H2O 723.4361
b4 741.4459
b5−H2O 852.4779
b5 870.4885
b6 1017.5589
A P T K E F Y A
b2 b3 b4 b5 b6 y2
Modification
Acetyl (Protein N−term)
GlyGly (K) no new terminus
Oxidation (M)

20200921_KIZ_MUb4_GlyGly_10plex − Scan number: 6508

Gene Names	Charge	m/z	Mass	Retention time	PEP	Score	Precursor Intensity
BAG2	2	500.2827	998.5509	21.558	0.00076489	75.311	6769867

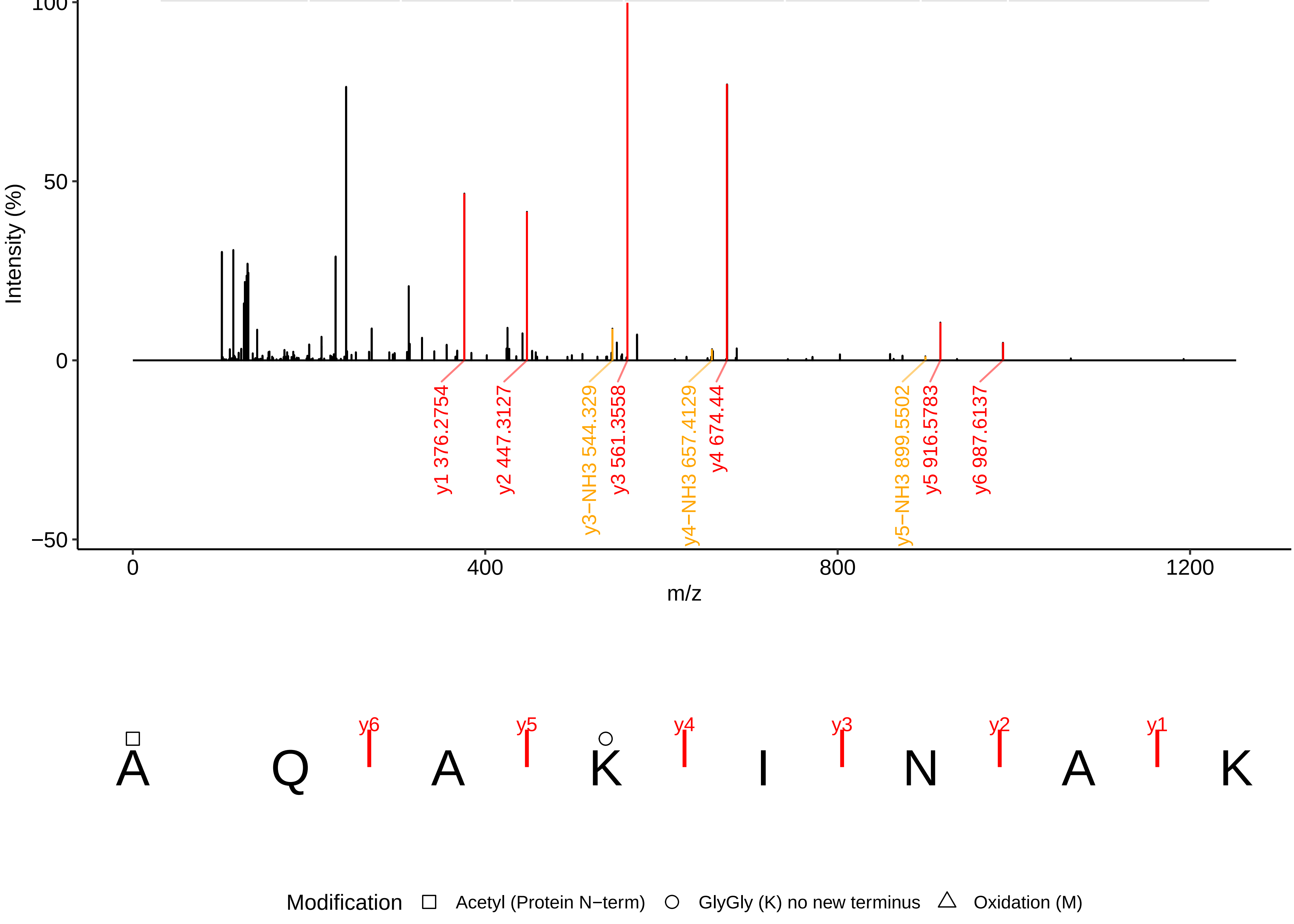

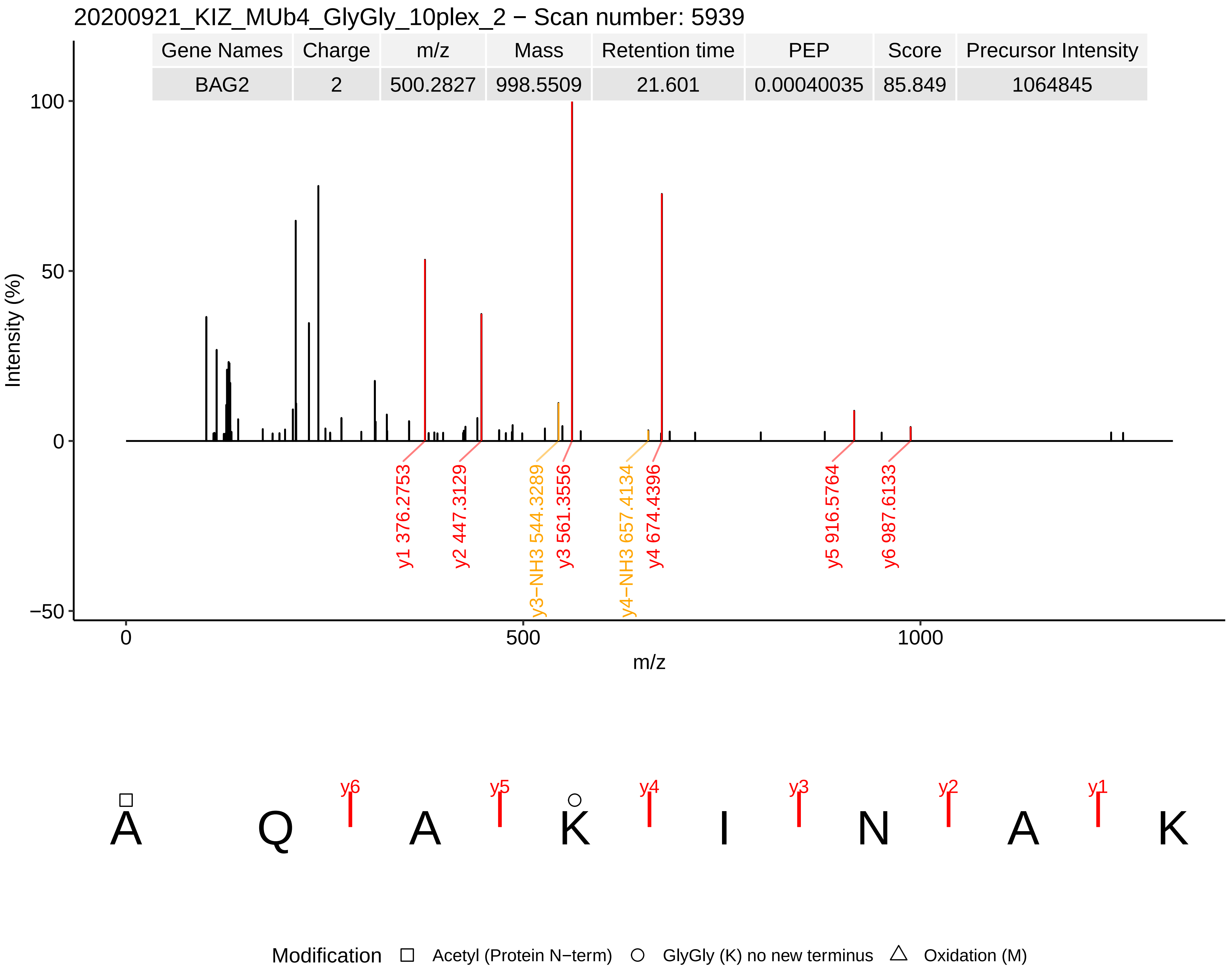

20200921_KIZ_MUb4_GlyGly_10plex_2 − Scan number: 5939
Gene Names | Charge | m/z | Mass | Retention time | PEP | Score | Precursor Intensity
BAG2 | 2 | 500.2827 | 998.5509 | 21.601 | 0.00040035 | 85.849 | 1064845
Intensity (%)
m/z
y1 376.2753
y2 447.3129
y3−NH3 544.3289
y3 561.3556
y4−NH3 657.4134
y4 674.4396
y5 916.5764
y6 987.6133
A Q A K I N A K
y6 y5 y4 y3 y2 y1
Modification
Acetyl (Protein N−term)
GlyGly (K) no new terminus
Oxidation (M)

20200924_KIZ_MUb5_GlyGly_1 − Scan number: 5668

Gene Names	Charge	m/z	Mass	Retention time	PEP	Score	Precursor Intensity
BAG2	2	500.2827	998.5509	17.685	0.00028391	88.734	4234881

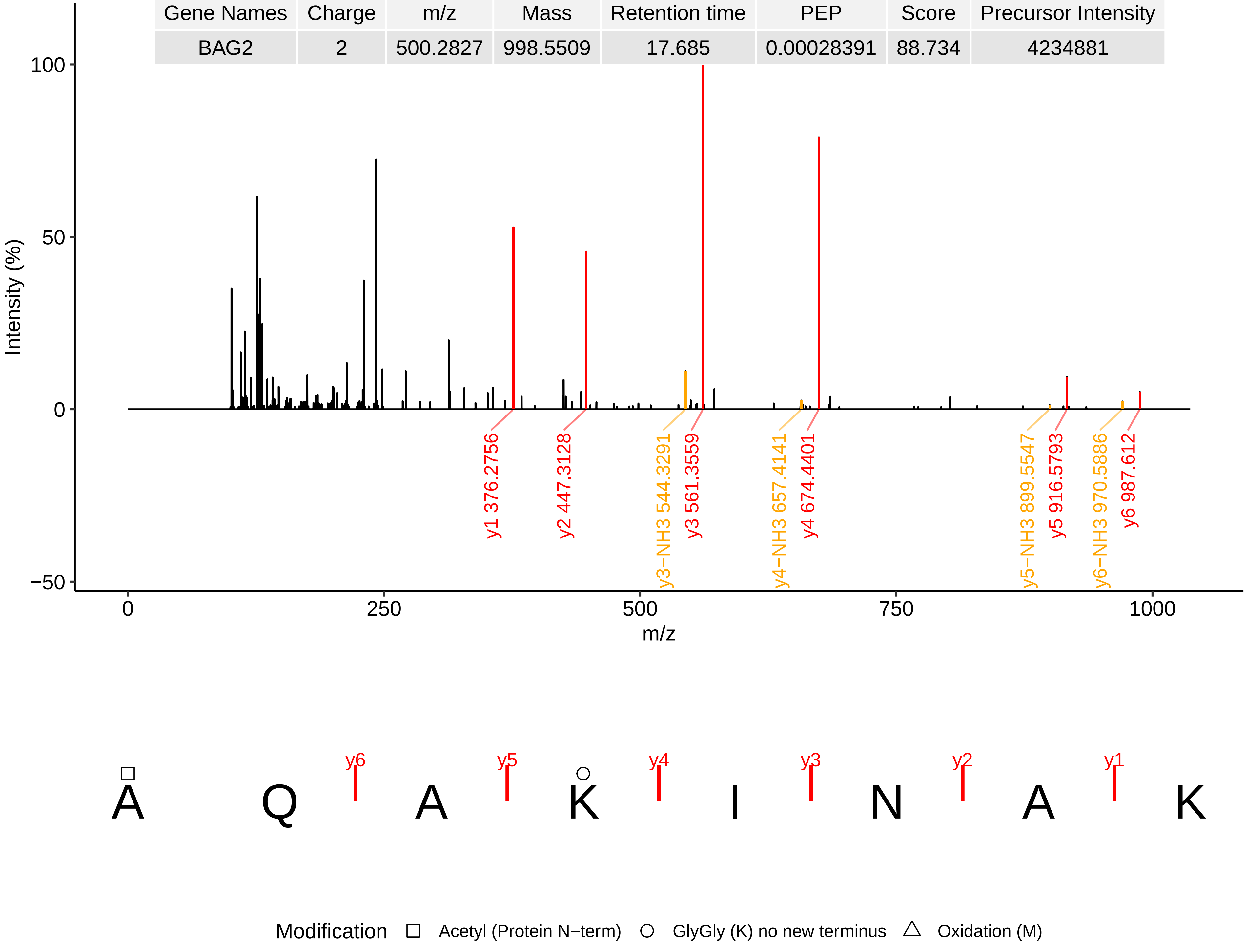

20200924_KIZ_MUb5_GlyGly_2 − Scan number: 5278

Gene Names	Charge	m/z	Mass	Retention time	PEP	Score	Precursor Intensity
BAG2	2	500.2827	998.5509	19.563	0.0005943	85.849	3434400

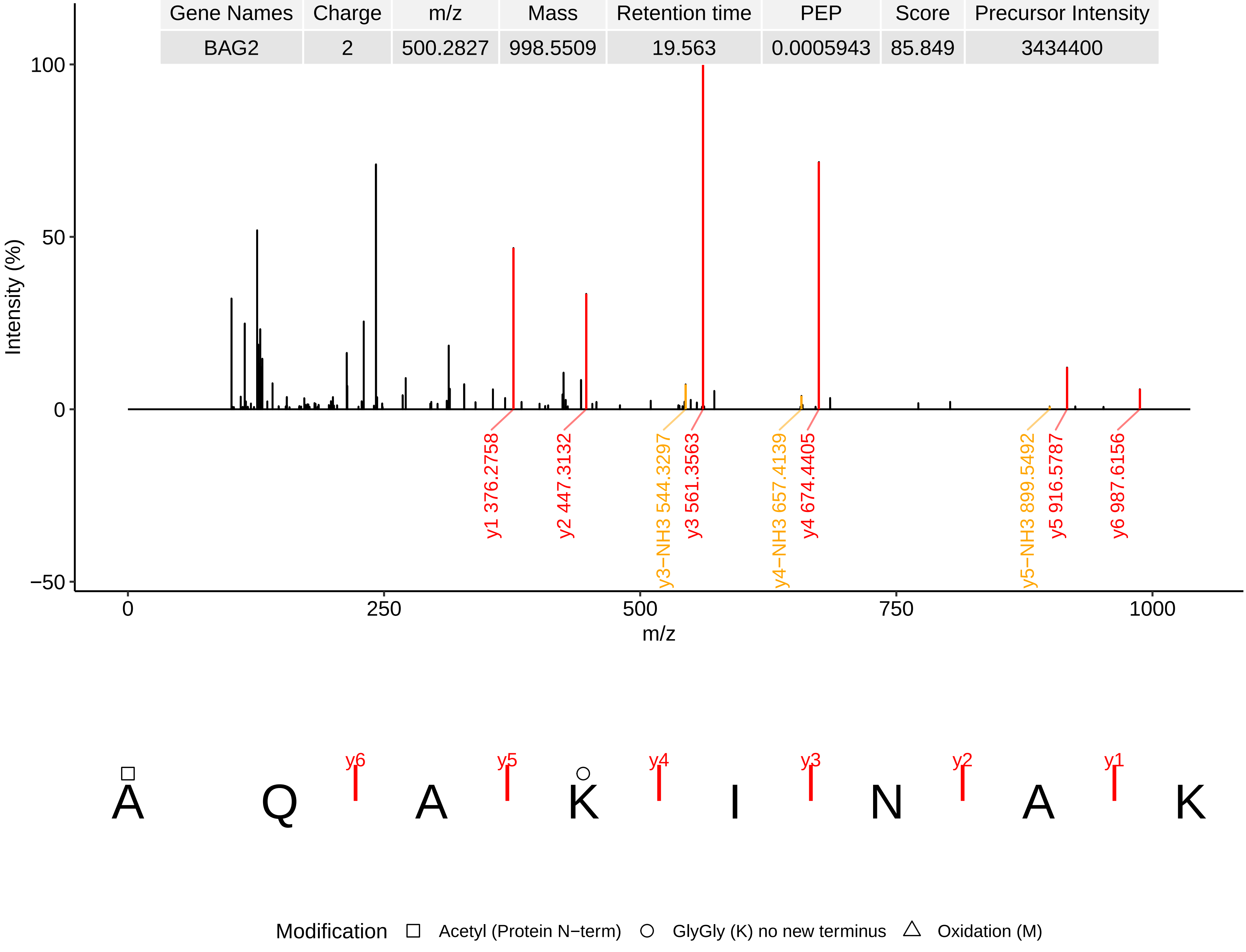

20201118_KIZ_MUb6_GlyGly_1 − Scan number: 5413

Gene Names	Charge	m/z	Mass	Retention time	PEP	Score	Precursor Intensity
BAG2	2	500.2827	998.5509	17.139	4.5263e−05	118.03	13808178

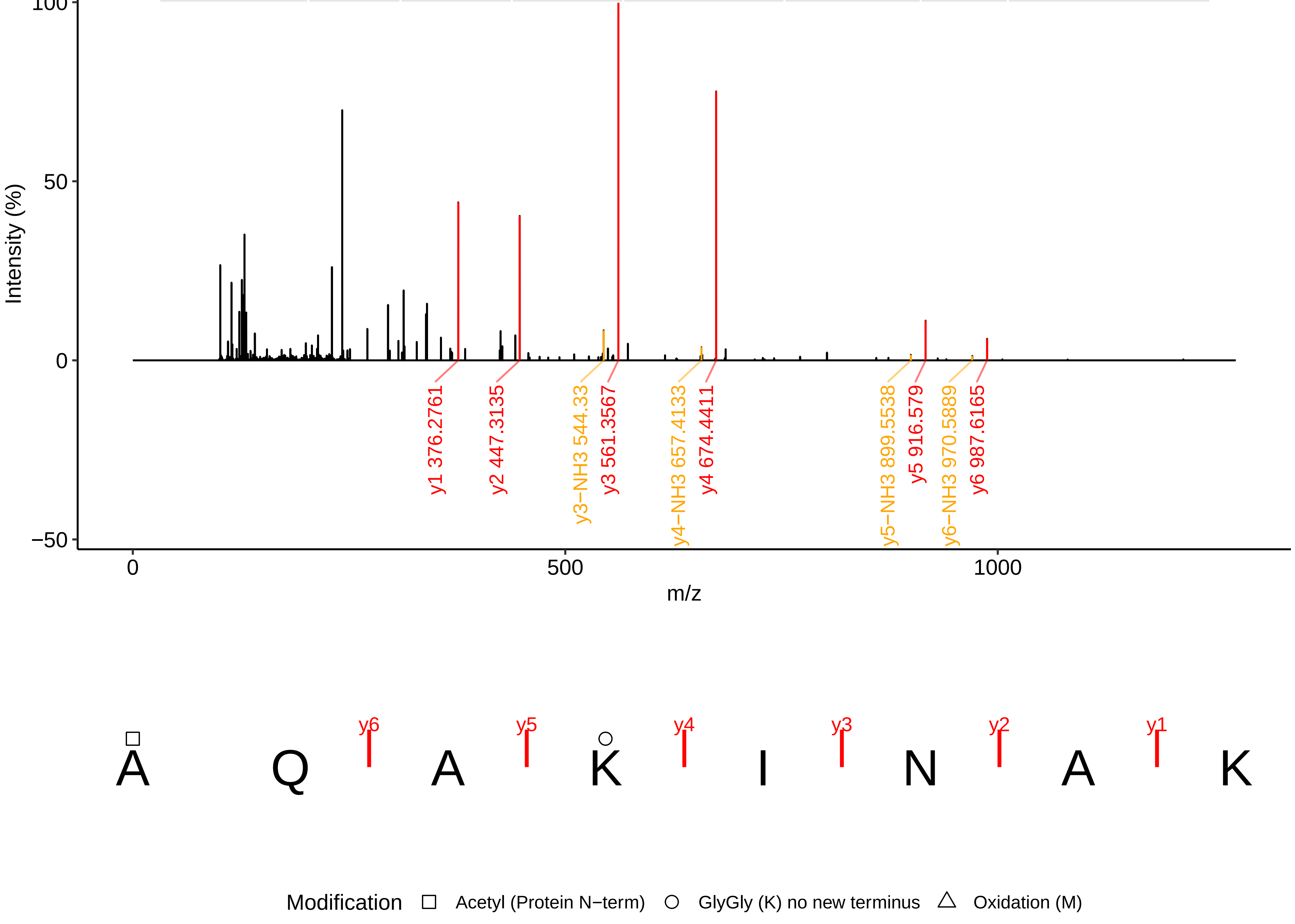

20201118_KIZ_MUb6_GlyGly_2 − Scan number: 6450

Gene Names	Charge	m/z	Mass	Retention time	PEP	Score	Precursor Intensity
BAG2	2	500.2827	998.5509	22.096	0.0024691	85.849	3493825

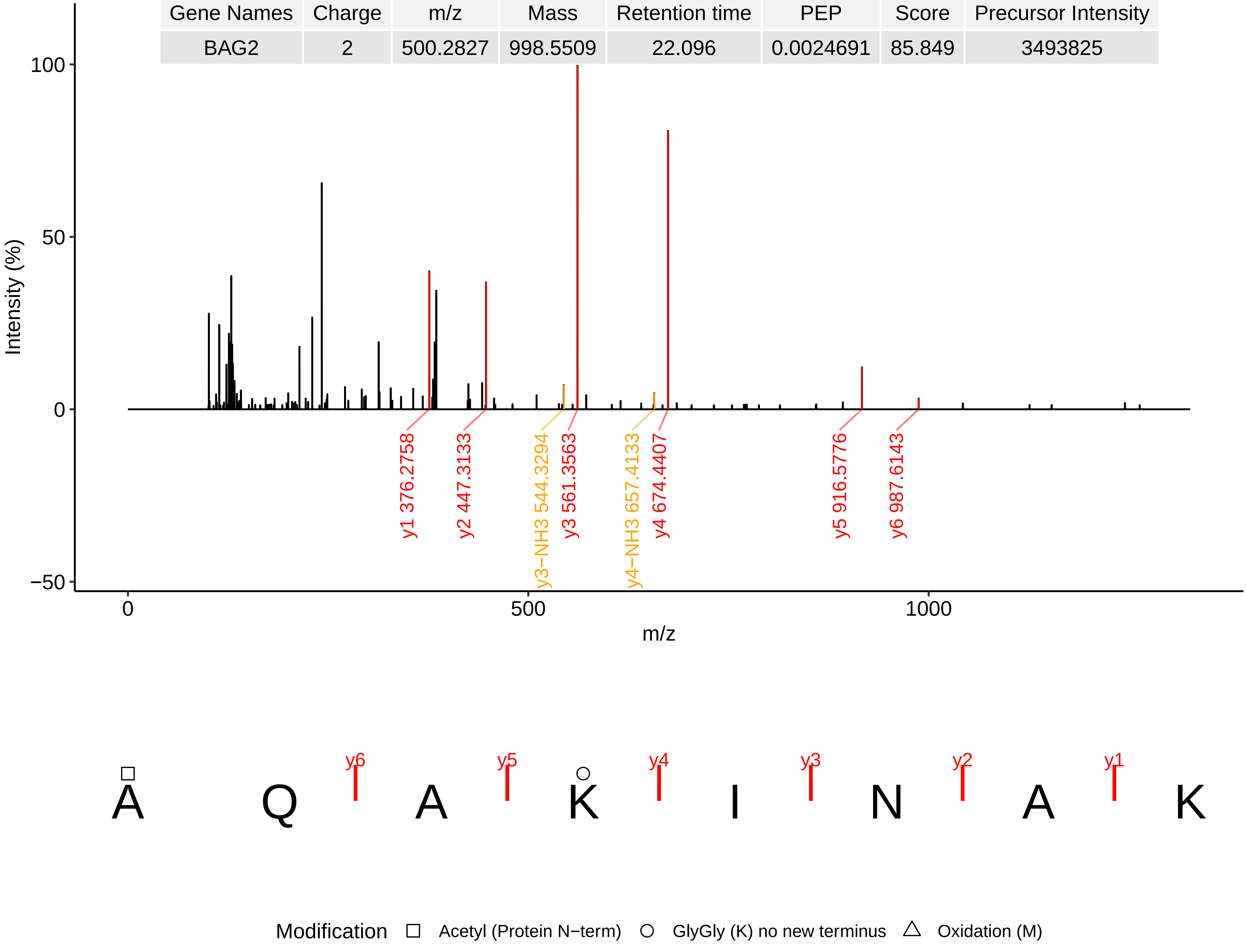

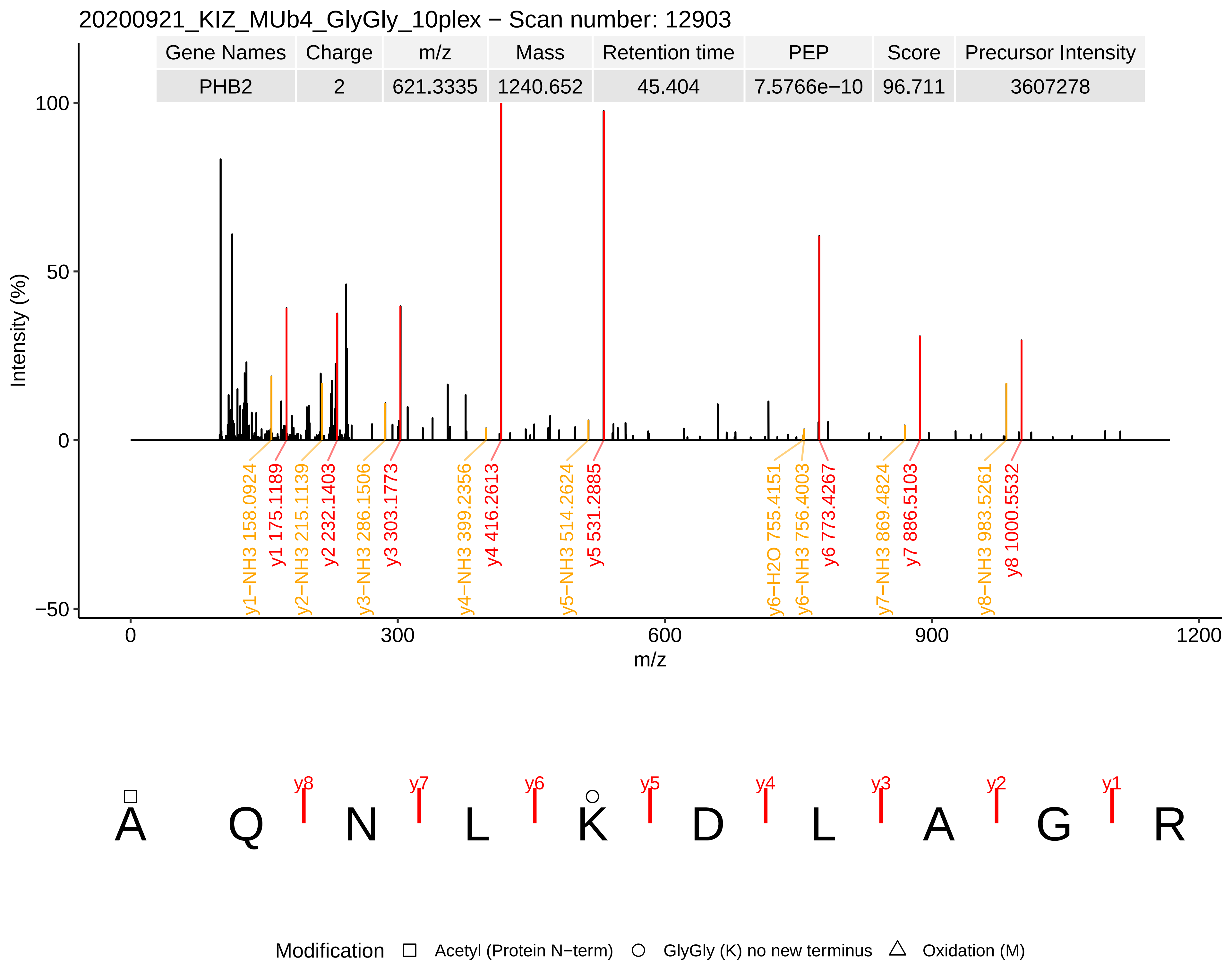
20200921_KIZ_MUb4_GlyGly_10plex − Scan number: 12903
Gene Names | Charge | m/z | Mass | Retention time | PEP | Score | Precursor Intensity
PHB2 | 2 | 621.3335 | 1240.652 | 45.404 | 7.5766e−10 | 96.711 | 3607278
Intensity (%)
m/z
y1−NH3 158.0924
y1 175.1189
y2−NH3 215.1139
y2 232.1403
y3−NH3 286.1506
y3 303.1773
y4−NH3 399.2356
y4 416.2613
y5−NH3 514.2624
y5 531.2885
y6−H2O 755.4151
y6−NH3 756.4003
y6 773.4267
y7−NH3 869.4824
y7 886.5103
y8−NH3 983.5261
y8 1000.5532
A Q N L K D L A G R
y8 y7 y6 y5 y4 y3 y2 y1
Modification
Acetyl (Protein N−term)
GlyGly (K) no new terminus
Oxidation (M)

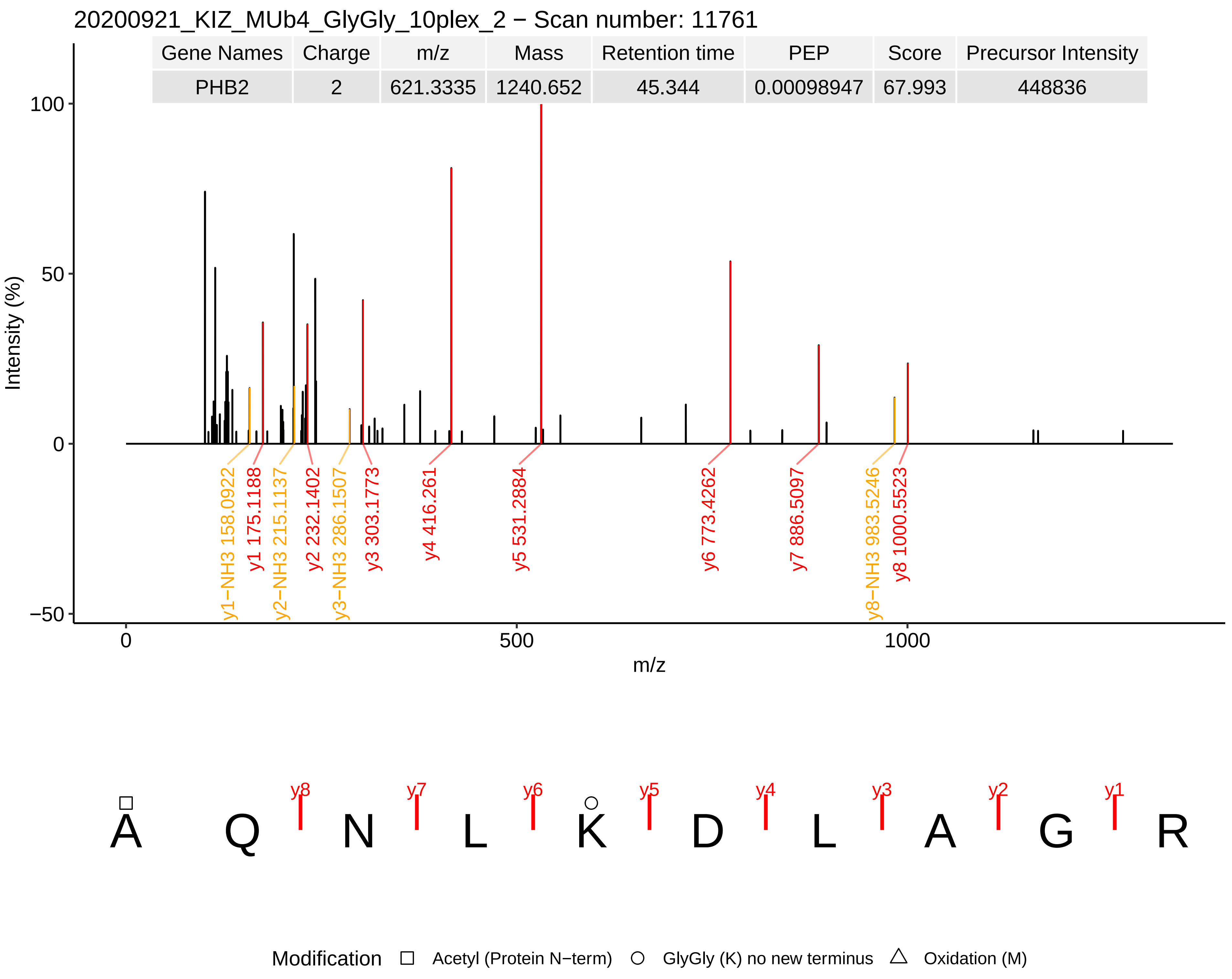
20200921_KIZ_MUb4_GlyGly_10plex_2 − Scan number: 11761
Gene Names | Charge | m/z | Mass | Retention time | PEP | Score | Precursor Intensity
PHB2 | 2 | 621.3335 | 1240.652 | 45.344 | 0.00098947 | 67.993 | 448836
Intensity (%)
m/z
y1−NH3 158.0922
y1 175.1188
y2−NH3 215.1137
y2 232.1402
y3−NH3 286.1507
y3 303.1773
y4 416.261
y5 531.2884
y6 773.4262
y7 886.5097
y8−NH3 983.5246
y8 1000.5523
A Q N L K D L A G R
y8 y7 y6 y5 y4 y3 y2 y1
Modification
Acetyl (Protein N−term)
GlyGly (K) no new terminus
Oxidation (M)

20200924_KIZ_MUb5_GlyGly_1 − Scan number: 12018

Gene Names	Charge	m/z	Mass	Retention time	PEP	Score	Precursor Intensity
PHB2	2	621.3335	1240.652	39.039	1.678e−06	95.741	13418270

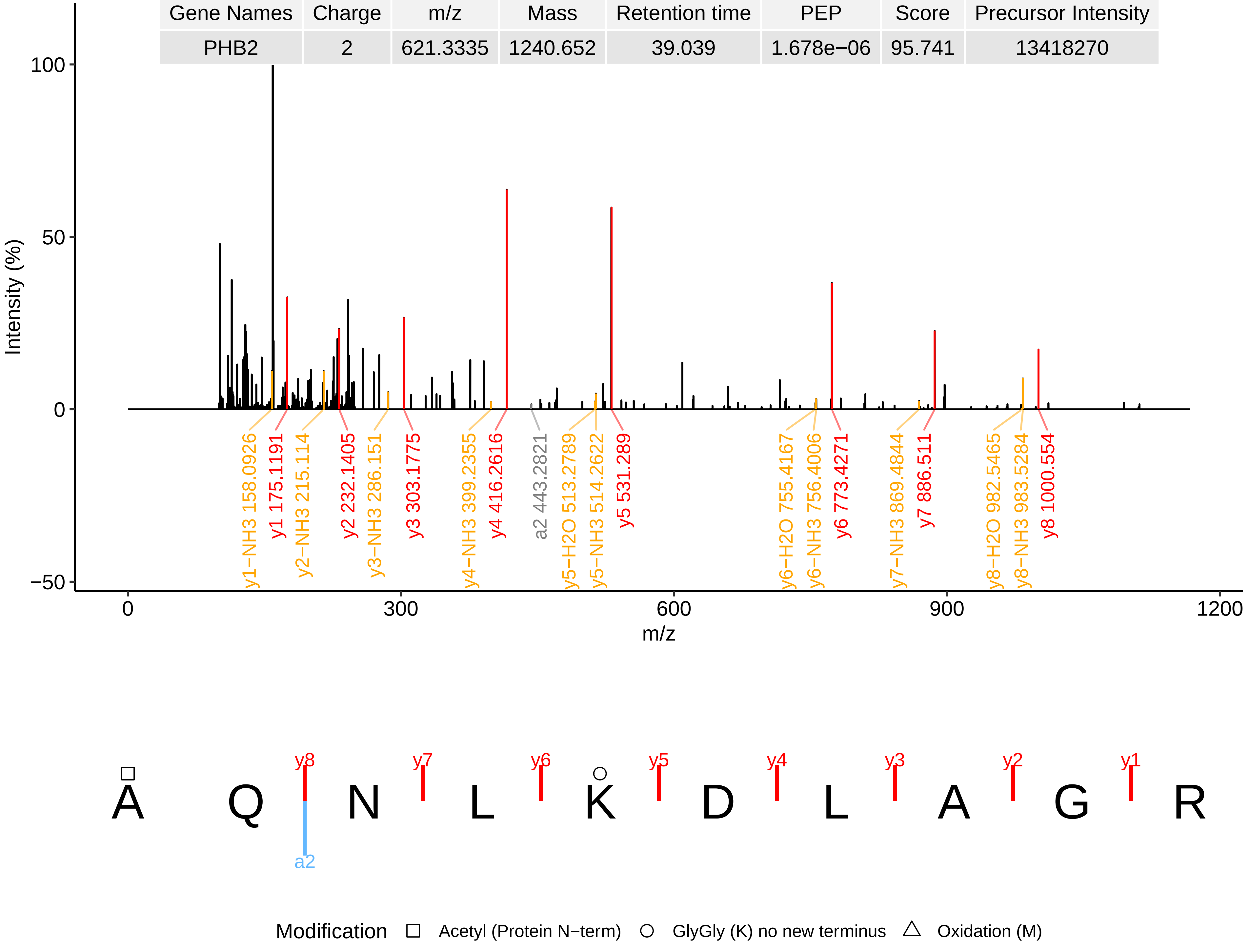

20200924_KIZ_MUb5_GlyGly_2 − Scan number: 11261

Gene Names	Charge	m/z	Mass	Retention time	PEP	Score	Precursor Intensity
PHB2	2	621.3335	1240.652	42.069	5.5155e−10	116.37	5233087

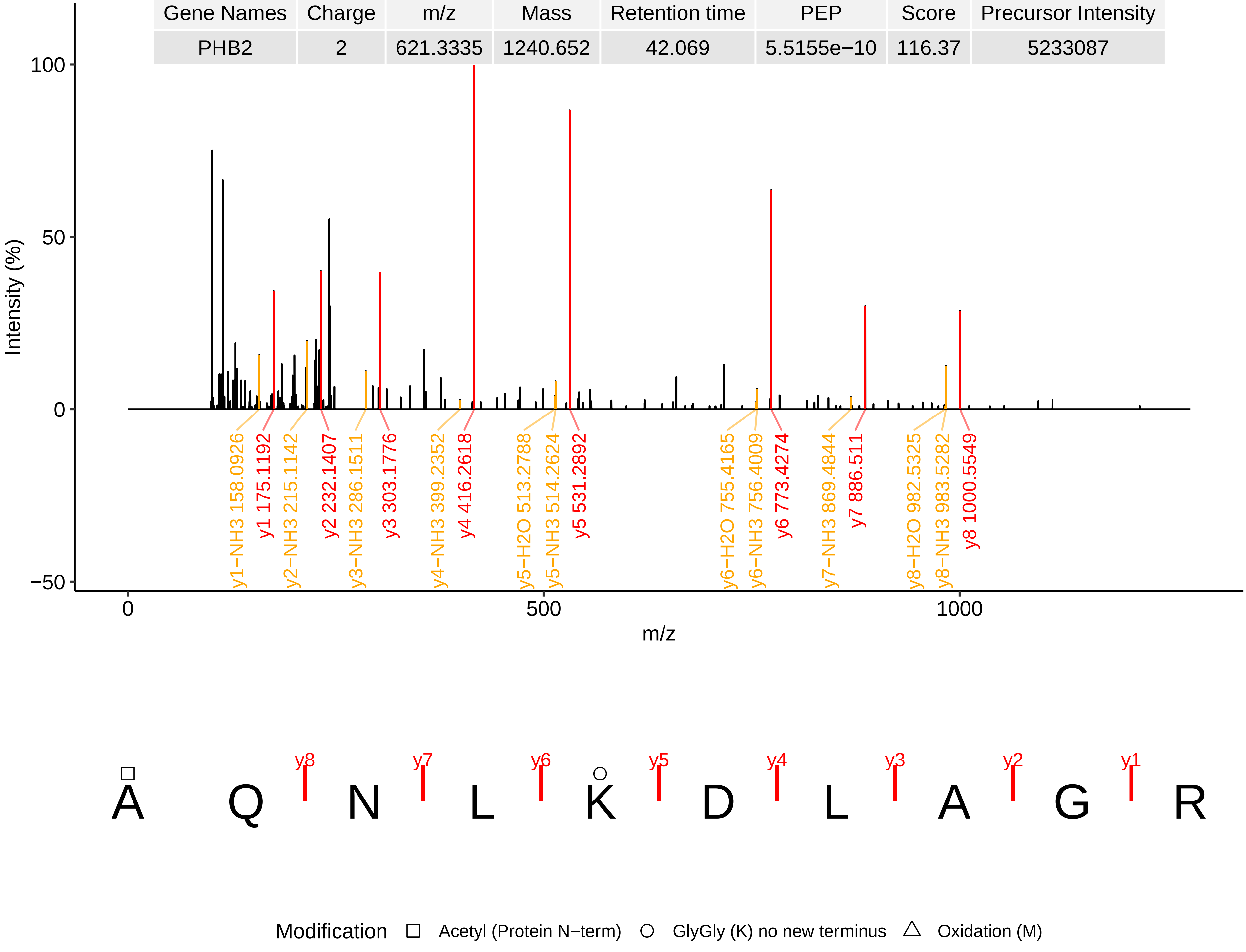

20200921_KIZ_MUb4_GlyGly_10plex − Scan number: 2595

Gene Names	Charge	m/z	Mass	Retention time	PEP	Score	Precursor Intensity
OGFR	3	341.1773	1020.51	8.1668	0.0060077	139.92	6414159

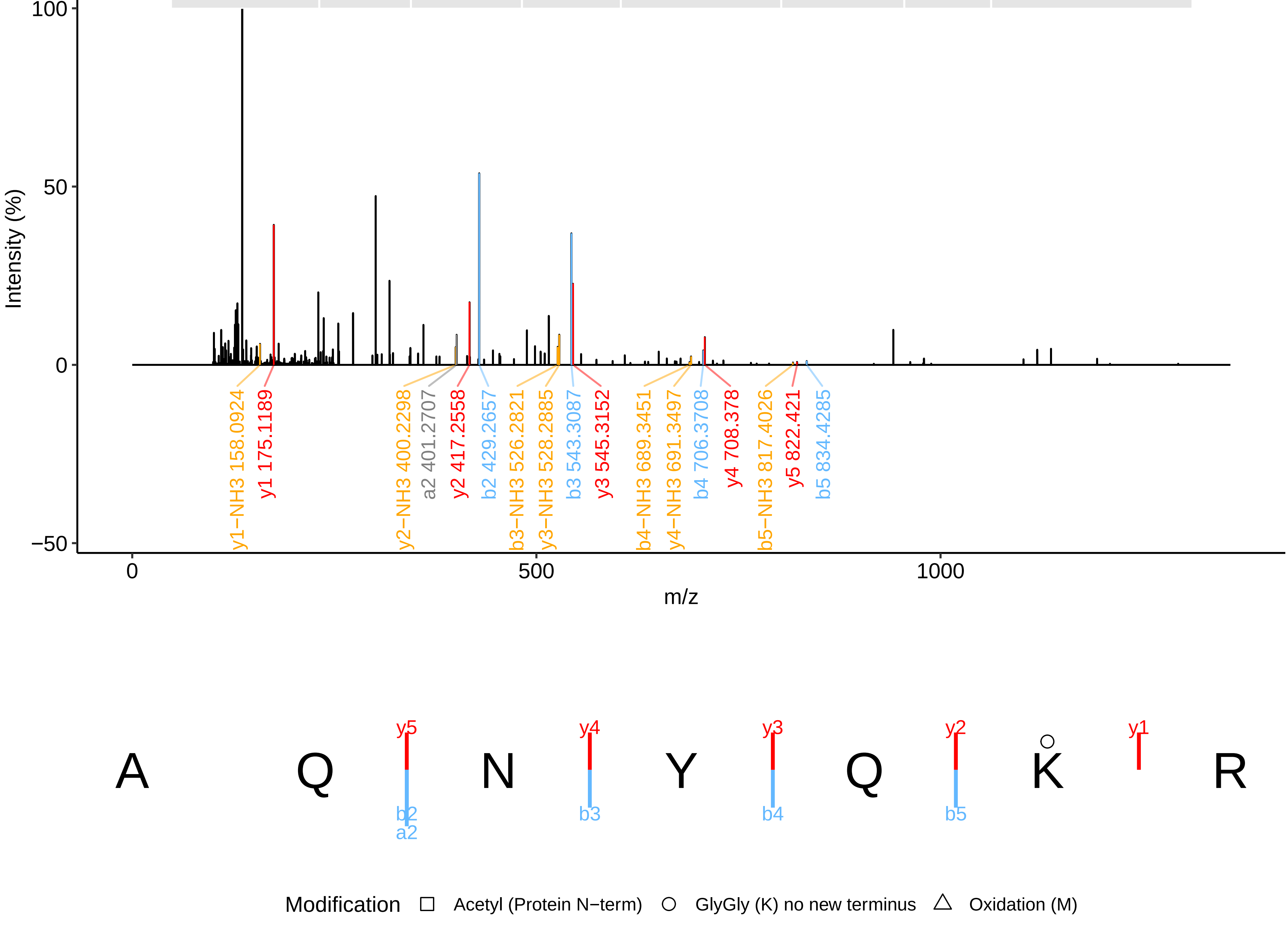

20200921_KIZ_MUb4_GlyGly_10plex_2 − Scan number: 3963

Gene Names	Charge	m/z	Mass	Retention time	PEP	Score	Precursor Intensity
ODR4	3	334.1893	999.5461	13.653	0.03999	87.251	1699530

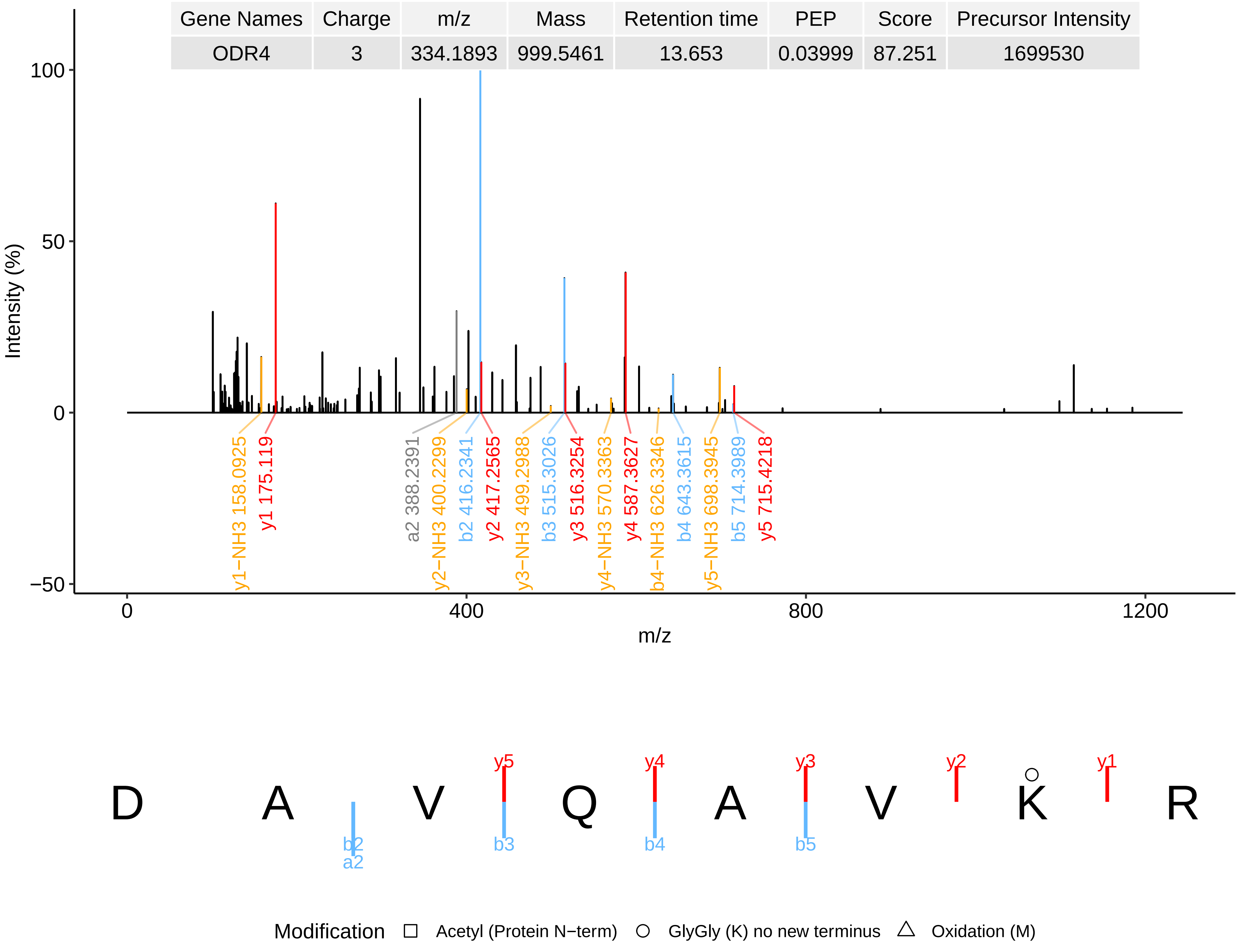

20200924_KIZ_MUb5_GlyGly_1 − Scan number: 2543

Gene Names	Charge	m/z	Mass	Retention time	PEP	Score	Precursor Intensity
EHBP1L1	3	342.837	1025.489	7.9035	0.098621	58.815	4018725

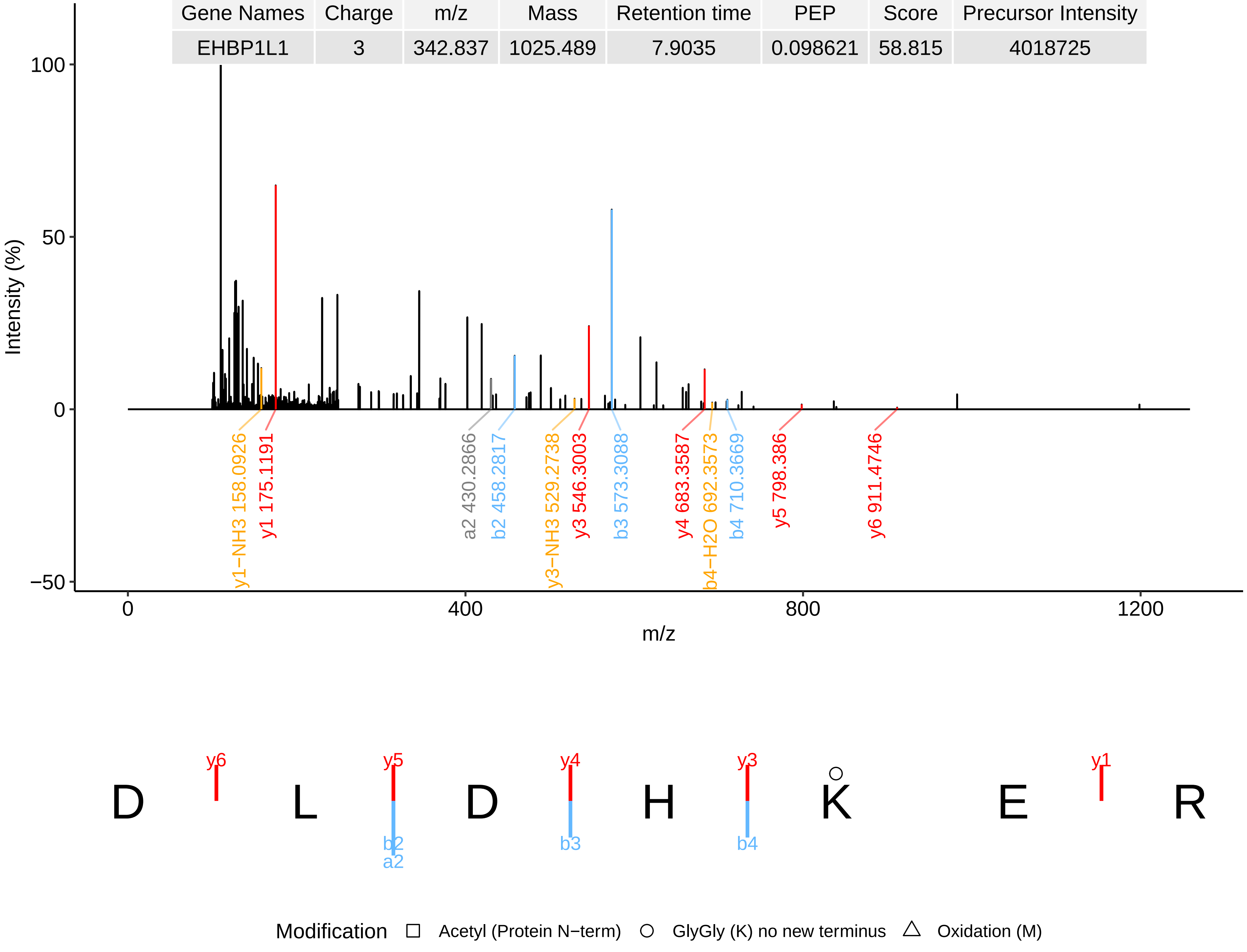

20200921_KIZ_MUb4_GlyGly_10plex − Scan number: 5994

Gene Names	Charge	m/z	Mass	Retention time	PEP	Score	Precursor Intensity
AAAS	3	333.1857	996.5352	19.686	0.011676	78.516	5198760

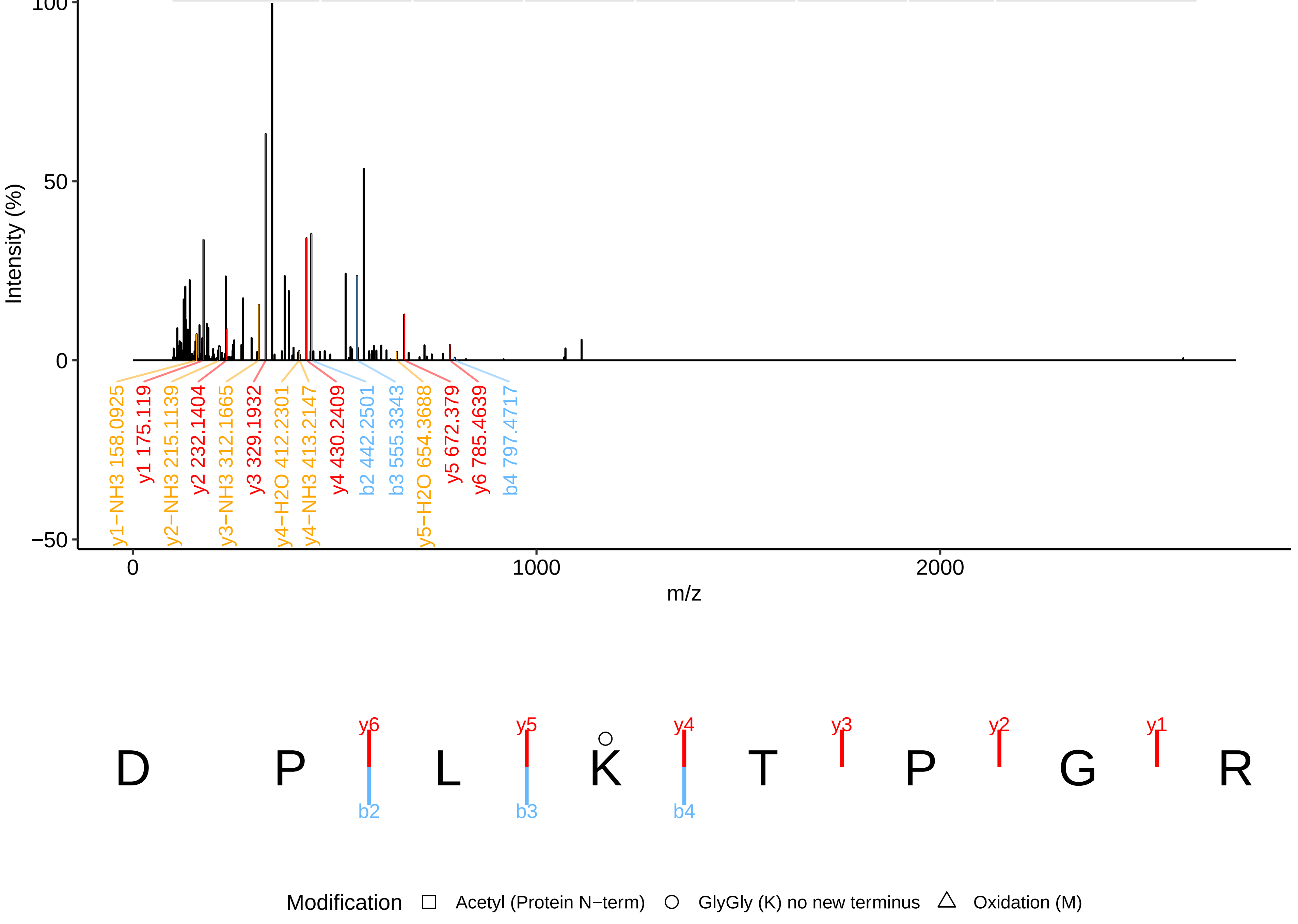

20200921_KIZ_MUb4_GlyGly_10plex_2 − Scan number: 5473

Gene Names	Charge	m/z	Mass	Retention time	PEP	Score	Precursor Intensity
AAAS	3	333.1857	996.5352	19.694	0.0018296	101.54	767423.8

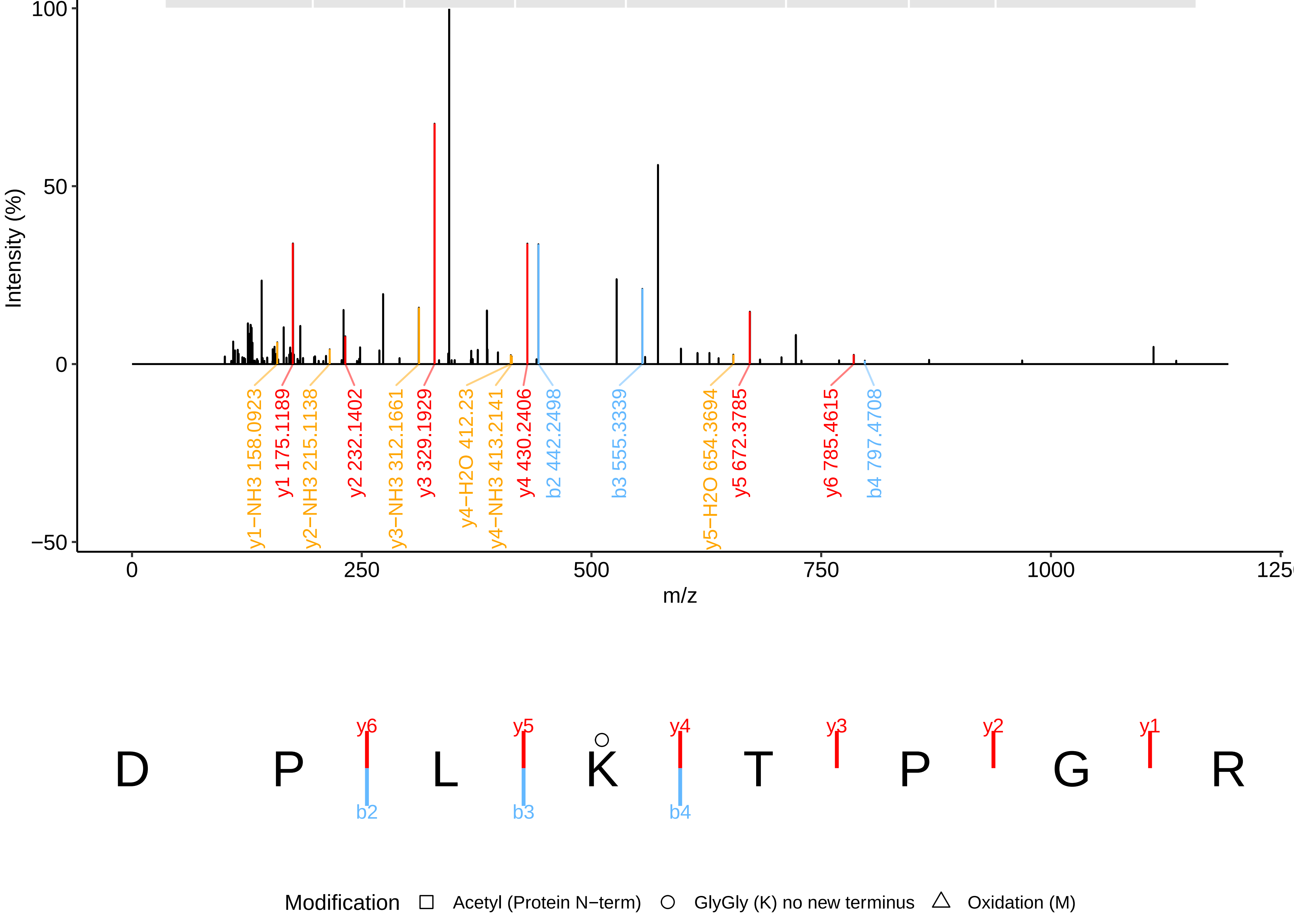

20200924_KIZ_MUb5_GlyGly_1 − Scan number: 5186

Gene Names	Charge	m/z	Mass	Retention time	PEP	Score	Precursor Intensity
AAAS	3	333.1857	996.5352	16.096	0.12138	51.066	10306021

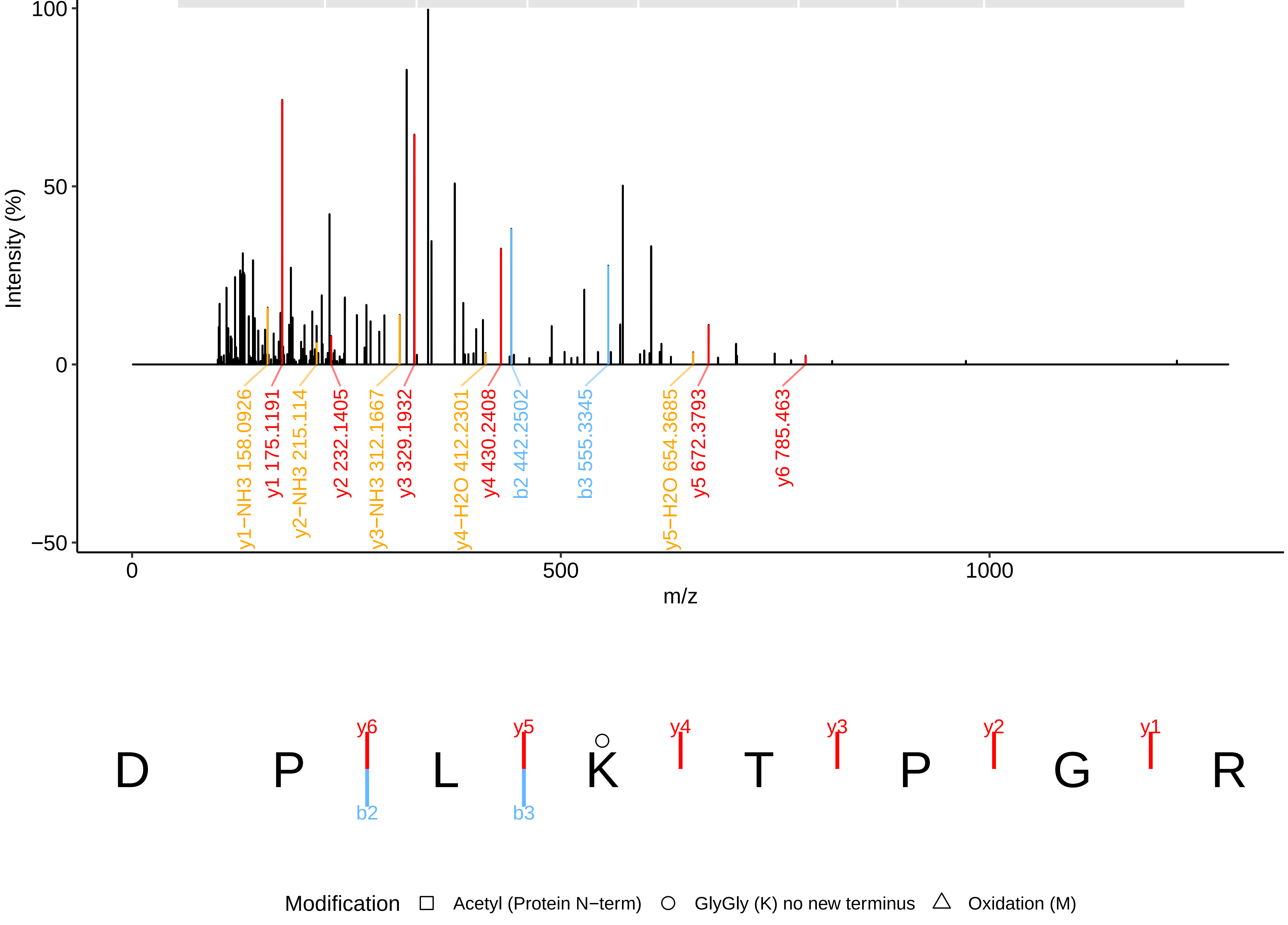

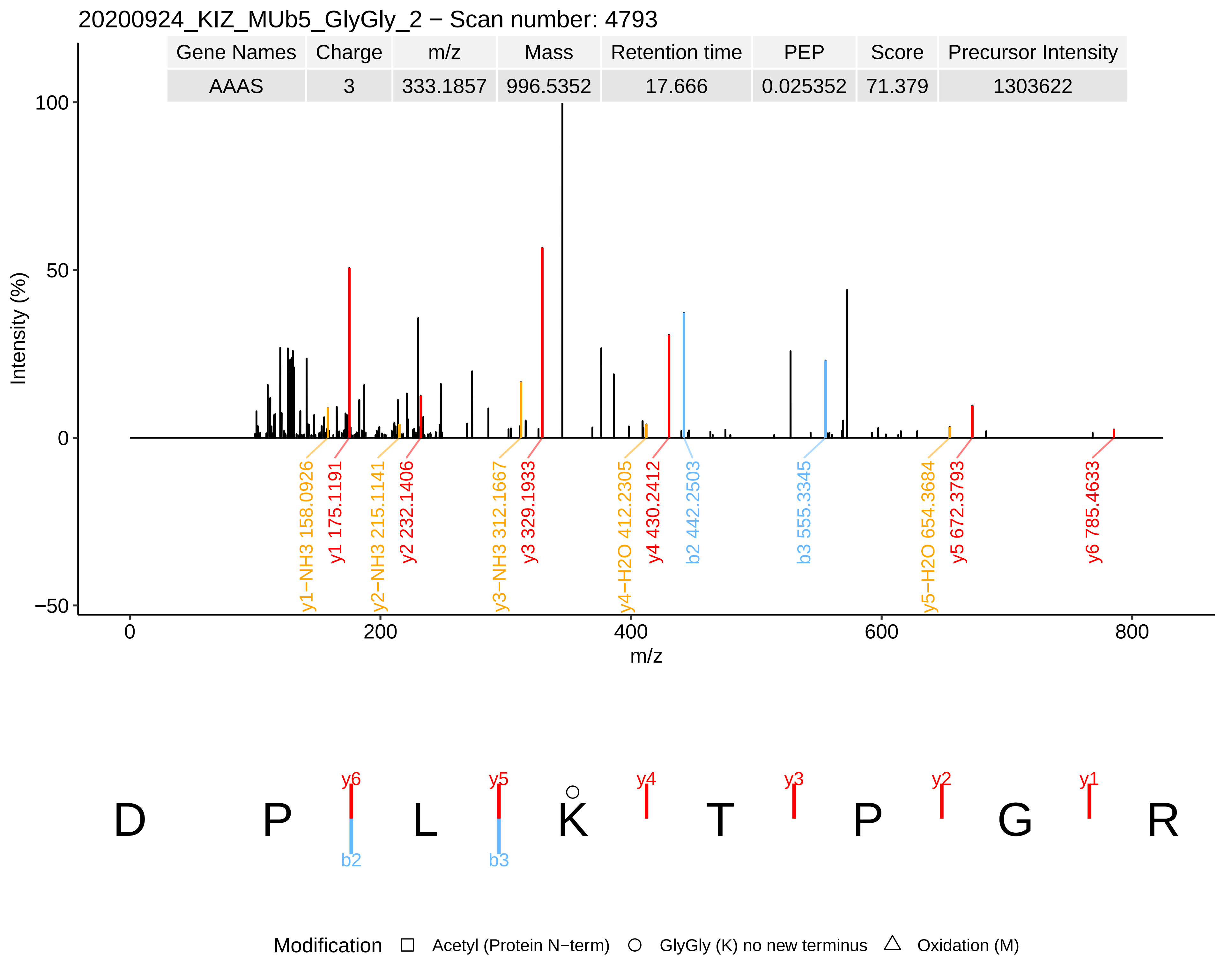

20200924_KIZ_MUb5_GlyGly_2 − Scan number: 4793
Gene Names | Charge | m/z | Mass | Retention time | PEP | Score | Precursor Intensity
AAAS | 3 | 333.1857 | 996.5352 | 17.666 | 0.025352 | 71.379 | 1303622
Intensity (%)
100
50
0
−50
0
200
400
600
800
m/z
y1−NH3 158.0926
y1 175.1191
y2−NH3 215.1141
y2 232.1406
y3−NH3 312.1667
y3 329.1933
y4−H2O 412.2305
y4 430.2412
b2 442.2503
b3 555.3345
y5−H2O 654.3684
y5 672.3793
y6 785.4633
D P L K T P G R
y6 y5 y4 y3 y2 y1
b2 b3
Modification
Acetyl (Protein N−term)
GlyGly (K) no new terminus
Oxidation (M)

20200921_KIZ_MUb4_GlyGly_10plex − Scan number: 8984

Gene Names	Charge	m/z	Mass	Retention time	PEP	Score	Precursor Intensity
USP5	3	343.5331	1027.577	30.672	0.020566	71.379	1192490

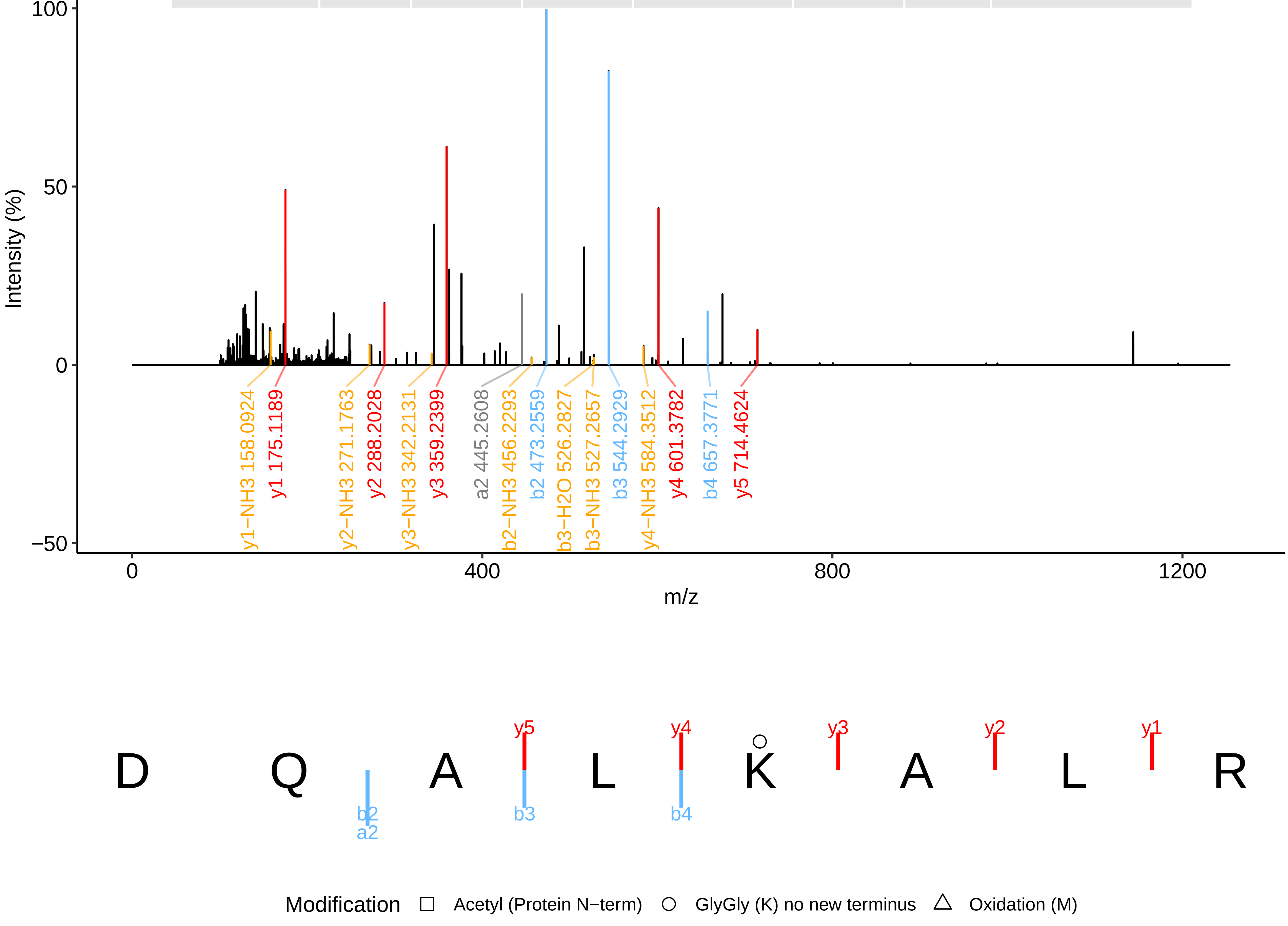

20200921_KIZ_MUb4_GlyGly_10plex − Scan number: 9019

Gene Names	Charge	m/z	Mass	Retention time	PEP	Score	Precursor Intensity
USP5	2	514.796	1027.577	30.81	0.00040492	136.27	6019712

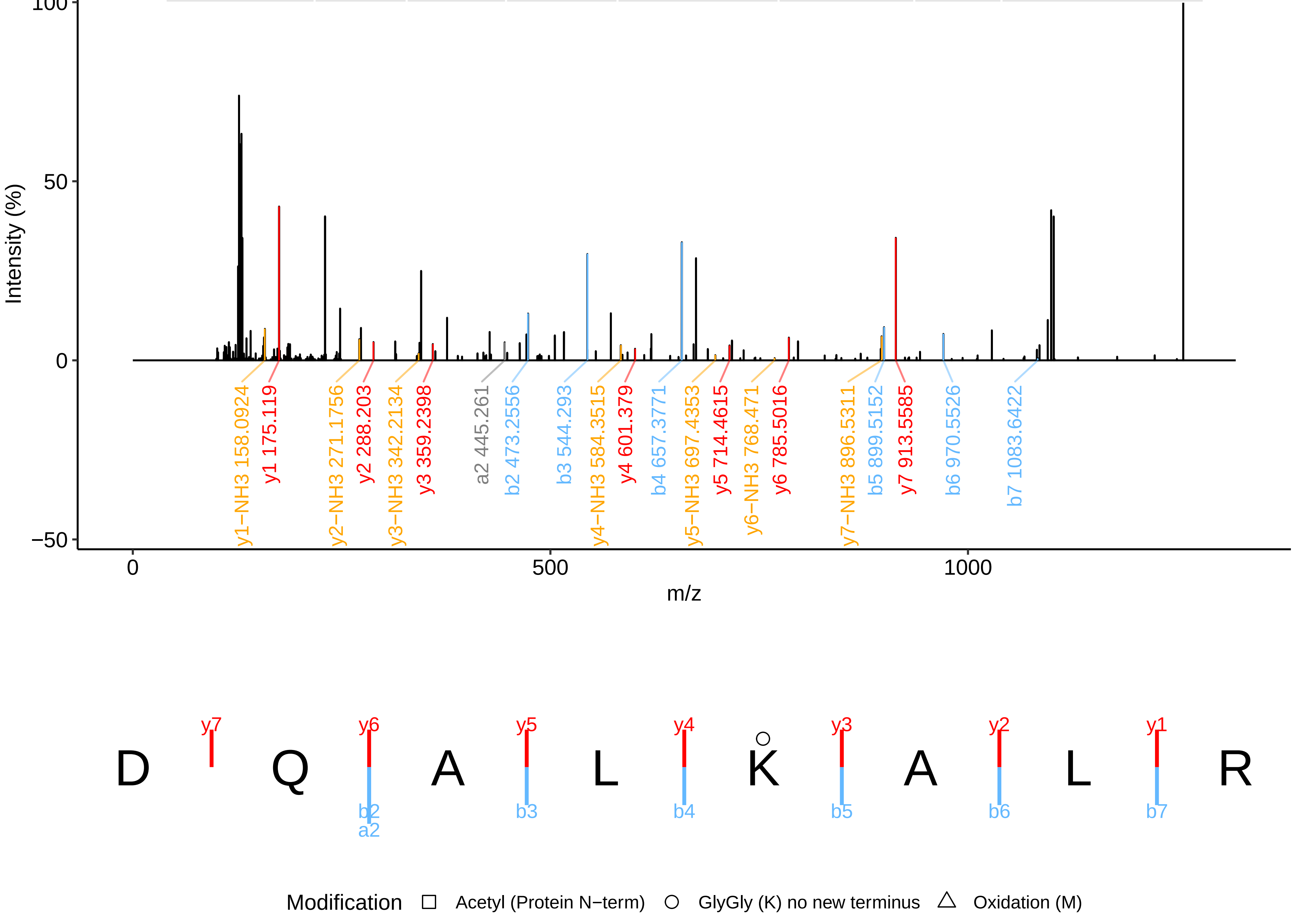

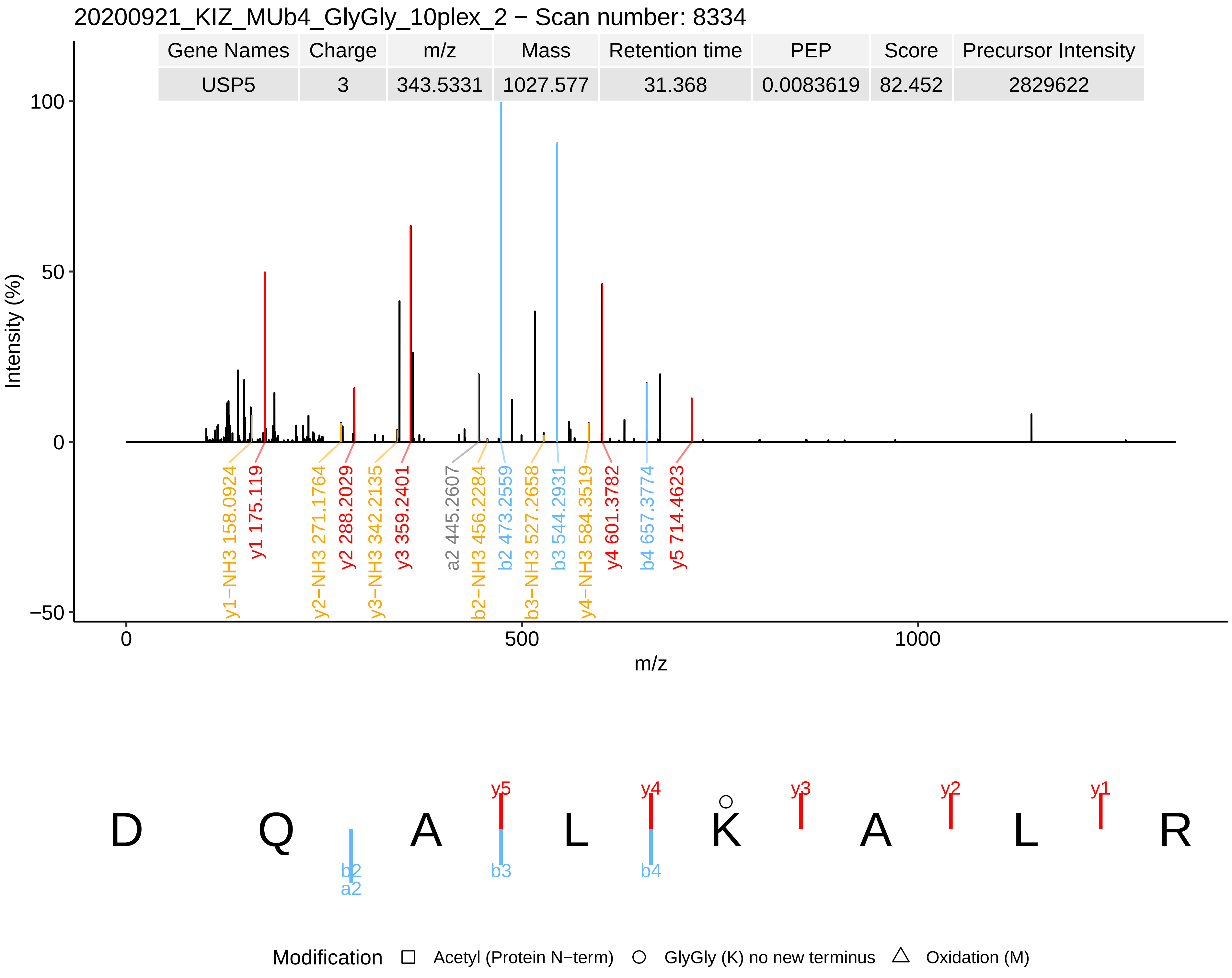
20200921_KIZ_MUb4_GlyGly_10plex_2 − Scan number: 8334
Gene Names
Charge
m/z
Mass
Retention time
PEP
Score
Precursor Intensity
USP5
3
343.5331
1027.577
31.368
0.0083619
82.452
2829622
Intensity (%)
100
50
0
−50
0
500
1000
m/z
y1−NH3 158.0924
y1 175.119
y2−NH3 271.1764
y2 288.2029
y3−NH3 342.2135
y3 359.2401
a2 445.2607
b2−NH3 456.2284
b2 473.2559
b3−NH3 527.2658
b3 544.2931
y4−NH3 584.3519
y4 601.3782
b4 657.3774
y5 714.4623
D
Q
A
L
K
A
L
R
y5
y4
y3
y2
y1
b2
a2
b3
b4
Modification
Acetyl (Protein N−term)
GlyGly (K) no new terminus
Oxidation (M)

20200921_KIZ_MUb4_GlyGly_10plex_2 − Scan number: 8373

Gene Names	Charge	m/z	Mass	Retention time	PEP	Score	Precursor Intensity
USP5	2	514.796	1027.577	31.525	0.00064208	126.52	1304580

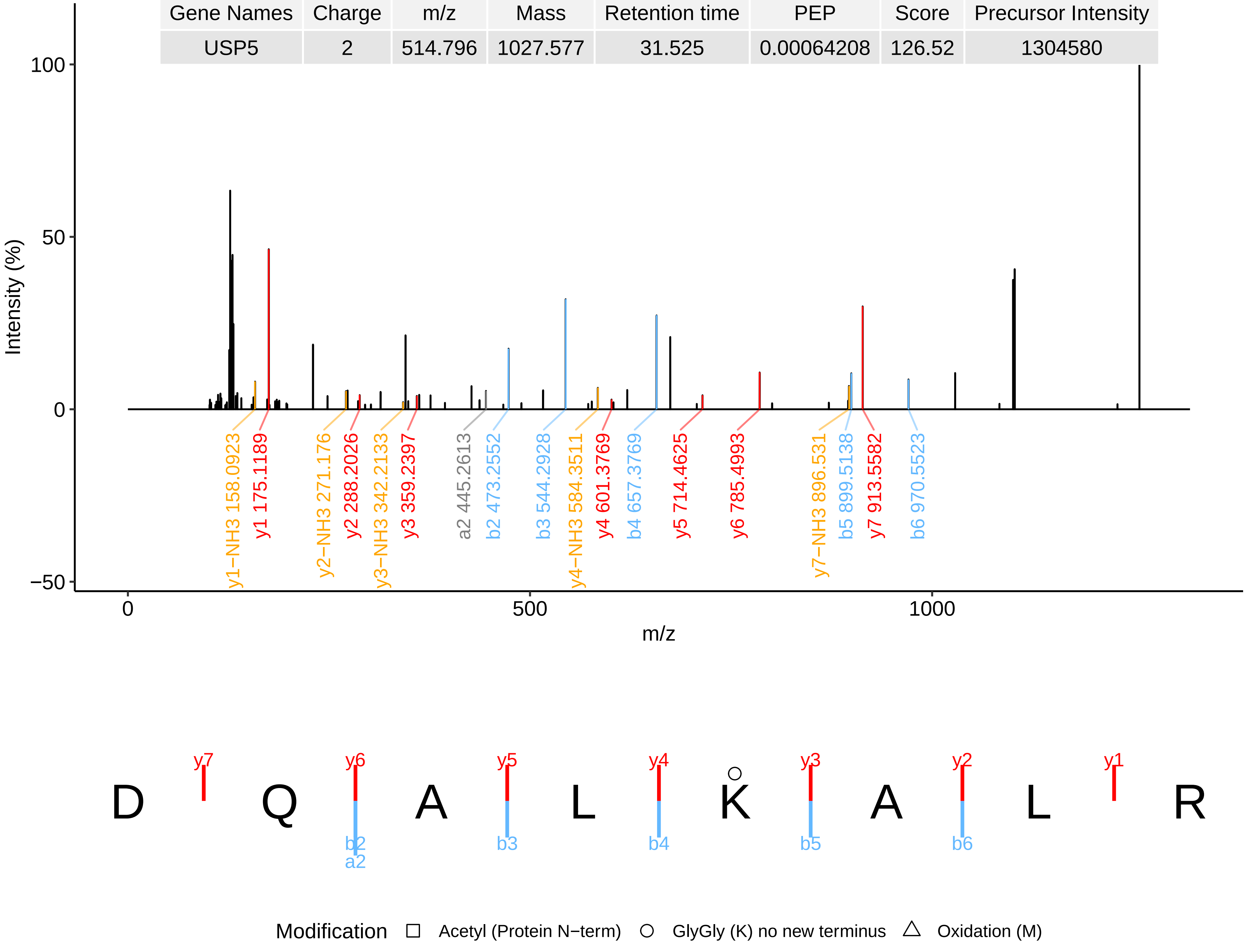

20200924_KIZ_MUb5_GlyGly_1 − Scan number: 7759

Gene Names	Charge	m/z	Mass	Retention time	PEP	Score	Precursor Intensity
USP5	2	514.796	1027.577	24.705	0.00084275	118.06	7663759

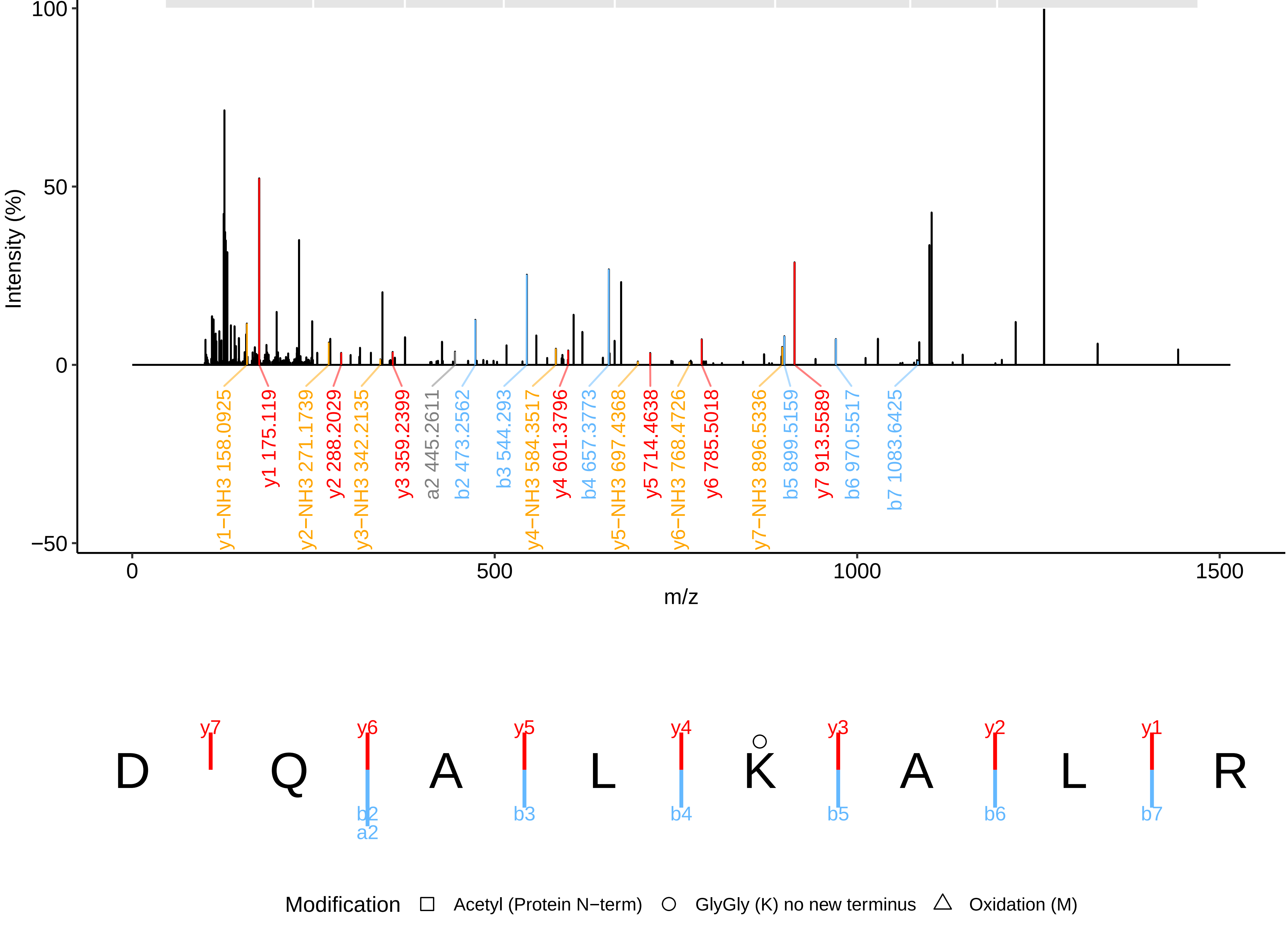

20200924_KIZ_MUb5_GlyGly_1 − Scan number: 7918

Gene Names	Charge	m/z	Mass	Retention time	PEP	Score	Precursor Intensity
USP5	2	514.796	1027.577	25.227	0.00034082	138.78	8930384

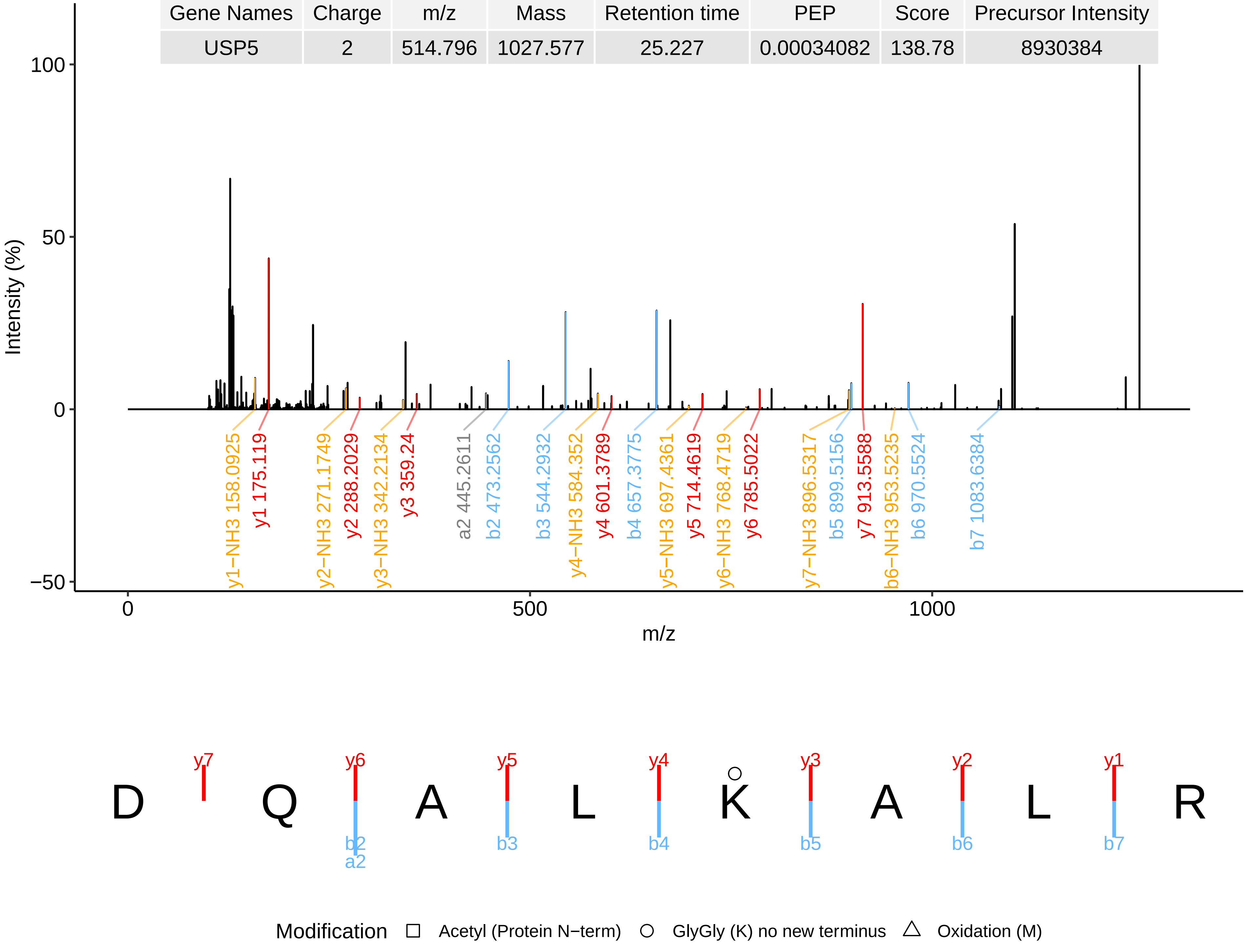

20200924_KIZ_MUb5_GlyGly_1 − Scan number: 7819

Gene Names	Charge	m/z	Mass	Retention time	PEP	Score	Precursor Intensity
USP5	3	343.5331	1027.577	24.906	0.00014578	132.08	78510064

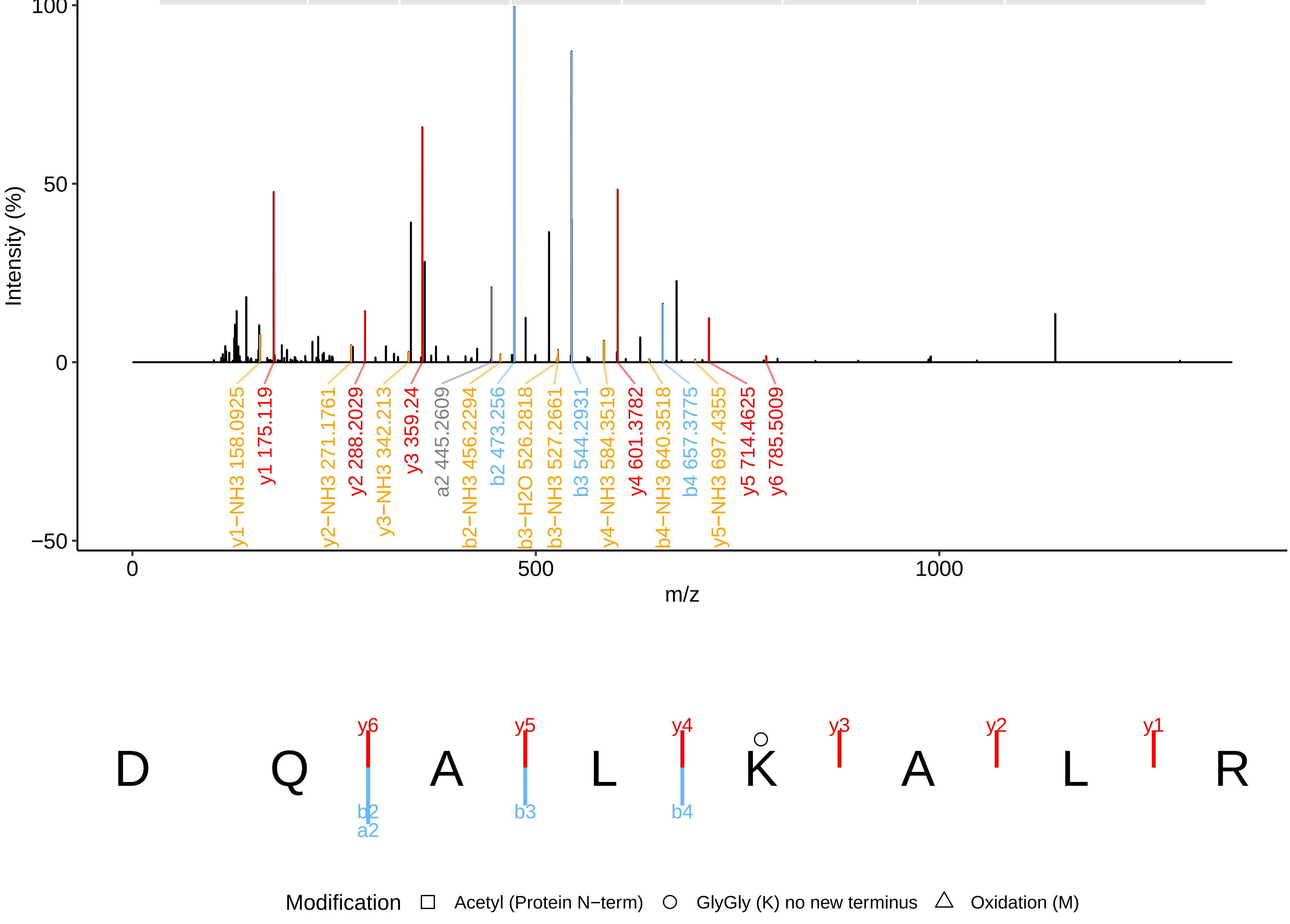

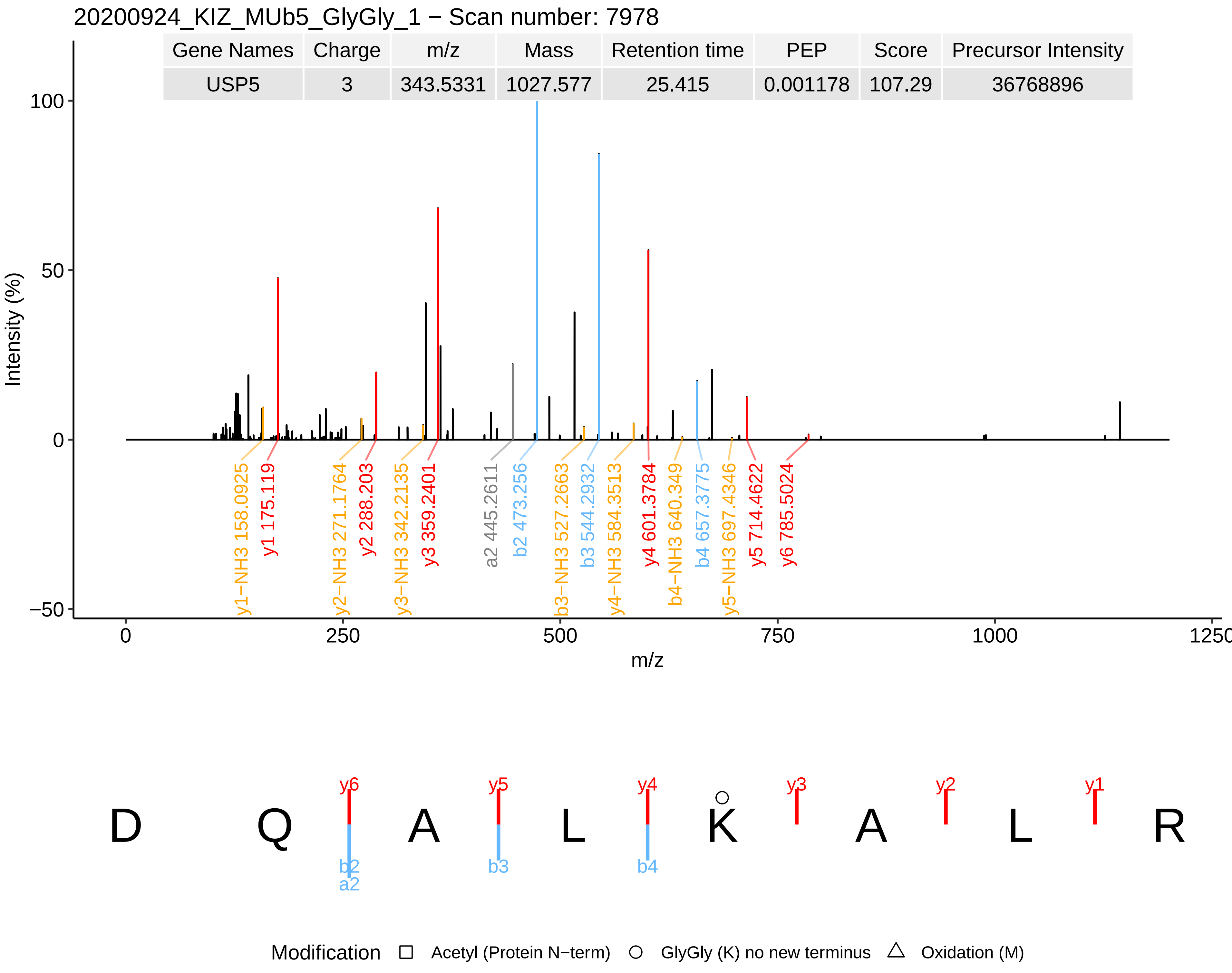
20200924_KIZ_MUb5_GlyGly_1 − Scan number: 7978
Gene Names | Charge | m/z | Mass | Retention time | PEP | Score | Precursor Intensity
USP5 | 3 | 343.5331 | 1027.577 | 25.415 | 0.001178 | 107.29 | 36768896
Intensity (%)
m/z
y1−NH3 158.0925
y1 175.119
y2−NH3 271.1764
y2 288.203
y3−NH3 342.2135
y3 359.2401
a2 445.2611
b2 473.256
b3−NH3 527.2663
b3 544.2932
y4−NH3 584.3513
y4 601.3784
b4−NH3 640.349
b4 657.3775
y5−NH3 697.4346
y5 714.4622
y6 785.5024
D Q A L K A L R
y6 y5 y4 y3 y2 y1
b2 a2 b3 b4
Modification
Acetyl (Protein N−term)
GlyGly (K) no new terminus
Oxidation (M)

20200924_KIZ_MUb5_GlyGly_2 − Scan number: 7323

Gene Names	Charge	m/z	Mass	Retention time	PEP	Score	Precursor Intensity
USP5	3	343.5331	1027.577	27.328	0.0031211	95.741	7021997

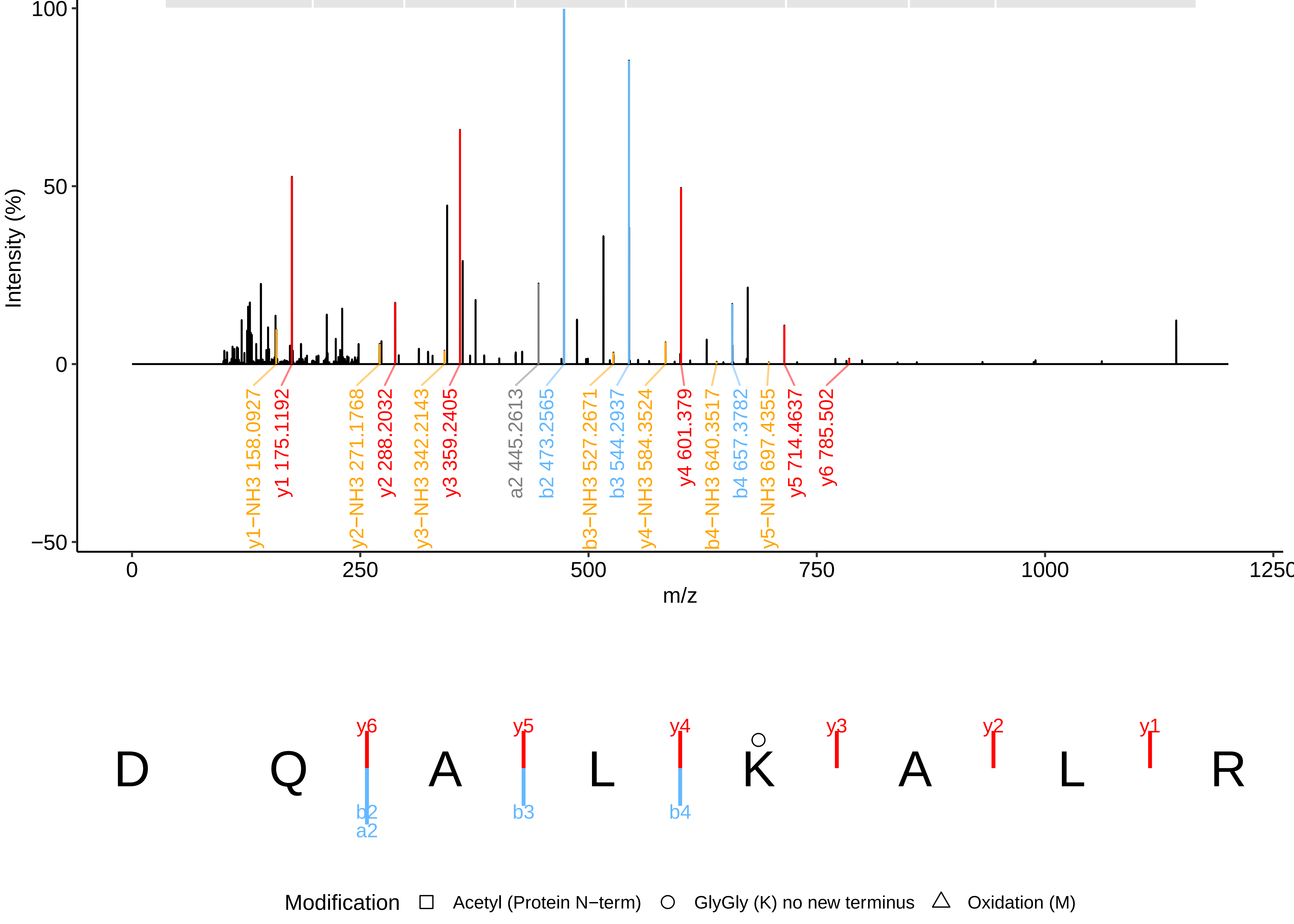

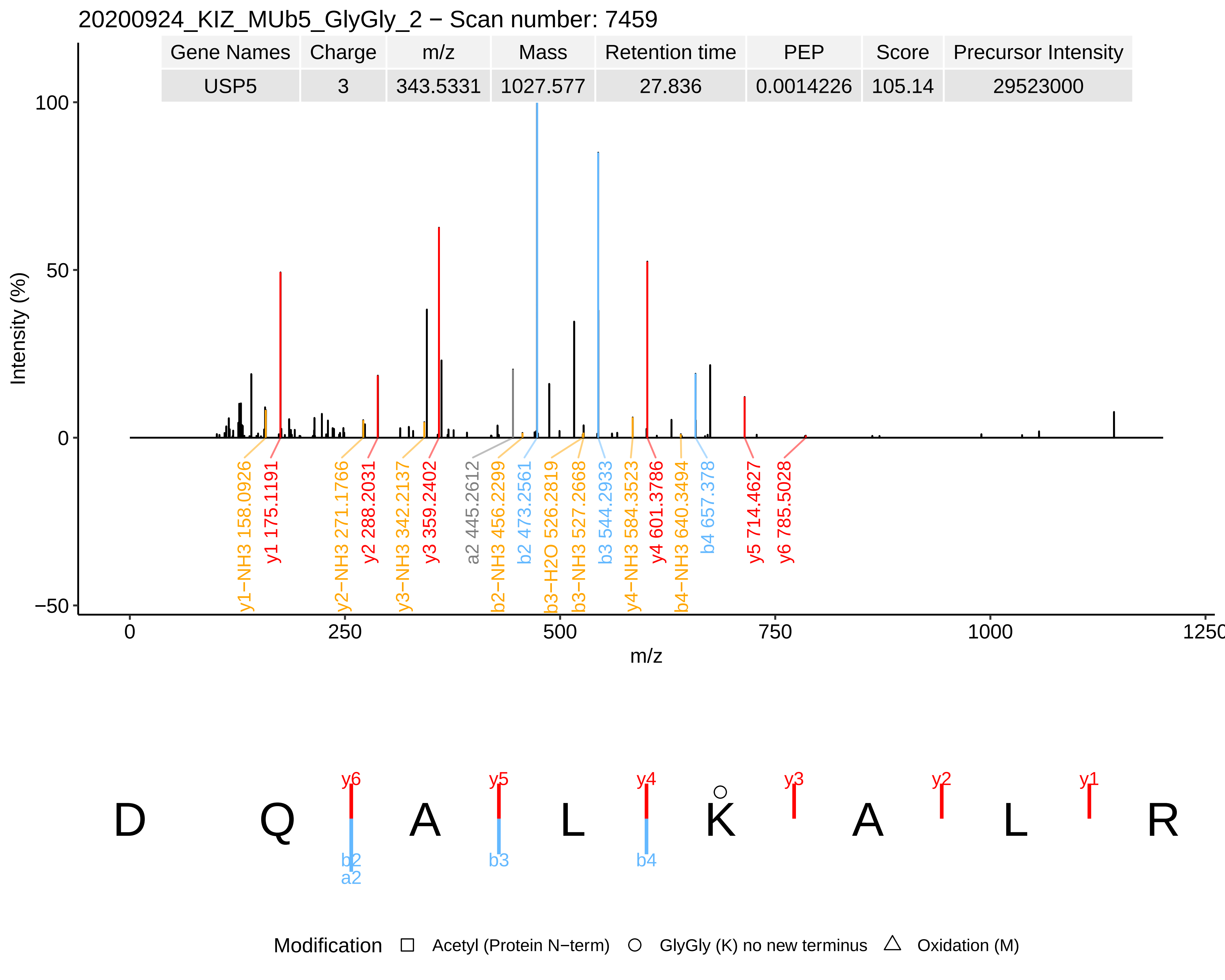
20200924_KIZ_MUb5_GlyGly_2 − Scan number: 7459
Gene Names
Charge
m/z
Mass
Retention time
PEP
Score
Precursor Intensity
USP5
3
343.5331
1027.577
27.836
0.0014226
105.14
29523000
Intensity (%)
100
50
0
−50
0
250
500
750
1000
1250
m/z
y1−NH3 158.0926
y1 175.1191
y2−NH3 271.1766
y2 288.2031
y3−NH3 342.2137
y3 359.2402
a2 445.2612
b2−NH3 456.2299
b2 473.2561
b3−H2O 526.2819
b3−NH3 527.2668
b3 544.2933
y4−NH3 584.3523
y4 601.3786
b4−NH3 640.3494
b4 657.378
y5 714.4627
y6 785.5028
D Q A L K A L R
y6
y5
y4
y3
y2
y1
b2
a2
b3
b4
Modification
Acetyl (Protein N−term)
GlyGly (K) no new terminus
Oxidation (M)

20200924_KIZ_MUb5_GlyGly_2 − Scan number: 7593

Gene Names	Charge	m/z	Mass	Retention time	PEP	Score	Precursor Intensity
USP5	3	343.5331	1027.577	28.354	0.0049656	91.064	6964381

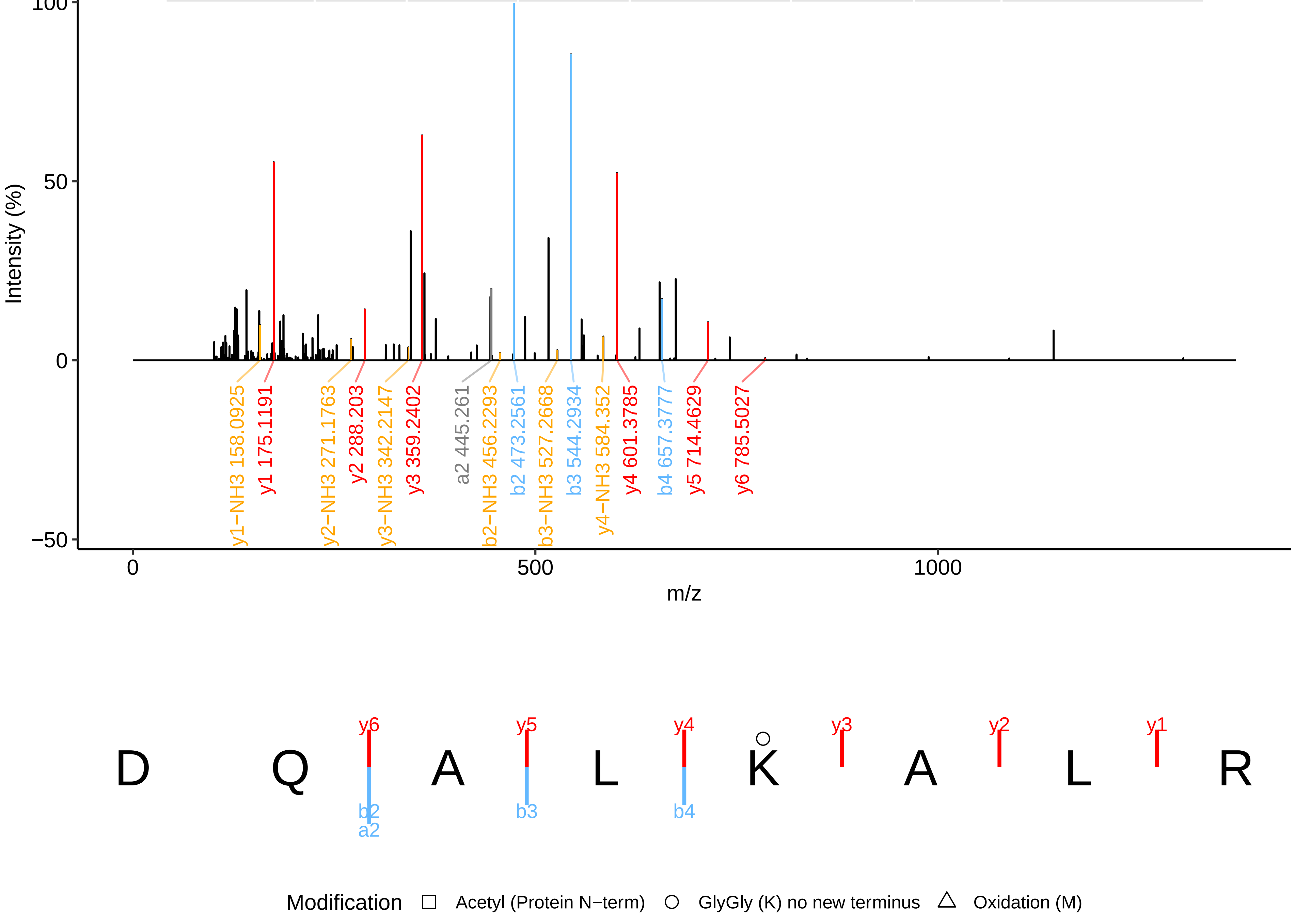

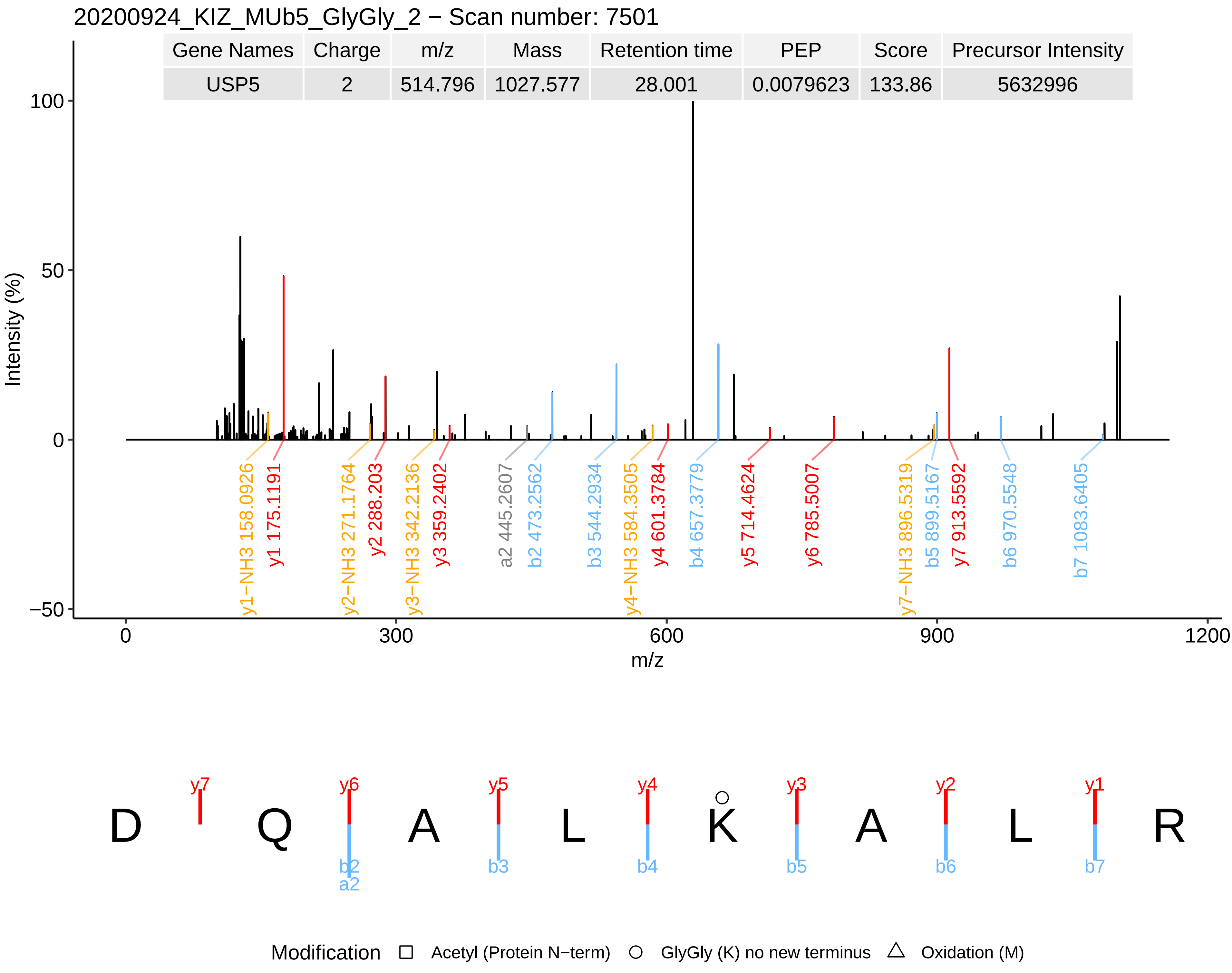
20200924_KIZ_MUb5_GlyGly_2 − Scan number: 7501
Gene Names | Charge | m/z | Mass | Retention time | PEP | Score | Precursor Intensity
USP5 | 2 | 514.796 | 1027.577 | 28.001 | 0.0079623 | 133.86 | 5632996
Intensity (%)
m/z
y1−NH3 158.0926
y1 175.1191
y2−NH3 271.1764
y2 288.203
y3−NH3 342.2136
y3 359.2402
a2 445.2607
b2 473.2562
b3 544.2934
y4−NH3 584.3505
y4 601.3784
b4 657.3779
y5 714.4624
y6 785.5007
y7−NH3 896.5319
b5 899.5167
y7 913.5592
b6 970.5548
b7 1083.6405
D Q A L K A L R
Modification □ Acetyl (Protein N−term) ○ GlyGly (K) no new terminus △ Oxidation (M)

20201118_KIZ_MUb6_GlyGly_1 − Scan number: 7415

Gene Names	Charge	m/z	Mass	Retention time	PEP	Score	Precursor Intensity
USP5	3	343.5331	1027.577	23.506	0.0099441	79.906	24107416

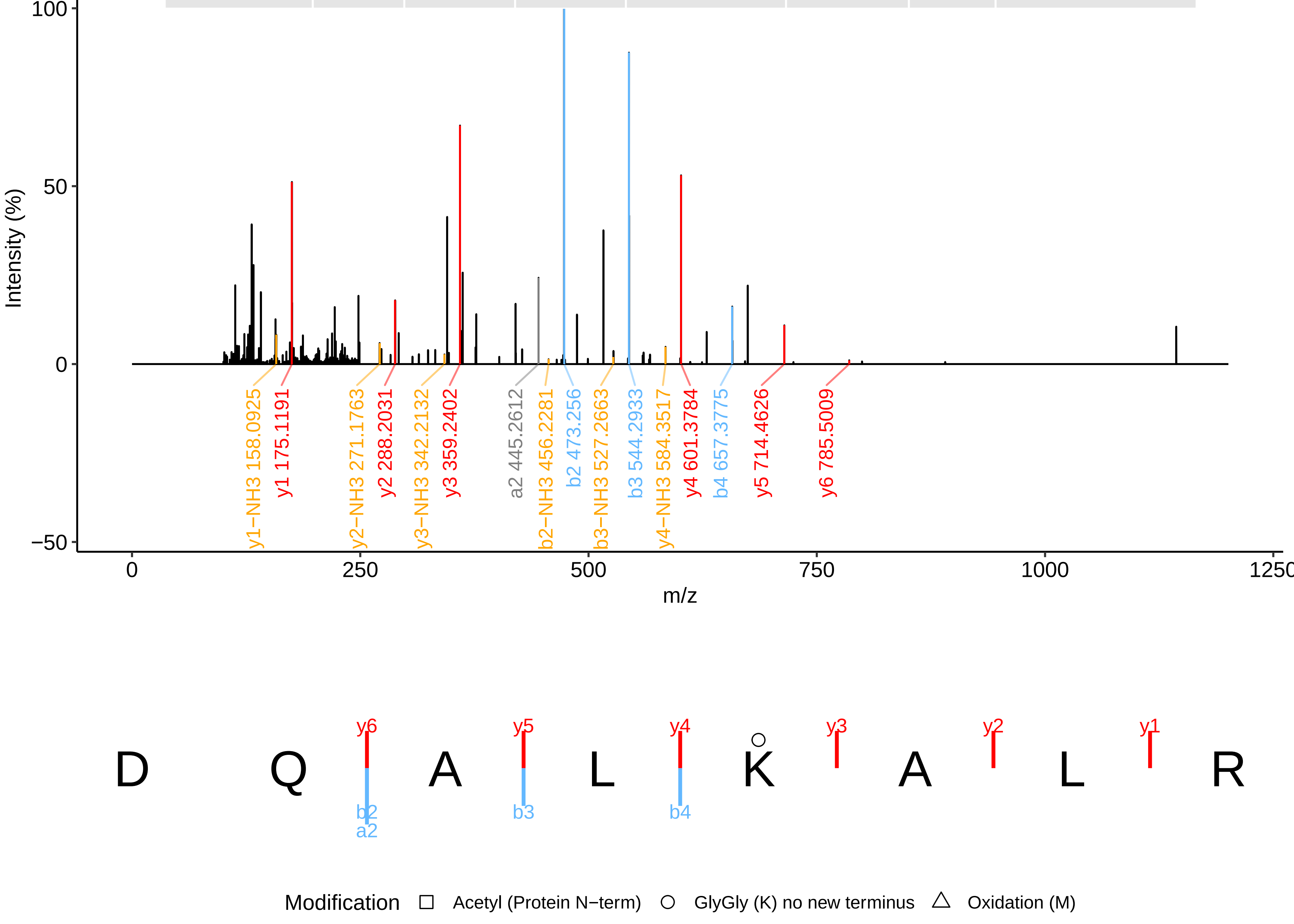

20201118_KIZ_MUb6_GlyGly_2 − Scan number: 9448

Gene Names	Charge	m/z	Mass	Retention time	PEP	Score	Precursor Intensity
USP5	3	343.5331	1027.577	32.034	0.0074585	84.743	8071441

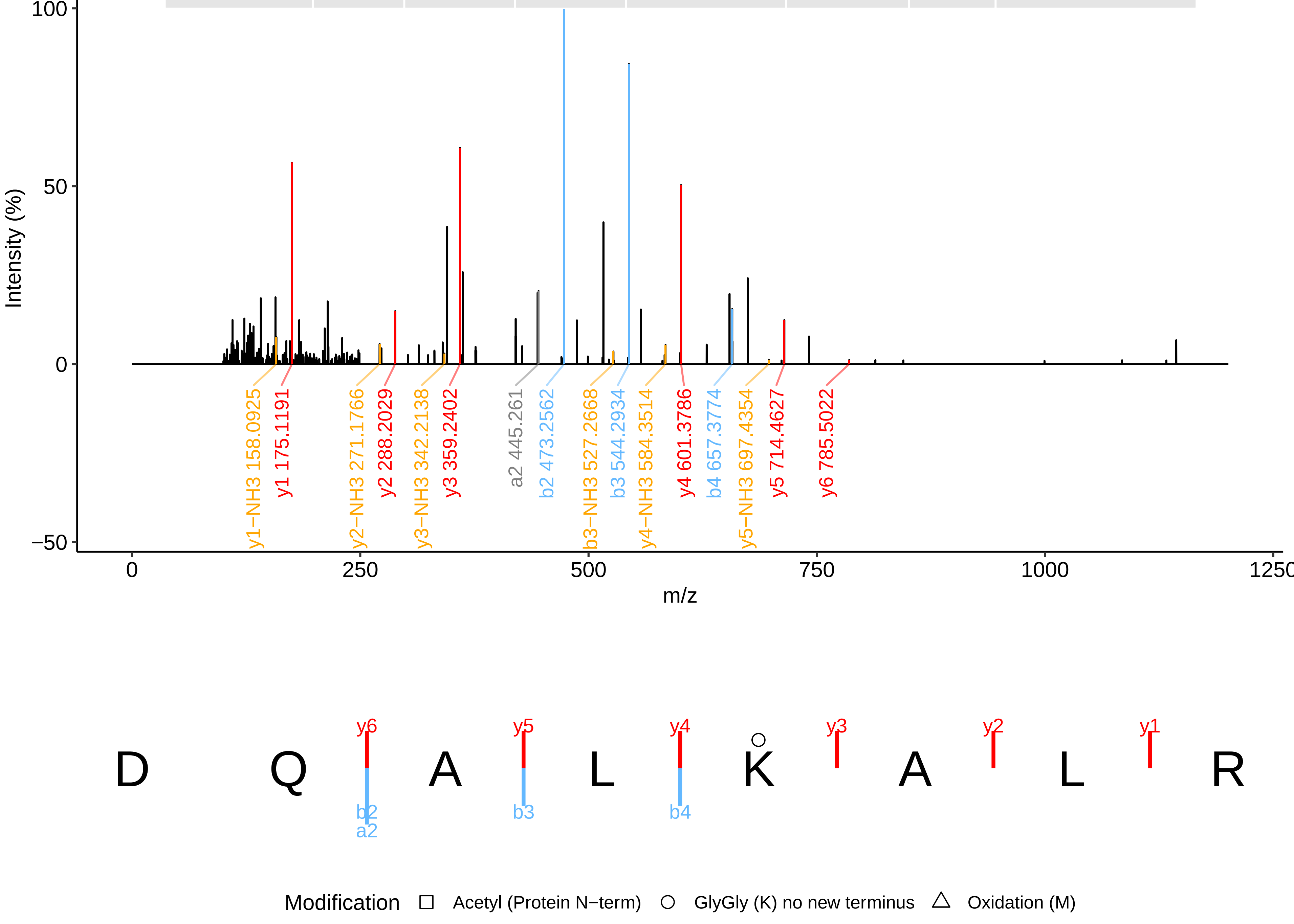

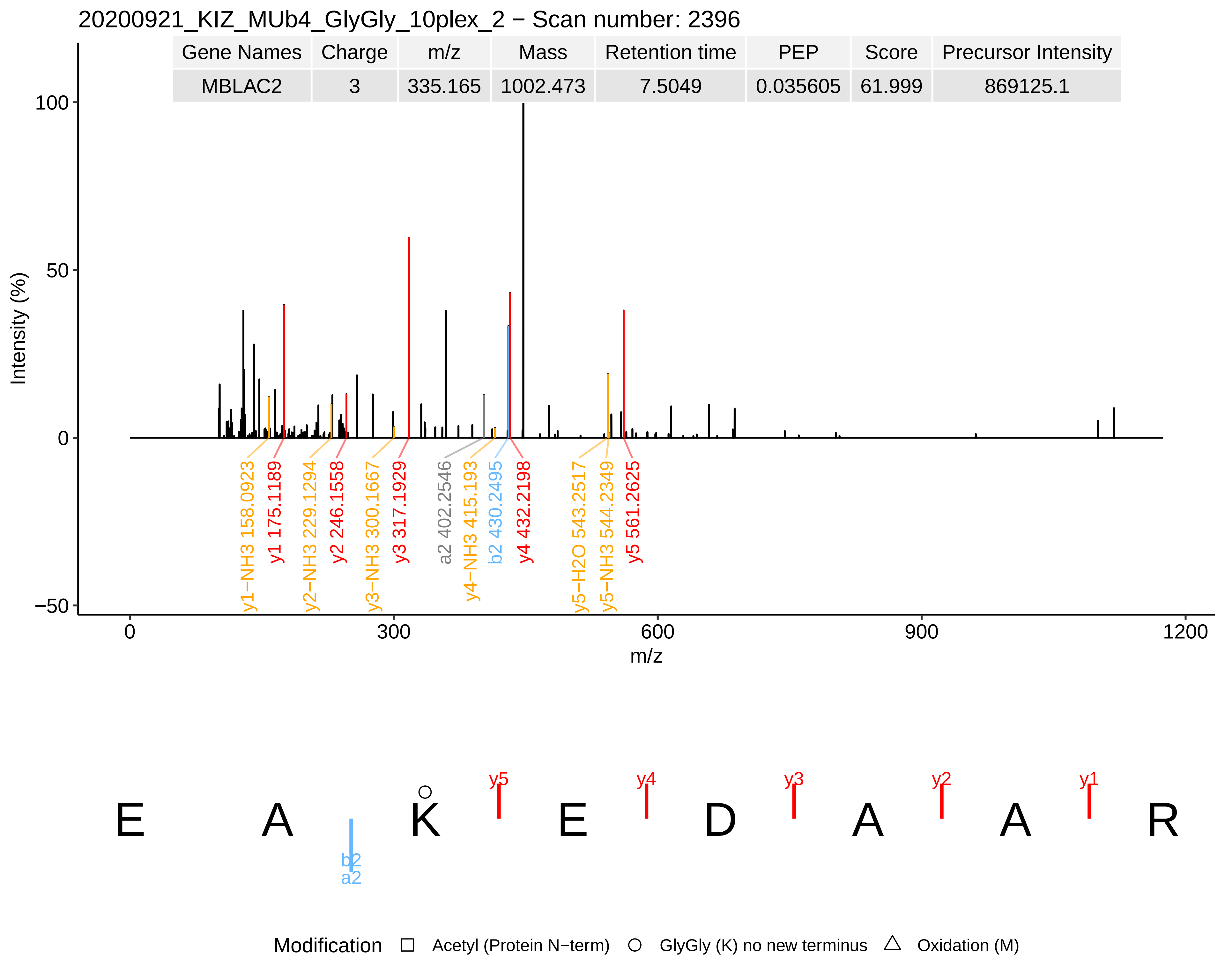

20200921_KIZ_MUb4_GlyGly_10plex_2 − Scan number: 2396
Gene Names | Charge | m/z | Mass | Retention time | PEP | Score | Precursor Intensity
MBLAC2 | 3 | 335.165 | 1002.473 | 7.5049 | 0.035605 | 61.999 | 869125.1
Intensity (%)
m/z
y1−NH3 158.0923
y1 175.1189
y2−NH3 229.1294
y2 246.1558
y3−NH3 300.1667
y3 317.1929
a2 402.2546
y4−NH3 415.193
b2 430.2495
y4 432.2198
y5−H2O 543.2517
y5−NH3 544.2349
y5 561.2625
E A K E D A A R
y5 y4 y3 y2 y1
b2
a2
Modification
Acetyl (Protein N−term)
GlyGly (K) no new terminus
Oxidation (M)

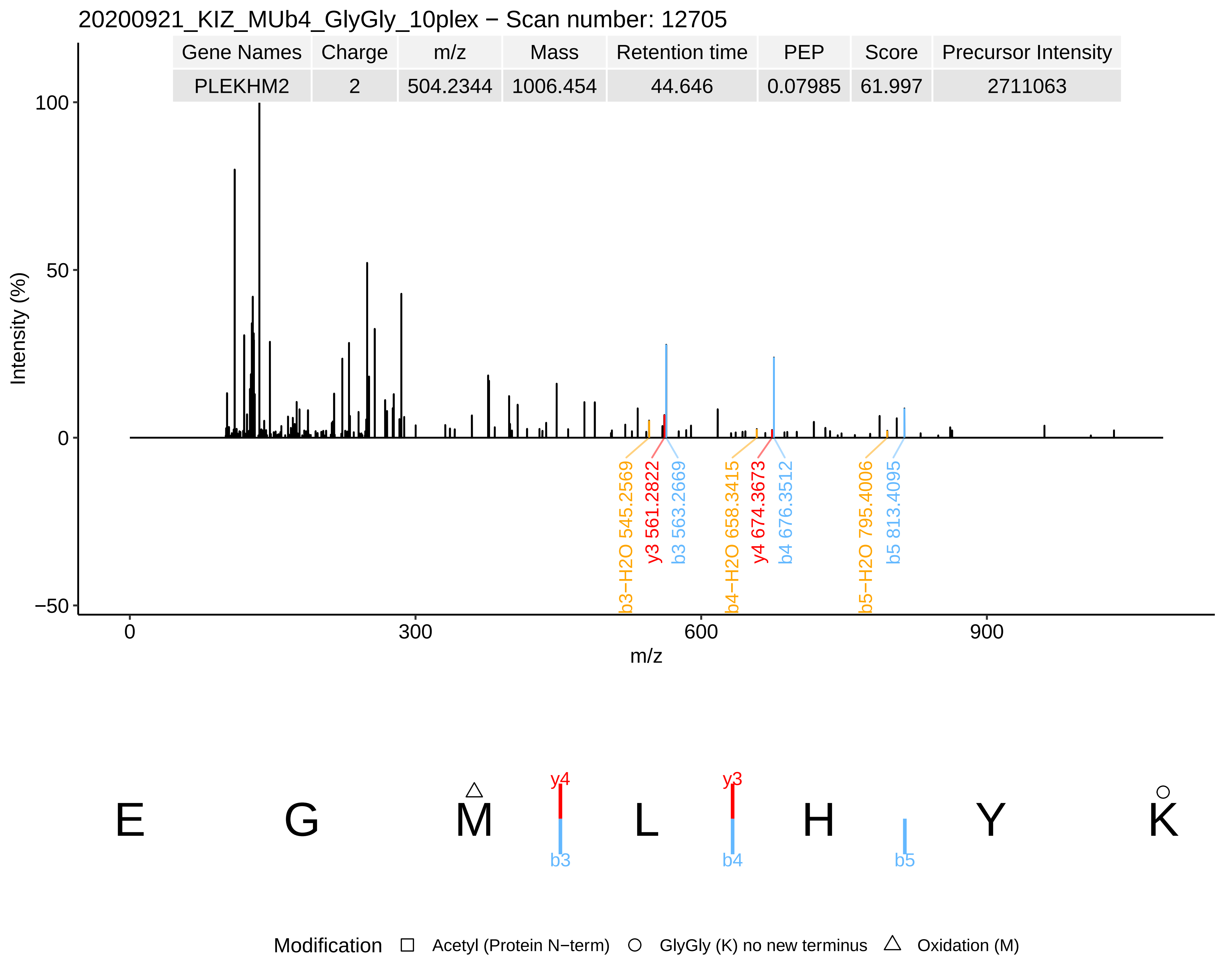
20200921_KIZ_MUb4_GlyGly_10plex − Scan number: 12705
Gene Names | Charge | m/z | Mass | Retention time | PEP | Score | Precursor Intensity
PLEKHM2 | 2 | 504.2344 | 1006.454 | 44.646 | 0.07985 | 61.997 | 2711063
Intensity (%)
100
50
0
−50
0
300
600
900
m/z
b3−H2O 545.2569
y3 561.2822
b3 563.2669
b4−H2O 658.3415
y4 674.3673
b4 676.3512
b5−H2O 795.4006
b5 813.4095
E G M L H Y K
y4
y3
b3
b4
b5
Modification
Acetyl (Protein N−term)
GlyGly (K) no new terminus
Oxidation (M)

20200921_KIZ_MUb4_GlyGly_10plex − Scan number: 8913

Gene Names	Charge	m/z	Mass	Retention time	PEP	Score	Precursor Intensity
VCP	3	331.5102	991.5087	30.401	0.014959	87.284	1279944

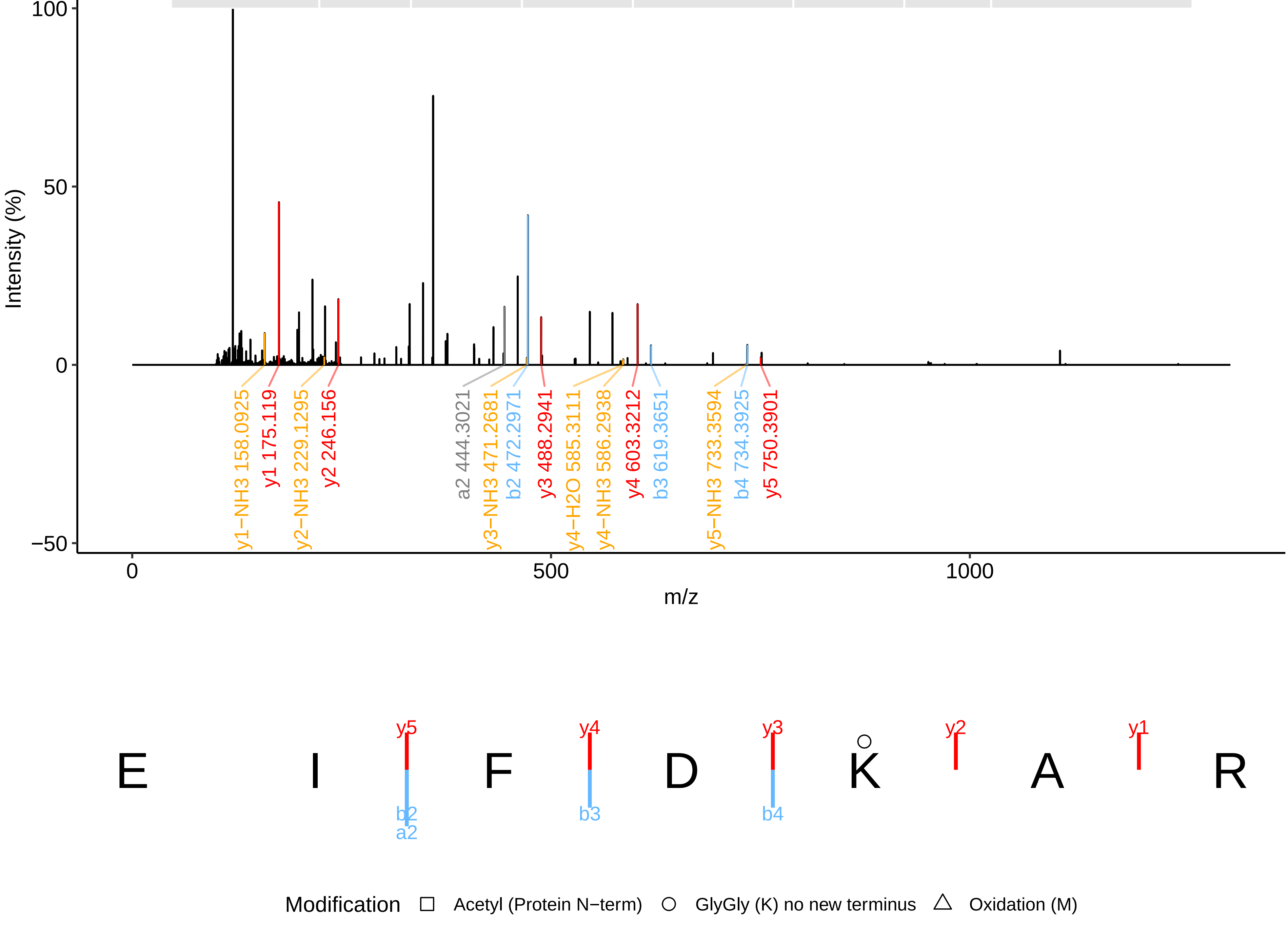

20200921_KIZ_MUb4_GlyGly_10plex − Scan number: 5713

Gene Names	Charge	m/z	Mass	Retention time	PEP	Score	Precursor Intensity
ZWINT	3	332.1821	993.5243	18.738	0.086606	58.687	14031581

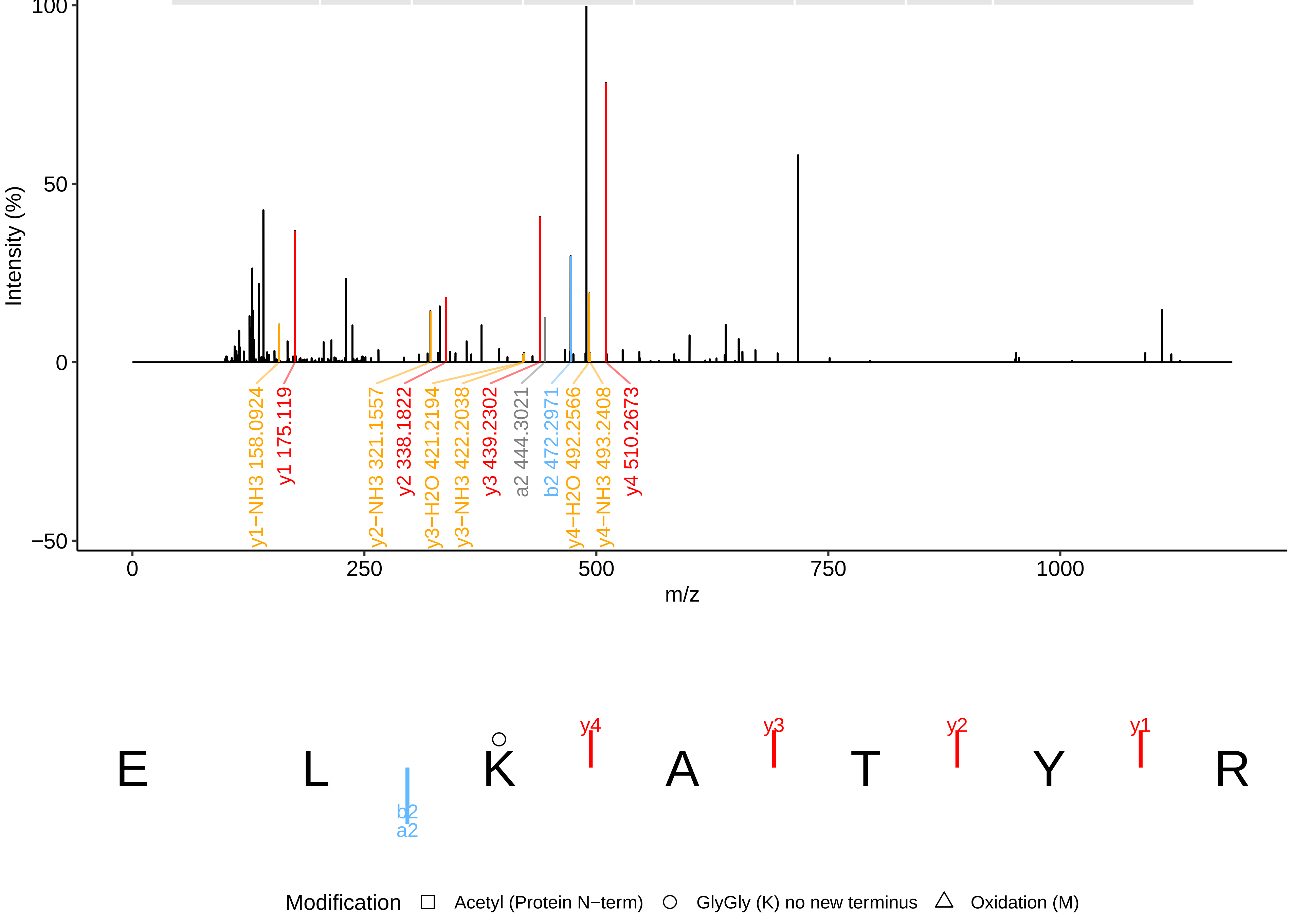

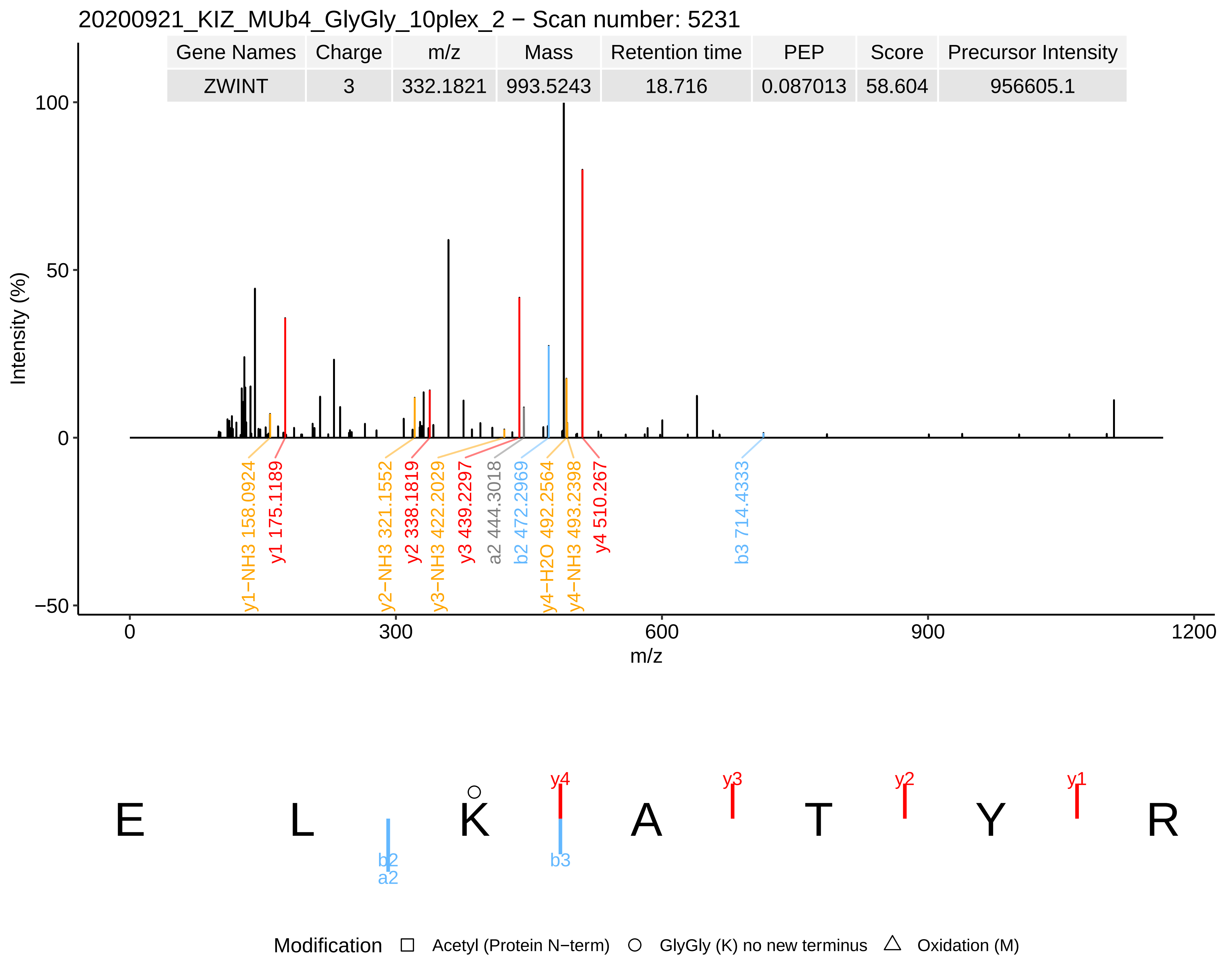

20200921_KIZ_MUb4_GlyGly_10plex_2 − Scan number: 5231
Gene Names | Charge | m/z | Mass | Retention time | PEP | Score | Precursor Intensity
ZWINT | 3 | 332.1821 | 993.5243 | 18.716 | 0.087013 | 58.604 | 956605.1
Intensity (%)
100
50
0
−50
0
300
600
900
1200
m/z
y1−NH3 158.0924
y1 175.1189
y2−NH3 321.1552
y2 338.1819
y3−NH3 422.2029
y3 439.2297
a2 444.3018
b2 472.2969
y4−H2O 492.2564
y4−NH3 493.2398
y4 510.267
b3 714.4333
E L K A T Y R
y4 y3 y2 y1
b2 a2 b3
Modification
Acetyl (Protein N−term)
GlyGly (K) no new terminus
Oxidation (M)

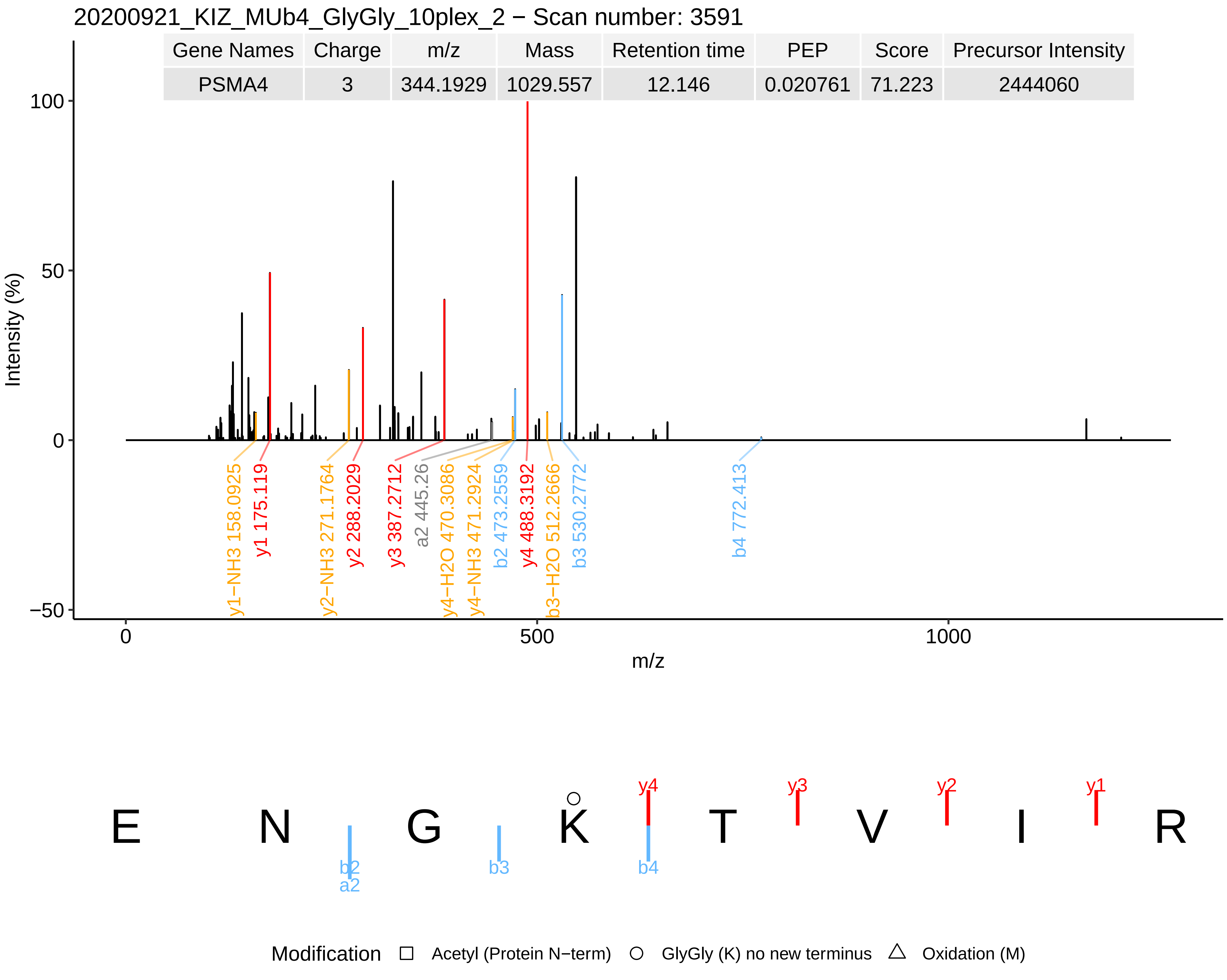

20200921_KIZ_MUb4_GlyGly_10plex_2 − Scan number: 3591
Gene Names | Charge | m/z | Mass | Retention time | PEP | Score | Precursor Intensity
PSMA4 | 3 | 344.1929 | 1029.557 | 12.146 | 0.020761 | 71.223 | 2444060
Intensity (%)
m/z
y1−NH3 158.0925
y1 175.119
y2−NH3 271.1764
y2 288.2029
y3 387.2712
a2 445.26
y4−H2O 470.3086
y4−NH3 471.2924
b2 473.2559
y4 488.3192
b3−H2O 512.2666
b3 530.2772
b4 772.413
E N G K T V I R
y4 y3 y2 y1
b2 a2 b3 b4
Modification
Acetyl (Protein N−term)
GlyGly (K) no new terminus
Oxidation (M)

20200924_KIZ_MUb5_GlyGly_1 − Scan number: 3372

Gene Names	Charge	m/z	Mass	Retention time	PEP	Score	Precursor Intensity
PSMA4	3	344.1929	1029.557	10.333	0.12322	50.805	15733627

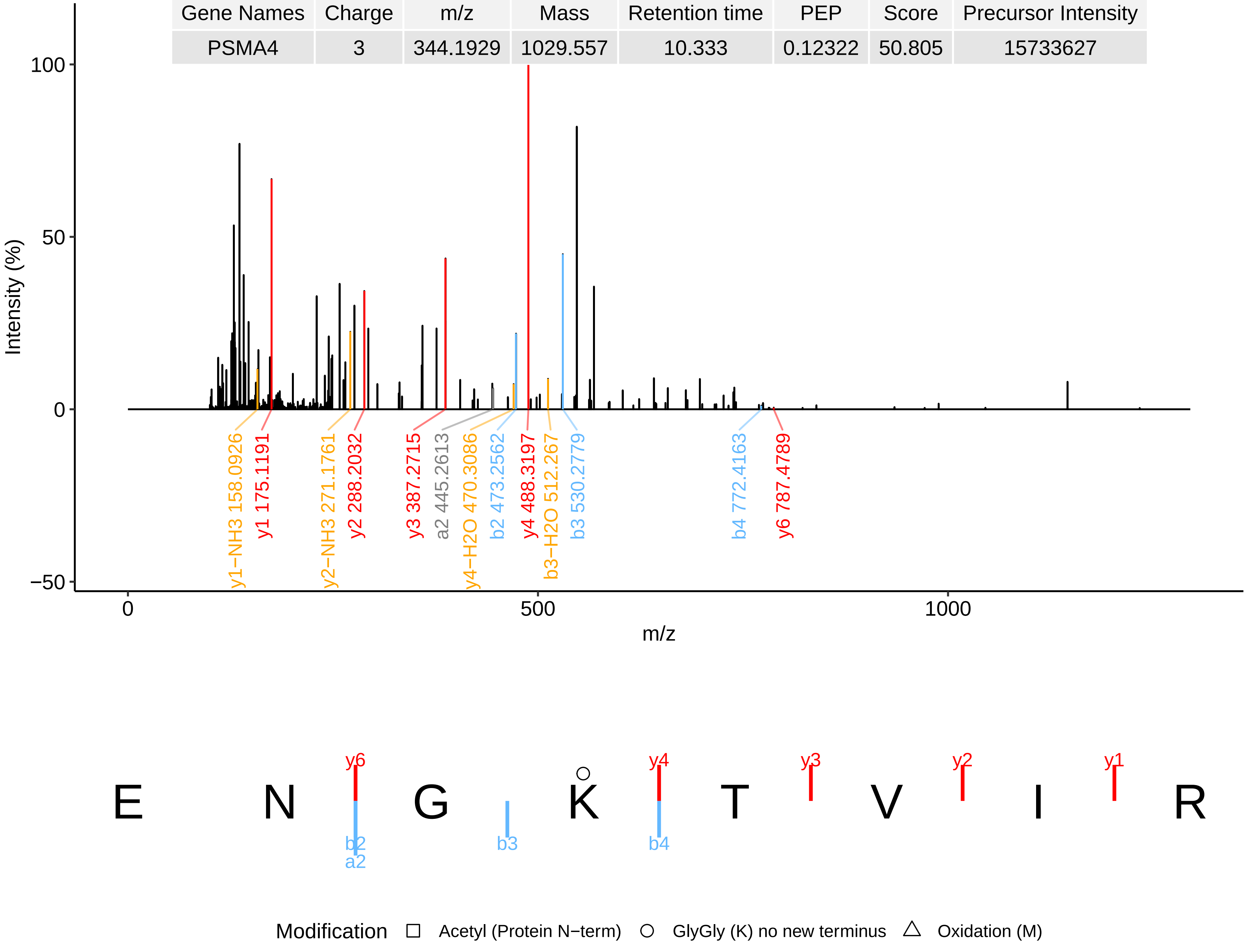

20201118_KIZ_MUb6_GlyGly_1 − Scan number: 3252

Gene Names	Charge	m/z	Mass	Retention time	PEP	Score	Precursor Intensity
PSMA4	3	344.1929	1029.557	9.999	0.02539	67.507	22297676

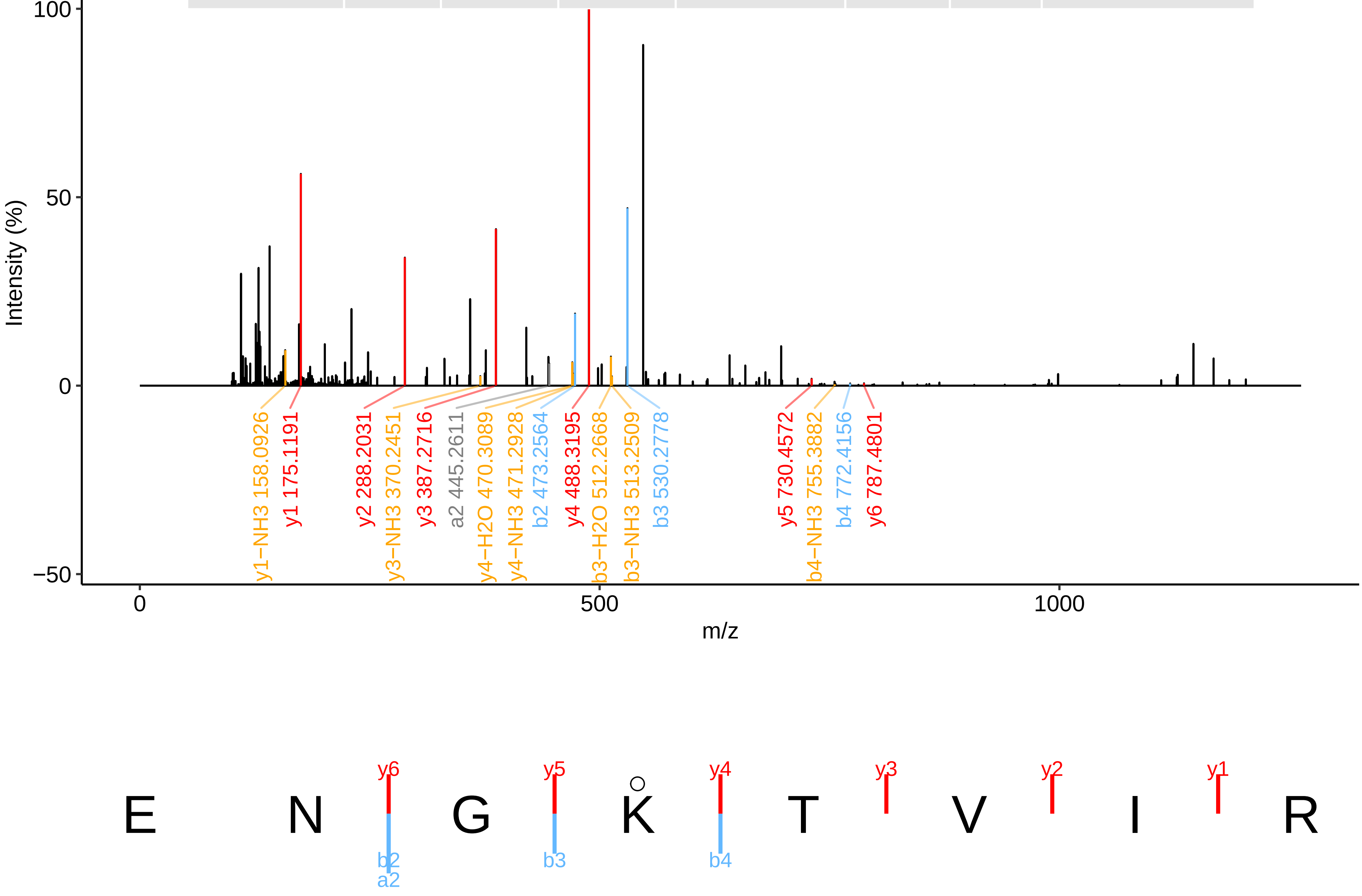

Modification □ Acetyl (Protein N−term) ○ GlyGly (K) no new terminus △ Oxidation (M)

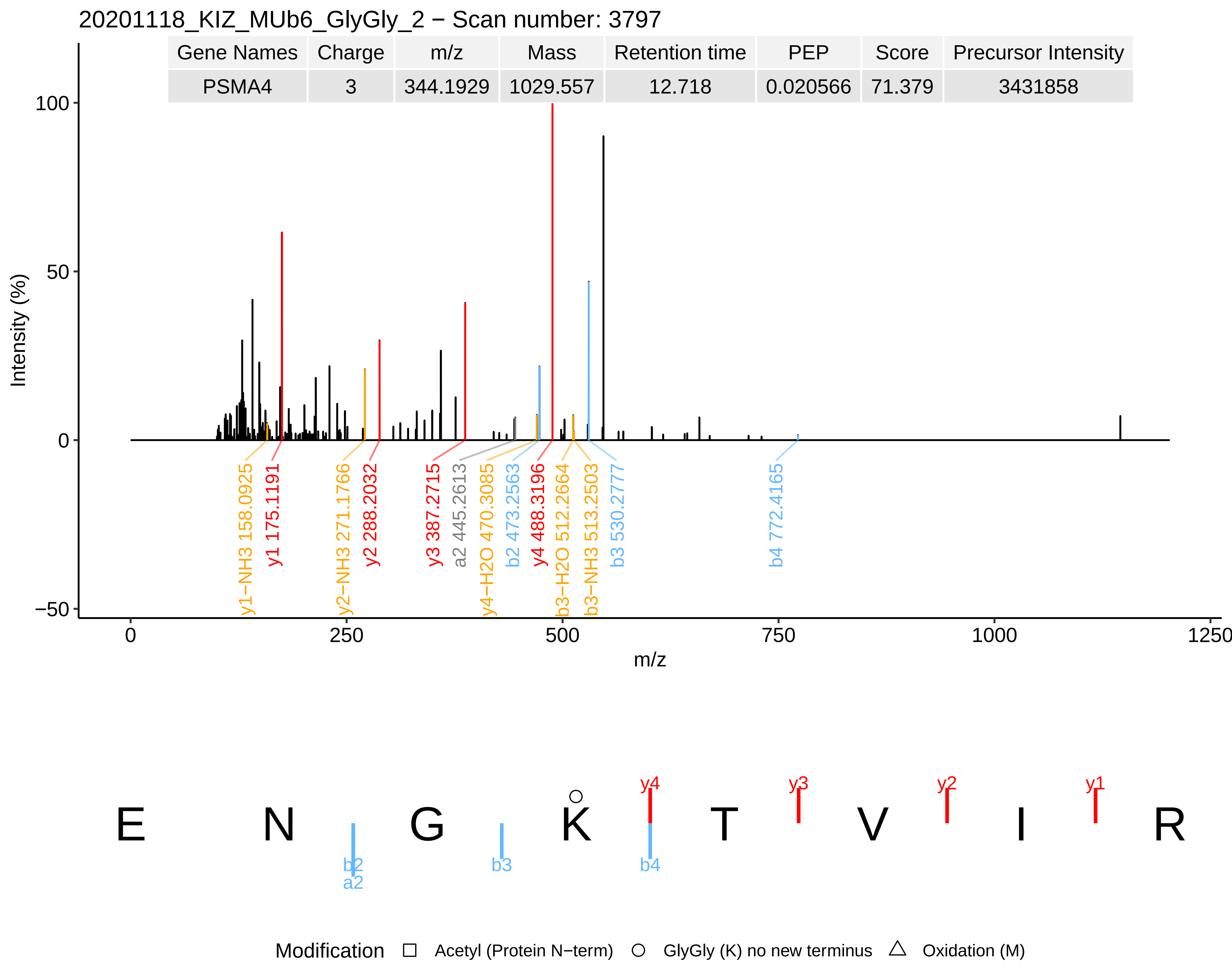

20201118_KIZ_MUb6_GlyGly_2 − Scan number: 3797
Gene Names | Charge | m/z | Mass | Retention time | PEP | Score | Precursor Intensity
PSMA4 | 3 | 344.1929 | 1029.557 | 12.718 | 0.020566 | 71.379 | 3431858
Intensity (%)
m/z
y1−NH3 158.0925
y1 175.1191
y2−NH3 271.1766
y2 288.2032
y3 387.2715
a2 445.2613
y4−H2O 470.3085
b2 473.2563
y4 488.3196
b3−H2O 512.2664
b3−NH3 513.2503
b3 530.2777
b4 772.4165
E N G K T V I R
y4 y3 y2 y1
b2 a2 b3 b4
Modification
Acetyl (Protein N−term)
GlyGly (K) no new terminus
Oxidation (M)

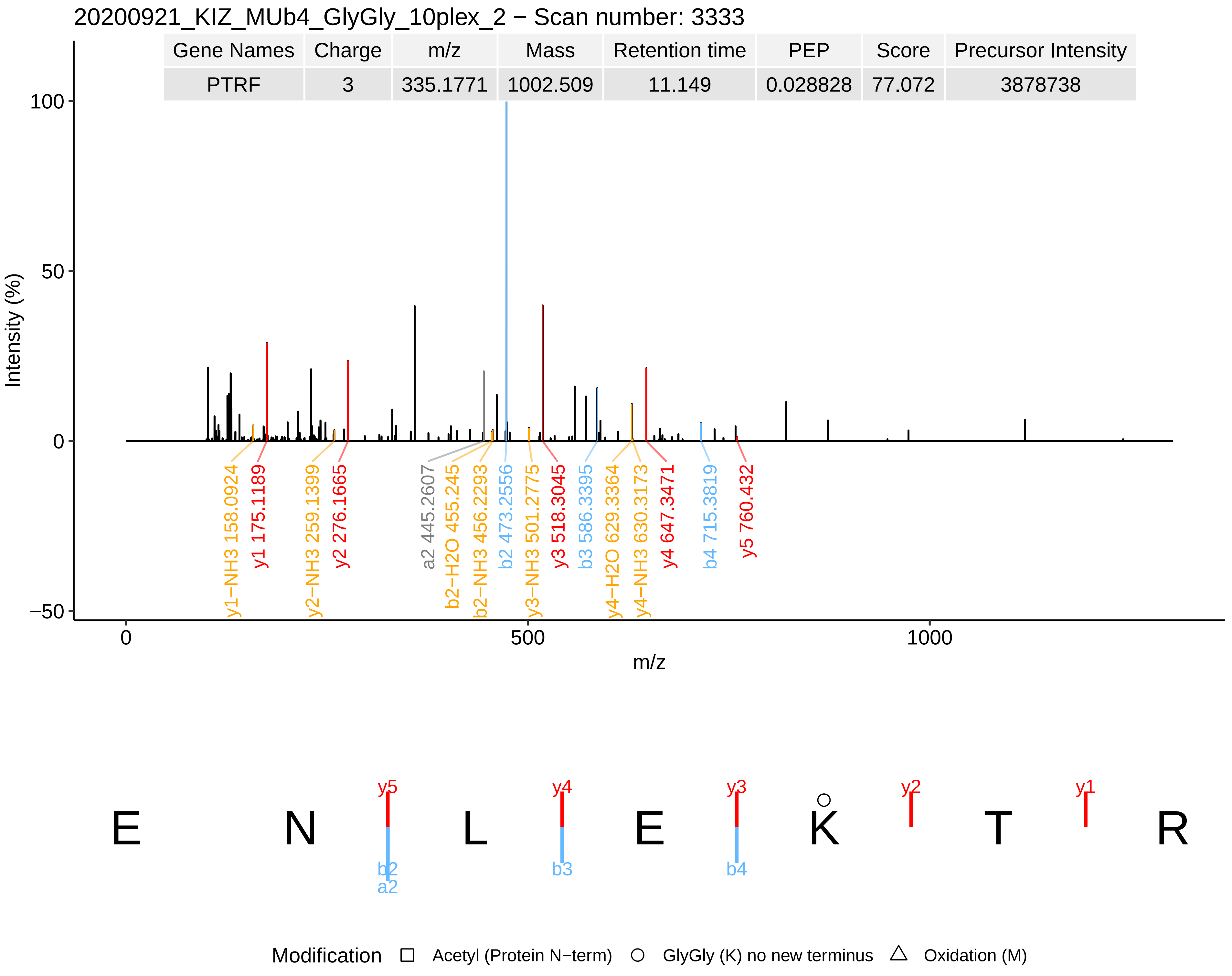
20200921_KIZ_MUb4_GlyGly_10plex_2 − Scan number: 3333
Gene Names | Charge | m/z | Mass | Retention time | PEP | Score | Precursor Intensity
PTRF | 3 | 335.1771 | 1002.509 | 11.149 | 0.028828 | 77.072 | 3878738
Intensity (%)
m/z
y1−NH3 158.0924
y1 175.1189
y2−NH3 259.1399
y2 276.1665
a2 445.2607
b2−H2O 455.245
b2−NH3 456.2293
b2 473.2556
y3−NH3 501.2775
y3 518.3045
b3 586.3395
y4−H2O 629.3364
y4−NH3 630.3173
y4 647.3471
b4 715.3819
y5 760.432
E N L E K T R
Modification
Acetyl (Protein N−term)
GlyGly (K) no new terminus
Oxidation (M)

20200924_KIZ_MUb5_GlyGly_1 − Scan number: 3210

Gene Names	Charge	m/z	Mass	Retention time	PEP	Score	Precursor Intensity
PTRF	3	335.1771	1002.509	9.8924	0.056028	66.457	39038680

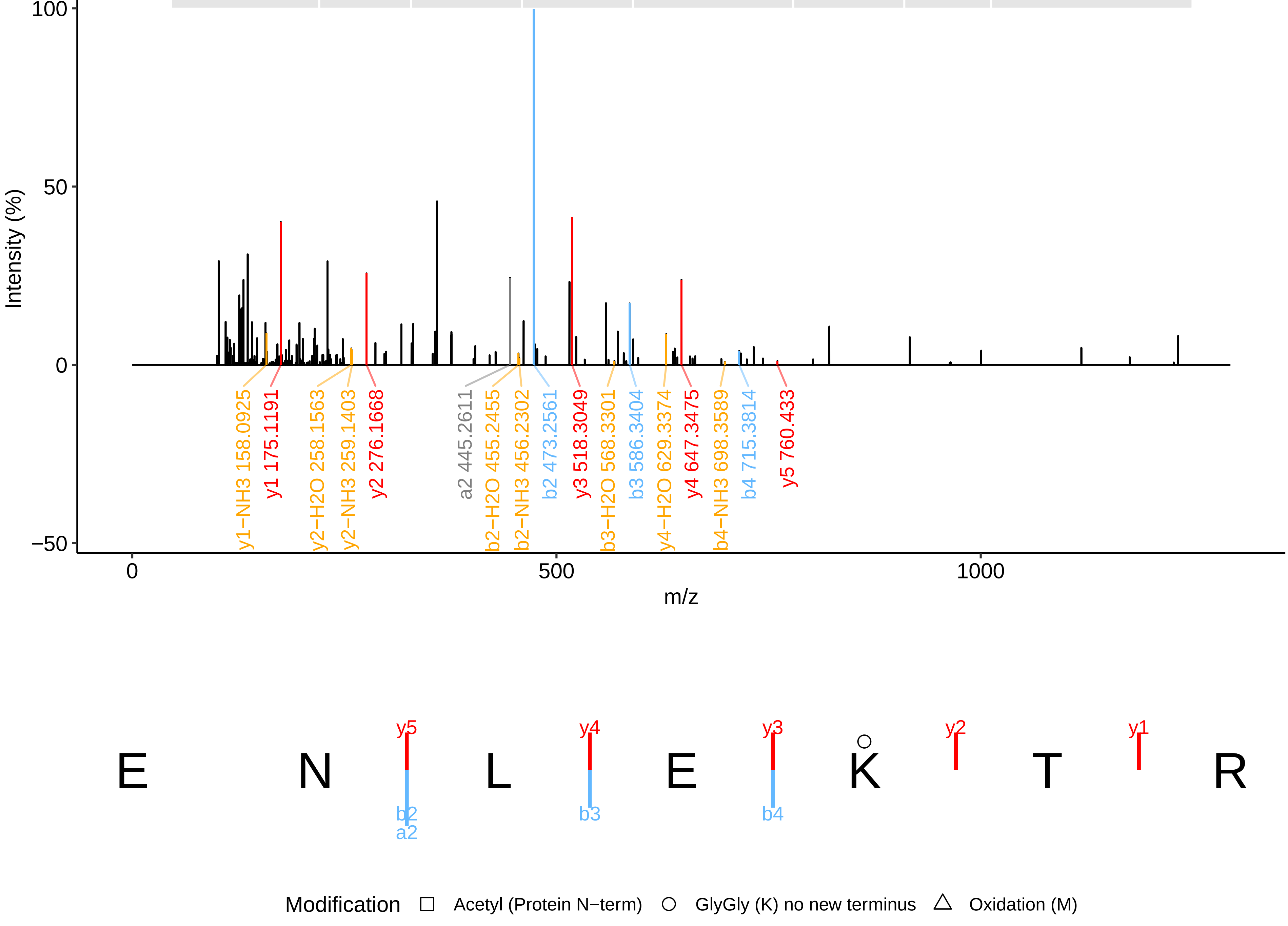

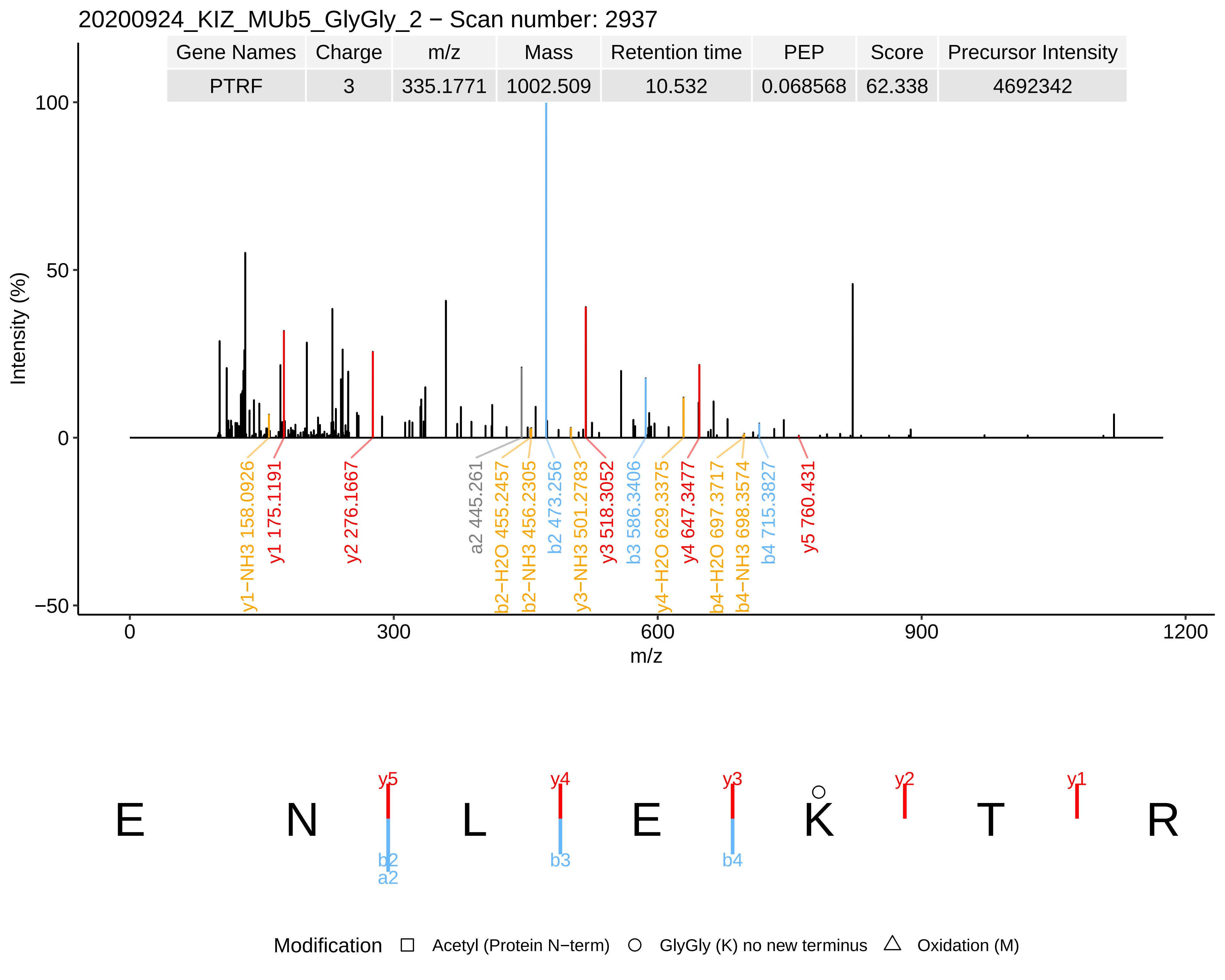
20200924_KIZ_MUb5_GlyGly_2 − Scan number: 2937
Gene Names | Charge | m/z | Mass | Retention time | PEP | Score | Precursor Intensity
PTRF | 3 | 335.1771 | 1002.509 | 10.532 | 0.068568 | 62.338 | 4692342
Intensity (%)
m/z
y1−NH3 158.0926
y1 175.1191
y2 276.1667
a2 445.261
b2−H2O 455.2457
b2−NH3 456.2305
b2 473.256
y3−NH3 501.2783
y3 518.3052
b3 586.3406
y4−H2O 629.3375
y4 647.3477
b4−H2O 697.3717
b4−NH3 698.3574
b4 715.3827
y5 760.431
E N L E K T R
y5 y4 y3 y2 y1
b2 a2 b3 b4
Modification
Acetyl (Protein N−term)
GlyGly (K) no new terminus
Oxidation (M)

20200921_KIZ_MUb4_GlyGly_10plex − Scan number: 6973

Gene Names	Charge	m/z	Mass	Retention time	PEP	Score	Precursor Intensity
PSMC2	3	343.8611	1028.562	23.27	0.016464	85.212	5730148

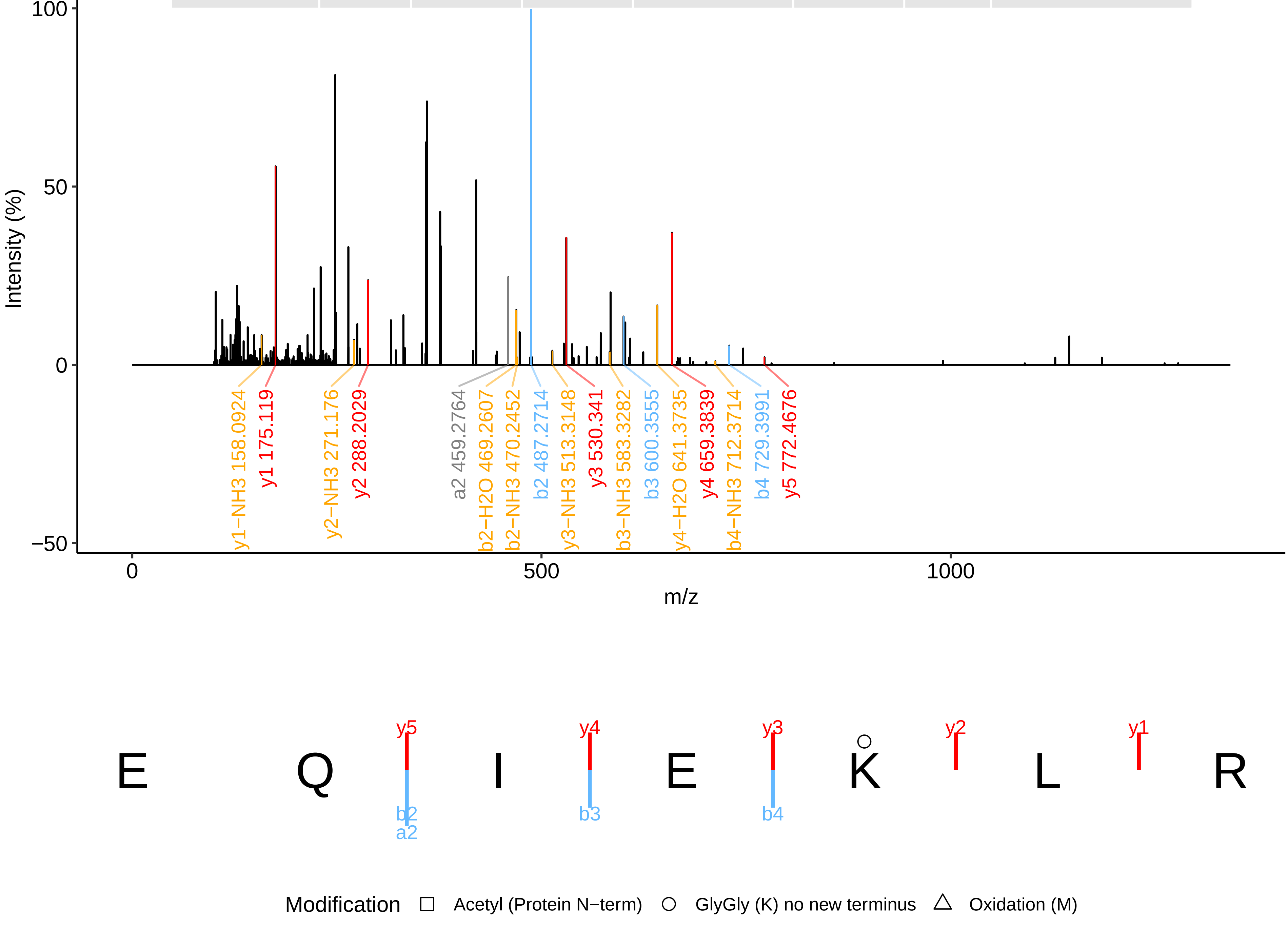

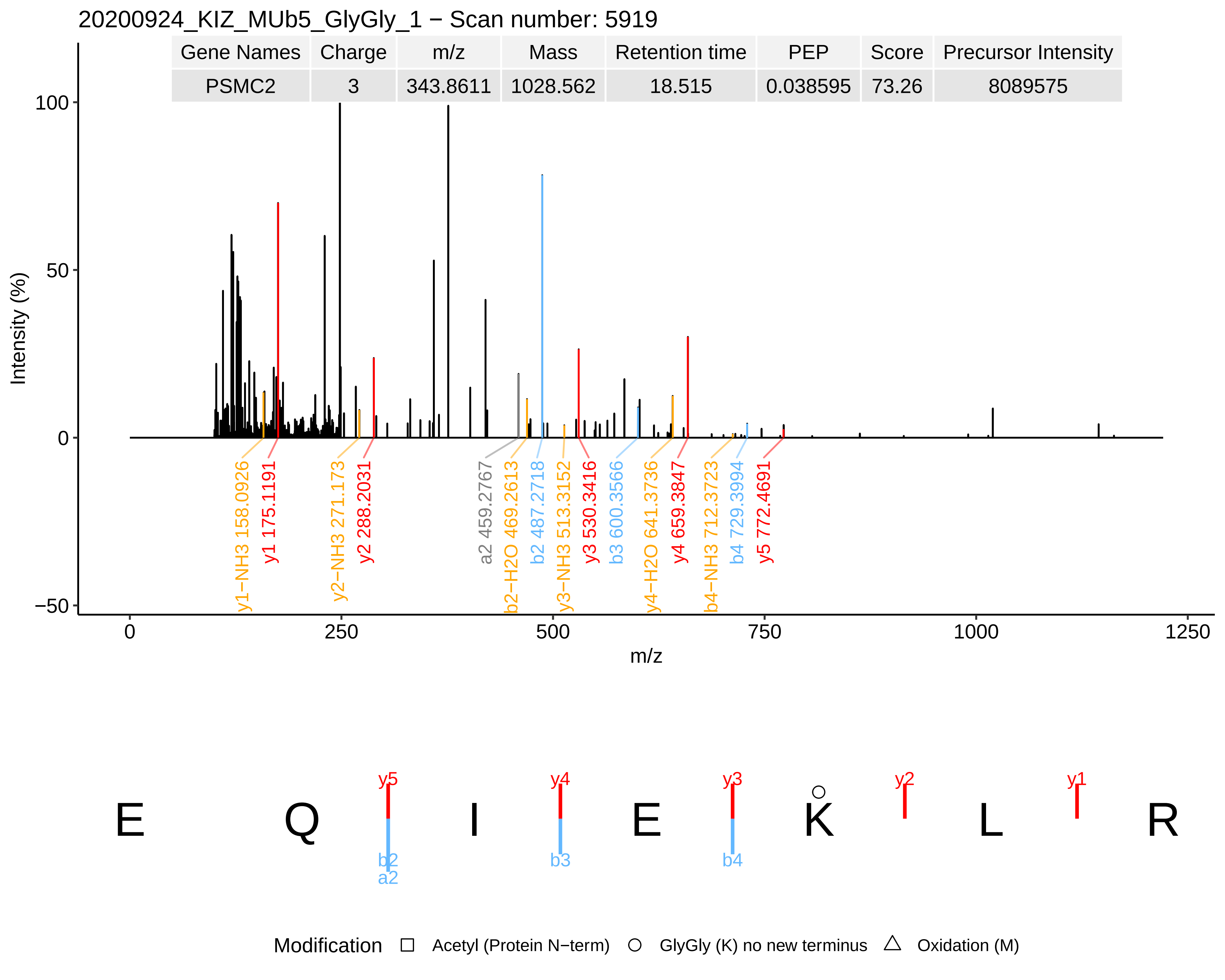

20200924_KIZ_MUb5_GlyGly_1 − Scan number: 5919
Gene Names | Charge | m/z | Mass | Retention time | PEP | Score | Precursor Intensity
PSMC2 | 3 | 343.8611 | 1028.562 | 18.515 | 0.038595 | 73.26 | 8089575
Intensity (%)
m/z
y1−NH3 158.0926
y1 175.1191
y2−NH3 271.173
y2 288.2031
a2 459.2767
b2−H2O 469.2613
b2 487.2718
y3−NH3 513.3152
y3 530.3416
b3 600.3566
y4−H2O 641.3736
y4 659.3847
b4−NH3 712.3723
b4 729.3994
y5 772.4691
E Q I E K L R
y5 y4 y3 y2 y1
b2 a2 b3 b4
Modification
Acetyl (Protein N−term)
GlyGly (K) no new terminus
Oxidation (M)

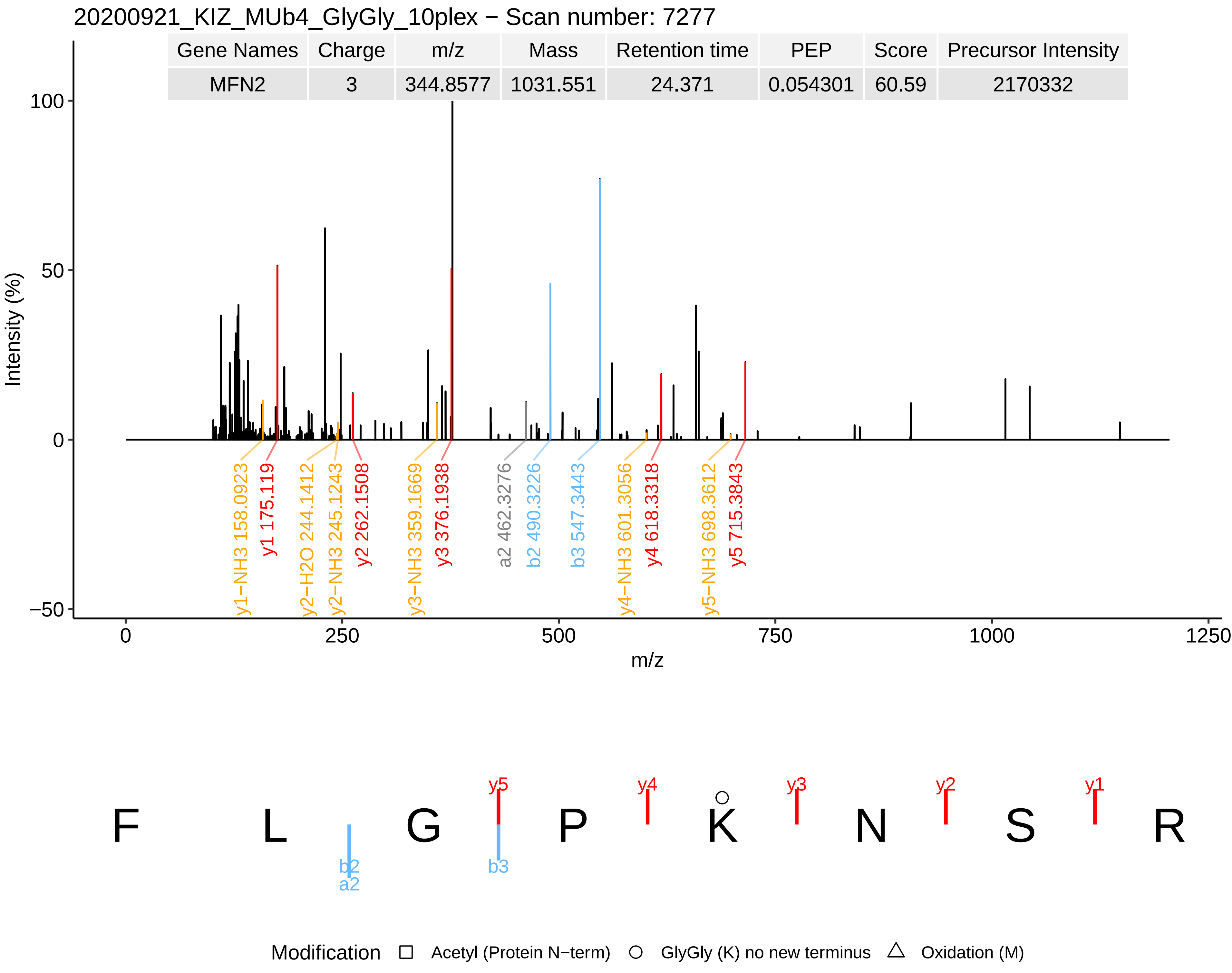
20200921_KIZ_MUb4_GlyGly_10plex − Scan number: 7277
Gene Names | Charge | m/z | Mass | Retention time | PEP | Score | Precursor Intensity
MFN2 | 3 | 344.8577 | 1031.551 | 24.371 | 0.054301 | 60.59 | 2170332
Intensity (%)
m/z
y1−NH3 158.0923
y1 175.119
y2−H2O 244.1412
y2−NH3 245.1243
y2 262.1508
y3−NH3 359.1669
y3 376.1938
a2 462.3276
b2 490.3226
b3 547.3443
y4−NH3 601.3056
y4 618.3318
y5−NH3 698.3612
y5 715.3843
F L G P K N S R
y5 y4 y3 y2 y1
b2 a2 b3
Modification
Acetyl (Protein N−term)
GlyGly (K) no new terminus
Oxidation (M)

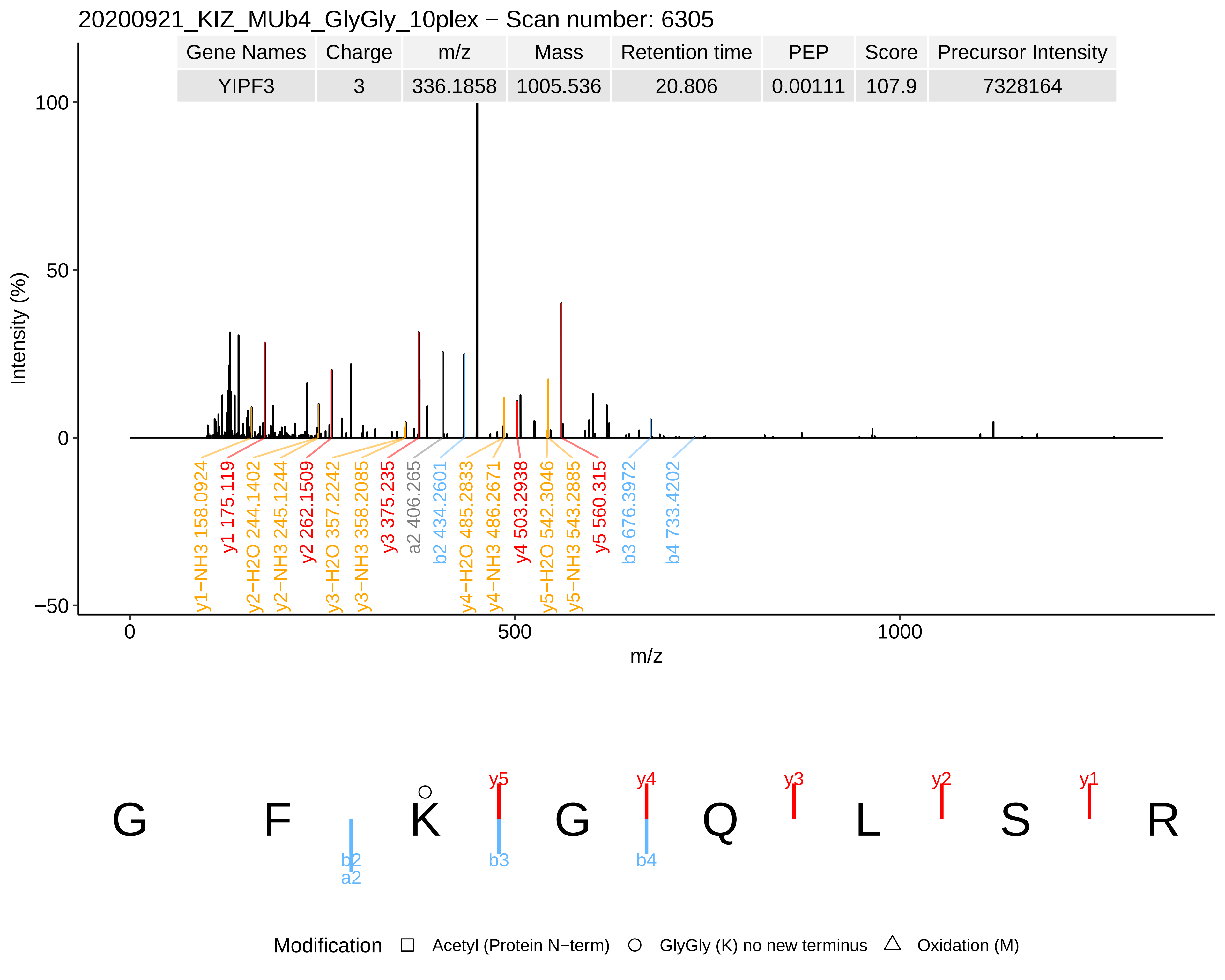

20200921_KIZ_MUb4_GlyGly_10plex − Scan number: 6305
Gene Names | Charge | m/z | Mass | Retention time | PEP | Score | Precursor Intensity
YIPF3 | 3 | 336.1858 | 1005.536 | 20.806 | 0.00111 | 107.9 | 7328164
Intensity (%)
m/z
y1−NH3 158.0924
y1 175.119
y2−H2O 244.1402
y2−NH3 245.1244
y2 262.1509
y3−H2O 357.2242
y3−NH3 358.2085
y3 375.235
a2 406.265
b2 434.2601
y4−H2O 485.2833
y4−NH3 486.2671
y4 503.2938
y5−H2O 542.3046
y5−NH3 543.2885
y5 560.315
b3 676.3972
b4 733.4202
G F K G Q L S R
y5 y4 y3 y2 y1
b2 a2 b3 b4
Modification
Acetyl (Protein N−term)
GlyGly (K) no new terminus
Oxidation (M)

20200921_KIZ_MUb4_GlyGly_10plex_2 − Scan number: 5810

Gene Names	Charge	m/z	Mass	Retention time	PEP	Score	Precursor Intensity
YIPF3	3	336.1858	1005.536	21.068	0.055397	57.177	1004890

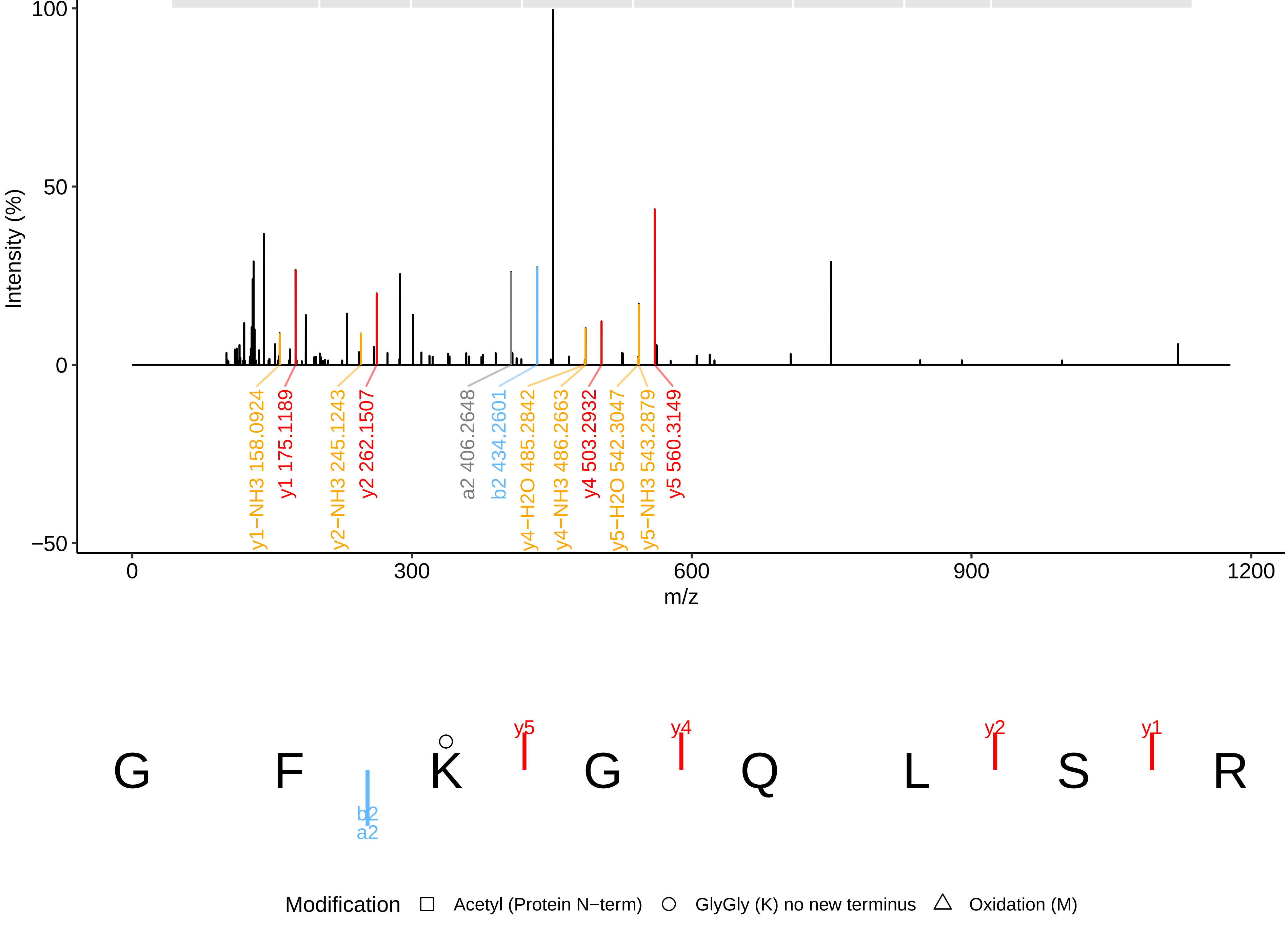

20200921_KIZ_MUb4_GlyGly_10plex_2 − Scan number: 4826

Gene Names	Charge	m/z	Mass	Retention time	PEP	Score	Precursor Intensity
DNAJC13	3	335.515	1003.523	17.096	0.010802	81.548	966096.3

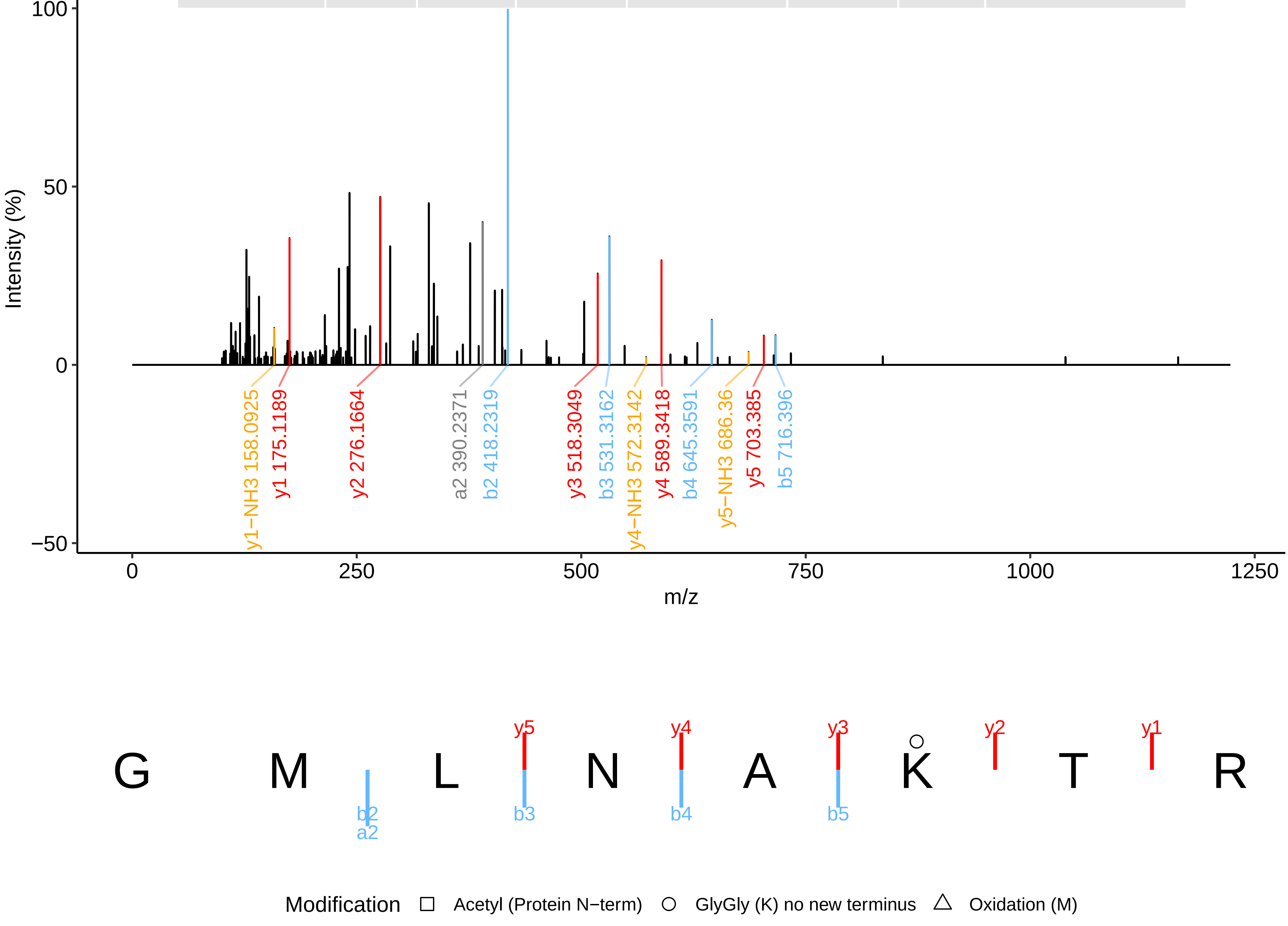

20200921_KIZ_MUb4_GlyGly_10plex − Scan number: 3966

Gene Names	Charge	m/z	Mass	Retention time	PEP	Score	Precursor Intensity
ZDHHC9	3	340.5054	1018.494	12.851	0.0056631	89.296	4106867

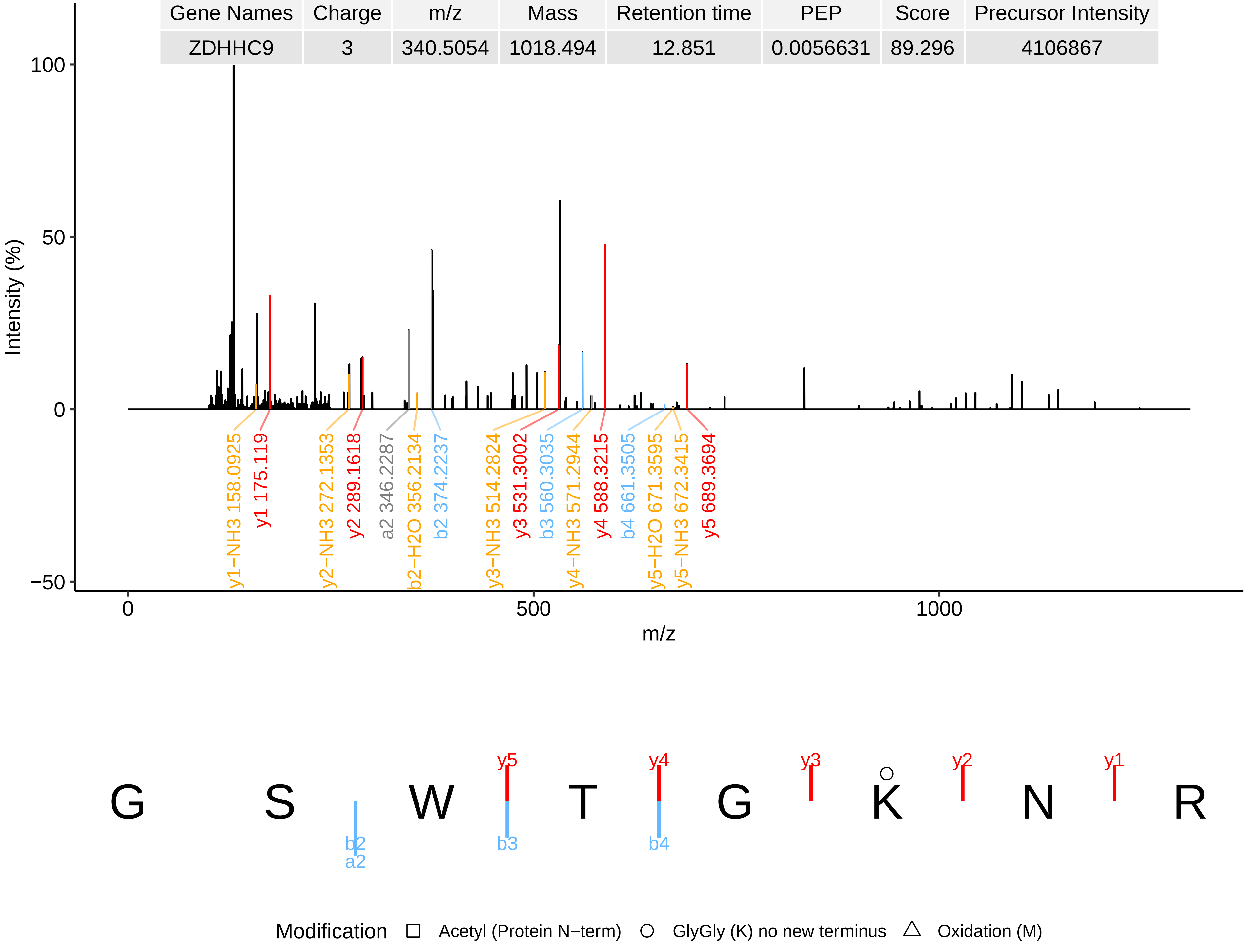

20200921_KIZ_MUb4_GlyGly_10plex − Scan number: 9141

Gene Names	Charge	m/z	Mass	Retention time	PEP	Score	Precursor Intensity
HSP90AA1;HSP90AA4P;HSP90AB1	3	348.8709	1043.591	31.265	0.0021834	98.418	23683860

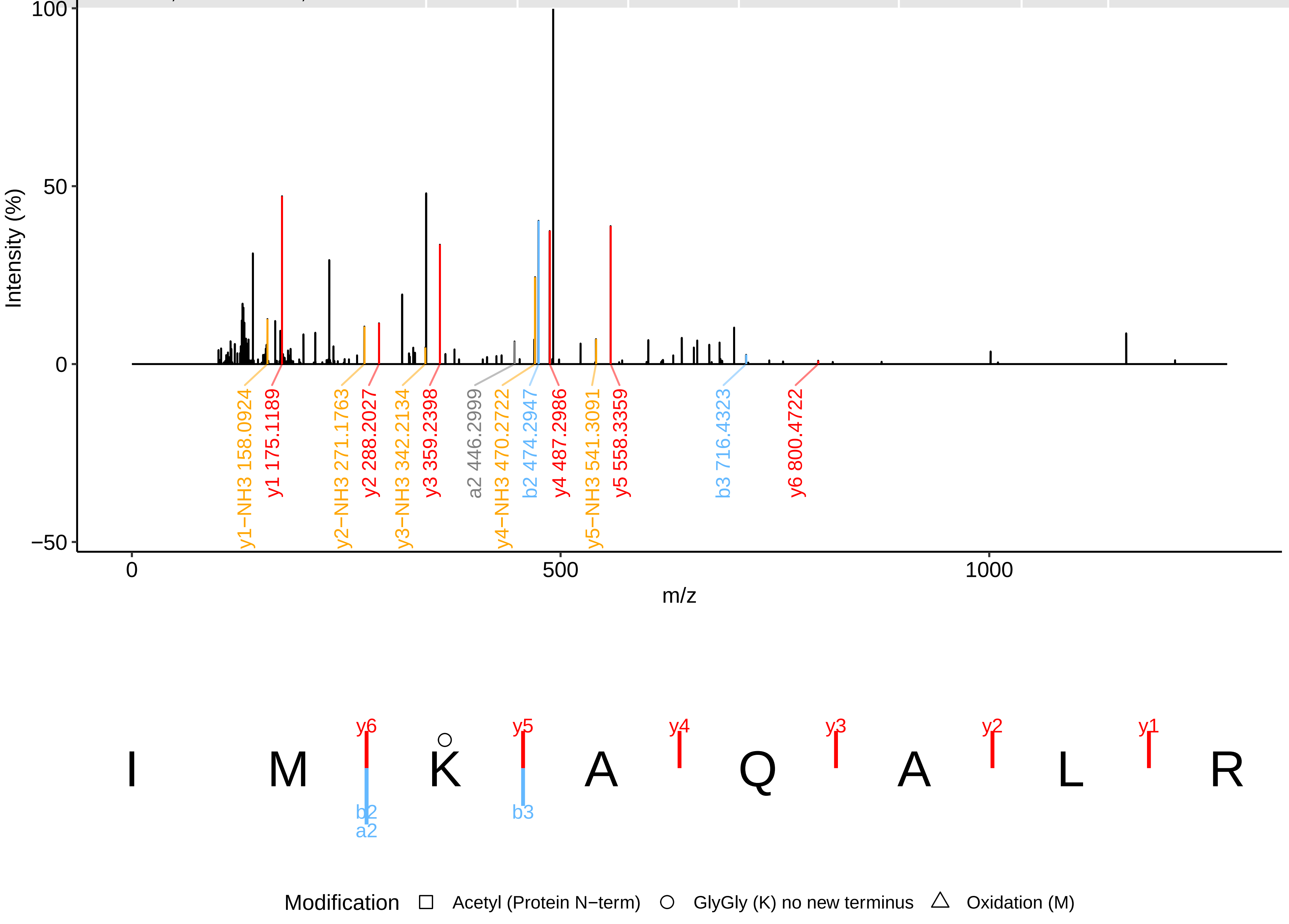

20200921_KIZ_MUb4_GlyGly_10plex_2 − Scan number: 8476

Gene Names	Charge	m/z	Mass	Retention time	PEP	Score	Precursor Intensity
HSP90AA1;HSP90AA4P;HSP90AB1	3	348.8709	1043.591	31.944	0.010511	79.451	2435405

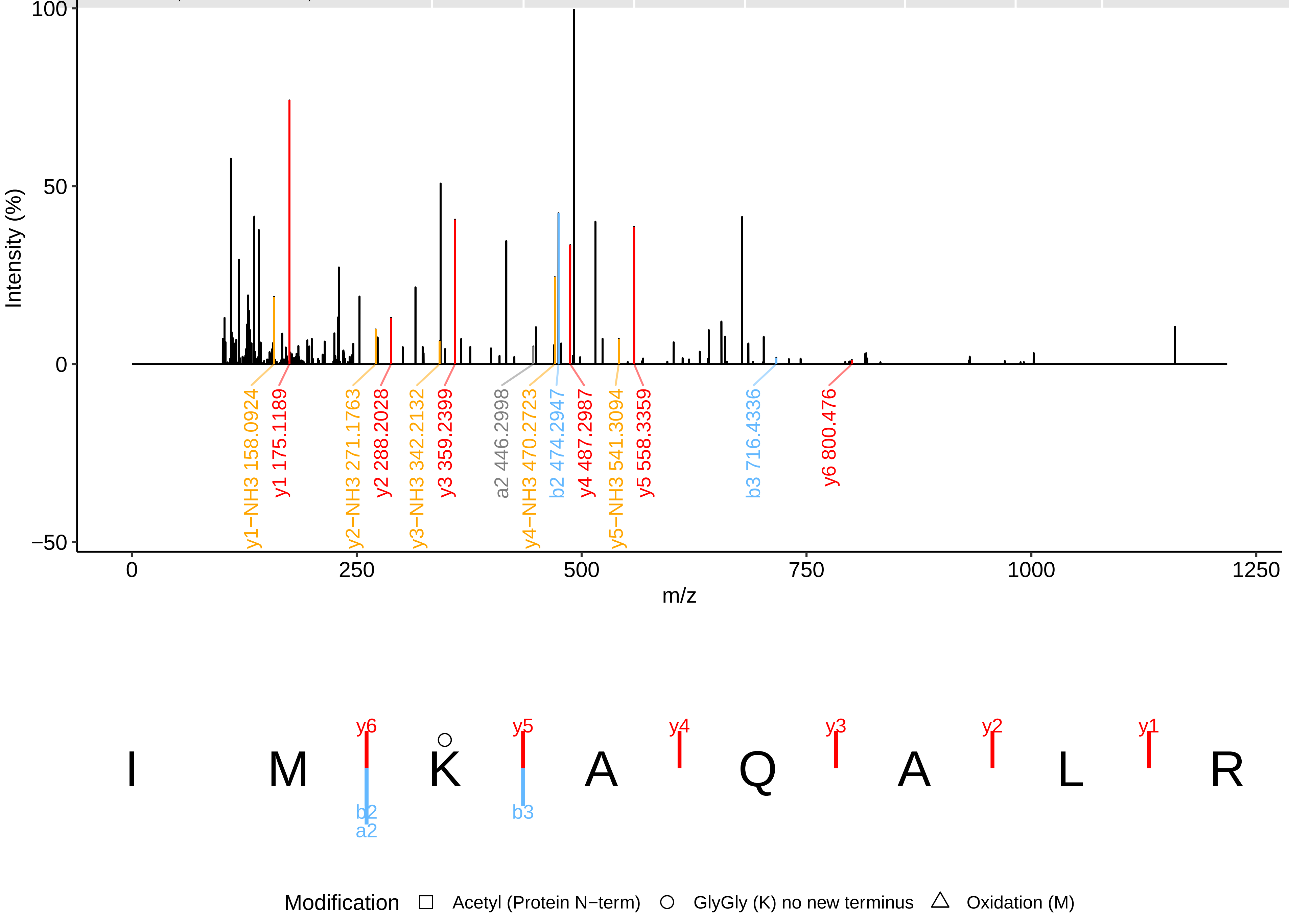

20200924_KIZ_MUb5_GlyGly_2 − Scan number: 7527

Gene Names	Charge	m/z	Mass	Retention time	PEP	Score	Precursor Intensity
HSP90AA1;HSP90AA4P;HSP90AB1	3	348.8709	1043.591	28.099	0.084729	56.27	3312806

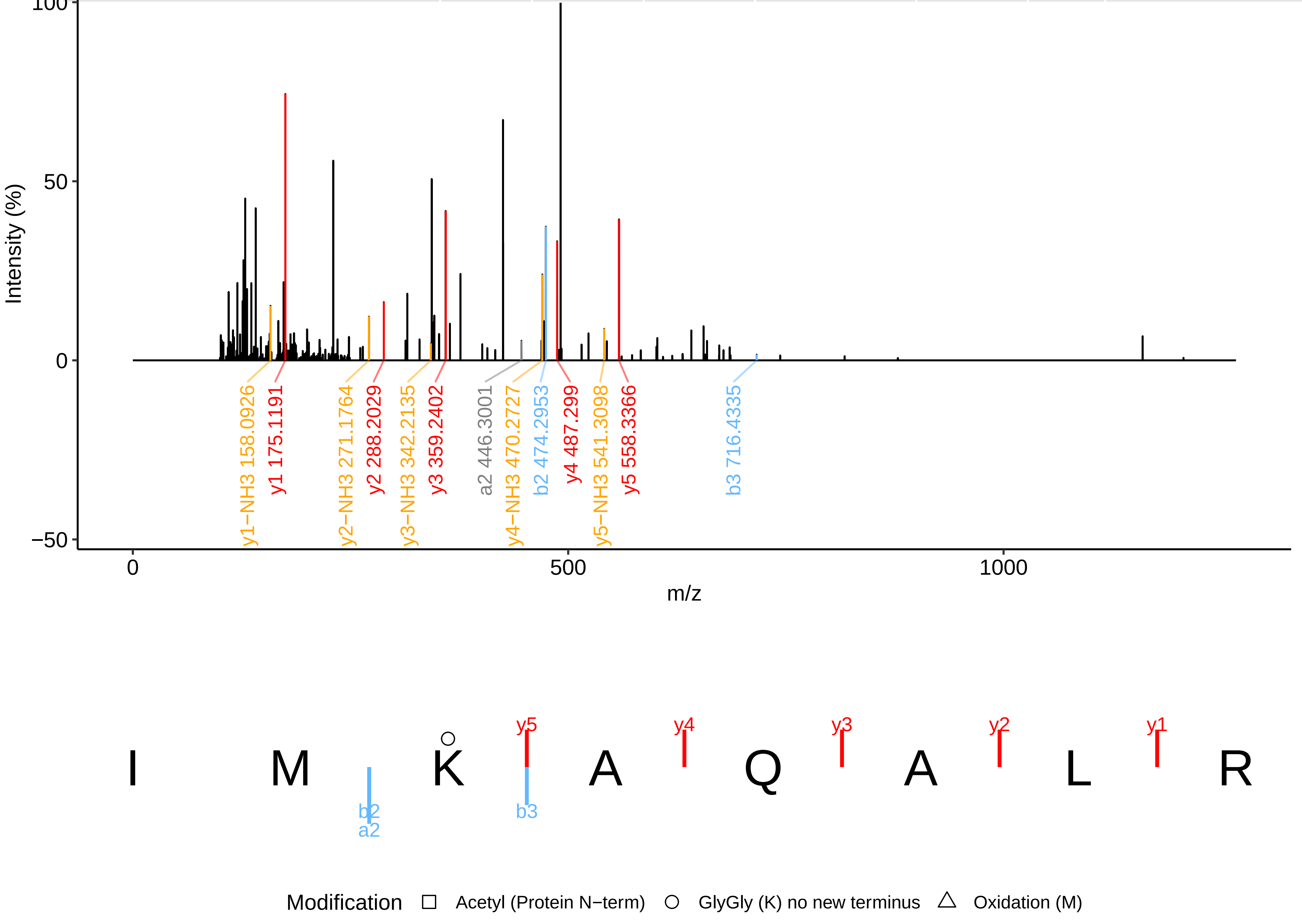

20201118_KIZ_MUb6_GlyGly_1 − Scan number: 7512

Gene Names	Charge	m/z	Mass	Retention time	PEP	Score	Precursor Intensity
HSP90AA1;HSP90AA4P;HSP90AB1	3	348.8709	1043.591	23.813	0.0070367	85.813	12122472

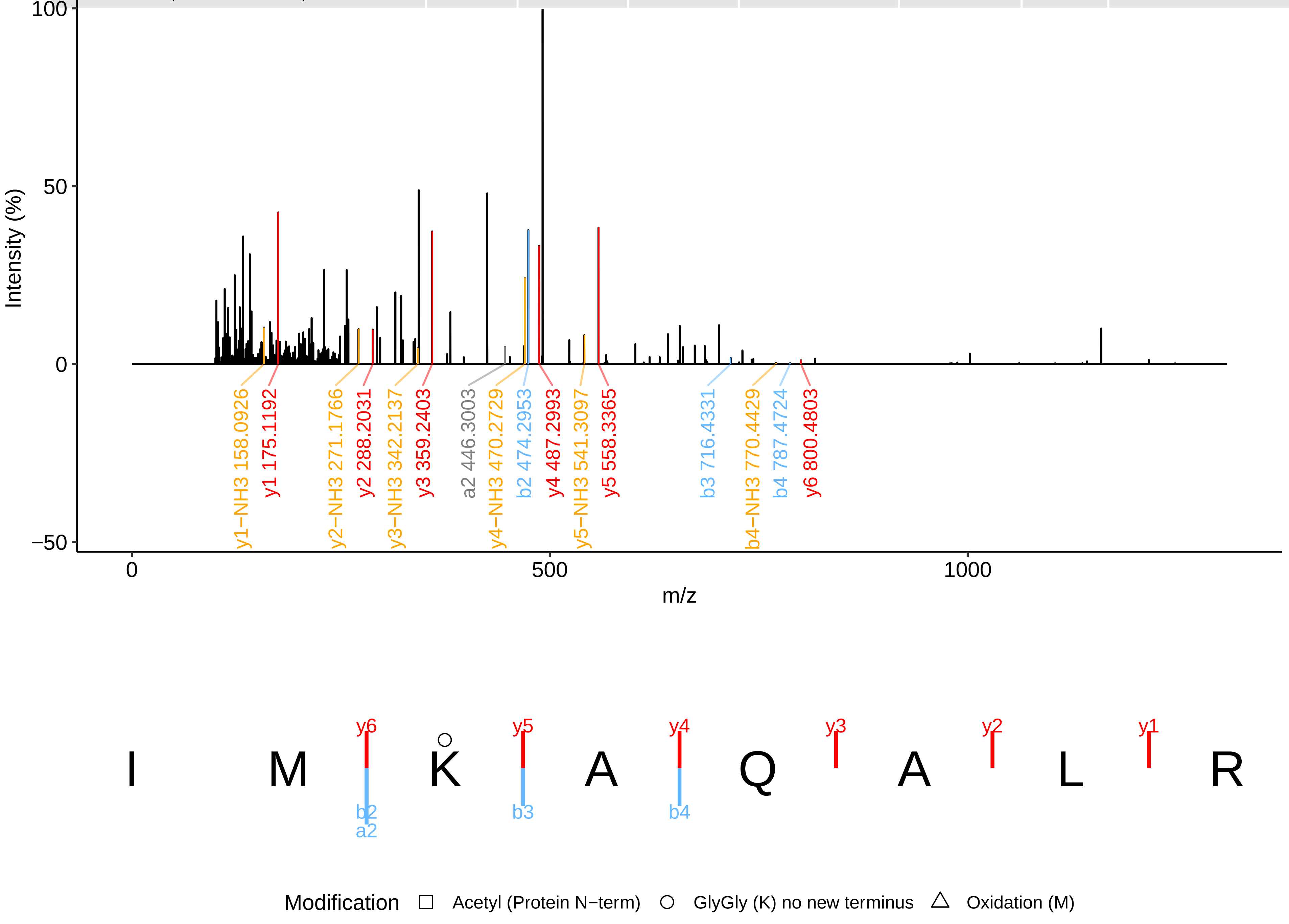

20201118_KIZ_MUb6_GlyGly_1 − Scan number: 4657

Gene Names	Charge	m/z	Mass	Retention time	PEP	Score	Precursor Intensity
SLC25A6	3	335.1892	1002.546	14.647	0.015753	75.243	12707282

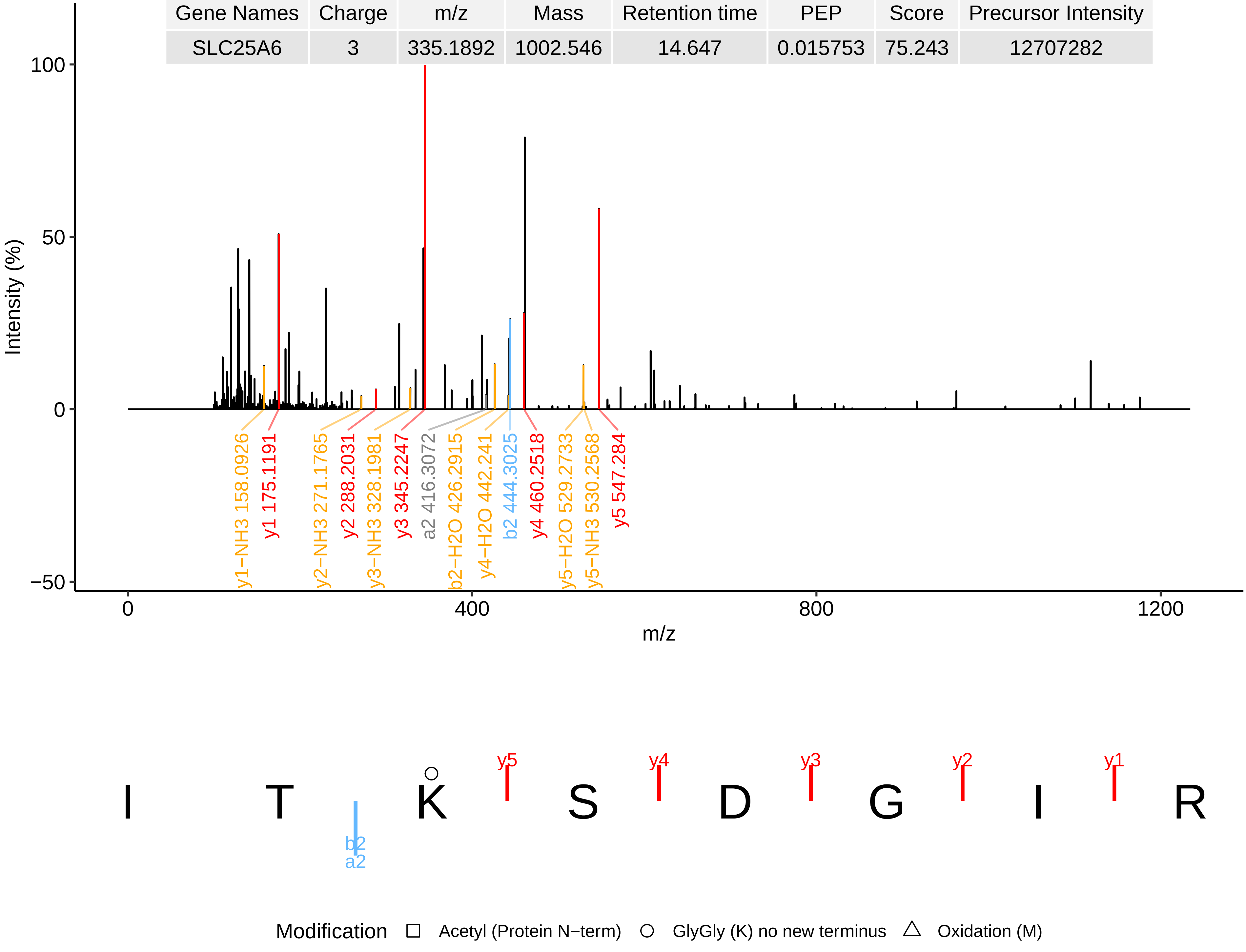

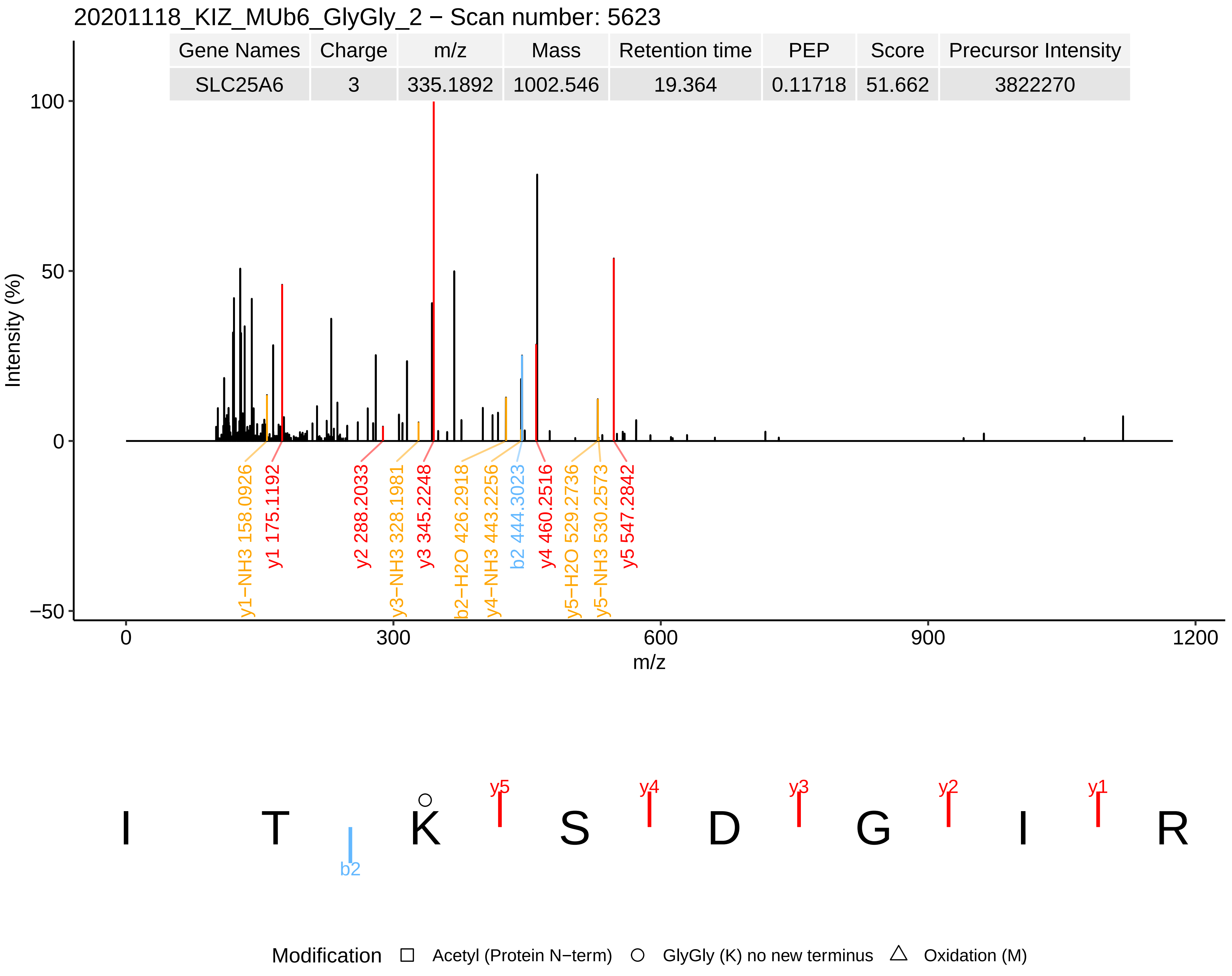
20201118_KIZ_MUb6_GlyGly_2 − Scan number: 5623
Gene Names | Charge | m/z | Mass | Retention time | PEP | Score | Precursor Intensity
SLC25A6 | 3 | 335.1892 | 1002.546 | 19.364 | 0.11718 | 51.662 | 3822270
Intensity (%)
m/z
y1−NH3 158.0926
y1 175.1192
y2 288.2033
y3−NH3 328.1981
y3 345.2248
b2−H2O 426.2918
y4−NH3 443.2256
b2 444.3023
y4 460.2516
y5−H2O 529.2736
y5−NH3 530.2573
y5 547.2842
I T K S D G I R
b2 y5 y4 y3 y2 y1
Modification □ Acetyl (Protein N−term) ○ GlyGly (K) no new terminus △ Oxidation (M)

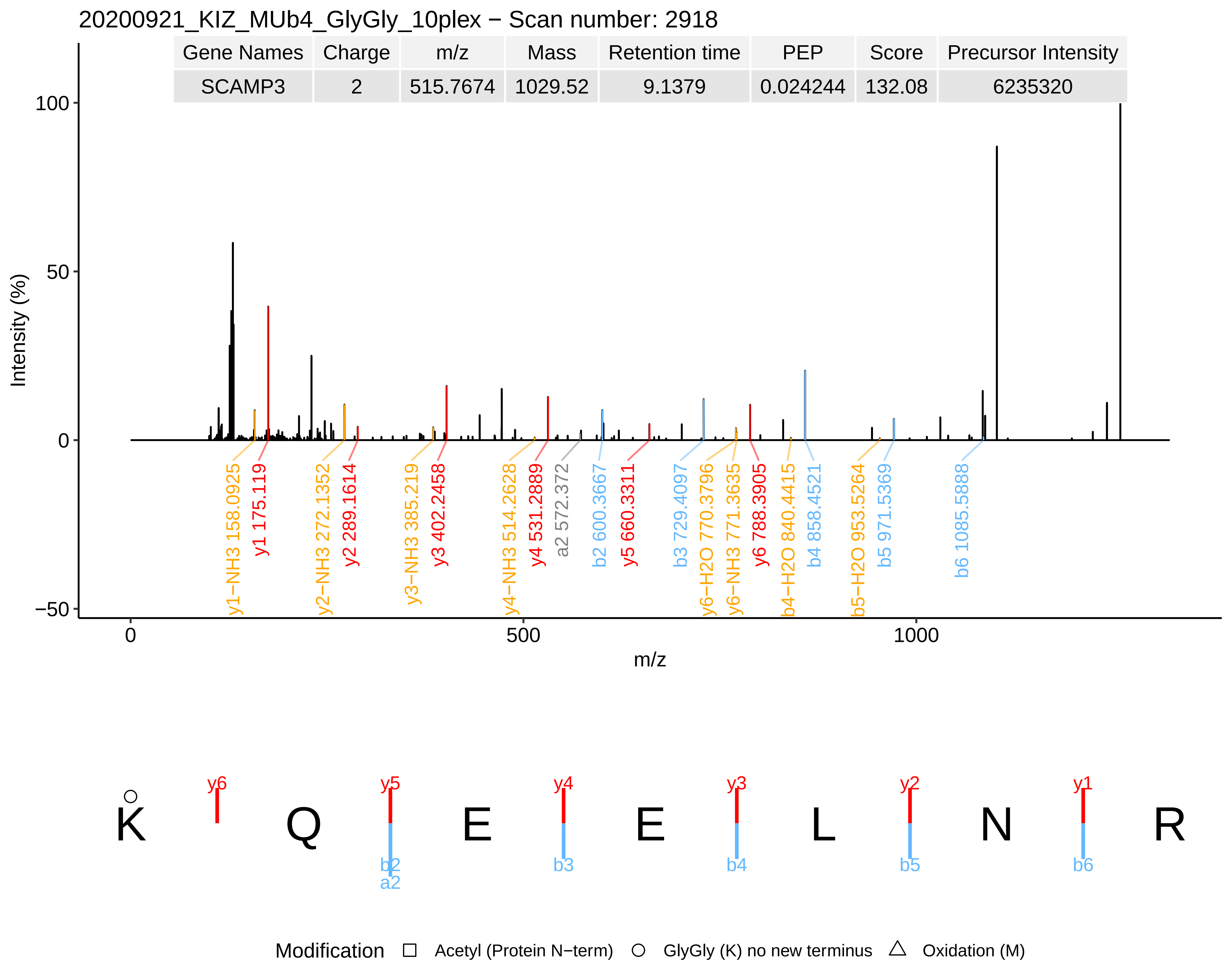

20200921_KIZ_MUb4_GlyGly_10plex − Scan number: 2918

Gene Names	Charge	m/z	Mass	Retention time	PEP	Score	Precursor Intensity
SCAMP3	2	515.7674	1029.52	9.1379	0.024244	132.08	6235320

Intensity (%)
m/z
y1−NH3 158.0925
y1 175.119
y2−NH3 272.1352
y2 289.1614
y3−NH3 385.219
y3 402.2458
y4−NH3 514.2628
y4 531.2889
a2 572.372
b2 600.3667
y5 660.3311
b3 729.4097
y6−H2O 770.3796
y6−NH3 771.3635
y6 788.3905
b4−H2O 840.4415
b4 858.4521
b5−H2O 953.5264
b5 971.5369
b6 1085.5888
K Q E E L N R
y6 y5 y4 y3 y2 y1
b2 a2 b3 b4 b5 b6
Modification □ Acetyl (Protein N−term) ○ GlyGly (K) no new terminus △ Oxidation (M)

20200921_KIZ_MUb4_GlyGly_10plex_2 − Scan number: 2857

Gene Names	Charge	m/z	Mass	Retention time	PEP	Score	Precursor Intensity
SCAMP3	3	344.1807	1029.52	9.2527	0.071716	91.906	8373688

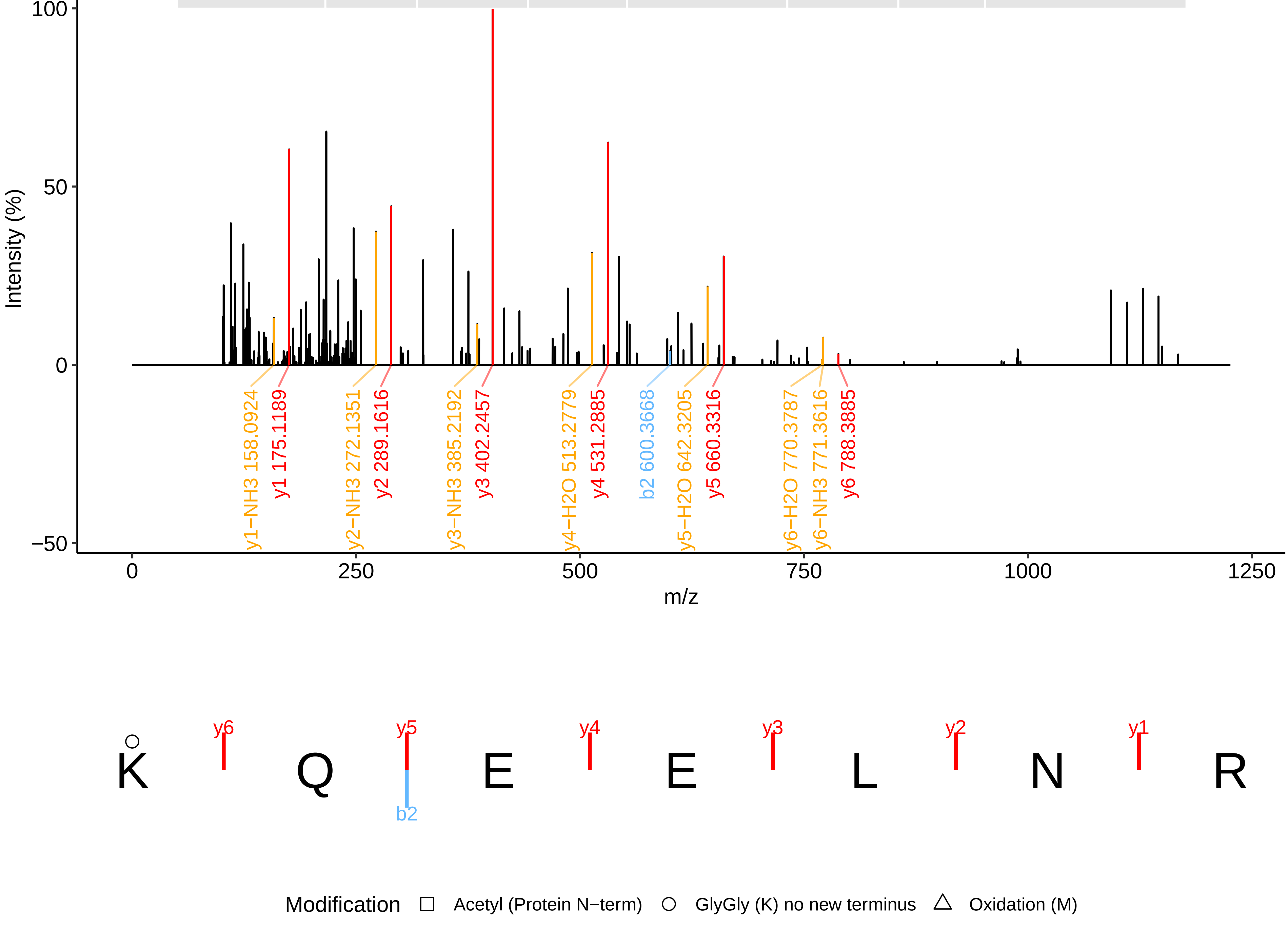

20200924_KIZ_MUb5_GlyGly_1 − Scan number: 2804

Gene Names	Charge	m/z	Mass	Retention time	PEP	Score	Precursor Intensity
SCAMP3	3	344.1807	1029.52	8.6588	0.093952	86.953	10462005

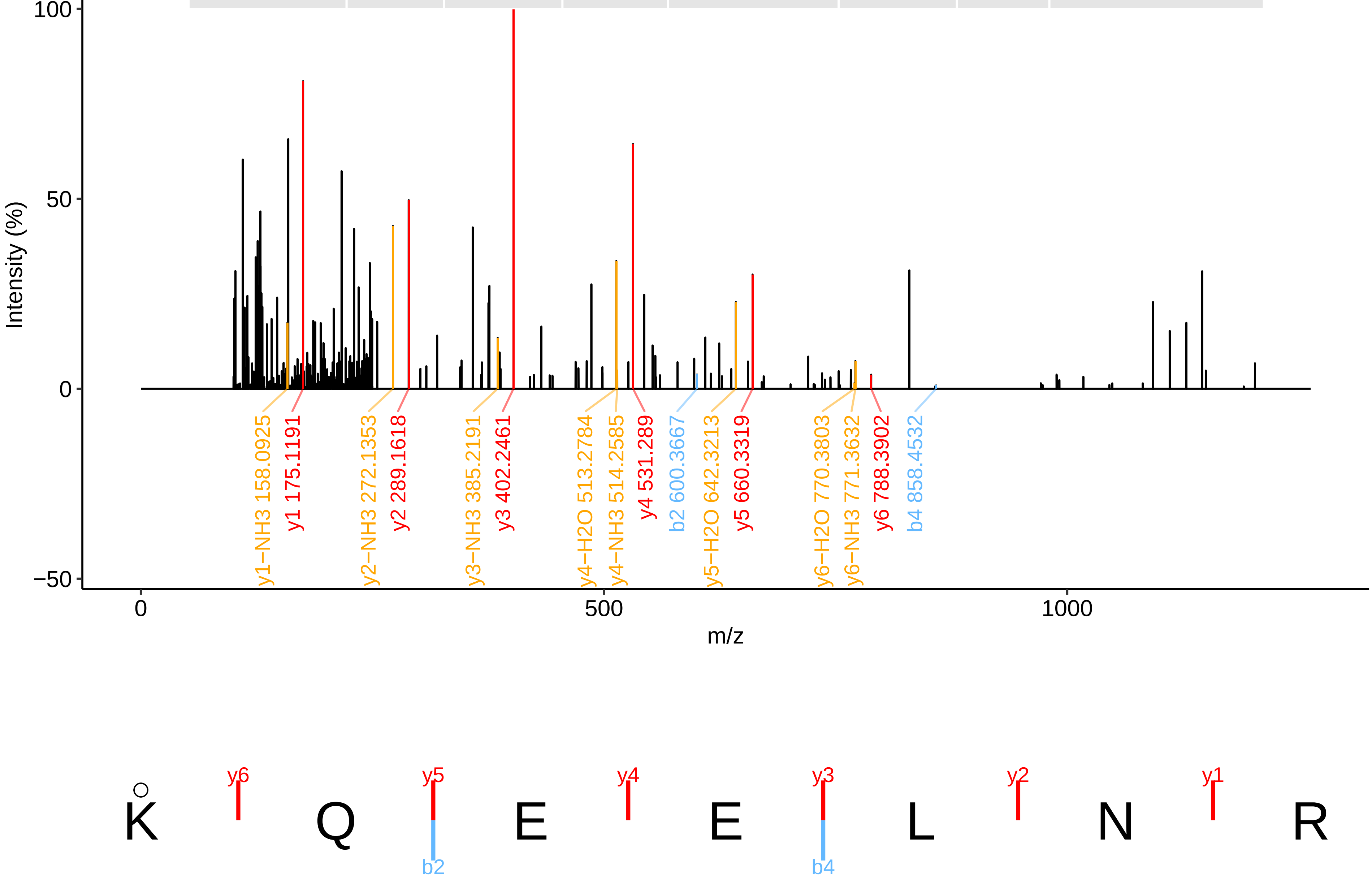

Modification □ Acetyl (Protein N−term) ○ GlyGly (K) no new terminus △ Oxidation (M)

20200924_KIZ_MUb5_GlyGly_1 − Scan number: 2826

Gene Names	Charge	m/z	Mass	Retention time	PEP	Score	Precursor Intensity
SCAMP3	2	515.7674	1029.52	8.7273	0.091043	93.429	NA

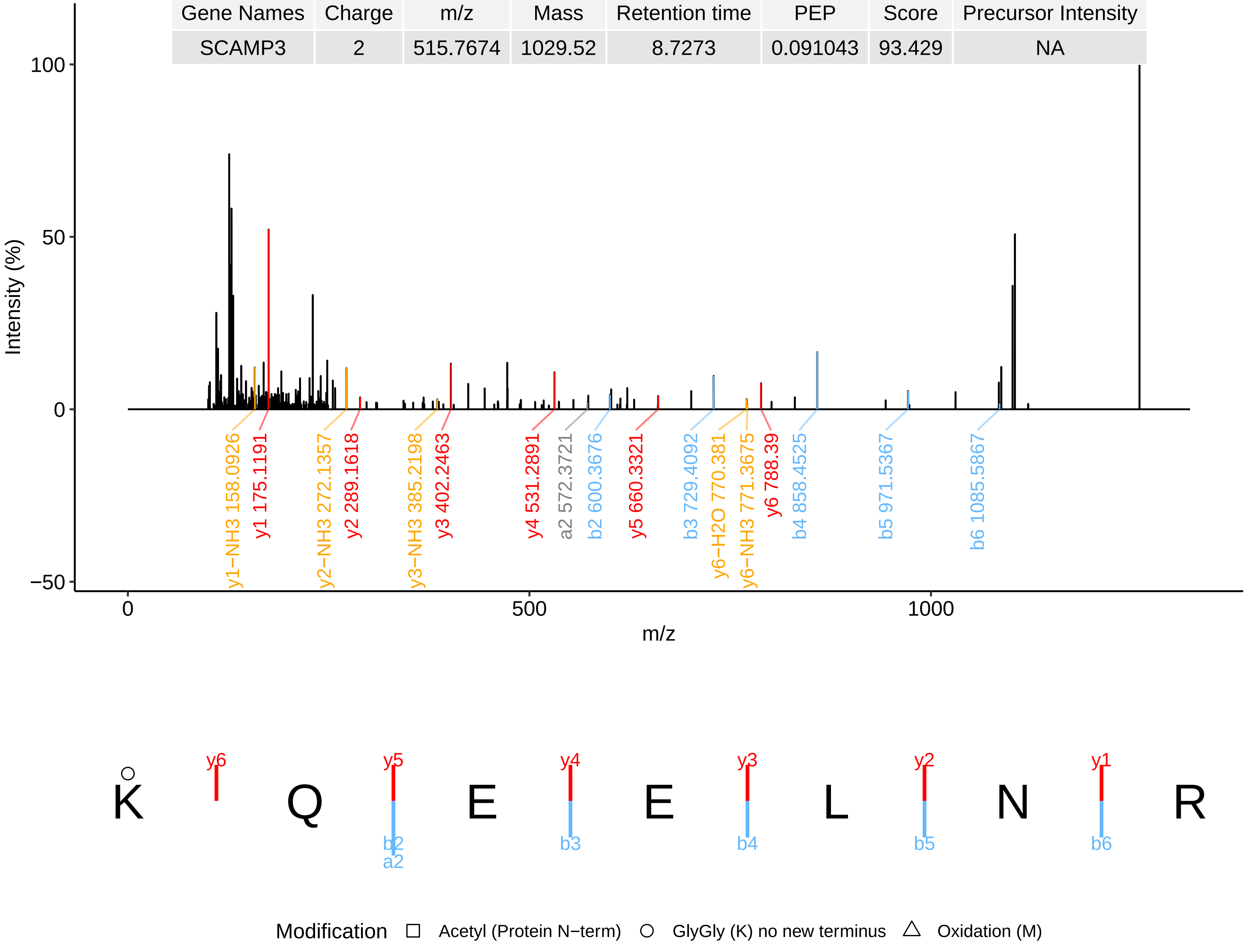

20201118_KIZ_MUb6_GlyGly_1 − Scan number: 2825

Gene Names	Charge	m/z	Mass	Retention time	PEP	Score	Precursor Intensity
SCAMP3	3	344.1807	1029.52	8.6474	0.0487	97.033	32331766

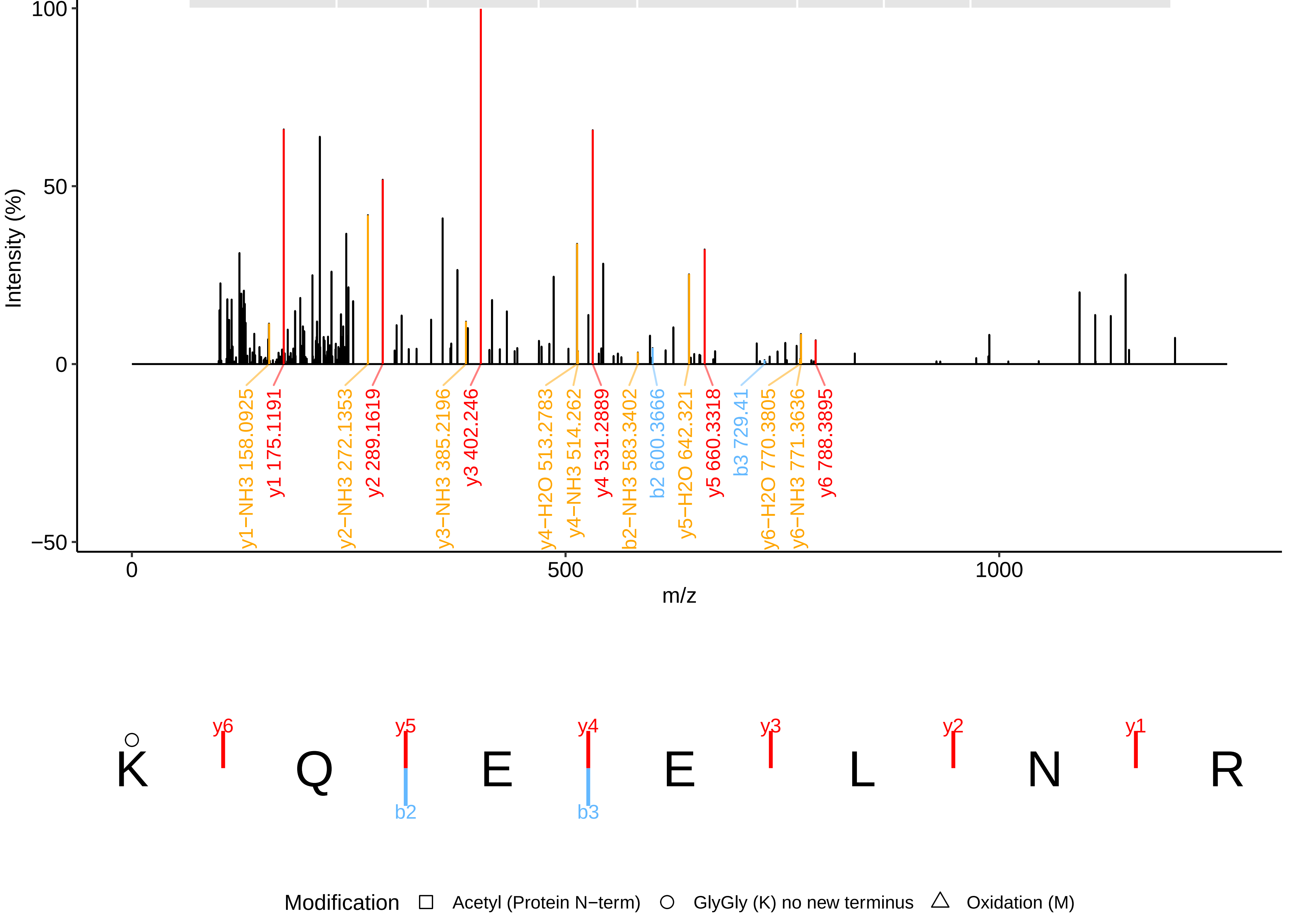

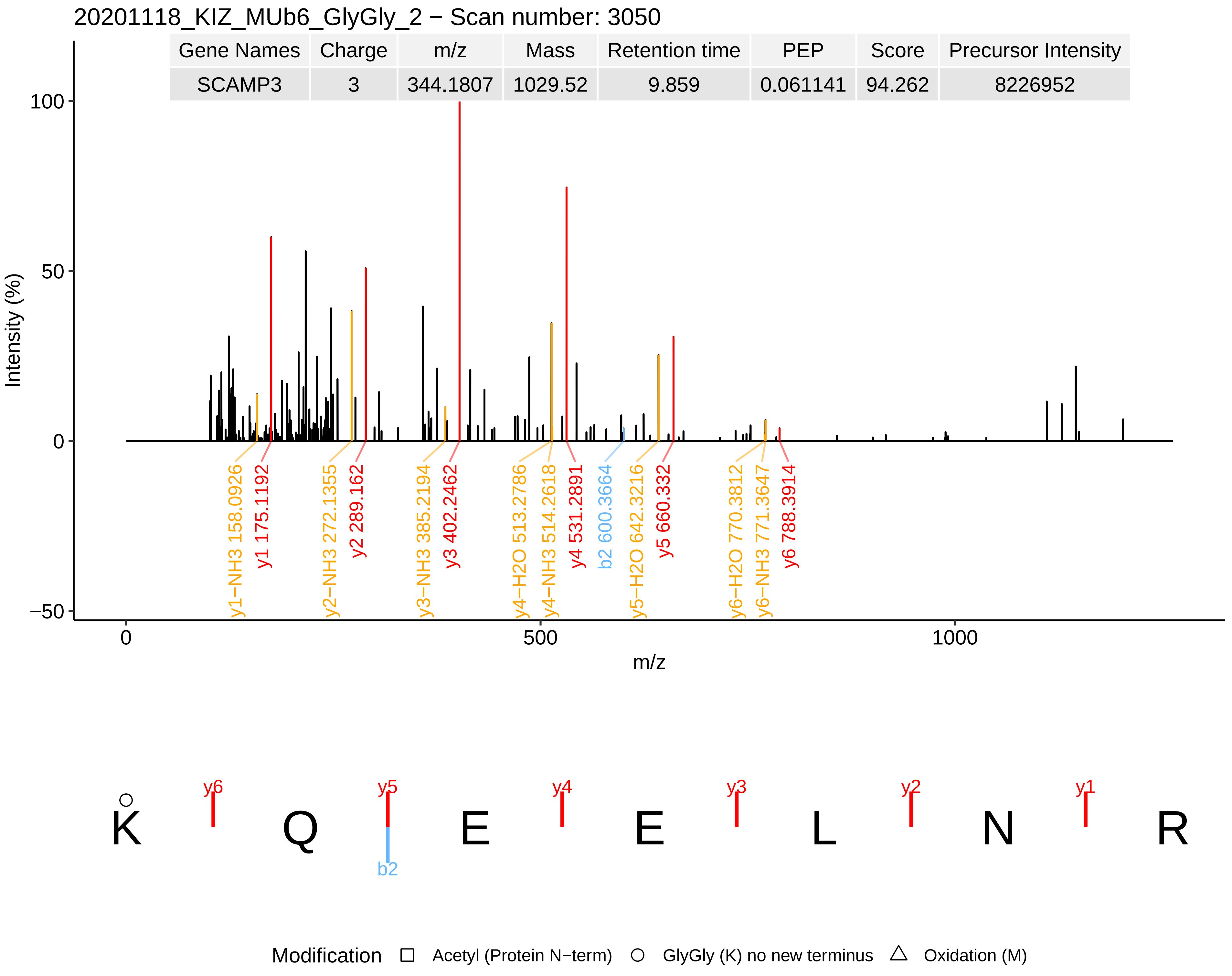

20201118_KIZ_MUb6_GlyGly_2 − Scan number: 3050
Gene Names | Charge | m/z | Mass | Retention time | PEP | Score | Precursor Intensity
SCAMP3 | 3 | 344.1807 | 1029.52 | 9.859 | 0.061141 | 94.262 | 8226952
Intensity (%)
m/z
y1−NH3 158.0926
y1 175.1192
y2−NH3 272.1355
y2 289.162
y3−NH3 385.2194
y3 402.2462
y4−H2O 513.2786
y4−NH3 514.2618
y4 531.2891
b2 600.3664
y5−H2O 642.3216
y5 660.332
y6−H2O 770.3812
y6−NH3 771.3647
y6 788.3914
K Q E E L N R
y6 y5 y4 y3 y2 y1
b2
Modification
Acetyl (Protein N−term)
GlyGly (K) no new terminus
Oxidation (M)

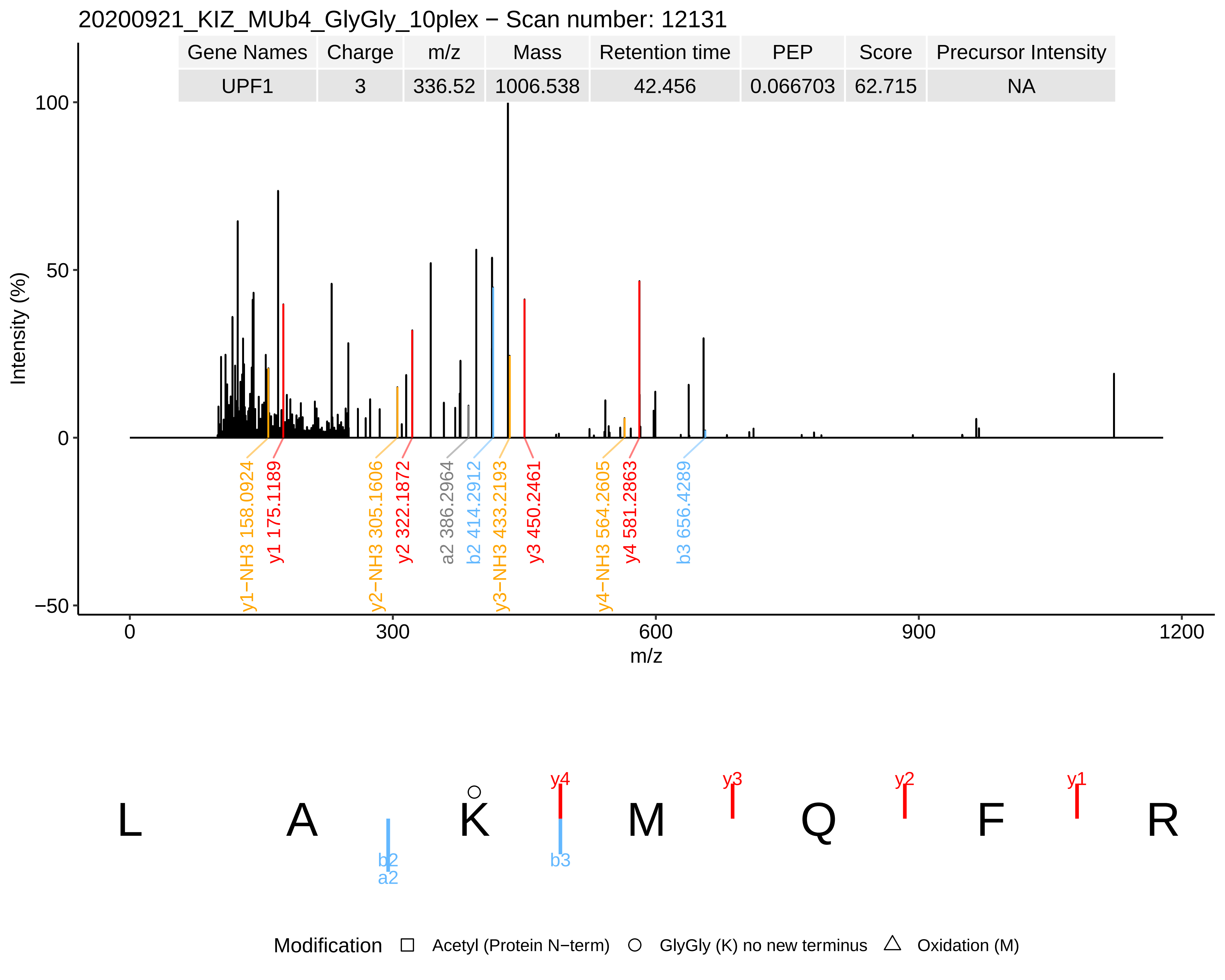

Gene Names	Charge	m/z	Mass	Retention time	PEP	Score	Precursor Intensity
UPF1	3	336.52	1006.538	42.456	0.066703	62.715	NA

20200924_KIZ_MUb5_GlyGly_1 − Scan number: 15860

Gene Names	Charge	m/z	Mass	Retention time	PEP	Score	Precursor Intensity
TRIM28	3	338.5537	1012.639	51.586	0.0068615	107.9	24339478

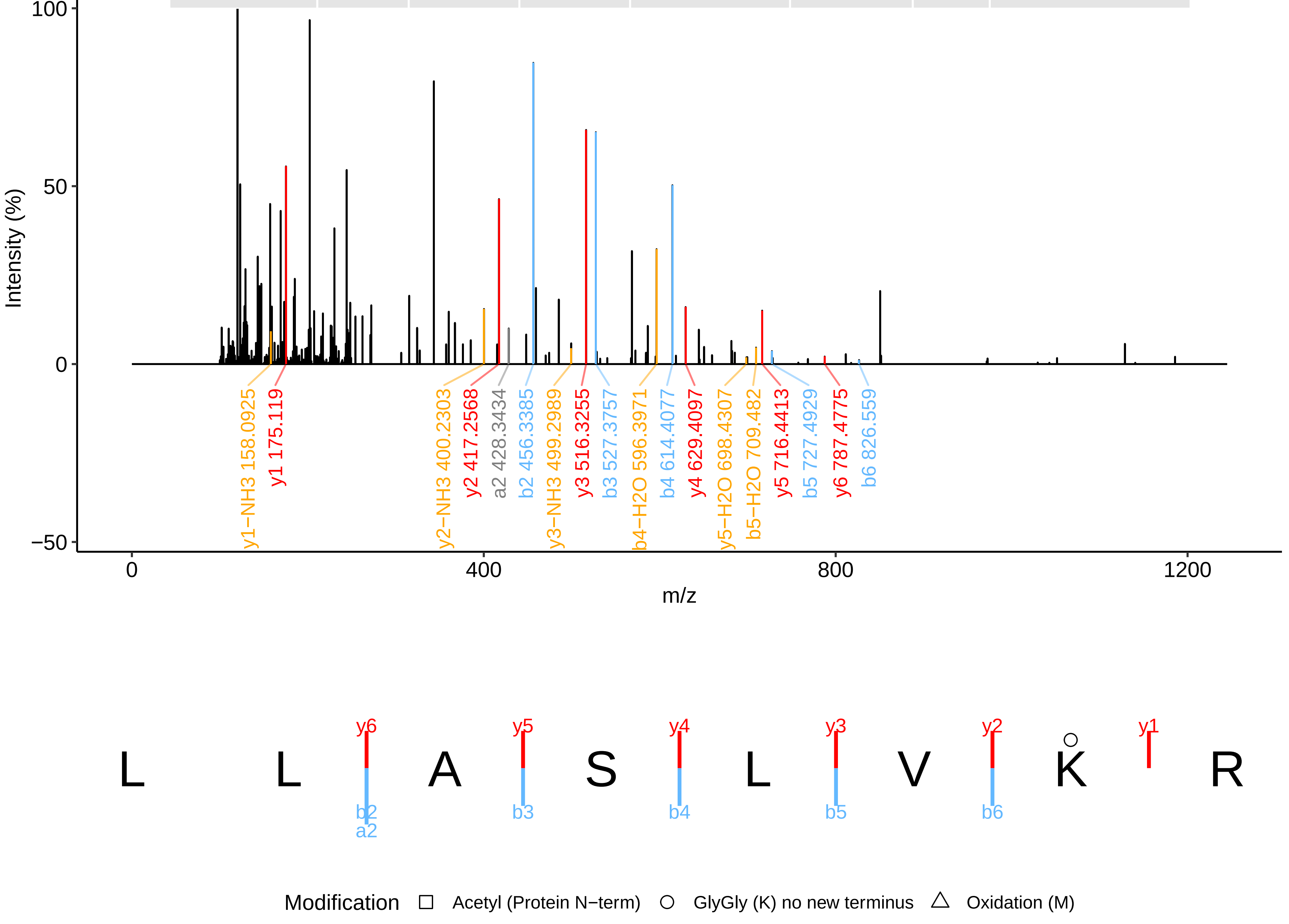

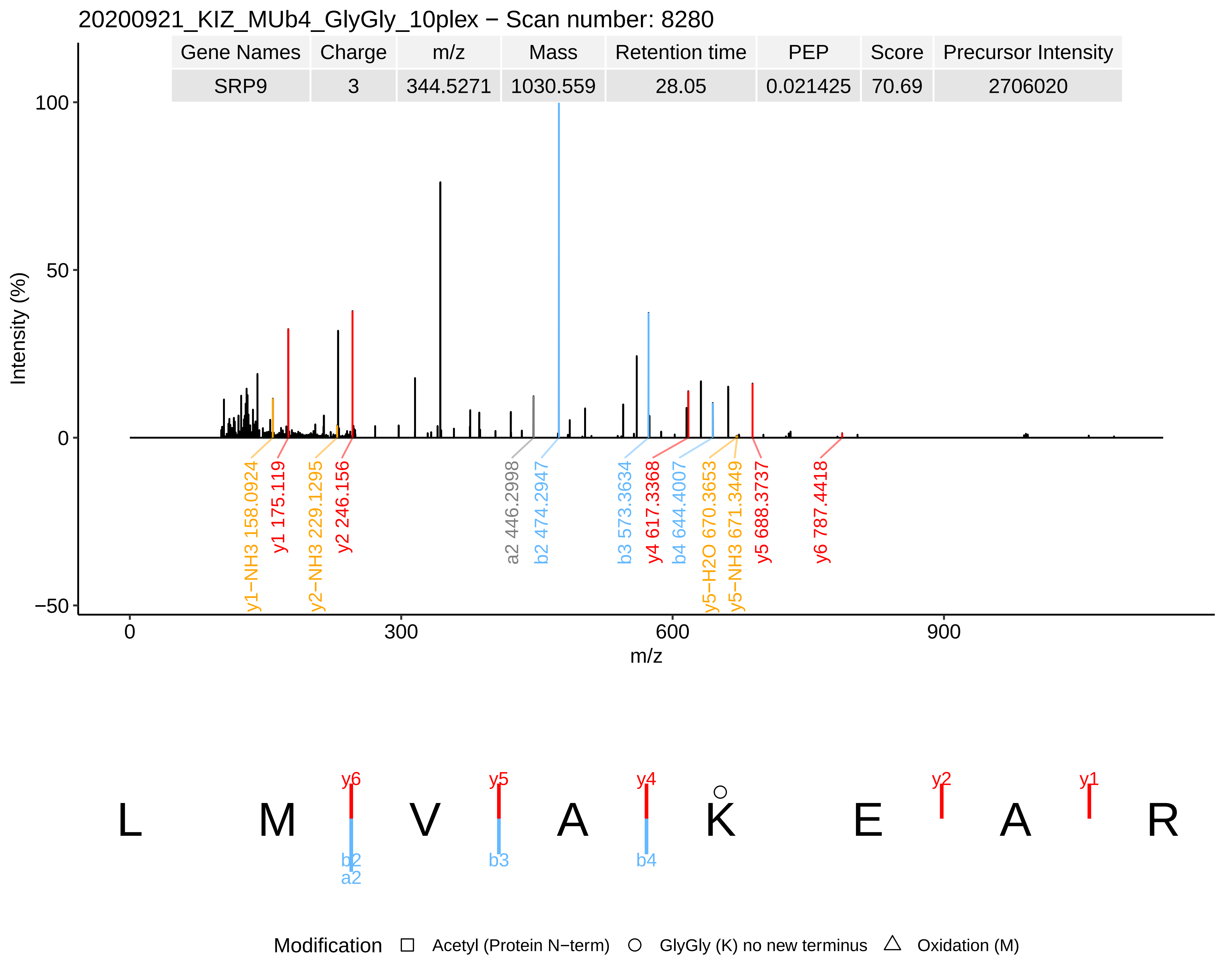
20200921_KIZ_MUb4_GlyGly_10plex − Scan number: 8280
Gene Names | Charge | m/z | Mass | Retention time | PEP | Score | Precursor Intensity
SRP9 | 3 | 344.5271 | 1030.559 | 28.05 | 0.021425 | 70.69 | 2706020
Intensity (%)
100
50
0
−50
0
300
600
900
m/z
y1−NH3 158.0924
y1 175.119
y2−NH3 229.1295
y2 246.156
a2 446.2998
b2 474.2947
b3 573.3634
y4 617.3368
b4 644.4007
y5−H2O 670.3653
y5−NH3 671.3449
y5 688.3737
y6 787.4418
L M V A K E A R
y6 y5 y4 y2 y1
b2 a2 b3 b4
Modification
Acetyl (Protein N−term)
GlyGly (K) no new terminus
Oxidation (M)

20200924_KIZ_MUb5_GlyGly_1 − Scan number: 7146

Gene Names	Charge	m/z	Mass	Retention time	PEP	Score	Precursor Intensity
SRP9	3	344.5271	1030.559	22.636	0.022394	73.248	15914776

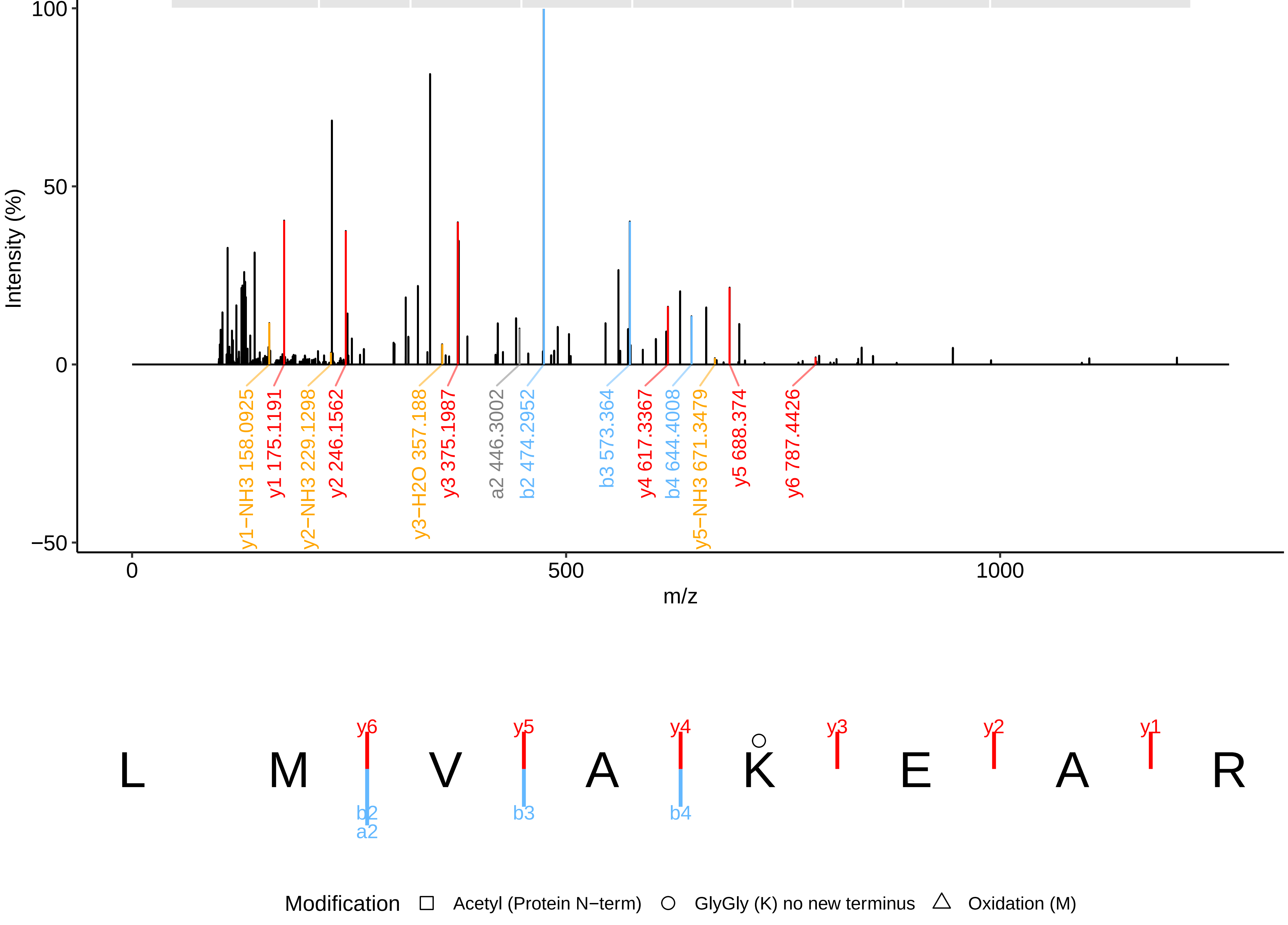

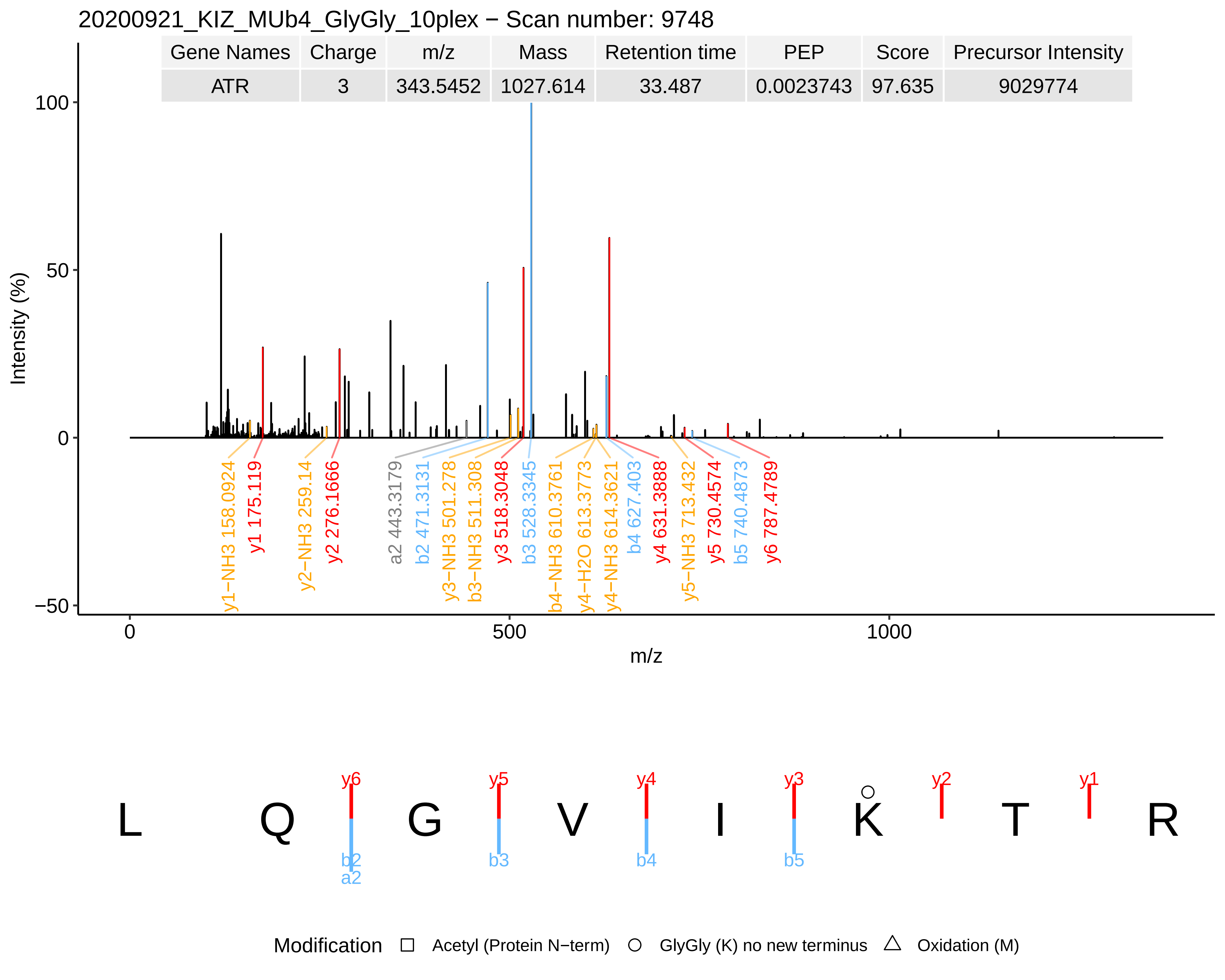
20200921_KIZ_MUb4_GlyGly_10plex − Scan number: 9748
Gene Names | Charge | m/z | Mass | Retention time | PEP | Score | Precursor Intensity
ATR | 3 | 343.5452 | 1027.614 | 33.487 | 0.0023743 | 97.635 | 9029774
Intensity (%)
100
50
0
−50
0
500
1000
m/z
y1−NH3 158.0924
y1 175.119
y2−NH3 259.14
y2 276.1666
a2 443.3179
b2 471.3131
y3−NH3 501.278
b3−NH3 511.308
y3 518.3048
b3 528.3345
b4−NH3 610.3761
y4−H2O 613.3773
y4−NH3 614.3621
b4 627.403
y4 631.3888
y5−NH3 713.432
y5 730.4574
b5 740.4873
y6 787.4789
L Q G V I K T R
y6 y5 y4 y3 y2 y1
b2 a2 b3 b4 b5
Modification
Acetyl (Protein N−term)
GlyGly (K) no new terminus
Oxidation (M)

20200921_KIZ_MUb4_GlyGly_10plex_2 − Scan number: 9007

Gene Names	Charge	m/z	Mass	Retention time	PEP	Score	Precursor Intensity
ATR	3	343.5452	1027.614	34.117	0.0066985	86.67	2673320

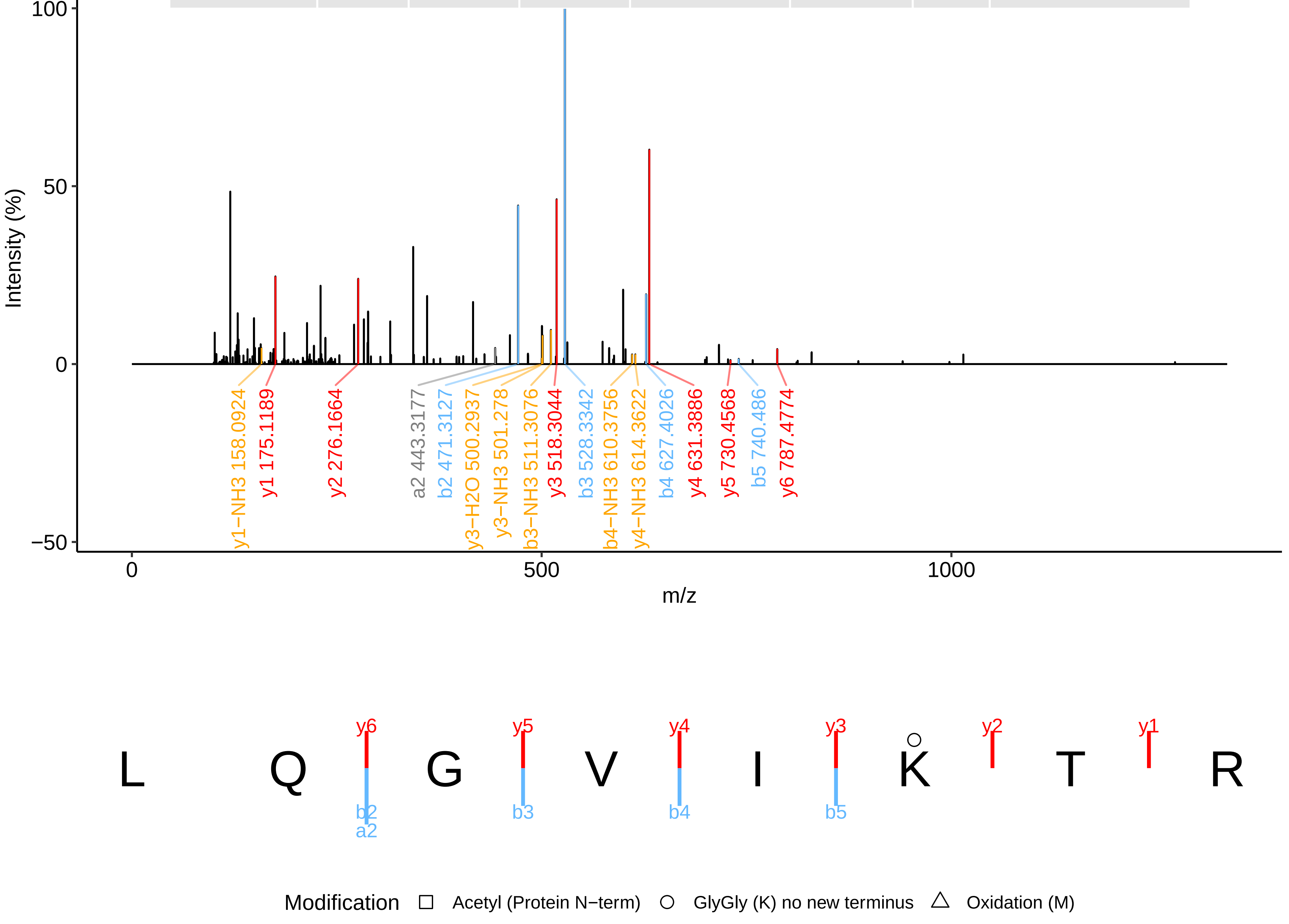

20200921_KIZ_MUb4_GlyGly_10plex_2 − Scan number: 10156

Gene Names	Charge	m/z	Mass	Retention time	PEP	Score	Precursor Intensity
ACO2	3	333.5417	997.6033	38.785	0.084338	50.127	367645.1

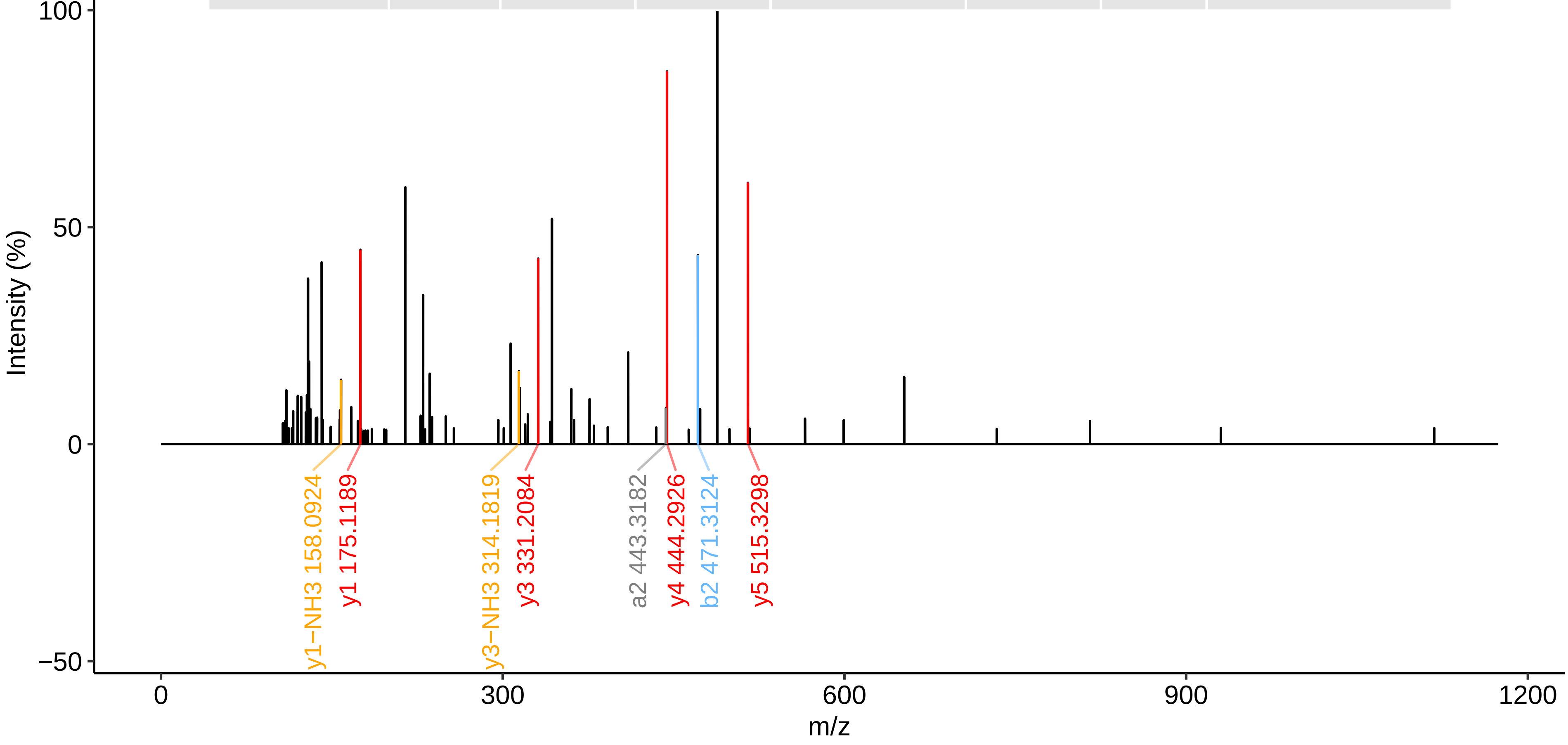

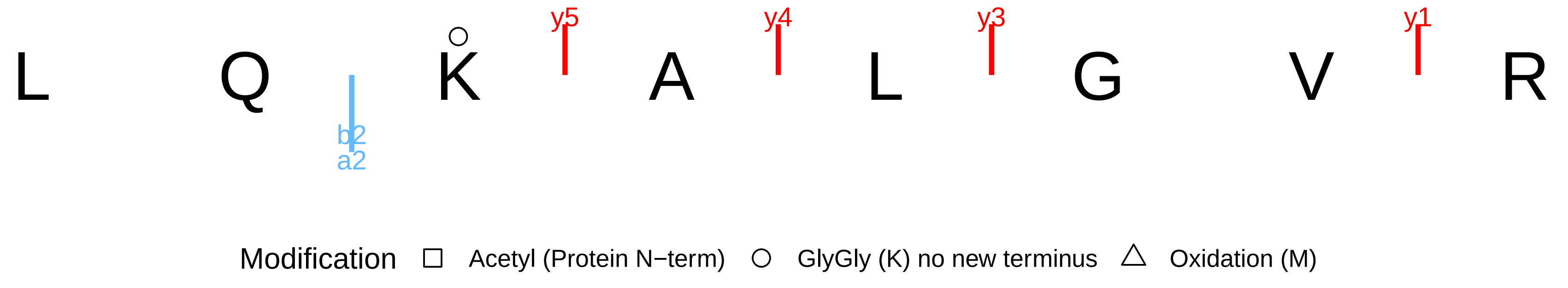

20200921_KIZ_MUb4_GlyGly_10plex_2 − Scan number: 11361

Gene Names	Charge	m/z	Mass	Retention time	PEP	Score	Precursor Intensity
ST13;ST13P5	2	496.7616	991.5087	43.714	0.11439	60.973	4212921

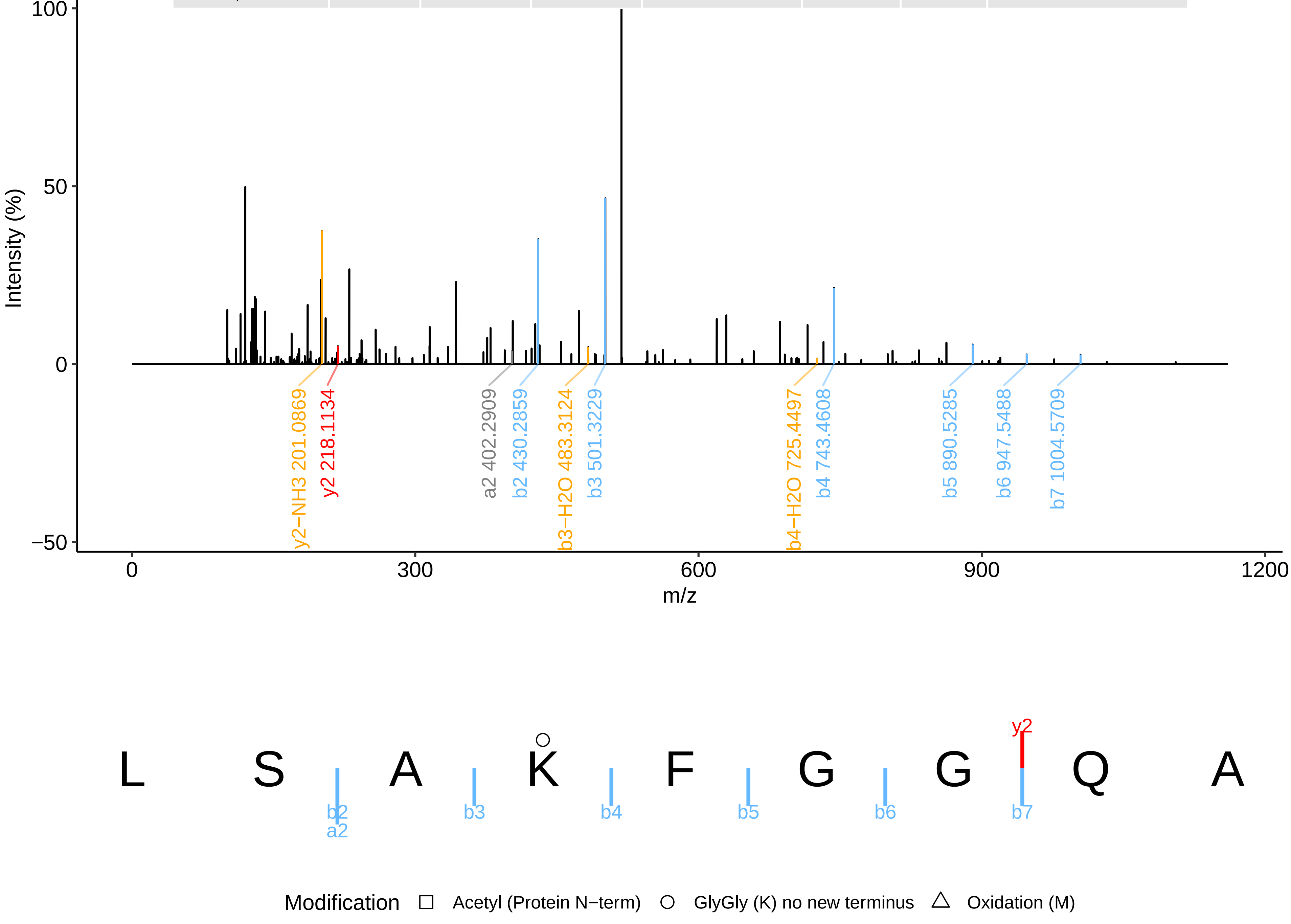

20201118_KIZ_MUb6_GlyGly_2 − Scan number: 12836

Gene Names	Charge	m/z	Mass	Retention time	PEP	Score	Precursor Intensity
ST13;ST13P5	2	496.7616	991.5087	43.207	0.11439	60.973	7595048

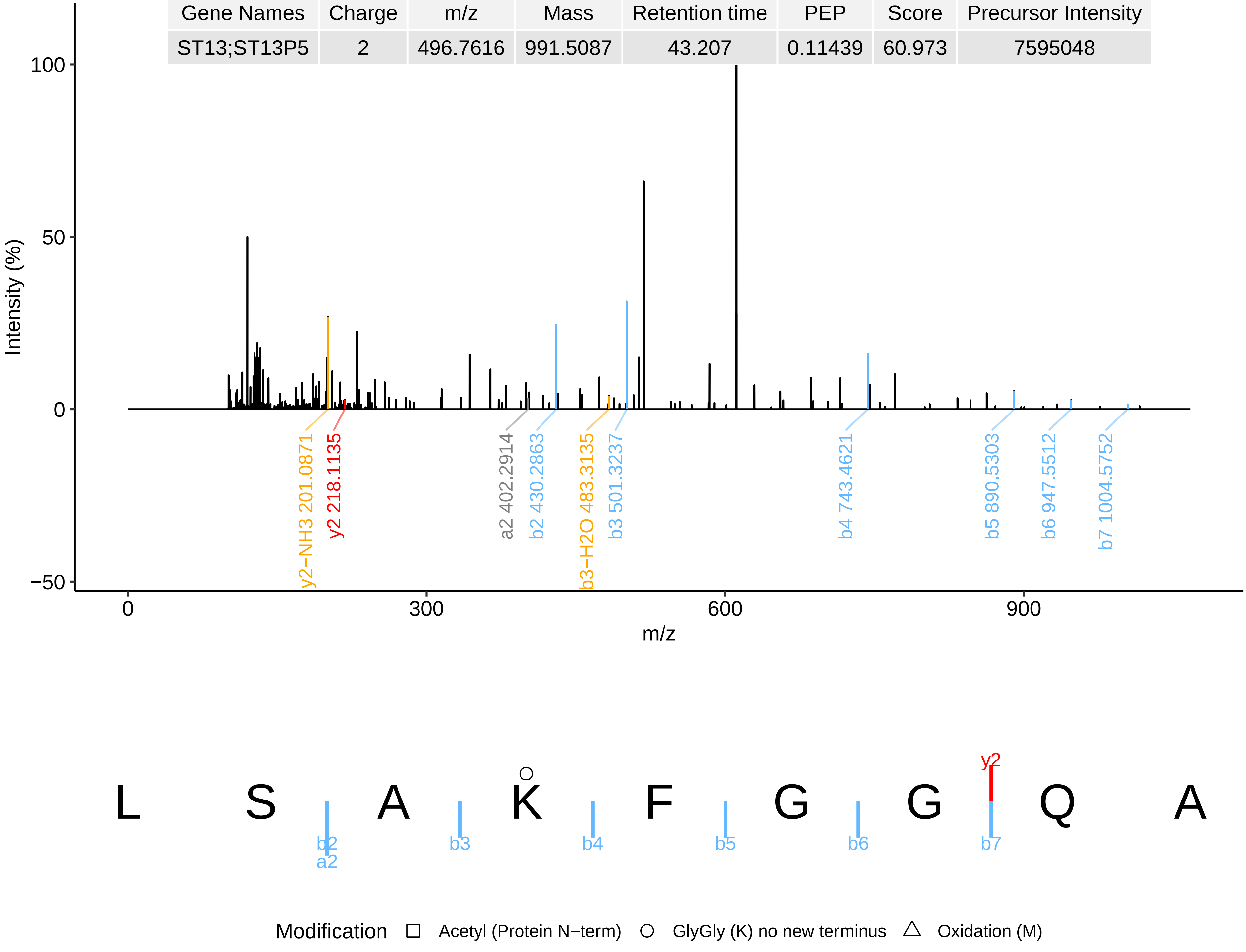

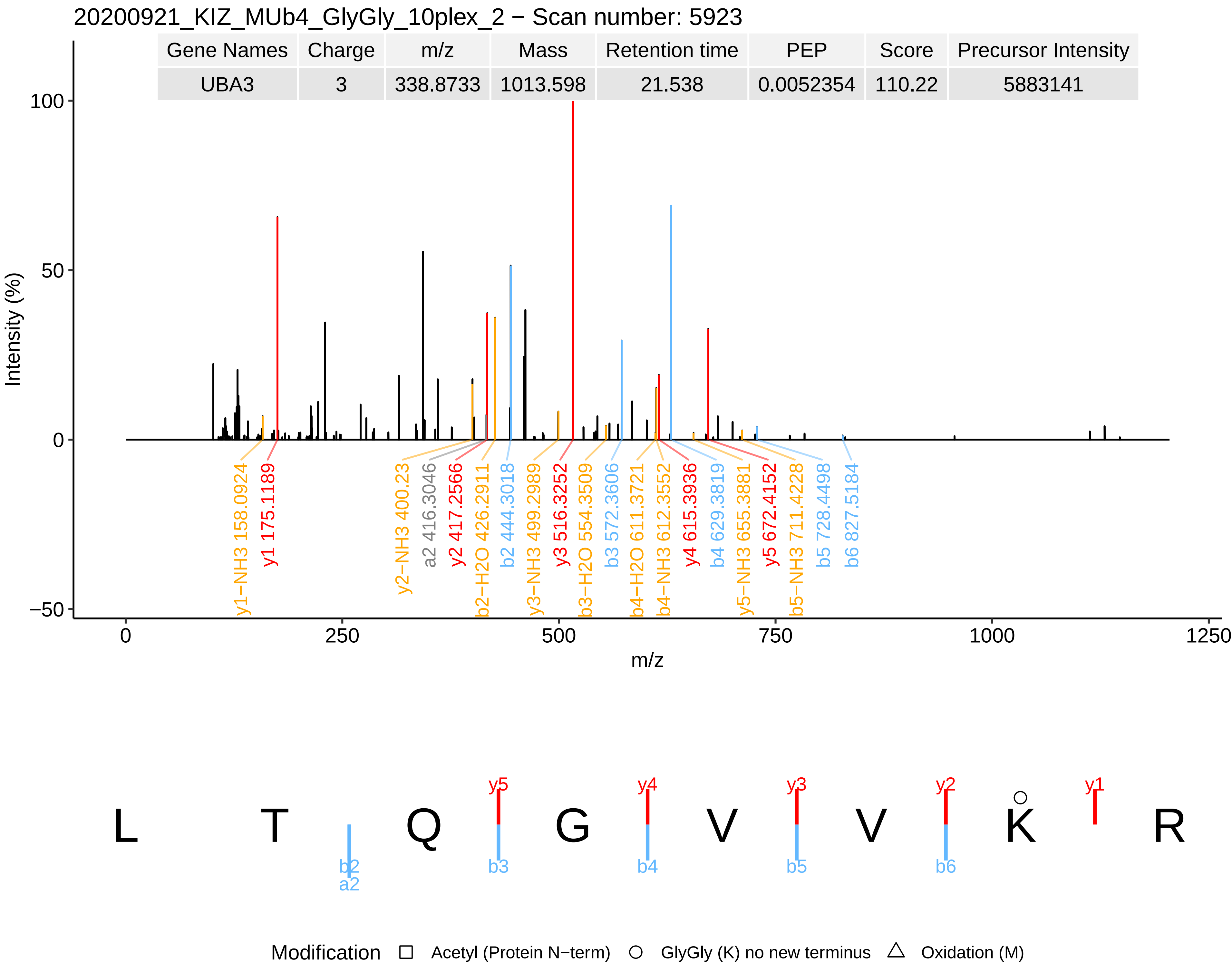

20200921_KIZ_MUb4_GlyGly_10plex_2 − Scan number: 5923
Gene Names | Charge | m/z | Mass | Retention time | PEP | Score | Precursor Intensity
UBA3 | 3 | 338.8733 | 1013.598 | 21.538 | 0.0052354 | 110.22 | 5883141
Intensity (%)
m/z
y1−NH3 158.0924
y1 175.1189
y2−NH3 400.23
a2 416.3046
y2 417.2566
b2−H2O 426.2911
b2 444.3018
y3−NH3 499.2989
y3 516.3252
b3−H2O 554.3509
b3 572.3606
b4−H2O 611.3721
b4−NH3 612.3552
y4 615.3936
b4 629.3819
y5−NH3 655.3881
y5 672.4152
b5−NH3 711.4228
b5 728.4498
b6 827.5184
L T Q G V V K R
y5 y4 y3 y2 y1
b2 a2 b3 b4 b5 b6
Modification
Acetyl (Protein N−term)
GlyGly (K) no new terminus
Oxidation (M)

20200924_KIZ_MUb5_GlyGly_1 − Scan number: 5454

Gene Names	Charge	m/z	Mass	Retention time	PEP	Score	Precursor Intensity
UBA3	3	338.8733	1013.598	16.975	0.024931	93.429	4403240

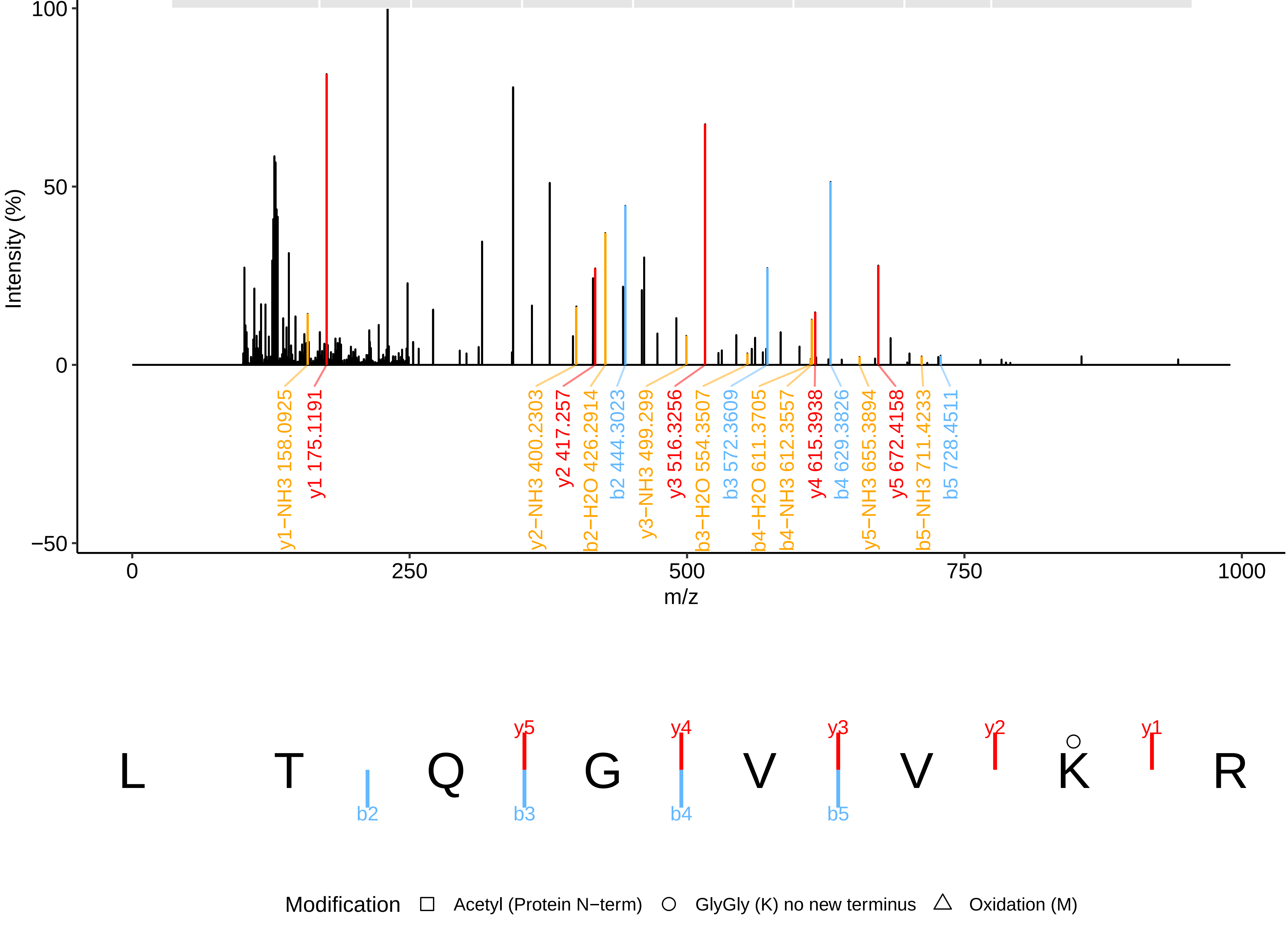

20200924_KIZ_MUb5_GlyGly_2 − Scan number: 5071

Gene Names	Charge	m/z	Mass	Retention time	PEP	Score	Precursor Intensity
UBA3	3	338.8733	1013.598	18.765	0.046104	84.743	2773897

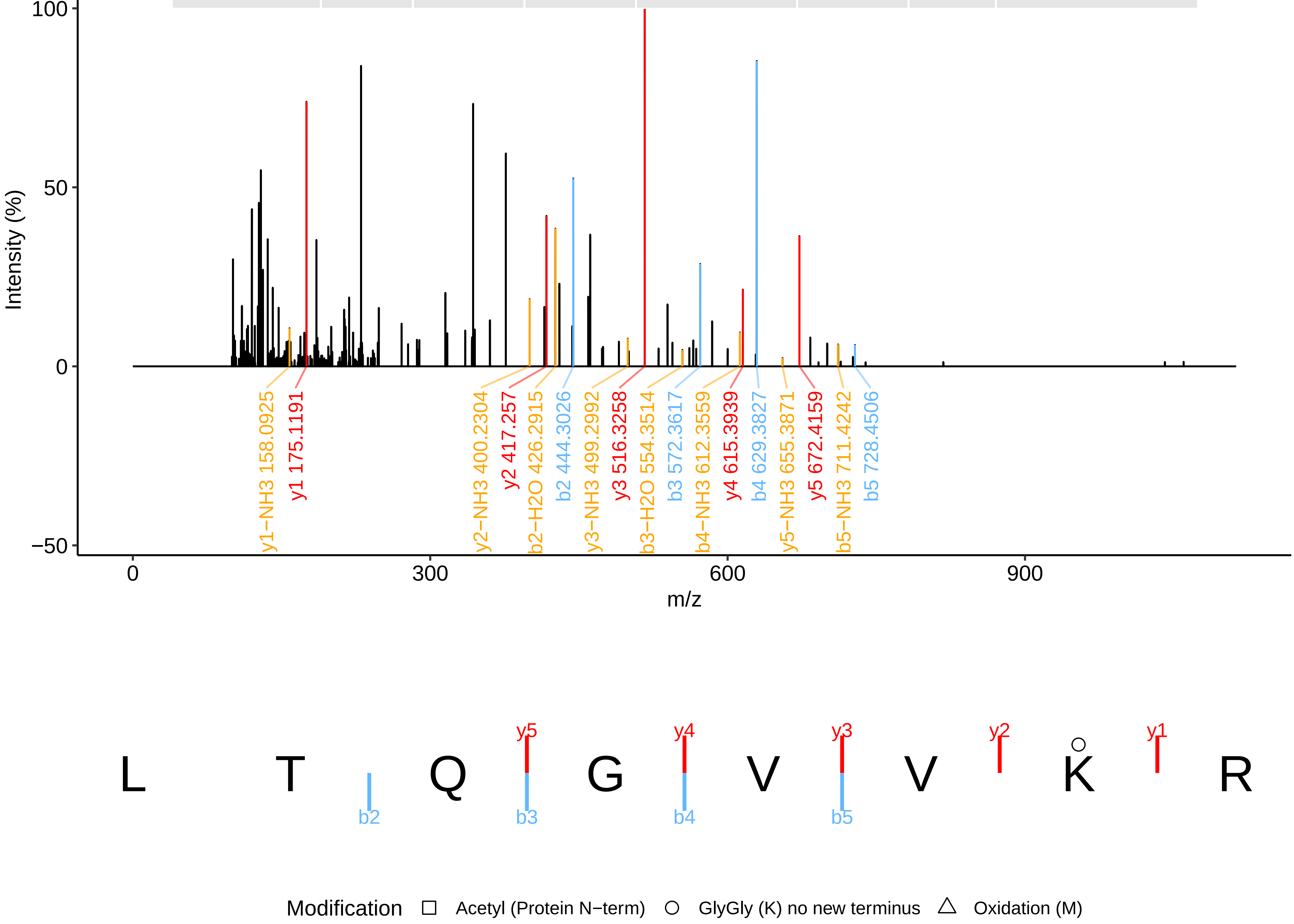

20200924_KIZ_MUb5_GlyGly_2 − Scan number: 5226

Gene Names	Charge	m/z	Mass	Retention time	PEP	Score	Precursor Intensity
UBA3	3	338.8733	1013.598	19.368	0.03633	82.279	3699097

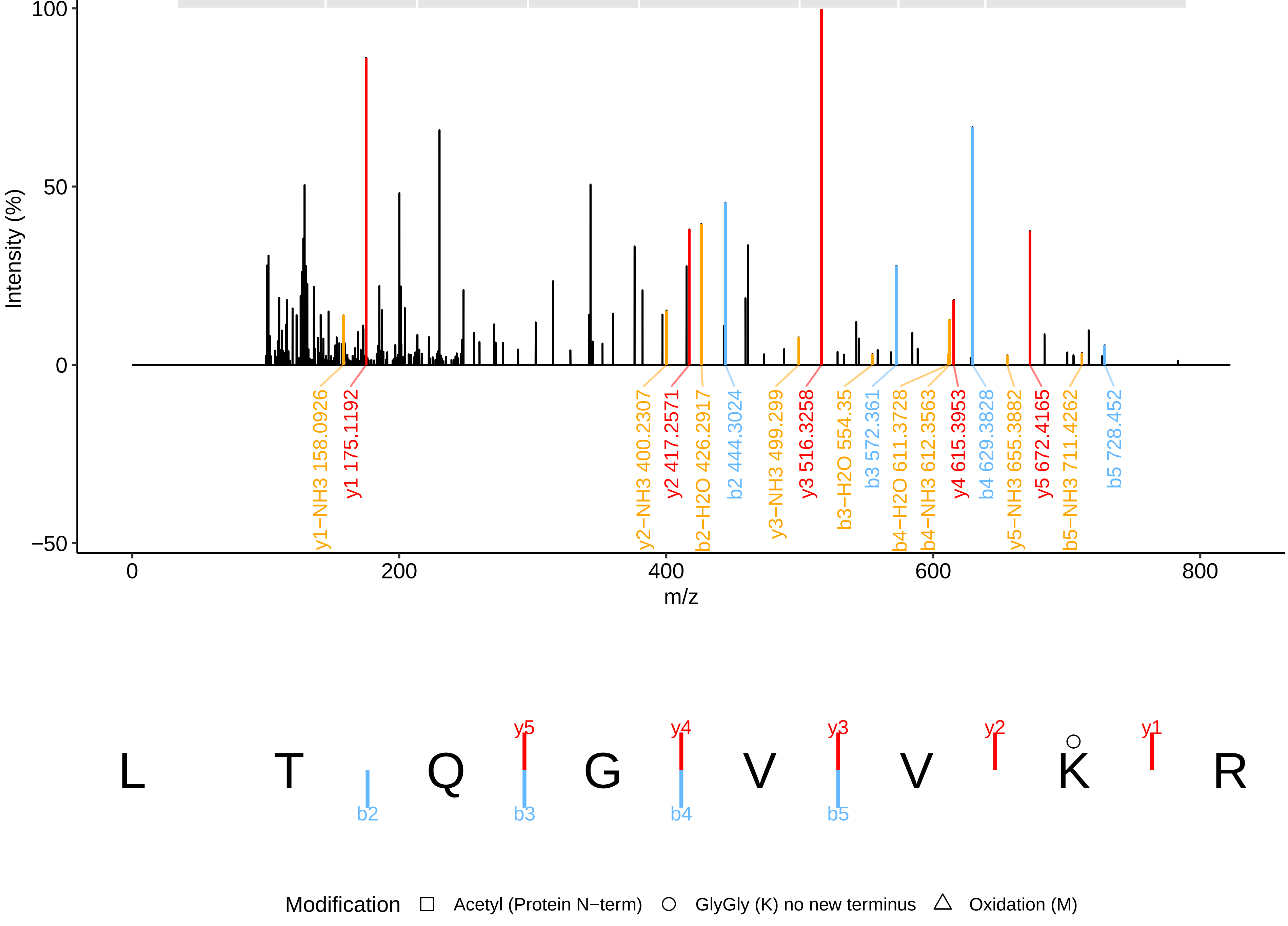

20200921_KIZ_MUb4_GlyGly_10plex − Scan number: 20302

Gene Names	Charge	m/z	Mass	Retention time	PEP	Score	Precursor Intensity
ERGIC3	2	523.2892	1044.564	73.369	0.0073757	57.103	3935106

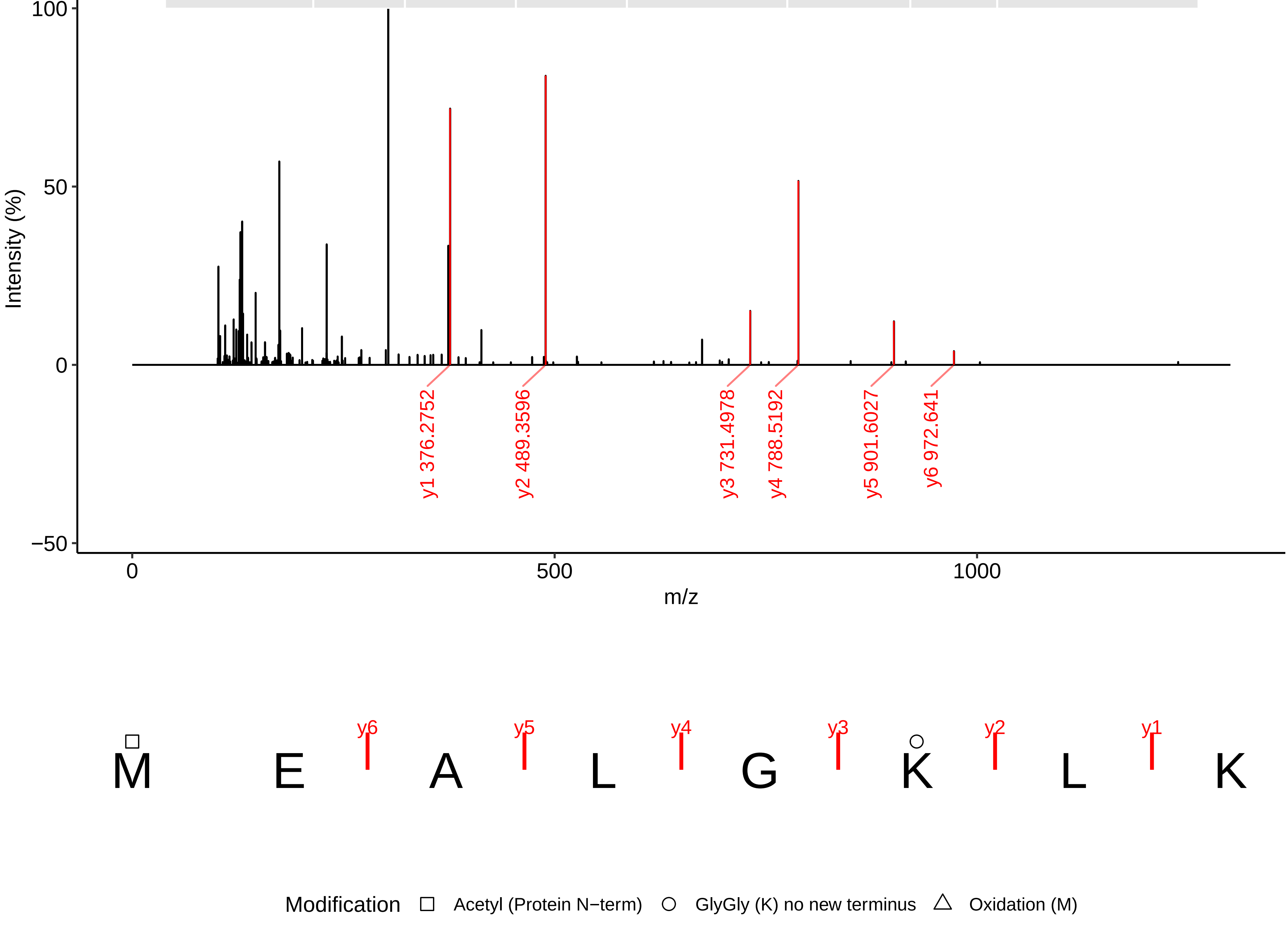

20200921_KIZ_MUb4_GlyGly_10plex_2 − Scan number: 8723

Gene Names	Charge	m/z	Mass	Retention time	PEP	Score	Precursor Intensity
YWHAQ;YWHAB	3	344.164	1029.47	32.962	0.04461	70.912	1747724

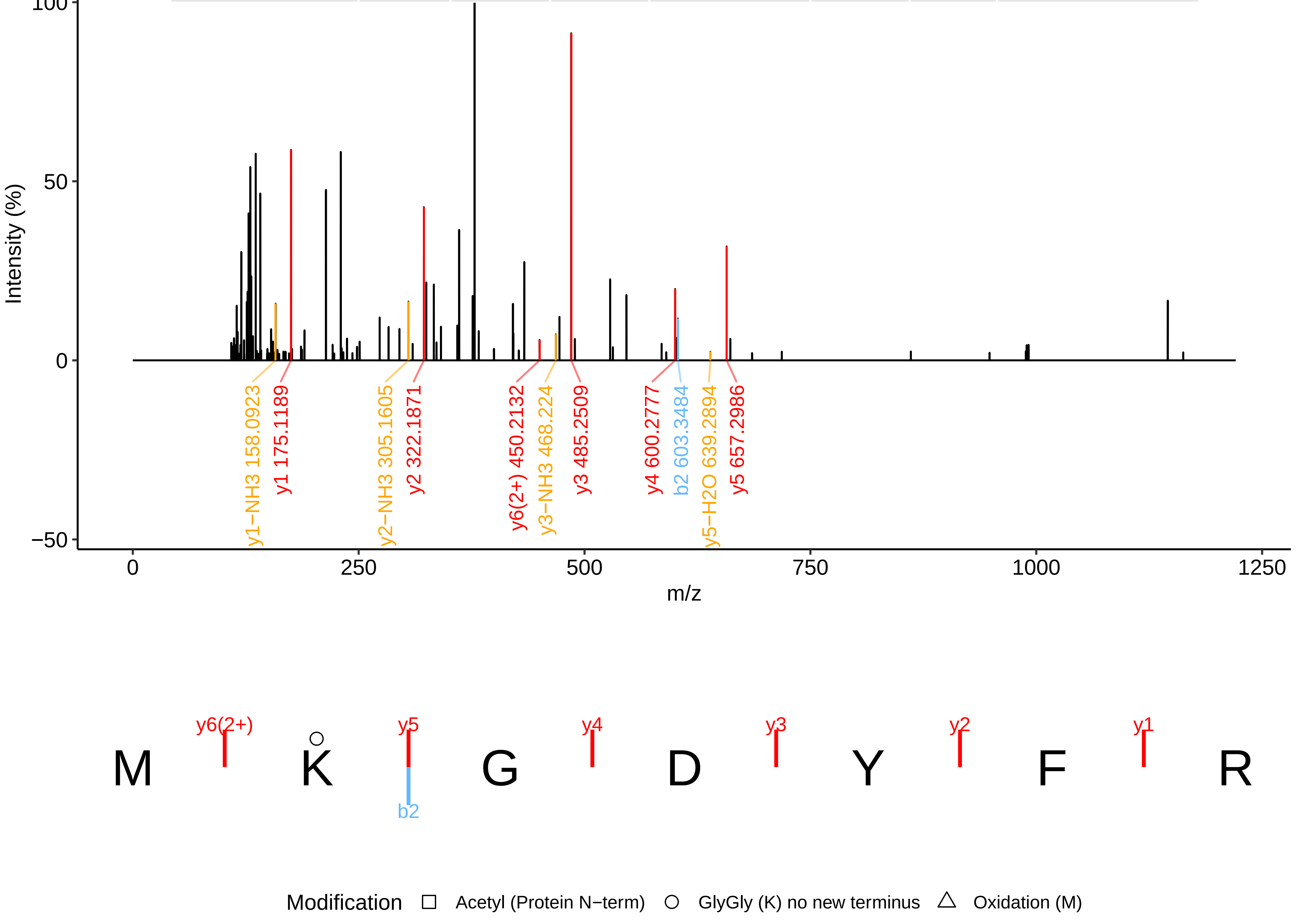

20200921_KIZ_MUb4_GlyGly_10plex − Scan number: 3747

Gene Names	Charge	m/z	Mass	Retention time	PEP	Score	Precursor Intensity
RHOB;RHOC	3	334.5114	1000.512	12.056	0.062697	64.236	11464935

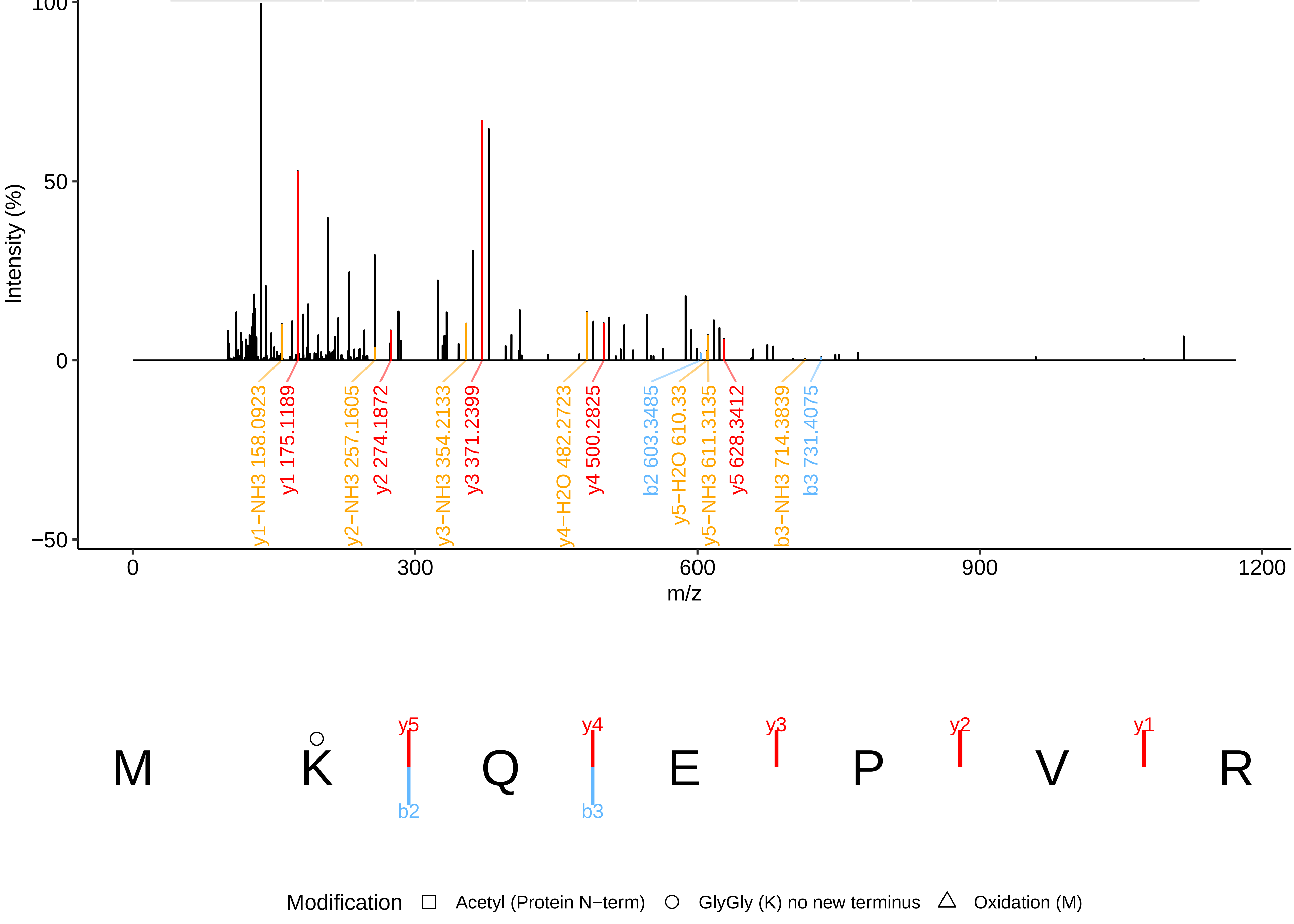

20200921_KIZ_MUb4_GlyGly_10plex_2 − Scan number: 3562

Gene Names	Charge	m/z	Mass	Retention time	PEP	Score	Precursor Intensity
RHOB;RHOC	3	334.5114	1000.512	12.034	0.038432	73.324	4187835

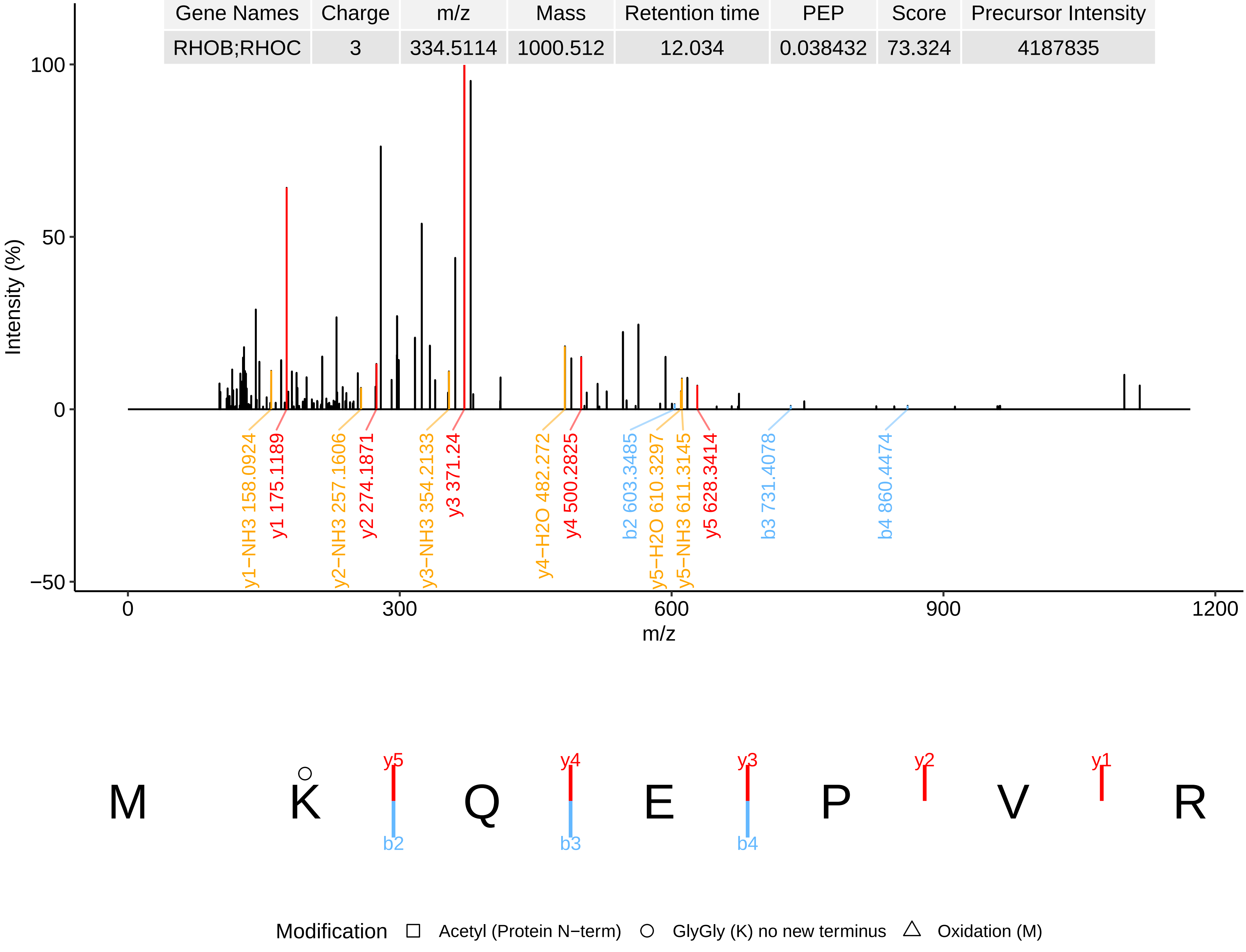

20200924_KIZ_MUb5_GlyGly_1 − Scan number: 3340

Gene Names	Charge	m/z	Mass	Retention time	PEP	Score	Precursor Intensity
RHOB;RHOC	3	334.5114	1000.512	10.251	0.076363	60.76	17518668

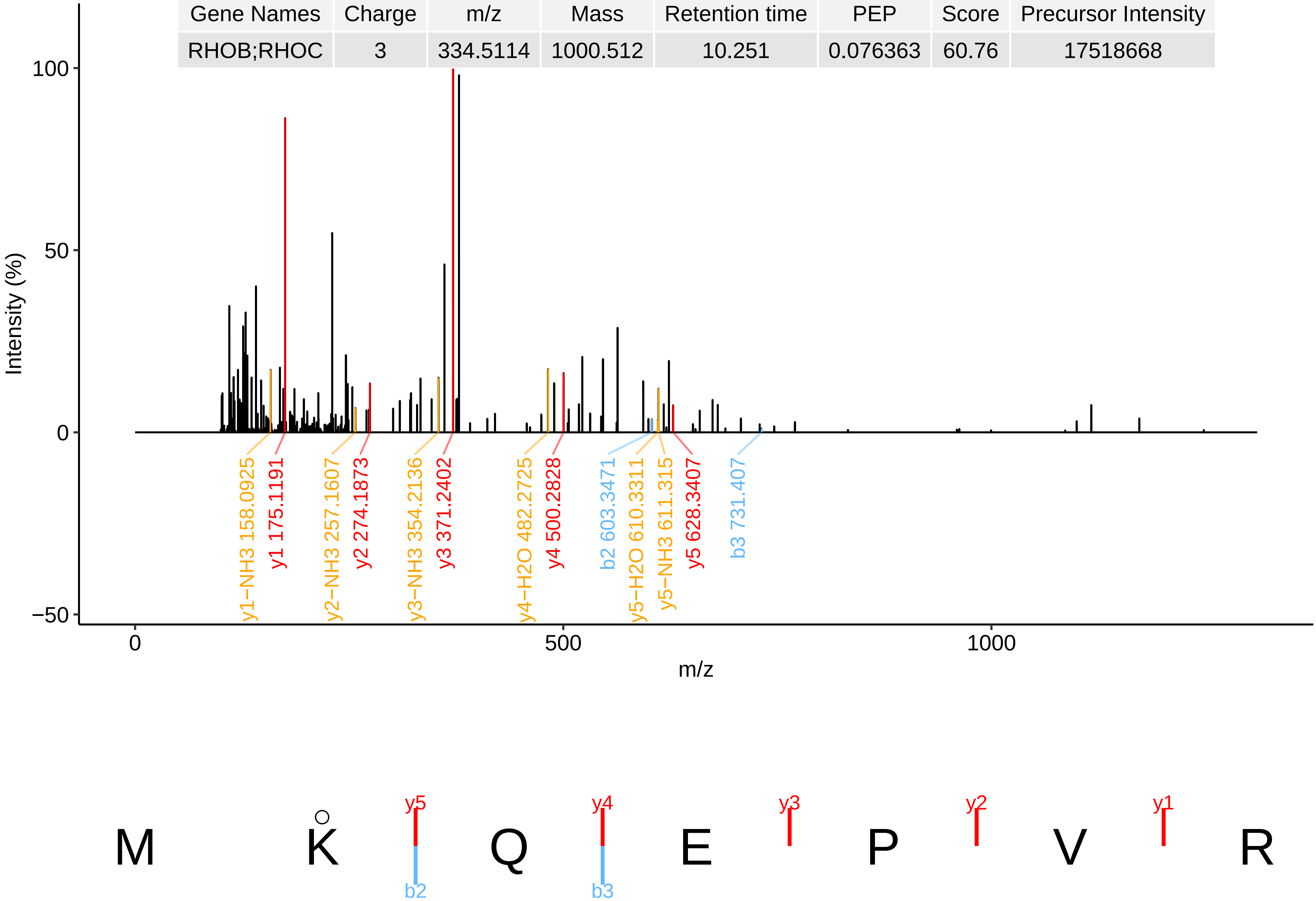

Modification □ Acetyl (Protein N−term) ○ GlyGly (K) no new terminus △ Oxidation (M)

20201118_KIZ_MUb6_GlyGly_1 − Scan number: 3224

Gene Names	Charge	m/z	Mass	Retention time	PEP	Score	Precursor Intensity
RHOB;RHOC	3	334.5114	1000.512	9.9254	0.041469	72.138	25881168

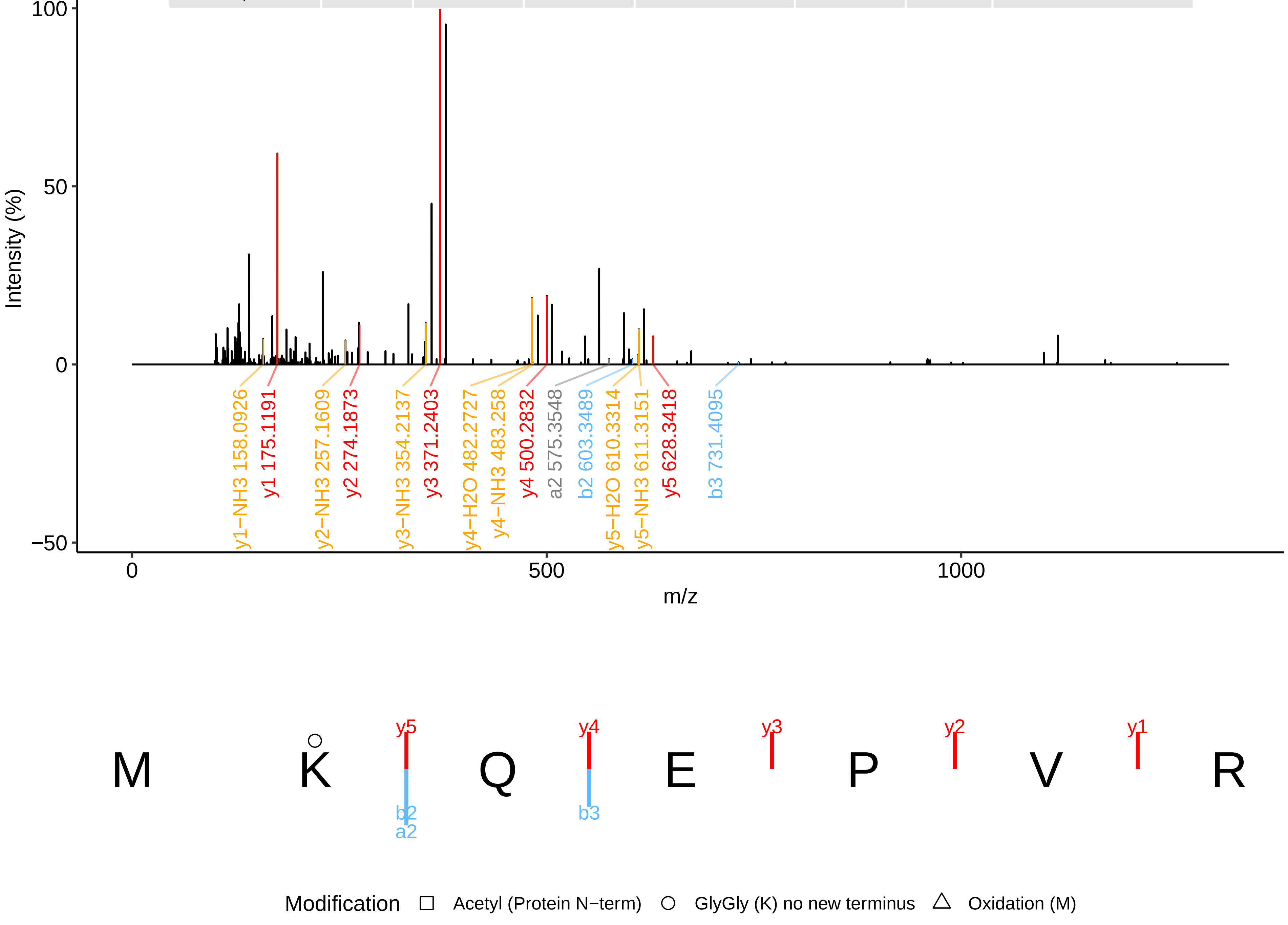

20201118_KIZ_MUb6_GlyGly_2 − Scan number: 3716

Gene Names	Charge	m/z	Mass	Retention time	PEP	Score	Precursor Intensity
RHOB;RHOC	3	334.5114	1000.512	12.428	0.085152	58.981	5367697

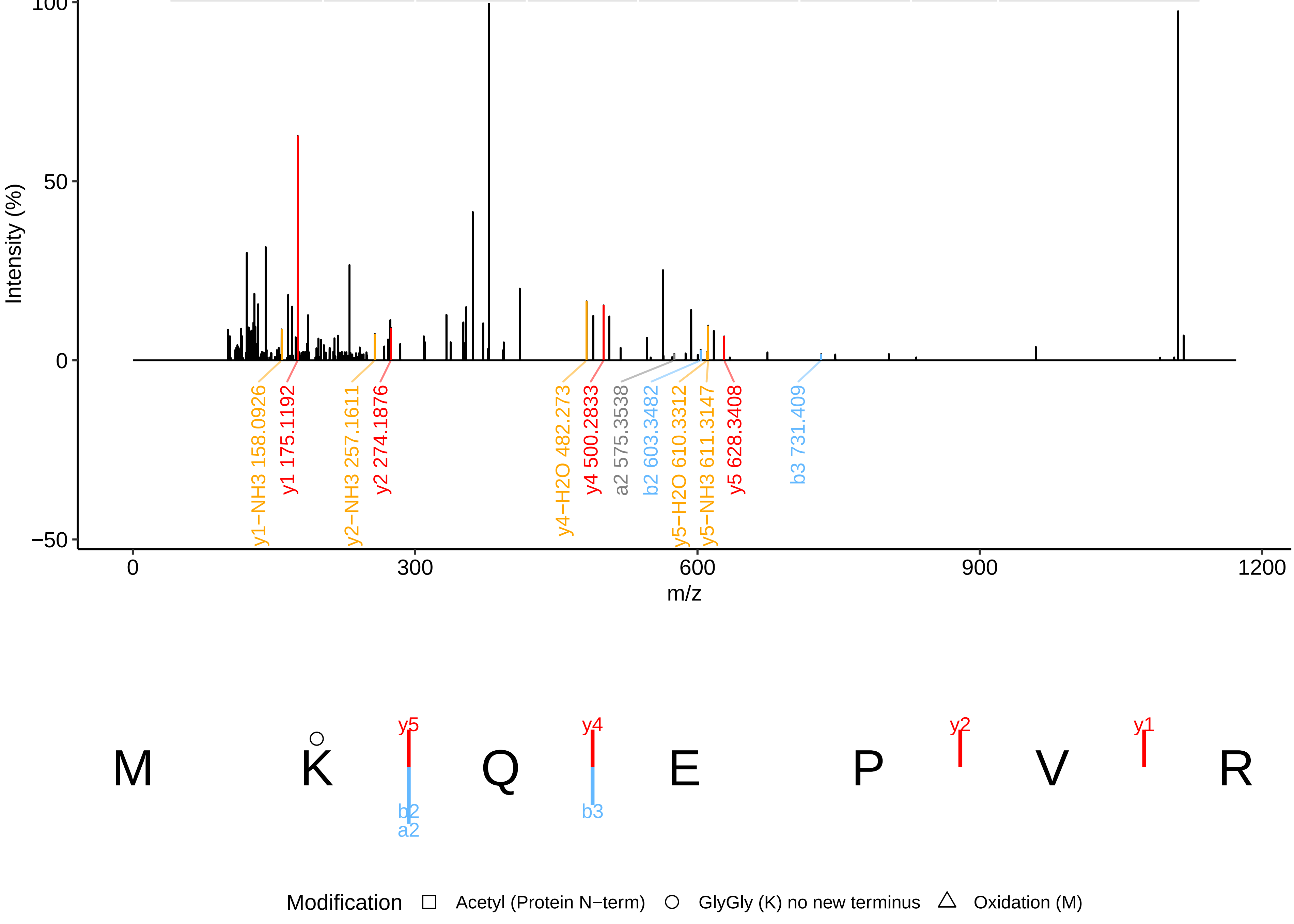

20200921_KIZ_MUb4_GlyGly_10plex − Scan number: 4056

Gene Names	Charge	m/z	Mass	Retention time	PEP	Score	Precursor Intensity
ATP6AP2	3	335.515	1003.523	13.19	0.0052195	104.76	7305614

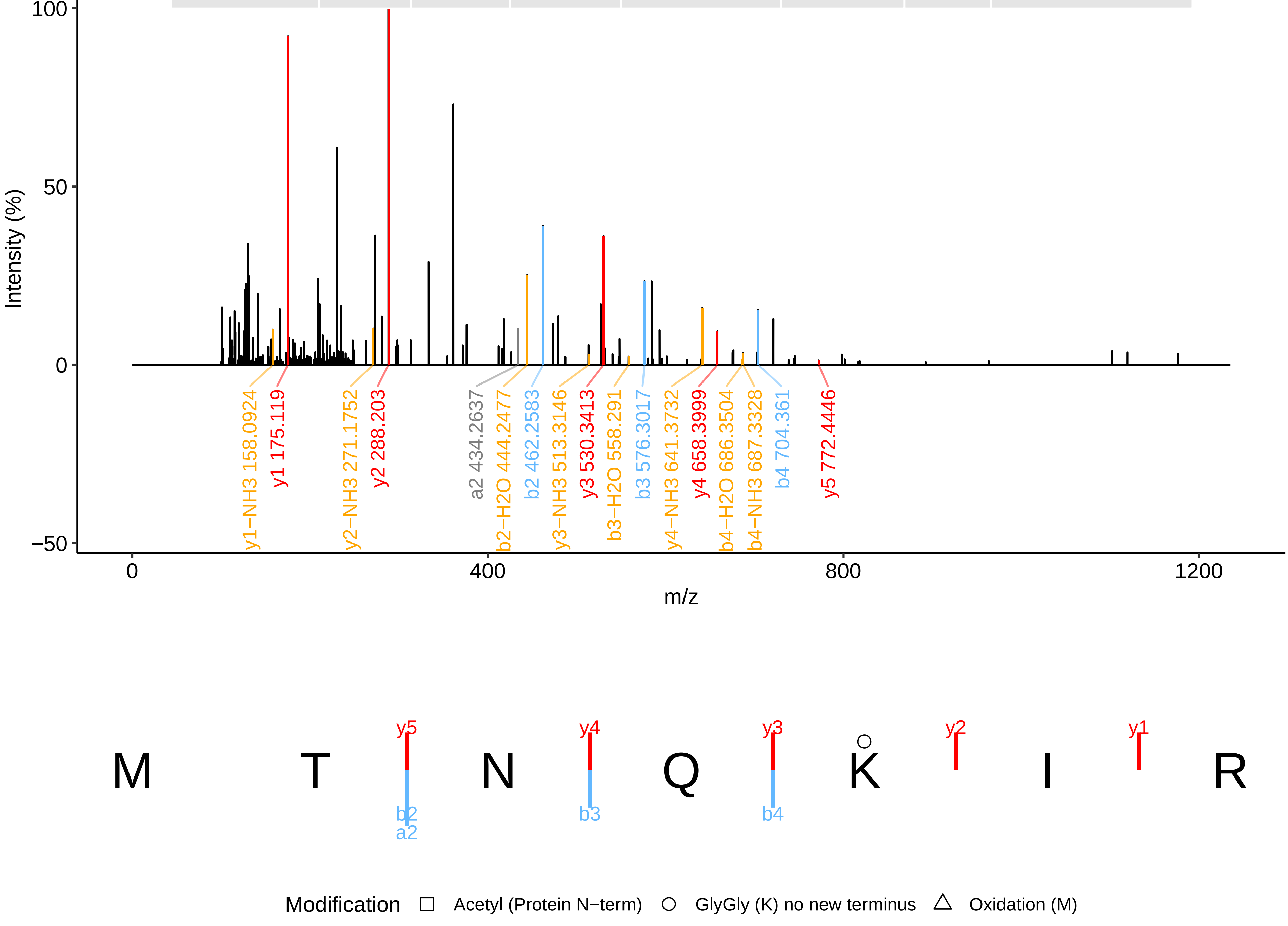

20200921_KIZ_MUb4_GlyGly_10plex_2 − Scan number: 3815

Gene Names	Charge	m/z	Mass	Retention time	PEP	Score	Precursor Intensity
ATP6AP2	3	335.515	1003.523	13.062	0.0063833	100.74	4038328

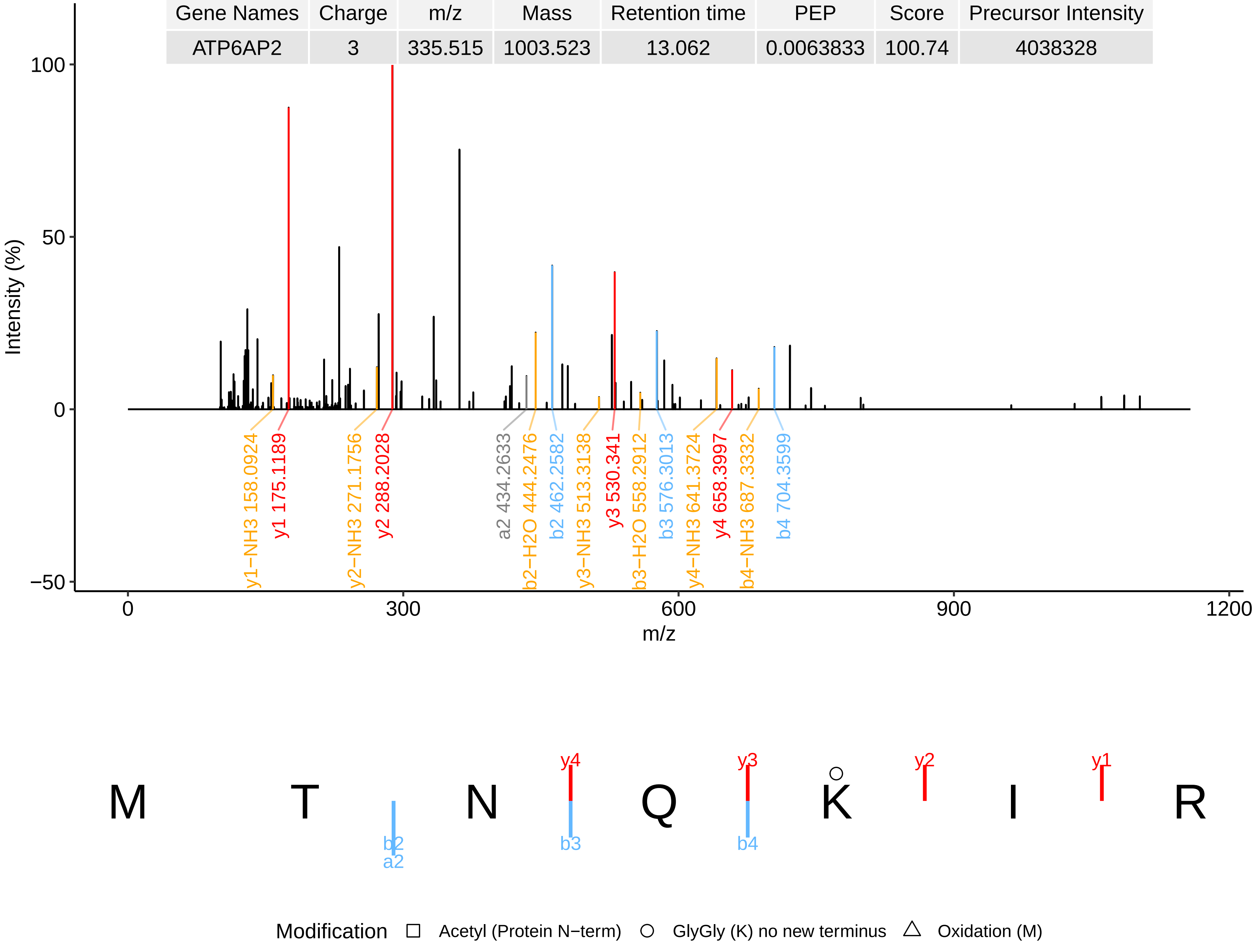

20200924_KIZ_MUb5_GlyGly_2 − Scan number: 3317

Gene Names	Charge	m/z	Mass	Retention time	PEP	Score	Precursor Intensity
ATP6AP2	3	335.515	1003.523	11.978	0.018954	81.784	2535321

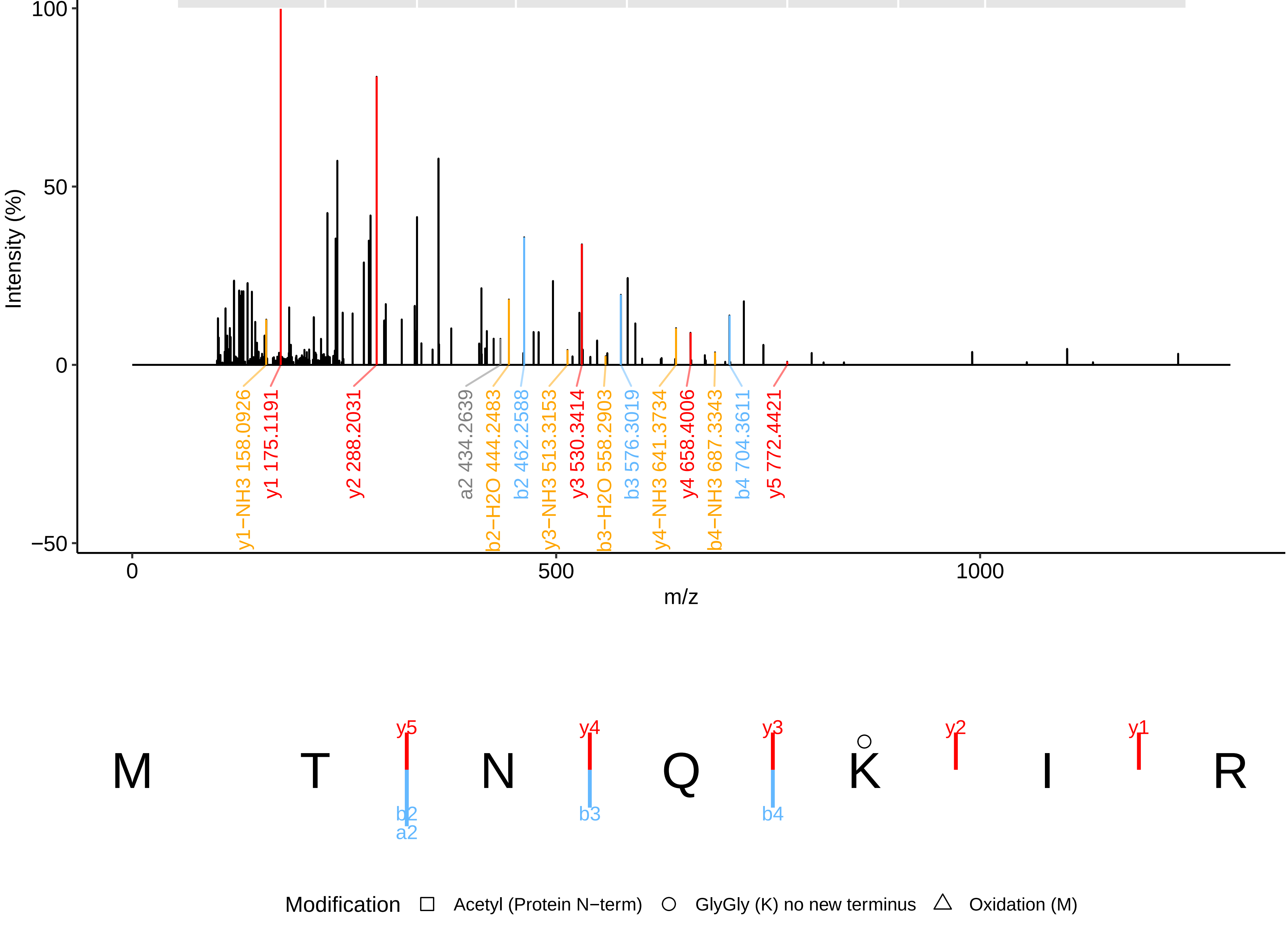

20200921_KIZ_MUb4_GlyGly_10plex_2 − Scan number: 12021

Gene Names	Charge	m/z	Mass	Retention time	PEP	Score	Precursor Intensity
SLC30A1	3	333.8697	998.5873	46.417	0.027015	78.616	944403.1

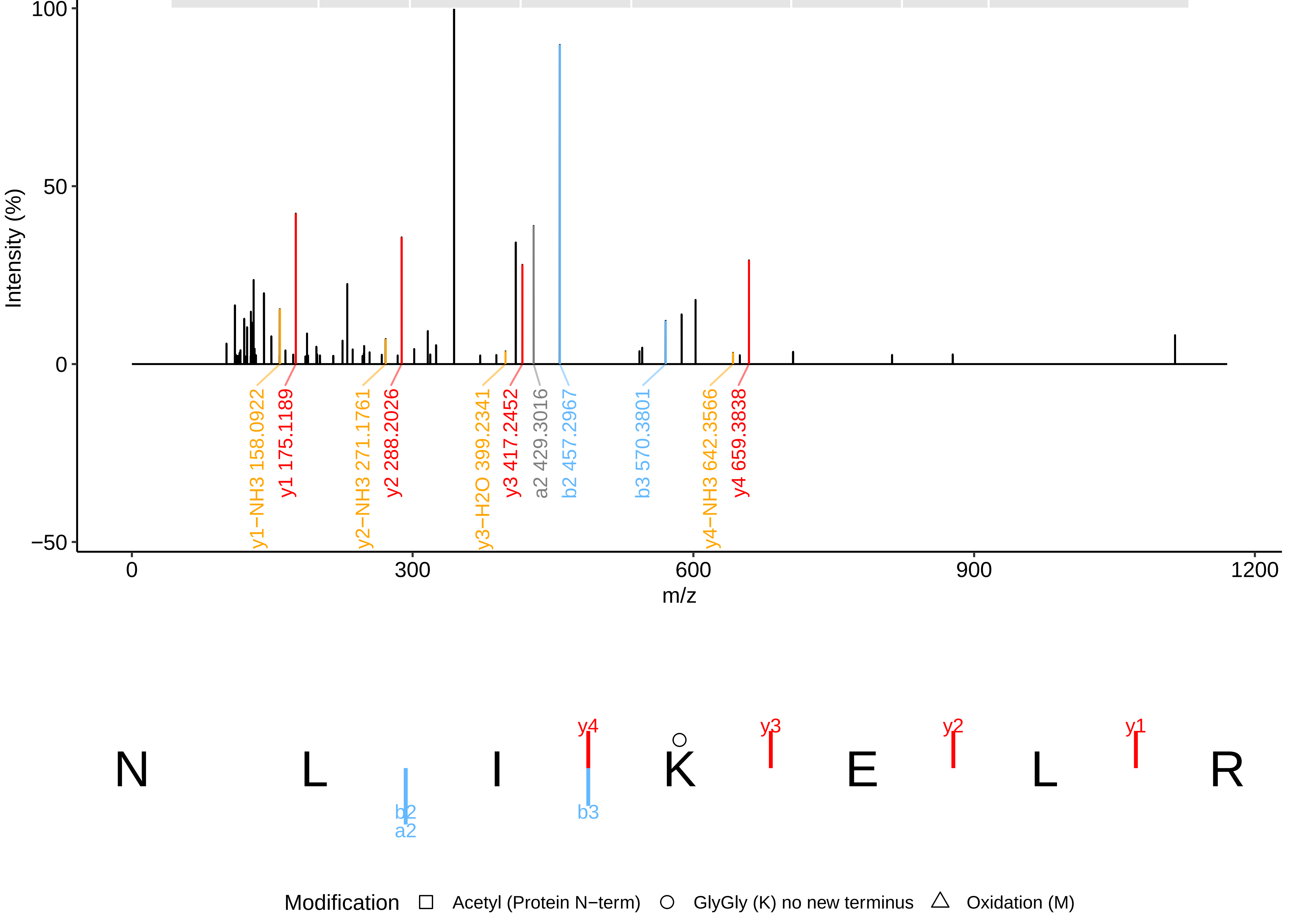

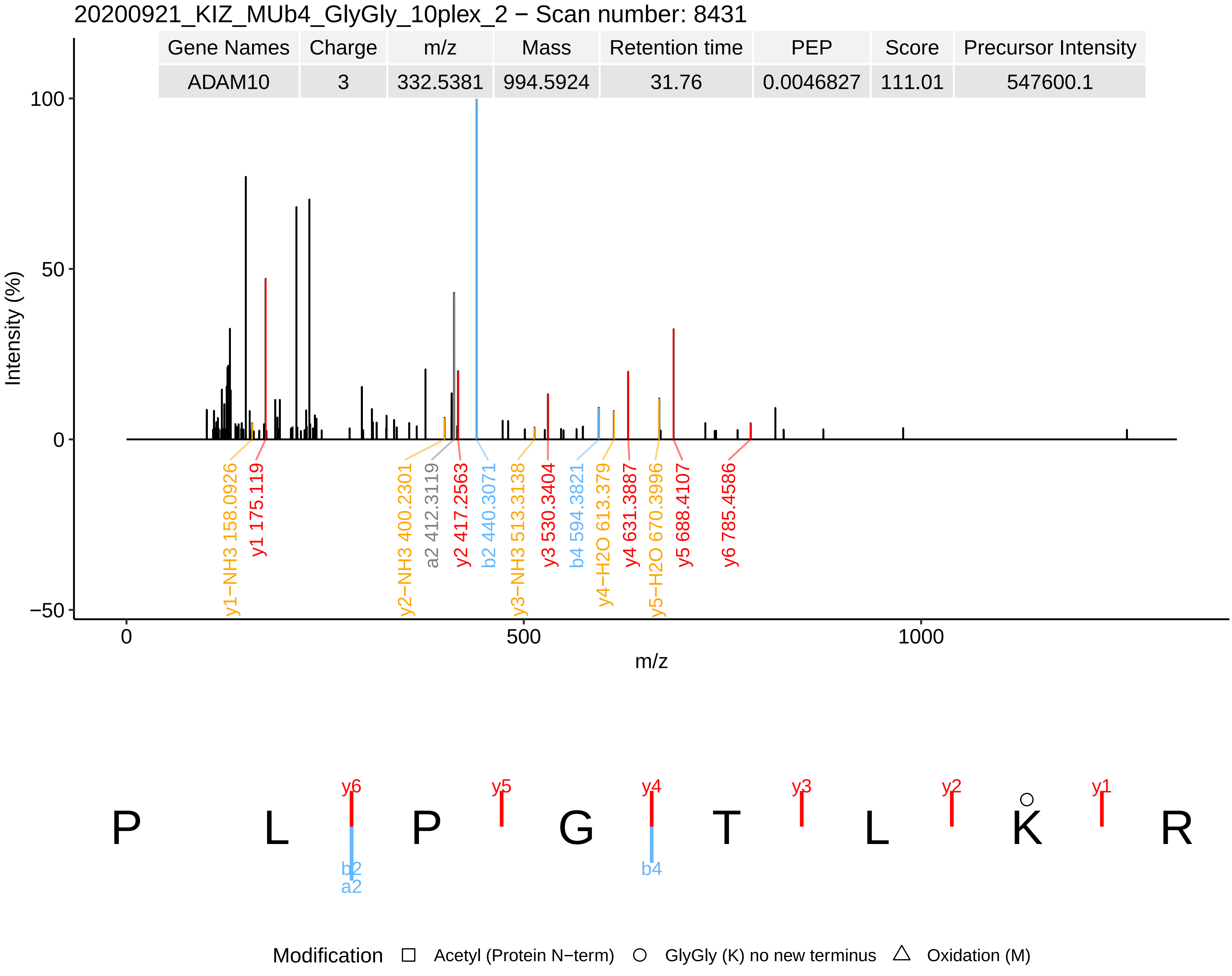
20200921_KIZ_MUb4_GlyGly_10plex_2 − Scan number: 8431
Gene Names
Charge
m/z
Mass
Retention time
PEP
Score
Precursor Intensity
ADAM10
3
332.5381
994.5924
31.76
0.0046827
111.01
547600.1
Intensity (%)
100
50
0
−50
0
500
1000
m/z
y1−NH3 158.0926
y1 175.119
y2−NH3 400.2301
a2 412.3119
y2 417.2563
b2 440.3071
y3−NH3 513.3138
y3 530.3404
b4 594.3821
y4−H2O 613.379
y4 631.3887
y5−H2O 670.3996
y5 688.4107
y6 785.4586
P L P G T L K R
y6 y5 y4 y3 y2 y1
b2 a2 b4
Modification
Acetyl (Protein N−term)
GlyGly (K) no new terminus
Oxidation (M)

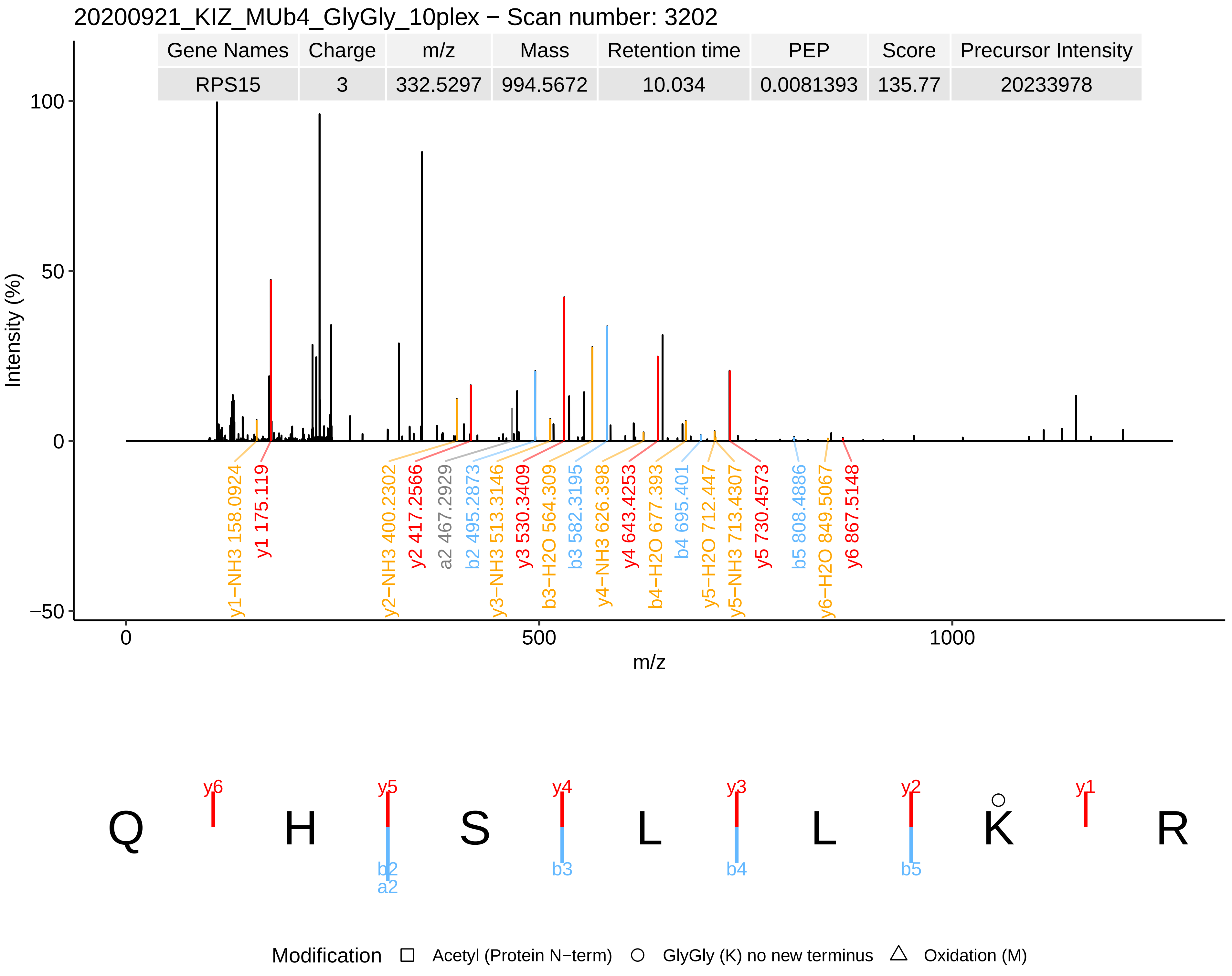

20200921_KIZ_MUb4_GlyGly_10plex − Scan number: 3202
Gene Names | Charge | m/z | Mass | Retention time | PEP | Score | Precursor Intensity
RPS15 | 3 | 332.5297 | 994.5672 | 10.034 | 0.0081393 | 135.77 | 20233978
Intensity (%)
m/z
y1−NH3 158.0924
y1 175.119
y2−NH3 400.2302
y2 417.2566
a2 467.2929
b2 495.2873
y3−NH3 513.3146
y3 530.3409
b3−H2O 564.309
b3 582.3195
y4−NH3 626.398
y4 643.4253
b4−H2O 677.393
b4 695.401
y5−H2O 712.447
y5−NH3 713.4307
y5 730.4573
b5 808.4886
y6−H2O 849.5067
y6 867.5148
Q H S L L K R
y6 y5 y4 y3 y2 y1
b2 a2 b3 b4 b5
Modification
Acetyl (Protein N−term)
GlyGly (K) no new terminus
Oxidation (M)

20200921_KIZ_MUb4_GlyGly_10plex_2 − Scan number: 3152

Gene Names	Charge	m/z	Mass	Retention time	PEP	Score	Precursor Intensity
RPS15	3	332.5297	994.5672	10.428	0.010564	128.37	4232585

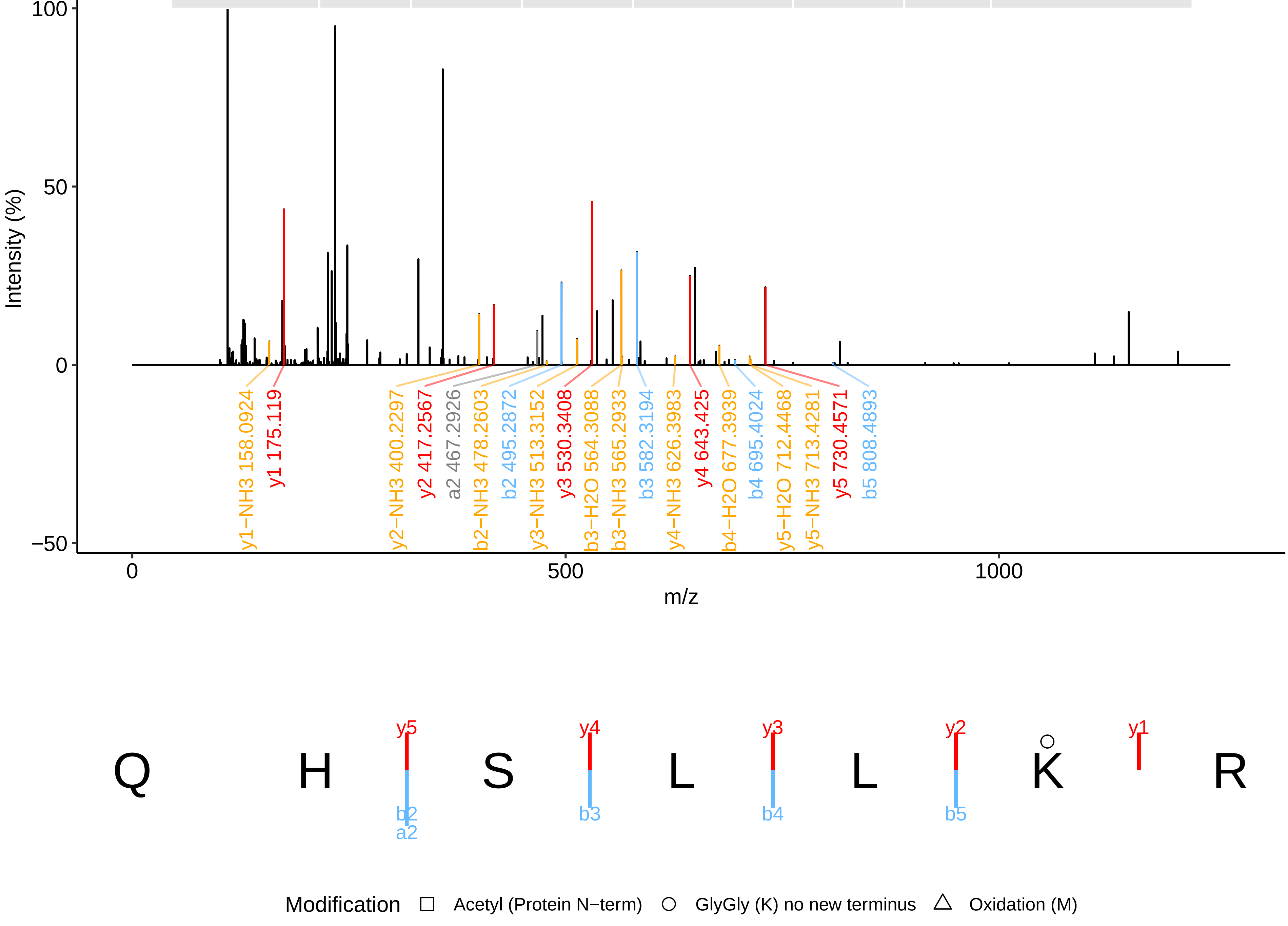

20201118_KIZ_MUb6_GlyGly_1 − Scan number: 2970

Gene Names	Charge	m/z	Mass	Retention time	PEP	Score	Precursor Intensity
RPS15	3	332.5297	994.5672	9.1099	0.016796	113.41	6311558

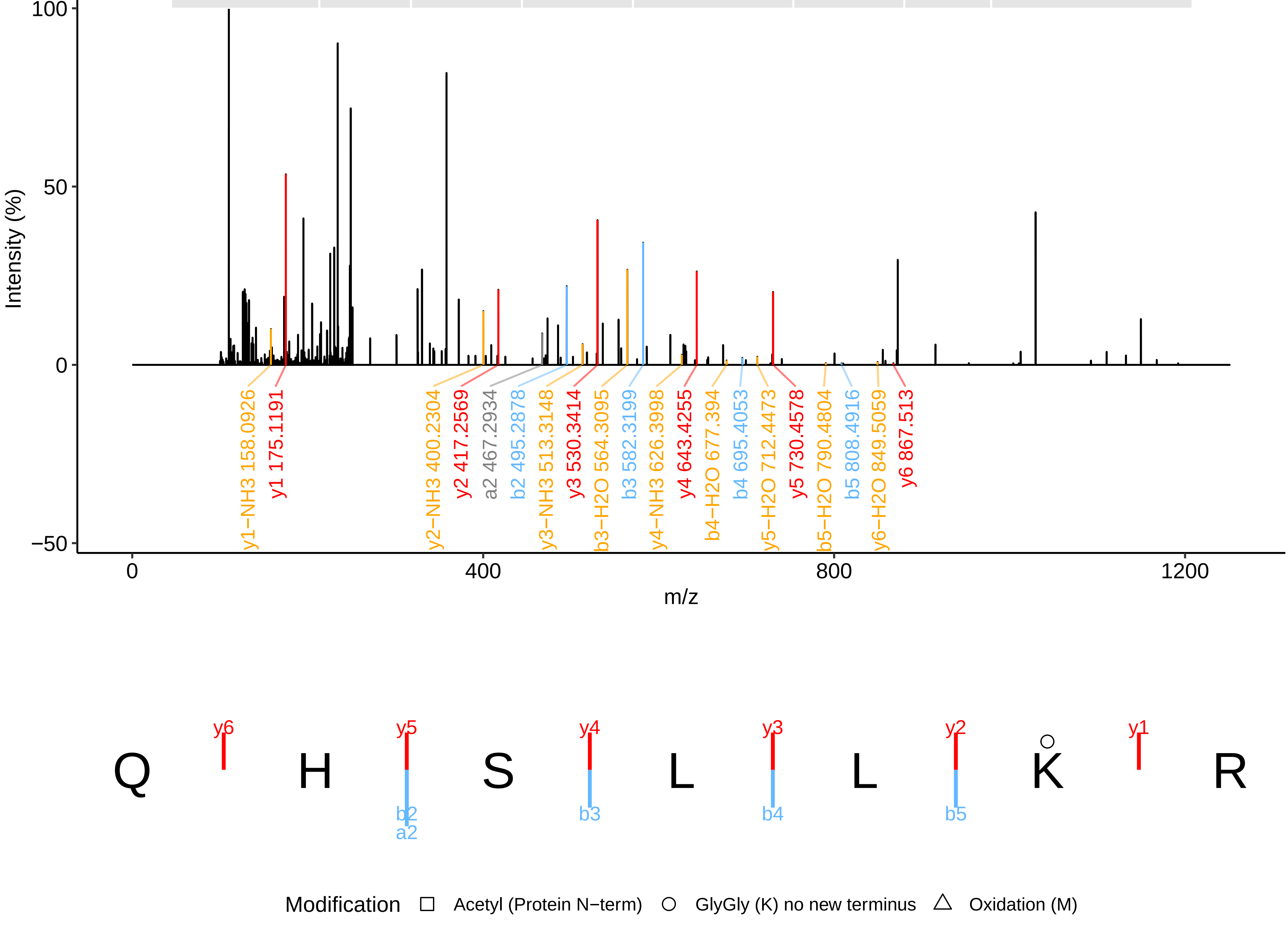

20200921_KIZ_MUb4_GlyGly_10plex − Scan number: 9390

Gene Names	Charge	m/z	Mass	Retention time	PEP	Score	Precursor Intensity
MFF	3	333.5417	997.6033	32.143	0.013224	89.673	42060908

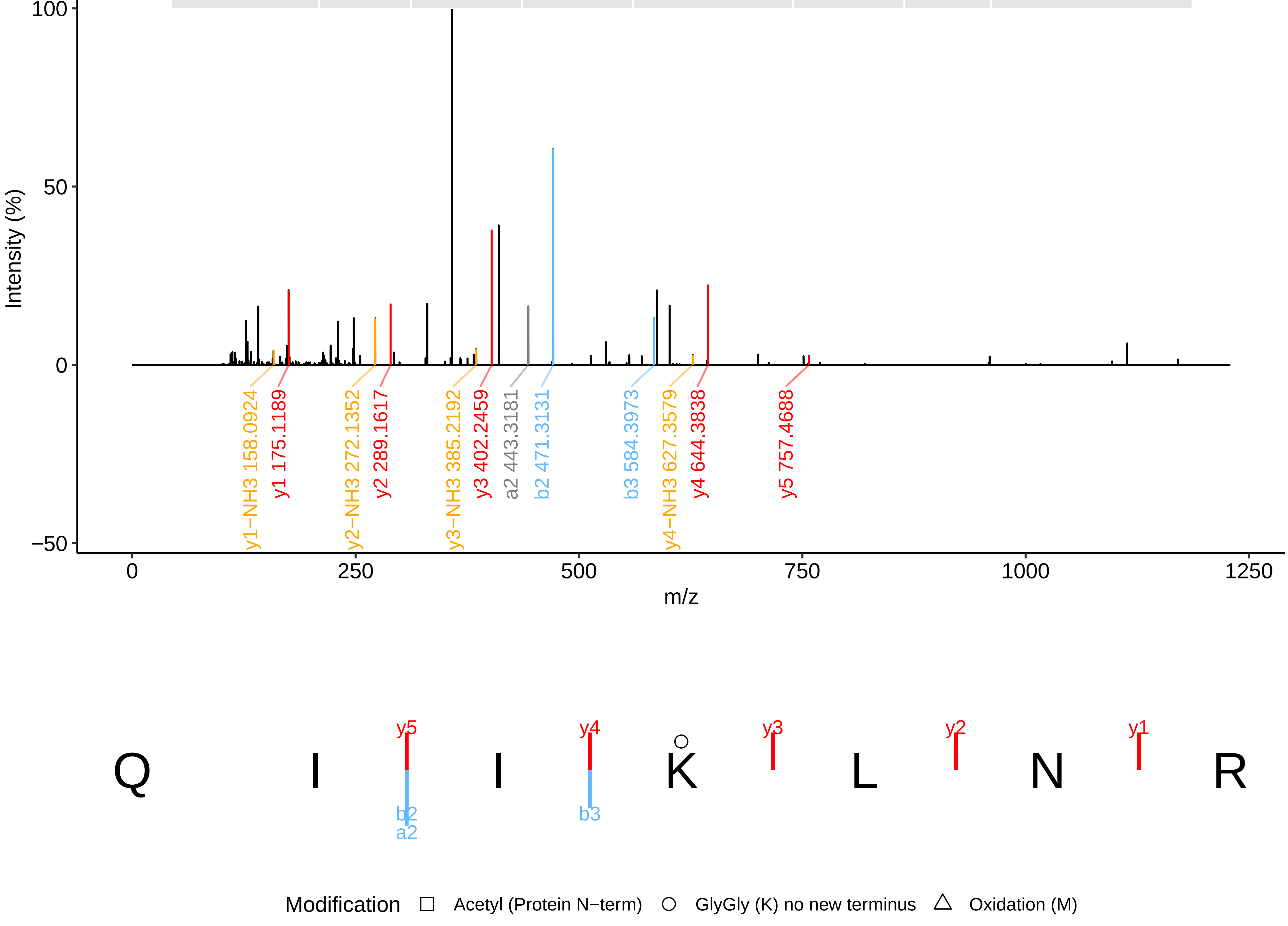

20200921_KIZ_MUb4_GlyGly_10plex_2 − Scan number: 8639

Gene Names	Charge	m/z	Mass	Retention time	PEP	Score	Precursor Intensity
MFF	3	333.5417	997.6033	32.609	0.013224	89.673	1150655

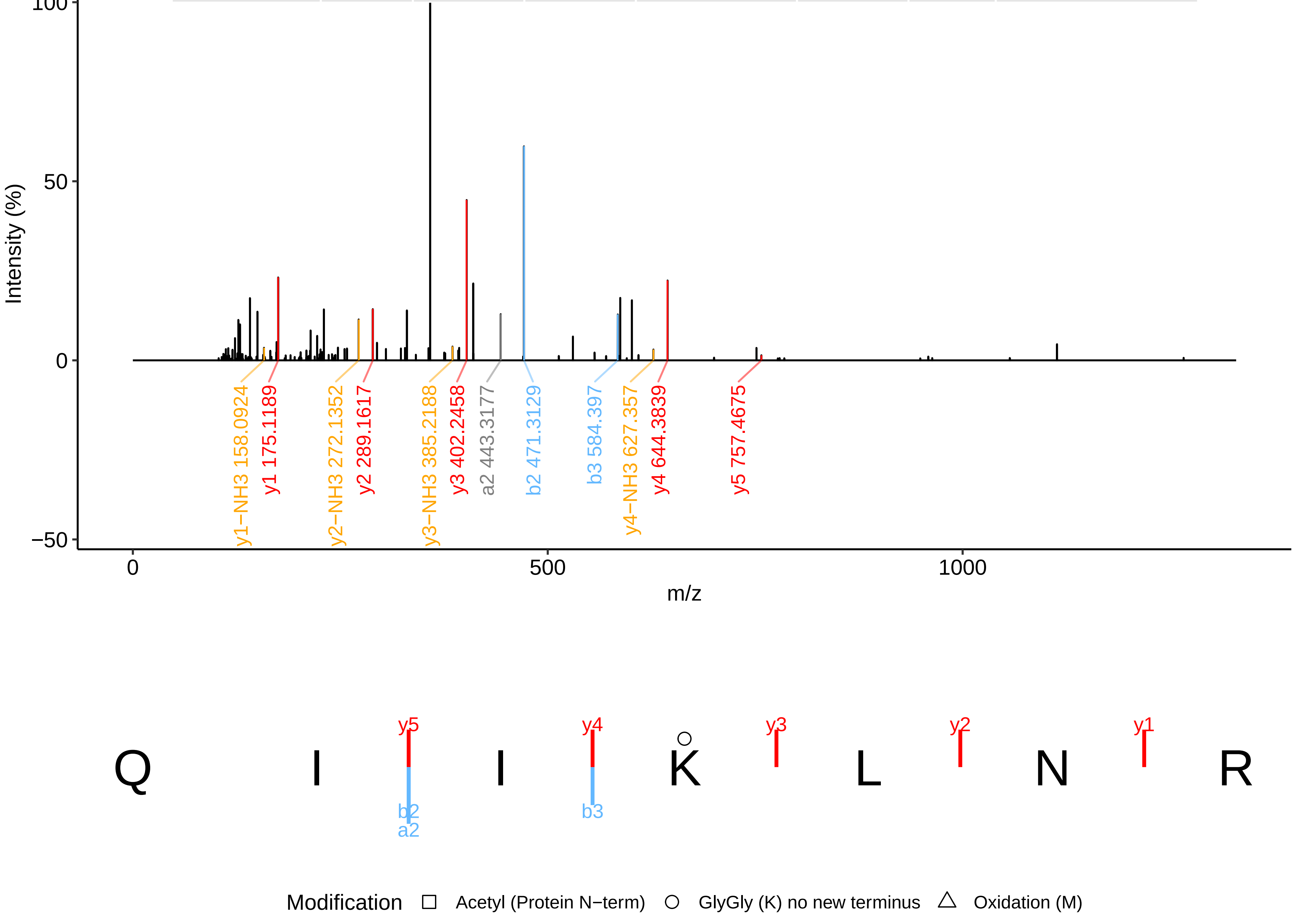

20200924_KIZ_MUb5_GlyGly_1 − Scan number: 8264

Gene Names	Charge	m/z	Mass	Retention time	PEP	Score	Precursor Intensity
MFF	3	333.5417	997.6033	26.385	0.013224	89.673	15701981

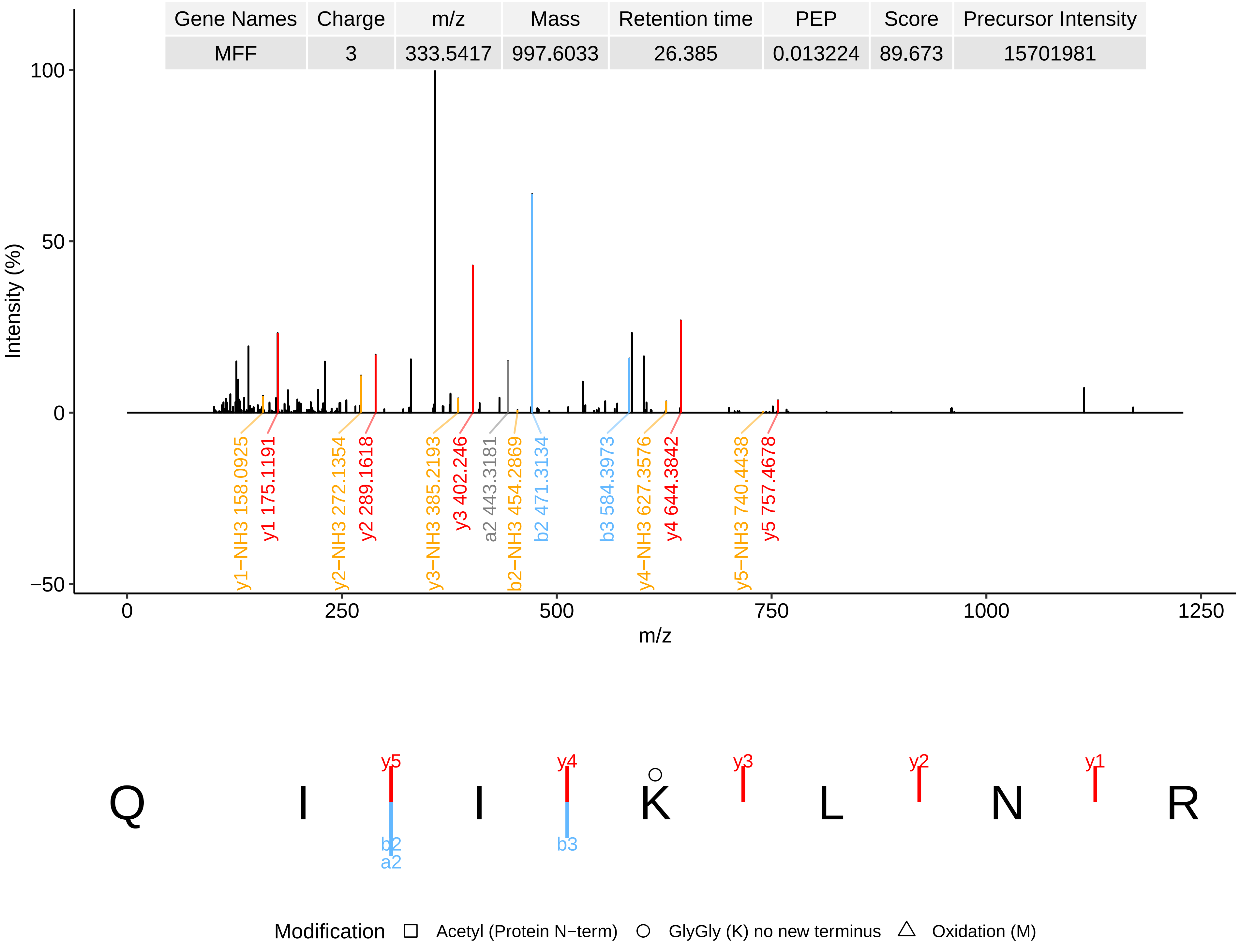

20200924_KIZ_MUb5_GlyGly_2 − Scan number: 7739

Gene Names	Charge	m/z	Mass	Retention time	PEP	Score	Precursor Intensity
MFF	3	333.5417	997.6033	28.906	0.012271	90.986	6770766

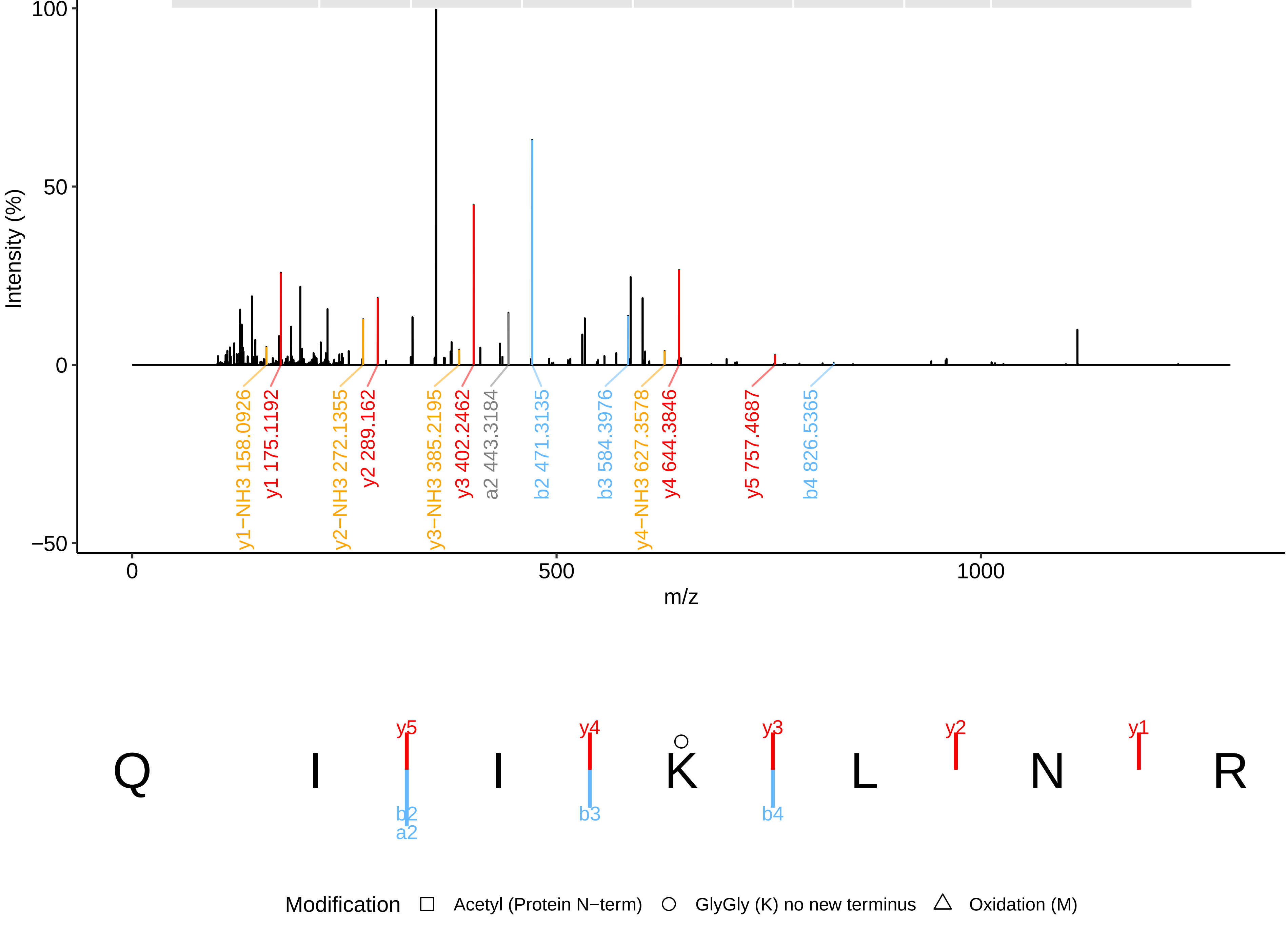

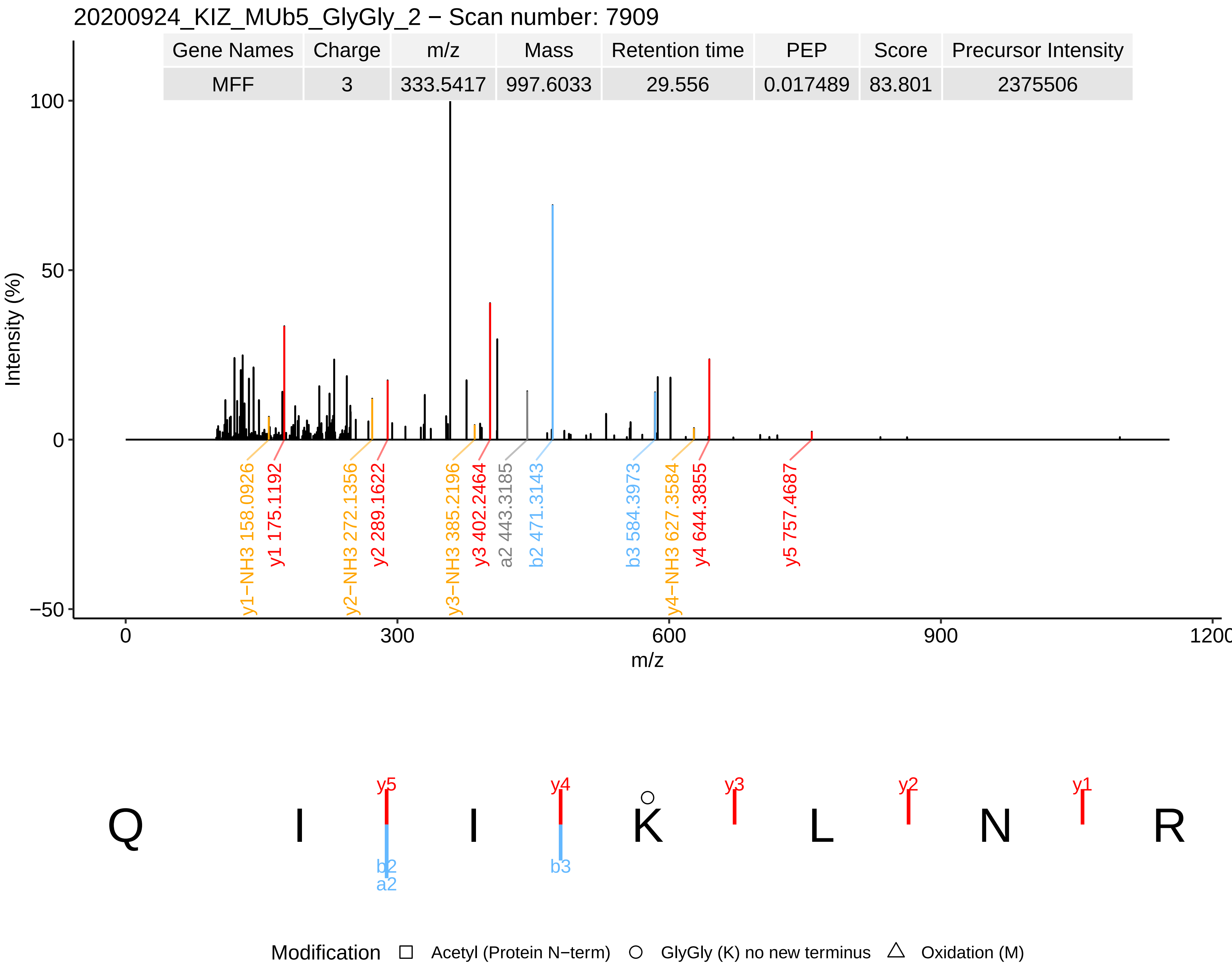

20200924_KIZ_MUb5_GlyGly_2 − Scan number: 7909
Gene Names | Charge | m/z | Mass | Retention time | PEP | Score | Precursor Intensity
MFF | 3 | 333.5417 | 997.6033 | 29.556 | 0.017489 | 83.801 | 2375506
Intensity (%)
m/z
y1−NH3 158.0926
y1 175.1192
y2−NH3 272.1356
y2 289.1622
y3−NH3 385.2196
y3 402.2464
a2 443.3185
b2 471.3143
b3 584.3973
y4−NH3 627.3584
y4 644.3855
y5 757.4687
Q I I K L N R
y5 y4 y3 y2 y1
b2 a2 b3
Modification
Acetyl (Protein N−term)
GlyGly (K) no new terminus
Oxidation (M)

20200921_KIZ_MUb4_GlyGly_10plex − Scan number: 2708

Gene Names	Charge	m/z	Mass	Retention time	PEP	Score	Precursor Intensity
RAD18	3	339.8527	1016.536	8.5266	0.0072938	85.161	9735960

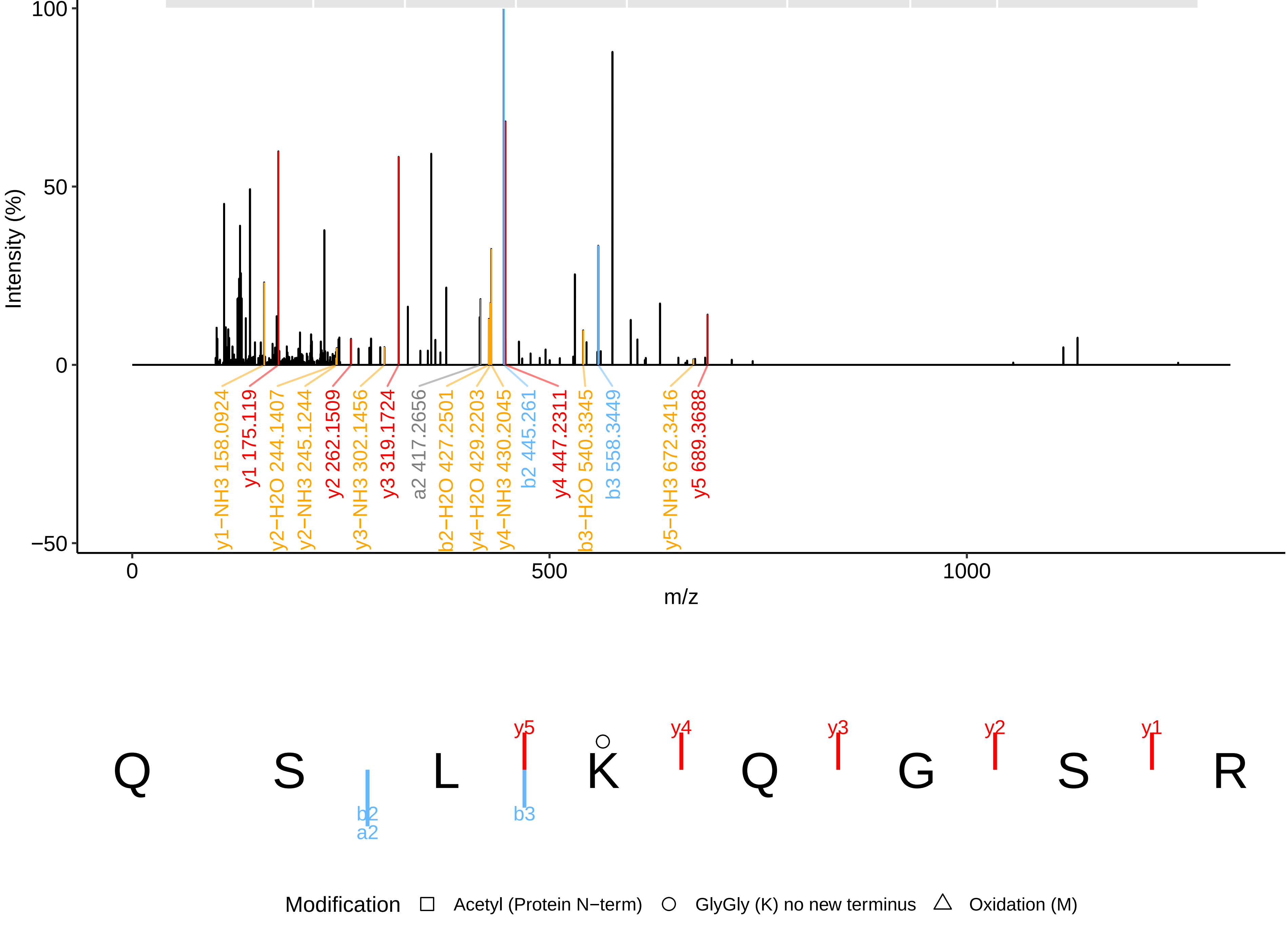

20200921_KIZ_MUb4_GlyGly_10plex_2 − Scan number: 2658

Gene Names	Charge	m/z	Mass	Retention time	PEP	Score	Precursor Intensity
RAD18	3	339.8527	1016.536	8.5159	0.0015017	104.44	914398.9

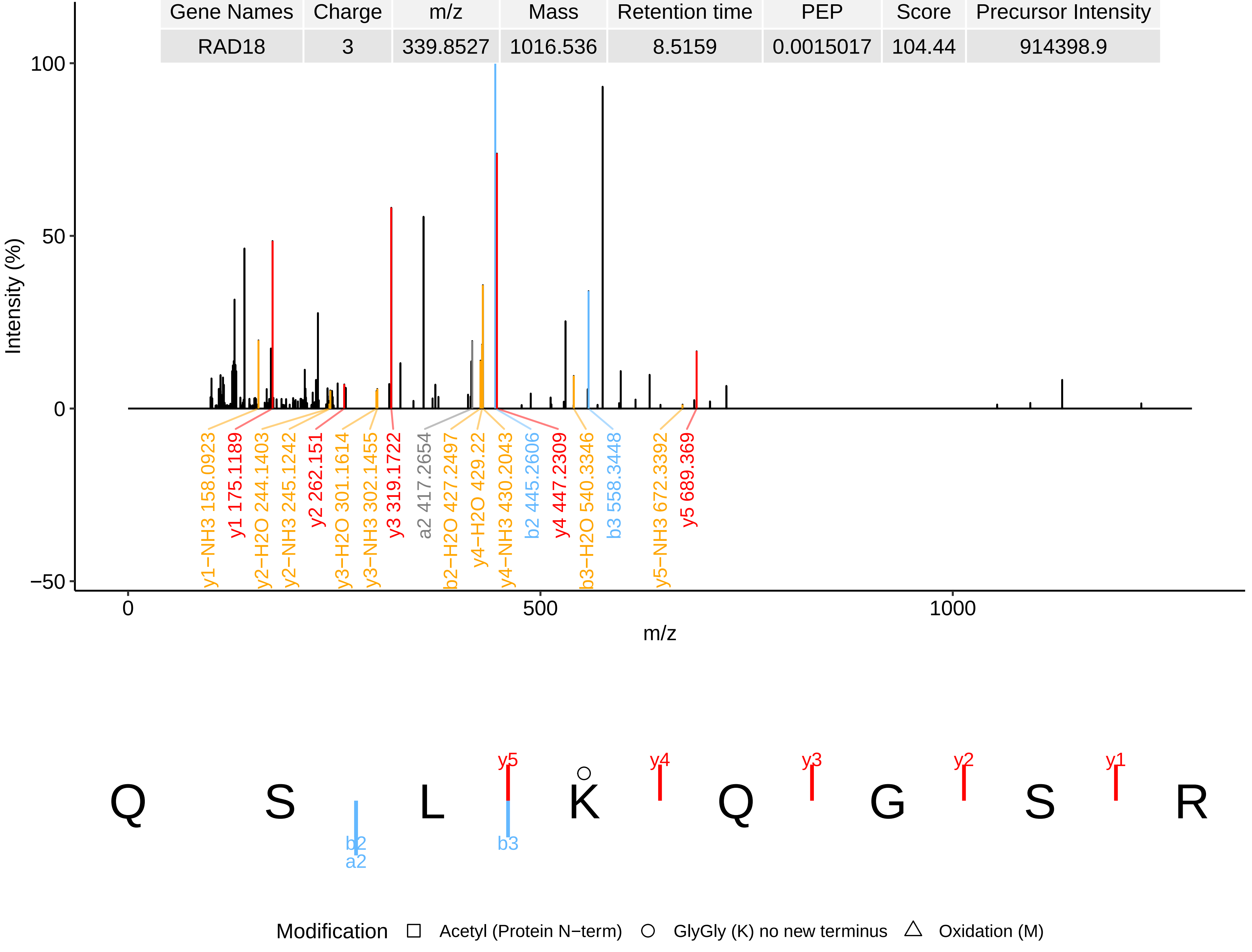

20200924_KIZ_MUb5_GlyGly_1 − Scan number: 2594

Gene Names	Charge	m/z	Mass	Retention time	PEP	Score	Precursor Intensity
RAD18	3	339.8527	1016.536	8.0491	0.0012106	107.01	13931763

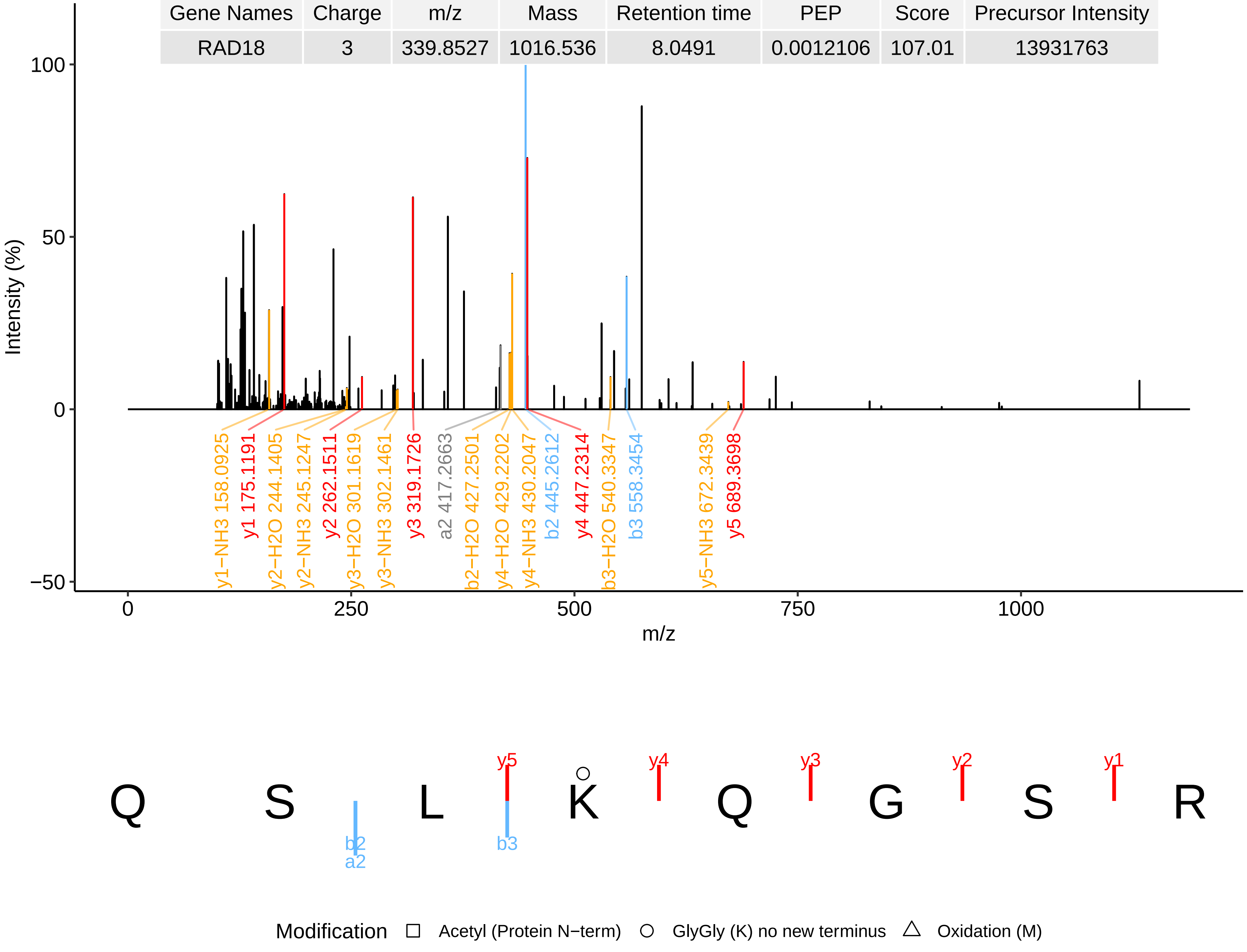

20201118_KIZ_MUb6_GlyGly_2 − Scan number: 2895

Gene Names	Charge	m/z	Mass	Retention time	PEP	Score	Precursor Intensity
RAD18	3	339.8527	1016.536	9.2661	0.001616	110.41	6516489

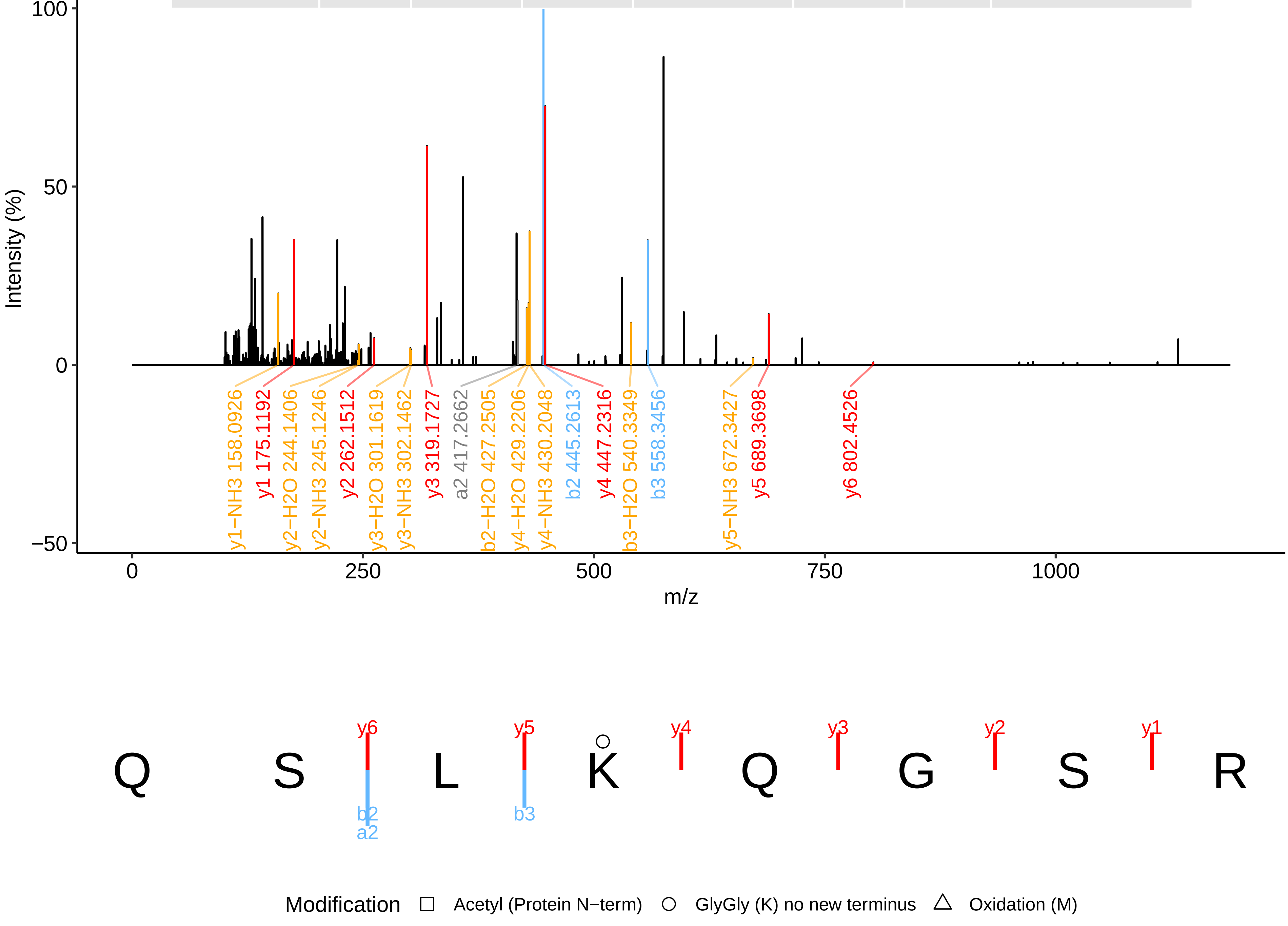

20200921_KIZ_MUb4_GlyGly_10plex_2 − Scan number: 8357

Gene Names	Charge	m/z	Mass	Retention time	PEP	Score	Precursor Intensity
RPL30	3	335.1954	1002.564	31.461	0.024872	78.616	1408457

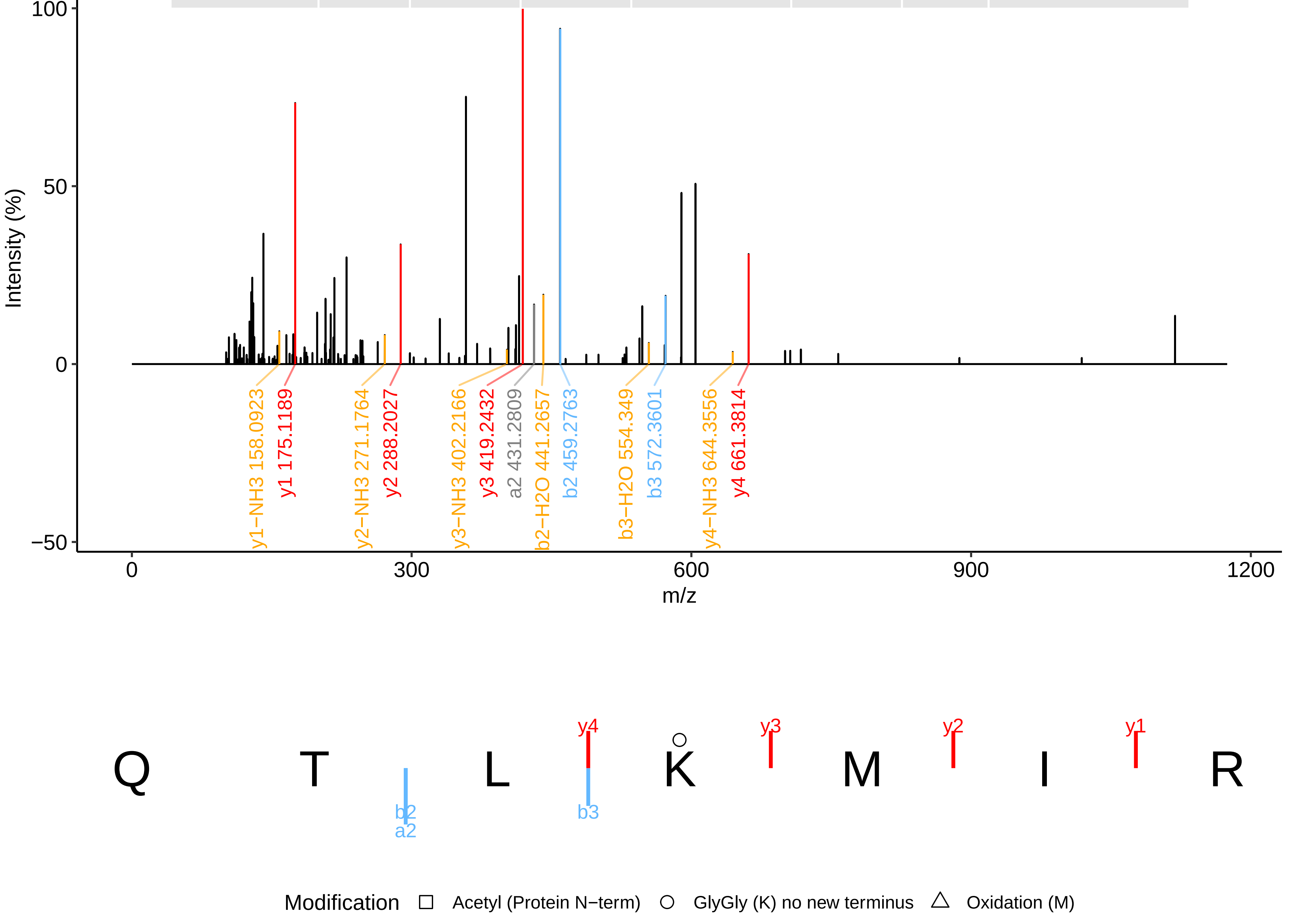

20200921_KIZ_MUb4_GlyGly_10plex − Scan number: 4873

Gene Names	Charge	m/z	Mass	Retention time	PEP	Score	Precursor Intensity
TPD52L2	3	343.5331	1027.577	15.876	0.056471	56.916	18206110

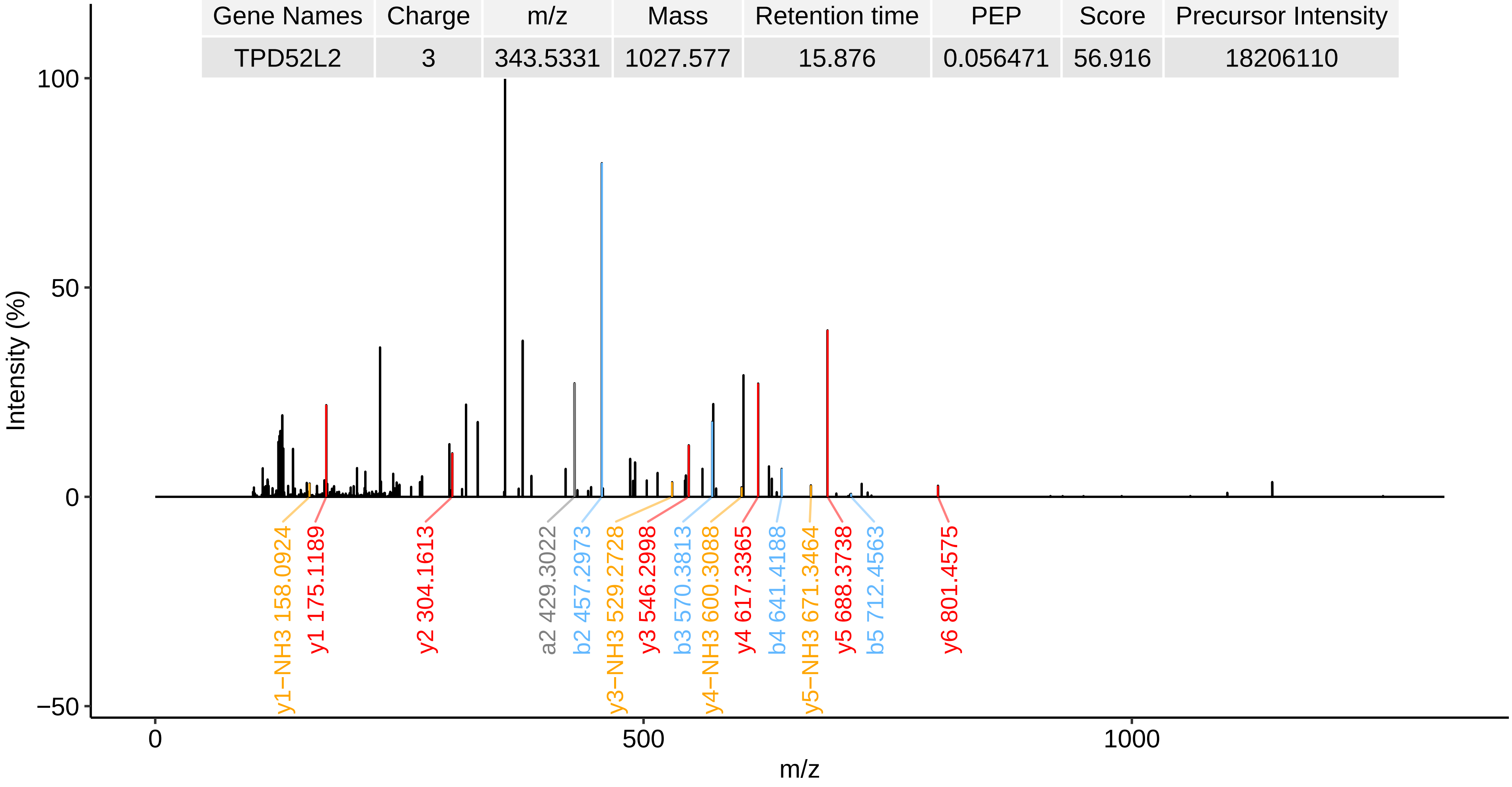

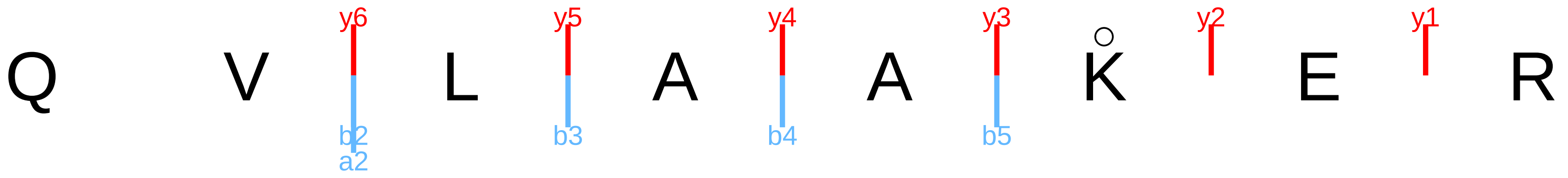

Modification □ Acetyl (Protein N−term) ○ GlyGly (K) no new terminus △ Oxidation (M)

20200924_KIZ_MUb5_GlyGly_1 − Scan number: 4338

Gene Names	Charge	m/z	Mass	Retention time	PEP	Score	Precursor Intensity
TPD52L2	3	343.5331	1027.577	13.378	0.069491	58.433	25005044

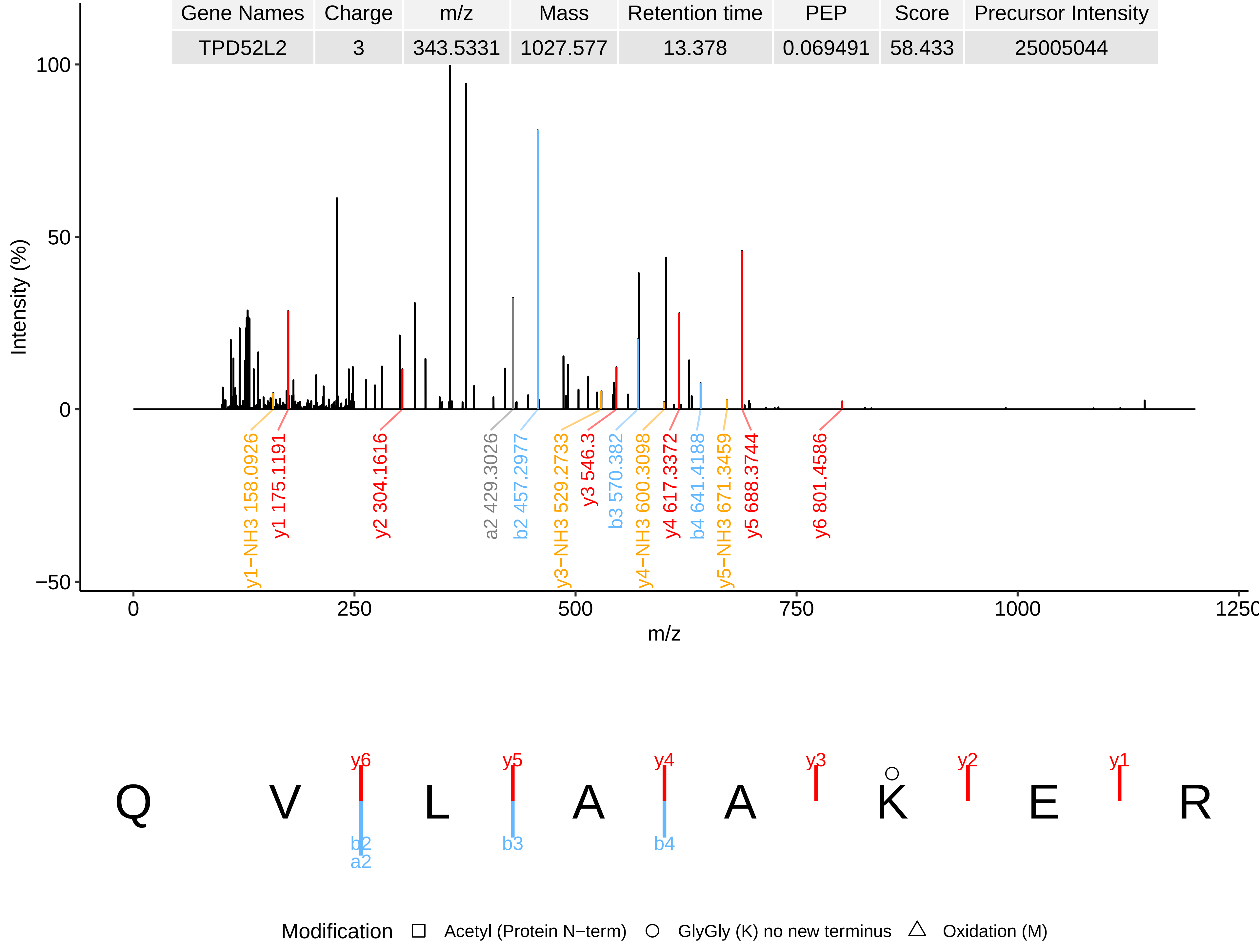

20200924_KIZ_MUb5_GlyGly_2 − Scan number: 3956

Gene Names	Charge	m/z	Mass	Retention time	PEP	Score	Precursor Intensity
TPD52L2	3	343.5331	1027.577	14.46	0.08018	56.916	6060479

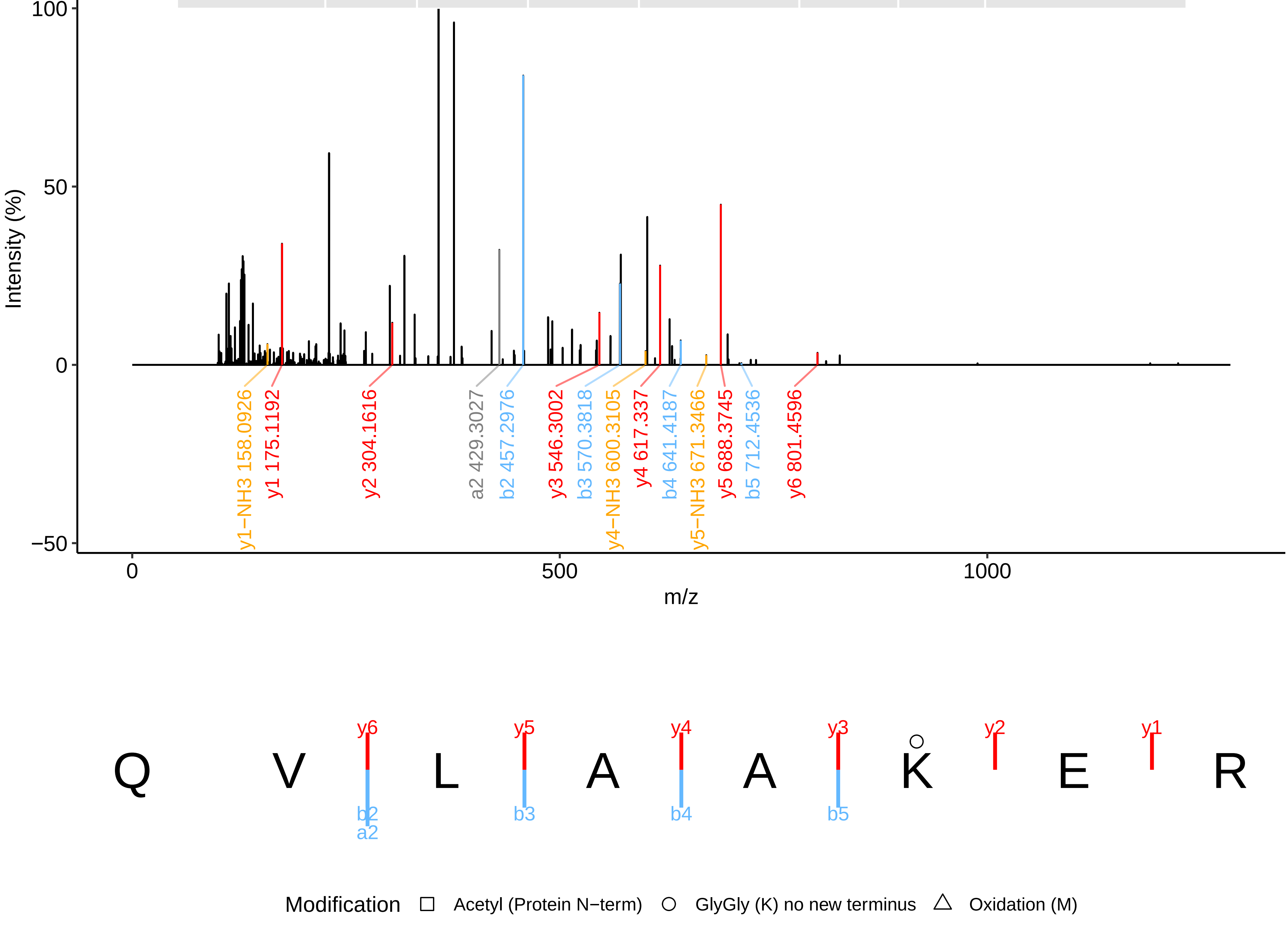

20200924_KIZ_MUb5_GlyGly_1 − Scan number: 4158

Gene Names	Charge	m/z	Mass	Retention time	PEP	Score	Precursor Intensity
TPD52L1	3	348.8647	1043.572	12.786	0.073539	57.859	10767219

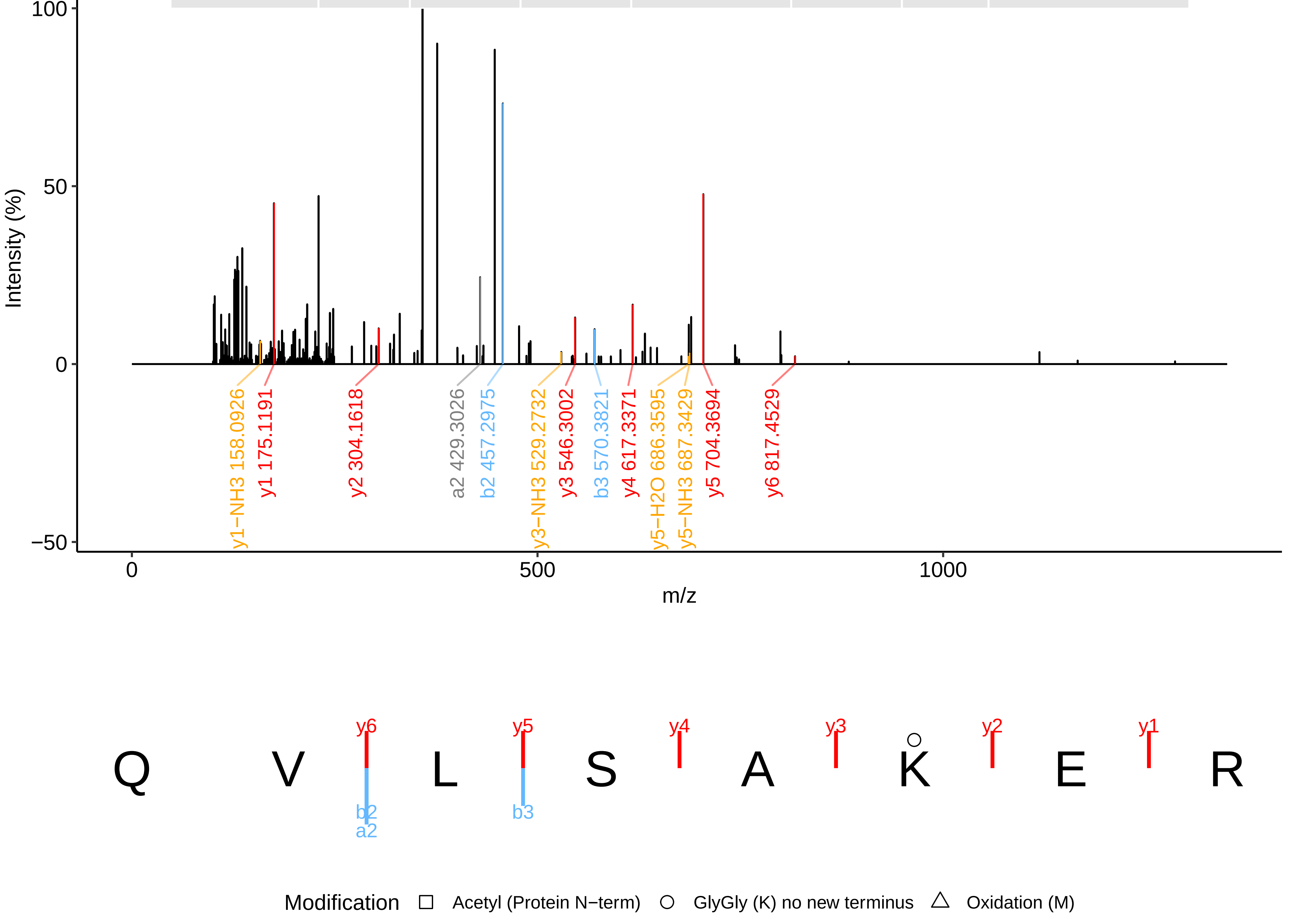

20200921_KIZ_MUb4_GlyGly_10plex − Scan number: 3849

Gene Names	Charge	m/z	Mass	Retention time	PEP	Score	Precursor Intensity
AIFM1	3	344.5054	1030.494	12.422	0.014617	87.754	6151795

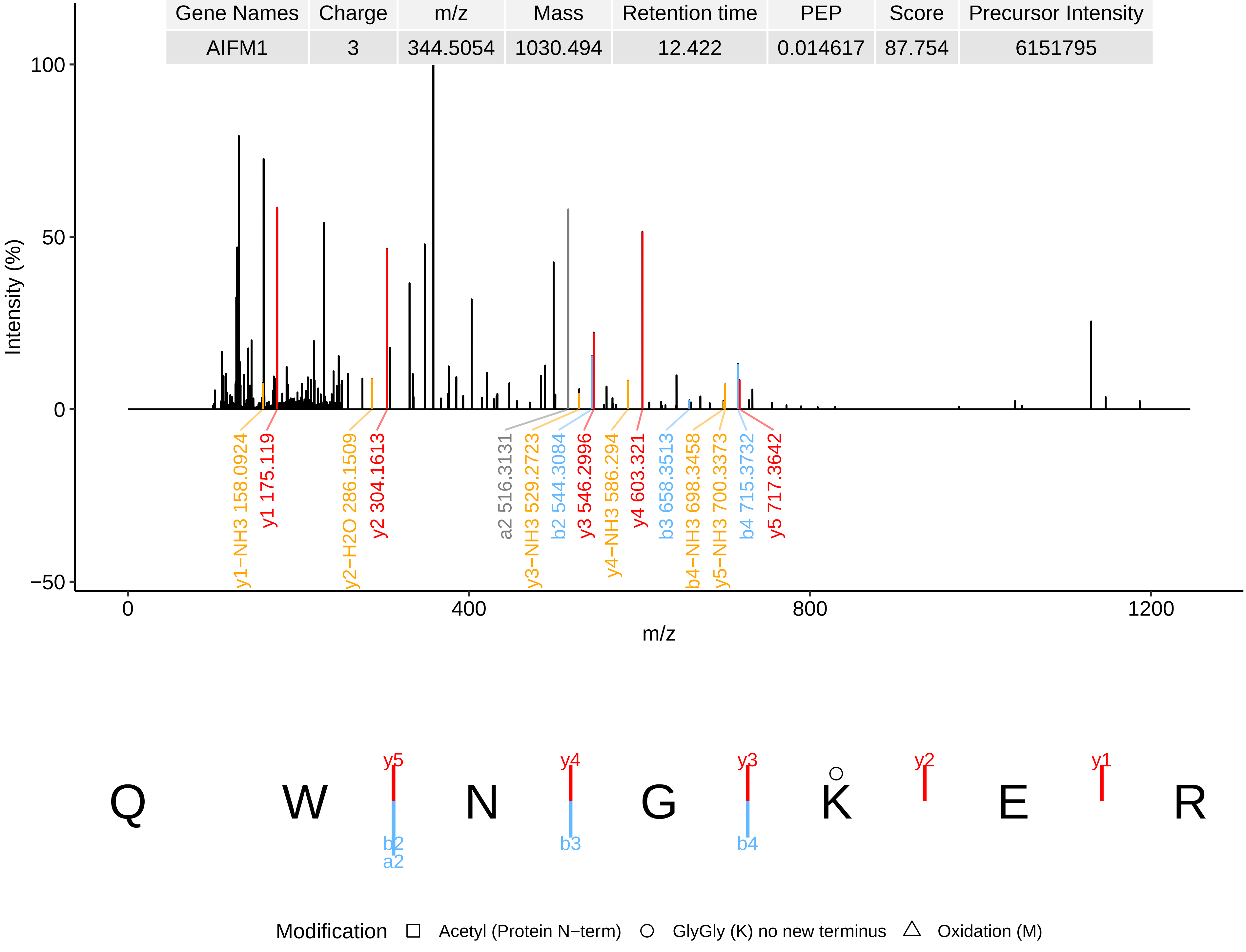

20200924_KIZ_MUb5_GlyGly_1 − Scan number: 2380

Gene Names	Charge	m/z	Mass	Retention time	PEP	Score	Precursor Intensity
PTTG1IP	3	335.8468	1004.519	7.4177	0.0025592	130.28	12688660

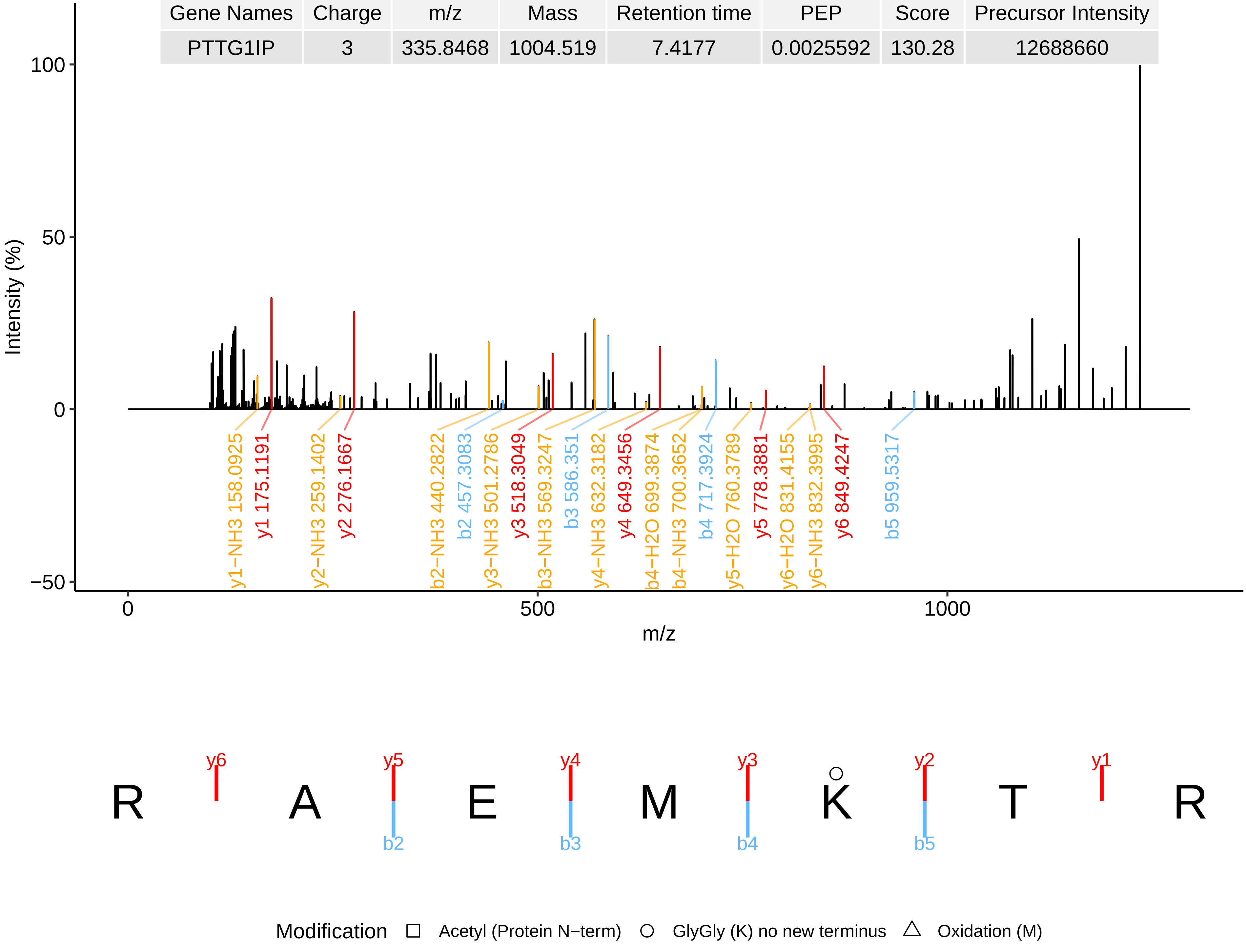

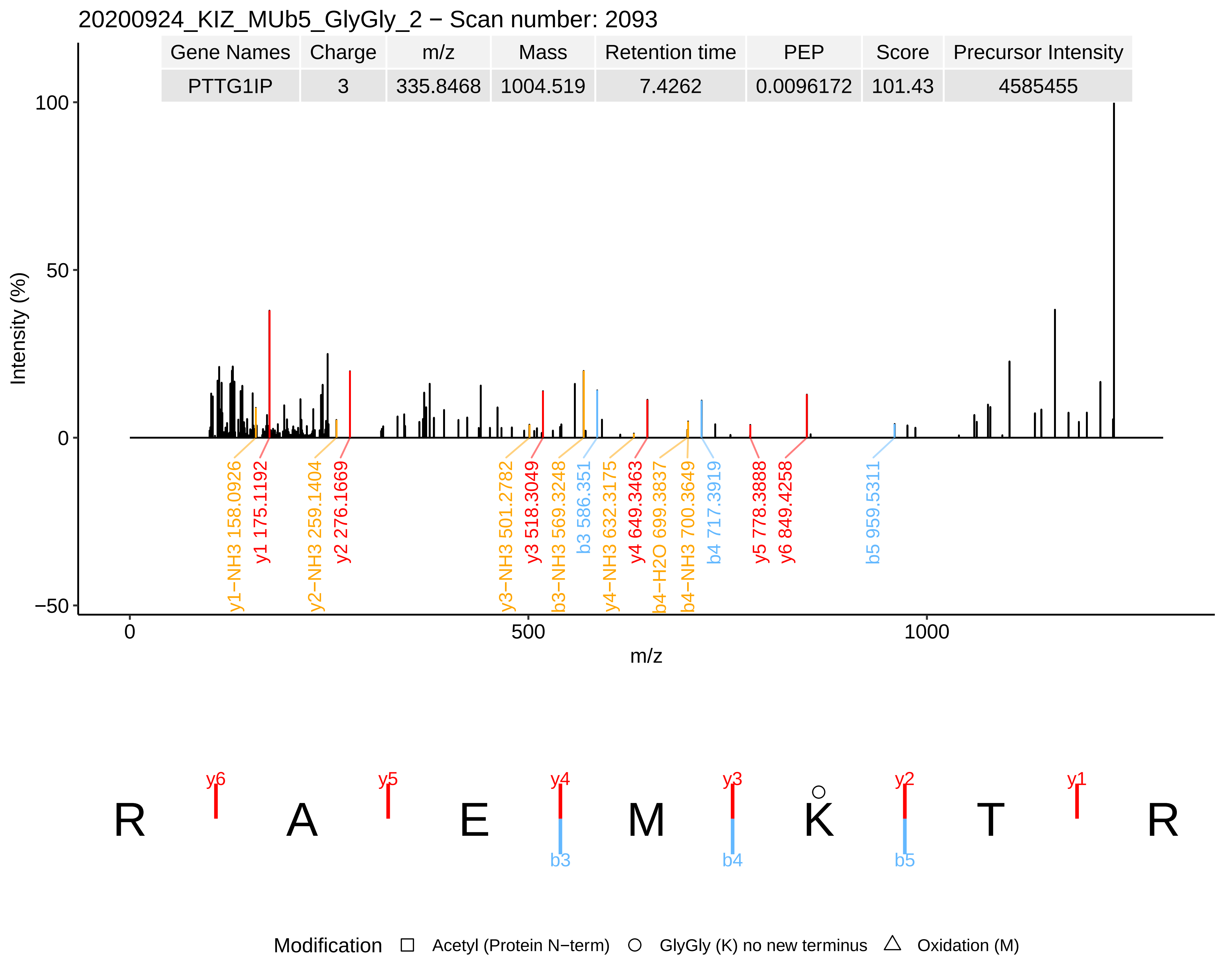

20200924_KIZ_MUb5_GlyGly_2 − Scan number: 2093
Gene Names | Charge | m/z | Mass | Retention time | PEP | Score | Precursor Intensity
PTTG1IP | 3 | 335.8468 | 1004.519 | 7.4262 | 0.0096172 | 101.43 | 4585455
Intensity (%)
m/z
y1−NH3 158.0926
y1 175.1192
y2−NH3 259.1404
y2 276.1669
y3−NH3 501.2782
y3 518.3049
b3−NH3 569.3248
b3 586.351
y4−NH3 632.3175
y4 649.3463
b4−H2O 699.3837
b4−NH3 700.3649
b4 717.3919
y5 778.3888
y6 849.4258
b5 959.5311
R A E M K T R
y6 y5 y4 y3 y2 y1
b3 b4 b5
Modification
Acetyl (Protein N−term)
GlyGly (K) no new terminus
Oxidation (M)

20201118_KIZ_MUb6_GlyGly_1 − Scan number: 2483

Gene Names	Charge	m/z	Mass	Retention time	PEP	Score	Precursor Intensity
PTTG1IP	3	335.8468	1004.519	7.6113	0.0033213	115.49	22427972

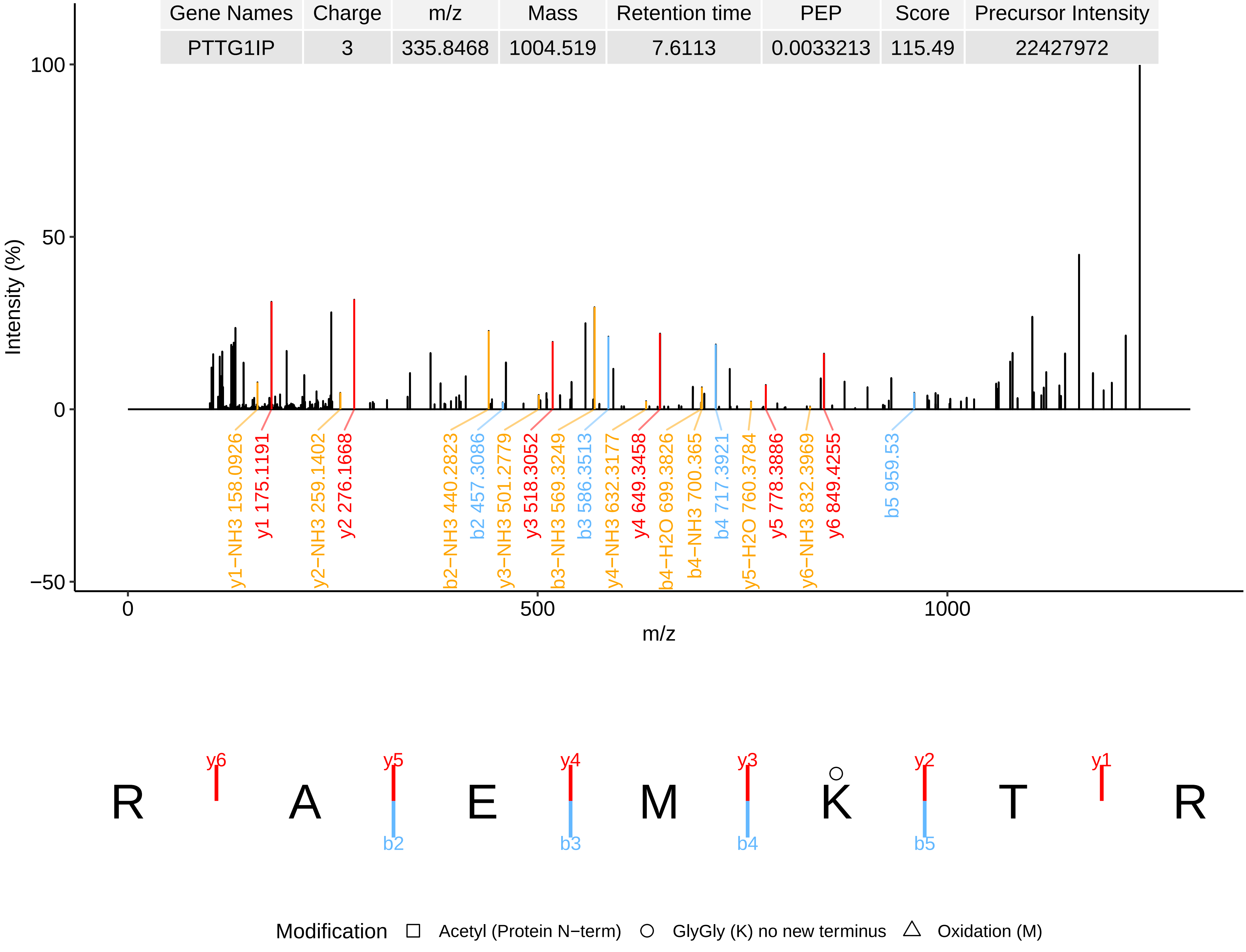

20201118_KIZ_MUb6_GlyGly_2 − Scan number: 2584

Gene Names	Charge	m/z	Mass	Retention time	PEP	Score	Precursor Intensity
PTTG1IP	3	335.8468	1004.519	8.0924	0.00017607	150.4	19980738

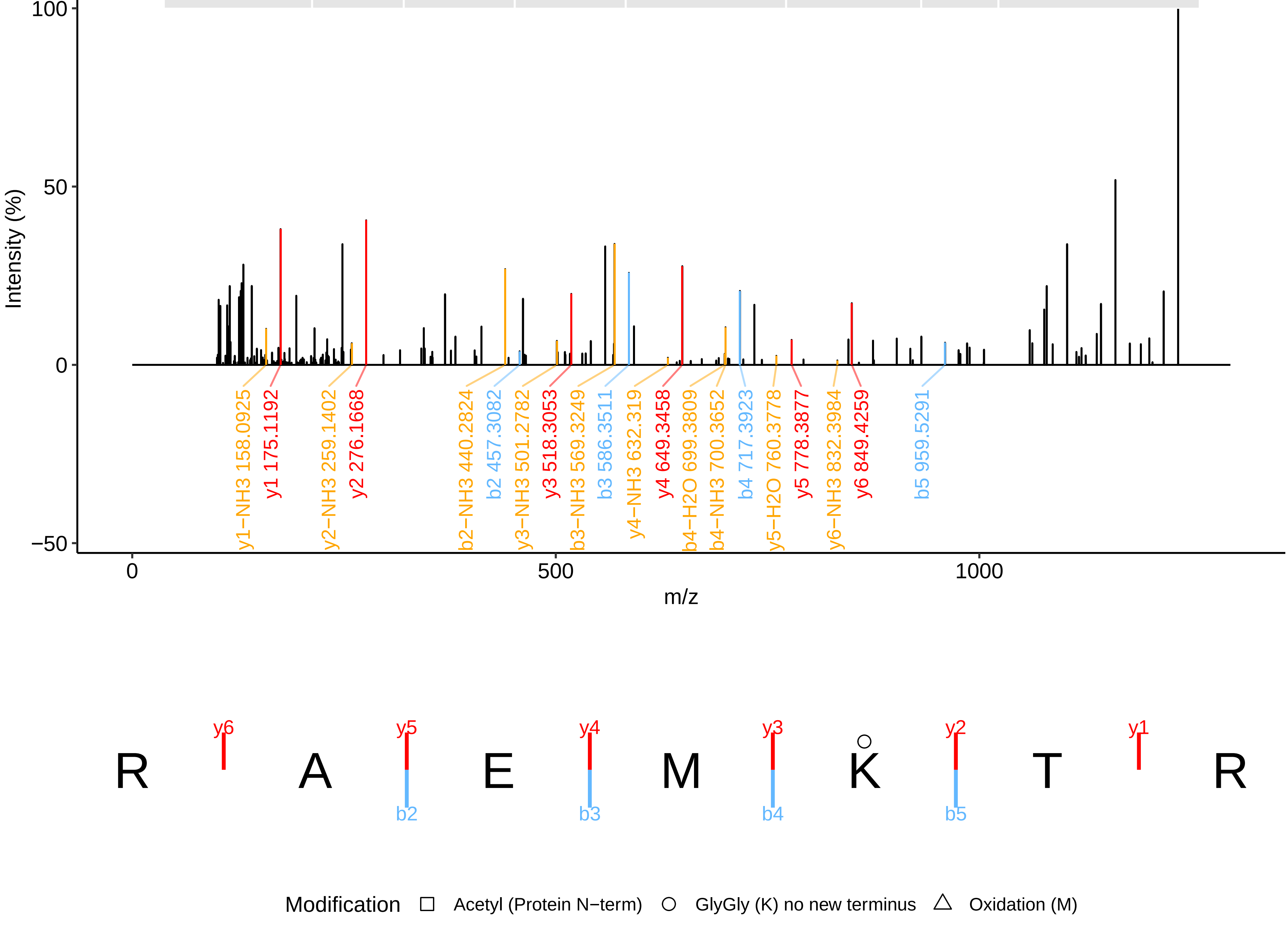

20201118_KIZ_MUb6_GlyGly_1 − Scan number: 3074

Gene Names	Charge	m/z	Mass	Retention time	PEP	Score	Precursor Intensity
RPL24	3	340.2017	1017.583	9.4652	0.064905	73.039	NA

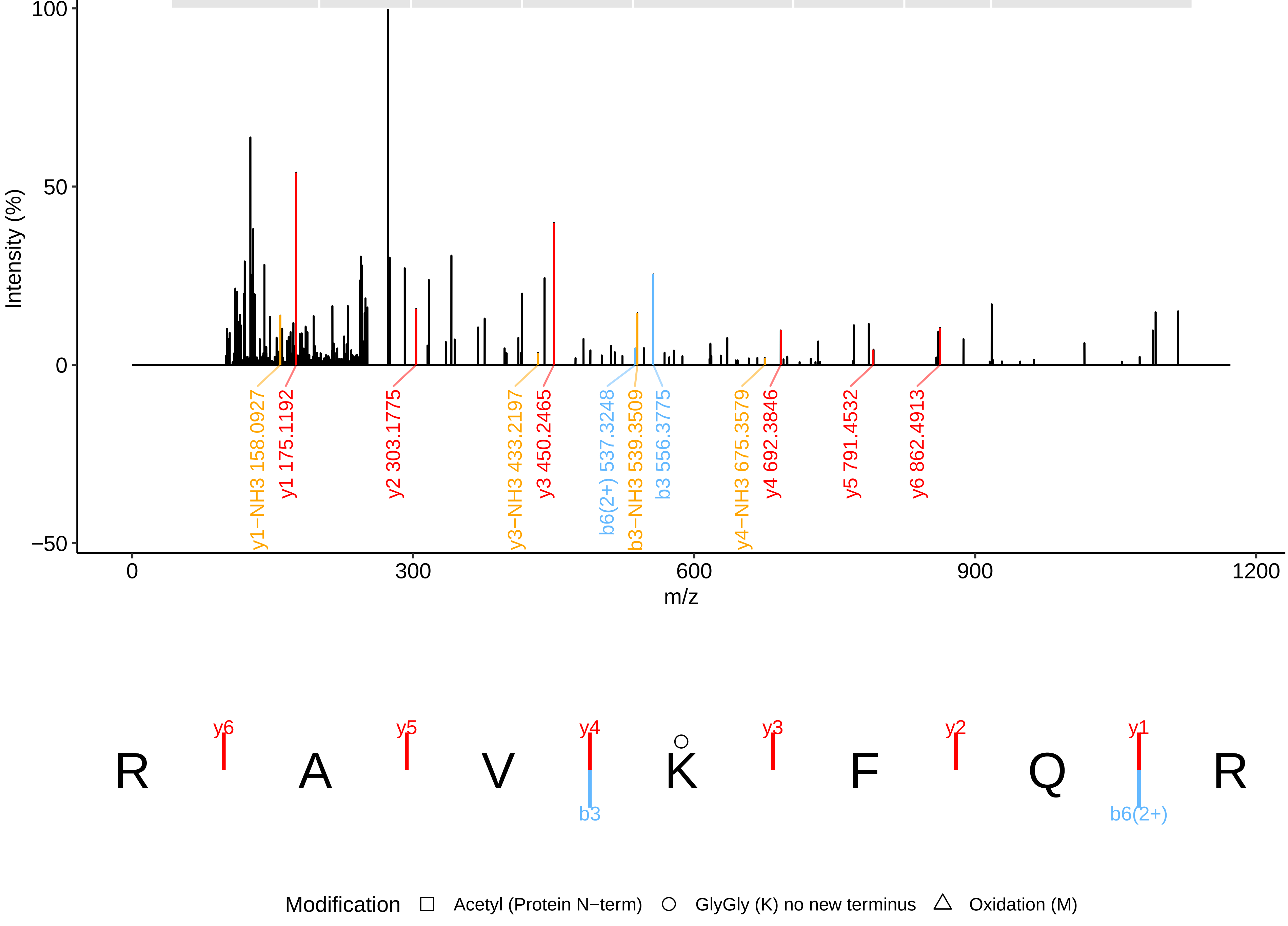

20200921_KIZ_MUb4_GlyGly_10plex − Scan number: 8015

Gene Names	Charge	m/z	Mass	Retention time	PEP	Score	Precursor Intensity
ANKFY1	3	334.5295	1000.567	27.082	0.011552	98.523	7338539

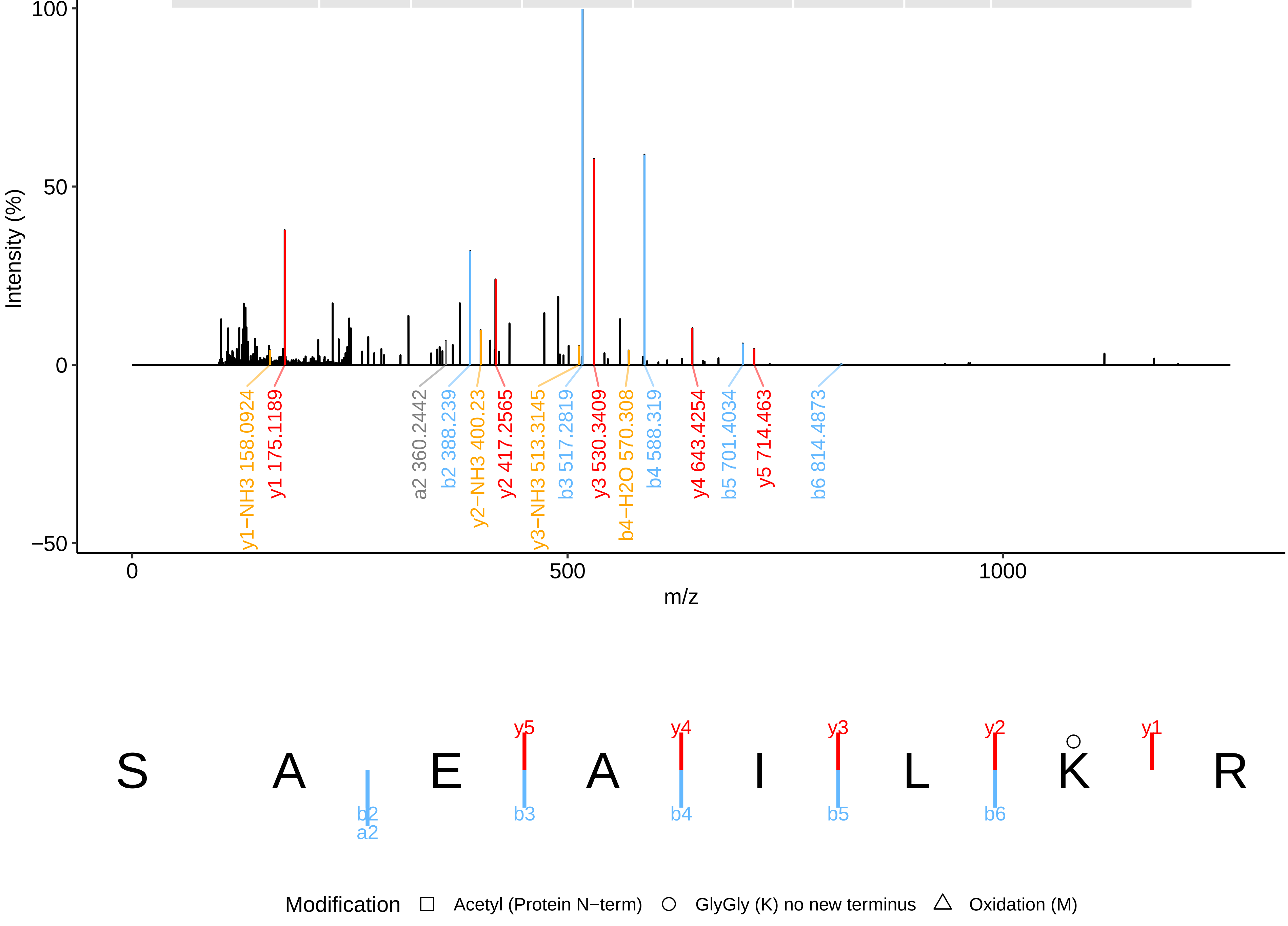

20200921_KIZ_MUb4_GlyGly_10plex_2 − Scan number: 7419

Gene Names	Charge	m/z	Mass	Retention time	PEP	Score	Precursor Intensity
ANKFY1	3	334.5295	1000.567	27.676	0.029161	86.794	284370

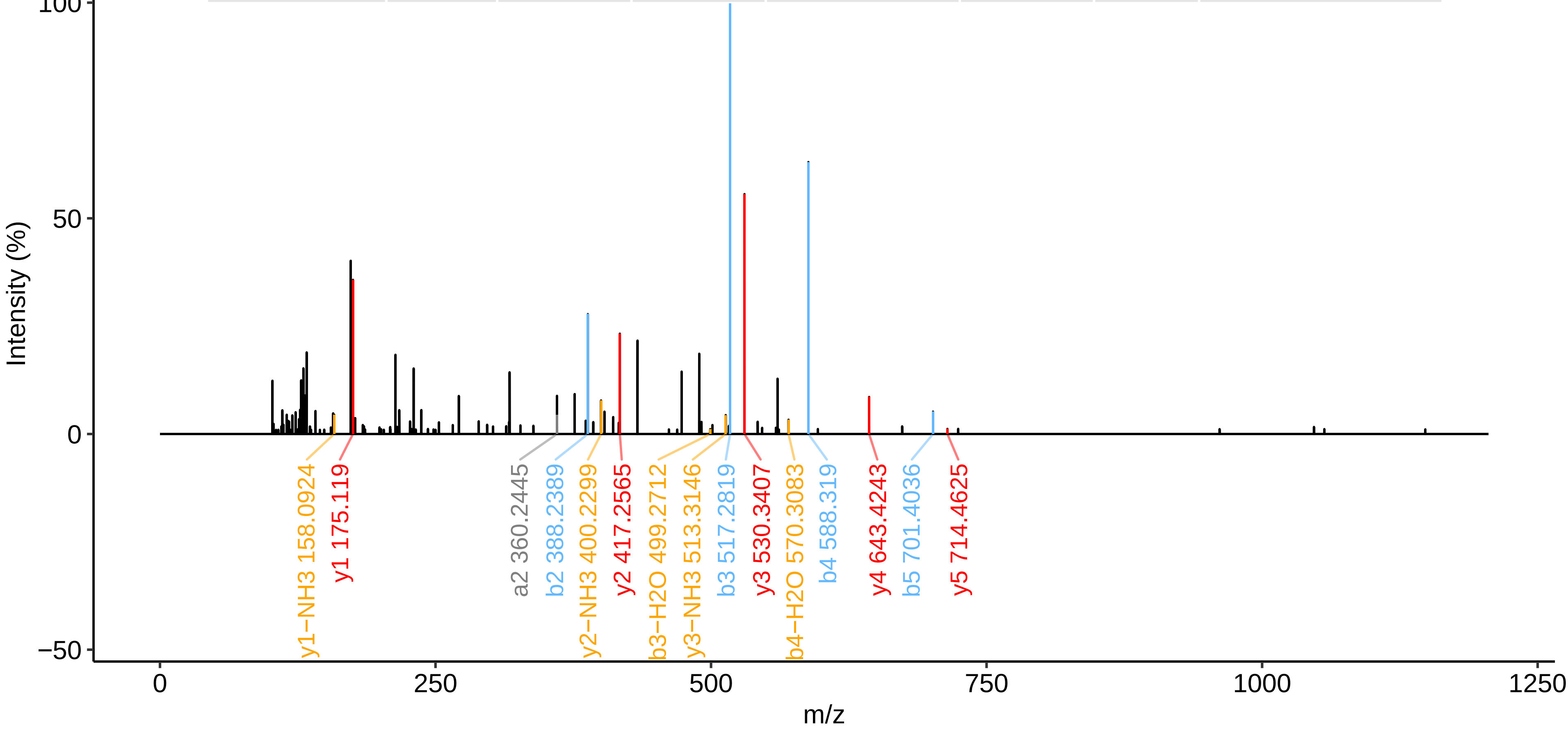

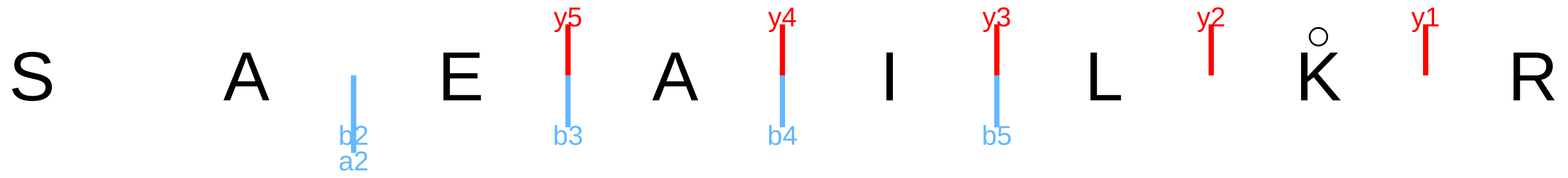

Modification □ Acetyl (Protein N−term) ○ GlyGly (K) no new terminus △ Oxidation (M)

20200924_KIZ_MUb5_GlyGly_1 − Scan number: 5372

Gene Names	Charge	m/z	Mass	Retention time	PEP	Score	Precursor Intensity
KATNAL1	2	503.2516	1004.489	16.694	0.031006	81.191	7059394

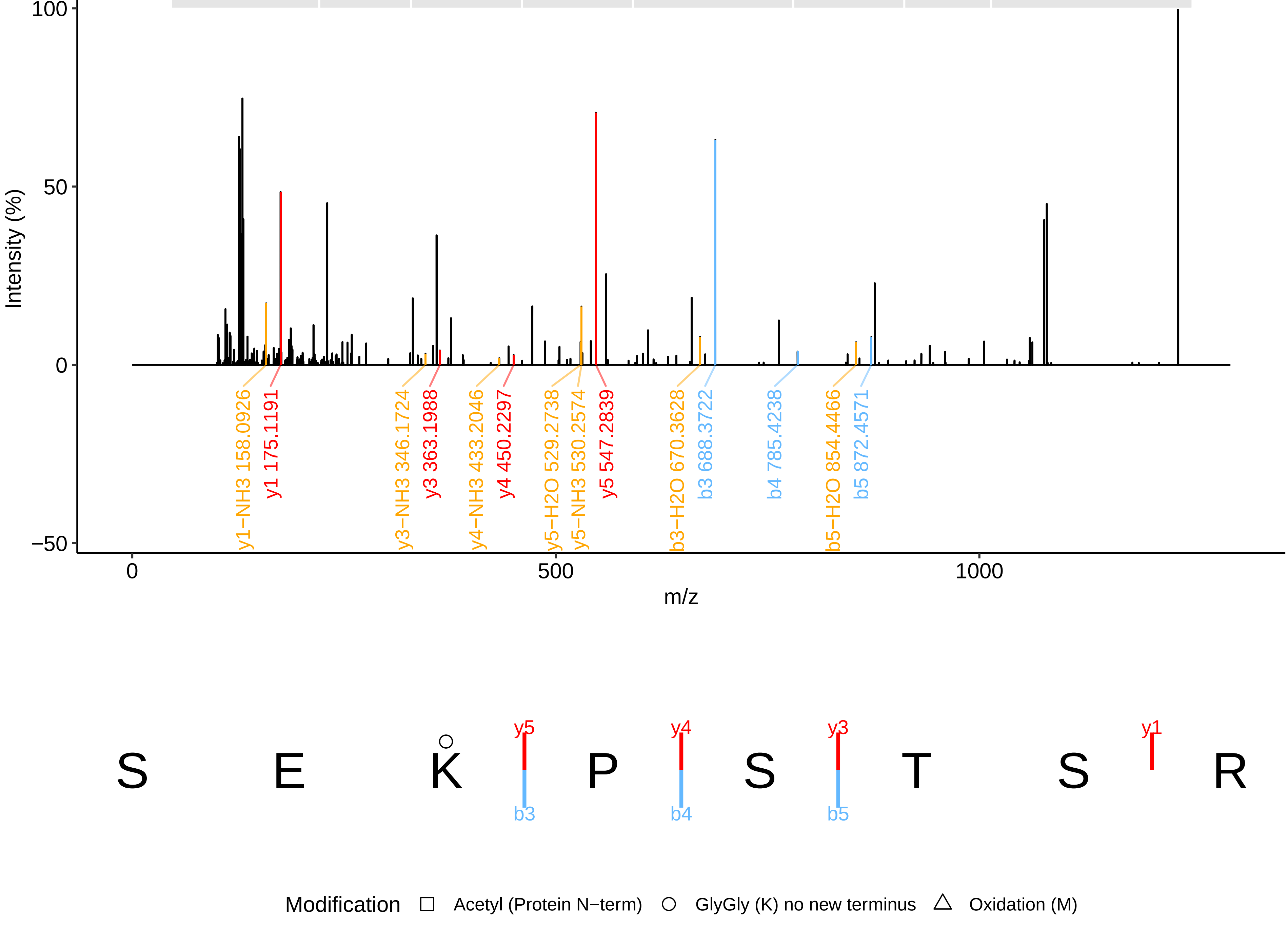

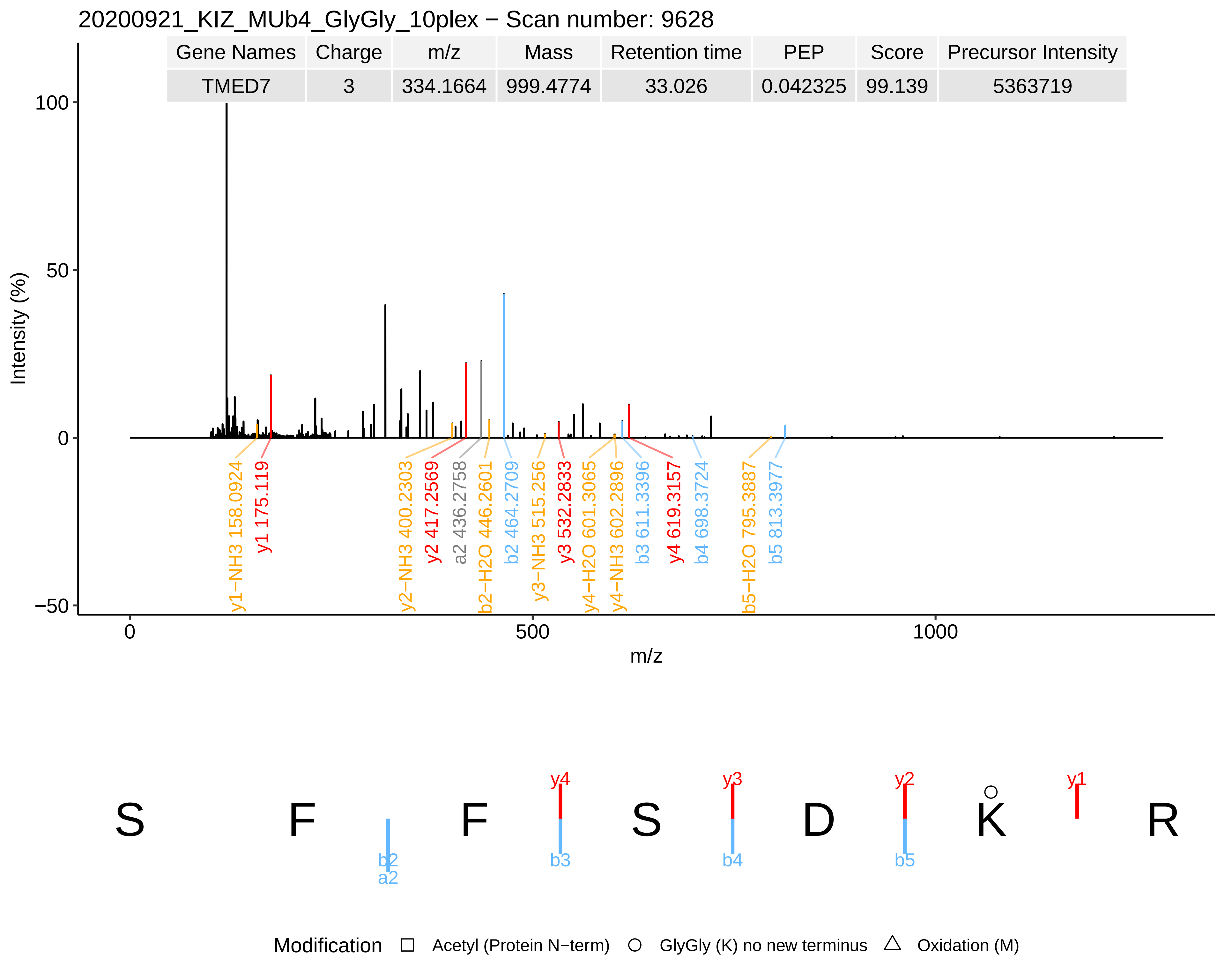

20200921_KIZ_MUb4_GlyGly_10plex − Scan number: 9628
Gene Names | Charge | m/z | Mass | Retention time | PEP | Score | Precursor Intensity
TMED7 | 3 | 334.1664 | 999.4774 | 33.026 | 0.042325 | 99.139 | 5363719
Intensity (%)
100
50
0
−50
0
500
1000
m/z
y1−NH3 158.0924
y1 175.119
y2−NH3 400.2303
y2 417.2569
a2 436.2758
b2−H2O 446.2601
b2 464.2709
y3−NH3 515.256
y3 532.2833
y4−H2O 601.3065
y4−NH3 602.2896
b3 611.3396
y4 619.3157
b4 698.3724
b5−H2O 795.3887
b5 813.3977
S F F S D K R
y4 y3 y2 y1
b2 a2 b3 b4 b5
Modification
Acetyl (Protein N−term)
GlyGly (K) no new terminus
Oxidation (M)

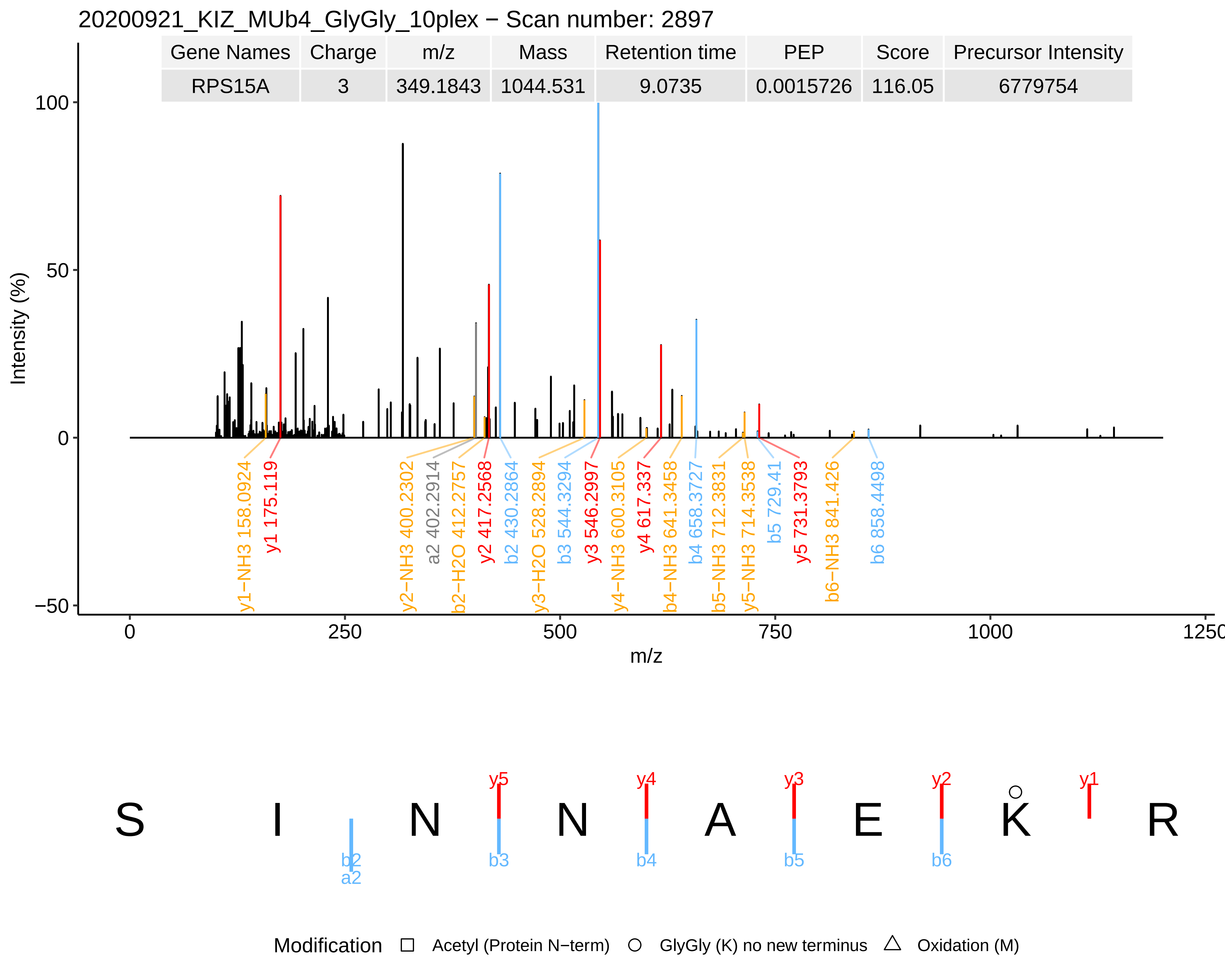

20200921_KIZ_MUb4_GlyGly_10plex − Scan number: 2897
Gene Names
Charge
m/z
Mass
Retention time
PEP
Score
Precursor Intensity
RPS15A
3
349.1843
1044.531
9.0735
0.0015726
116.05
6779754
Intensity (%)
m/z
y1−NH3 158.0924
y1 175.119
y2−NH3 400.2302
a2 402.2914
b2−H2O 412.2757
y2 417.2568
b2 430.2864
y3−H2O 528.2894
b3 544.3294
y3 546.2997
y4−NH3 600.3105
y4 617.337
b4−NH3 641.3458
b4 658.3727
b5−NH3 712.3831
y5−NH3 714.3538
b5 729.41
y5 731.3793
b6−NH3 841.426
b6 858.4498
S I N N A E K R
y5 y4 y3 y2 y1
b2 a2 b3 b4 b5 b6
Modification
Acetyl (Protein N−term)
GlyGly (K) no new terminus
Oxidation (M)

20200924_KIZ_MUb5_GlyGly_1 − Scan number: 2801

Gene Names	Charge	m/z	Mass	Retention time	PEP	Score	Precursor Intensity
RPS15A	3	349.1843	1044.531	8.6517	0.010563	102.61	14056476

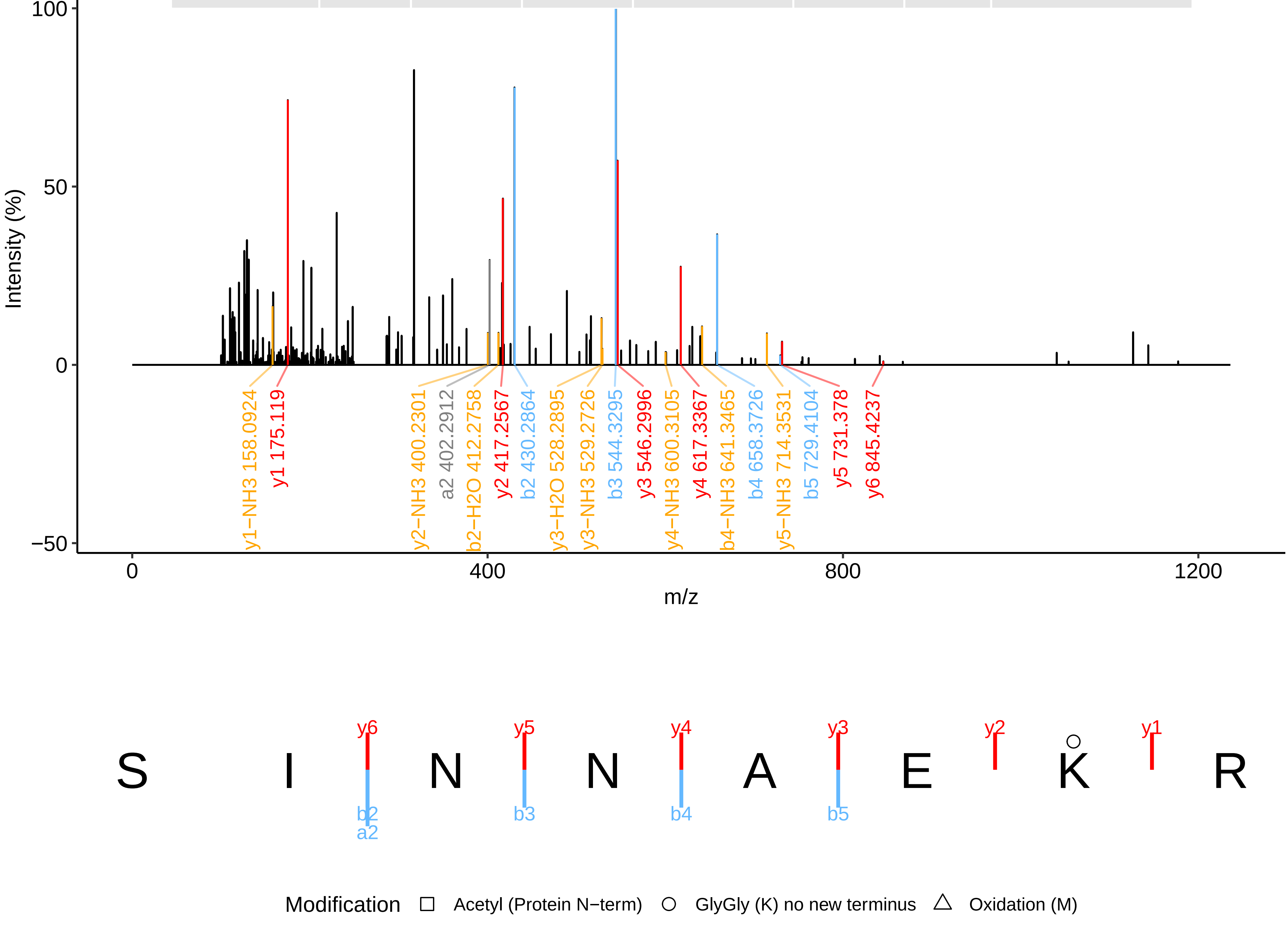

20200921_KIZ_MUb4_GlyGly_10plex_2 − Scan number: 2787

Gene Names	Charge	m/z	Mass	Retention time	PEP	Score	Precursor Intensity
SLC4A7	3	348.8526	1043.536	8.9765	0.0080294	105.57	2661696

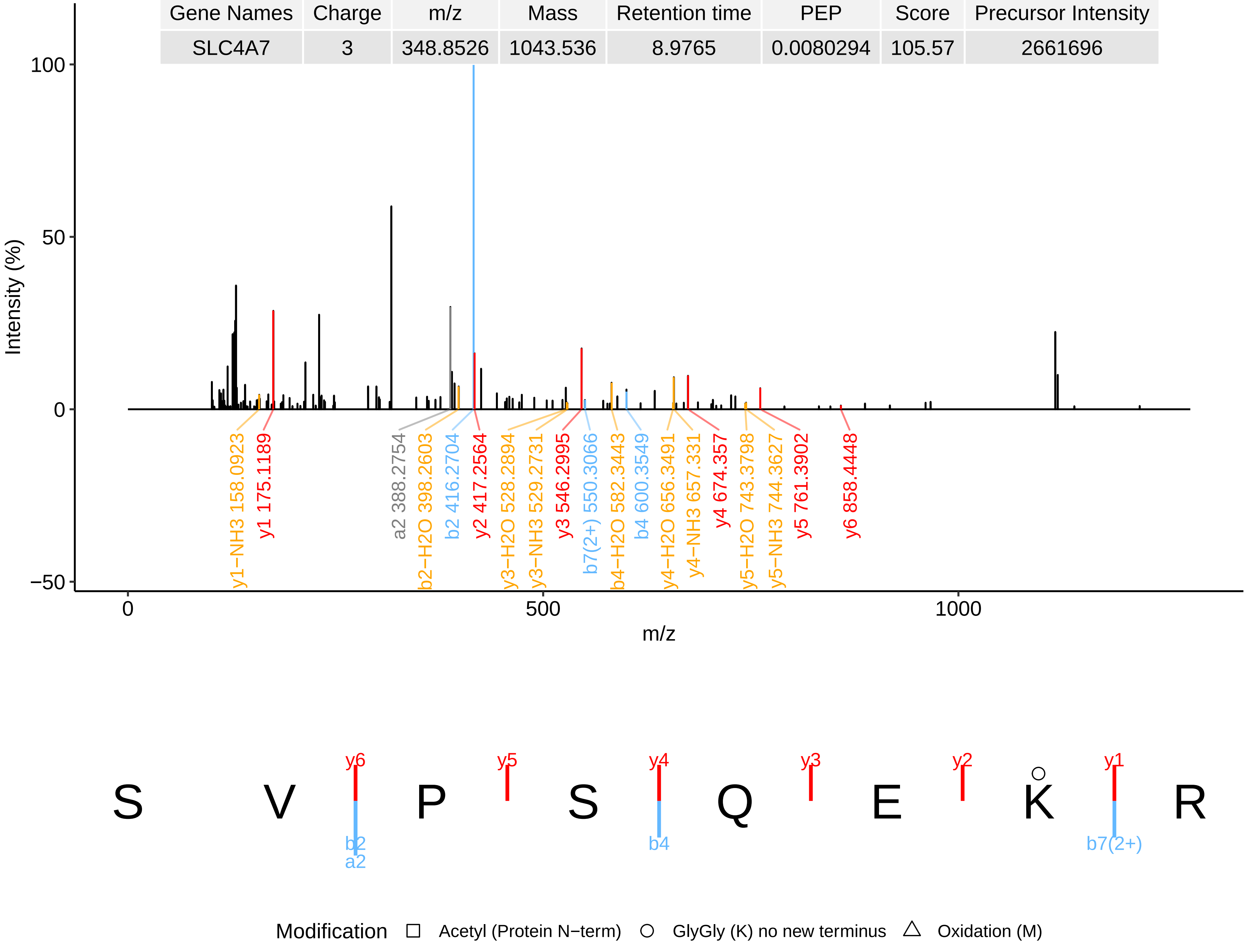

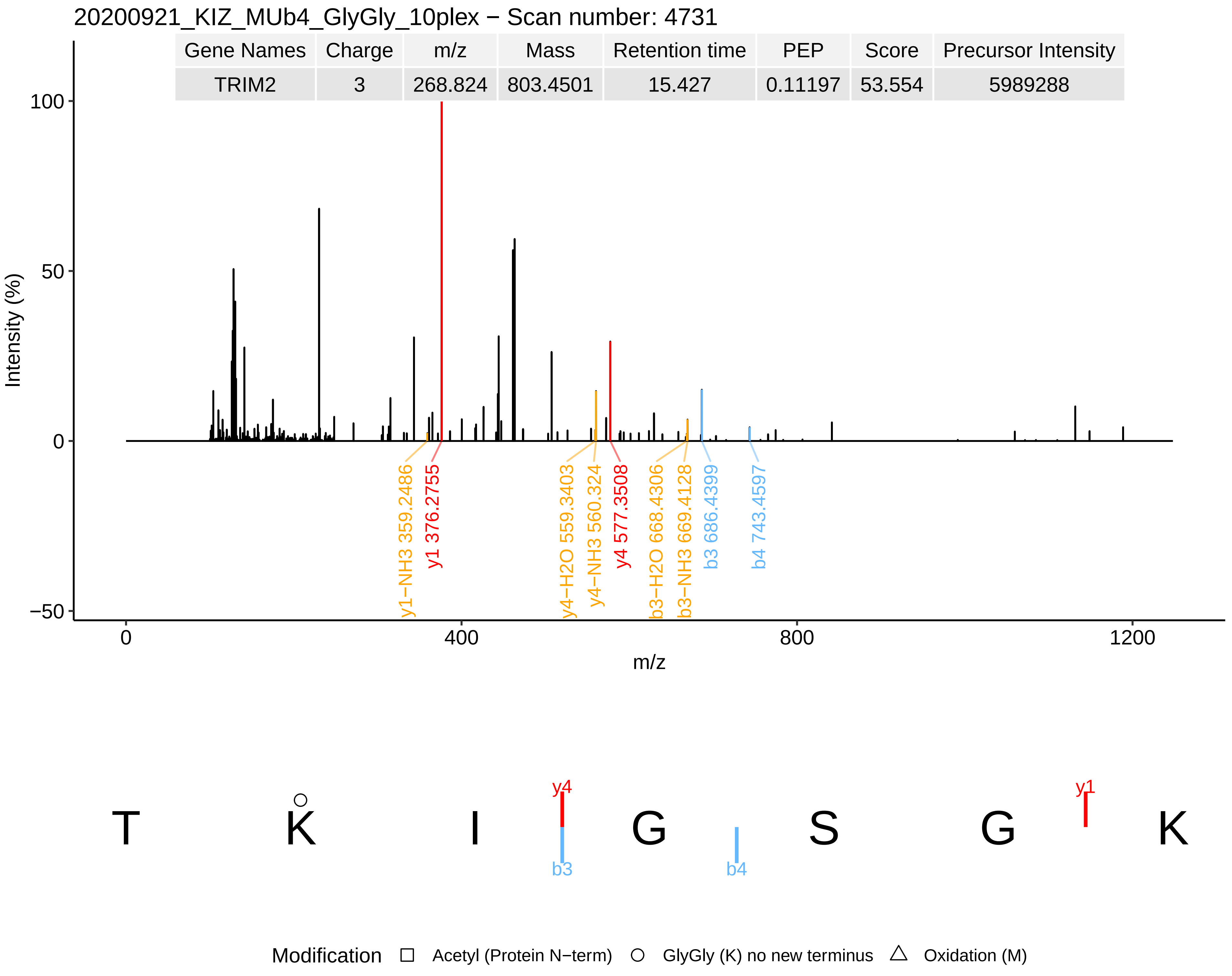

20200921_KIZ_MUb4_GlyGly_10plex − Scan number: 4731
Gene Names | Charge | m/z | Mass | Retention time | PEP | Score | Precursor Intensity
TRIM2 | 3 | 268.824 | 803.4501 | 15.427 | 0.11197 | 53.554 | 5989288
Intensity (%)
m/z
y1−NH3 359.2486
y1 376.2755
y4−H2O 559.3403
y4−NH3 560.324
y4 577.3508
b3−H2O 668.4306
b3−NH3 669.4128
b3 686.4399
b4 743.4597
T K I G S G K
y4
y1
b3
b4
Modification
Acetyl (Protein N−term)
GlyGly (K) no new terminus
Oxidation (M)

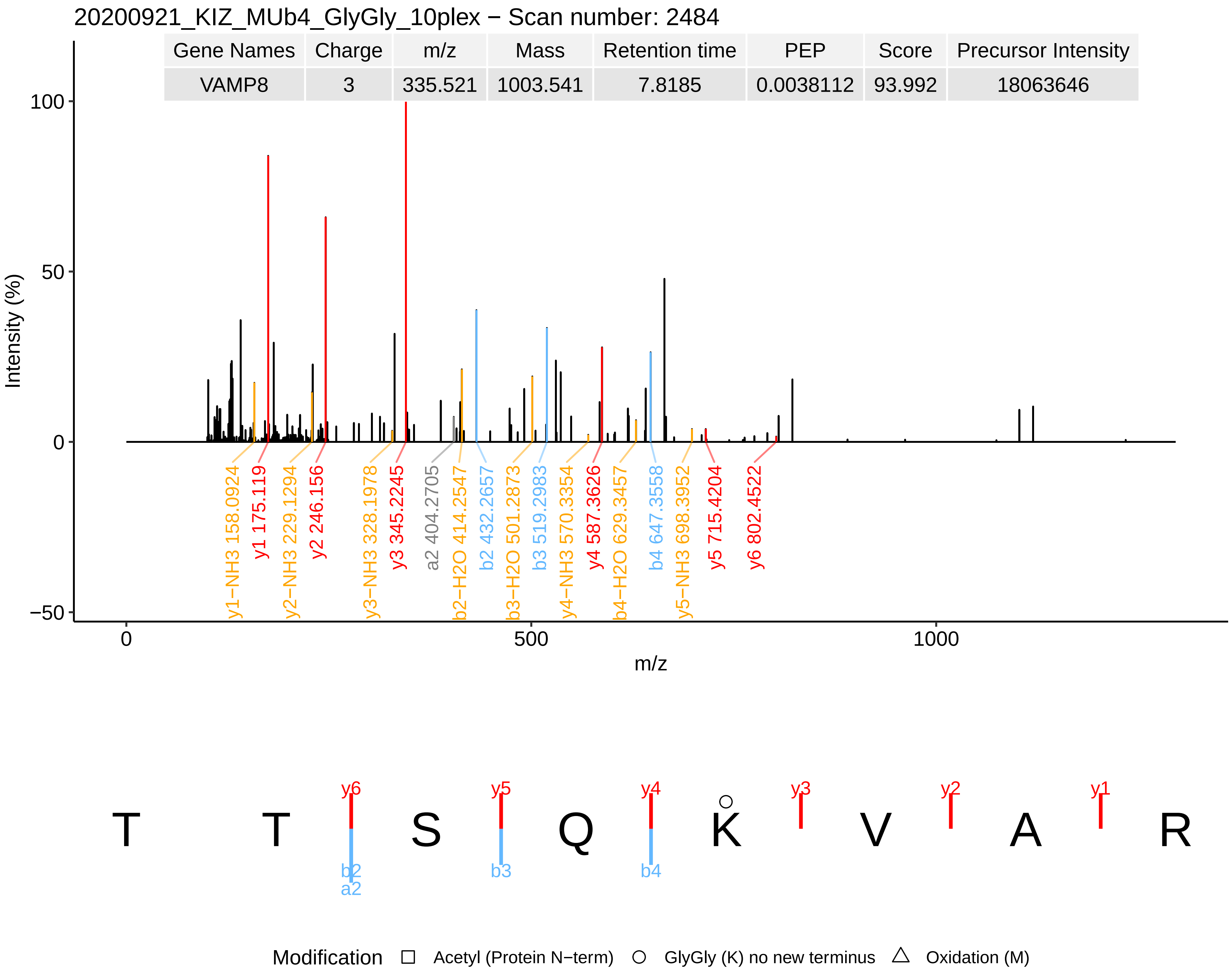
20200921_KIZ_MUb4_GlyGly_10plex − Scan number: 2484
Gene Names | Charge | m/z | Mass | Retention time | PEP | Score | Precursor Intensity
VAMP8 | 3 | 335.521 | 1003.541 | 7.8185 | 0.0038112 | 93.992 | 18063646
Intensity (%)
m/z
y1−NH3 158.0924
y1 175.119
y2−NH3 229.1294
y2 246.156
y3−NH3 328.1978
y3 345.2245
a2 404.2705
b2−H2O 414.2547
b2 432.2657
b3−H2O 501.2873
b3 519.2983
y4−NH3 570.3354
y4 587.3626
b4−H2O 629.3457
b4 647.3558
y5−NH3 698.3952
y5 715.4204
y6 802.4522
T T S Q K V A R
Modification
Acetyl (Protein N−term)
GlyGly (K) no new terminus
Oxidation (M)

20200921_KIZ_MUb4_GlyGly_10plex_2 − Scan number: 2451

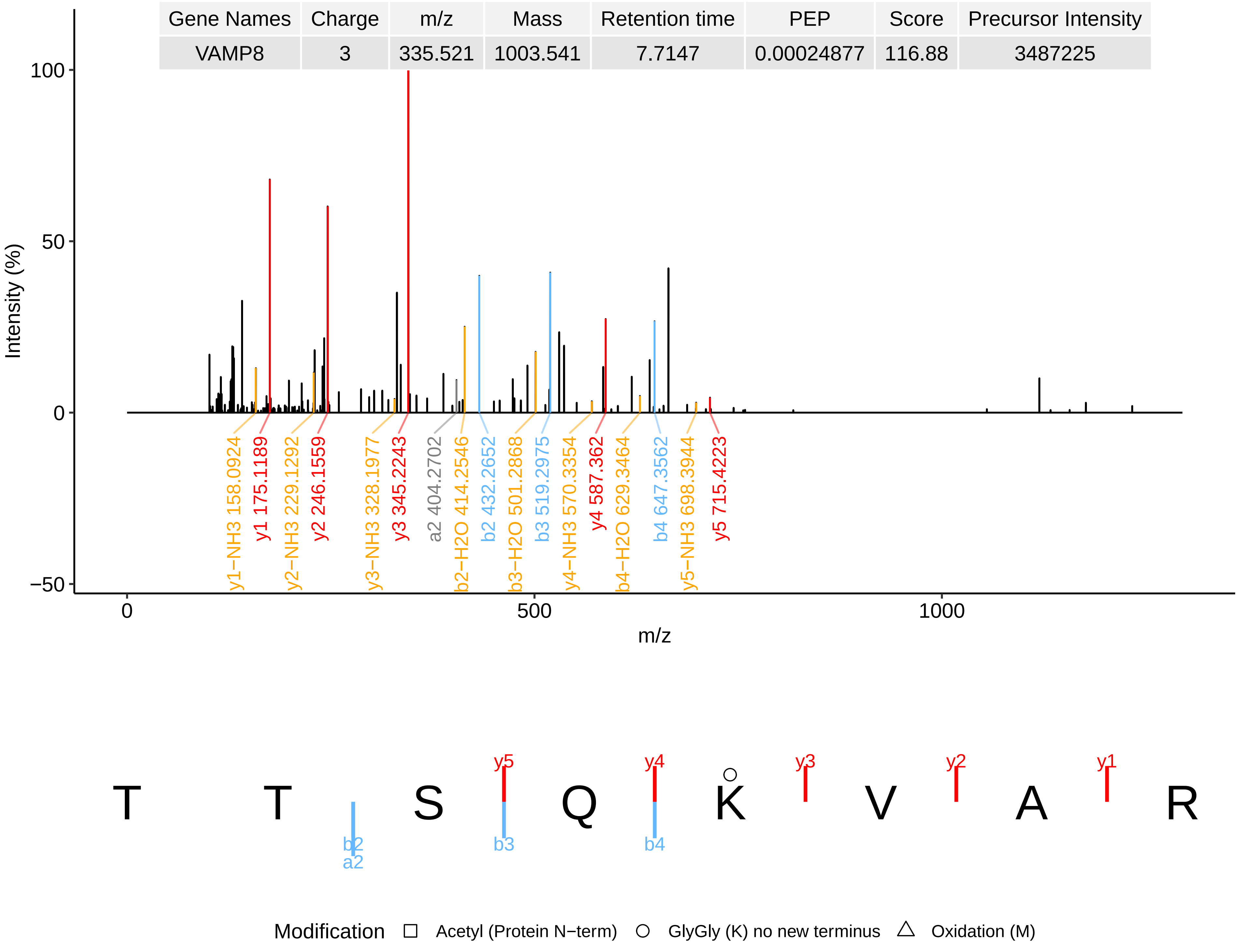

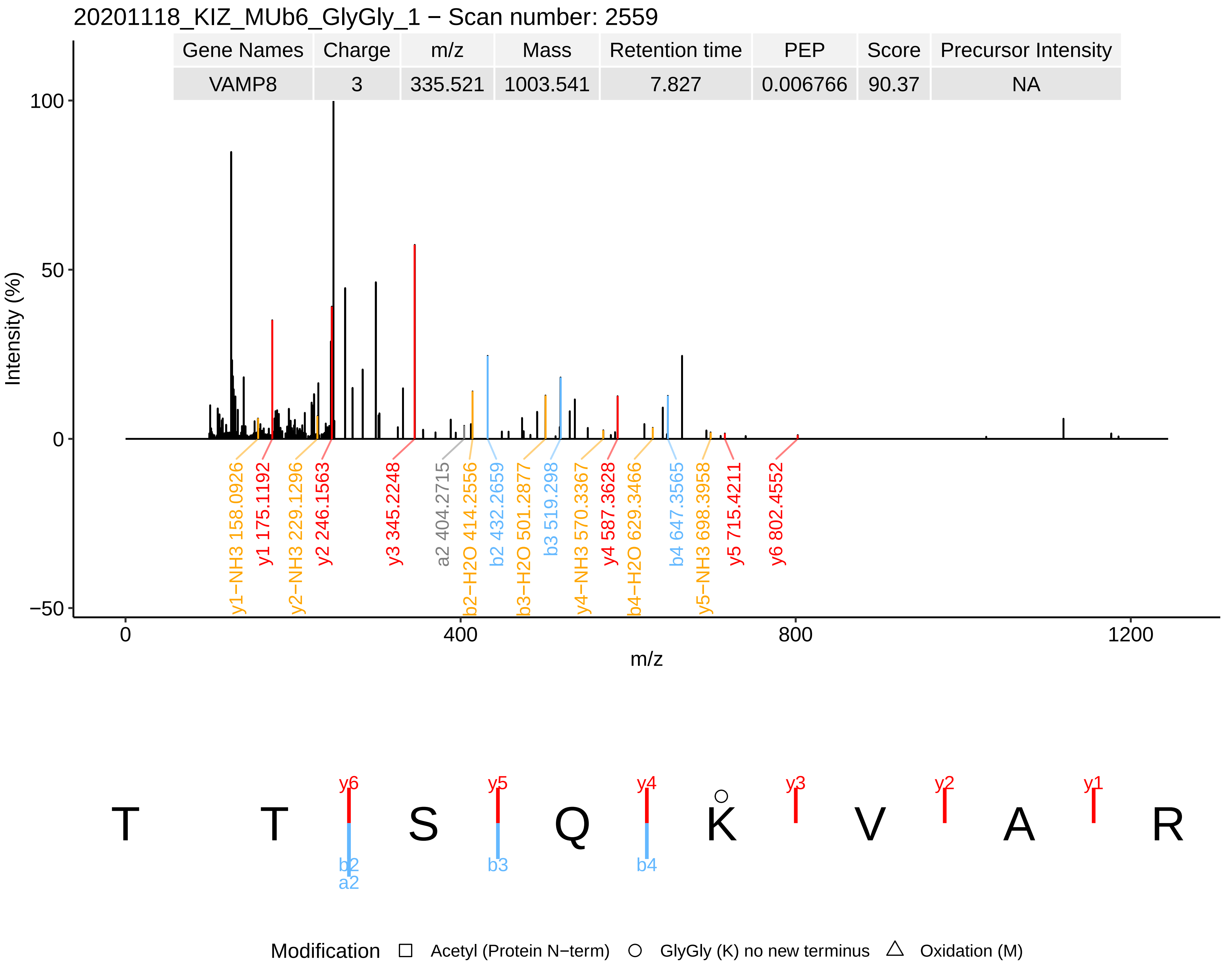

20201118_KIZ_MUb6_GlyGly_1 − Scan number: 2559
Gene Names | Charge | m/z | Mass | Retention time | PEP | Score | Precursor Intensity
VAMP8 | 3 | 335.521 | 1003.541 | 7.827 | 0.006766 | 90.37 | NA
Intensity (%)
m/z
y1−NH3 158.0926
y1 175.1192
y2−NH3 229.1296
y2 246.1563
y3 345.2248
a2 404.2715
b2−H2O 414.2556
b2 432.2659
b3−H2O 501.2877
b3 519.298
y4−NH3 570.3367
y4 587.3628
b4−H2O 629.3466
b4 647.3565
y5−NH3 698.3958
y5 715.4211
y6 802.4552
T T S Q K V A R
y6 y5 y4 y3 y2 y1
b2 a2 b3 b4
Modification
Acetyl (Protein N−term)
GlyGly (K) no new terminus
Oxidation (M)

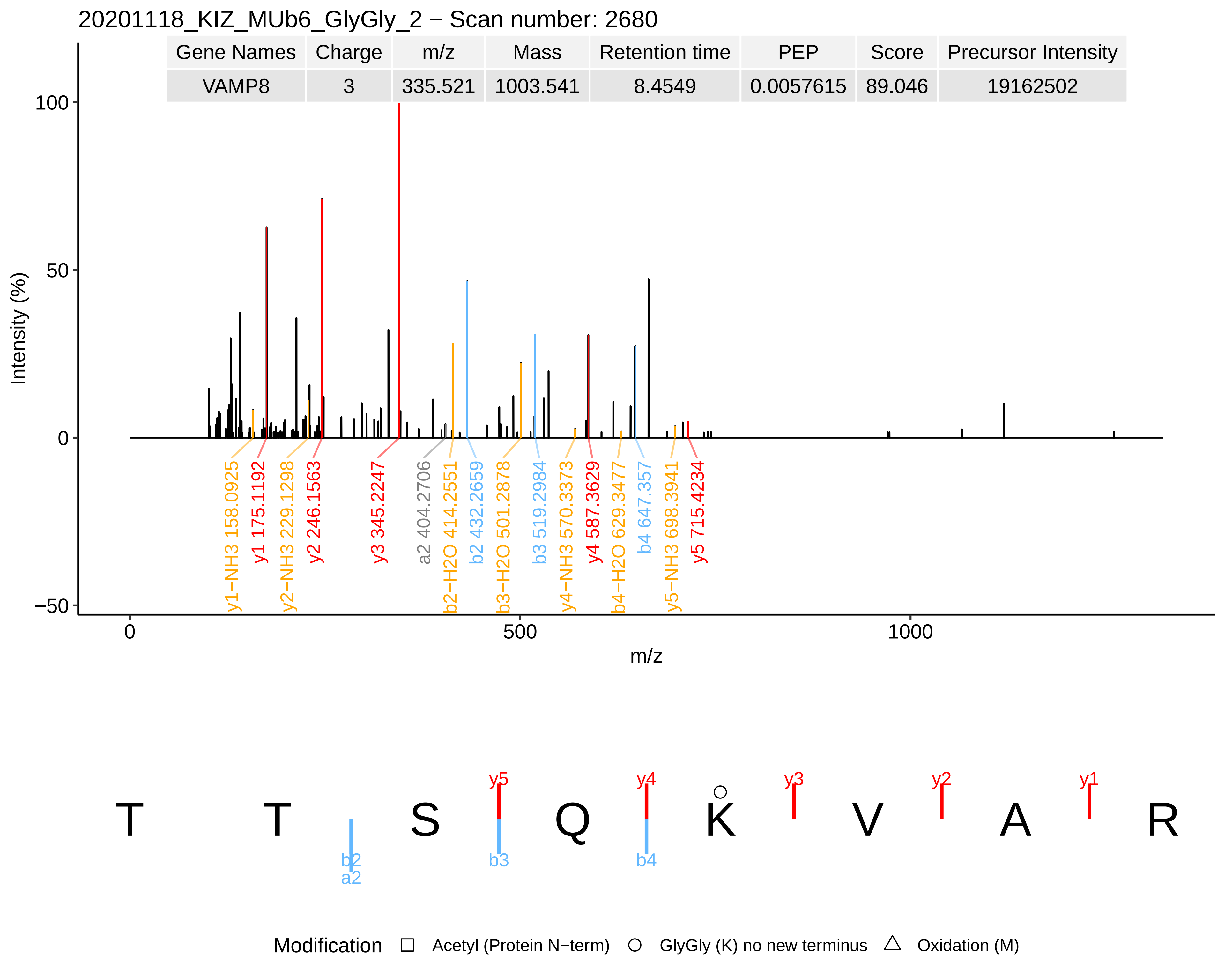

20201118_KIZ_MUb6_GlyGly_2 − Scan number: 2680
Gene Names | Charge | m/z | Mass | Retention time | PEP | Score | Precursor Intensity
VAMP8 | 3 | 335.521 | 1003.541 | 8.4549 | 0.0057615 | 89.046 | 19162502
Intensity (%)
m/z
y1−NH3 158.0925
y1 175.1192
y2−NH3 229.1298
y2 246.1563
y3 345.2247
a2 404.2706
b2−H2O 414.2551
b2 432.2659
b3−H2O 501.2878
b3 519.2984
y4−NH3 570.3373
y4 587.3629
b4−H2O 629.3477
b4 647.357
y5−NH3 698.3941
y5 715.4234
T T S Q K V A R
Modification
Acetyl (Protein N−term)
GlyGly (K) no new terminus
Oxidation (M)

20200921_KIZ_MUb4_GlyGly_10plex_2 − Scan number: 8411

Gene Names	Charge	m/z	Mass	Retention time	PEP	Score	Precursor Intensity
SRSF2;SRSF8	3	338.1785	1011.514	31.679	0.060333	64.776	1271989

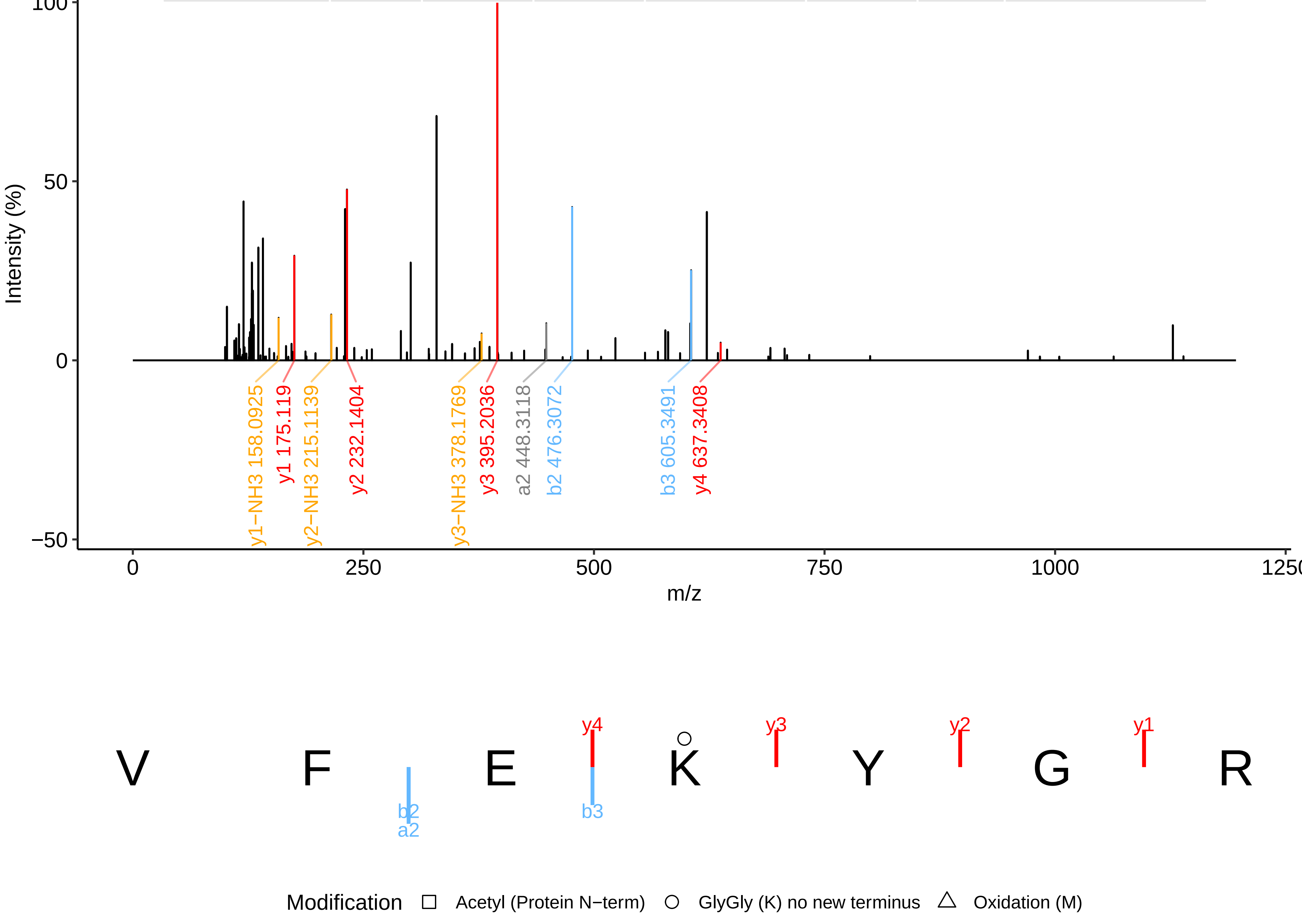

20200924_KIZ_MUb5_GlyGly_1 − Scan number: 7764

Gene Names	Charge	m/z	Mass	Retention time	PEP	Score	Precursor Intensity
SRSF2;SRSF8	3	338.1785	1011.514	24.725	0.061357	64.776	4674998

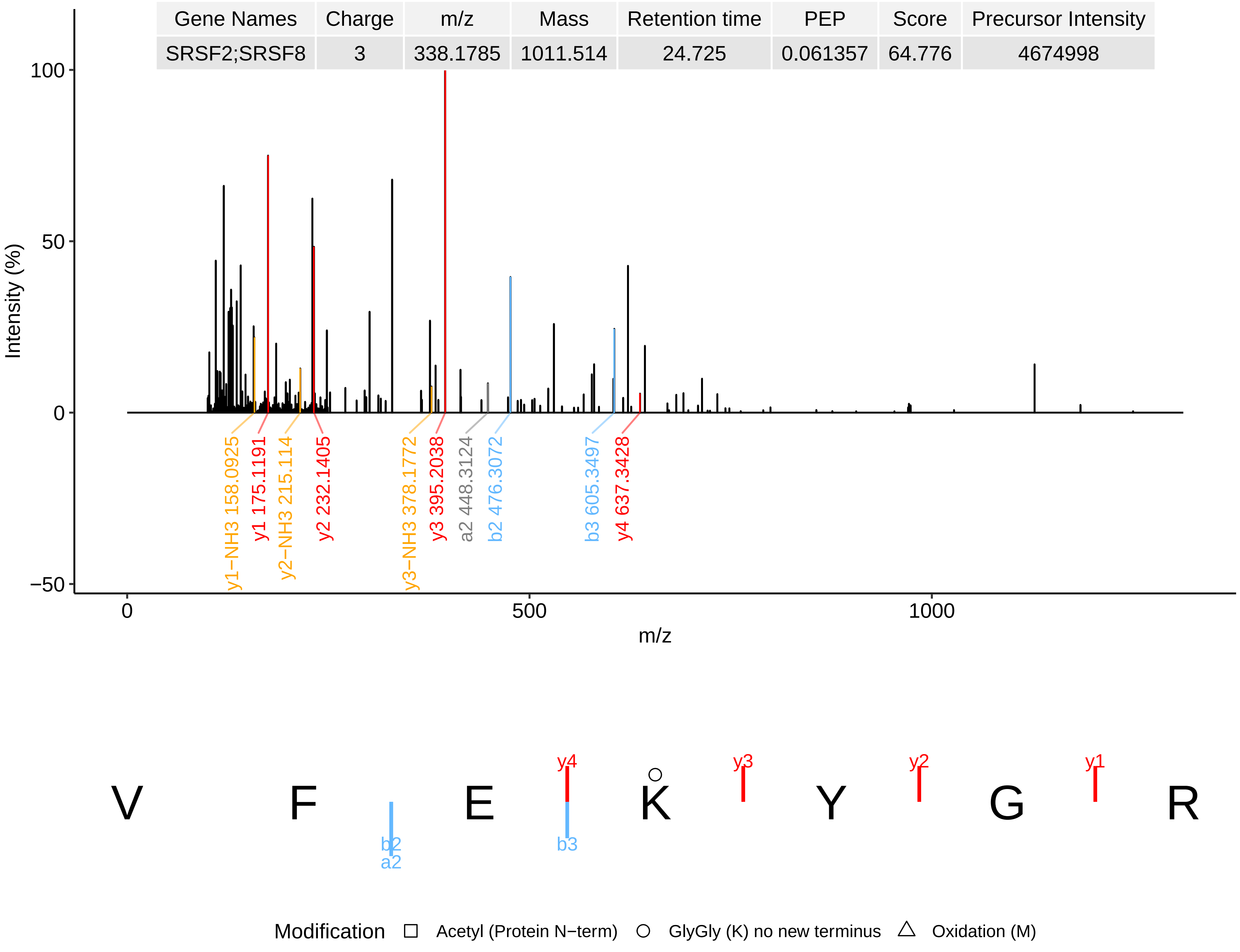

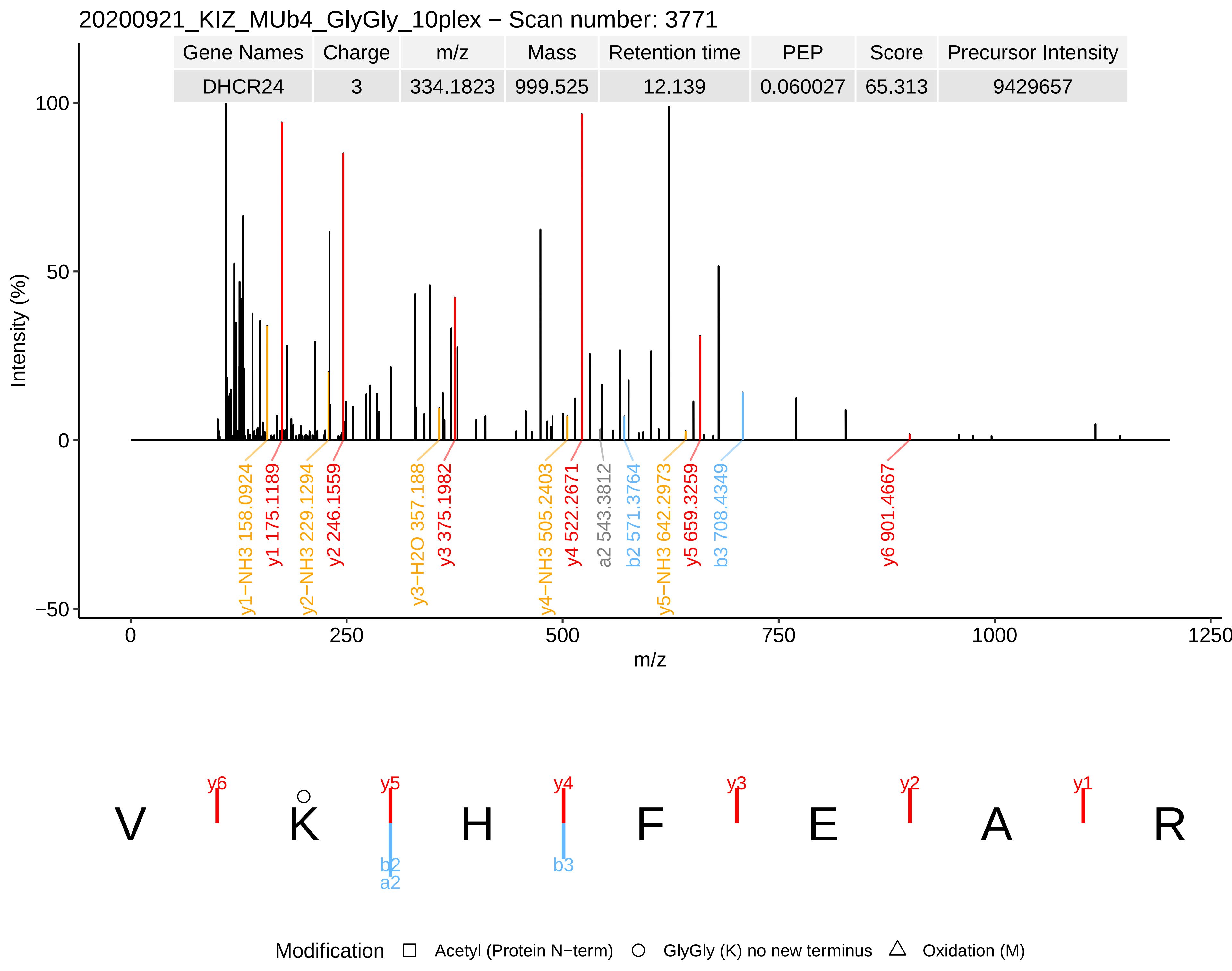
20200921_KIZ_MUb4_GlyGly_10plex − Scan number: 3771
Gene Names | Charge | m/z | Mass | Retention time | PEP | Score | Precursor Intensity
DHCR24 | 3 | 334.1823 | 999.525 | 12.139 | 0.060027 | 65.313 | 9429657
Intensity (%)
m/z
y1−NH3 158.0924
y1 175.1189
y2−NH3 229.1294
y2 246.1559
y3−H2O 357.188
y3 375.1982
y4−NH3 505.2403
y4 522.2671
a2 543.3812
b2 571.3764
y5−NH3 642.2973
y5 659.3259
b3 708.4349
y6 901.4667
V K H F E A R
y6 y5 y4 y3 y2 y1
b2 a2 b3
Modification
Acetyl (Protein N−term)
GlyGly (K) no new terminus
Oxidation (M)

20200921_KIZ_MUb4_GlyGly_10plex_2 − Scan number: 8649

Gene Names	Charge	m/z	Mass	Retention time	PEP	Score	Precursor Intensity
PLCXD1	3	348.5451	1042.613	32.651	0.039818	60.973	3394412

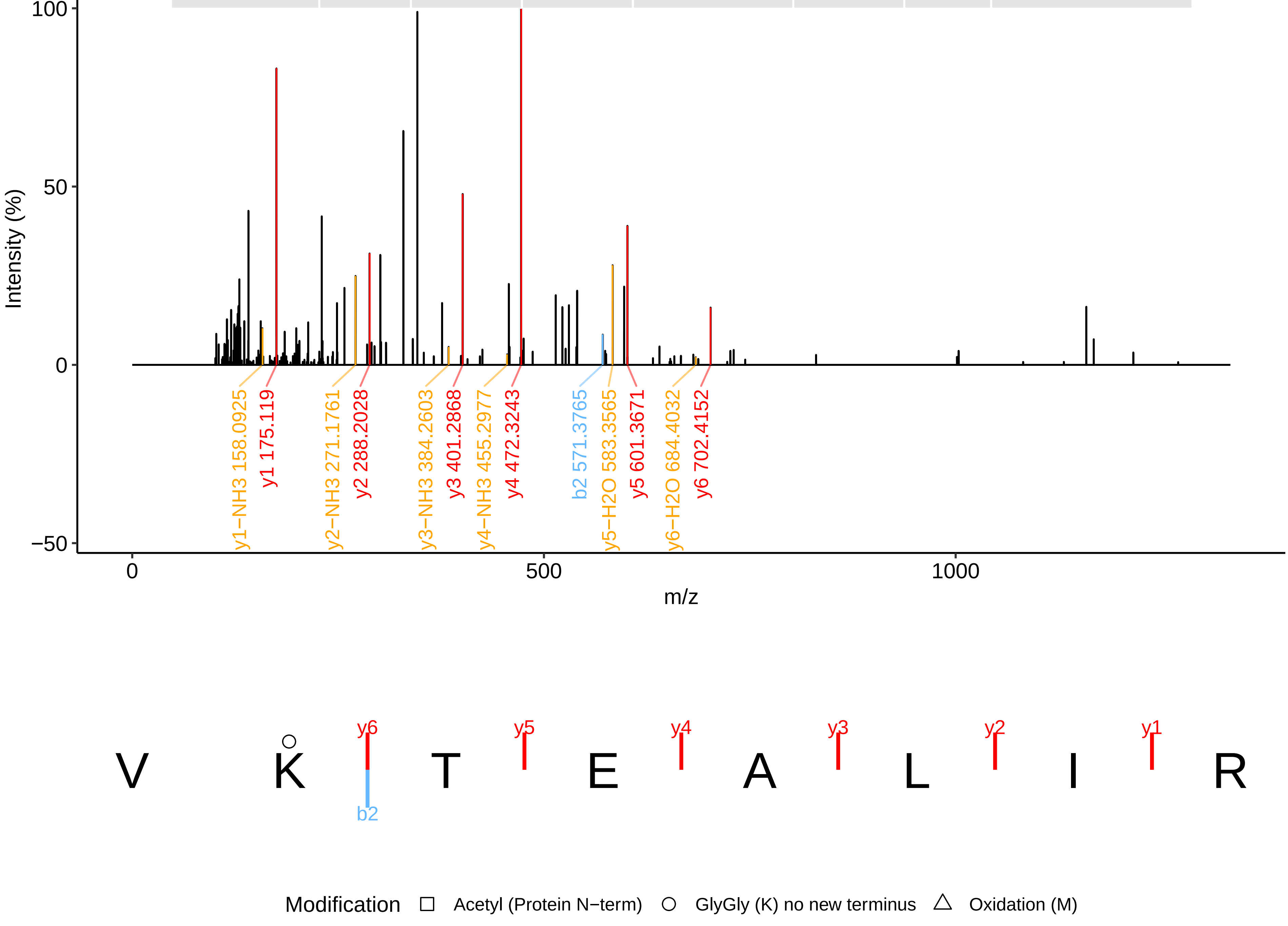

20200921_KIZ_MUb4_GlyGly_10plex_2 − Scan number: 8675

Gene Names	Charge	m/z	Mass	Retention time	PEP	Score	Precursor Intensity
PLCXD1	2	522.314	1042.613	32.759	0.080503	69.423	1055345

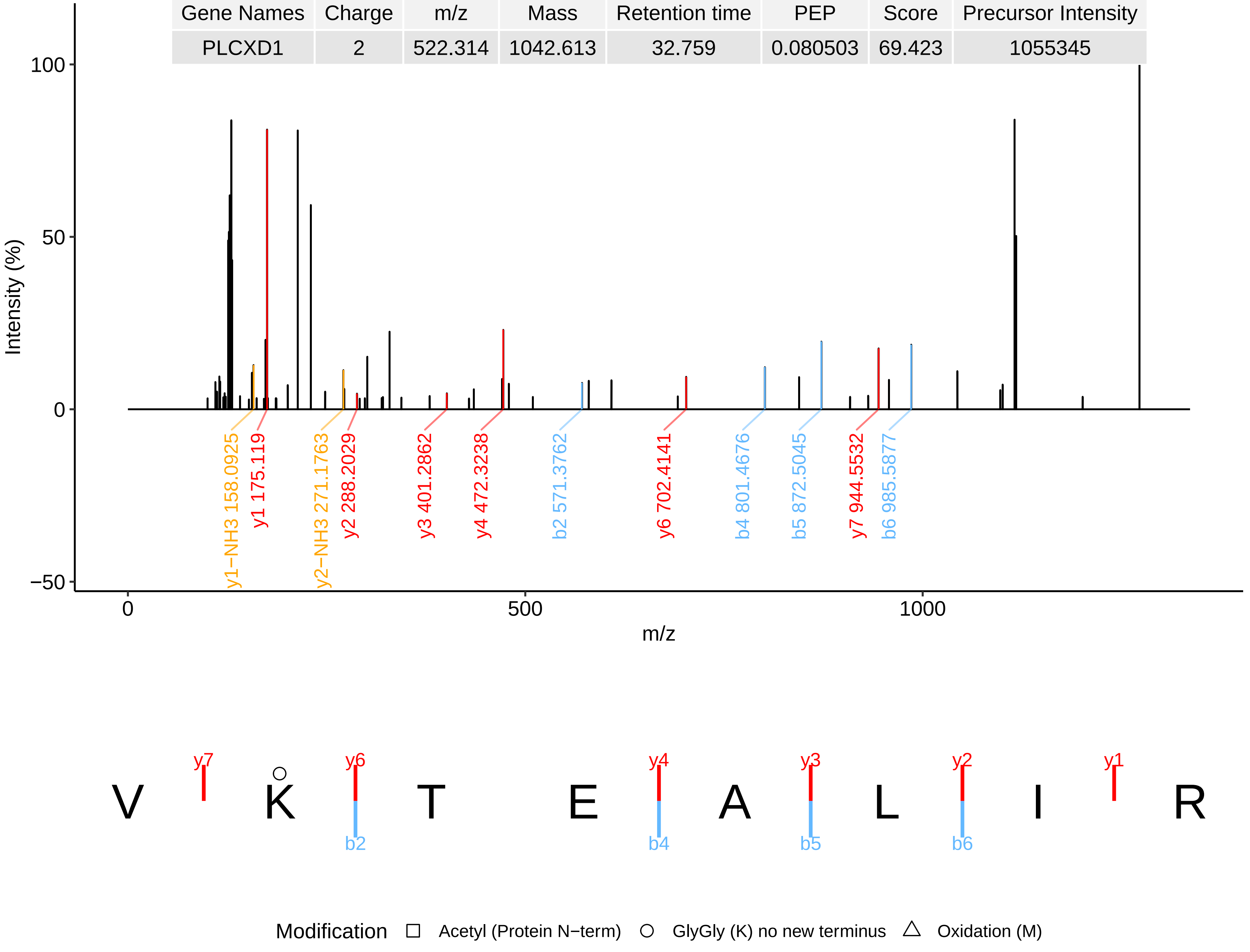

20200924_KIZ_MUb5_GlyGly_1 − Scan number: 8146

Gene Names	Charge	m/z	Mass	Retention time	PEP	Score	Precursor Intensity
PLCXD1	3	348.5451	1042.613	25.976	0.035605	61.999	13274903

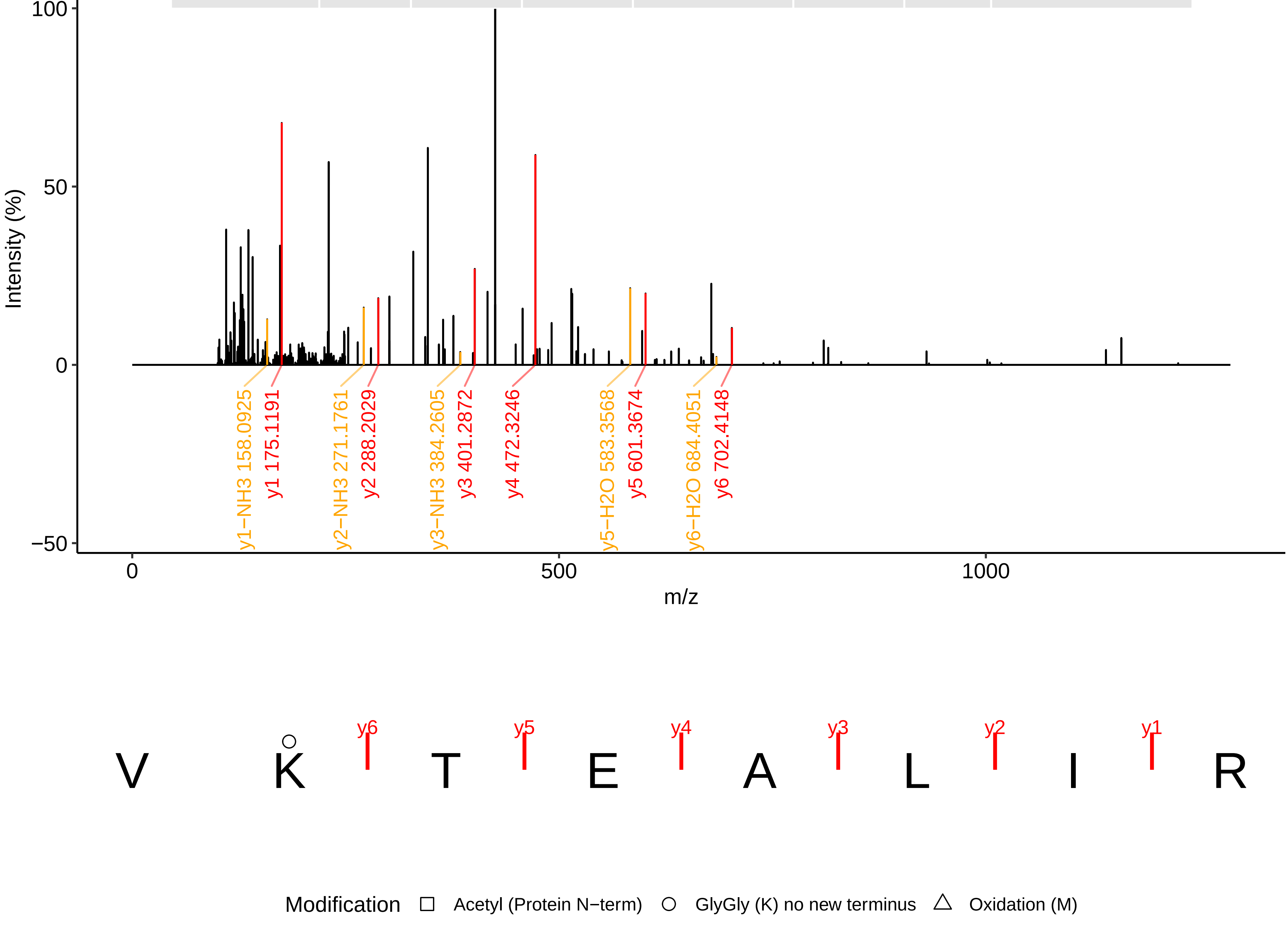

20200924_KIZ_MUb5_GlyGly_1 − Scan number: 8296

Gene Names	Charge	m/z	Mass	Retention time	PEP	Score	Precursor Intensity
PLCXD1	3	348.5451	1042.613	26.492	0.04139	60.59	17617162

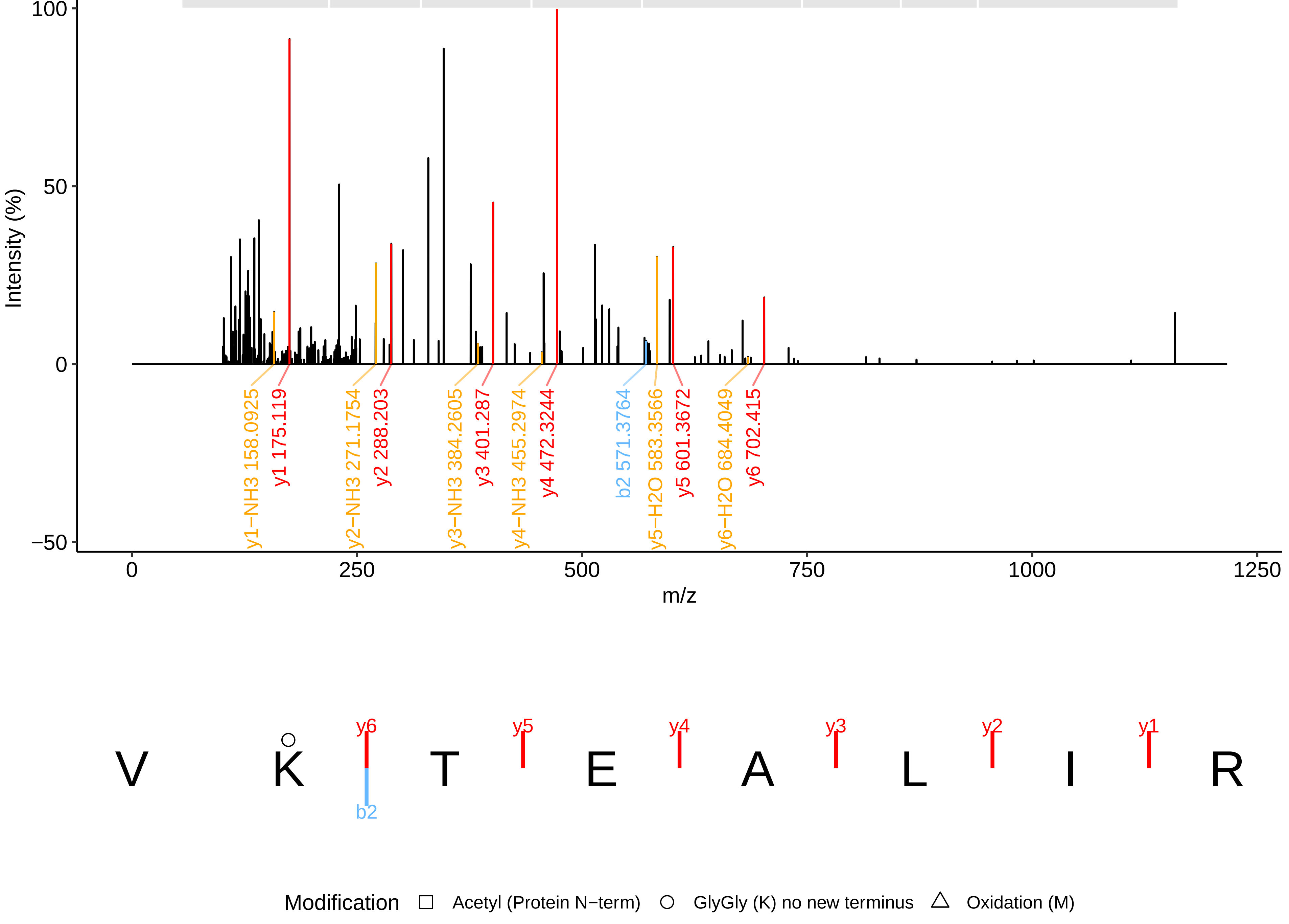

20200924_KIZ_MUb5_GlyGly_2 − Scan number: 7615

Gene Names	Charge	m/z	Mass	Retention time	PEP	Score	Precursor Intensity
PLCXD1	3	348.5451	1042.613	28.44	0.079334	51.346	2527394

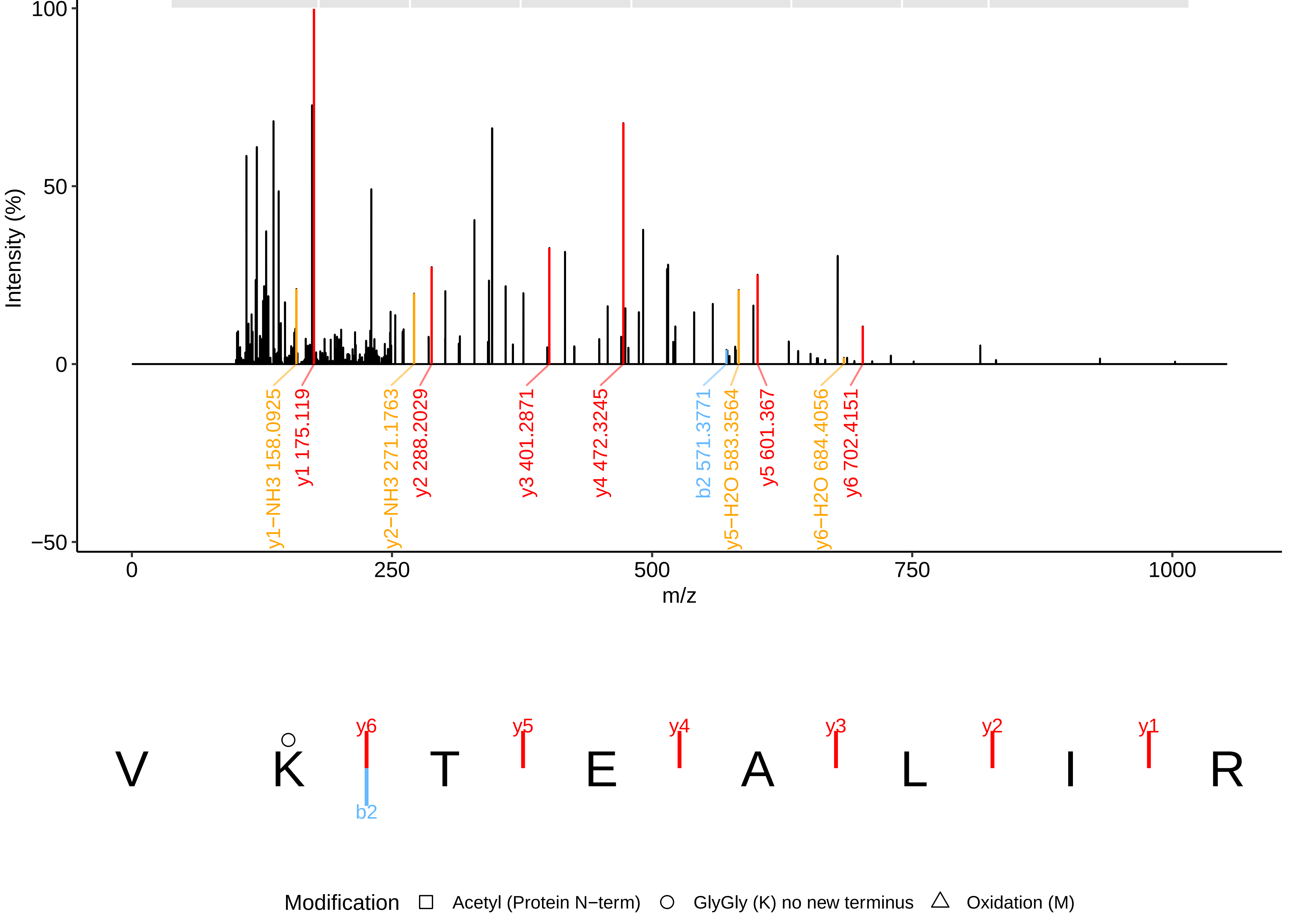

20200924_KIZ_MUb5_GlyGly_2 − Scan number: 7750

Gene Names	Charge	m/z	Mass	Retention time	PEP	Score	Precursor Intensity
PLCXD1	3	348.5451	1042.613	28.945	0.05727	56.721	2727619

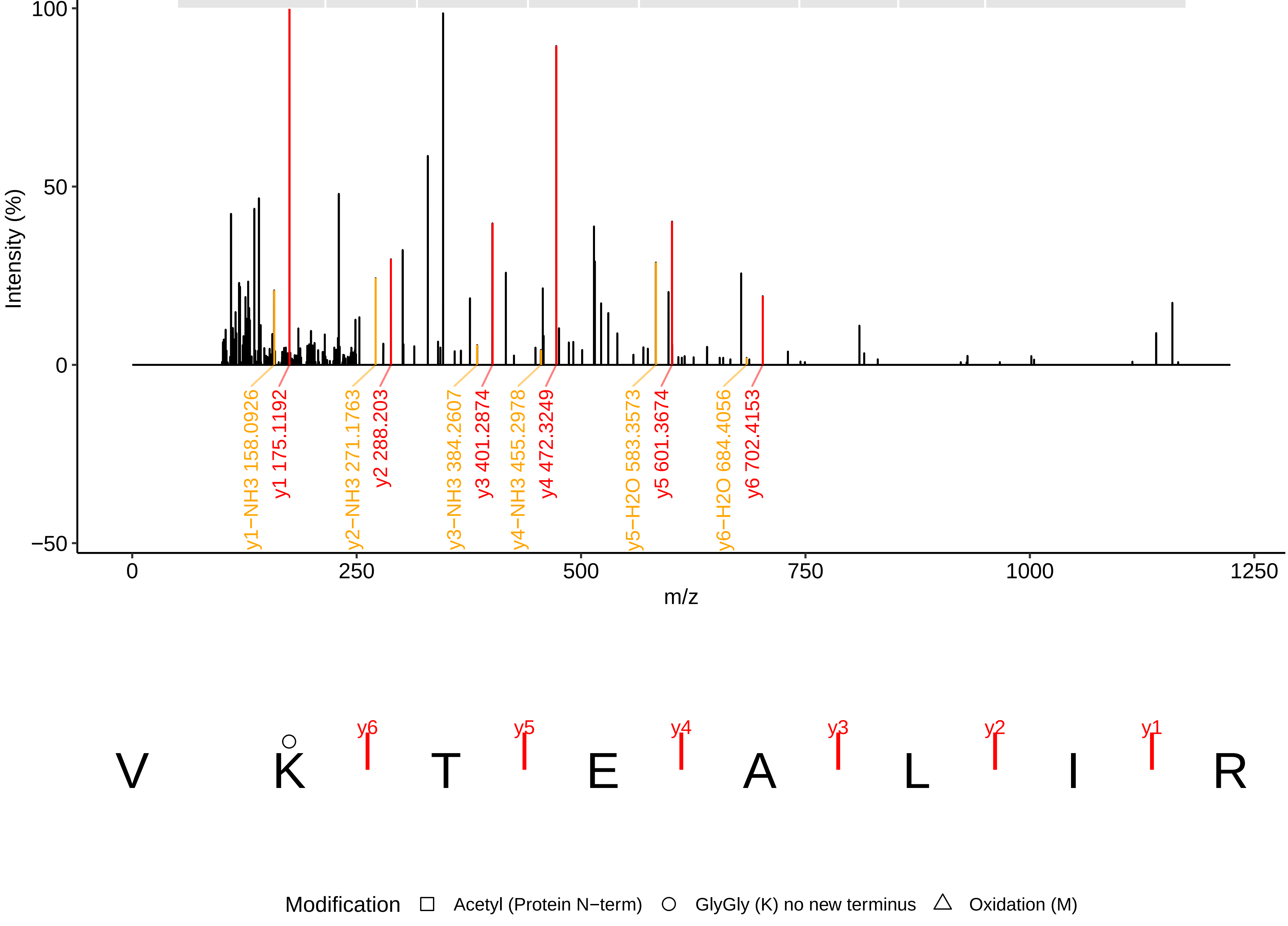

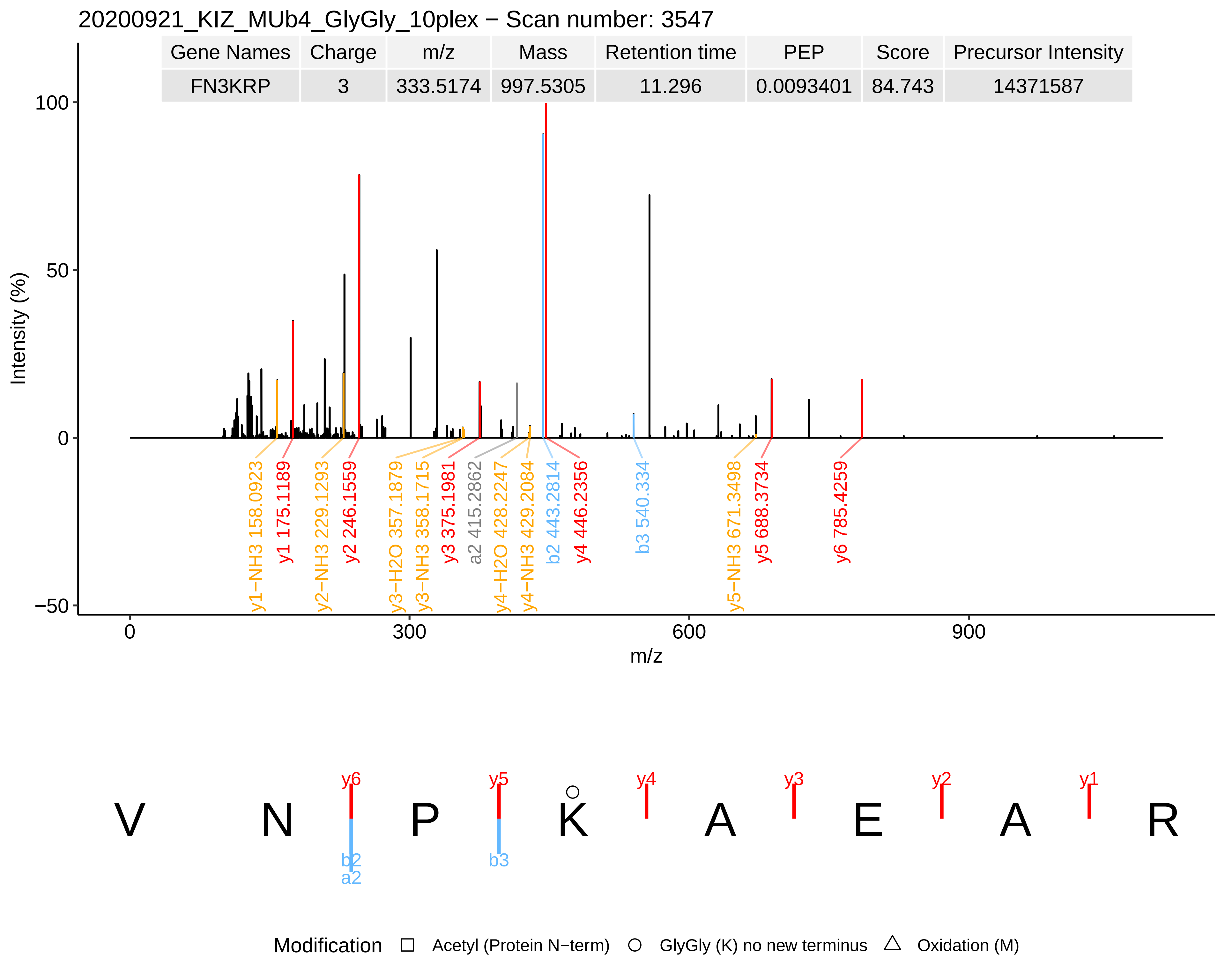

20200921_KIZ_MUb4_GlyGly_10plex − Scan number: 3547
Gene Names | Charge | m/z | Mass | Retention time | PEP | Score | Precursor Intensity
FN3KRP | 3 | 333.5174 | 997.5305 | 11.296 | 0.0093401 | 84.743 | 14371587
Intensity (%)
100
50
0
−50
0
300
600
900
m/z
y1−NH3 158.0923
y1 175.1189
y2−NH3 229.1293
y2 246.1559
y3−H2O 357.1879
y3−NH3 358.1715
y3 375.1981
a2 415.2862
y4−H2O 428.2247
y4−NH3 429.2084
b2 443.2814
y4 446.2356
b3 540.334
y5−NH3 671.3498
y5 688.3734
y6 785.4259
V N P K A E A R
y6 y5 y4 y3 y2 y1
b2 a2 b3
Modification
Acetyl (Protein N−term)
GlyGly (K) no new terminus
Oxidation (M)

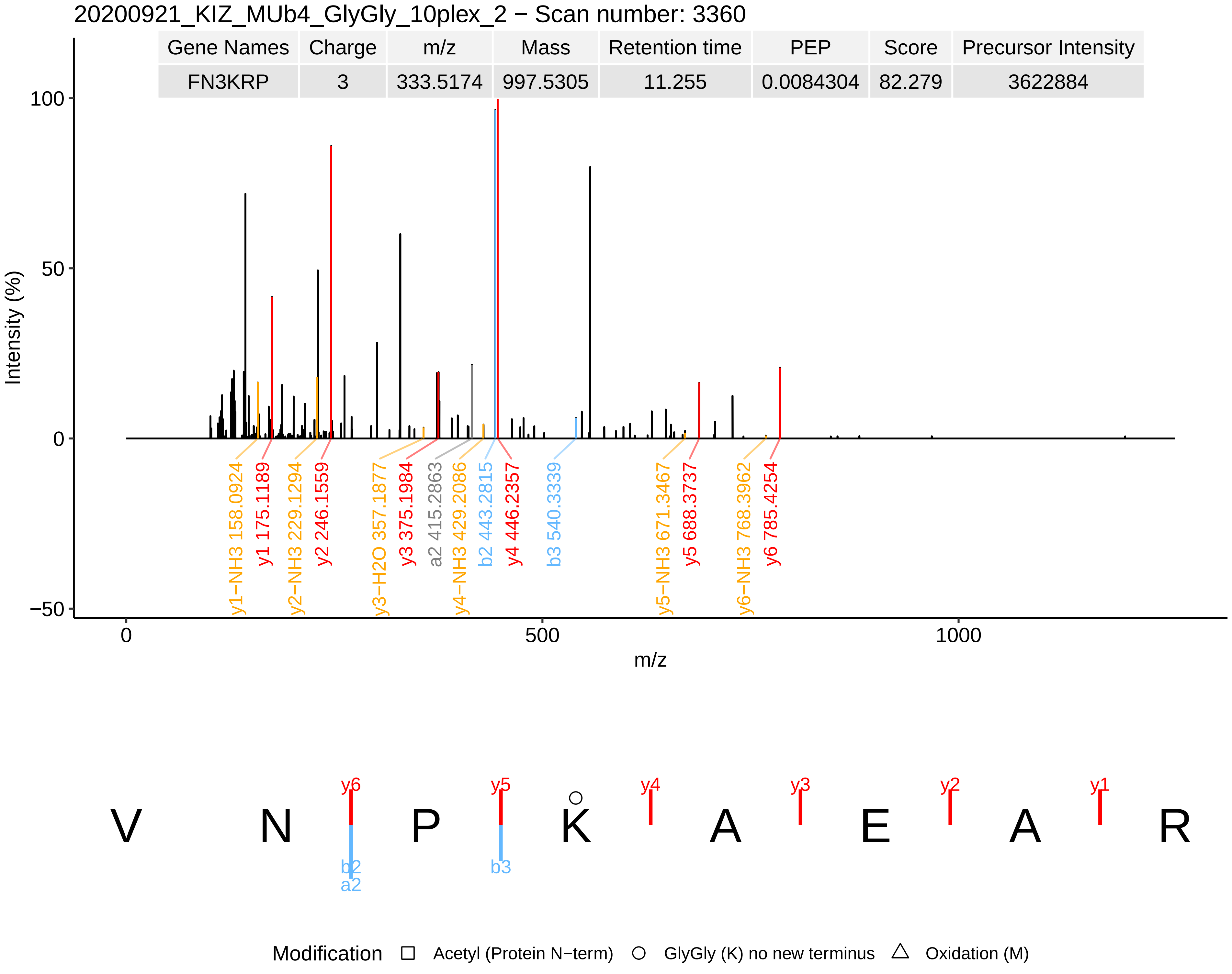
20200921_KIZ_MUb4_GlyGly_10plex_2 − Scan number: 3360
Gene Names | Charge | m/z | Mass | Retention time | PEP | Score | Precursor Intensity
FN3KRP | 3 | 333.5174 | 997.5305 | 11.255 | 0.0084304 | 82.279 | 3622884
Intensity (%)
m/z
y1−NH3 158.0924
y1 175.1189
y2−NH3 229.1294
y2 246.1559
y3−H2O 357.1877
y3 375.1984
a2 415.2863
y4−NH3 429.2086
b2 443.2815
y4 446.2357
b3 540.3339
y5−NH3 671.3467
y5 688.3737
y6−NH3 768.3962
y6 785.4254
V N P K A E A R
y6 y5 y4 y3 y2 y1
b2 a2 b3
Modification
Acetyl (Protein N−term)
GlyGly (K) no new terminus
Oxidation (M)

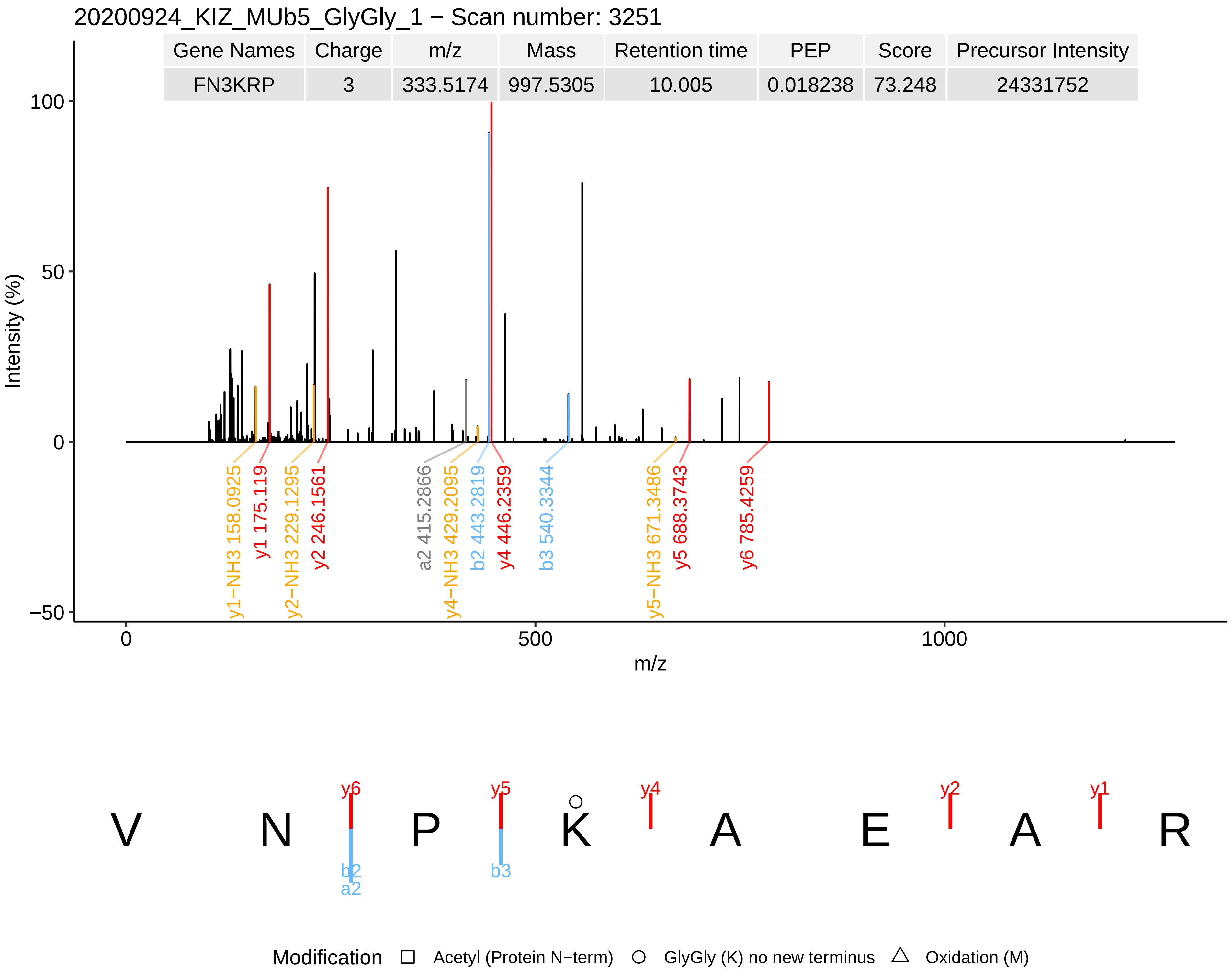
20200924_KIZ_MUb5_GlyGly_1 − Scan number: 3251
Gene Names | Charge | m/z | Mass | Retention time | PEP | Score | Precursor Intensity
FN3KRP | 3 | 333.5174 | 997.5305 | 10.005 | 0.018238 | 73.248 | 24331752
Intensity (%)
m/z
y1−NH3 158.0925
y1 175.119
y2−NH3 229.1295
y2 246.1561
a2 415.2866
y4−NH3 429.2095
b2 443.2819
y4 446.2359
b3 540.3344
y5−NH3 671.3486
y5 688.3743
y6 785.4259
V N P K A E A R
y6 y5 y4 y2 y1
b2 a2 b3
Modification
Acetyl (Protein N−term)
GlyGly (K) no new terminus
Oxidation (M)

20201118_KIZ_MUb6_GlyGly_1 − Scan number: 3168

Gene Names	Charge	m/z	Mass	Retention time	PEP	Score	Precursor Intensity
FN3KRP	3	333.5174	997.5305	9.7645	0.013169	77.318	17782822

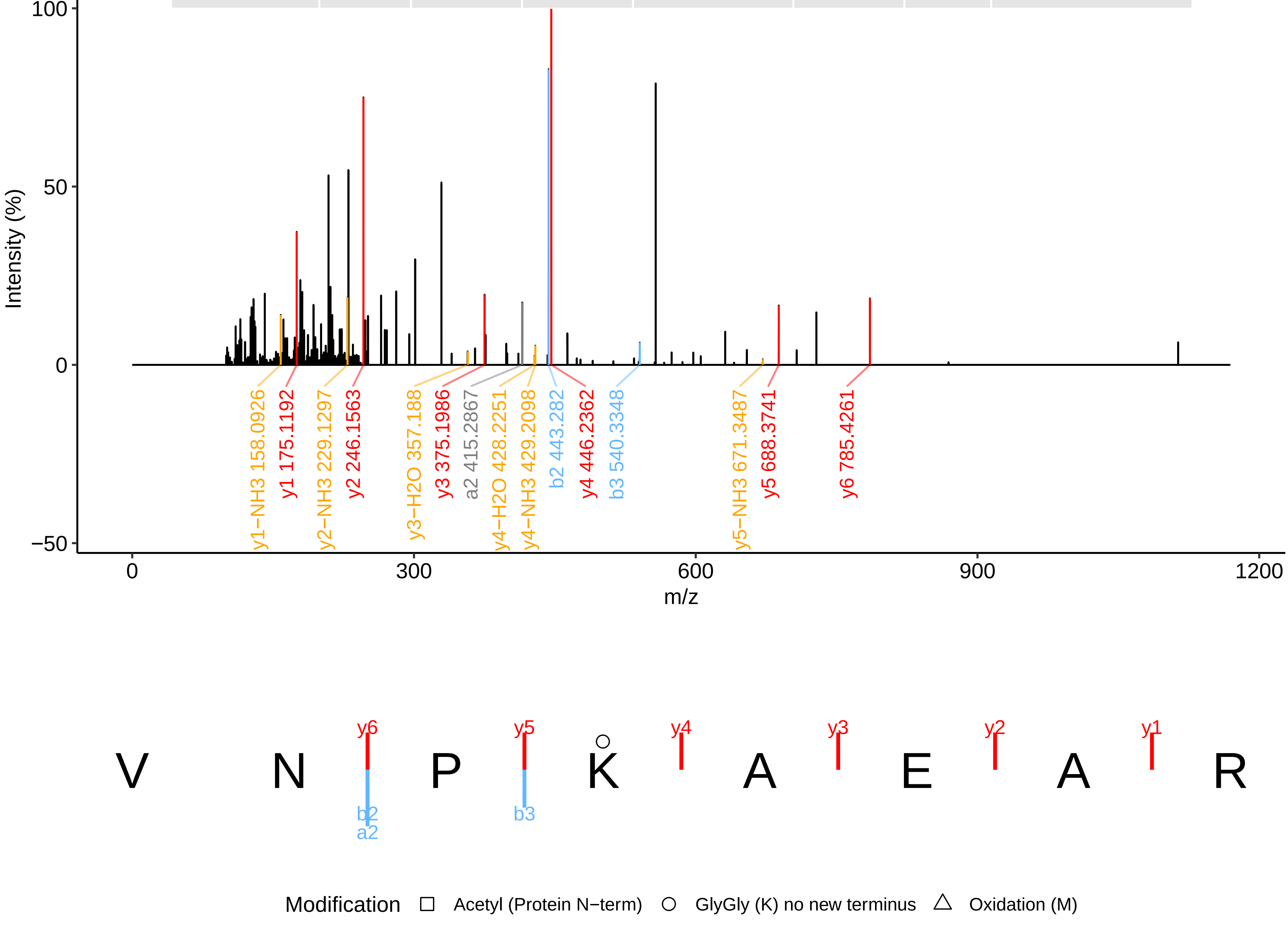

20200921_KIZ_MUb4_GlyGly_10plex − Scan number: 10402

Gene Names	Charge	m/z	Mass	Retention time	PEP	Score	Precursor Intensity
SCD	3	348.2171	1041.63	35.993	0.10491	46.001	10378132

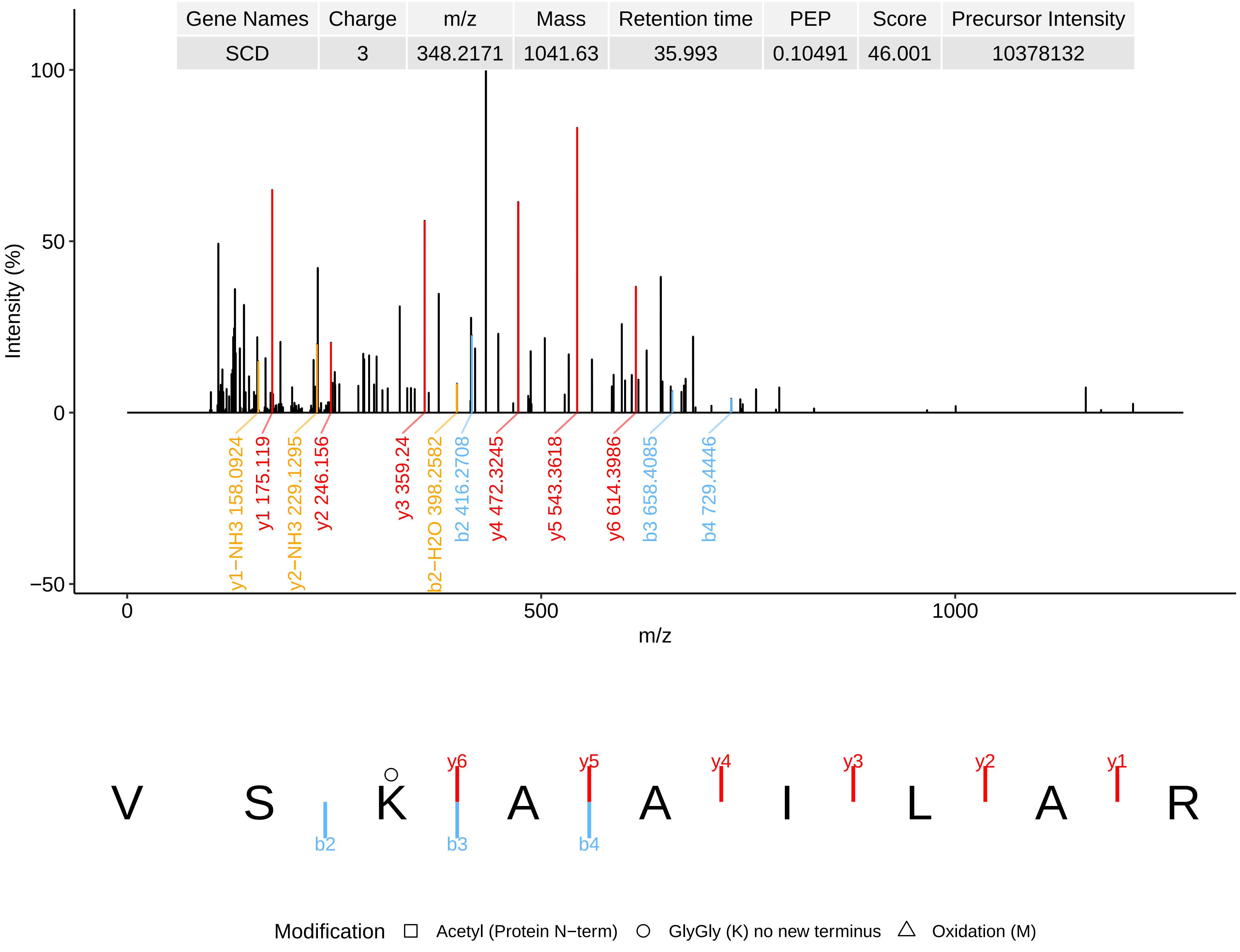

20200921_KIZ_MUb4_GlyGly_10plex_2 − Scan number: 9618

Gene Names	Charge	m/z	Mass	Retention time	PEP	Score	Precursor Intensity
SCD	3	348.2171	1041.63	36.623	0.067499	53.756	1478326

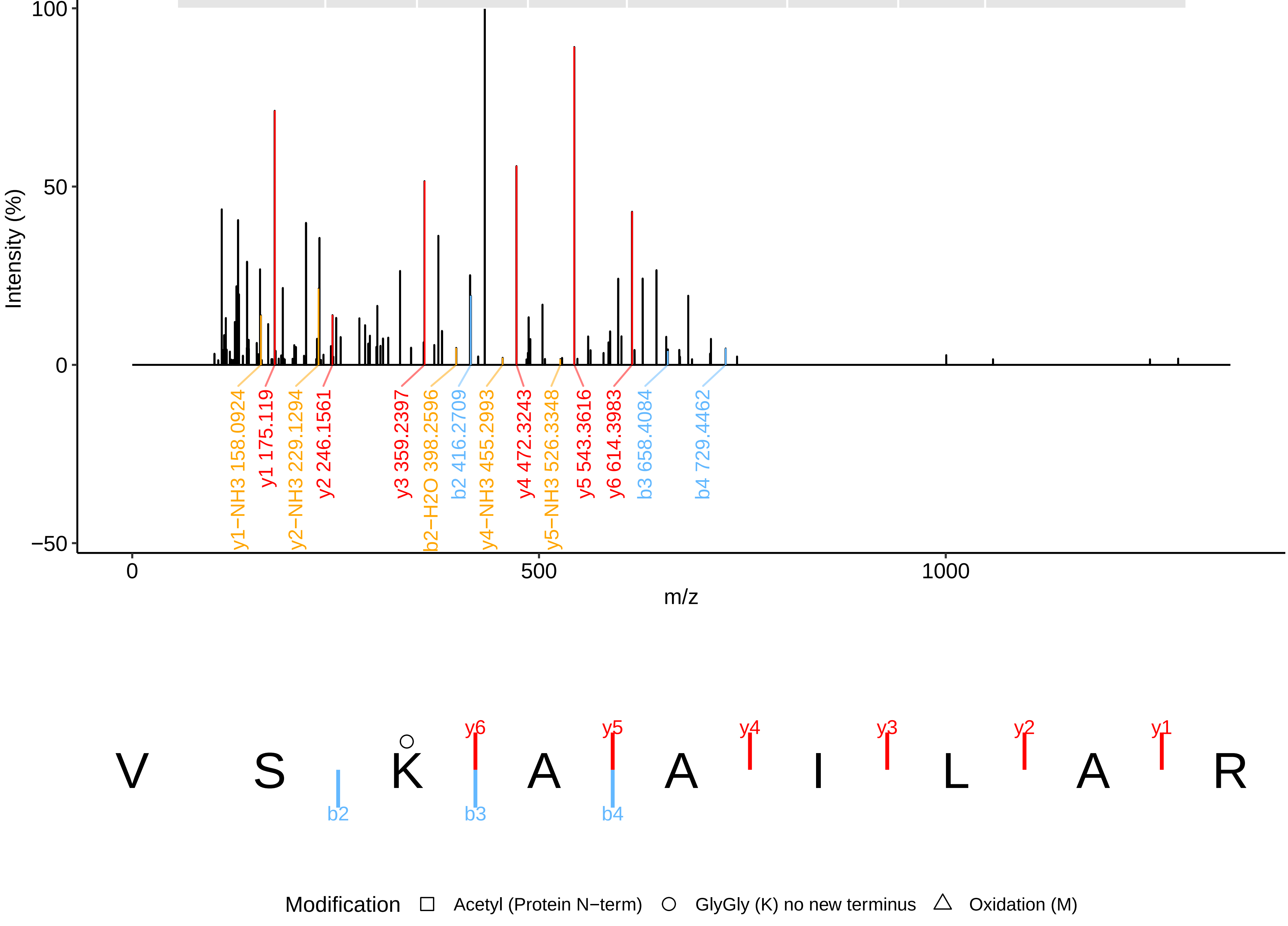

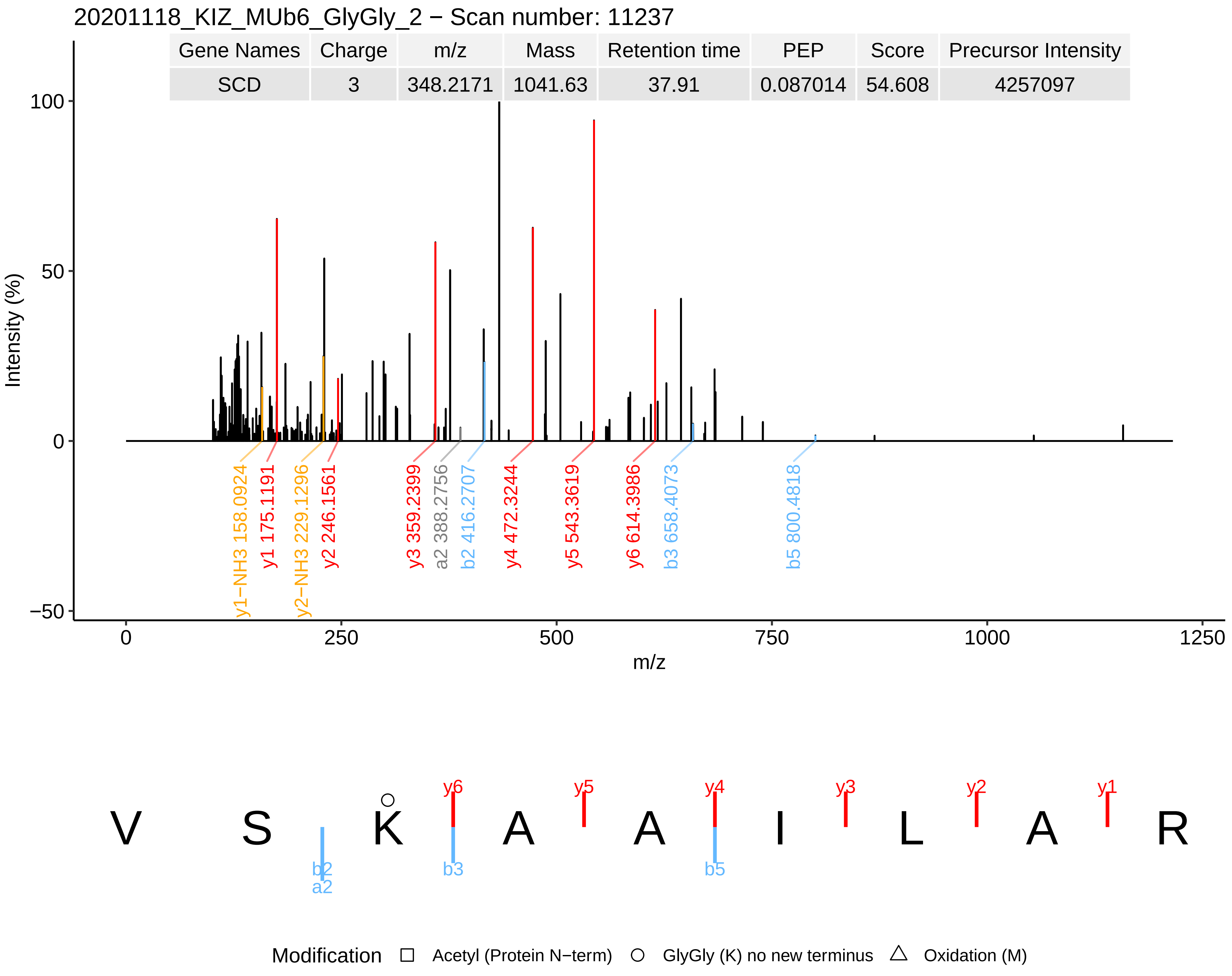
20201118_KIZ_MUb6_GlyGly_2 − Scan number: 11237
Gene Names | Charge | m/z | Mass | Retention time | PEP | Score | Precursor Intensity
SCD | 3 | 348.2171 | 1041.63 | 37.91 | 0.087014 | 54.608 | 4257097
Intensity (%)
100
50
0
−50
y1−NH3 158.0924
y1 175.1191
y2−NH3 229.1296
y2 246.1561
y3 359.2399
a2 388.2756
b2 416.2707
y4 472.3244
y5 543.3619
y6 614.3986
b3 658.4073
b5 800.4818
0
250
500
750
1000
1250
m/z
V S K A A I L A R
y6 y5 y4 y3 y2 y1
b2
a2
b3
b5
Modification
Acetyl (Protein N−term)
GlyGly (K) no new terminus
Oxidation (M)

20200921_KIZ_MUb4_GlyGly_10plex − Scan number: 8463

Gene Names	Charge	m/z	Mass	Retention time	PEP	Score	Precursor Intensity
COX7B	3	343.854	1028.54	28.725	0.031485	76.035	2986739

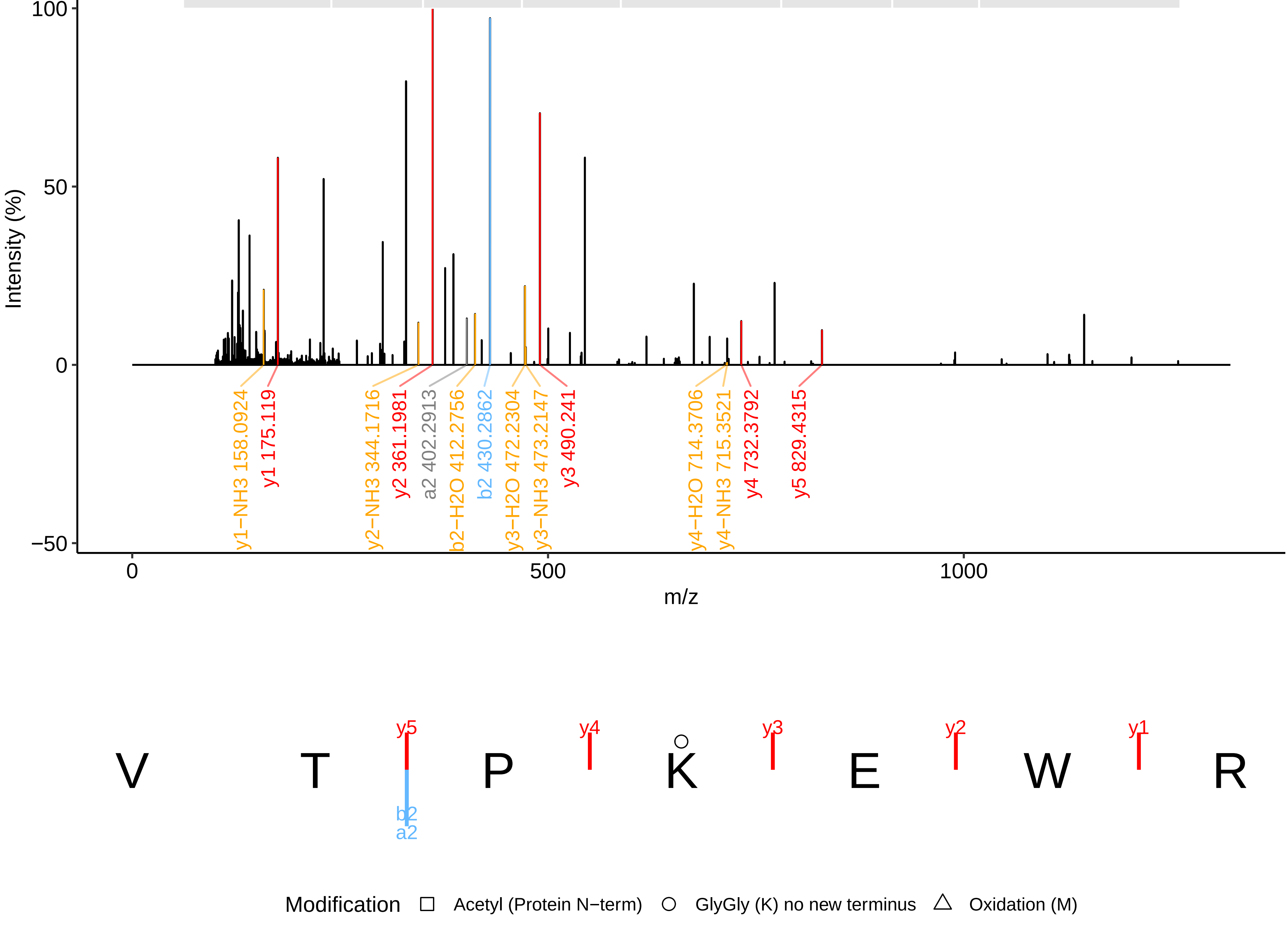

20200921_KIZ_MUb4_GlyGly_10plex_2 − Scan number: 7865

Gene Names	Charge	m/z	Mass	Retention time	PEP	Score	Precursor Intensity
COX7B	3	343.854	1028.54	29.484	0.02014	80.462	4501253

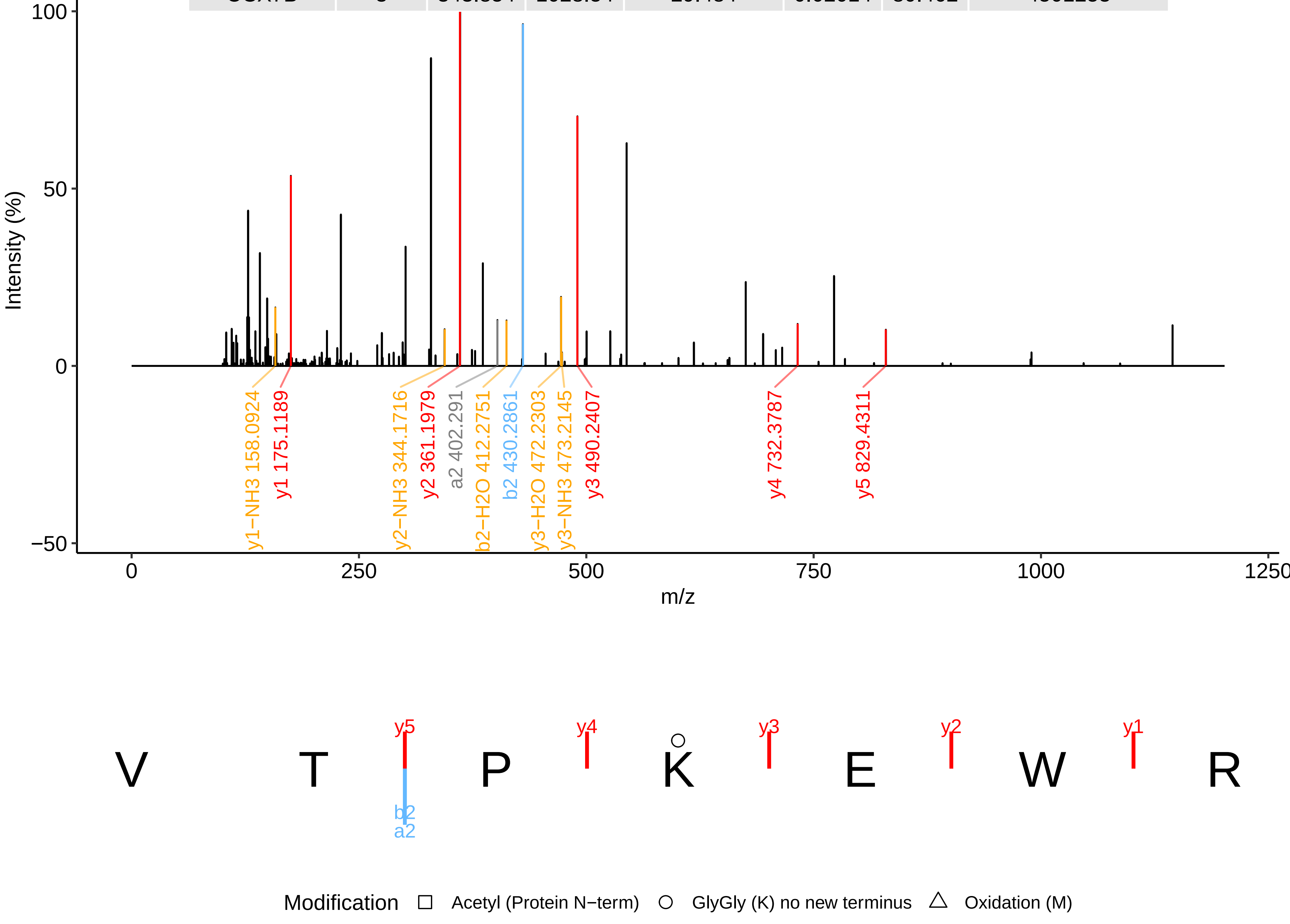

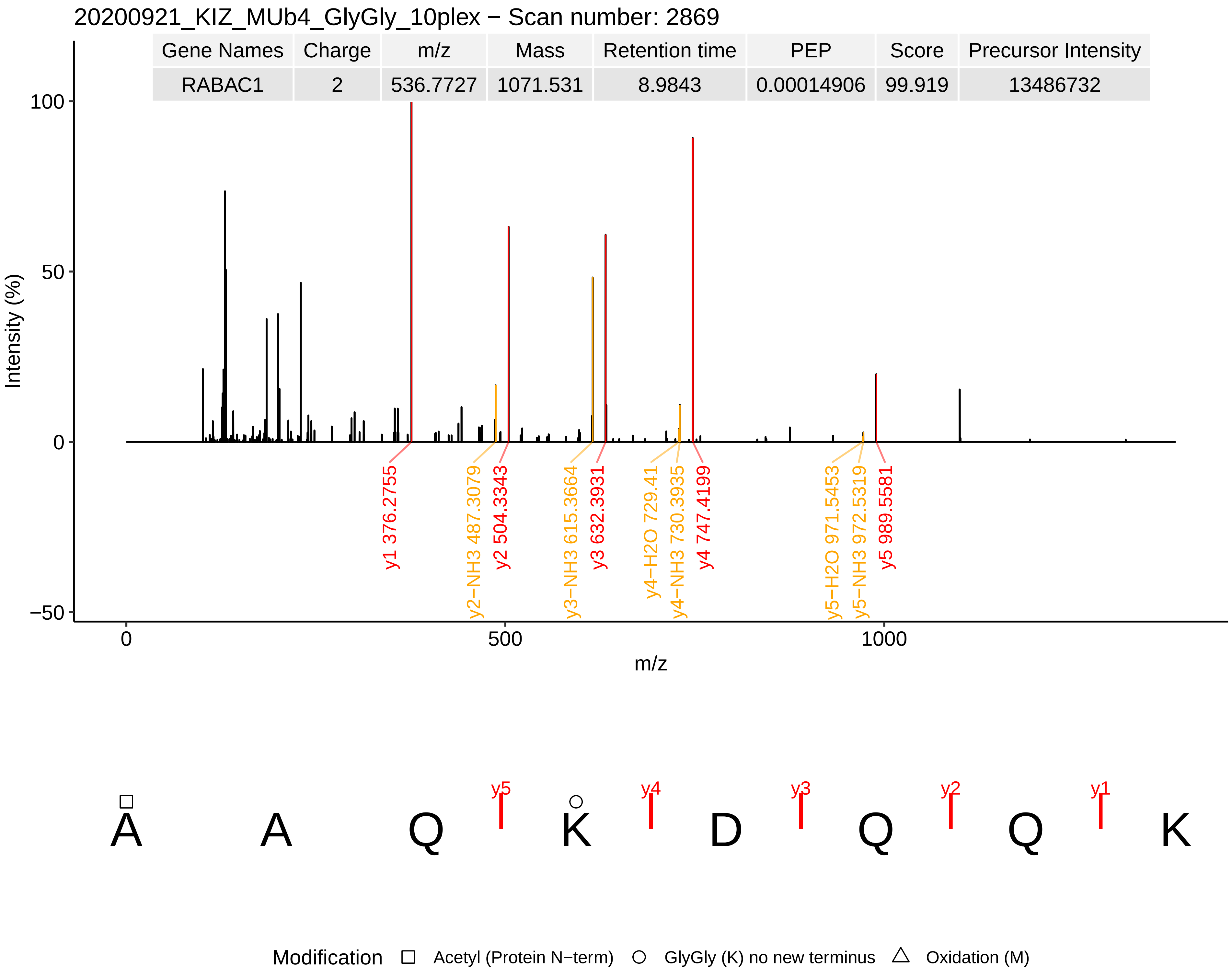

20200921_KIZ_MUb4_GlyGly_10plex − Scan number: 2869
Gene Names | Charge | m/z | Mass | Retention time | PEP | Score | Precursor Intensity
RABAC1 | 2 | 536.7727 | 1071.531 | 8.9843 | 0.00014906 | 99.919 | 13486732
Intensity (%)
m/z
y1 376.2755
y2−NH3 487.3079
y2 504.3343
y3−NH3 615.3664
y3 632.3931
y4−H2O 729.41
y4−NH3 730.3935
y4 747.4199
y5−H2O 971.5453
y5−NH3 972.5319
y5 989.5581
A A Q K D Q Q K
y5 y4 y3 y2 y1
Modification
Acetyl (Protein N−term)
GlyGly (K) no new terminus
Oxidation (M)

20200921_KIZ_MUb4_GlyGly_10plex_2 − Scan number: 2812

Gene Names	Charge	m/z	Mass	Retention time	PEP	Score	Precursor Intensity
RABAC1	2	536.7727	1071.531	9.0715	0.0151	69.344	1267354

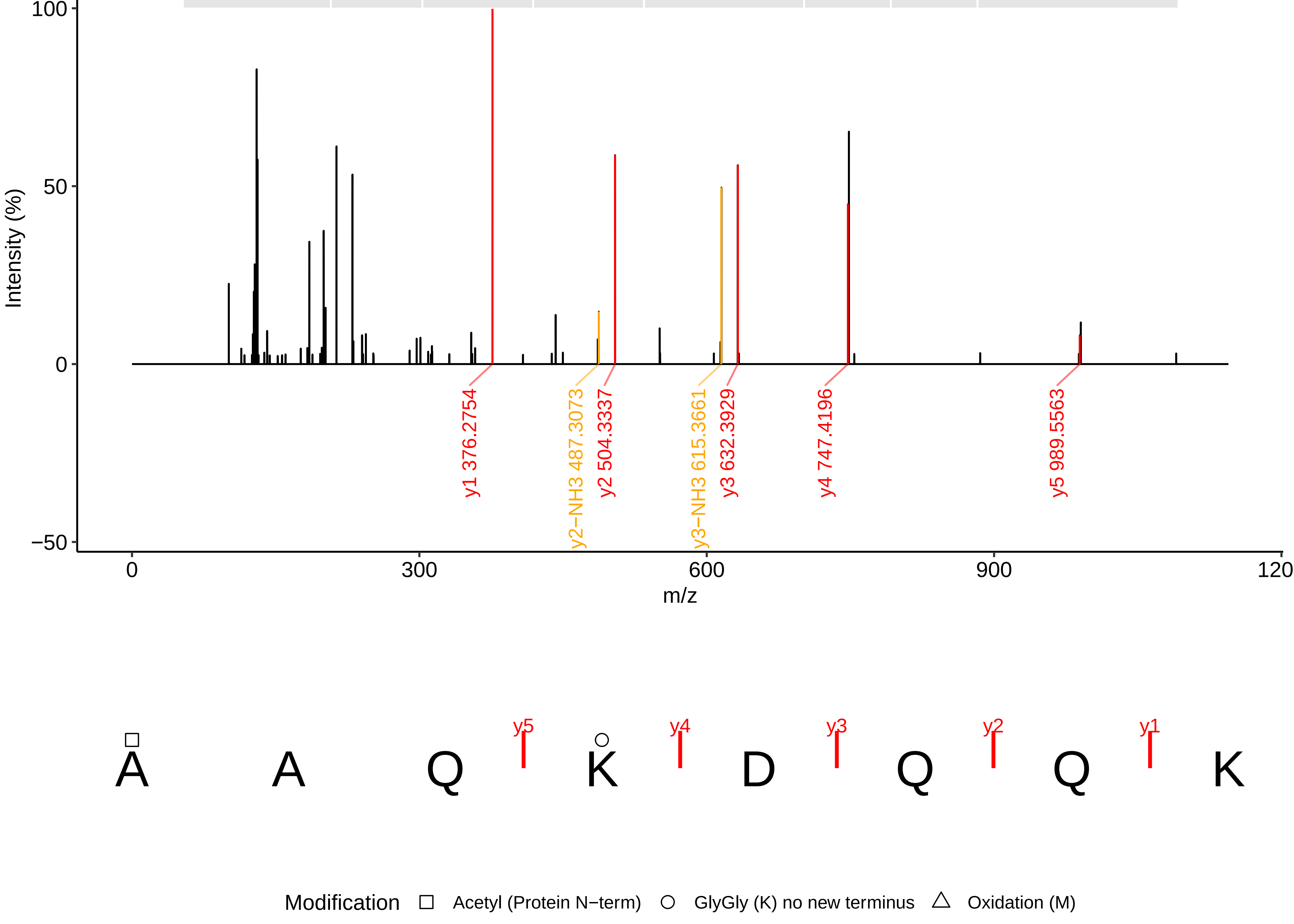

20200924_KIZ_MUb5_GlyGly_1 − Scan number: 2790

Gene Names	Charge	m/z	Mass	Retention time	PEP	Score	Precursor Intensity
RABAC1	2	536.7727	1071.531	8.6171	0.00029706	99.919	9715290

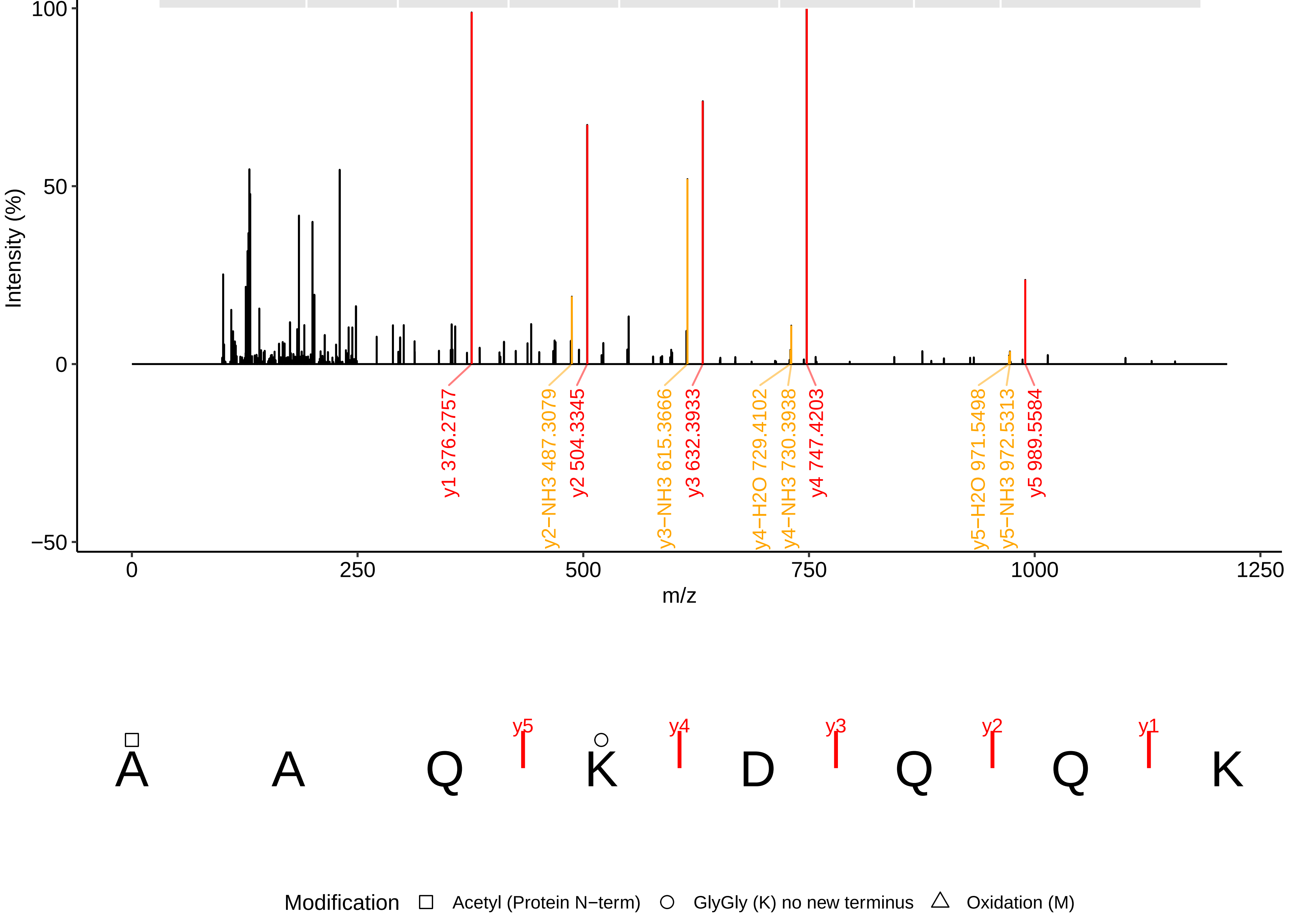

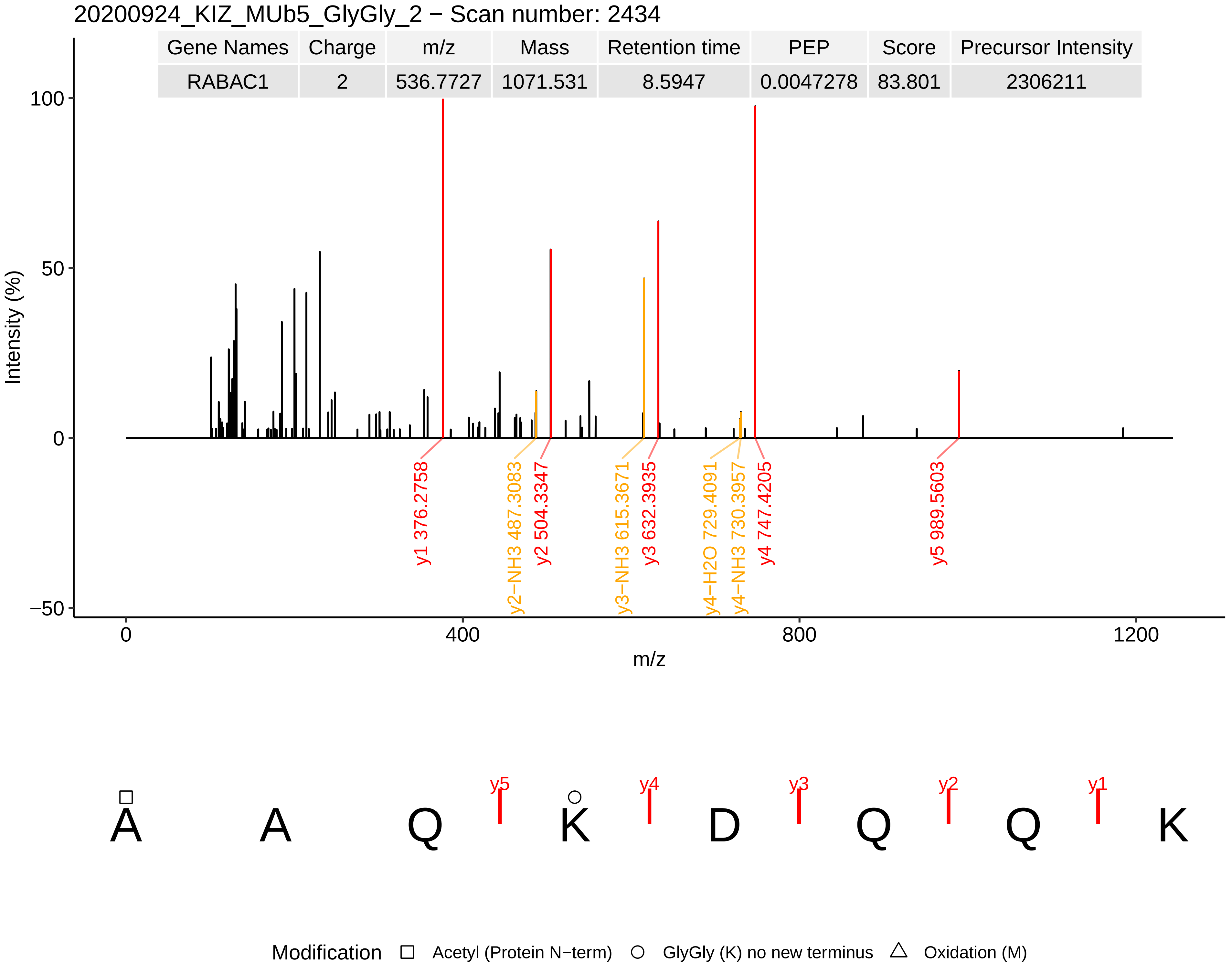
20200924_KIZ_MUb5_GlyGly_2 − Scan number: 2434
Gene Names | Charge | m/z | Mass | Retention time | PEP | Score | Precursor Intensity
RABAC1 | 2 | 536.7727 | 1071.531 | 8.5947 | 0.0047278 | 83.801 | 2306211
Intensity (%)
m/z
y1 376.2758
y2−NH3 487.3083
y2 504.3347
y3−NH3 615.3671
y3 632.3935
y4−H2O 729.4091
y4−NH3 730.3957
y4 747.4205
y5 989.5603
A A Q K D Q Q K
y5 y4 y3 y2 y1
Modification
Acetyl (Protein N−term)
GlyGly (K) no new terminus
Oxidation (M)

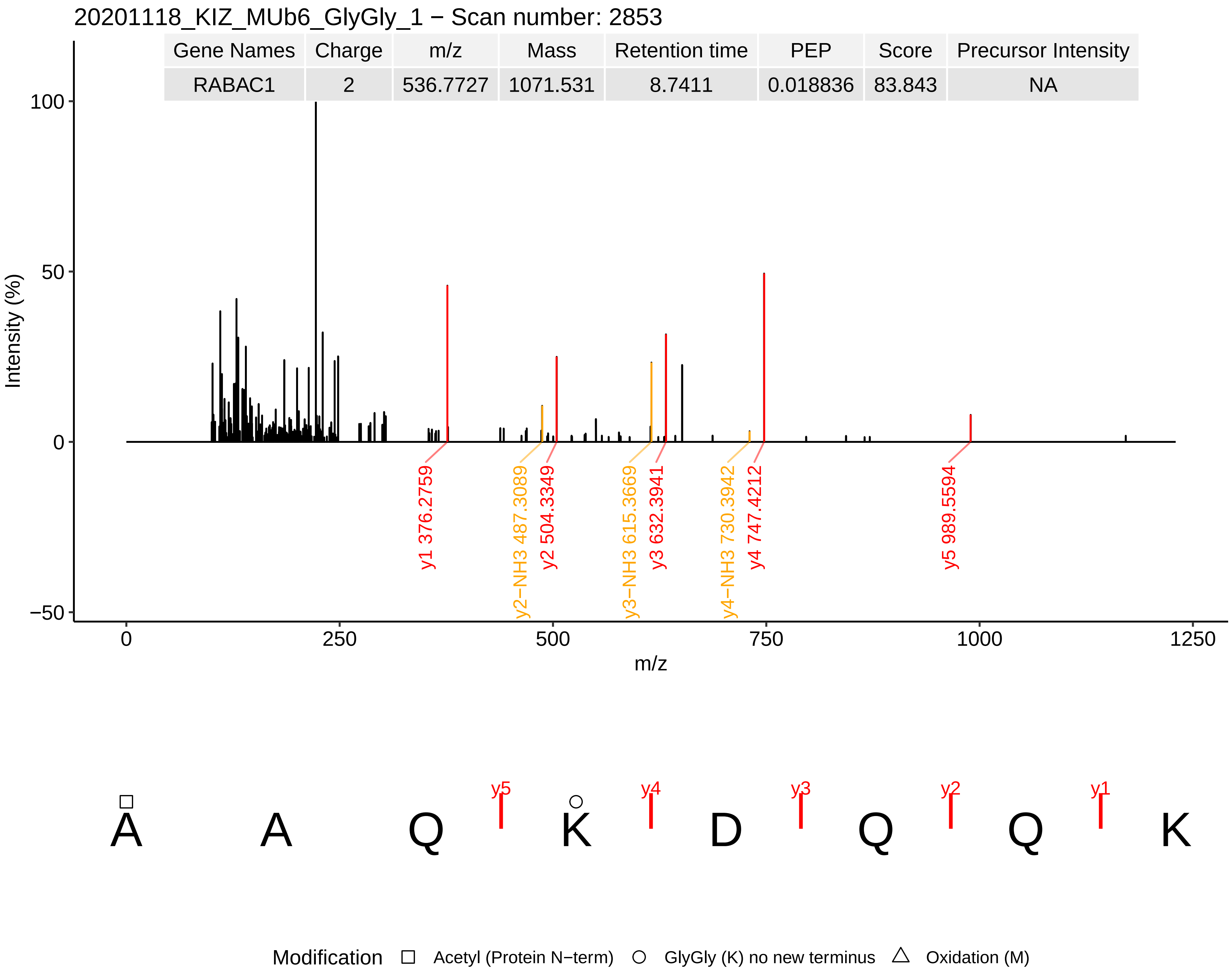

20201118_KIZ_MUb6_GlyGly_1 − Scan number: 2853
Gene Names | Charge | m/z | Mass | Retention time | PEP | Score | Precursor Intensity
RABAC1 | 2 | 536.7727 | 1071.531 | 8.7411 | 0.018836 | 83.843 | NA
Intensity (%)
m/z
y1 376.2759
y2−NH3 487.3089
y2 504.3349
y3−NH3 615.3669
y3 632.3941
y4−NH3 730.3942
y4 747.4212
y5 989.5594
A A Q K D Q Q K
y5 y4 y3 y2 y1
Modification
Acetyl (Protein N−term)
GlyGly (K) no new terminus
Oxidation (M)

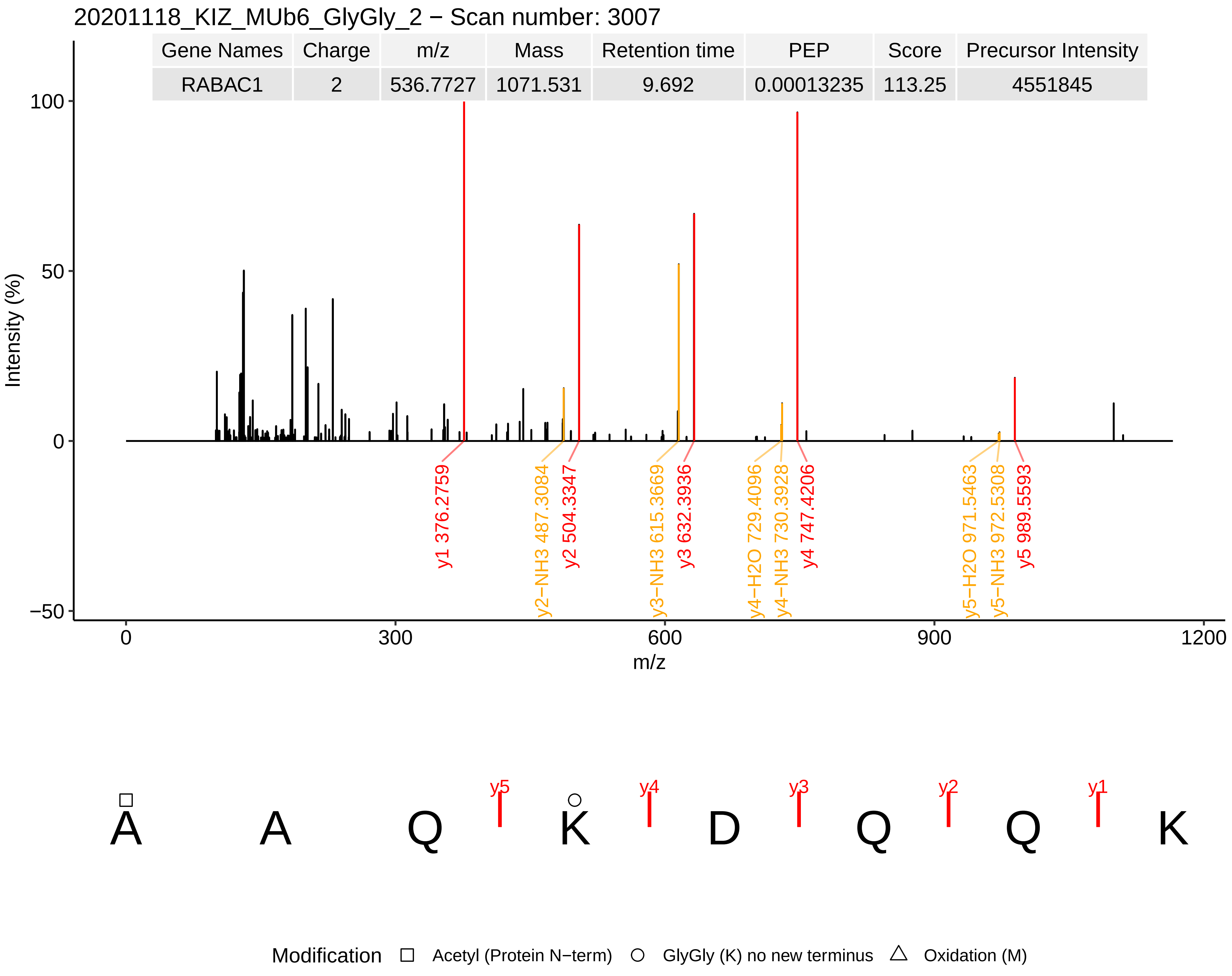

20201118_KIZ_MUb6_GlyGly_2 − Scan number: 3007
Gene Names | Charge | m/z | Mass | Retention time | PEP | Score | Precursor Intensity
RABAC1 | 2 | 536.7727 | 1071.531 | 9.692 | 0.00013235 | 113.25 | 4551845
Intensity (%)
m/z
y1 376.2759
y2−NH3 487.3084
y2 504.3347
y3−NH3 615.3669
y3 632.3936
y4−H2O 729.4096
y4−NH3 730.3928
y4 747.4206
y5−H2O 971.5463
y5−NH3 972.5308
y5 989.5593
A A Q K D Q Q K
y5 y4 y3 y2 y1
Modification
Acetyl (Protein N−term)
GlyGly (K) no new terminus
Oxidation (M)

20200921_KIZ_MUb4_GlyGly_10plex − Scan number: 9734

Gene Names	Charge	m/z	Mass	Retention time	PEP	Score	Precursor Intensity
PLLP	2	639.3279	1276.641	33.438	1.3406e−10	135.79	8011590

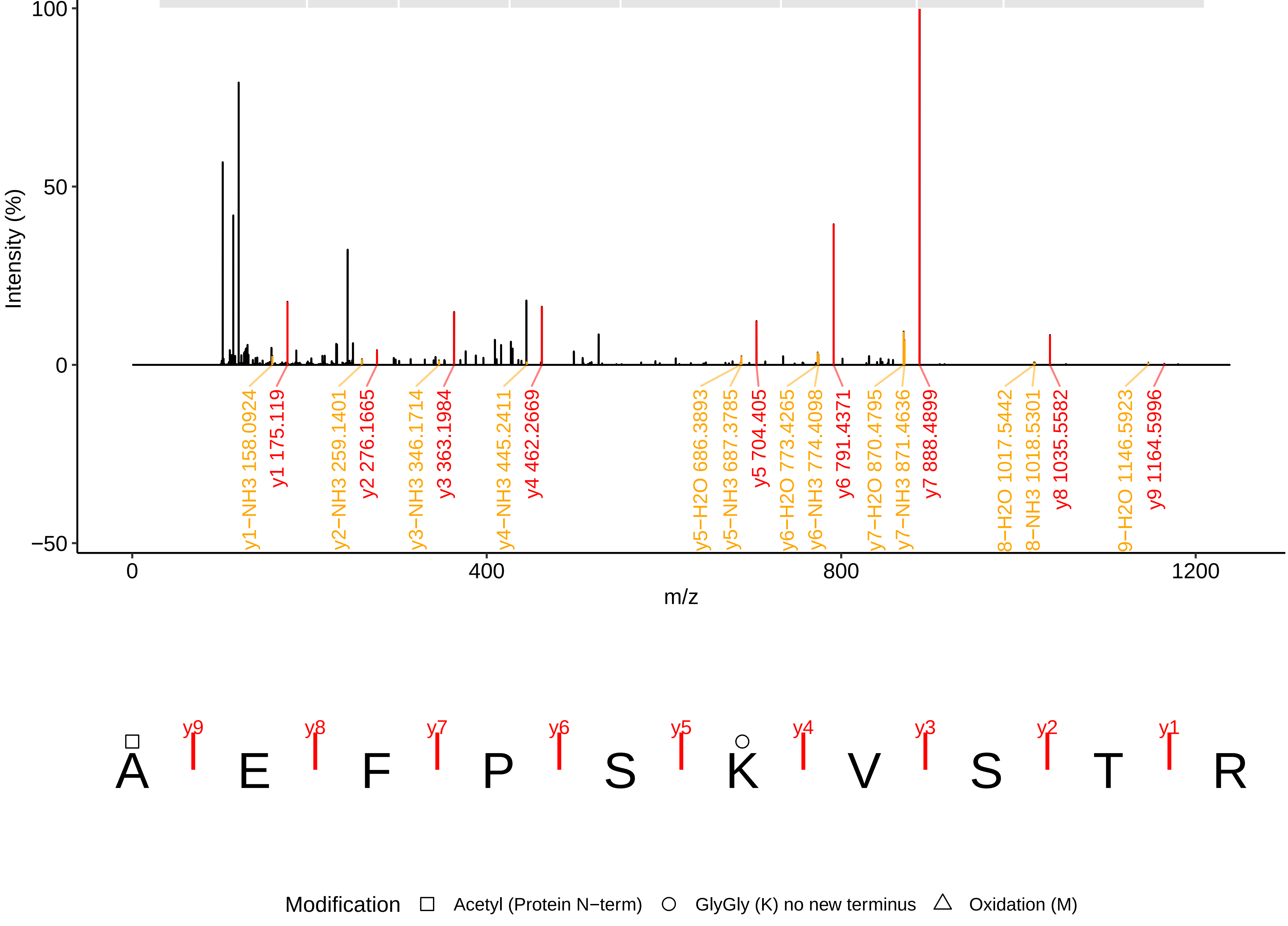

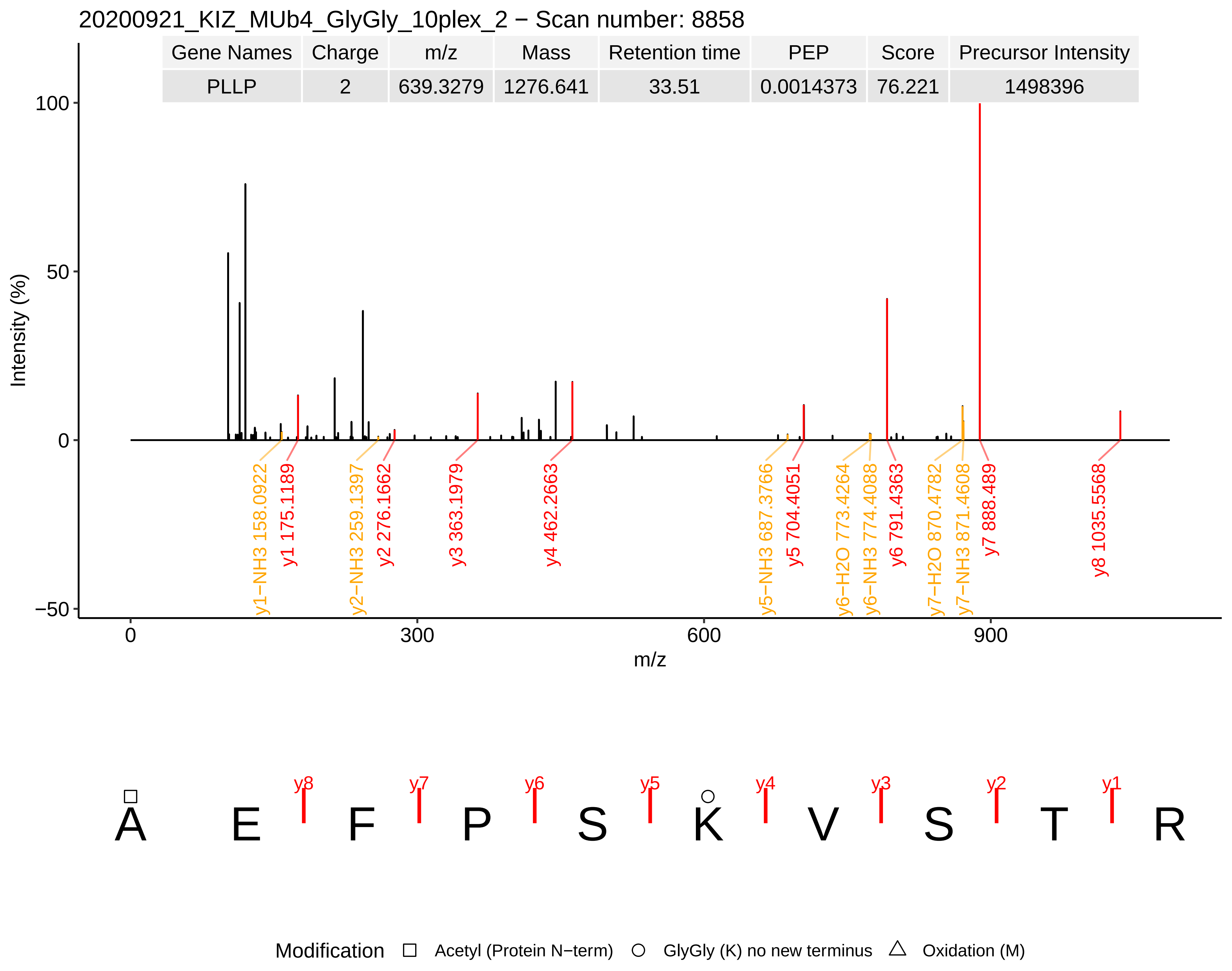
20200921_KIZ_MUb4_GlyGly_10plex_2 − Scan number: 8858
Gene Names | Charge | m/z | Mass | Retention time | PEP | Score | Precursor Intensity
PLLP | 2 | 639.3279 | 1276.641 | 33.51 | 0.0014373 | 76.221 | 1498396
Intensity (%)
100
50
0
−50
0
300
600
900
m/z
y1−NH3 158.0922
y1 175.1189
y2−NH3 259.1397
y2 276.1662
y3 363.1979
y4 462.2663
y5−NH3 687.3766
y5 704.4051
y6−H2O 773.4264
y6−NH3 774.4088
y6 791.4363
y7−H2O 870.4782
y7−NH3 871.4608
y7 888.489
y8 1035.5568
A E F P S K V S T R
y8 y7 y6 y5 y4 y3 y2 y1
Modification
Acetyl (Protein N−term)
GlyGly (K) no new terminus
Oxidation (M)

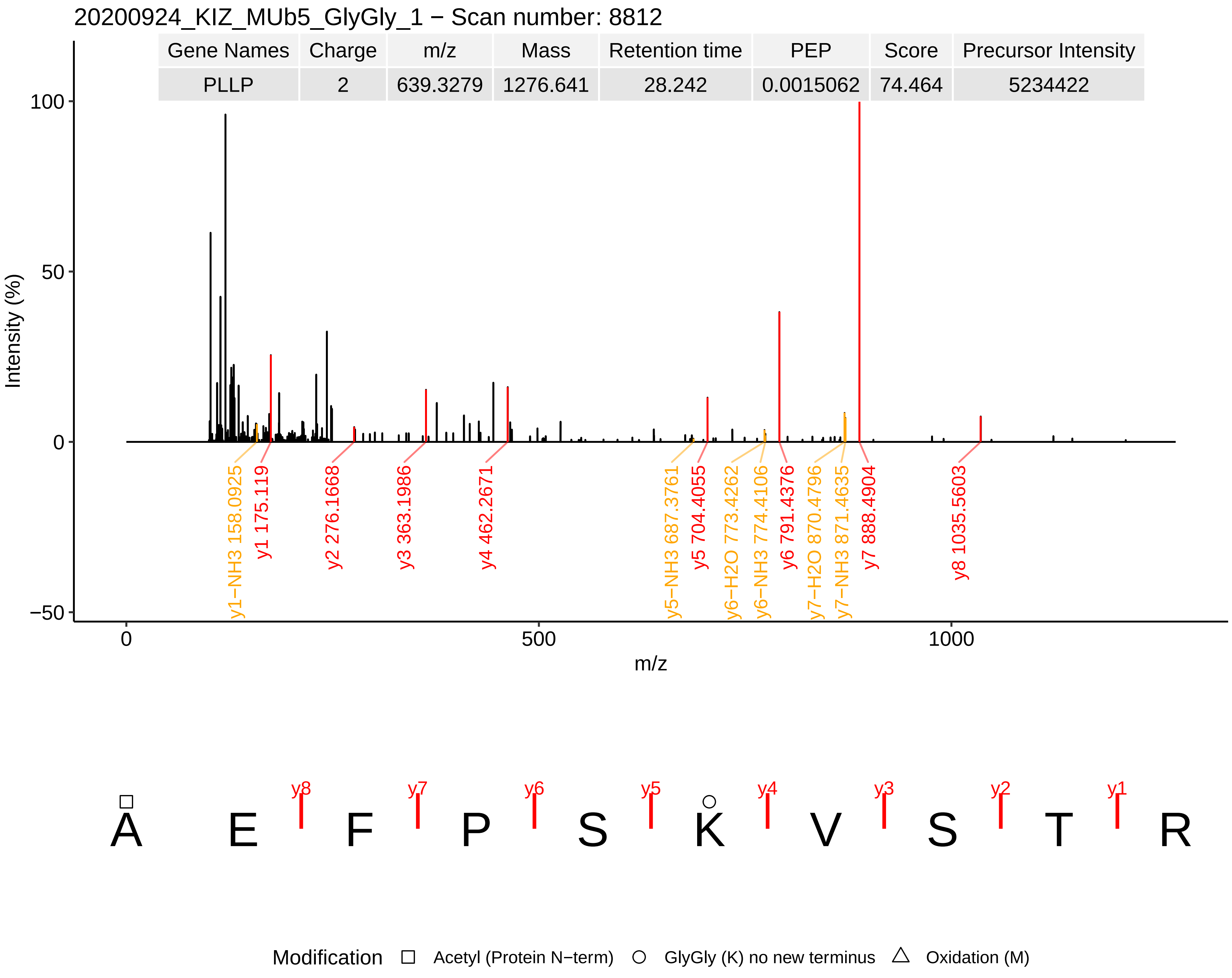
20200924_KIZ_MUb5_GlyGly_1 − Scan number: 8812
Gene Names | Charge | m/z | Mass | Retention time | PEP | Score | Precursor Intensity
PLLP | 2 | 639.3279 | 1276.641 | 28.242 | 0.0015062 | 74.464 | 5234422
Intensity (%)
100
50
0
−50
0
500
1000
m/z
y1−NH3 158.0925
y1 175.119
y2 276.1668
y3 363.1986
y4 462.2671
y5−NH3 687.3761
y5 704.4055
y6−H2O 773.4262
y6−NH3 774.4106
y6 791.4376
y7−H2O 870.4796
y7−NH3 871.4635
y7 888.4904
y8 1035.5603
A E F P S K V S T R
y8 y7 y6 y5 y4 y3 y2 y1
Modification
Acetyl (Protein N−term)
GlyGly (K) no new terminus
Oxidation (M)

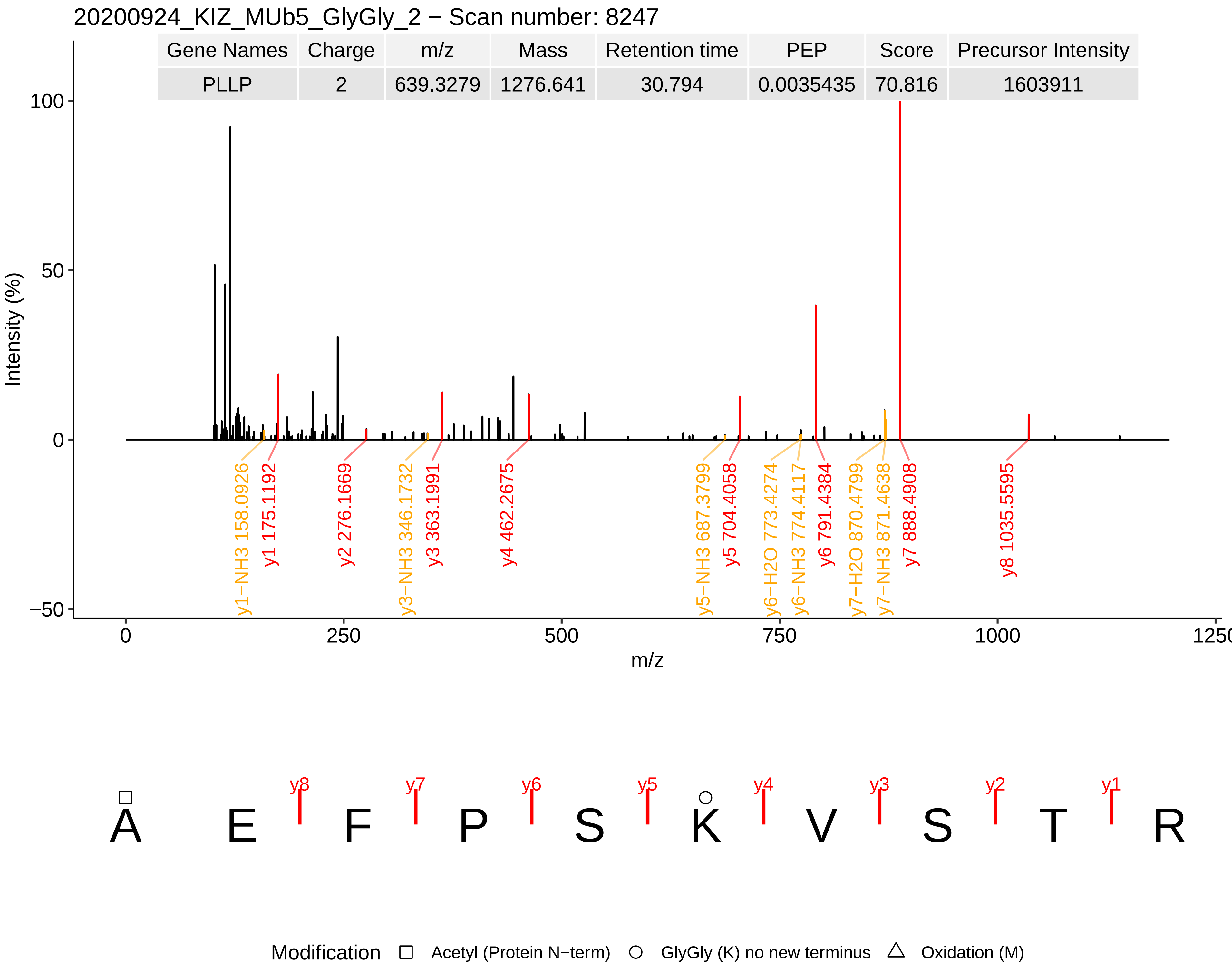

20200924_KIZ_MUb5_GlyGly_2 − Scan number: 8247
Gene Names	Charge	m/z	Mass	Retention time	PEP	Score	Precursor Intensity
PLLP	2	639.3279	1276.641	30.794	0.0035435	70.816	1603911
Intensity (%)
m/z
y1−NH3 158.0926
y1 175.1192
y2 276.1669
y3−NH3 346.1732
y3 363.1991
y4 462.2675
y5−NH3 687.3799
y5 704.4058
y6−H2O 773.4274
y6−NH3 774.4117
y6 791.4384
y7−H2O 870.4799
y7−NH3 871.4638
y7 888.4908
y8 1035.5595
A E F P S K V S T R
y8 y7 y6 y5 y4 y3 y2 y1
Modification
Acetyl (Protein N−term)
GlyGly (K) no new terminus
Oxidation (M)

20200921_KIZ_MUb4_GlyGly_10plex_2 − Scan number: 3343

Gene Names	Charge	m/z	Mass	Retention time	PEP	Score	Precursor Intensity
PGK1	3	287.1592	858.4559	11.189	0.041469	72.138	1176800

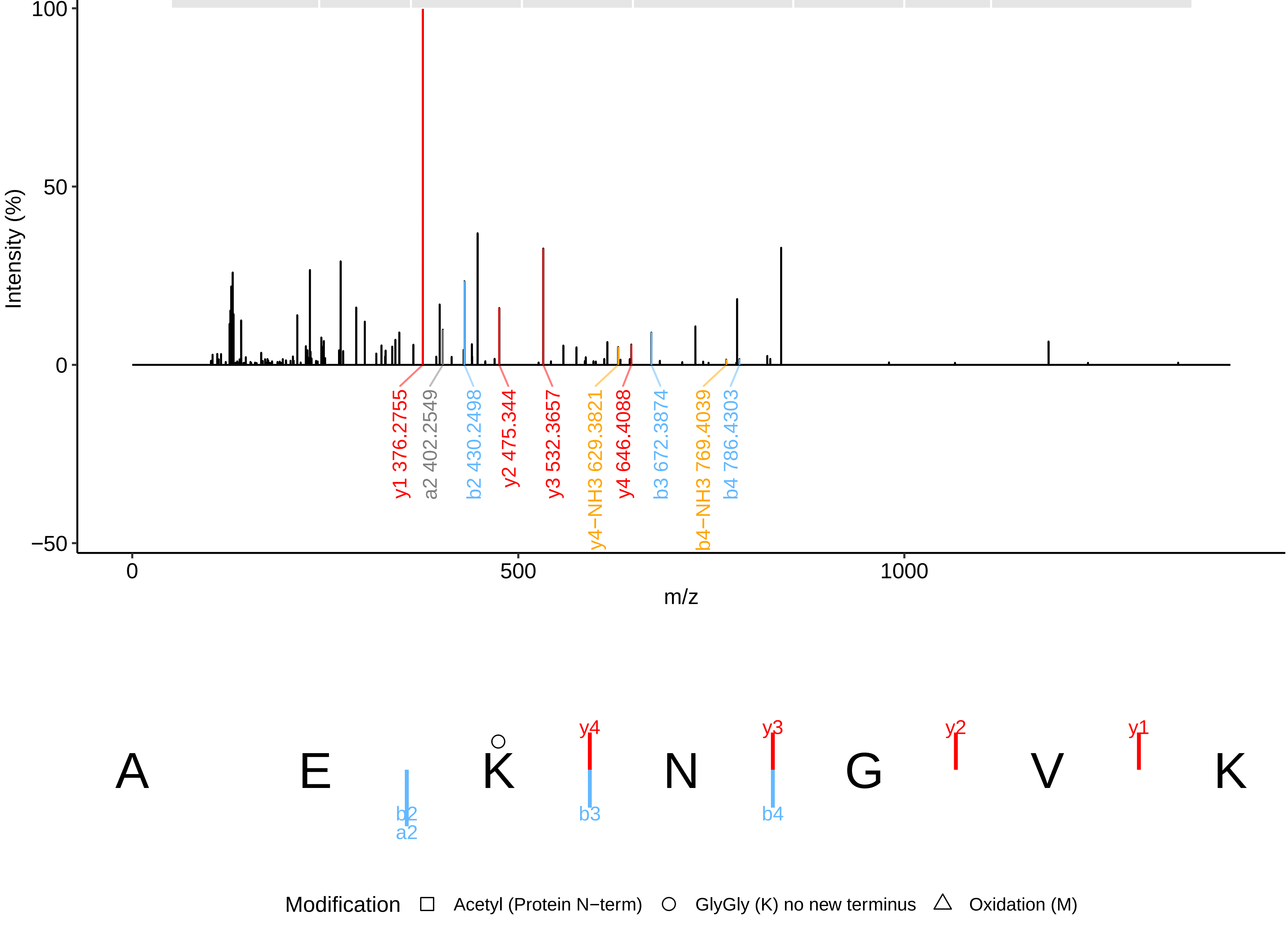

20200921_KIZ_MUb4_GlyGly_10plex − Scan number: 14721

Gene Names	Charge	m/z	Mass	Retention time	PEP	Score	Precursor Intensity
TPM3	2	657.8724	1313.73	52.363	2.1903e−12	152.63	NA

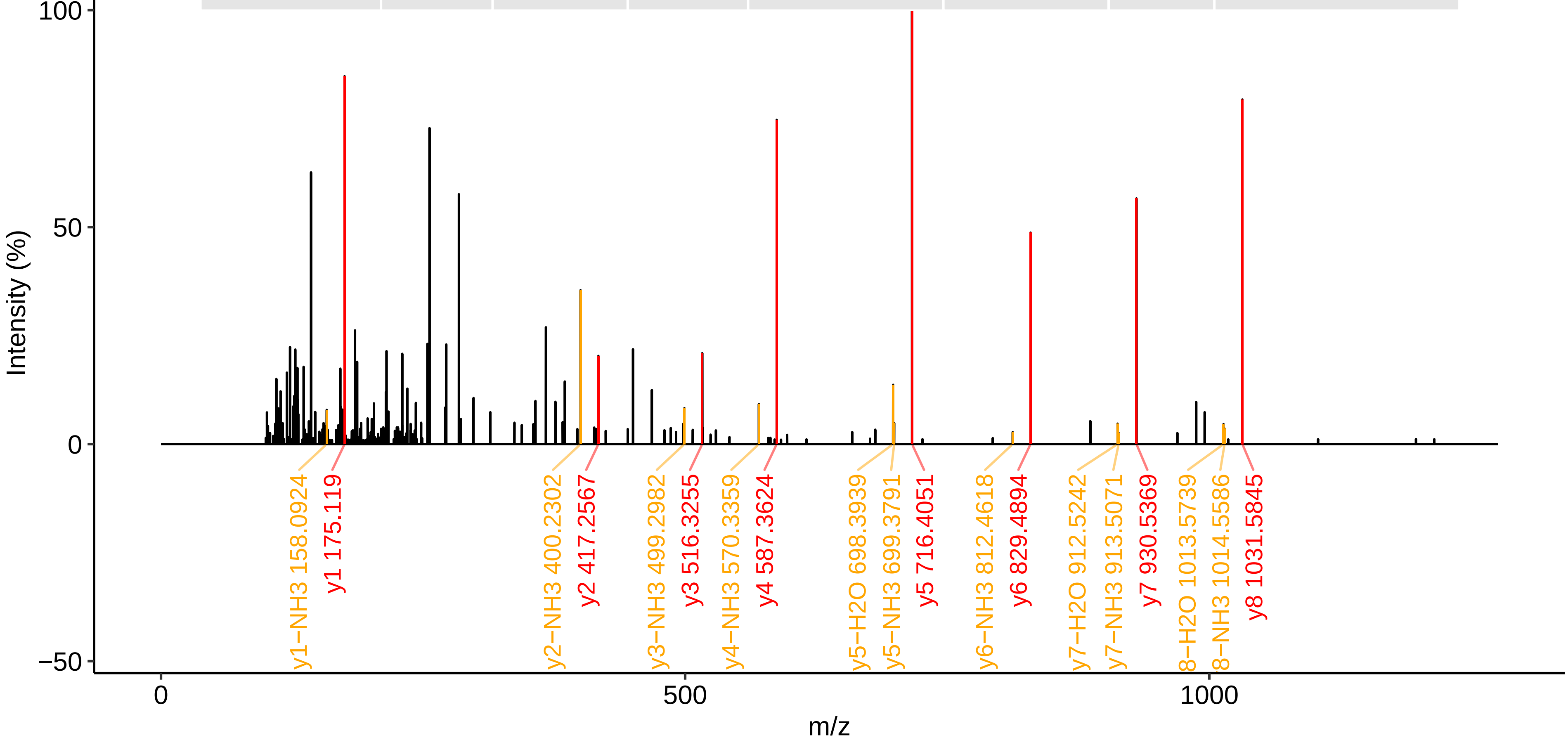

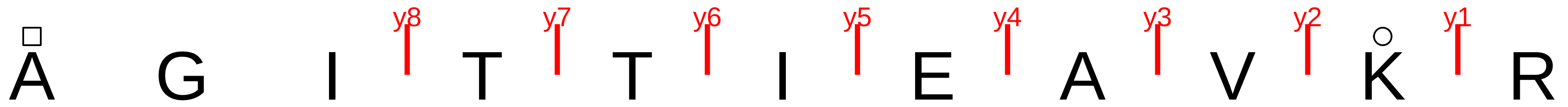

Modification □ Acetyl (Protein N−term) ○ GlyGly (K) no new terminus △ Oxidation (M)

20200921_KIZ_MUb4_GlyGly_10plex_2 − Scan number: 13485

Gene Names	Charge	m/z	Mass	Retention time	PEP	Score	Precursor Intensity
TPM3	2	657.8724	1313.73	52.368	0.010663	57.598	441939.8

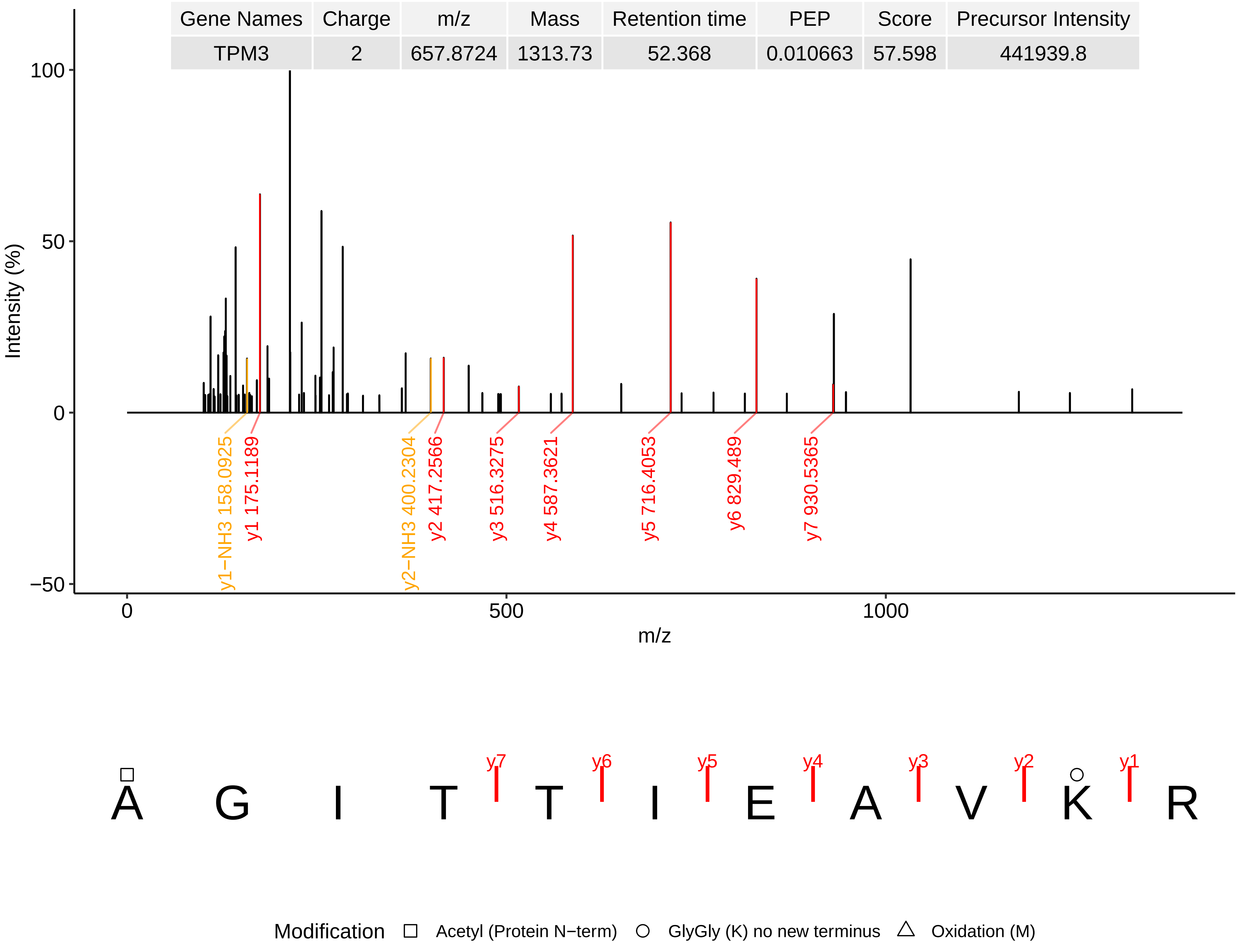

20200921_KIZ_MUb4_GlyGly_10plex − Scan number: 6803

Gene Names	Charge	m/z	Mass	Retention time	PEP	Score	Precursor Intensity
RAN	3	357.8734	1070.599	22.642	0.03277	66.692	6174083

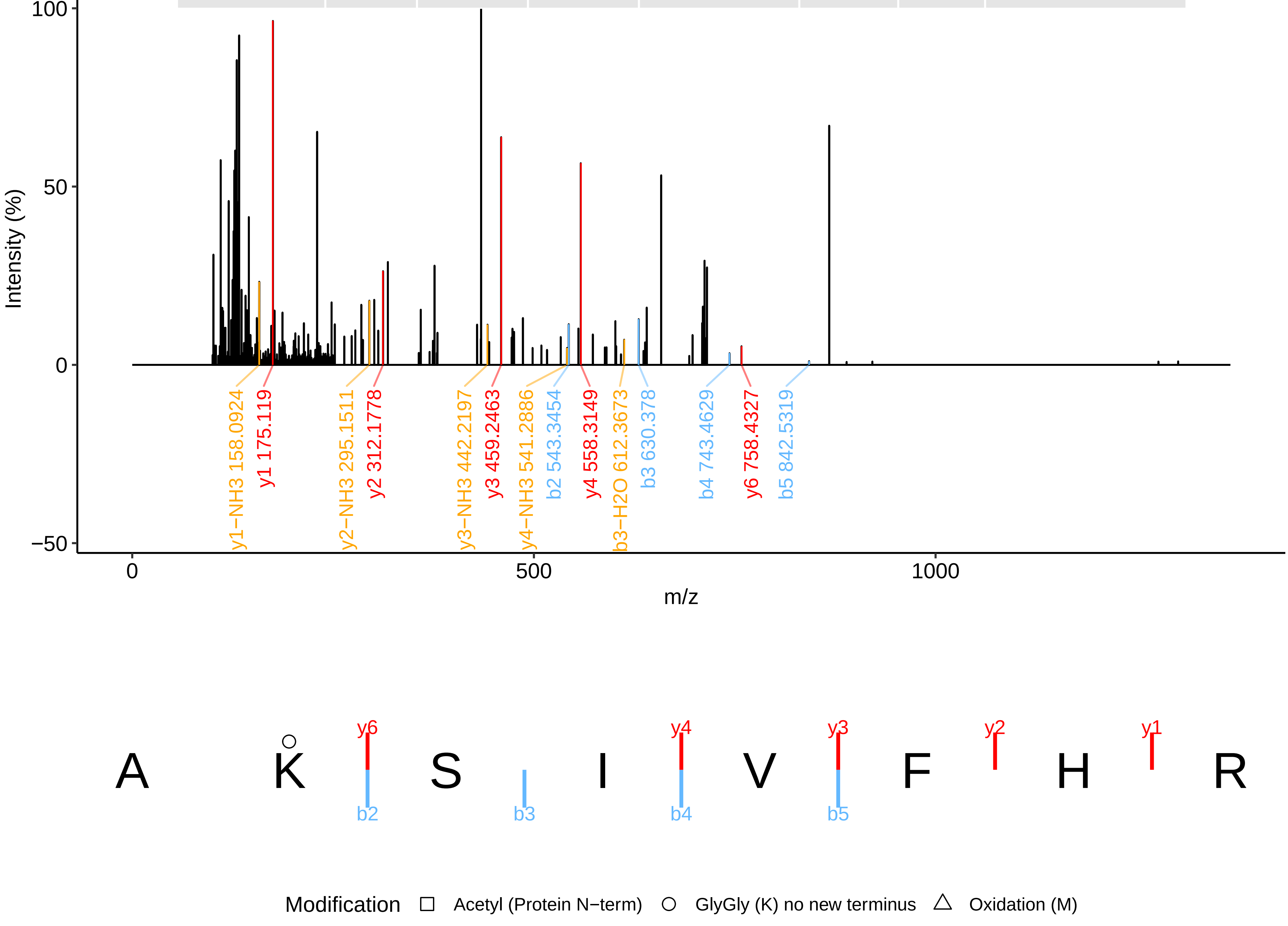

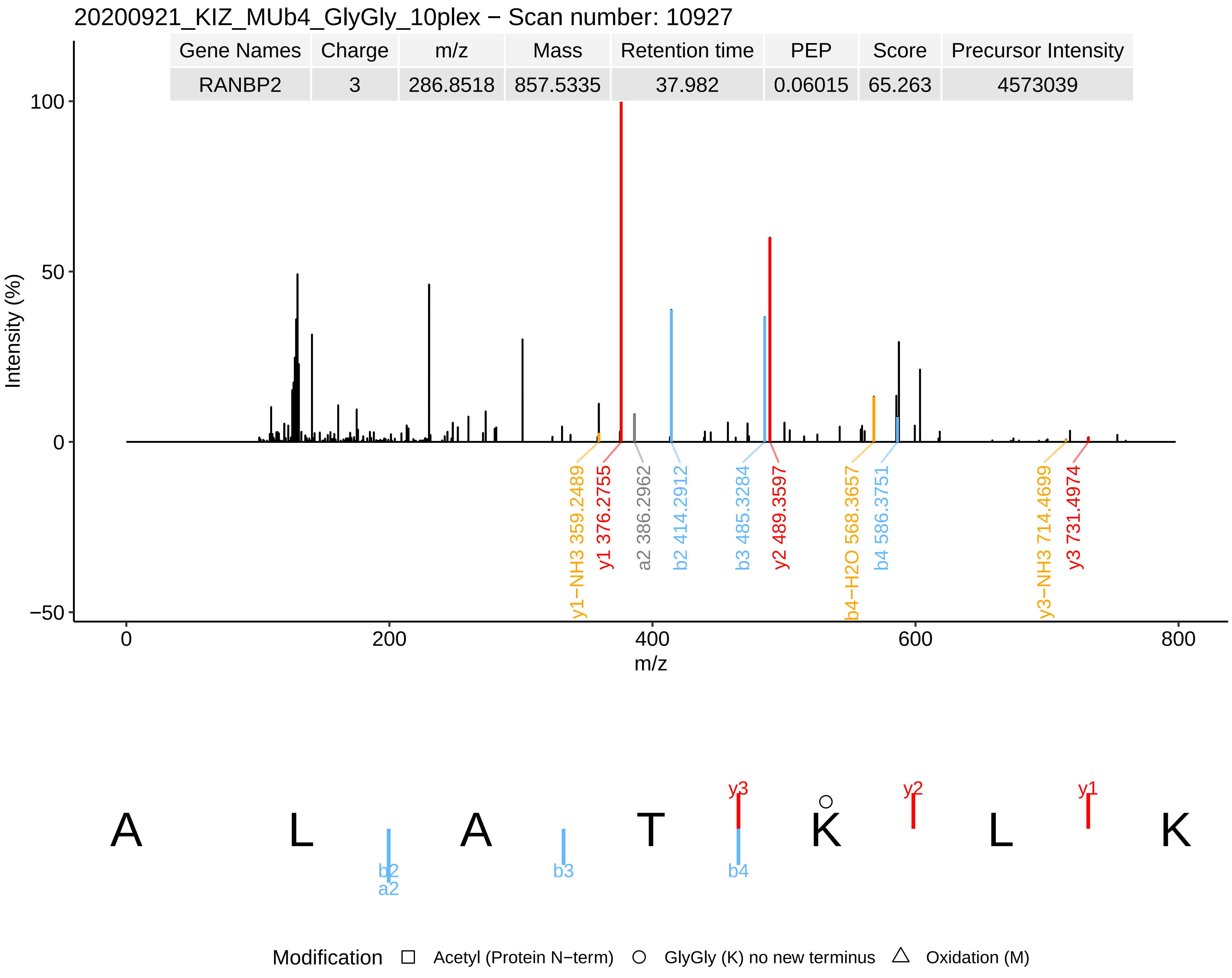
20200921_KIZ_MUb4_GlyGly_10plex − Scan number: 10927
Gene Names | Charge | m/z | Mass | Retention time | PEP | Score | Precursor Intensity
RANBP2 | 3 | 286.8518 | 857.5335 | 37.982 | 0.06015 | 65.263 | 4573039
Intensity (%)
m/z
y1−NH3 359.2489
y1 376.2755
a2 386.2962
b2 414.2912
b3 485.3284
y2 489.3597
b4−H2O 568.3657
b4 586.3751
y3−NH3 714.4699
y3 731.4974
A L A T K L K
y3 y2 y1
b2 a2 b3 b4
Modification □ Acetyl (Protein N−term) ○ GlyGly (K) no new terminus △ Oxidation (M)

20200921_KIZ_MUb4_GlyGly_10plex_2 − Scan number: 3393

Gene Names	Charge	m/z	Mass	Retention time	PEP	Score	Precursor Intensity
HUS1	3	362.872	1085.594	11.384	0.0013476	93.429	880595

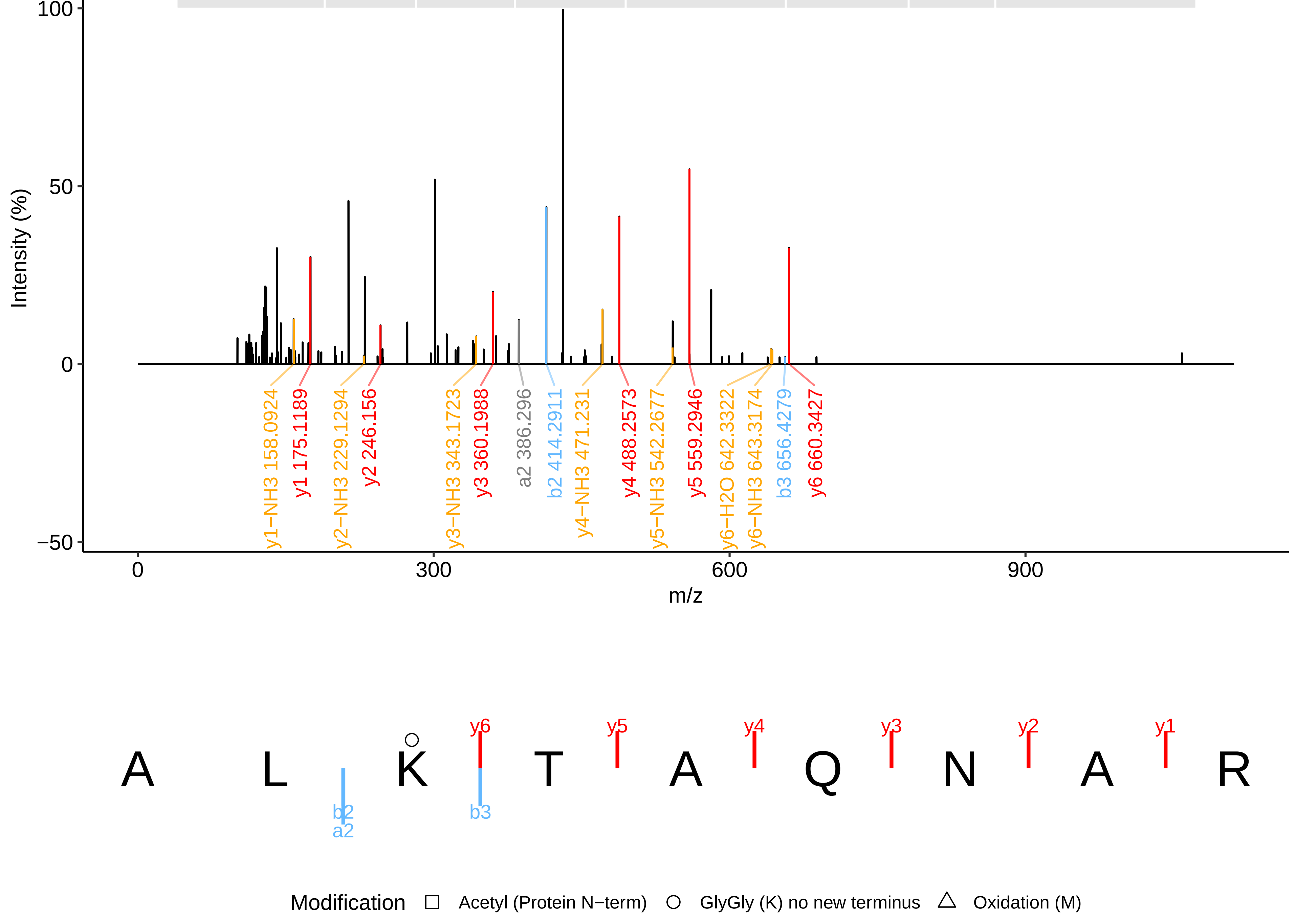

20201118_KIZ_MUb6_GlyGly_2 − Scan number: 3596

Gene Names	Charge	m/z	Mass	Retention time	PEP	Score	Precursor Intensity
HUS1	3	362.872	1085.594	11.973	0.020746	64.42	2461731

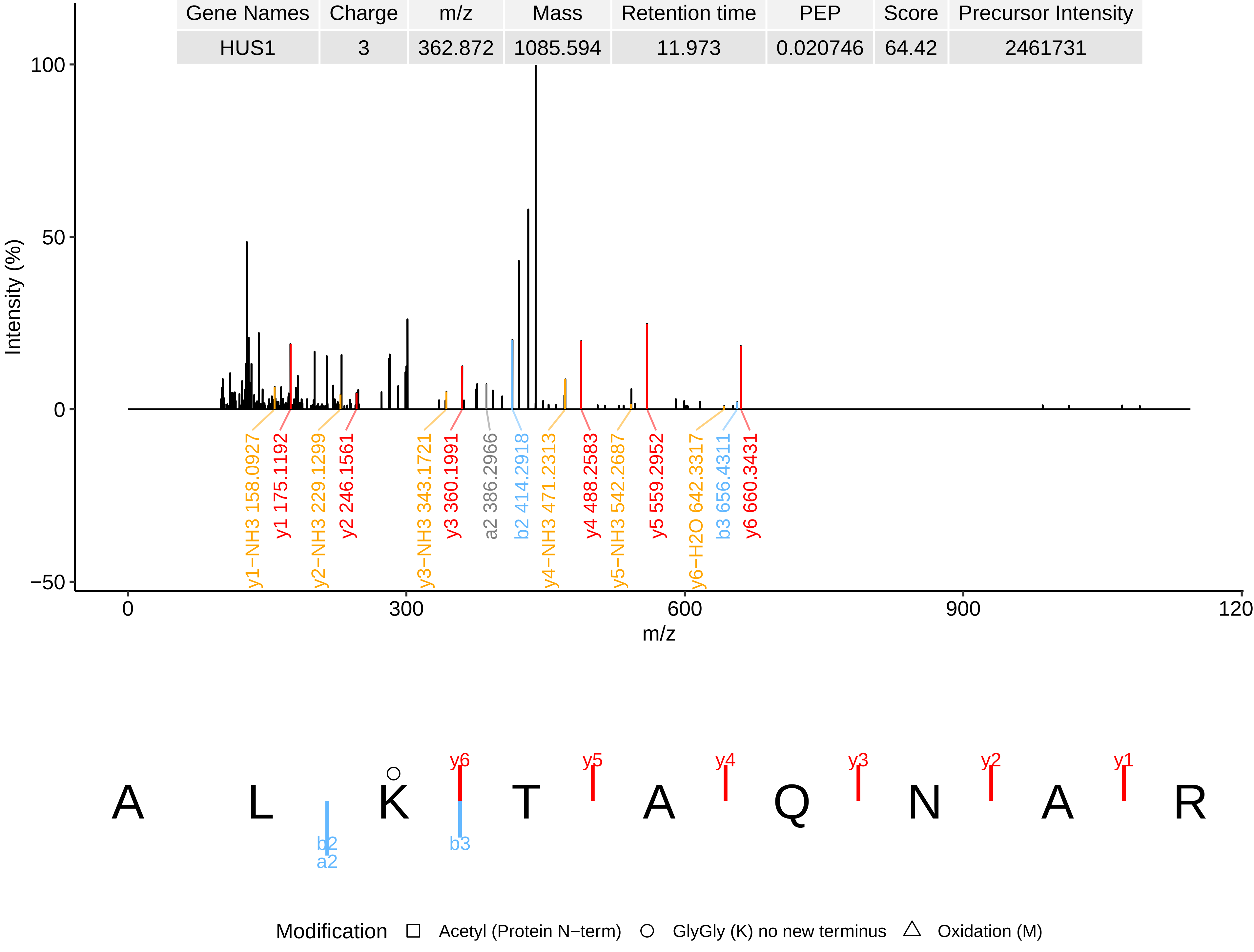

20200921_KIZ_MUb4_GlyGly_10plex_2 − Scan number: 5819

Gene Names	Charge	m/z	Mass	Retention time	PEP	Score	Precursor Intensity
SKIV2L	3	359.8648	1076.573	21.107	0.01255	79.469	403899.2

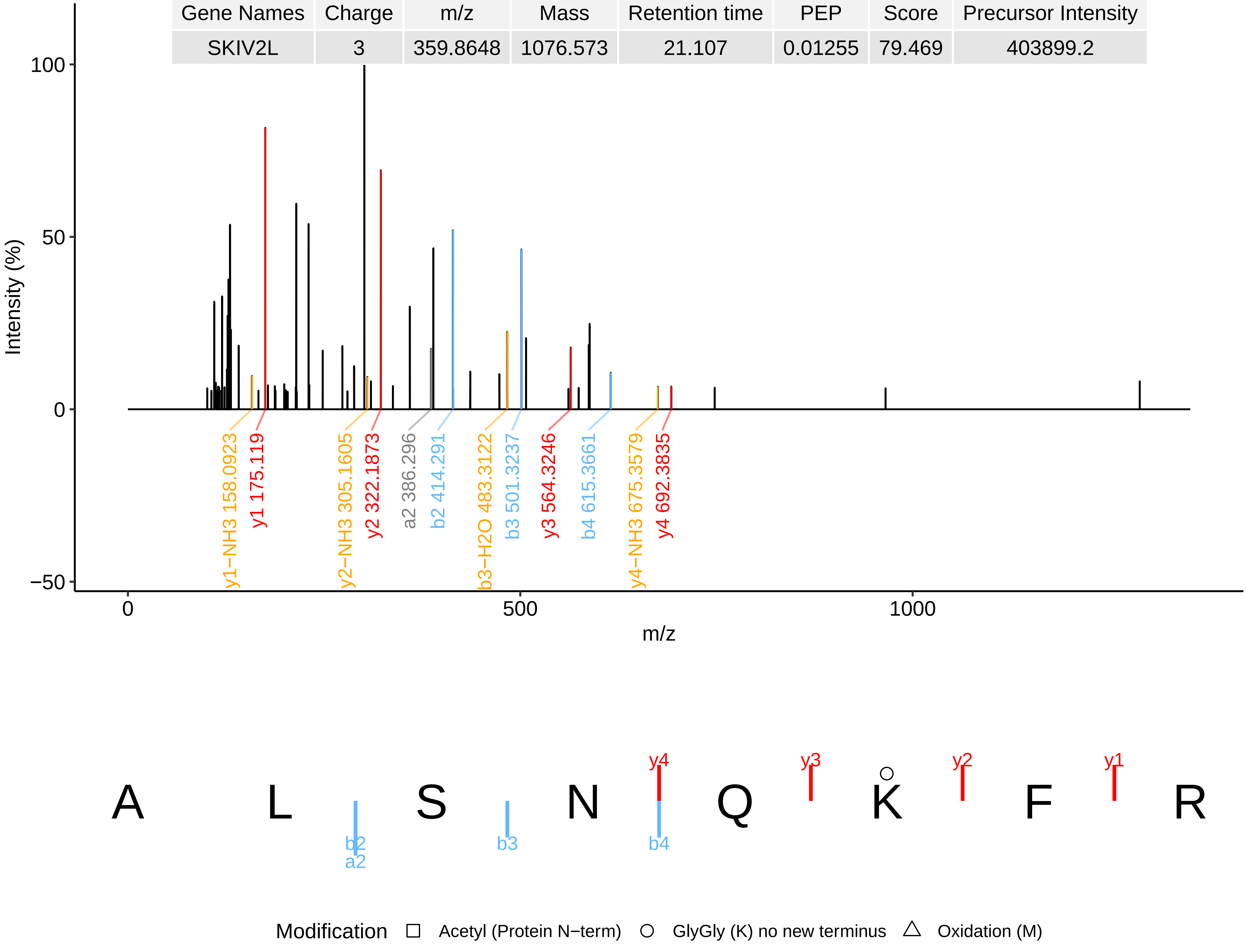

20200921_KIZ_MUb4_GlyGly_10plex_2 − Scan number: 4014

Gene Names	Charge	m/z	Mass	Retention time	PEP	Score	Precursor Intensity
SKIV2L2	3	365.1965	1092.568	13.855	0.00062575	112.17	269707.1

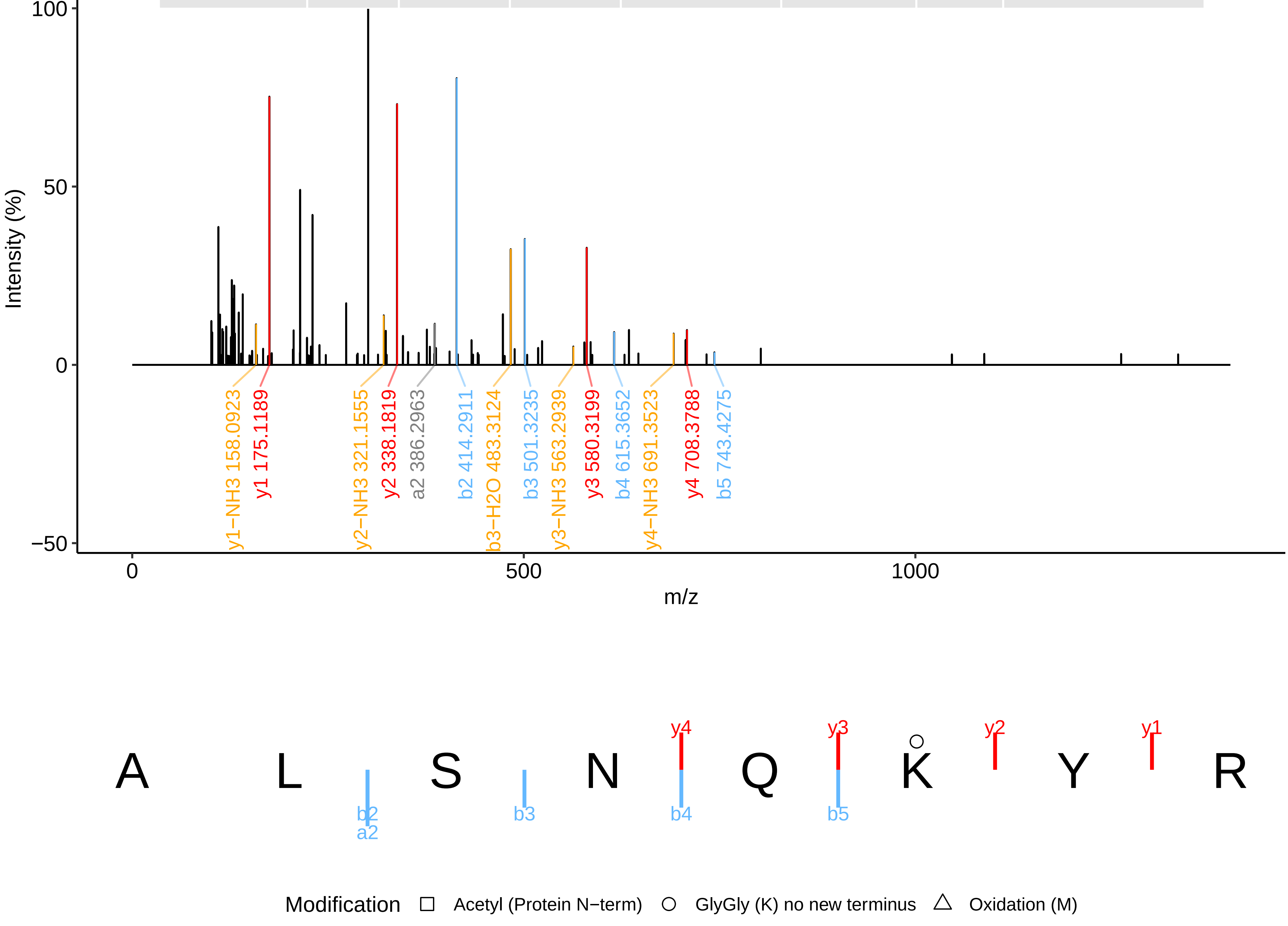

20200921_KIZ_MUb4_GlyGly_10plex_2 − Scan number: 3978

Gene Names	Charge	m/z	Mass	Retention time	PEP	Score	Precursor Intensity
BTAF1	3	356.1807	1065.52	13.71	0.10771	44.377	16924692

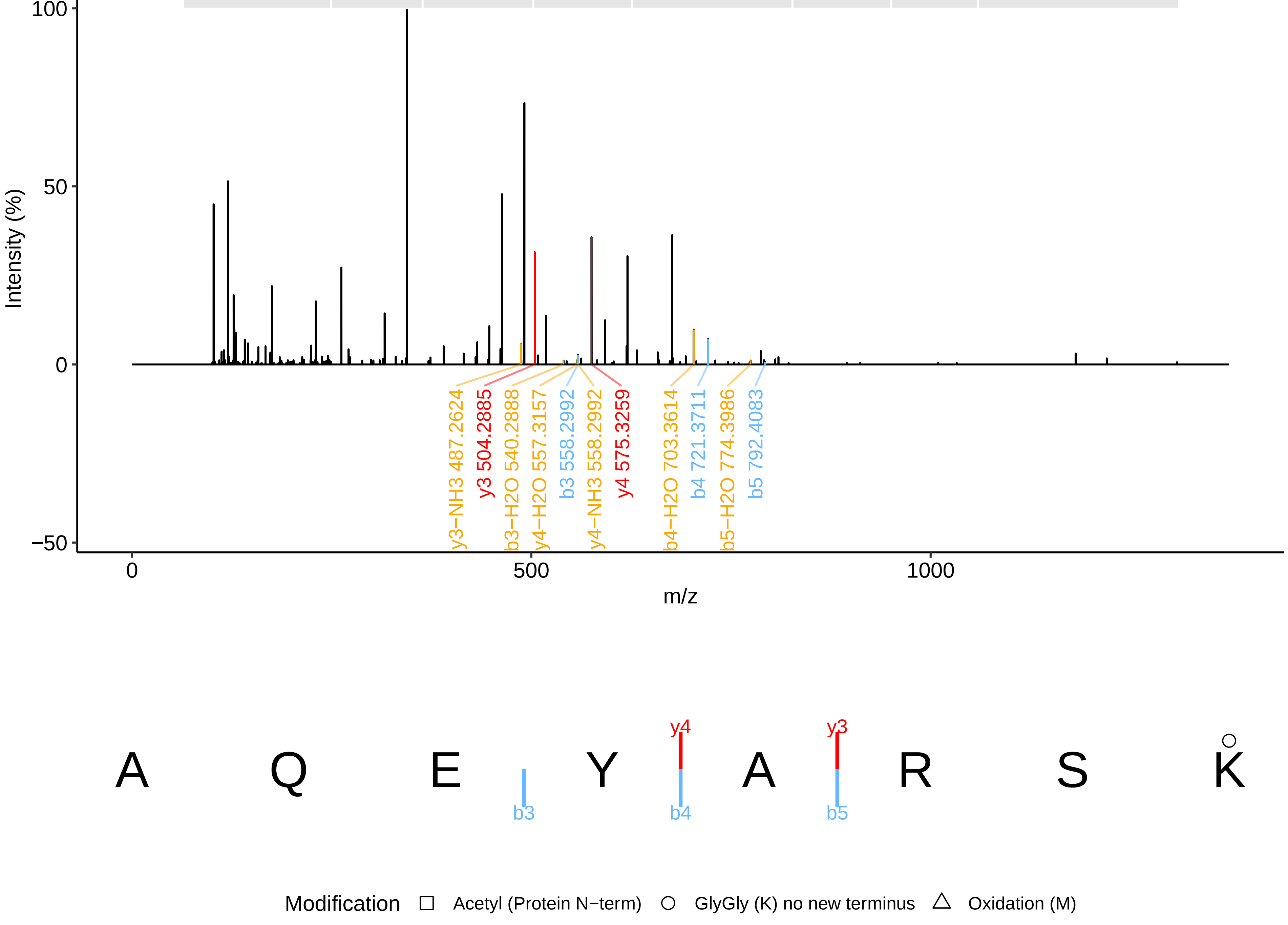

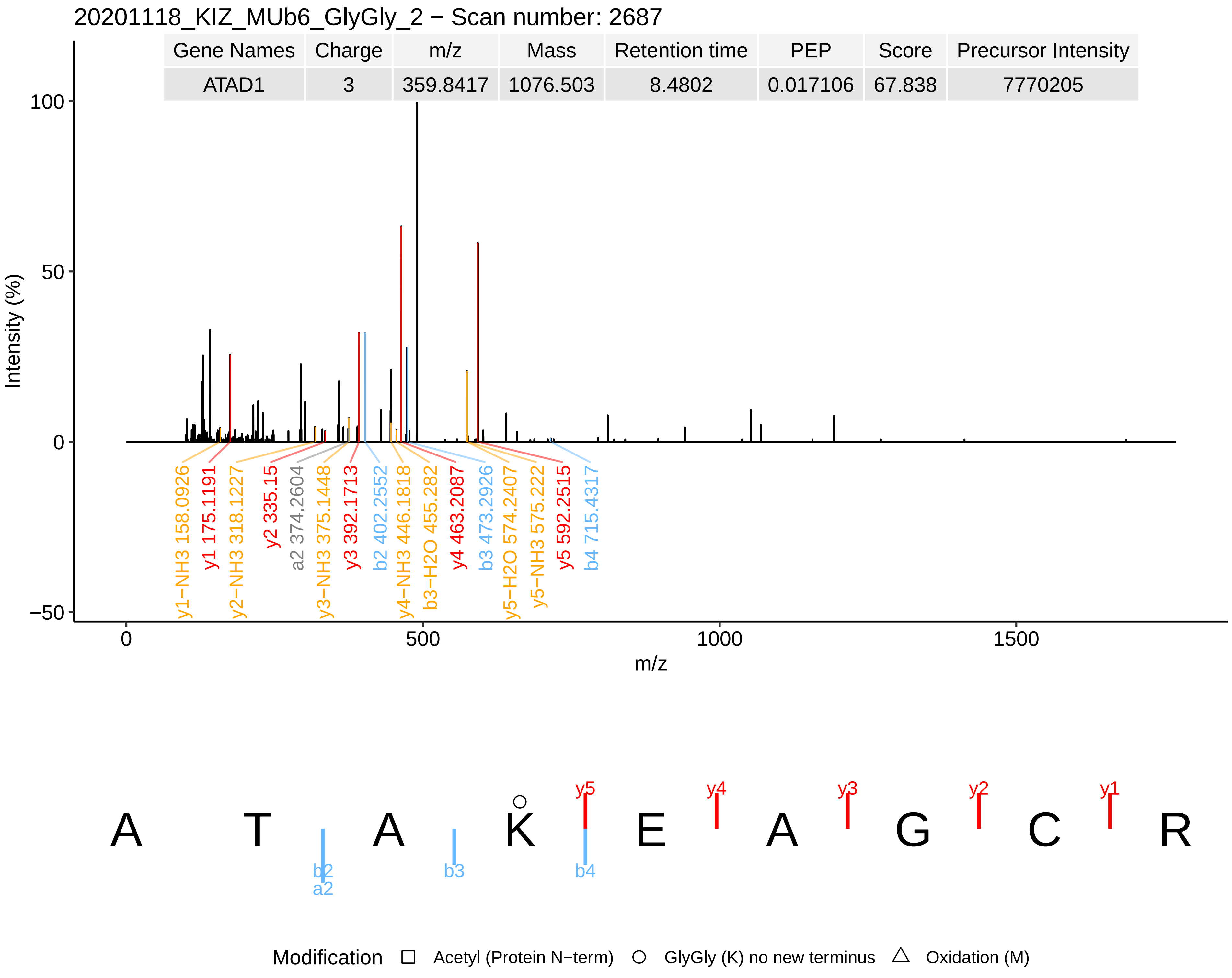

20201118_KIZ_MUb6_GlyGly_2 − Scan number: 2687
Gene Names | Charge | m/z | Mass | Retention time | PEP | Score | Precursor Intensity
ATAD1 | 3 | 359.8417 | 1076.503 | 8.4802 | 0.017106 | 67.838 | 7770205
Intensity (%)
100
50
0
−50
y1−NH3 158.0926
y1 175.1191
y2−NH3 318.1227
y2 335.15
a2 374.2604
y3−NH3 375.1448
y3 392.1713
b2 402.2552
y4−NH3 446.1818
b3−H2O 455.282
y4 463.2087
b3 473.2926
y5−H2O 574.2407
y5−NH3 575.222
y5 592.2515
b4 715.4317
0
500
1000
1500
m/z
A T A K E A G C R
y5 y4 y3 y2 y1
b2 a2 b3 b4
Modification
Acetyl (Protein N−term)
GlyGly (K) no new terminus
Oxidation (M)

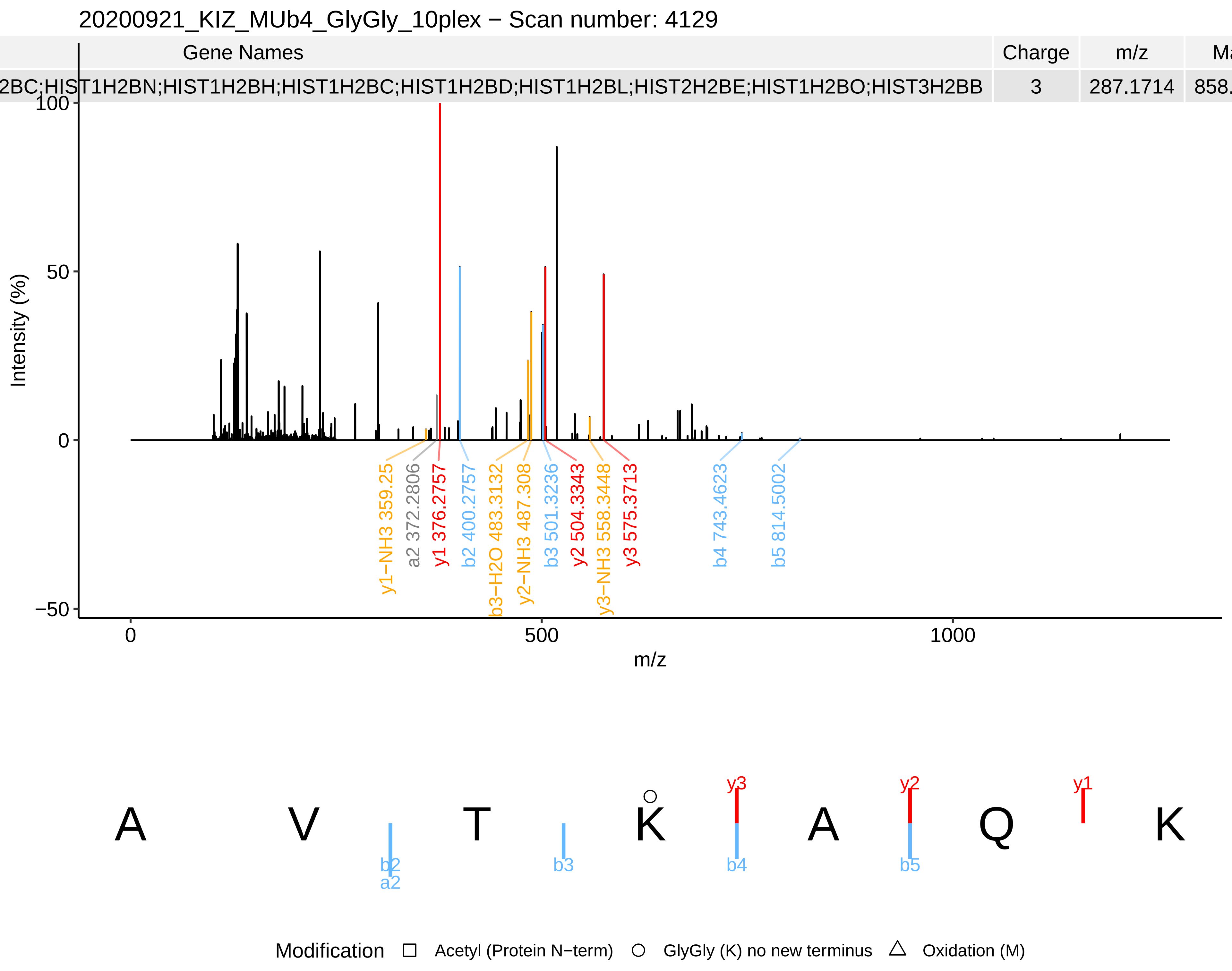

20200921_KIZ_MUb4_GlyGly_10plex − Scan number: 4129
Gene Names
Charge
m/z
2BC;HIST1H2BN;HIST1H2BH;HIST1H2BC;HIST1H2BD;HIST1H2BL;HIST2H2BE;HIST1H2BO;HIST3H2BB
3
287.1714
858.
Intensity (%)
m/z
y1−NH3 359.25
a2 372.2806
y1 376.2757
b2 400.2757
b3−H2O 483.3132
y2−NH3 487.308
b3 501.3236
y2 504.3343
y3−NH3 558.3448
y3 575.3713
b4 743.4623
b5 814.5002
A V T K A Q K
Modification
Acetyl (Protein N−term)
GlyGly (K) no new terminus
Oxidation (M)

20200921_KIZ_MUb4_GlyGly_10plex_2 − Scan number: 3867

Gene Names	Charge	m/z	Ma
2BC;HIST1H2BN;HIST1H2BH;HIST1H2BC;HIST1H2BD;HIST1H2BL;HIST2H2BE;HIST1H2BO;HIST3H2BB	3	287.1714	858.4

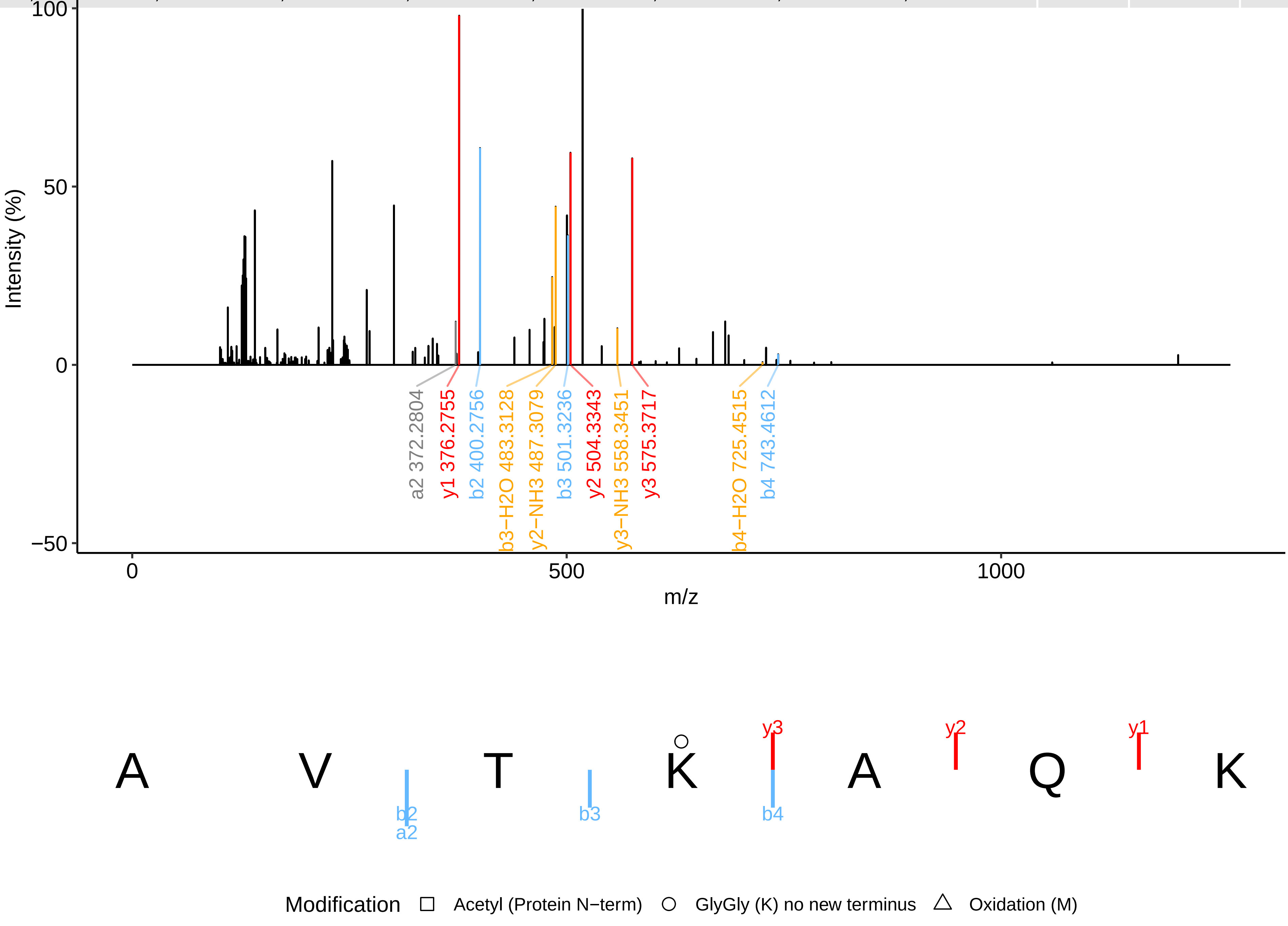

20200921_KIZ_MUb4_GlyGly_10plex − Scan number: 4051

Gene Names	Charge	m/z	Mass	Retention time	PEP	Score	Precursor Intensity
HDAC2	2	526.262	1050.509	13.17	0.00206	78.496	4463856

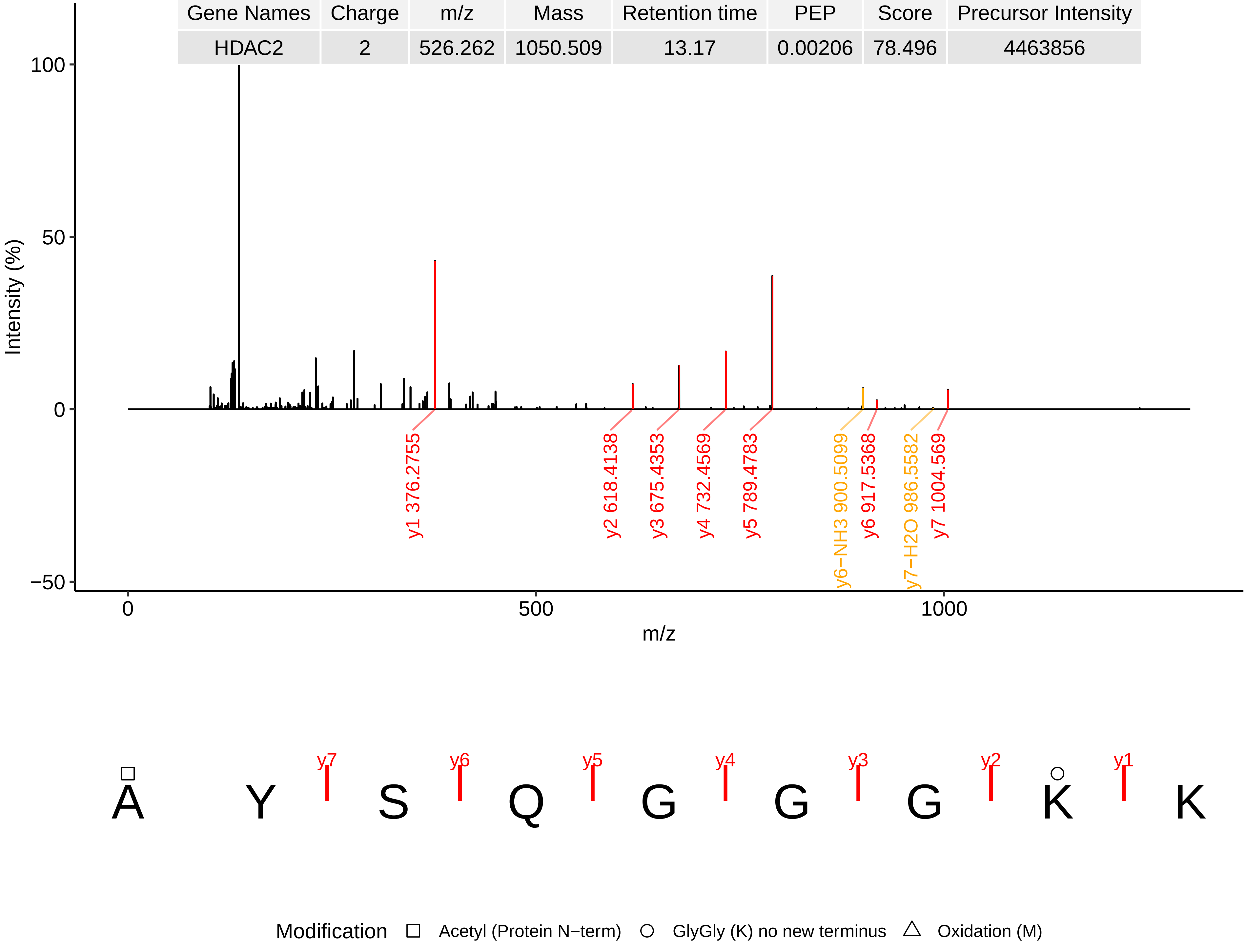

20200921_KIZ_MUb4_GlyGly_10plex_2 − Scan number: 3795

Gene Names	Charge	m/z	Mass	Retention time	PEP	Score	Precursor Intensity
HDAC2	2	526.262	1050.509	12.98	0.00039244	78.496	791641

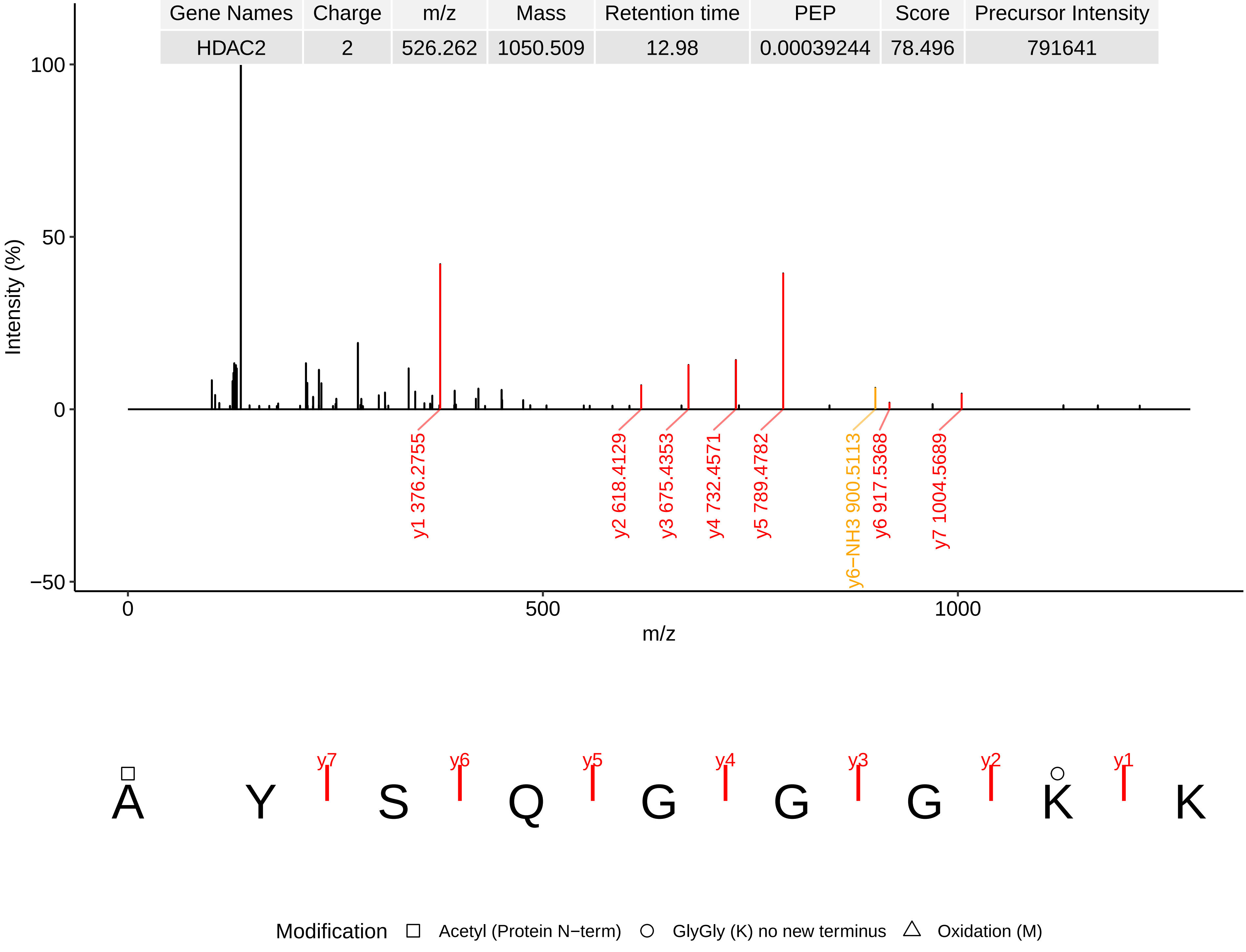

20200924_KIZ_MUb5_GlyGly_1 − Scan number: 3556

Gene Names	Charge	m/z	Mass	Retention time	PEP	Score	Precursor Intensity
HDAC2	2	526.262	1050.509	10.881	0.00053369	55.676	7106774

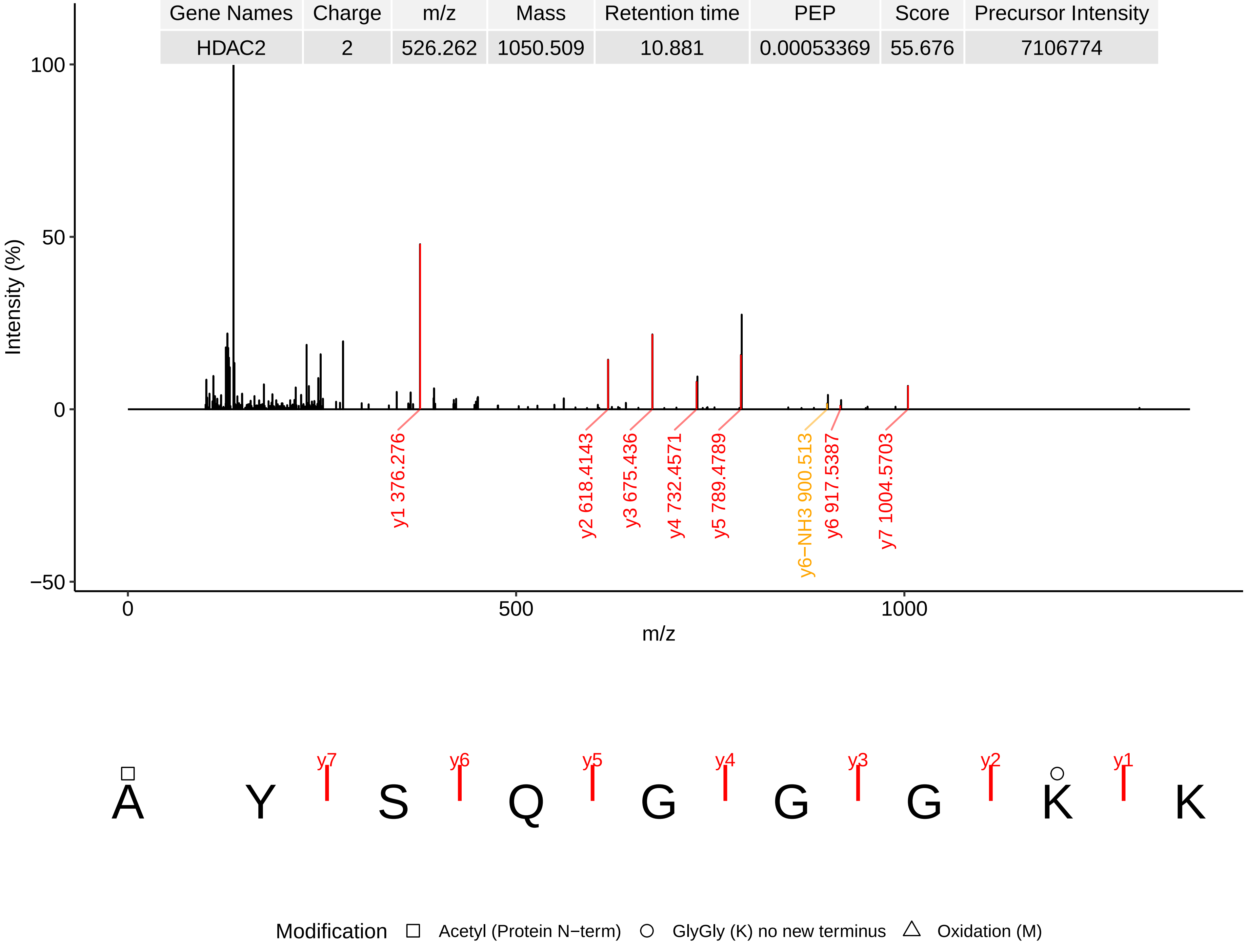

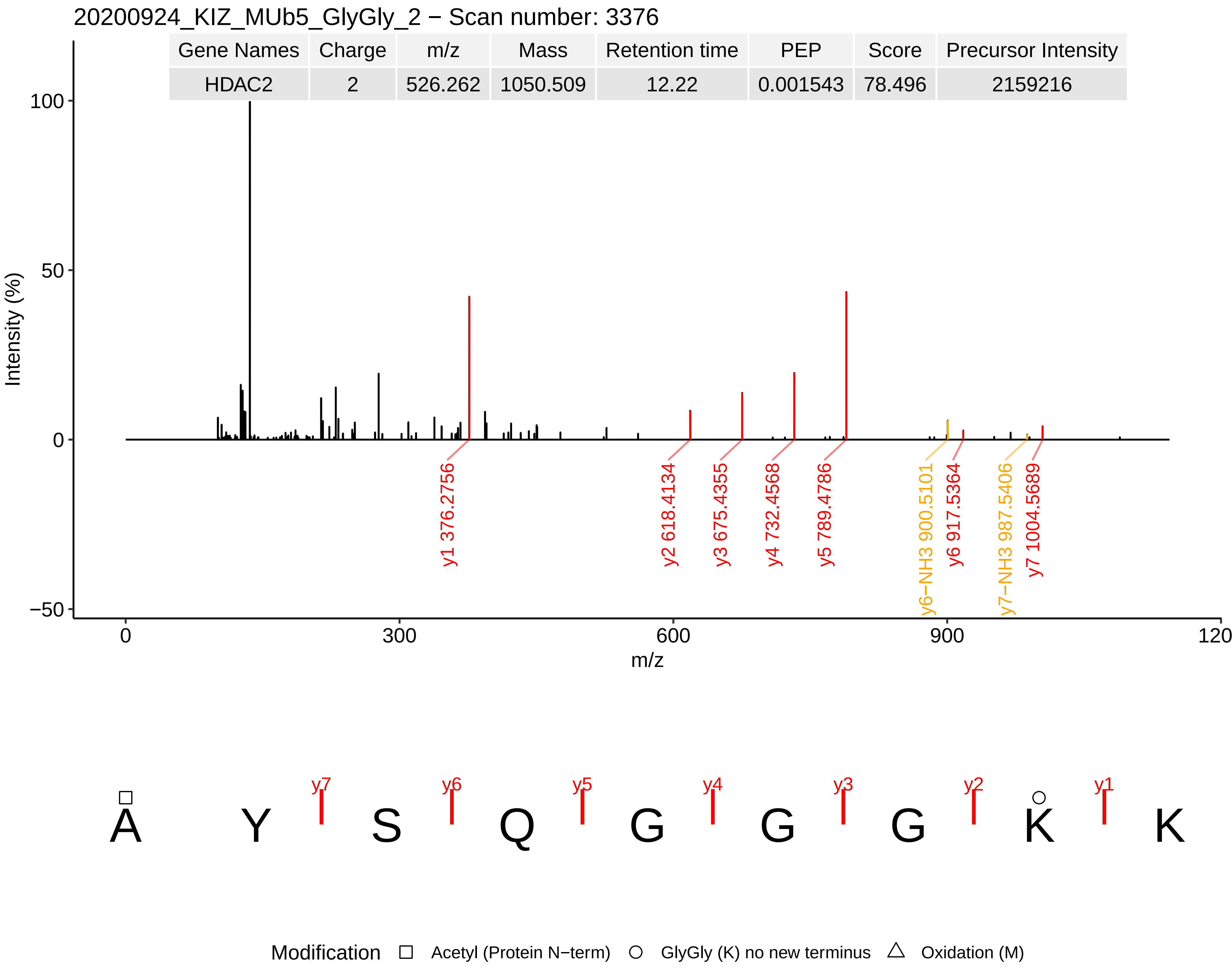

20200924_KIZ_MUb5_GlyGly_2 − Scan number: 3376
Gene Names
Charge
m/z
Mass
Retention time
PEP
Score
Precursor Intensity
HDAC2
2
526.262
1050.509
12.22
0.001543
78.496
2159216
Intensity (%)
m/z
y1 376.2756
y2 618.4134
y3 675.4355
y4 732.4568
y5 789.4786
y6−NH3 900.5101
y6 917.5364
y7−NH3 987.5406
y7 1004.5689
A Y S Q G G G K K
y7
y6
y5
y4
y3
y2
y1
Modification
Acetyl (Protein N−term)
GlyGly (K) no new terminus
Oxidation (M)

20200921_KIZ_MUb4_GlyGly_10plex_2 − Scan number: 5062

Gene Names	Charge	m/z	Mass	Retention time	PEP	Score	Precursor Intensity
HDAC2;HDAC1	3	287.1592	858.4559	18.027	0.030301	63.565	1232198

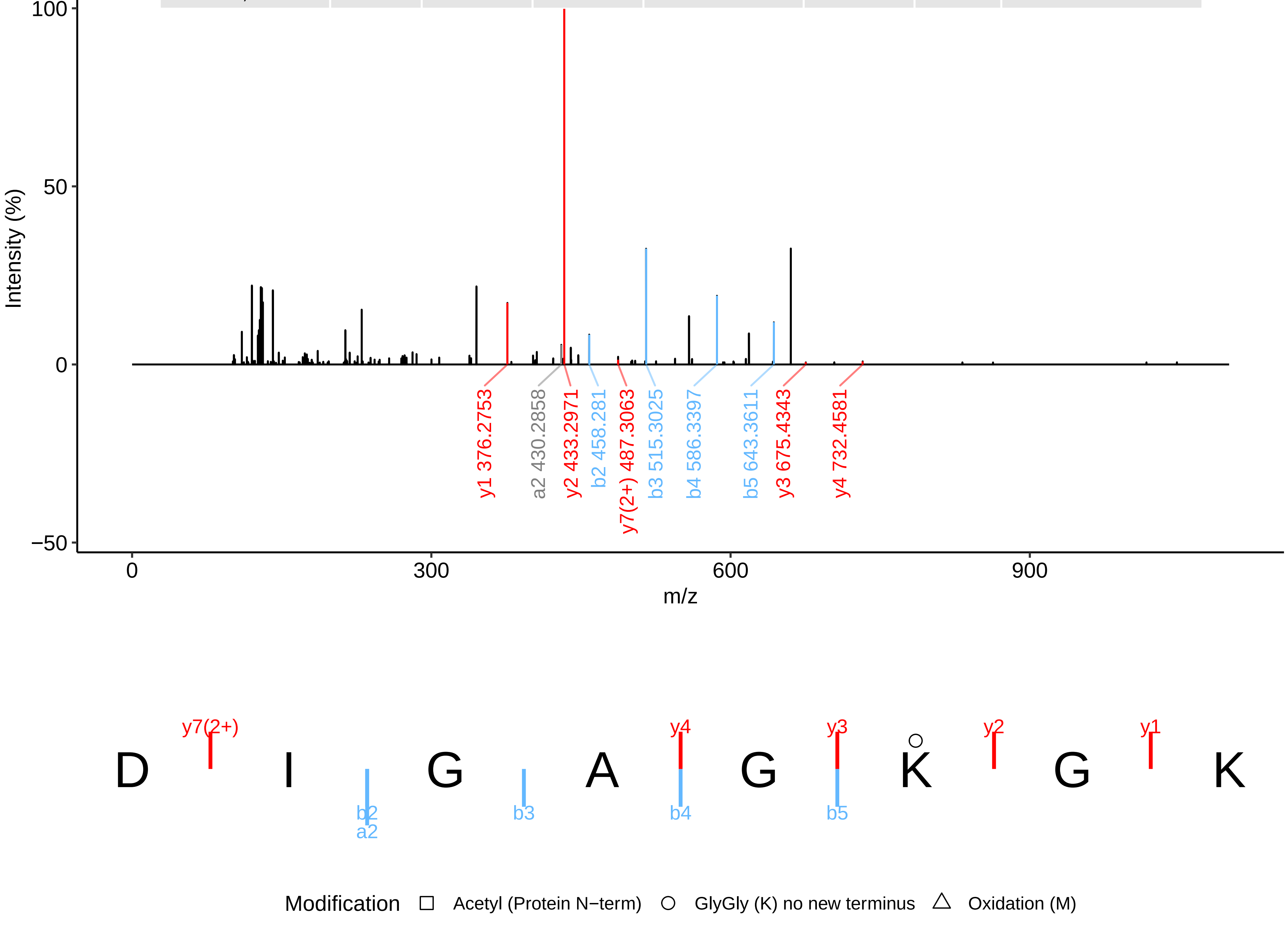

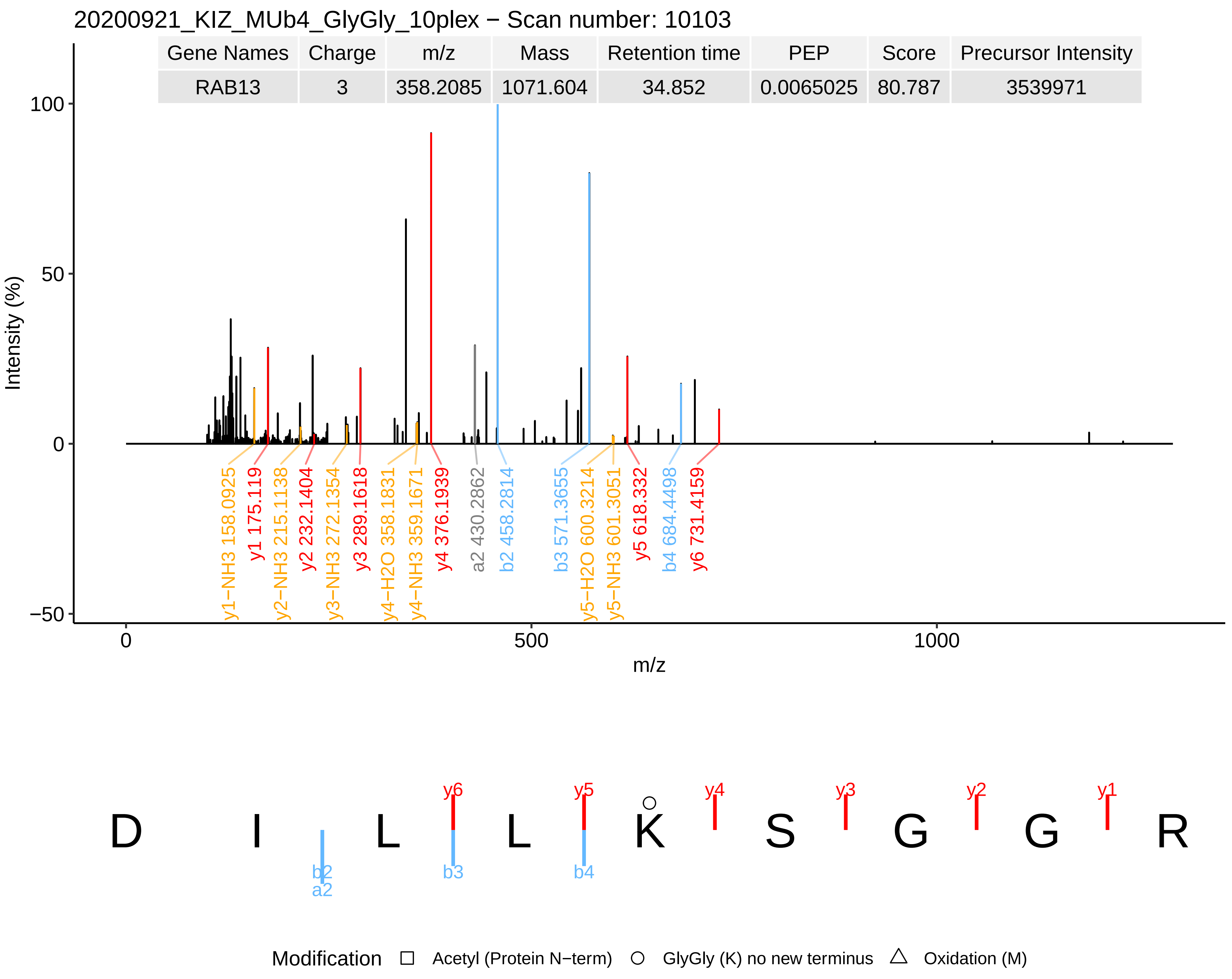

20200921_KIZ_MUb4_GlyGly_10plex − Scan number: 10103
Gene Names | Charge | m/z | Mass | Retention time | PEP | Score | Precursor Intensity
RAB13 | 3 | 358.2085 | 1071.604 | 34.852 | 0.0065025 | 80.787 | 3539971
Intensity (%)
m/z
y1−NH3 158.0925
y1 175.119
y2−NH3 215.1138
y2 232.1404
y3−NH3 272.1354
y3 289.1618
y4−H2O 358.1831
y4−NH3 359.1671
y4 376.1939
a2 430.2862
b2 458.2814
b3 571.3655
y5−H2O 600.3214
y5−NH3 601.3051
y5 618.332
b4 684.4498
y6 731.4159
D I L L K S G G R
Modification
Acetyl (Protein N−term)
GlyGly (K) no new terminus
Oxidation (M)

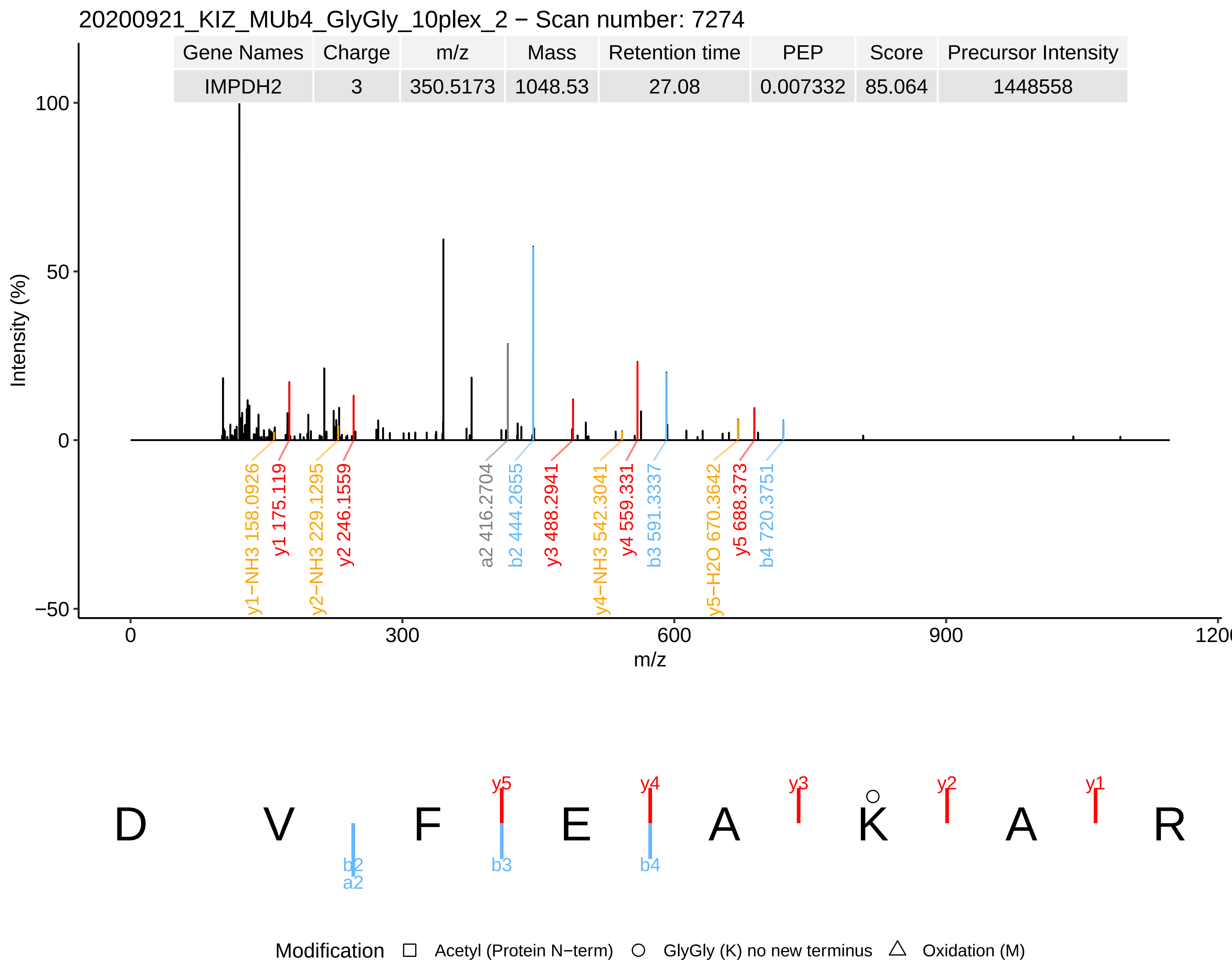

20200921_KIZ_MUb4_GlyGly_10plex_2 − Scan number: 7274
Gene Names | Charge | m/z | Mass | Retention time | PEP | Score | Precursor Intensity
IMPDH2 | 3 | 350.5173 | 1048.53 | 27.08 | 0.007332 | 85.064 | 1448558
Intensity (%)
m/z
y1−NH3 158.0926
y1 175.119
y2−NH3 229.1295
y2 246.1559
a2 416.2704
b2 444.2655
y3 488.2941
y4−NH3 542.3041
y4 559.331
b3 591.3337
y5−H2O 670.3642
y5 688.373
b4 720.3751
D V F E A K A R
y5 y4 y3 y2 y1
b2 a2 b3 b4
Modification
Acetyl (Protein N−term)
GlyGly (K) no new terminus
Oxidation (M)

20200921_KIZ_MUb4_GlyGly_10plex − Scan number: 11323

Gene Names	Charge	m/z	Mass	Retention time	PEP	Score	Precursor Intensity
MCL1	3	353.5296	1057.567	39.391	0.0033344	111.28	8514248

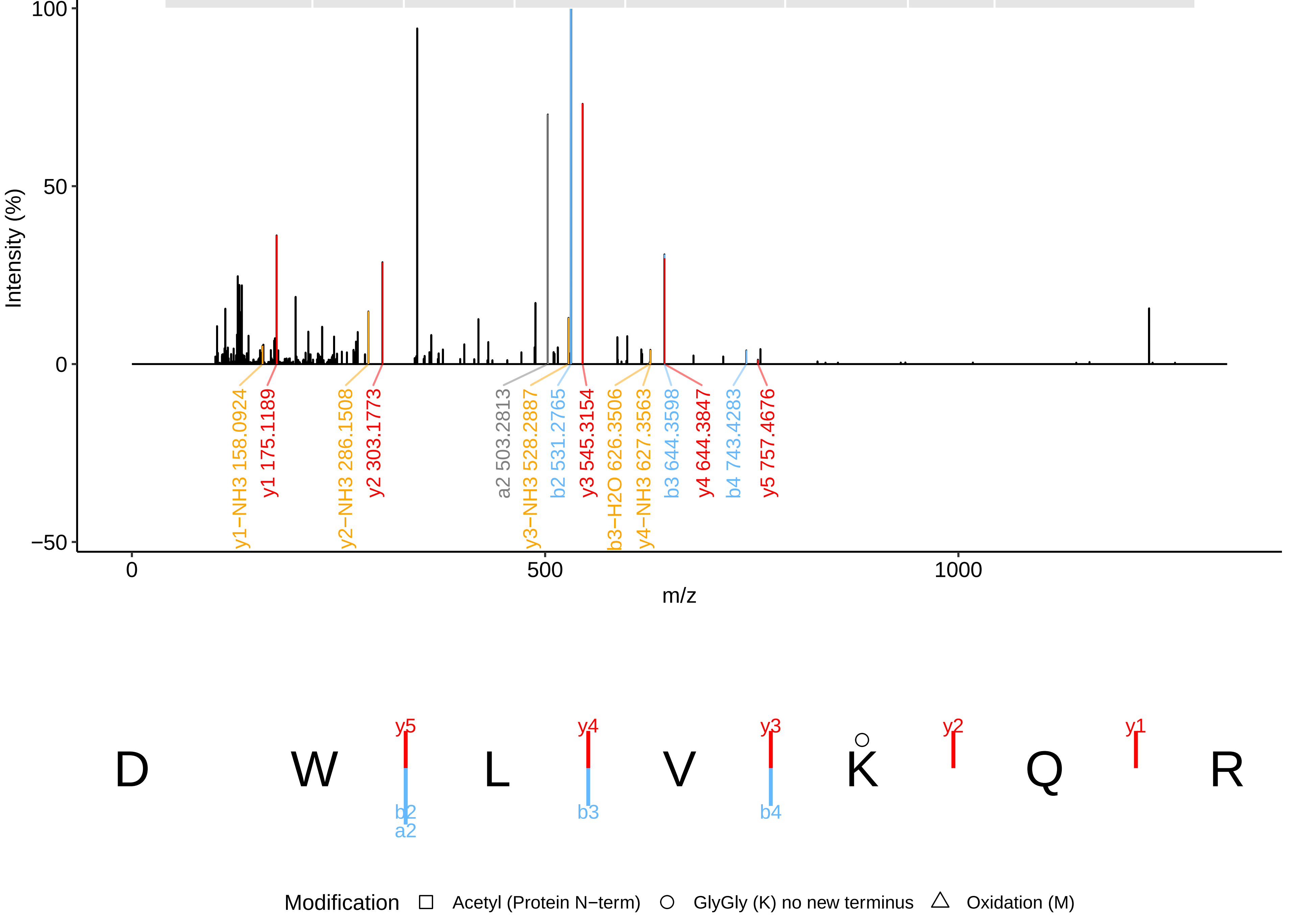

20200921_KIZ_MUb4_GlyGly_10plex_2 − Scan number: 5765

Gene Names	Charge	m/z	Mass	Retention time	PEP	Score	Precursor Intensity
EIF3A	3	362.8682	1085.583	20.883	0.017143	74.127	699298

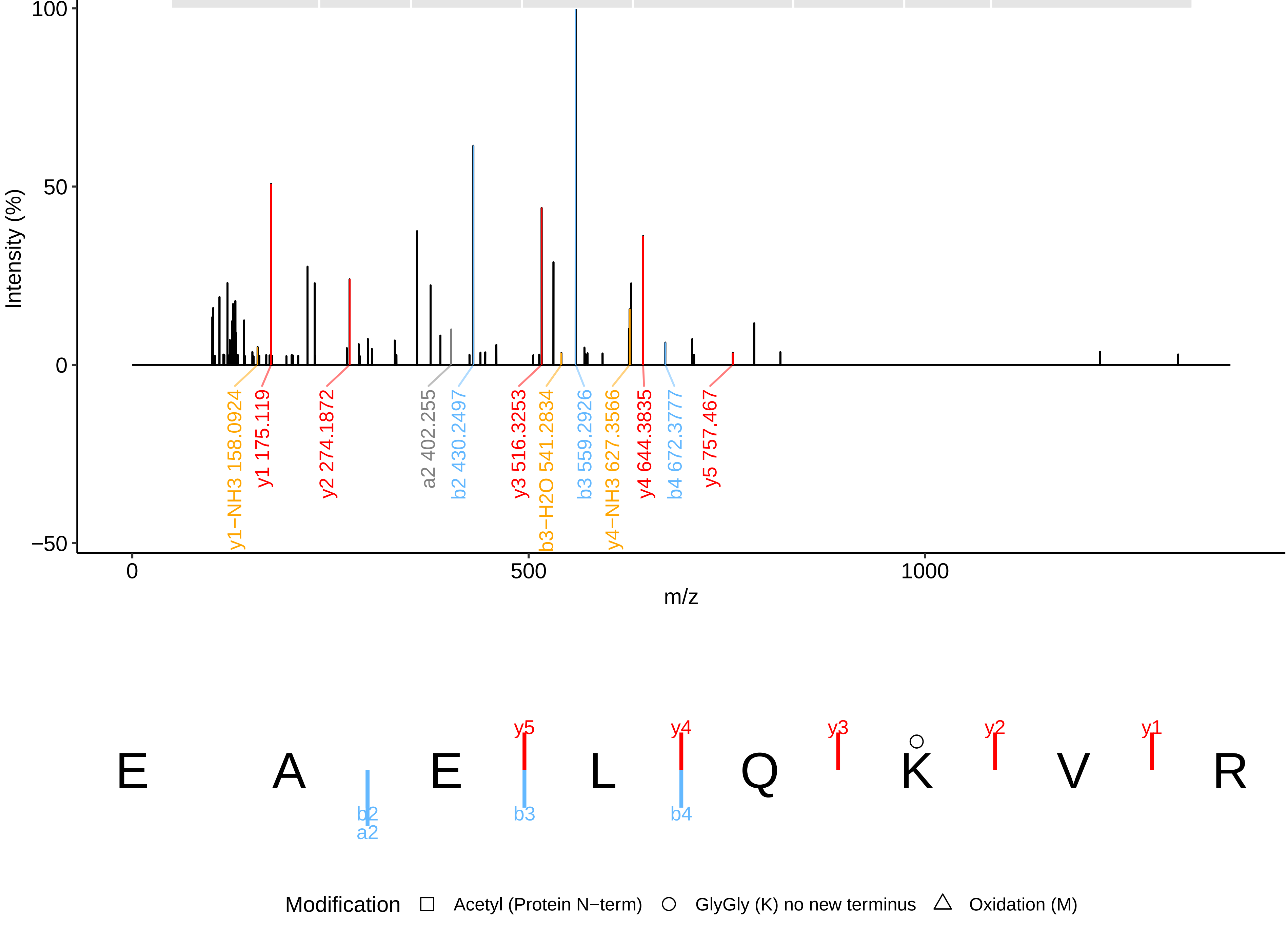

20200921_KIZ_MUb4_GlyGly_10plex − Scan number: 5305

Gene Names	Charge	m/z	Mass	Retention time	PEP	Score	Precursor Intensity
SLC12A6;SLC12A4	3	358.1964	1071.567	17.351	0.0051328	90.64	16800778

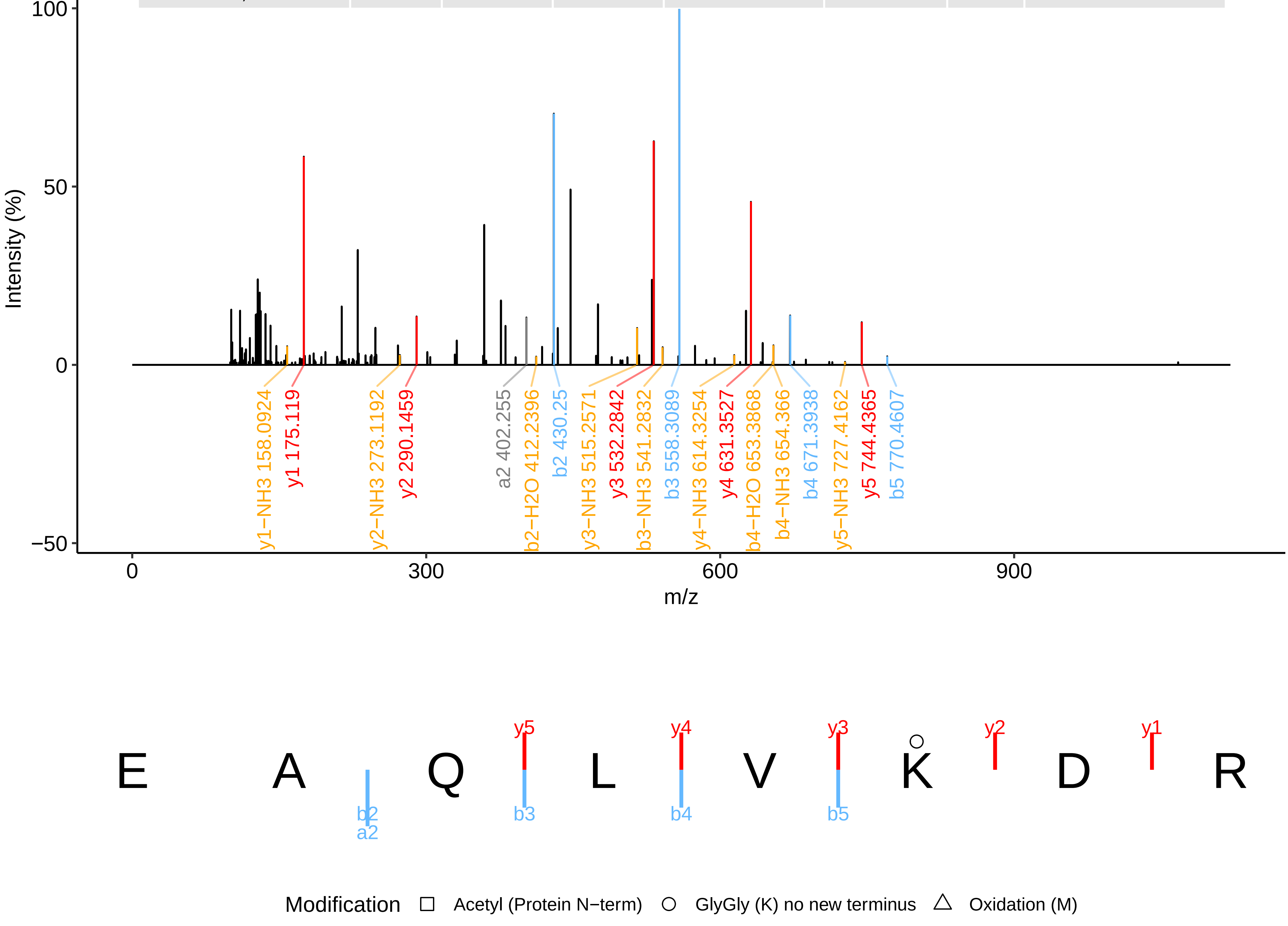

20200921_KIZ_MUb4_GlyGly_10plex_2 − Scan number: 4900

Gene Names	Charge	m/z	Mass	Retention time	PEP	Score	Precursor Intensity
SLC12A6;SLC12A4	3	358.1964	1071.567	17.383	0.050242	58.433	1648602

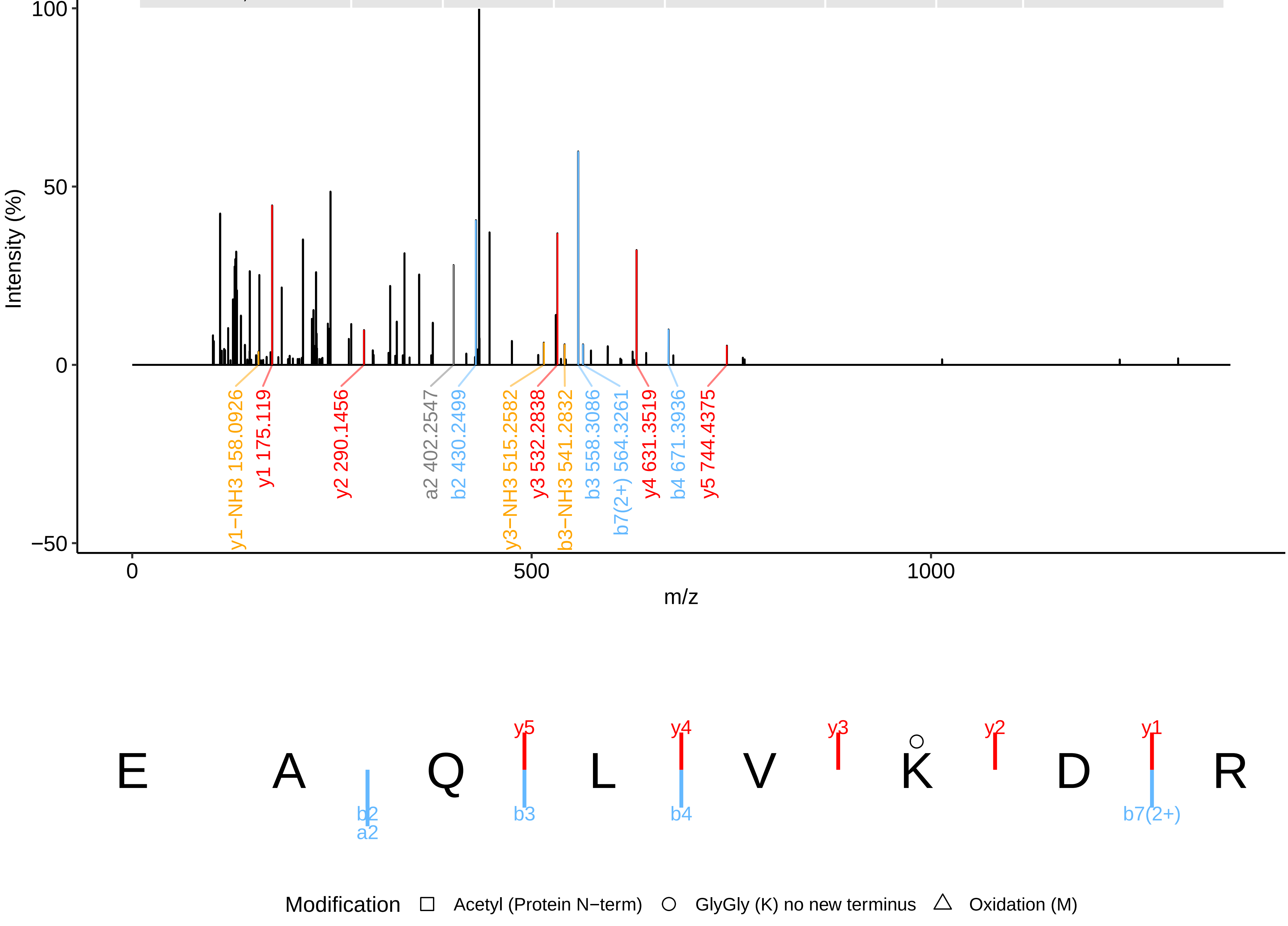

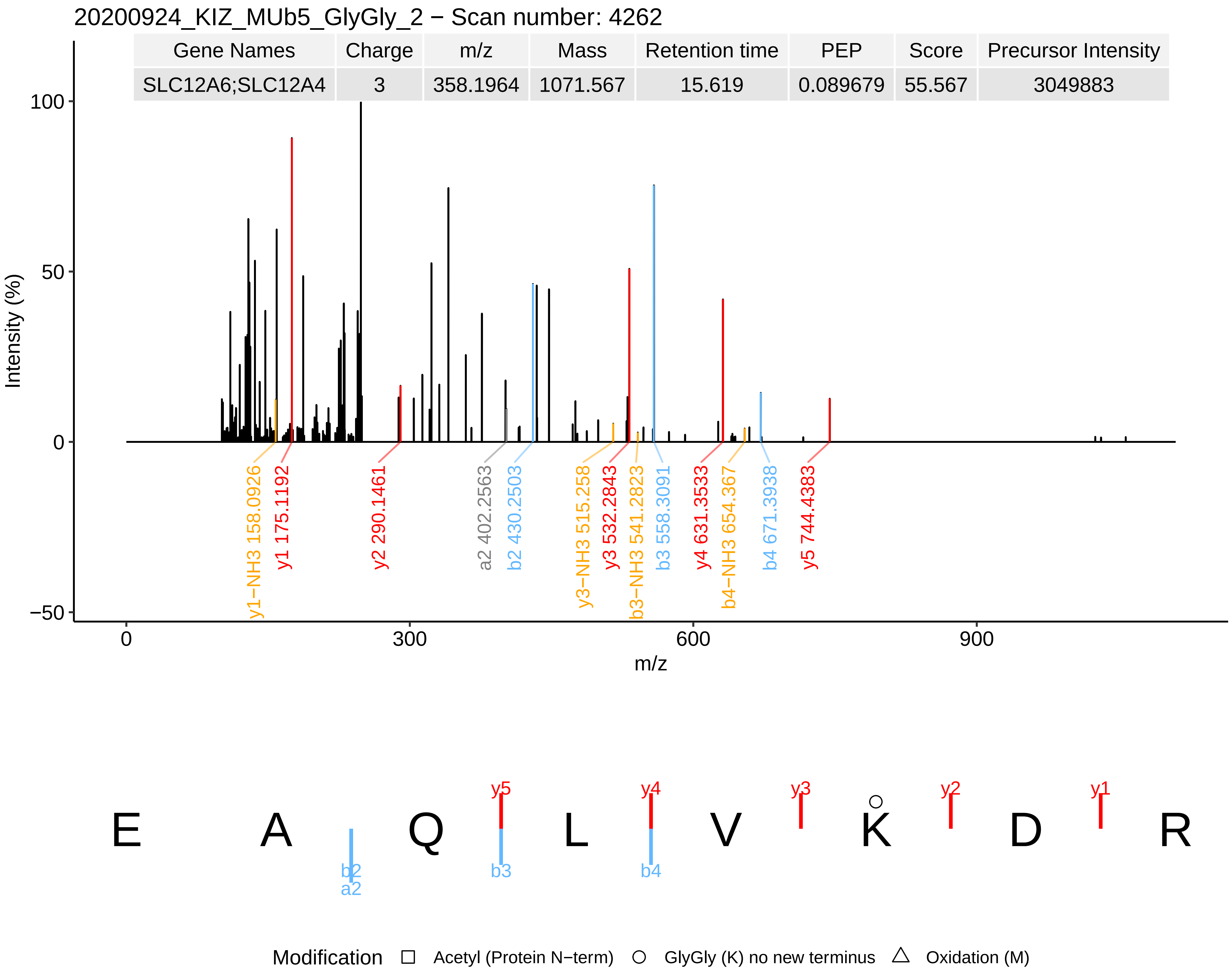

20200924_KIZ_MUb5_GlyGly_2 − Scan number: 4262
Gene Names | Charge | m/z | Mass | Retention time | PEP | Score | Precursor Intensity
SLC12A6;SLC12A4 | 3 | 358.1964 | 1071.567 | 15.619 | 0.089679 | 55.567 | 3049883
Intensity (%)
m/z
y1−NH3 158.0926
y1 175.1192
y2 290.1461
a2 402.2563
b2 430.2503
y3−NH3 515.258
y3 532.2843
b3−NH3 541.2823
b3 558.3091
y4 631.3533
b4−NH3 654.367
b4 671.3938
y5 744.4383
E A Q L V K D R
y5 y4 y3 y2 y1
b2 a2 b3 b4
Modification
Acetyl (Protein N−term)
GlyGly (K) no new terminus
Oxidation (M)

20201118_KIZ_MUb6_GlyGly_1 − Scan number: 4427

Gene Names	Charge	m/z	Mass	Retention time	PEP	Score	Precursor Intensity
SLC12A6;SLC12A4	3	358.1964	1071.567	13.842	0.017882	76.1	5932410

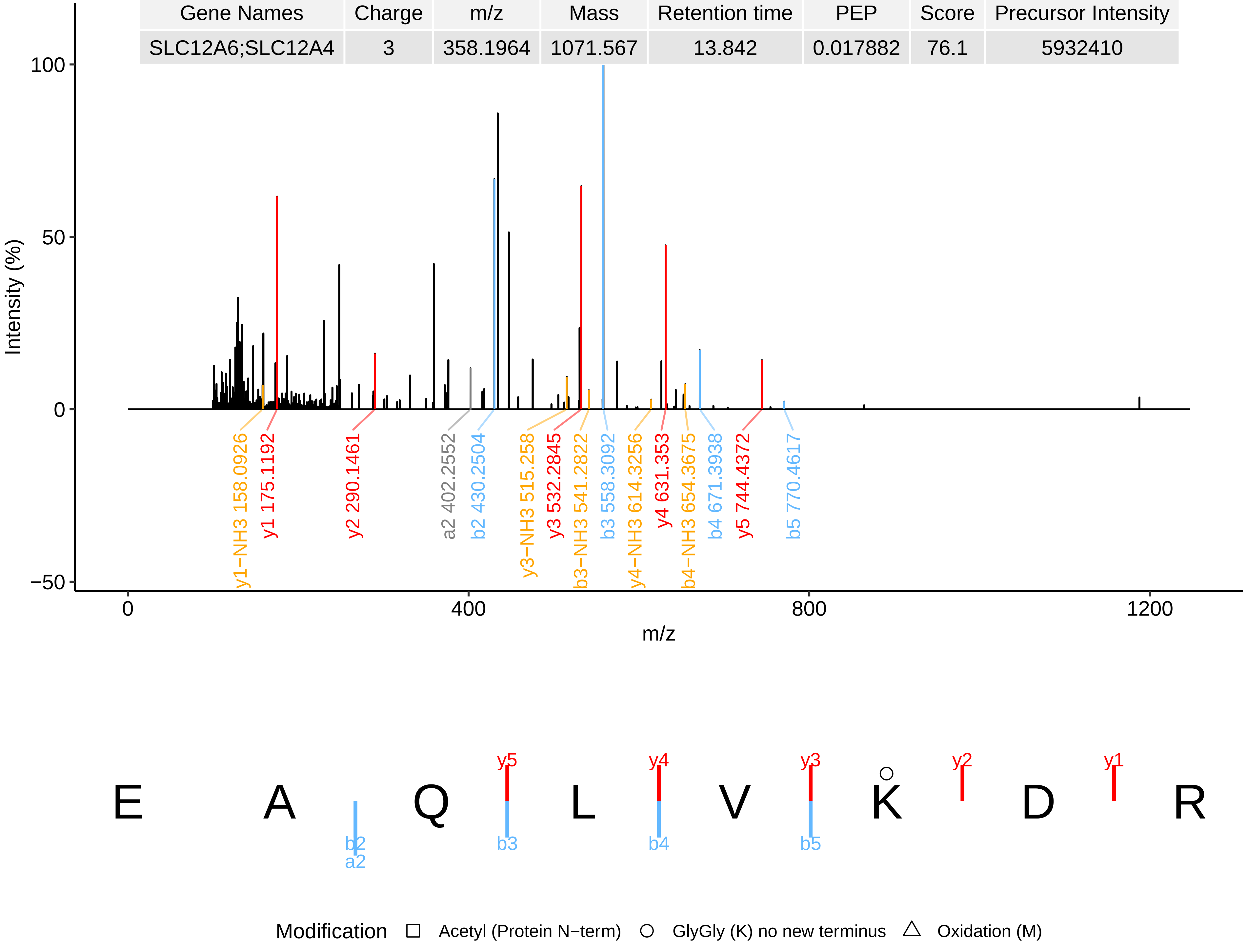

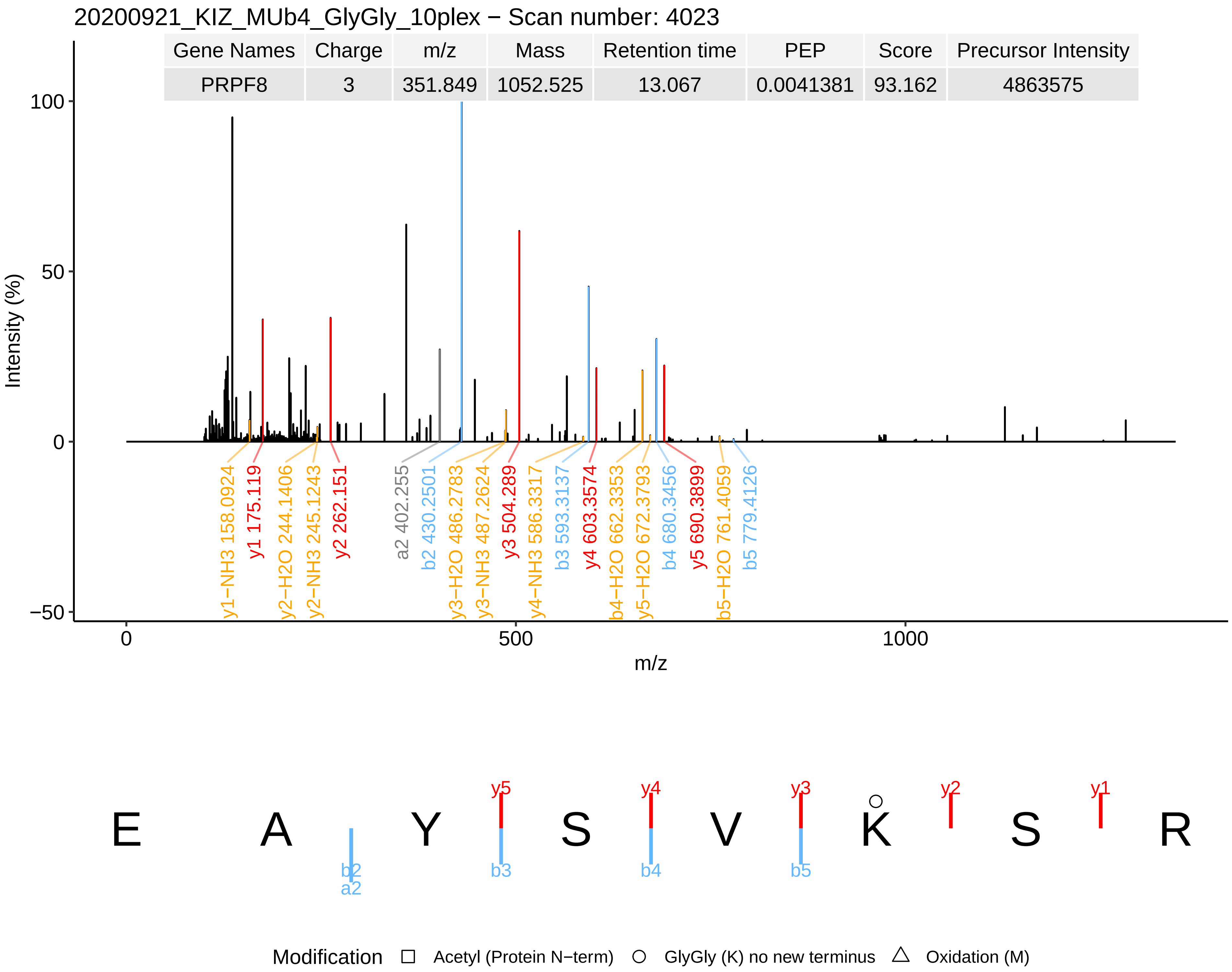

20200921_KIZ_MUb4_GlyGly_10plex − Scan number: 4023
Gene Names | Charge | m/z | Mass | Retention time | PEP | Score | Precursor Intensity
PRPF8 | 3 | 351.849 | 1052.525 | 13.067 | 0.0041381 | 93.162 | 4863575
Intensity (%)
m/z
y1−NH3 158.0924
y1 175.119
y2−H2O 244.1406
y2−NH3 245.1243
y2 262.151
a2 402.255
b2 430.2501
y3−H2O 486.2783
y3−NH3 487.2624
y3 504.289
y4−NH3 586.3317
b3 593.3137
y4 603.3574
b4−H2O 662.3353
y5−H2O 672.3793
b4 680.3456
y5 690.3899
b5−H2O 761.4059
b5 779.4126
E A Y S V K S R
y5 y4 y3 y2 y1
b2 a2 b3 b4 b5
Modification
Acetyl (Protein N−term)
GlyGly (K) no new terminus
Oxidation (M)

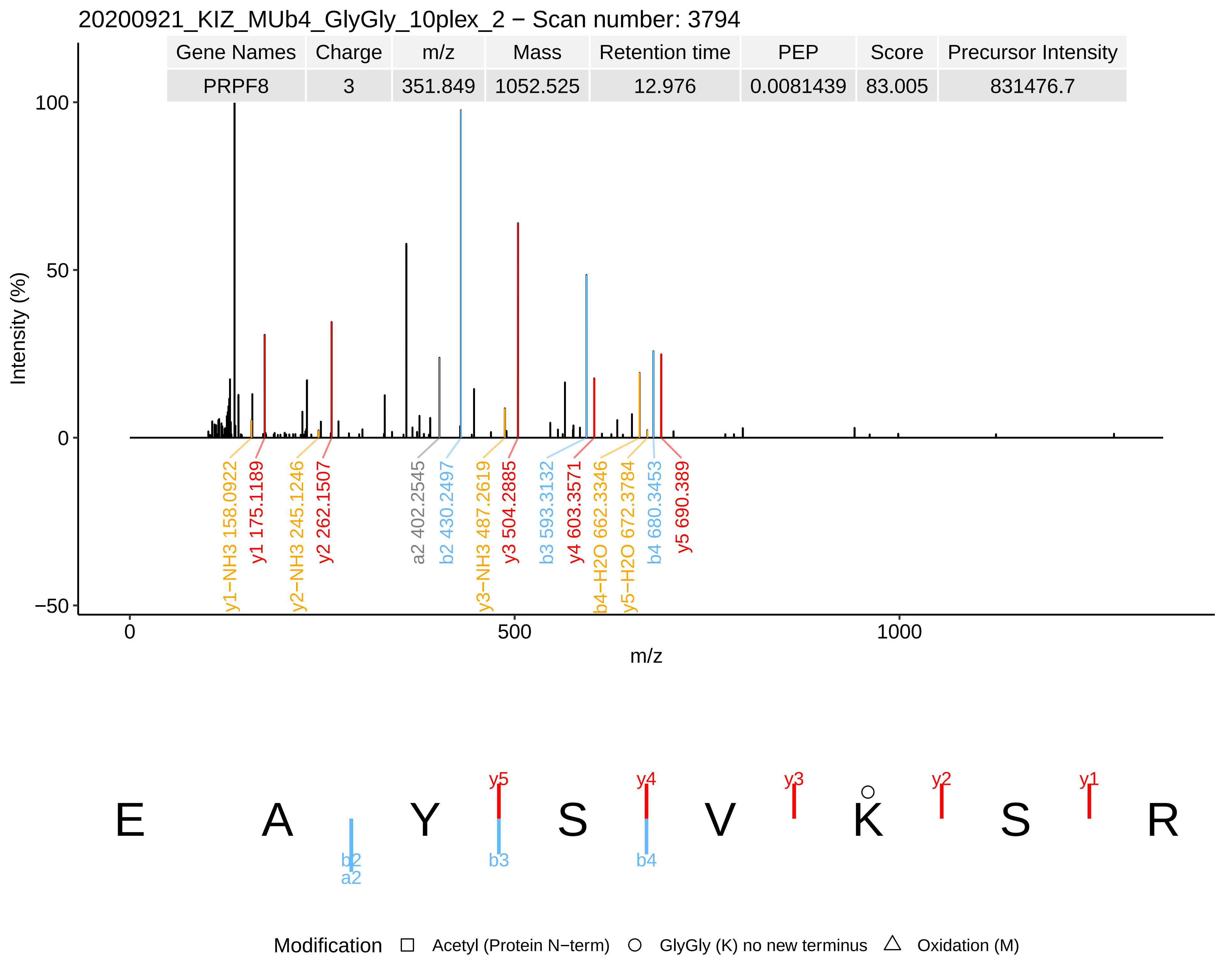
20200921_KIZ_MUb4_GlyGly_10plex_2 − Scan number: 3794
Gene Names | Charge | m/z | Mass | Retention time | PEP | Score | Precursor Intensity
PRPF8 | 3 | 351.849 | 1052.525 | 12.976 | 0.0081439 | 83.005 | 831476.7
Intensity (%)
m/z
y1−NH3 158.0922
y1 175.1189
y2−NH3 245.1246
y2 262.1507
a2 402.2545
b2 430.2497
y3−NH3 487.2619
y3 504.2885
b3 593.3132
y4 603.3571
b4−H2O 662.3346
y5−H2O 672.3784
b4 680.3453
y5 690.389
E A Y S V K S R
y5 y4 y3 y2 y1
b2 a2 b3 b4
Modification
Acetyl (Protein N−term)
GlyGly (K) no new terminus
Oxidation (M)

20200924_KIZ_MUb5_GlyGly_1 − Scan number: 3500

Gene Names	Charge	m/z	Mass	Retention time	PEP	Score	Precursor Intensity
PRPF8	3	351.849	1052.525	10.71	0.0059917	92.062	NA

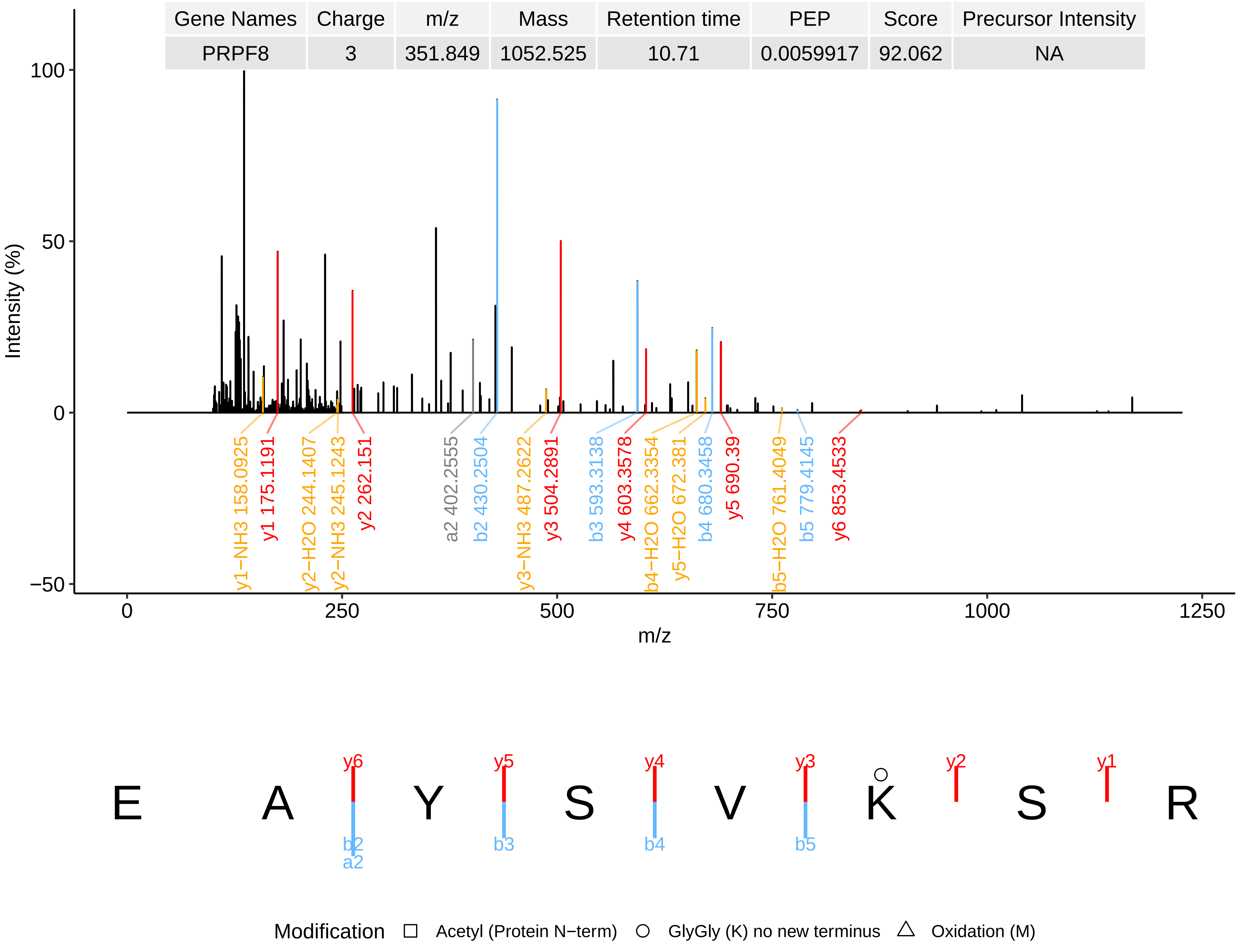

20200921_KIZ_MUb4_GlyGly_10plex − Scan number: 5591

Gene Names	Charge	m/z	Mass	Retention time	PEP	Score	Precursor Intensity
YWHAG	3	362.5403	1084.599	18.341	0.0020675	99.442	5195339

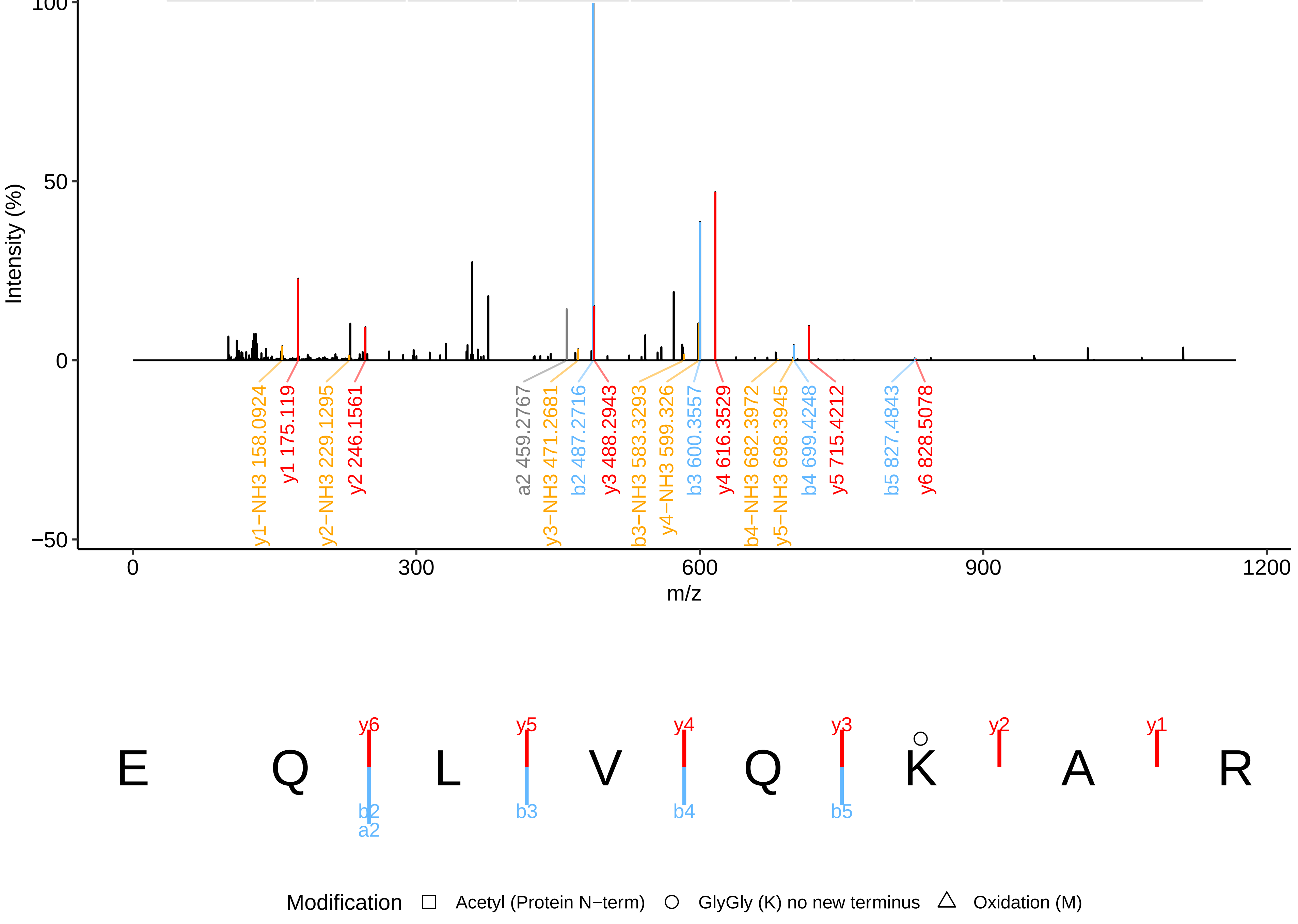

20200921_KIZ_MUb4_GlyGly_10plex_2 − Scan number: 5121

Gene Names	Charge	m/z	Mass	Retention time	PEP	Score	Precursor Intensity
YWHAG	3	362.5403	1084.599	18.268	0.0051264	90.657	1930069

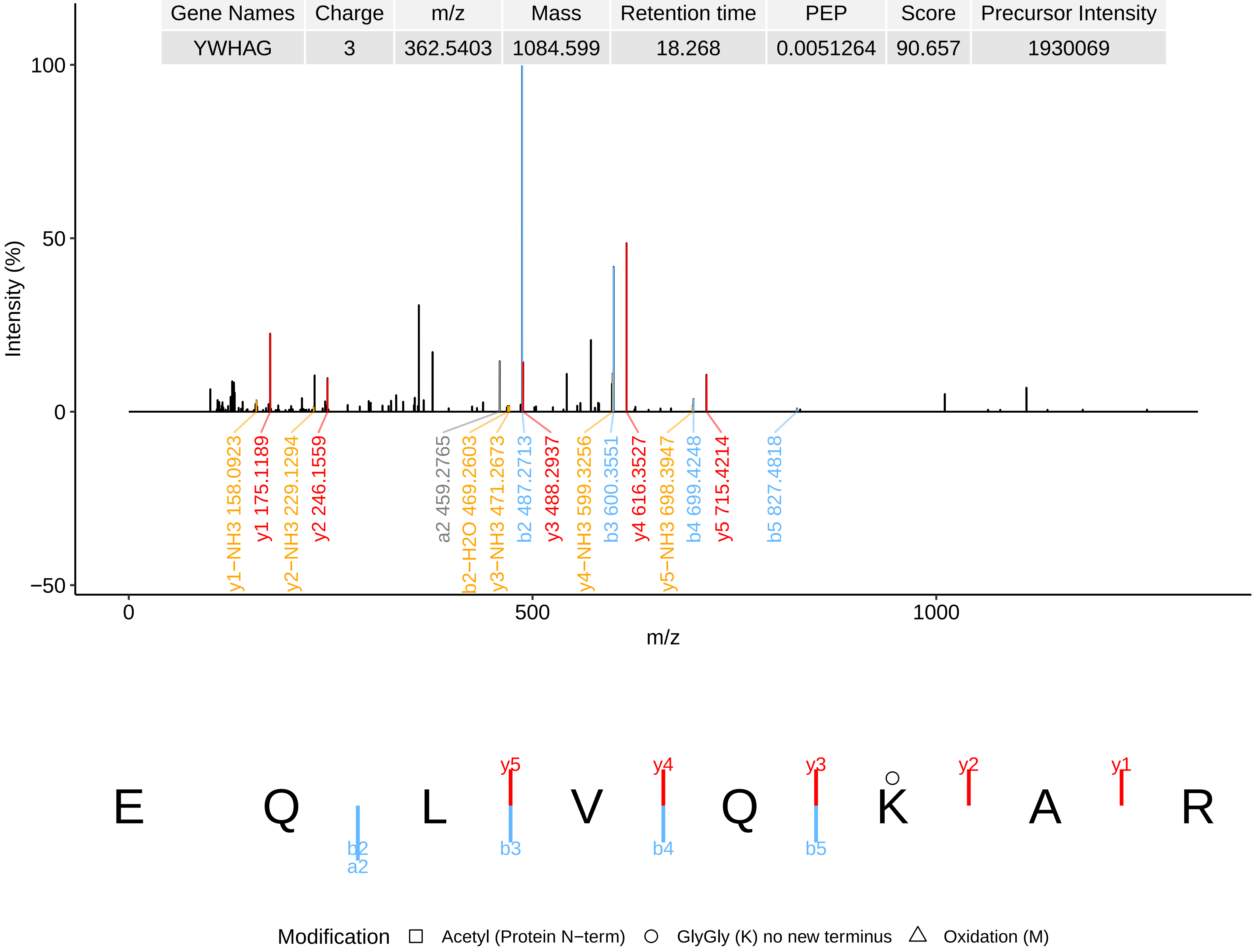

20201118_KIZ_MUb6_GlyGly_2 − Scan number: 2635

Gene Names	Charge	m/z	Mass	Retention time	PEP	Score	Precursor Intensity
ZNF330	3	358.2001	1071.579	8.2888	0.055418	74.415	2536203

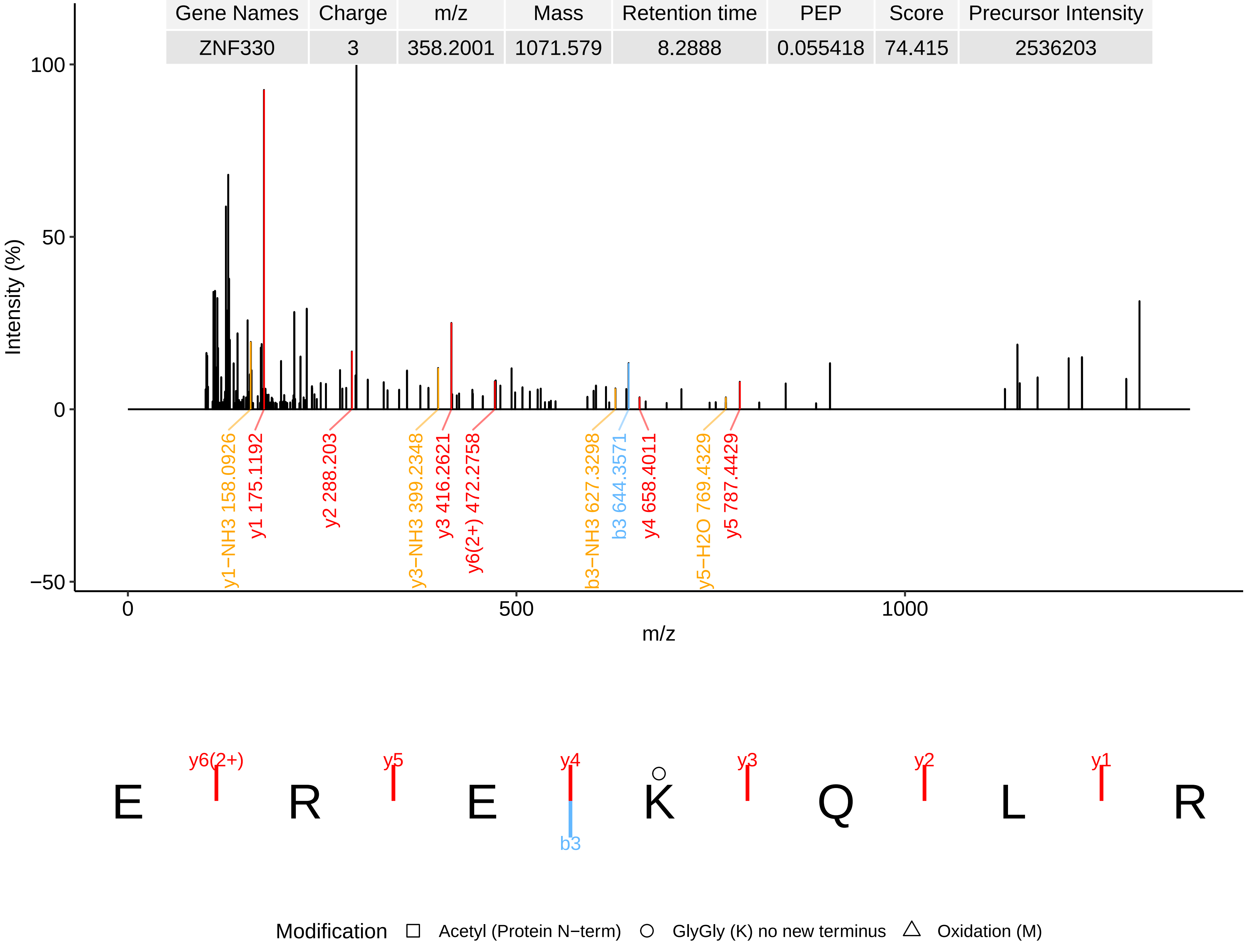

20200921_KIZ_MUb4_GlyGly_10plex − Scan number: 4540

Gene Names	Charge	m/z	Mass	Retention time	PEP	Score	Precursor Intensity
PRKCD;PRKCQ	3	277.1679	828.4818	14.801	0.03584	74.335	25941490

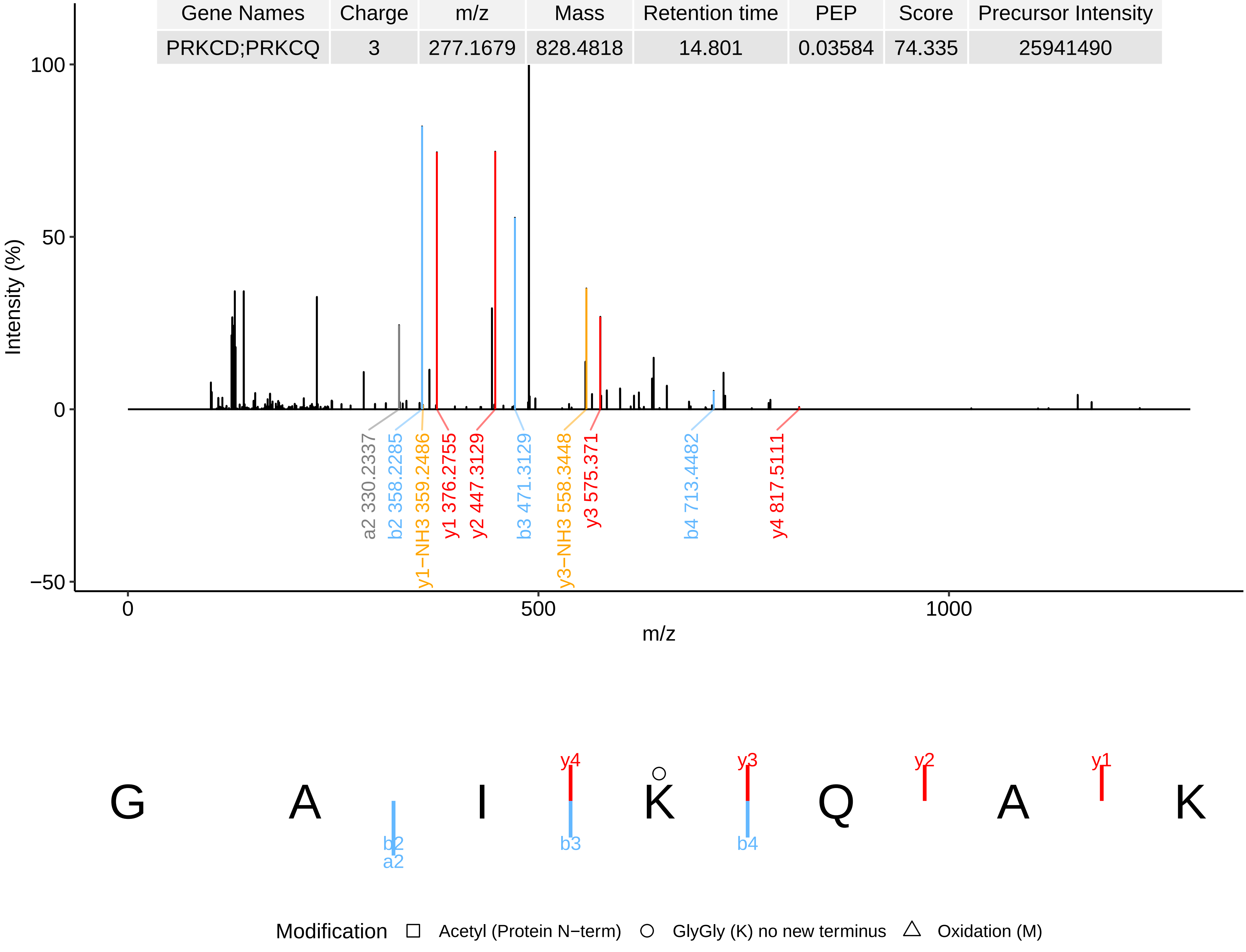

20200921_KIZ_MUb4_GlyGly_10plex − Scan number: 4570

Gene Names	Charge	m/z	Mass	Retention time	PEP	Score	Precursor Intensity
PRKCD;PRKCQ	2	415.2482	828.4818	14.897	0.036406	132.22	3514847

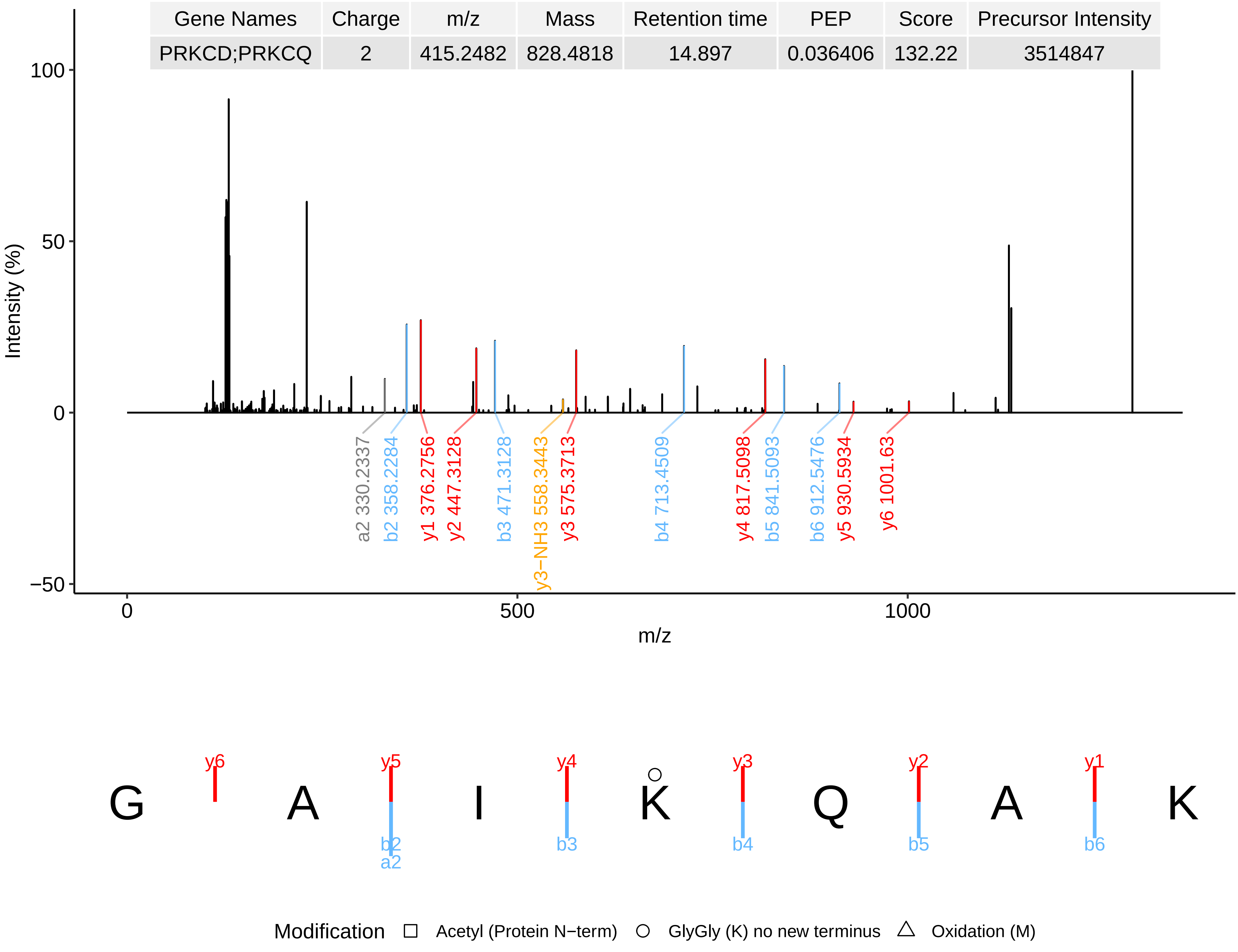

20200921_KIZ_MUb4_GlyGly_10plex_2 − Scan number: 4243

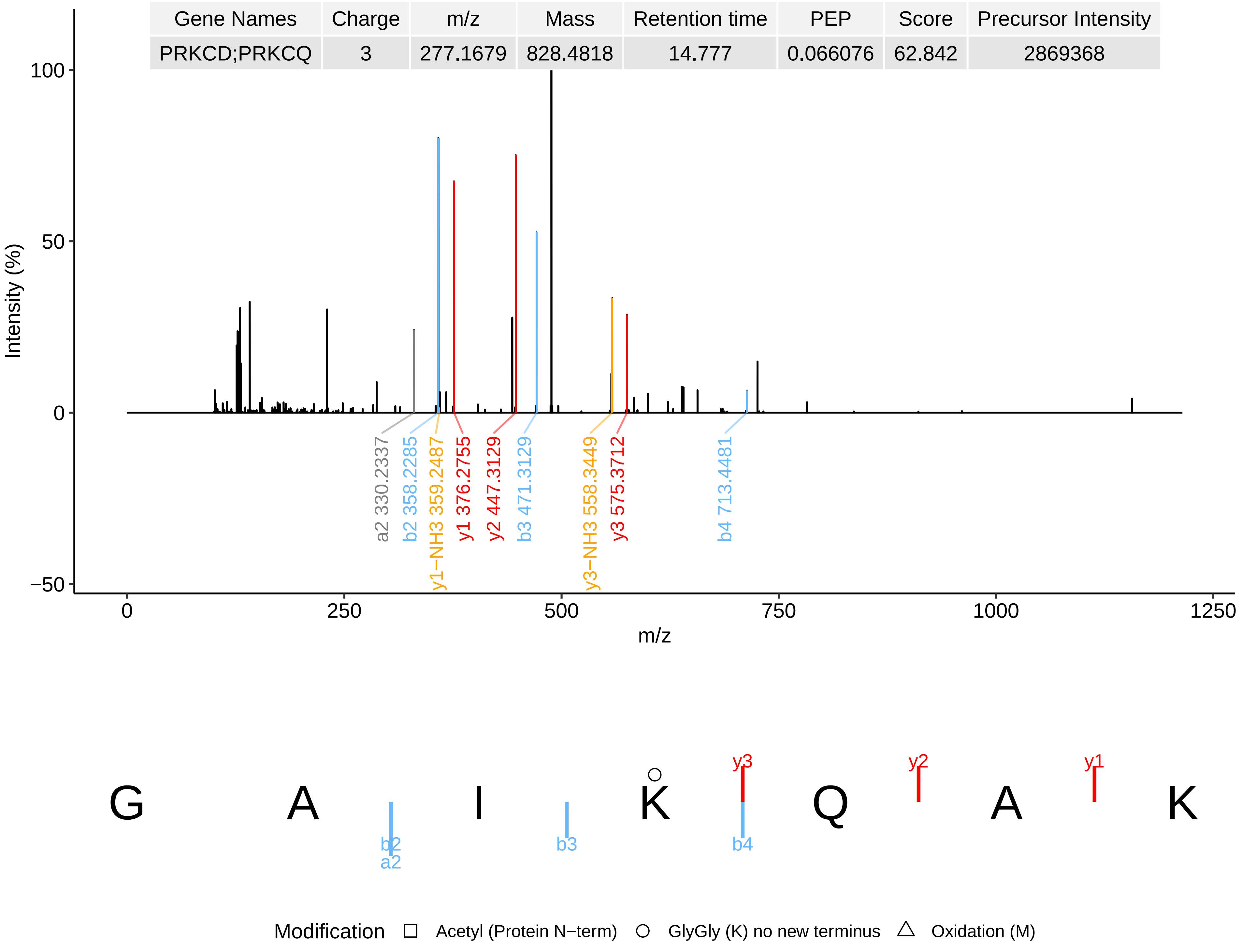

20200921_KIZ_MUb4_GlyGly_10plex_2 − Scan number: 4249

Gene Names	Charge	m/z	Mass	Retention time	PEP	Score	Precursor Intensity
PRKCD;PRKCQ	2	415.2482	828.4818	14.801	0.0073743	116.35	677668.4

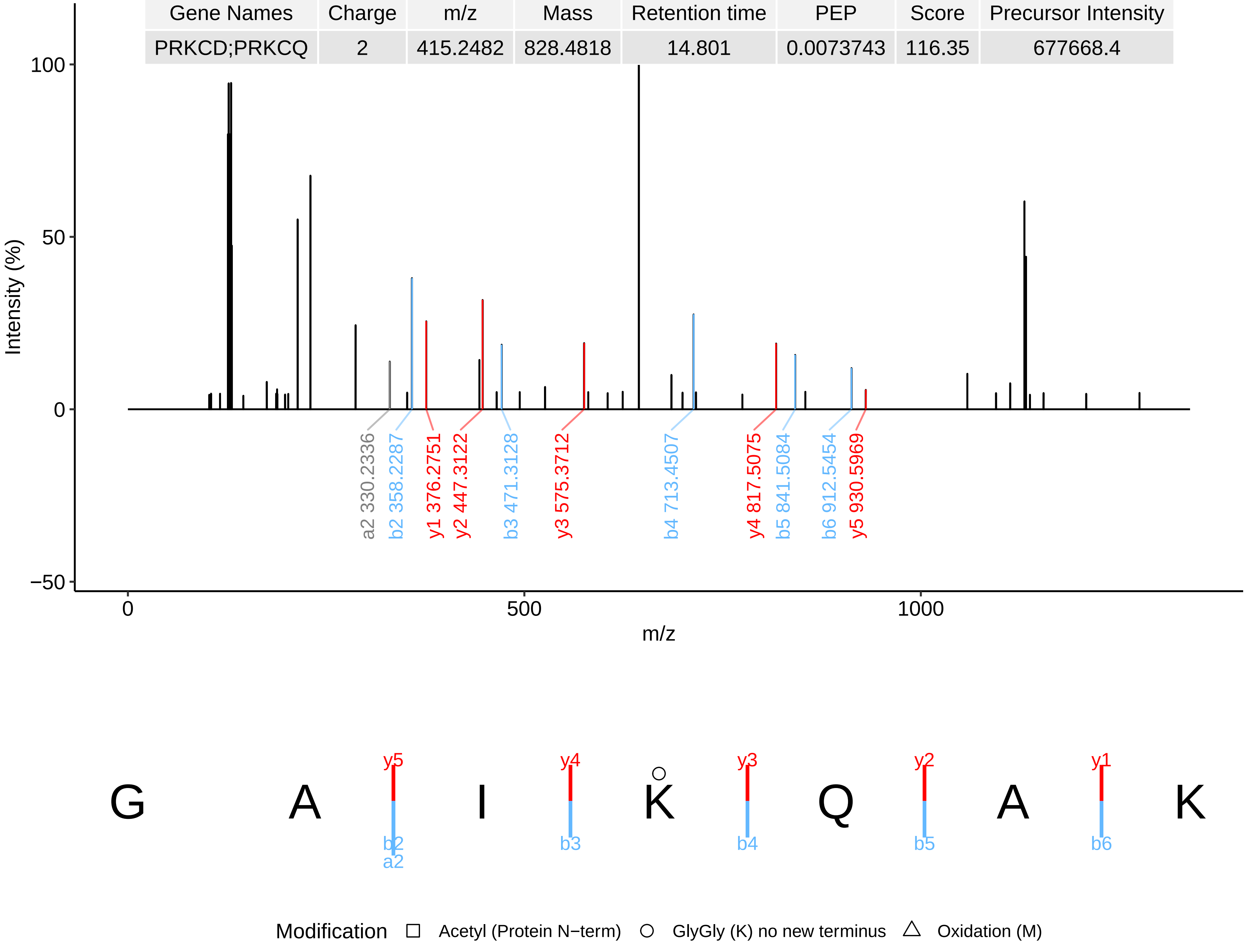

20200924_KIZ_MUb5_GlyGly_1 − Scan number: 4097

Gene Names	Charge	m/z	Mass	Retention time	PEP	Score	Precursor Intensity
PRKCD;PRKCQ	3	277.1679	828.4818	12.584	0.091872	59.641	8422930

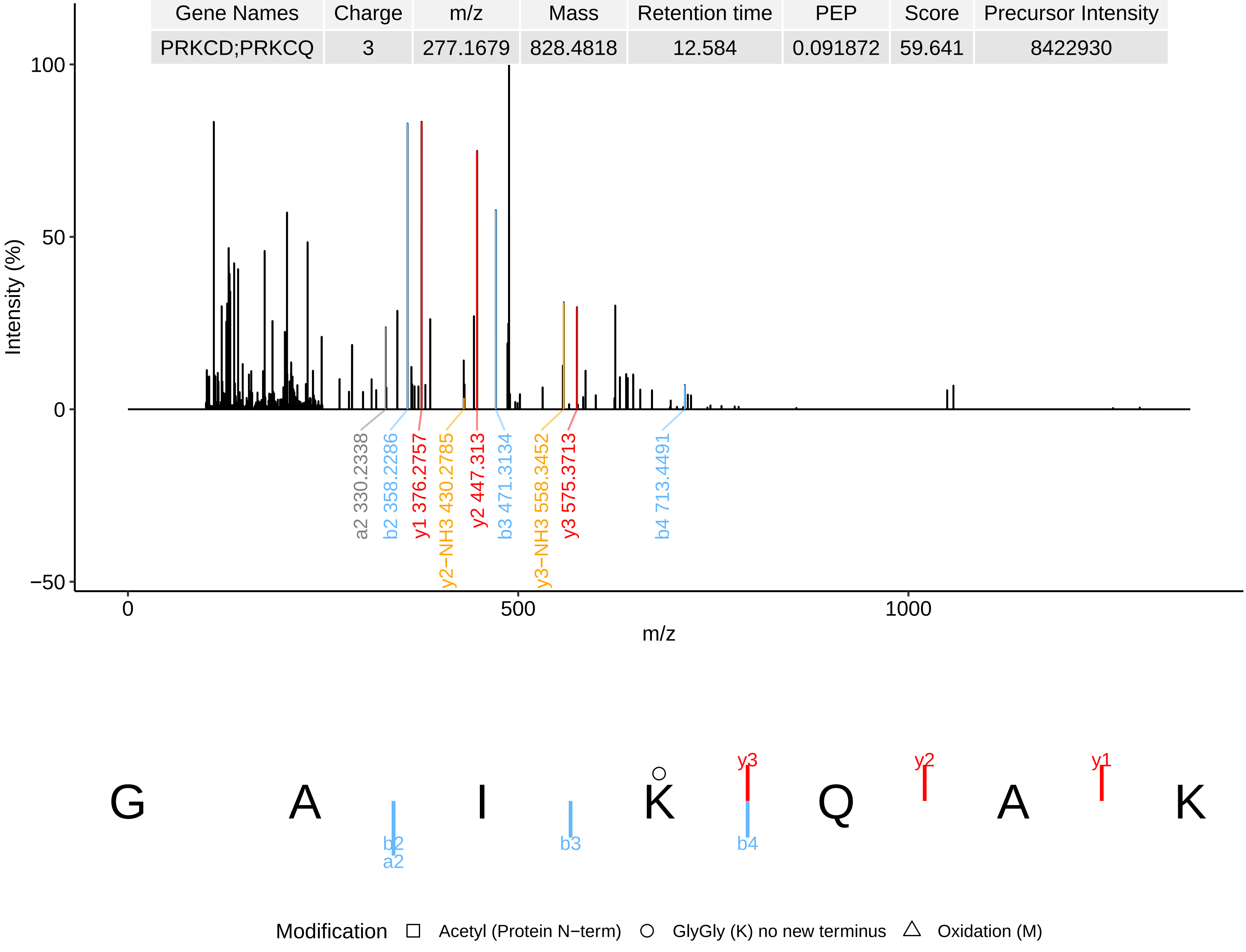

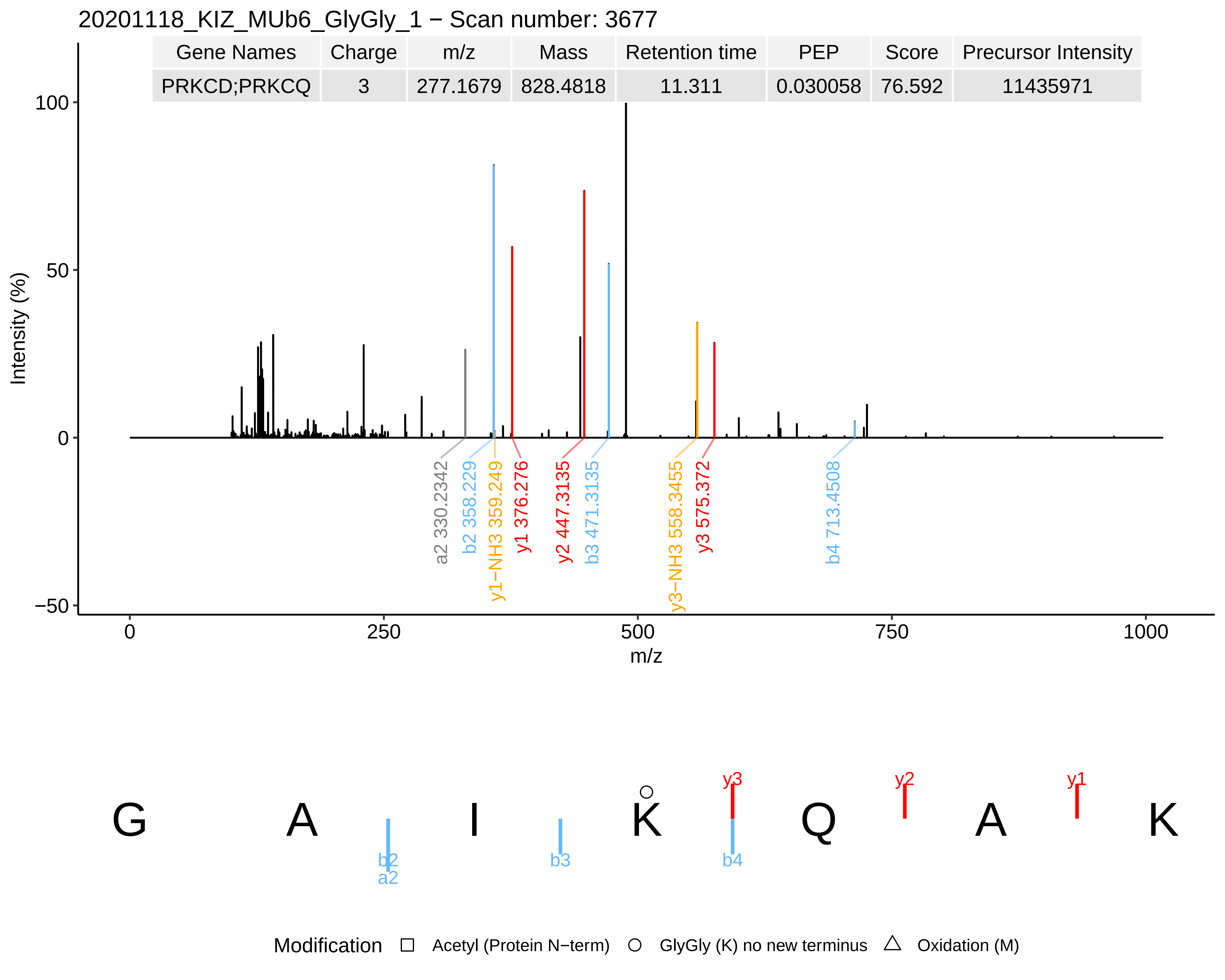

20201118_KIZ_MUb6_GlyGly_1 − Scan number: 3677
Gene Names | Charge | m/z | Mass | Retention time | PEP | Score | Precursor Intensity
PRKCD;PRKCQ | 3 | 277.1679 | 828.4818 | 11.311 | 0.030058 | 76.592 | 11435971
Intensity (%)
m/z
a2 330.2342
b2 358.229
y1−NH3 359.249
y1 376.276
y2 447.3135
b3 471.3135
y3−NH3 558.3455
y3 575.372
b4 713.4508
G A I K Q A K
b2 a2 b3 b4 y3 y2 y1
Modification
Acetyl (Protein N−term)
GlyGly (K) no new terminus
Oxidation (M)

20201118_KIZ_MUb6_GlyGly_2 − Scan number: 4760

Gene Names	Charge	m/z	Mass	Retention time	PEP	Score	Precursor Intensity
PRKCD;PRKCQ	3	277.1679	828.4818	16.353	0.054652	66.994	6908427

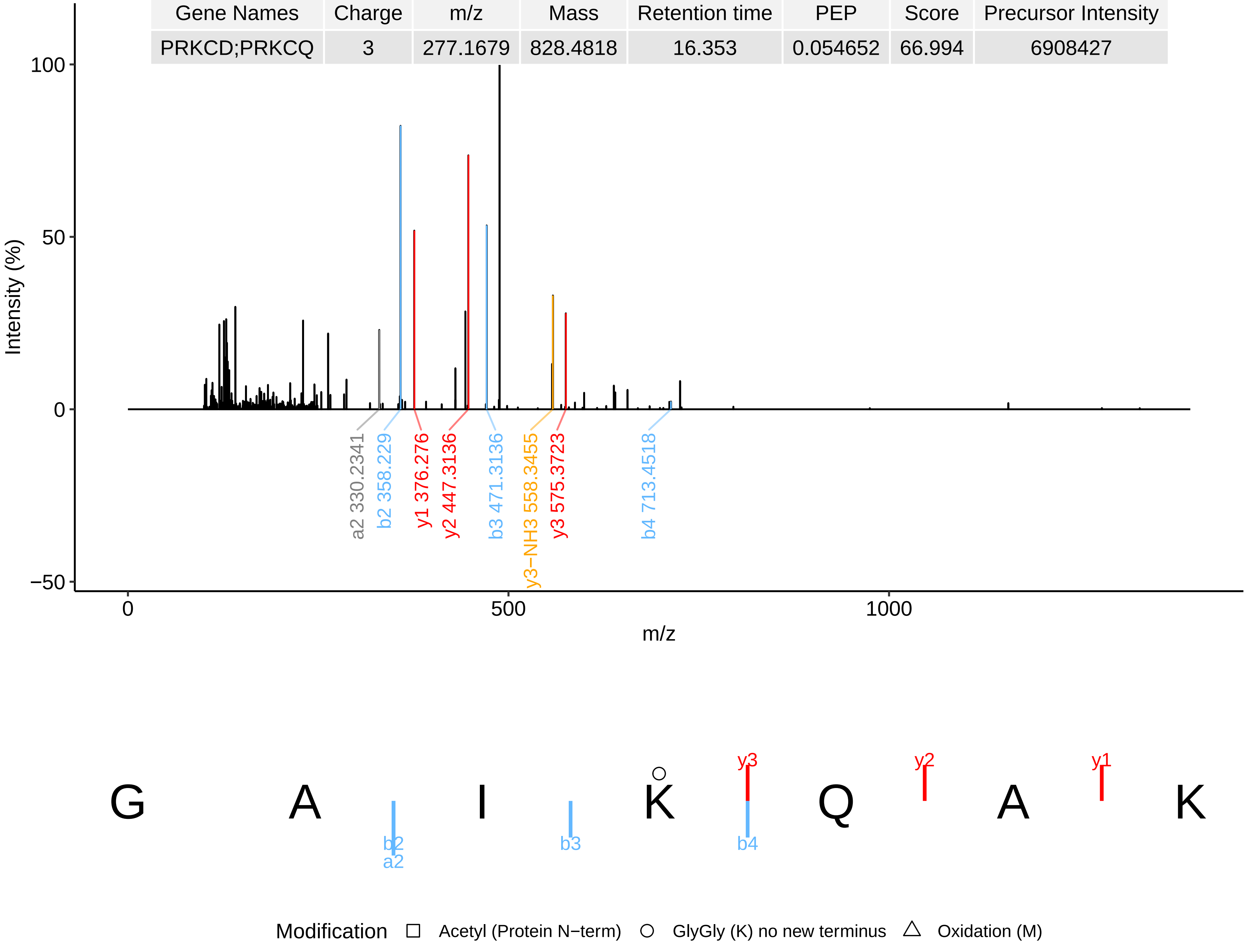

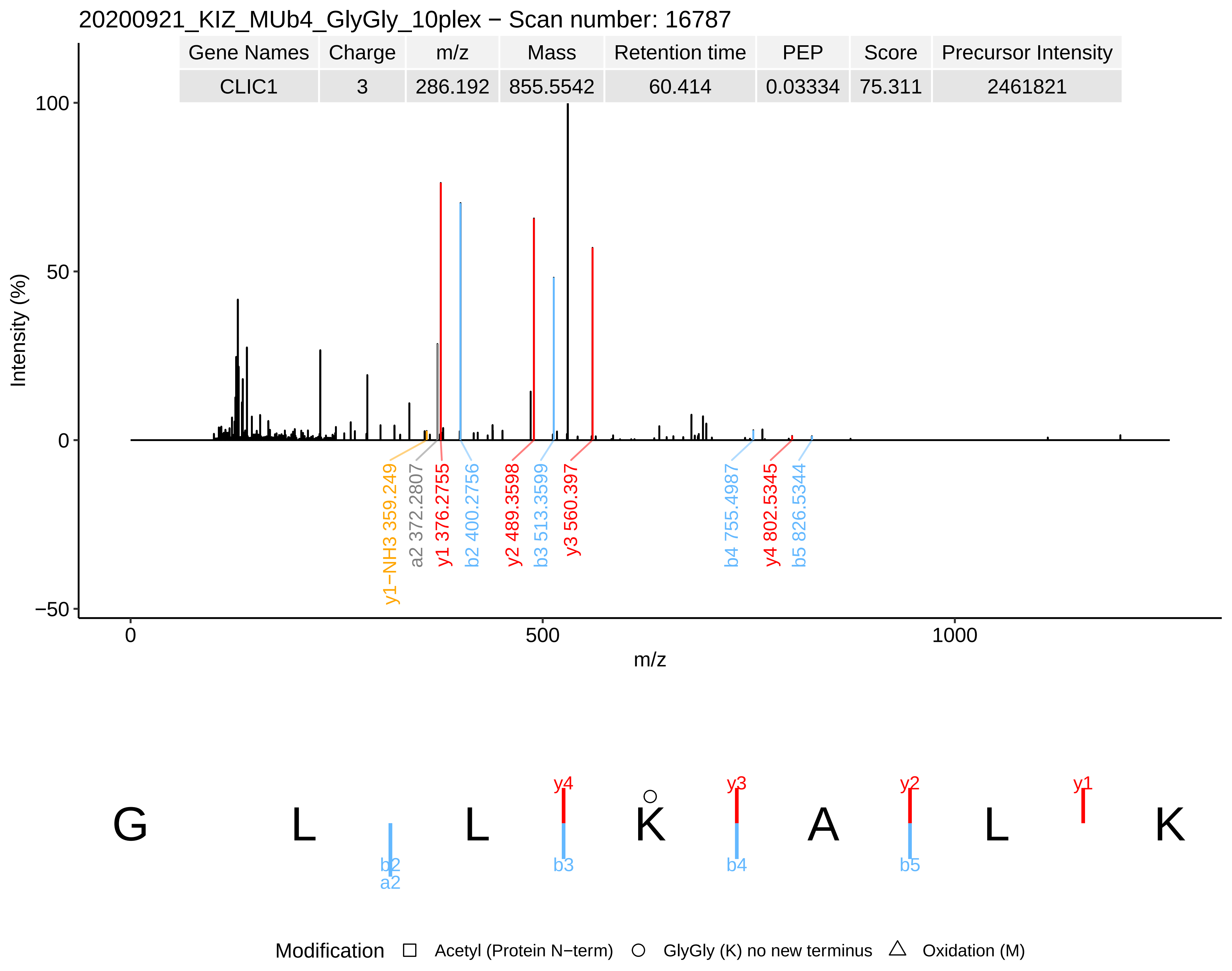

20200921_KIZ_MUb4_GlyGly_10plex − Scan number: 16787
Gene Names | Charge | m/z | Mass | Retention time | PEP | Score | Precursor Intensity
CLIC1 | 3 | 286.192 | 855.5542 | 60.414 | 0.03334 | 75.311 | 2461821
Intensity (%)
100
50
0
−50
y1−NH3 359.249
a2 372.2807
y1 376.2755
b2 400.2756
y2 489.3598
b3 513.3599
y3 560.397
b4 755.4987
y4 802.5345
b5 826.5344
0
500
1000
m/z
G L L K A L K
y4 y3 y2 y1
b2 a2 b3 b4 b5
Modification
Acetyl (Protein N−term)
GlyGly (K) no new terminus
Oxidation (M)

20200921_KIZ_MUb4_GlyGly_10plex − Scan number: 6658

Gene Names	Charge	m/z	Mass	Retention time	PEP	Score	Precursor Intensity
ATP9A	3	362.8804	1085.619	22.118	0.0035147	80.676	3806552

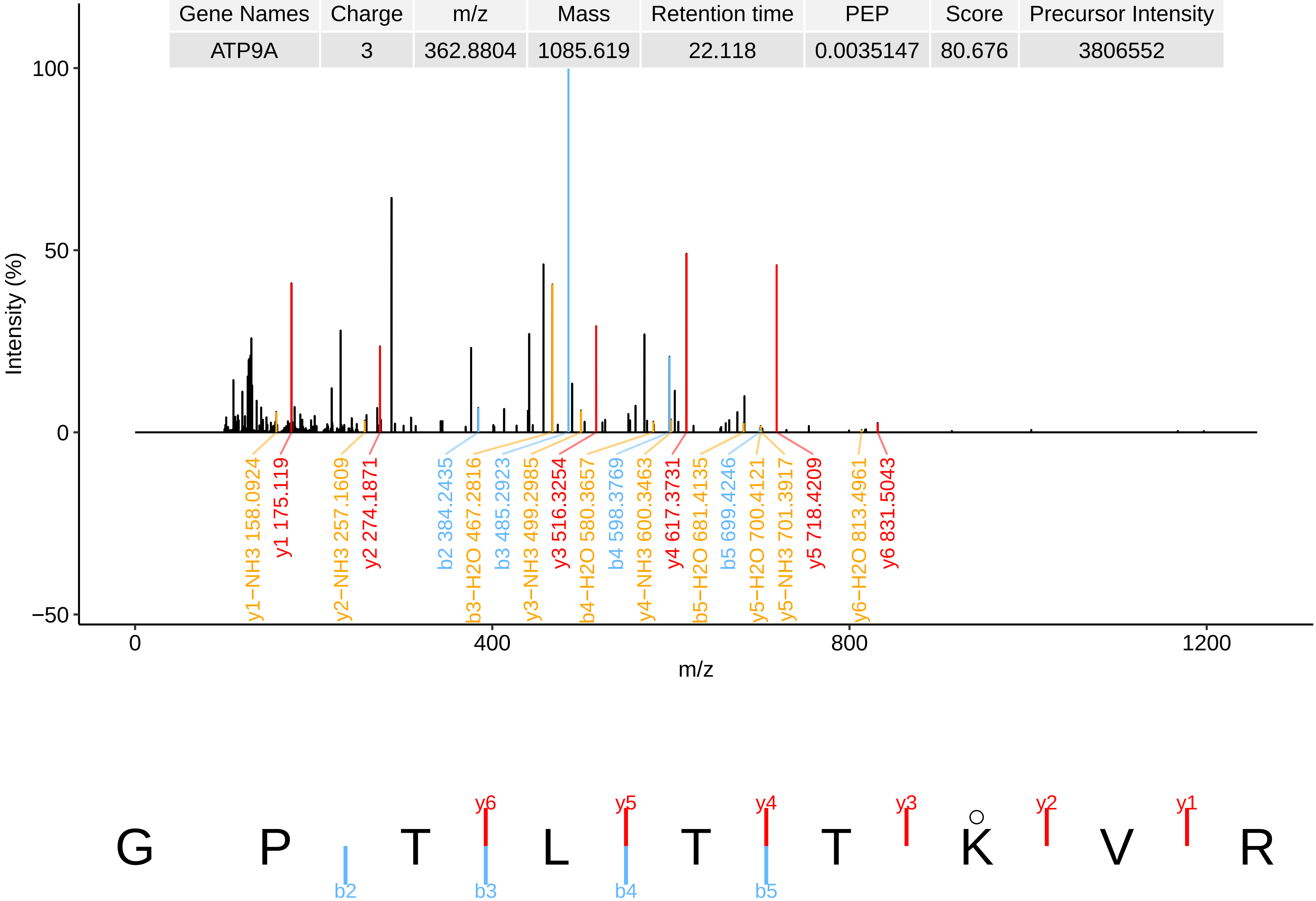

Modification □ Acetyl (Protein N−term) ○ GlyGly (K) no new terminus △ Oxidation (M)

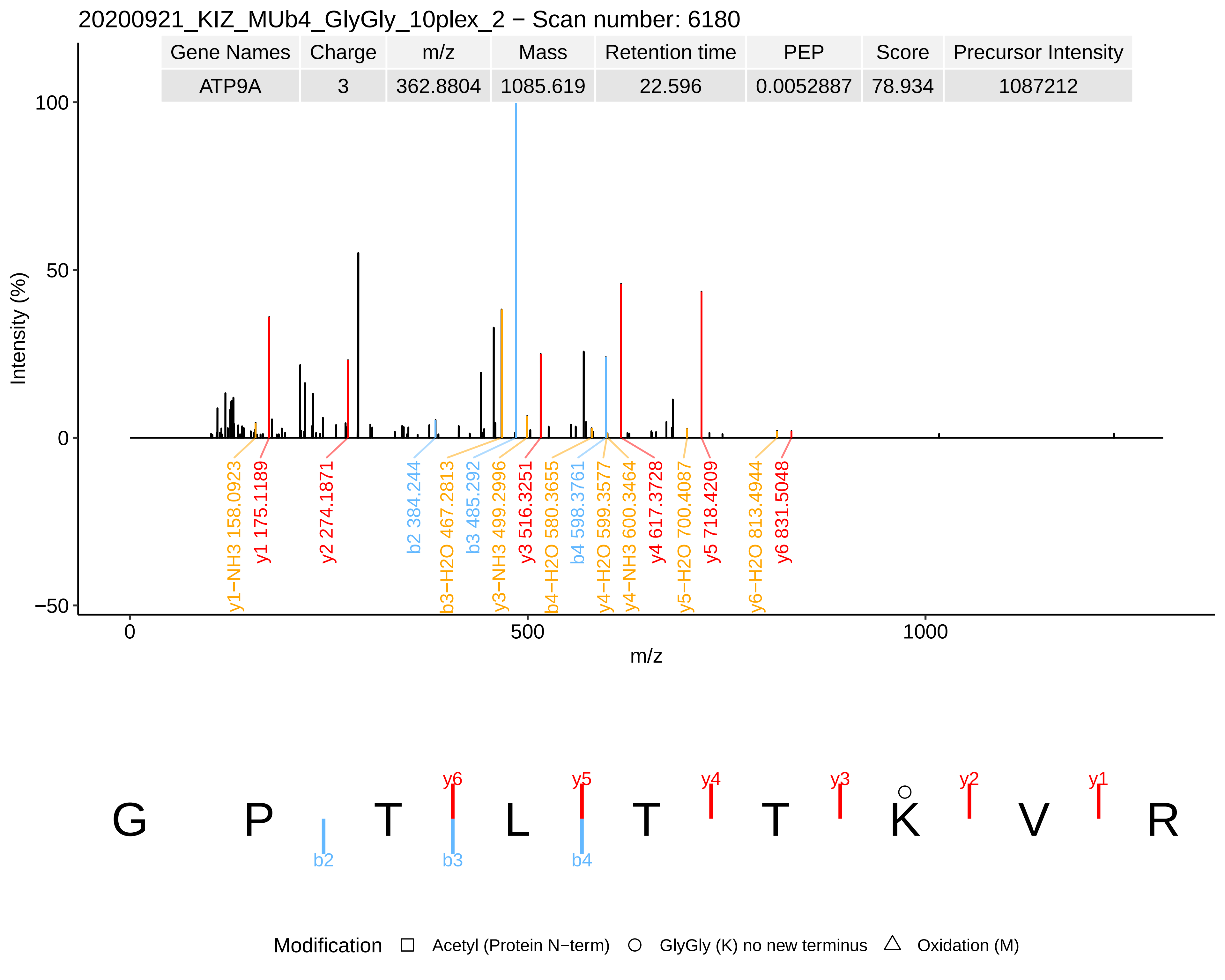

20200921_KIZ_MUb4_GlyGly_10plex_2 − Scan number: 6180
Gene Names | Charge | m/z | Mass | Retention time | PEP | Score | Precursor Intensity
ATP9A | 3 | 362.8804 | 1085.619 | 22.596 | 0.0052887 | 78.934 | 1087212
Intensity (%)
m/z
y1−NH3 158.0923
y1 175.1189
y2 274.1871
b2 384.244
b3−H2O 467.2813
b3 485.292
y3−NH3 499.2996
y3 516.3251
b4−H2O 580.3655
b4 598.3761
y4−H2O 599.3577
y4−NH3 600.3464
y4 617.3728
y5−H2O 700.4087
y5 718.4209
y6−H2O 813.4944
y6 831.5048
G P T L T T K V R
y6 y5 y4 y3 y2 y1
b2 b3 b4
Modification
Acetyl (Protein N−term)
GlyGly (K) no new terminus
Oxidation (M)

20200924_KIZ_MUb5_GlyGly_1 − Scan number: 2416

Gene Names	Charge	m/z	Mass	Retention time	PEP	Score	Precursor Intensity
ZSWIM6	3	364.8685	1091.584	7.5136	0.017711	84.498	30823560

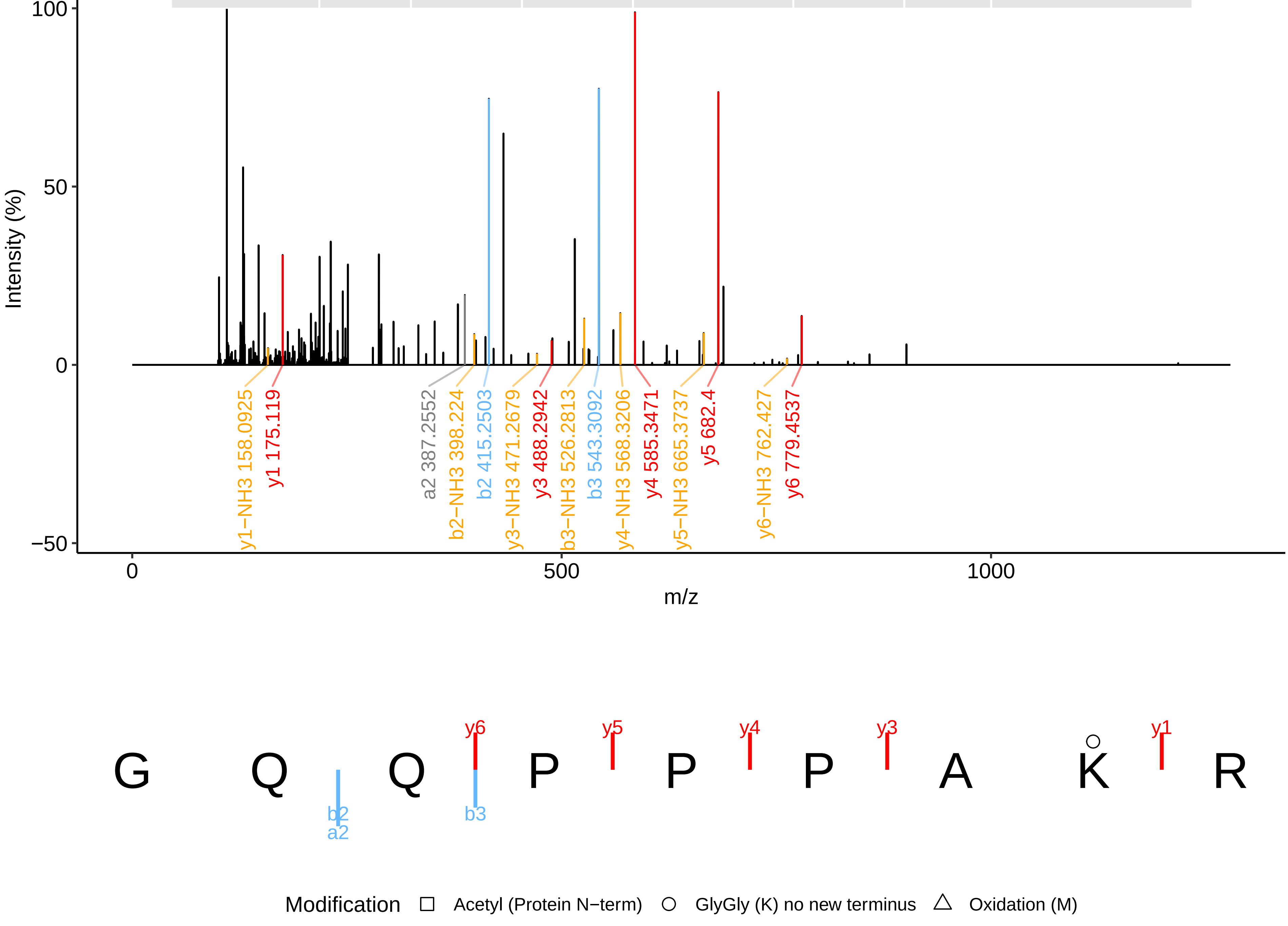

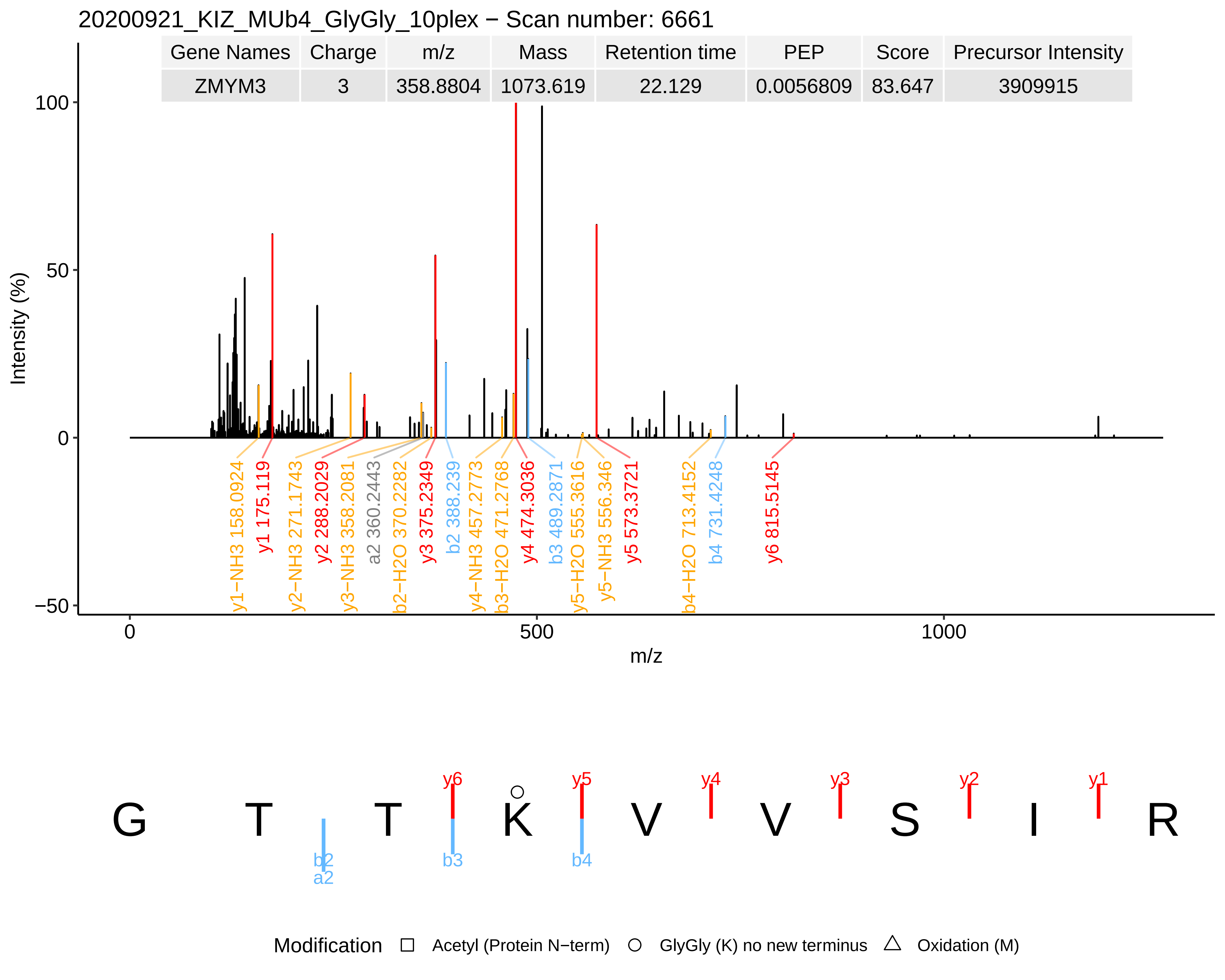

20200921_KIZ_MUb4_GlyGly_10plex − Scan number: 6661
Gene Names | Charge | m/z | Mass | Retention time | PEP | Score | Precursor Intensity
ZMYM3 | 3 | 358.8804 | 1073.619 | 22.129 | 0.0056809 | 83.647 | 3909915
Intensity (%)
m/z
y1−NH3 158.0924
y1 175.119
y2−NH3 271.1743
y2 288.2029
y3−NH3 358.2081
a2 360.2443
b2−H2O 370.2282
y3 375.2349
b2 388.239
y4−NH3 457.2773
b3−H2O 471.2768
y4 474.3036
b3 489.2871
y5−H2O 555.3616
y5−NH3 556.346
y5 573.3721
b4−H2O 713.4152
b4 731.4248
y6 815.5145
G T T K V V S I R
y6 y5 y4 y3 y2 y1
b2 a2 b3 b4
Modification
Acetyl (Protein N−term)
GlyGly (K) no new terminus
Oxidation (M)

20200921_KIZ_MUb4_GlyGly_10plex_2 − Scan number: 6192

Gene Names	Charge	m/z	Mass	Retention time	PEP	Score	Precursor Intensity
ZMYM3	3	358.8804	1073.619	22.643	0.028221	63.216	NA

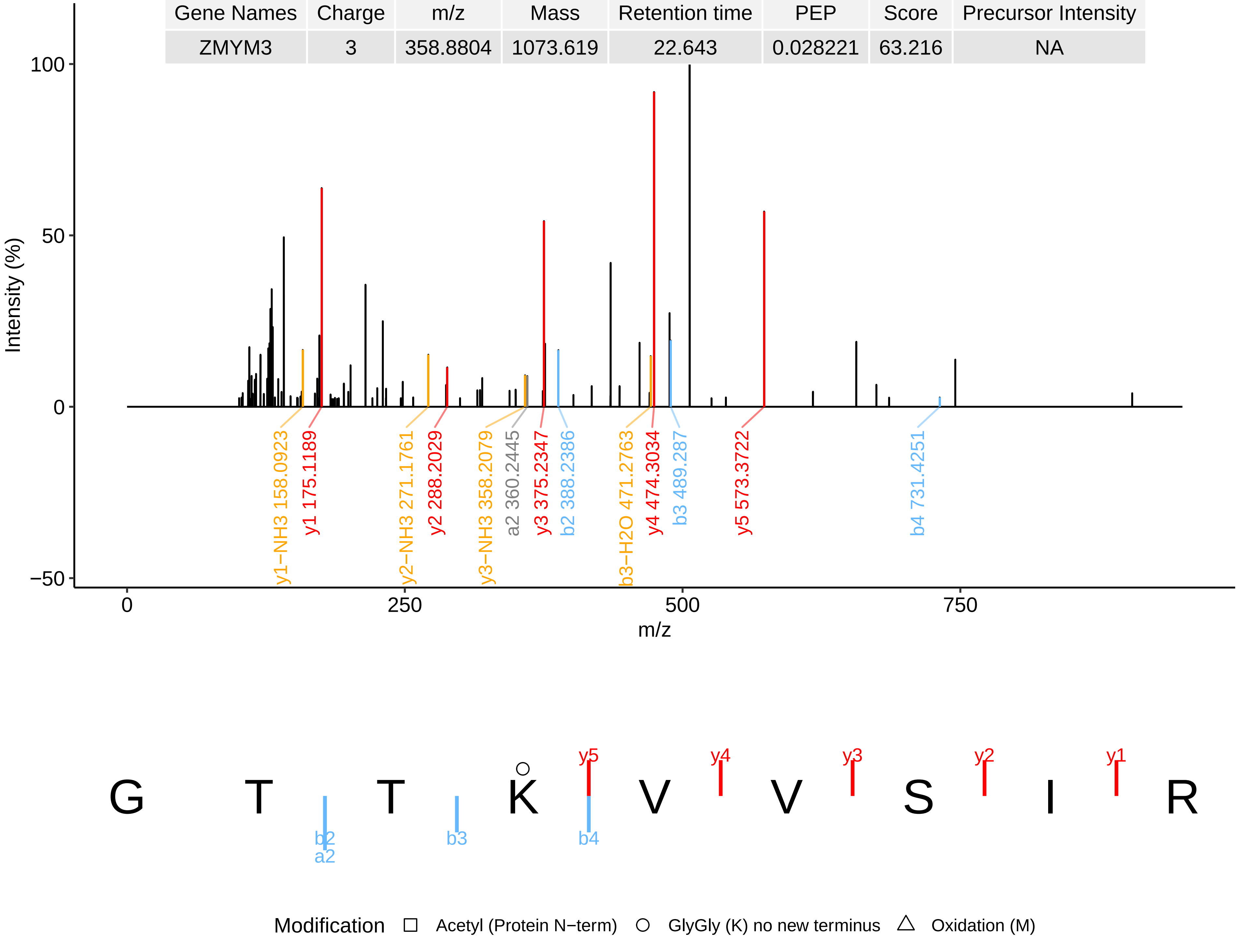

20200921_KIZ_MUb4_GlyGly_10plex − Scan number: 7093

Gene Names	Charge	m/z	Mass	Retention time	PEP	Score	Precursor Intensity
HNRNPL	3	364.533	1090.577	23.705	0.022785	69.598	3265330

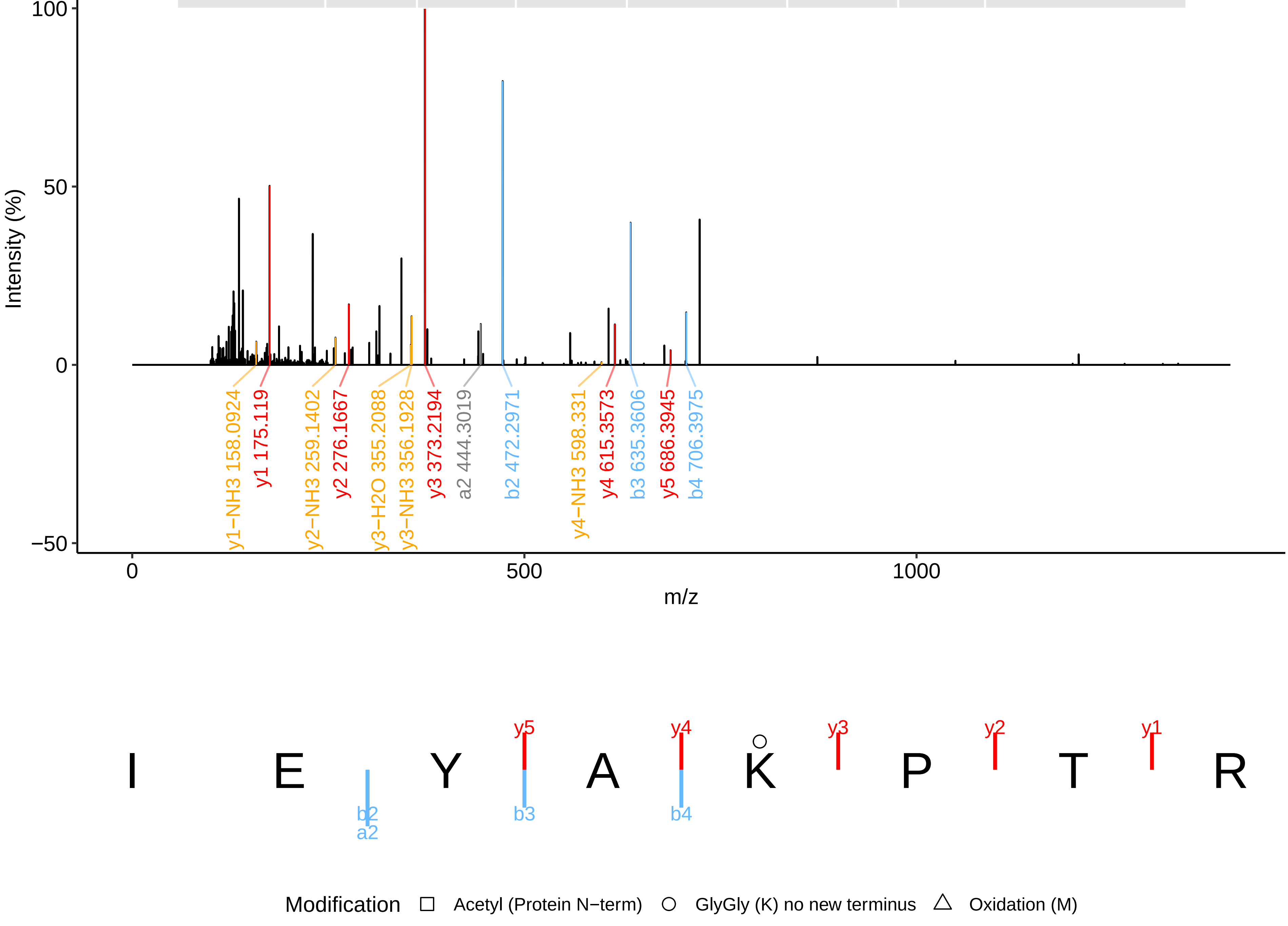

20200921_KIZ_MUb4_GlyGly_10plex_2 − Scan number: 6522

Gene Names	Charge	m/z	Mass	Retention time	PEP	Score	Precursor Intensity
HNRNPL	3	364.533	1090.577	24.013	0.0092817	80.438	1763427

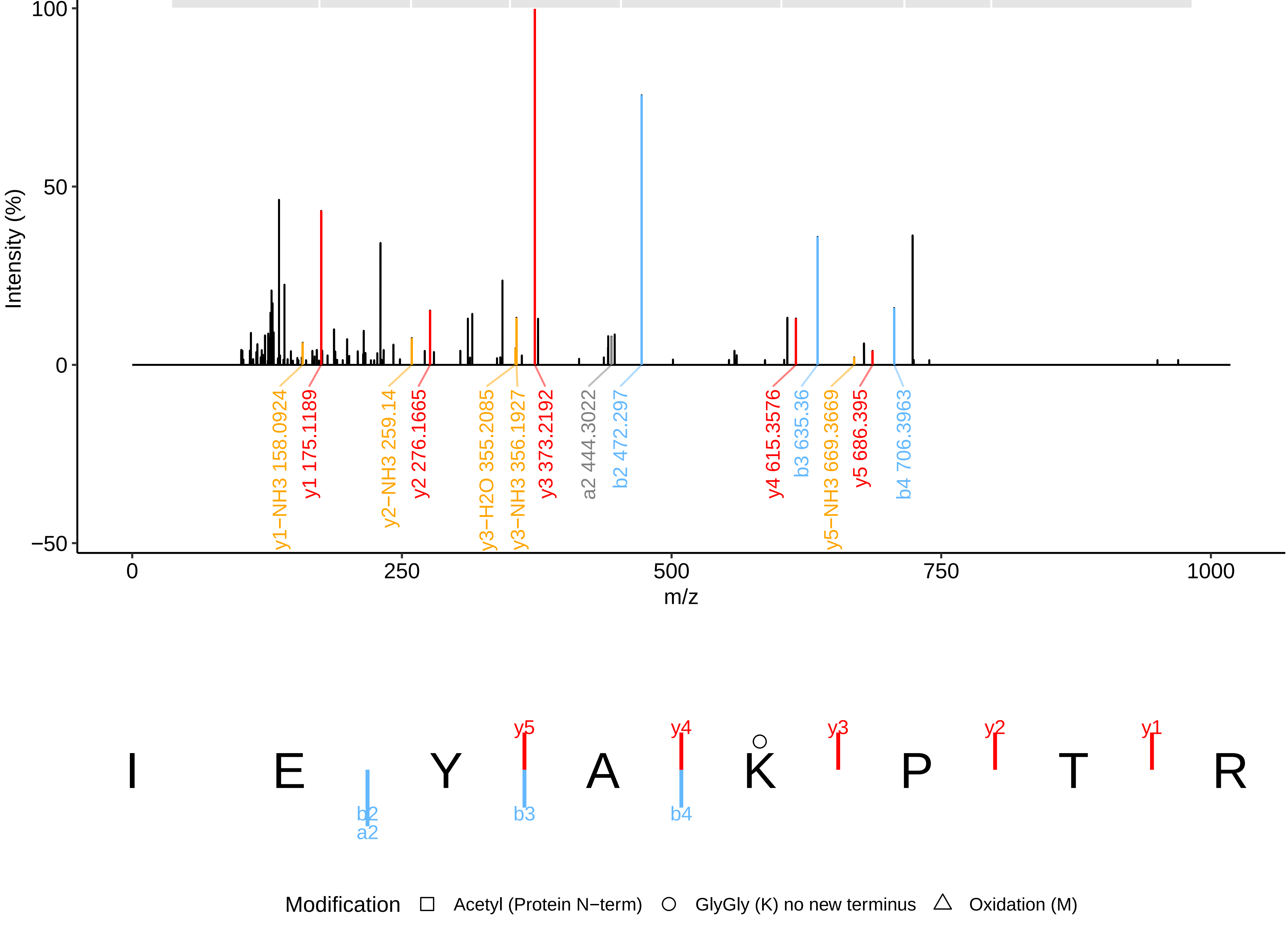

20200921_KIZ_MUb4_GlyGly_10plex_2 − Scan number: 7108

Gene Names	Charge	m/z	Mass	Retention time	PEP	Score	Precursor Intensity
EIF3B	3	350.5173	1048.53	26.402	0.095432	59.205	838240

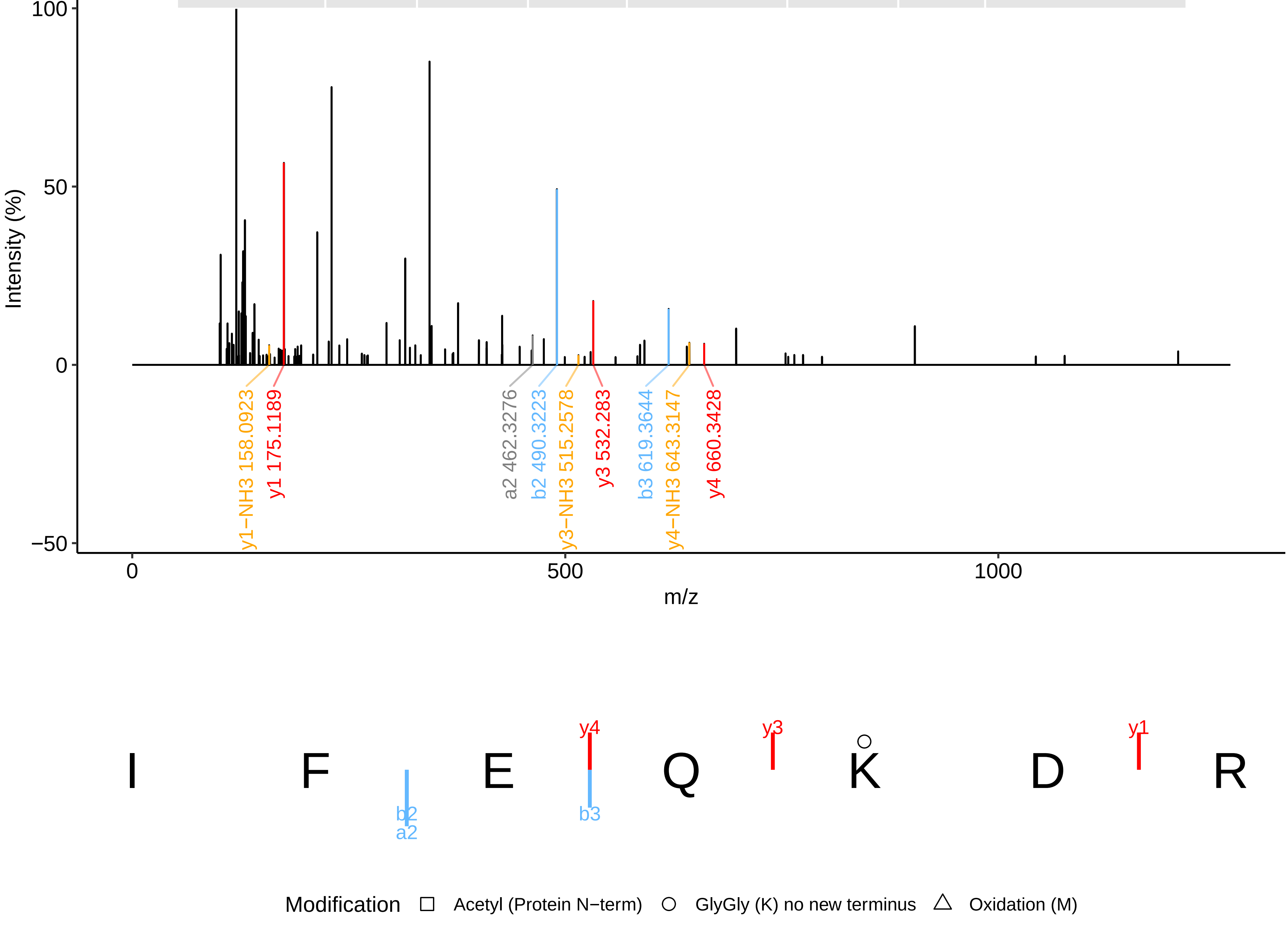

20200921_KIZ_MUb4_GlyGly_10plex − Scan number: 9851

Gene Names	Charge	m/z	Mass	Retention time	PEP	Score	Precursor Intensity
GART	3	362.8804	1085.619	33.886	0.0072074	85.38	4224918

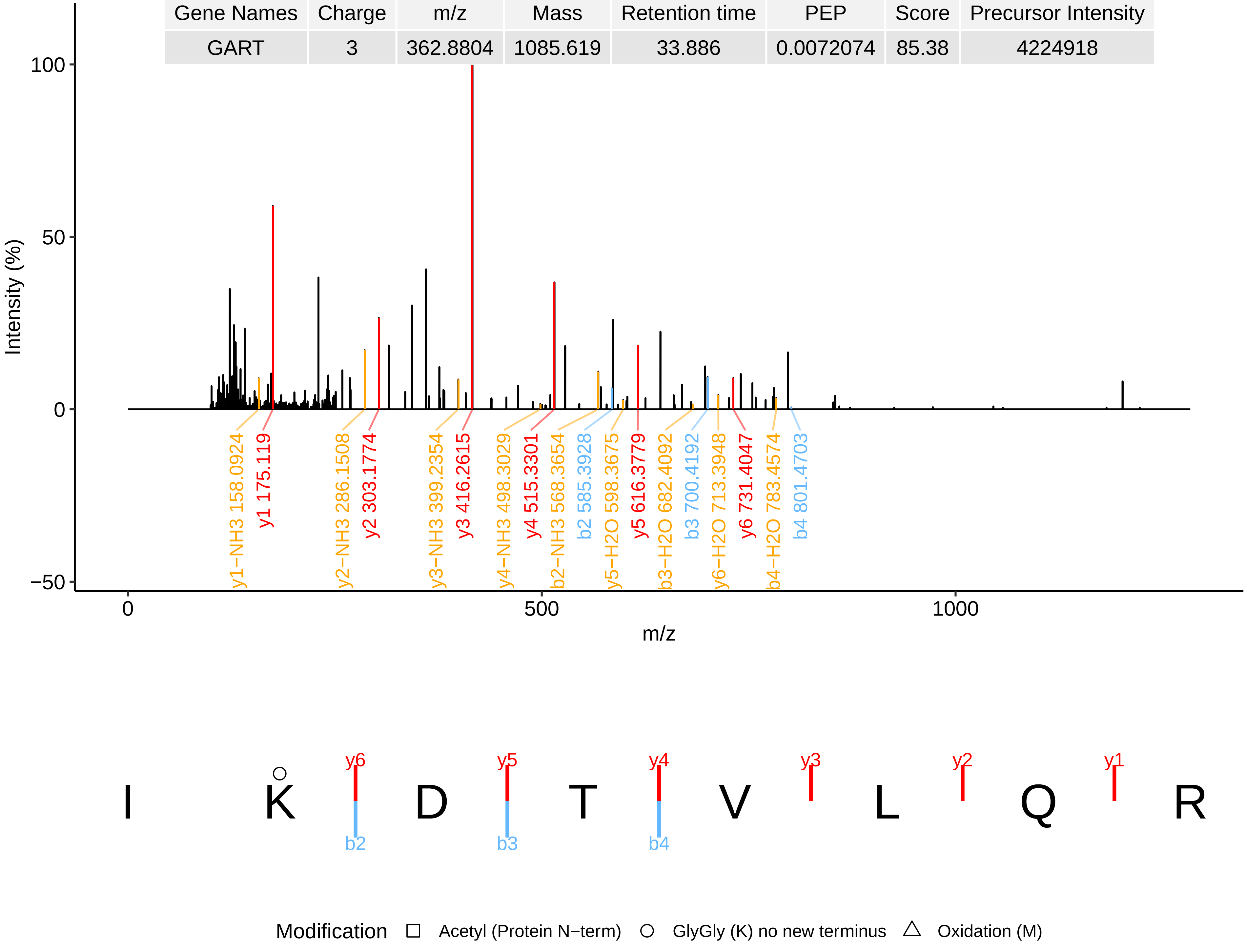

20200921_KIZ_MUb4_GlyGly_10plex_2 − Scan number: 9087

Gene Names	Charge	m/z	Mass	Retention time	PEP	Score	Precursor Intensity
GART	3	362.8804	1085.619	34.445	0.069364	53.775	1508005

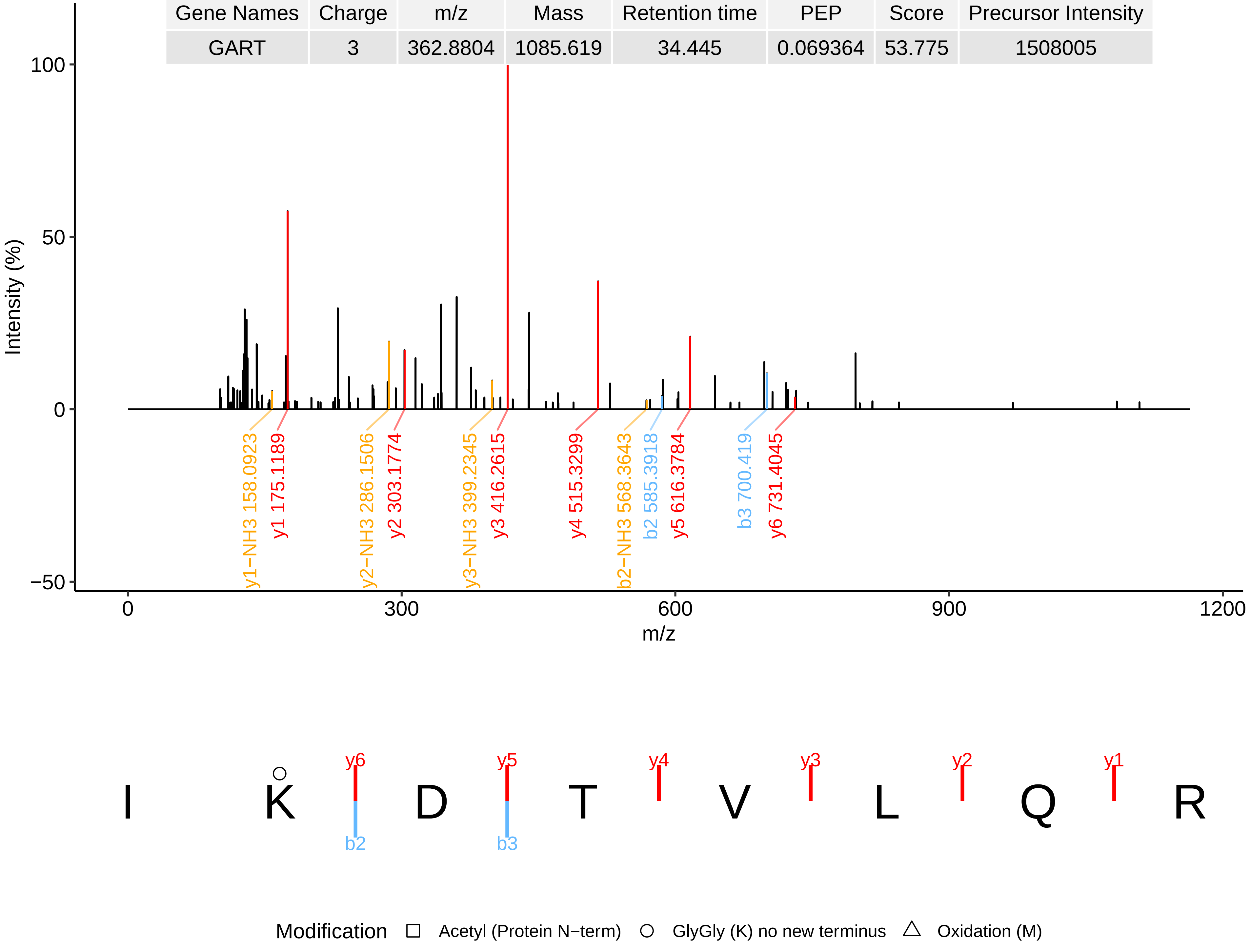

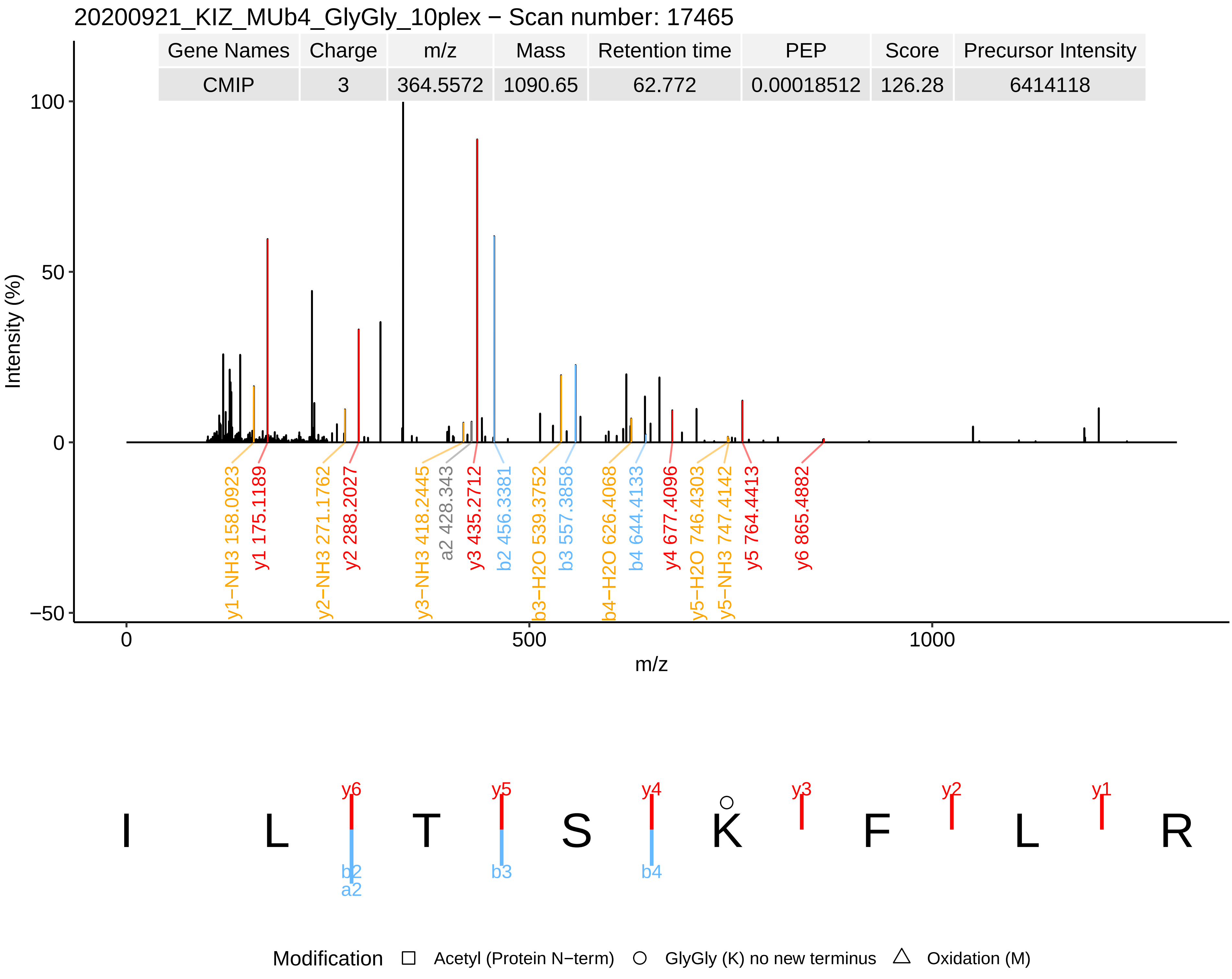
20200921_KIZ_MUb4_GlyGly_10plex − Scan number: 17465
Gene Names | Charge | m/z | Mass | Retention time | PEP | Score | Precursor Intensity
CMIP | 3 | 364.5572 | 1090.65 | 62.772 | 0.00018512 | 126.28 | 6414118
Intensity (%)
100
50
0
−50
0
500
1000
m/z
y1−NH3 158.0923
y1 175.1189
y2−NH3 271.1762
y2 288.2027
y3−NH3 418.2445
a2 428.343
y3 435.2712
b2 456.3381
b3−H2O 539.3752
b3 557.3858
b4−H2O 626.4068
b4 644.4133
y4 677.4096
y5−H2O 746.4303
y5−NH3 747.4142
y5 764.4413
y6 865.4882
I L T S K F L R
y6 y5 y4 y3 y2 y1
b2 a2 b3 b4
Modification
Acetyl (Protein N−term)
GlyGly (K) no new terminus
Oxidation (M)

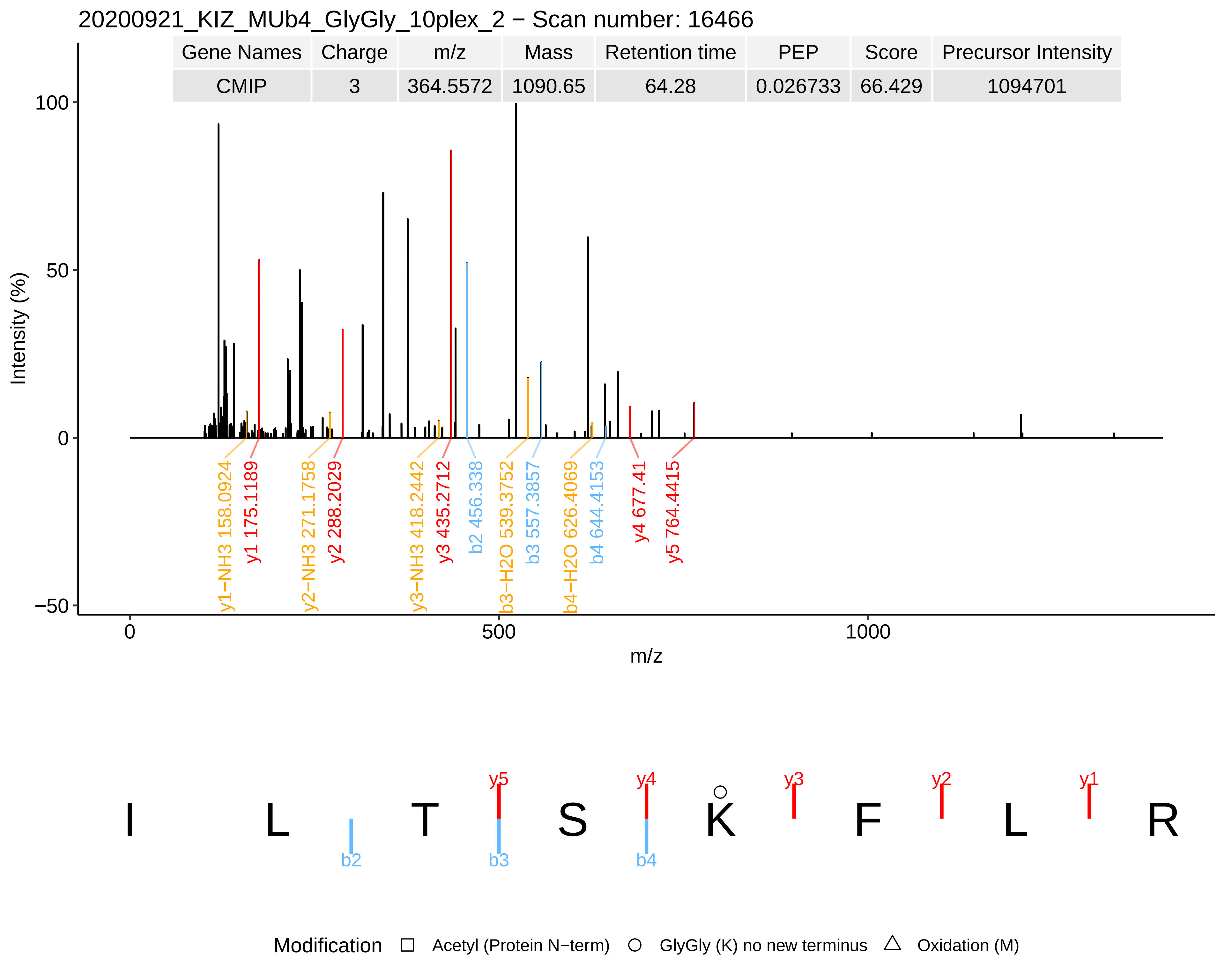
20200921_KIZ_MUb4_GlyGly_10plex_2 − Scan number: 16466
Gene Names | Charge | m/z | Mass | Retention time | PEP | Score | Precursor Intensity
CMIP | 3 | 364.5572 | 1090.65 | 64.28 | 0.026733 | 66.429 | 1094701
Intensity (%)
100
50
0
−50
0
500
1000
m/z
y1−NH3 158.0924
y1 175.1189
y2−NH3 271.1758
y2 288.2029
y3−NH3 418.2442
y3 435.2712
b2 456.338
b3−H2O 539.3752
b3 557.3857
b4−H2O 626.4069
b4 644.4153
y4 677.41
y5 764.4415
I L T S K F L R
y5 y4 y3 y2 y1
b2 b3 b4
Modification
Acetyl (Protein N−term)
GlyGly (K) no new terminus
Oxidation (M)

20200921_KIZ_MUb4_GlyGly_10plex − Scan number: 6313

Gene Names	Charge	m/z	Mass	Retention time	PEP	Score	Precursor Intensity
HSP90AA1;HSP90AA4P;HSP90AB1	3	354.2026	1059.586	20.832	0.010099	77.741	6071959

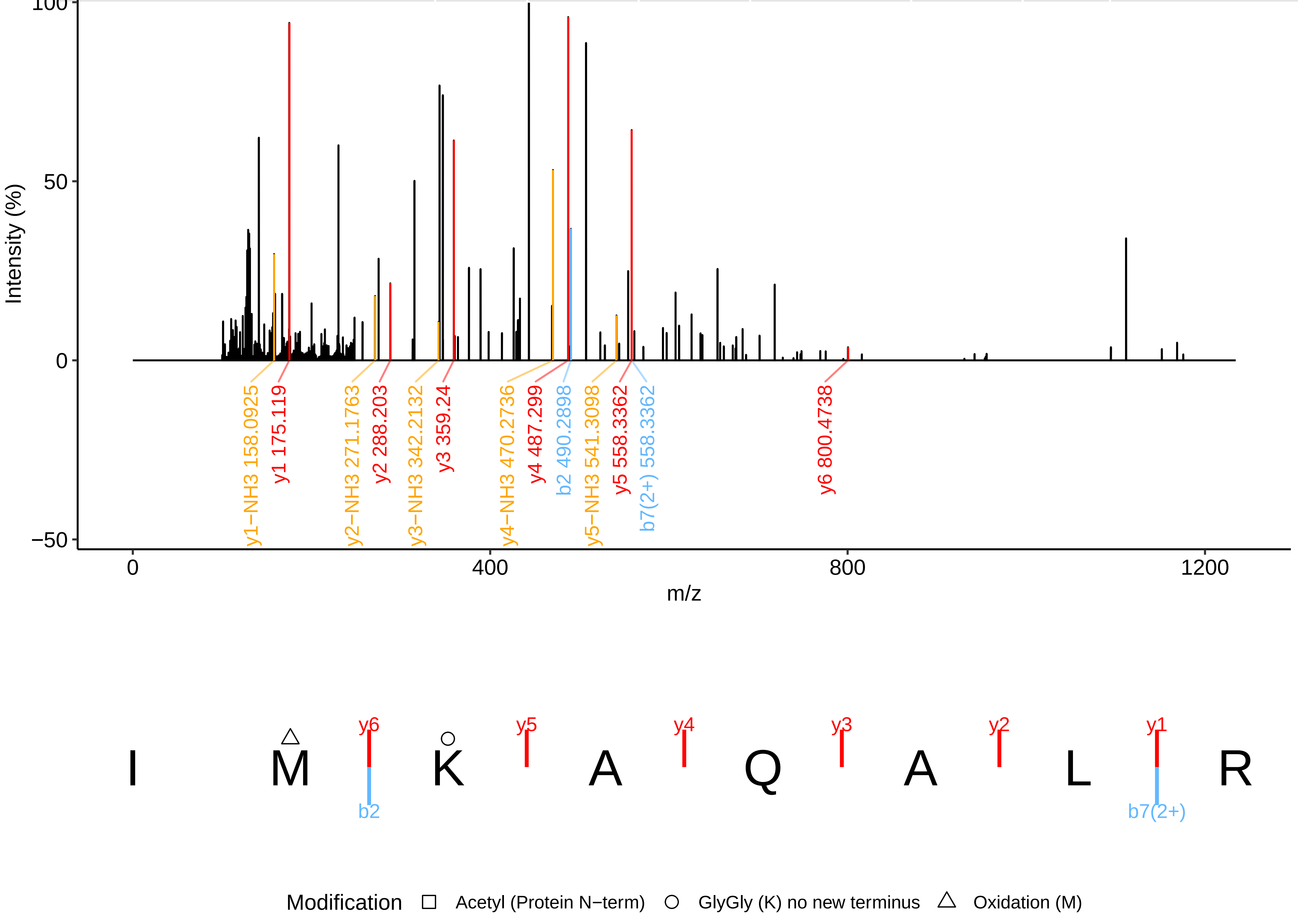

20200921_KIZ_MUb4_GlyGly_10plex_2 − Scan number: 5822

Gene Names	Charge	m/z	Mass	Retention time	PEP	Score	Precursor Intensity
HSP90AA1;HSP90AA4P;HSP90AB1	3	354.2026	1059.586	21.12	0.099519	40.098	1331141

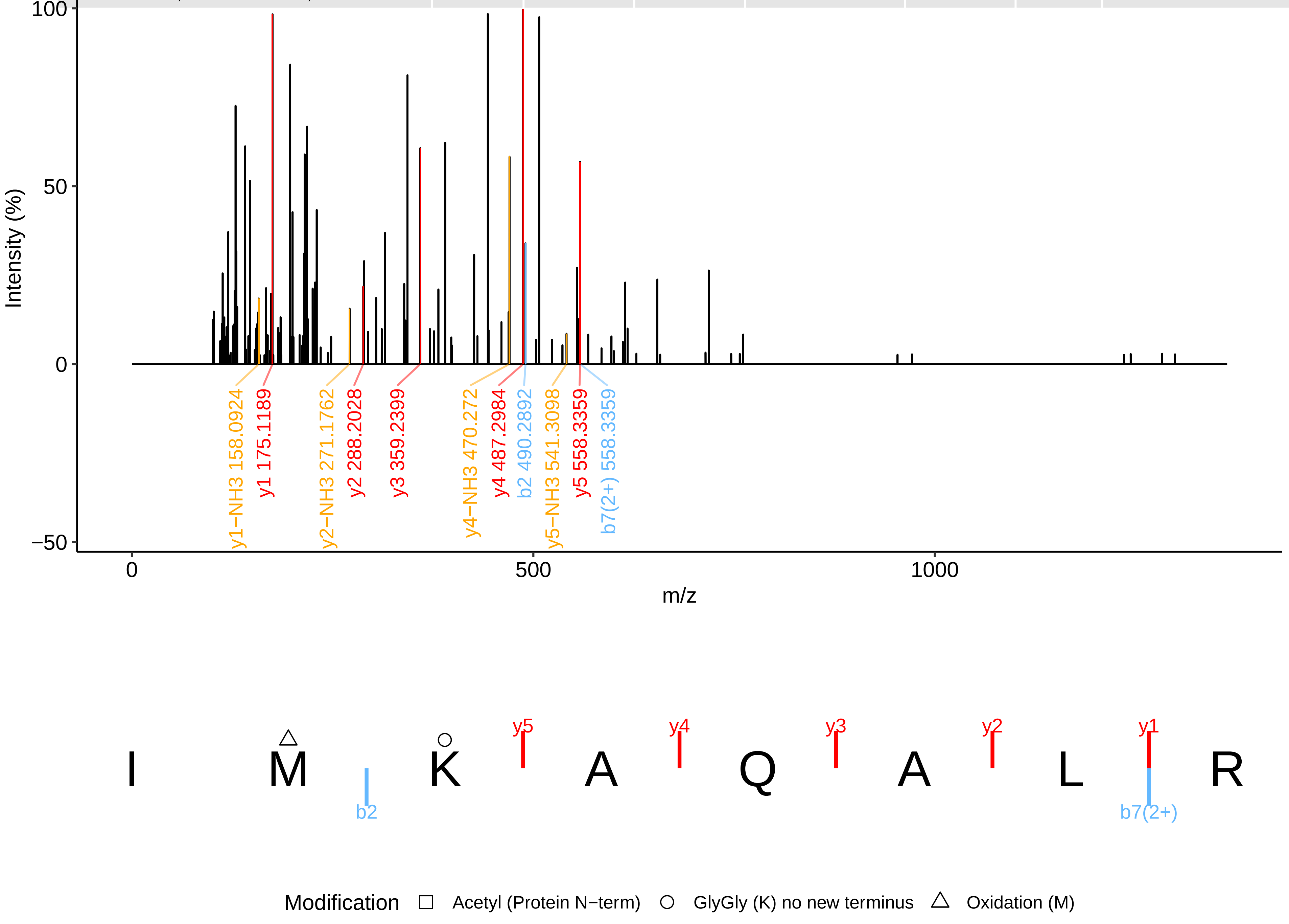

20200921_KIZ_MUb4_GlyGly_10plex − Scan number: 11223

Gene Names	Charge	m/z	Mass	Retention time	PEP	Score	Precursor Intensity
PSMC3	3	363.8743	1088.601	39.046	0.0069253	86.095	25033864

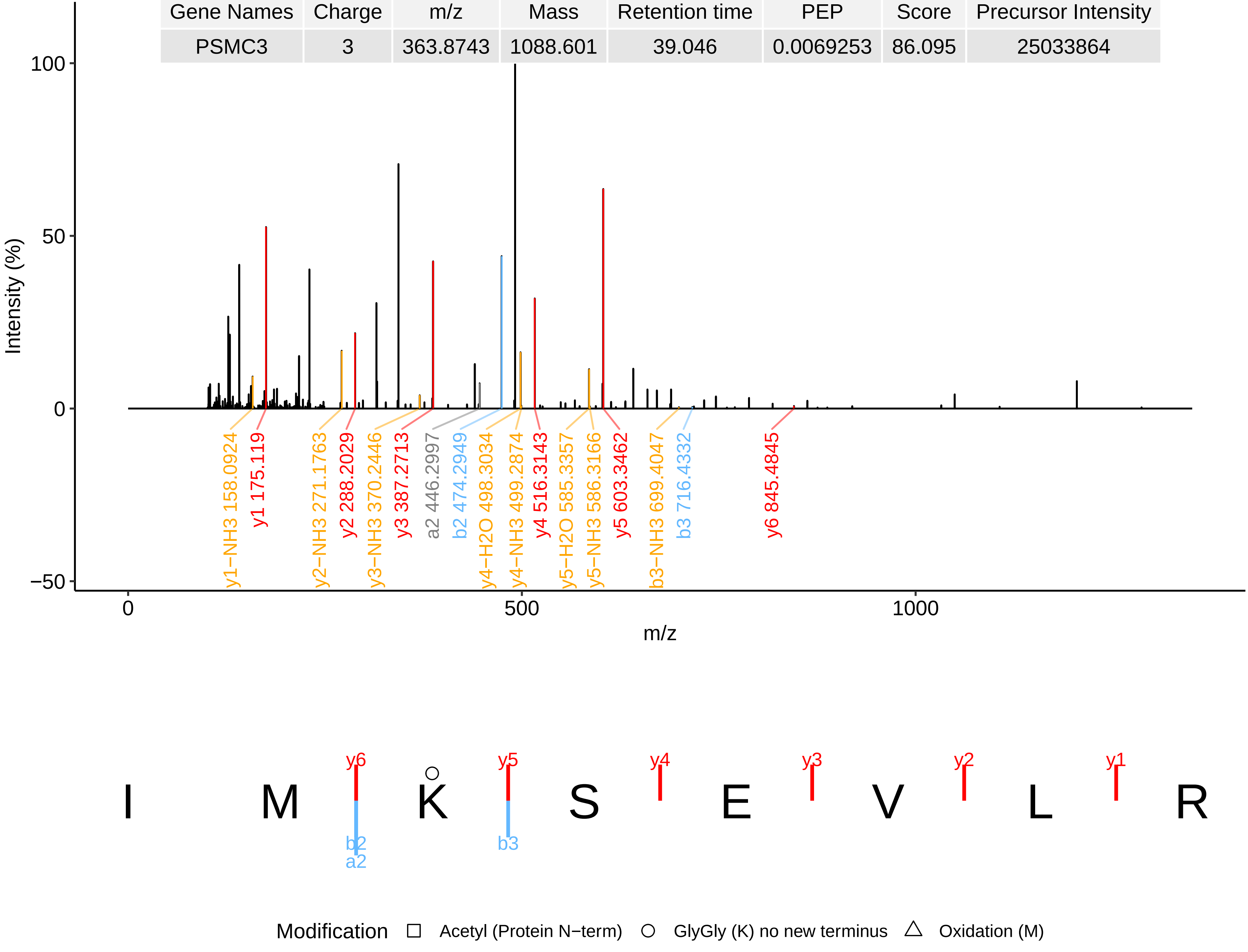

20200921_KIZ_MUb4_GlyGly_10plex − Scan number: 11298

Gene Names	Charge	m/z	Mass	Retention time	PEP	Score	Precursor Intensity
PSMC3	2	545.3079	1088.601	39.301	0.0072236	120.15	6049421

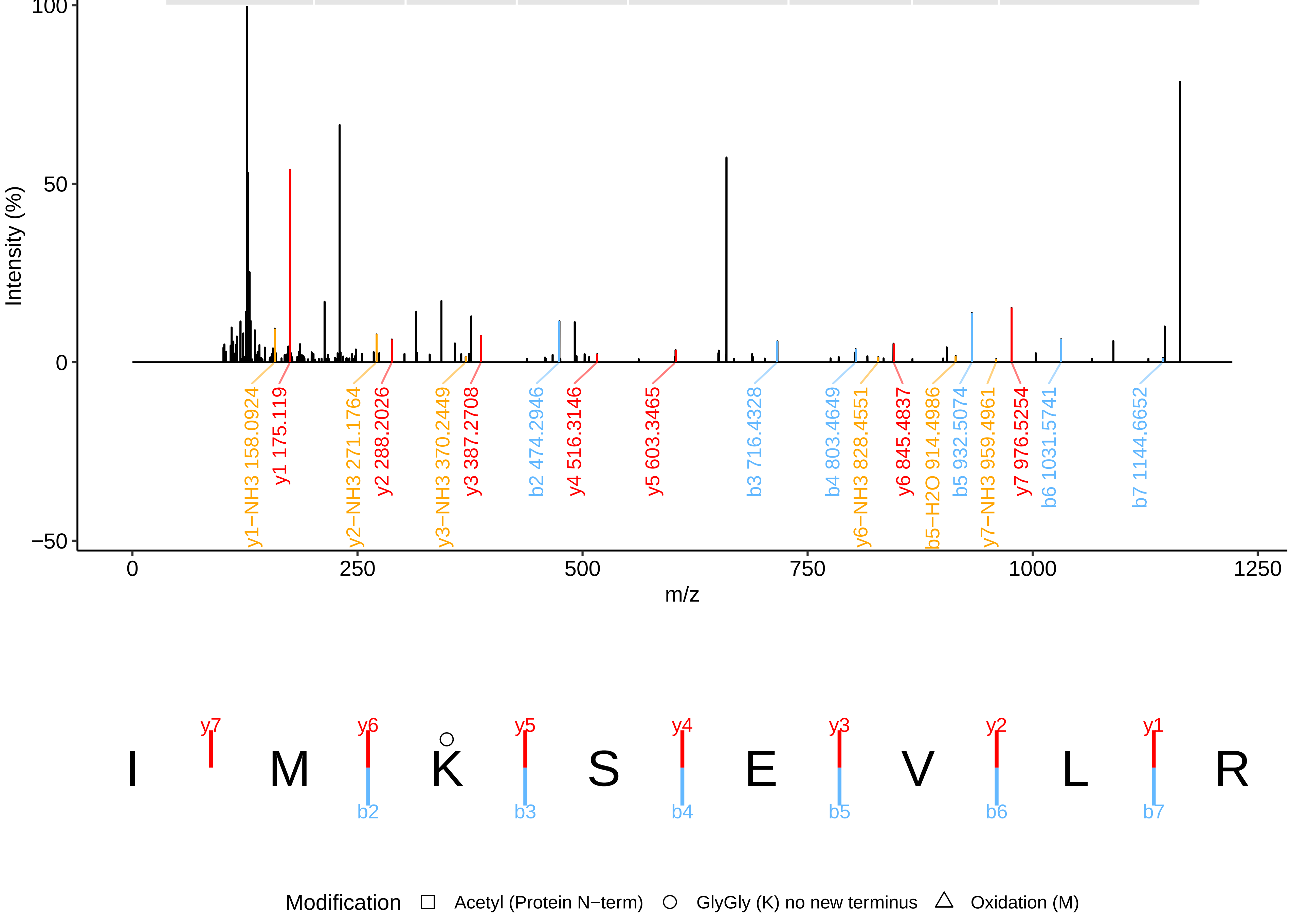

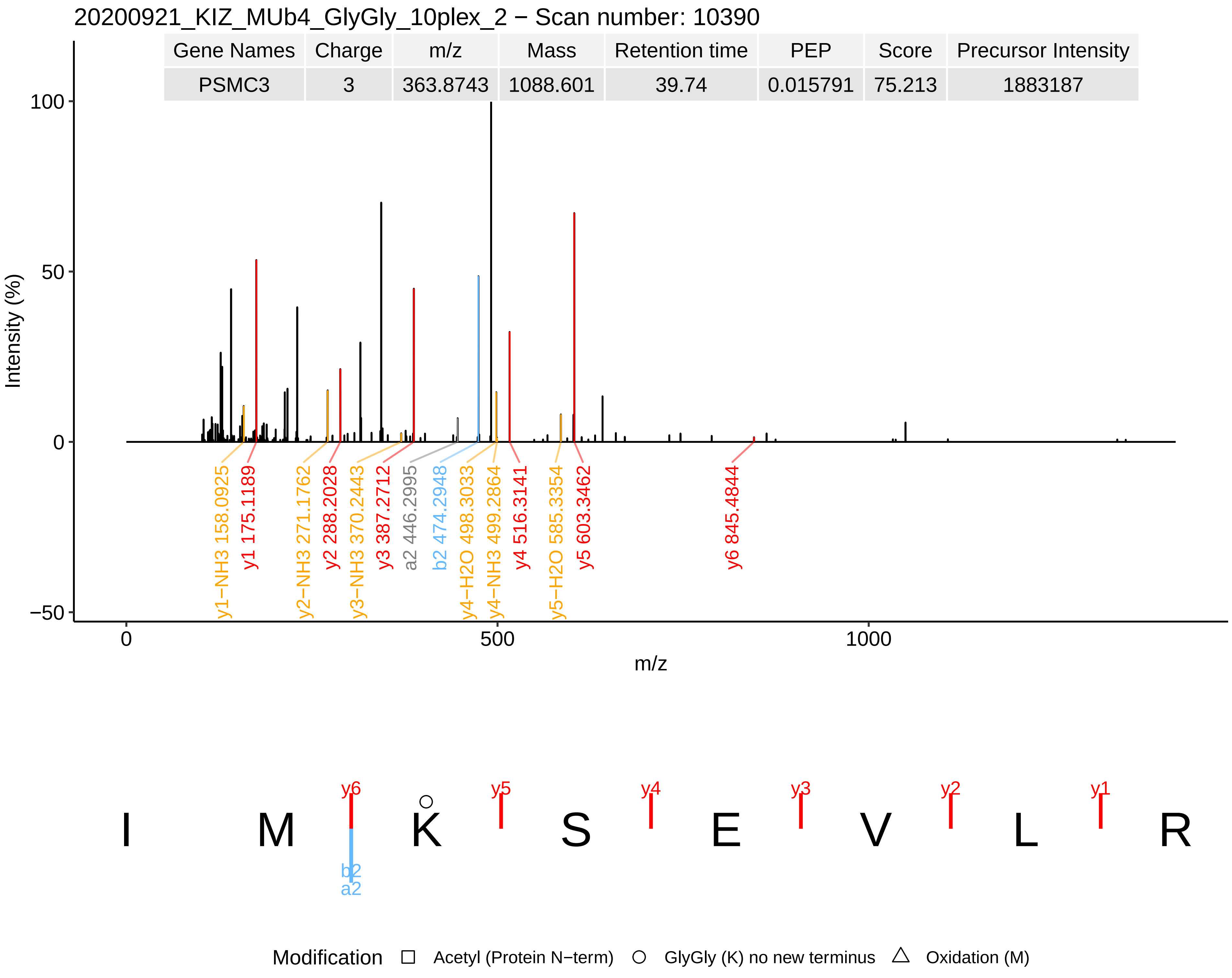

20200921_KIZ_MUb4_GlyGly_10plex_2 − Scan number: 10390
Gene Names | Charge | m/z | Mass | Retention time | PEP | Score | Precursor Intensity
PSMC3 | 3 | 363.8743 | 1088.601 | 39.74 | 0.015791 | 75.213 | 1883187
Intensity (%)
m/z
y1−NH3 158.0925
y1 175.1189
y2−NH3 271.1762
y2 288.2028
y3−NH3 370.2443
y3 387.2712
a2 446.2995
b2 474.2948
y4−H2O 498.3033
y4−NH3 499.2864
y4 516.3141
y5−H2O 585.3354
y5 603.3462
y6 845.4844
I M K S E V L R
y6 y5 y4 y3 y2 y1
b2
a2
Modification
Acetyl (Protein N−term)
GlyGly (K) no new terminus
Oxidation (M)

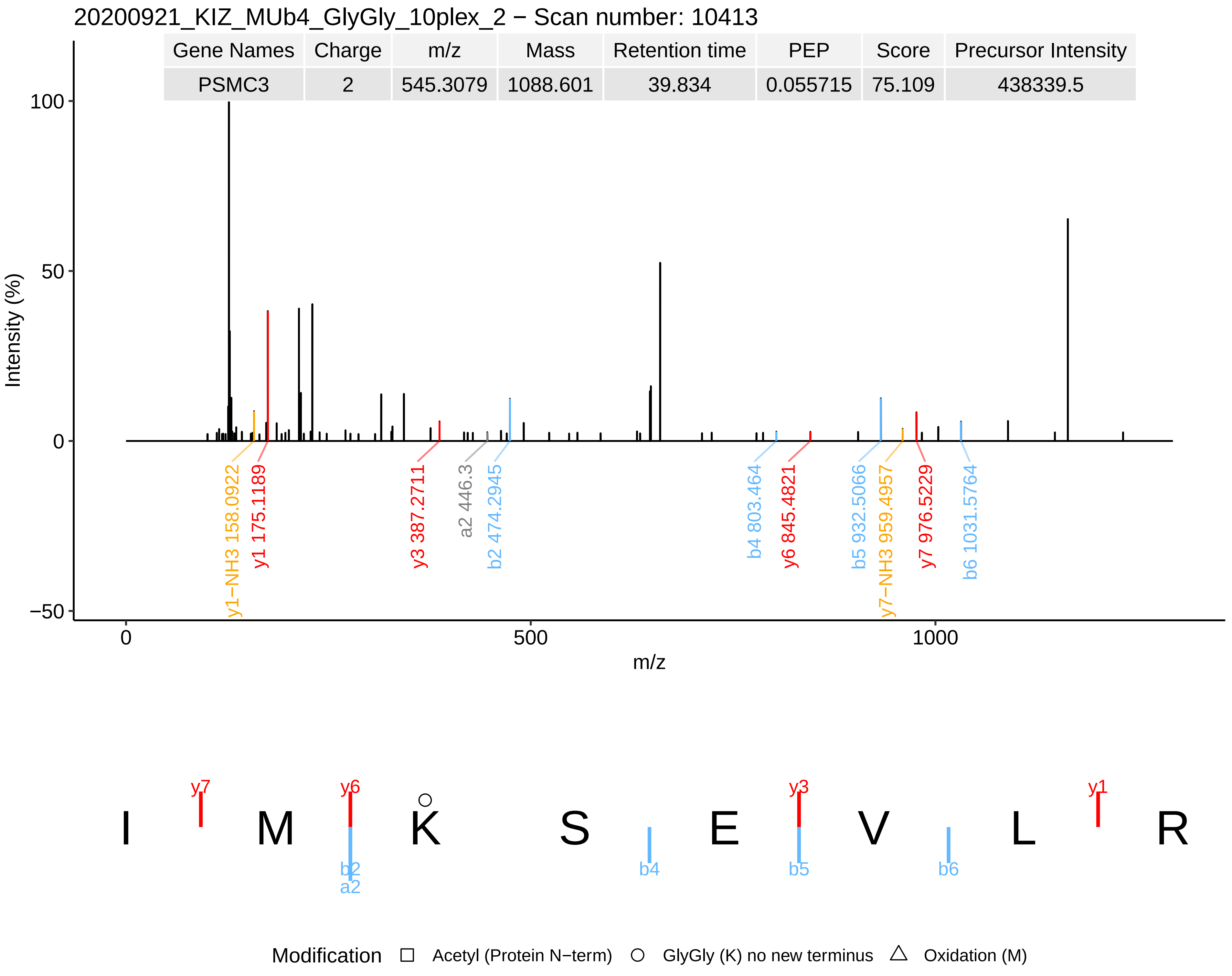

20200921_KIZ_MUb4_GlyGly_10plex_2 − Scan number: 10413
Gene Names | Charge | m/z | Mass | Retention time | PEP | Score | Precursor Intensity
PSMC3 | 2 | 545.3079 | 1088.601 | 39.834 | 0.055715 | 75.109 | 438339.5
Intensity (%)
m/z
y1−NH3 158.0922
y1 175.1189
y3 387.2711
a2 446.3
b2 474.2945
b4 803.464
y6 845.4821
b5 932.5066
y7−NH3 959.4957
y7 976.5229
b6 1031.5764
I M K S E V L R
y7 y6 y3 y1
b2 a2 b4 b5 b6
Modification
Acetyl (Protein N−term)
GlyGly (K) no new terminus
Oxidation (M)

20200924_KIZ_MUb5_GlyGly_1 − Scan number: 9930

Gene Names	Charge	m/z	Mass	Retention time	PEP	Score	Precursor Intensity
PSMC3	2	545.3079	1088.601	31.991	2.7066e−05	158.07	NA

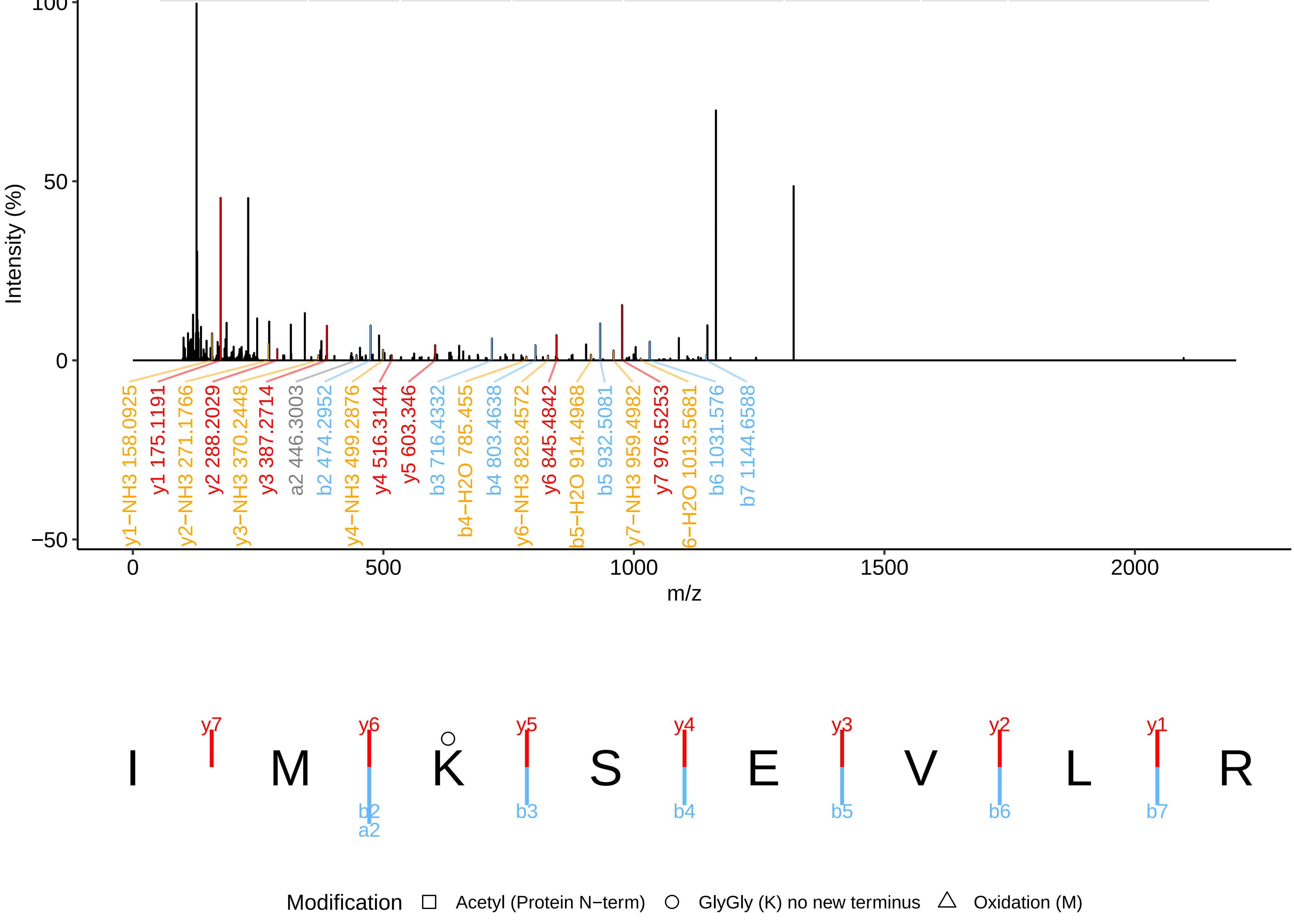

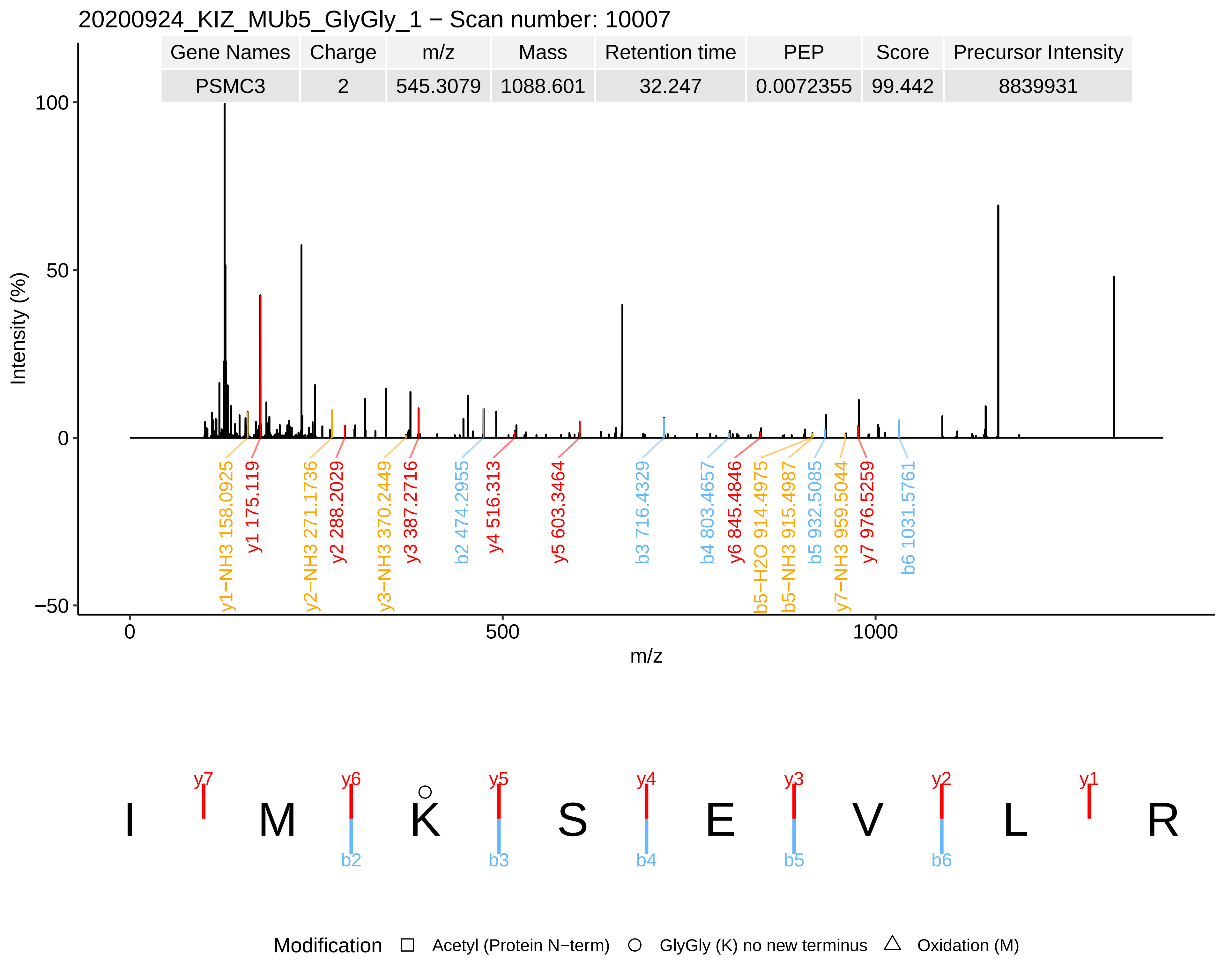

20200924_KIZ_MUb5_GlyGly_1 − Scan number: 10007
Gene Names | Charge | m/z | Mass | Retention time | PEP | Score | Precursor Intensity
PSMC3 | 2 | 545.3079 | 1088.601 | 32.247 | 0.0072355 | 99.442 | 8839931
Intensity (%)
m/z
y1−NH3 158.0925
y1 175.119
y2−NH3 271.1736
y2 288.2029
y3−NH3 370.2449
y3 387.2716
b2 474.2955
y4 516.313
y5 603.3464
b3 716.4329
b4 803.4657
y6 845.4846
b5−H2O 914.4975
b5−NH3 915.4987
b5 932.5085
y7−NH3 959.5044
y7 976.5259
b6 1031.5761
I M K S E V L R
y7 y6 y5 y4 y3 y2 y1
b2 b3 b4 b5 b6
Modification
Acetyl (Protein N−term)
GlyGly (K) no new terminus
Oxidation (M)

20200924_KIZ_MUb5_GlyGly_1 − Scan number: 10019

Gene Names	Charge	m/z	Mass	Retention time	PEP	Score	Precursor Intensity
PSMC3	3	363.8743	1088.601	32.287	0.011916	78.324	82874264

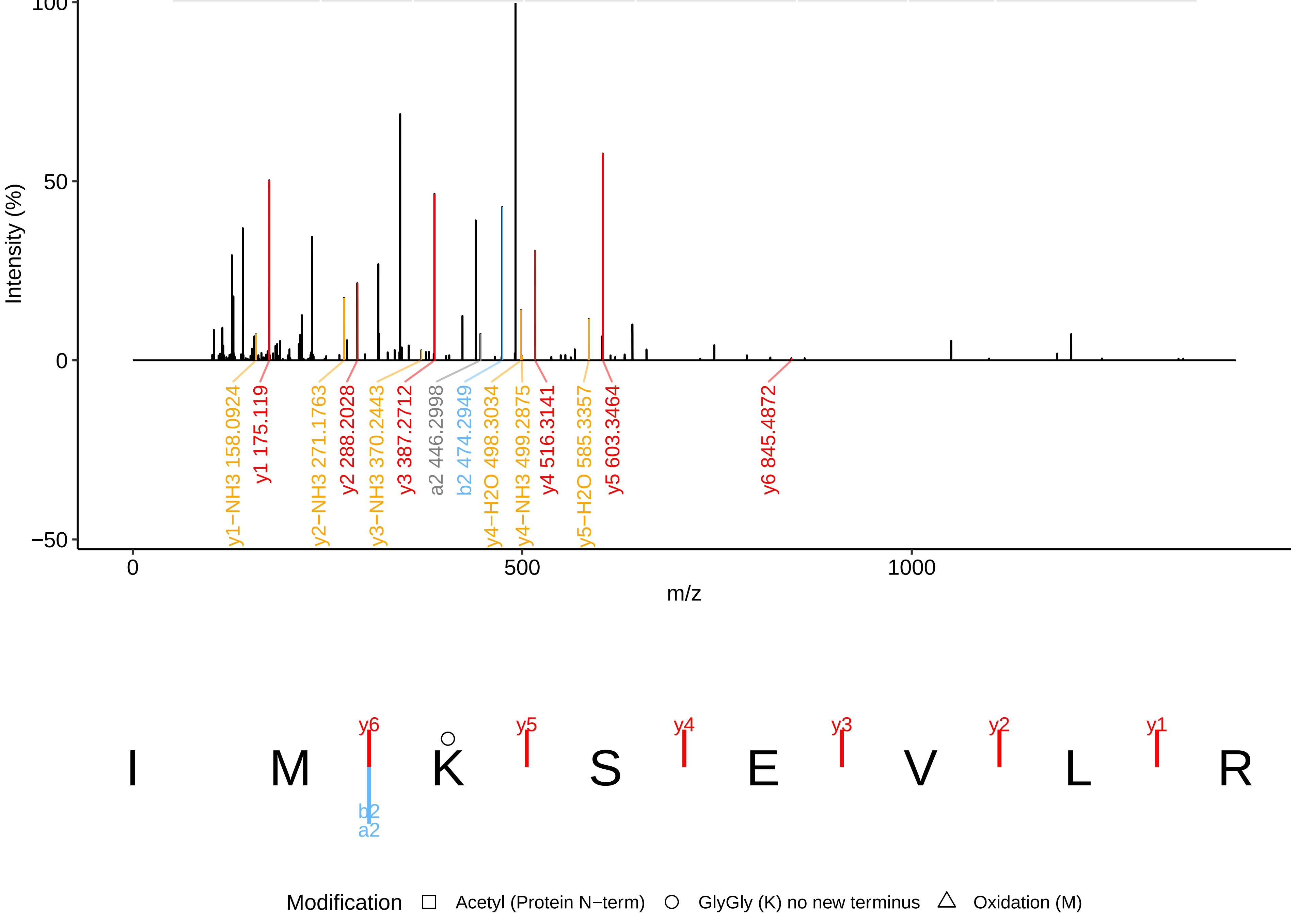

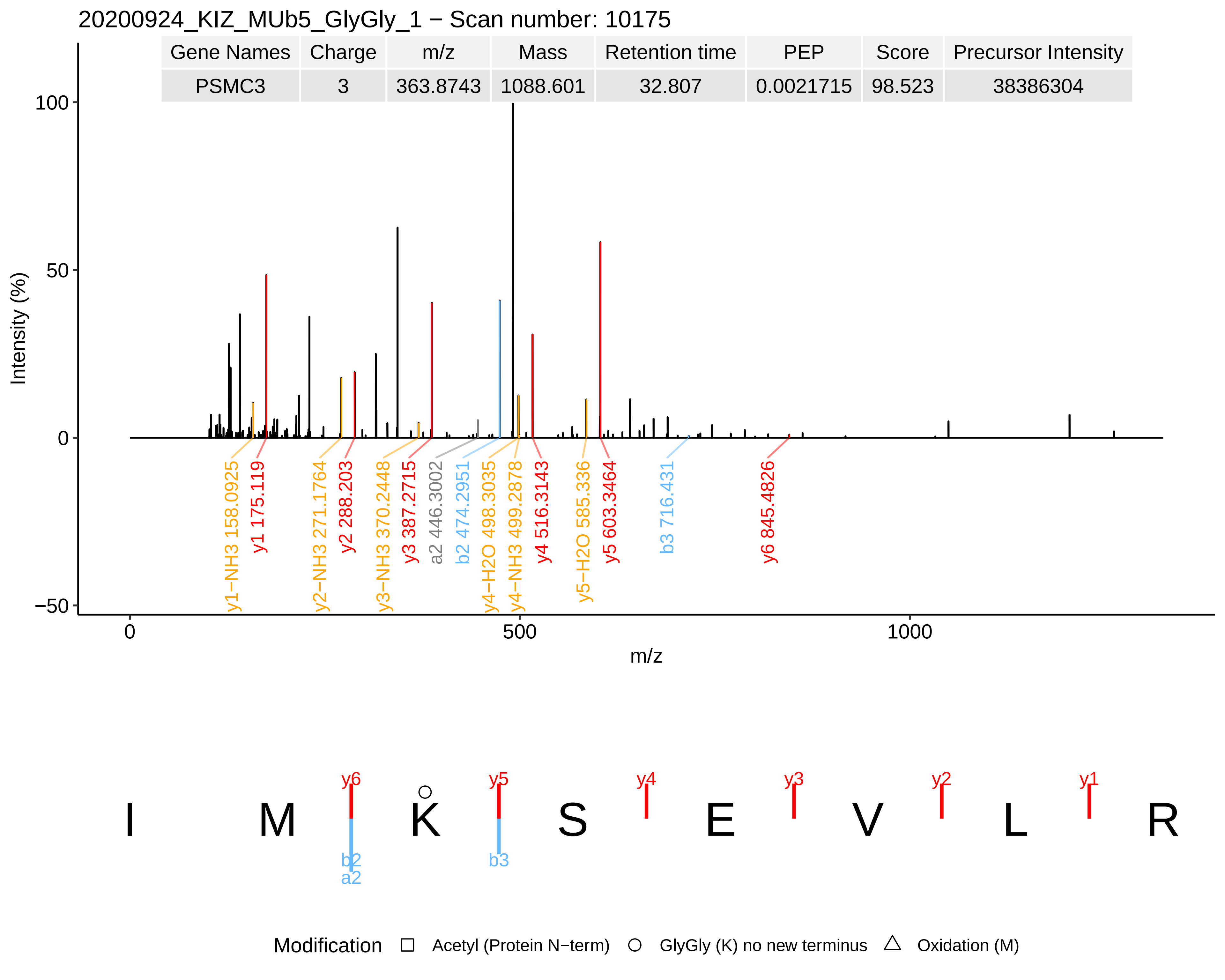

20200924_KIZ_MUb5_GlyGly_1 − Scan number: 10175
Gene Names | Charge | m/z | Mass | Retention time | PEP | Score | Precursor Intensity
PSMC3 | 3 | 363.8743 | 1088.601 | 32.807 | 0.0021715 | 98.523 | 38386304
Intensity (%)
m/z
y1−NH3 158.0925
y1 175.119
y2−NH3 271.1764
y2 288.203
y3−NH3 370.2448
y3 387.2715
a2 446.3002
b2 474.2951
y4−H2O 498.3035
y4−NH3 499.2878
y4 516.3143
y5−H2O 585.336
y5 603.3464
b3 716.431
y6 845.4826
I M K S E V L R
y6 y5 y4 y3 y2 y1
b2 a2 b3
Modification
Acetyl (Protein N−term)
GlyGly (K) no new terminus
Oxidation (M)

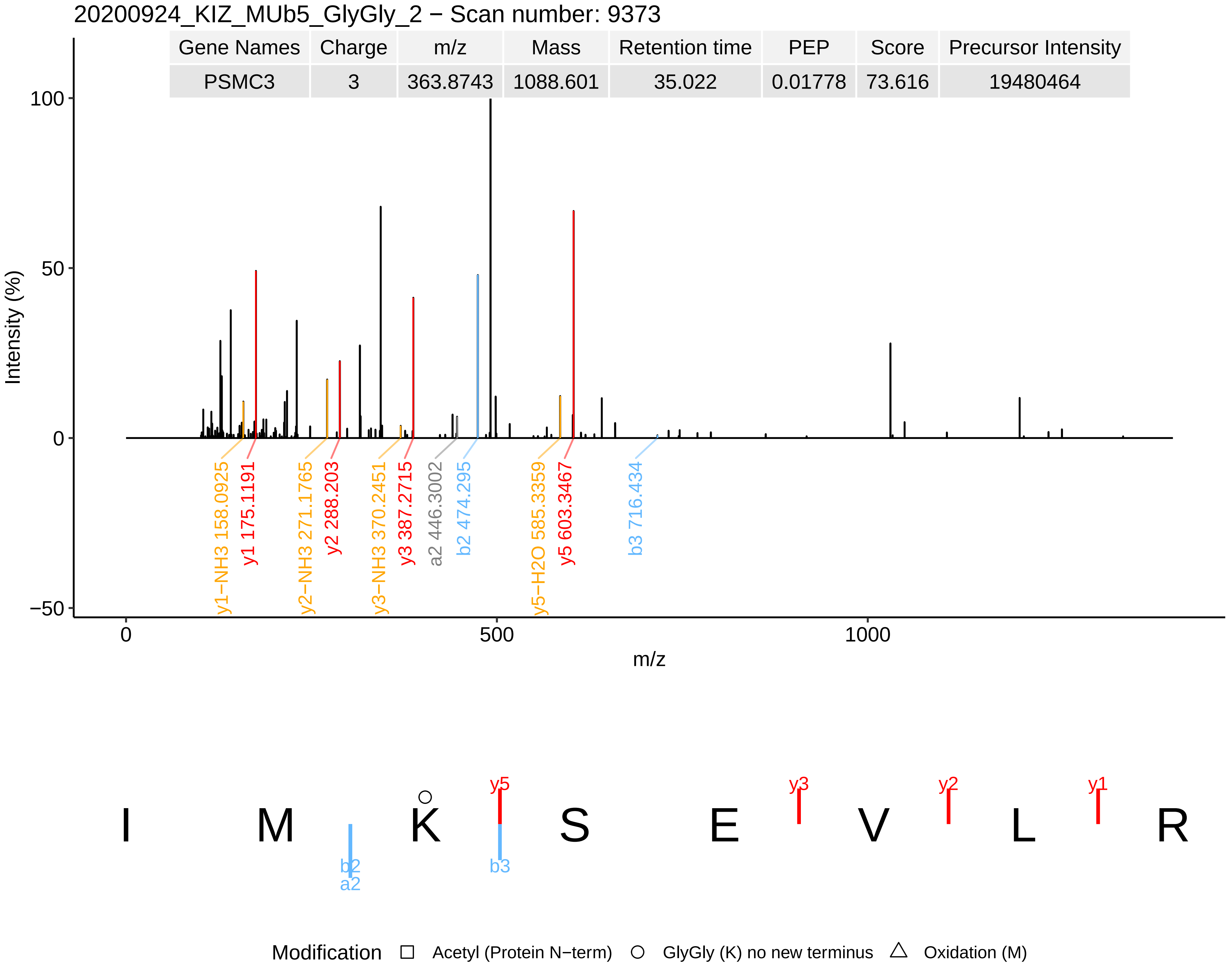

20200924_KIZ_MUb5_GlyGly_2 − Scan number: 9373
Gene Names | Charge | m/z | Mass | Retention time | PEP | Score | Precursor Intensity
PSMC3 | 3 | 363.8743 | 1088.601 | 35.022 | 0.01778 | 73.616 | 19480464
Intensity (%)
m/z
y1−NH3 158.0925
y1 175.1191
y2−NH3 271.1765
y2 288.203
y3−NH3 370.2451
y3 387.2715
a2 446.3002
b2 474.295
y5−H2O 585.3359
y5 603.3467
b3 716.434
I M K S E V L R
y5 y3 y2 y1
b2 a2 b3
Modification
Acetyl (Protein N−term)
GlyGly (K) no new terminus
Oxidation (M)

20200924_KIZ_MUb5_GlyGly_2 − Scan number: 9507

Gene Names	Charge	m/z	Mass	Retention time	PEP	Score	Precursor Intensity
PSMC3	3	363.8743	1088.601	35.529	0.0018539	101.33	15483554

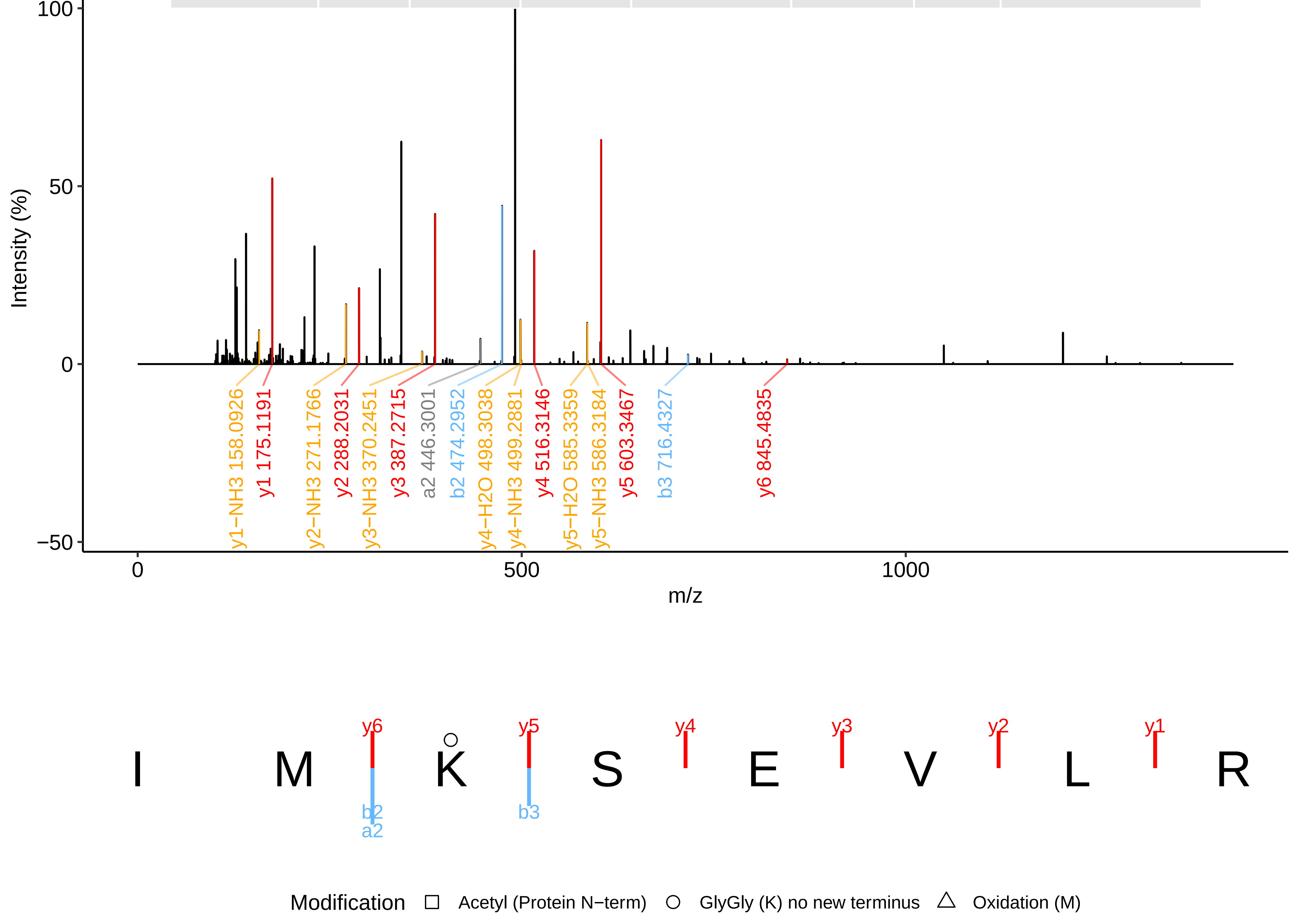

20200924_KIZ_MUb5_GlyGly_2 − Scan number: 9457

Gene Names	Charge	m/z	Mass	Retention time	PEP	Score	Precursor Intensity
PSMC3	2	545.3079	1088.601	35.352	0.019575	103.83	NA

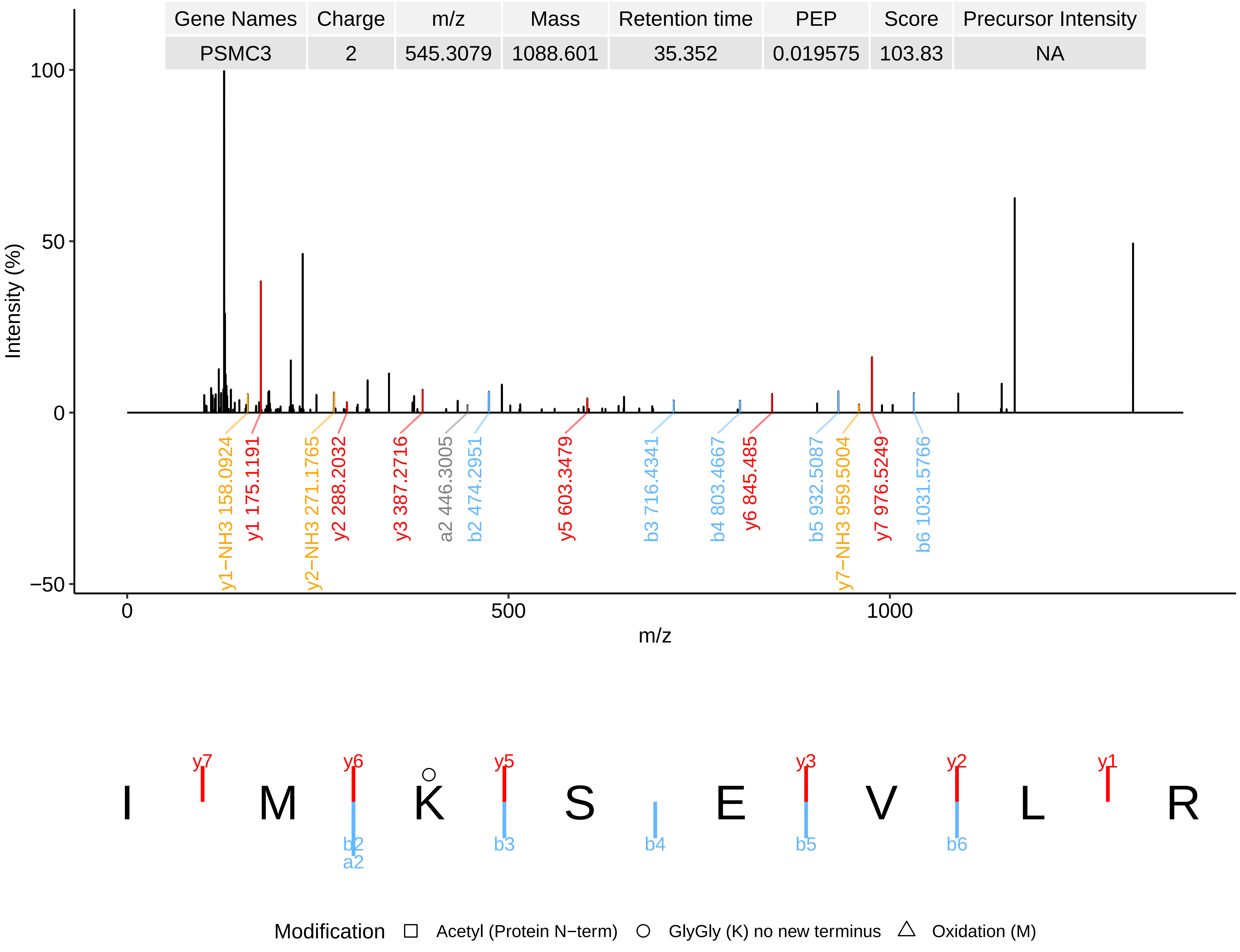

20201118_KIZ_MUb6_GlyGly_2 − Scan number: 12212

Gene Names	Charge	m/z	Mass	Retention time	PEP	Score	Precursor Intensity
PSMC3	3	363.8743	1088.601	41.092	0.015791	75.213	9829852

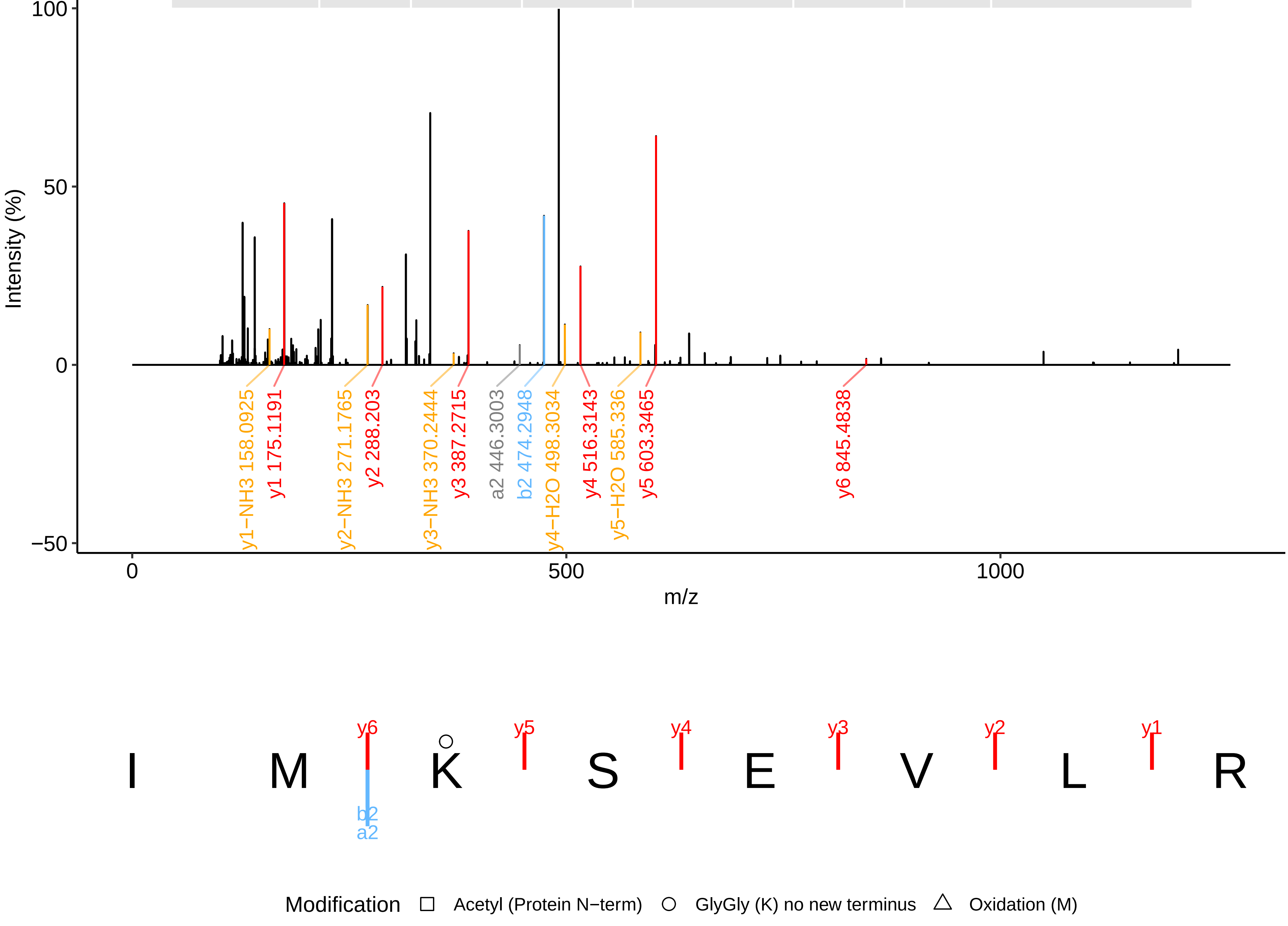

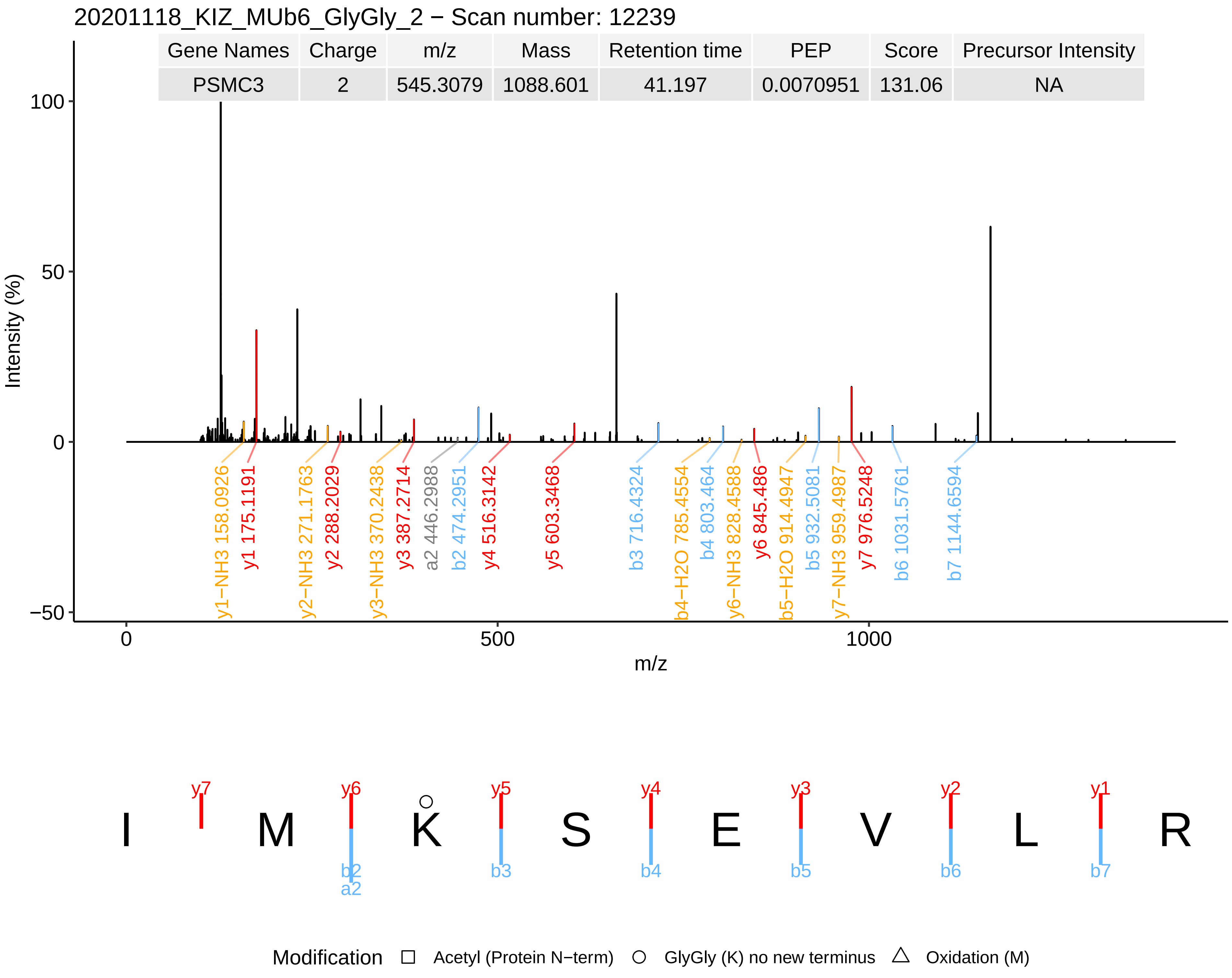

20201118_KIZ_MUb6_GlyGly_2 − Scan number: 12239
Gene Names | Charge | m/z | Mass | Retention time | PEP | Score | Precursor Intensity
PSMC3 | 2 | 545.3079 | 1088.601 | 41.197 | 0.0070951 | 131.06 | NA
Intensity (%)
m/z
y1−NH3 158.0926
y1 175.1191
y2−NH3 271.1763
y2 288.2029
y3−NH3 370.2438
y3 387.2714
a2 446.2988
b2 474.2951
y4 516.3142
y5 603.3468
b3 716.4324
b4−H2O 785.4554
b4 803.464
y6−NH3 828.4588
y6 845.486
b5−H2O 914.4947
b5 932.5081
y7−NH3 959.4987
y7 976.5248
b6 1031.5761
b7 1144.6594
I M K S E V L R
y7 y6 y5 y4 y3 y2 y1
b2 a2 b3 b4 b5 b6 b7
Modification
Acetyl (Protein N−term)
GlyGly (K) no new terminus
Oxidation (M)

20200921_KIZ_MUb4_GlyGly_10plex − Scan number: 3449

Gene Names	Charge	m/z	Mass	Retention time	PEP	Score	Precursor Intensity
BAG2	3	362.8599	1085.558	10.935	0.013085	71.614	6287420

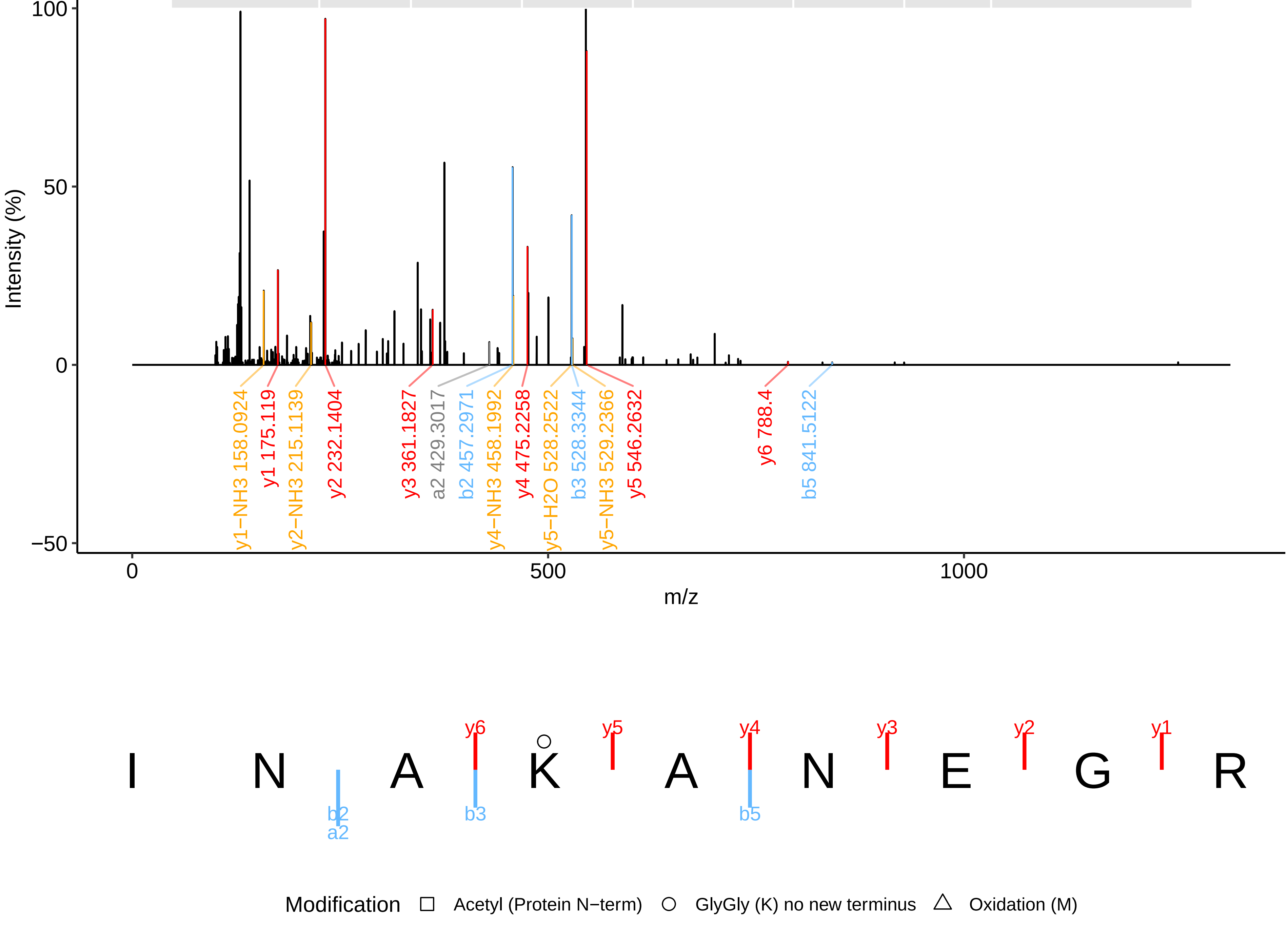

20200924_KIZ_MUb5_GlyGly_1 − Scan number: 3222

Gene Names	Charge	m/z	Mass	Retention time	PEP	Score	Precursor Intensity
BAG2	3	362.8599	1085.558	9.9232	0.014304	74.173	29247360

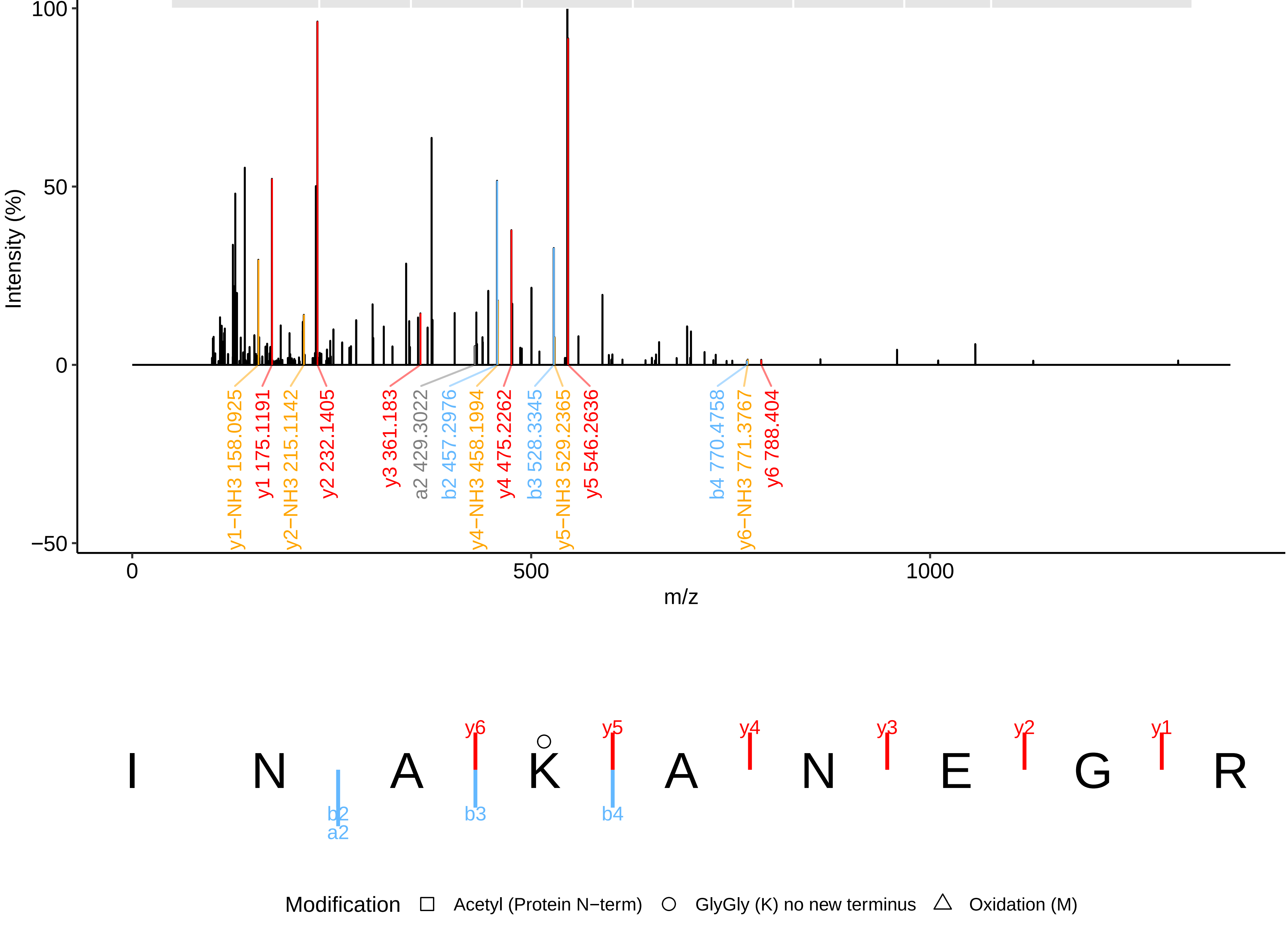

20200924_KIZ_MUb5_GlyGly_2 − Scan number: 2896

Gene Names	Charge	m/z	Mass	Retention time	PEP	Score	Precursor Intensity
BAG2	3	362.8599	1085.558	10.384	0.016651	72.325	2195033

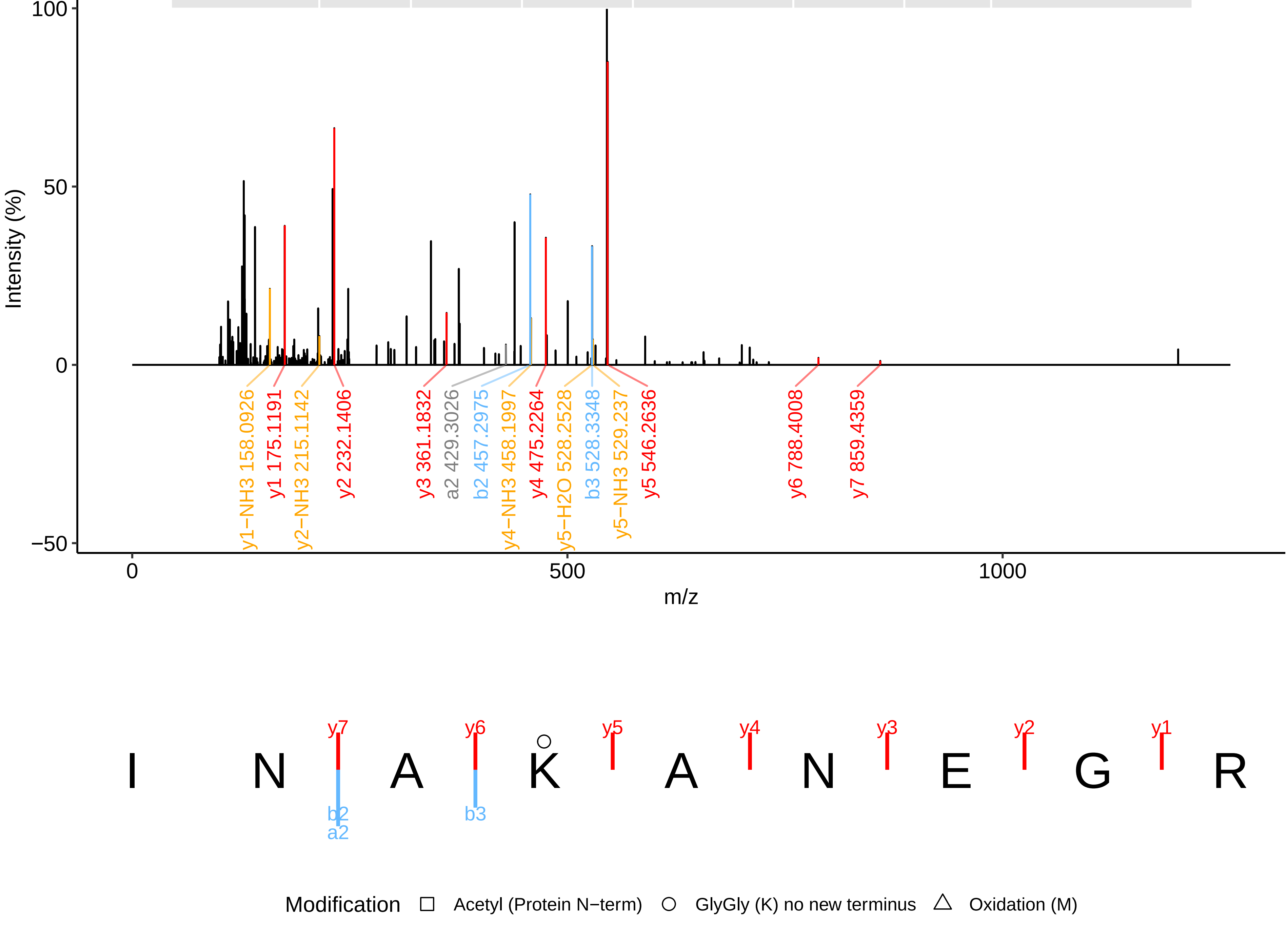

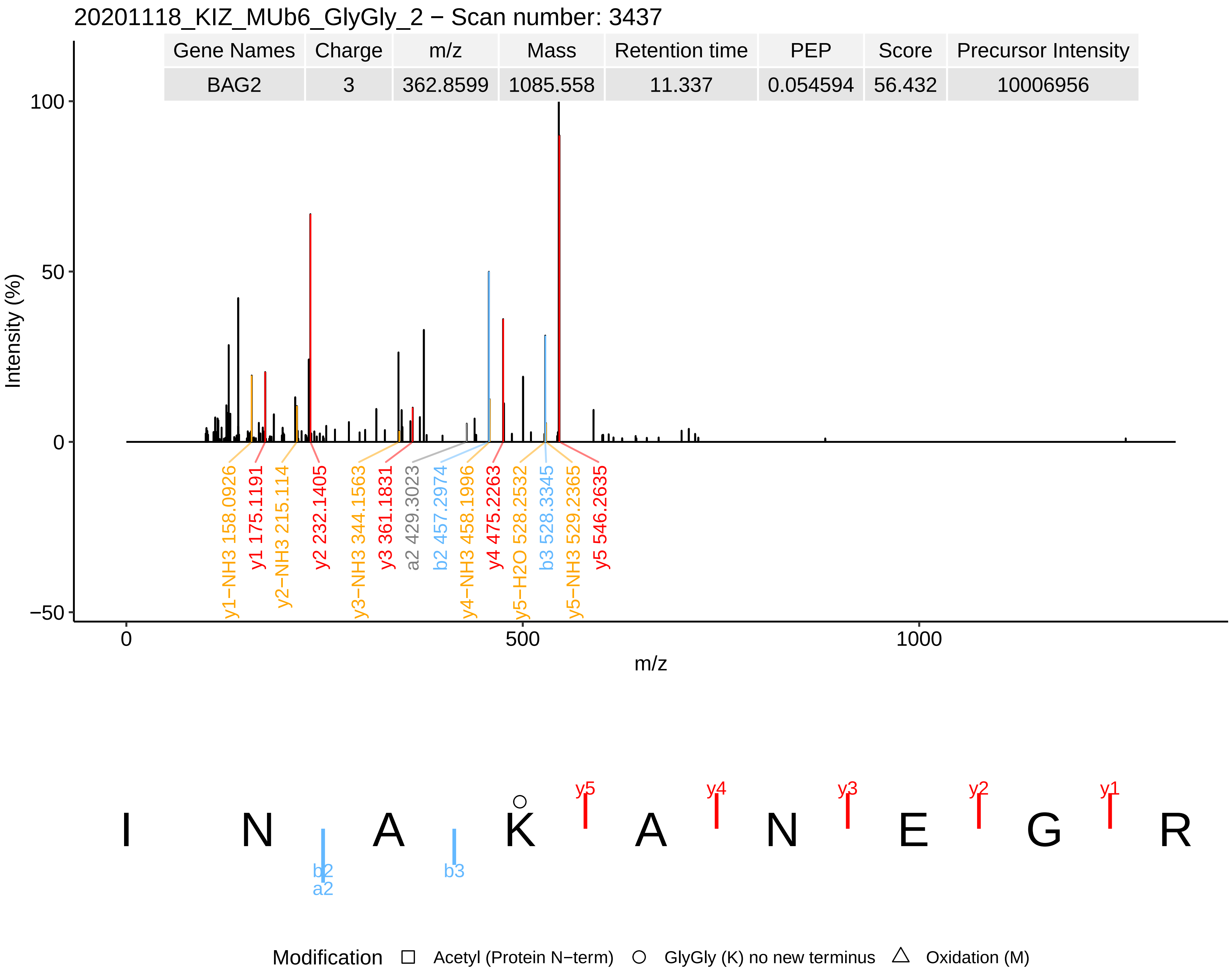

20201118_KIZ_MUb6_GlyGly_2 − Scan number: 3437

Gene Names	Charge	m/z	Mass	Retention time	PEP	Score	Precursor Intensity
BAG2	3	362.8599	1085.558	11.337	0.054594	56.432	10006956

Intensity (%)
100
50
0
−50
0
500
1000
m/z
y1−NH3 158.0926
y1 175.1191
y2−NH3 215.114
y2 232.1405
y3−NH3 344.1563
y3 361.1831
a2 429.3023
b2 457.2974
y4−NH3 458.1996
y4 475.2263
y5−H2O 528.2532
b3 528.3345
y5−NH3 529.2365
y5 546.2635
I N A K A N E G R
y5 y4 y3 y2 y1
b2 a2 b3
Modification
Acetyl (Protein N−term)
GlyGly (K) no new terminus
Oxidation (M)

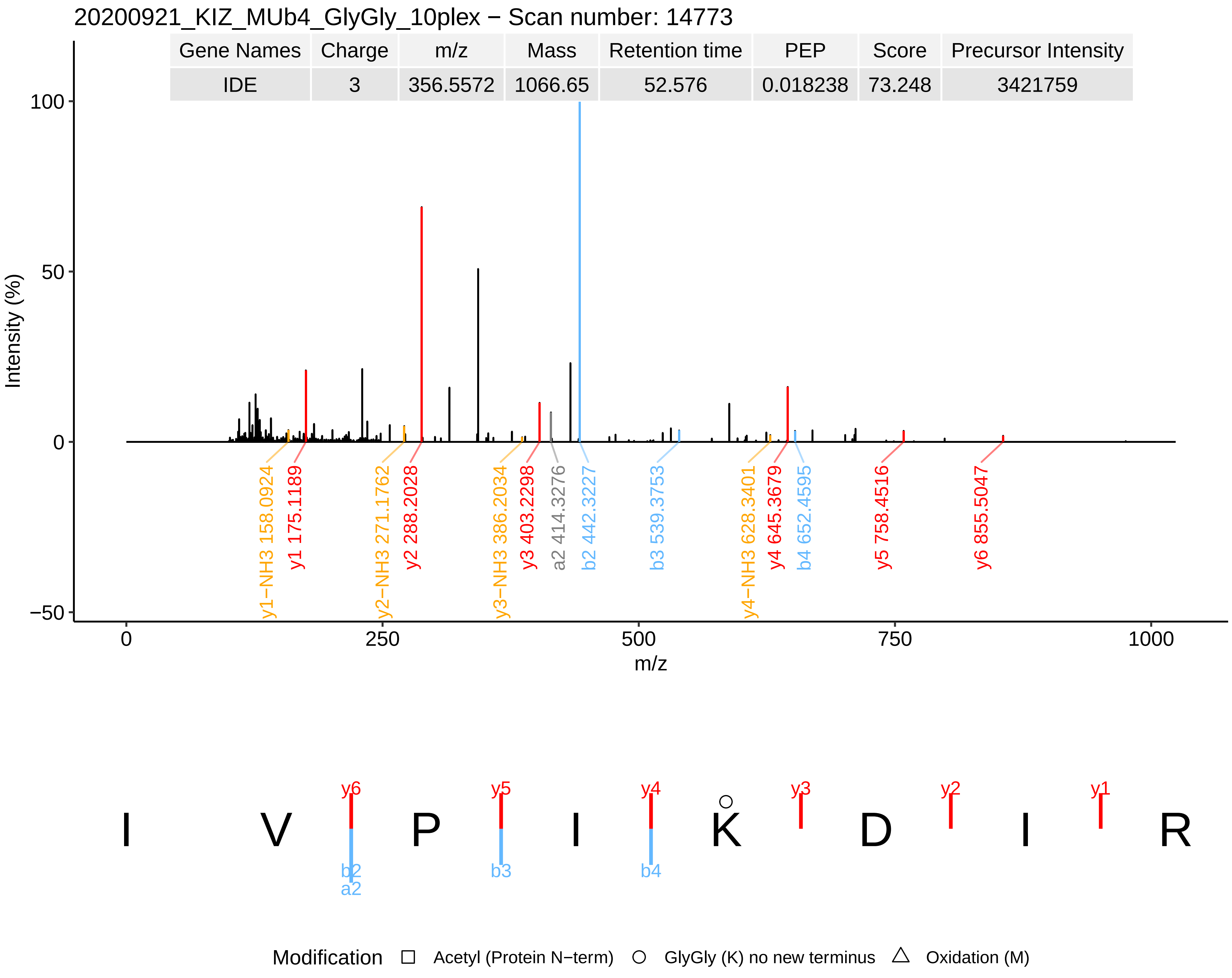
20200921_KIZ_MUb4_GlyGly_10plex − Scan number: 14773
Gene Names | Charge | m/z | Mass | Retention time | PEP | Score | Precursor Intensity
IDE | 3 | 356.5572 | 1066.65 | 52.576 | 0.018238 | 73.248 | 3421759
Intensity (%)
m/z
y1−NH3 158.0924
y1 175.1189
y2−NH3 271.1762
y2 288.2028
y3−NH3 386.2034
y3 403.2298
a2 414.3276
b2 442.3227
b3 539.3753
y4−NH3 628.3401
y4 645.3679
b4 652.4595
y5 758.4516
y6 855.5047
I V P I K D I R
y6 y5 y4 y3 y2 y1
b2 a2 b3 b4
Modification
Acetyl (Protein N−term)
GlyGly (K) no new terminus
Oxidation (M)

20200921_KIZ_MUb4_GlyGly_10plex_2 − Scan number: 13656

Gene Names	Charge	m/z	Mass	Retention time	PEP	Score	Precursor Intensity
IDE	3	356.5572	1066.65	53.066	0.0032042	95.531	1178133

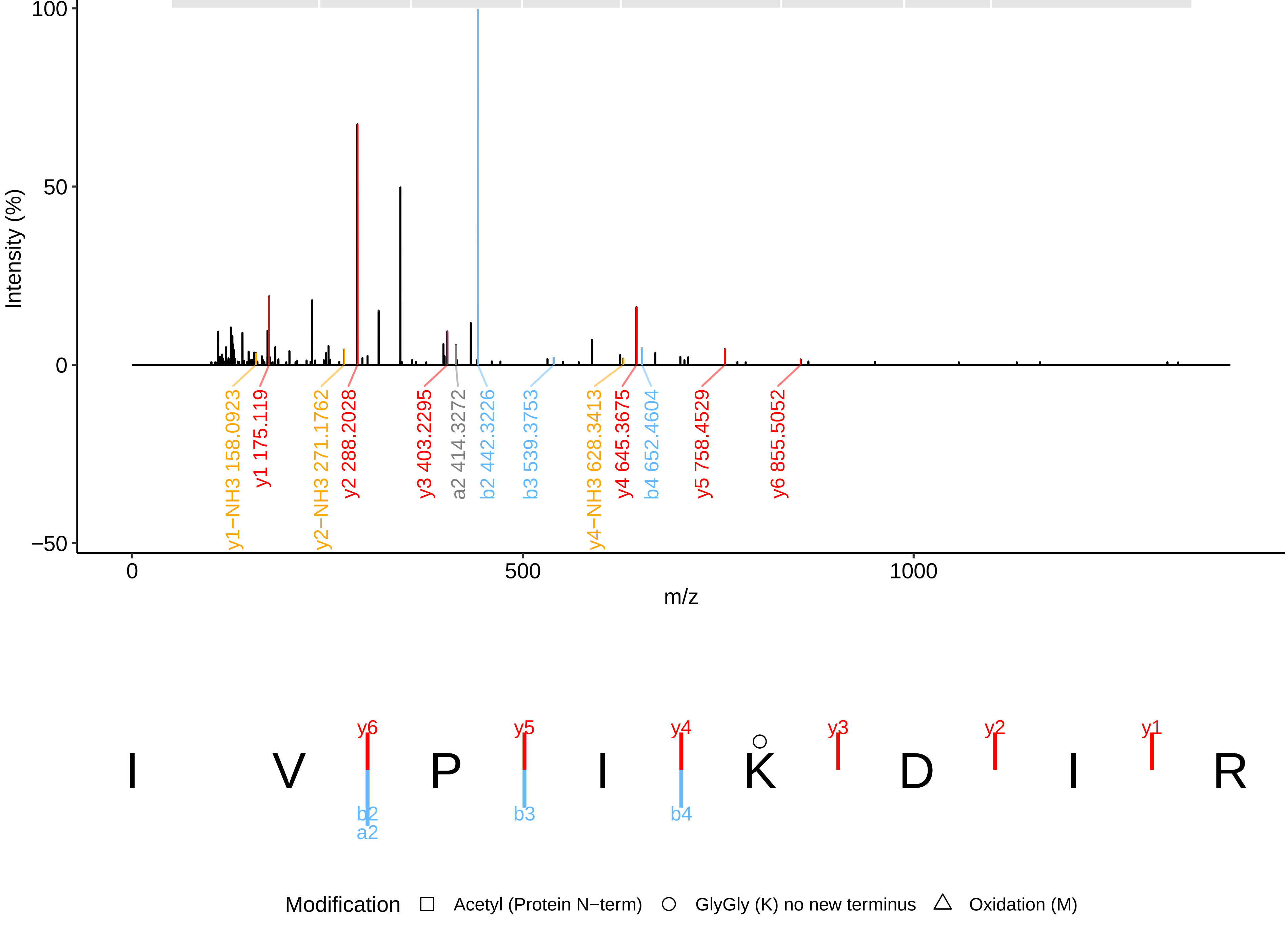

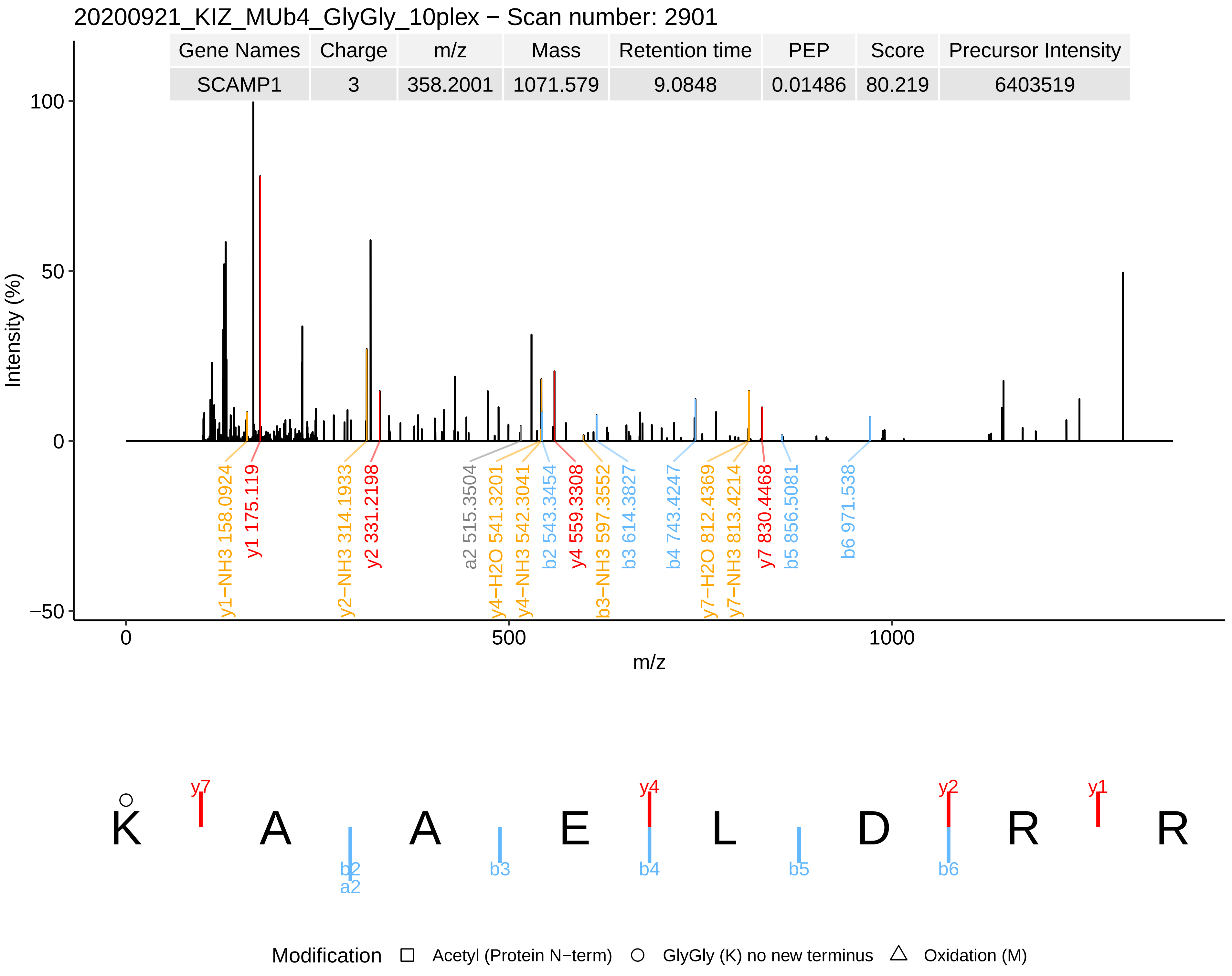
20200921_KIZ_MUb4_GlyGly_10plex − Scan number: 2901
Gene Names
Charge
m/z
Mass
Retention time
PEP
Score
Precursor Intensity
SCAMP1
3
358.2001
1071.579
9.0848
0.01486
80.219
6403519
Intensity (%)
100
50
0
−50
0
500
1000
m/z
y1−NH3 158.0924
y1 175.119
y2−NH3 314.1933
y2 331.2198
a2 515.3504
y4−H2O 541.3201
y4−NH3 542.3041
b2 543.3454
y4 559.3308
b3−NH3 597.3552
b3 614.3827
b4 743.4247
y7−H2O 812.4369
y7−NH3 813.4214
y7 830.4468
b5 856.5081
b6 971.538
K A A E L D R R
y7
y4
y2
y1
b2
a2
b3
b4
b5
b6
Modification
Acetyl (Protein N−term)
GlyGly (K) no new terminus
Oxidation (M)

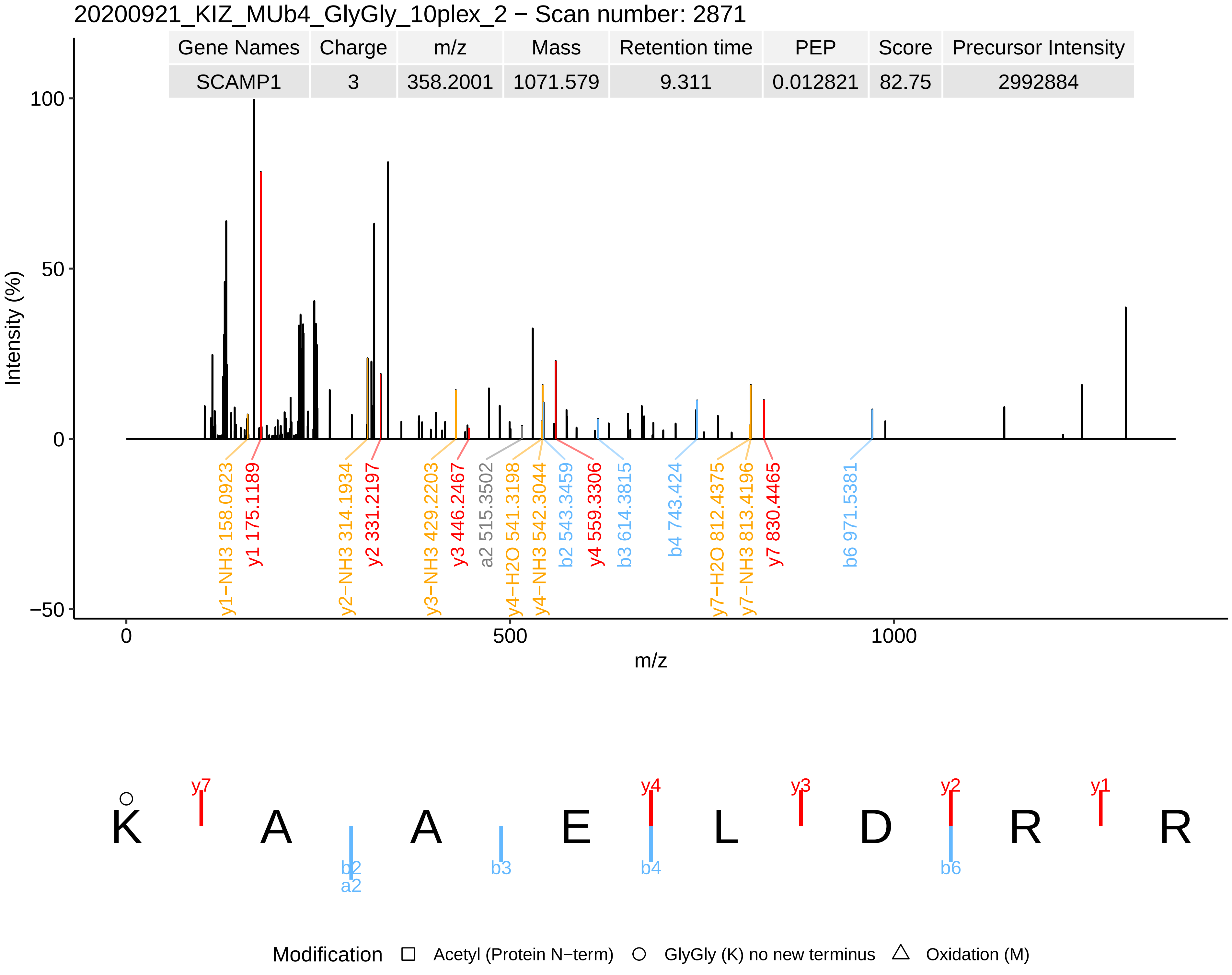

20200921_KIZ_MUb4_GlyGly_10plex_2 − Scan number: 2871
Gene Names | Charge | m/z | Mass | Retention time | PEP | Score | Precursor Intensity
SCAMP1 | 3 | 358.2001 | 1071.579 | 9.311 | 0.012821 | 82.75 | 2992884
Intensity (%)
m/z
y1−NH3 158.0923
y1 175.1189
y2−NH3 314.1934
y2 331.2197
y3−NH3 429.2203
y3 446.2467
a2 515.3502
y4−H2O 541.3198
y4−NH3 542.3044
b2 543.3459
y4 559.3306
b3 614.3815
b4 743.424
y7−H2O 812.4375
y7−NH3 813.4196
y7 830.4465
b6 971.5381
K A A E L D R R
y7 y4 y3 y2 y1
b2 a2 b3 b4 b6
Modification
Acetyl (Protein N−term)
GlyGly (K) no new terminus
Oxidation (M)

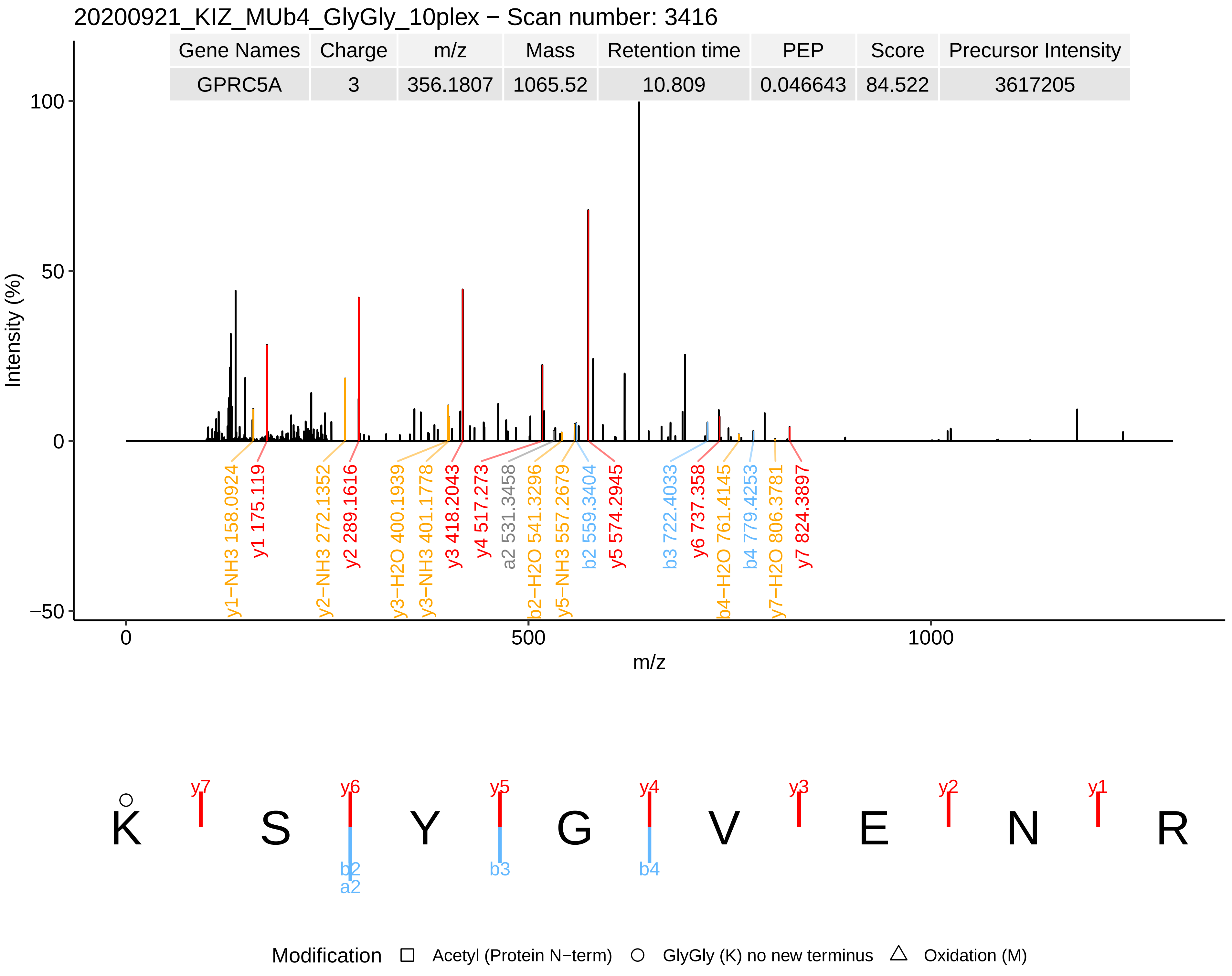
20200921_KIZ_MUb4_GlyGly_10plex − Scan number: 3416
Gene Names | Charge | m/z | Mass | Retention time | PEP | Score | Precursor Intensity
GPRC5A | 3 | 356.1807 | 1065.52 | 10.809 | 0.046643 | 84.522 | 3617205
Intensity (%)
100
50
0
−50
0
500
1000
m/z
y1−NH3 158.0924
y1 175.119
y2−NH3 272.1352
y2 289.1616
y3−H2O 400.1939
y3−NH3 401.1778
y3 418.2043
y4 517.273
a2 531.3458
b2−H2O 541.3296
y5−NH3 557.2679
b2 559.3404
y5 574.2945
b3 722.4033
y6 737.358
b4−H2O 761.4145
b4 779.4253
y7−H2O 806.3781
y7 824.3897
K S Y G V E N R
y7 y6 y5 y4 y3 y2 y1
b2 a2 b3 b4
Modification
Acetyl (Protein N−term)
GlyGly (K) no new terminus
Oxidation (M)

20200921_KIZ_MUb4_GlyGly_10plex − Scan number: 3434

Gene Names	Charge	m/z	Mass	Retention time	PEP	Score	Precursor Intensity
GPRC5A	3	356.1807	1065.52	10.876	0.0046434	111.06	30282092

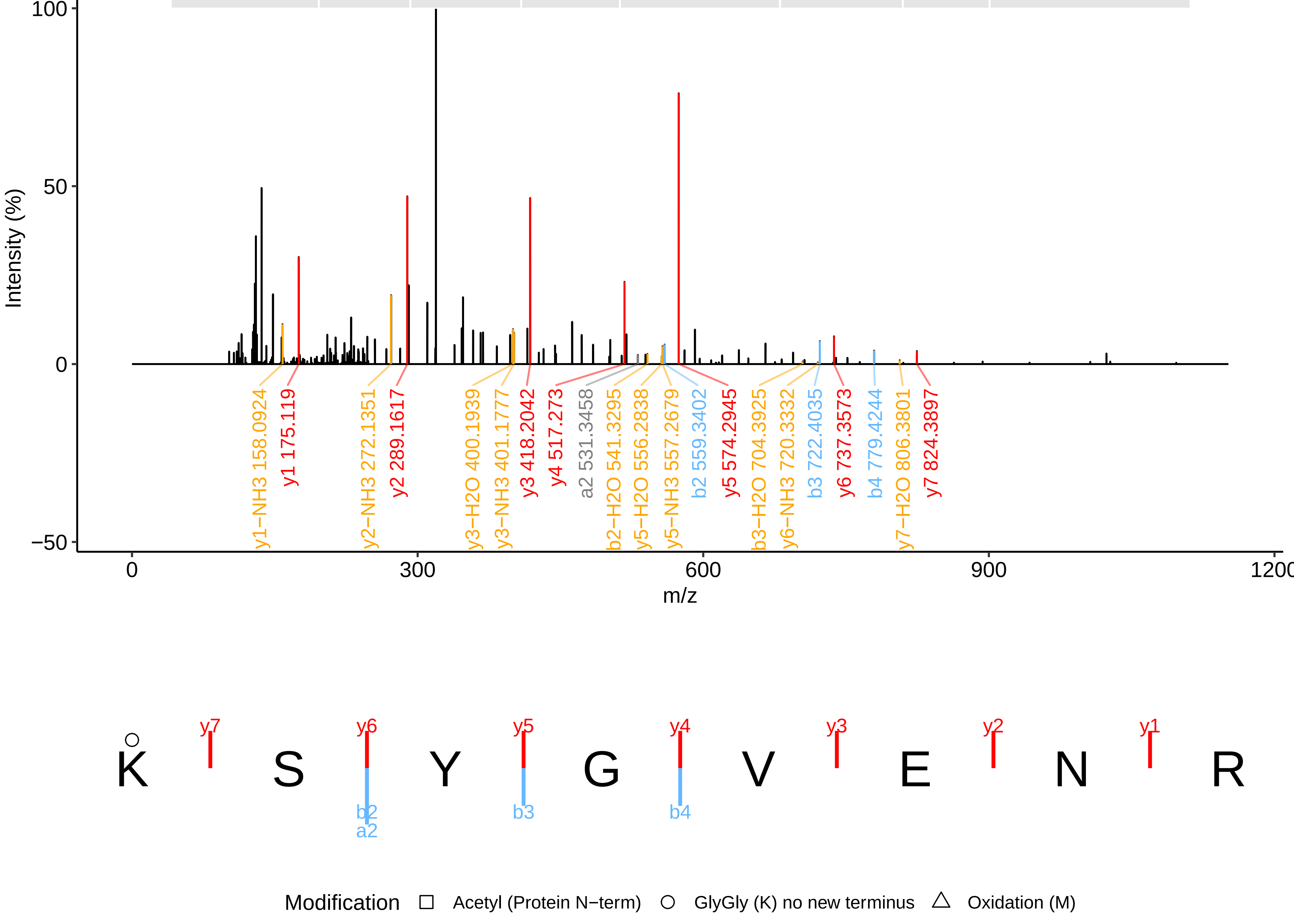

20200921_KIZ_MUb4_GlyGly_10plex − Scan number: 3427

Gene Names	Charge	m/z	Mass	Retention time	PEP	Score	Precursor Intensity
GPRC5A	2	533.7674	1065.52	10.849	0.0036366	118.5	1320577

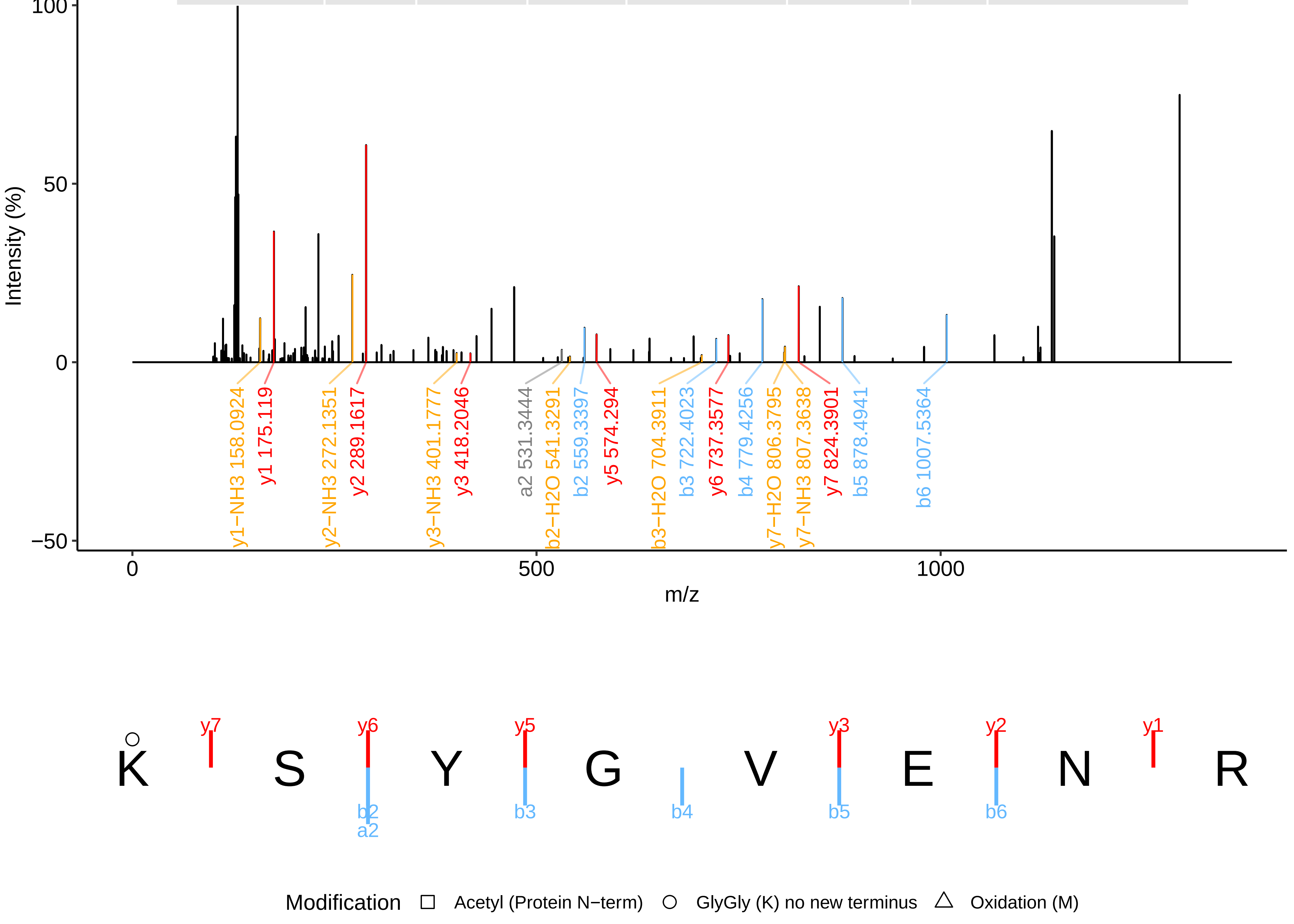

20200921_KIZ_MUb4_GlyGly_10plex_2 − Scan number: 3275

Gene Names	Charge	m/z	Mass	Retention time	PEP	Score	Precursor Intensity
GPRC5A	3	356.1807	1065.52	10.921	0.031048	90.919	3548436

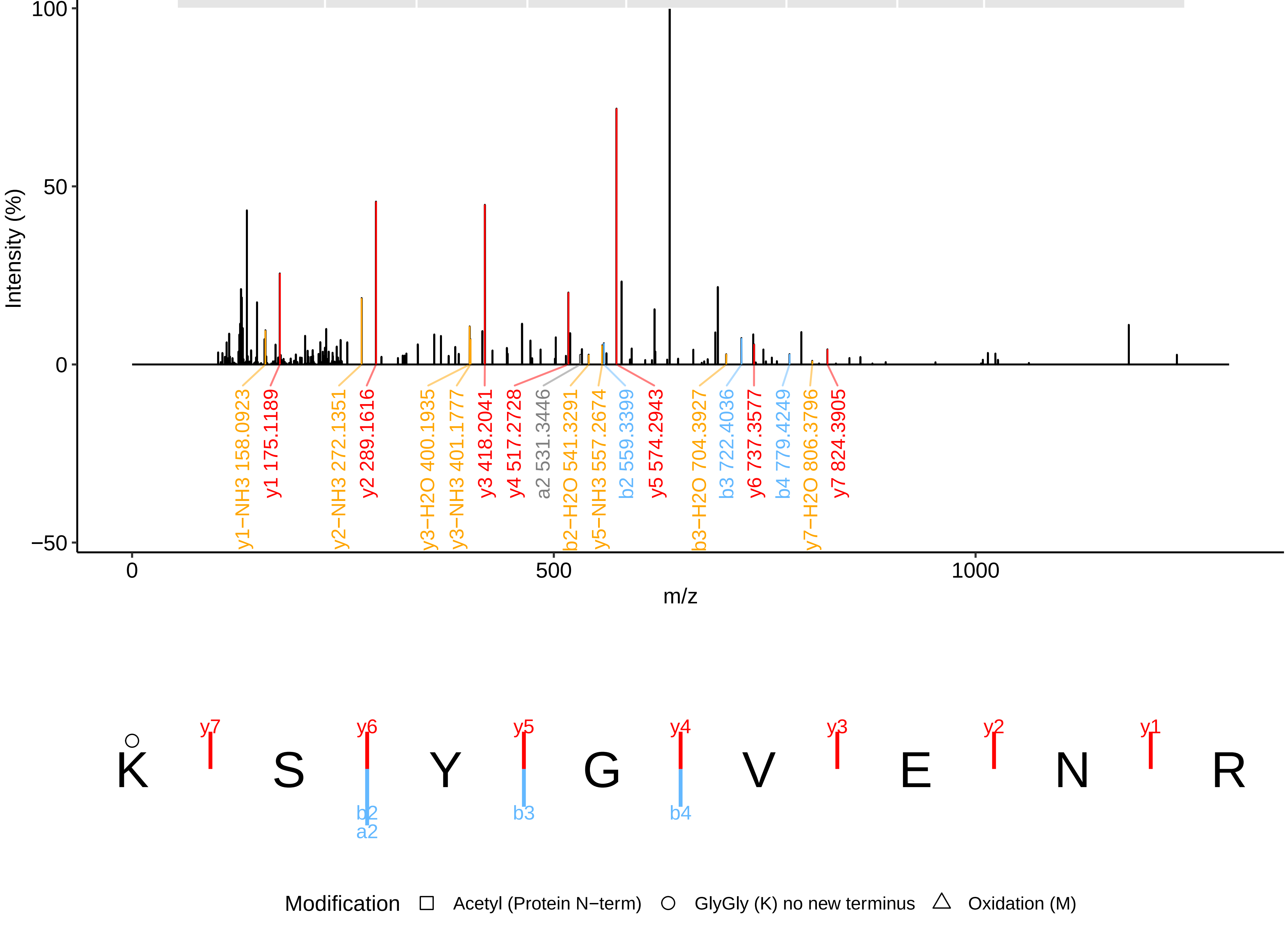

20200924_KIZ_MUb5_GlyGly_1 − Scan number: 3143

Gene Names	Charge	m/z	Mass	Retention time	PEP	Score	Precursor Intensity
GPRC5A	3	356.1807	1065.52	9.6994	0.075395	78.098	3510000

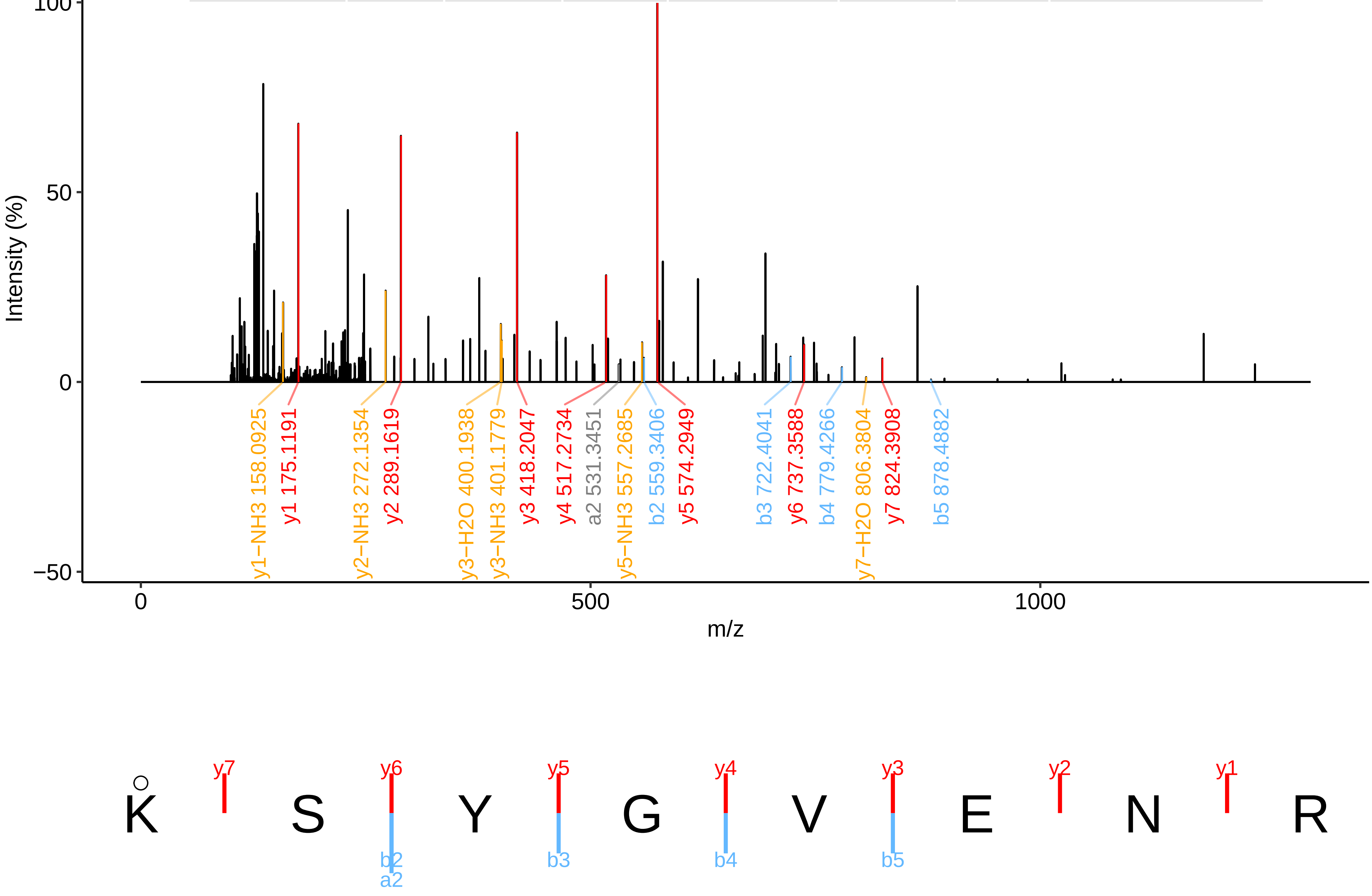

Modification □ Acetyl (Protein N−term) ○ GlyGly (K) no new terminus △ Oxidation (M)

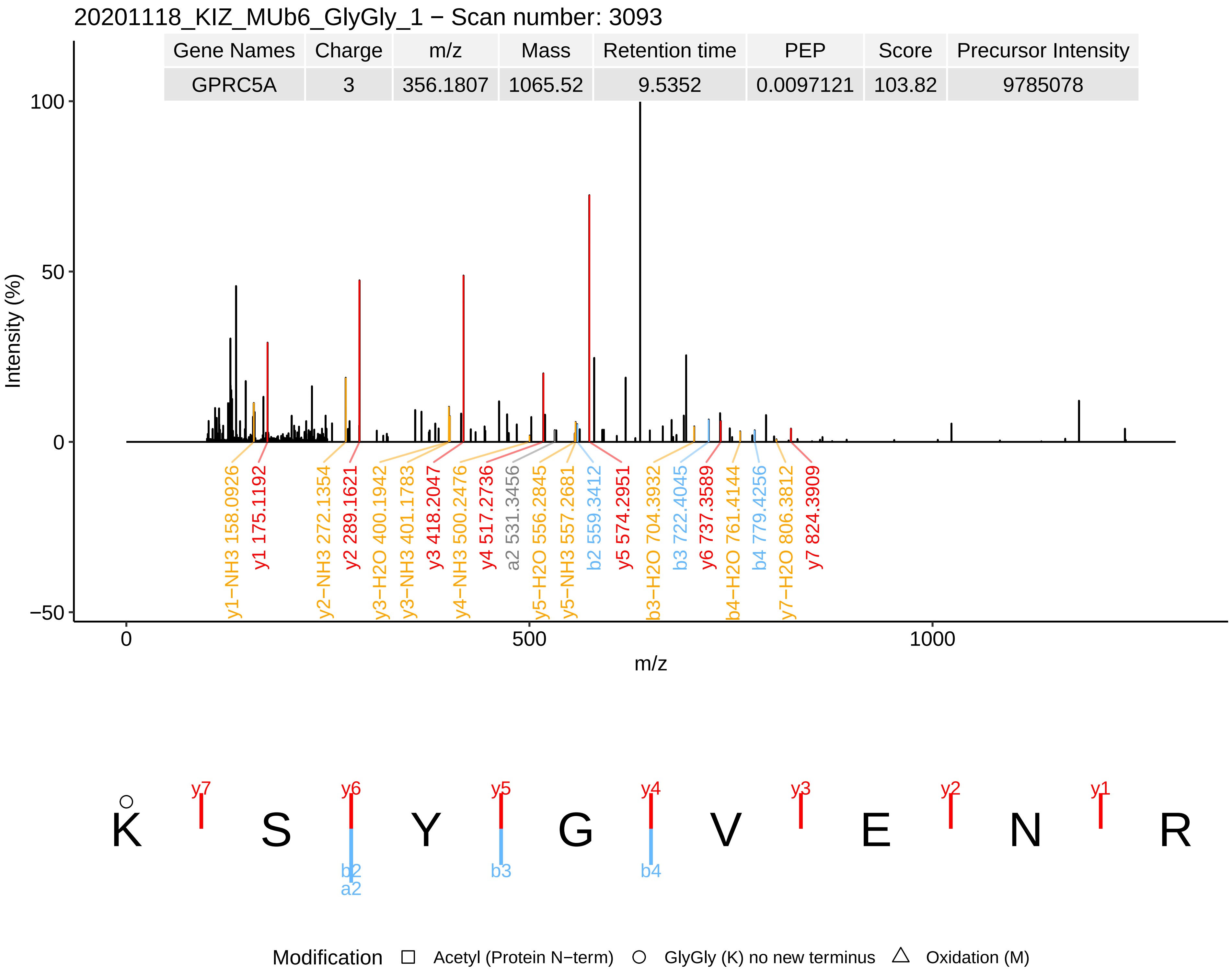

20201118_KIZ_MUb6_GlyGly_1 − Scan number: 3093
Gene Names | Charge | m/z | Mass | Retention time | PEP | Score | Precursor Intensity
GPRC5A | 3 | 356.1807 | 1065.52 | 9.5352 | 0.0097121 | 103.82 | 9785078
Intensity (%)
m/z
y1−NH3 158.0926
y1 175.1192
y2−NH3 272.1354
y2 289.1621
y3−H2O 400.1942
y3−NH3 401.1783
y3 418.2047
y4−NH3 500.2476
y4 517.2736
a2 531.3456
y5−H2O 556.2845
y5−NH3 557.2681
b2 559.3412
y5 574.2951
b3−H2O 704.3932
b3 722.4045
y6 737.3589
b4−H2O 761.4144
b4 779.4256
y7−H2O 806.3812
y7 824.3909
K S Y G V E N R
y7 y6 y5 y4 y3 y2 y1
b2 a2 b3 b4
Modification
Acetyl (Protein N−term)
GlyGly (K) no new terminus
Oxidation (M)

20201118_KIZ_MUb6_GlyGly_2 − Scan number: 3378

Gene Names	Charge	m/z	Mass	Retention time	PEP	Score	Precursor Intensity
GPRC5A	3	356.1807	1065.52	11.105	0.0095791	104.01	5084937

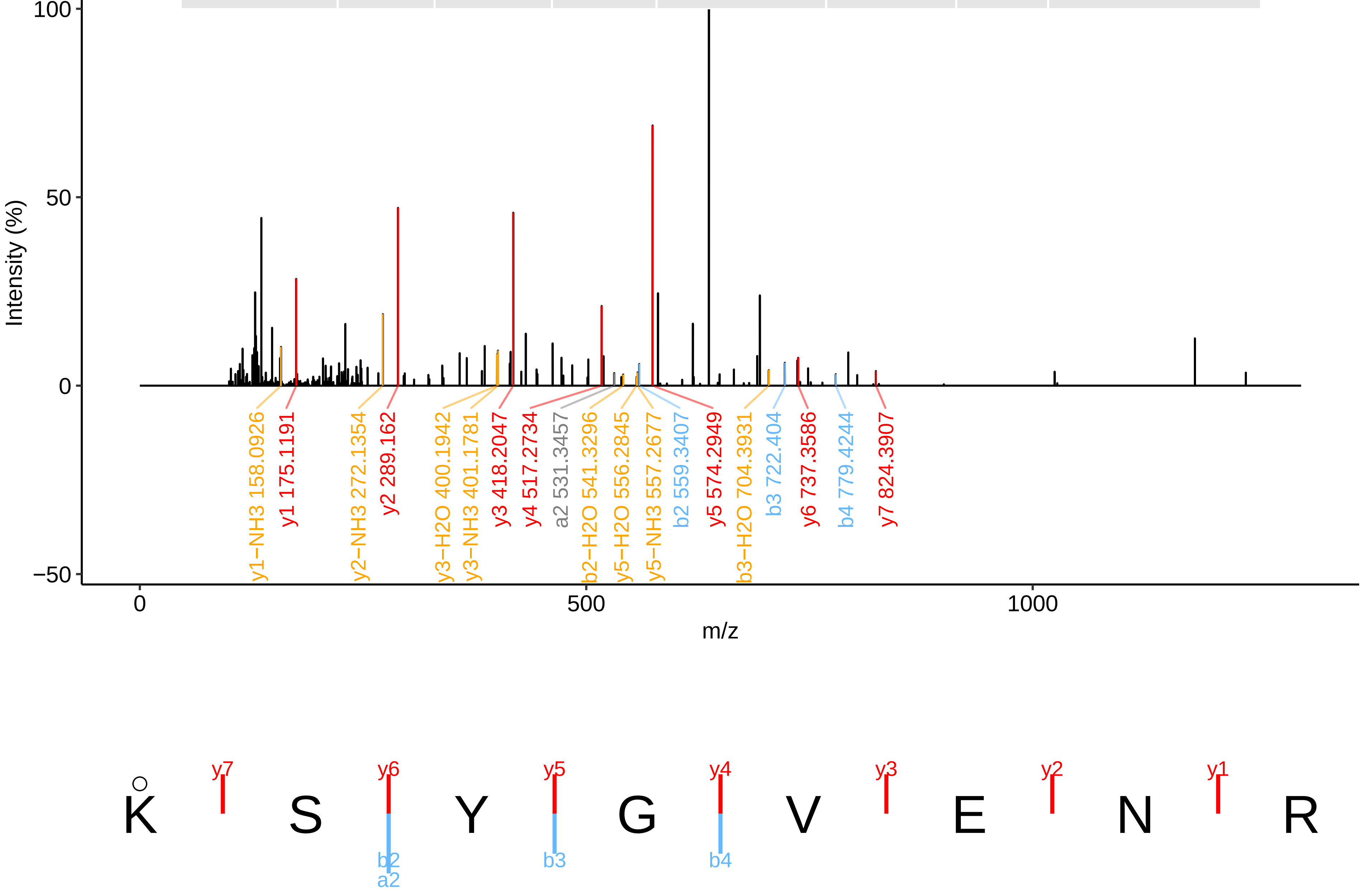

Modification □ Acetyl (Protein N−term) ○ GlyGly (K) no new terminus △ Oxidation (M)

20200921_KIZ_MUb4_GlyGly_10plex − Scan number: 5304

Gene Names	Charge	m/z	Mass	Retention time	PEP	Score	Precursor Intensity
TCP1	3	354.1904	1059.55	17.349	0.012415	77.923	25361370

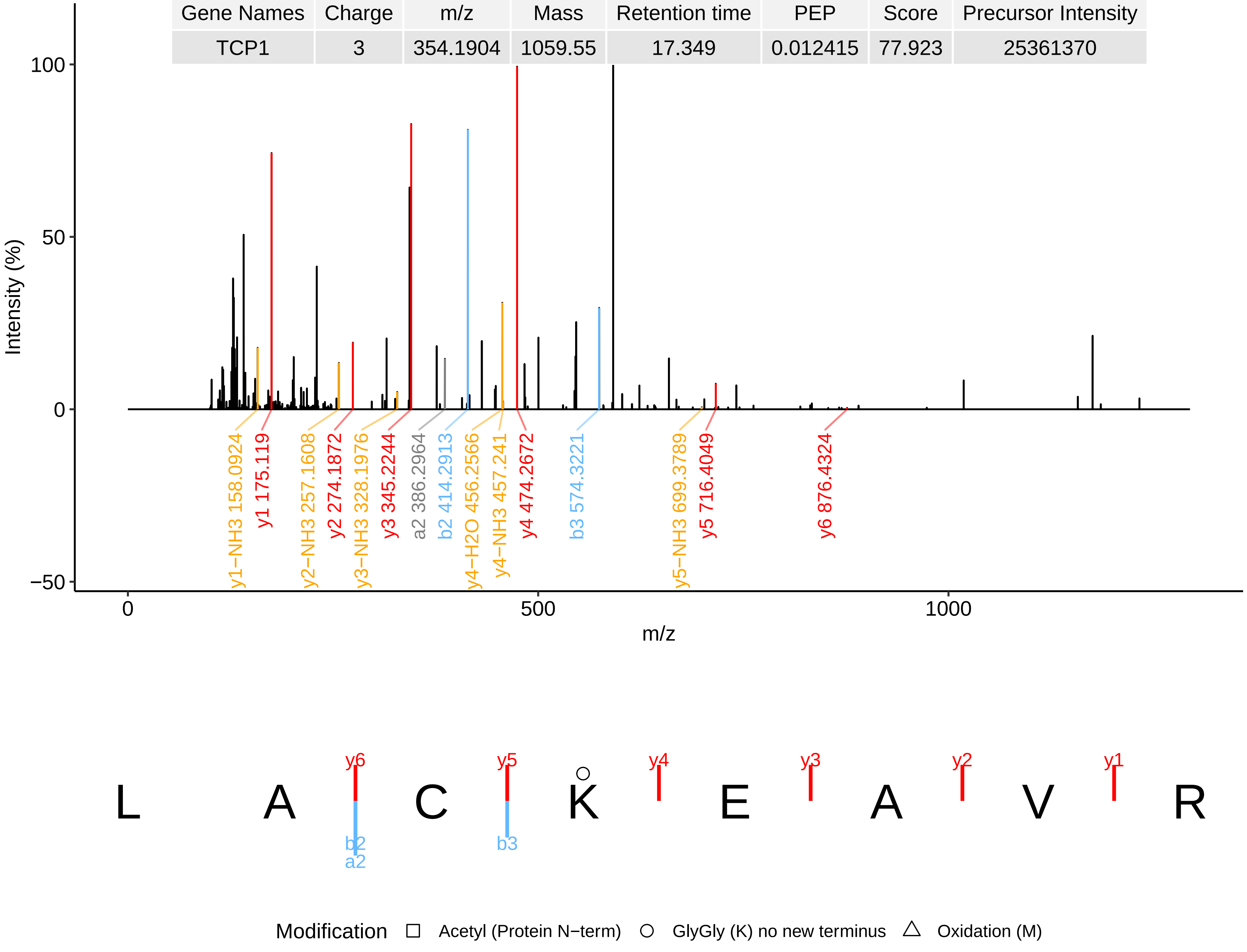

20200921_KIZ_MUb4_GlyGly_10plex_2 − Scan number: 4881

Gene Names	Charge	m/z	Mass	Retention time	PEP	Score	Precursor Intensity
TCP1	3	354.1904	1059.55	17.31	0.0248	67.981	2896359

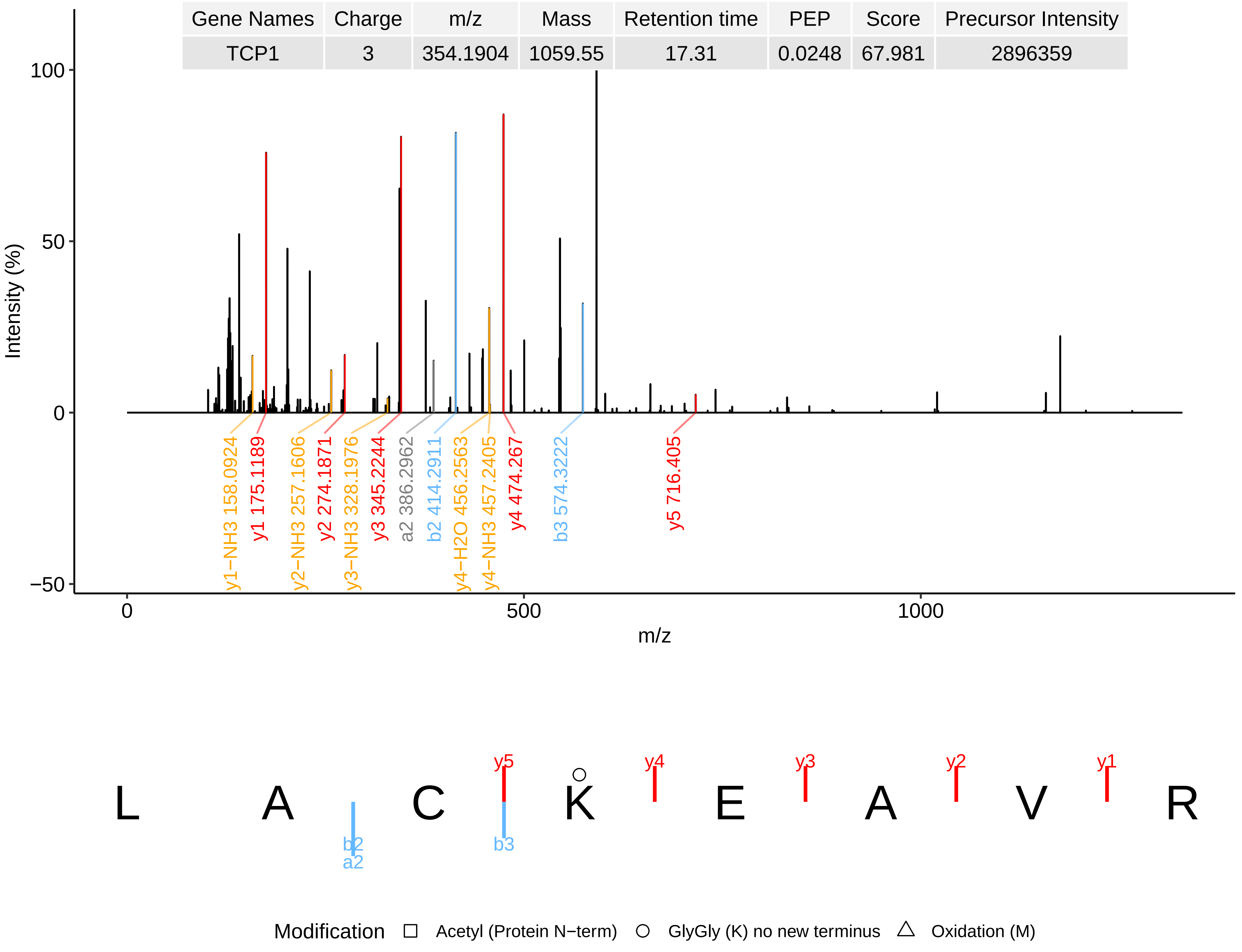

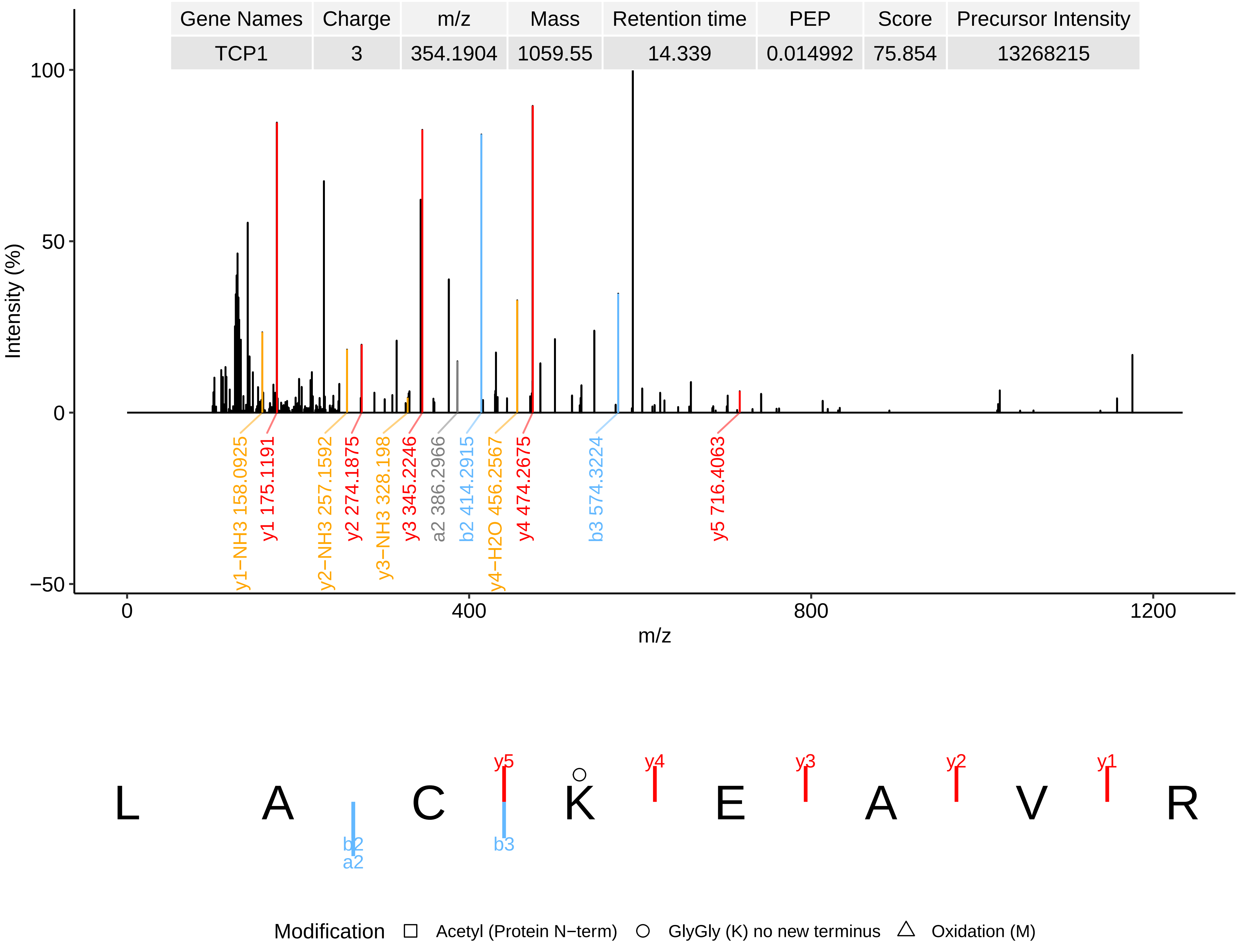

Gene Names	Charge	m/z	Mass	Retention time	PEP	Score	Precursor Intensity
TCP1	3	354.1904	1059.55	14.339	0.014992	75.854	13268215

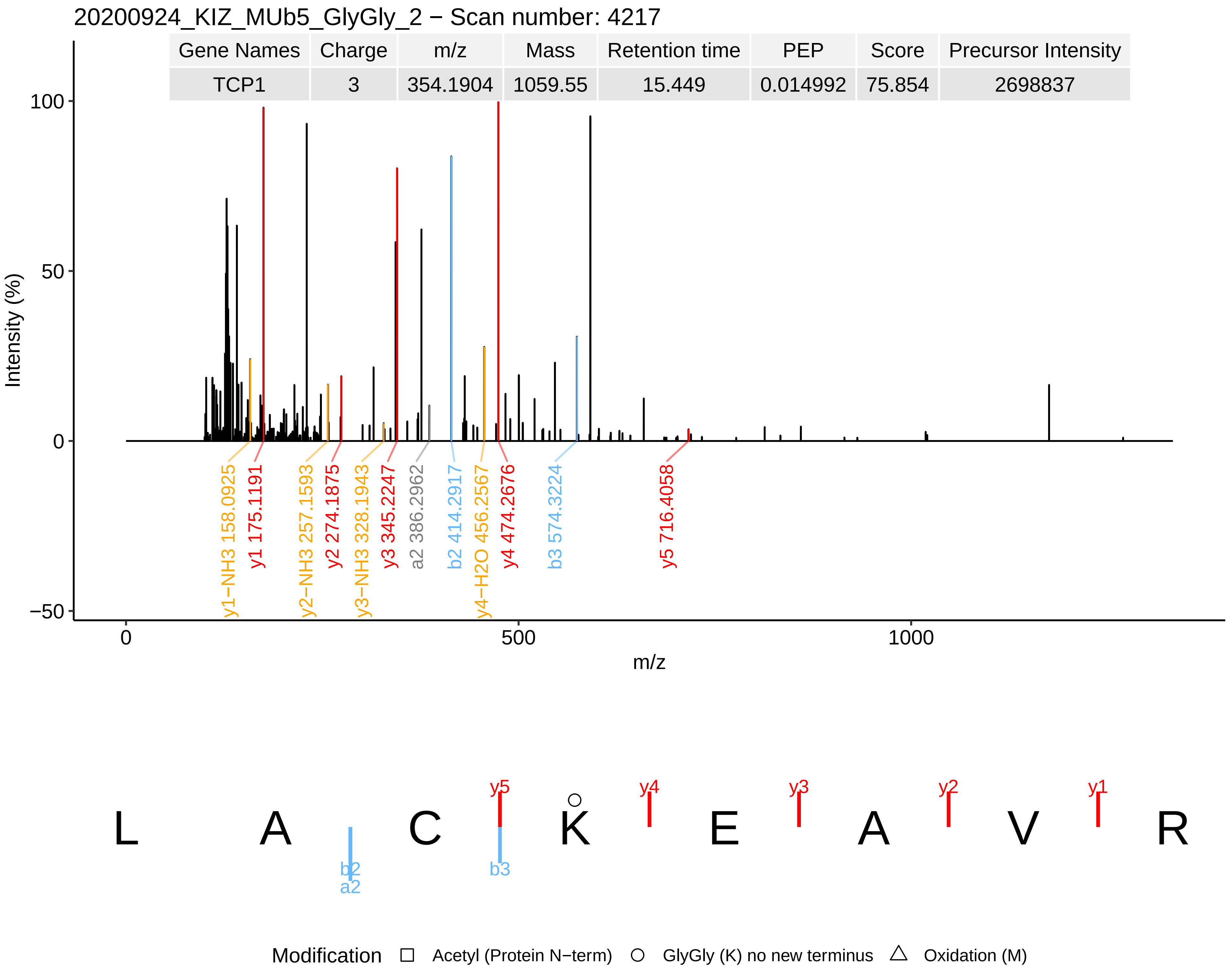
20200924_KIZ_MUb5_GlyGly_2 − Scan number: 4217
Gene Names | Charge | m/z | Mass | Retention time | PEP | Score | Precursor Intensity
TCP1 | 3 | 354.1904 | 1059.55 | 15.449 | 0.014992 | 75.854 | 2698837
Intensity (%)
100
50
0
−50
0
500
1000
m/z
y1−NH3 158.0925
y1 175.1191
y2−NH3 257.1593
y2 274.1875
y3−NH3 328.1943
y3 345.2247
a2 386.2962
b2 414.2917
y4−H2O 456.2567
y4 474.2676
b3 574.3224
y5 716.4058
L A C K E A V R
y5 y4 y3 y2 y1
b2
a2
b3
Modification
Acetyl (Protein N−term)
GlyGly (K) no new terminus
Oxidation (M)

20201118_KIZ_MUb6_GlyGly_1 − Scan number: 4404

Gene Names	Charge	m/z	Mass	Retention time	PEP	Score	Precursor Intensity
TCP1	3	354.1904	1059.55	13.769	0.007332	85.064	8990933

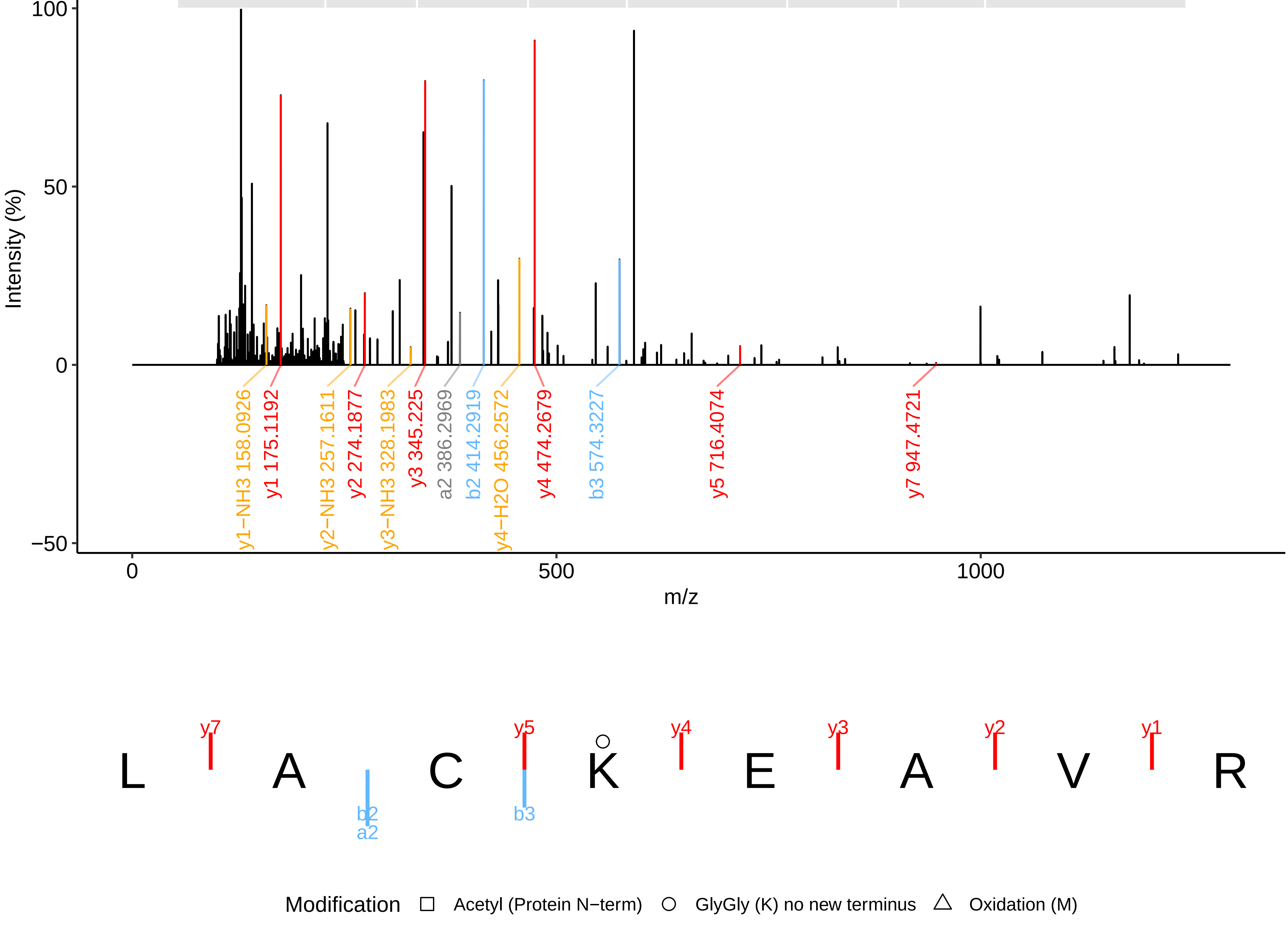

20200921_KIZ_MUb4_GlyGly_10plex − Scan number: 6135

Gene Names	Charge	m/z	Mass	Retention time	PEP	Score	Precursor Intensity
EMG1	3	352.8685	1055.584	20.193	0.0027293	80.455	7868467

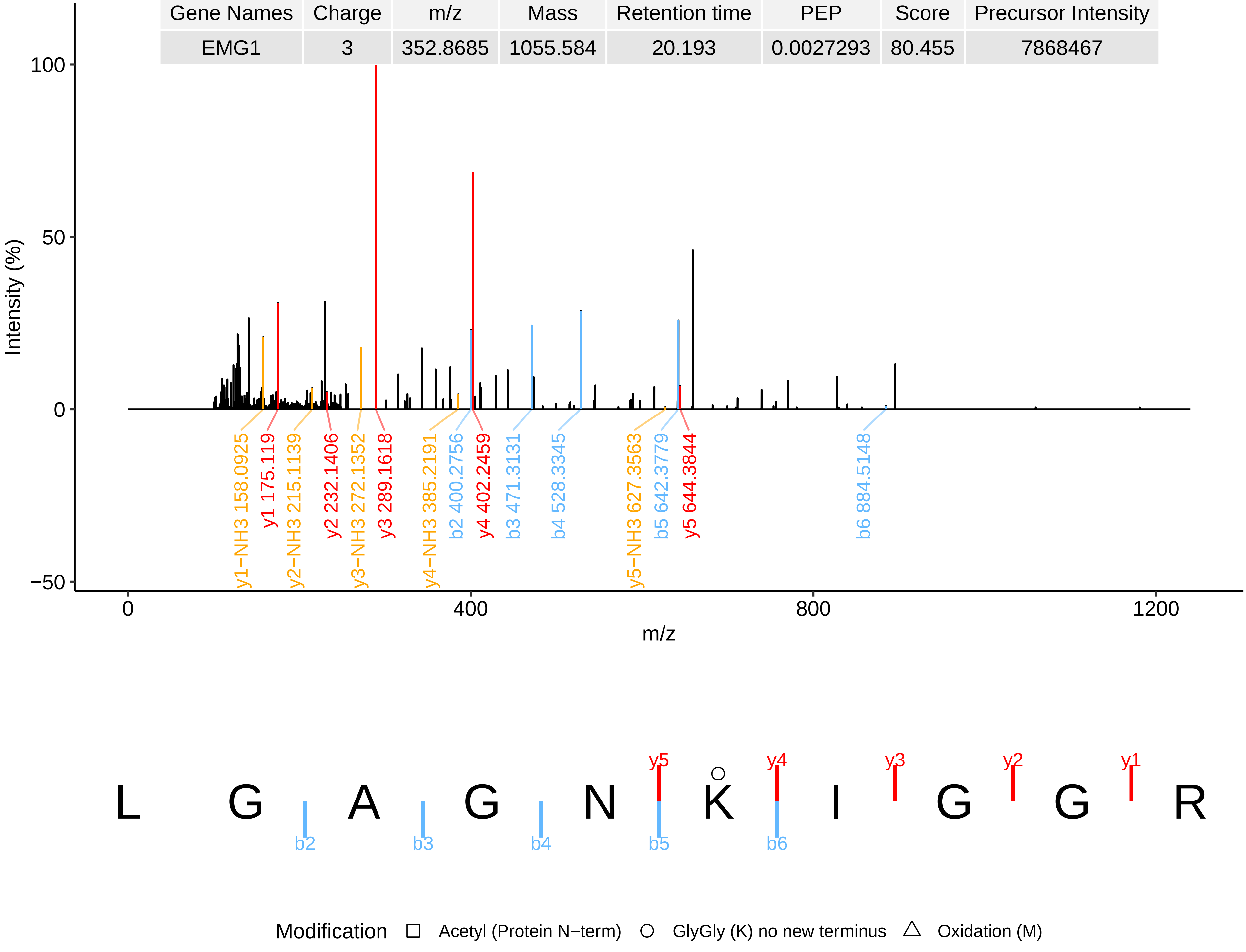

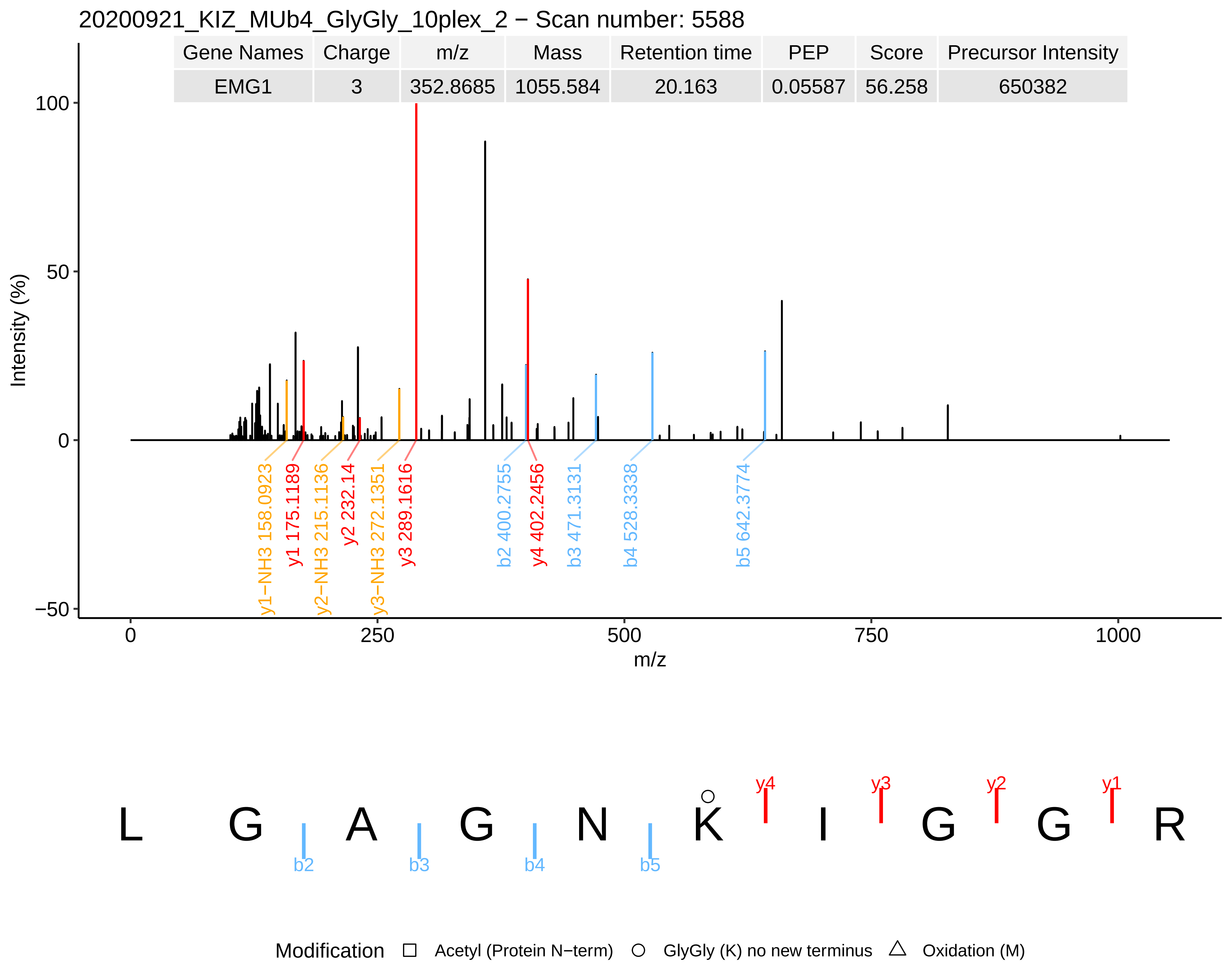

20200921_KIZ_MUb4_GlyGly_10plex_2 − Scan number: 5588
Gene Names | Charge | m/z | Mass | Retention time | PEP | Score | Precursor Intensity
EMG1 | 3 | 352.8685 | 1055.584 | 20.163 | 0.05587 | 56.258 | 650382
Intensity (%)
m/z
y1−NH3 158.0923
y1 175.1189
y2−NH3 215.1136
y2 232.14
y3−NH3 272.1351
y3 289.1616
b2 400.2755
y4 402.2456
b3 471.3131
b4 528.3338
b5 642.3774
L G A G N K I G G R
y4 y3 y2 y1
b2 b3 b4 b5
Modification
Acetyl (Protein N−term)
GlyGly (K) no new terminus
Oxidation (M)

20200921_KIZ_MUb4_GlyGly_10plex − Scan number: 9071

Gene Names	Charge	m/z	Mass	Retention time	PEP	Score	Precursor Intensity
RPS4X	3	360.533	1078.577	31.011	9.709e−05	138.83	6059457

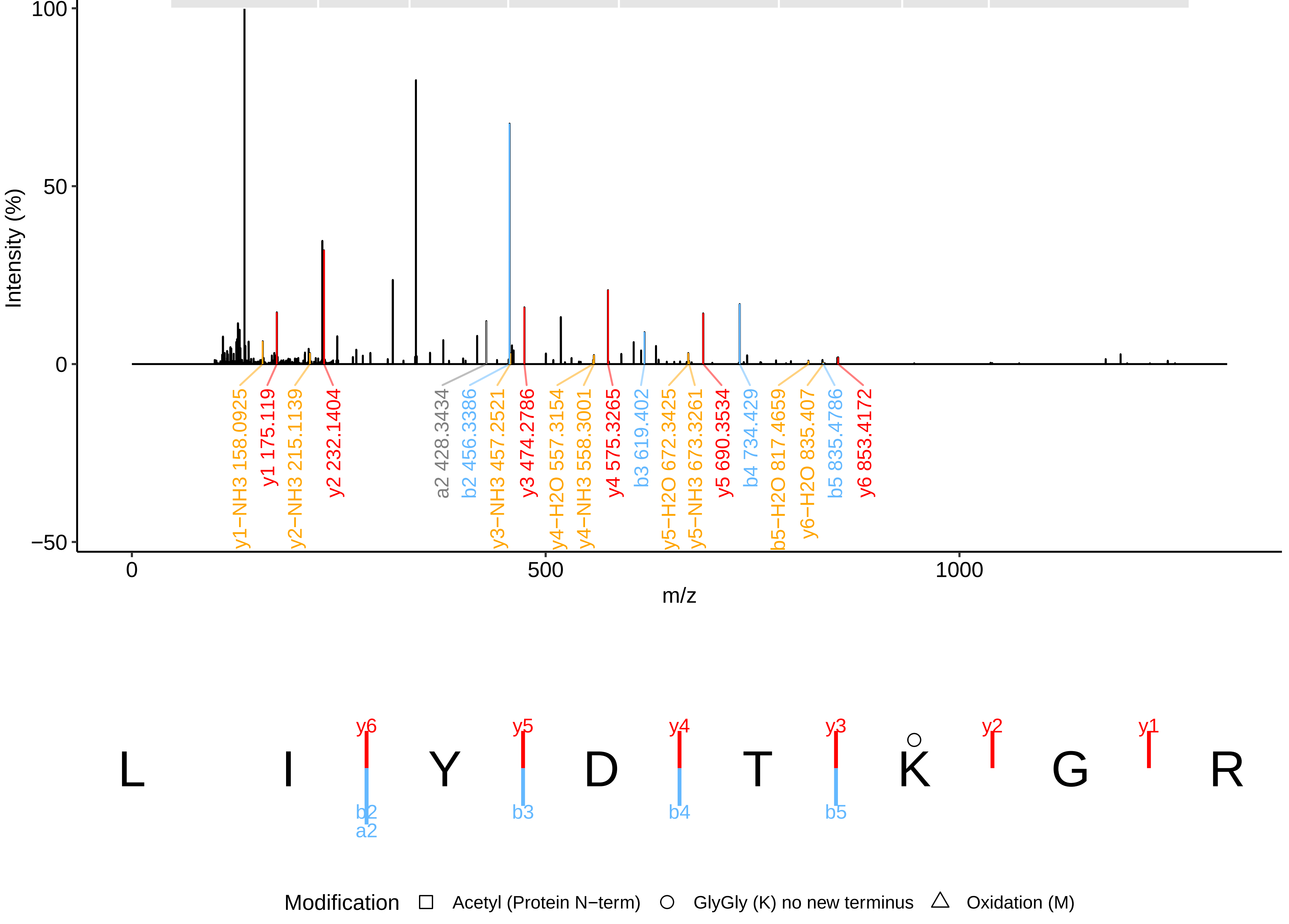

20200921_KIZ_MUb4_GlyGly_10plex_2 − Scan number: 8395

Gene Names	Charge	m/z	Mass	Retention time	PEP	Score	Precursor Intensity
RPS4X	3	360.533	1078.577	31.613	0.0078492	83.753	1202592

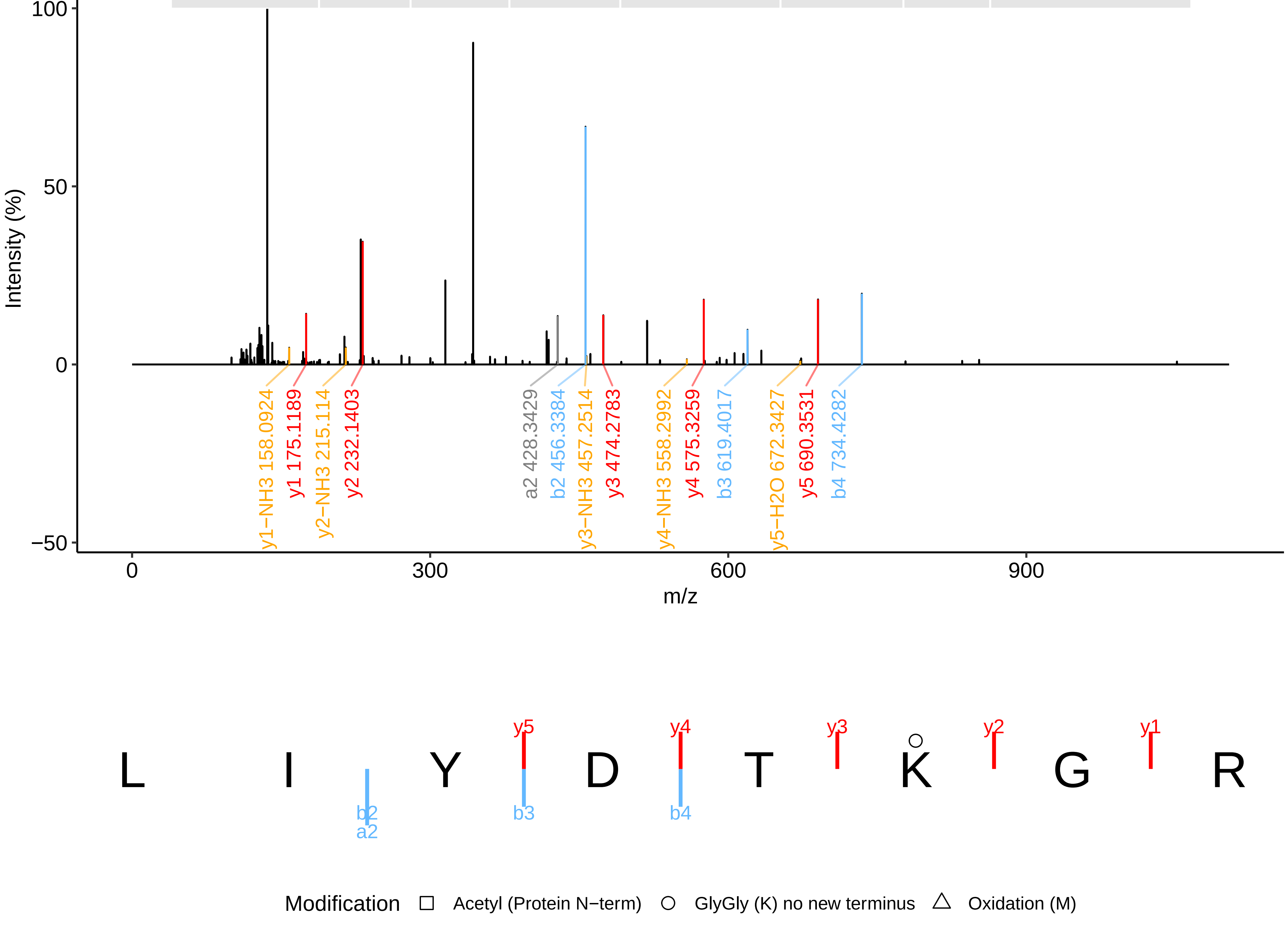

20200924_KIZ_MUb5_GlyGly_1 − Scan number: 7914

Gene Names	Charge	m/z	Mass	Retention time	PEP	Score	Precursor Intensity
RPS4X	3	360.533	1078.577	25.212	0.0018539	101.33	13087015

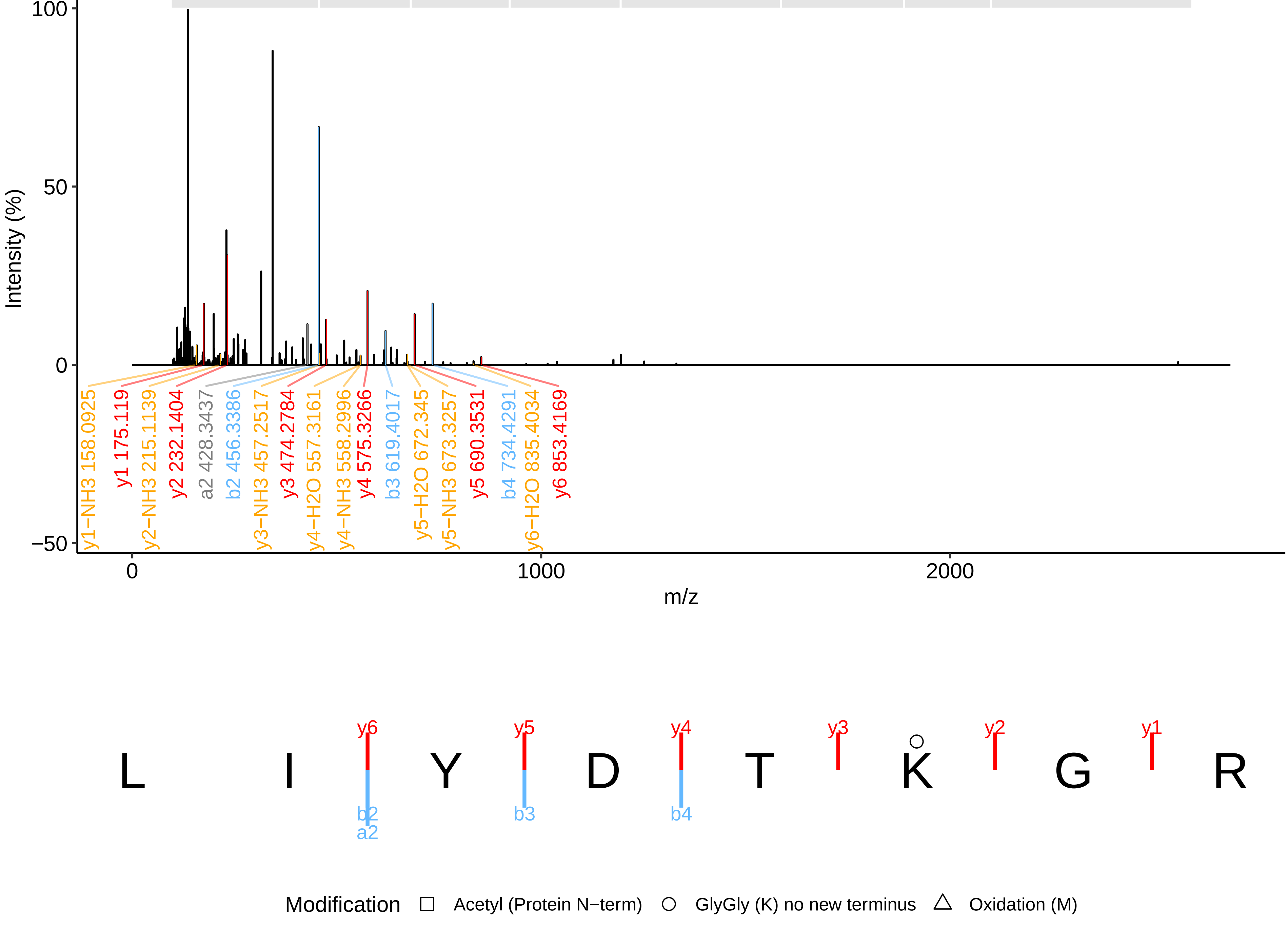

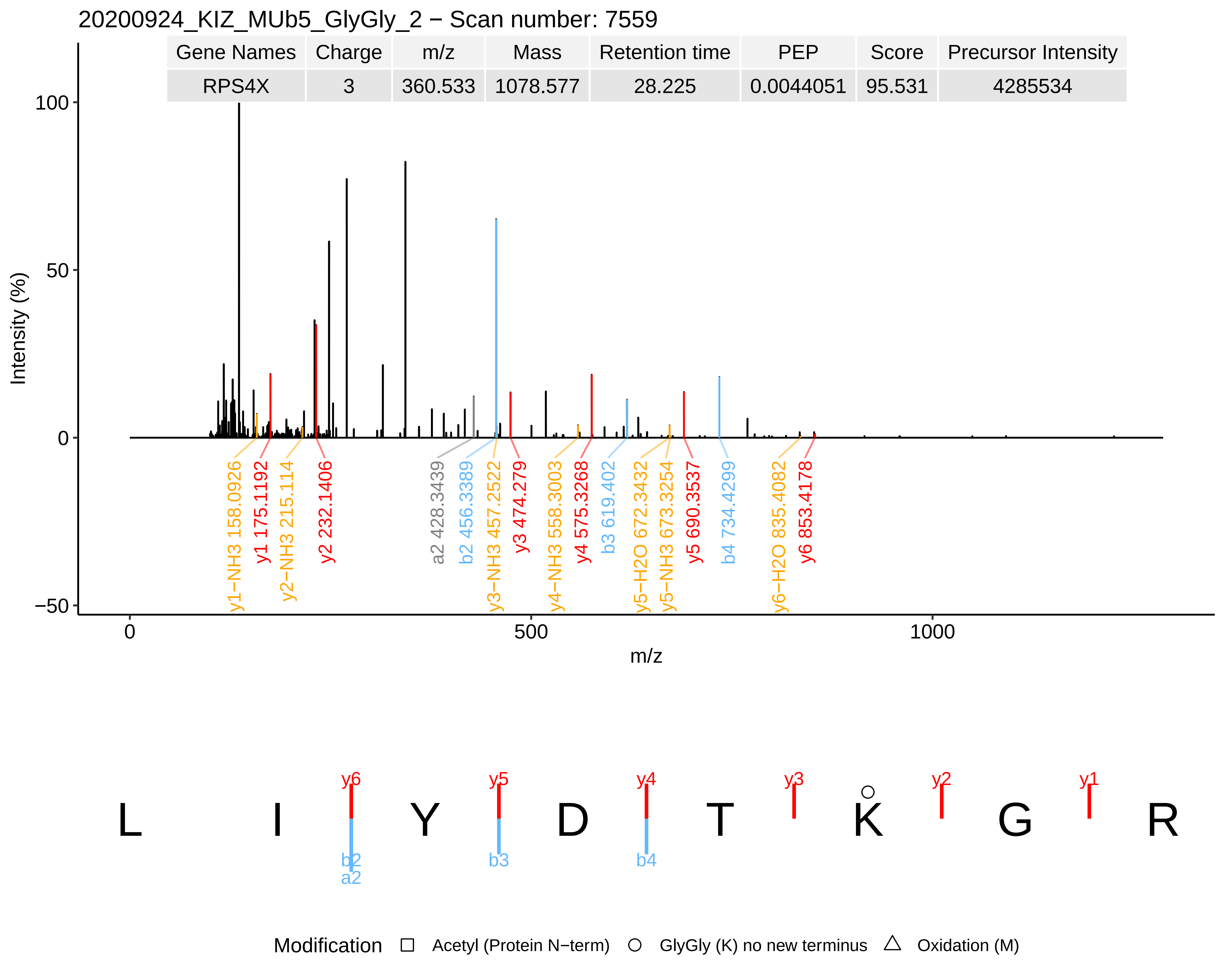

20200924_KIZ_MUb5_GlyGly_2 − Scan number: 7559
Gene Names | Charge | m/z | Mass | Retention time | PEP | Score | Precursor Intensity
RPS4X | 3 | 360.533 | 1078.577 | 28.225 | 0.0044051 | 95.531 | 4285534
Intensity (%)
100
50
0
−50
0
500
1000
m/z
y1−NH3 158.0926
y1 175.1192
y2−NH3 215.114
y2 232.1406
a2 428.3439
b2 456.3389
y3−NH3 457.2522
y3 474.279
y4−NH3 558.3003
y4 575.3268
b3 619.402
y5−H2O 672.3432
y5−NH3 673.3254
y5 690.3537
b4 734.4299
y6−H2O 835.4082
y6 853.4178
L I Y D T K G R
y6 y5 y4 y3 y2 y1
b2 a2 b3 b4
Modification
Acetyl (Protein N−term)
GlyGly (K) no new terminus
Oxidation (M)

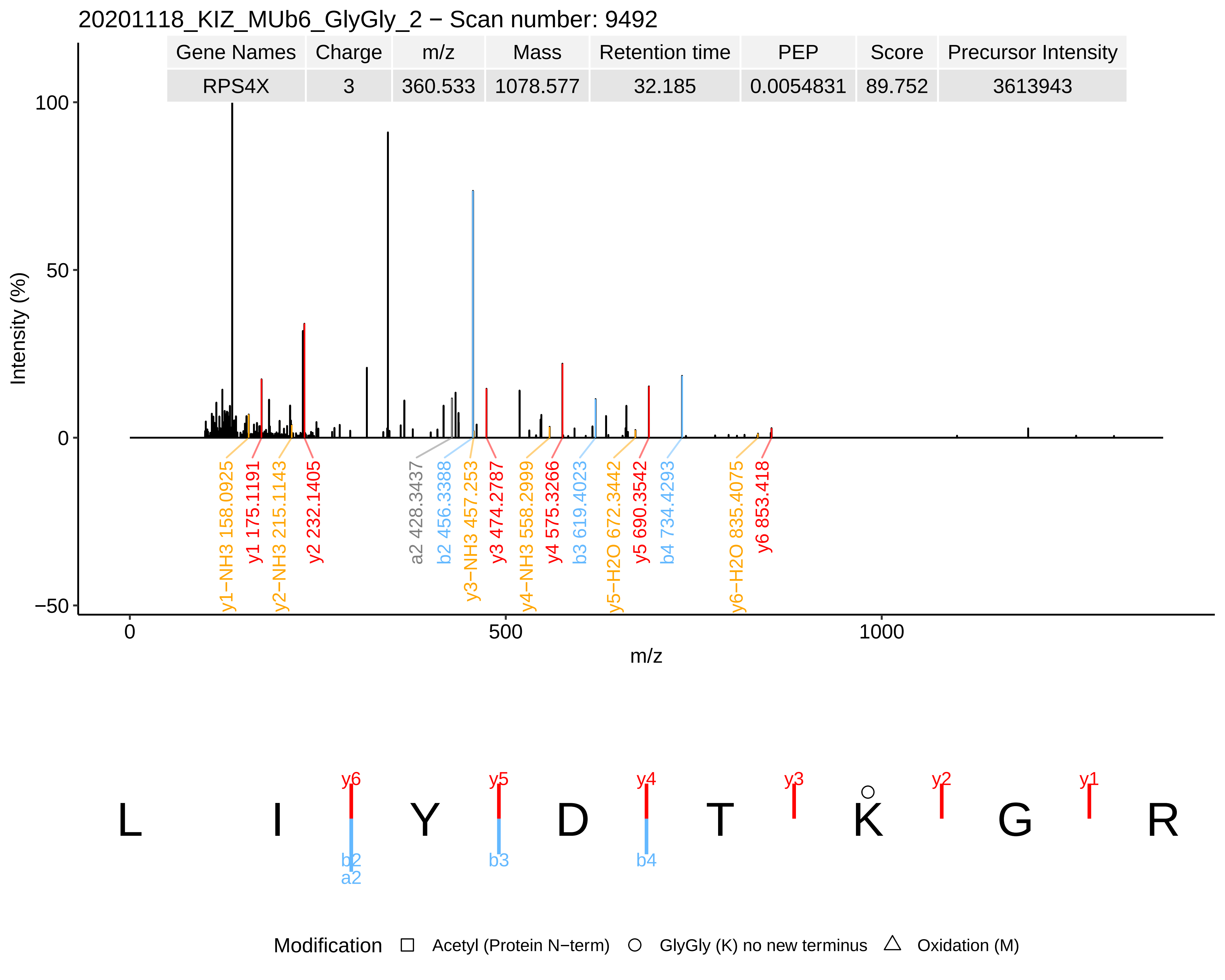
20201118_KIZ_MUb6_GlyGly_2 − Scan number: 9492
Gene Names | Charge | m/z | Mass | Retention time | PEP | Score | Precursor Intensity
RPS4X | 3 | 360.533 | 1078.577 | 32.185 | 0.0054831 | 89.752 | 3613943
Intensity (%)
100
50
0
−50
0
500
1000
m/z
y1−NH3 158.0925
y1 175.1191
y2−NH3 215.1143
y2 232.1405
a2 428.3437
b2 456.3388
y3−NH3 457.253
y3 474.2787
y4−NH3 558.2999
y4 575.3266
b3 619.4023
y5−H2O 672.3442
y5 690.3542
b4 734.4293
y6−H2O 835.4075
y6 853.418
L I Y D T K G R
y6 y5 y4 y3 y2 y1
b2 a2 b3 b4
Modification
Acetyl (Protein N−term)
GlyGly (K) no new terminus
Oxidation (M)

20201118_KIZ_MUb6_GlyGly_2 − Scan number: 9508

Gene Names	Charge	m/z	Mass	Retention time	PEP	Score	Precursor Intensity
RPS4X	3	360.533	1078.577	32.243	0.013846	76.774	3405045

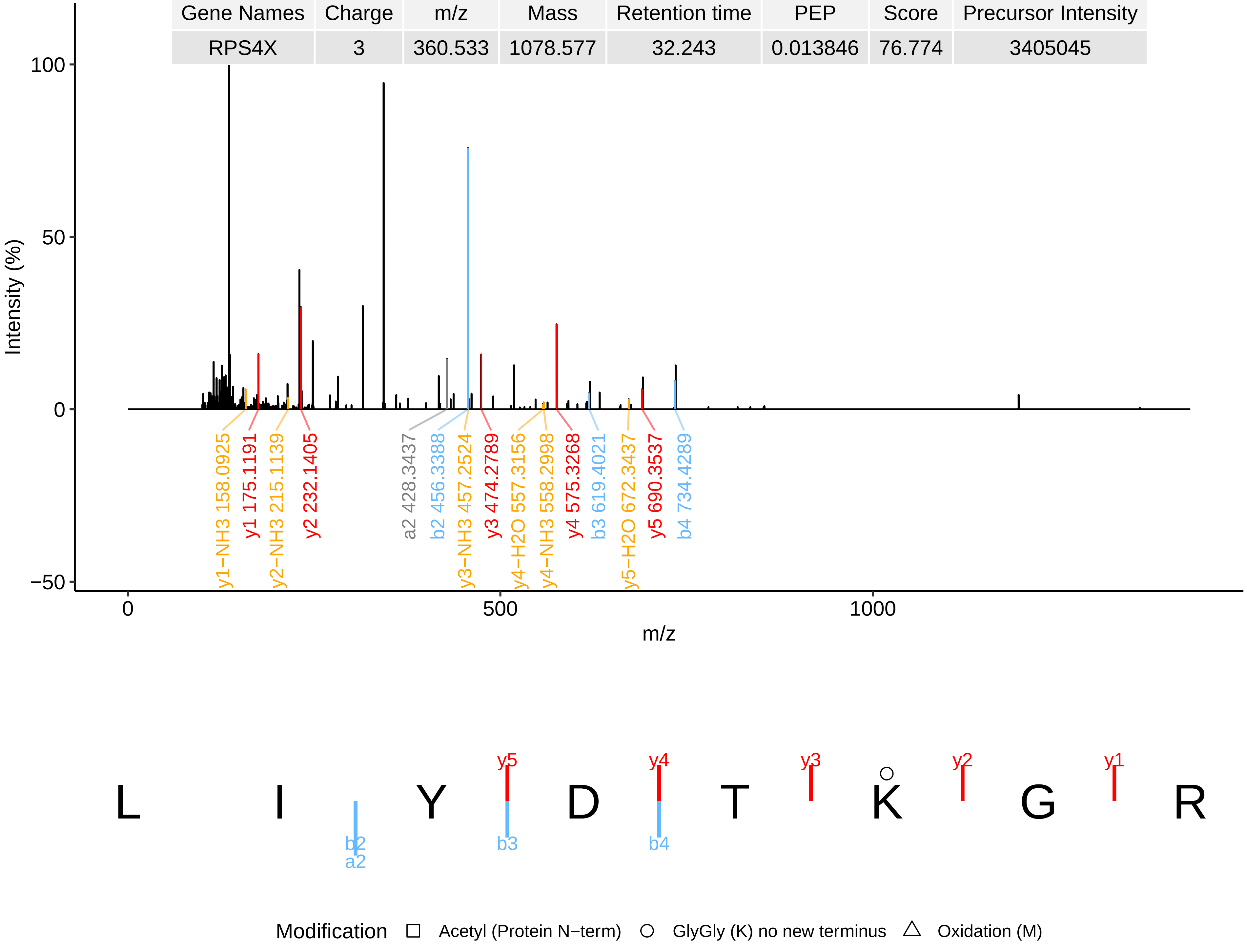

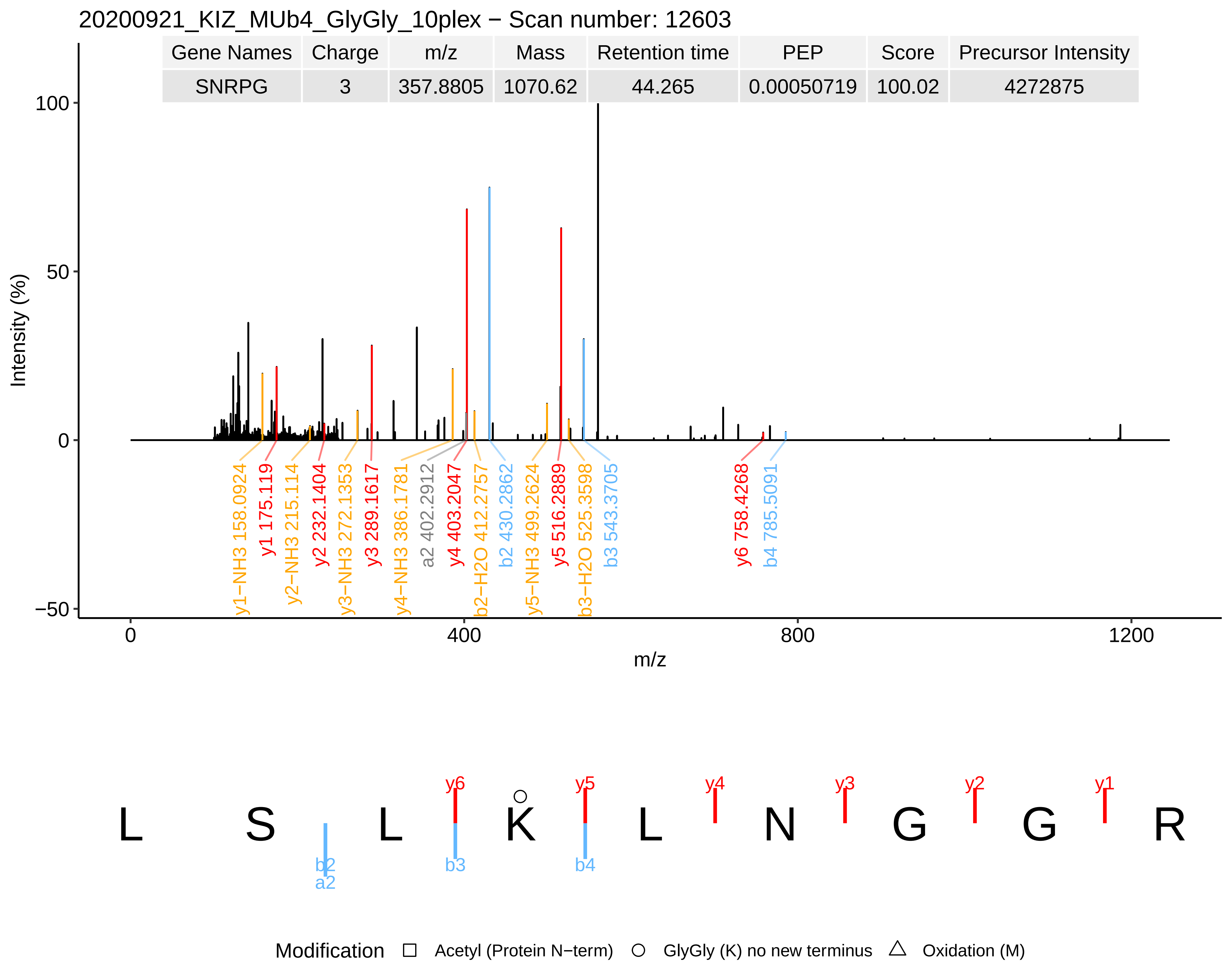

20200921_KIZ_MUb4_GlyGly_10plex − Scan number: 12603
Gene Names | Charge | m/z | Mass | Retention time | PEP | Score | Precursor Intensity
SNRPG | 3 | 357.8805 | 1070.62 | 44.265 | 0.00050719 | 100.02 | 4272875
Intensity (%)
100
50
0
−50
0
400
800
1200
m/z
y1−NH3 158.0924
y1 175.119
y2−NH3 215.114
y2 232.1404
y3−NH3 272.1353
y3 289.1617
y4−NH3 386.1781
a2 402.2912
y4 403.2047
b2−H2O 412.2757
b2 430.2862
y5−NH3 499.2624
y5 516.2889
b3−H2O 525.3598
b3 543.3705
y6 758.4268
b4 785.5091
L S L K L N G G R
y6 y5 y4 y3 y2 y1
b2 a2 b3 b4
Modification
Acetyl (Protein N−term)
GlyGly (K) no new terminus
Oxidation (M)

20200921_KIZ_MUb4_GlyGly_10plex_2 − Scan number: 11601

Gene Names	Charge	m/z	Mass	Retention time	PEP	Score	Precursor Intensity
SNRPG	3	357.8805	1070.62	44.668	0.012804	71.877	236872.5

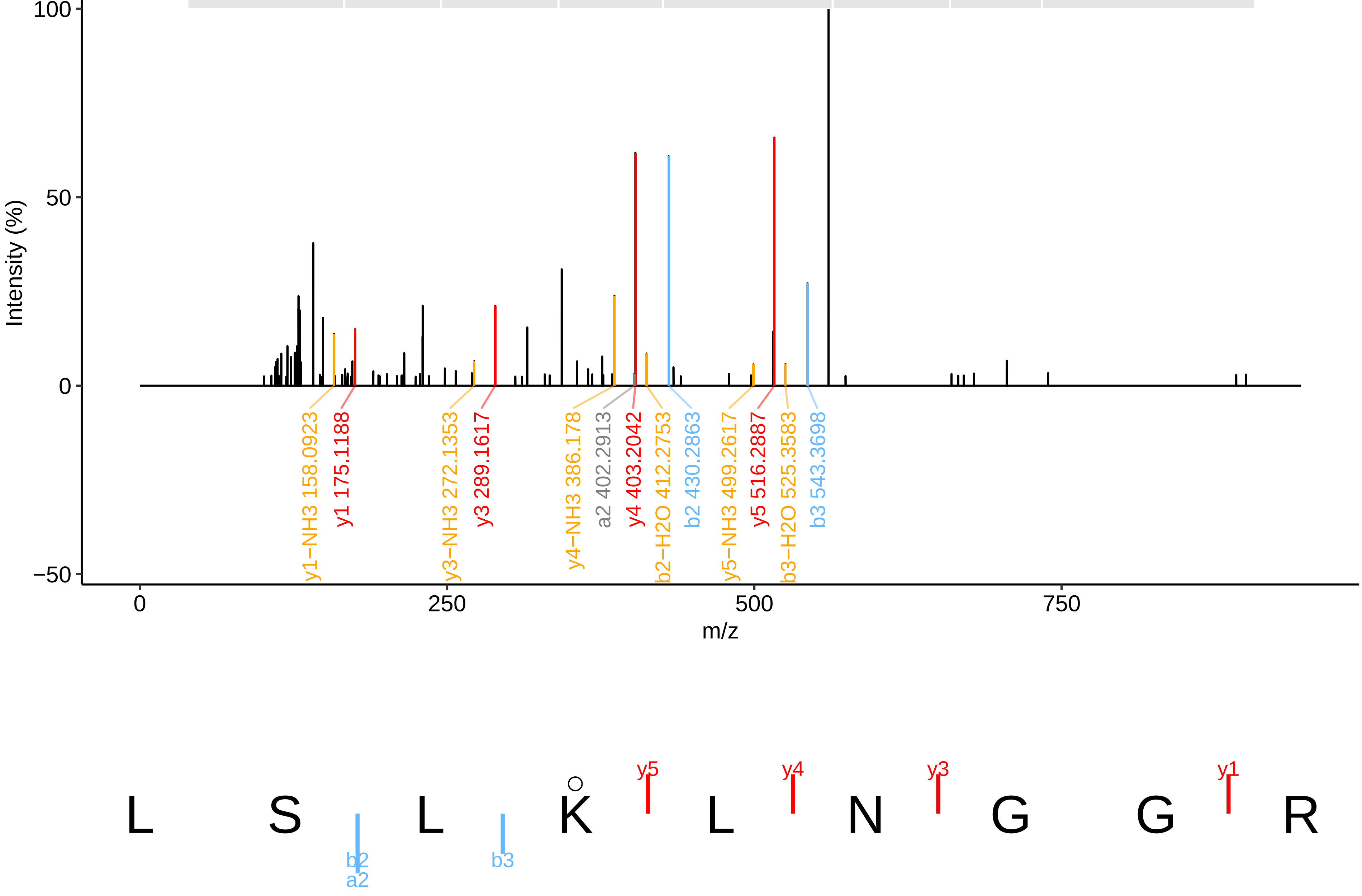

Modification □ Acetyl (Protein N−term) ○ GlyGly (K) no new terminus △ Oxidation (M)

20201118_KIZ_MUb6_GlyGly_2 − Scan number: 13476

Gene Names	Charge	m/z	Mass	Retention time	PEP	Score	Precursor Intensity
SNRPG	3	357.8805	1070.62	45.482	0.0015591	100.02	3090847

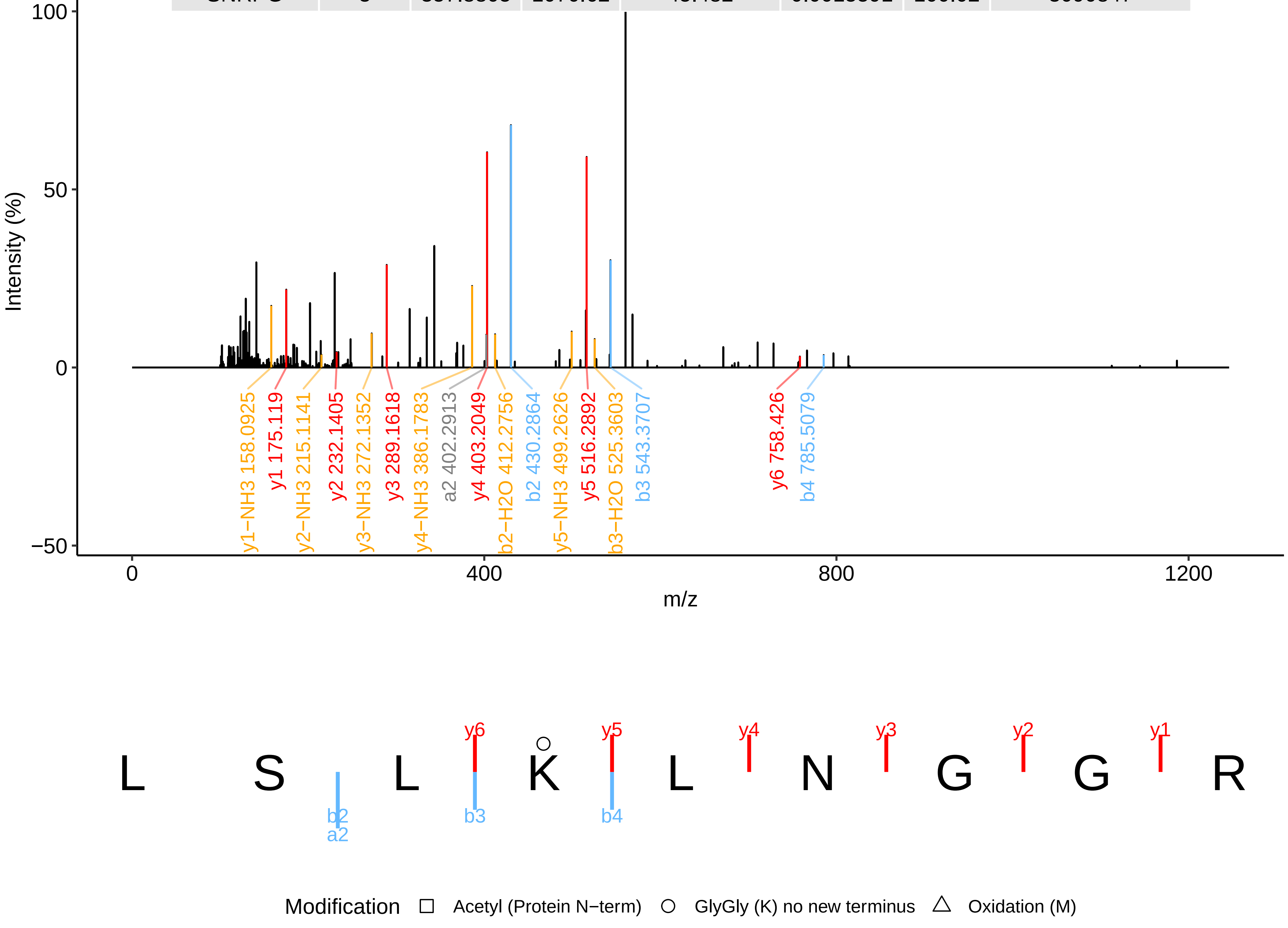

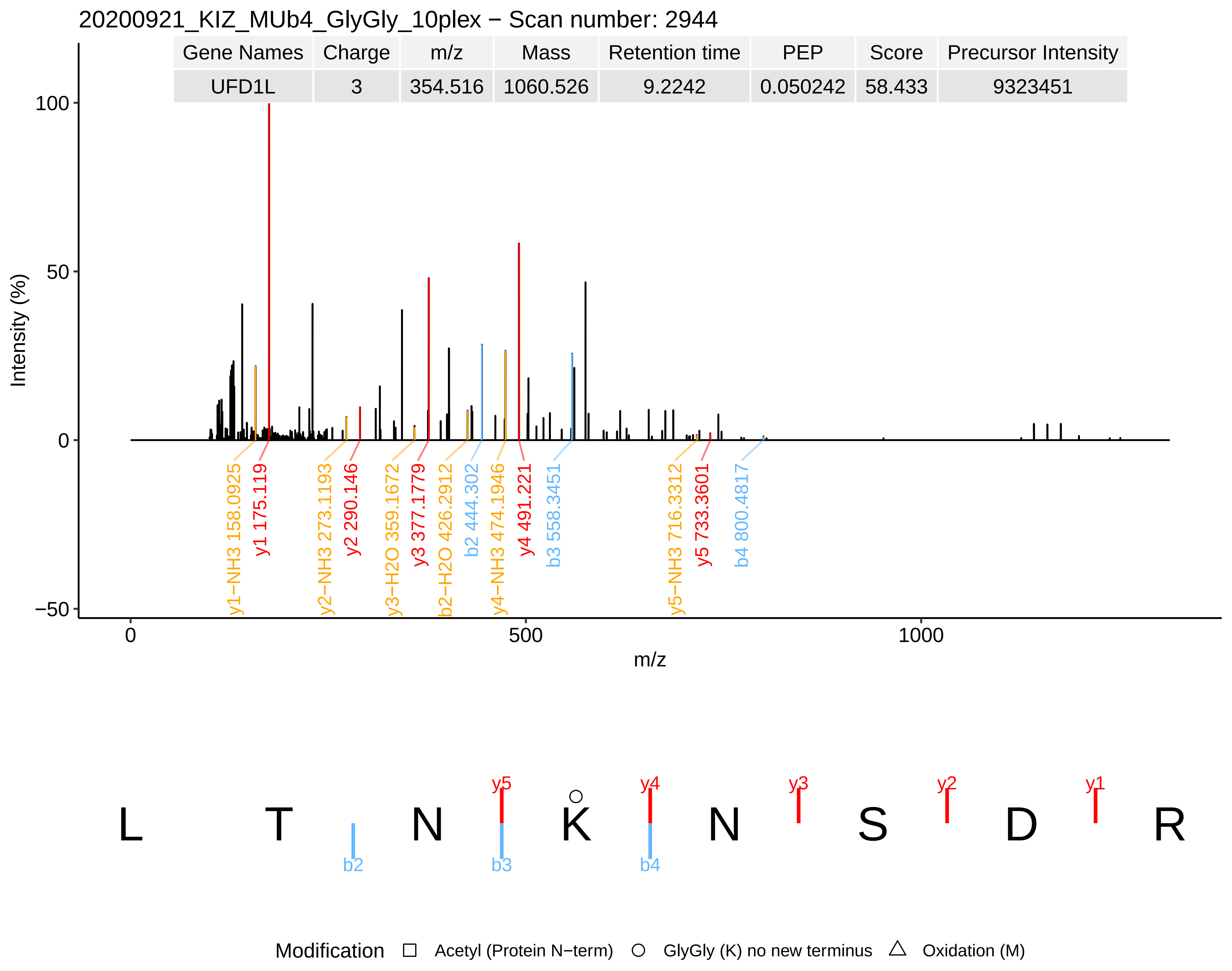

20200921_KIZ_MUb4_GlyGly_10plex − Scan number: 2944
Gene Names | Charge | m/z | Mass | Retention time | PEP | Score | Precursor Intensity
UFD1L | 3 | 354.516 | 1060.526 | 9.2242 | 0.050242 | 58.433 | 9323451
Intensity (%)
m/z
y1−NH3 158.0925
y1 175.119
y2−NH3 273.1193
y2 290.146
y3−H2O 359.1672
y3 377.1779
b2−H2O 426.2912
b2 444.302
y4−NH3 474.1946
y4 491.221
b3 558.3451
y5−NH3 716.3312
y5 733.3601
b4 800.4817
L T N K N S D R
y5 y4 y3 y2 y1
b2 b3 b4
Modification
Acetyl (Protein N−term)
GlyGly (K) no new terminus
Oxidation (M)

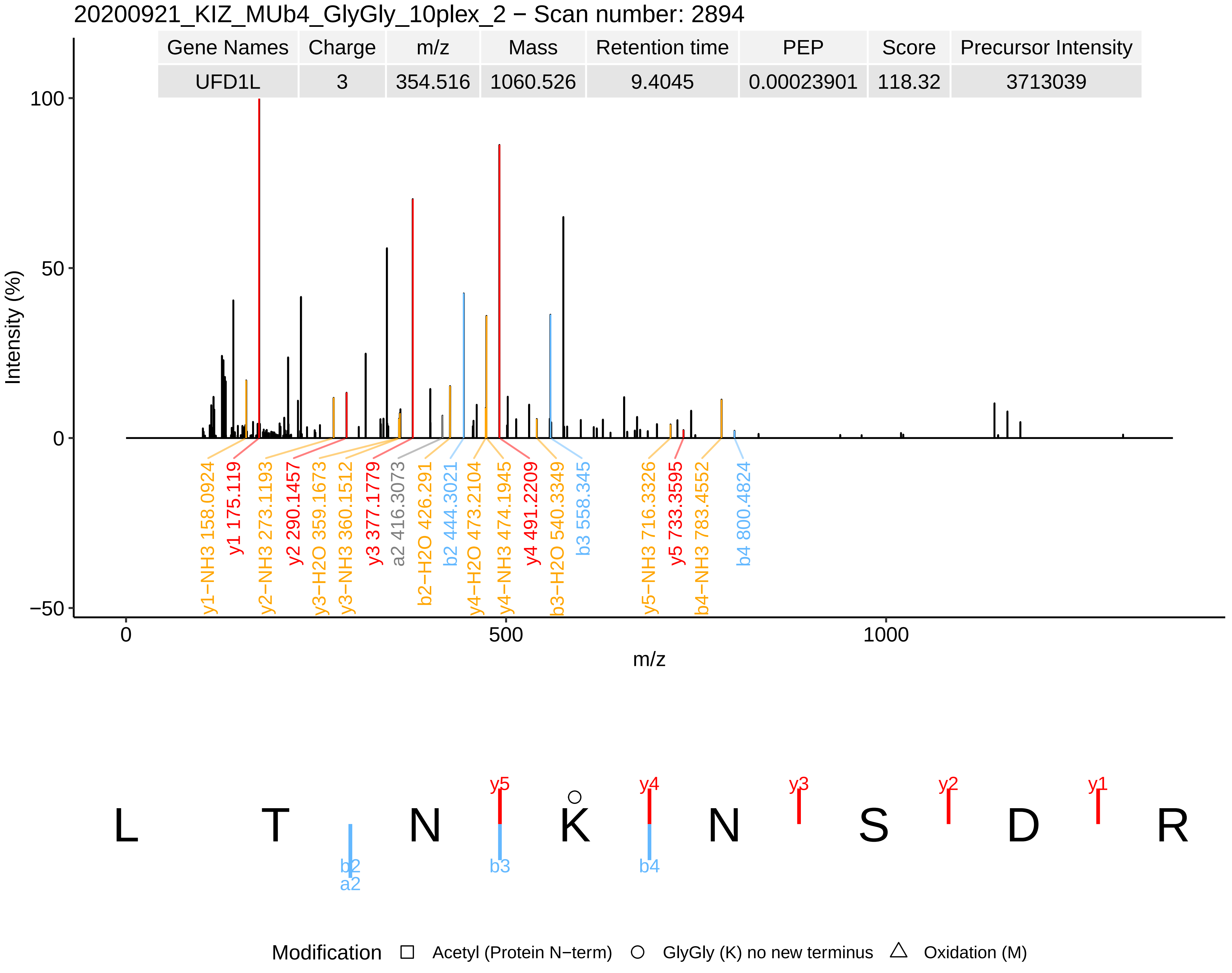

20200921_KIZ_MUb4_GlyGly_10plex_2 − Scan number: 2894
Gene Names | Charge | m/z | Mass | Retention time | PEP | Score | Precursor Intensity
UFD1L | 3 | 354.516 | 1060.526 | 9.4045 | 0.00023901 | 118.32 | 3713039
Intensity (%)
m/z
y1−NH3 158.0924
y1 175.119
y2−NH3 273.1193
y2 290.1457
y3−H2O 359.1673
y3−NH3 360.1512
y3 377.1779
a2 416.3073
b2−H2O 426.291
b2 444.3021
y4−H2O 473.2104
y4−NH3 474.1945
y4 491.2209
b3−H2O 540.3349
b3 558.345
y5−NH3 716.3326
y5 733.3595
b4−NH3 783.4552
b4 800.4824
L T N K N S D R
y5 y4 y3 y2 y1
b2 a2 b3 b4
Modification
Acetyl (Protein N−term)
GlyGly (K) no new terminus
Oxidation (M)

20201118_KIZ_MUb6_GlyGly_1 − Scan number: 2854

Gene Names	Charge	m/z	Mass	Retention time	PEP	Score	Precursor Intensity
UFD1L	3	354.516	1060.526	8.7433	0.0030085	96.027	22526466

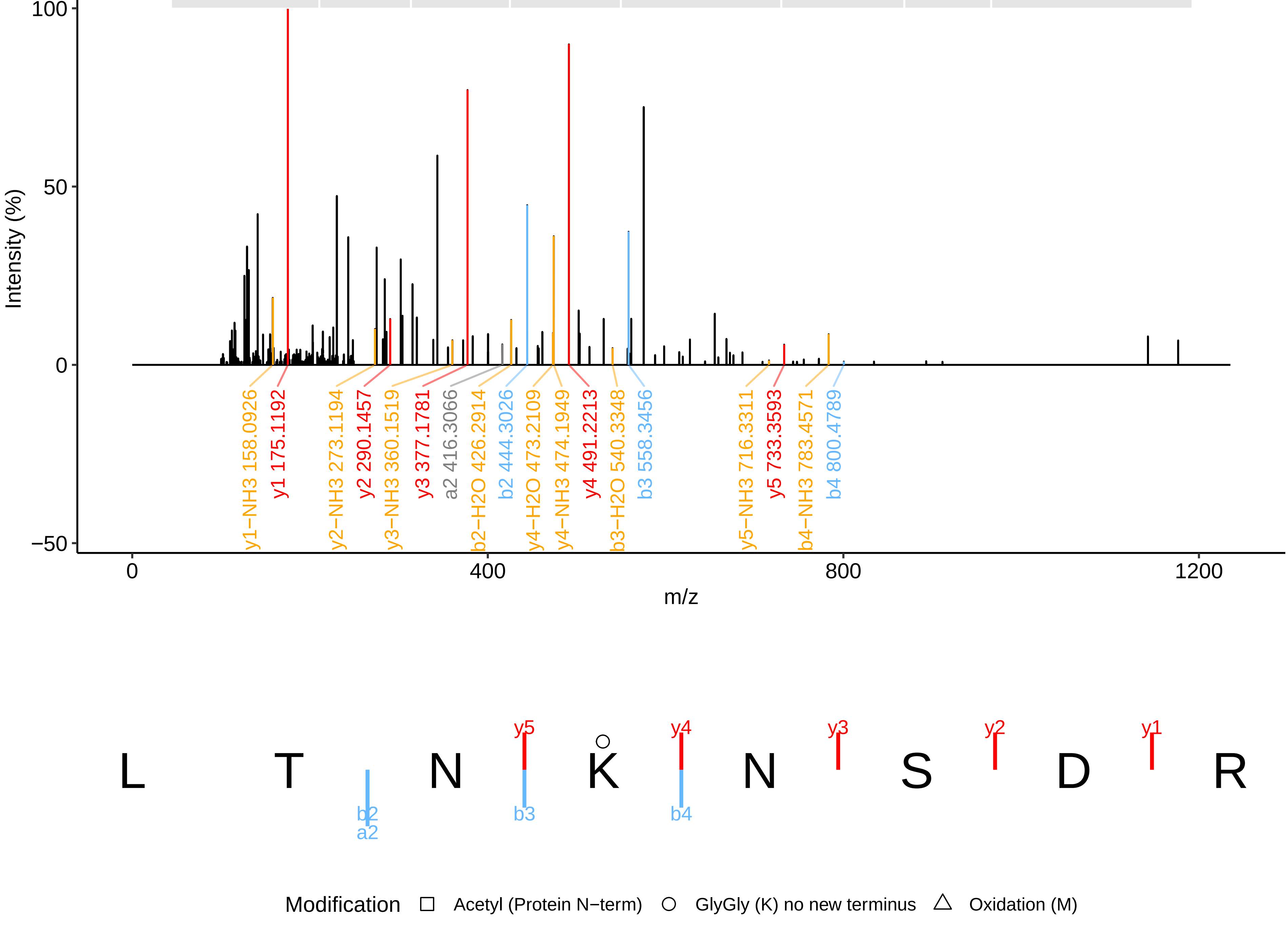

20201118_KIZ_MUb6_GlyGly_2 − Scan number: 3109

Gene Names	Charge	m/z	Mass	Retention time	PEP	Score	Precursor Intensity
UFD1L	3	354.516	1060.526	10.084	0.0041457	93.143	13128213

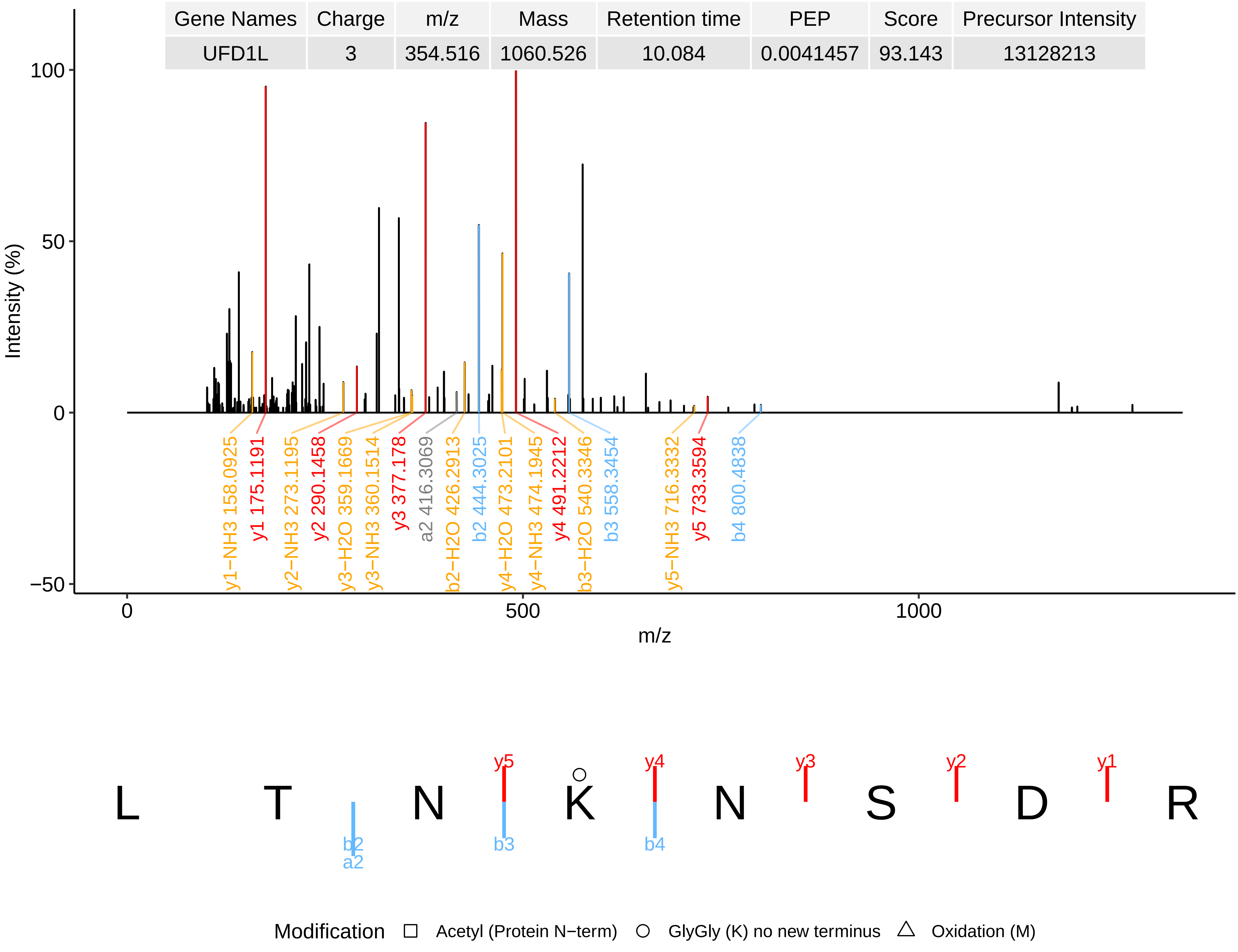

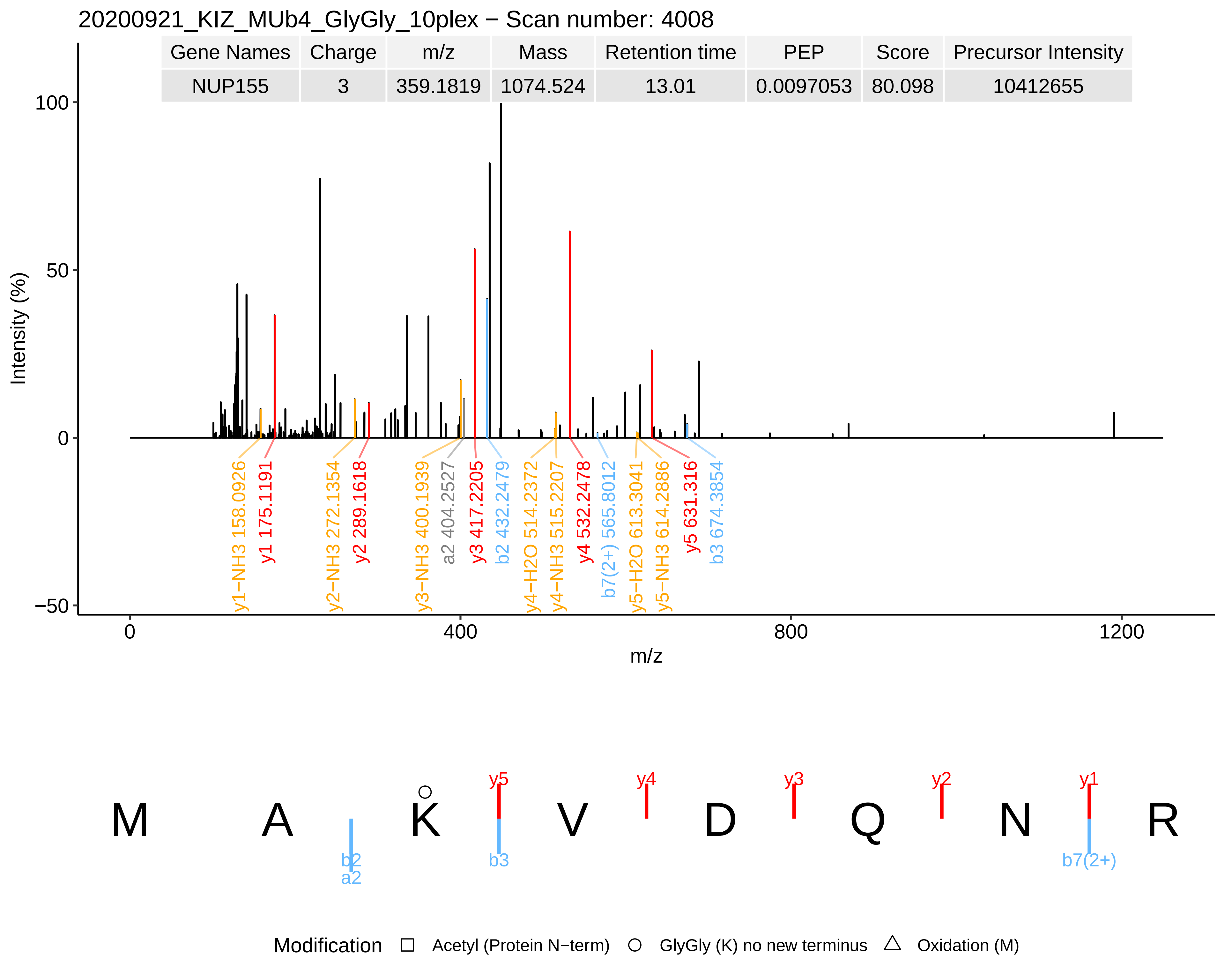

20200921_KIZ_MUb4_GlyGly_10plex − Scan number: 4008
Gene Names | Charge | m/z | Mass | Retention time | PEP | Score | Precursor Intensity
NUP155 | 3 | 359.1819 | 1074.524 | 13.01 | 0.0097053 | 80.098 | 10412655
Intensity (%)
100
50
0
−50
0
400
800
1200
m/z
y1−NH3 158.0926
y1 175.1191
y2−NH3 272.1354
y2 289.1618
y3−NH3 400.1939
a2 404.2527
y3 417.2205
b2 432.2479
y4−H2O 514.2372
y4−NH3 515.2207
y4 532.2478
b7(2+) 565.8012
y5−H2O 613.3041
y5−NH3 614.2886
y5 631.316
b3 674.3854
M A K V D Q N R
y5 y4 y3 y2 y1
b2 a2 b3 b7(2+)
Modification
Acetyl (Protein N−term)
GlyGly (K) no new terminus
Oxidation (M)

20200921_KIZ_MUb4_GlyGly_10plex_2 − Scan number: 5387

Gene Names	Charge	m/z	Mass	Retention time	PEP	Score	Precursor Intensity
YWHAG;SFN;YWHAZ;YWHAH	3	349.4957	1045.465	19.342	0.026489	77.984	2231734

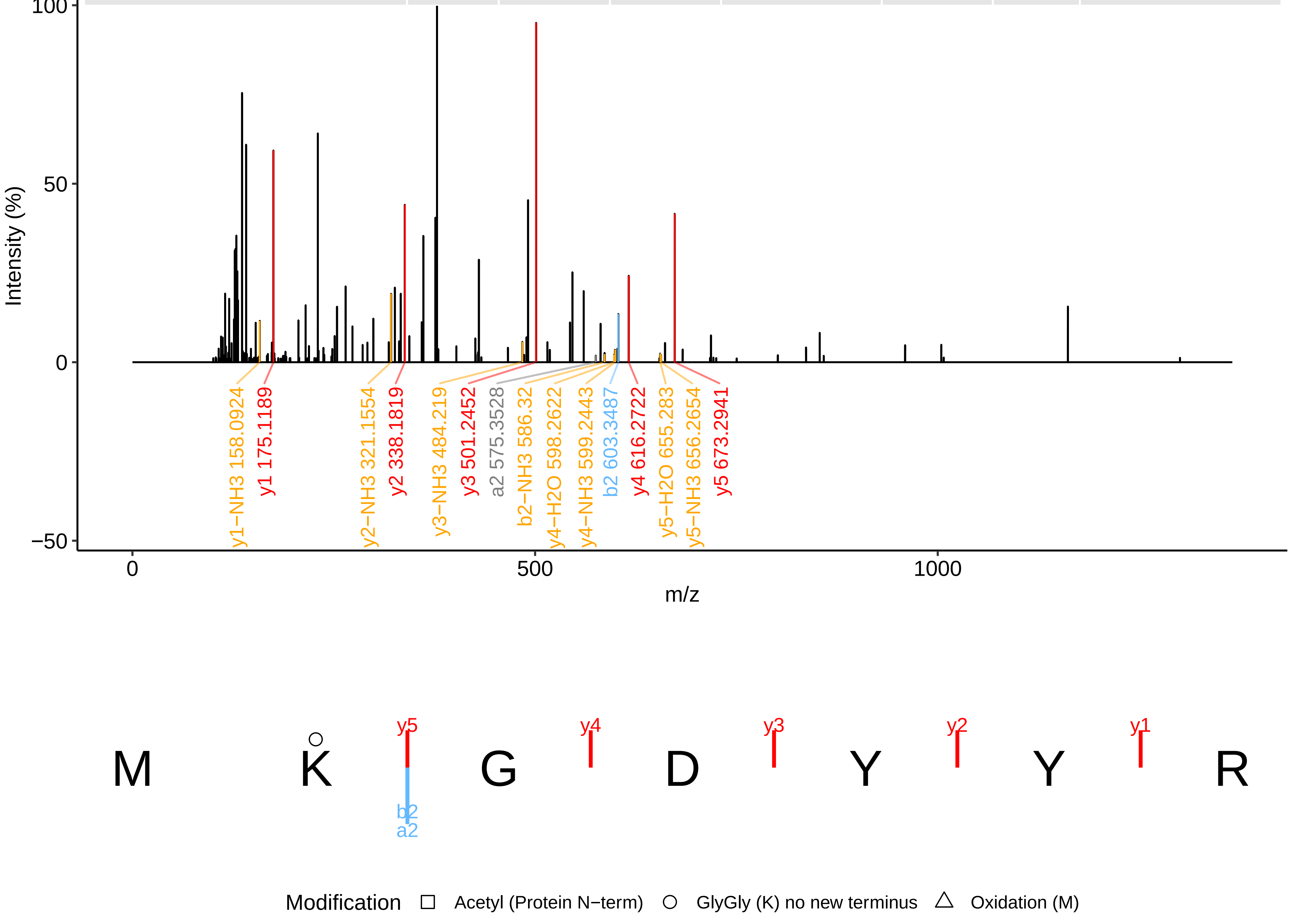

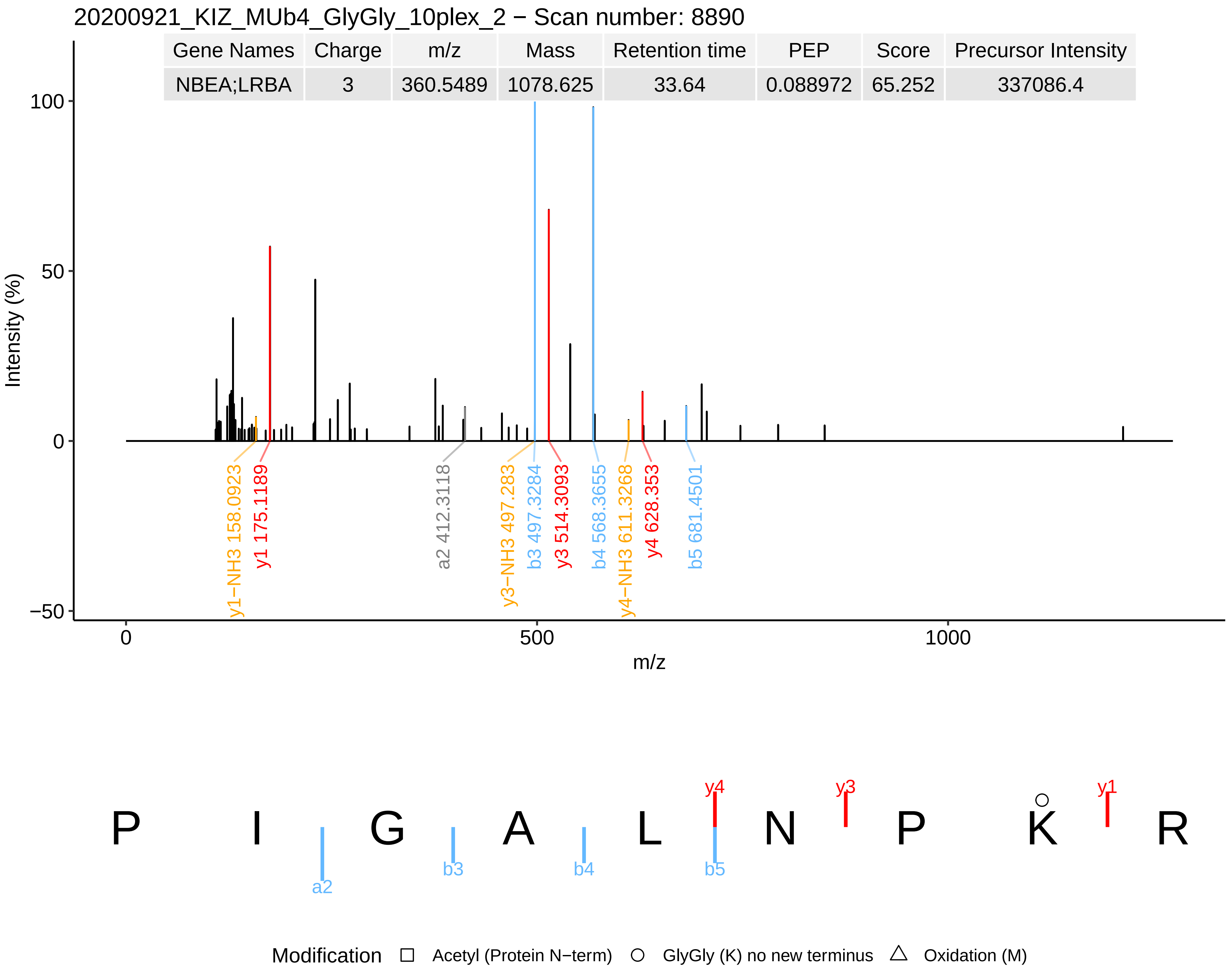

20200921_KIZ_MUb4_GlyGly_10plex_2 − Scan number: 8890
Gene Names | Charge | m/z | Mass | Retention time | PEP | Score | Precursor Intensity
NBEA;LRBA | 3 | 360.5489 | 1078.625 | 33.64 | 0.088972 | 65.252 | 337086.4
Intensity (%)
m/z
y1−NH3 158.0923
y1 175.1189
a2 412.3118
y3−NH3 497.283
b3 497.3284
y3 514.3093
b4 568.3655
y4−NH3 611.3268
y4 628.353
b5 681.4501
P I G A L N P K R
a2 b3 b4 b5
y4 y3 y1
Modification
Acetyl (Protein N−term)
GlyGly (K) no new terminus
Oxidation (M)

20200921_KIZ_MUb4_GlyGly_10plex − Scan number: 6152

Gene Names	Charge	m/z	Mass	Retention time	PEP	Score	Precursor Intensity
PKN2	3	364.5367	1090.588	20.253	0.006497	87.181	3331605

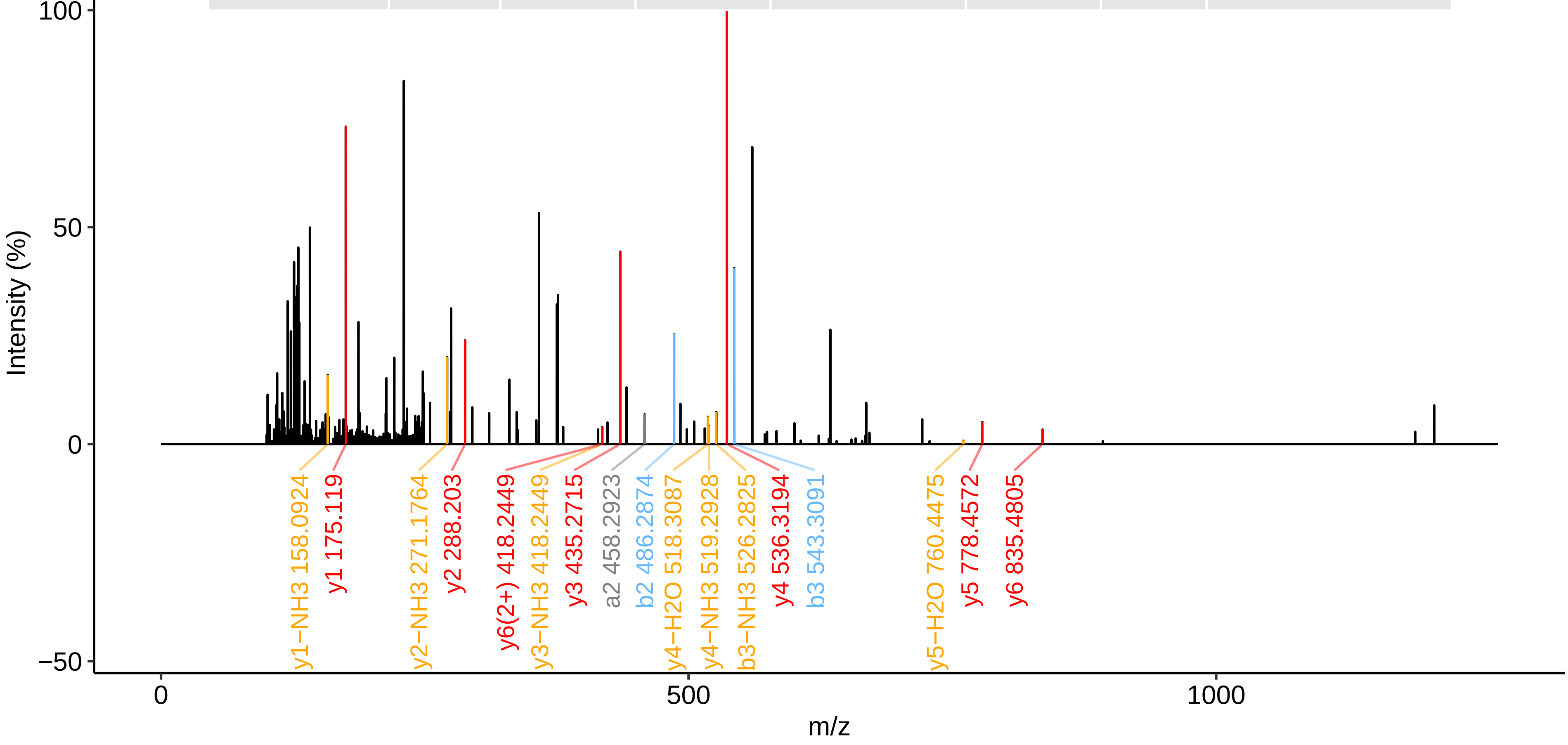

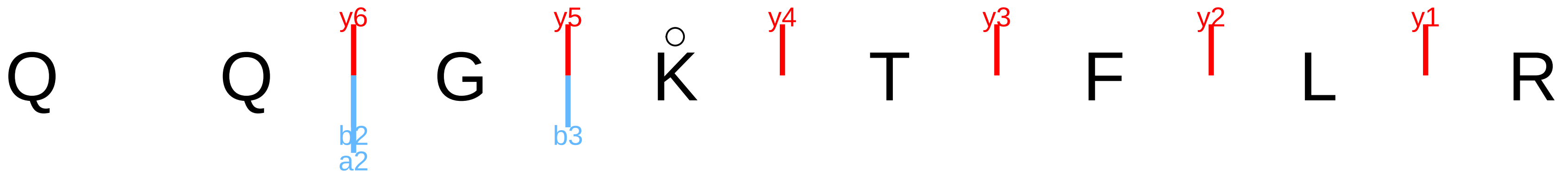

Modification □ Acetyl (Protein N−term) ○ GlyGly (K) no new terminus △ Oxidation (M)

20200921_KIZ_MUb4_GlyGly_10plex_2 − Scan number: 5643

Gene Names	Charge	m/z	Mass	Retention time	PEP	Score	Precursor Intensity
PKN2	3	364.5367	1090.588	20.388	0.035757	61.962	1252308

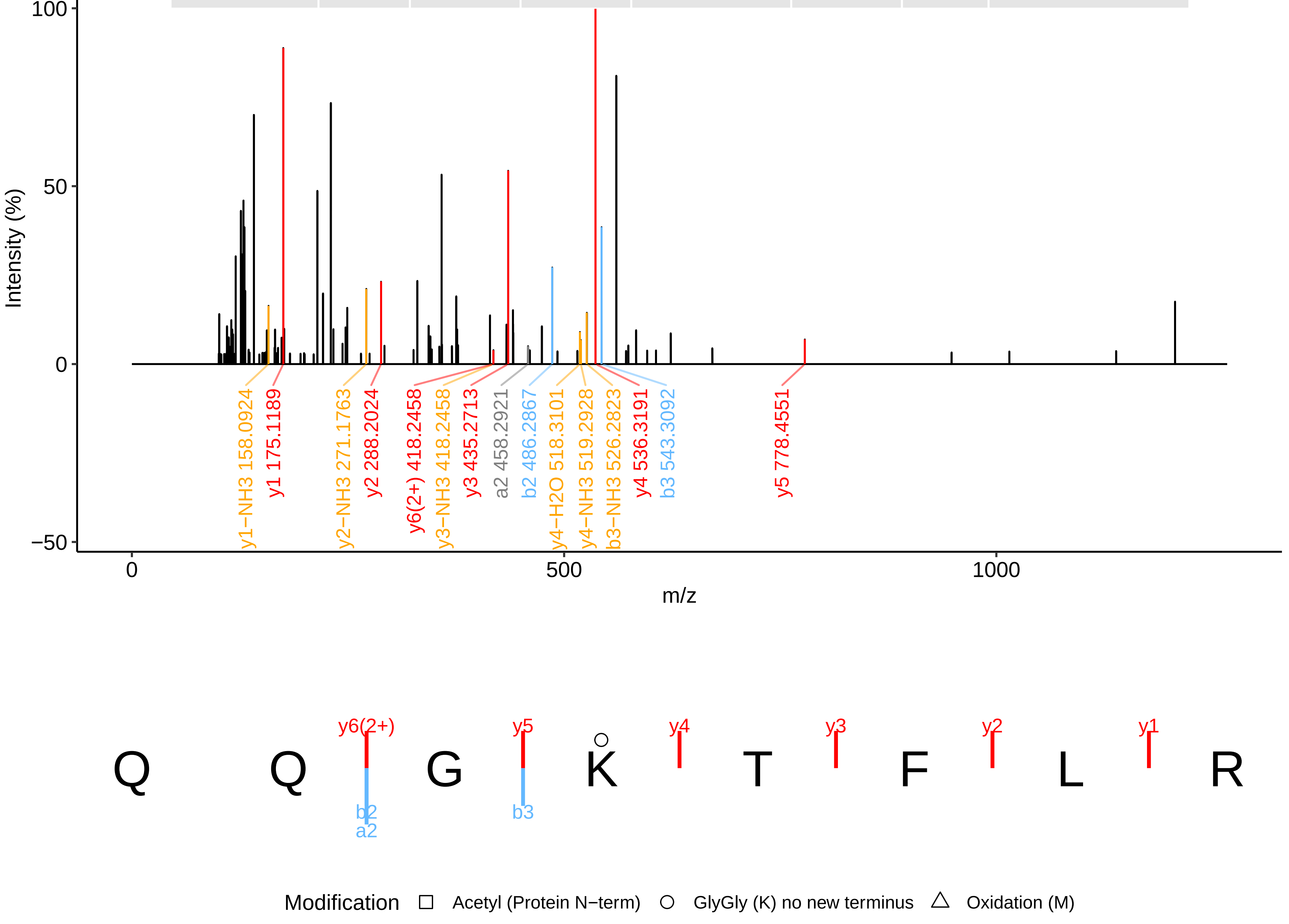

20200921_KIZ_MUb4_GlyGly_10plex − Scan number: 6725

Gene Names	Charge	m/z	Mass	Retention time	PEP	Score	Precursor Intensity
GCN1L1	3	349.5307	1045.57	22.359	0.0031441	99.802	NA

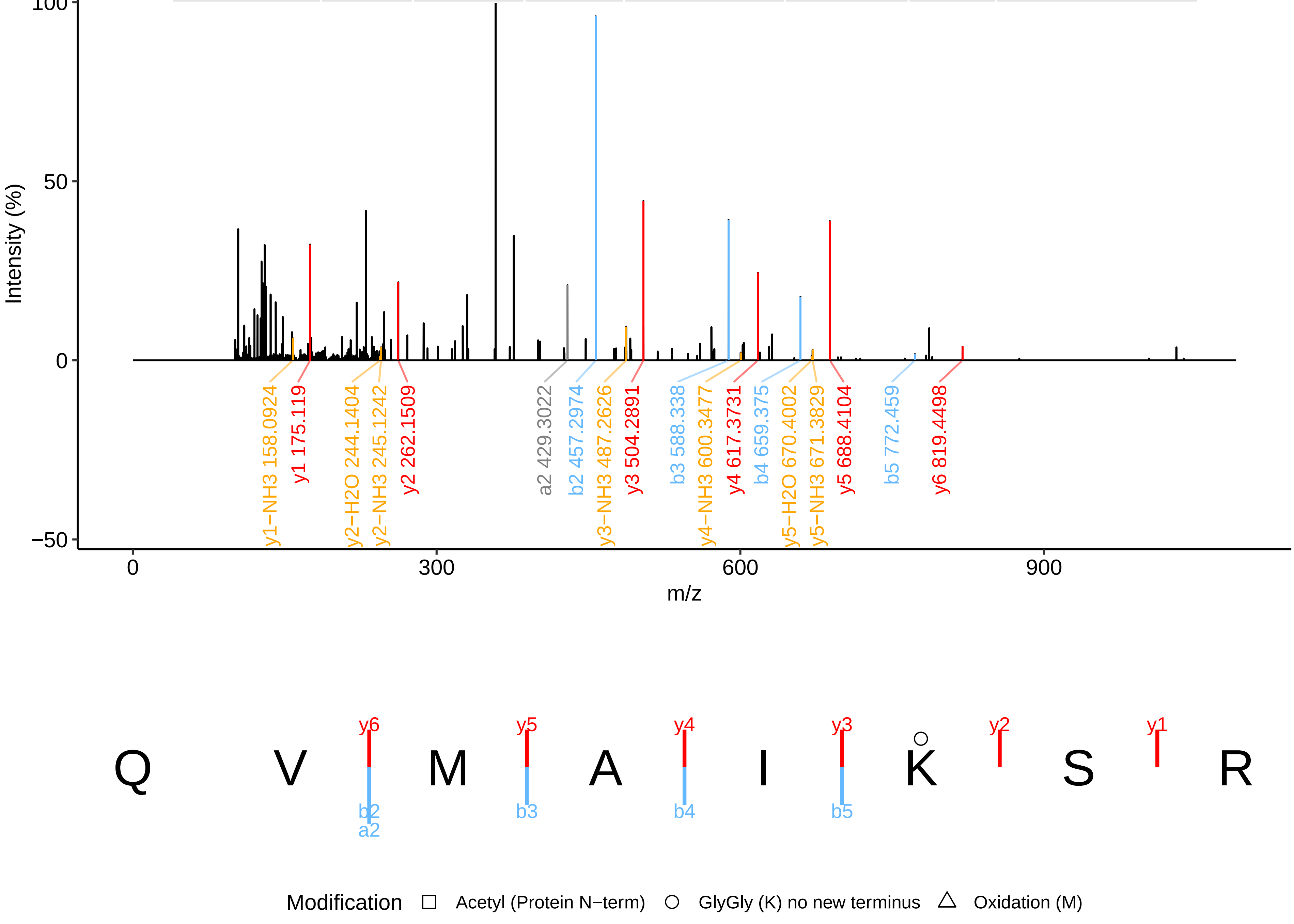

20200921_KIZ_MUb4_GlyGly_10plex − Scan number: 3218

Gene Names	Charge	m/z	Mass	Retention time	PEP	Score	Precursor Intensity
TRIM28	3	358.5281	1072.562	10.078	0.0013156	122.19	18759570

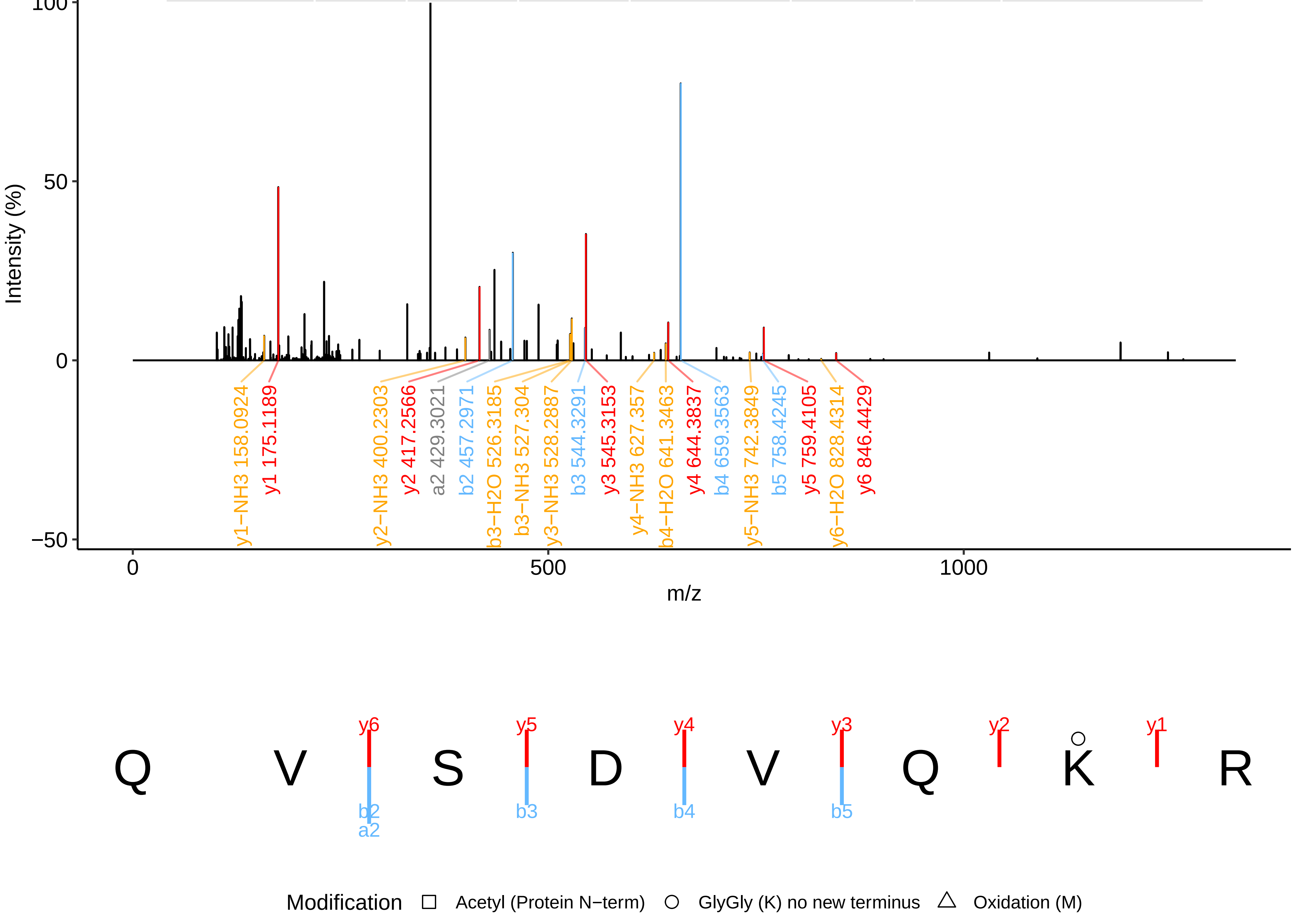

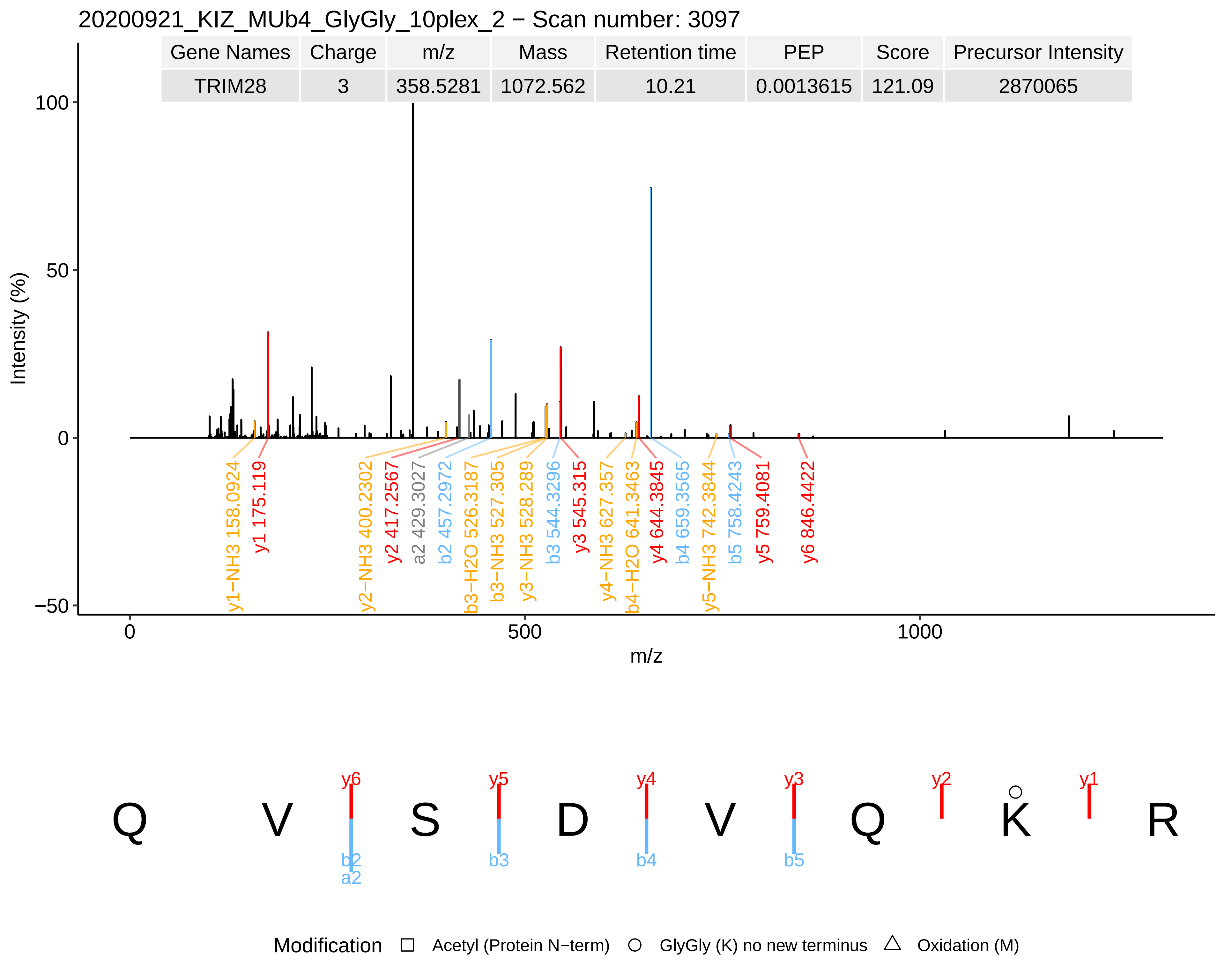
20200921_KIZ_MUb4_GlyGly_10plex_2 − Scan number: 3097
Gene Names
Charge
m/z
Mass
Retention time
PEP
Score
Precursor Intensity
TRIM28
3
358.5281
1072.562
10.21
0.0013615
121.09
2870065
Intensity (%)
100
50
0
−50
0
500
1000
m/z
y1−NH3 158.0924
y1 175.119
y2−NH3 400.2302
y2 417.2567
a2 429.3027
b2 457.2972
b3−H2O 526.3187
b3−NH3 527.305
y3−NH3 528.289
b3 544.3296
y3 545.315
y4−NH3 627.357
b4−H2O 641.3463
y4 644.3845
b4 659.3565
y5−NH3 742.3844
b5 758.4243
y5 759.4081
y6 846.4422
Q
V
S
D
V
Q
K
R
y6
y5
y4
y3
y2
y1
b2
a2
b3
b4
b5
Modification
Acetyl (Protein N−term)
GlyGly (K) no new terminus
Oxidation (M)

20200924_KIZ_MUb5_GlyGly_2 − Scan number: 2729

Gene Names	Charge	m/z	Mass	Retention time	PEP	Score	Precursor Intensity
TRIM28	3	358.5281	1072.562	9.7261	0.0051026	110.41	4291173

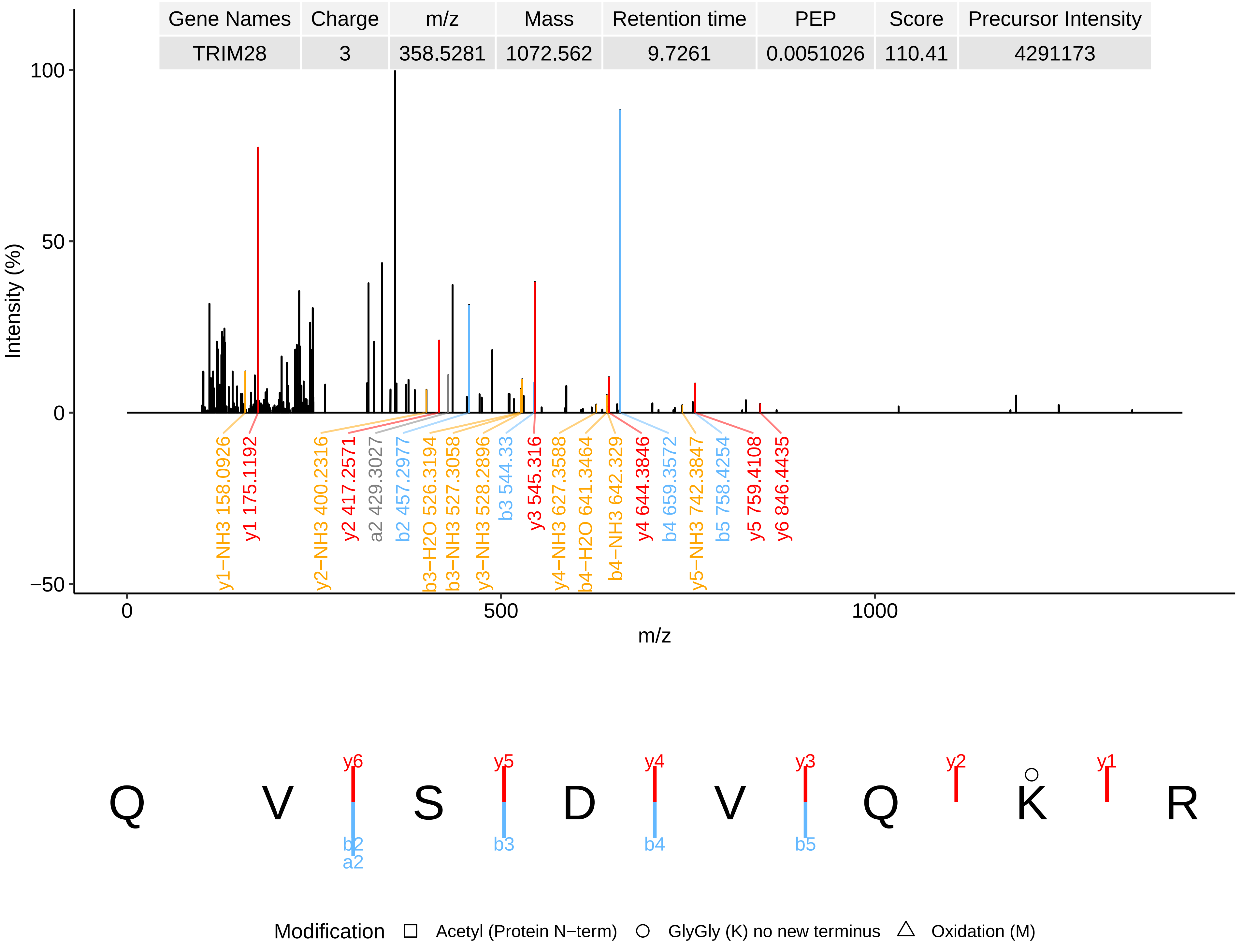

20201118_KIZ_MUb6_GlyGly_1 − Scan number: 3020

Gene Names	Charge	m/z	Mass	Retention time	PEP	Score	Precursor Intensity
TRIM28	3	358.5281	1072.562	9.2807	7.2608e−05	153.08	15638619

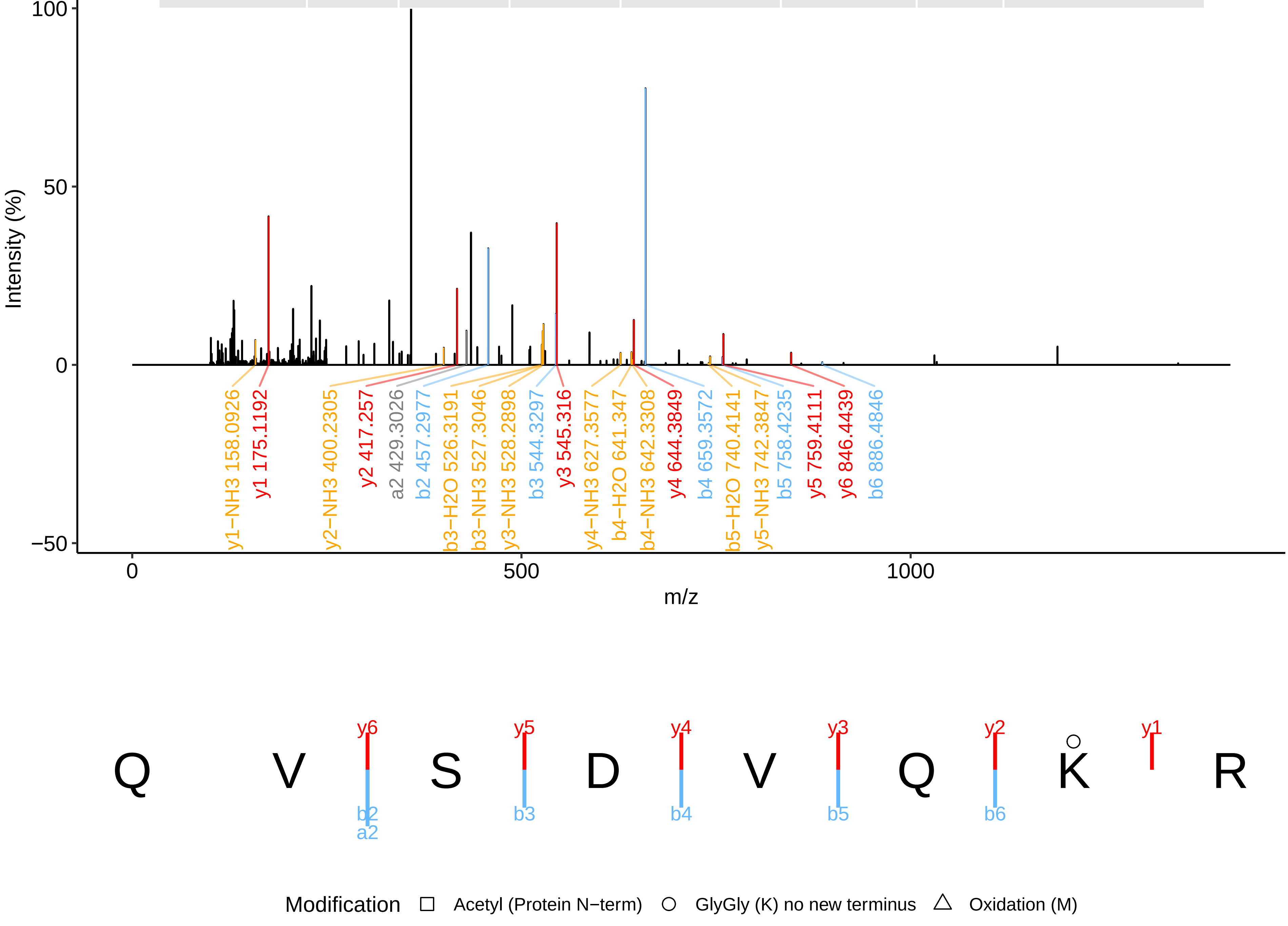

20201118_KIZ_MUb6_GlyGly_2 − Scan number: 3229

Gene Names	Charge	m/z	Mass	Retention time	PEP	Score	Precursor Intensity
TRIM28	3	358.5281	1072.562	10.514	0.0098944	103.56	6323987

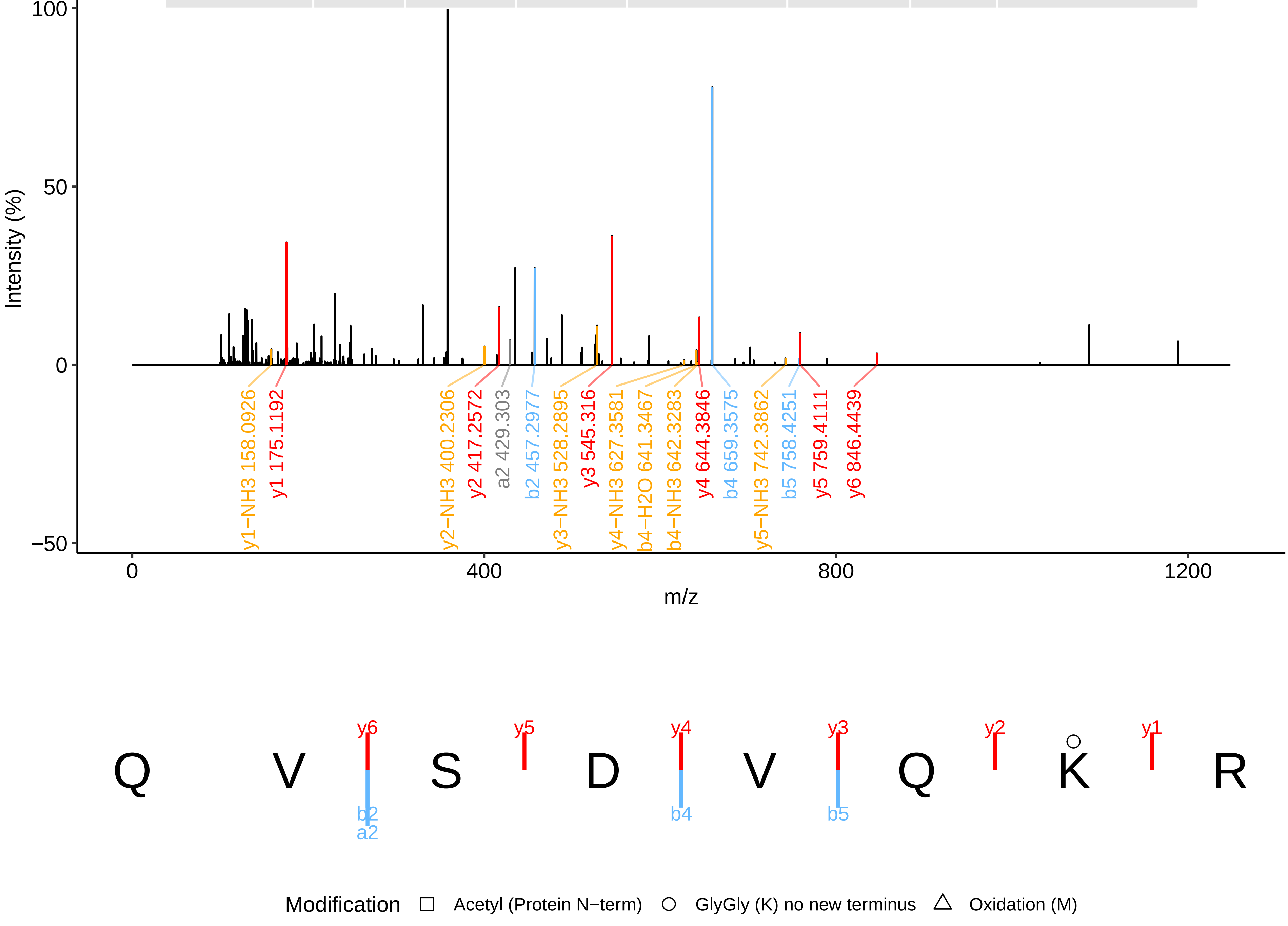

20200921_KIZ_MUb4_GlyGly_10plex − Scan number: 3914

Gene Names	Charge	m/z	Mass	Retention time	PEP	Score	Precursor Intensity
TOMM20	3	364.5125	1090.516	12.655	0.0013199	106.04	17089580

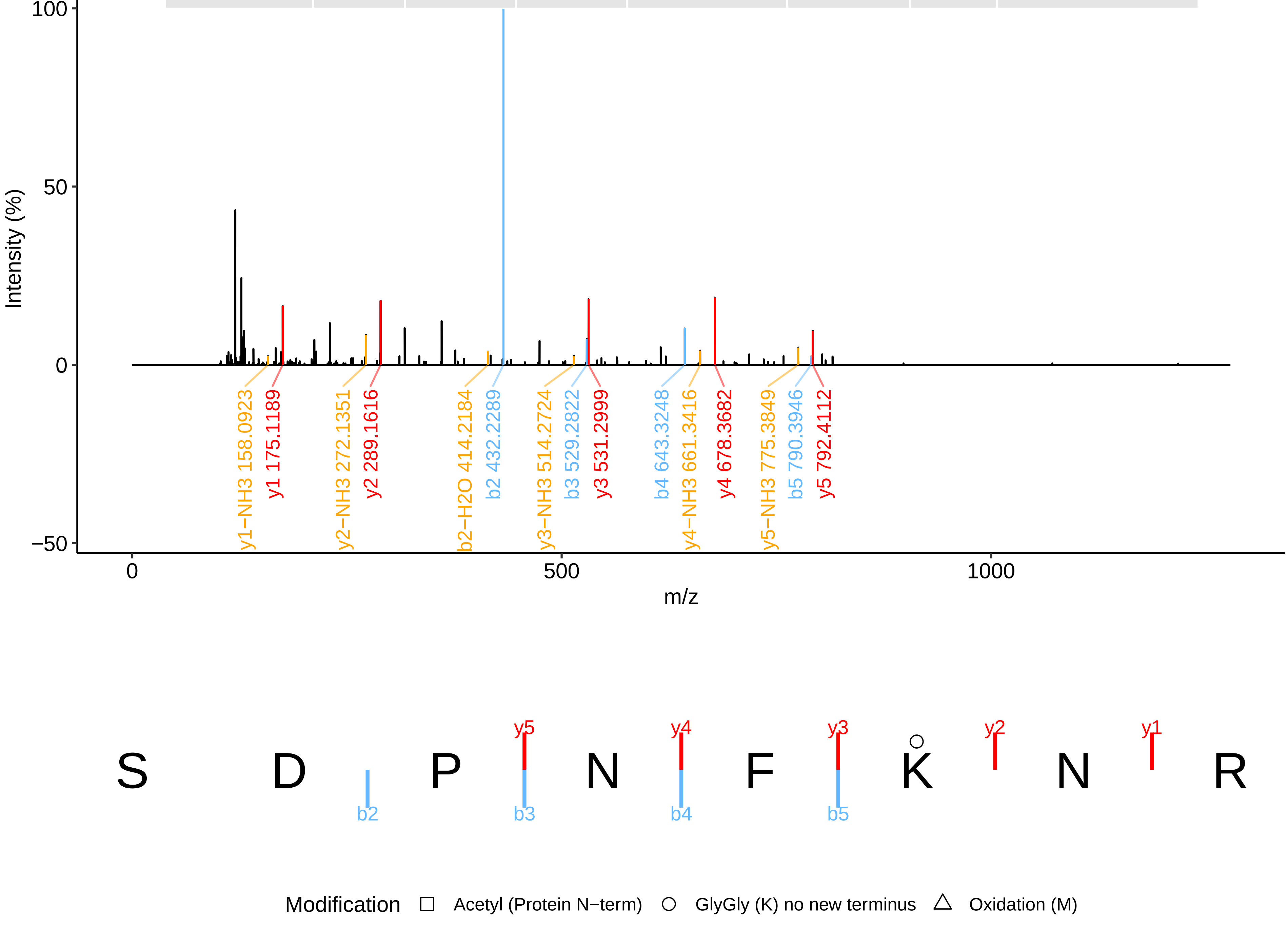

20200921_KIZ_MUb4_GlyGly_10plex_2 − Scan number: 3682

Gene Names	Charge	m/z	Mass	Retention time	PEP	Score	Precursor Intensity
TOMM20	3	364.5125	1090.516	12.522	0.018224	73.26	210622.2

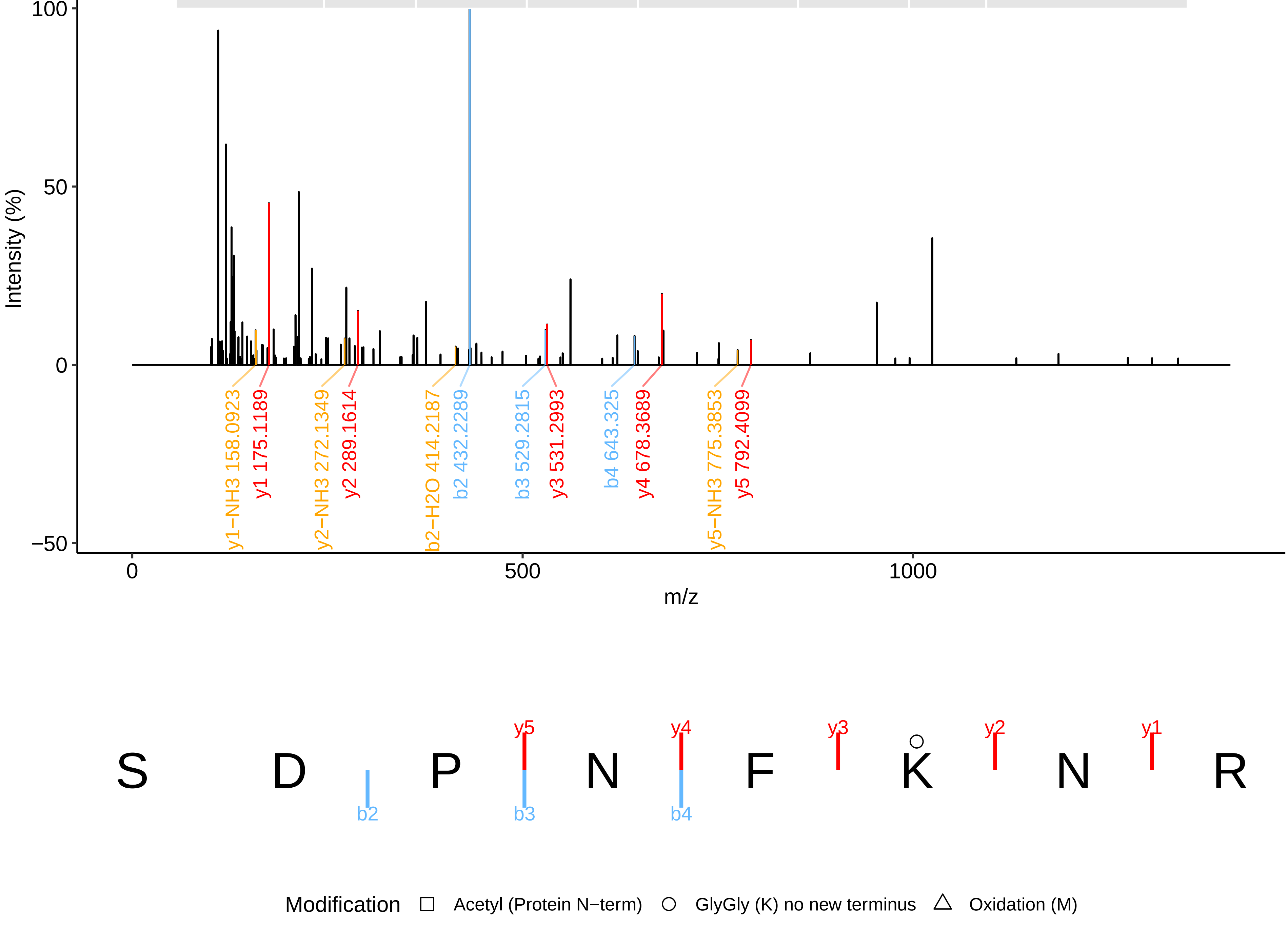

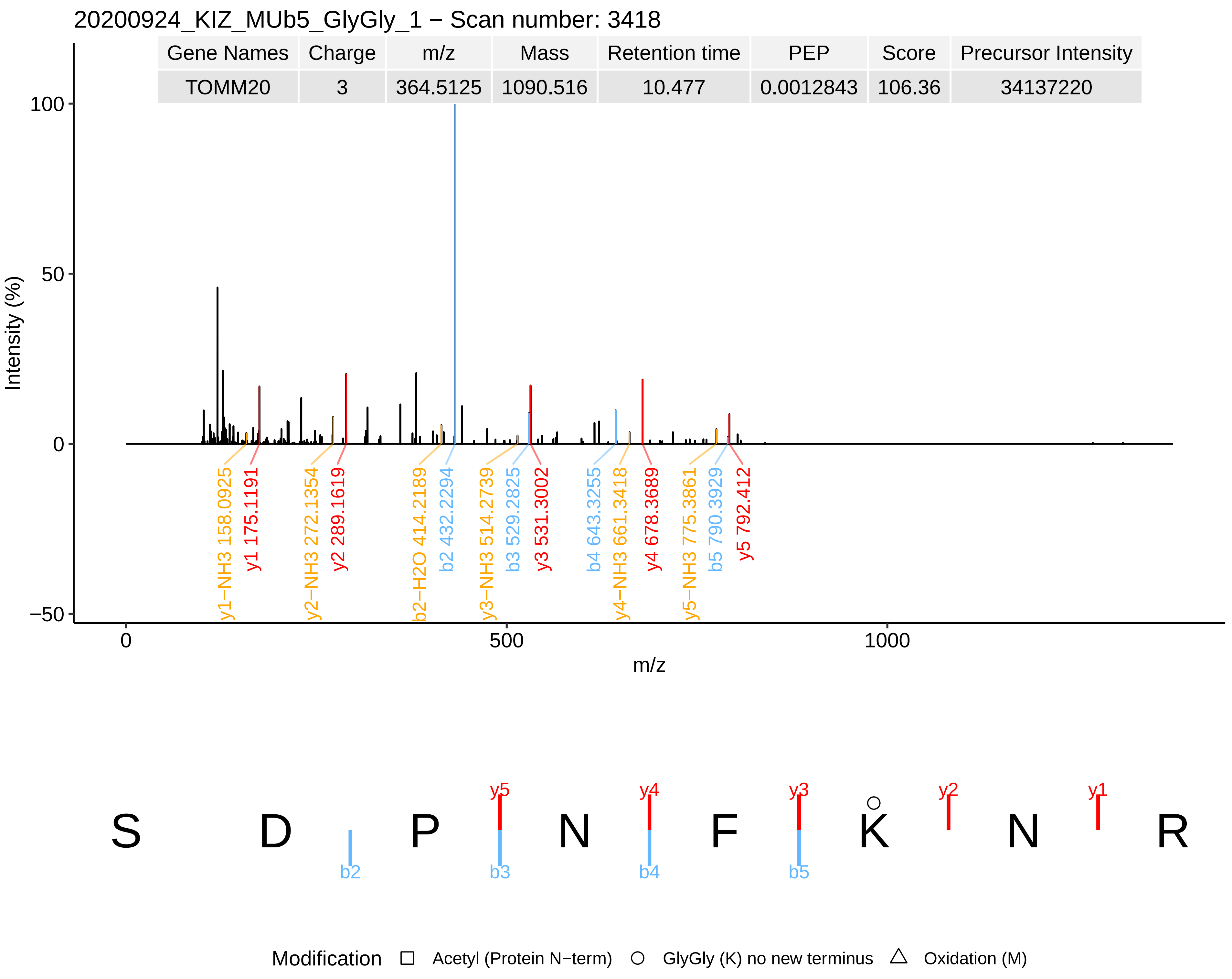

20200924_KIZ_MUb5_GlyGly_1 − Scan number: 3418

Gene Names	Charge	m/z	Mass	Retention time	PEP	Score	Precursor Intensity
TOMM20	3	364.5125	1090.516	10.477	0.0012843	106.36	34137220

Intensity (%)
m/z
y1−NH3 158.0925
y1 175.1191
y2−NH3 272.1354
y2 289.1619
b2−H2O 414.2189
b2 432.2294
y3−NH3 514.2739
b3 529.2825
y3 531.3002
b4 643.3255
y4−NH3 661.3418
y4 678.3689
y5−NH3 775.3861
b5 790.3929
y5 792.412
S D P N F K N R
y5 y4 y3 y2 y1
b2 b3 b4 b5
Modification
Acetyl (Protein N−term)
GlyGly (K) no new terminus
Oxidation (M)

20200924_KIZ_MUb5_GlyGly_2 − Scan number: 3193

Gene Names	Charge	m/z	Mass	Retention time	PEP	Score	Precursor Intensity
TOMM20	3	364.5125	1090.516	11.491	0.010186	79.713	1173031

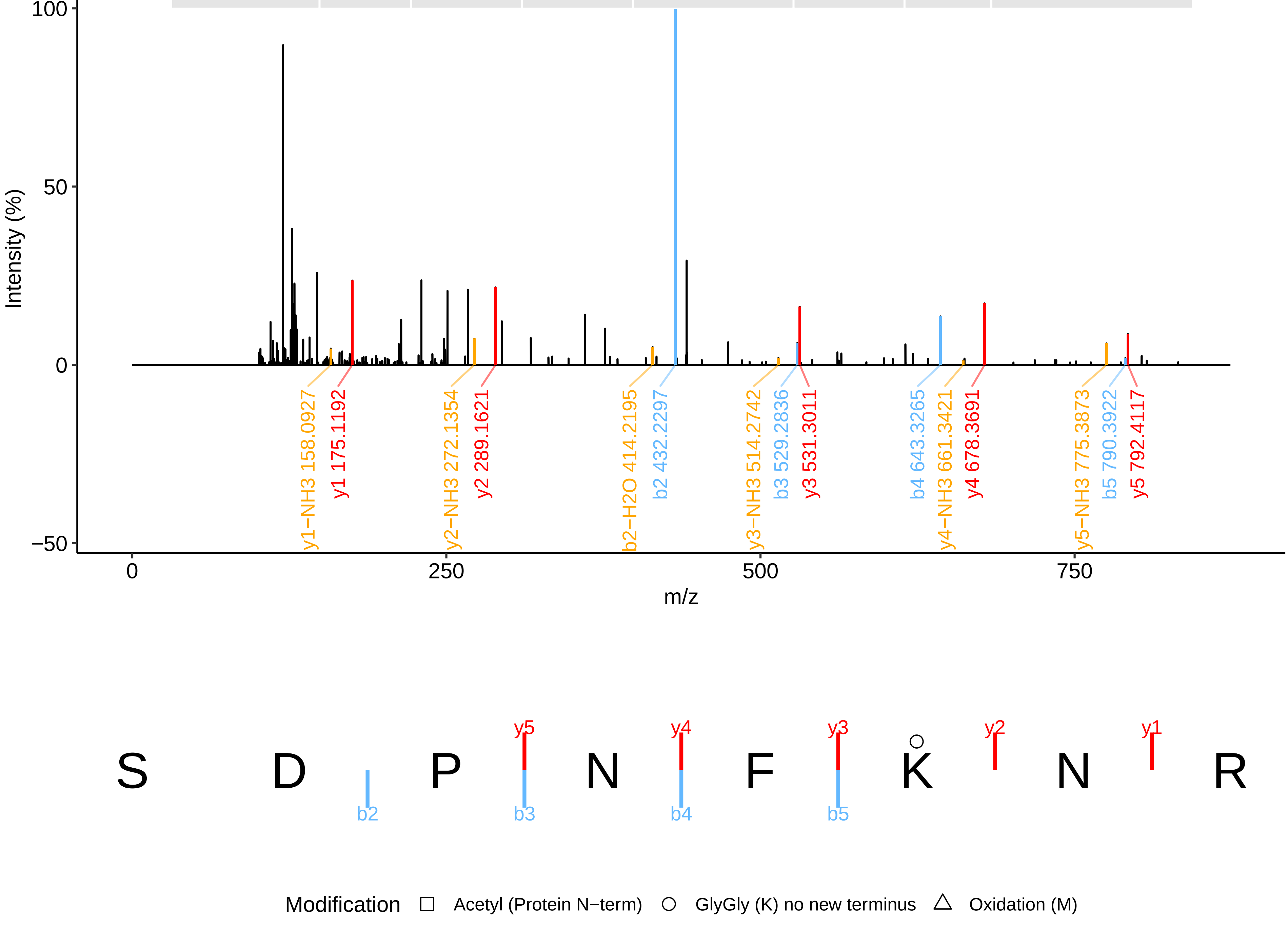

20201118_KIZ_MUb6_GlyGly_1 − Scan number: 3309

Gene Names	Charge	m/z	Mass	Retention time	PEP	Score	Precursor Intensity
TOMM20	3	364.5125	1090.516	10.161	0.0012843	106.36	16995742

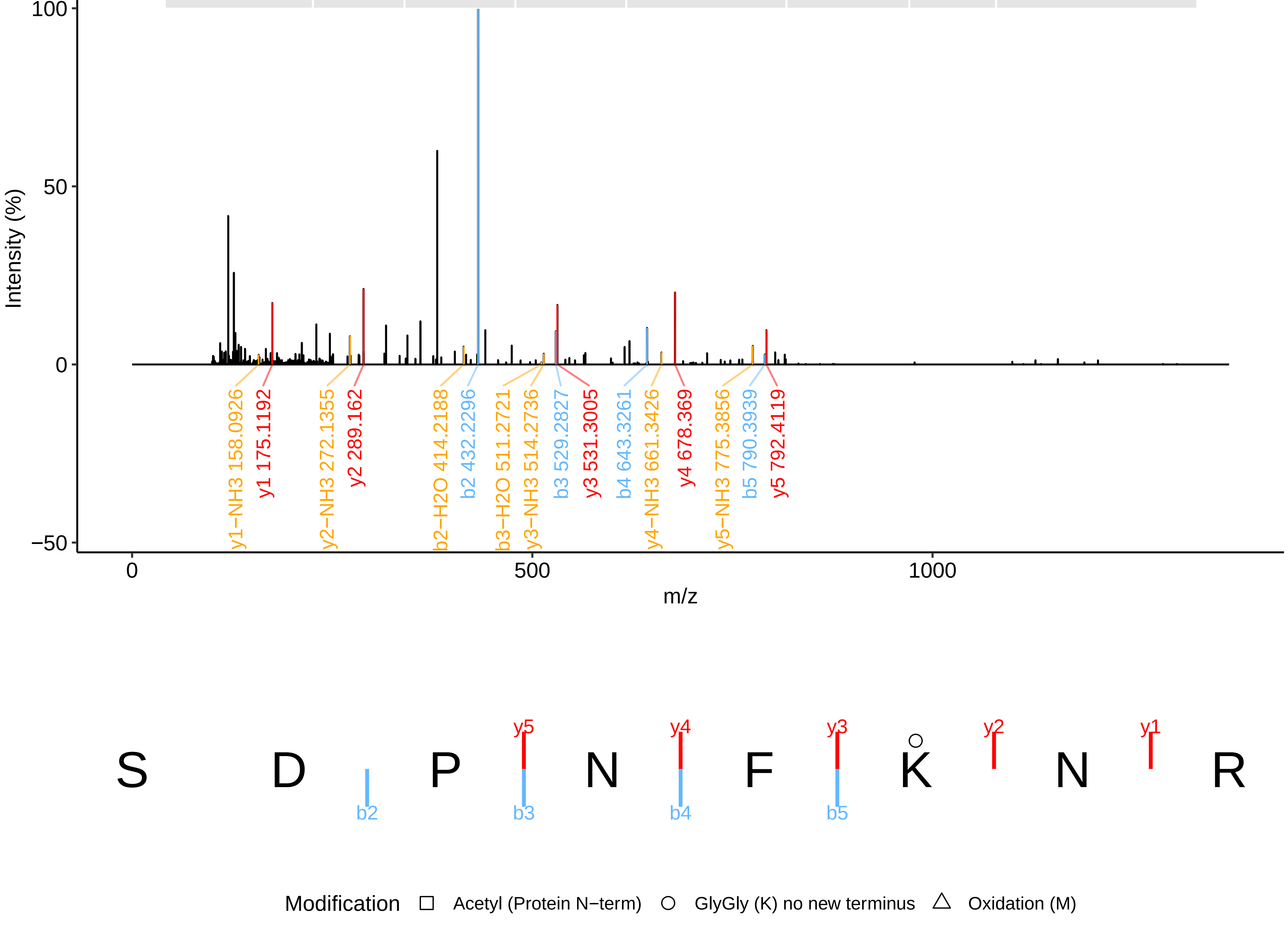

20201118_KIZ_MUb6_GlyGly_2 − Scan number: 3795

Gene Names	Charge	m/z	Mass	Retention time	PEP	Score	Precursor Intensity
TOMM20	3	364.5125	1090.516	12.709	0.0037627	94.114	1878894

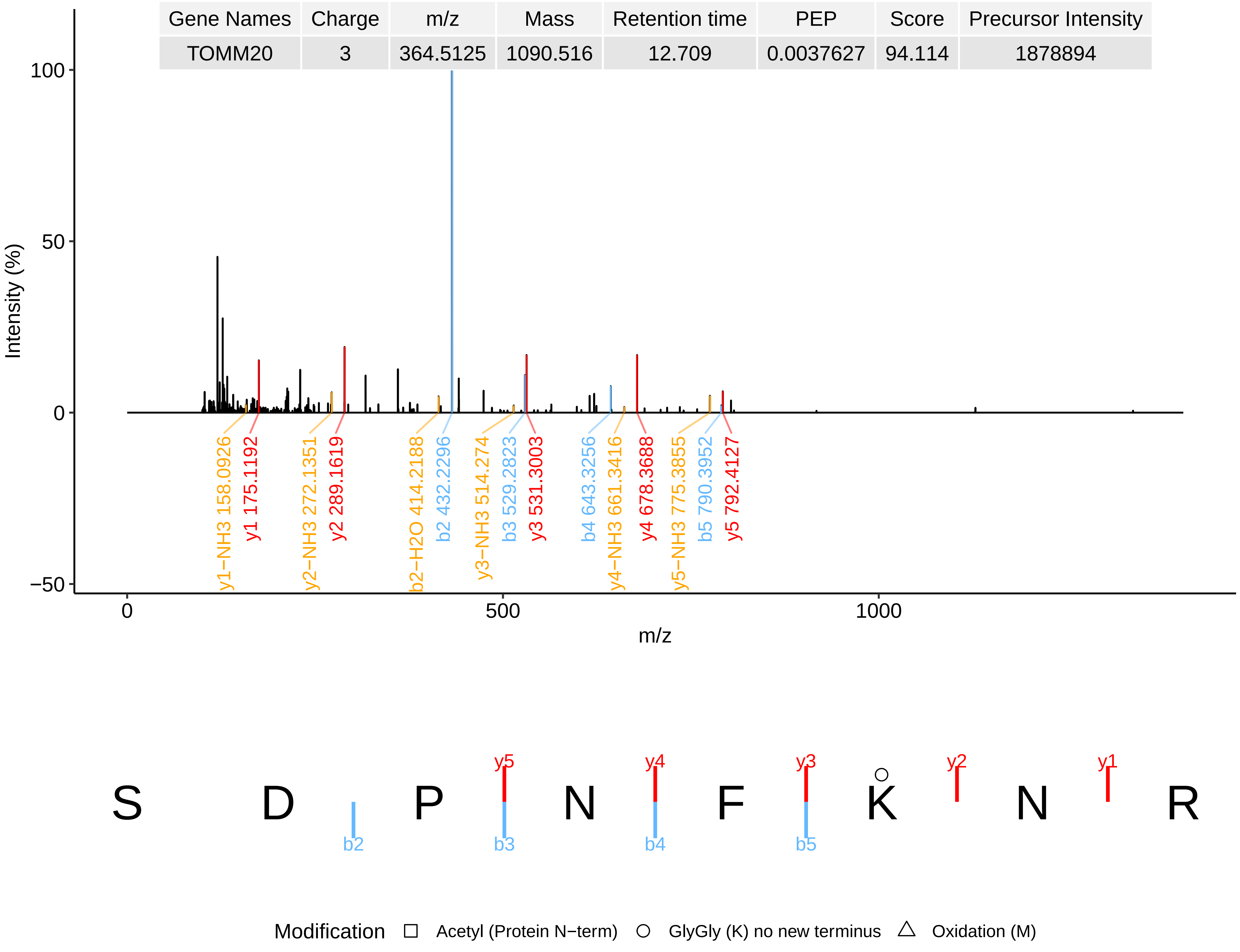

20200924_KIZ_MUb5_GlyGly_1 − Scan number: 7710

Gene Names	Charge	m/z	Mass	Retention time	PEP	Score	Precursor Intensity
UBA1	2	415.7584	829.5022	24.531	0.089024	96.253	NA

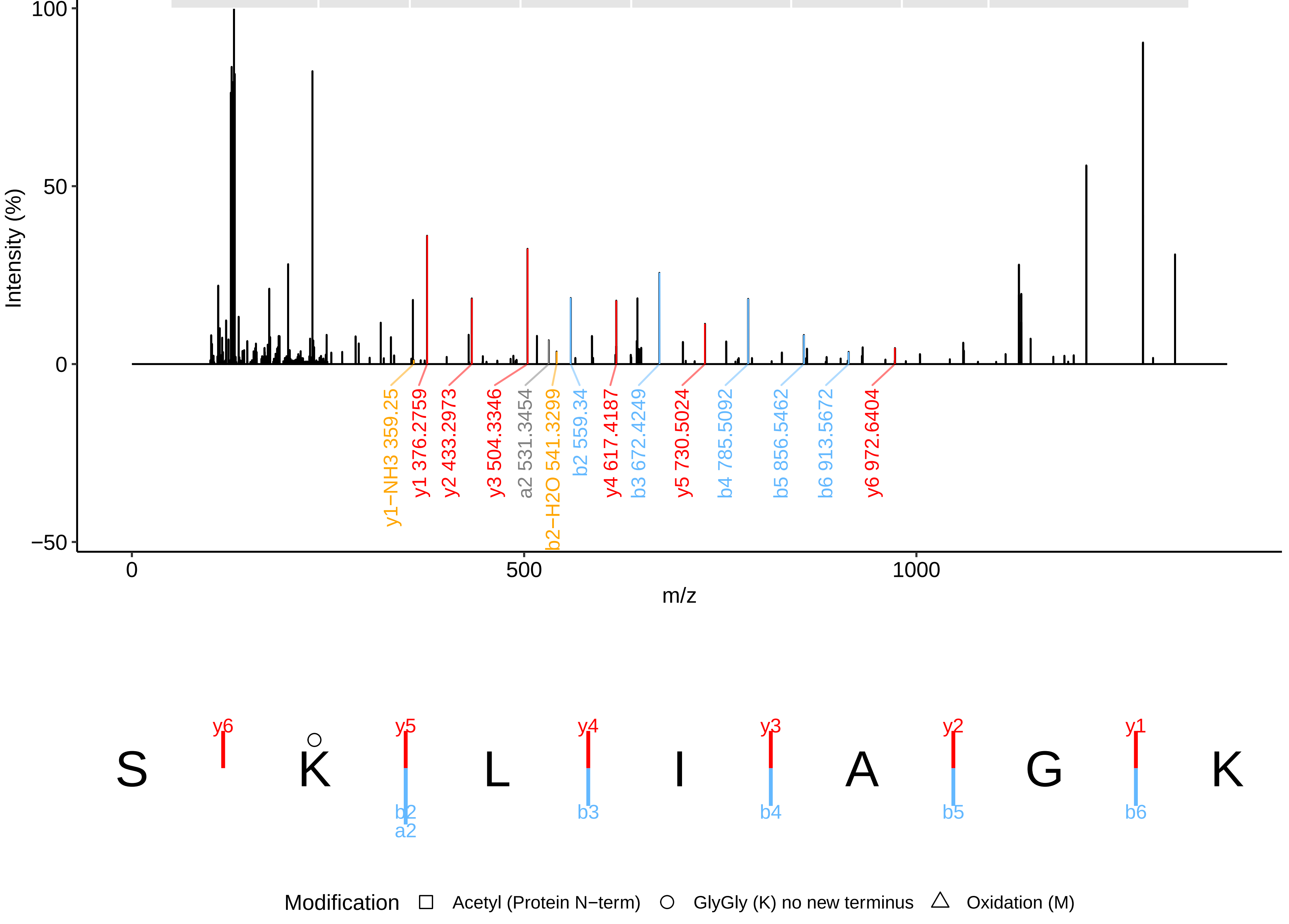

20200924_KIZ_MUb5_GlyGly_2 − Scan number: 19980

Gene Names	Charge	m/z	Mass	Retention time	PEP	Score	Precursor Intensity
TPTE2	2	547.2871	1092.56	74.059	0.057918	67.981	6517558

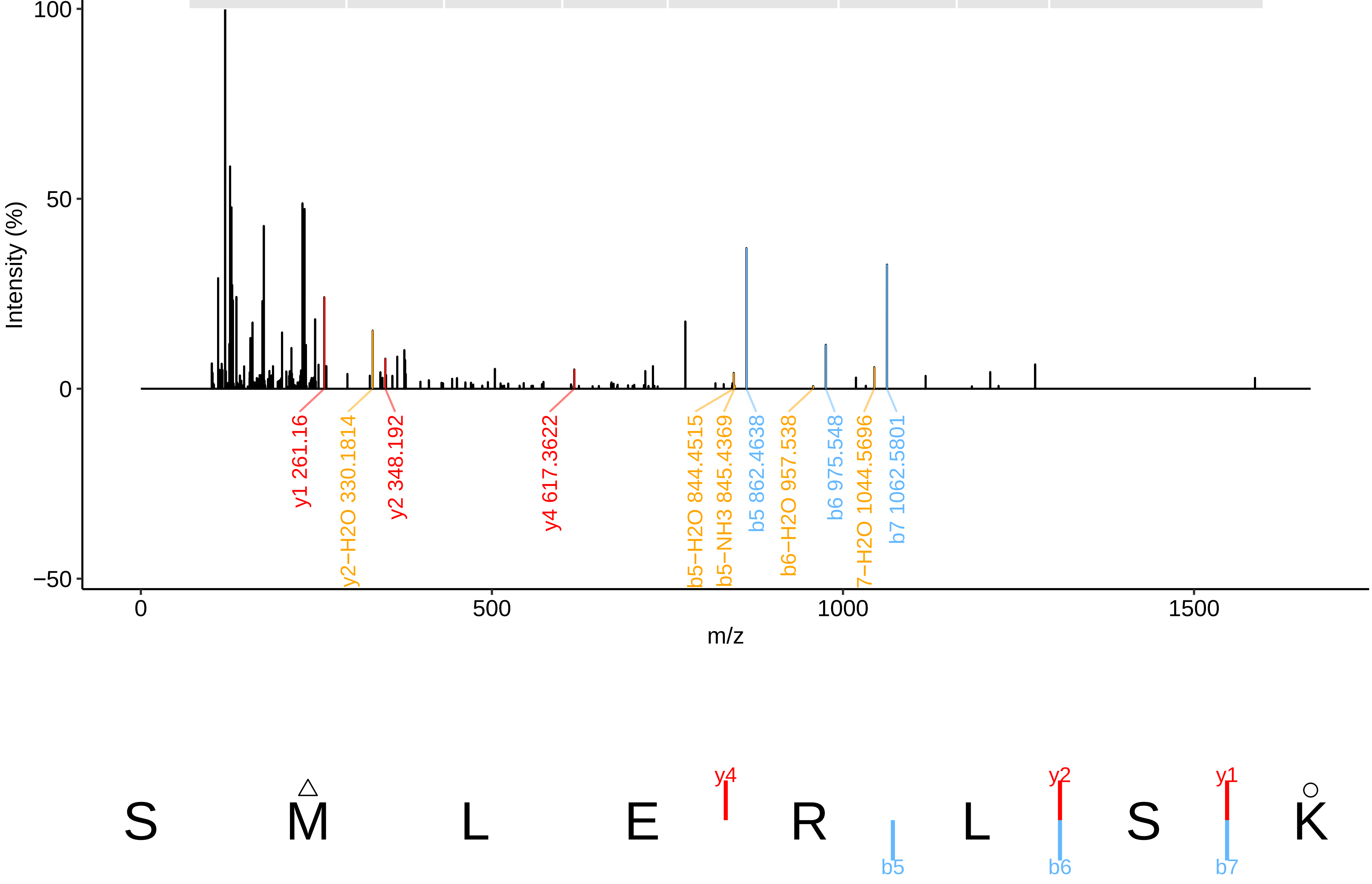

Modification □ Acetyl (Protein N−term) ○ GlyGly (K) no new terminus △ Oxidation (M)

20201118_KIZ_MUb6_GlyGly_1 − Scan number: 21961

Gene Names	Charge	m/z	Mass	Retention time	PEP	Score	Precursor Intensity
TPTE2	2	547.2871	1092.56	68.366	0.010298	88.942	14649878

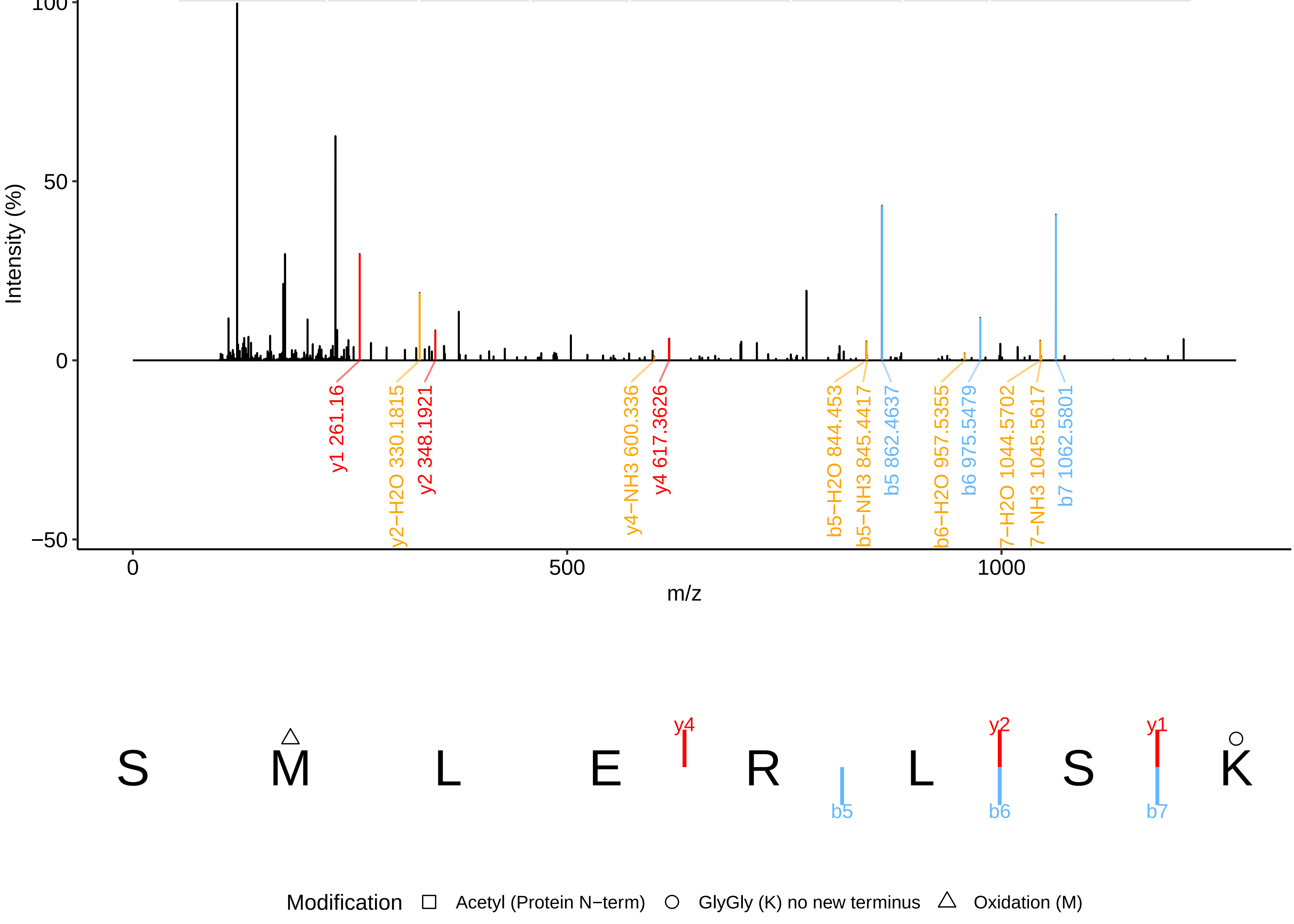

20200921_KIZ_MUb4_GlyGly_10plex − Scan number: 6731

Gene Names	Charge	m/z	Mass	Retention time	PEP	Score	Precursor Intensity
GNAS;GNAL	3	359.5342	1075.581	22.383	0.0025092	104.21	4462949

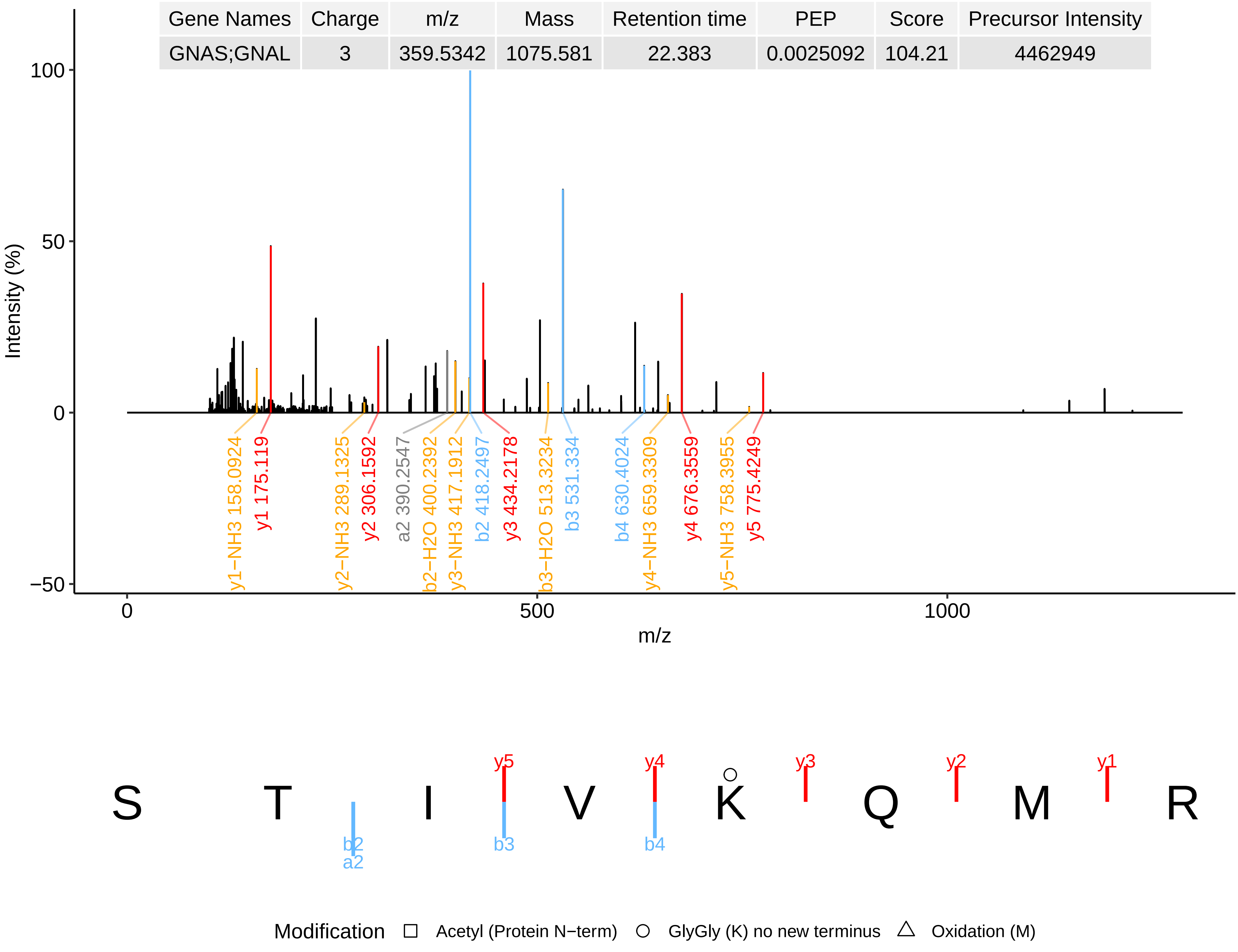

20200921_KIZ_MUb4_GlyGly_10plex − Scan number: 12314

Gene Names	Charge	m/z	Mass	Retention time	PEP	Score	Precursor Intensity
TECR	3	352.8539	1055.54	43.124	0.018575	82.305	11149502

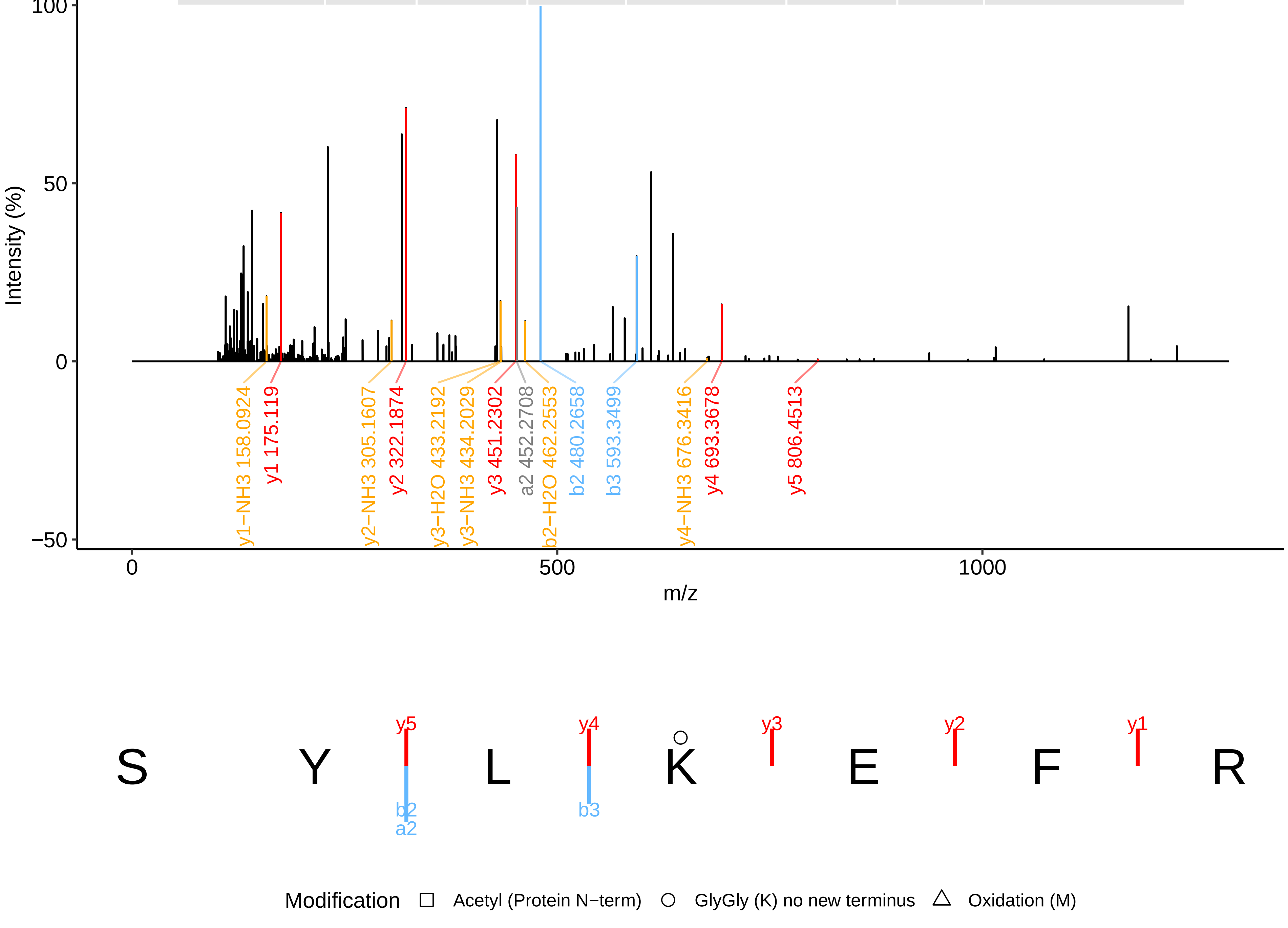

20200921_KIZ_MUb4_GlyGly_10plex − Scan number: 8462

Gene Names	Charge	m/z	Mass	Retention time	PEP	Score	Precursor Intensity
PTGES2	3	365.1747	1092.502	28.721	0.015829	86.086	4469190

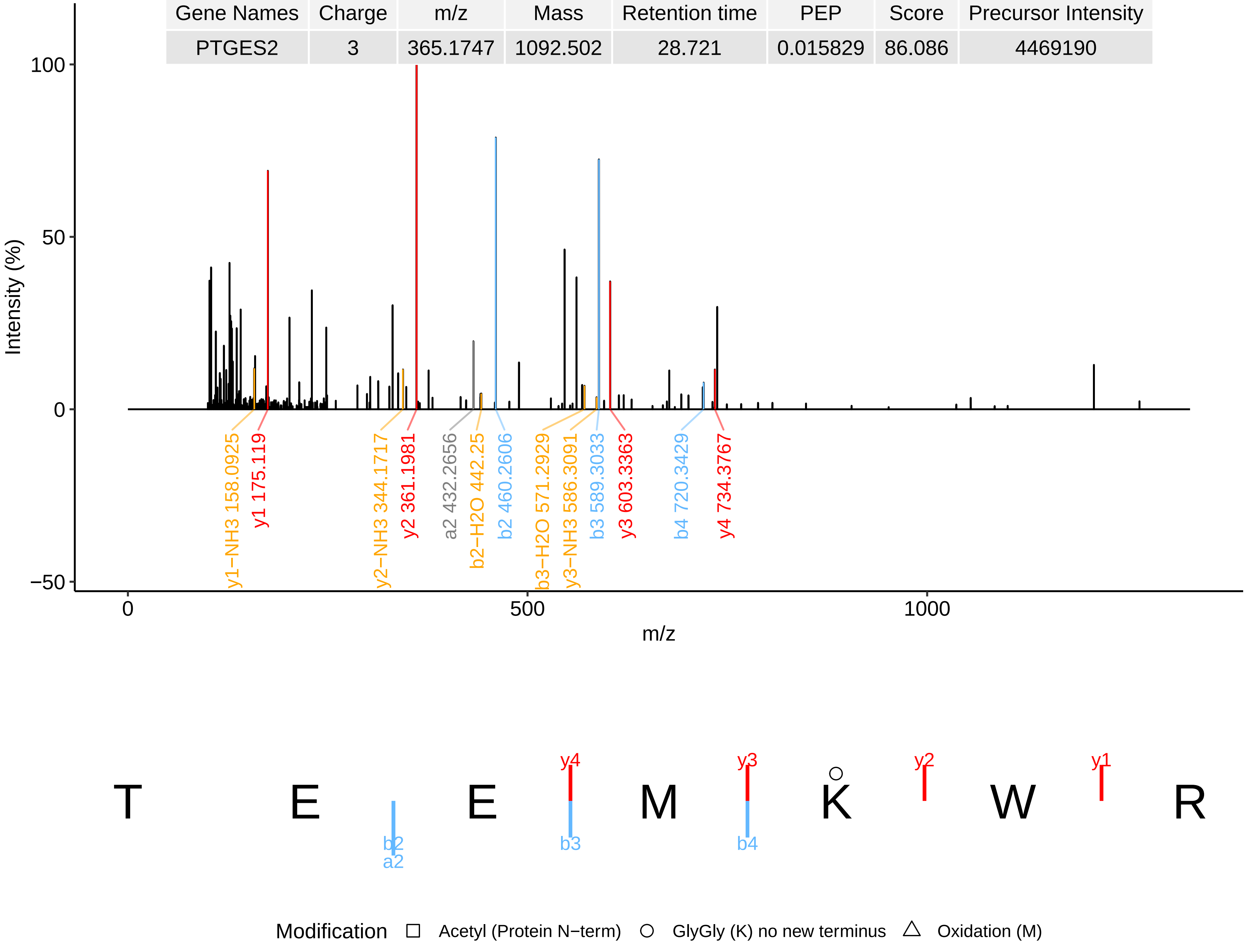

20200921_KIZ_MUb4_GlyGly_10plex_2 − Scan number: 7808

Gene Names	Charge	m/z	Mass	Retention time	PEP	Score	Precursor Intensity
PTGES2	3	365.1747	1092.502	29.256	0.060264	64.803	561465.8

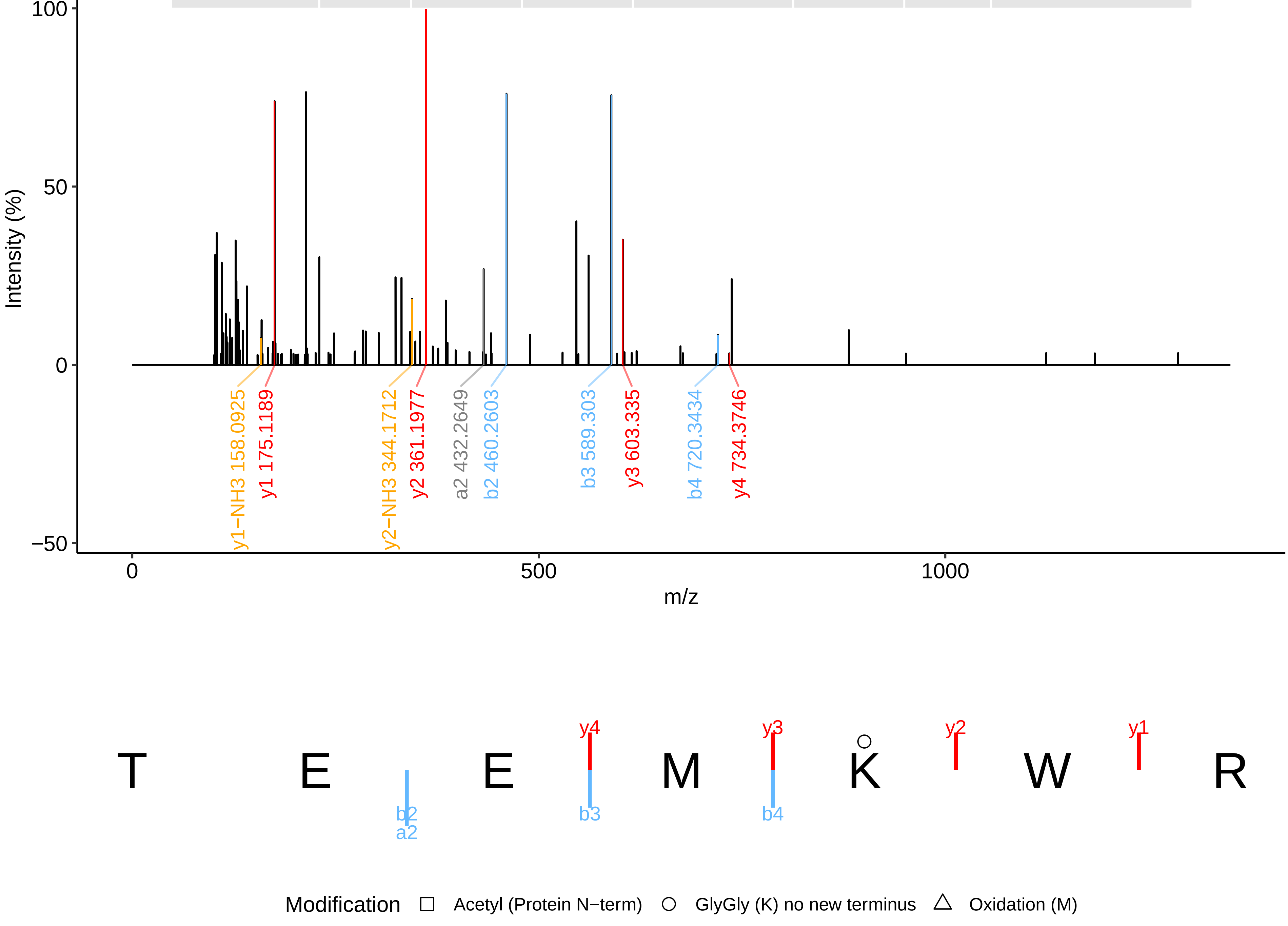

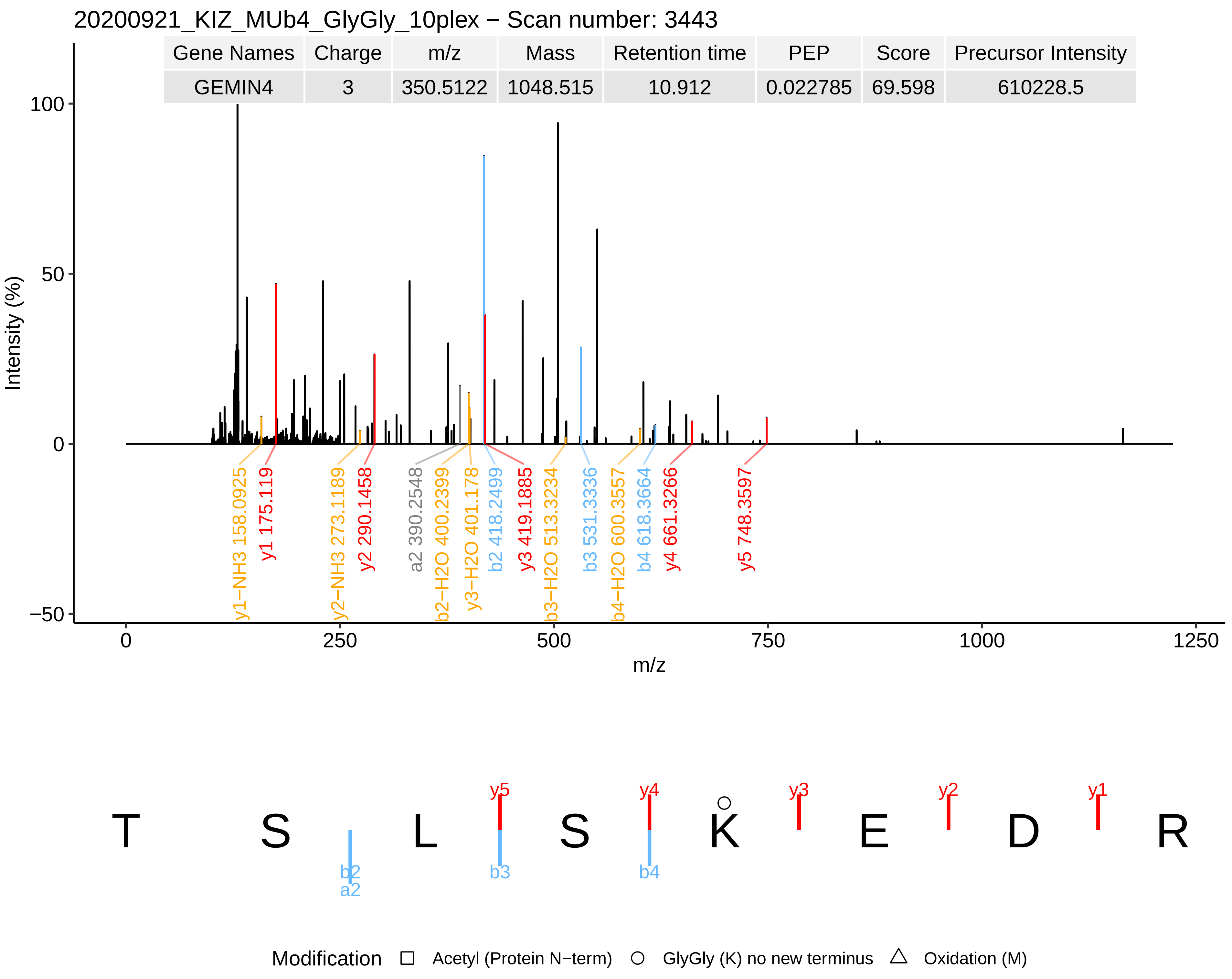

20200921_KIZ_MUb4_GlyGly_10plex − Scan number: 3443
Gene Names | Charge | m/z | Mass | Retention time | PEP | Score | Precursor Intensity
GEMIN4 | 3 | 350.5122 | 1048.515 | 10.912 | 0.022785 | 69.598 | 610228.5
Intensity (%)
m/z
y1−NH3 158.0925
y1 175.119
y2−NH3 273.1189
y2 290.1458
a2 390.2548
b2−H2O 400.2399
y3−H2O 401.178
b2 418.2499
y3 419.1885
b3−H2O 513.3234
b3 531.3336
b4−H2O 600.3557
b4 618.3664
y4 661.3266
y5 748.3597
T S L S K E D R
y5 y4 y3 y2 y1
b2 a2 b3 b4
Modification
Acetyl (Protein N−term)
GlyGly (K) no new terminus
Oxidation (M)

20200924_KIZ_MUb5_GlyGly_2 − Scan number: 4957

Gene Names	Charge	m/z	Mass	Retention time	PEP	Score	Precursor Intensity
FXR1;FMR1;FXR2	3	277.1679	828.4818	18.309	0.13367	54.525	998889.6

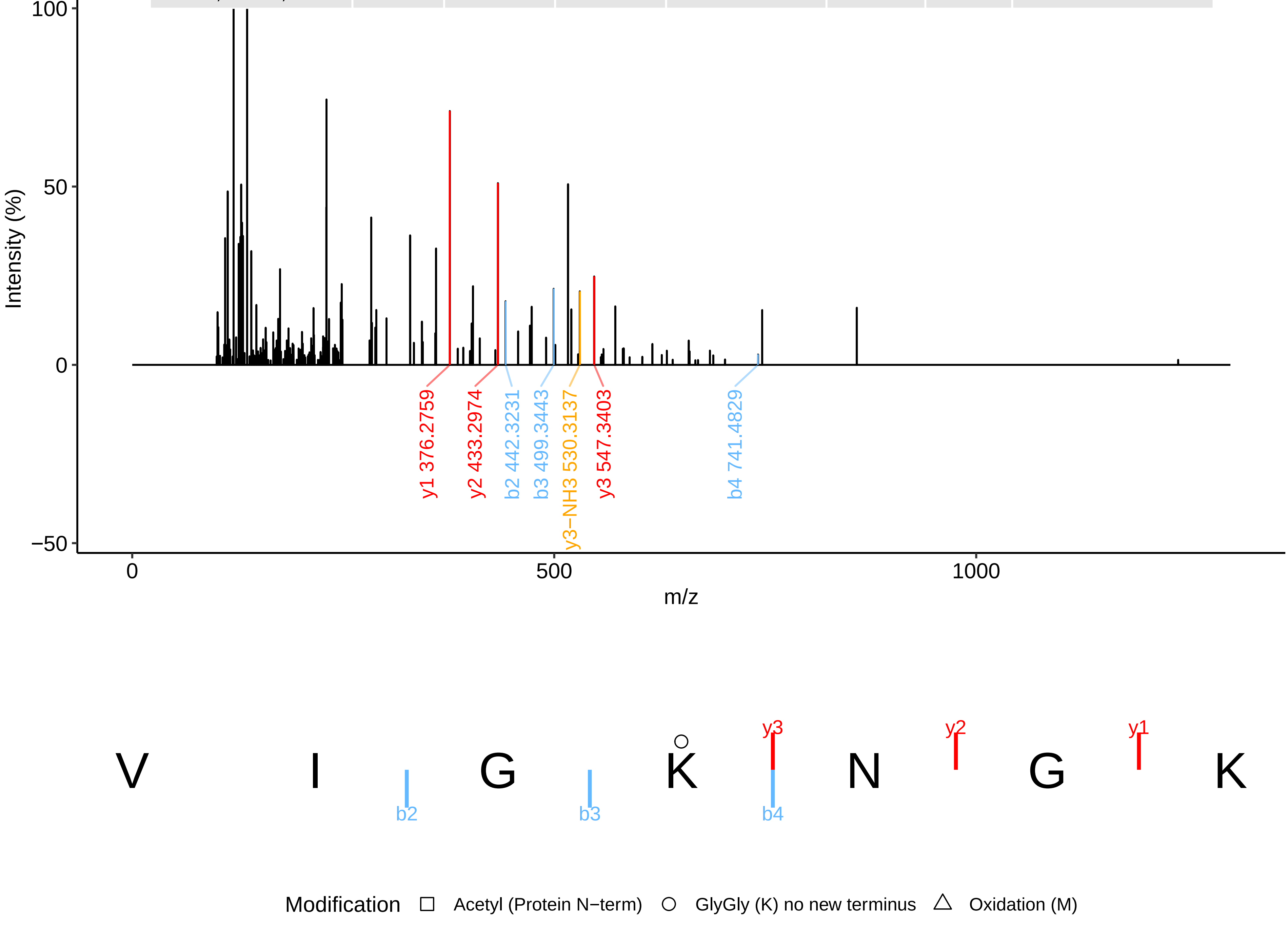

20200921_KIZ_MUb4_GlyGly_10plex − Scan number: 5076

Gene Names	Charge	m/z	Mass	Retention time	PEP	Score	Precursor Intensity
PGK1	3	286.1678	855.4814	16.567	0.10705	54.549	6461131

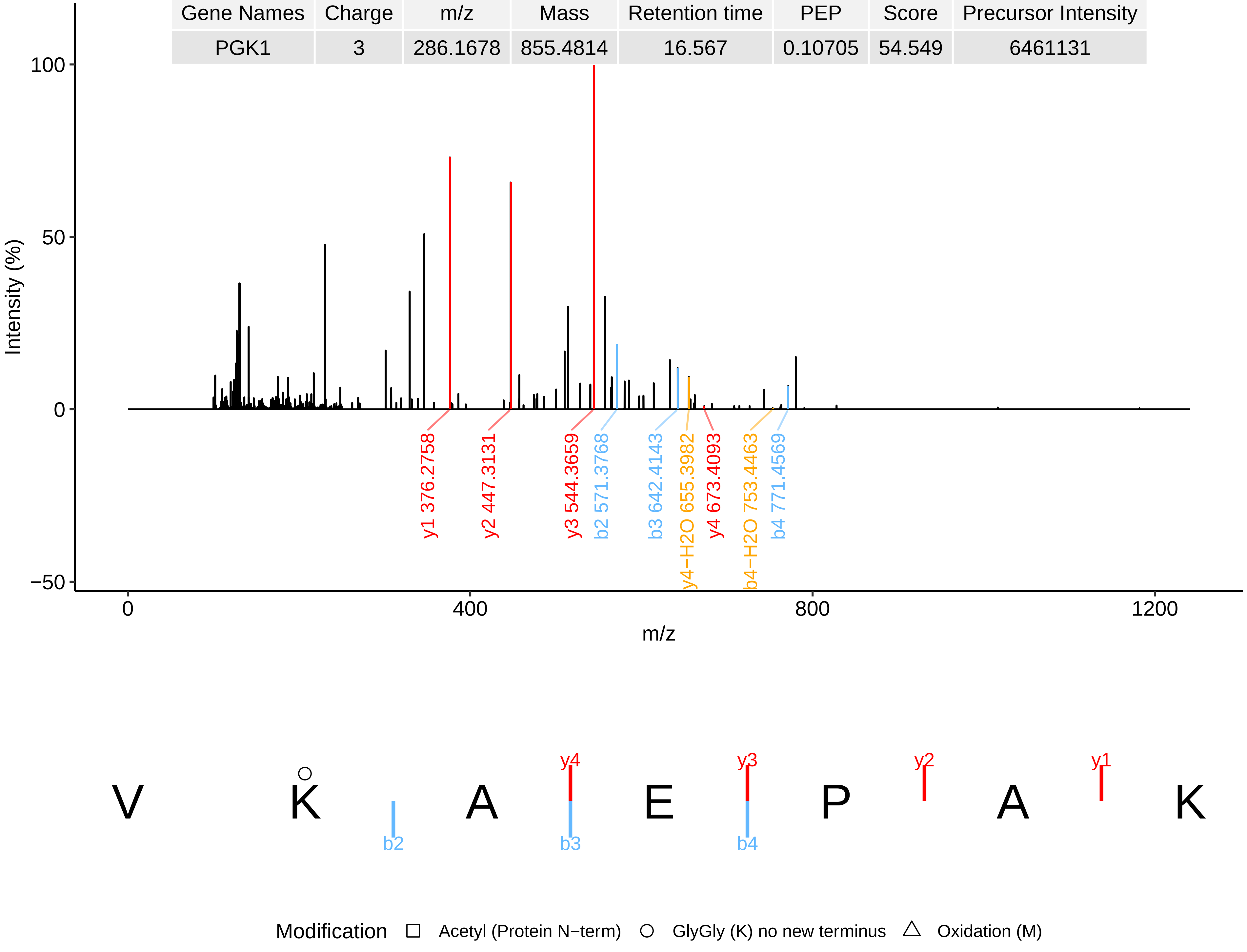

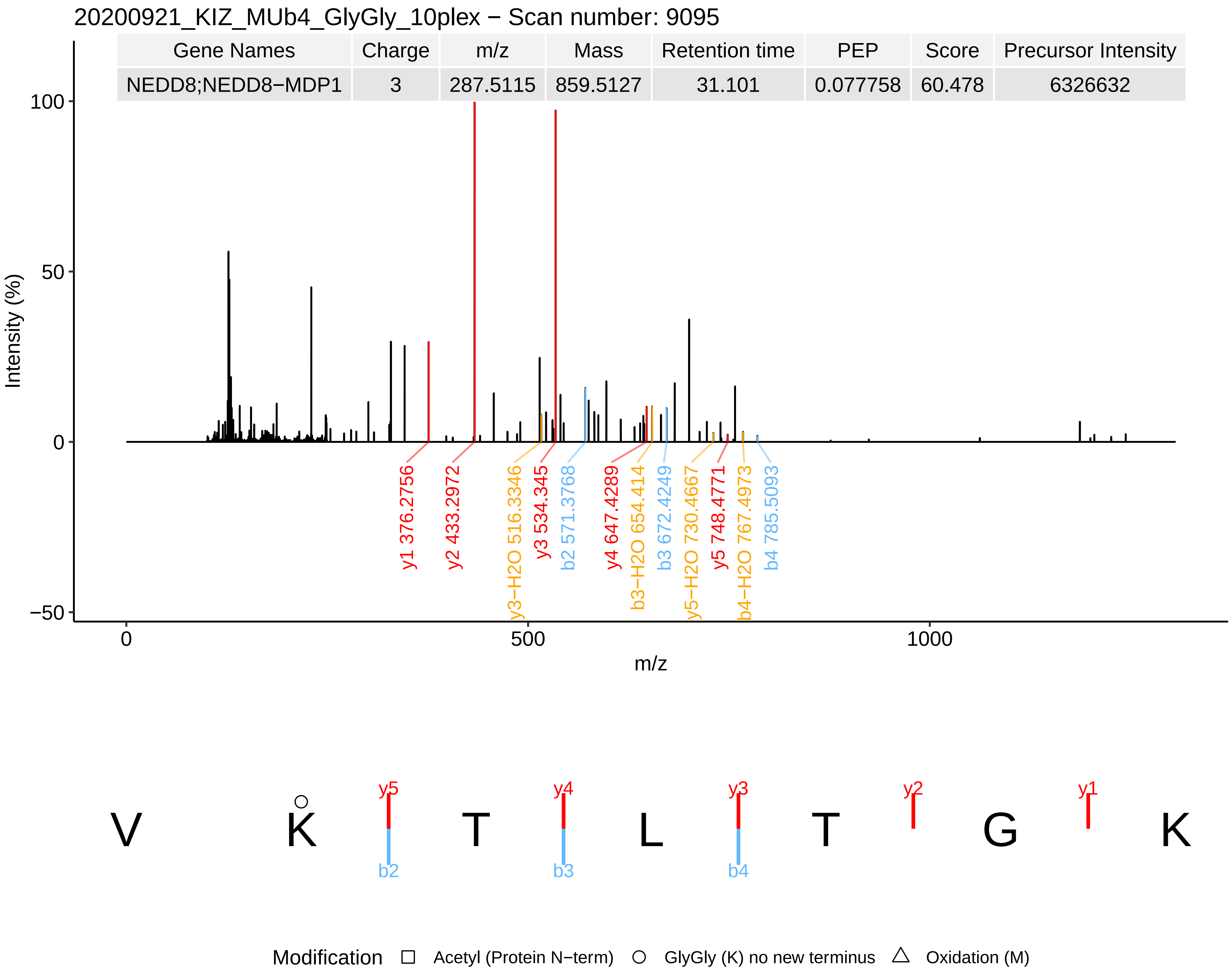
20200921_KIZ_MUb4_GlyGly_10plex − Scan number: 9095
Gene Names
Charge
m/z
Mass
Retention time
PEP
Score
Precursor Intensity
NEDD8;NEDD8−MDP1
3
287.5115
859.5127
31.101
0.077758
60.478
6326632
Intensity (%)
100
50
0
−50
0
500
1000
m/z
y1 376.2756
y2 433.2972
y3−H2O 516.3346
y3 534.345
b2 571.3768
y4 647.4289
b3−H2O 654.414
b3 672.4249
y5−H2O 730.4667
y5 748.4771
b4−H2O 767.4973
b4 785.5093
V
K
T
L
T
G
K
y5
y4
y3
y2
y1
b2
b3
b4
Modification
Acetyl (Protein N−term)
GlyGly (K) no new terminus
Oxidation (M)

20200921_KIZ_MUb4_GlyGly_10plex_2 − Scan number: 8453

Gene Names	Charge	m/z	Mass	Retention time	PEP	Score	Precursor Intensity
NEDD8;NEDD8−MDP1	3	287.5115	859.5127	31.85	0.061645	64.265	2337760

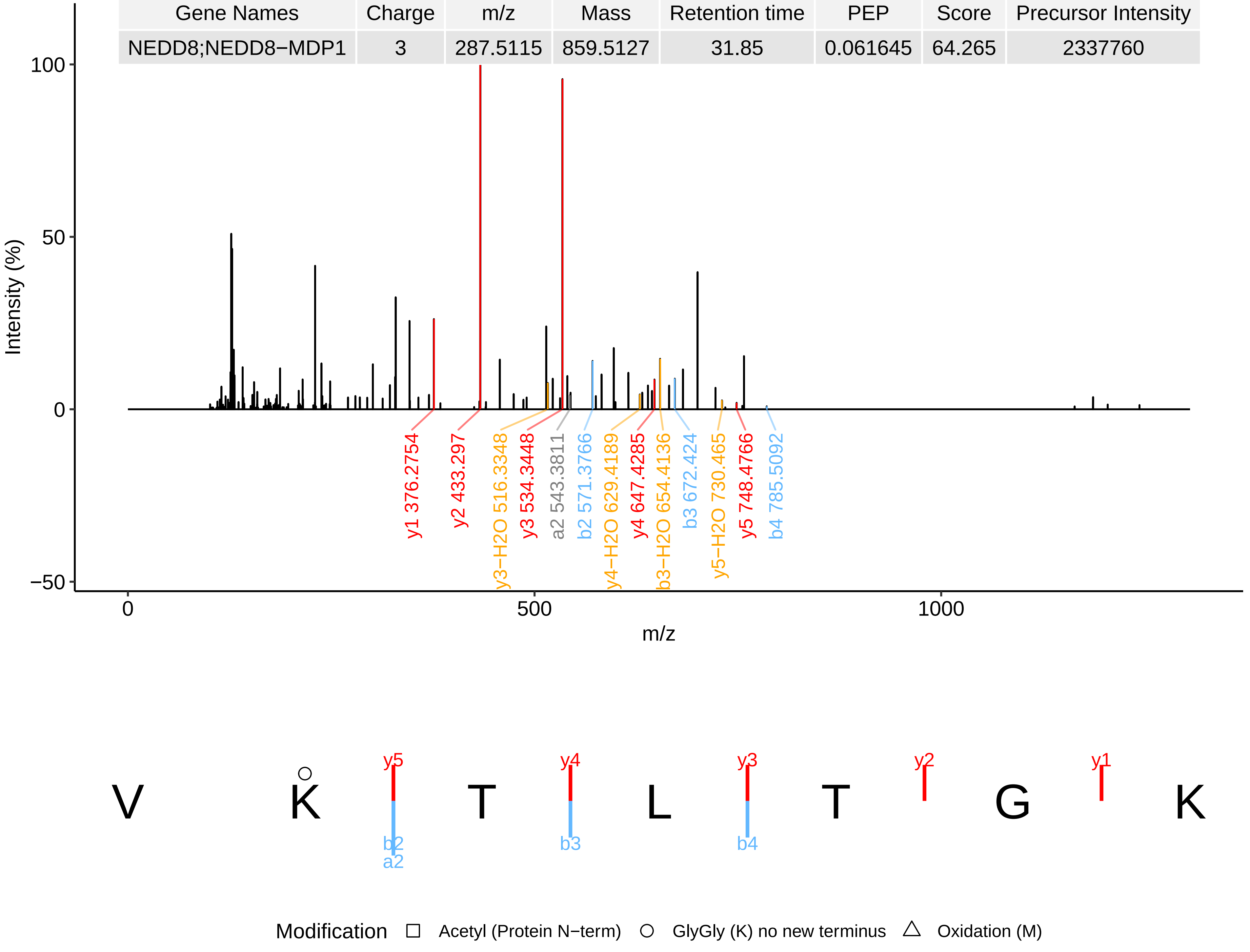

20201118_KIZ_MUb6_GlyGly_1 − Scan number: 7557

Gene Names	Charge	m/z	Mass	Retention time	PEP	Score	Precursor Intensity
NEDD8;NEDD8−MDP1	3	287.5115	859.5127	23.959	0.061791	64.207	16357001

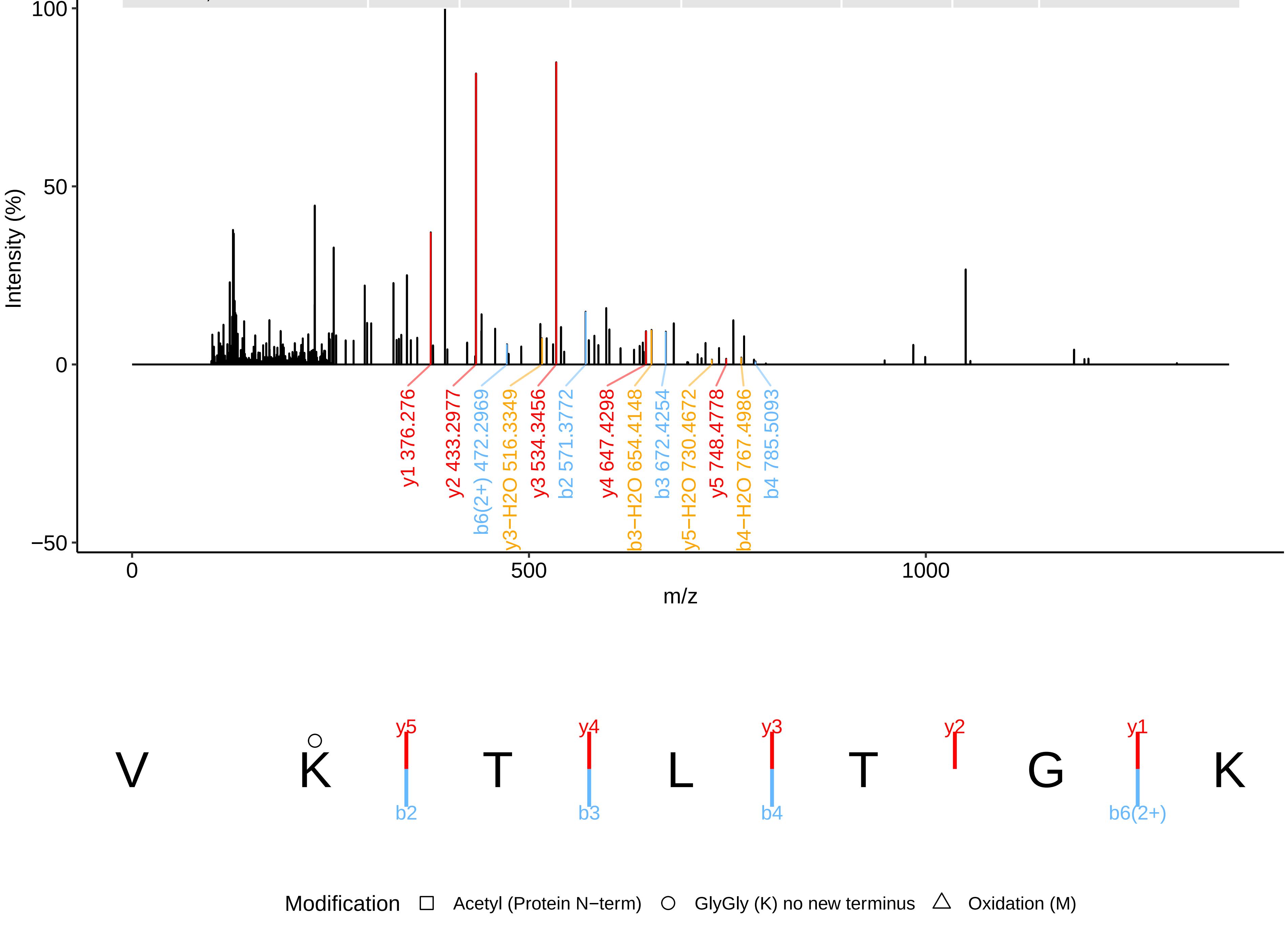

20200921_KIZ_MUb4_GlyGly_10plex − Scan number: 6829

Gene Names	Charge	m/z	Mass	Retention time	PEP	Score	Precursor Intensity
GNAS;GNAL	3	272.8361	815.4865	22.735	0.060264	64.803	14906236

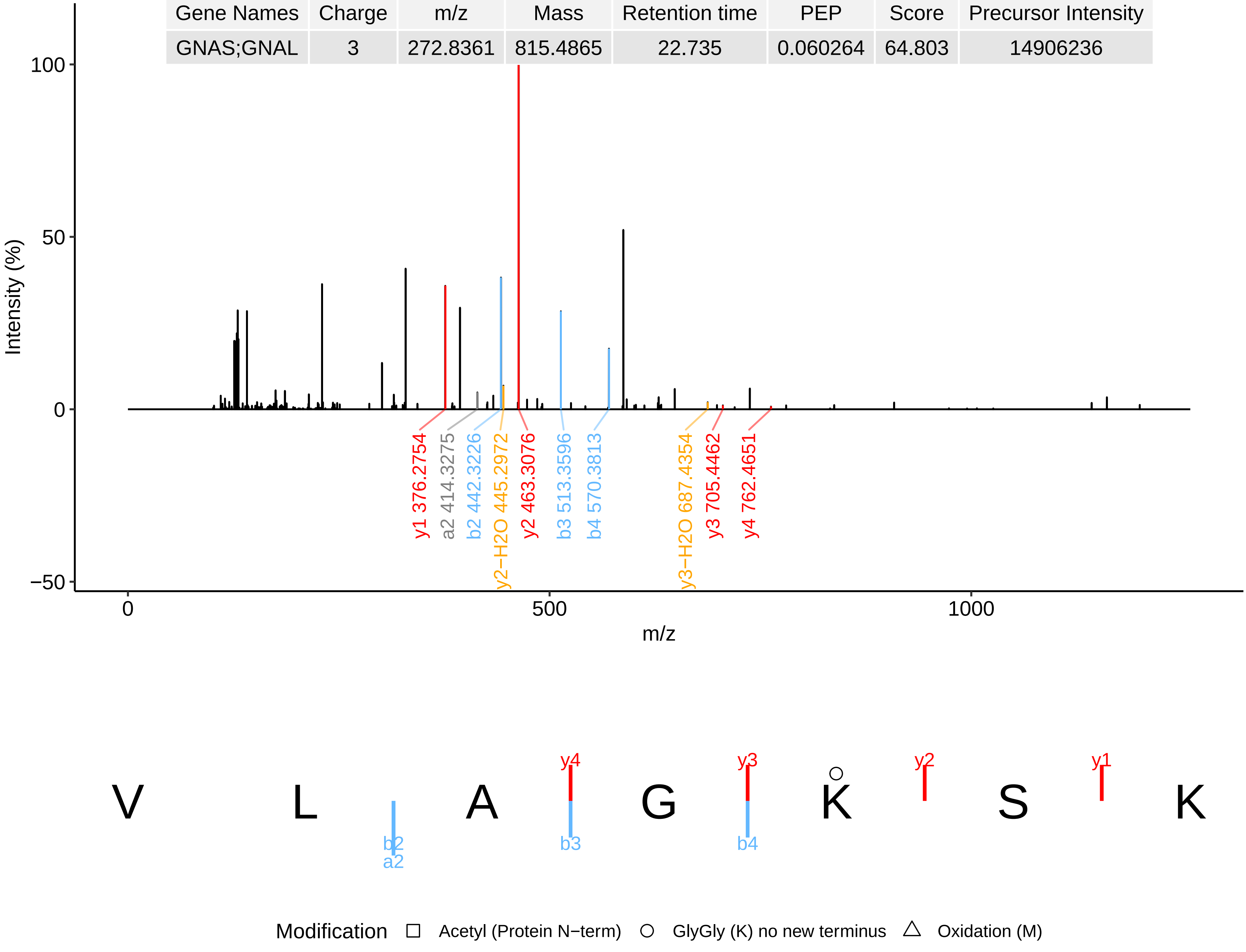

20200921_KIZ_MUb4_GlyGly_10plex_2 − Scan number: 10444

Gene Names	Charge	m/z	Mass	Retention time	PEP	Score	Precursor Intensity
MMP15	3	355.8673	1064.58	39.958	0.094037	86.935	5743608

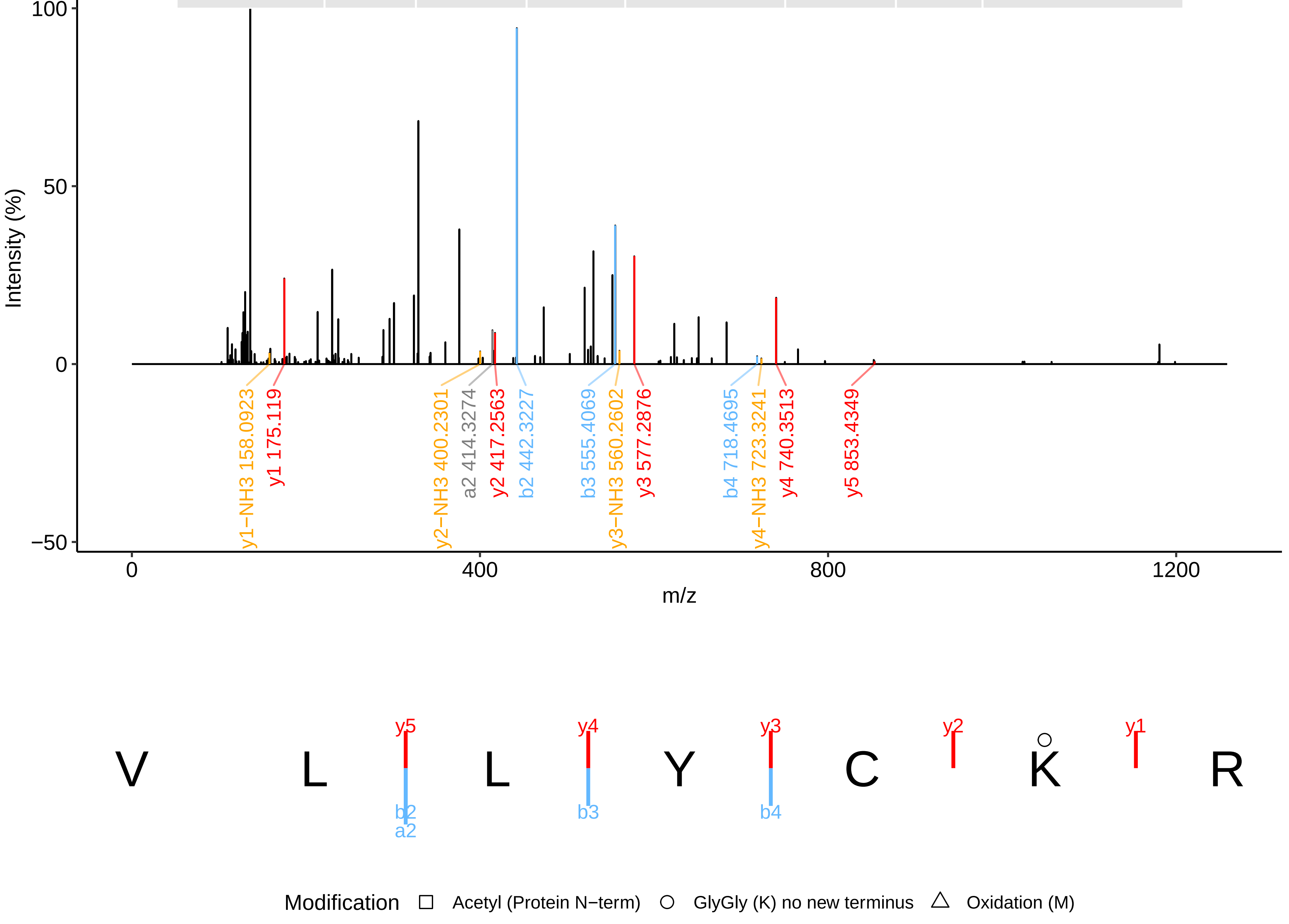

20201118_KIZ_MUb6_GlyGly_1 − Scan number: 9446

Gene Names	Charge	m/z	Mass	Retention time	PEP	Score	Precursor Intensity
MMP15	3	355.8673	1064.58	29.895	0.038408	101.33	23433092

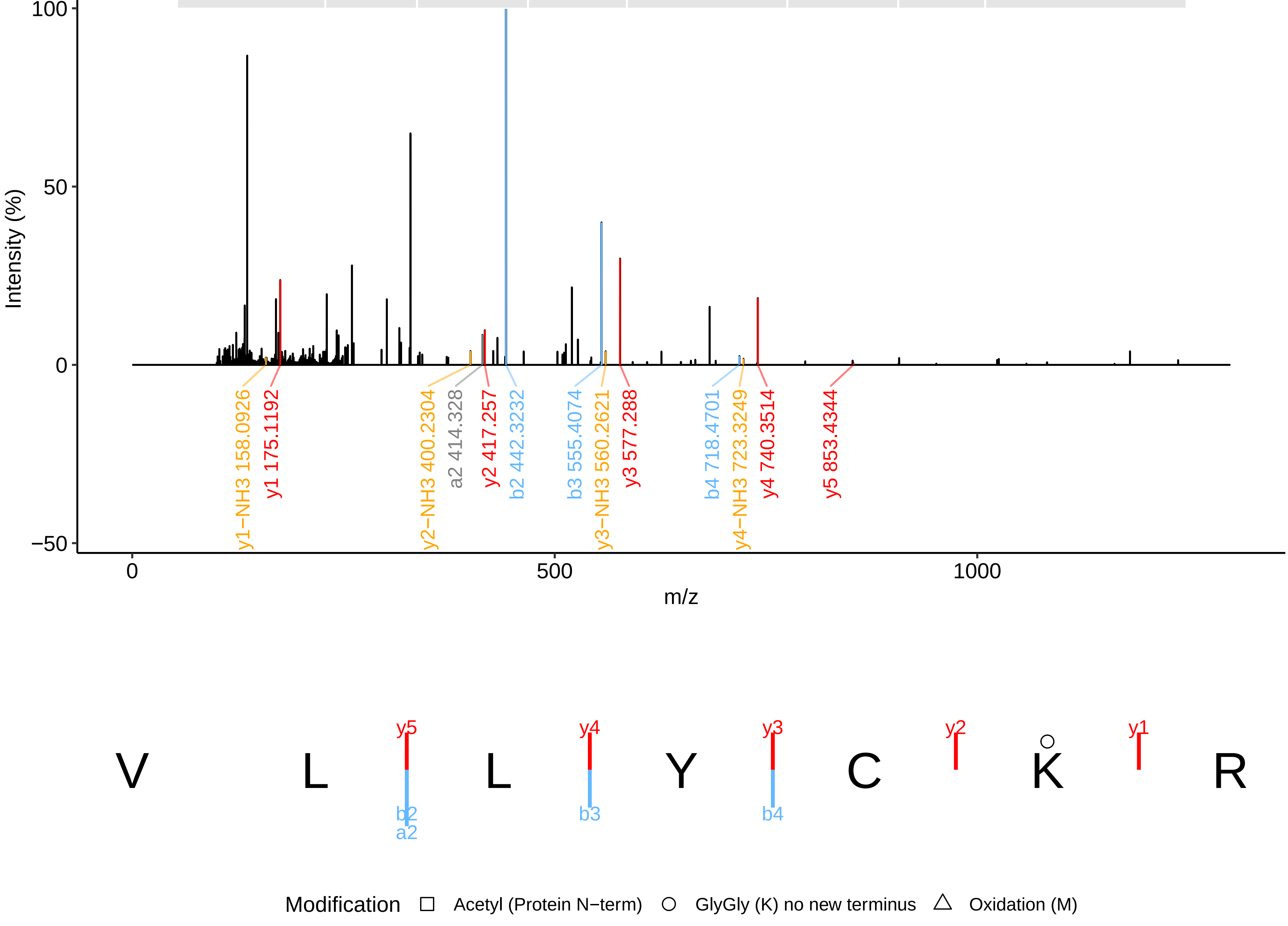

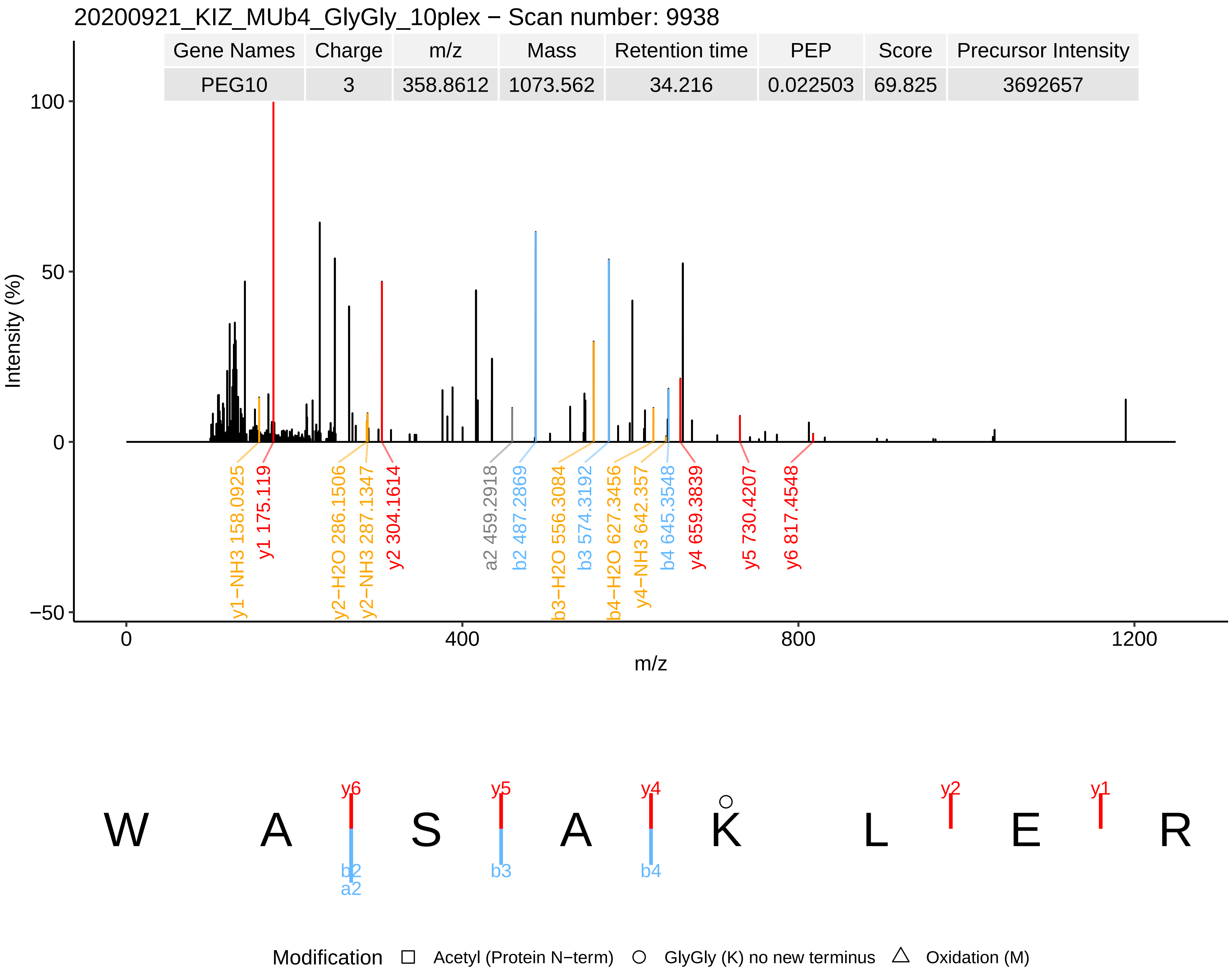
20200921_KIZ_MUb4_GlyGly_10plex − Scan number: 9938
Gene Names | Charge | m/z | Mass | Retention time | PEP | Score | Precursor Intensity
PEG10 | 3 | 358.8612 | 1073.562 | 34.216 | 0.022503 | 69.825 | 3692657
Intensity (%)
m/z
y1−NH3 158.0925
y1 175.119
y2−H2O 286.1506
y2−NH3 287.1347
y2 304.1614
a2 459.2918
b2 487.2869
b3−H2O 556.3084
b3 574.3192
b4−H2O 627.3456
y4−NH3 642.357
b4 645.3548
y4 659.3839
y5 730.4207
y6 817.4548
W A S A K L E R
y6 y5 y4 y2 y1
b2 a2 b3 b4
Modification
Acetyl (Protein N−term)
GlyGly (K) no new terminus
Oxidation (M)

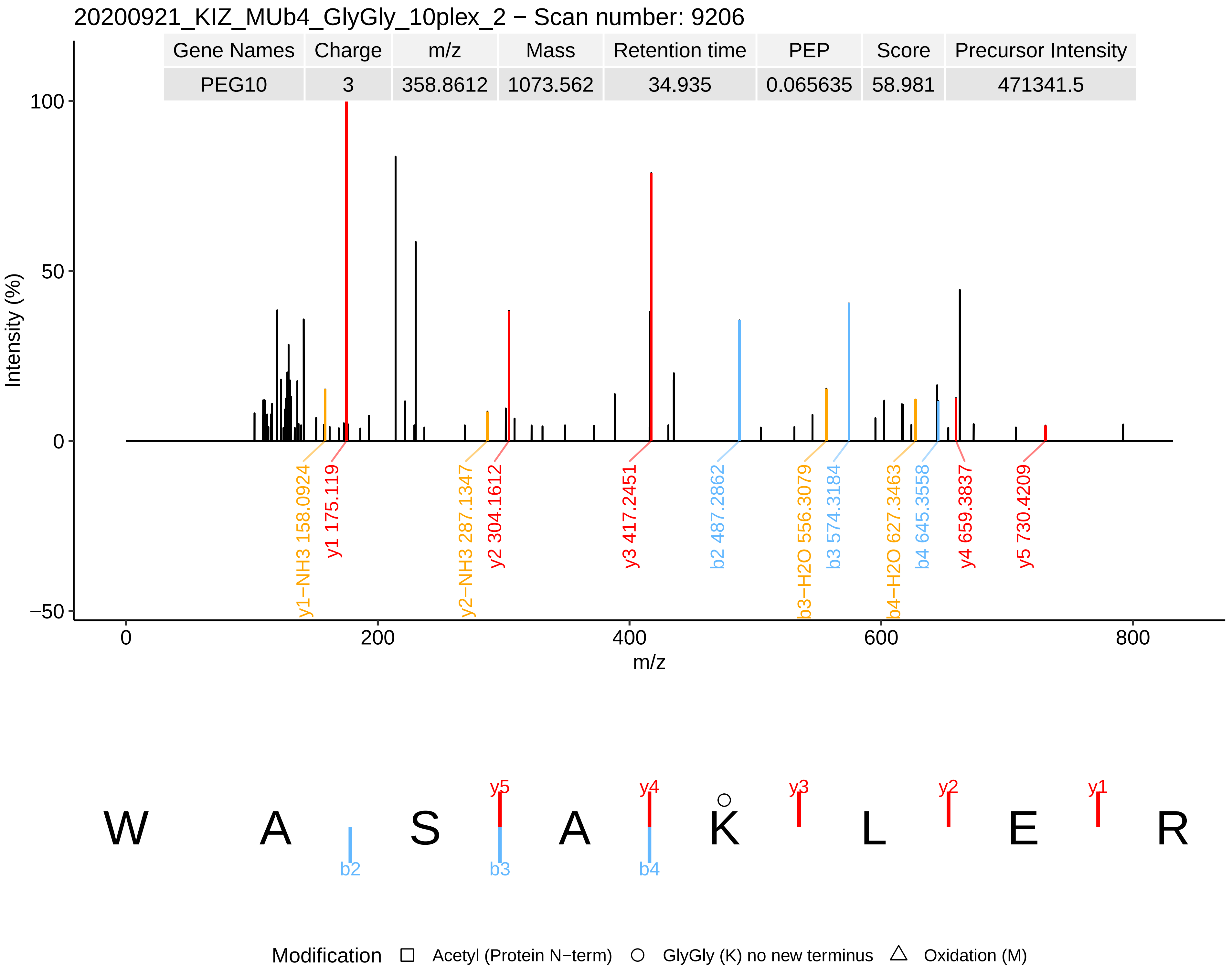
20200921_KIZ_MUb4_GlyGly_10plex_2 − Scan number: 9206
Gene Names | Charge | m/z | Mass | Retention time | PEP | Score | Precursor Intensity
PEG10 | 3 | 358.8612 | 1073.562 | 34.935 | 0.065635 | 58.981 | 471341.5
Intensity (%)
m/z
y1−NH3 158.0924
y1 175.119
y2−NH3 287.1347
y2 304.1612
y3 417.2451
b2 487.2862
b3−H2O 556.3079
b3 574.3184
b4−H2O 627.3463
b4 645.3558
y4 659.3837
y5 730.4209
W A S A K L E R
Modification
Acetyl (Protein N−term)
GlyGly (K) no new terminus
Oxidation (M)

20200924_KIZ_MUb5_GlyGly_1 − Scan number: 10774

Gene Names	Charge	m/z	Mass	Retention time	PEP	Score	Precursor Intensity
SPI1	2	536.786	1071.557	34.826	0.1125	62.842	NA

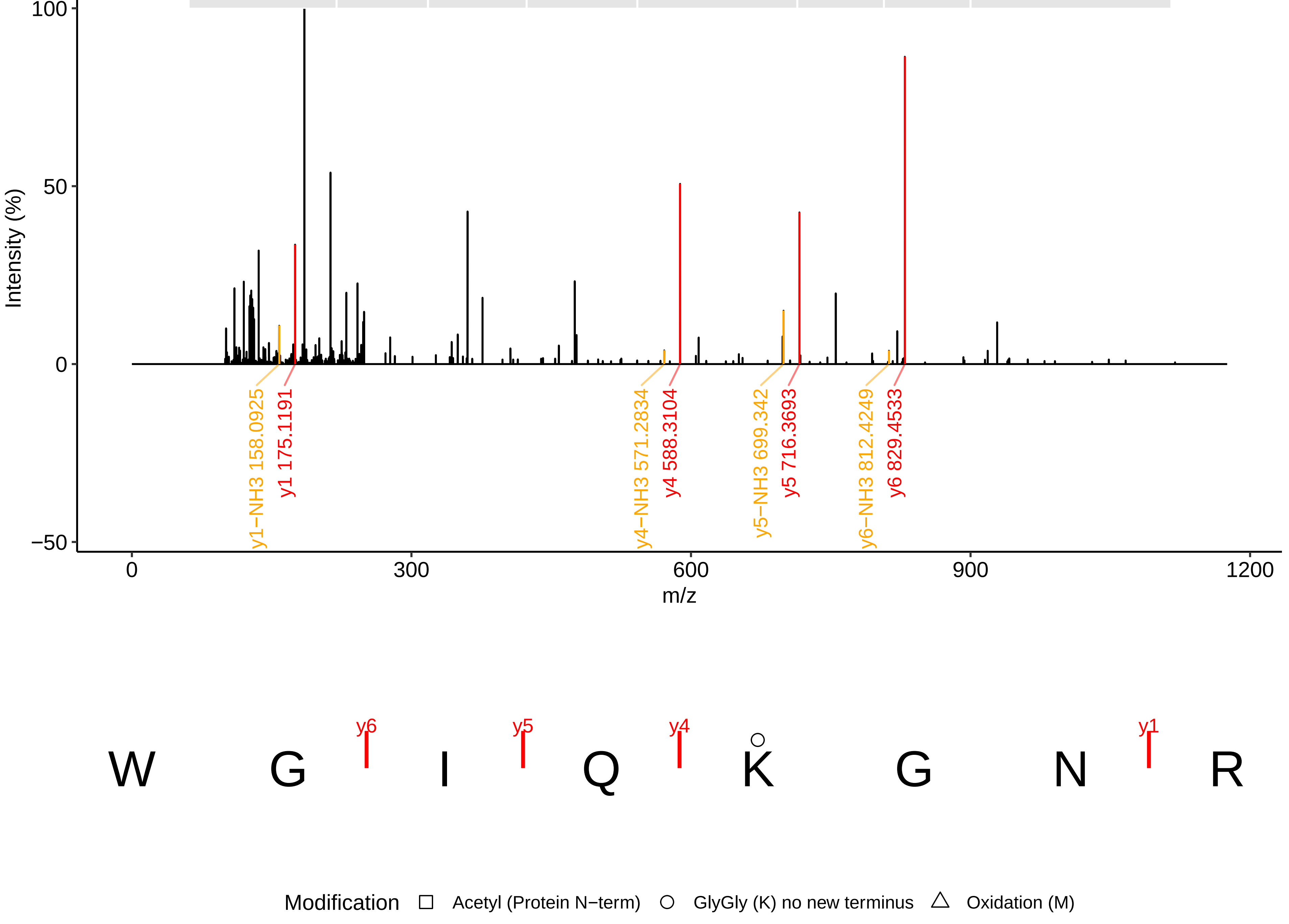

20200924_KIZ_MUb5_GlyGly_2 − Scan number: 7372

Gene Names	Charge	m/z	Mass	Retention time	PEP	Score	Precursor Intensity
CHRDL1	2	541.2495	1080.485	27.512	0.12299	49.347	NA

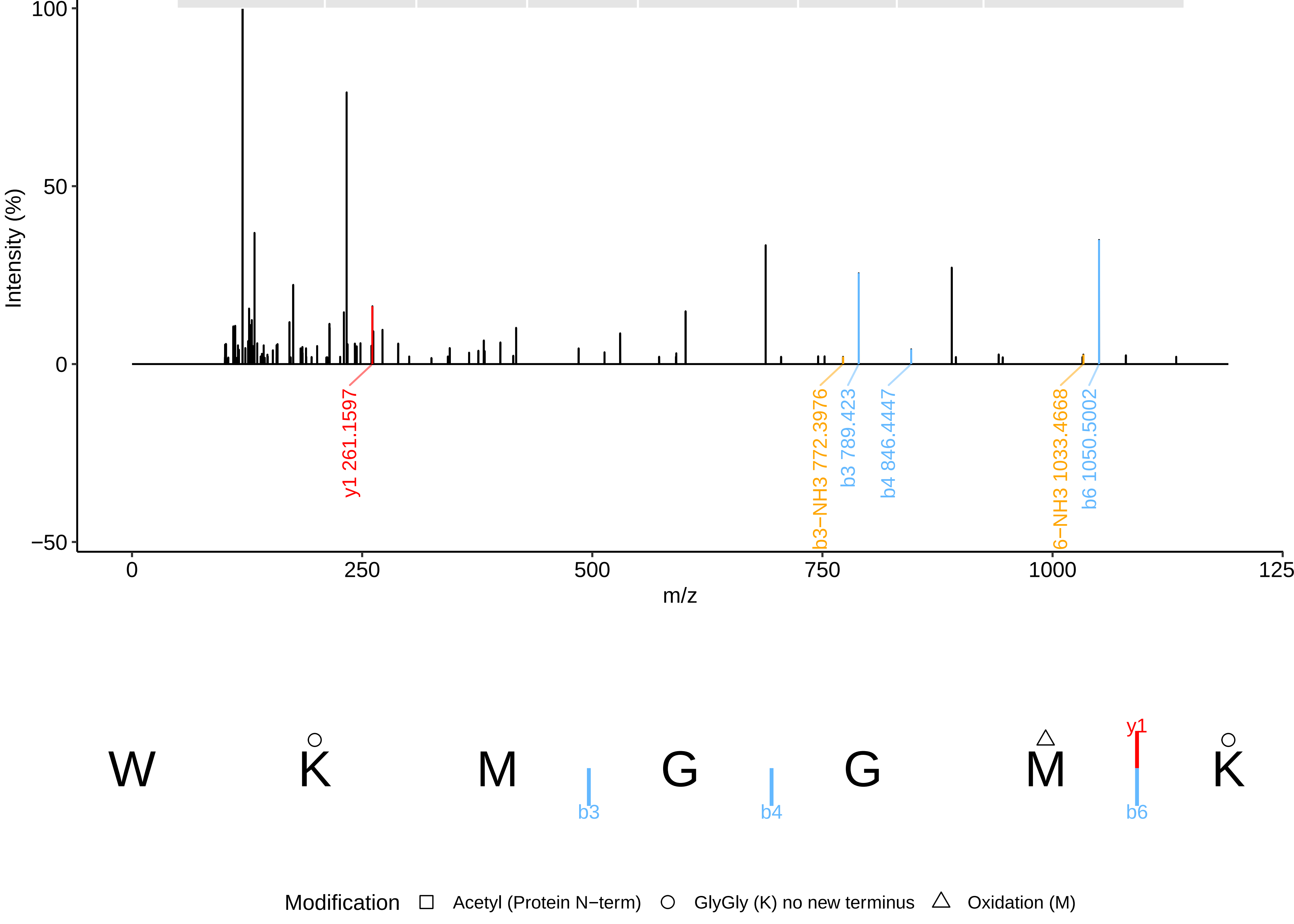

20200921_KIZ_MUb4_GlyGly_10plex − Scan number: 6381

Gene Names	Charge	m/z	Mass	Retention time	PEP	Score	Precursor Intensity
MARCH5	3	358.8491	1073.525	21.089	0.031485	76.035	5459321

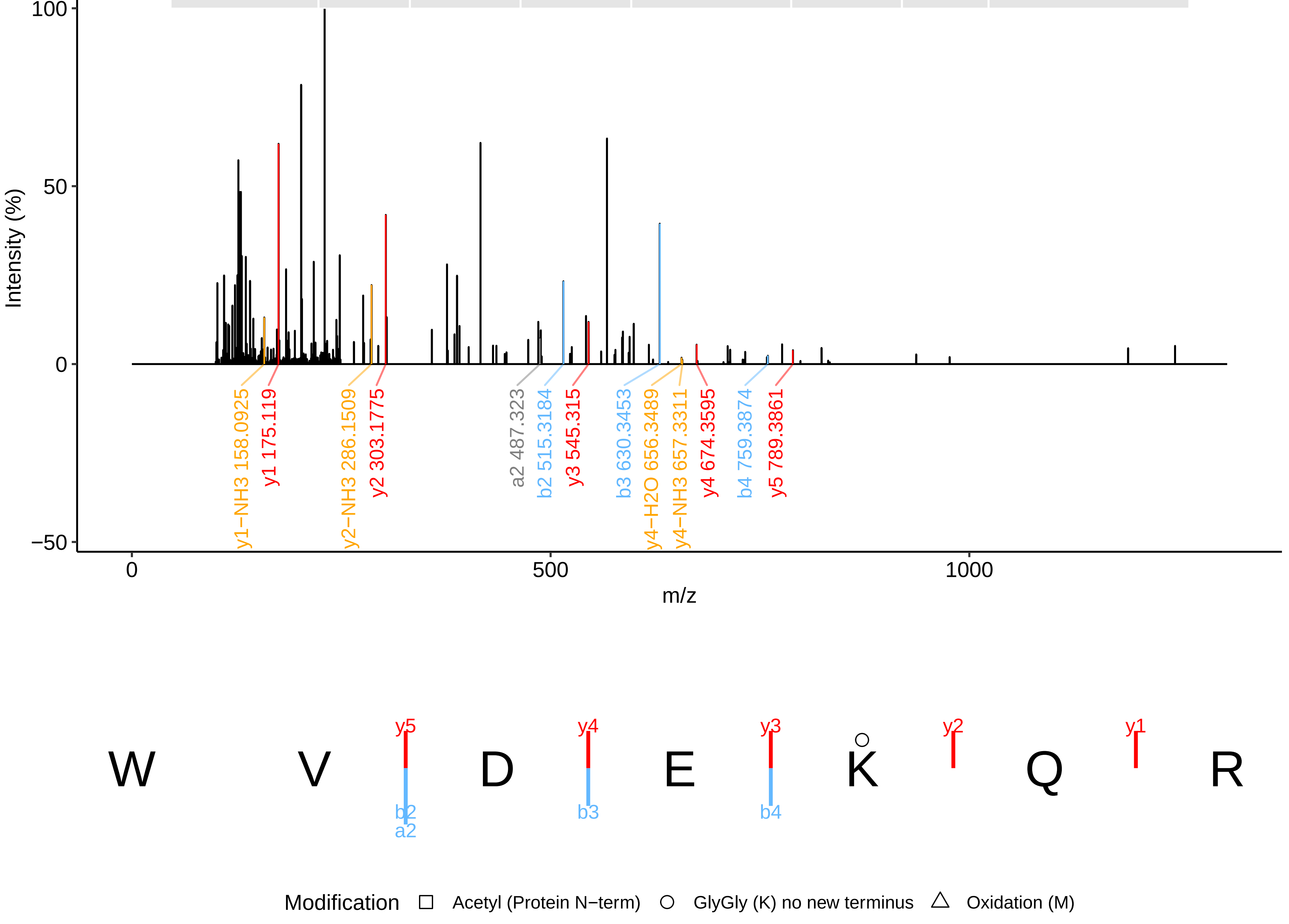

20200921_KIZ_MUb4_GlyGly_10plex_2 − Scan number: 5910

Gene Names	Charge	m/z	Mass	Retention time	PEP	Score	Precursor Intensity
MARCH5	3	358.8491	1073.525	21.488	0.05044	69.176	1646167

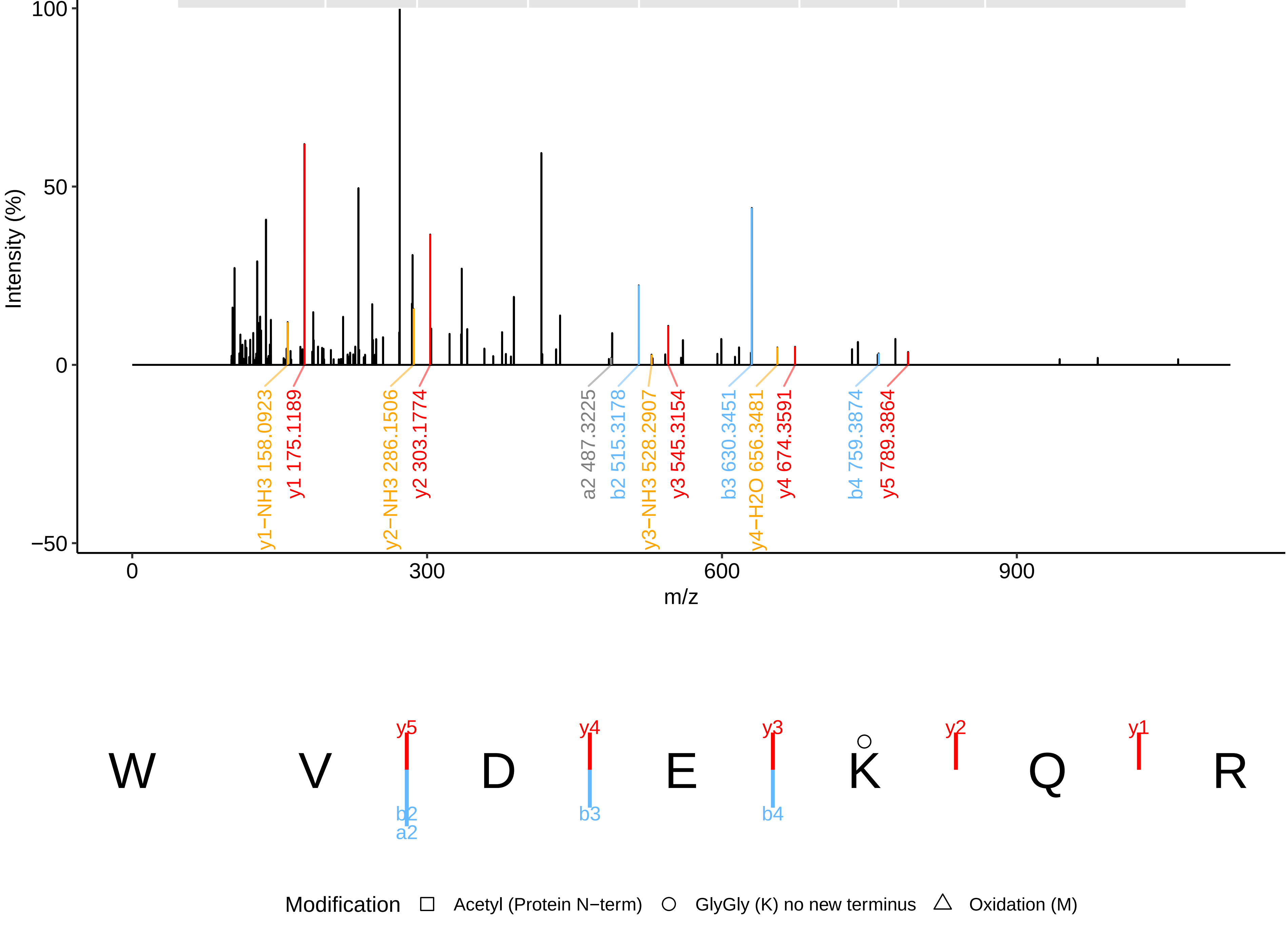

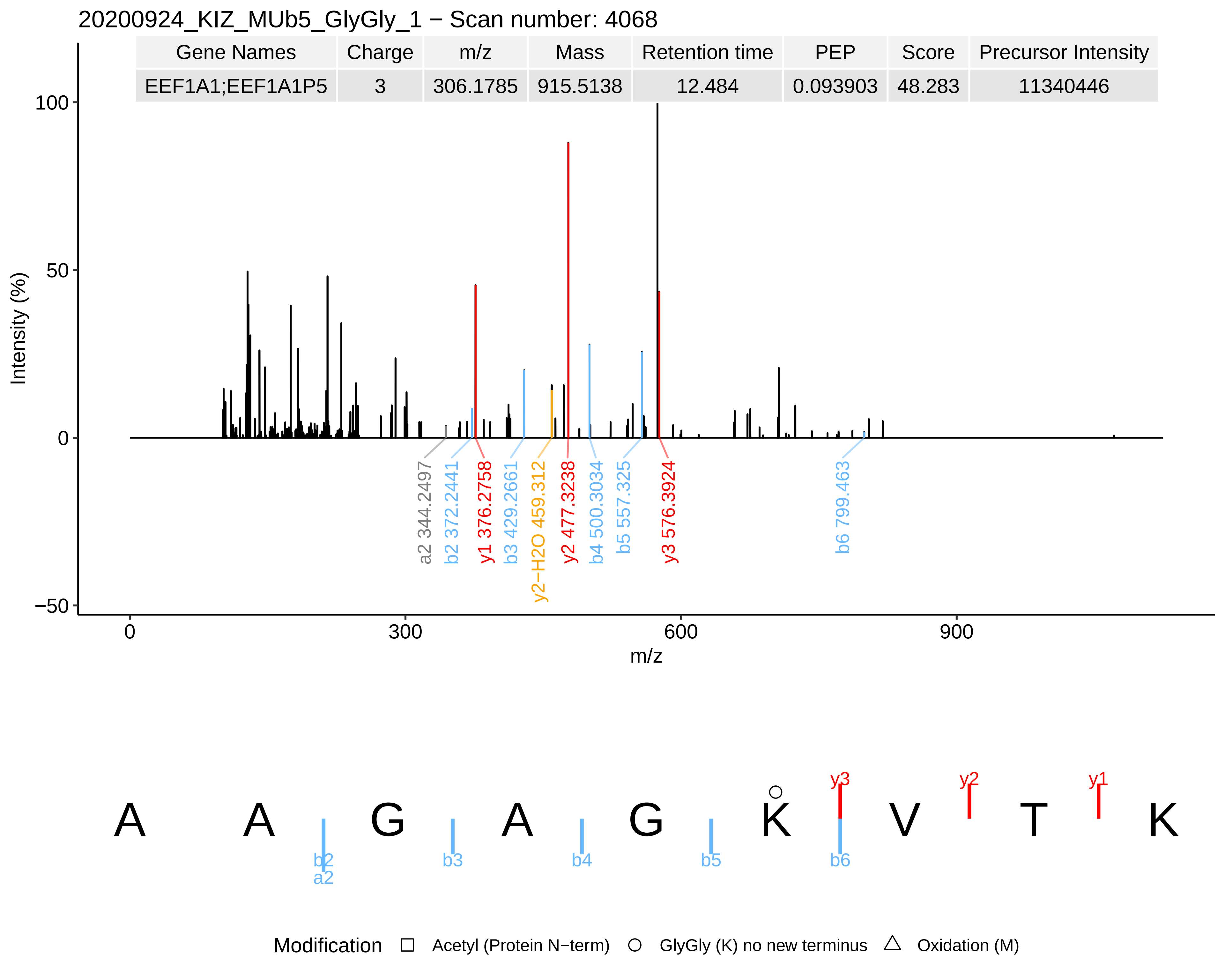
20200924_KIZ_MUb5_GlyGly_1 − Scan number: 4068
Gene Names | Charge | m/z | Mass | Retention time | PEP | Score | Precursor Intensity
EEF1A1;EEF1A1P5 | 3 | 306.1785 | 915.5138 | 12.484 | 0.093903 | 48.283 | 11340446
Intensity (%)
100
50
0
−50
a2 344.2497
b2 372.2441
y1 376.2758
b3 429.2661
y2−H2O 459.312
y2 477.3238
b4 500.3034
b5 557.325
y3 576.3924
b6 799.463
0
300
600
900
m/z
A A G A G K V T K
y3 y2 y1
b2 a2 b3 b4 b5 b6
Modification
Acetyl (Protein N−term)
GlyGly (K) no new terminus
Oxidation (M)

20200924_KIZ_MUb5_GlyGly_2 − Scan number: 3745

Gene Names	Charge	m/z	Mass	Retention time	PEP	Score	Precursor Intensity
EEF1A1;EEF1A1P5	3	306.1785	915.5138	13.639	0.085426	50.04	2733125

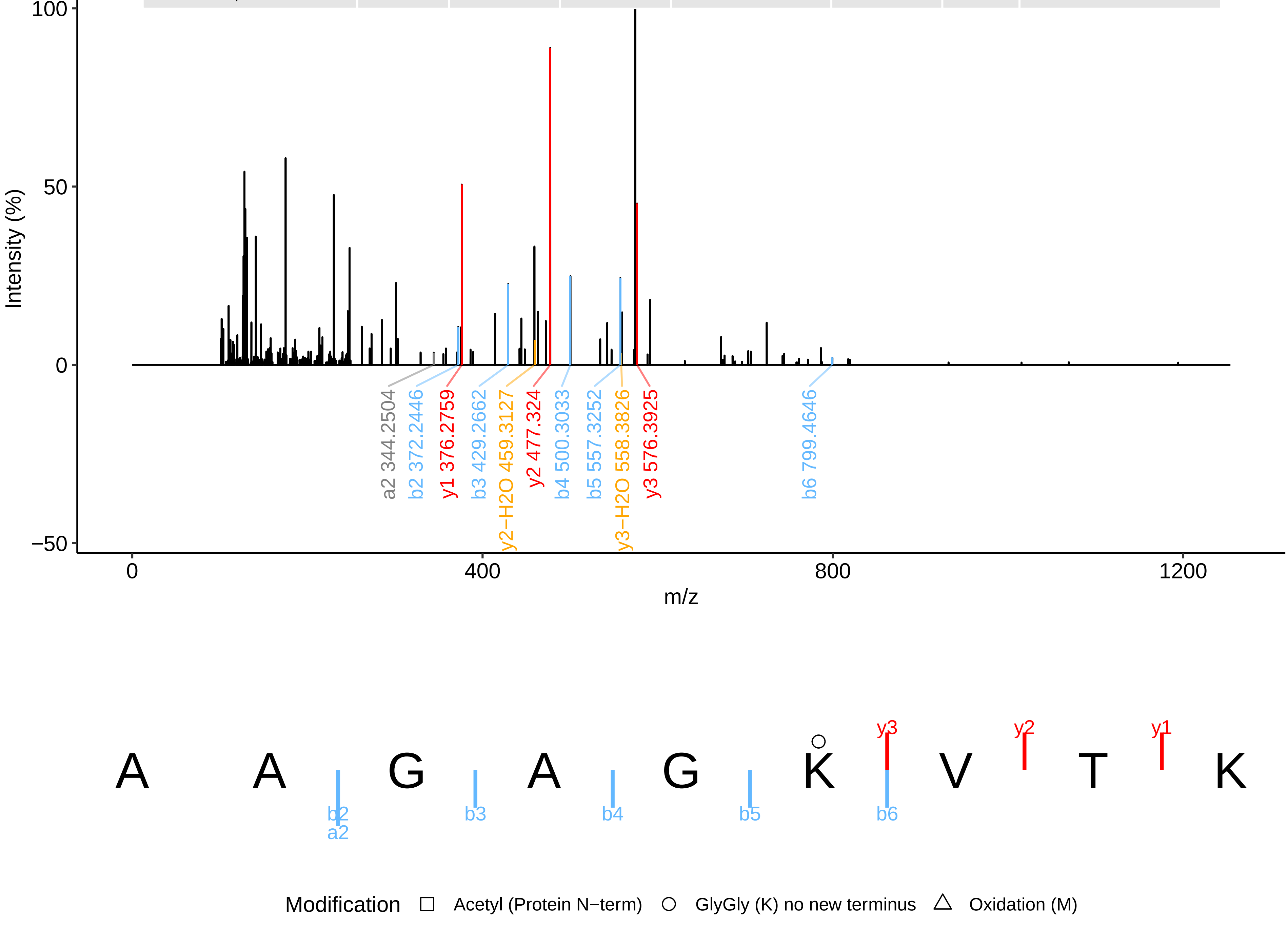

20200921_KIZ_MUb4_GlyGly_10plex − Scan number: 11549

Gene Names	Charge	m/z	Mass	Retention time	PEP	Score	Precursor Intensity
CCT6A	2	549.3193	1096.624	40.278	1.335e−05	91.041	9752084

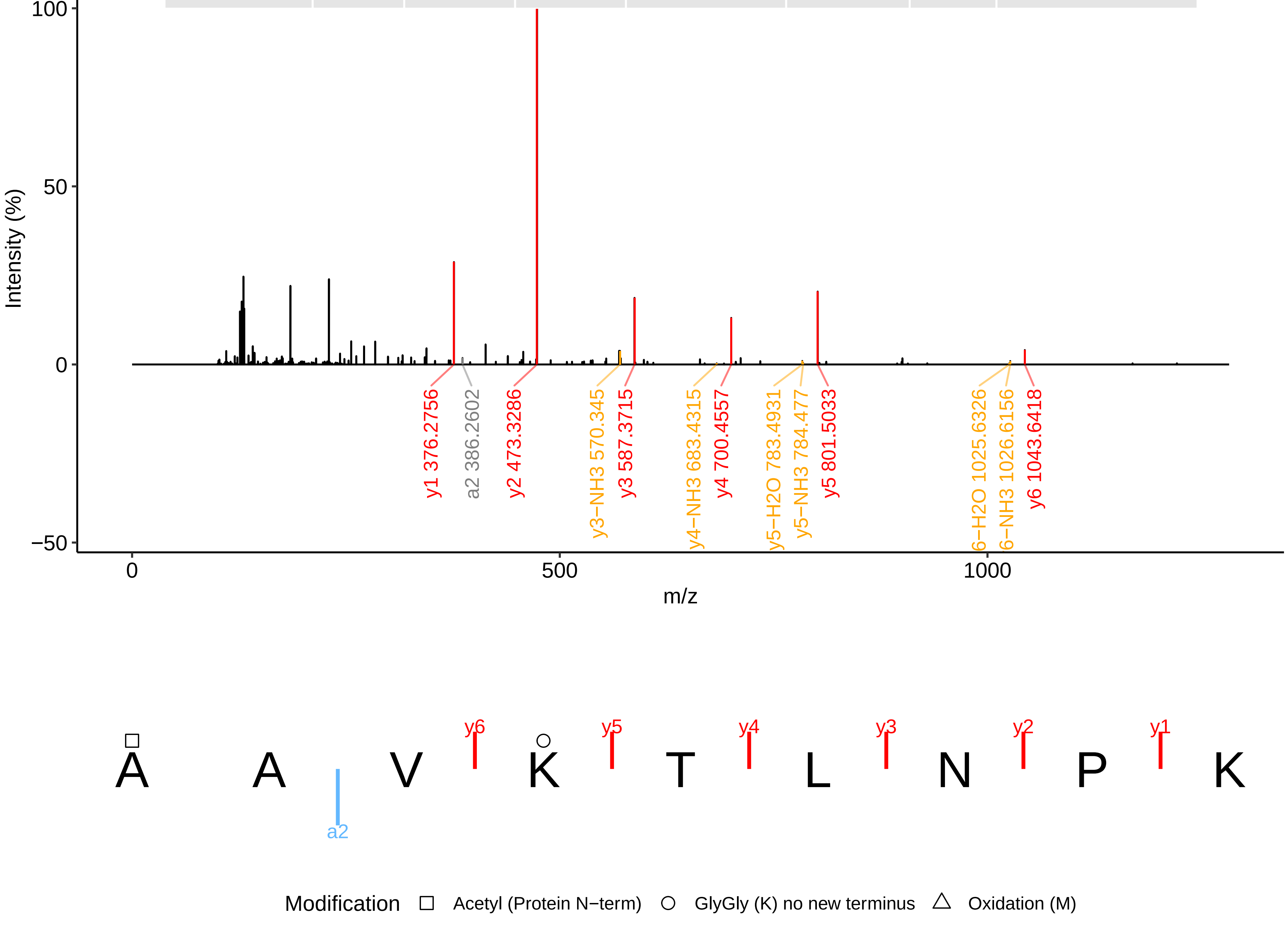

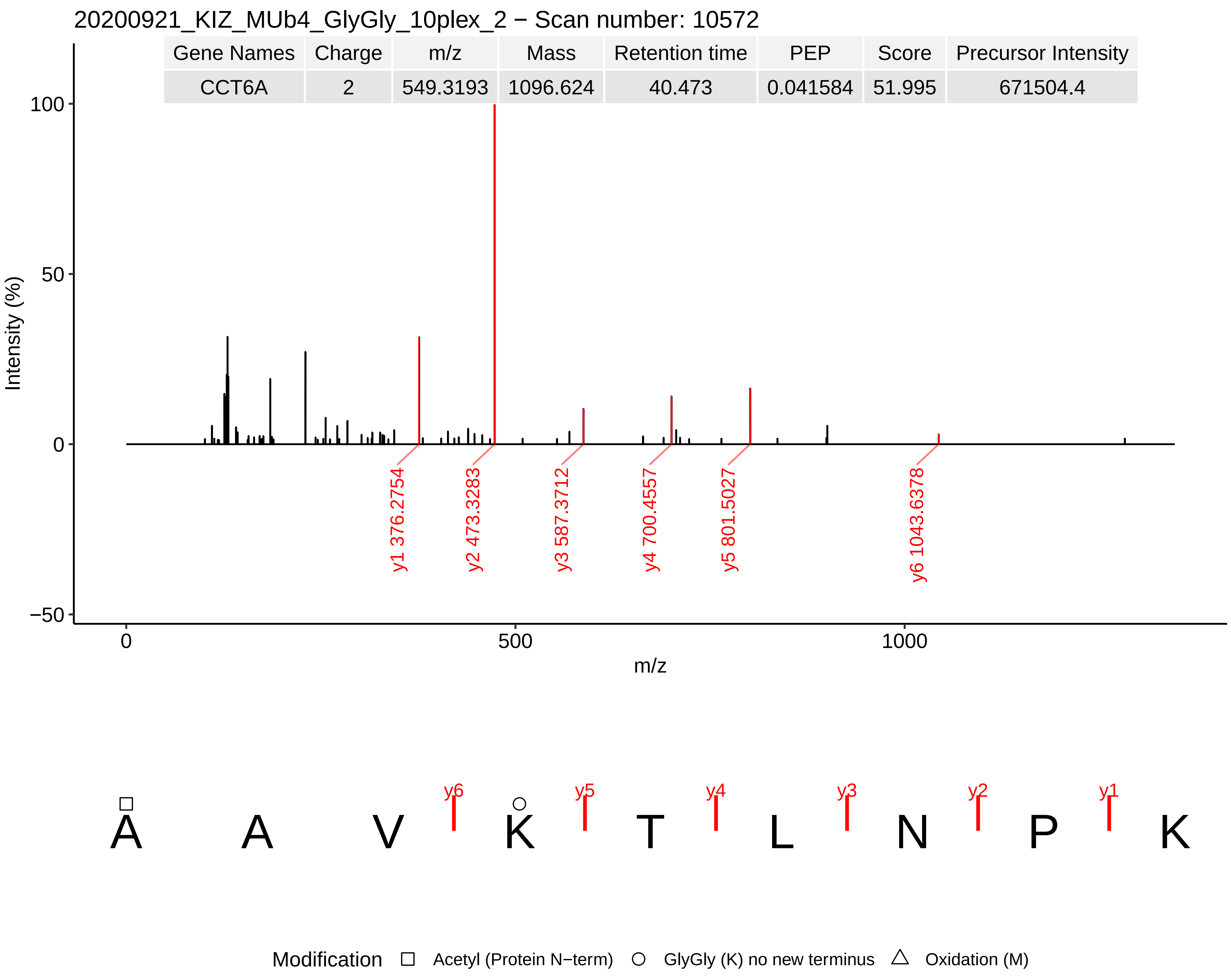

20200921_KIZ_MUb4_GlyGly_10plex_2 − Scan number: 10572
Gene Names | Charge | m/z | Mass | Retention time | PEP | Score | Precursor Intensity
CCT6A | 2 | 549.3193 | 1096.624 | 40.473 | 0.041584 | 51.995 | 671504.4
Intensity (%)
m/z
y1 376.2754
y2 473.3283
y3 587.3712
y4 700.4557
y5 801.5027
y6 1043.6378
A A V K T L N P K
y6 y5 y4 y3 y2 y1
Modification
Acetyl (Protein N−term)
GlyGly (K) no new terminus
Oxidation (M)

20200924_KIZ_MUb5_GlyGly_2 − Scan number: 9853

Gene Names	Charge	m/z	Mass	Retention time	PEP	Score	Precursor Intensity
CCT6A	2	549.3193	1096.624	36.826	0.015502	65.038	1859500

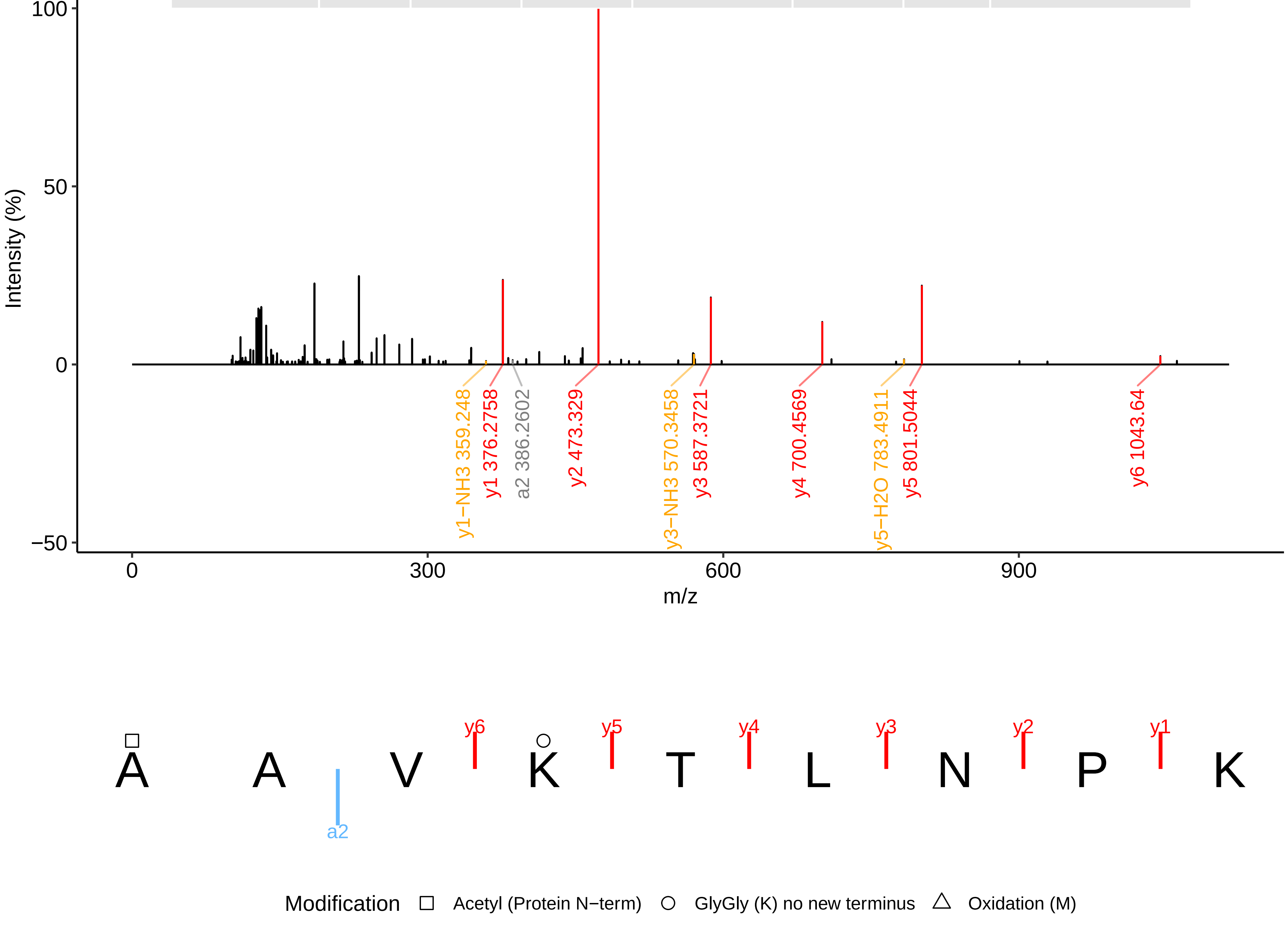

20200921_KIZ_MUb4_GlyGly_10plex − Scan number: 6322

Gene Names	Charge	m/z	Mass	Retention time	PEP	Score	Precursor Intensity
MCM6	3	372.2158	1113.626	20.867	0.00093662	92.051	5439904

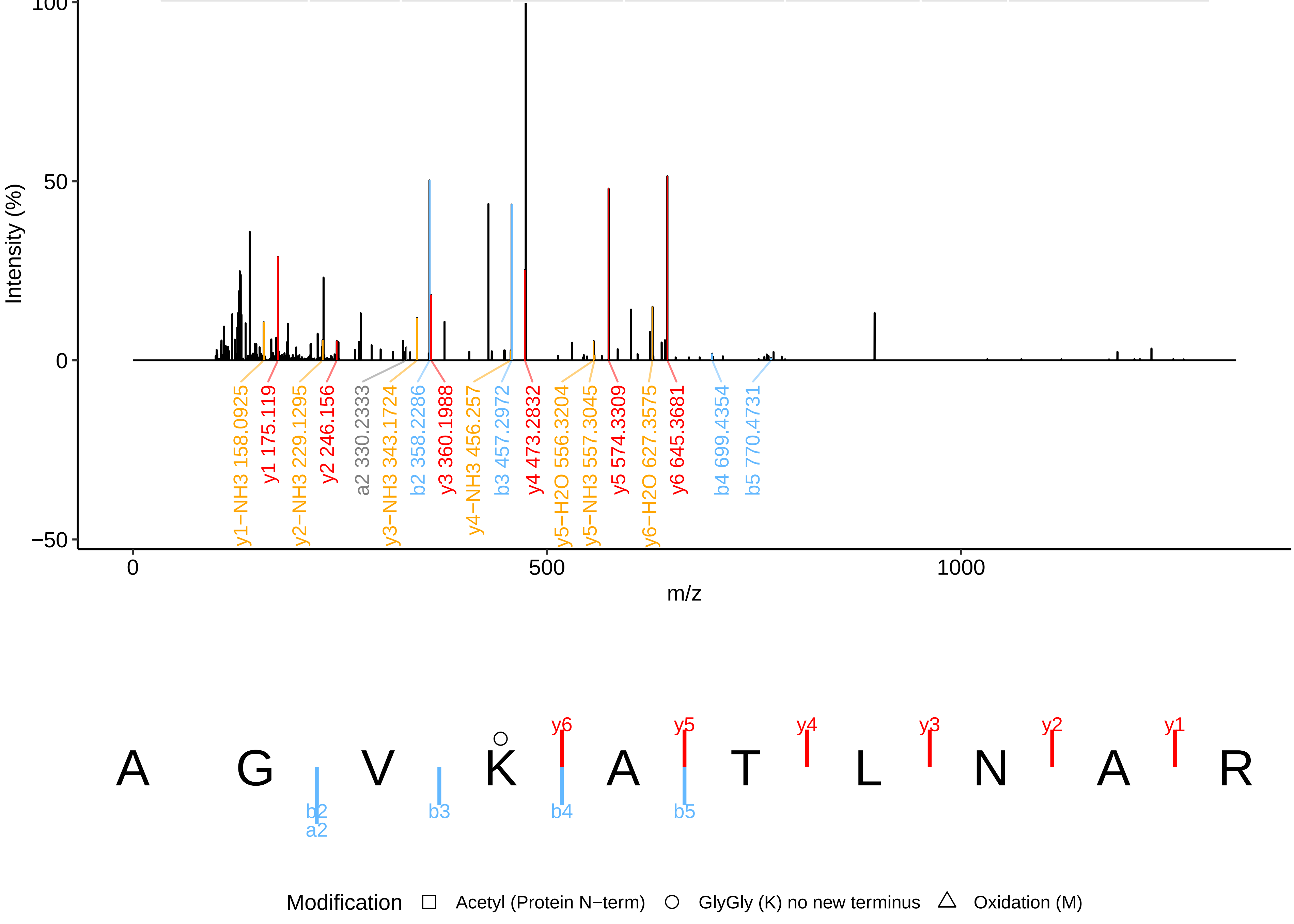

20200921_KIZ_MUb4_GlyGly_10plex − Scan number: 3694

Gene Names	Charge	m/z	Mass	Retention time	PEP	Score	Precursor Intensity
EPN1	3	294.8434	881.5083	11.854	0.019308	81.297	6935650

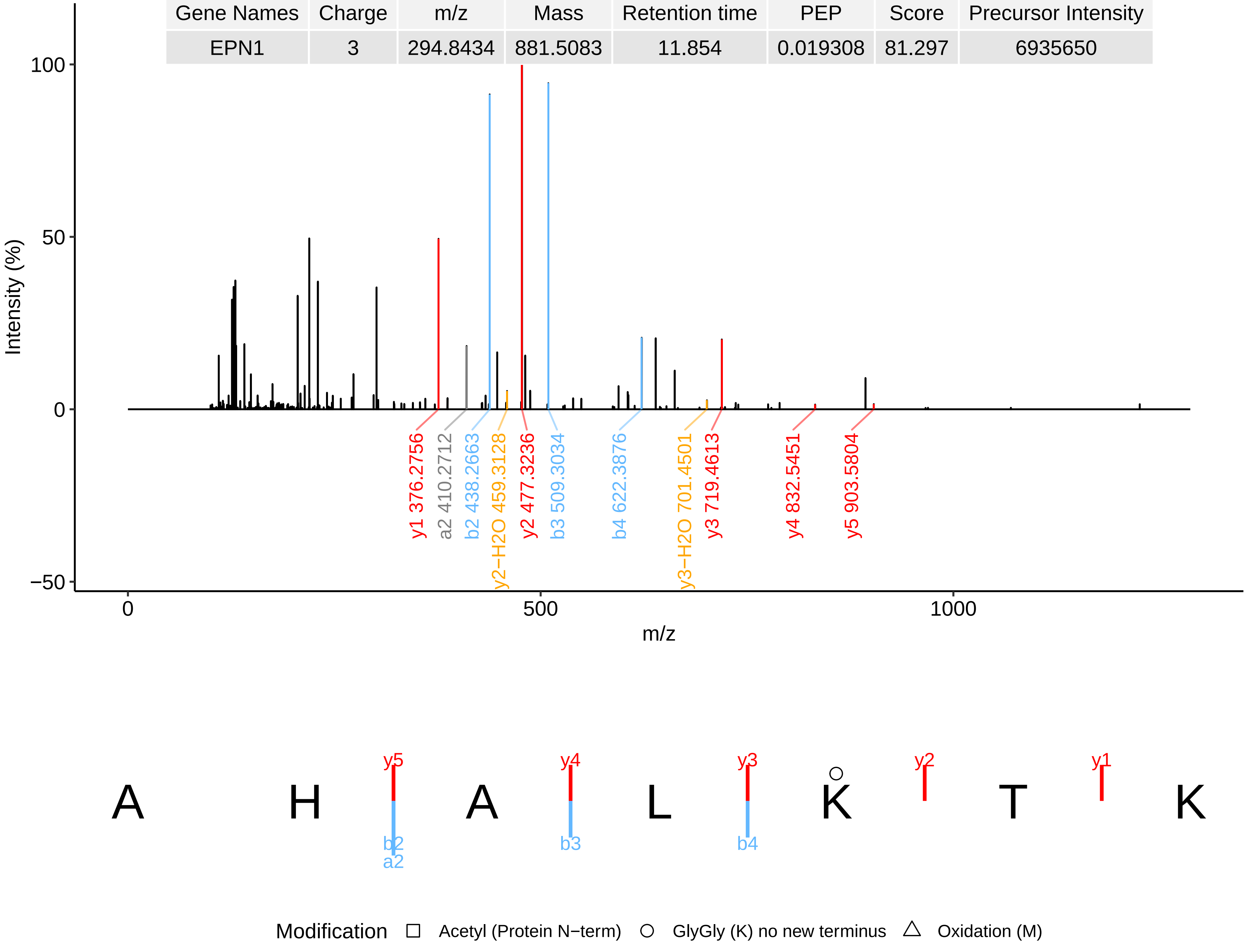

20200921_KIZ_MUb4_GlyGly_10plex_2 − Scan number: 3574

Gene Names	Charge	m/z	Mass	Retention time	PEP	Score	Precursor Intensity
EPN1	3	294.8434	881.5083	12.079	0.076225	60.788	1164217

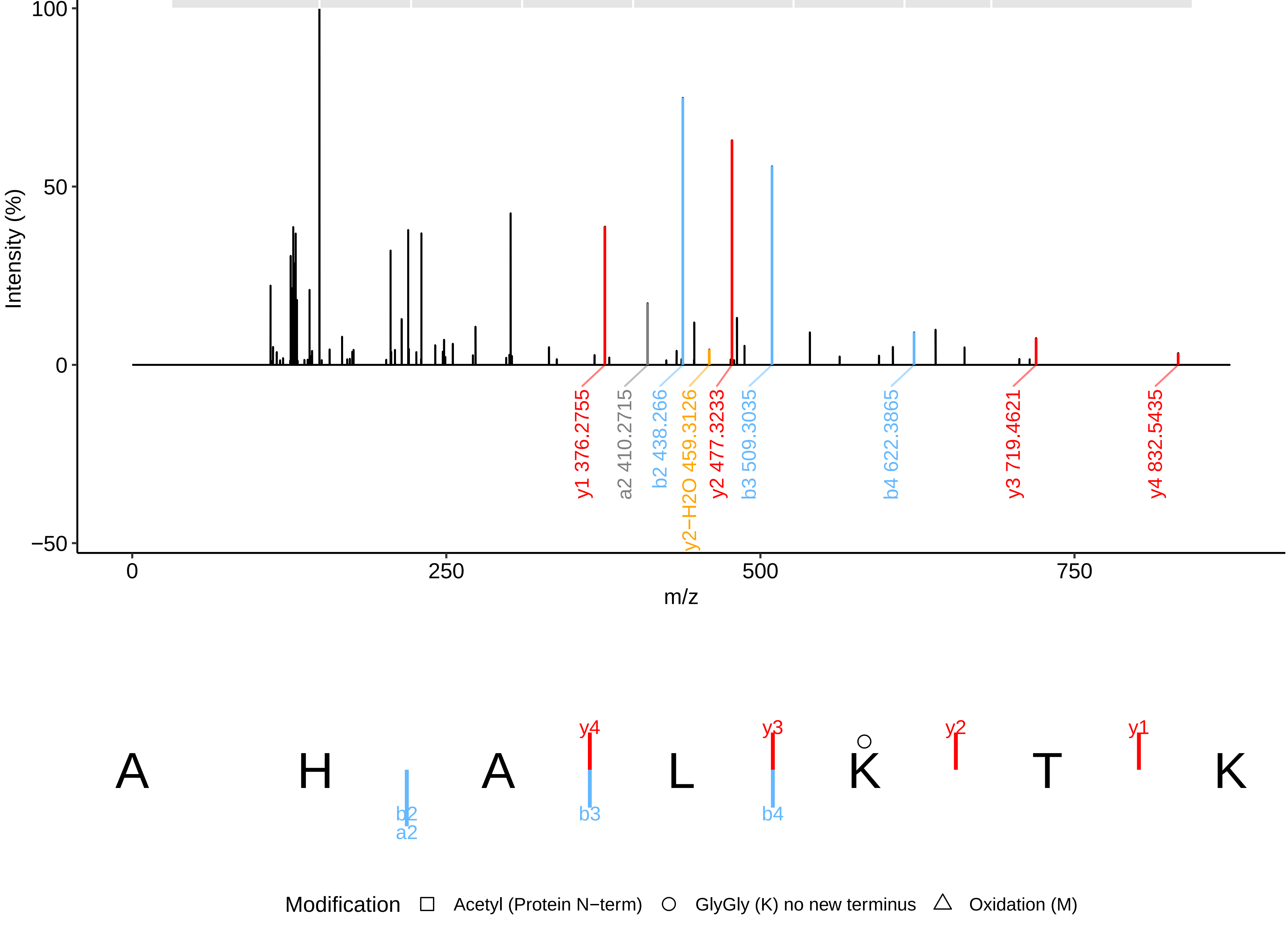

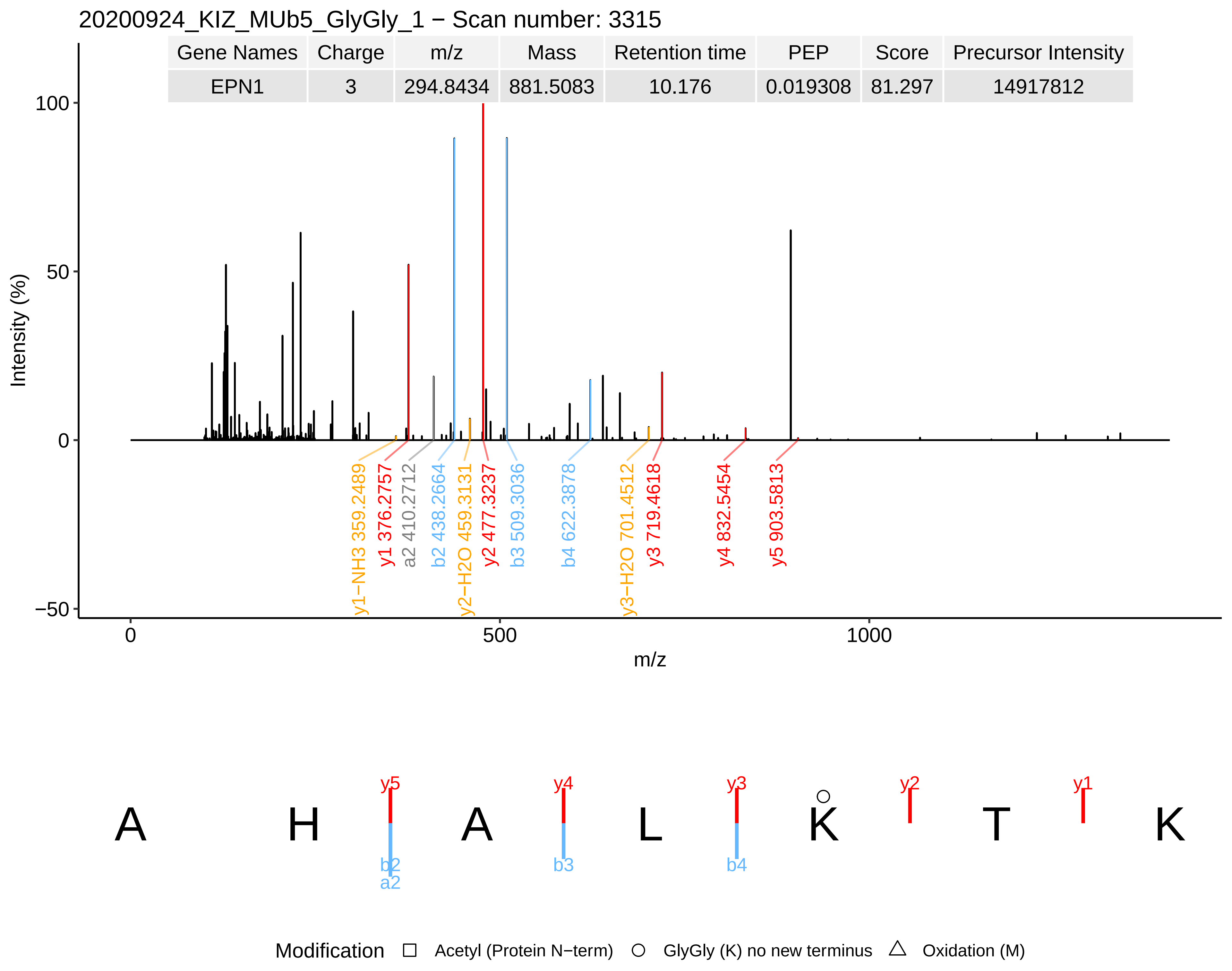

20200924_KIZ_MUb5_GlyGly_1 − Scan number: 3315
Gene Names | Charge | m/z | Mass | Retention time | PEP | Score | Precursor Intensity
EPN1 | 3 | 294.8434 | 881.5083 | 10.176 | 0.019308 | 81.297 | 14917812
Intensity (%)
m/z
y1−NH3 359.2489
y1 376.2757
a2 410.2712
b2 438.2664
y2−H2O 459.3131
y2 477.3237
b3 509.3036
b4 622.3878
y3−H2O 701.4512
y3 719.4618
y4 832.5454
y5 903.5813
A H A L K T K
y5 y4 y3 y2 y1
b2 a2 b3 b4
Modification
Acetyl (Protein N−term)
GlyGly (K) no new terminus
Oxidation (M)

20200924_KIZ_MUb5_GlyGly_2 − Scan number: 3035

Gene Names	Charge	m/z	Mass	Retention time	PEP	Score	Precursor Intensity
EPN1	3	294.8434	881.5083	10.895	0.041469	72.138	1506971

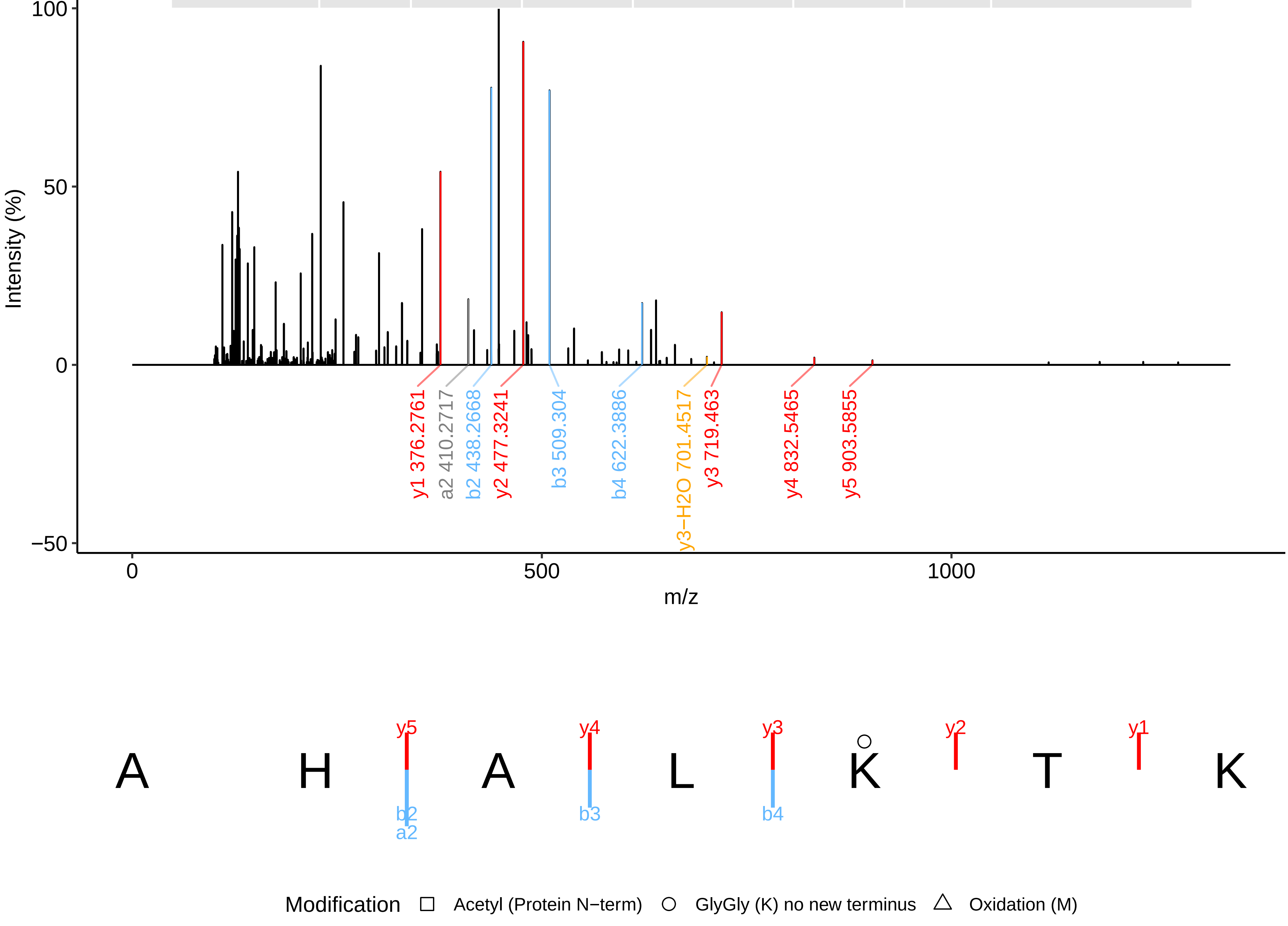

20200921_KIZ_MUb4_GlyGly_10plex − Scan number: 4532

Gene Names	Charge	m/z	Mass	Retention time	PEP	Score	Precursor Intensity
SPG20	3	306.1785	915.5138	14.778	0.10075	46.129	9952031

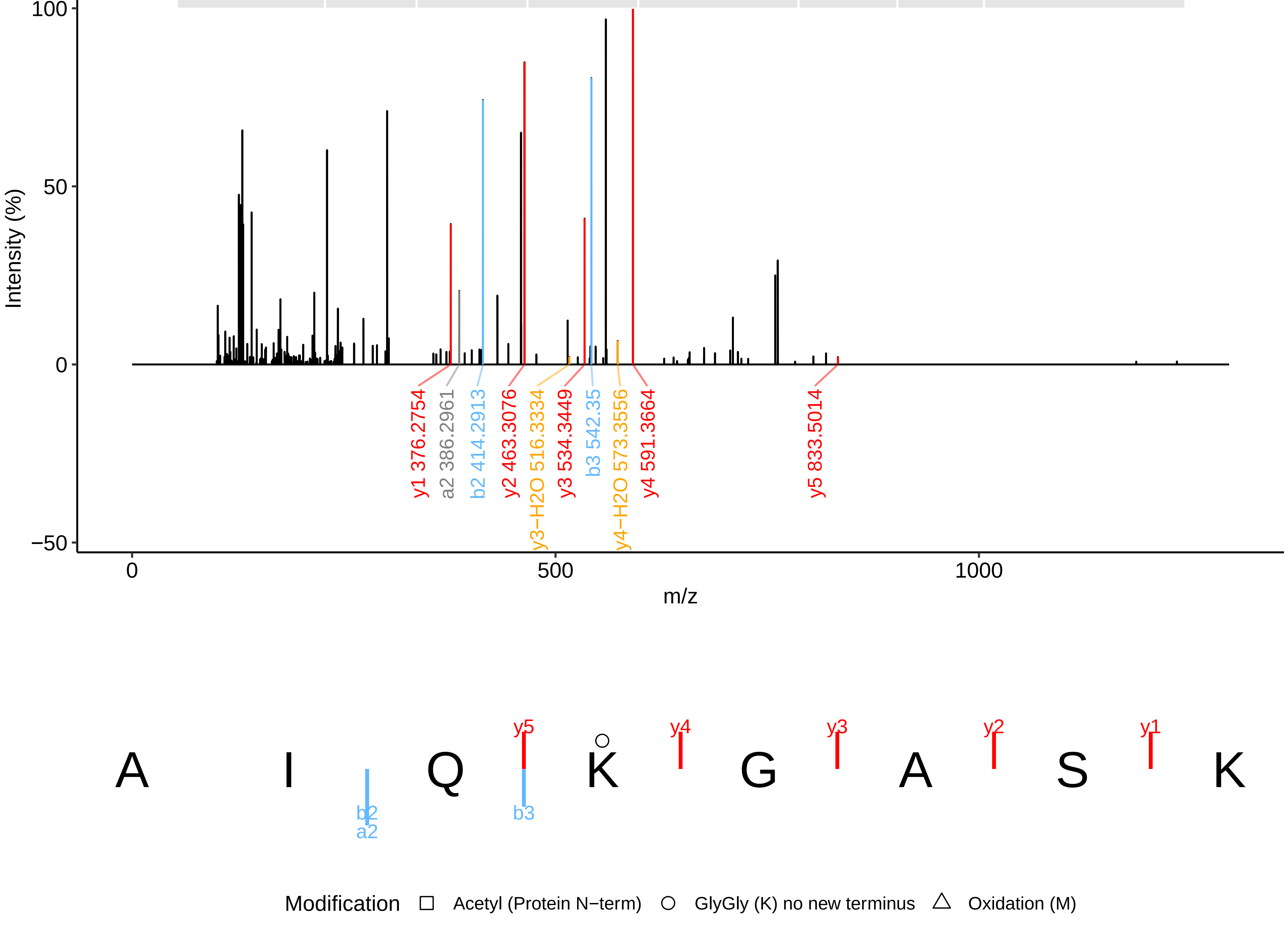

20200921_KIZ_MUb4_GlyGly_10plex_2 − Scan number: 4236

Gene Names	Charge	m/z	Mass	Retention time	PEP	Score	Precursor Intensity
SPG20	3	306.1785	915.5138	14.747	0.062816	55.37	703561.9

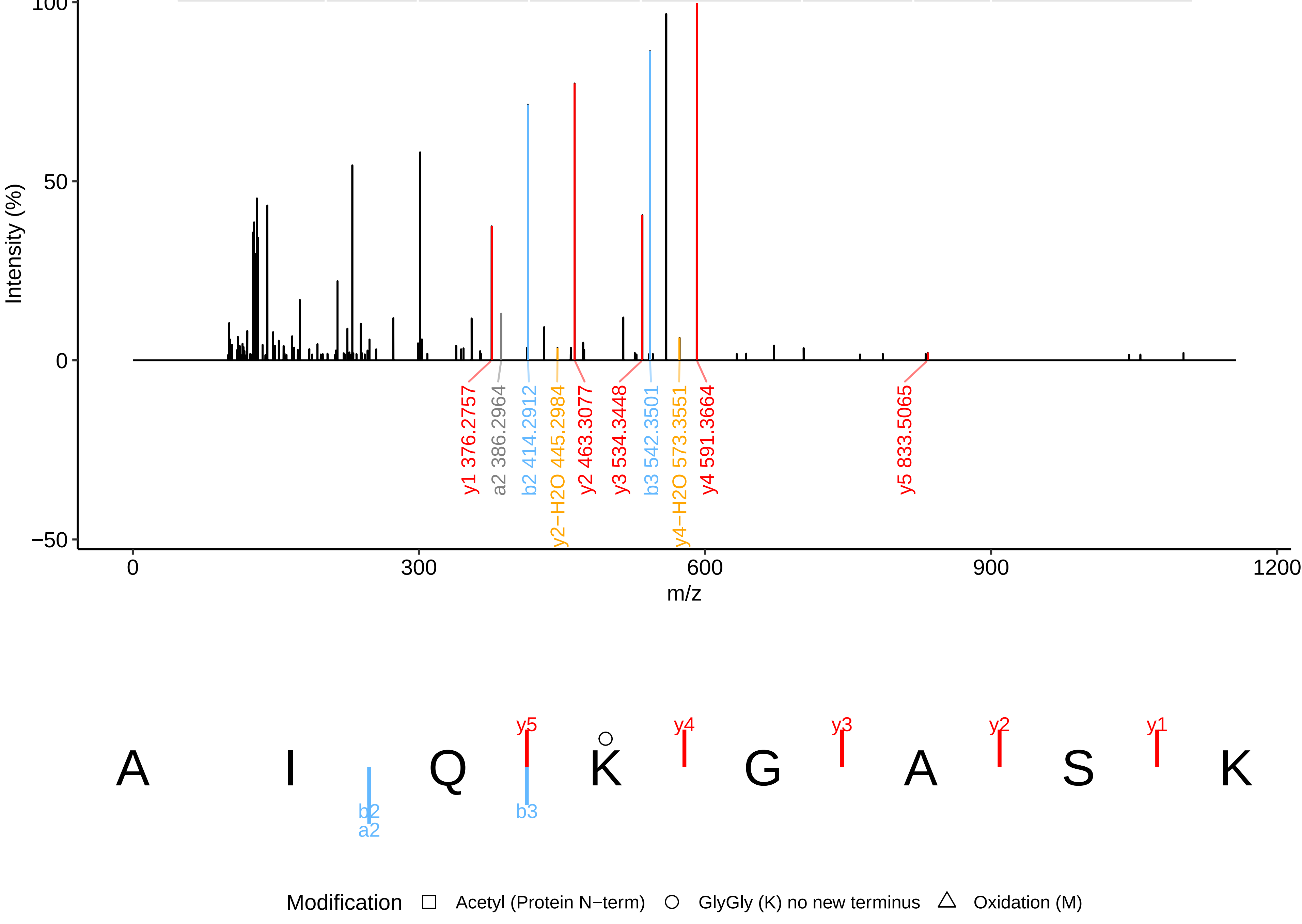

20200921_KIZ_MUb4_GlyGly_10plex − Scan number: 5436

Gene Names	Charge	m/z	Mass	Retention time	PEP	Score	Precursor Intensity
S100A11	2	687.8466	1373.679	17.803	1.4215e−12	128.86	9309026

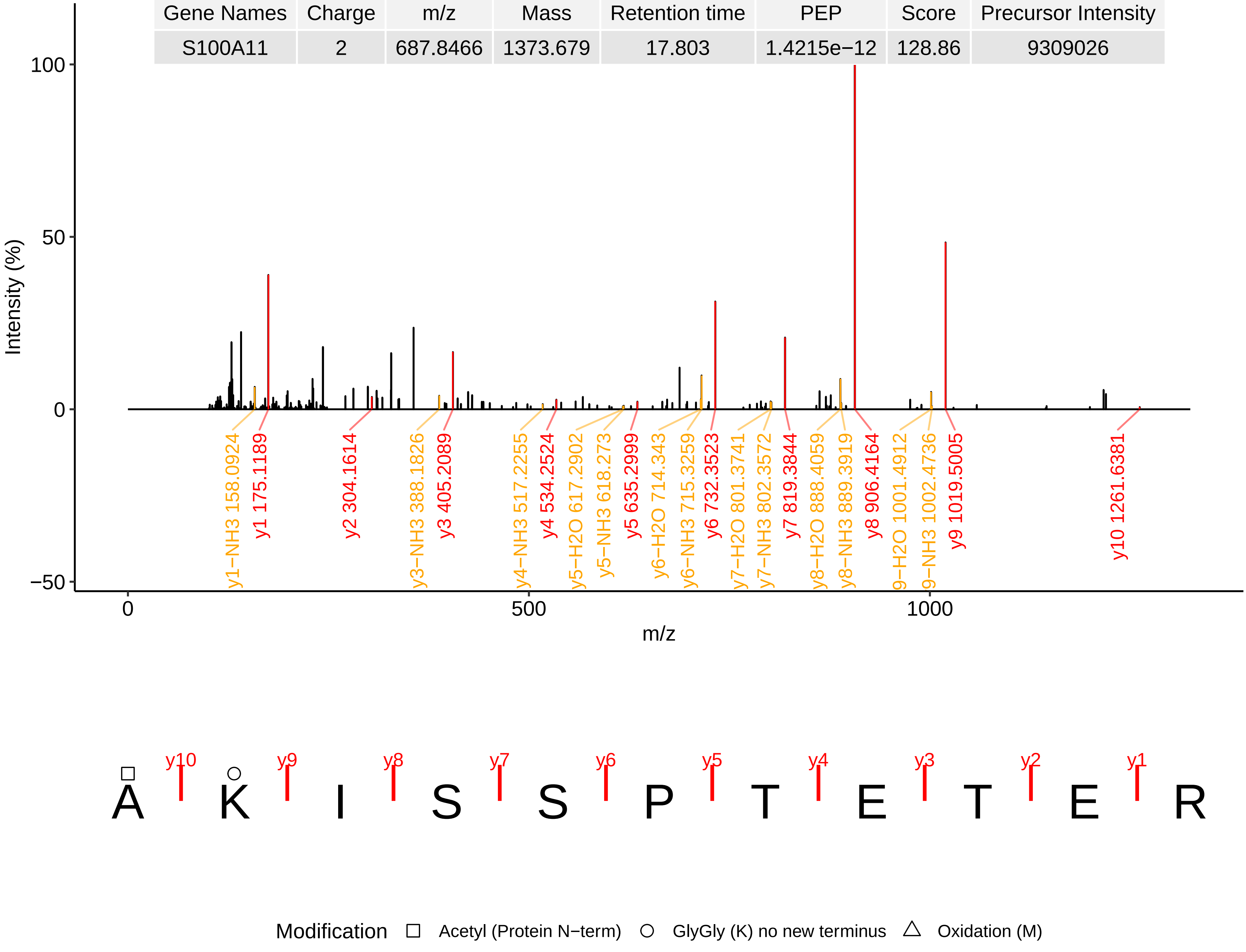

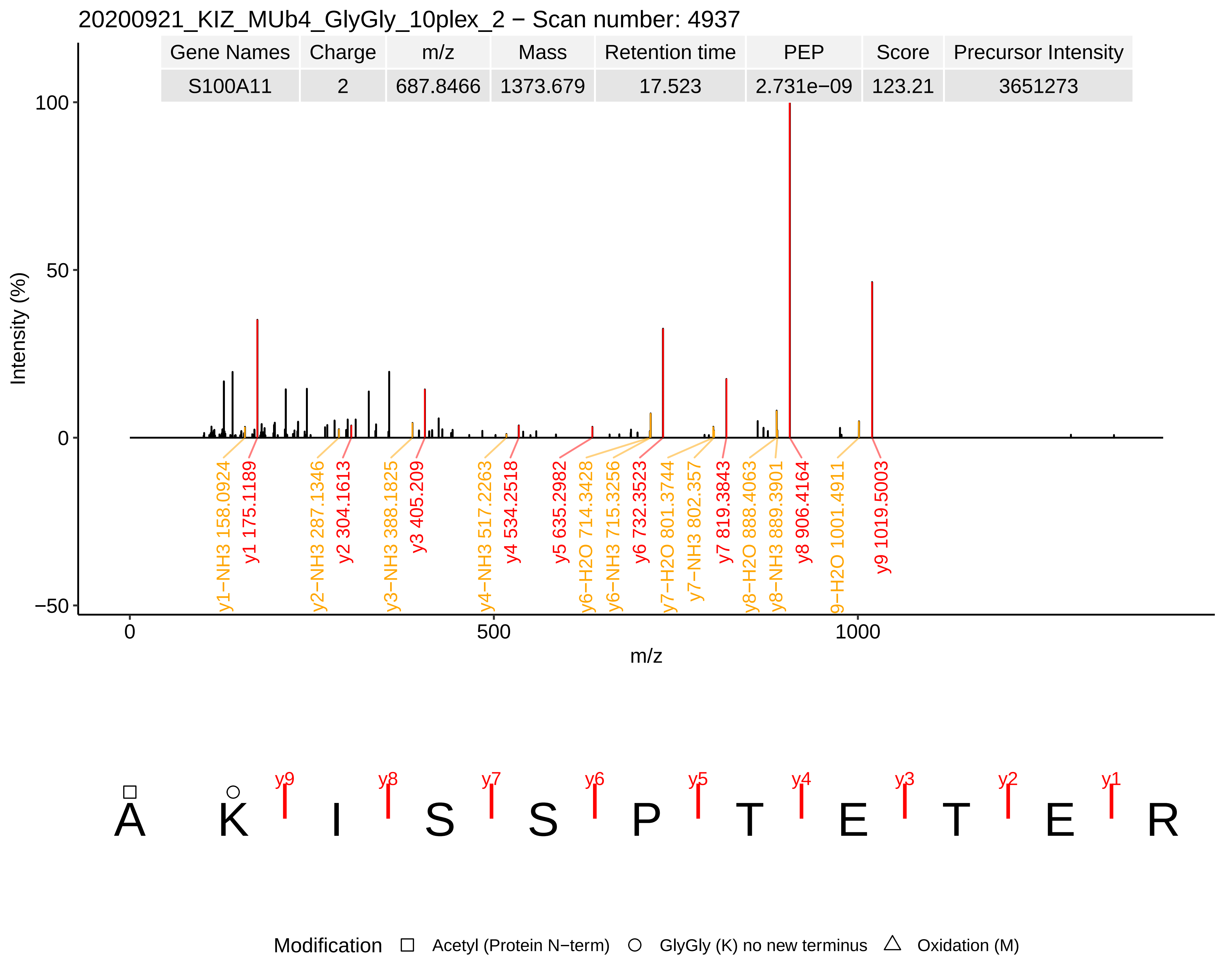

20200921_KIZ_MUb4_GlyGly_10plex_2 − Scan number: 4937
Gene Names | Charge | m/z | Mass | Retention time | PEP | Score | Precursor Intensity
S100A11 | 2 | 687.8466 | 1373.679 | 17.523 | 2.731e−09 | 123.21 | 3651273
Intensity (%)
m/z
y1−NH3 158.0924
y1 175.1189
y2−NH3 287.1346
y2 304.1613
y3−NH3 388.1825
y3 405.209
y4−NH3 517.2263
y4 534.2518
y5 635.2982
y6−H2O 714.3428
y6−NH3 715.3256
y6 732.3523
y7−H2O 801.3744
y7−NH3 802.357
y7 819.3843
y8−H2O 888.4063
y8−NH3 889.3901
y8 906.4164
y9−H2O 1001.4911
y9 1019.5003
A K I S S P T E T E R
y9 y8 y7 y6 y5 y4 y3 y2 y1
Modification
Acetyl (Protein N−term)
GlyGly (K) no new terminus
Oxidation (M)

20200921_KIZ_MUb4_GlyGly_10plex − Scan number: 4708

Gene Names	Charge	m/z	Mass	Retention time	PEP	Score	Precursor Intensity
YWHAQ;YWHAB;SFN;YWHAZ	3	377.2035	1128.589	15.353	0.0029697	83.883	4603983

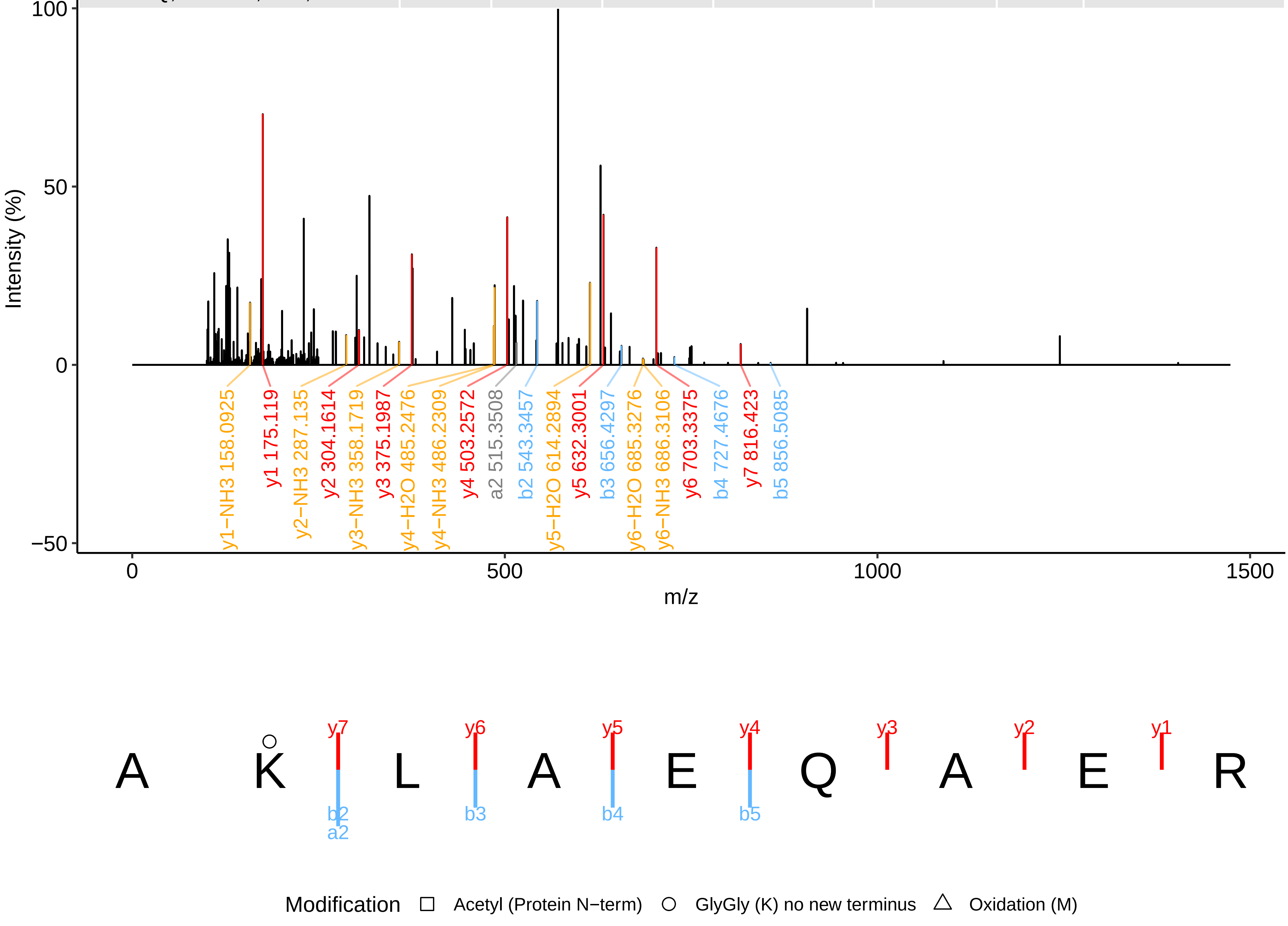

20200921_KIZ_MUb4_GlyGly_10plex_2 − Scan number: 4372

Gene Names	Charge	m/z	Mass	Retention time	PEP	Score	Precursor Intensity
YWHAQ;YWHAB;SFN;YWHAZ	3	377.2035	1128.589	15.305	0.012381	72.275	1524865

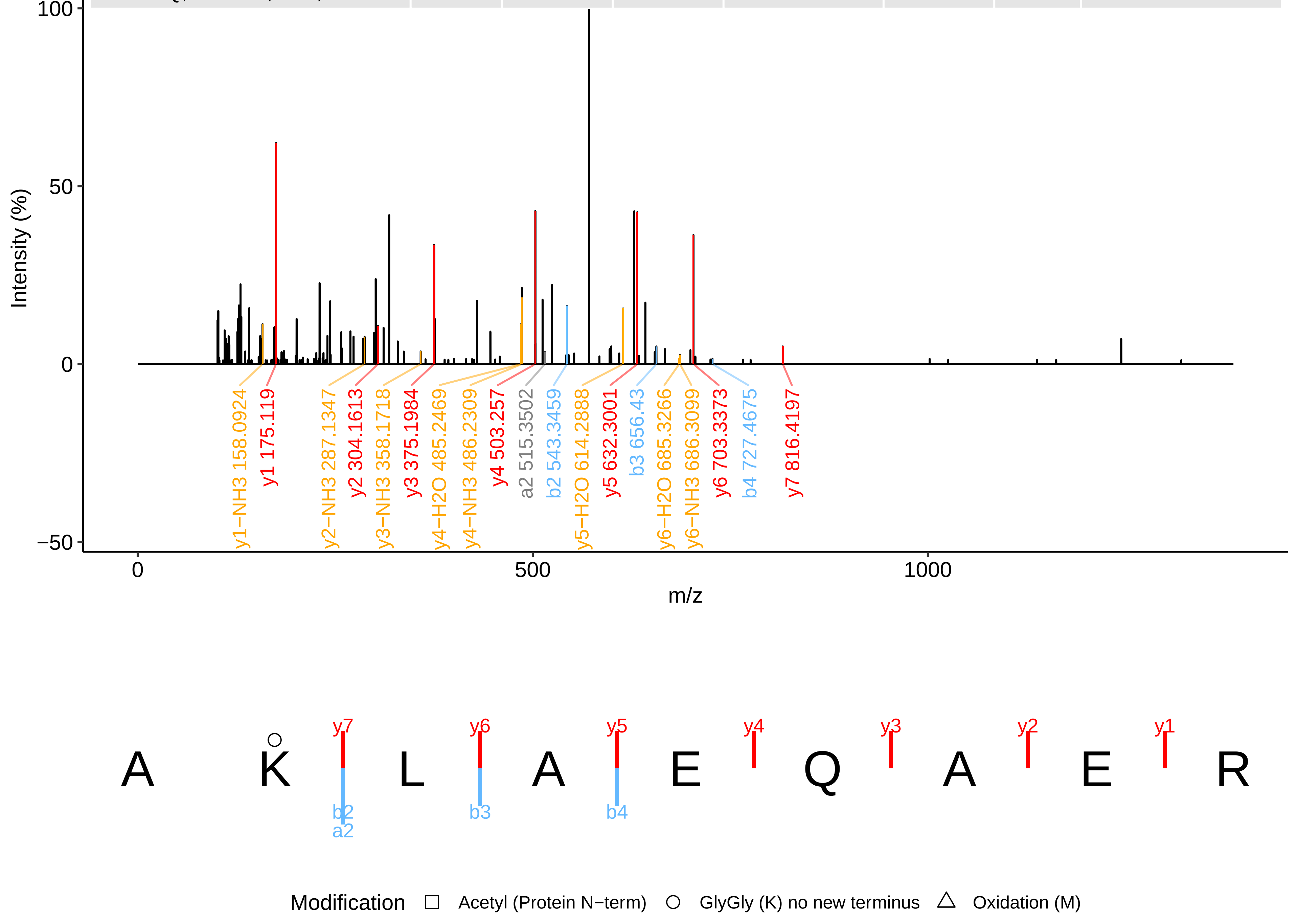

20200924_KIZ_MUb5_GlyGly_1 − Scan number: 4152

Gene Names	Charge	m/z	Mass	Retention time	PEP	Score	Precursor Intensity
YWHAQ;YWHAB;SFN;YWHAZ	3	377.2035	1128.589	12.77	0.003059	83.358	12692326

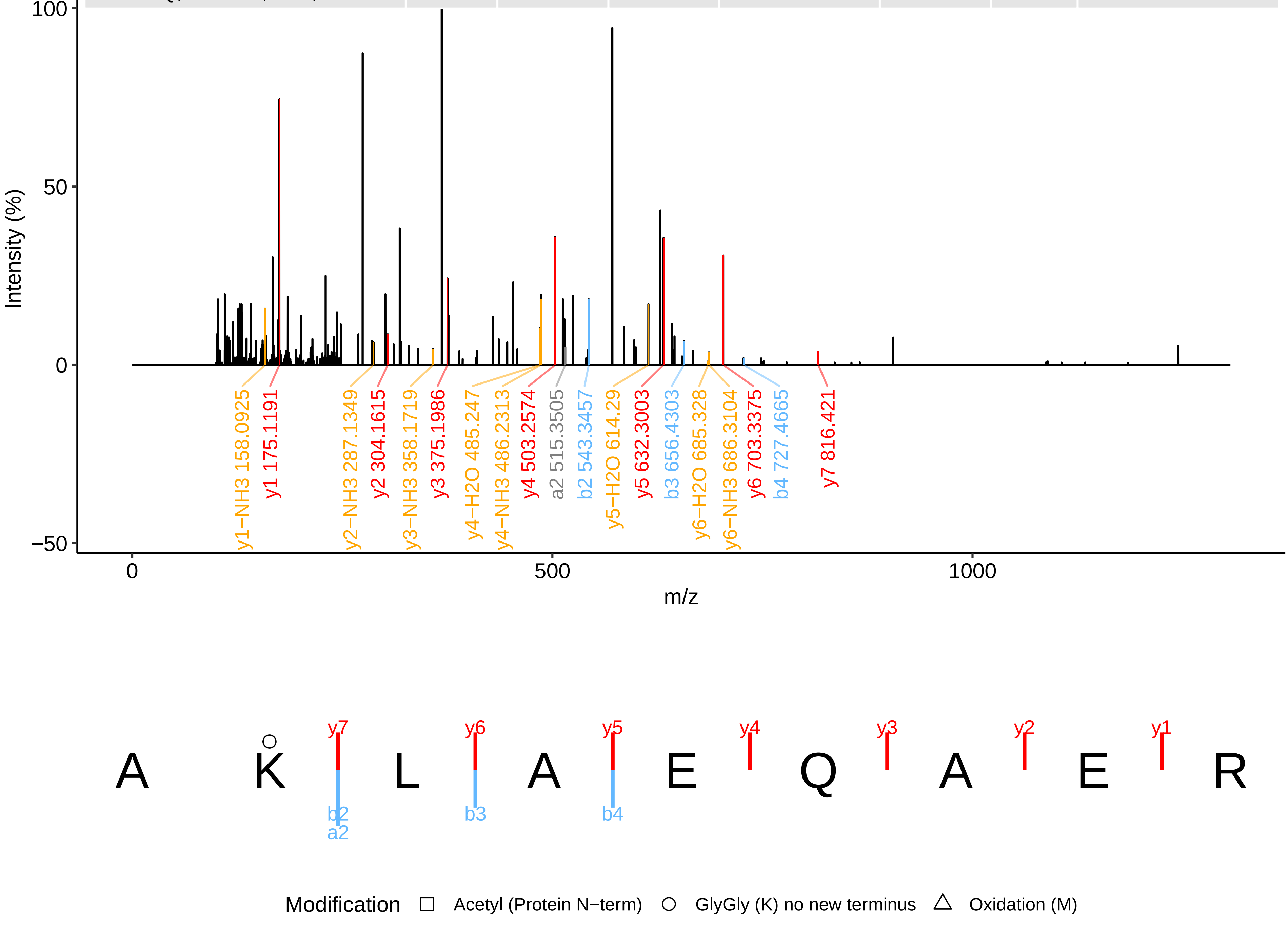

20200924_KIZ_MUb5_GlyGly_2 − Scan number: 3833

Gene Names	Charge	m/z	Mass	Retention time	PEP	Score	Precursor Intensity
YWHAQ;YWHAB;SFN;YWHAZ	3	377.2035	1128.589	13.98	0.0097217	74.772	2405024

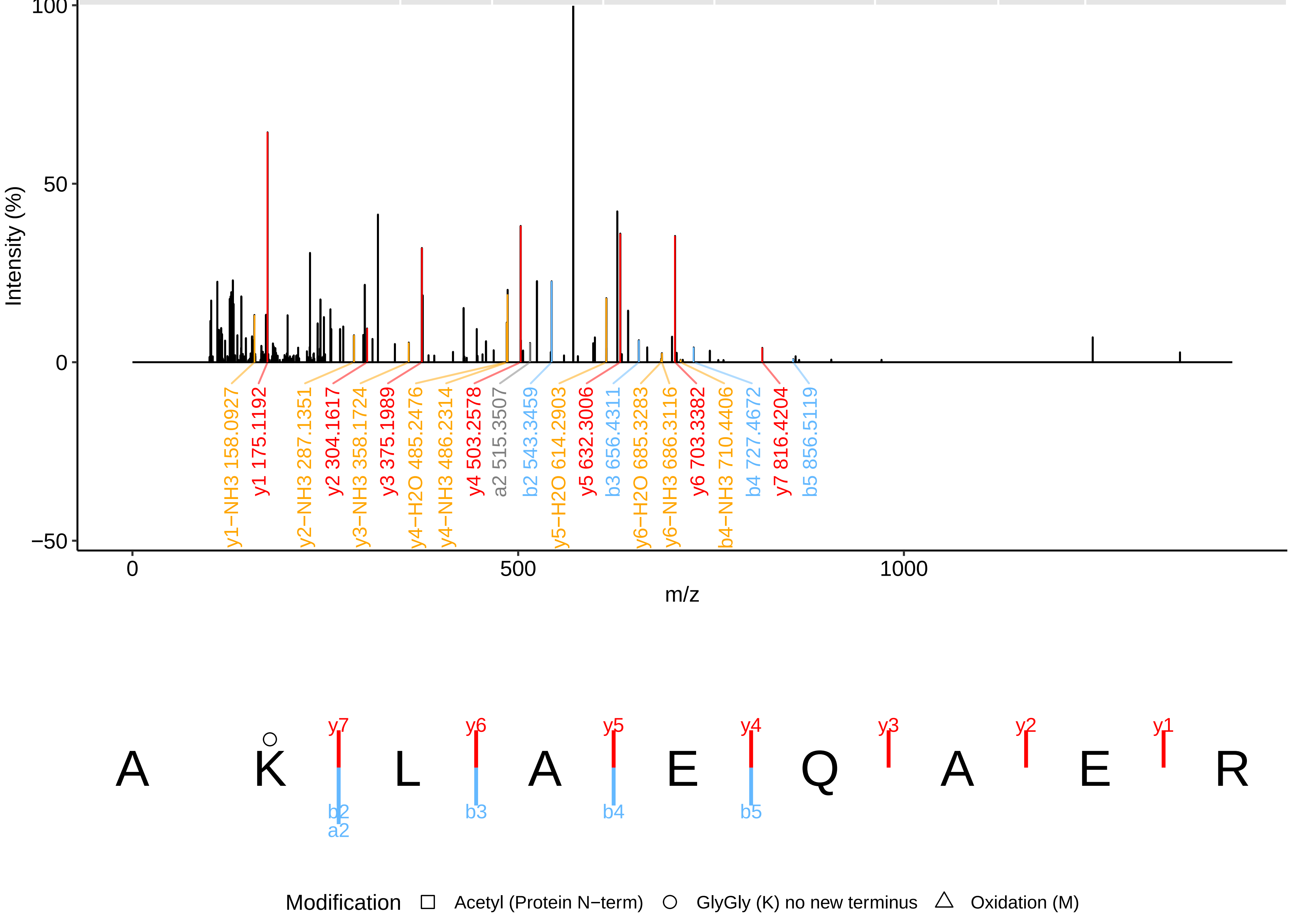

20201118_KIZ_MUb6_GlyGly_2 − Scan number: 4782

Gene Names	Charge	m/z	Mass	Retention time	PEP	Score	Precursor Intensity
YWHAQ;YWHAB;SFN;YWHAZ	3	377.2035	1128.589	16.437	0.083615	55.084	2178794

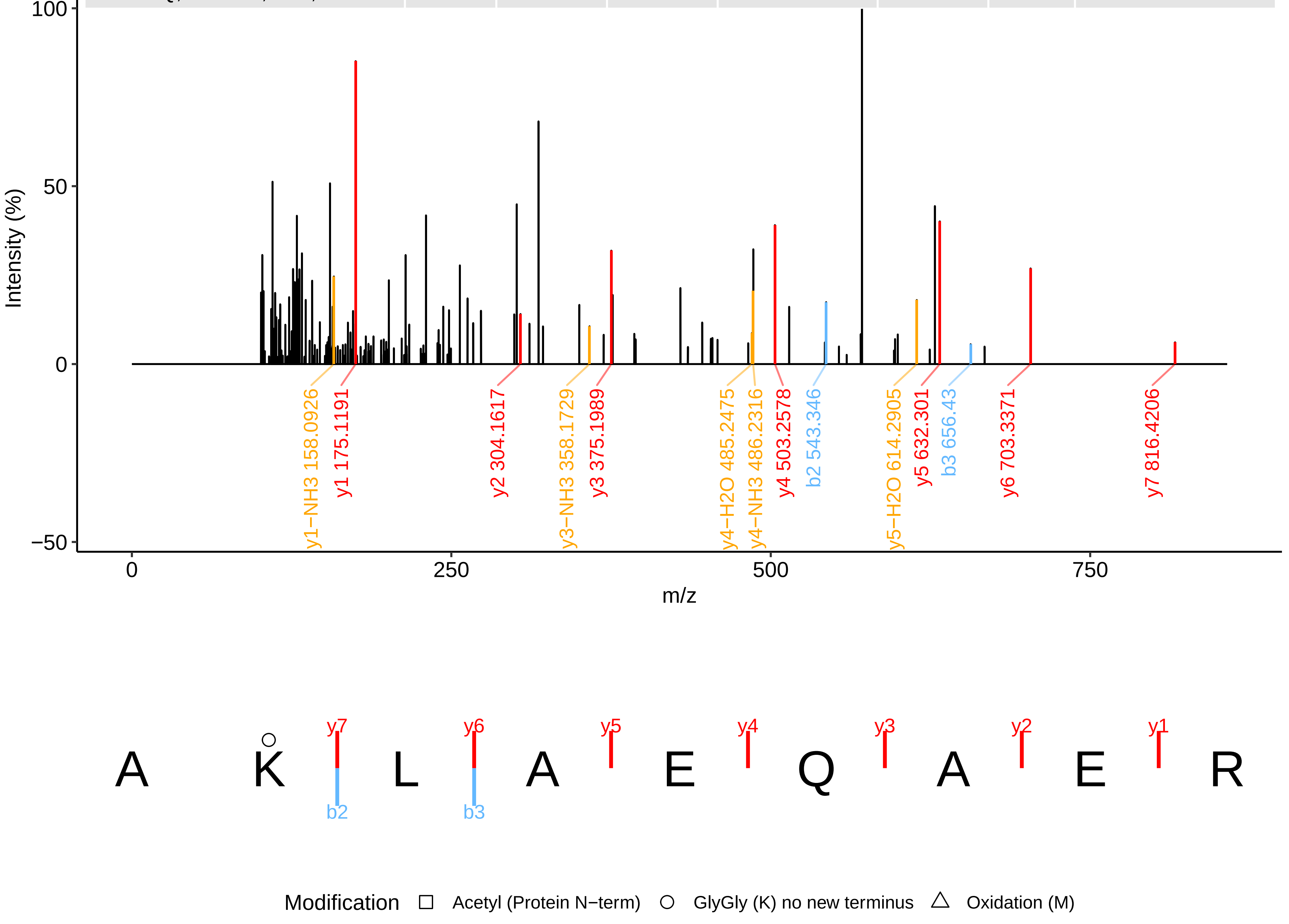

20200921_KIZ_MUb4_GlyGly_10plex − Scan number: 9541

Gene Names	Charge	m/z	Mass	Retention time	PEP	Score	Precursor Intensity
LMBRD1	3	378.8682	1133.583	32.693	0.04202	60.436	NA

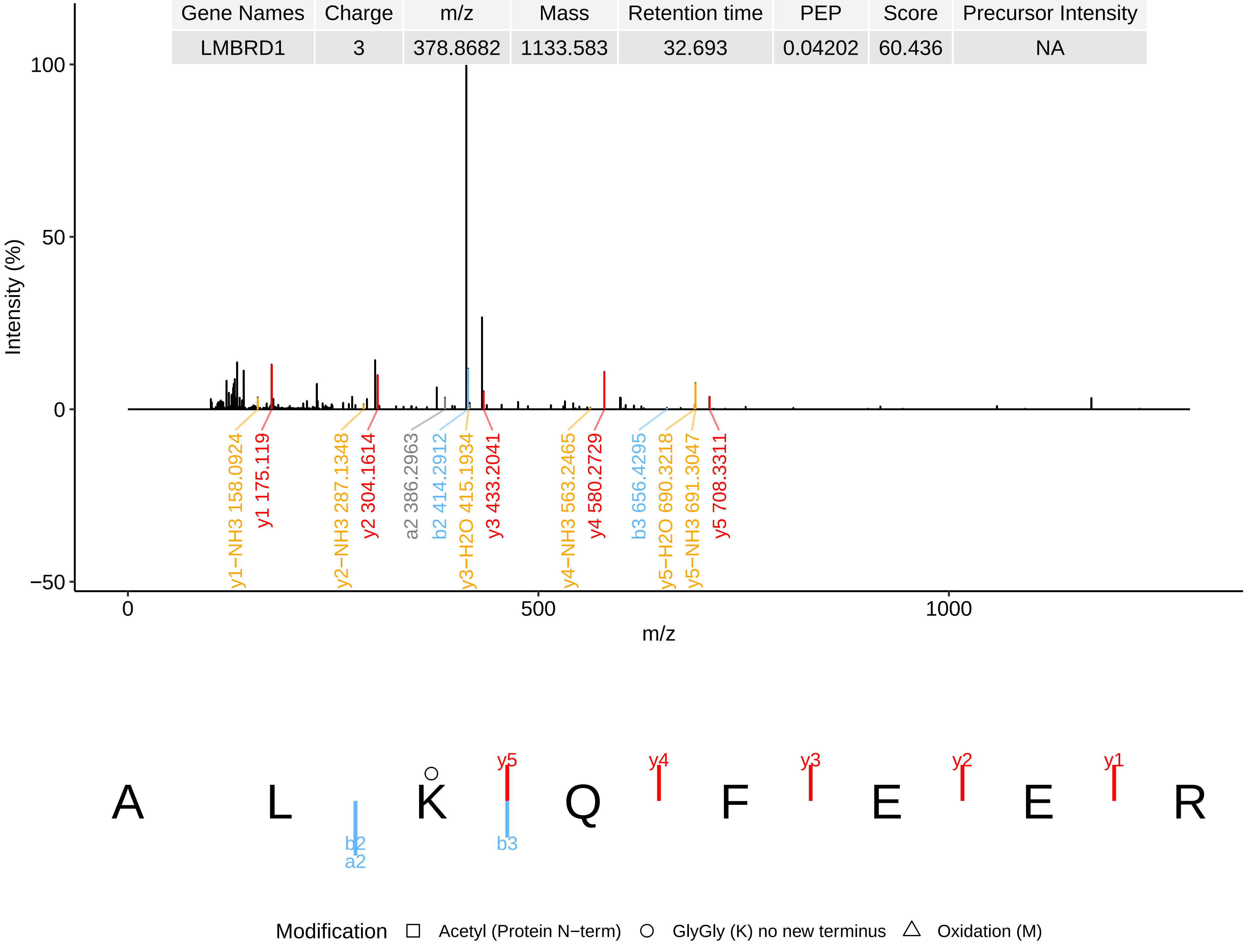

20200921_KIZ_MUb4_GlyGly_10plex_2 − Scan number: 8830

Gene Names	Charge	m/z	Mass	Retention time	PEP	Score	Precursor Intensity
LMBRD1	3	378.8682	1133.583	33.397	0.018224	73.26	1485769

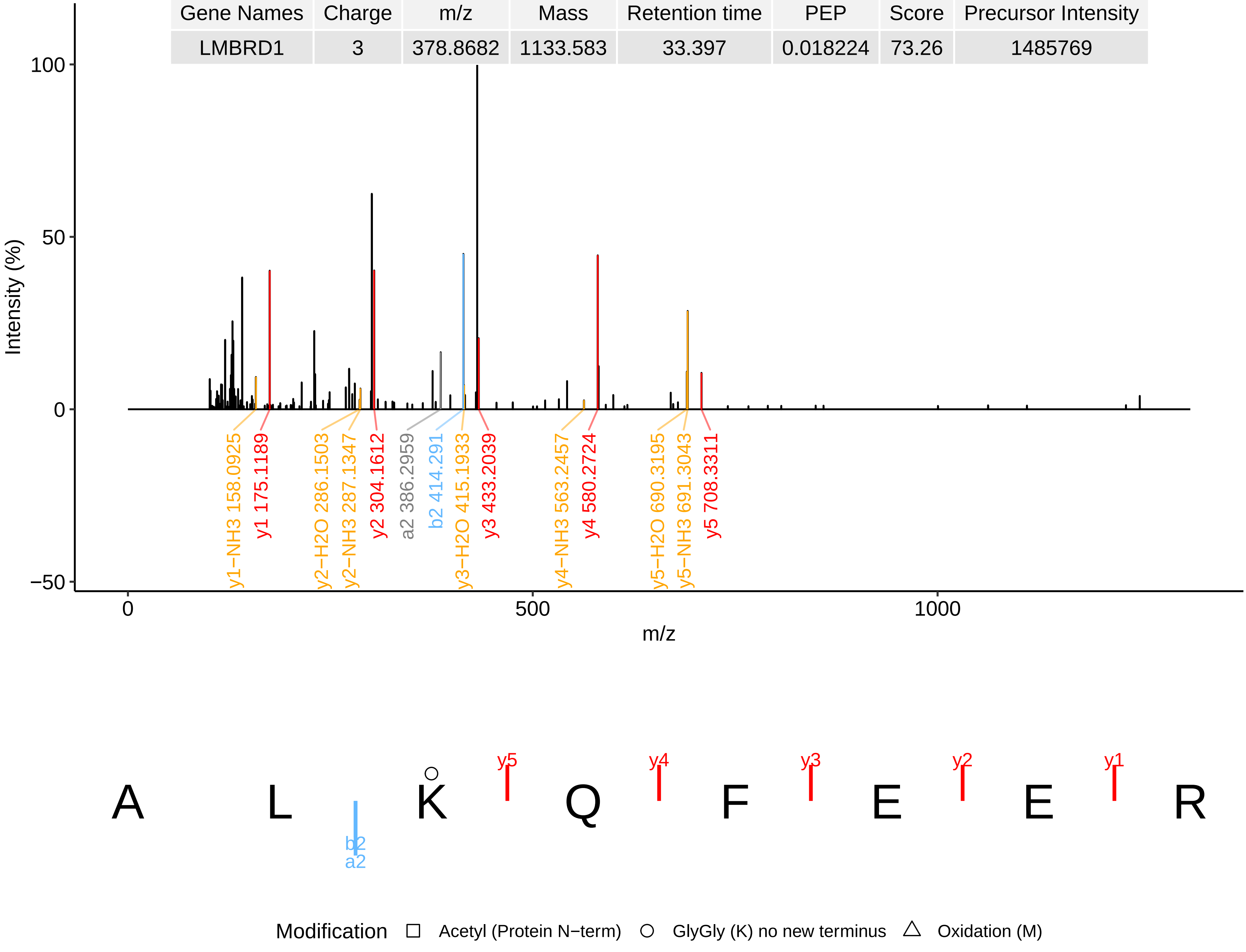

20200921_KIZ_MUb4_GlyGly_10plex − Scan number: 5339

Gene Names	Charge	m/z	Mass	Retention time	PEP	Score	Precursor Intensity
EP400	3	381.5595	1141.657	17.47	0.11527	43.635	8318001

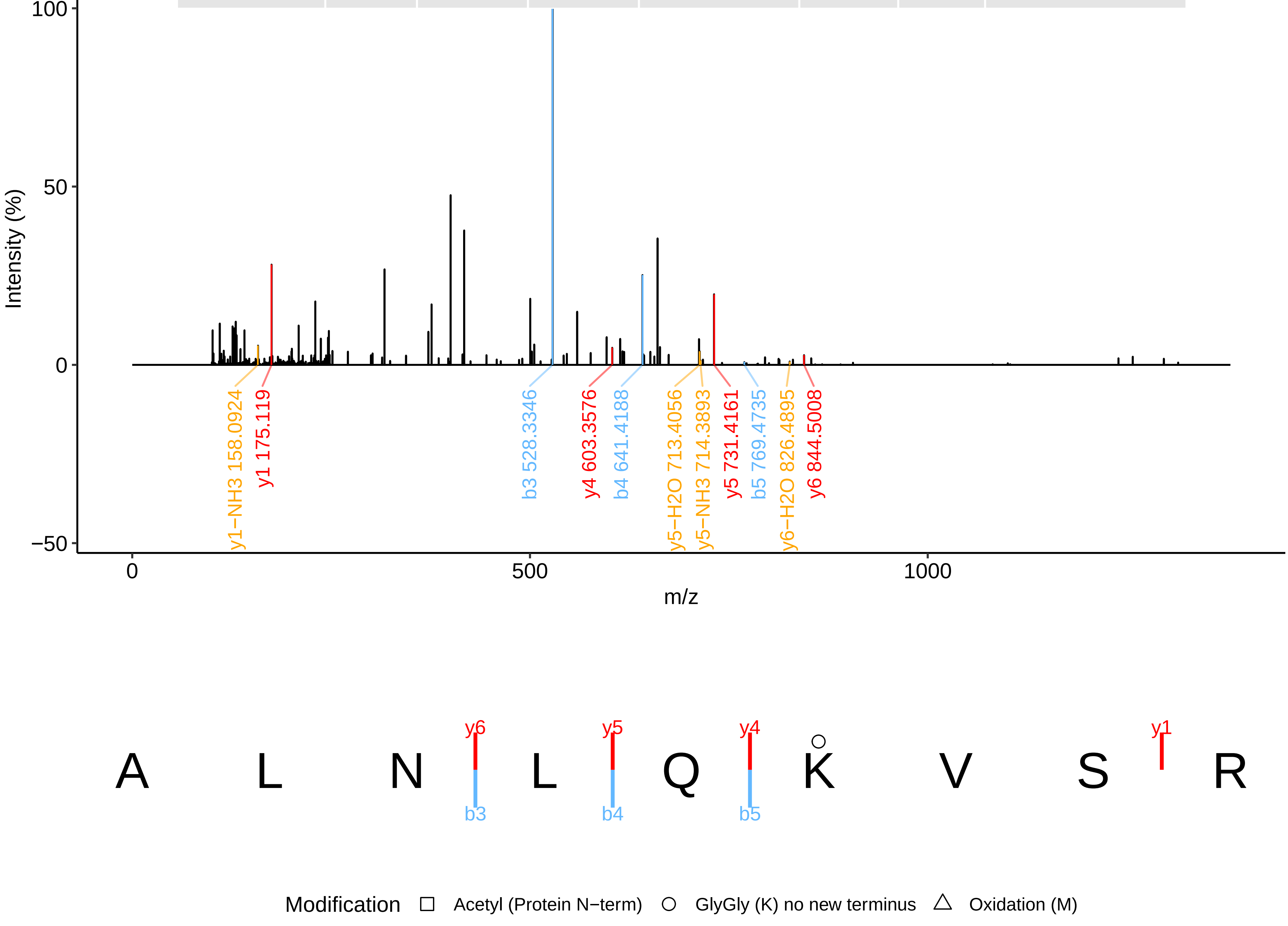

20201118_KIZ_MUb6_GlyGly_1 − Scan number: 4563

Gene Names	Charge	m/z	Mass	Retention time	PEP	Score	Precursor Intensity
EP400	3	381.5595	1141.657	14.324	0.078793	51.415	8727081

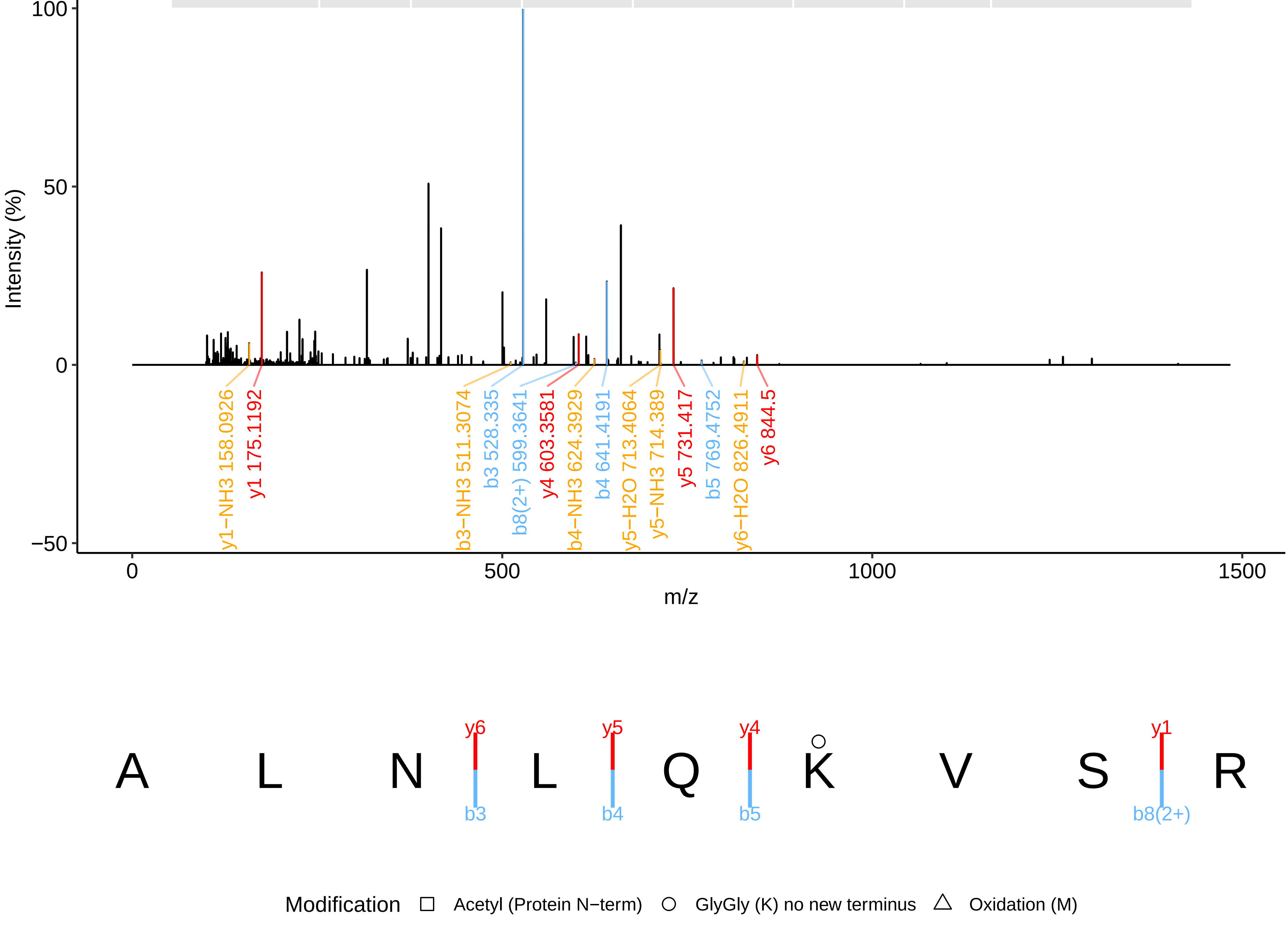

20200921_KIZ_MUb4_GlyGly_10plex − Scan number: 4759

Gene Names	Charge	m/z	Mass	Retention time	PEP	Score	Precursor Intensity
TFRC	3	291.1714	870.4923	15.517	0.022357	79.597	17421750

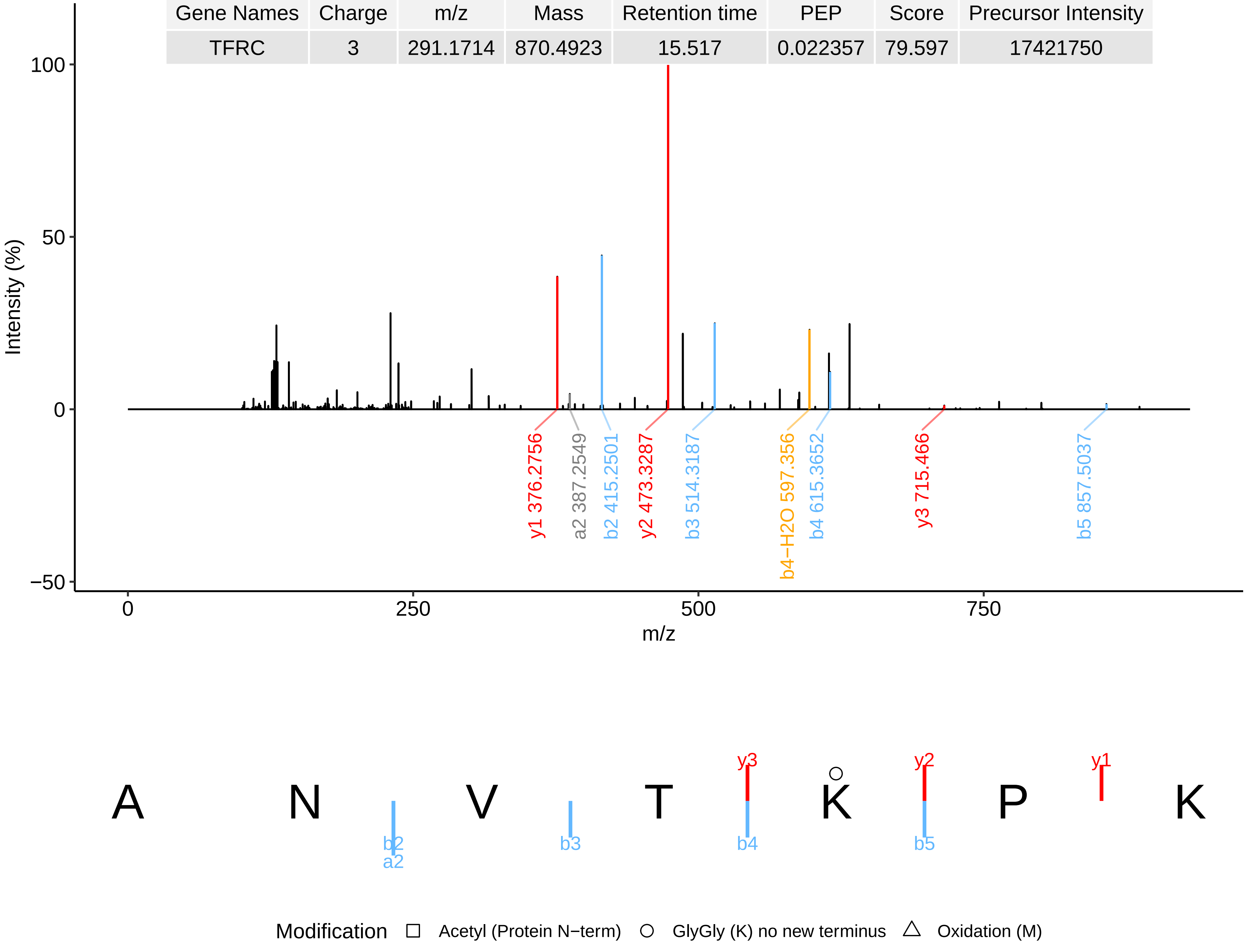

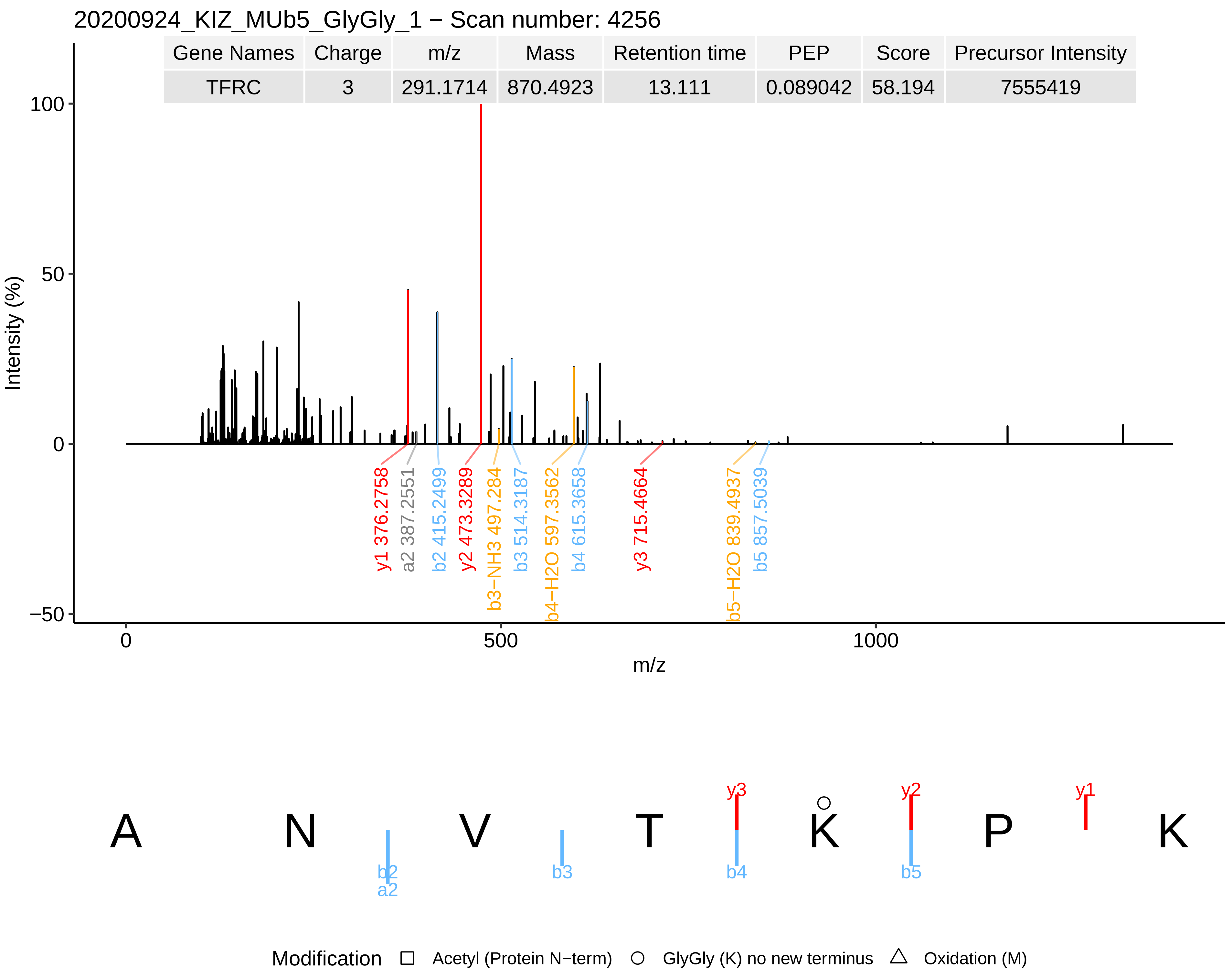

20200924_KIZ_MUb5_GlyGly_1 − Scan number: 4256
Gene Names | Charge | m/z | Mass | Retention time | PEP | Score | Precursor Intensity
TFRC | 3 | 291.1714 | 870.4923 | 13.111 | 0.089042 | 58.194 | 7555419
Intensity (%)
m/z
y1 376.2758
a2 387.2551
b2 415.2499
y2 473.3289
b3−NH3 497.284
b3 514.3187
b4−H2O 597.3562
b4 615.3658
y3 715.4664
b5−H2O 839.4937
b5 857.5039
A N V T K P K
y3 y2 y1
b2 a2 b3 b4 b5
Modification
Acetyl (Protein N−term)
GlyGly (K) no new terminus
Oxidation (M)

20201118_KIZ_MUb6_GlyGly_1 − Scan number: 3841

Gene Names	Charge	m/z	Mass	Retention time	PEP	Score	Precursor Intensity
TFRC	3	291.1714	870.4923	11.884	0.031947	75.854	4856816

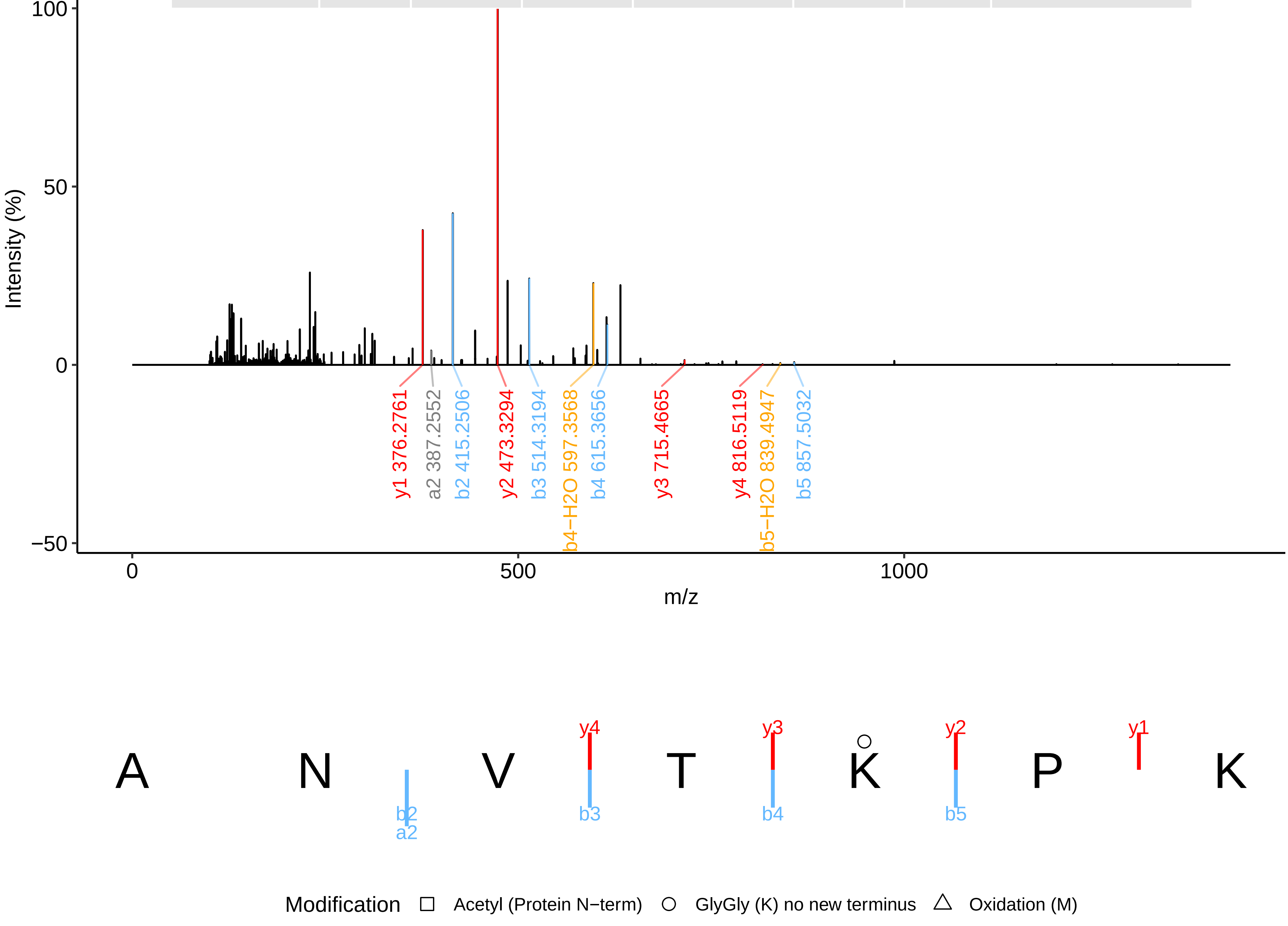

20200921_KIZ_MUb4_GlyGly_10plex − Scan number: 11521

Gene Names	Charge	m/z	Mass	Retention time	PEP	Score	Precursor Intensity
METTL7A	3	298.8396	893.4971	40.174	0.017797	83.377	3223248

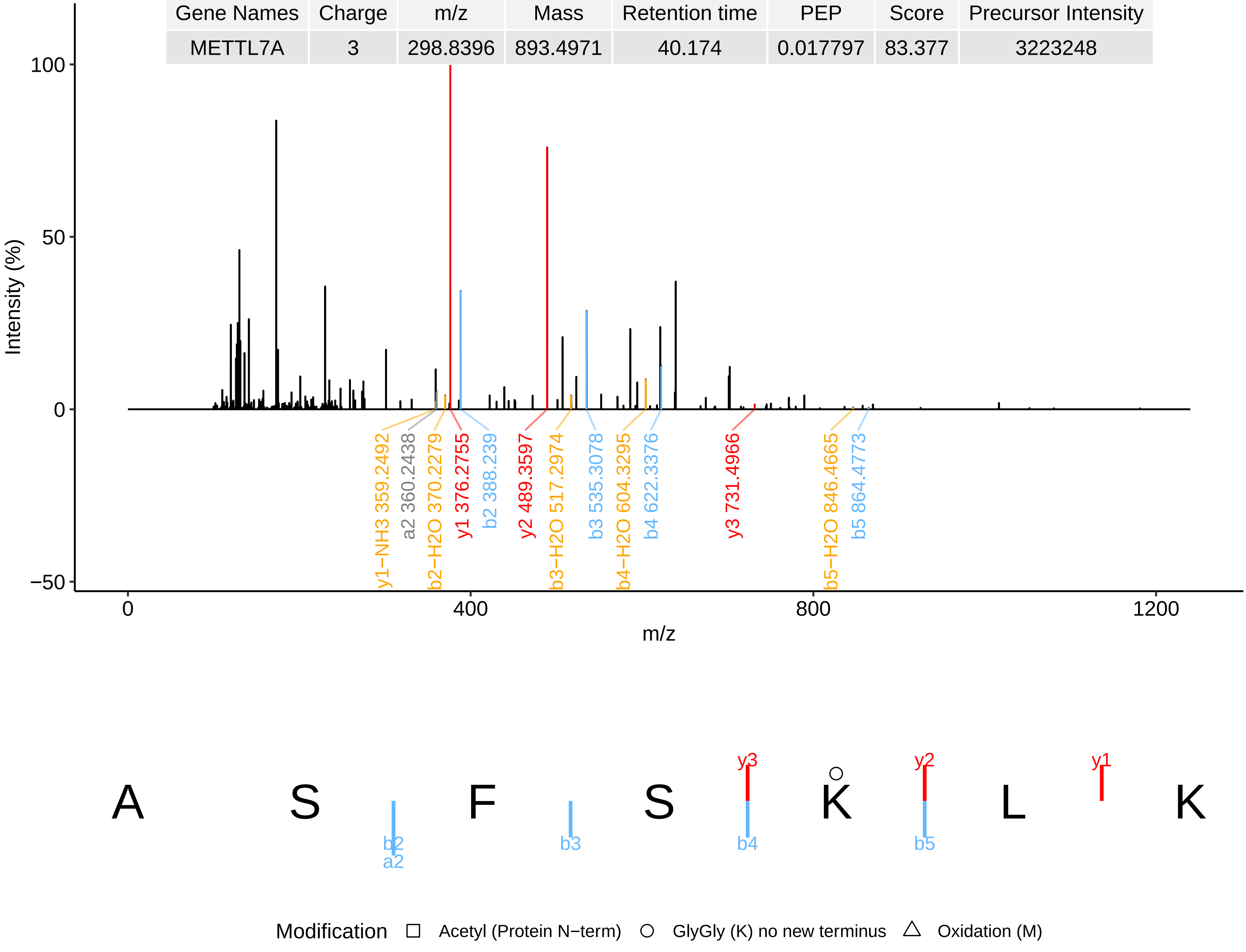

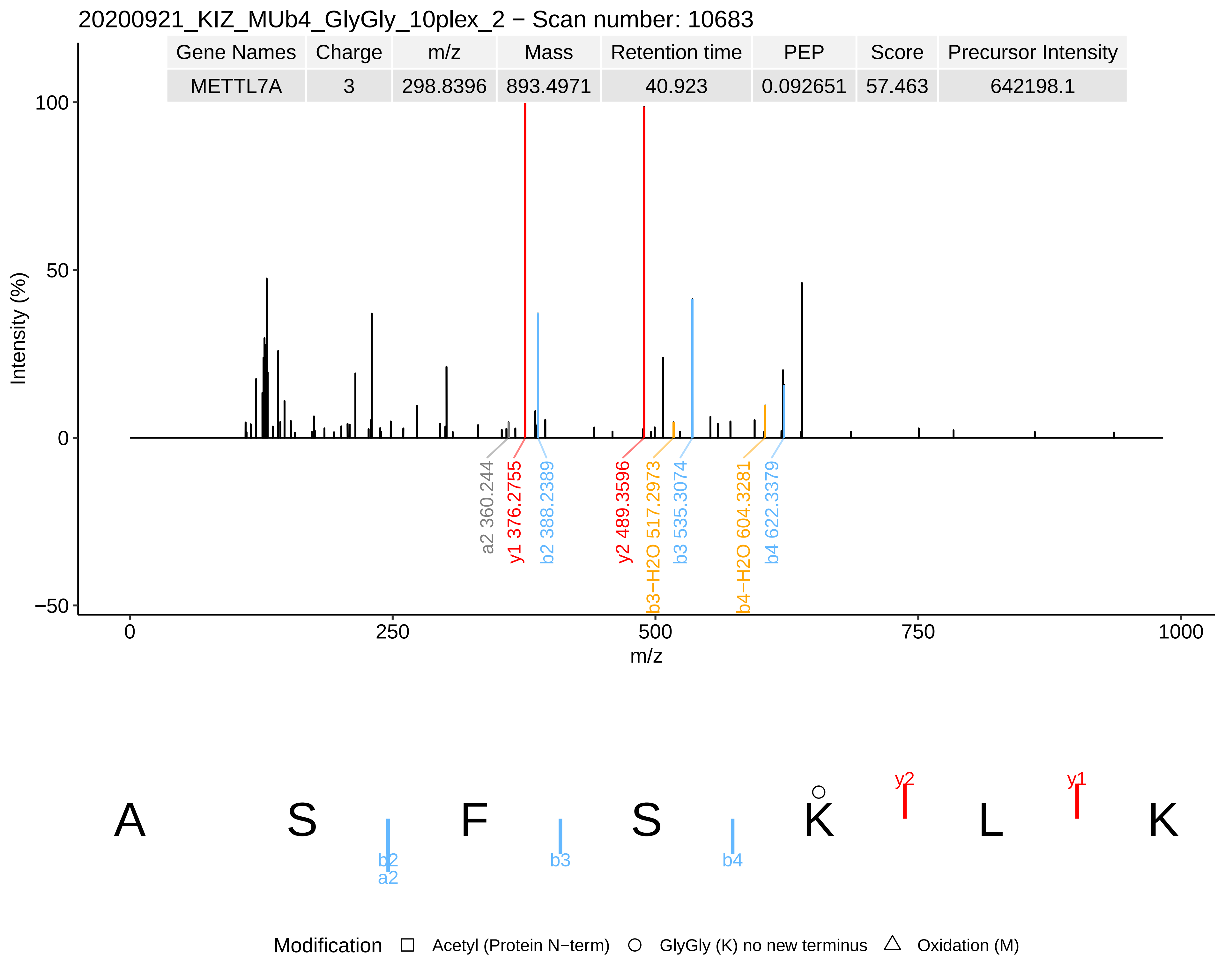
20200921_KIZ_MUb4_GlyGly_10plex_2 − Scan number: 10683
Gene Names | Charge | m/z | Mass | Retention time | PEP | Score | Precursor Intensity
METTL7A | 3 | 298.8396 | 893.4971 | 40.923 | 0.092651 | 57.463 | 642198.1
Intensity (%)
m/z
a2 360.244
y1 376.2755
b2 388.2389
y2 489.3596
b3−H2O 517.2973
b3 535.3074
b4−H2O 604.3281
b4 622.3379
A S F S K L K
b2
a2
b3
b4
y2
y1
Modification
Acetyl (Protein N−term)
GlyGly (K) no new terminus
Oxidation (M)

20200921_KIZ_MUb4_GlyGly_10plex − Scan number: 6654

Gene Names	Charge	m/z	Mass	Retention time	PEP	Score	Precursor Intensity
SLC16A1	3	306.5065	916.4978	22.105	0.041012	60.682	18139916

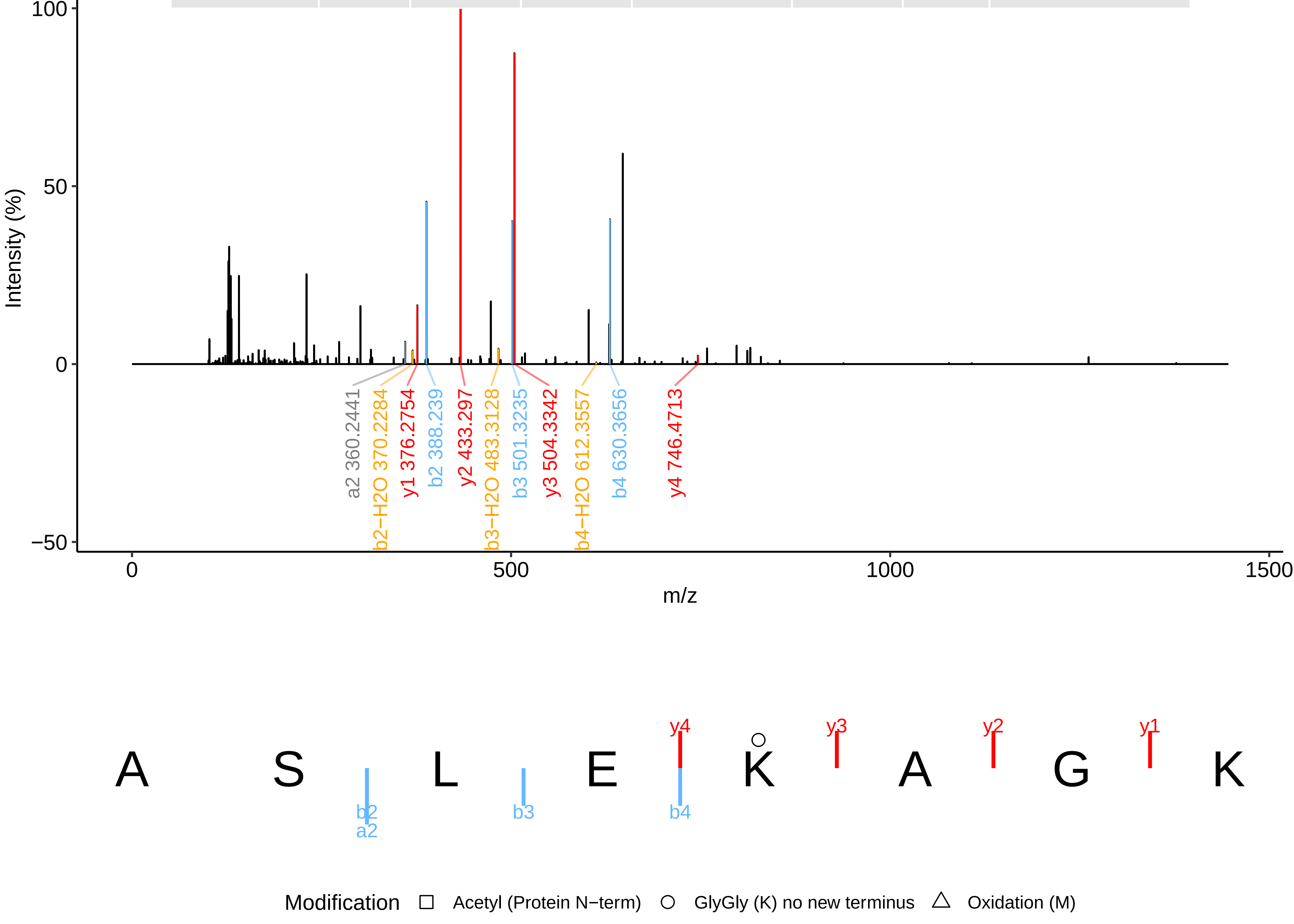

20200921_KIZ_MUb4_GlyGly_10plex − Scan number: 6673

Gene Names	Charge	m/z	Mass	Retention time	PEP	Score	Precursor Intensity
SLC16A1	2	459.2562	916.4978	22.175	0.0072131	121.05	NA

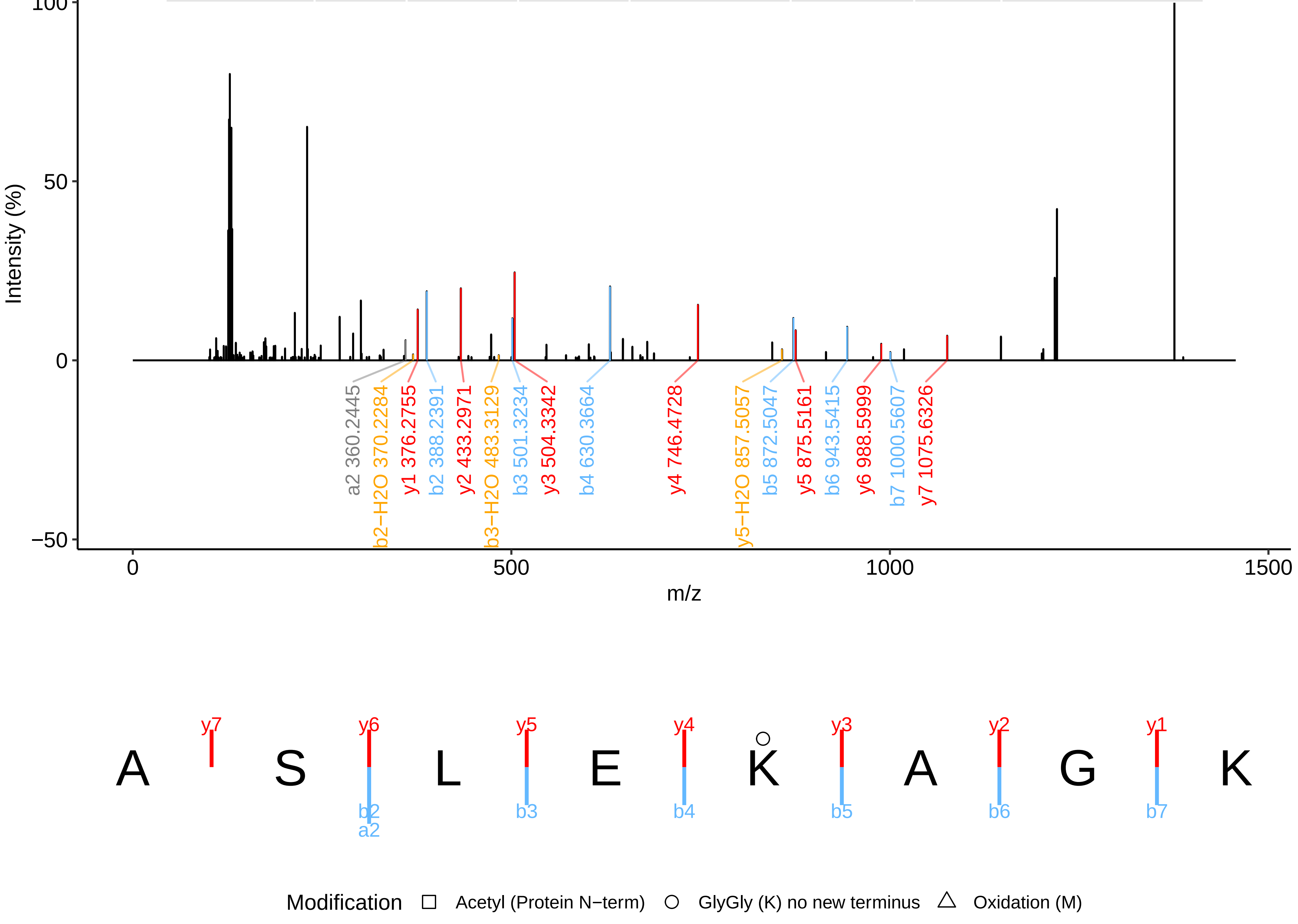

20200921_KIZ_MUb4_GlyGly_10plex_2 − Scan number: 6162

Gene Names	Charge	m/z	Mass	Retention time	PEP	Score	Precursor Intensity
SLC16A1	3	306.5065	916.4978	22.52	0.10494	45.081	730956.9

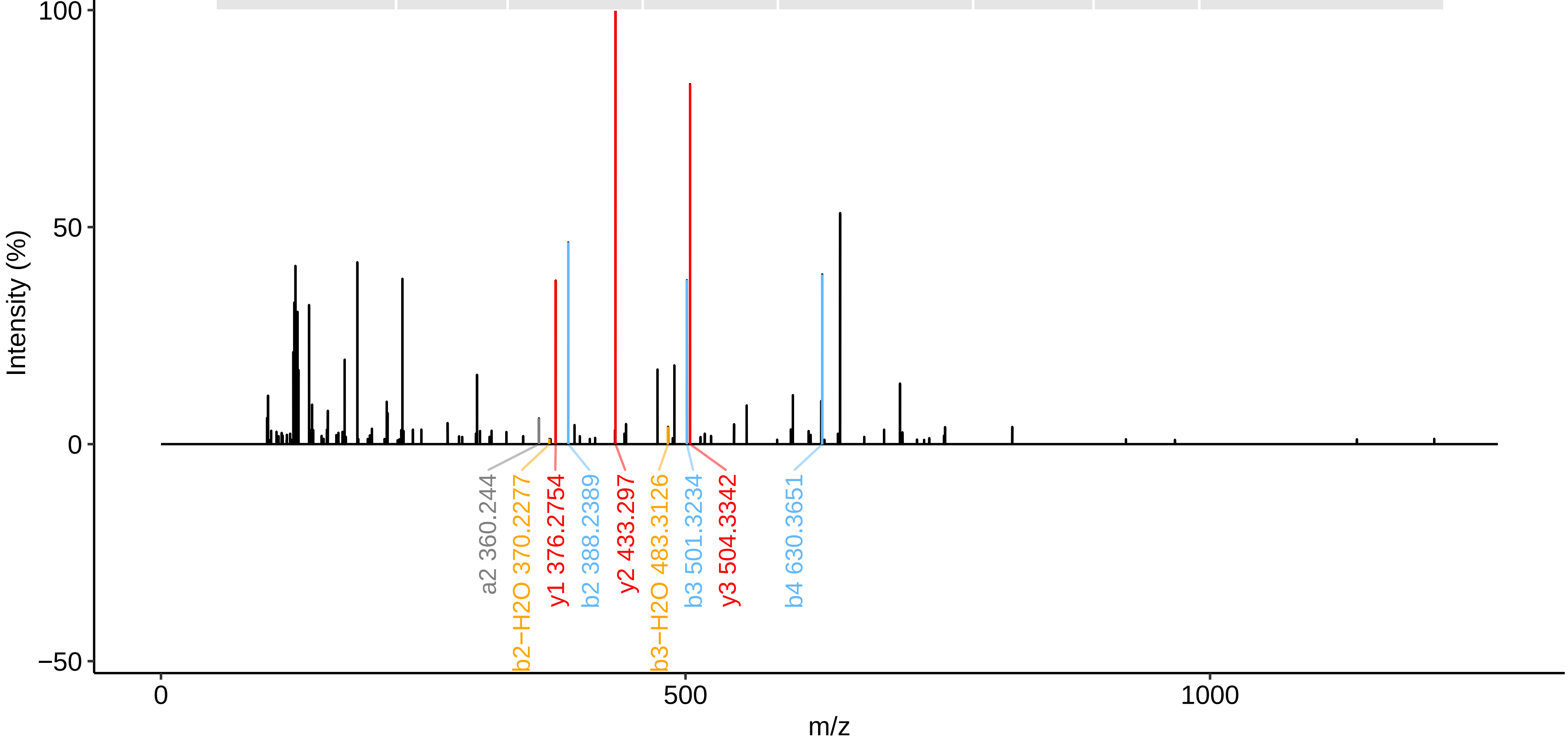

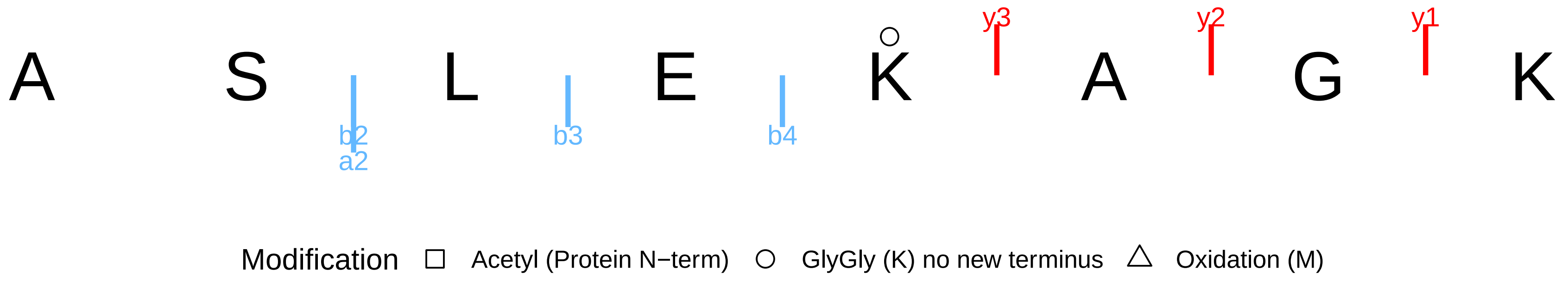

20200924_KIZ_MUb5_GlyGly_1 − Scan number: 5678

Gene Names	Charge	m/z	Mass	Retention time	PEP	Score	Precursor Intensity
SLC16A1	3	306.5065	916.4978	17.715	0.090763	48.561	28659264

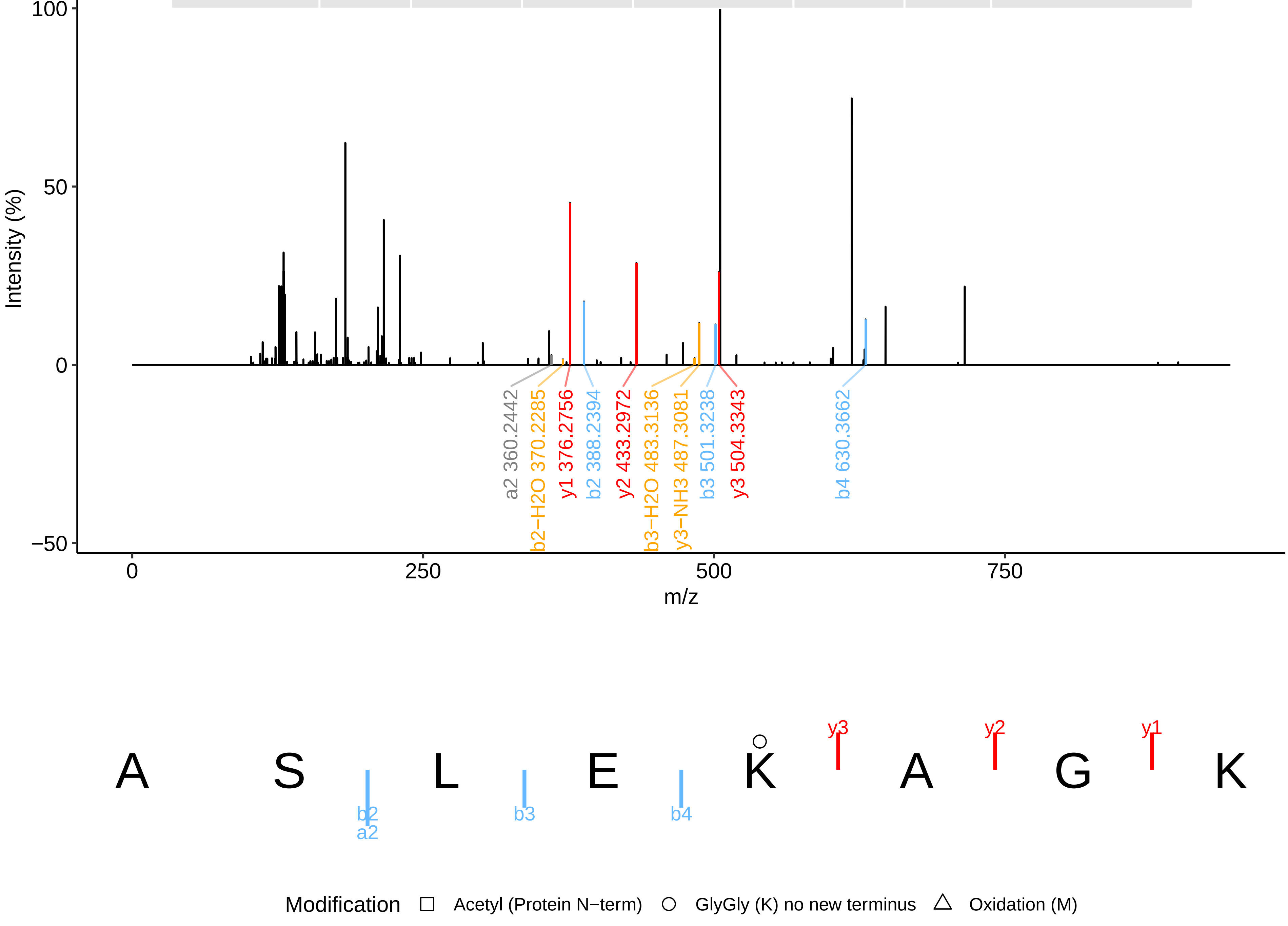

20200924_KIZ_MUb5_GlyGly_1 − Scan number: 5790

Gene Names	Charge	m/z	Mass	Retention time	PEP	Score	Precursor Intensity
SLC16A1	3	306.5065	916.4978	18.079	0.0248	67.981	33239492

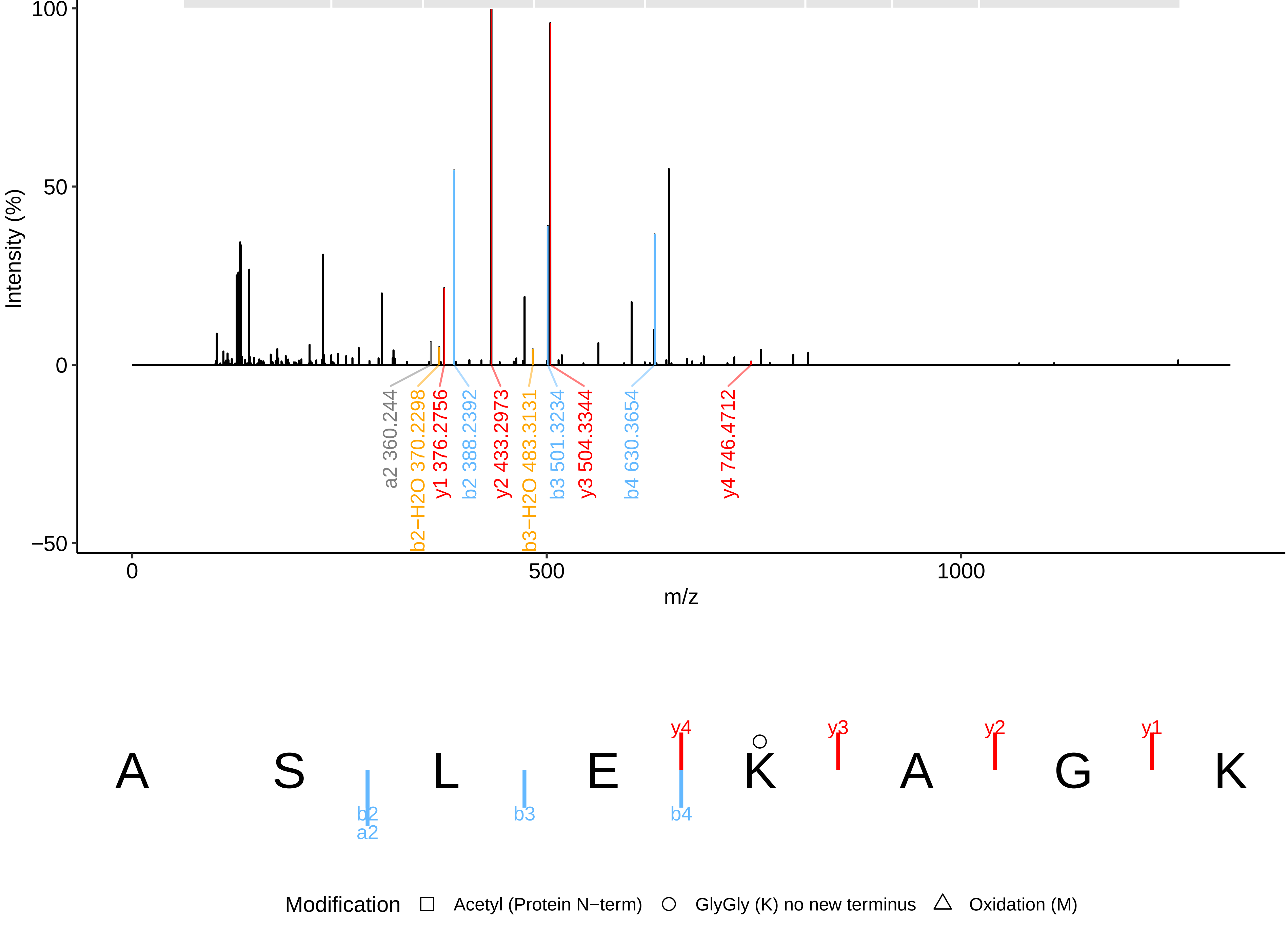

20200924_KIZ_MUb5_GlyGly_2 − Scan number: 5335

Gene Names	Charge	m/z	Mass	Retention time	PEP	Score	Precursor Intensity
SLC16A1	3	306.5065	916.4978	19.784	0.06593	54.611	3660863

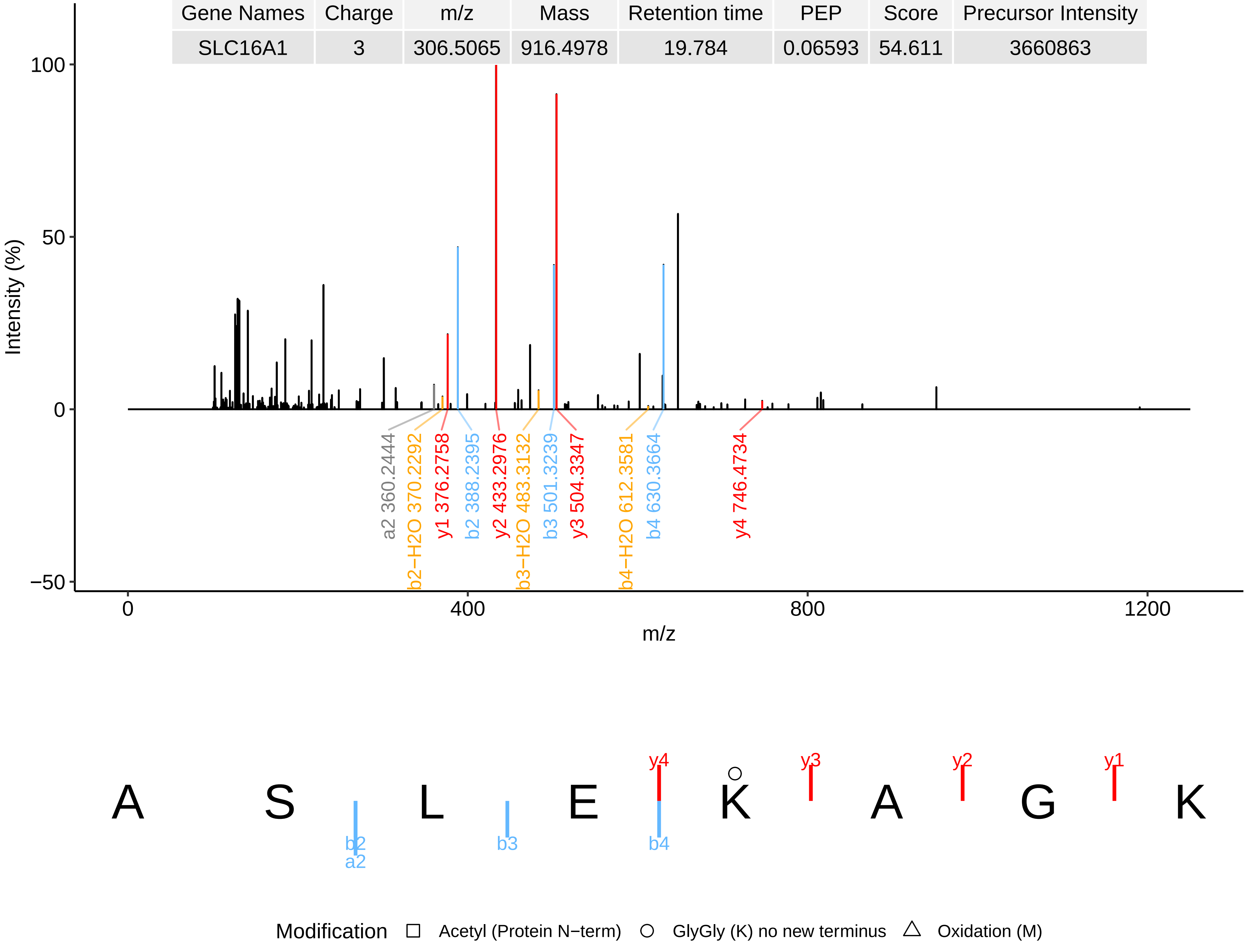

20200924_KIZ_MUb5_GlyGly_2 − Scan number: 5470

Gene Names	Charge	m/z	Mass	Retention time	PEP	Score	Precursor Intensity
SLC16A1	3	306.5065	916.4978	20.303	0.096836	47.082	5119456

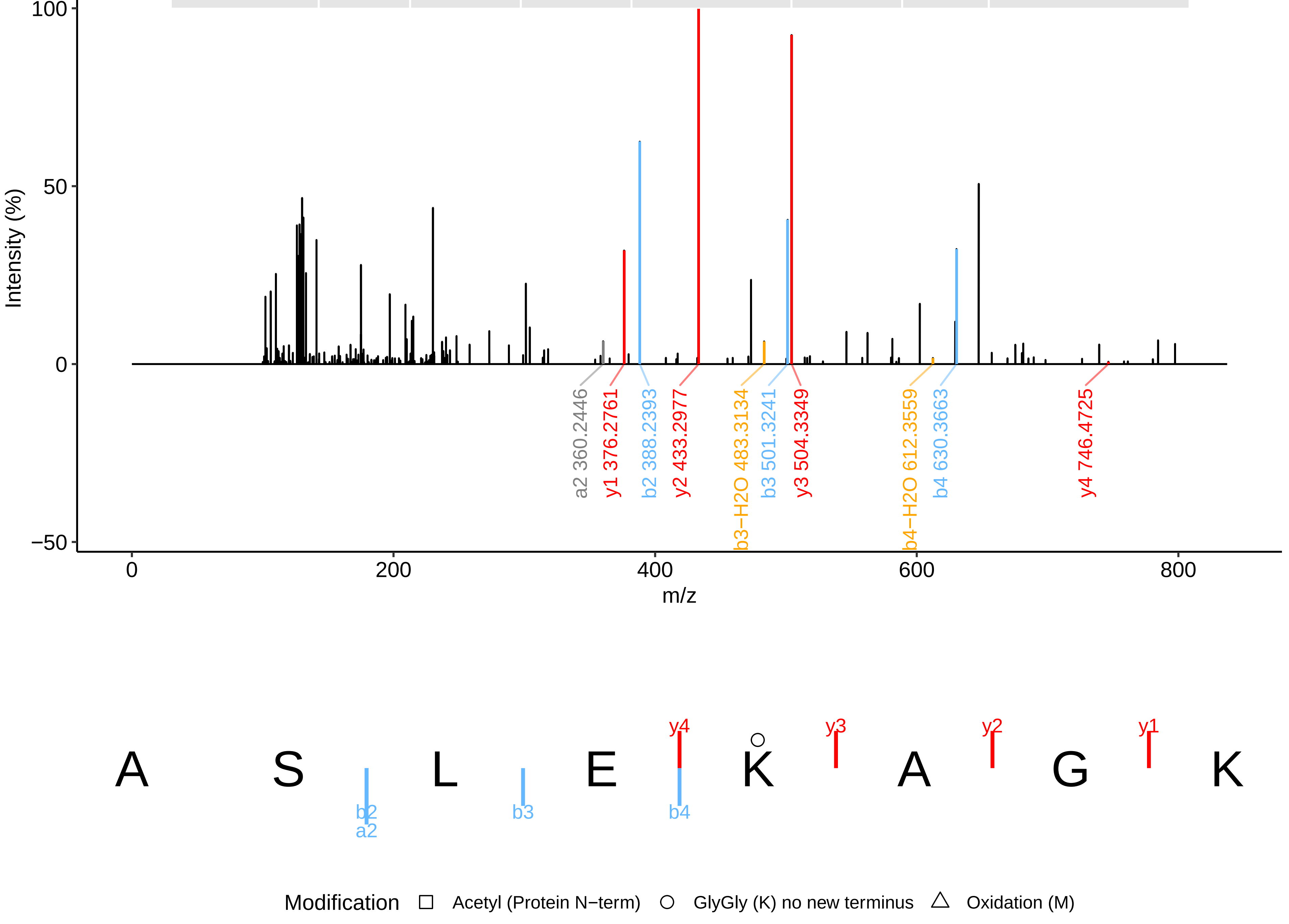

20201118_KIZ_MUb6_GlyGly_1 − Scan number: 5426

Gene Names	Charge	m/z	Mass	Retention time	PEP	Score	Precursor Intensity
SLC16A1	3	306.5065	916.4978	17.178	0.0248	67.981	18882308

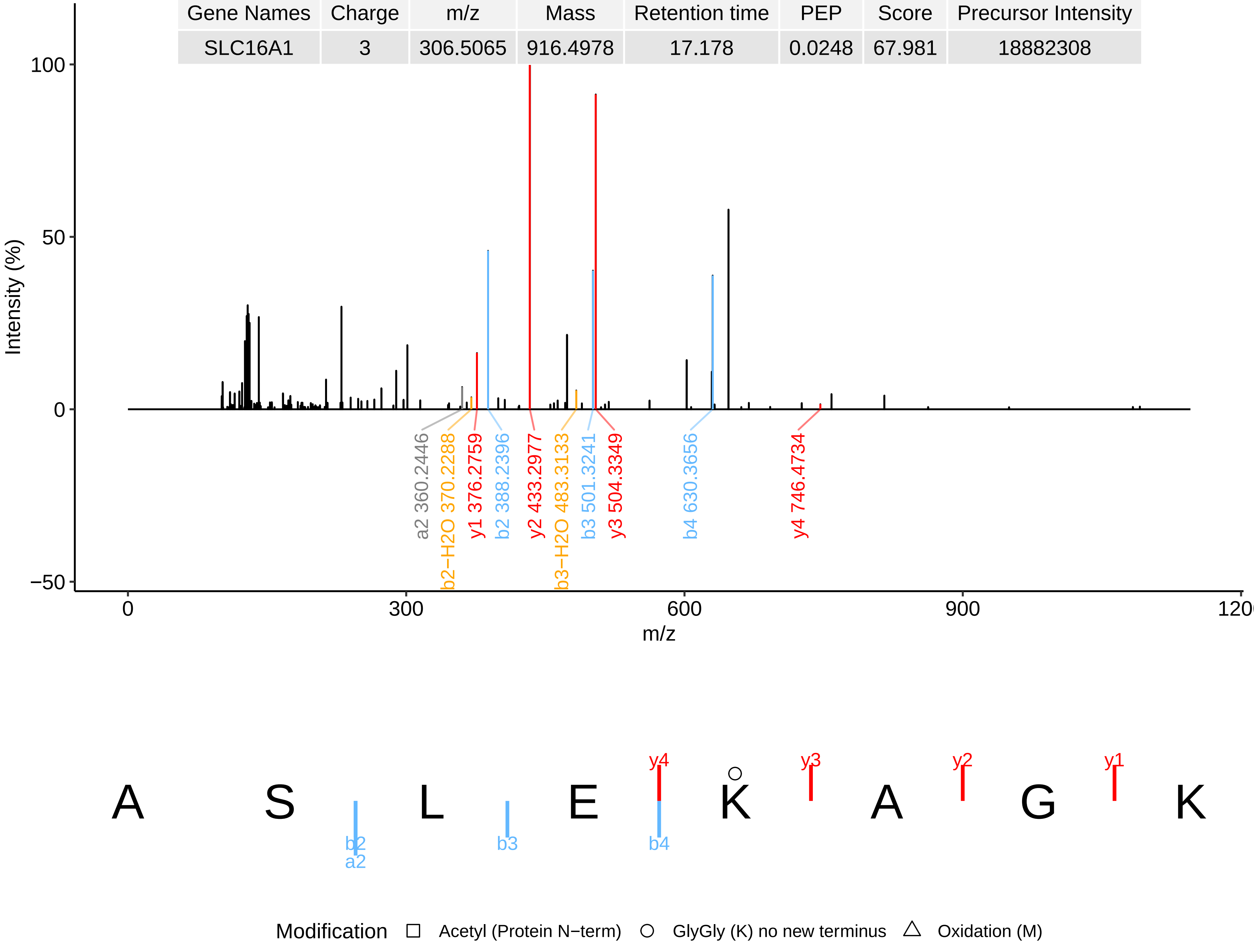

20201118_KIZ_MUb6_GlyGly_2 − Scan number: 6933

Gene Names	Charge	m/z	Mass	Retention time	PEP	Score	Precursor Intensity
SLC16A1	3	306.5065	916.4978	23.659	0.036132	64.567	7861150

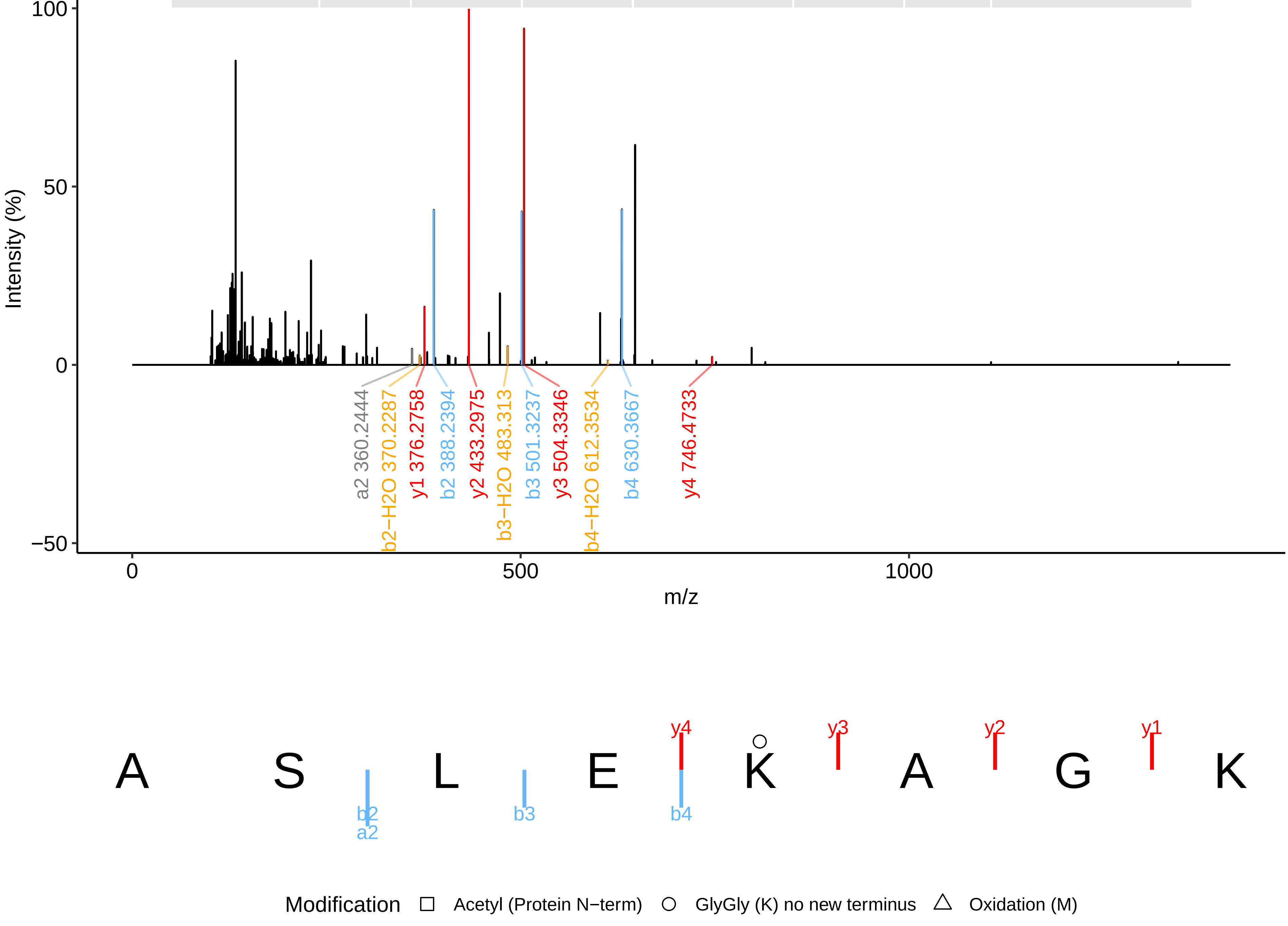

20200921_KIZ_MUb4_GlyGly_10plex − Scan number: 4087

Gene Names	Charge	m/z	Mass	Retention time	PEP	Score	Precursor Intensity
HNRNPC	2	679.8366	1357.659	13.295	2.537e−05	57.598	7336632

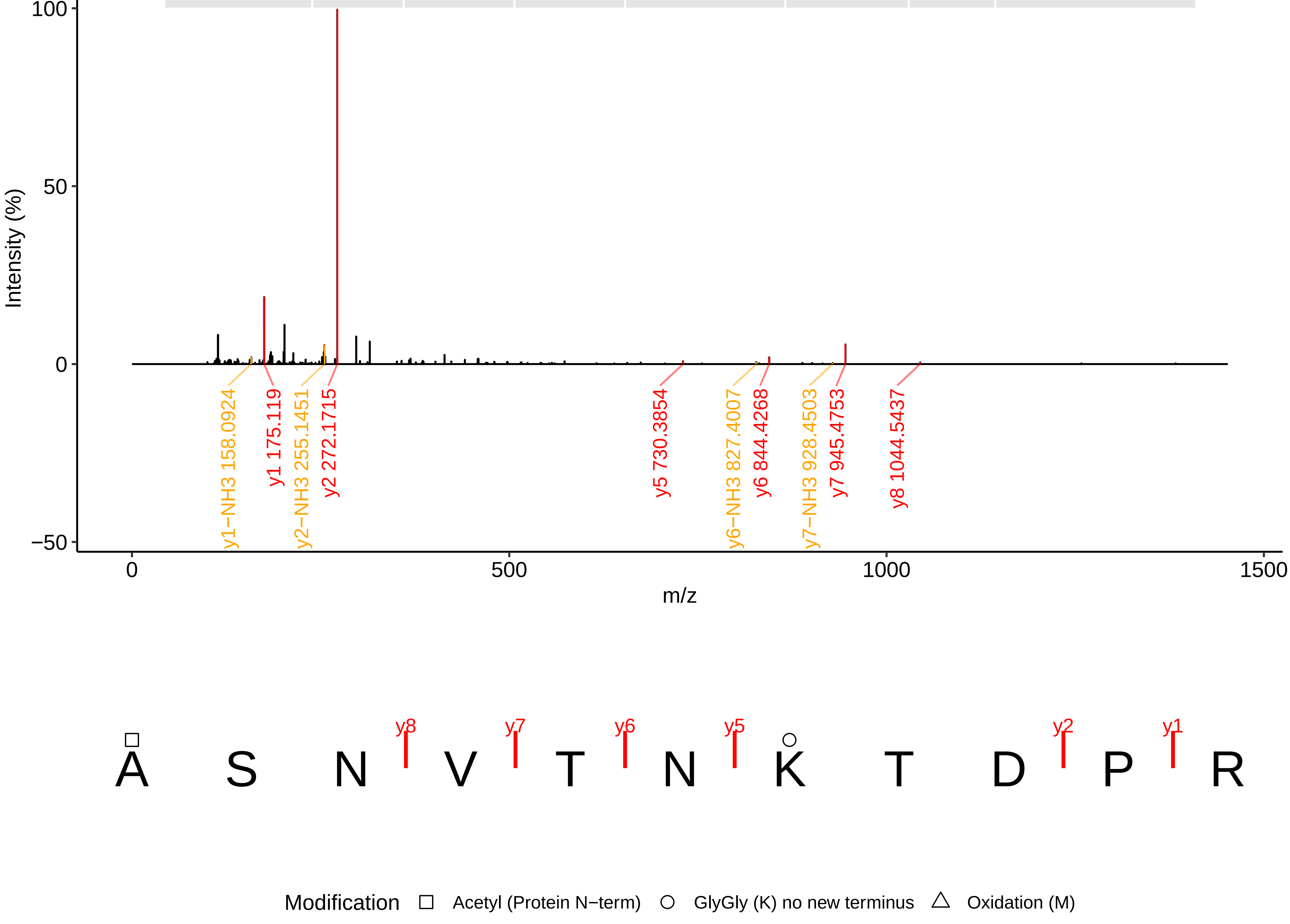

20200921_KIZ_MUb4_GlyGly_10plex_2 − Scan number: 3805

Gene Names	Charge	m/z	Mass	Retention time	PEP	Score	Precursor Intensity
HNRNPC	2	679.8366	1357.659	13.022	0.010483	46.158	1019125

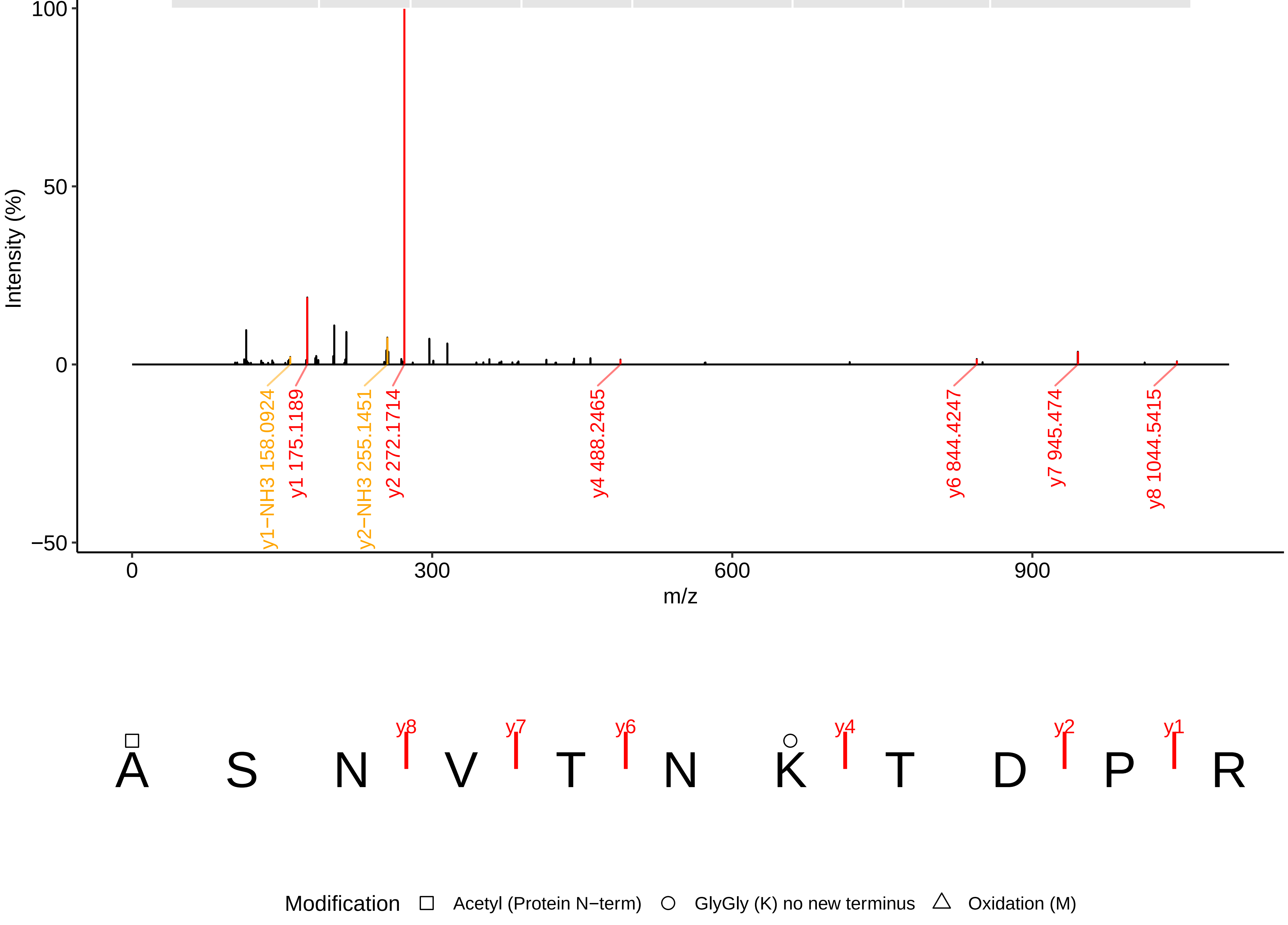

20200924_KIZ_MUb5_GlyGly_1 − Scan number: 3571

Gene Names	Charge	m/z	Mass	Retention time	PEP	Score	Precursor Intensity
HNRNPC	2	679.8366	1357.659	10.929	3.34e−08	72.928	3645574

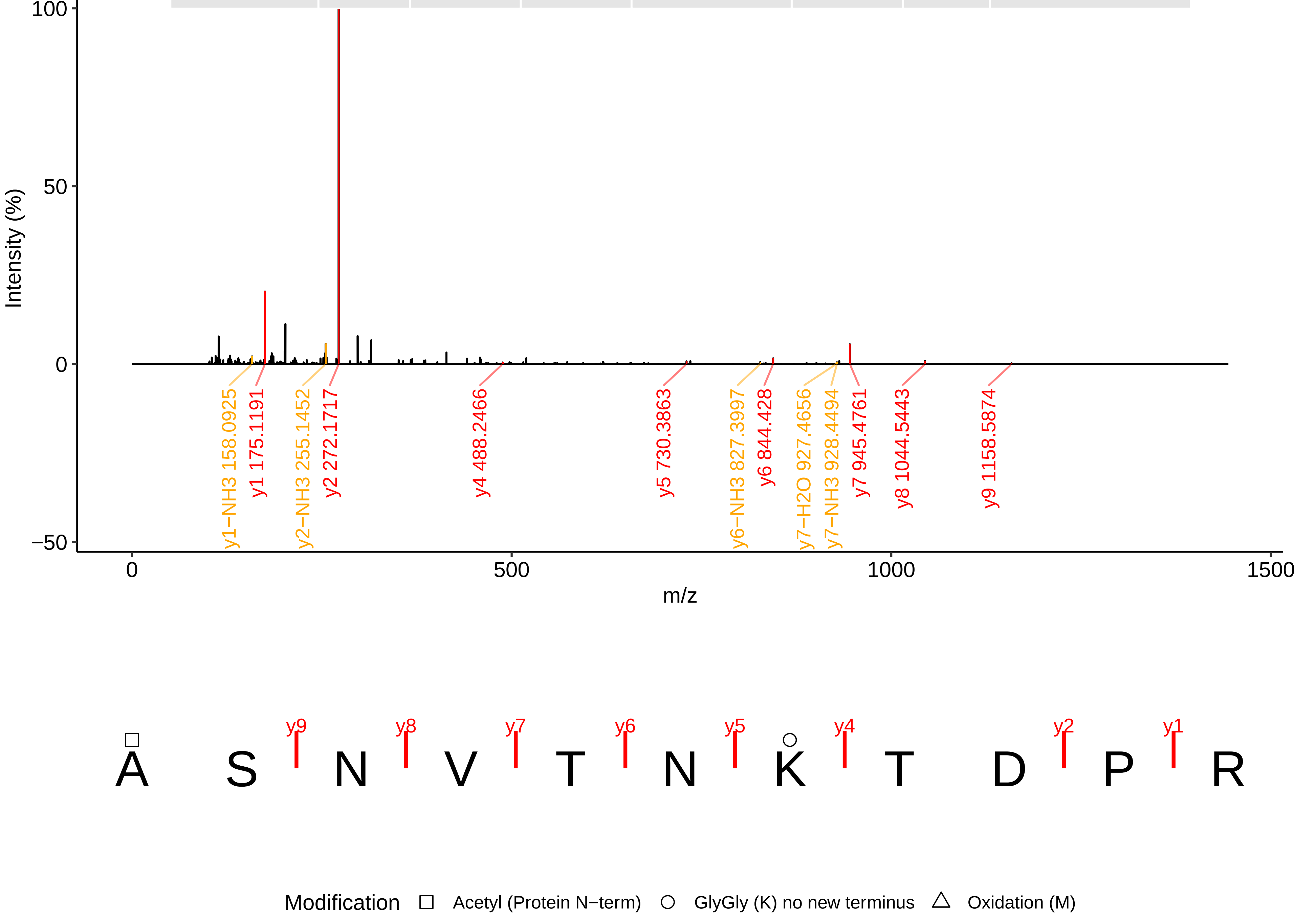

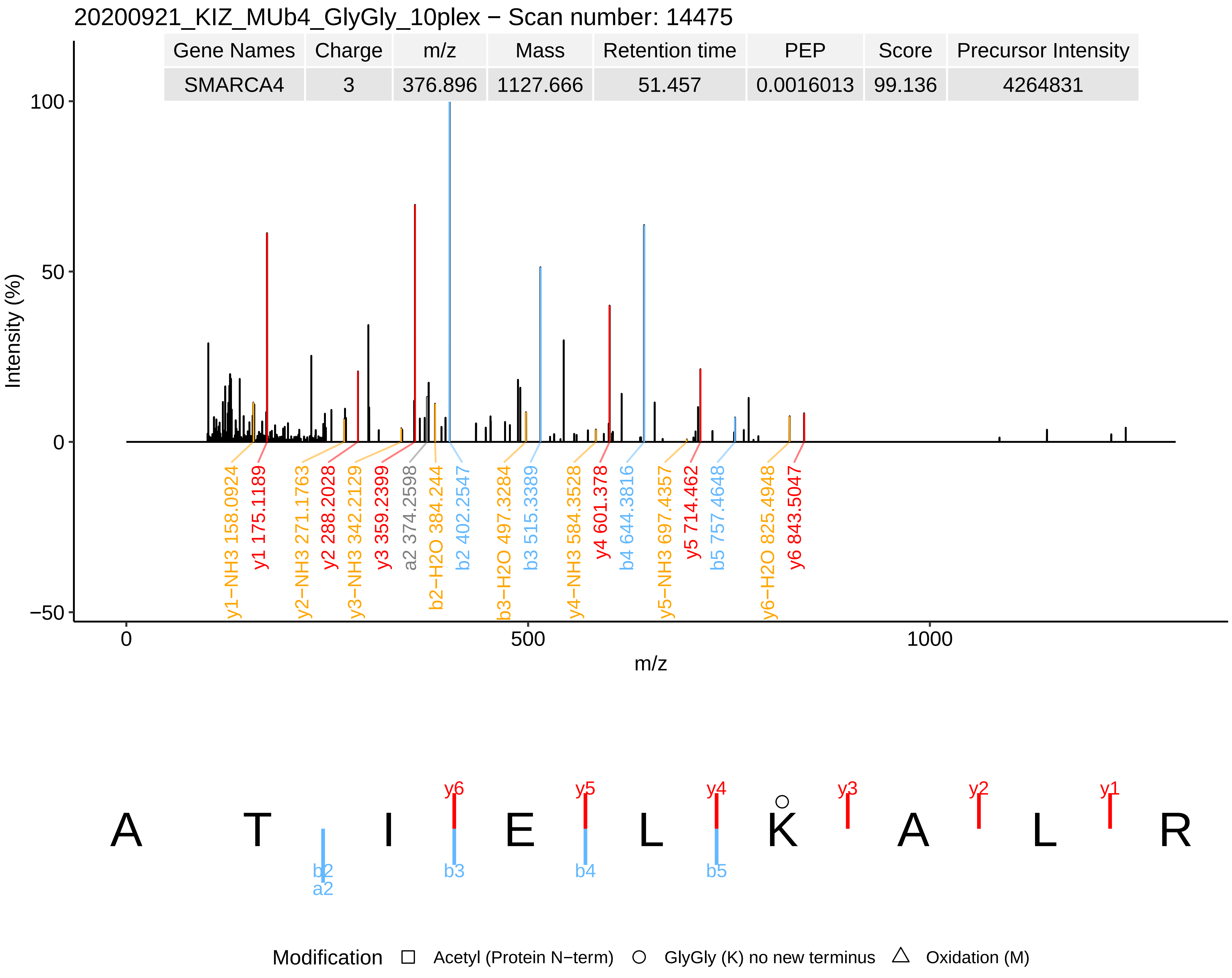

20200921_KIZ_MUb4_GlyGly_10plex − Scan number: 14475
Gene Names | Charge | m/z | Mass | Retention time | PEP | Score | Precursor Intensity
SMARCA4 | 3 | 376.896 | 1127.666 | 51.457 | 0.0016013 | 99.136 | 4264831
Intensity (%)
m/z
y1−NH3 158.0924
y1 175.1189
y2−NH3 271.1763
y2 288.2028
y3−NH3 342.2129
y3 359.2399
a2 374.2598
b2−H2O 384.244
b2 402.2547
b3−H2O 497.3284
b3 515.3389
y4−NH3 584.3528
y4 601.378
b4 644.3816
y5−NH3 697.4357
y5 714.462
b5 757.4648
y6−H2O 825.4948
y6 843.5047
A T I E L K A L R
y6 y5 y4 y3 y2 y1
b2 a2 b3 b4 b5
Modification
Acetyl (Protein N−term)
GlyGly (K) no new terminus
Oxidation (M)

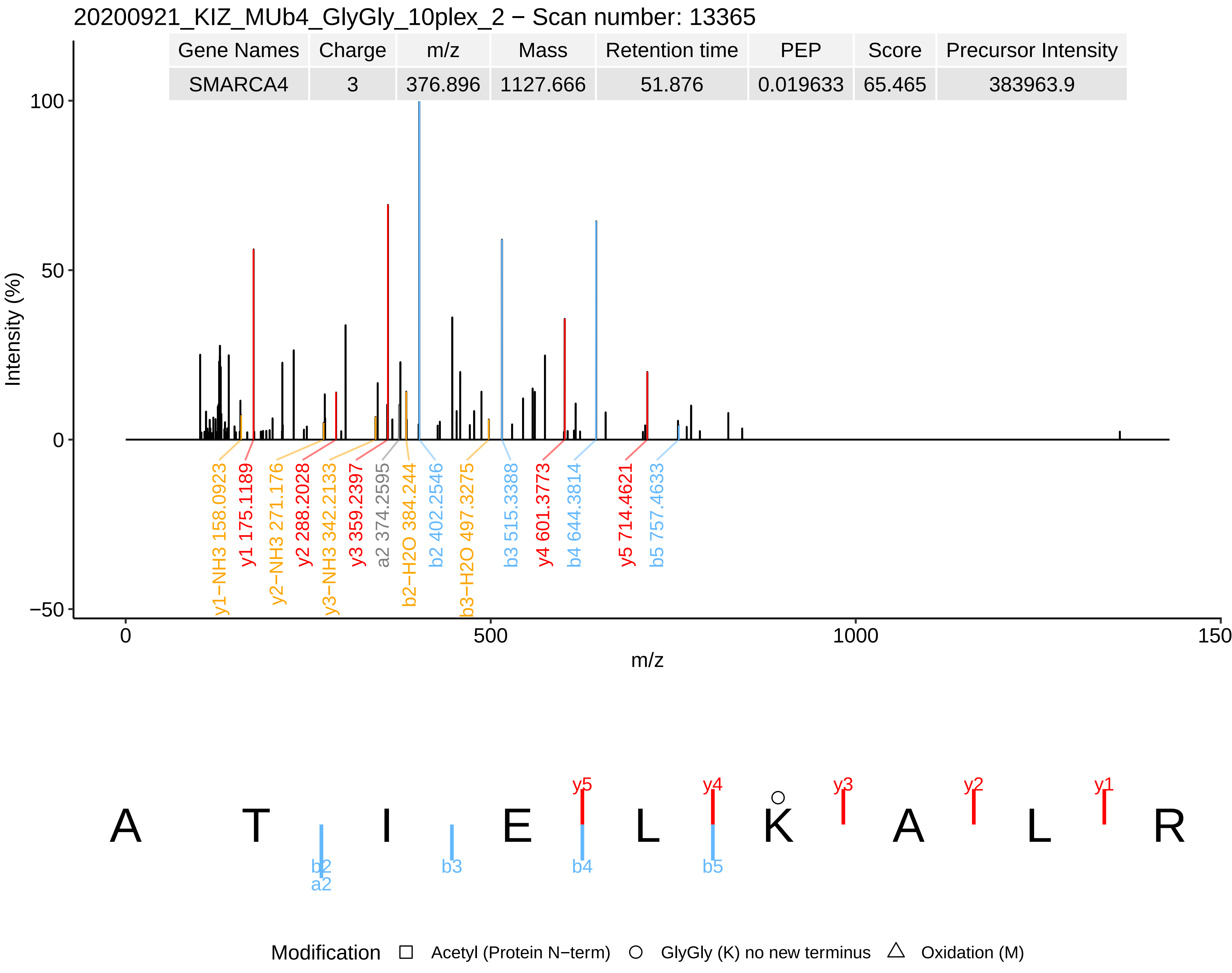

20200921_KIZ_MUb4_GlyGly_10plex_2 − Scan number: 13365
Gene Names | Charge | m/z | Mass | Retention time | PEP | Score | Precursor Intensity
SMARCA4 | 3 | 376.896 | 1127.666 | 51.876 | 0.019633 | 65.465 | 383963.9
Intensity (%)
100
50
0
−50
0
500
1000
150
m/z
y1−NH3 158.0923
y1 175.1189
y2−NH3 271.176
y2 288.2028
y3−NH3 342.2133
y3 359.2397
a2 374.2595
b2−H2O 384.244
b2 402.2546
b3−H2O 497.3275
b3 515.3388
y4 601.3773
b4 644.3814
y5 714.4621
b5 757.4633
A T I E L K A L R
y5 y4 y3 y2 y1
b2 a2 b3 b4 b5
Modification
Acetyl (Protein N−term)
GlyGly (K) no new terminus
Oxidation (M)

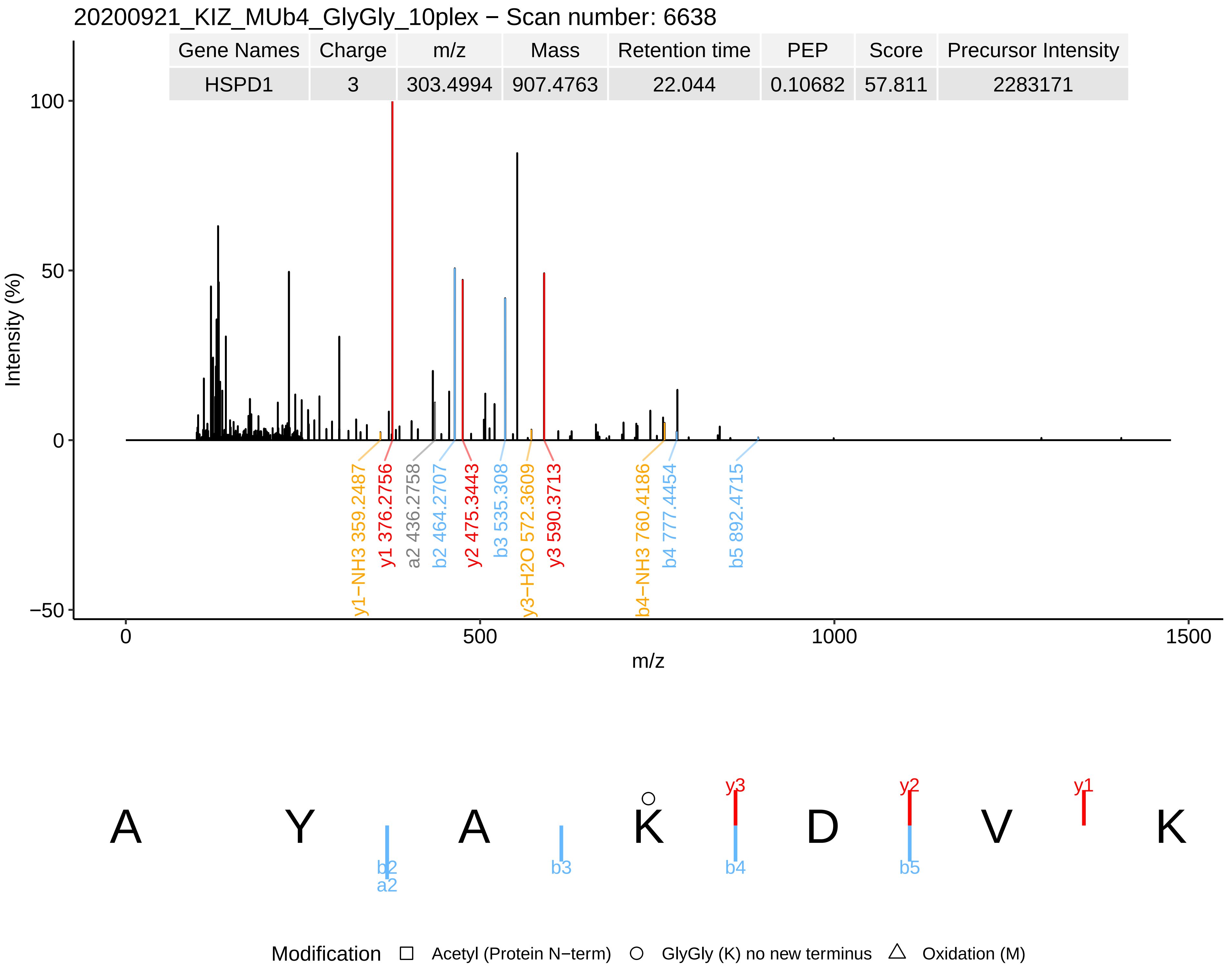

20200921_KIZ_MUb4_GlyGly_10plex − Scan number: 6638
Gene Names | Charge | m/z | Mass | Retention time | PEP | Score | Precursor Intensity
HSPD1 | 3 | 303.4994 | 907.4763 | 22.044 | 0.10682 | 57.811 | 2283171
Intensity (%)
m/z
y1−NH3 359.2487
y1 376.2756
a2 436.2758
b2 464.2707
y2 475.3443
b3 535.308
y3−H2O 572.3609
y3 590.3713
b4−NH3 760.4186
b4 777.4454
b5 892.4715
A Y A K D V K
y3 y2 y1
b2 a2 b3 b4 b5
Modification
Acetyl (Protein N−term)
GlyGly (K) no new terminus
Oxidation (M)

20200921_KIZ_MUb4_GlyGly_10plex − Scan number: 5223

Gene Names	Charge	m/z	Mass	Retention time	PEP	Score	Precursor Intensity
SRSF1	3	381.8791	1142.616	17.072	0.044186	69.825	10760202

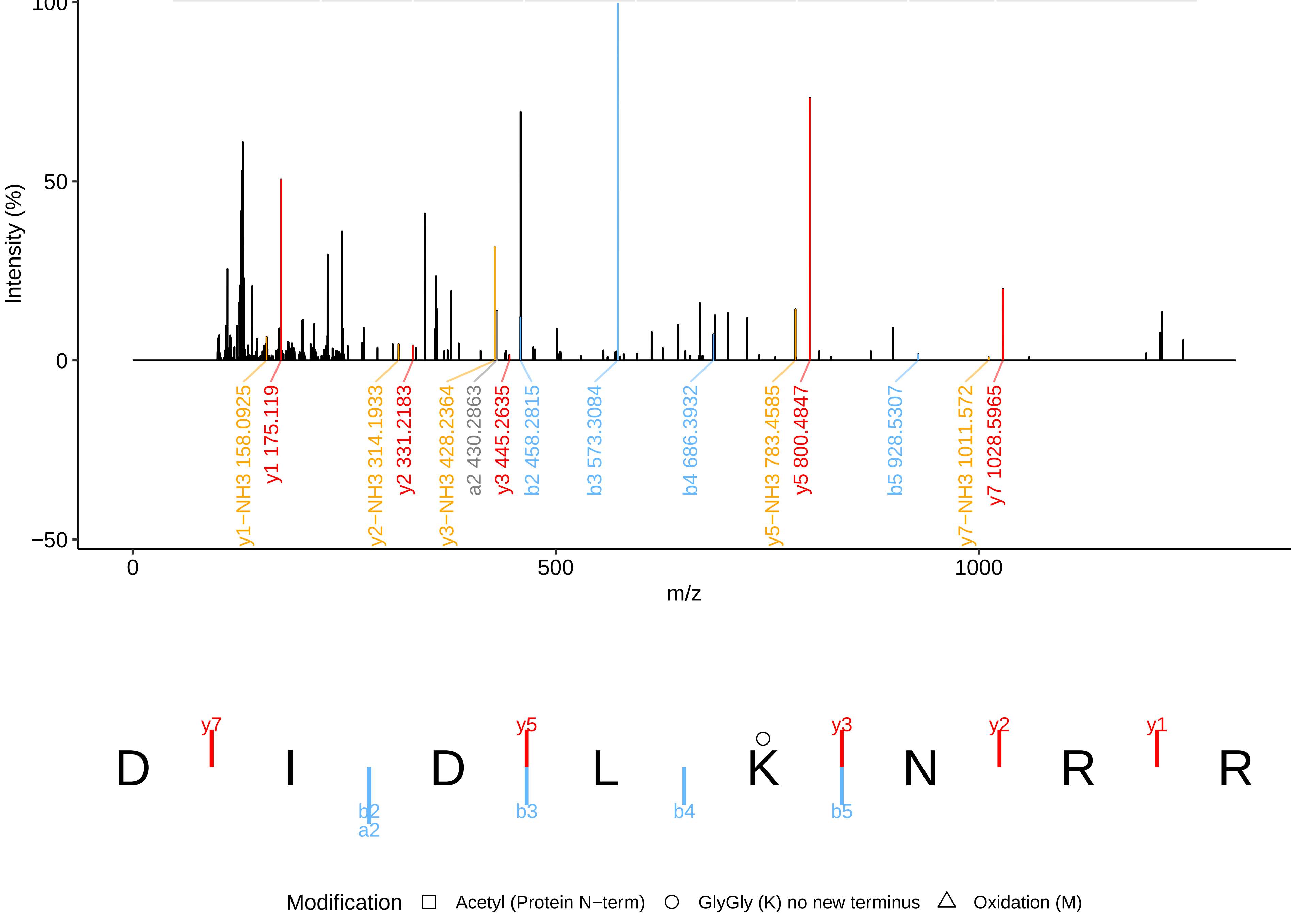

20200921_KIZ_MUb4_GlyGly_10plex_2 − Scan number: 6815

Gene Names	Charge	m/z	Mass	Retention time	PEP	Score	Precursor Intensity
LGR4	3	307.1604	918.4593	25.213	0.076225	60.788	2244116

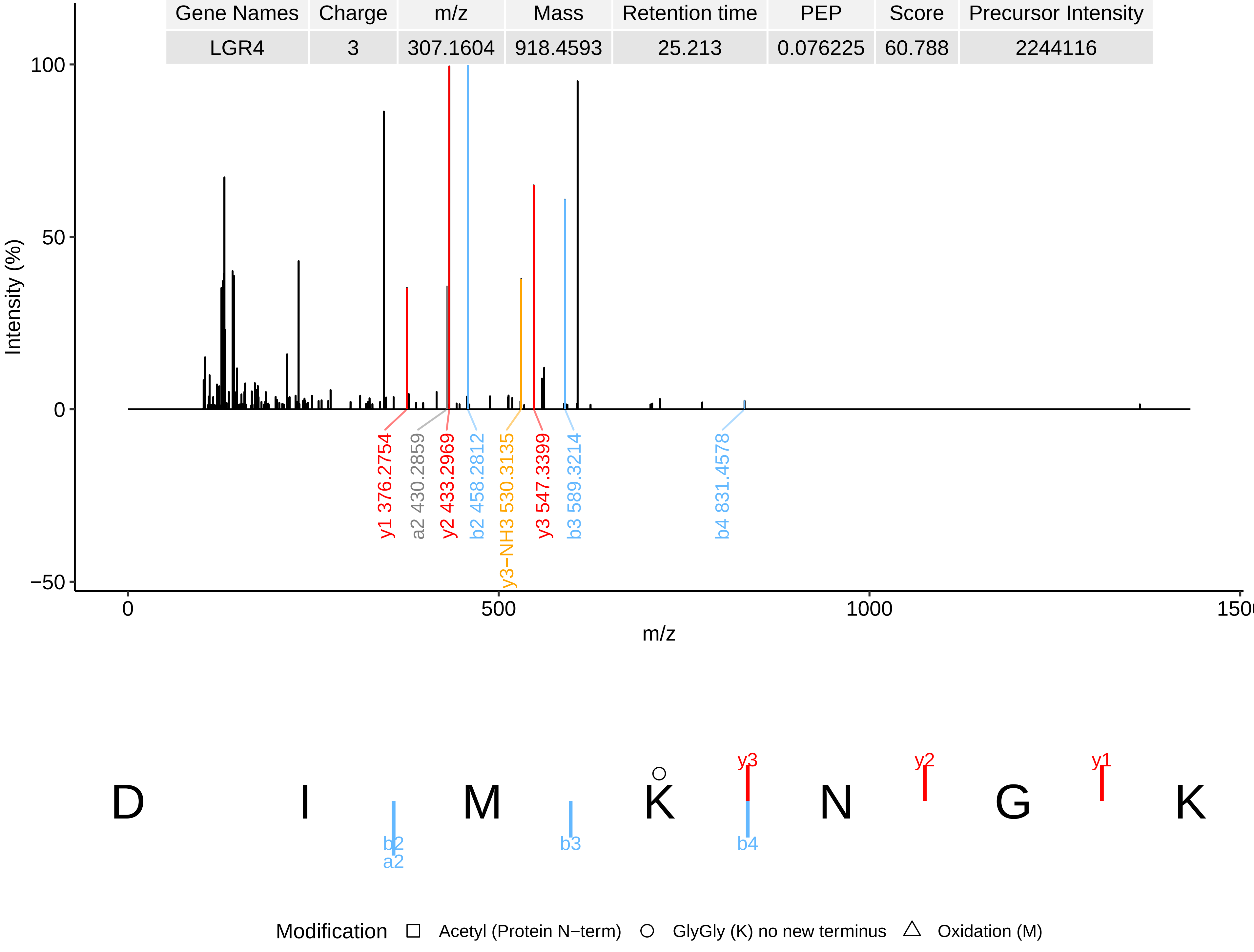

20200924_KIZ_MUb5_GlyGly_1 − Scan number: 6422

Gene Names	Charge	m/z	Mass	Retention time	PEP	Score	Precursor Intensity
LGR4	3	307.1604	918.4593	20.188	0.039162	73.039	9320036

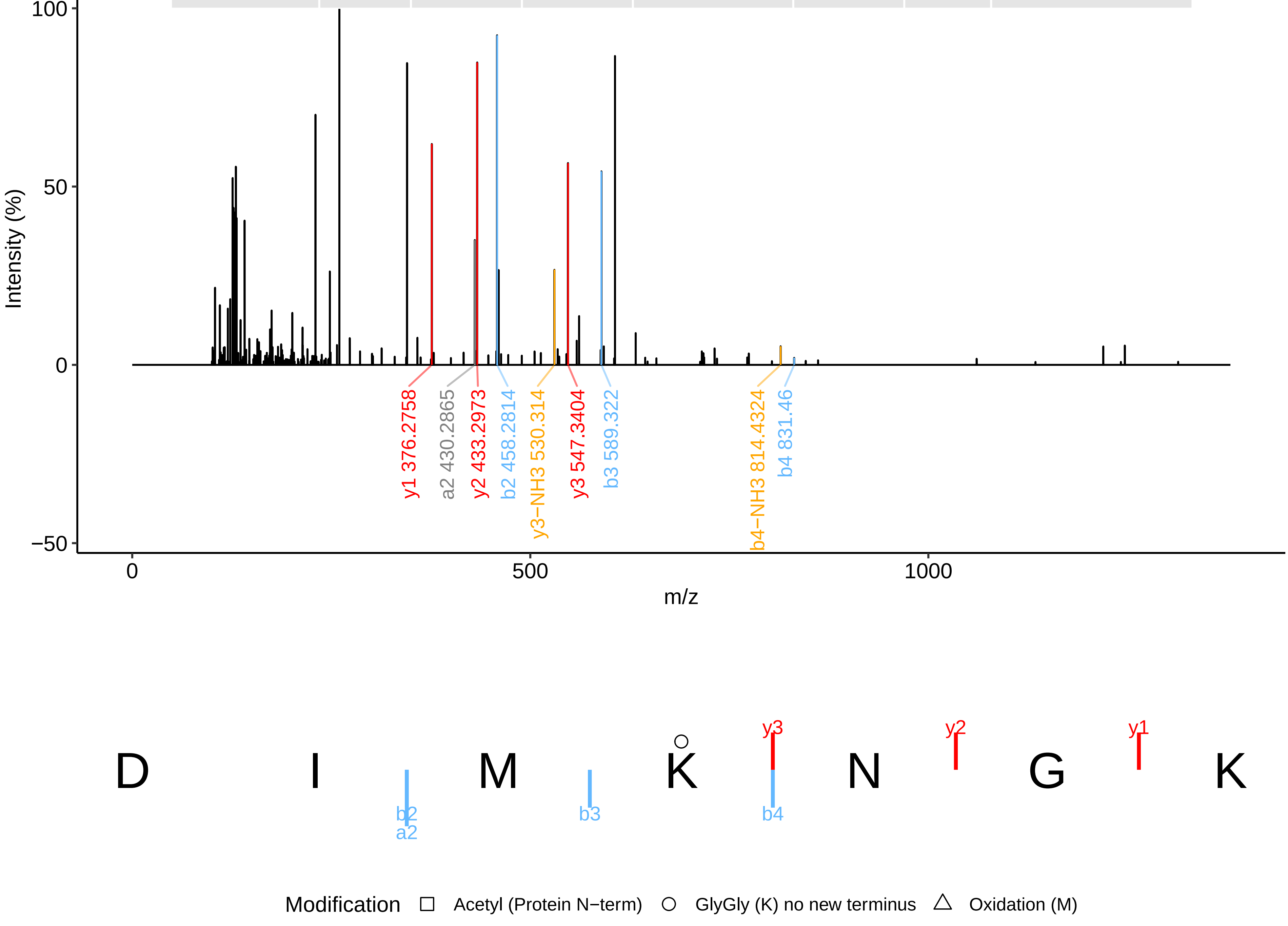

20200924_KIZ_MUb5_GlyGly_2 − Scan number: 5947

Gene Names	Charge	m/z	Mass	Retention time	PEP	Score	Precursor Intensity
LGR4	3	307.1604	918.4593	22.13	0.097263	58.981	2409137

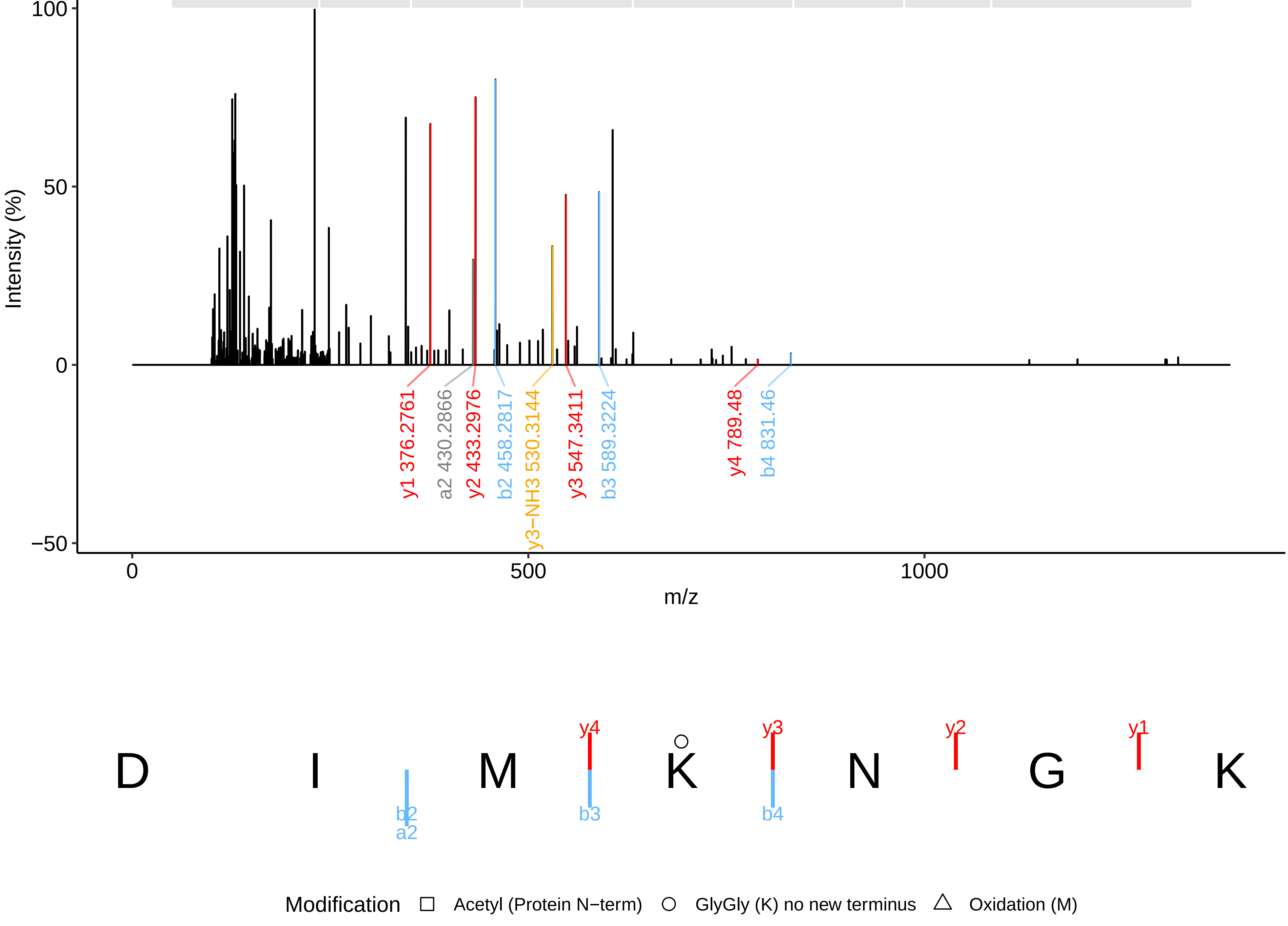

20200921_KIZ_MUb4_GlyGly_10plex − Scan number: 14382

Gene Names	Charge	m/z	Mass	Retention time	PEP	Score	Precursor Intensity
PSMD4	3	300.8553	899.544	51.102	0.085152	58.981	13107122

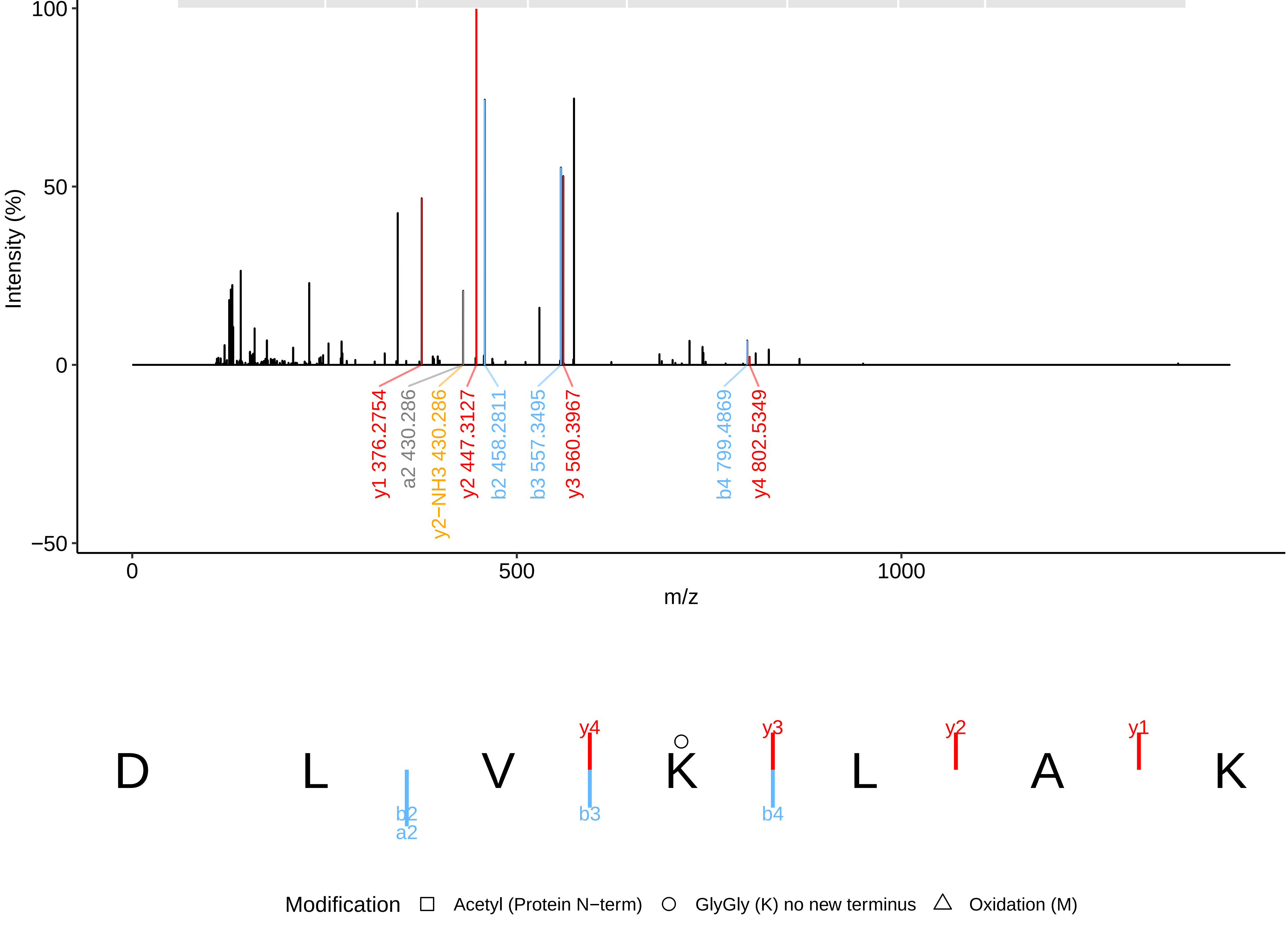

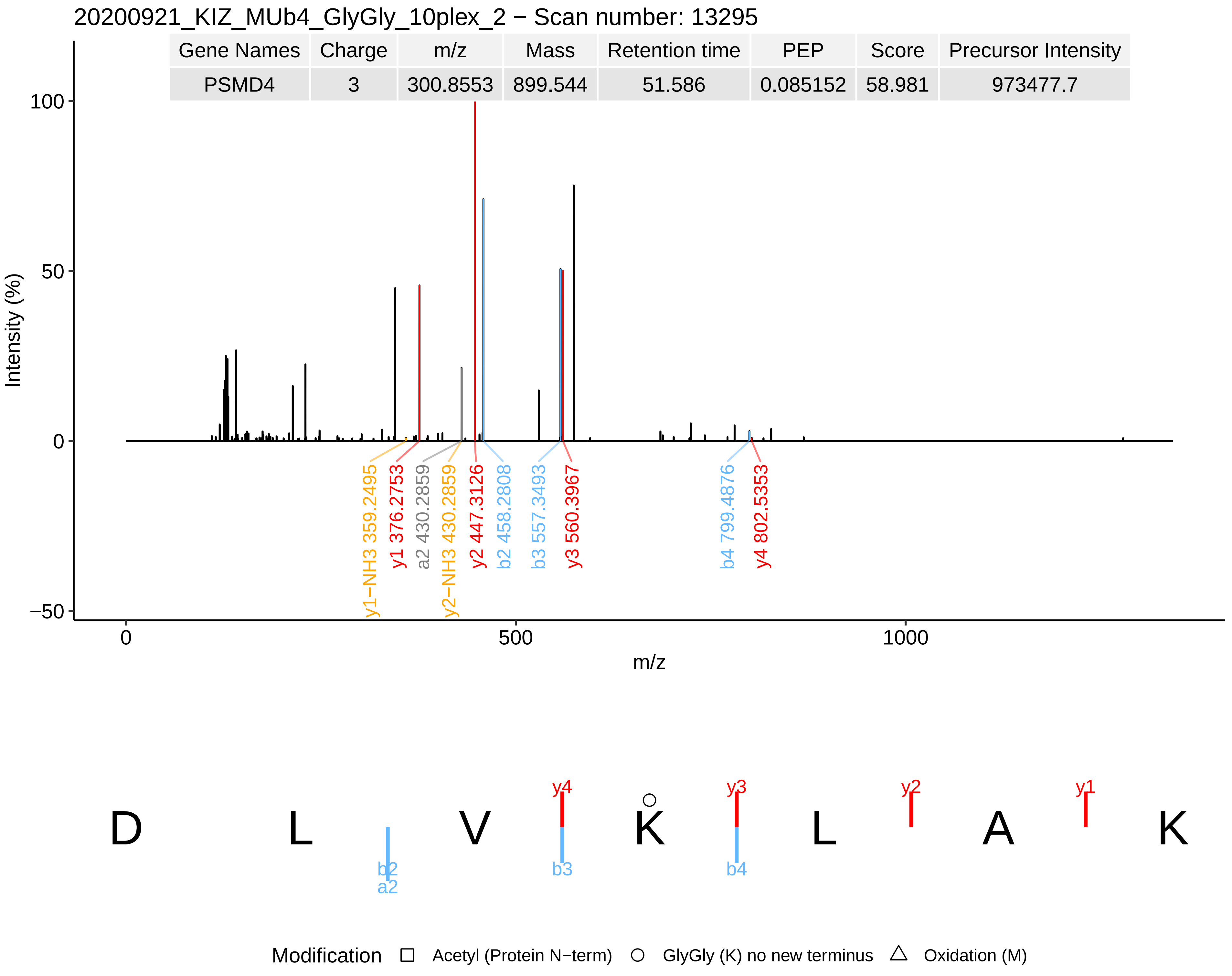
20200921_KIZ_MUb4_GlyGly_10plex_2 − Scan number: 13295
Gene Names | Charge | m/z | Mass | Retention time | PEP | Score | Precursor Intensity
PSMD4 | 3 | 300.8553 | 899.544 | 51.586 | 0.085152 | 58.981 | 973477.7
Intensity (%)
m/z
y1−NH3 359.2495
y1 376.2753
a2 430.2859
y2−NH3 430.2859
y2 447.3126
b2 458.2808
b3 557.3493
y3 560.3967
b4 799.4876
y4 802.5353
D L V K L A K
y4 y3 y2 y1
b2 a2 b3 b4
Modification
Acetyl (Protein N−term)
GlyGly (K) no new terminus
Oxidation (M)

20200924_KIZ_MUb5_GlyGly_1 − Scan number: 13289

Gene Names	Charge	m/z	Mass	Retention time	PEP	Score	Precursor Intensity
PSMD4	3	300.8553	899.544	43.305	0.099829	56.011	15661807

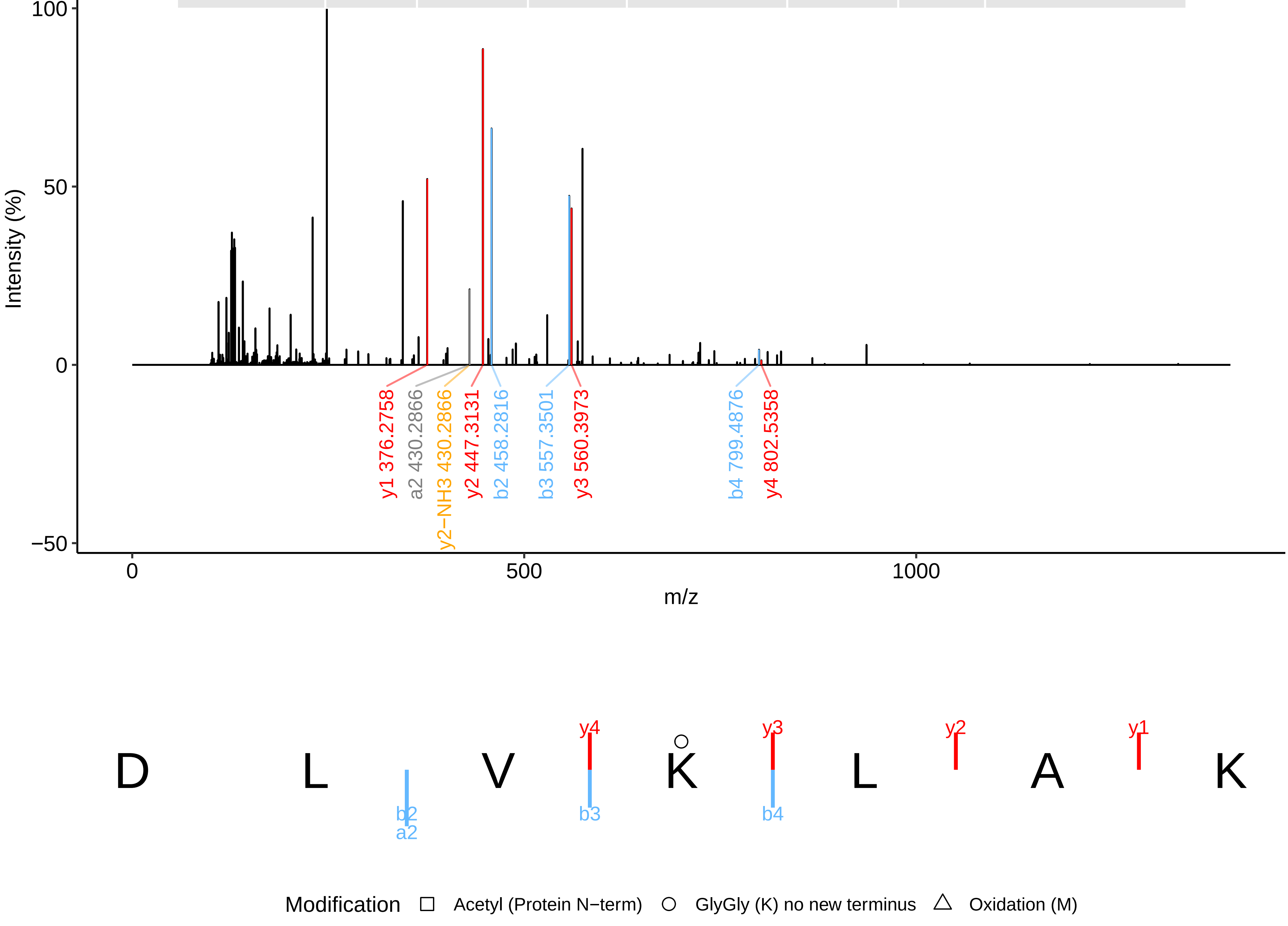

20200924_KIZ_MUb5_GlyGly_1 − Scan number: 13442

Gene Names	Charge	m/z	Mass	Retention time	PEP	Score	Precursor Intensity
PSMD4	3	300.8553	899.544	43.829	0.099829	56.011	24954678

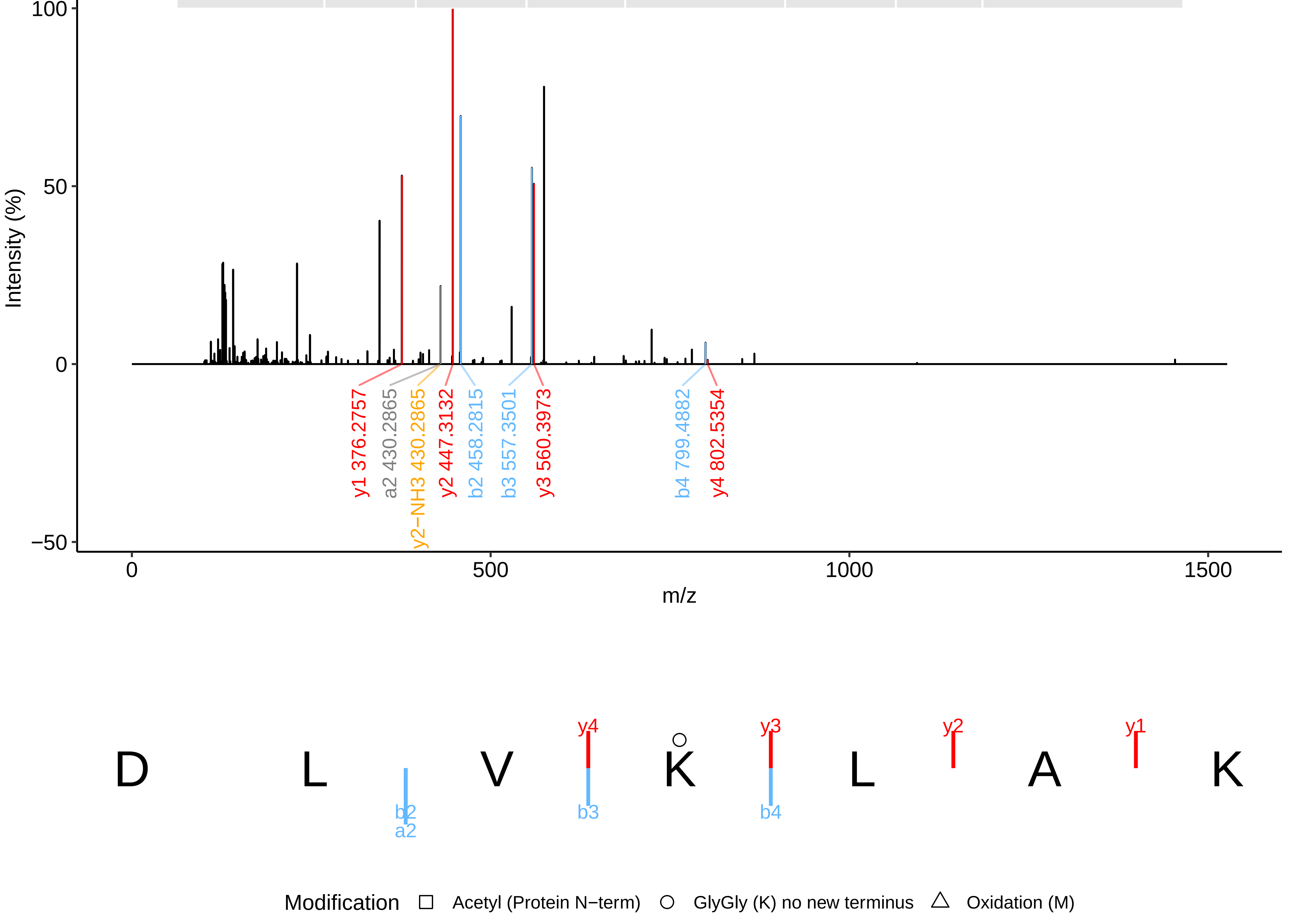

20201118_KIZ_MUb6_GlyGly_1 − Scan number: 13282

Gene Names	Charge	m/z	Mass	Retention time	PEP	Score	Precursor Intensity
PSMD4	3	300.8553	899.544	42.03	0.099829	56.011	12434184

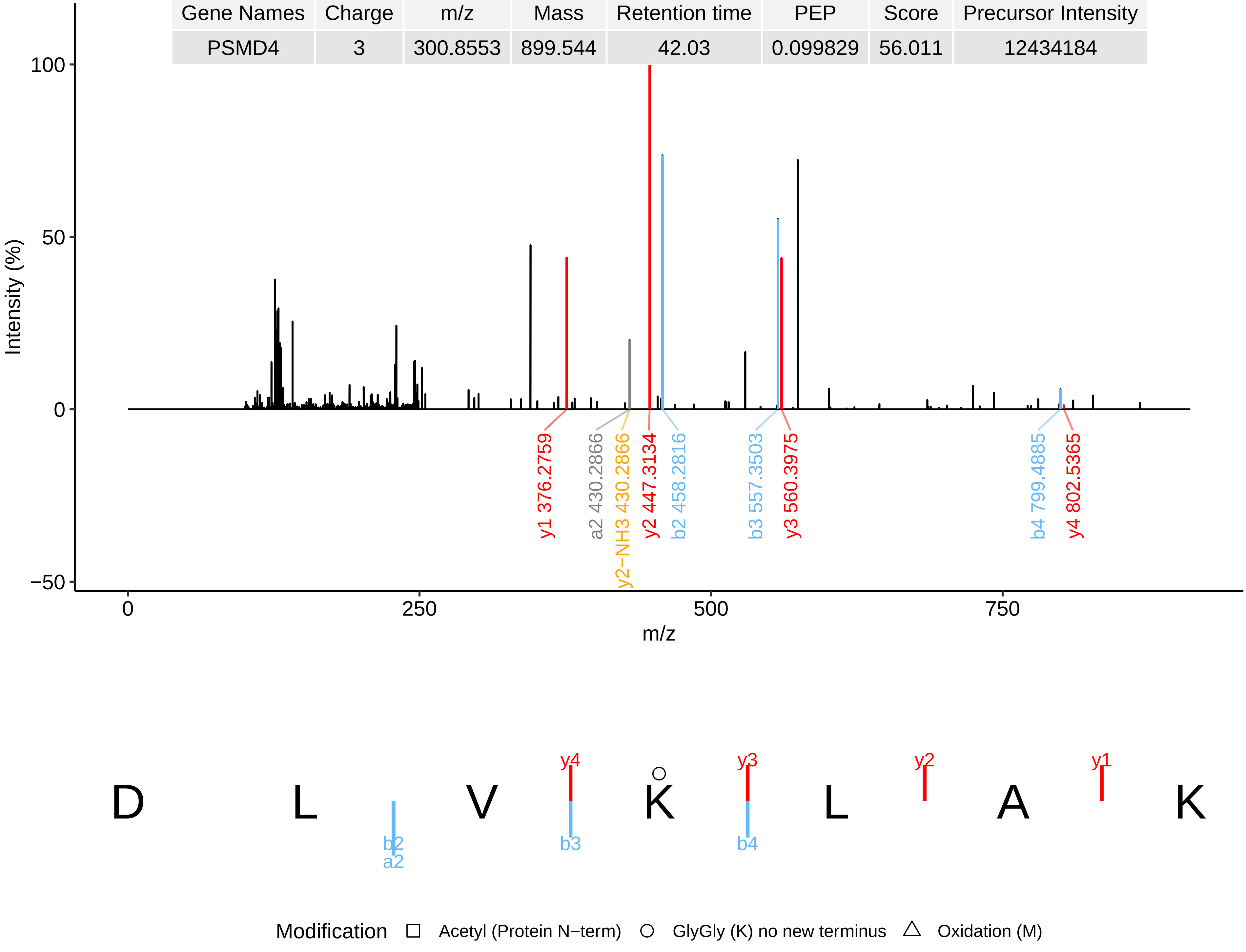

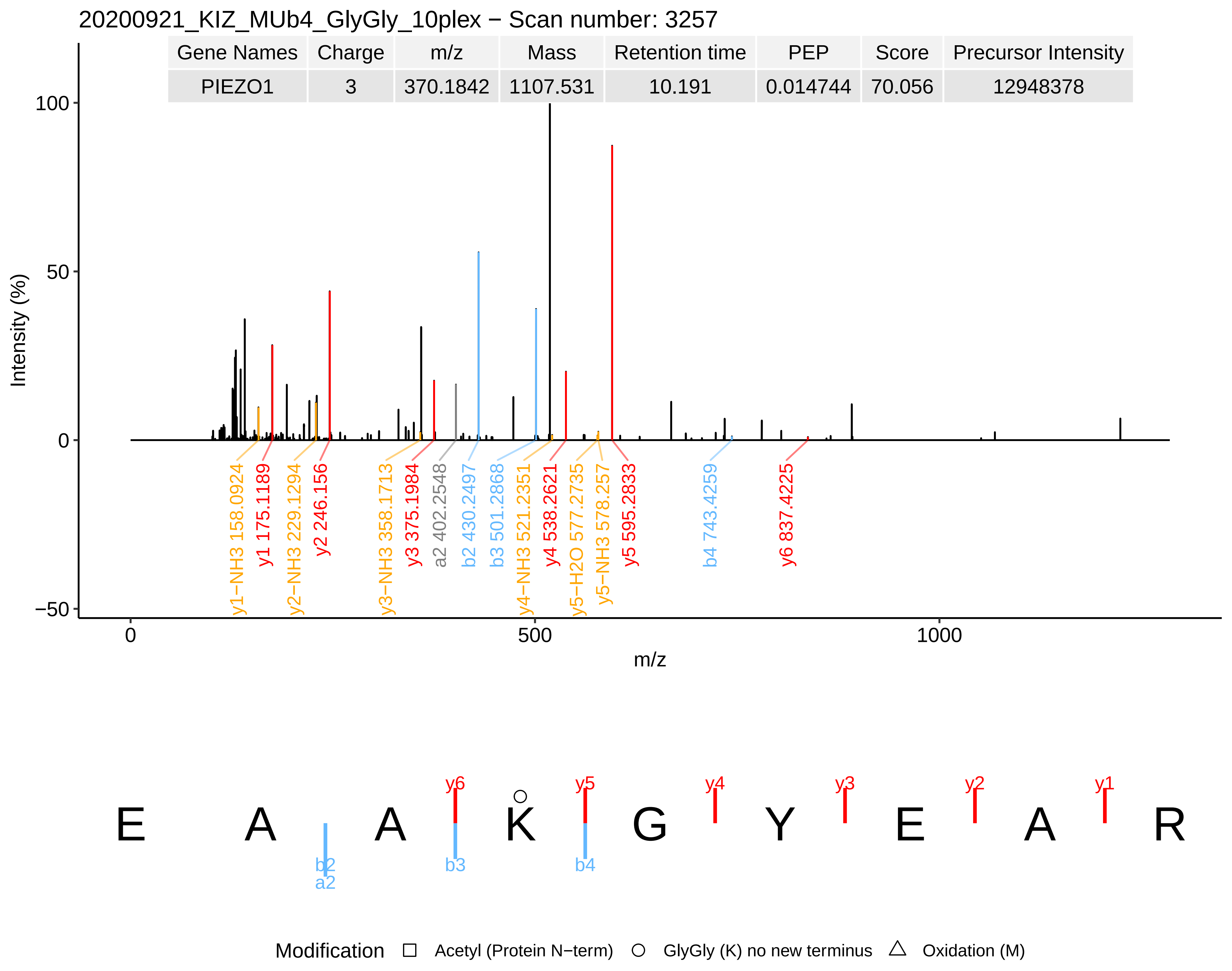

20200921_KIZ_MUb4_GlyGly_10plex − Scan number: 3257
Gene Names | Charge | m/z | Mass | Retention time | PEP | Score | Precursor Intensity
PIEZO1 | 3 | 370.1842 | 1107.531 | 10.191 | 0.014744 | 70.056 | 12948378
Intensity (%)
100
50
0
−50
0
500
1000
m/z
y1−NH3 158.0924
y1 175.1189
y2−NH3 229.1294
y2 246.156
y3−NH3 358.1713
y3 375.1984
a2 402.2548
b2 430.2497
b3 501.2868
y4−NH3 521.2351
y4 538.2621
y5−H2O 577.2735
y5−NH3 578.257
y5 595.2833
b4 743.4259
y6 837.4225
E A A K G Y E A R
y6 y5 y4 y3 y2 y1
b2 a2 b3 b4
Modification
Acetyl (Protein N−term)
GlyGly (K) no new terminus
Oxidation (M)

20200921_KIZ_MUb4_GlyGly_10plex_2 − Scan number: 3142

Gene Names	Charge	m/z	Mass	Retention time	PEP	Score	Precursor Intensity
PIEZO1	3	370.1842	1107.531	10.39	0.0031624	82.749	4459585

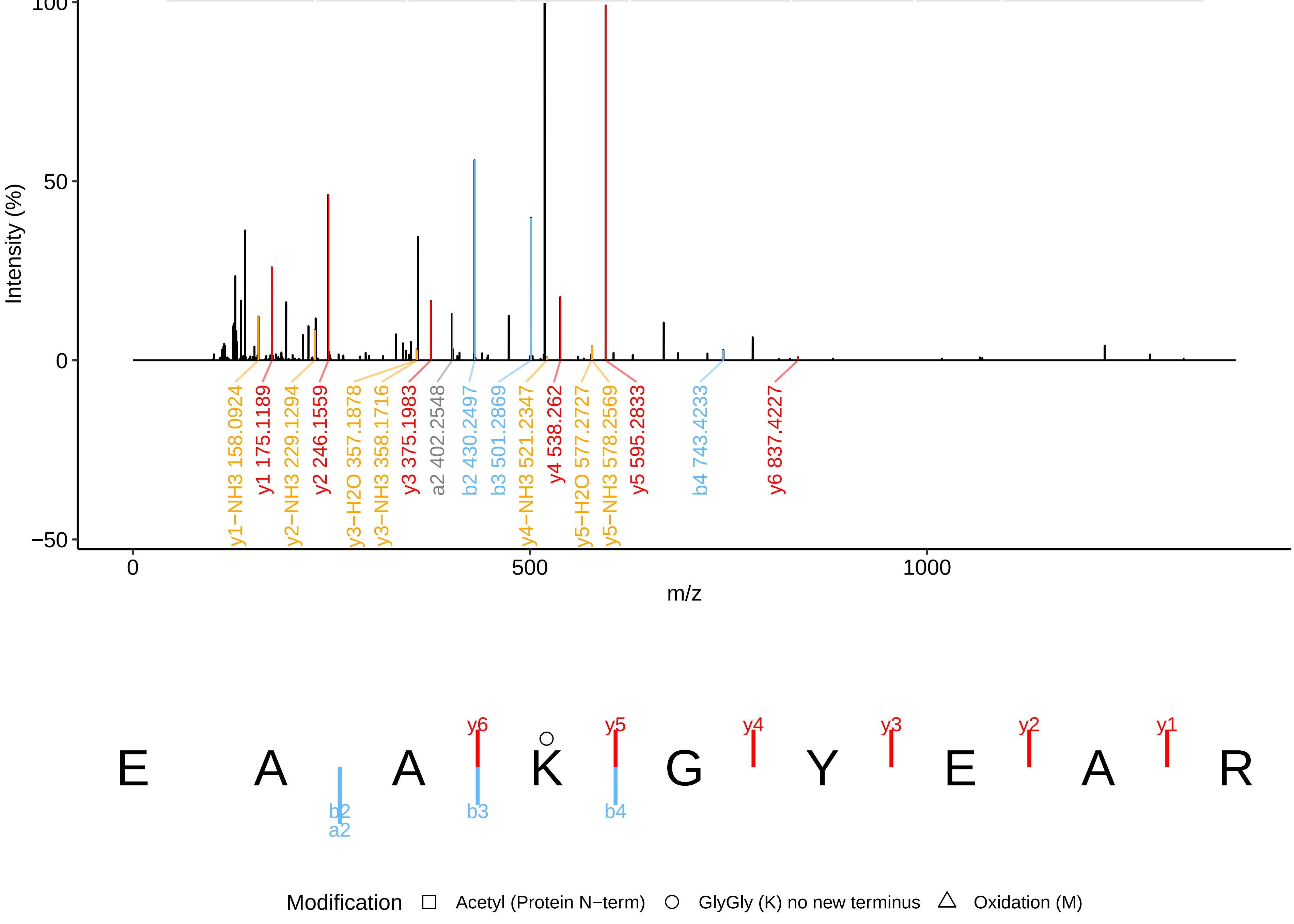

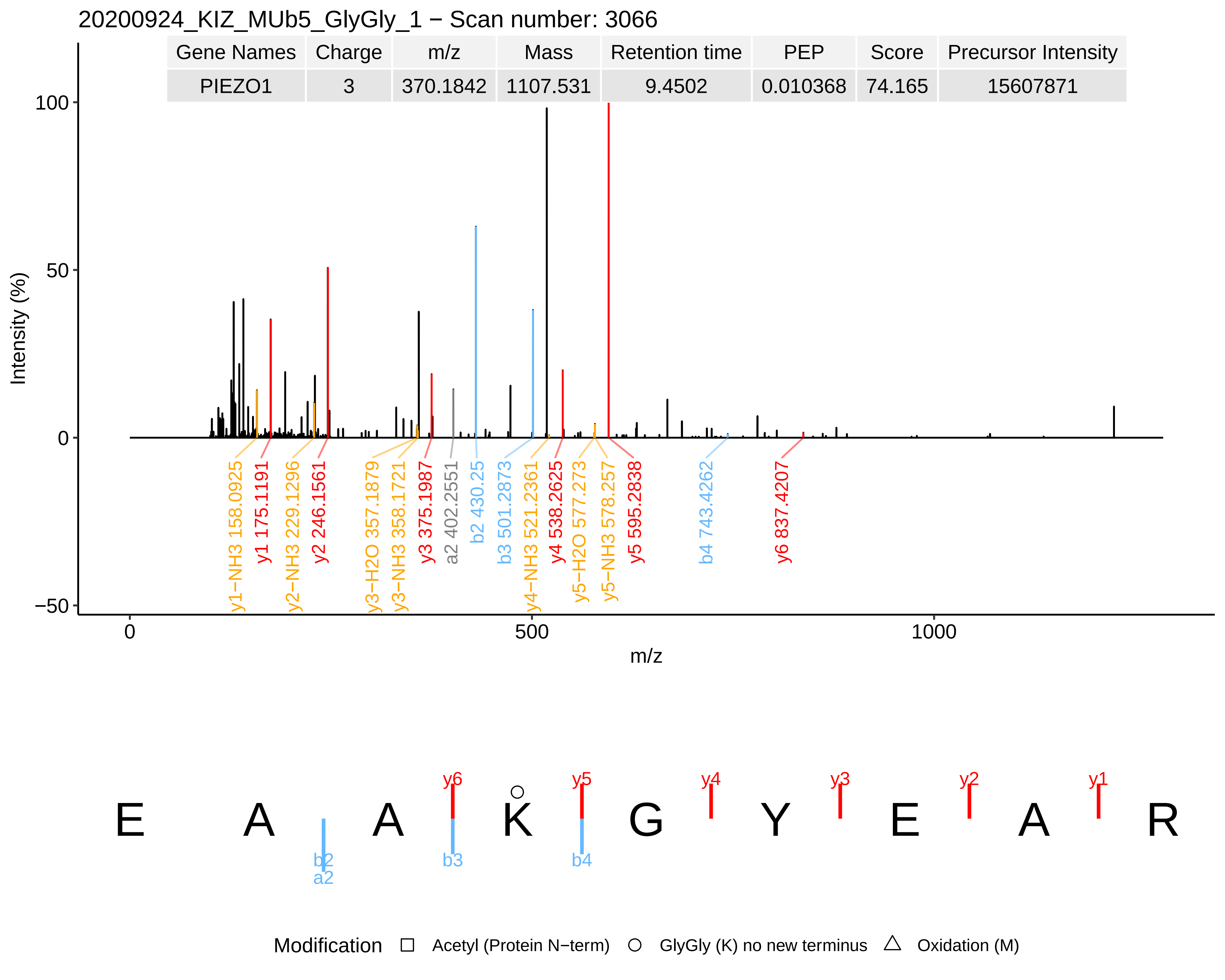
20200924_KIZ_MUb5_GlyGly_1 − Scan number: 3066
Gene Names | Charge | m/z | Mass | Retention time | PEP | Score | Precursor Intensity
PIEZO1 | 3 | 370.1842 | 1107.531 | 9.4502 | 0.010368 | 74.165 | 15607871
Intensity (%)
m/z
y1−NH3 158.0925
y1 175.1191
y2−NH3 229.1296
y2 246.1561
y3−H2O 357.1879
y3−NH3 358.1721
y3 375.1987
a2 402.2551
b2 430.25
b3 501.2873
y4−NH3 521.2361
y4 538.2625
y5−H2O 577.273
y5−NH3 578.257
y5 595.2838
b4 743.4262
y6 837.4207
E A A K G Y E A R
Modification
Acetyl (Protein N−term)
GlyGly (K) no new terminus
Oxidation (M)

20200924_KIZ_MUb5_GlyGly_2 − Scan number: 2790

Gene Names	Charge	m/z	Mass	Retention time	PEP	Score	Precursor Intensity
PIEZO1	3	370.1842	1107.531	9.9677	0.0492	57.55	2618667

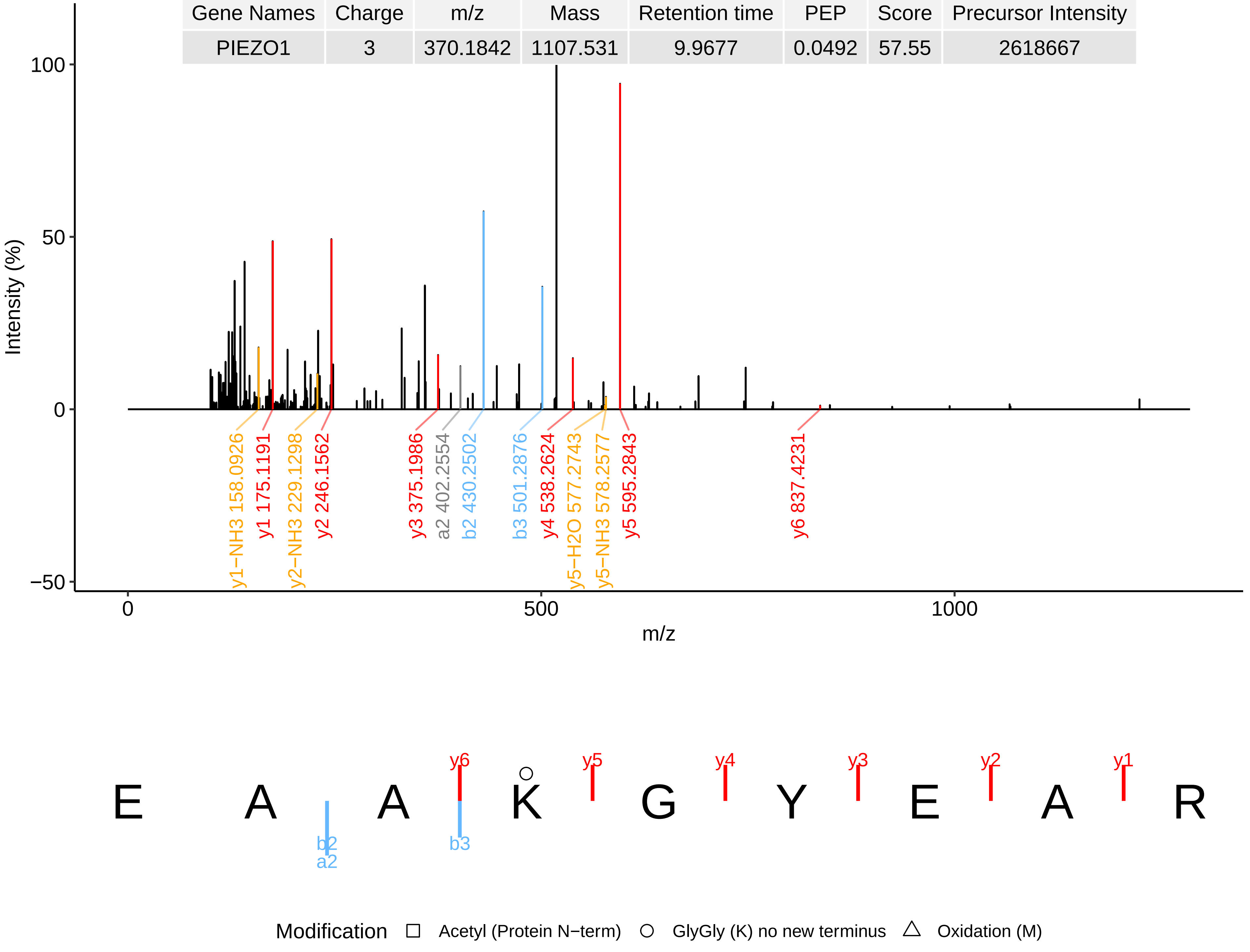

20201118_KIZ_MUb6_GlyGly_1 − Scan number: 3033

Gene Names	Charge	m/z	Mass	Retention time	PEP	Score	Precursor Intensity
PIEZO1	3	370.1842	1107.531	9.3238	0.014744	70.056	12204581

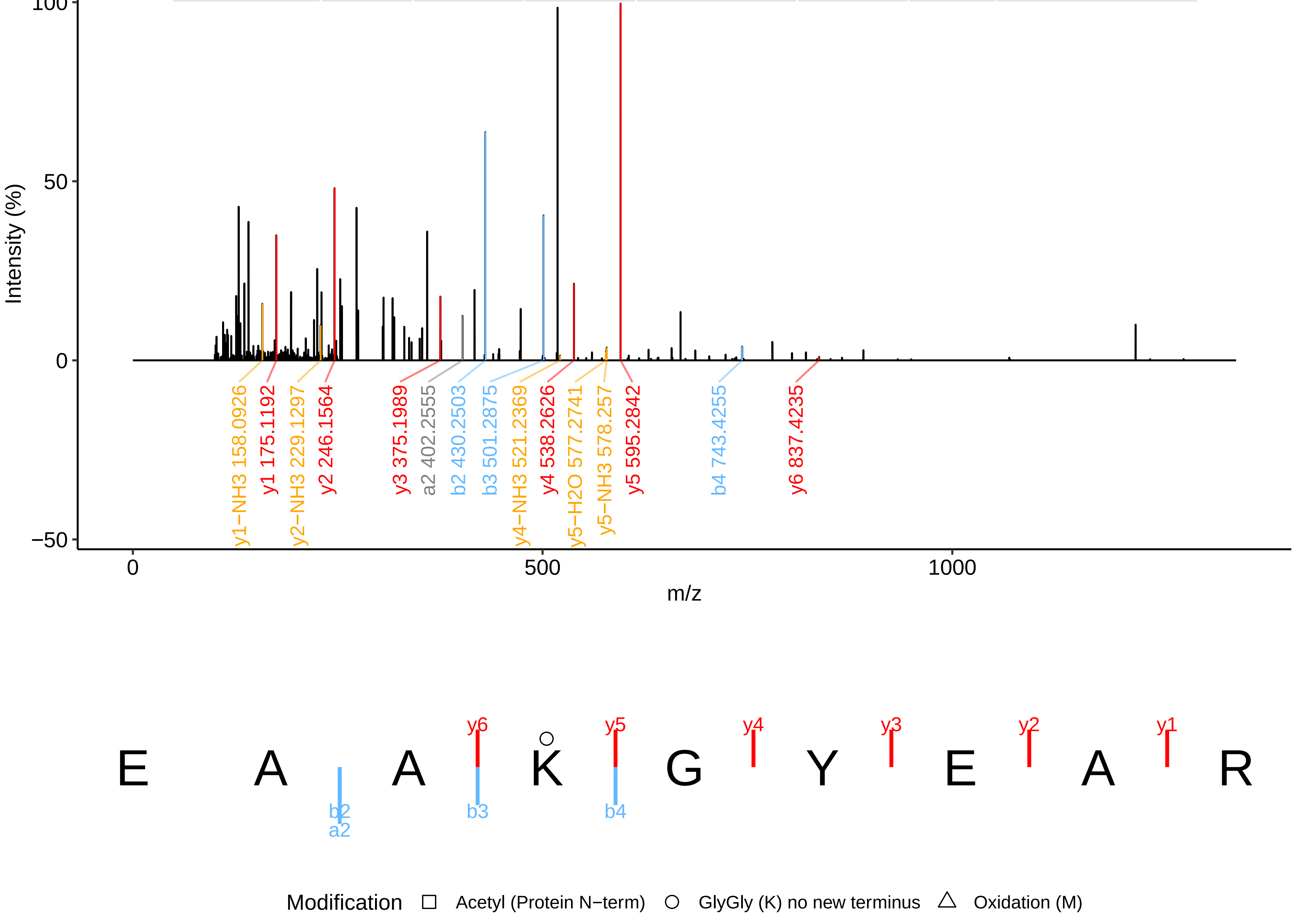

20201118_KIZ_MUb6_GlyGly_2 − Scan number: 3257

Gene Names	Charge	m/z	Mass	Retention time	PEP	Score	Precursor Intensity
PIEZO1	3	370.1842	1107.531	10.616	0.033077	60.892	2728251

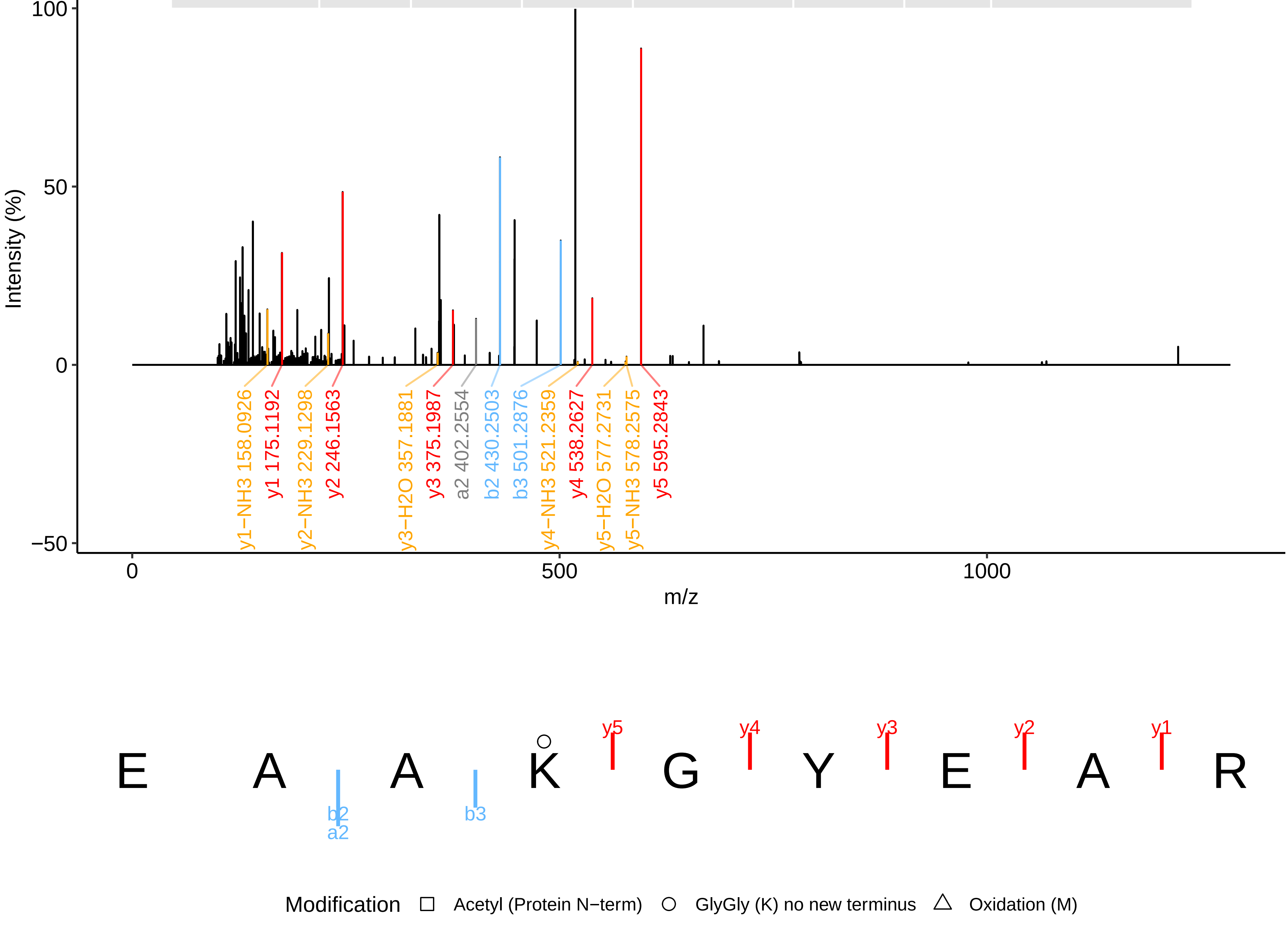

20200921_KIZ_MUb4_GlyGly_10plex − Scan number: 8029

Gene Names	Charge	m/z	Mass	Retention time	PEP	Score	Precursor Intensity
UBR5	3	372.8718	1115.593	27.137	0.0022394	88.181	8269474

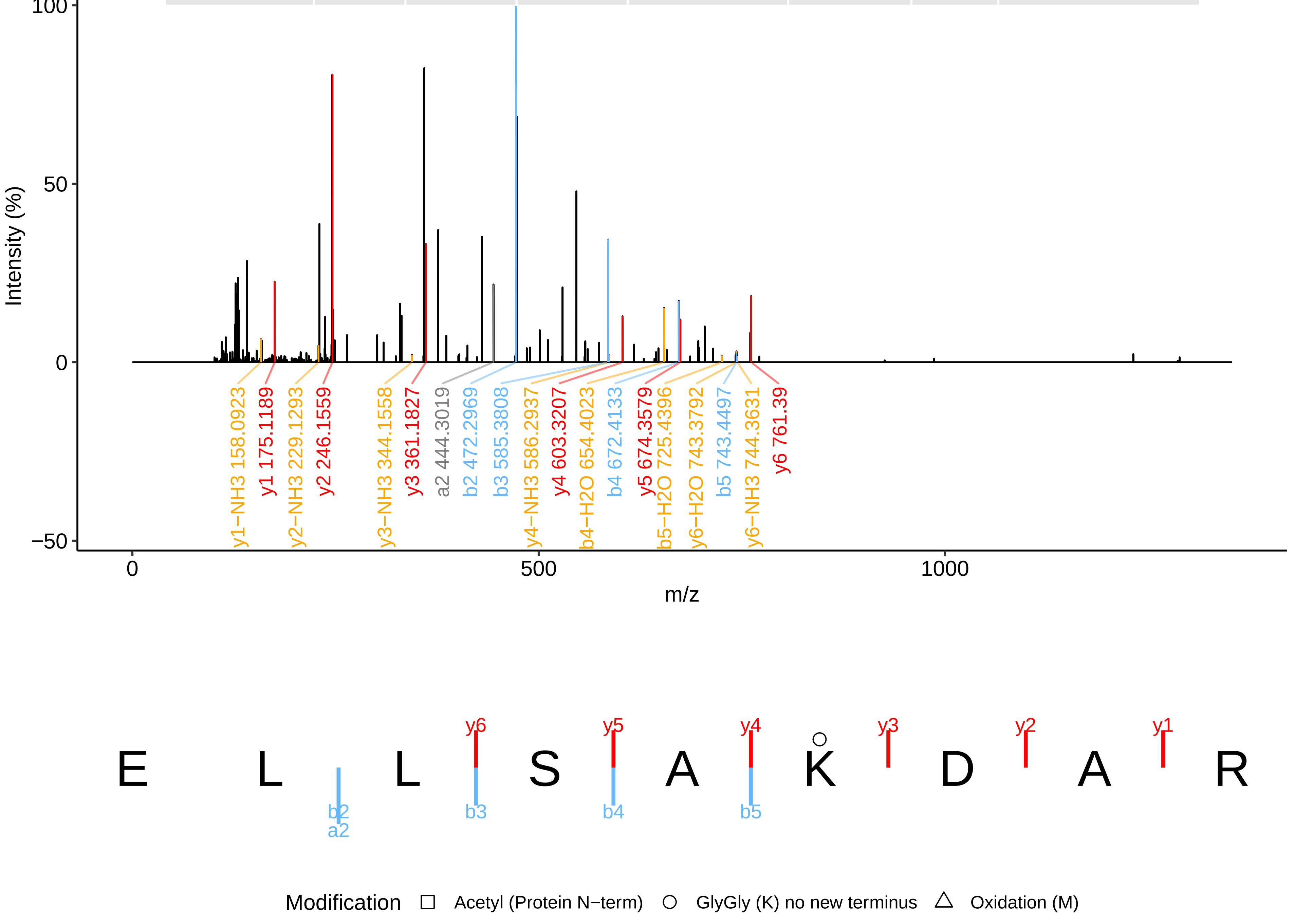

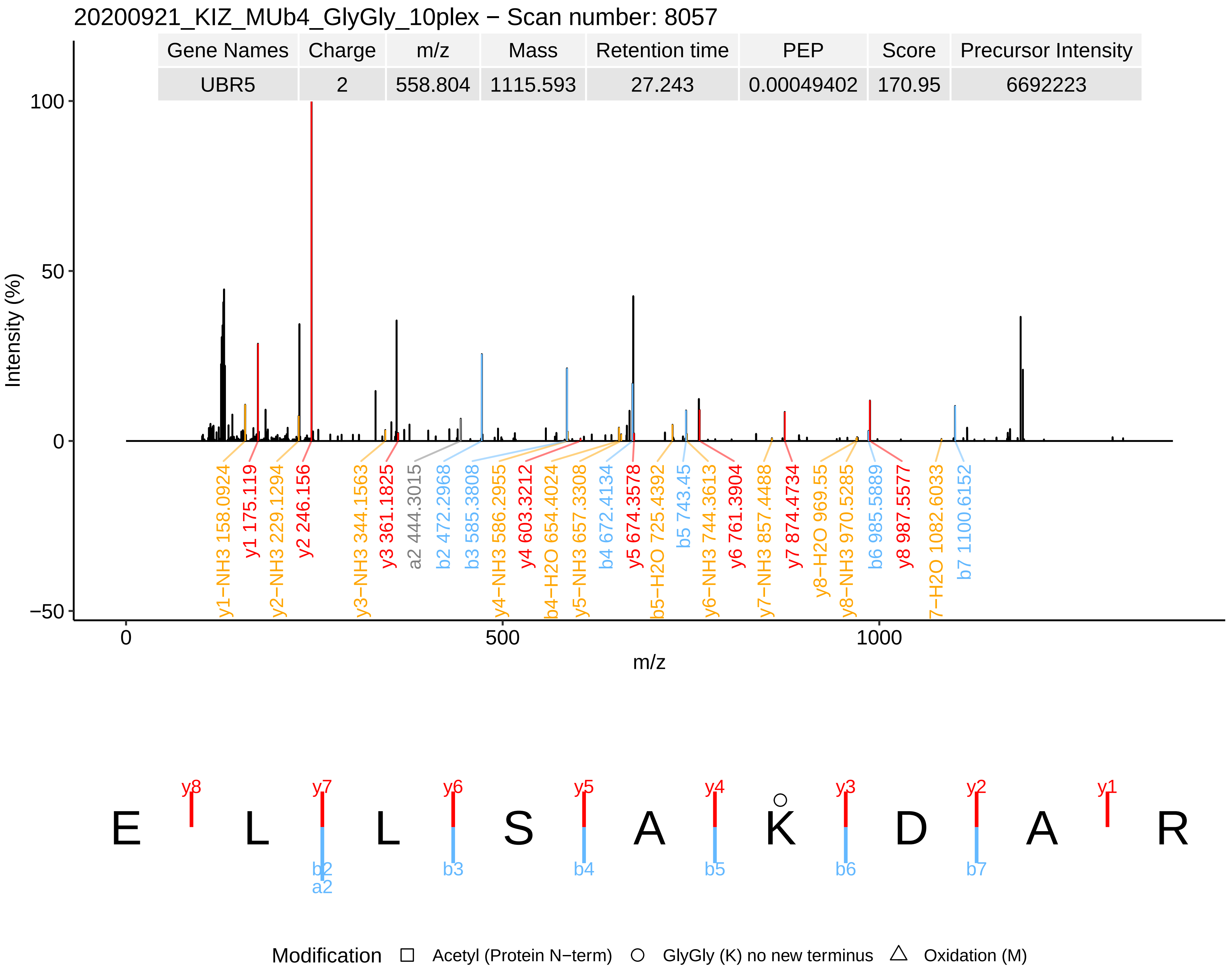

20200921_KIZ_MUb4_GlyGly_10plex − Scan number: 8057
Gene Names | Charge | m/z | Mass | Retention time | PEP | Score | Precursor Intensity
UBR5 | 2 | 558.804 | 1115.593 | 27.243 | 0.00049402 | 170.95 | 6692223
Intensity (%)
m/z
y1−NH3 158.0924
y1 175.119
y2−NH3 229.1294
y2 246.156
y3−NH3 344.1563
y3 361.1825
a2 444.3015
b2 472.2968
b3 585.3808
y4−NH3 586.2955
y4 603.3212
b4−H2O 654.4024
y5−NH3 657.3308
b4 672.4134
y5 674.3578
b5−H2O 725.4392
b5 743.45
y6−NH3 744.3613
y6 761.3904
y7−NH3 857.4488
y7 874.4734
y8−H2O 969.55
y8−NH3 970.5285
b6 985.5889
y8 987.5577
7−H2O 1082.6033
b7 1100.6152
E L L S A K D A R
y8 y7 y6 y5 y4 y3 y2 y1
b2 a2 b3 b4 b5 b6 b7
Modification
Acetyl (Protein N−term)
GlyGly (K) no new terminus
Oxidation (M)

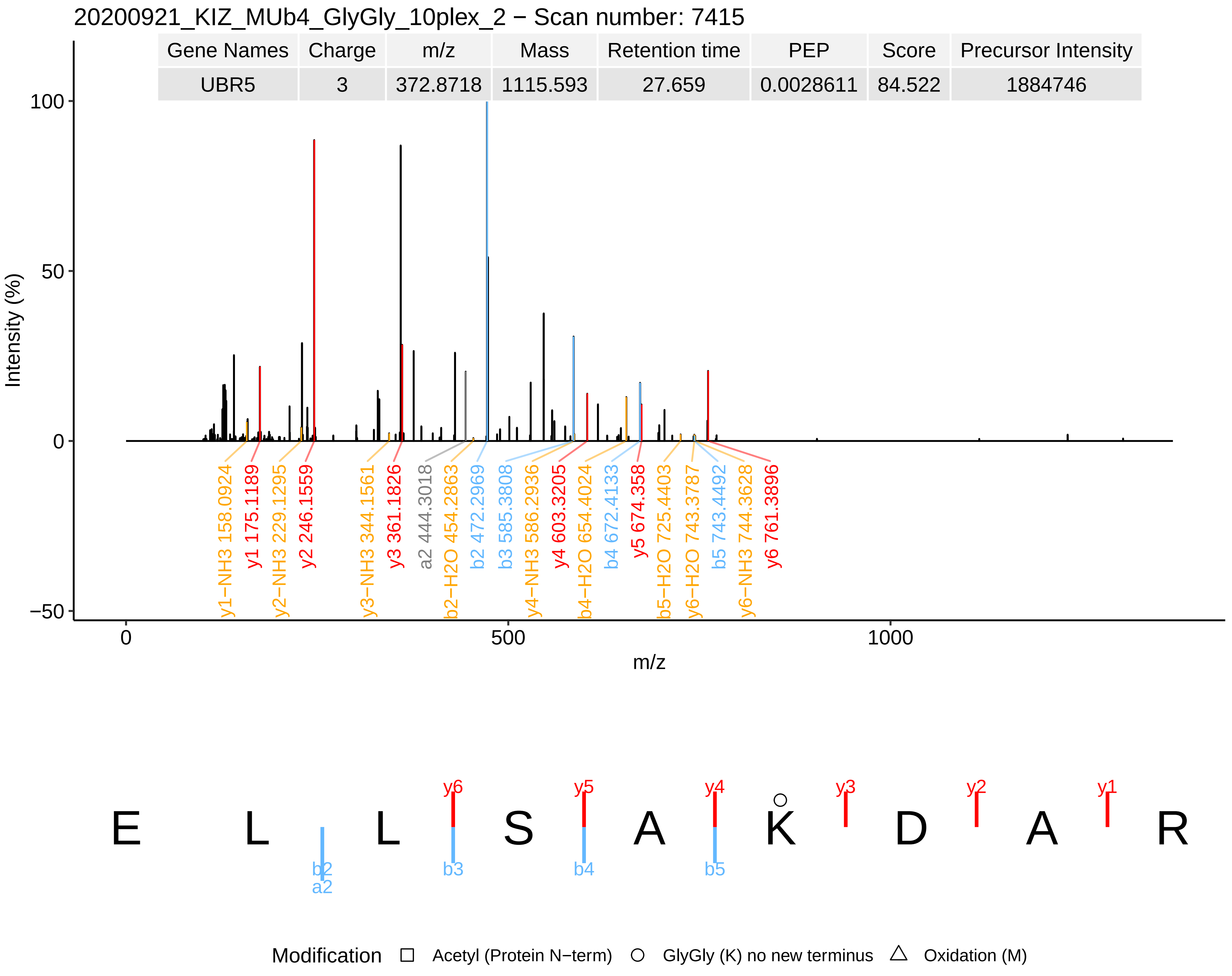
20200921_KIZ_MUb4_GlyGly_10plex_2 − Scan number: 7415
Gene Names
Charge
m/z
Mass
Retention time
PEP
Score
Precursor Intensity
UBR5
3
372.8718
1115.593
27.659
0.0028611
84.522
1884746
Intensity (%)
m/z
y1−NH3 158.0924
y1 175.1189
y2−NH3 229.1295
y2 246.1559
y3−NH3 344.1561
y3 361.1826
a2 444.3018
b2−H2O 454.2863
b2 472.2969
b3 585.3808
y4−NH3 586.2936
y4 603.3205
b4−H2O 654.4024
b4 672.4133
y5 674.358
b5−H2O 725.4403
y6−H2O 743.3787
b5 743.4492
y6−NH3 744.3628
y6 761.3896
E L L S A K D A R
Modification
Acetyl (Protein N−term)
GlyGly (K) no new terminus
Oxidation (M)

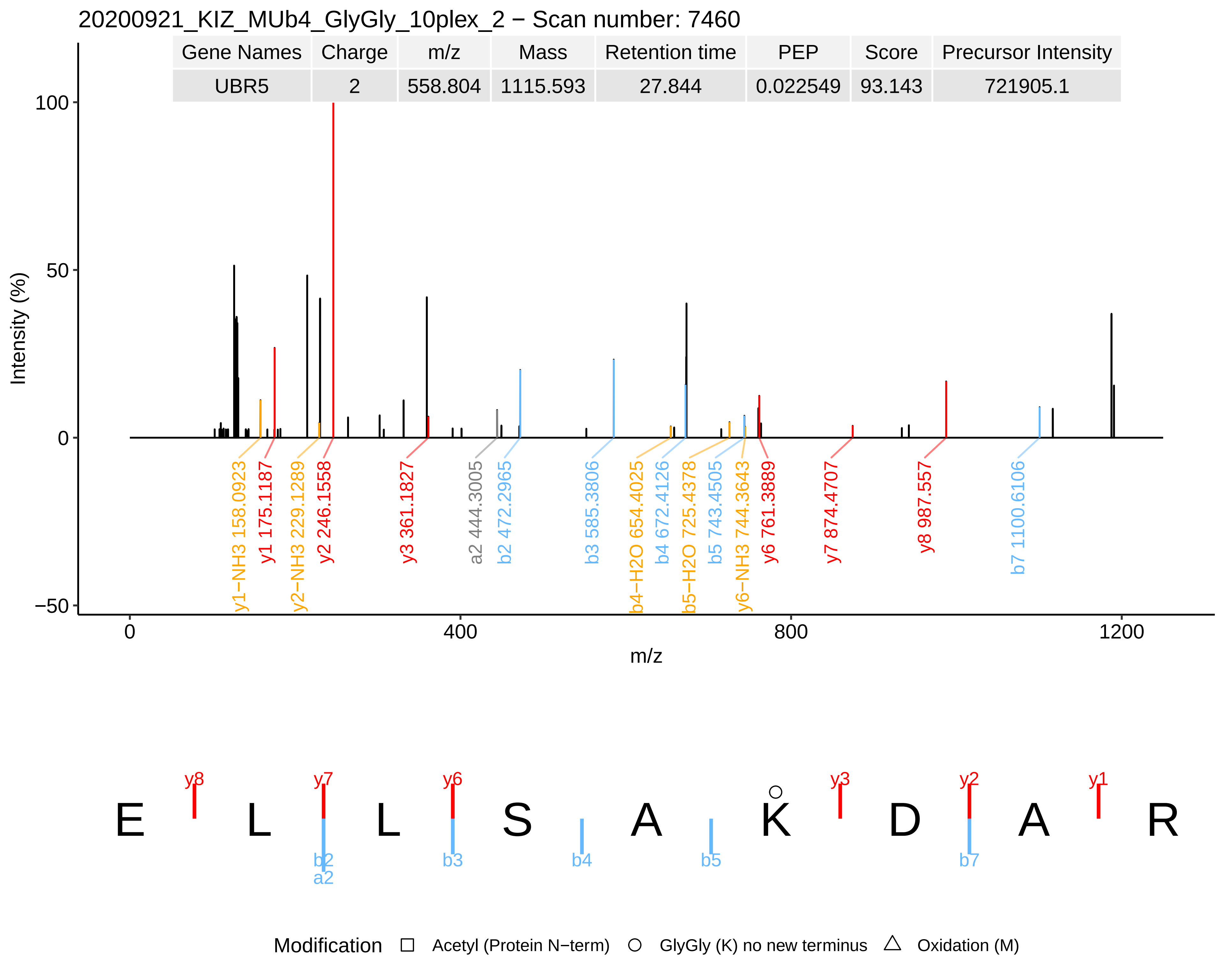

20200921_KIZ_MUb4_GlyGly_10plex_2 − Scan number: 7460
Gene Names | Charge | m/z | Mass | Retention time | PEP | Score | Precursor Intensity
UBR5 | 2 | 558.804 | 1115.593 | 27.844 | 0.022549 | 93.143 | 721905.1
Intensity (%)
m/z
y1−NH3 158.0923
y1 175.1187
y2−NH3 229.1289
y2 246.1558
y3 361.1827
a2 444.3005
b2 472.2965
b3 585.3806
b4−H2O 654.4025
b4 672.4126
b5−H2O 725.4378
b5 743.4505
y6−NH3 744.3643
y6 761.3889
y7 874.4707
y8 987.557
b7 1100.6106
E L L S A K D A R
Modification
Acetyl (Protein N−term)
GlyGly (K) no new terminus
Oxidation (M)

20200924_KIZ_MUb5_GlyGly_1 − Scan number: 6879

Gene Names	Charge	m/z	Mass	Retention time	PEP	Score	Precursor Intensity
UBR5	3	372.8718	1115.593	21.746	0.0031624	82.749	11988751

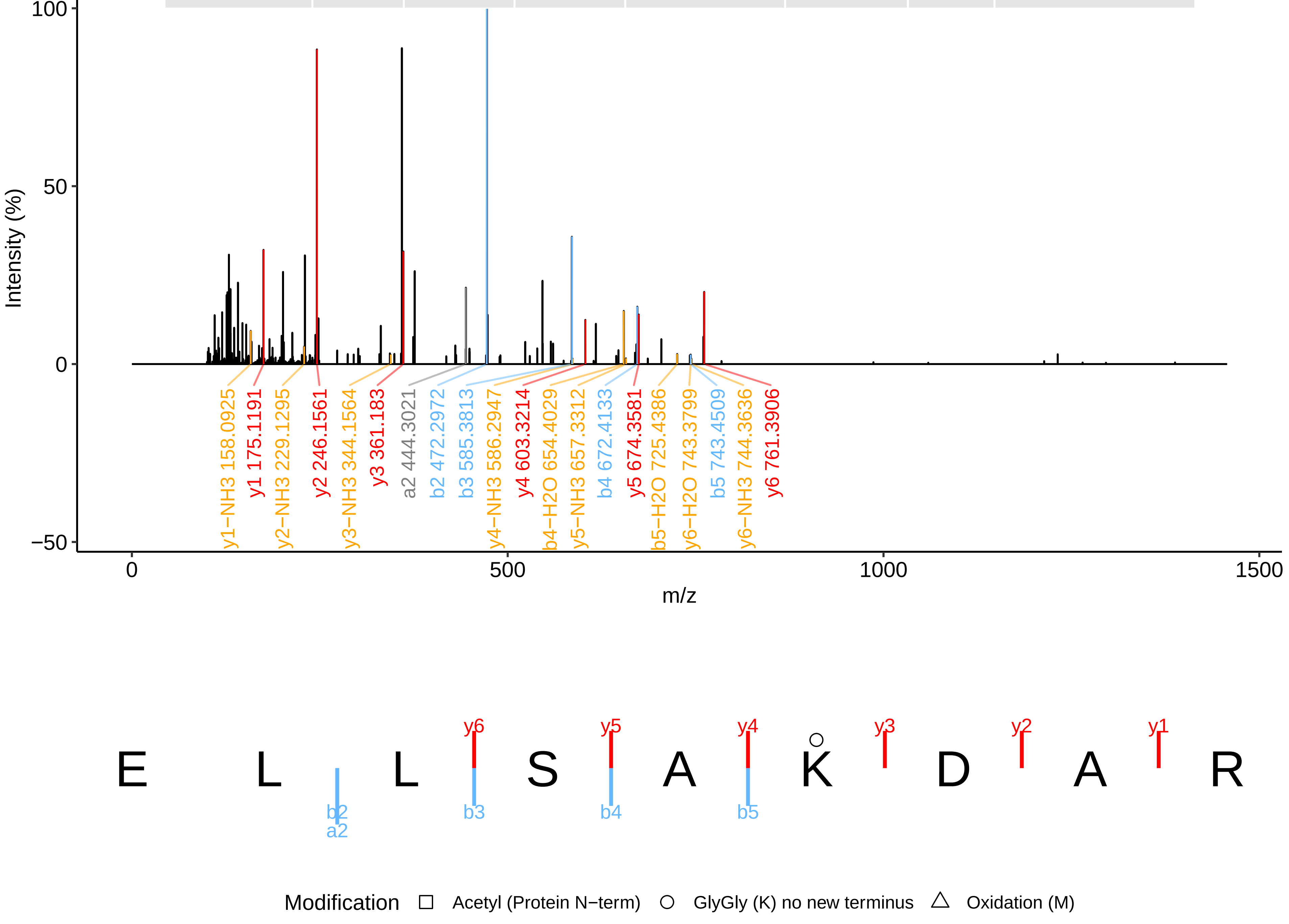

20200924_KIZ_MUb5_GlyGly_1 − Scan number: 7033

Gene Names	Charge	m/z	Mass	Retention time	PEP	Score	Precursor Intensity
UBR5	3	372.8718	1115.593	22.263	0.0016611	91.584	59555636

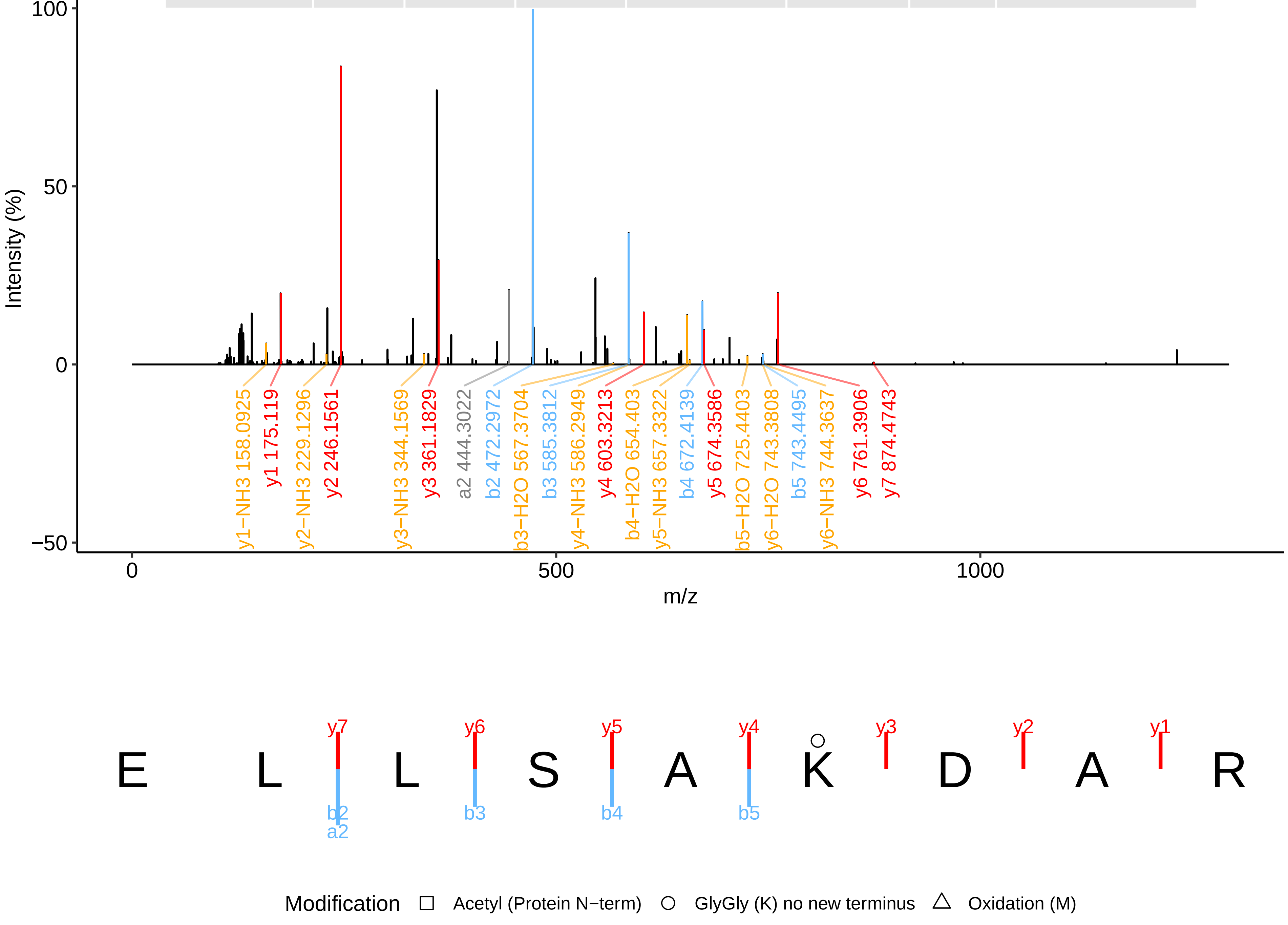

20200924_KIZ_MUb5_GlyGly_1 − Scan number: 6951

Gene Names	Charge	m/z	Mass	Retention time	PEP	Score	Precursor Intensity
UBR5	2	558.804	1115.593	21.988	0.0021629	128.36	6012714

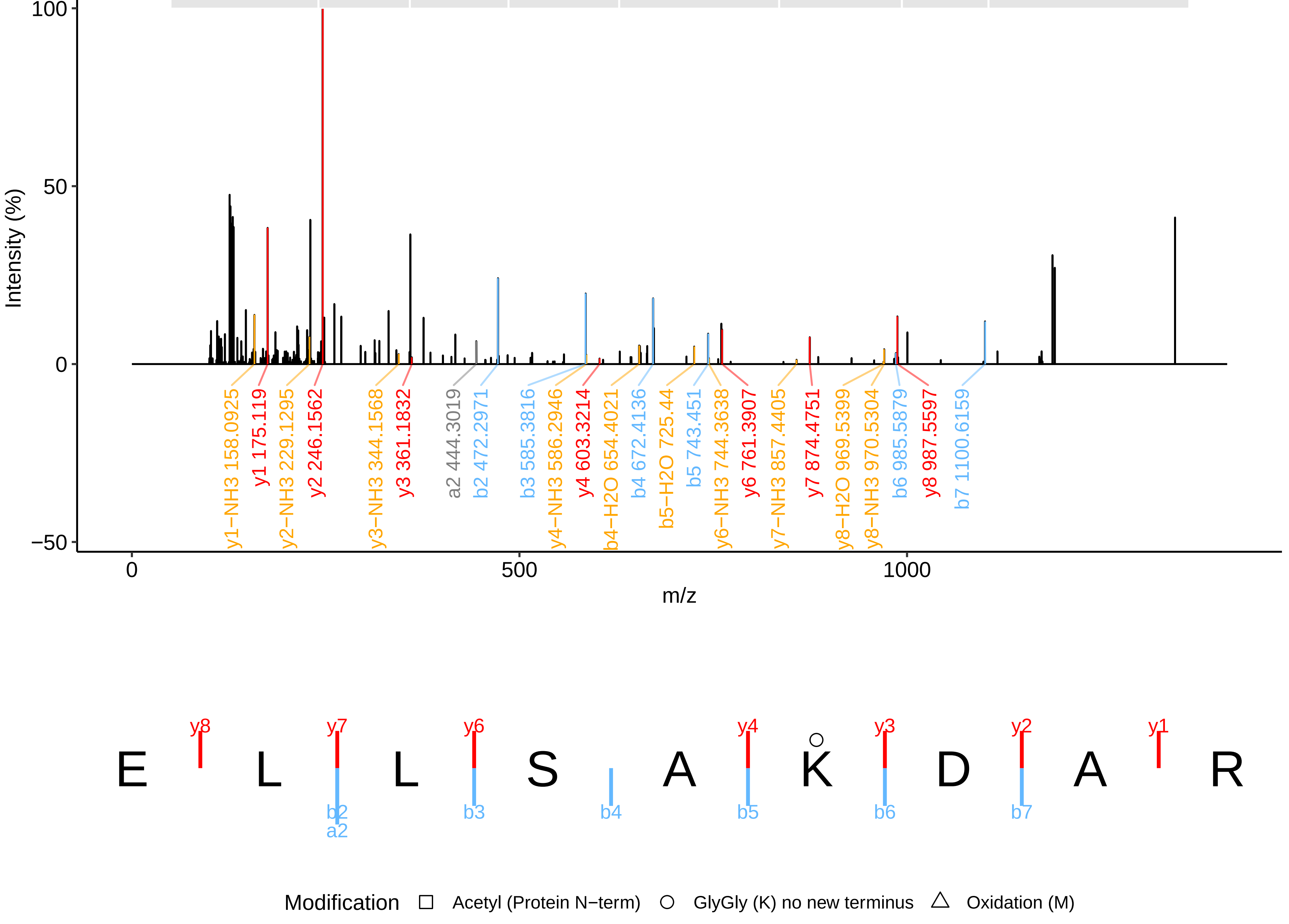

20200924_KIZ_MUb5_GlyGly_2 − Scan number: 6488

Gene Names	Charge	m/z	Mass	Retention time	PEP	Score	Precursor Intensity
UBR5	3	372.8718	1115.593	24.201	0.001812	90.696	5073433

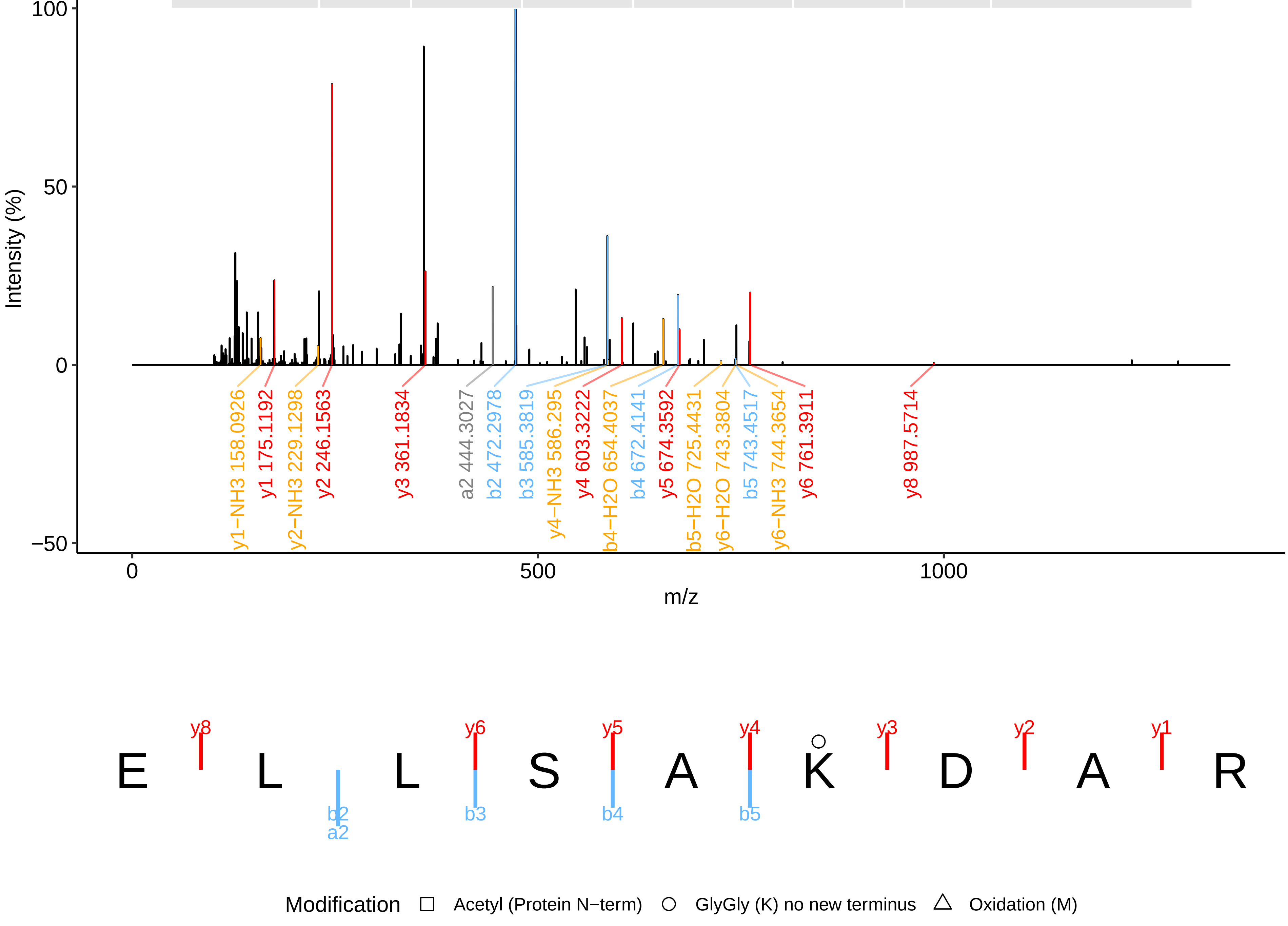

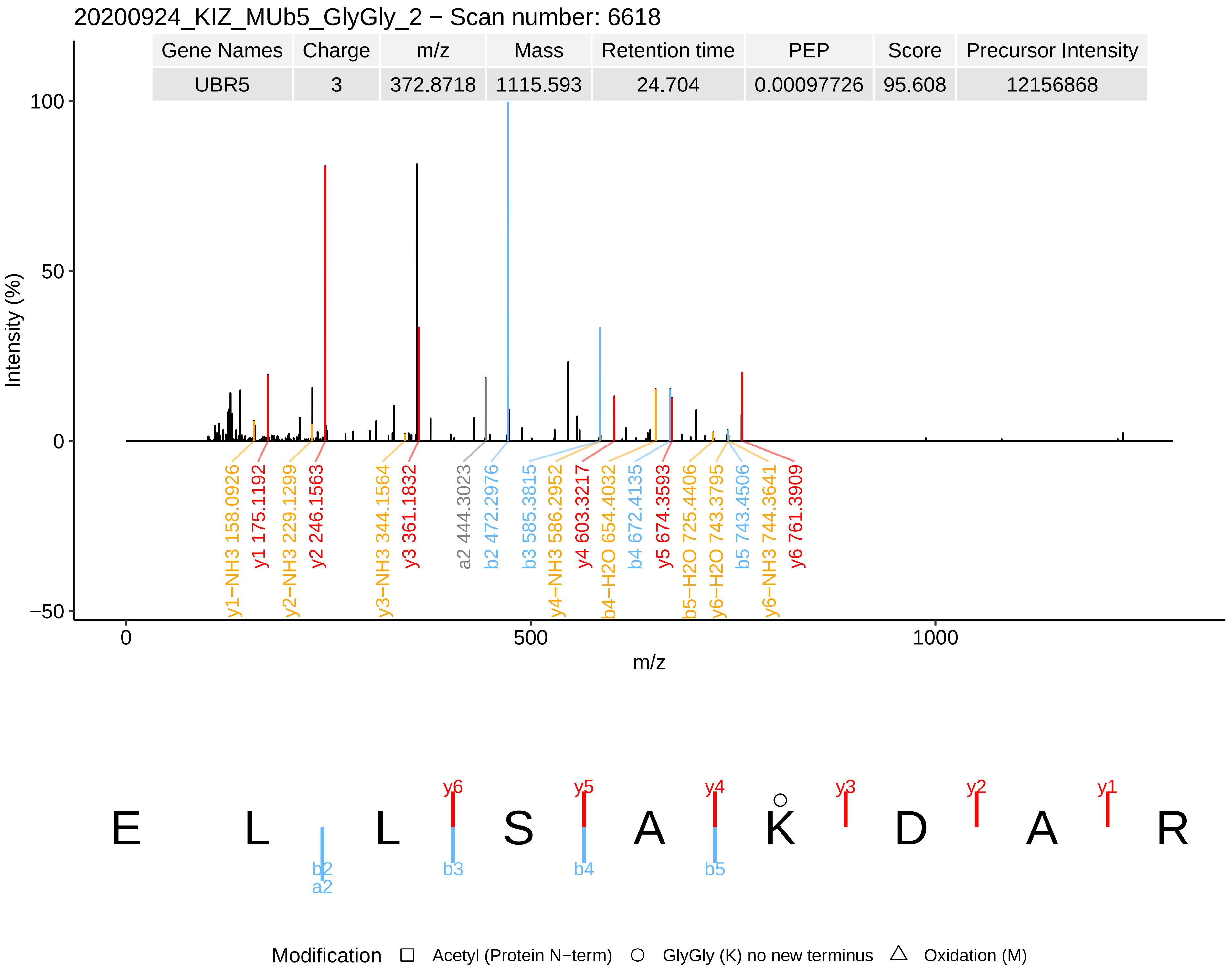

20200924_KIZ_MUb5_GlyGly_2 − Scan number: 6618
Gene Names | Charge | m/z | Mass | Retention time | PEP | Score | Precursor Intensity
UBR5 | 3 | 372.8718 | 1115.593 | 24.704 | 0.00097726 | 95.608 | 12156868
Intensity (%)
m/z
y1−NH3 158.0926
y1 175.1192
y2−NH3 229.1299
y2 246.1563
y3−NH3 344.1564
y3 361.1832
a2 444.3023
b2 472.2976
b3 585.3815
y4−NH3 586.2952
y4 603.3217
b4−H2O 654.4032
b4 672.4135
y5 674.3593
b5−H2O 725.4406
y6−H2O 743.3795
b5 743.4506
y6−NH3 744.3641
y6 761.3909
E L L S A K D A R
Modification
Acetyl (Protein N−term)
GlyGly (K) no new terminus
Oxidation (M)

20201118_KIZ_MUb6_GlyGly_1 − Scan number: 6574

Gene Names	Charge	m/z	Mass	Retention time	PEP	Score	Precursor Intensity
UBR5	3	372.8718	1115.593	20.817	0.00056492	121.64	15425315

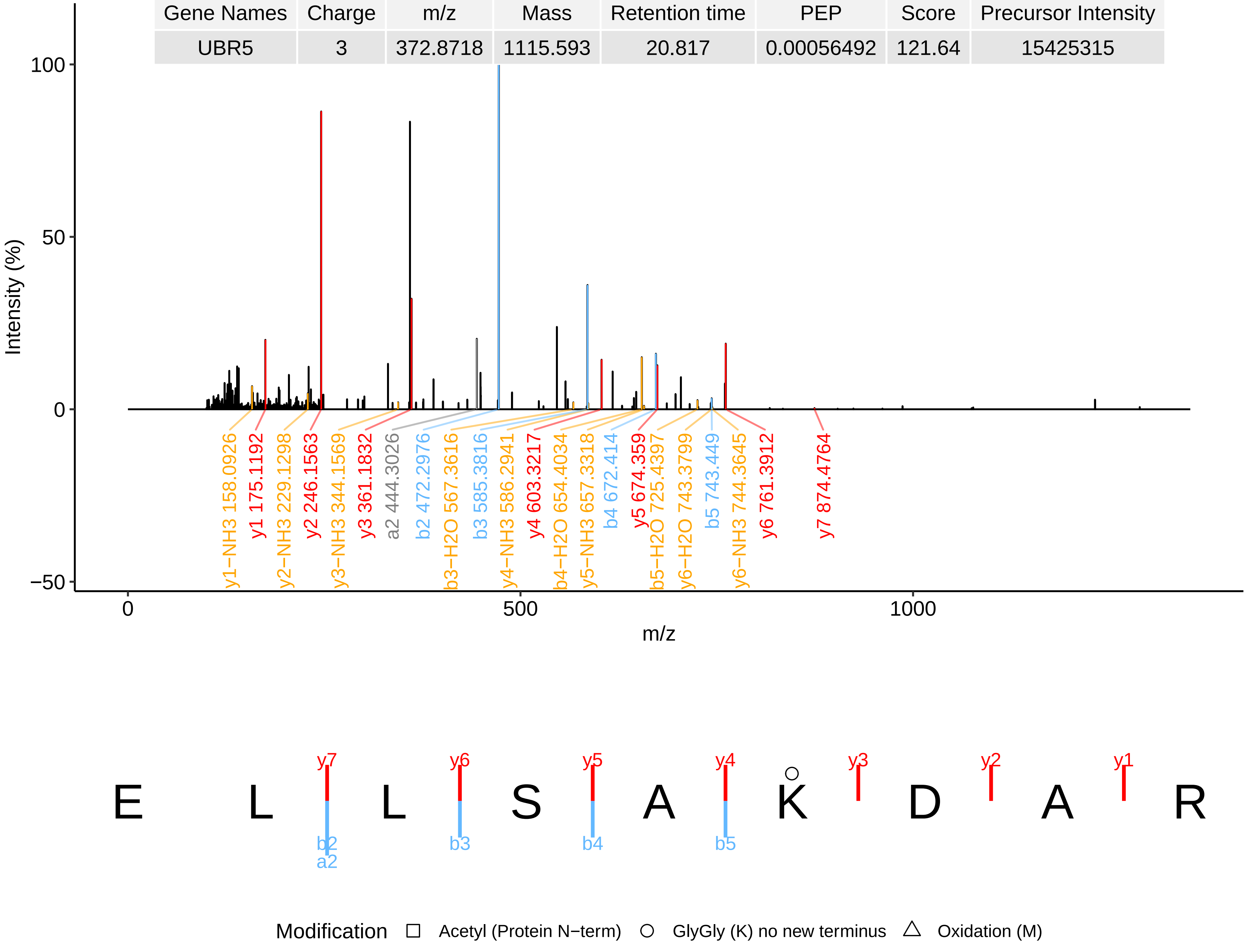

20201118_KIZ_MUb6_GlyGly_2 − Scan number: 8416

Gene Names	Charge	m/z	Mass	Retention time	PEP	Score	Precursor Intensity
UBR5	3	372.8718	1115.593	28.578	0.00032018	105.94	4644488

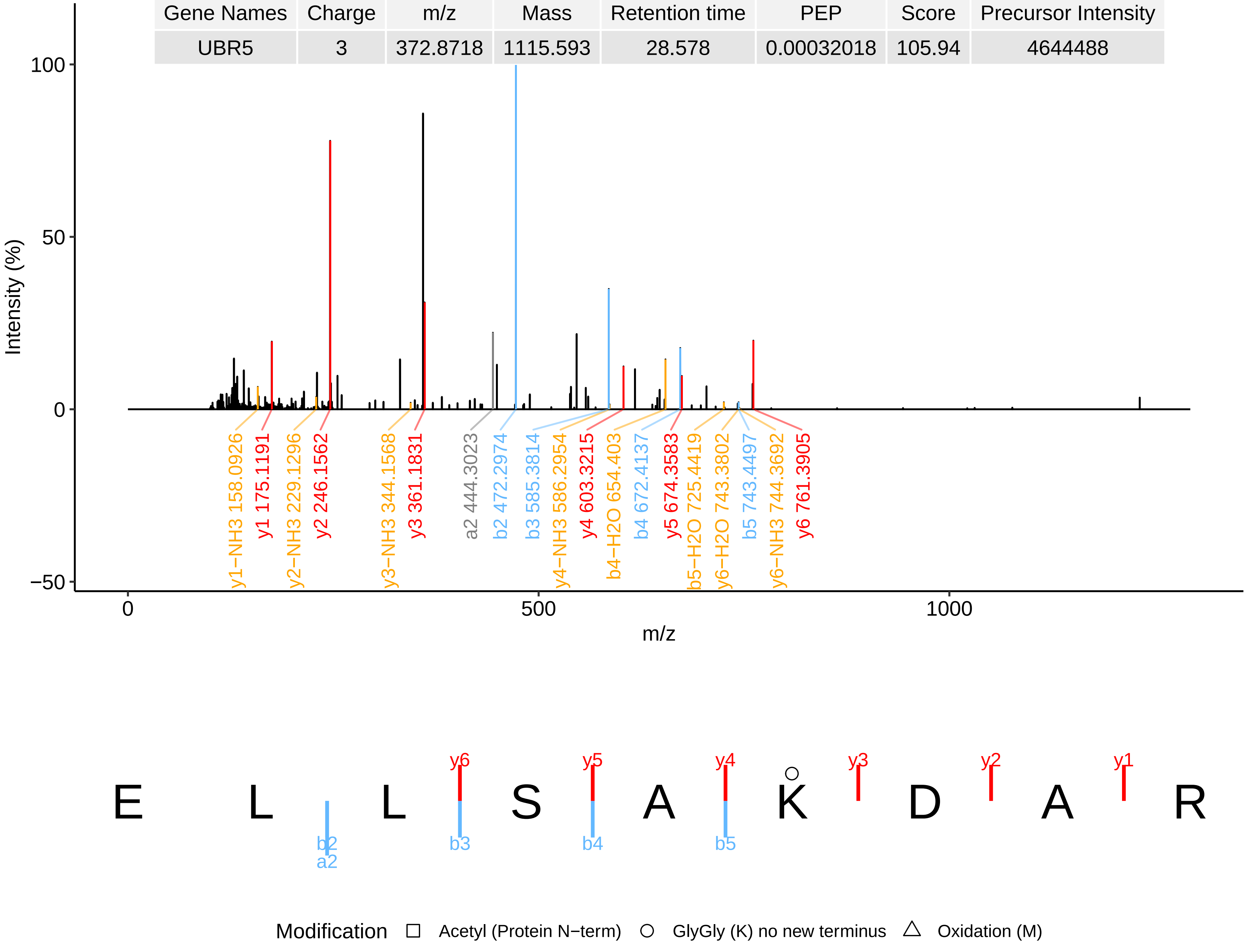

20200924_KIZ_MUb5_GlyGly_2 − Scan number: 18767

Gene Names	Charge	m/z	Mass	Retention time	PEP	Score	Precursor Intensity
	2	552.7951	1103.576	69.609	0.11429	62.717	1387068

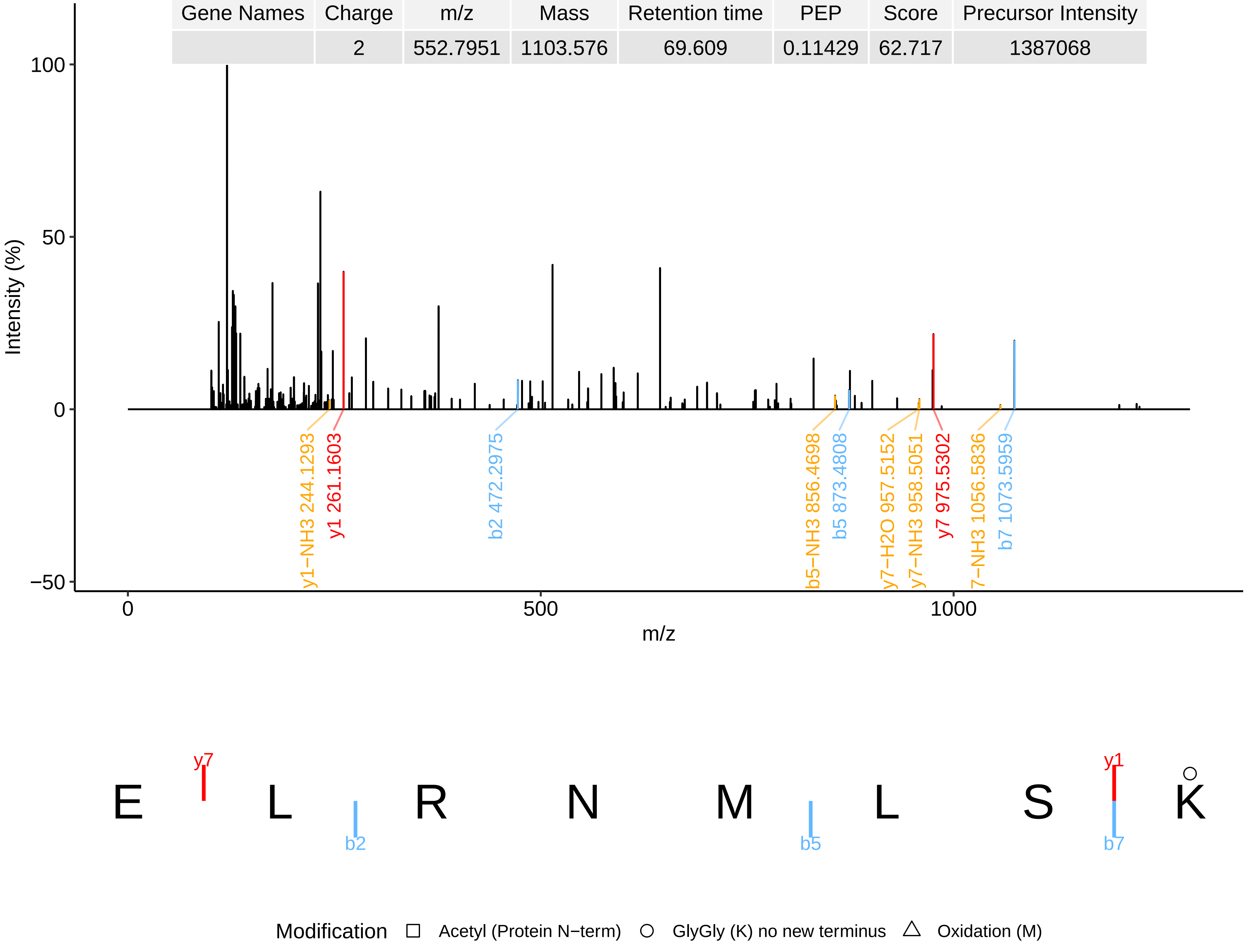

20200921_KIZ_MUb4_GlyGly_10plex − Scan number: 5133

Gene Names	Charge	m/z	Mass	Retention time	PEP	Score	Precursor Intensity
DCUN1D1	3	368.8596	1103.557	16.759	0.0082329	76.17	9362216

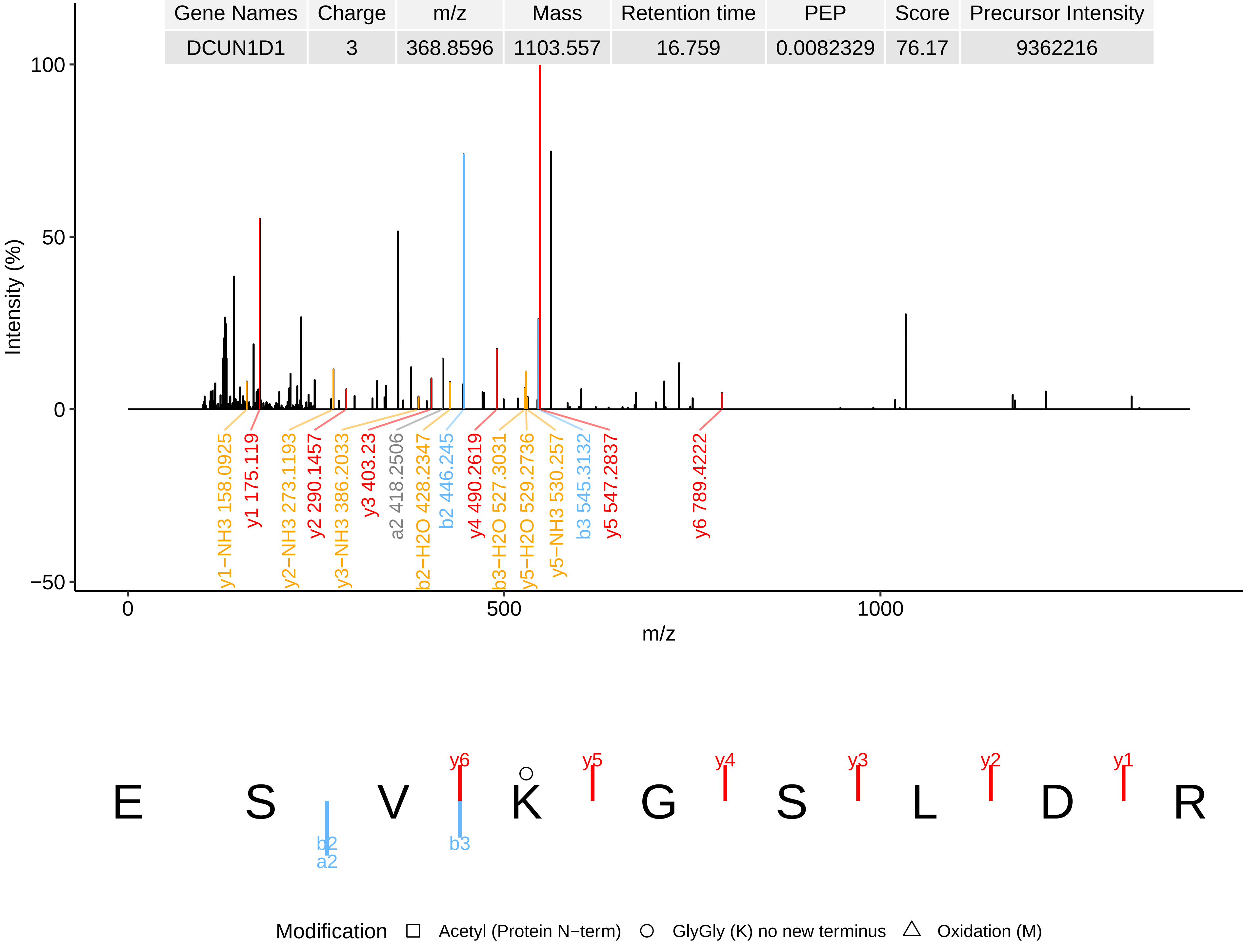

20200921_KIZ_MUb4_GlyGly_10plex_2 − Scan number: 4707

Gene Names	Charge	m/z	Mass	Retention time	PEP	Score	Precursor Intensity
DCUN1D1	3	368.8596	1103.557	16.623	0.015469	69.375	4578914

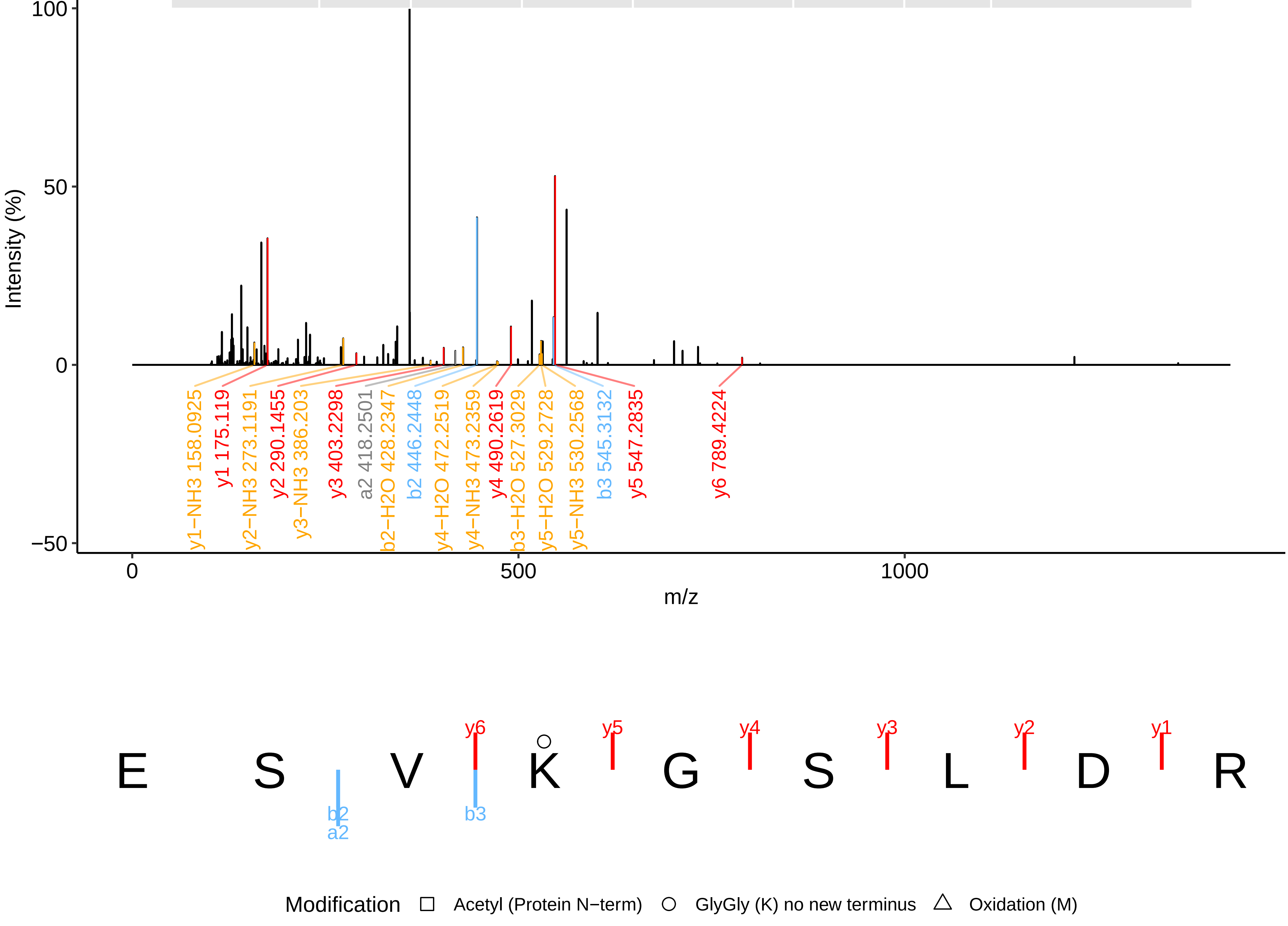

20200921_KIZ_MUb4_GlyGly_10plex_2 − Scan number: 10254

Gene Names	Charge	m/z	Mass	Retention time	PEP	Score	Precursor Intensity
TAX1BP1	3	372.54	1114.598	39.18	0.0016946	109.86	1558840

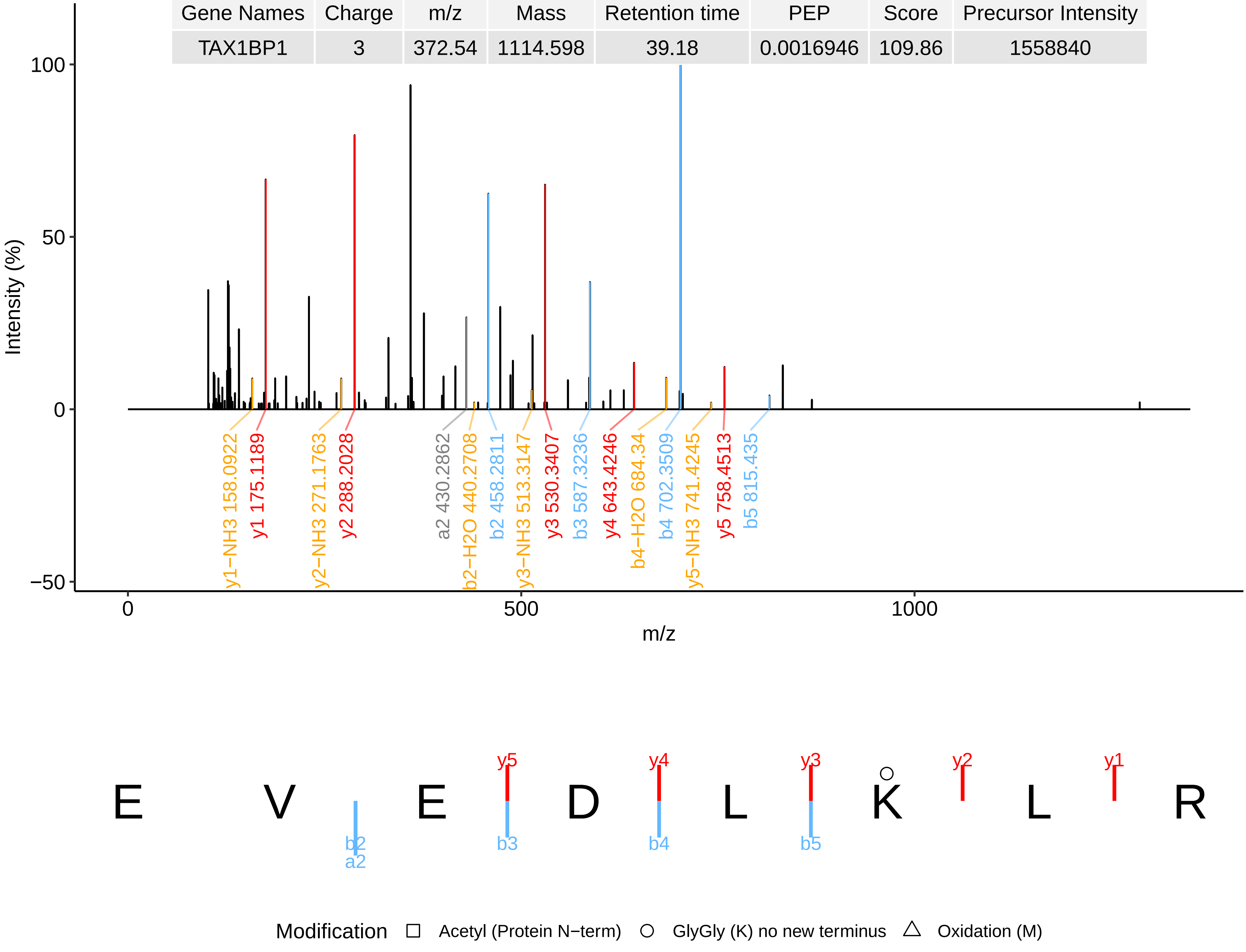

20200924_KIZ_MUb5_GlyGly_1 − Scan number: 10079

Gene Names	Charge	m/z	Mass	Retention time	PEP	Score	Precursor Intensity
TAX1BP1	3	372.54	1114.598	32.484	0.094814	47.574	27787040

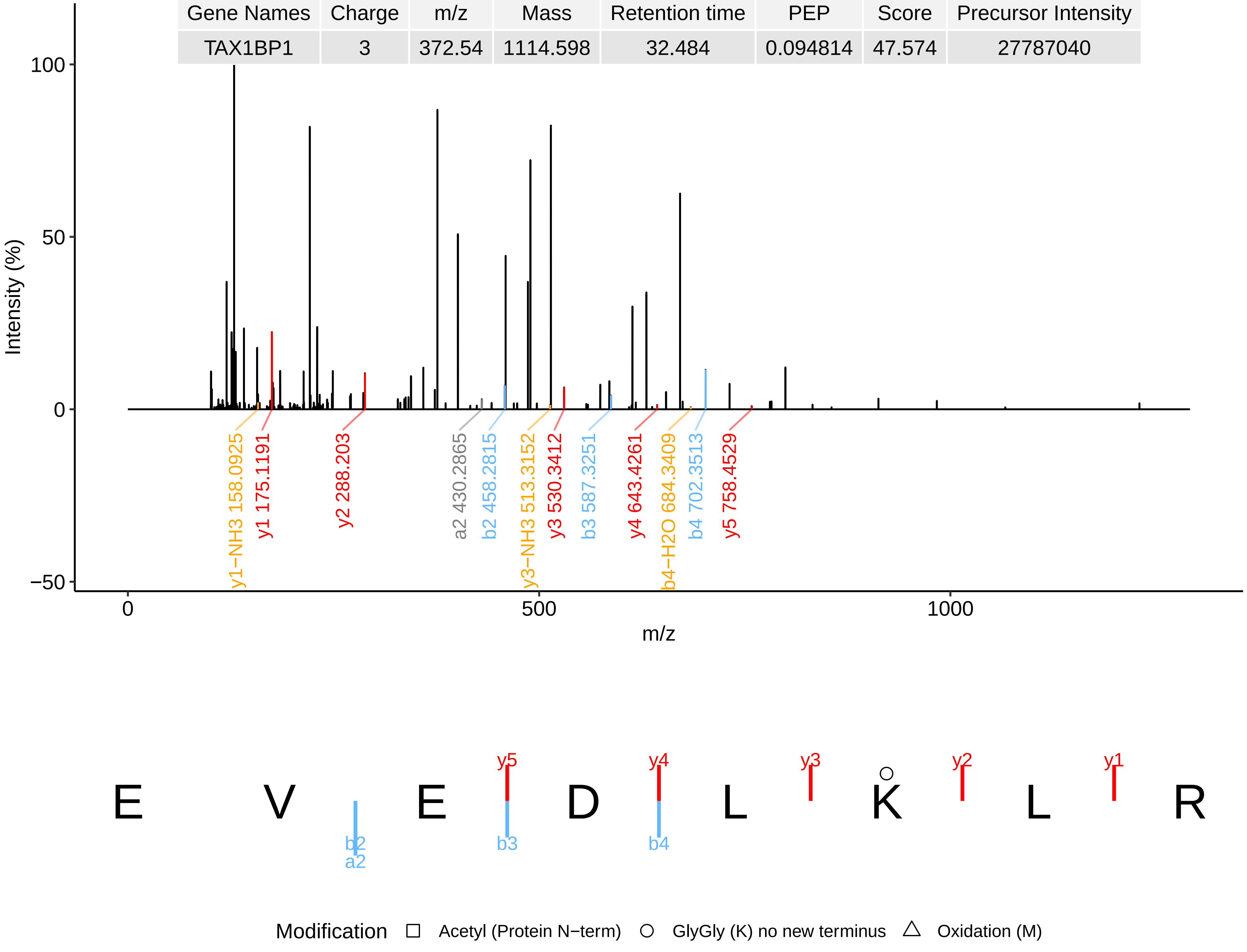

20200921_KIZ_MUb4_GlyGly_10plex − Scan number: 19294

Gene Names	Charge	m/z	Mass	Retention time	PEP	Score	Precursor Intensity
ACSL4	3	372.5572	1114.65	69.53	0.00024785	117.02	6439255

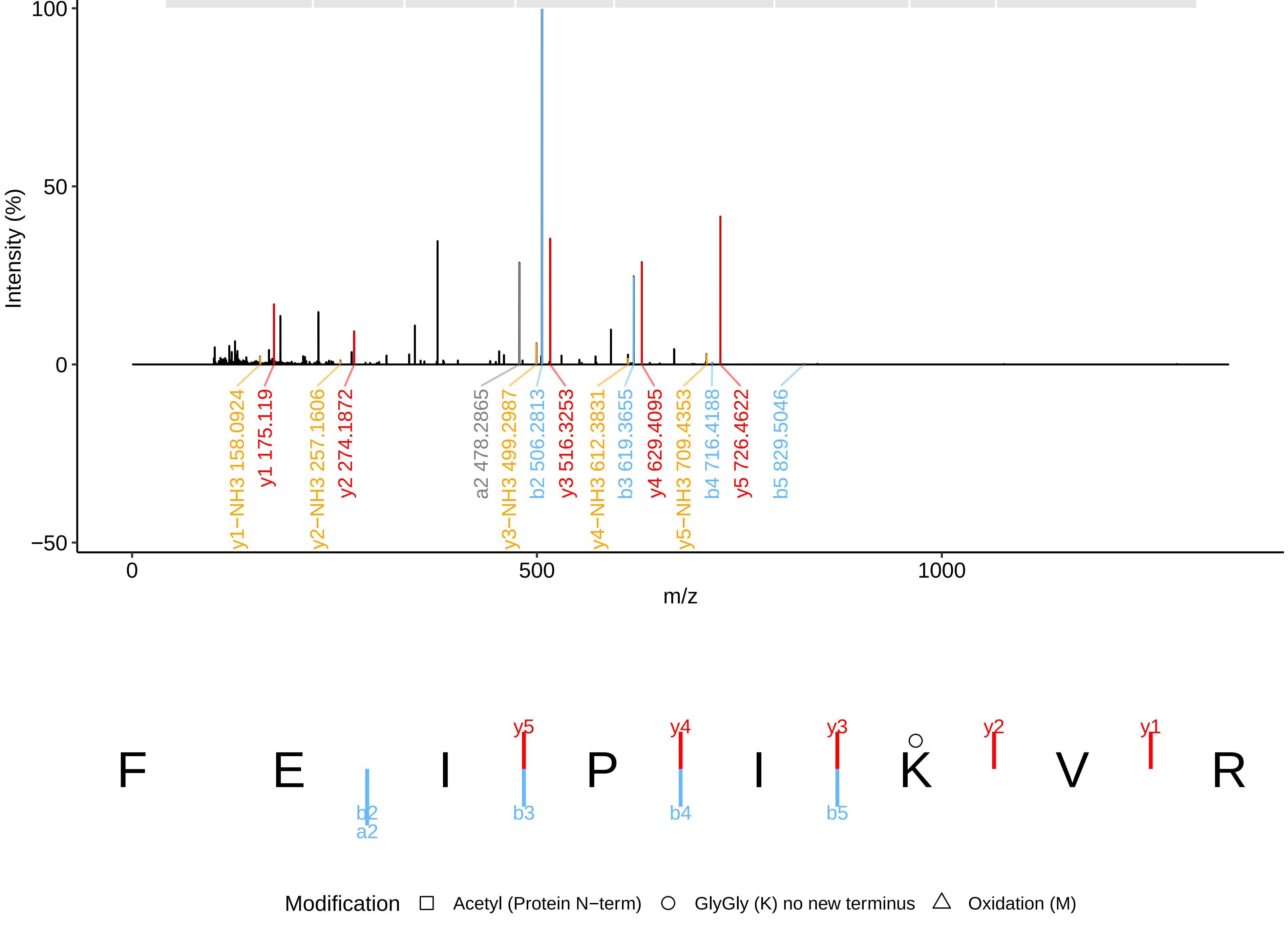

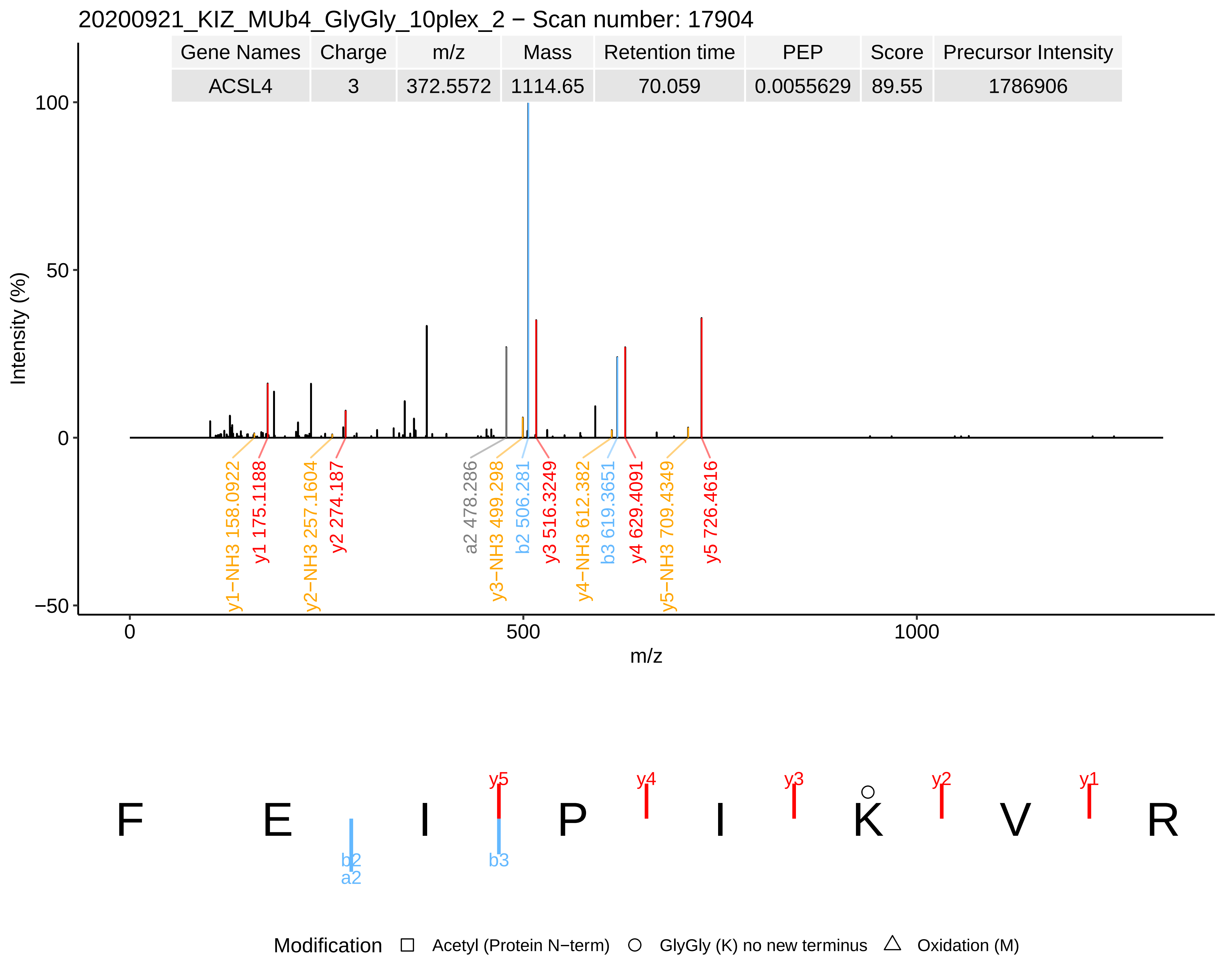

20200921_KIZ_MUb4_GlyGly_10plex_2 − Scan number: 17904
Gene Names | Charge | m/z | Mass | Retention time | PEP | Score | Precursor Intensity
ACSL4 | 3 | 372.5572 | 1114.65 | 70.059 | 0.0055629 | 89.55 | 1786906
Intensity (%)
m/z
y1−NH3 158.0922
y1 175.1188
y2−NH3 257.1604
y2 274.187
a2 478.286
y3−NH3 499.298
b2 506.281
y3 516.3249
y4−NH3 612.382
b3 619.3651
y4 629.4091
y5−NH3 709.4349
y5 726.4616
F E I P I K V R
y5 y4 y3 y2 y1
b2 a2 b3
Modification
Acetyl (Protein N−term)
GlyGly (K) no new terminus
Oxidation (M)

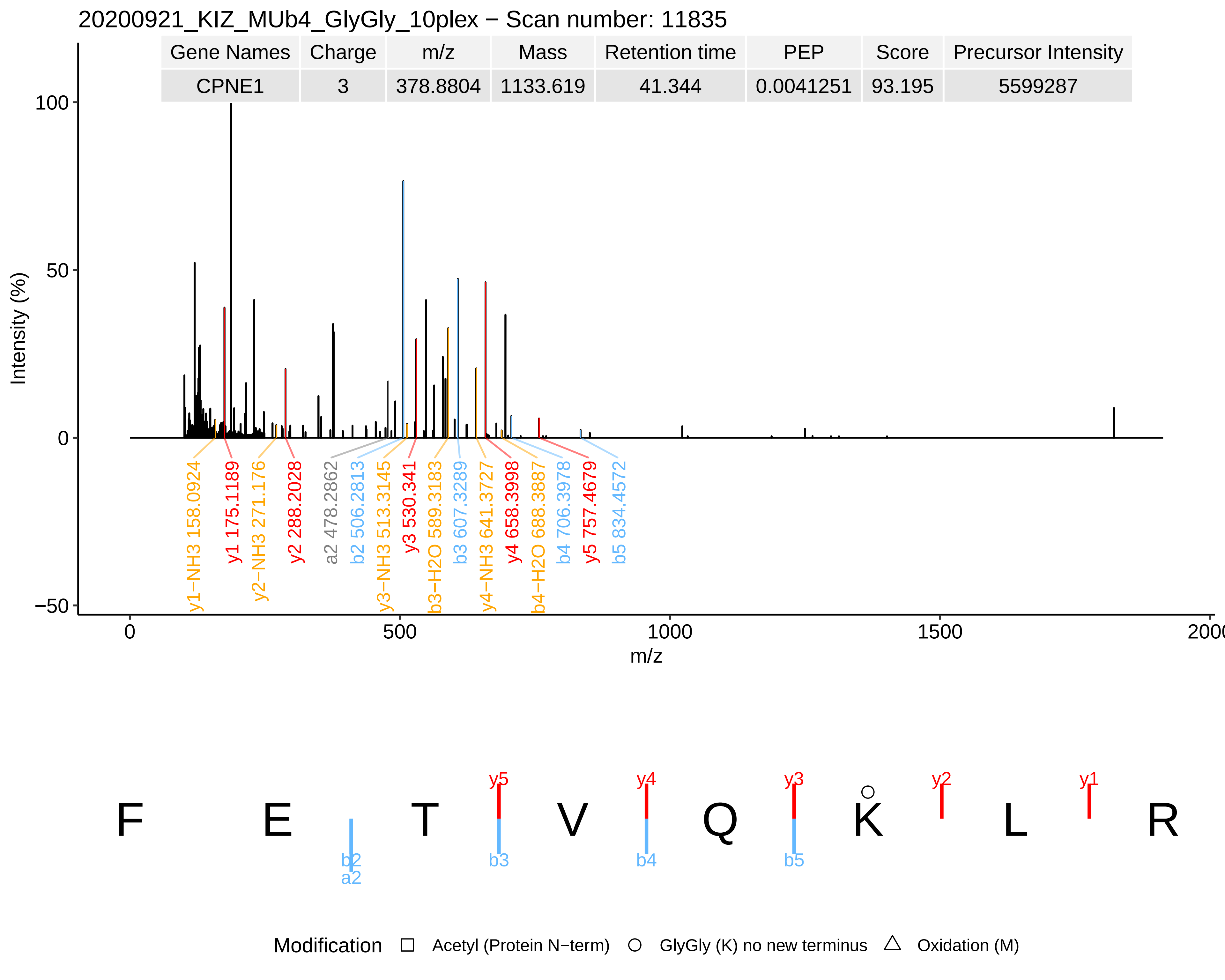
20200921_KIZ_MUb4_GlyGly_10plex − Scan number: 11835
Gene Names | Charge | m/z | Mass | Retention time | PEP | Score | Precursor Intensity
CPNE1 | 3 | 378.8804 | 1133.619 | 41.344 | 0.0041251 | 93.195 | 5599287
Intensity (%)
m/z
y1−NH3 158.0924
y1 175.1189
y2−NH3 271.176
y2 288.2028
a2 478.2862
b2 506.2813
y3−NH3 513.3145
y3 530.341
b3−H2O 589.3183
b3 607.3289
y4−NH3 641.3727
y4 658.3998
b4−H2O 688.3887
b4 706.3978
y5 757.4679
b5 834.4572
F E T V Q K L R
y5 y4 y3 y2 y1
b2 a2 b3 b4 b5
Modification
Acetyl (Protein N−term)
GlyGly (K) no new terminus
Oxidation (M)

20200921_KIZ_MUb4_GlyGly_10plex − Scan number: 15198

Gene Names	Charge	m/z	Mass	Retention time	PEP	Score	Precursor Intensity
PMAIP1	3	370.8612	1109.562	54.228	0.06593	54.611	2302429

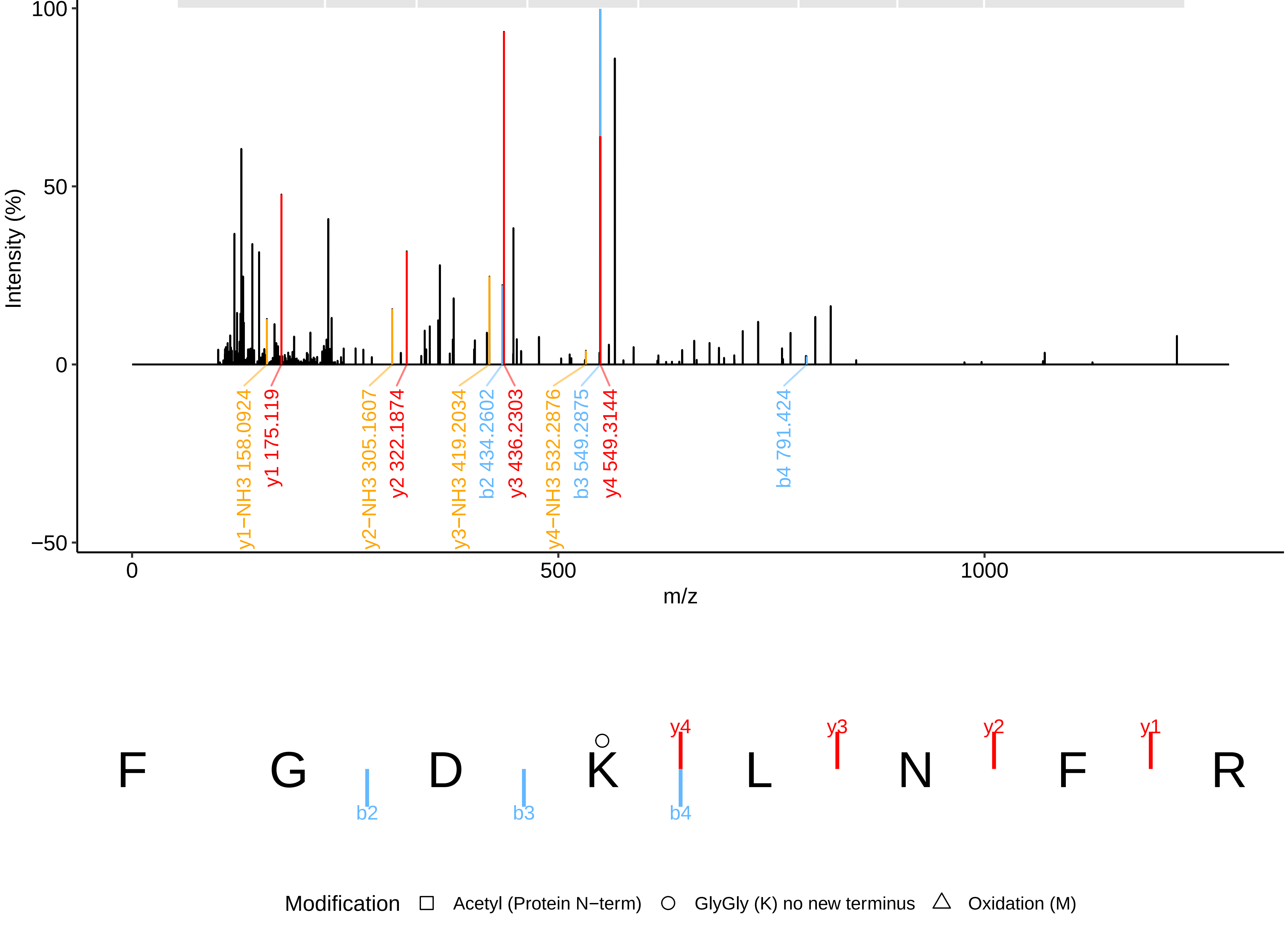

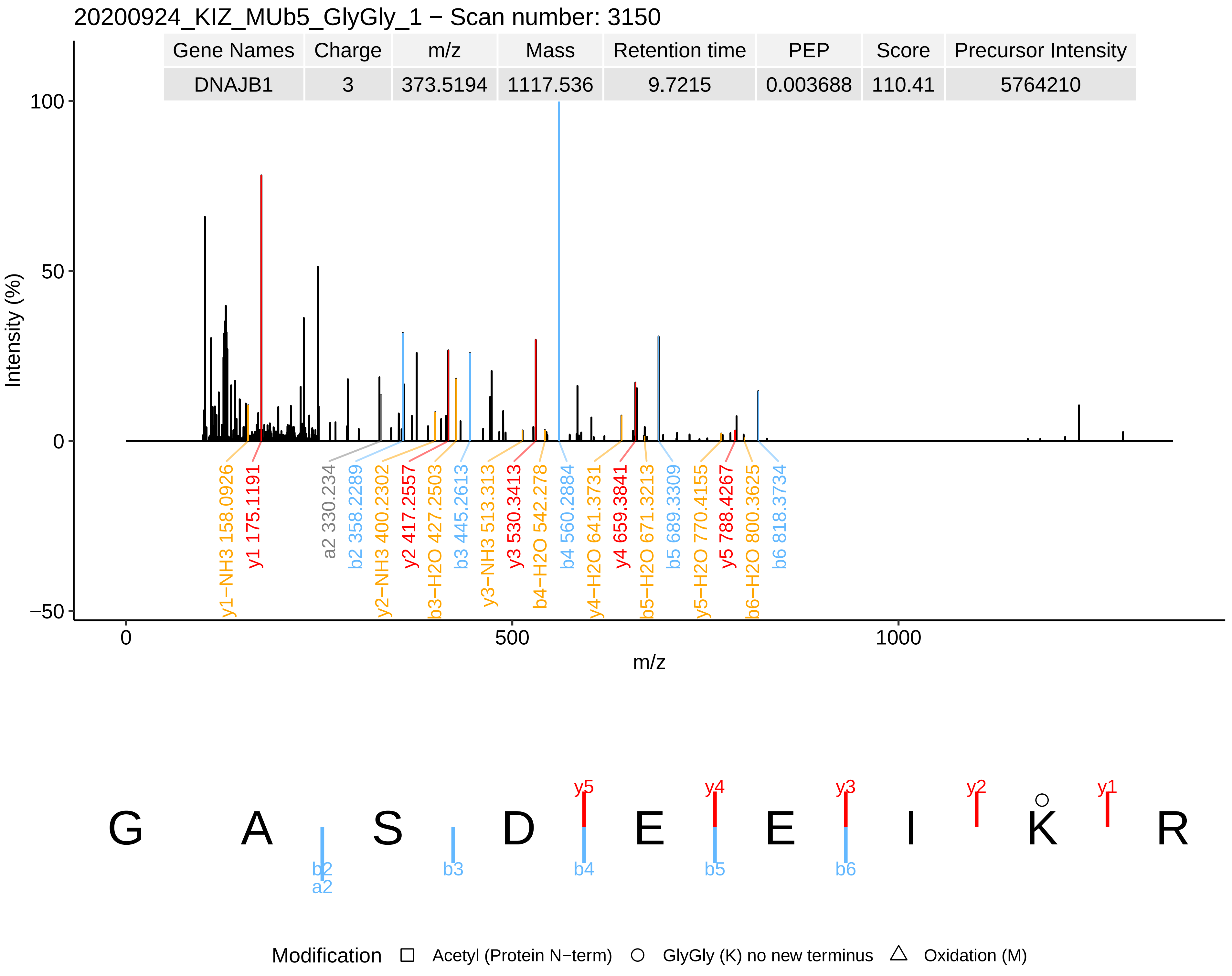

20200924_KIZ_MUb5_GlyGly_1 − Scan number: 3150
Gene Names | Charge | m/z | Mass | Retention time | PEP | Score | Precursor Intensity
DNAJB1 | 3 | 373.5194 | 1117.536 | 9.7215 | 0.003688 | 110.41 | 5764210
Intensity (%)
m/z
y1−NH3 158.0926
y1 175.1191
a2 330.234
b2 358.2289
y2−NH3 400.2302
y2 417.2557
b3−H2O 427.2503
b3 445.2613
y3−NH3 513.313
y3 530.3413
b4−H2O 542.278
b4 560.2884
y4−H2O 641.3731
y4 659.3841
b5−H2O 671.3213
b5 689.3309
y5−H2O 770.4155
y5 788.4267
b6−H2O 800.3625
b6 818.3734
G A S D E E I K R
y5 y4 y3 y2 y1
b2 a2 b3 b4 b5 b6
Modification
Acetyl (Protein N−term)
GlyGly (K) no new terminus
Oxidation (M)

20200921_KIZ_MUb4_GlyGly_10plex − Scan number: 11765

Gene Names	Charge	m/z	Mass	Retention time	PEP	Score	Precursor Intensity
AUP1	3	382.2155	1143.625	41.087	0.0099706	91.867	4975187

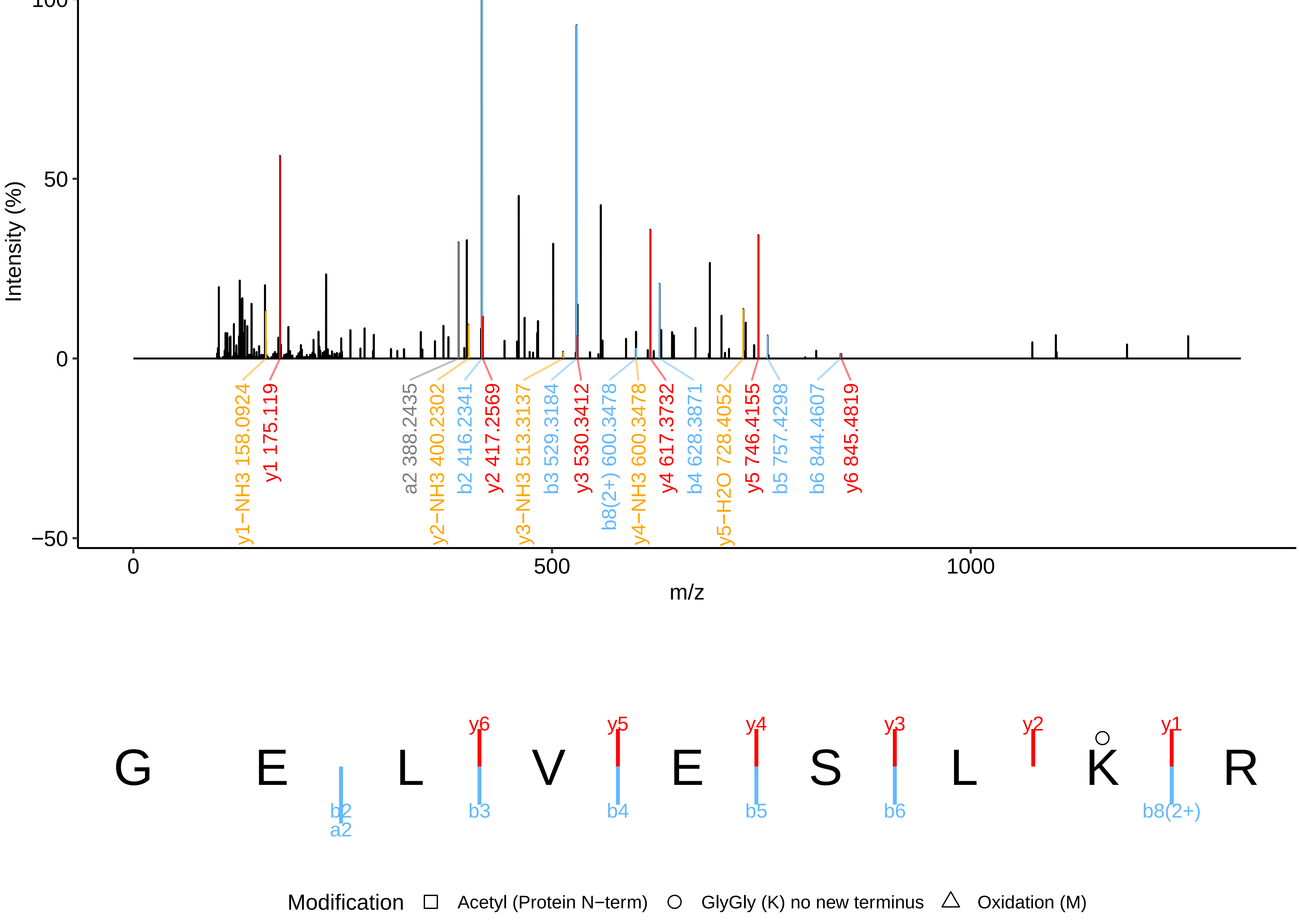

20200921_KIZ_MUb4_GlyGly_10plex_2 − Scan number: 10861

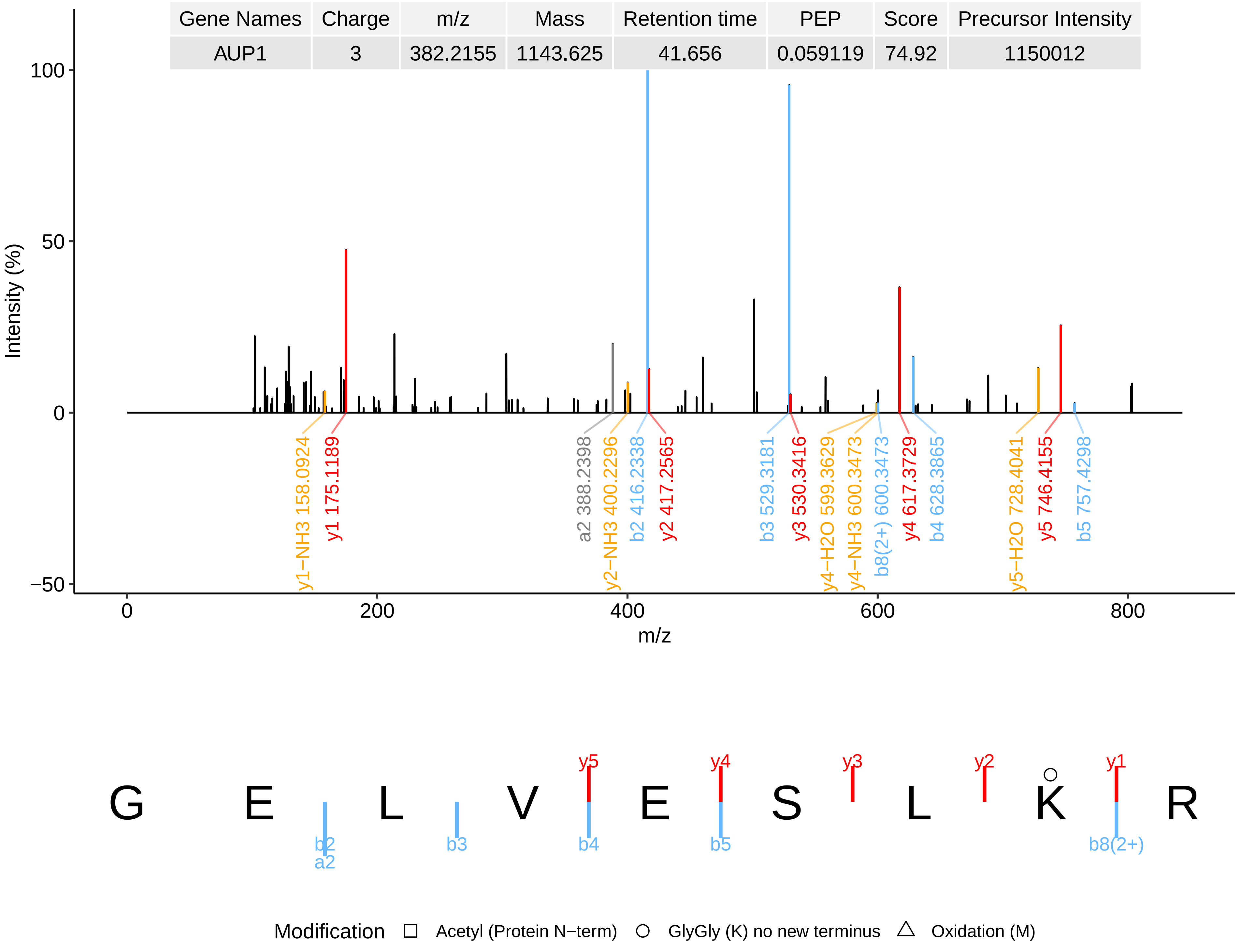

20200921_KIZ_MUb4_GlyGly_10plex − Scan number: 12764

Gene Names	Charge	m/z	Mass	Retention time	PEP	Score	Precursor Intensity
RPL5	3	297.5201	889.5385	44.864	0.066758	62.714	7671206

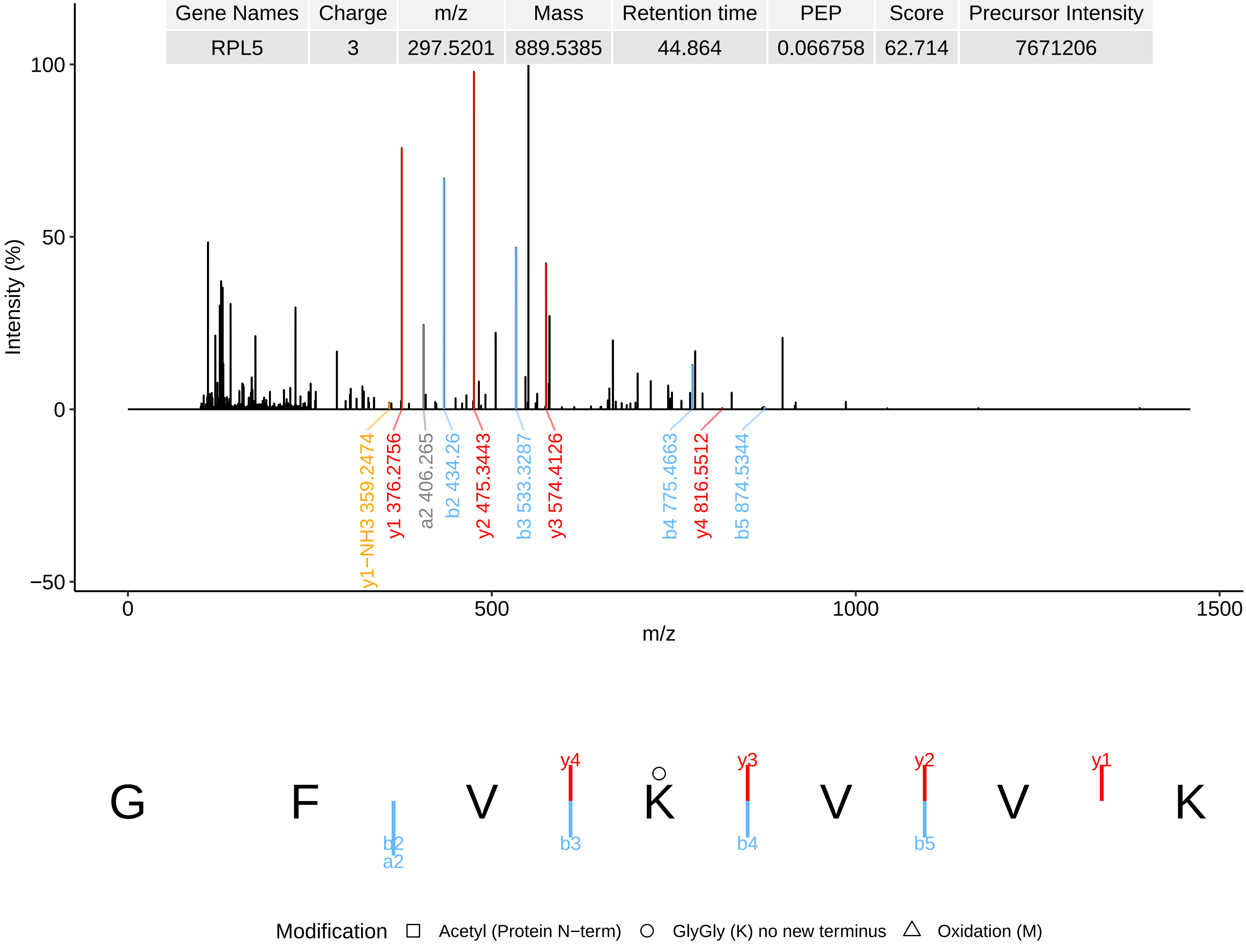

20200921_KIZ_MUb4_GlyGly_10plex_2 − Scan number: 11833

Gene Names	Charge	m/z	Mass	Retention time	PEP	Score	Precursor Intensity
RPL5	3	297.5201	889.5385	45.642	0.066076	62.842	NA

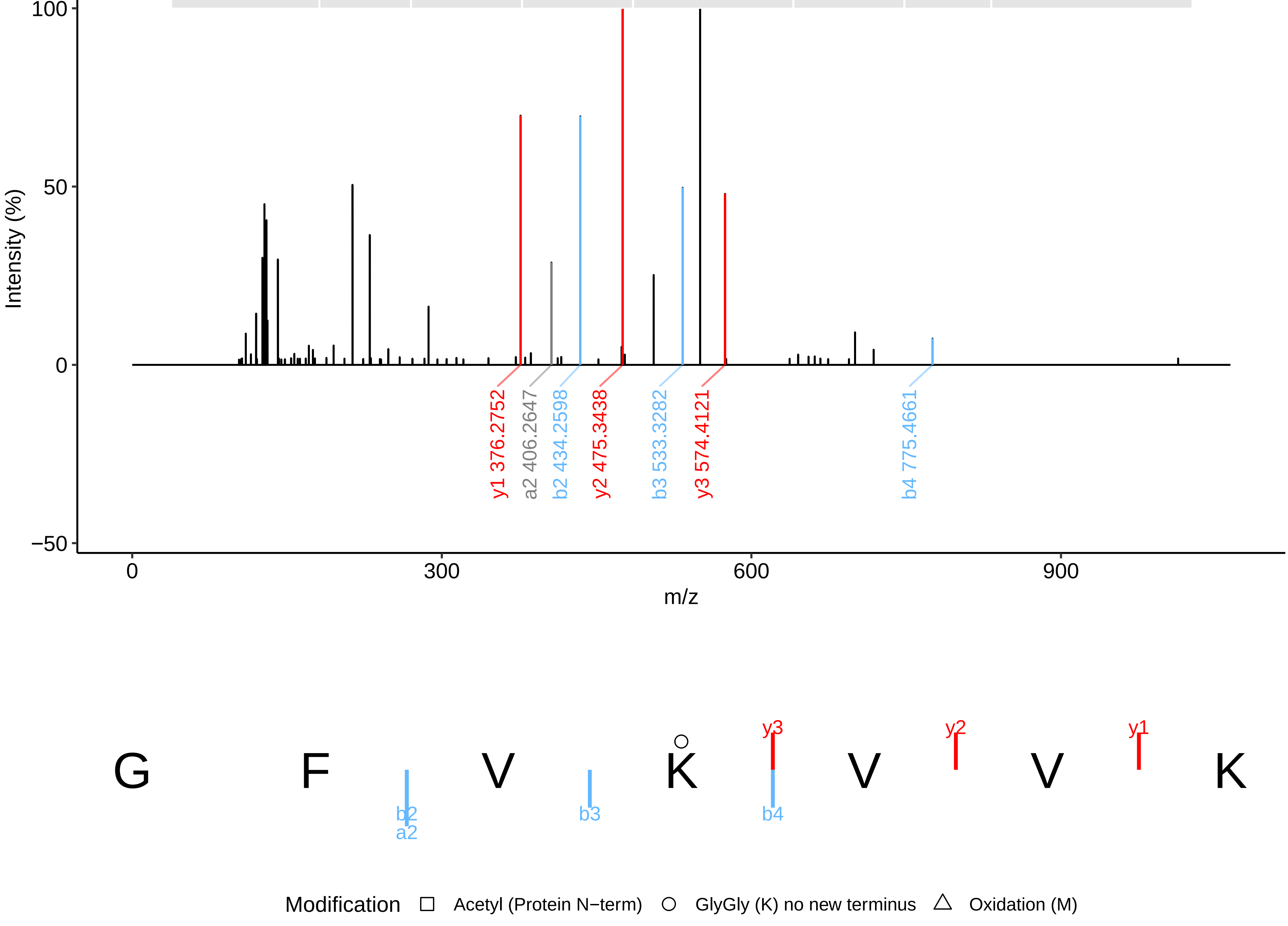

20201118_KIZ_MUb6_GlyGly_2 − Scan number: 14164

Gene Names	Charge	m/z	Mass	Retention time	PEP	Score	Precursor Intensity
RPL5	3	297.5201	889.5385	47.704	0.037637	74.335	NA

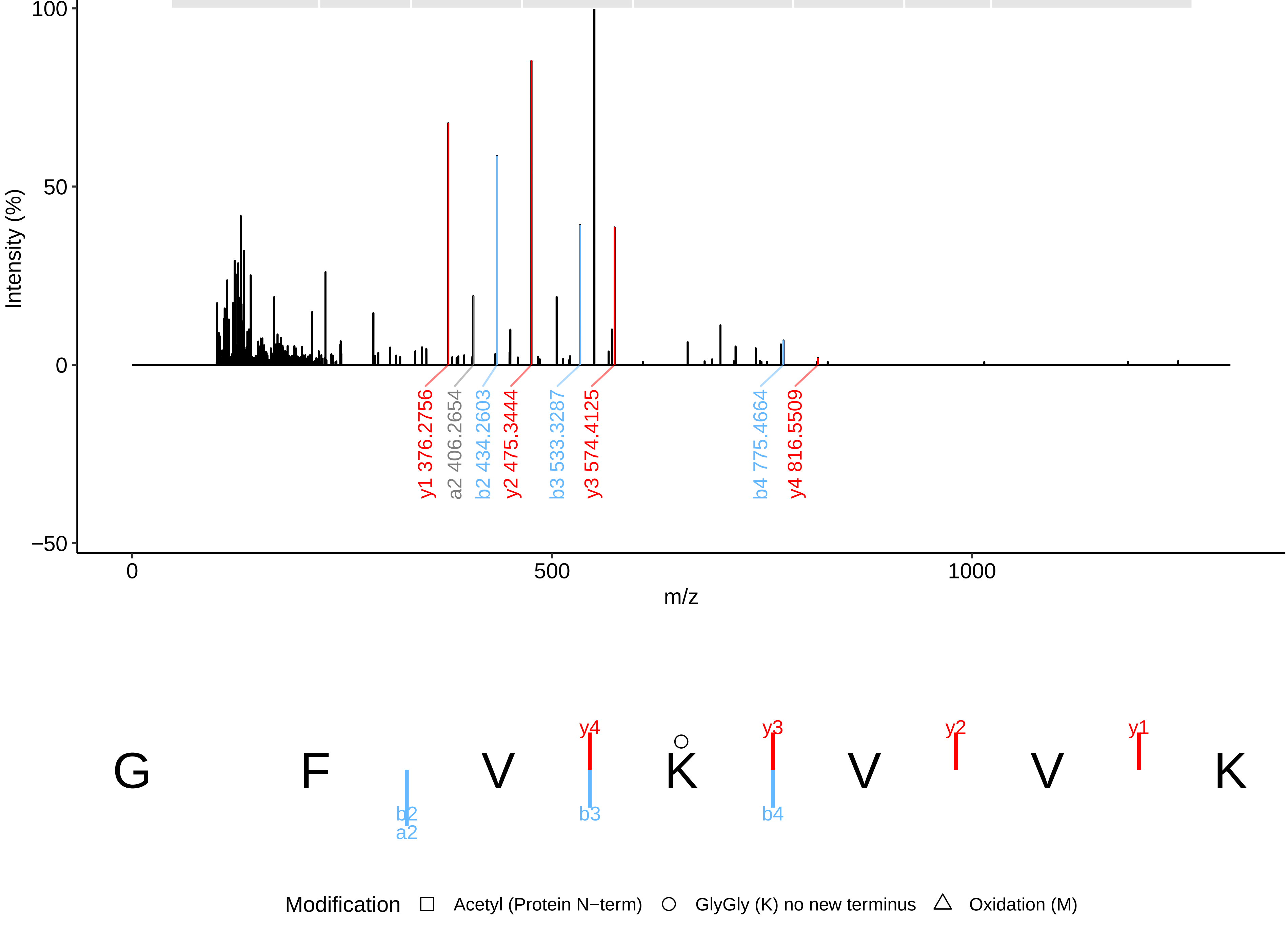

20200921_KIZ_MUb4_GlyGly_10plex − Scan number: 6023

Gene Names	Charge	m/z	Mass	Retention time	PEP	Score	Precursor Intensity
HTATIP2	3	369.1965	1104.568	19.784	0.070392	53.432	4881258

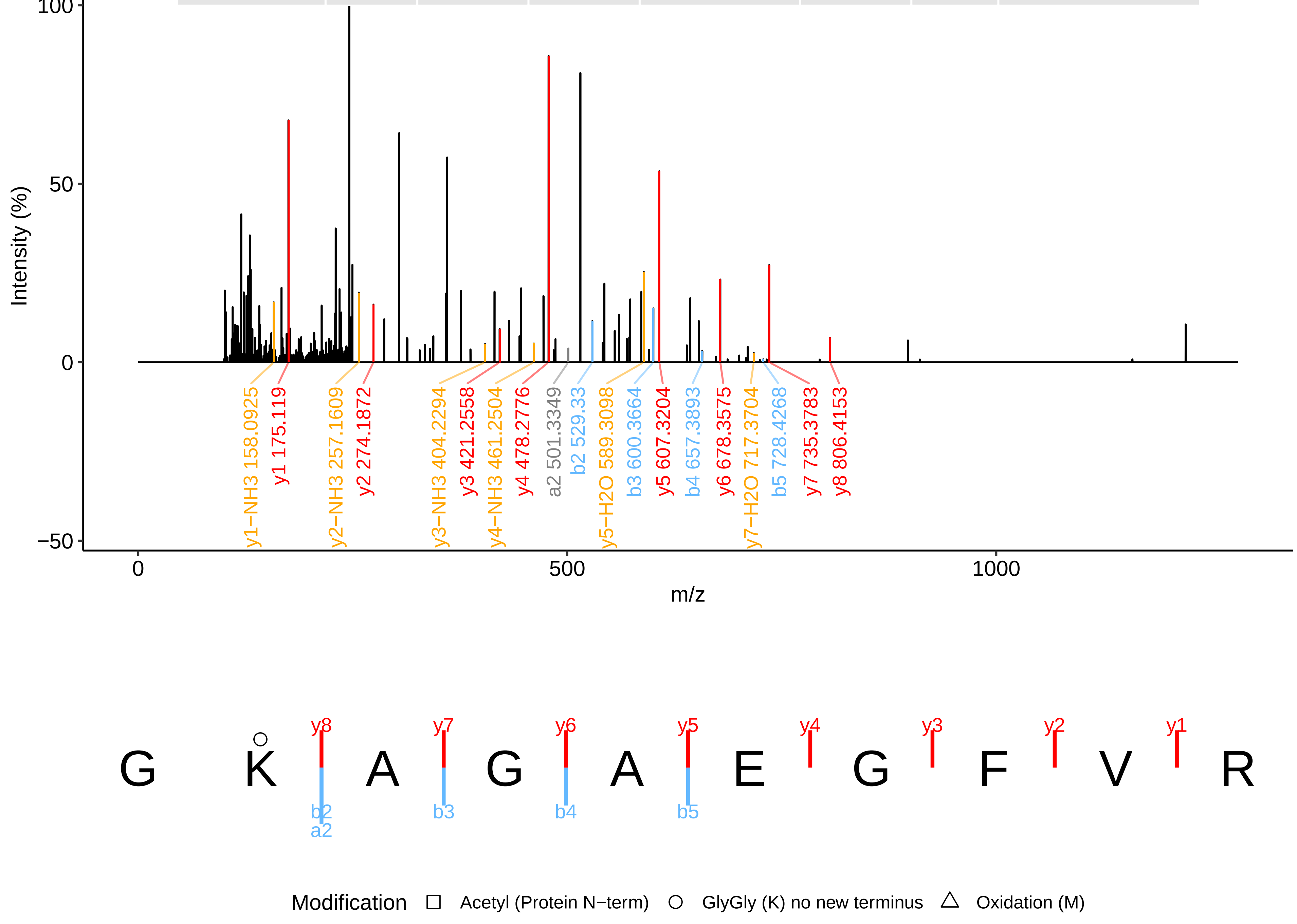

20200921_KIZ_MUb4_GlyGly_10plex − Scan number: 2592

Gene Names	Charge	m/z	Mass	Retention time	PEP	Score	Precursor Intensity
RNF5	3	376.1915	1125.553	8.1577	0.02139	63.816	3842318

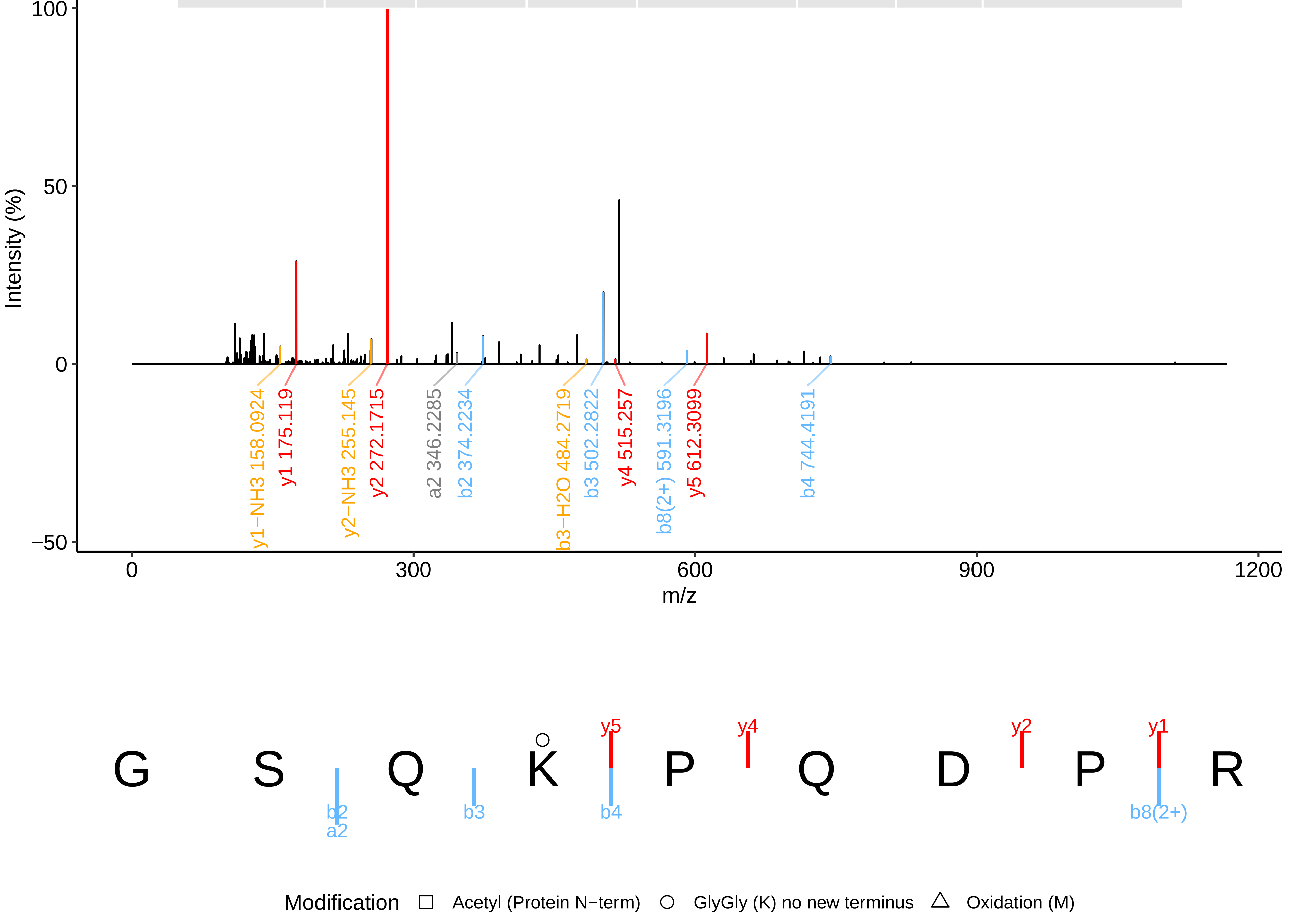

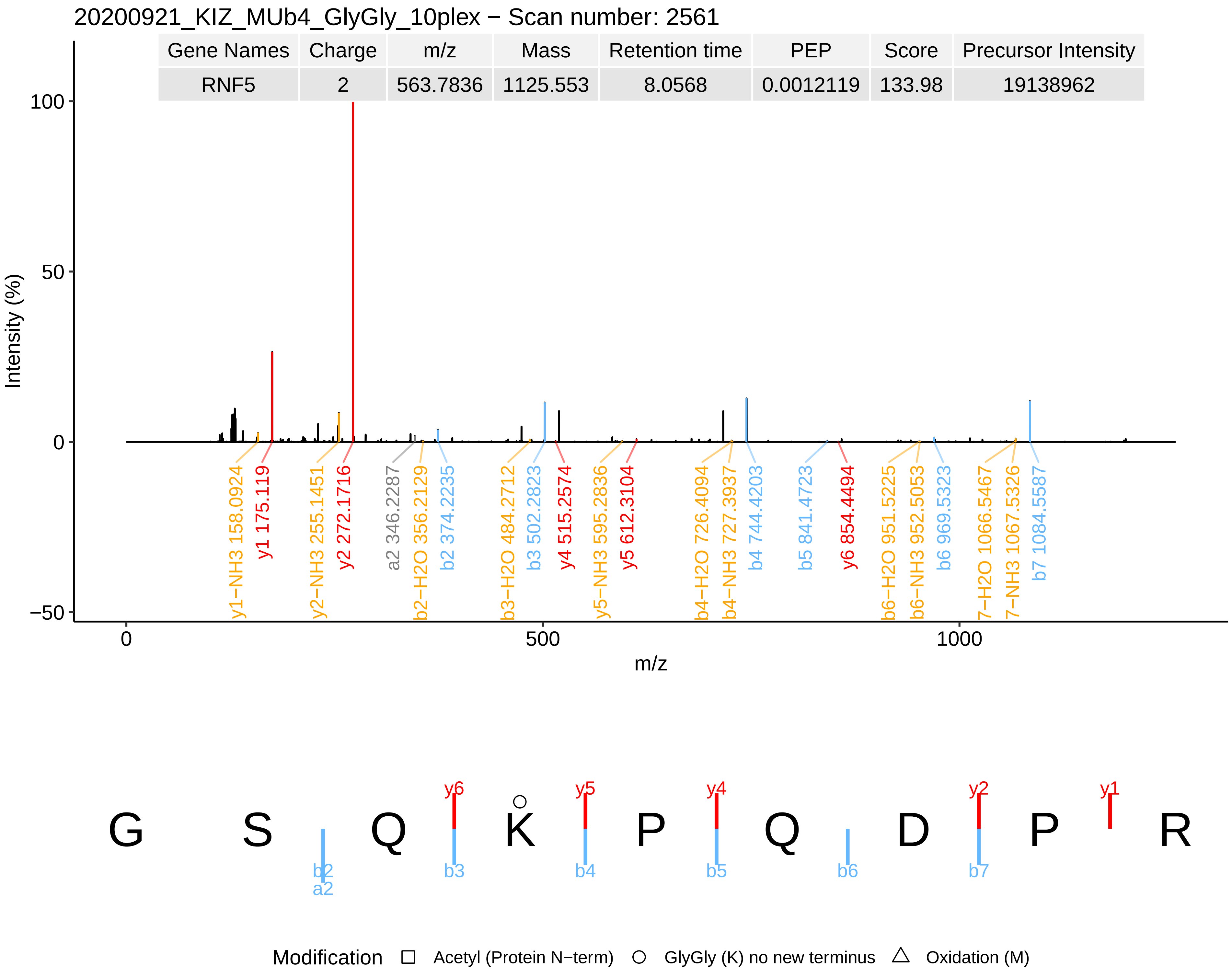

20200921_KIZ_MUb4_GlyGly_10plex − Scan number: 2561
Gene Names | Charge | m/z | Mass | Retention time | PEP | Score | Precursor Intensity
RNF5 | 2 | 563.7836 | 1125.553 | 8.0568 | 0.0012119 | 133.98 | 19138962
Intensity (%)
100
50
0
−50
0
500
1000
m/z
y1−NH3 158.0924
y1 175.119
y2−NH3 255.1451
y2 272.1716
a2 346.2287
b2−H2O 356.2129
b2 374.2235
b3−H2O 484.2712
b3 502.2823
y4 515.2574
y5−NH3 595.2836
y5 612.3104
b4−H2O 726.4094
b4−NH3 727.3937
b4 744.4203
b5 841.4723
y6 854.4494
b6−H2O 951.5225
b6−NH3 952.5053
b6 969.5323
7−H2O 1066.5467
7−NH3 1067.5326
b7 1084.5587
G S Q K P Q D P R
y6 y5 y4 y2 y1
b2 a2 b3 b4 b5 b6 b7
Modification
Acetyl (Protein N−term)
GlyGly (K) no new terminus
Oxidation (M)

20200921_KIZ_MUb4_GlyGly_10plex_2 − Scan number: 2489

Gene Names	Charge	m/z	Mass	Retention time	PEP	Score	Precursor Intensity
RNF5	3	376.1915	1125.553	7.8688	0.063391	54.608	2793423

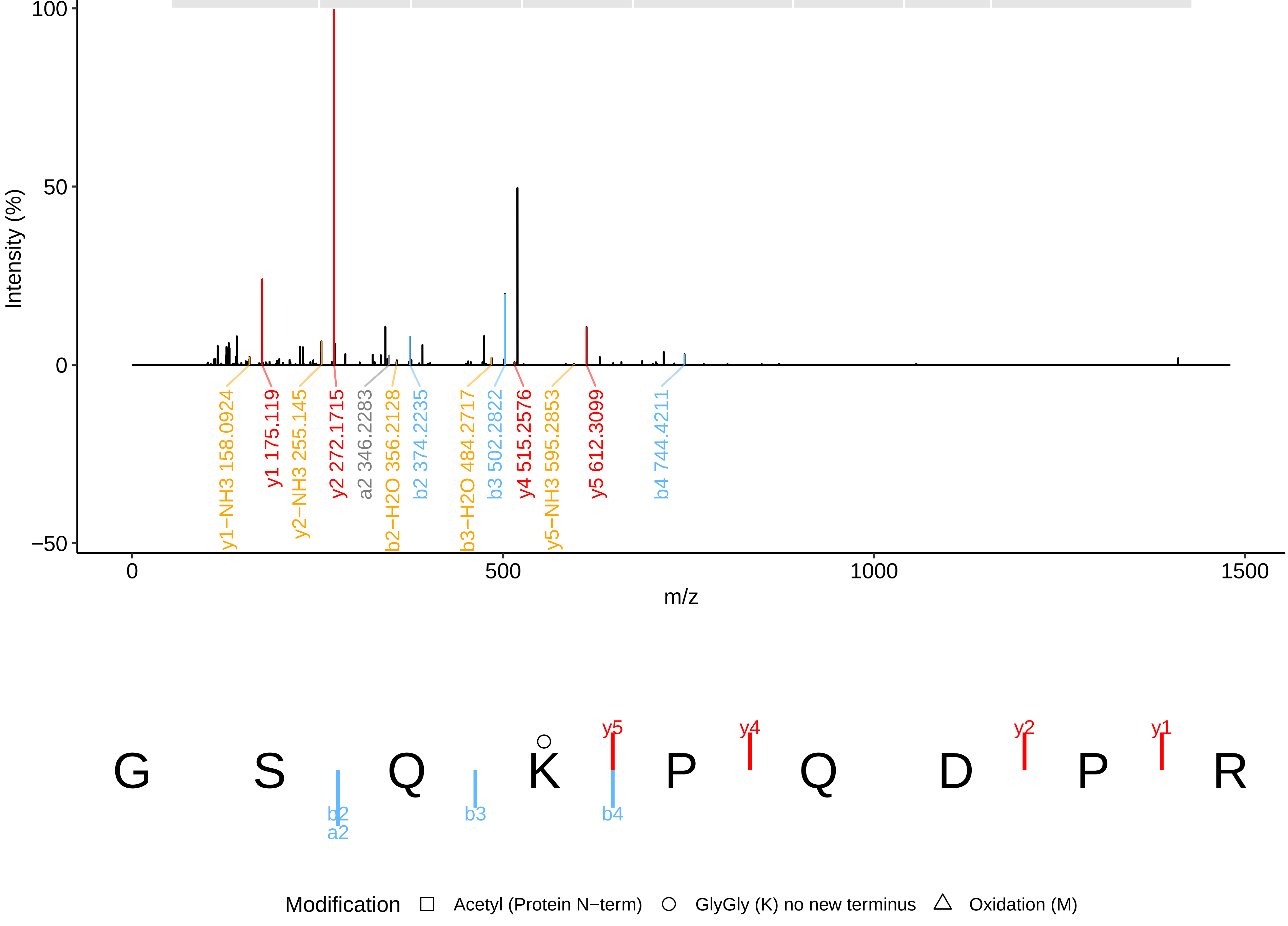

20200921_KIZ_MUb4_GlyGly_10plex_2 − Scan number: 2543

Gene Names	Charge	m/z	Mass	Retention time	PEP	Score	Precursor Intensity
RNF5	2	563.7836	1125.553	8.047	0.0013829	103.56	2096209

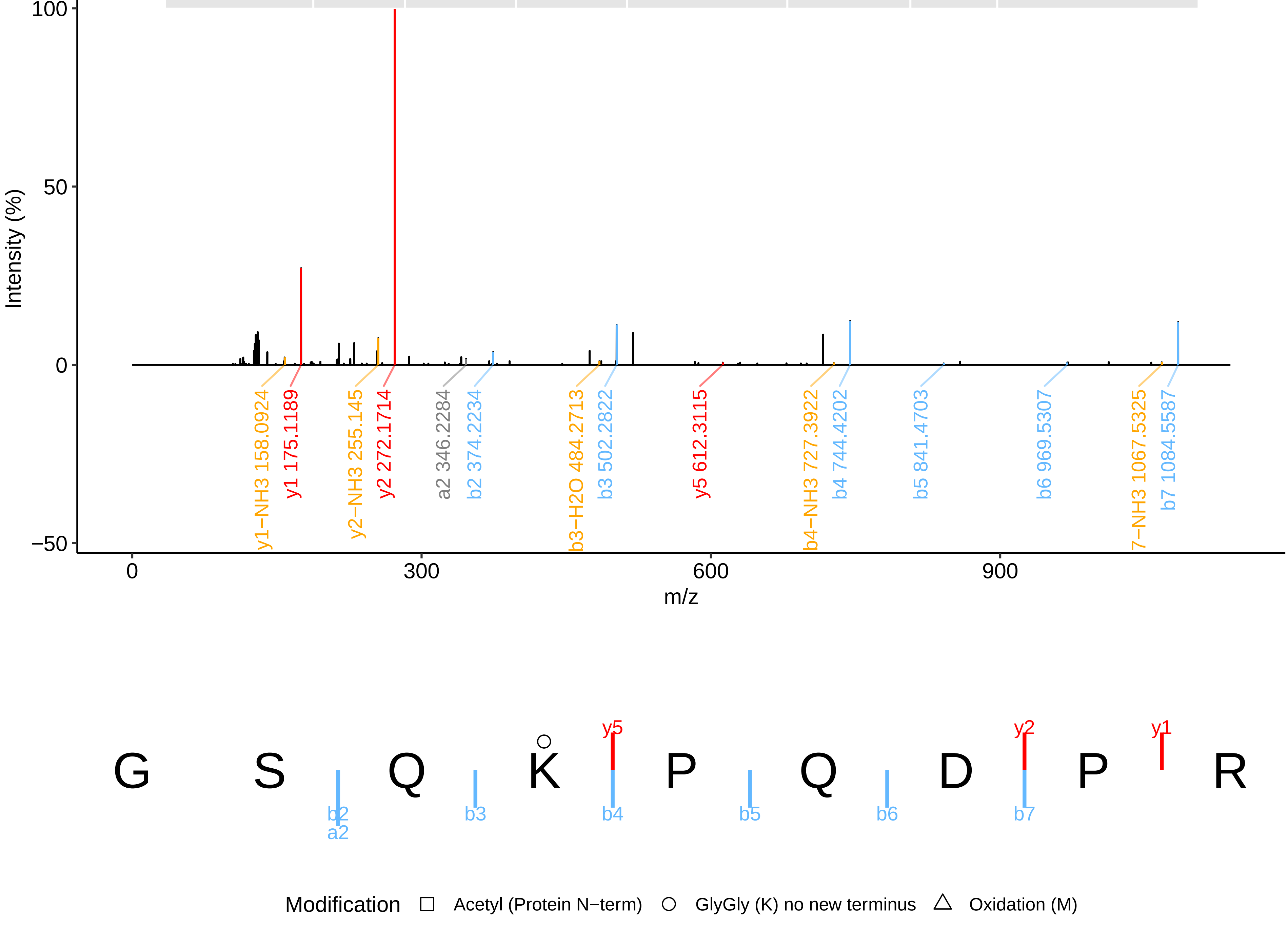

20200924_KIZ_MUb5_GlyGly_1 − Scan number: 2433

Gene Names	Charge	m/z	Mass	Retention time	PEP	Score	Precursor Intensity
RNF5	3	376.1915	1125.553	7.5581	0.02139	63.816	21656798

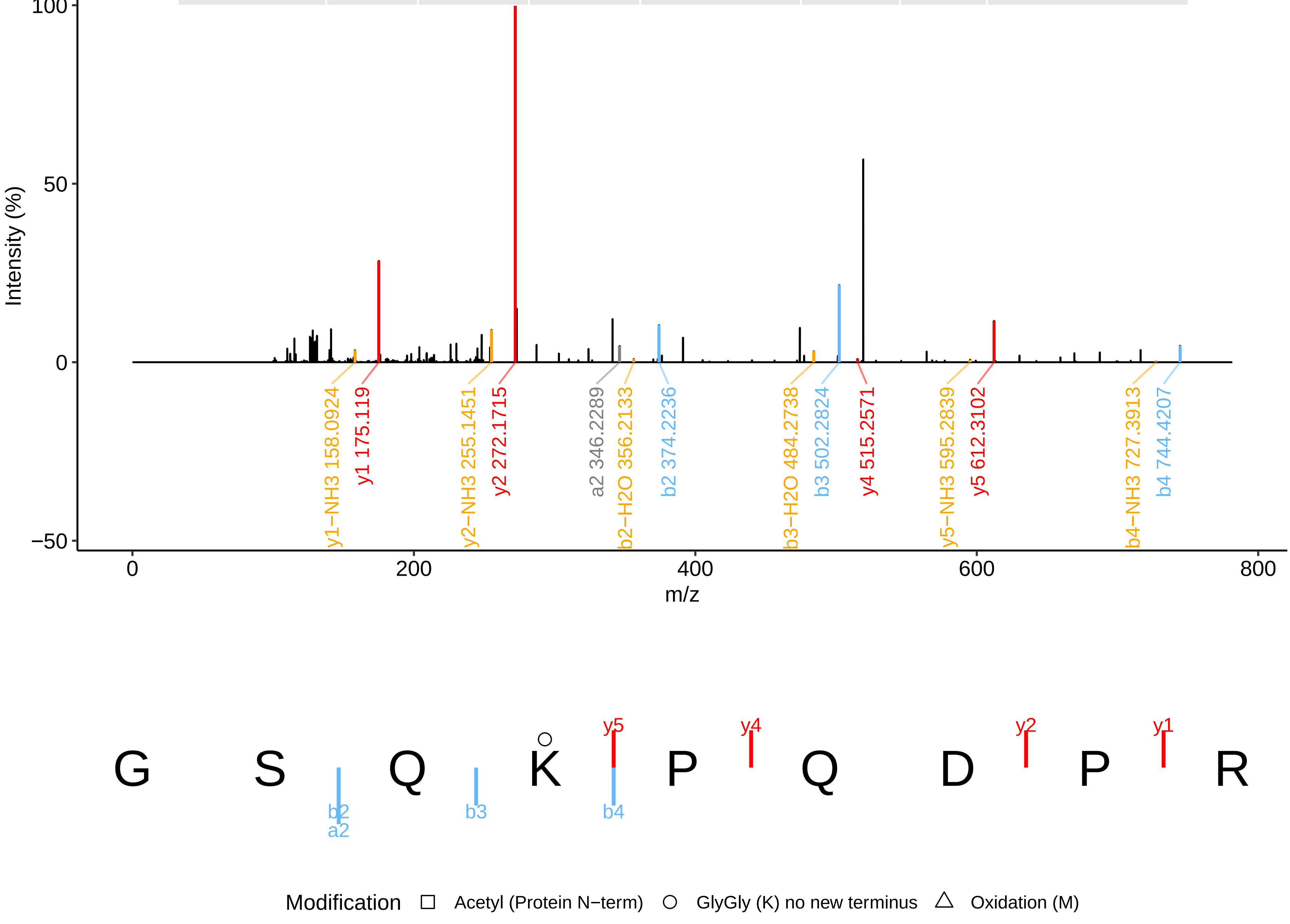

20200924_KIZ_MUb5_GlyGly_2 − Scan number: 2135

Gene Names	Charge	m/z	Mass	Retention time	PEP	Score	Precursor Intensity
RNF5	3	376.1915	1125.553	7.5664	0.057117	55.908	19544936

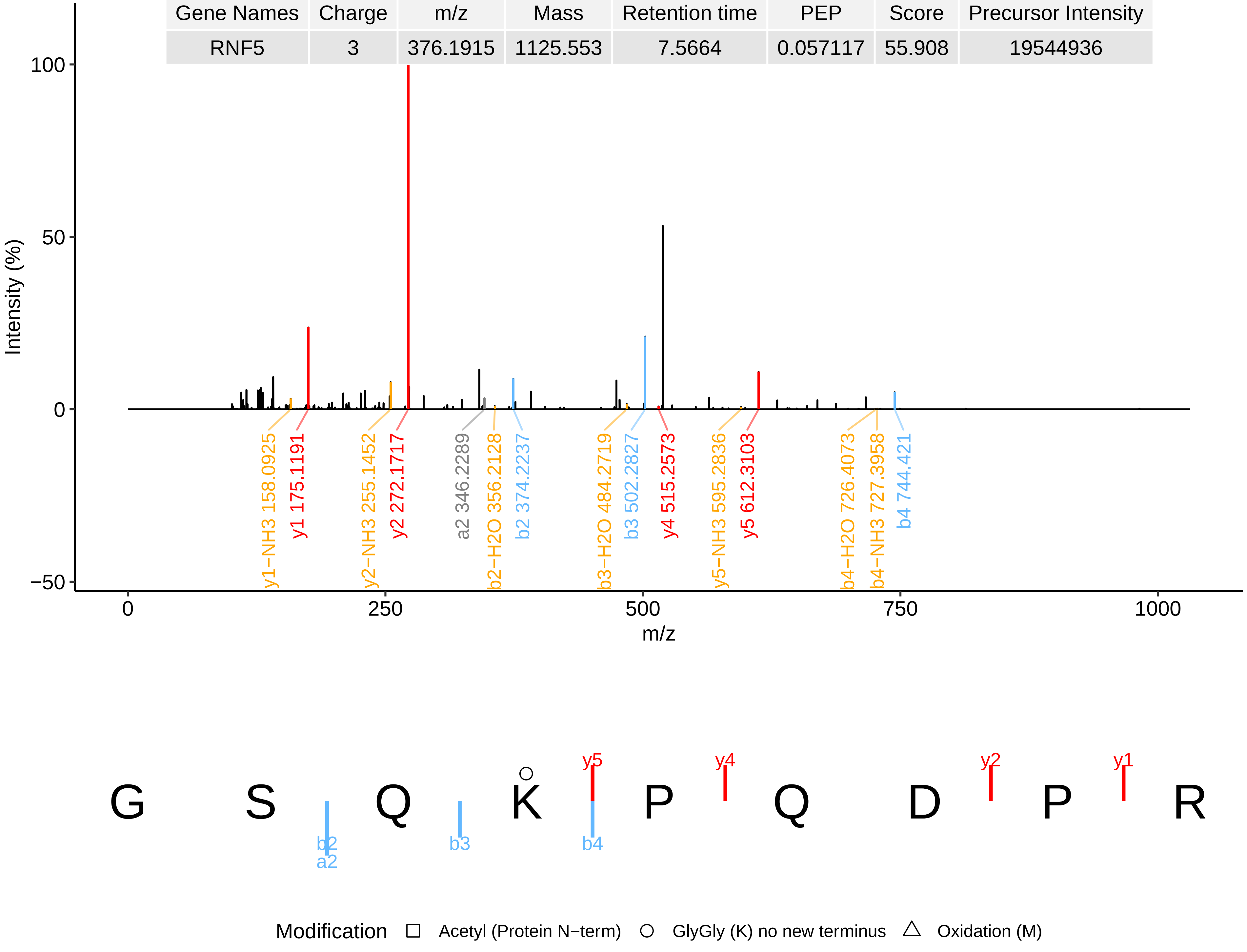

20200921_KIZ_MUb4_GlyGly_10plex − Scan number: 8175

Gene Names	Charge	m/z	Mass	Retention time	PEP	Score	Precursor Intensity
SLC25A3	3	377.8875	1130.641	27.675	1.632e−06	123.88	5460236

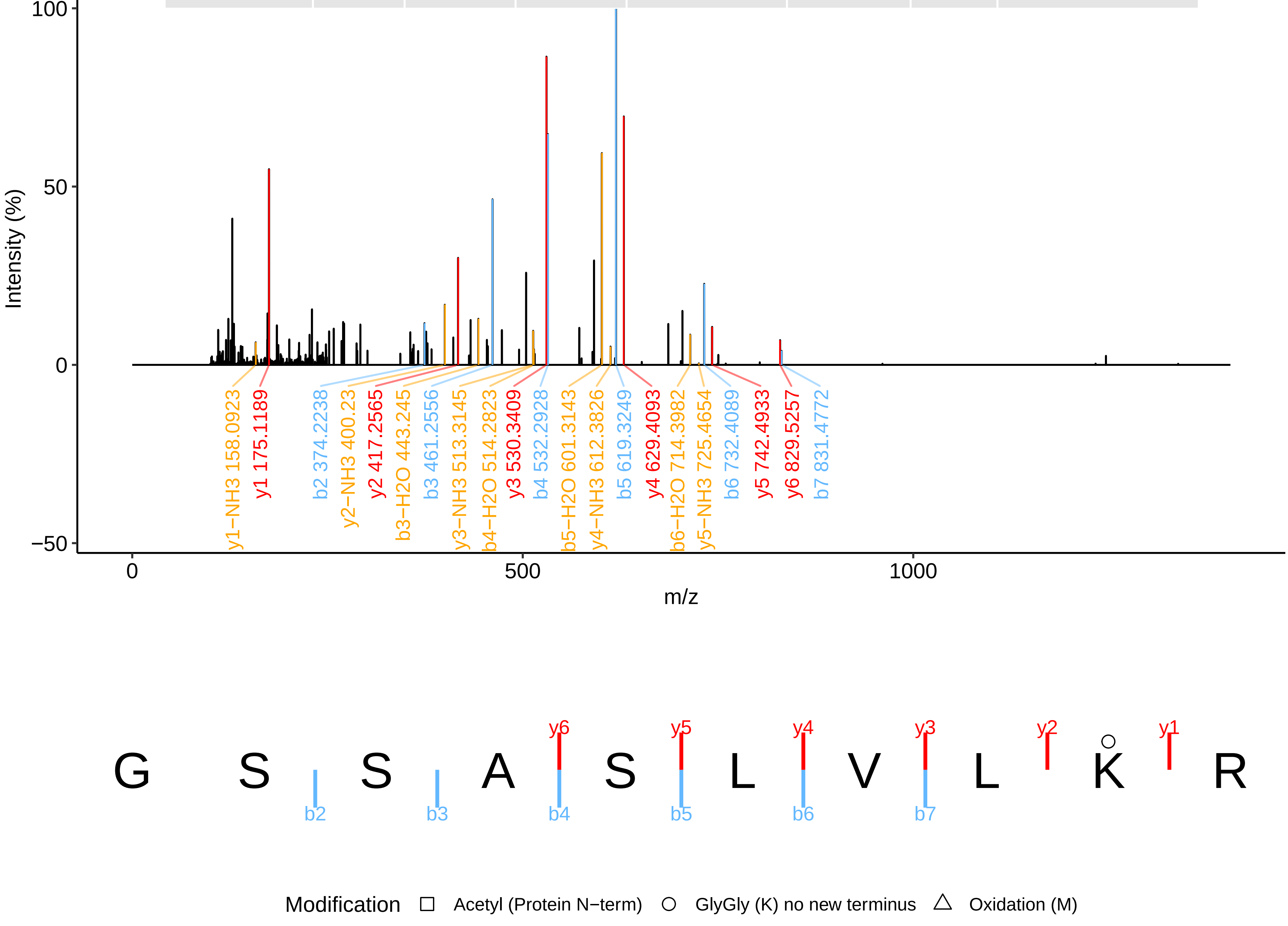

20200921_KIZ_MUb4_GlyGly_10plex − Scan number: 13534

Gene Names	Charge	m/z	Mass	Retention time	PEP	Score	Precursor Intensity
WRNIP1	3	370.8612	1109.562	47.784	0.00088723	109.86	10706192

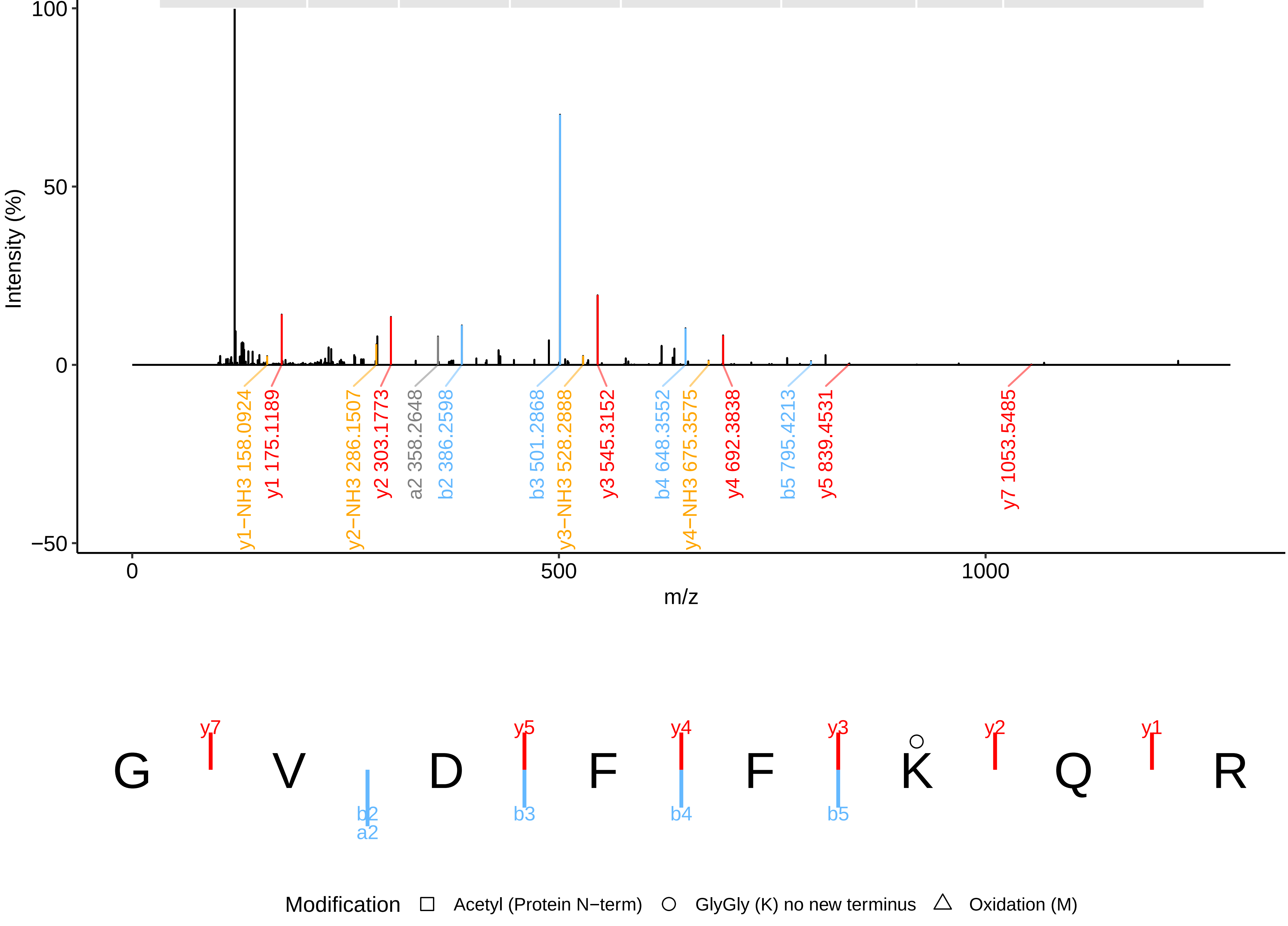

20200921_KIZ_MUb4_GlyGly_10plex_2 − Scan number: 12485

Gene Names	Charge	m/z	Mass	Retention time	PEP	Score	Precursor Intensity
WRNIP1	3	370.8612	1109.562	48.307	0.010929	79.116	2548010

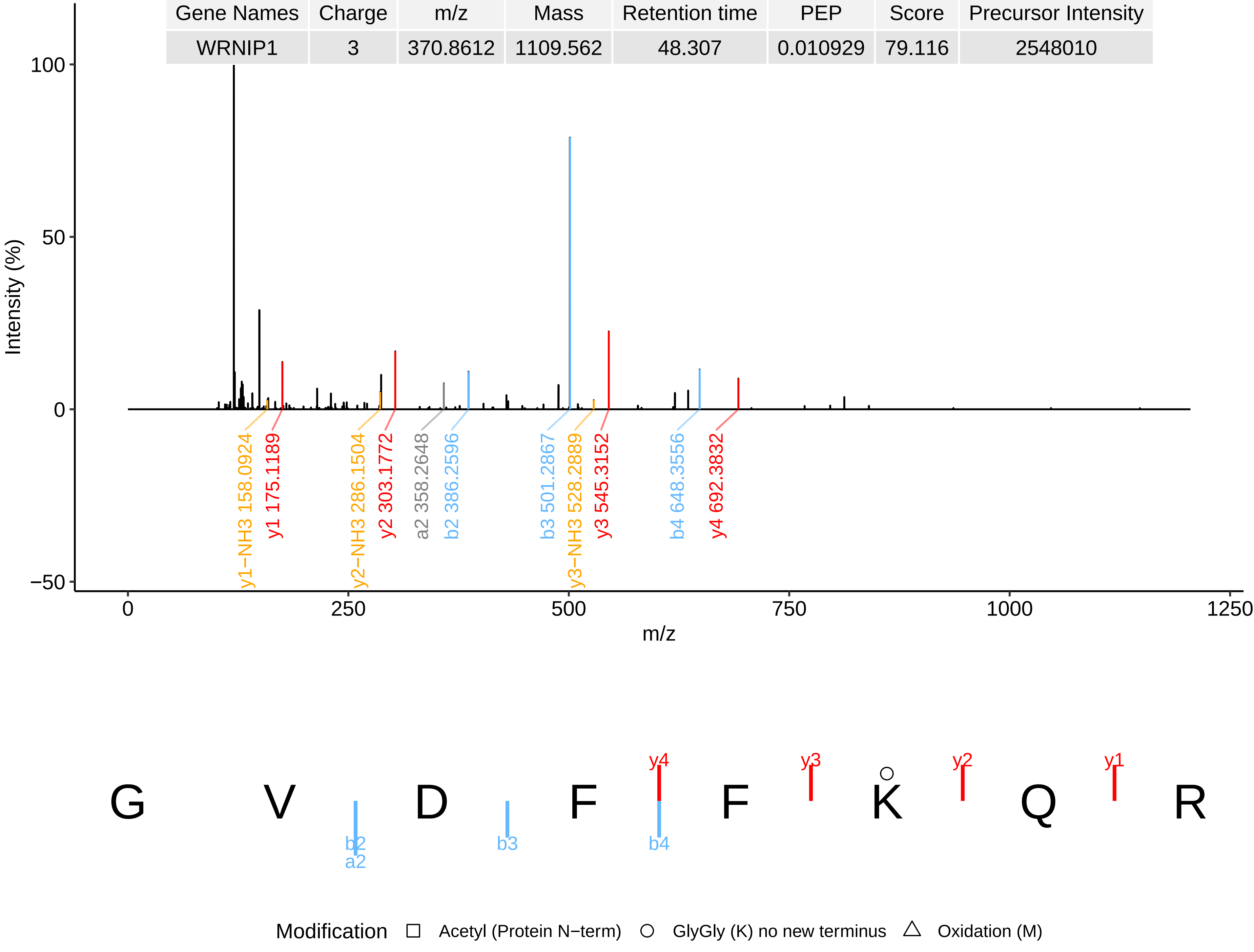

20200924_KIZ_MUb5_GlyGly_2 − Scan number: 11570

Gene Names	Charge	m/z	Mass	Retention time	PEP	Score	Precursor Intensity
WRNIP1	3	370.8612	1109.562	43.263	0.082778	56.547	2367771

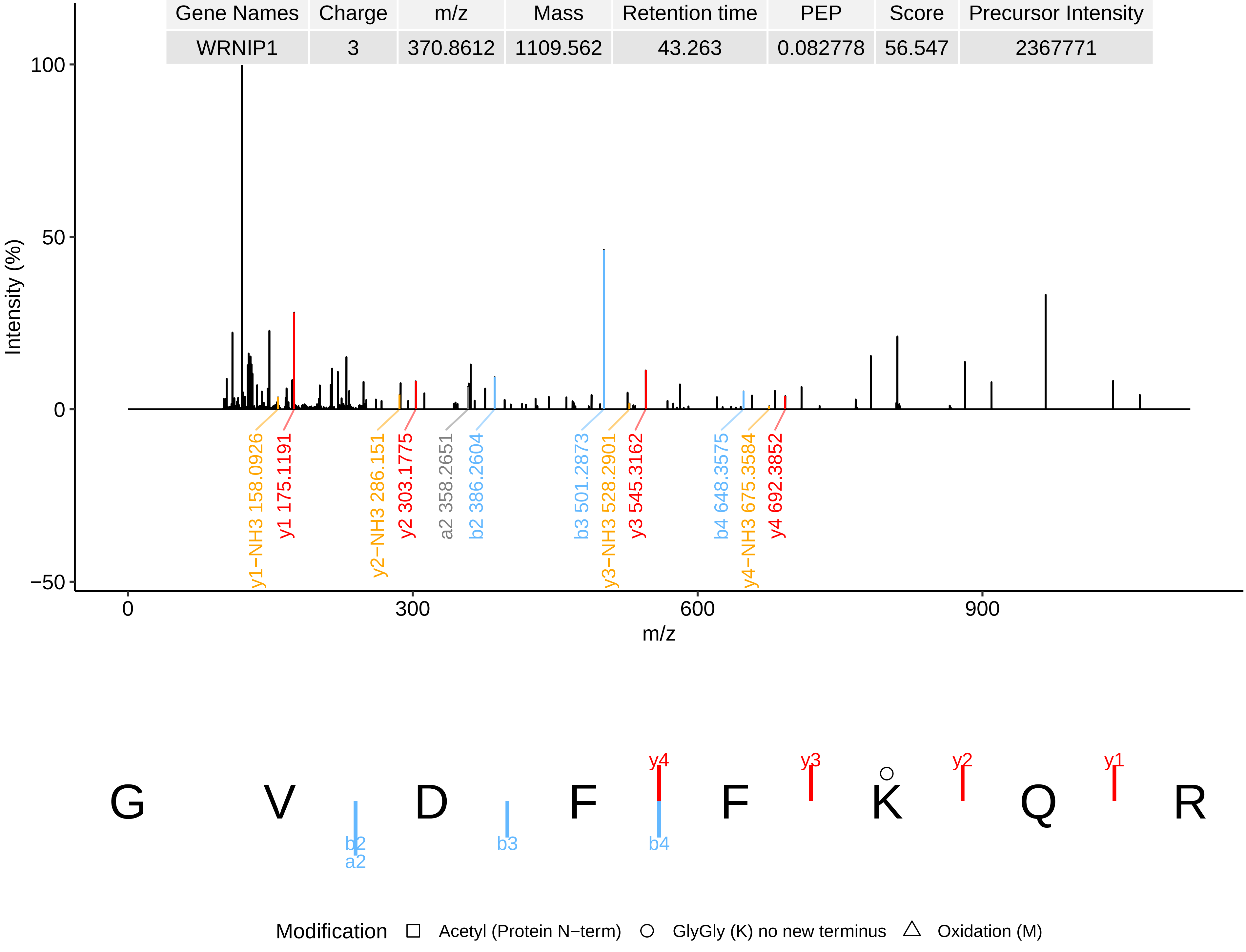

20200921_KIZ_MUb4_GlyGly_10plex_2 − Scan number: 7340

Gene Names	Charge	m/z	Mass	Retention time	PEP	Score	Precursor Intensity
DDI2	3	381.5595	1141.657	27.353	4.3339e−05	105.52	962144.1

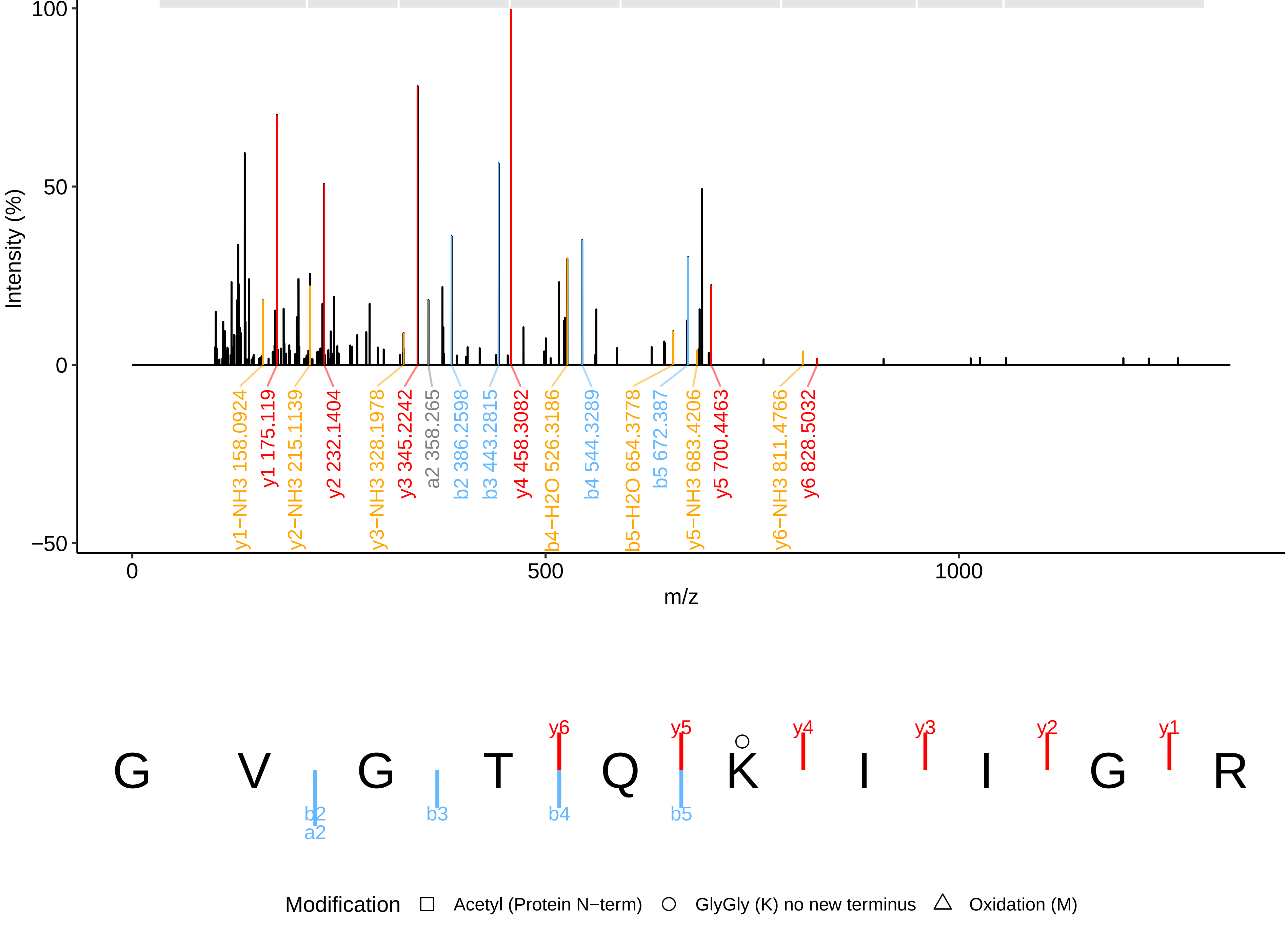

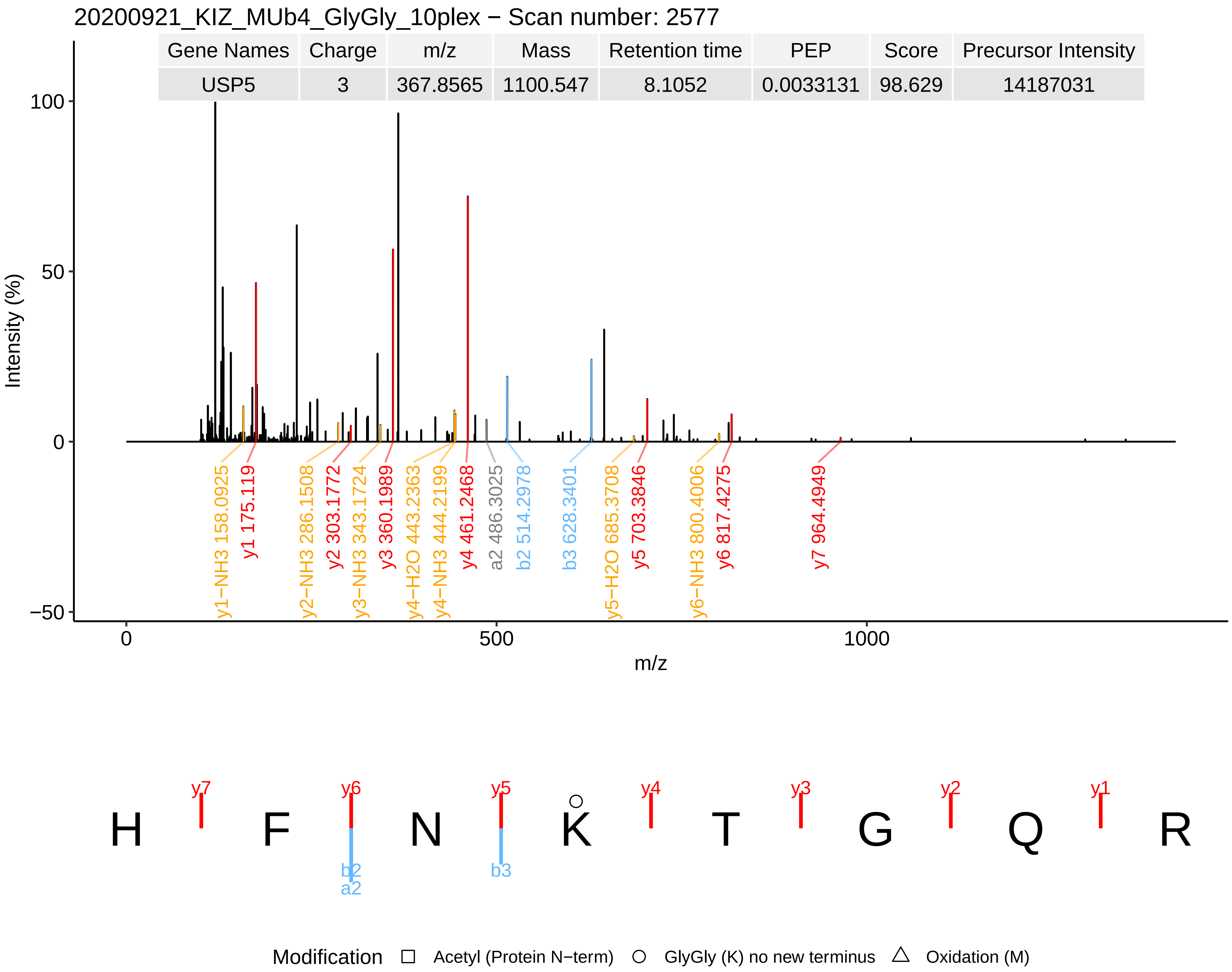

20200921_KIZ_MUb4_GlyGly_10plex − Scan number: 2577
Gene Names | Charge | m/z | Mass | Retention time | PEP | Score | Precursor Intensity
USP5 | 3 | 367.8565 | 1100.547 | 8.1052 | 0.0033131 | 98.629 | 14187031
Intensity (%)
100
50
0
−50
0
500
1000
m/z
y1−NH3 158.0925
y1 175.119
y2−NH3 286.1508
y2 303.1772
y3−NH3 343.1724
y3 360.1989
y4−H2O 443.2363
y4−NH3 444.2199
y4 461.2468
a2 486.3025
b2 514.2978
b3 628.3401
y5−H2O 685.3708
y5 703.3846
y6−NH3 800.4006
y6 817.4275
y7 964.4949
H F N K T G Q R
y7 y6 y5 y4 y3 y2 y1
b2 a2 b3
Modification
Acetyl (Protein N−term)
GlyGly (K) no new terminus
Oxidation (M)

20200921_KIZ_MUb4_GlyGly_10plex − Scan number: 8820

Gene Names	Charge	m/z	Mass	Retention time	PEP	Score	Precursor Intensity
TRIP4	3	296.8431	887.5076	30.054	0.10705	54.549	7598530

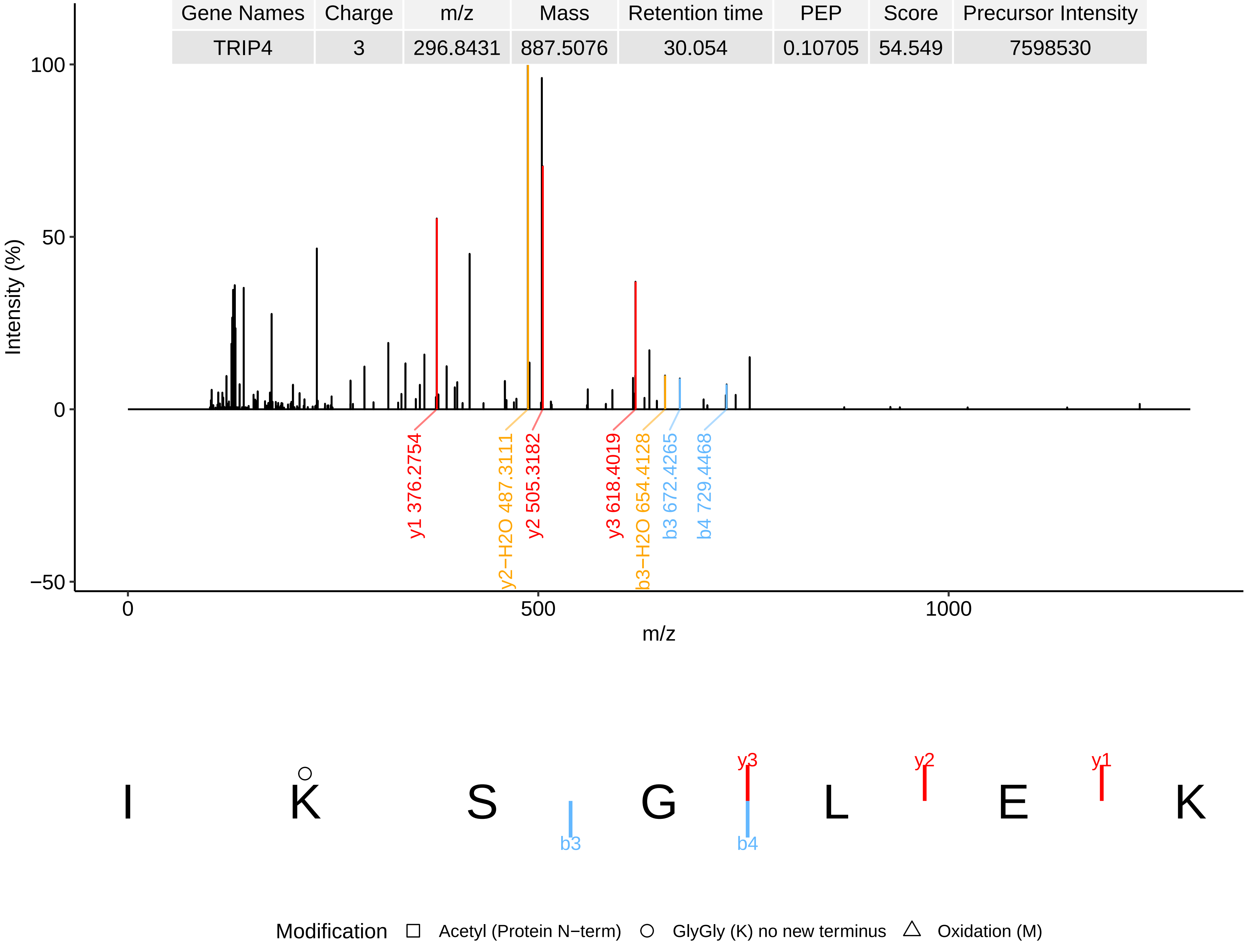

20200921_KIZ_MUb4_GlyGly_10plex − Scan number: 8450

Gene Names	Charge	m/z	Mass	Retention time	PEP	Score	Precursor Intensity
PSMC3	3	369.206	1104.596	28.673	0.039207	58.716	11361730

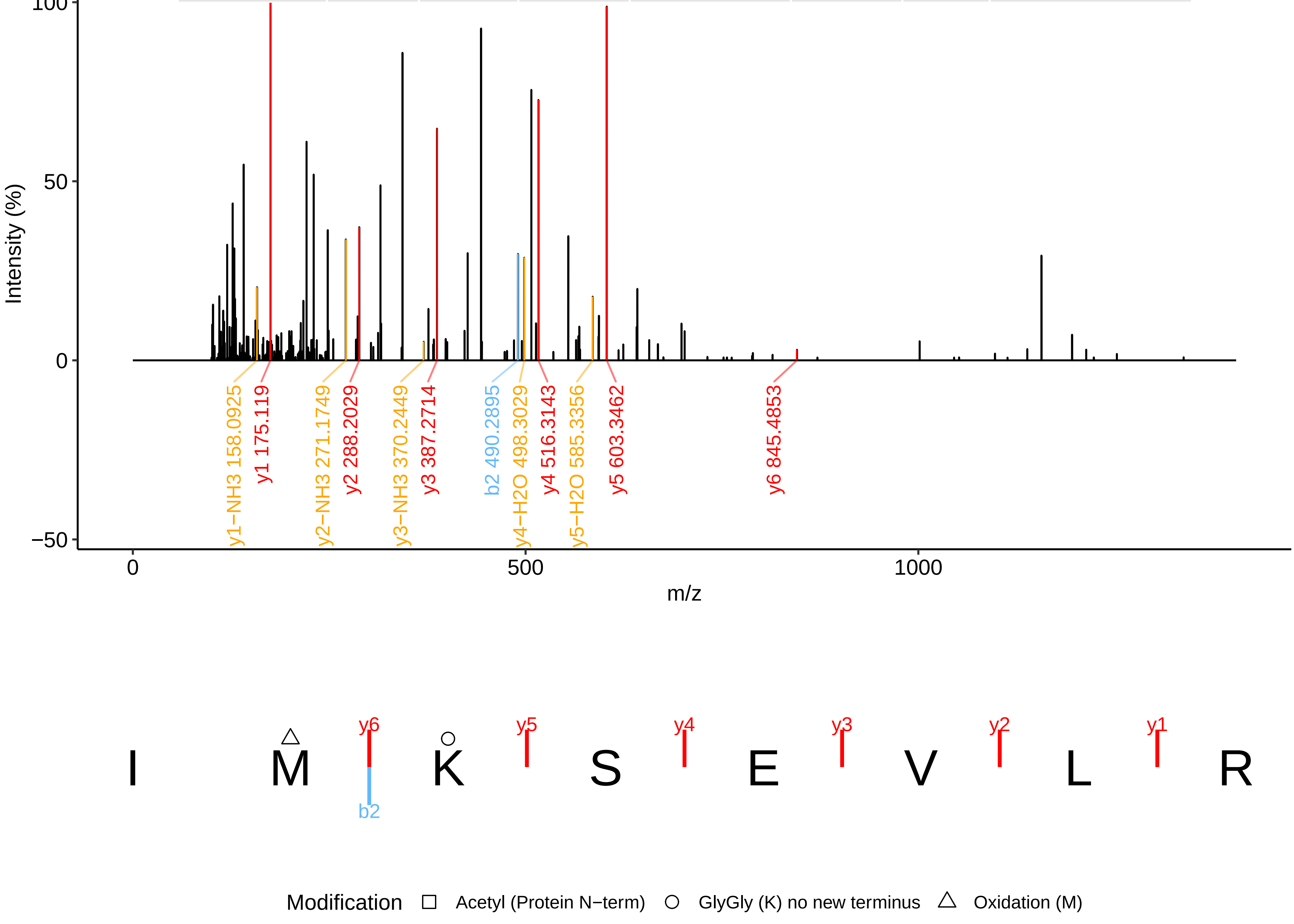

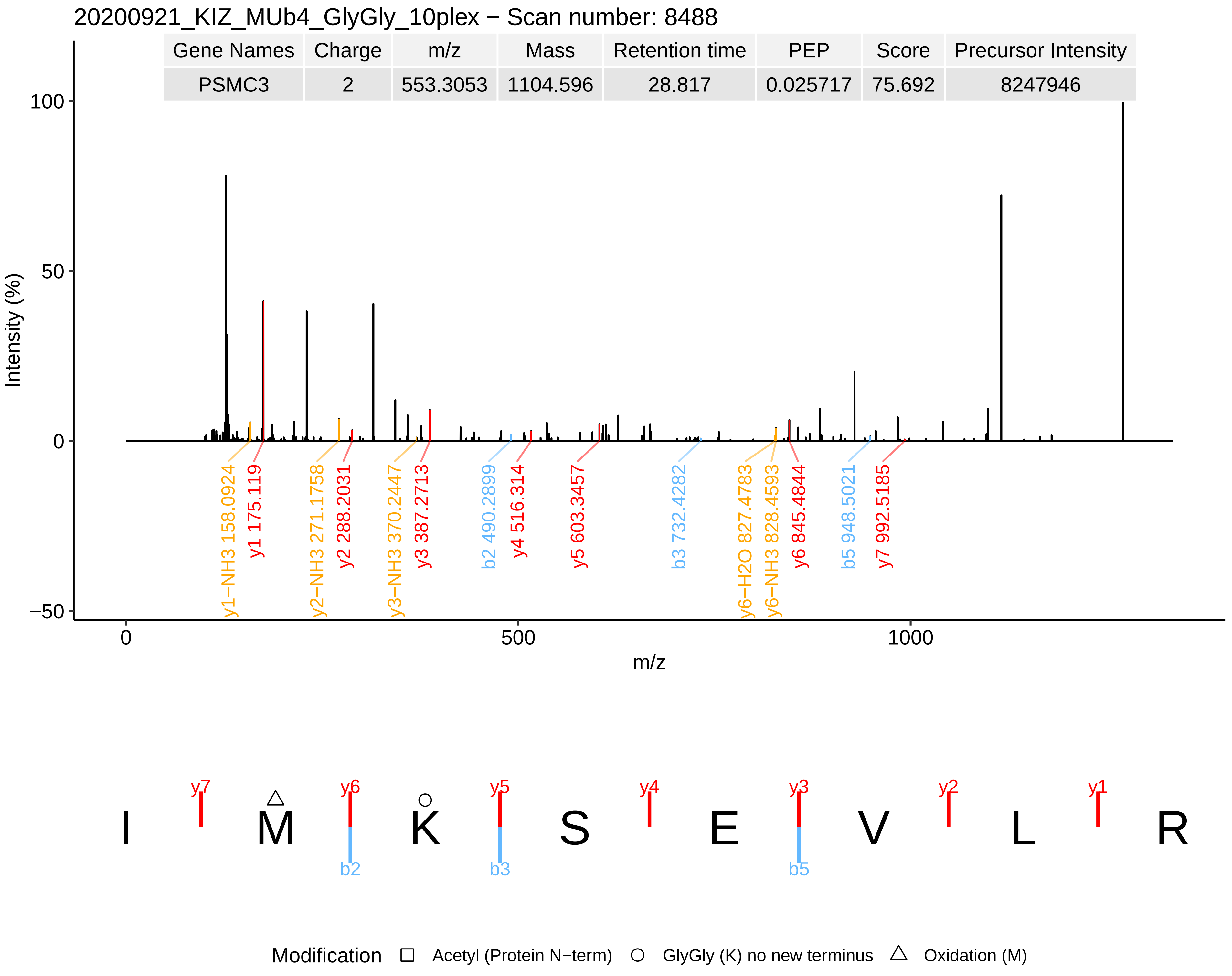

20200921_KIZ_MUb4_GlyGly_10plex − Scan number: 8488
Gene Names | Charge | m/z | Mass | Retention time | PEP | Score | Precursor Intensity
PSMC3 | 2 | 553.3053 | 1104.596 | 28.817 | 0.025717 | 75.692 | 8247946
Intensity (%)
100
50
0
−50
0
500
1000
m/z
y1−NH3 158.0924
y1 175.119
y2−NH3 271.1758
y2 288.2031
y3−NH3 370.2447
y3 387.2713
b2 490.2899
y4 516.314
y5 603.3457
b3 732.4282
y6−H2O 827.4783
y6−NH3 828.4593
y6 845.4844
b5 948.5021
y7 992.5185
I M K S E V L R
y7 y6 y5 y4 y3 y2 y1
b2 b3 b5
Modification
Acetyl (Protein N−term)
GlyGly (K) no new terminus
Oxidation (M)

20200921_KIZ_MUb4_GlyGly_10plex_2 − Scan number: 7829

Gene Names	Charge	m/z	Mass	Retention time	PEP	Score	Precursor Intensity
PSMC3	3	369.206	1104.596	29.338	0.028563	61.962	9560130

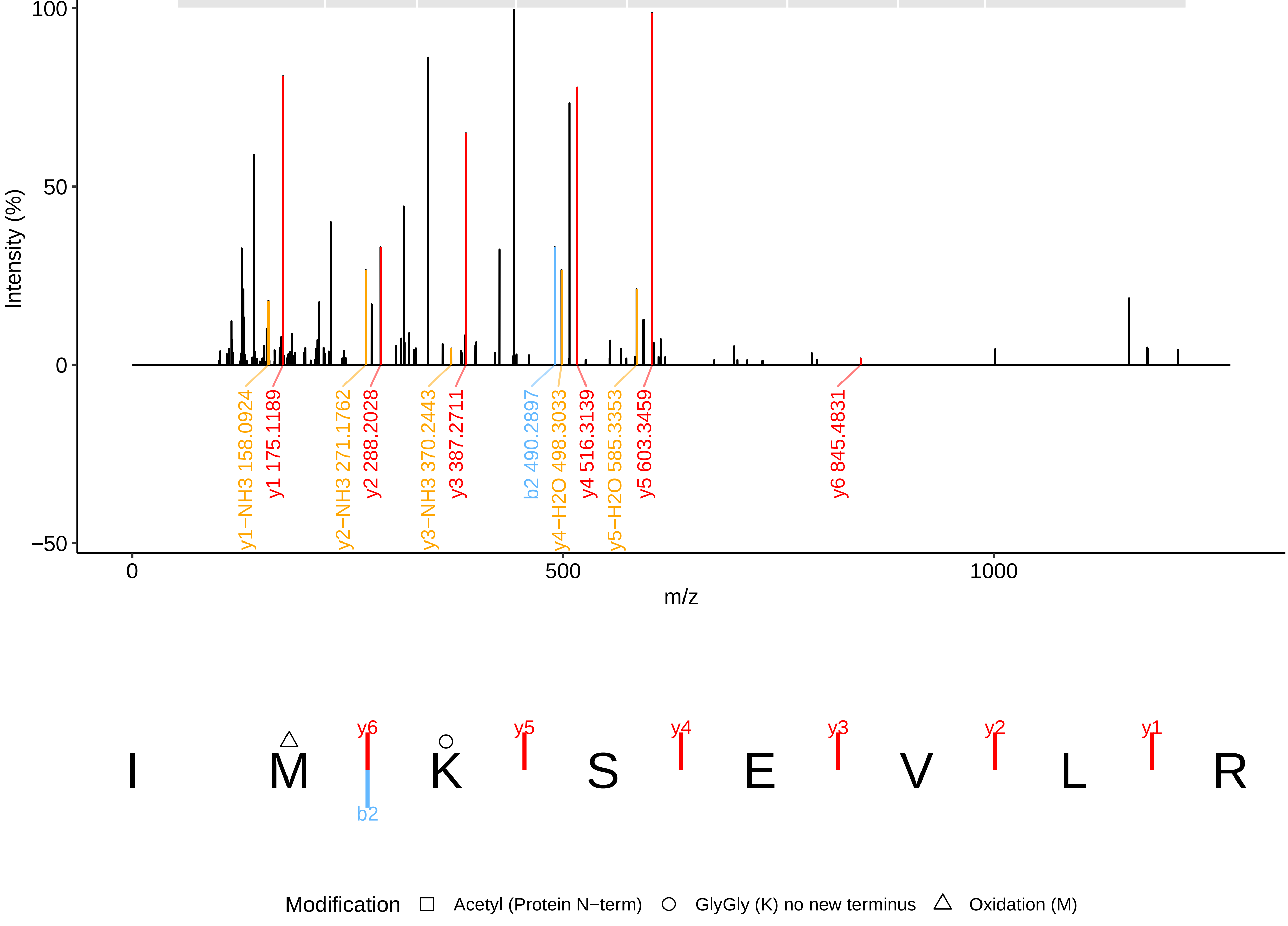

20200924_KIZ_MUb5_GlyGly_2 − Scan number: 6854

Gene Names	Charge	m/z	Mass	Retention time	PEP	Score	Precursor Intensity
PSMC3	3	369.206	1104.596	25.569	0.028563	61.962	6477598

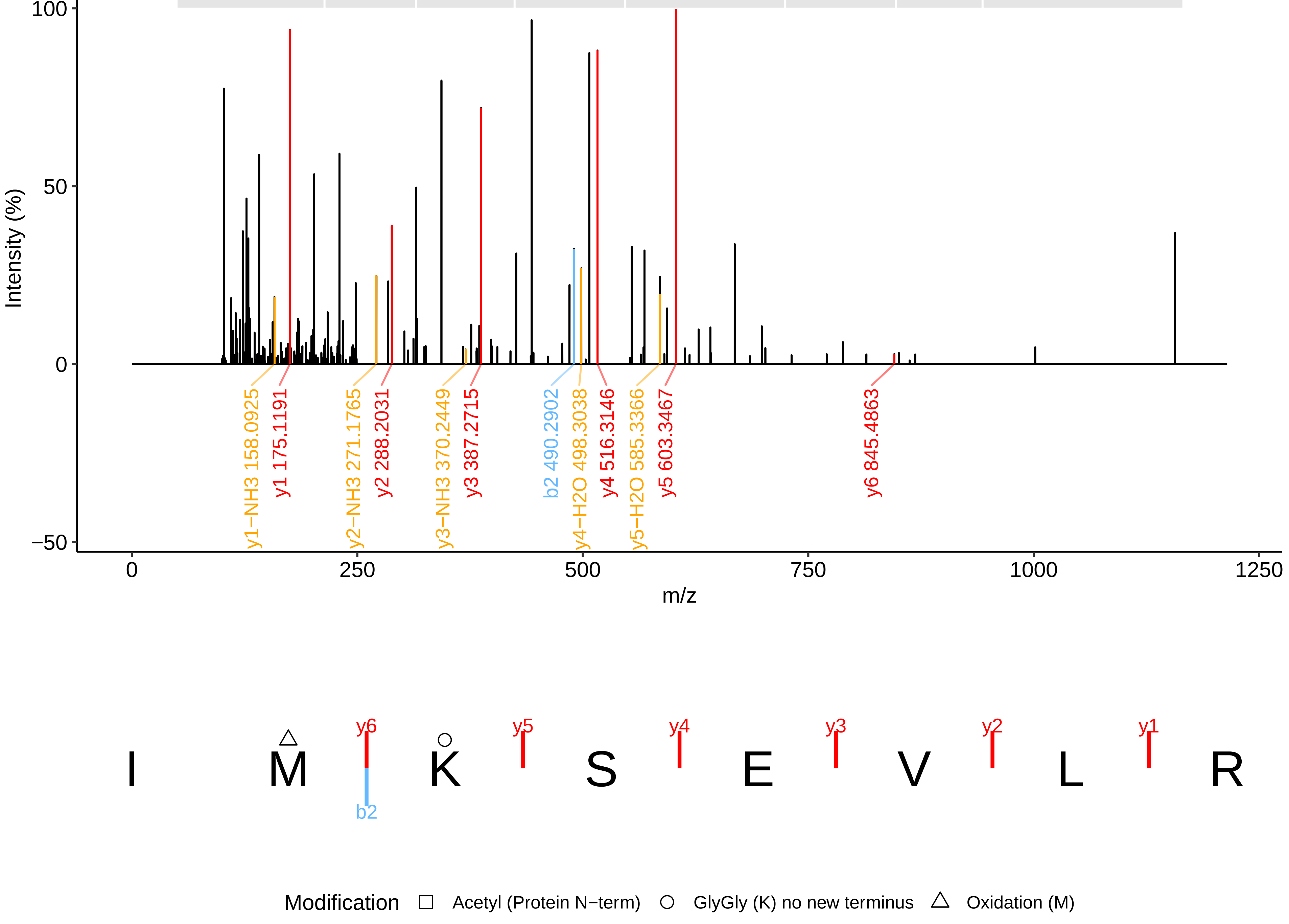

20200924_KIZ_MUb5_GlyGly_2 − Scan number: 7064

Gene Names	Charge	m/z	Mass	Retention time	PEP	Score	Precursor Intensity
PSMC3	3	369.206	1104.596	26.342	0.028563	61.962	4654472

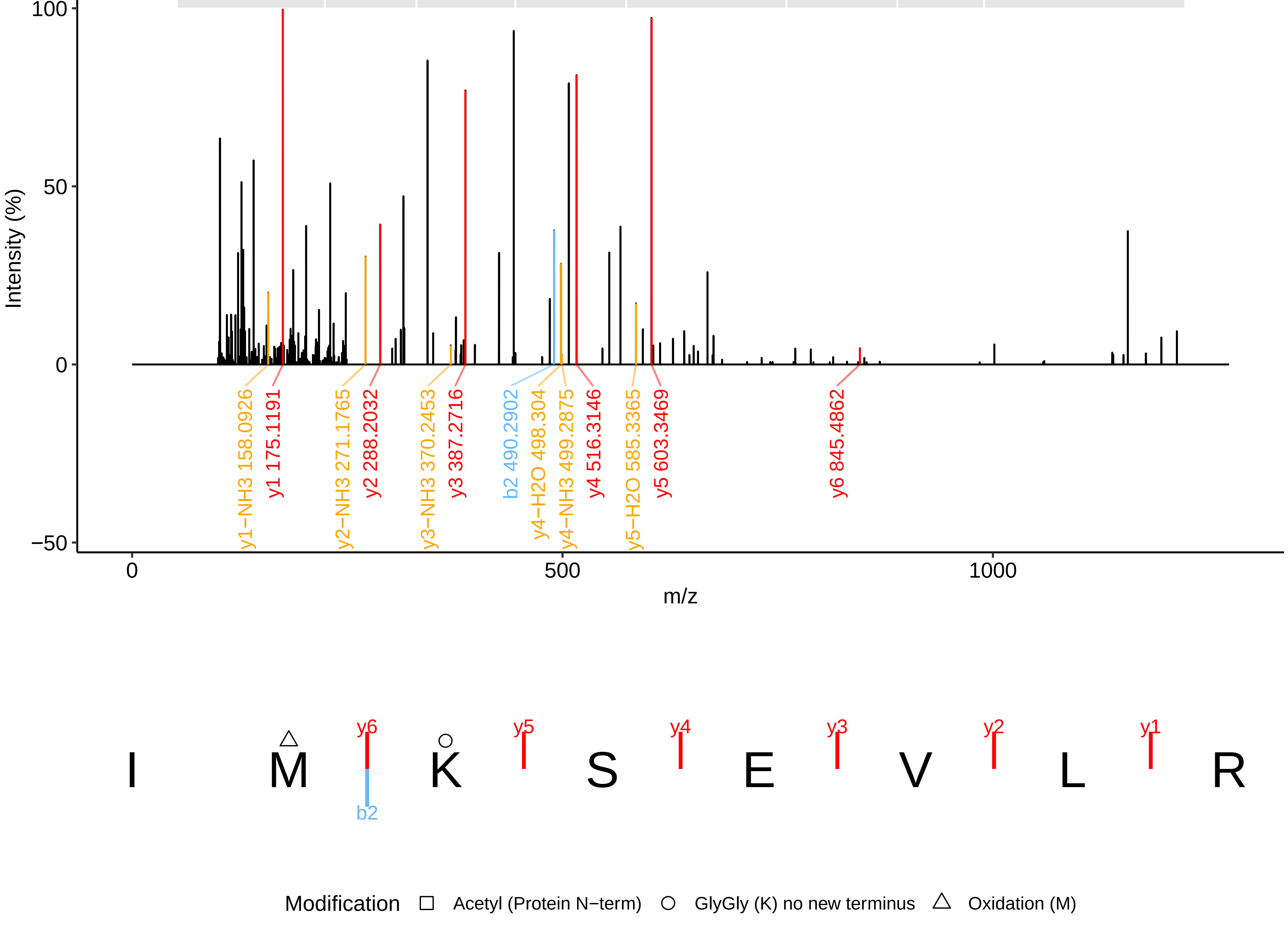

20201118_KIZ_MUb6_GlyGly_1 − Scan number: 7047

Gene Names	Charge	m/z	Mass	Retention time	PEP	Score	Precursor Intensity
PSMC3	3	369.206	1104.596	22.337	0.019257	77.923	14030207

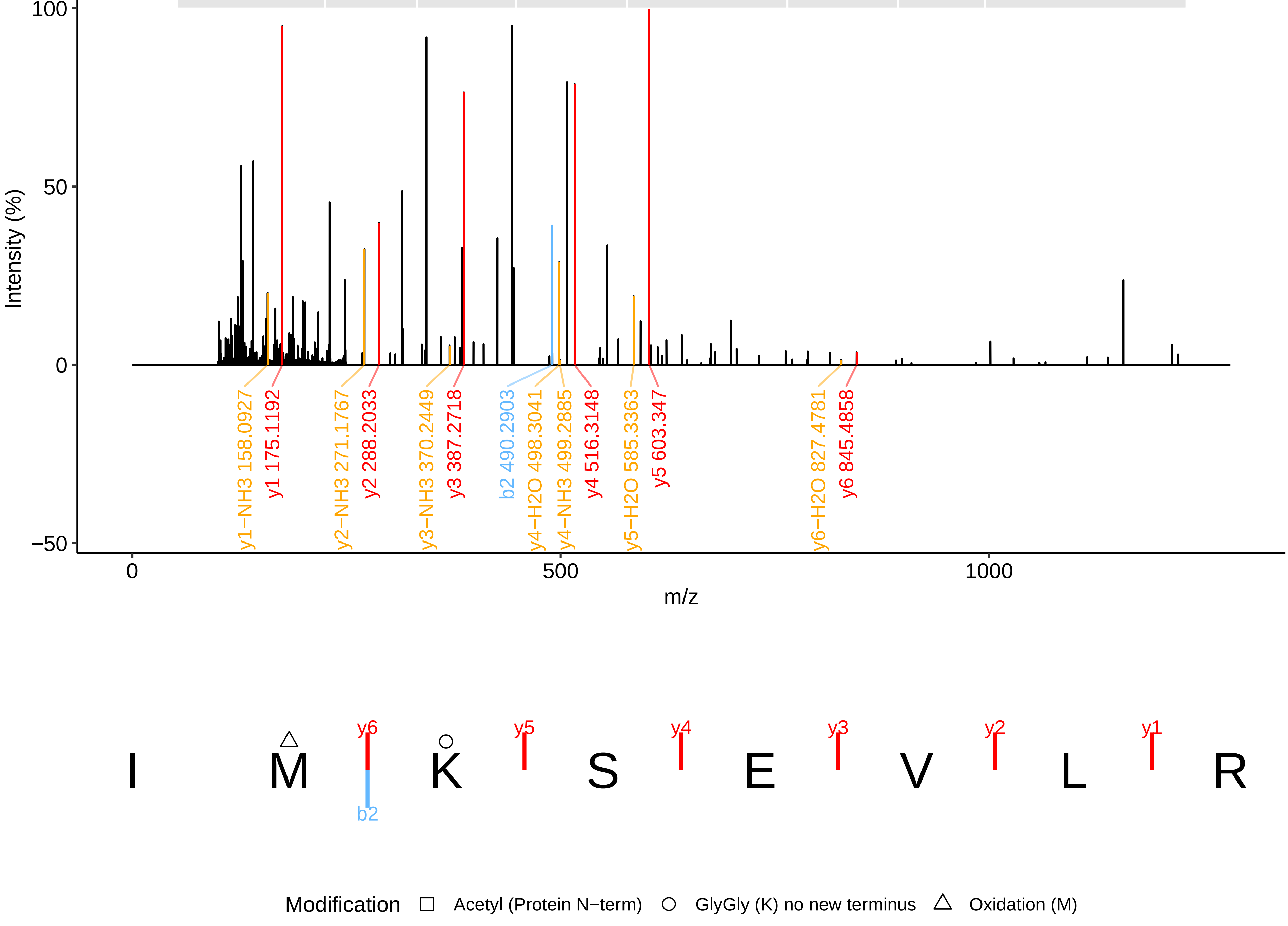

20201118_KIZ_MUb6_GlyGly_2 − Scan number: 8957

Gene Names	Charge	m/z	Mass	Retention time	PEP	Score	Precursor Intensity
PSMC3	3	369.206	1104.596	30.354	0.034289	70.525	4611638

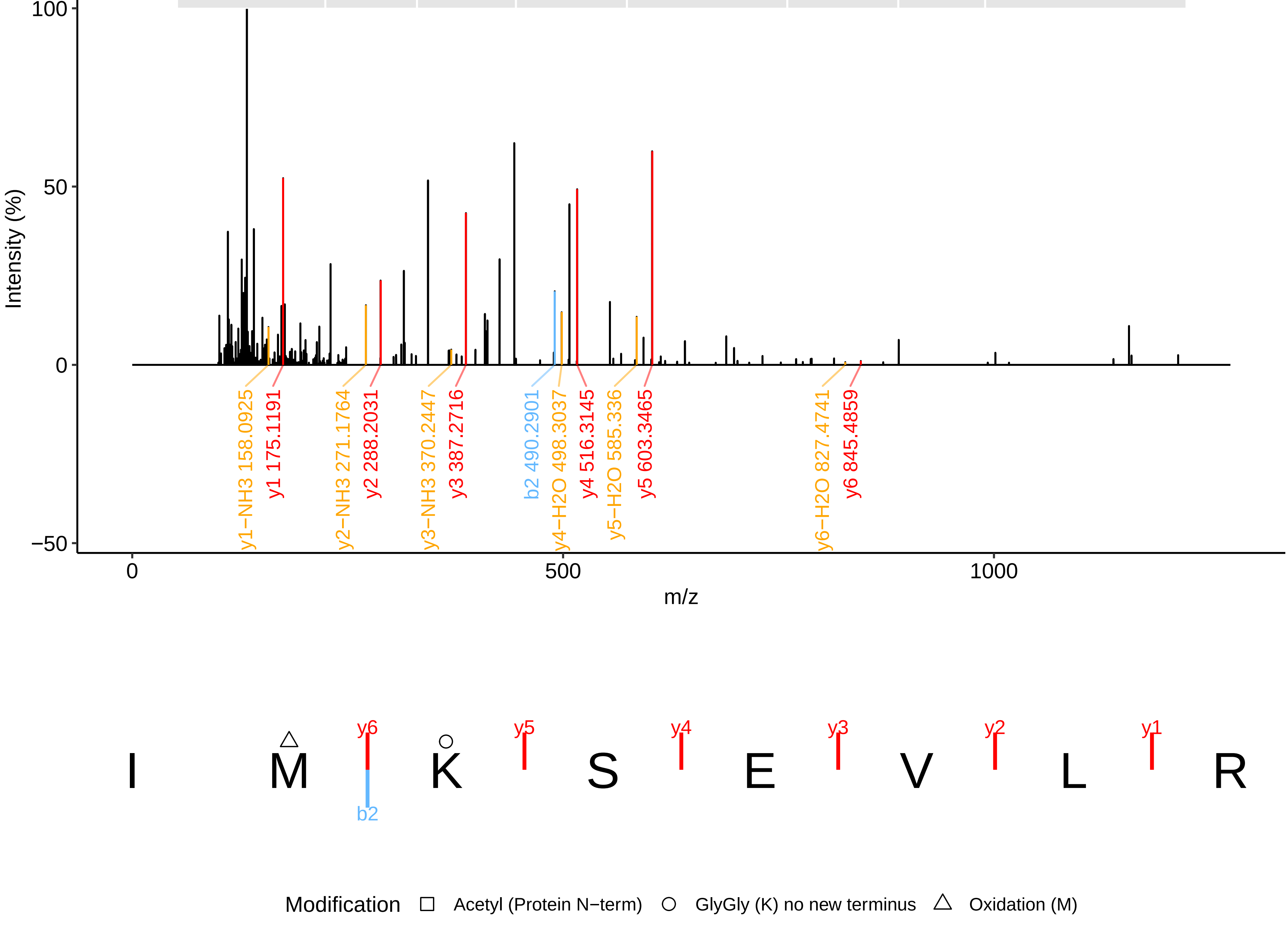

20200921_KIZ_MUb4_GlyGly_10plex − Scan number: 6992

Gene Names	Charge	m/z	Mass	Retention time	PEP	Score	Precursor I
HSPA8;HSPA1B;HSPA1A;HSPA1L;HSPA6;HSPA2	3	377.8754	1130.604	23.34	1.8385e−10	169.21	53051

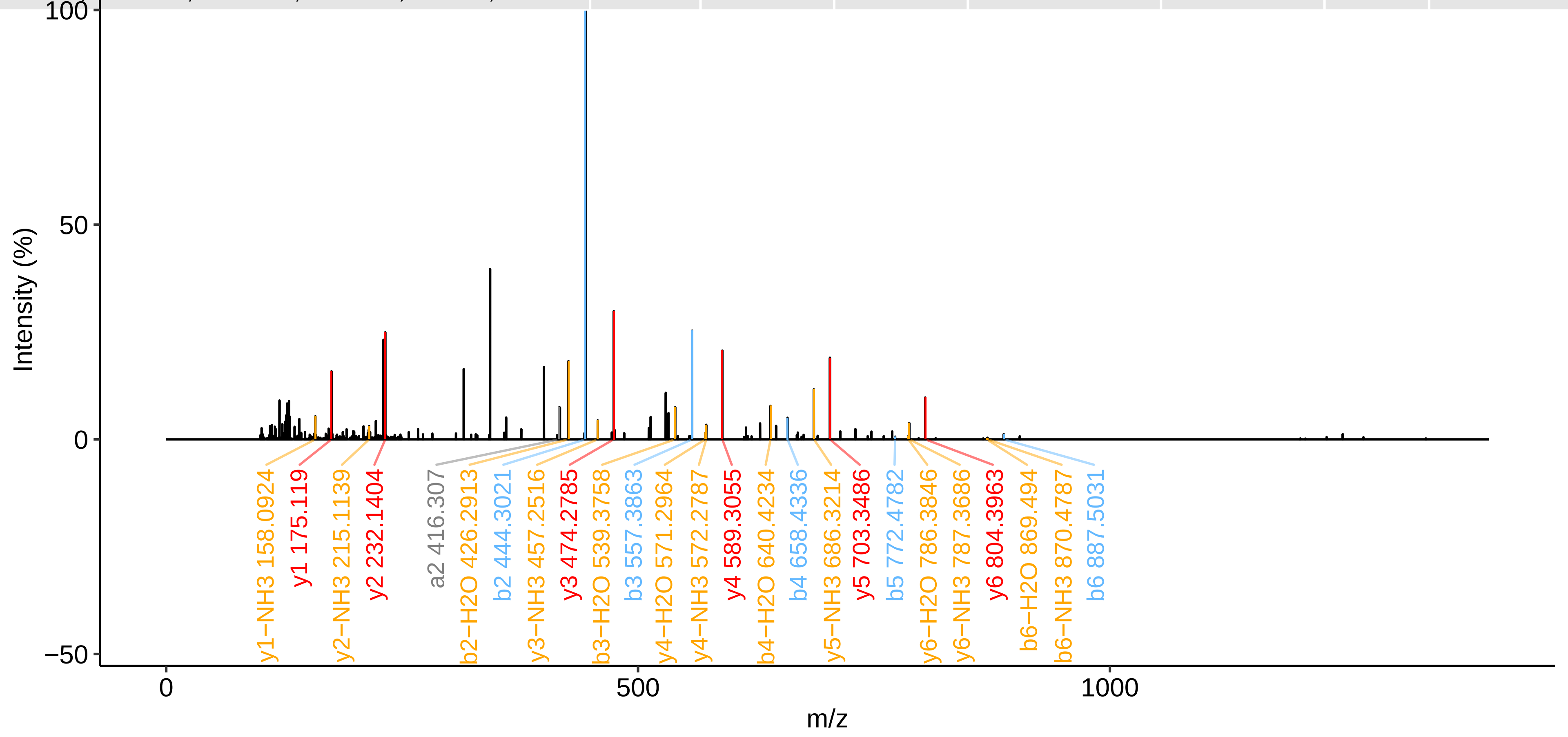

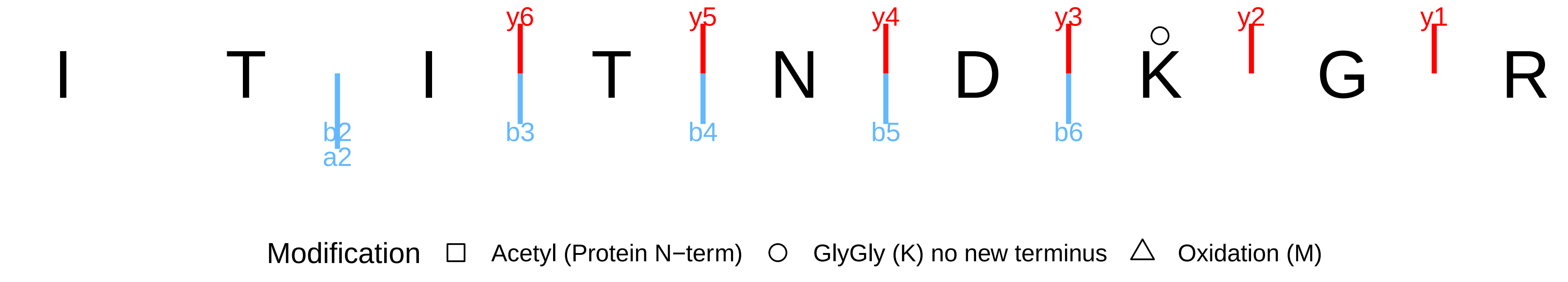

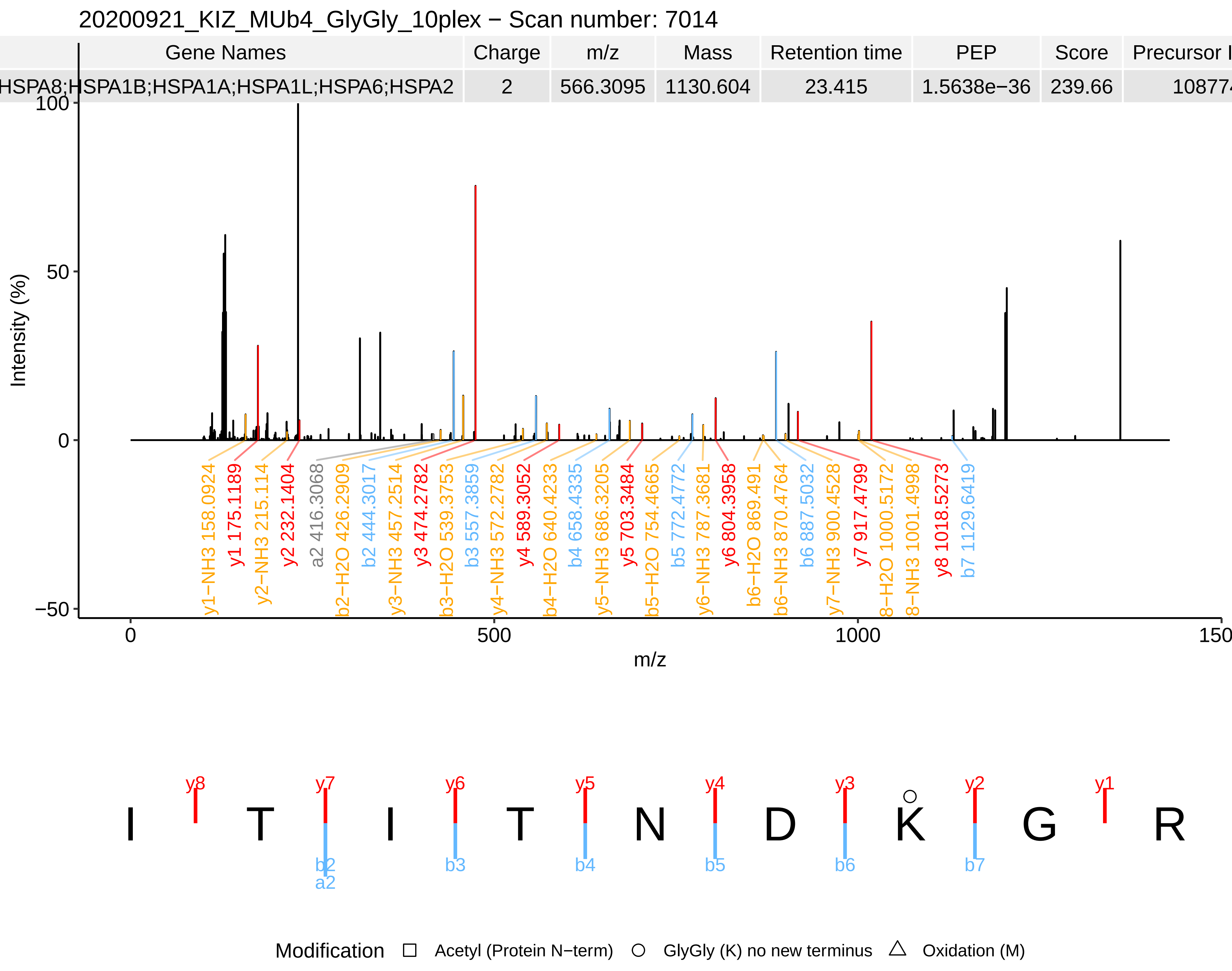

20200921_KIZ_MUb4_GlyGly_10plex − Scan number: 7014
Gene Names
Charge
m/z
Mass
Retention time
PEP
Score
Precursor I
HSPA8;HSPA1B;HSPA1A;HSPA1L;HSPA6;HSPA2
2
566.3095
1130.604
23.415
1.5638e−36
239.66
10877
Intensity (%)
100
50
0
−50
m/z
0
500
1000
150
y1−NH3 158.0924
y1 175.1189
y2−NH3 215.114
y2 232.1404
a2 416.3068
b2−H2O 426.2909
b2 444.3017
y3−NH3 457.2514
y3 474.2782
b3−H2O 539.3753
b3 557.3859
y4−NH3 572.2782
y4 589.3052
b4−H2O 640.4233
b4 658.4335
y5−NH3 686.3205
y5 703.3484
b5−H2O 754.4665
b5 772.4772
y6−NH3 787.3681
y6 804.3958
b6−H2O 869.491
b6−NH3 870.4764
b6 887.5032
y7−NH3 900.4528
y7 917.4799
8−H2O 1000.5172
8−NH3 1001.4998
y8 1018.5273
b7 1129.6419
y8
y7
y6
y5
y4
y3
y2
y1
I
T
I
T
N
D
K
G
R
b2
a2
b3
b4
b5
b6
b7
Modification
Acetyl (Protein N−term)
GlyGly (K) no new terminus
Oxidation (M)

20200921_KIZ_MUb4_GlyGly_10plex_2 − Scan number: 6454

Gene Names	Charge	m/z	Mass	Retention time	PEP	Score	Precursor In
HSPA8;HSPA1B;HSPA1A;HSPA1L;HSPA6;HSPA2	3	377.8754	1130.604	23.723	1.8777e−06	131.5	12465

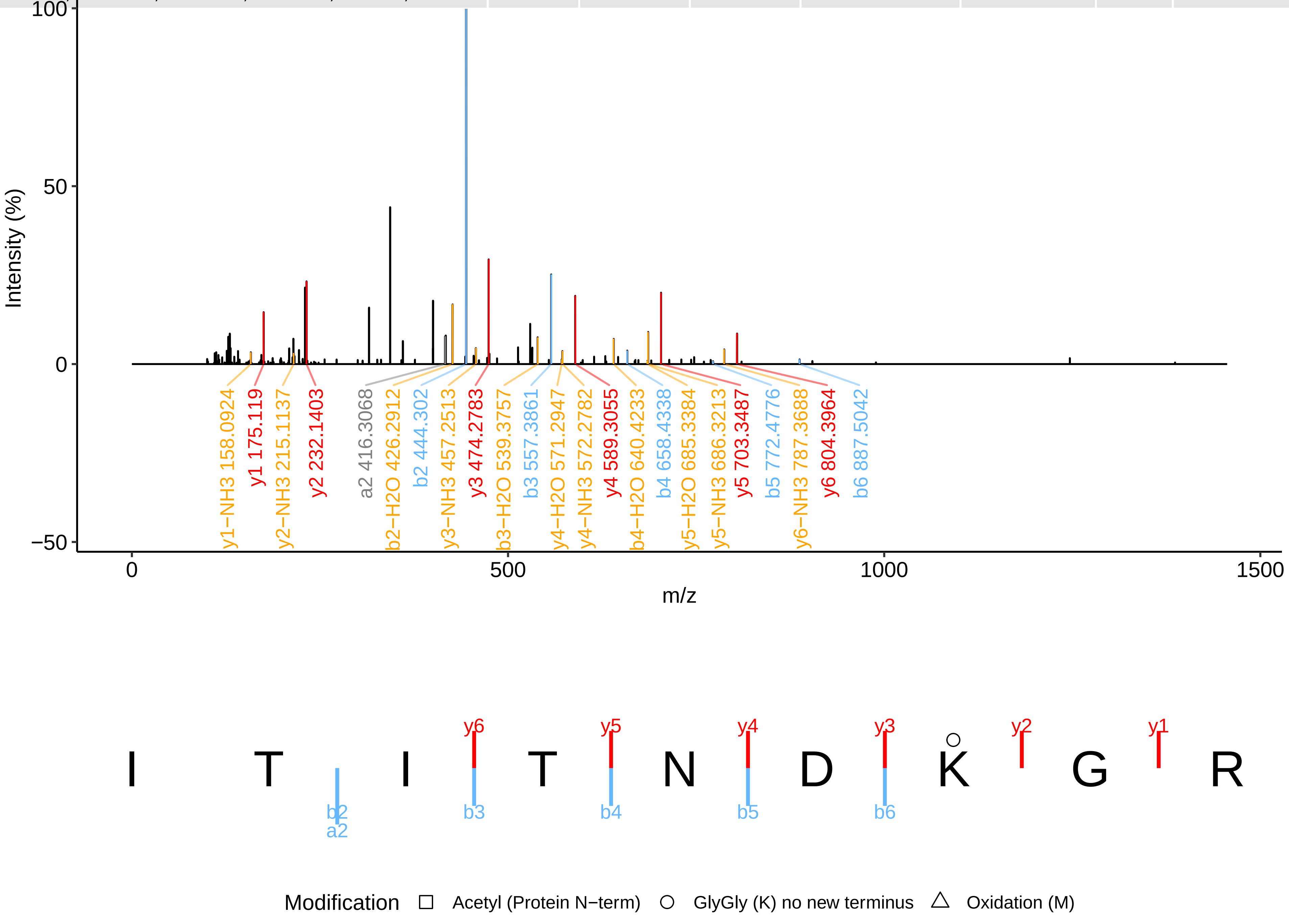

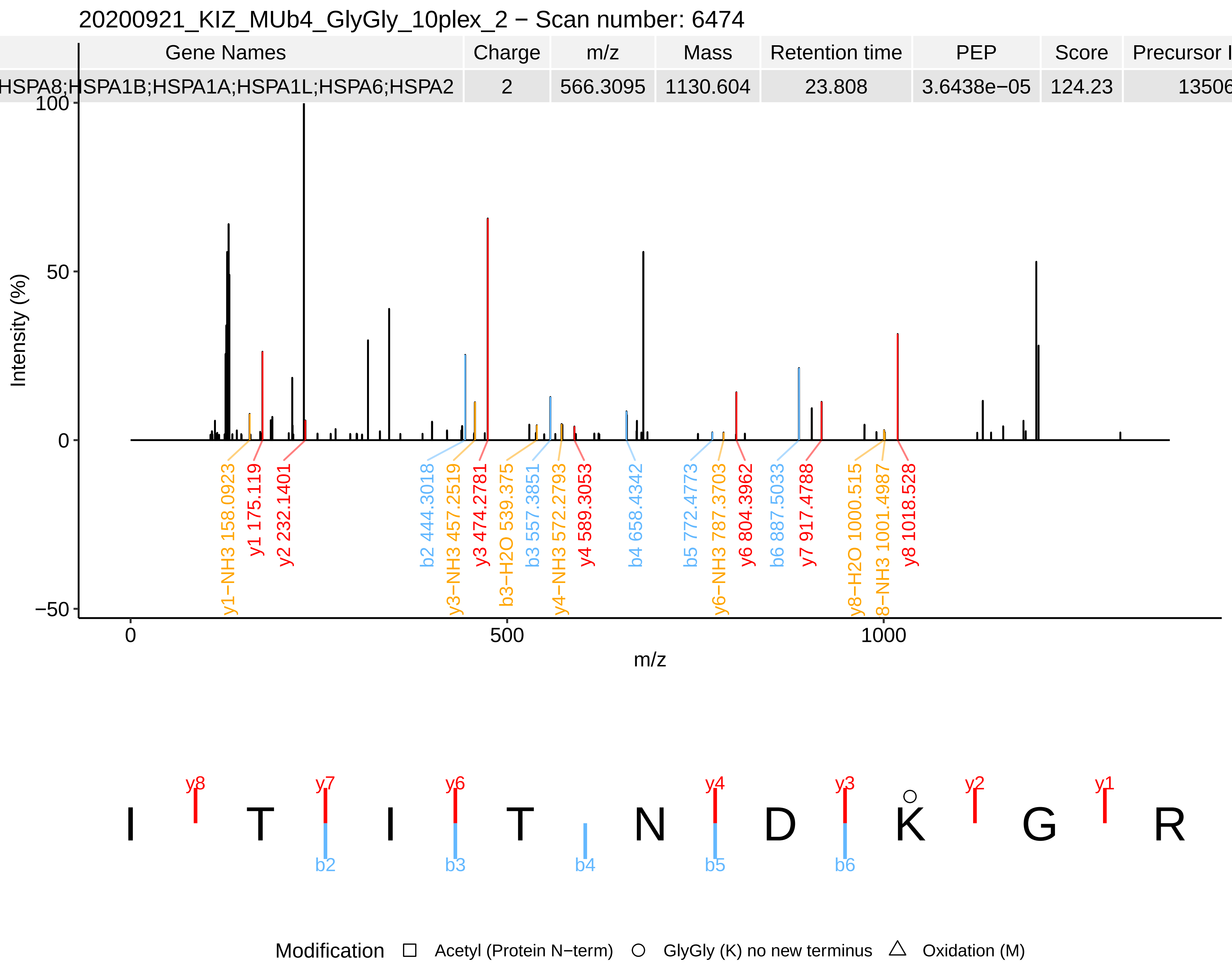

20200921_KIZ_MUb4_GlyGly_10plex_2 − Scan number: 6474
Gene Names
Charge
m/z
Mass
Retention time
PEP
Score
Precursor I
HSPA8;HSPA1B;HSPA1A;HSPA1L;HSPA6;HSPA2
2
566.3095
1130.604
23.808
3.6438e−05
124.23
13506
Intensity (%)
m/z
y1−NH3 158.0923
y1 175.119
y2 232.1401
b2 444.3018
y3−NH3 457.2519
y3 474.2781
b3−H2O 539.375
b3 557.3851
y4−NH3 572.2793
y4 589.3053
b4 658.4342
b5 772.4773
y6−NH3 787.3703
y6 804.3962
b6 887.5033
y7 917.4788
y8−H2O 1000.515
8−NH3 1001.4987
y8 1018.528
I T I T N D K G R
y8 y7 y6 y4 y3 y2 y1
b2 b3 b4 b5 b6
Modification
Acetyl (Protein N−term)
GlyGly (K) no new terminus
Oxidation (M)

20200924_KIZ_MUb5_GlyGly_1 − Scan number: 5939

Gene Names	Charge	m/z	Mass	Retention time	PEP	Score	Precursor I
HSPA8;HSPA1B;HSPA1A;HSPA1L;HSPA6;HSPA2	3	377.8754	1130.604	18.574	2.9825e−07	134.66	267348

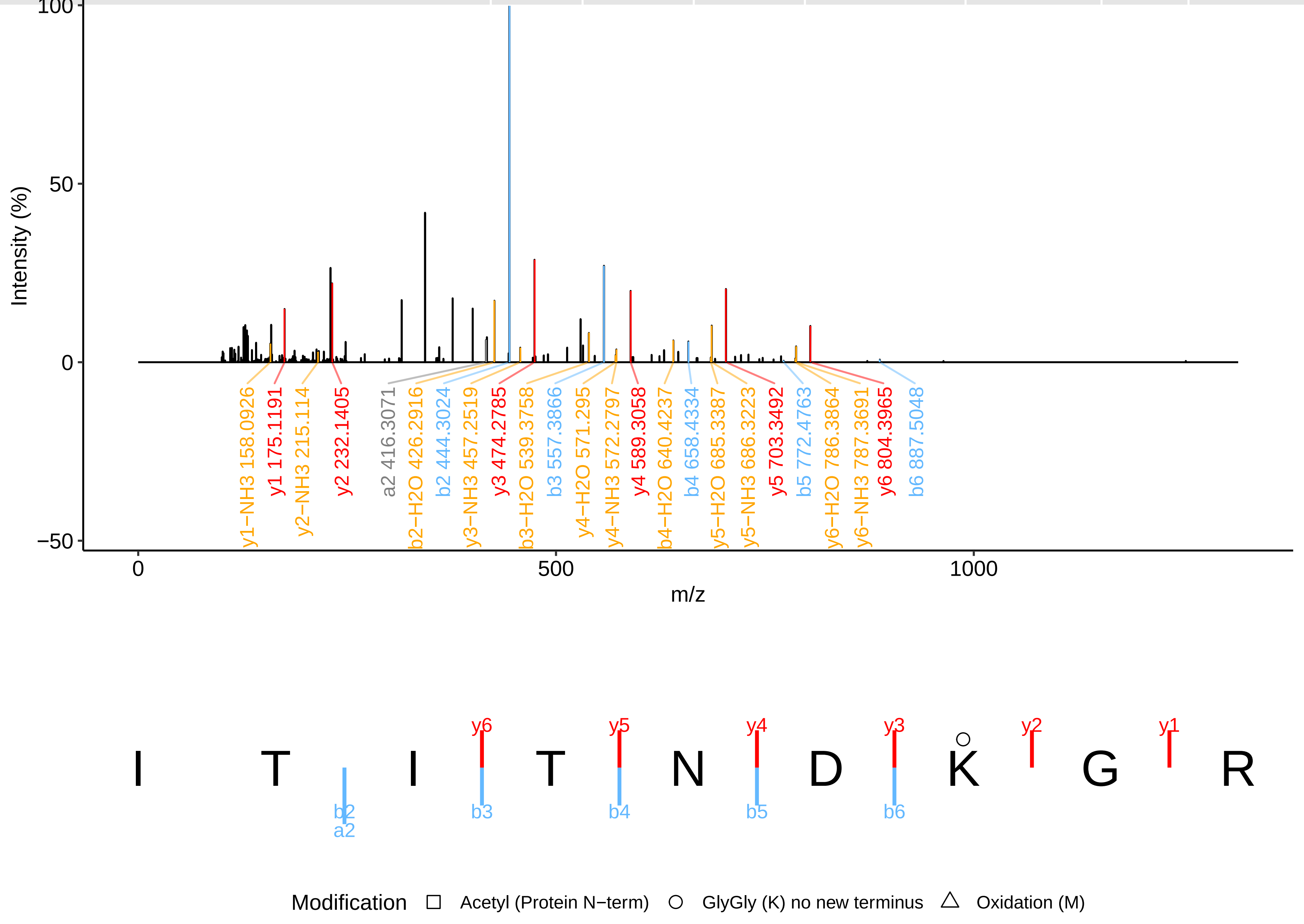

20200924_KIZ_MUb5_GlyGly_1 − Scan number: 6088

Gene Names	Charge	m/z	Mass	Retention time	PEP	Score	Precursor I
HSPA8;HSPA1B;HSPA1A;HSPA1L;HSPA6;HSPA2	3	377.8754	1130.604	19.075	1.3404e−08	162.88	182115

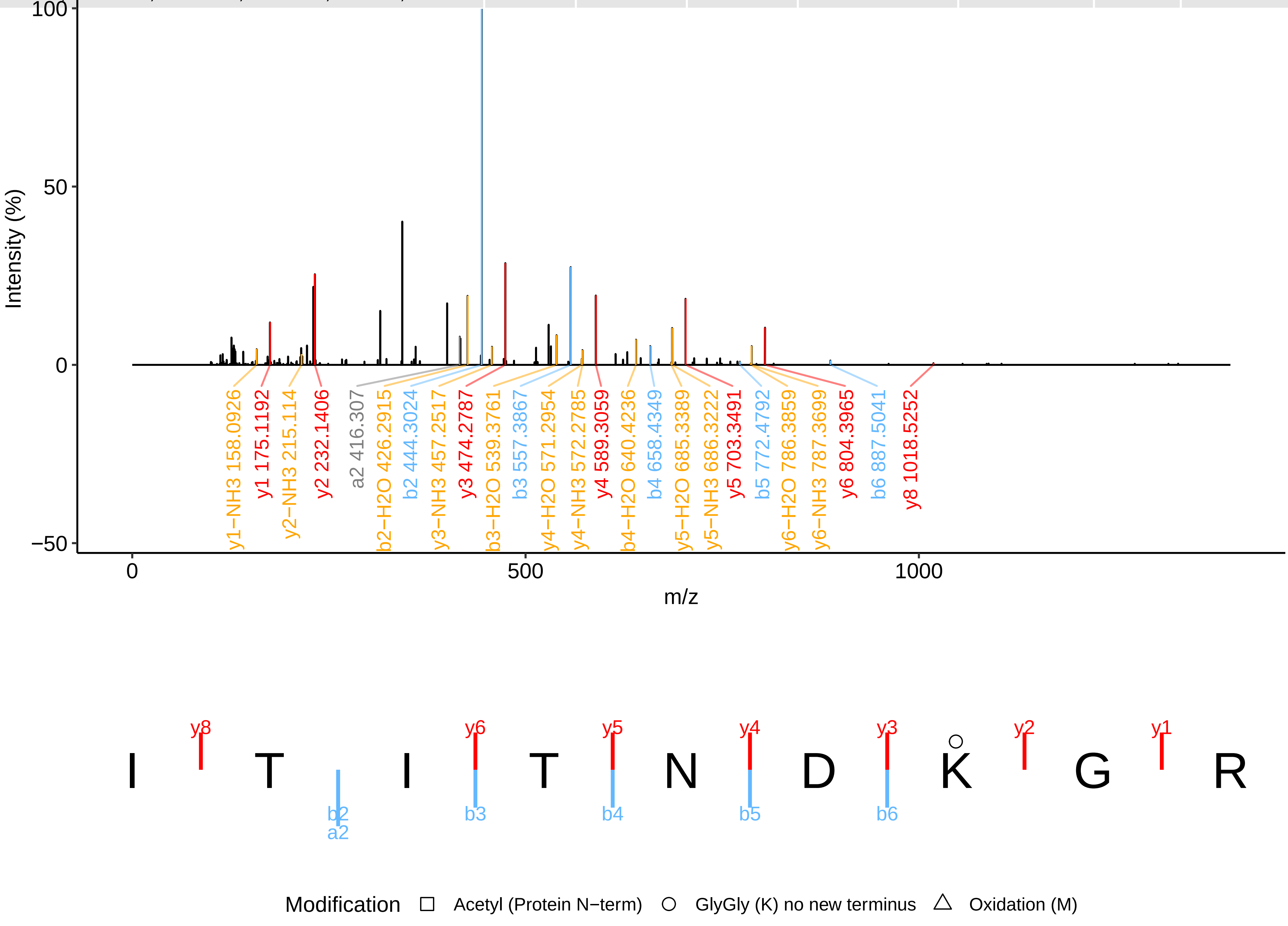

20200924_KIZ_MUb5_GlyGly_1 − Scan number: 6241

Gene Names	Charge	m/z	Mass	Retention time	PEP	Score	Precursor In[illegible]
HSPA8;HSPA1B;HSPA1A;HSPA1L;HSPA6;HSPA2	3	377.8754	1130.604	19.588	2.586e−07	137.13	73811[illegible]

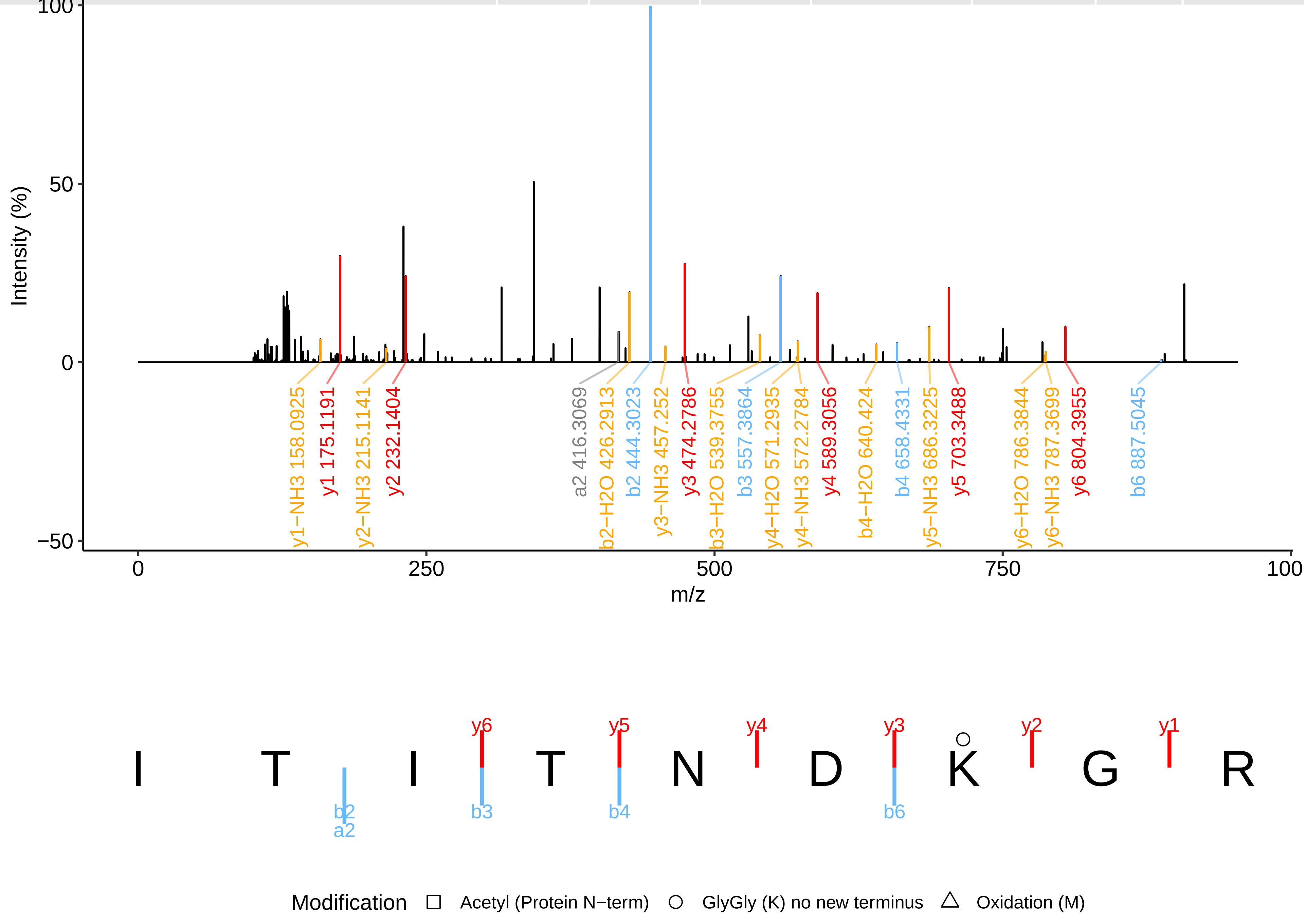

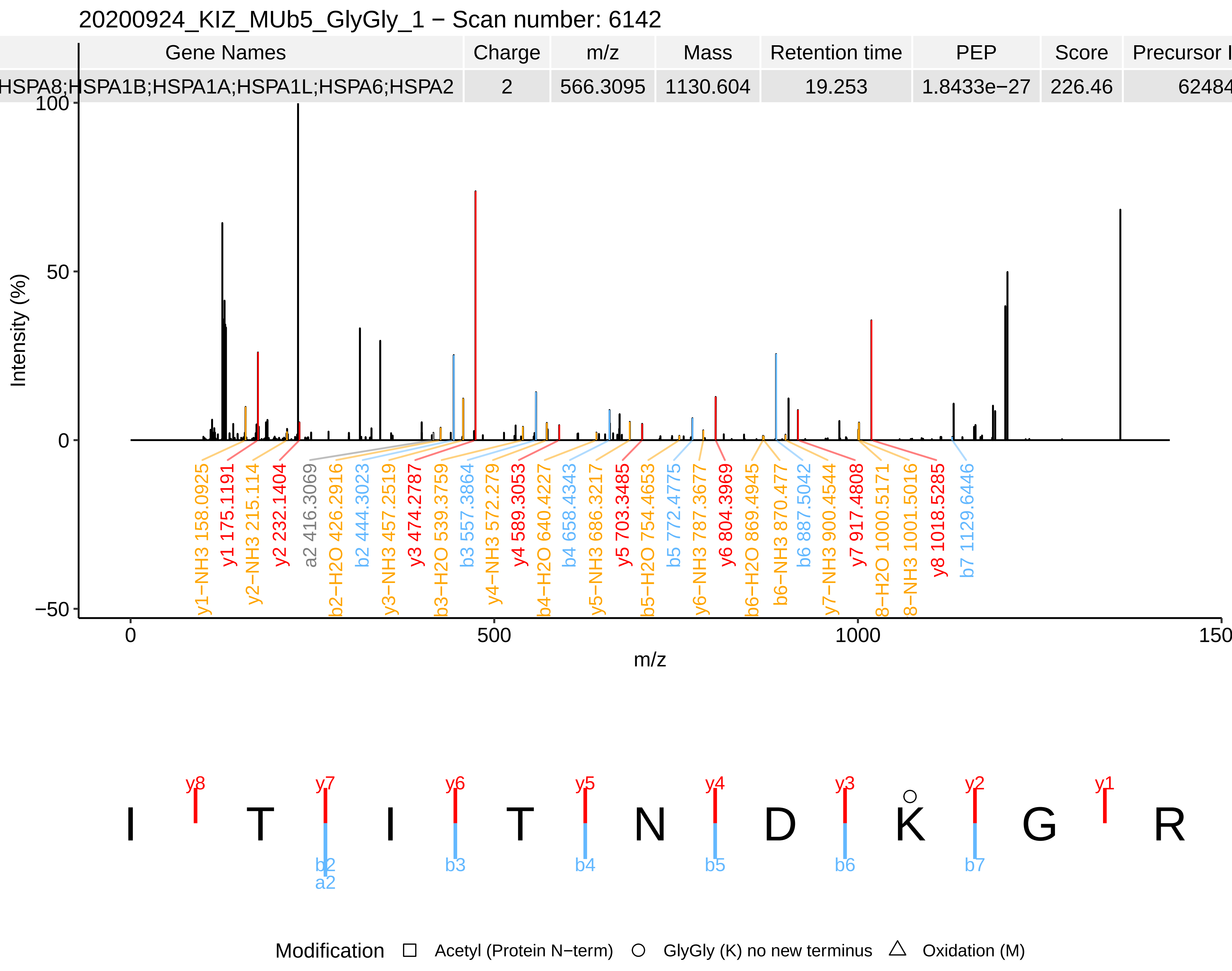
20200924_KIZ_MUb5_GlyGly_1 − Scan number: 6142
Gene Names | Charge | m/z | Mass | Retention time | PEP | Score | Precursor I
HSPA8;HSPA1B;HSPA1A;HSPA1L;HSPA6;HSPA2 | 2 | 566.3095 | 1130.604 | 19.253 | 1.8433e−27 | 226.46 | 62484
Intensity (%)
100
50
0
−50
0
500
1000
150
m/z
y1−NH3 158.0925
y1 175.1191
y2−NH3 215.114
y2 232.1404
a2 416.3069
b2−H2O 426.2916
b2 444.3023
y3−NH3 457.2519
y3 474.2787
b3−H2O 539.3759
b3 557.3864
y4−NH3 572.279
y4 589.3053
b4−H2O 640.4227
b4 658.4343
y5−NH3 686.3217
y5 703.3485
b5−H2O 754.4653
b5 772.4775
y6−NH3 787.3677
y6 804.3969
b6−H2O 869.4945
b6−NH3 870.477
b6 887.5042
y7−NH3 900.4544
y7 917.4808
8−H2O 1000.5171
8−NH3 1001.5016
y8 1018.5285
b7 1129.6446
I T I T N D K G R
y8 y7 y6 y5 y4 y3 y2 y1
b2 a2 b3 b4 b5 b6 b7
Modification
Acetyl (Protein N−term)
GlyGly (K) no new terminus
Oxidation (M)

20200924_KIZ_MUb5_GlyGly_1 − Scan number: 5991

Gene Names	Charge	m/z	Mass	Retention time	PEP	Score	Precursor In[illegible]
HSPA8;HSPA1B;HSPA1A;HSPA1L;HSPA6;HSPA2	2	566.3095	1130.604	18.749	7.8143e−12	218.2	73738[illegible]

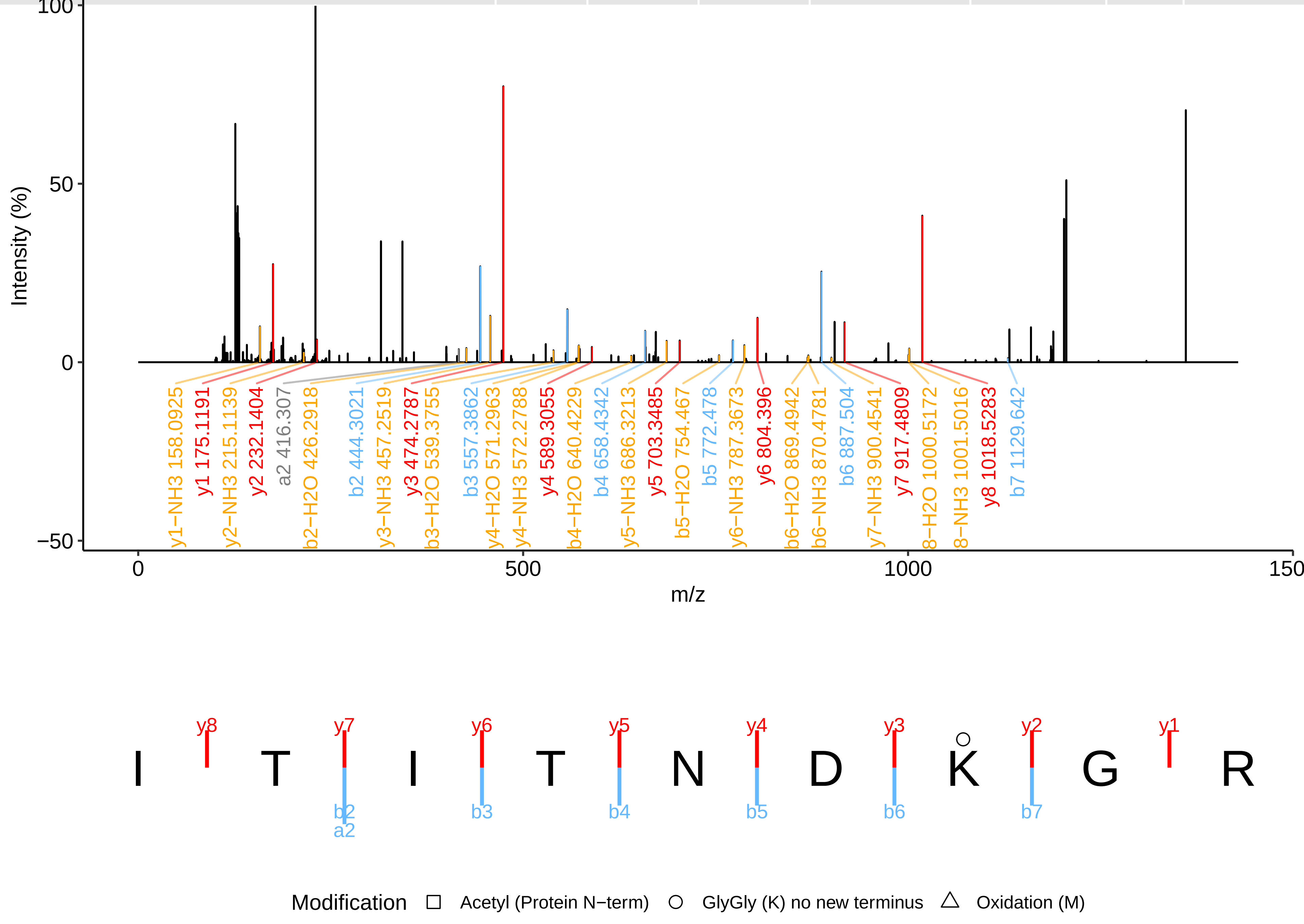

20200924_KIZ_MUb5_GlyGly_2 − Scan number: 5562

Gene Names	Charge	m/z	Mass	Retention time	PEP	Score	Precursor I
HSPA8;HSPA1B;HSPA1A;HSPA1L;HSPA6;HSPA2	3	377.8754	1130.604	20.654	3.1462e−08	156.35	10193

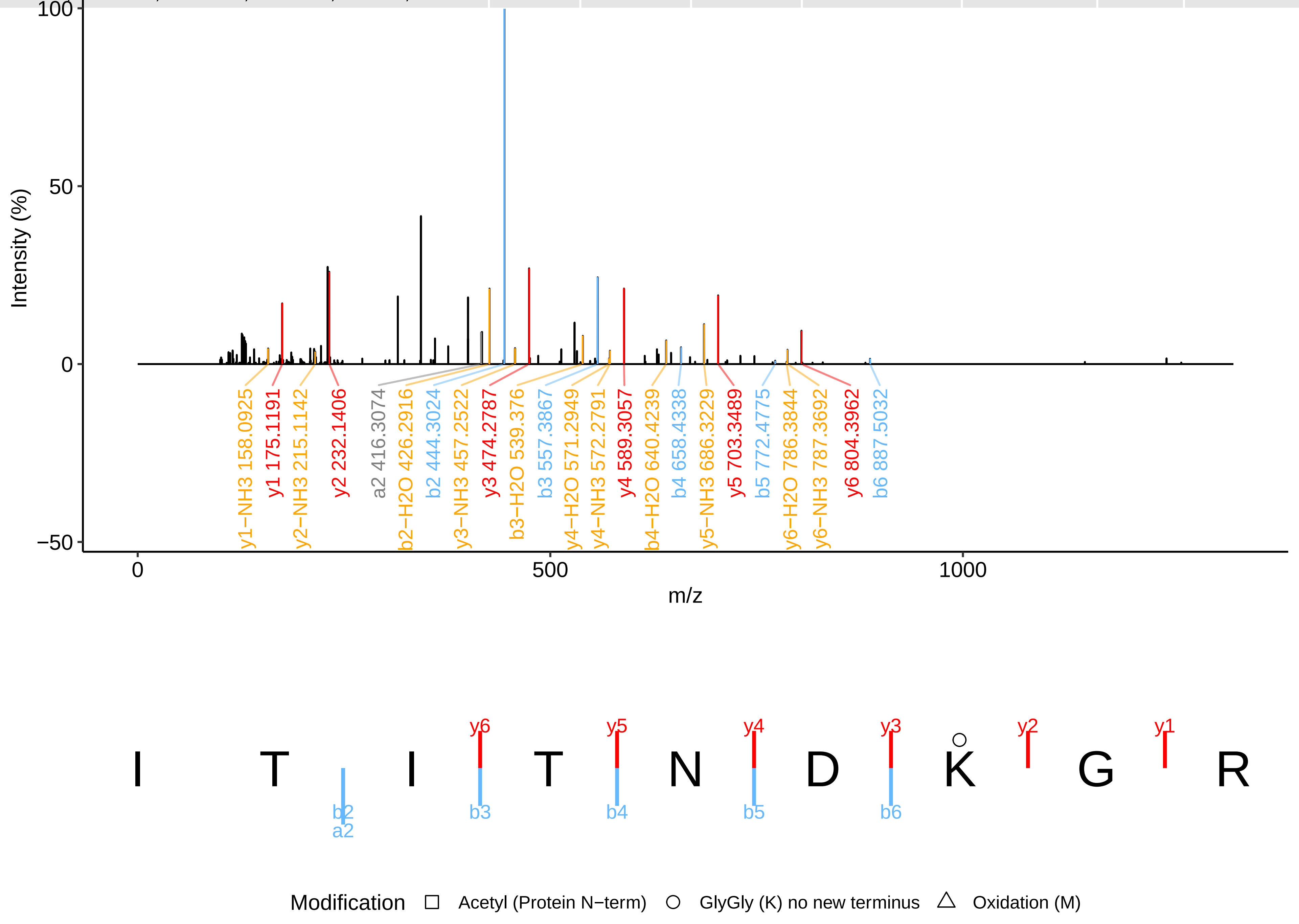

20200924_KIZ_MUb5_GlyGly_2 − Scan number: 5706

Gene Names	Charge	m/z	Mass	Retention time	PEP	Score	Precursor I
HSPA8;HSPA1B;HSPA1A;HSPA1L;HSPA6;HSPA2	2	566.3095	1130.604	21.215	2.6796e−08	164.96	36066

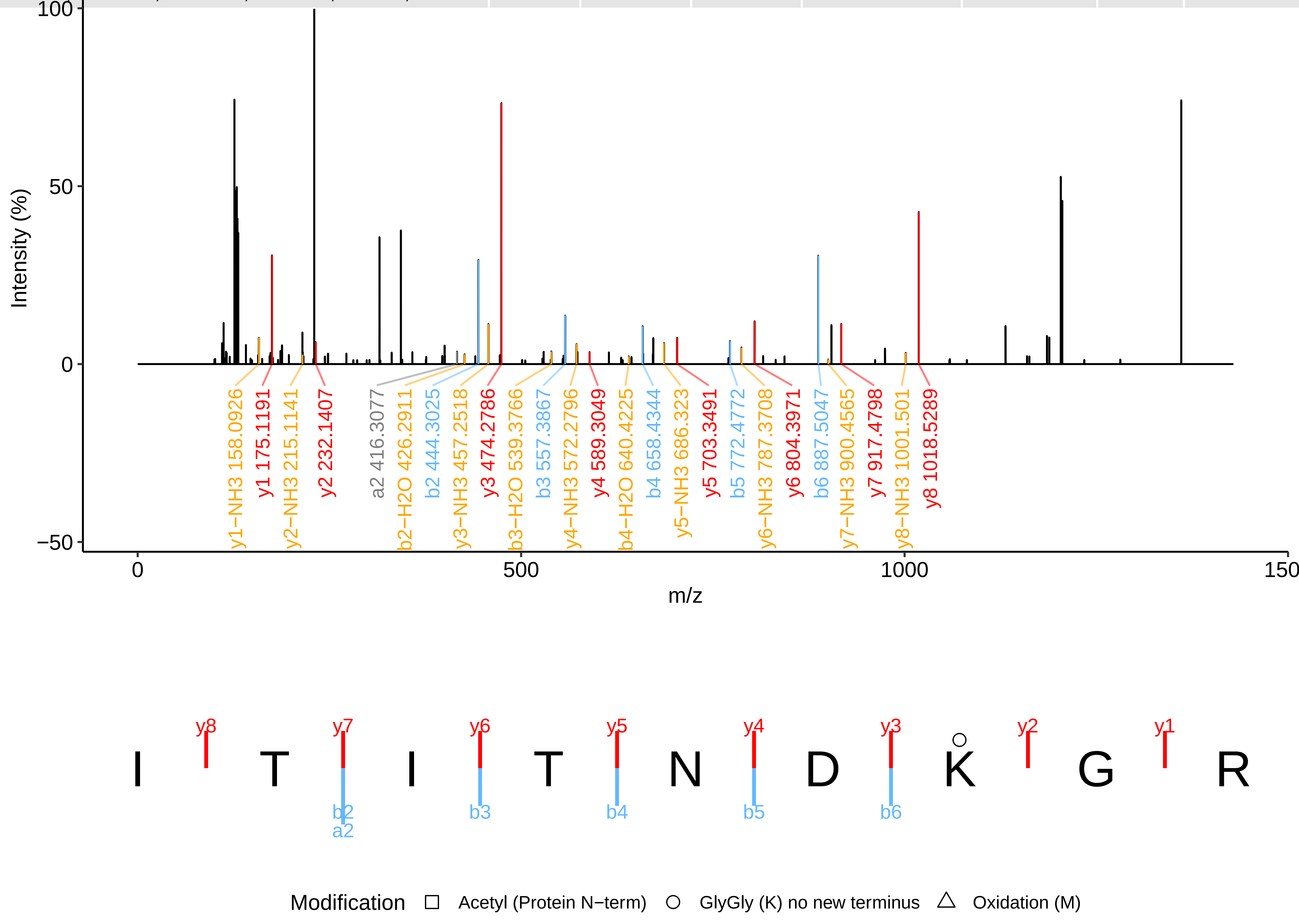

20201118_KIZ_MUb6_GlyGly_1 − Scan number: 5618

Gene Names	Charge	m/z	Mass	Retention time	PEP	Score	Precursor I
HSPA8;HSPA1B;HSPA1A;HSPA1L;HSPA6;HSPA2	3	377.8754	1130.604	17.799	7.4639e−05	113.71	37961

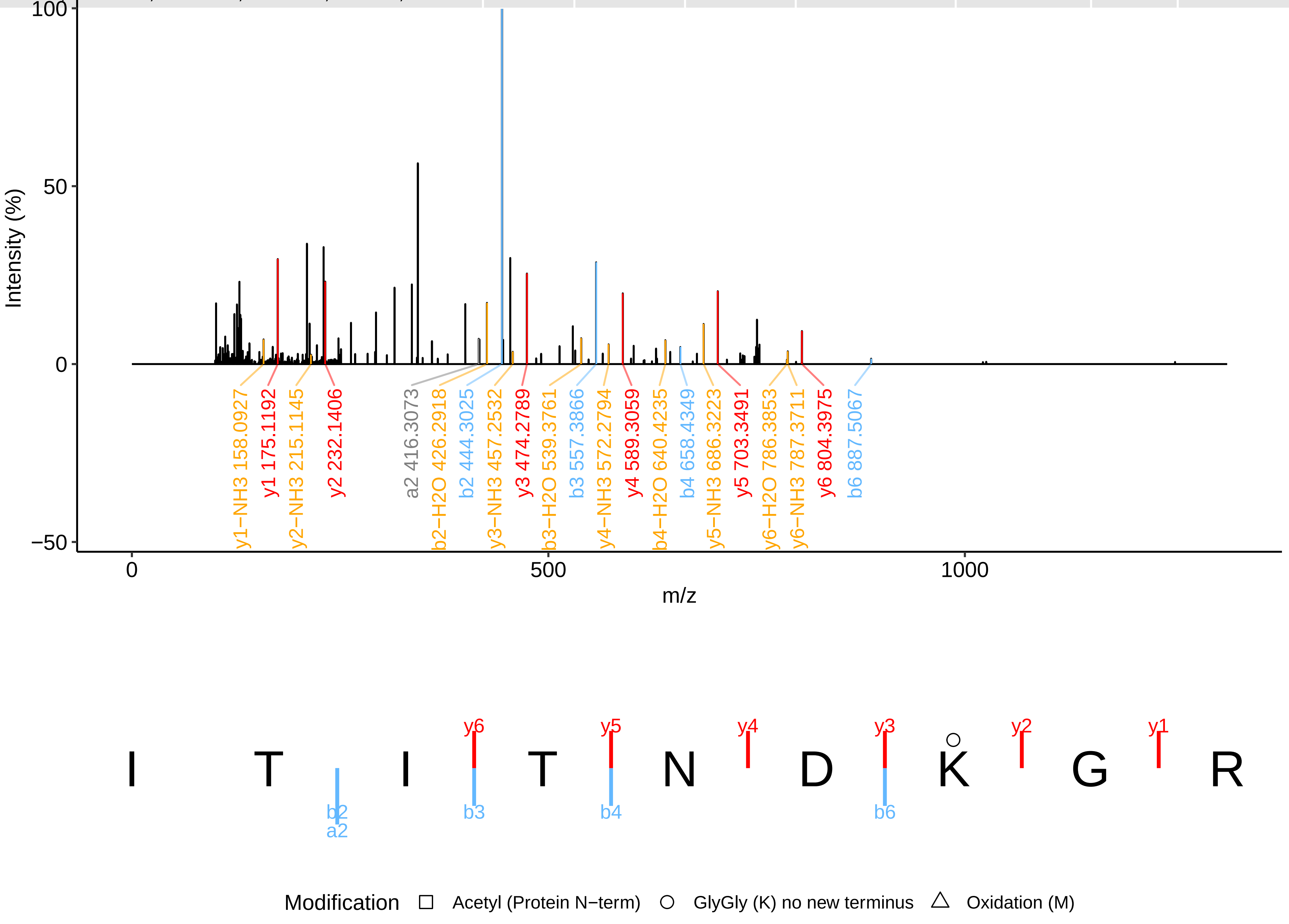

20201118_KIZ_MUb6_GlyGly_2 − Scan number: 7150

Gene Names	Charge	m/z	Mass	Retention time	PEP	Score	Precursor I
HSPA8;HSPA1B;HSPA1A;HSPA1L;HSPA6;HSPA2	3	377.8754	1130.604	24.415	8.5499e−06	125.82	59934

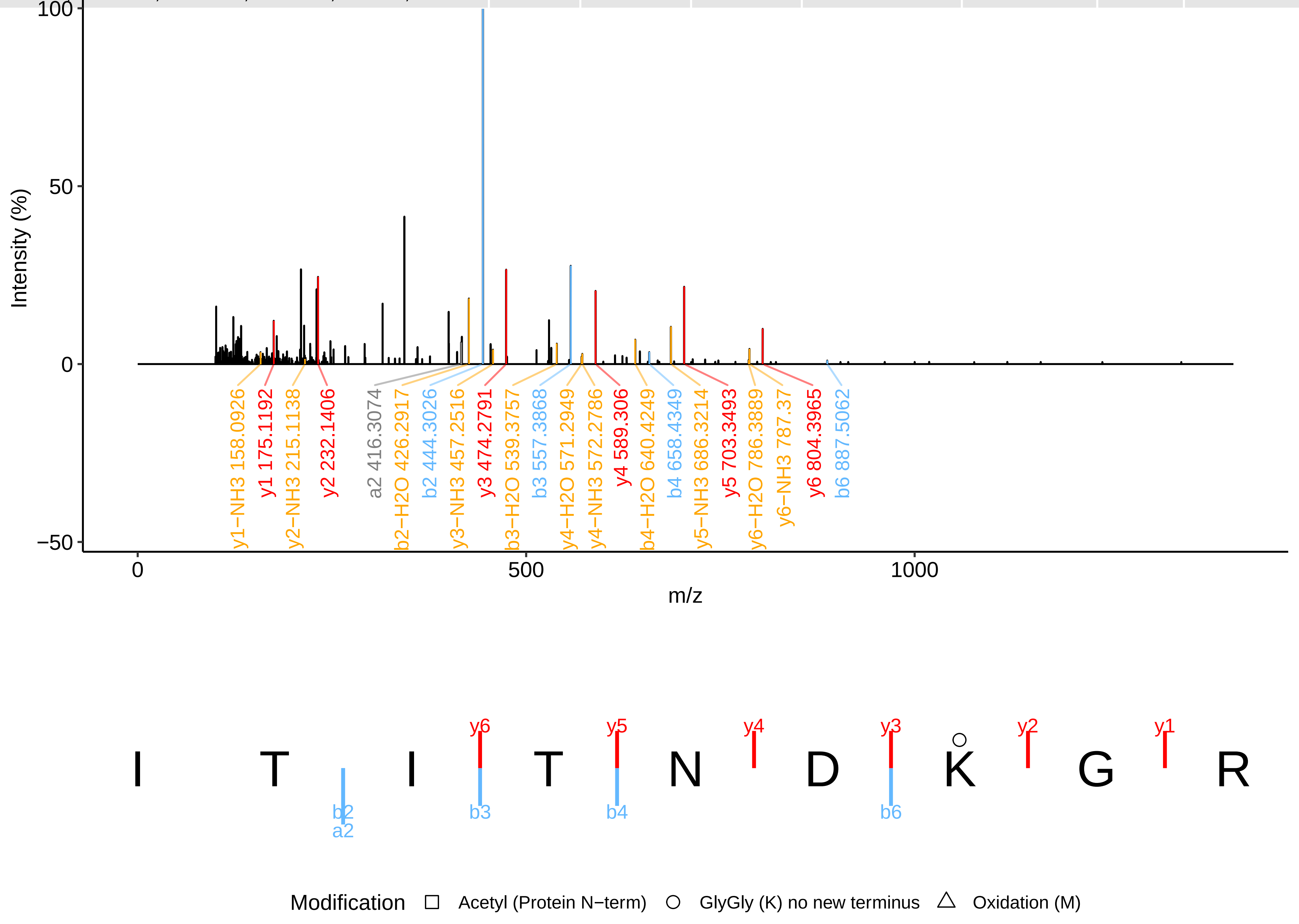

20200921_KIZ_MUb4_GlyGly_10plex − Scan number: 3864

Gene Names	Charge	m/z	Mass	Retention time	PEP	Score	Precursor Intensity
RAB8A	3	367.2	1098.578	12.478	0.0089439	103.74	2904861

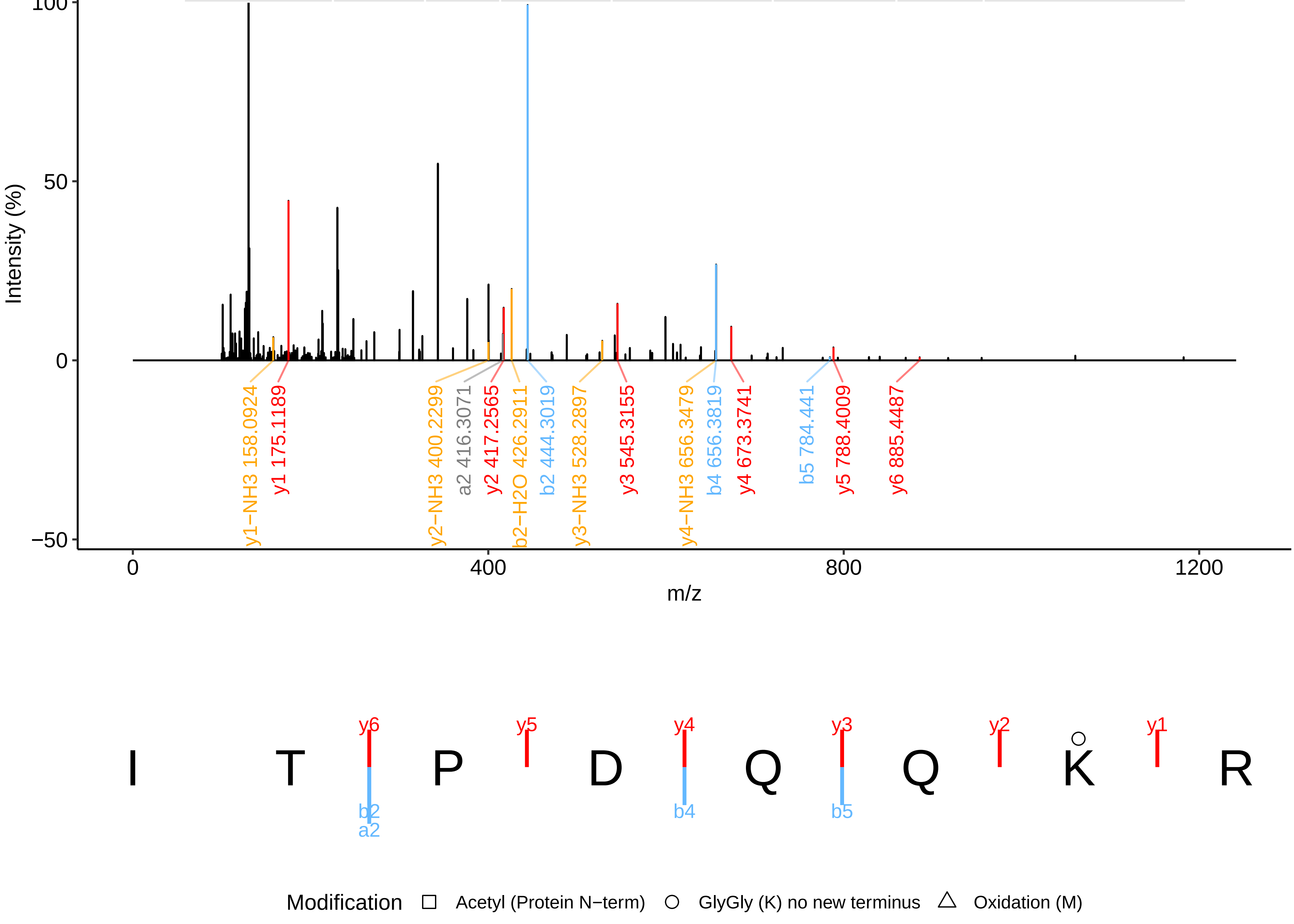

20200924_KIZ_MUb5_GlyGly_1 − Scan number: 2802

Gene Names	Charge	m/z	Mass	Retention time	PEP	Score	Precursor Intensity
SCAMP3	3	377.5353	1129.584	8.654	0.074641	58.981	10420323

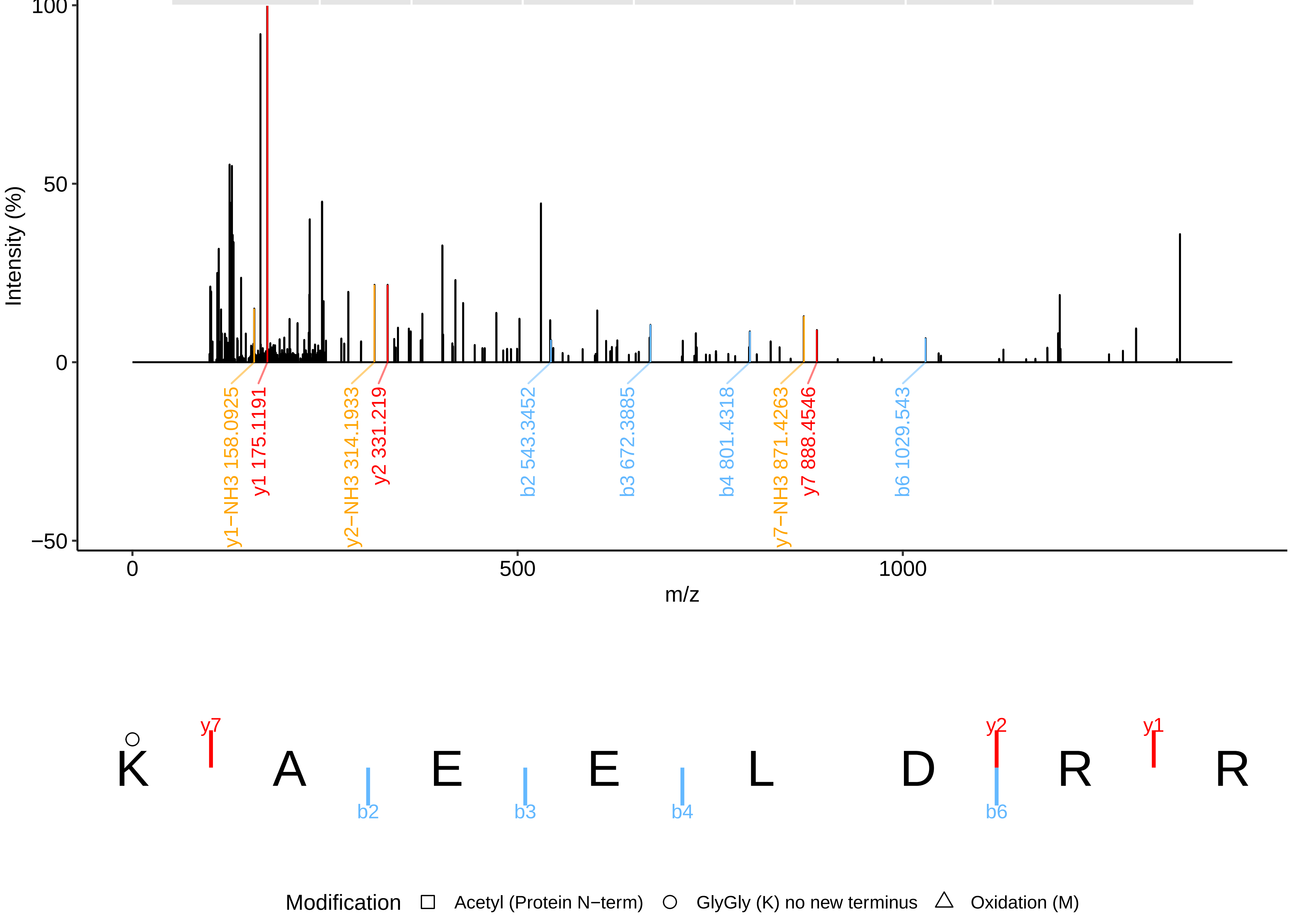

20200924_KIZ_MUb5_GlyGly_2 − Scan number: 2421

Gene Names	Charge	m/z	Mass	Retention time	PEP	Score	Precursor Intensity
SCAMP3	3	377.5353	1129.584	8.5415	0.081804	57.859	1804416

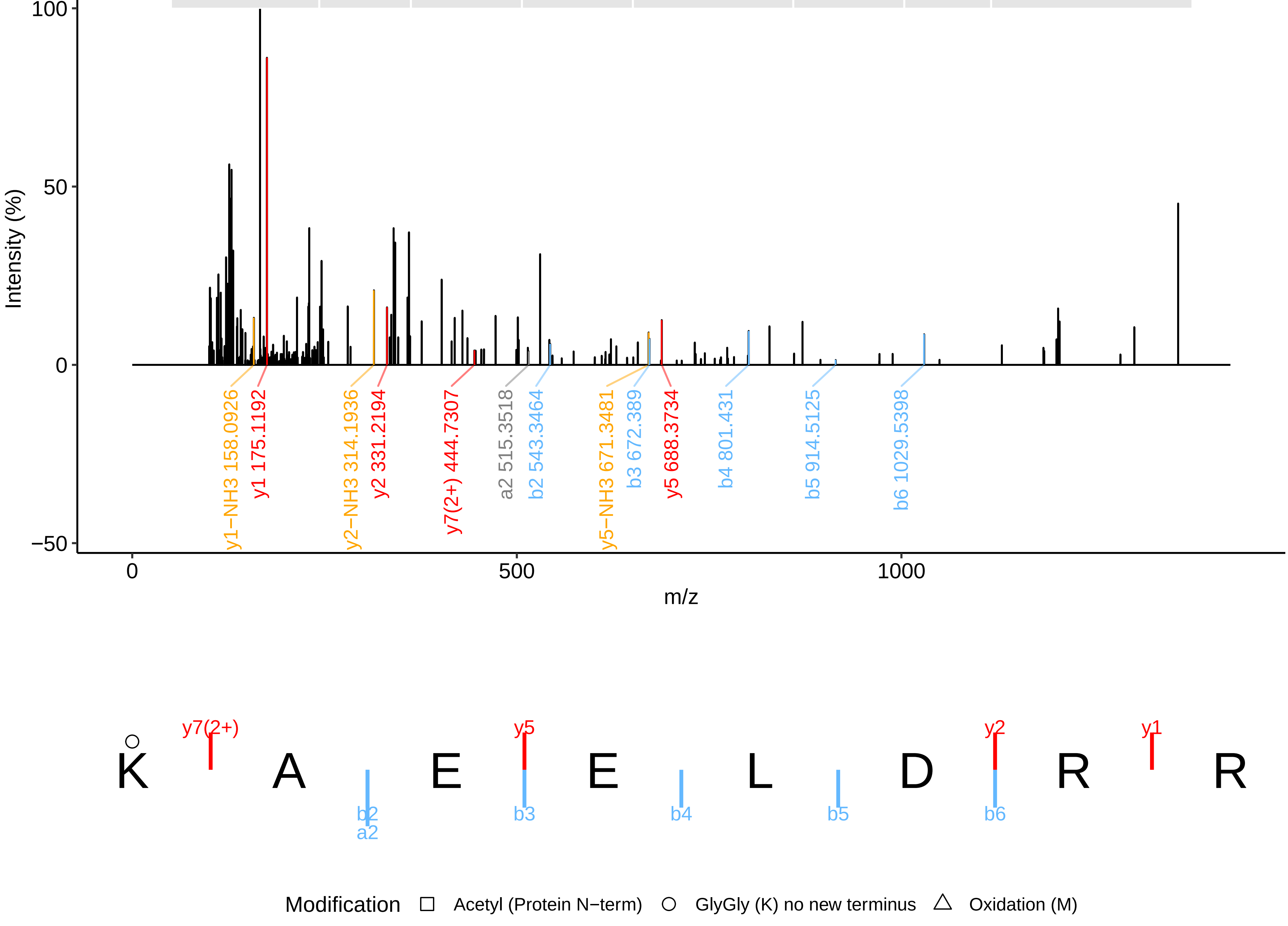

20201118_KIZ_MUb6_GlyGly_1 − Scan number: 2822

Gene Names	Charge	m/z	Mass	Retention time	PEP	Score	Precursor Intensity
SCAMP3	3	377.5353	1129.584	8.6405	0.062324	60.91	13566051

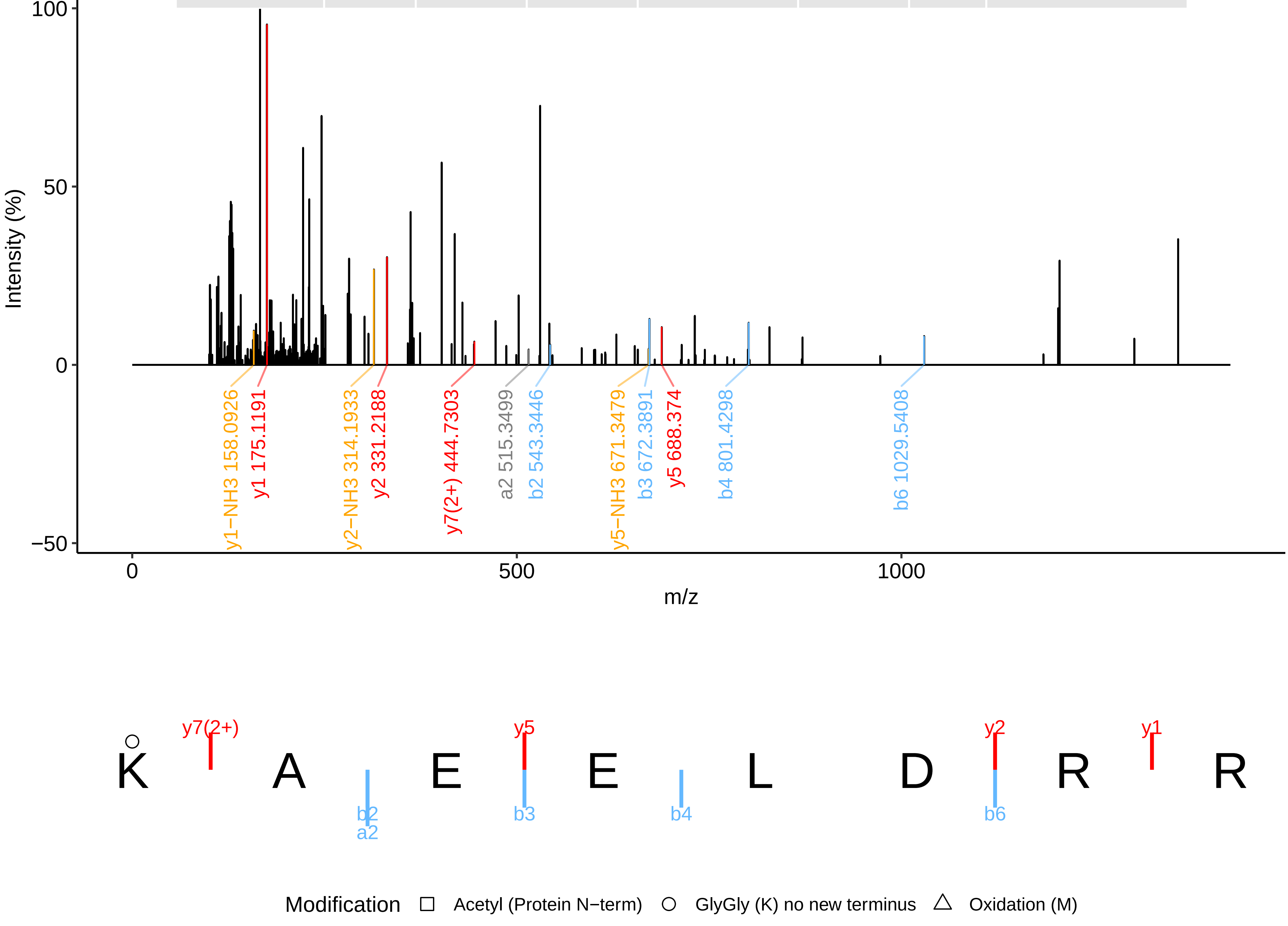

20201118_KIZ_MUb6_GlyGly_2 − Scan number: 3102

Gene Names	Charge	m/z	Mass	Retention time	PEP	Score	Precursor Intensity
SCAMP3	3	377.5353	1129.584	10.059	0.00039566	116.05	12355954

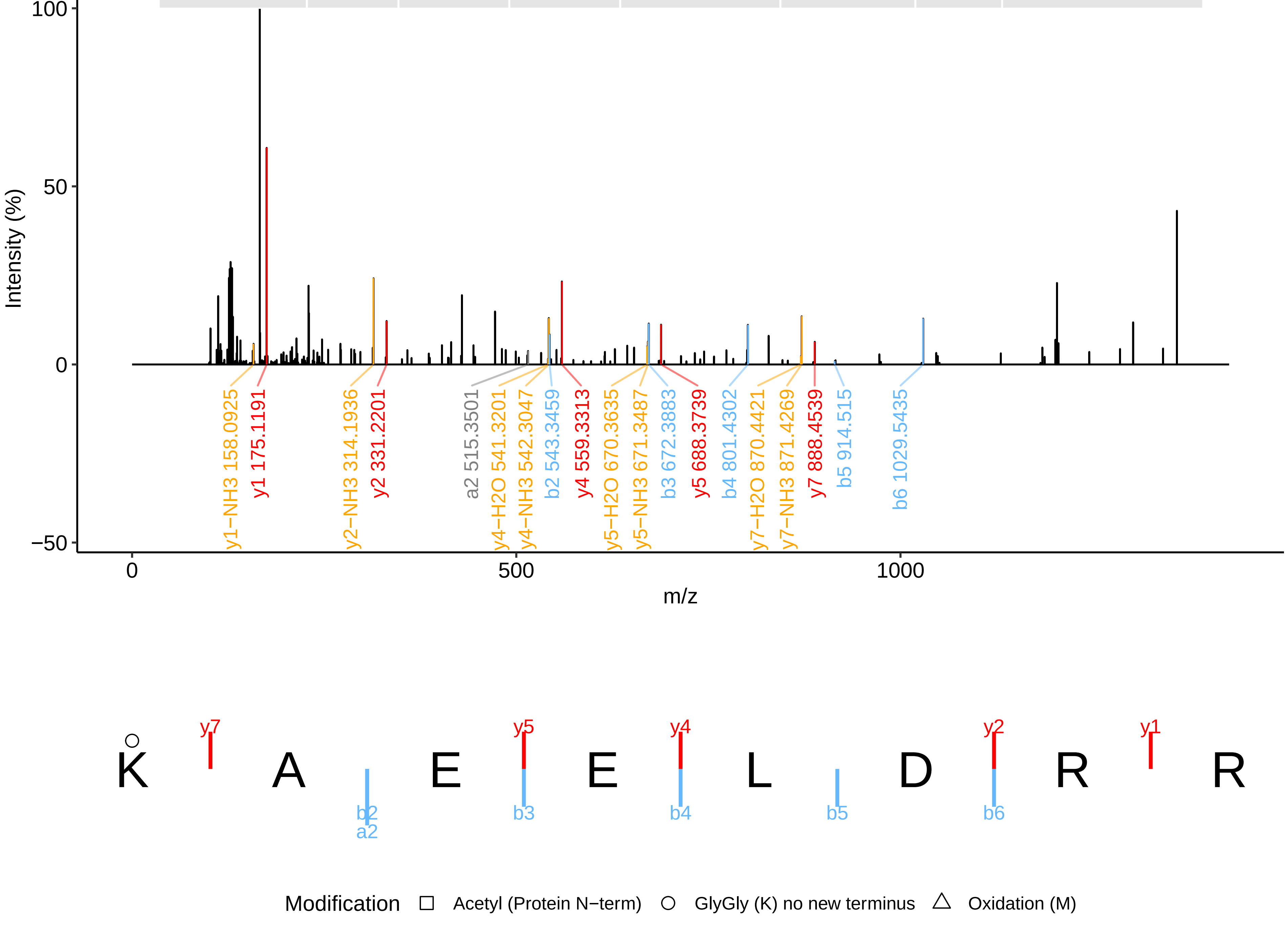

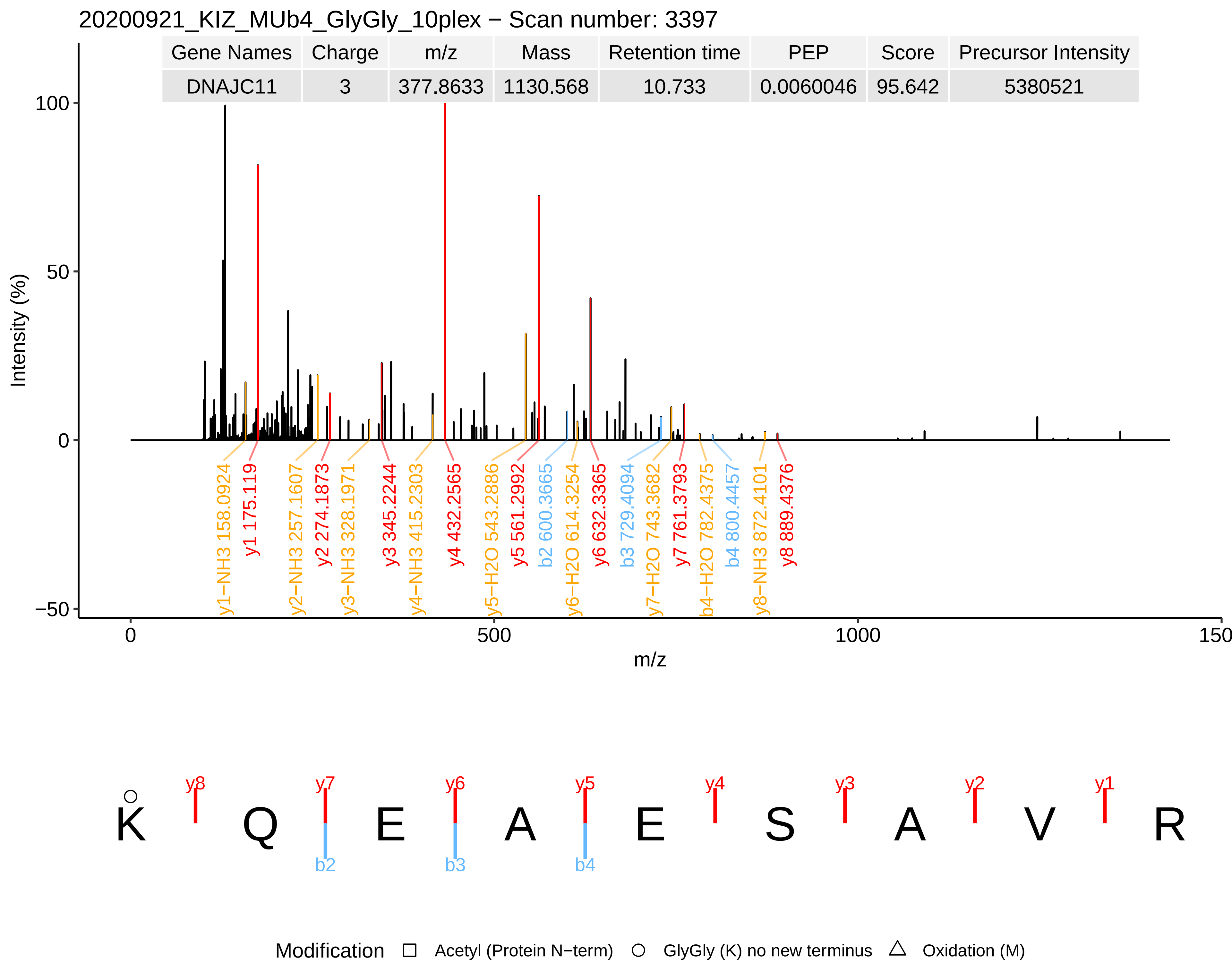

20200921_KIZ_MUb4_GlyGly_10plex − Scan number: 3397
Gene Names | Charge | m/z | Mass | Retention time | PEP | Score | Precursor Intensity
DNAJC11 | 3 | 377.8633 | 1130.568 | 10.733 | 0.0060046 | 95.642 | 5380521
Intensity (%)
m/z
y1−NH3 158.0924
y1 175.119
y2−NH3 257.1607
y2 274.1873
y3−NH3 328.1971
y3 345.2244
y4−NH3 415.2303
y4 432.2565
y5−H2O 543.2886
y5 561.2992
b2 600.3665
y6−H2O 614.3254
y6 632.3365
b3 729.4094
y7−H2O 743.3682
y7 761.3793
b4−H2O 782.4375
b4 800.4457
y8−NH3 872.4101
y8 889.4376
K Q E A E S A V R
y8 y7 y6 y5 y4 y3 y2 y1
b2 b3 b4
Modification □ Acetyl (Protein N−term) ○ GlyGly (K) no new terminus △ Oxidation (M)

20201118_KIZ_MUb6_GlyGly_2 − Scan number: 3307

Gene Names	Charge	m/z	Mass	Retention time	PEP	Score	Precursor Intensity
DNAJC11	3	377.8633	1130.568	10.814	0.016414	85.733	4268060

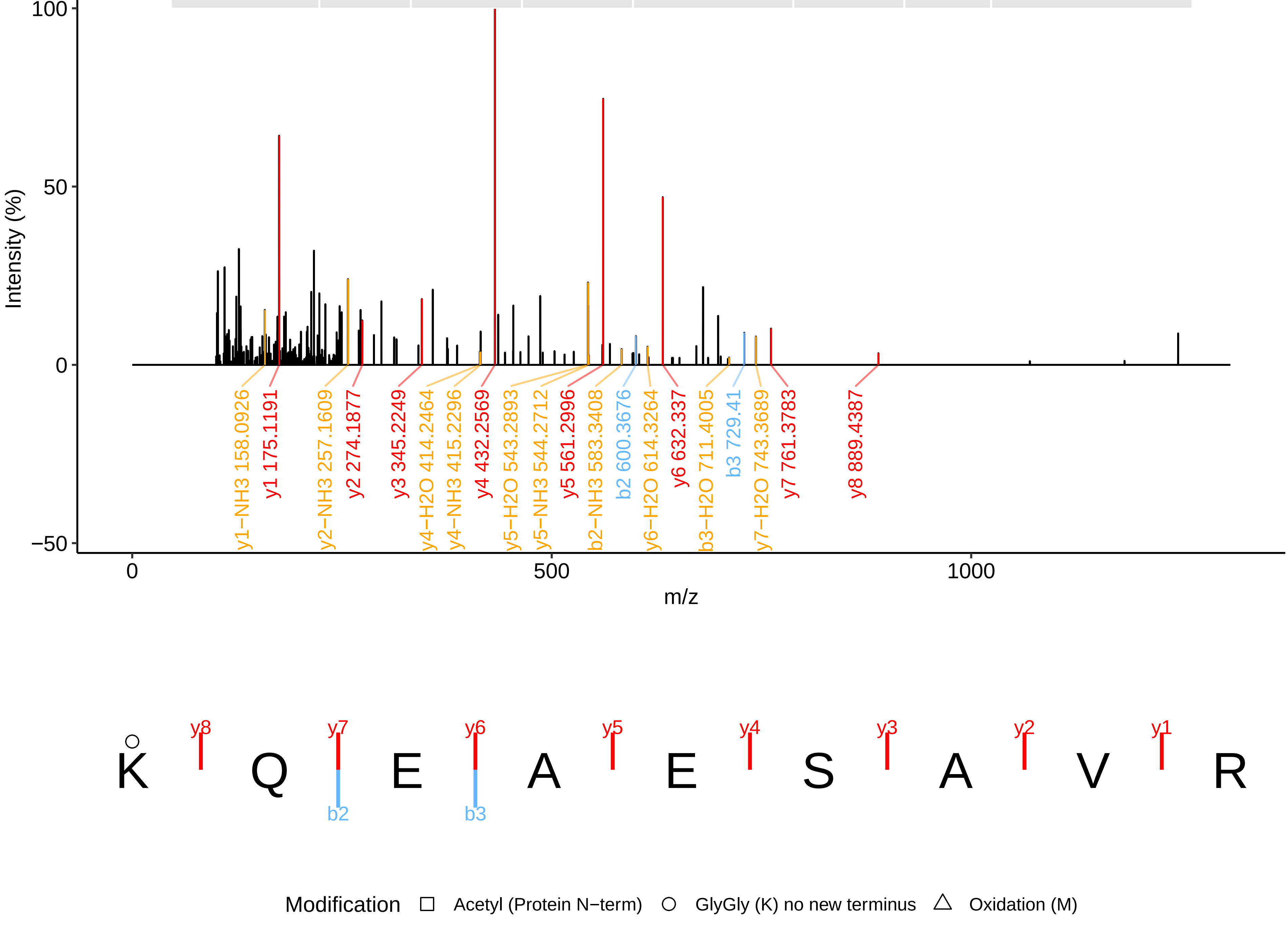

20200921_KIZ_MUb4_GlyGly_10plex − Scan number: 14822

Gene Names	Charge	m/z	Mass	Retention time	PEP	Score	Precursor Intensity
PCNA	3	305.5272	913.5597	52.772	0.12307	75.213	4255333

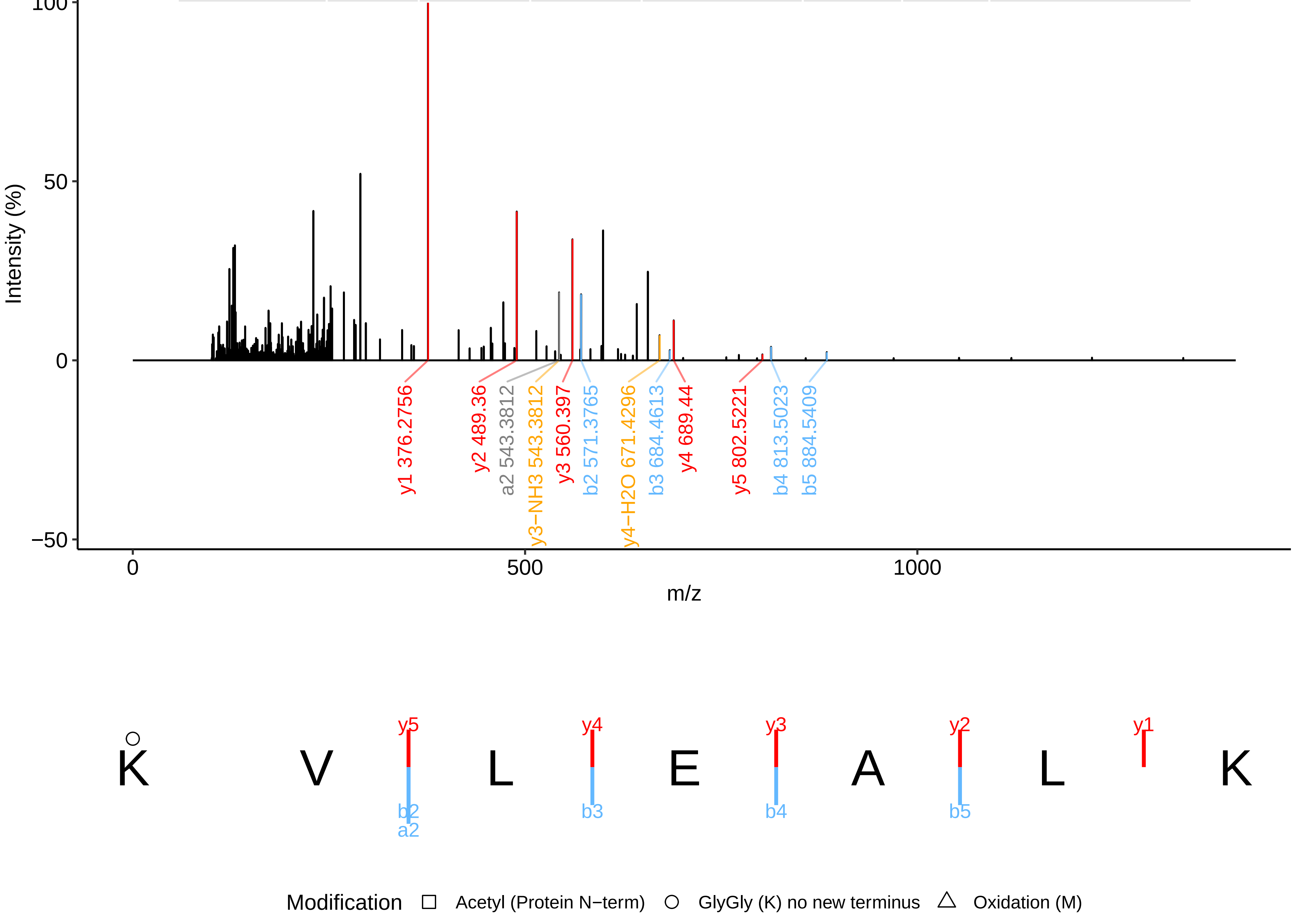

20200921_KIZ_MUb4_GlyGly_10plex − Scan number: 5712

Gene Names	Charge	m/z	Mass	Retention time	PEP	Score	Precursor Intensity
LINC00116	3	291.8433	872.508	18.735	0.069946	84.359	15392262

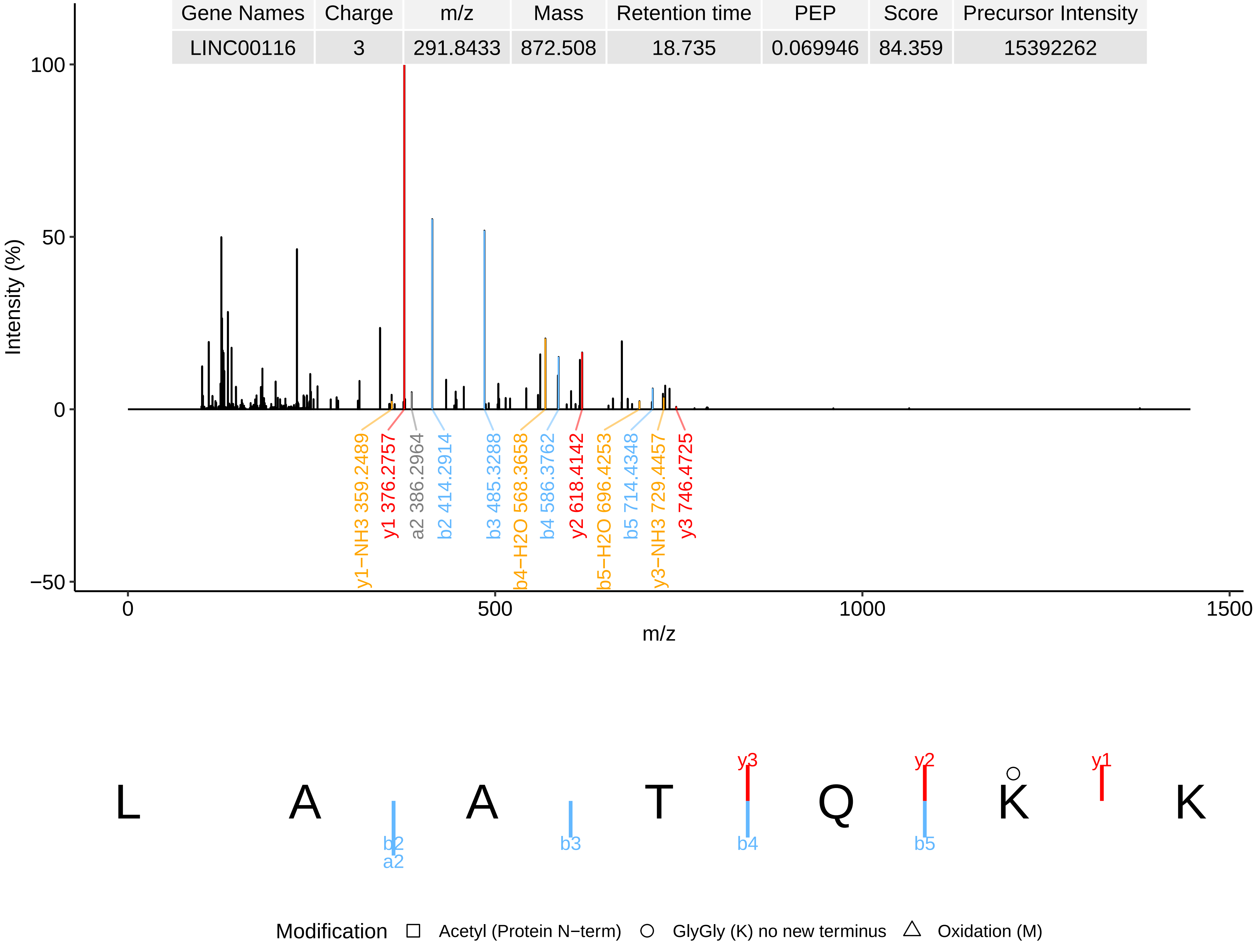

20200921_KIZ_MUb4_GlyGly_10plex_2 − Scan number: 8564

Gene Names	Charge	m/z	Mass	Retention time	PEP	Score	Precursor Intensity
TOMM5	3	377.8536	1130.539	32.304	0.11274	73.248	644506.9

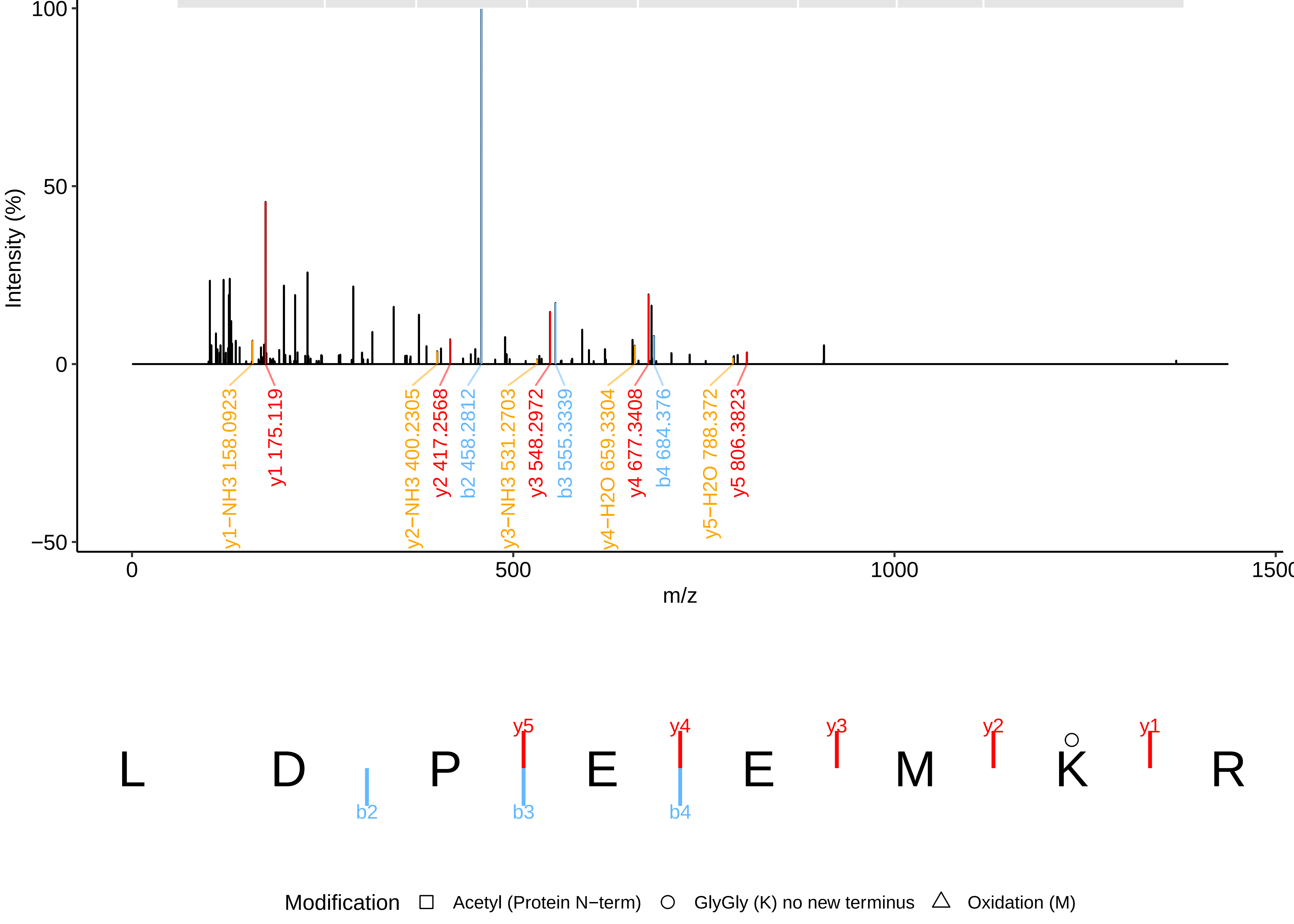

20200924_KIZ_MUb5_GlyGly_1 − Scan number: 8218

Gene Names	Charge	m/z	Mass	Retention time	PEP	Score	Precursor Intensity
TOMM5	3	377.8536	1130.539	26.226	0.075439	73.665	9141881

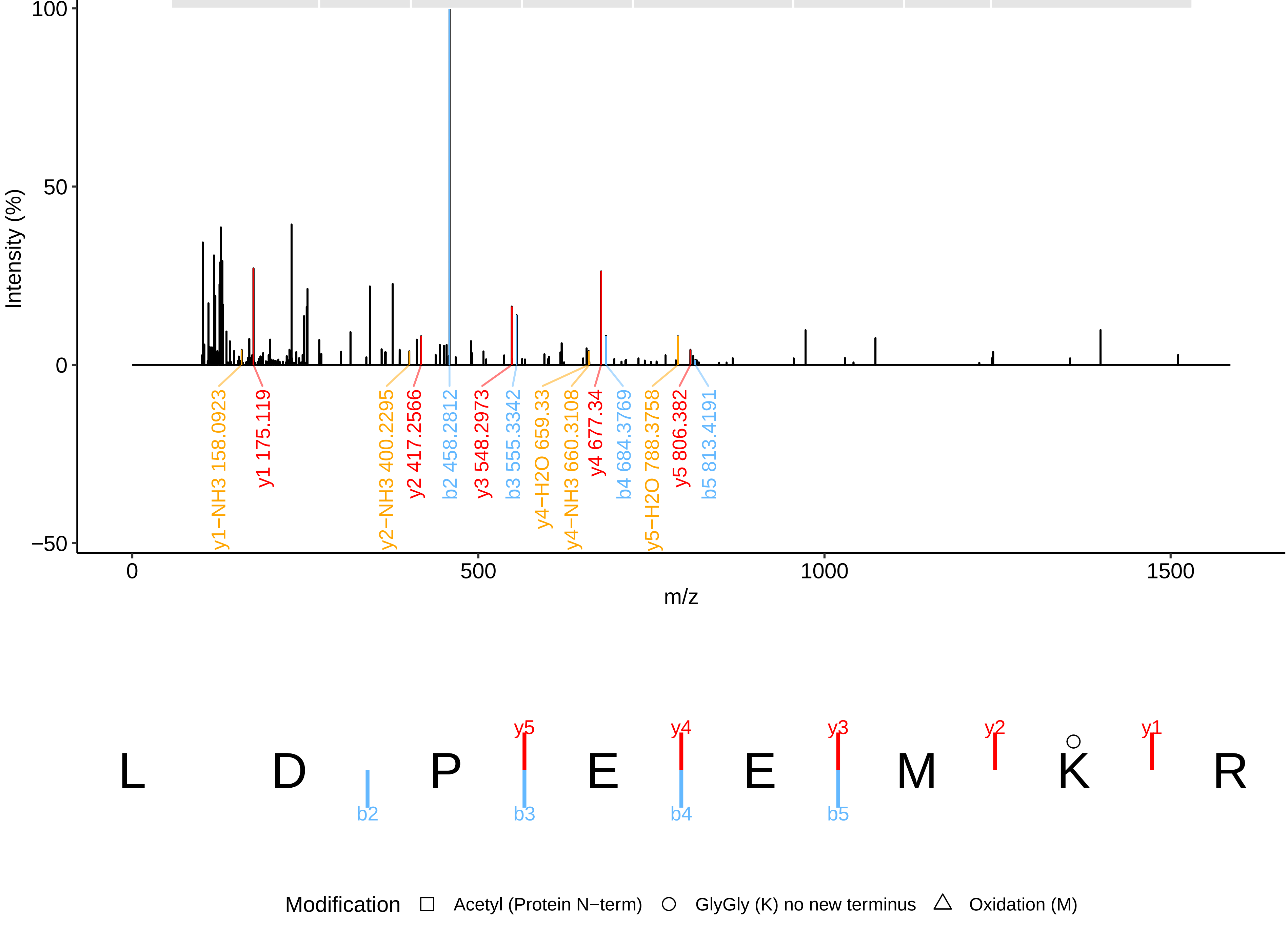

20200924_KIZ_MUb5_GlyGly_2 − Scan number: 7753

Gene Names	Charge	m/z	Mass	Retention time	PEP	Score	Precursor Intensity
TOMM5	3	377.8536	1130.539	28.956	0.075439	73.665	3134107

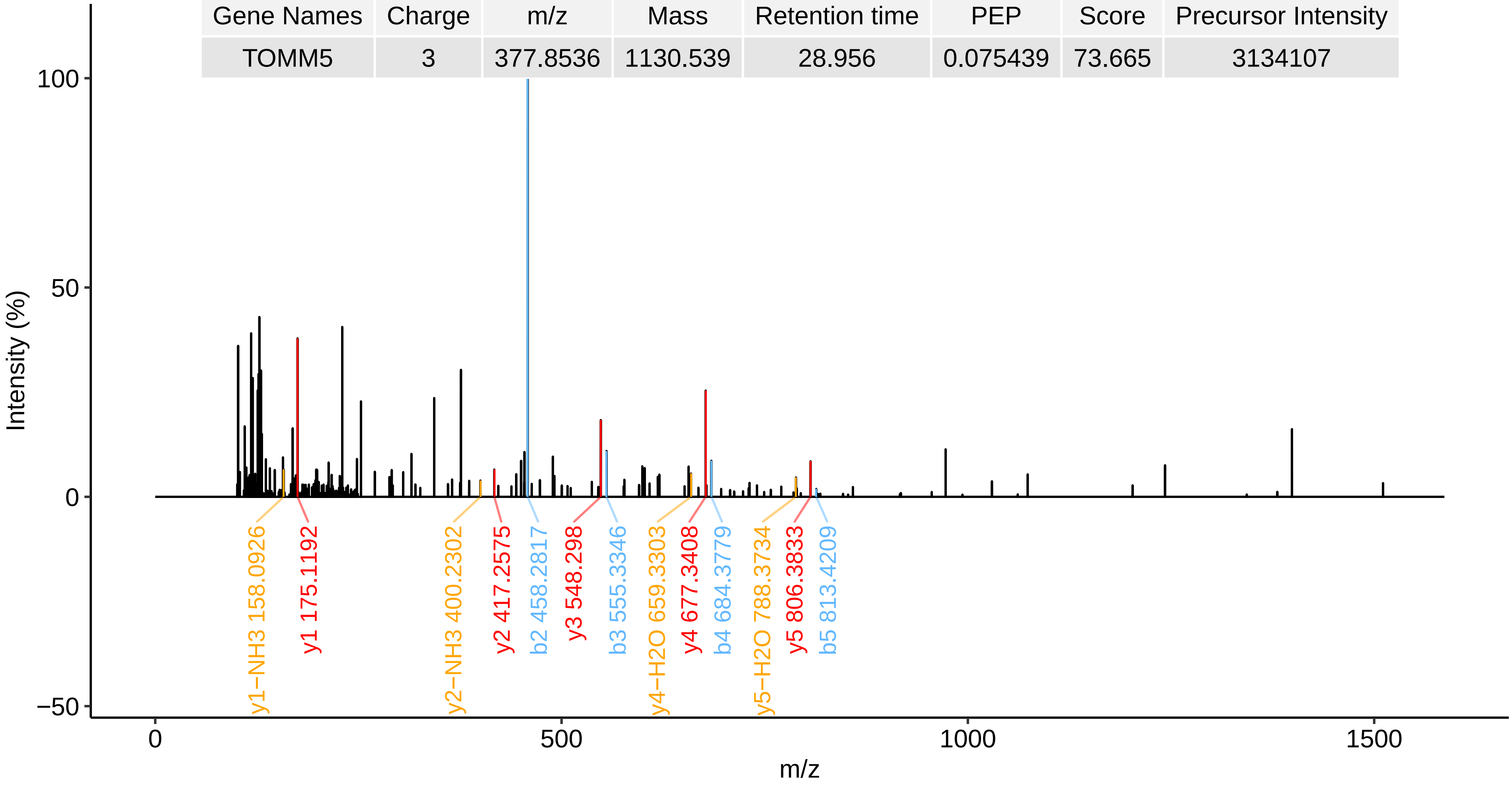

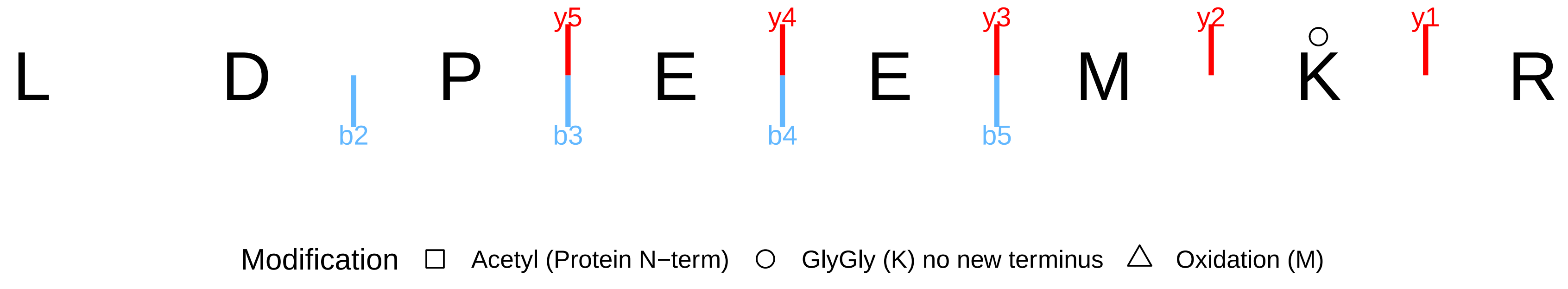

20200921_KIZ_MUb4_GlyGly_10plex − Scan number: 11600

Gene Names	Charge	m/z	Mass	Retention time	PEP	Score	Precursor Intensity
CYP51A1	3	382.8682	1145.583	40.477	0.013122	77.355	4060569

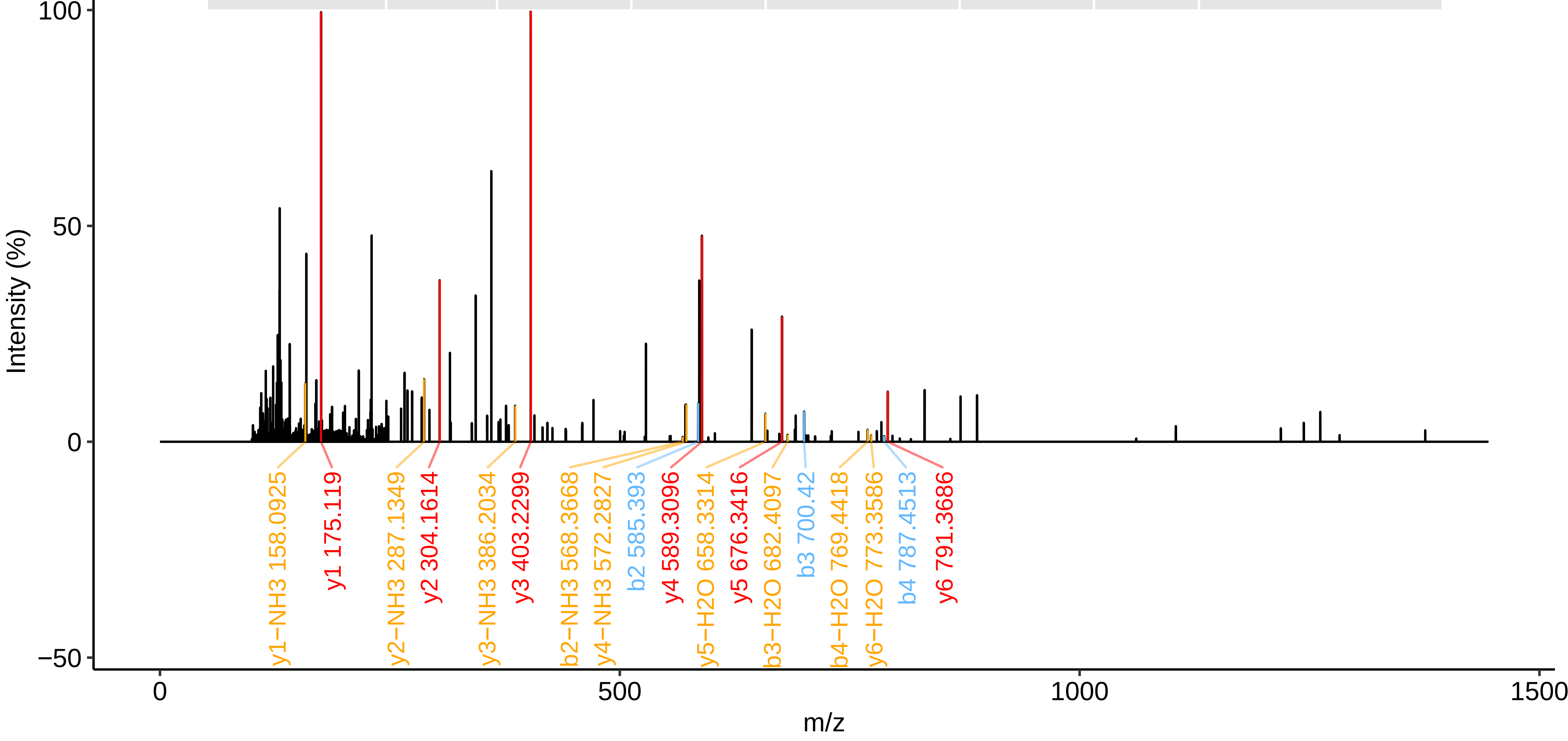

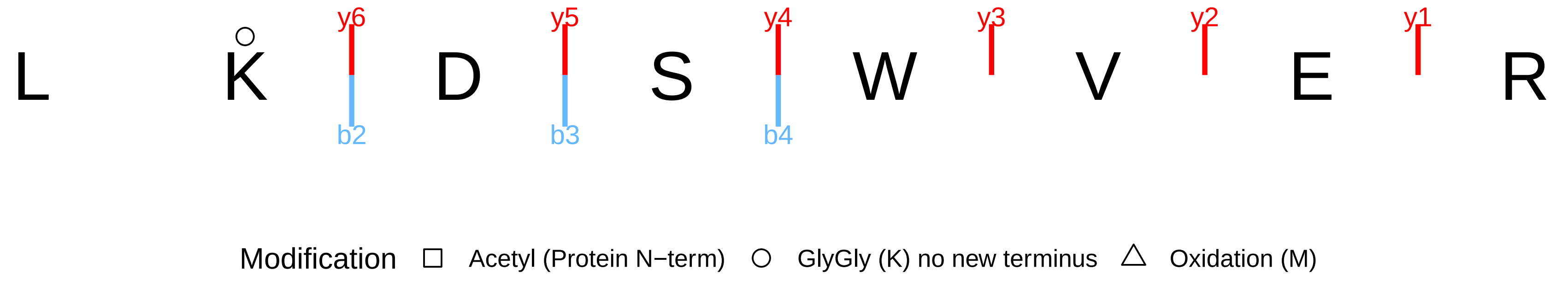

20200921_KIZ_MUb4_GlyGly_10plex_2 − Scan number: 10735

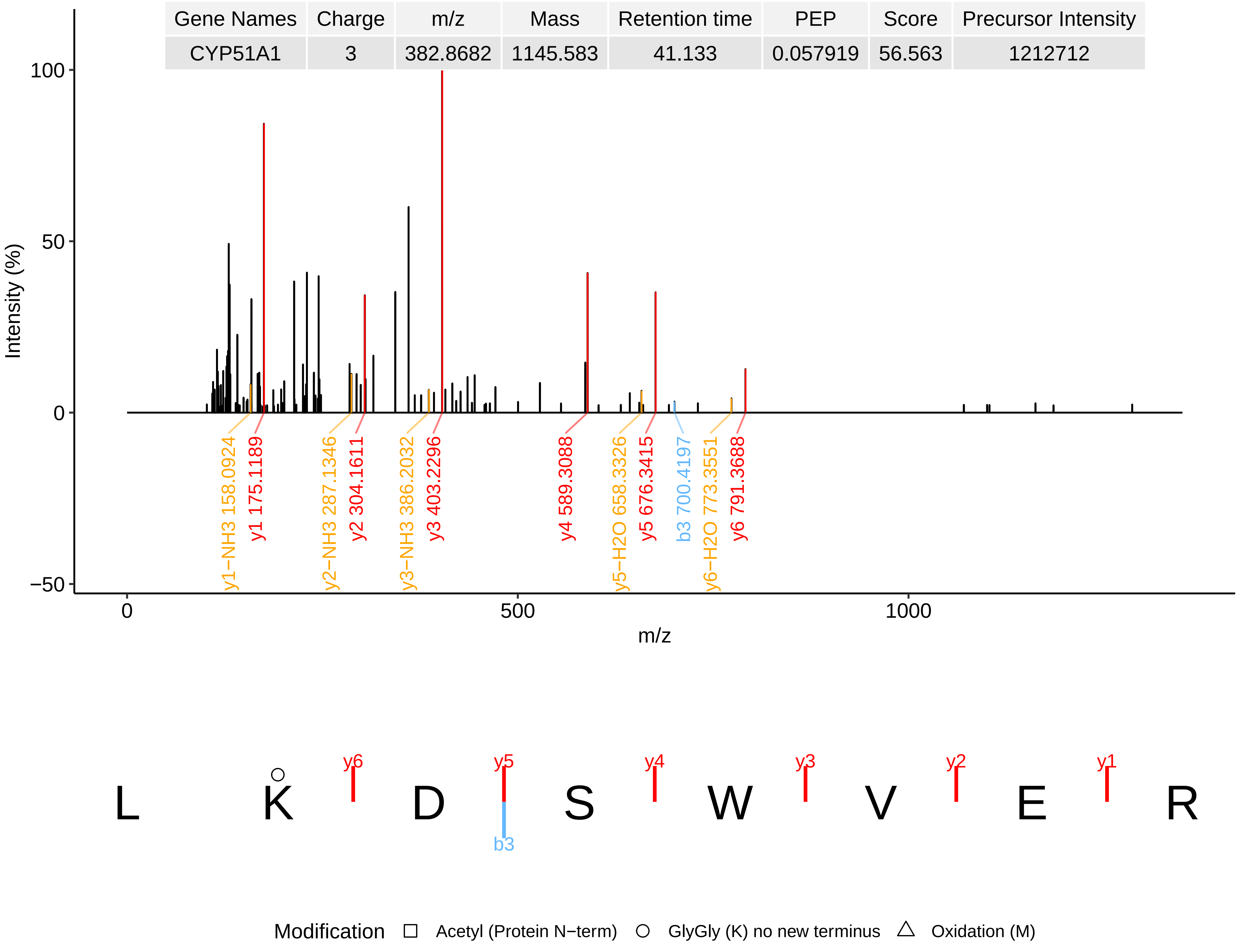

20200921_KIZ_MUb4_GlyGly_10plex − Scan number: 13745

Gene Names	Charge	m/z	Mass	Retention time	PEP	Score	Precursor Intensity
TNIP1	3	296.1955	885.5648	48.597	0.039162	73.039	4501100

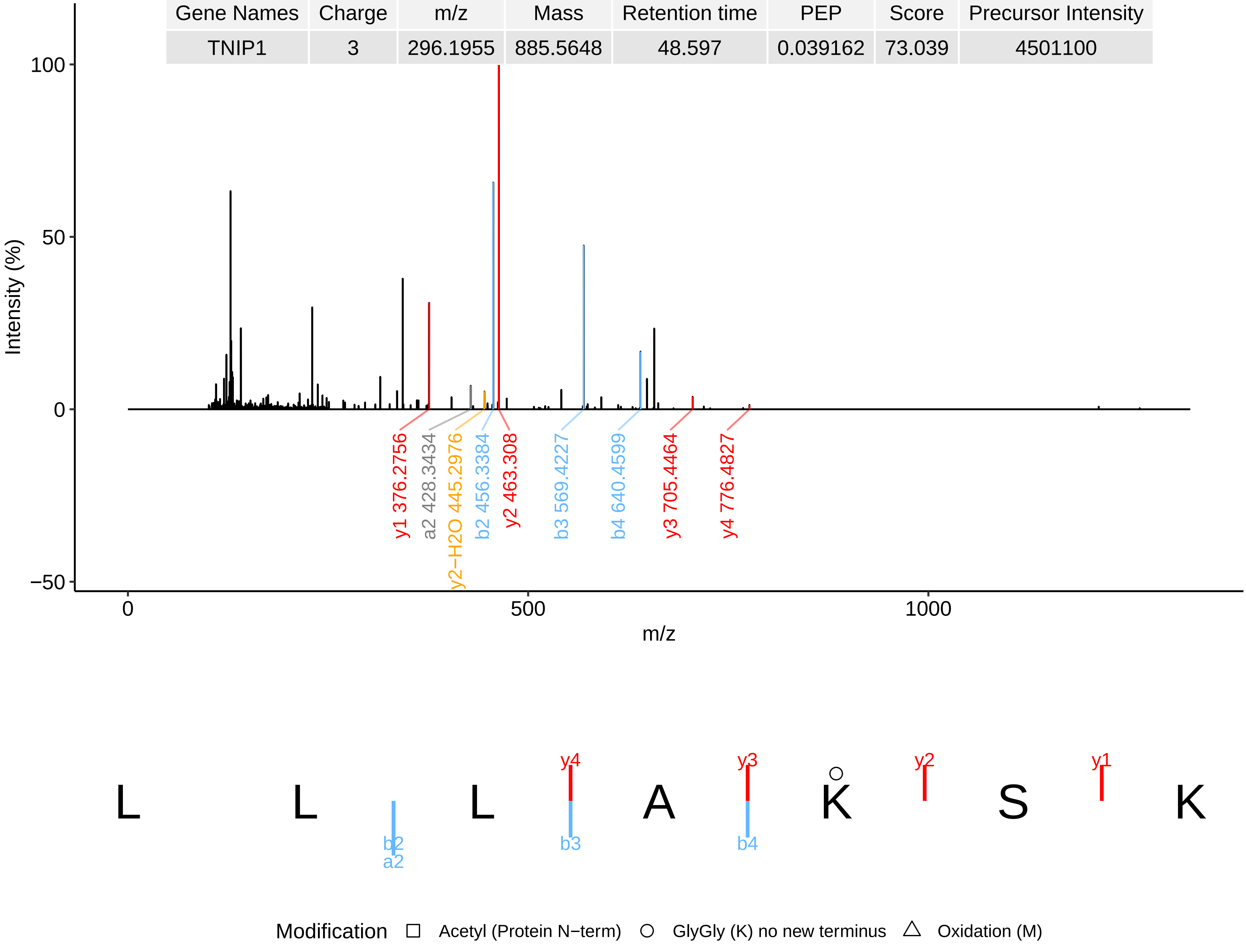

20200921_KIZ_MUb4_GlyGly_10plex − Scan number: 7699

Gene Names	Charge	m/z	Mass	Retention time	PEP	Score	Precursor Intensity
HSPA1B;HSPA1A;HSPA1L	3	373.1998	1116.578	25.93	0.018238	73.248	3263951

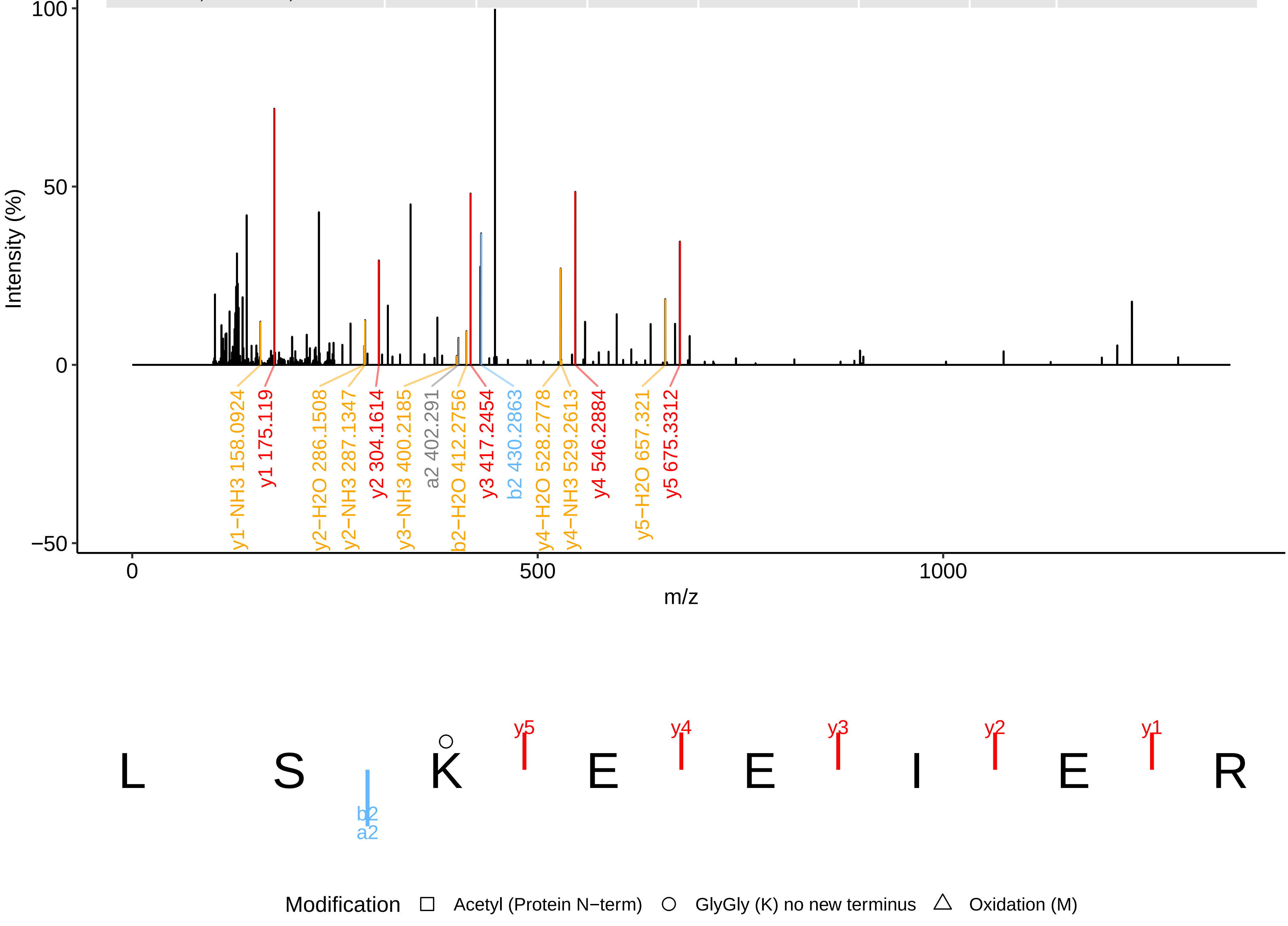

20200921_KIZ_MUb4_GlyGly_10plex_2 − Scan number: 7091

Gene Names	Charge	m/z	Mass	Retention time	PEP	Score	Precursor Intensity
HSPA1B;HSPA1A;HSPA1L	3	373.1998	1116.578	26.332	0.018238	73.248	1737673

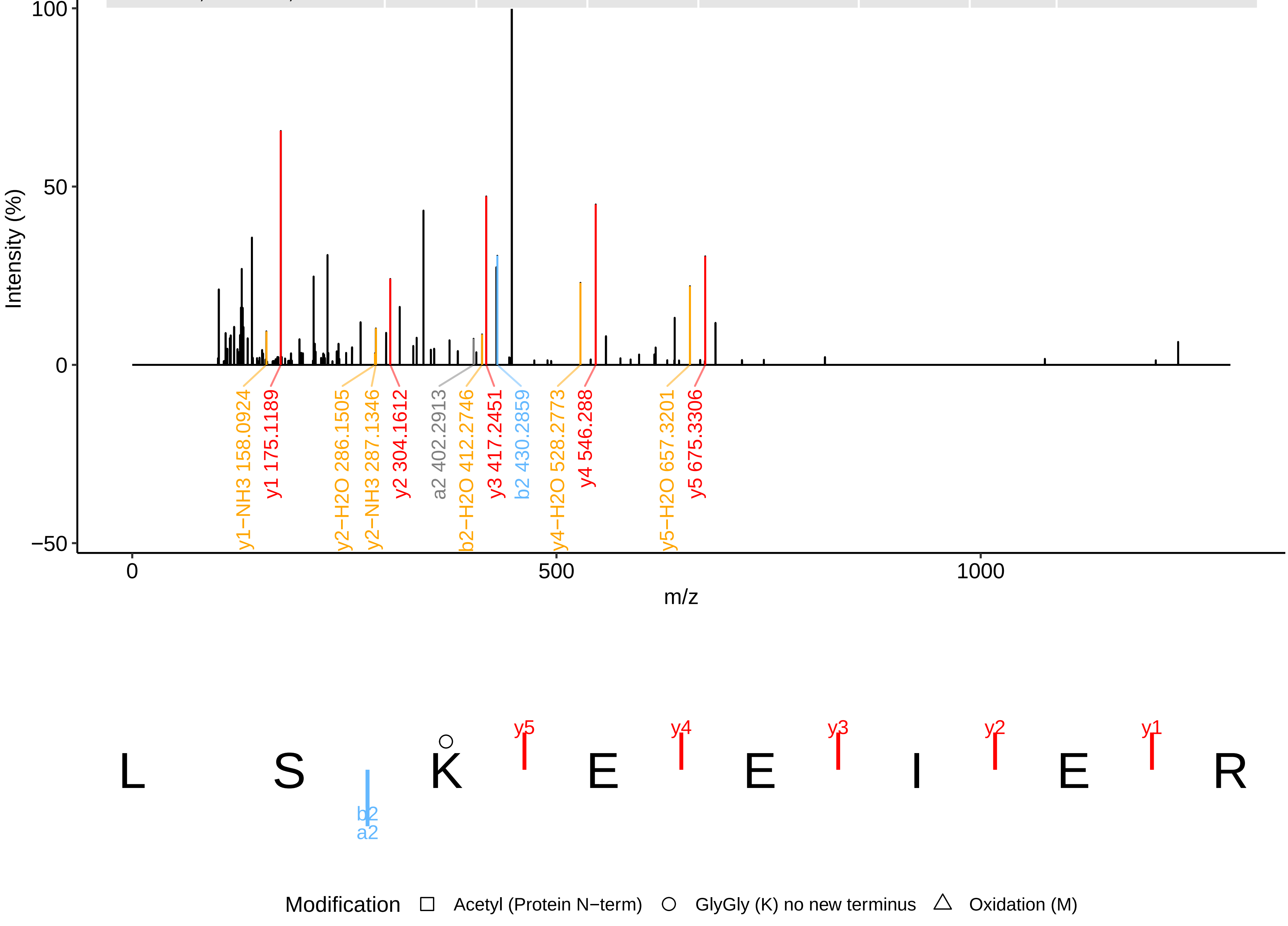

20200924_KIZ_MUb5_GlyGly_1 − Scan number: 6686

Gene Names	Charge	m/z	Mass	Retention time	PEP	Score	Precursor Intensity
HSPA1B;HSPA1A;HSPA1L	3	373.1998	1116.578	21.099	0.035058	65.246	3208341

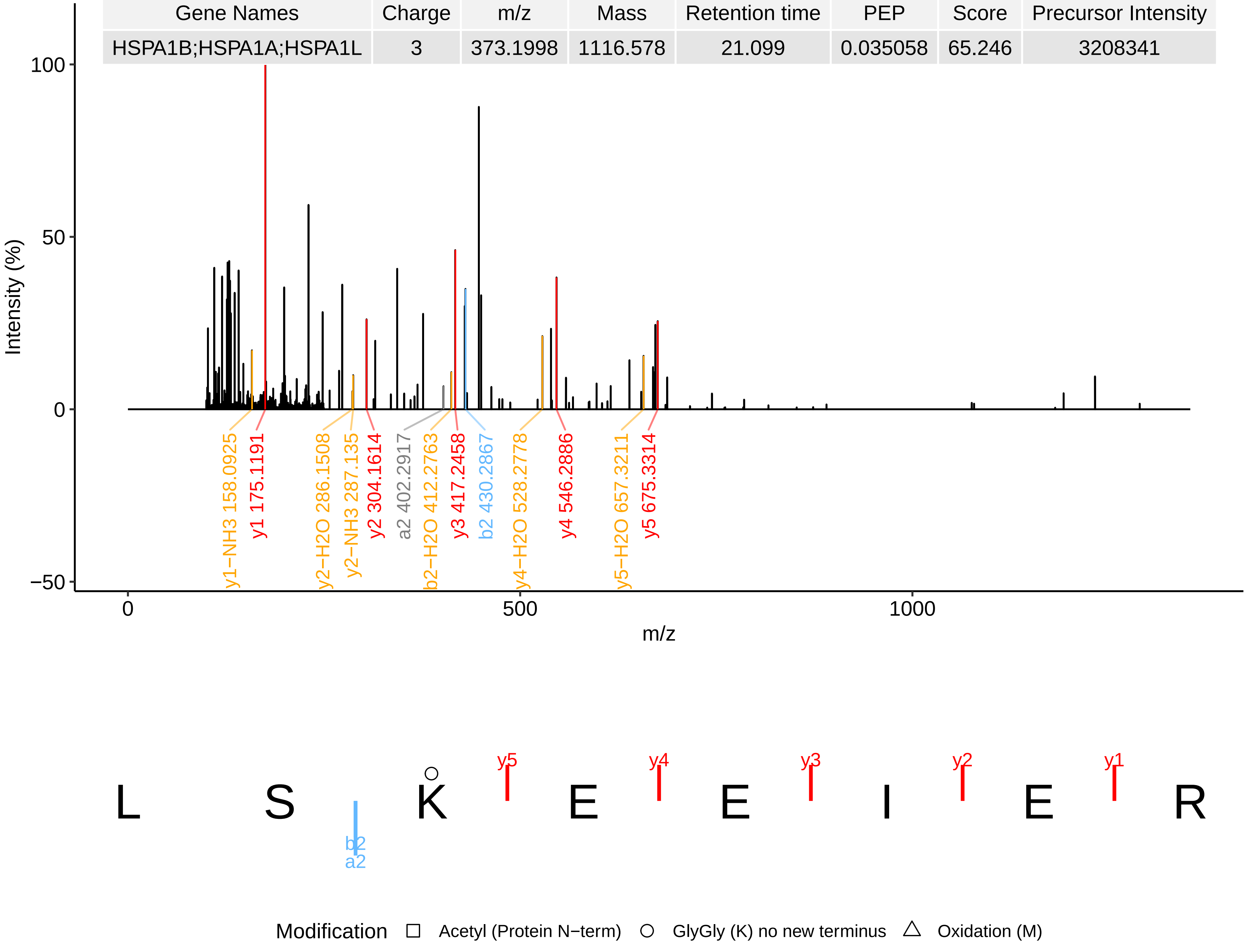

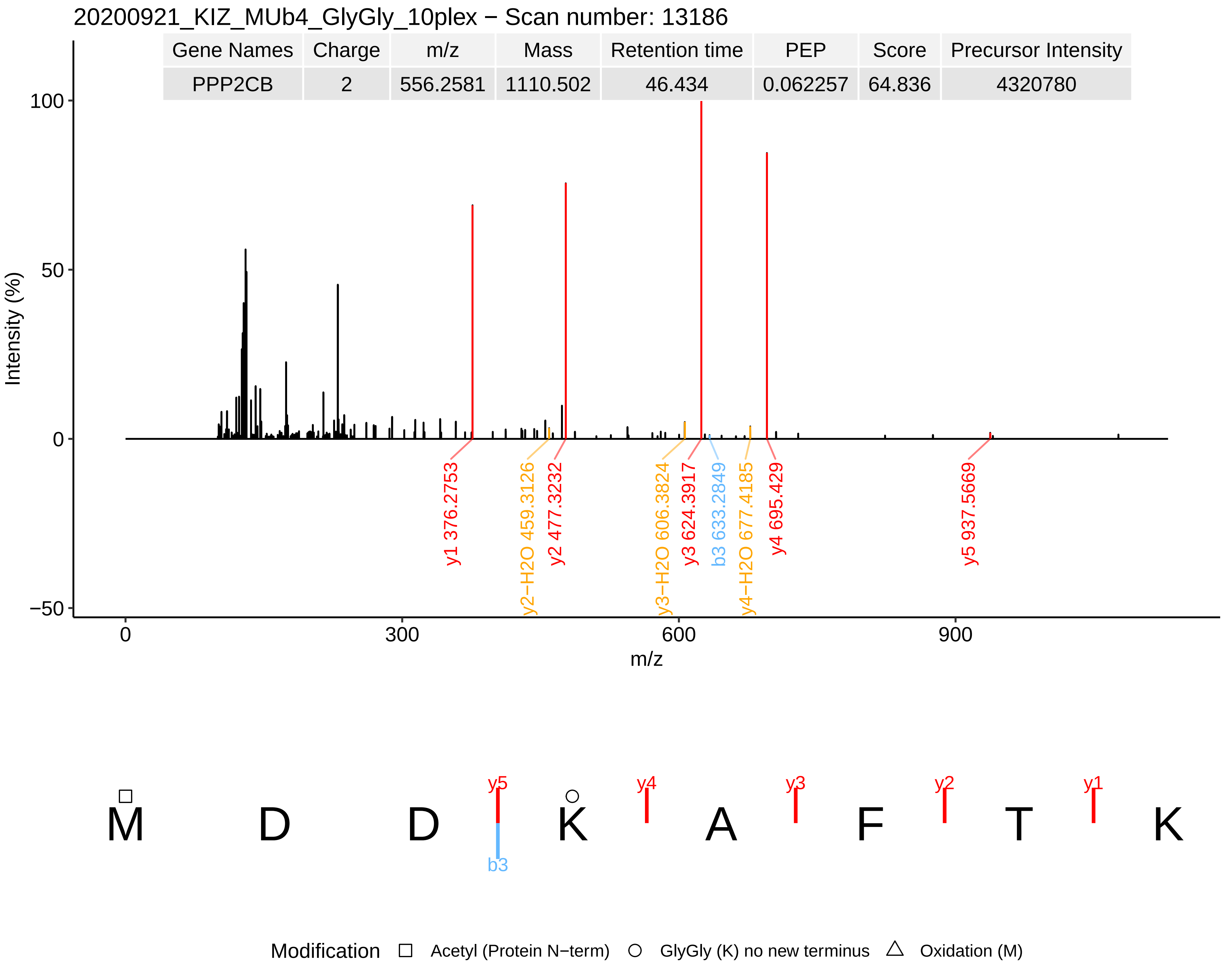

20200921_KIZ_MUb4_GlyGly_10plex − Scan number: 13186
Gene Names | Charge | m/z | Mass | Retention time | PEP | Score | Precursor Intensity
PPP2CB | 2 | 556.2581 | 1110.502 | 46.434 | 0.062257 | 64.836 | 4320780
Intensity (%)
100
50
0
−50
0
300
600
900
m/z
y1 376.2753
y2−H2O 459.3126
y2 477.3232
y3−H2O 606.3824
y3 624.3917
b3 633.2849
y4−H2O 677.4185
y4 695.429
y5 937.5669
M D D K A F T K
y5 y4 y3 y2 y1
b3
Modification
Acetyl (Protein N−term)
GlyGly (K) no new terminus
Oxidation (M)

20200924_KIZ_MUb5_GlyGly_1 − Scan number: 12306

Gene Names	Charge	m/z	Mass	Retention time	PEP	Score	Precursor Intensity
PPP2CB	2	556.2581	1110.502	40.023	0.065189	63.48	NA

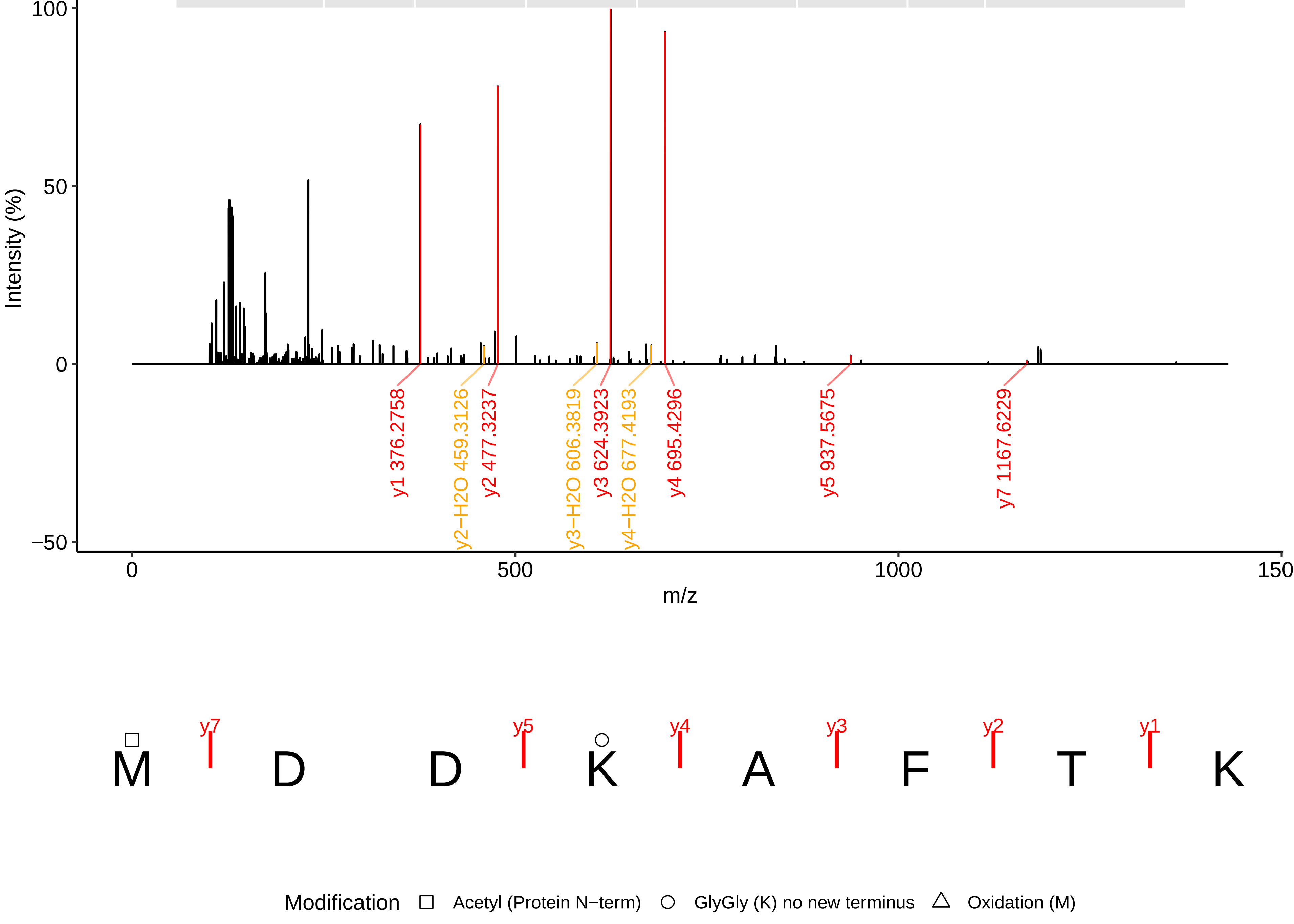

20200921_KIZ_MUb4_GlyGly_10plex_2 − Scan number: 8876

Gene Names	Charge	m/z	Mass	Retention time	PEP	Score	Precursor Intensity
RPL7	2	553.2633	1104.512	33.581	0.010321	67.757	829859.6

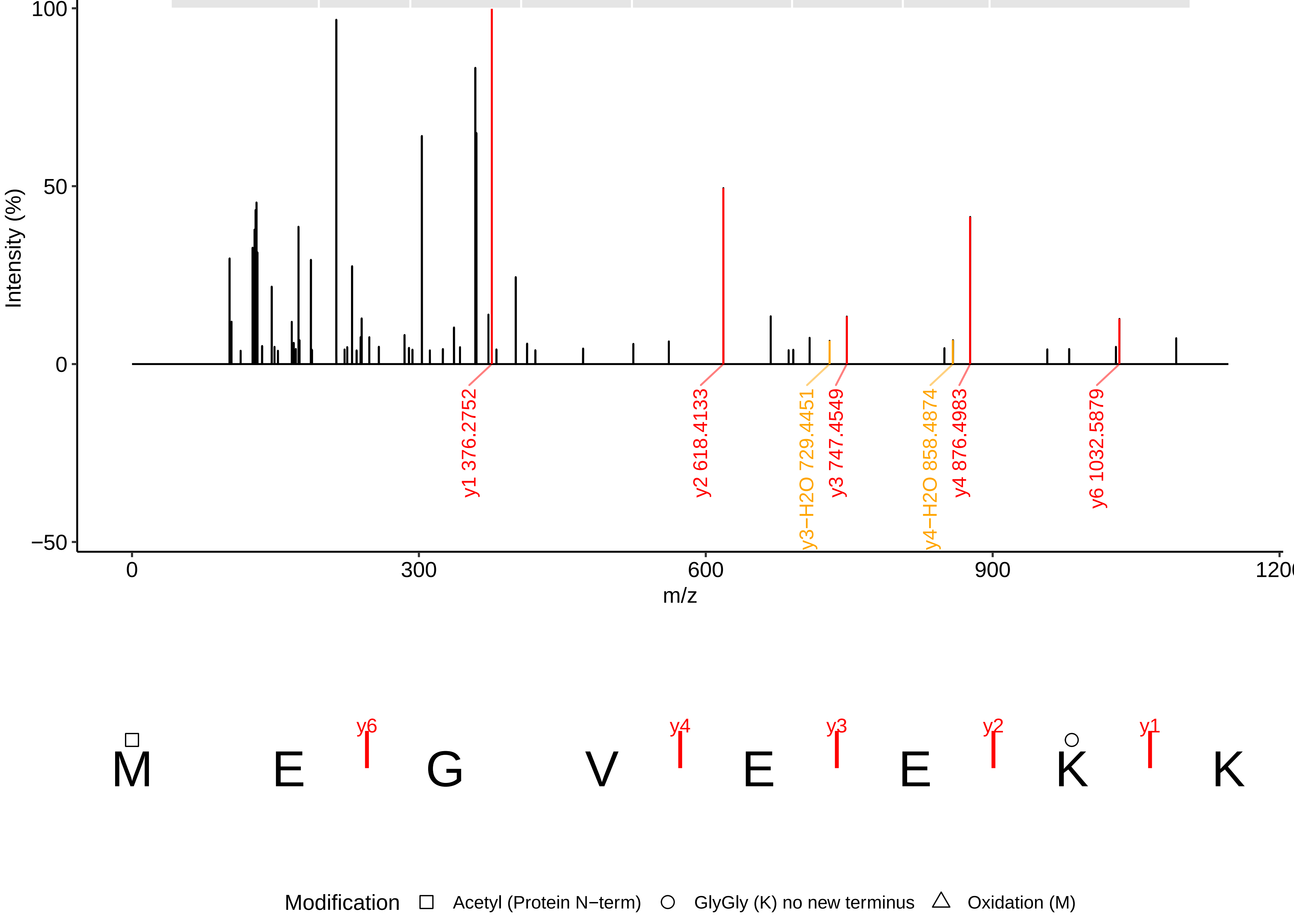

20200924_KIZ_MUb5_GlyGly_2 − Scan number: 8332

Gene Names	Charge	m/z	Mass	Retention time	PEP	Score	Precursor Intensity
RPL7	2	553.2633	1104.512	31.133	0.010633	56.011	1164656

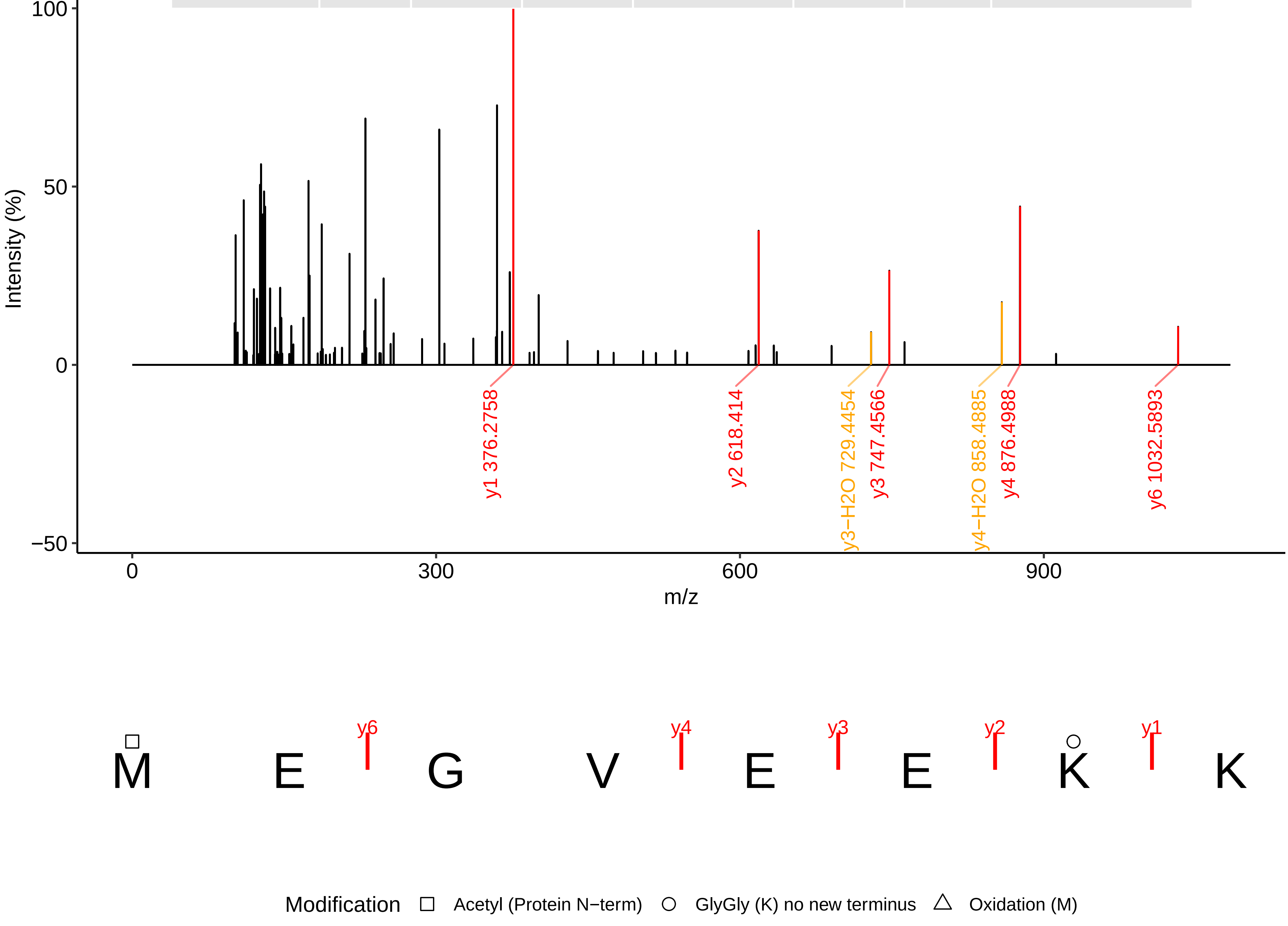

20200921_KIZ_MUb4_GlyGly_10plex − Scan number: 12539

Gene Names	Charge	m/z	Mass	Retention time	PEP	Score	Precursor Intensity
FGF13	2	680.3561	1358.698	44.004	0.079273	44.692	NA

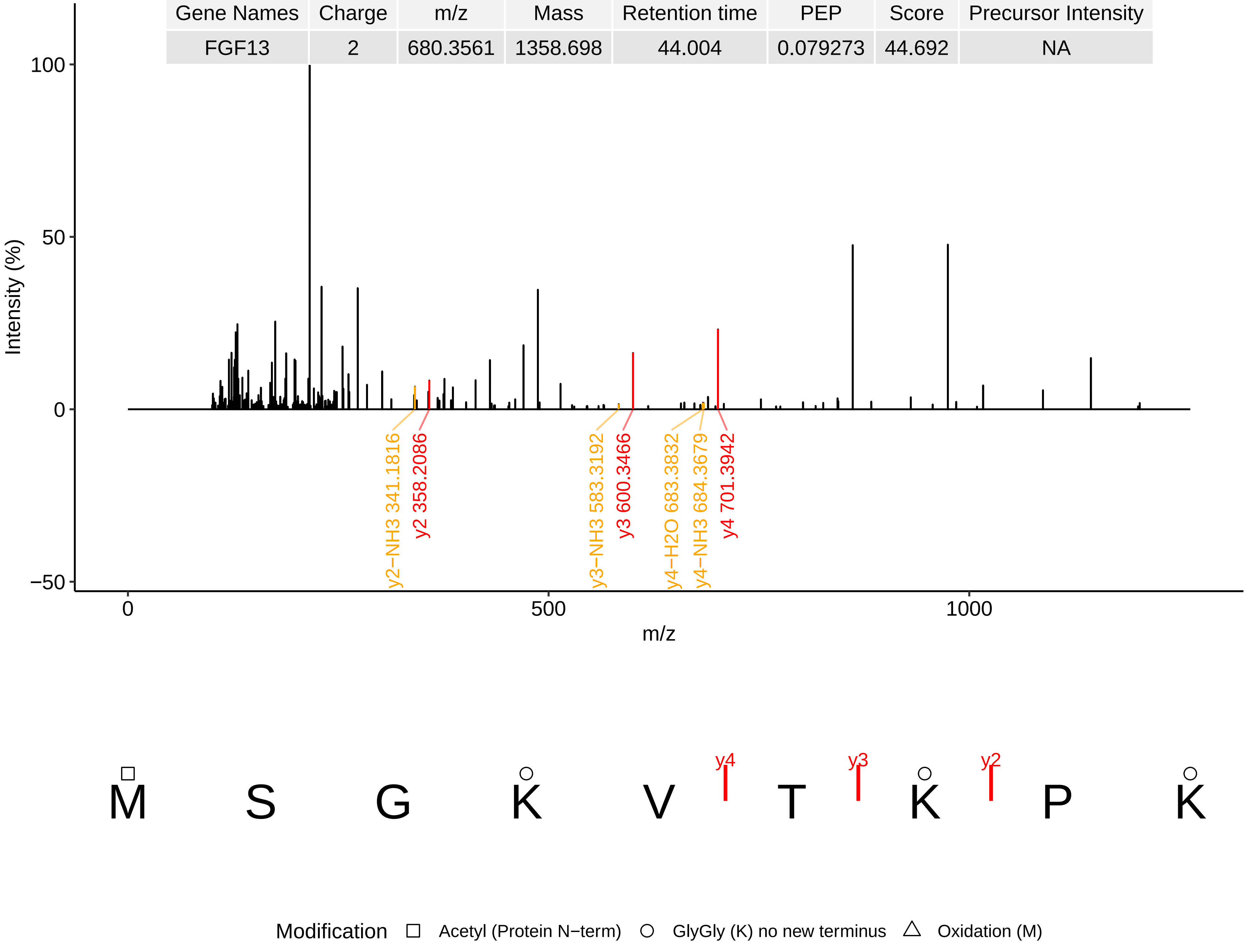

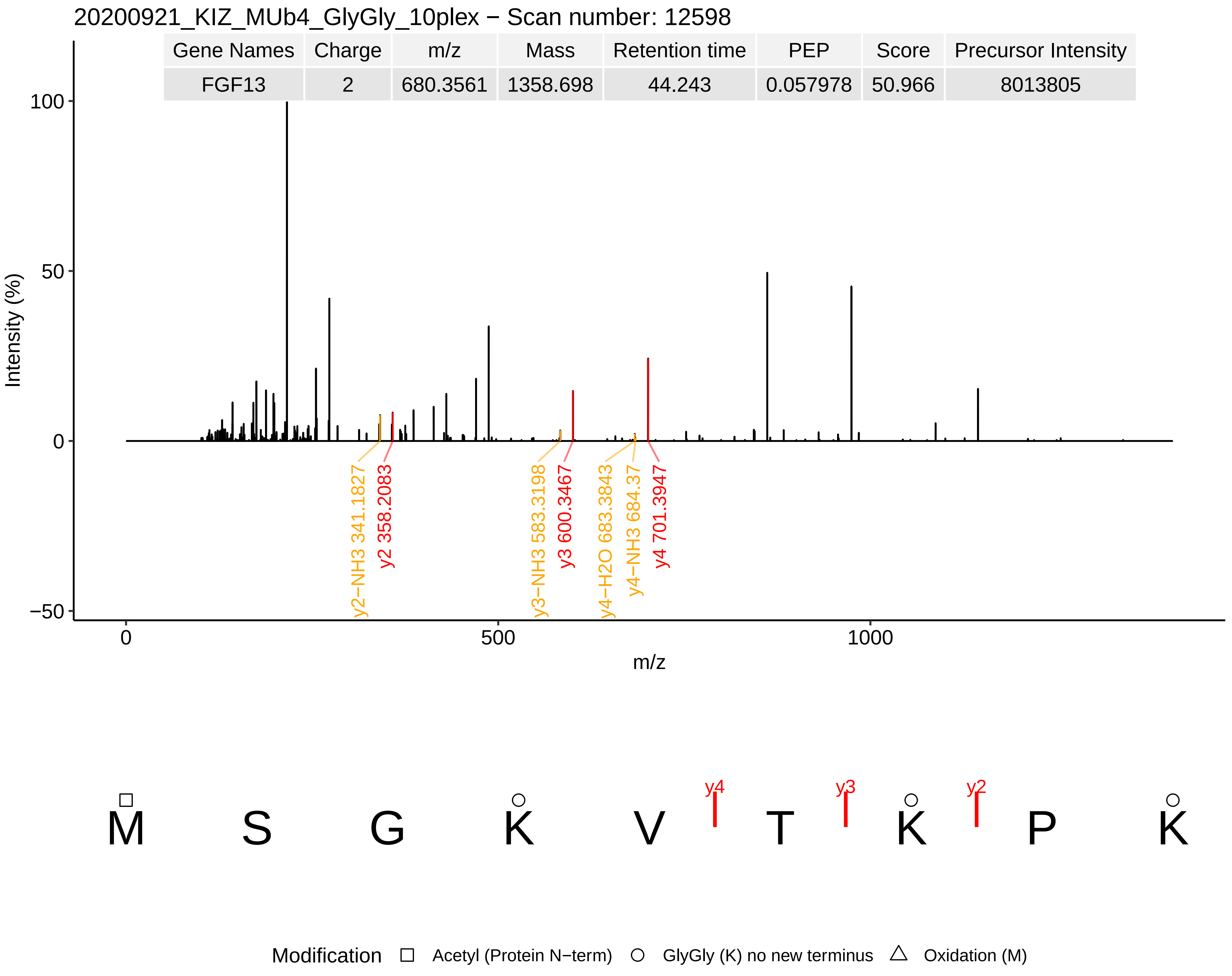

20200921_KIZ_MUb4_GlyGly_10plex − Scan number: 12598
Gene Names | Charge | m/z | Mass | Retention time | PEP | Score | Precursor Intensity
FGF13 | 2 | 680.3561 | 1358.698 | 44.243 | 0.057978 | 50.966 | 8013805
Intensity (%)
100
50
0
−50
0
500
1000
m/z
y2−NH3 341.1827
y2 358.2083
y3−NH3 583.3198
y3 600.3467
y4−H2O 683.3843
y4−NH3 684.37
y4 701.3947
M
S
G
K
V
y4
T
y3
K
y2
P
K
Modification
Acetyl (Protein N−term)
GlyGly (K) no new terminus
Oxidation (M)

20200921_KIZ_MUb4_GlyGly_10plex − Scan number: 11066

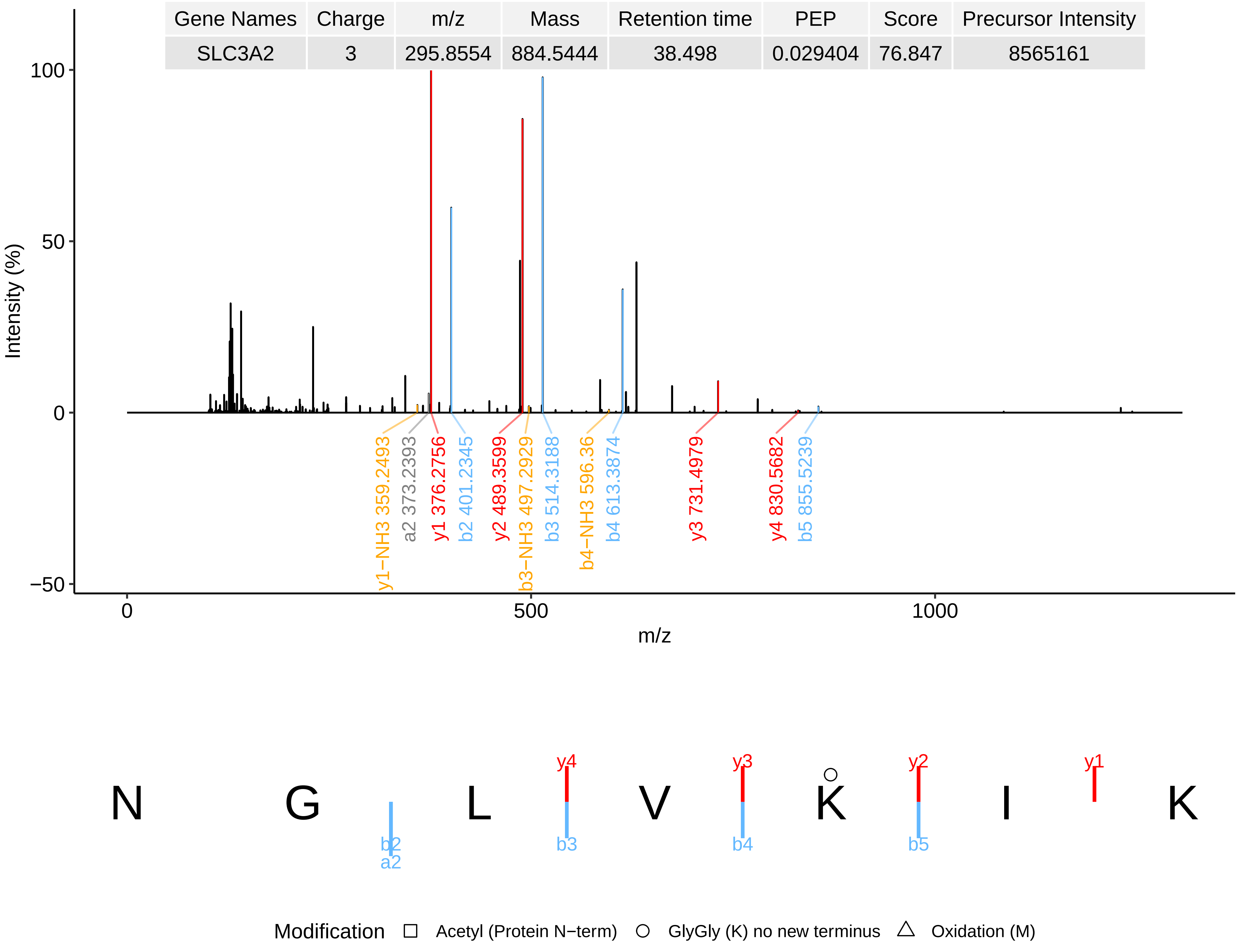

20200921_KIZ_MUb4_GlyGly_10plex_2 − Scan number: 10272

Gene Names	Charge	m/z	Mass	Retention time	PEP	Score	Precursor Intensity
SLC3A2	3	295.8554	884.5444	39.252	0.048628	69.344	2338458

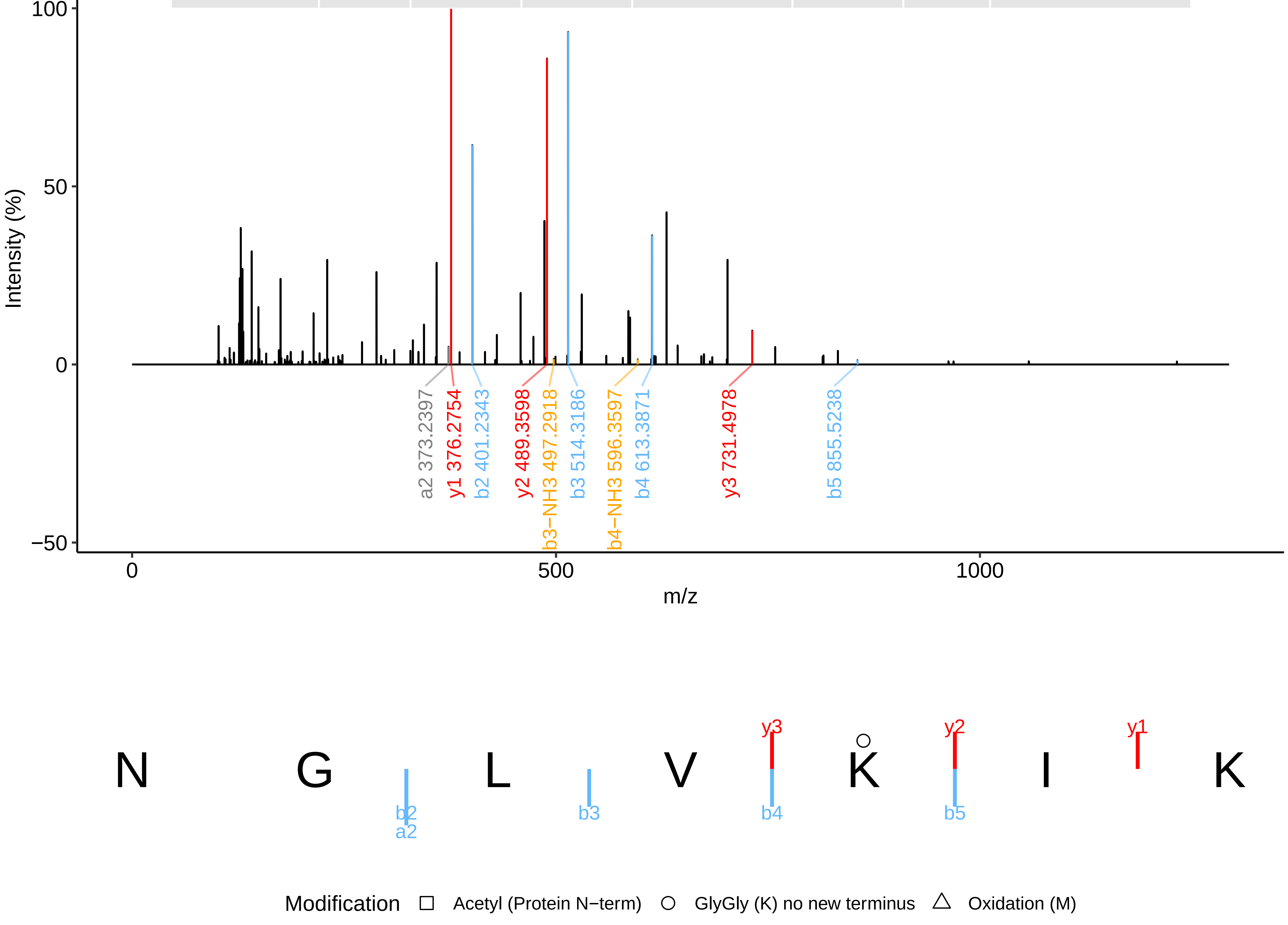

20200924_KIZ_MUb5_GlyGly_1 − Scan number: 10048

Gene Names	Charge	m/z	Mass	Retention time	PEP	Score	Precursor Intensity
SLC3A2	3	295.8554	884.5444	32.38	0.094431	57.103	16476226

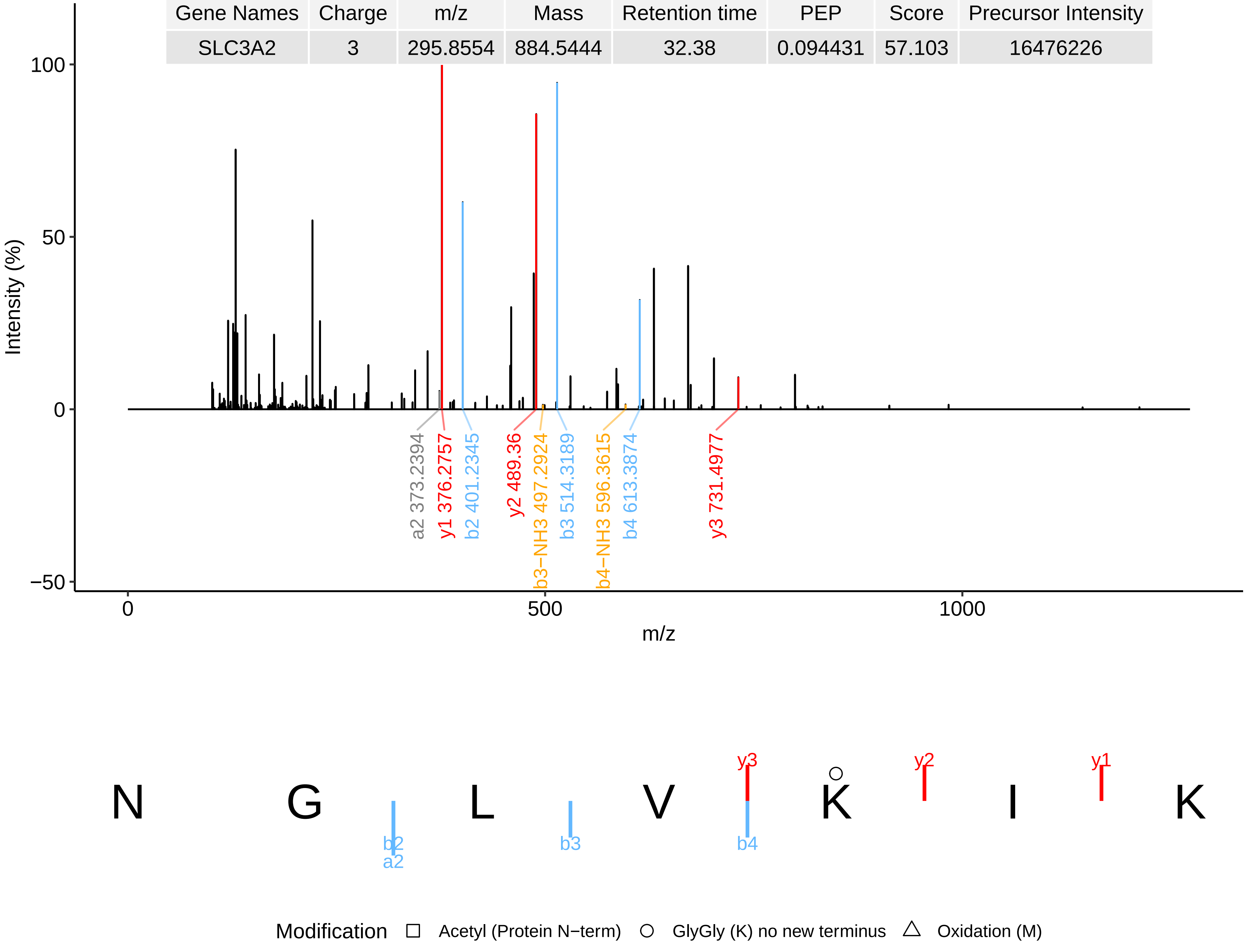

20200924_KIZ_MUb5_GlyGly_2 − Scan number: 9349

Gene Names	Charge	m/z	Mass	Retention time	PEP	Score	Precursor Intensity
SLC3A2	3	295.8554	884.5444	34.938	0.048628	69.344	8669515

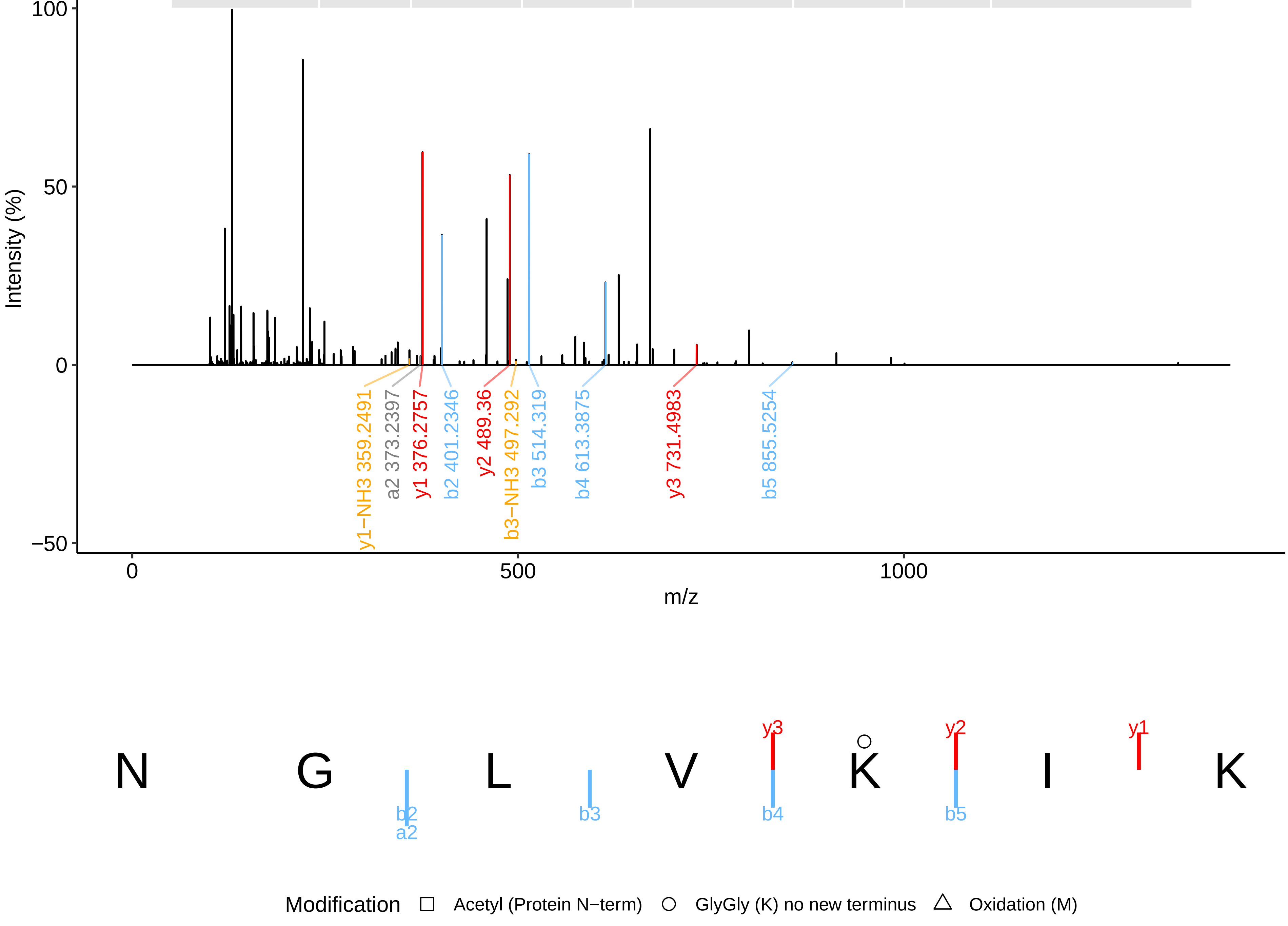

20201118_KIZ_MUb6_GlyGly_1 − Scan number: 9664

Gene Names	Charge	m/z	Mass	Retention time	PEP	Score	Precursor Intensity
SLC3A2	3	295.8554	884.5444	30.564	0.052695	67.757	24166008

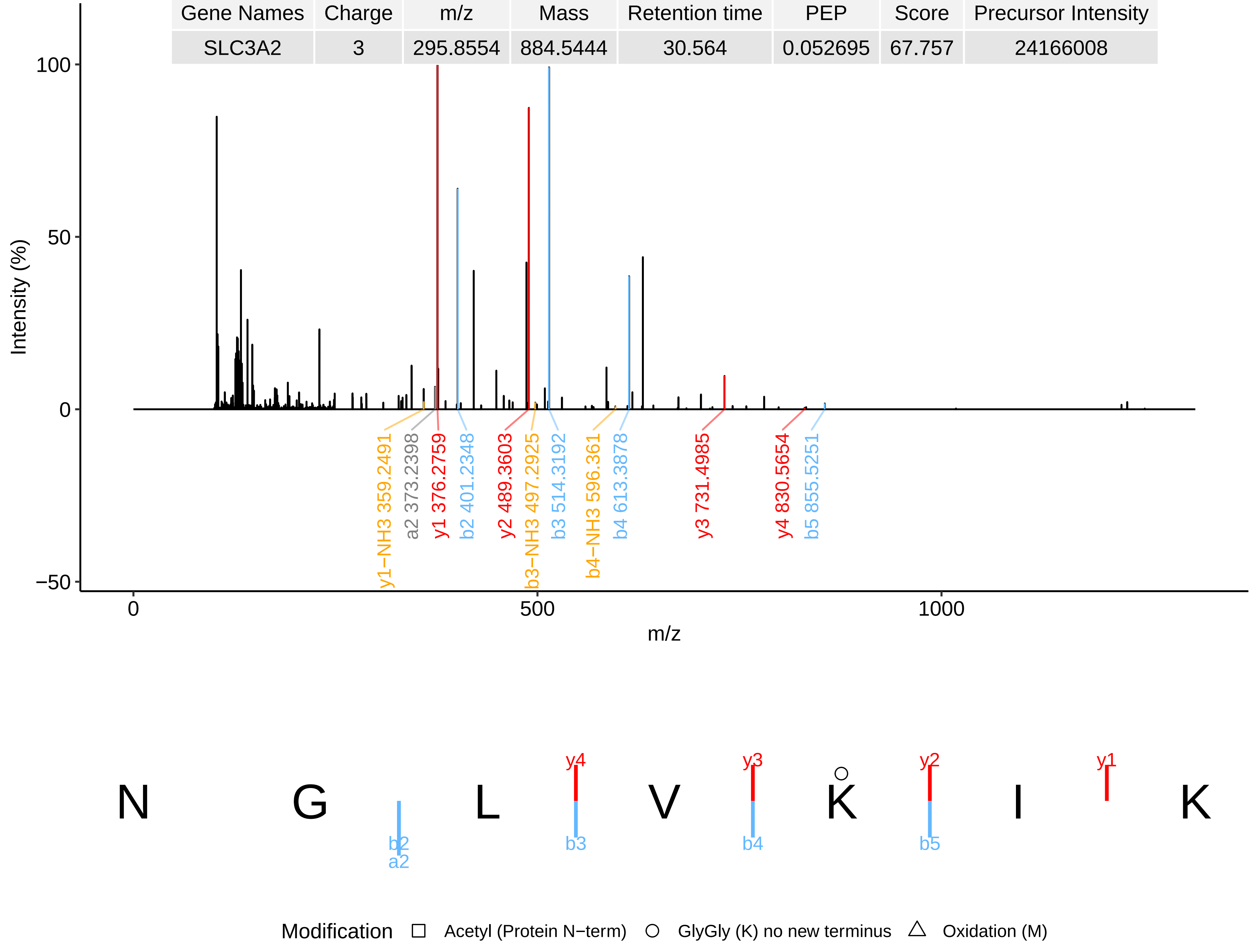

20201118_KIZ_MUb6_GlyGly_2 − Scan number: 12175

Gene Names	Charge	m/z	Mass	Retention time	PEP	Score	Precursor Intensity
SLC3A2	3	295.8554	884.5444	40.96	0.017624	83.614	12202212

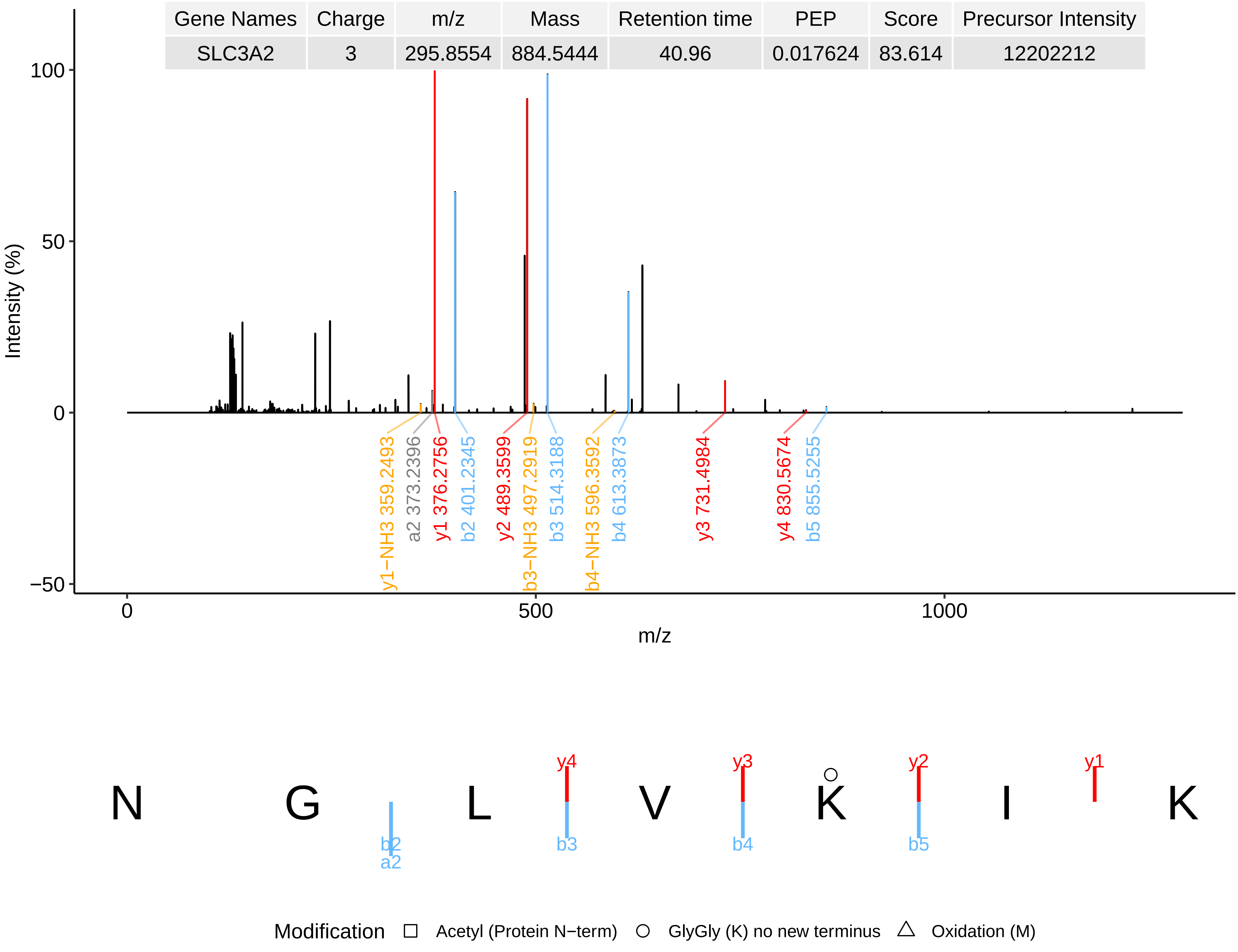

20200921_KIZ_MUb4_GlyGly_10plex − Scan number: 9442

Gene Names	Charge	m/z	Mass	Retention time	PEP	Score	Precursor Intensity
RPS3A	3	372.5559	1114.646	32.325	0.0039074	80.231	3426628

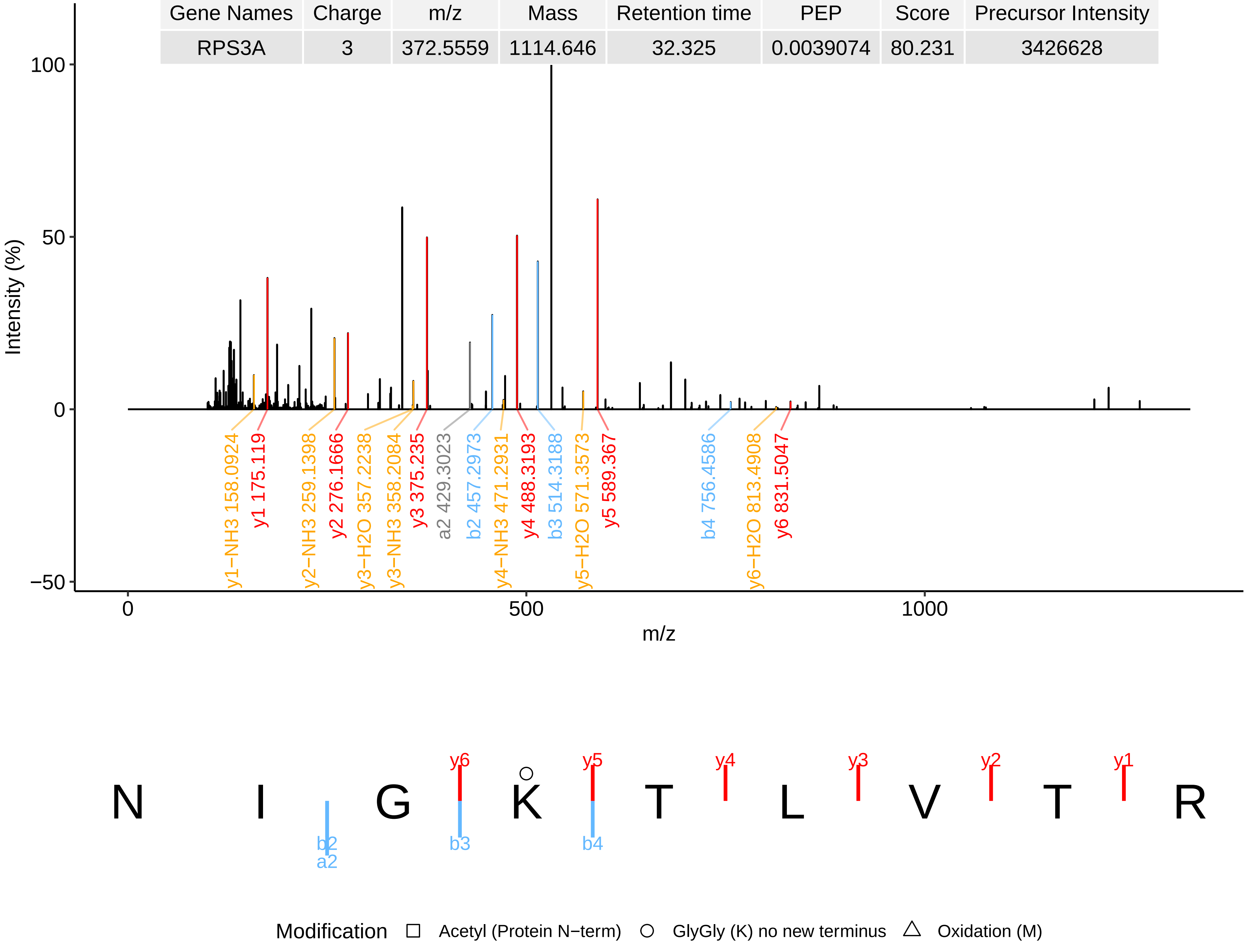

20200921_KIZ_MUb4_GlyGly_10plex_2 − Scan number: 8722

Gene Names	Charge	m/z	Mass	Retention time	PEP	Score	Precursor Intensity
RPS3A	3	372.5559	1114.646	32.958	0.001613	91.867	3320136

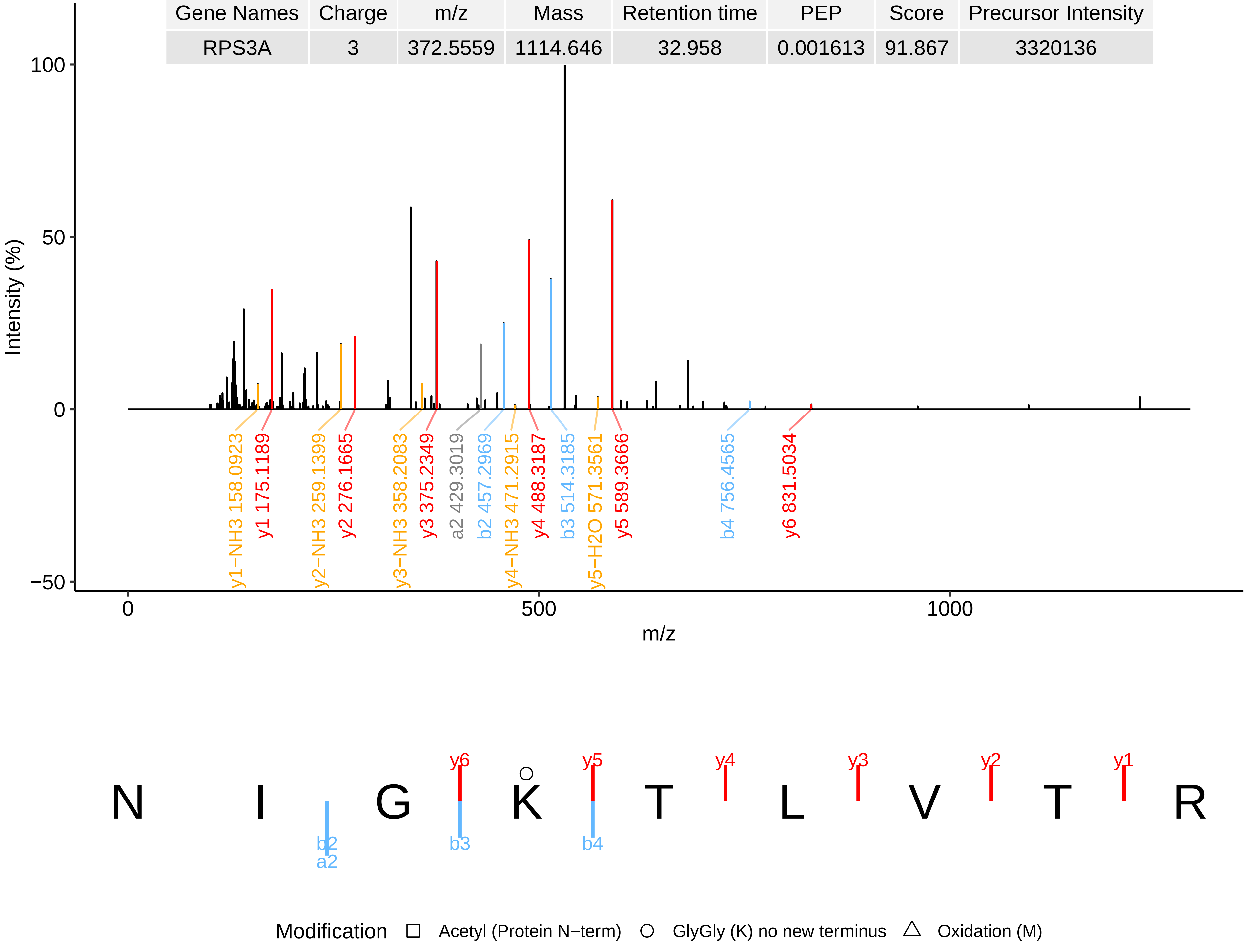

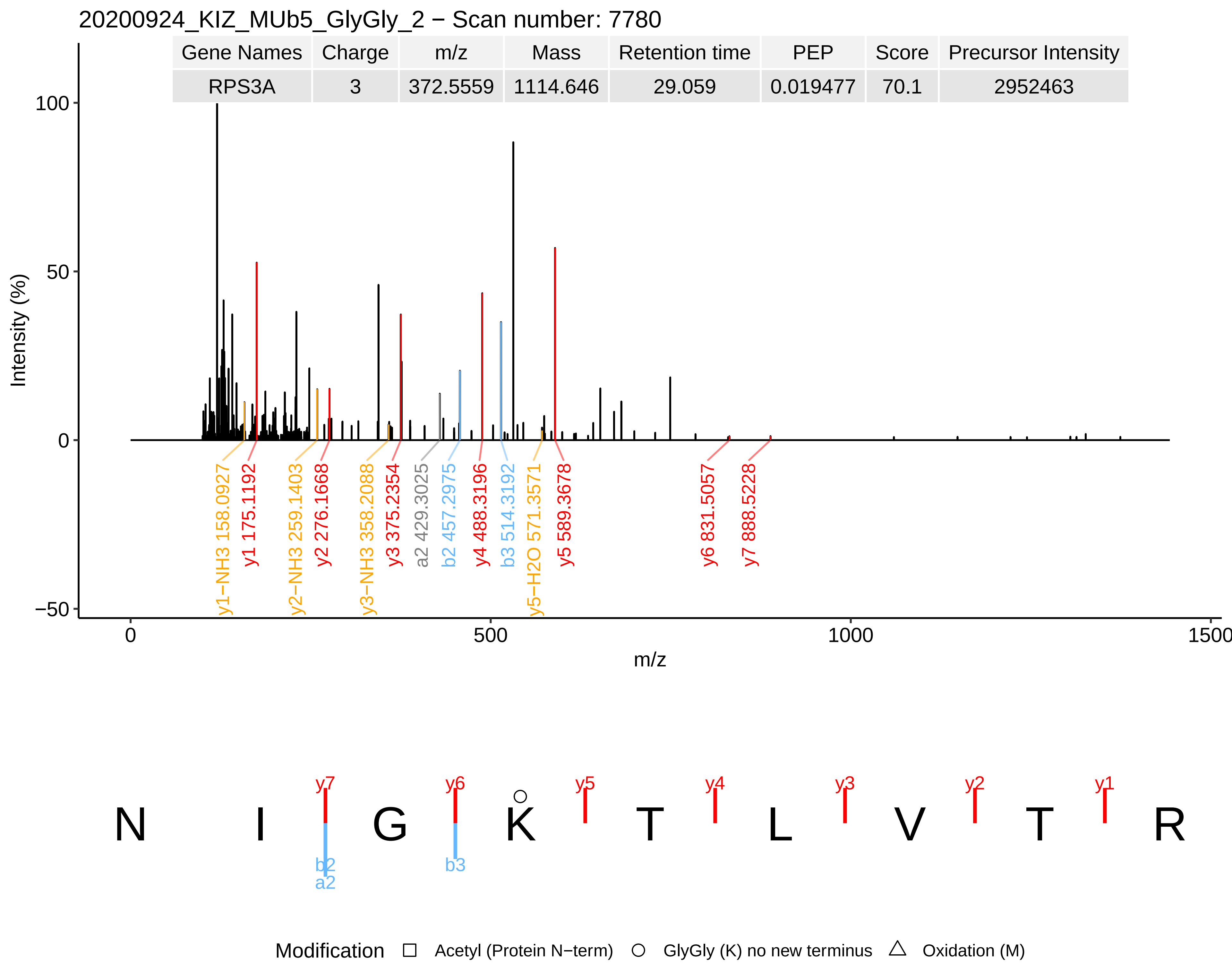

20200924_KIZ_MUb5_GlyGly_2 − Scan number: 7780

Gene Names	Charge	m/z	Mass	Retention time	PEP	Score	Precursor Intensity
RPS3A	3	372.5559	1114.646	29.059	0.019477	70.1	2952463

Intensity (%)
m/z
y1−NH3 158.0927
y1 175.1192
y2−NH3 259.1403
y2 276.1668
y3−NH3 358.2088
y3 375.2354
a2 429.3025
b2 457.2975
y4 488.3196
b3 514.3192
y5−H2O 571.3571
y5 589.3678
y6 831.5057
y7 888.5228
N I G K T L V T R
y7 y6 y5 y4 y3 y2 y1
b2 a2 b3
Modification
Acetyl (Protein N−term)
GlyGly (K) no new terminus
Oxidation (M)

20200921_KIZ_MUb4_GlyGly_10plex − Scan number: 5835

Gene Names	Charge	m/z	Mass	Retention time	PEP	Score	Precursor Intensity
PBRM1	3	306.5065	916.4978	19.138	0.080184	80.229	7050831

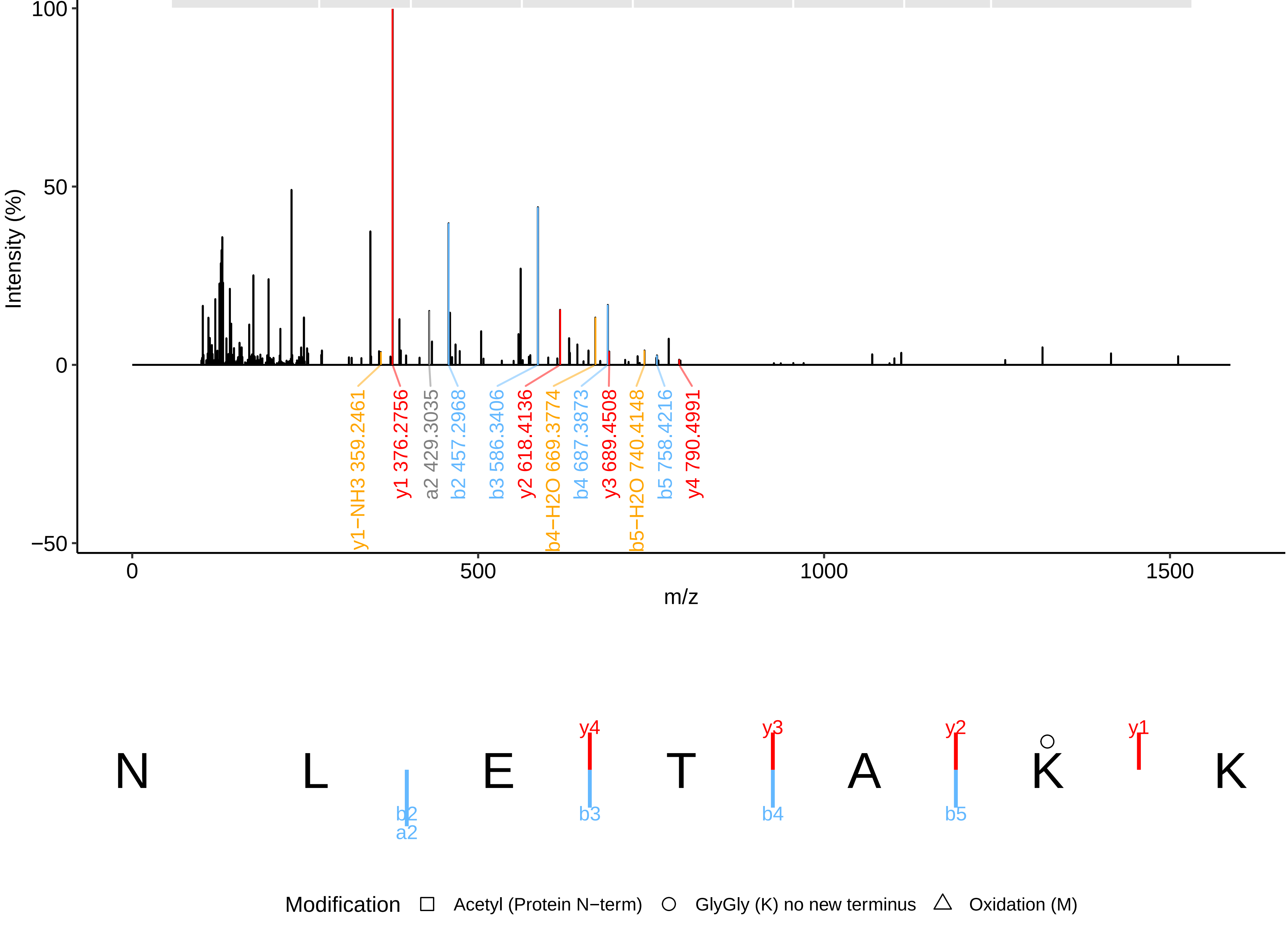

20200921_KIZ_MUb4_GlyGly_10plex − Scan number: 11906

Gene Names	Charge	m/z	Mass	Retention time	PEP	Score	Precursor Intensity
RHOT1	3	381.8816	1142.623	41.594	0.02139	63.816	14222136

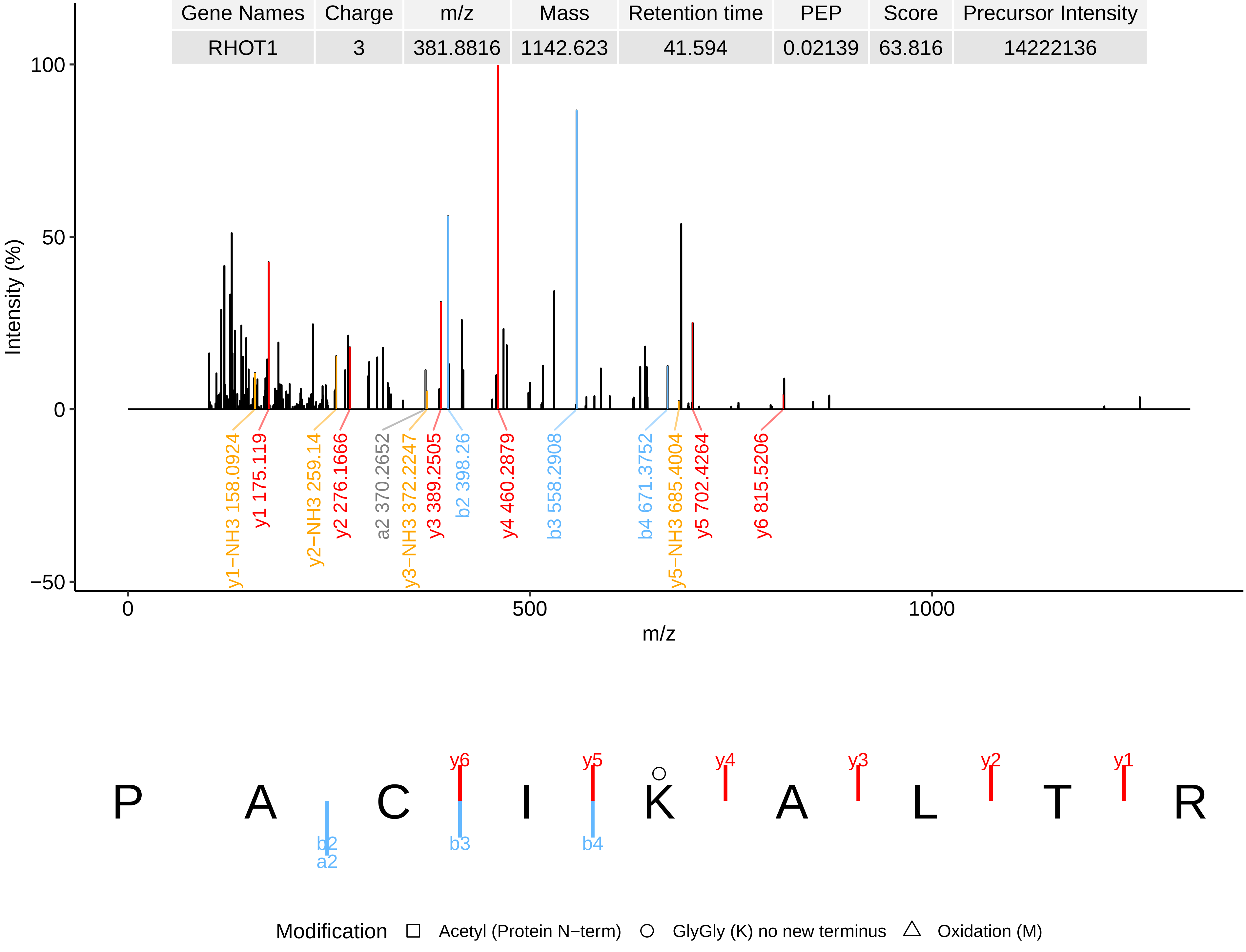

20200921_KIZ_MUb4_GlyGly_10plex_2 − Scan number: 10992

Gene Names	Charge	m/z	Mass	Retention time	PEP	Score	Precursor Intensity
RHOT1	3	381.8816	1142.623	42.207	0.0081778	76.221	3117974

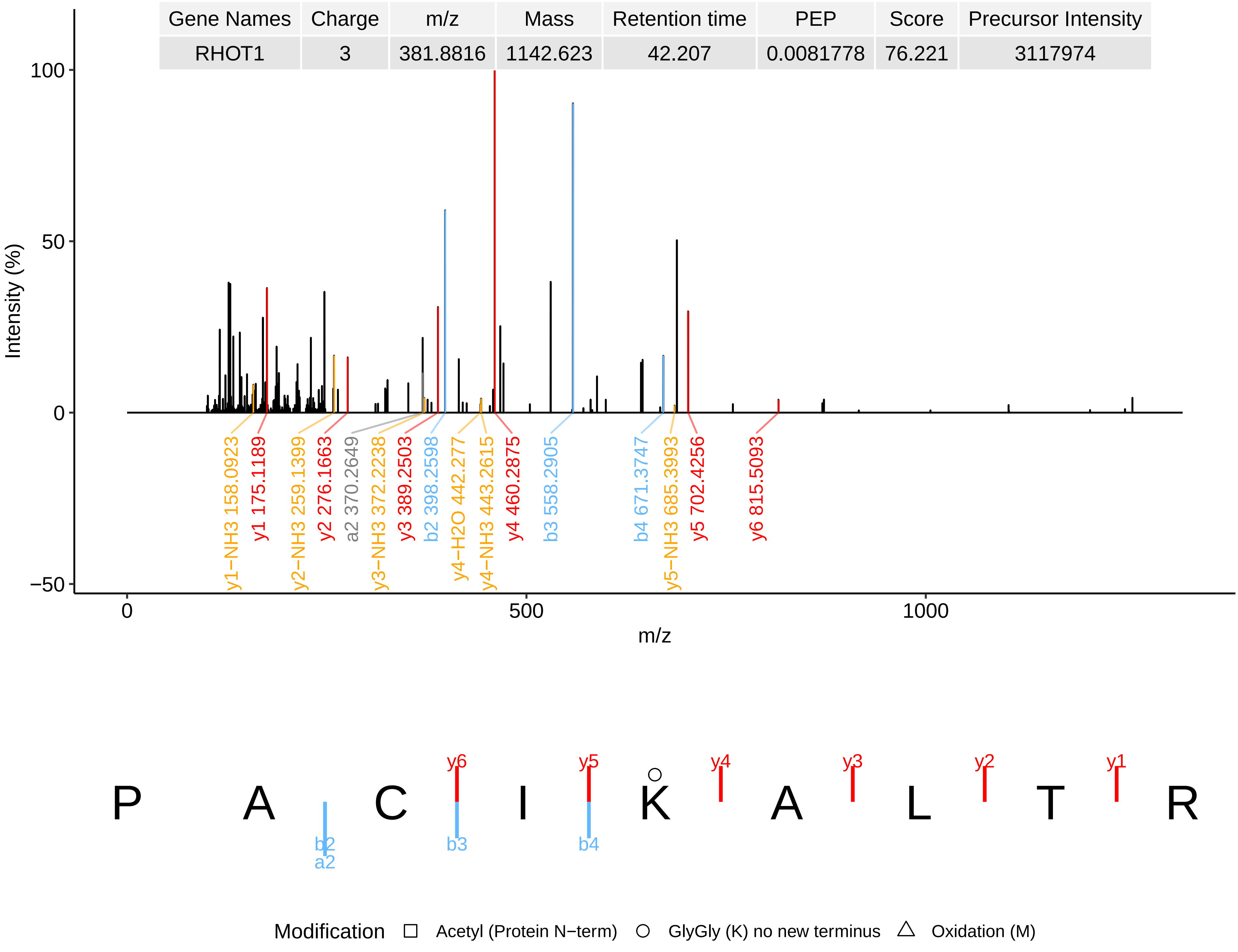

20200921_KIZ_MUb4_GlyGly_10plex_2 − Scan number: 11031

Gene Names	Charge	m/z	Mass	Retention time	PEP	Score	Precursor Intensity
RHOT1	3	381.8816	1142.623	42.355	0.0090758	75.378	2144596

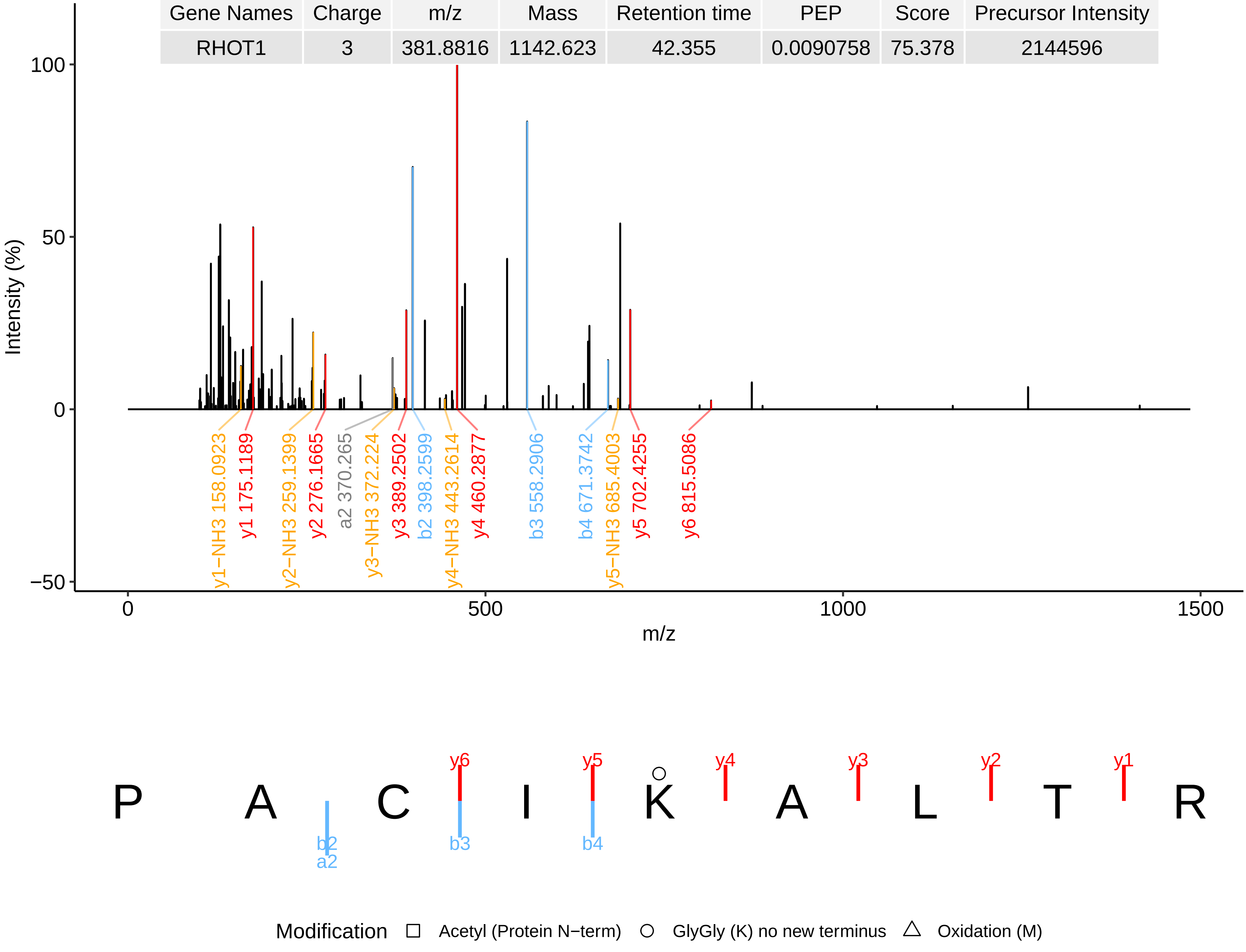

20200924_KIZ_MUb5_GlyGly_1 − Scan number: 10725

Gene Names	Charge	m/z	Mass	Retention time	PEP	Score	Precursor Intensity
RHOT1	3	381.8816	1142.623	34.651	0.012773	75.378	9221308

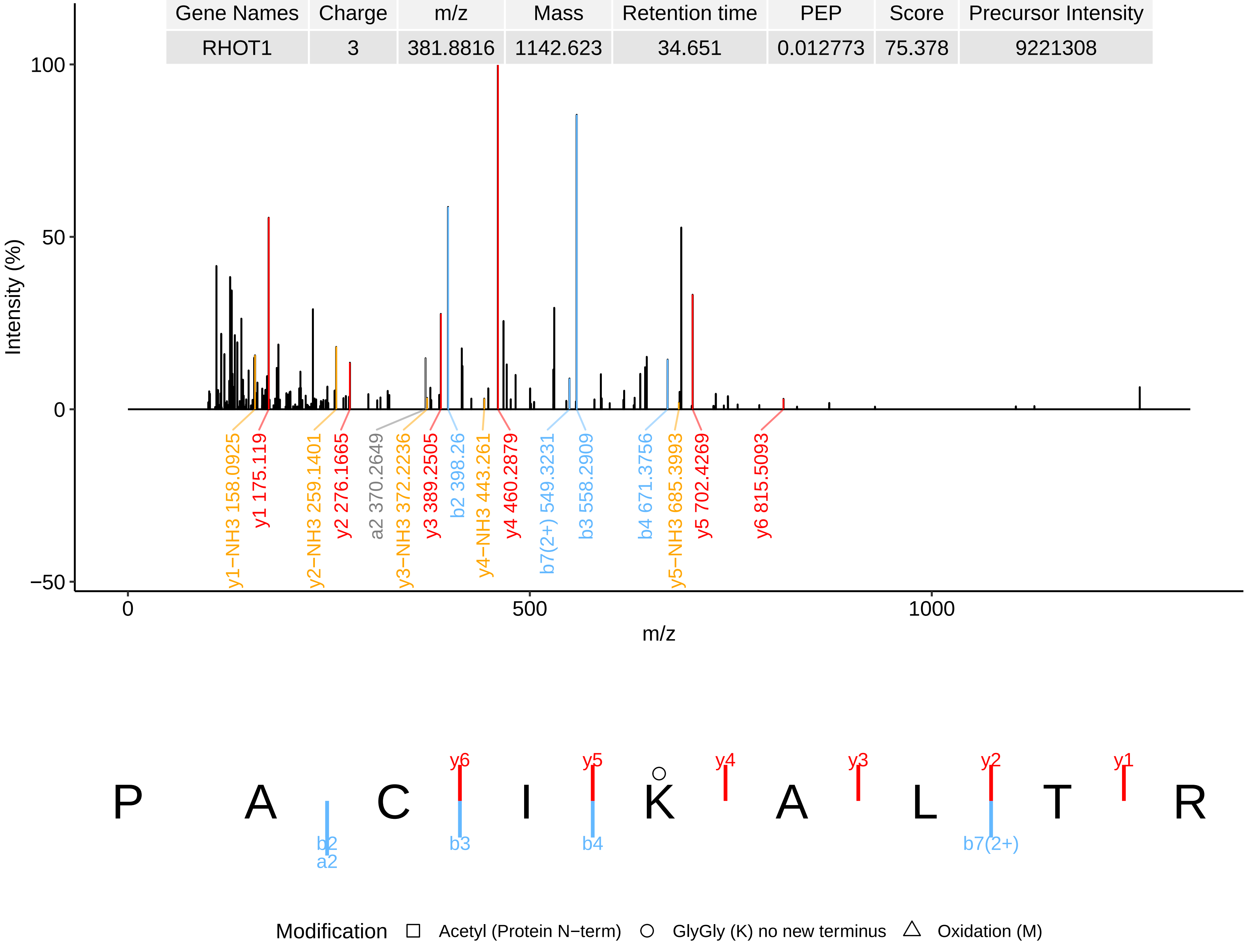

20200921_KIZ_MUb4_GlyGly_10plex_2 − Scan number: 5070

Gene Names	Charge	m/z	Mass	Retention time	PEP	Score	Precursor Intensity
PCBP2	3	381.5474	1141.62	18.061	0.0029501	83.998	2364166

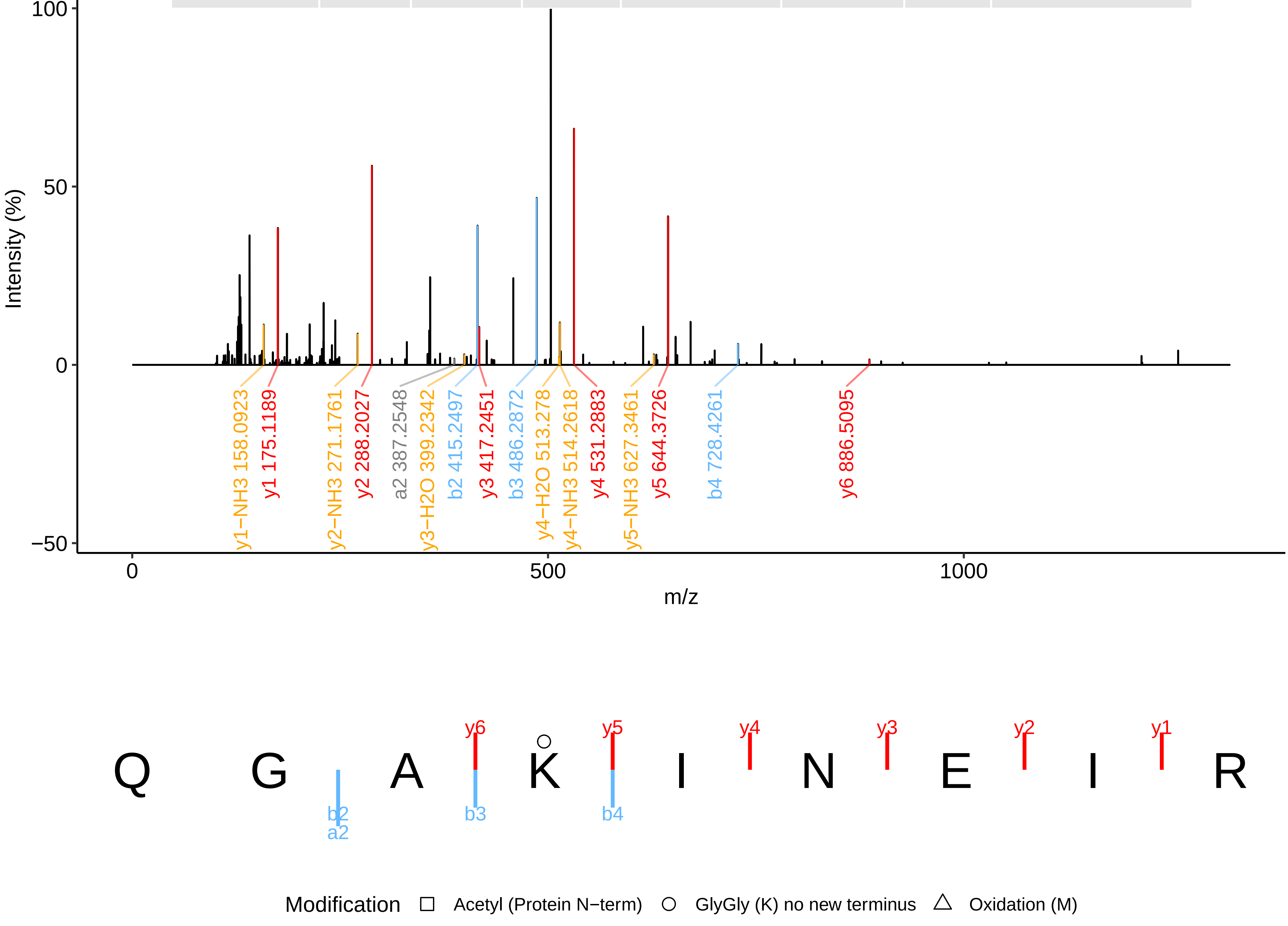

20200921_KIZ_MUb4_GlyGly_10plex − Scan number: 5020

Gene Names	Charge	m/z	Mass	Retention time	PEP	Score	Precursor Intensity
TRABD	3	367.556	1099.646	16.372	0.018669	79.974	4645253

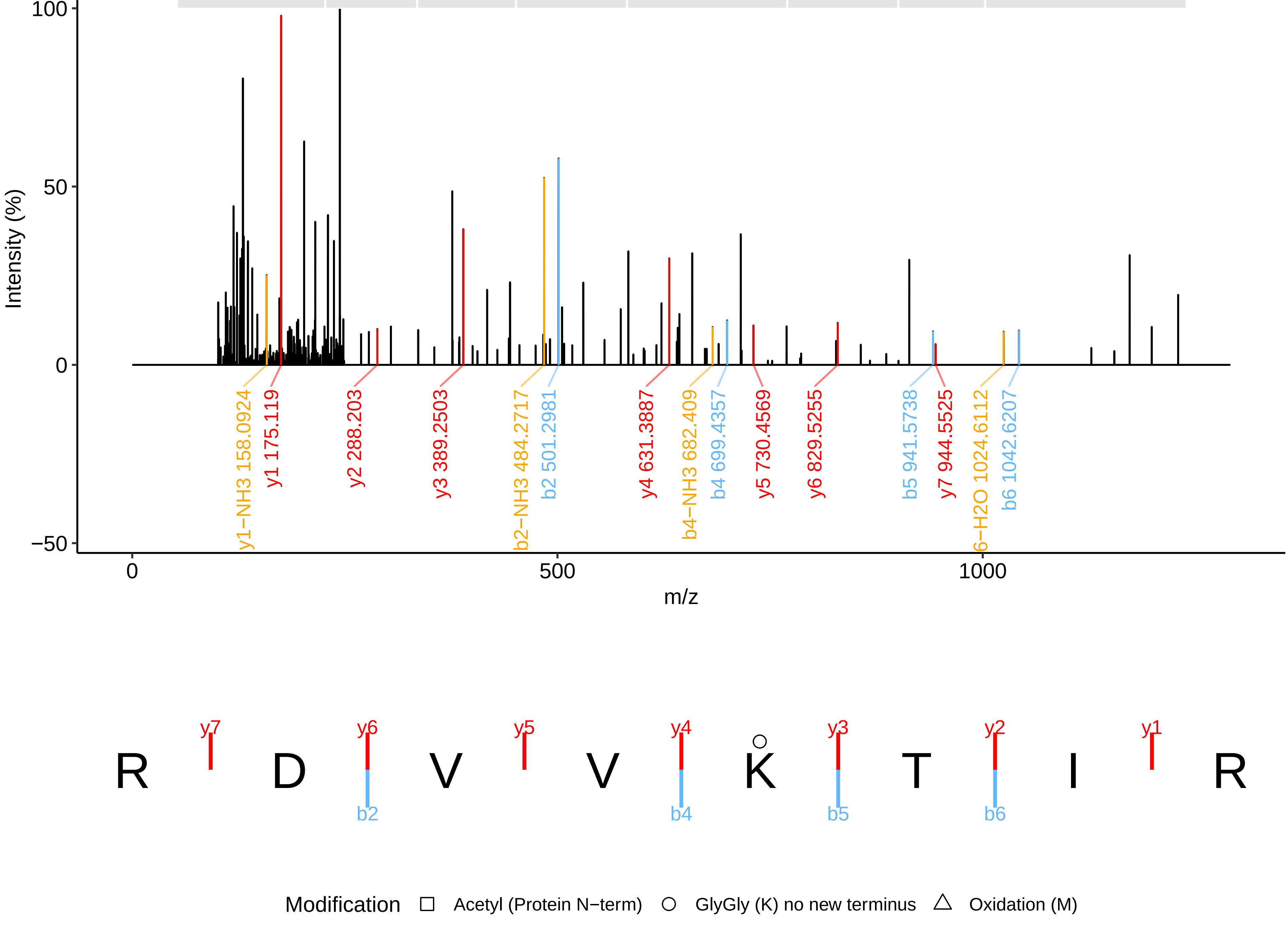

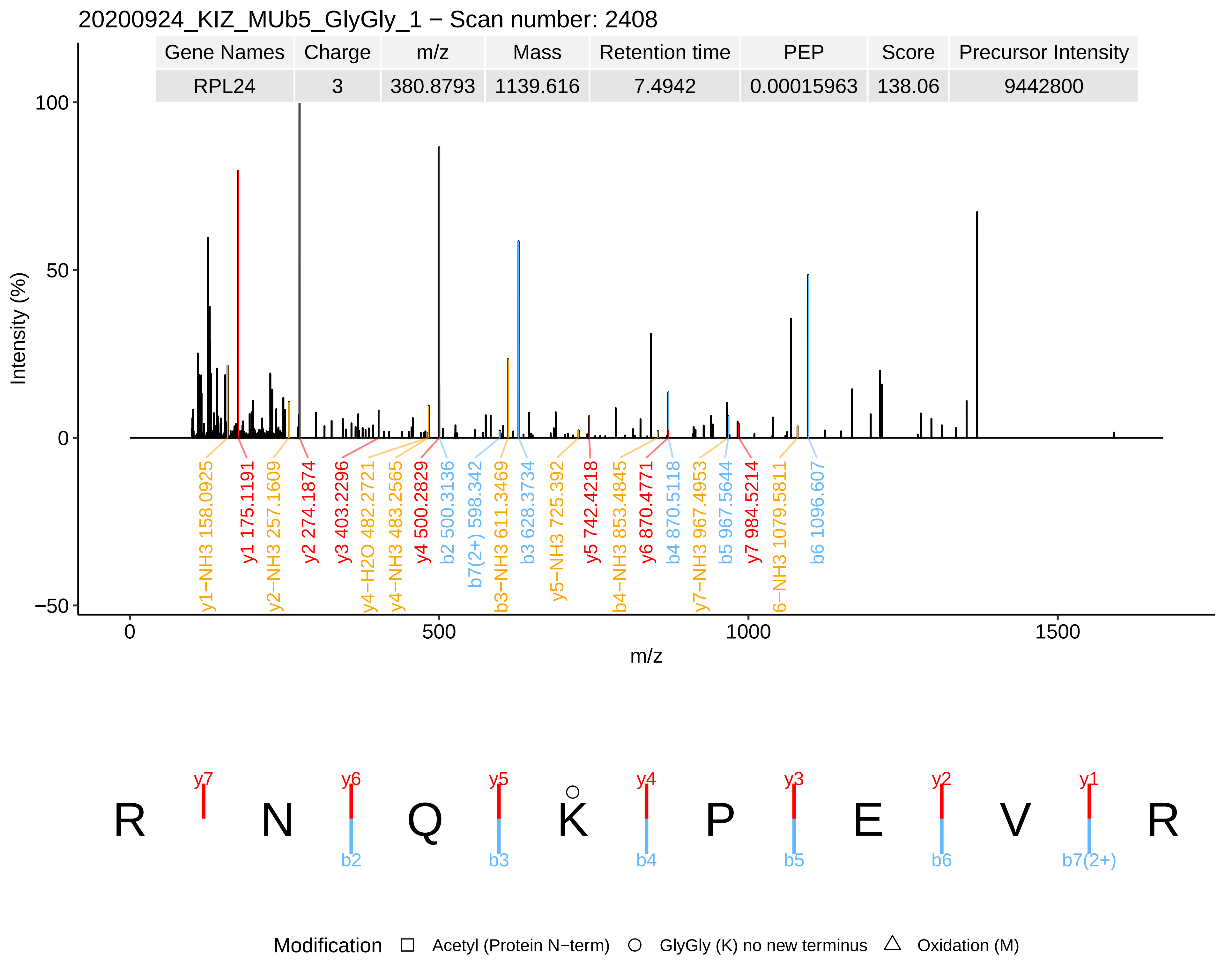

20200924_KIZ_MUb5_GlyGly_1 − Scan number: 2408
Gene Names | Charge | m/z | Mass | Retention time | PEP | Score | Precursor Intensity
RPL24 | 3 | 380.8793 | 1139.616 | 7.4942 | 0.00015963 | 138.06 | 9442800
Intensity (%)
m/z
y1−NH3 158.0925
y1 175.1191
y2−NH3 257.1609
y2 274.1874
y3 403.2296
y4−H2O 482.2721
y4−NH3 483.2565
y4 500.2829
b2 500.3136
b7(2+) 598.342
b3−NH3 611.3469
b3 628.3734
y5−NH3 725.392
y5 742.4218
b4−NH3 853.4845
y6 870.4771
b4 870.5118
y7−NH3 967.4953
b5 967.5644
y7 984.5214
6−NH3 1079.5811
b6 1096.607
R N Q K P E V R
y7 y6 y5 y4 y3 y2 y1
b2 b3 b4 b5 b6 b7(2+)
Modification
Acetyl (Protein N−term)
GlyGly (K) no new terminus
Oxidation (M)

20200924_KIZ_MUb5_GlyGly_2 − Scan number: 2118

Gene Names	Charge	m/z	Mass	Retention time	PEP	Score	Precursor Intensity
RPL24	3	380.8793	1139.616	7.513	0.055549	65.305	NA

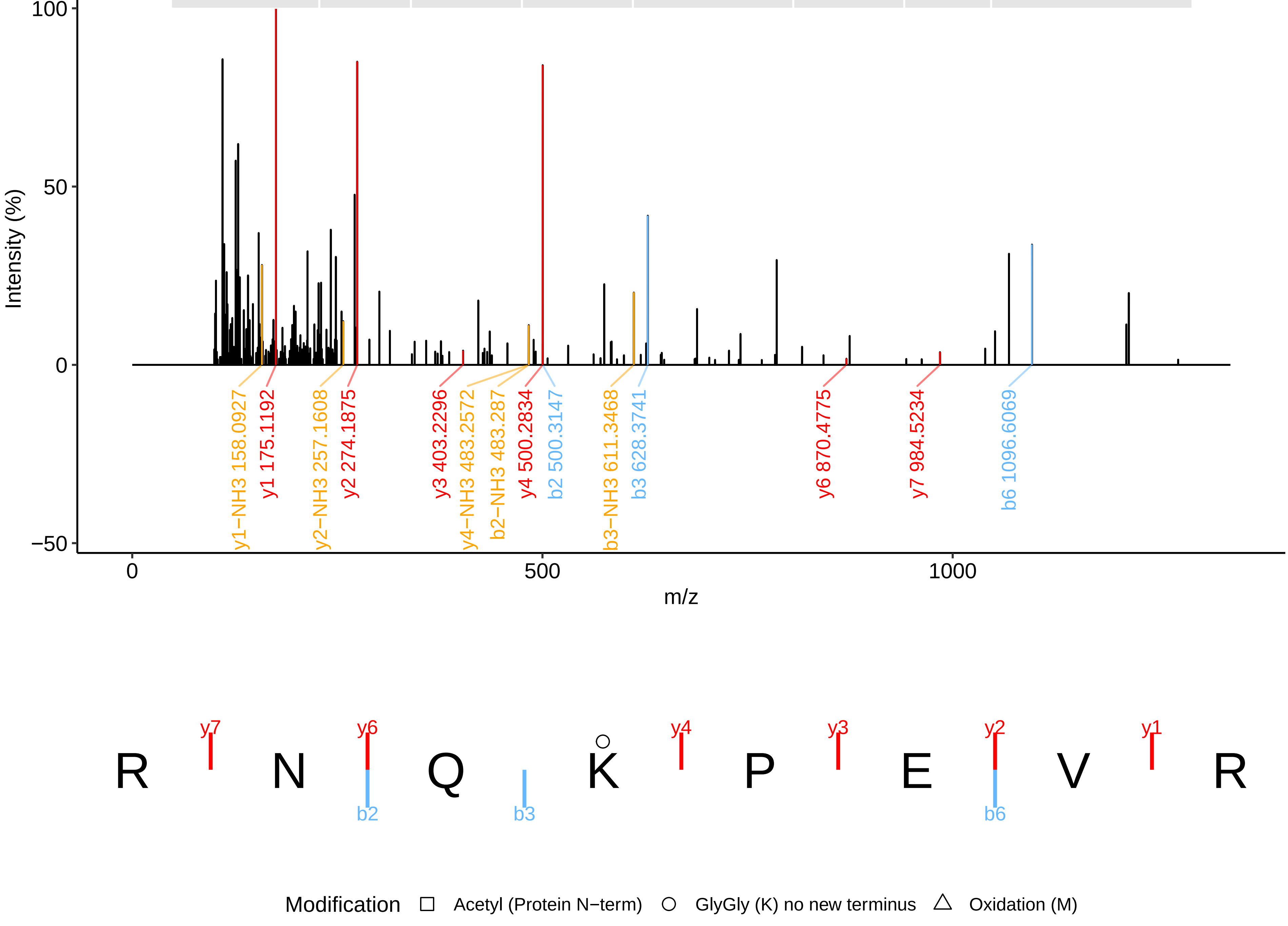

20200921_KIZ_MUb4_GlyGly_10plex_2 − Scan number: 8977

Gene Names	Charge	m/z	Mass	Retention time	PEP	Score	Precursor Intensity
RPS7	3	373.8805	1118.62	33.995	0.030301	63.565	443762.2

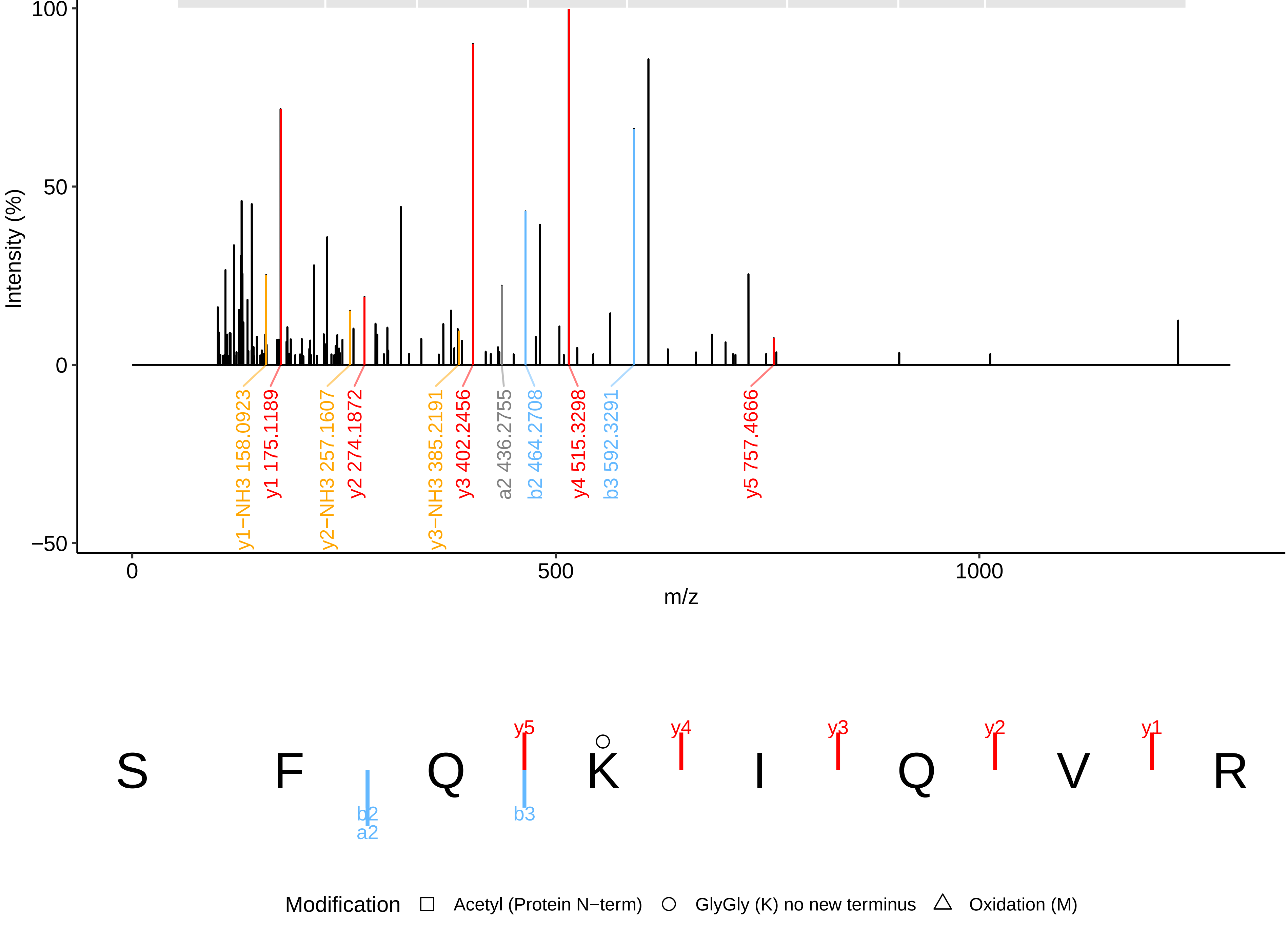

20200921_KIZ_MUb4_GlyGly_10plex_2 − Scan number: 5212

Gene Names	Charge	m/z	Mass	Retention time	PEP	Score	Precursor Intensity
SLC16A1	3	292.831	875.4713	18.638	0.11227	53.493	3672561

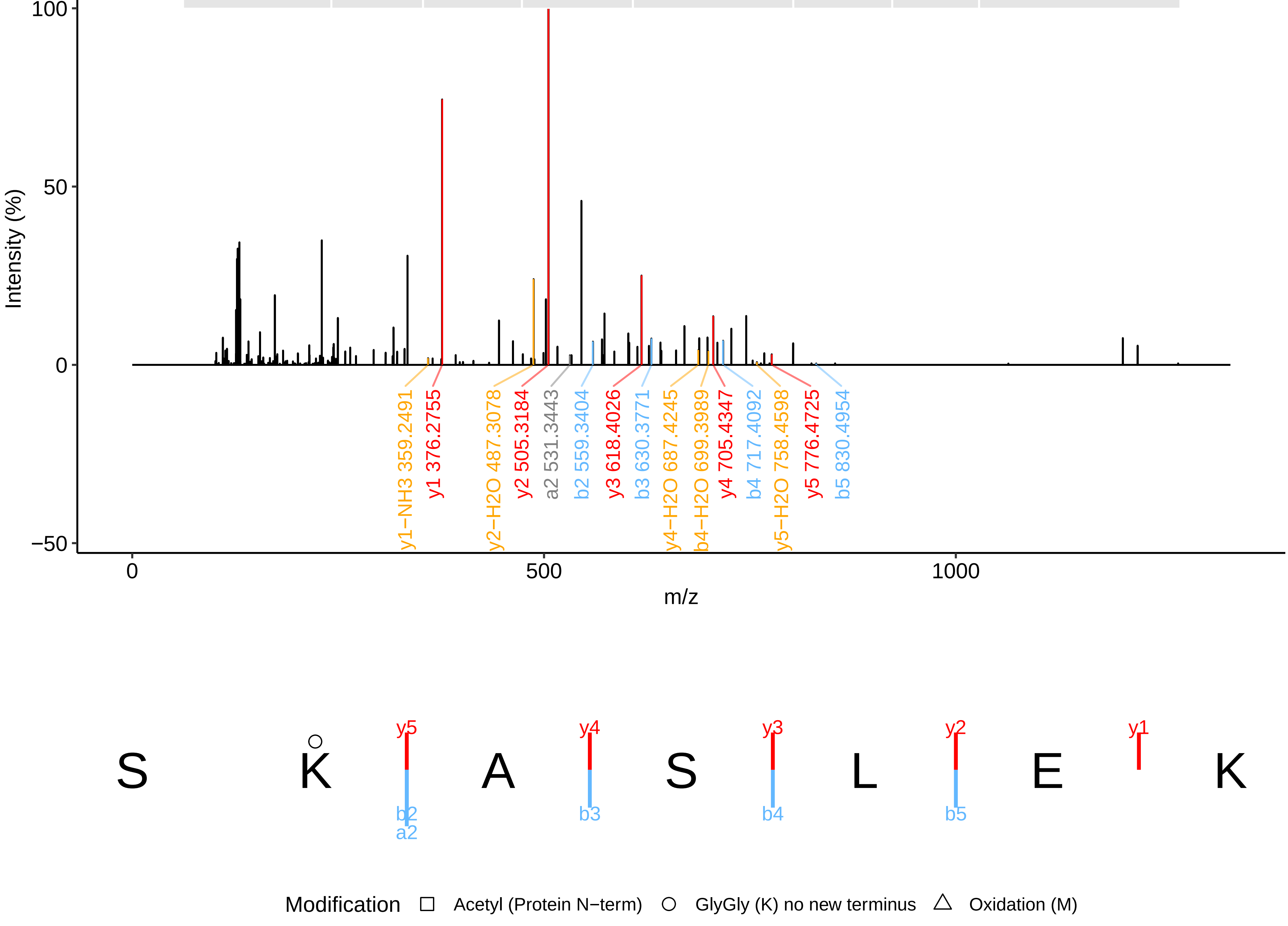

20200924_KIZ_MUb5_GlyGly_1 − Scan number: 4925

Gene Names	Charge	m/z	Mass	Retention time	PEP	Score	Precursor Intensity
SLC16A1	3	292.831	875.4713	15.264	0.10624	54.712	31908762

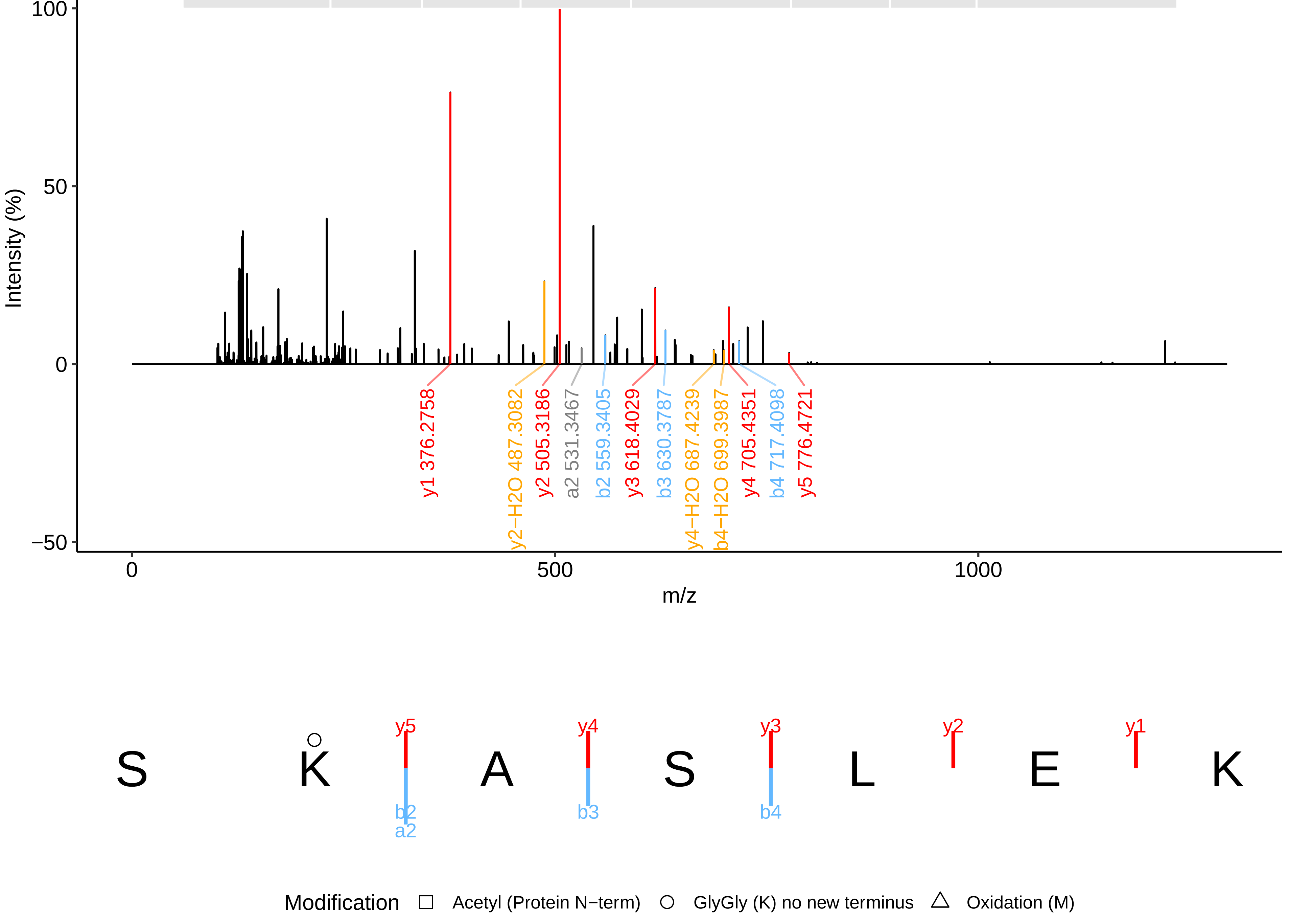

20200924_KIZ_MUb5_GlyGly_2 − Scan number: 4557

Gene Names	Charge	m/z	Mass	Retention time	PEP	Score	Precursor Intensity
SLC16A1	3	292.831	875.4713	16.741	0.087895	58.426	8095733

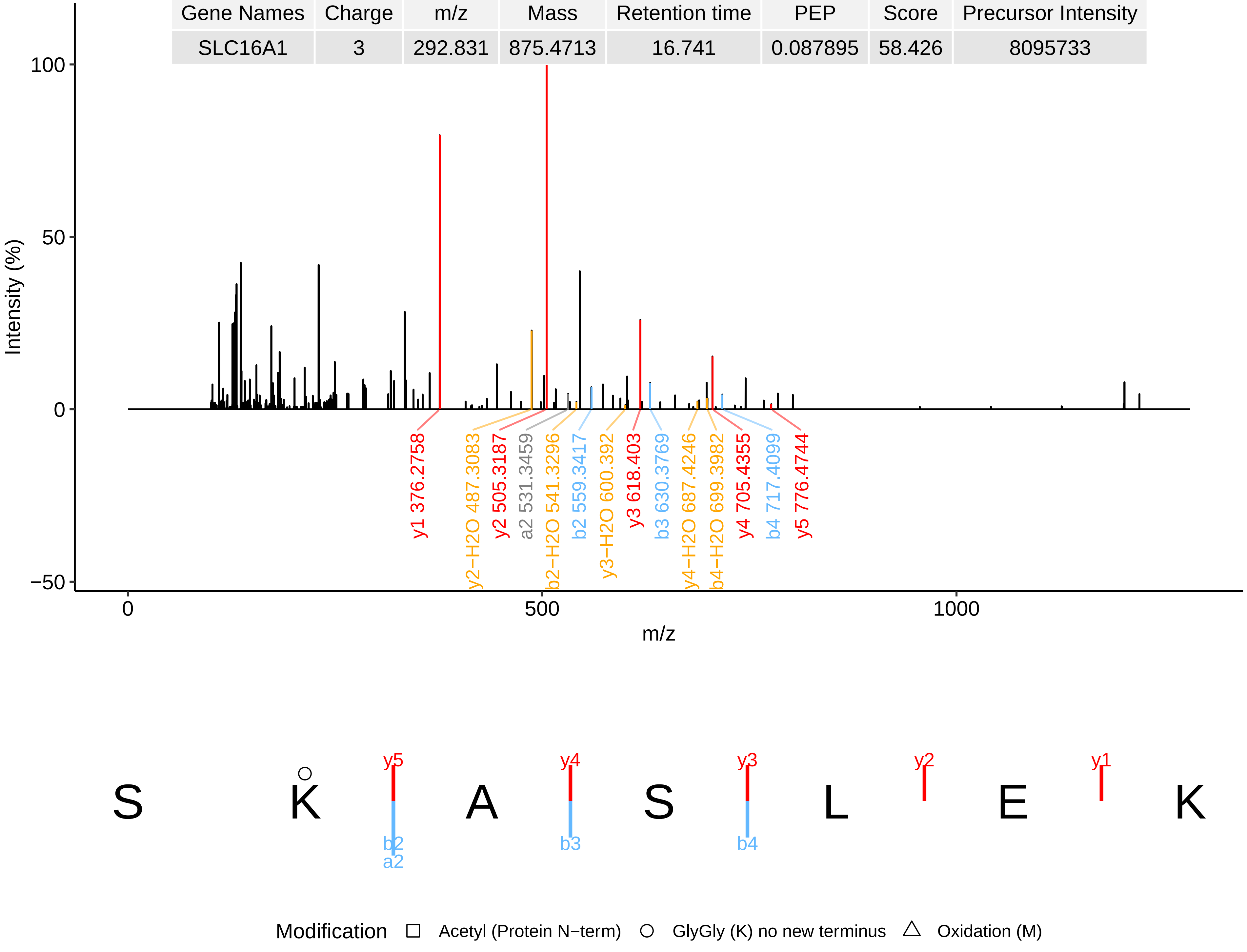

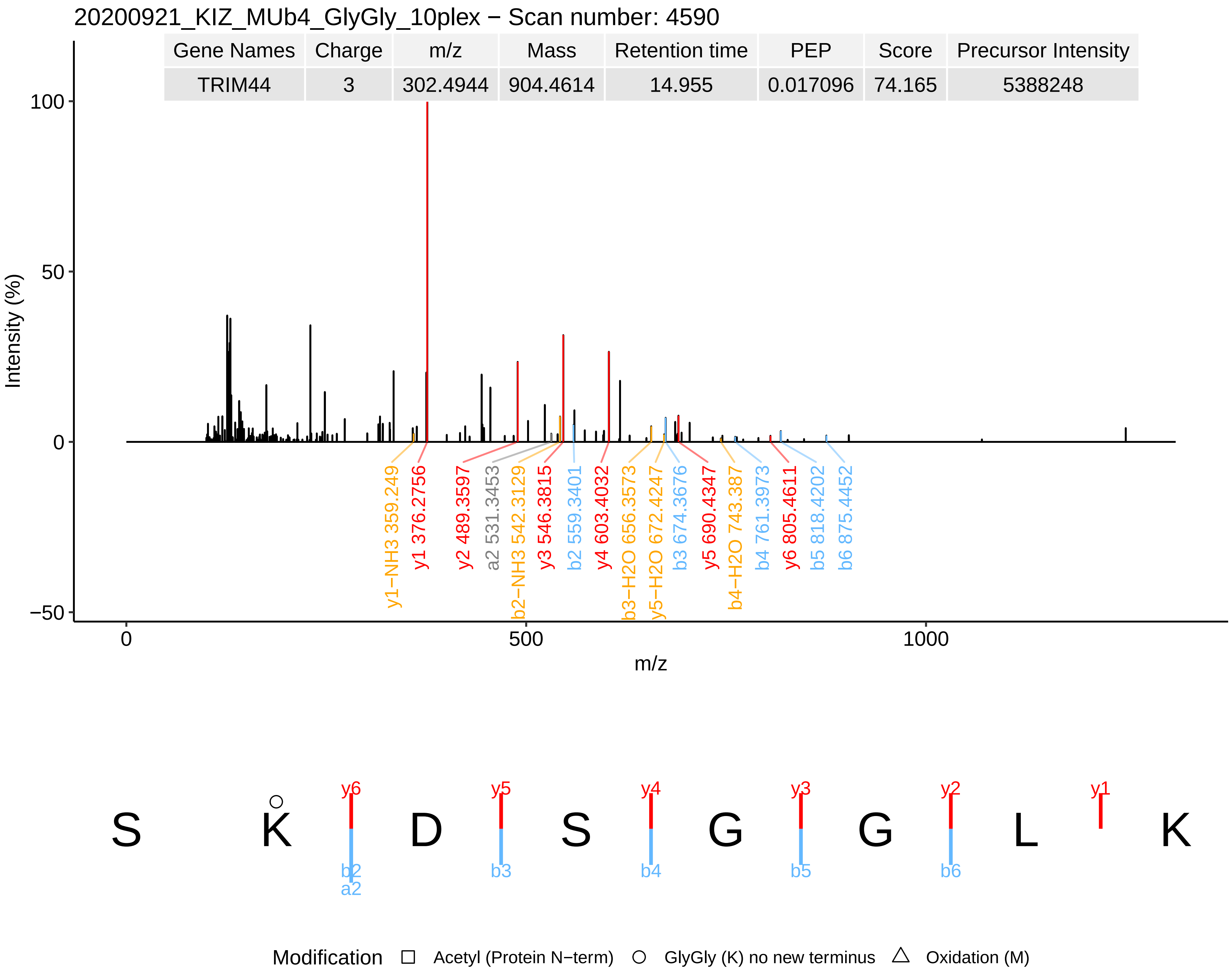
20200921_KIZ_MUb4_GlyGly_10plex − Scan number: 4590
Gene Names | Charge | m/z | Mass | Retention time | PEP | Score | Precursor Intensity
TRIM44 | 3 | 302.4944 | 904.4614 | 14.955 | 0.017096 | 74.165 | 5388248
Intensity (%)
m/z
y1−NH3 359.249
y1 376.2756
y2 489.3597
a2 531.3453
b2−NH3 542.3129
y3 546.3815
b2 559.3401
y4 603.4032
b3−H2O 656.3573
y5−H2O 672.4247
b3 674.3676
y5 690.4347
b4−H2O 743.387
b4 761.3973
y6 805.4611
b5 818.4202
b6 875.4452
S K D S G G L K
Modification
Acetyl (Protein N−term)
GlyGly (K) no new terminus
Oxidation (M)

20200921_KIZ_MUb4_GlyGly_10plex_2 − Scan number: 4276

Gene Names	Charge	m/z	Mass	Retention time	PEP	Score	Precursor Intensity
TRIM44	3	302.4944	904.4614	14.916	0.032224	62.823	553088.4

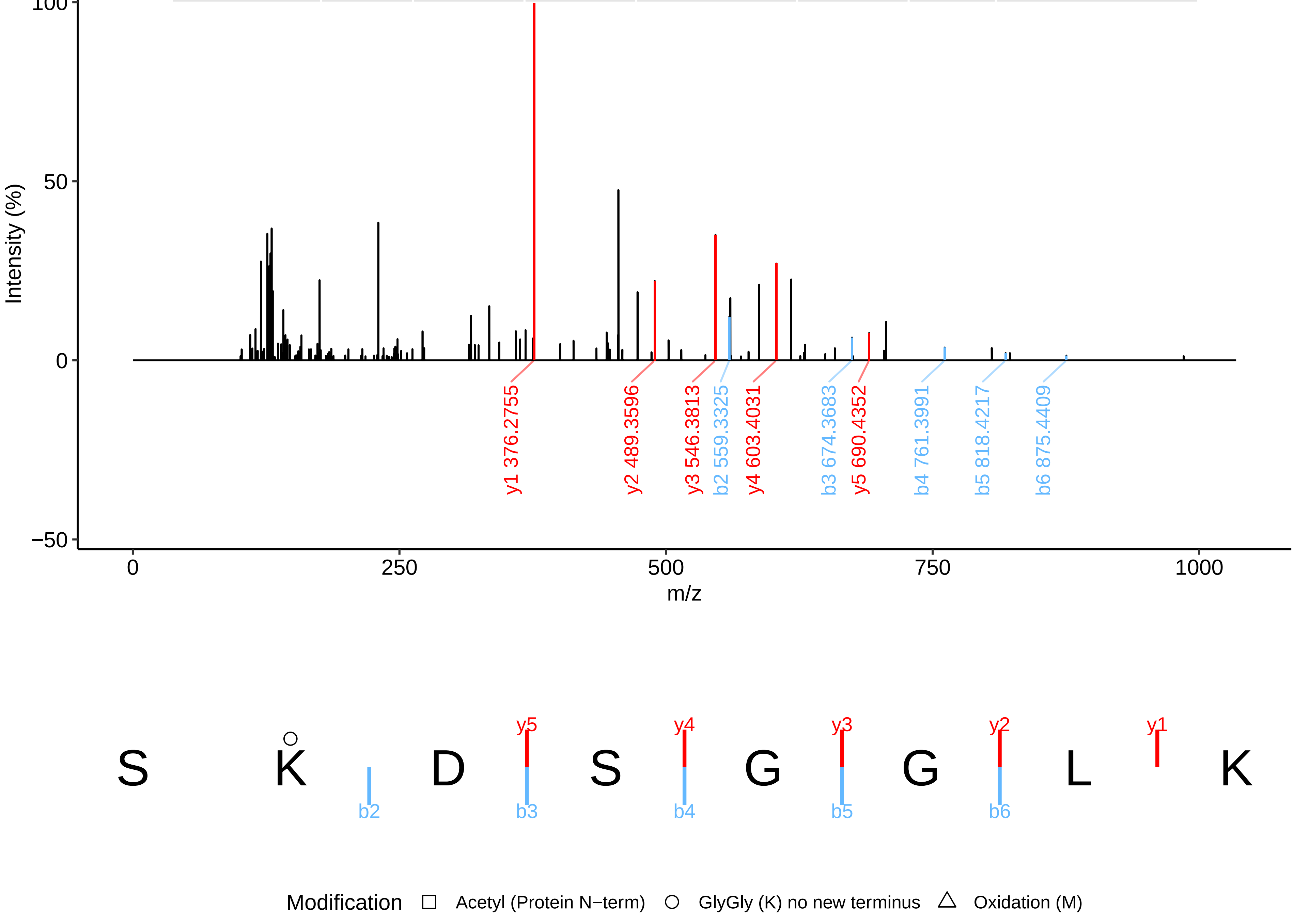

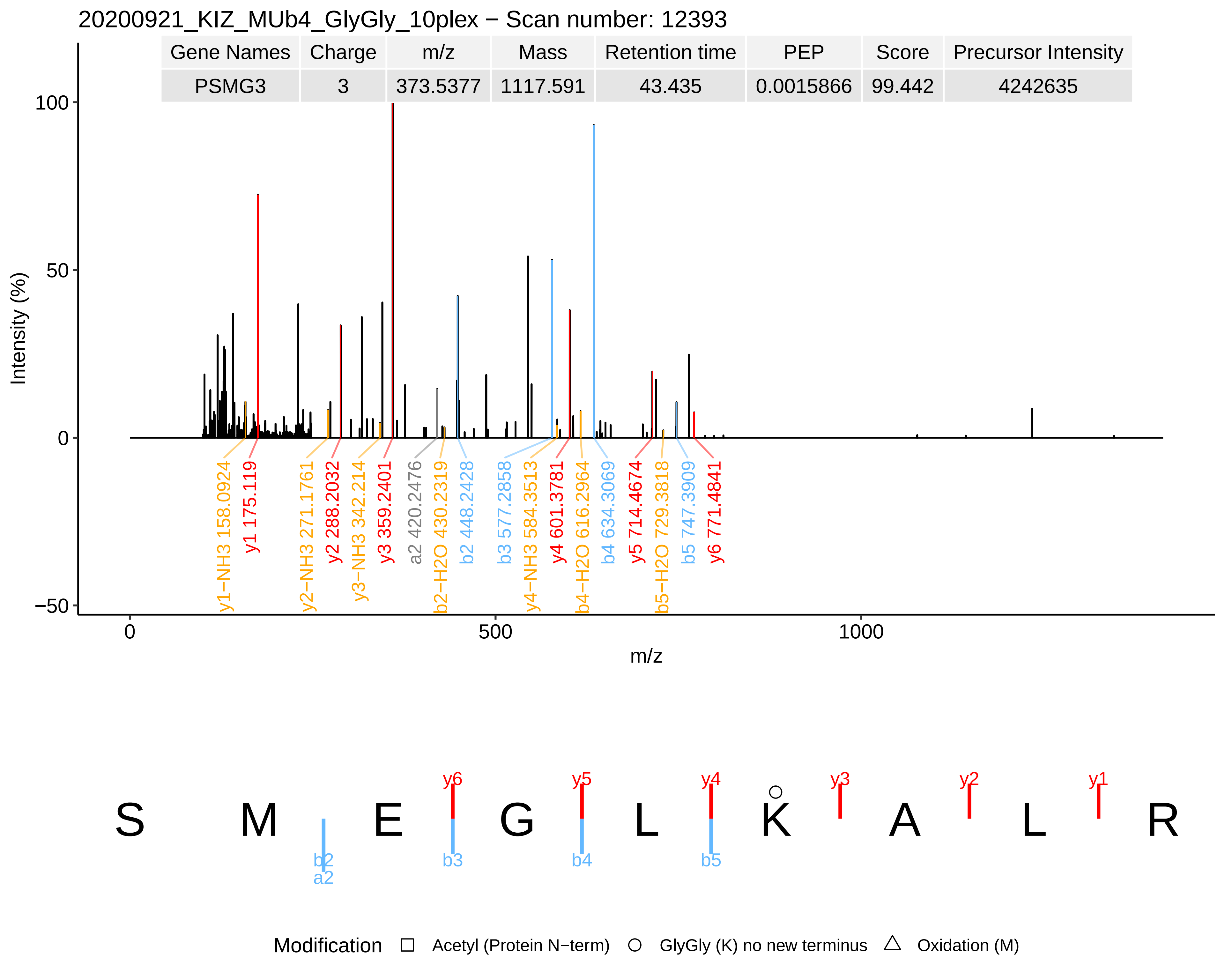
20200921_KIZ_MUb4_GlyGly_10plex − Scan number: 12393
Gene Names | Charge | m/z | Mass | Retention time | PEP | Score | Precursor Intensity
PSMG3 | 3 | 373.5377 | 1117.591 | 43.435 | 0.0015866 | 99.442 | 4242635
Intensity (%)
m/z
y1−NH3 158.0924
y1 175.119
y2−NH3 271.1761
y2 288.2032
y3−NH3 342.214
y3 359.2401
a2 420.2476
b2−H2O 430.2319
b2 448.2428
b3 577.2858
y4−NH3 584.3513
y4 601.3781
b4−H2O 616.2964
b4 634.3069
y5 714.4674
b5−H2O 729.3818
b5 747.3909
y6 771.4841
S M E G L K A L R
Modification
Acetyl (Protein N−term)
GlyGly (K) no new terminus
Oxidation (M)

20200924_KIZ_MUb5_GlyGly_1 − Scan number: 10998

Gene Names	Charge	m/z	Mass	Retention time	PEP	Score	Precursor Intensity
PSMG3	3	373.5377	1117.591	35.613	0.018327	66.692	8727079

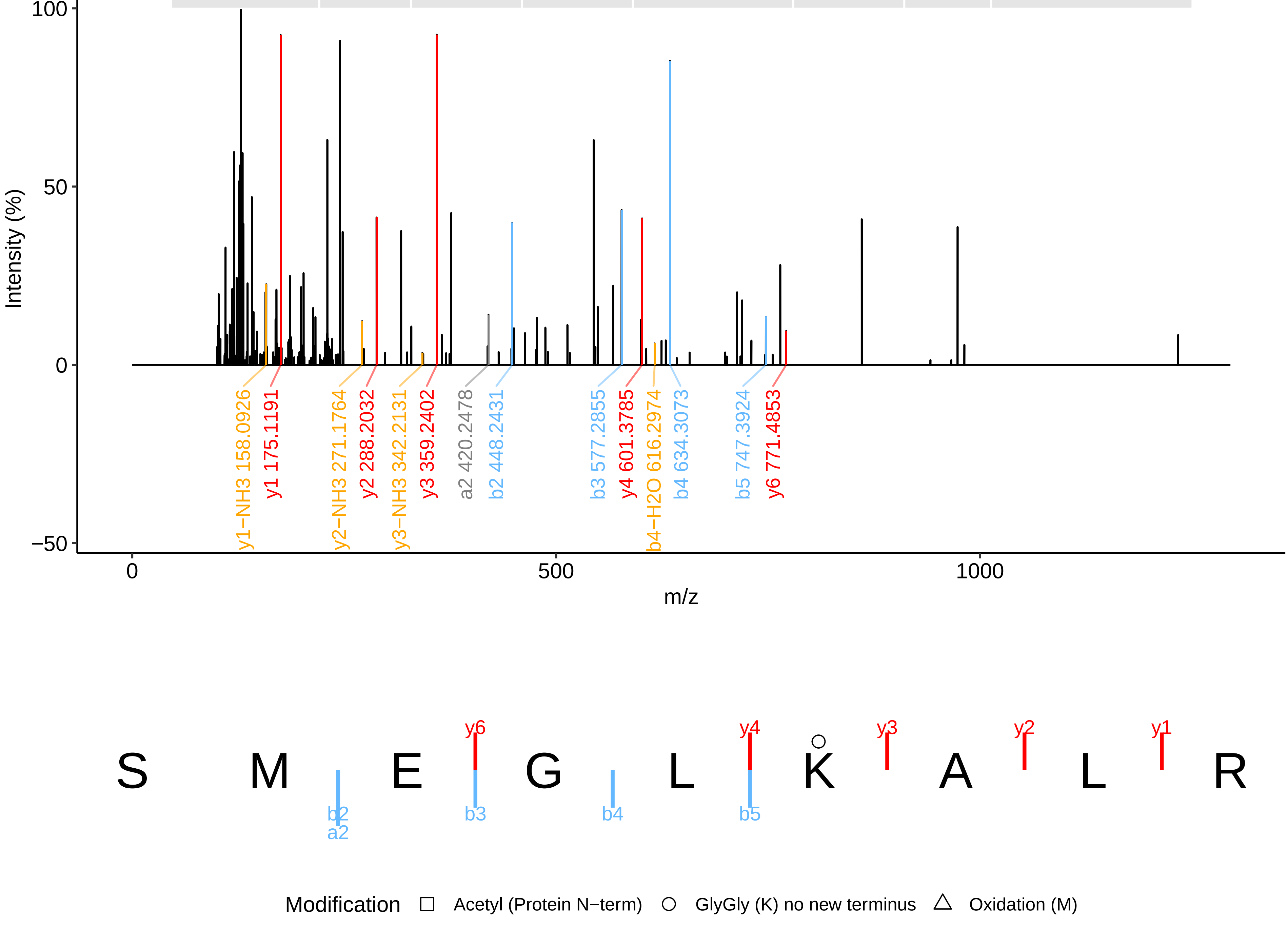

20200921_KIZ_MUb4_GlyGly_10plex − Scan number: 5033

Gene Names	Charge	m/z	Mass	Retention time	PEP	Score	Precursor Intensity
RPL30	2	560.7506	1119.487	16.418	4.7824e−07	156.75	2430033

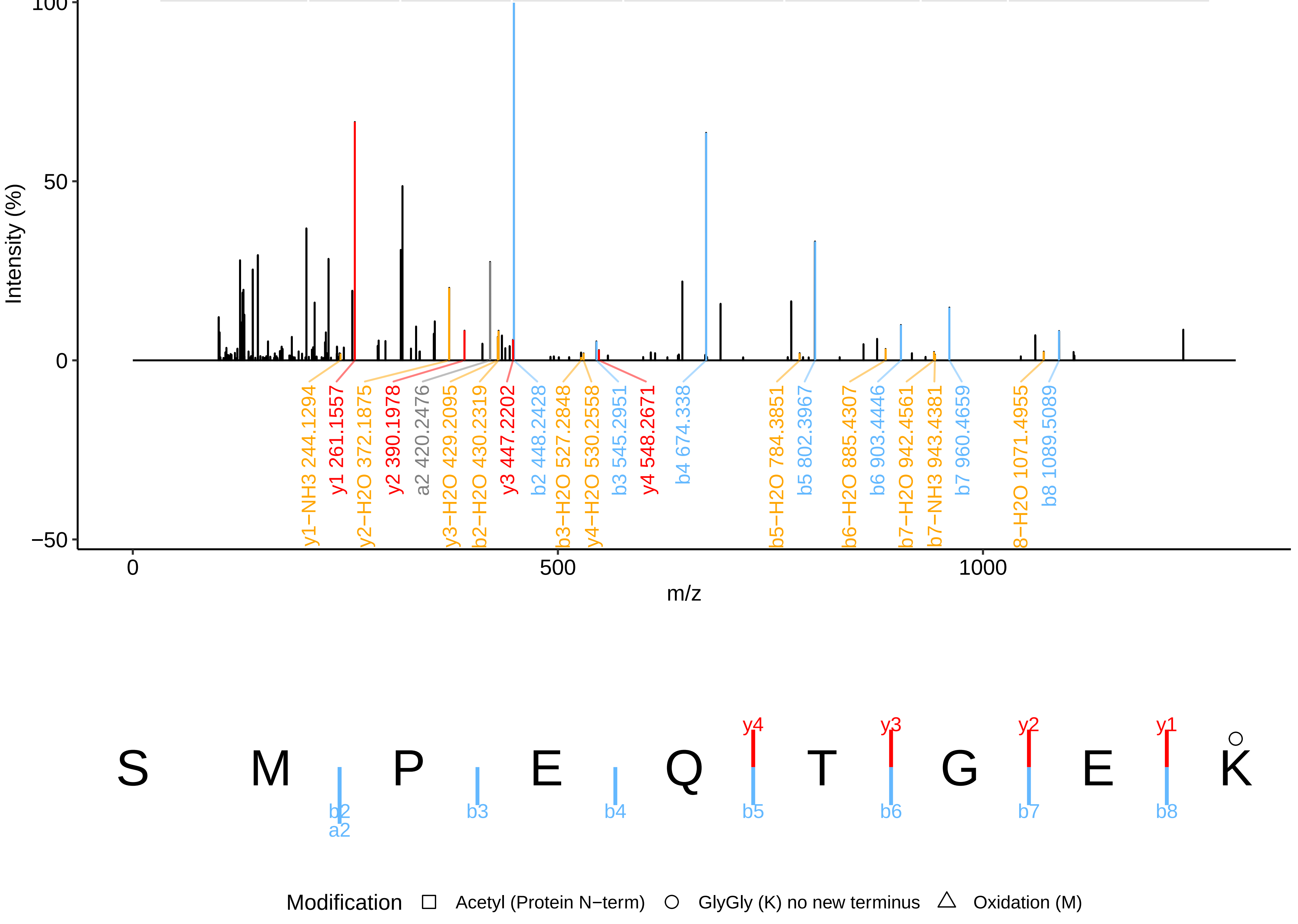

20200921_KIZ_MUb4_GlyGly_10plex − Scan number: 2890

Gene Names	Charge	m/z	Mass	Retention time	PEP	Score	Precursor Intensity
FLOT1	3	377.5353	1129.584	9.0546	0.044171	58.592	3412197

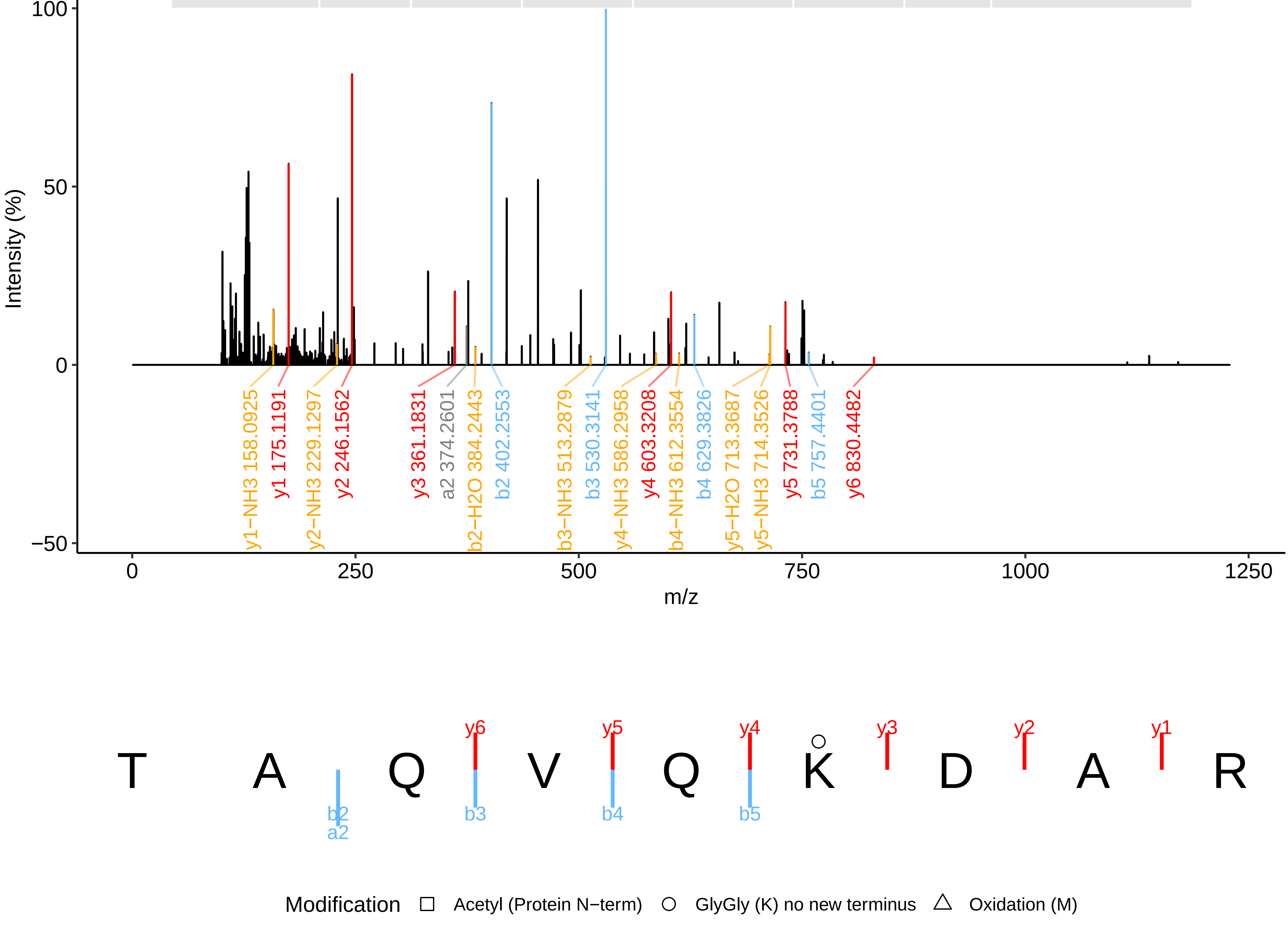

20200921_KIZ_MUb4_GlyGly_10plex_2 − Scan number: 2834

Gene Names	Charge	m/z	Mass	Retention time	PEP	Score	Precursor Intensity
FLOT1	3	377.5353	1129.584	9.1629	0.008171	76.228	902254.9

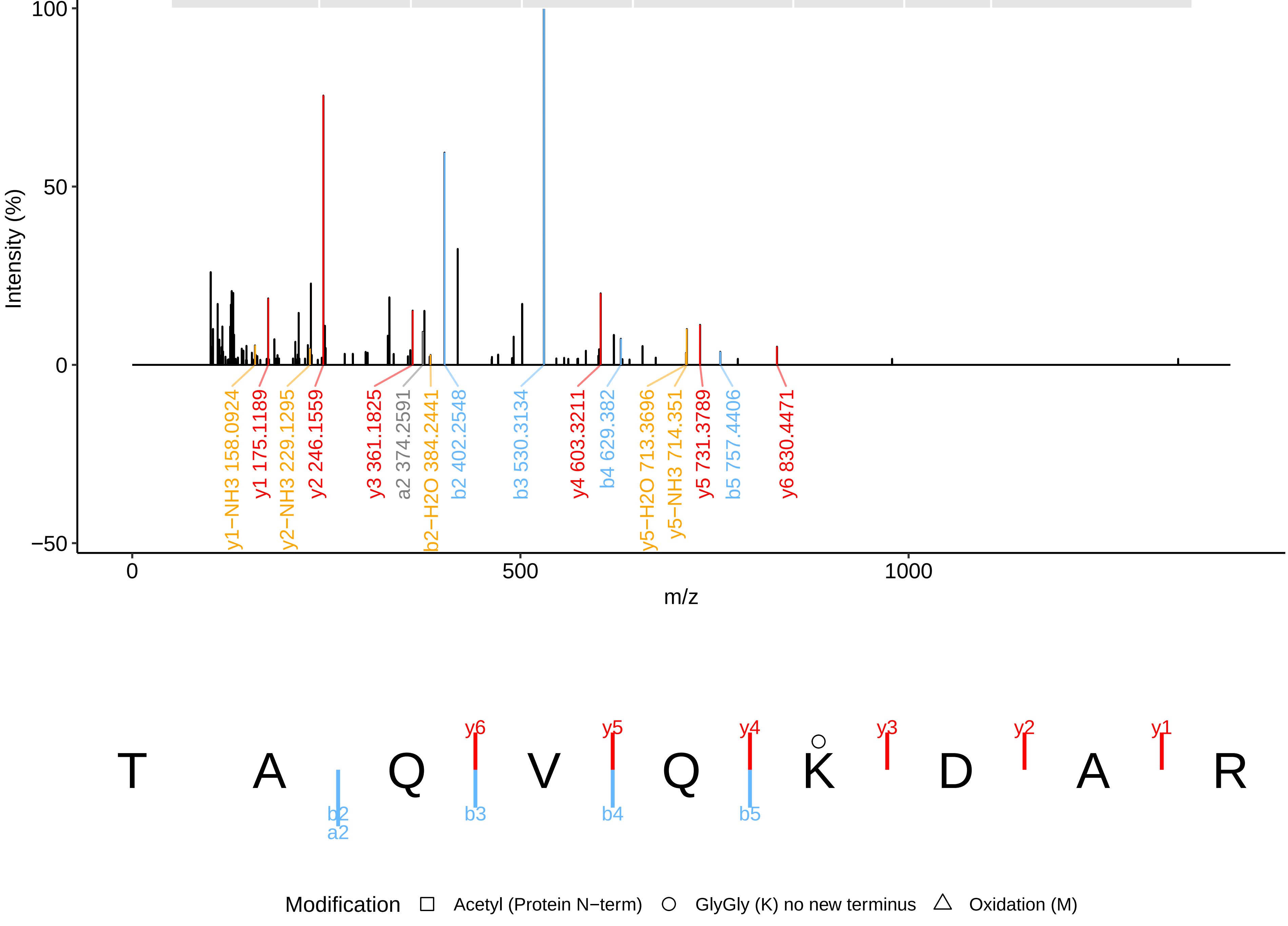

20200921_KIZ_MUb4_GlyGly_10plex − Scan number: 6996

Gene Names	Charge	m/z	Mass	Retention time	PEP	Score	Precursor Intensity
IMMT	3	372.8718	1115.593	23.353	2.0541e−07	140.45	15322904

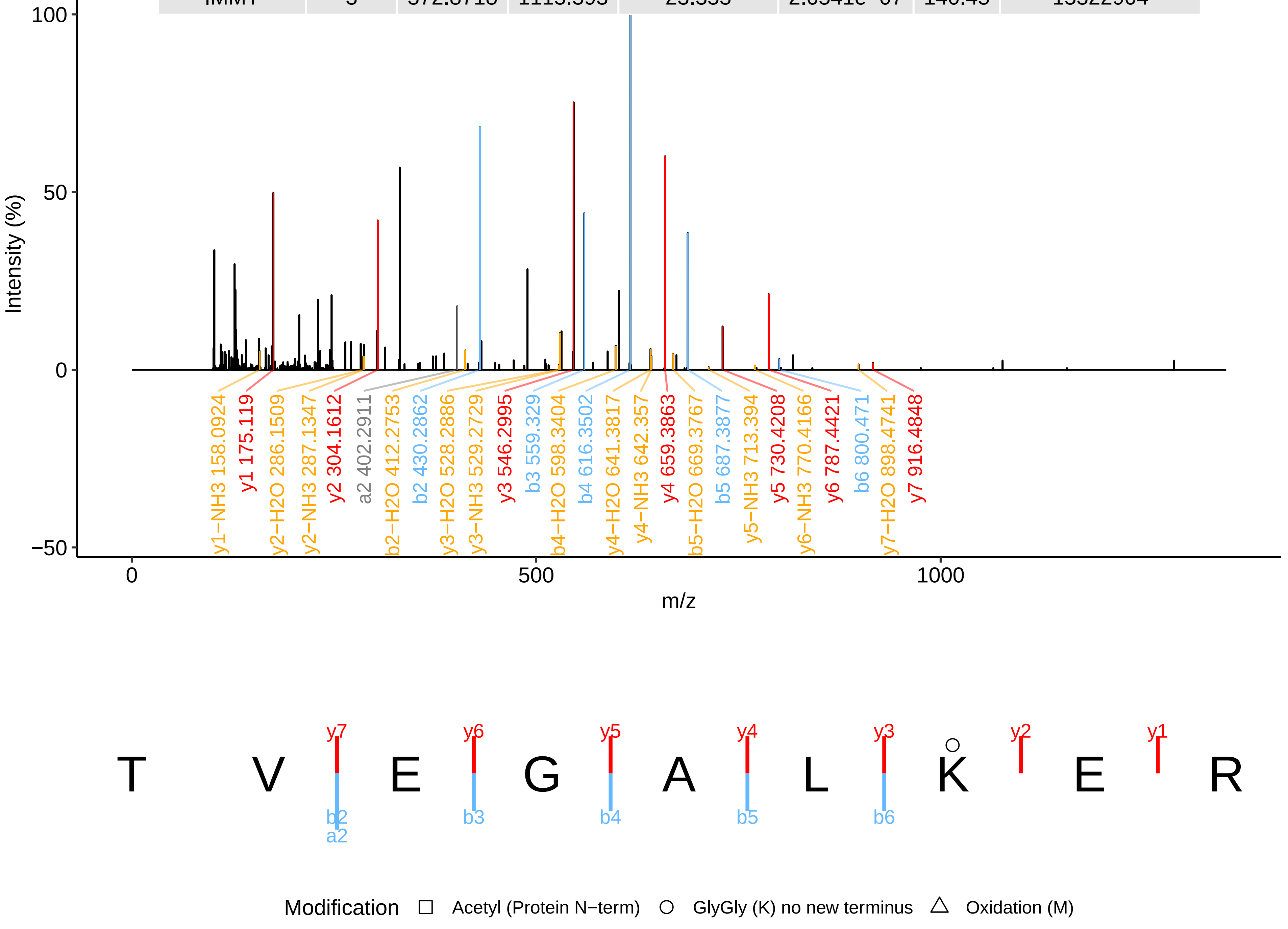

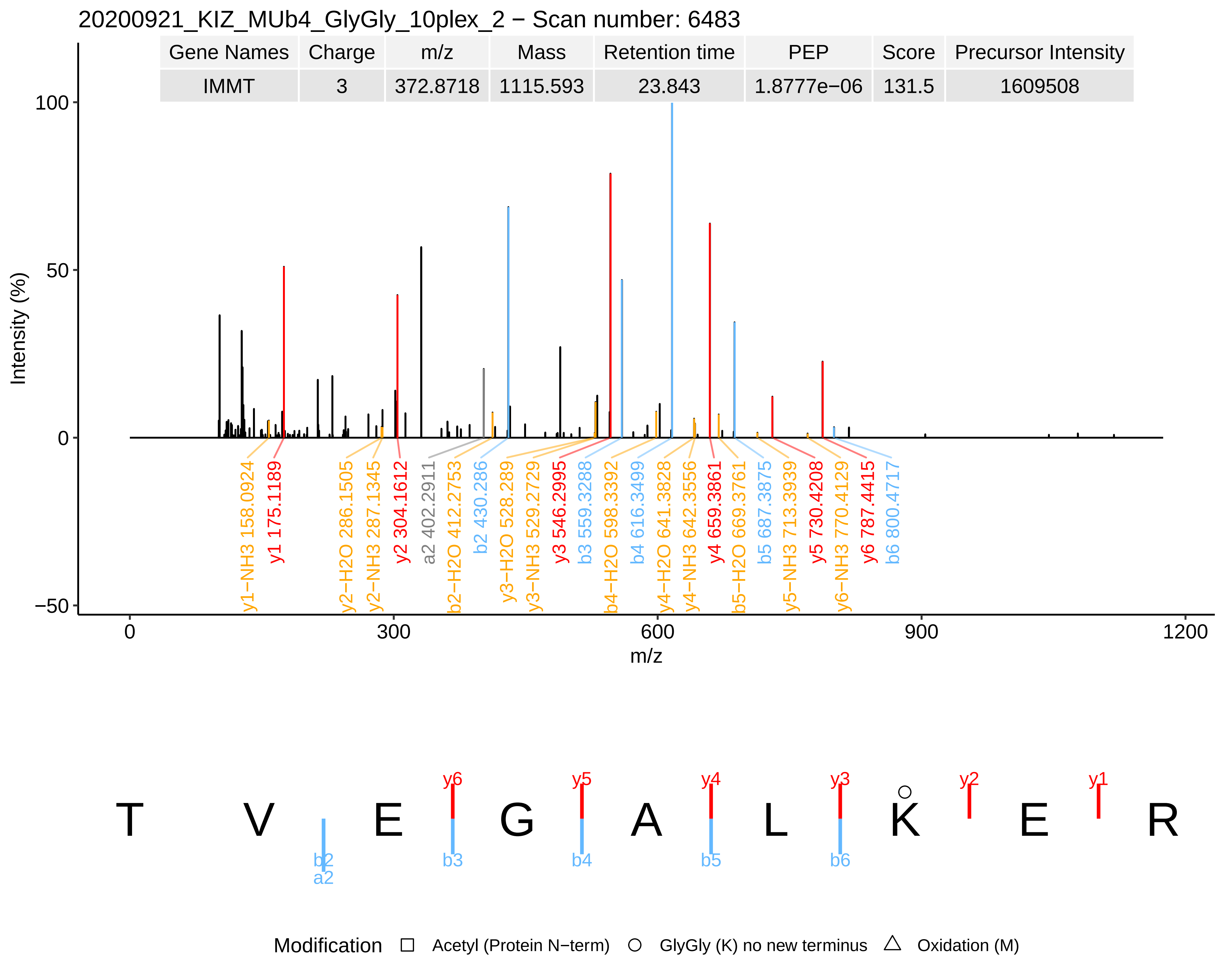

20200921_KIZ_MUb4_GlyGly_10plex_2 − Scan number: 6483
Gene Names | Charge | m/z | Mass | Retention time | PEP | Score | Precursor Intensity
IMMT | 3 | 372.8718 | 1115.593 | 23.843 | 1.8777e−06 | 131.5 | 1609508
Intensity (%)
m/z
y1−NH3 158.0924
y1 175.1189
y2−H2O 286.1505
y2−NH3 287.1345
y2 304.1612
a2 402.2911
b2−H2O 412.2753
b2 430.286
y3−H2O 528.289
y3−NH3 529.2729
y3 546.2995
b3 559.3288
b4−H2O 598.3392
b4 616.3499
y4−H2O 641.3828
y4−NH3 642.3556
y4 659.3861
b5−H2O 669.3761
b5 687.3875
y5−NH3 713.3939
y5 730.4208
y6−NH3 770.4129
y6 787.4415
b6 800.4717
T V E G A L K E R
Modification
Acetyl (Protein N−term)
GlyGly (K) no new terminus
Oxidation (M)

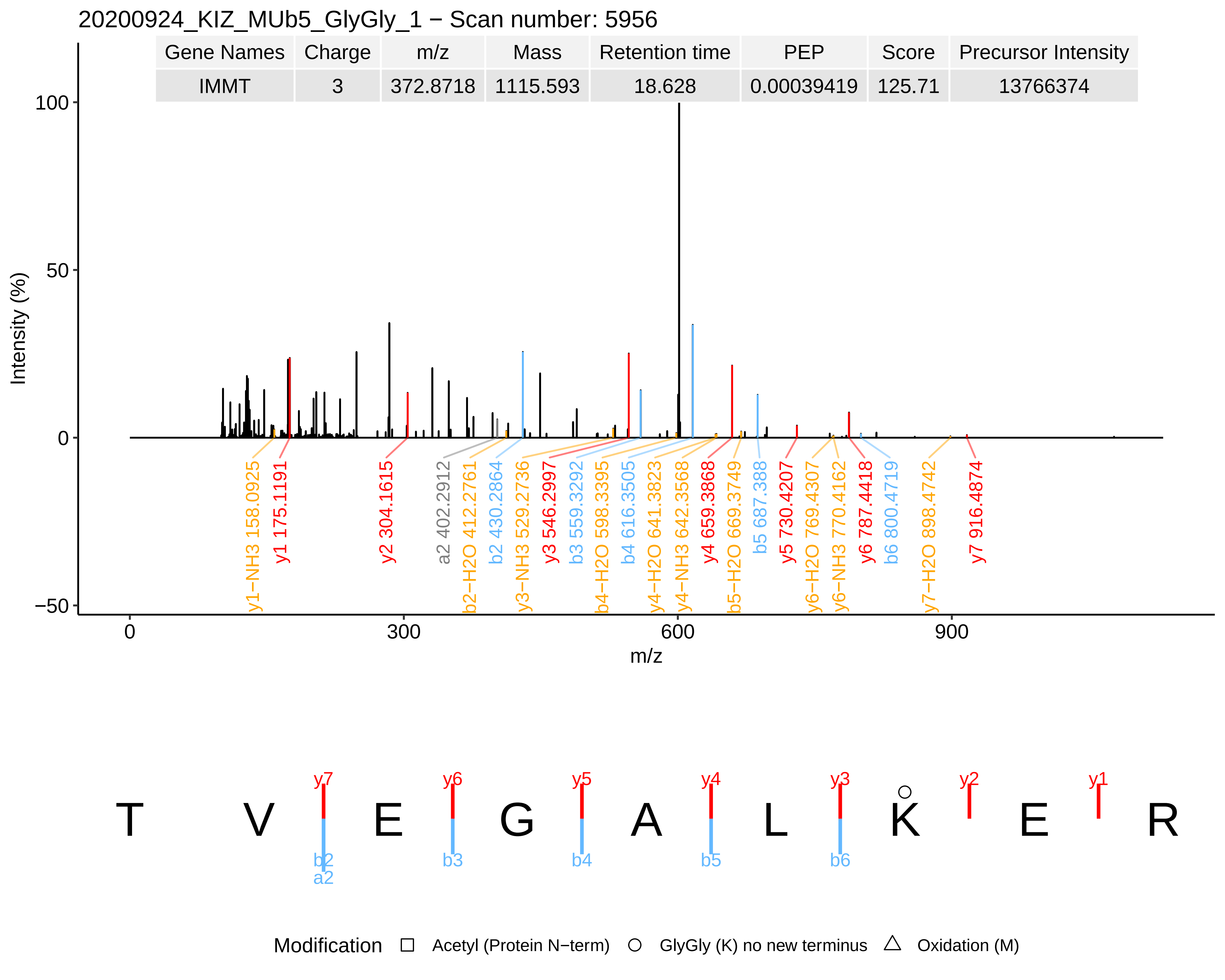

20200924_KIZ_MUb5_GlyGly_1 − Scan number: 5956
Gene Names | Charge | m/z | Mass | Retention time | PEP | Score | Precursor Intensity
IMMT | 3 | 372.8718 | 1115.593 | 18.628 | 0.00039419 | 125.71 | 13766374
Intensity (%)
m/z
y1−NH3 158.0925
y1 175.1191
y2 304.1615
a2 402.2912
b2−H2O 412.2761
b2 430.2864
y3−NH3 529.2736
y3 546.2997
b3 559.3292
b4−H2O 598.3395
b4 616.3505
y4−H2O 641.3823
y4−NH3 642.3568
y4 659.3868
b5−H2O 669.3749
b5 687.388
y5 730.4207
y6−H2O 769.4307
y6−NH3 770.4162
y6 787.4418
b6 800.4719
y7−H2O 898.4742
y7 916.4874
T V E G A L K E R
y7 y6 y5 y4 y3 y2 y1
b2 a2 b3 b4 b5 b6
Modification
Acetyl (Protein N−term)
GlyGly (K) no new terminus
Oxidation (M)

20200921_KIZ_MUb4_GlyGly_10plex − Scan number: 15553

Gene Names	Charge	m/z	Mass	Retention time	PEP	Score	Precursor Intensity
VCP	3	371.2364	1110.687	55.585	0.00016624	110.81	14757654

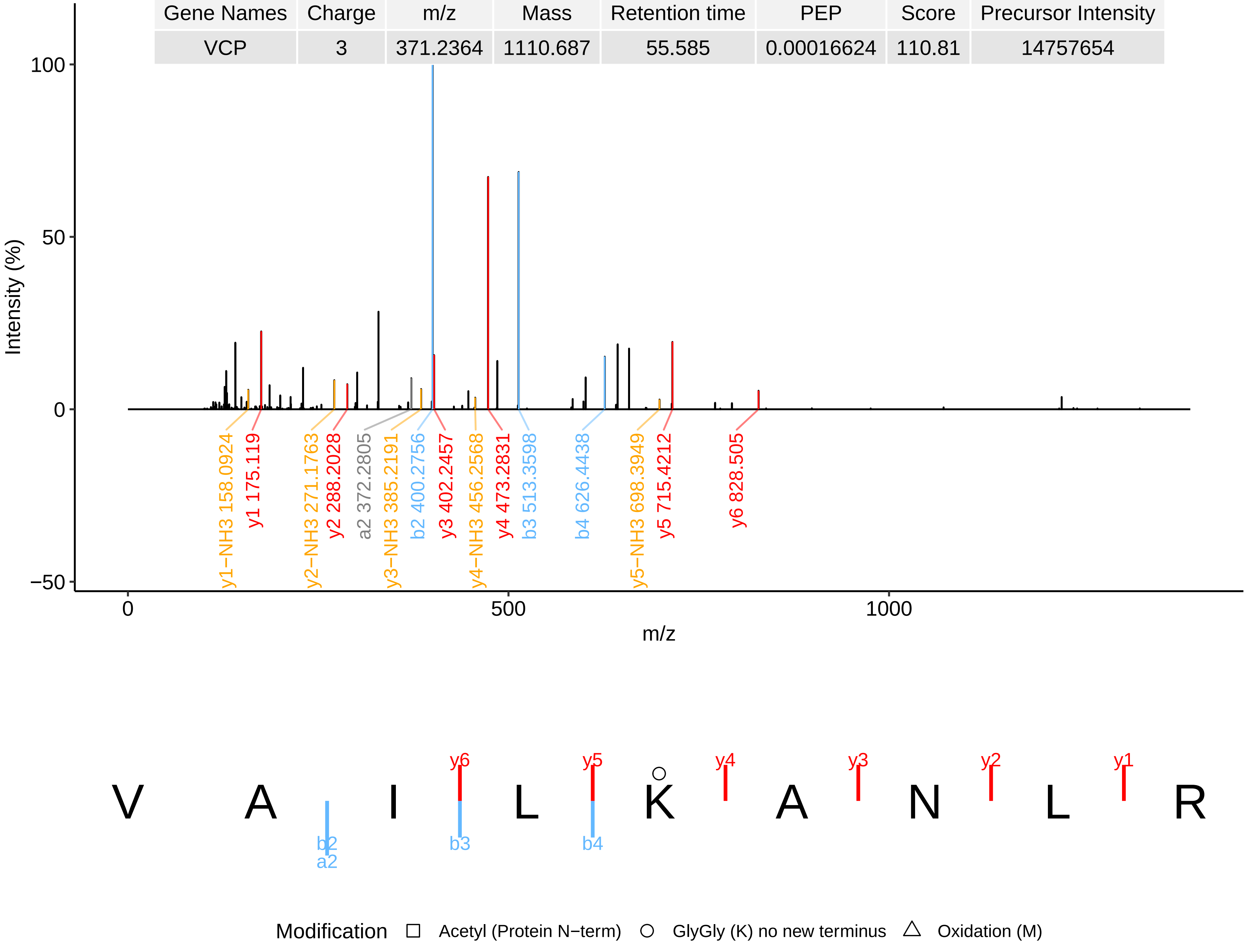

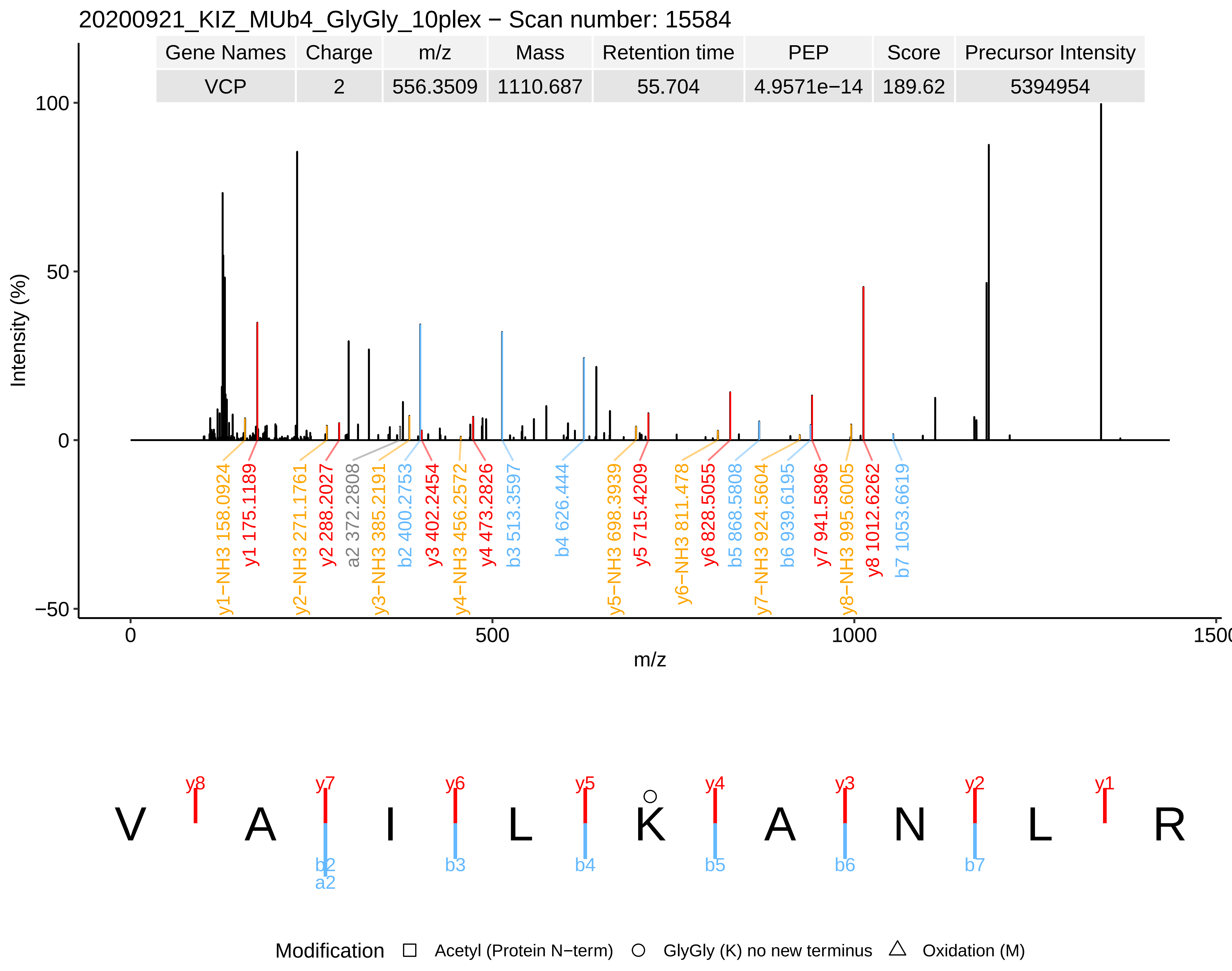

20200921_KIZ_MUb4_GlyGly_10plex − Scan number: 15584
Gene Names | Charge | m/z | Mass | Retention time | PEP | Score | Precursor Intensity
VCP | 2 | 556.3509 | 1110.687 | 55.704 | 4.9571e−14 | 189.62 | 5394954
Intensity (%)
m/z
y1−NH3 158.0924
y1 175.1189
y2−NH3 271.1761
y2 288.2027
a2 372.2808
y3−NH3 385.2191
b2 400.2753
y3 402.2454
y4−NH3 456.2572
y4 473.2826
b3 513.3597
b4 626.444
y5−NH3 698.3939
y5 715.4209
y6−NH3 811.478
y6 828.5055
b5 868.5808
y7−NH3 924.5604
b6 939.6195
y7 941.5896
y8−NH3 995.6005
y8 1012.6262
b7 1053.6619
V A I L K A N L R
y8 y7 y6 y5 y4 y3 y2 y1
b2 a2 b3 b4 b5 b6 b7
Modification
Acetyl (Protein N−term)
GlyGly (K) no new terminus
Oxidation (M)

20200921_KIZ_MUb4_GlyGly_10plex − Scan number: 15709

Gene Names	Charge	m/z	Mass	Retention time	PEP	Score	Precursor Intensity
VCP	3	371.2364	1110.687	56.193	0.012375	75.692	1355844

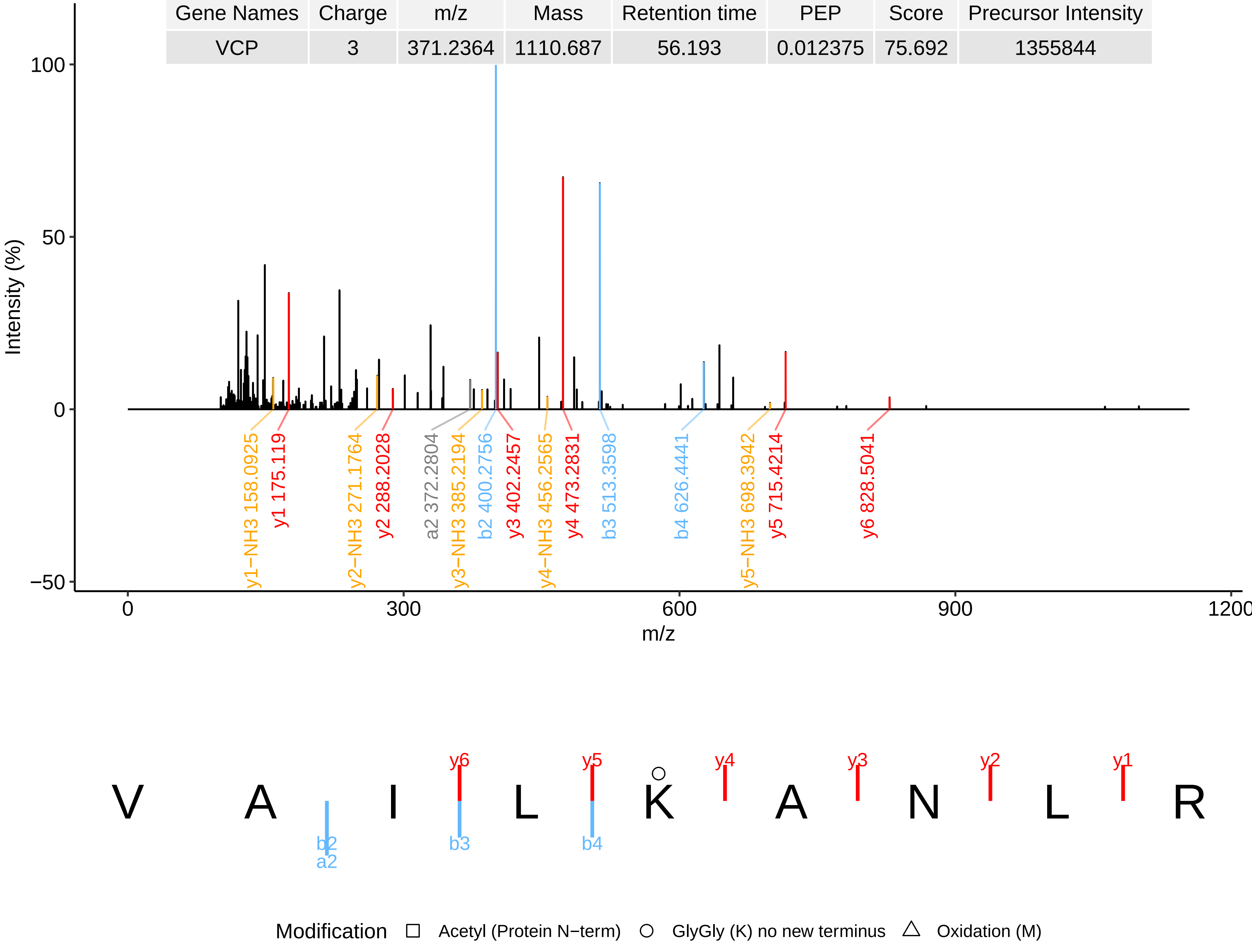

20200921_KIZ_MUb4_GlyGly_10plex_2 − Scan number: 14444

Gene Names	Charge	m/z	Mass	Retention time	PEP	Score	Precursor Intensity
VCP	3	371.2364	1110.687	56.318	1.2878e−05	122.13	8257270

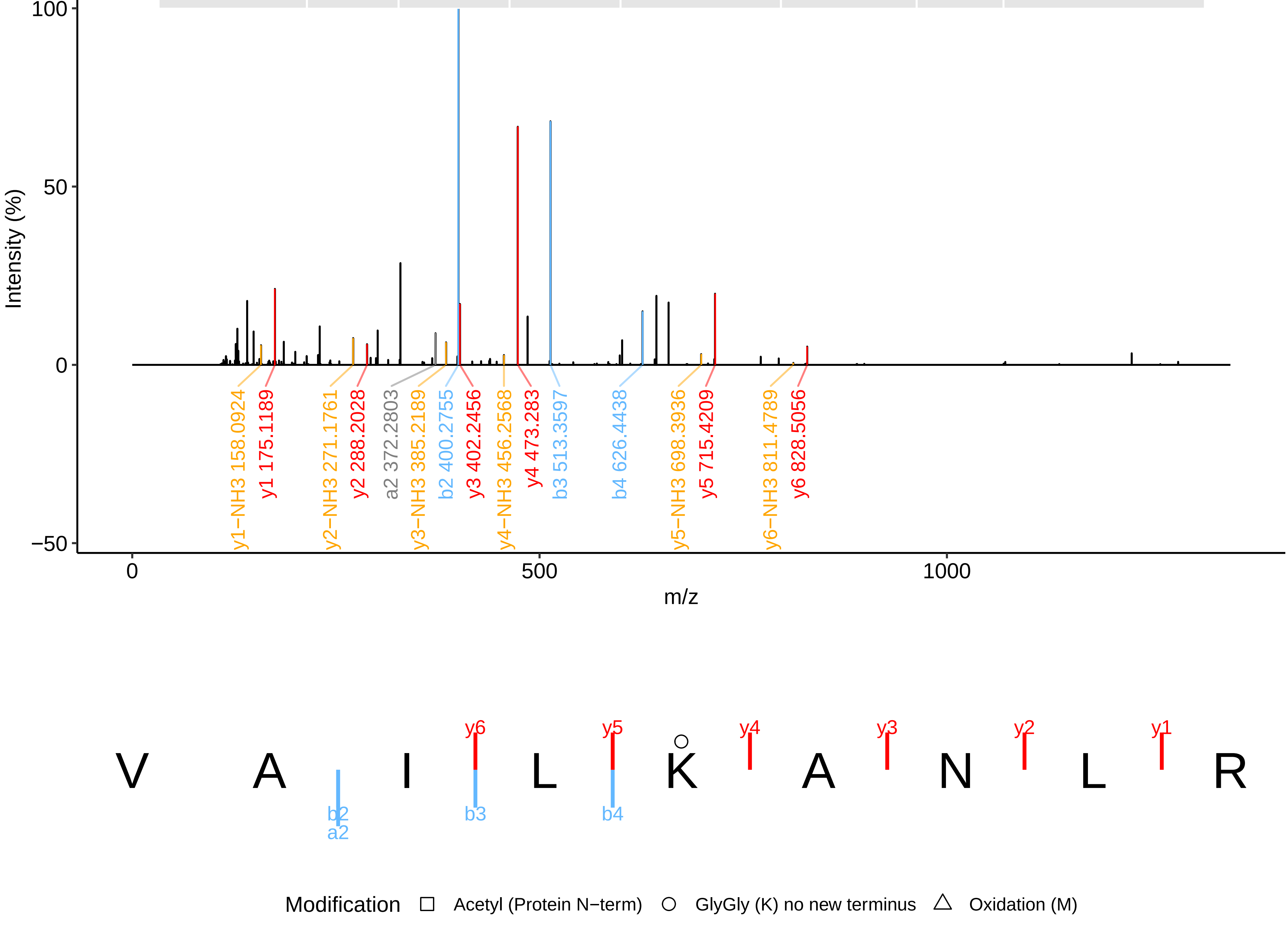

20200921_KIZ_MUb4_GlyGly_10plex_2 − Scan number: 14473

Gene Names	Charge	m/z	Mass	Retention time	PEP	Score	Precursor Intensity
VCP	2	556.3509	1110.687	56.434	0.054881	69.176	829367.2

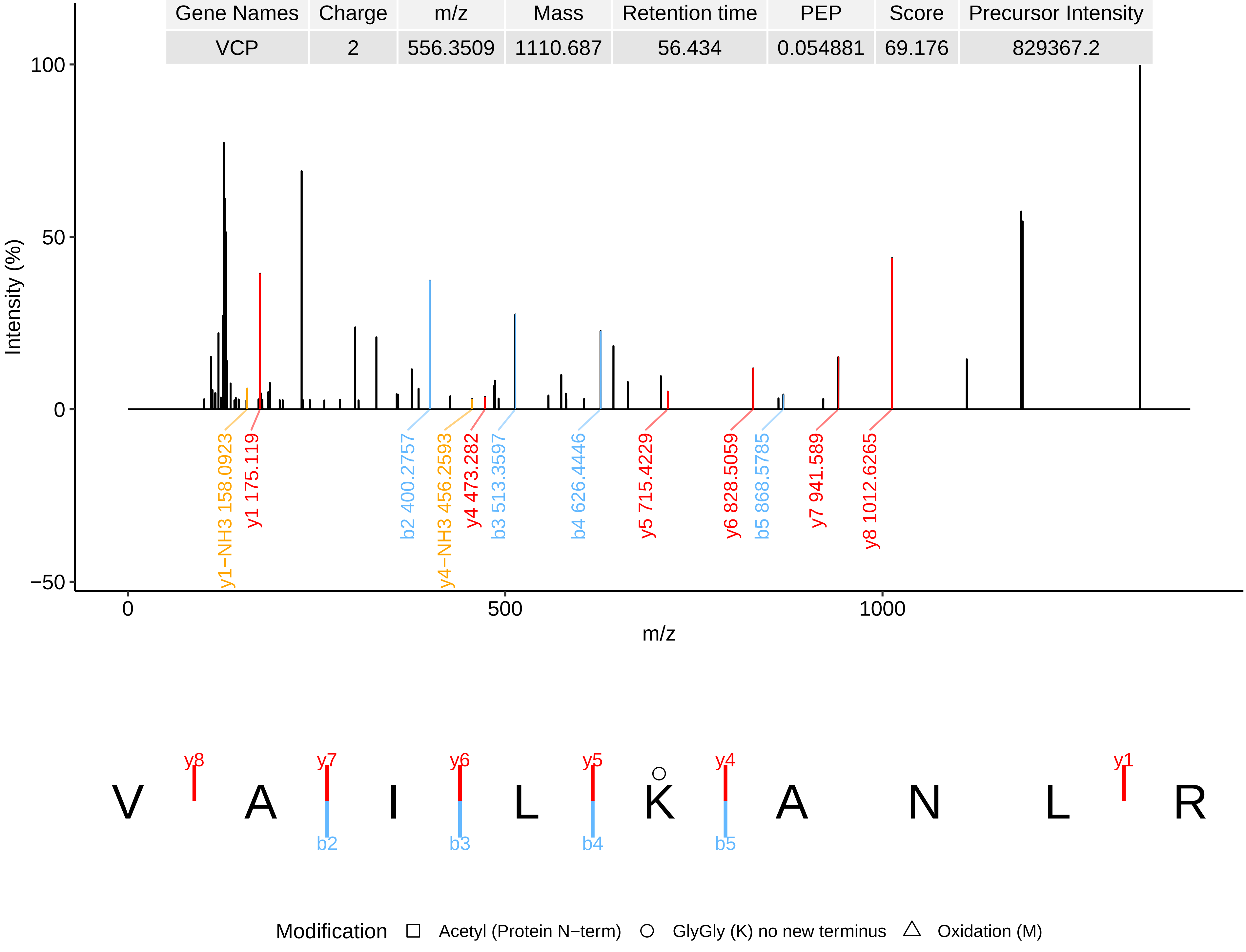

20200924_KIZ_MUb5_GlyGly_1 − Scan number: 14383

Gene Names	Charge	m/z	Mass	Retention time	PEP	Score	Precursor Intensity
VCP	3	371.2364	1110.687	46.966	3.1523e−07	133.6	19492112

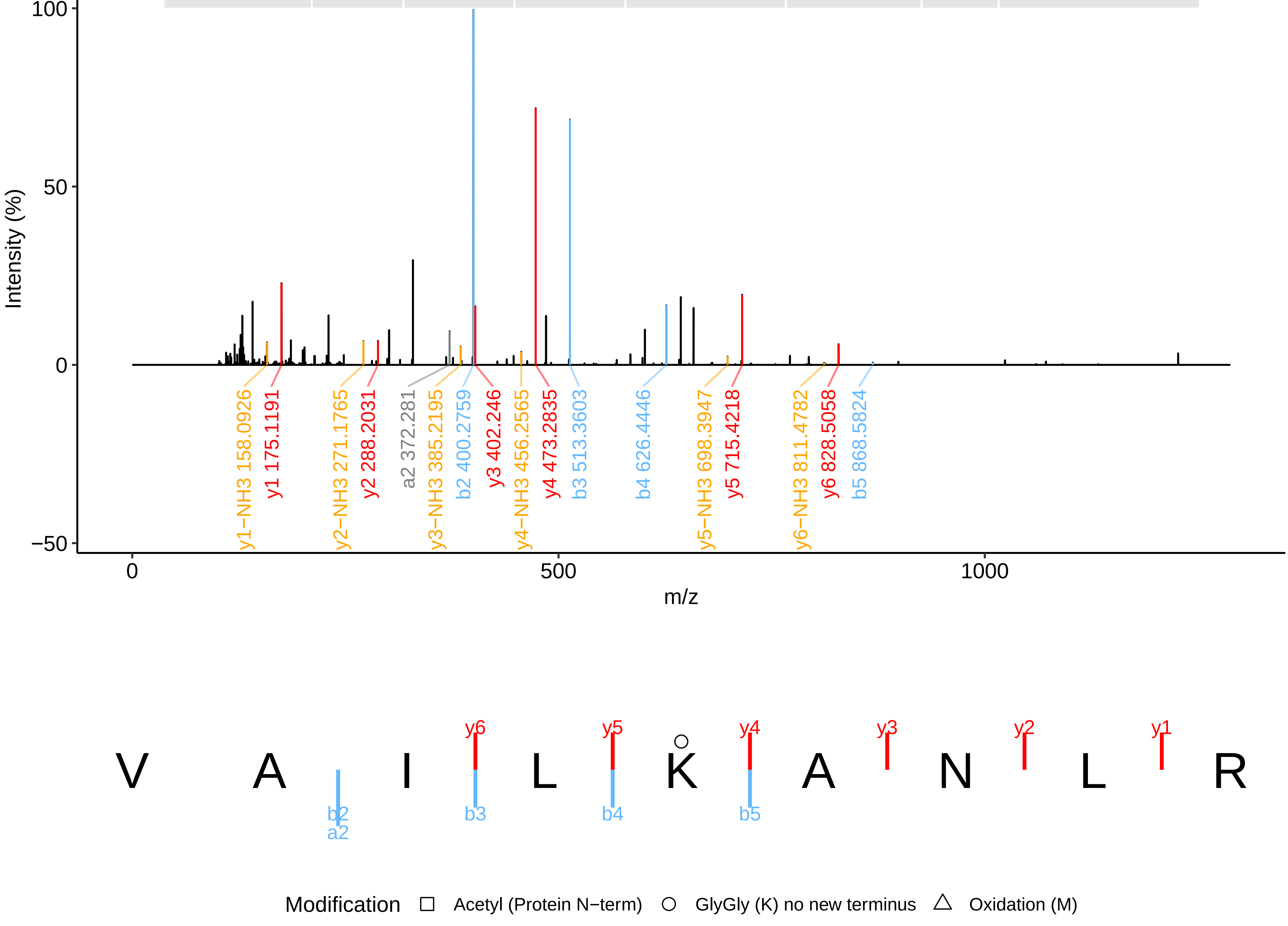

20200924_KIZ_MUb5_GlyGly_1 − Scan number: 14230

Gene Names	Charge	m/z	Mass	Retention time	PEP	Score	Precursor Intensity
VCP	3	371.2364	1110.687	46.448	0.0049776	86.095	NA

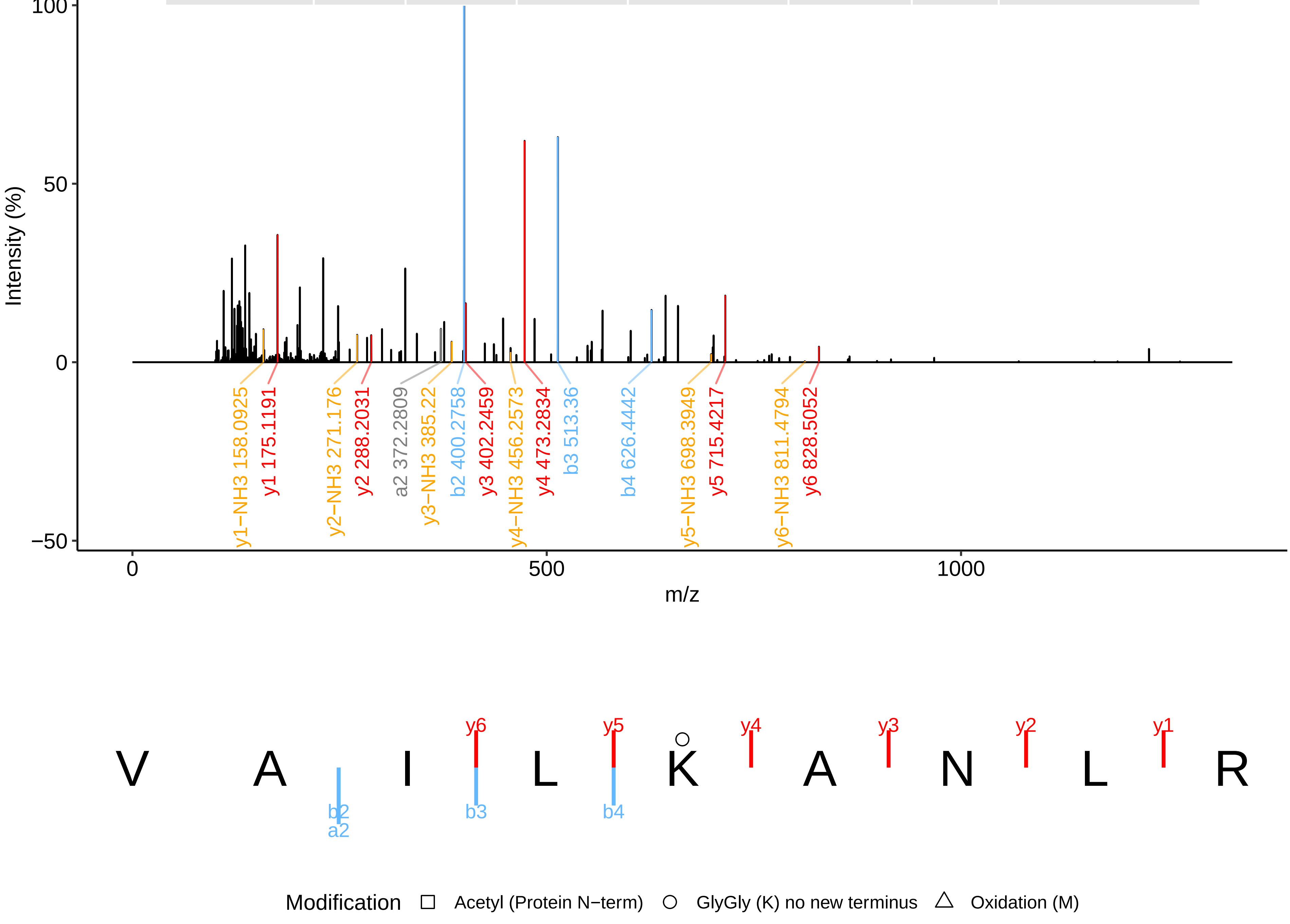

20200924_KIZ_MUb5_GlyGly_1 − Scan number: 14540

Gene Names	Charge	m/z	Mass	Retention time	PEP	Score	Precursor Intensity
VCP	3	371.2364	1110.687	47.479	0.0011293	109.01	11394930

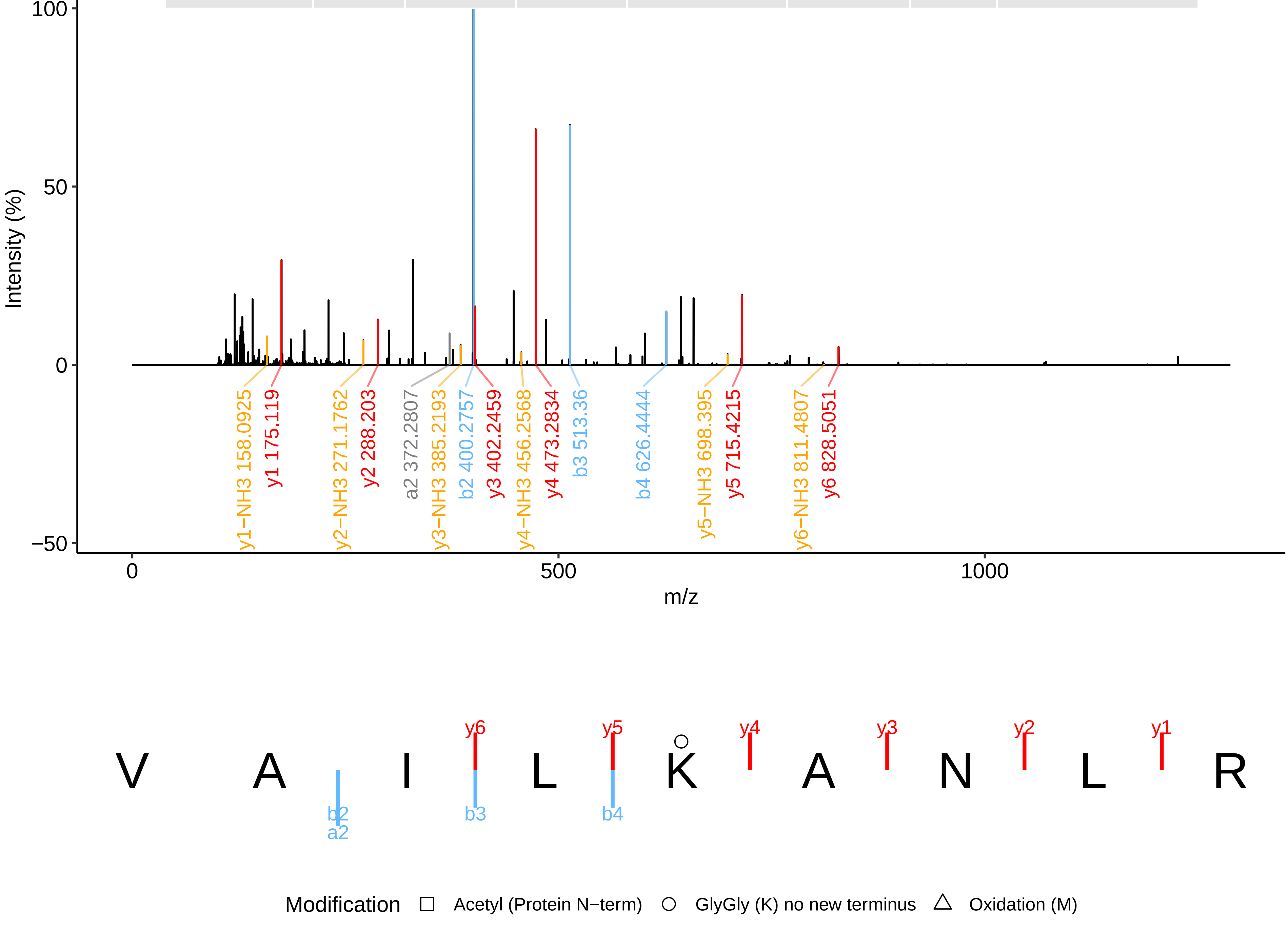

20200924_KIZ_MUb5_GlyGly_2 − Scan number: 13607

Gene Names	Charge	m/z	Mass	Retention time	PEP	Score	Precursor Intensity
VCP	3	371.2364	1110.687	50.882	8.1188e−05	113.5	5350808

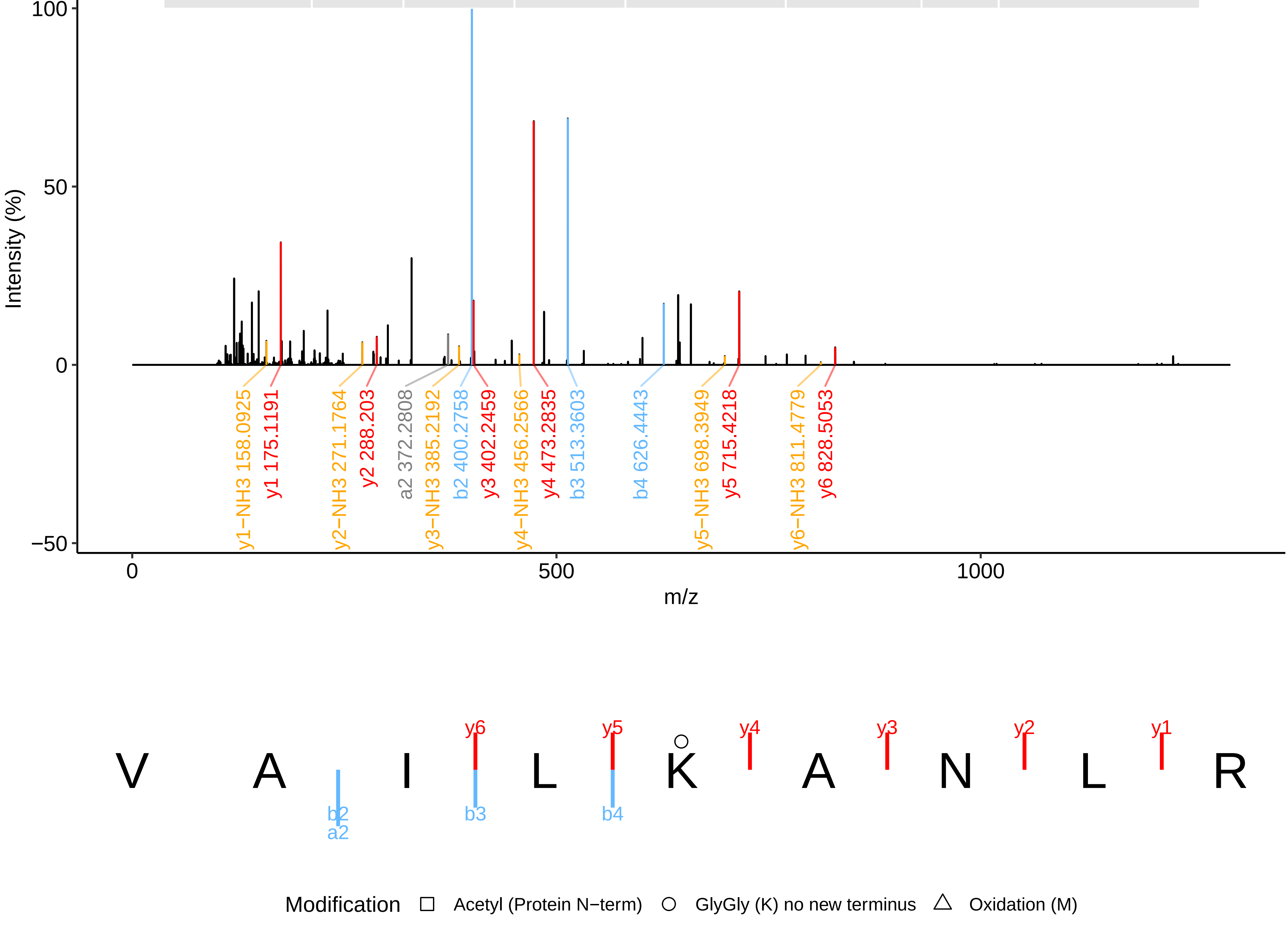

20201118_KIZ_MUb6_GlyGly_1 − Scan number: 14536

Gene Names	Charge	m/z	Mass	Retention time	PEP	Score	Precursor Intensity
VCP	3	371.2364	1110.687	45.981	0.00022296	109.01	17044904

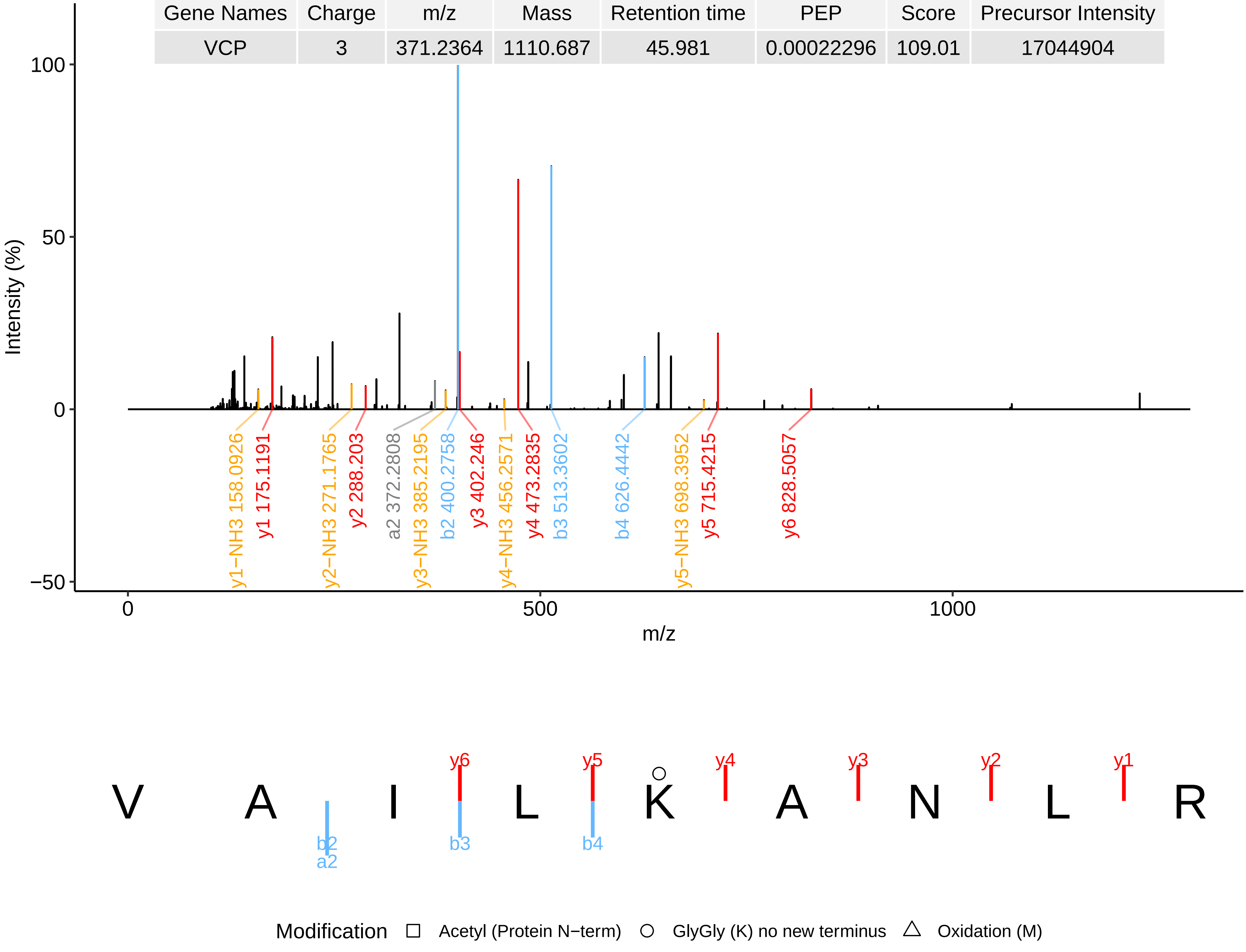

20201118_KIZ_MUb6_GlyGly_2 − Scan number: 17101

Gene Names	Charge	m/z	Mass	Retention time	PEP	Score	Precursor Intensity
VCP	3	371.2364	1110.687	57.812	1.2878e−05	122.13	11095701

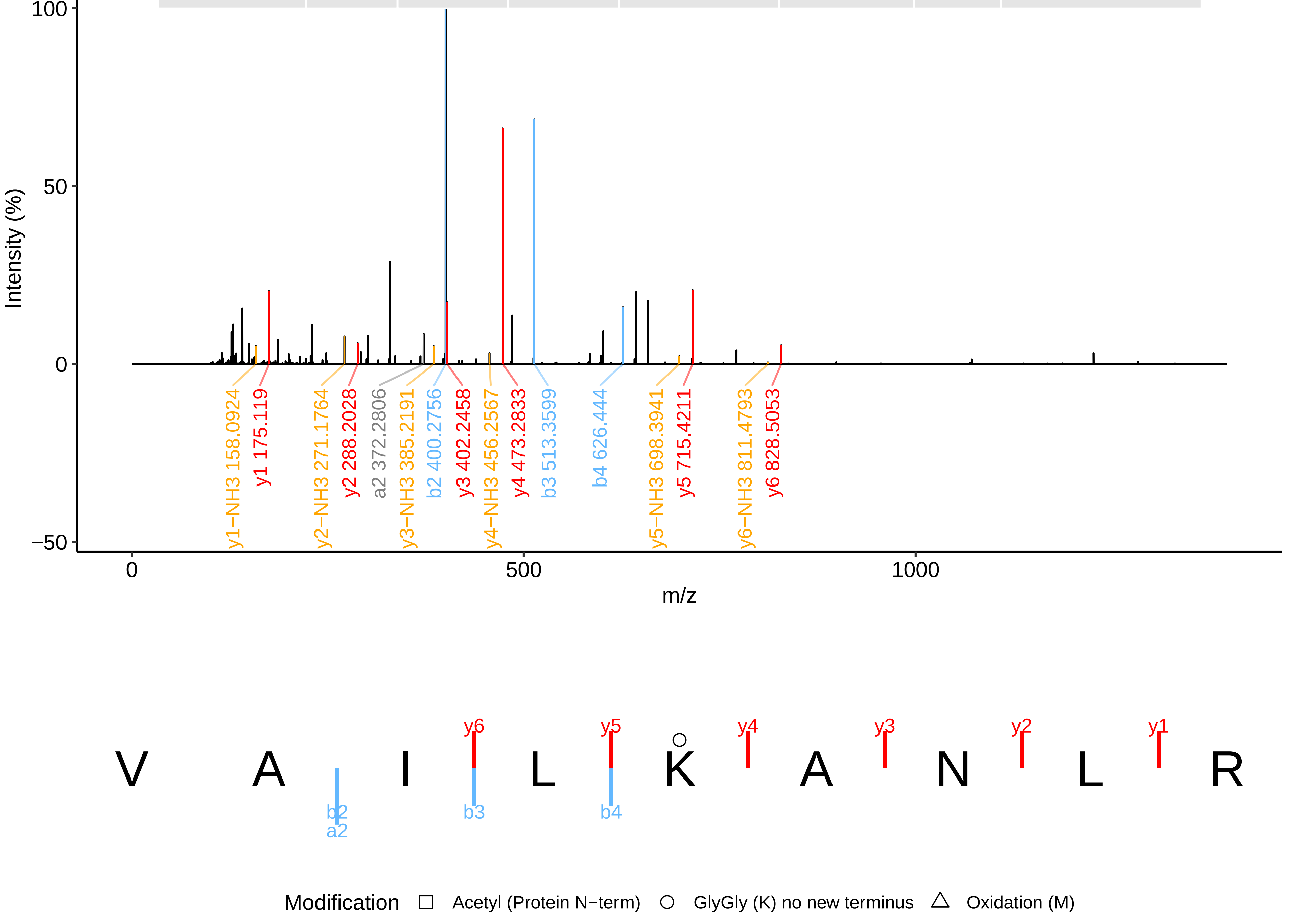

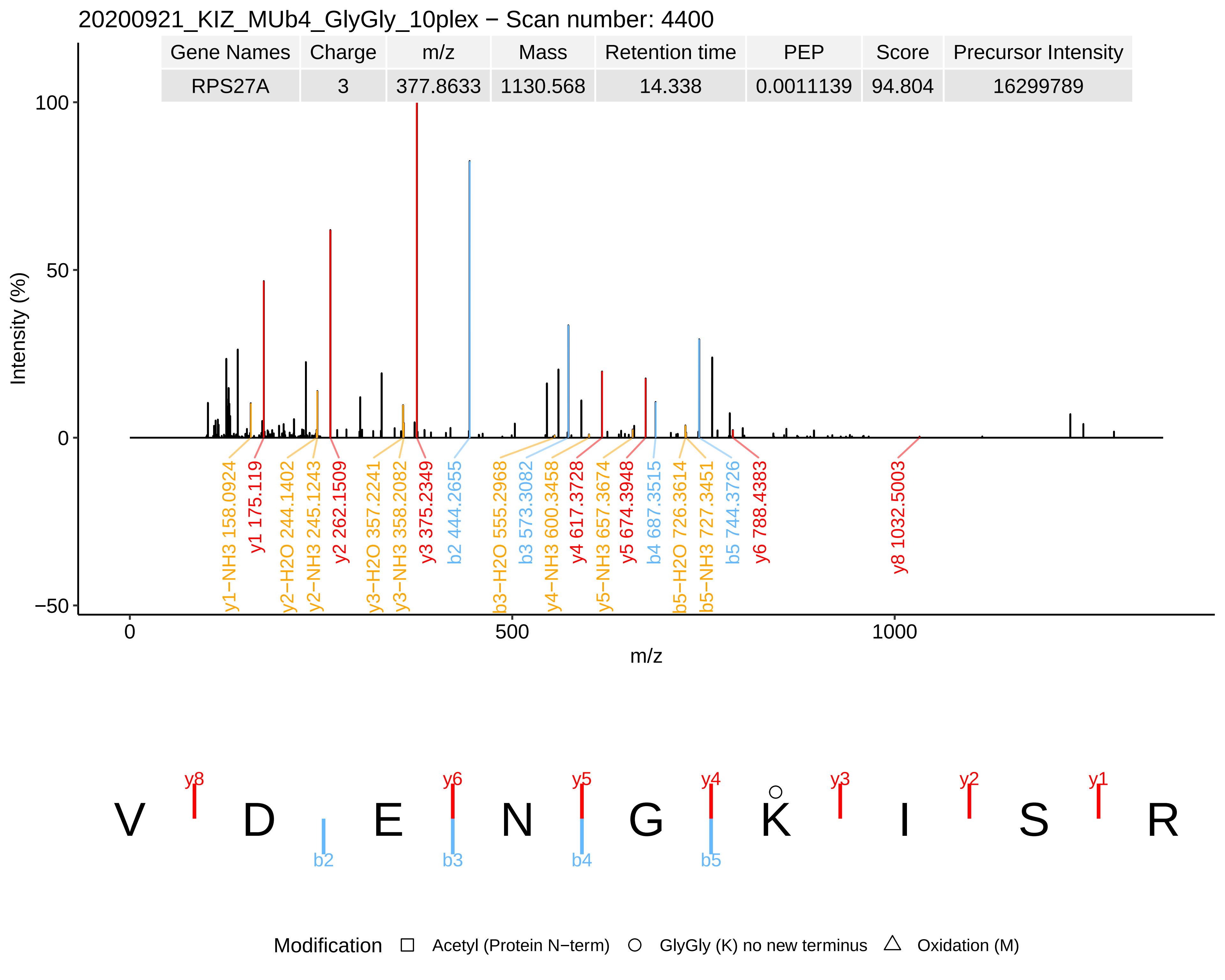
20200921_KIZ_MUb4_GlyGly_10plex − Scan number: 4400
Gene Names | Charge | m/z | Mass | Retention time | PEP | Score | Precursor Intensity
RPS27A | 3 | 377.8633 | 1130.568 | 14.338 | 0.0011139 | 94.804 | 16299789
Intensity (%)
m/z
y1−NH3 158.0924
y1 175.119
y2−H2O 244.1402
y2−NH3 245.1243
y2 262.1509
y3−H2O 357.2241
y3−NH3 358.2082
y3 375.2349
b2 444.2655
b3−H2O 555.2968
b3 573.3082
y4−NH3 600.3458
y4 617.3728
y5−NH3 657.3674
y5 674.3948
b4 687.3515
b5−H2O 726.3614
b5−NH3 727.3451
b5 744.3726
y6 788.4383
y8 1032.5003
V D E N G K I S R
y8 y6 y5 y4 y3 y2 y1
b2 b3 b4 b5
Modification
Acetyl (Protein N−term)
GlyGly (K) no new terminus
Oxidation (M)

20200921_KIZ_MUb4_GlyGly_10plex − Scan number: 4409

Gene Names	Charge	m/z	Mass	Retention time	PEP	Score	Precursor Intensity
RPS27A	2	566.2913	1130.568	14.37	4.1252e−07	145.9	8885492

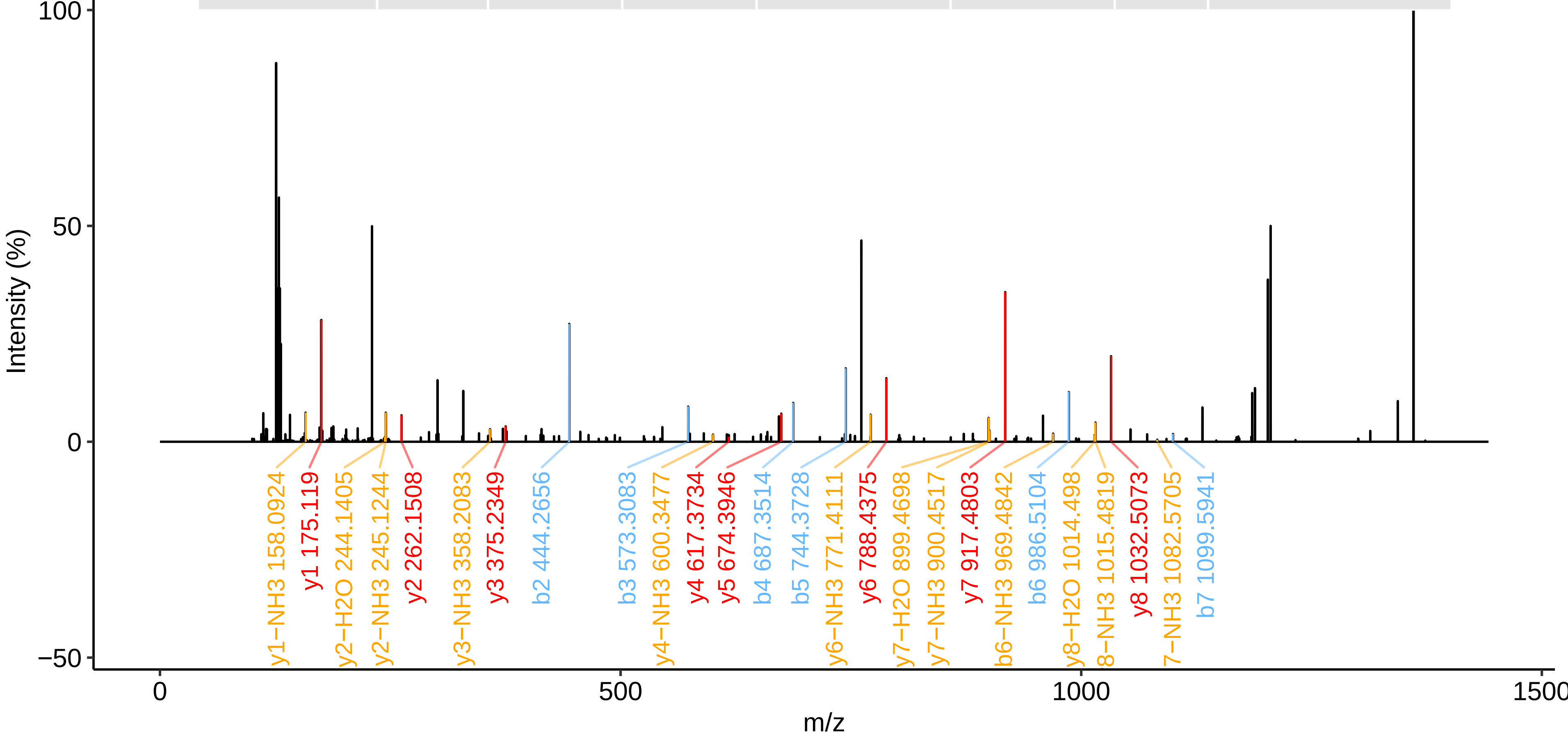

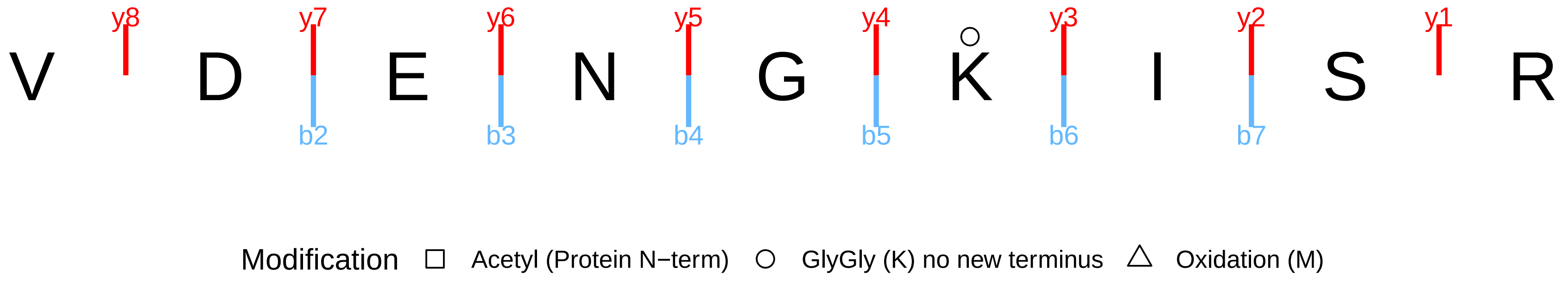

20200921_KIZ_MUb4_GlyGly_10plex_2 − Scan number: 4095

Gene Names	Charge	m/z	Mass	Retention time	PEP	Score	Precursor Intensity
RPS27A	3	377.8633	1130.568	14.174	0.0019118	90.108	23321502

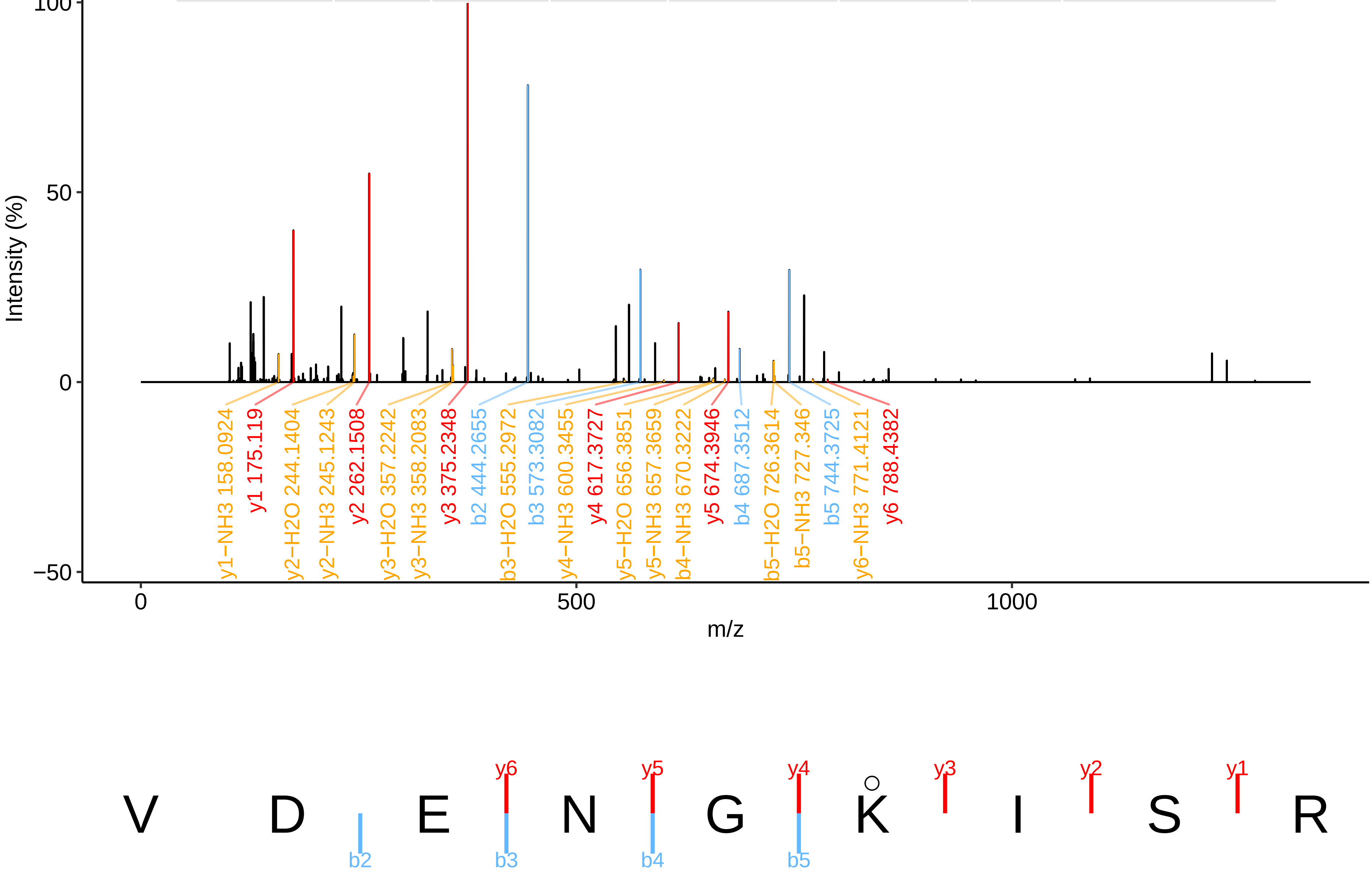

Modification □ Acetyl (Protein N−term) ○ GlyGly (K) no new terminus △ Oxidation (M)

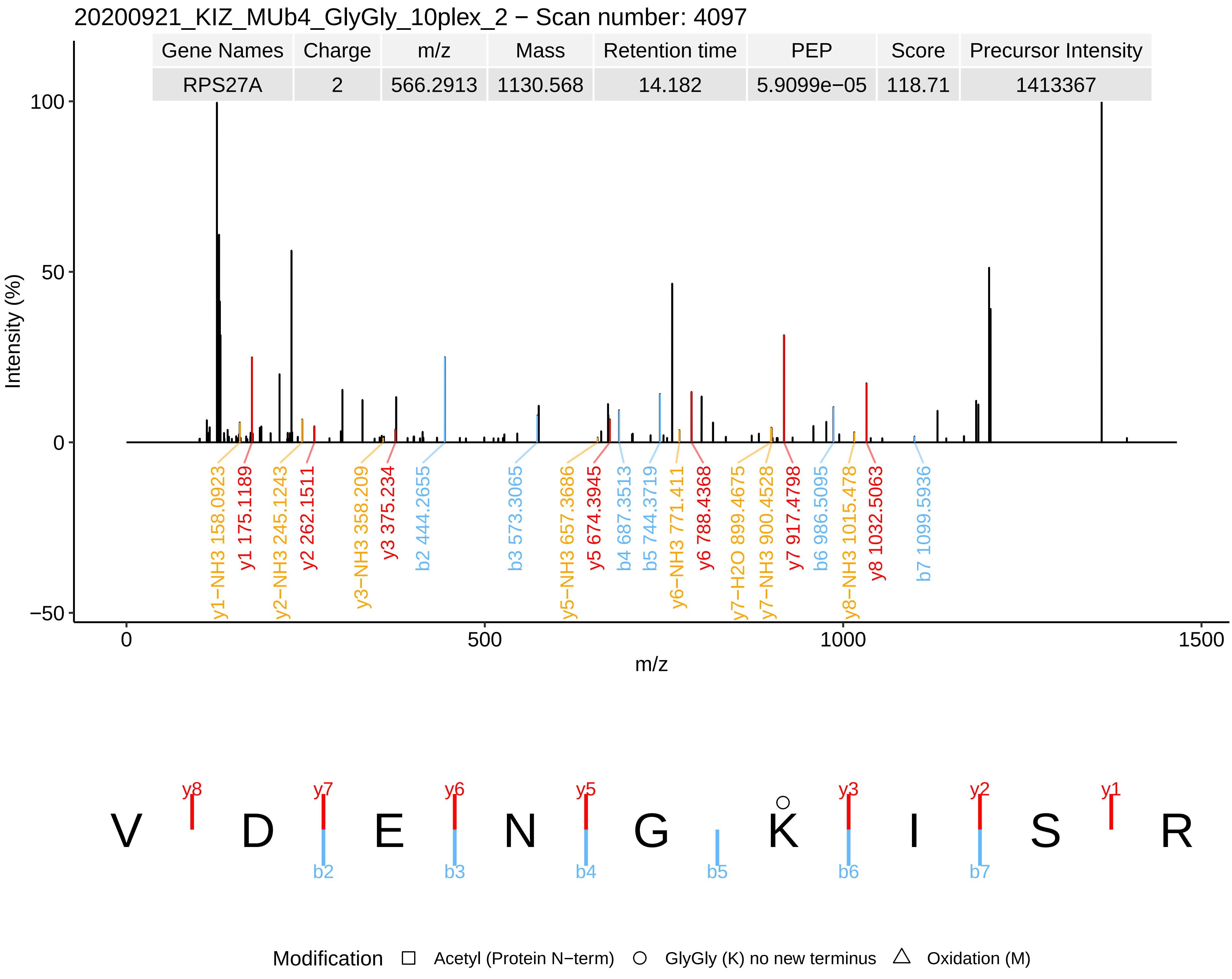

20200921_KIZ_MUb4_GlyGly_10plex_2 − Scan number: 4097
Gene Names | Charge | m/z | Mass | Retention time | PEP | Score | Precursor Intensity
RPS27A | 2 | 566.2913 | 1130.568 | 14.182 | 5.9099e−05 | 118.71 | 1413367
Intensity (%)
100
50
0
−50
y1−NH3 158.0923
y1 175.1189
y2−NH3 245.1243
y2 262.1511
y3−NH3 358.209
y3 375.234
b2 444.2655
b3 573.3065
y5−NH3 657.3686
y5 674.3945
b4 687.3513
b5 744.3719
y6−NH3 771.411
y6 788.4368
y7−H2O 899.4675
y7−NH3 900.4528
y7 917.4798
b6 986.5095
y8−NH3 1015.478
y8 1032.5063
b7 1099.5936
0
500
1000
1500
m/z
V D E N G K I S R
y8 y7 y6 y5 y3 y2 y1
b2 b3 b4 b5 b6 b7
Modification
Acetyl (Protein N−term)
GlyGly (K) no new terminus
Oxidation (M)

20200924_KIZ_MUb5_GlyGly_1 − Scan number: 3754

Gene Names	Charge	m/z	Mass	Retention time	PEP	Score	Precursor Intensity
RPS27A	3	377.8633	1130.568	11.469	0.008171	76.228	25313508

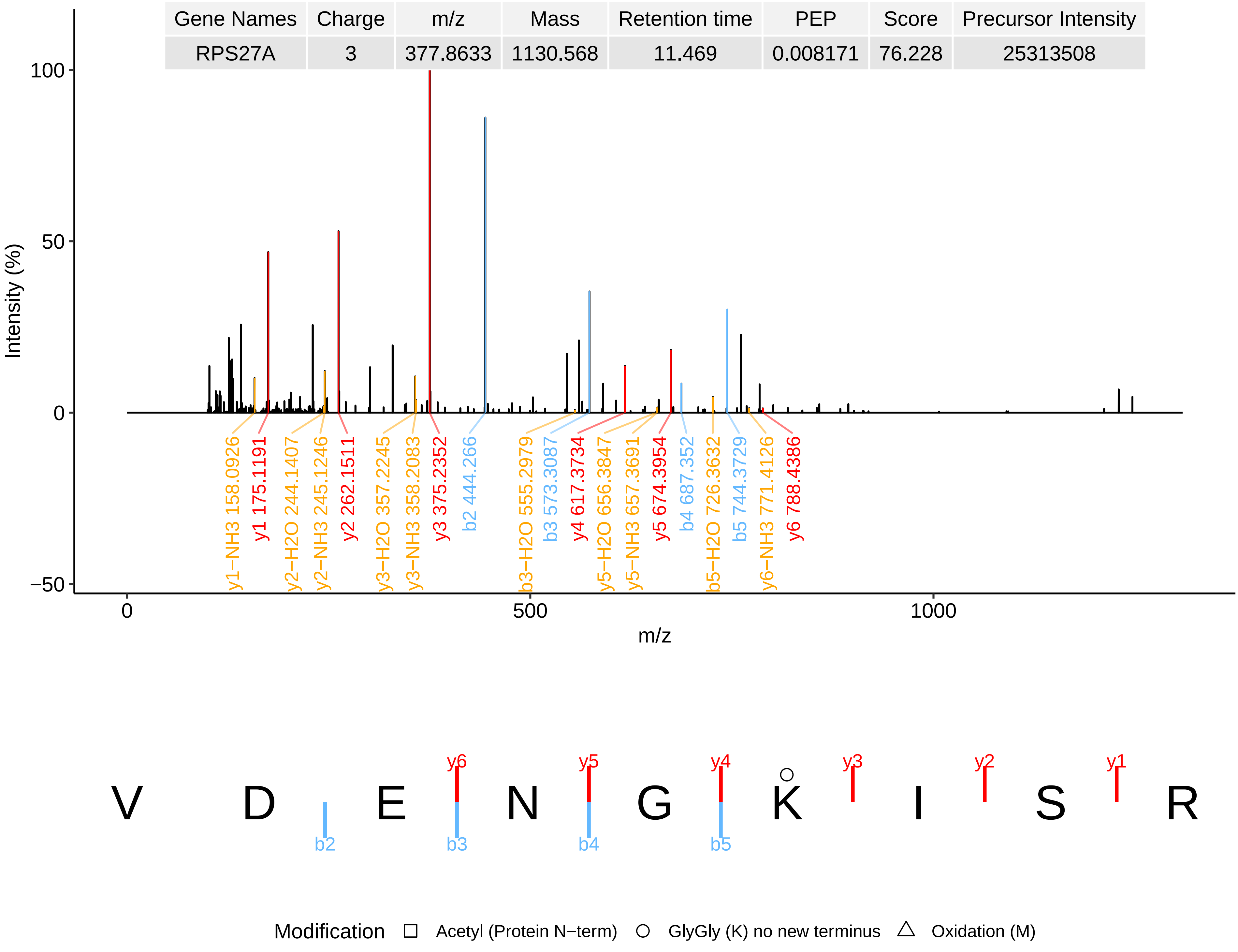

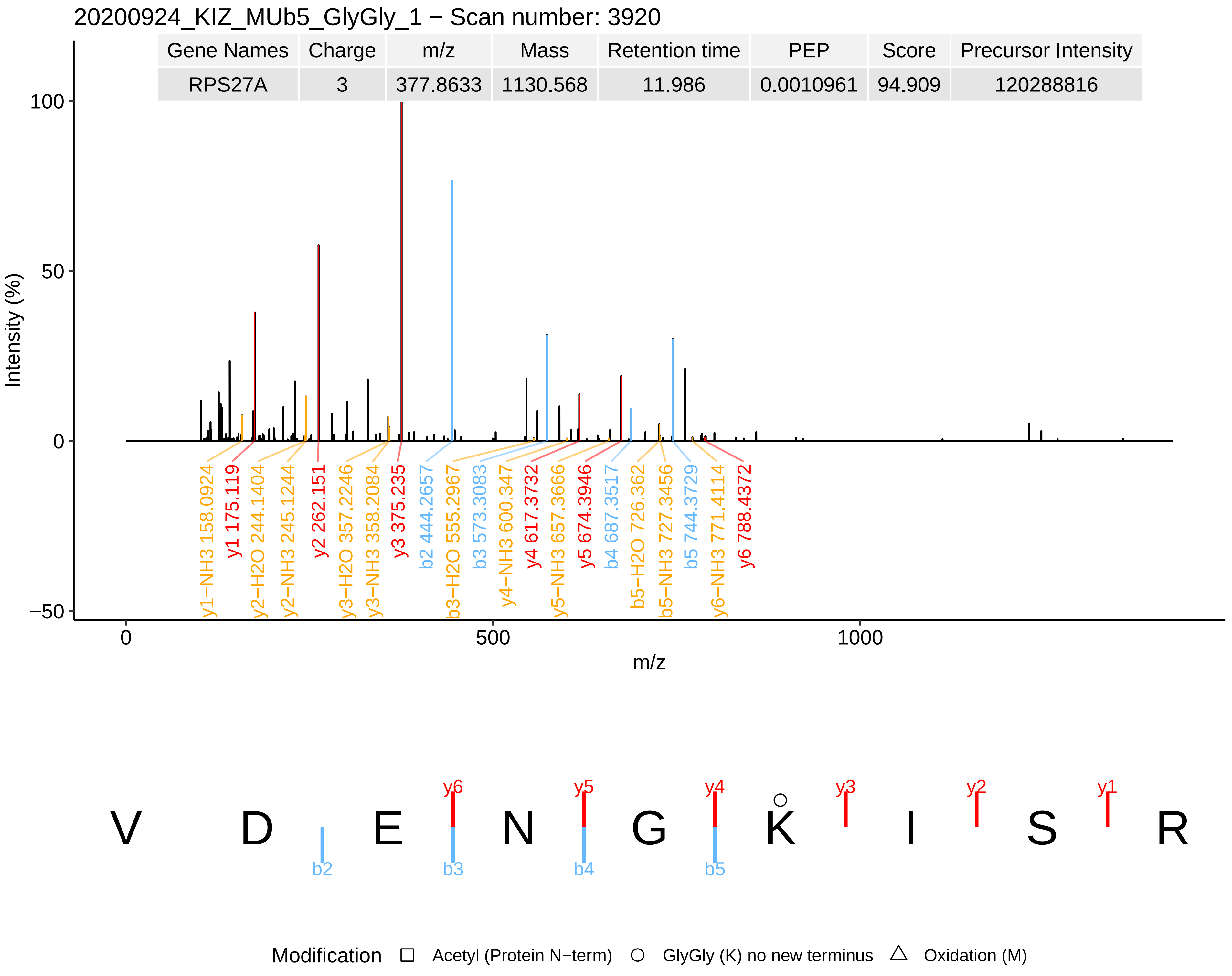
20200924_KIZ_MUb5_GlyGly_1 − Scan number: 3920
Gene Names | Charge | m/z | Mass | Retention time | PEP | Score | Precursor Intensity
RPS27A | 3 | 377.8633 | 1130.568 | 11.986 | 0.0010961 | 94.909 | 120288816
Intensity (%)
100
50
0
−50
m/z
0
500
1000
y1−NH3 158.0924
y1 175.119
y2−H2O 244.1404
y2−NH3 245.1244
y2 262.151
y3−H2O 357.2246
y3−NH3 358.2084
y3 375.235
b2 444.2657
b3−H2O 555.2967
b3 573.3083
y4−NH3 600.347
y4 617.3732
y5−NH3 657.3666
y5 674.3946
b4 687.3517
b5−H2O 726.362
b5−NH3 727.3456
b5 744.3729
y6−NH3 771.4114
y6 788.4372
V D E N G K I S R
y6 y5 y4 y3 y2 y1
b2 b3 b4 b5
Modification
Acetyl (Protein N−term)
GlyGly (K) no new terminus
Oxidation (M)

20200924_KIZ_MUb5_GlyGly_1 − Scan number: 3777

Gene Names	Charge	m/z	Mass	Retention time	PEP	Score	Precursor Intensity
RPS27A	2	566.2913	1130.568	11.538	9.8077e−08	157.59	12248054

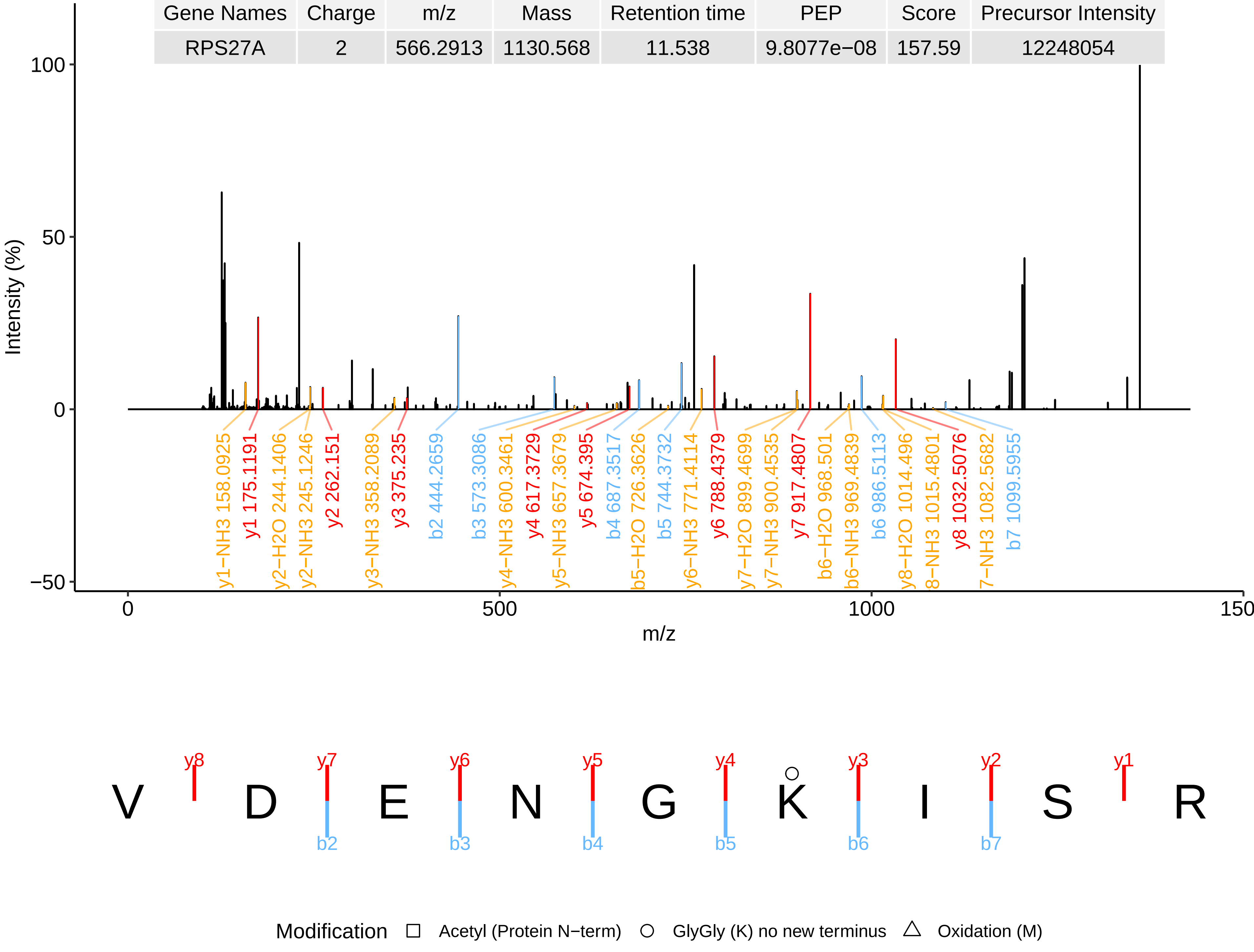

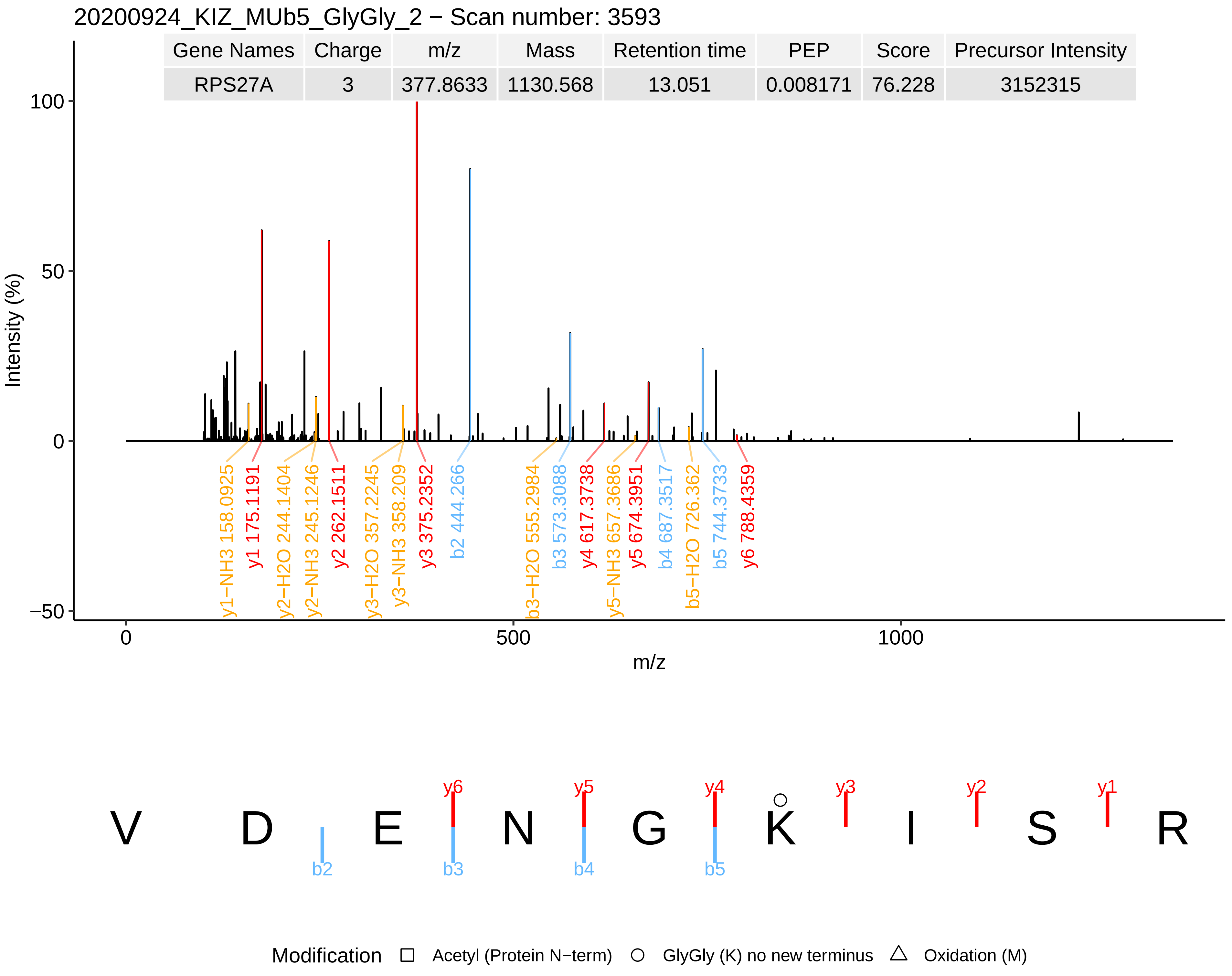
20200924_KIZ_MUb5_GlyGly_2 − Scan number: 3593
Gene Names | Charge | m/z | Mass | Retention time | PEP | Score | Precursor Intensity
RPS27A | 3 | 377.8633 | 1130.568 | 13.051 | 0.008171 | 76.228 | 3152315
Intensity (%)
m/z
y1−NH3 158.0925
y1 175.1191
y2−H2O 244.1404
y2−NH3 245.1246
y2 262.1511
y3−H2O 357.2245
y3−NH3 358.209
y3 375.2352
b2 444.266
b3−H2O 555.2984
b3 573.3088
y4 617.3738
y5−NH3 657.3686
y5 674.3951
b4 687.3517
b5−H2O 726.362
b5 744.3733
y6 788.4359
V D E N G K I S R
Modification
Acetyl (Protein N−term)
GlyGly (K) no new terminus
Oxidation (M)

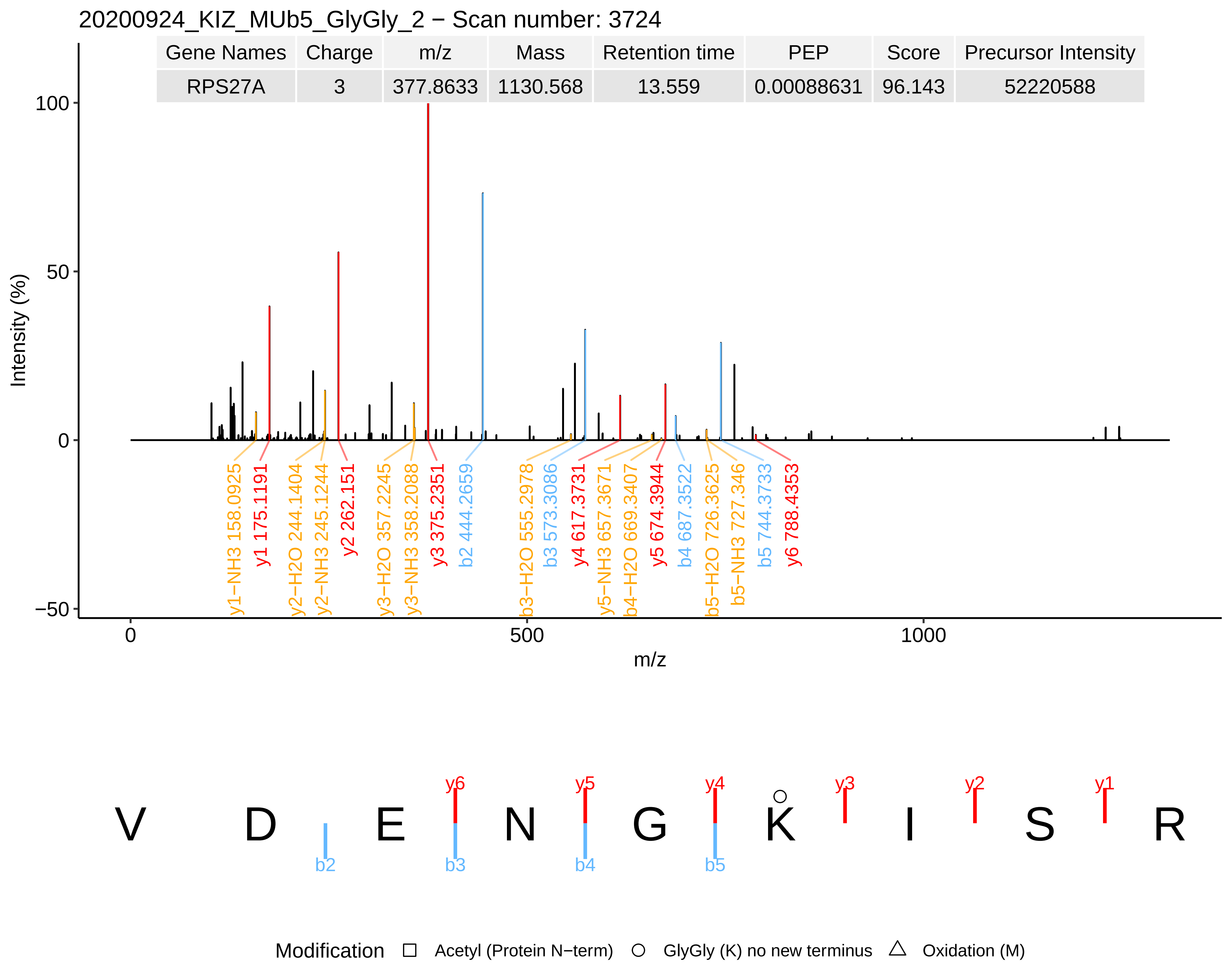

20200924_KIZ_MUb5_GlyGly_2 − Scan number: 3724
Gene Names | Charge | m/z | Mass | Retention time | PEP | Score | Precursor Intensity
RPS27A | 3 | 377.8633 | 1130.568 | 13.559 | 0.00088631 | 96.143 | 52220588
Intensity (%)
m/z
y1−NH3 158.0925
y1 175.1191
y2−H2O 244.1404
y2−NH3 245.1244
y2 262.151
y3−H2O 357.2245
y3−NH3 358.2088
y3 375.2351
b2 444.2659
b3−H2O 555.2978
b3 573.3086
y4 617.3731
y5−NH3 657.3671
b4−H2O 669.3407
y5 674.3944
b4 687.3522
b5−H2O 726.3625
b5−NH3 727.346
b5 744.3733
y6 788.4353
V D E N G K I S R
y6 y5 y4 y3 y2 y1
b2 b3 b4 b5
Modification
Acetyl (Protein N−term)
GlyGly (K) no new terminus
Oxidation (M)

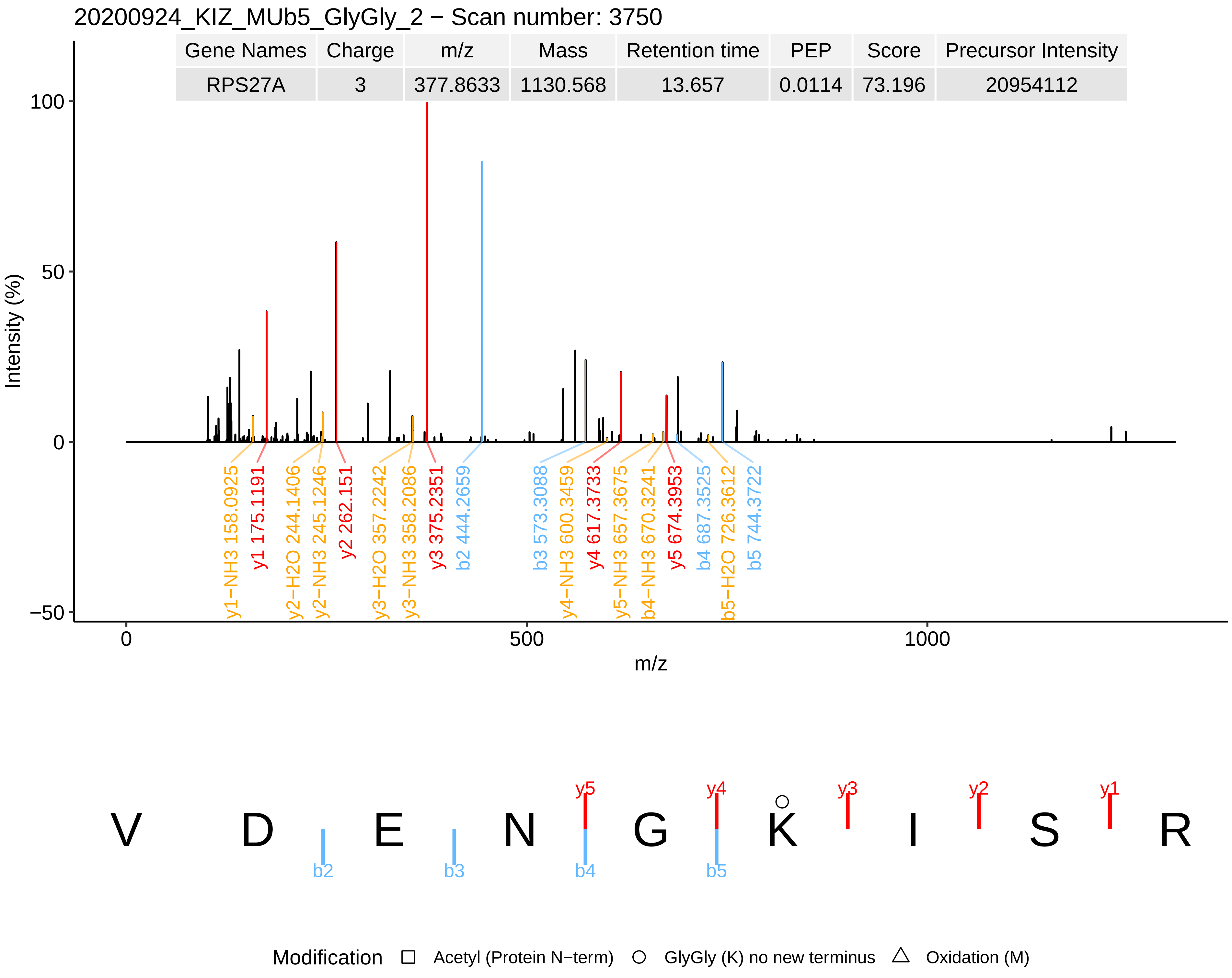
20200924_KIZ_MUb5_GlyGly_2 − Scan number: 3750
Gene Names
RPS27A
Charge
3
m/z
377.8633
Mass
1130.568
Retention time
13.657
PEP
0.0114
Score
73.196
Precursor Intensity
20954112
Intensity (%)
100
50
0
−50
0
500
1000
m/z
y1−NH3 158.0925
y1 175.1191
y2−H2O 244.1406
y2−NH3 245.1246
y2 262.151
y3−H2O 357.2242
y3−NH3 358.2086
y3 375.2351
b2 444.2659
b3 573.3088
y4−NH3 600.3459
y4 617.3733
y5−NH3 657.3675
b4−NH3 670.3241
y5 674.3953
b4 687.3525
b5−H2O 726.3612
b5 744.3722
V D E N G K I S R
y5 y4 y3 y2 y1
b2 b3 b4 b5
Modification
Acetyl (Protein N−term)
GlyGly (K) no new terminus
Oxidation (M)

20200924_KIZ_MUb5_GlyGly_2 − Scan number: 3615

Gene Names	Charge	m/z	Mass	Retention time	PEP	Score	Precursor Intensity
RPS27A	2	566.2913	1130.568	13.138	0.0010161	106.88	2487844

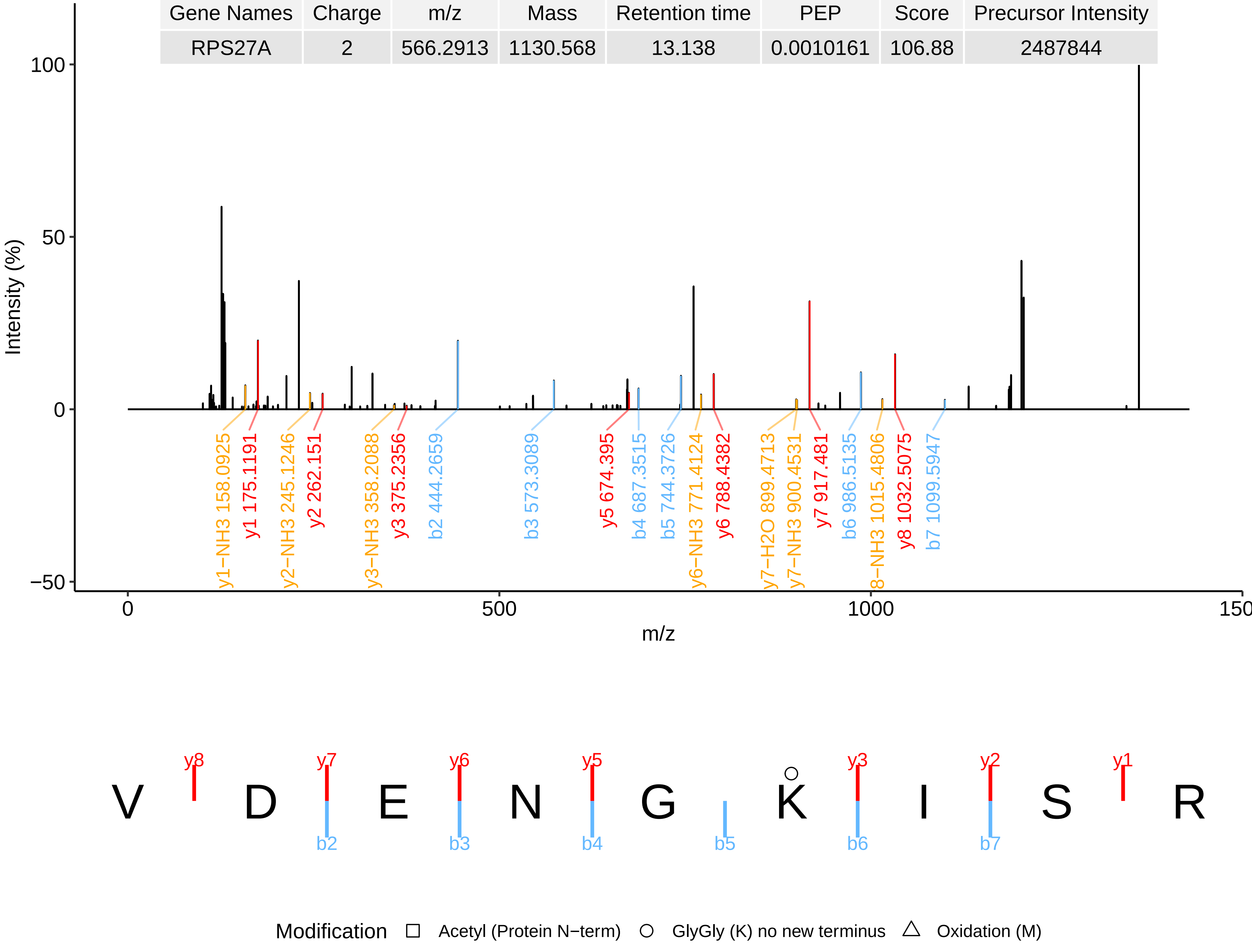

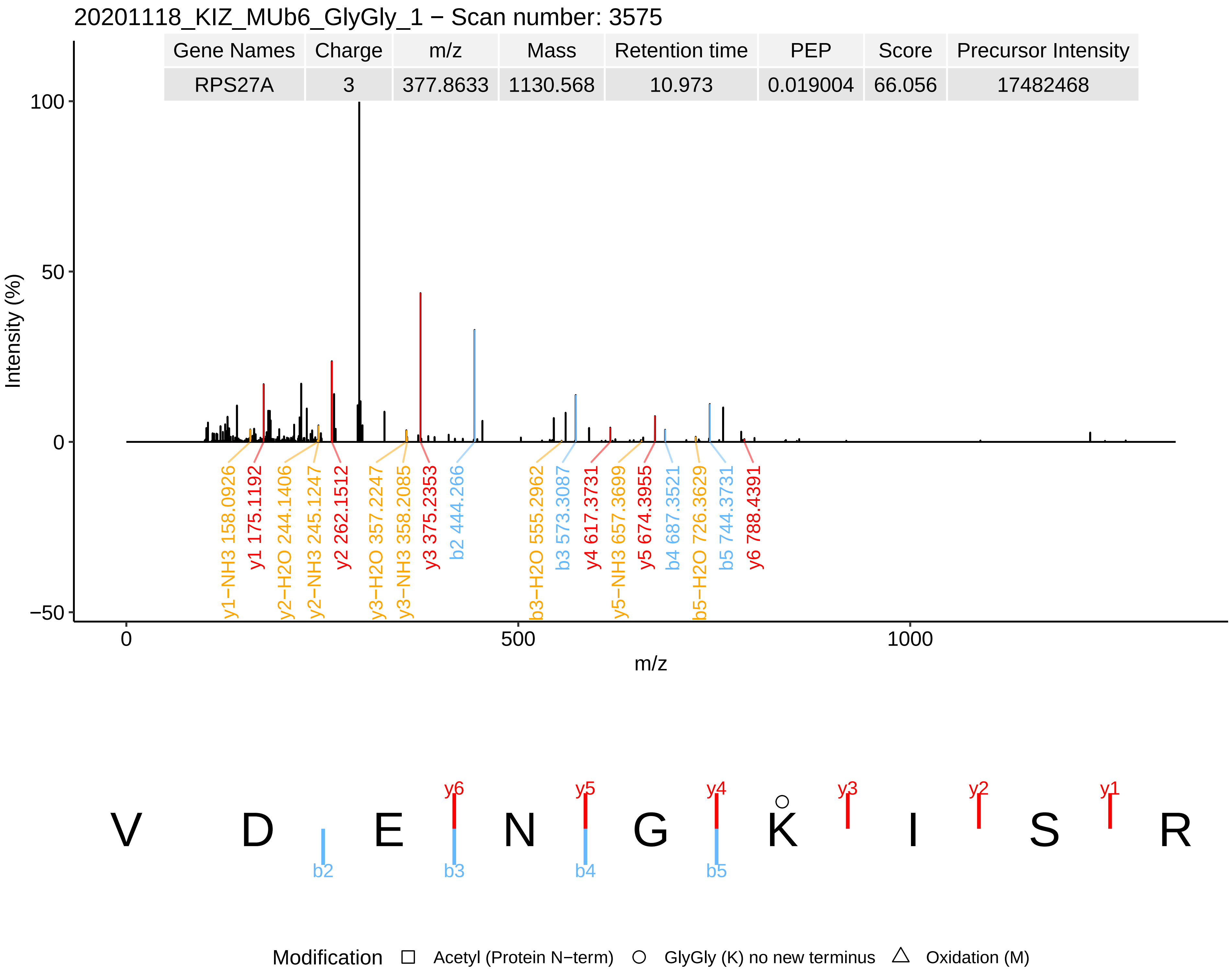

20201118_KIZ_MUb6_GlyGly_1 − Scan number: 3575
Gene Names | Charge | m/z | Mass | Retention time | PEP | Score | Precursor Intensity
RPS27A | 3 | 377.8633 | 1130.568 | 10.973 | 0.019004 | 66.056 | 17482468
Intensity (%)
m/z
y1−NH3 158.0926
y1 175.1192
y2−H2O 244.1406
y2−NH3 245.1247
y2 262.1512
y3−H2O 357.2247
y3−NH3 358.2085
y3 375.2353
b2 444.266
b3−H2O 555.2962
b3 573.3087
y4 617.3731
y5−NH3 657.3699
y5 674.3955
b4 687.3521
b5−H2O 726.3629
b5 744.3731
y6 788.4391
V D E N G K I S R
y6 y5 y4 y3 y2 y1
b2 b3 b4 b5
Modification
Acetyl (Protein N−term)
GlyGly (K) no new terminus
Oxidation (M)

20201118_KIZ_MUb6_GlyGly_1 − Scan number: 3585

Gene Names	Charge	m/z	Mass	Retention time	PEP	Score	Precursor Intensity
RPS27A	2	566.2913	1130.568	11.009	4.1252e−07	145.9	7057206

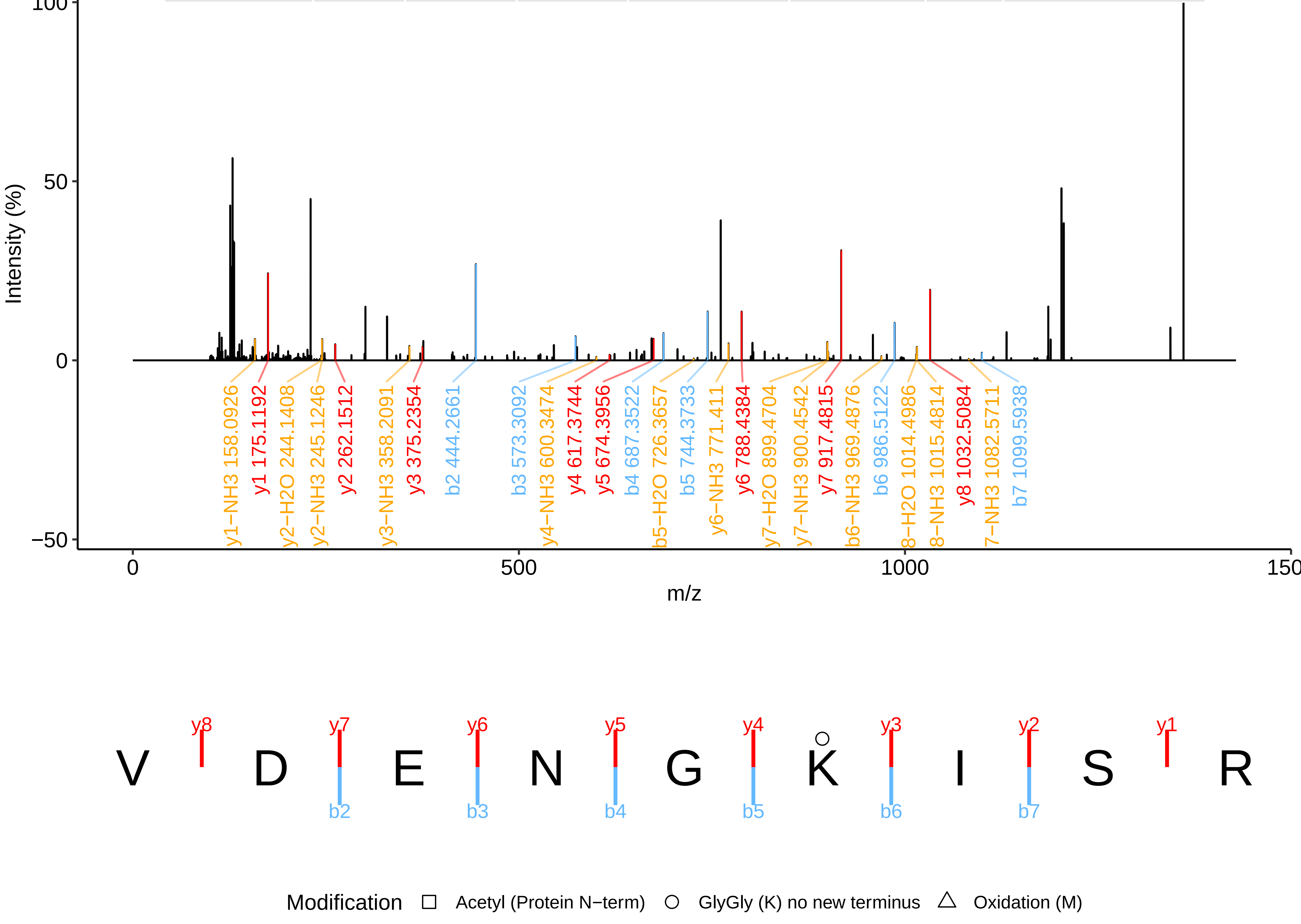

20201118_KIZ_MUb6_GlyGly_2 − Scan number: 4371

Gene Names	Charge	m/z	Mass	Retention time	PEP	Score	Precursor Intensity
RPS27A	3	377.8633	1130.568	14.899	0.019363	65.719	3619842

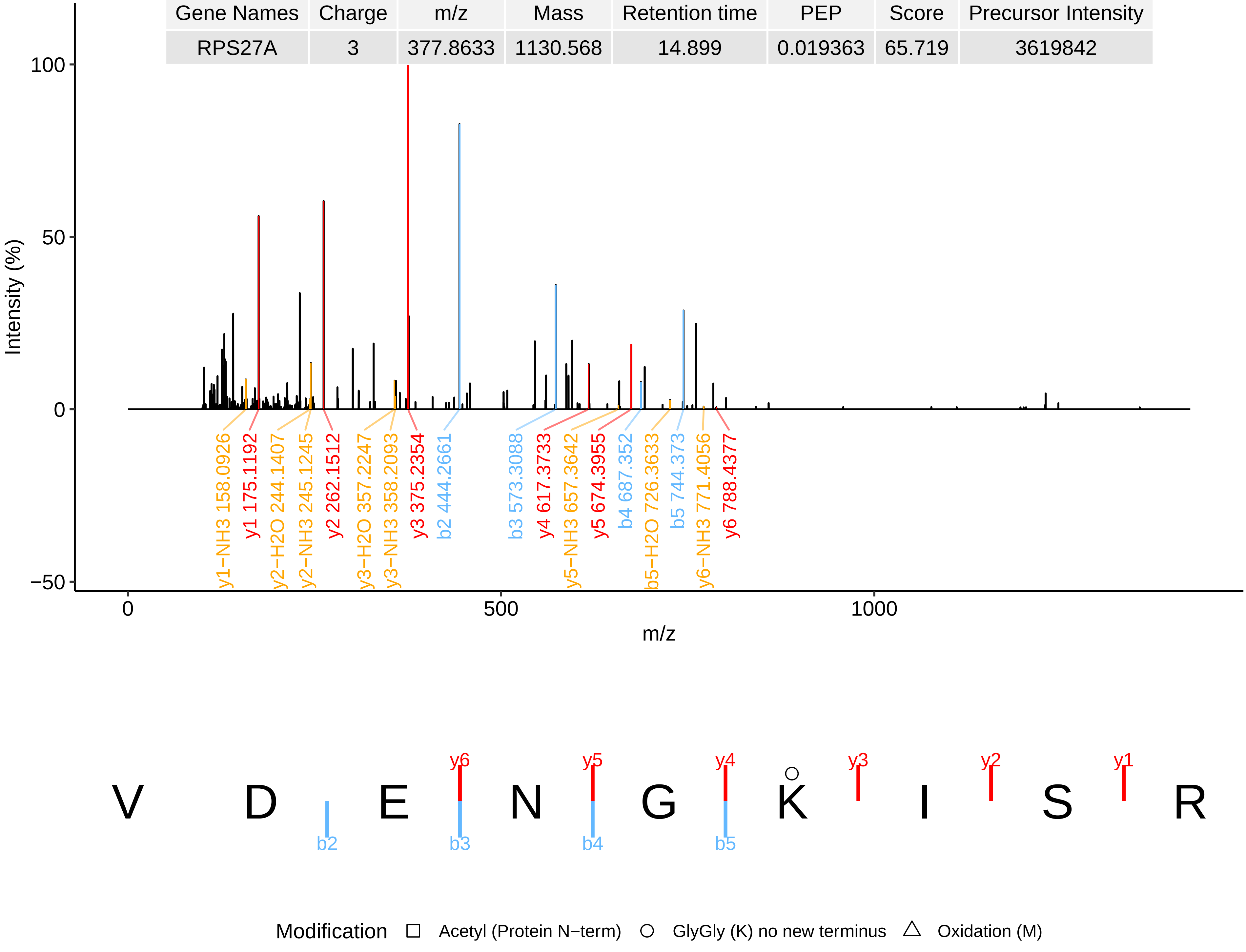

20201118_KIZ_MUb6_GlyGly_2 − Scan number: 4402

Gene Names	Charge	m/z	Mass	Retention time	PEP	Score	Precursor Intensity
RPS27A	3	377.8633	1130.568	15.014	0.014216	70.552	36681328

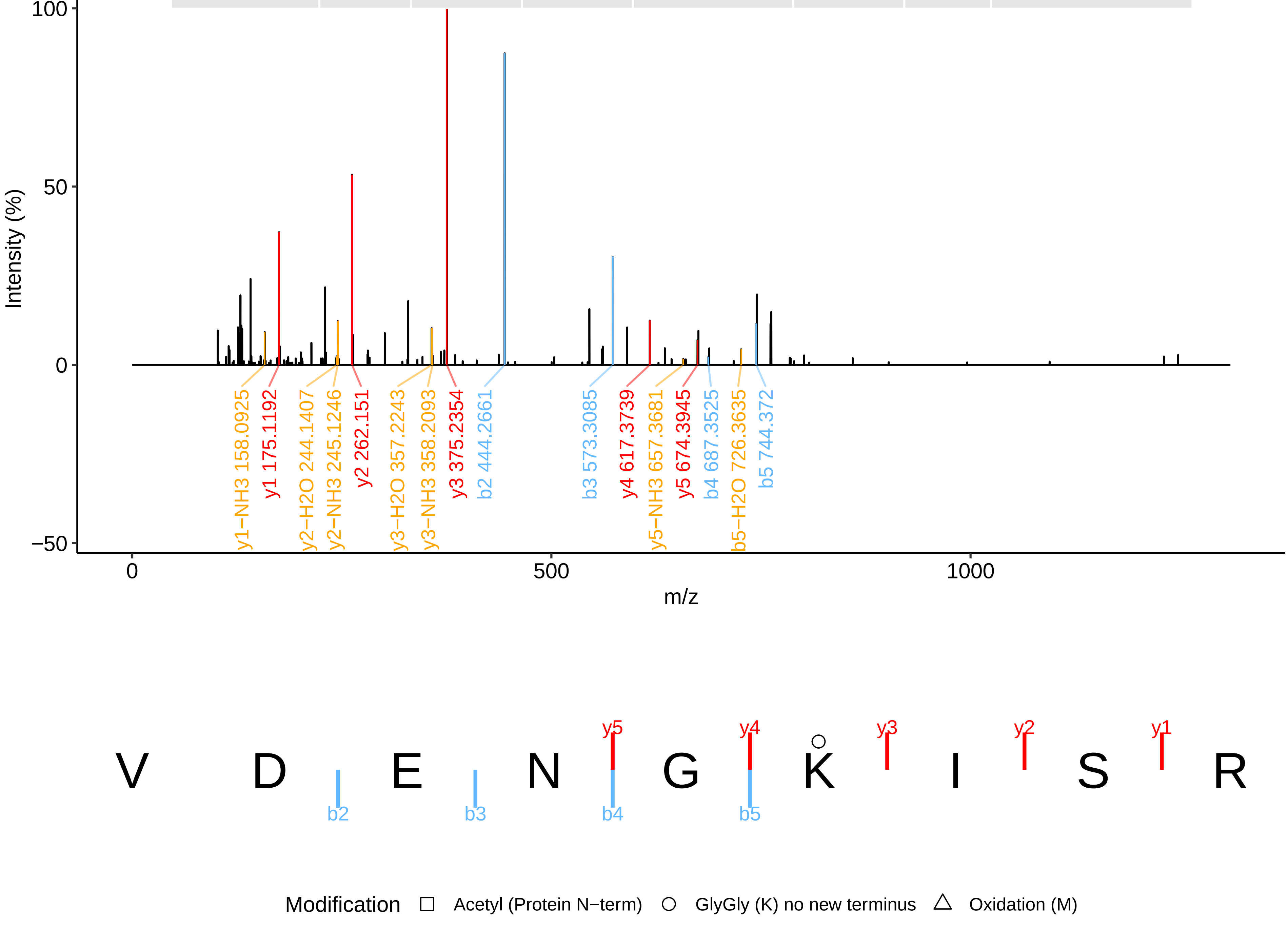

20201118_KIZ_MUb6_GlyGly_2 − Scan number: 4400

Gene Names	Charge	m/z	Mass	Retention time	PEP	Score	Precursor Intensity
RPS27A	2	566.2913	1130.568	15.009	1.0852e−10	182.39	5870615

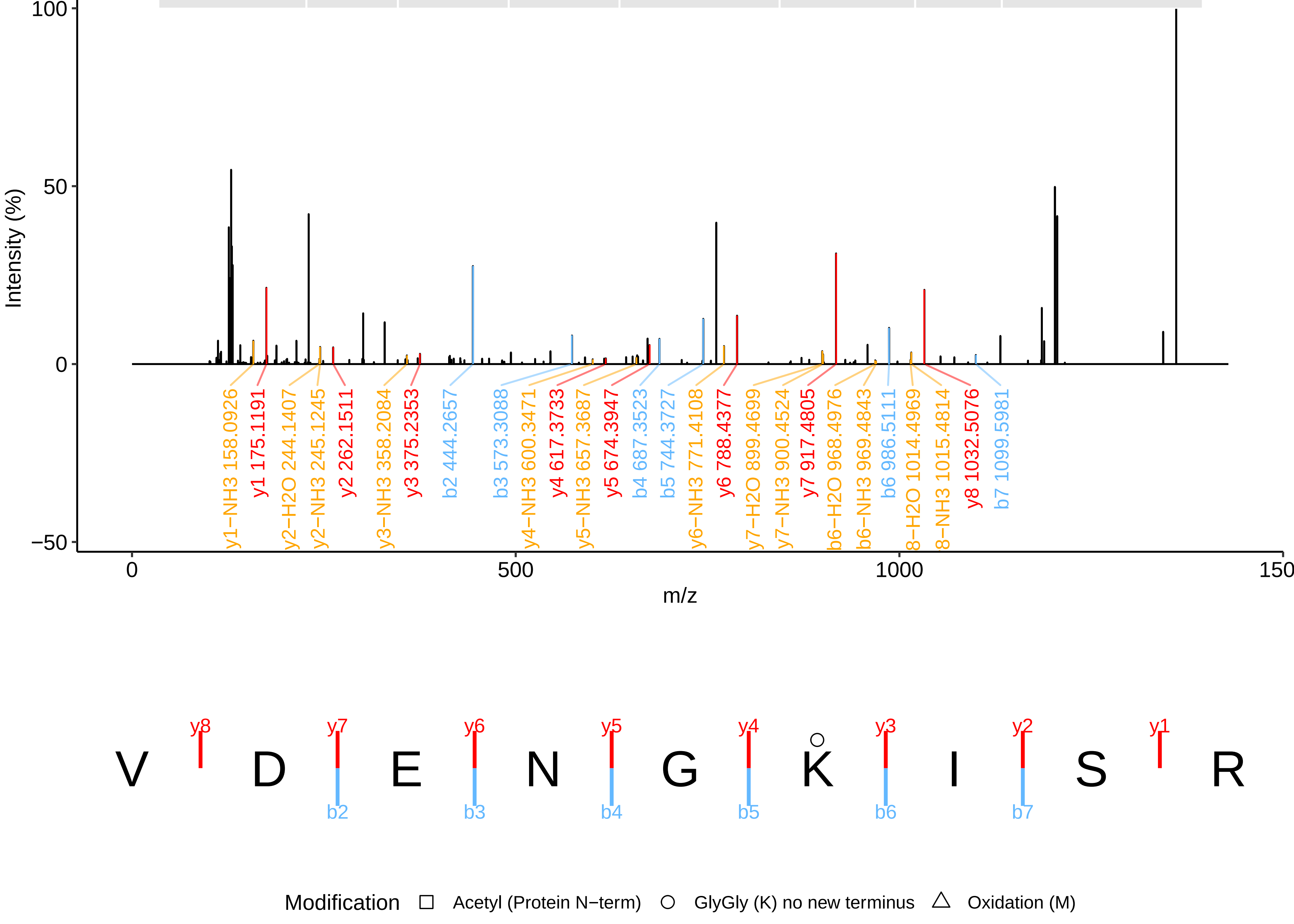

20200921_KIZ_MUb4_GlyGly_10plex − Scan number: 16568

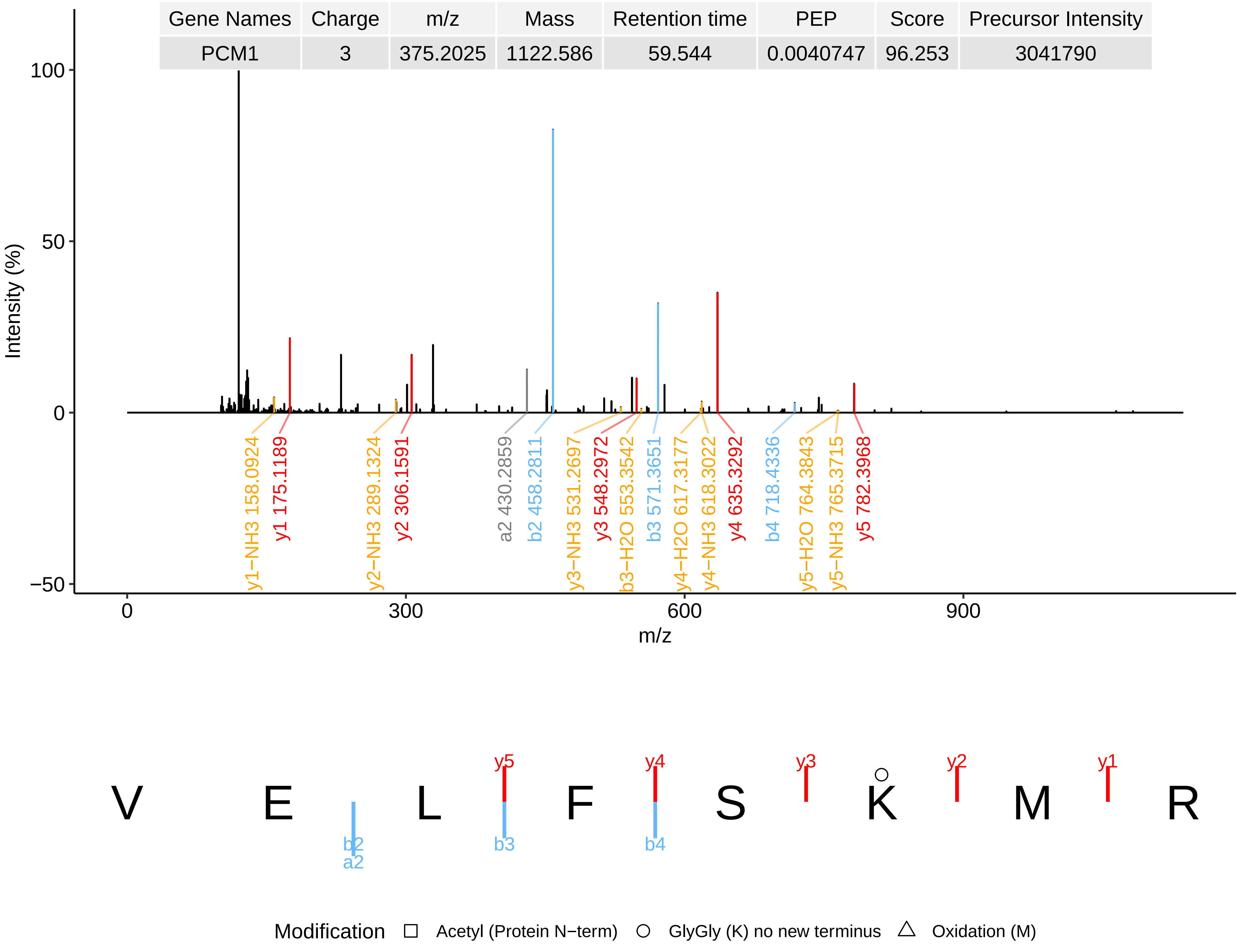

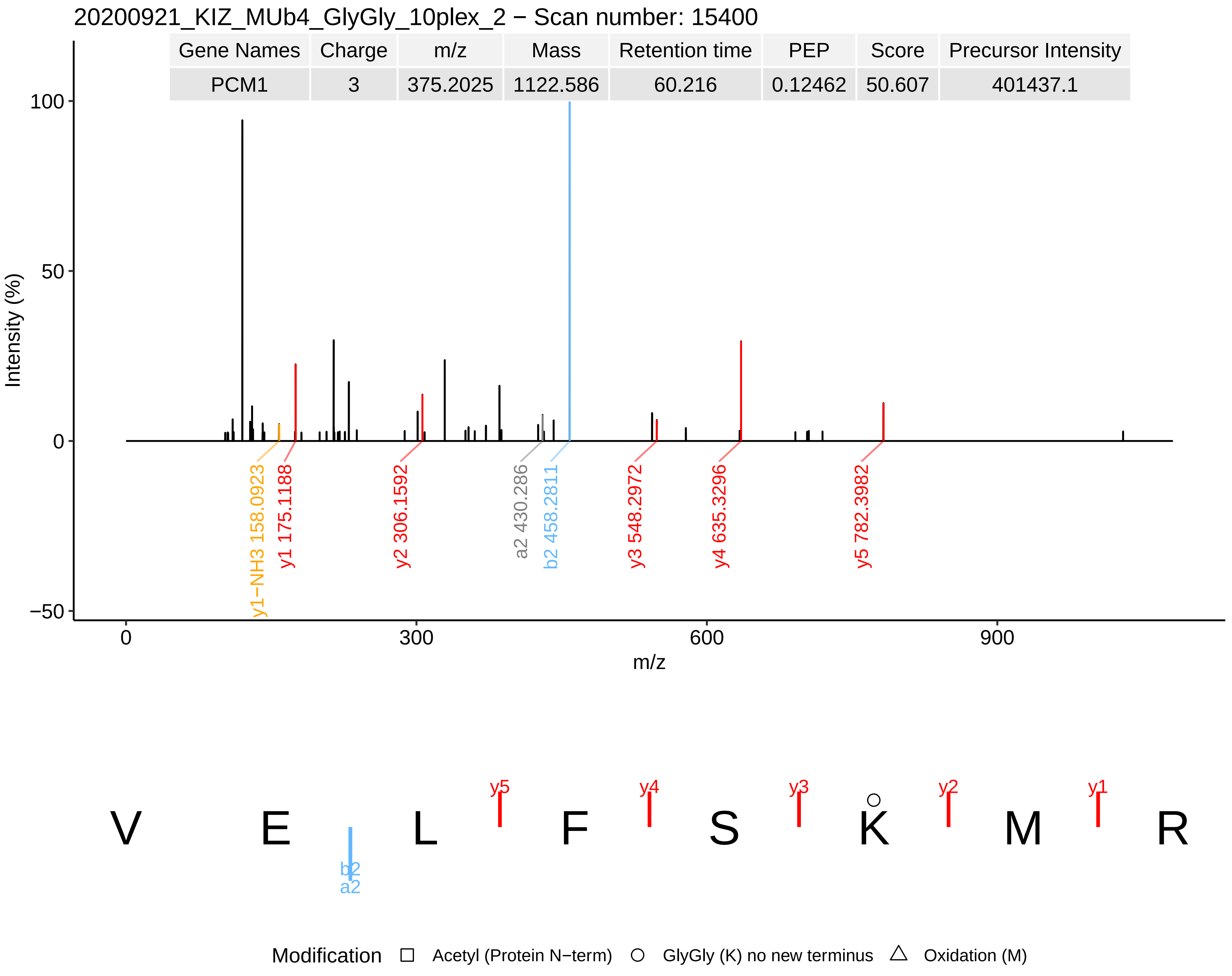

20200921_KIZ_MUb4_GlyGly_10plex_2 − Scan number: 15400
Gene Names | Charge | m/z | Mass | Retention time | PEP | Score | Precursor Intensity
PCM1 | 3 | 375.2025 | 1122.586 | 60.216 | 0.12462 | 50.607 | 401437.1
Intensity (%)
m/z
y1−NH3 158.0923
y1 175.1188
y2 306.1592
a2 430.286
b2 458.2811
y3 548.2972
y4 635.3296
y5 782.3982
V E L F S K M R
y5 y4 y3 y2 y1
b2 a2
Modification
Acetyl (Protein N−term)
GlyGly (K) no new terminus
Oxidation (M)

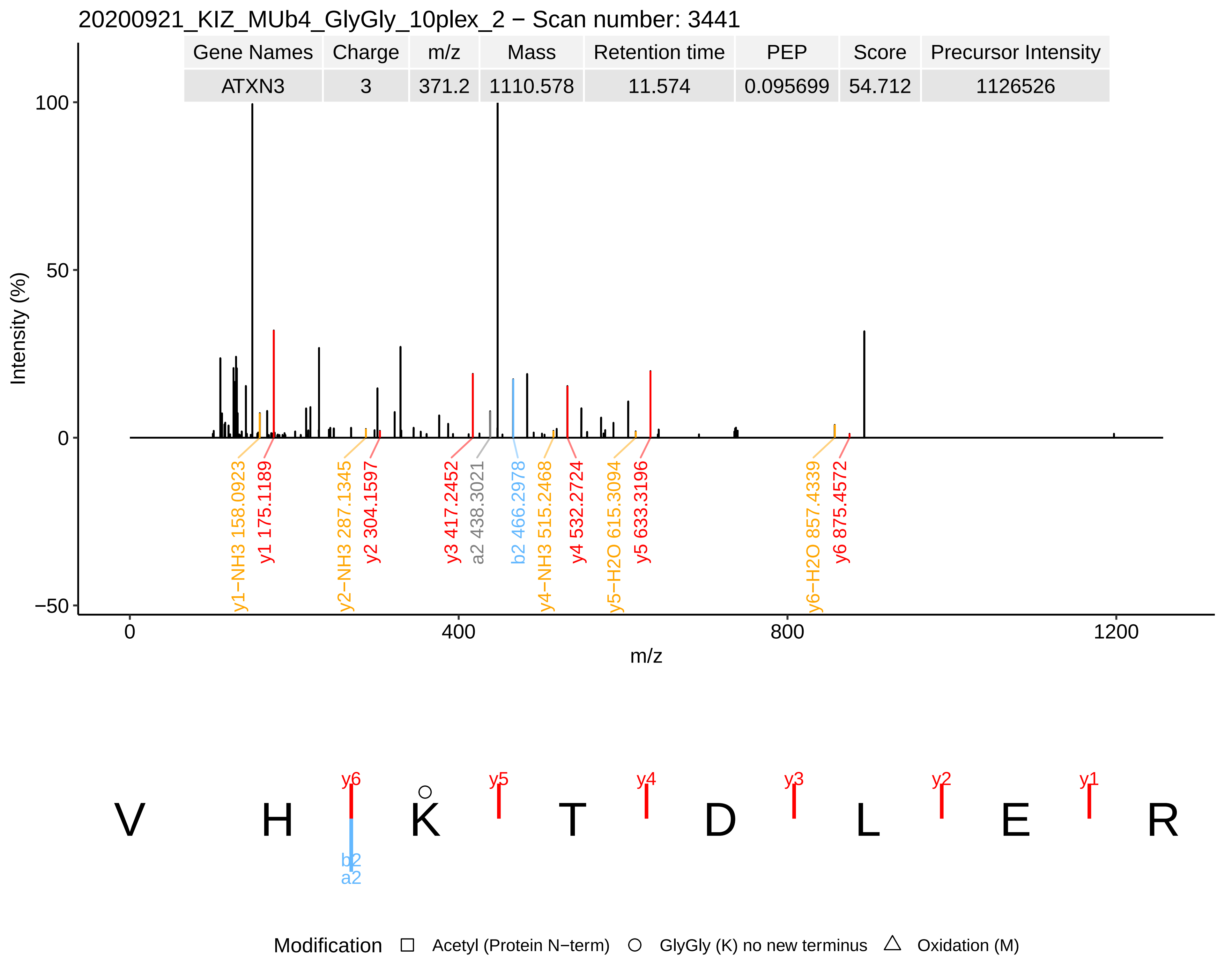
20200921_KIZ_MUb4_GlyGly_10plex_2 − Scan number: 3441
Gene Names
Charge
m/z
Mass
Retention time
PEP
Score
Precursor Intensity
ATXN3
3
371.2
1110.578
11.574
0.095699
54.712
1126526
Intensity (%)
100
50
0
−50
0
400
800
1200
m/z
y1−NH3 158.0923
y1 175.1189
y2−NH3 287.1345
y2 304.1597
y3 417.2452
a2 438.3021
b2 466.2978
y4−NH3 515.2468
y4 532.2724
y5−H2O 615.3094
y5 633.3196
y6−H2O 857.4339
y6 875.4572
V H K T D L E R
y6 y5 y4 y3 y2 y1
b2
a2
Modification
Acetyl (Protein N−term)
GlyGly (K) no new terminus
Oxidation (M)

20200921_KIZ_MUb4_GlyGly_10plex − Scan number: 15085

Gene Names	Charge	m/z	Mass	Retention time	PEP	Score	Precursor Intensity
PARK2	3	382.8925	1145.656	53.803	2.2076e−06	131.22	19926288

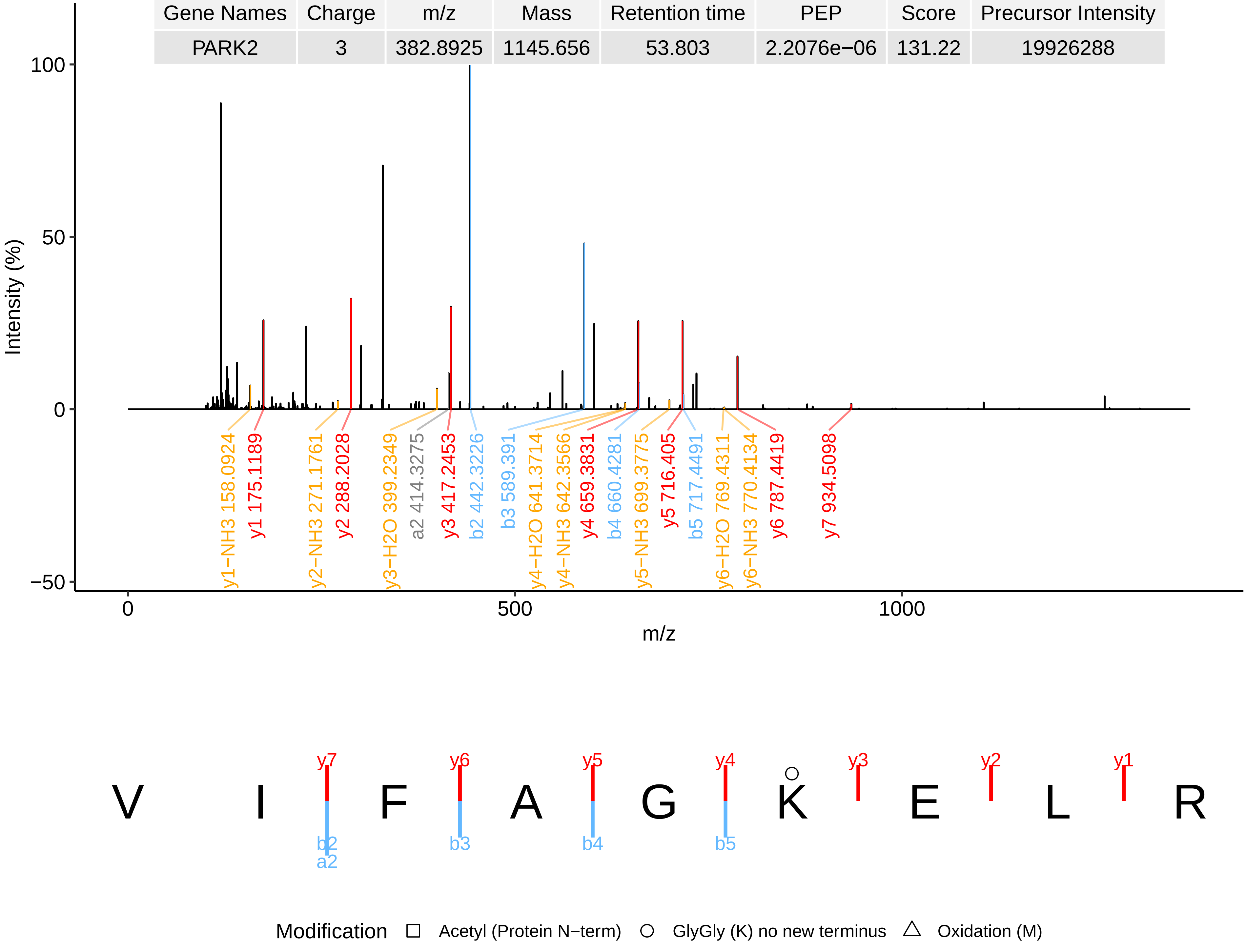

20200921_KIZ_MUb4_GlyGly_10plex_2 − Scan number: 13950

Gene Names	Charge	m/z	Mass	Retention time	PEP	Score	Precursor Intensity
PARK2	3	382.8925	1145.656	54.279	0.0026667	85.666	909318.4

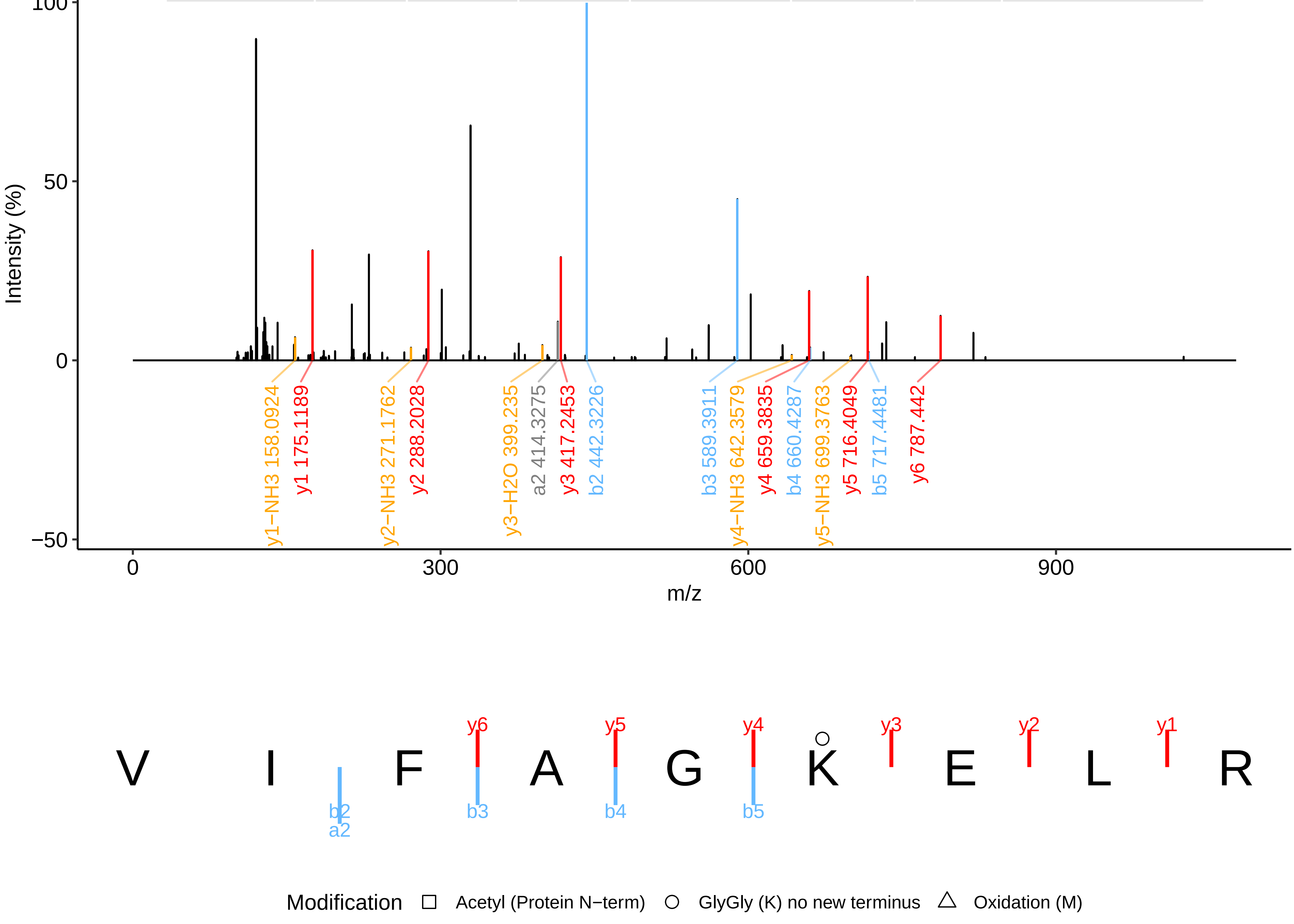

20200924_KIZ_MUb5_GlyGly_1 − Scan number: 13858

Gene Names	Charge	m/z	Mass	Retention time	PEP	Score	Precursor Intensity
PARK2	3	382.8925	1145.656	45.212	0.0011061	94.85	17119746

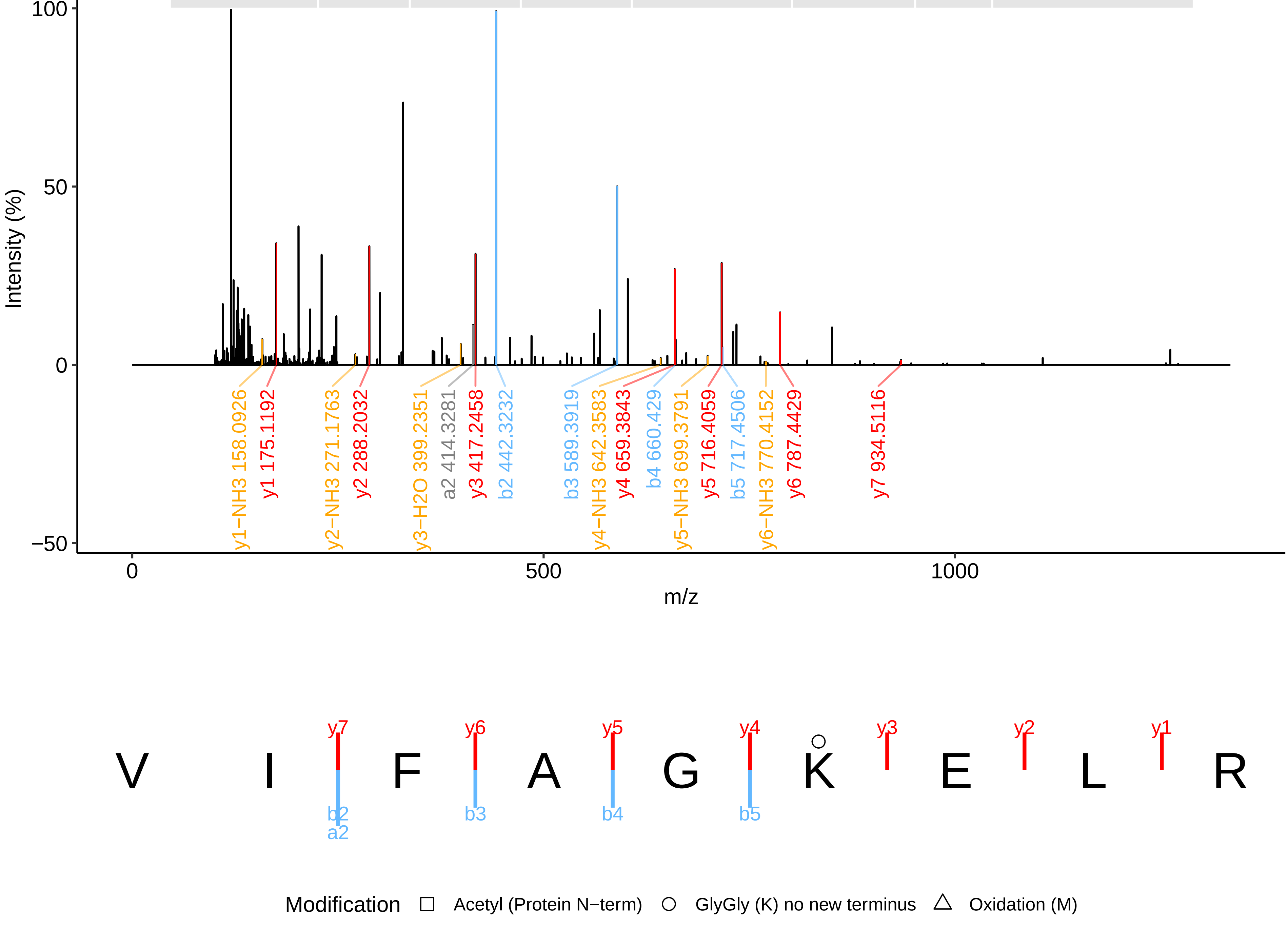

20200924_KIZ_MUb5_GlyGly_2 − Scan number: 13077

Gene Names	Charge	m/z	Mass	Retention time	PEP	Score	Precursor Intensity
PARK2	3	382.8925	1145.656	48.912	0.00037954	104.06	4911045

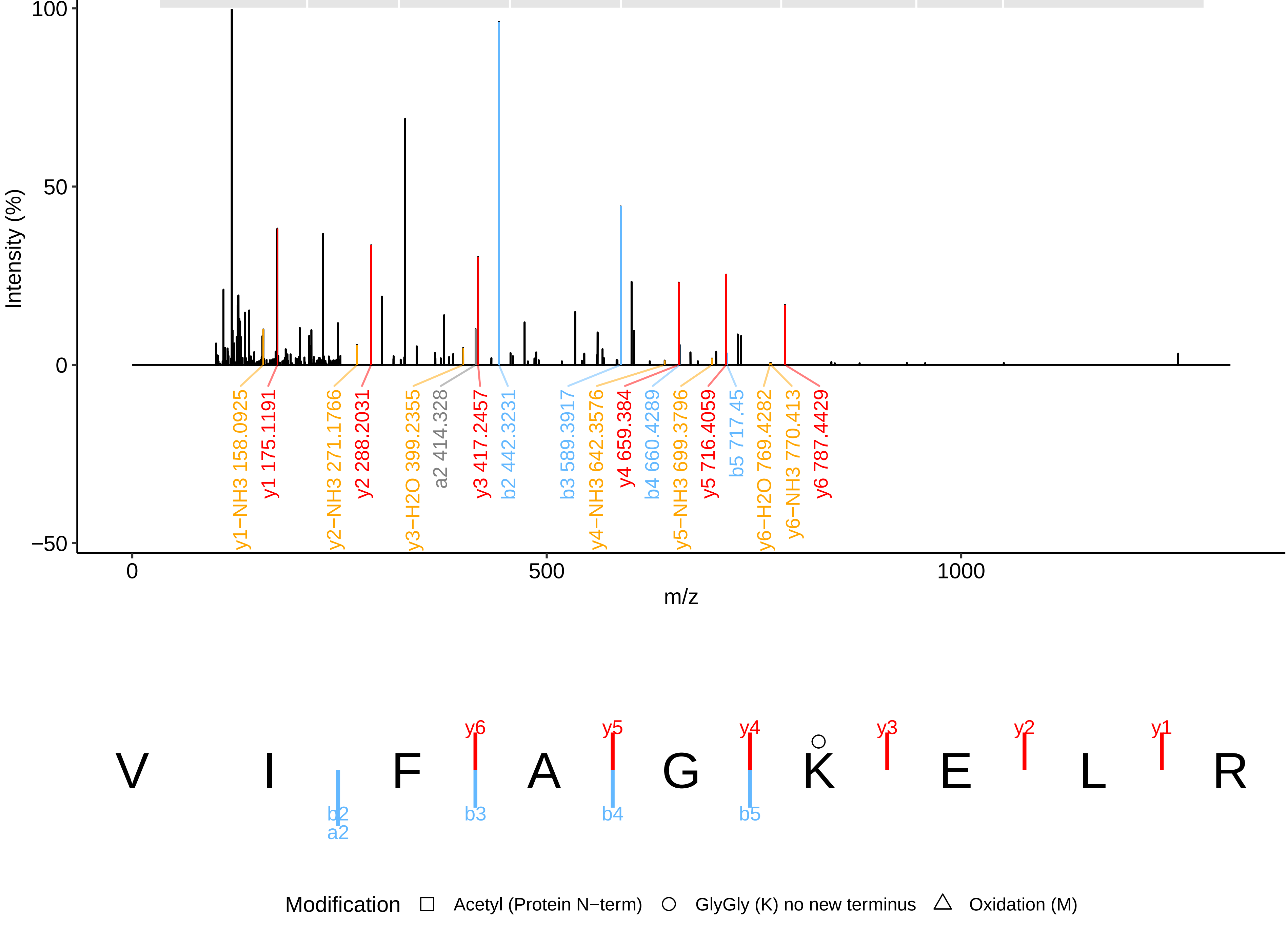

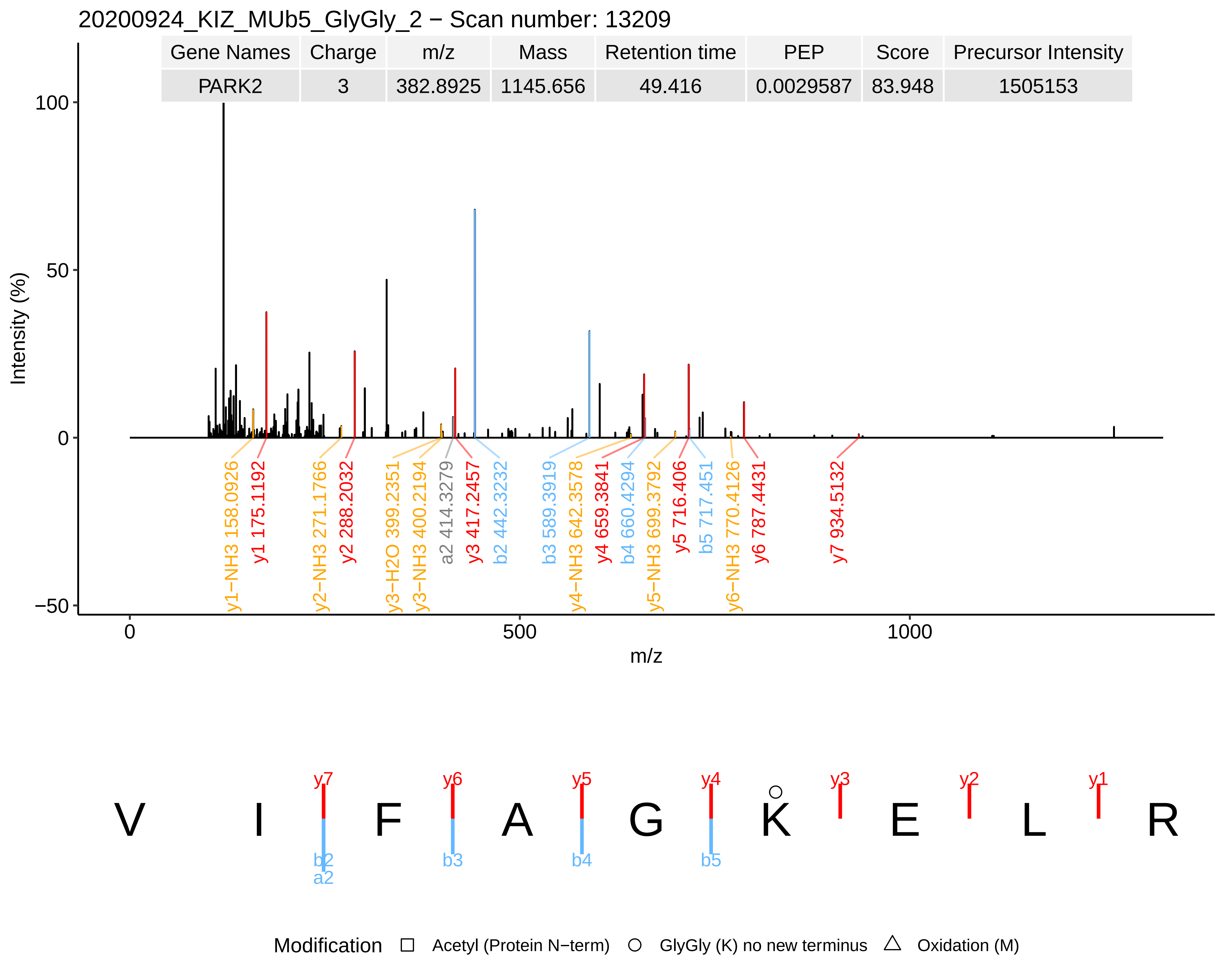
20200924_KIZ_MUb5_GlyGly_2 − Scan number: 13209
Gene Names | Charge | m/z | Mass | Retention time | PEP | Score | Precursor Intensity
PARK2 | 3 | 382.8925 | 1145.656 | 49.416 | 0.0029587 | 83.948 | 1505153
Intensity (%)
100
50
0
−50
0
500
1000
m/z
y1−NH3 158.0926
y1 175.1192
y2−NH3 271.1766
y2 288.2032
y3−H2O 399.2351
y3−NH3 400.2194
a2 414.3279
y3 417.2457
b2 442.3232
b3 589.3919
y4−NH3 642.3578
y4 659.3841
b4 660.4294
y5−NH3 699.3792
y5 716.406
b5 717.451
y6−NH3 770.4126
y6 787.4431
y7 934.5132
V I F A G K E L R
y7 y6 y5 y4 y3 y2 y1
b2 a2 b3 b4 b5
Modification
Acetyl (Protein N−term)
GlyGly (K) no new terminus
Oxidation (M)

20200921_KIZ_MUb4_GlyGly_10plex − Scan number: 10351

Gene Names	Charge	m/z	Mass	Retention time	PEP	Score	Precursor Intensity
RPL19	3	302.5116	904.5131	35.805	0.060264	64.803	9434750

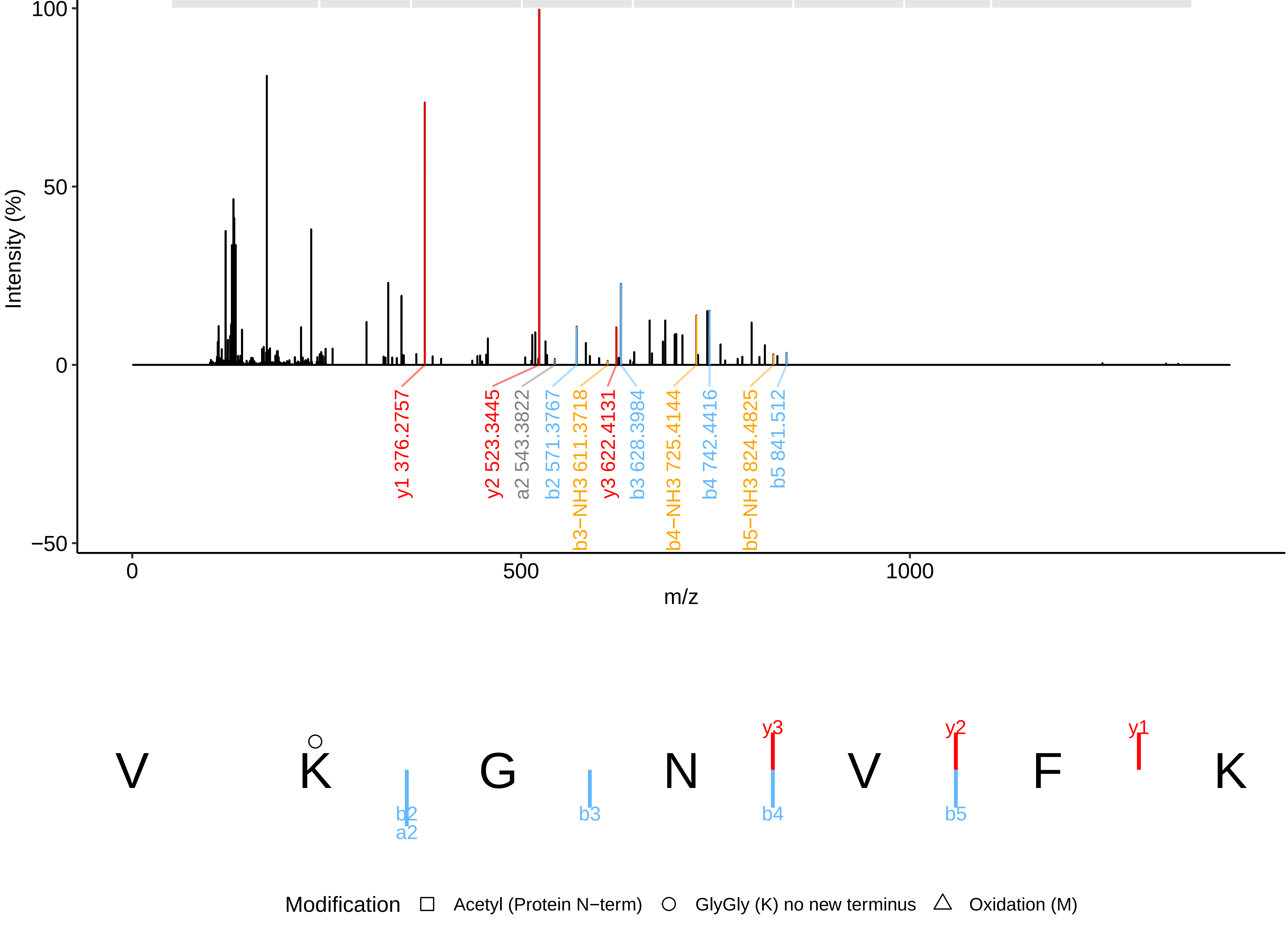

20201118_KIZ_MUb6_GlyGly_1 − Scan number: 8823

Gene Names	Charge	m/z	Mass	Retention time	PEP	Score	Precursor Intensity
RPL19	3	302.5116	904.5131	27.891	0.093552	57.281	9679400

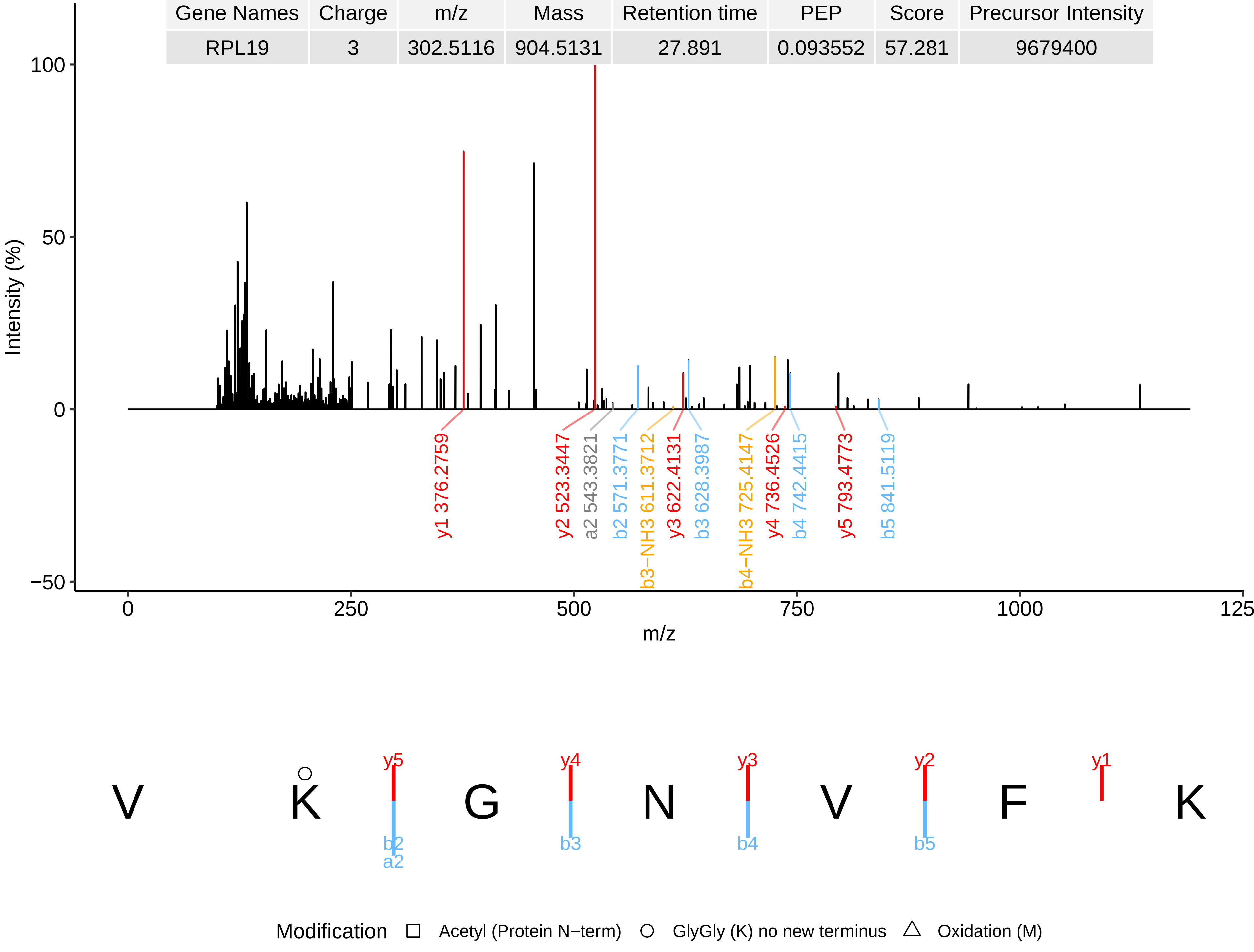

20200921_KIZ_MUb4_GlyGly_10plex − Scan number: 7591

Gene Names	Charge	m/z	Mass	Retention time	PEP	Score	Precursor Intensity
CCT7	3	367.1963	1098.567	25.533	0.04101	86.833	5702623

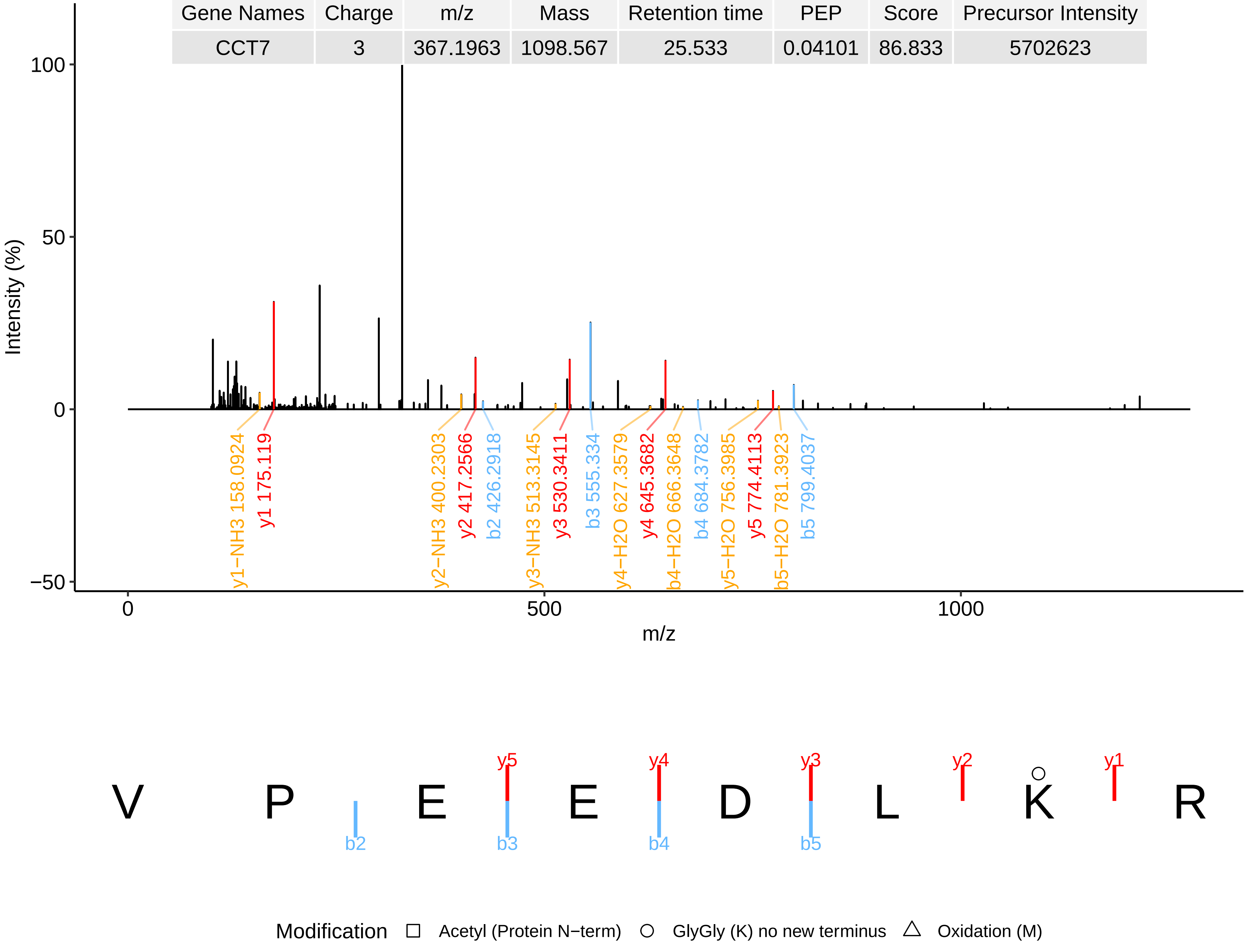

20200921_KIZ_MUb4_GlyGly_10plex − Scan number: 8755

Gene Names	Charge	m/z	Mass	Retention time	PEP	Score	Precursor Intensity
PTRF	3	372.5559	1114.646	29.809	0.00048976	100.57	5709122

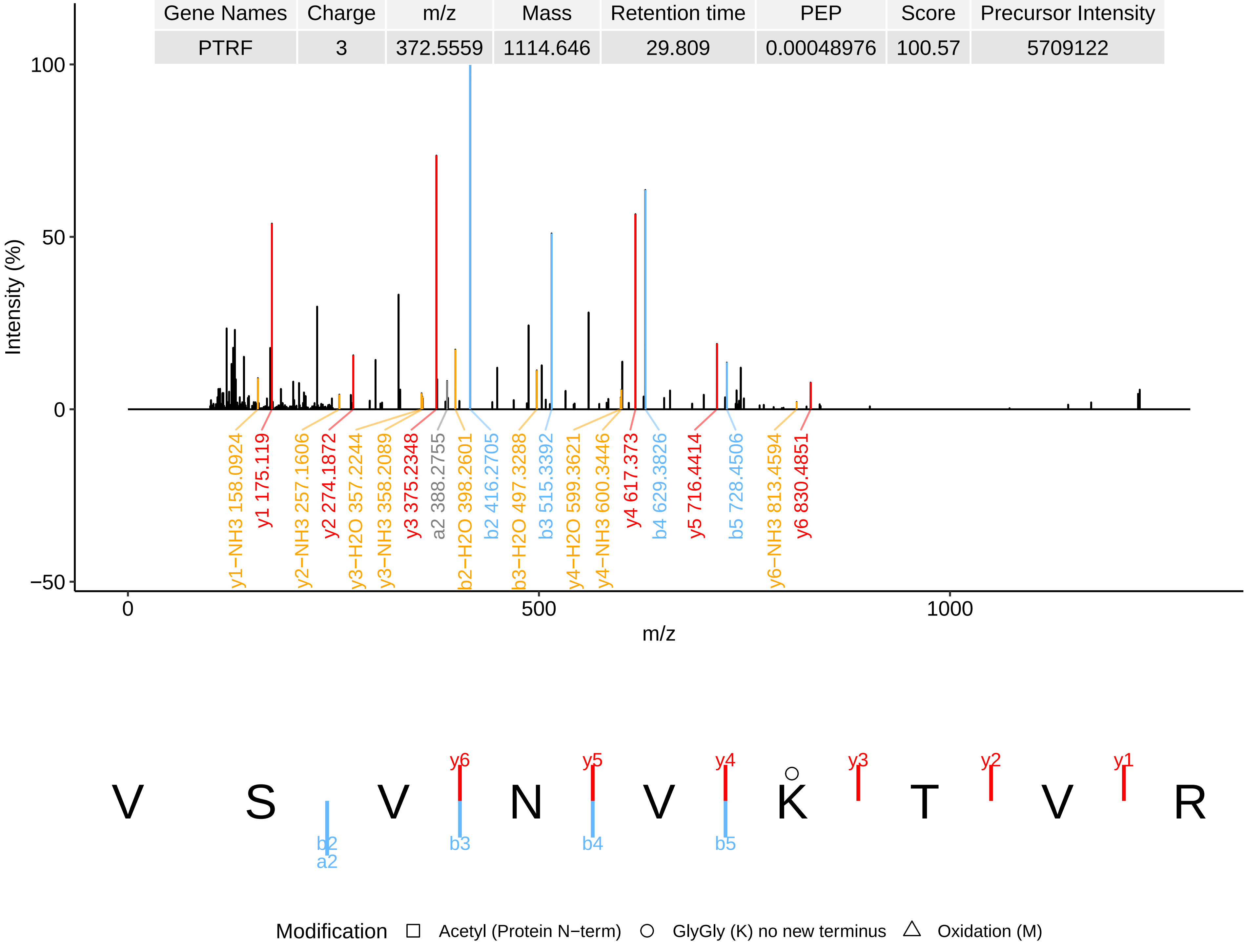

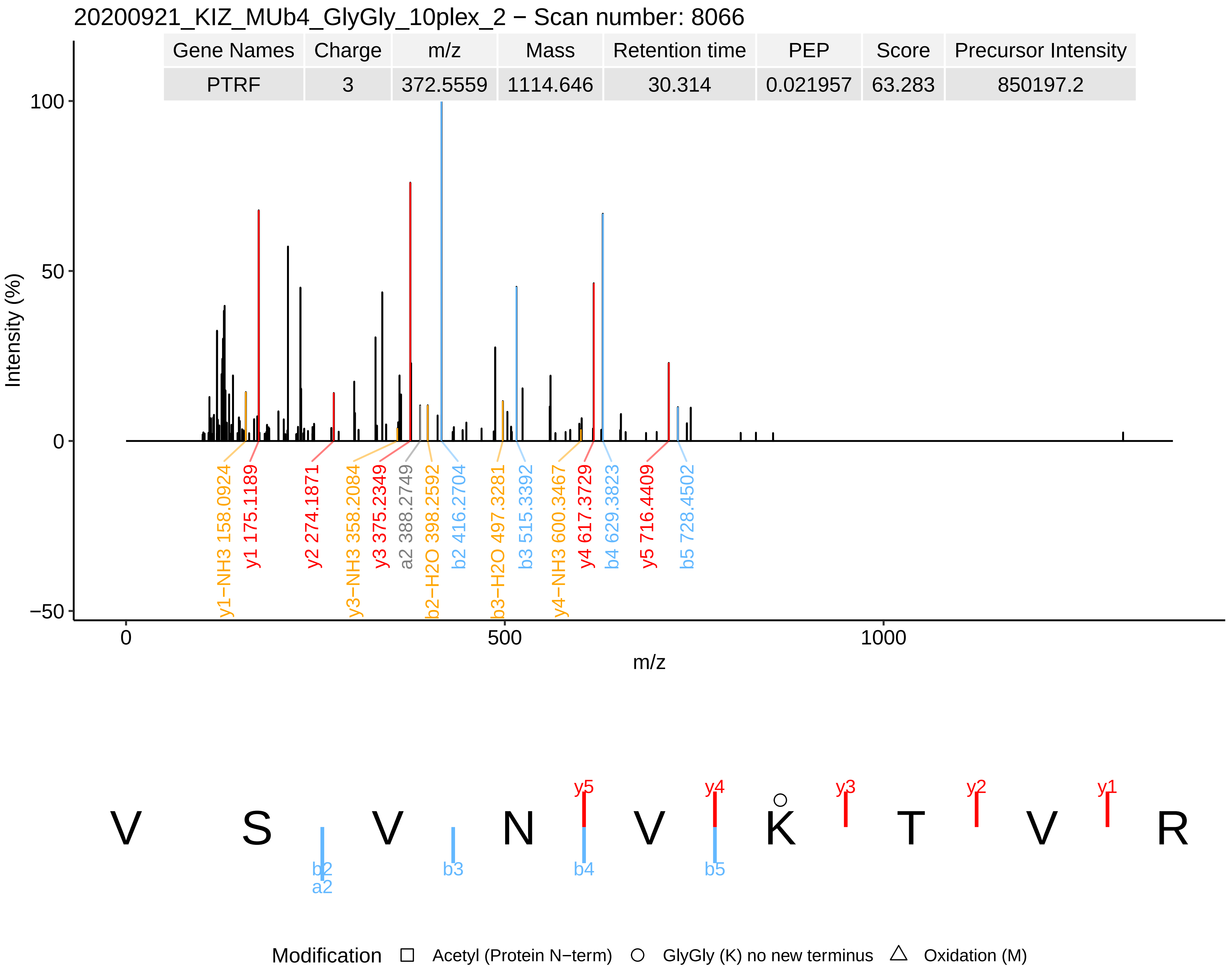
20200921_KIZ_MUb4_GlyGly_10plex_2 − Scan number: 8066

Gene Names	Charge	m/z	Mass	Retention time	PEP	Score	Precursor Intensity
PTRF	3	372.5559	1114.646	30.314	0.021957	63.283	850197.2

Intensity (%)
m/z
y1−NH3 158.0924
y1 175.1189
y2 274.1871
y3−NH3 358.2084
y3 375.2349
a2 388.2749
b2−H2O 398.2592
b2 416.2704
b3−H2O 497.3281
b3 515.3392
y4−NH3 600.3467
y4 617.3729
b4 629.3823
y5 716.4409
b5 728.4502
V S V N V K T V R
y5 y4 y3 y2 y1
b2 a2 b3 b4 b5
Modification □ Acetyl (Protein N−term) ○ GlyGly (K) no new terminus △ Oxidation (M)

20200924_KIZ_MUb5_GlyGly_1 − Scan number: 7752

Gene Names	Charge	m/z	Mass	Retention time	PEP	Score	Precursor Intensity
PTRF	3	372.5559	1114.646	24.682	0.0069362	79.974	8972879

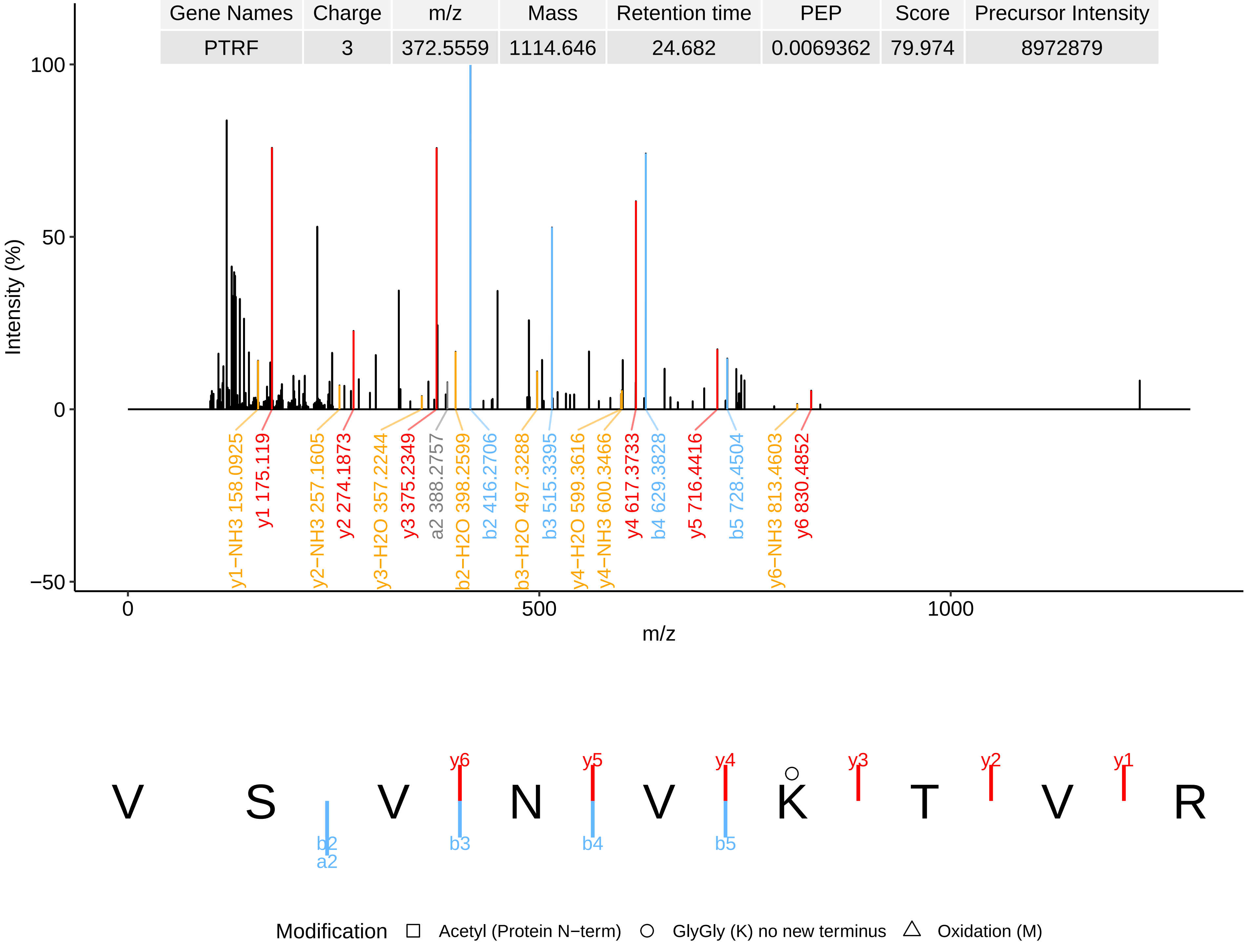

20200924_KIZ_MUb5_GlyGly_2 − Scan number: 7121

Gene Names	Charge	m/z	Mass	Retention time	PEP	Score	Precursor Intensity
PTRF	3	372.5559	1114.646	26.547	0.0046435	87.258	4145500

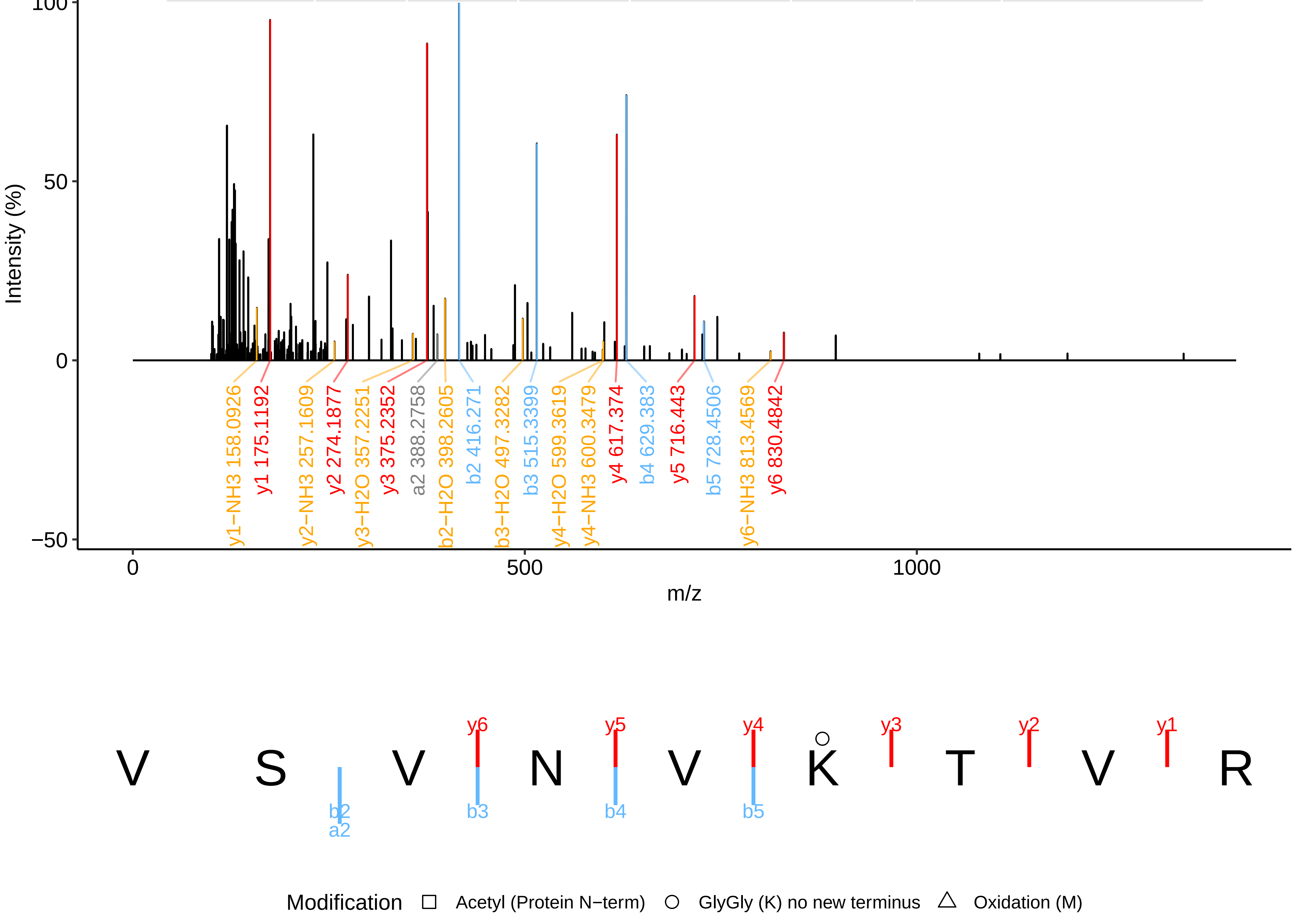

20201118_KIZ_MUb6_GlyGly_1 − Scan number: 7374

Gene Names	Charge	m/z	Mass	Retention time	PEP	Score	Precursor Intensity
PTRF	3	372.5559	1114.646	23.374	0.0063657	81.263	5986451

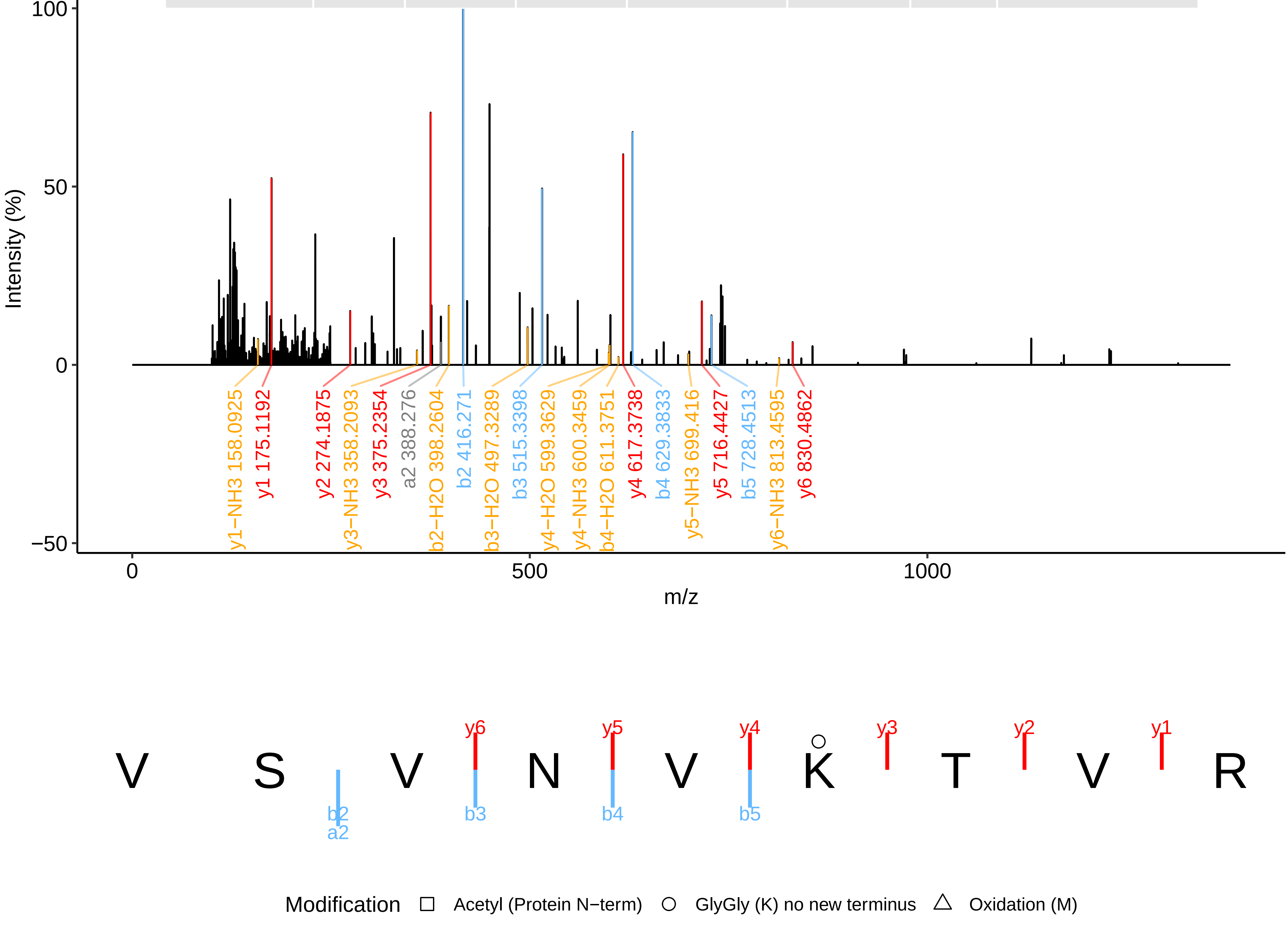

20200924_KIZ_MUb5_GlyGly_1 − Scan number: 6283

Gene Names	Charge	m/z	Mass	Retention time	PEP	Score	Precursor Intensity
KPNA2	3	317.8347	950.4821	19.726	0.12427	55.676	4847067

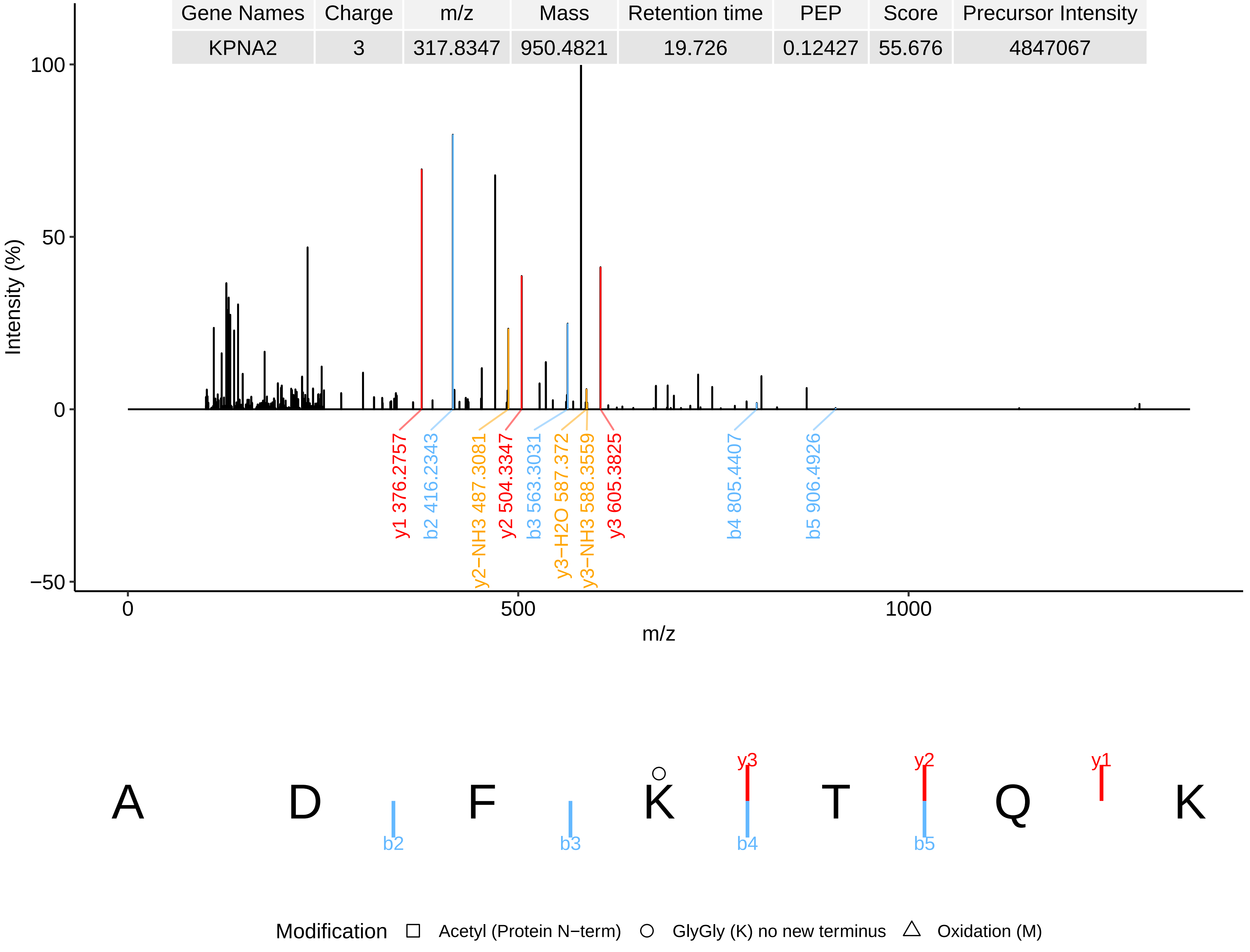

20200921_KIZ_MUb4_GlyGly_10plex_2 − Scan number: 2551

Gene Names	Charge	m/z	Mass	Retention time	PEP	Score	Precursor Intensity
GAPDH	4	295.664	1178.627	8.0797	0.0536	51.445	1163327

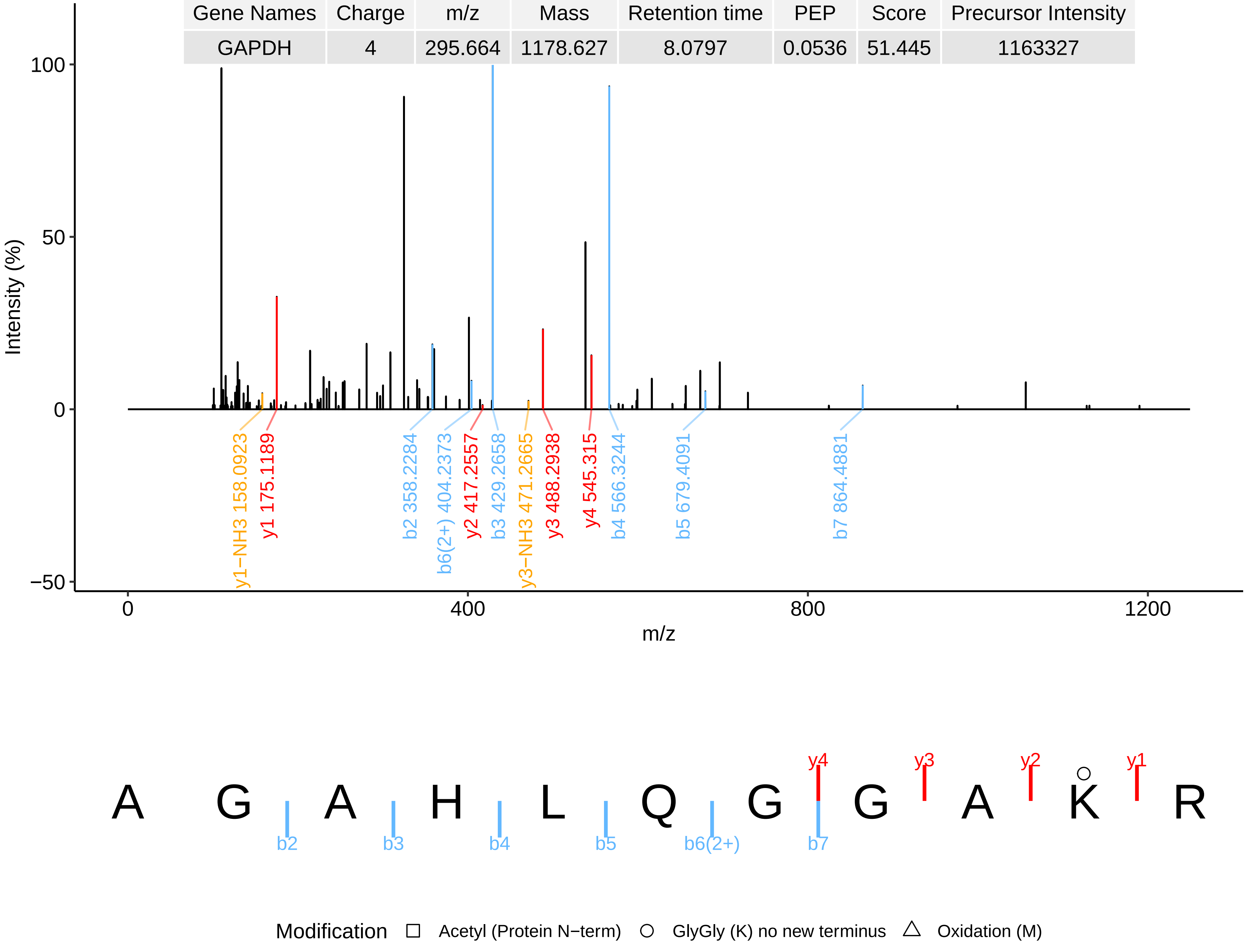

20201118_KIZ_MUb6_GlyGly_2 − Scan number: 2806

Gene Names	Charge	m/z	Mass	Retention time	PEP	Score	Precursor Intensity
GAPDH	4	295.664	1178.627	8.9366	0.016321	61.765	2867672

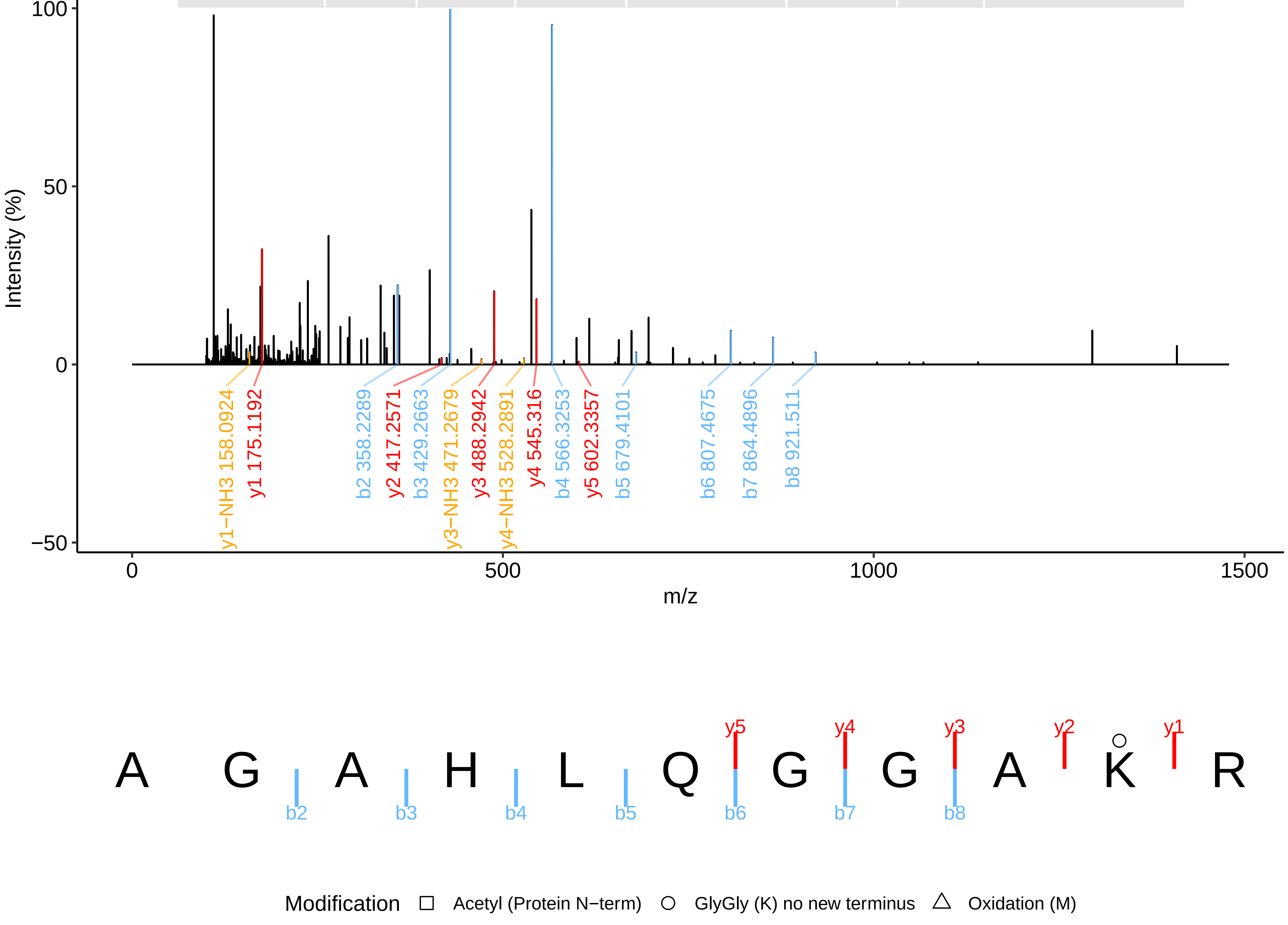

20200921_KIZ_MUb4_GlyGly_10plex − Scan number: 5377

Gene Names	Charge	m/z	Mass	Retention time	PEP	Score	Precursor Intensity
UQCRB	2	580.3069	1158.599	17.6	0.00020349	122.13	8064290

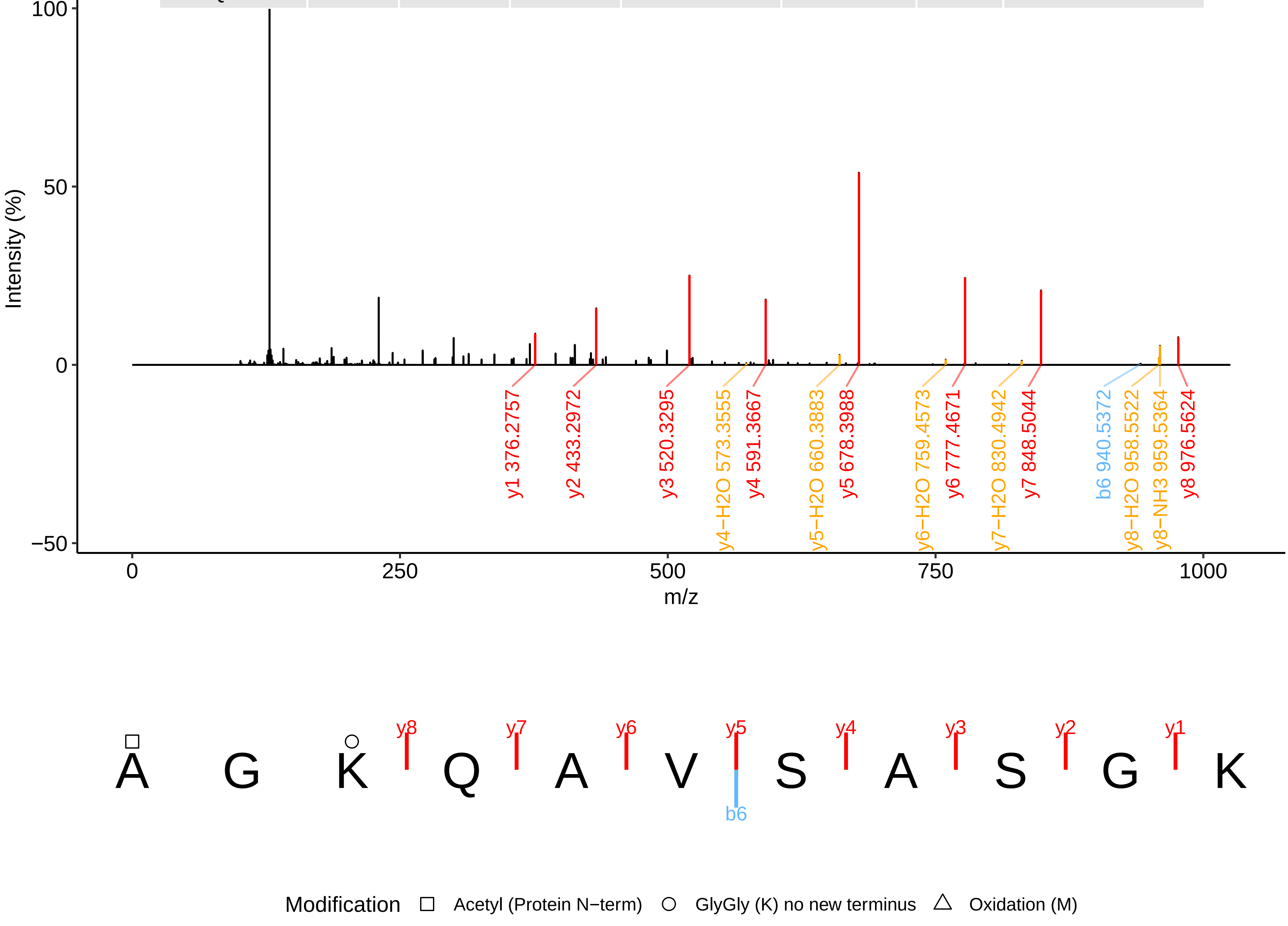

20200921_KIZ_MUb4_GlyGly_10plex_2 − Scan number: 4899

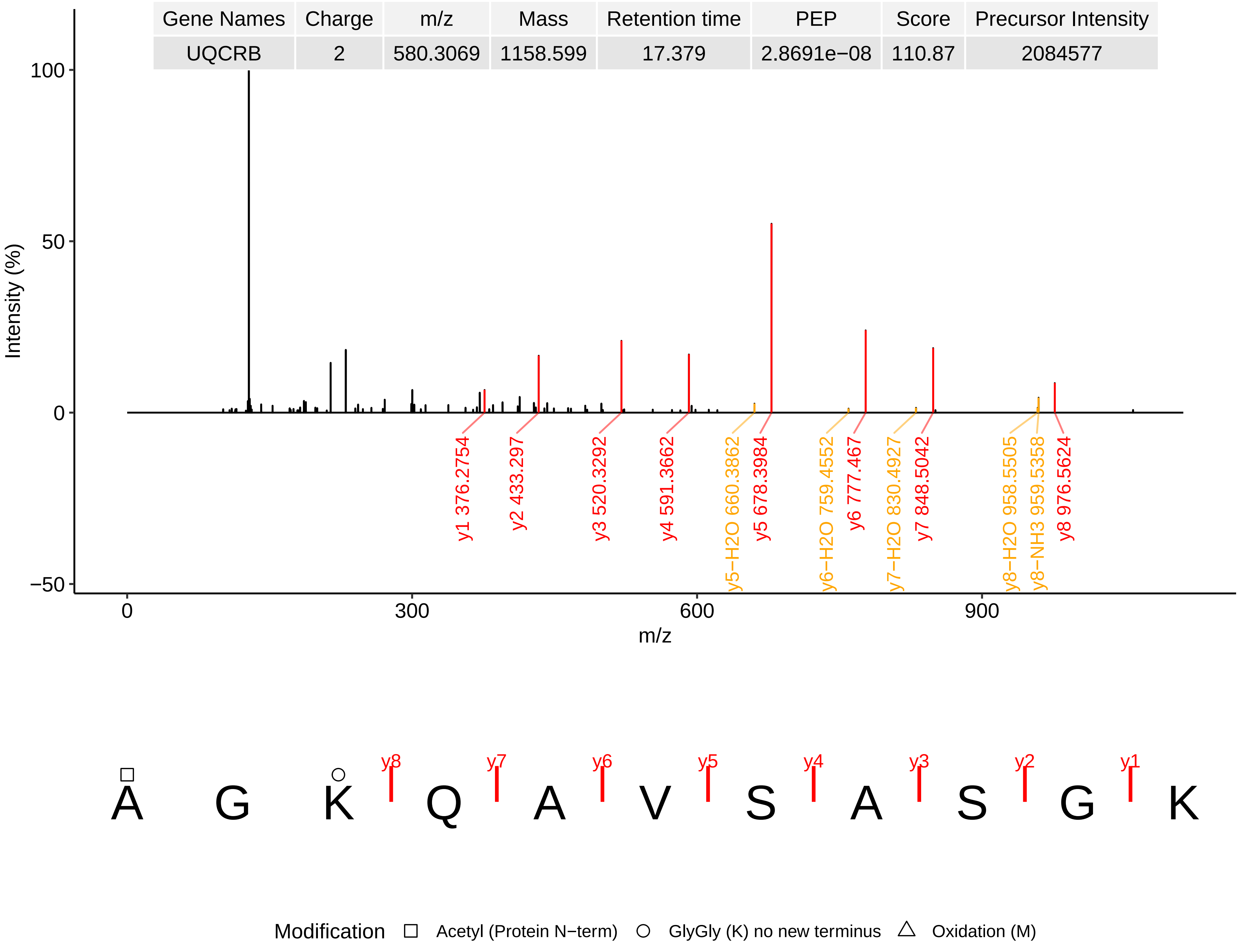

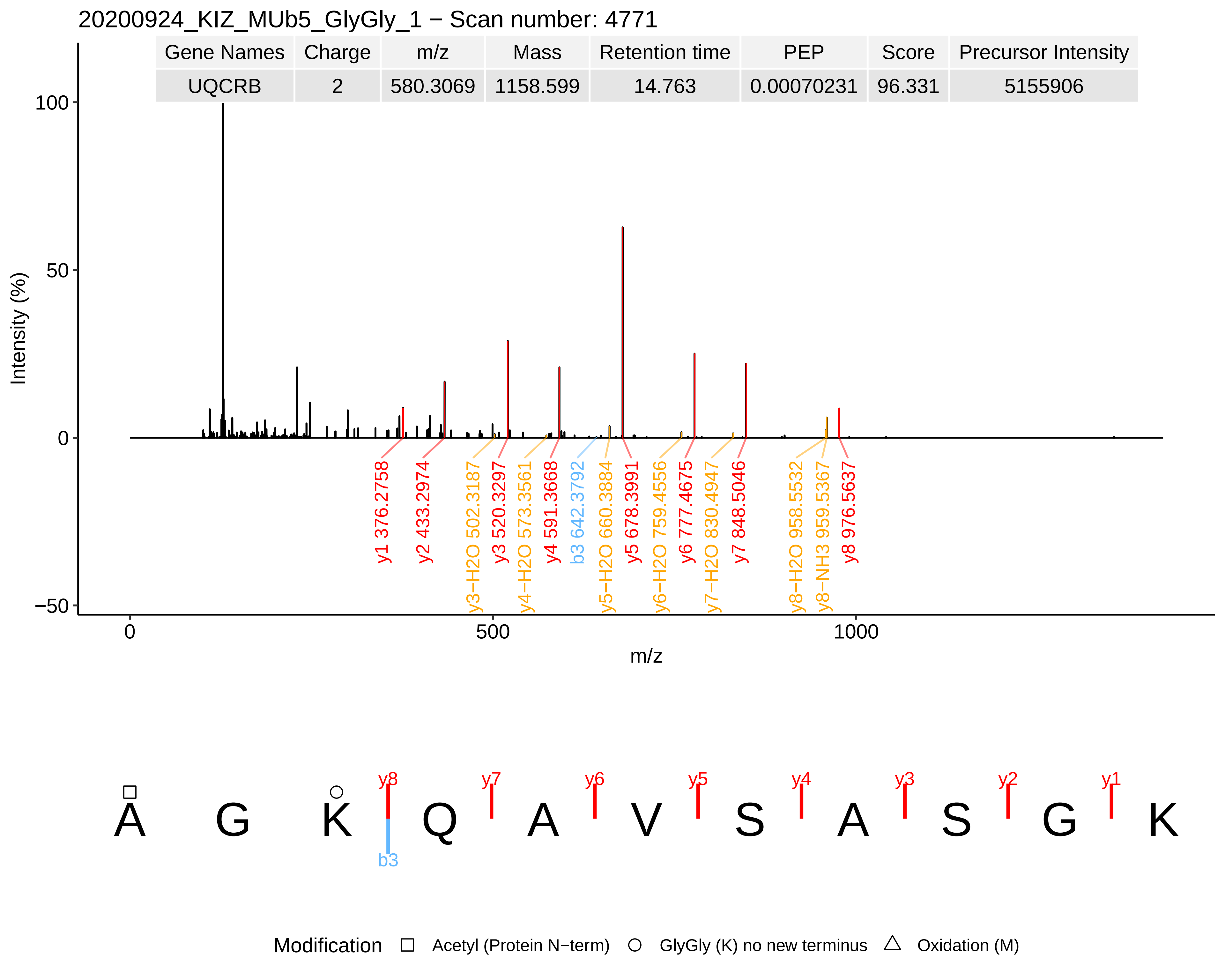

20200924_KIZ_MUb5_GlyGly_1 − Scan number: 4771
Gene Names | Charge | m/z | Mass | Retention time | PEP | Score | Precursor Intensity
UQCRB | 2 | 580.3069 | 1158.599 | 14.763 | 0.00070231 | 96.331 | 5155906
Intensity (%)
m/z
y1 376.2758
y2 433.2974
y3−H2O 502.3187
y3 520.3297
y4−H2O 573.3561
y4 591.3668
b3 642.3792
y5−H2O 660.3884
y5 678.3991
y6−H2O 759.4556
y6 777.4675
y7−H2O 830.4947
y7 848.5046
y8−H2O 958.5532
y8−NH3 959.5367
y8 976.5637
A G K Q A V S A S G K
y8 y7 y6 y5 y4 y3 y2 y1
b3
Modification
Acetyl (Protein N−term)
GlyGly (K) no new terminus
Oxidation (M)

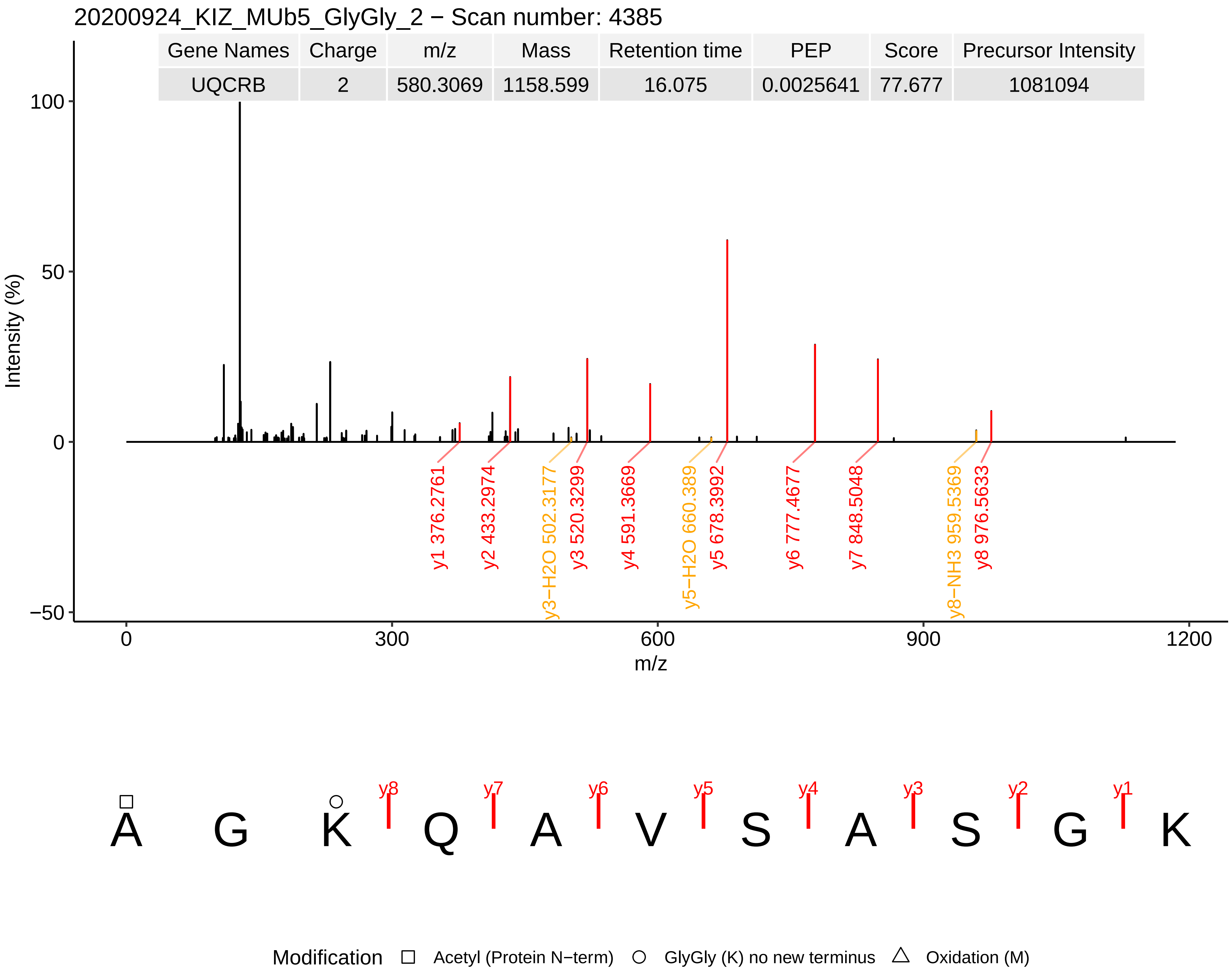
20200924_KIZ_MUb5_GlyGly_2 − Scan number: 4385
Gene Names | Charge | m/z | Mass | Retention time | PEP | Score | Precursor Intensity
UQCRB | 2 | 580.3069 | 1158.599 | 16.075 | 0.0025641 | 77.677 | 1081094
Intensity (%)
m/z
y1 376.2761
y2 433.2974
y3−H2O 502.3177
y3 520.3299
y4 591.3669
y5−H2O 660.389
y5 678.3992
y6 777.4677
y7 848.5048
y8−NH3 959.5369
y8 976.5633
A G K Q A V S A S G K
y8 y7 y6 y5 y4 y3 y2 y1
Modification
Acetyl (Protein N−term)
GlyGly (K) no new terminus
Oxidation (M)

20201118_KIZ_MUb6_GlyGly_2 − Scan number: 5137

Gene Names	Charge	m/z	Mass	Retention time	PEP	Score	Precursor Intensity
UQCRB	2	580.3069	1158.599	17.702	1.5235e−05	98.033	2454458

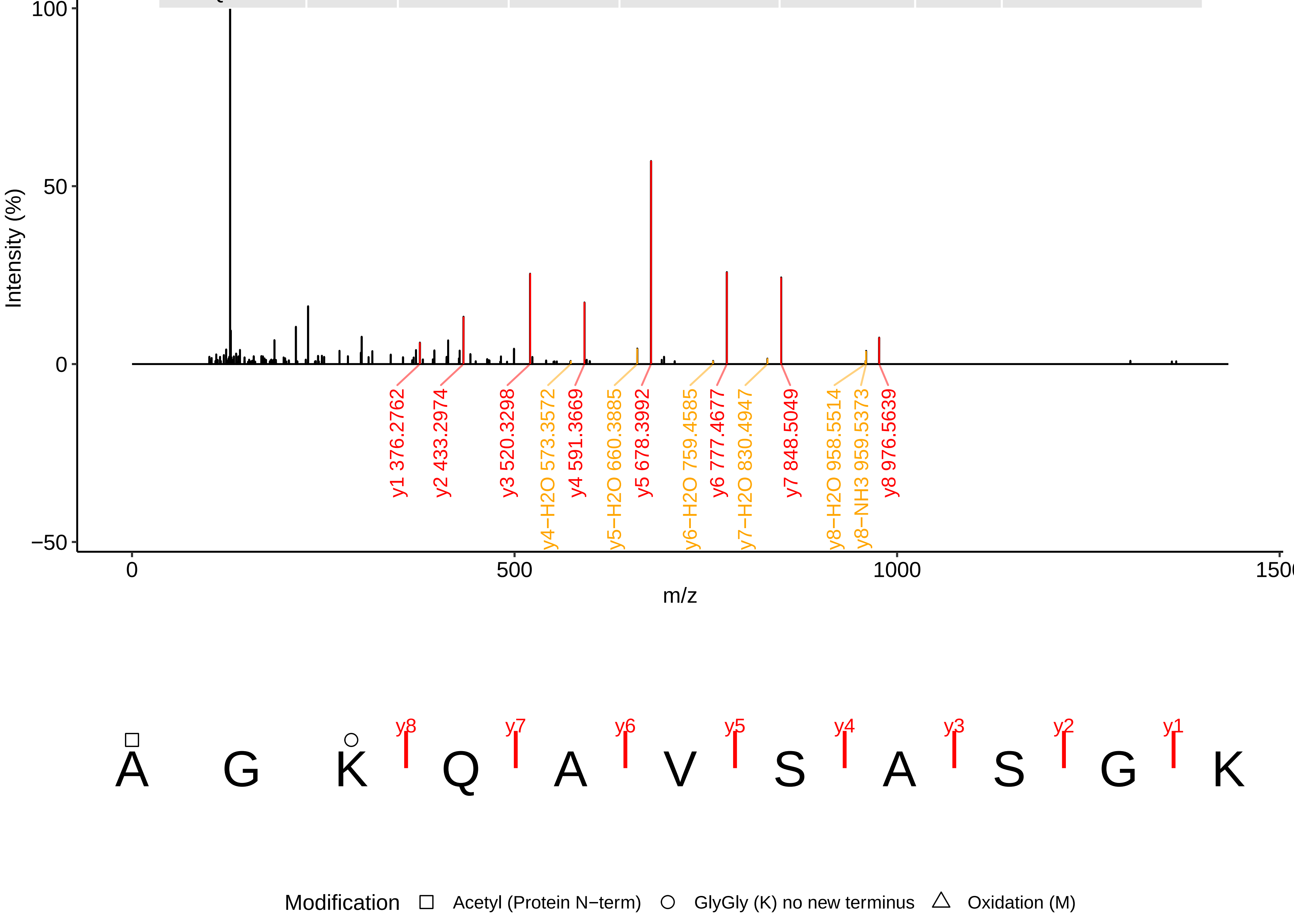

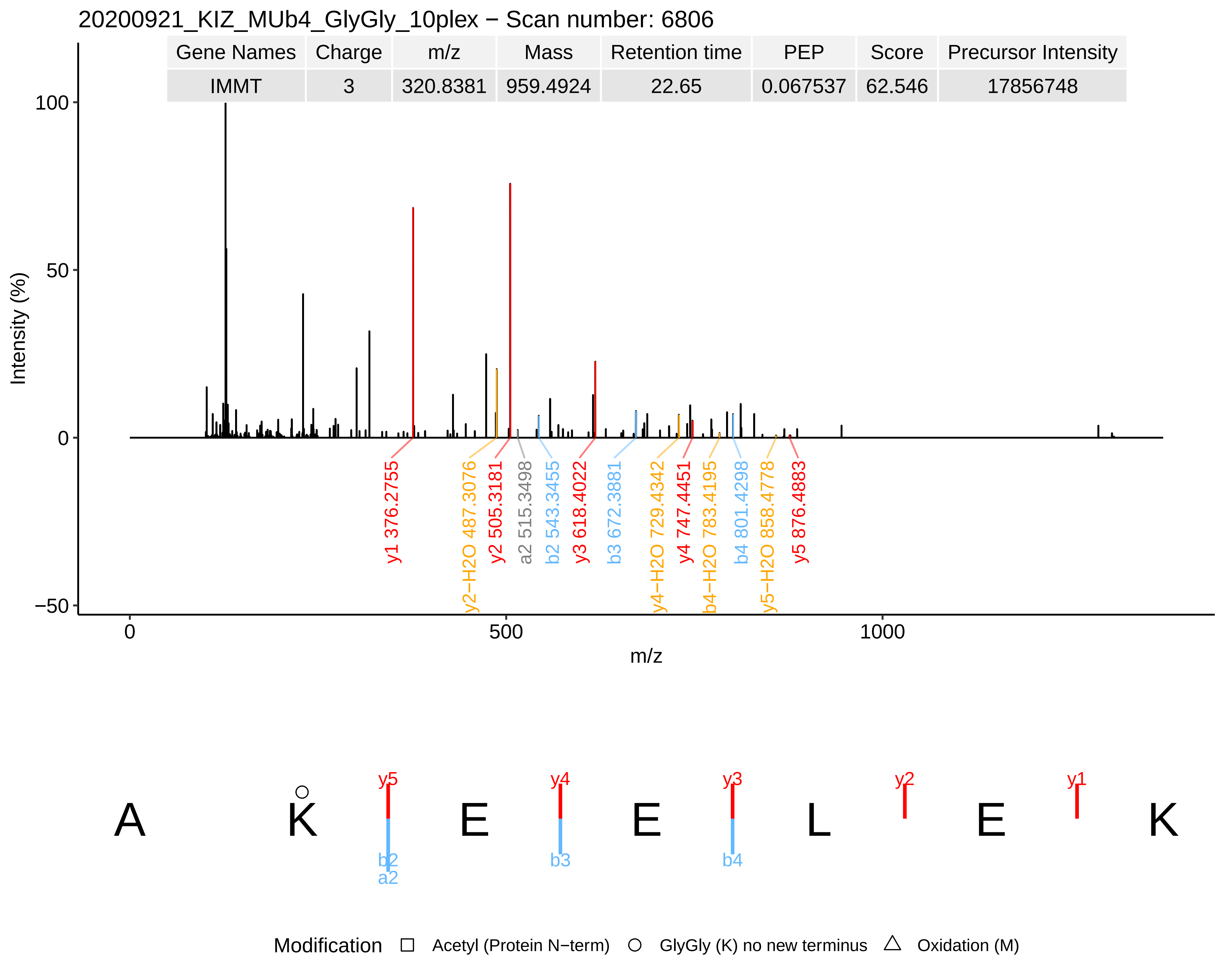

20200921_KIZ_MUb4_GlyGly_10plex − Scan number: 6806
Gene Names | Charge | m/z | Mass | Retention time | PEP | Score | Precursor Intensity
IMMT | 3 | 320.8381 | 959.4924 | 22.65 | 0.067537 | 62.546 | 17856748
Intensity (%)
m/z
y1 376.2755
y2−H2O 487.3076
y2 505.3181
a2 515.3498
b2 543.3455
y3 618.4022
b3 672.3881
y4−H2O 729.4342
y4 747.4451
b4−H2O 783.4195
b4 801.4298
y5−H2O 858.4778
y5 876.4883
A K E E L E K
y5 y4 y3 y2 y1
b2 a2 b3 b4
Modification
Acetyl (Protein N−term)
GlyGly (K) no new terminus
Oxidation (M)

20200921_KIZ_MUb4_GlyGly_10plex_2 − Scan number: 6314

Gene Names	Charge	m/z	Mass	Retention time	PEP	Score	Precursor Intensity
IMMT	3	320.8381	959.4924	23.148	0.11502	52.937	3042121

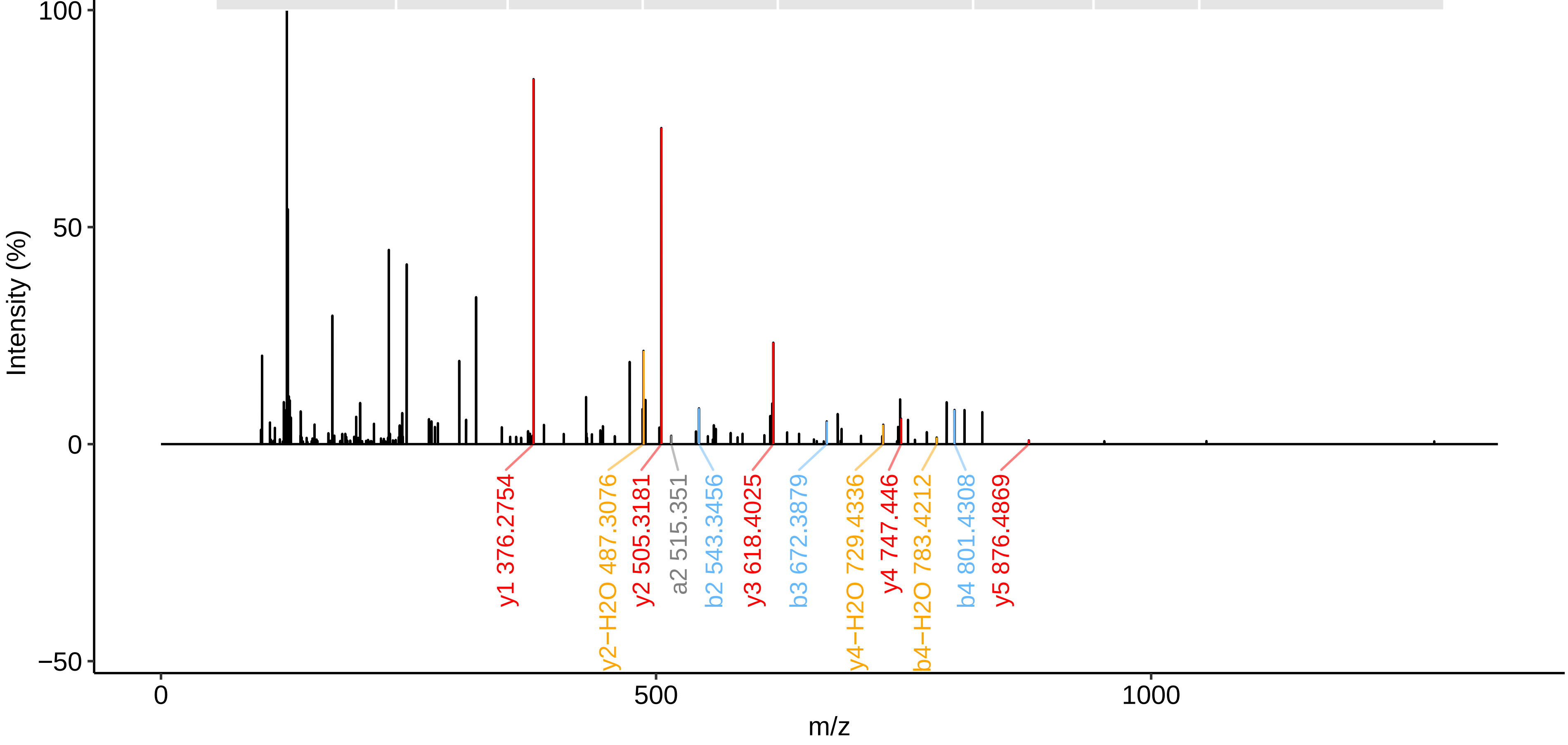

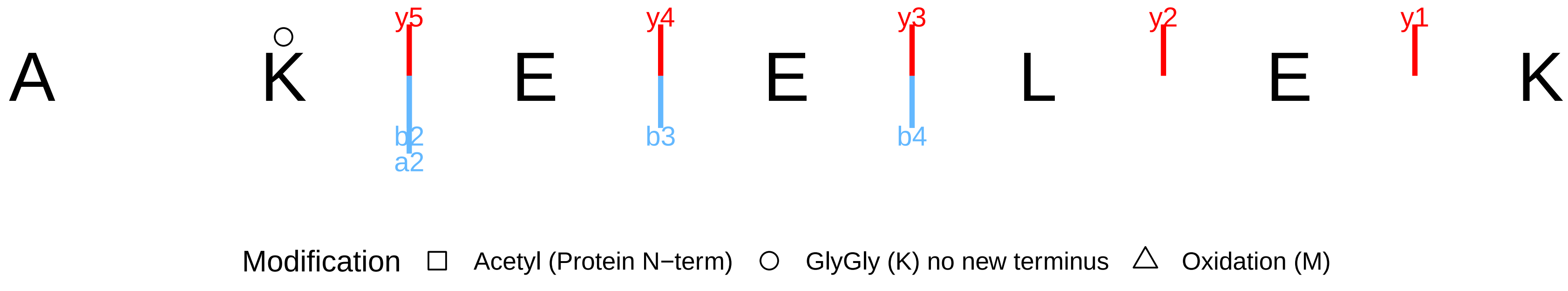

20200924_KIZ_MUb5_GlyGly_1 − Scan number: 5785

Gene Names	Charge	m/z	Mass	Retention time	PEP	Score	Precursor Intensity
IMMT	3	320.8381	959.4924	18.065	0.083848	59.245	18458288

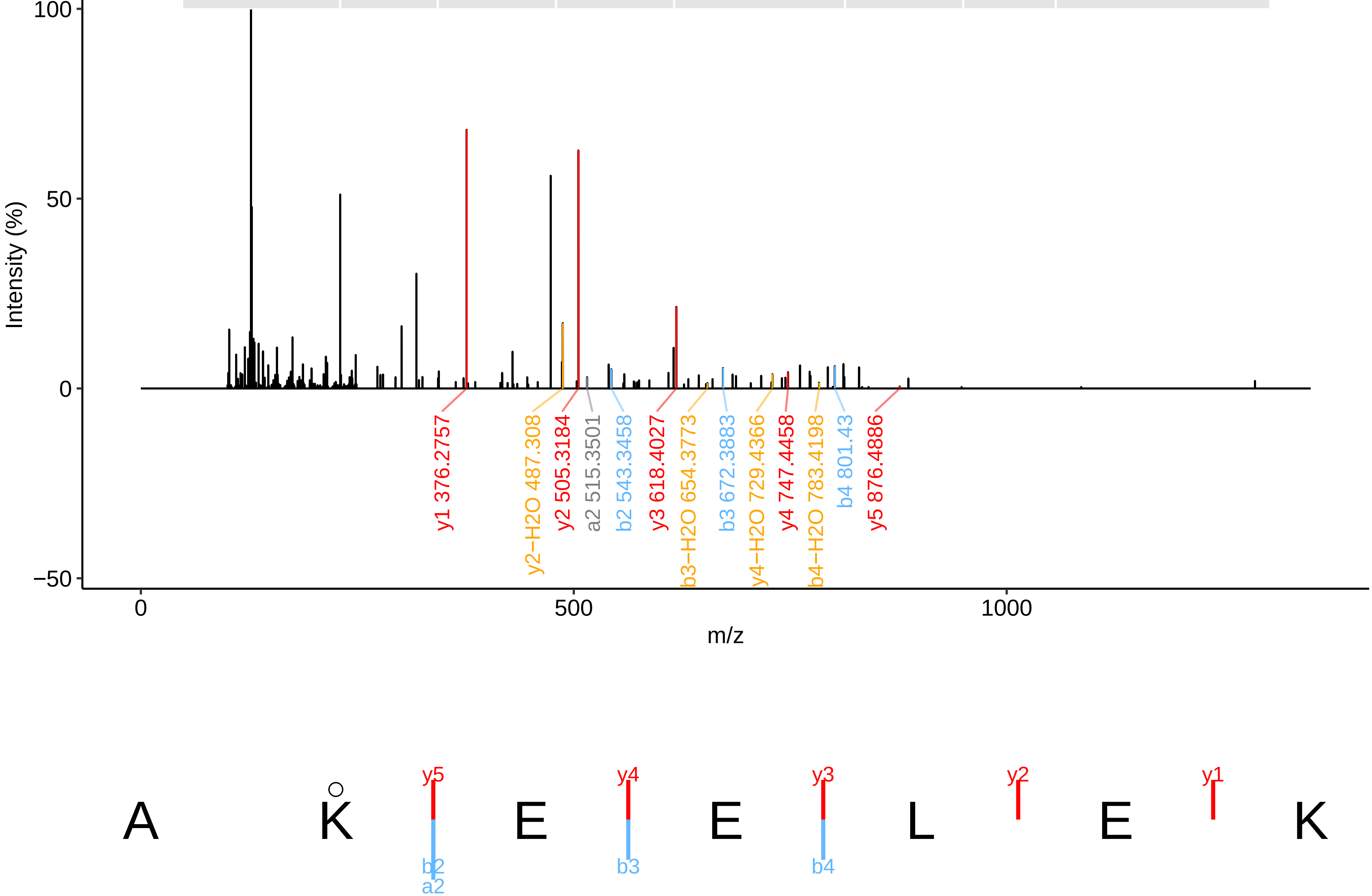

Modification □ Acetyl (Protein N−term) ○ GlyGly (K) no new terminus △ Oxidation (M)

20200924_KIZ_MUb5_GlyGly_1 − Scan number: 5878

Gene Names	Charge	m/z	Mass	Retention time	PEP	Score	Precursor Intensity
IMMT	2	480.7535	959.4924	18.374	0.047254	121.96	NA

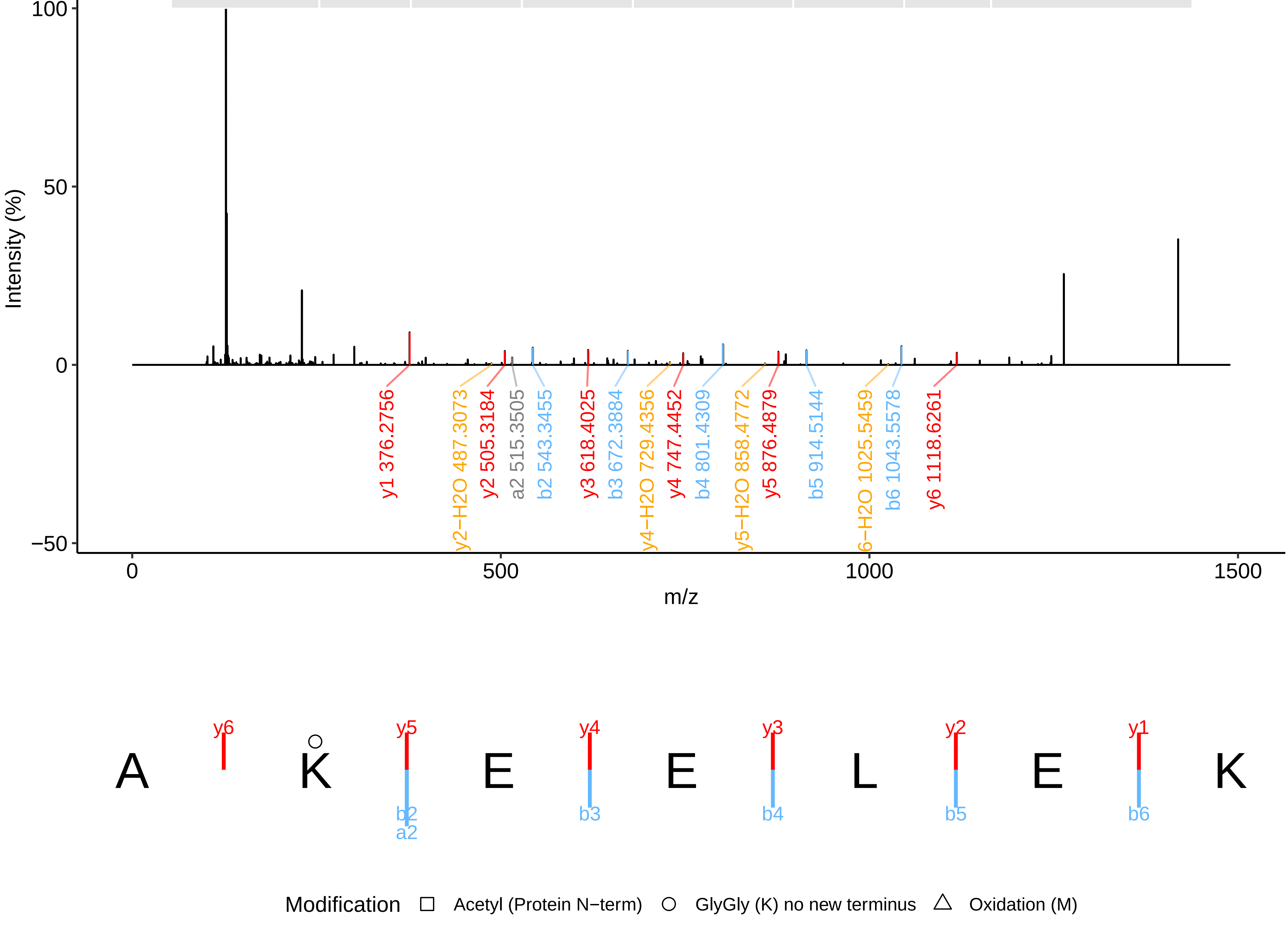

20200924_KIZ_MUb5_GlyGly_2 − Scan number: 5489

Gene Names	Charge	m/z	Mass	Retention time	PEP	Score	Precursor Intensity
IMMT	3	320.8381	959.4924	20.379	0.034155	75.738	7926130

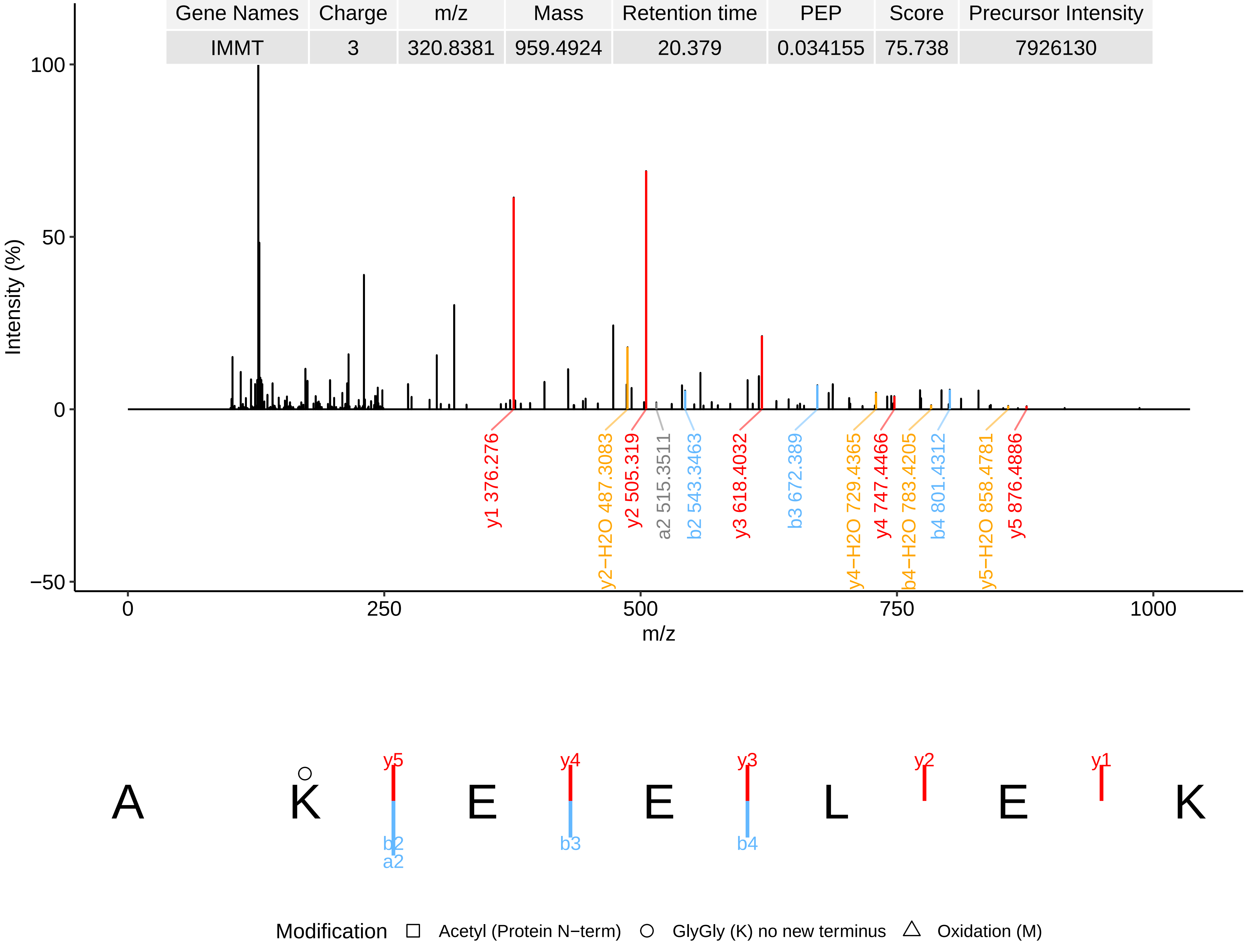

20200921_KIZ_MUb4_GlyGly_10plex_2 − Scan number: 5353

Gene Names	Charge	m/z	Mass	Retention time	PEP	Score	Precursor Intensity
TPM3	3	316.1662	945.4767	19.205	0.04613	70.912	423060.5

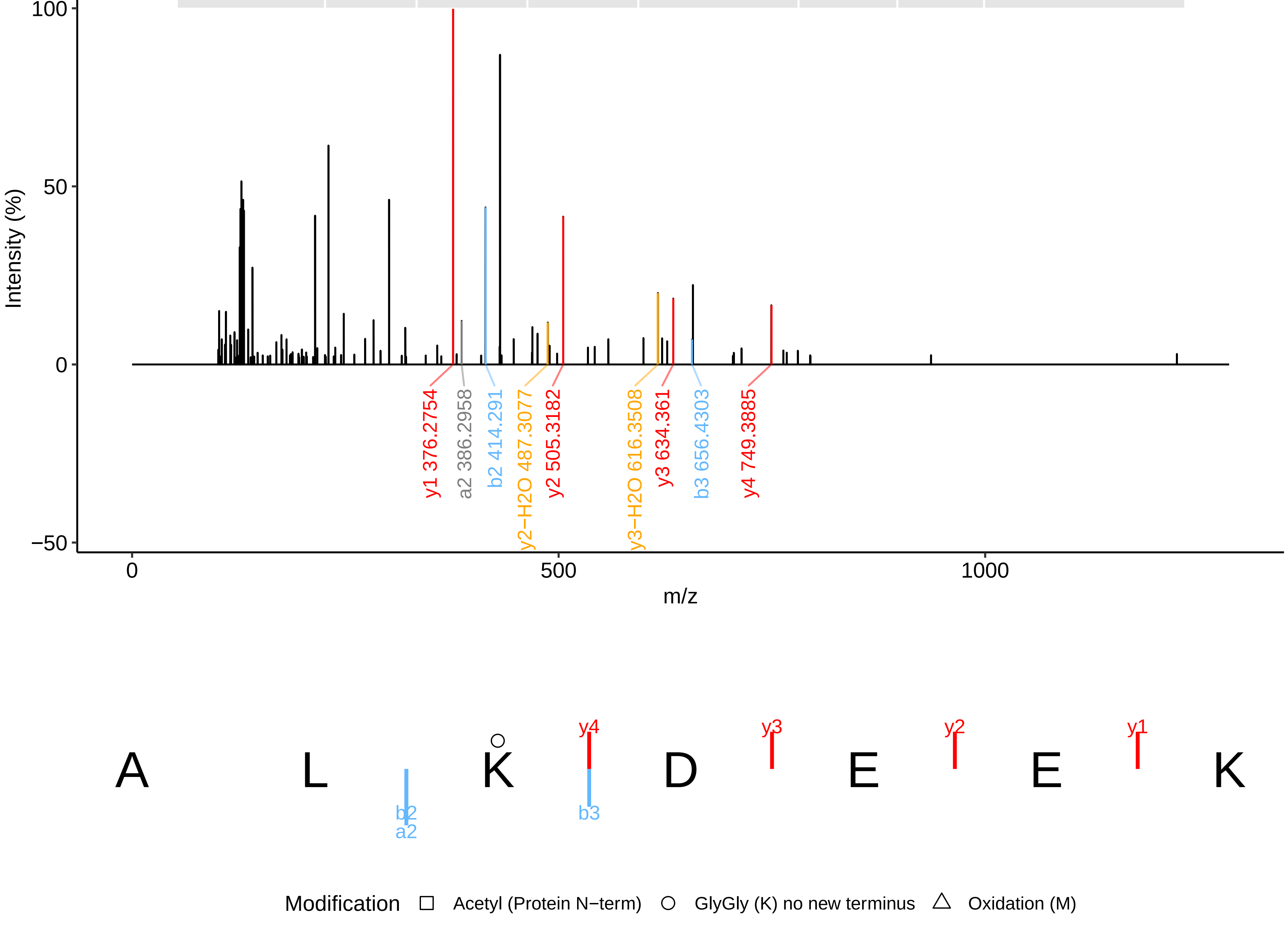

20200921_KIZ_MUb4_GlyGly_10plex − Scan number: 6250

Gene Names	Charge	m/z	Mass	Retention time	PEP	Score	Precursor Intensity
AIMP1	2	575.7797	1149.545	20.609	1.4522e−08	136.57	3802055

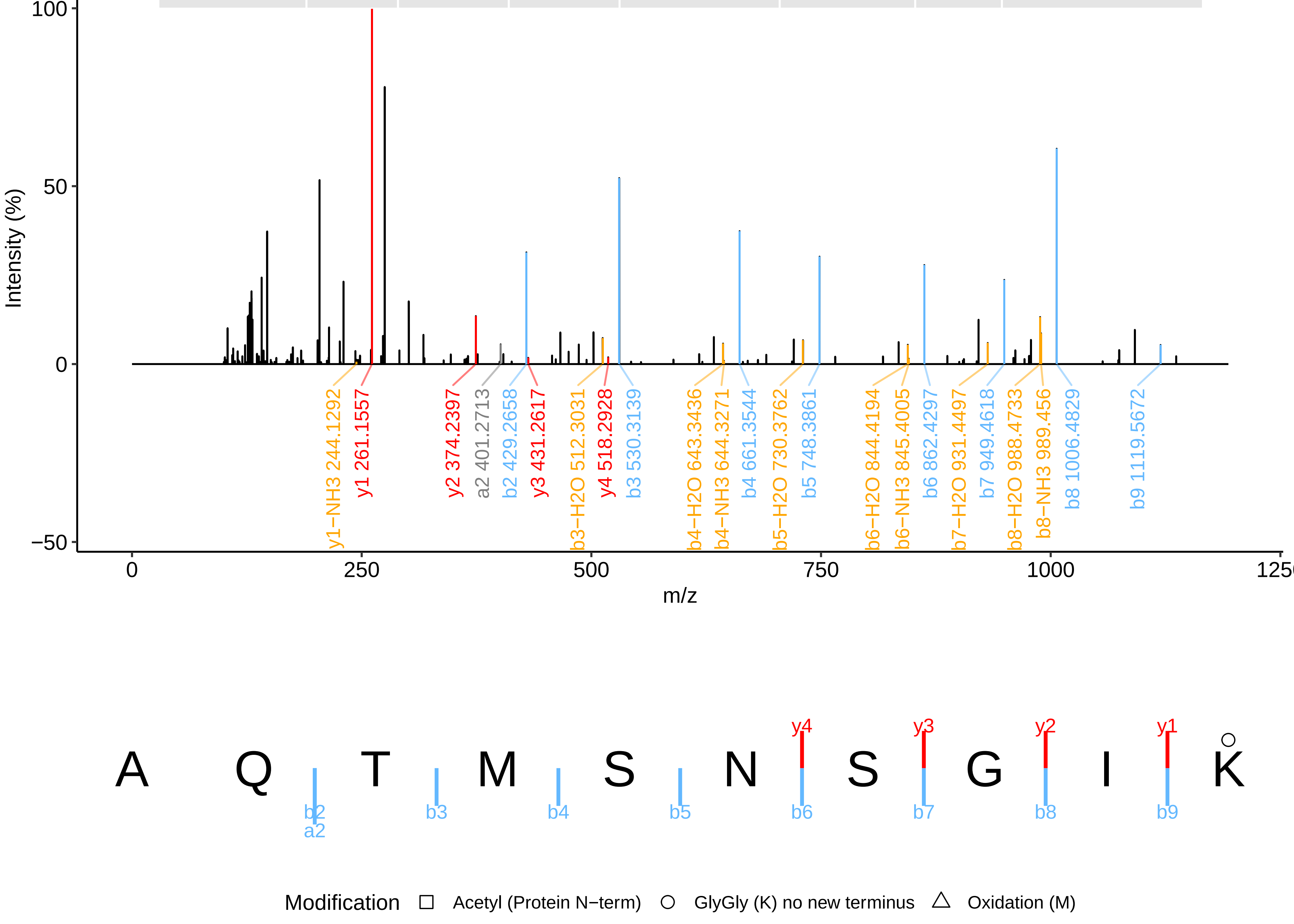

20200921_KIZ_MUb4_GlyGly_10plex_2 − Scan number: 5659

Gene Names	Charge	m/z	Mass	Retention time	PEP	Score	Precursor Intensity
AIMP1	2	575.7797	1149.545	20.45	4.796e−06	122.52	444214.6

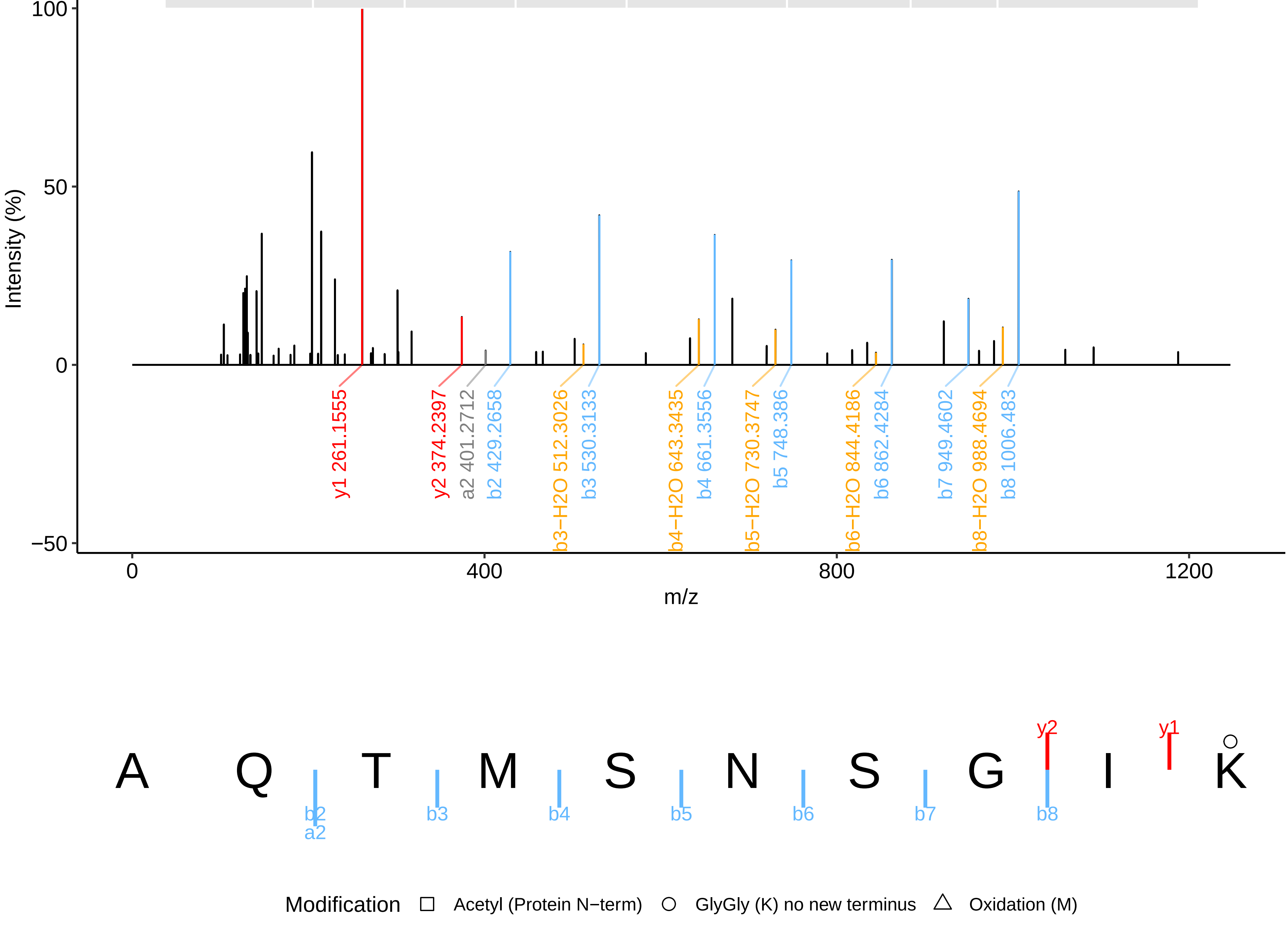

20200924_KIZ_MUb5_GlyGly_1 − Scan number: 5452

Gene Names	Charge	m/z	Mass	Retention time	PEP	Score	Precursor Intensity
AIMP1	2	575.7797	1149.545	16.967	0.015187	91.867	10705075

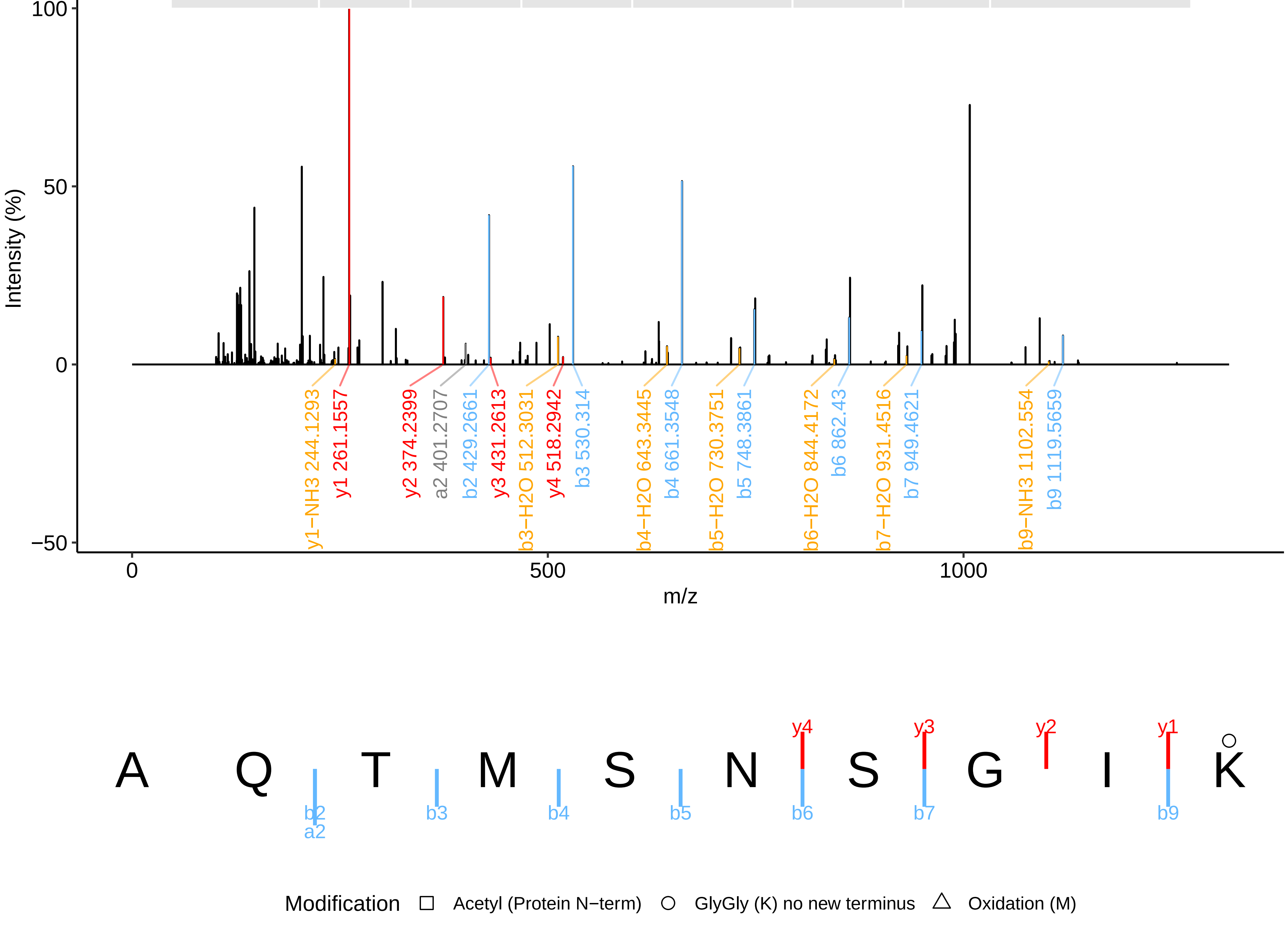

20200924_KIZ_MUb5_GlyGly_2 − Scan number: 5043

Gene Names	Charge	m/z	Mass	Retention time	PEP	Score	Precursor Intensity
AIMP1	2	575.7797	1149.545	18.652	6.5731e−09	144.09	2268587

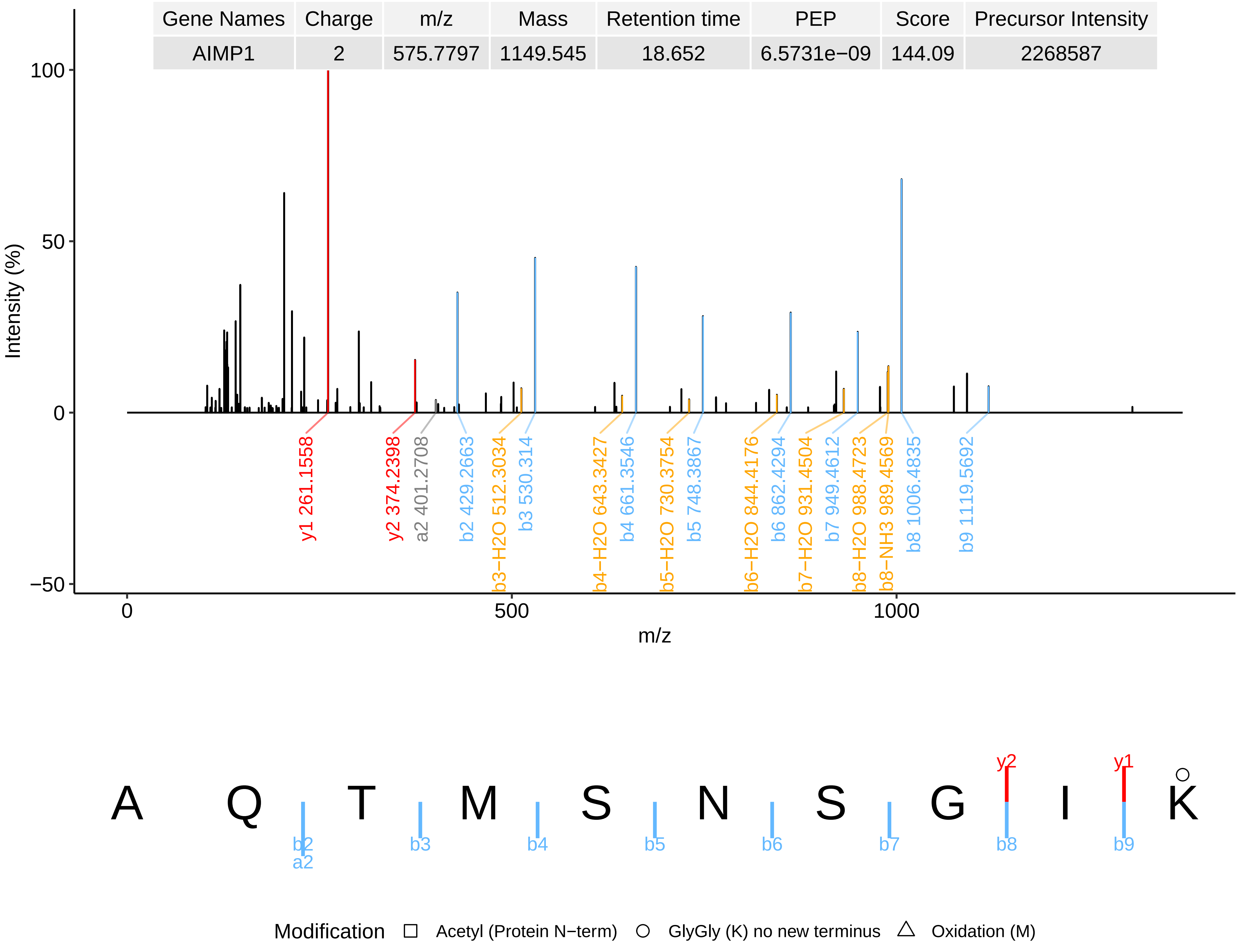

20201118_KIZ_MUb6_GlyGly_1 − Scan number: 5177

Gene Names	Charge	m/z	Mass	Retention time	PEP	Score	Precursor Intensity
AIMP1	2	575.7797	1149.545	16.381	0.0068937	95.502	11181631

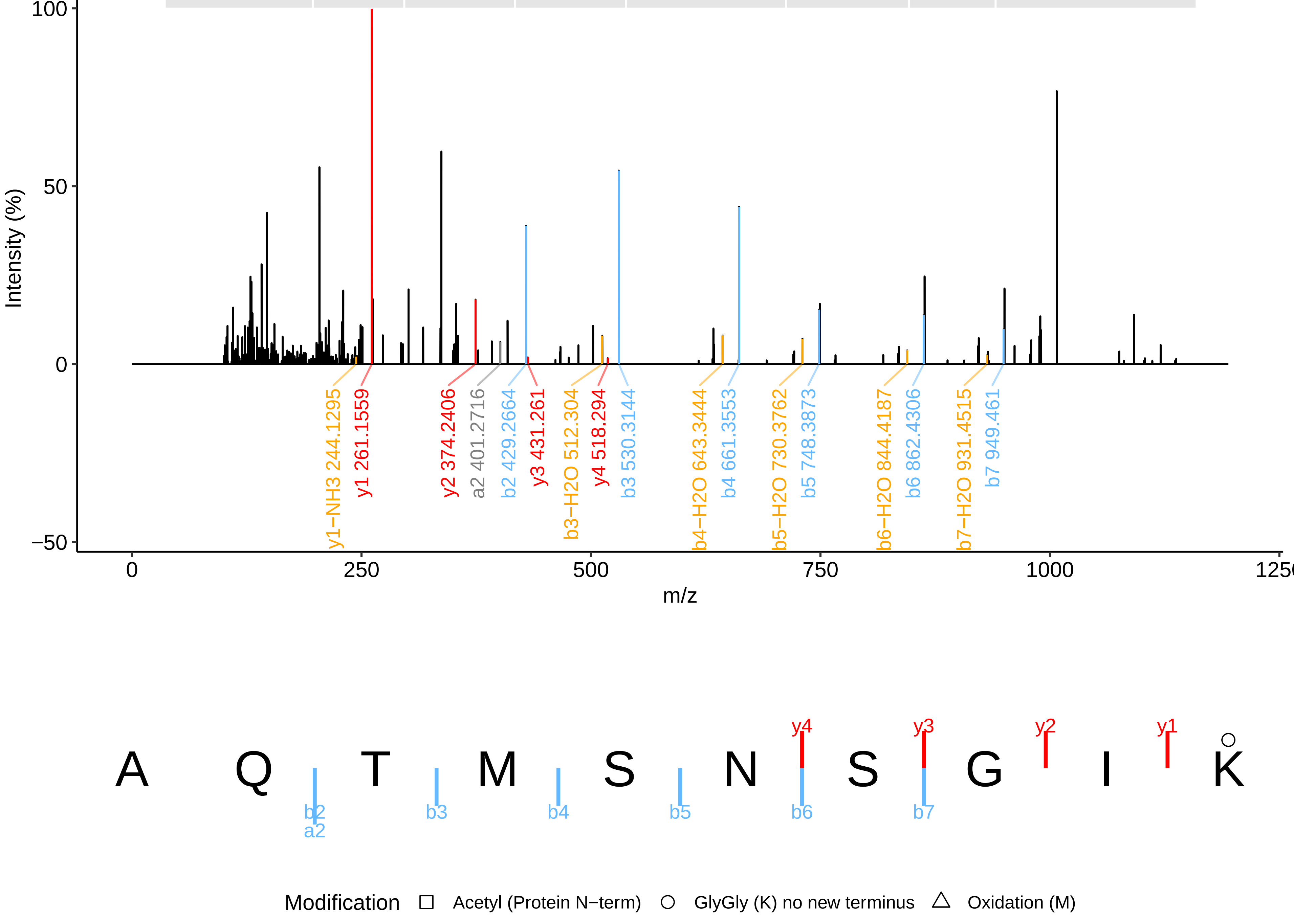

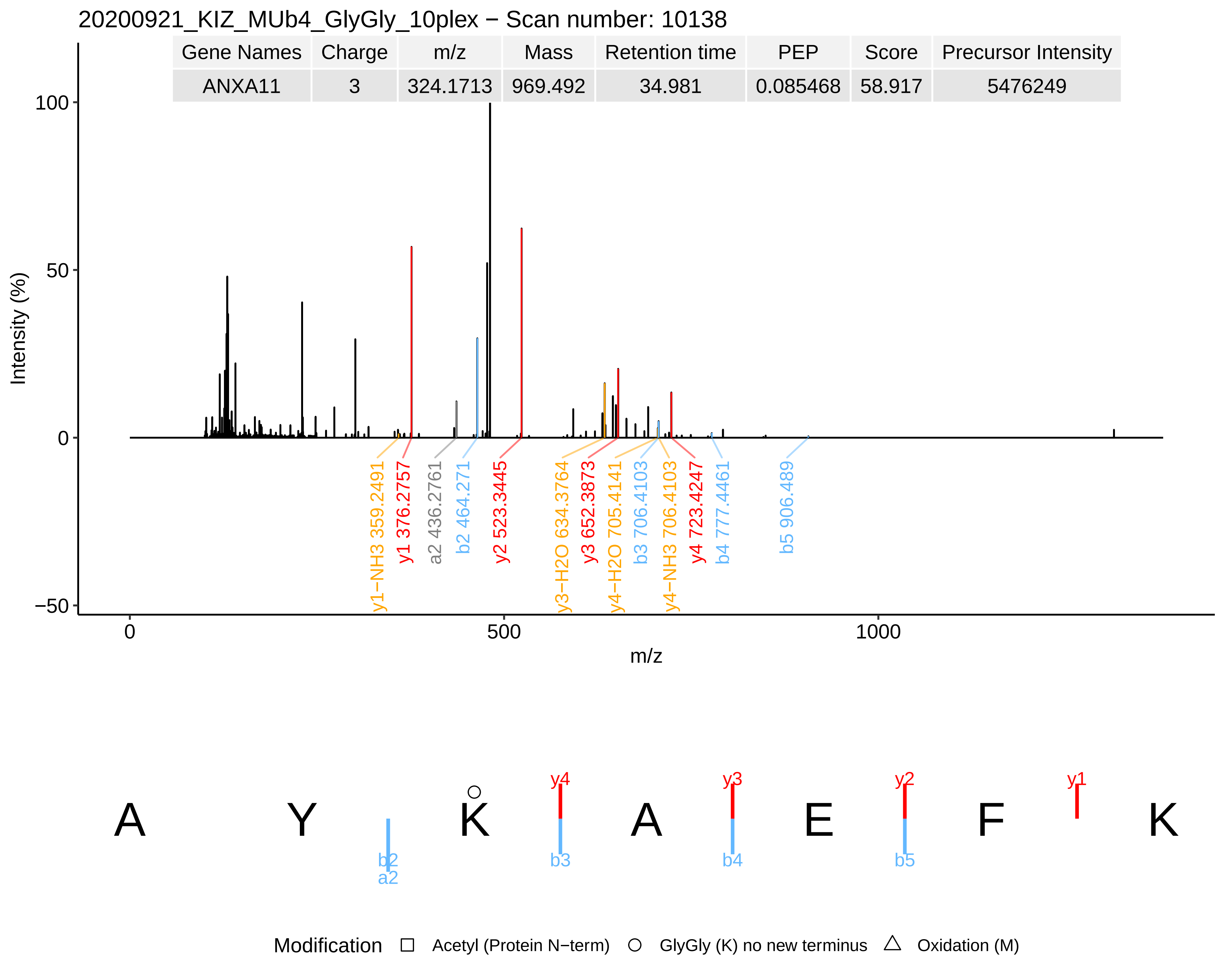
20200921_KIZ_MUb4_GlyGly_10plex − Scan number: 10138
Gene Names | Charge | m/z | Mass | Retention time | PEP | Score | Precursor Intensity
ANXA11 | 3 | 324.1713 | 969.492 | 34.981 | 0.085468 | 58.917 | 5476249
Intensity (%)
100
50
0
−50
0
500
1000
m/z
y1−NH3 359.2491
y1 376.2757
a2 436.2761
b2 464.271
y2 523.3445
y3−H2O 634.3764
y3 652.3873
y4−H2O 705.4141
b3 706.4103
y4−NH3 706.4103
y4 723.4247
b4 777.4461
b5 906.489
A Y K A E F K
y4 y3 y2 y1
b2 a2 b3 b4 b5
Modification
Acetyl (Protein N−term)
GlyGly (K) no new terminus
Oxidation (M)

20200921_KIZ_MUb4_GlyGly_10plex_2 − Scan number: 9372

Gene Names	Charge	m/z	Mass	Retention time	PEP	Score	Precursor Intensity
ANXA11	3	324.1713	969.492	35.607	0.046262	70.268	1419138

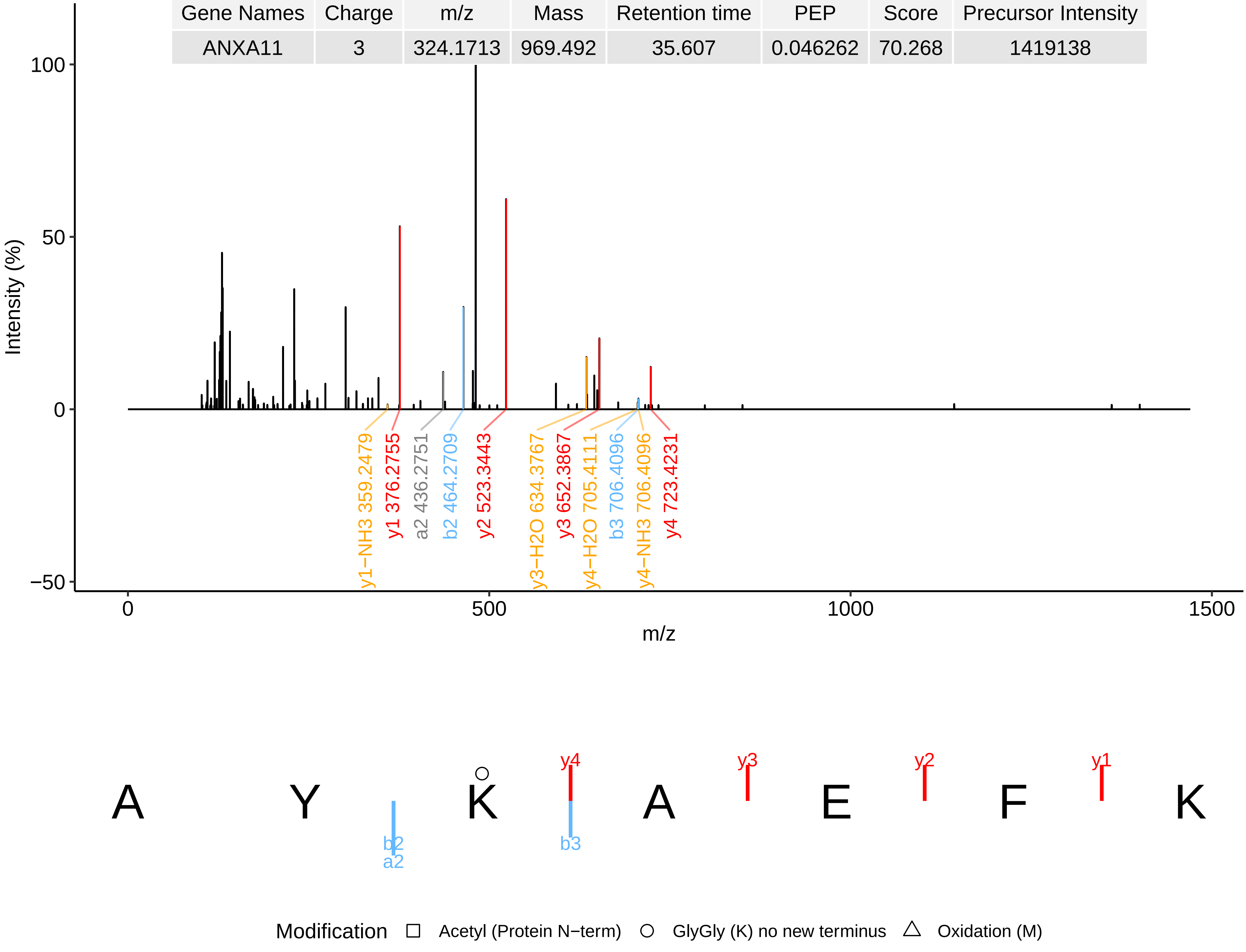

20200921_KIZ_MUb4_GlyGly_10plex − Scan number: 3510

Gene Names	Charge	m/z	Mass	Retention time	PEP	Score	Precursor Intensity
PGK1	3	311.498	931.4723	11.165	0.096836	47.082	1418014

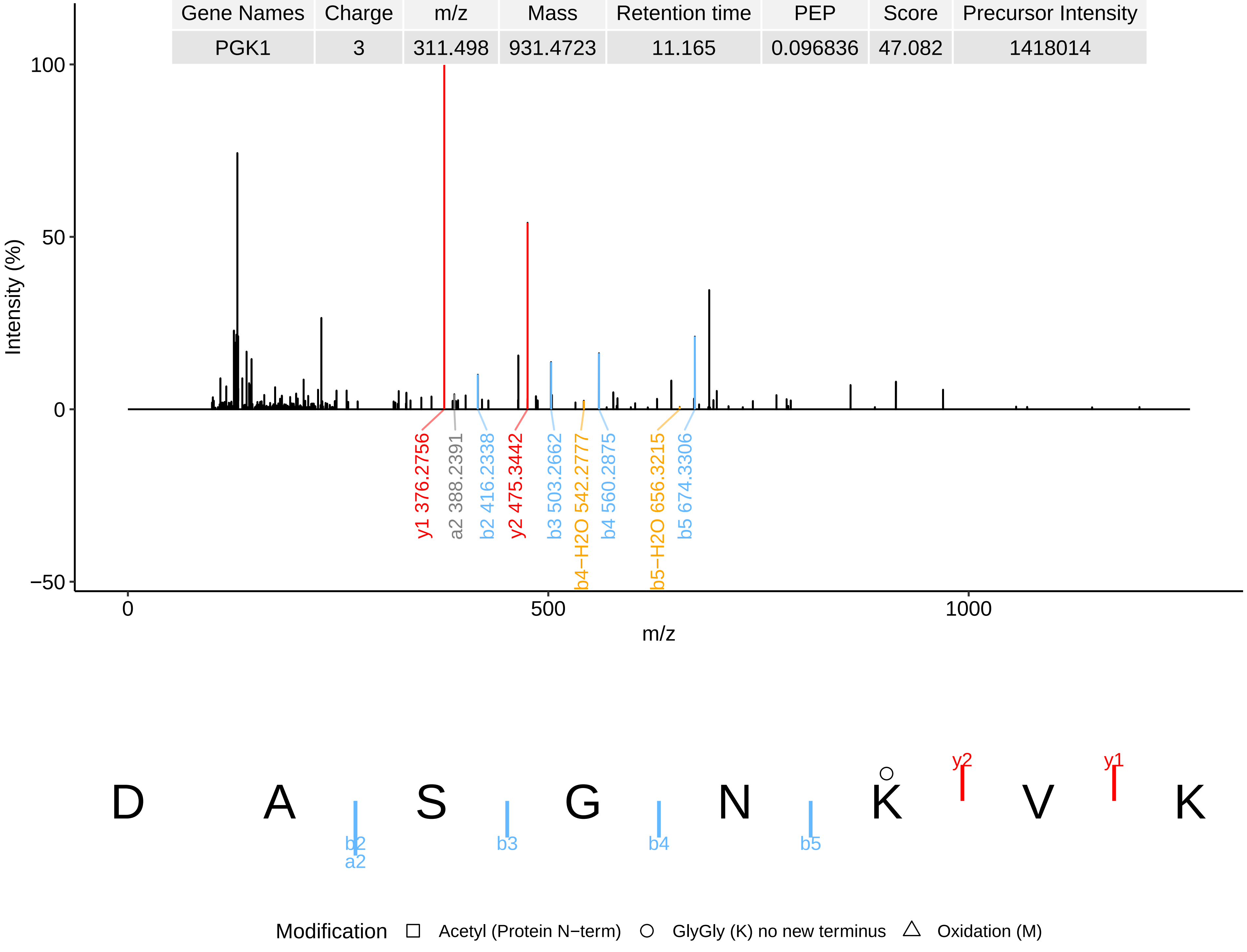

20200921_KIZ_MUb4_GlyGly_10plex − Scan number: 12665

Gene Names	Charge	m/z	Mass	Retention time	PEP	Score	Precursor Intensity
ALDOA;ALDOC	3	393.8562	1178.547	44.491	0.00099529	95.502	6650348

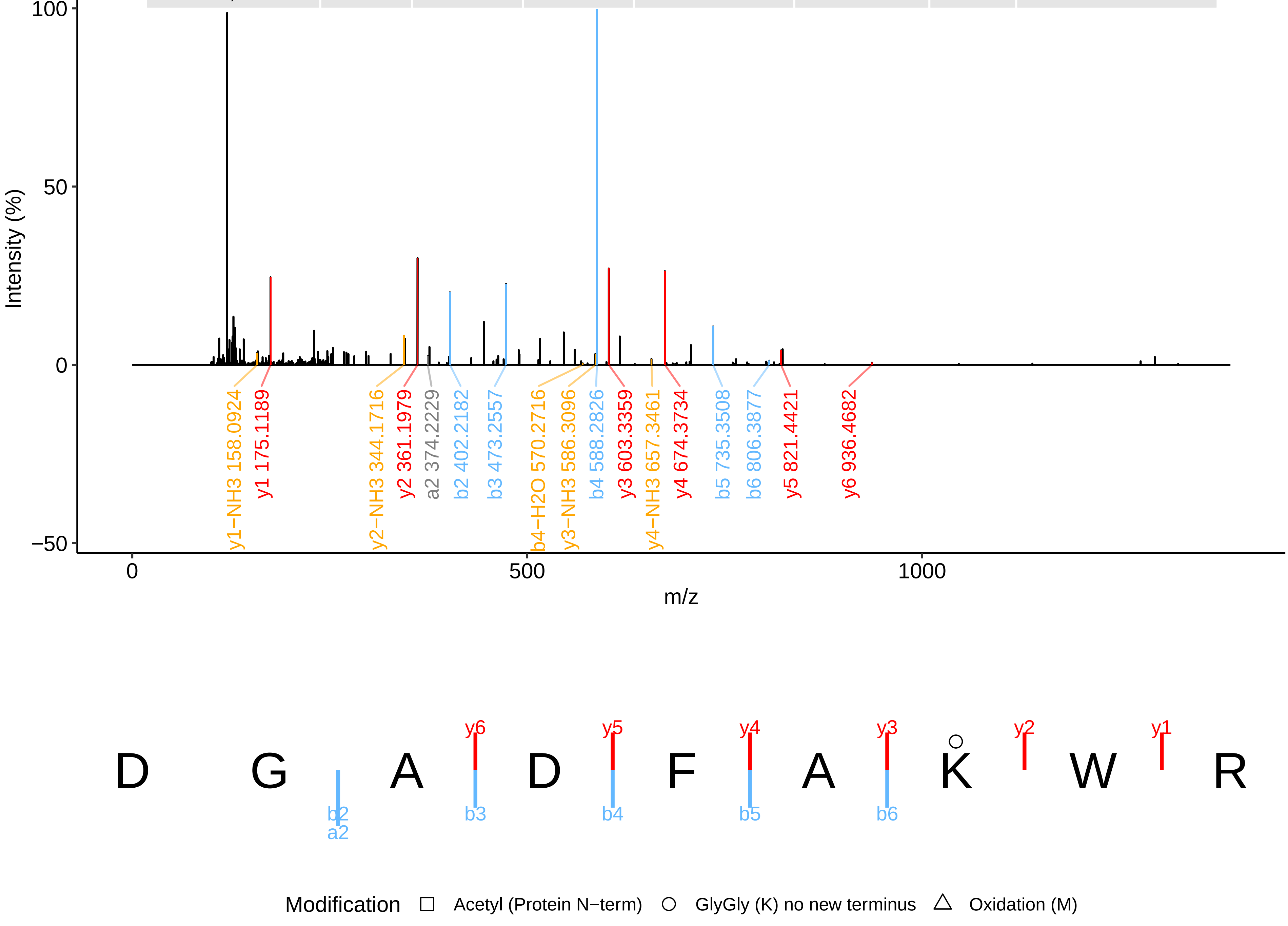

20200921_KIZ_MUb4_GlyGly_10plex_2 − Scan number: 11613

Gene Names	Charge	m/z	Mass	Retention time	PEP	Score	Precursor Intensity
ALDOA;ALDOC	3	393.8562	1178.547	44.72	0.048964	57.598	403918.2

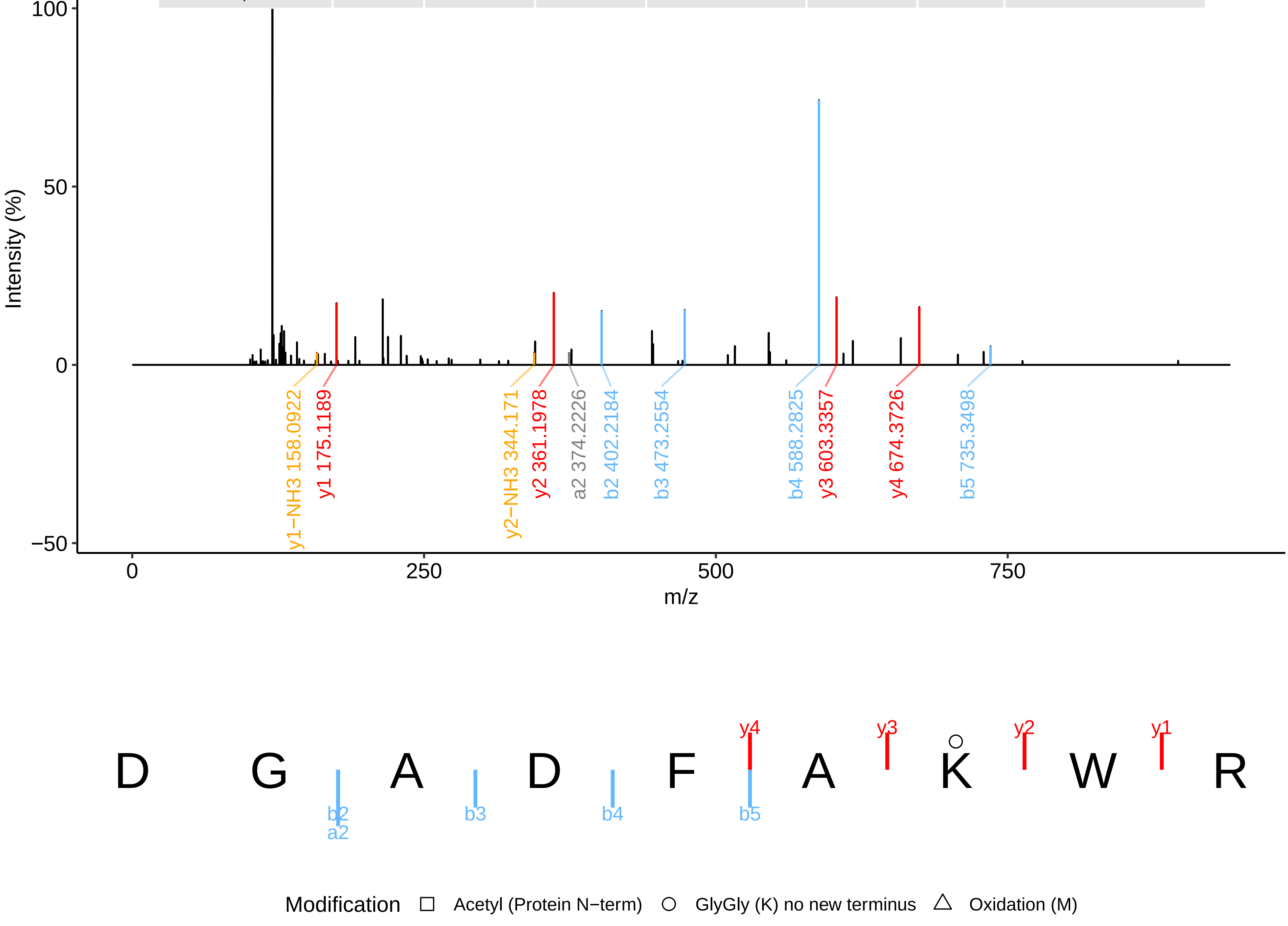

20200924_KIZ_MUb5_GlyGly_1 − Scan number: 11361

Gene Names	Charge	m/z	Mass	Retention time	PEP	Score	Precursor Intensity
ALDOA;ALDOC	3	393.8562	1178.547	36.799	0.014543	73.985	2077891

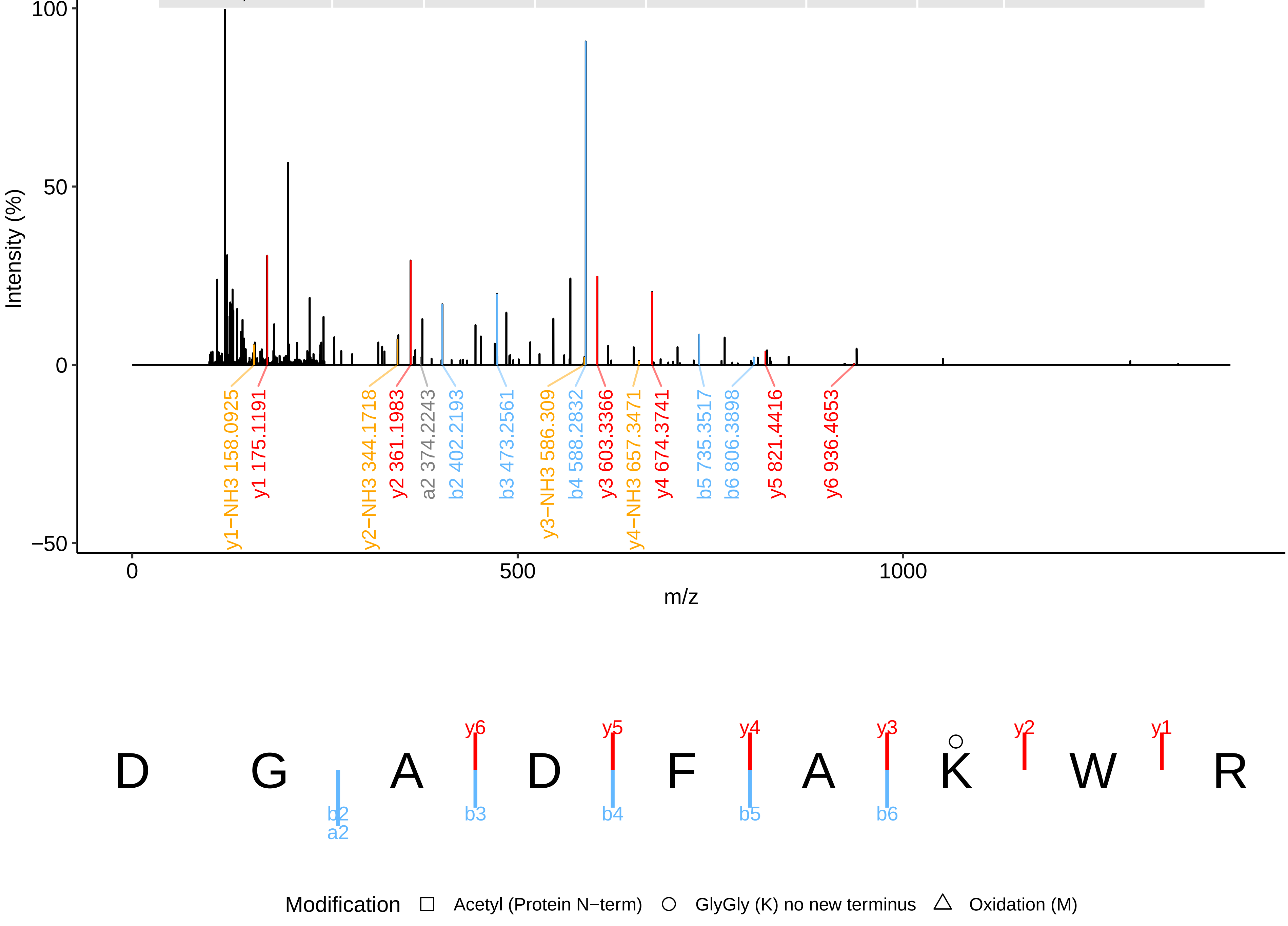

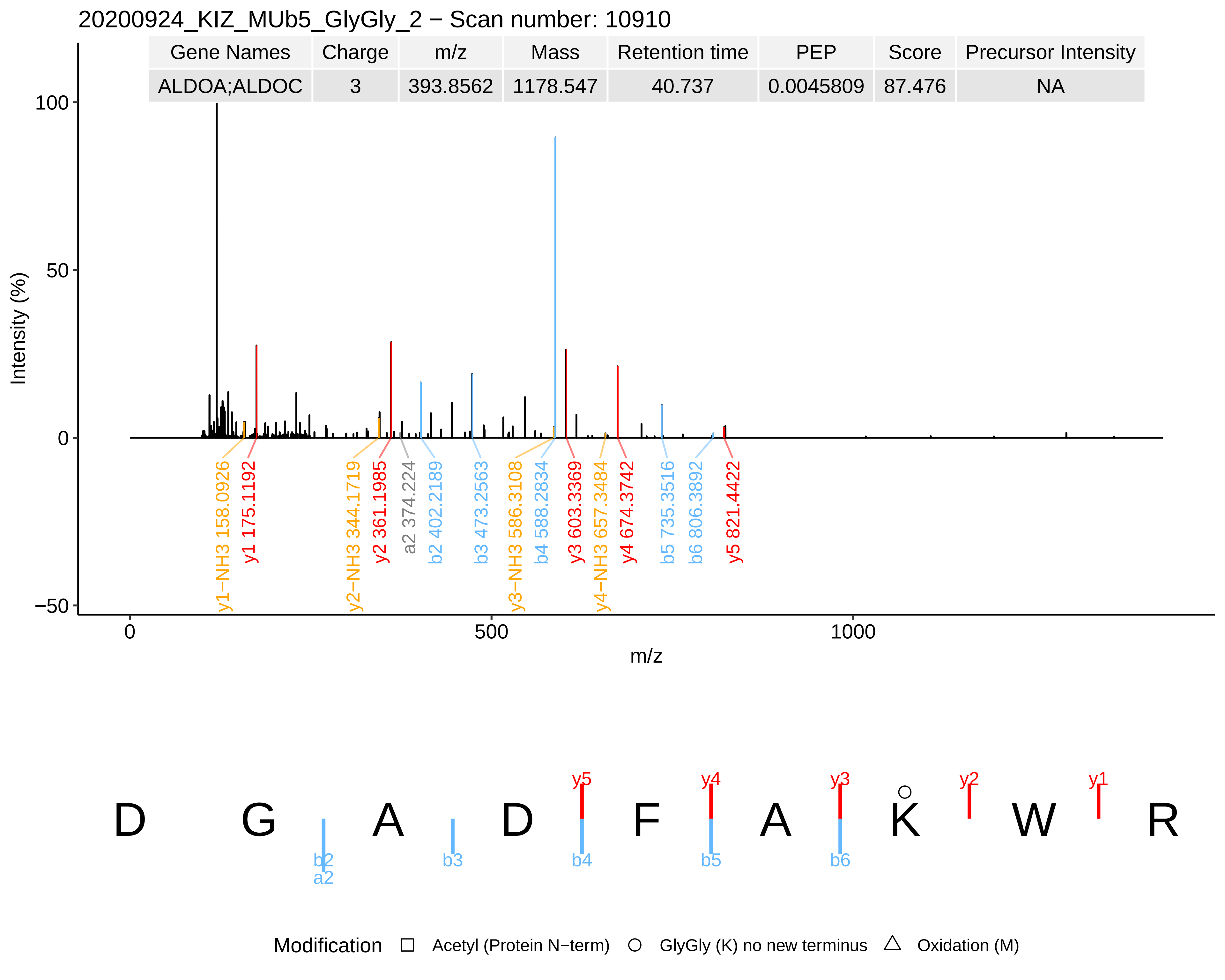
20200924_KIZ_MUb5_GlyGly_2 − Scan number: 10910
Gene Names | Charge | m/z | Mass | Retention time | PEP | Score | Precursor Intensity
ALDOA;ALDOC | 3 | 393.8562 | 1178.547 | 40.737 | 0.0045809 | 87.476 | NA
Intensity (%)
100
50
0
−50
0
500
1000
m/z
y1−NH3 158.0926
y1 175.1192
y2−NH3 344.1719
y2 361.1985
a2 374.224
b2 402.2189
b3 473.2563
y3−NH3 586.3108
b4 588.2834
y3 603.3369
y4−NH3 657.3484
y4 674.3742
b5 735.3516
b6 806.3892
y5 821.4422
D G A D F A K W R
y5 y4 y3 y2 y1
b2 a2 b3 b4 b5 b6
Modification
Acetyl (Protein N−term)
GlyGly (K) no new terminus
Oxidation (M)

20200921_KIZ_MUb4_GlyGly_10plex − Scan number: 8666

Gene Names	Charge	m/z	Mass	Retention time	PEP	Score	Precursor Intensity
GGCX	3	400.5508	1198.631	29.486	0.016968	66.486	6071279

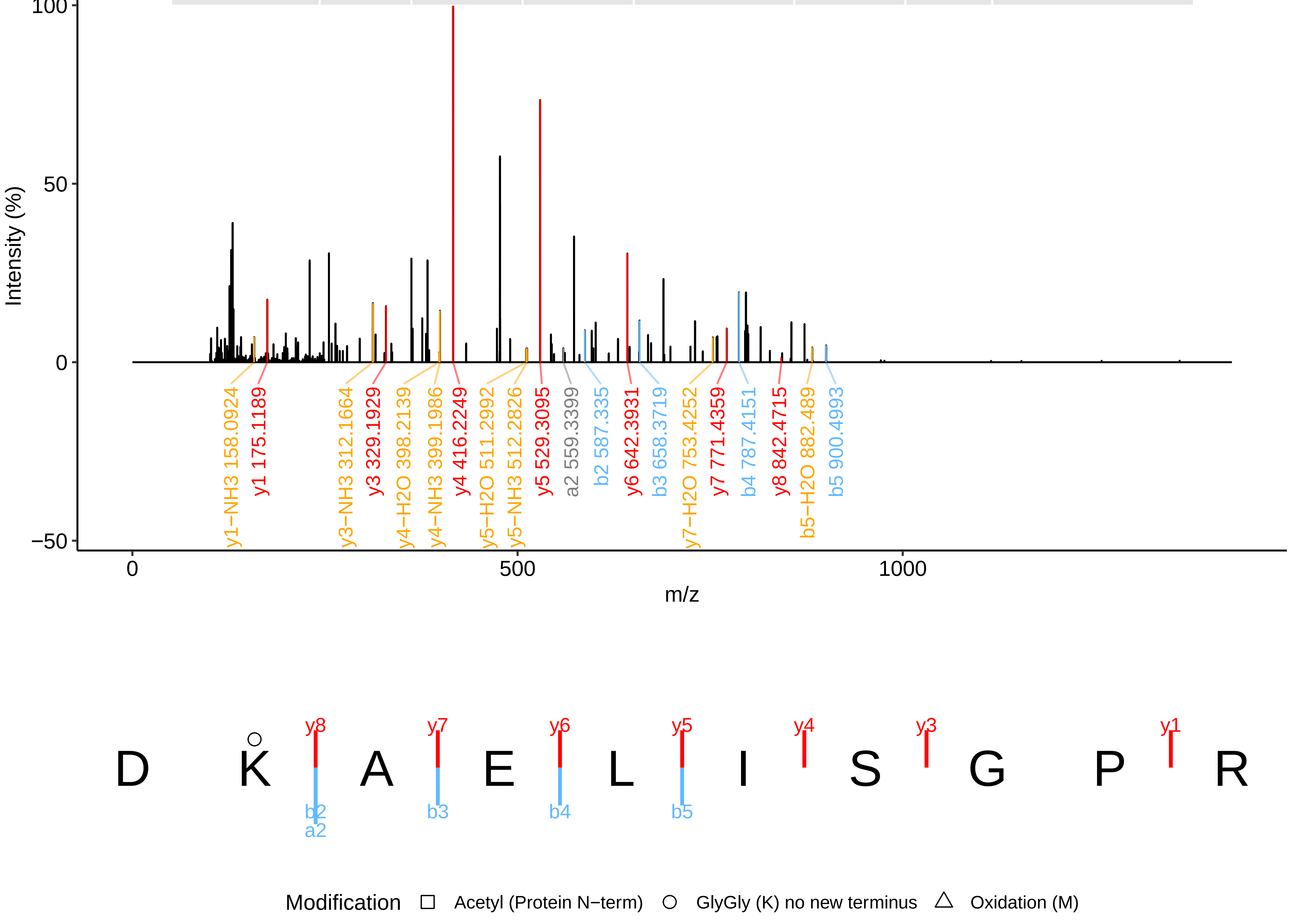

20200921_KIZ_MUb4_GlyGly_10plex − Scan number: 8305

Gene Names	Charge	m/z	Mass	Retention time	PEP	Score	Precursor Intensity
ANXA2	3	384.1999	1149.578	28.143	0.056101	75.378	9688346

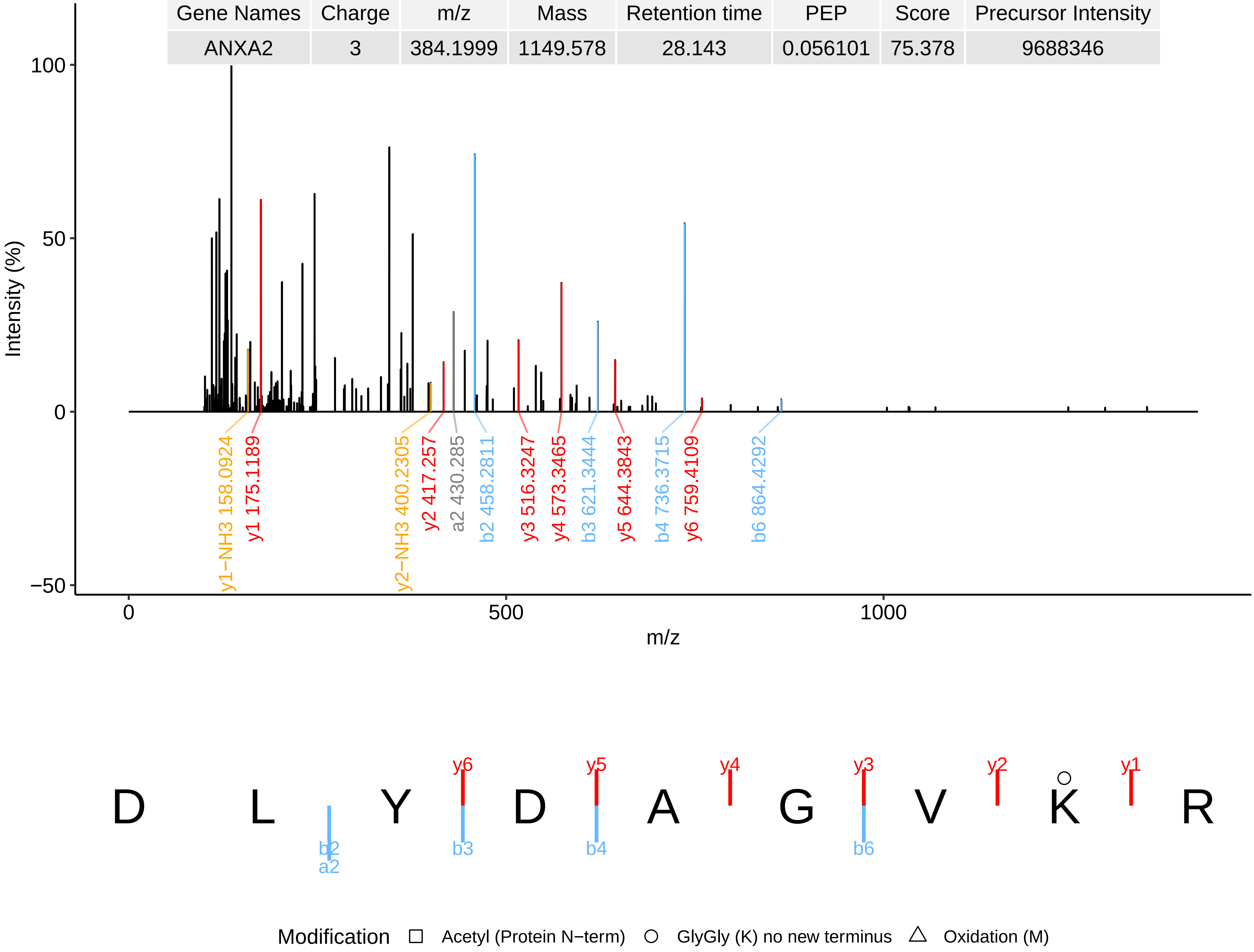

20200921_KIZ_MUb4_GlyGly_10plex_2 − Scan number: 4844

Gene Names	Charge	m/z	Mass	Retention time	PEP	Score	Precursor Intensity
ABCD1;ABCD2	3	401.5423	1201.605	17.168	4.0101e−07	119.22	1871349

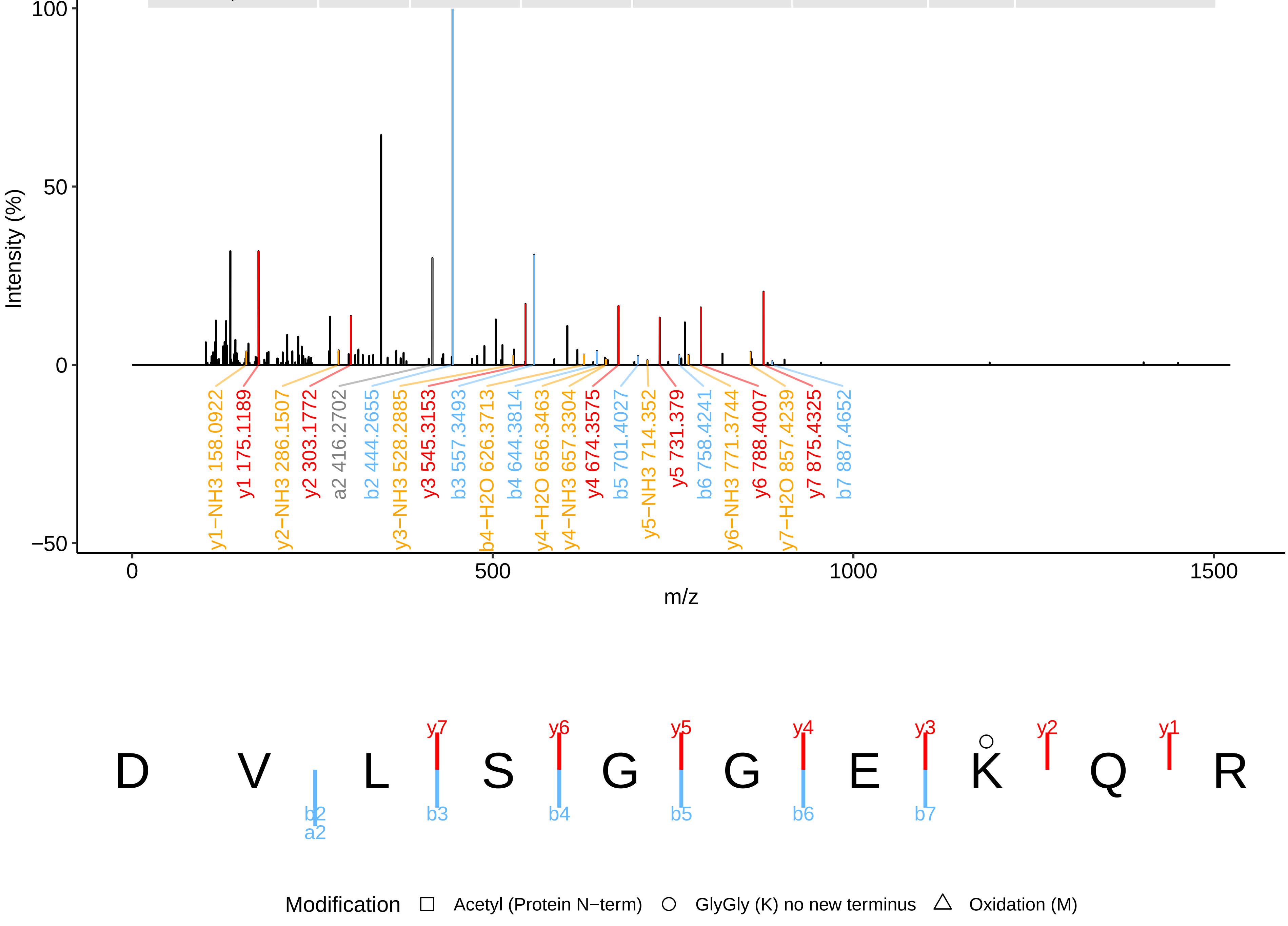

20200924_KIZ_MUb5_GlyGly_2 − Scan number: 4207

Gene Names	Charge	m/z	Mass	Retention time	PEP	Score	Precursor Intensity
ABCD1;ABCD2	3	401.5423	1201.605	15.408	0.0023535	82.261	1597153

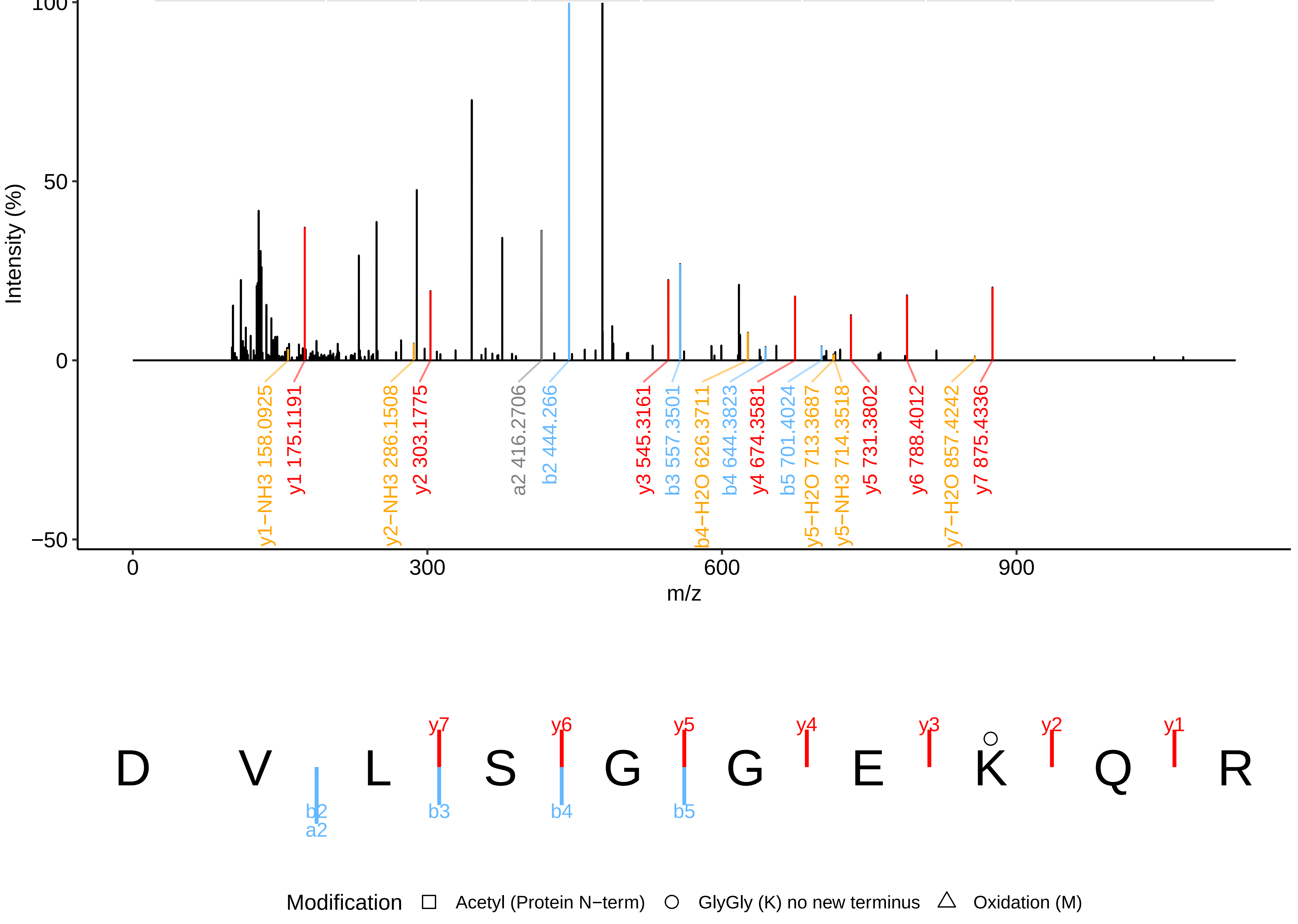

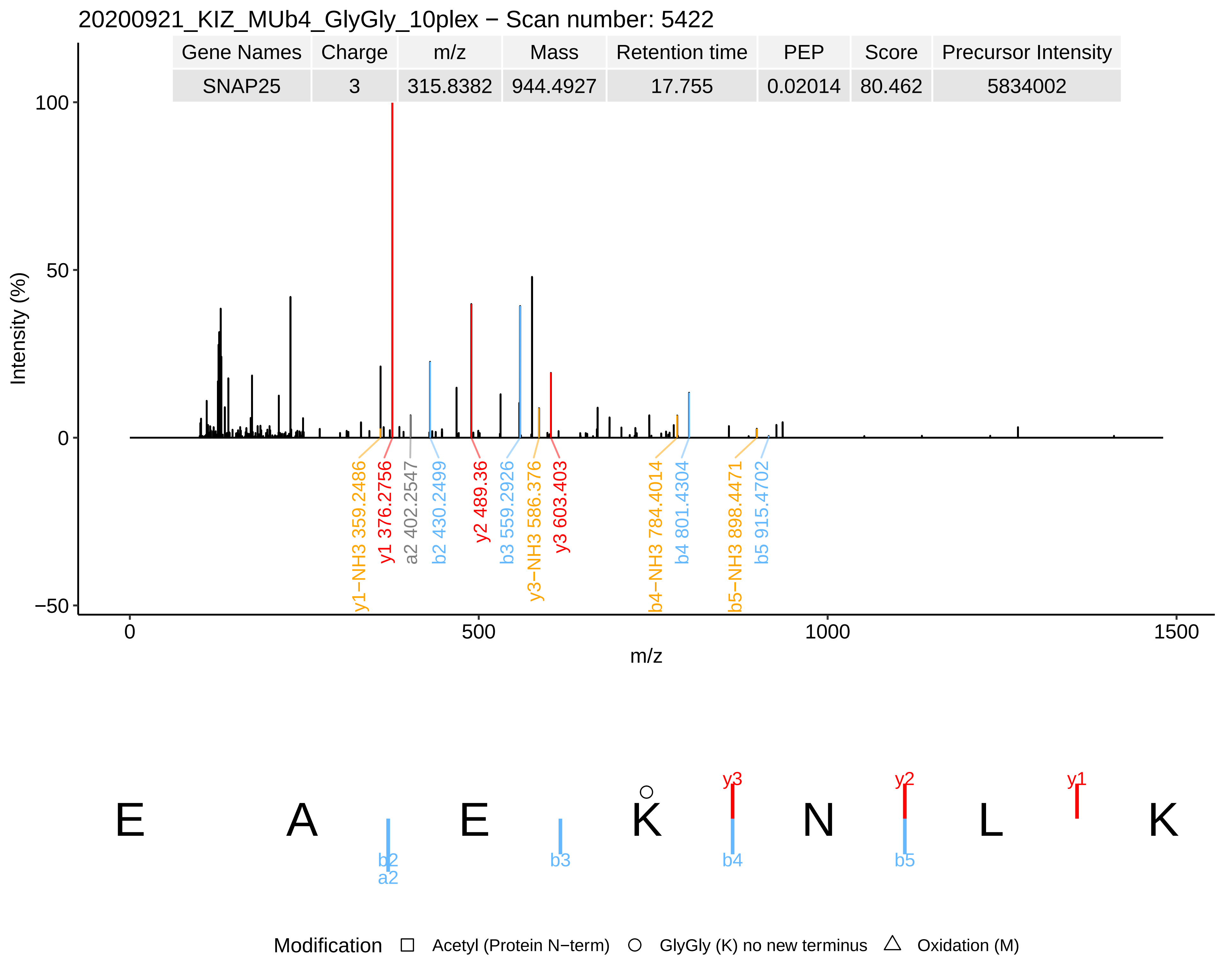
20200921_KIZ_MUb4_GlyGly_10plex − Scan number: 5422
Gene Names | Charge | m/z | Mass | Retention time | PEP | Score | Precursor Intensity
SNAP25 | 3 | 315.8382 | 944.4927 | 17.755 | 0.02014 | 80.462 | 5834002
Intensity (%)
m/z
y1−NH3 359.2486
y1 376.2756
a2 402.2547
b2 430.2499
y2 489.36
b3 559.2926
y3−NH3 586.376
y3 603.403
b4−NH3 784.4014
b4 801.4304
b5−NH3 898.4471
b5 915.4702
E A E K N L K
y3 y2 y1
b2 a2 b3 b4 b5
Modification
Acetyl (Protein N−term)
GlyGly (K) no new terminus
Oxidation (M)

20200921_KIZ_MUb4_GlyGly_10plex − Scan number: 5209

Gene Names	Charge	m/z	Mass	Retention time	PEP	Score	Precursor Intensity
FYN	3	397.5386	1189.594	17.018	0.0032945	81.972	7634688

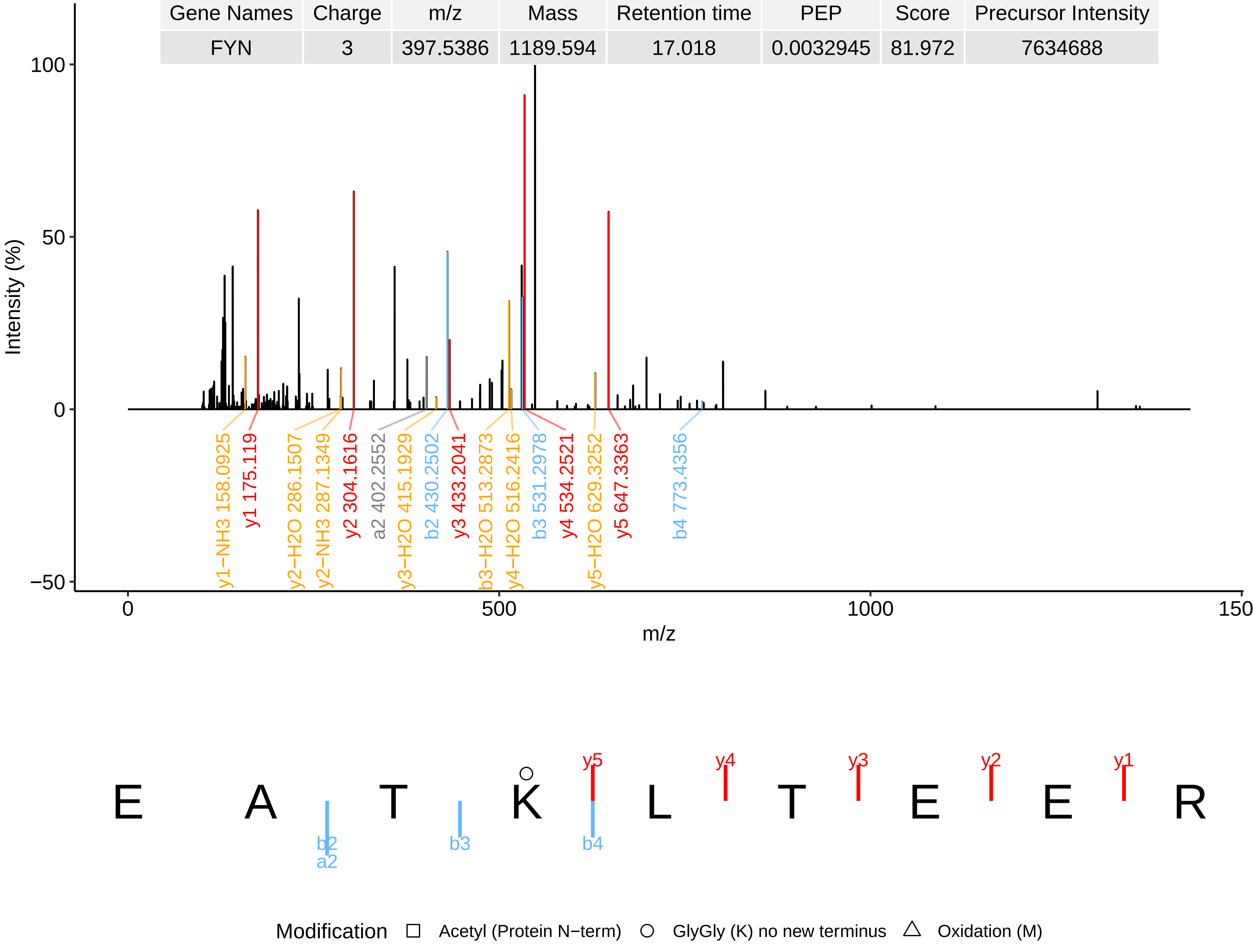

20200921_KIZ_MUb4_GlyGly_10plex_2 − Scan number: 4798

Gene Names	Charge	m/z	Mass	Retention time	PEP	Score	Precursor Intensity
FYN	3	397.5386	1189.594	16.988	0.00045571	101.65	2467462

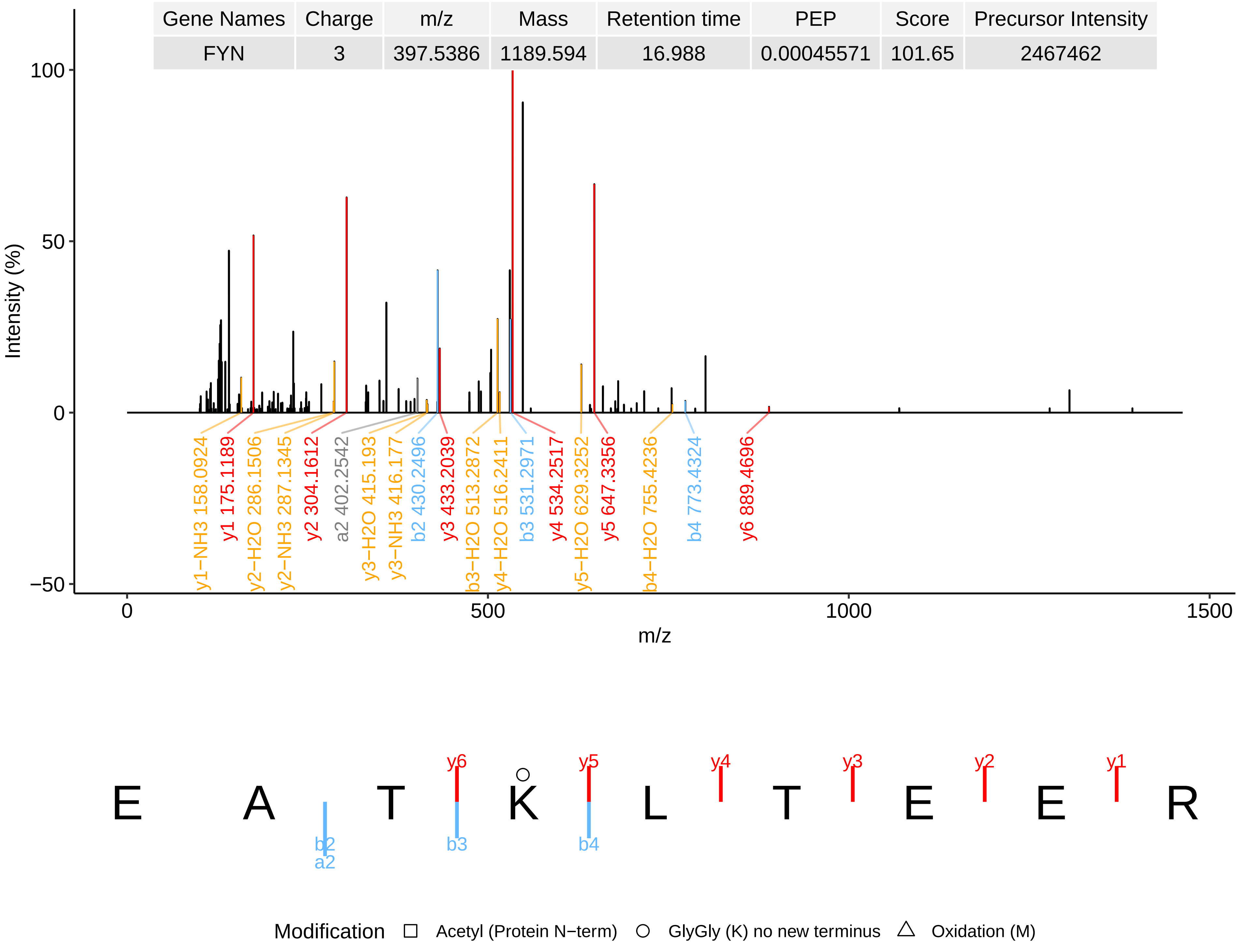

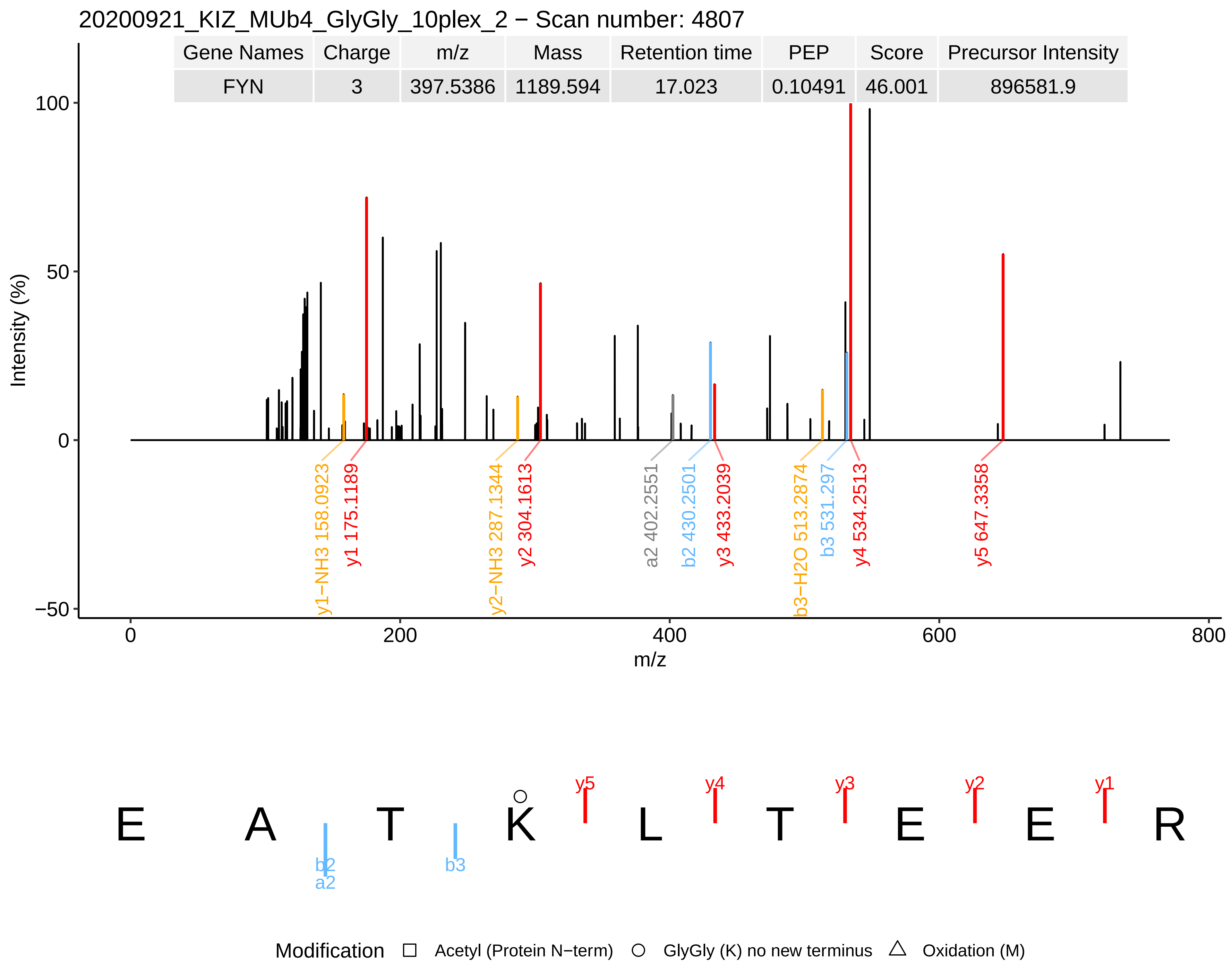

20200921_KIZ_MUb4_GlyGly_10plex_2 − Scan number: 4807
Gene Names | Charge | m/z | Mass | Retention time | PEP | Score | Precursor Intensity
FYN | 3 | 397.5386 | 1189.594 | 17.023 | 0.10491 | 46.001 | 896581.9
Intensity (%)
m/z
y1−NH3 158.0923
y1 175.1189
y2−NH3 287.1344
y2 304.1613
a2 402.2551
b2 430.2501
y3 433.2039
b3−H2O 513.2874
b3 531.297
y4 534.2513
y5 647.3358
E A T K L T E E R
y5 y4 y3 y2 y1
b2 a2 b3
Modification
Acetyl (Protein N−term)
GlyGly (K) no new terminus
Oxidation (M)

20201118_KIZ_MUb6_GlyGly_1 − Scan number: 4196

Gene Names	Charge	m/z	Mass	Retention time	PEP	Score	Precursor Intensity
FYN	3	397.5386	1189.594	13.088	0.020524	69.276	10272609

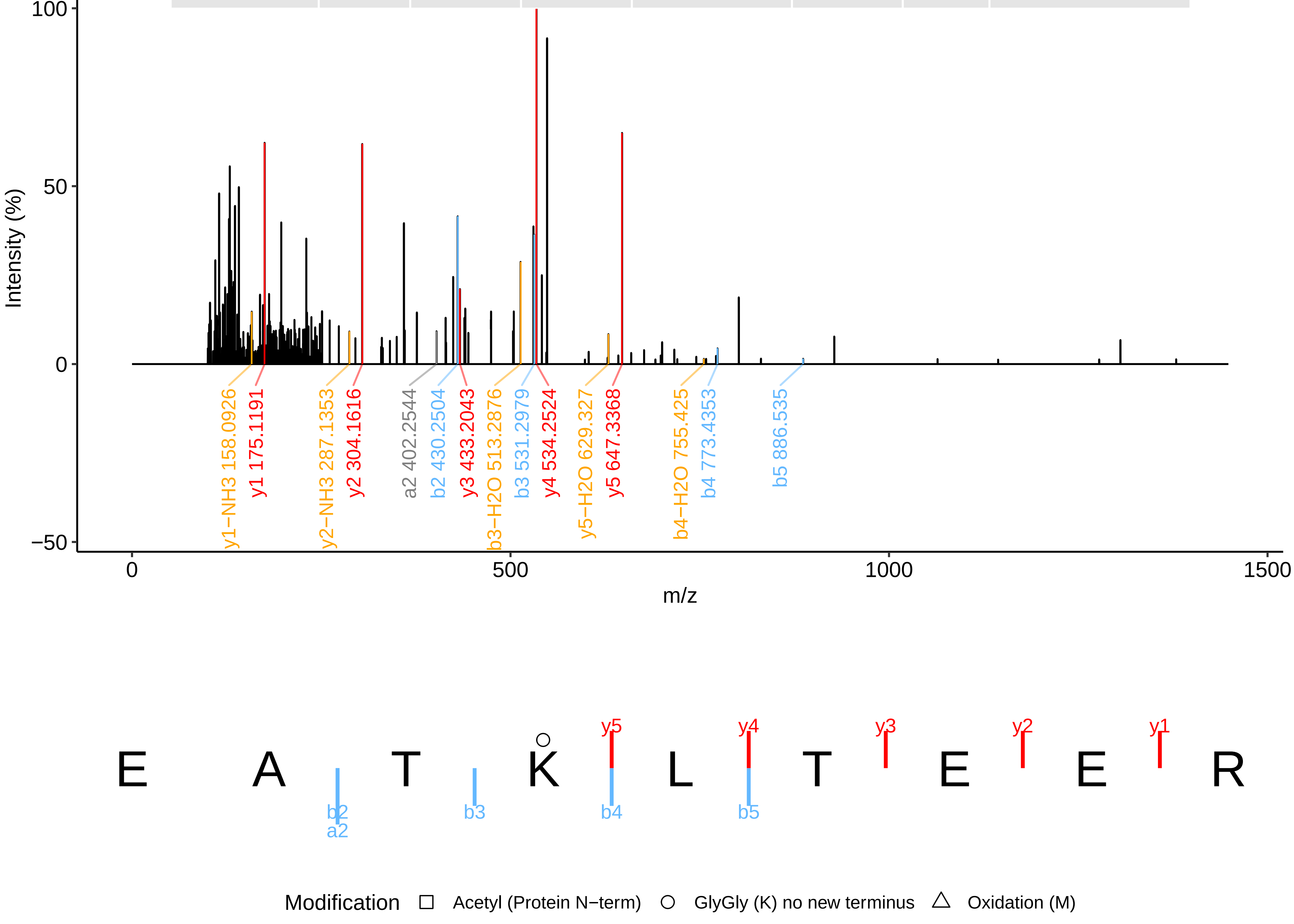

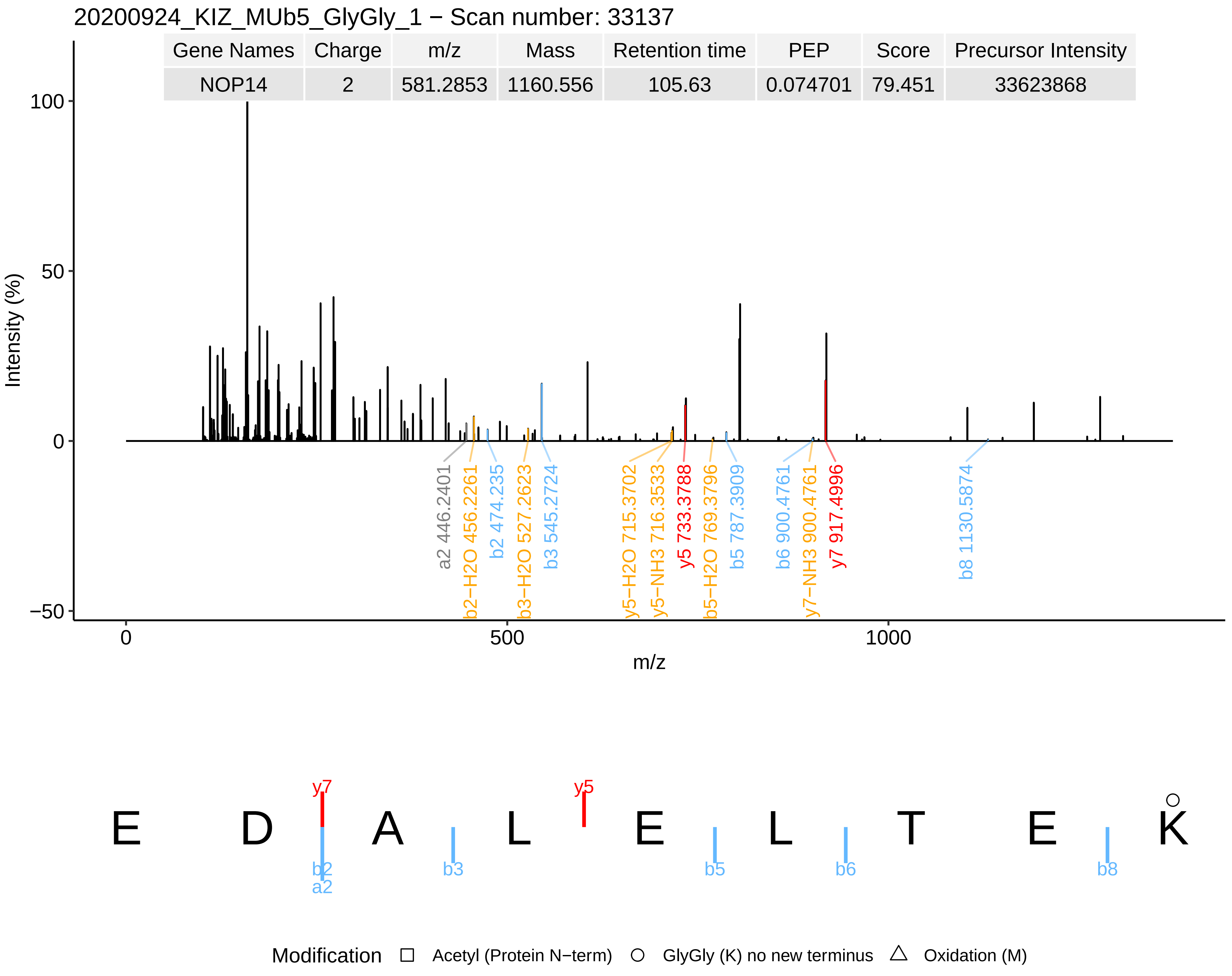

20200924_KIZ_MUb5_GlyGly_1 − Scan number: 33137
Gene Names | Charge | m/z | Mass | Retention time | PEP | Score | Precursor Intensity
NOP14 | 2 | 581.2853 | 1160.556 | 105.63 | 0.074701 | 79.451 | 33623868
Intensity (%)
m/z
a2 446.2401
b2−H2O 456.2261
b2 474.235
b3−H2O 527.2623
b3 545.2724
y5−H2O 715.3702
y5−NH3 716.3533
y5 733.3788
b5−H2O 769.3796
b5 787.3909
b6 900.4761
y7−NH3 900.4761
y7 917.4996
b8 1130.5874
E D A L E L T E K
y7 y5
b2 a2 b3 b5 b6 b8
Modification □ Acetyl (Protein N−term) ○ GlyGly (K) no new terminus △ Oxidation (M)

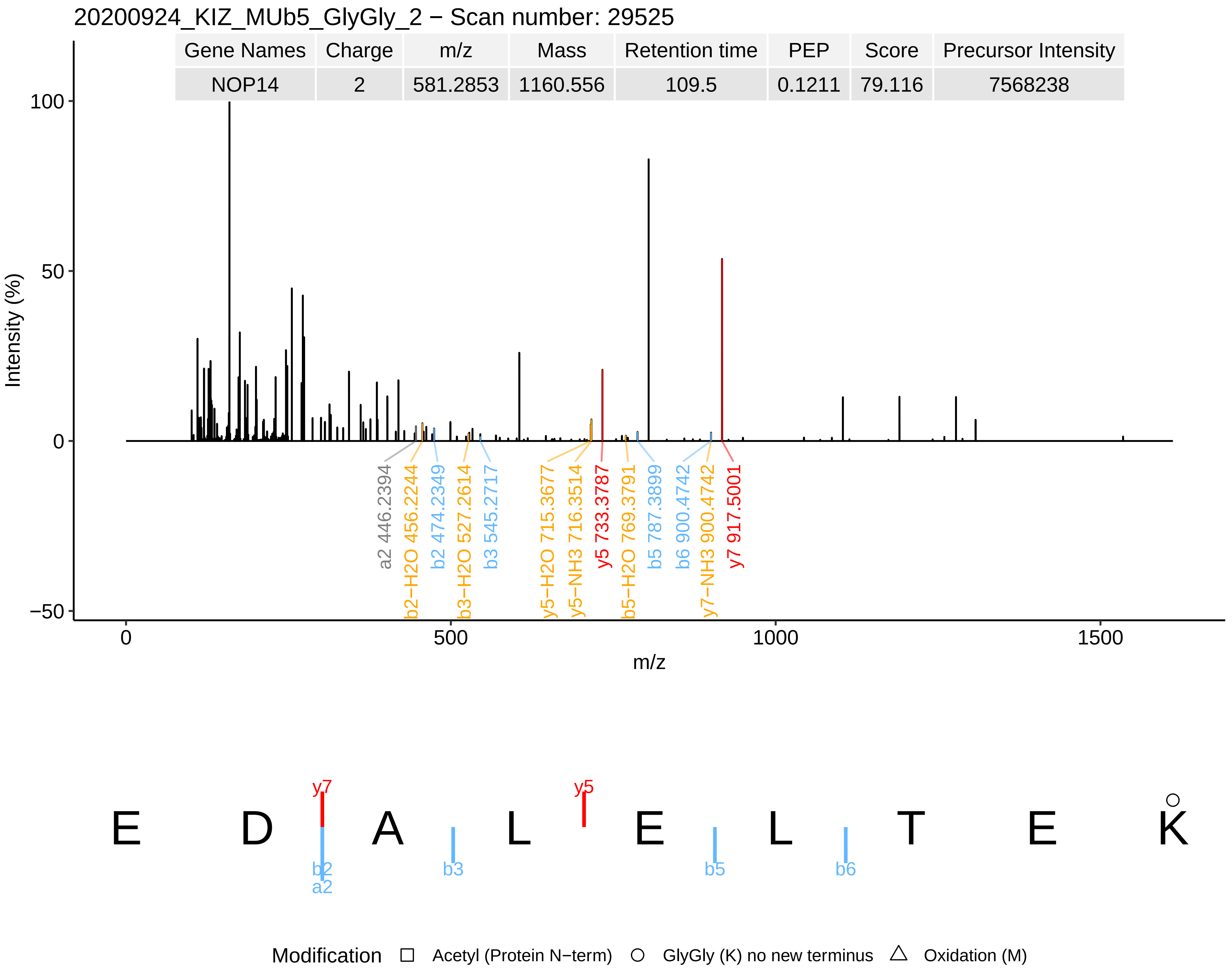
20200924_KIZ_MUb5_GlyGly_2 − Scan number: 29525
Gene Names
Charge
m/z
Mass
Retention time
PEP
Score
Precursor Intensity
NOP14
2
581.2853
1160.556
109.5
0.1211
79.116
7568238
Intensity (%)
100
50
0
−50
m/z
0
500
1000
1500
a2 446.2394
b2−H2O 456.2244
b2 474.2349
b3−H2O 527.2614
b3 545.2717
y5−H2O 715.3677
y5−NH3 716.3514
y5 733.3787
b5−H2O 769.3791
b5 787.3899
b6 900.4742
y7−NH3 900.4742
y7 917.5001
E D A L E L T E K
y7
y5
b2
a2
b3
b5
b6
Modification
Acetyl (Protein N−term)
GlyGly (K) no new terminus
Oxidation (M)

20200921_KIZ_MUb4_GlyGly_10plex − Scan number: 3536

Gene Names	Charge	m/z	Mass	Retention time	PEP	Score	Precursor Intensity
GABARAP	3	395.8598	1184.557	11.258	0.019807	95.531	15351934

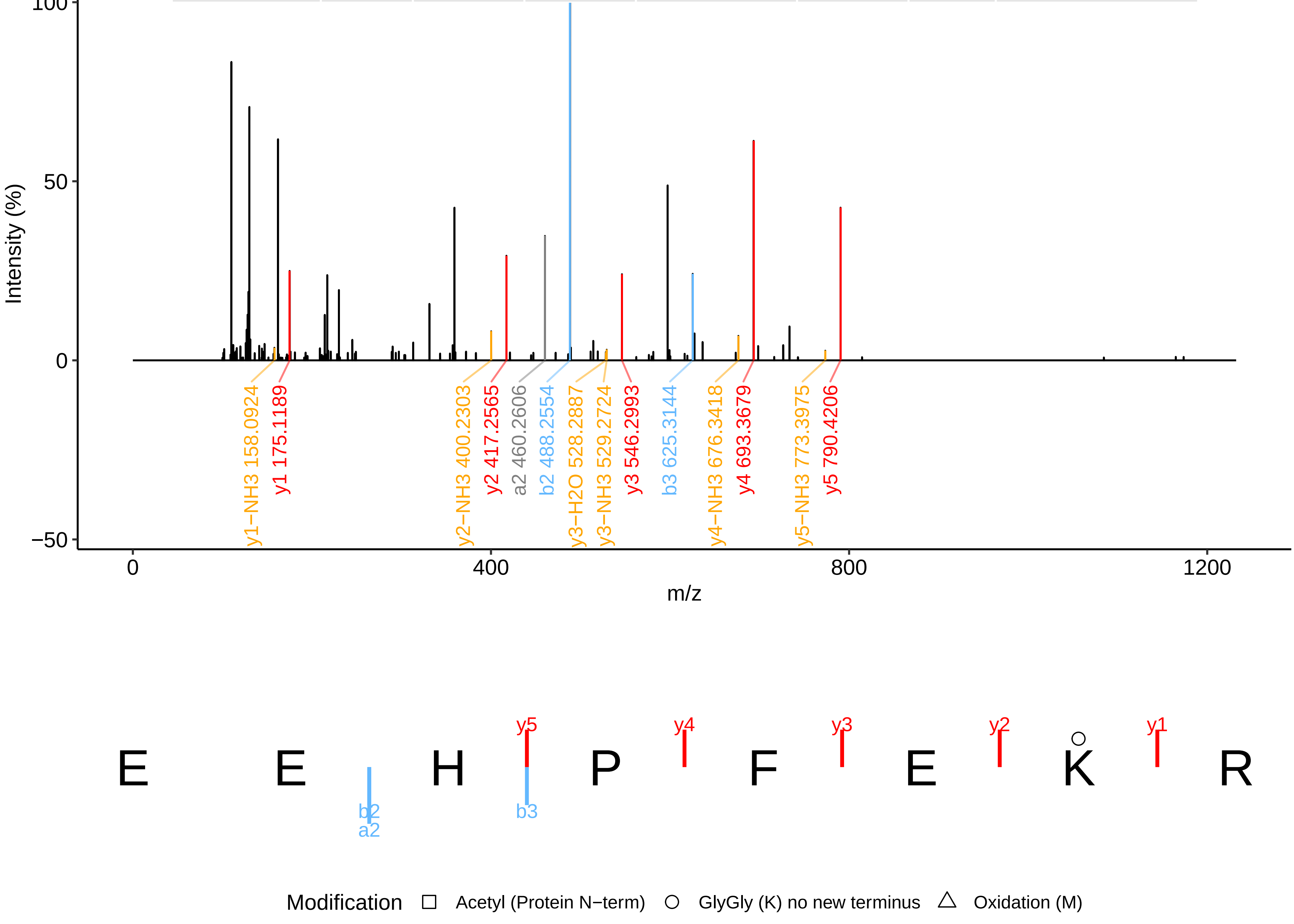

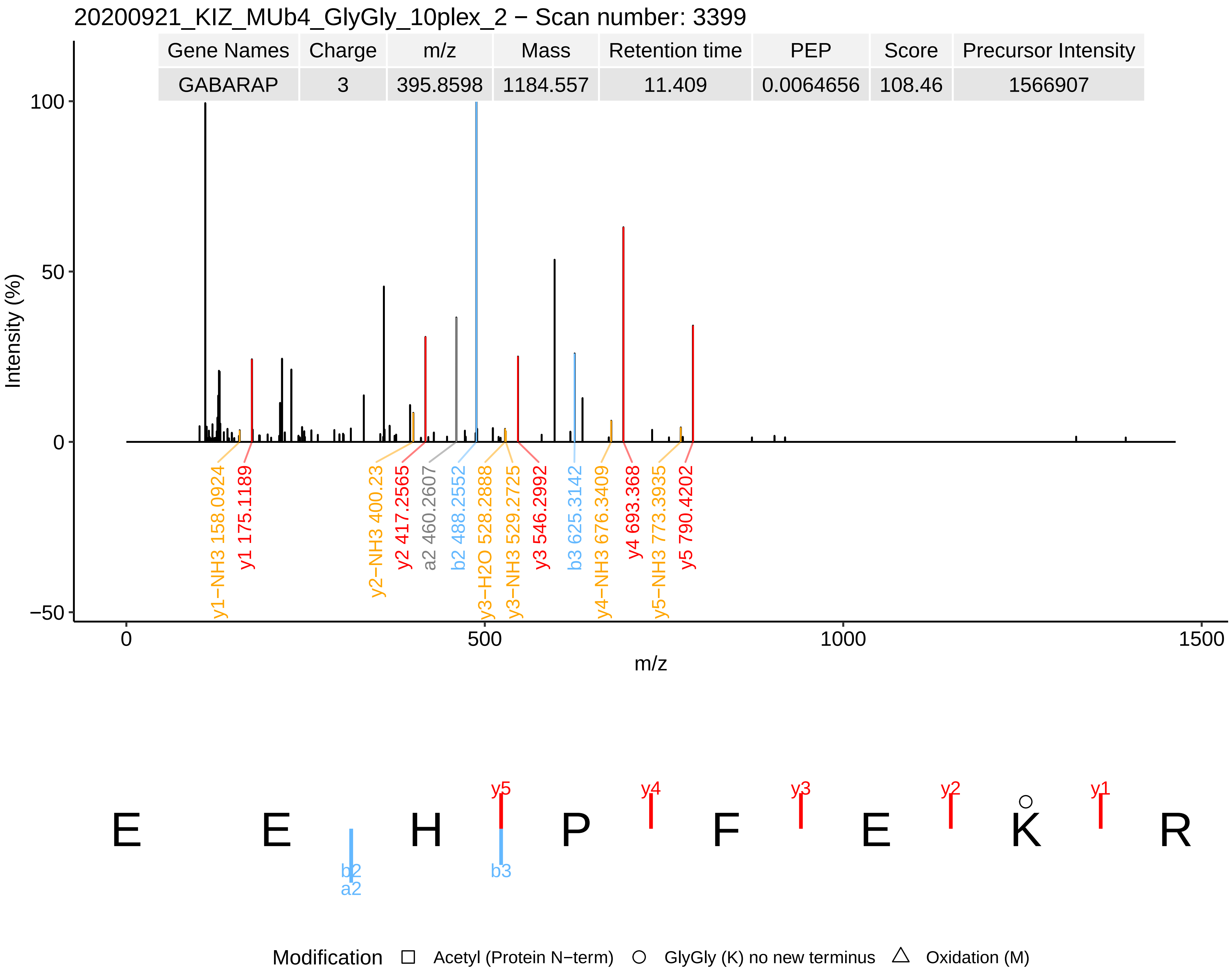

20200921_KIZ_MUb4_GlyGly_10plex_2 − Scan number: 3399
Gene Names
GABARAP
Charge
3
m/z
395.8598
Mass
1184.557
Retention time
11.409
PEP
0.0064656
Score
108.46
Precursor Intensity
1566907
Intensity (%)
m/z
y1−NH3 158.0924
y1 175.1189
y2−NH3 400.23
y2 417.2565
a2 460.2607
b2 488.2552
y3−H2O 528.2888
y3−NH3 529.2725
y3 546.2992
b3 625.3142
y4−NH3 676.3409
y4 693.368
y5−NH3 773.3935
y5 790.4202
E E H P F E K R
y5 y4 y3 y2 y1
b2 a2 b3
Modification
Acetyl (Protein N−term)
GlyGly (K) no new terminus
Oxidation (M)

20200921_KIZ_MUb4_GlyGly_10plex − Scan number: 7693

Gene Names	Charge	m/z	Mass	Retention time	PEP	Score	Precursor Intensity
RBBP7	3	325.1819	972.524	25.907	0.062079	55.549	5935111

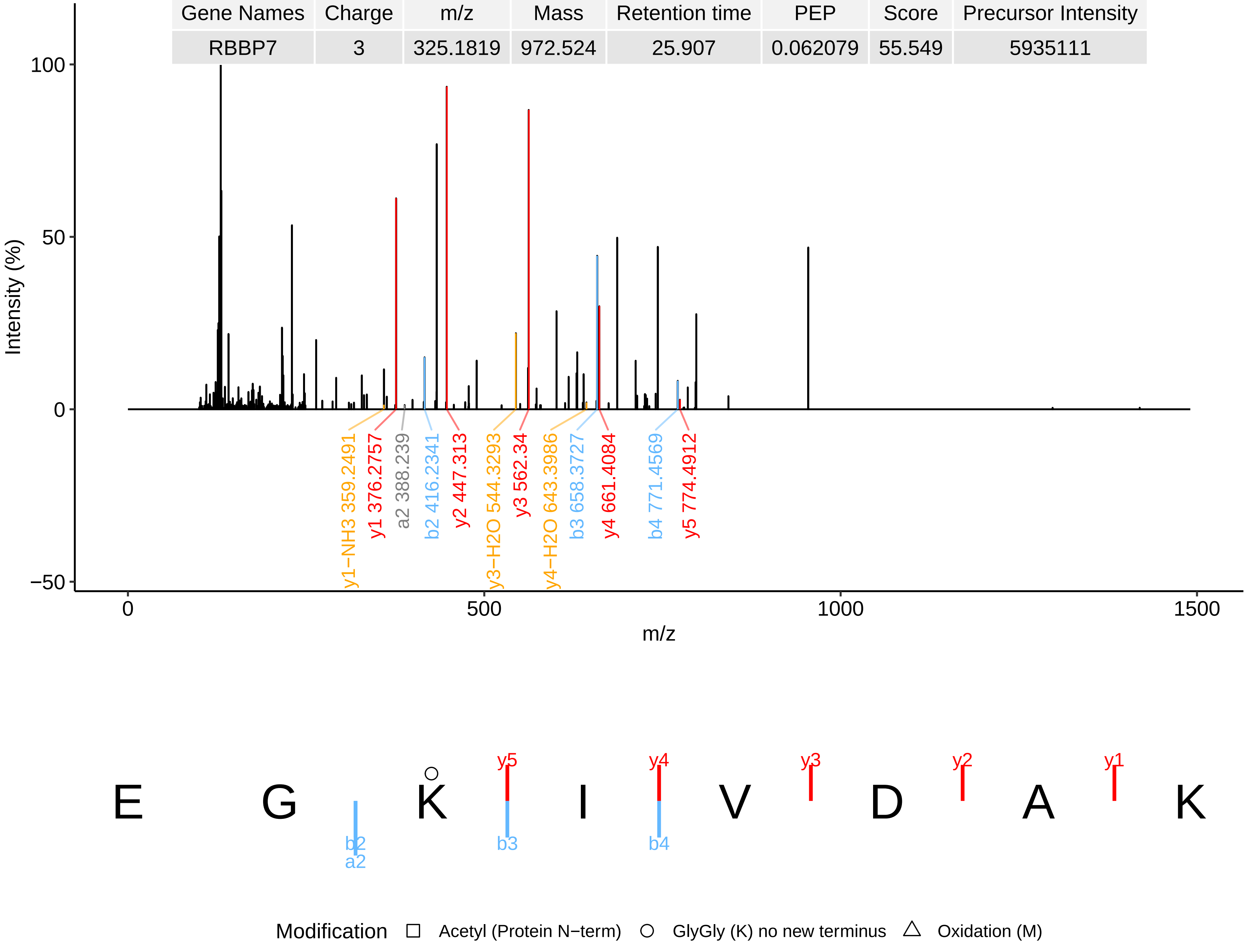

20200921_KIZ_MUb4_GlyGly_10plex_2 − Scan number: 7096

Gene Names	Charge	m/z	Mass	Retention time	PEP	Score	Precursor Intensity
RBBP7	3	325.1819	972.524	26.354	0.089807	48.794	2218309

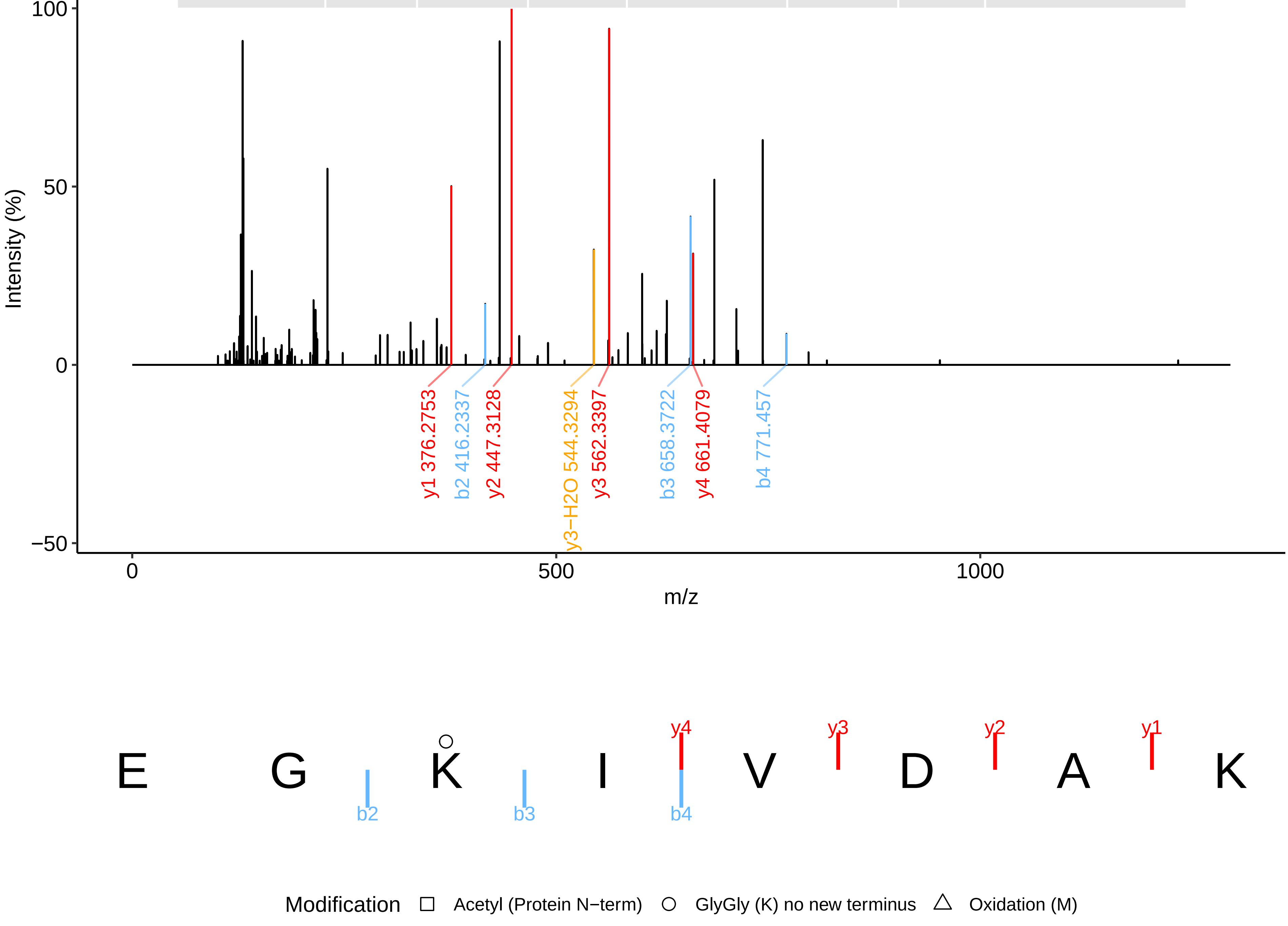

20200921_KIZ_MUb4_GlyGly_10plex − Scan number: 5091

Gene Names	Charge	m/z	Mass	Retention time	PEP	Score	Precursor Intensity
RMDN3	3	396.5387	1186.594	16.617	0.0096479	74.841	5119063

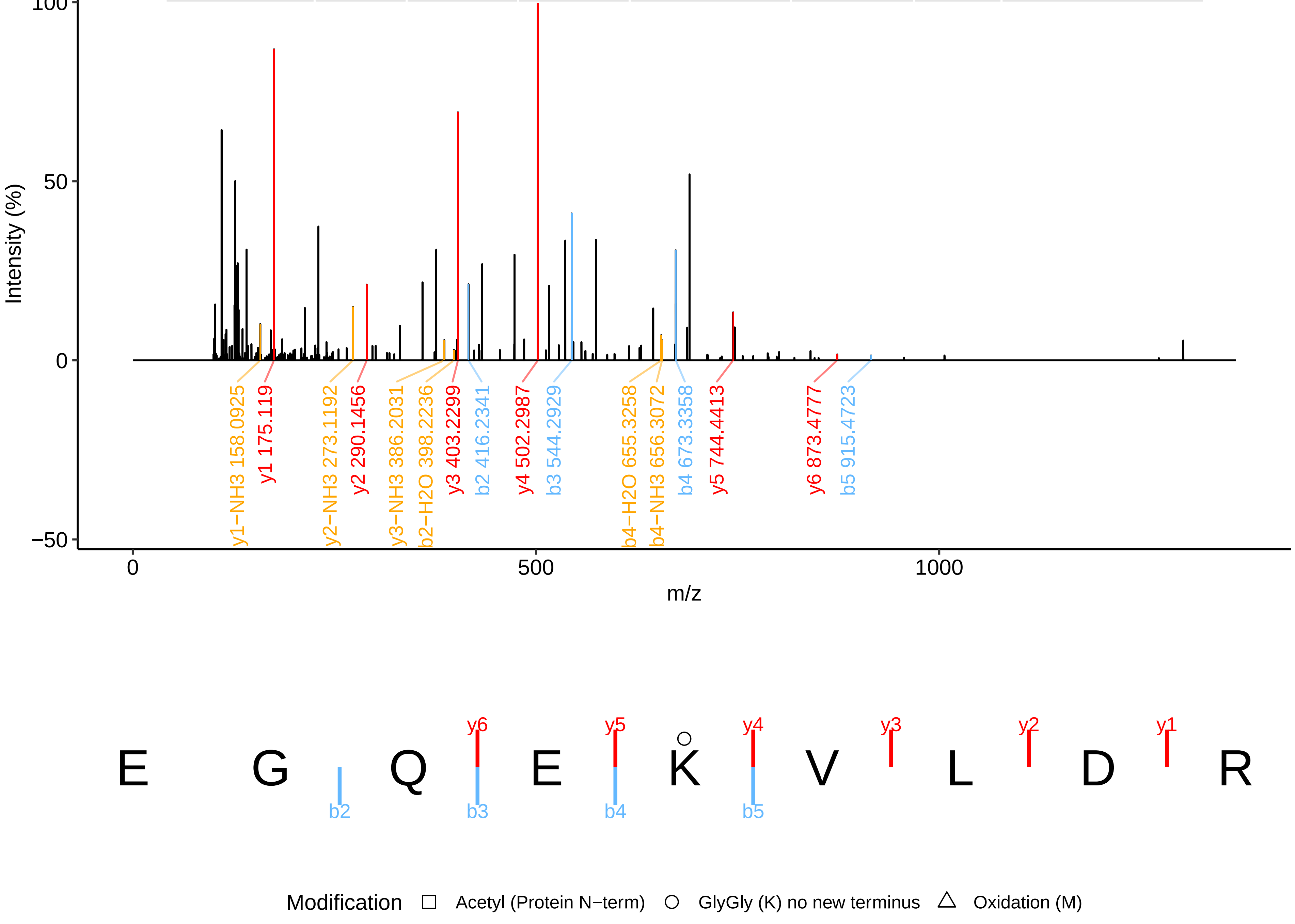

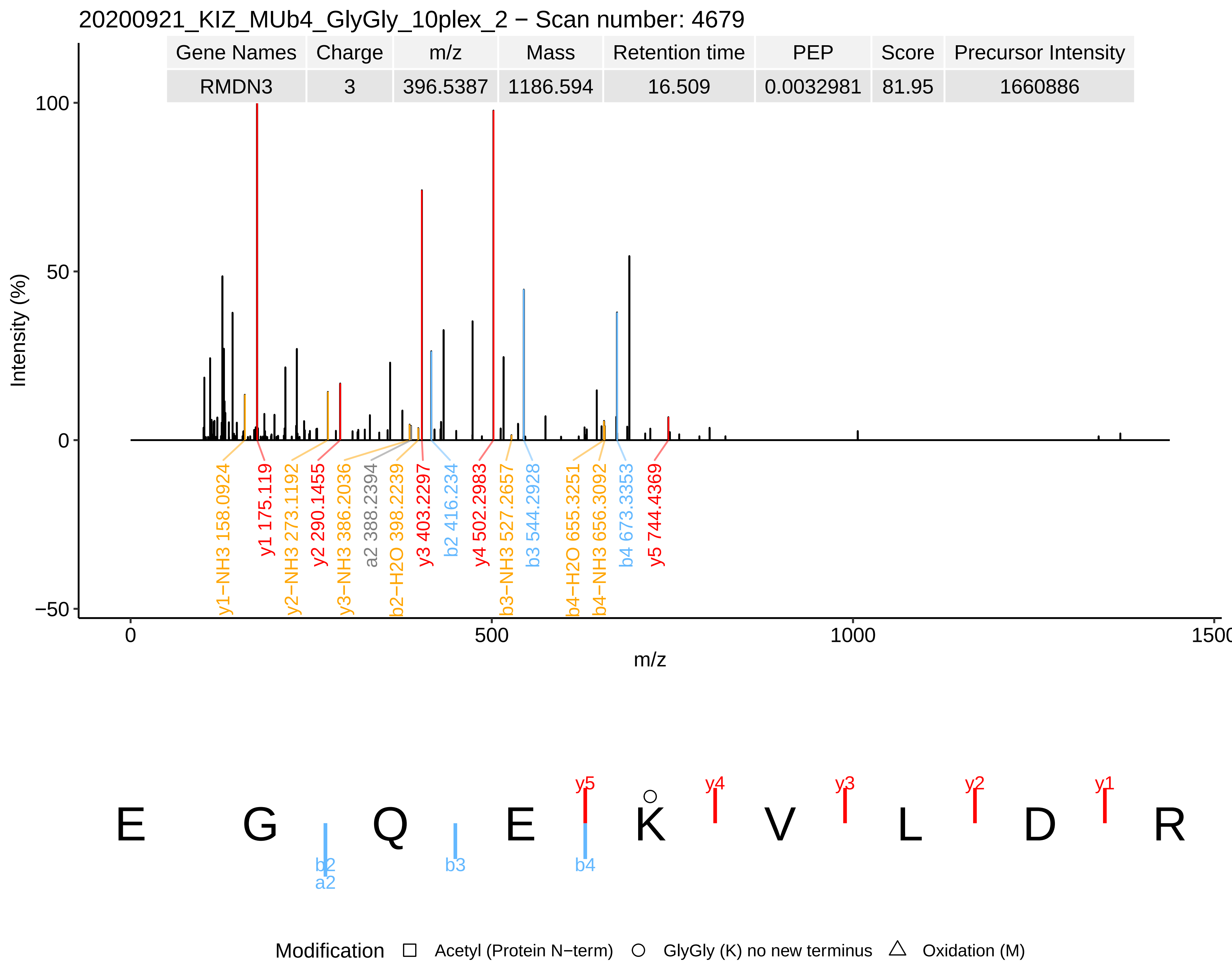

20200921_KIZ_MUb4_GlyGly_10plex_2 − Scan number: 4679
Gene Names | Charge | m/z | Mass | Retention time | PEP | Score | Precursor Intensity
RMDN3 | 3 | 396.5387 | 1186.594 | 16.509 | 0.0032981 | 81.95 | 1660886
Intensity (%)
m/z
y1−NH3 158.0924
y1 175.119
y2−NH3 273.1192
y2 290.1455
y3−NH3 386.2036
a2 388.2394
b2−H2O 398.2239
y3 403.2297
b2 416.234
y4 502.2983
b3−NH3 527.2657
b3 544.2928
b4−H2O 655.3251
b4−NH3 656.3092
b4 673.3353
y5 744.4369
E G Q E K V L D R
y5 y4 y3 y2 y1
b2 a2 b3 b4
Modification □ Acetyl (Protein N−term) ○ GlyGly (K) no new terminus △ Oxidation (M)

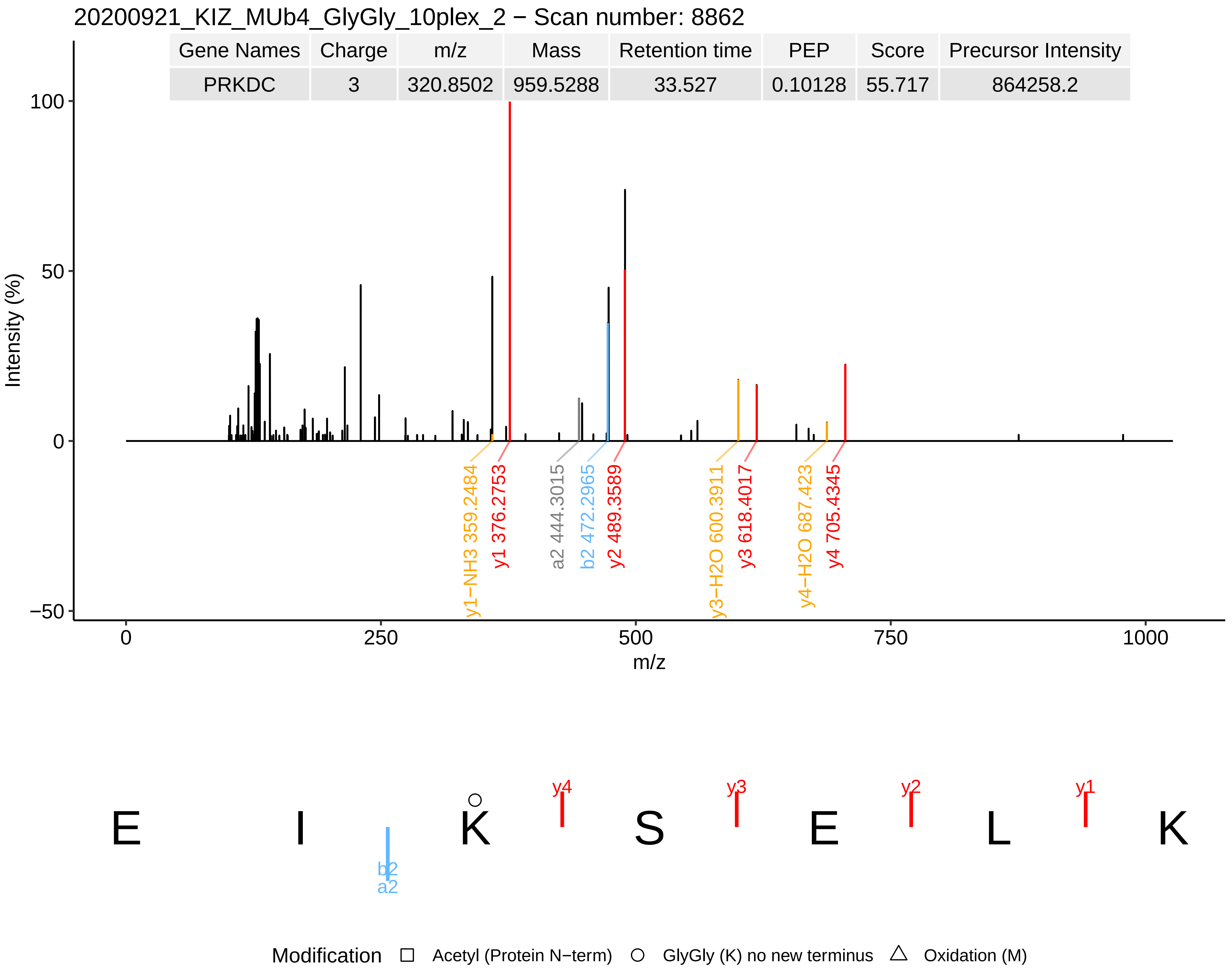

20200921_KIZ_MUb4_GlyGly_10plex_2 − Scan number: 8862
Gene Names | Charge | m/z | Mass | Retention time | PEP | Score | Precursor Intensity
PRKDC | 3 | 320.8502 | 959.5288 | 33.527 | 0.10128 | 55.717 | 864258.2
Intensity (%)
100
50
0
−50
0
250
500
750
1000
m/z
y1−NH3 359.2484
y1 376.2753
a2 444.3015
b2 472.2965
y2 489.3589
y3−H2O 600.3911
y3 618.4017
y4−H2O 687.423
y4 705.4345
E I K S E L K
y4
y3
y2
y1
b2
a2
Modification
Acetyl (Protein N−term)
GlyGly (K) no new terminus
Oxidation (M)

20201118_KIZ_MUb6_GlyGly_2 − Scan number: 17149

Gene Names	Charge	m/z	Mass	Retention time	PEP	Score	Precursor Intensity
SMG1	3	401.2047	1200.592	57.975	0.018037	67.981	5860180

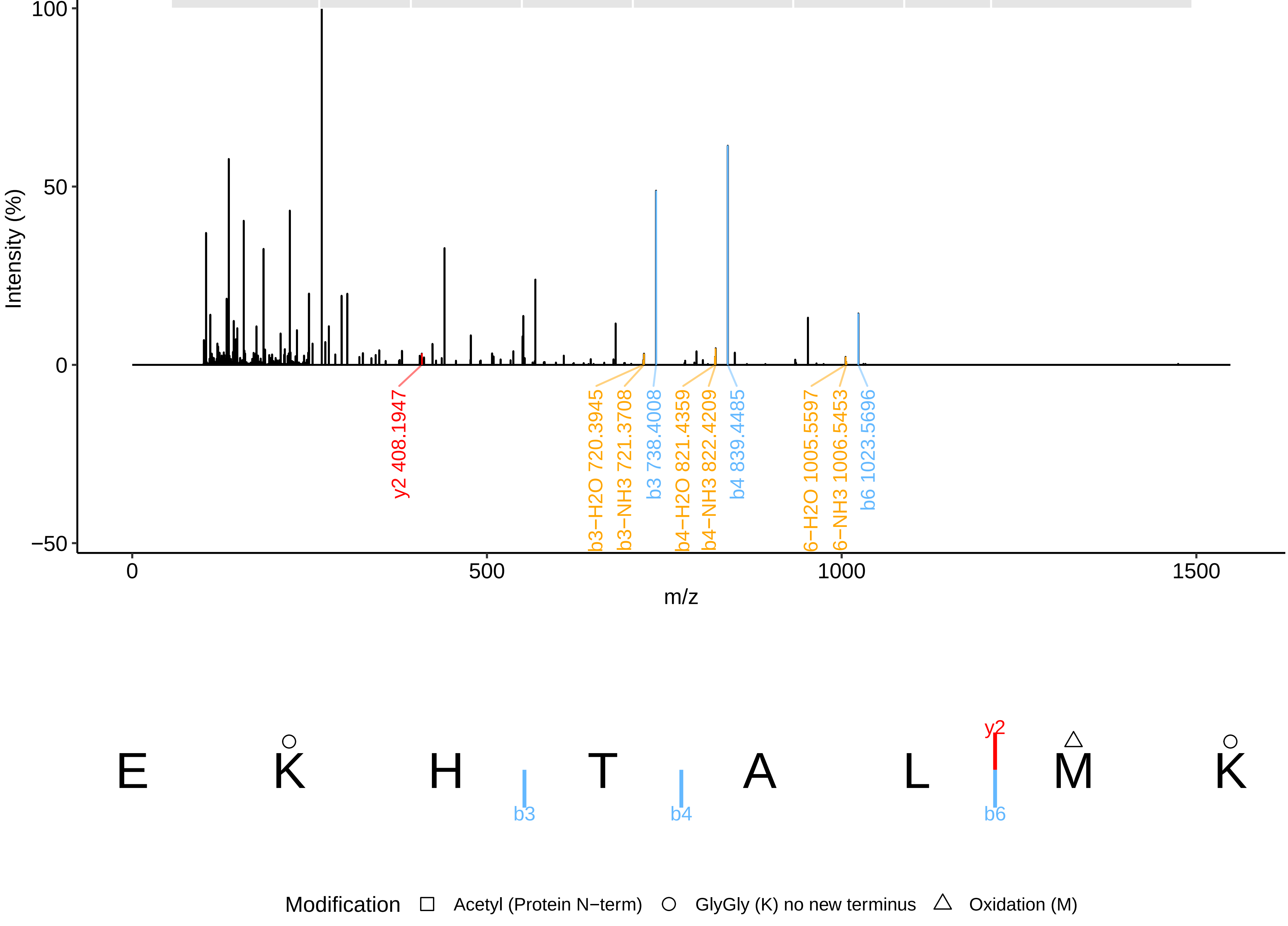

20200921_KIZ_MUb4_GlyGly_10plex_2 − Scan number: 10056

Gene Names	Charge	m/z	Mass	Retention time	PEP	Score	Precursor Intensity
RNF5	3	392.2242	1173.651	38.374	0.11377	43.982	2067831

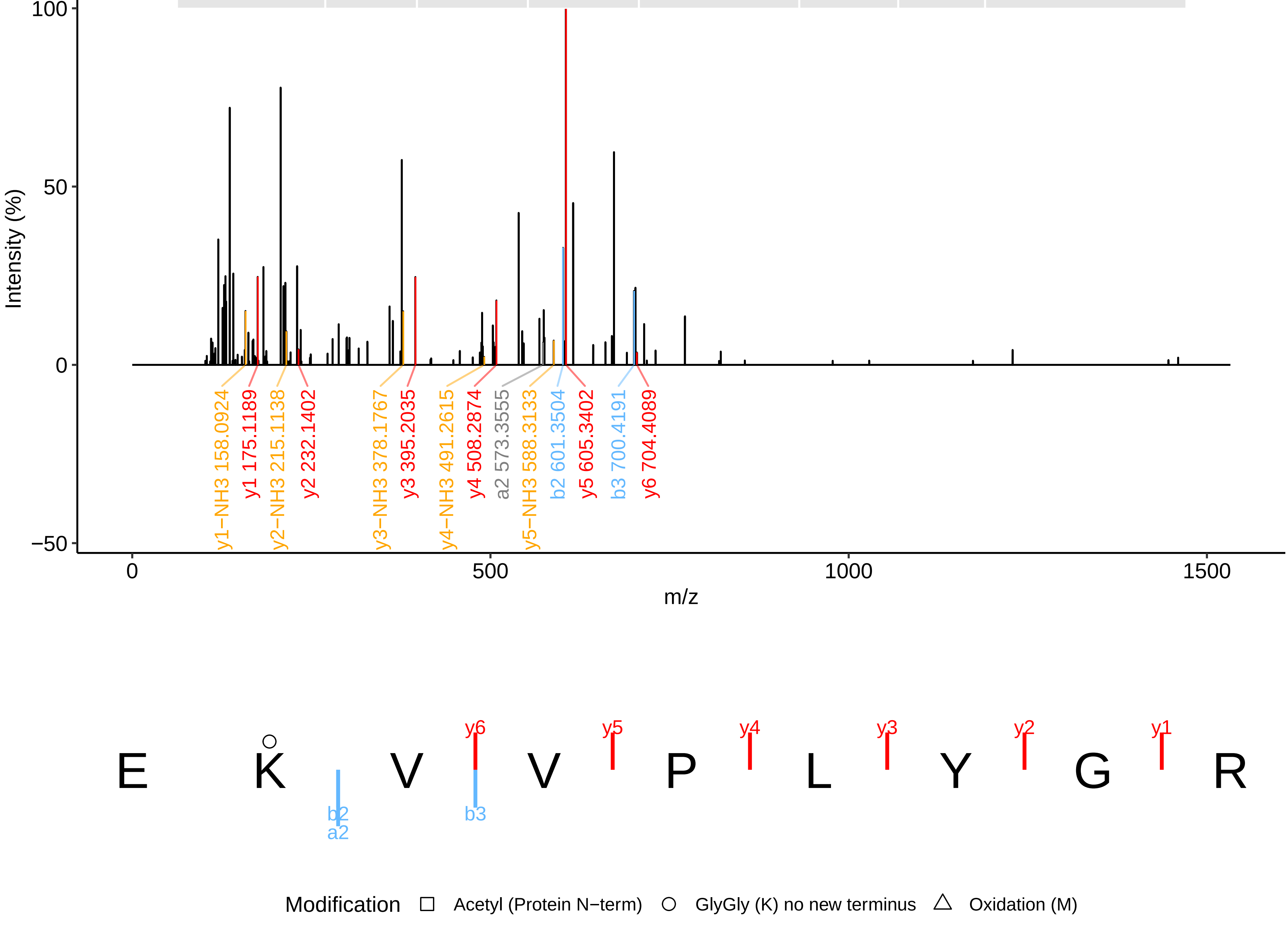

20200921_KIZ_MUb4_GlyGly_10plex − Scan number: 16633

Gene Names	Charge	m/z	Mass	Retention time	PEP	Score	Precursor Intensity
S100A4	3	387.5523	1159.635	59.808	0.00011035	117.89	3373869

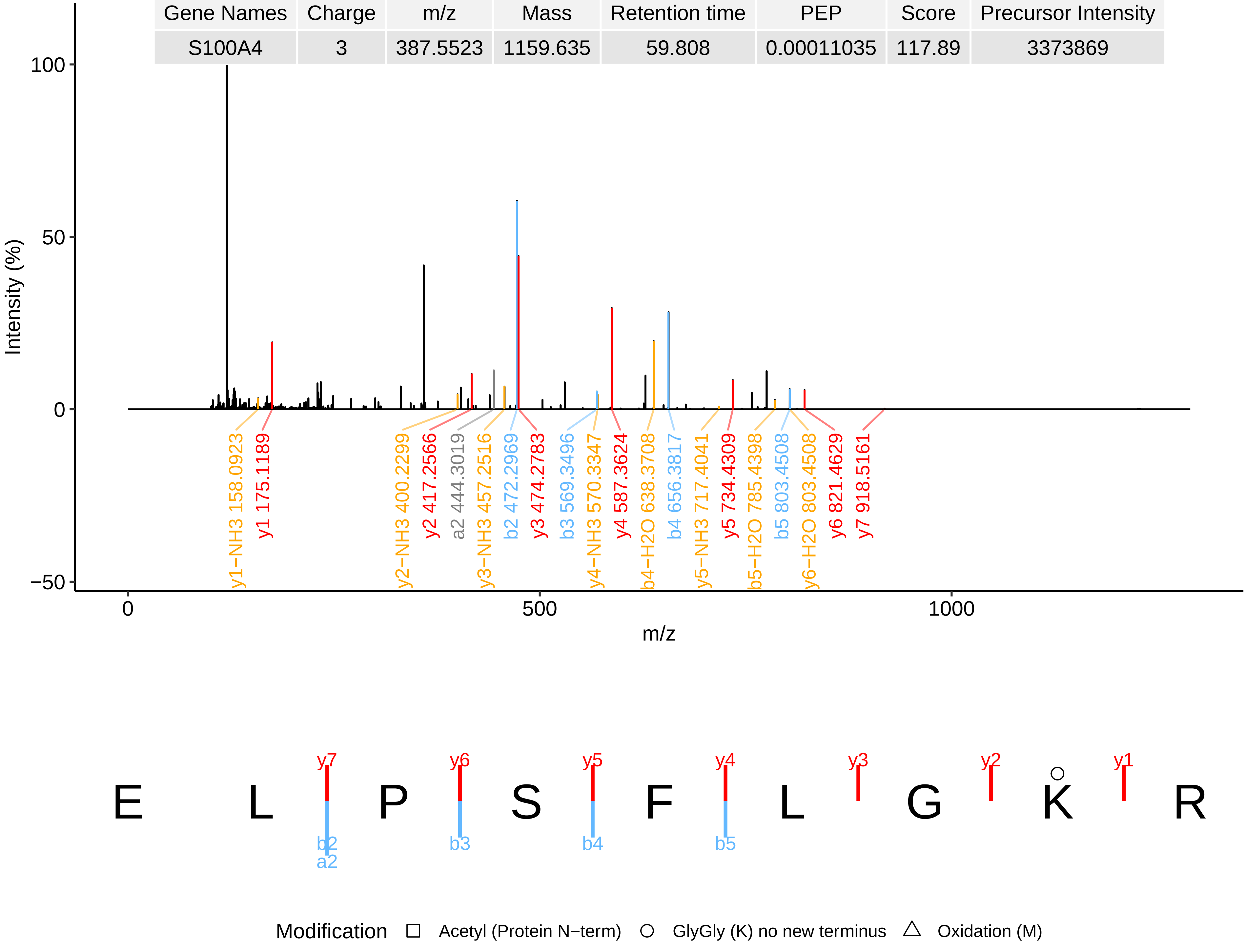

20200921_KIZ_MUb4_GlyGly_10plex − Scan number: 16760

Gene Names	Charge	m/z	Mass	Retention time	PEP	Score	Precursor Intensity
S100A4	3	387.5523	1159.635	60.312	8.7121e−05	121.09	28591878

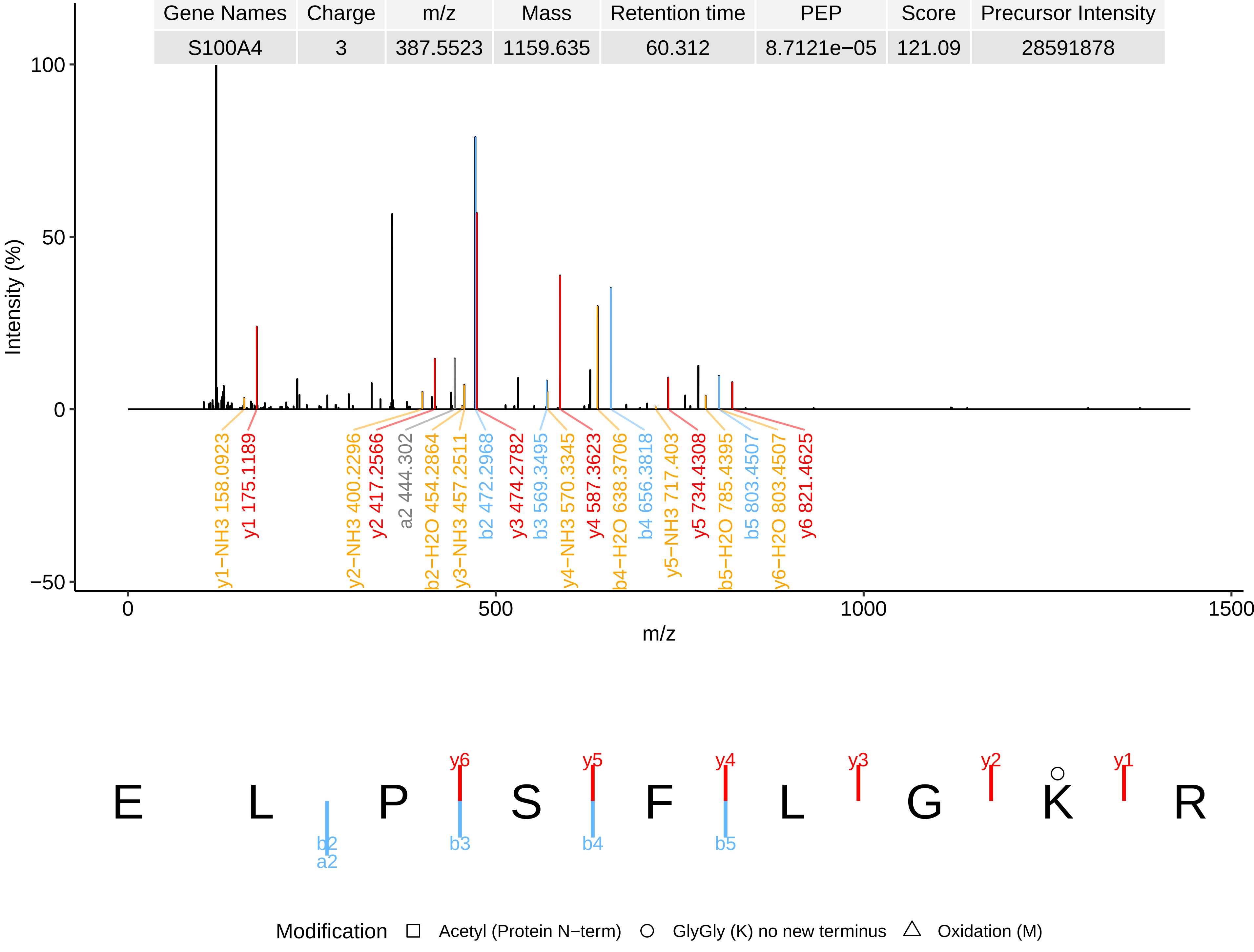

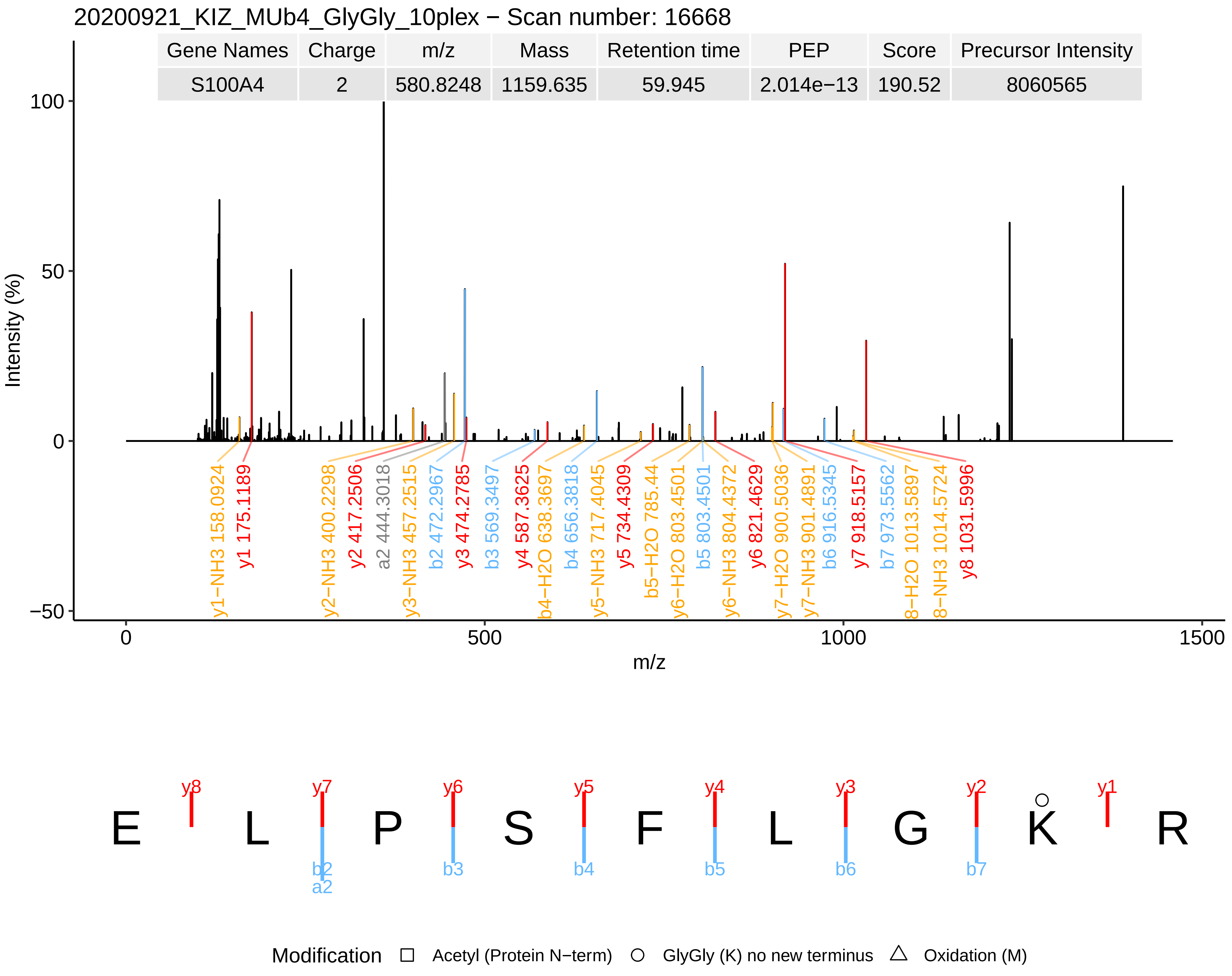
20200921_KIZ_MUb4_GlyGly_10plex − Scan number: 16668
Gene Names | Charge | m/z | Mass | Retention time | PEP | Score | Precursor Intensity
S100A4 | 2 | 580.8248 | 1159.635 | 59.945 | 2.014e−13 | 190.52 | 8060565
Intensity (%)
100
50
0
−50
0
500
1000
1500
m/z
y1−NH3 158.0924
y1 175.1189
y2−NH3 400.2298
y2 417.2506
a2 444.3018
y3−NH3 457.2515
b2 472.2967
y3 474.2785
b3 569.3497
y4 587.3625
b4−H2O 638.3697
b4 656.3818
y5−NH3 717.4045
y5 734.4309
b5−H2O 785.44
y6−H2O 803.4501
b5 803.4501
y6−NH3 804.4372
y6 821.4629
y7−H2O 900.5036
y7−NH3 901.4891
b6 916.5345
y7 918.5157
b7 973.5562
8−H2O 1013.5897
8−NH3 1014.5724
y8 1031.5996
E L P S F L G K R
y8 y7 y6 y5 y4 y3 y2 y1
b2 a2 b3 b4 b5 b6 b7
Modification
Acetyl (Protein N−term)
GlyGly (K) no new terminus
Oxidation (M)

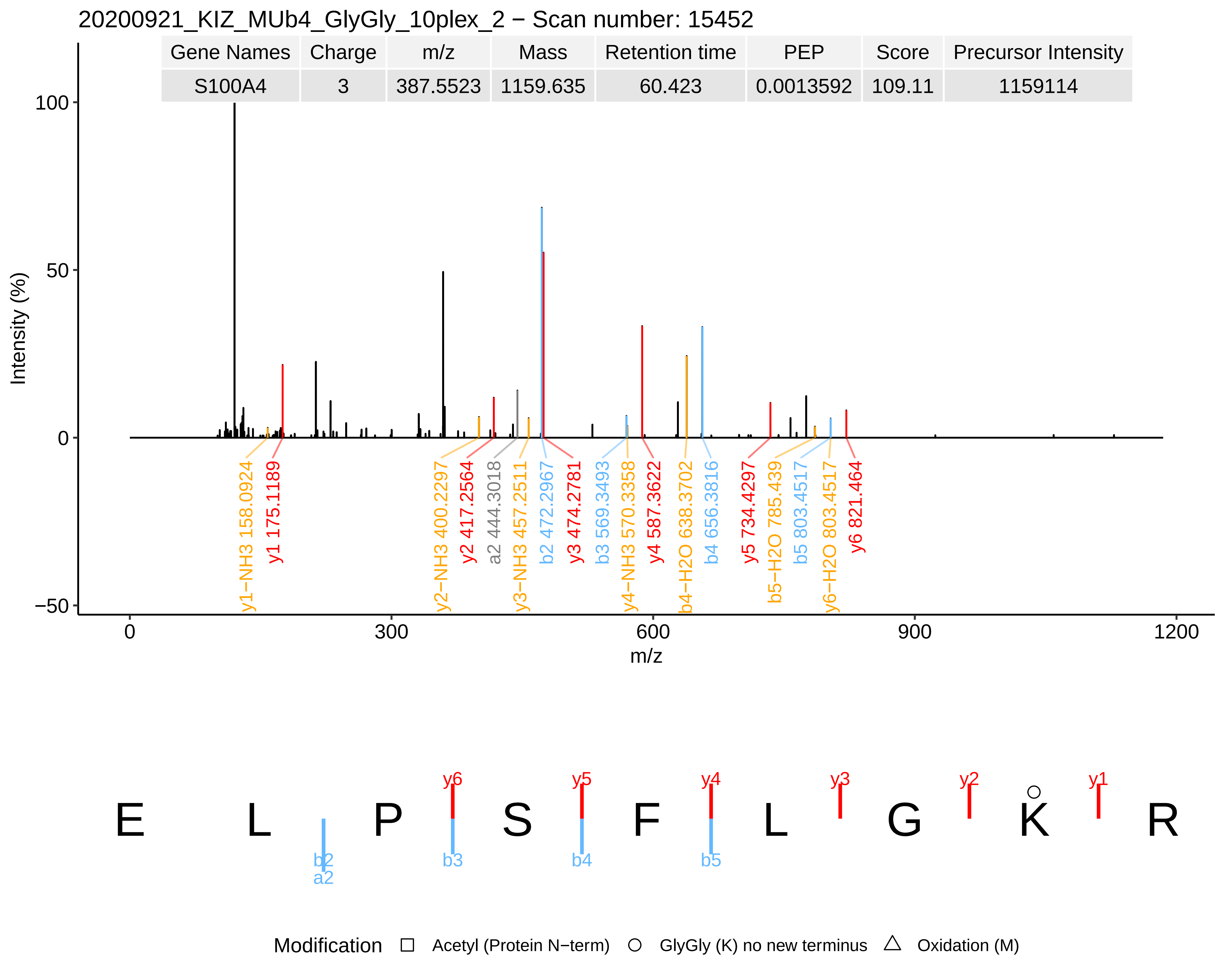
20200921_KIZ_MUb4_GlyGly_10plex_2 − Scan number: 15452
Gene Names | Charge | m/z | Mass | Retention time | PEP | Score | Precursor Intensity
S100A4 | 3 | 387.5523 | 1159.635 | 60.423 | 0.0013592 | 109.11 | 1159114
Intensity (%)
100
50
0
−50
0
300
600
900
1200
m/z
y1−NH3 158.0924
y1 175.1189
y2−NH3 400.2297
y2 417.2564
a2 444.3018
y3−NH3 457.2511
b2 472.2967
y3 474.2781
b3 569.3493
y4−NH3 570.3358
y4 587.3622
b4−H2O 638.3702
b4 656.3816
y5 734.4297
b5−H2O 785.439
b5 803.4517
y6−H2O 803.4517
y6 821.464
E L P S F L G K R
y6 y5 y4 y3 y2 y1
b2 a2 b3 b4 b5
Modification
Acetyl (Protein N−term)
GlyGly (K) no new terminus
Oxidation (M)

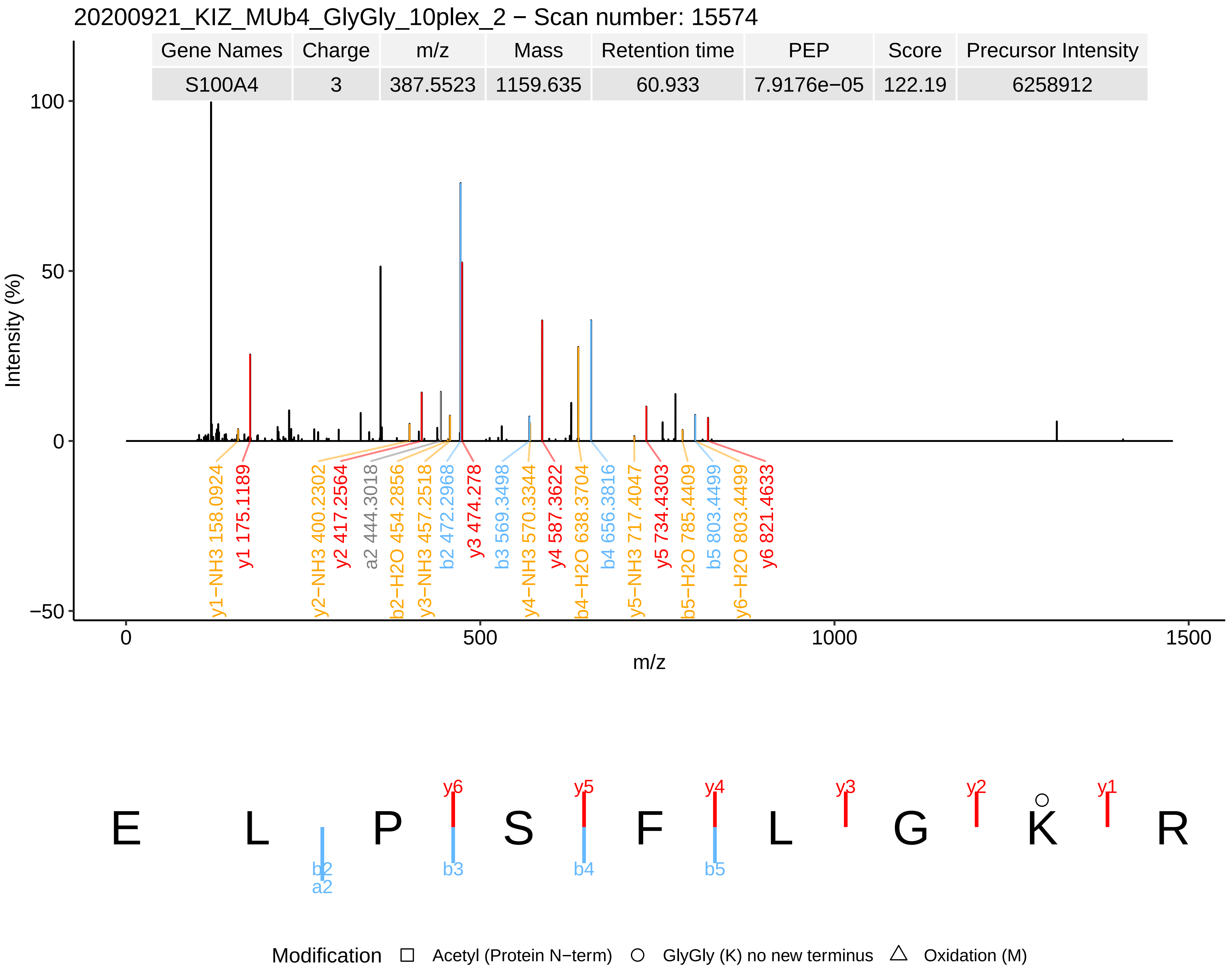
20200921_KIZ_MUb4_GlyGly_10plex_2 − Scan number: 15574
Gene Names	Charge	m/z	Mass	Retention time	PEP	Score	Precursor Intensity
S100A4	3	387.5523	1159.635	60.933	7.9176e−05	122.19	6258912
Intensity (%)
m/z
y1−NH3 158.0924
y1 175.1189
y2−NH3 400.2302
y2 417.2564
a2 444.3018
b2−H2O 454.2856
y3−NH3 457.2518
b2 472.2968
y3 474.278
b3 569.3498
y4−NH3 570.3344
y4 587.3622
b4−H2O 638.3704
b4 656.3816
y5−NH3 717.4047
y5 734.4303
b5−H2O 785.4409
b5 803.4499
y6−H2O 803.4499
y6 821.4633
E L P S F L G K R
y6 y5 y4 y3 y2 y1
b2 a2 b3 b4 b5
Modification
Acetyl (Protein N−term)
GlyGly (K) no new terminus
Oxidation (M)

20200921_KIZ_MUb4_GlyGly_10plex_2 − Scan number: 15498

Gene Names	Charge	m/z	Mass	Retention time	PEP	Score	Precursor Intensity
S100A4	2	580.8248	1159.635	60.618	0.00019604	122.18	942365.6

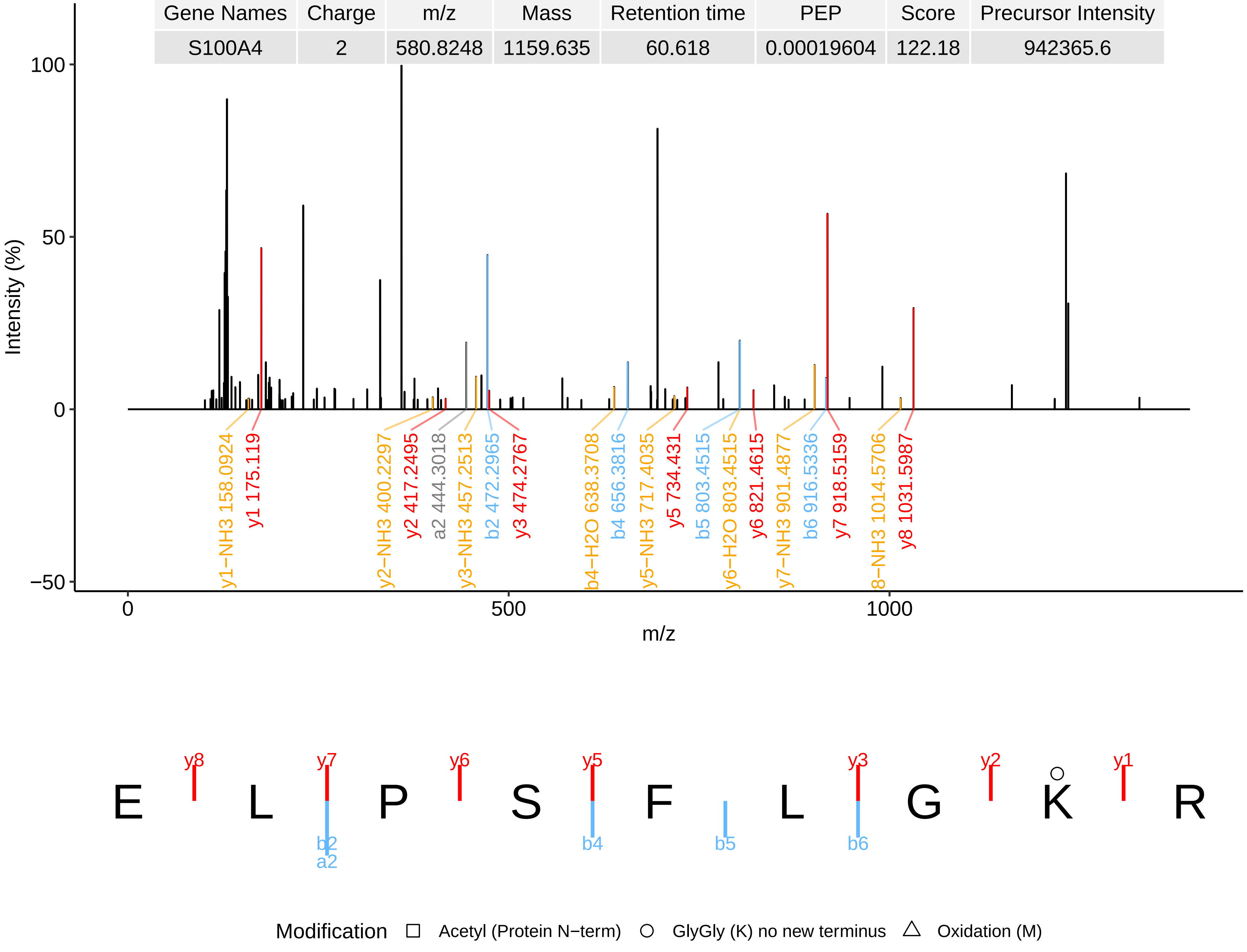

20200924_KIZ_MUb5_GlyGly_2 − Scan number: 14780

Gene Names	Charge	m/z	Mass	Retention time	PEP	Score	Precursor Intensity
S100A4	3	387.5523	1159.635	55.221	0.0033073	99.136	5508420

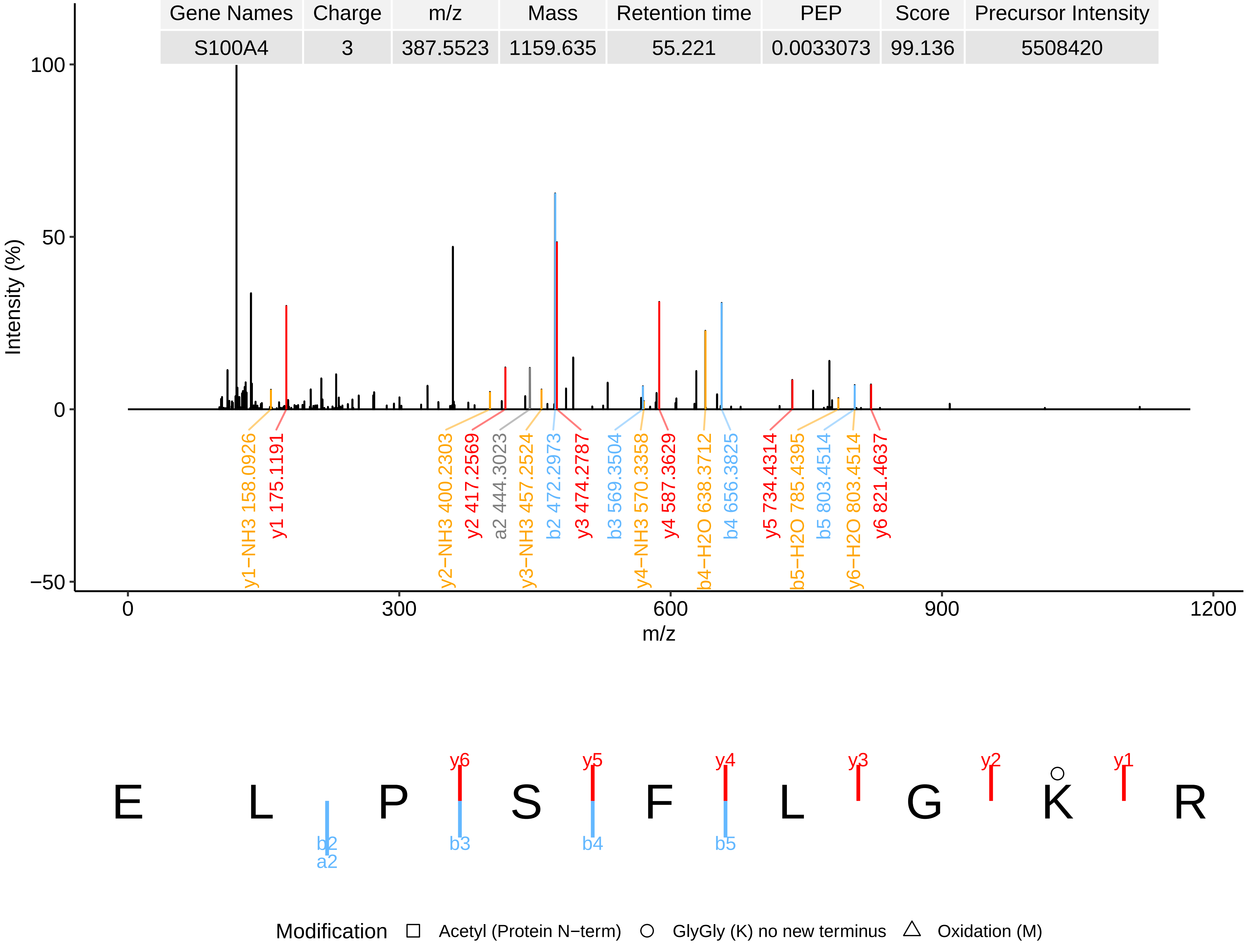

20201118_KIZ_MUb6_GlyGly_2 − Scan number: 18050

Gene Names	Charge	m/z	Mass	Retention time	PEP	Score	Precursor Intensity
S100A4	3	387.5523	1159.635	61.059	0.0039033	109.11	9995990

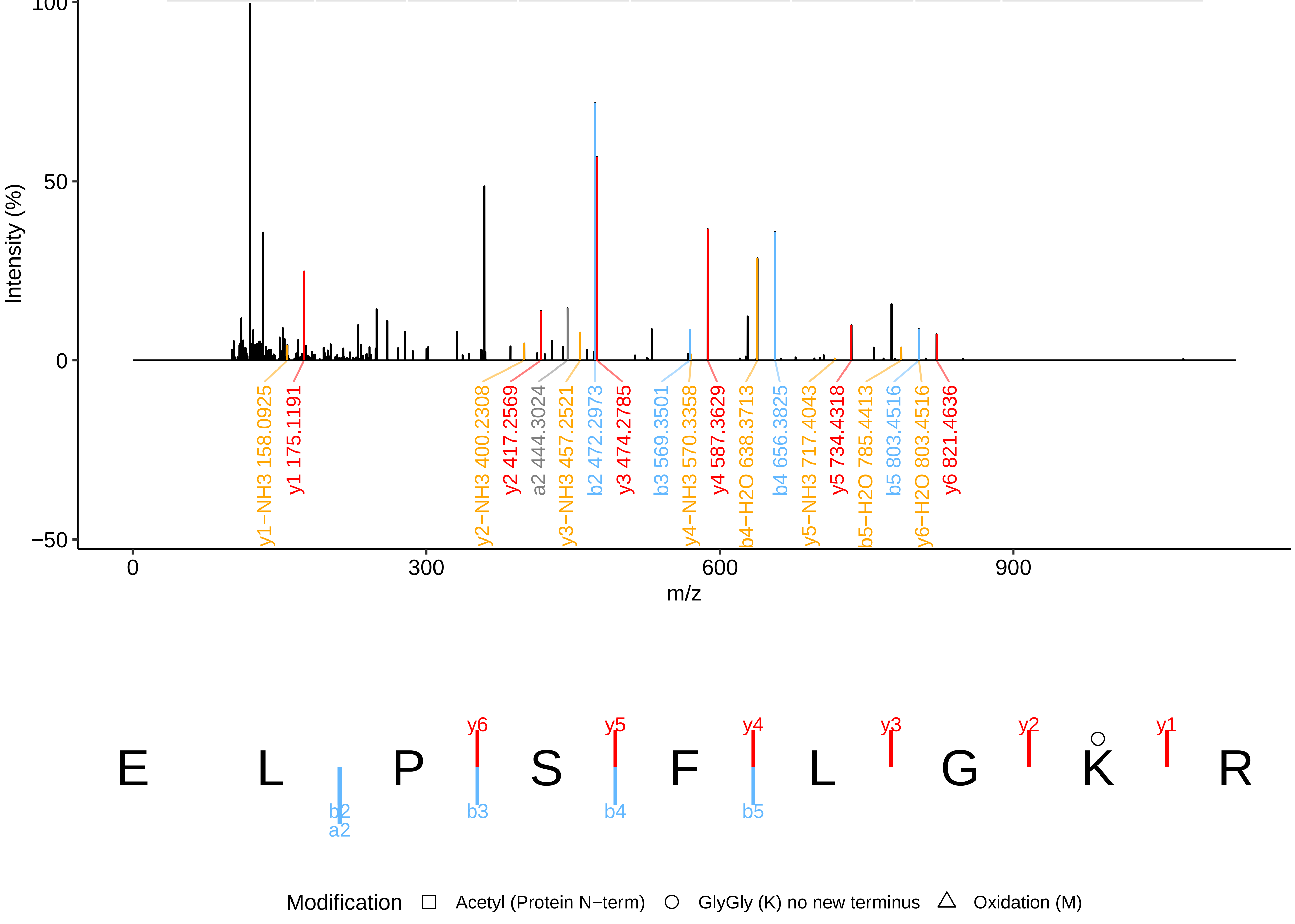

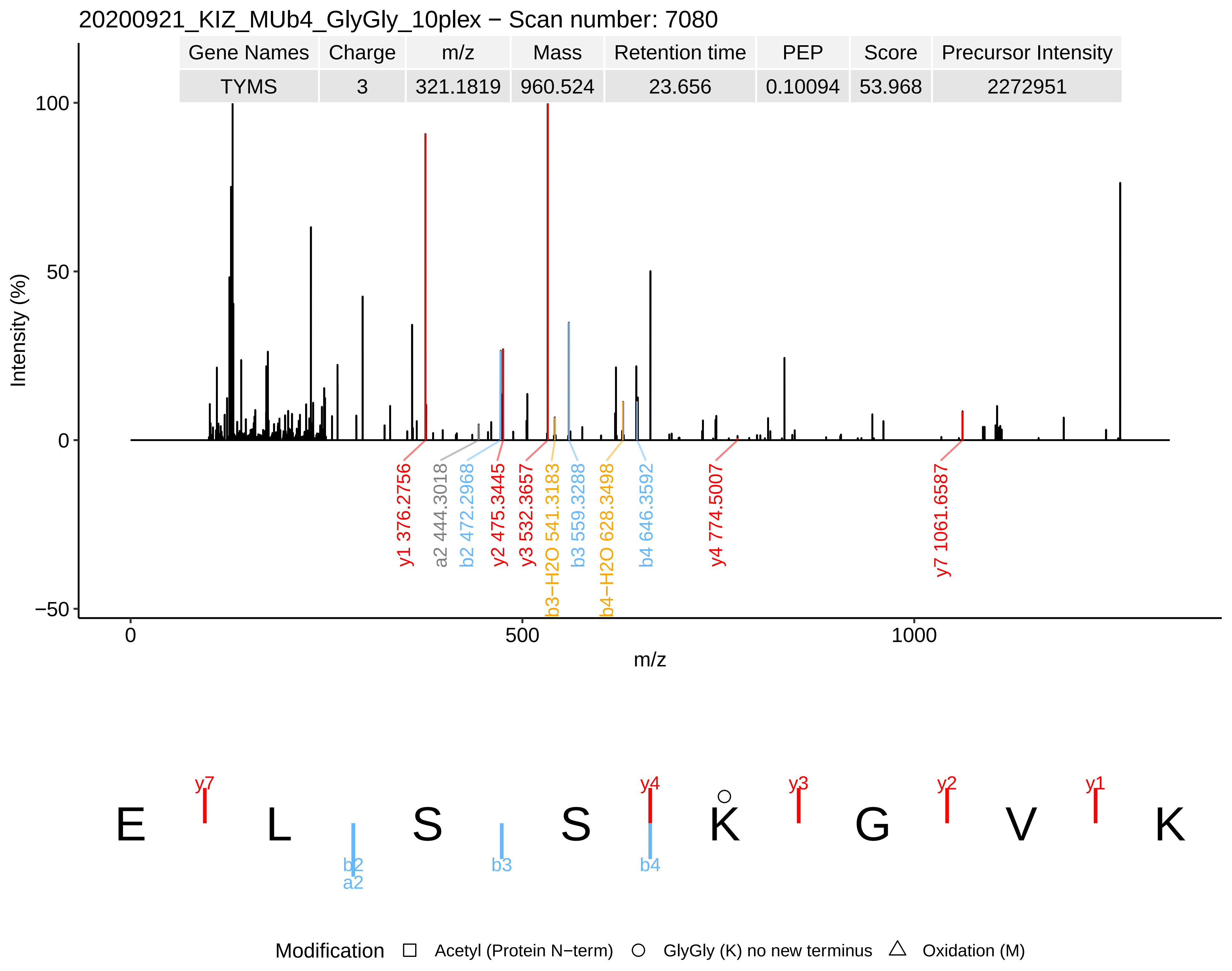

20200921_KIZ_MUb4_GlyGly_10plex − Scan number: 7080
Gene Names | Charge | m/z | Mass | Retention time | PEP | Score | Precursor Intensity
TYMS | 3 | 321.1819 | 960.524 | 23.656 | 0.10094 | 53.968 | 2272951
Intensity (%)
m/z
y1 376.2756
a2 444.3018
b2 472.2968
y2 475.3445
y3 532.3657
b3−H2O 541.3183
b3 559.3288
b4−H2O 628.3498
b4 646.3592
y4 774.5007
y7 1061.6587
E L S S K G V K
y7 y4 y3 y2 y1
b2 a2 b3 b4
Modification
Acetyl (Protein N−term)
GlyGly (K) no new terminus
Oxidation (M)

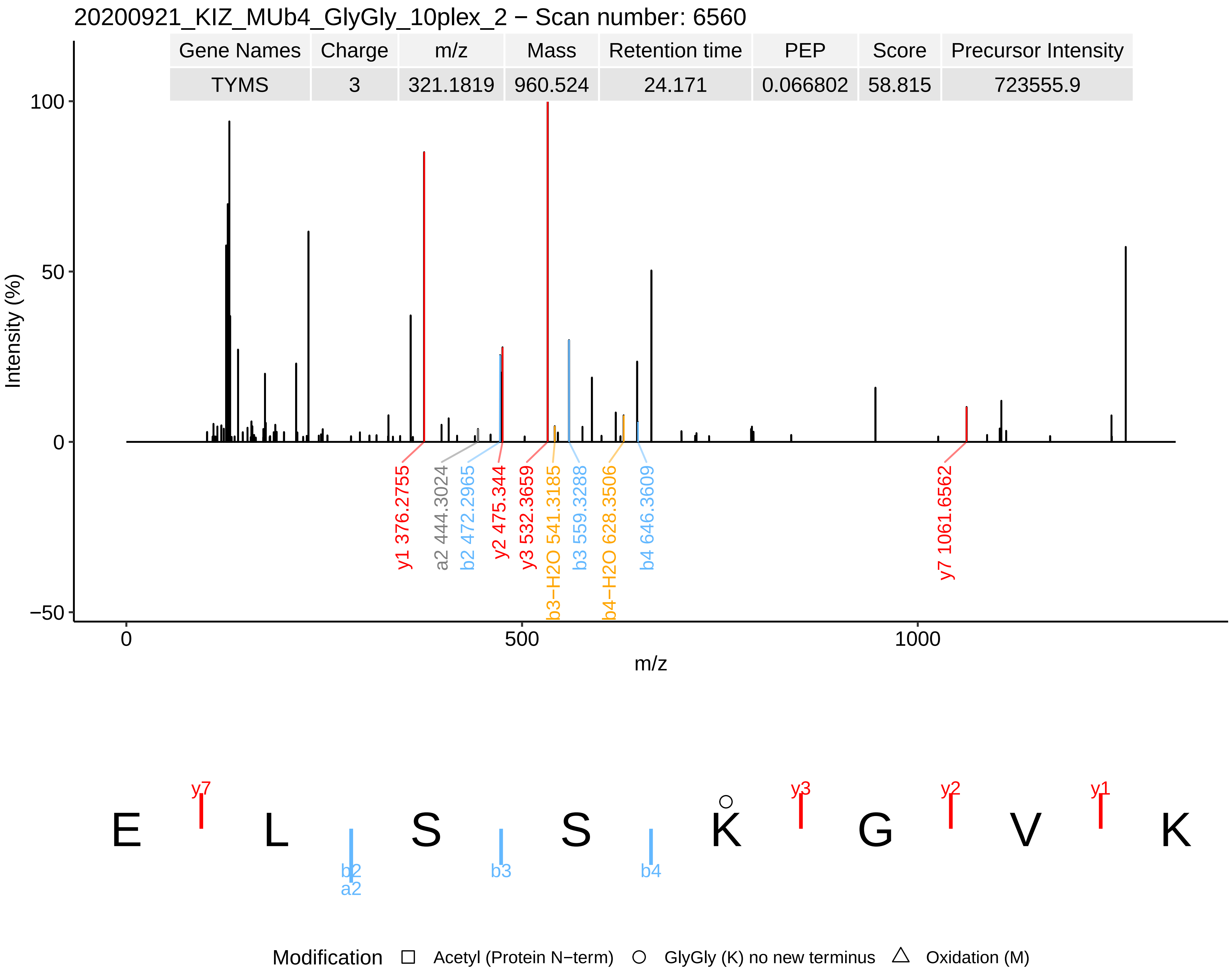

20200921_KIZ_MUb4_GlyGly_10plex_2 − Scan number: 6560
Gene Names | Charge | m/z | Mass | Retention time | PEP | Score | Precursor Intensity
TYMS | 3 | 321.1819 | 960.524 | 24.171 | 0.066802 | 58.815 | 723555.9
Intensity (%)
m/z
y1 376.2755
a2 444.3024
b2 472.2965
y2 475.344
y3 532.3659
b3−H2O 541.3185
b3 559.3288
b4−H2O 628.3506
b4 646.3609
y7 1061.6562
E L S S K G V K
y7 y3 y2 y1
b2 a2 b3 b4
Modification
Acetyl (Protein N−term)
GlyGly (K) no new terminus
Oxidation (M)

20200921_KIZ_MUb4_GlyGly_10plex − Scan number: 11312

Gene Names	Charge	m/z	Mass	Retention time	PEP	Score	Precursor Intensity
AARS	3	311.5186	931.5339	39.354	0.039801	72.789	3878141

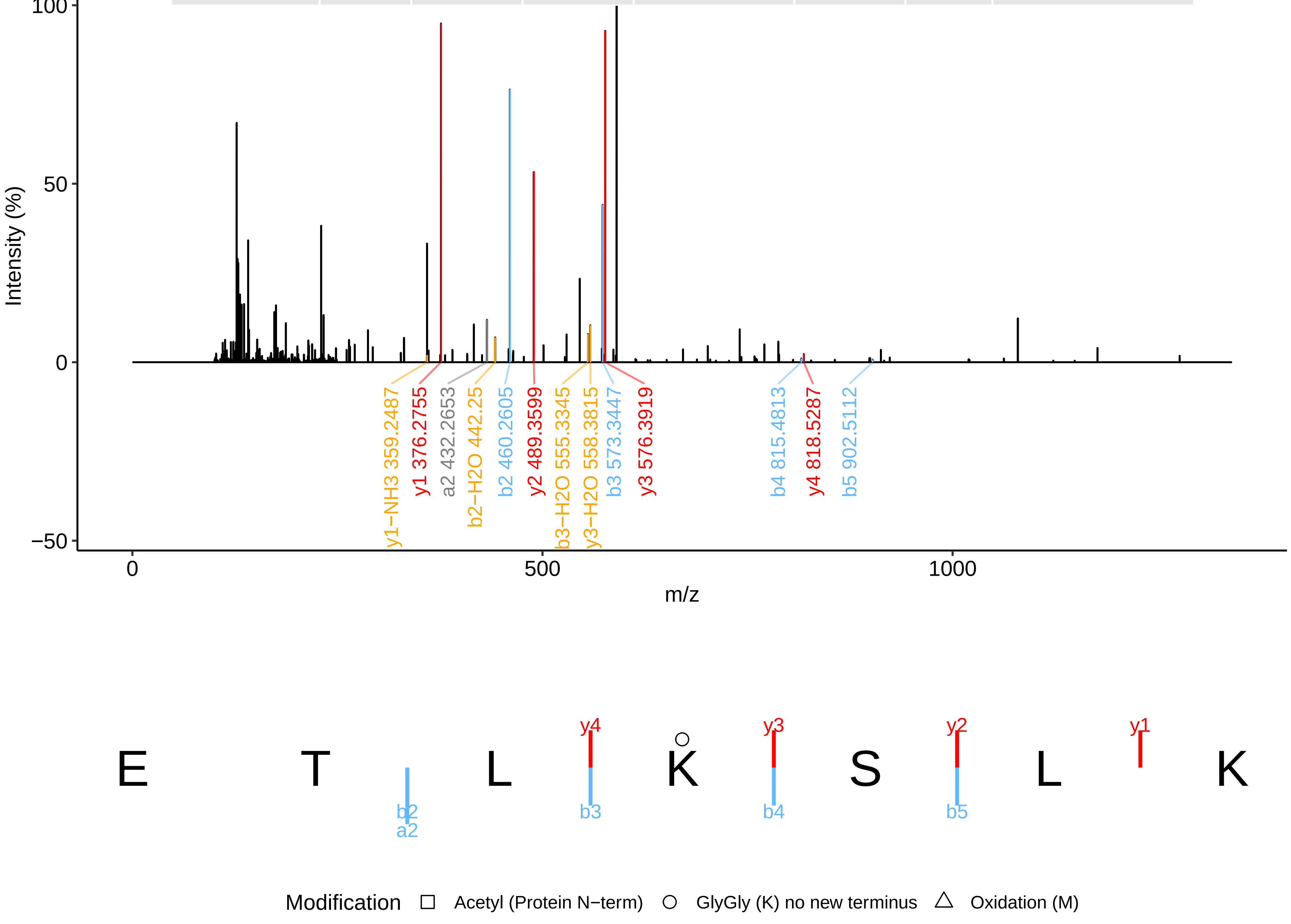

20200921_KIZ_MUb4_GlyGly_10plex_2 − Scan number: 10448

Gene Names	Charge	m/z	Mass	Retention time	PEP	Score	Precursor Intensity
AARS	3	311.5186	931.5339	39.975	0.10588	54.787	981817.2

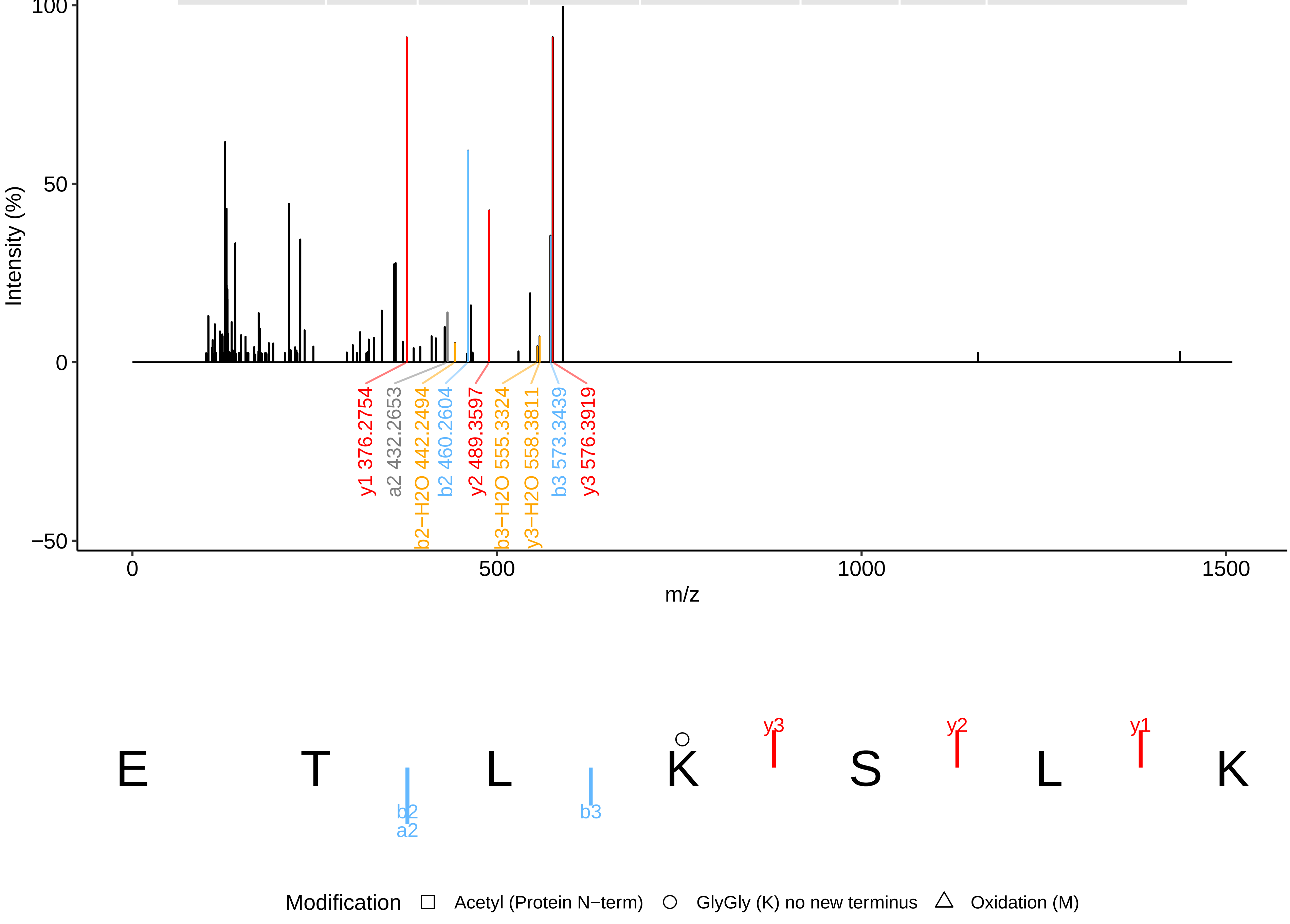

20200921_KIZ_MUb4_GlyGly_10plex − Scan number: 15766

Gene Names	Charge	m/z	Mass	Retention time	PEP	Score	Precursor Intensity
UBE2D3;UBE2D2;UBE2D1;UBE2D4	2	585.766	1169.517	56.419	0.054837	75.311	5401832

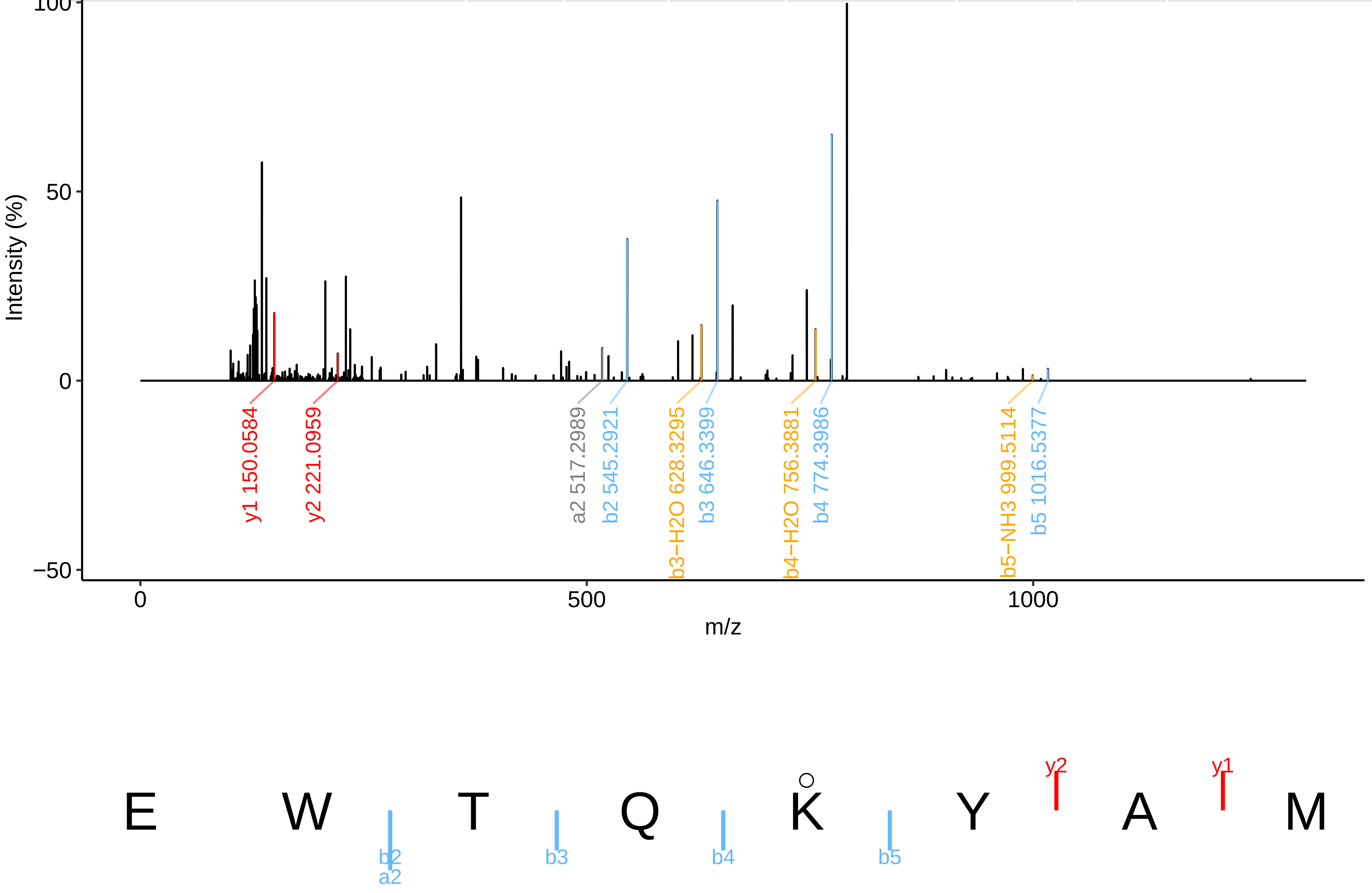

Modification □ Acetyl (Protein N−term) ○ GlyGly (K) no new terminus △ Oxidation (M)

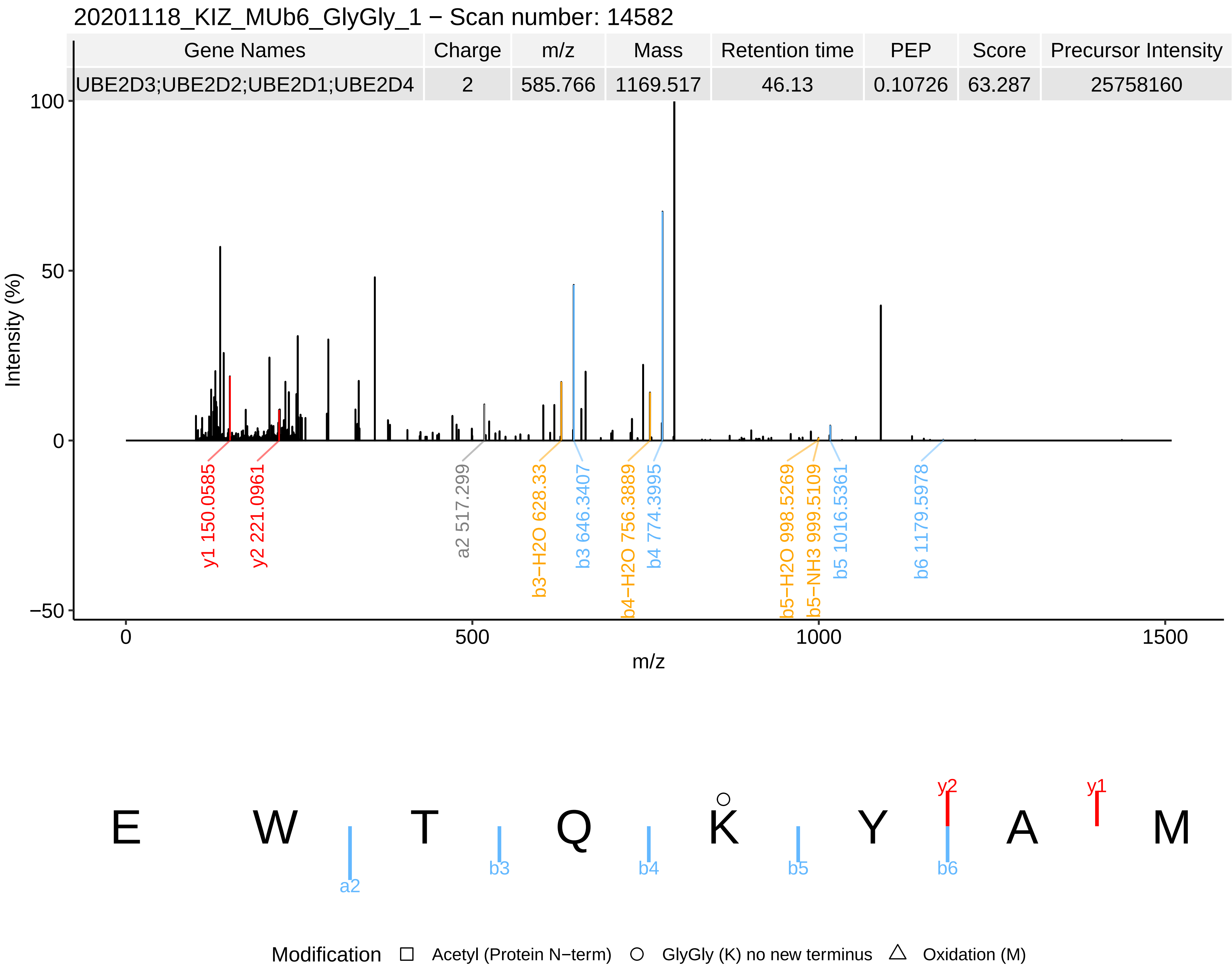

20201118_KIZ_MUb6_GlyGly_1 − Scan number: 14582
Gene Names | Charge | m/z | Mass | Retention time | PEP | Score | Precursor Intensity
UBE2D3;UBE2D2;UBE2D1;UBE2D4 | 2 | 585.766 | 1169.517 | 46.13 | 0.10726 | 63.287 | 25758160
Intensity (%)
m/z
y1 150.0585
y2 221.0961
a2 517.299
b3−H2O 628.33
b3 646.3407
b4−H2O 756.3889
b4 774.3995
b5−H2O 998.5269
b5−NH3 999.5109
b5 1016.5361
b6 1179.5978
E W T Q K Y A M
a2 b3 b4 b5 b6
y2 y1
Modification
Acetyl (Protein N−term)
GlyGly (K) no new terminus
Oxidation (M)

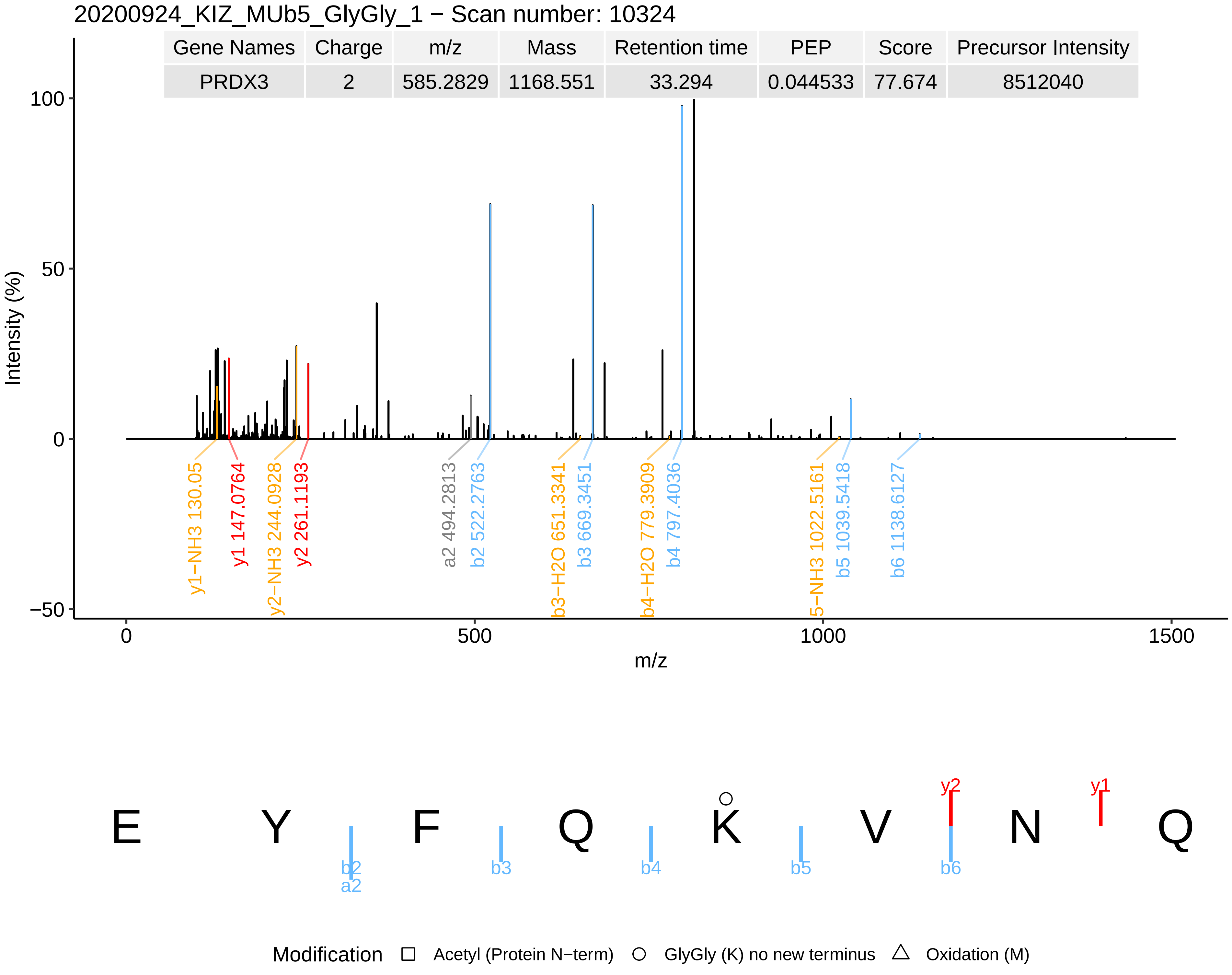

20200924_KIZ_MUb5_GlyGly_1 − Scan number: 10324
Gene Names | Charge | m/z | Mass | Retention time | PEP | Score | Precursor Intensity
PRDX3 | 2 | 585.2829 | 1168.551 | 33.294 | 0.044533 | 77.674 | 8512040
Intensity (%)
m/z
y1−NH3 130.05
y1 147.0764
y2−NH3 244.0928
y2 261.1193
a2 494.2813
b2 522.2763
b3−H2O 651.3341
b3 669.3451
b4−H2O 779.3909
b4 797.4036
5−NH3 1022.5161
b5 1039.5418
b6 1138.6127
E Y F Q K V N Q
y2 y1
b2 a2 b3 b4 b5 b6
Modification
Acetyl (Protein N−term)
GlyGly (K) no new terminus
Oxidation (M)

20201118_KIZ_MUb6_GlyGly_2 − Scan number: 11443

Gene Names	Charge	m/z	Mass	Retention time	PEP	Score	Precursor Intensity
PRDX3	2	585.2829	1168.551	38.638	0.048141	76.847	3864011

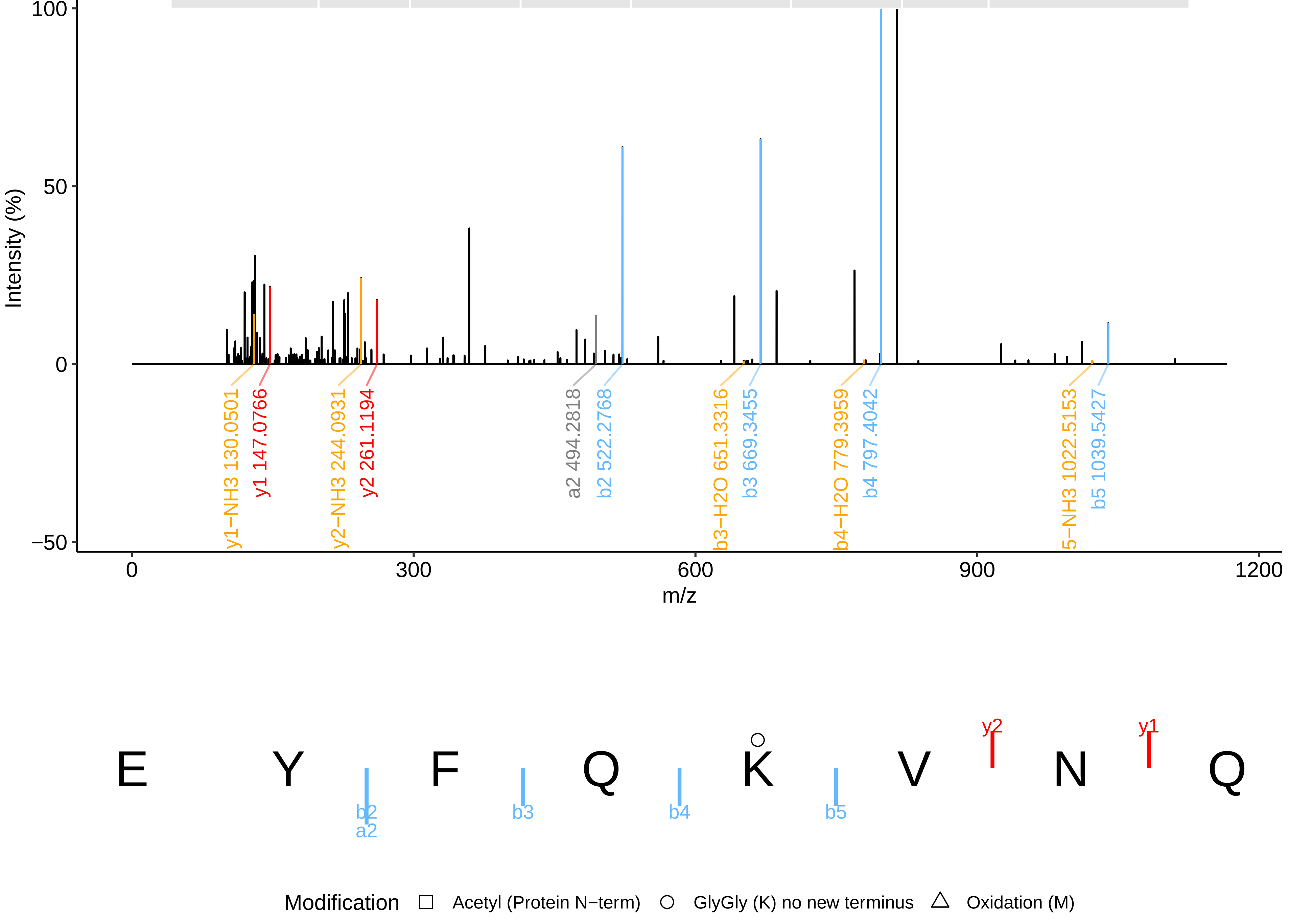

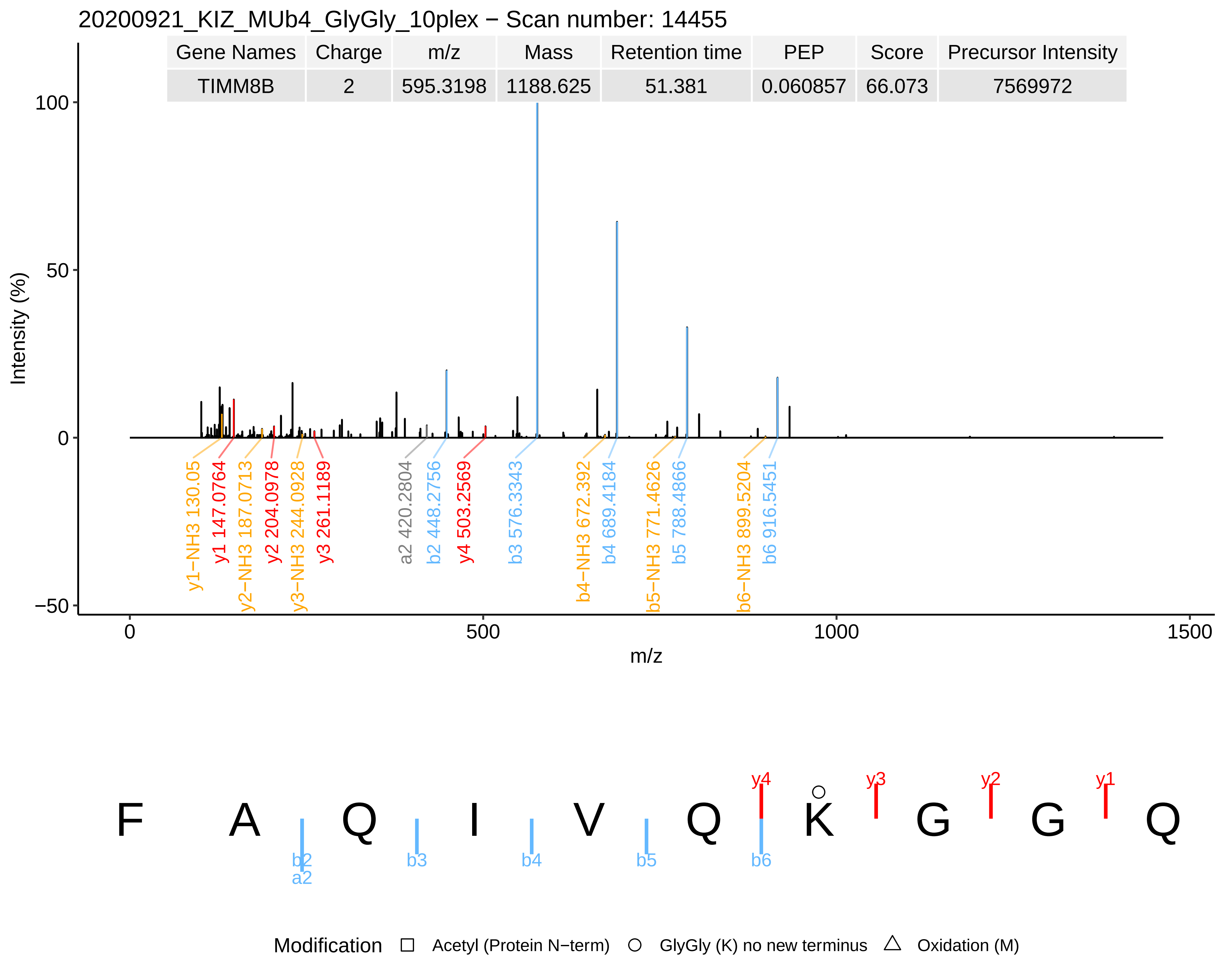

20200921_KIZ_MUb4_GlyGly_10plex − Scan number: 14455
Gene Names | Charge | m/z | Mass | Retention time | PEP | Score | Precursor Intensity
TIMM8B | 2 | 595.3198 | 1188.625 | 51.381 | 0.060857 | 66.073 | 7569972
Intensity (%)
m/z
y1−NH3 130.05
y1 147.0764
y2−NH3 187.0713
y2 204.0978
y3−NH3 244.0928
y3 261.1189
a2 420.2804
b2 448.2756
y4 503.2569
b3 576.3343
b4−NH3 672.392
b4 689.4184
b5−NH3 771.4626
b5 788.4866
b6−NH3 899.5204
b6 916.5451
F A Q I V Q K G G Q
y4 y3 y2 y1
b2 a2 b3 b4 b5 b6
Modification
Acetyl (Protein N−term)
GlyGly (K) no new terminus
Oxidation (M)

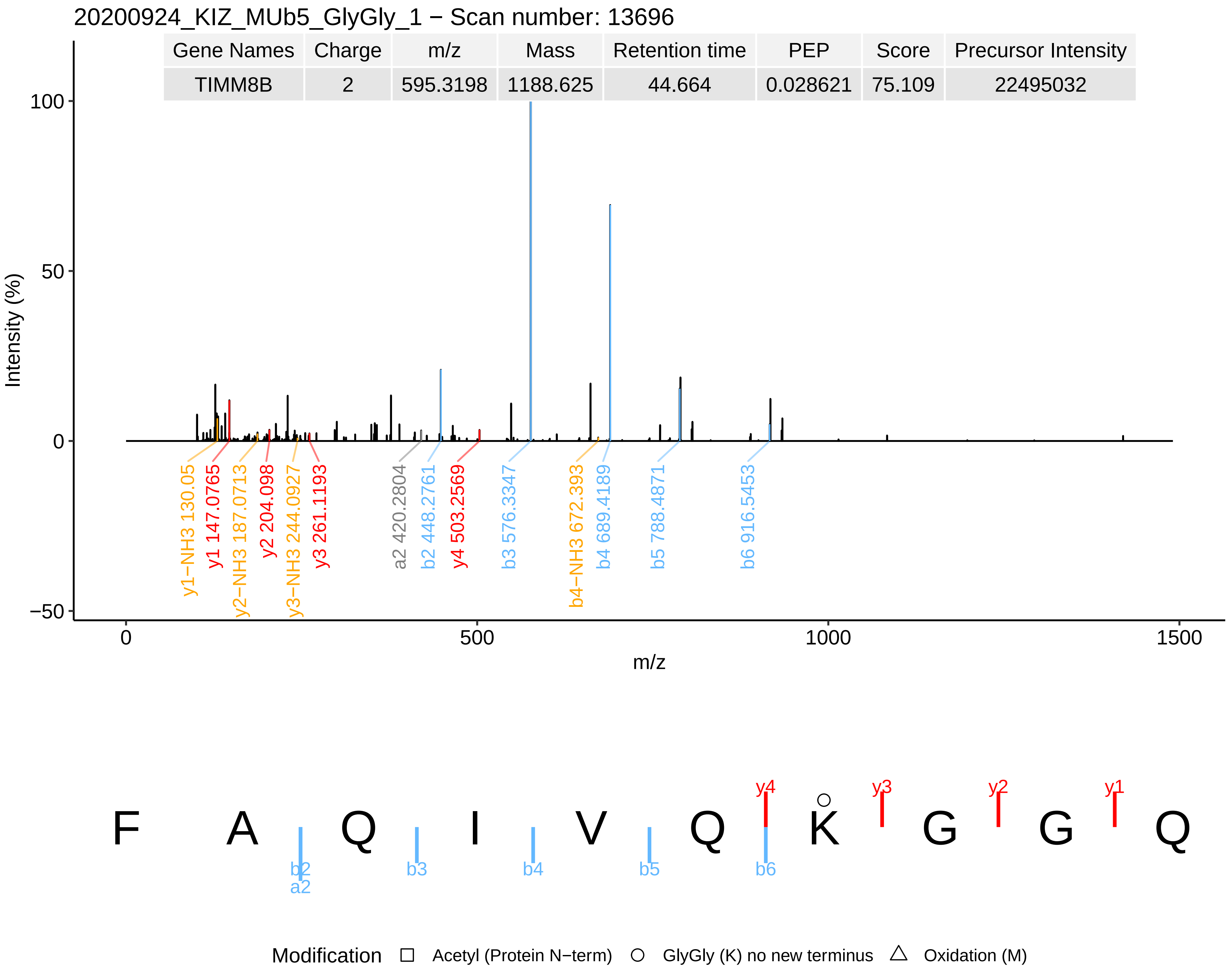

20200924_KIZ_MUb5_GlyGly_1 − Scan number: 13696
Gene Names | Charge | m/z | Mass | Retention time | PEP | Score | Precursor Intensity
TIMM8B | 2 | 595.3198 | 1188.625 | 44.664 | 0.028621 | 75.109 | 22495032
Intensity (%)
m/z
y1−NH3 130.05
y1 147.0765
y2−NH3 187.0713
y2 204.098
y3−NH3 244.0927
y3 261.1193
a2 420.2804
b2 448.2761
y4 503.2569
b3 576.3347
b4−NH3 672.393
b4 689.4189
b5 788.4871
b6 916.5453
F A Q I V Q K G G Q
y4 y3 y2 y1
b2 a2 b3 b4 b5 b6
Modification
Acetyl (Protein N−term)
GlyGly (K) no new terminus
Oxidation (M)

20200924_KIZ_MUb5_GlyGly_2 − Scan number: 12705

Gene Names	Charge	m/z	Mass	Retention time	PEP	Score	Precursor Intensity
TIMM8B	2	595.3198	1188.625	47.506	0.044976	70.525	4322933

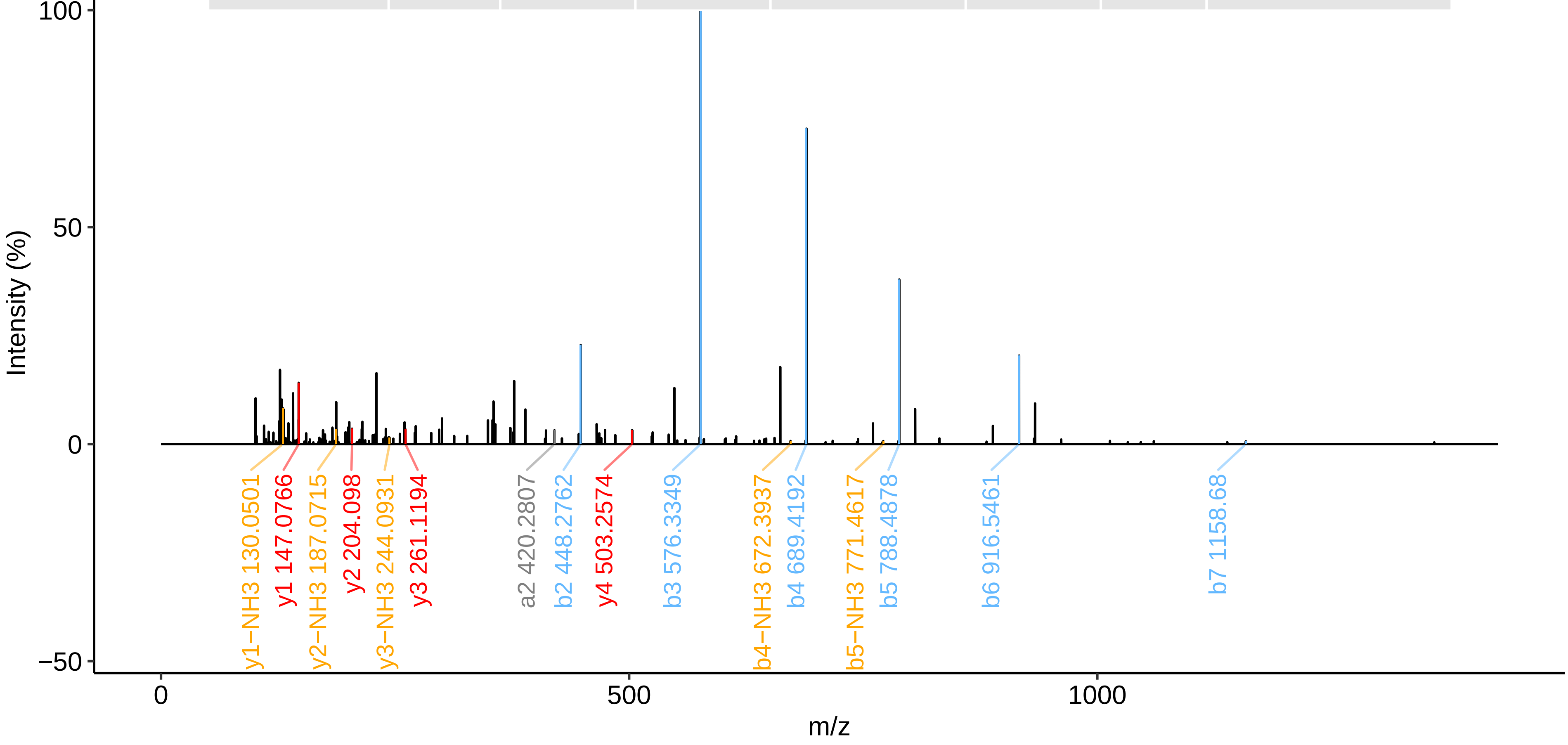

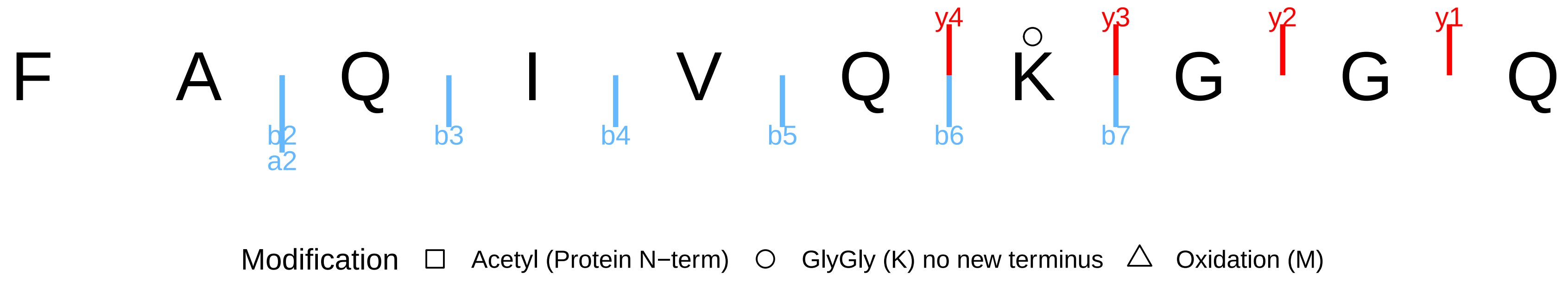

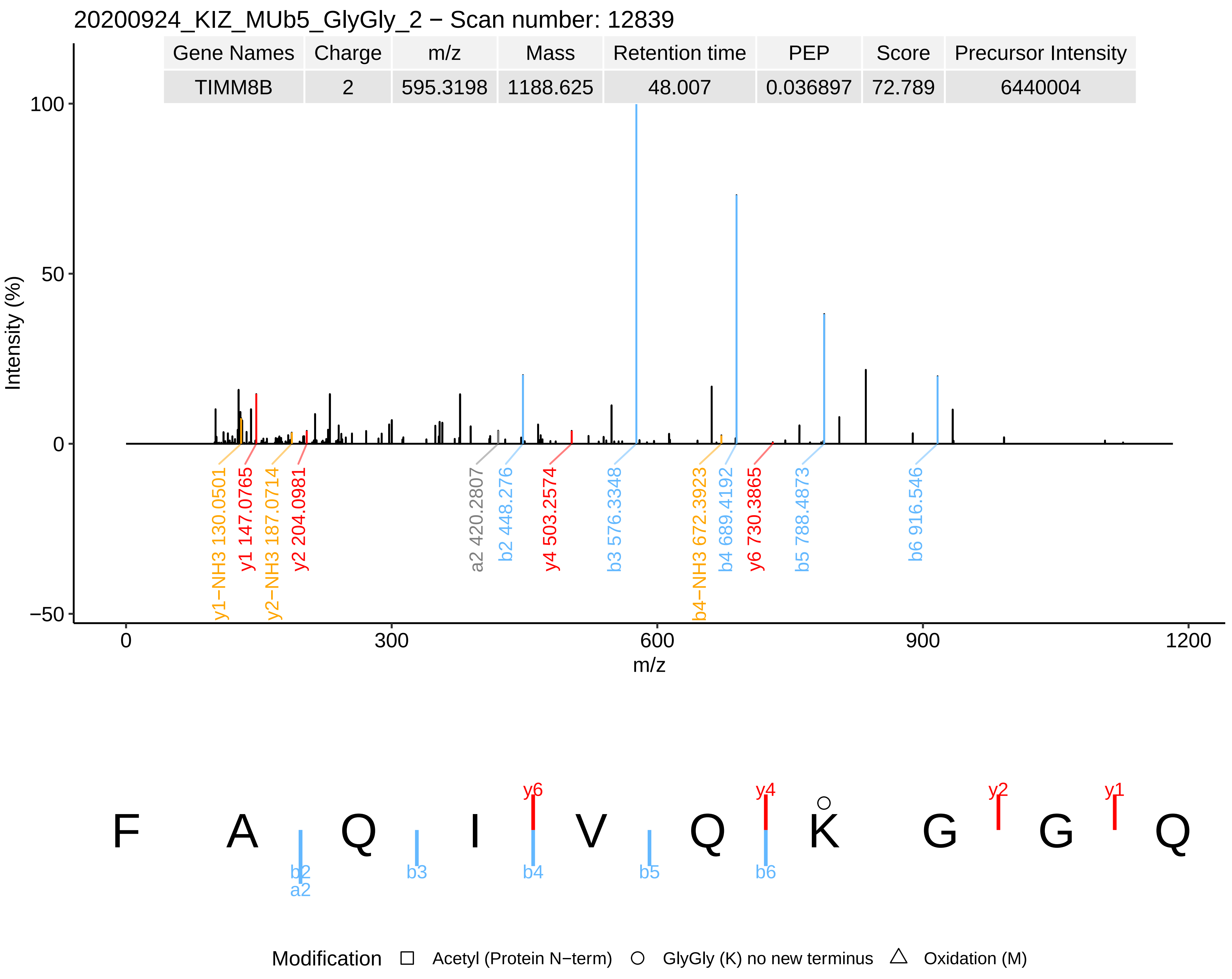
20200924_KIZ_MUb5_GlyGly_2 − Scan number: 12839
Gene Names | Charge | m/z | Mass | Retention time | PEP | Score | Precursor Intensity
TIMM8B | 2 | 595.3198 | 1188.625 | 48.007 | 0.036897 | 72.789 | 6440004
Intensity (%)
m/z
y1−NH3 130.0501
y1 147.0765
y2−NH3 187.0714
y2 204.0981
a2 420.2807
b2 448.276
y4 503.2574
b3 576.3348
b4−NH3 672.3923
b4 689.4192
y6 730.3865
b5 788.4873
b6 916.546
F A Q I V Q K G G Q
y6 y4 y2 y1
b2 a2 b3 b4 b5 b6
Modification
Acetyl (Protein N−term)
GlyGly (K) no new terminus
Oxidation (M)

20201118_KIZ_MUb6_GlyGly_1 − Scan number: 13334

Gene Names	Charge	m/z	Mass	Retention time	PEP	Score	Precursor Intensity
TIMM8B	2	595.3198	1188.625	42.193	0.023881	87.323	20027030

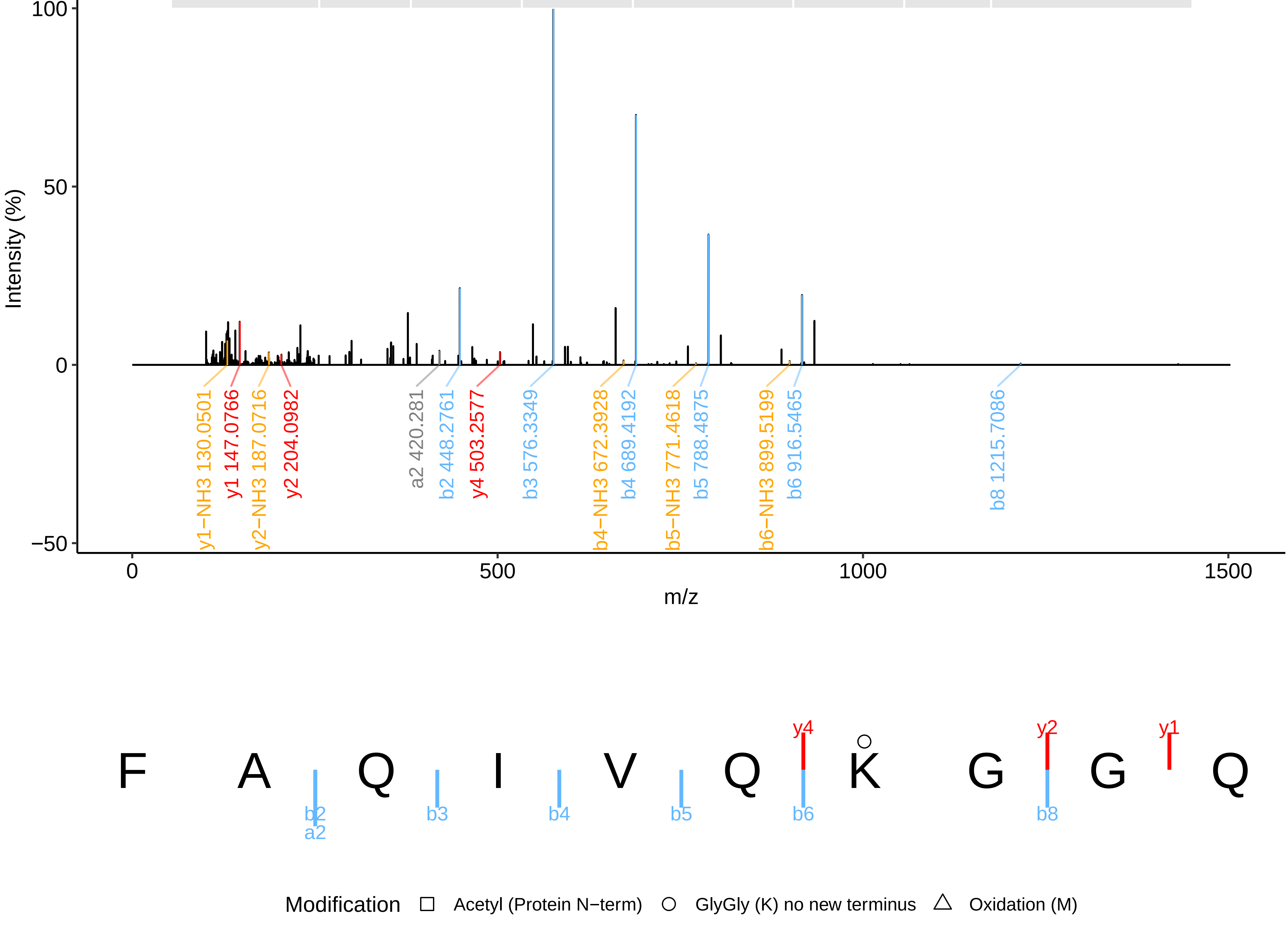

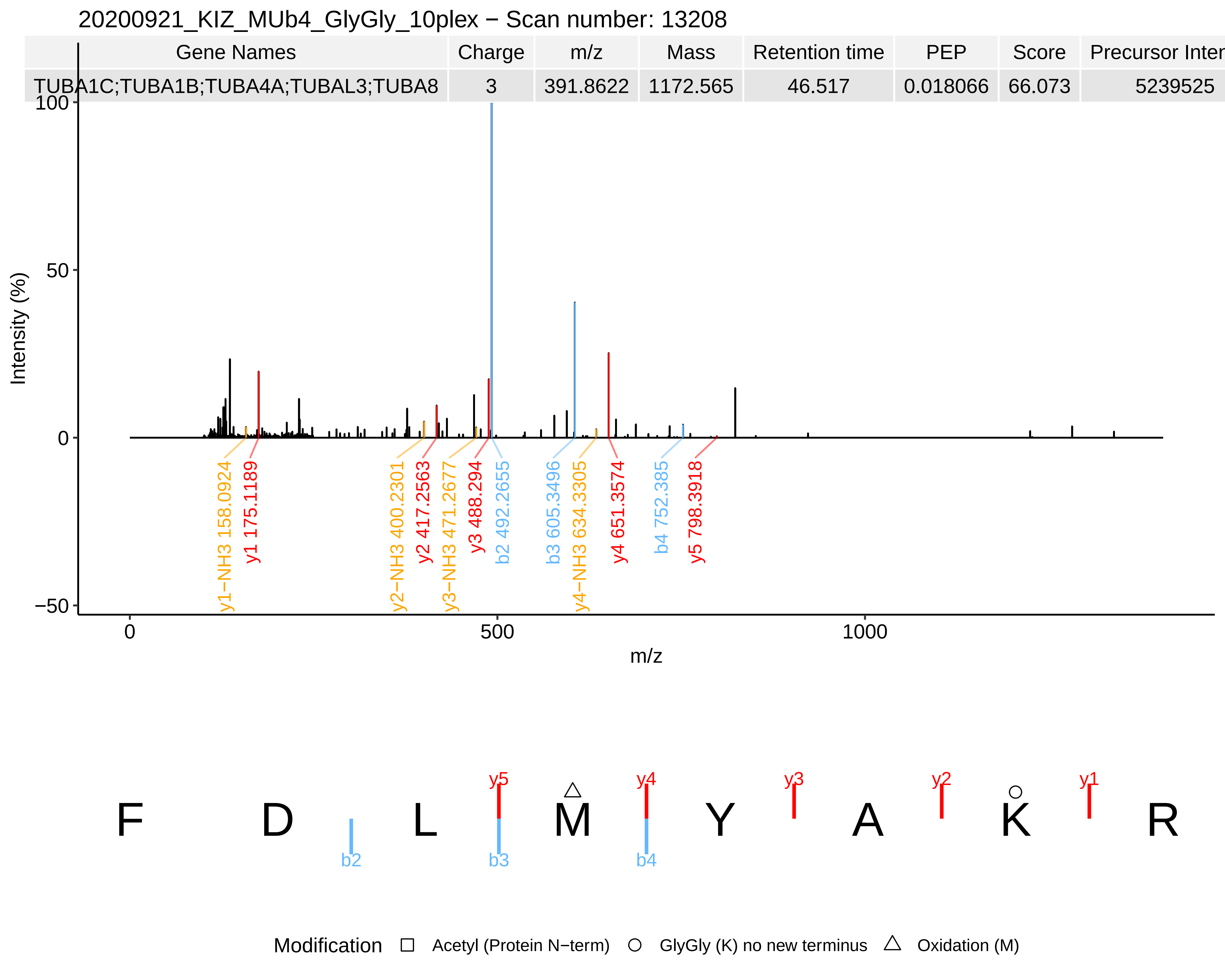

20200921_KIZ_MUb4_GlyGly_10plex − Scan number: 13208
Gene Names
Charge
m/z
Mass
Retention time
PEP
Score
Precursor Inter
TUBA1C;TUBA1B;TUBA4A;TUBAL3;TUBA8
3
391.8622
1172.565
46.517
0.018066
66.073
5239525
Intensity (%)
m/z
y1−NH3 158.0924
y1 175.1189
y2−NH3 400.2301
y2 417.2563
y3−NH3 471.2677
y3 488.294
b2 492.2655
b3 605.3496
y4−NH3 634.3305
y4 651.3574
b4 752.385
y5 798.3918
F D L M Y A K R
Modification
Acetyl (Protein N−term)
GlyGly (K) no new terminus
Oxidation (M)

20200921_KIZ_MUb4_GlyGly_10plex − Scan number: 13341

Gene Names	Charge	m/z	Mass	Retention time	PEP	Score	Precursor Inter
TUBA1C;TUBA1B;TUBA4A;TUBAL3;TUBA8	3	391.8622	1172.565	47.033	0.045067	54.157	5382786

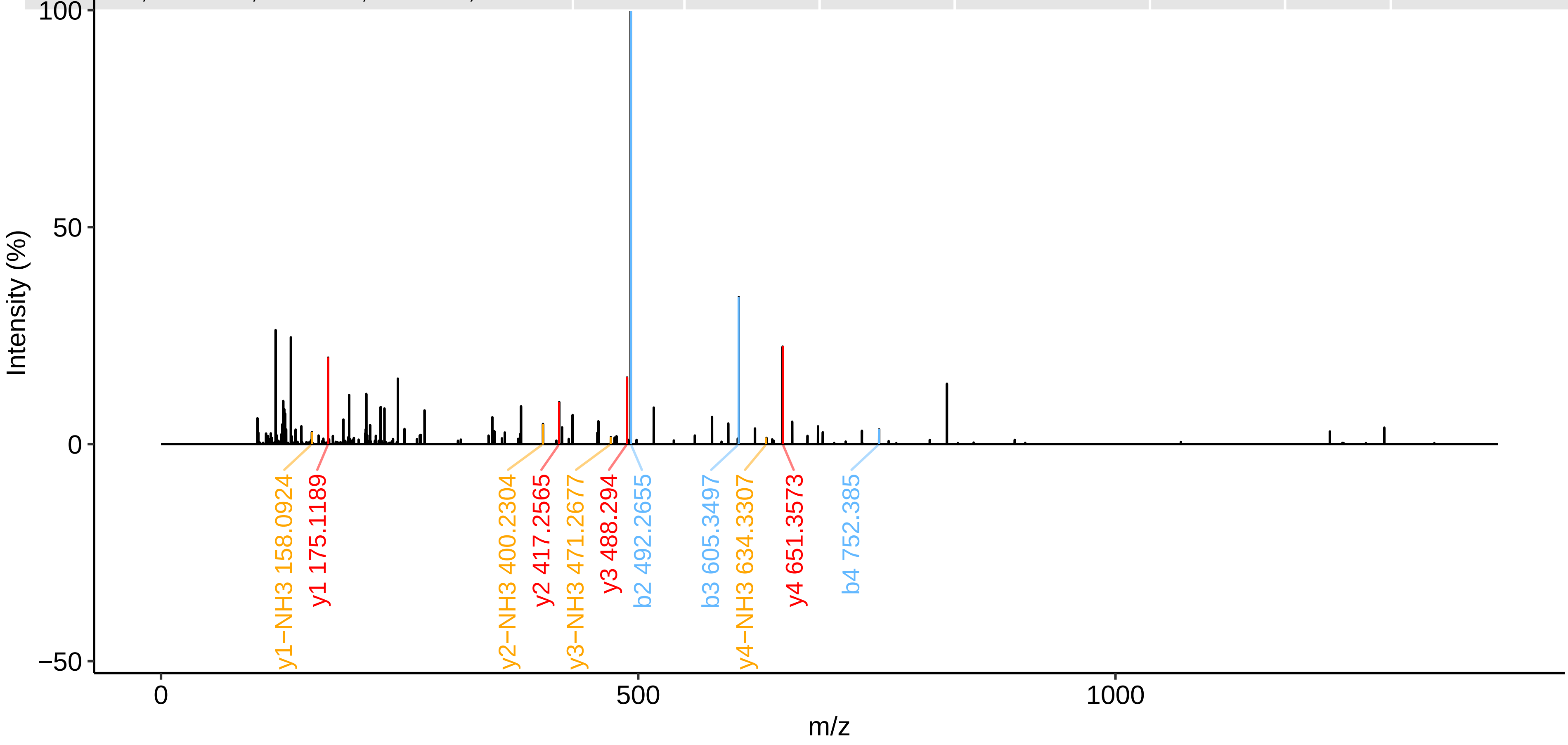

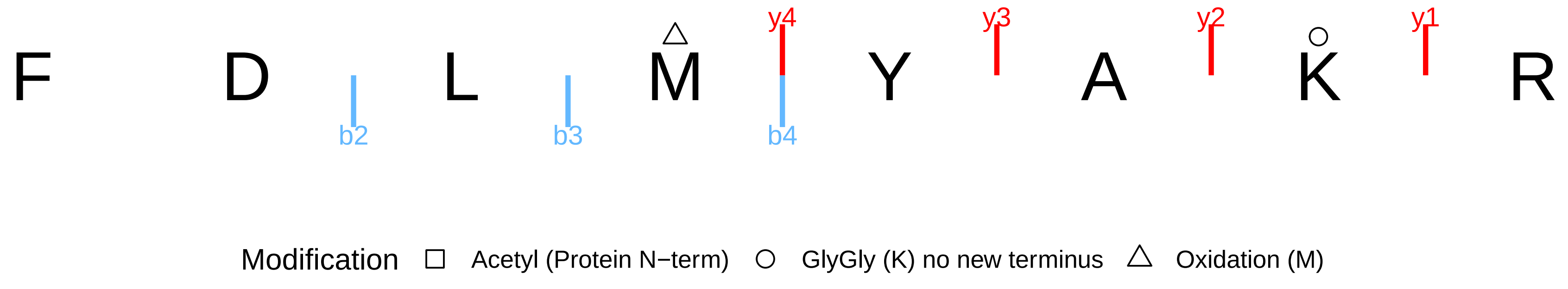

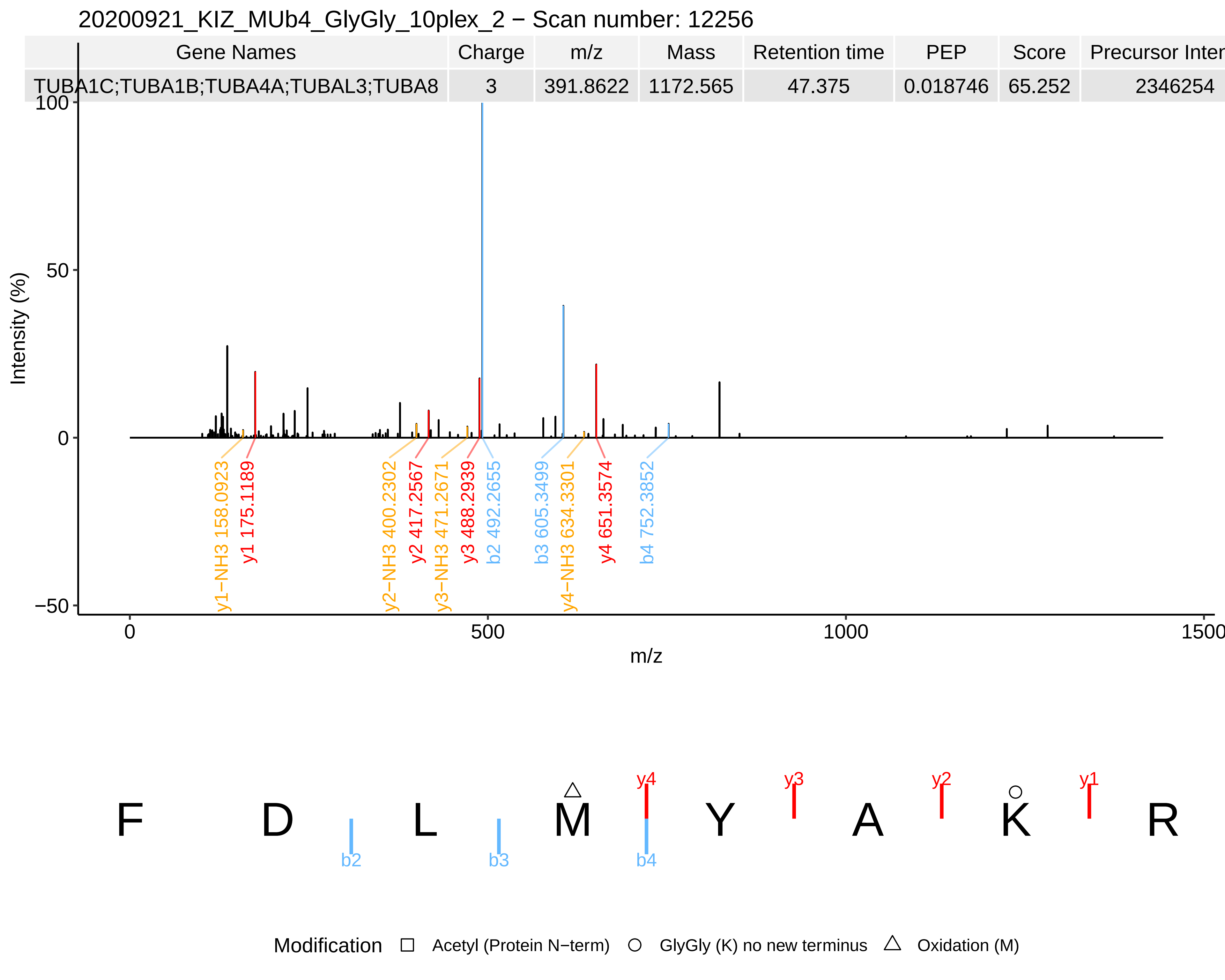
20200921_KIZ_MUb4_GlyGly_10plex_2 − Scan number: 12256
Gene Names
Charge
m/z
Mass
Retention time
PEP
Score
Precursor Inter
TUBA1C;TUBA1B;TUBA4A;TUBAL3;TUBA8
3
391.8622
1172.565
47.375
0.018746
65.252
2346254
Intensity (%)
100
50
0
−50
y1−NH3 158.0923
y1 175.1189
y2−NH3 400.2302
y2 417.2567
y3−NH3 471.2671
y3 488.2939
b2 492.2655
b3 605.3499
y4−NH3 634.3301
y4 651.3574
b4 752.3852
0
500
1000
1500
m/z
F D L M Y A K R
y4 y3 y2 y1
b2 b3 b4
Modification
Acetyl (Protein N−term)
GlyGly (K) no new terminus
Oxidation (M)

20200924_KIZ_MUb5_GlyGly_1 − Scan number: 12031

Gene Names	Charge	m/z	Mass	Retention time	PEP	Score	Precursor Inter
TUBA1C;TUBA1B;TUBA4A;TUBAL3;TUBA8	3	391.8622	1172.565	39.075	0.020143	63.565	9603542

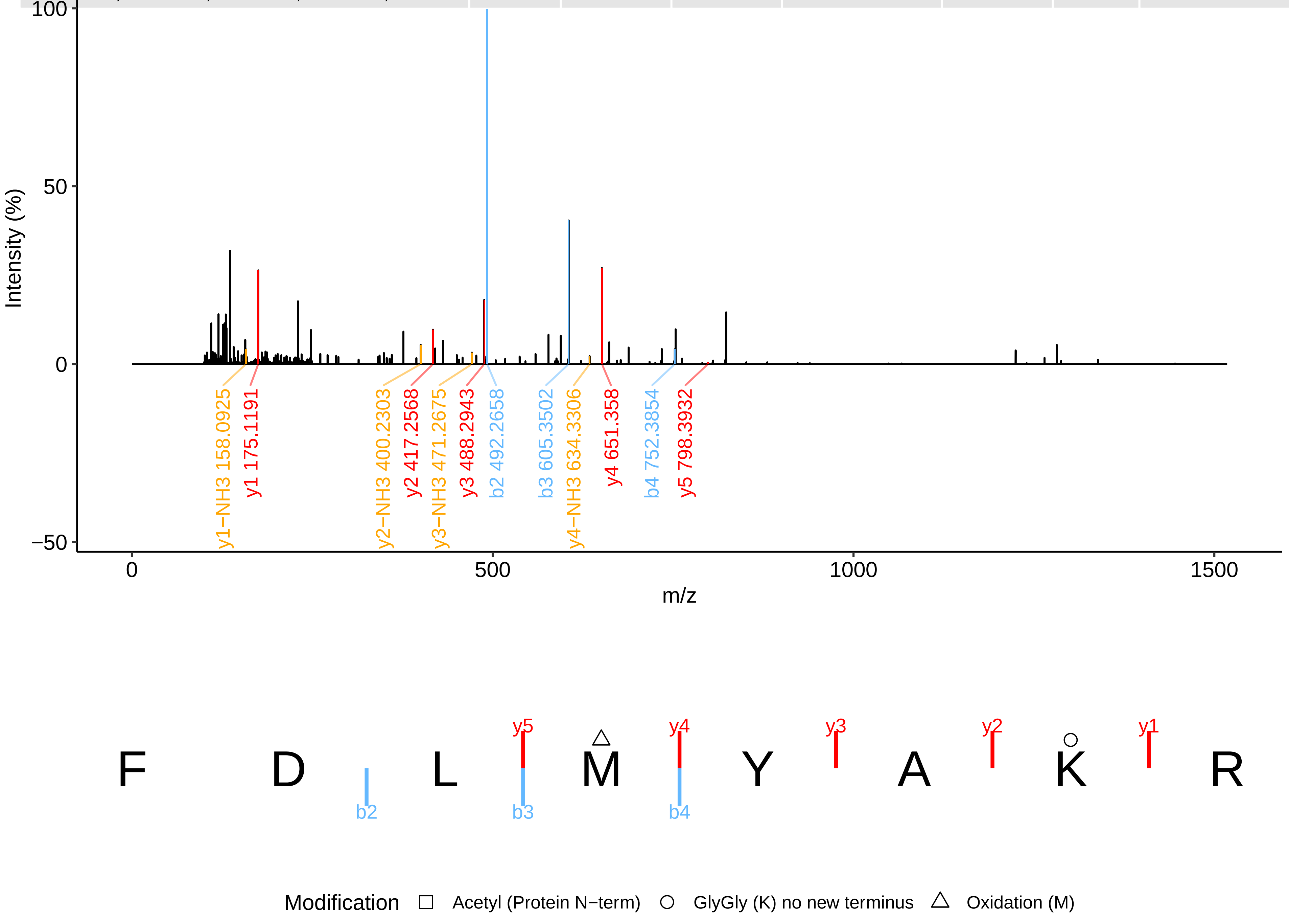

20200924_KIZ_MUb5_GlyGly_1 − Scan number: 12179

Gene Names	Charge	m/z	Mass	Retention time	PEP	Score	Precursor Inter
TUBA1C;TUBA1B;TUBA4A;TUBAL3;TUBA8	3	391.8622	1172.565	39.582	0.019359	63.565	7998767

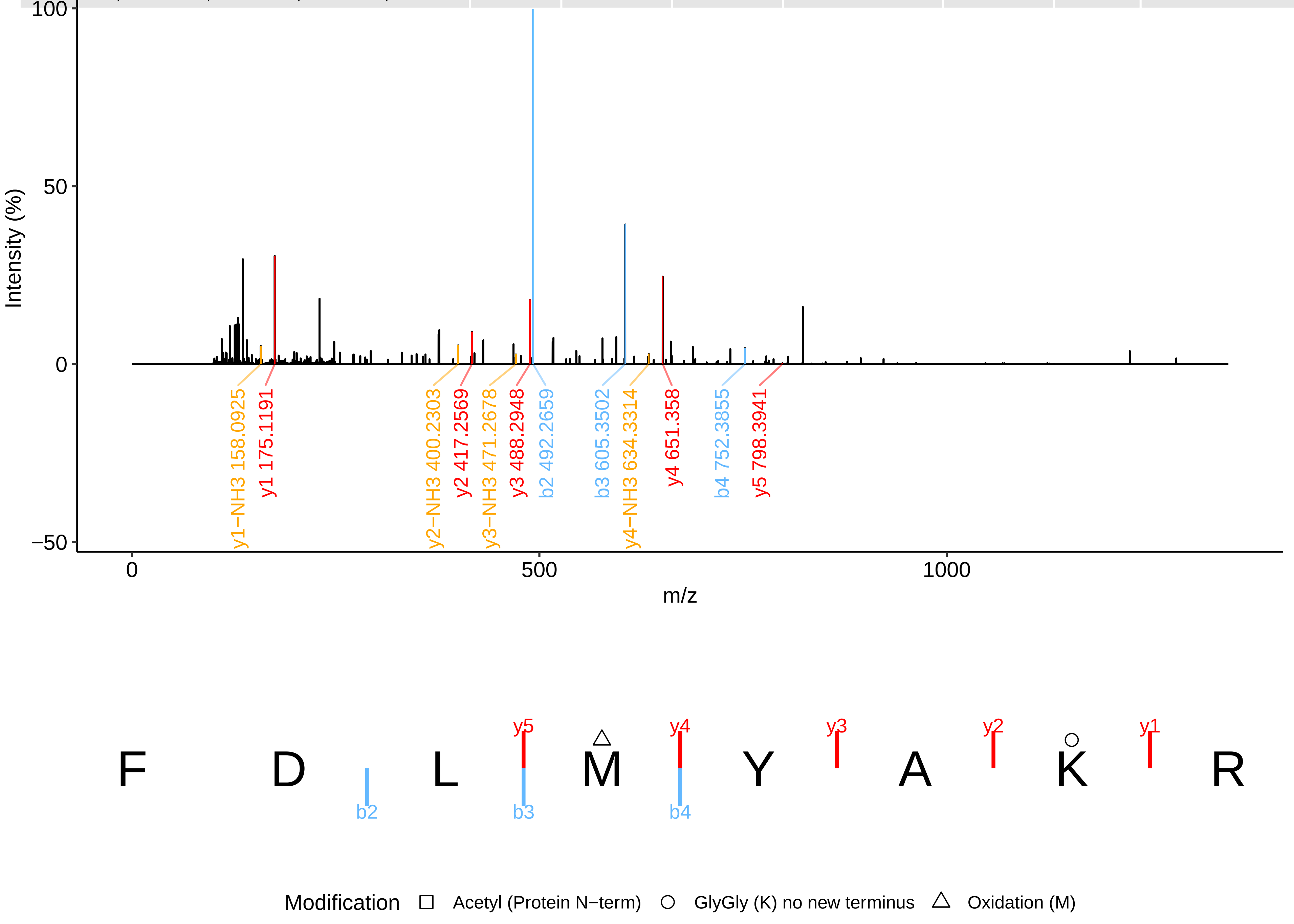

20200924_KIZ_MUb5_GlyGly_2 − Scan number: 11303

Gene Names	Charge	m/z	Mass	Retention time	PEP	Score	Precursor Inter
TUBA1C;TUBA1B;TUBA4A;TUBAL3;TUBA8	3	391.8622	1172.565	42.231	0.051351	51.854	NA

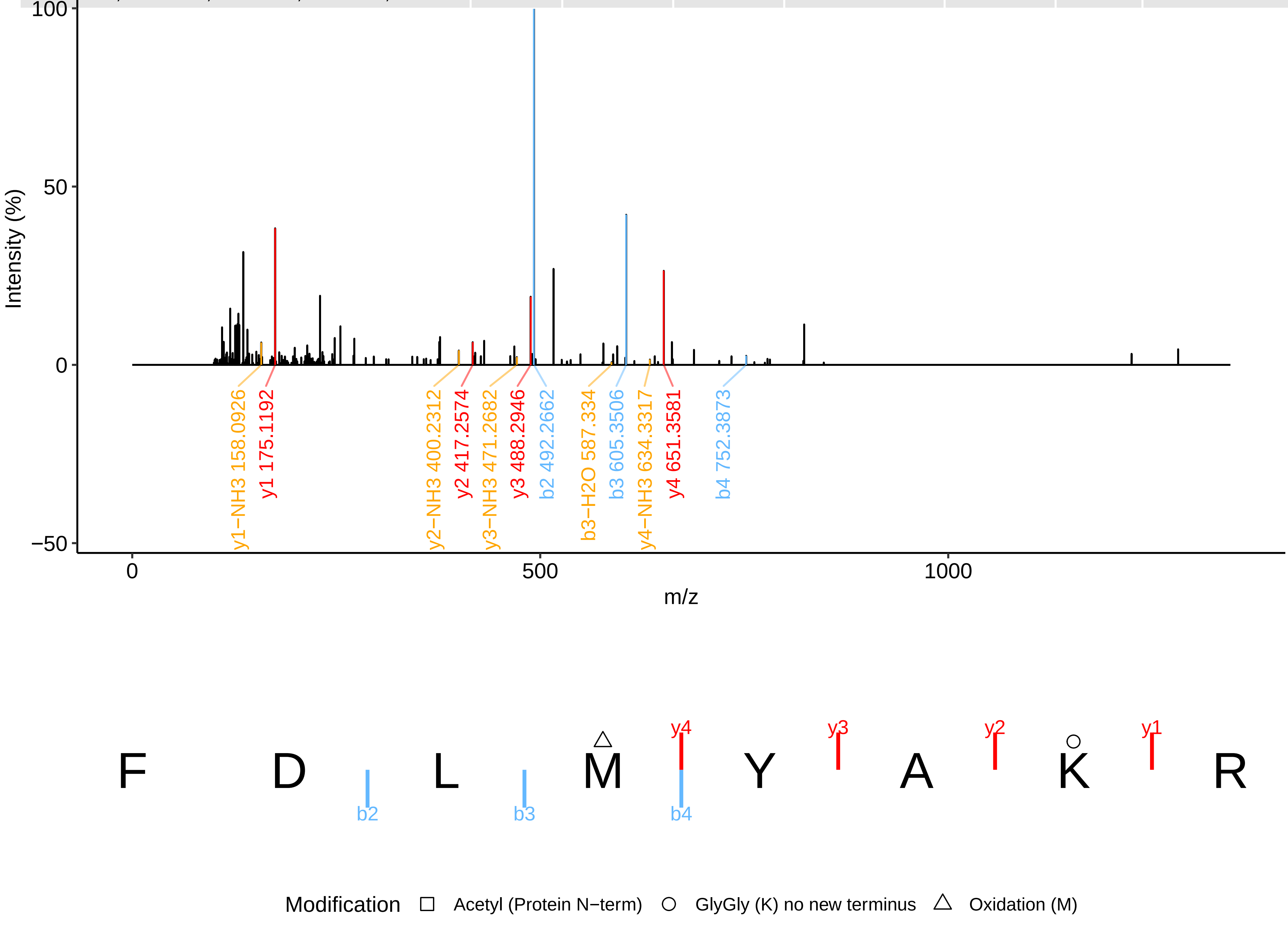

20200924_KIZ_MUb5_GlyGly_2 − Scan number: 11433

Gene Names	Charge	m/z	Mass	Retention time	PEP	Score	Precursor Inter
TUBA1C;TUBA1B;TUBA4A;TUBAL3;TUBA8	3	391.8622	1172.565	42.736	0.038072	56.721	3002330

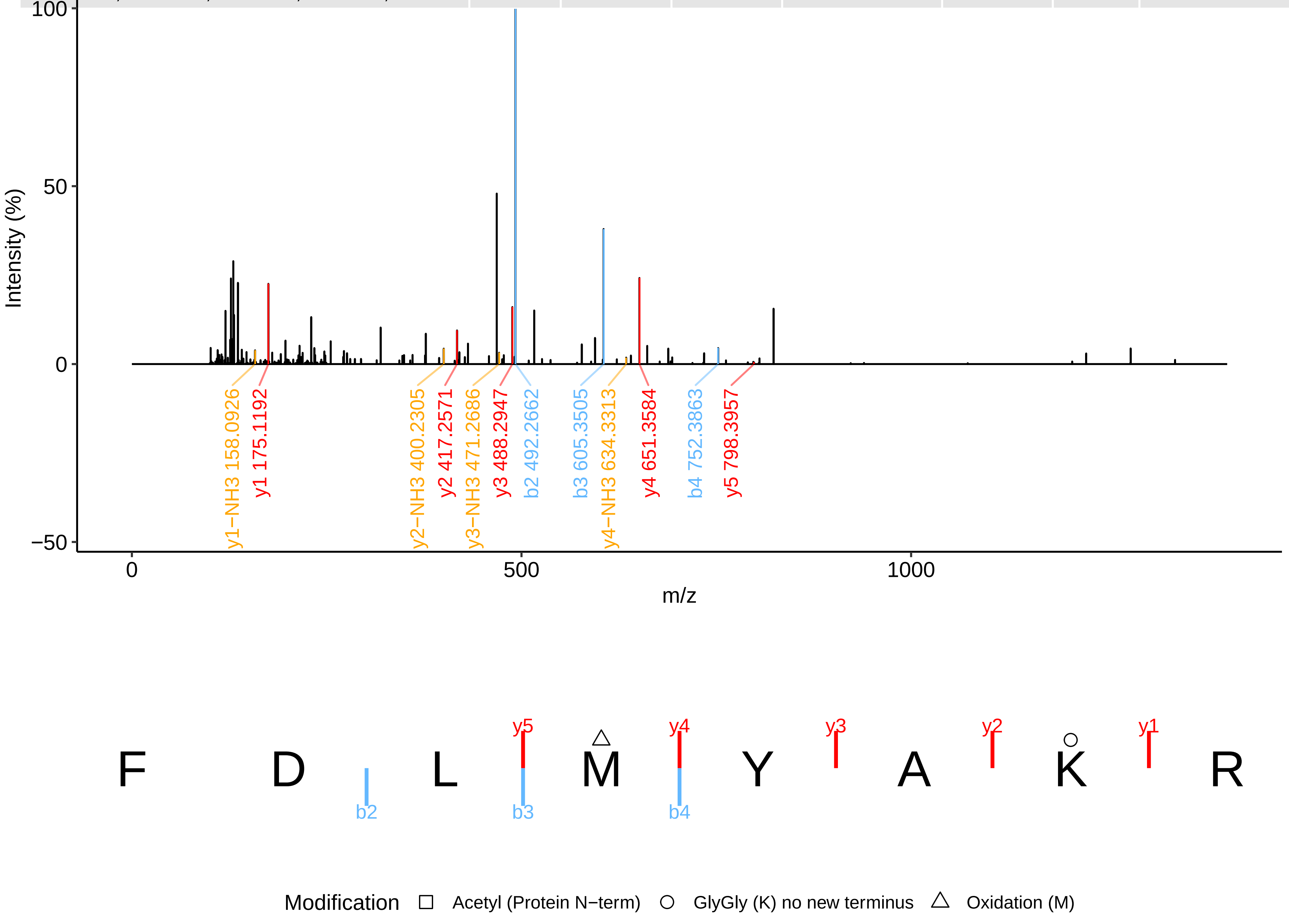

20200921_KIZ_MUb4_GlyGly_10plex − Scan number: 16420

Gene Names	Charge	m/z	Mass	Retention time	PEP	Score	Precursor Intens
TUBA1C;TUBA1B;TUBA4A;TUBAL3;TUBA8	3	386.5306	1156.57	58.963	0.01358	98.299	4835408

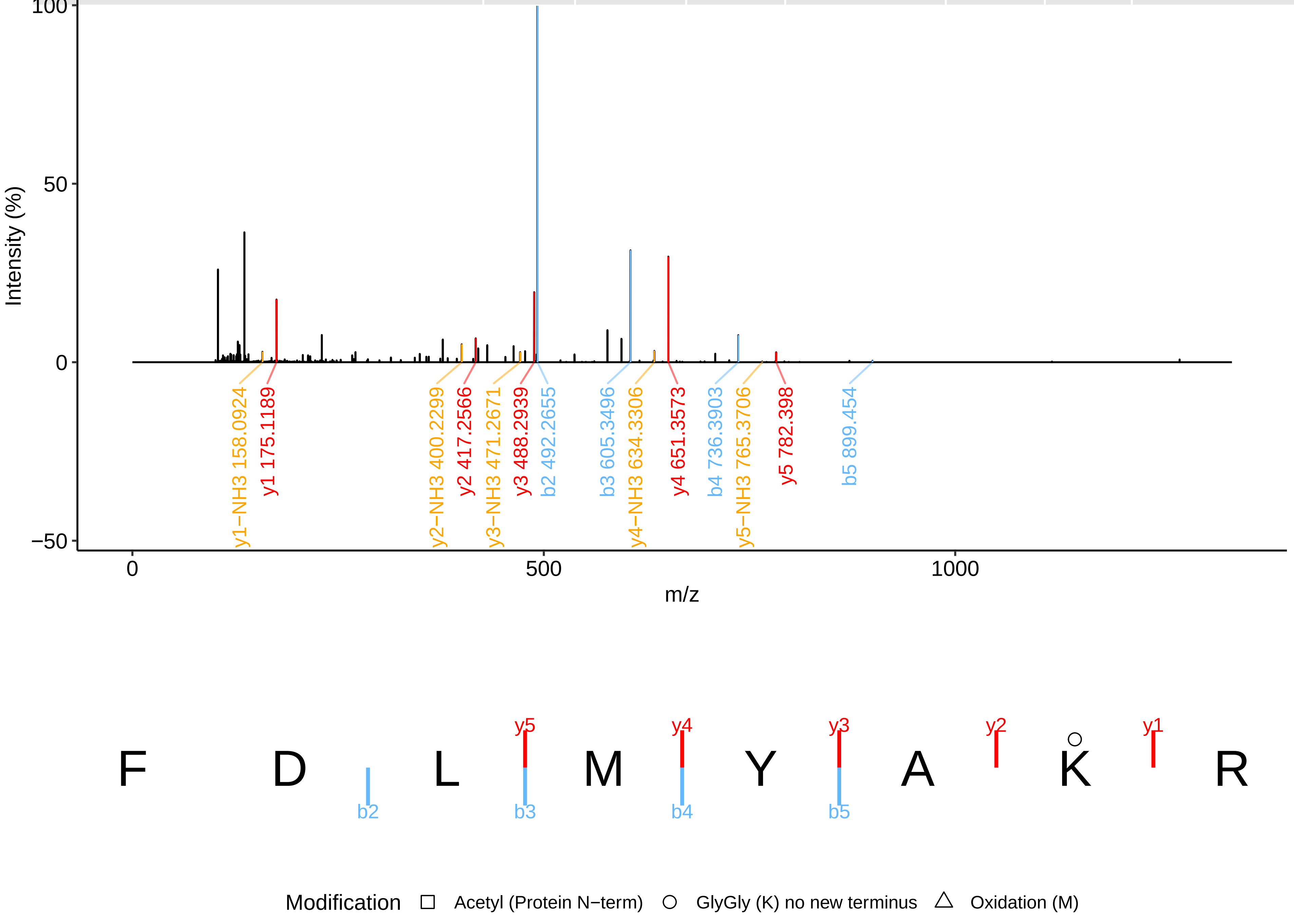

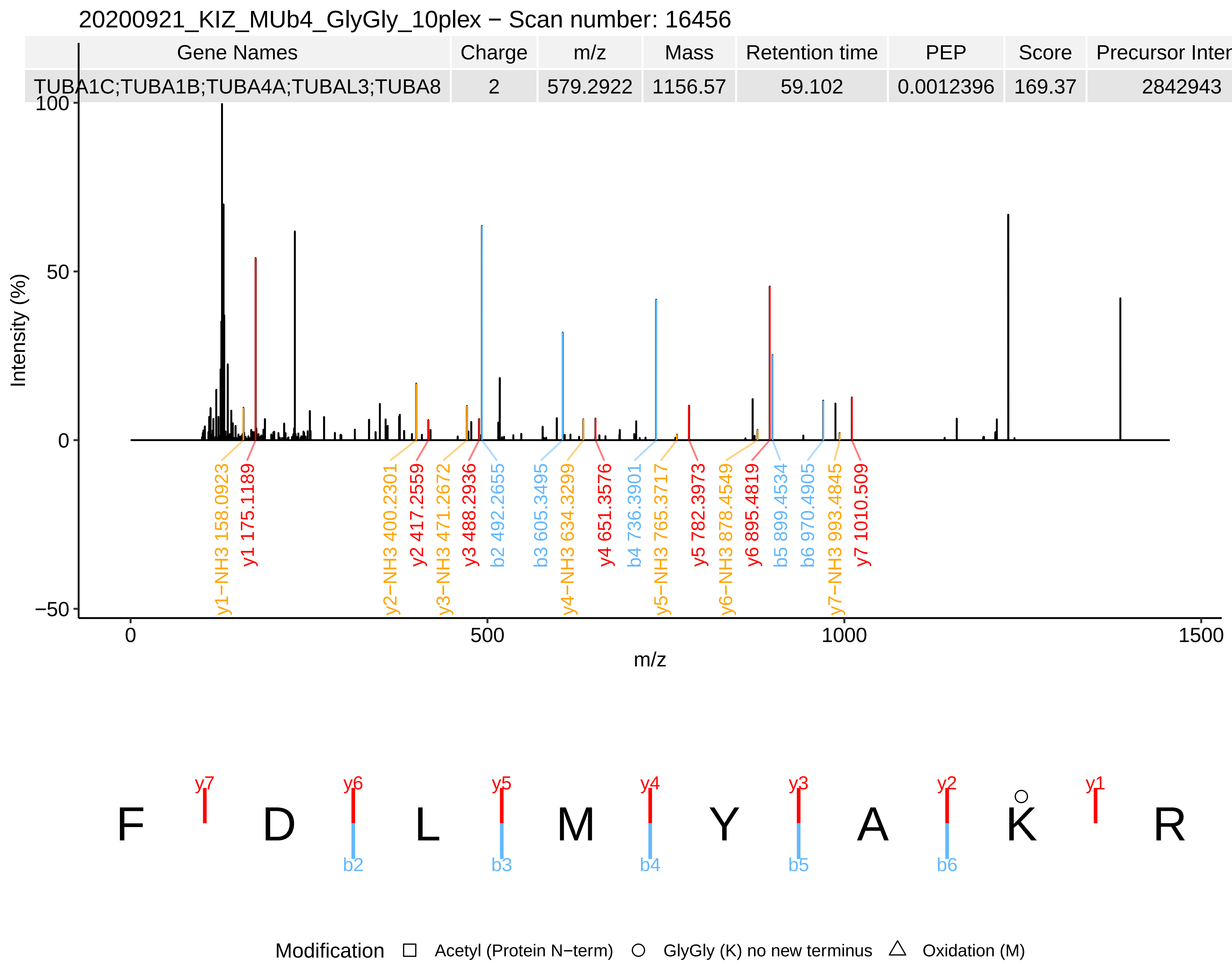

20200921_KIZ_MUb4_GlyGly_10plex − Scan number: 16456
Gene Names | Charge | m/z | Mass | Retention time | PEP | Score | Precursor Inter
TUBA1C;TUBA1B;TUBA4A;TUBAL3;TUBA8 | 2 | 579.2922 | 1156.57 | 59.102 | 0.0012396 | 169.37 | 2842943
Intensity (%)
m/z
y1−NH3 158.0923
y1 175.1189
y2−NH3 400.2301
y2 417.2559
y3−NH3 471.2672
y3 488.2936
b2 492.2655
b3 605.3495
y4−NH3 634.3299
y4 651.3576
b4 736.3901
y5−NH3 765.3717
y5 782.3973
y6−NH3 878.4549
y6 895.4819
b5 899.4534
b6 970.4905
y7−NH3 993.4845
y7 1010.509
F D L M Y A K R
Modification
Acetyl (Protein N−term)
GlyGly (K) no new terminus
Oxidation (M)

20200921_KIZ_MUb4_GlyGly_10plex_2 − Scan number: 15239

Gene Names	Charge	m/z	Mass	Retention time	PEP	Score	Precursor Inten
TUBA1C;TUBA1B;TUBA4A;TUBAL3;TUBA8	3	386.5306	1156.57	59.555	0.044962	85.212	644096.1

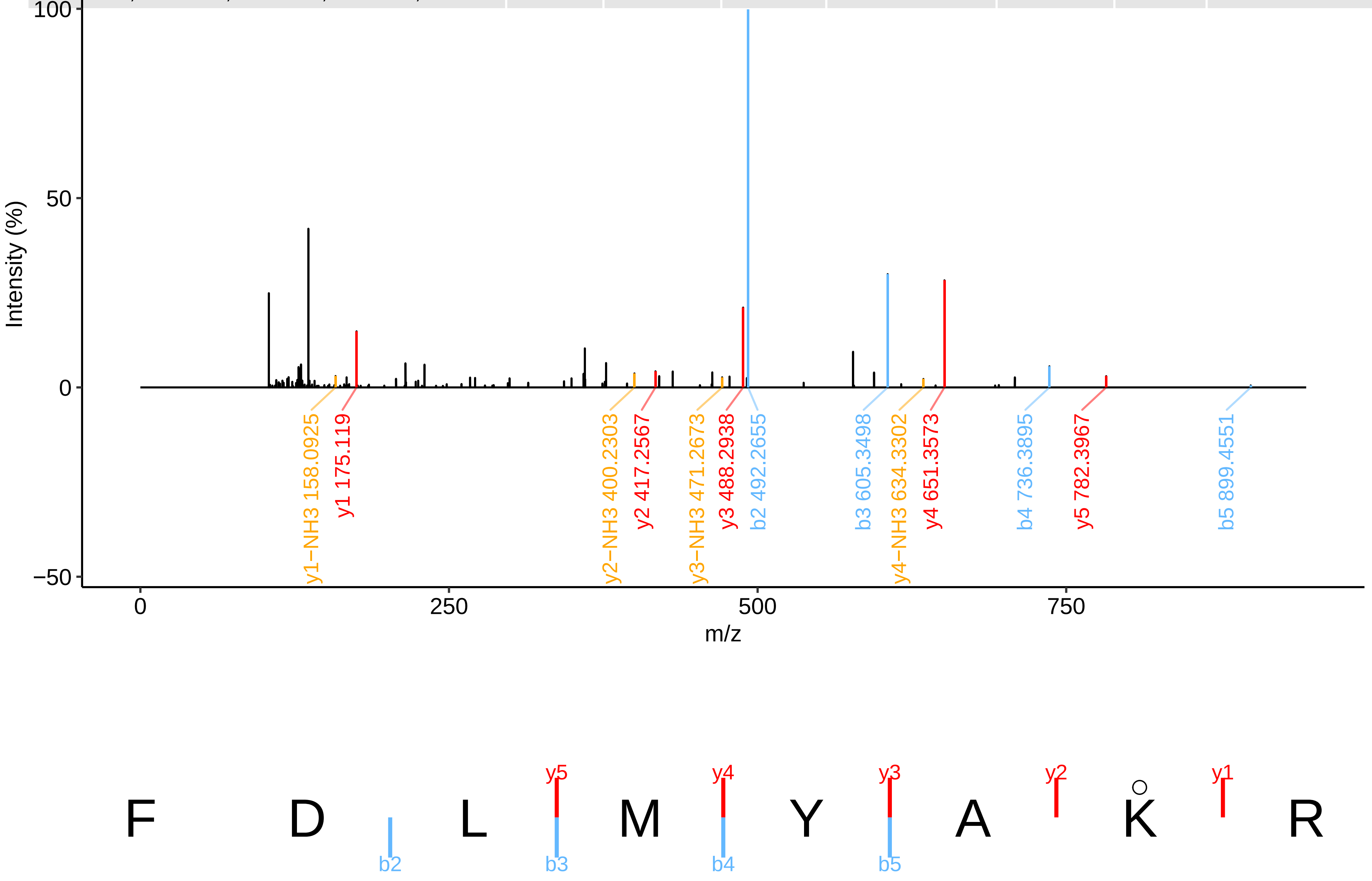

Modification □ Acetyl (Protein N−term) ○ GlyGly (K) no new terminus △ Oxidation (M)

20200924_KIZ_MUb5_GlyGly_1 − Scan number: 15259

Gene Names	Charge	m/z	Mass	Retention time	PEP	Score	Precursor Inten
TUBA1C;TUBA1B;TUBA4A;TUBAL3;TUBA8	3	386.5306	1156.57	49.828	0.019807	95.531	13898156

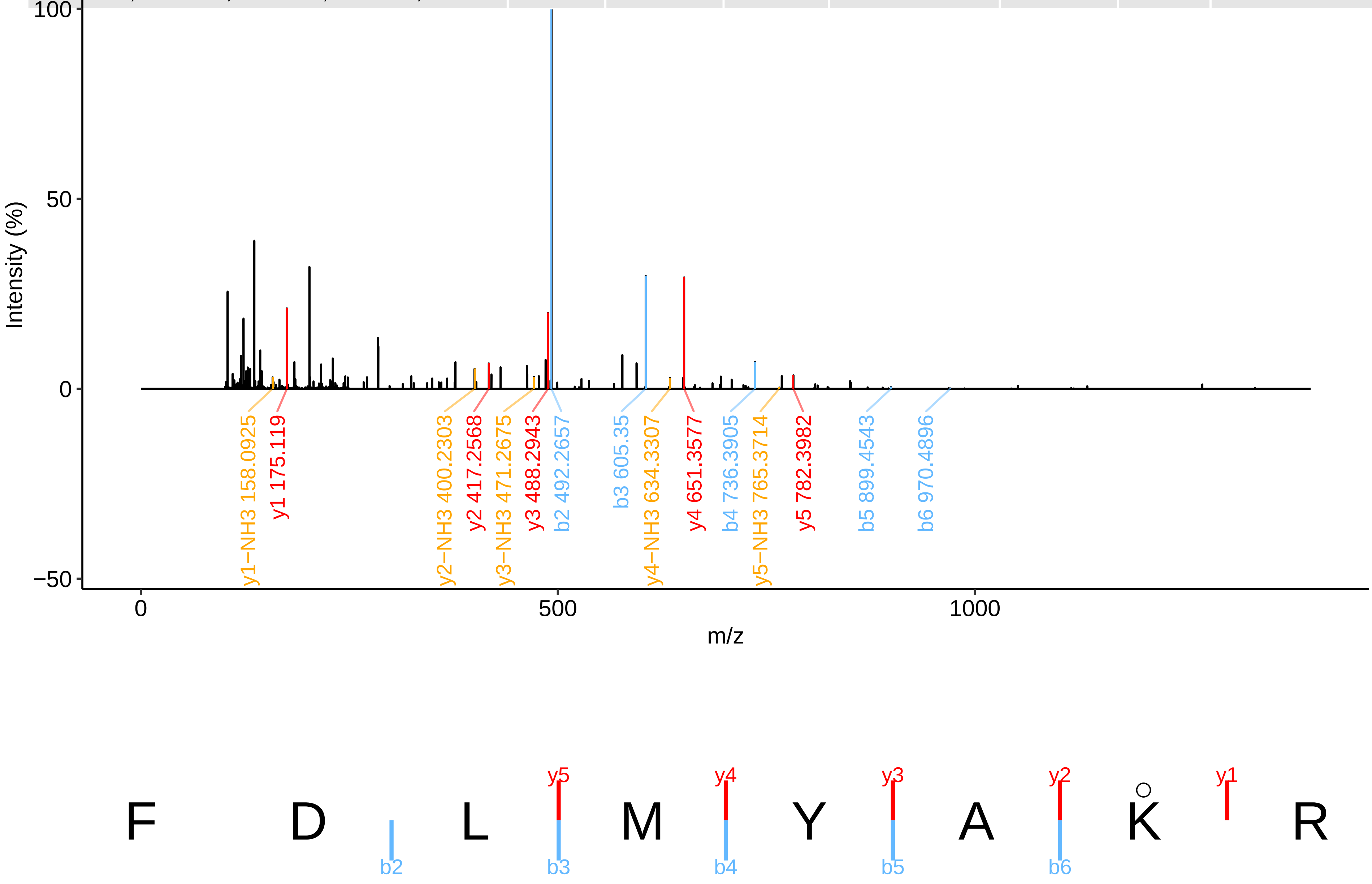

Modification □ Acetyl (Protein N−term) ○ GlyGly (K) no new terminus △ Oxidation (M)

20200924_KIZ_MUb5_GlyGly_1 − Scan number: 15419

Gene Names	Charge	m/z	Mass	Retention time	PEP	Score	Precursor Inten
TUBA1C;TUBA1B;TUBA4A;TUBAL3;TUBA8	3	386.5306	1156.57	50.343	0.003868	112.17	93356064

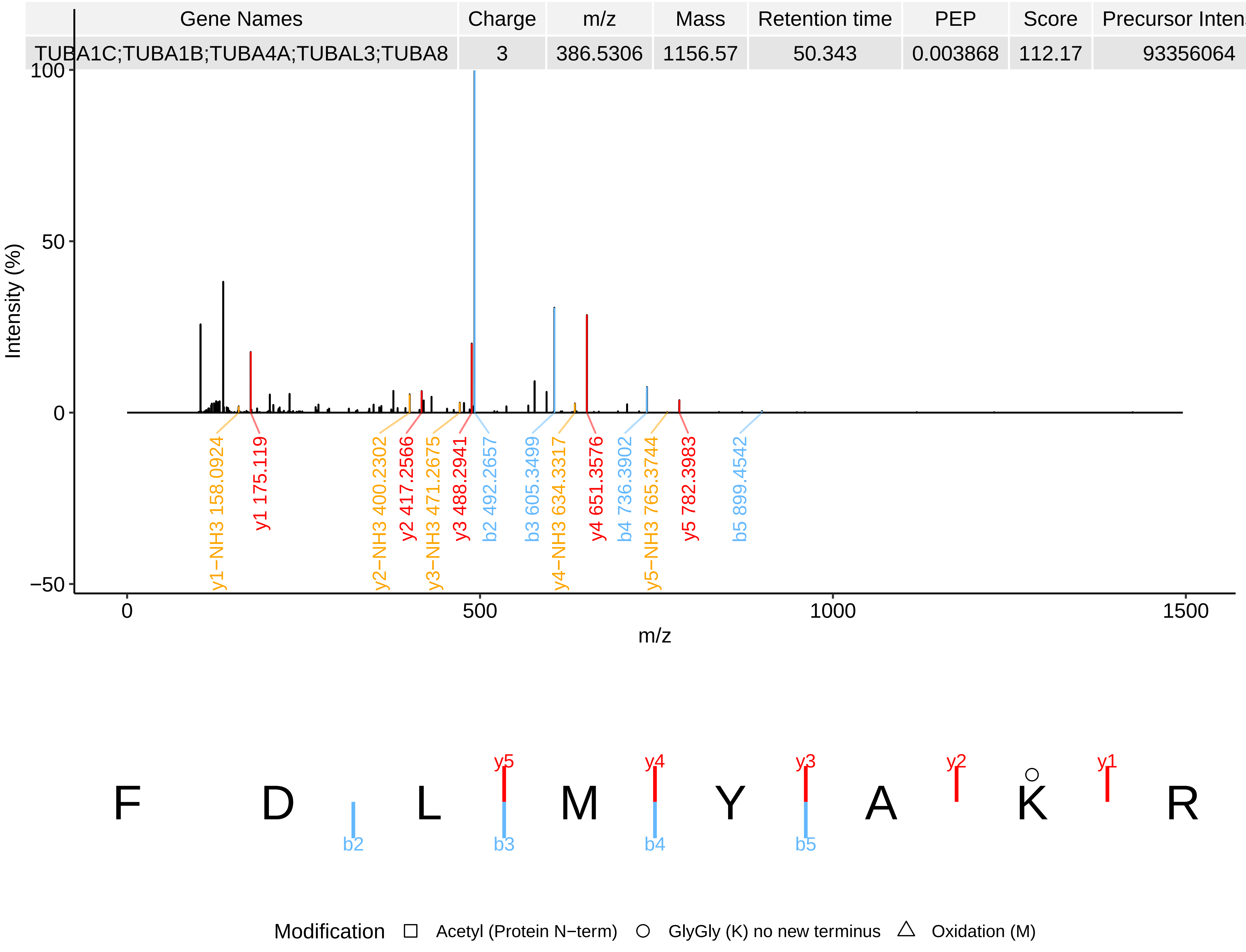

20200924_KIZ_MUb5_GlyGly_1 − Scan number: 15401

Gene Names	Charge	m/z	Mass	Retention time	PEP	Score	Precursor Inter
TUBA1C;TUBA1B;TUBA4A;TUBAL3;TUBA8	2	579.2922	1156.57	50.281	0.0012396	169.37	9586533

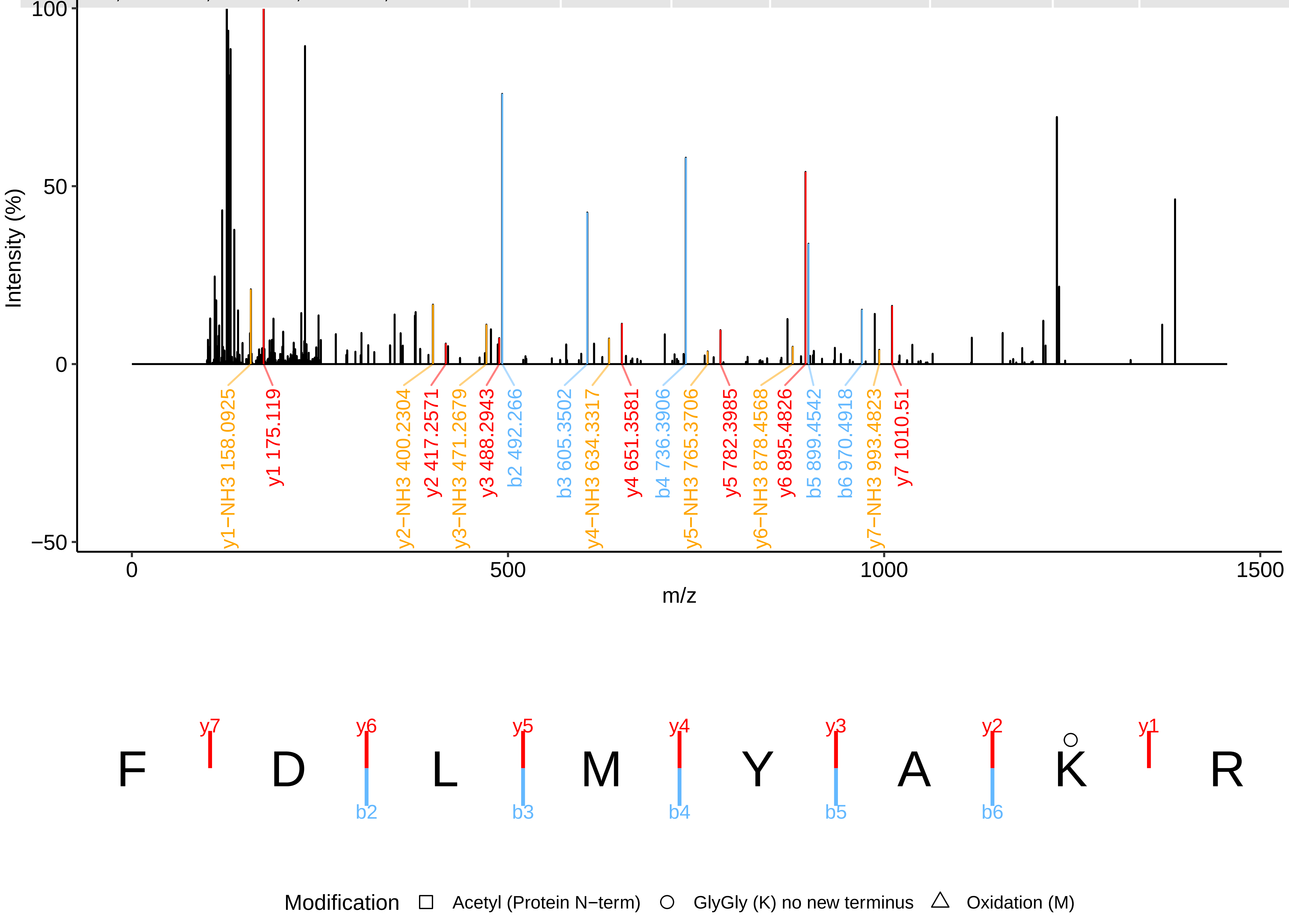

20200924_KIZ_MUb5_GlyGly_2 − Scan number: 14498

Gene Names	Charge	m/z	Mass	Retention time	PEP	Score	Precursor Inten
TUBA1C;TUBA1B;TUBA4A;TUBAL3;TUBA8	3	386.5306	1156.57	54.189	0.098408	75.109	9467367

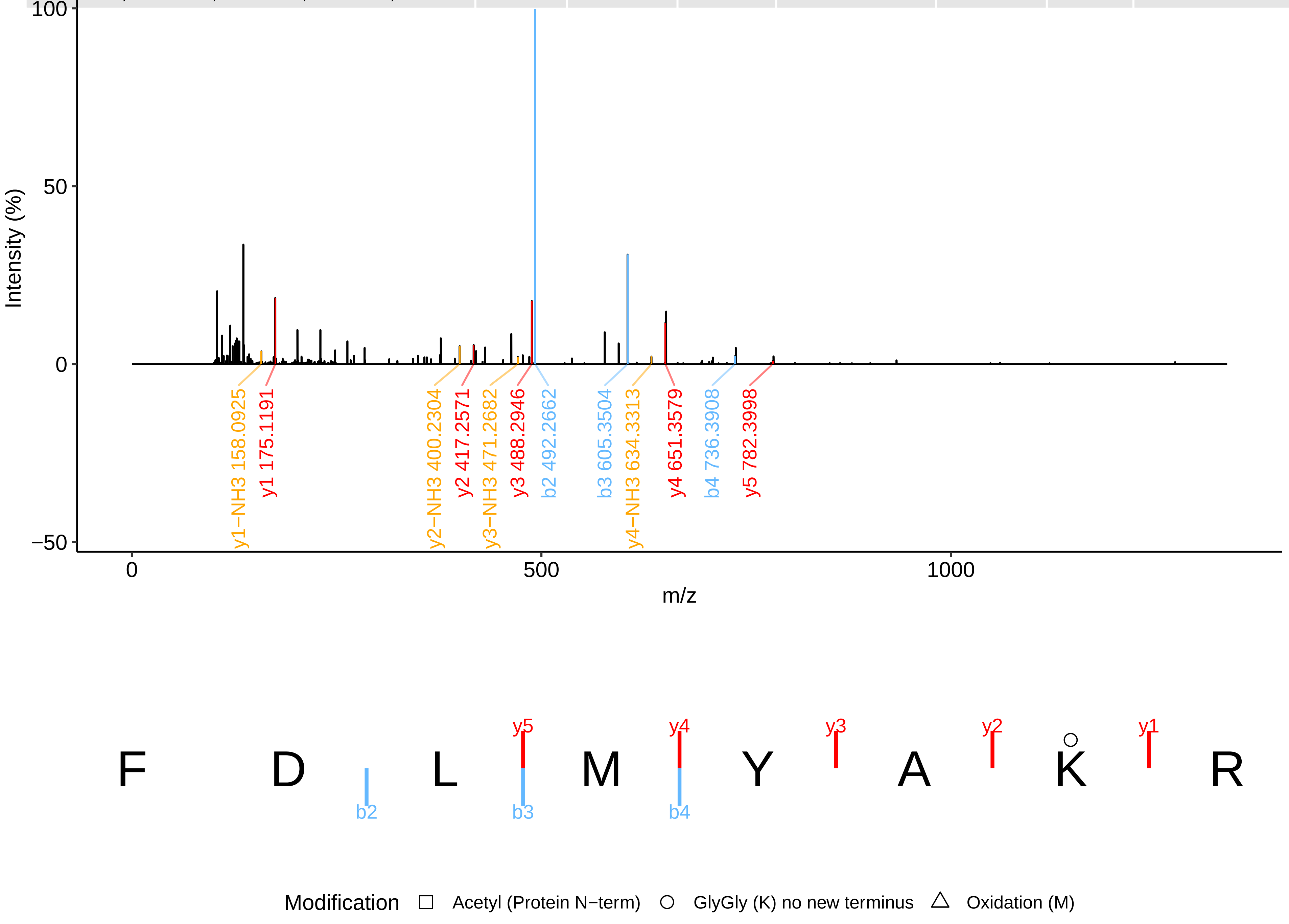

20200924_KIZ_MUb5_GlyGly_2 − Scan number: 14635

Gene Names	Charge	m/z	Mass	Retention time	PEP	Score	Precursor Intens
TUBA1C;TUBA1B;TUBA4A;TUBAL3;TUBA8	3	386.5306	1156.57	54.689	0.01358	98.299	33108270

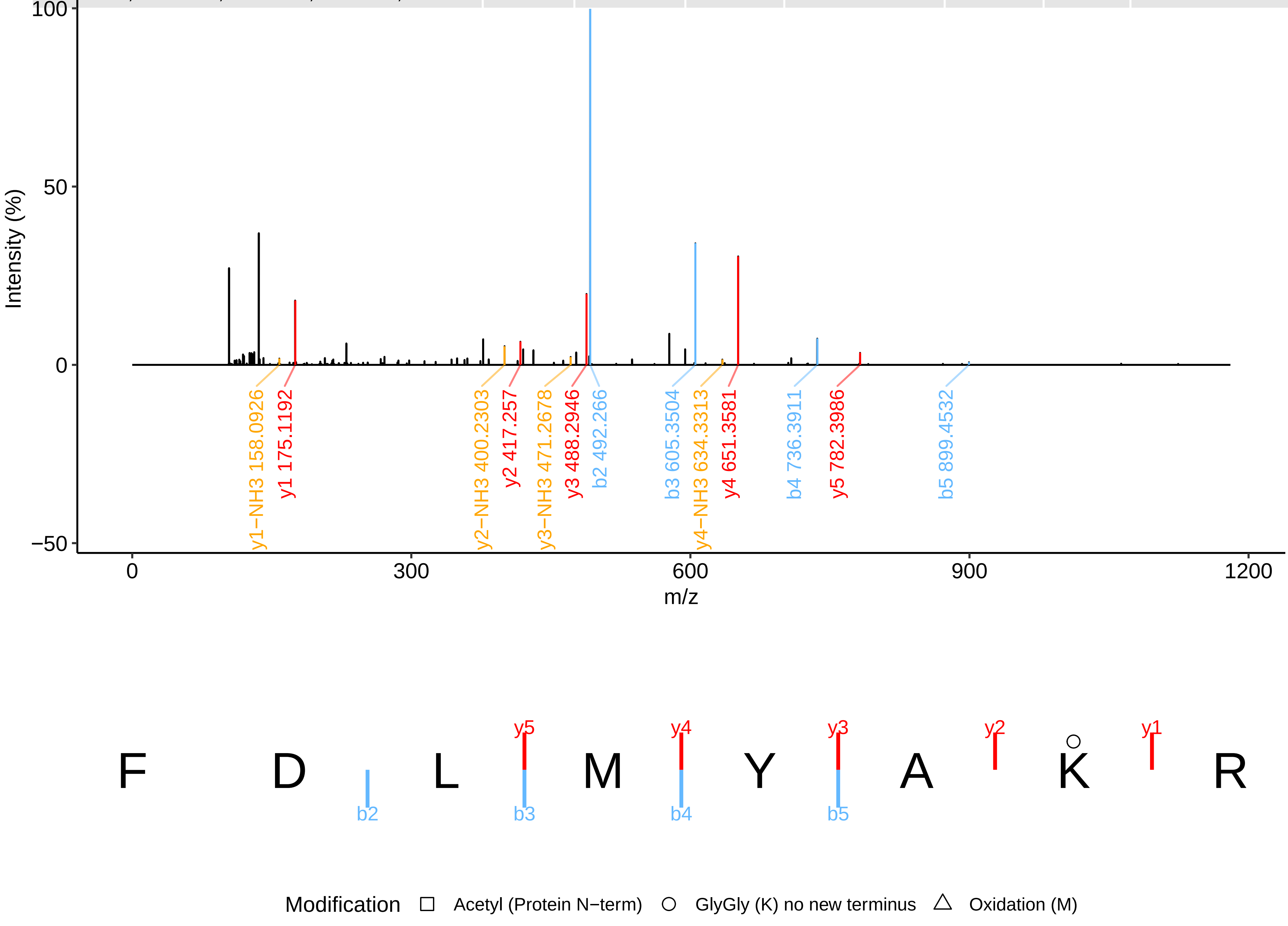

20201118_KIZ_MUb6_GlyGly_1 − Scan number: 15282

Gene Names	Charge	m/z	Mass	Retention time	PEP	Score	Precursor Inten[illegible]
TUBA1C;TUBA1B;TUBA4A;TUBAL3;TUBA8	3	386.5306	1156.57	48.352	0.044962	85.212	19040334

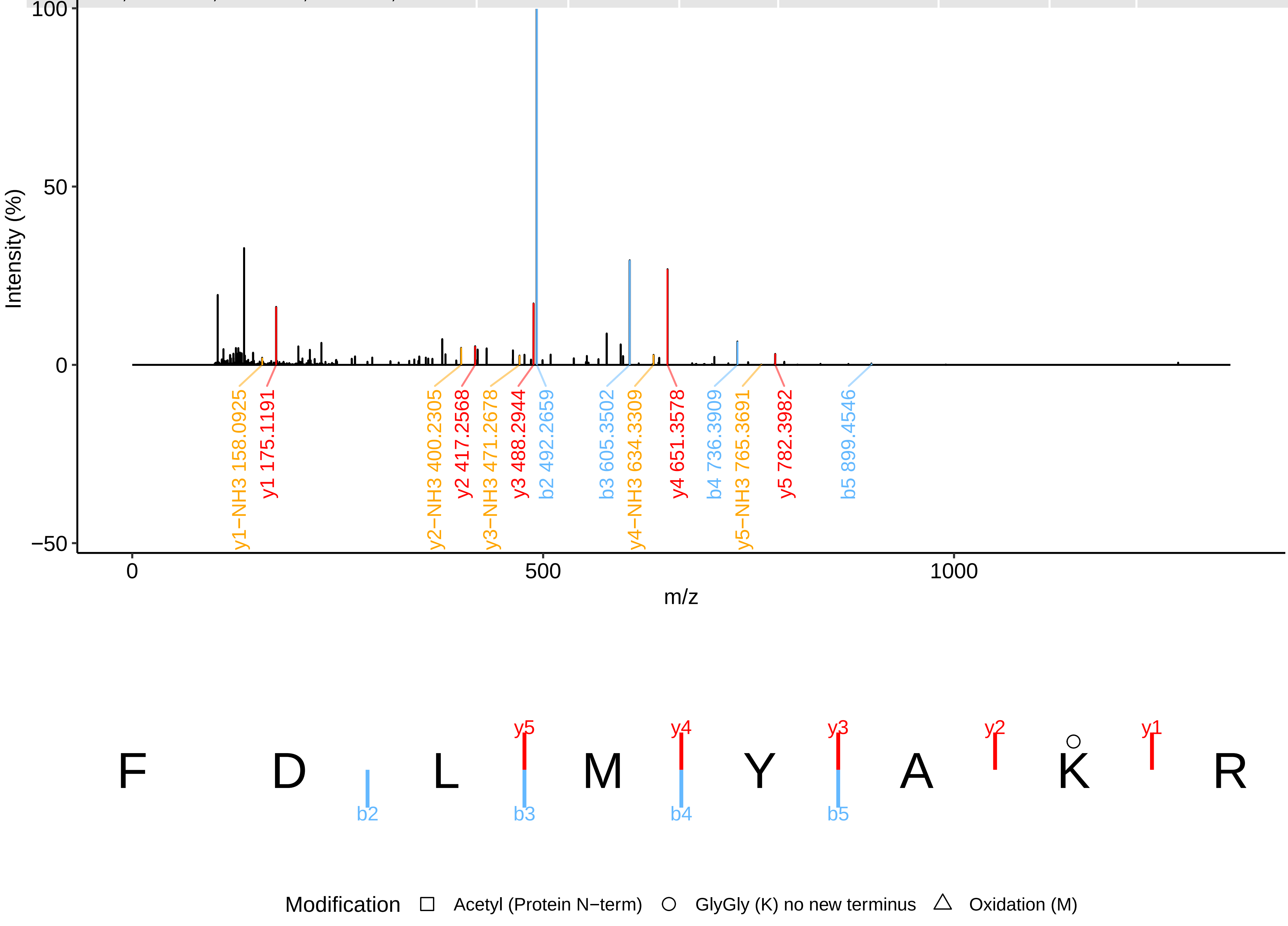

20201118_KIZ_MUb6_GlyGly_2 − Scan number: 17659

Gene Names	Charge	m/z	Mass	Retention time	PEP	Score	Precursor Intens
TUBA1C;TUBA1B;TUBA4A;TUBAL3;TUBA8	3	386.5306	1156.57	59.694	0.01358	98.299	6217627

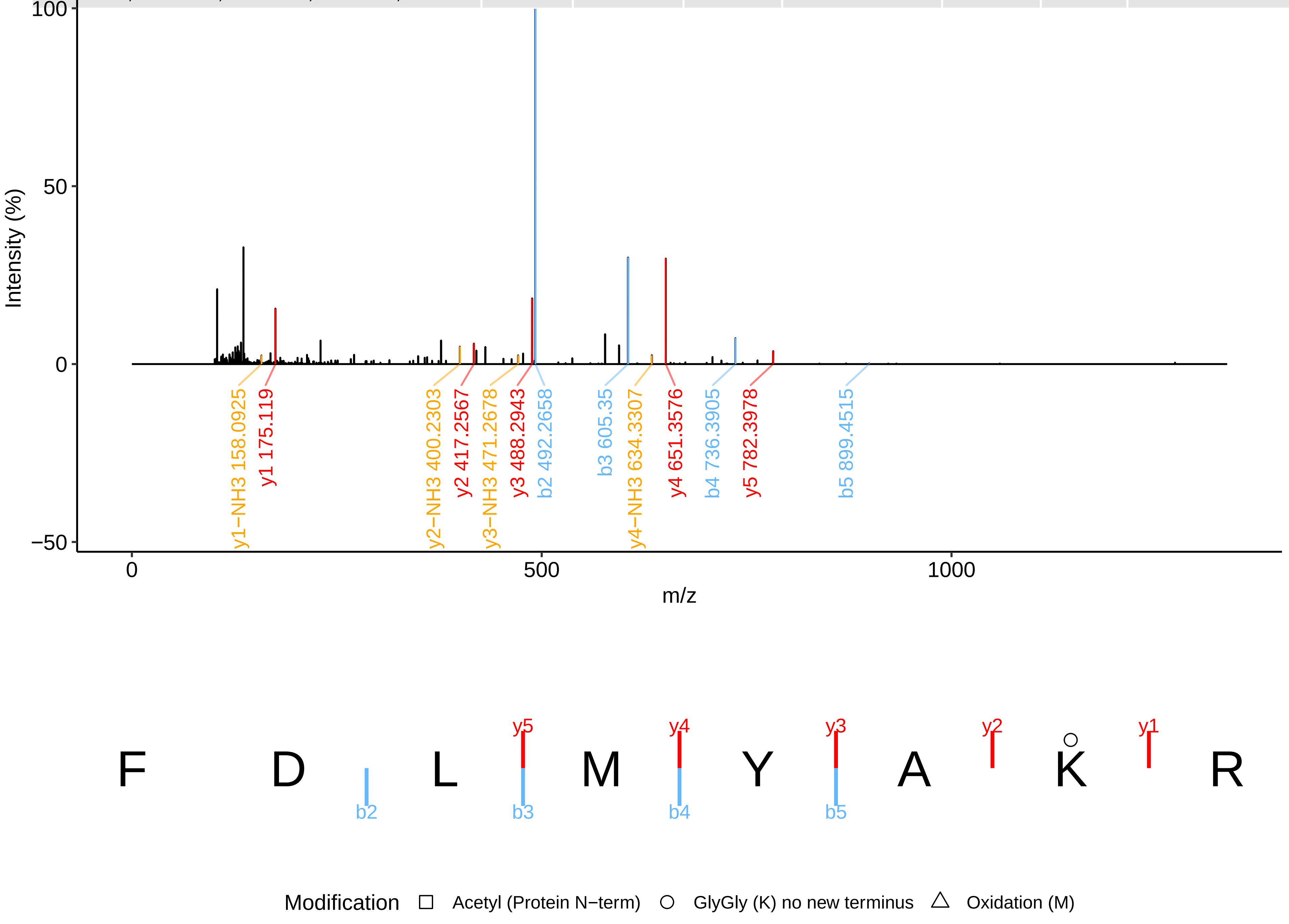

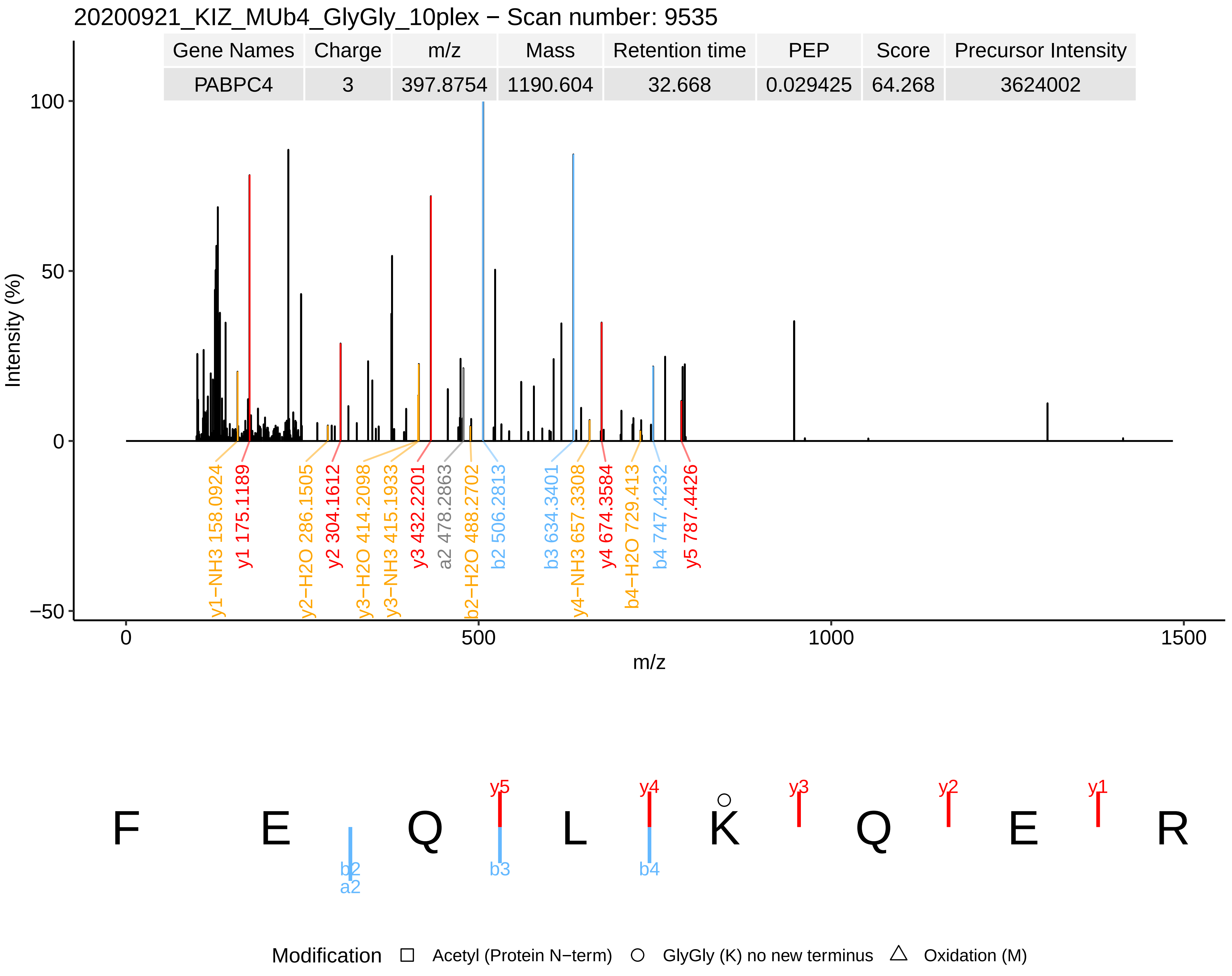

20200921_KIZ_MUb4_GlyGly_10plex − Scan number: 9535
Gene Names | Charge | m/z | Mass | Retention time | PEP | Score | Precursor Intensity
PABPC4 | 3 | 397.8754 | 1190.604 | 32.668 | 0.029425 | 64.268 | 3624002
Intensity (%)
m/z
y1−NH3 158.0924
y1 175.1189
y2−H2O 286.1505
y2 304.1612
y3−H2O 414.2098
y3−NH3 415.1933
y3 432.2201
a2 478.2863
b2−H2O 488.2702
b2 506.2813
b3 634.3401
y4−NH3 657.3308
y4 674.3584
b4−H2O 729.413
b4 747.4232
y5 787.4426
F E Q L K Q E R
y5 y4 y3 y2 y1
b2 a2 b3 b4
Modification
Acetyl (Protein N−term)
GlyGly (K) no new terminus
Oxidation (M)

20200921_KIZ_MUb4_GlyGly_10plex_2 − Scan number: 8801

Gene Names	Charge	m/z	Mass	Retention time	PEP	Score	Precursor Intensity
PABPC4	3	397.8754	1190.604	33.283	0.069491	58.433	775175.6

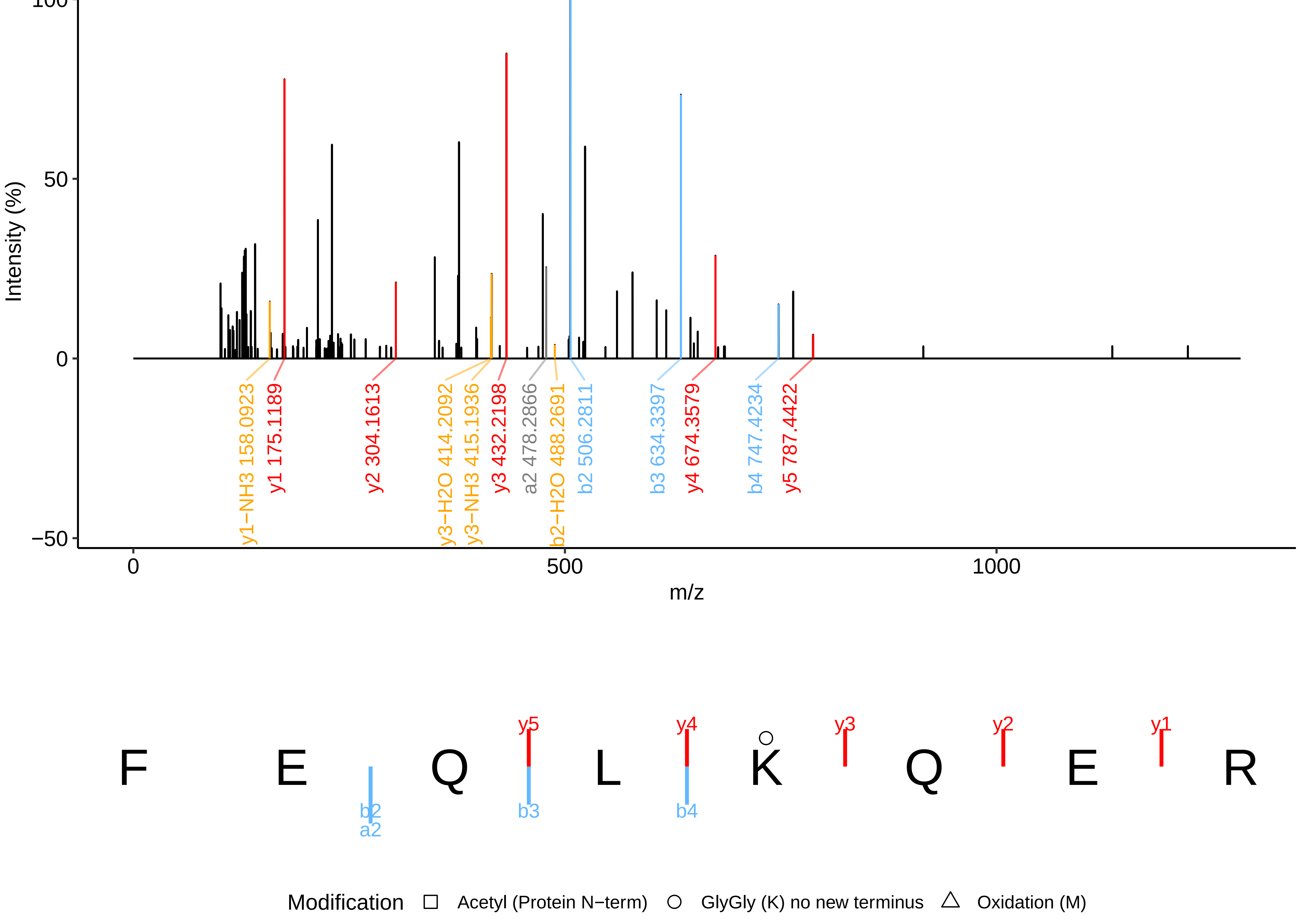

20200921_KIZ_MUb4_GlyGly_10plex − Scan number: 7362

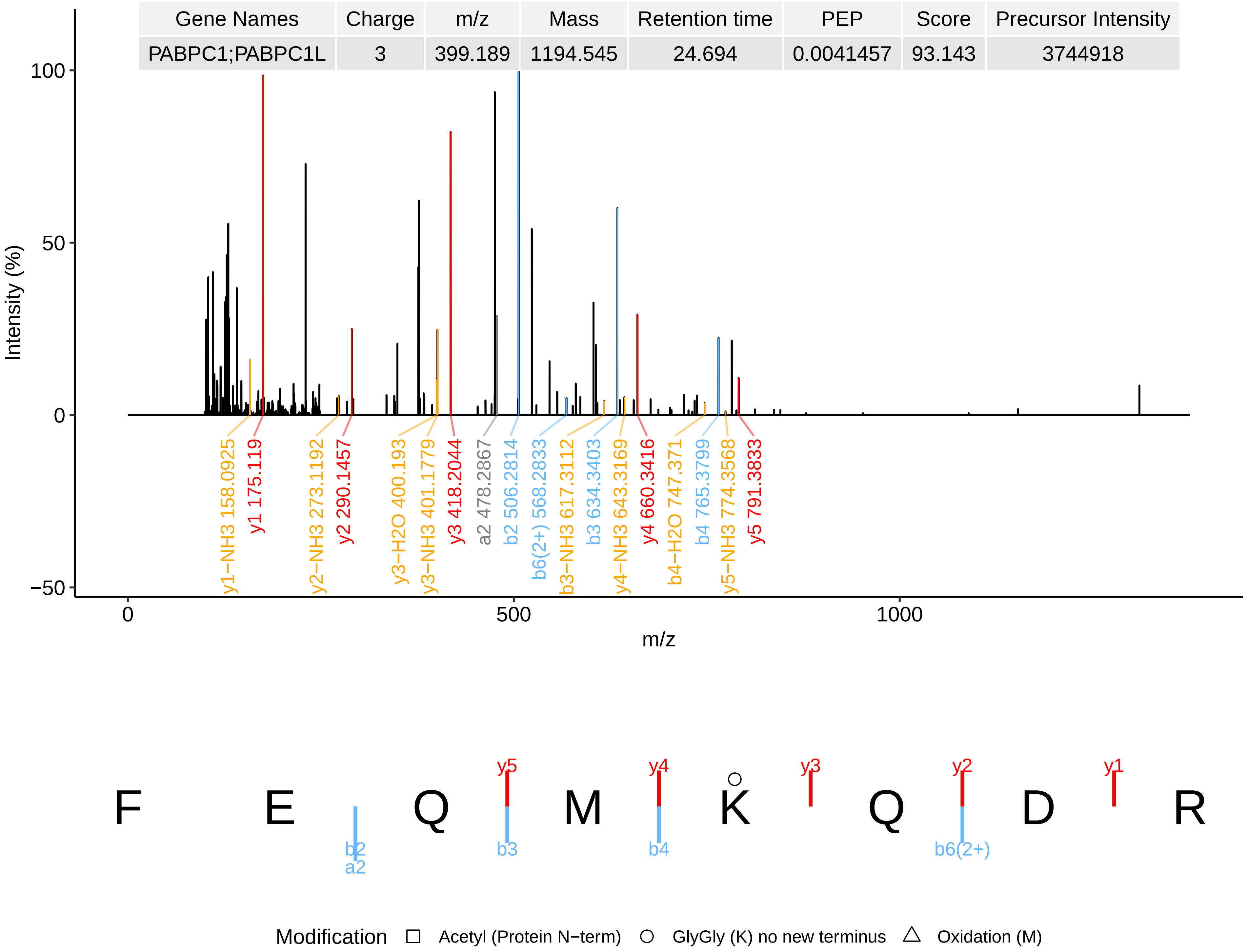

20200921_KIZ_MUb4_GlyGly_10plex_2 − Scan number: 6788

Gene Names	Charge	m/z	Mass	Retention time	PEP	Score	Precursor Intensity
PABPC1;PABPC1L	3	399.189	1194.545	25.102	0.0058512	88.819	990031.2

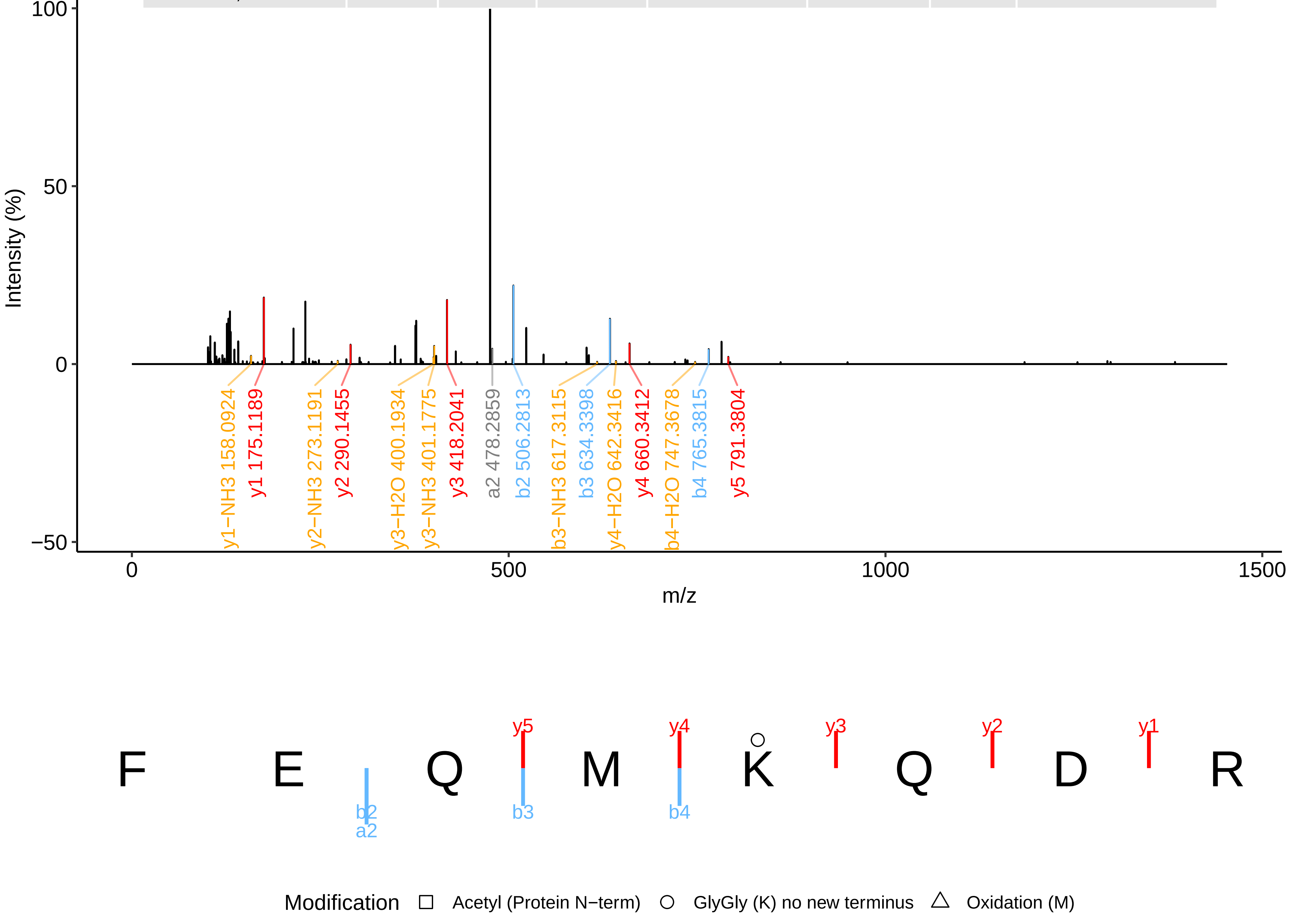

20200924_KIZ_MUb5_GlyGly_1 − Scan number: 6292

Gene Names	Charge	m/z	Mass	Retention time	PEP	Score	Precursor Intensity
PABPC1;PABPC1L	3	399.189	1194.545	19.756	0.0058512	88.819	8525241

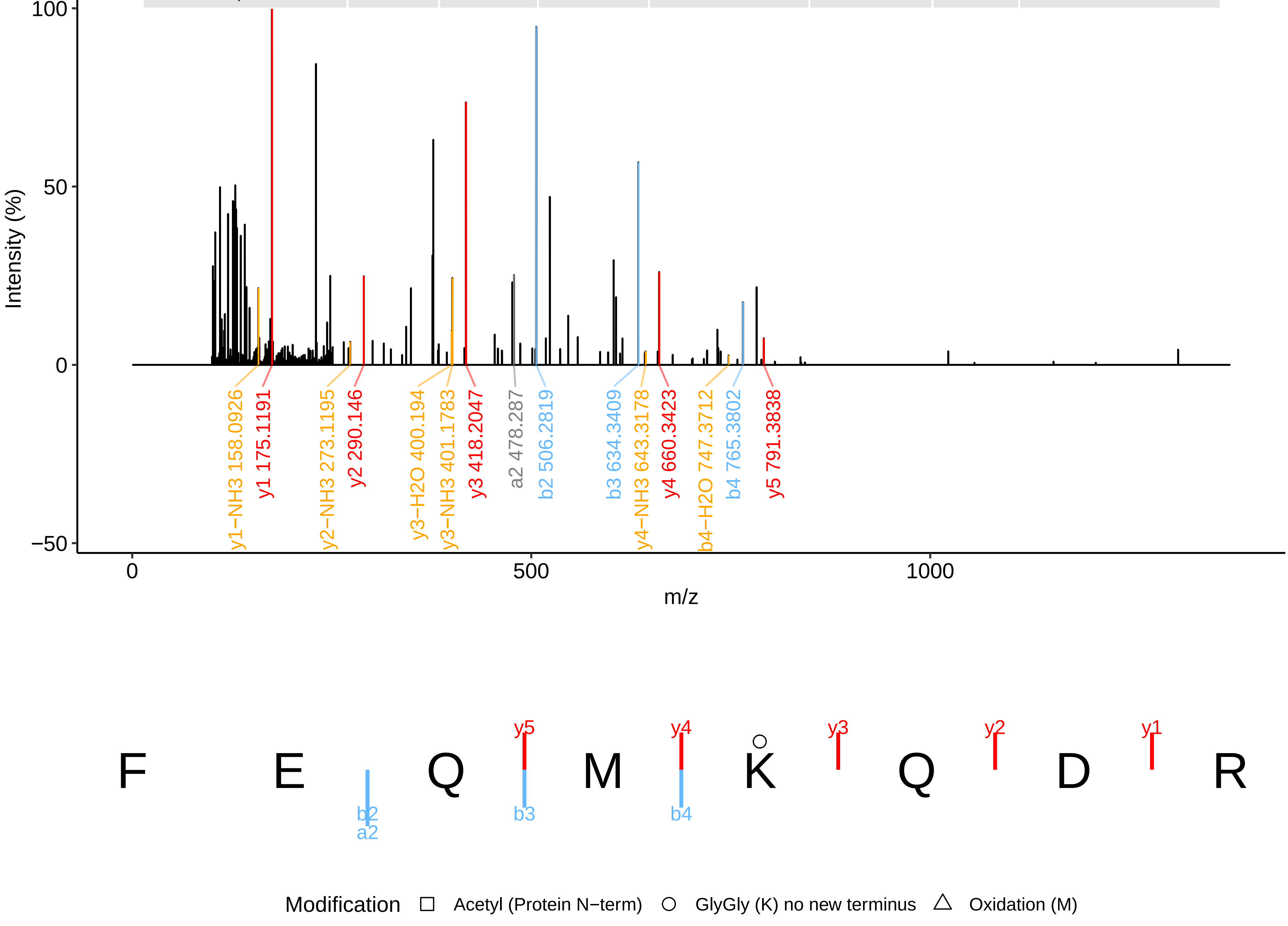

20200924_KIZ_MUb5_GlyGly_2 − Scan number: 5997

Gene Names	Charge	m/z	Mass	Retention time	PEP	Score	Precursor Intensity
PABPC1;PABPC1L	3	399.189	1194.545	22.322	0.018698	72.879	2668427

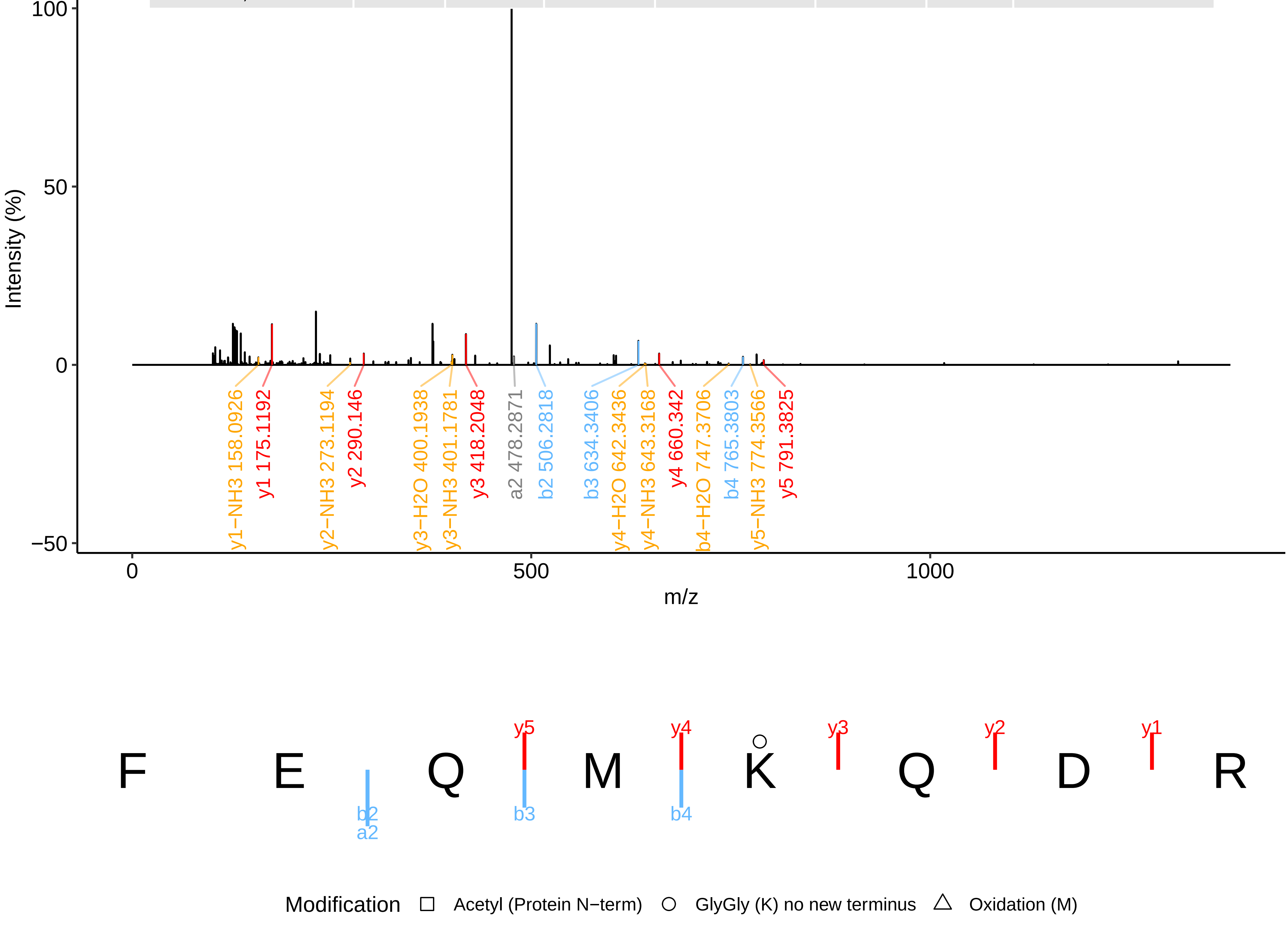

20200921_KIZ_MUb4_GlyGly_10plex − Scan number: 10817

Gene Names	Charge	m/z	Mass	Retention time	PEP	Score	Precursor Intensity
STK26	3	317.187	948.5393	37.567	0.029404	76.847	3925734

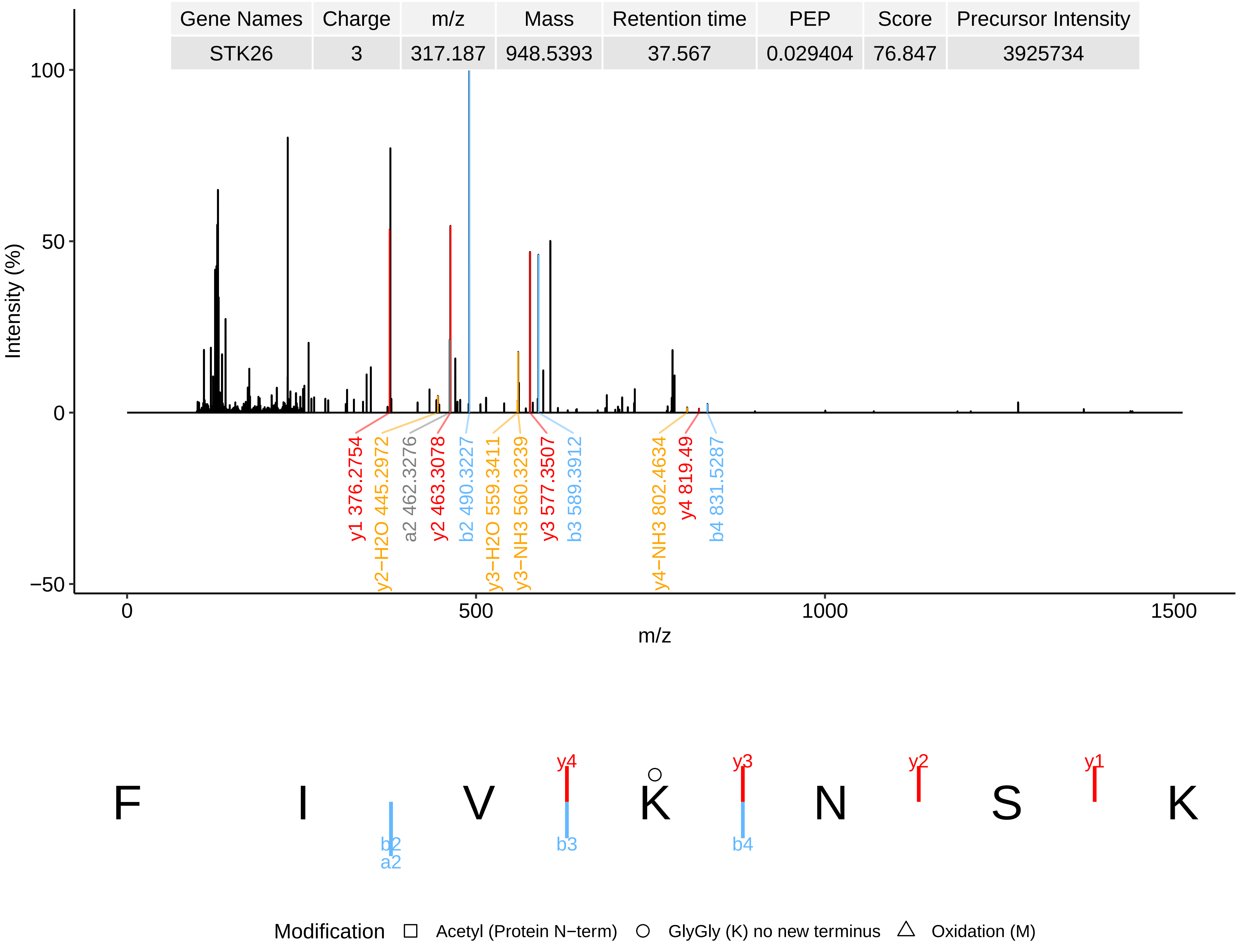

20200921_KIZ_MUb4_GlyGly_10plex − Scan number: 6107

Gene Names	Charge	m/z	Mass	Retention time	PEP	Score	Precursor Intensity
CD151	3	322.4944	964.4614	20.096	0.09247	87.284	3927248

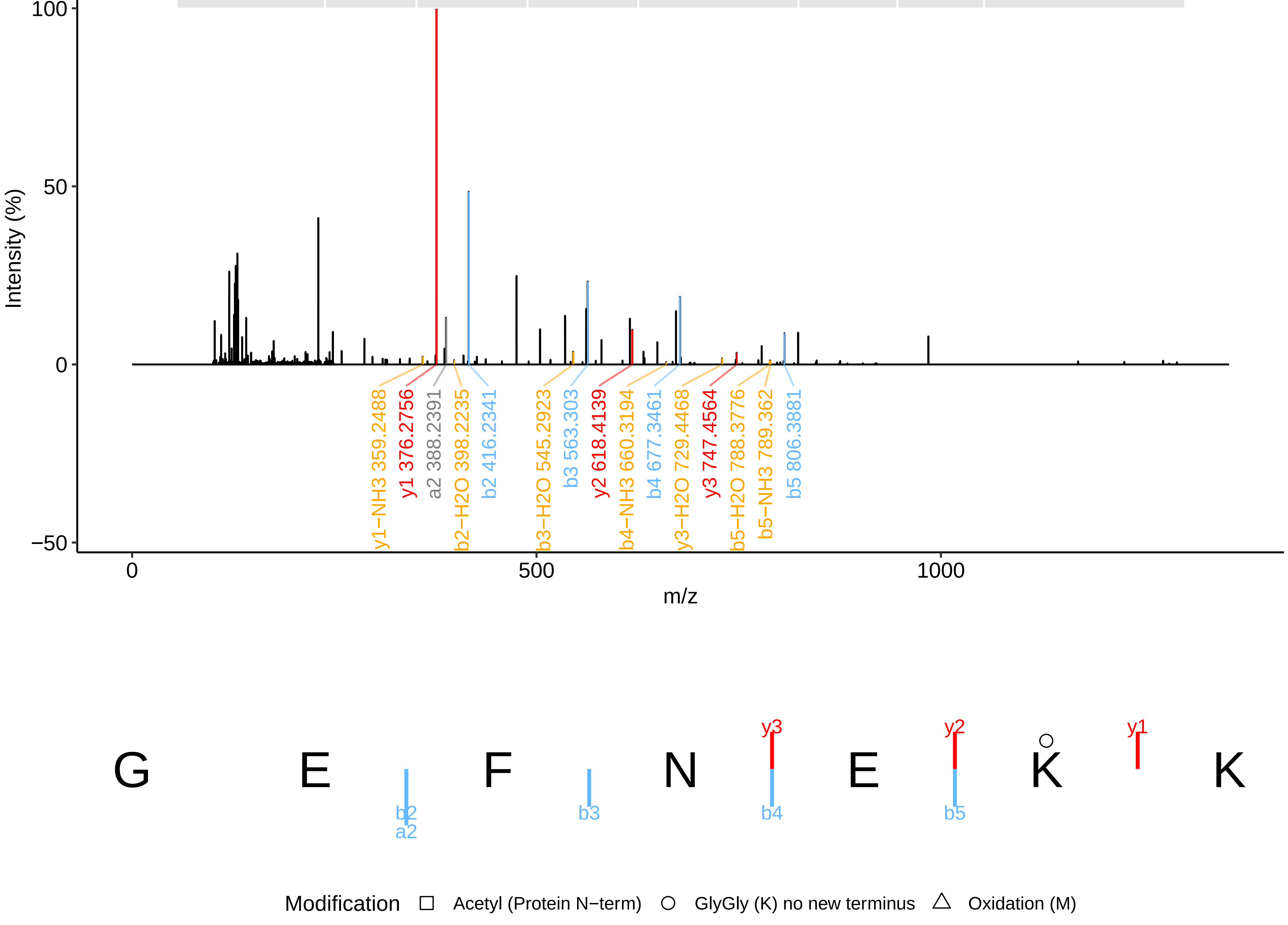

20200924_KIZ_MUb5_GlyGly_2 − Scan number: 4990

Gene Names	Charge	m/z	Mass	Retention time	PEP	Score	Precursor Intensity
CD151	3	322.4944	964.4614	18.442	0.094316	86.872	3843989

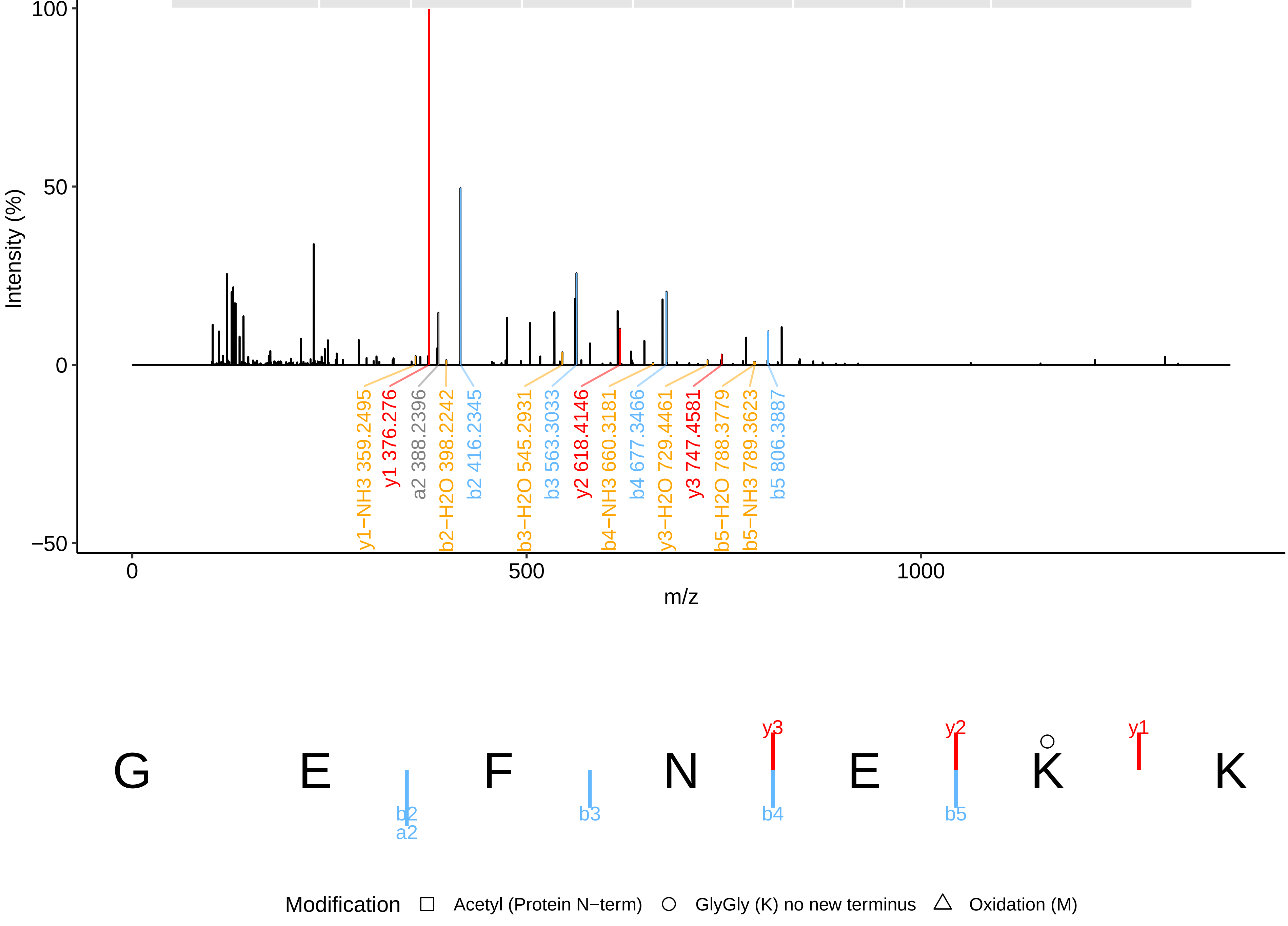

20201118_KIZ_MUb6_GlyGly_1 − Scan number: 5055

Gene Names	Charge	m/z	Mass	Retention time	PEP	Score	Precursor Intensity
CD151	3	322.4944	964.4614	15.986	0.043463	98.503	36524040

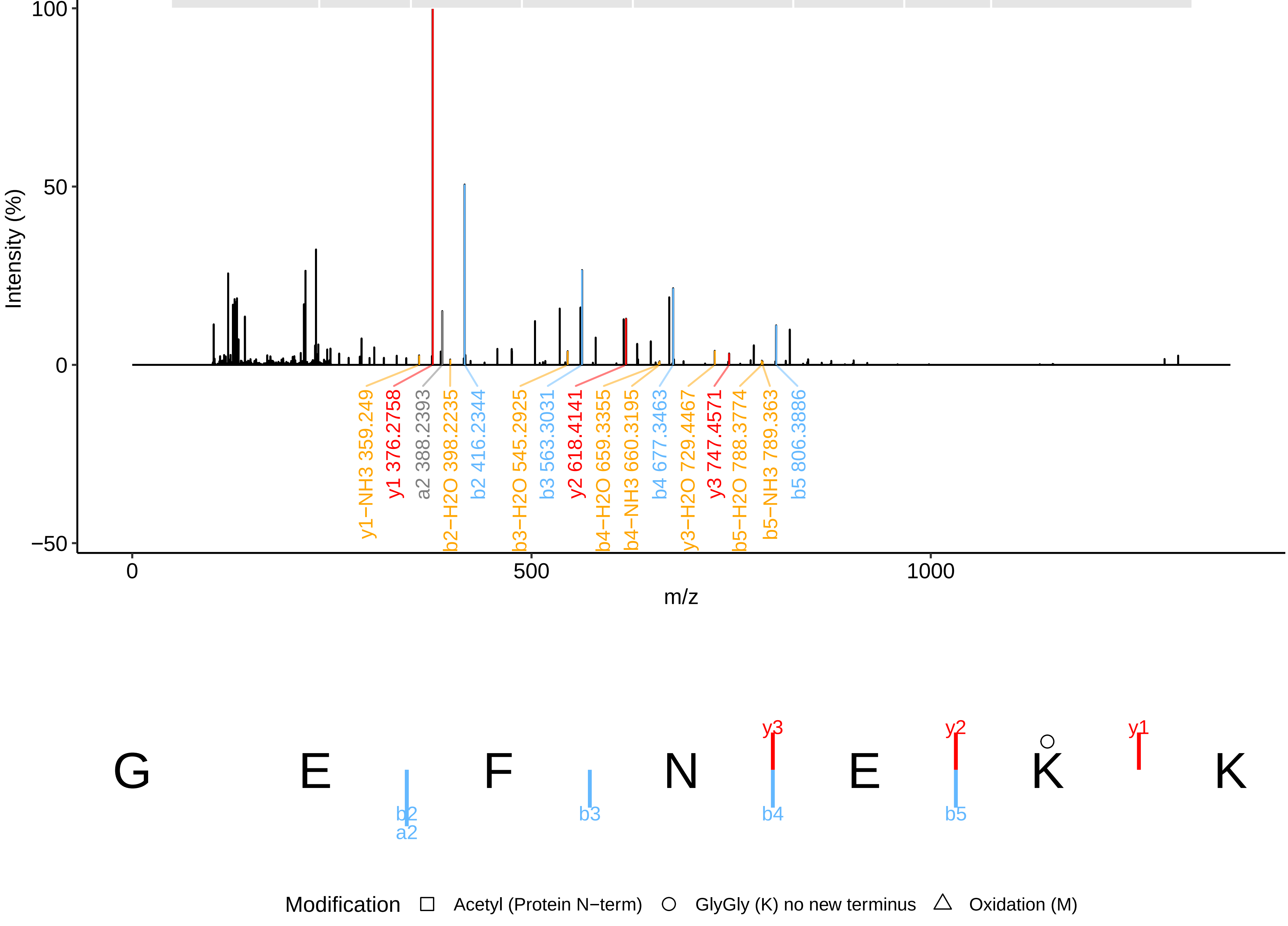

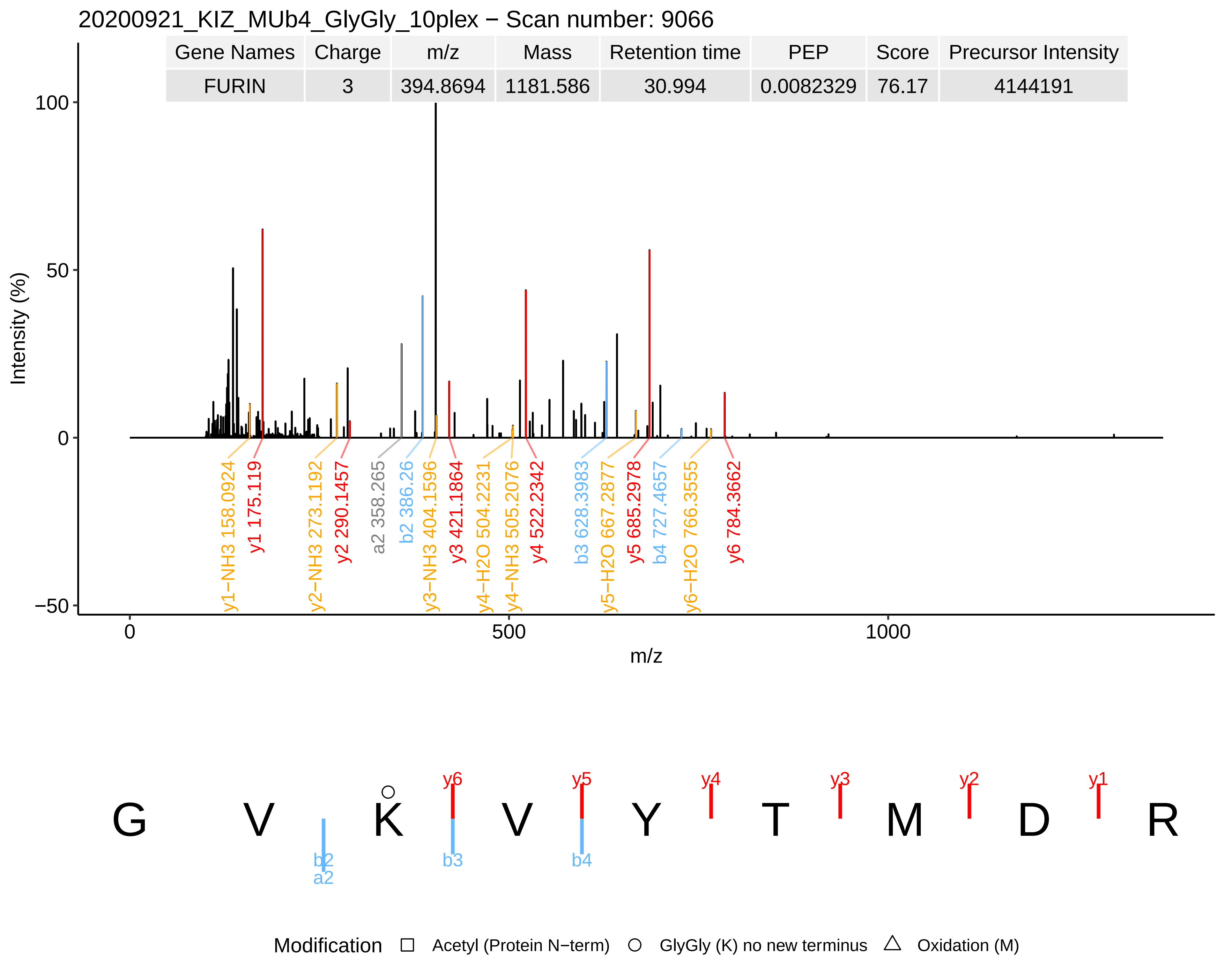
20200921_KIZ_MUb4_GlyGly_10plex − Scan number: 9066
Gene Names | Charge | m/z | Mass | Retention time | PEP | Score | Precursor Intensity
FURIN | 3 | 394.8694 | 1181.586 | 30.994 | 0.0082329 | 76.17 | 4144191
Intensity (%)
m/z
y1−NH3 158.0924
y1 175.119
y2−NH3 273.1192
y2 290.1457
a2 358.265
b2 386.26
y3−NH3 404.1596
y3 421.1864
y4−H2O 504.2231
y4−NH3 505.2076
y4 522.2342
b3 628.3983
y5−H2O 667.2877
y5 685.2978
b4 727.4657
y6−H2O 766.3555
y6 784.3662
G V K V Y T M D R
Modification
Acetyl (Protein N−term)
GlyGly (K) no new terminus
Oxidation (M)

20200921_KIZ_MUb4_GlyGly_10plex − Scan number: 4144

Gene Names	Charge	m/z	Mass	Retention time	PEP	Score	Precursor Intensity
NASP	3	397.1984	1188.573	13.485	0.00016786	110.76	8927233

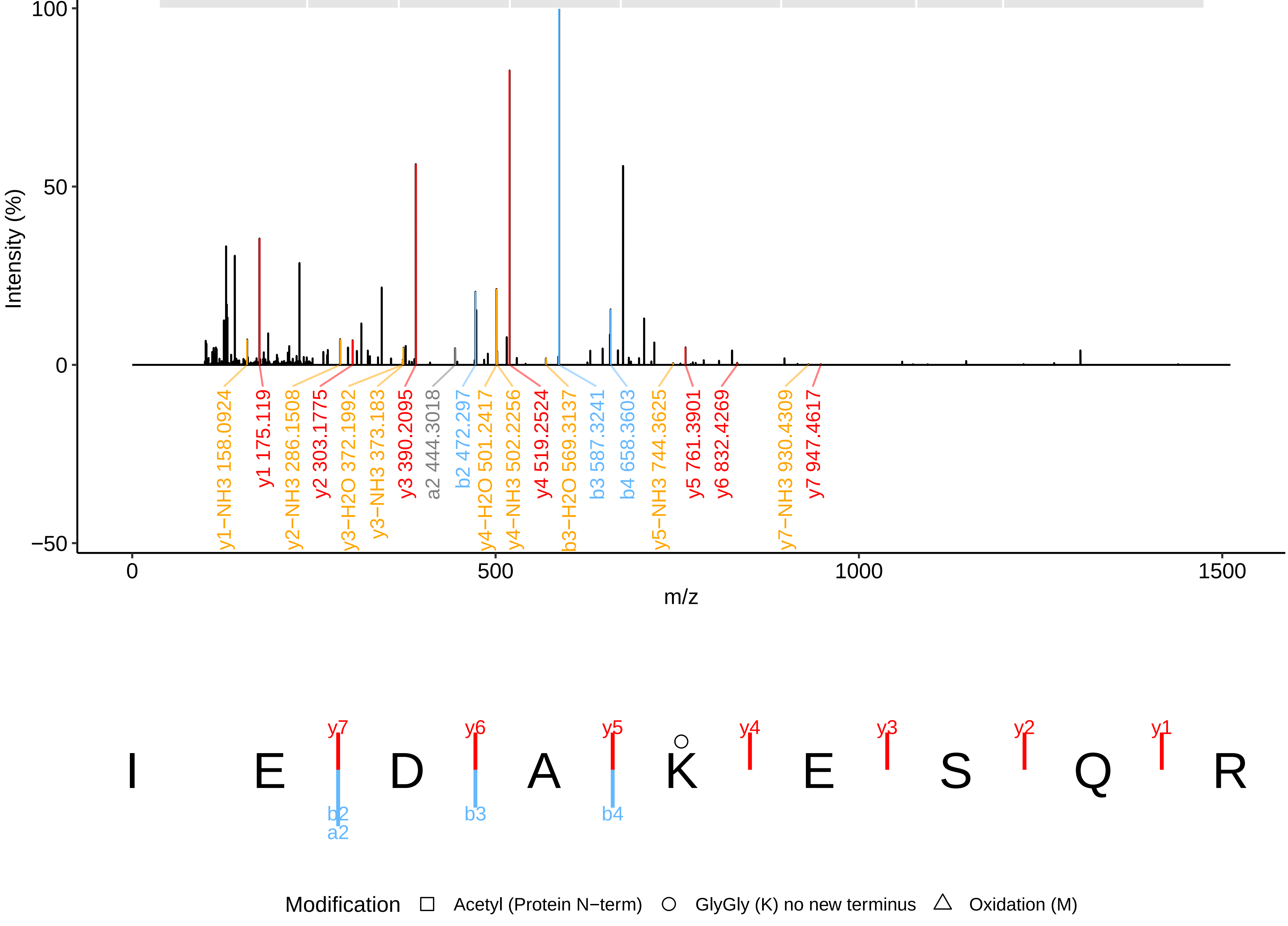

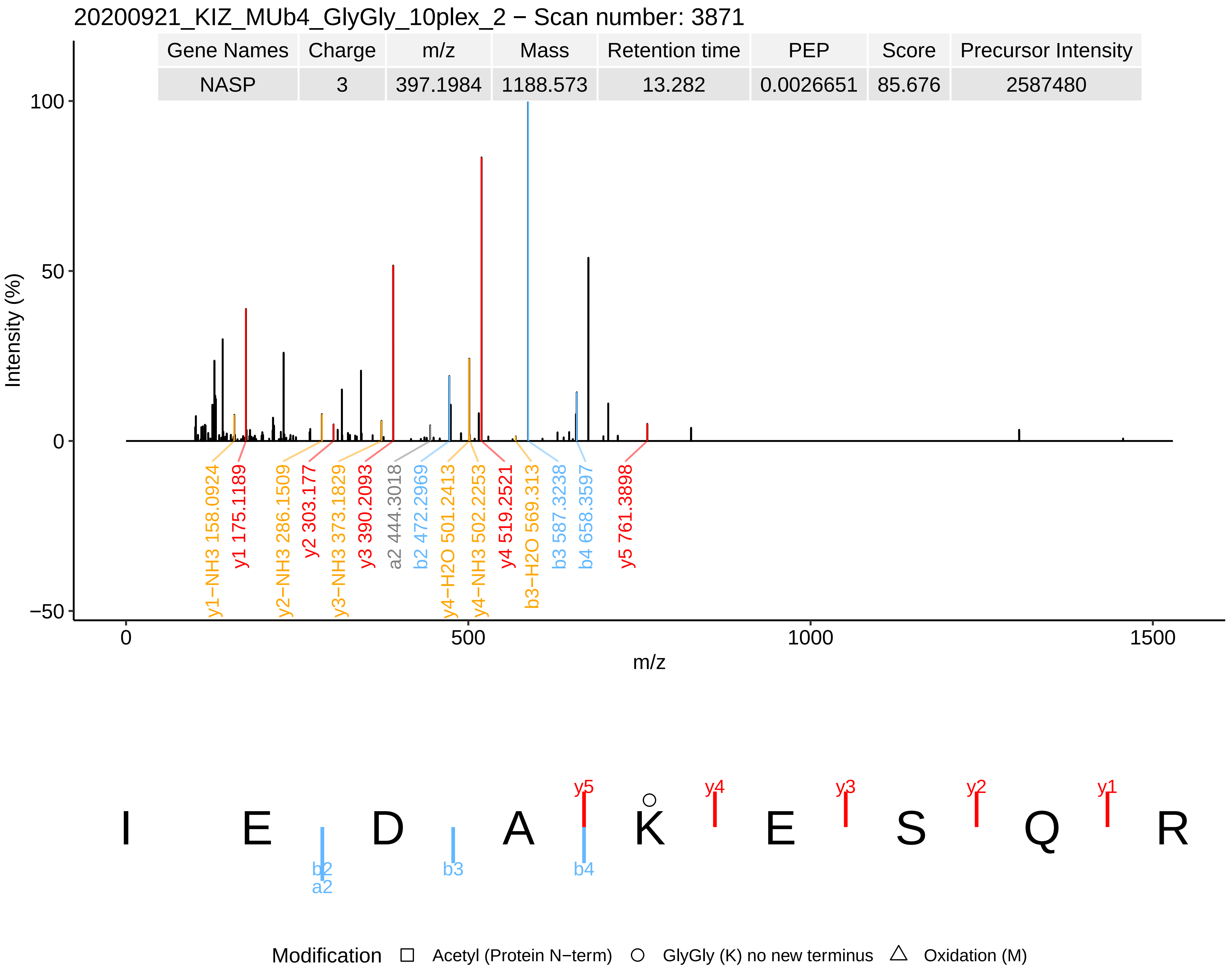
20200921_KIZ_MUb4_GlyGly_10plex_2 − Scan number: 3871
Gene Names | Charge | m/z | Mass | Retention time | PEP | Score | Precursor Intensity
NASP | 3 | 397.1984 | 1188.573 | 13.282 | 0.0026651 | 85.676 | 2587480
Intensity (%)
m/z
y1−NH3 158.0924
y1 175.1189
y2−NH3 286.1509
y2 303.177
y3−NH3 373.1829
y3 390.2093
a2 444.3018
b2 472.2969
y4−H2O 501.2413
y4−NH3 502.2253
y4 519.2521
b3−H2O 569.313
b3 587.3238
b4 658.3597
y5 761.3898
I E D A K E S Q R
y5 y4 y3 y2 y1
b2 a2 b3 b4
Modification
Acetyl (Protein N−term)
GlyGly (K) no new terminus
Oxidation (M)

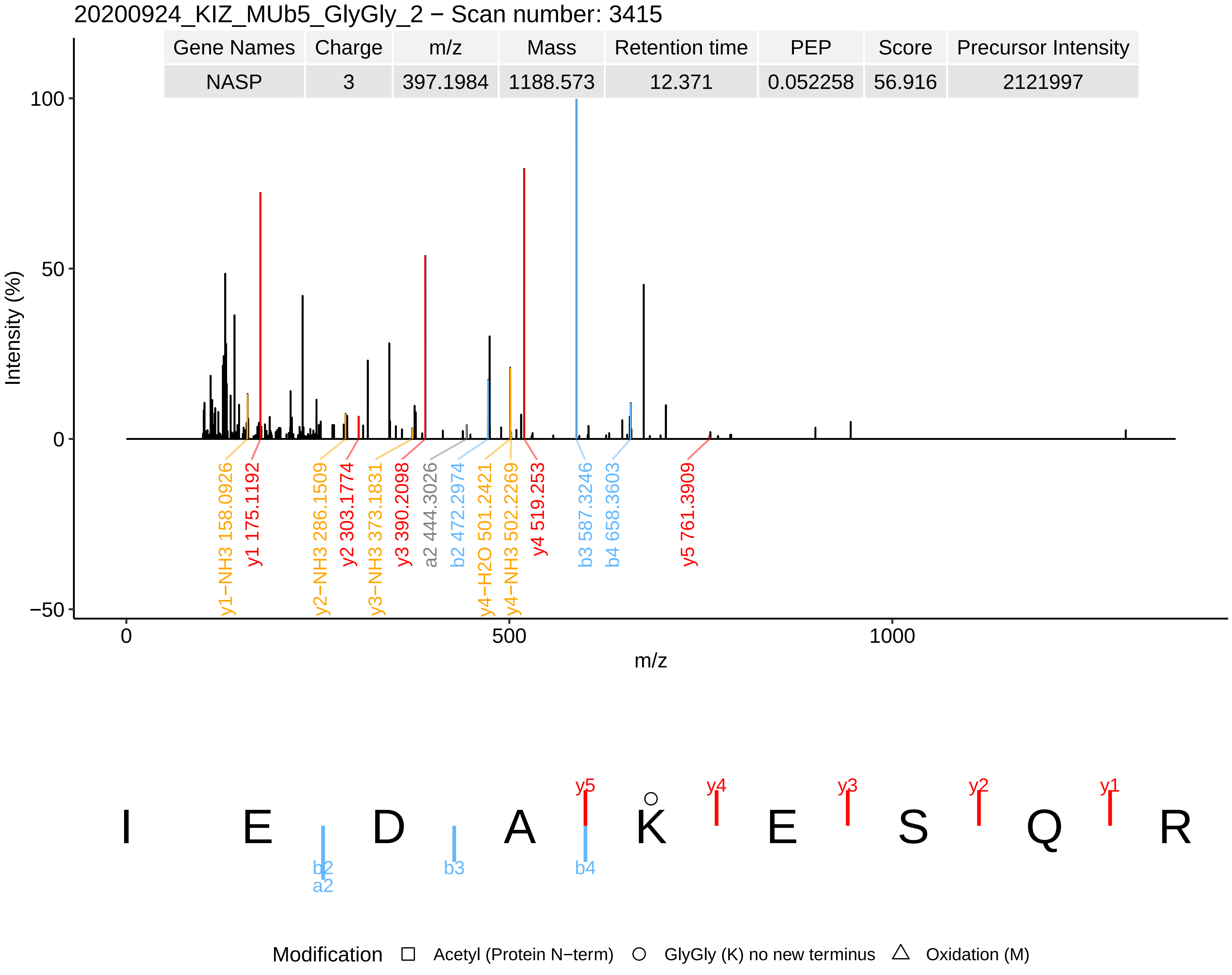
20200924_KIZ_MUb5_GlyGly_2 − Scan number: 3415
Gene Names | Charge | m/z | Mass | Retention time | PEP | Score | Precursor Intensity
NASP | 3 | 397.1984 | 1188.573 | 12.371 | 0.052258 | 56.916 | 2121997
Intensity (%)
m/z
y1−NH3 158.0926
y1 175.1192
y2−NH3 286.1509
y2 303.1774
y3−NH3 373.1831
y3 390.2098
a2 444.3026
b2 472.2974
y4−H2O 501.2421
y4−NH3 502.2269
y4 519.253
b3 587.3246
b4 658.3603
y5 761.3909
I E D A K E S Q R
y5 y4 y3 y2 y1
b2 a2 b3 b4
Modification
Acetyl (Protein N−term)
GlyGly (K) no new terminus
Oxidation (M)

20201118_KIZ_MUb6_GlyGly_1 − Scan number: 16048

Gene Names	Charge	m/z	Mass	Retention time	PEP	Score	Precursor Intensity
EIF3B	3	314.1799	939.5178	50.708	0.10264	55.441	44153912

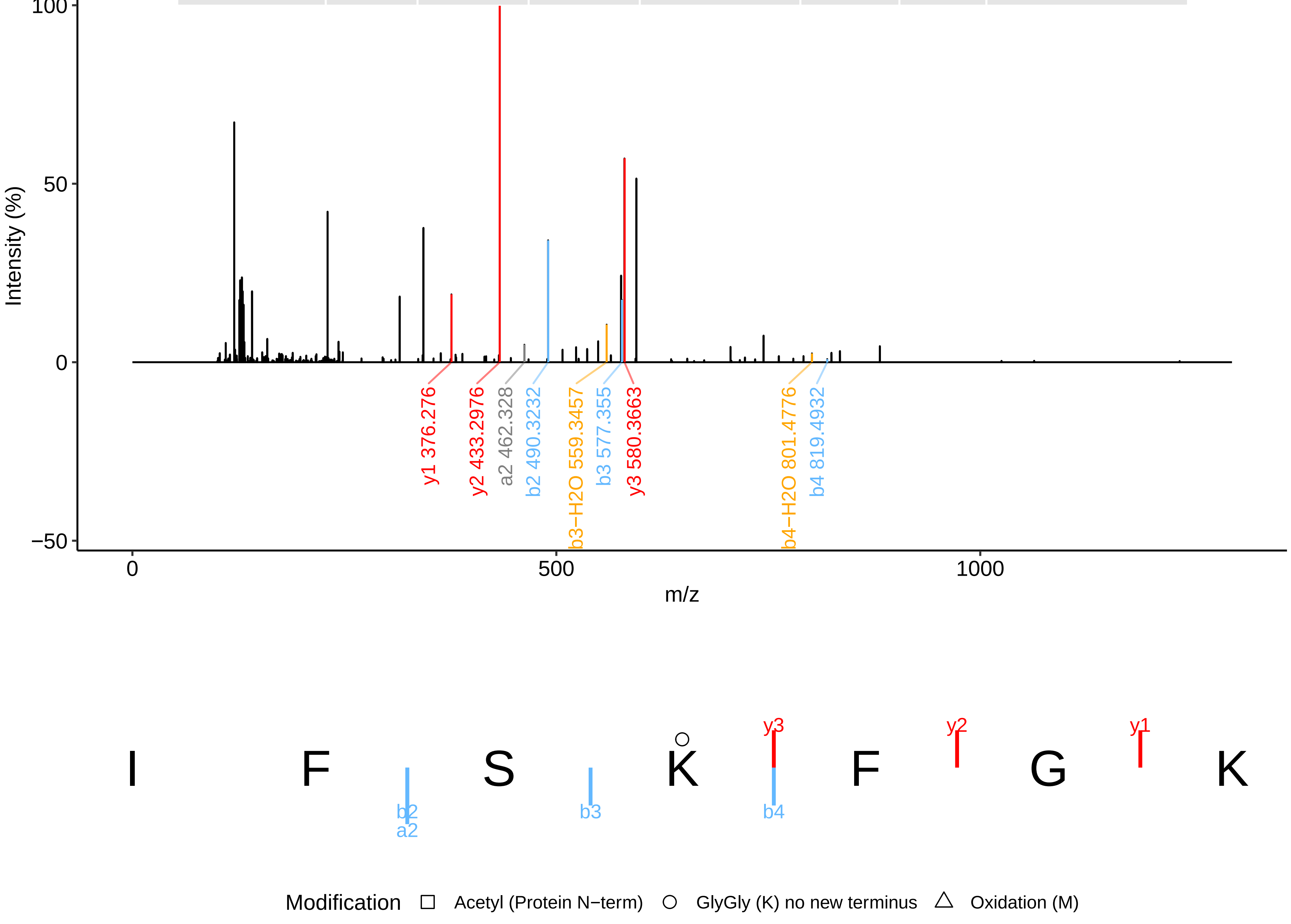

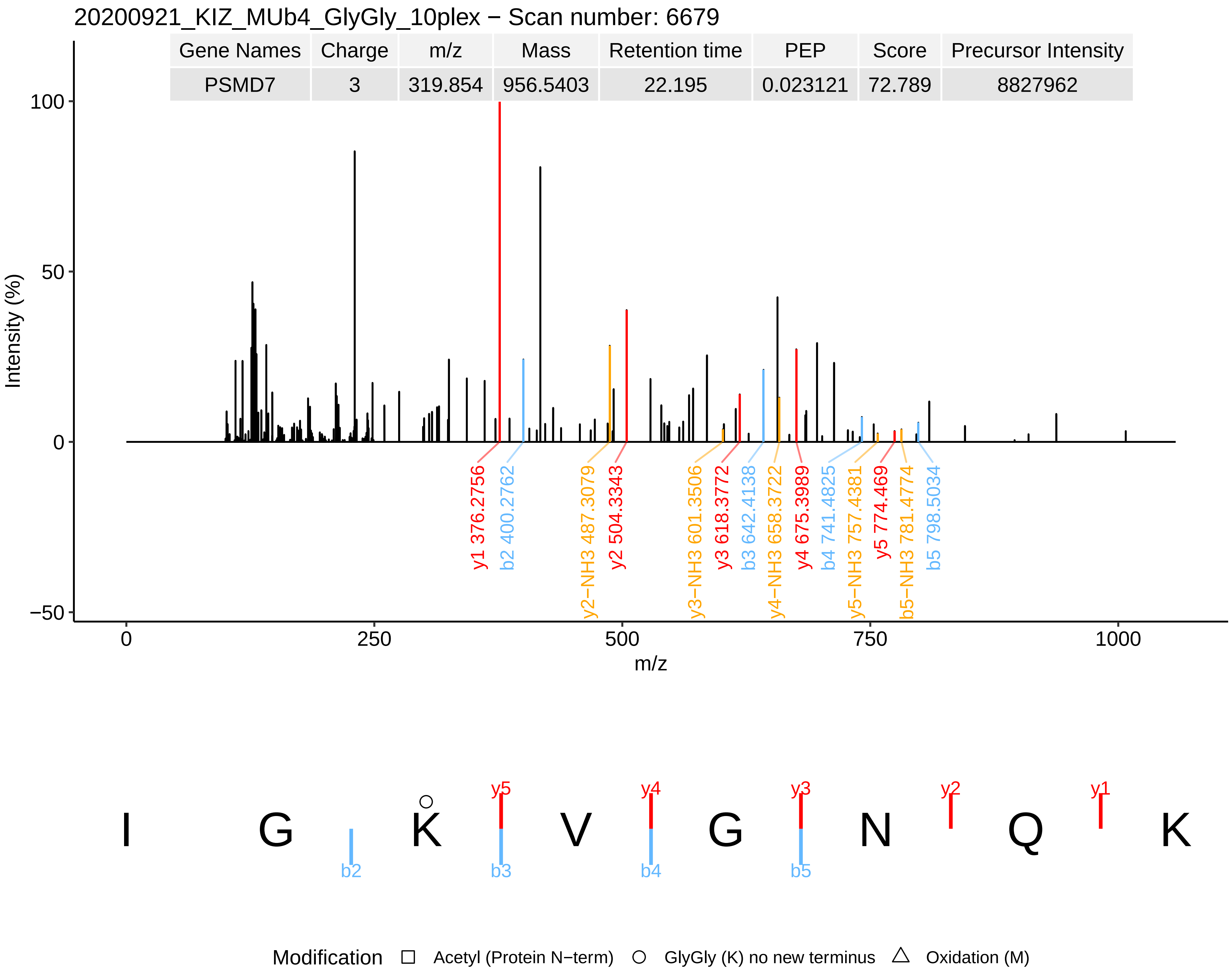

20200921_KIZ_MUb4_GlyGly_10plex − Scan number: 6679
Gene Names | Charge | m/z | Mass | Retention time | PEP | Score | Precursor Intensity
PSMD7 | 3 | 319.854 | 956.5403 | 22.195 | 0.023121 | 72.789 | 8827962
Intensity (%)
m/z
y1 376.2756
b2 400.2762
y2−NH3 487.3079
y2 504.3343
y3−NH3 601.3506
y3 618.3772
b3 642.4138
y4−NH3 658.3722
y4 675.3989
b4 741.4825
y5−NH3 757.4381
y5 774.469
b5−NH3 781.4774
b5 798.5034
I G K V G N Q K
y5 y4 y3 y2 y1
b2 b3 b4 b5
Modification
Acetyl (Protein N−term)
GlyGly (K) no new terminus
Oxidation (M)

20200921_KIZ_MUb4_GlyGly_10plex_2 − Scan number: 6212

Gene Names	Charge	m/z	Mass	Retention time	PEP	Score	Precursor Intensity
PSMD7	3	319.854	956.5403	22.726	0.011405	80.229	1447917

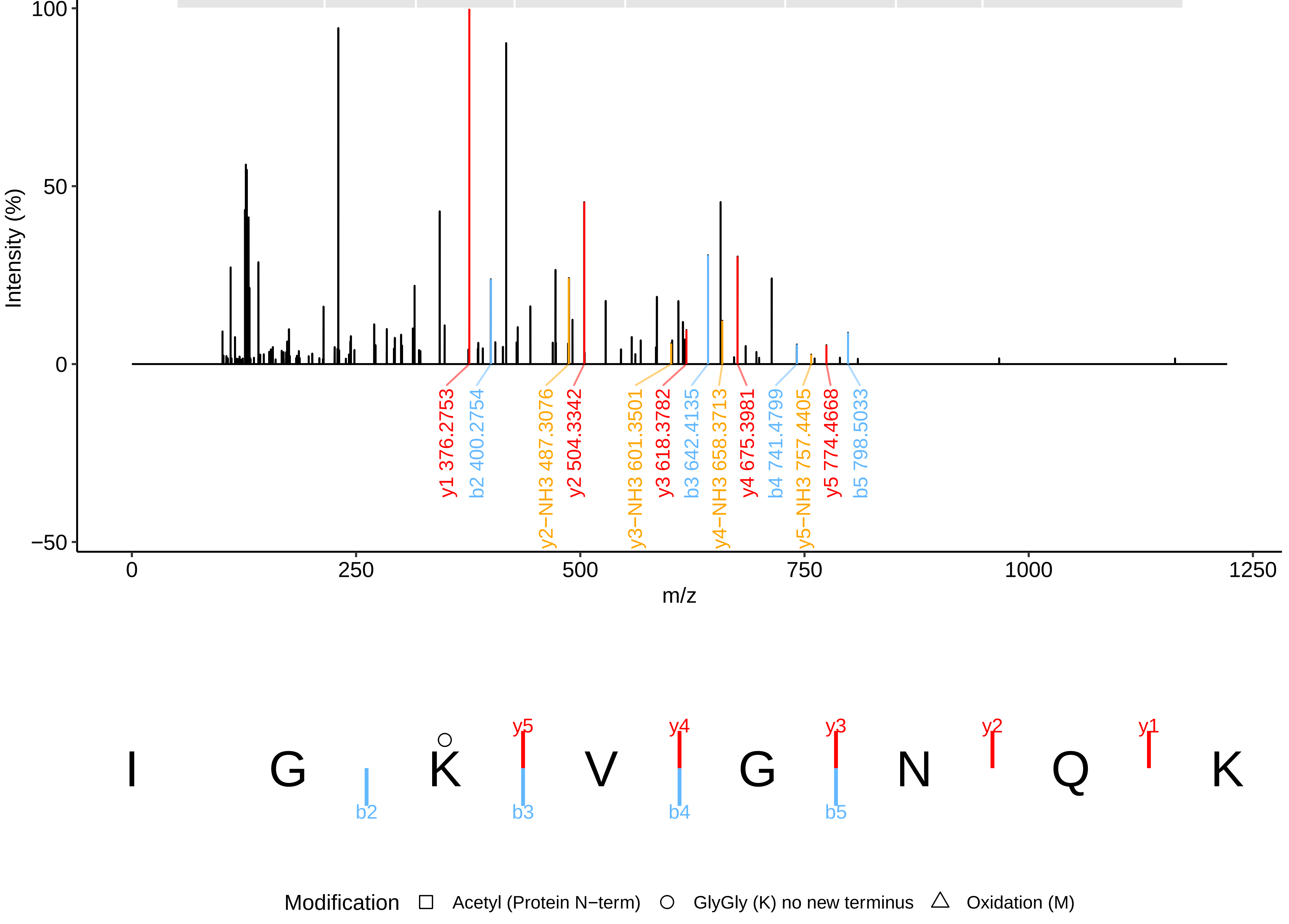

20200924_KIZ_MUb5_GlyGly_1 − Scan number: 5716

Gene Names	Charge	m/z	Mass	Retention time	PEP	Score	Precursor Intensity
PSMD7	3	319.854	956.5403	17.841	0.019285	75.213	NA

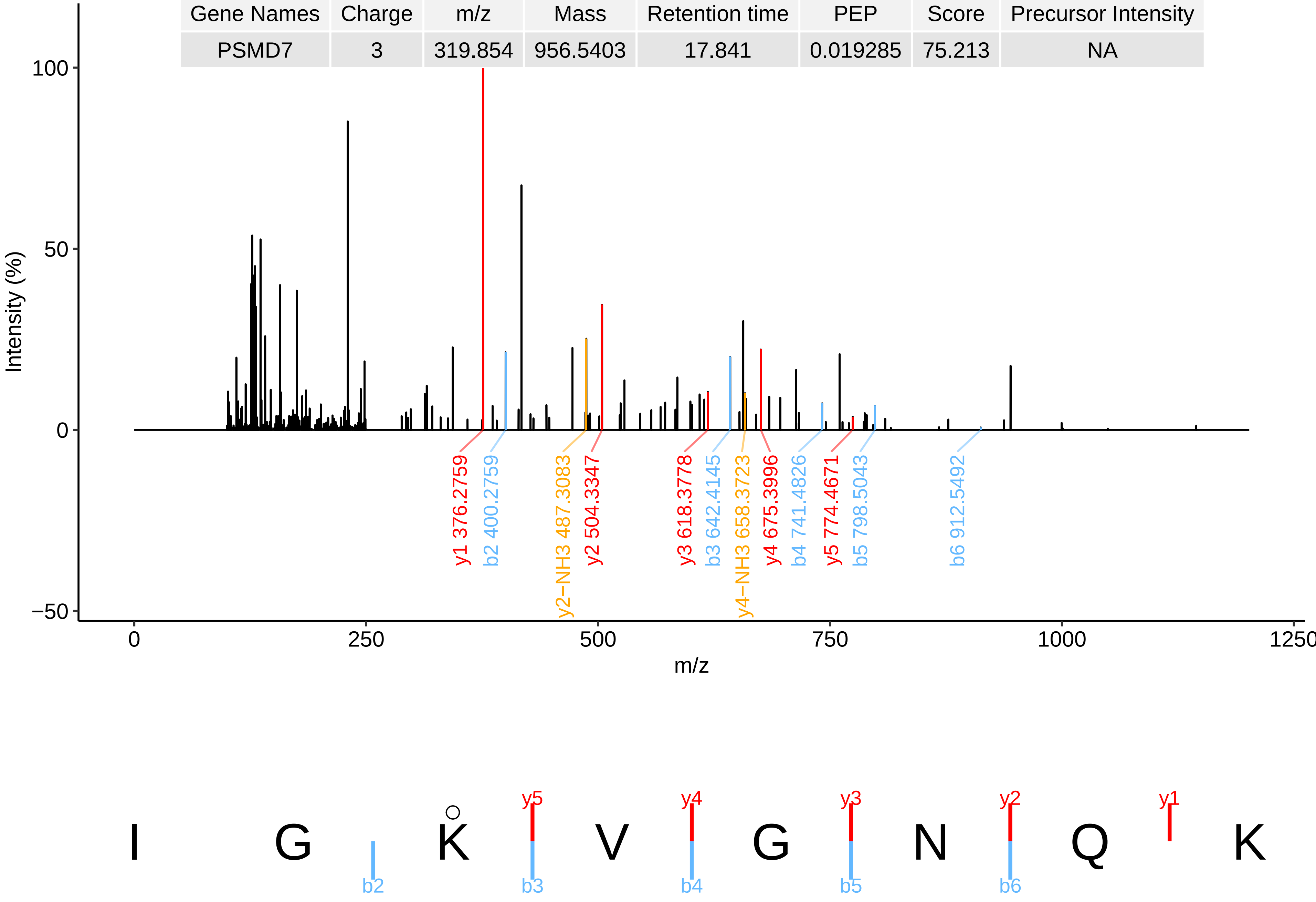

Modification □ Acetyl (Protein N−term) ○ GlyGly (K) no new terminus △ Oxidation (M)

20200921_KIZ_MUb4_GlyGly_10plex − Scan number: 14710

Gene Names	Charge	m/z	Mass	Retention time	PEP	Score	Precursor Intensity
MYH9	3	399.2243	1194.651	52.321	0.015232	69.598	5371811

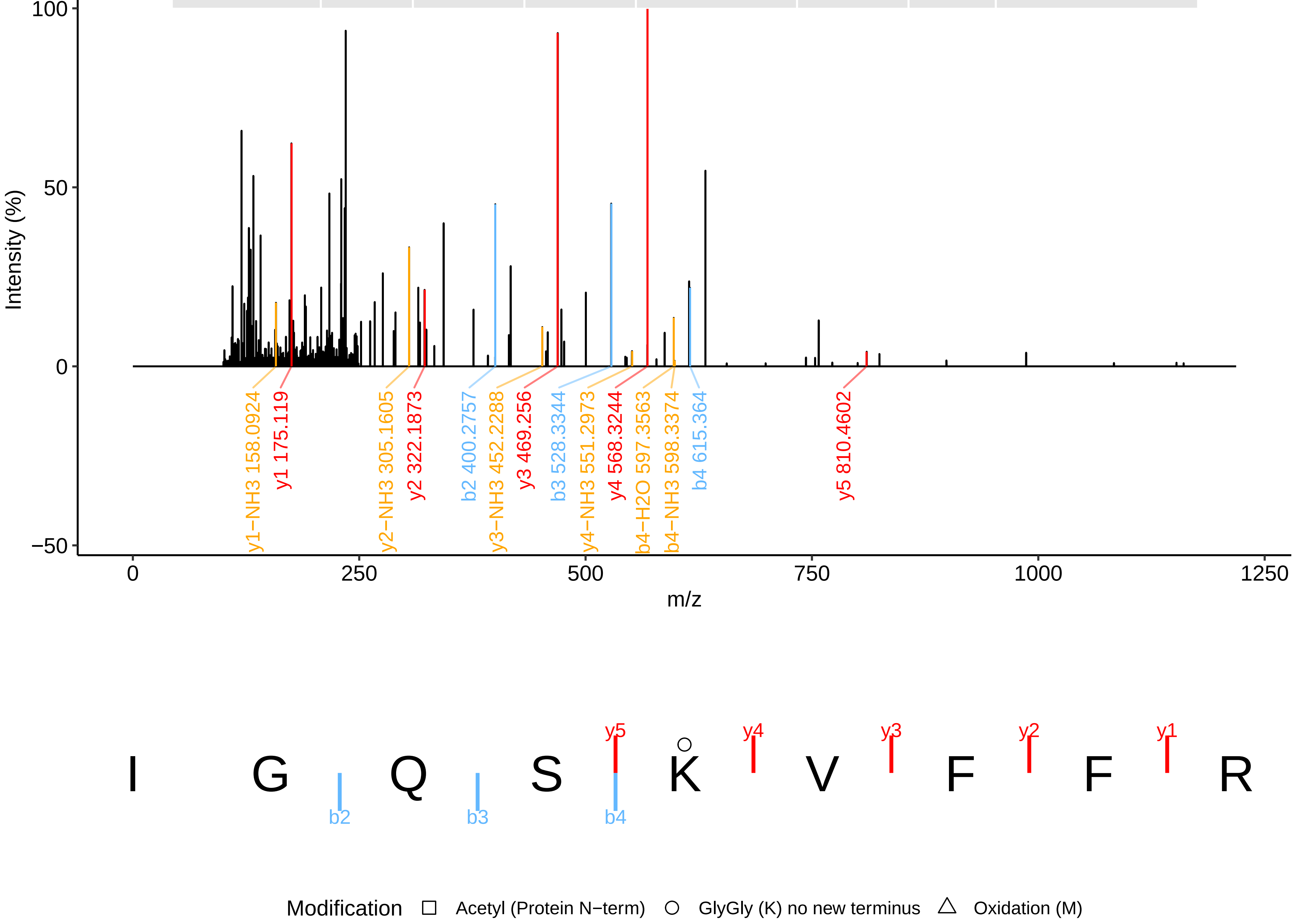

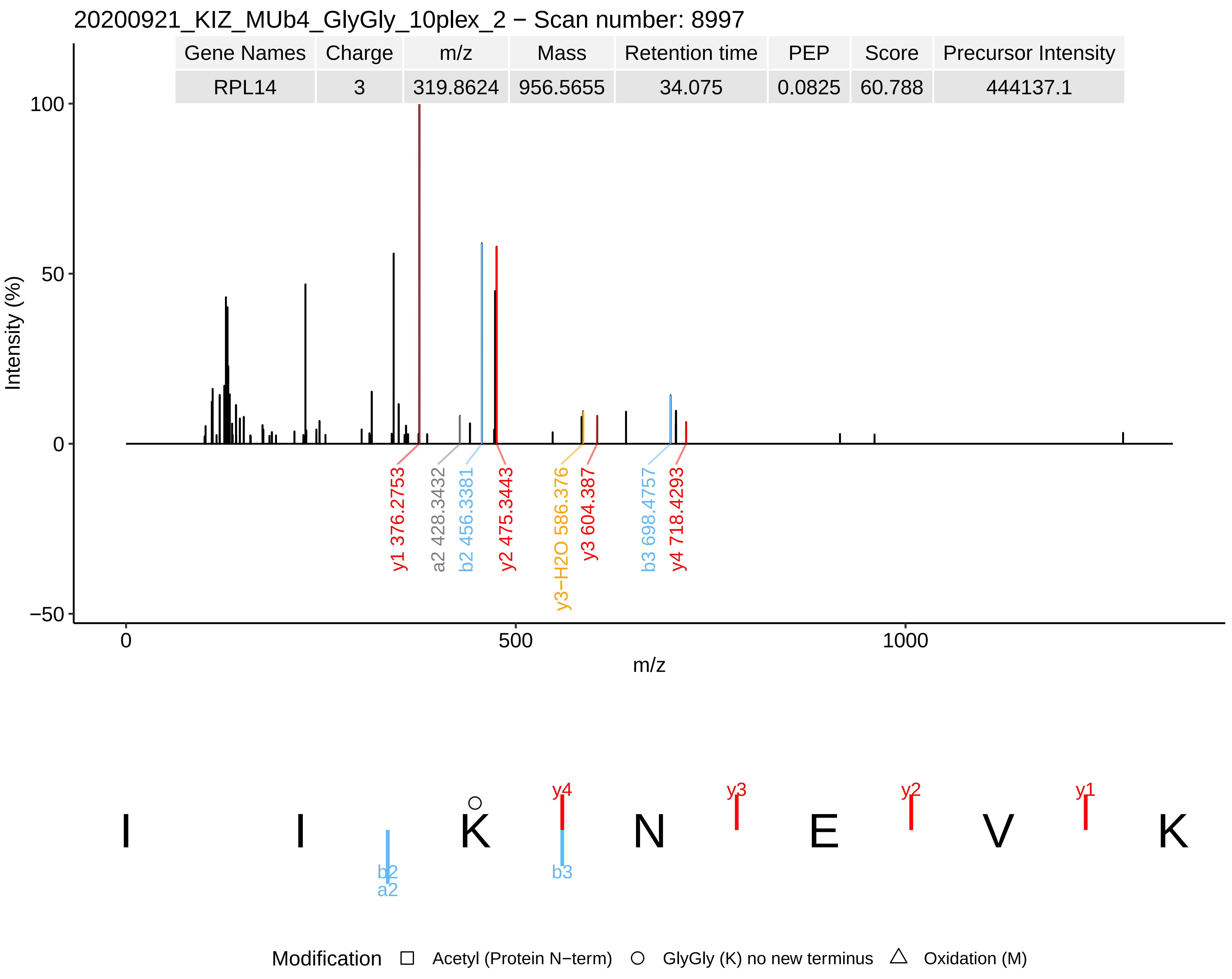

20200921_KIZ_MUb4_GlyGly_10plex_2 − Scan number: 8997
Gene Names | Charge | m/z | Mass | Retention time | PEP | Score | Precursor Intensity
RPL14 | 3 | 319.8624 | 956.5655 | 34.075 | 0.0825 | 60.788 | 444137.1
Intensity (%)
m/z
y1 376.2753
a2 428.3432
b2 456.3381
y2 475.3443
y3−H2O 586.376
y3 604.387
b3 698.4757
y4 718.4293
I I K N E V K
y4 y3 y2 y1
b2 a2 b3
Modification
Acetyl (Protein N−term)
GlyGly (K) no new terminus
Oxidation (M)

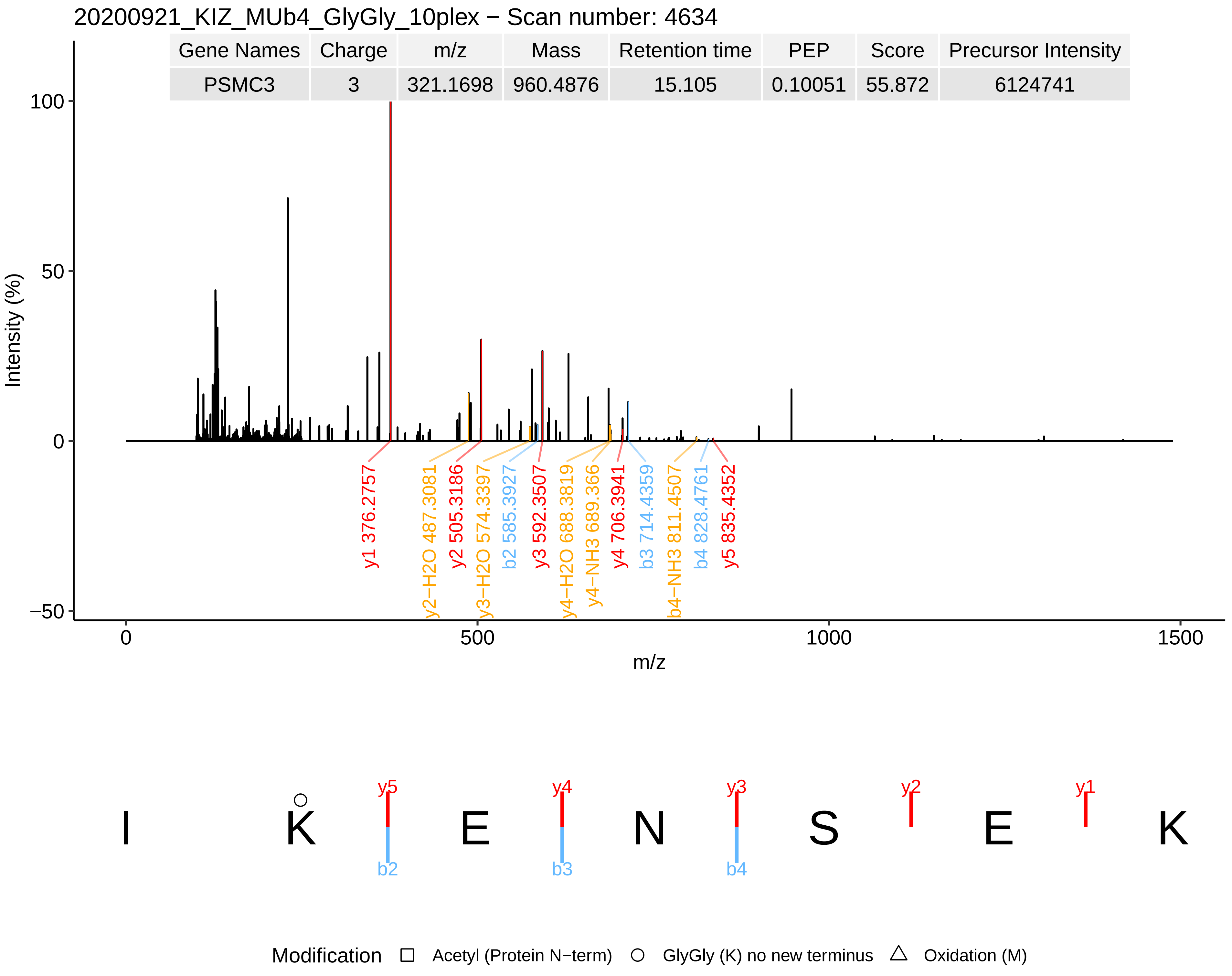

20200921_KIZ_MUb4_GlyGly_10plex − Scan number: 4634
Gene Names | Charge | m/z | Mass | Retention time | PEP | Score | Precursor Intensity
PSMC3 | 3 | 321.1698 | 960.4876 | 15.105 | 0.10051 | 55.872 | 6124741
Intensity (%)
100
50
0
−50
0
500
1000
1500
m/z
y1 376.2757
y2−H2O 487.3081
y2 505.3186
y3−H2O 574.3397
b2 585.3927
y3 592.3507
y4−H2O 688.3819
y4−NH3 689.366
y4 706.3941
b3 714.4359
b4−NH3 811.4507
b4 828.4761
y5 835.4352
I K E N S E K
y5 y4 y3 y2 y1
b2 b3 b4
Modification
Acetyl (Protein N−term)
GlyGly (K) no new terminus
Oxidation (M)

20200924_KIZ_MUb5_GlyGly_2 − Scan number: 9251

Gene Names	Charge	m/z	Mass	Retention time	PEP	Score	Precursor Intensity
RPL4	3	399.9032	1196.688	34.569	0.025442	65.404	2173961

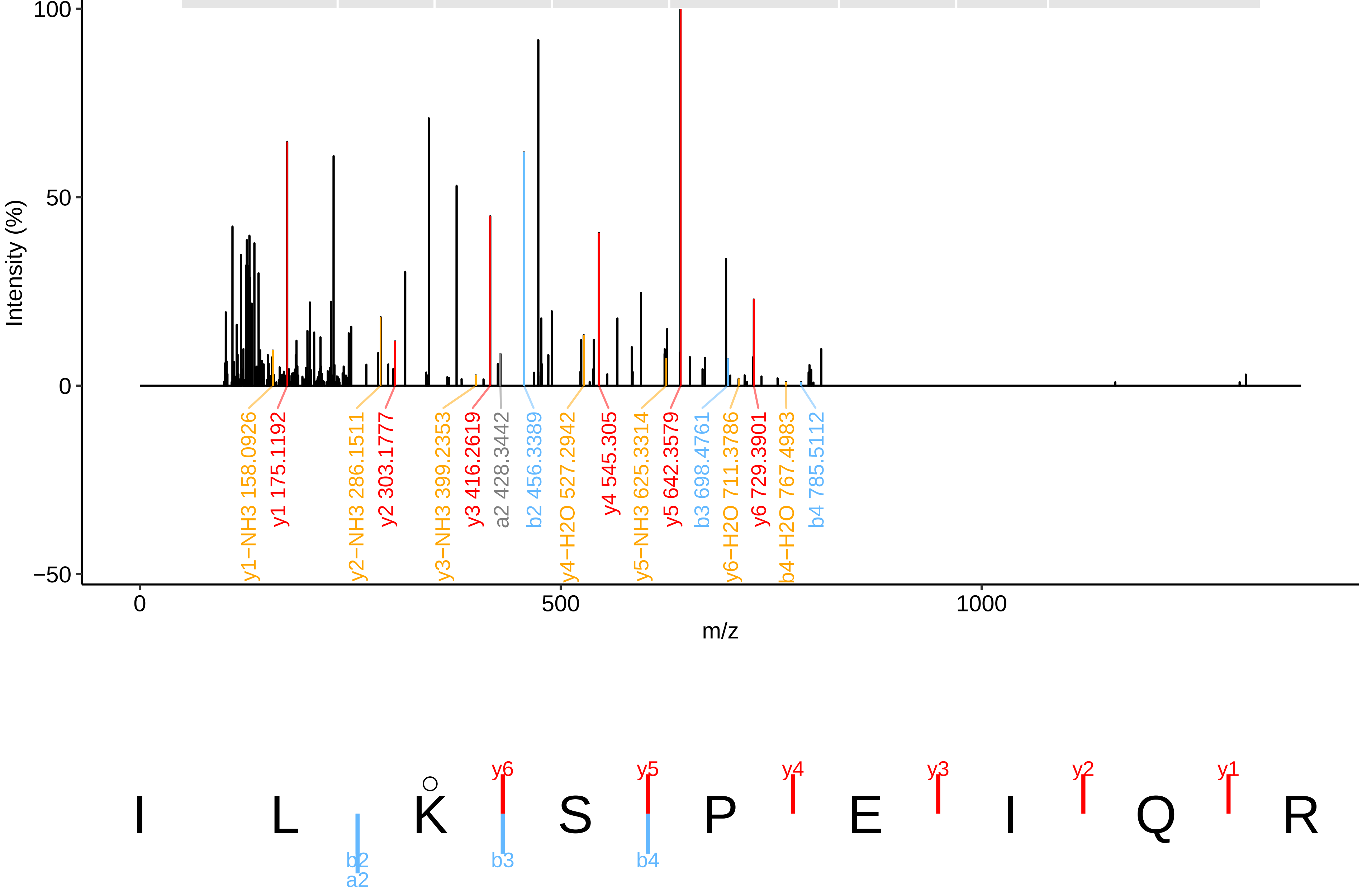

Modification □ Acetyl (Protein N−term) ○ GlyGly (K) no new terminus △ Oxidation (M)

20200921_KIZ_MUb4_GlyGly_10plex − Scan number: 16477

Gene Names	Charge	m/z	Mass	Retention time	PEP	Score	Precursor Intensity
CETN2;CETN1	3	321.8589	962.5549	59.187	0.0018243	124.89	4522285

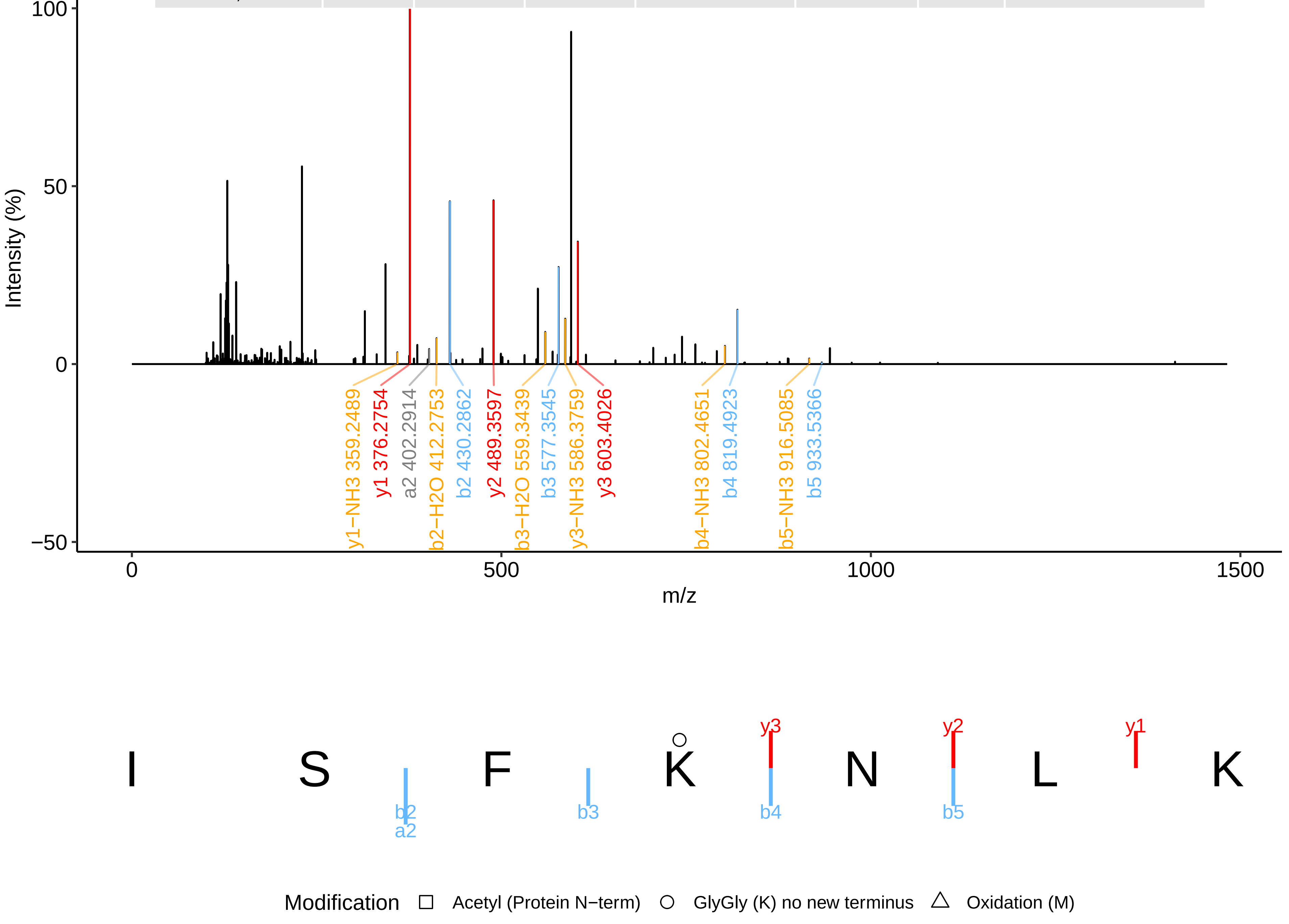

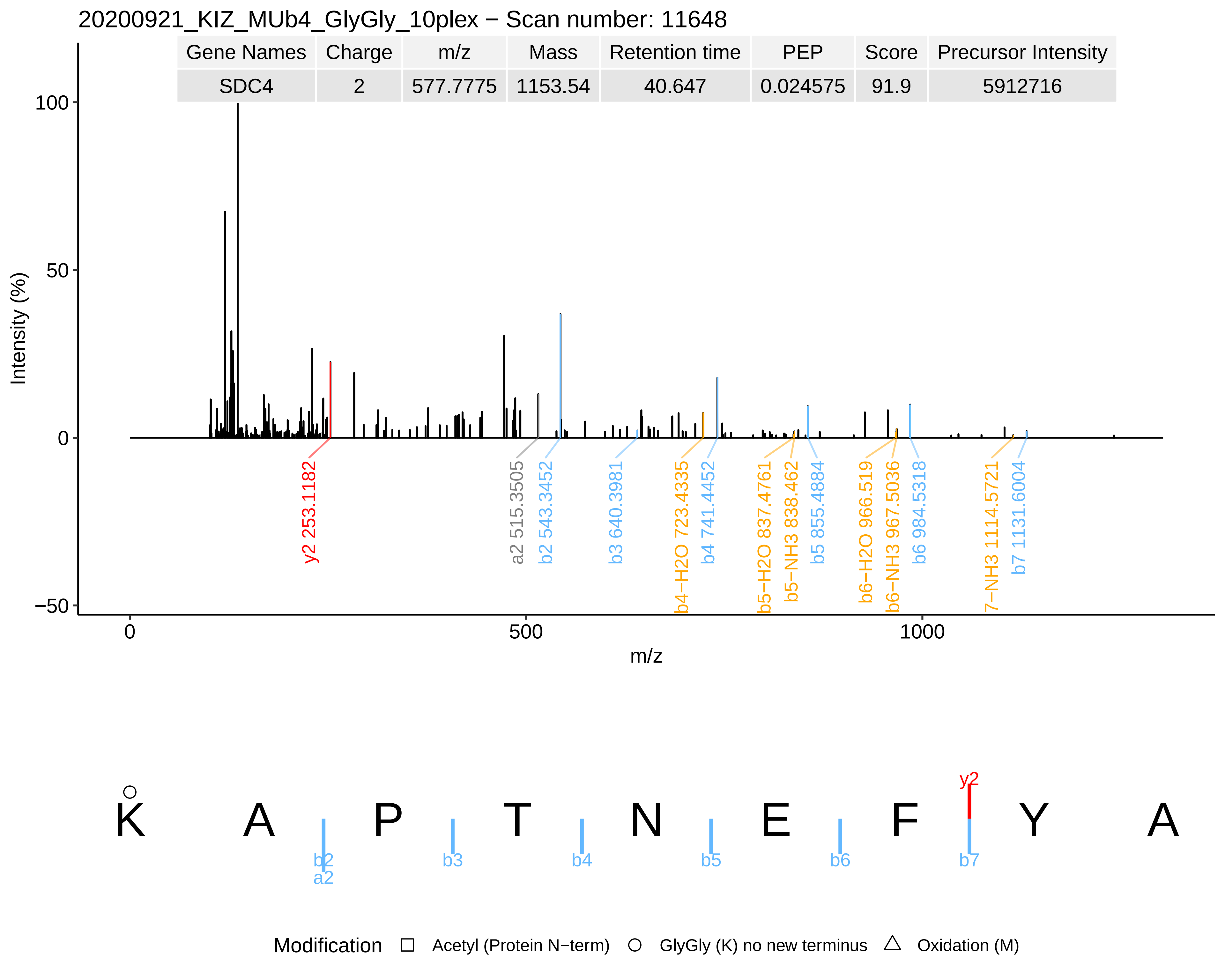
20200921_KIZ_MUb4_GlyGly_10plex − Scan number: 11648
Gene Names | Charge | m/z | Mass | Retention time | PEP | Score | Precursor Intensity
SDC4 | 2 | 577.7775 | 1153.54 | 40.647 | 0.024575 | 91.9 | 5912716
Intensity (%)
m/z
y2 253.1182
a2 515.3505
b2 543.3452
b3 640.3981
b4−H2O 723.4335
b4 741.4452
b5−H2O 837.4761
b5−NH3 838.462
b5 855.4884
b6−H2O 966.519
b6−NH3 967.5036
b6 984.5318
7−NH3 1114.5721
b7 1131.6004
K A P T N E F Y A
y2
b2 a2 b3 b4 b5 b6 b7
Modification
Acetyl (Protein N−term)
GlyGly (K) no new terminus
Oxidation (M)

20200921_KIZ_MUb4_GlyGly_10plex − Scan number: 3523

Gene Names	Charge	m/z	Mass	Retention time	PEP	Score	Precursor Intensity
AQP3	4	237.395	945.5508	11.211	0.10931	49.347	13851343

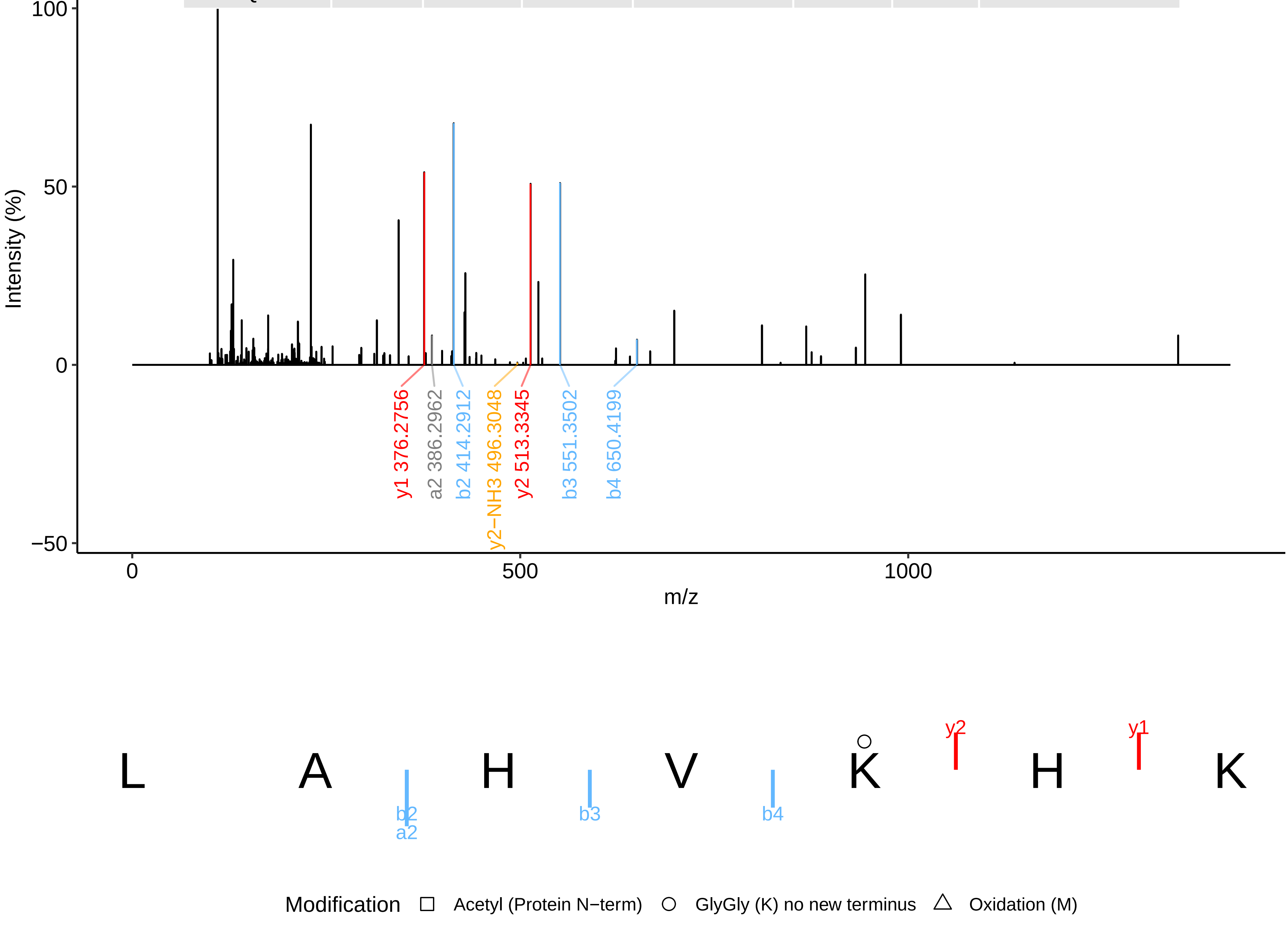

20200924_KIZ_MUb5_GlyGly_1 − Scan number: 3175

Gene Names	Charge	m/z	Mass	Retention time	PEP	Score	Precursor Intensity
AQP3	4	237.395	945.5508	9.7957	0.10617	50.12	10847495

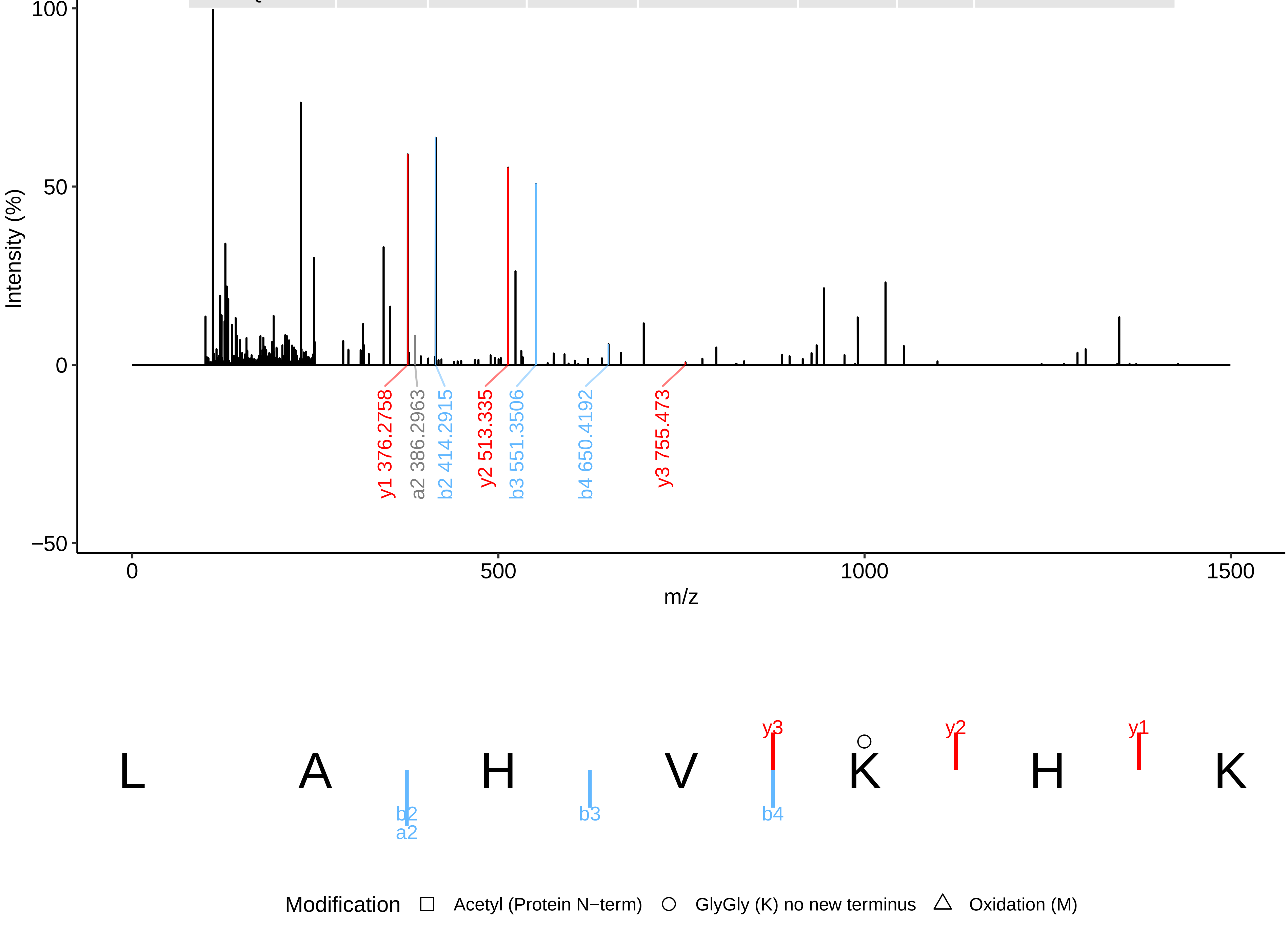

20200924_KIZ_MUb5_GlyGly_1 − Scan number: 3242

Gene Names	Charge	m/z	Mass	Retention time	PEP	Score	Precursor Intensity
AQP3	3	316.1909	945.5508	9.9776	0.01038	99.171	NA

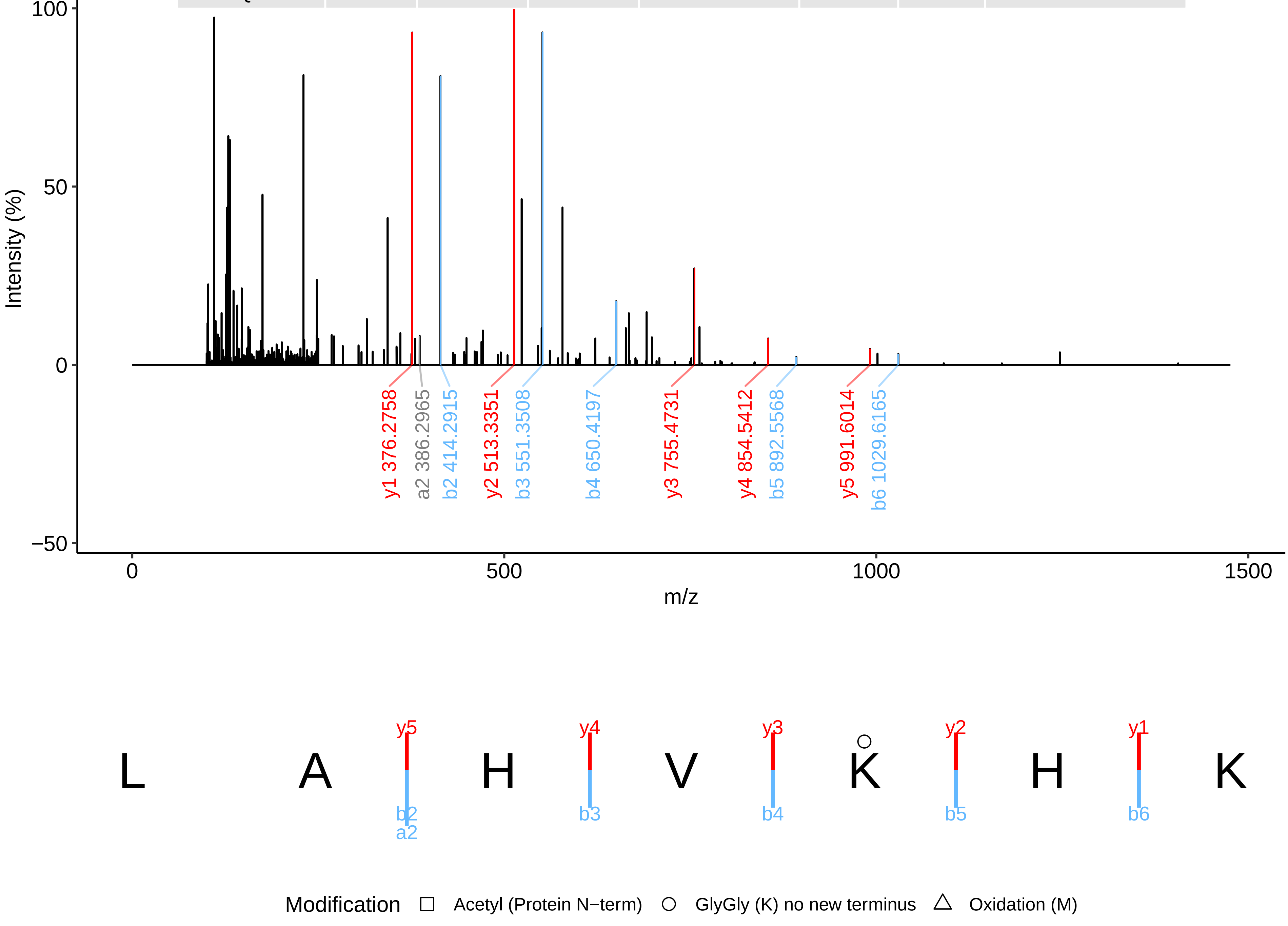

20201118_KIZ_MUb6_GlyGly_1 − Scan number: 3095

Gene Names	Charge	m/z	Mass	Retention time	PEP	Score	Precursor Intensity
AQP3	4	237.395	945.5508	9.5409	0.080169	56.51	23560278

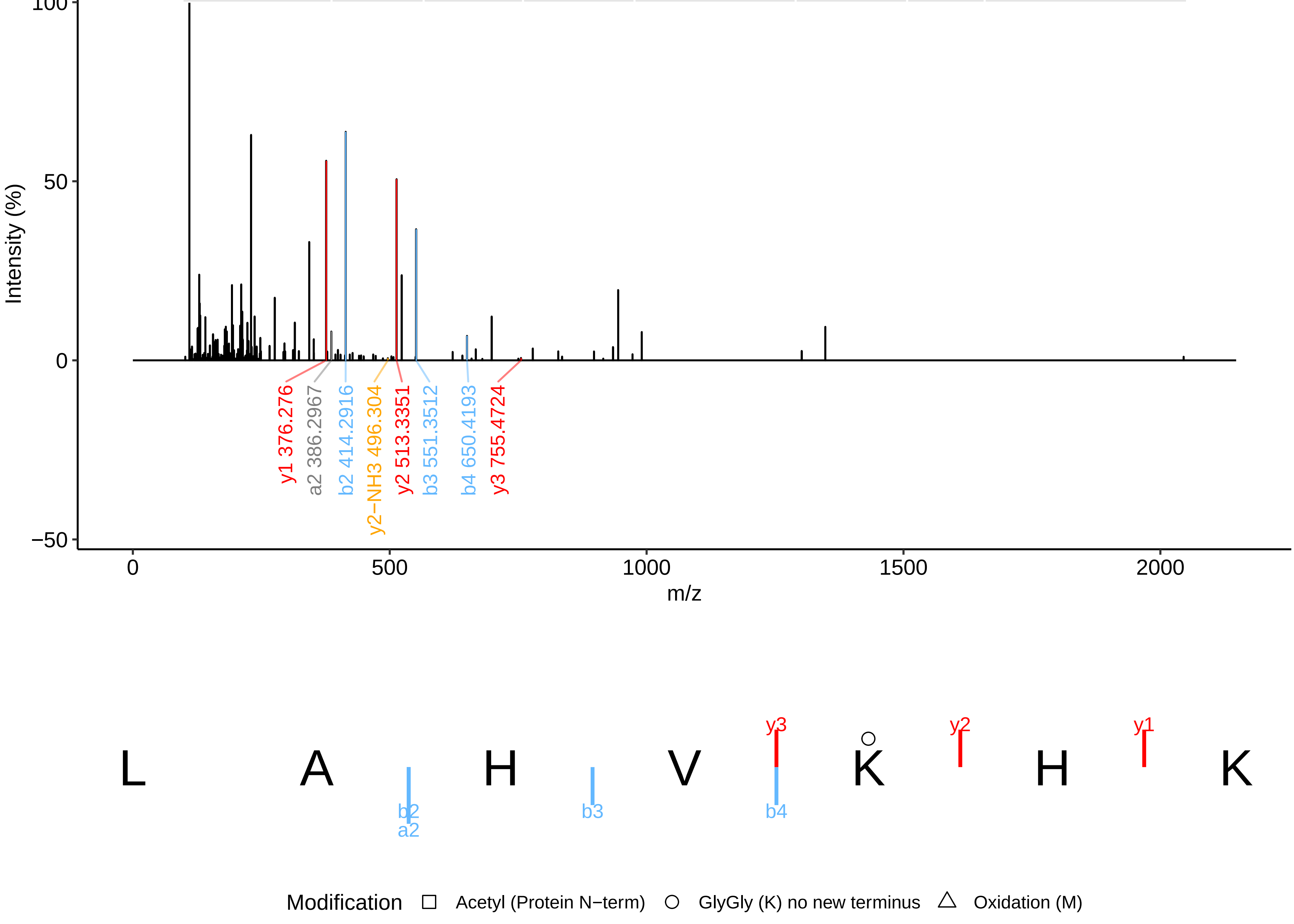

20201118_KIZ_MUb6_GlyGly_2 − Scan number: 3920

Gene Names	Charge	m/z	Mass	Retention time	PEP	Score	Precursor Intensity
AQP3	4	237.395	945.5508	13.182	0.058497	61.837	3176651

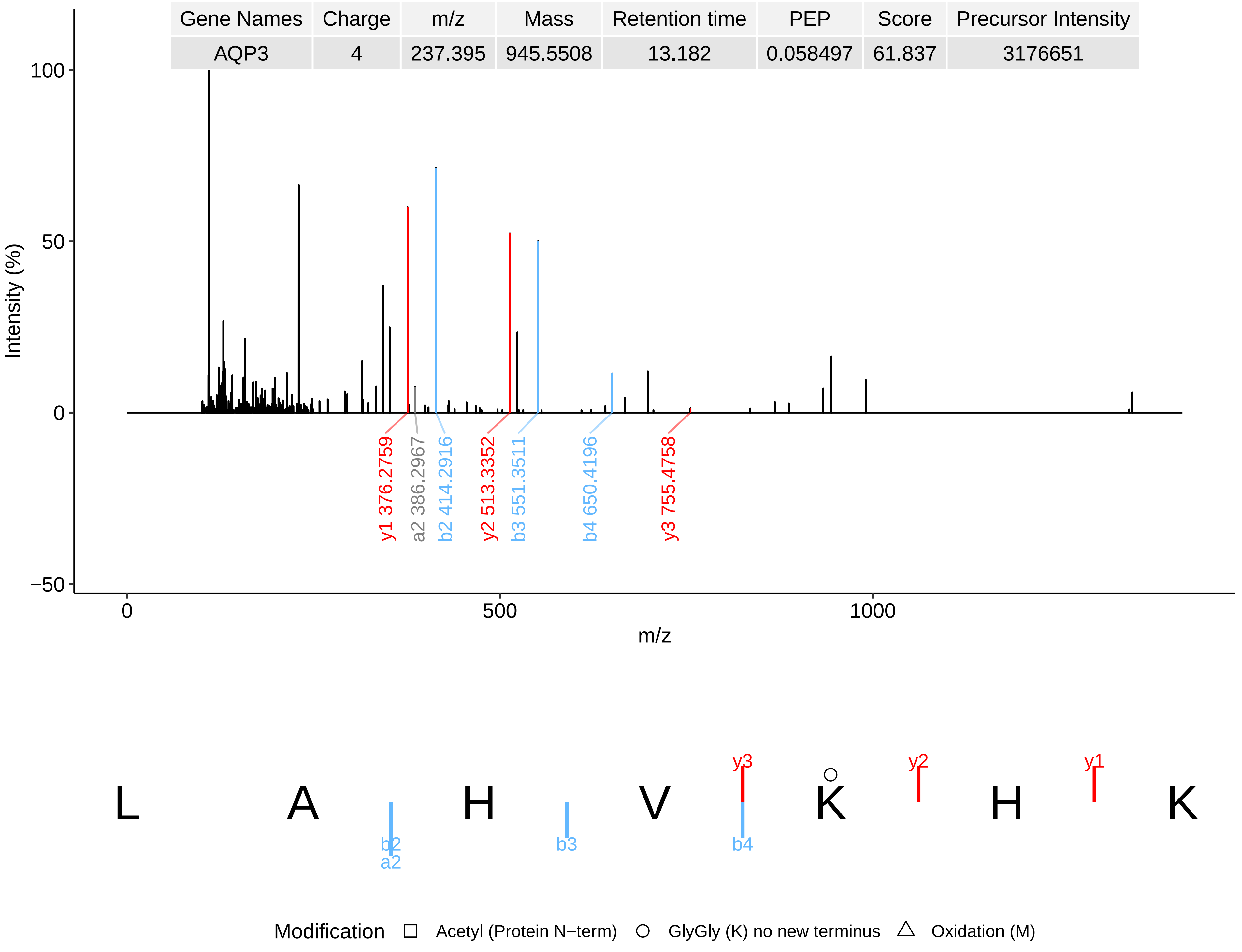

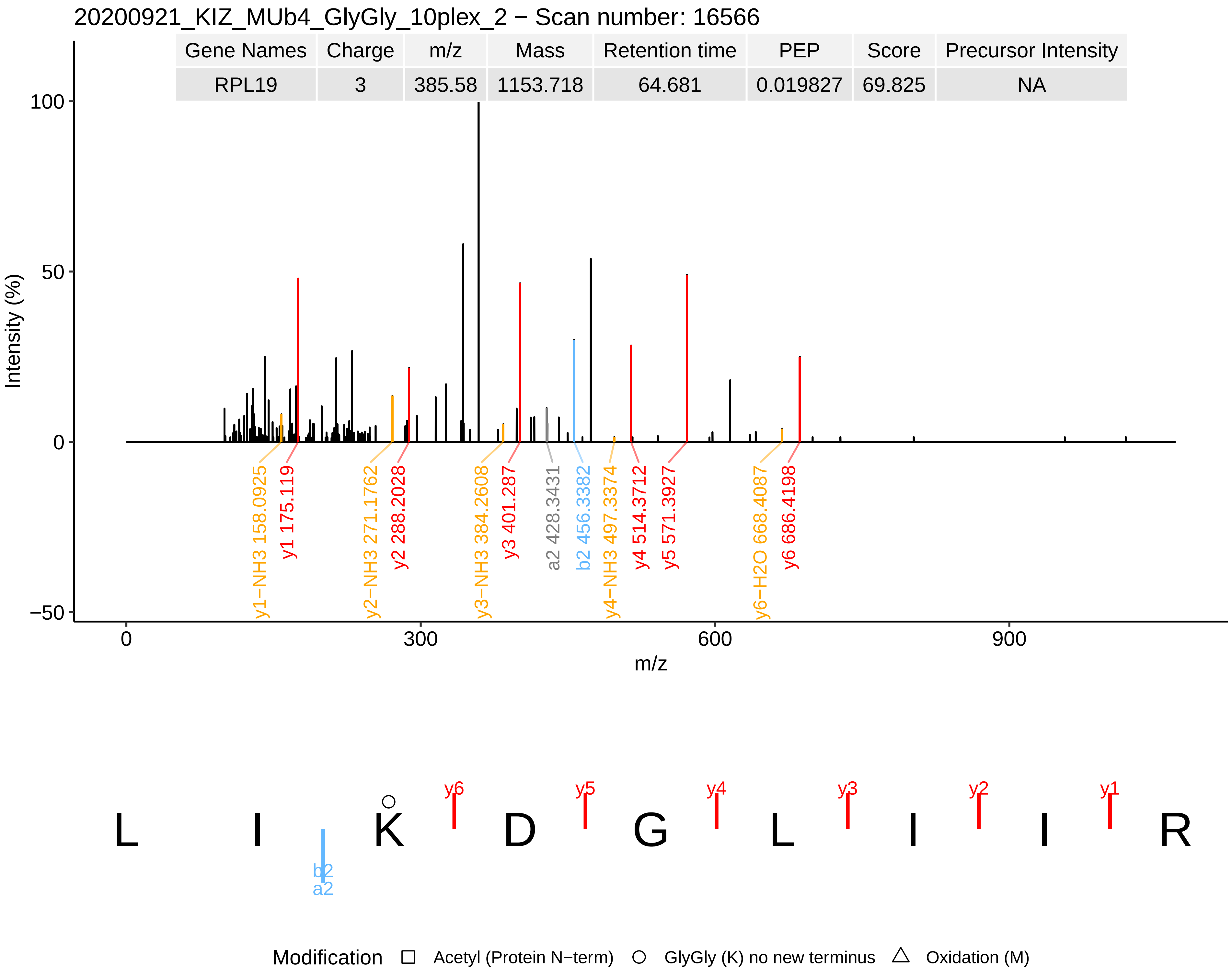
20200921_KIZ_MUb4_GlyGly_10plex_2 − Scan number: 16566
Gene Names | Charge | m/z | Mass | Retention time | PEP | Score | Precursor Intensity
RPL19 | 3 | 385.58 | 1153.718 | 64.681 | 0.019827 | 69.825 | NA
Intensity (%)
100
50
0
−50
0
300
600
900
m/z
y1−NH3 158.0925
y1 175.119
y2−NH3 271.1762
y2 288.2028
y3−NH3 384.2608
y3 401.287
a2 428.3431
b2 456.3382
y4−NH3 497.3374
y4 514.3712
y5 571.3927
y6−H2O 668.4087
y6 686.4198
L I K D G L I I R
y6 y5 y4 y3 y2 y1
b2
a2
Modification
Acetyl (Protein N−term)
GlyGly (K) no new terminus
Oxidation (M)

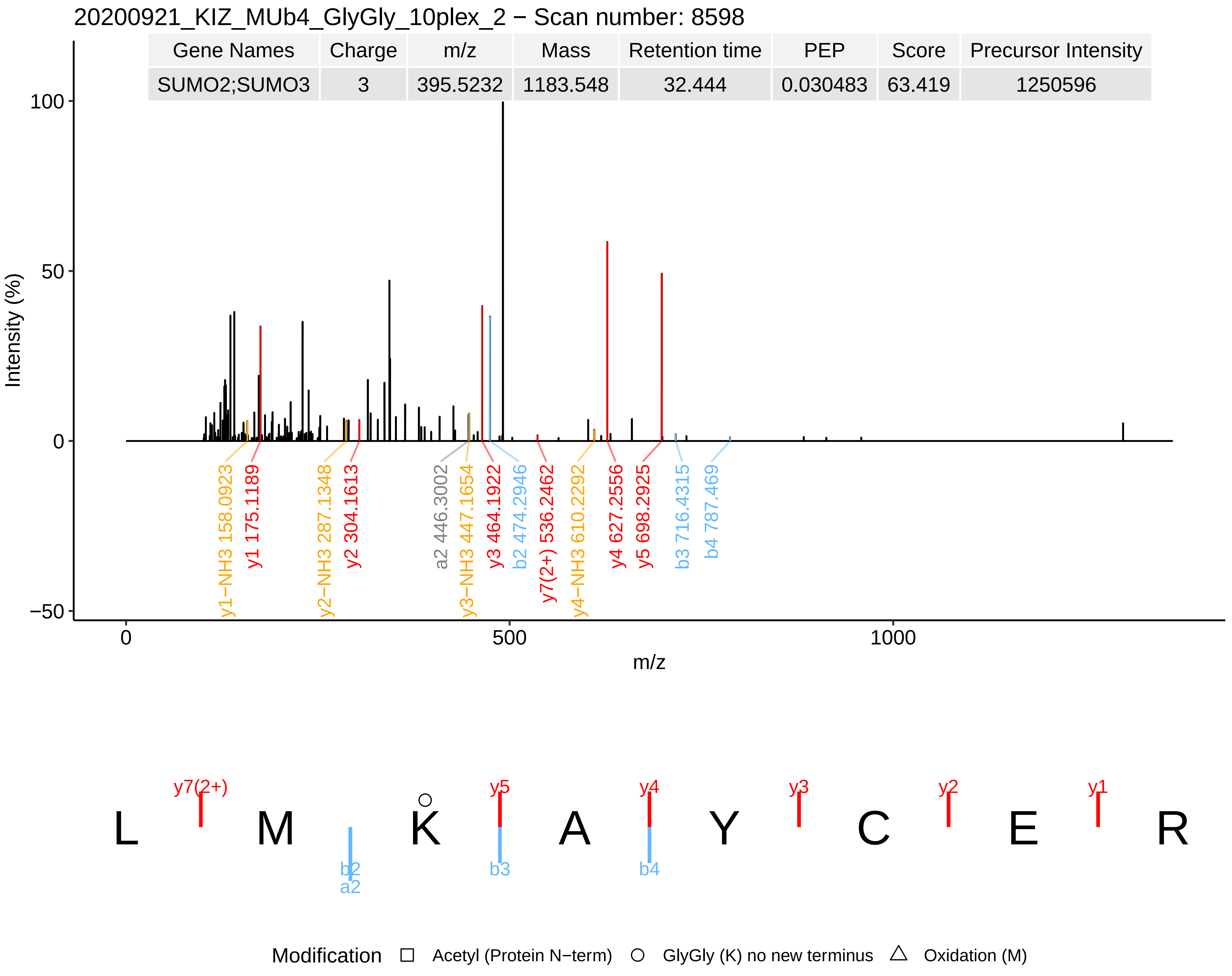
20200921_KIZ_MUb4_GlyGly_10plex_2 − Scan number: 8598
Gene Names | Charge | m/z | Mass | Retention time | PEP | Score | Precursor Intensity
SUMO2;SUMO3 | 3 | 395.5232 | 1183.548 | 32.444 | 0.030483 | 63.419 | 1250596
Intensity (%)
100
50
0
−50
0
500
1000
m/z
y1−NH3 158.0923
y1 175.1189
y2−NH3 287.1348
y2 304.1613
a2 446.3002
y3−NH3 447.1654
y3 464.1922
b2 474.2946
y7(2+) 536.2462
y4−NH3 610.2292
y4 627.2556
y5 698.2925
b3 716.4315
b4 787.469
L M K A Y C E R
y7(2+)
y5
y4
y3
y2
y1
b2
a2
b3
b4
Modification
Acetyl (Protein N−term)
GlyGly (K) no new terminus
Oxidation (M)

20200921_KIZ_MUb4_GlyGly_10plex − Scan number: 9673

Gene Names	Charge	m/z	Mass	Retention time	PEP	Score	Precursor Intensity
S100A4	3	315.8503	944.5291	33.201	0.011606	91.9	7121013

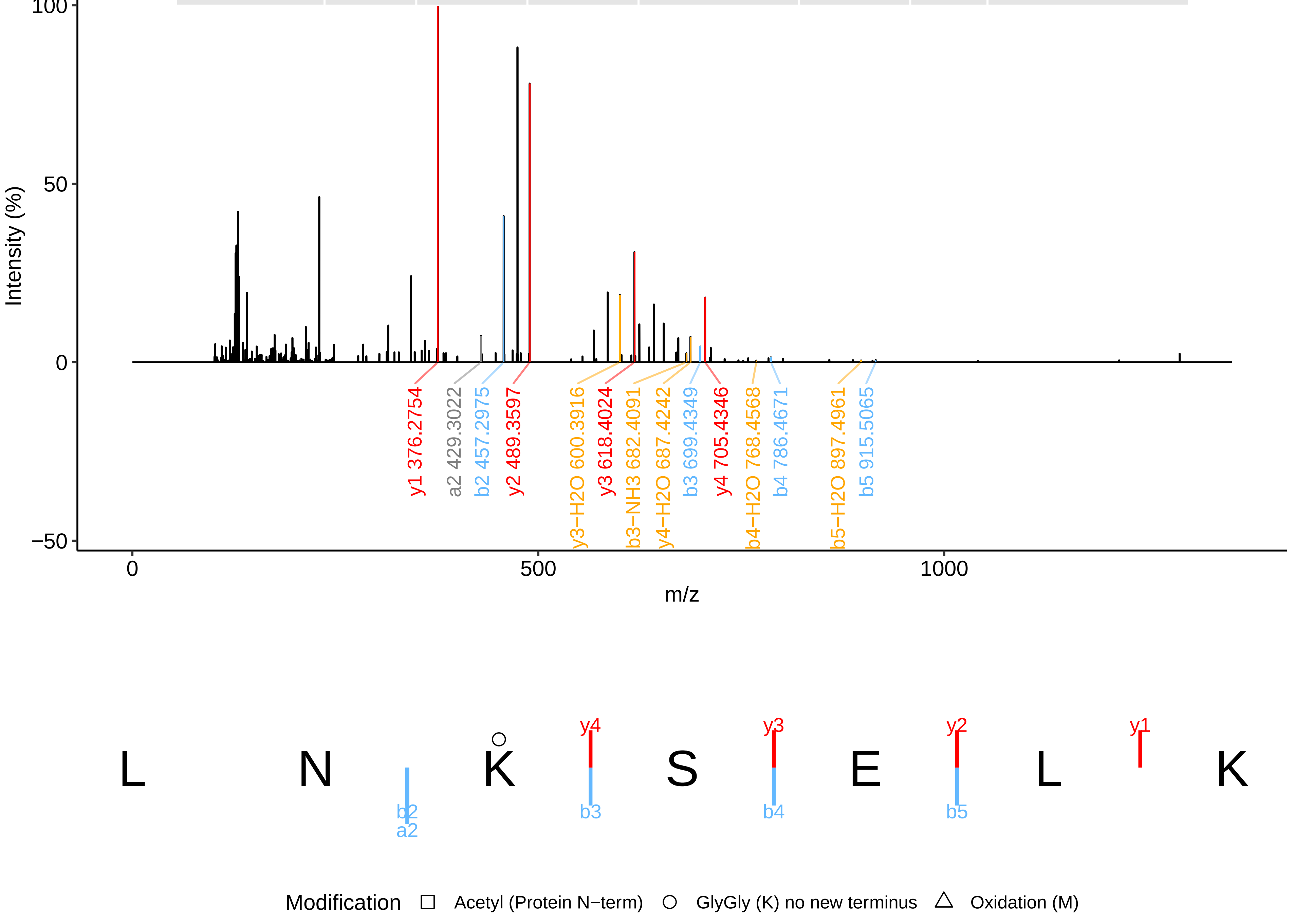

20200921_KIZ_MUb4_GlyGly_10plex − Scan number: 16268

Gene Names	Charge	m/z	Mass	Retention time	PEP	Score	Precursor Intensity
YWHAQ	3	388.2242	1161.651	58.385	0.0017195	102.51	5045058

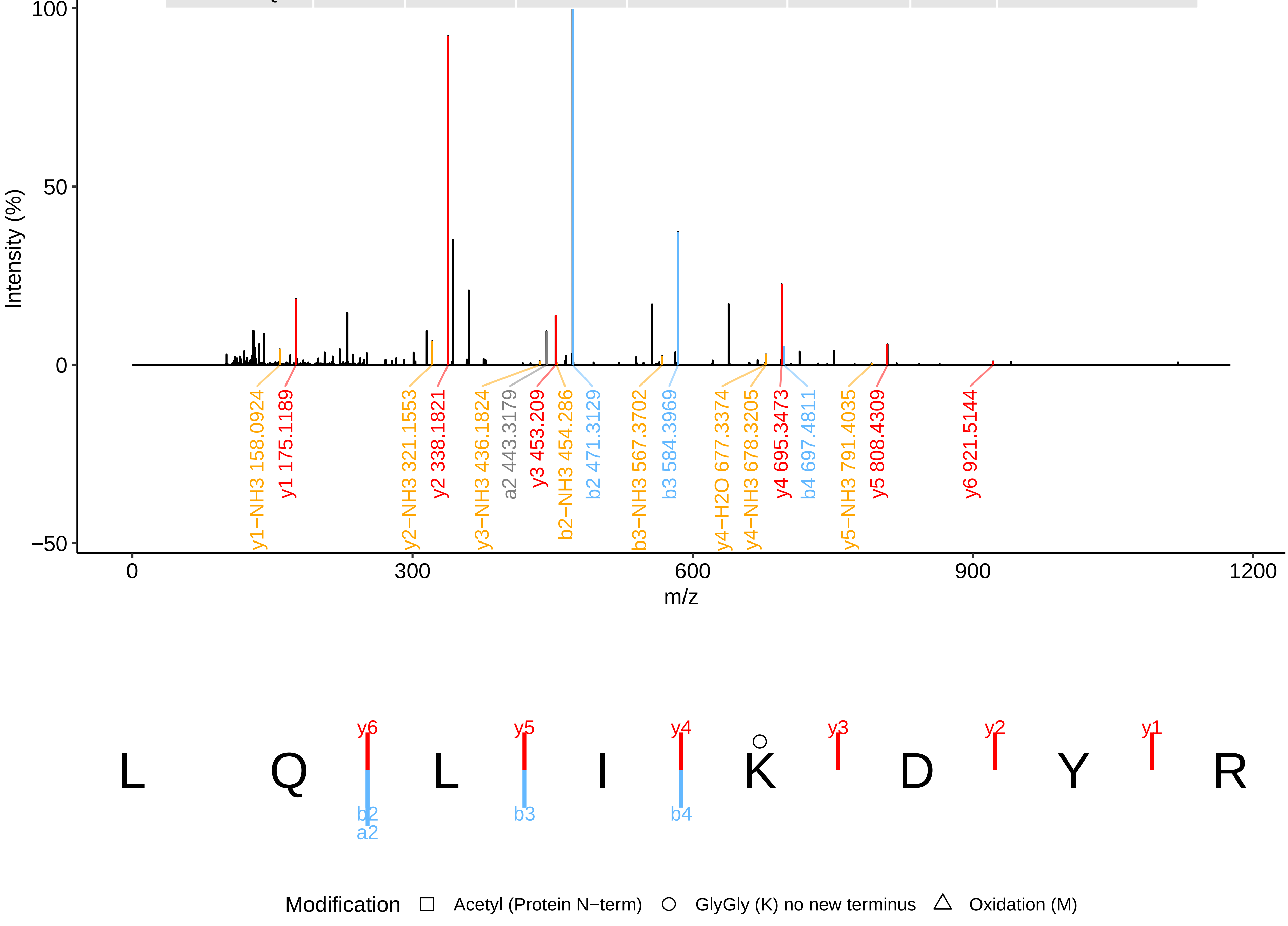

20200921_KIZ_MUb4_GlyGly_10plex_2 − Scan number: 15114

Gene Names	Charge	m/z	Mass	Retention time	PEP	Score	Precursor Intensity
YWHAQ	3	388.2242	1161.651	59.041	0.0037627	94.114	600515.1

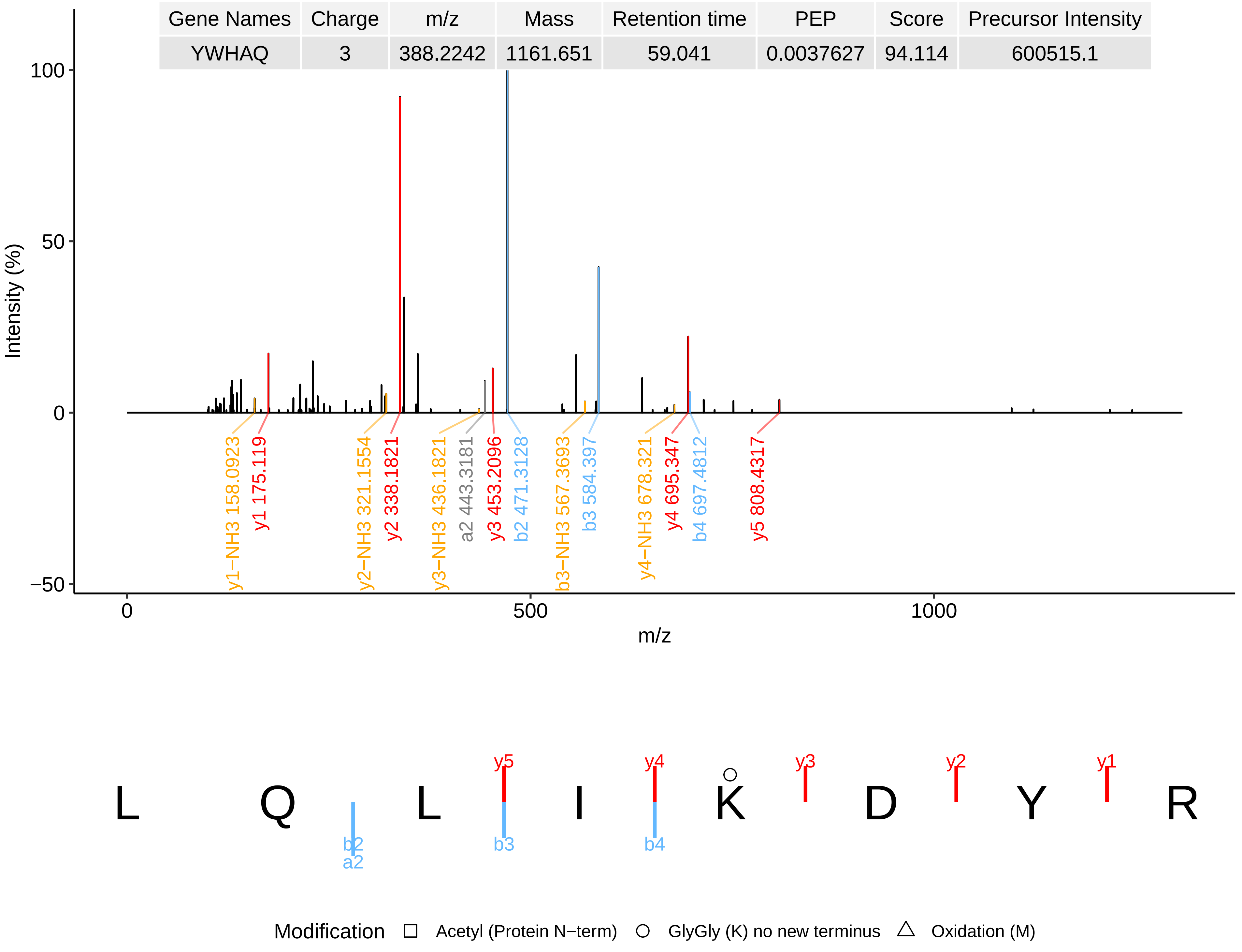

20200924_KIZ_MUb5_GlyGly_1 − Scan number: 15145

Gene Names	Charge	m/z	Mass	Retention time	PEP	Score	Precursor Intensity
YWHAQ	3	388.2242	1161.651	49.473	0.0029095	101.43	33738464

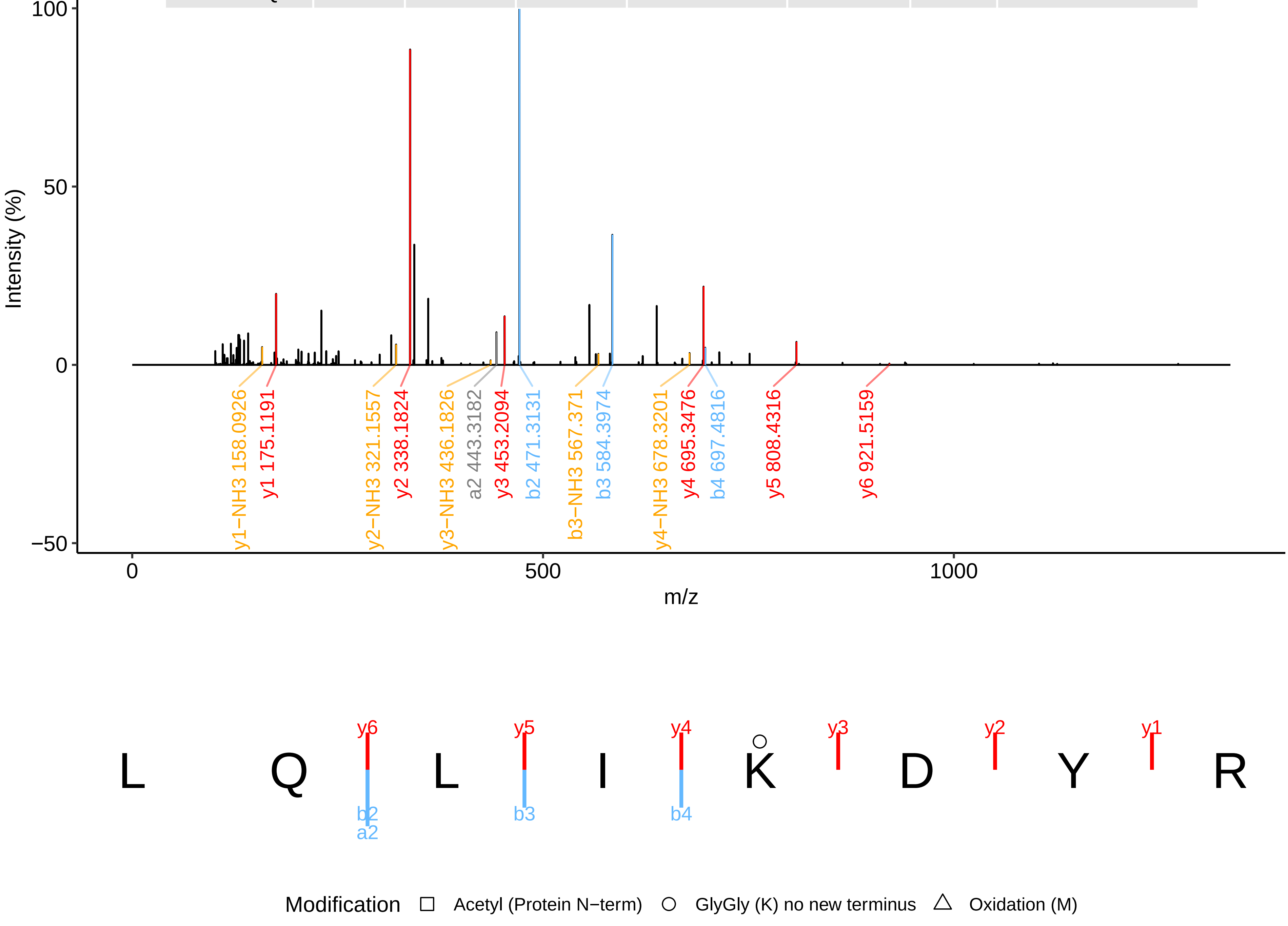

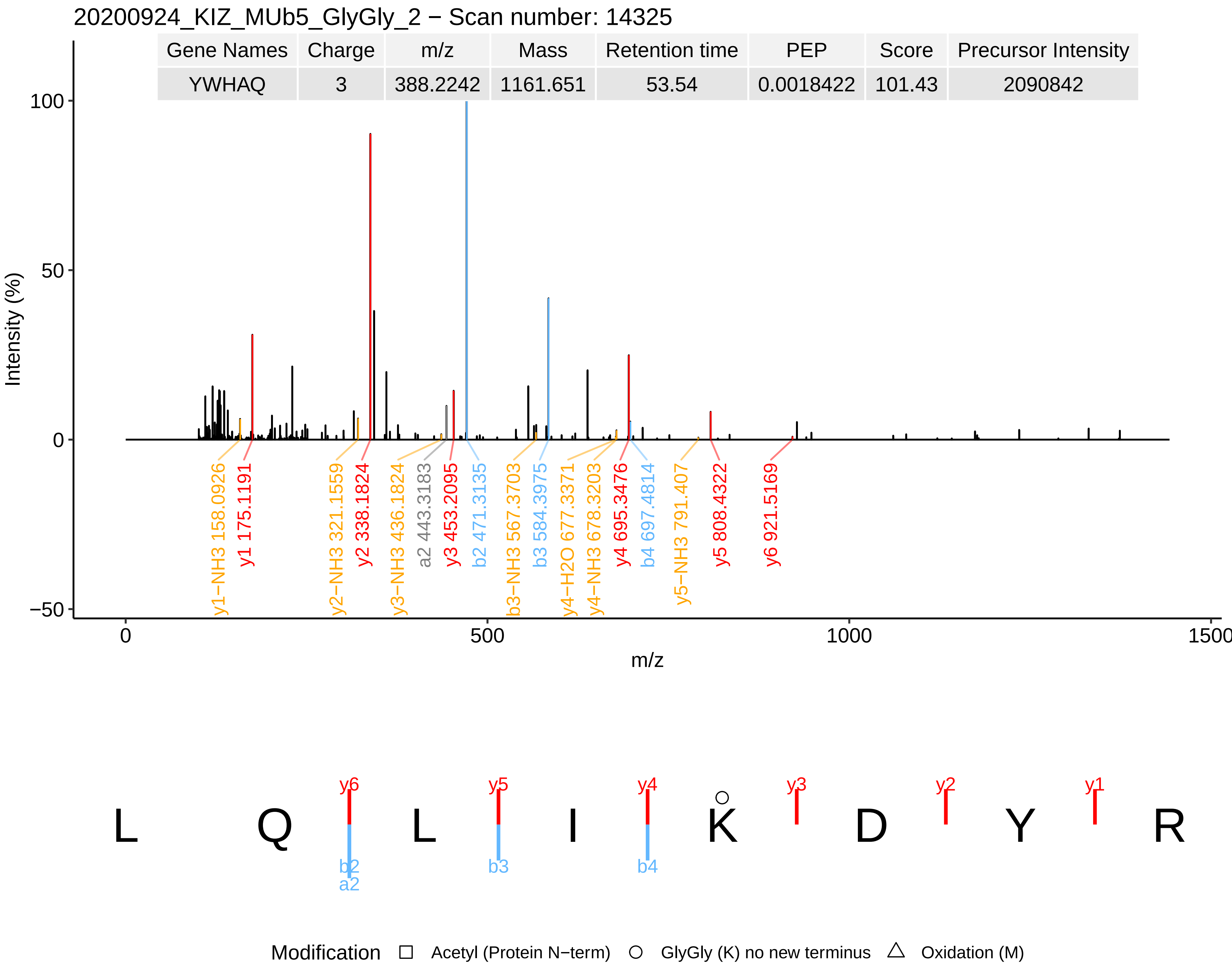
20200924_KIZ_MUb5_GlyGly_2 − Scan number: 14325
Gene Names | Charge | m/z | Mass | Retention time | PEP | Score | Precursor Intensity
YWHAQ | 3 | 388.2242 | 1161.651 | 53.54 | 0.0018422 | 101.43 | 2090842
Intensity (%)
m/z
y1−NH3 158.0926
y1 175.1191
y2−NH3 321.1559
y2 338.1824
y3−NH3 436.1824
a2 443.3183
y3 453.2095
b2 471.3135
b3−NH3 567.3703
b3 584.3975
y4−H2O 677.3371
y4−NH3 678.3203
y4 695.3476
b4 697.4814
y5−NH3 791.407
y5 808.4322
y6 921.5169
L Q L I K D Y R
y6 y5 y4 y3 y2 y1
b2 a2 b3 b4
Modification
Acetyl (Protein N−term)
GlyGly (K) no new terminus
Oxidation (M)

20200924_KIZ_MUb5_GlyGly_2 − Scan number: 14462

Gene Names	Charge	m/z	Mass	Retention time	PEP	Score	Precursor Intensity
YWHAQ	3	388.2242	1161.651	54.053	0.00045188	113.71	8667329

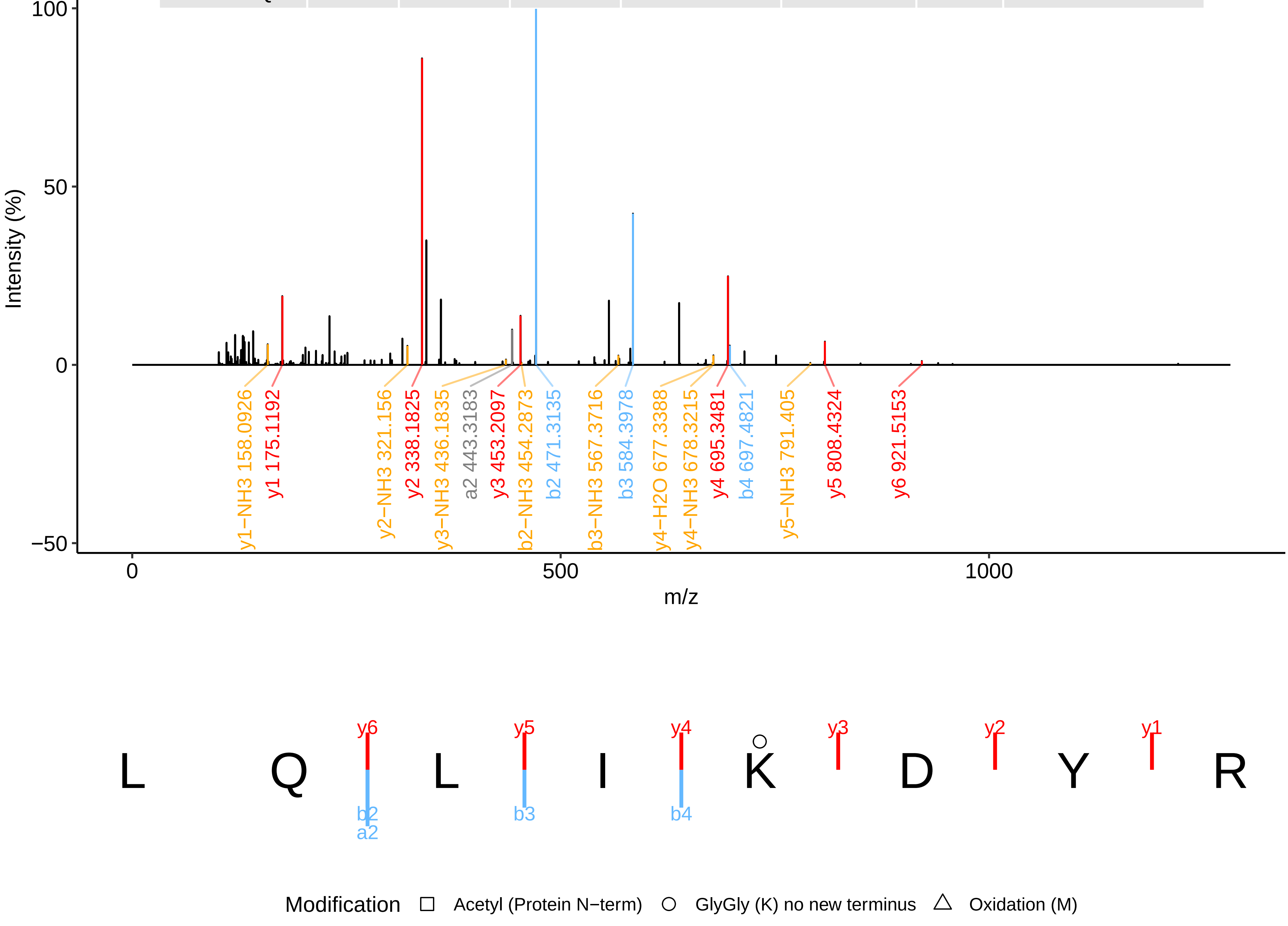

20201118_KIZ_MUb6_GlyGly_2 − Scan number: 17741

Gene Names	Charge	m/z	Mass	Retention time	PEP	Score	Precursor Intensity
YWHAQ	3	388.2242	1161.651	59.98	0.00045188	113.71	11544957

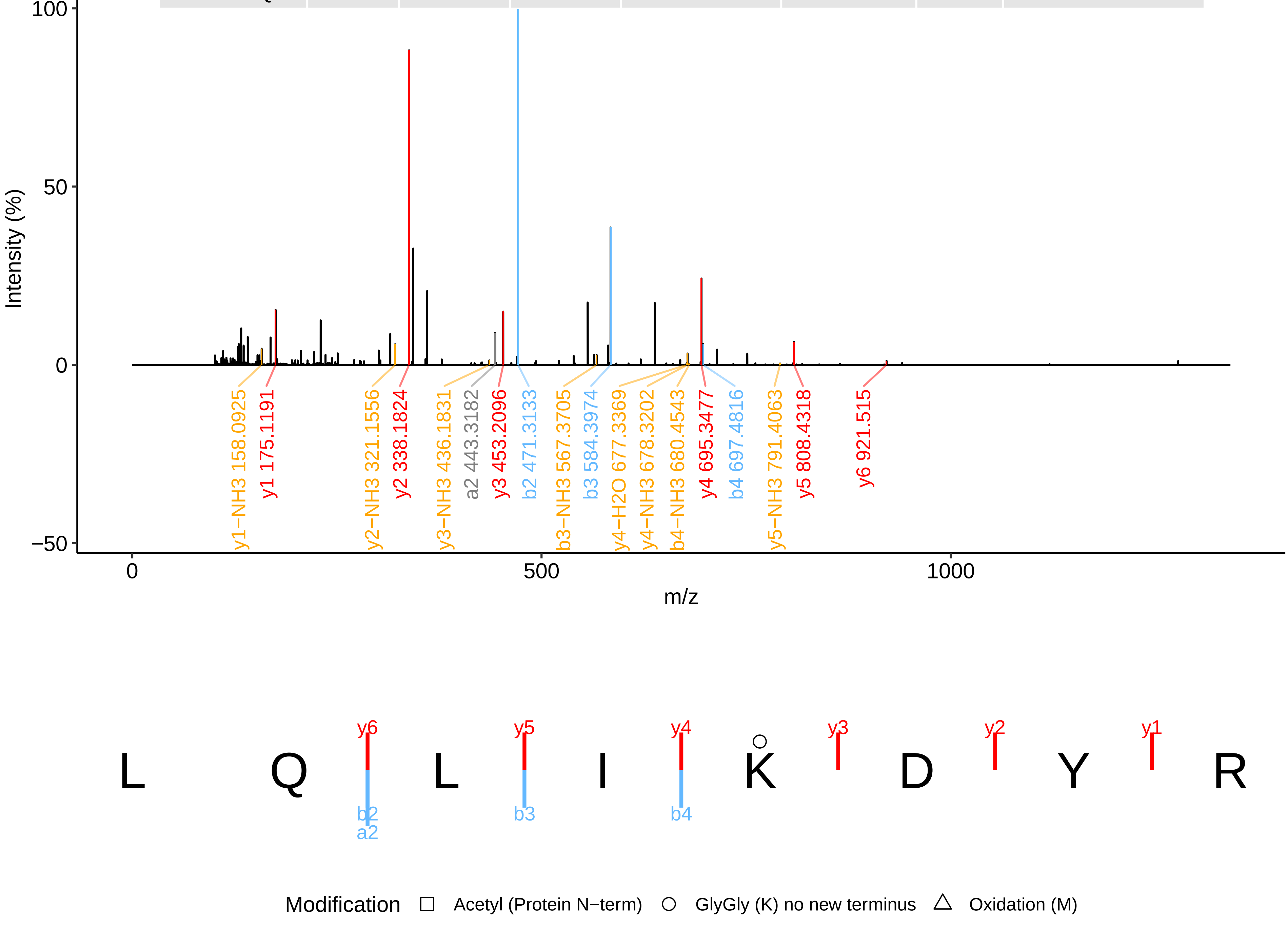

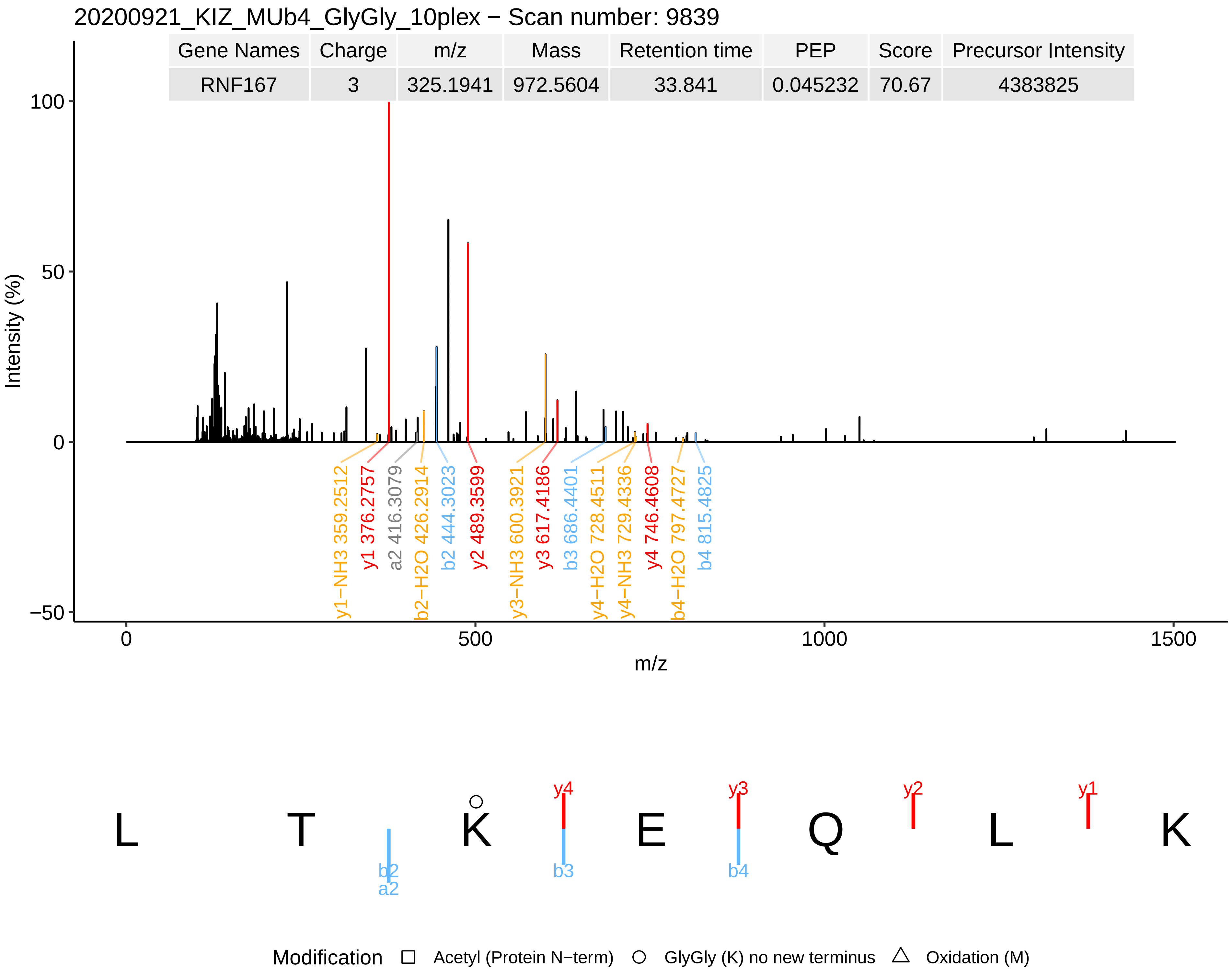
20200921_KIZ_MUb4_GlyGly_10plex − Scan number: 9839
Gene Names | Charge | m/z | Mass | Retention time | PEP | Score | Precursor Intensity
RNF167 | 3 | 325.1941 | 972.5604 | 33.841 | 0.045232 | 70.67 | 4383825
Intensity (%)
100
50
0
−50
y1−NH3 359.2512
y1 376.2757
a2 416.3079
b2−H2O 426.2914
b2 444.3023
y2 489.3599
y3−NH3 600.3921
y3 617.4186
b3 686.4401
y4−H2O 728.4511
y4−NH3 729.4336
y4 746.4608
b4−H2O 797.4727
b4 815.4825
0
500
1000
1500
m/z
L T K E Q L K
y4 y3 y2 y1
b2 a2 b3 b4
Modification
Acetyl (Protein N−term)
GlyGly (K) no new terminus
Oxidation (M)

20200921_KIZ_MUb4_GlyGly_10plex_2 − Scan number: 9095

Gene Names	Charge	m/z	Mass	Retention time	PEP	Score	Precursor Intensity
RNF167	3	325.1941	972.5604	34.477	0.027284	77.674	896531.6

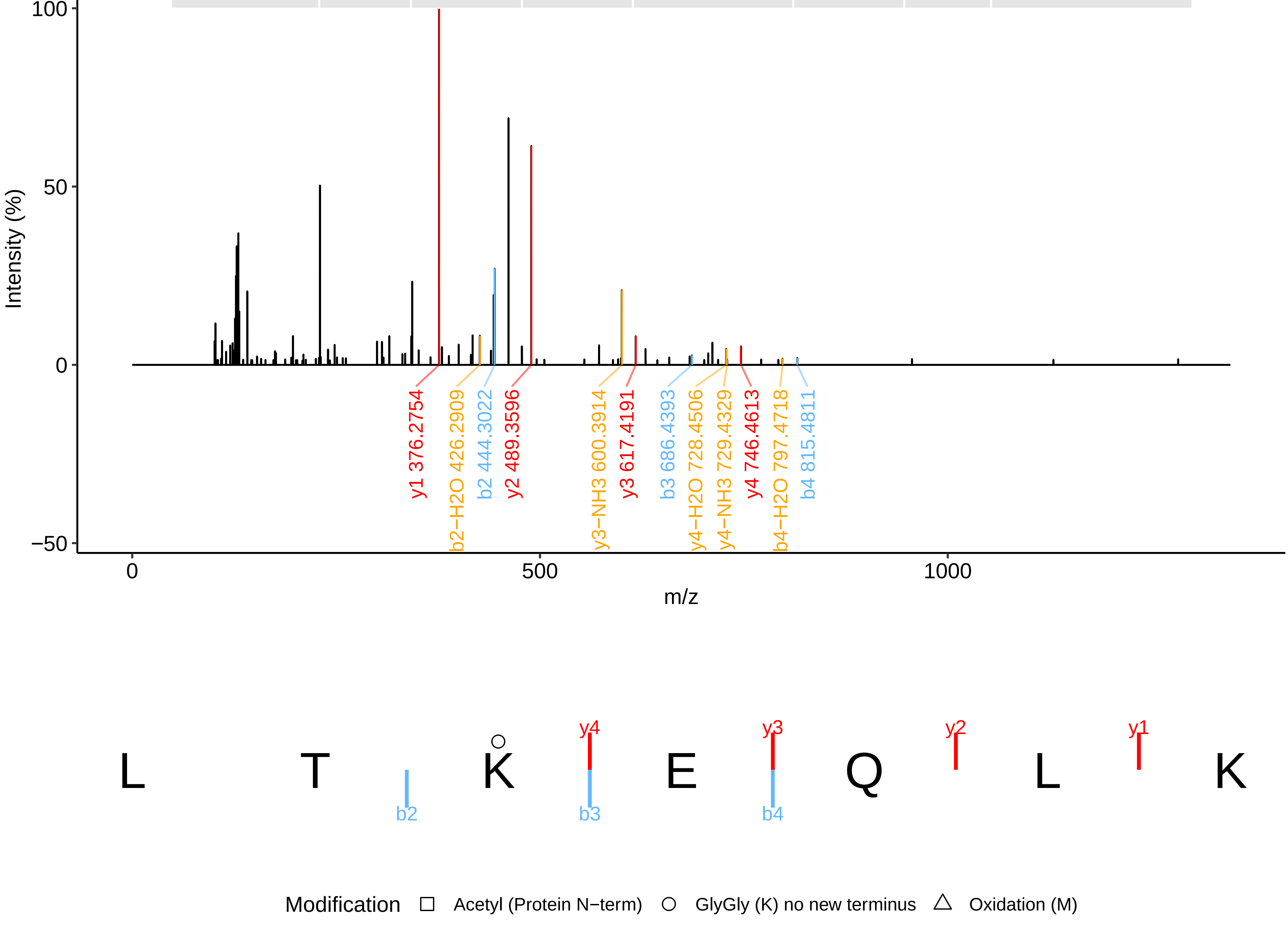

20200921_KIZ_MUb4_GlyGly_10plex − Scan number: 15958

Gene Names	Charge	m/z	Mass	Retention time	PEP	Score	Precursor Intensity
PPP2CA	2	577.2815	1152.549	57.175	0.01843	53.17	5966785

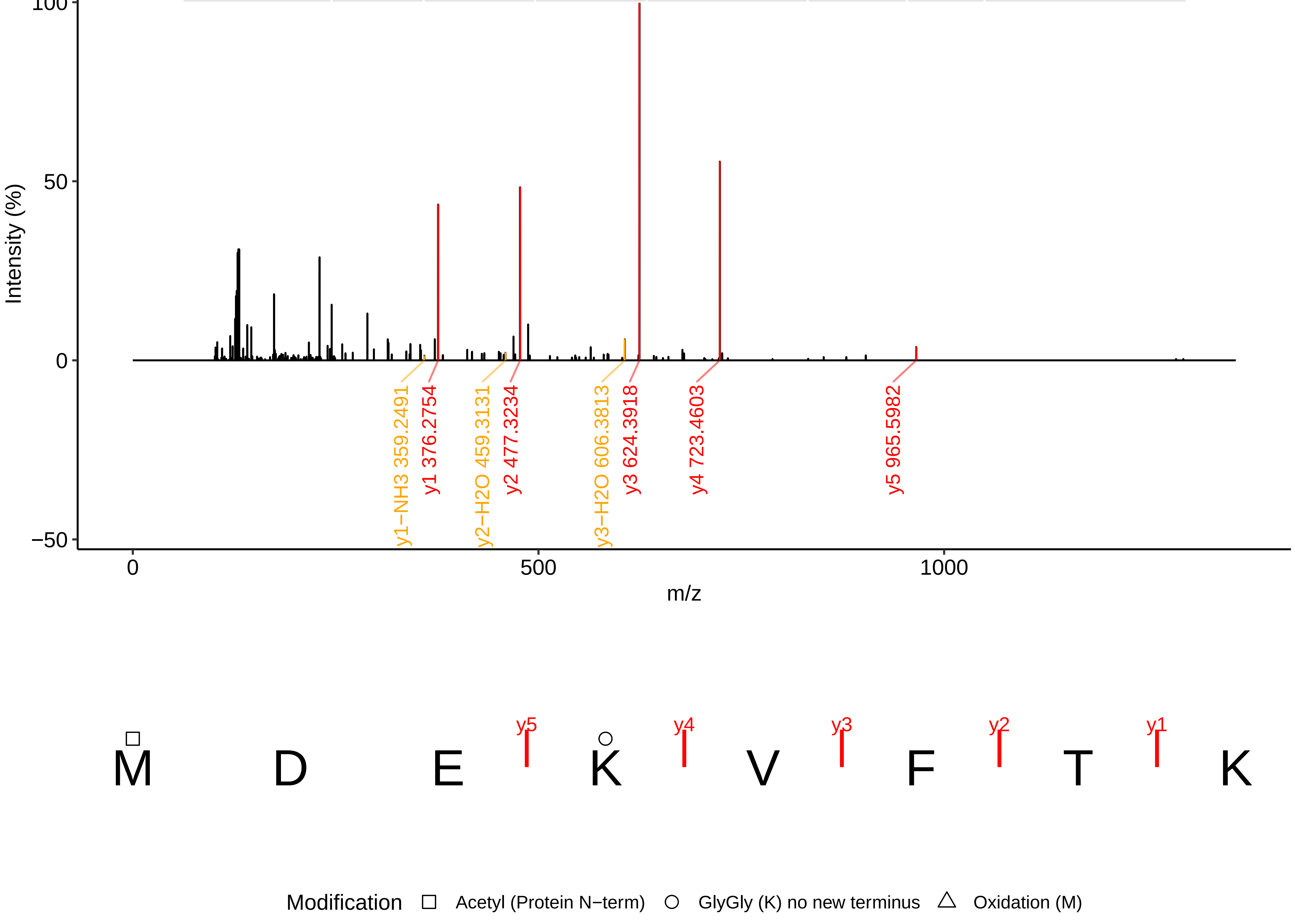

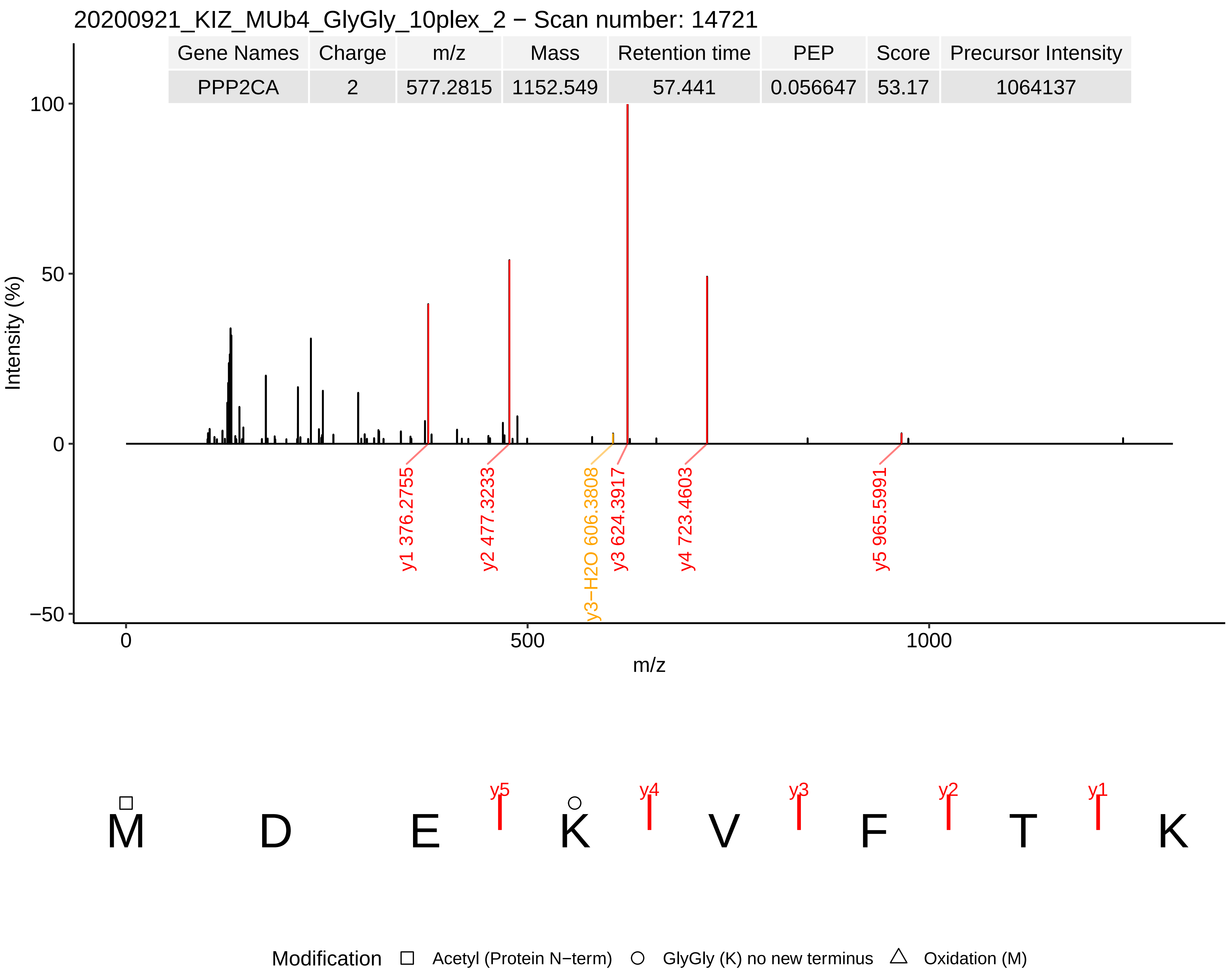
20200921_KIZ_MUb4_GlyGly_10plex_2 − Scan number: 14721
Gene Names | Charge | m/z | Mass | Retention time | PEP | Score | Precursor Intensity
PPP2CA | 2 | 577.2815 | 1152.549 | 57.441 | 0.056647 | 53.17 | 1064137
Intensity (%)
100
50
0
−50
0
500
1000
m/z
y1 376.2755
y2 477.3233
y3−H2O 606.3808
y3 624.3917
y4 723.4603
y5 965.5991
M D E y5 K y4 V y3 F y2 T y1 K
Modification
Acetyl (Protein N−term)
GlyGly (K) no new terminus
Oxidation (M)

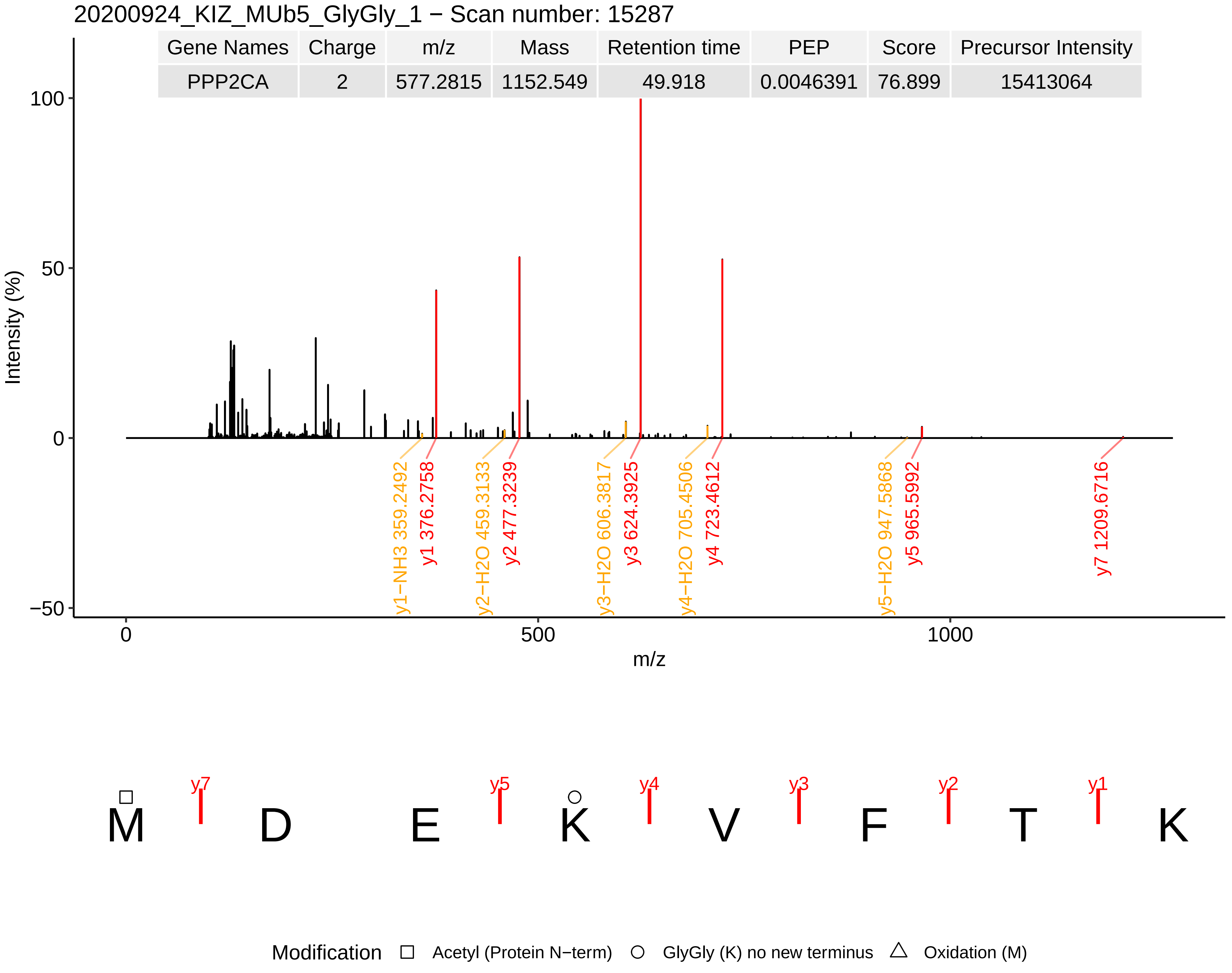

20200924_KIZ_MUb5_GlyGly_1 − Scan number: 15287
Gene Names | Charge | m/z | Mass | Retention time | PEP | Score | Precursor Intensity
PPP2CA | 2 | 577.2815 | 1152.549 | 49.918 | 0.0046391 | 76.899 | 15413064
Intensity (%)
m/z
y1−NH3 359.2492
y1 376.2758
y2−H2O 459.3133
y2 477.3239
y3−H2O 606.3817
y3 624.3925
y4−H2O 705.4506
y4 723.4612
y5−H2O 947.5868
y5 965.5992
y7 1209.6716
M D E K V F T K
y7 y5 y4 y3 y2 y1
Modification
Acetyl (Protein N−term)
GlyGly (K) no new terminus
Oxidation (M)

20200924_KIZ_MUb5_GlyGly_2 − Scan number: 14304

Gene Names	Charge	m/z	Mass	Retention time	PEP	Score	Precursor Intensity
PPP2CA	2	577.2815	1152.549	53.463	0.080095	43.027	1812064

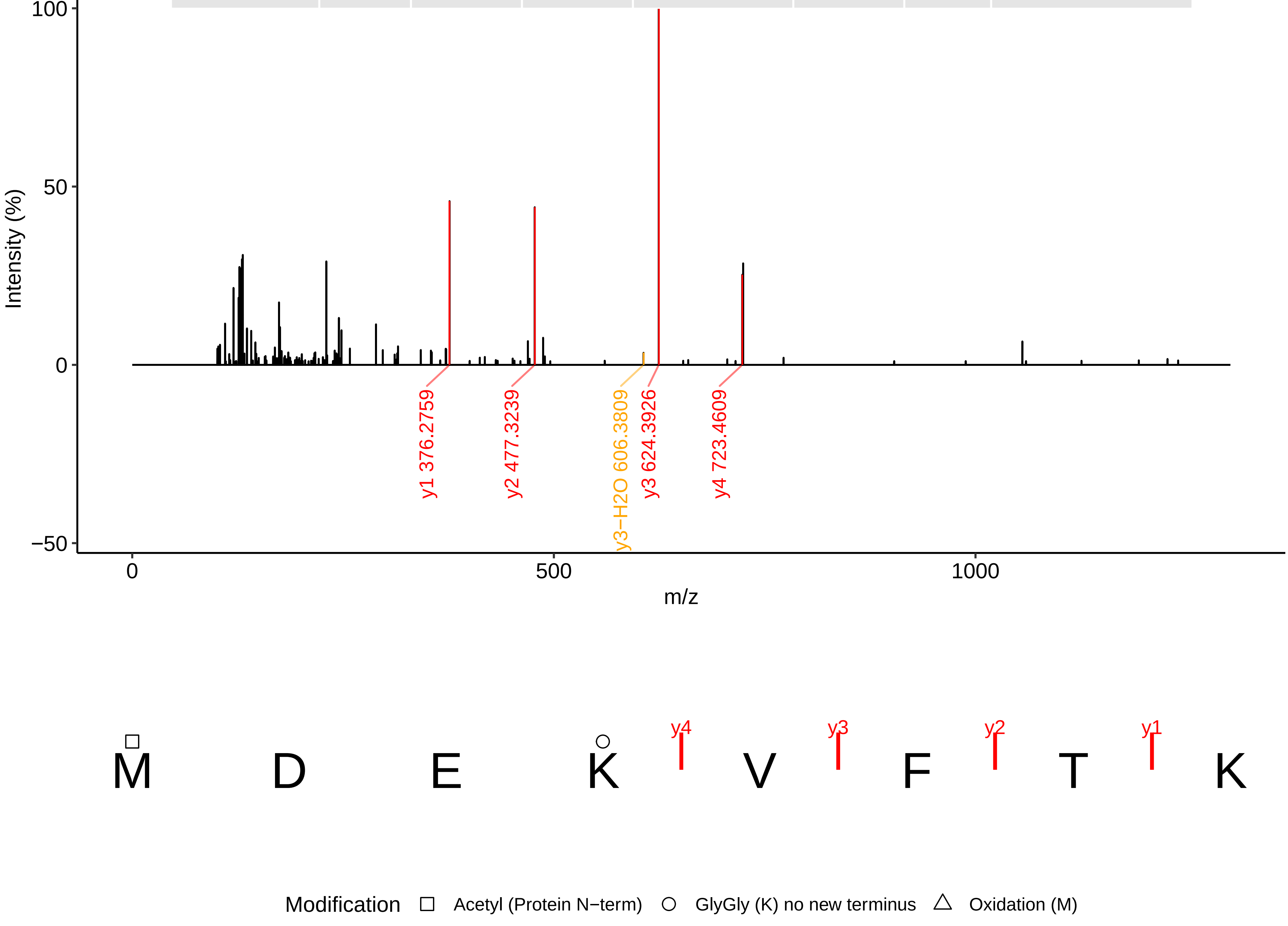

20200924_KIZ_MUb5_GlyGly_2 − Scan number: 9782

Gene Names	Charge	m/z	Mass	Retention time	PEP	Score	Precursor Intensity
PPP2CA	2	585.279	1168.543	36.558	0.04883	53.17	NA

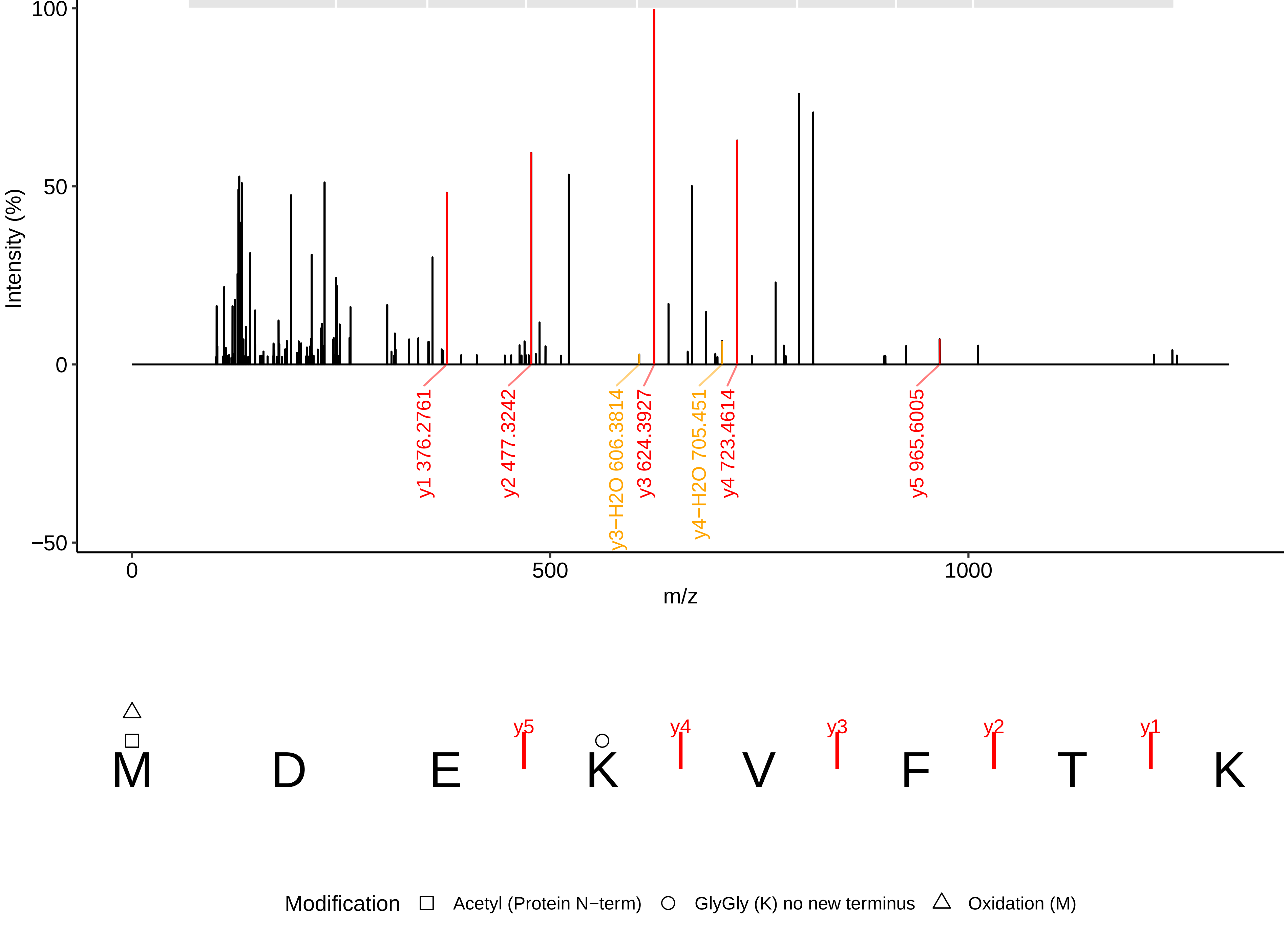

20200921_KIZ_MUb4_GlyGly_10plex_2 − Scan number: 22415

Gene Names	Charge	m/z	Mass	Retention time	PEP	Score	Precursor Intensity
UBE2M	2	576.3283	1150.642	88.356	0.079616	41.979	563725.3

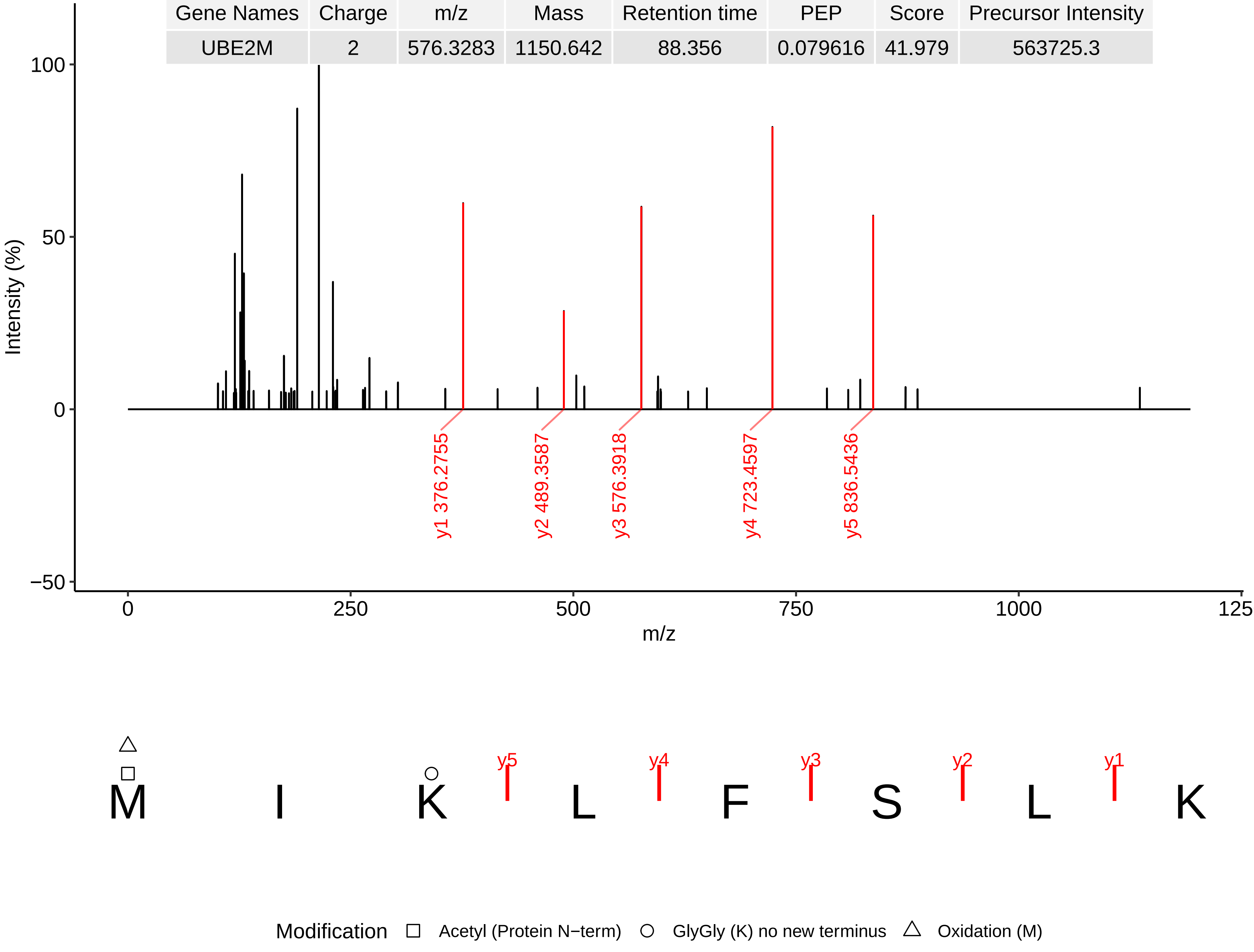

20201118_KIZ_MUb6_GlyGly_1 − Scan number: 5513

Gene Names	Charge	m/z	Mass	Retention time	PEP	Score	Precursor Intensity
CD9	3	311.1725	930.4957	17.475	0.049574	58.596	15987397

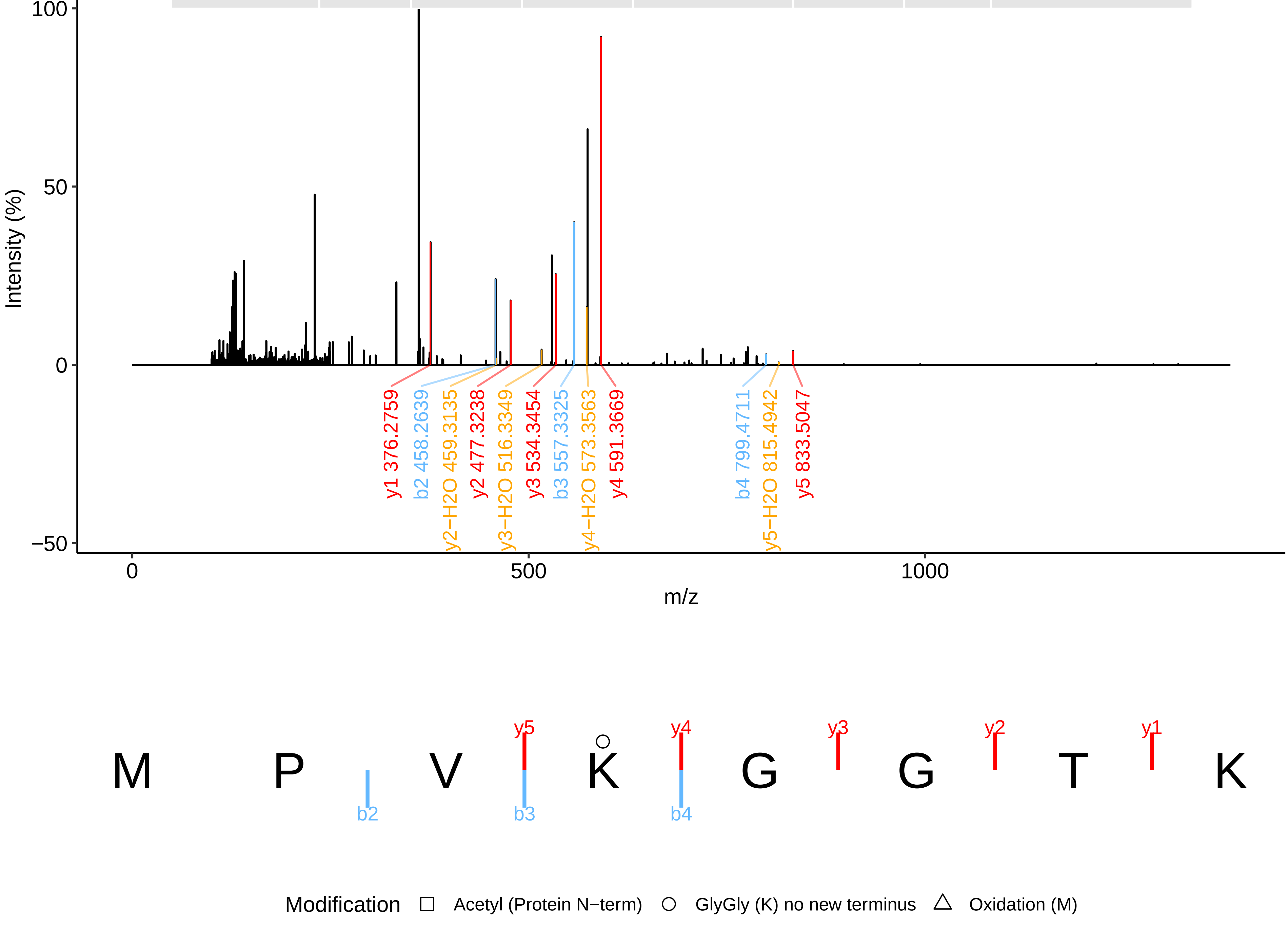

20200921_KIZ_MUb4_GlyGly_10plex − Scan number: 8339

Gene Names	Charge	m/z	Mass	Retention time	PEP	Score	Precursor Intensity
RPS20	3	311.1784	930.5135	28.27	0.018309	82.671	9777098

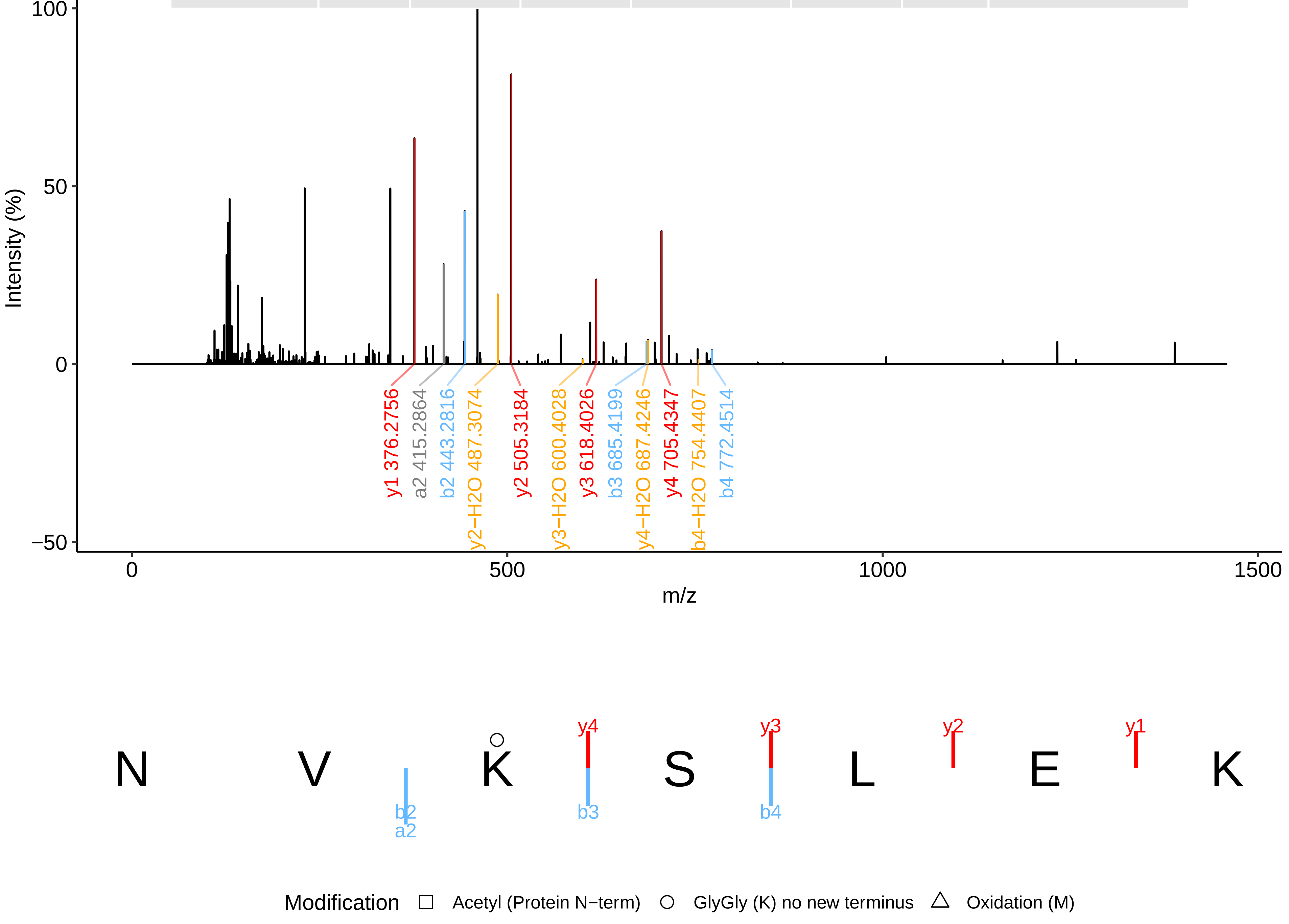

20200921_KIZ_MUb4_GlyGly_10plex_2 − Scan number: 7722

Gene Names	Charge	m/z	Mass	Retention time	PEP	Score	Precursor Intensity
RPS20	3	311.1784	930.5135	28.903	0.018309	82.671	3353006

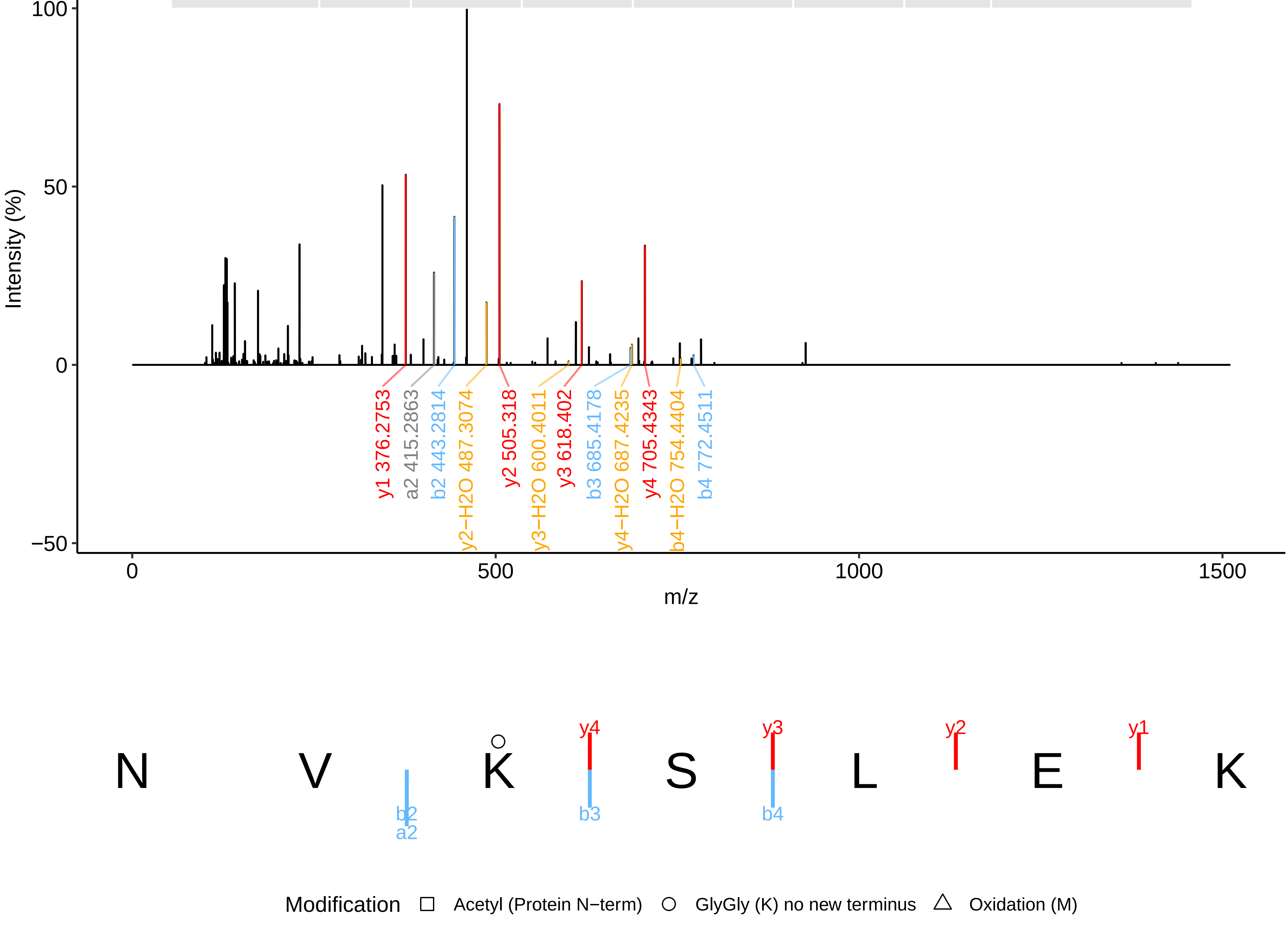

20200924_KIZ_MUb5_GlyGly_1 − Scan number: 7139

Gene Names	Charge	m/z	Mass	Retention time	PEP	Score	Precursor Intensity
RPS20	3	311.1784	930.5135	22.617	0.077758	60.478	11347528

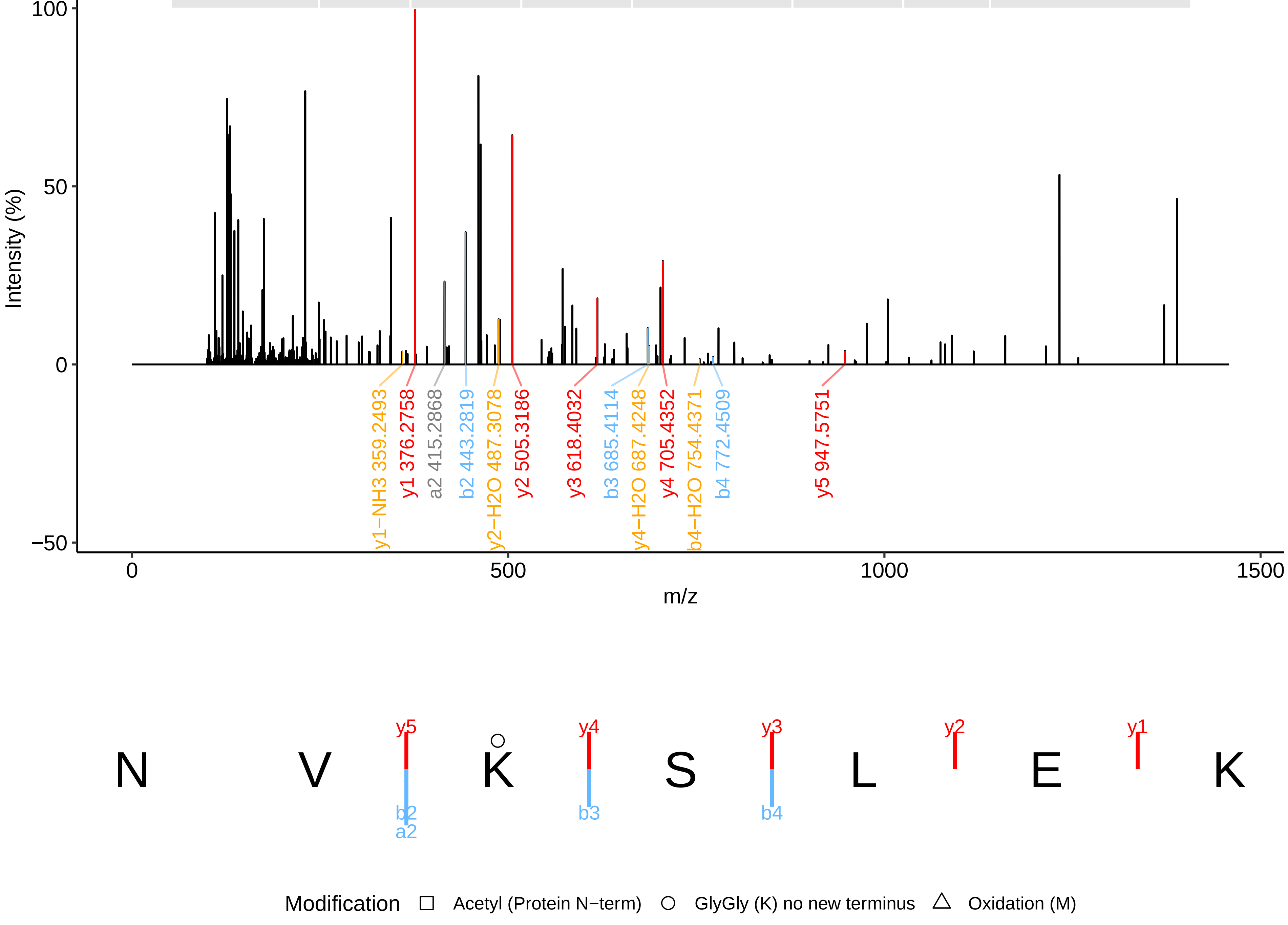

20200924_KIZ_MUb5_GlyGly_1 − Scan number: 7292

Gene Names	Charge	m/z	Mass	Retention time	PEP	Score	Precursor Intensity
RPS20	3	311.1784	930.5135	23.119	0.052122	67.981	16000760

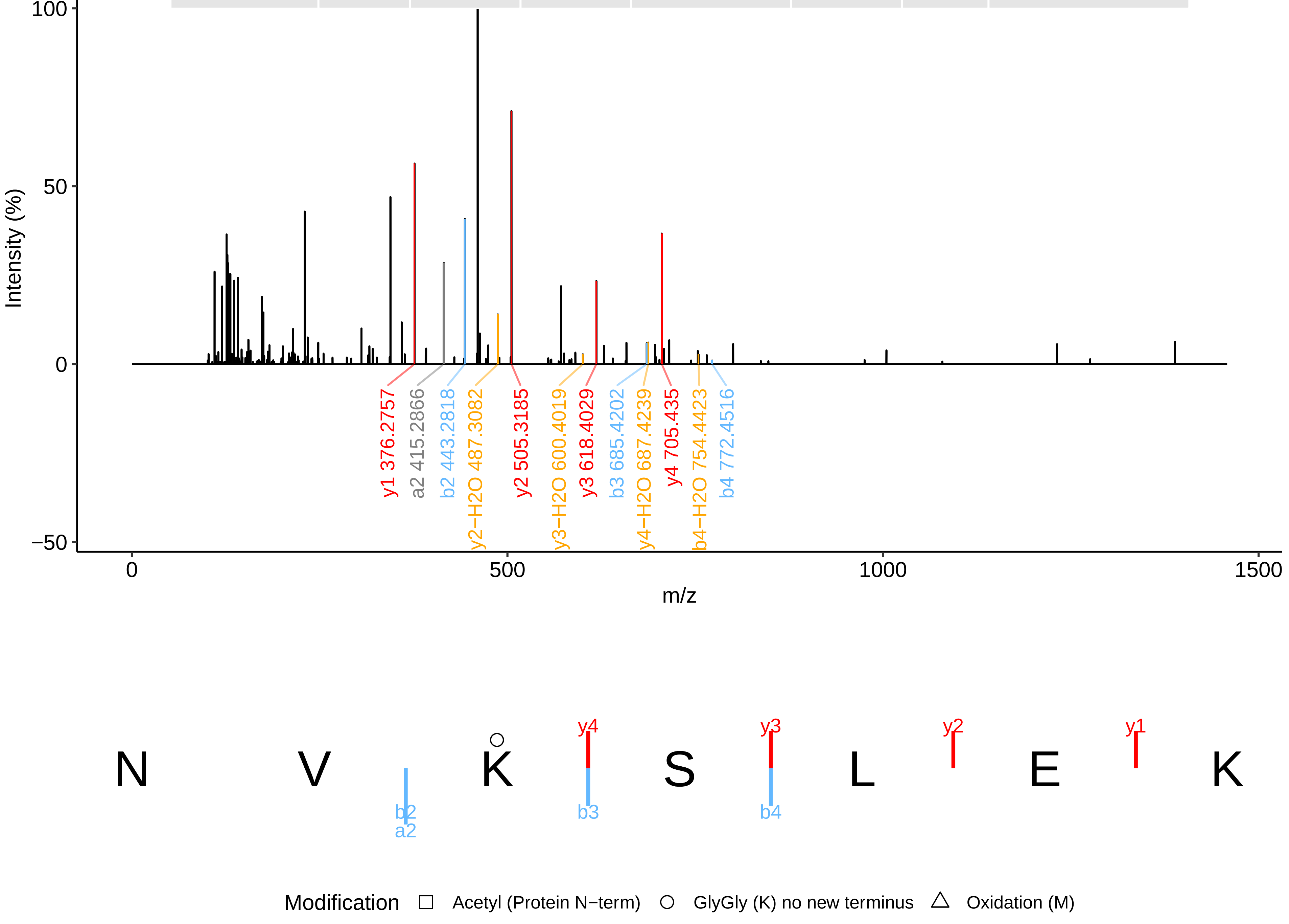

20200924_KIZ_MUb5_GlyGly_2 − Scan number: 6894

Gene Names	Charge	m/z	Mass	Retention time	PEP	Score	Precursor Intensity
RPS20	3	311.1784	930.5135	25.716	0.052122	67.981	5632966

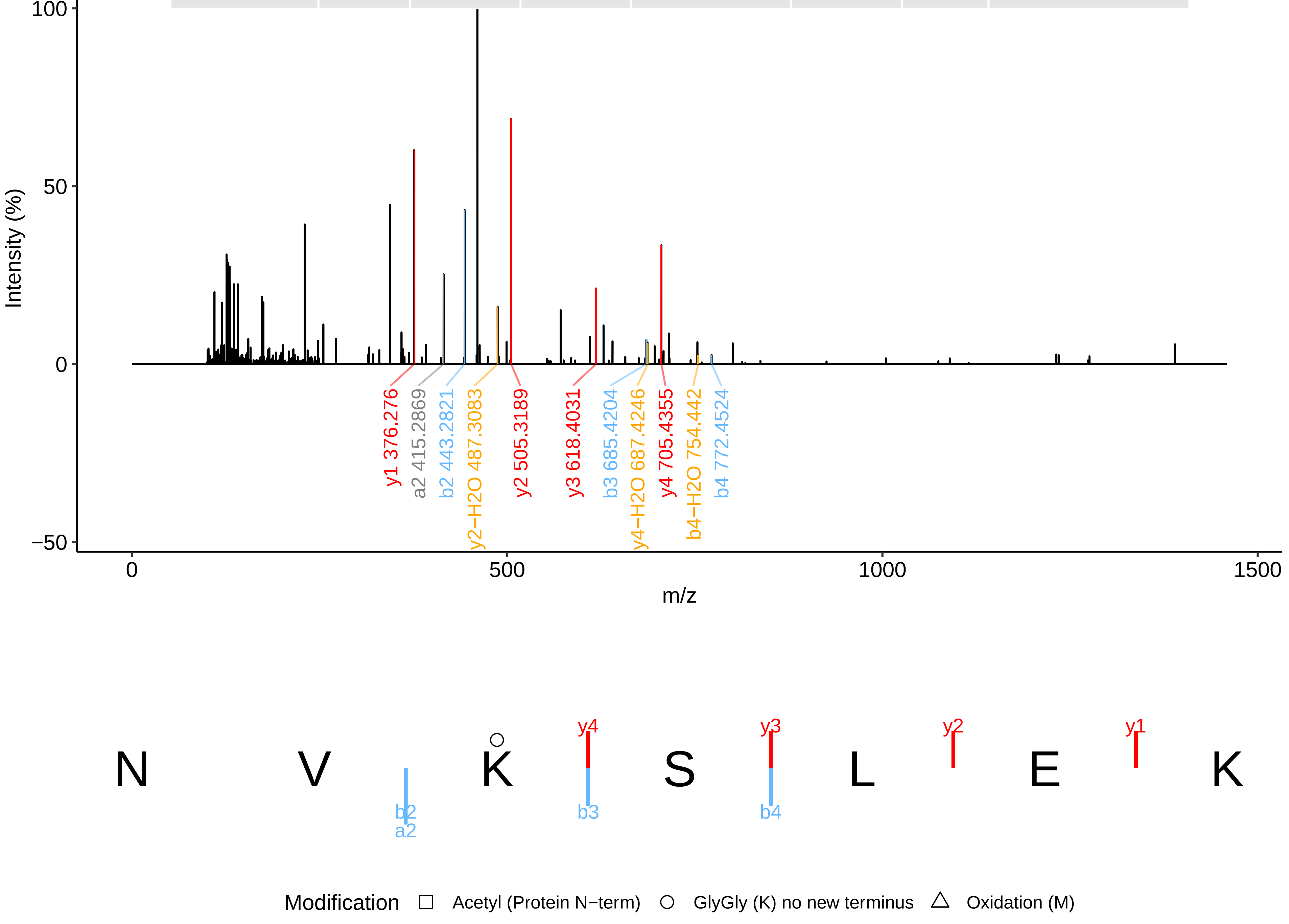

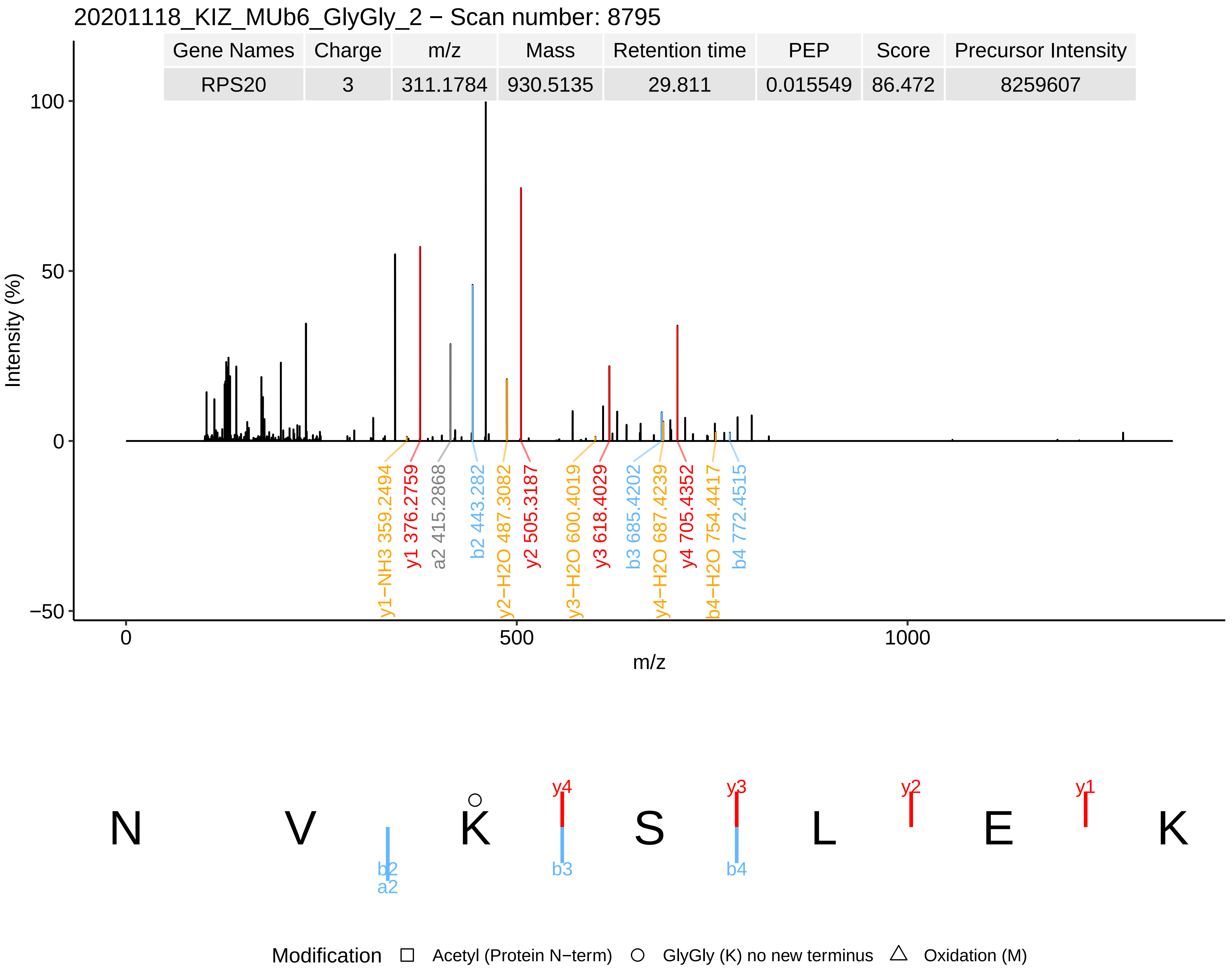
20201118_KIZ_MUb6_GlyGly_2 − Scan number: 8795
Gene Names | Charge | m/z | Mass | Retention time | PEP | Score | Precursor Intensity
RPS20 | 3 | 311.1784 | 930.5135 | 29.811 | 0.015549 | 86.472 | 8259607
Intensity (%)
m/z
y1−NH3 359.2494
y1 376.2759
a2 415.2868
b2 443.282
y2−H2O 487.3082
y2 505.3187
y3−H2O 600.4019
y3 618.4029
b3 685.4202
y4−H2O 687.4239
y4 705.4352
b4−H2O 754.4417
b4 772.4515
N V K S L E K
y4 y3 y2 y1
b2 a2 b3 b4
Modification □ Acetyl (Protein N−term) ○ GlyGly (K) no new terminus △ Oxidation (M)

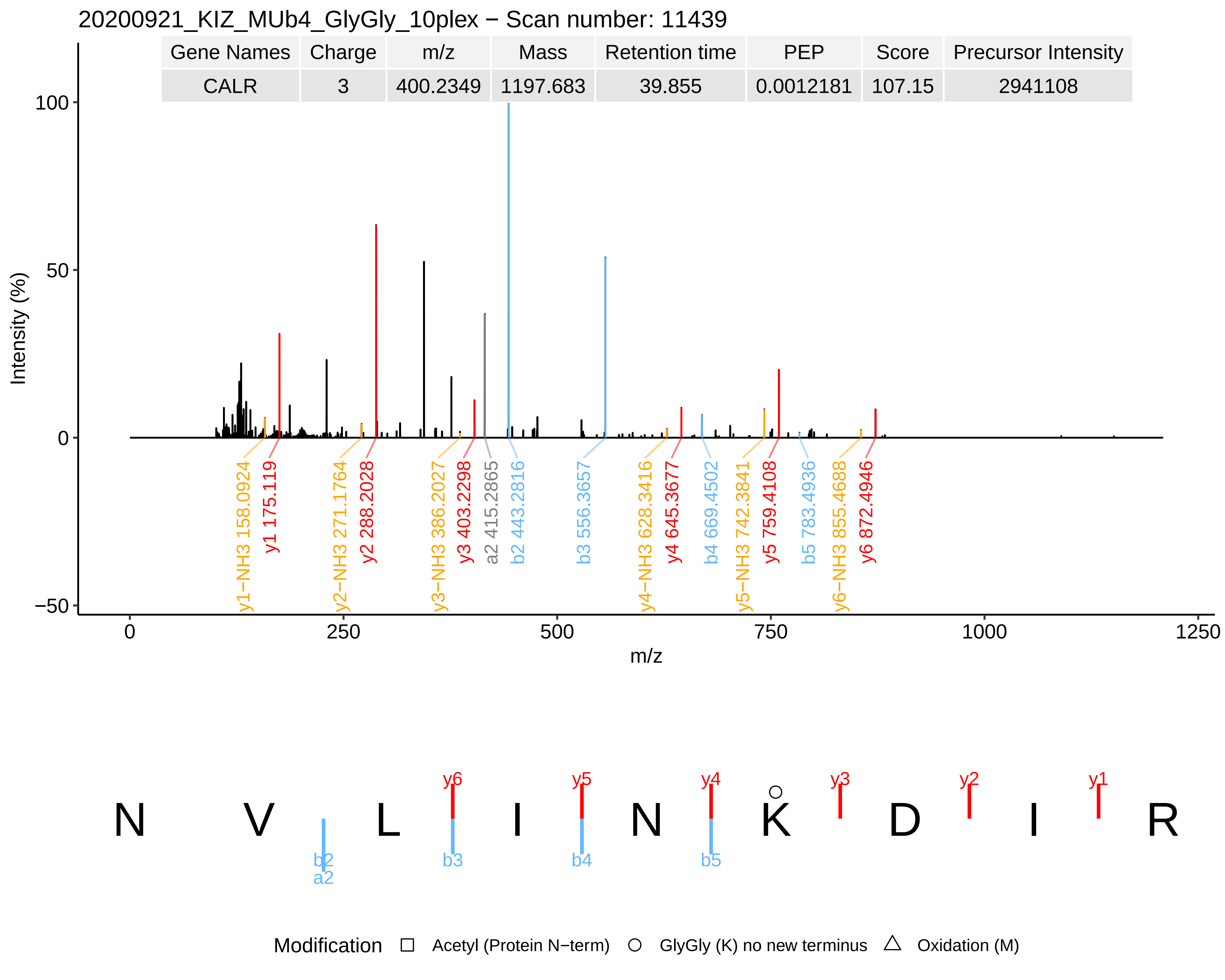
20200921_KIZ_MUb4_GlyGly_10plex − Scan number: 11439
Gene Names | Charge | m/z | Mass | Retention time | PEP | Score | Precursor Intensity
CALR | 3 | 400.2349 | 1197.683 | 39.855 | 0.0012181 | 107.15 | 2941108
Intensity (%)
m/z
y1−NH3 158.0924
y1 175.119
y2−NH3 271.1764
y2 288.2028
y3−NH3 386.2027
y3 403.2298
a2 415.2865
b2 443.2816
b3 556.3657
y4−NH3 628.3416
y4 645.3677
b4 669.4502
y5−NH3 742.3841
y5 759.4108
b5 783.4936
y6−NH3 855.4688
y6 872.4946
N V L I N K D I R
y6 y5 y4 y3 y2 y1
b2 a2 b3 b4 b5
Modification
Acetyl (Protein N−term)
GlyGly (K) no new terminus
Oxidation (M)

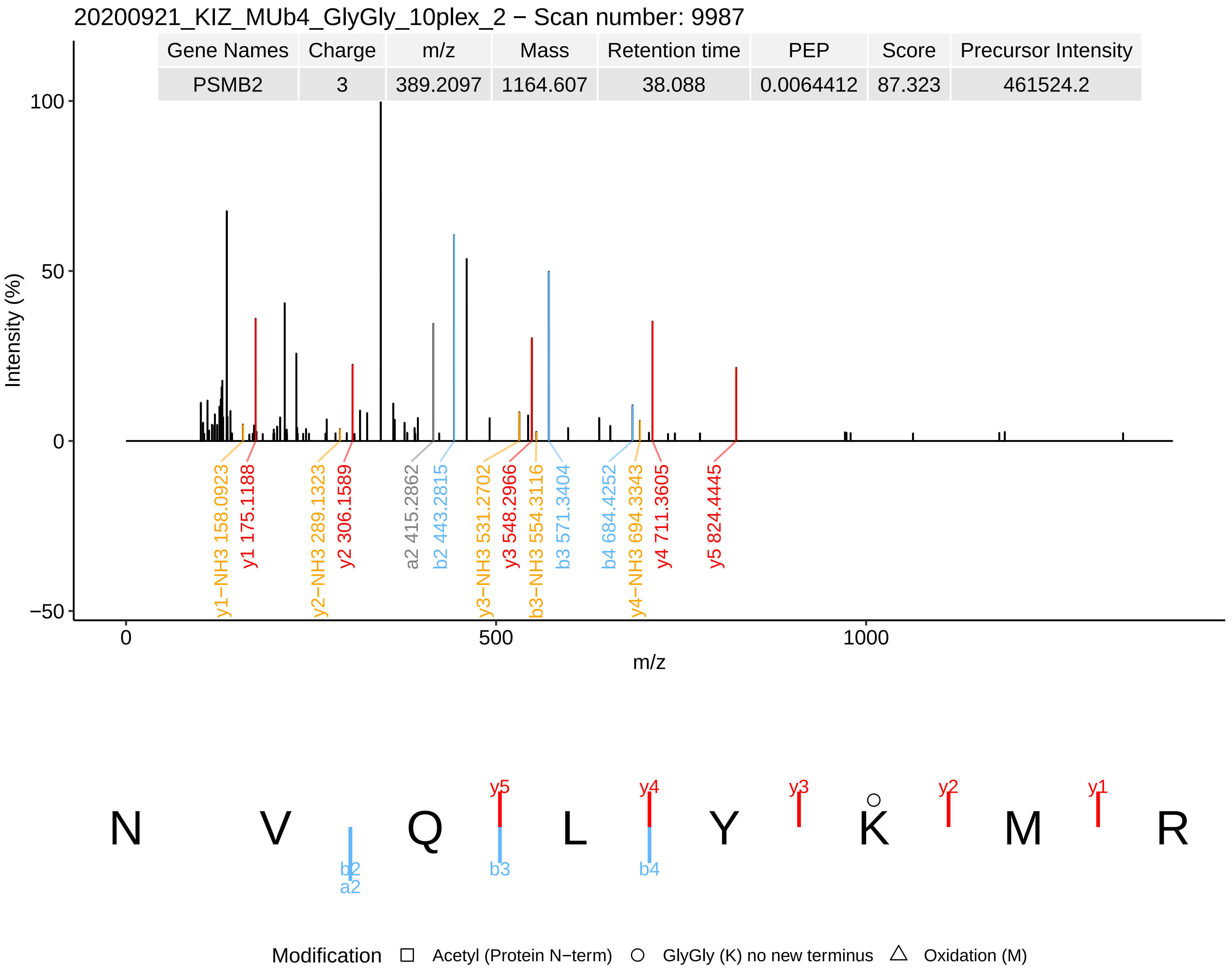
20200921_KIZ_MUb4_GlyGly_10plex_2 − Scan number: 9987
Gene Names | Charge | m/z | Mass | Retention time | PEP | Score | Precursor Intensity
PSMB2 | 3 | 389.2097 | 1164.607 | 38.088 | 0.0064412 | 87.323 | 461524.2
Intensity (%)
m/z
y1−NH3 158.0923
y1 175.1188
y2−NH3 289.1323
y2 306.1589
a2 415.2862
b2 443.2815
y3−NH3 531.2702
y3 548.2966
b3−NH3 554.3116
b3 571.3404
b4 684.4252
y4−NH3 694.3343
y4 711.3605
y5 824.4445
N V Q L Y K M R
y5 y4 y3 y2 y1
b2 a2 b3 b4
Modification
Acetyl (Protein N−term)
GlyGly (K) no new terminus
Oxidation (M)

20200921_KIZ_MUb4_GlyGly_10plex − Scan number: 14348

Gene Names	Charge	m/z	Mass	Retention time	PEP	Score	Precursor Intensity
RAB5C;RAB5A	3	396.2158	1185.626	50.965	0.00022456	120.46	NA

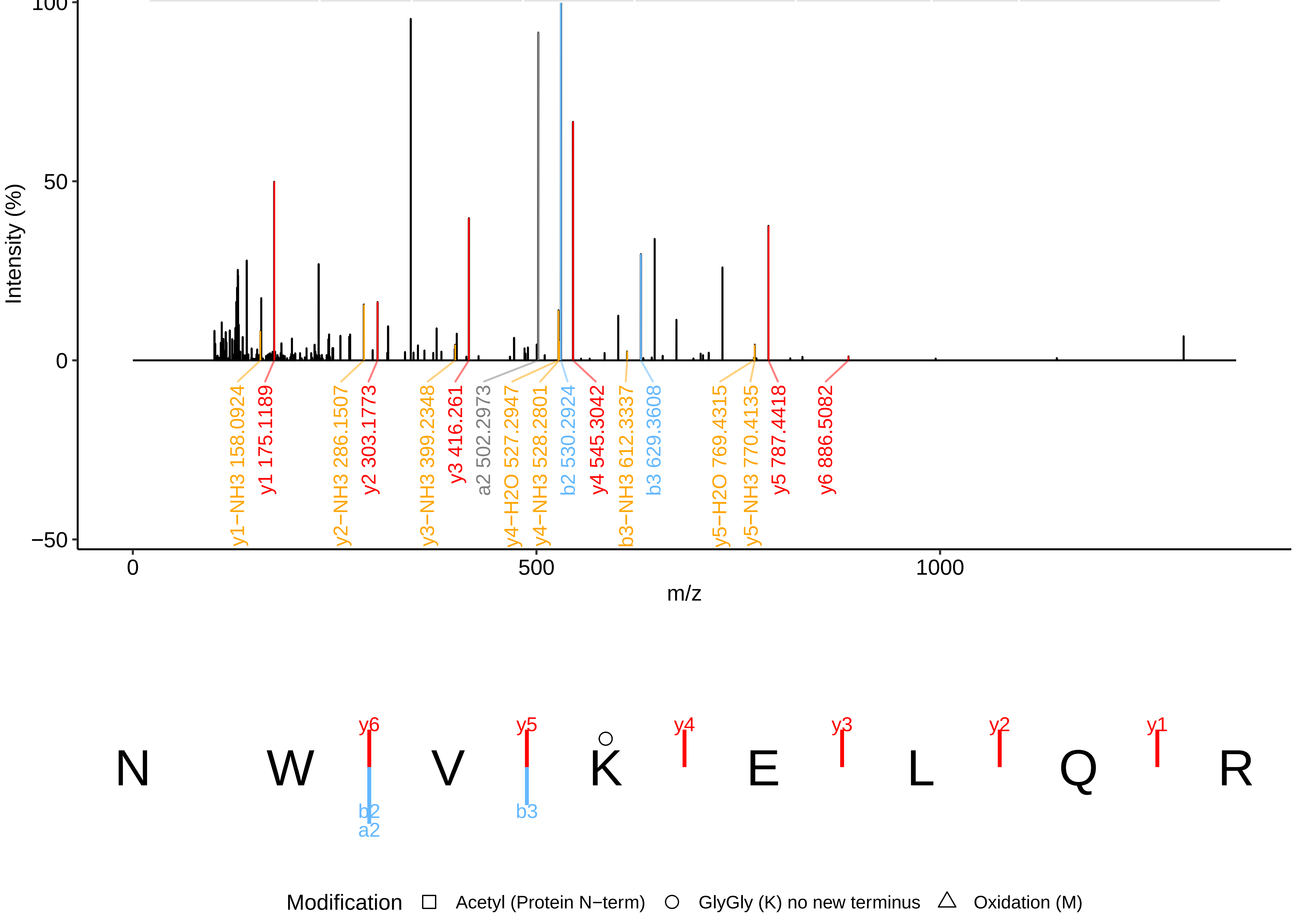

20200921_KIZ_MUb4_GlyGly_10plex_2 − Scan number: 13250

Gene Names	Charge	m/z	Mass	Retention time	PEP	Score	Precursor Intensity
RAB5C;RAB5A	3	396.2158	1185.626	51.404	0.012415	77.923	637510.4

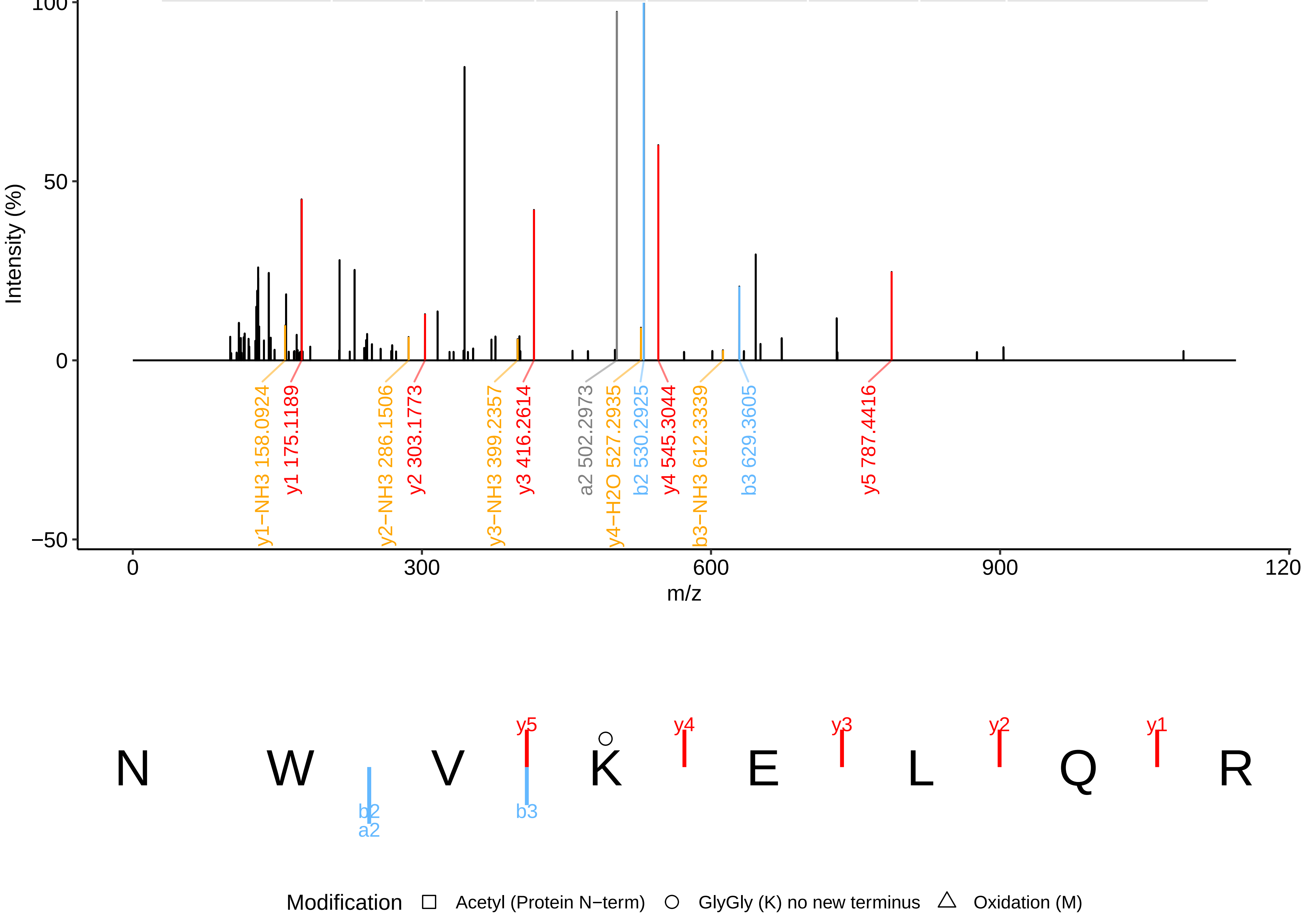

20200921_KIZ_MUb4_GlyGly_10plex − Scan number: 6584

Gene Names	Charge	m/z	Mass	Retention time	PEP	Score	Precursor Intensity
RABEP1	3	401.2106	1200.61	21.841	0.0025027	103.26	5285464

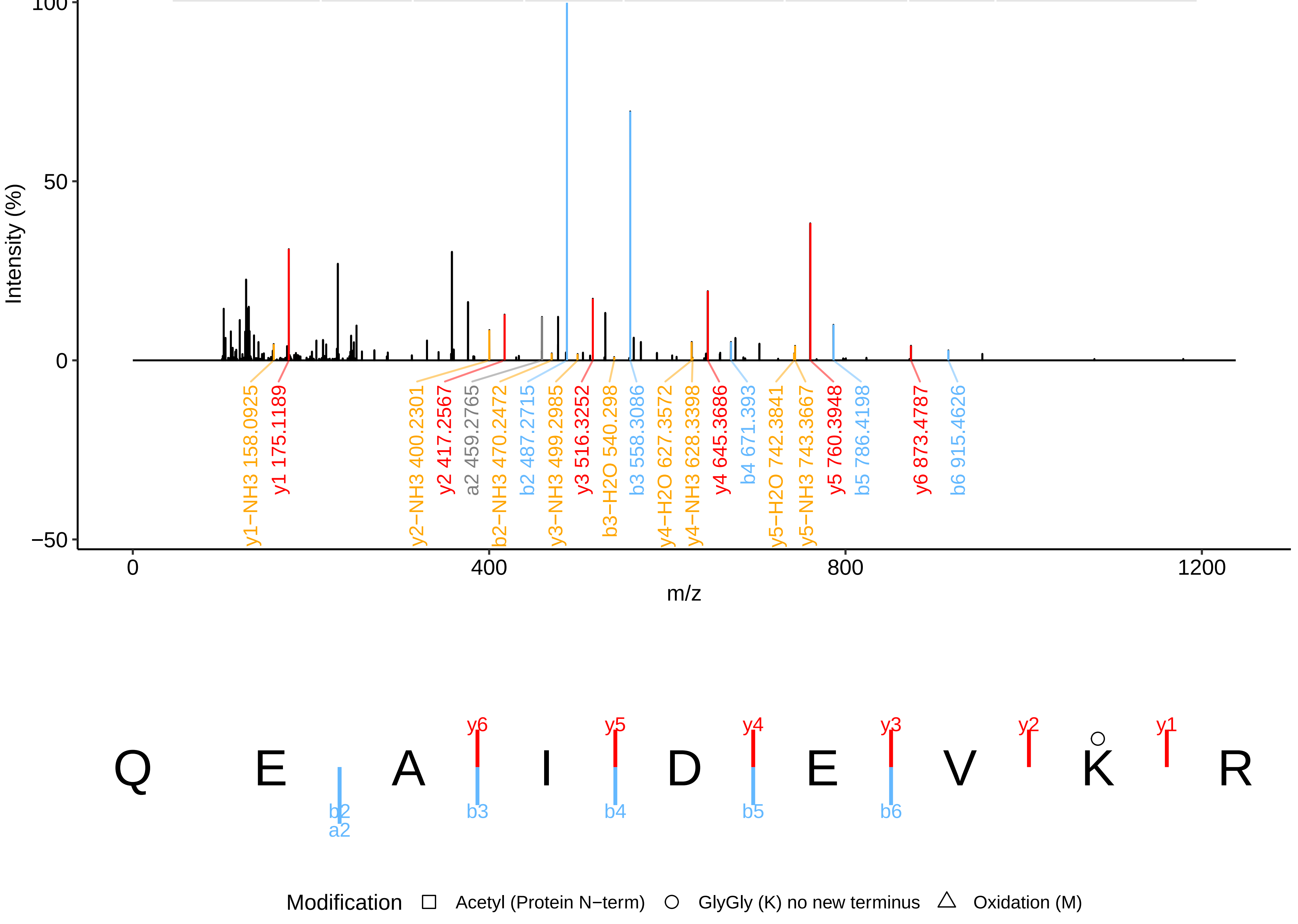

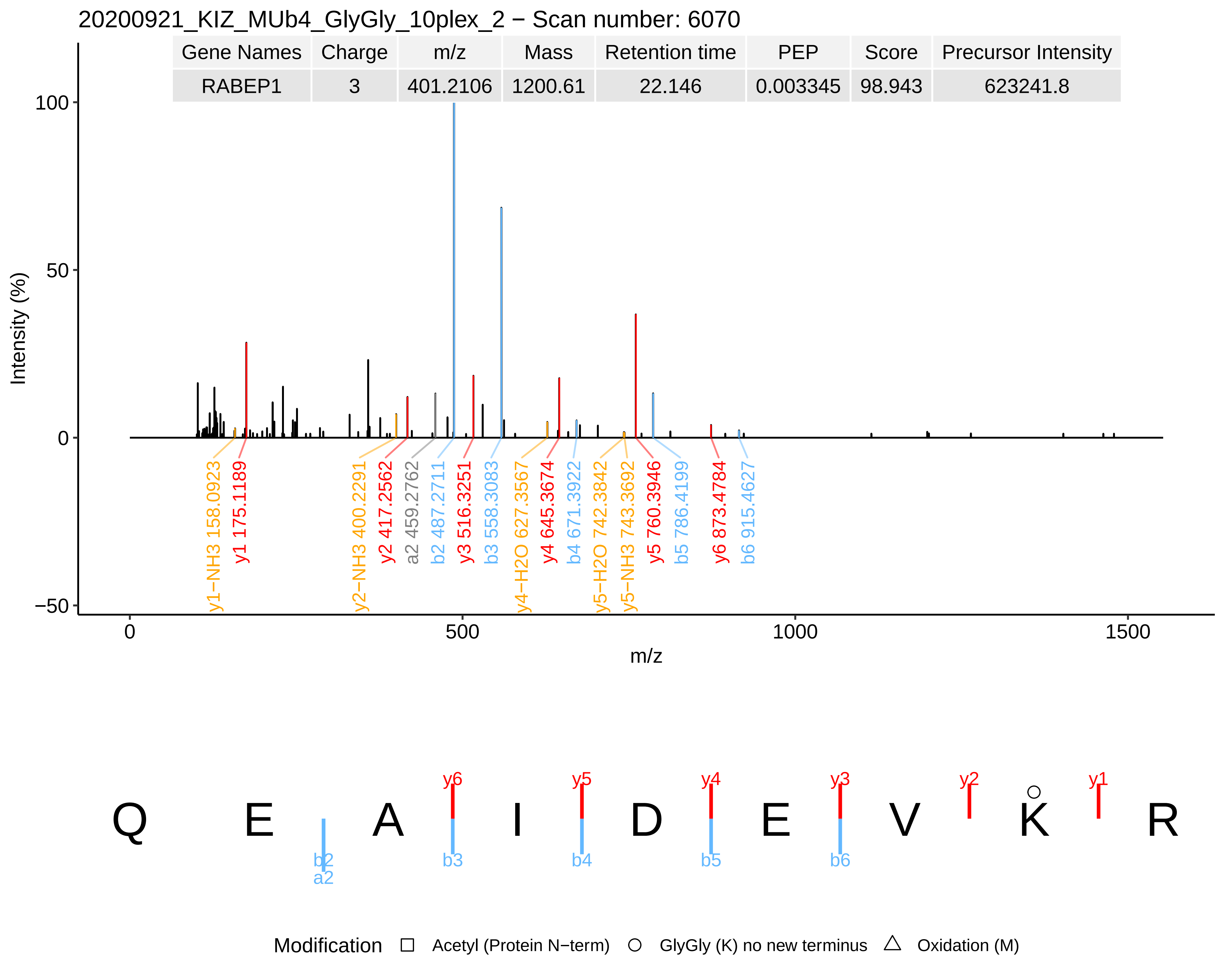

20200921_KIZ_MUb4_GlyGly_10plex_2 − Scan number: 6070
Gene Names | Charge | m/z | Mass | Retention time | PEP | Score | Precursor Intensity
RABEP1 | 3 | 401.2106 | 1200.61 | 22.146 | 0.003345 | 98.943 | 623241.8
Intensity (%)
m/z
y1−NH3 158.0923
y1 175.1189
y2−NH3 400.2291
y2 417.2562
a2 459.2762
b2 487.2711
y3 516.3251
b3 558.3083
y4−H2O 627.3567
y4 645.3674
b4 671.3922
y5−H2O 742.3842
y5−NH3 743.3692
y5 760.3946
b5 786.4199
y6 873.4784
b6 915.4627
Q E A I D E V K R
y6 y5 y4 y3 y2 y1
b2 a2 b3 b4 b5 b6
Modification
Acetyl (Protein N−term)
GlyGly (K) no new terminus
Oxidation (M)

20201118_KIZ_MUb6_GlyGly_1 − Scan number: 5377

Gene Names	Charge	m/z	Mass	Retention time	PEP	Score	Precursor Intensity
RABEP1	3	401.2106	1200.61	17.014	0.051109	73.841	NA

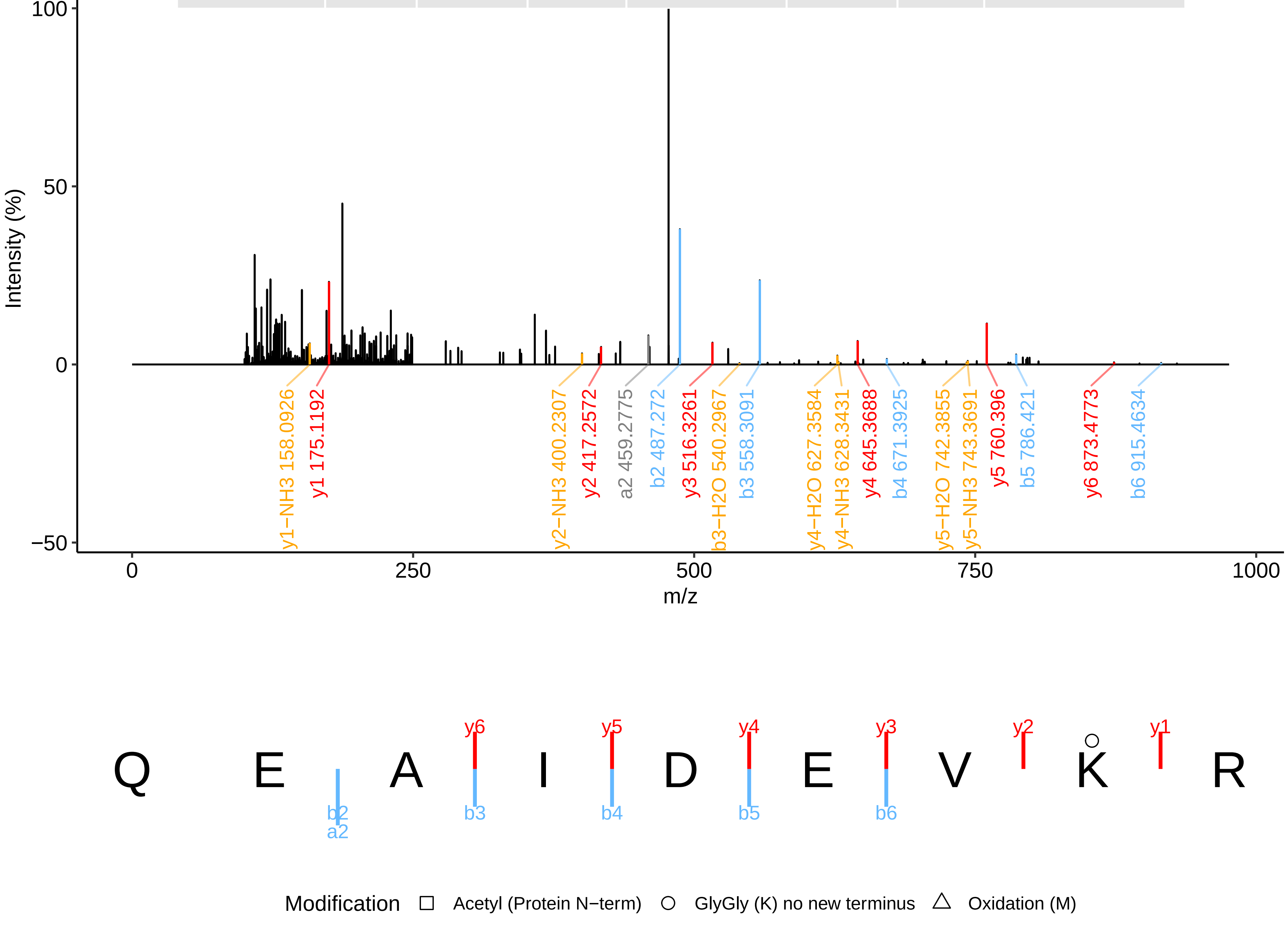

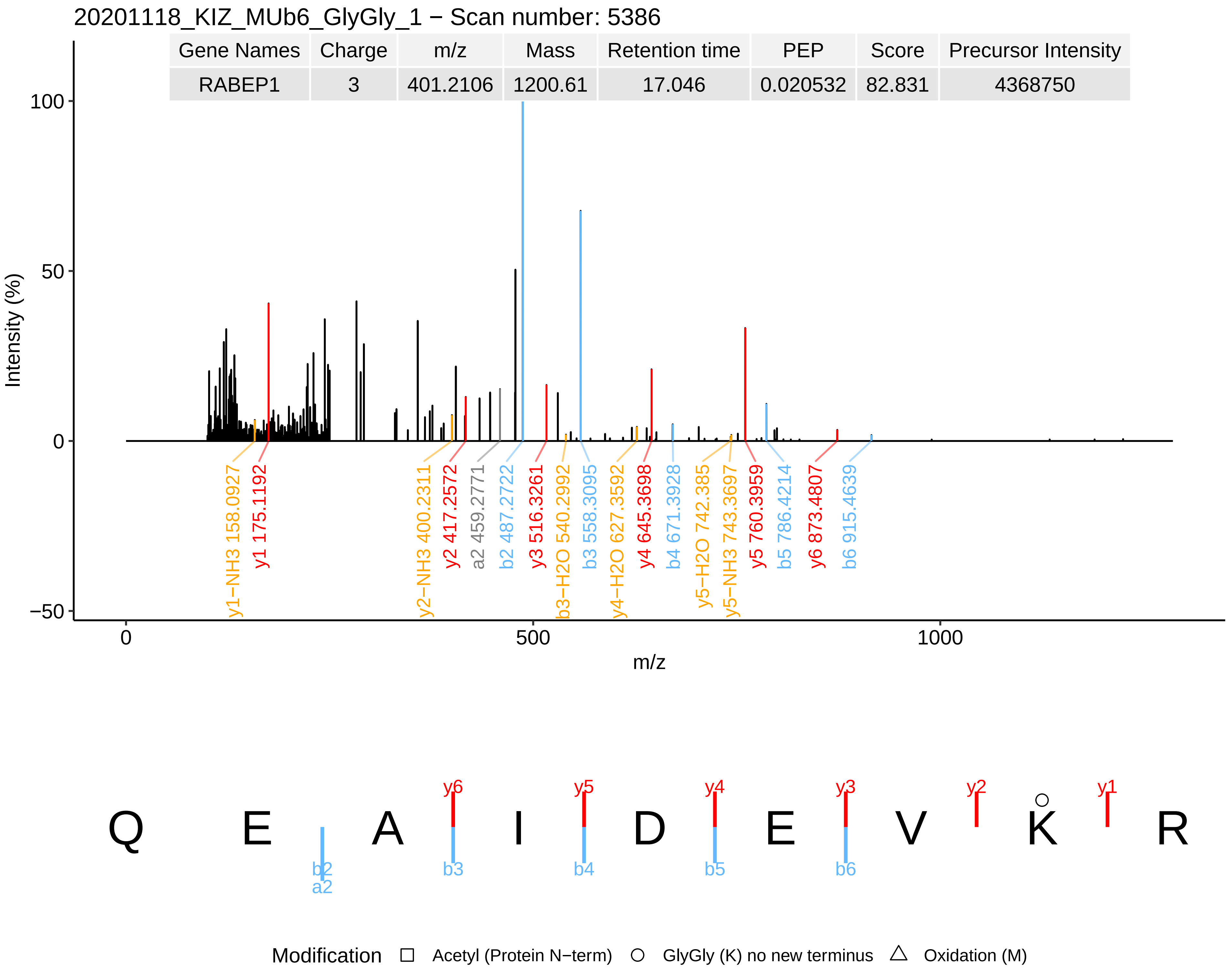
20201118_KIZ_MUb6_GlyGly_1 − Scan number: 5386
Gene Names | Charge | m/z | Mass | Retention time | PEP | Score | Precursor Intensity
RABEP1 | 3 | 401.2106 | 1200.61 | 17.046 | 0.020532 | 82.831 | 4368750
Intensity (%)
m/z
y1−NH3 158.0927
y1 175.1192
y2−NH3 400.2311
y2 417.2572
a2 459.2771
b2 487.2722
y3 516.3261
b3−H2O 540.2992
b3 558.3095
y4−H2O 627.3592
y4 645.3698
b4 671.3928
y5−H2O 742.385
y5−NH3 743.3697
y5 760.3959
b5 786.4214
y6 873.4807
b6 915.4639
Q E A I D E V K R
y6 y5 y4 y3 y2 y1
b2 a2 b3 b4 b5 b6
Modification
Acetyl (Protein N−term)
GlyGly (K) no new terminus
Oxidation (M)

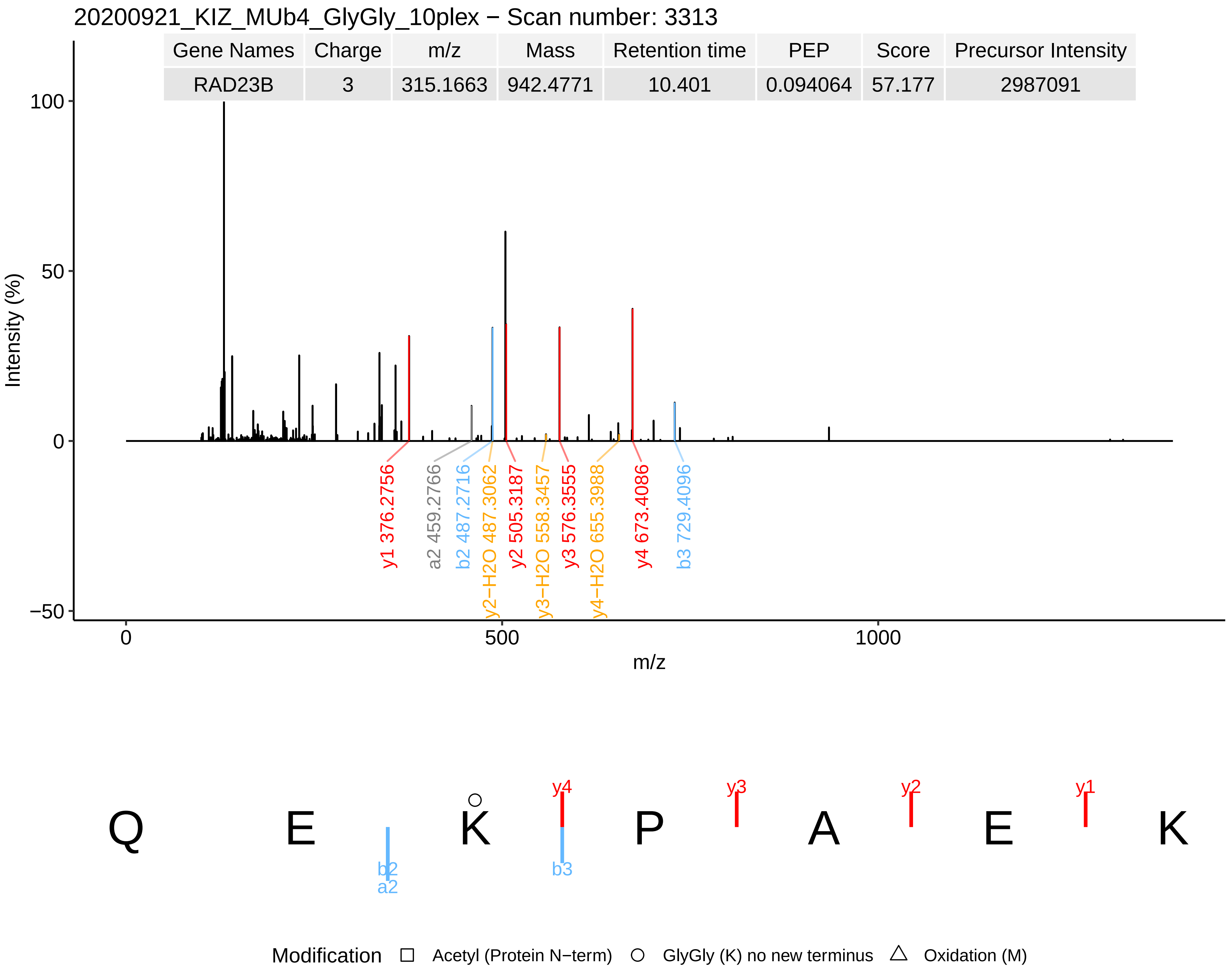

20200921_KIZ_MUb4_GlyGly_10plex − Scan number: 3313
Gene Names | Charge | m/z | Mass | Retention time | PEP | Score | Precursor Intensity
RAD23B | 3 | 315.1663 | 942.4771 | 10.401 | 0.094064 | 57.177 | 2987091
Intensity (%)
100
50
0
−50
0
500
1000
m/z
y1 376.2756
a2 459.2766
b2 487.2716
y2−H2O 487.3062
y2 505.3187
y3−H2O 558.3457
y3 576.3555
y4−H2O 655.3988
y4 673.4086
b3 729.4096
Q E K P A E K
y4 y3 y2 y1
b2 a2 b3
Modification
Acetyl (Protein N−term)
GlyGly (K) no new terminus
Oxidation (M)

20200924_KIZ_MUb5_GlyGly_1 − Scan number: 3105

Gene Names	Charge	m/z	Mass	Retention time	PEP	Score	Precursor Intensity
RAD23B	3	315.1663	942.4771	9.5779	0.076749	60.682	9650571

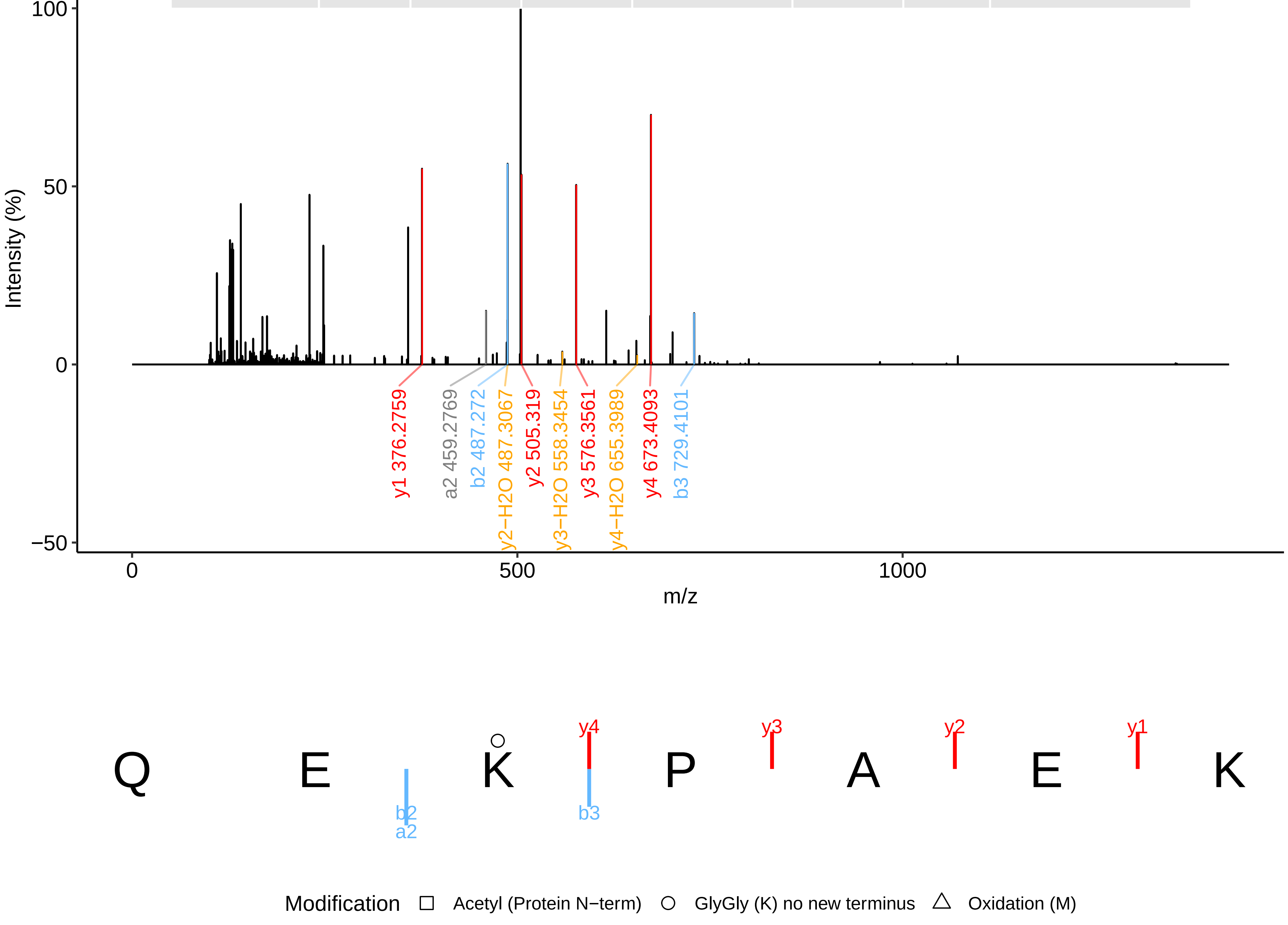

20200924_KIZ_MUb5_GlyGly_2 − Scan number: 2832

Gene Names	Charge	m/z	Mass	Retention time	PEP	Score	Precursor Intensity
RAD23B	3	315.1663	942.4771	10.137	0.112	57.177	1524340

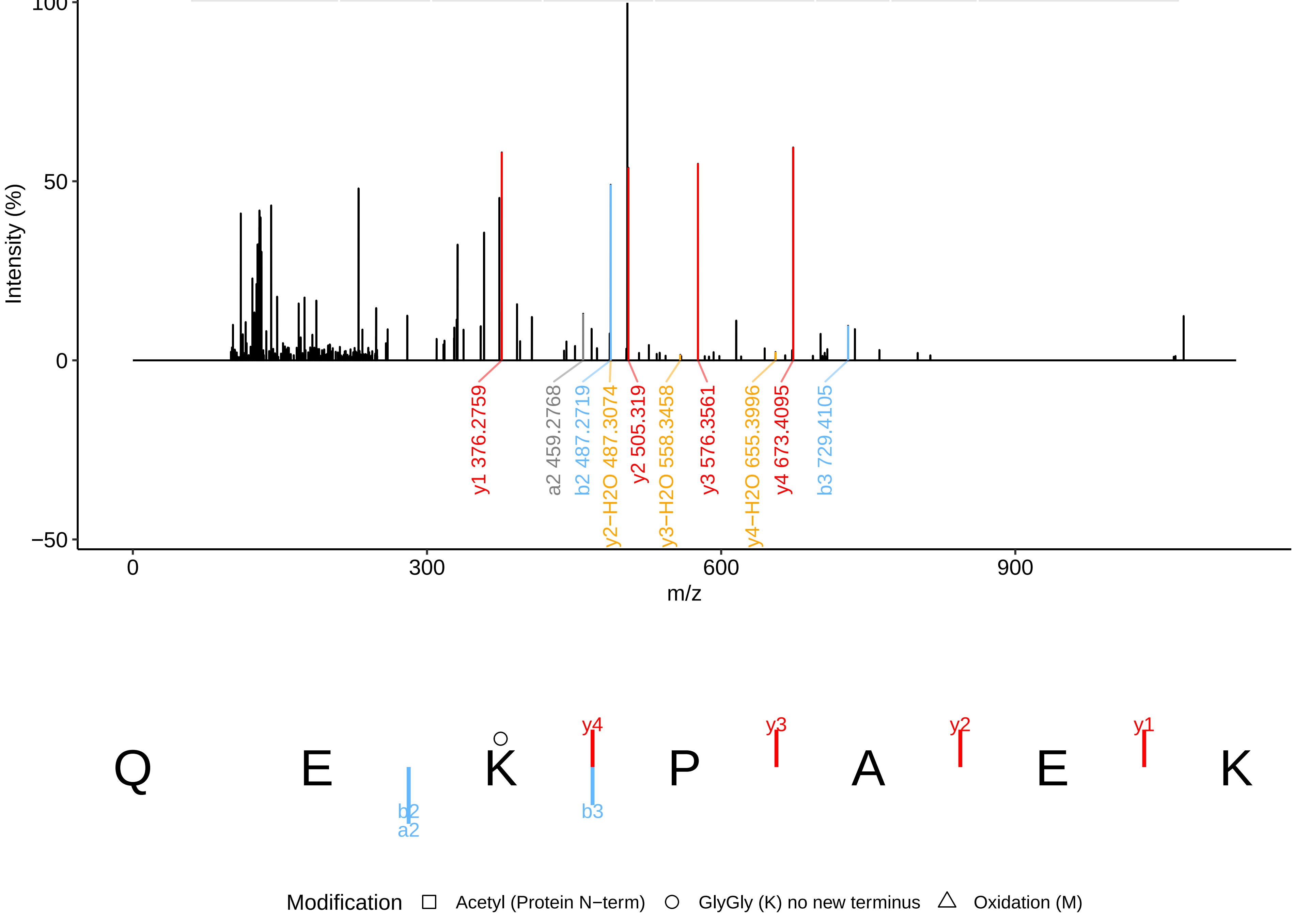

20200921_KIZ_MUb4_GlyGly_10plex − Scan number: 4700

Gene Names	Charge	m/z	Mass	Retention time	PEP	Score	Precursor Intensity
DDX24	3	389.5315	1165.573	15.32	0.018521	75.696	4211109

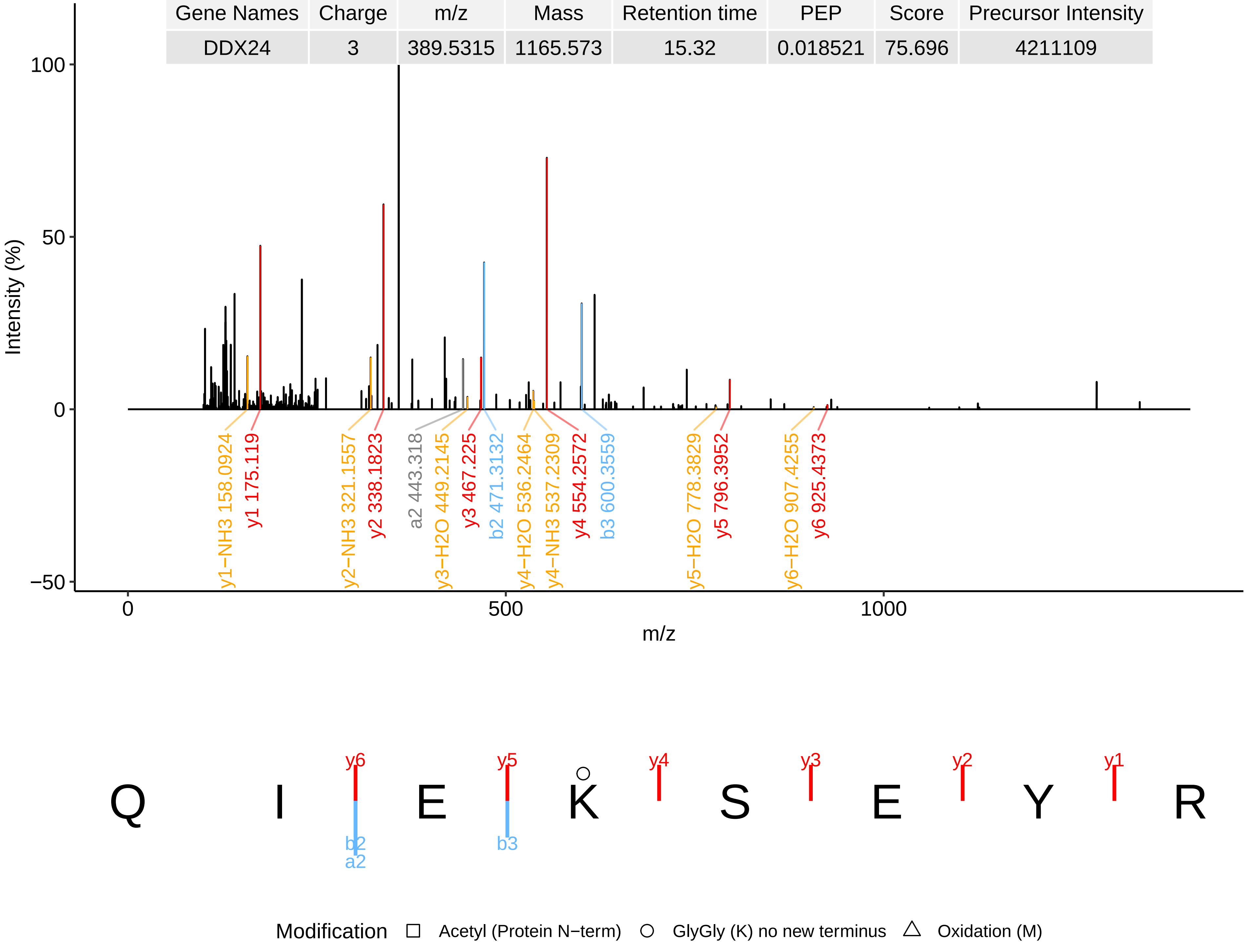

20200921_KIZ_MUb4_GlyGly_10plex − Scan number: 6550

Gene Names	Charge	m/z	Mass	Retention time	PEP	Score	Precursor Intensity
MFF	3	385.5754	1153.704	21.719	0.0049831	95.531	6935317

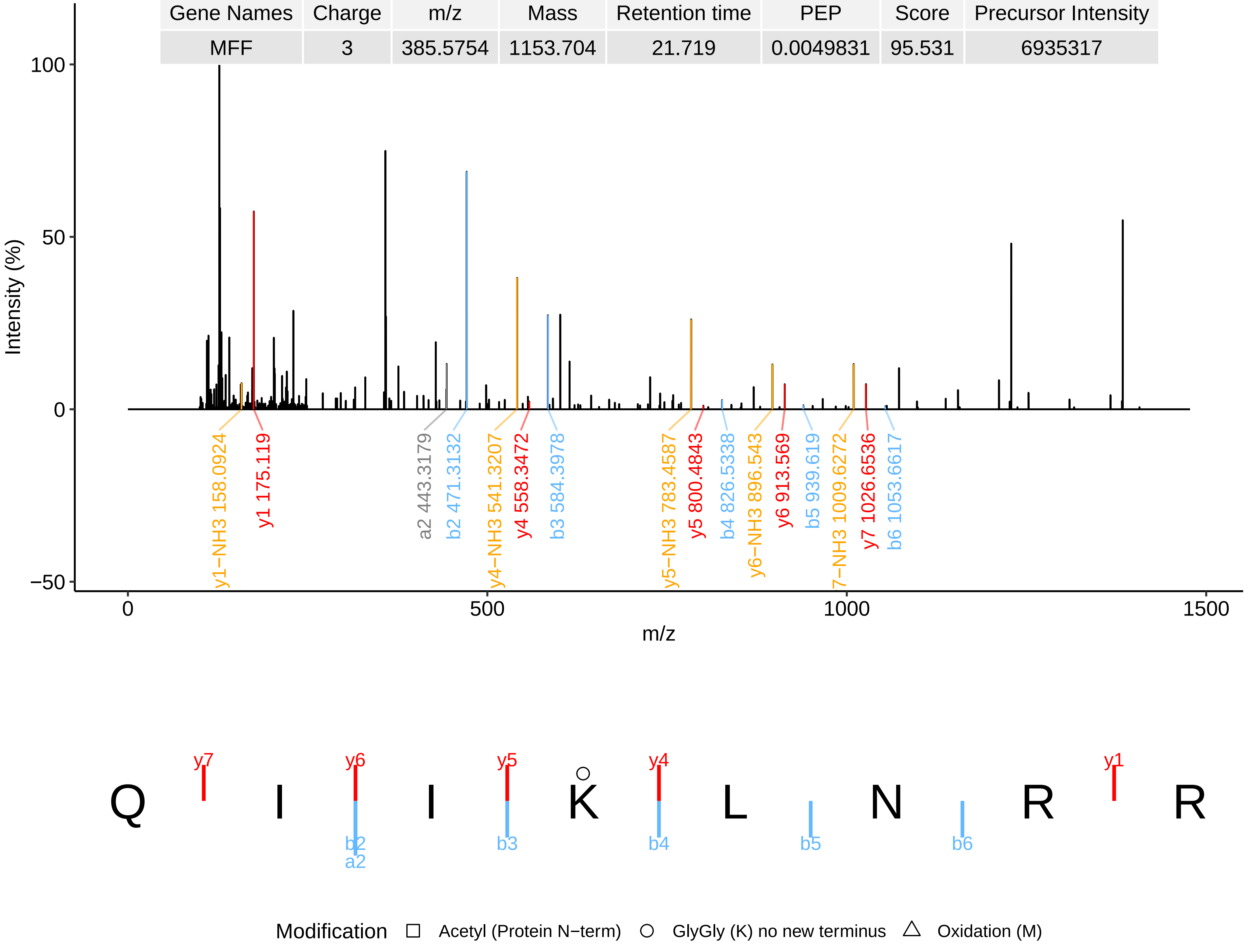

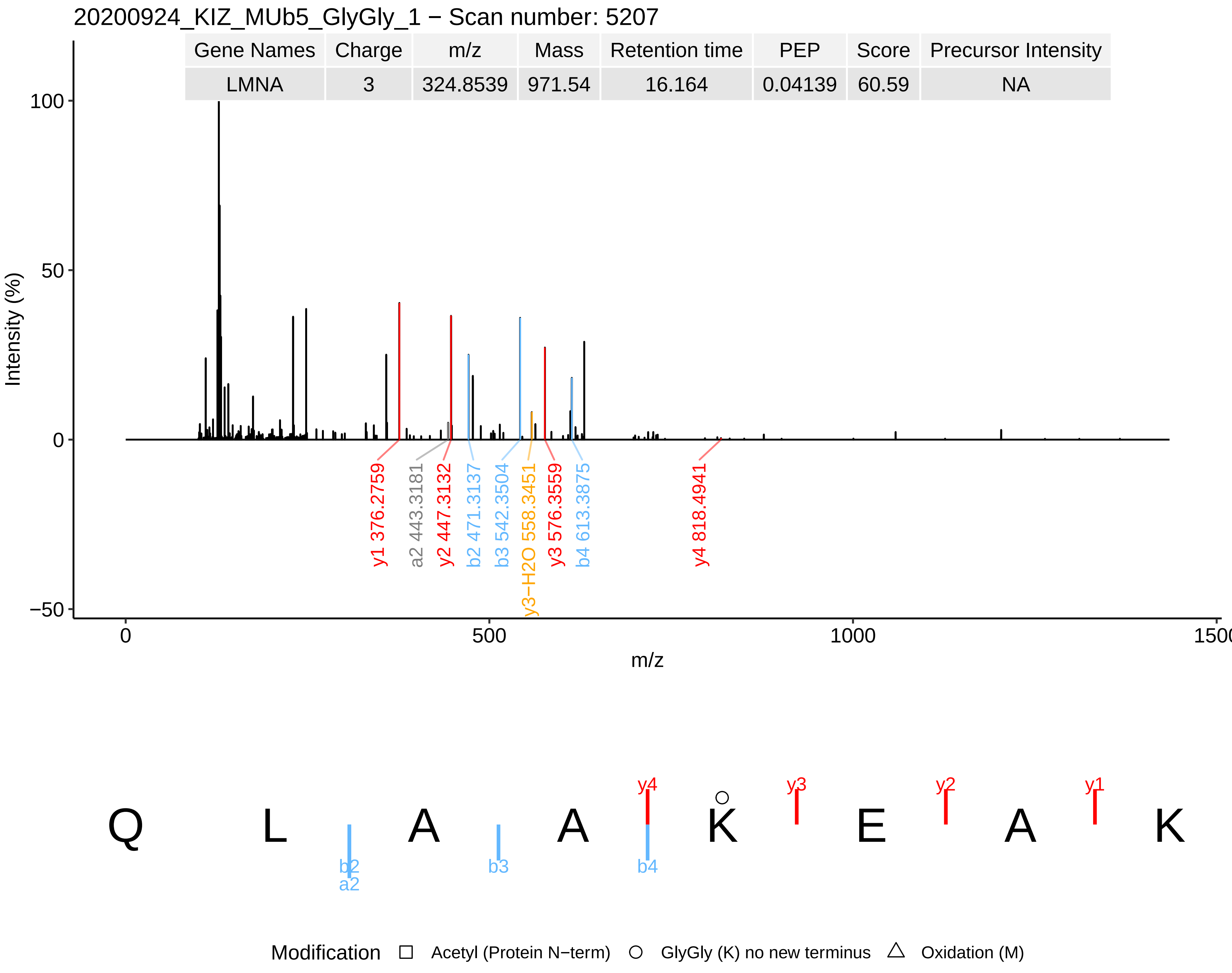

20200924_KIZ_MUb5_GlyGly_1 − Scan number: 5207

Gene Names	Charge	m/z	Mass	Retention time	PEP	Score	Precursor Intensity
LMNA	3	324.8539	971.54	16.164	0.04139	60.59	NA

Intensity (%)
m/z
y1 376.2759
a2 443.3181
y2 447.3132
b2 471.3137
b3 542.3504
y3−H2O 558.3451
y3 576.3559
b4 613.3875
y4 818.4941
Q L A A K E A K
y4 y3 y2 y1
b2 a2 b3 b4
Modification
Acetyl (Protein N−term)
GlyGly (K) no new terminus
Oxidation (M)

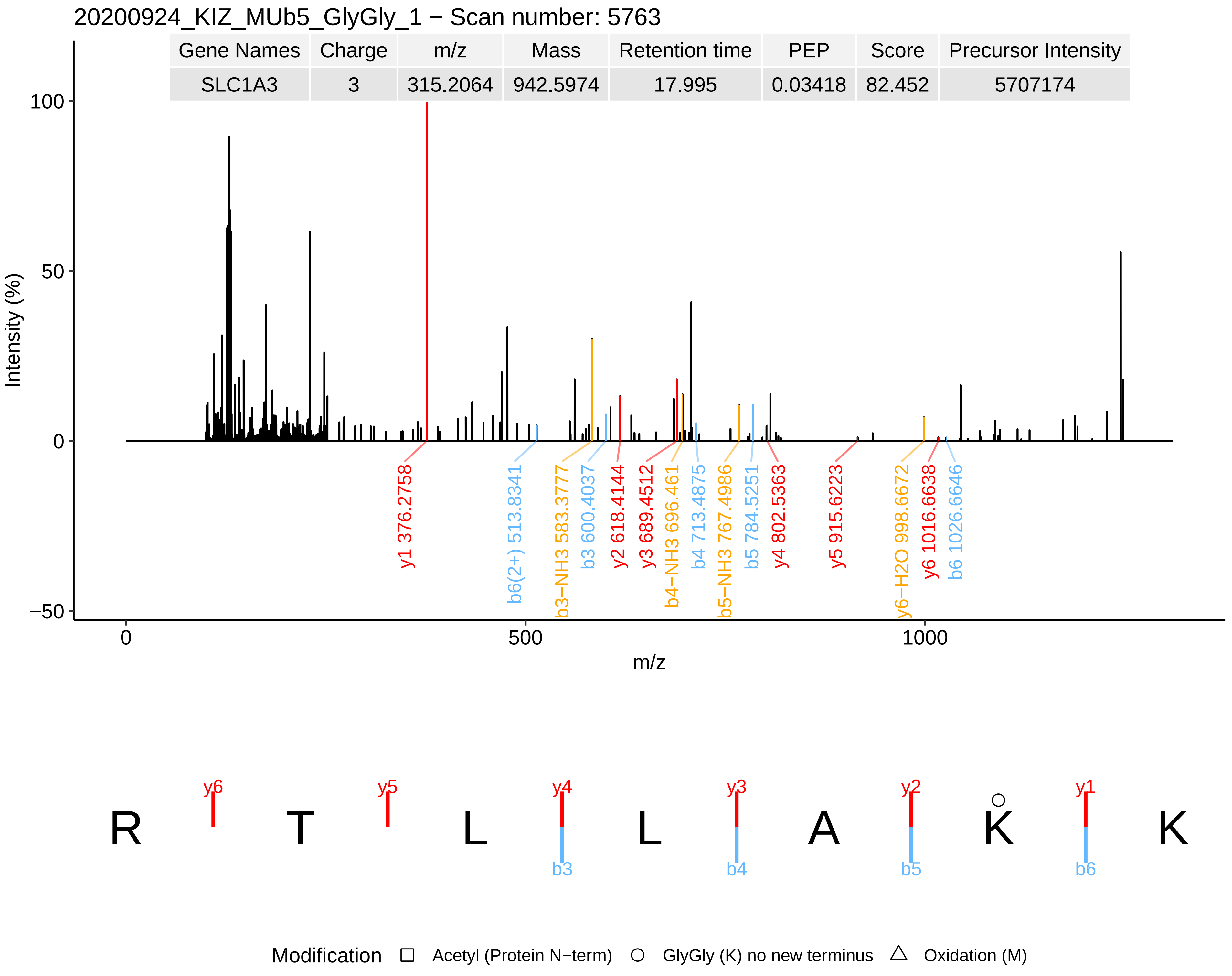

20200924_KIZ_MUb5_GlyGly_1 − Scan number: 5763
Gene Names | Charge | m/z | Mass | Retention time | PEP | Score | Precursor Intensity
SLC1A3 | 3 | 315.2064 | 942.5974 | 17.995 | 0.03418 | 82.452 | 5707174
Intensity (%)
m/z
y1 376.2758
b6(2+) 513.8341
b3−NH3 583.3777
b3 600.4037
y2 618.4144
y3 689.4512
b4−NH3 696.461
b4 713.4875
b5−NH3 767.4986
b5 784.5251
y4 802.5363
y5 915.6223
y6−H2O 998.6672
y6 1016.6638
b6 1026.6646
R T L L A K K
y6 y5 y4 y3 y2 y1
b3 b4 b5 b6
Modification
Acetyl (Protein N−term)
GlyGly (K) no new terminus
Oxidation (M)

20201118_KIZ_MUb6_GlyGly_1 − Scan number: 5400

Gene Names	Charge	m/z	Mass	Retention time	PEP	Score	Precursor Intensity
SLC1A3	3	315.2064	942.5974	17.093	0.028721	82.452	13846010

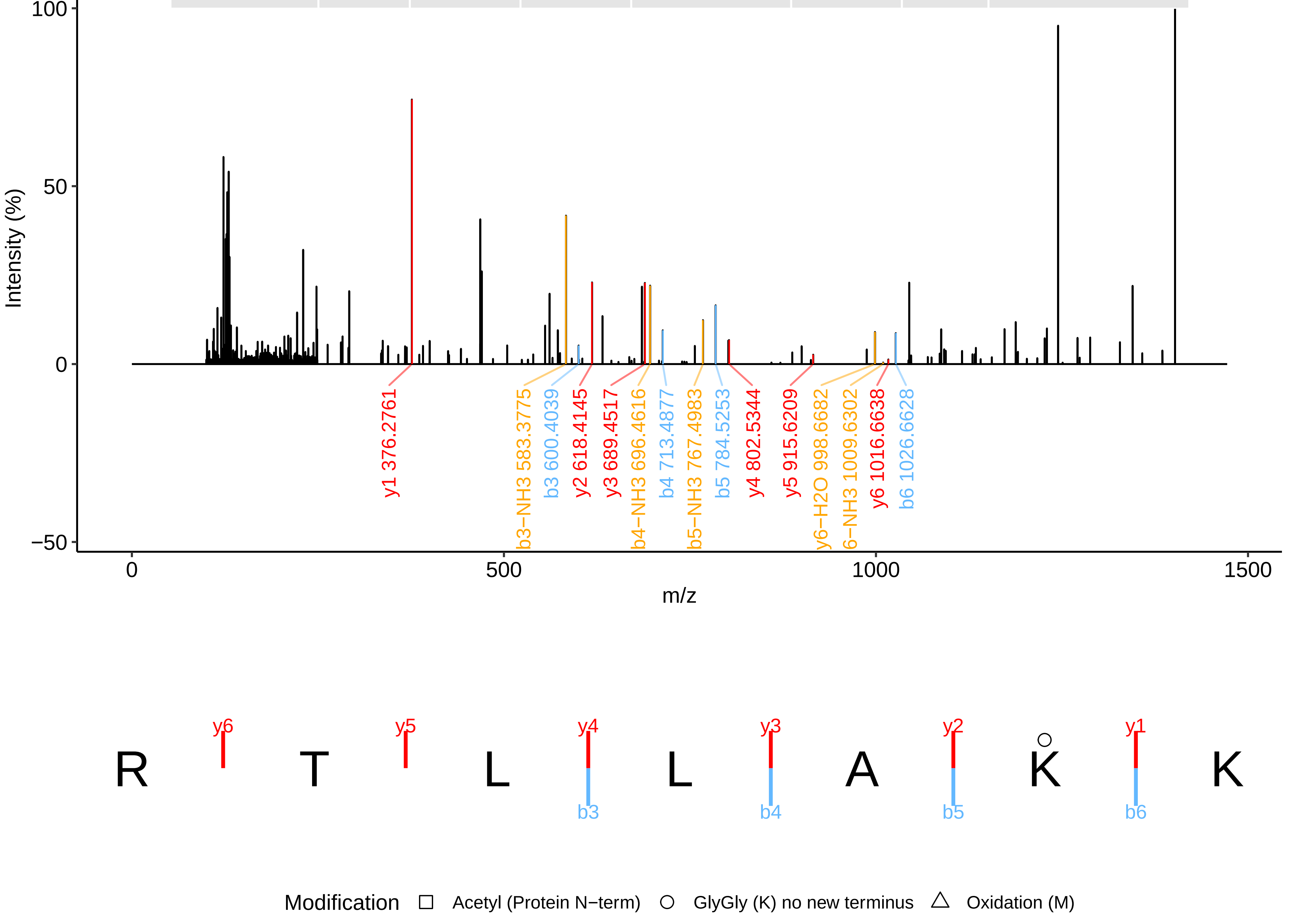

20200921_KIZ_MUb4_GlyGly_10plex − Scan number: 11078

Gene Names	Charge	m/z	Mass	Retention time	PEP	Score	Precursor Intensity
HSPD1	3	311.5064	931.4975	38.546	0.050993	68.422	8000114

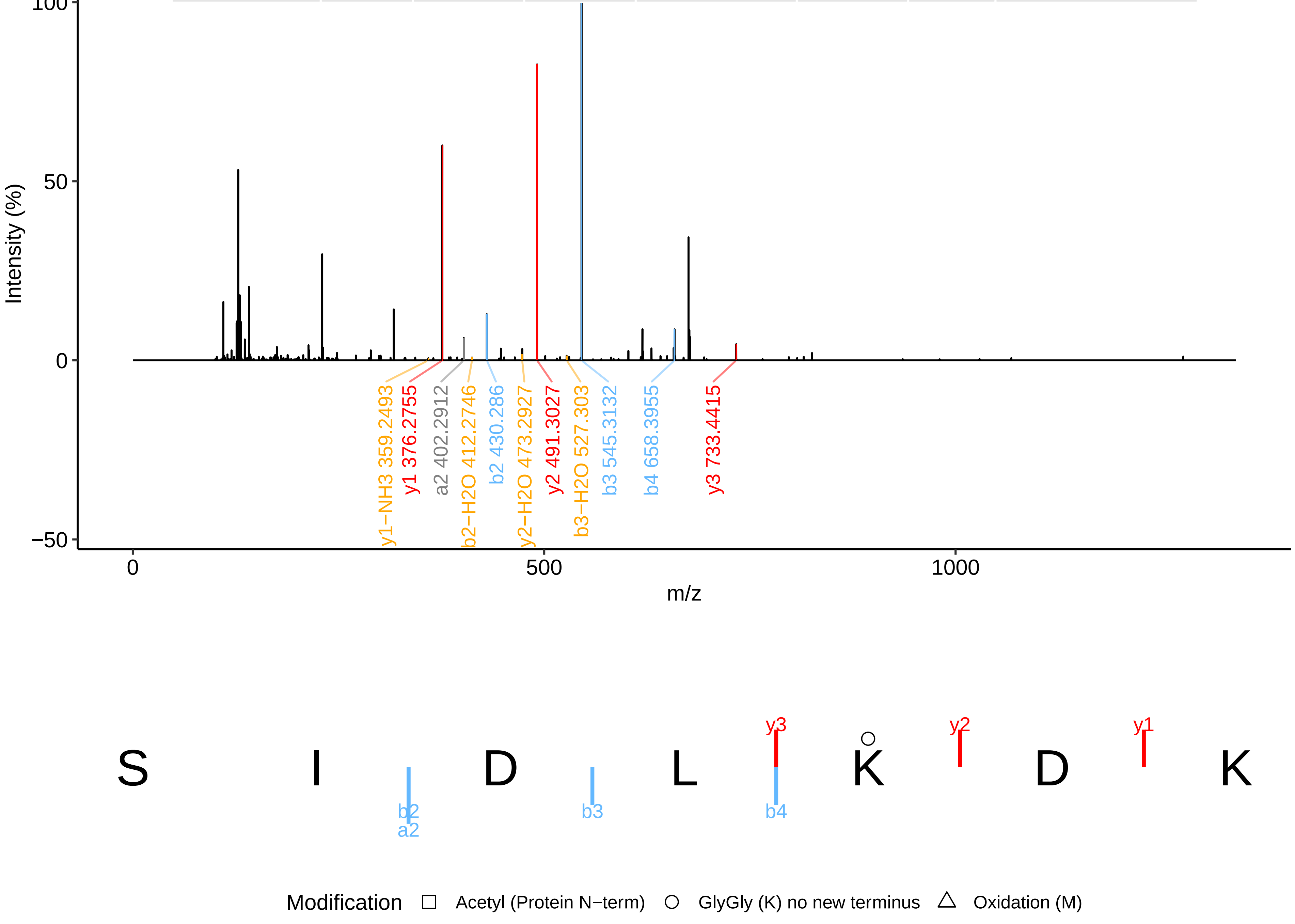

20200921_KIZ_MUb4_GlyGly_10plex − Scan number: 8248

Gene Names	Charge	m/z	Mass	Retention time	PEP	Score	Precursor Intensity
TBC1D15	3	396.5508	1186.631	27.939	0.001253	106.42	6276169

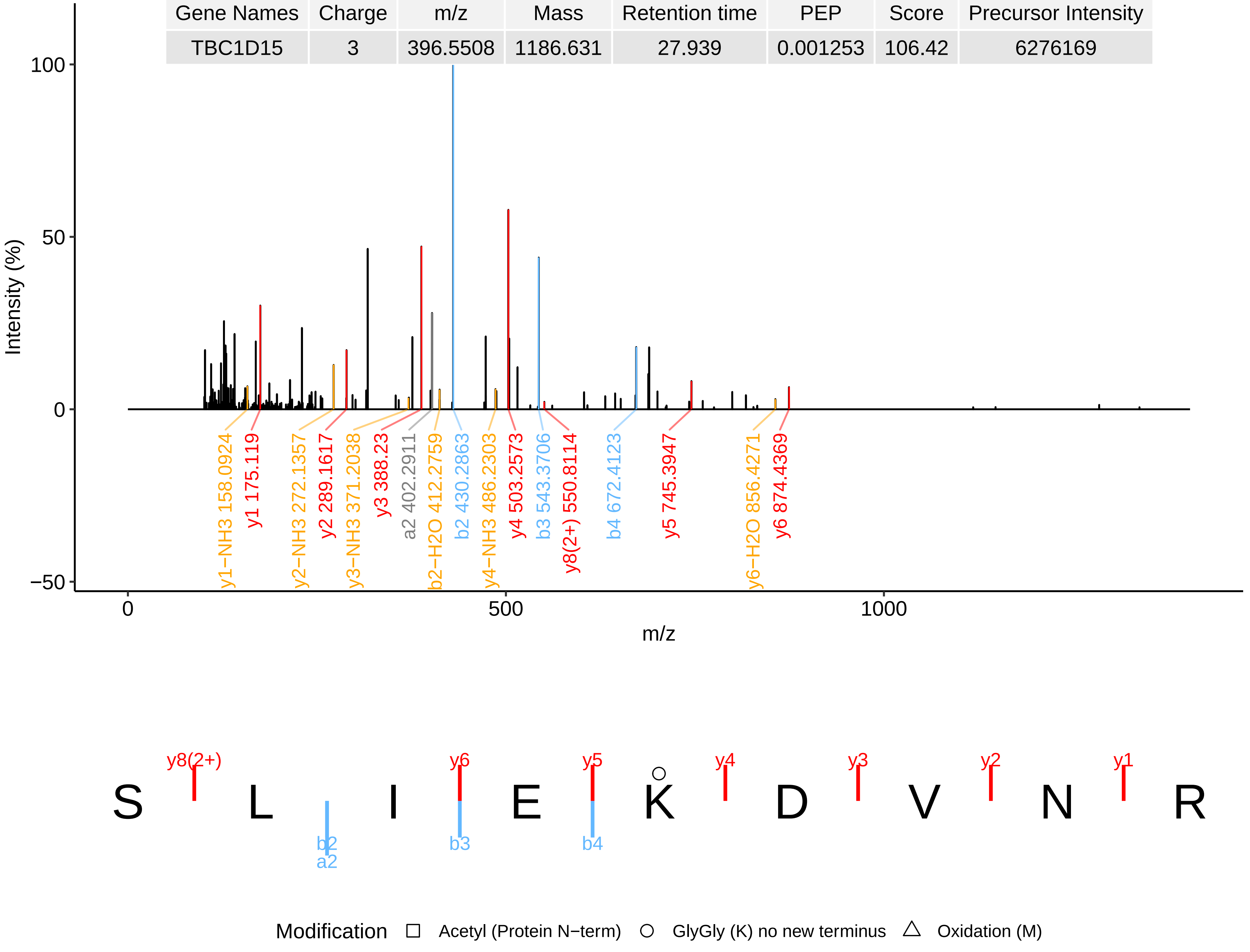

20200921_KIZ_MUb4_GlyGly_10plex_2 − Scan number: 7588

Gene Names	Charge	m/z	Mass	Retention time	PEP	Score	Precursor Intensity
TBC1D15	3	396.5508	1186.631	28.364	0.0056419	83.783	910039.9

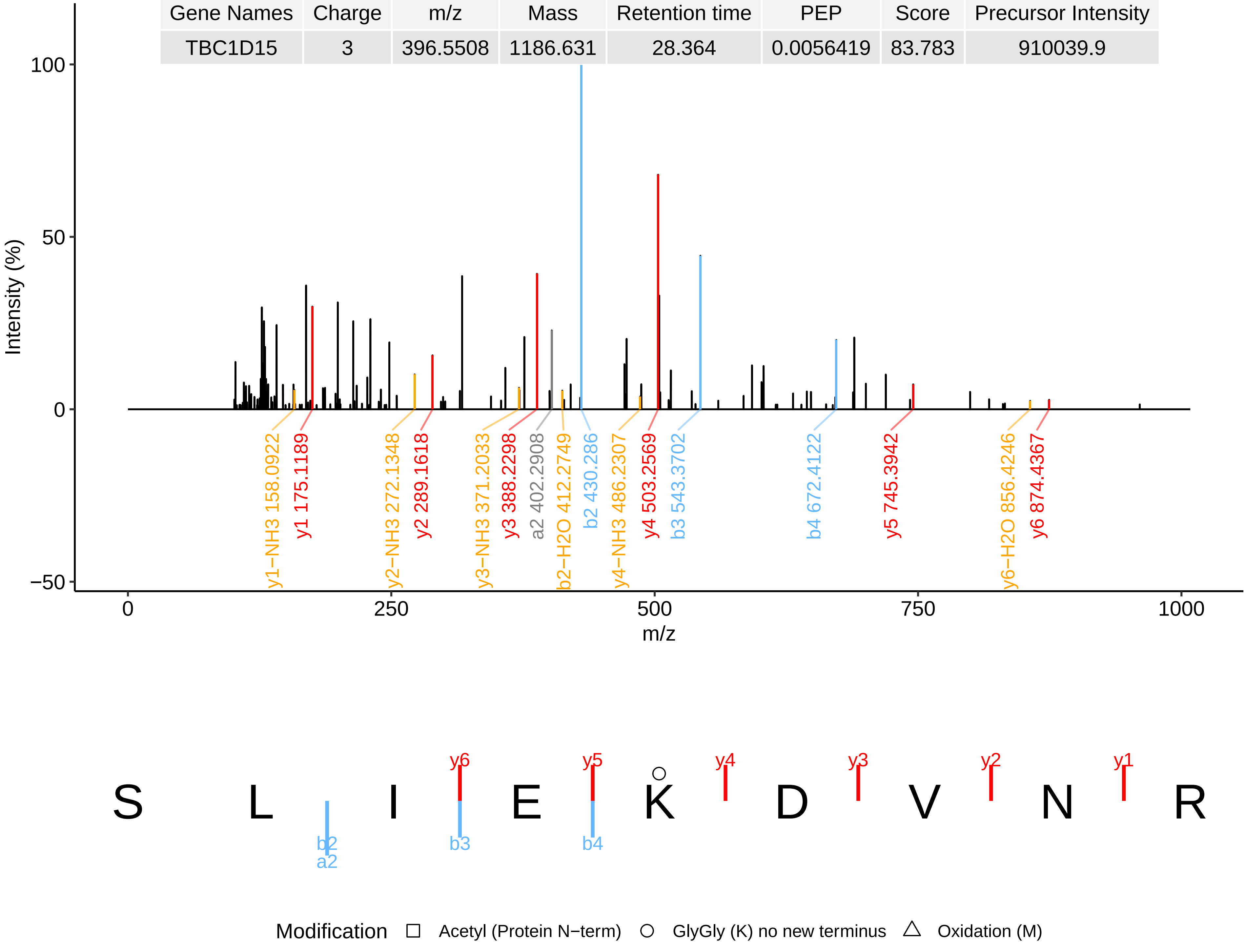

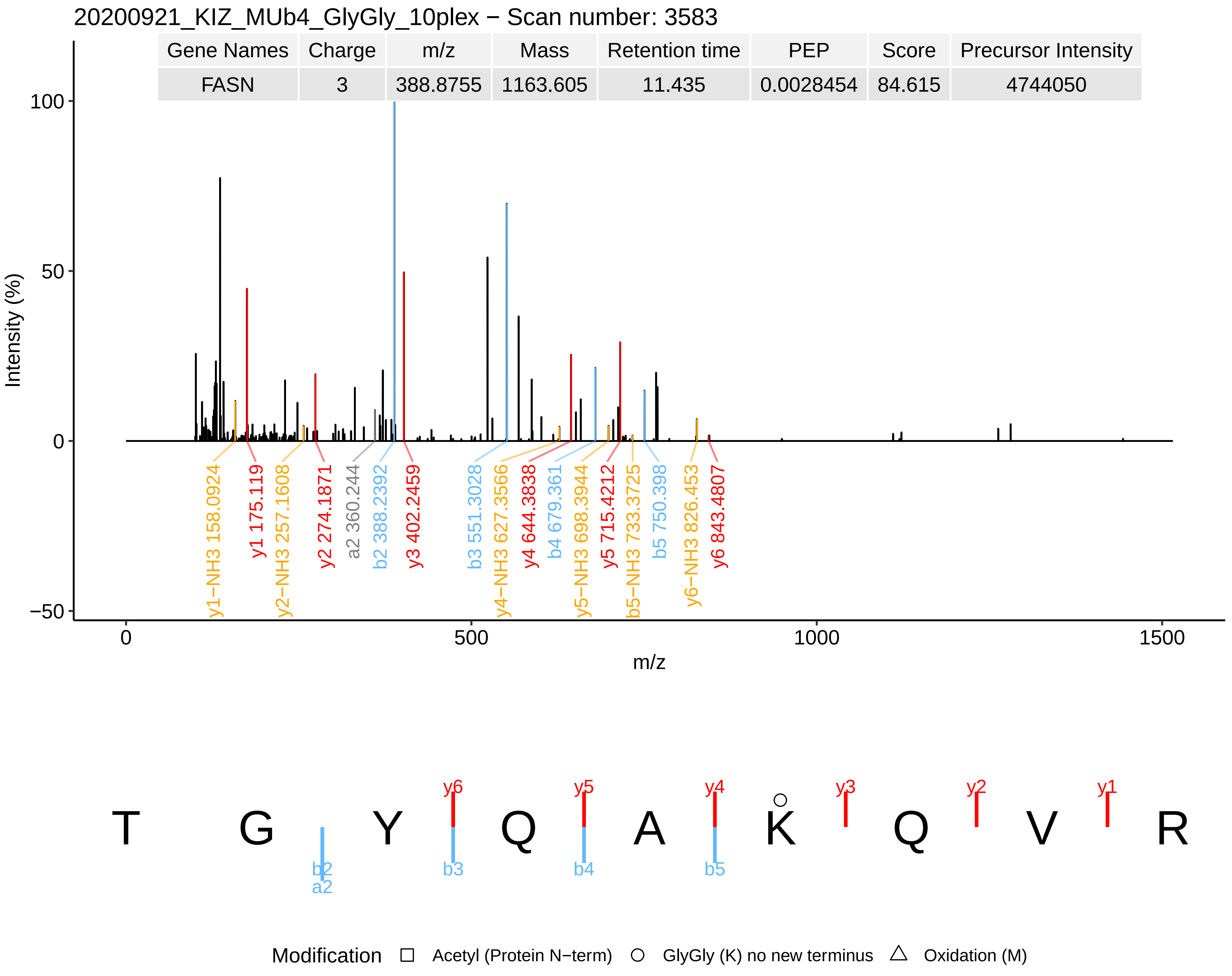
20200921_KIZ_MUb4_GlyGly_10plex − Scan number: 3583
Gene Names
Charge
m/z
Mass
Retention time
PEP
Score
Precursor Intensity
FASN
3
388.8755
1163.605
11.435
0.0028454
84.615
4744050
Intensity (%)
100
50
0
−50
0
500
1000
1500
m/z
y1−NH3 158.0924
y1 175.119
y2−NH3 257.1608
y2 274.1871
a2 360.244
b2 388.2392
y3 402.2459
b3 551.3028
y4−NH3 627.3566
y4 644.3838
b4 679.361
y5−NH3 698.3944
y5 715.4212
b5−NH3 733.3725
b5 750.398
y6−NH3 826.453
y6 843.4807
T G Y Q A K Q V R
y6 y5 y4 y3 y2 y1
b2 a2 b3 b4 b5
Modification
Acetyl (Protein N−term)
GlyGly (K) no new terminus
Oxidation (M)

20200921_KIZ_MUb4_GlyGly_10plex − Scan number: 5720

Gene Names	Charge	m/z	Mass	Retention time	PEP	Score	Precursor Intensity
PTRF	3	325.8417	974.5033	18.759	0.053447	67.464	5677660

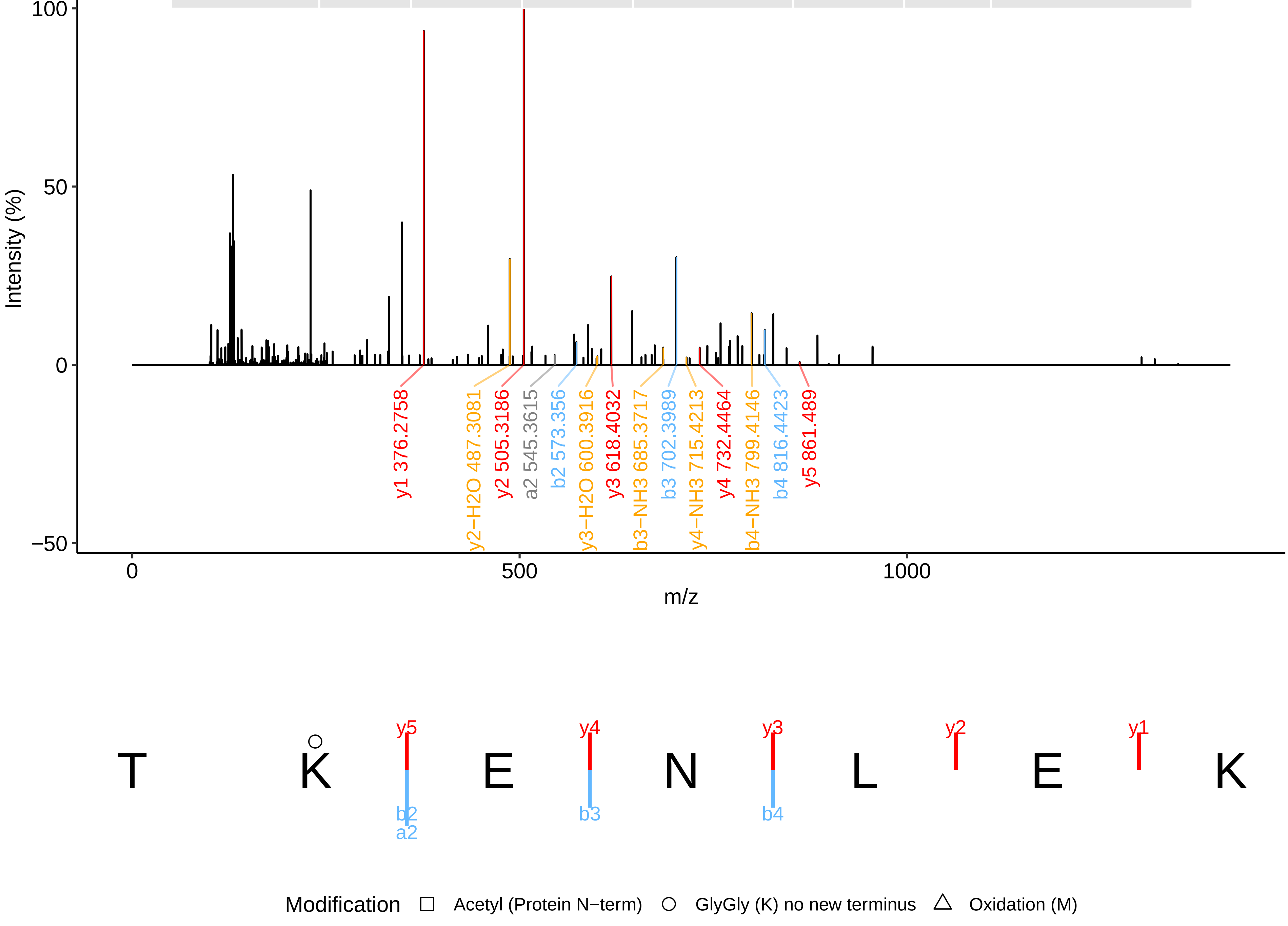

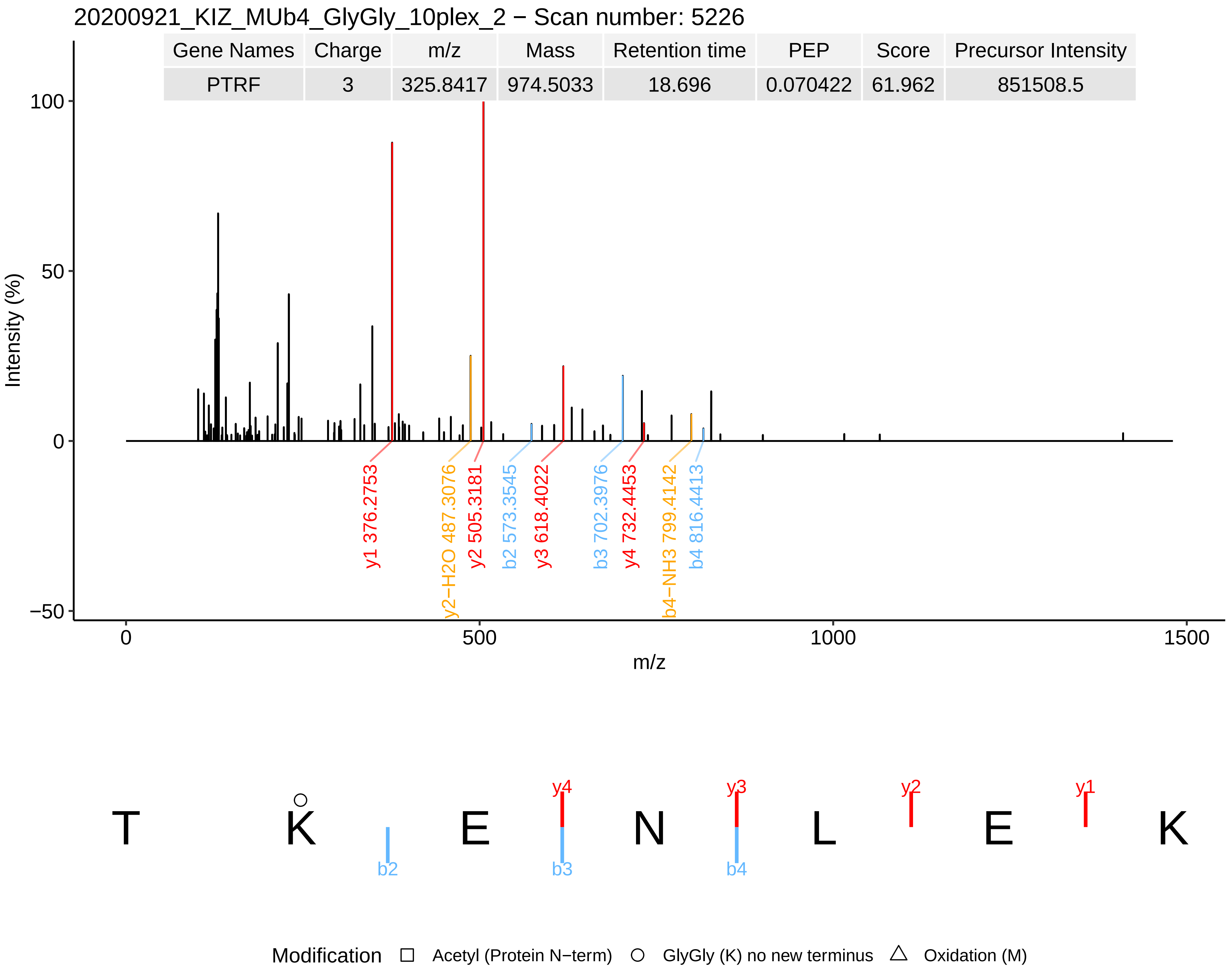
20200921_KIZ_MUb4_GlyGly_10plex_2 − Scan number: 5226
Gene Names | Charge | m/z | Mass | Retention time | PEP | Score | Precursor Intensity
PTRF | 3 | 325.8417 | 974.5033 | 18.696 | 0.070422 | 61.962 | 851508.5
Intensity (%)
m/z
y1 376.2753
y2−H2O 487.3076
y2 505.3181
b2 573.3545
y3 618.4022
b3 702.3976
y4 732.4453
b4−NH3 799.4142
b4 816.4413
T K E N L E K
y4 y3 y2 y1
b2 b3 b4
Modification
Acetyl (Protein N−term)
GlyGly (K) no new terminus
Oxidation (M)

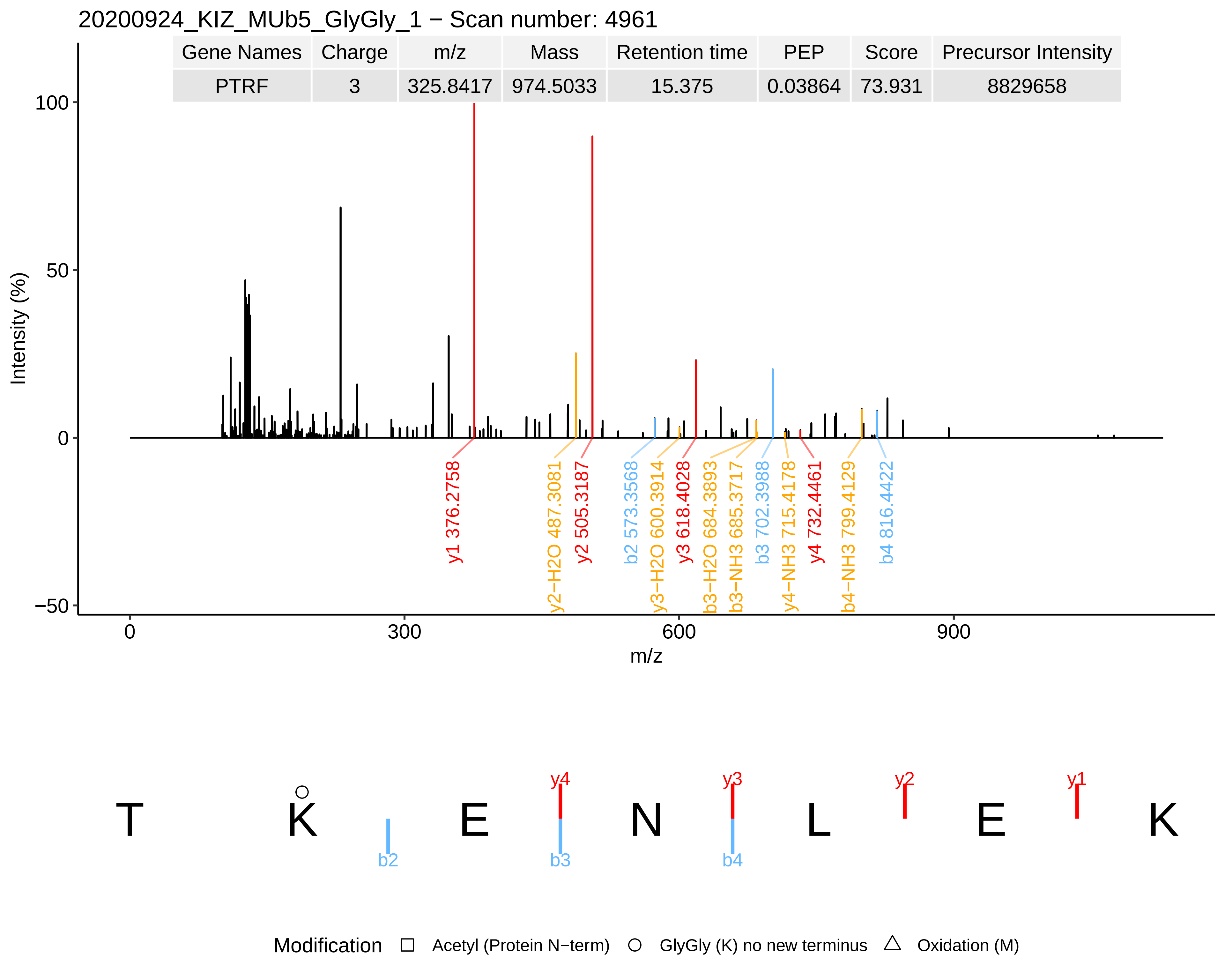

20200924_KIZ_MUb5_GlyGly_1 − Scan number: 4961

Gene Names	Charge	m/z	Mass	Retention time	PEP	Score	Precursor Intensity
PTRF	3	325.8417	974.5033	15.375	0.03864	73.931	8829658

Intensity (%)
m/z
y1 376.2758
y2−H2O 487.3081
y2 505.3187
b2 573.3568
y3−H2O 600.3914
y3 618.4028
b3−H2O 684.3893
b3−NH3 685.3717
b3 702.3988
y4−NH3 715.4178
y4 732.4461
b4−NH3 799.4129
b4 816.4422
T K E N L E K
y4 y3 y2 y1
b2 b3 b4
Modification
Acetyl (Protein N−term)
GlyGly (K) no new terminus
Oxidation (M)

20200921_KIZ_MUb4_GlyGly_10plex − Scan number: 5910

Gene Names	Charge	m/z	Mass	Retention time	PEP	Score	Precursor Intensity
PPP2CB	3	321.8358	962.4855	19.385	0.13463	54.408	6264693

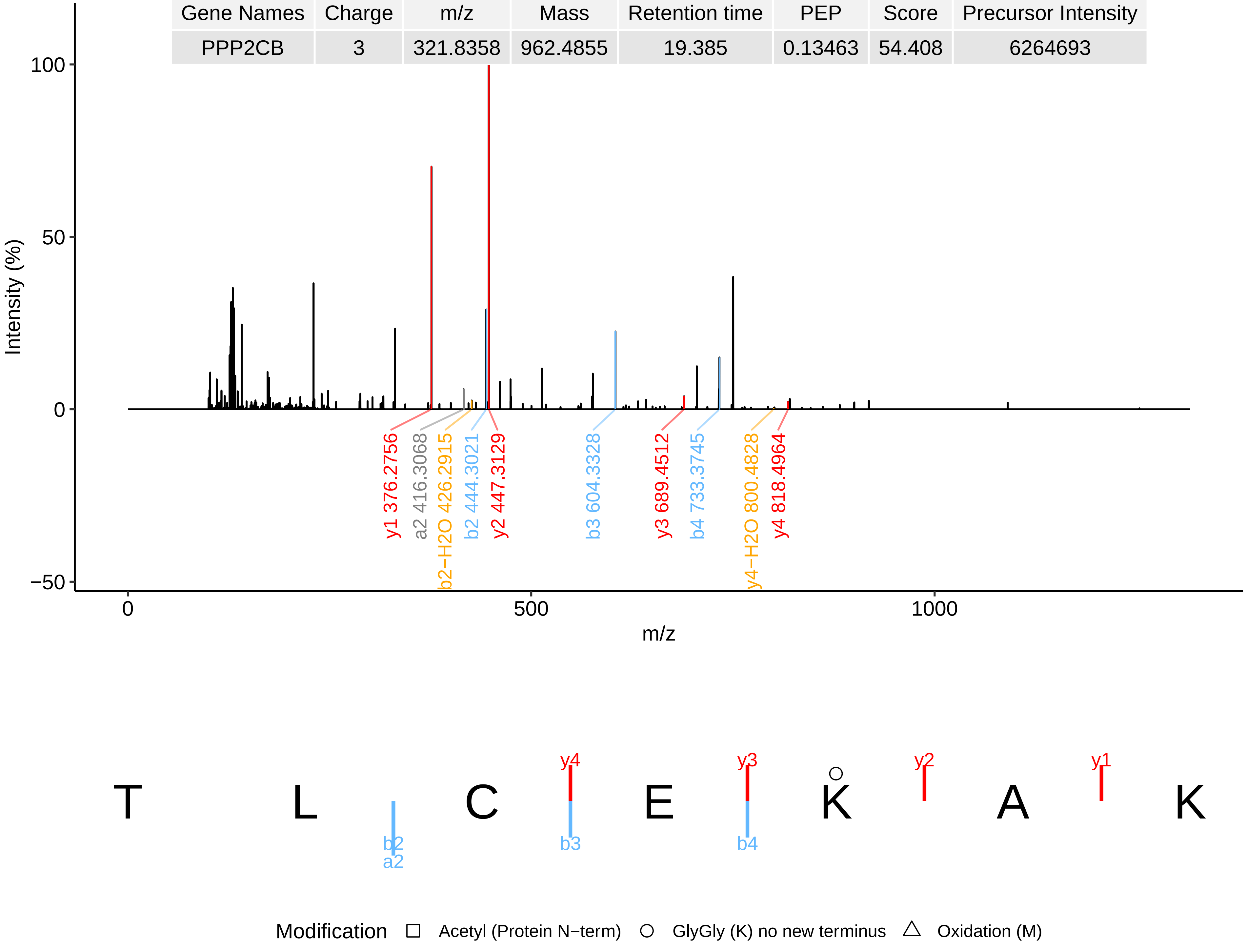

20200921_KIZ_MUb4_GlyGly_10plex_2 − Scan number: 7639

Gene Names	Charge	m/z	Mass	Retention time	PEP	Score	Precursor Intensity
BRCC3	3	401.2227	1200.646	28.572	0.01298	70.399	795315.1

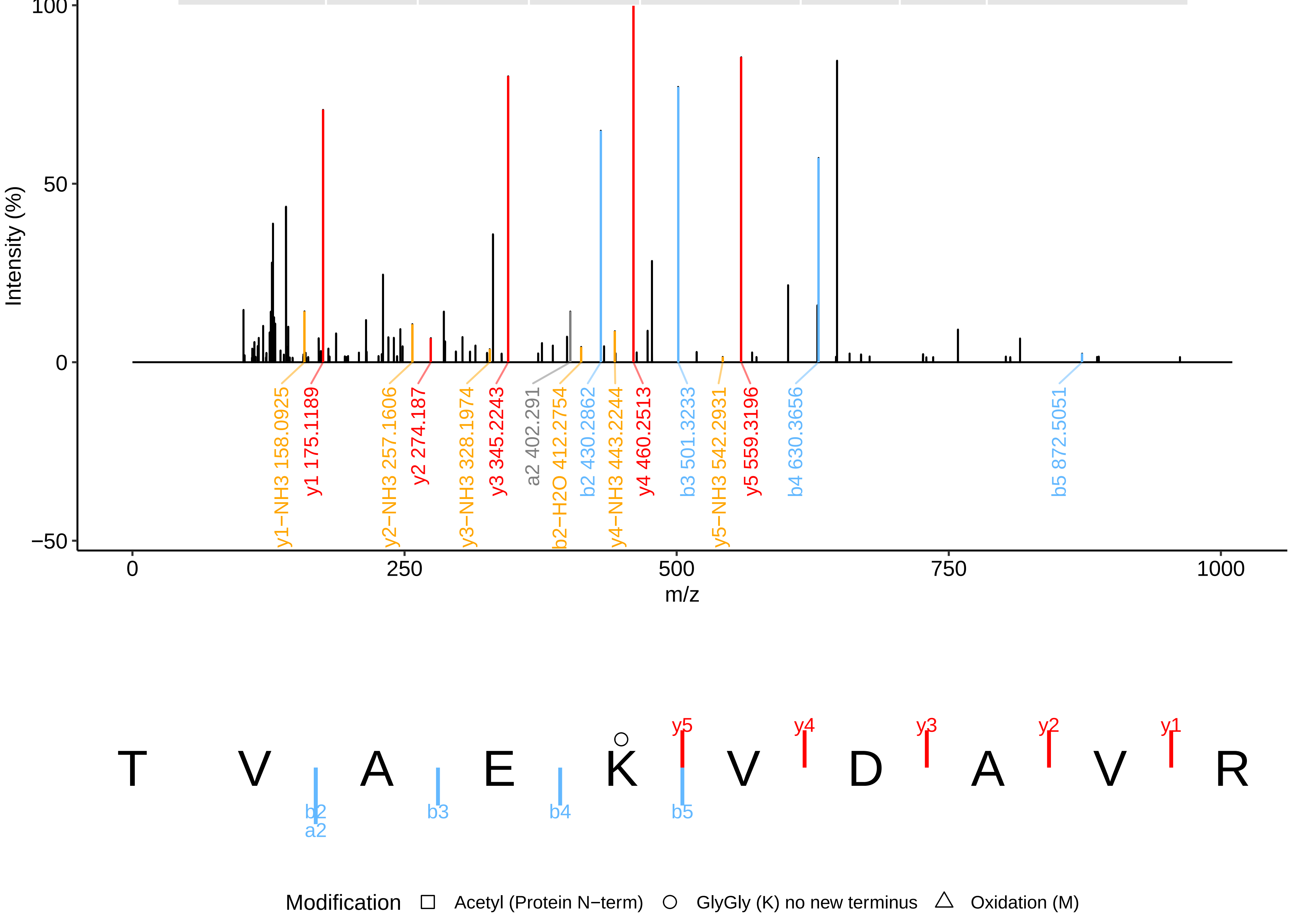

20200921_KIZ_MUb4_GlyGly_10plex − Scan number: 13569

Gene Names	Charge	m/z	Mass	Retention time	PEP	Score	Precursor Intensity
SLC43A3	2	602.3039	1202.593	47.915	0.0087251	81.296	5627181

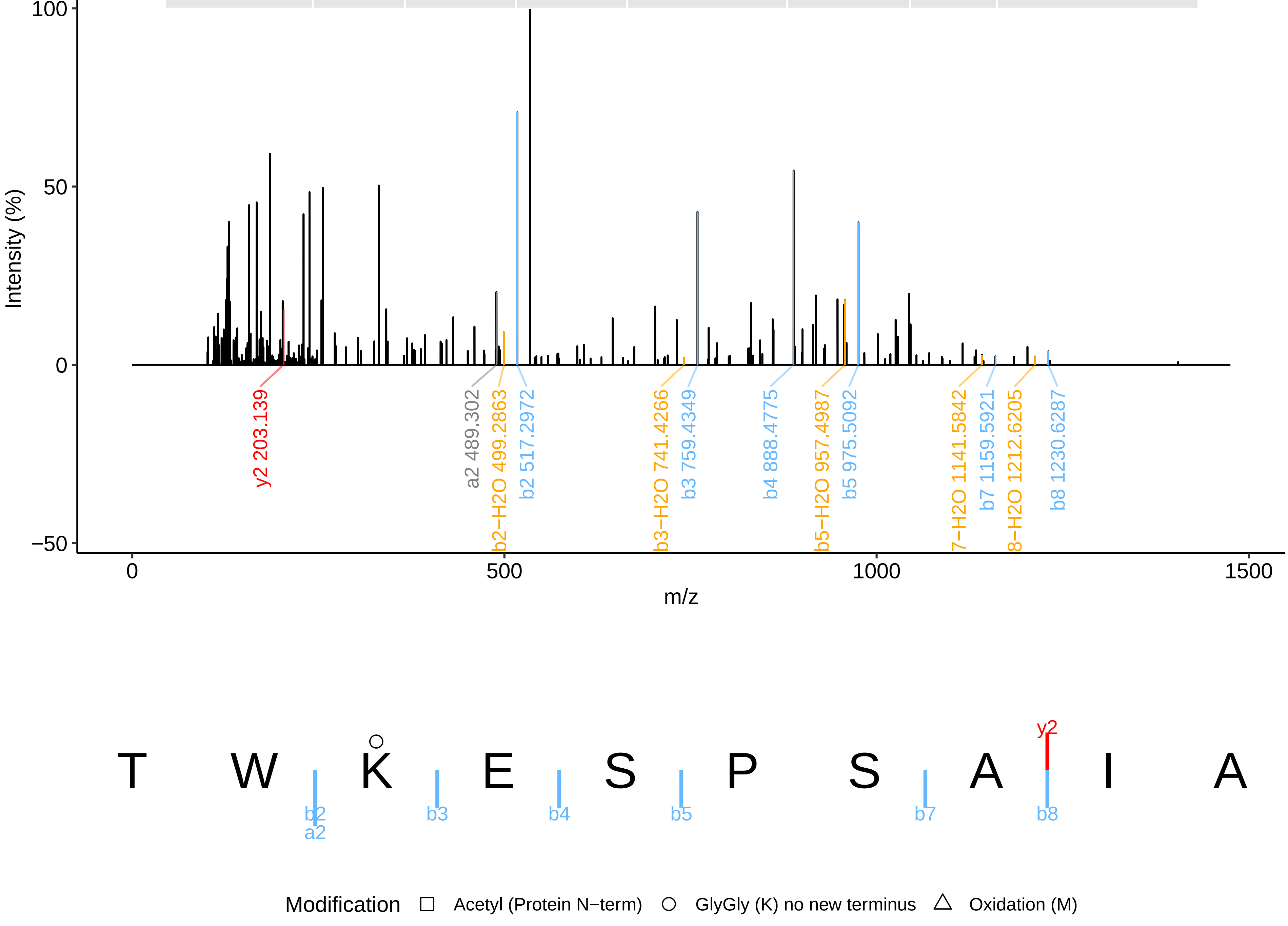

20200921_KIZ_MUb4_GlyGly_10plex − Scan number: 11594

Gene Names	Charge	m/z	Mass	Retention time	PEP	Score	Precursor Intensity
ATP6V0A1	3	399.9069	1196.699	40.454	0.00046486	95.311	6628784

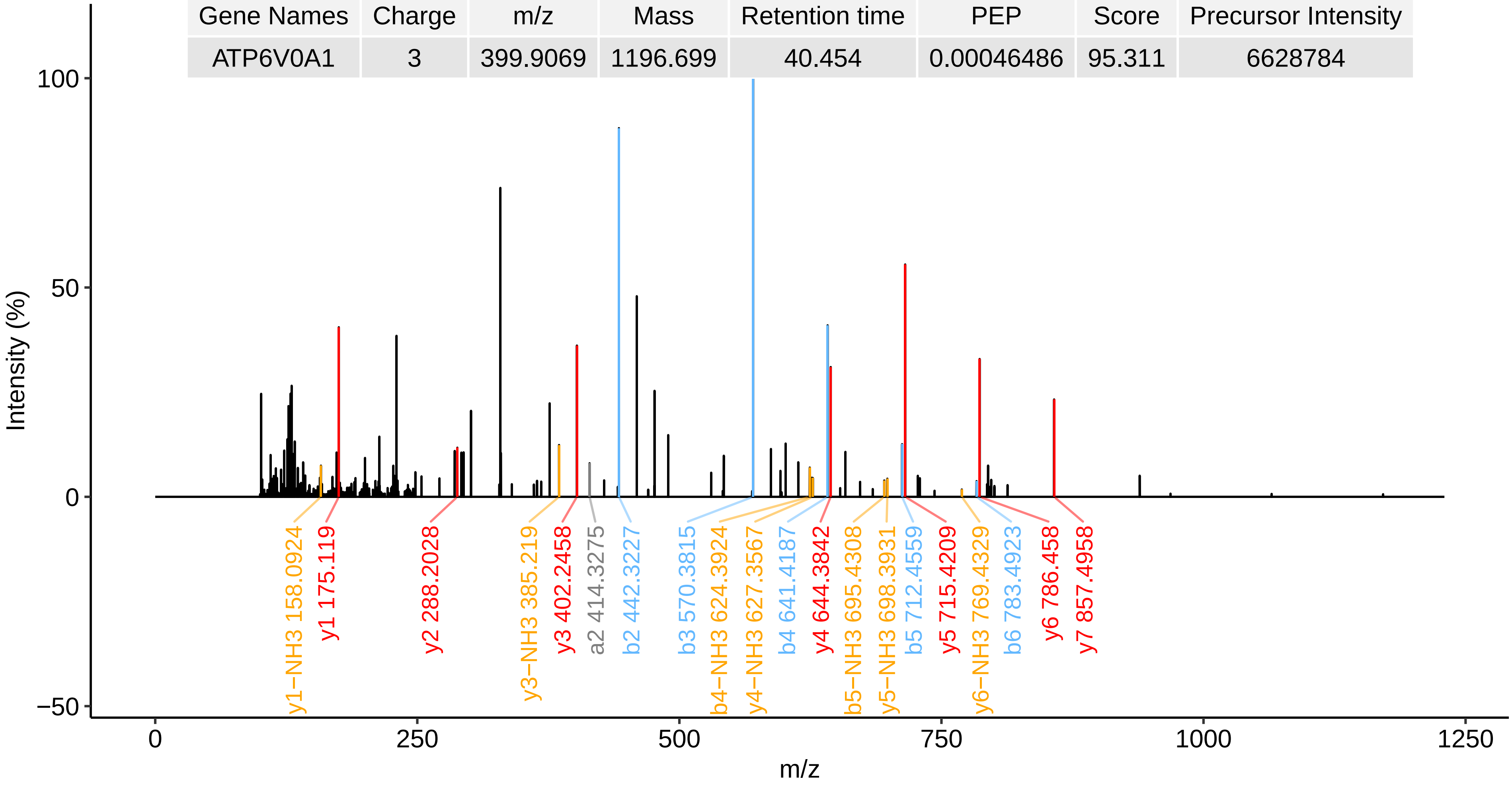

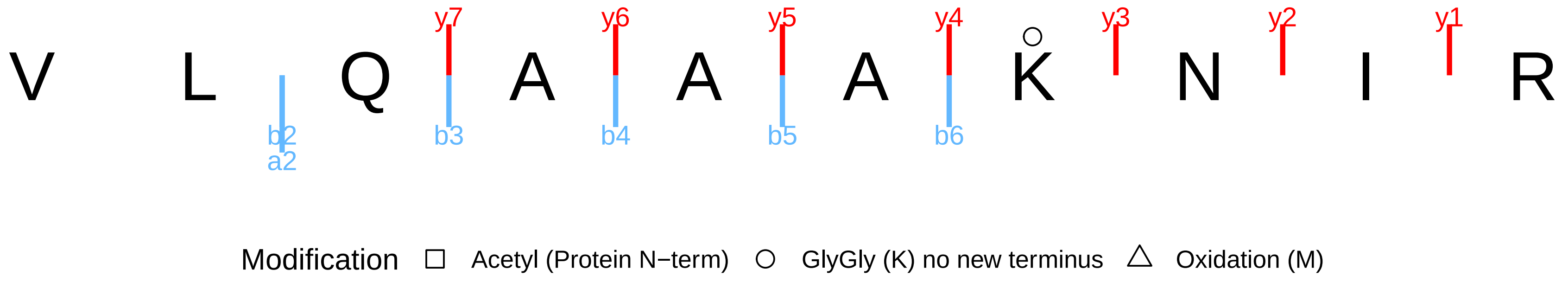

20200921_KIZ_MUb4_GlyGly_10plex − Scan number: 13958

Gene Names	Charge	m/z	Mass	Retention time	PEP	Score	Precursor Intensity
VAMP3;VAMP2;VAMP1	3	395.8911	1184.651	49.427	0.0012001	94.297	4609337

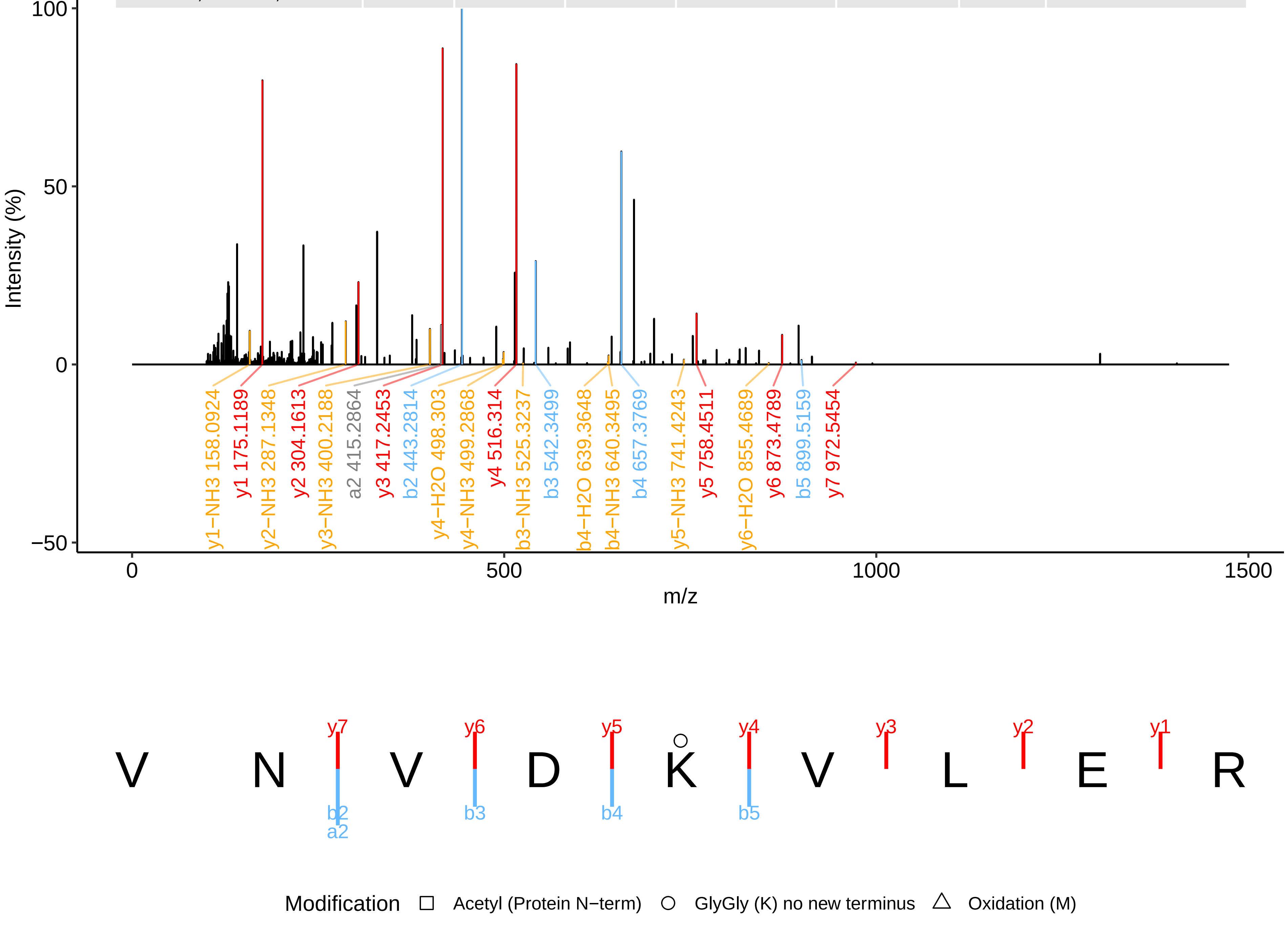

20200921_KIZ_MUb4_GlyGly_10plex_2 − Scan number: 12827

Gene Names	Charge	m/z	Mass	Retention time	PEP	Score	Precursor Intensity
VAMP3;VAMP2;VAMP1	3	395.8911	1184.651	49.714	0.01634	68.557	NA

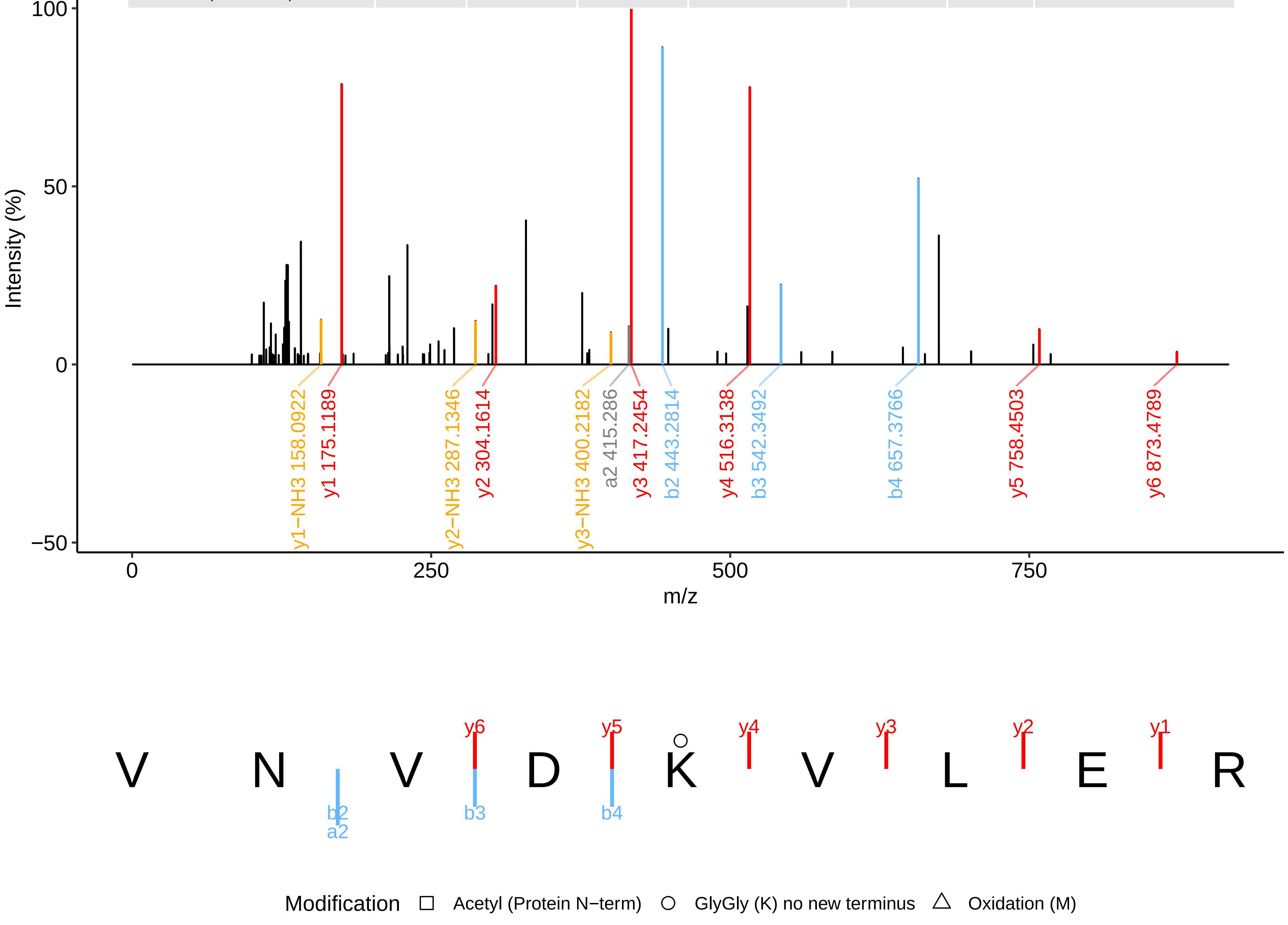

20200921_KIZ_MUb4_GlyGly_10plex − Scan number: 5946

Gene Names	Charge	m/z	Mass	Retention time	PEP	Score	Precursor Intensity
BST2	3	387.517	1159.529	19.513	0.0021421	105.1	7518939

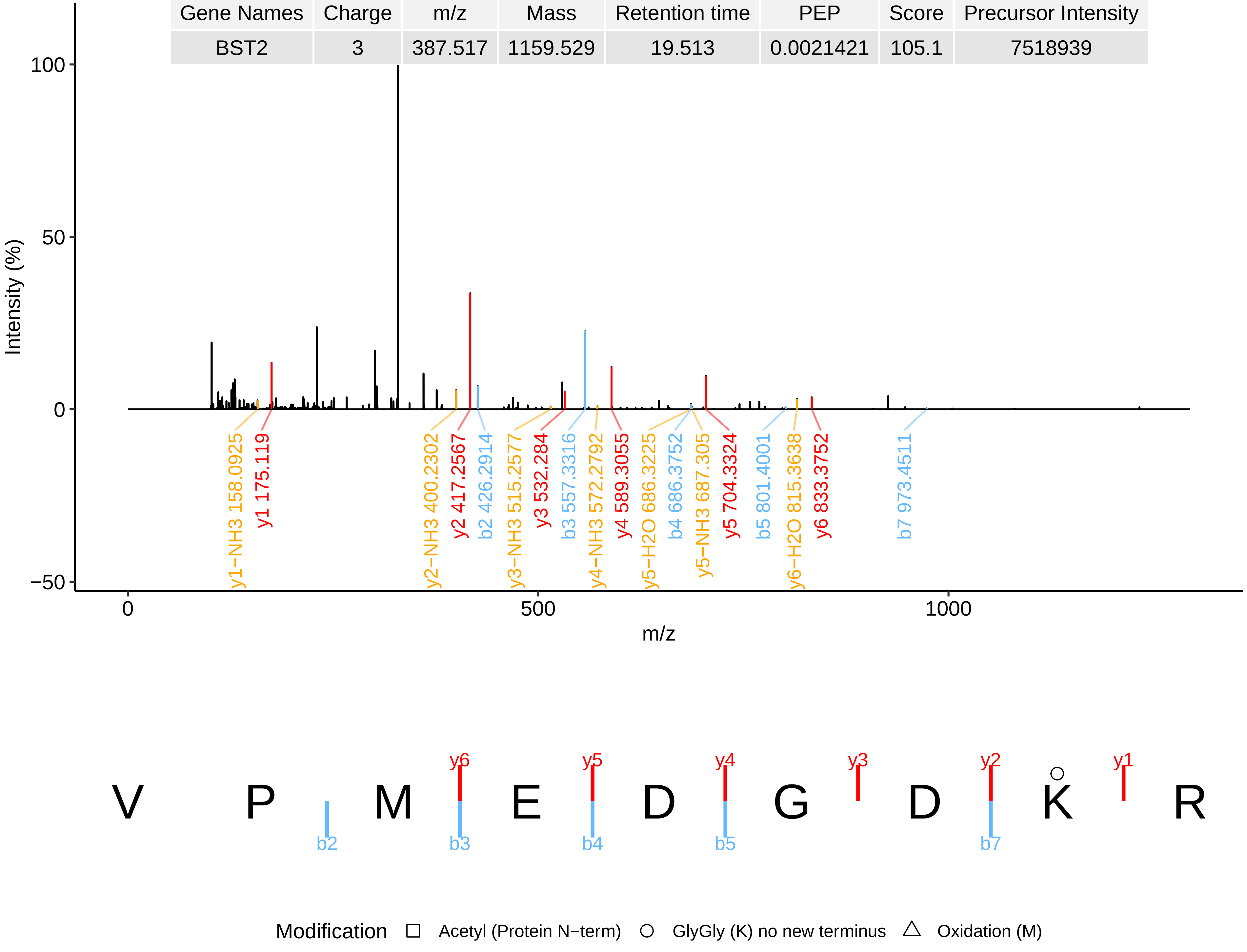

20200921_KIZ_MUb4_GlyGly_10plex_2 − Scan number: 5404

Gene Names	Charge	m/z	Mass	Retention time	PEP	Score	Precursor Intensity
BST2	3	387.517	1159.529	19.411	0.032691	78.934	1000005

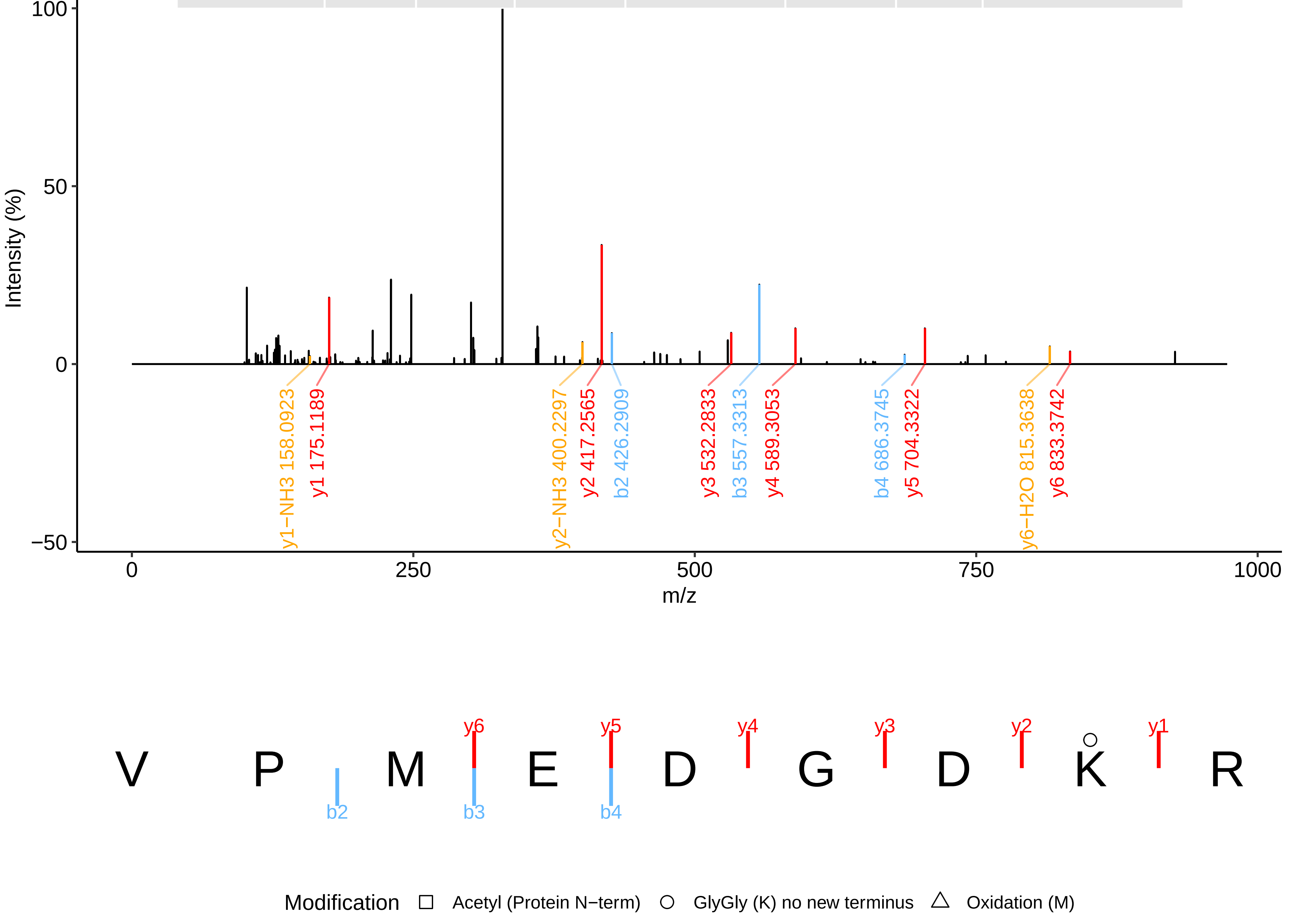

20200924_KIZ_MUb5_GlyGly_1 − Scan number: 5168

Gene Names	Charge	m/z	Mass	Retention time	PEP	Score	Precursor Intensity
BST2	3	387.517	1159.529	16.044	0.021031	81.338	10079704

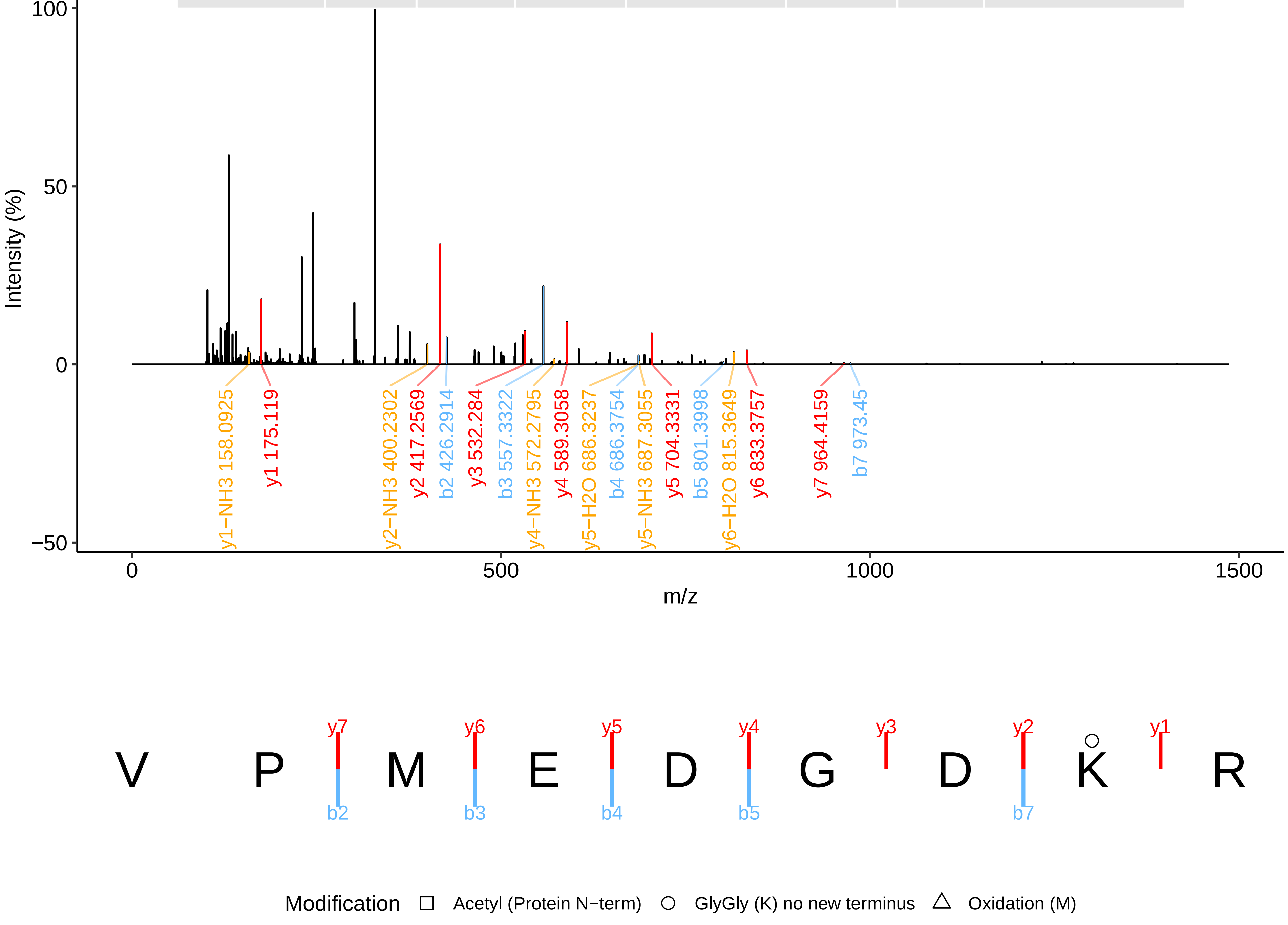

20200921_KIZ_MUb4_GlyGly_10plex − Scan number: 10890

Gene Names	Charge	m/z	Mass	Retention time	PEP	Score	Precursor Intensity
PPP2R1A	3	391.8911	1172.651	37.838	0.019121	64.374	17647250

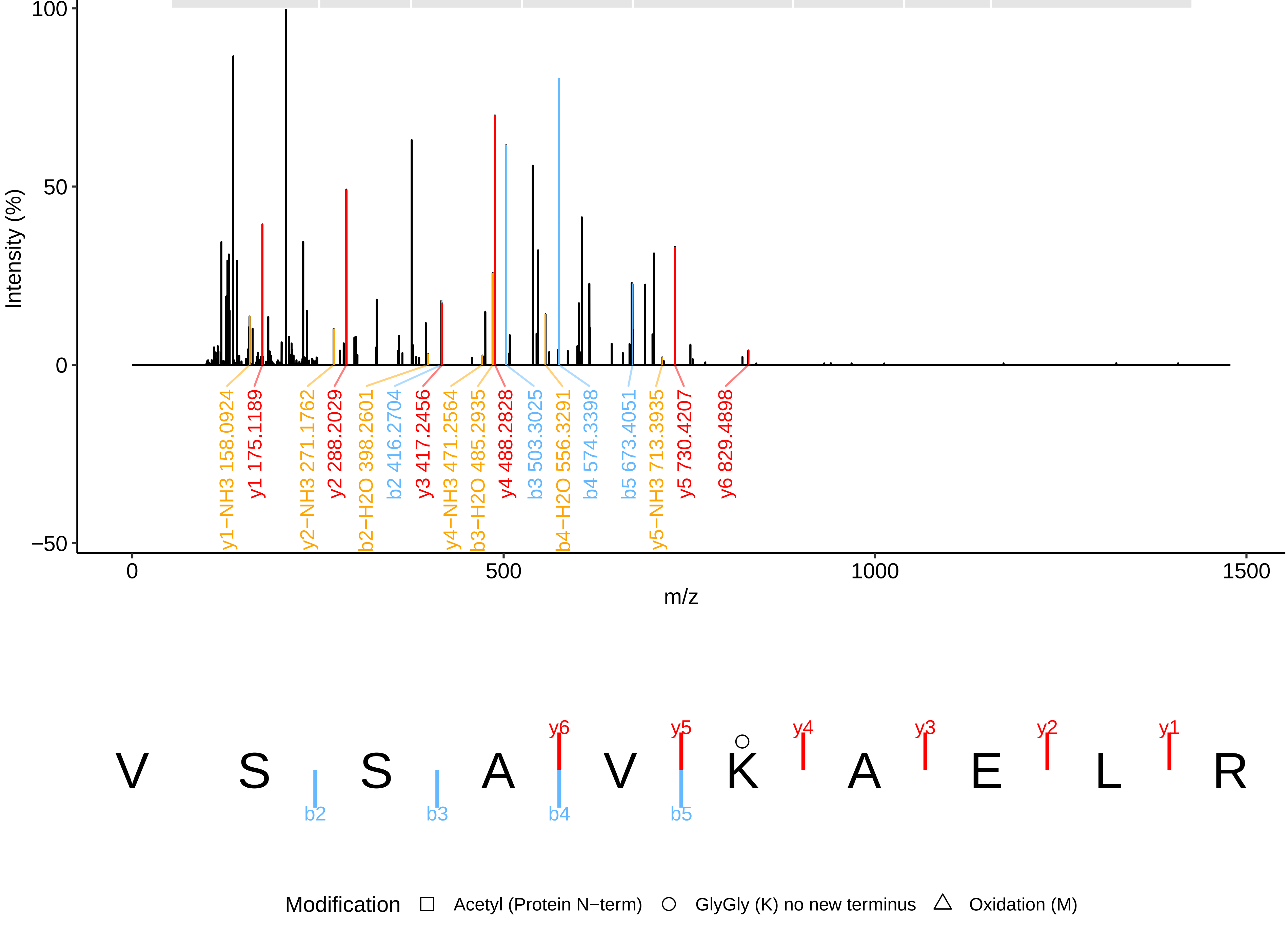

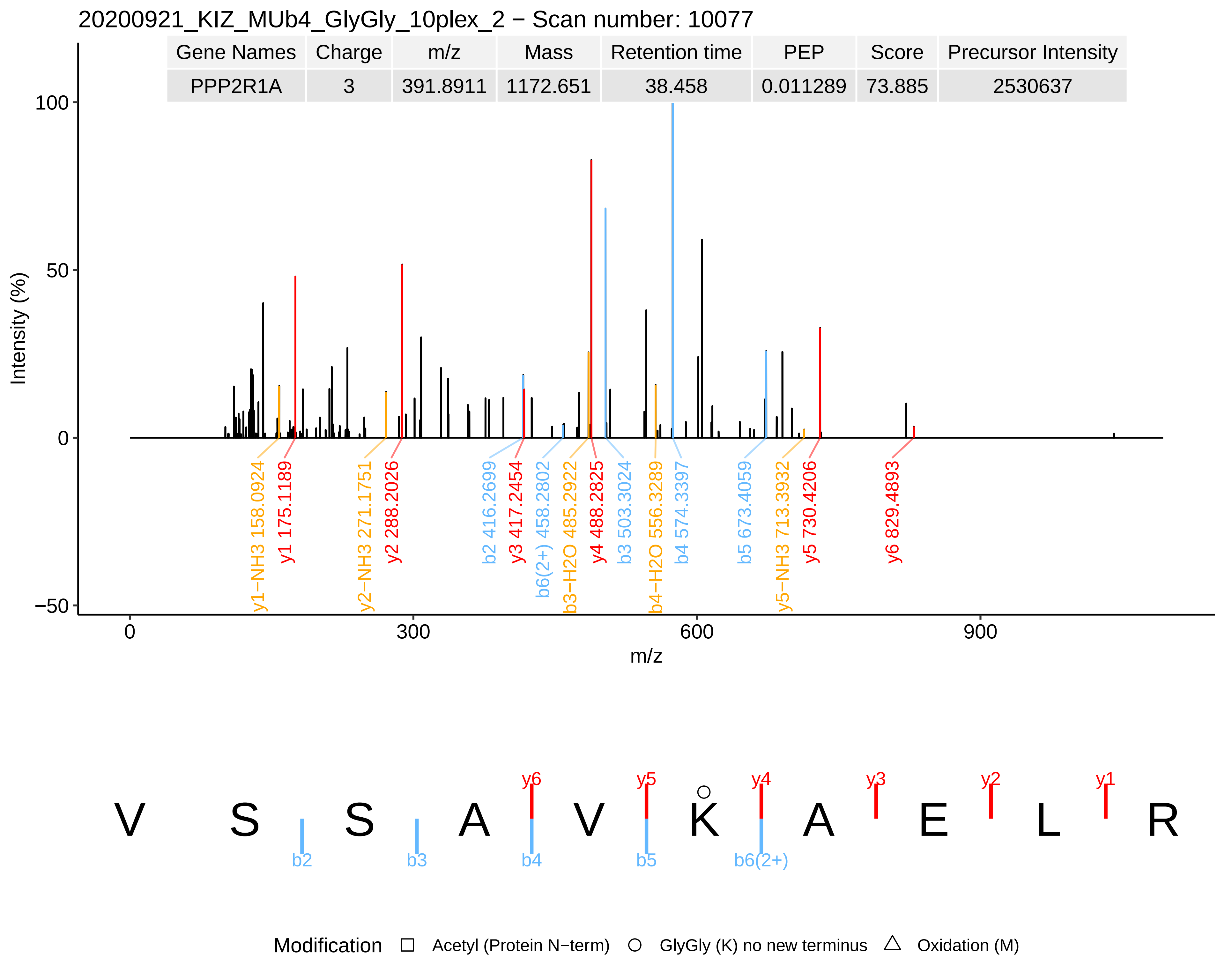

20200921_KIZ_MUb4_GlyGly_10plex_2 − Scan number: 10077
Gene Names | Charge | m/z | Mass | Retention time | PEP | Score | Precursor Intensity
PPP2R1A | 3 | 391.8911 | 1172.651 | 38.458 | 0.011289 | 73.885 | 2530637
Intensity (%)
m/z
y1−NH3 158.0924
y1 175.1189
y2−NH3 271.1751
y2 288.2026
b2 416.2699
y3 417.2454
b6(2+) 458.2802
b3−H2O 485.2922
y4 488.2825
b3 503.3024
b4−H2O 556.3289
b4 574.3397
b5 673.4059
y5−NH3 713.3932
y5 730.4206
y6 829.4893
V S S A V K A E L R
y6 y5 y4 y3 y2 y1
b2 b3 b4 b5 b6(2+)
Modification
Acetyl (Protein N−term)
GlyGly (K) no new terminus
Oxidation (M)

20200921_KIZ_MUb4_GlyGly_10plex − Scan number: 5742

Gene Names	Charge	m/z	Mass	Retention time	PEP	Score	Precursor Intensity
KRT8	3	323.1784	966.5135	18.832	0.06456	63.486	4453883

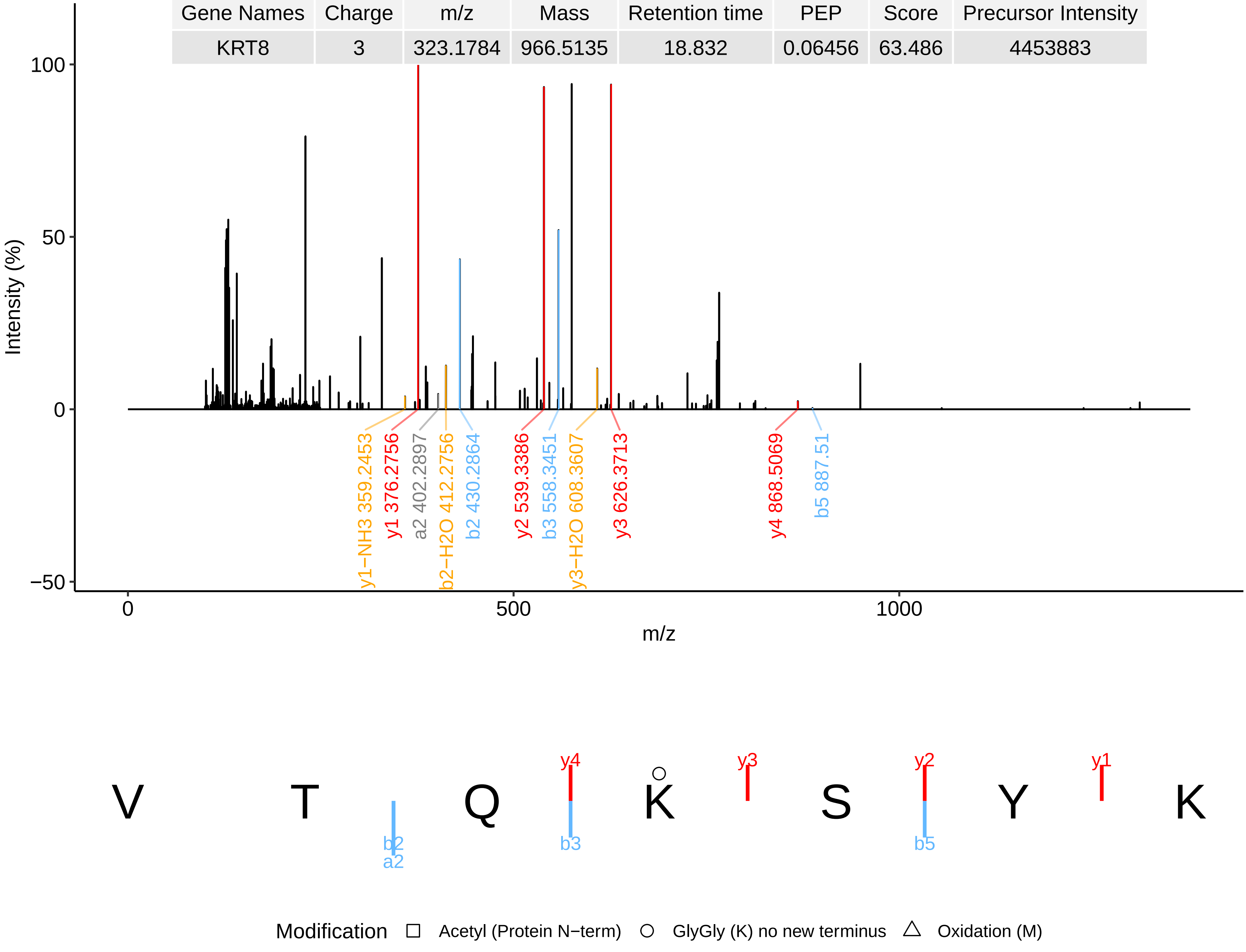

20200924_KIZ_MUb5_GlyGly_1 − Scan number: 4988

Gene Names	Charge	m/z	Mass	Retention time	PEP	Score	Precursor Intensity
KRT8	3	323.1784	966.5135	15.475	0.063656	63.48	9981230

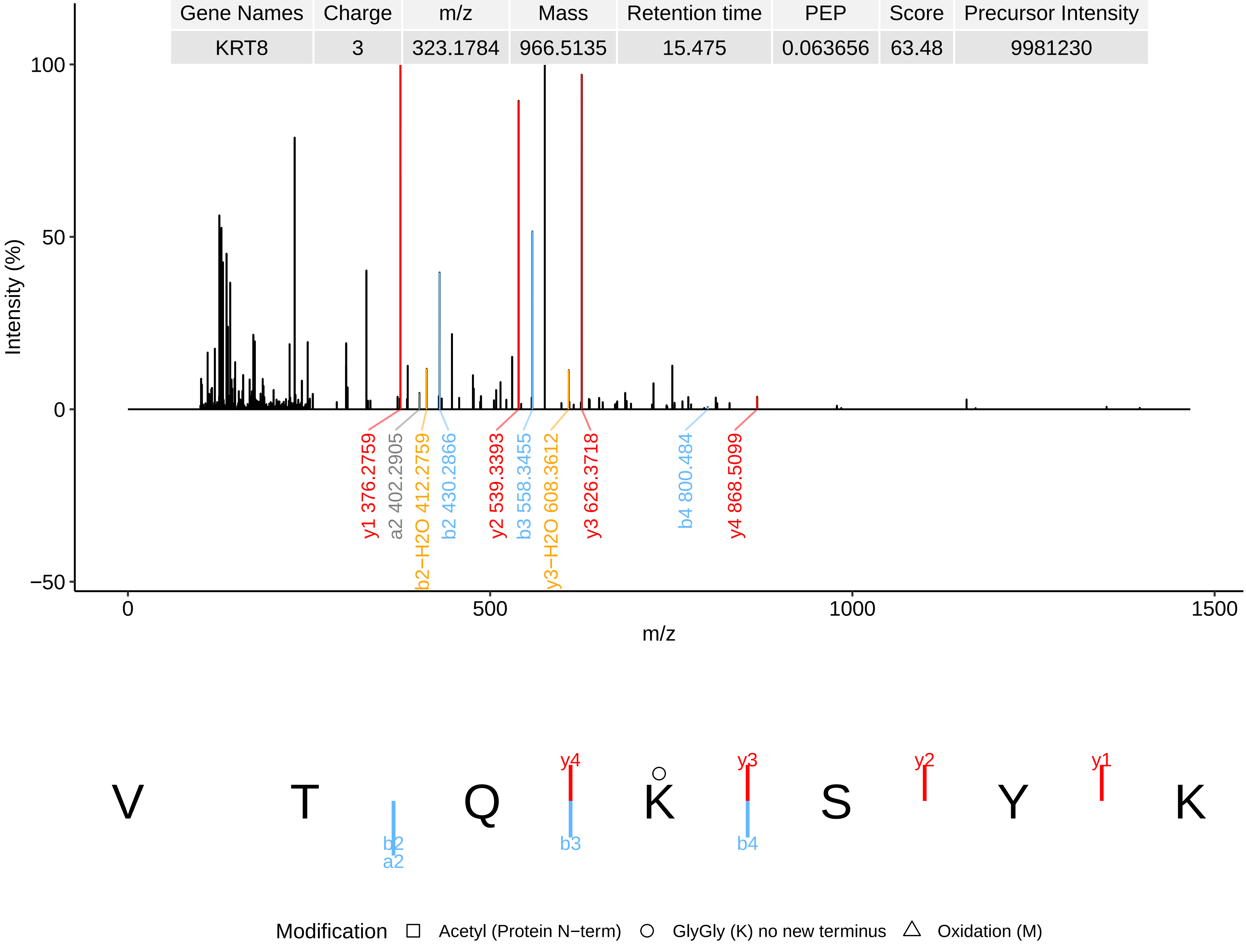

20200921_KIZ_MUb4_GlyGly_10plex − Scan number: 8802

Gene Names	Charge	m/z	Mass	Retention time	PEP	Score	Precursor Intensity
EPRS	3	393.2035	1176.589	29.986	0.00090489	96.034	5700468

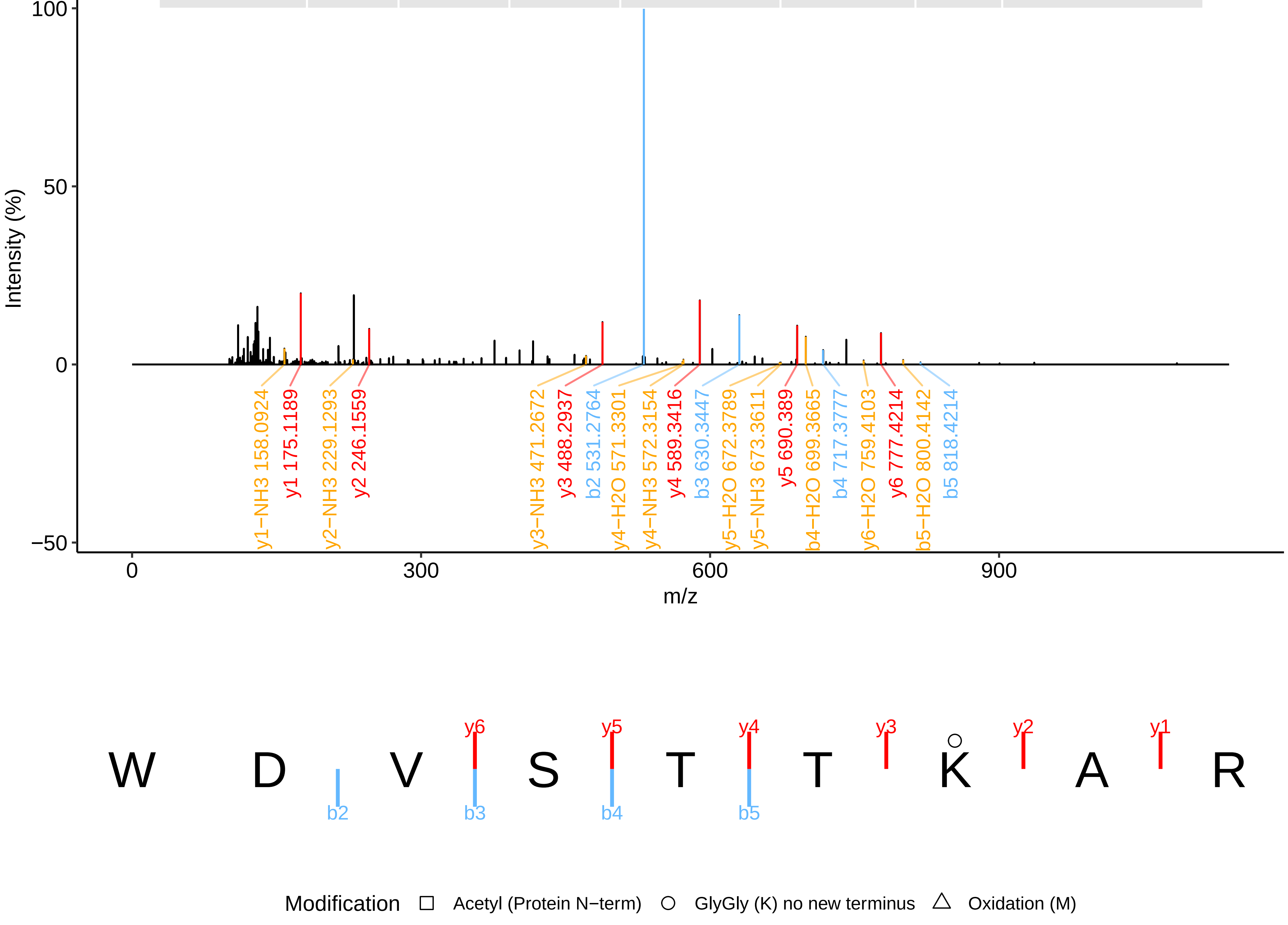

20200921_KIZ_MUb4_GlyGly_10plex_2 − Scan number: 8116

Gene Names	Charge	m/z	Mass	Retention time	PEP	Score	Precursor Intensity
EPRS	3	393.2035	1176.589	30.521	0.06957	53.327	NA

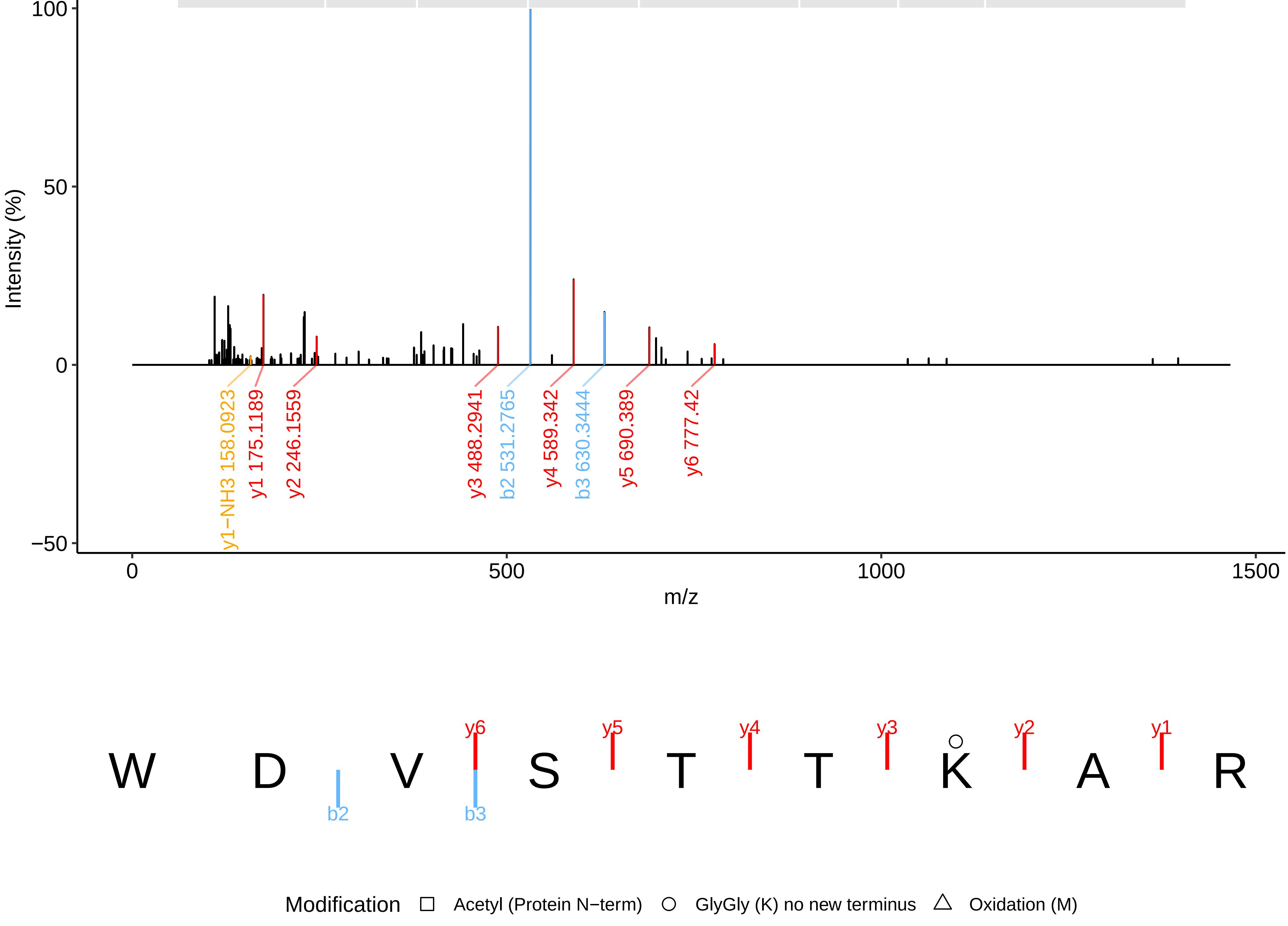

20200921_KIZ_MUb4_GlyGly_10plex_2 − Scan number: 13383

Gene Names	Charge	m/z	Mass	Retention time	PEP	Score	Precursor Intensity
SNRPD2	3	396.5462	1186.617	51.948	0.053998	54.205	1628834

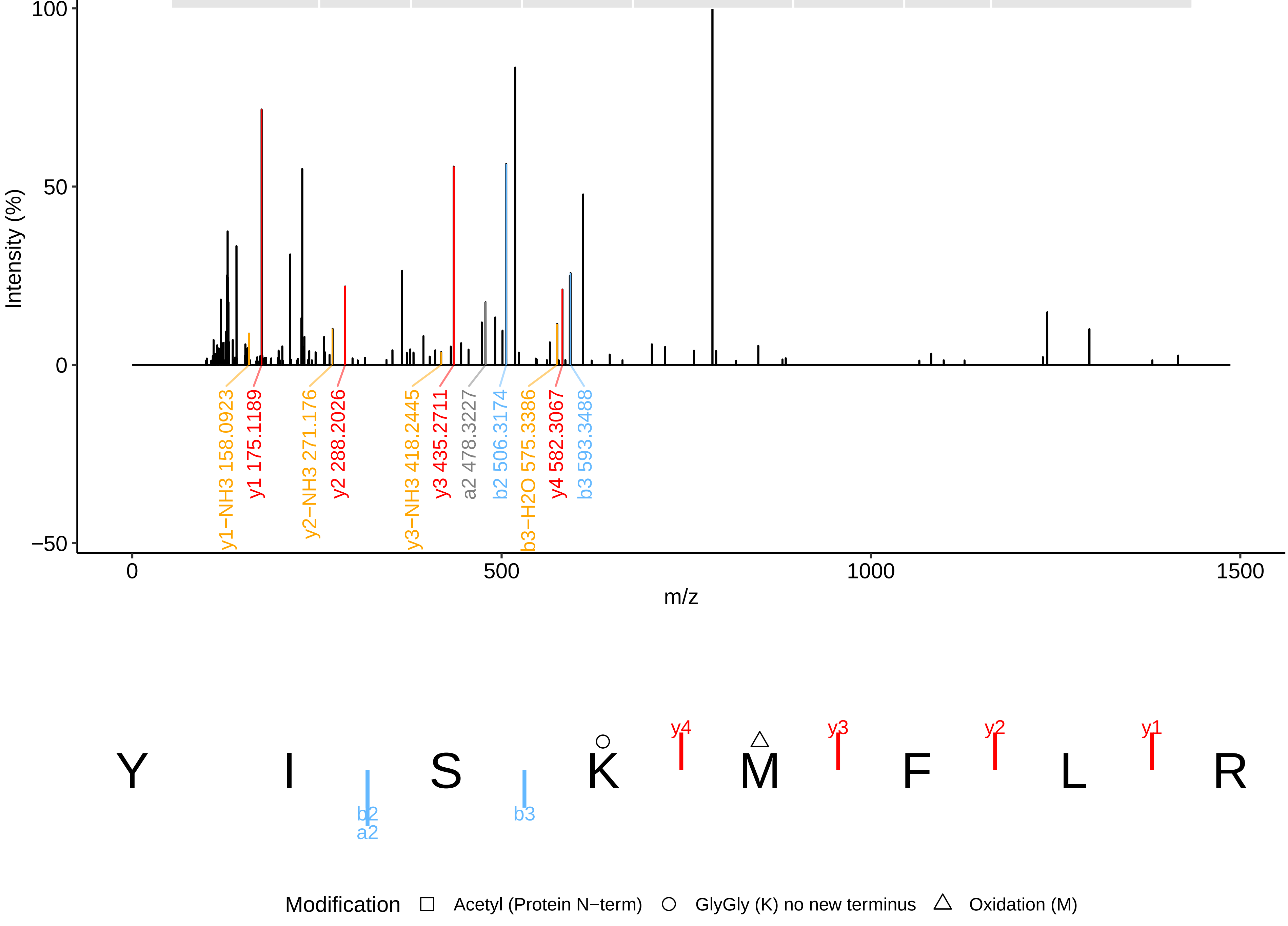

20200921_KIZ_MUb4_GlyGly_10plex − Scan number: 10814

Gene Names	Charge	m/z	Mass	Retention time	PEP	Score	Precursor Intensity
VDAC3	3	336.1821	1005.524	37.554	0.018289	73.208	4887123

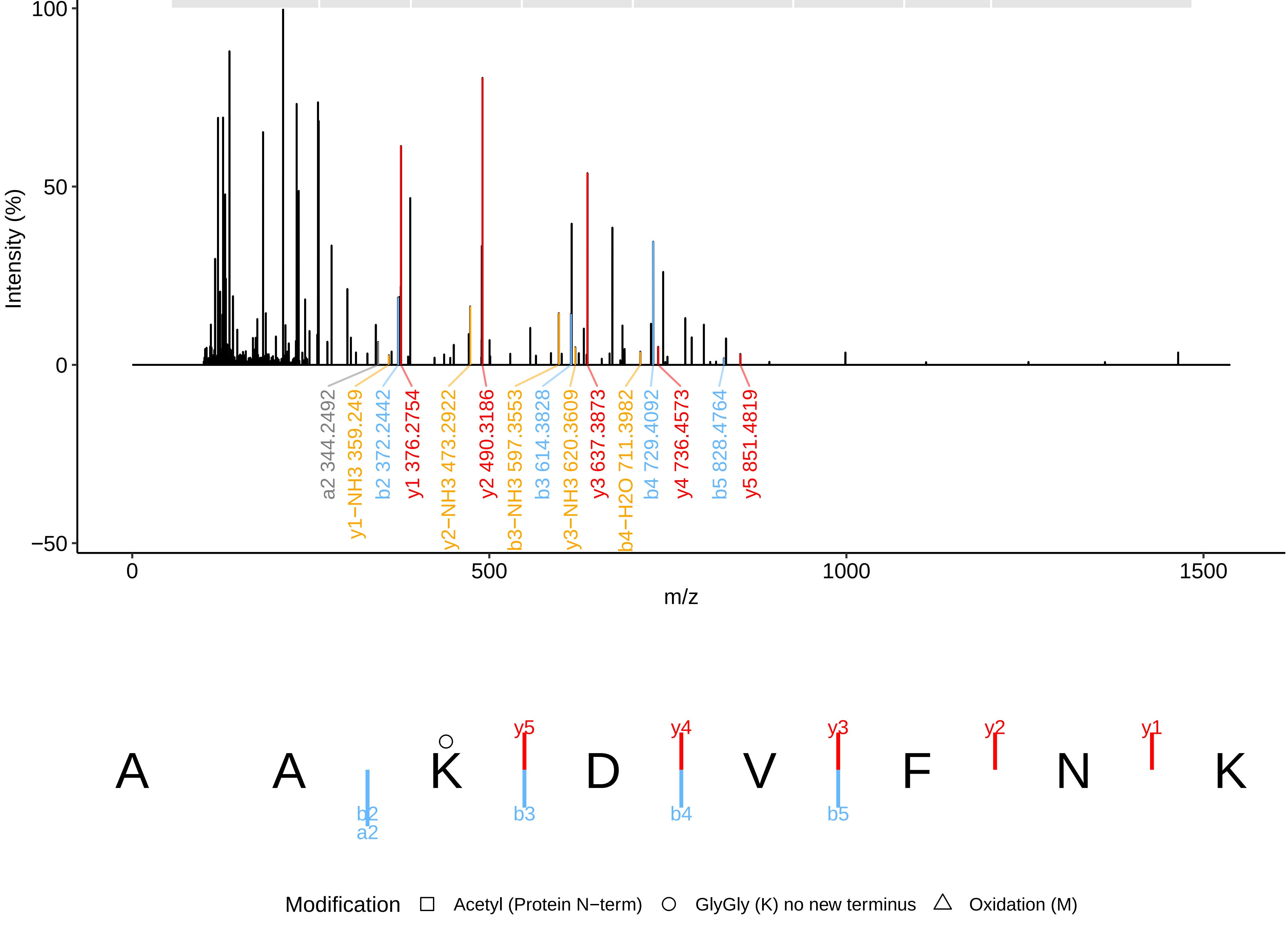

20200921_KIZ_MUb4_GlyGly_10plex − Scan number: 22075

Gene Names	Charge	m/z	Mass	Retention time	PEP	Score	Precursor Intensity
PHB	2	603.3299	1204.645	80.054	0.002852	84.476	4428550

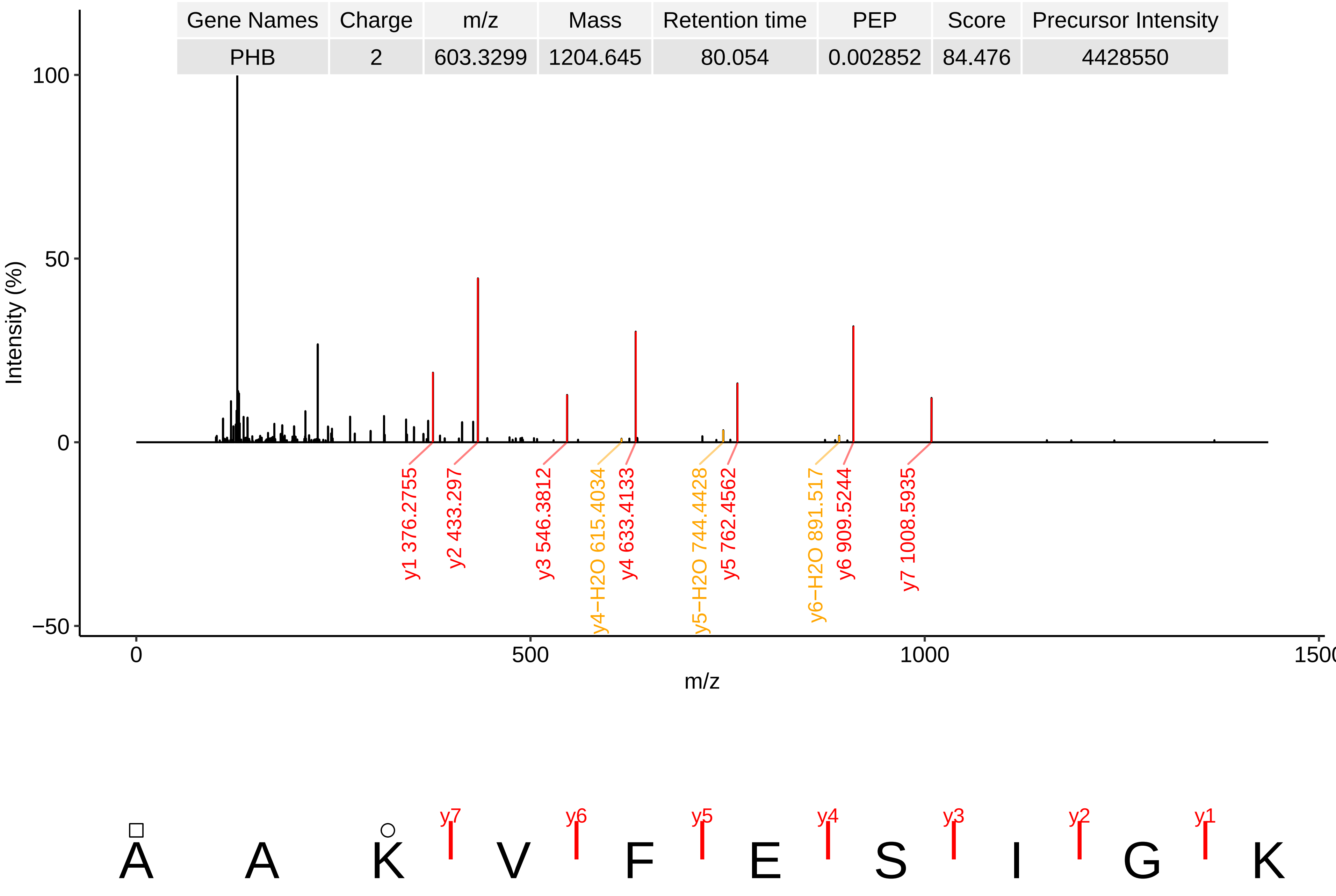

Modification □ Acetyl (Protein N−term) ○ GlyGly (K) no new terminus △ Oxidation (M)

20200921_KIZ_MUb4_GlyGly_10plex_2 − Scan number: 20409

Gene Names	Charge	m/z	Mass	Retention time	PEP	Score	Precursor Intensity
PHB	2	603.3299	1204.645	80.216	0.034976	58.676	1201431

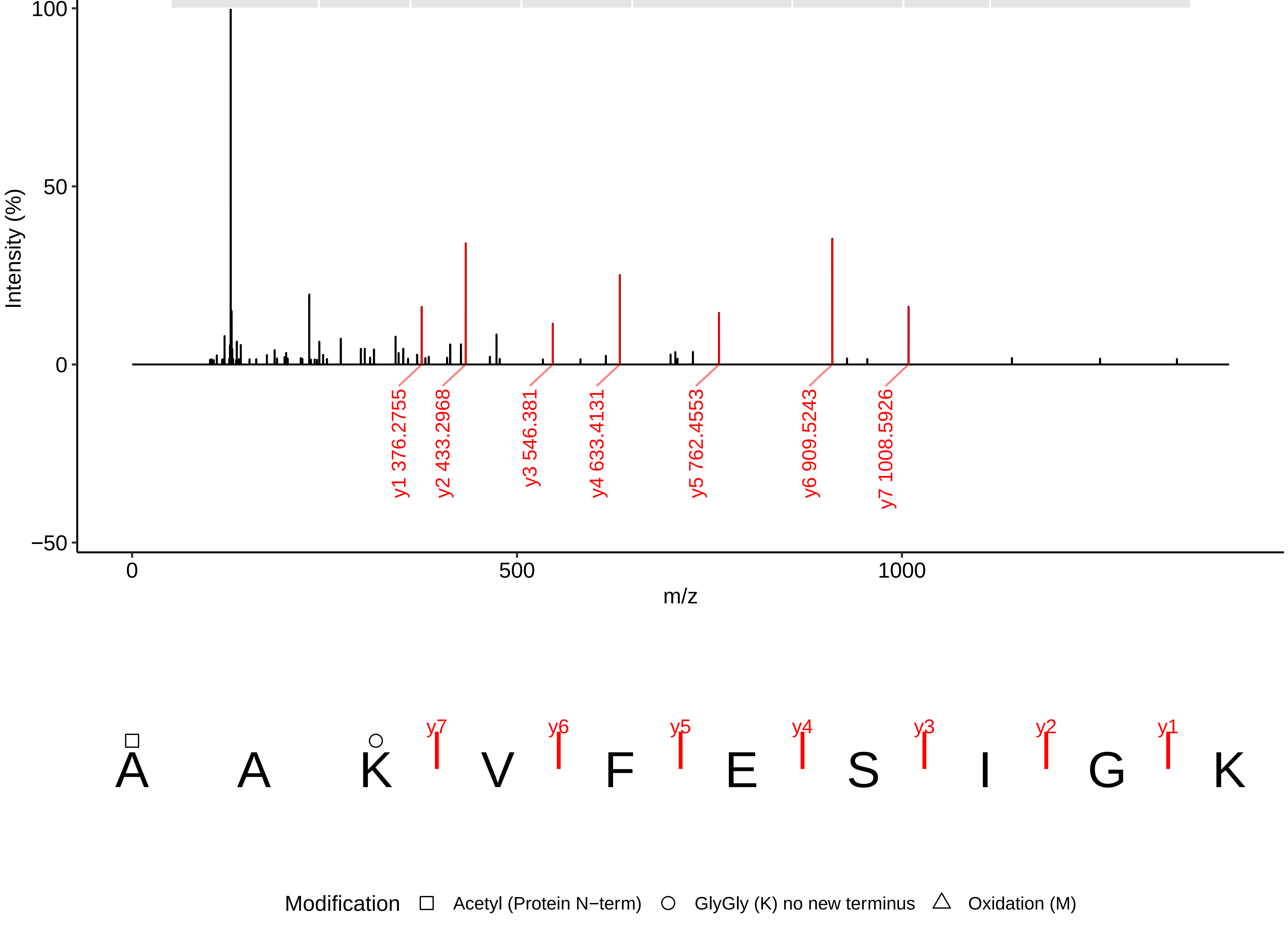

20200921_KIZ_MUb4_GlyGly_10plex − Scan number: 4423

Gene Names	Charge	m/z	Mass	Retention time	PEP	Score	Precursor Intensity
ALDOA	3	403.207	1206.599	14.417	7.1773e−05	123.21	16754652

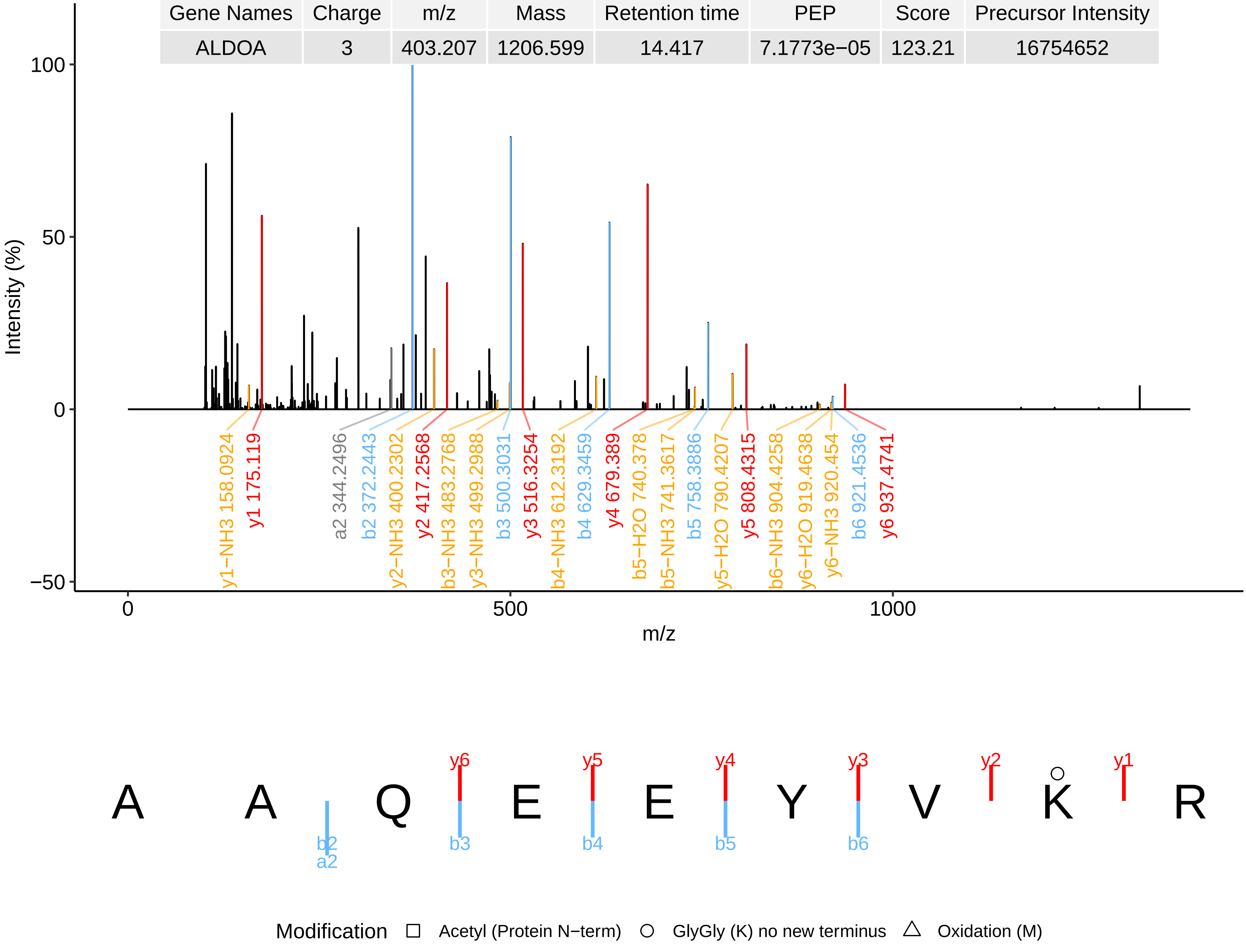

20200921_KIZ_MUb4_GlyGly_10plex_2 − Scan number: 4119

Gene Names	Charge	m/z	Mass	Retention time	PEP	Score	Precursor Intensity
ALDOA	3	403.207	1206.599	14.27	7.0709e−05	123.35	4442614

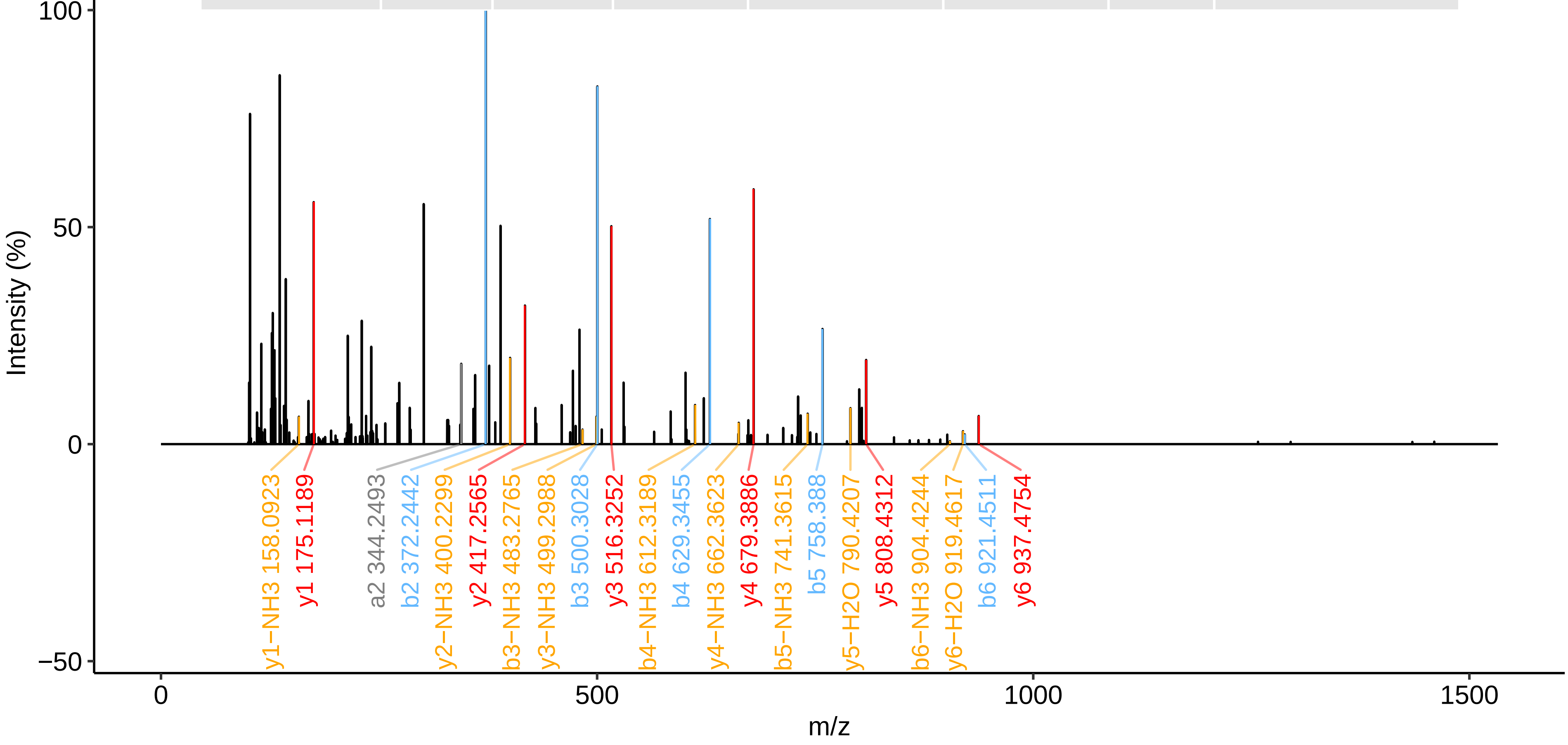

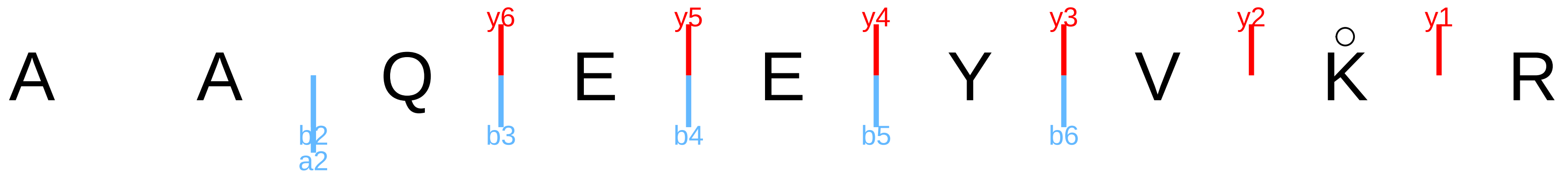

Modification □ Acetyl (Protein N−term) ○ GlyGly (K) no new terminus △ Oxidation (M)

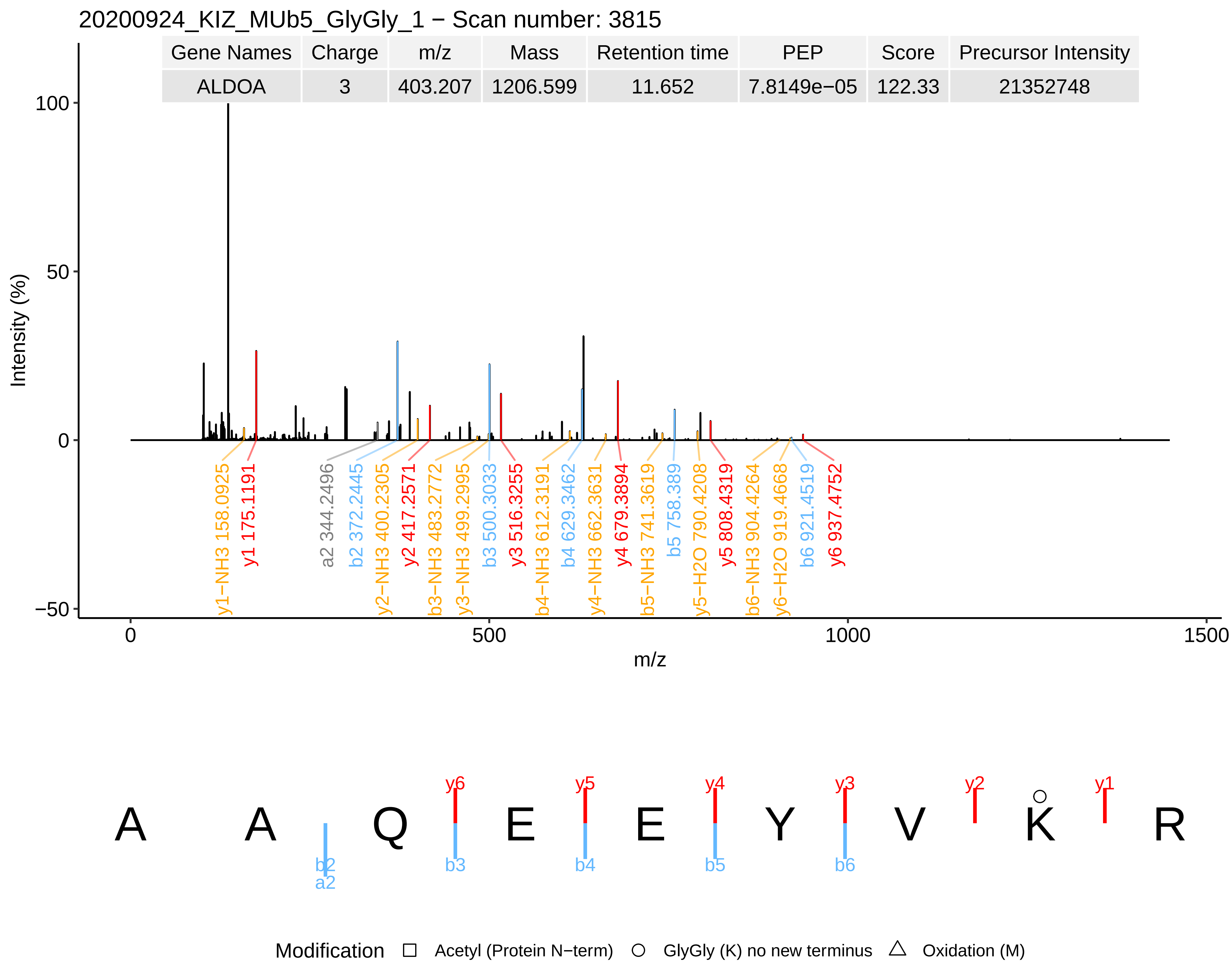

20200924_KIZ_MUb5_GlyGly_1 − Scan number: 3815
Gene Names | Charge | m/z | Mass | Retention time | PEP | Score | Precursor Intensity
ALDOA | 3 | 403.207 | 1206.599 | 11.652 | 7.8149e−05 | 122.33 | 21352748
Intensity (%)
100
50
0
−50
y1−NH3 158.0925
y1 175.1191
a2 344.2496
b2 372.2445
y2−NH3 400.2305
y2 417.2571
b3−NH3 483.2772
y3−NH3 499.2995
b3 500.3033
y3 516.3255
b4−NH3 612.3191
b4 629.3462
y4−NH3 662.3631
y4 679.3894
b5−NH3 741.3619
b5 758.389
y5−H2O 790.4208
y5 808.4319
b6−NH3 904.4264
y6−H2O 919.4668
b6 921.4519
y6 937.4752
0
500
1000
1500
m/z
A A Q E E Y V K R
y6 y5 y4 y3 y2 y1
b2 a2 b3 b4 b5 b6
Modification
Acetyl (Protein N−term)
GlyGly (K) no new terminus
Oxidation (M)

20200924_KIZ_MUb5_GlyGly_2 − Scan number: 3658

Gene Names	Charge	m/z	Mass	Retention time	PEP	Score	Precursor Intensity
ALDOA	3	403.207	1206.599	13.302	0.00057937	134.26	3574428

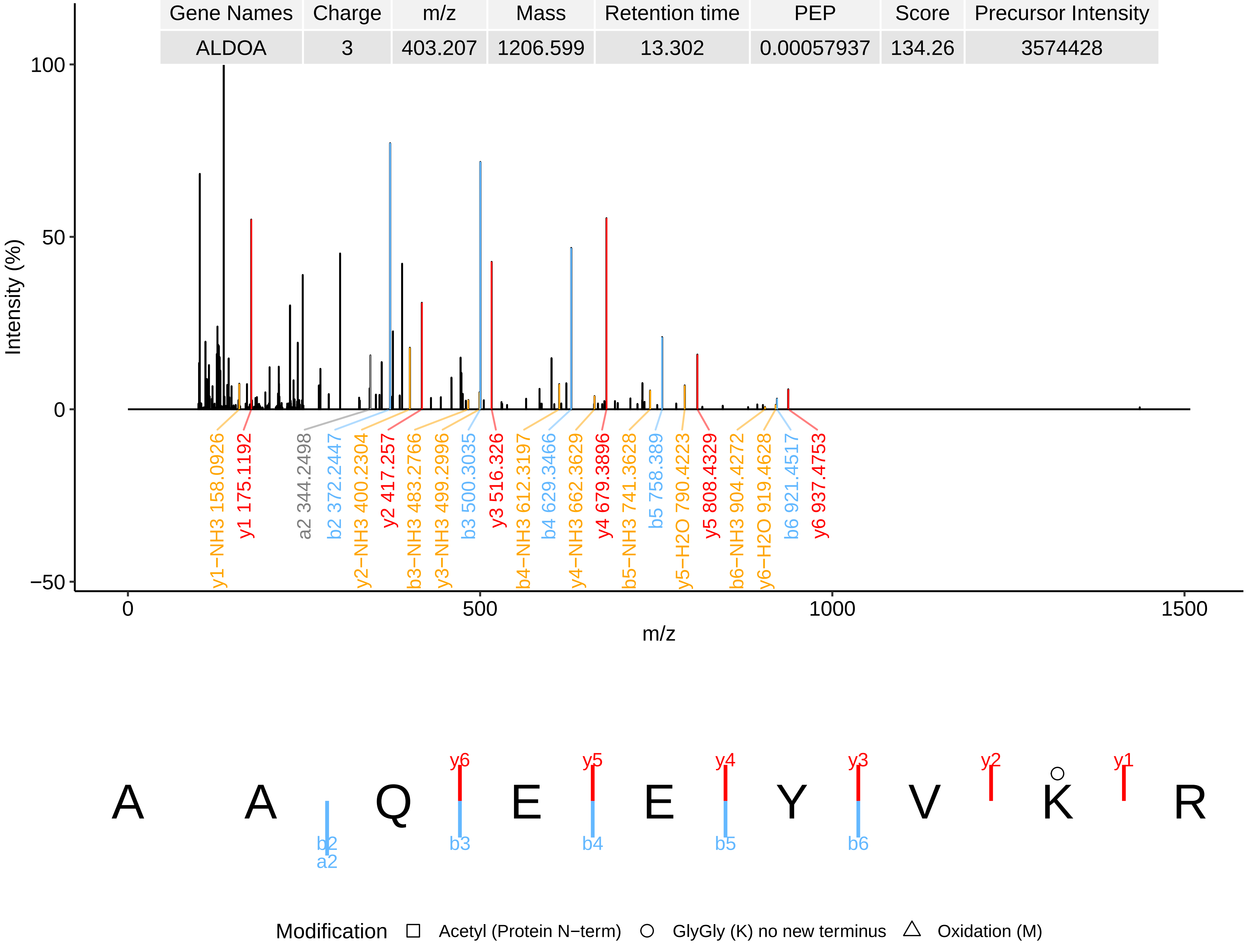

20201118_KIZ_MUb6_GlyGly_1 − Scan number: 3587

Gene Names	Charge	m/z	Mass	Retention time	PEP	Score	Precursor Intensity
ALDOA	3	403.207	1206.599	11.015	7.0709e−05	123.35	15419916

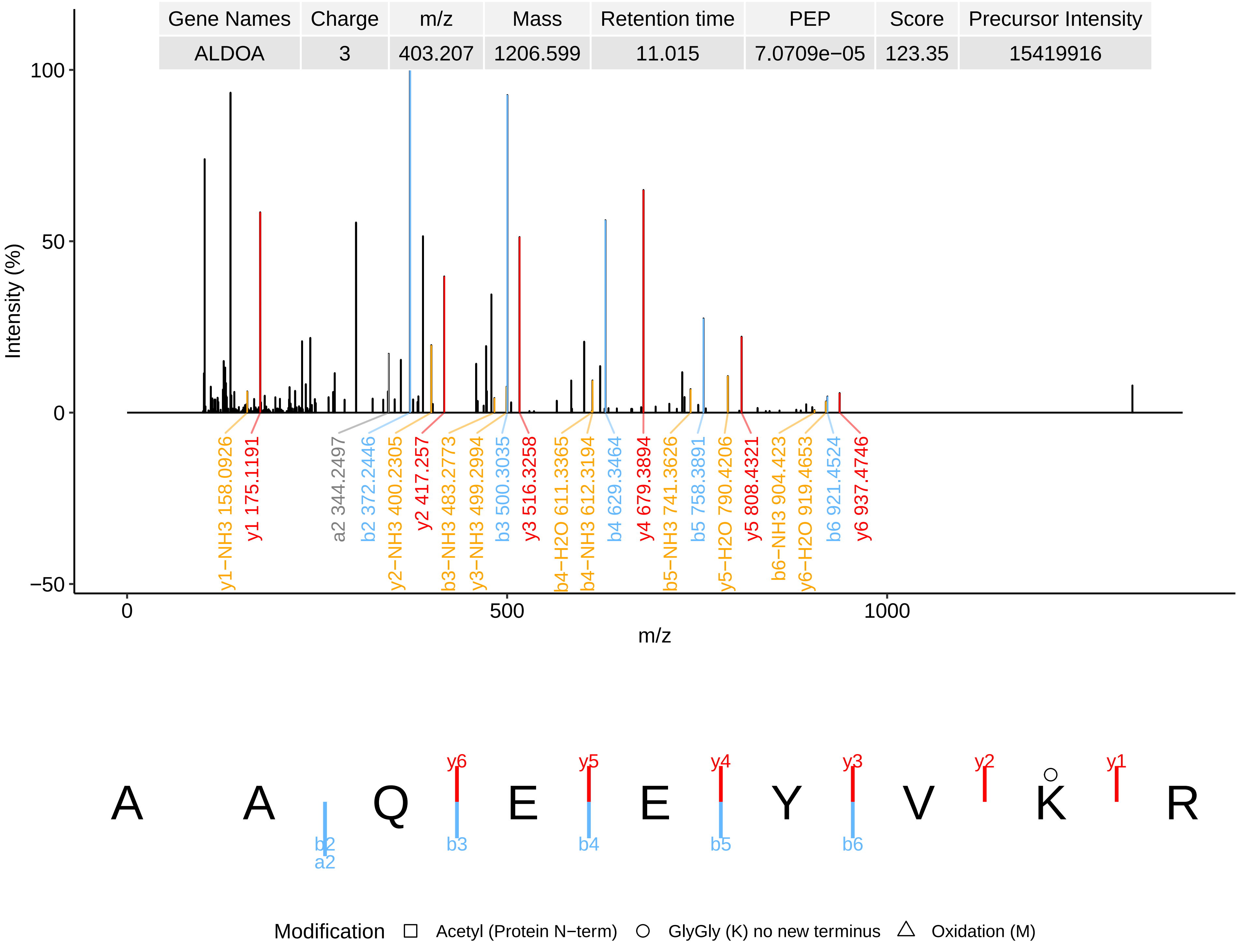

20201118_KIZ_MUb6_GlyGly_2 − Scan number: 4452

Gene Names	Charge	m/z	Mass	Retention time	PEP	Score	Precursor Intensity
ALDOA	3	403.207	1206.599	15.22	0.011774	90.15	5735089

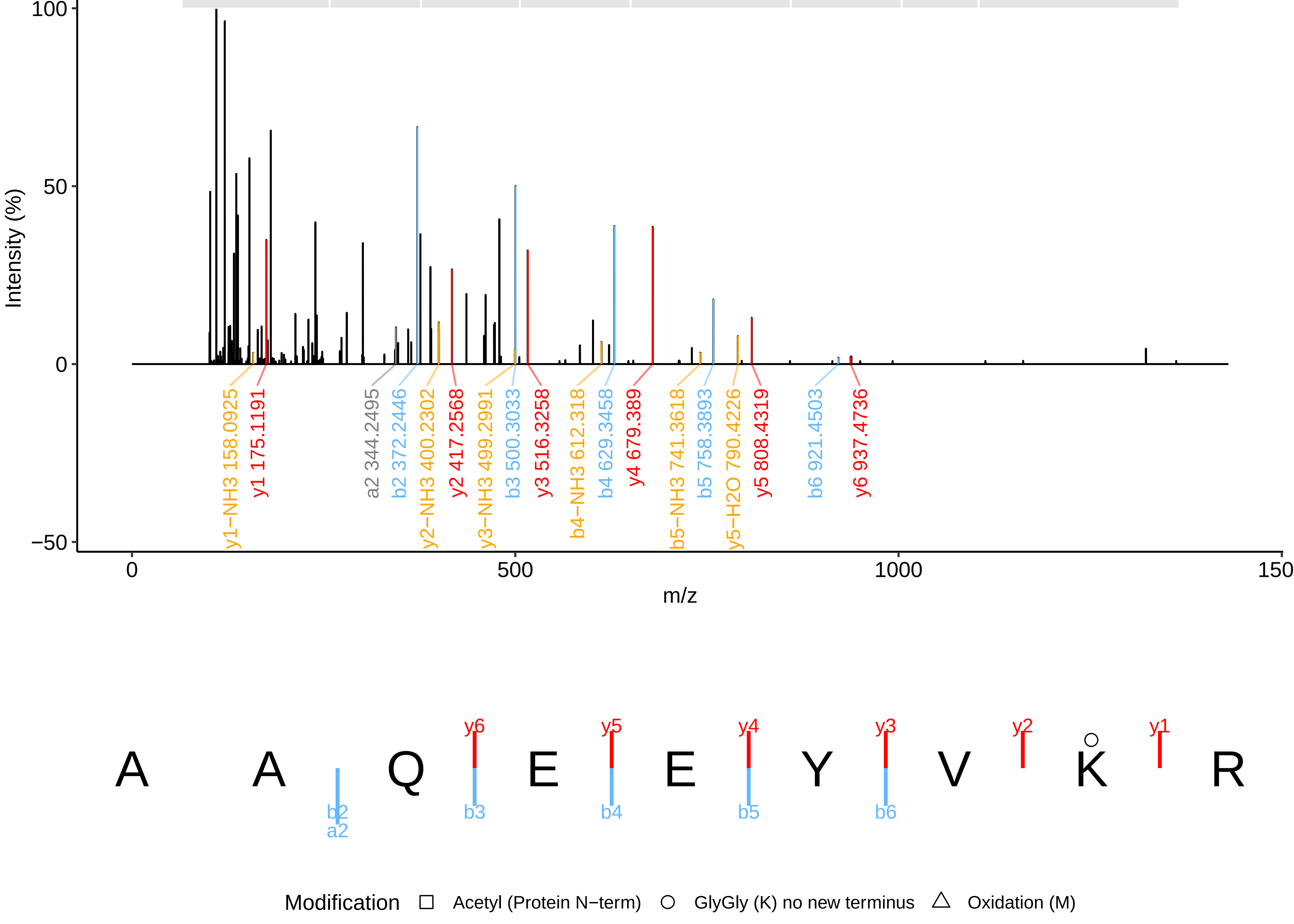

20200921_KIZ_MUb4_GlyGly_10plex − Scan number: 5955

Gene Names	Charge	m/z	Mass	Retention time	PEP	Score	Precursor Intensity
PRKDC	3	326.5103	976.509	19.545	0.0011442	107.59	5163442

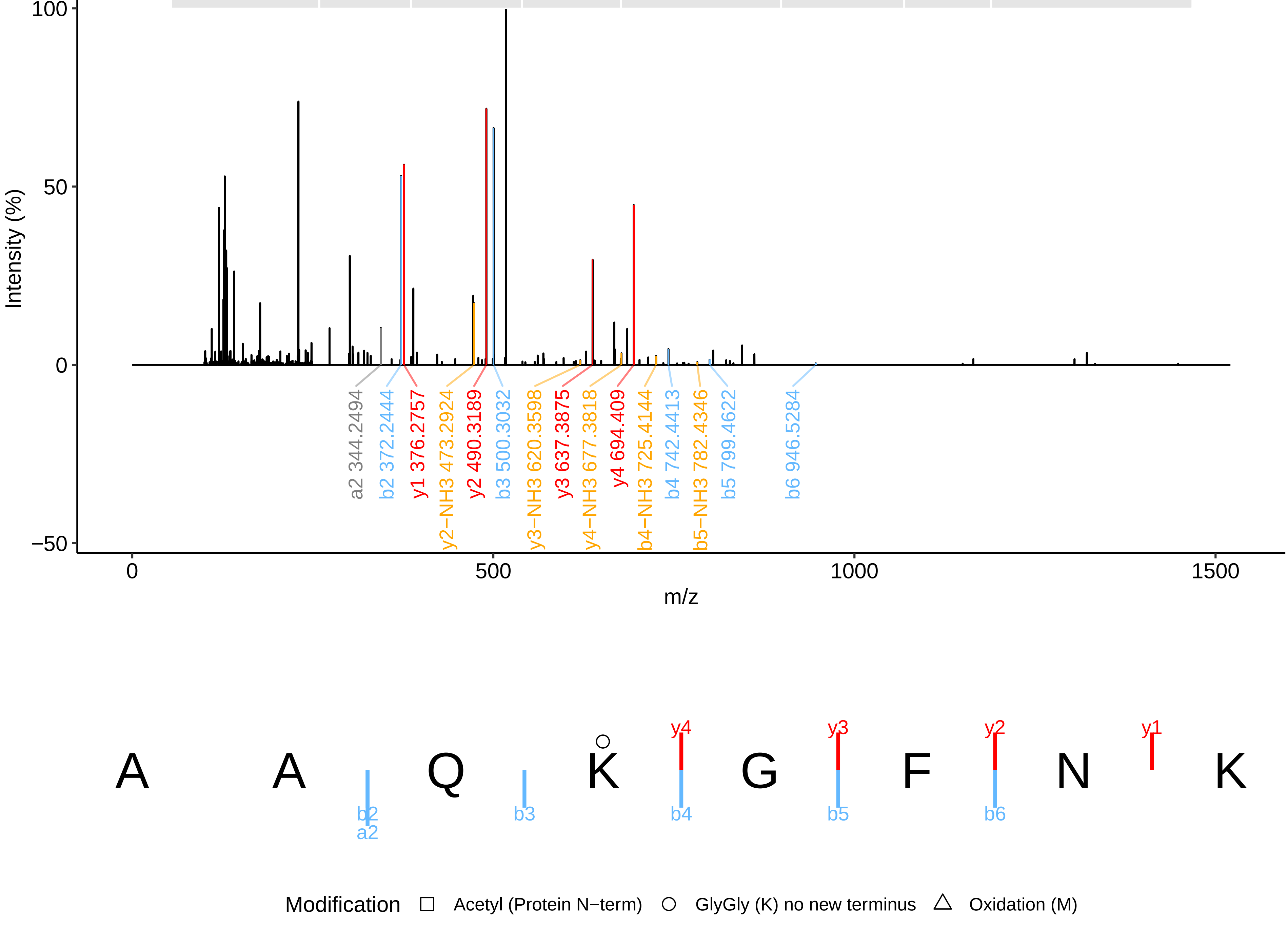

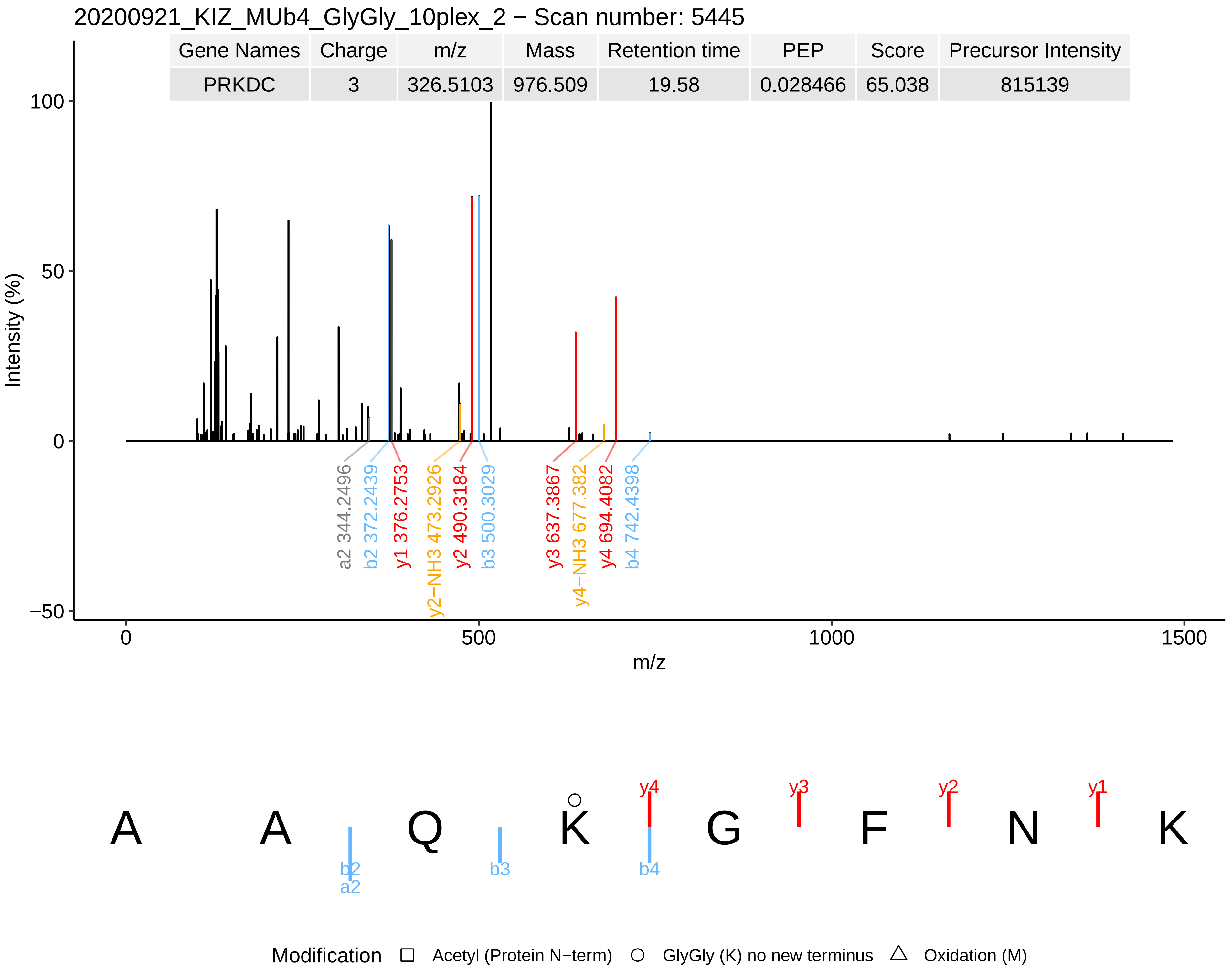
20200921_KIZ_MUb4_GlyGly_10plex_2 − Scan number: 5445
Gene Names | Charge | m/z | Mass | Retention time | PEP | Score | Precursor Intensity
PRKDC | 3 | 326.5103 | 976.509 | 19.58 | 0.028466 | 65.038 | 815139
Intensity (%)
m/z
a2 344.2496
b2 372.2439
y1 376.2753
y2−NH3 473.2926
y2 490.3184
b3 500.3029
y3 637.3867
y4−NH3 677.382
y4 694.4082
b4 742.4398
A A Q K G F N K
y4 y3 y2 y1
b2 a2 b3 b4
Modification
Acetyl (Protein N−term)
GlyGly (K) no new terminus
Oxidation (M)

20200924_KIZ_MUb5_GlyGly_1 − Scan number: 5138

Gene Names	Charge	m/z	Mass	Retention time	PEP	Score	Precursor Intensity
PRKDC	3	326.5103	976.509	15.953	0.067794	54.157	3971439

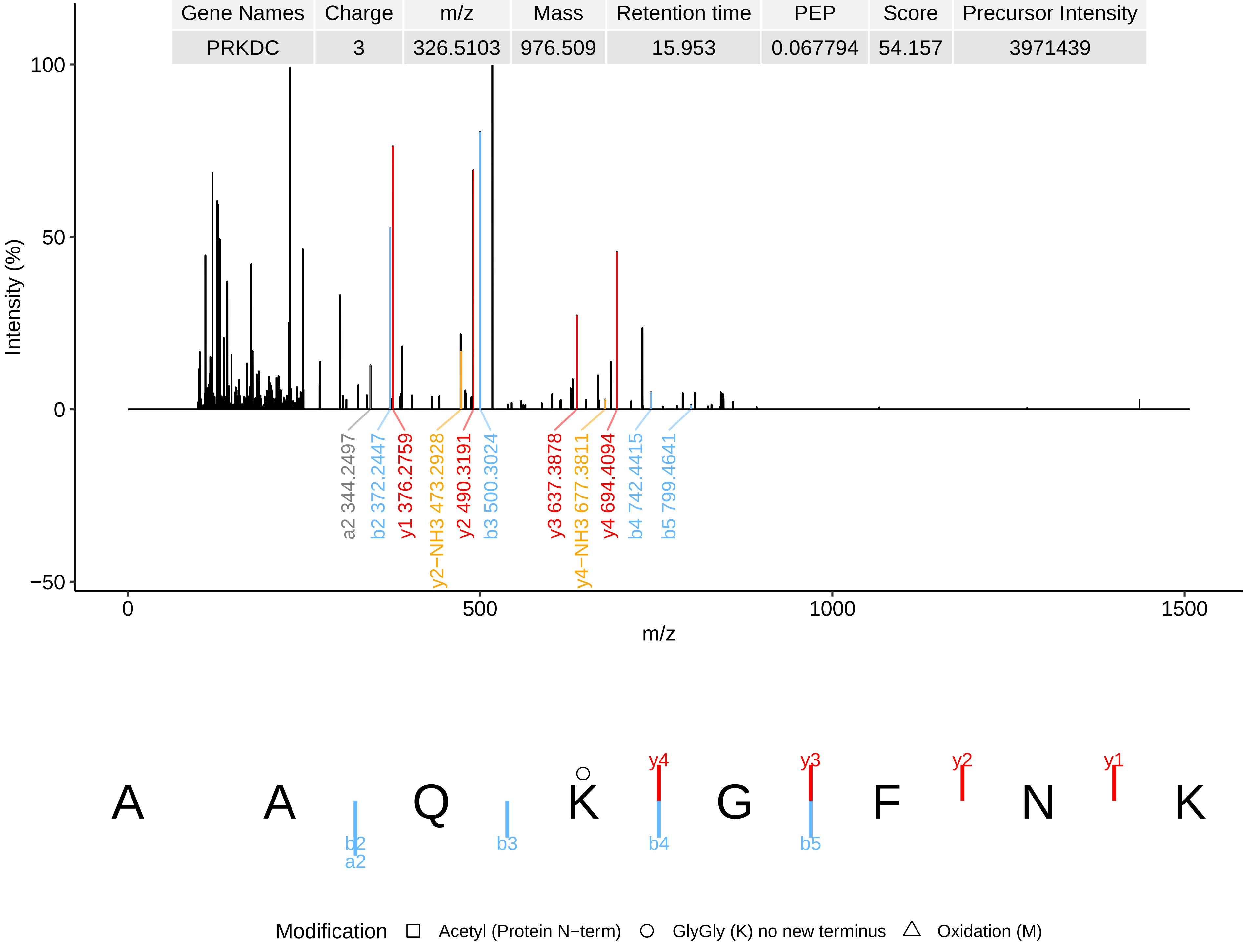

20200921_KIZ_MUb4_GlyGly_10plex − Scan number: 6688

Gene Names	Charge	m/z	Mass	Retention time	PEP	Score	Precursor Intensity
ATP1A1;ATP1A3;ATP1A2	3	419.8838	1256.63	22.225	4.0272e−08	120.09	4298746

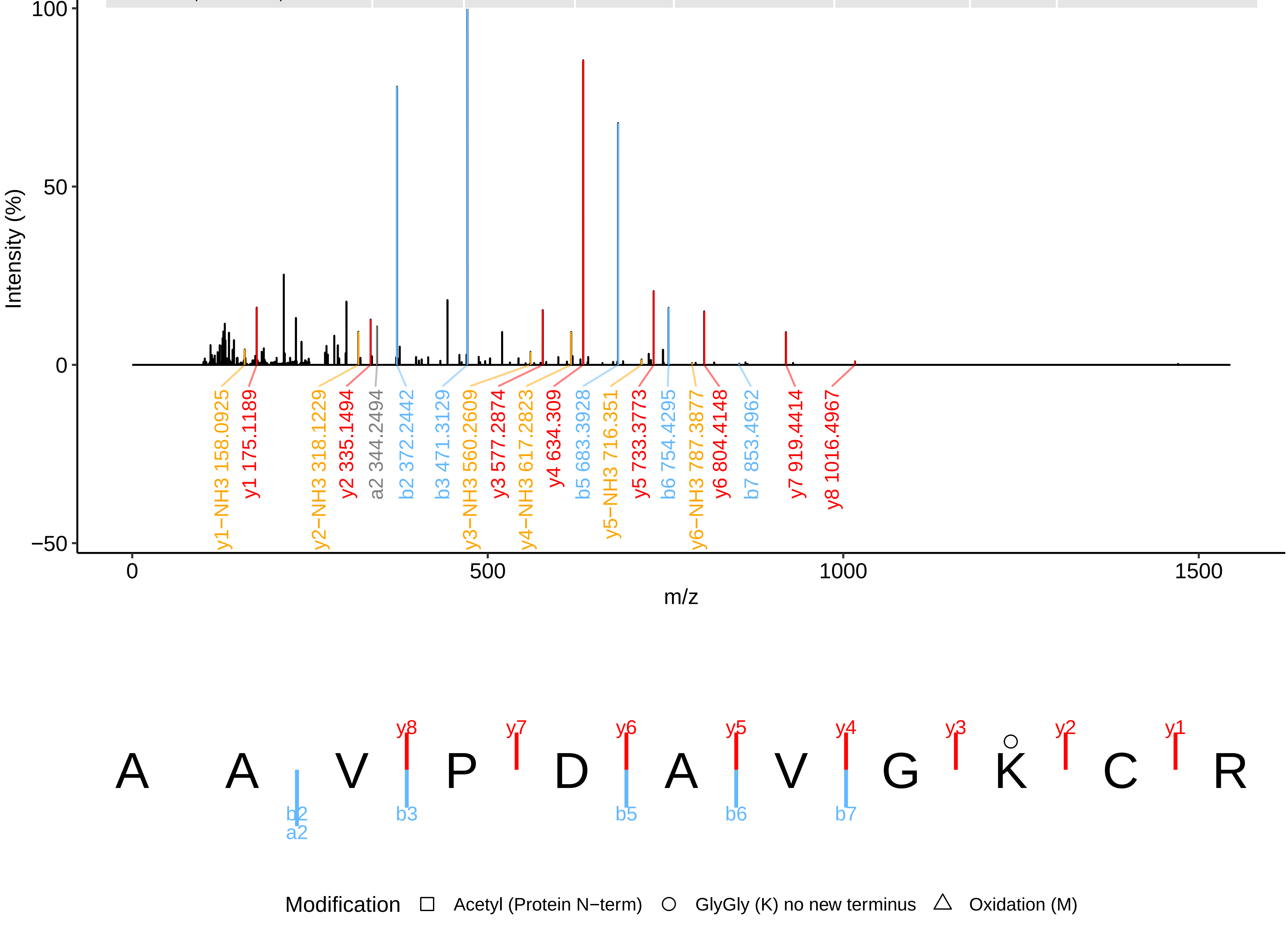

20200921_KIZ_MUb4_GlyGly_10plex_2 − Scan number: 6167

Gene Names	Charge	m/z	Mass	Retention time	PEP	Score	Precursor Intensity
ATP1A1;ATP1A3;ATP1A2	3	419.8838	1256.63	22.541	1.5499e−05	109.42	919380.7

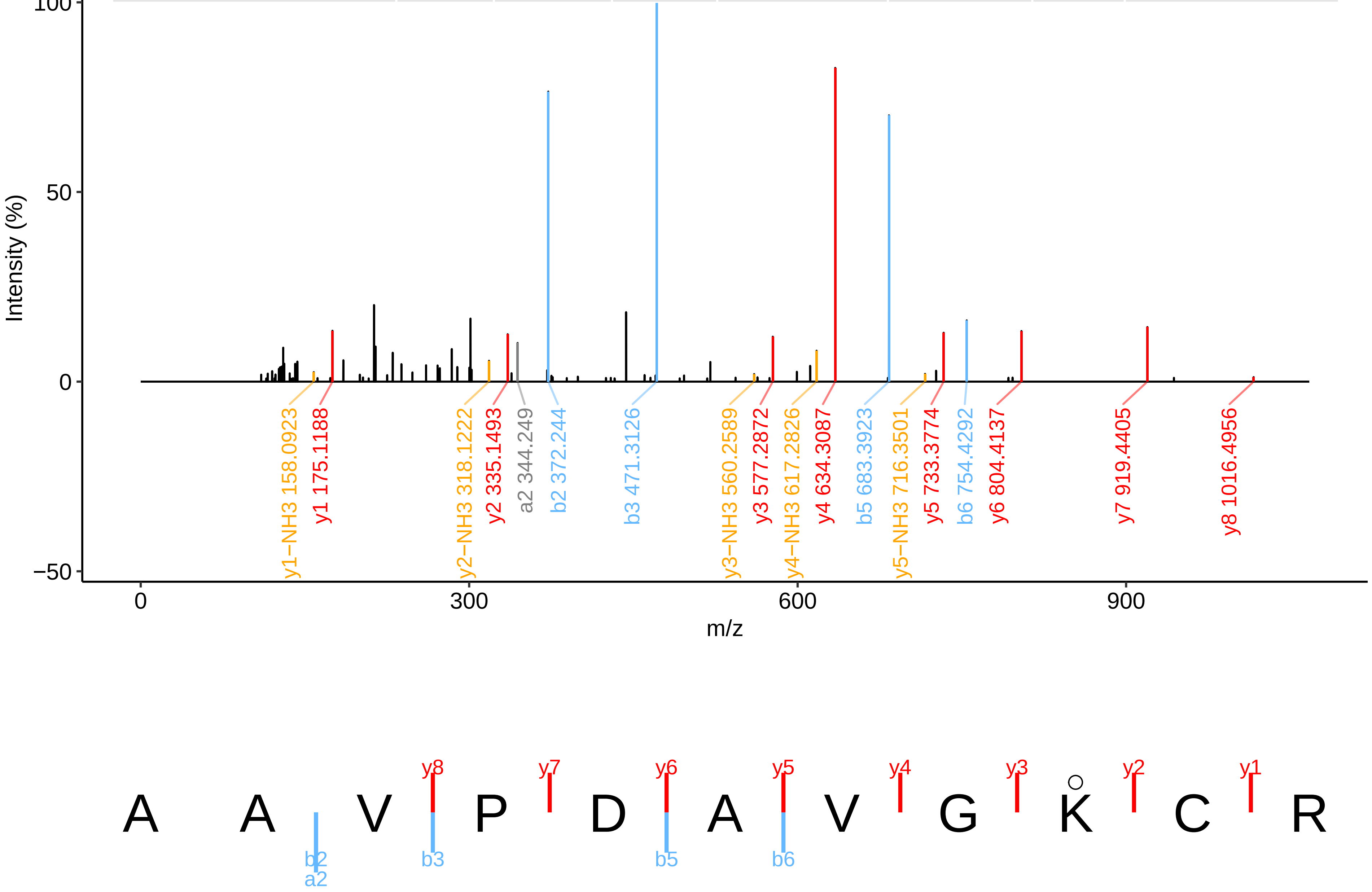

Modification □ Acetyl (Protein N−term) ○ GlyGly (K) no new terminus △ Oxidation (M)

20200924_KIZ_MUb5_GlyGly_1 − Scan number: 5807

Gene Names	Charge	m/z	Mass	Retention time	PEP	Score	Precursor Intensity
ATP1A1;ATP1A3;ATP1A2	3	419.8838	1256.63	18.136	2.8894e−05	104.22	23629342

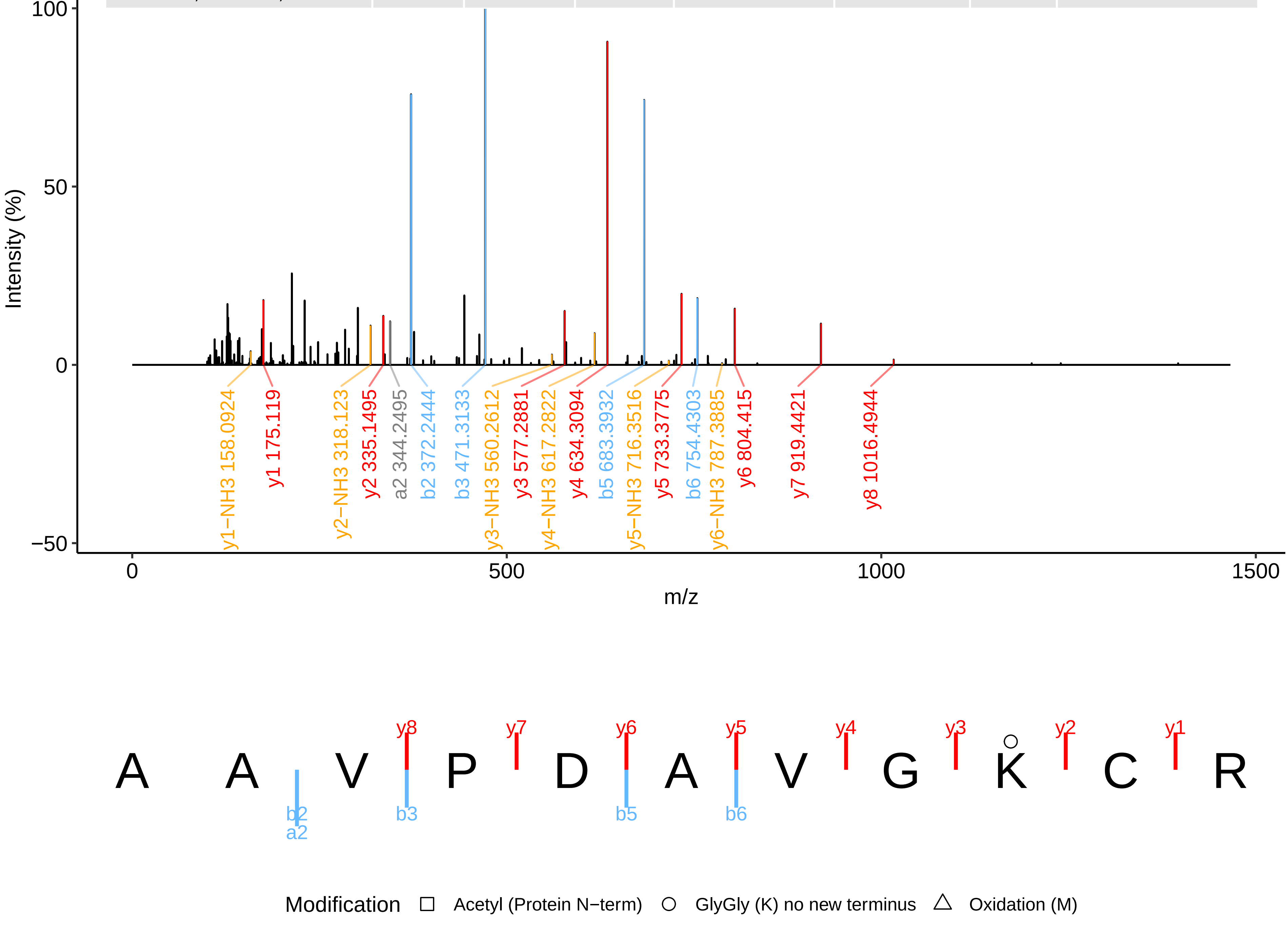

20201118_KIZ_MUb6_GlyGly_1 − Scan number: 5446

Gene Names	Charge	m/z	Mass	Retention time	PEP	Score	Precursor Intensity
ATP1A1;ATP1A3;ATP1A2	3	419.8838	1256.63	17.247	1.6003e−08	127.76	8082703

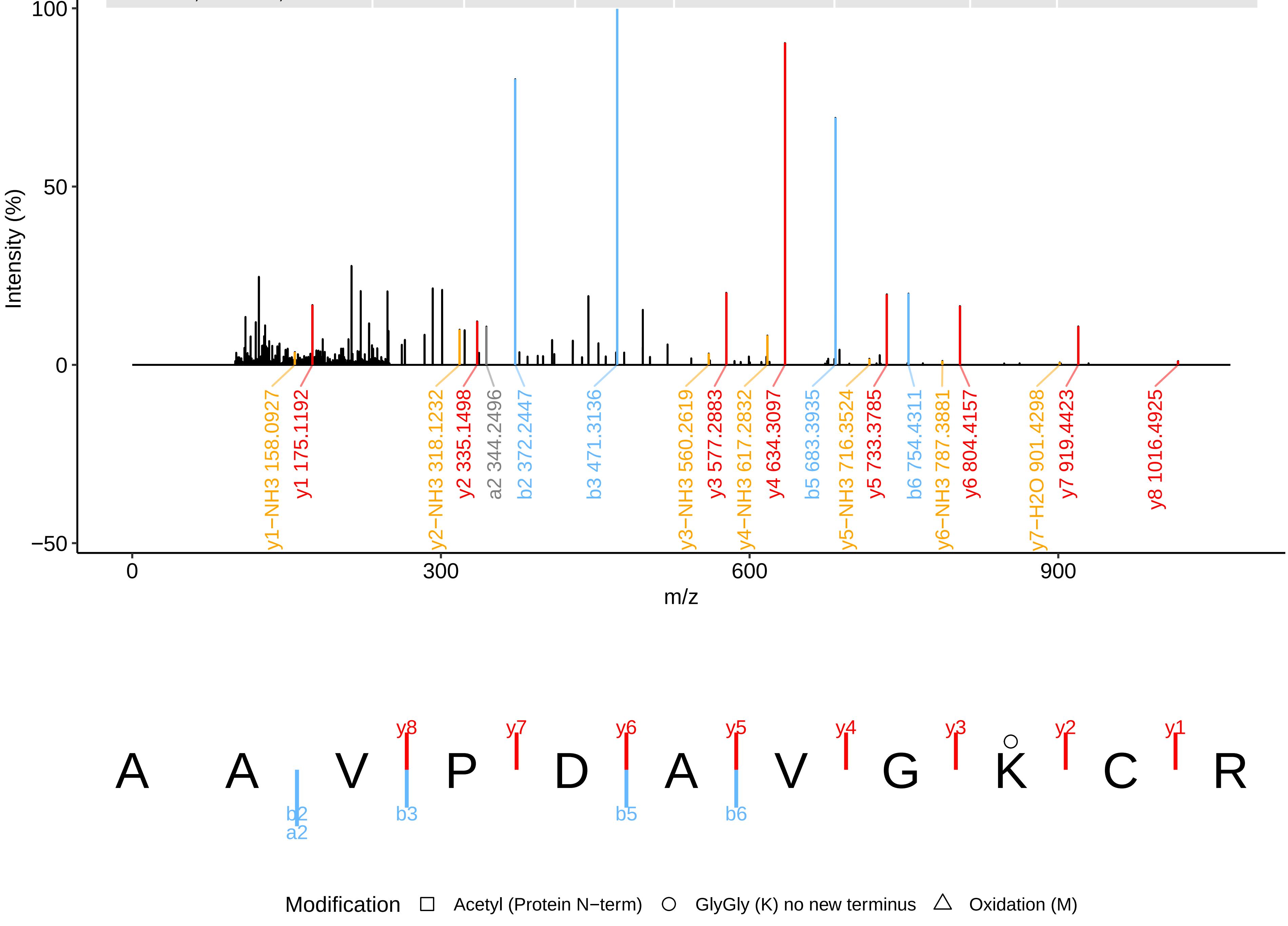

20200921_KIZ_MUb4_GlyGly_10plex − Scan number: 12852

Gene Names	Charge	m/z	Mass	Retention time	PEP	Score	Precursor Intensity
COX6B1	2	610.795	1219.575	45.21	0.00035917	76.899	5530059

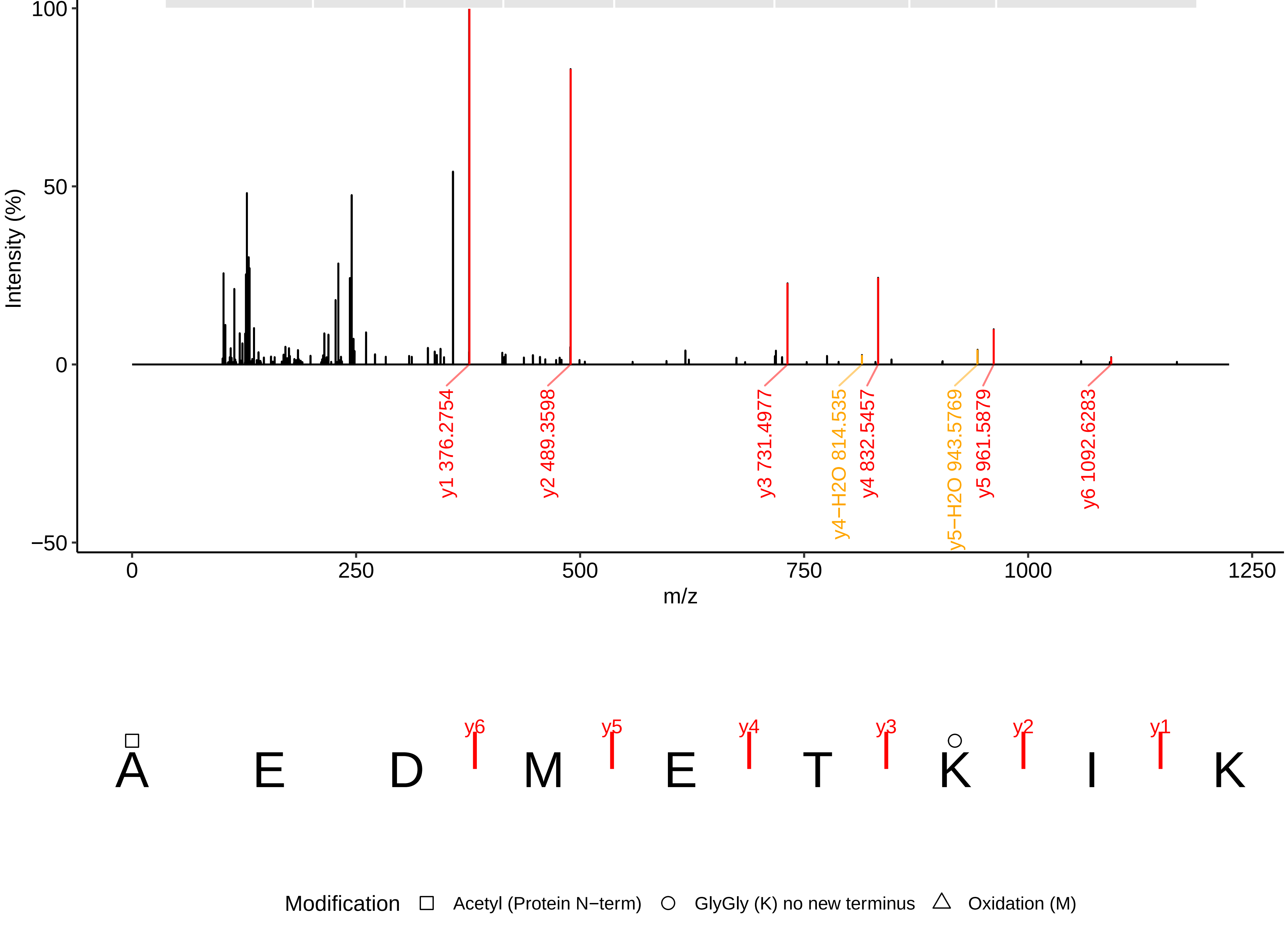

20200921_KIZ_MUb4_GlyGly_10plex_2 − Scan number: 11731

Gene Names	Charge	m/z	Mass	Retention time	PEP	Score	Precursor Intensity
COX6B1	2	610.795	1219.575	45.22	0.058112	40.987	433234.8

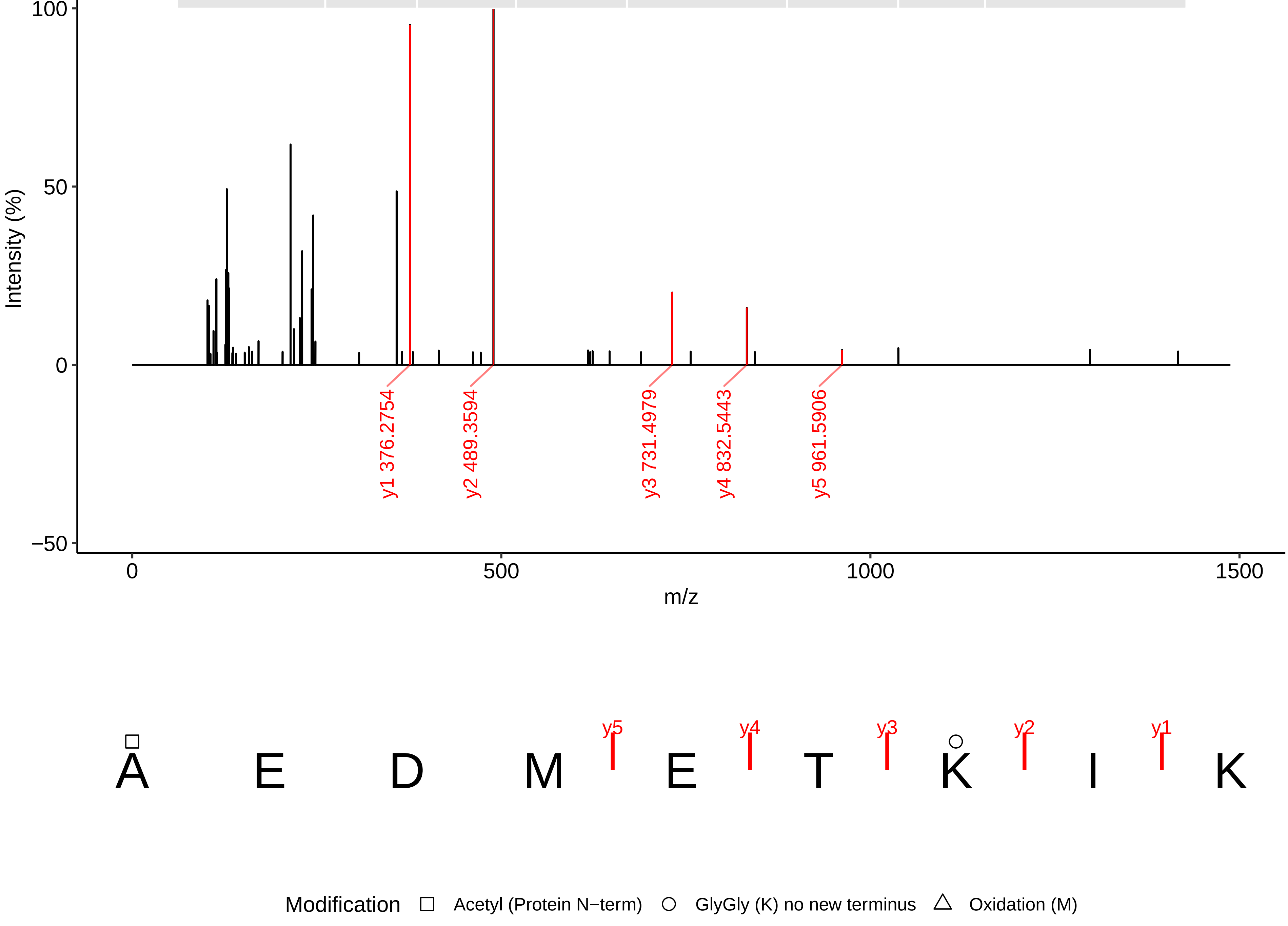

20200924_KIZ_MUb5_GlyGly_2 − Scan number: 11238

Gene Names	Charge	m/z	Mass	Retention time	PEP	Score	Precursor Intensity
COX6B1	2	610.795	1219.575	41.978	0.035612	51.854	2502287

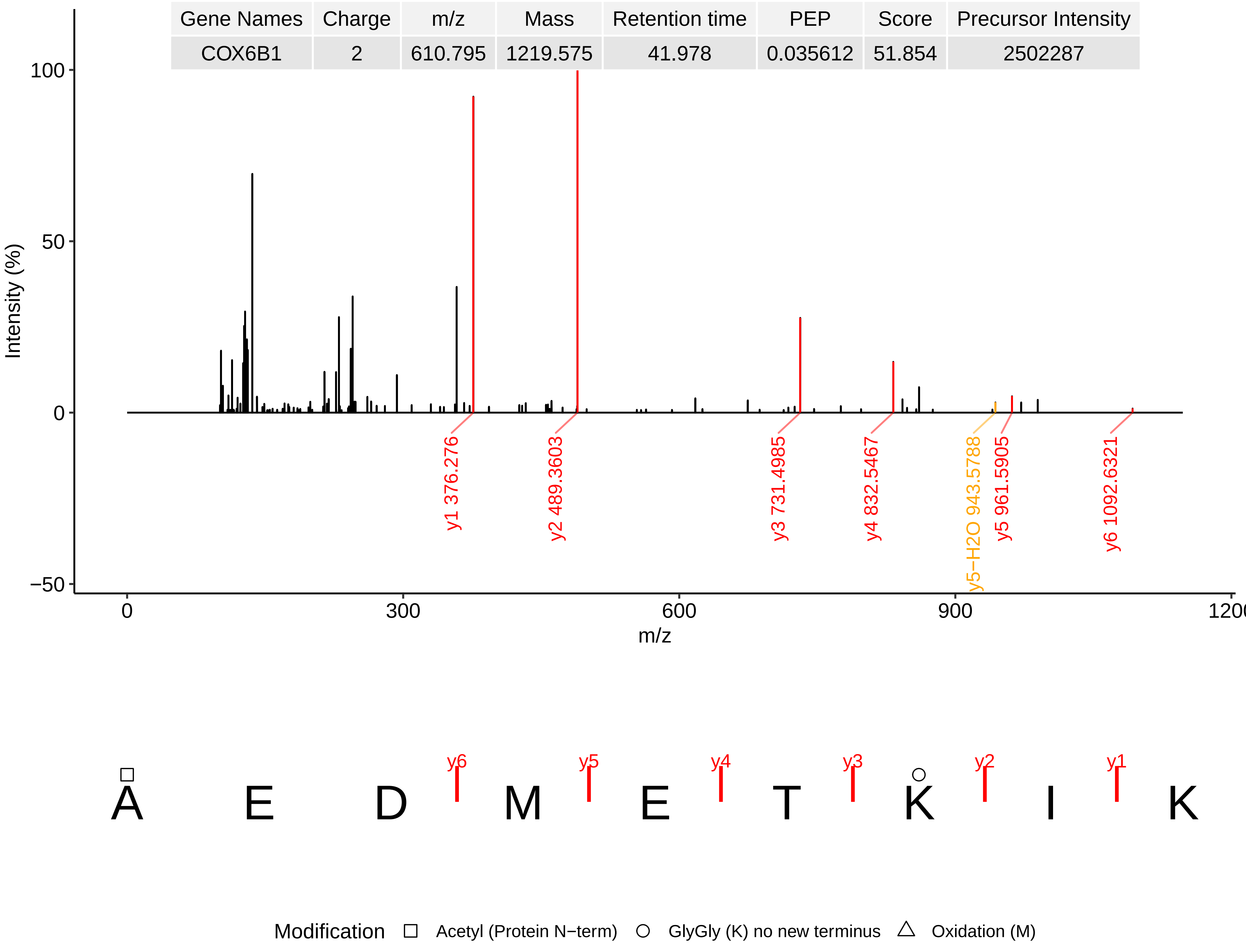

20200921_KIZ_MUb4_GlyGly_10plex − Scan number: 11666

Gene Names	Charge	m/z	Mass	Retention time	PEP	Score	Precursor Intensity
PTGES2	3	405.5437	1213.609	40.713	0.0032981	81.95	3173463

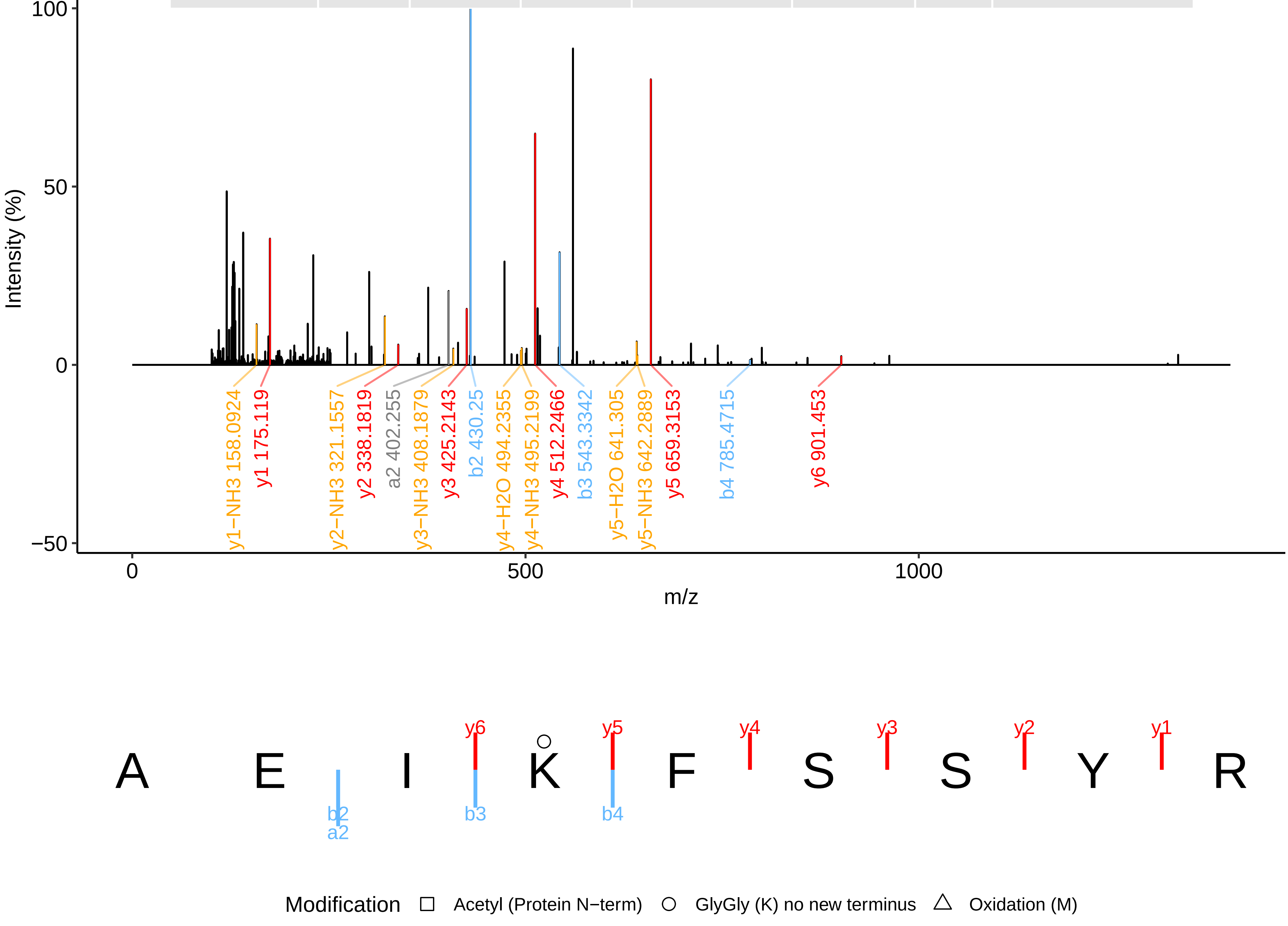

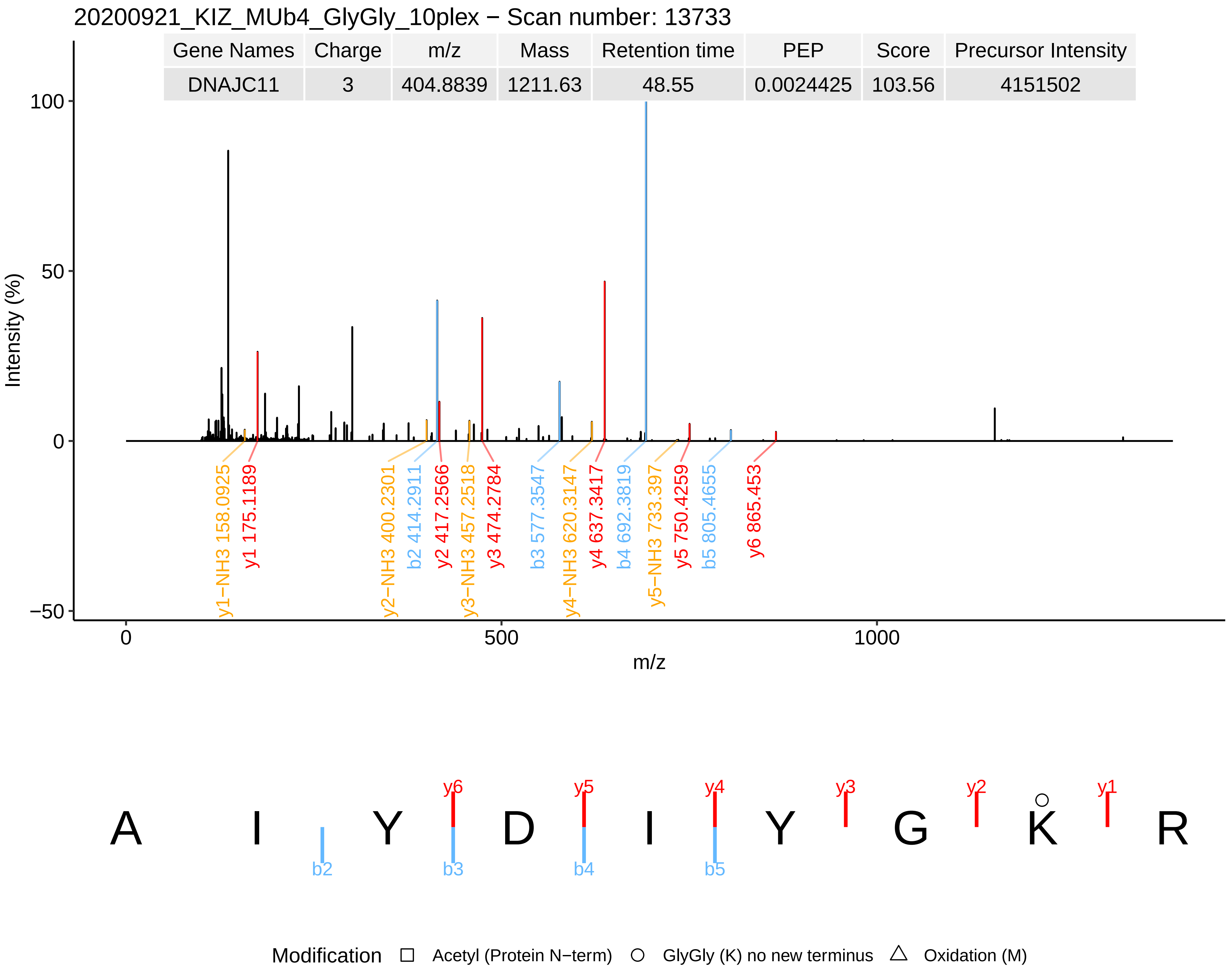

20200921_KIZ_MUb4_GlyGly_10plex − Scan number: 13733
Gene Names | Charge | m/z | Mass | Retention time | PEP | Score | Precursor Intensity
DNAJC11 | 3 | 404.8839 | 1211.63 | 48.55 | 0.0024425 | 103.56 | 4151502
Intensity (%)
m/z
y1−NH3 158.0925
y1 175.1189
y2−NH3 400.2301
b2 414.2911
y2 417.2566
y3−NH3 457.2518
y3 474.2784
b3 577.3547
y4−NH3 620.3147
y4 637.3417
b4 692.3819
y5−NH3 733.397
y5 750.4259
b5 805.4655
y6 865.453
A I Y D I Y G K R
y6 y5 y4 y3 y2 y1
b2 b3 b4 b5
Modification
Acetyl (Protein N−term)
GlyGly (K) no new terminus
Oxidation (M)

20200921_KIZ_MUb4_GlyGly_10plex_2 − Scan number: 12654

Gene Names	Charge	m/z	Mass	Retention time	PEP	Score	Precursor Intensity
DNAJC11	3	404.8839	1211.63	49	0.010809	91.069	974664.3

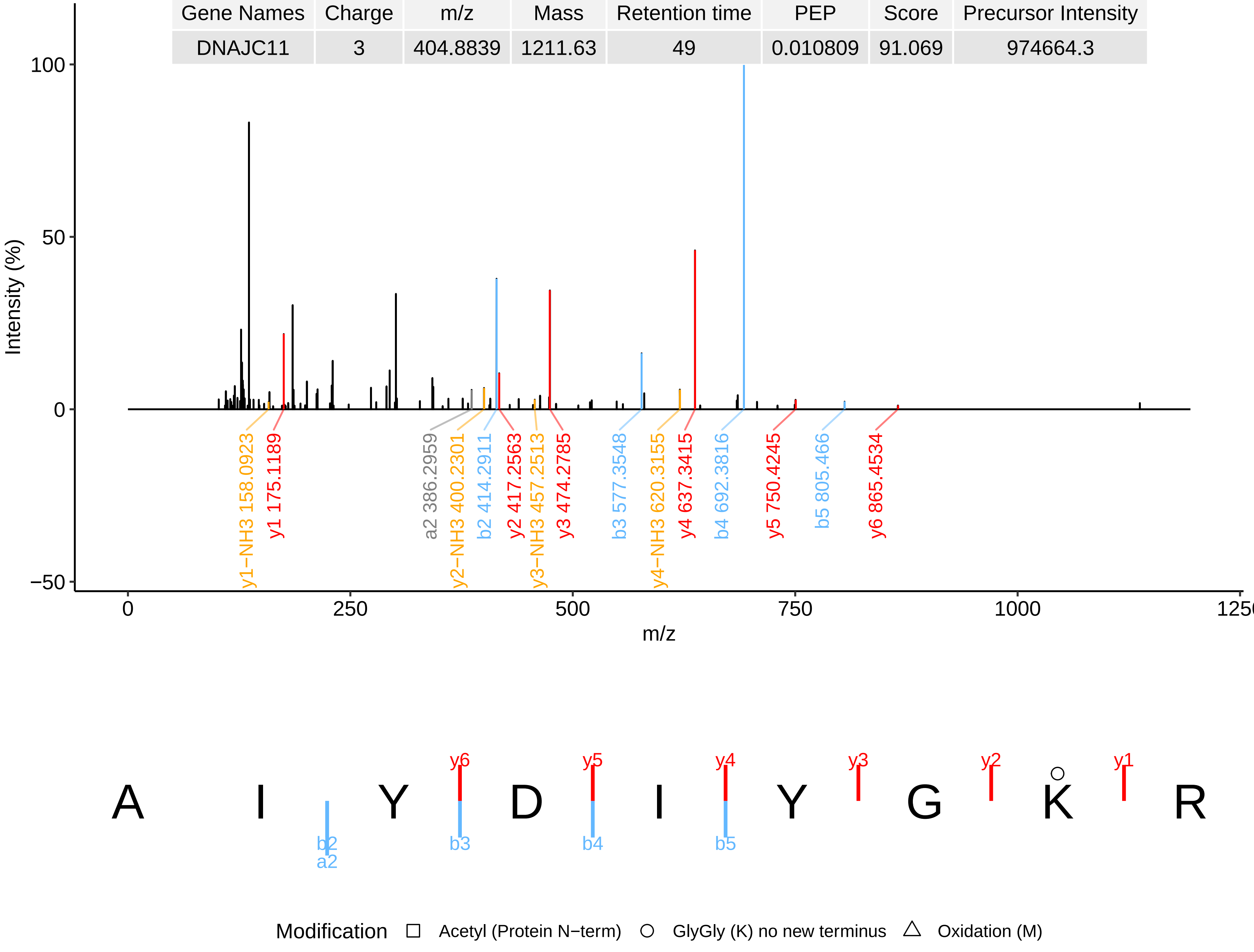

20200921_KIZ_MUb4_GlyGly_10plex − Scan number: 8154

Gene Names	Charge	m/z	Mass	Retention time	PEP	Score	Precursor Intensity
MYOF	3	334.8515	1001.533	27.593	0.049652	55.531	9796524

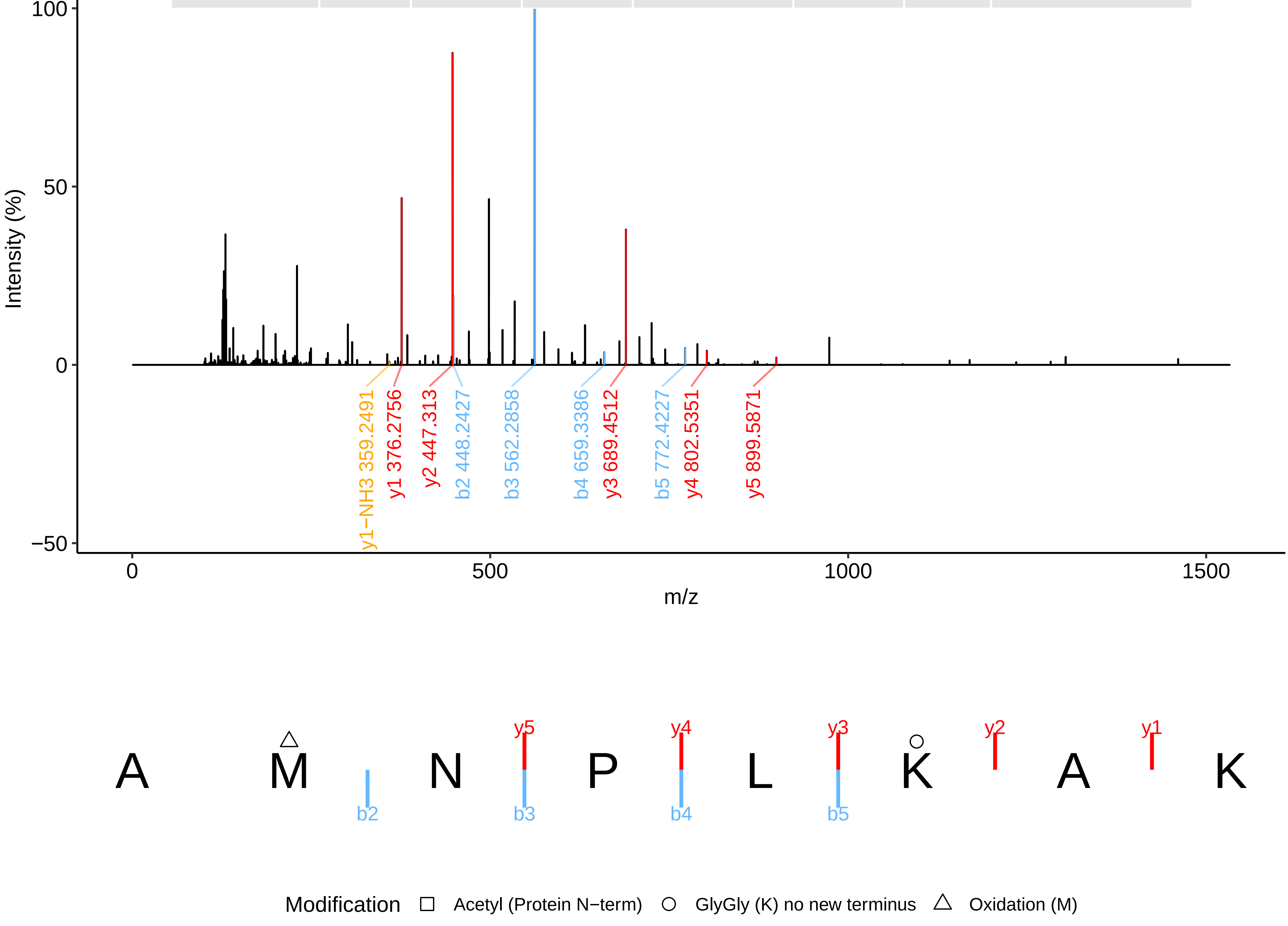

20200921_KIZ_MUb4_GlyGly_10plex_2 − Scan number: 7510

Gene Names	Charge	m/z	Mass	Retention time	PEP	Score	Precursor Intensity
MYOF	3	334.8515	1001.533	28.051	0.089604	43.246	626939.5

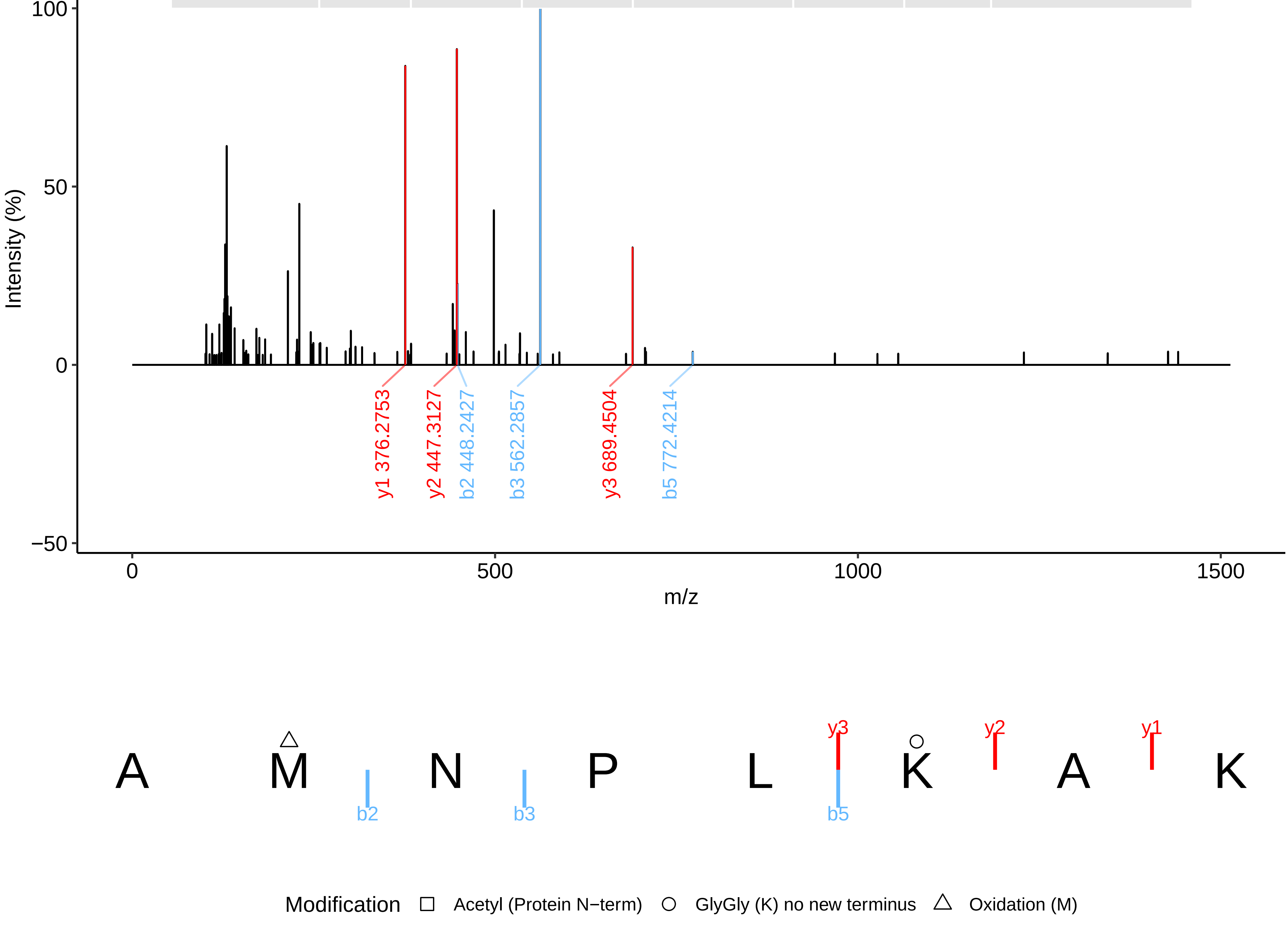

20200921_KIZ_MUb4_GlyGly_10plex − Scan number: 11424

Gene Names	Charge	m/z	Mass	Retention time	PEP	Score	Precursor Intensity
MYOF	3	329.5199	985.5379	39.796	0.013691	76.899	21427300

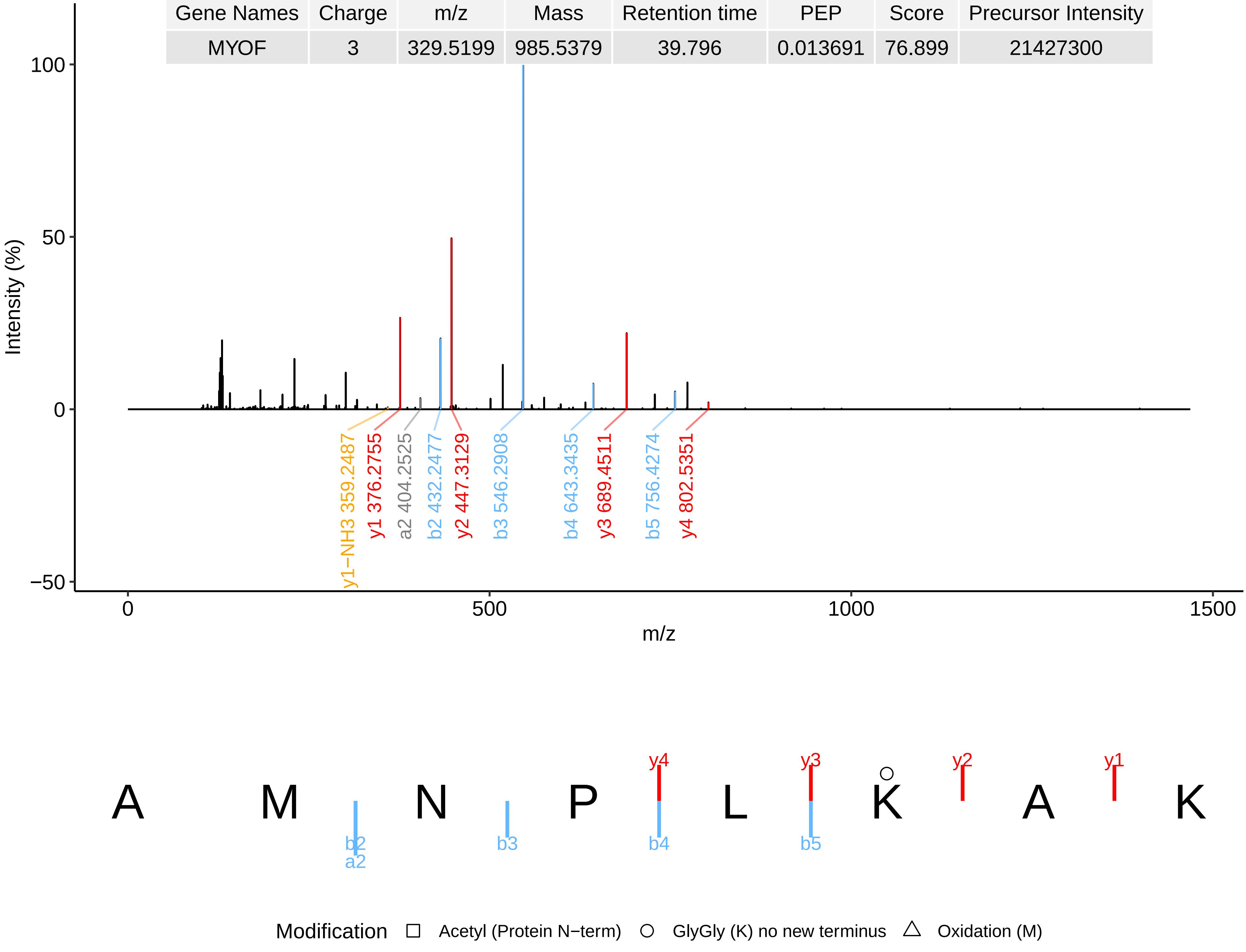

20200921_KIZ_MUb4_GlyGly_10plex_2 − Scan number: 10557

Gene Names	Charge	m/z	Mass	Retention time	PEP	Score	Precursor Intensity
MYOF	3	329.5199	985.5379	40.41	0.028717	64.836	903622.4

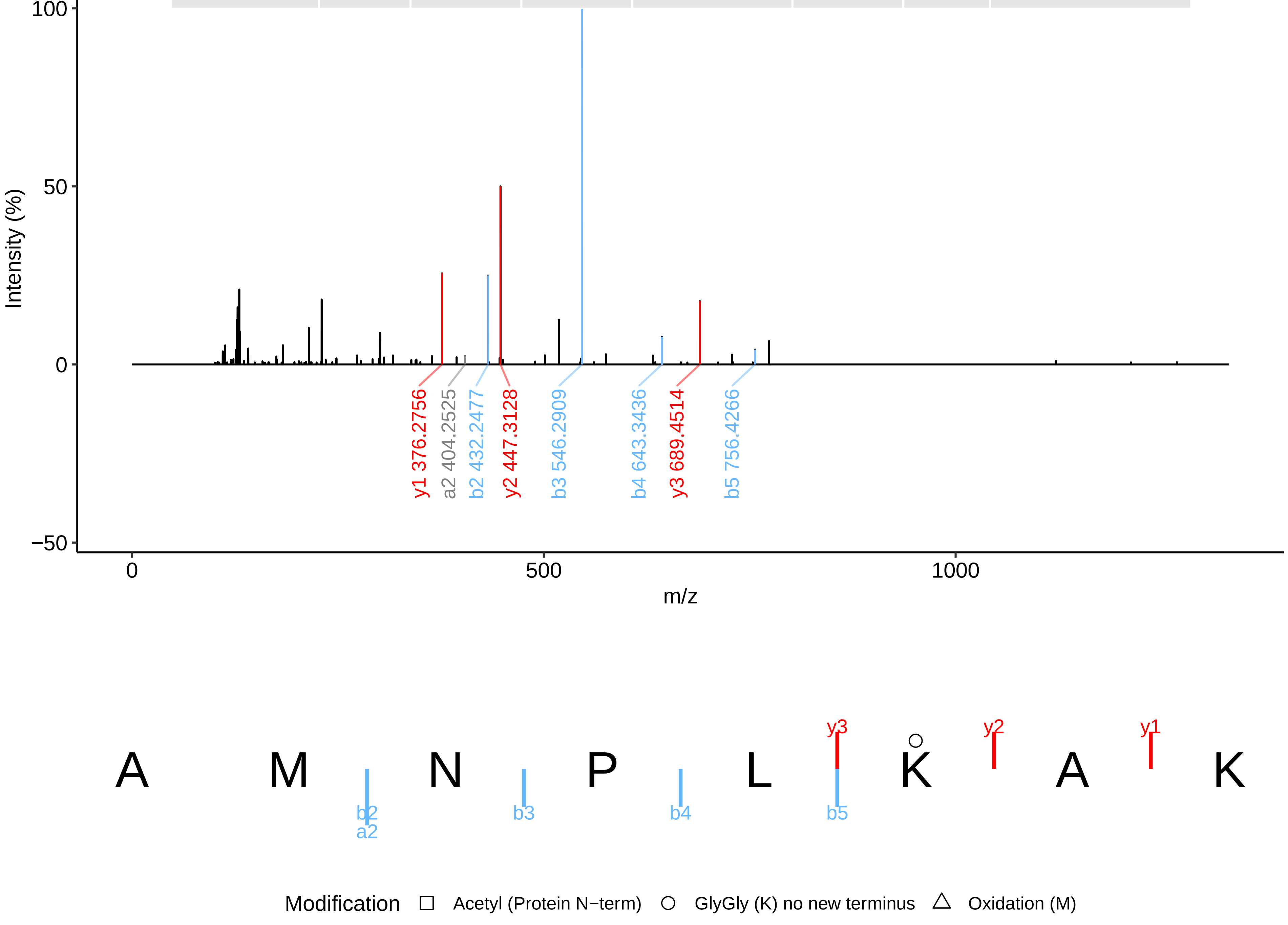

20200924_KIZ_MUb5_GlyGly_1 − Scan number: 10159

Gene Names	Charge	m/z	Mass	Retention time	PEP	Score	Precursor Intensity
MYOF	3	329.5199	985.5379	32.75	0.028199	65.252	13058945

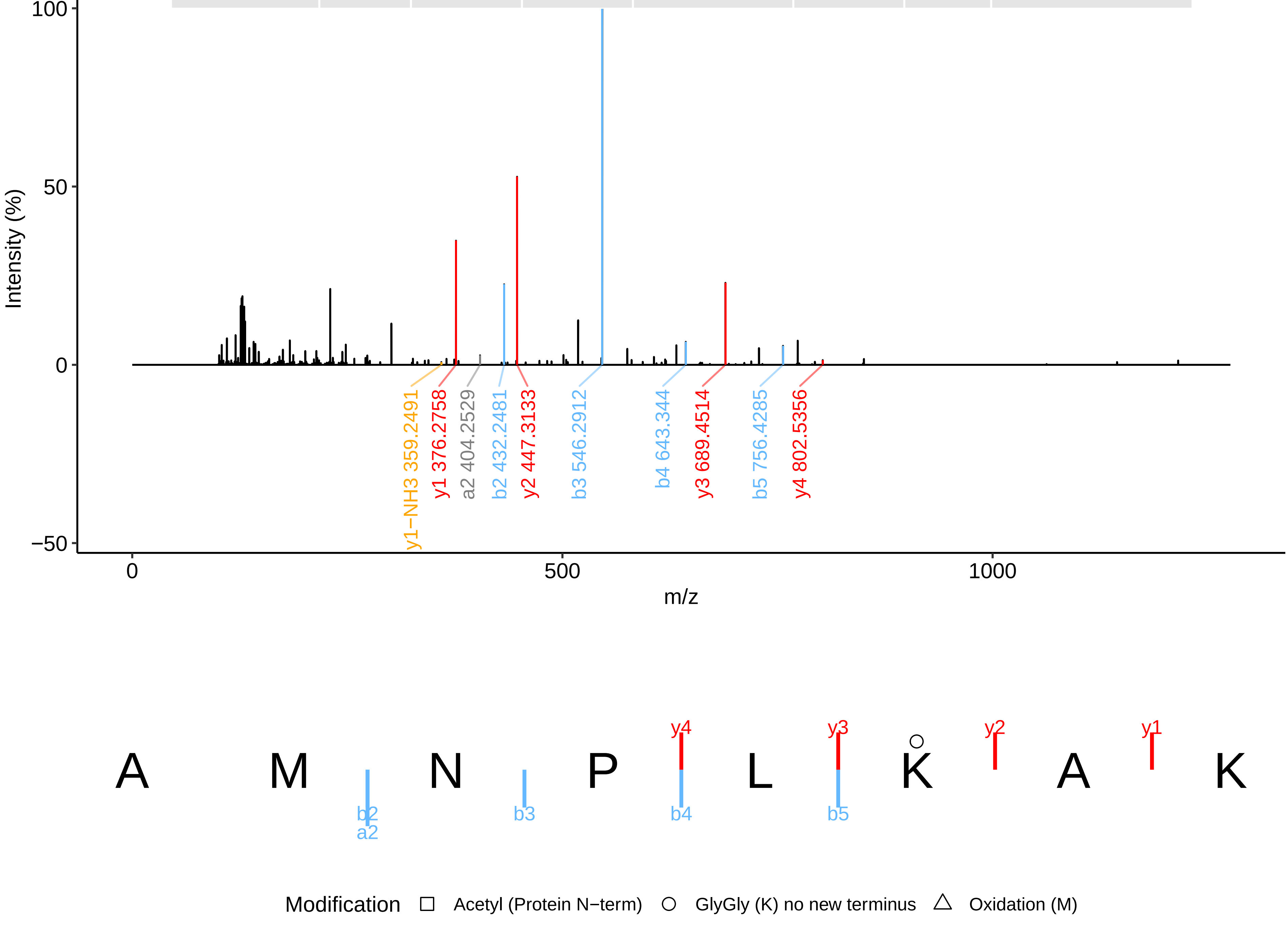

20200924_KIZ_MUb5_GlyGly_1 − Scan number: 10463

Gene Names	Charge	m/z	Mass	Retention time	PEP	Score	Precursor Intensity
MYOF	3	329.5199	985.5379	33.782	0.013213	77.282	11864203

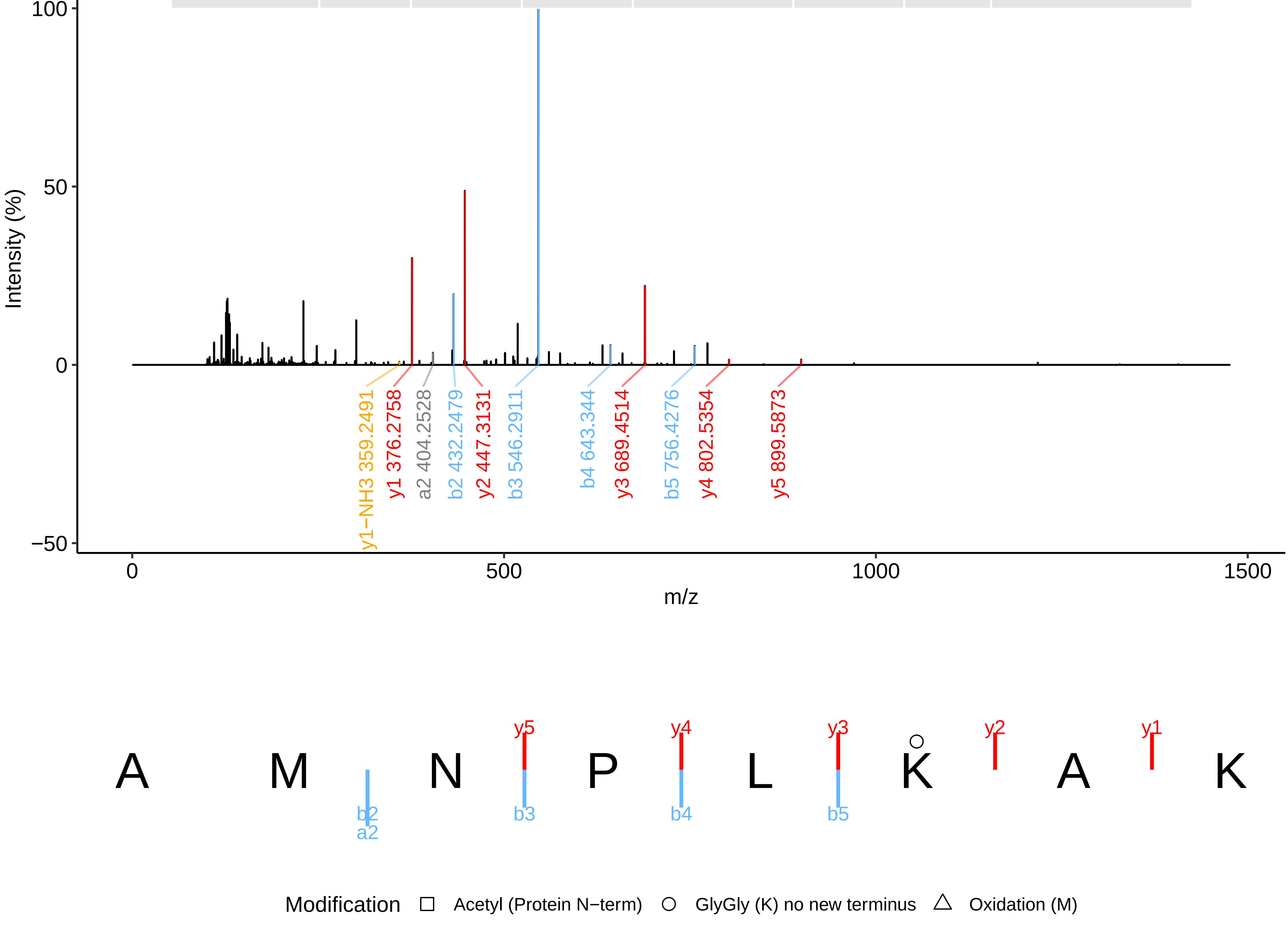

20200924_KIZ_MUb5_GlyGly_2 − Scan number: 9729

Gene Names	Charge	m/z	Mass	Retention time	PEP	Score	Precursor Intensity
MYOF	3	329.5199	985.5379	36.35	0.055397	57.177	3080366

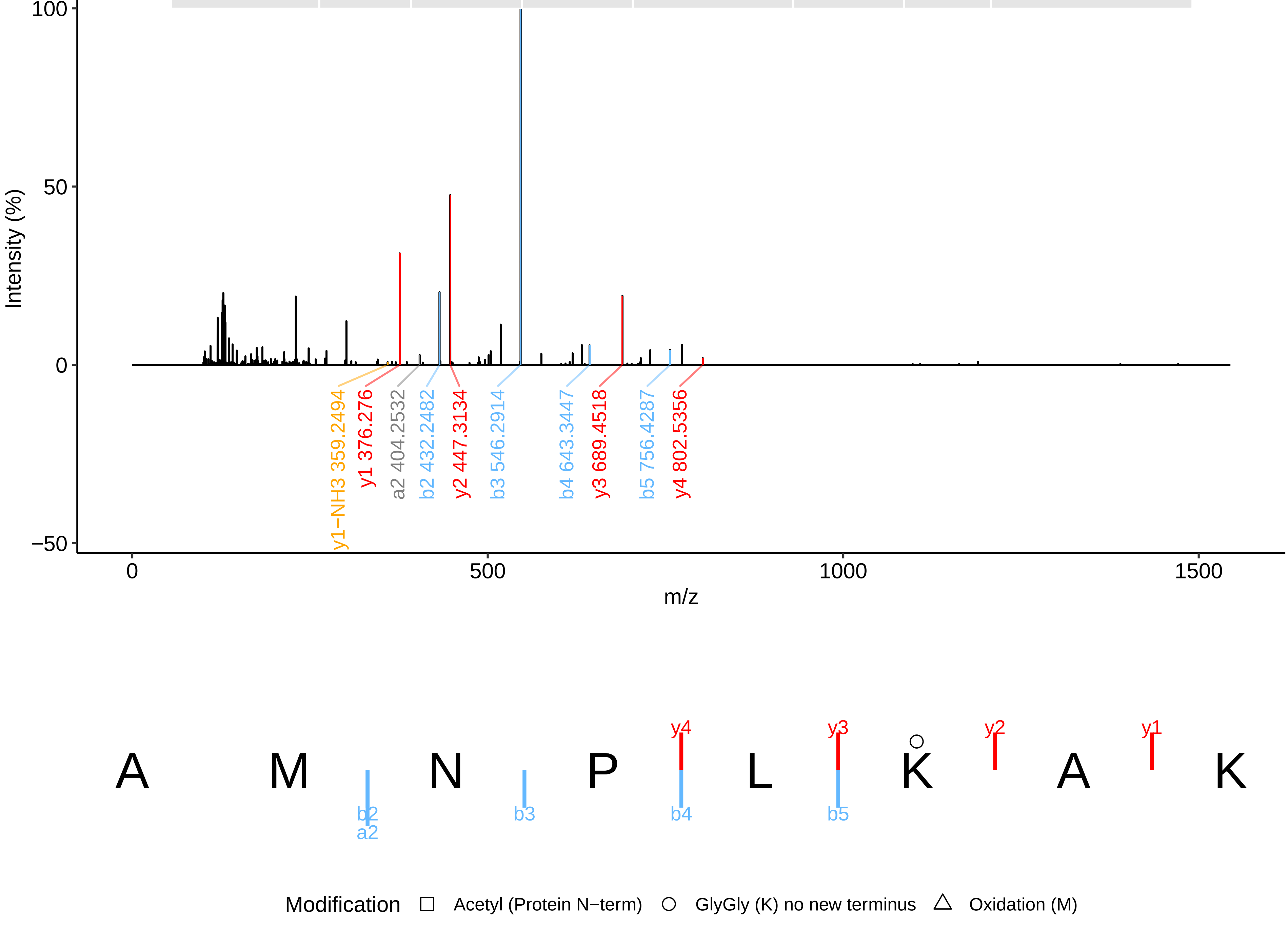

20200924_KIZ_MUb5_GlyGly_2 − Scan number: 9484

Gene Names	Charge	m/z	Mass	Retention time	PEP	Score	Precursor Intensity
MYOF	3	329.5199	985.5379	35.446	0.036132	64.567	4406612

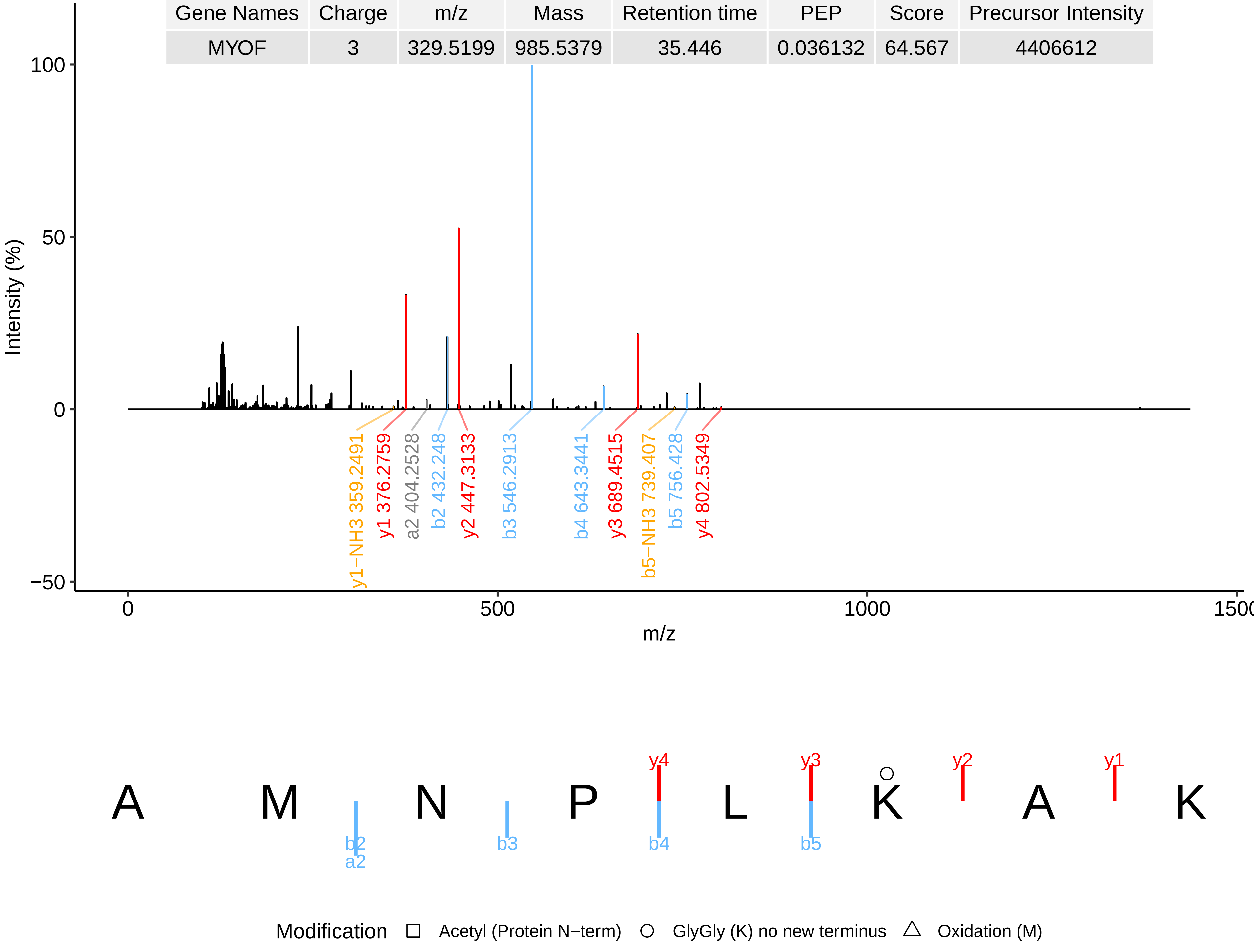

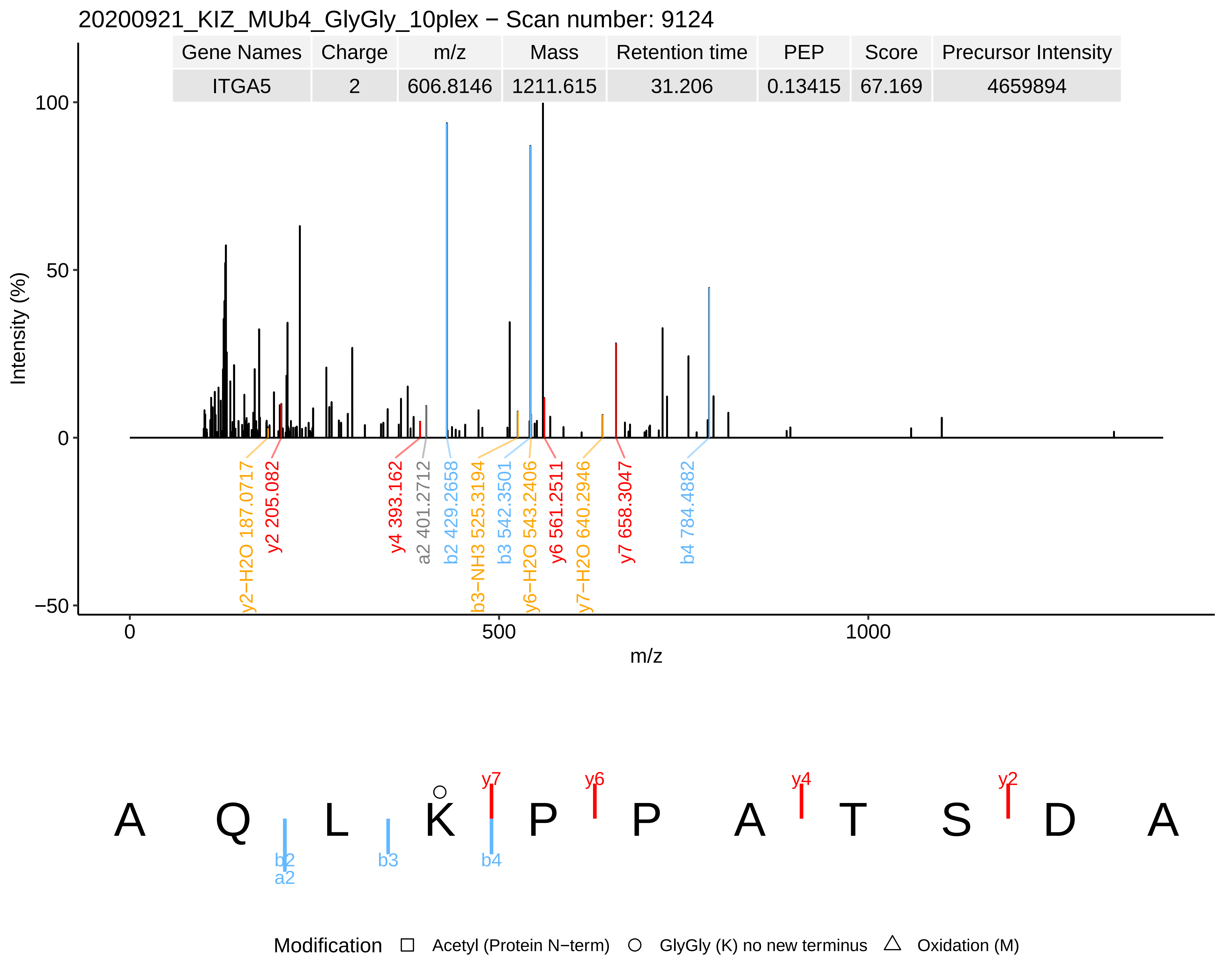
20200921_KIZ_MUb4_GlyGly_10plex − Scan number: 9124
Gene Names | Charge | m/z | Mass | Retention time | PEP | Score | Precursor Intensity
ITGA5 | 2 | 606.8146 | 1211.615 | 31.206 | 0.13415 | 67.169 | 4659894
Intensity (%)
m/z
y2−H2O 187.0717
y2 205.082
y4 393.162
a2 401.2712
b2 429.2658
b3−NH3 525.3194
b3 542.3501
y6−H2O 543.2406
y6 561.2511
y7−H2O 640.2946
y7 658.3047
b4 784.4882
A Q L K P P A T S D A
y7 y6 y4 y2
b2 a2 b3 b4
Modification
Acetyl (Protein N−term)
GlyGly (K) no new terminus
Oxidation (M)

20200921_KIZ_MUb4_GlyGly_10plex − Scan number: 6423

Gene Names	Charge	m/z	Mass	Retention time	PEP	Score	Precursor Intensity
ATAD3B;ATAD3A	3	402.879	1205.615	21.244	1.8818e−07	141.52	7252122

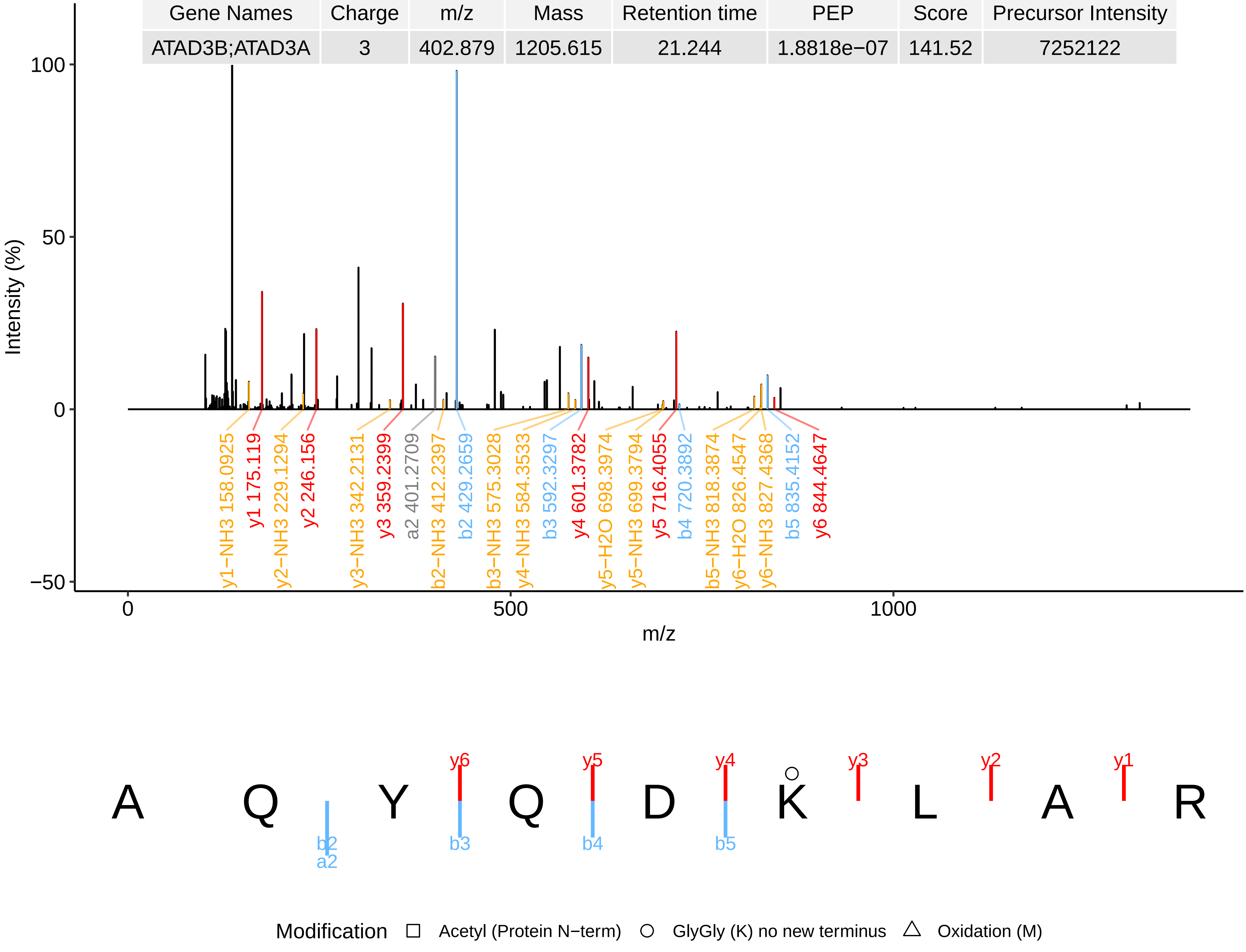

20200921_KIZ_MUb4_GlyGly_10plex_2 − Scan number: 5898

Gene Names	Charge	m/z	Mass	Retention time	PEP	Score	Precursor Intensity
ATAD3B;ATAD3A	3	402.879	1205.615	21.437	0.00022943	108.81	1155925

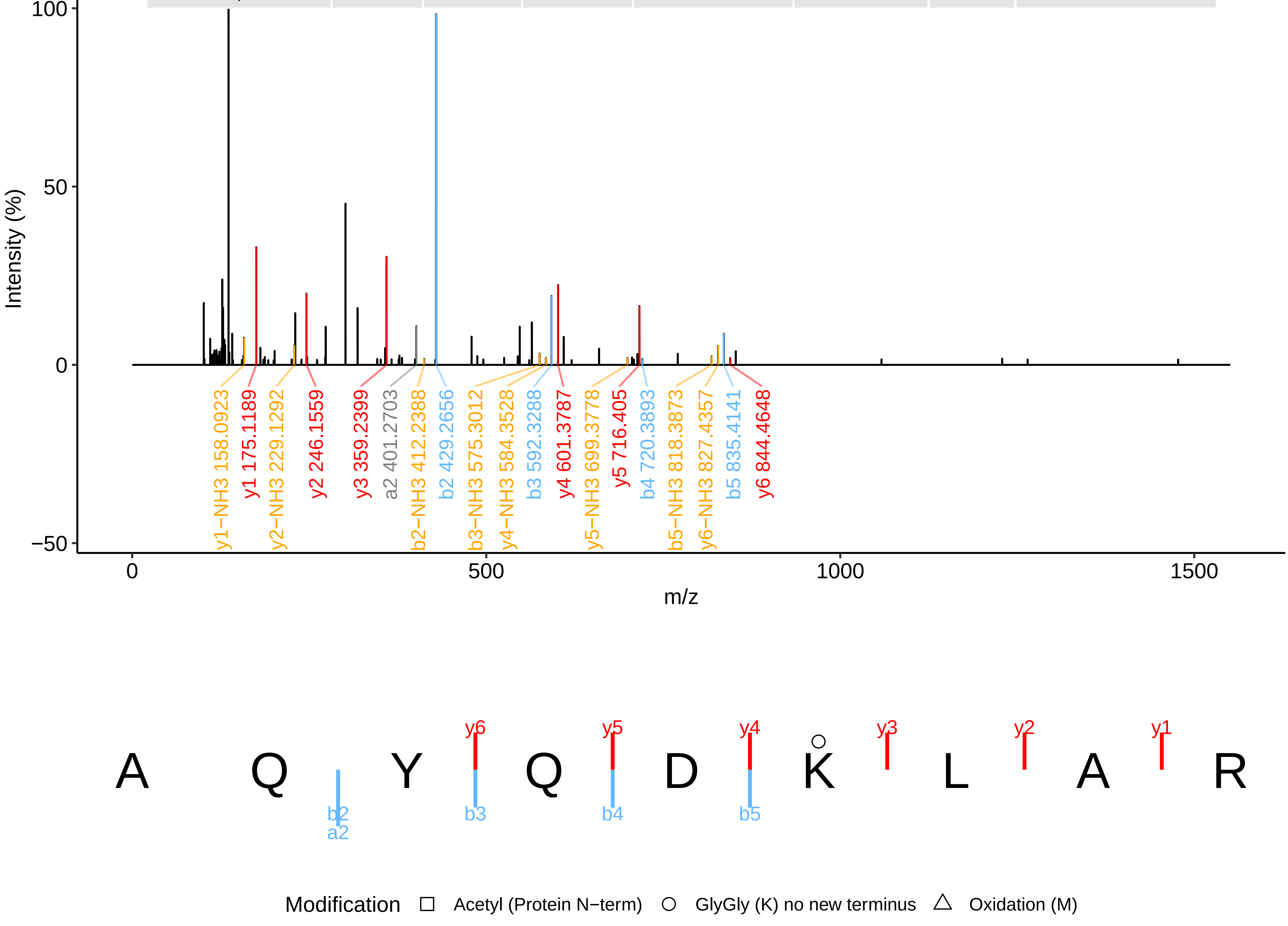

20200924_KIZ_MUb5_GlyGly_1 − Scan number: 5494

Gene Names	Charge	m/z	Mass	Retention time	PEP	Score	Precursor Intensity
ATAD3B;ATAD3A	3	402.879	1205.615	17.099	0.028221	63.216	5879909

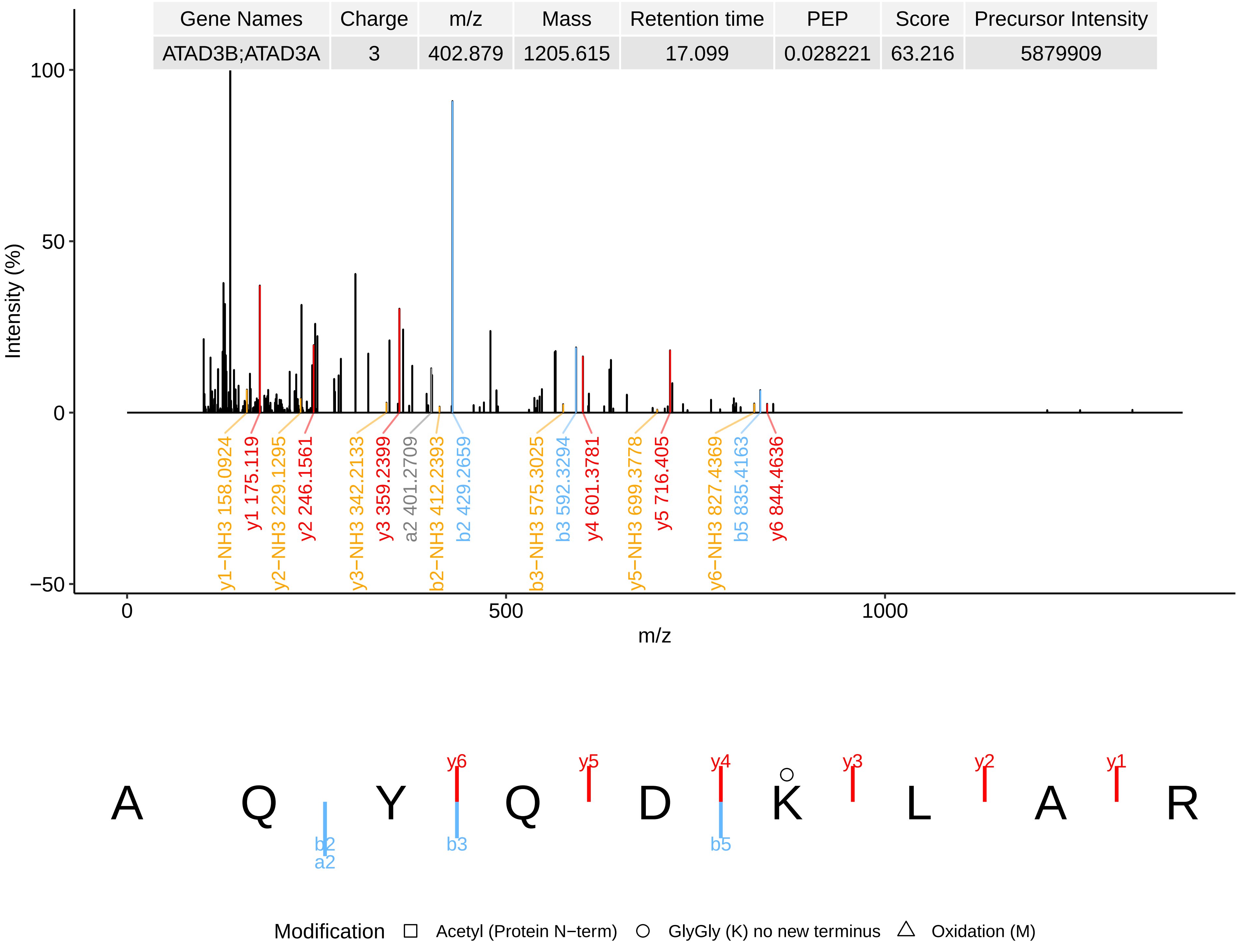

20200924_KIZ_MUb5_GlyGly_2 − Scan number: 5264

Gene Names	Charge	m/z	Mass	Retention time	PEP	Score	Precursor Intensity
ATAD3B;ATAD3A	3	402.879	1205.615	19.51	0.0029428	93.178	NA

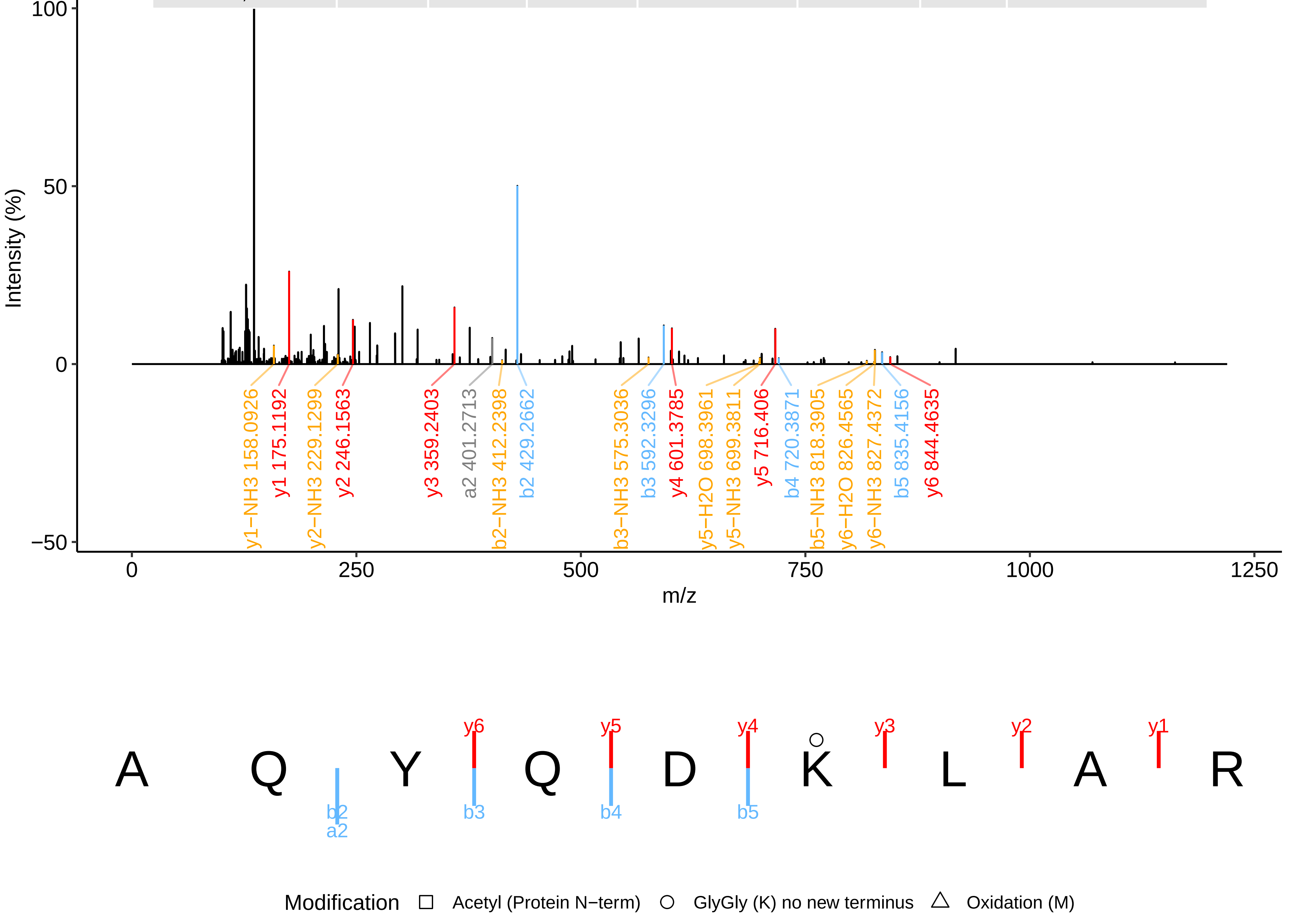

20200921_KIZ_MUb4_GlyGly_10plex − Scan number: 15428

Gene Names	Charge	m/z	Mass	Retention time	PEP	Score	Precursor Intensity
WBSCR22	3	329.8589	986.5549	55.108	0.14179	73.039	2231684

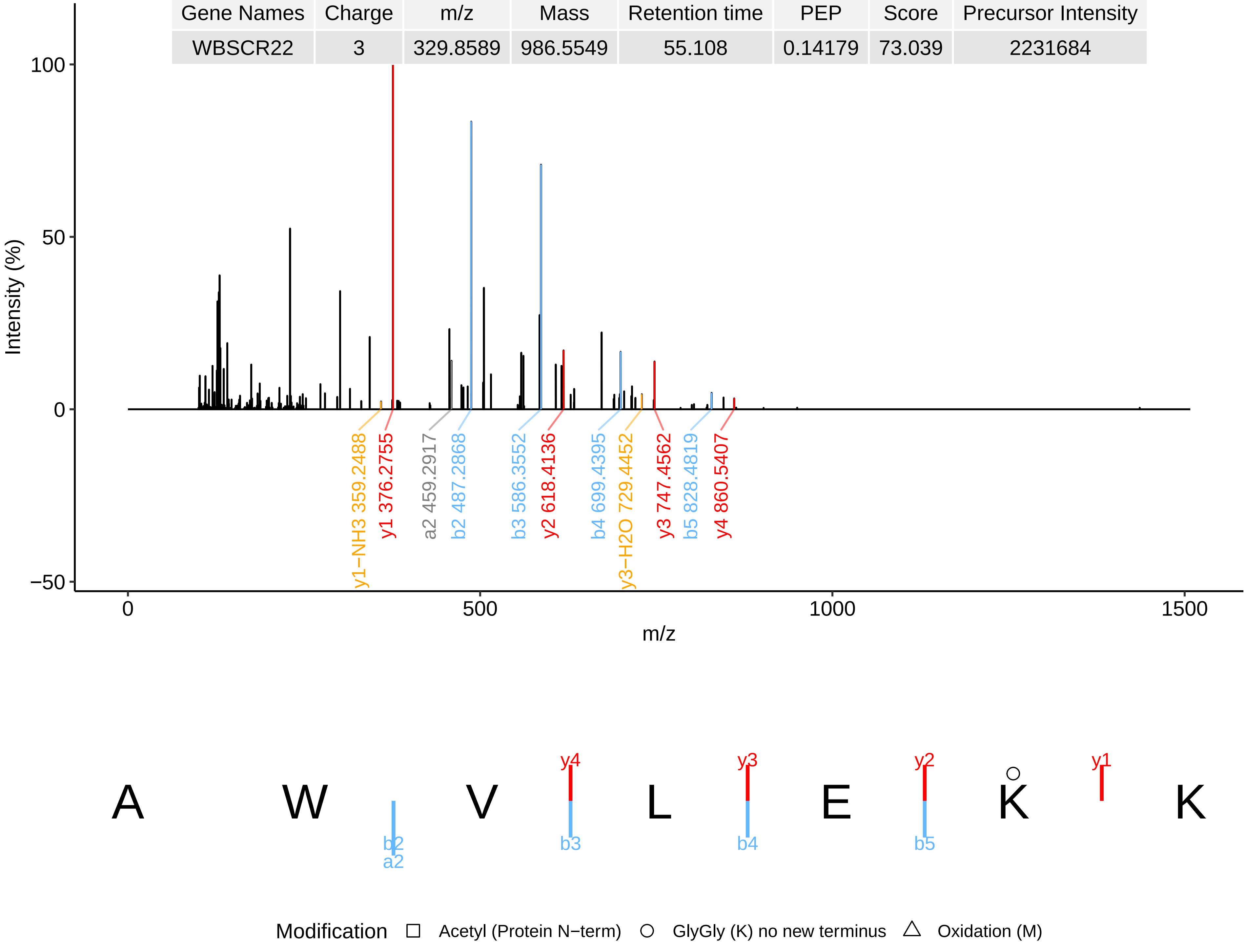

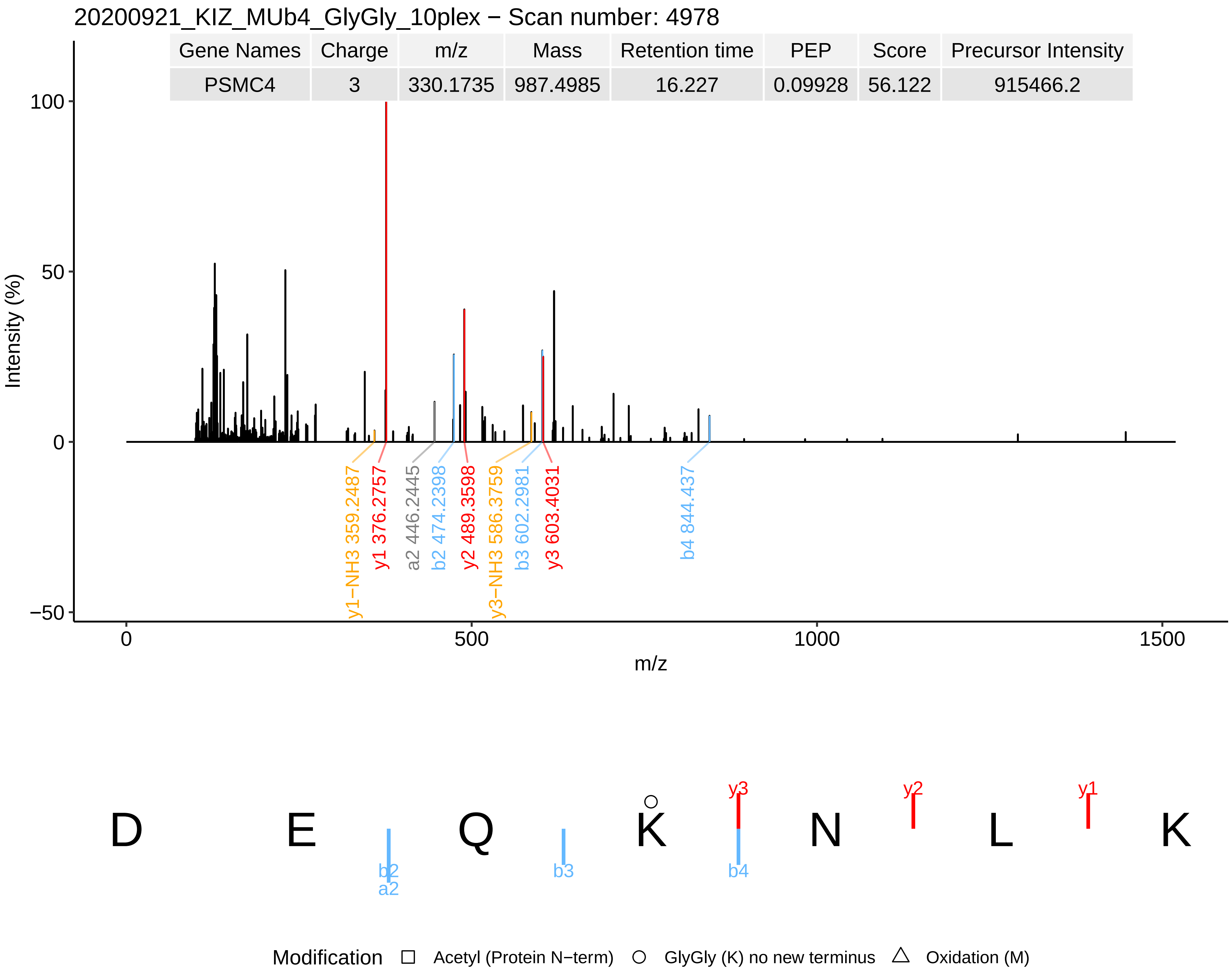

20200921_KIZ_MUb4_GlyGly_10plex − Scan number: 4978
Gene Names | Charge | m/z | Mass | Retention time | PEP | Score | Precursor Intensity
PSMC4 | 3 | 330.1735 | 987.4985 | 16.227 | 0.09928 | 56.122 | 915466.2
Intensity (%)
m/z
y1−NH3 359.2487
y1 376.2757
a2 446.2445
b2 474.2398
y2 489.3598
y3−NH3 586.3759
b3 602.2981
y3 603.4031
b4 844.437
D E Q K N L K
y3 y2 y1
b2 a2 b3 b4
Modification
Acetyl (Protein N−term)
GlyGly (K) no new terminus
Oxidation (M)

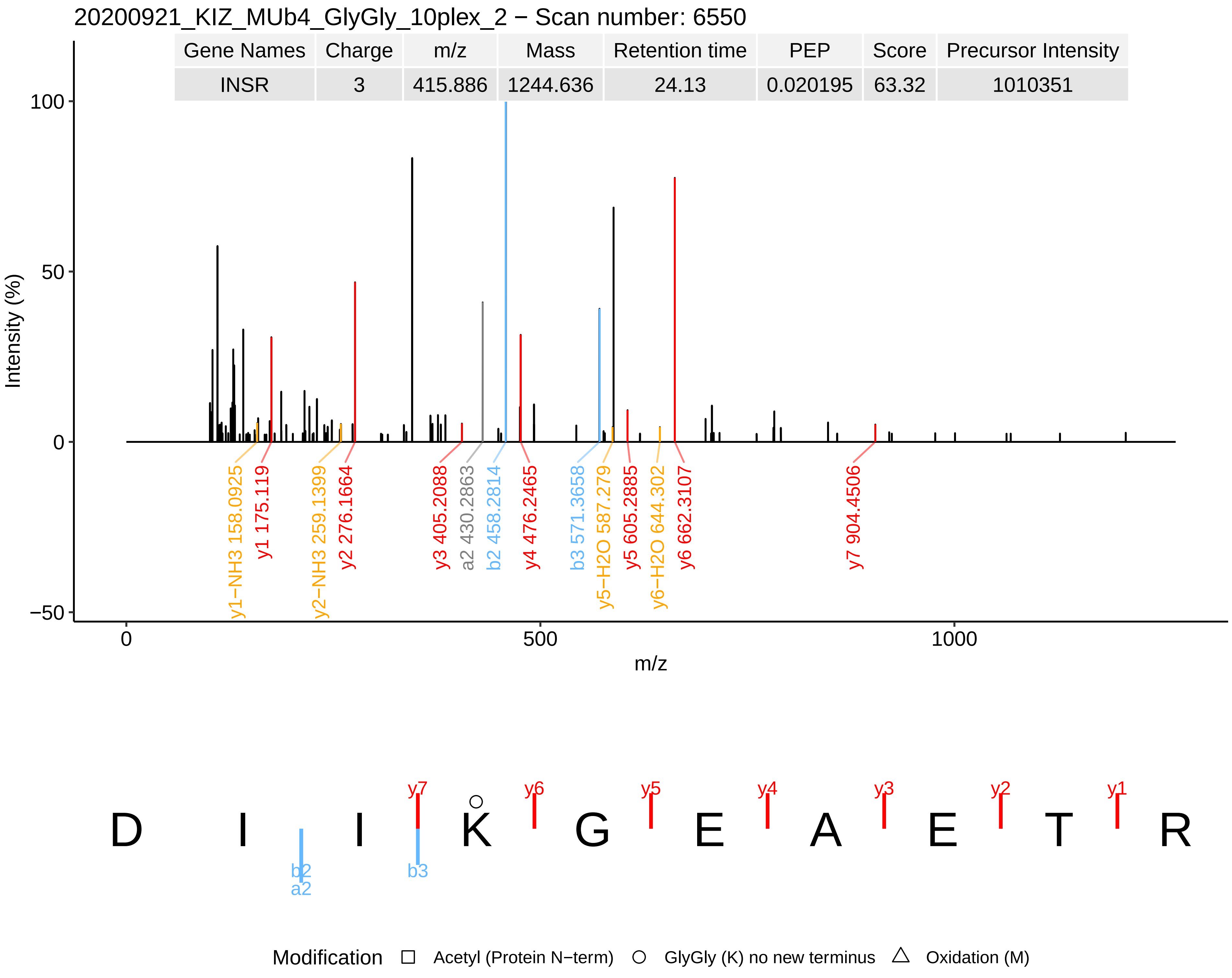
20200921_KIZ_MUb4_GlyGly_10plex_2 − Scan number: 6550
Gene Names | Charge | m/z | Mass | Retention time | PEP | Score | Precursor Intensity
INSR | 3 | 415.886 | 1244.636 | 24.13 | 0.020195 | 63.32 | 1010351
Intensity (%)
100
50
0
−50
0
500
1000
m/z
y1−NH3 158.0925
y1 175.119
y2−NH3 259.1399
y2 276.1664
y3 405.2088
a2 430.2863
b2 458.2814
y4 476.2465
b3 571.3658
y5−H2O 587.279
y5 605.2885
y6−H2O 644.302
y6 662.3107
y7 904.4506
D I I K G E A E T R
y7 y6 y5 y4 y3 y2 y1
b2 a2 b3
Modification
Acetyl (Protein N−term)
GlyGly (K) no new terminus
Oxidation (M)

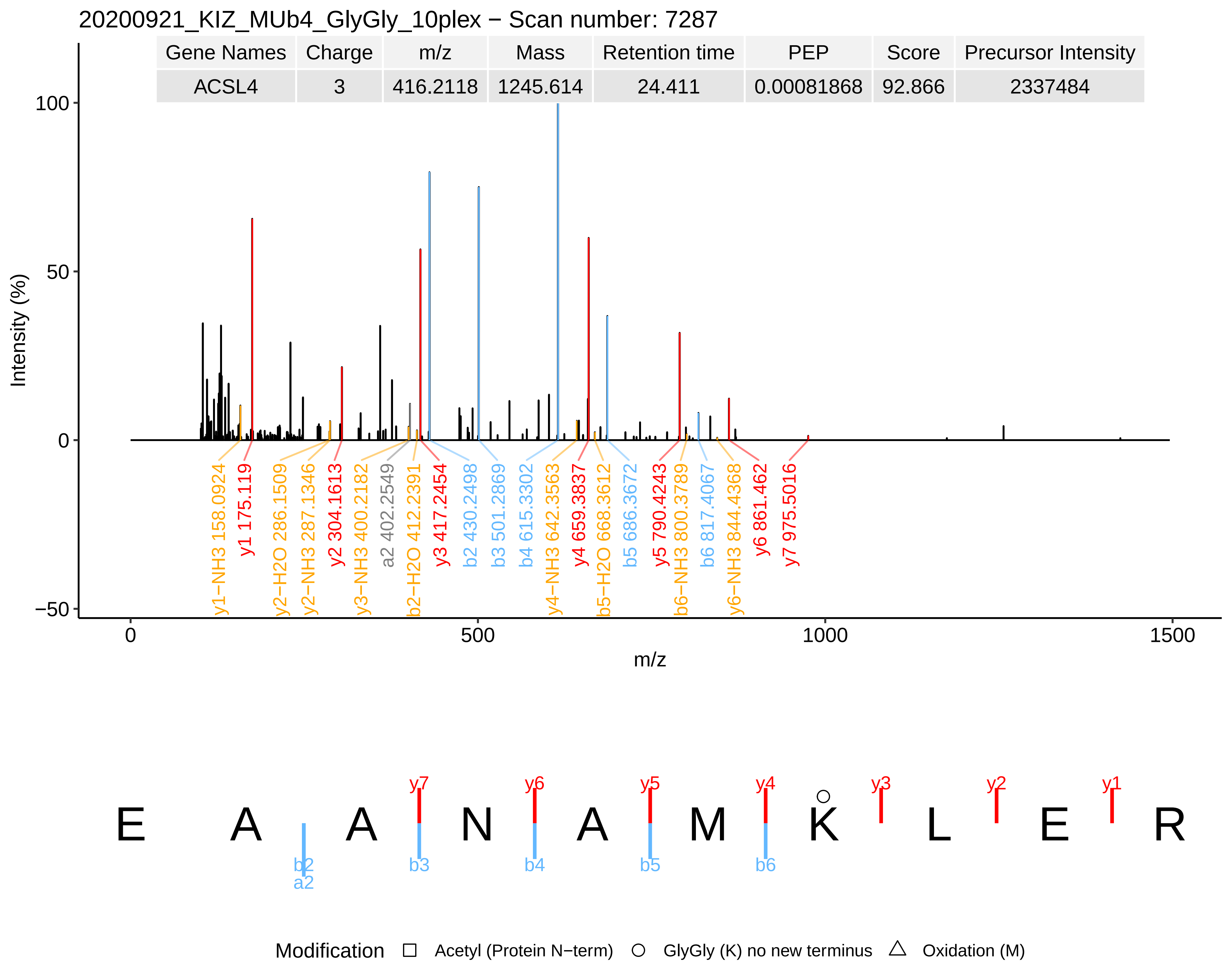

20200921_KIZ_MUb4_GlyGly_10plex − Scan number: 7287
Gene Names | Charge | m/z | Mass | Retention time | PEP | Score | Precursor Intensity
ACSL4 | 3 | 416.2118 | 1245.614 | 24.411 | 0.00081868 | 92.866 | 2337484
Intensity (%)
m/z
y1−NH3 158.0924
y1 175.119
y2−H2O 286.1509
y2−NH3 287.1346
y2 304.1613
y3−NH3 400.2182
a2 402.2549
b2−H2O 412.2391
y3 417.2454
b2 430.2498
b3 501.2869
b4 615.3302
y4−NH3 642.3563
y4 659.3837
b5−H2O 668.3612
b5 686.3672
y5 790.4243
b6−NH3 800.3789
b6 817.4067
y6−NH3 844.4368
y6 861.462
y7 975.5016
E A A N A M K L E R
Modification
Acetyl (Protein N−term)
GlyGly (K) no new terminus
Oxidation (M)

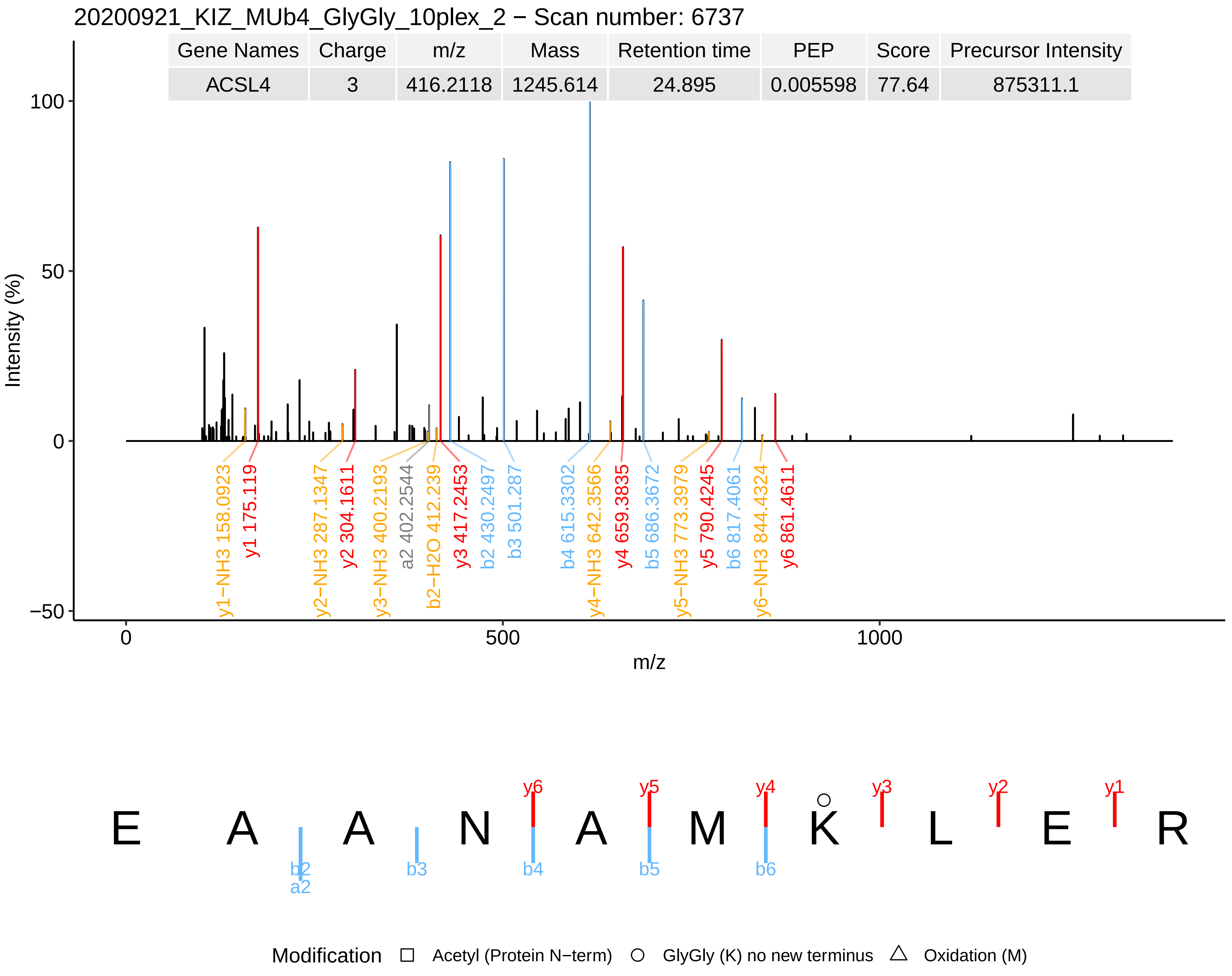

20200921_KIZ_MUb4_GlyGly_10plex_2 − Scan number: 6737
Gene Names | Charge | m/z | Mass | Retention time | PEP | Score | Precursor Intensity
ACSL4 | 3 | 416.2118 | 1245.614 | 24.895 | 0.005598 | 77.64 | 875311.1
Intensity (%)
m/z
y1−NH3 158.0923
y1 175.119
y2−NH3 287.1347
y2 304.1611
y3−NH3 400.2193
a2 402.2544
b2−H2O 412.239
y3 417.2453
b2 430.2497
b3 501.287
b4 615.3302
y4−NH3 642.3566
y4 659.3835
b5 686.3672
y5−NH3 773.3979
y5 790.4245
b6 817.4061
y6−NH3 844.4324
y6 861.4611
E A A N A M K L E R
Modification
Acetyl (Protein N−term)
GlyGly (K) no new terminus
Oxidation (M)

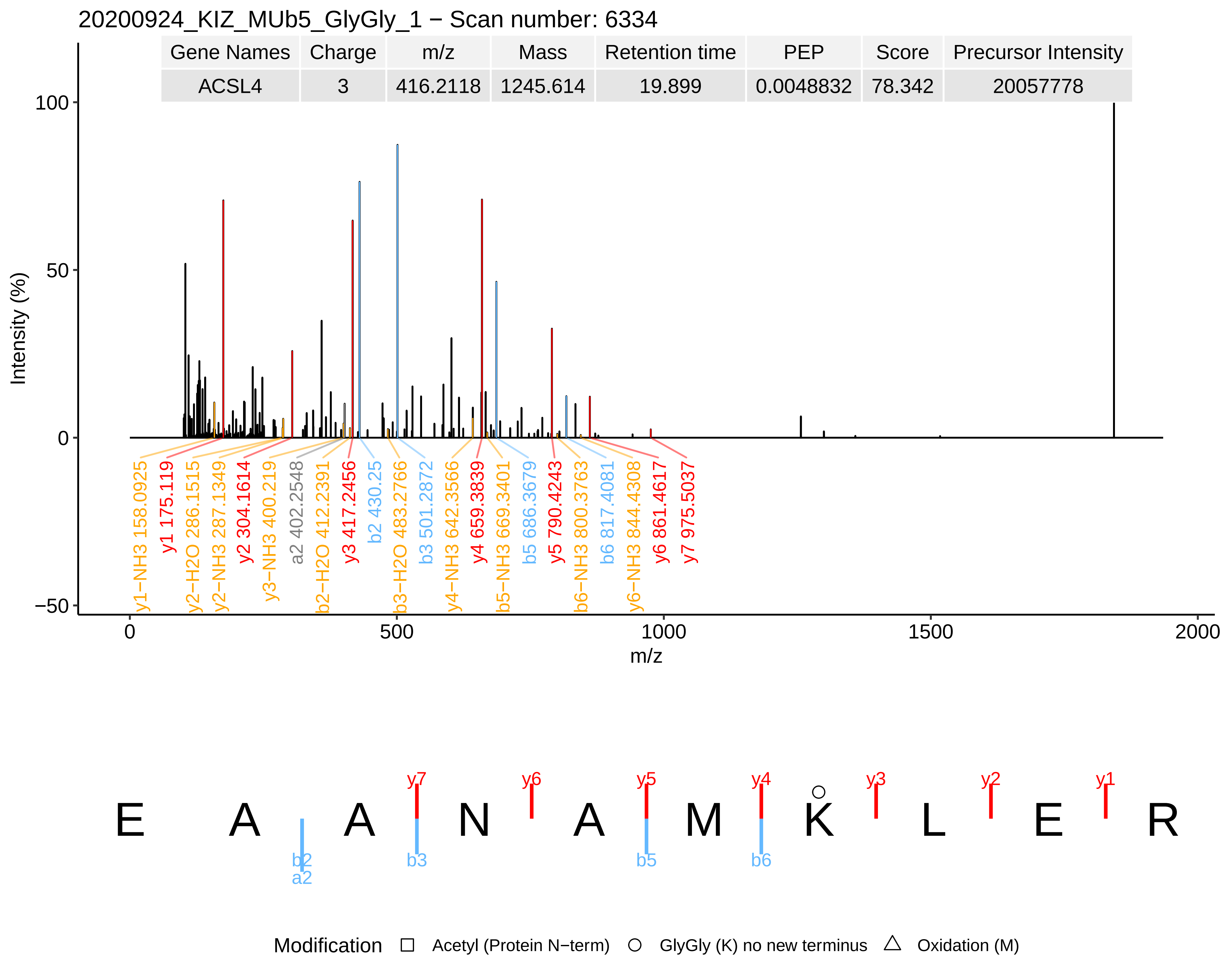

20200924_KIZ_MUb5_GlyGly_1 − Scan number: 6334
Gene Names | Charge | m/z | Mass | Retention time | PEP | Score | Precursor Intensity
ACSL4 | 3 | 416.2118 | 1245.614 | 19.899 | 0.0048832 | 78.342 | 20057778
Intensity (%)
100
50
0
−50
0
500
1000
1500
2000
m/z
y1−NH3 158.0925
y1 175.119
y2−H2O 286.1515
y2−NH3 287.1349
y2 304.1614
y3−NH3 400.219
a2 402.2548
b2−H2O 412.2391
y3 417.2456
b2 430.25
b3−H2O 483.2766
b3 501.2872
y4−NH3 642.3566
y4 659.3839
b5−NH3 669.3401
b5 686.3679
y5 790.4243
b6−NH3 800.3763
b6 817.4081
y6−NH3 844.4308
y6 861.4617
y7 975.5037
E A A N A M K L E R
y7 y6 y5 y4 y3 y2 y1
b2 a2 b3 b5 b6
Modification
Acetyl (Protein N−term)
GlyGly (K) no new terminus
Oxidation (M)

20200924_KIZ_MUb5_GlyGly_2 − Scan number: 5884

Gene Names	Charge	m/z	Mass	Retention time	PEP	Score	Precursor Intensity
ACSL4	3	416.2118	1245.614	21.891	0.011801	71.555	2512209

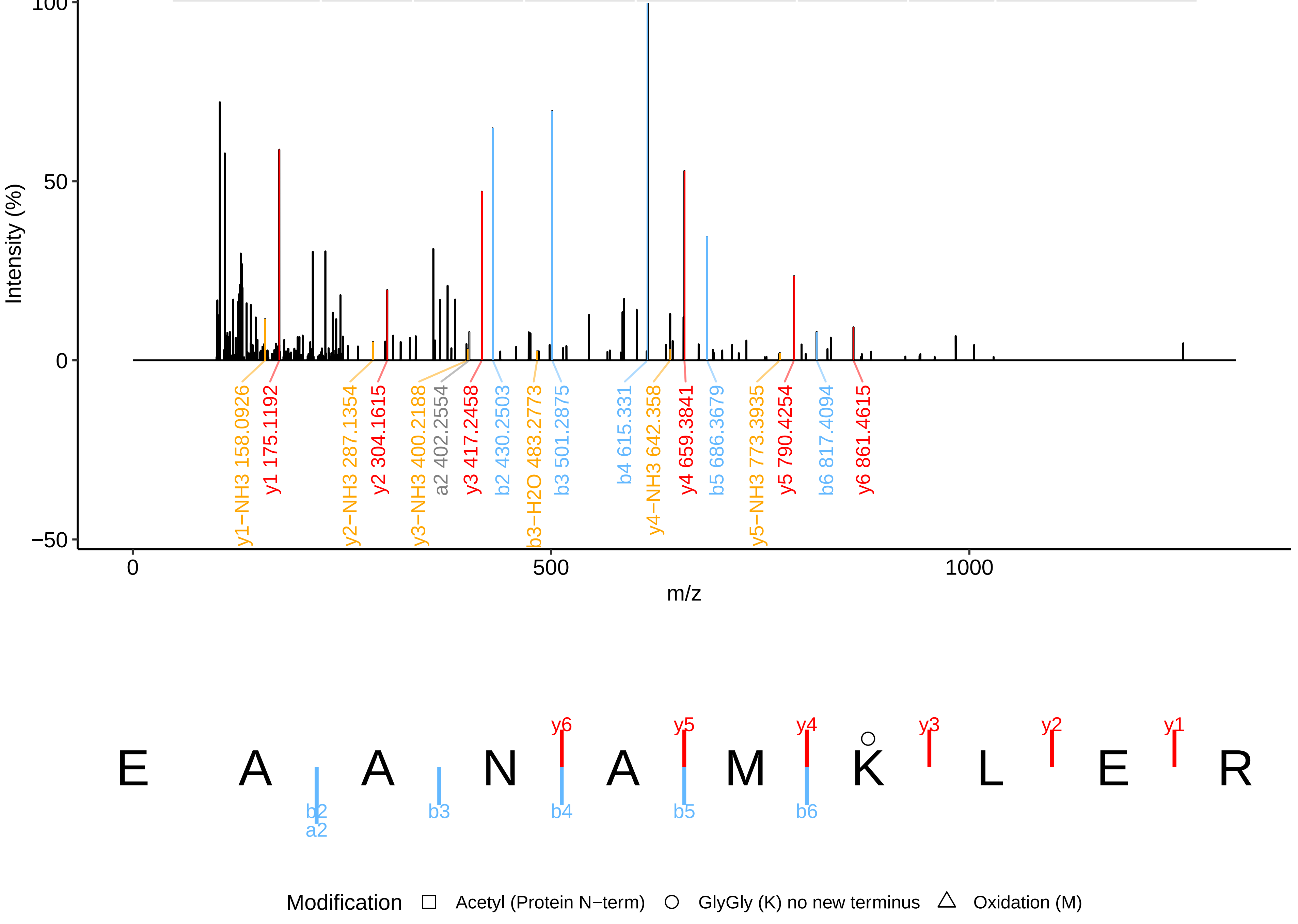

20200924_KIZ_MUb5_GlyGly_2 − Scan number: 5915

Gene Names	Charge	m/z	Mass	Retention time	PEP	Score	Precursor Intensity
ACSL4	3	416.2118	1245.614	22.012	0.069688	53.569	1456813

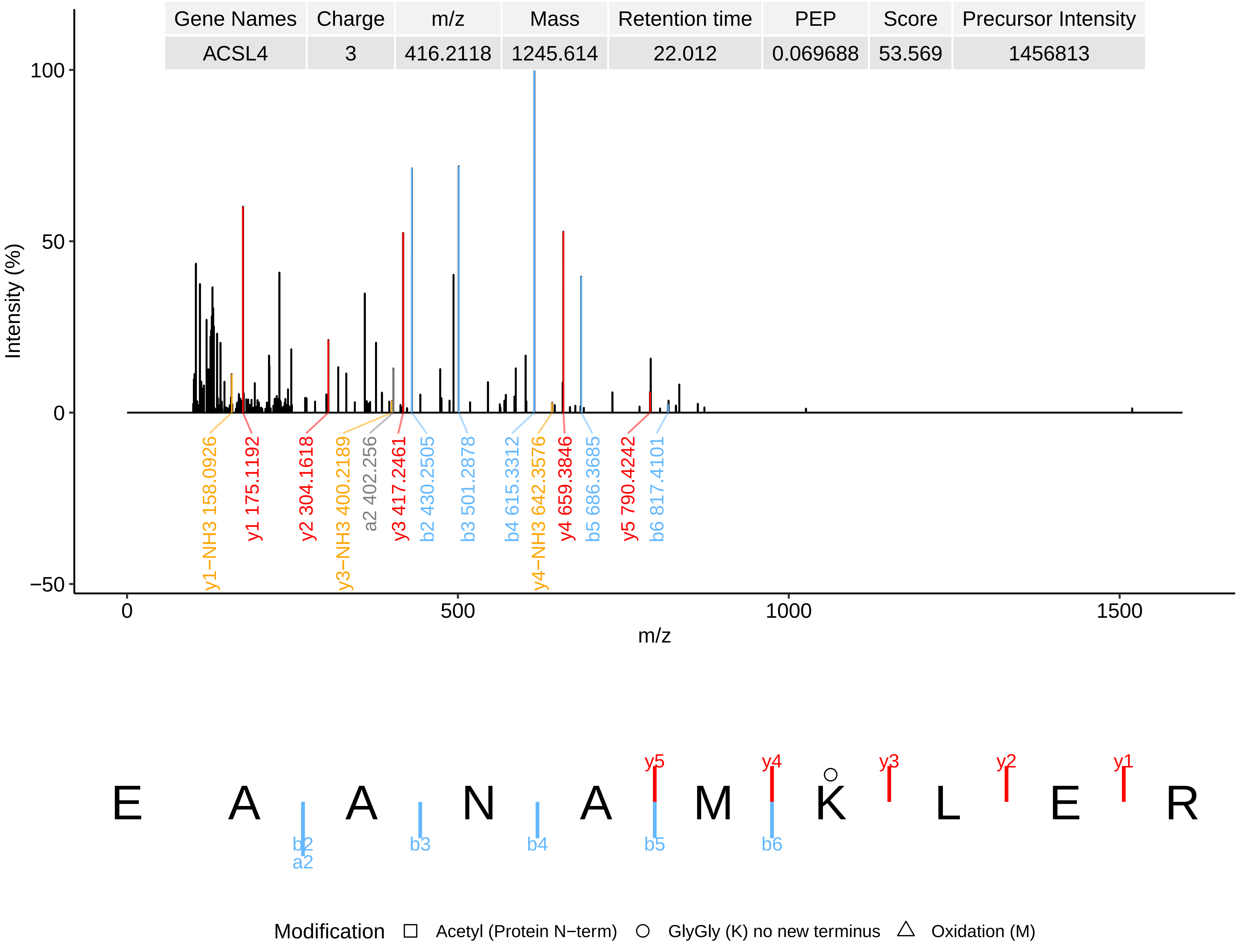

20201118_KIZ_MUb6_GlyGly_1 − Scan number: 5848

Gene Names	Charge	m/z	Mass	Retention time	PEP	Score	Precursor Intensity
ACSL4	3	416.2118	1245.614	18.515	0.0011013	90.913	11301819

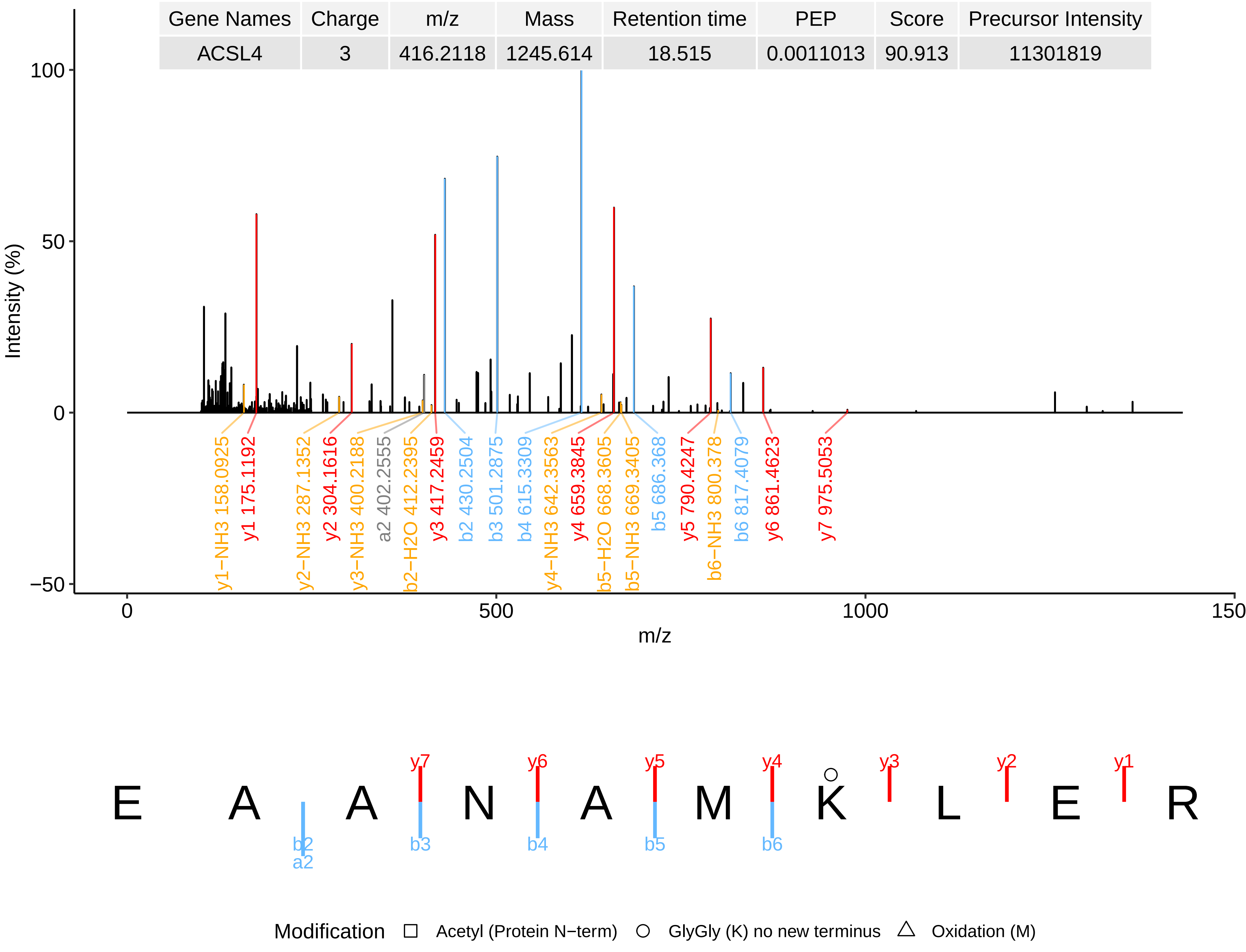

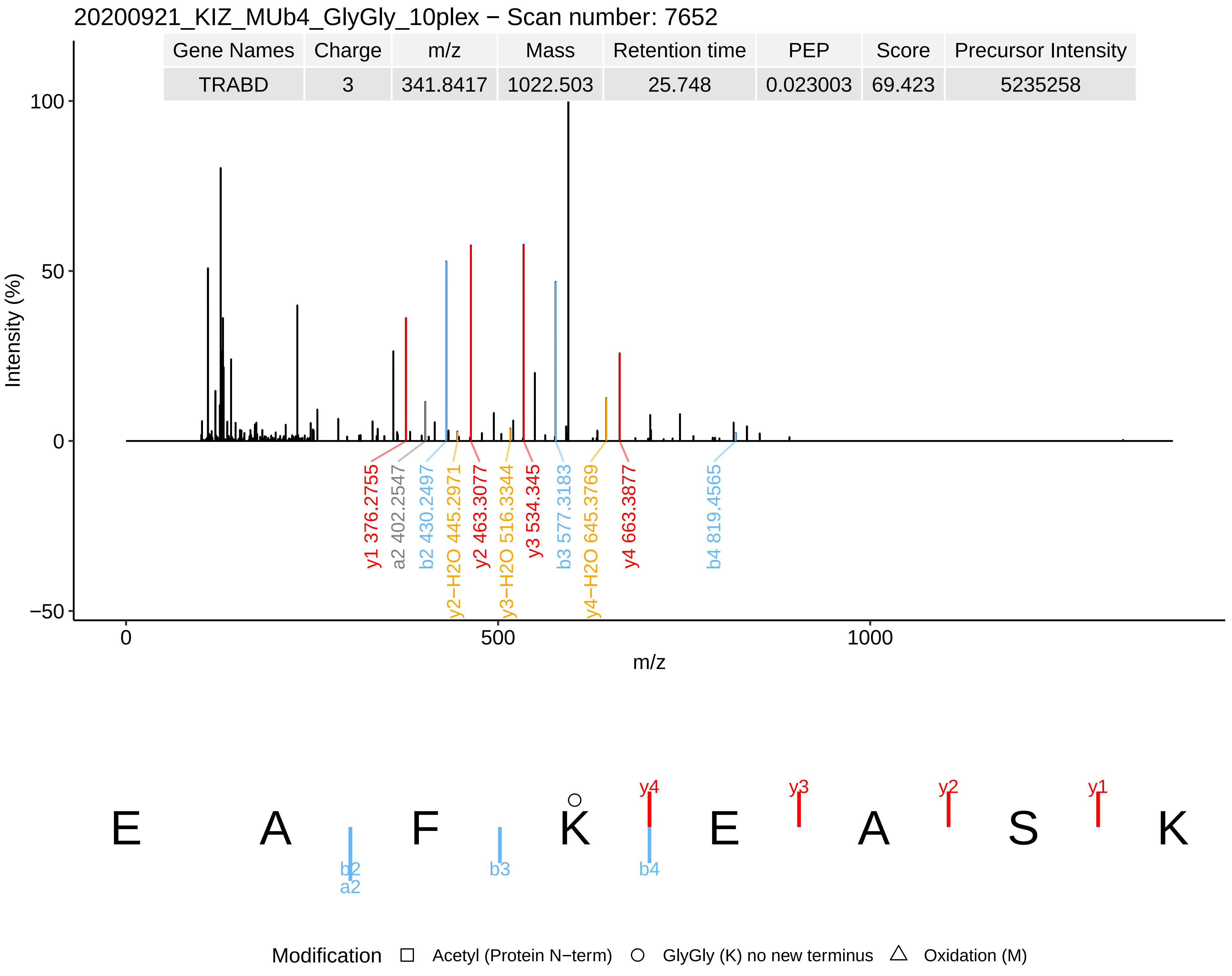

20200921_KIZ_MUb4_GlyGly_10plex − Scan number: 7652
Gene Names | Charge | m/z | Mass | Retention time | PEP | Score | Precursor Intensity
TRABD | 3 | 341.8417 | 1022.503 | 25.748 | 0.023003 | 69.423 | 5235258
Intensity (%)
100
50
0
−50
0
500
1000
m/z
y1 376.2755
a2 402.2547
b2 430.2497
y2−H2O 445.2971
y2 463.3077
y3−H2O 516.3344
y3 534.345
b3 577.3183
y4−H2O 645.3769
y4 663.3877
b4 819.4565
E A F K E A S K
y4
y3
y2
y1
b2
a2
b3
b4
Modification
Acetyl (Protein N−term)
GlyGly (K) no new terminus
Oxidation (M)

20200921_KIZ_MUb4_GlyGly_10plex_2 − Scan number: 7072

Gene Names	Charge	m/z	Mass	Retention time	PEP	Score	Precursor Intensity
TRABD	3	341.8417	1022.503	26.258	0.04139	60.59	943492.7

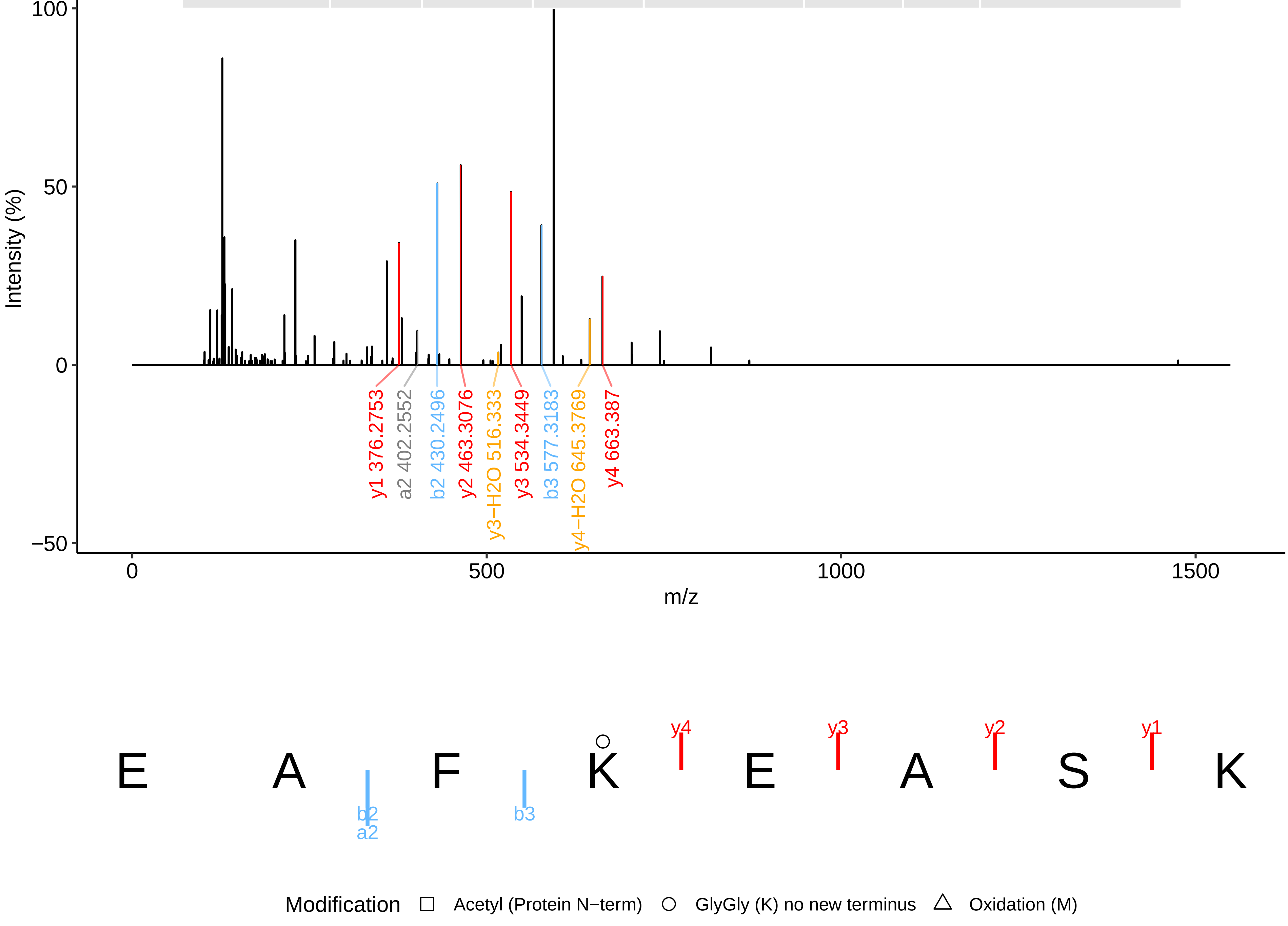

20200921_KIZ_MUb4_GlyGly_10plex − Scan number: 3848

Gene Names	Charge	m/z	Mass	Retention time	PEP	Score	Precursor Intensity
RAD18	3	339.5172	1015.53	12.419	0.001613	91.867	10146483

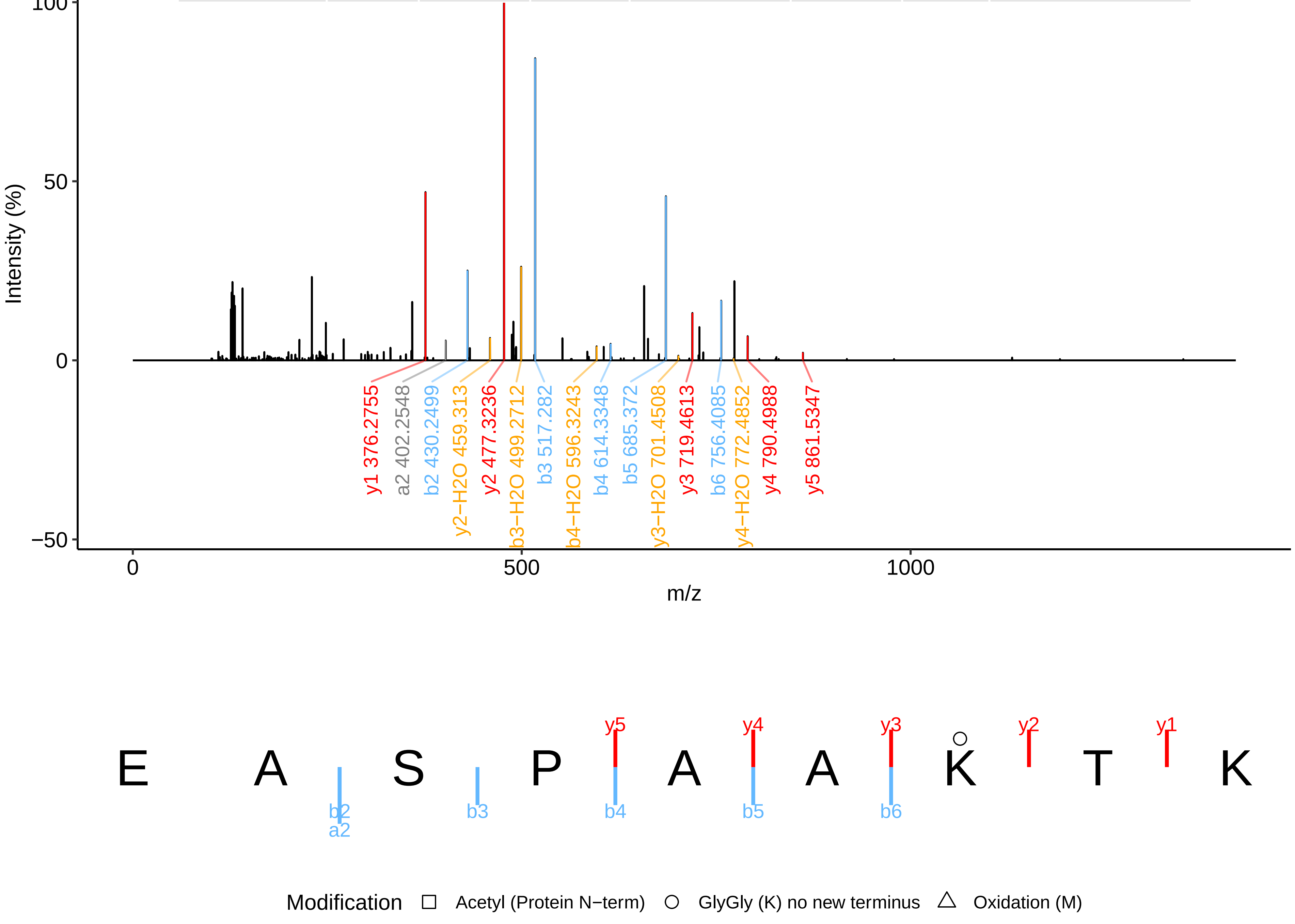

20200921_KIZ_MUb4_GlyGly_10plex_2 − Scan number: 3679

Gene Names	Charge	m/z	Mass	Retention time	PEP	Score	Precursor Intensity
RAD18	3	339.5172	1015.53	12.508	0.010143	74.376	1320178

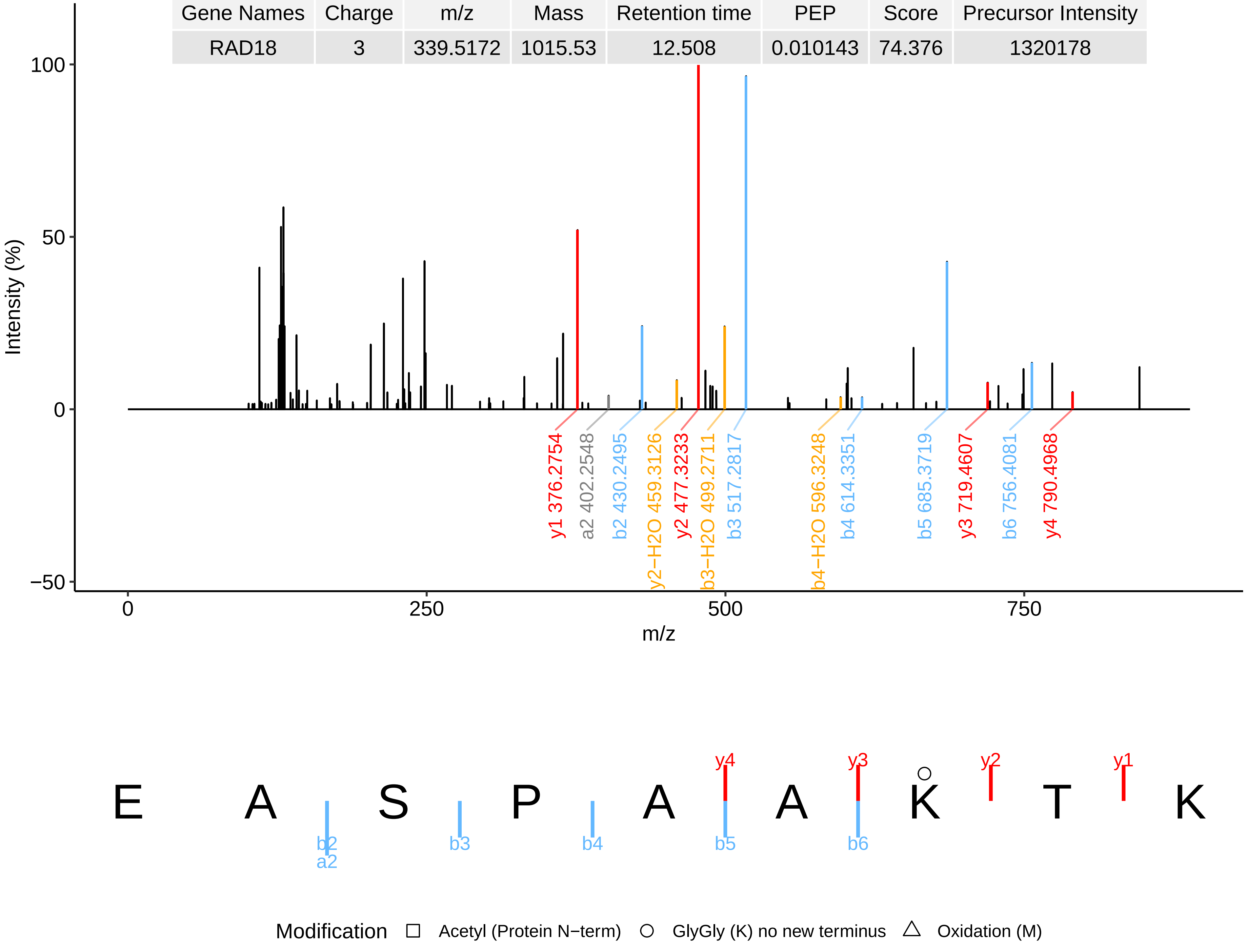

20200924_KIZ_MUb5_GlyGly_1 − Scan number: 3398

Gene Names	Charge	m/z	Mass	Retention time	PEP	Score	Precursor Intensity
RAD18	3	339.5172	1015.53	10.412	0.012327	72.325	28835264

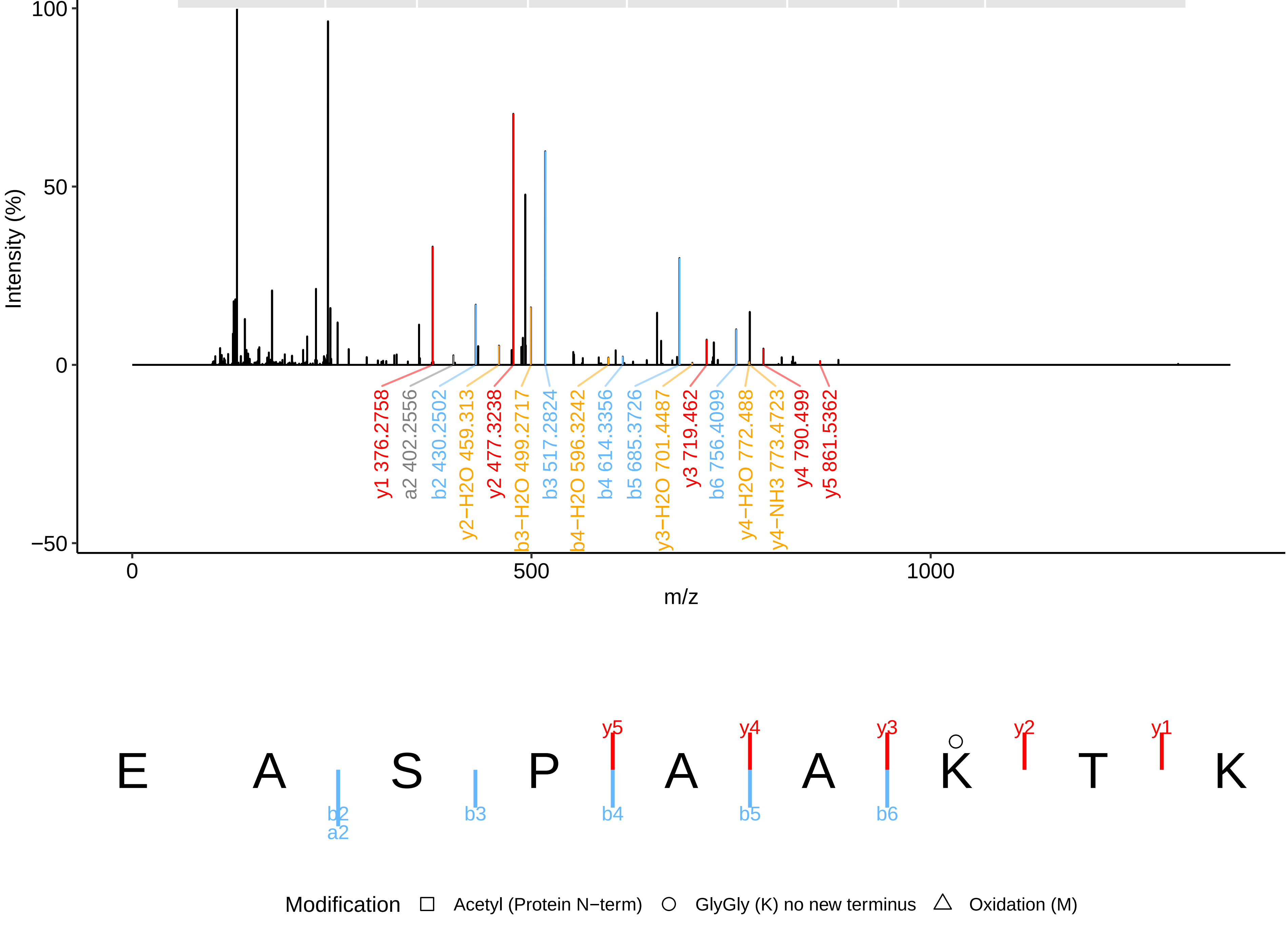

20200924_KIZ_MUb5_GlyGly_2 − Scan number: 3203

Gene Names	Charge	m/z	Mass	Retention time	PEP	Score	Precursor Intensity
RAD18	3	339.5172	1015.53	11.532	0.0035126	80.688	3247827

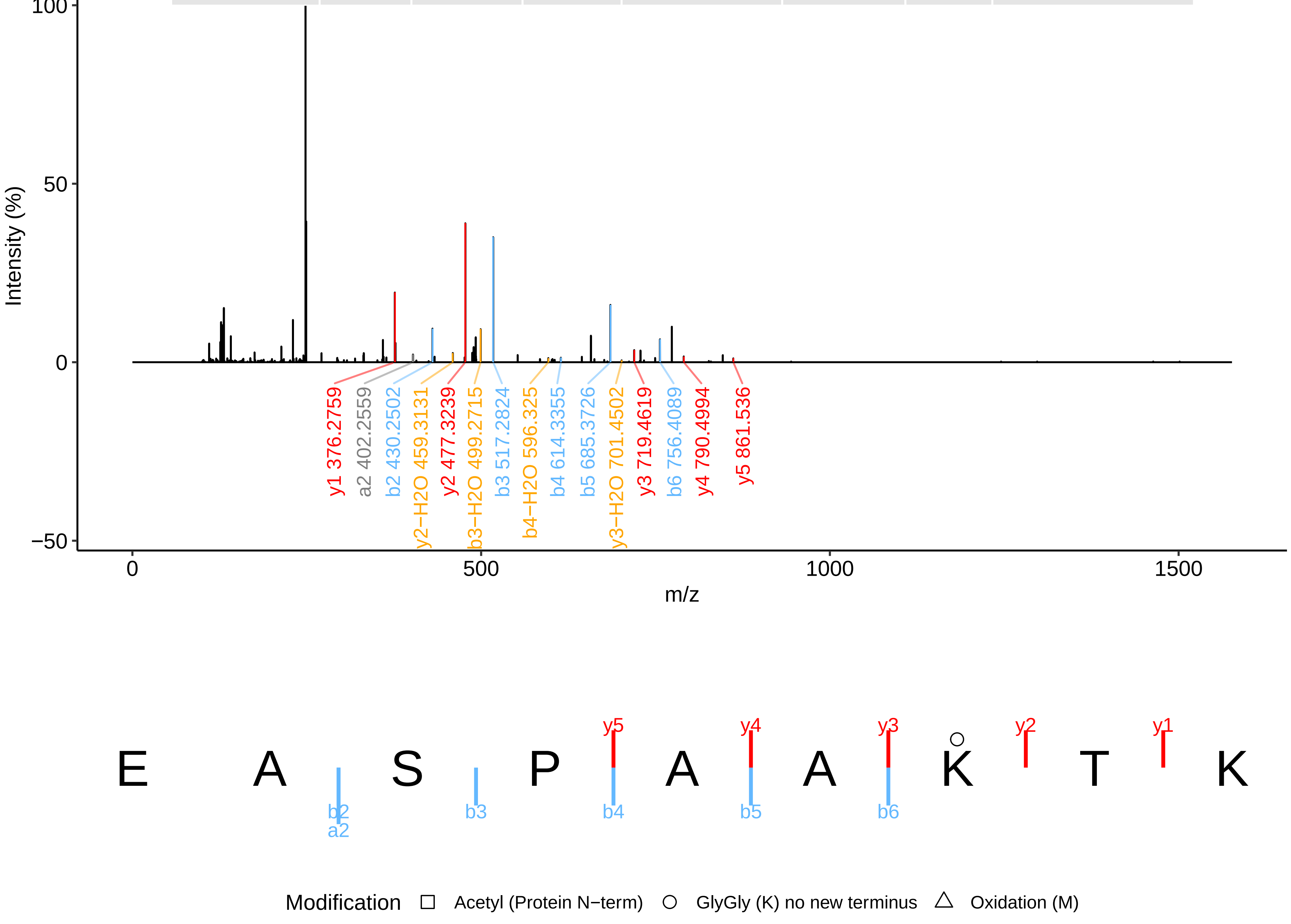

20201118_KIZ_MUb6_GlyGly_1 − Scan number: 3291

Gene Names	Charge	m/z	Mass	Retention time	PEP	Score	Precursor Intensity
RAD18	3	339.5172	1015.53	10.111	0.0011329	94.692	18581492

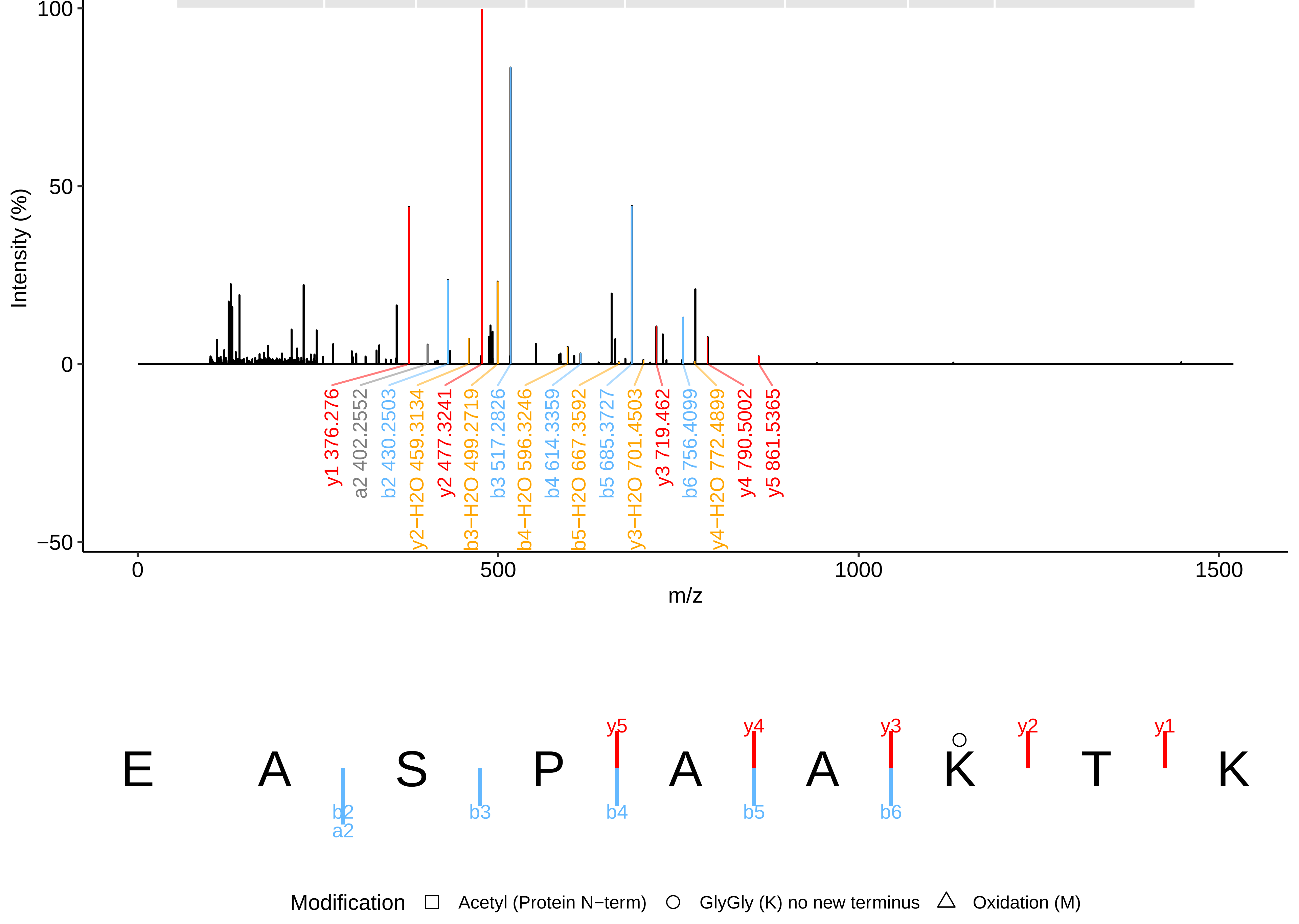

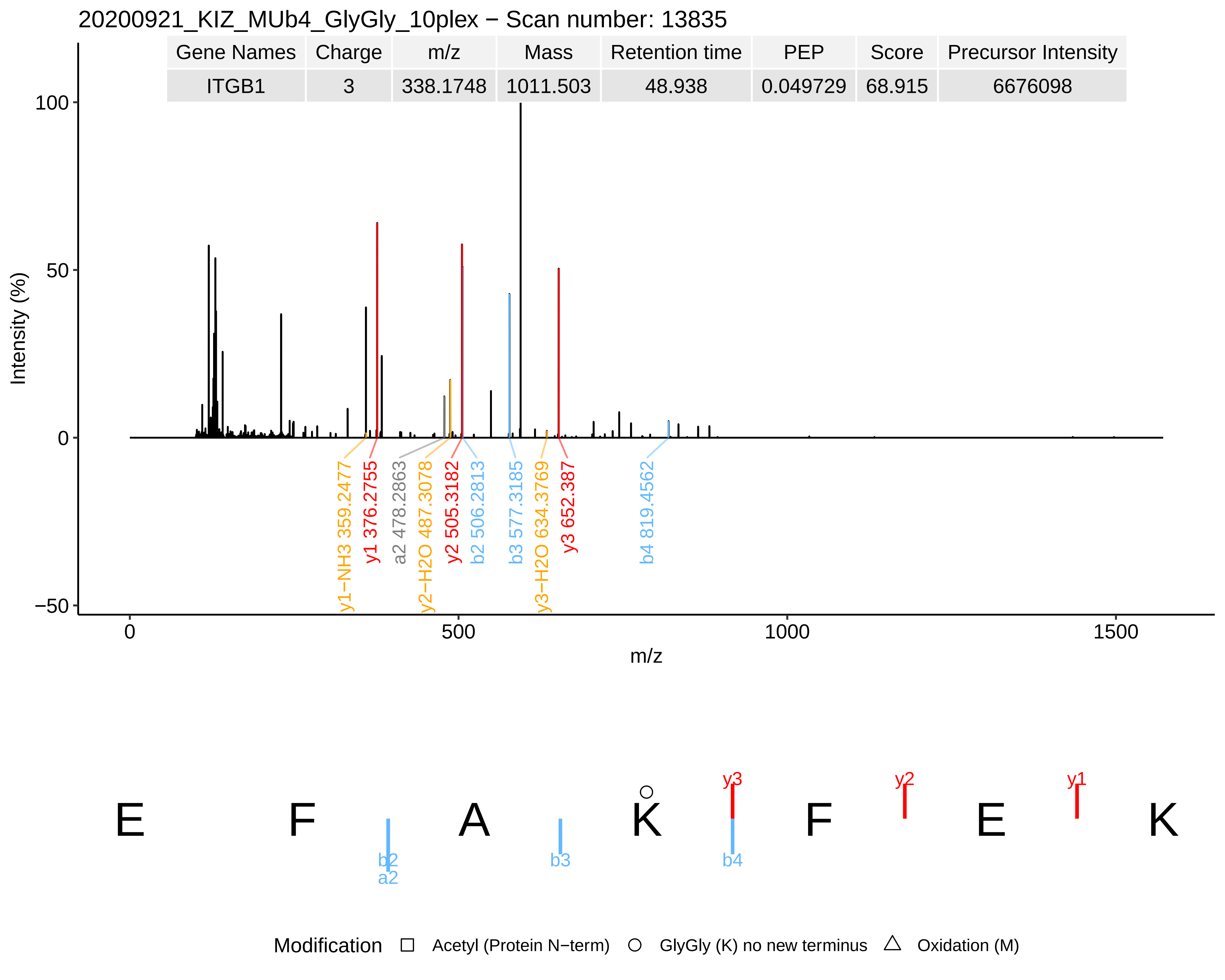

20200921_KIZ_MUb4_GlyGly_10plex − Scan number: 13835
Gene Names
Charge
m/z
Mass
Retention time
PEP
Score
Precursor Intensity
ITGB1
3
338.1748
1011.503
48.938
0.049729
68.915
6676098
Intensity (%)
100
50
0
−50
0
500
1000
1500
m/z
y1−NH3 359.2477
y1 376.2755
a2 478.2863
y2−H2O 487.3078
y2 505.3182
b2 506.2813
b3 577.3185
y3−H2O 634.3769
y3 652.387
b4 819.4562
E
F
A
K
F
E
K
y3
y2
y1
b2
a2
b3
b4
Modification
Acetyl (Protein N−term)
GlyGly (K) no new terminus
Oxidation (M)

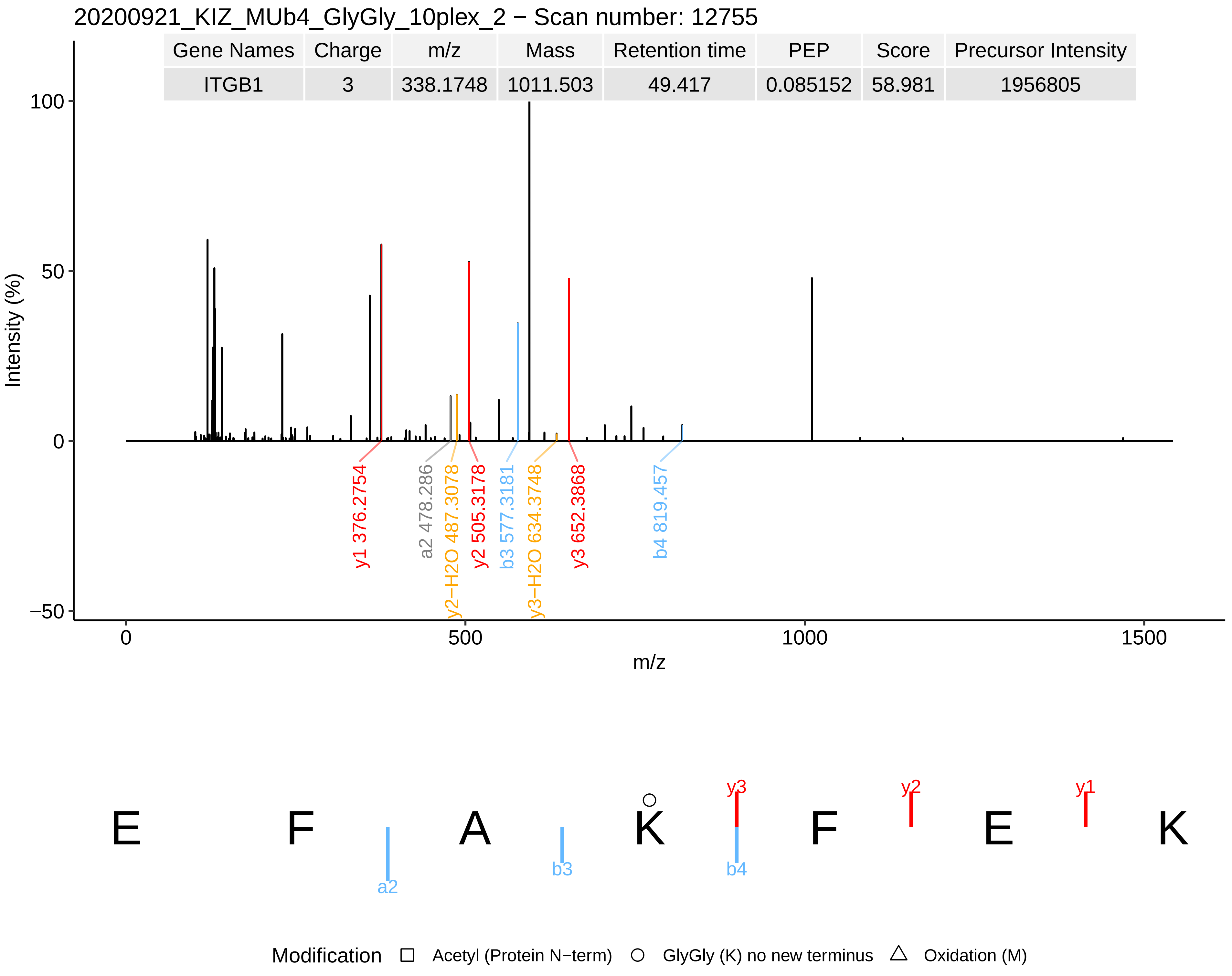

20200921_KIZ_MUb4_GlyGly_10plex_2 − Scan number: 12755
Gene Names | Charge | m/z | Mass | Retention time | PEP | Score | Precursor Intensity
ITGB1 | 3 | 338.1748 | 1011.503 | 49.417 | 0.085152 | 58.981 | 1956805
Intensity (%)
100
50
0
−50
0
500
1000
1500
m/z
y1 376.2754
a2 478.286
y2−H2O 487.3078
y2 505.3178
b3 577.3181
y3−H2O 634.3748
y3 652.3868
b4 819.457
E F A K F E K
y3
y2
y1
a2
b3
b4
Modification
Acetyl (Protein N−term)
GlyGly (K) no new terminus
Oxidation (M)

20200924_KIZ_MUb5_GlyGly_1 − Scan number: 12636

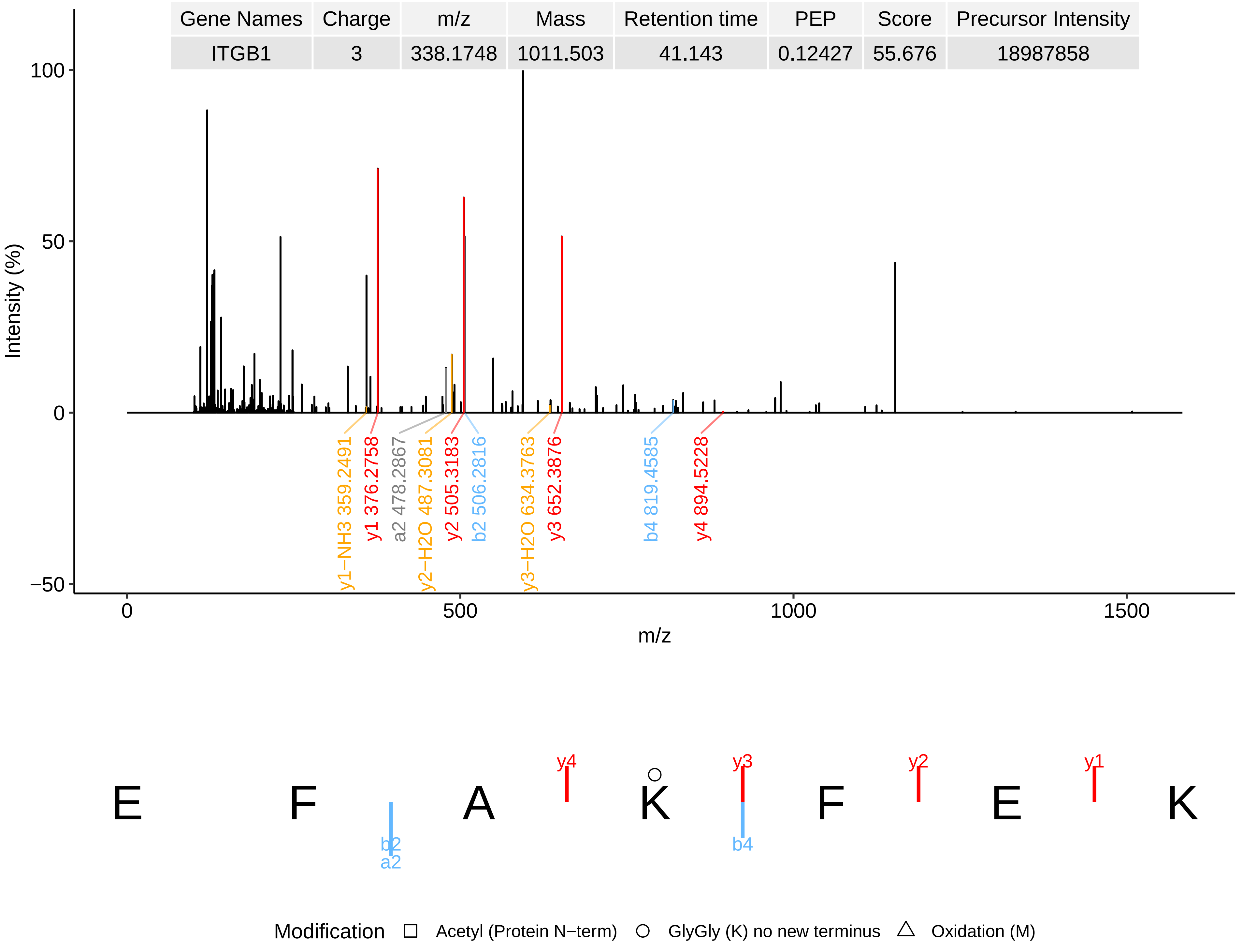

20200921_KIZ_MUb4_GlyGly_10plex − Scan number: 10073

Gene Names	Charge	m/z	Mass	Retention time	PEP	Score	Precursor Intensity
ITGB5	3	419.207	1254.599	34.731	0.014304	74.173	3353072

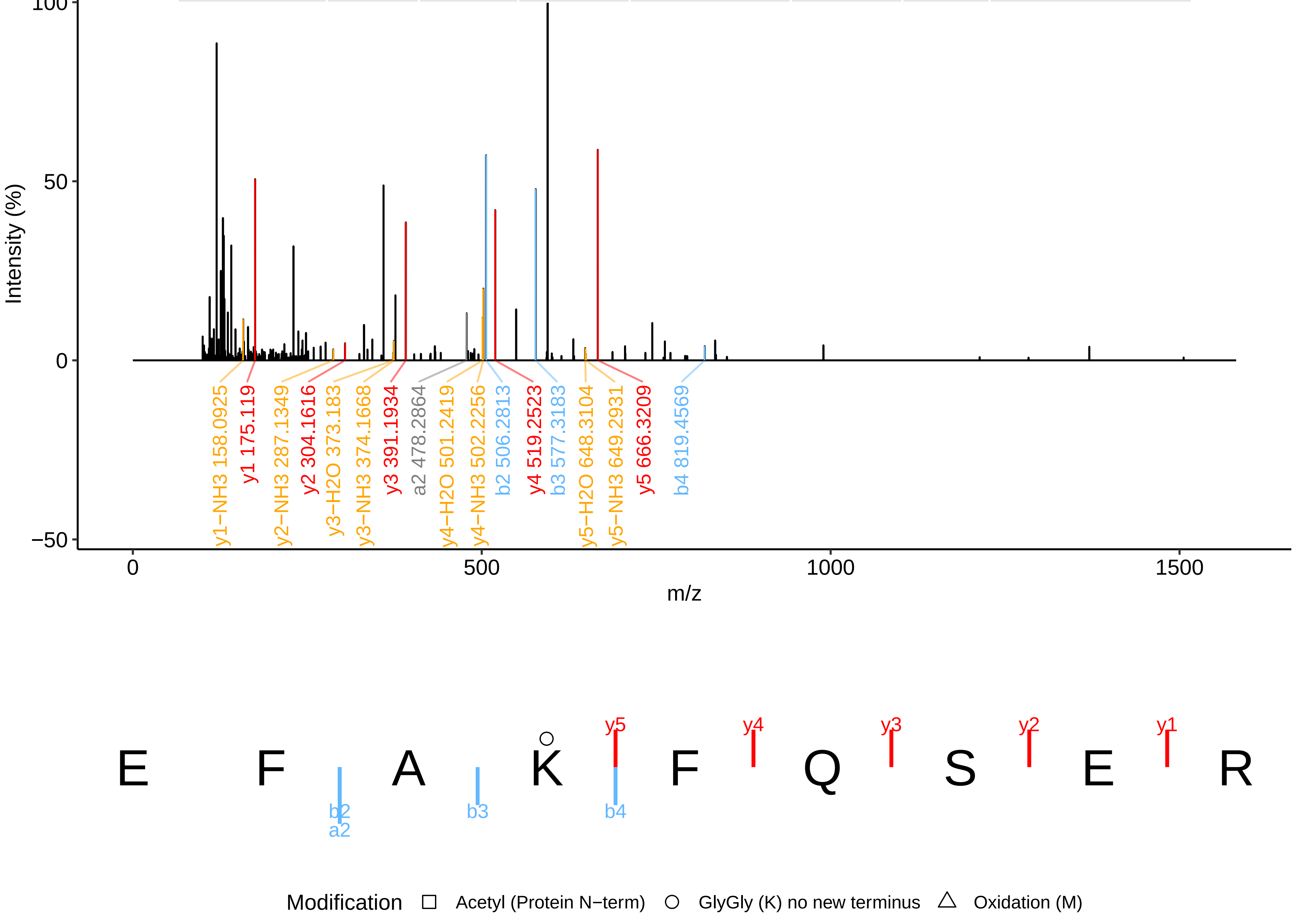

20200921_KIZ_MUb4_GlyGly_10plex_2 − Scan number: 9297

Gene Names	Charge	m/z	Mass	Retention time	PEP	Score	Precursor Intensity
ITGB5	3	419.207	1254.599	35.303	0.11416	50.805	786050.6

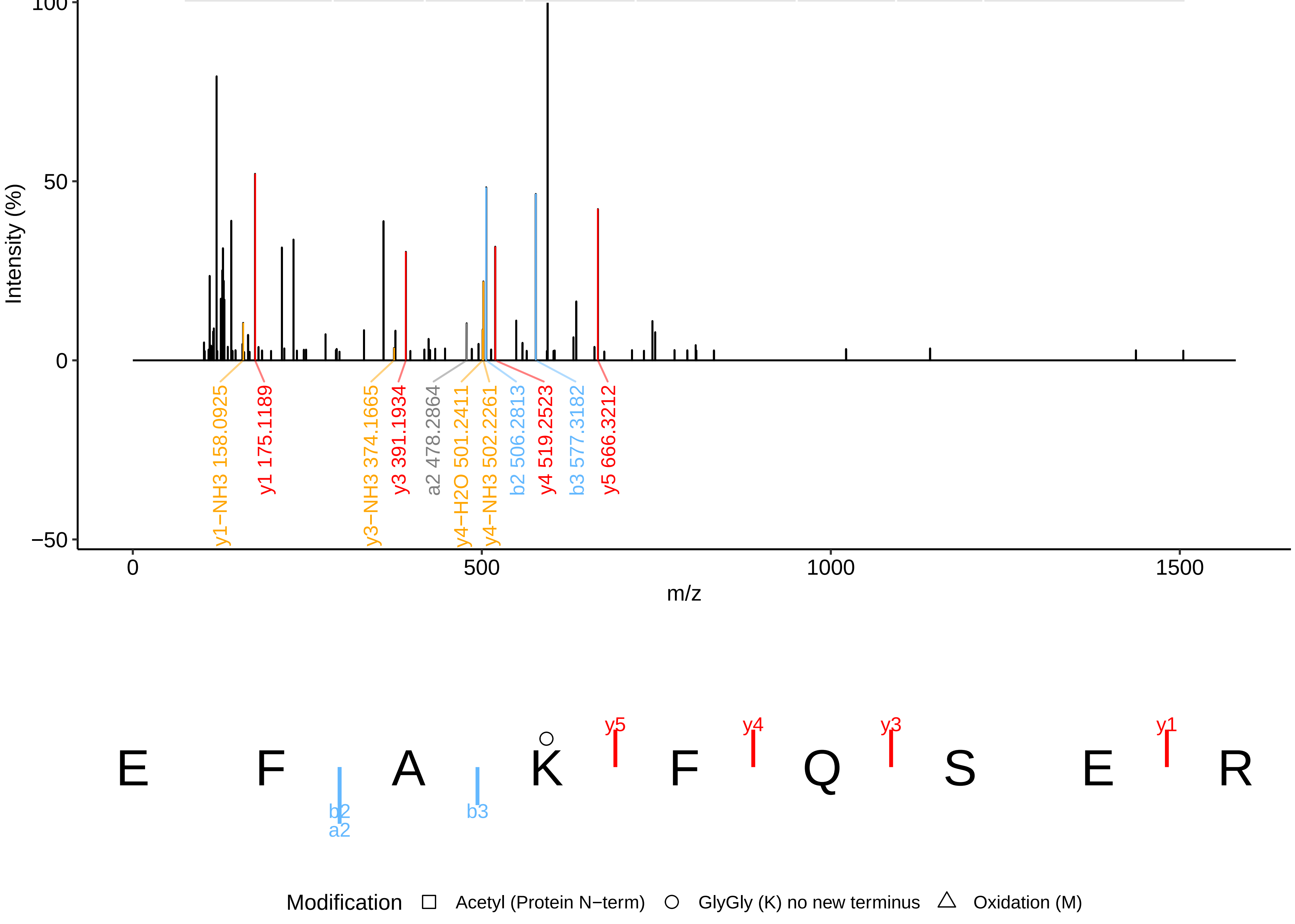

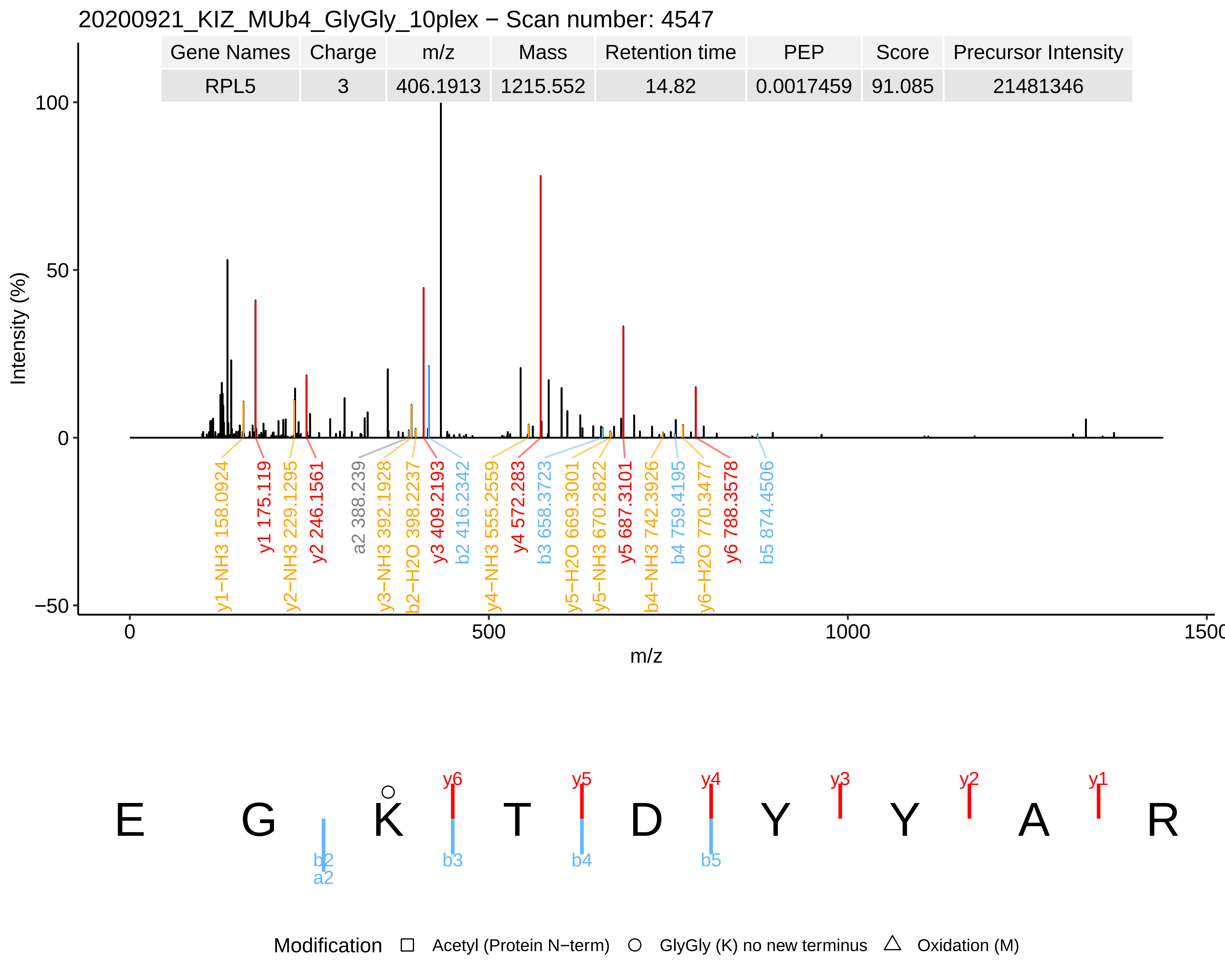
20200921_KIZ_MUb4_GlyGly_10plex − Scan number: 4547
Gene Names | Charge | m/z | Mass | Retention time | PEP | Score | Precursor Intensity
RPL5 | 3 | 406.1913 | 1215.552 | 14.82 | 0.0017459 | 91.085 | 21481346
Intensity (%)
100
50
0
−50
0
500
1000
1500
m/z
y1−NH3 158.0924
y1 175.119
y2−NH3 229.1295
y2 246.1561
a2 388.239
y3−NH3 392.1928
b2−H2O 398.2237
y3 409.2193
b2 416.2342
y4−NH3 555.2559
y4 572.283
b3 658.3723
y5−H2O 669.3001
y5−NH3 670.2822
y5 687.3101
b4−NH3 742.3926
b4 759.4195
y6−H2O 770.3477
y6 788.3578
b5 874.4506
E G K T D Y Y A R
y6 y5 y4 y3 y2 y1
b2 a2 b3 b4 b5
Modification
Acetyl (Protein N−term)
GlyGly (K) no new terminus
Oxidation (M)

20200921_KIZ_MUb4_GlyGly_10plex_2 − Scan number: 4217

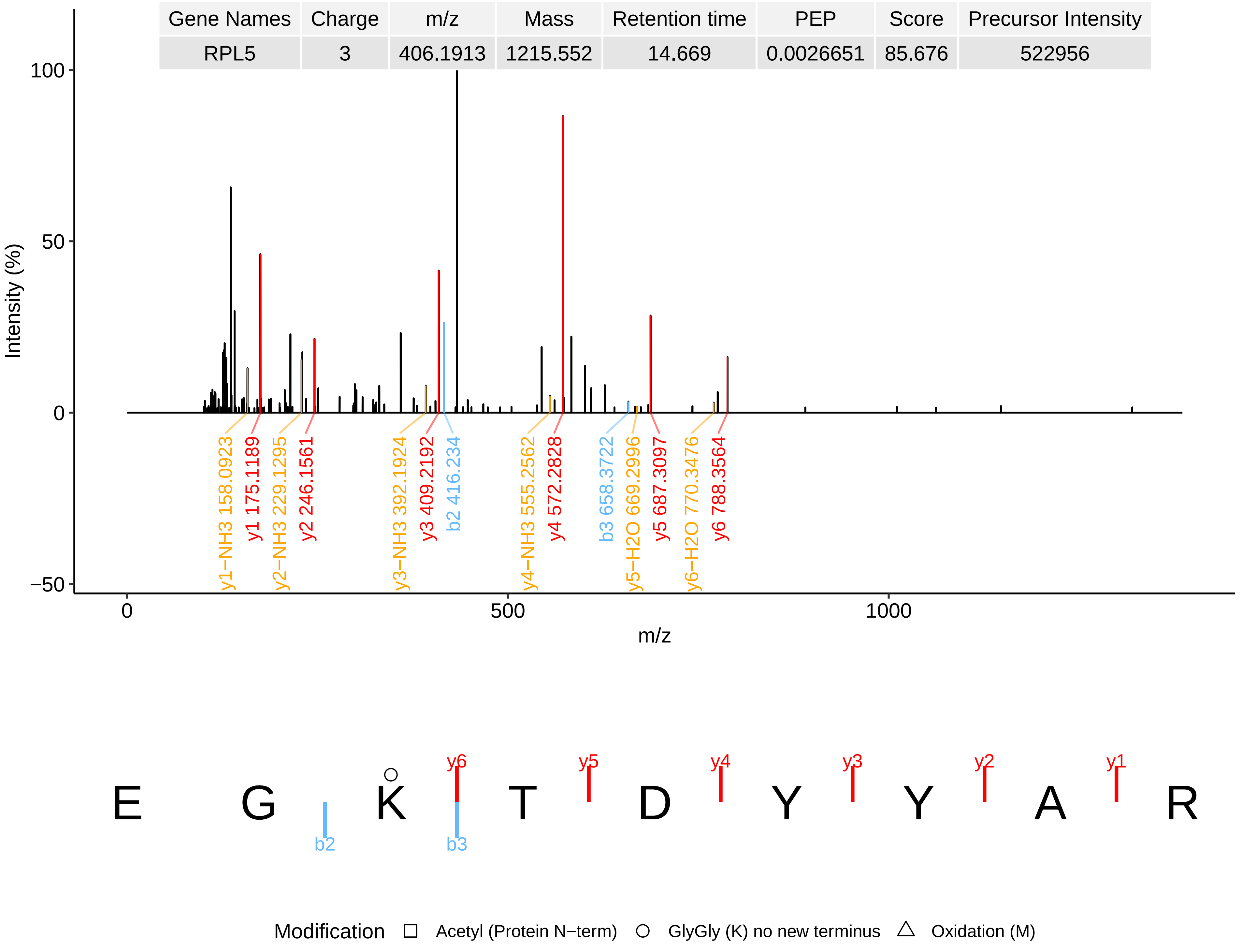

20200924_KIZ_MUb5_GlyGly_1 − Scan number: 3861

Gene Names	Charge	m/z	Mass	Retention time	PEP	Score	Precursor Intensity
RPL5	3	406.1913	1215.552	11.801	0.067499	53.756	9105497

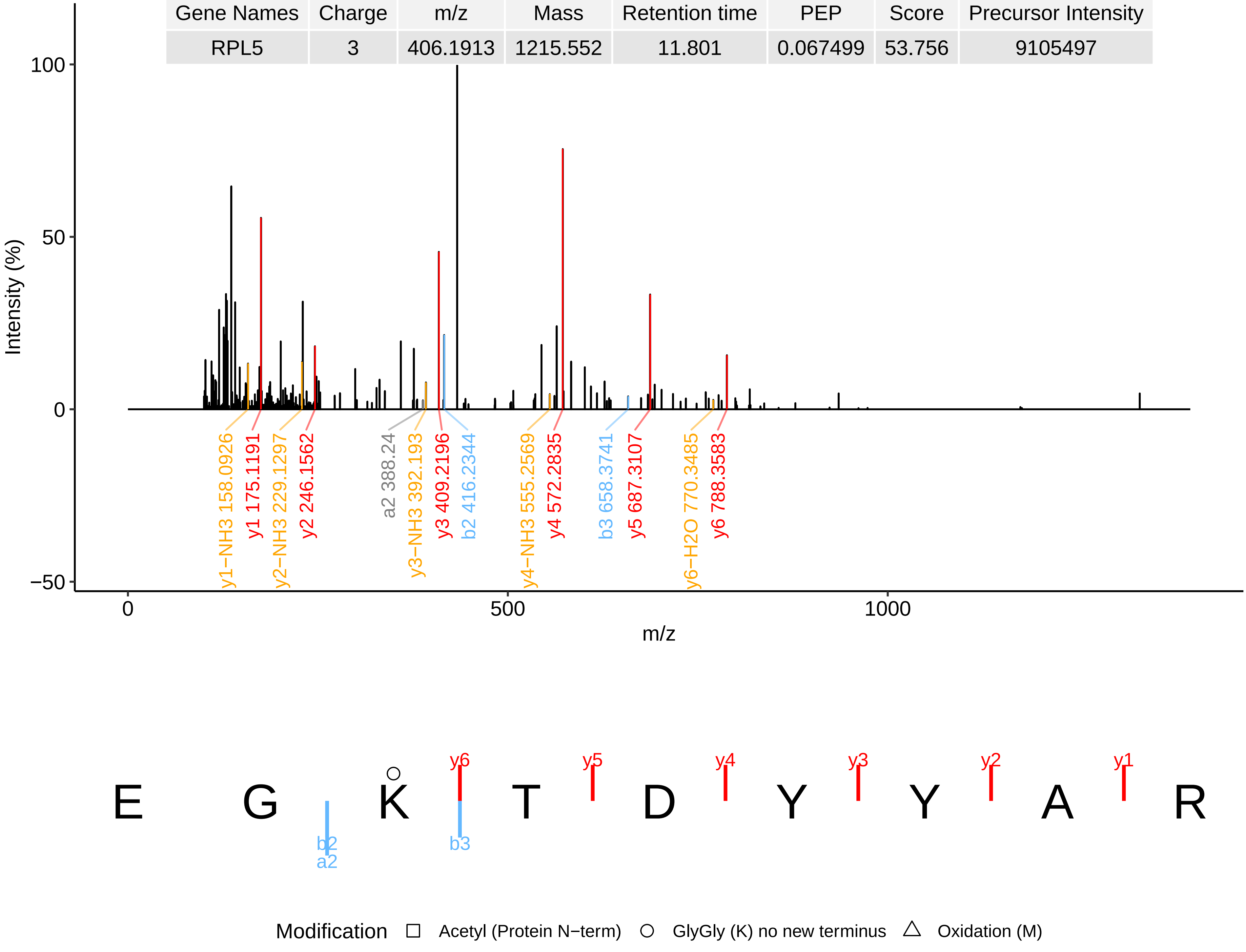

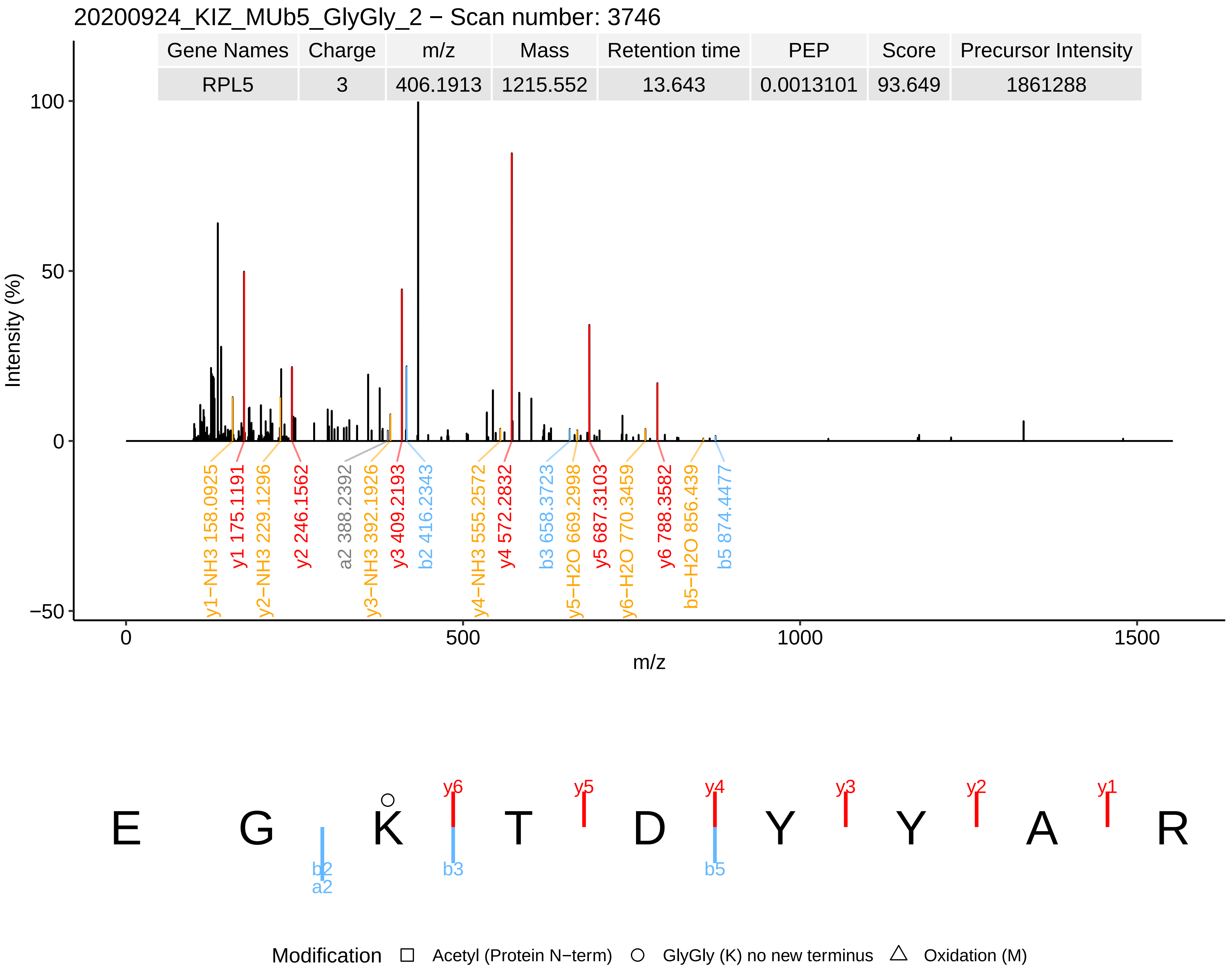
20200924_KIZ_MUb5_GlyGly_2 − Scan number: 3746
Gene Names | Charge | m/z | Mass | Retention time | PEP | Score | Precursor Intensity
RPL5 | 3 | 406.1913 | 1215.552 | 13.643 | 0.0013101 | 93.649 | 1861288
Intensity (%)
m/z
y1−NH3 158.0925
y1 175.1191
y2−NH3 229.1296
y2 246.1562
a2 388.2392
y3−NH3 392.1926
y3 409.2193
b2 416.2343
y4−NH3 555.2572
y4 572.2832
b3 658.3723
y5−H2O 669.2998
y5 687.3103
y6−H2O 770.3459
y6 788.3582
b5−H2O 856.439
b5 874.4477
E G K T D Y Y A R
y6 y5 y4 y3 y2 y1
b2 a2 b3 b5
Modification
Acetyl (Protein N−term)
GlyGly (K) no new terminus
Oxidation (M)

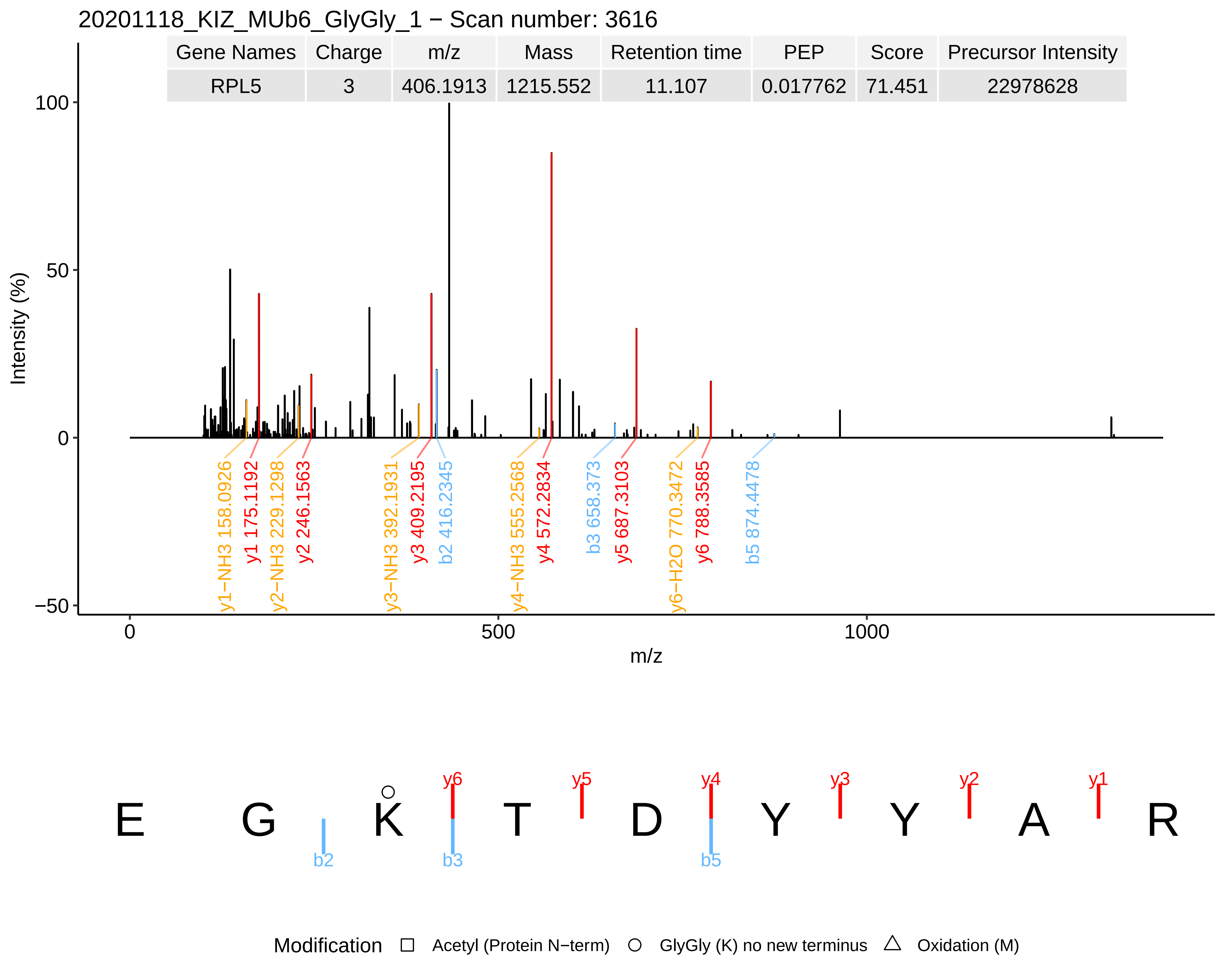
20201118_KIZ_MUb6_GlyGly_1 − Scan number: 3616
Gene Names | Charge | m/z | Mass | Retention time | PEP | Score | Precursor Intensity
RPL5 | 3 | 406.1913 | 1215.552 | 11.107 | 0.017762 | 71.451 | 22978628
Intensity (%)
100
50
0
−50
0
500
1000
m/z
y1−NH3 158.0926
y1 175.1192
y2−NH3 229.1298
y2 246.1563
y3−NH3 392.1931
y3 409.2195
b2 416.2345
y4−NH3 555.2568
y4 572.2834
b3 658.373
y5 687.3103
y6−H2O 770.3472
y6 788.3585
b5 874.4478
E G K T D Y Y A R
y6 y5 y4 y3 y2 y1
b2 b3 b5
Modification
Acetyl (Protein N−term)
GlyGly (K) no new terminus
Oxidation (M)

20200921_KIZ_MUb4_GlyGly_10plex − Scan number: 7502

Gene Names	Charge	m/z	Mass	Retention time	PEP	Score	Precursor Intensity
GPR108	3	330.1856	987.5349	25.194	0.027177	66.073	6084137

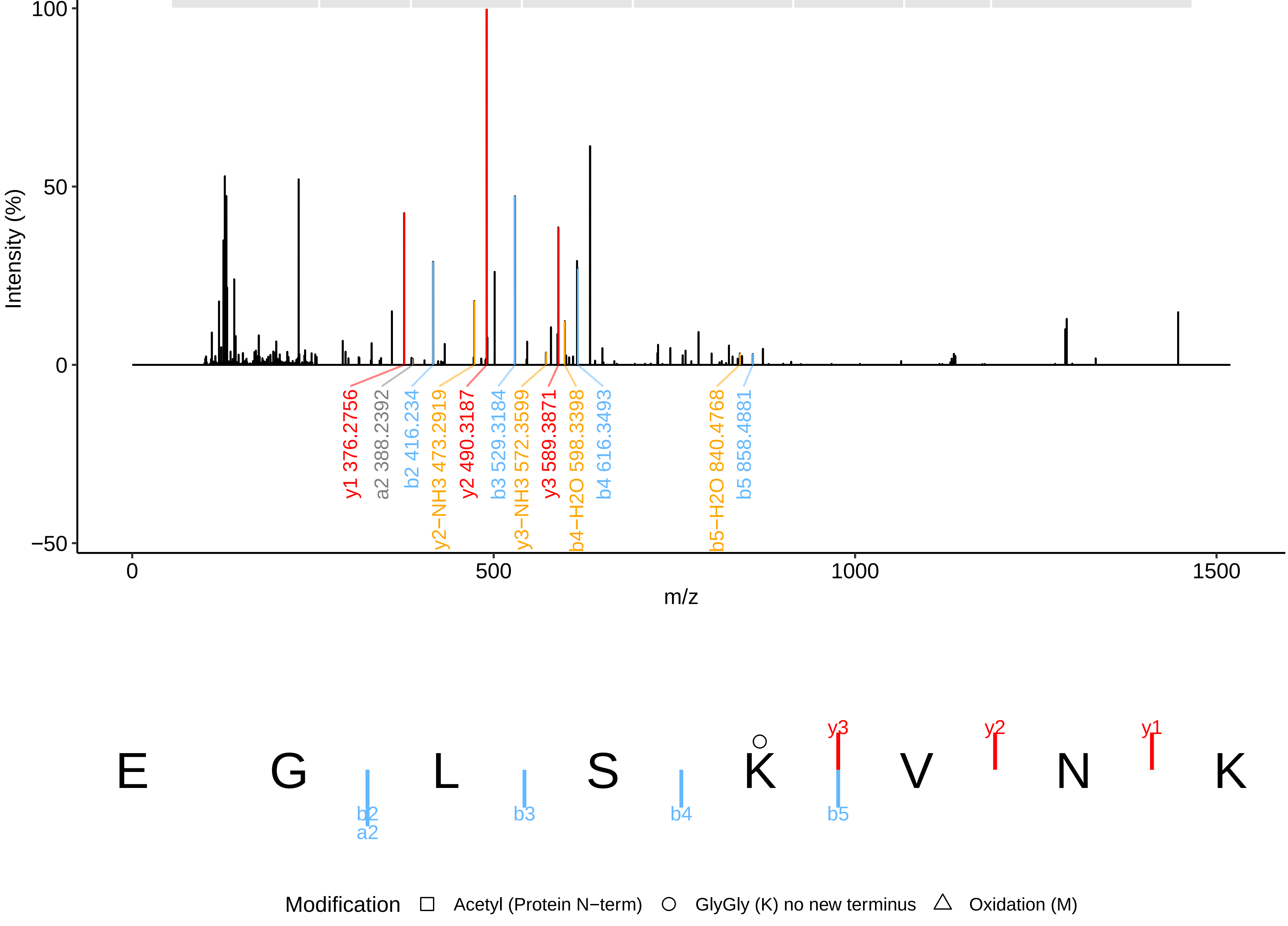

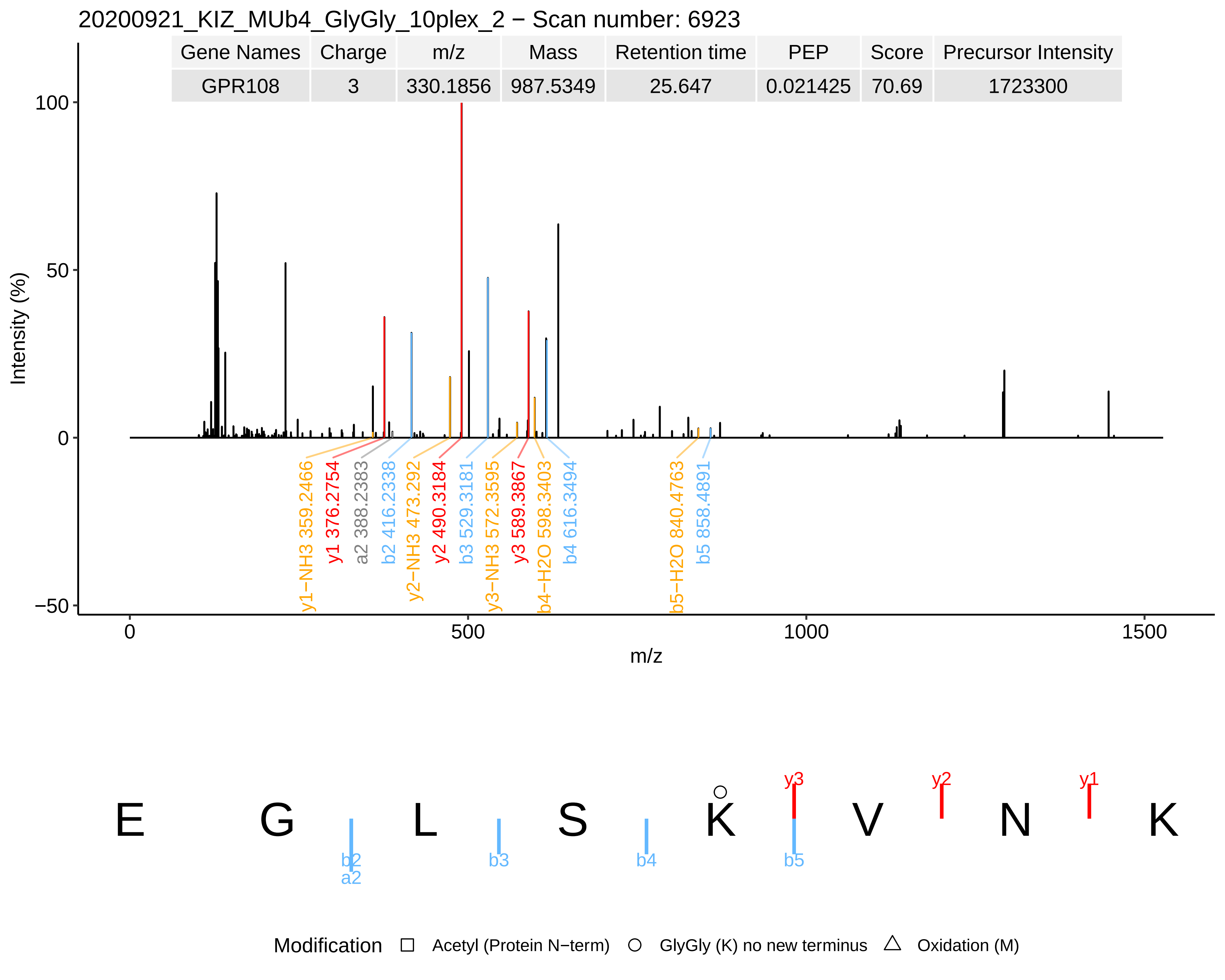

20200921_KIZ_MUb4_GlyGly_10plex_2 − Scan number: 6923
Gene Names
Charge
m/z
Mass
Retention time
PEP
Score
Precursor Intensity
GPR108
3
330.1856
987.5349
25.647
0.021425
70.69
1723300
Intensity (%)
100
50
0
−50
0
500
1000
1500
m/z
y1−NH3 359.2466
y1 376.2754
a2 388.2383
b2 416.2338
y2−NH3 473.292
y2 490.3184
b3 529.3181
y3−NH3 572.3595
y3 589.3867
b4−H2O 598.3403
b4 616.3494
b5−H2O 840.4763
b5 858.4891
E G L S K V N K
y3
y2
y1
b2
a2
b3
b4
b5
Modification
Acetyl (Protein N−term)
GlyGly (K) no new terminus
Oxidation (M)

20200924_KIZ_MUb5_GlyGly_1 − Scan number: 6512

Gene Names	Charge	m/z	Mass	Retention time	PEP	Score	Precursor Intensity
GPR108	3	330.1856	987.5349	20.484	0.062157	55.531	3456277

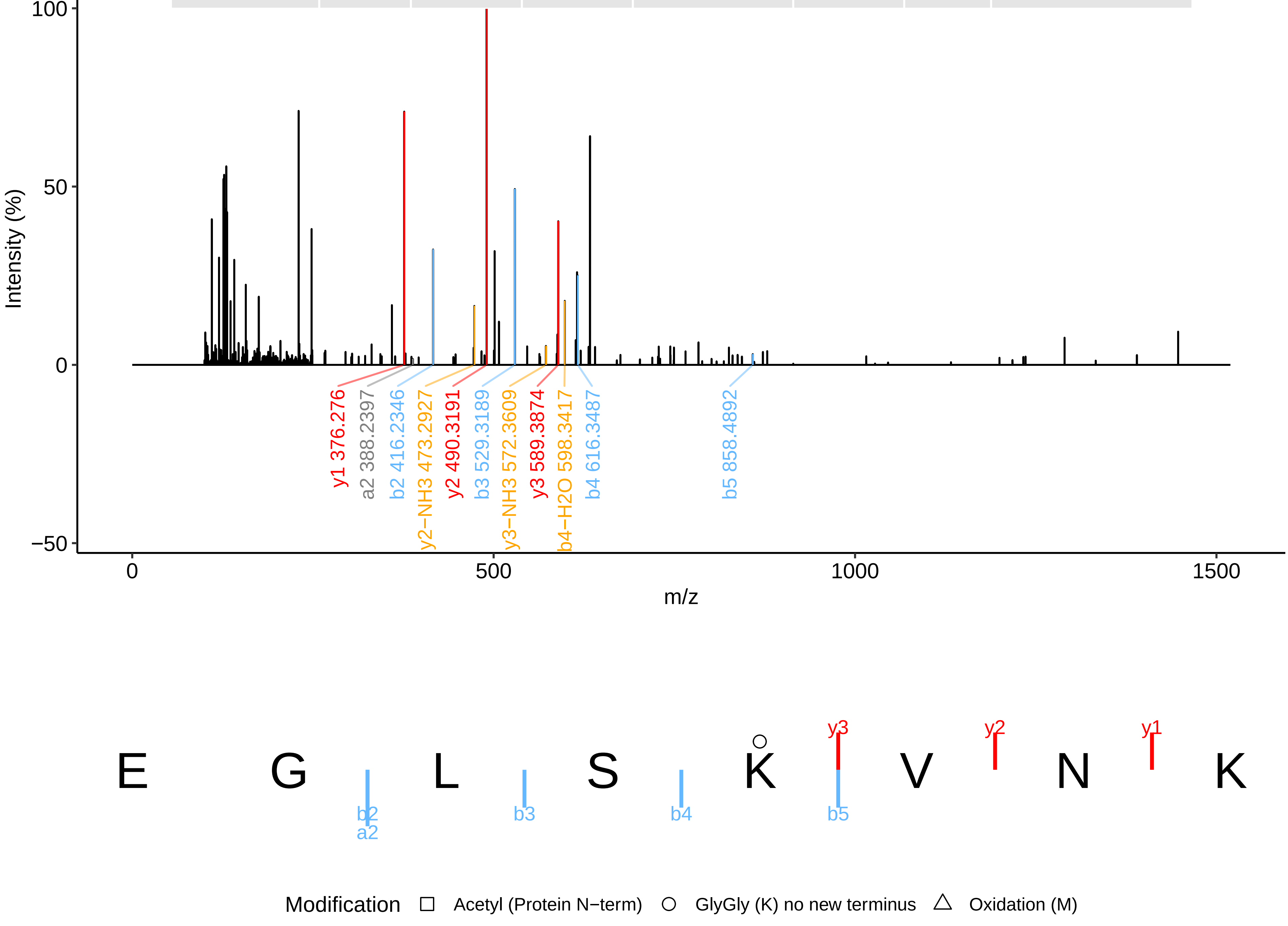

20200924_KIZ_MUb5_GlyGly_2 − Scan number: 6076

Gene Names	Charge	m/z	Mass	Retention time	PEP	Score	Precursor Intensity
GPR108	3	330.1856	987.5349	22.629	0.037718	63.565	NA

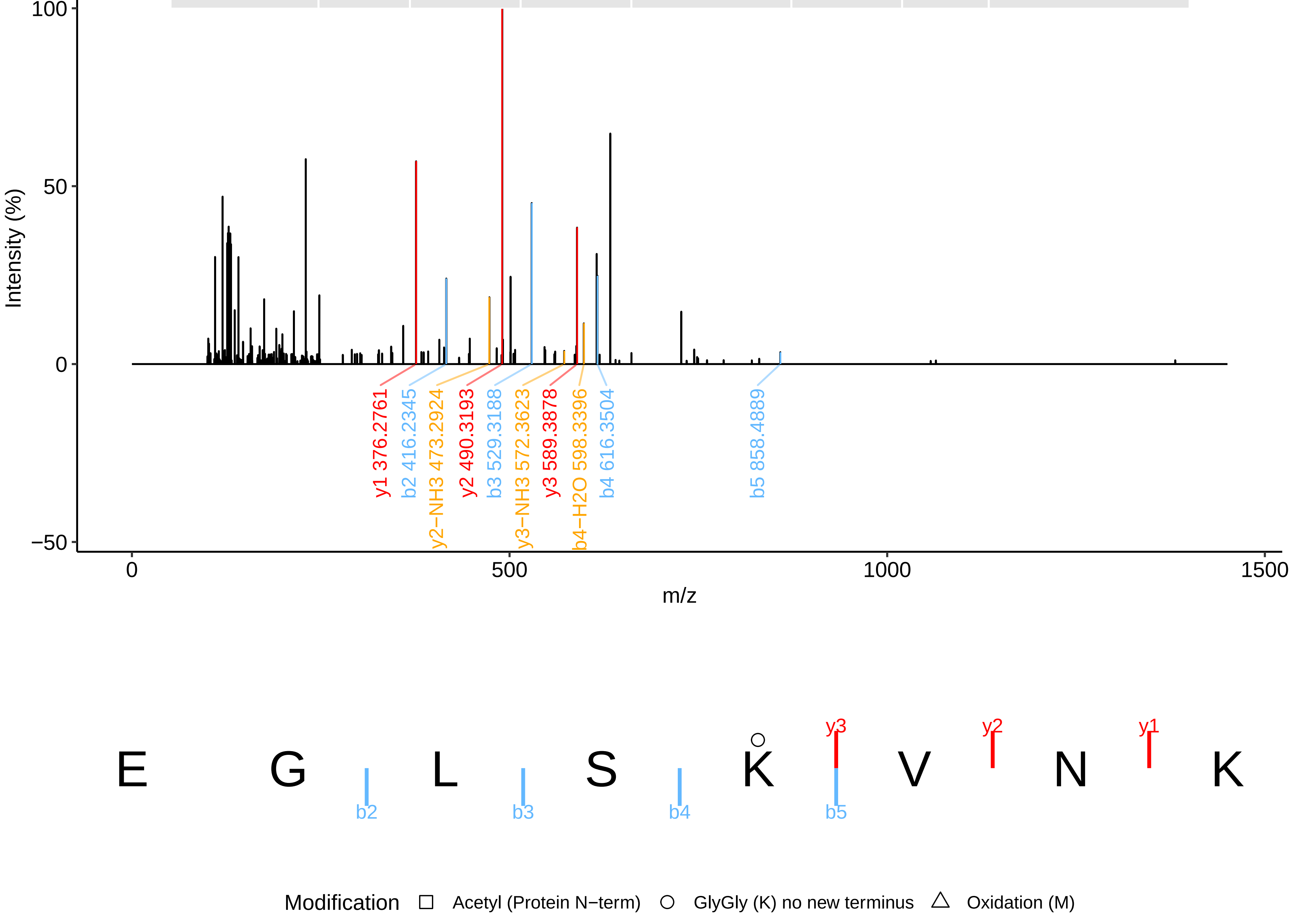

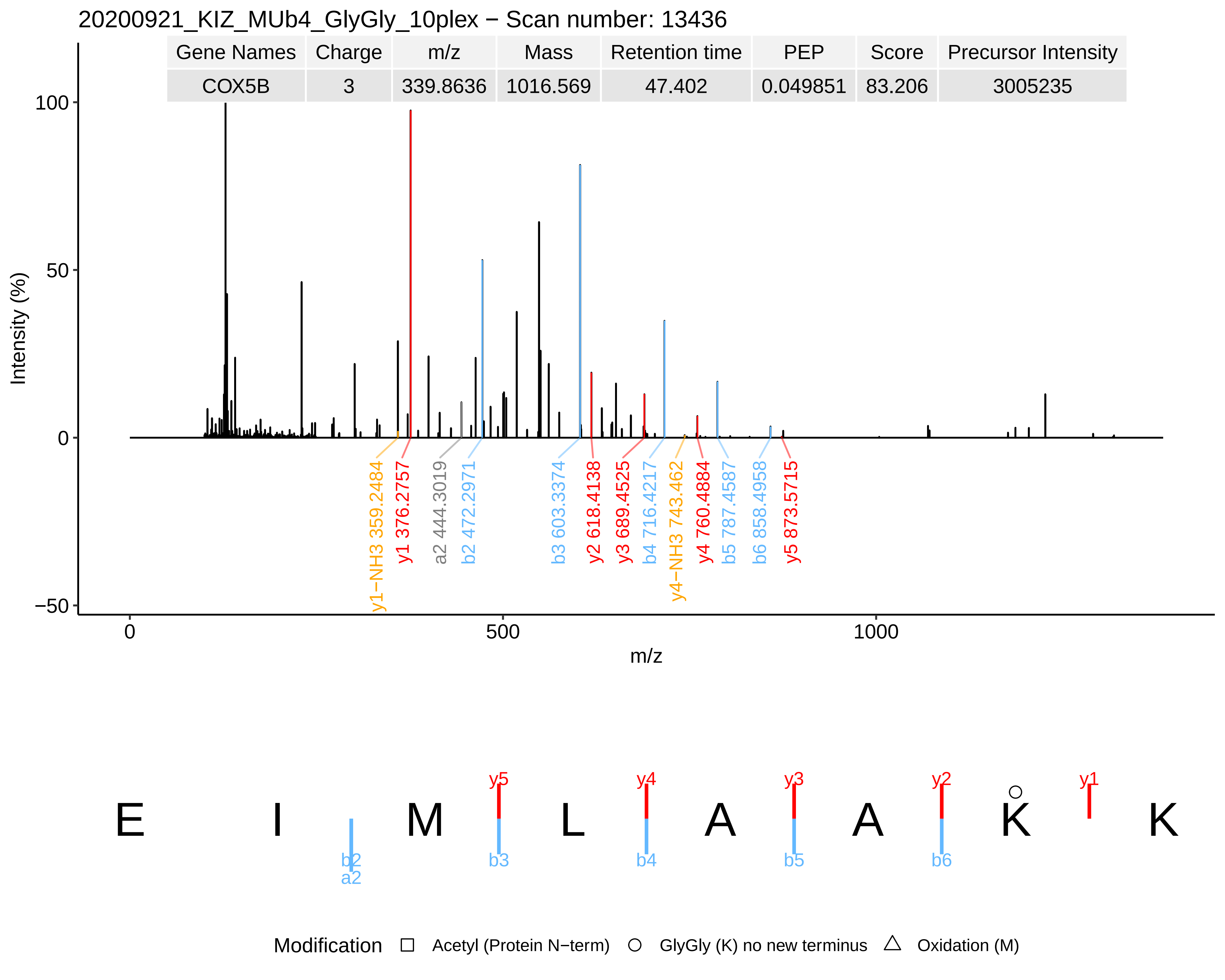

20200921_KIZ_MUb4_GlyGly_10plex − Scan number: 13436
Gene Names
Charge
m/z
Mass
Retention time
PEP
Score
Precursor Intensity
COX5B
3
339.8636
1016.569
47.402
0.049851
83.206
3005235
Intensity (%)
m/z
y1−NH3 359.2484
y1 376.2757
a2 444.3019
b2 472.2971
b3 603.3374
y2 618.4138
y3 689.4525
b4 716.4217
y4−NH3 743.462
y4 760.4884
b5 787.4587
b6 858.4958
y5 873.5715
E I M L A A K K
Modification
Acetyl (Protein N−term)
GlyGly (K) no new terminus
Oxidation (M)

20200921_KIZ_MUb4_GlyGly_10plex_2 − Scan number: 12420

Gene Names	Charge	m/z	Mass	Retention time	PEP	Score	Precursor Intensity
COX5B	3	339.8636	1016.569	48.046	0.098408	75.109	902161.1

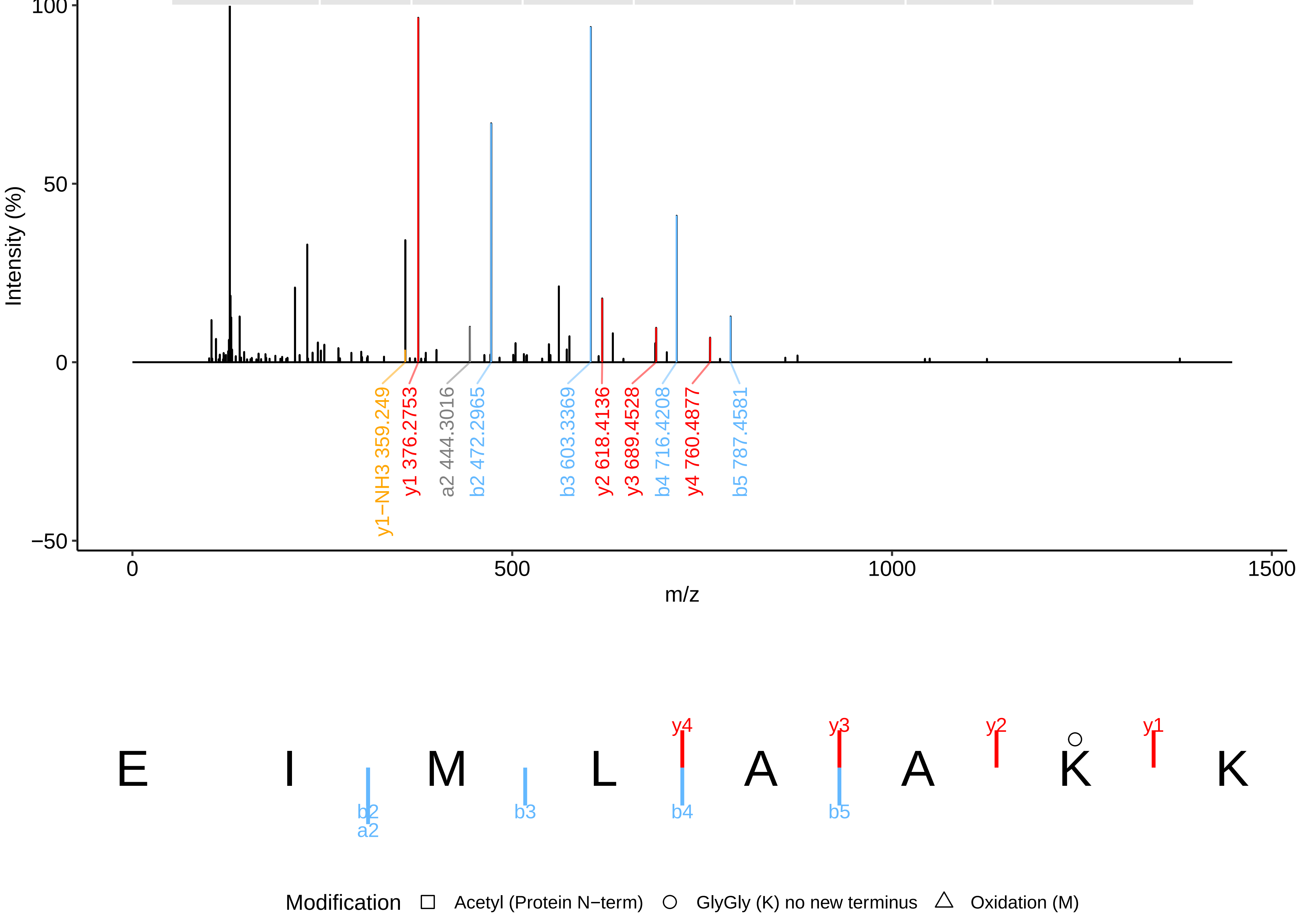

20201118_KIZ_MUb6_GlyGly_1 − Scan number: 12354

Gene Names	Charge	m/z	Mass	Retention time	PEP	Score	Precursor Intensity
COX5B	3	339.8636	1016.569	38.997	0.034765	83.265	NA

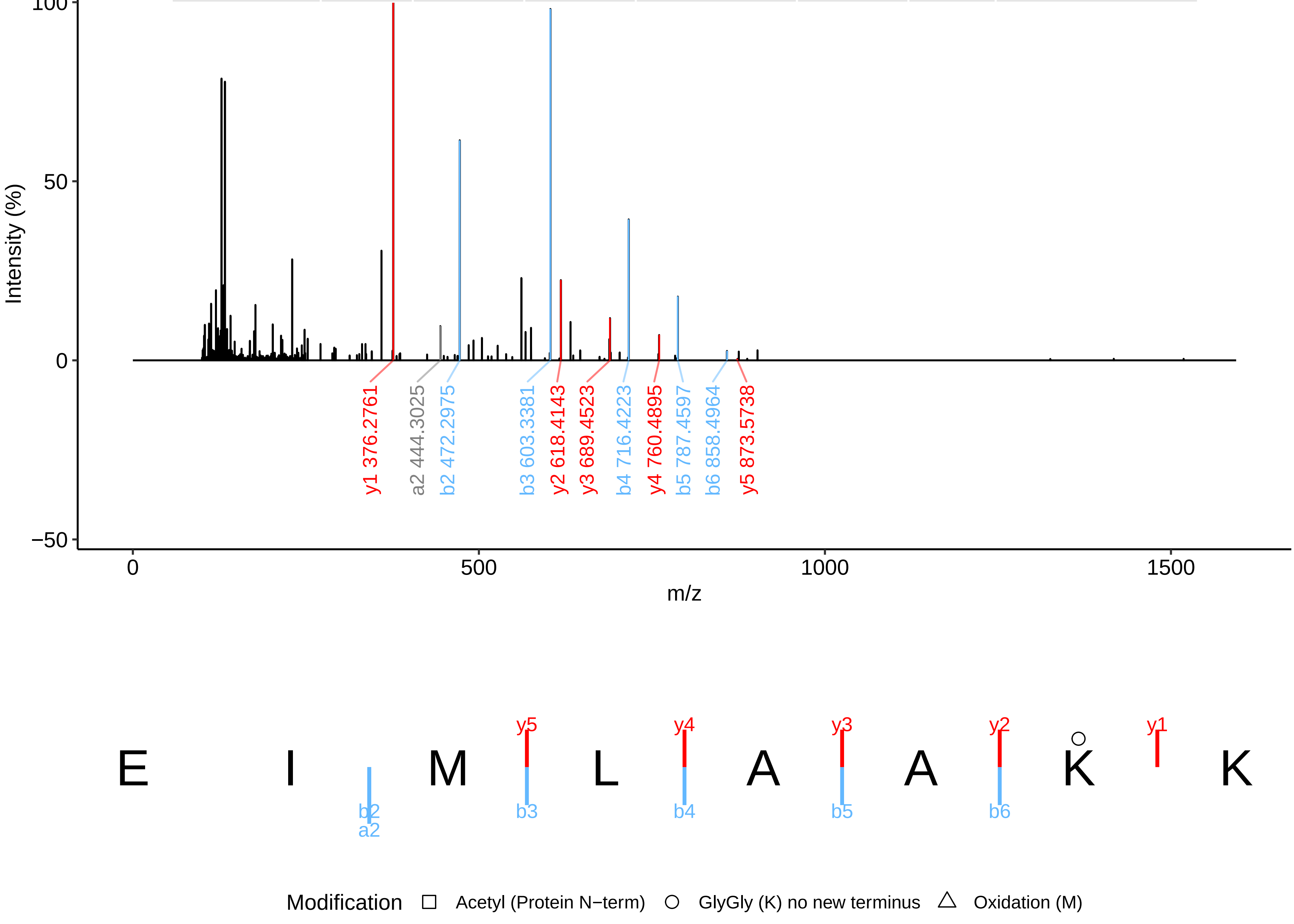

20200921_KIZ_MUb4_GlyGly_10plex_2 − Scan number: 18646

Gene Names	Charge	m/z	Mass	Retention time	PEP	Score	Precursor Intensity
CDC2;CDK1	3	405.575	1213.703	73.073	1.5672e−05	119.75	1572683

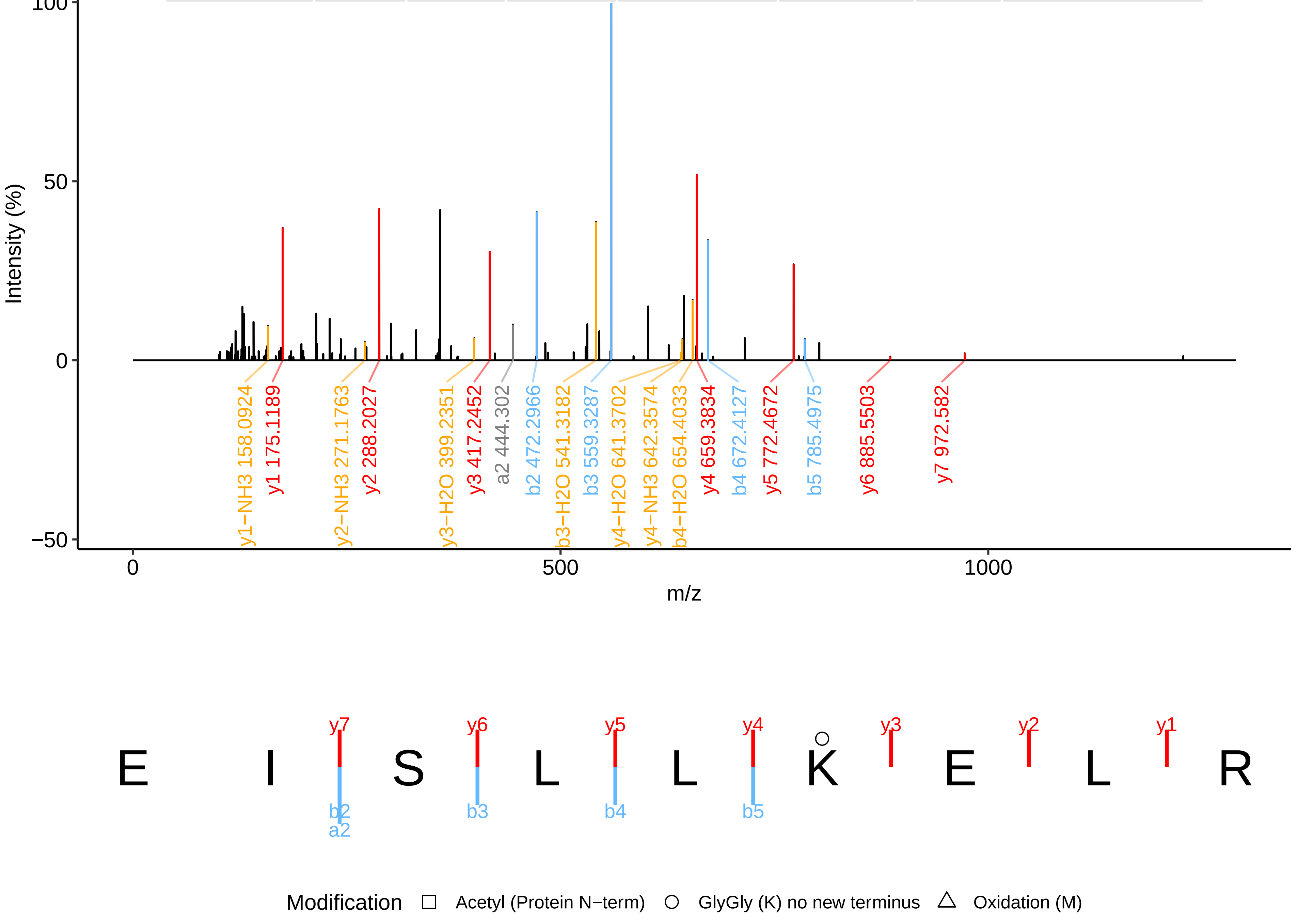

20200921_KIZ_MUb4_GlyGly_10plex − Scan number: 15748

Gene Names	Charge	m/z	Mass	Retention time	PEP	Score	Precursor Intensity
ATP5I	2	623.8117	1245.609	56.345	1.544e−06	129.53	12558368

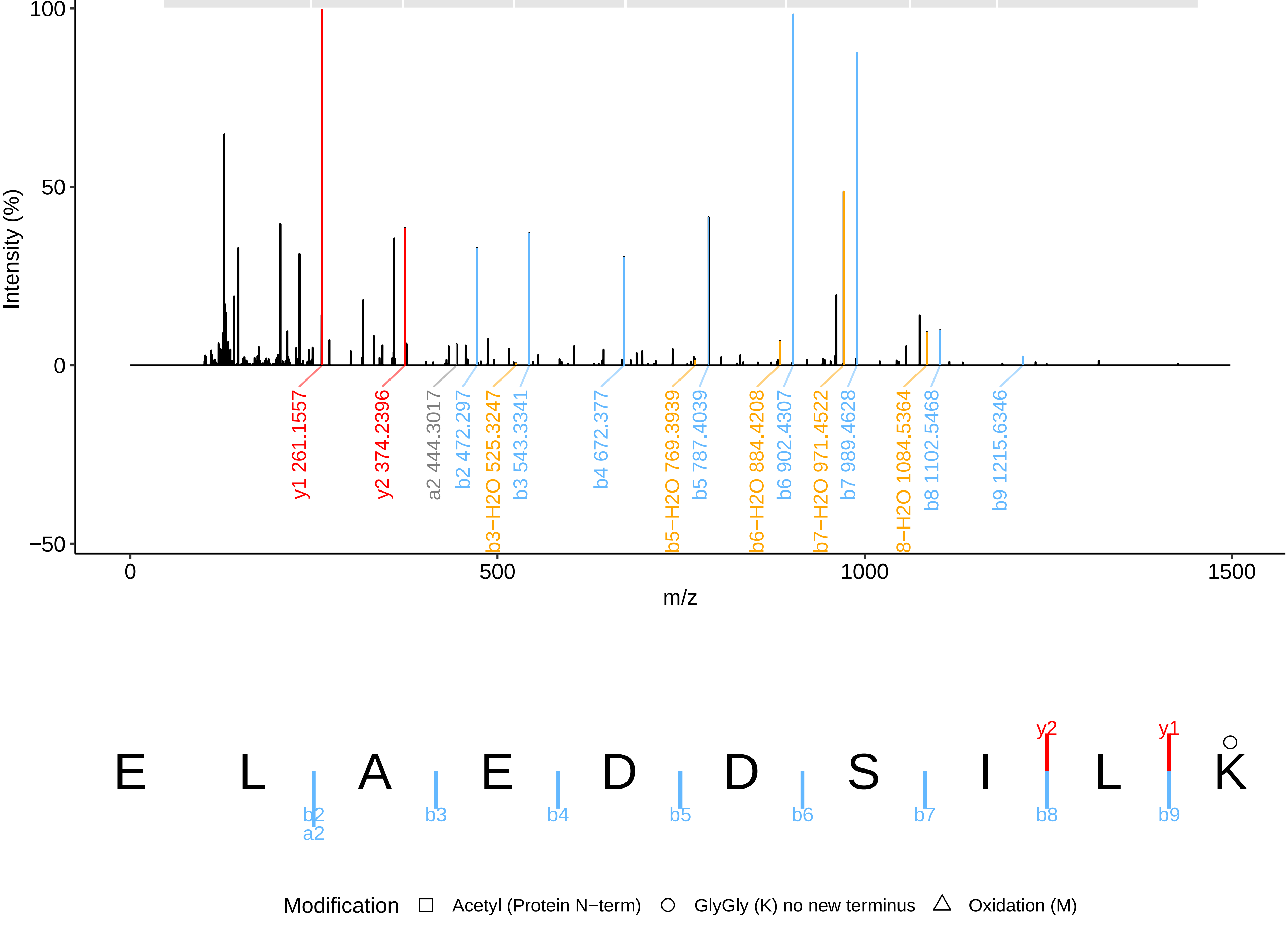

20200921_KIZ_MUb4_GlyGly_10plex − Scan number: 15878

Gene Names	Charge	m/z	Mass	Retention time	PEP	Score	Precursor Intensity
ATP5I	2	623.8117	1245.609	56.865	4.1807e−09	146.35	4763182

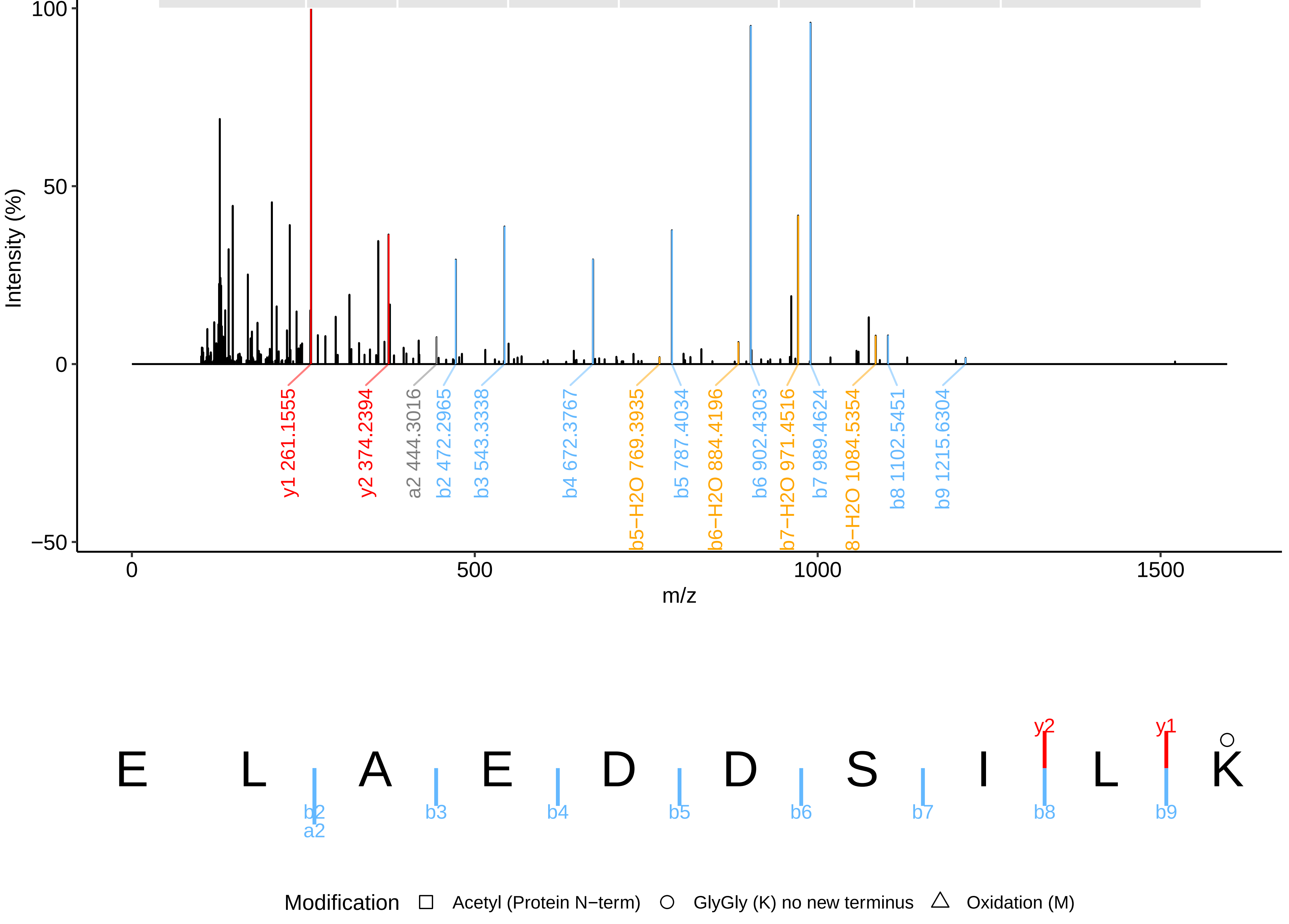

20200921_KIZ_MUb4_GlyGly_10plex_2 − Scan number: 14494

Gene Names	Charge	m/z	Mass	Retention time	PEP	Score	Precursor Intensity
ATP5I	2	623.8117	1245.609	56.522	1.5001e−08	136.11	4739736

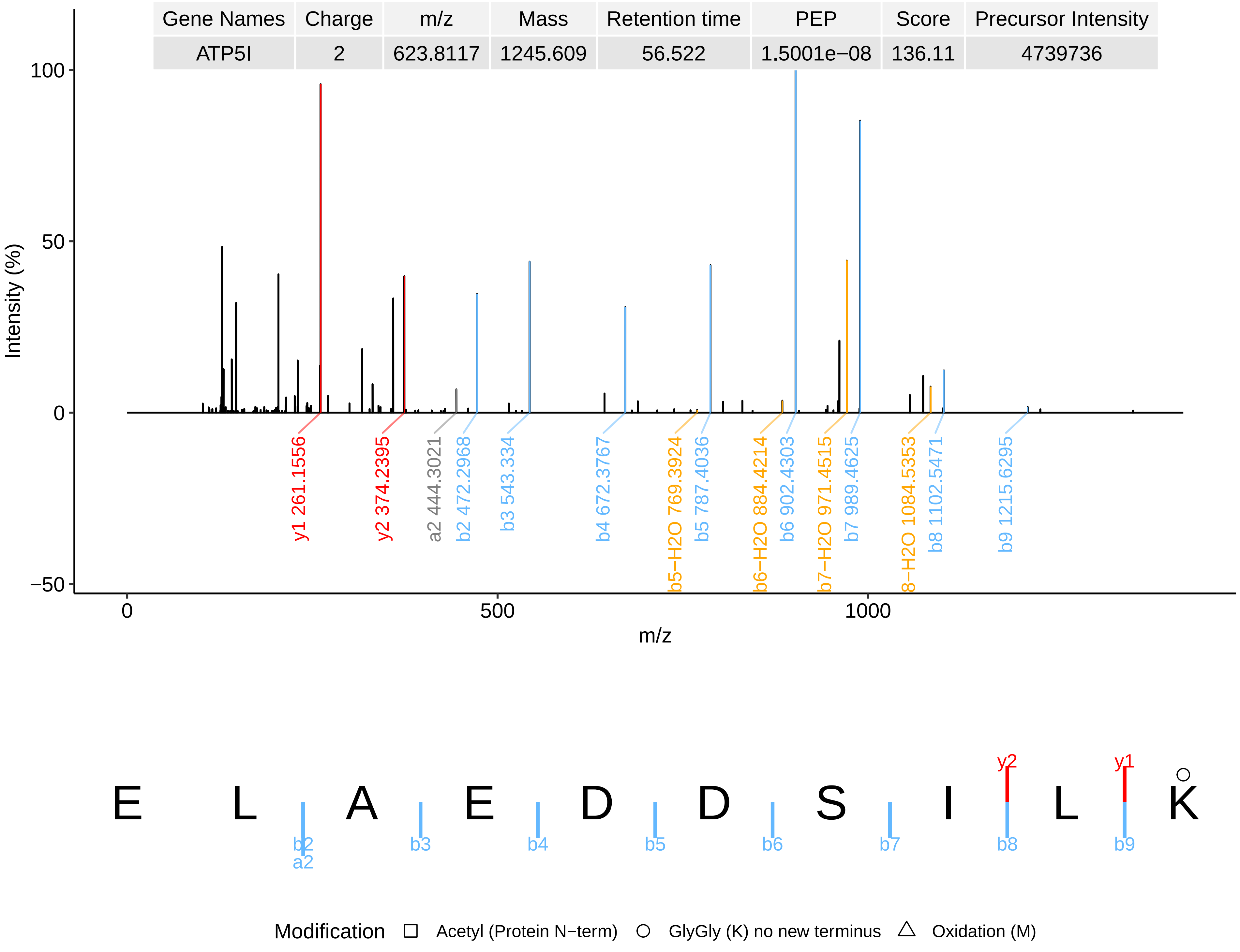

20200924_KIZ_MUb5_GlyGly_2 − Scan number: 14060

Gene Names	Charge	m/z	Mass	Retention time	PEP	Score	Precursor Intensity
ATP5I	2	623.8117	1245.609	52.577	1.5001e−08	136.11	3657927

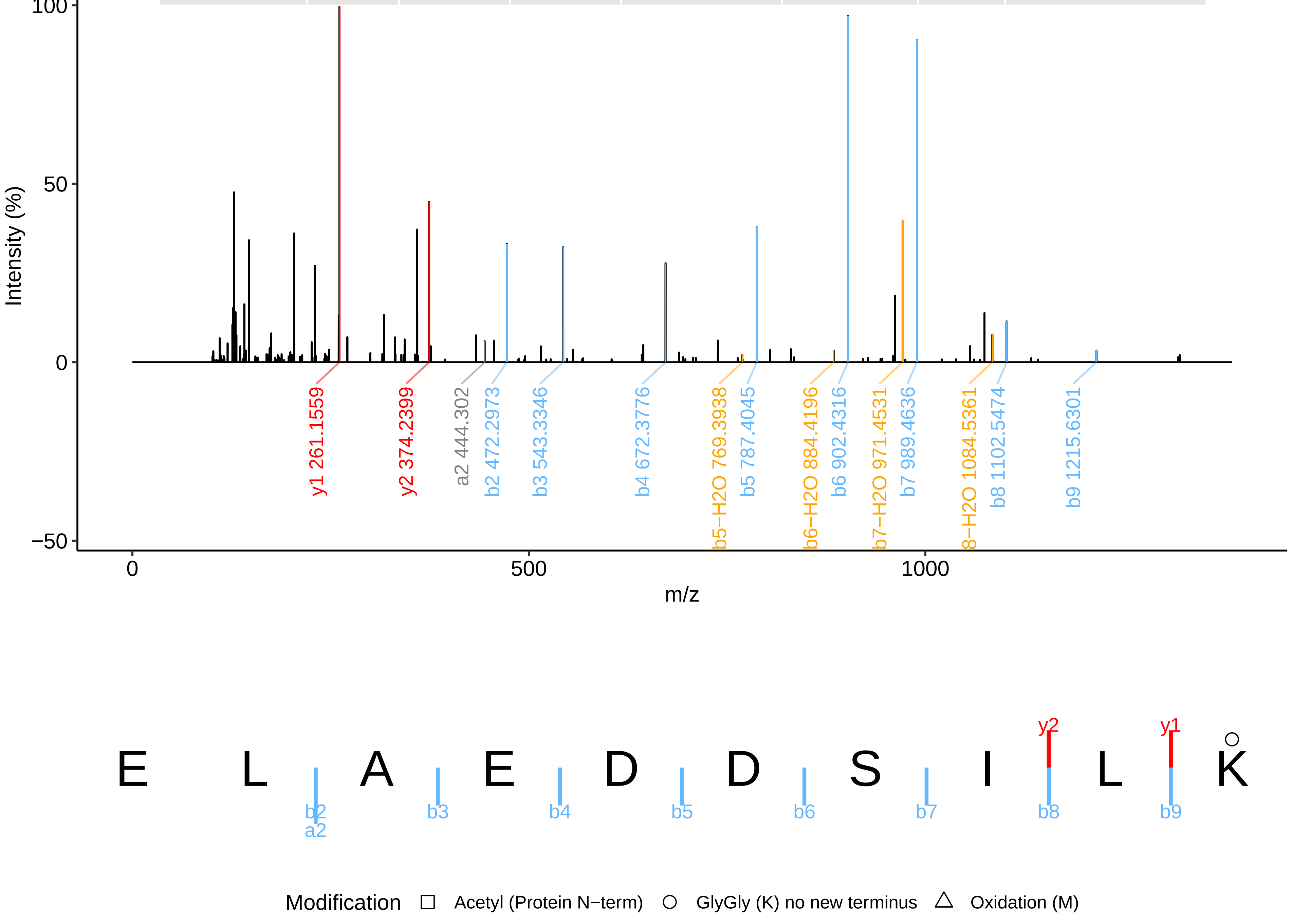

20200924_KIZ_MUb5_GlyGly_2 − Scan number: 14202

Gene Names	Charge	m/z	Mass	Retention time	PEP	Score	Precursor Intensity
ATP5I	2	623.8117	1245.609	53.089	1.2061e−08	138.9	3113823

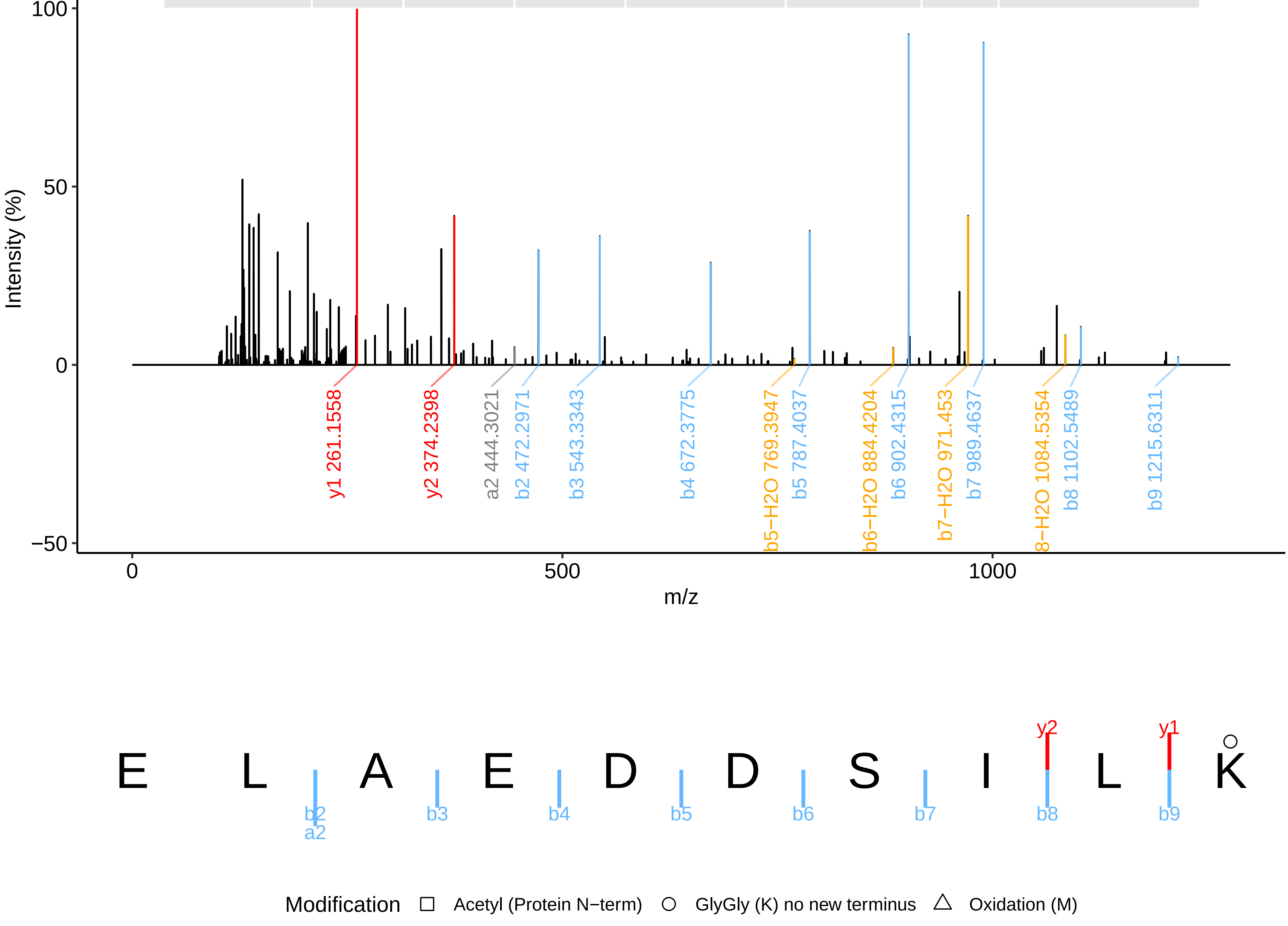

20200921_KIZ_MUb4_GlyGly_10plex − Scan number: 5671

Gene Names	Charge	m/z	Mass	Retention time	PEP	Score	Precursor Intensity
SEC62	3	330.5015	988.4825	18.598	0.093552	57.281	14641791

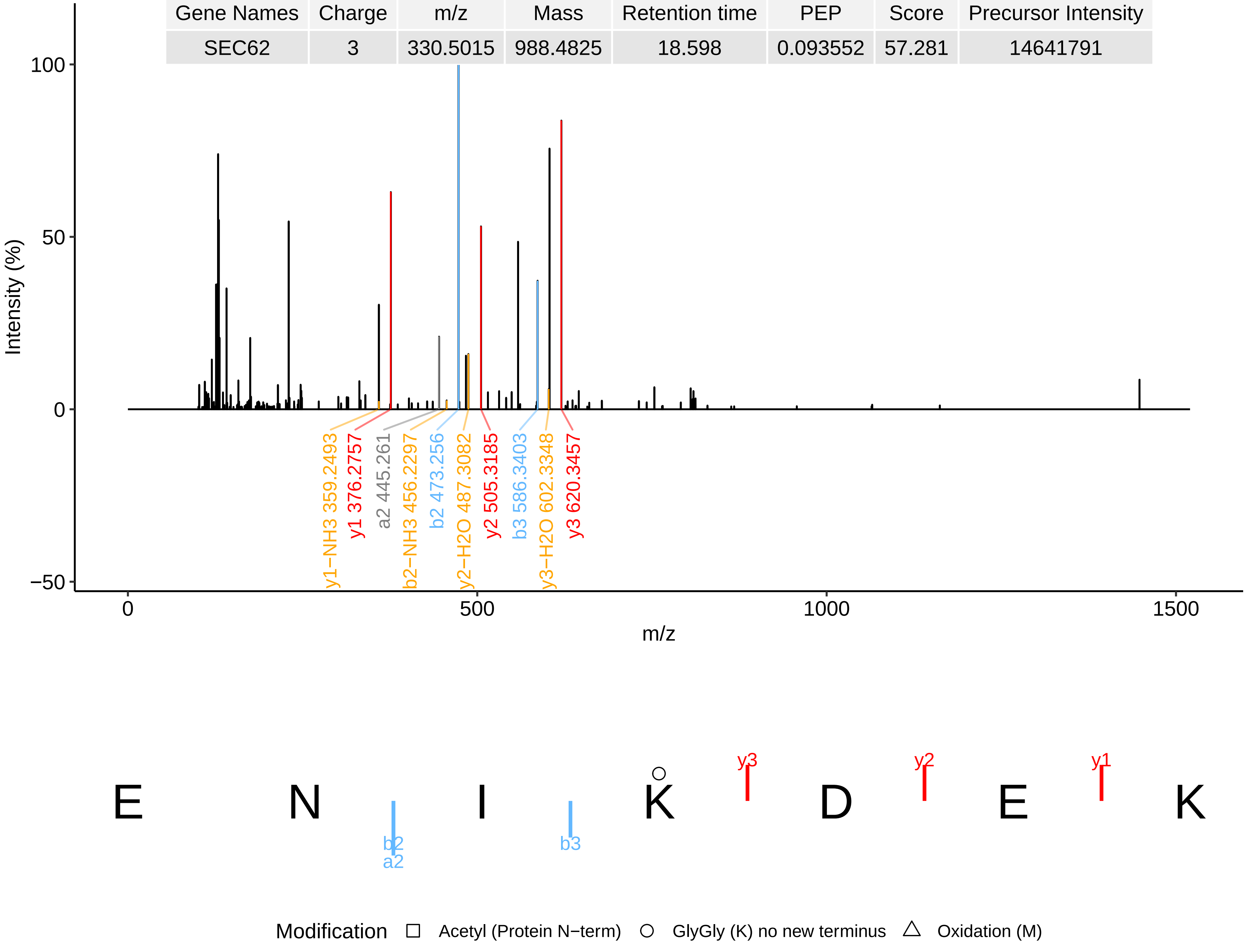

20200921_KIZ_MUb4_GlyGly_10plex_2 − Scan number: 5157

Gene Names	Charge	m/z	Mass	Retention time	PEP	Score	Precursor Intensity
SEC62	3	330.5015	988.4825	18.412	0.10899	54.157	897239

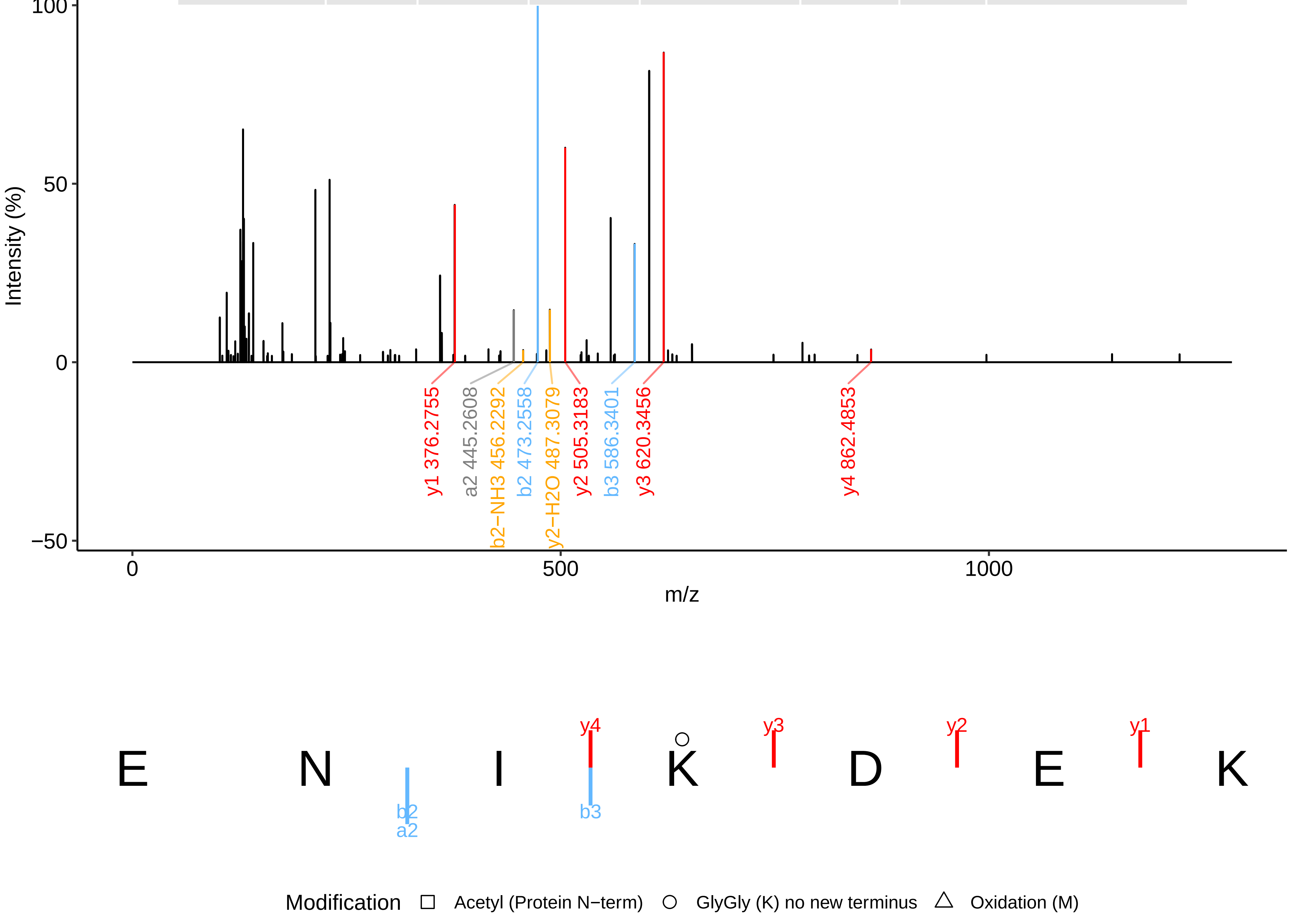

20200921_KIZ_MUb4_GlyGly_10plex − Scan number: 5665

Gene Names	Charge	m/z	Mass	Retention time	PEP	Score	Precursor Intensity
YES1	3	334.1856	999.5349	18.582	0.063036	59.35	NA

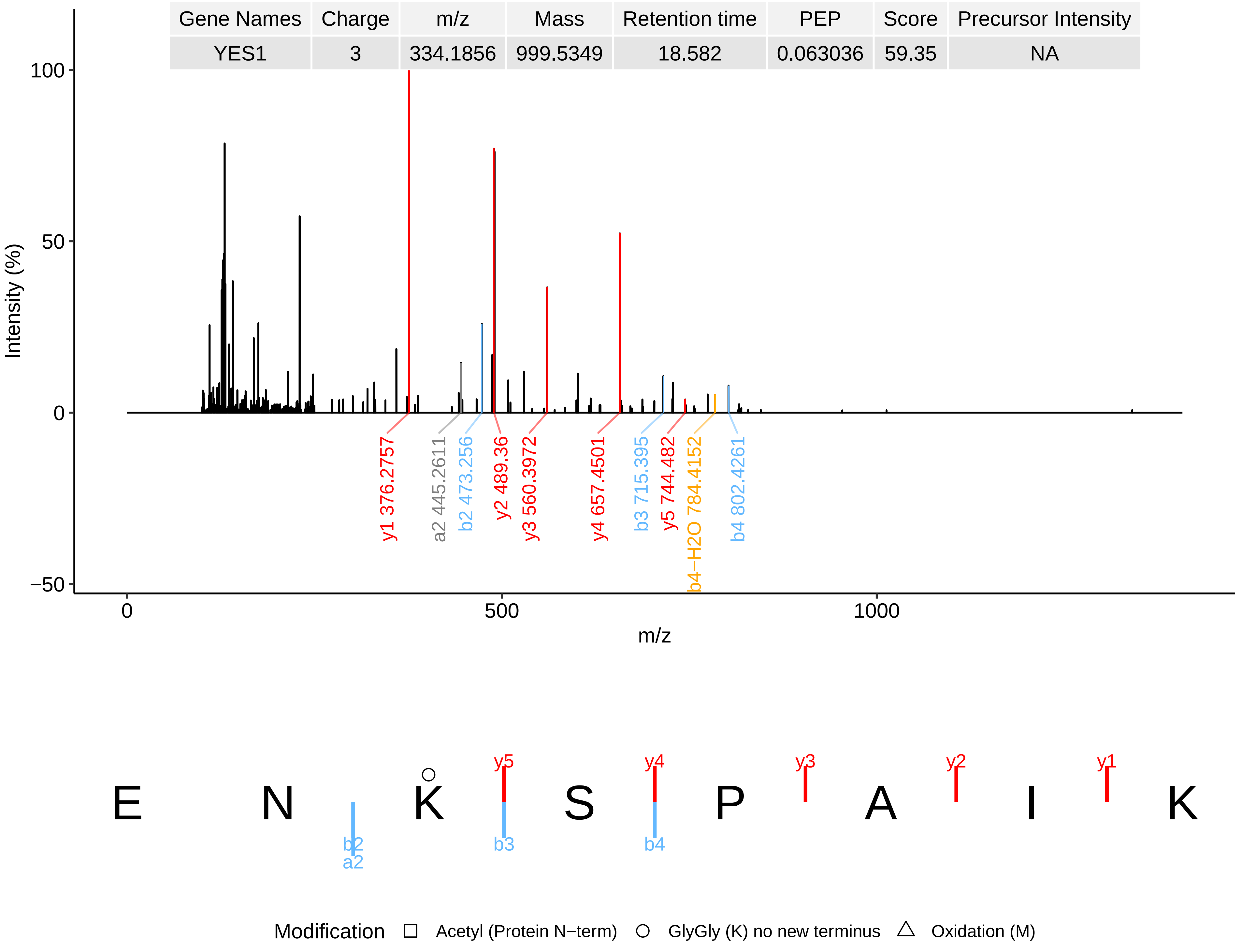

20200921_KIZ_MUb4_GlyGly_10plex − Scan number: 7379

Gene Names	Charge	m/z	Mass	Retention time	PEP	Score	Precursor Intensity
TMEM87A	3	415.5181	1243.533	24.752	0.019909	62.582	5286298

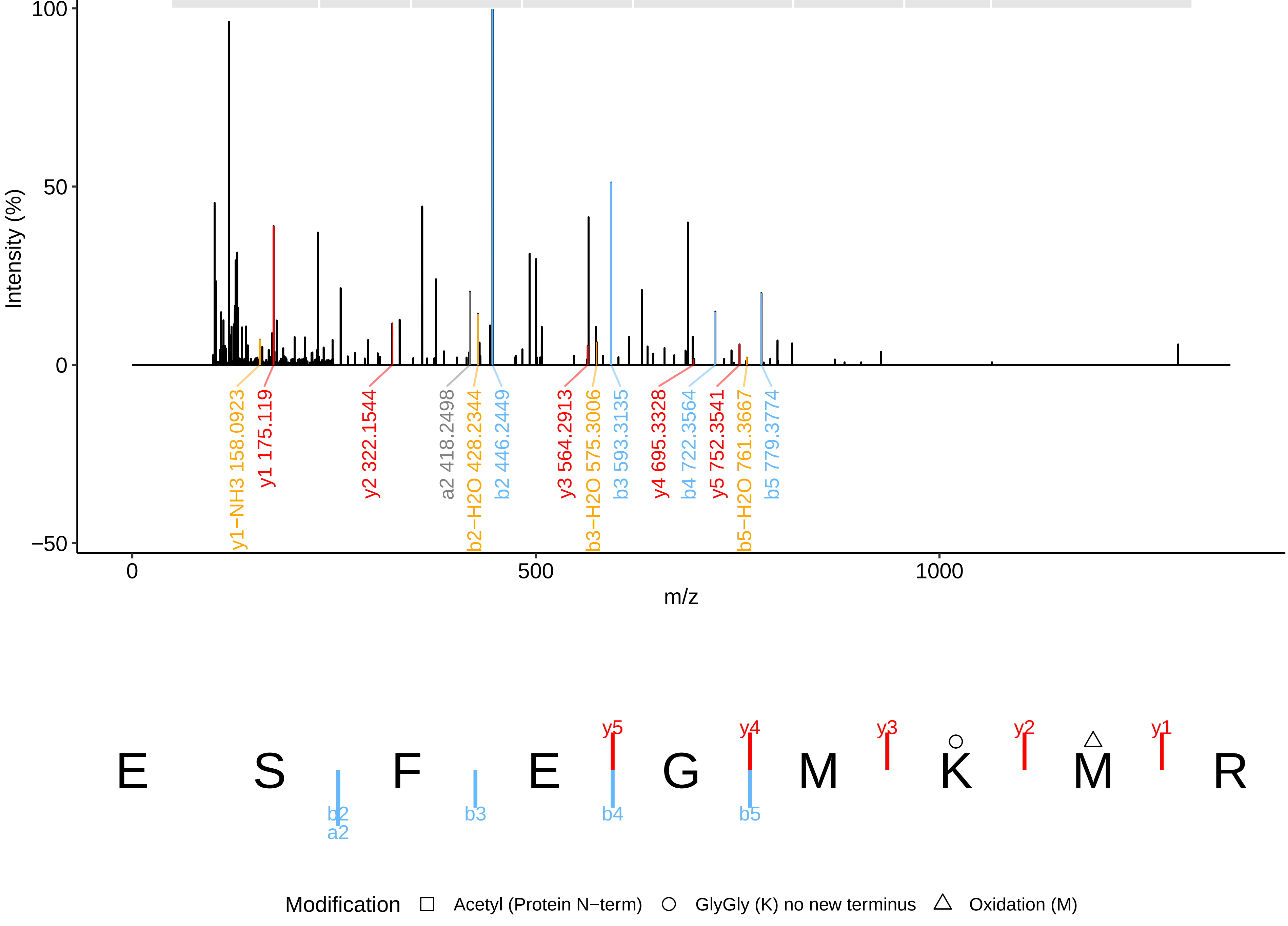

20200921_KIZ_MUb4_GlyGly_10plex_2 − Scan number: 5731

Gene Names	Charge	m/z	Mass	Retention time	PEP	Score	Precursor Intensity
TMEM87A	3	415.5181	1243.533	20.744	0.033787	58.981	569419.5

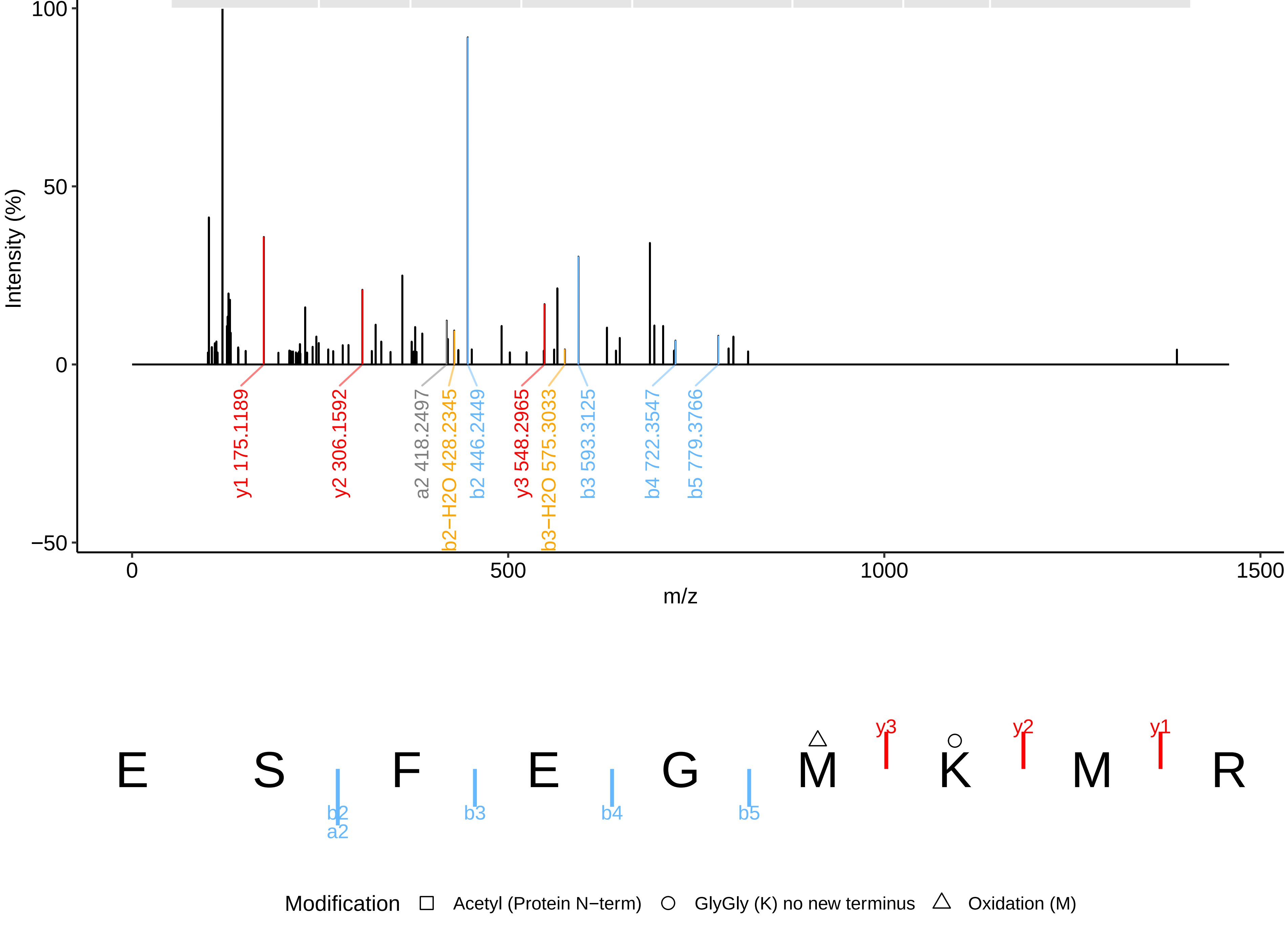

20200921_KIZ_MUb4_GlyGly_10plex_2 − Scan number: 6798

Gene Names	Charge	m/z	Mass	Retention time	PEP	Score	Precursor Intensity
TMEM87A	3	415.5181	1243.533	25.142	0.076608	47.869	841081.7

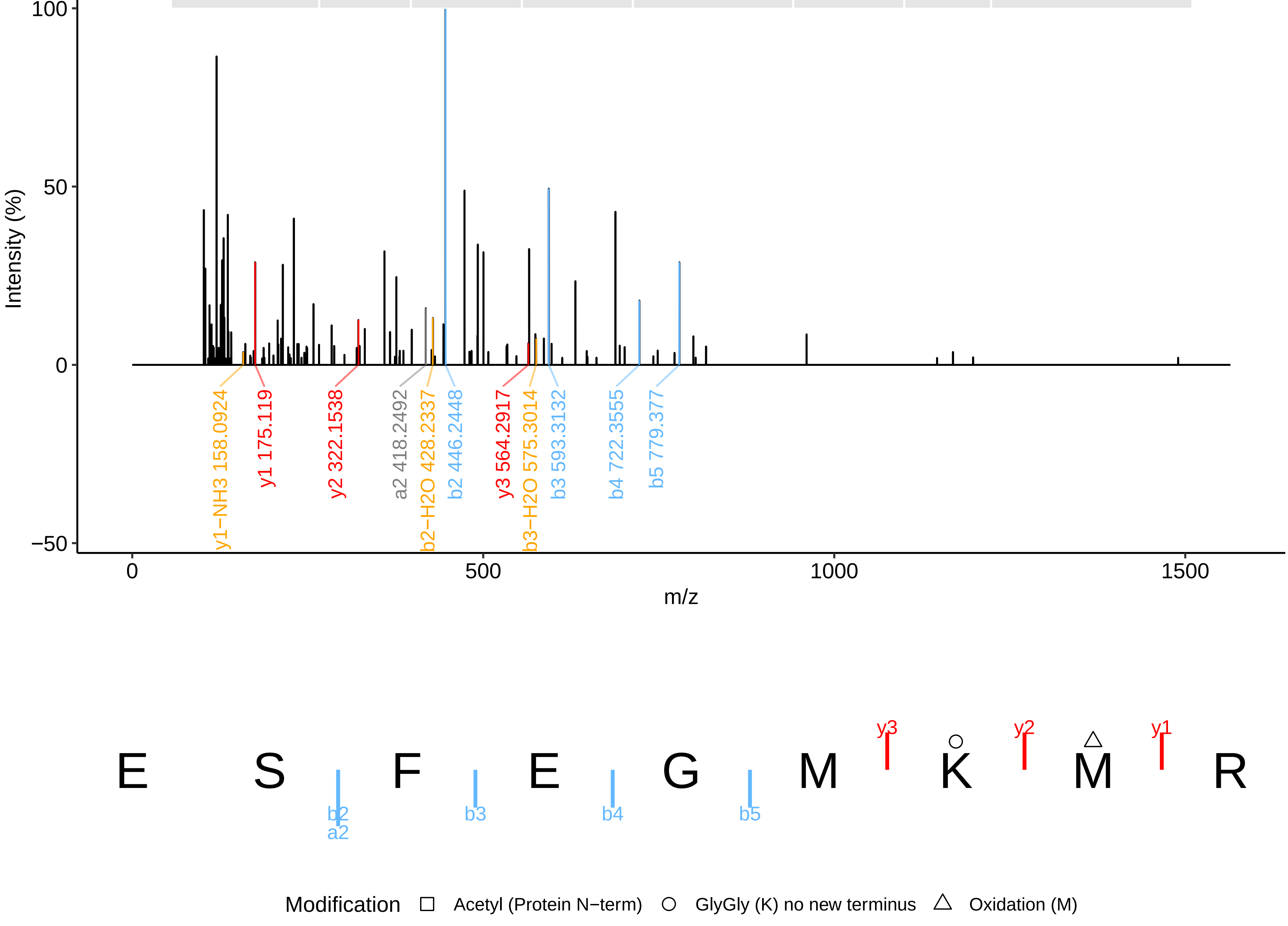

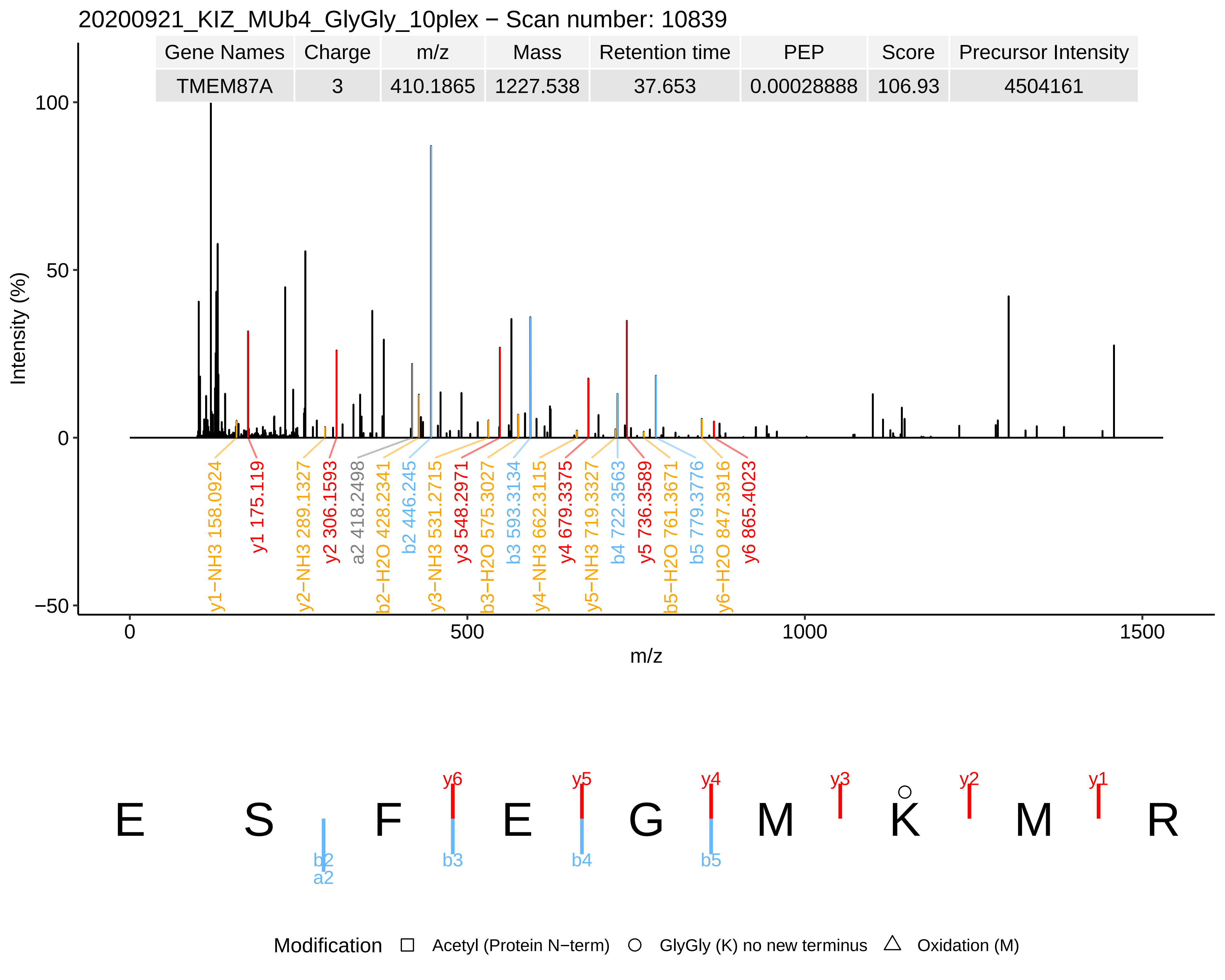
20200921_KIZ_MUb4_GlyGly_10plex − Scan number: 10839
Gene Names | Charge | m/z | Mass | Retention time | PEP | Score | Precursor Intensity
TMEM87A | 3 | 410.1865 | 1227.538 | 37.653 | 0.00028888 | 106.93 | 4504161
Intensity (%)
100
50
0
−50
0
500
1000
1500
m/z
y1−NH3 158.0924
y1 175.119
y2−NH3 289.1327
y2 306.1593
a2 418.2498
b2−H2O 428.2341
b2 446.245
y3−NH3 531.2715
y3 548.2971
b3−H2O 575.3027
b3 593.3134
y4−NH3 662.3115
y4 679.3375
y5−NH3 719.3327
b4 722.3563
y5 736.3589
b5−H2O 761.3671
b5 779.3776
y6−H2O 847.3916
y6 865.4023
E S F E G M K M R
y6 y5 y4 y3 y2 y1
b2 a2 b3 b4 b5
Modification
Acetyl (Protein N−term)
GlyGly (K) no new terminus
Oxidation (M)

20200921_KIZ_MUb4_GlyGly_10plex − Scan number: 19917

Gene Names	Charge	m/z	Mass	Retention time	PEP	Score	Precursor Intensity
MSN	3	417.5679	1249.682	71.898	2.6395e−07	136.8	3984950

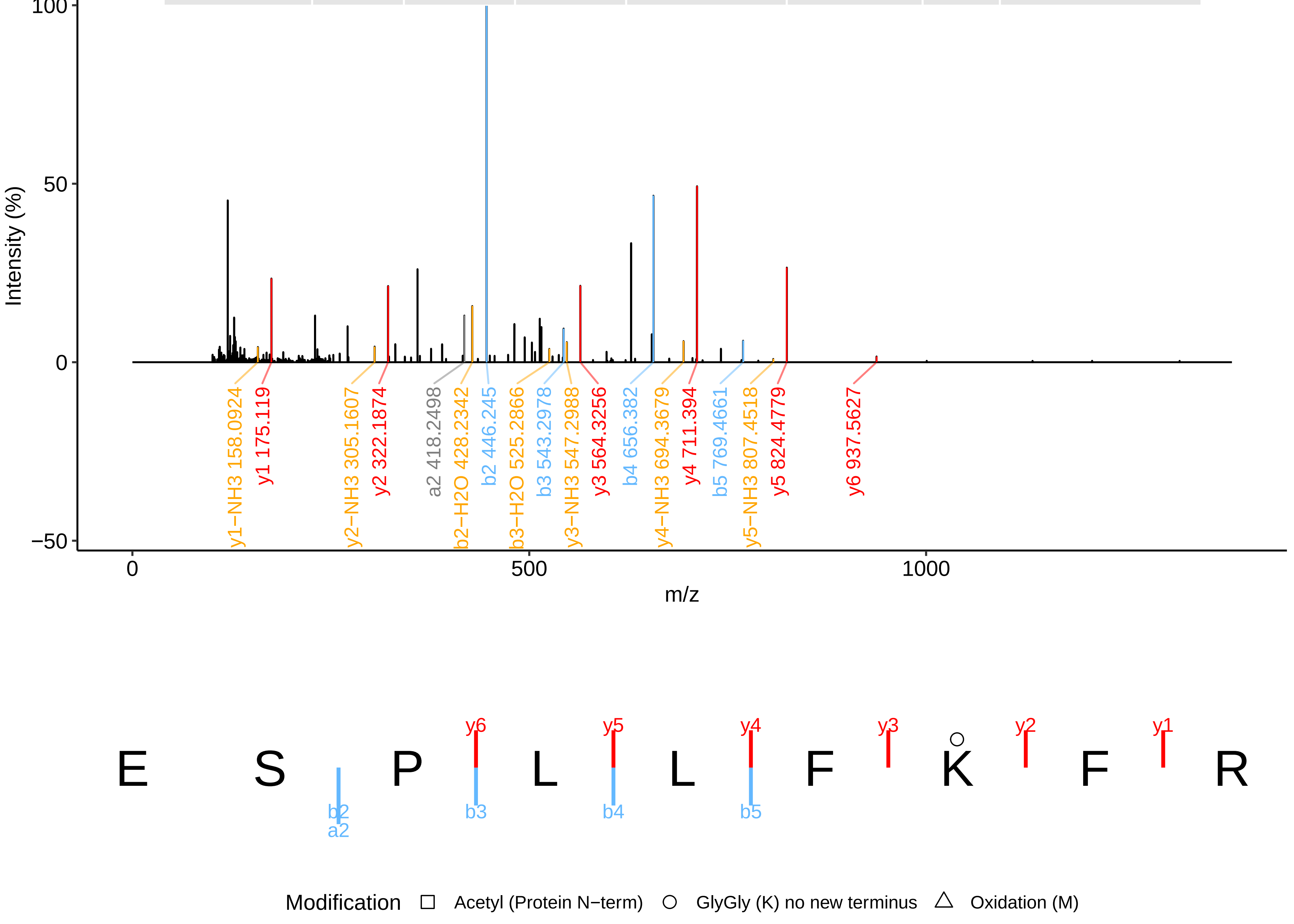

20200921_KIZ_MUb4_GlyGly_10plex_2 − Scan number: 18391

Gene Names	Charge	m/z	Mass	Retention time	PEP	Score	Precursor Intensity
MSN	3	417.5679	1249.682	72.011	0.00045579	101.64	1420927

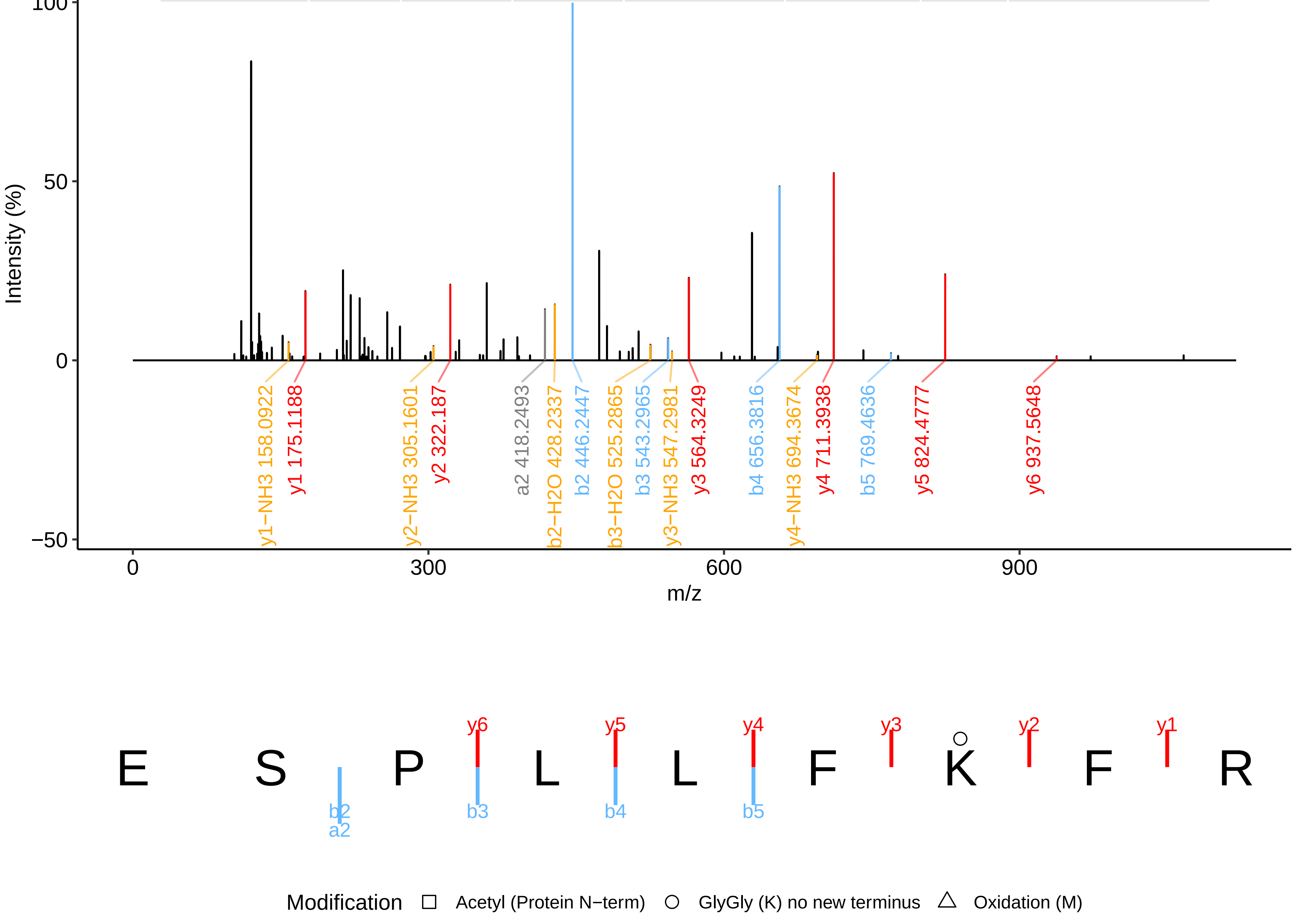

20200924_KIZ_MUb5_GlyGly_1 − Scan number: 19261

Gene Names	Charge	m/z	Mass	Retention time	PEP	Score	Precursor Intensity
MSN	3	417.5679	1249.682	62.247	0.00042477	124.98	9209080

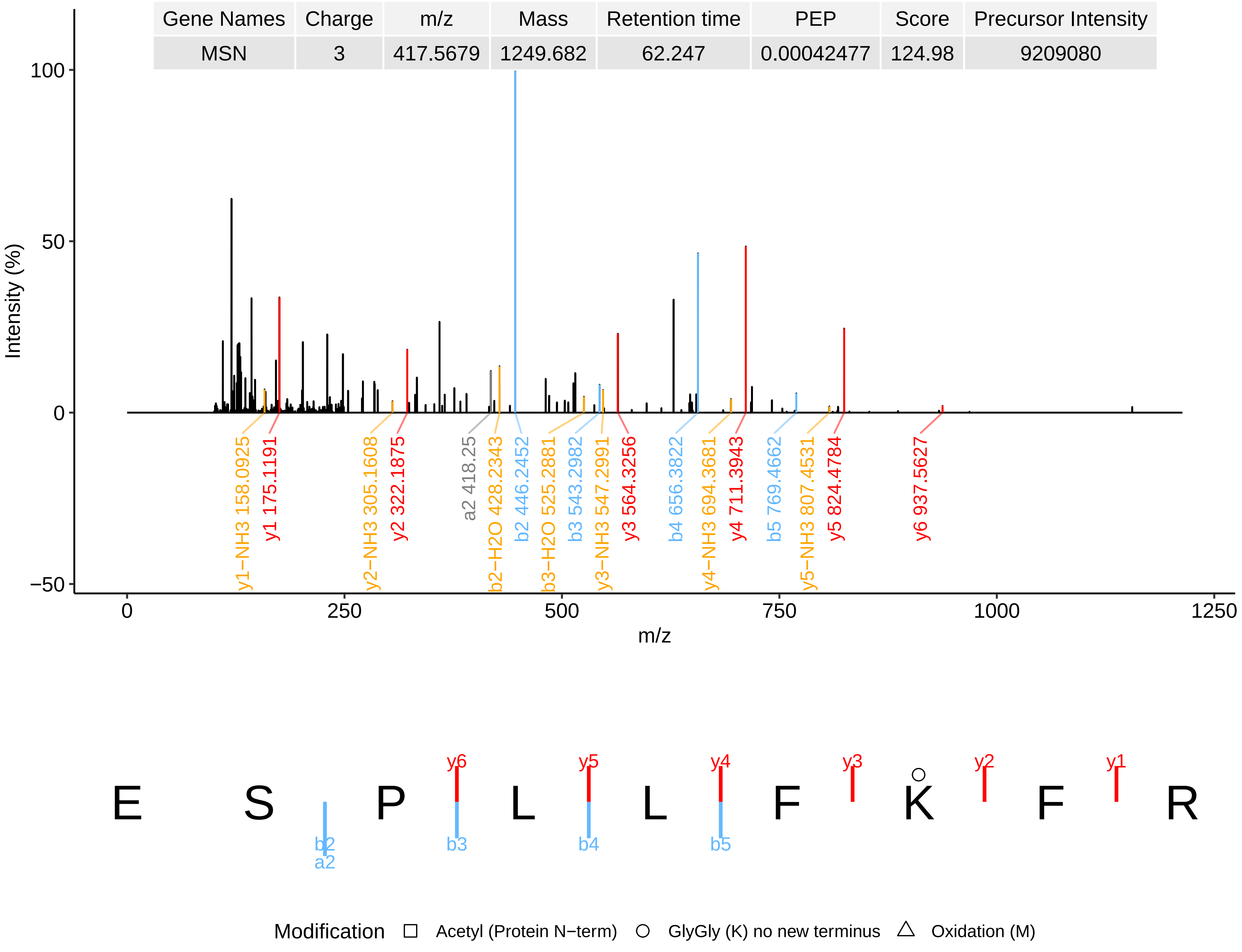

20200924_KIZ_MUb5_GlyGly_2 − Scan number: 18038

Gene Names	Charge	m/z	Mass	Retention time	PEP	Score	Precursor Intensity
MSN	3	417.5679	1249.682	66.869	2.6395e−07	136.8	2041061

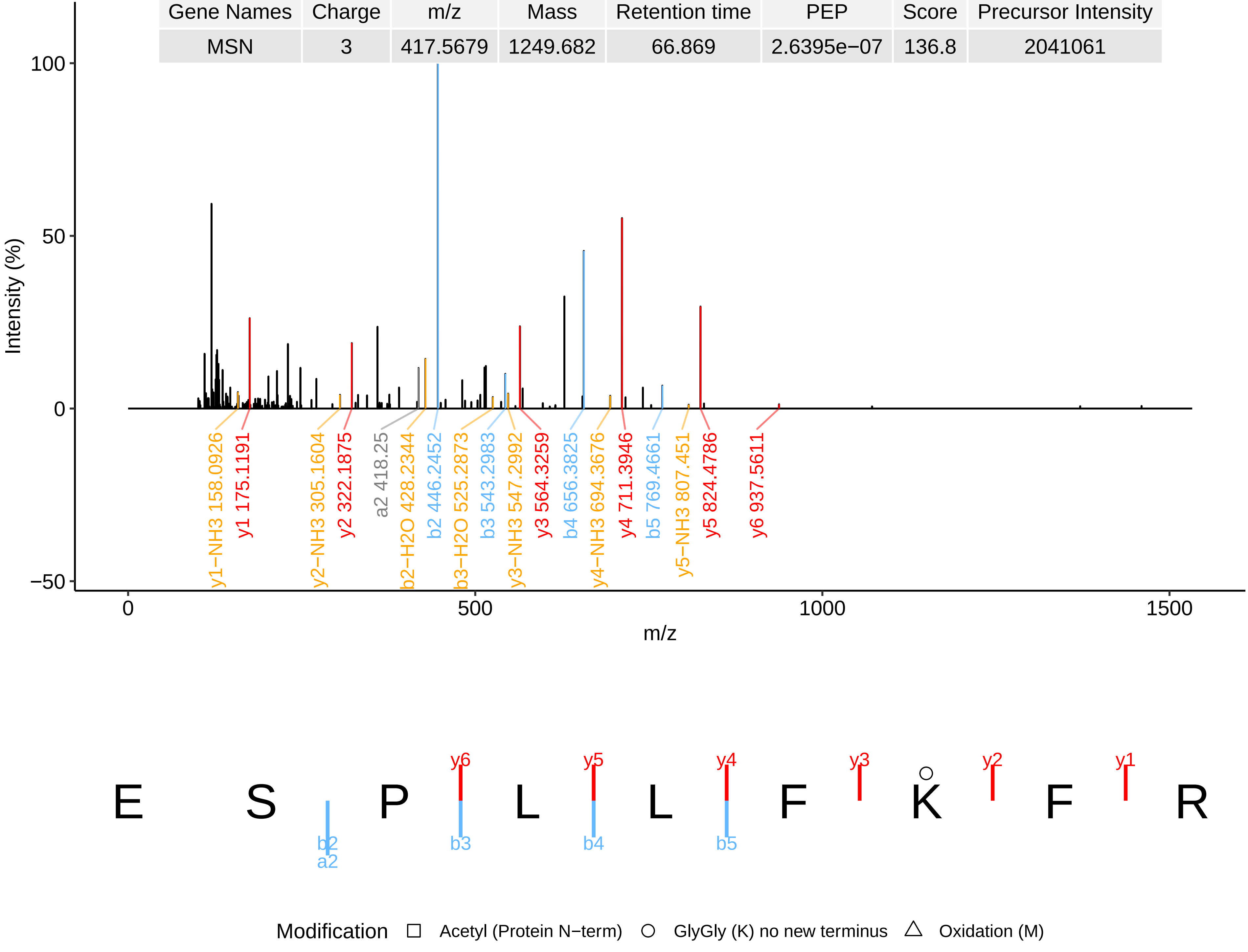

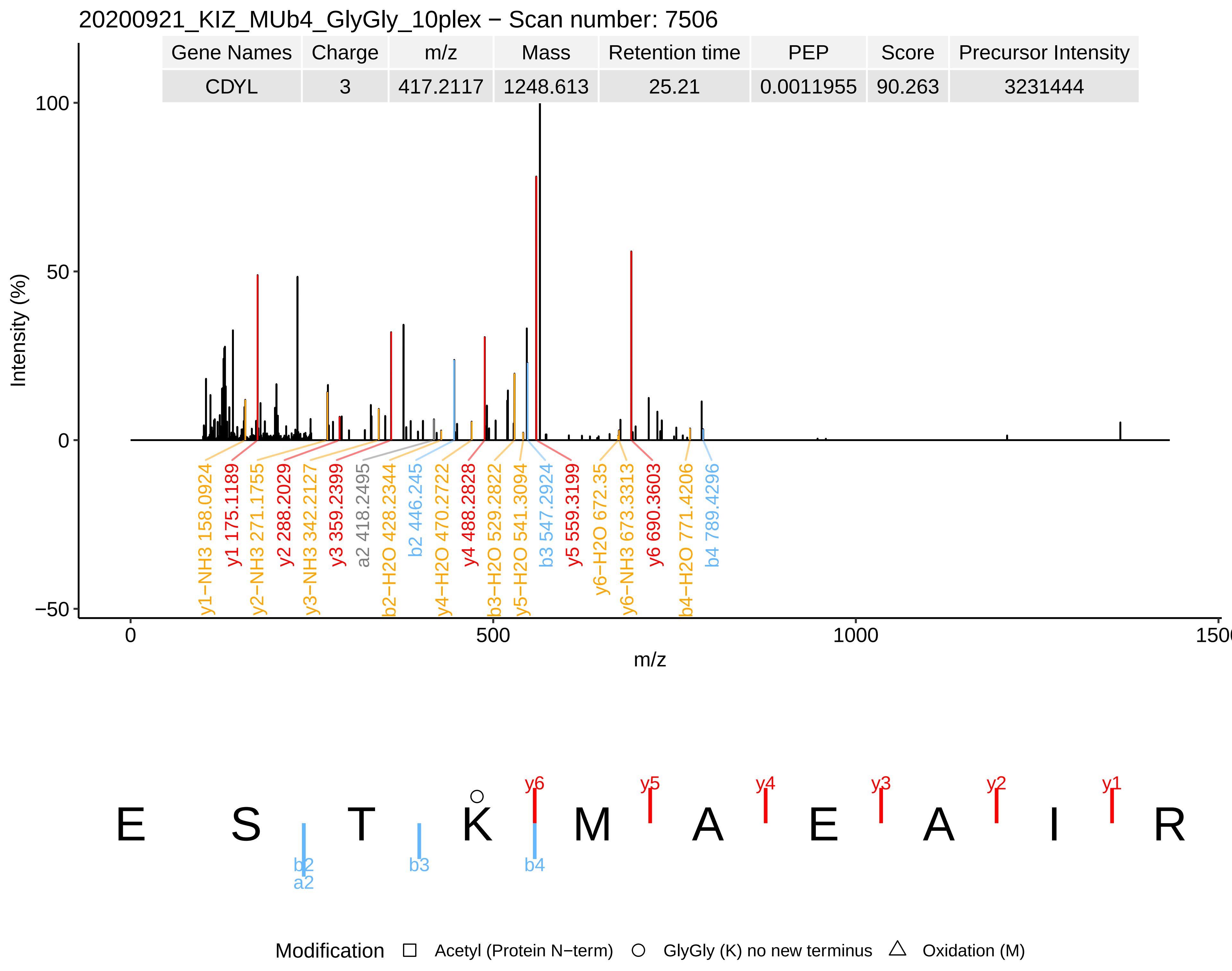

20200921_KIZ_MUb4_GlyGly_10plex − Scan number: 7506
Gene Names | Charge | m/z | Mass | Retention time | PEP | Score | Precursor Intensity
CDYL | 3 | 417.2117 | 1248.613 | 25.21 | 0.0011955 | 90.263 | 3231444
Intensity (%)
m/z
y1−NH3 158.0924
y1 175.1189
y2−NH3 271.1755
y2 288.2029
y3−NH3 342.2127
y3 359.2399
a2 418.2495
b2−H2O 428.2344
b2 446.245
y4−H2O 470.2722
y4 488.2828
b3−H2O 529.2822
y5−H2O 541.3094
b3 547.2924
y5 559.3199
y6−H2O 672.35
y6−NH3 673.3313
y6 690.3603
b4−H2O 771.4206
b4 789.4296
E S T K M A E A I R
y6 y5 y4 y3 y2 y1
b2 a2 b3 b4
Modification
Acetyl (Protein N−term)
GlyGly (K) no new terminus
Oxidation (M)

20200921_KIZ_MUb4_GlyGly_10plex − Scan number: 7549

Gene Names	Charge	m/z	Mass	Retention time	PEP	Score	Precursor Intensity
CDYL	3	417.2117	1248.613	25.371	0.043879	58.592	2143967

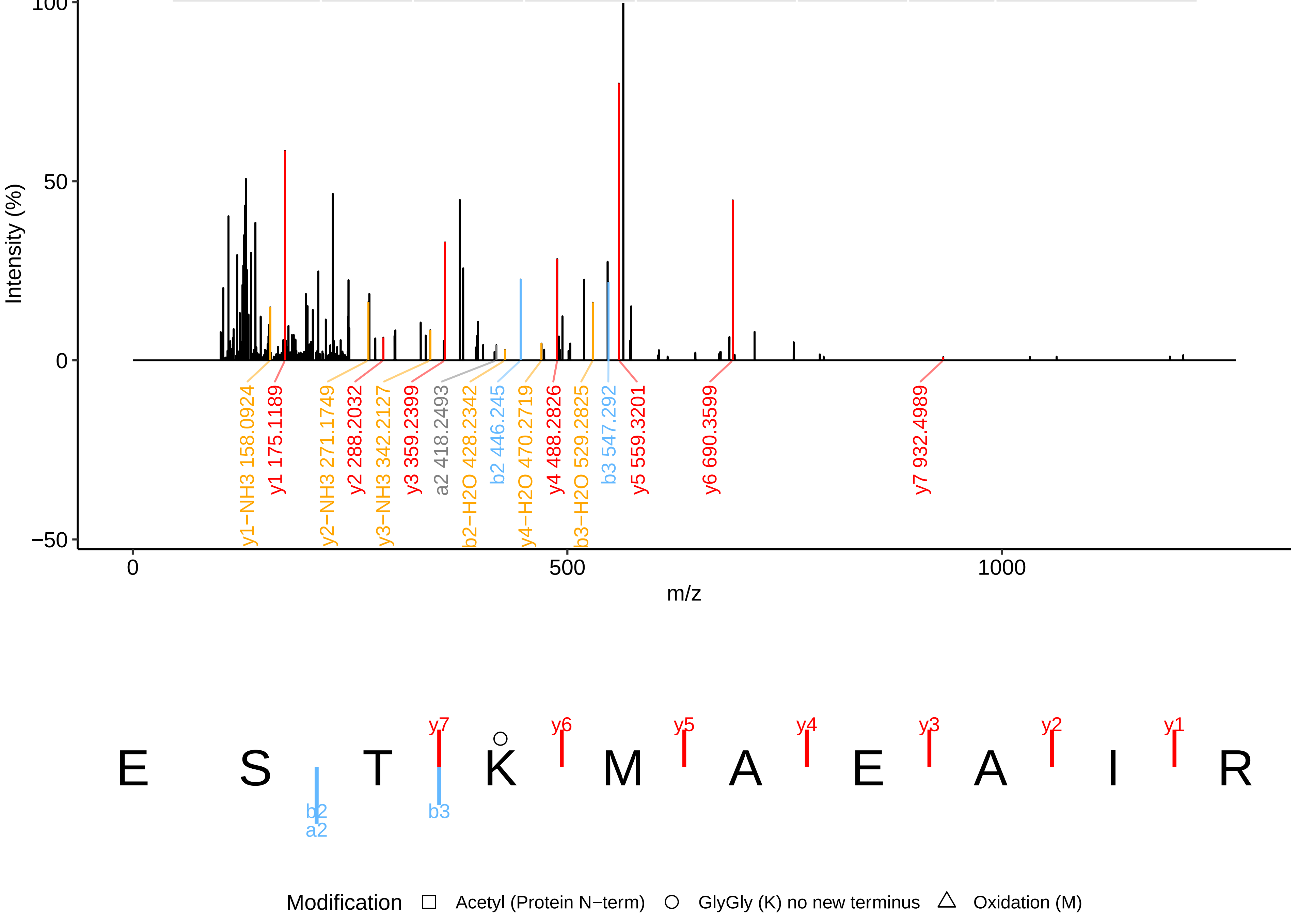

20200924_KIZ_MUb5_GlyGly_1 − Scan number: 6491

Gene Names	Charge	m/z	Mass	Retention time	PEP	Score	Precursor Intensity
CDYL	3	417.2117	1248.613	20.418	0.019735	66.19	NA

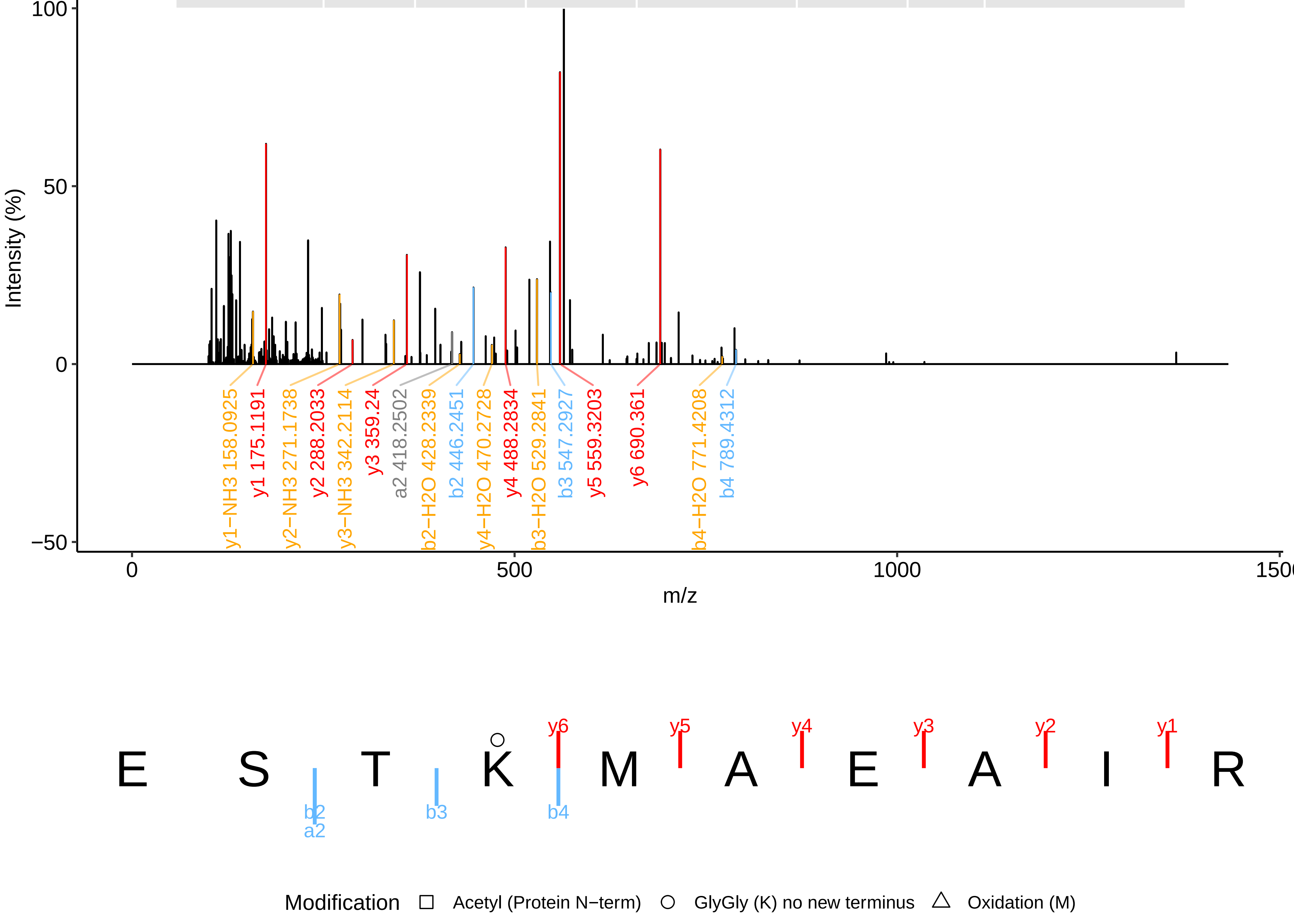

20200921_KIZ_MUb4_GlyGly_10plex − Scan number: 5892

Gene Names	Charge	m/z	Mass	Retention time	PEP	Score	Precursor Intensity
PLD1	3	404.2069	1209.599	19.327	0.00013681	111.74	4335288

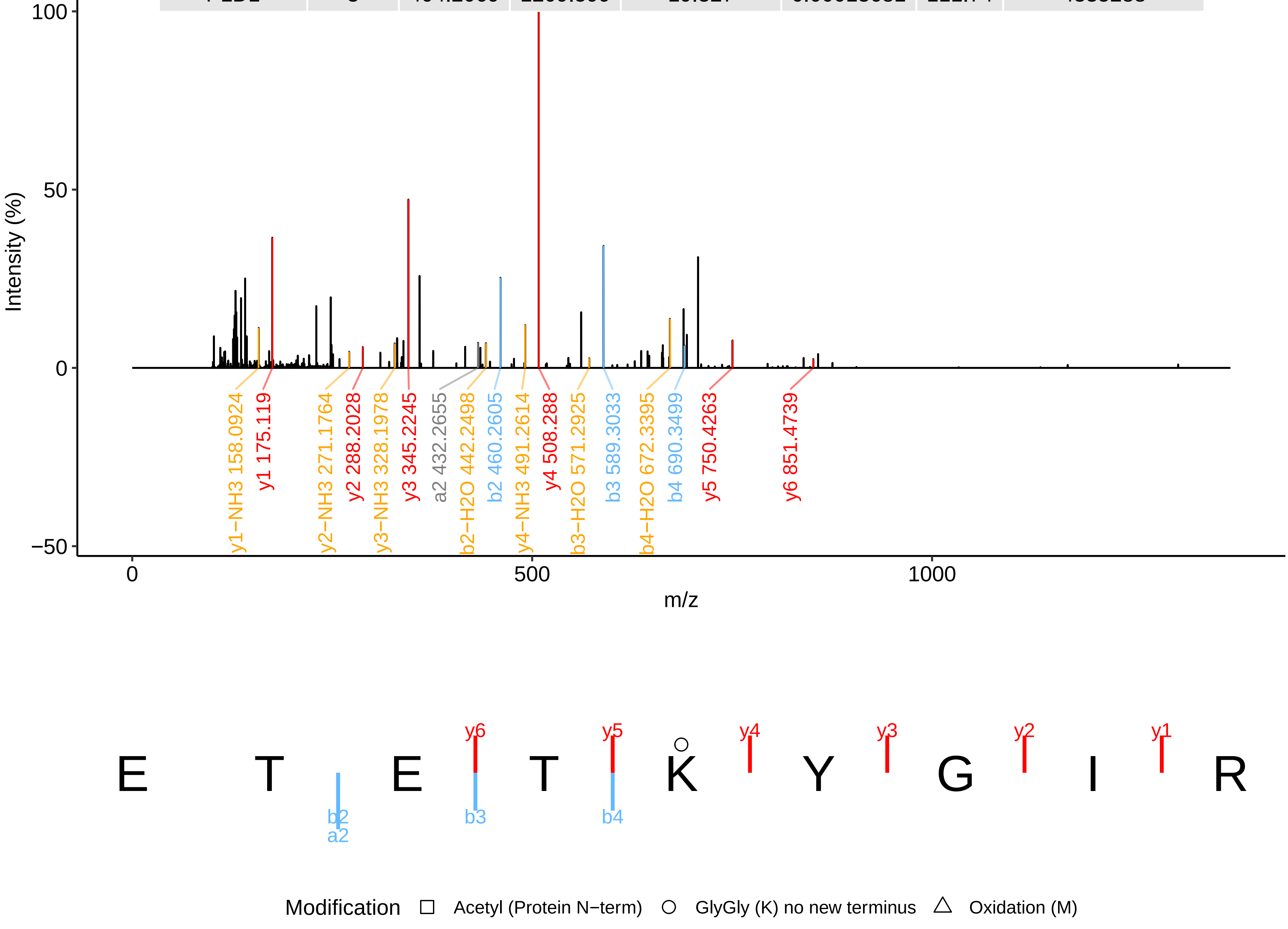

20200921_KIZ_MUb4_GlyGly_10plex_2 − Scan number: 5382

Gene Names	Charge	m/z	Mass	Retention time	PEP	Score	Precursor Intensity
PLD1	3	404.2069	1209.599	19.323	1.0702e−05	123.98	609307.9

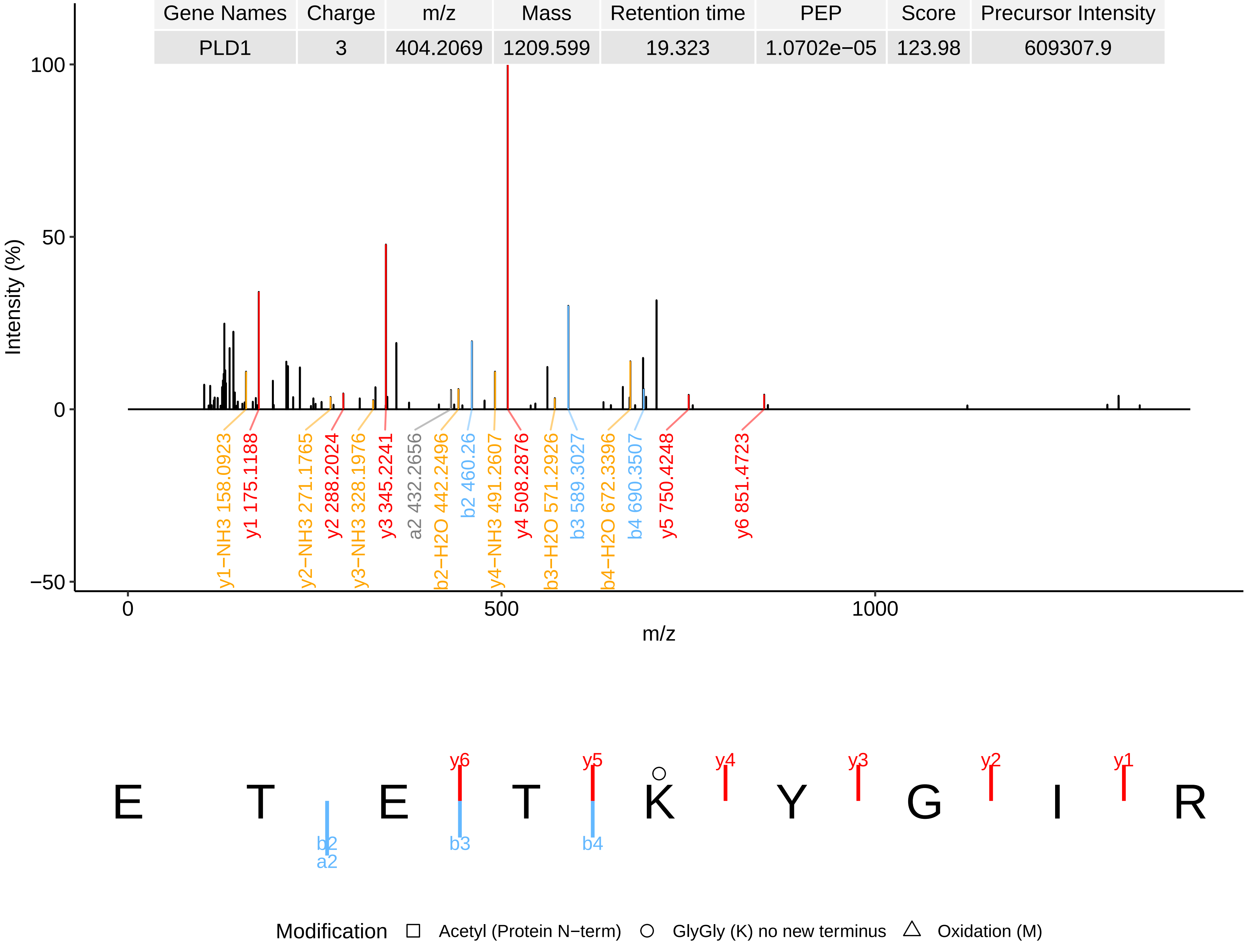

20200924_KIZ_MUb5_GlyGly_1 − Scan number: 5077

Gene Names	Charge	m/z	Mass	Retention time	PEP	Score	Precursor Intensity
PLD1	3	404.2069	1209.599	15.765	0.020908	64.268	16282857

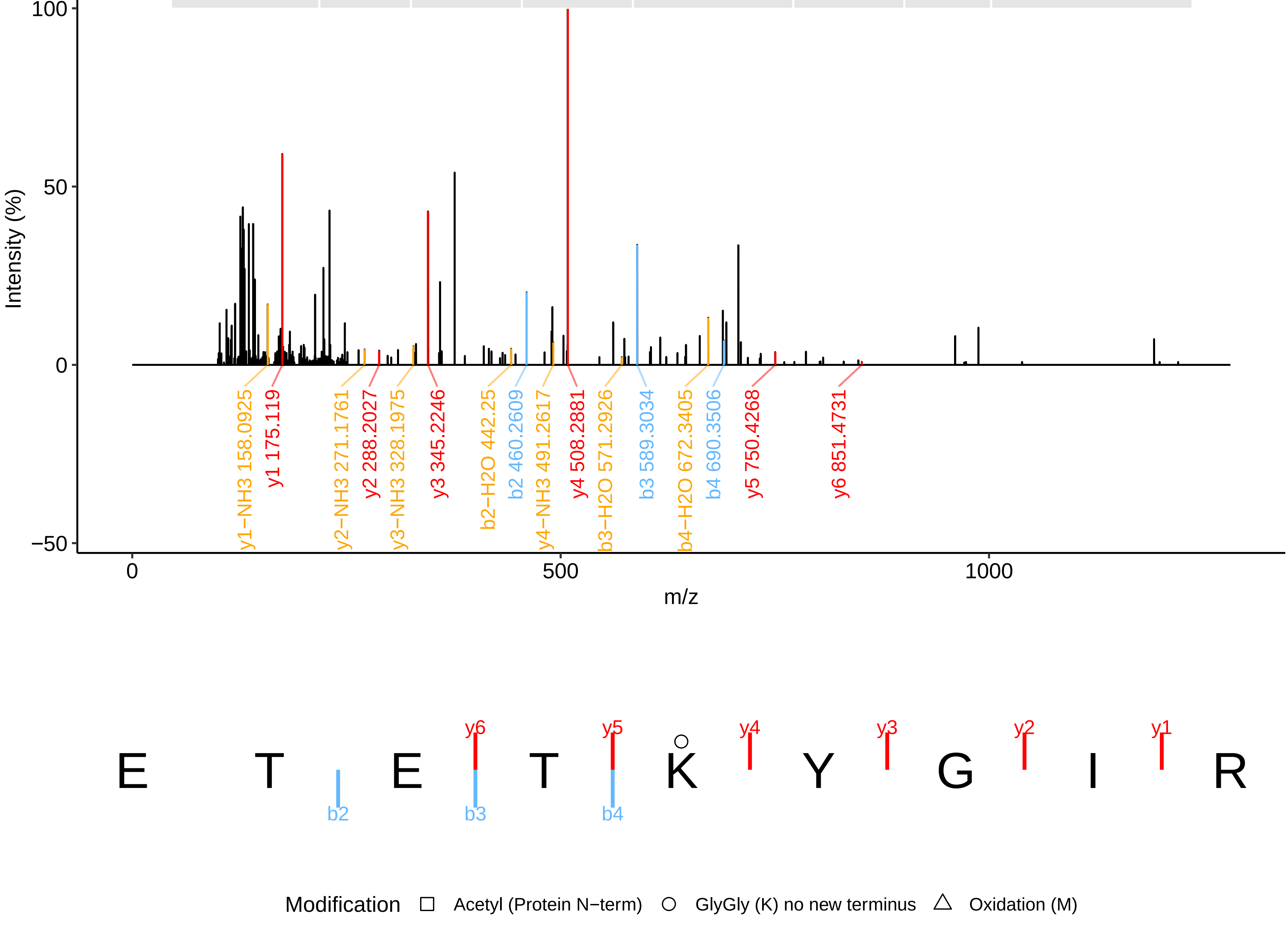

20200921_KIZ_MUb4_GlyGly_10plex_2 − Scan number: 8050

Gene Names	Charge	m/z	Mass	Retention time	PEP	Score	Precursor Intensity
AZIN1	3	404.2048	1209.592	30.25	0.017698	71.501	915480.3

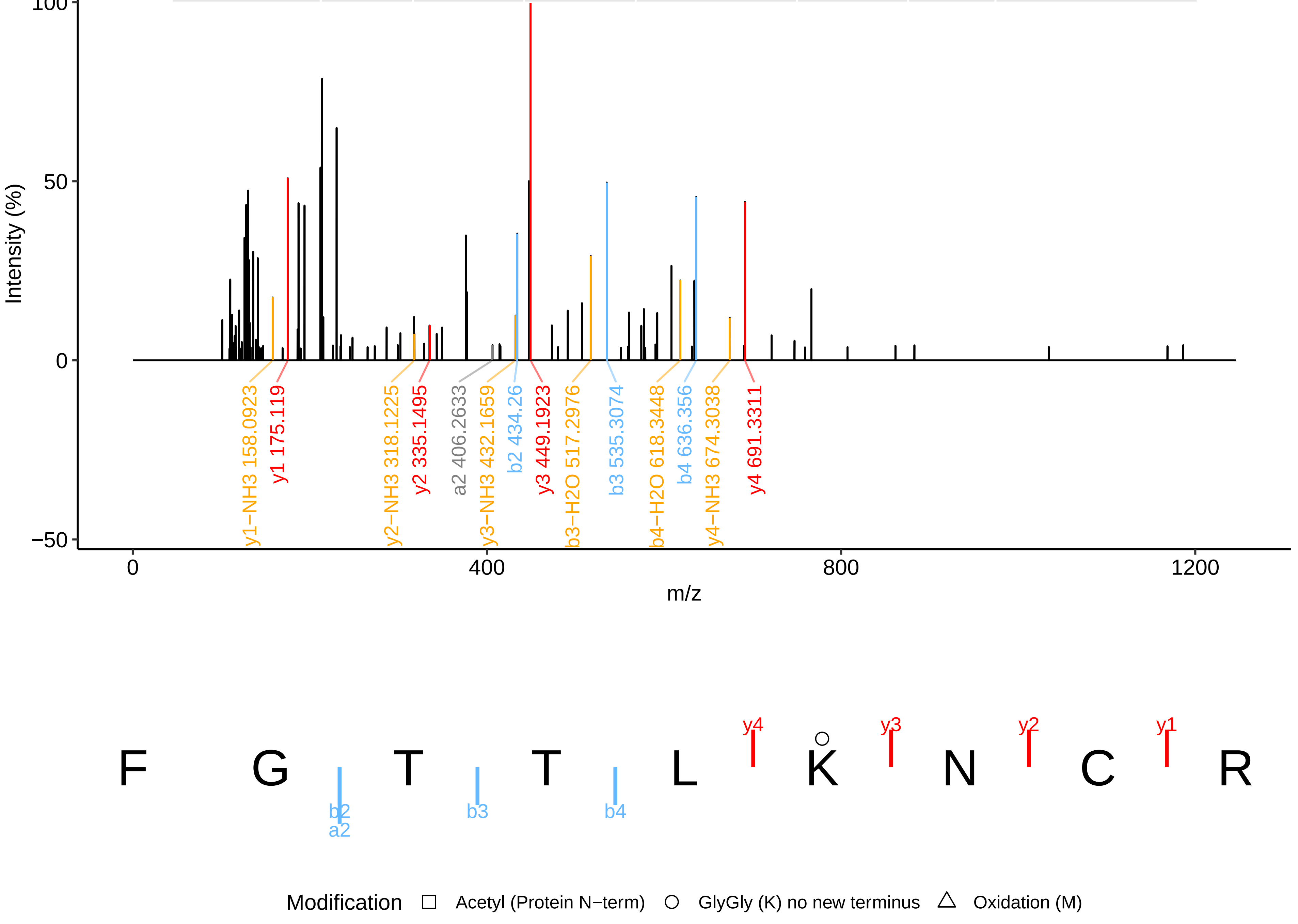

20201118_KIZ_MUb6_GlyGly_1 − Scan number: 7093

Gene Names	Charge	m/z	Mass	Retention time	PEP	Score	Precursor Intensity
AZIN1	3	404.2048	1209.592	22.479	0.003155	92.439	NA

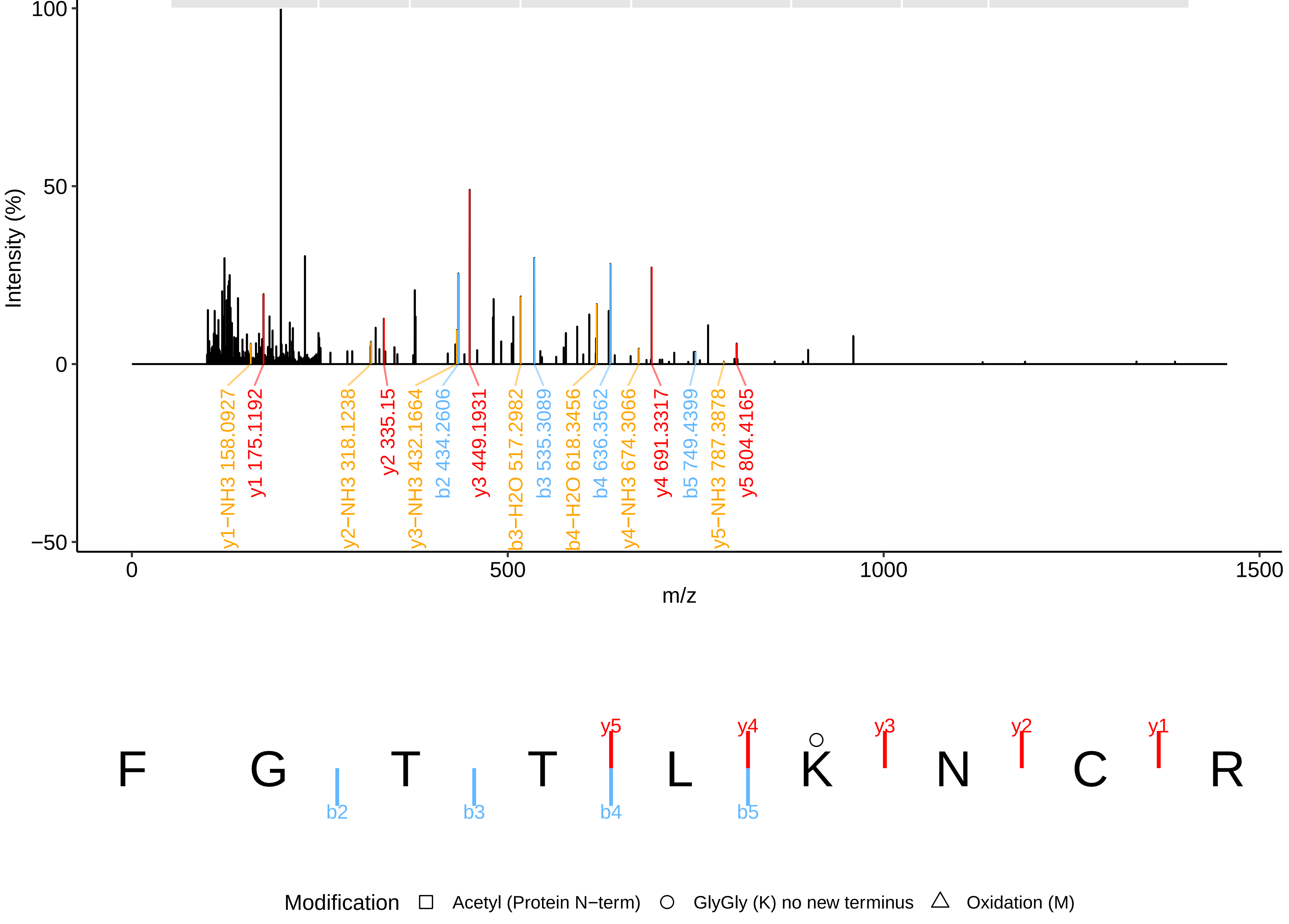

20200921_KIZ_MUb4_GlyGly_10plex − Scan number: 13471

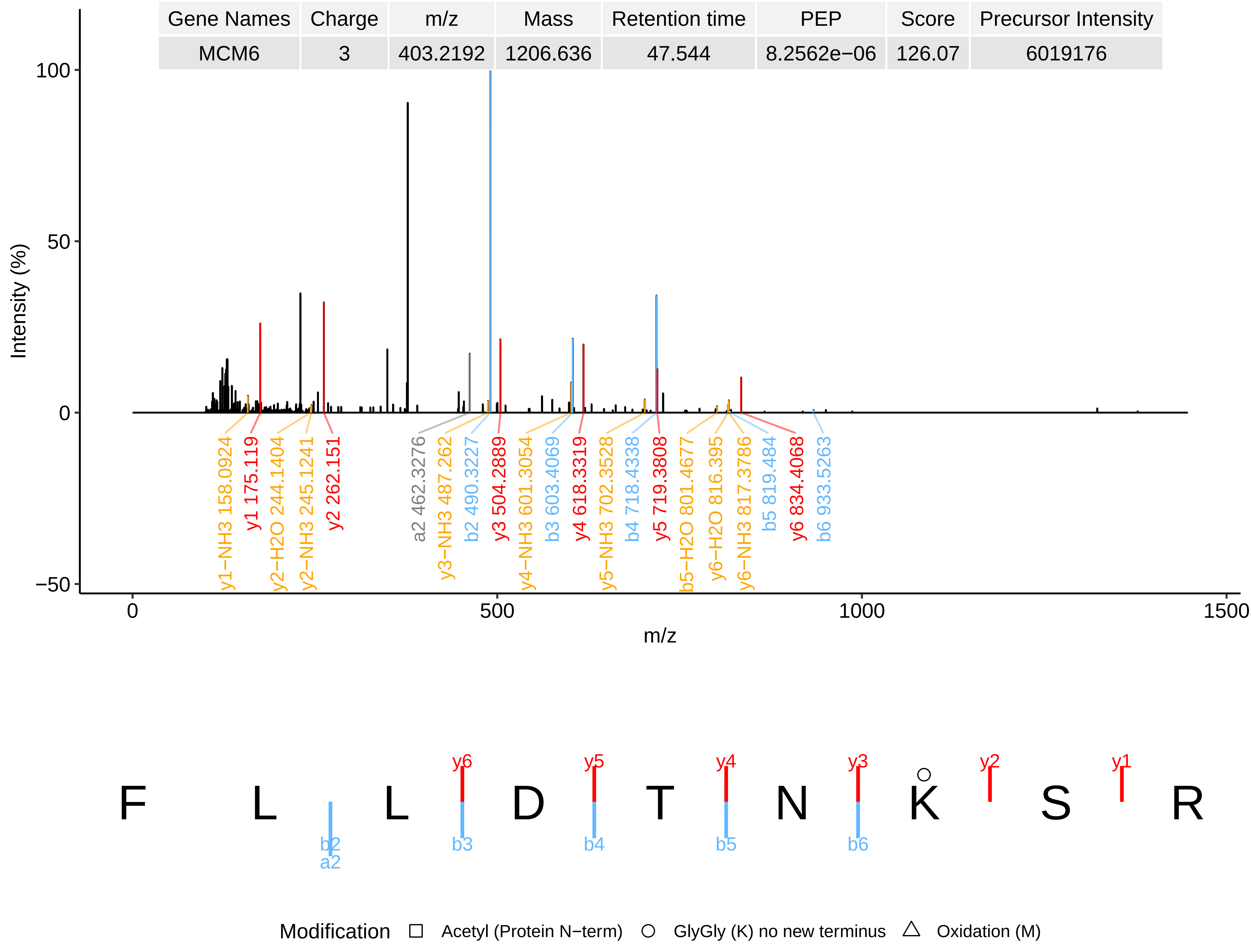

20200921_KIZ_MUb4_GlyGly_10plex_2 − Scan number: 12458

Gene Names	Charge	m/z	Mass	Retention time	PEP	Score	Precursor Intensity
MCM6	3	403.2192	1206.636	48.2	0.0010404	110.87	904081.2

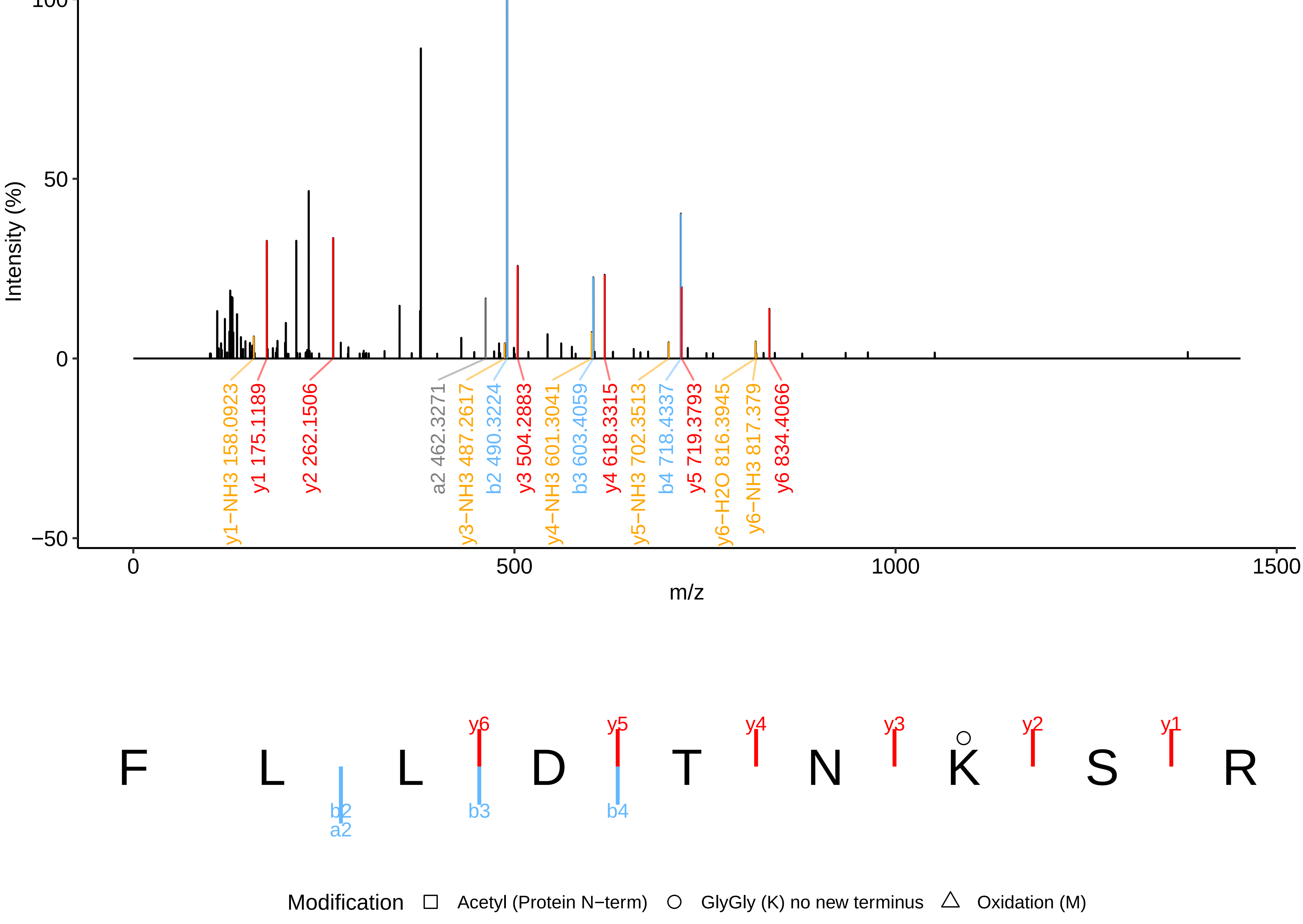

20200921_KIZ_MUb4_GlyGly_10plex − Scan number: 15699

Gene Names	Charge	m/z	Mass	Retention time	PEP	Score	Precursor Intensity
IMMT	3	403.8961	1208.667	56.151	0.0011991	94.302	4082146

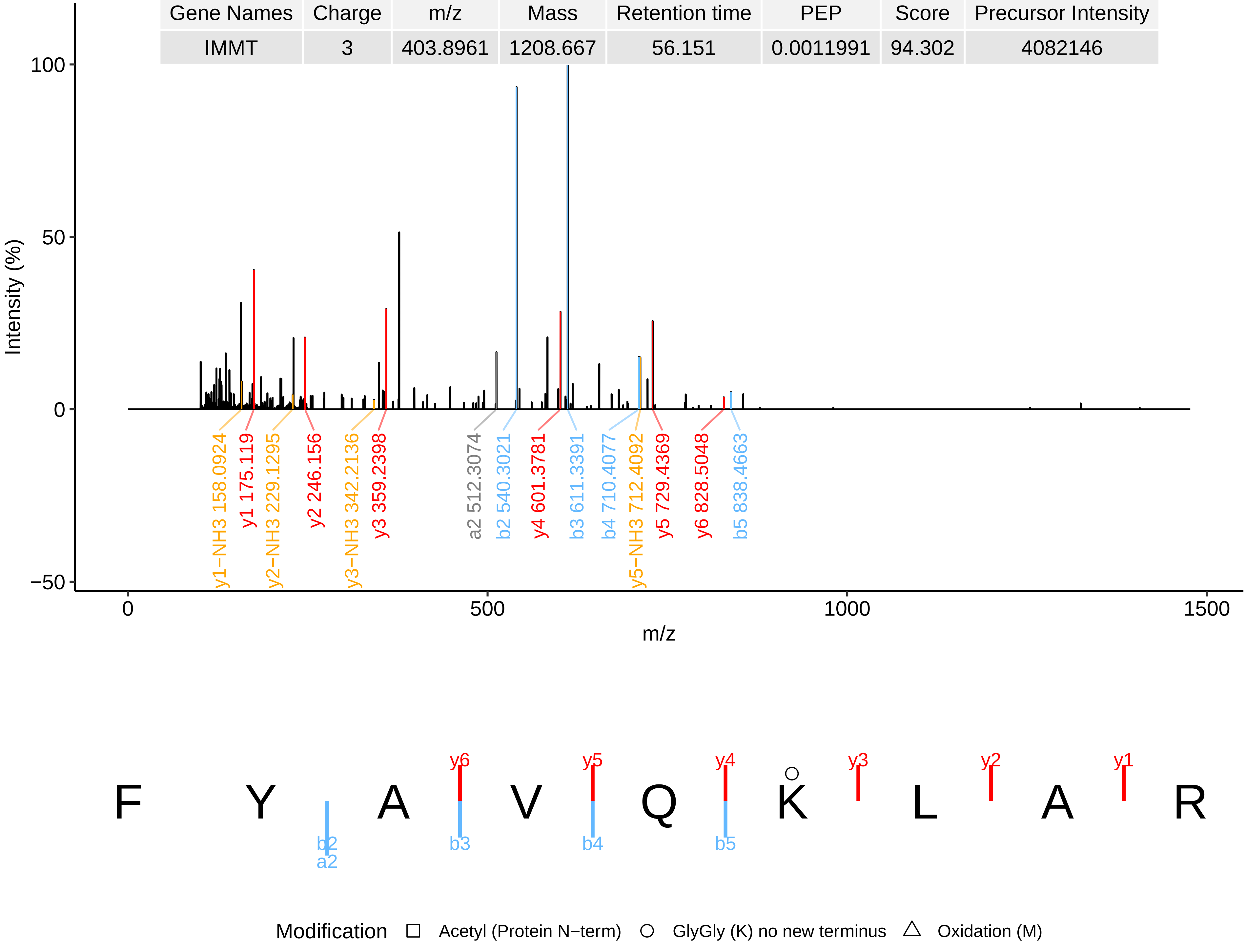

20200921_KIZ_MUb4_GlyGly_10plex_2 − Scan number: 14618

Gene Names	Charge	m/z	Mass	Retention time	PEP	Score	Precursor Intensity
IMMT	3	403.8961	1208.667	57.016	0.00099038	95.531	932368.3

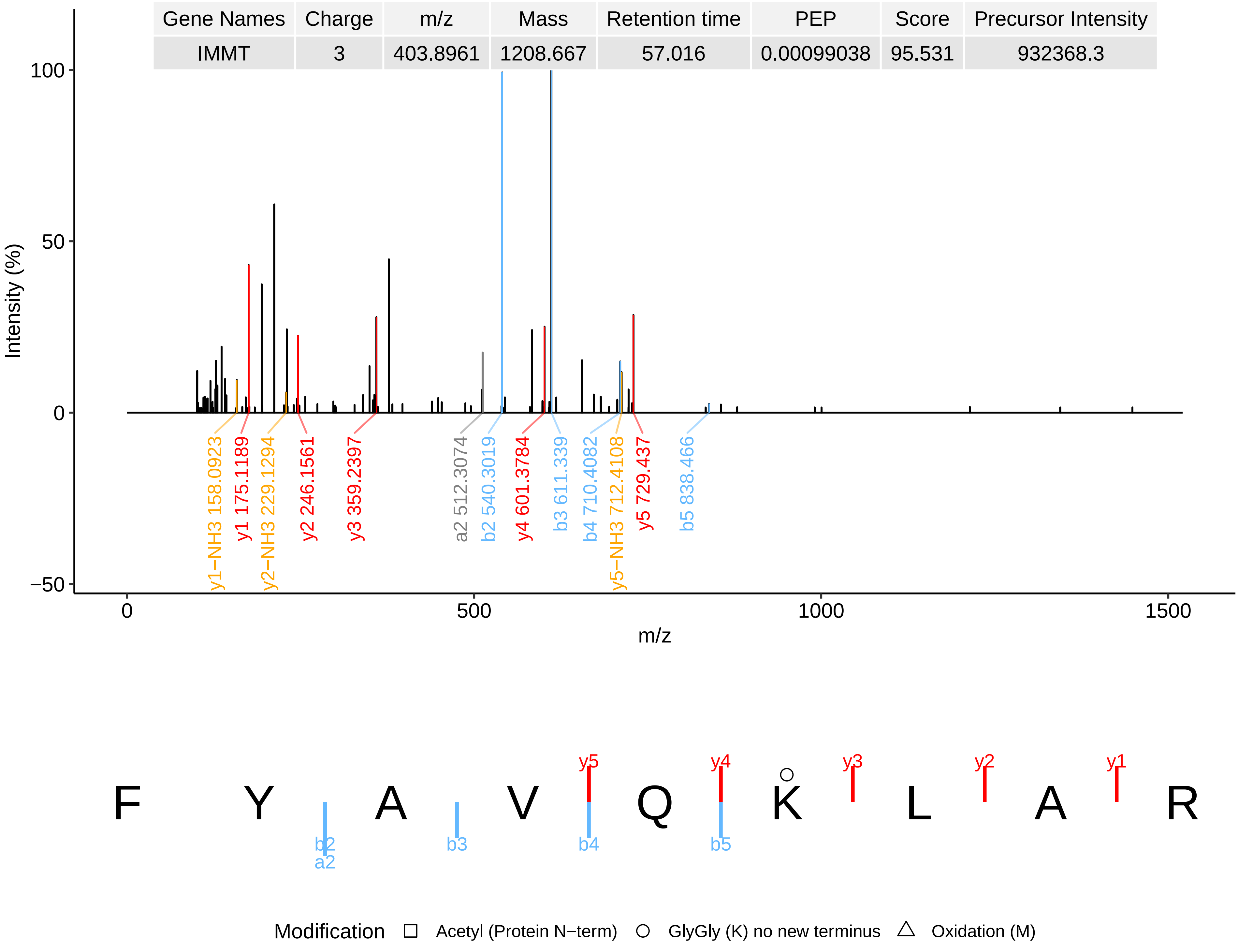

20200924_KIZ_MUb5_GlyGly_1 − Scan number: 5125

Gene Names	Charge	m/z	Mass	Retention time	PEP	Score	Precursor Intensity
HSPD1	3	334.8453	1001.514	15.91	0.035996	64.654	4494457

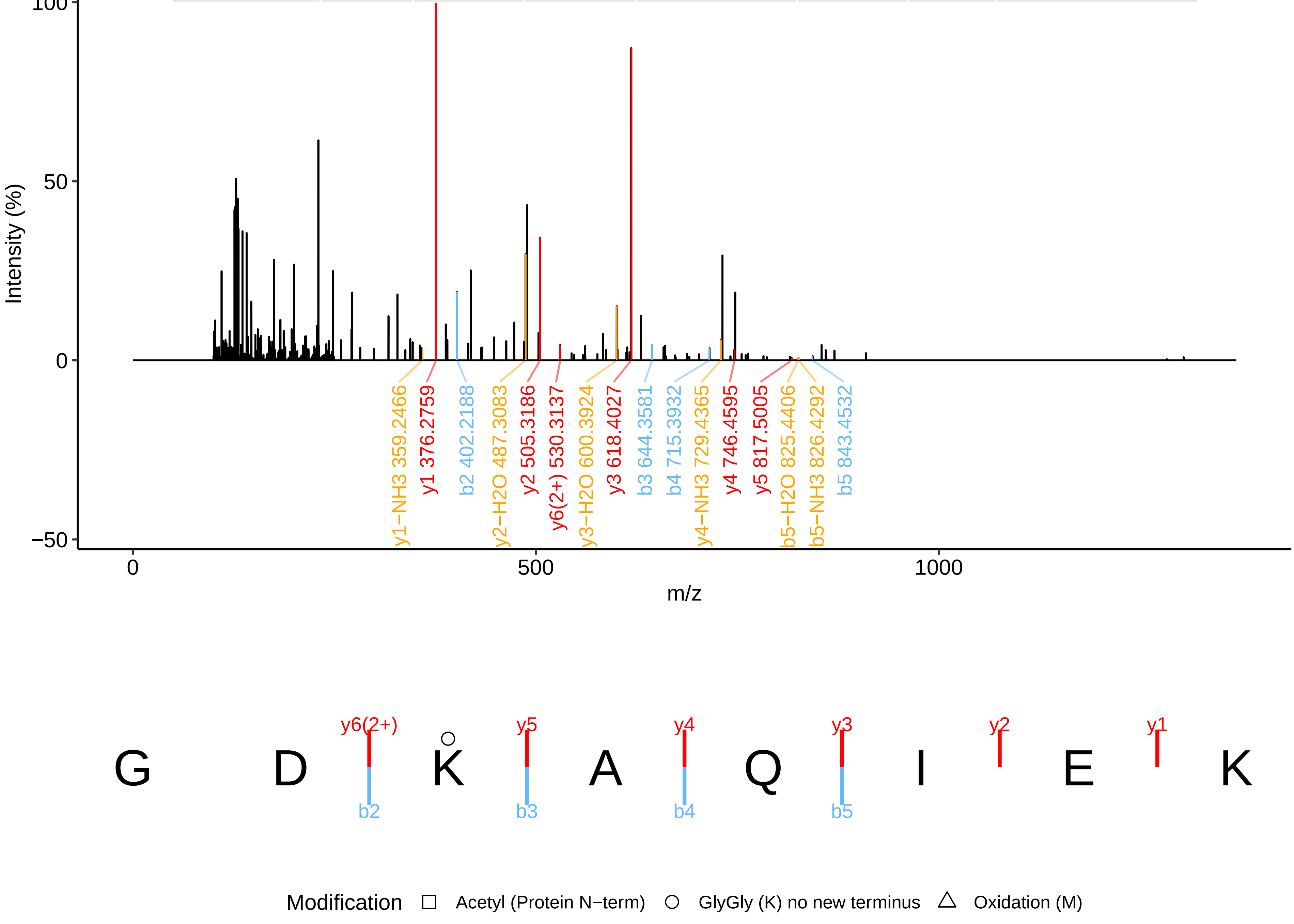

20200921_KIZ_MUb4_GlyGly_10plex_2 − Scan number: 13417

Gene Names	Charge	m/z	Mass	Retention time	PEP	Score	Precursor Intensity
PRKDC	3	340.2063	1017.597	52.089	0.11217	43.246	563879.7

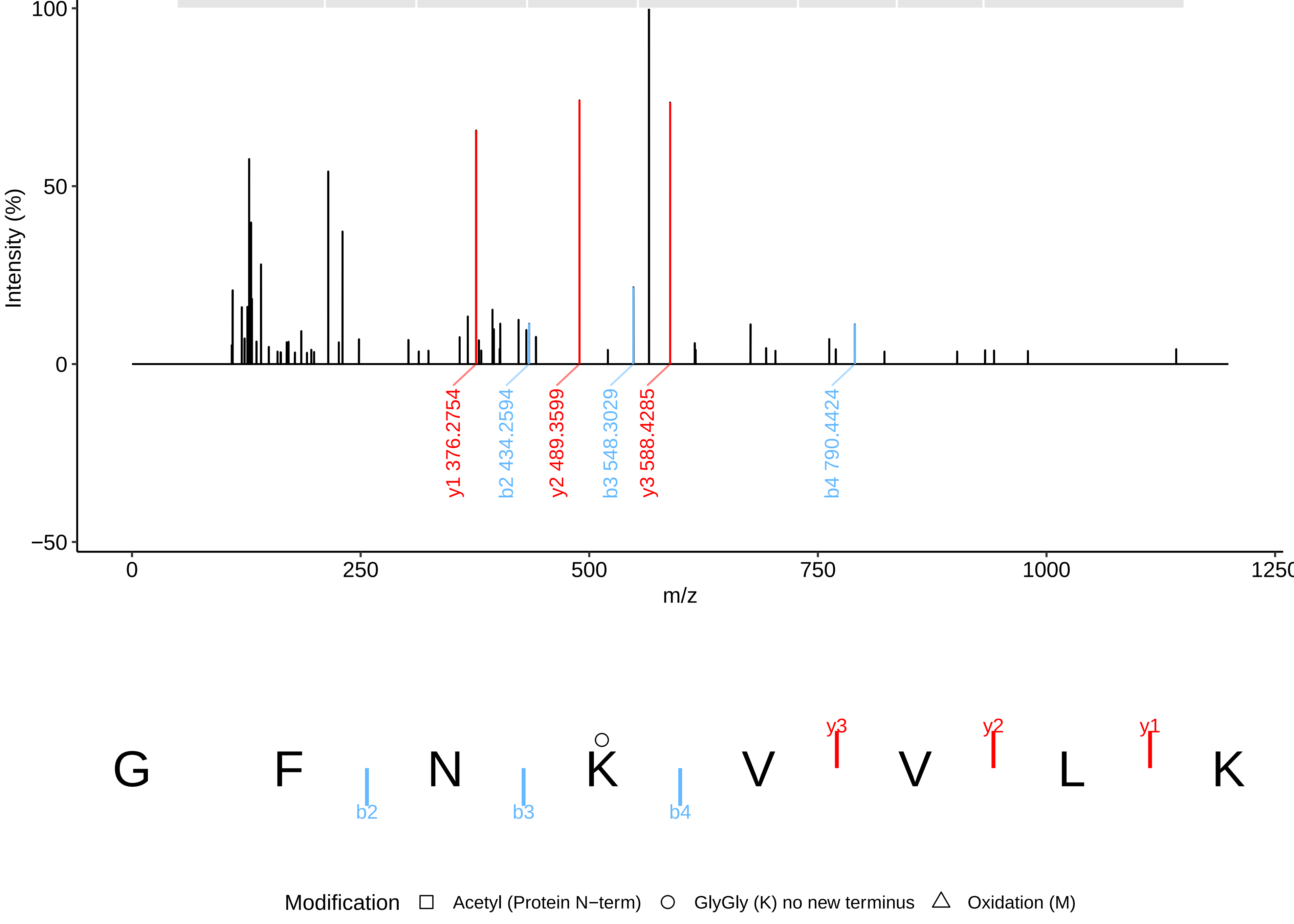

20200921_KIZ_MUb4_GlyGly_10plex_2 − Scan number: 9507

Gene Names	Charge	m/z	Mass	Retention time	PEP	Score	Precursor Intensity
CKB	3	418.2083	1251.603	36.156	0.0060541	78.655	NA

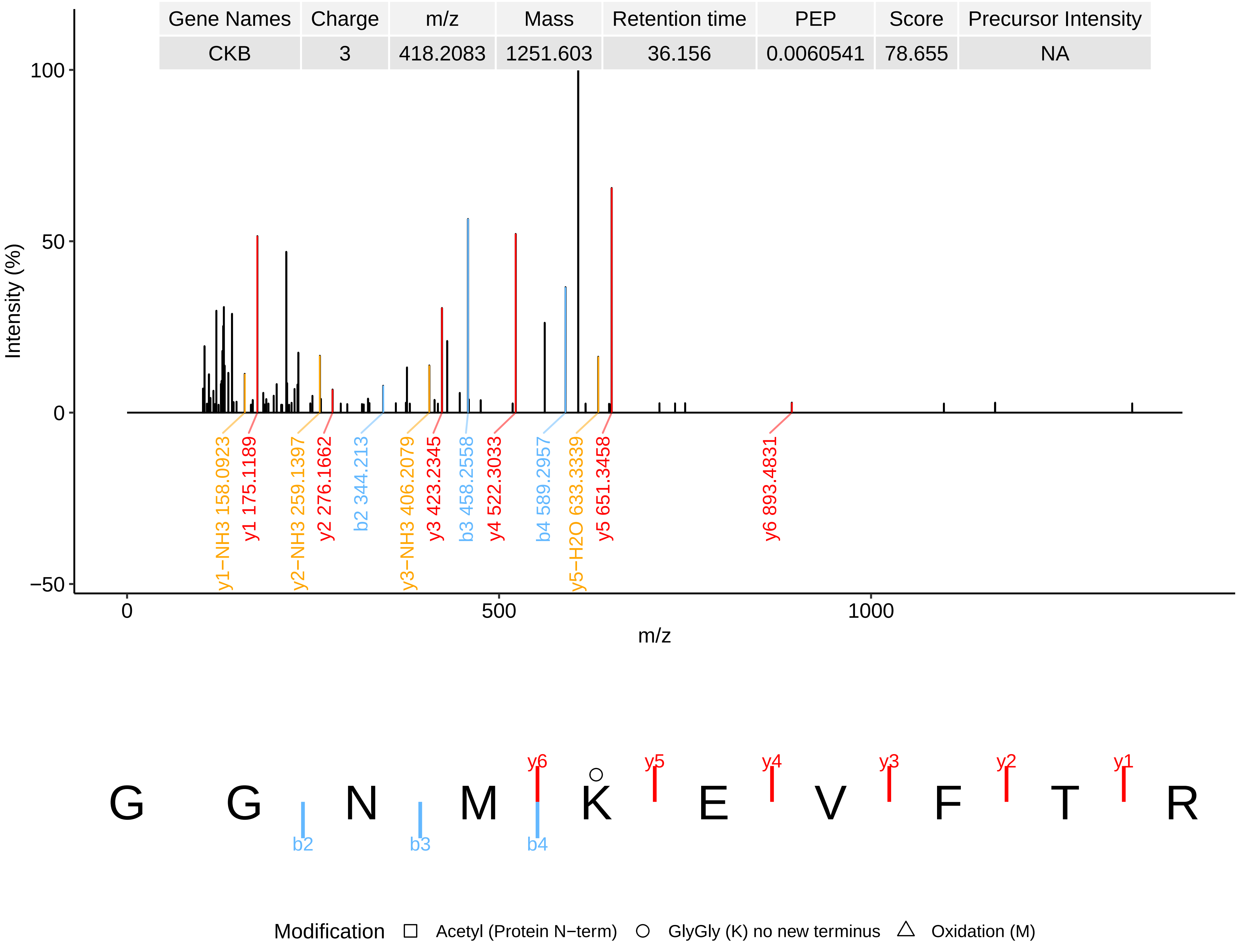

20200921_KIZ_MUb4_GlyGly_10plex − Scan number: 14555

Gene Names	Charge	m/z	Mass	Retention time	PEP	Score	Precursor Intensity
CMAS	3	332.8782	995.6128	51.768	0.023636	68.915	5630012

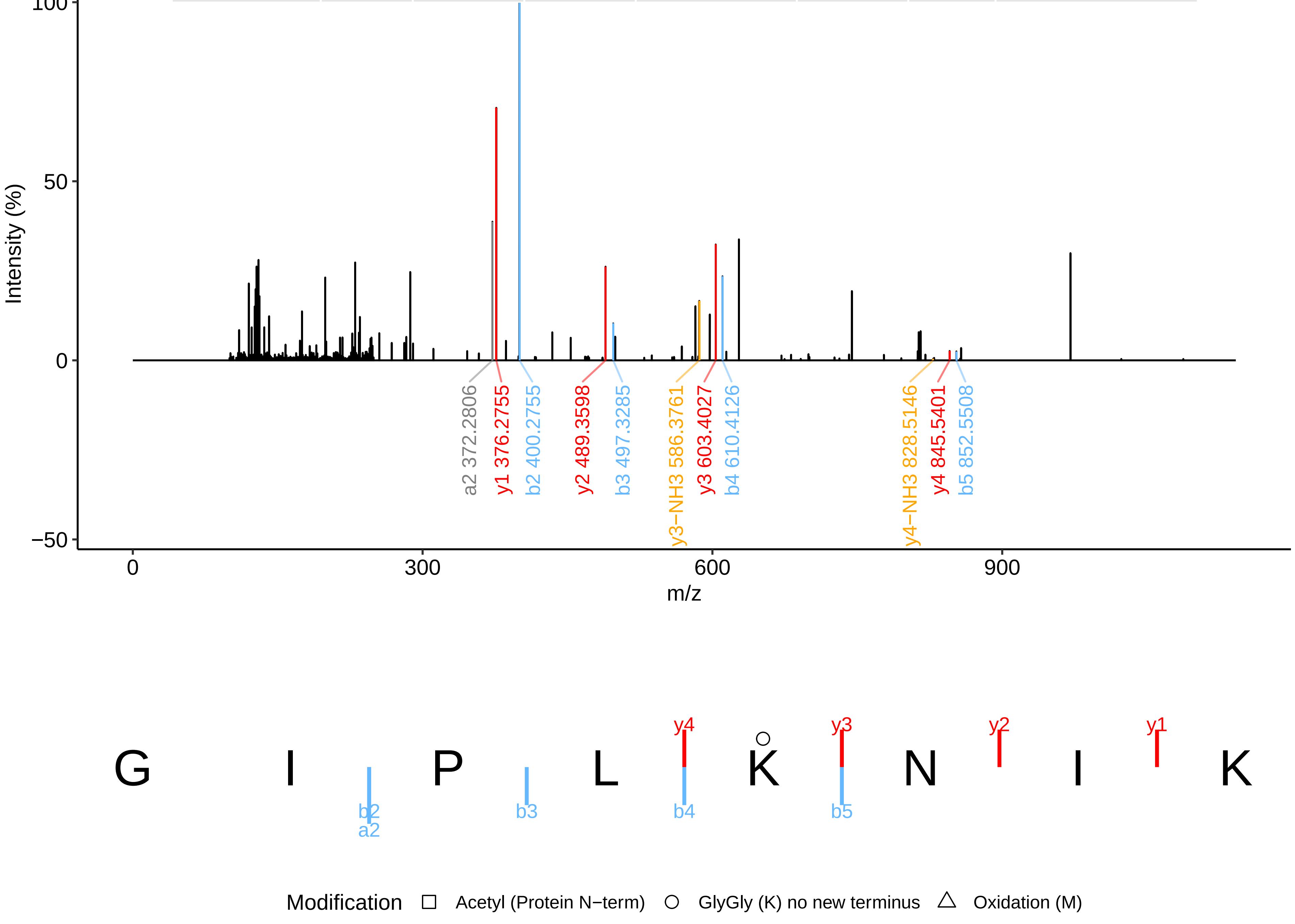

20200921_KIZ_MUb4_GlyGly_10plex − Scan number: 13170

Gene Names	Charge	m/z	Mass	Retention time	PEP	Score	Precursor Intensity
RPLP0	3	339.5356	1015.585	46.371	0.081739	50.805	11425947

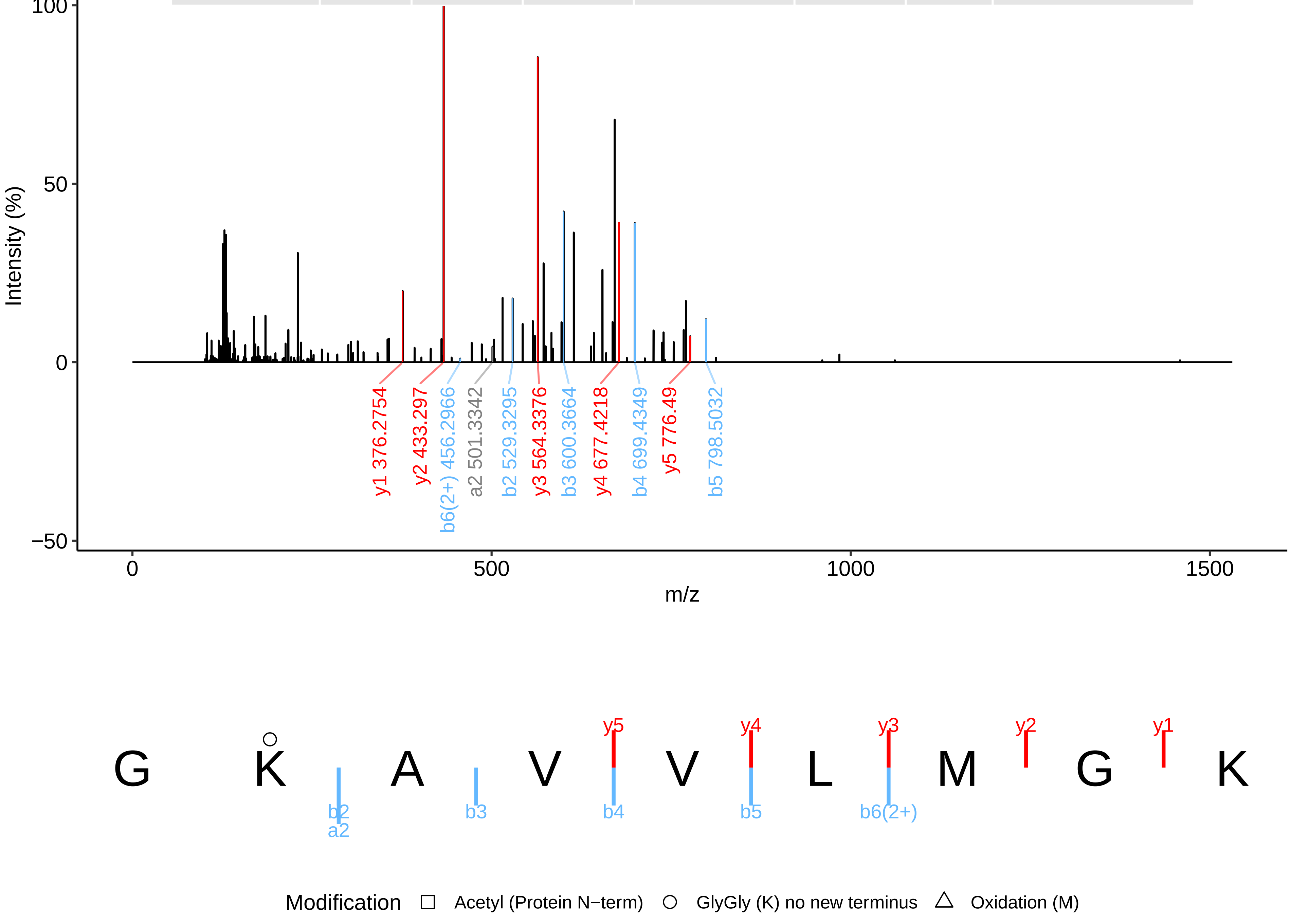

20200921_KIZ_MUb4_GlyGly_10plex_2 − Scan number: 12175

Gene Names	Charge	m/z	Mass	Retention time	PEP	Score	Precursor Intensity
RPLP0	3	339.5356	1015.585	47.049	0.018402	66.621	2066958

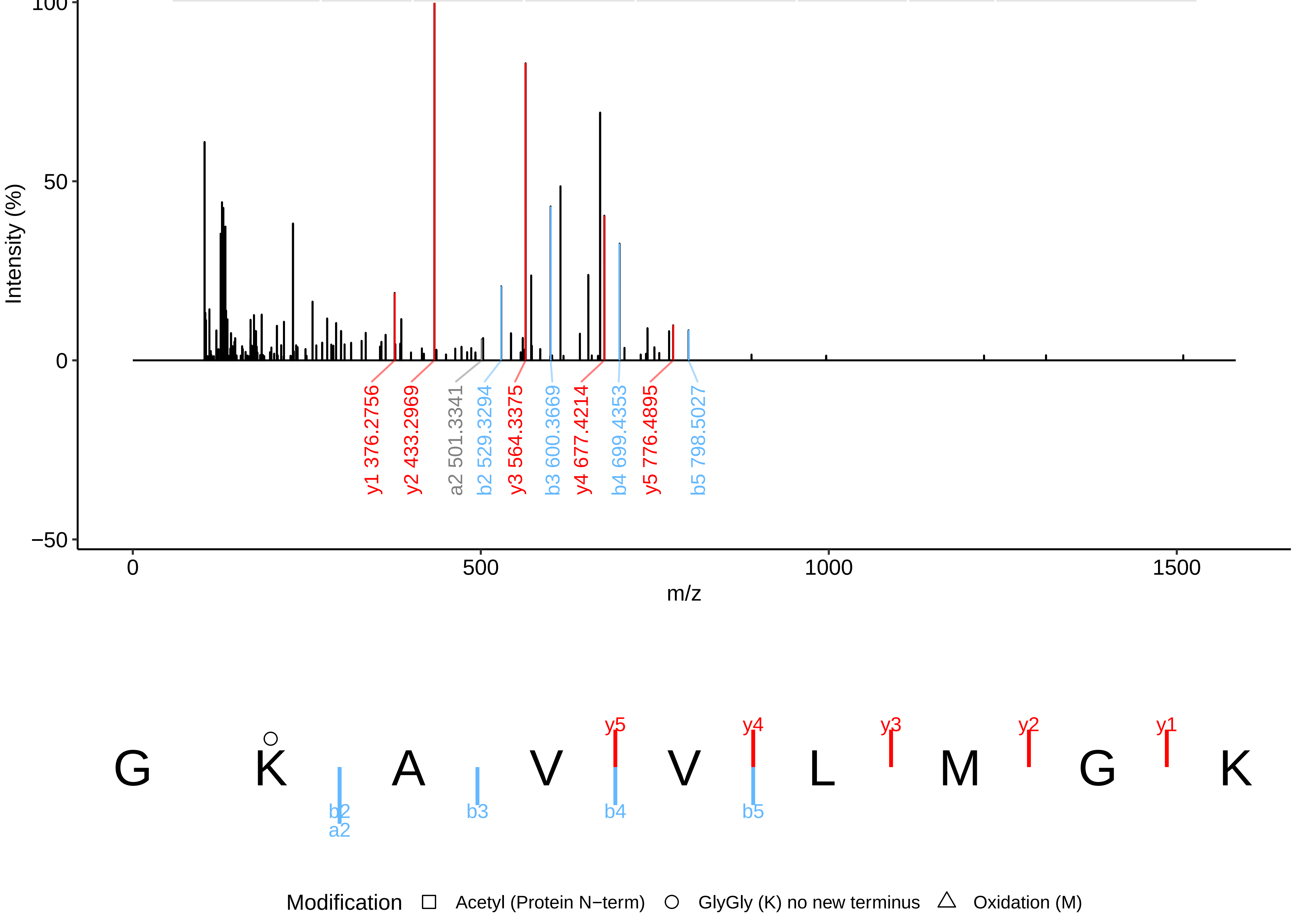

20200921_KIZ_MUb4_GlyGly_10plex − Scan number: 9655

Gene Names	Charge	m/z	Mass	Retention time	PEP	Score	Precursor Intensity
ABCB7;ABCB6	3	334.8575	1001.551	33.134	0.040148	59.426	3796151

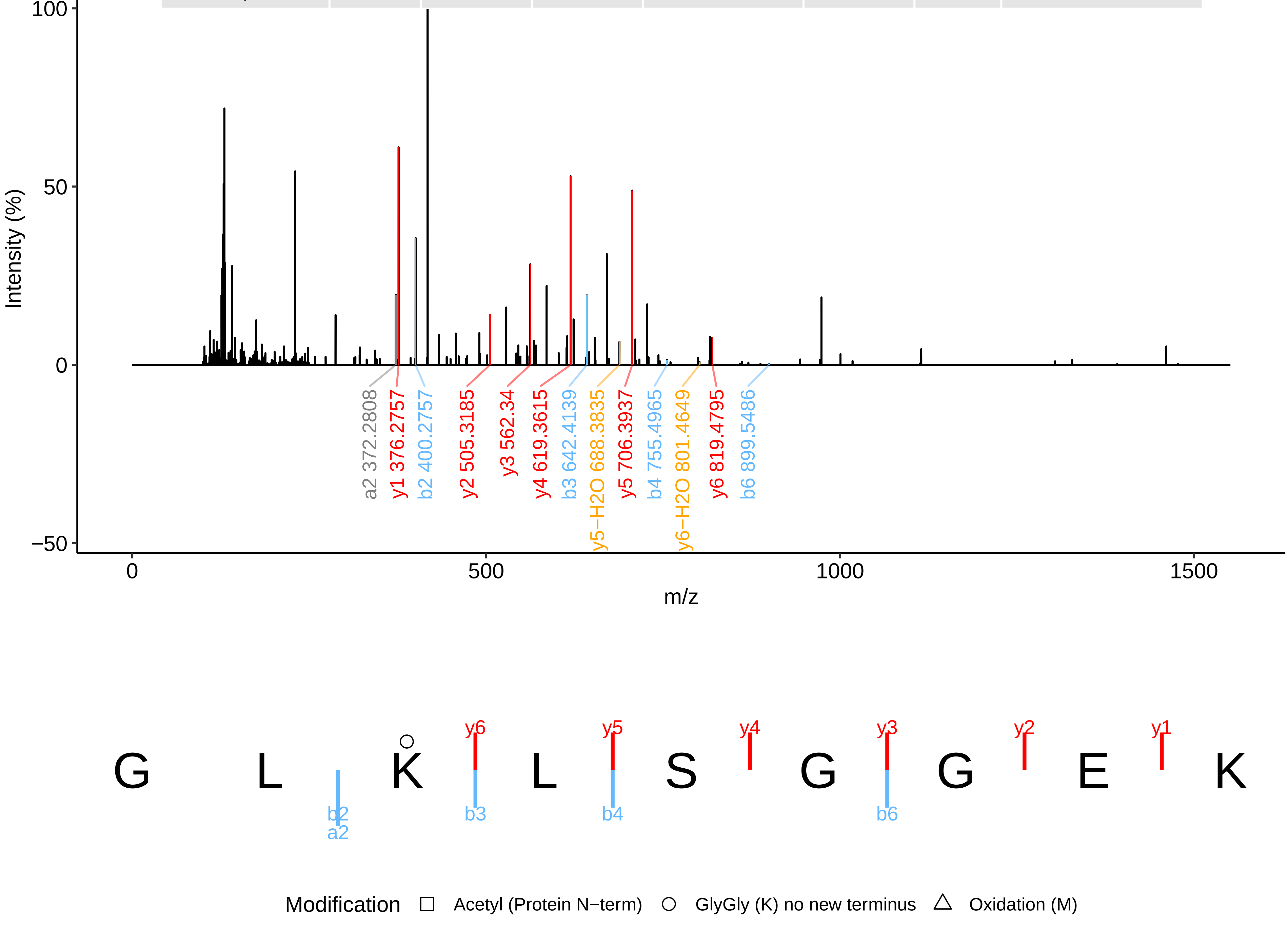

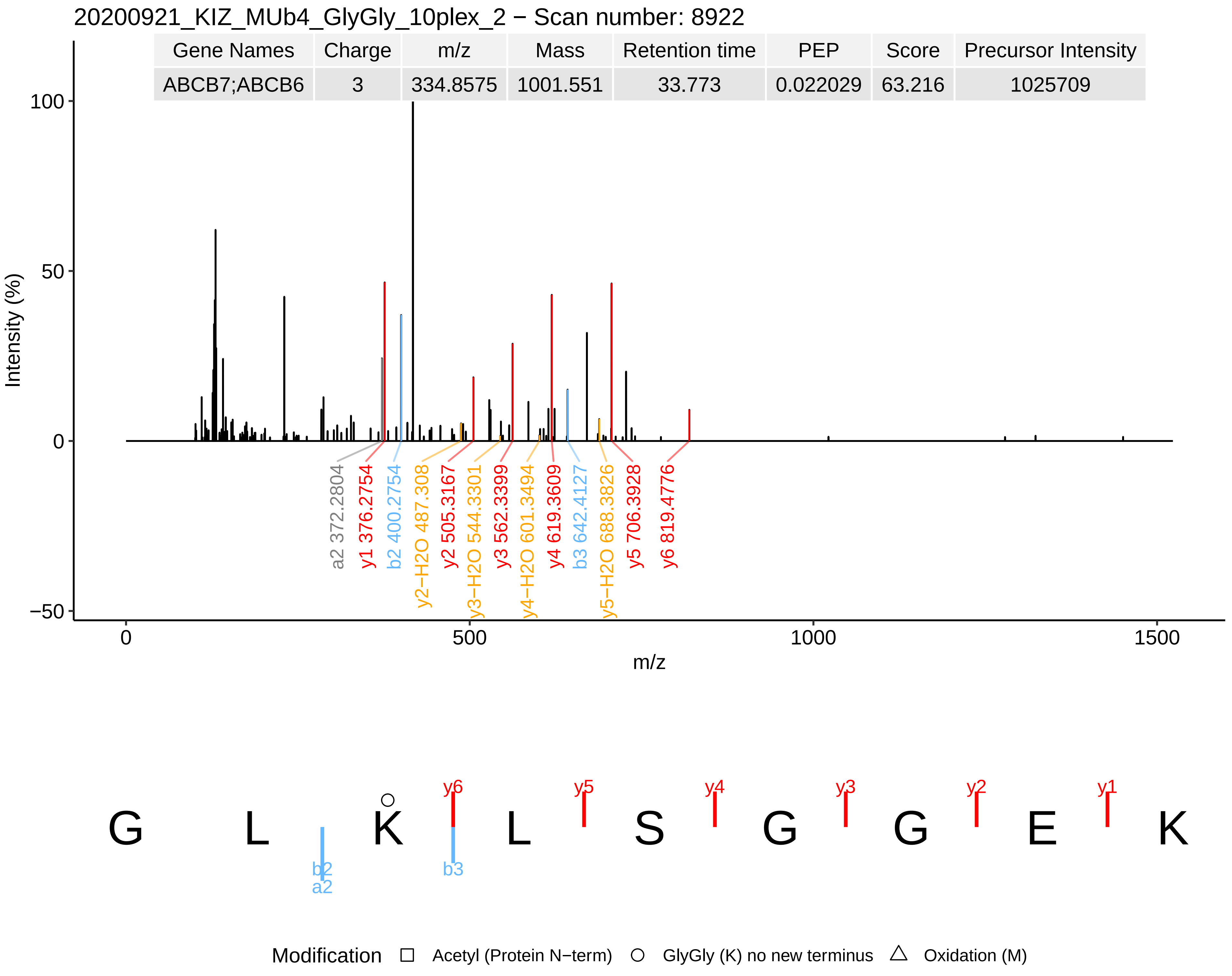

20200921_KIZ_MUb4_GlyGly_10plex_2 − Scan number: 8922
Gene Names | Charge | m/z | Mass | Retention time | PEP | Score | Precursor Intensity
ABCB7;ABCB6 | 3 | 334.8575 | 1001.551 | 33.773 | 0.022029 | 63.216 | 1025709
Intensity (%)
m/z
a2 372.2804
y1 376.2754
b2 400.2754
y2−H2O 487.308
y2 505.3167
y3−H2O 544.3301
y3 562.3399
y4−H2O 601.3494
y4 619.3609
b3 642.4127
y5−H2O 688.3826
y5 706.3928
y6 819.4776
G L K L S G G E K
y6 y5 y4 y3 y2 y1
b2 a2 b3
Modification
Acetyl (Protein N−term)
GlyGly (K) no new terminus
Oxidation (M)

20200924_KIZ_MUb5_GlyGly_1 − Scan number: 8441

Gene Names	Charge	m/z	Mass	Retention time	PEP	Score	Precursor Intensity
ABCB7;ABCB6	3	334.8575	1001.551	26.987	0.093129	53.751	NA

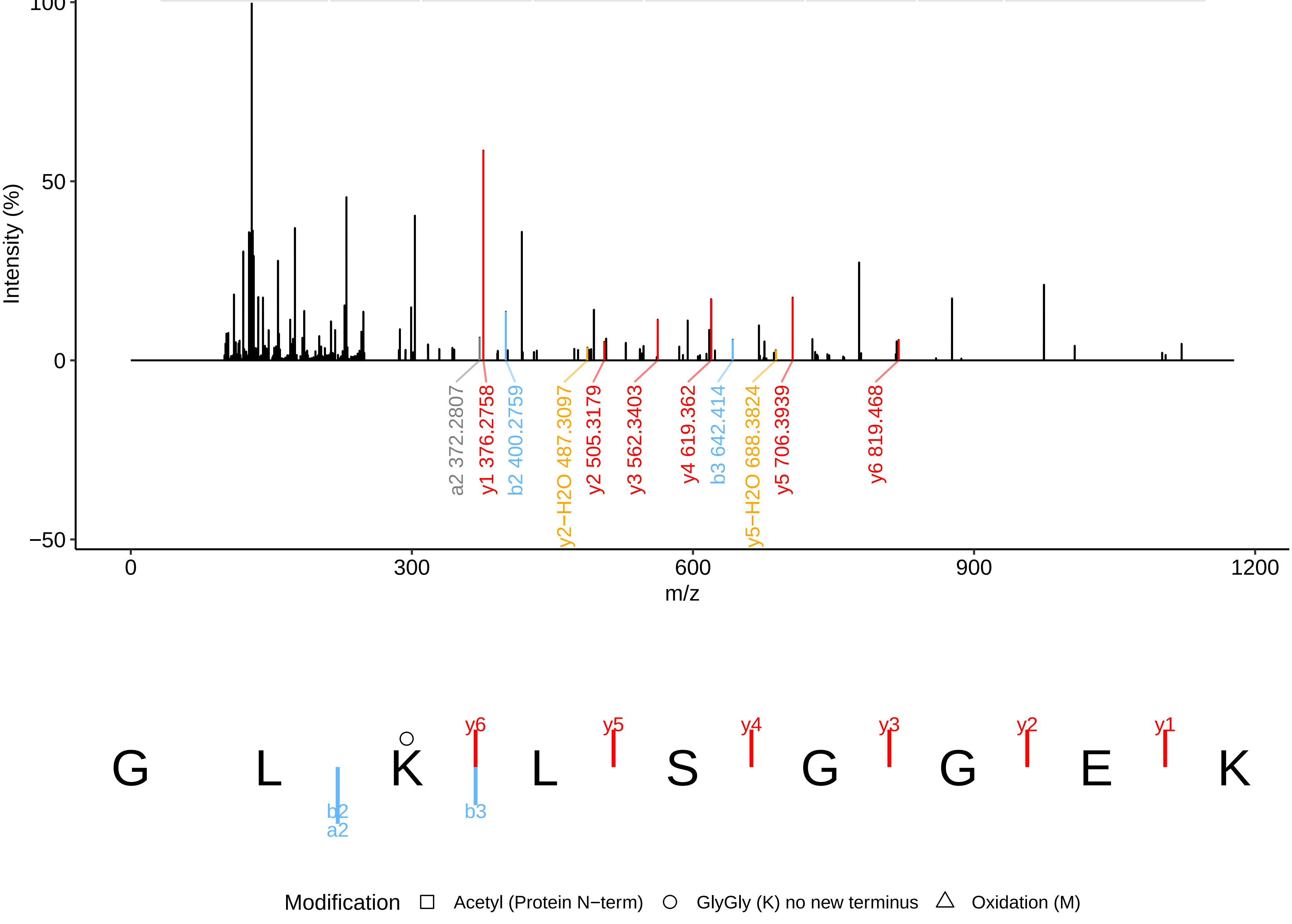

20200924_KIZ_MUb5_GlyGly_1 − Scan number: 8544

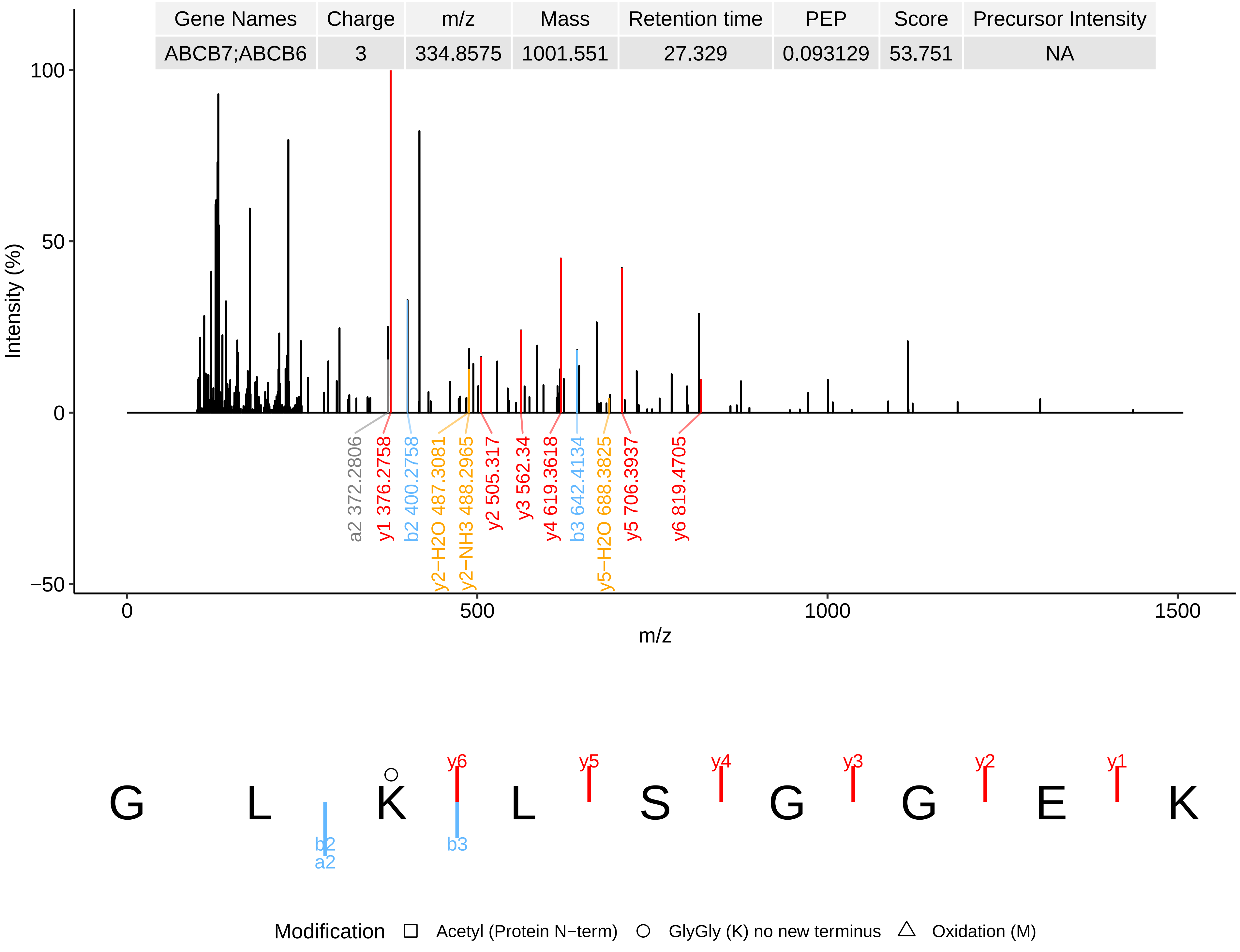

20200921_KIZ_MUb4_GlyGly_10plex_2 − Scan number: 10936

Gene Names	Charge	m/z	Mass	Retention time	PEP	Score	Precursor Intensity
MET	3	337.8479	1010.522	41.971	0.052185	57.96	944650.1

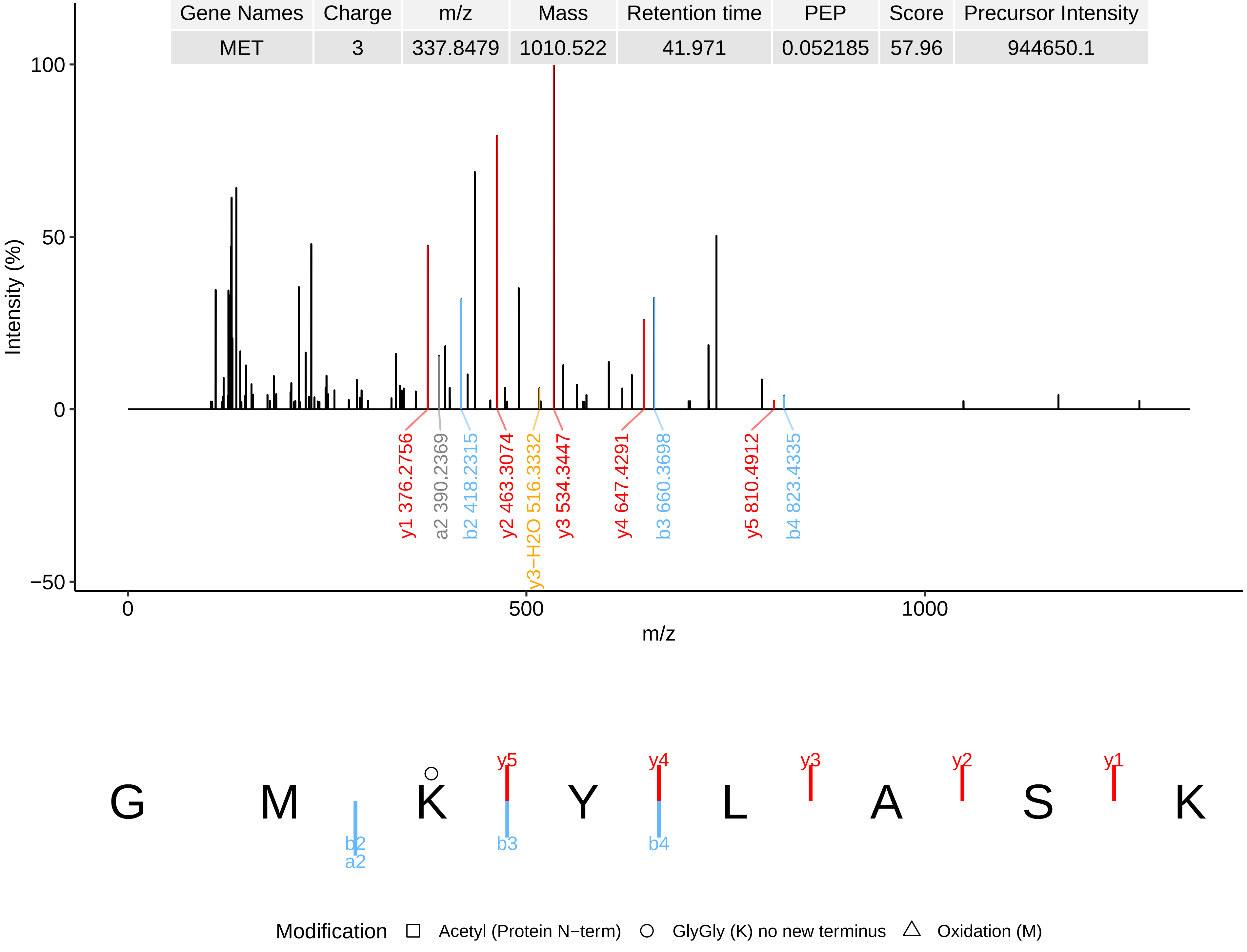

20200921_KIZ_MUb4_GlyGly_10plex − Scan number: 9053

Gene Names	Charge	m/z	Mass	Retention time	PEP	Score	Precursor Intensity
PHB2	3	340.1891	1017.545	30.945	0.006569	77.732	9671120

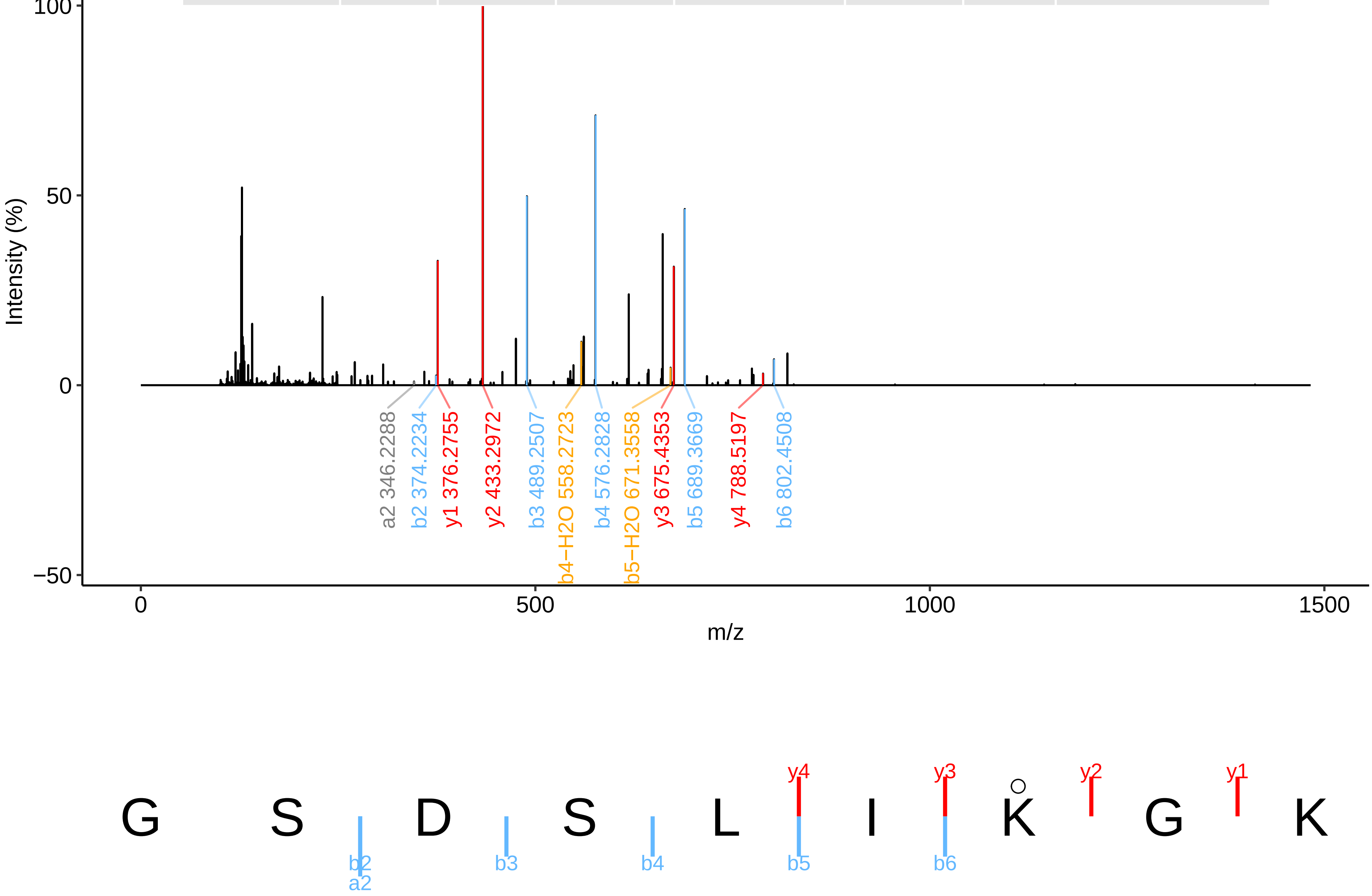

Modification □ Acetyl (Protein N−term) ○ GlyGly (K) no new terminus △ Oxidation (M)

20200921_KIZ_MUb4_GlyGly_10plex_2 − Scan number: 8376

Gene Names	Charge	m/z	Mass	Retention time	PEP	Score	Precursor Intensity
PHB2	3	340.1891	1017.545	31.538	0.017043	67.897	1469734

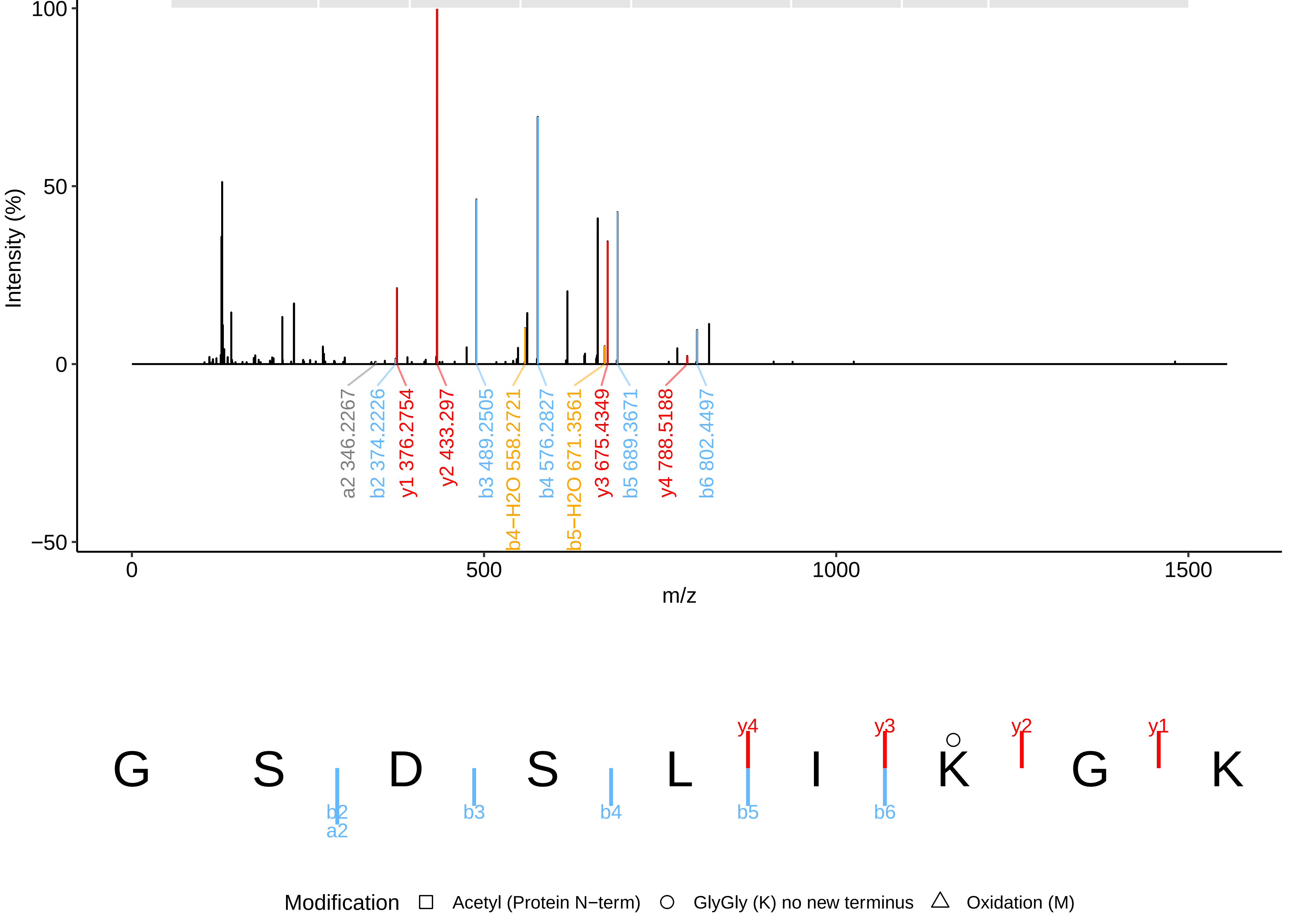

20200924_KIZ_MUb5_GlyGly_1 − Scan number: 7922

Gene Names	Charge	m/z	Mass	Retention time	PEP	Score	Precursor Intensity
PHB2	3	340.1891	1017.545	25.238	0.074742	52.255	4601581

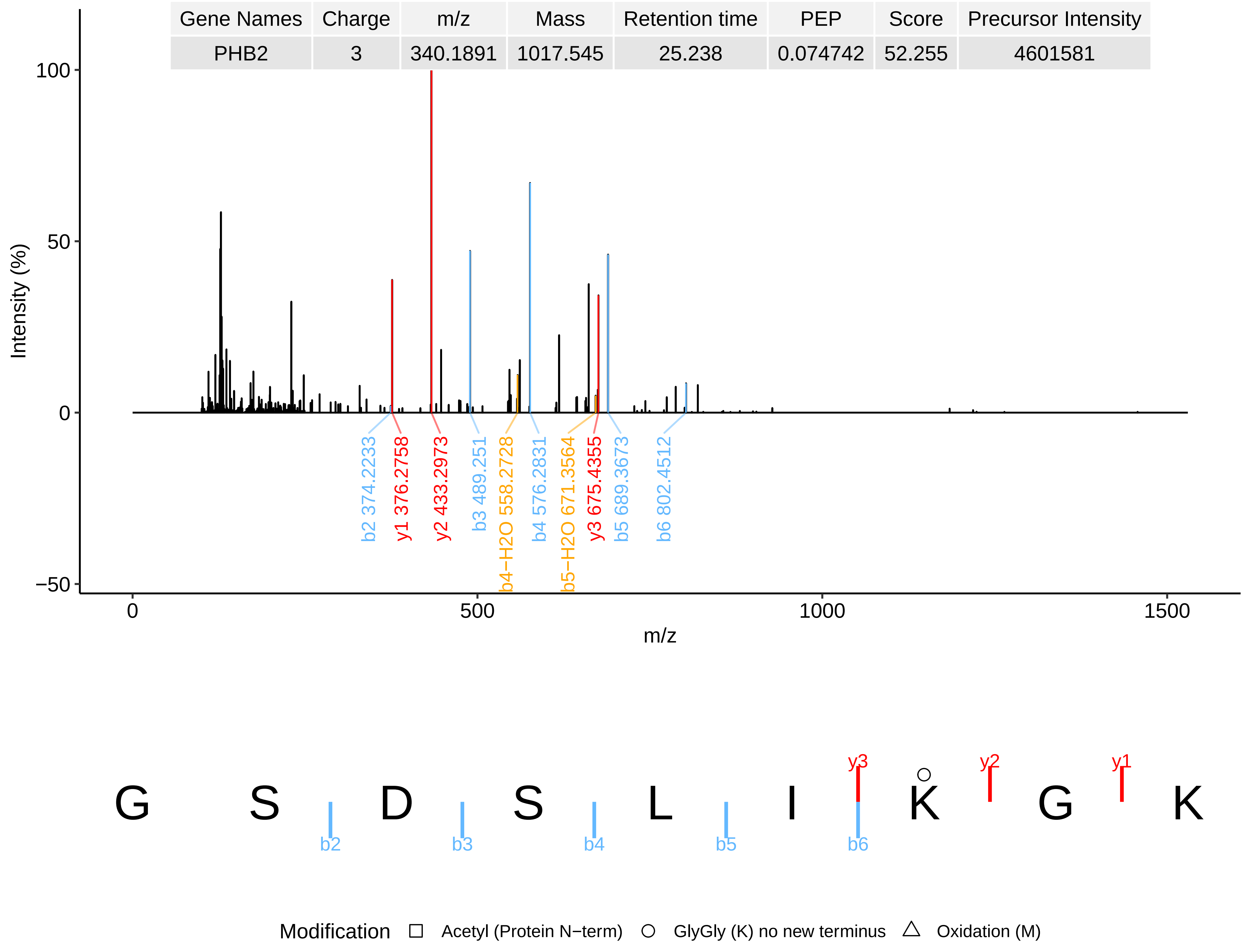

20200924_KIZ_MUb5_GlyGly_1 − Scan number: 8079

Gene Names	Charge	m/z	Mass	Retention time	PEP	Score	Precursor Intensity
PHB2	3	340.1891	1017.545	25.746	0.017043	67.897	15960857

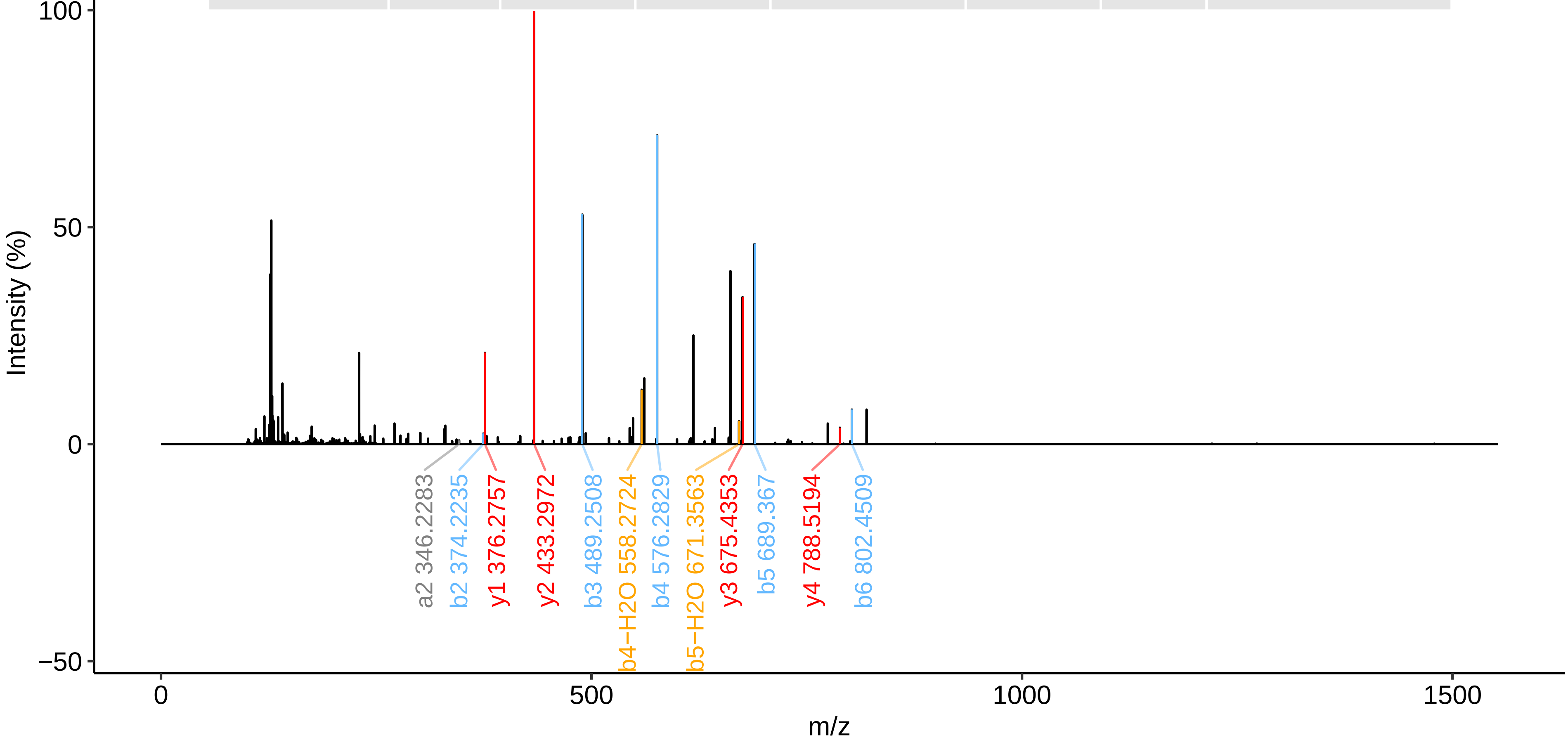

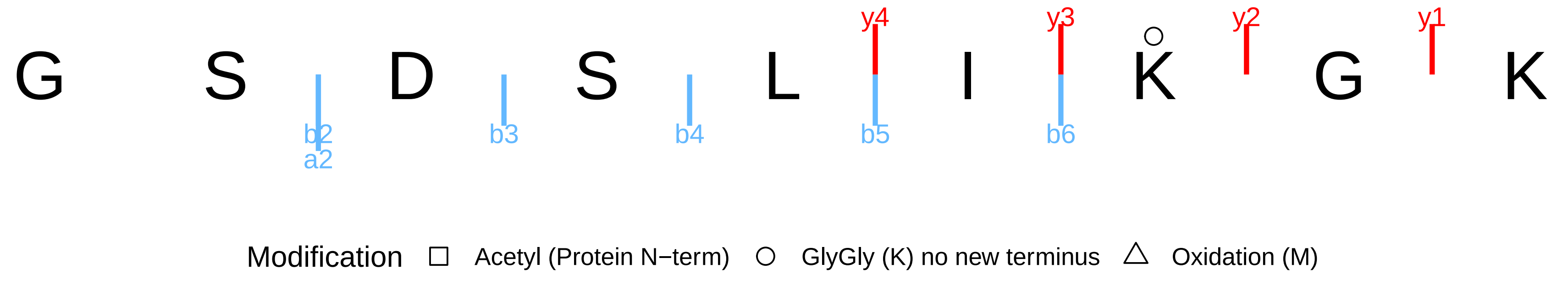

20200924_KIZ_MUb5_GlyGly_2 − Scan number: 7470

Gene Names	Charge	m/z	Mass	Retention time	PEP	Score	Precursor Intensity
PHB2	3	340.1891	1017.545	27.881	0.021861	68.224	2611358

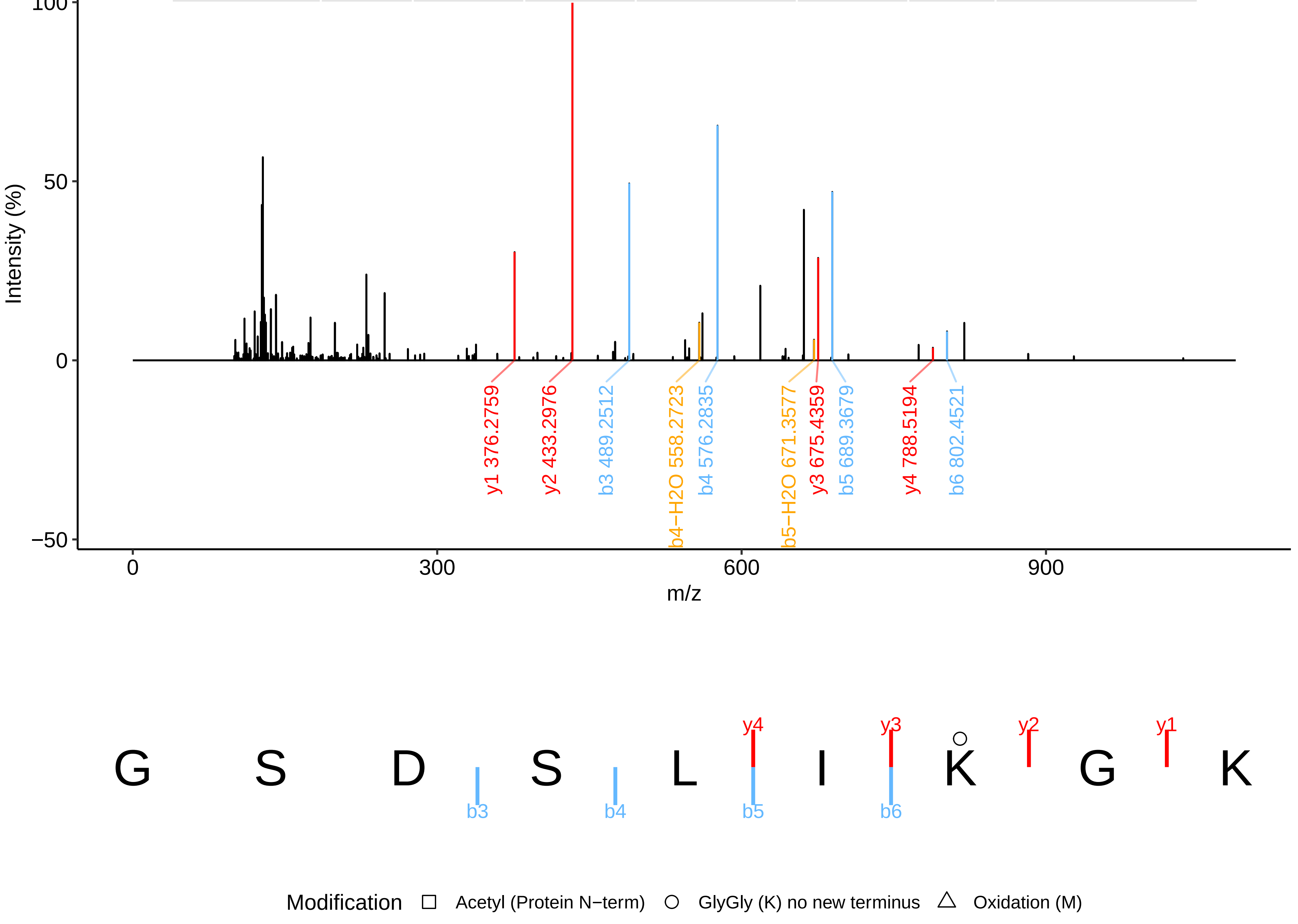

20200924_KIZ_MUb5_GlyGly_2 − Scan number: 7602

Gene Names	Charge	m/z	Mass	Retention time	PEP	Score	Precursor Intensity
PHB2	3	340.1891	1017.545	28.391	0.0097835	77.732	8966791

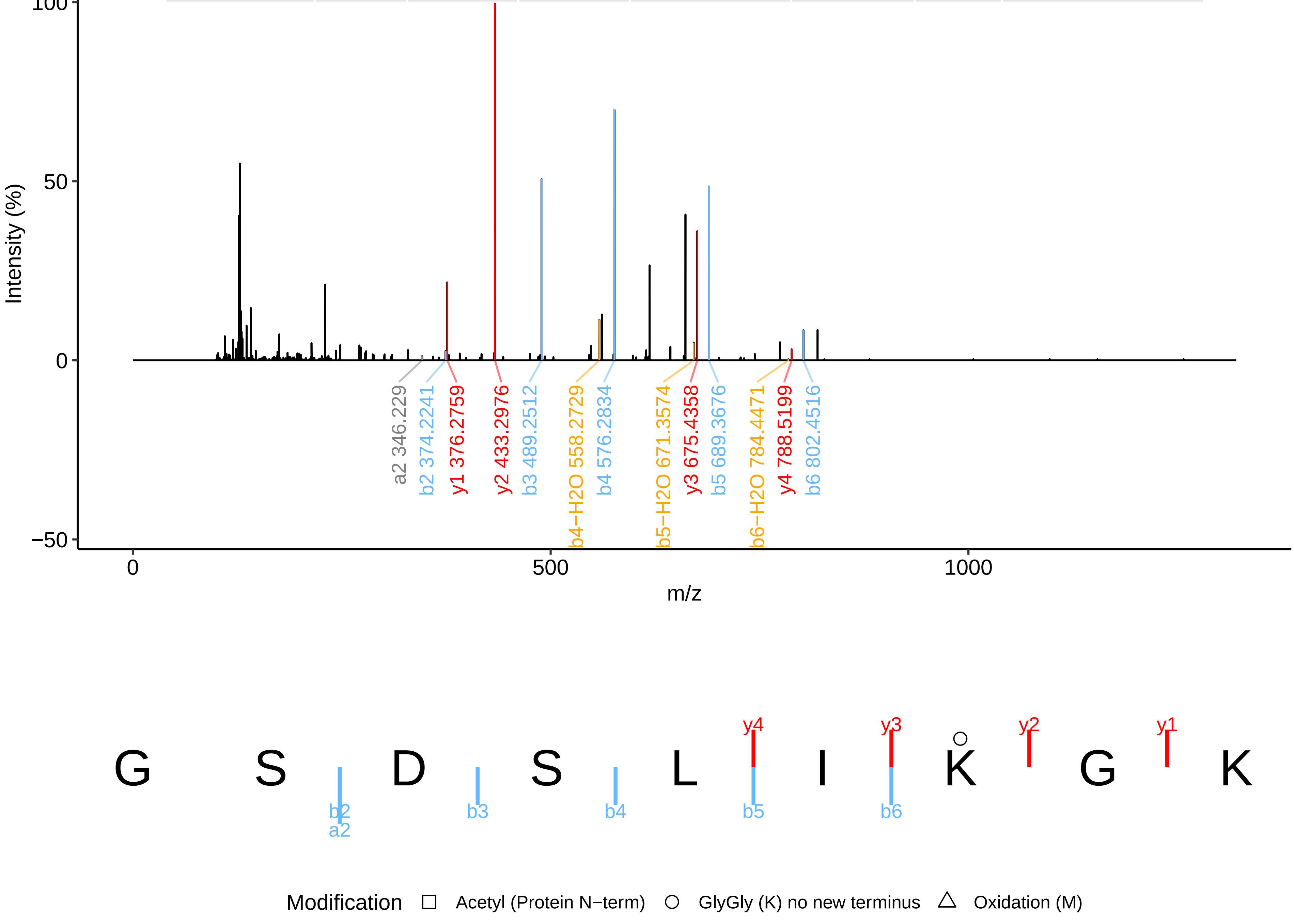

20201118_KIZ_MUb6_GlyGly_2 − Scan number: 9563

Gene Names	Charge	m/z	Mass	Retention time	PEP	Score	Precursor Intensity
PHB2	3	340.1891	1017.545	32.429	0.006569	77.732	5999962

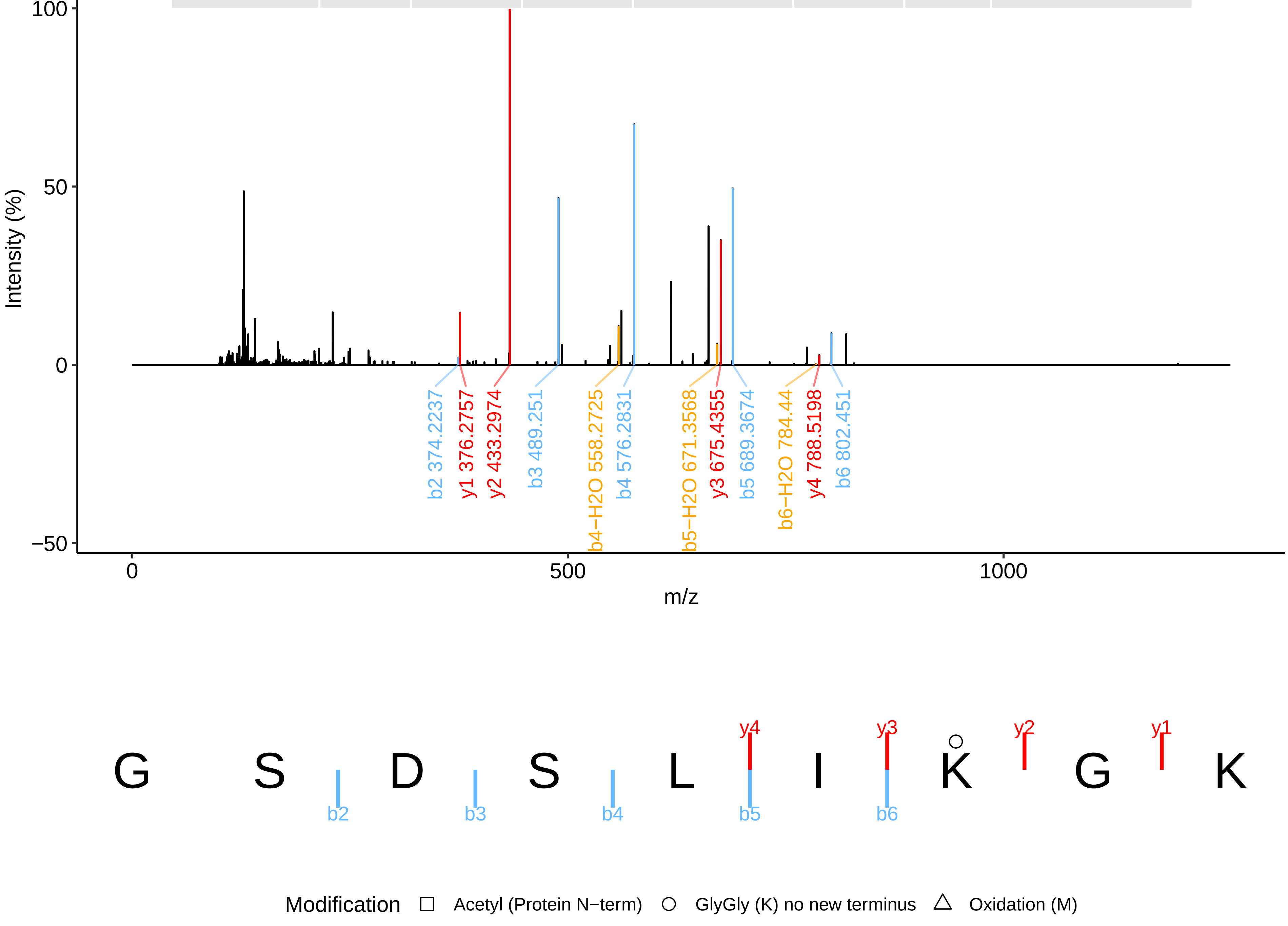

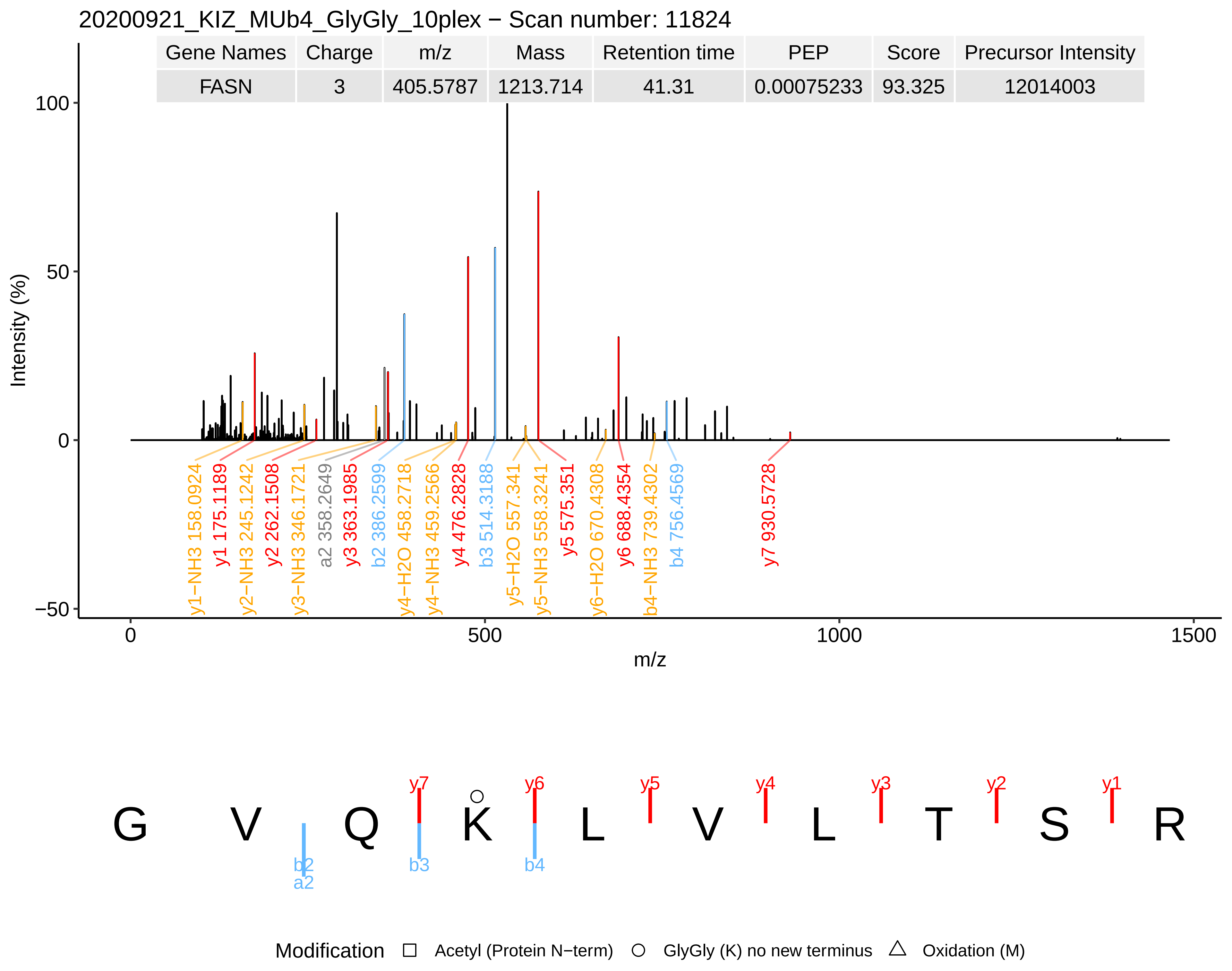

20200921_KIZ_MUb4_GlyGly_10plex − Scan number: 11824
Gene Names
Charge
m/z
Mass
Retention time
PEP
Score
Precursor Intensity
FASN
3
405.5787
1213.714
41.31
0.00075233
93.325
12014003
Intensity (%)
m/z
y1−NH3 158.0924
y1 175.1189
y2−NH3 245.1242
y2 262.1508
y3−NH3 346.1721
a2 358.2649
y3 363.1985
b2 386.2599
y4−H2O 458.2718
y4−NH3 459.2566
y4 476.2828
b3 514.3188
y5−H2O 557.341
y5−NH3 558.3241
y5 575.351
y6−H2O 670.4308
y6 688.4354
b4−NH3 739.4302
b4 756.4569
y7 930.5728
G V Q K L V L T S R
y7 y6 y5 y4 y3 y2 y1
b2 a2 b3 b4
Modification
Acetyl (Protein N−term)
GlyGly (K) no new terminus
Oxidation (M)

20200921_KIZ_MUb4_GlyGly_10plex_2 − Scan number: 10939

Gene Names	Charge	m/z	Mass	Retention time	PEP	Score	Precursor Intensity
FASN	3	405.5787	1213.714	41.984	1.3738e−05	112.36	1342157

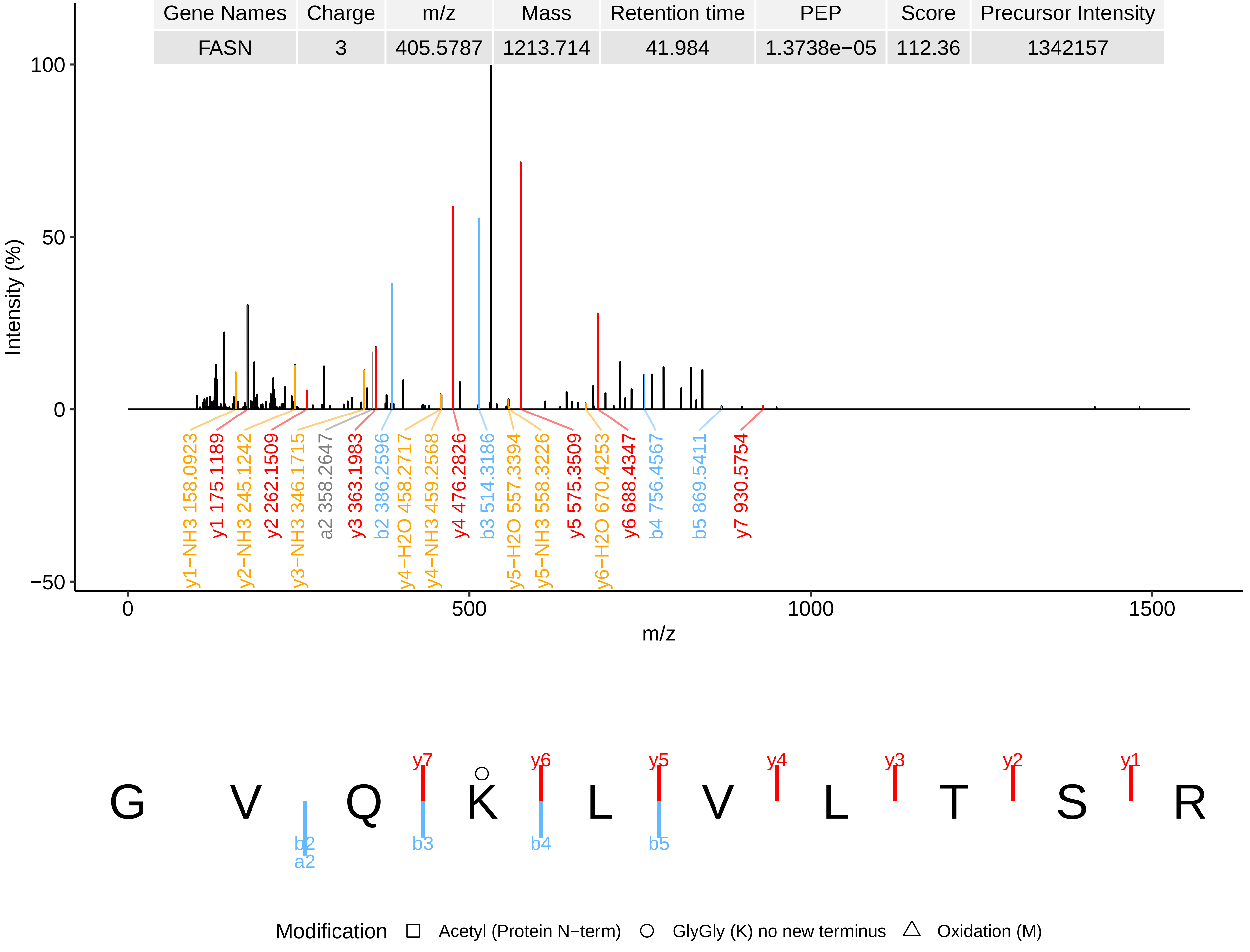

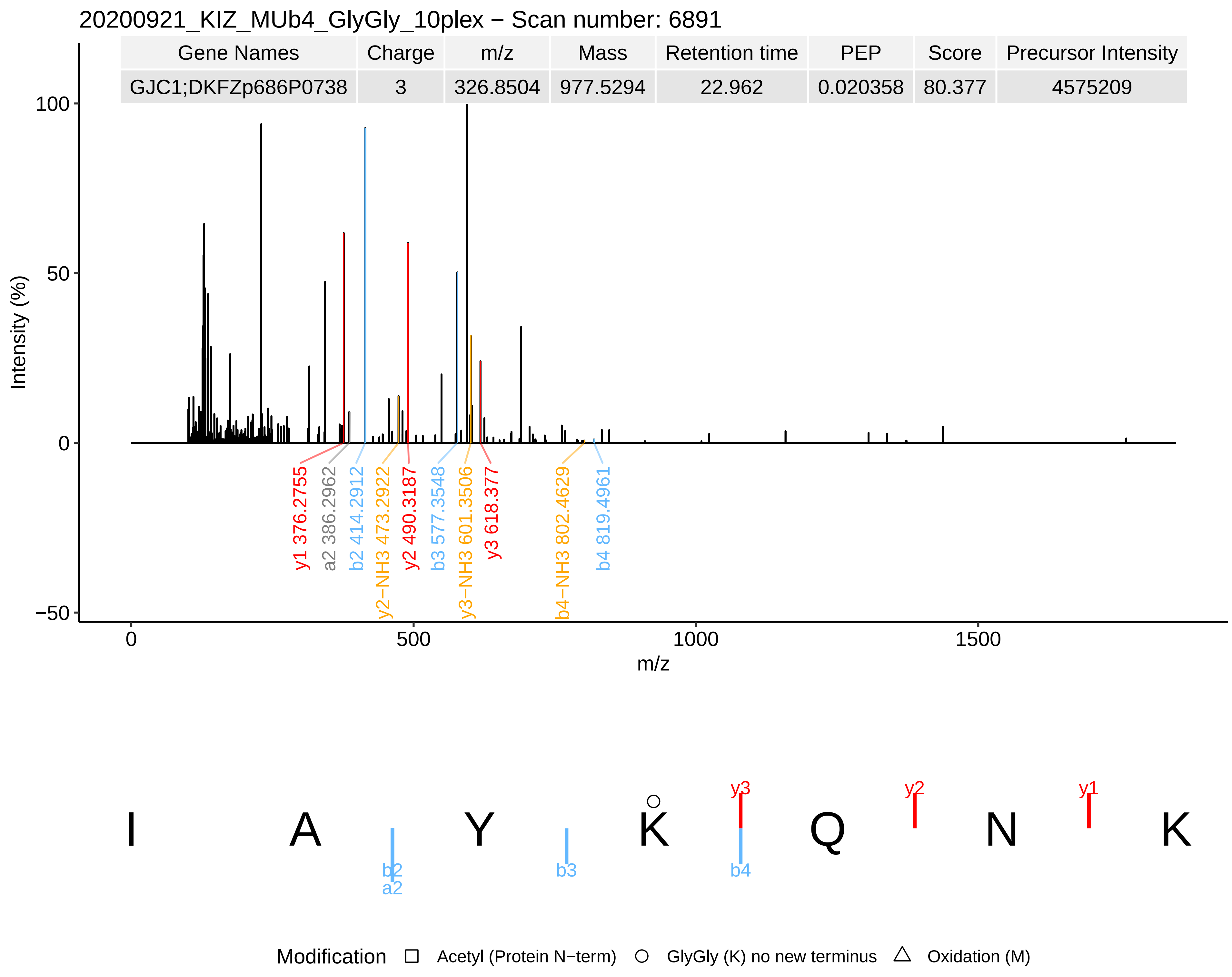

Gene Names	Charge	m/z	Mass	Retention time	PEP	Score	Precursor Intensity
GJC1;DKFZp686P0738	3	326.8504	977.5294	22.962	0.020358	80.377	4575209

20200921_KIZ_MUb4_GlyGly_10plex_2 − Scan number: 6394

Gene Names	Charge	m/z	Mass	Retention time	PEP	Score	Precursor Intensity
GJC1;DKFZp686P0738	3	326.8504	977.5294	23.476	0.068563	62.338	1026636

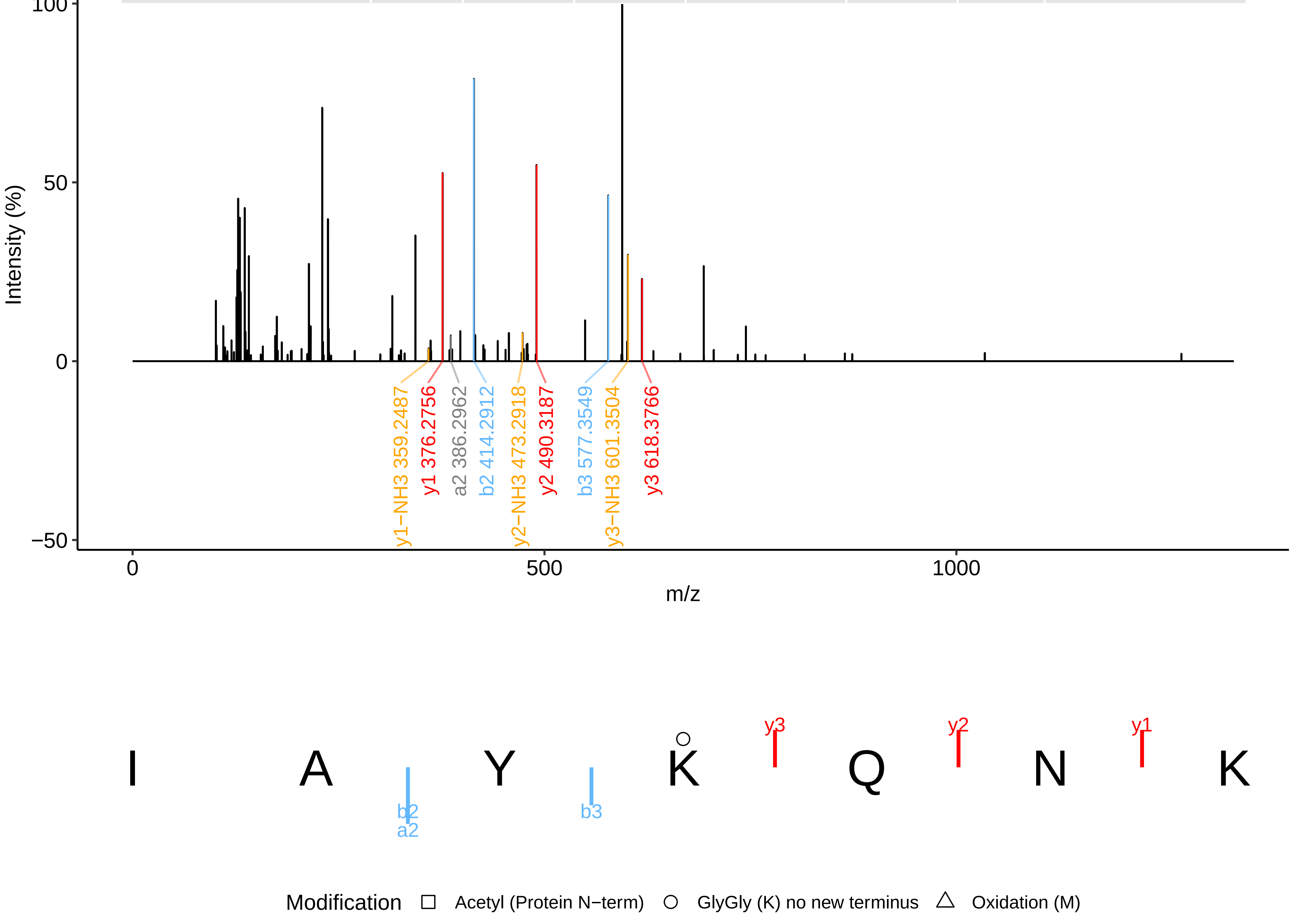

20200921_KIZ_MUb4_GlyGly_10plex − Scan number: 8596

Gene Names	Charge	m/z	Mass	Retention time	PEP	Score	Precursor Intensity
PRKAR2A;PRKAR2B	3	338.5416	1012.603	29.224	0.0088177	81.297	5935172

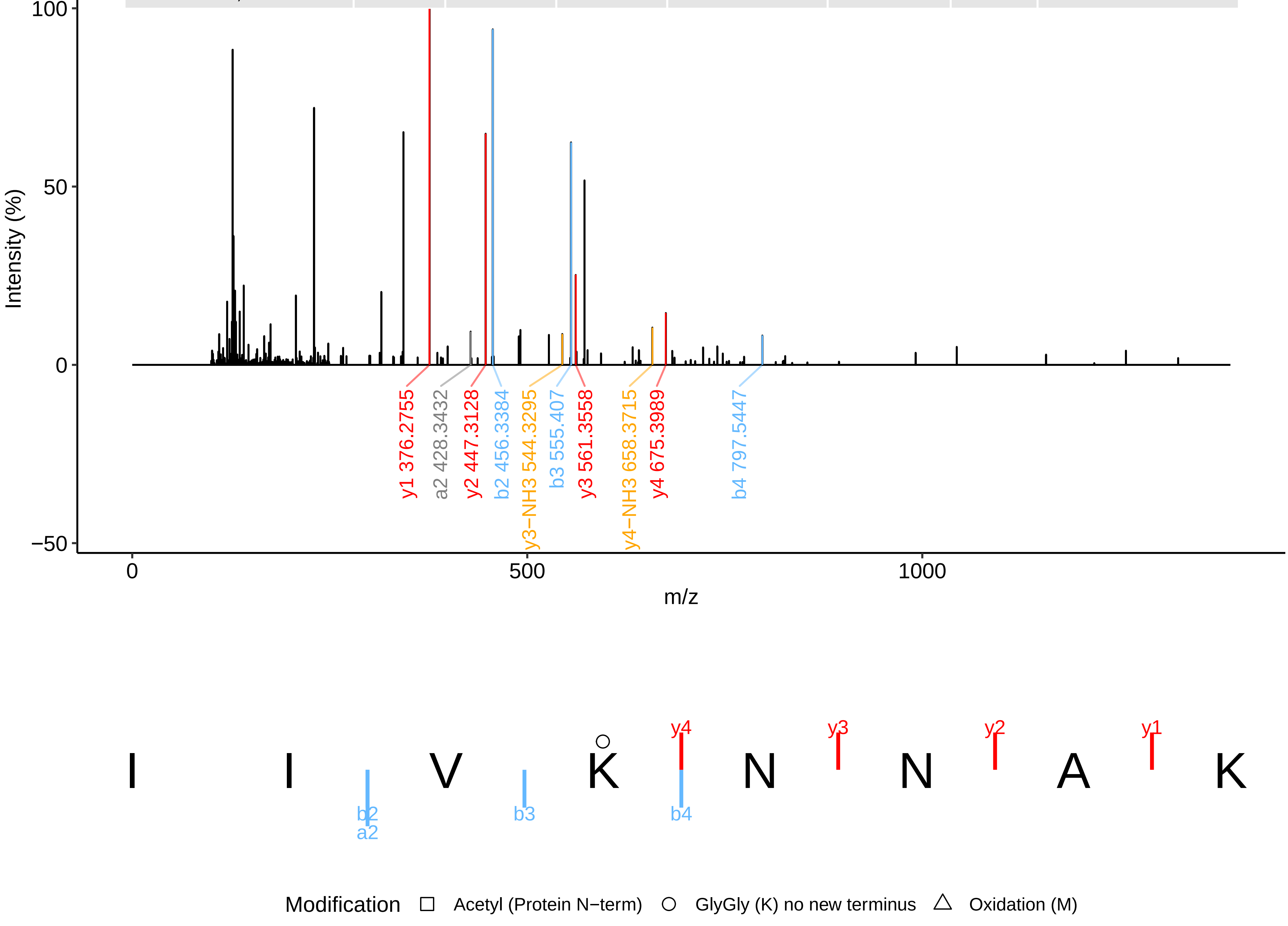

20200921_KIZ_MUb4_GlyGly_10plex_2 − Scan number: 7966

Gene Names	Charge	m/z	Mass	Retention time	PEP	Score	Precursor Intensity
PRKAR2A;PRKAR2B	3	338.5416	1012.603	29.9	0.0088177	81.297	752766.4

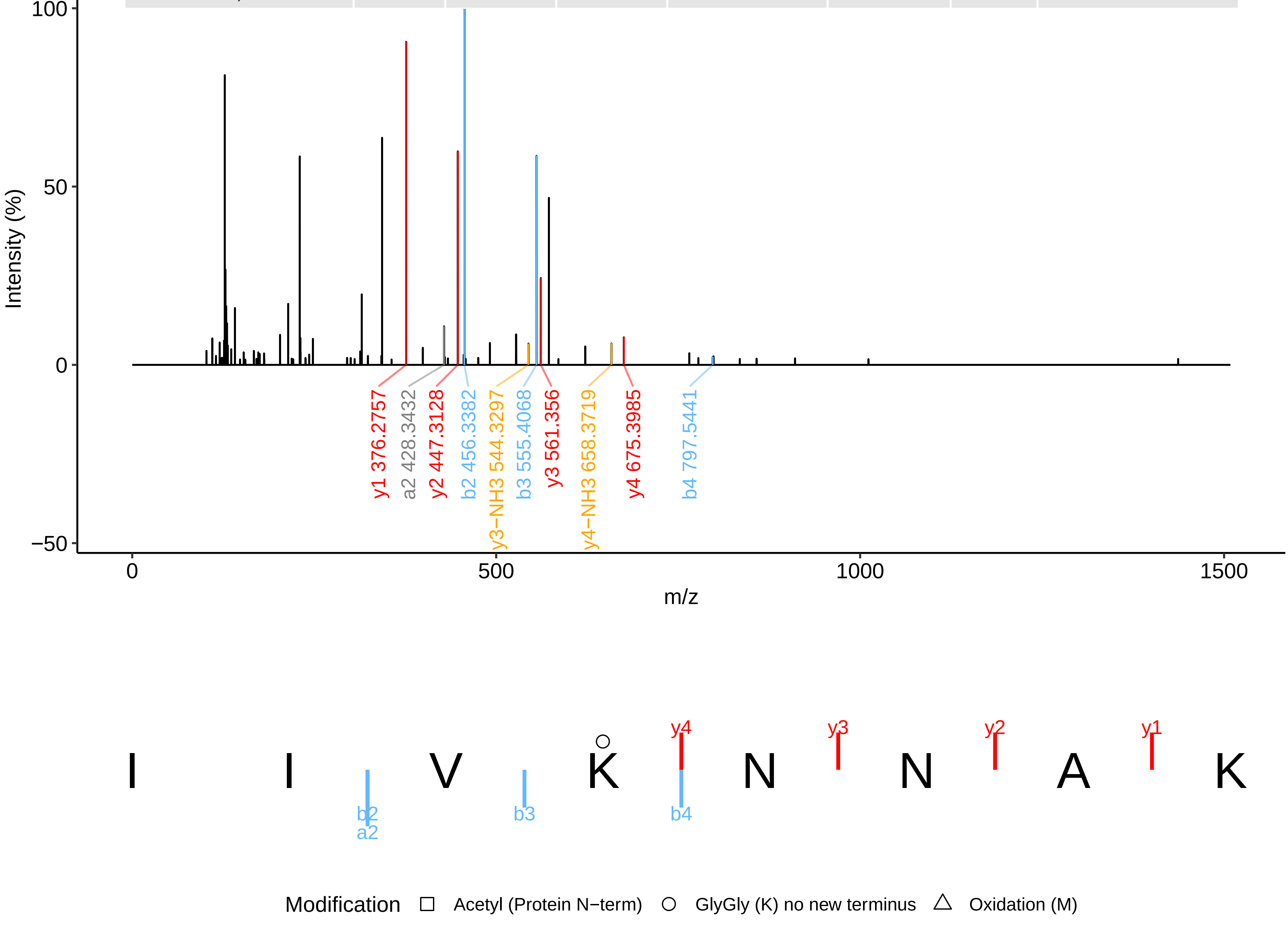

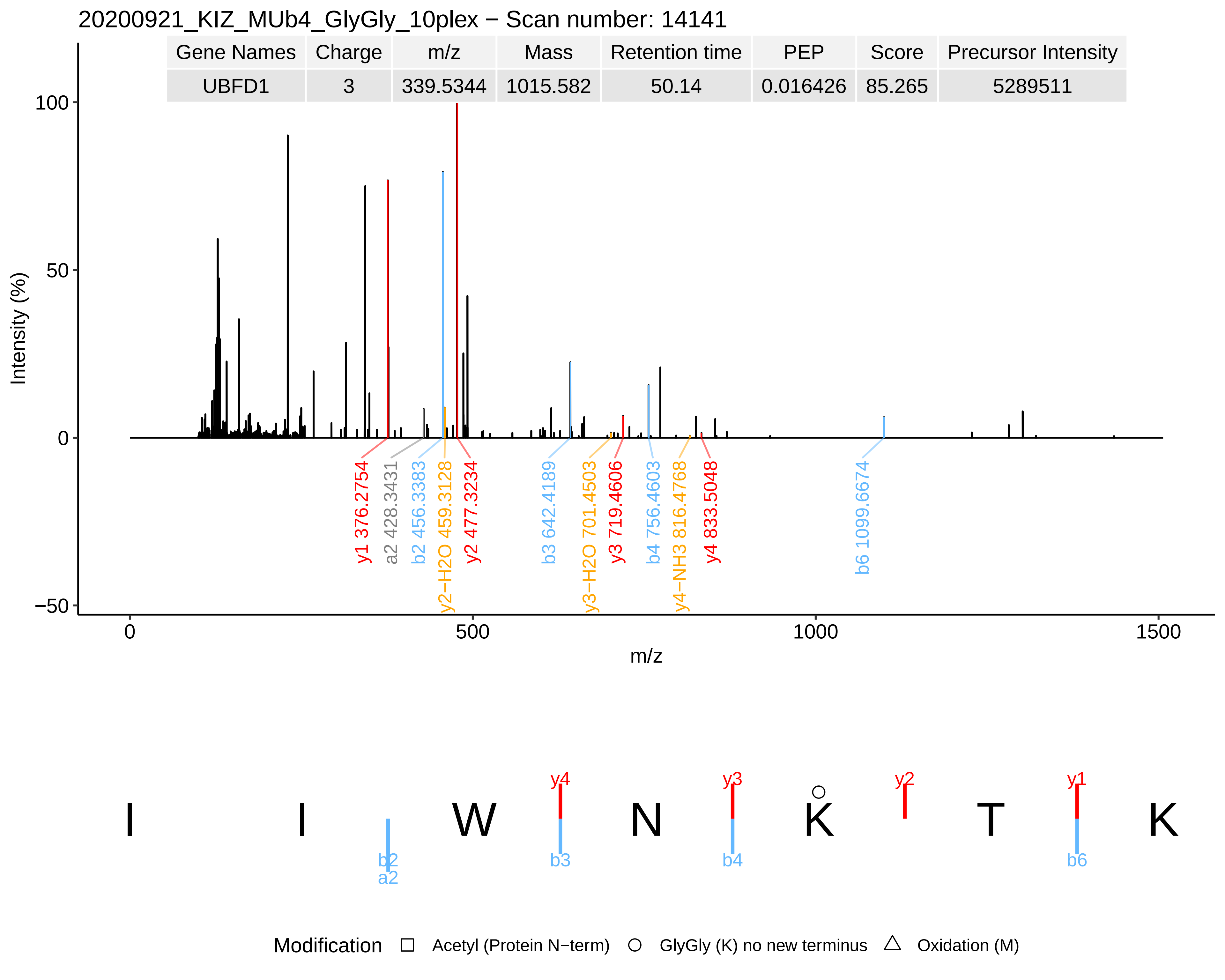
20200921_KIZ_MUb4_GlyGly_10plex − Scan number: 14141
Gene Names | Charge | m/z | Mass | Retention time | PEP | Score | Precursor Intensity
UBFD1 | 3 | 339.5344 | 1015.582 | 50.14 | 0.016426 | 85.265 | 5289511
Intensity (%)
100
50
0
−50
0
500
1000
1500
m/z
y1 376.2754
a2 428.3431
b2 456.3383
y2−H2O 459.3128
y2 477.3234
b3 642.4189
y3−H2O 701.4503
y3 719.4606
b4 756.4603
y4−NH3 816.4768
y4 833.5048
b6 1099.6674
I
I
W
N
K
T
K
y4
y3
y2
y1
b2
a2
b3
b4
b6
Modification
Acetyl (Protein N−term)
GlyGly (K) no new terminus
Oxidation (M)

20200921_KIZ_MUb4_GlyGly_10plex − Scan number: 5827

Gene Names	Charge	m/z	Mass	Retention time	PEP	Score	Precursor Intensity
CUL9	4	253.1502	1008.572	19.114	0.021253	67.169	11035289

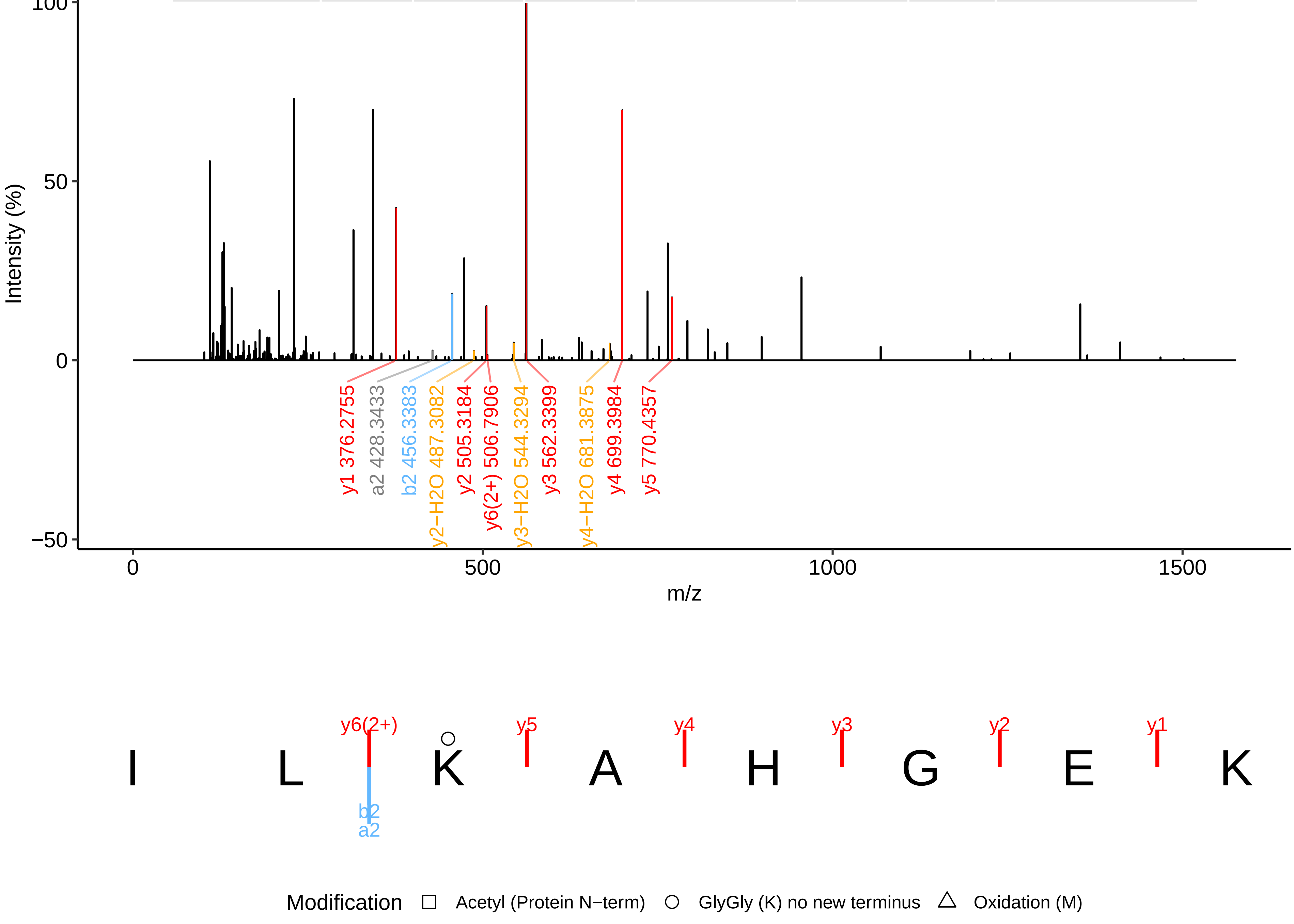

20200921_KIZ_MUb4_GlyGly_10plex − Scan number: 5842

Gene Names	Charge	m/z	Mass	Retention time	PEP	Score	Precursor Intensity
CUL9	3	337.1978	1008.572	19.16	0.001379	105.52	8823004

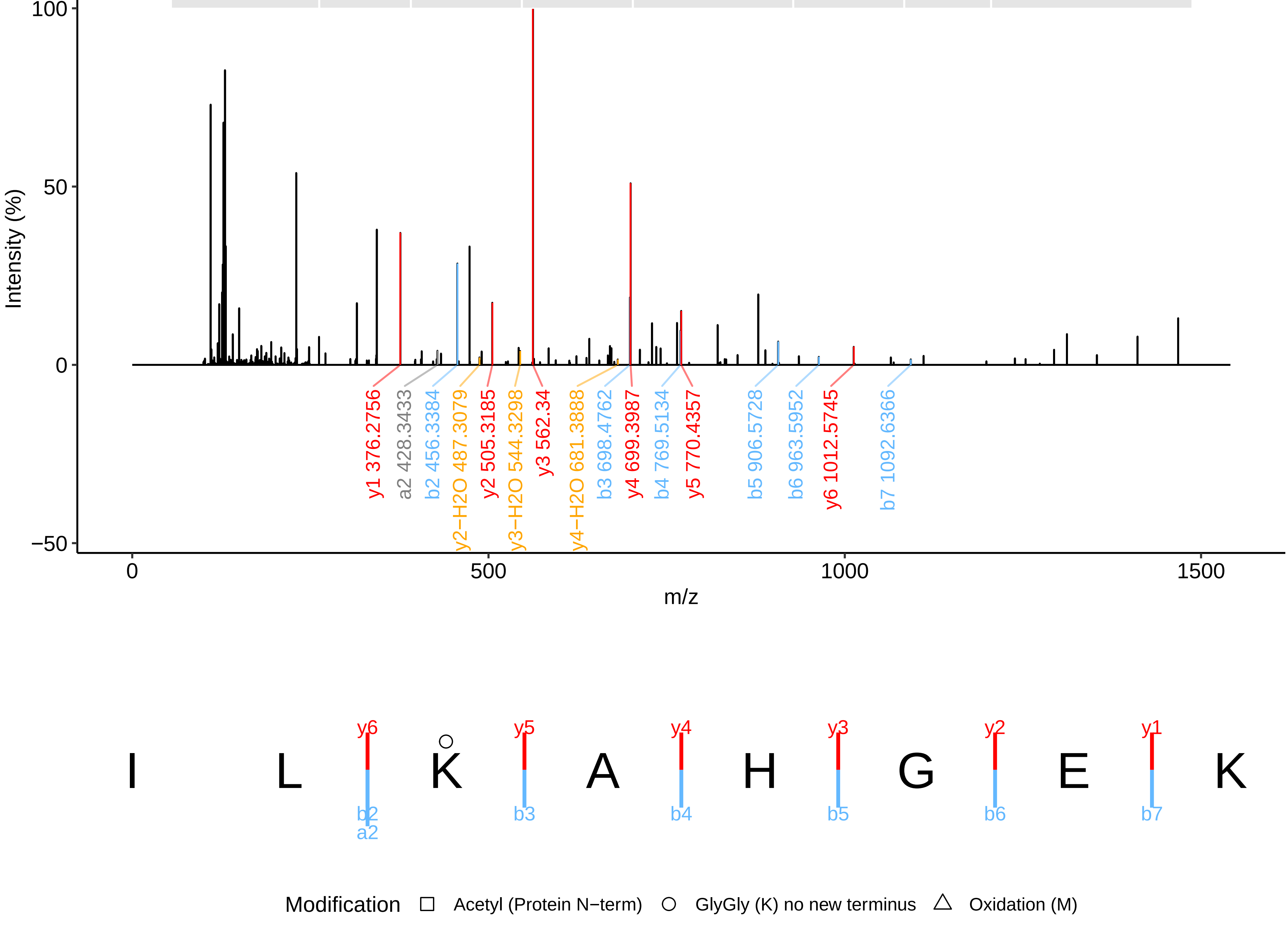

20200921_KIZ_MUb4_GlyGly_10plex_2 − Scan number: 5414

Gene Names	Charge	m/z	Mass	Retention time	PEP	Score	Precursor Intensity
CUL9	3	337.1978	1008.572	19.451	0.047995	58.981	849561.9

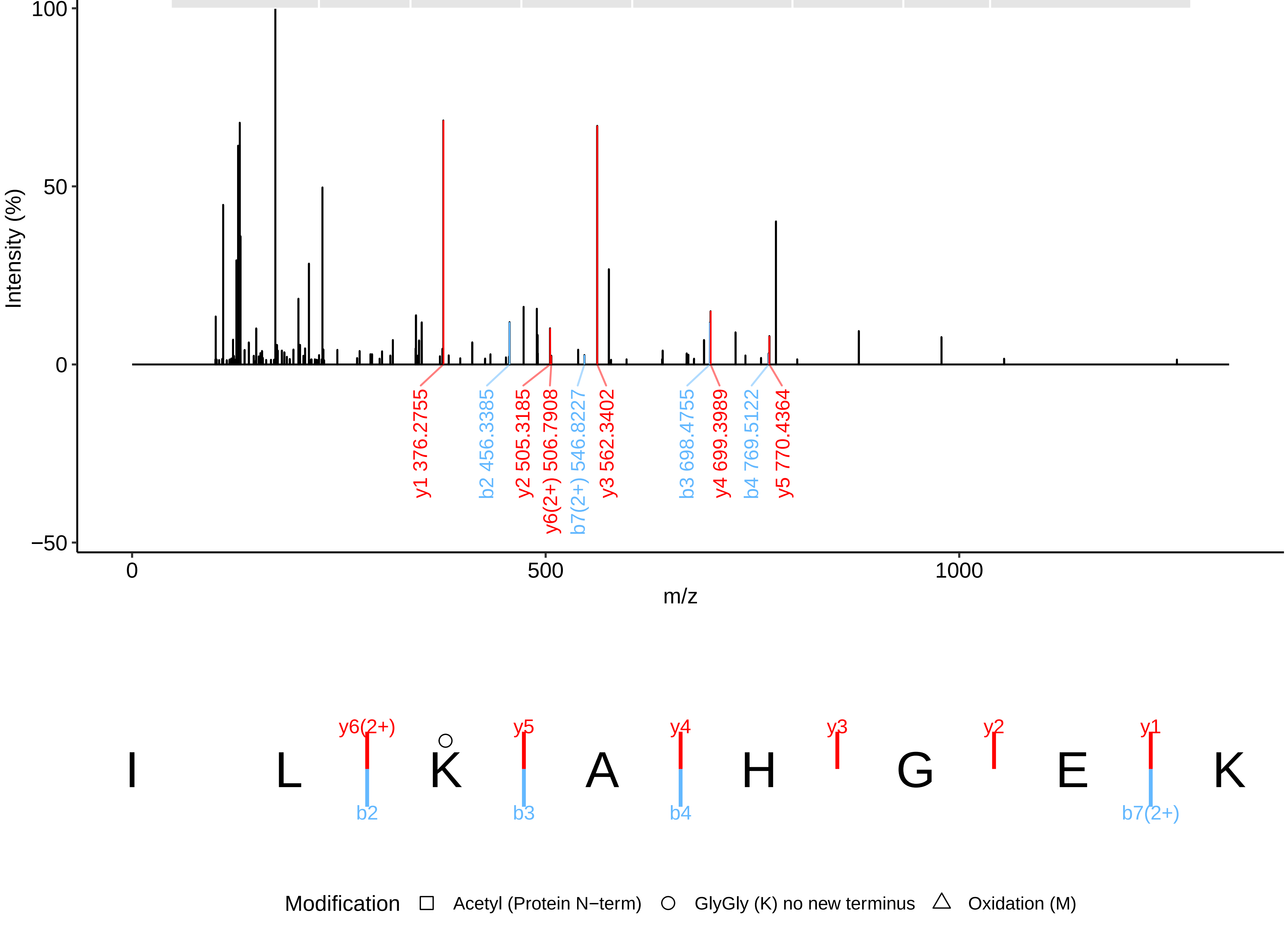

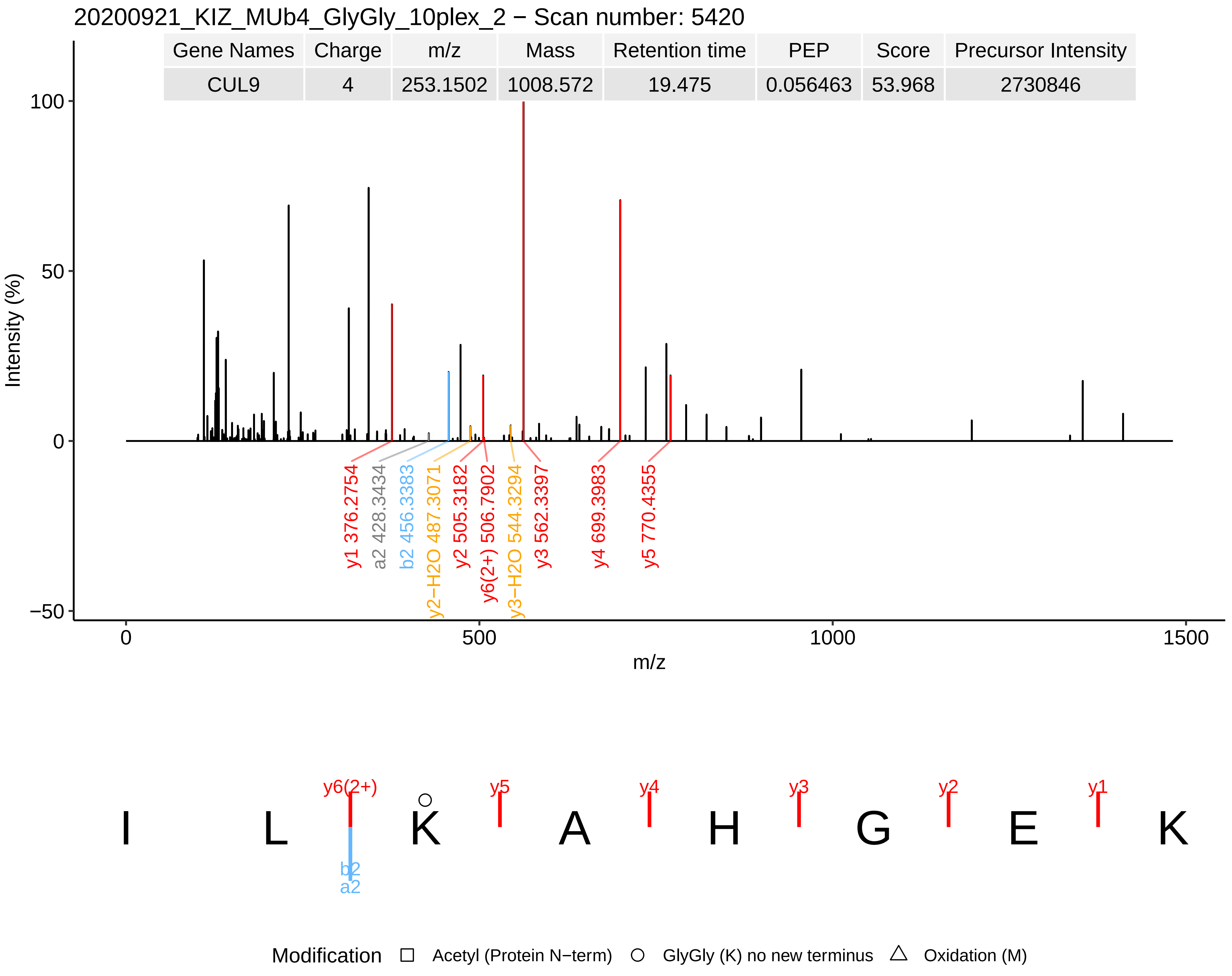
20200921_KIZ_MUb4_GlyGly_10plex_2 − Scan number: 5420
Gene Names | Charge | m/z | Mass | Retention time | PEP | Score | Precursor Intensity
CUL9 | 4 | 253.1502 | 1008.572 | 19.475 | 0.056463 | 53.968 | 2730846
Intensity (%)
100
50
0
−50
y1 376.2754
a2 428.3434
b2 456.3383
y2−H2O 487.3071
y2 505.3182
y6(2+) 506.7902
y3−H2O 544.3294
y3 562.3397
y4 699.3983
y5 770.4355
0
500
1000
1500
m/z
I L K A H G E K
y6(2+)
y5
y4
y3
y2
y1
b2
a2
Modification
Acetyl (Protein N−term)
GlyGly (K) no new terminus
Oxidation (M)

20200924_KIZ_MUb5_GlyGly_1 − Scan number: 4930

Gene Names	Charge	m/z	Mass	Retention time	PEP	Score	Precursor Intensity
CUL9	3	337.1978	1008.572	15.282	0.0076678	84.213	8683114

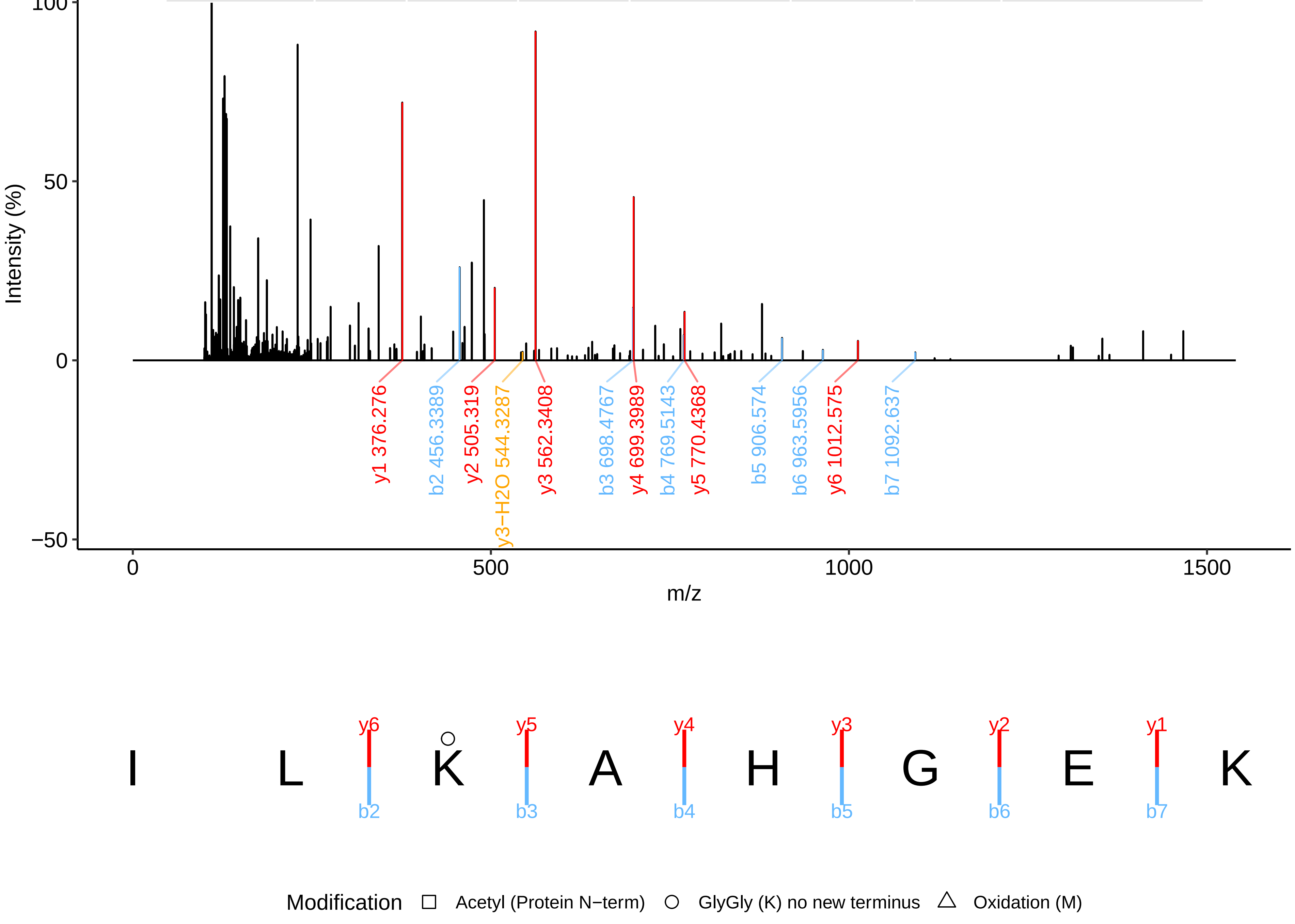

20200924_KIZ_MUb5_GlyGly_1 − Scan number: 5013

Gene Names	Charge	m/z	Mass	Retention time	PEP	Score	Precursor Intensity
CUL9	4	253.1502	1008.572	15.559	0.071841	49.418	3211806

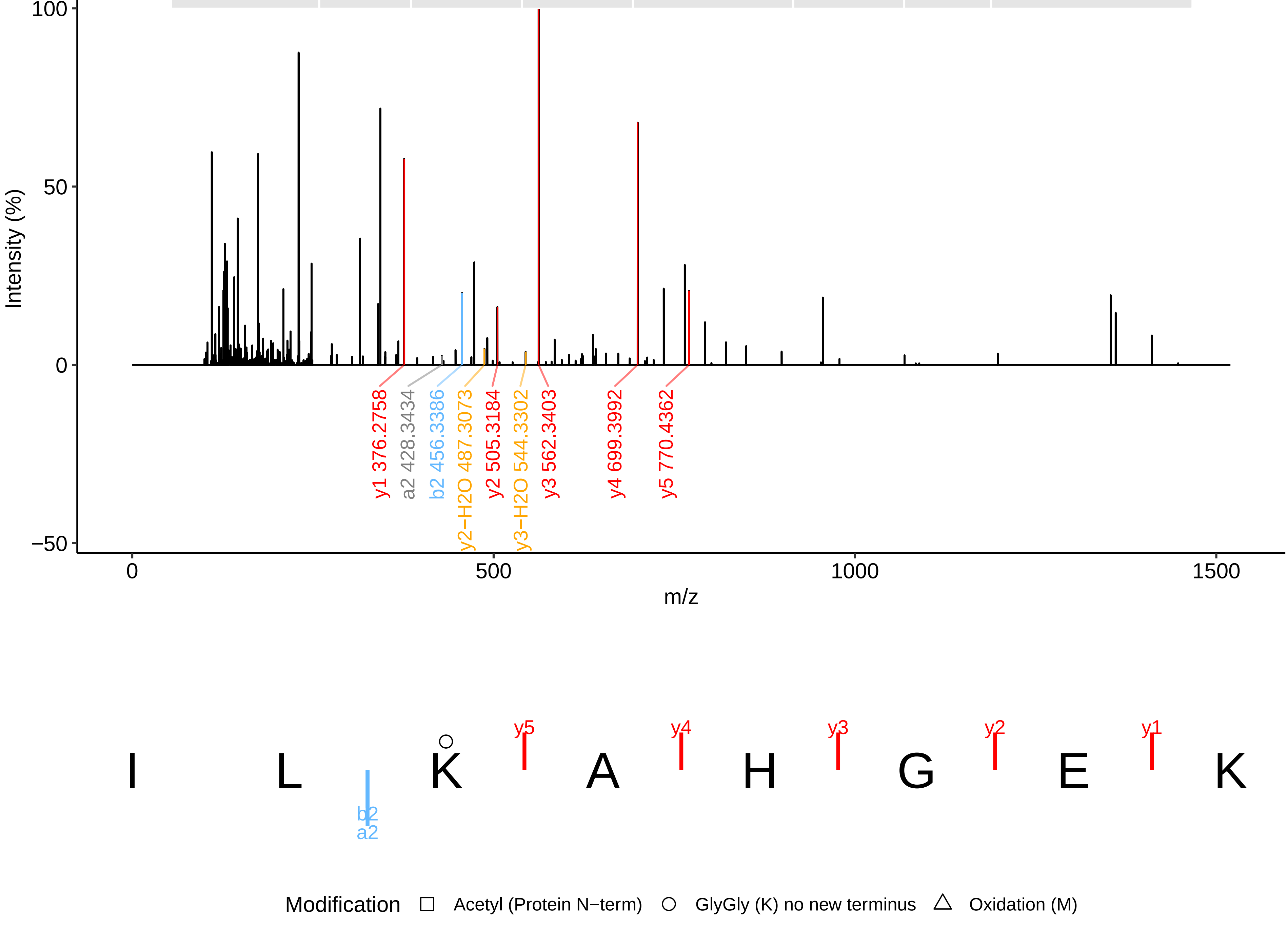

20200924_KIZ_MUb5_GlyGly_2 − Scan number: 4561

Gene Names	Charge	m/z	Mass	Retention time	PEP	Score	Precursor Intensity
CUL9	4	253.1502	1008.572	16.755	0.088784	44.349	535398.1

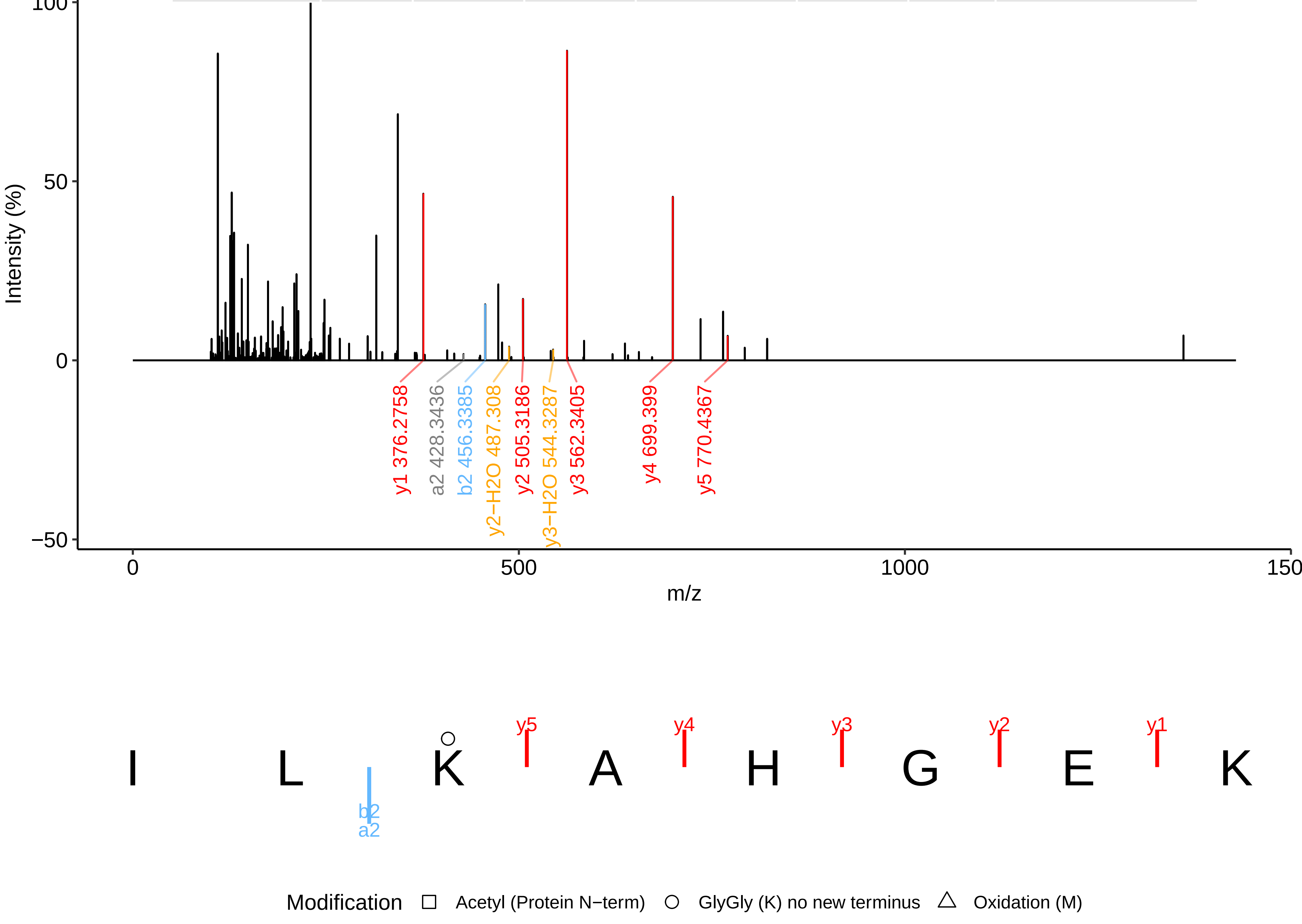

20200924_KIZ_MUb5_GlyGly_2 − Scan number: 4663

Gene Names	Charge	m/z	Mass	Retention time	PEP	Score	Precursor Intensity
CUL9	4	253.1502	1008.572	17.16	0.065325	51.346	2130617

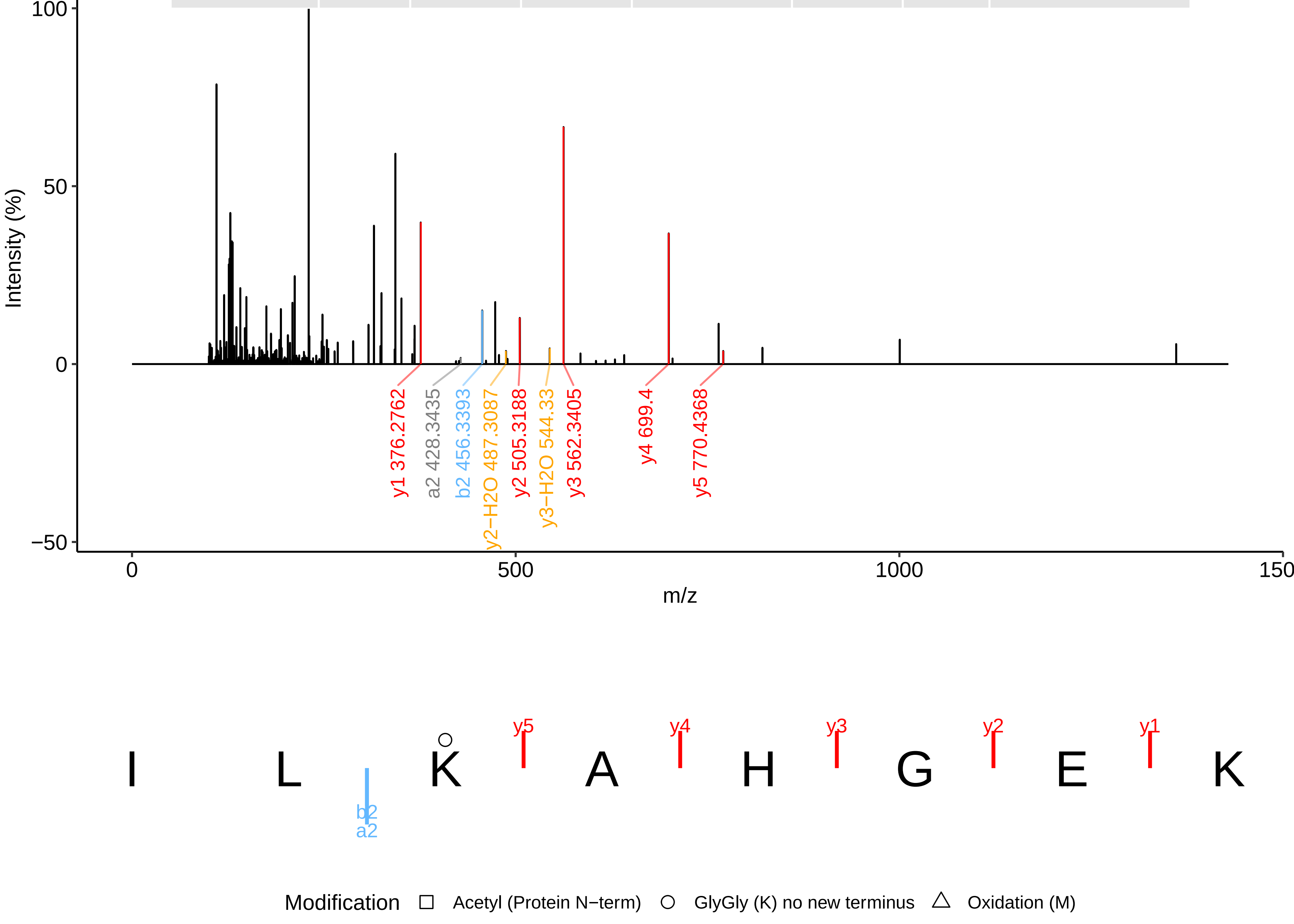

20200924_KIZ_MUb5_GlyGly_2 − Scan number: 4693

Gene Names	Charge	m/z	Mass	Retention time	PEP	Score	Precursor Intensity
CUL9	4	253.1502	1008.572	17.276	0.098202	41.448	1941197

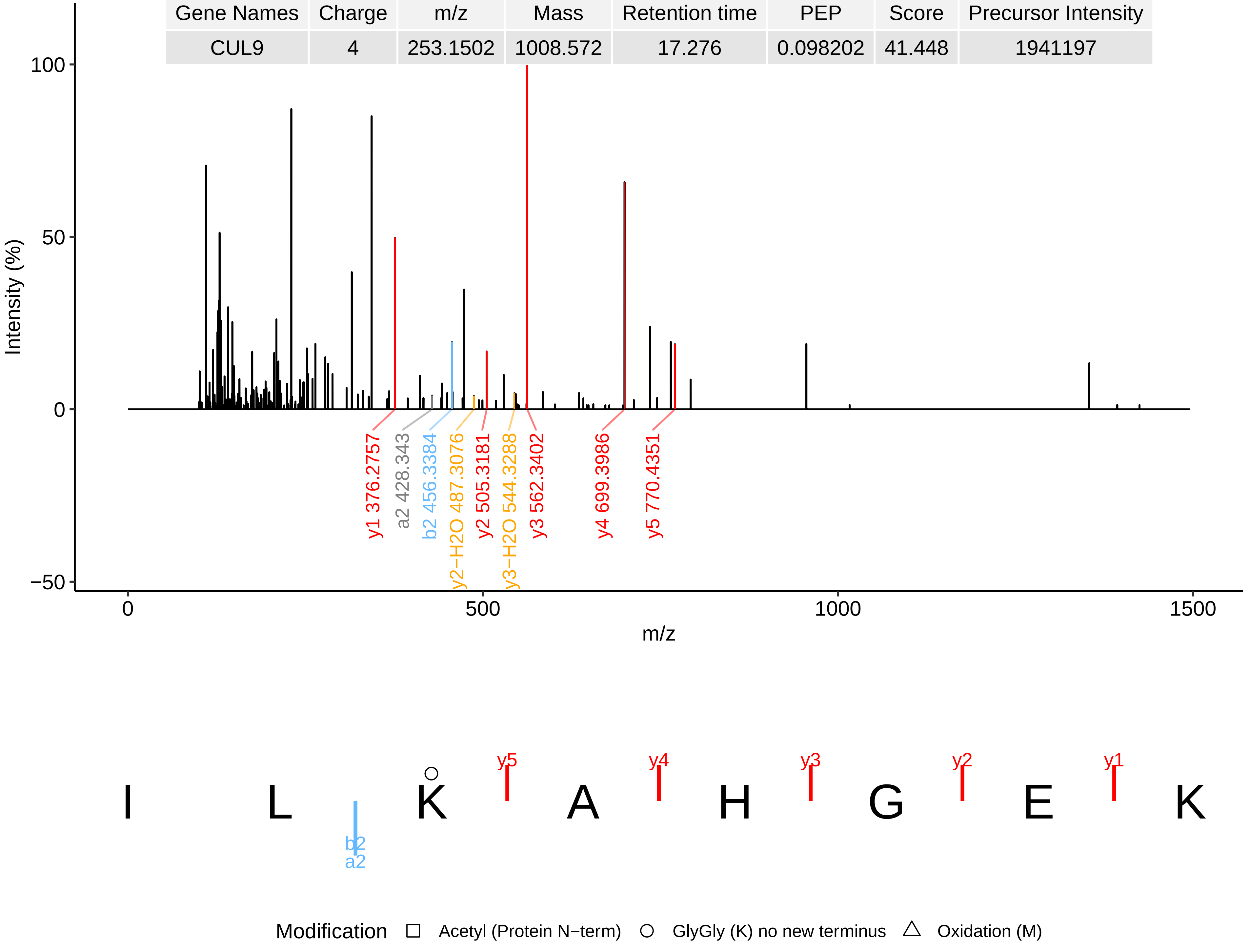

20201118_KIZ_MUb6_GlyGly_1 − Scan number: 4766

Gene Names	Charge	m/z	Mass	Retention time	PEP	Score	Precursor Intensity
CUL9	4	253.1502	1008.572	15.02	0.016935	71.379	11538517

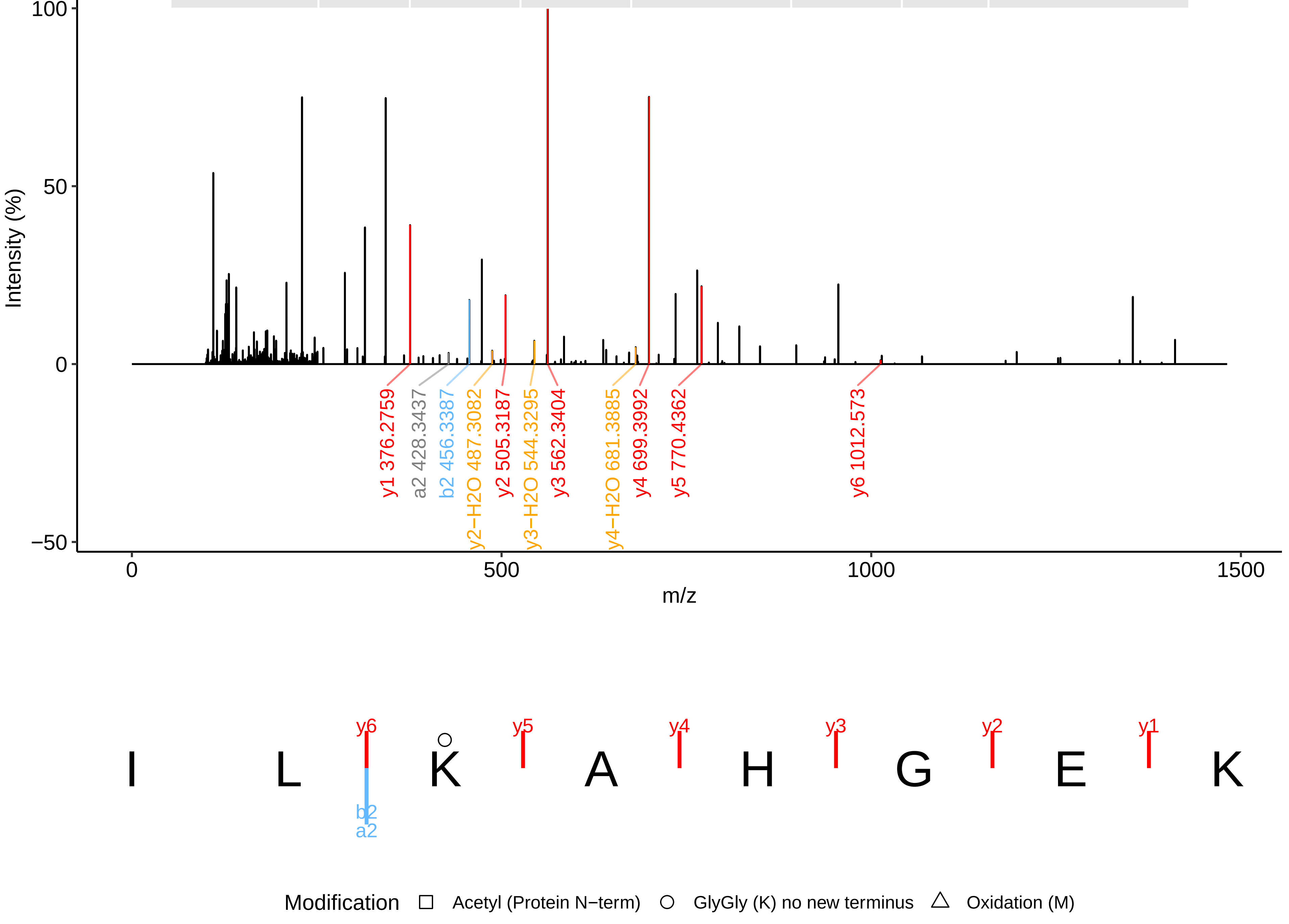

20200921_KIZ_MUb4_GlyGly_10plex_2 − Scan number: 4774

Gene Names	Charge	m/z	Mass	Retention time	PEP	Score	Precursor Intensity
DPY30	3	330.8453	989.5142	16.891	0.057919	56.563	759937.4

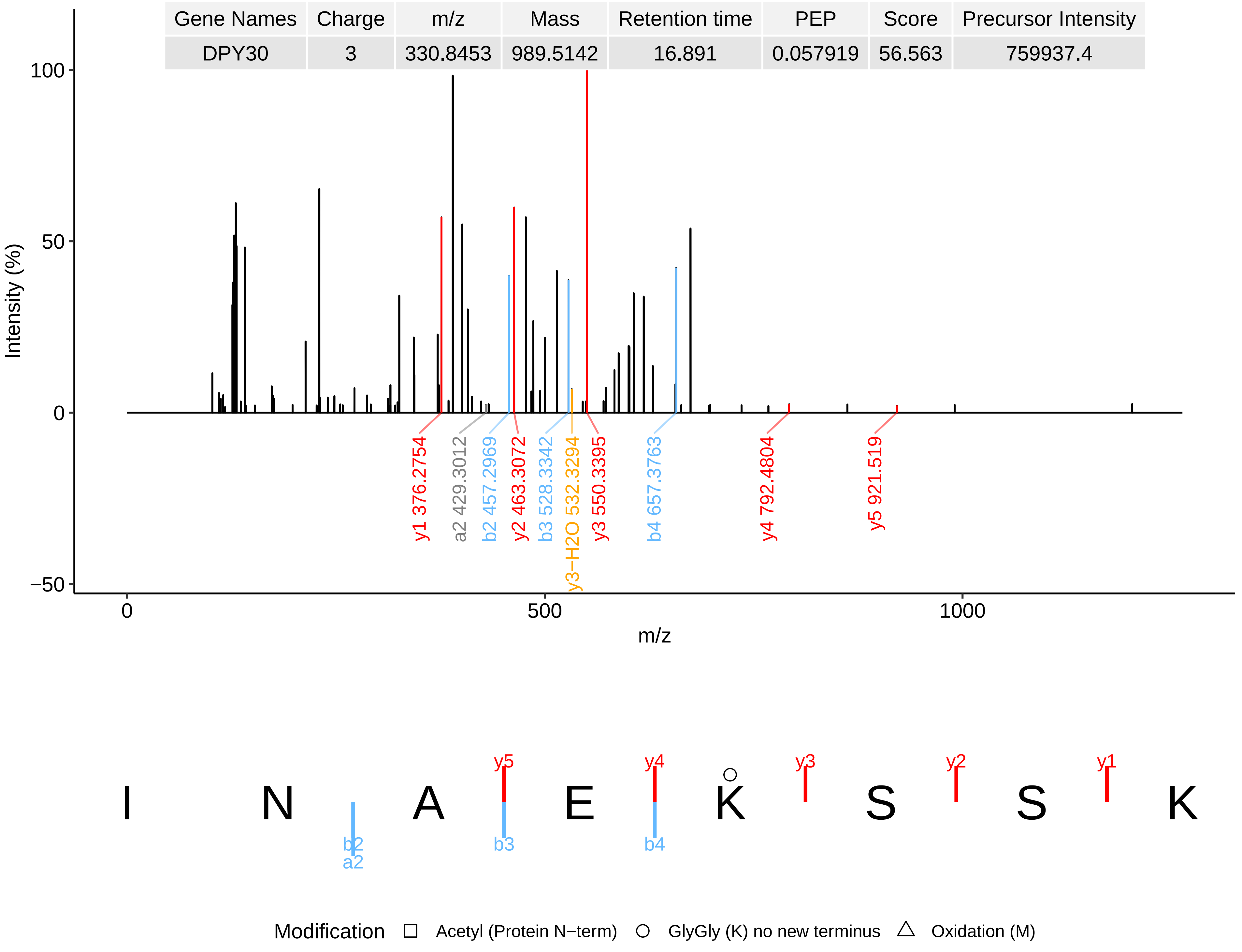

20200921_KIZ_MUb4_GlyGly_10plex − Scan number: 17054

Gene Names	Charge	m/z	Mass	Retention time	PEP	Score	Precursor Intensity
EPRS	3	340.859	1019.555	61.47	0.032812	103.71	9703497

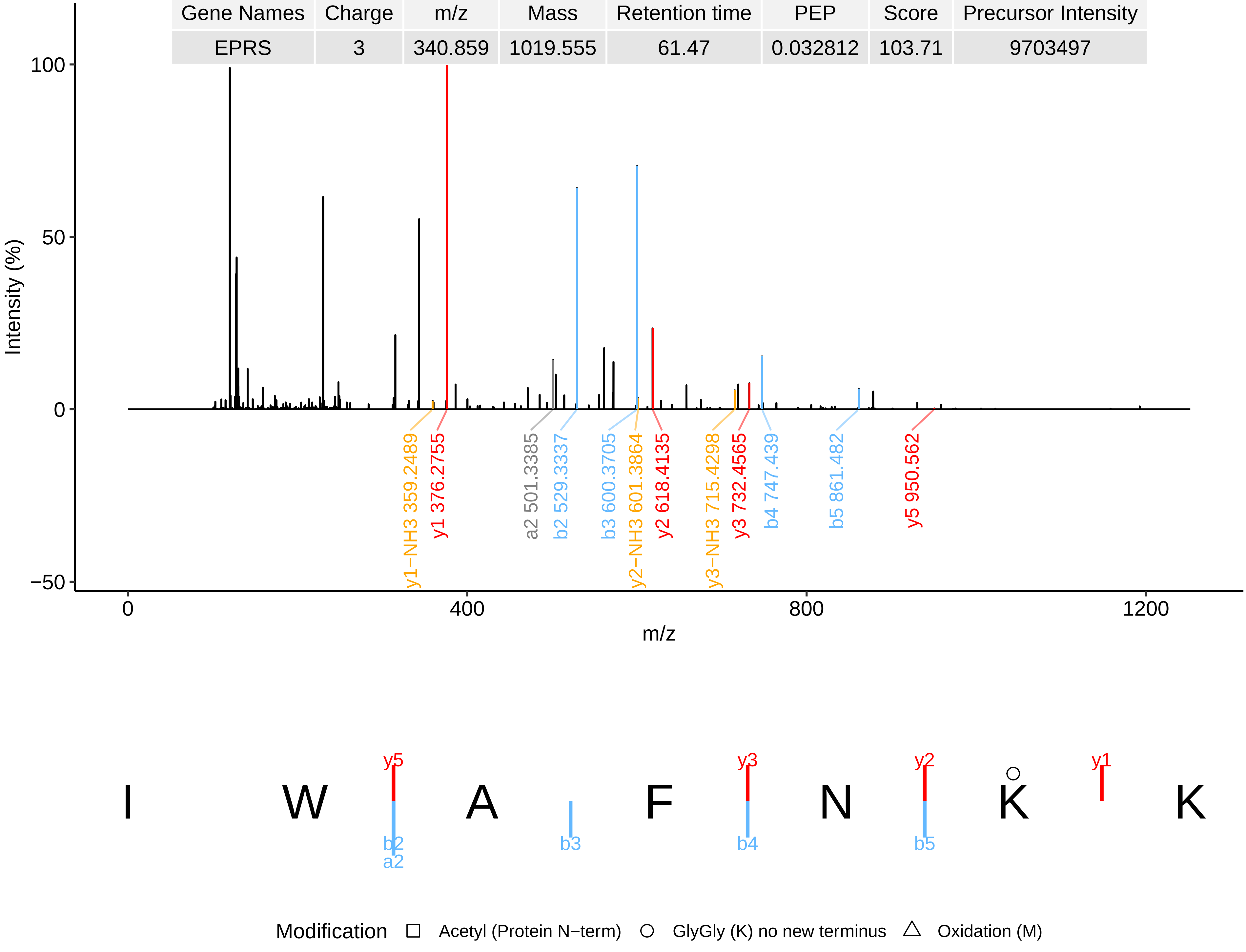

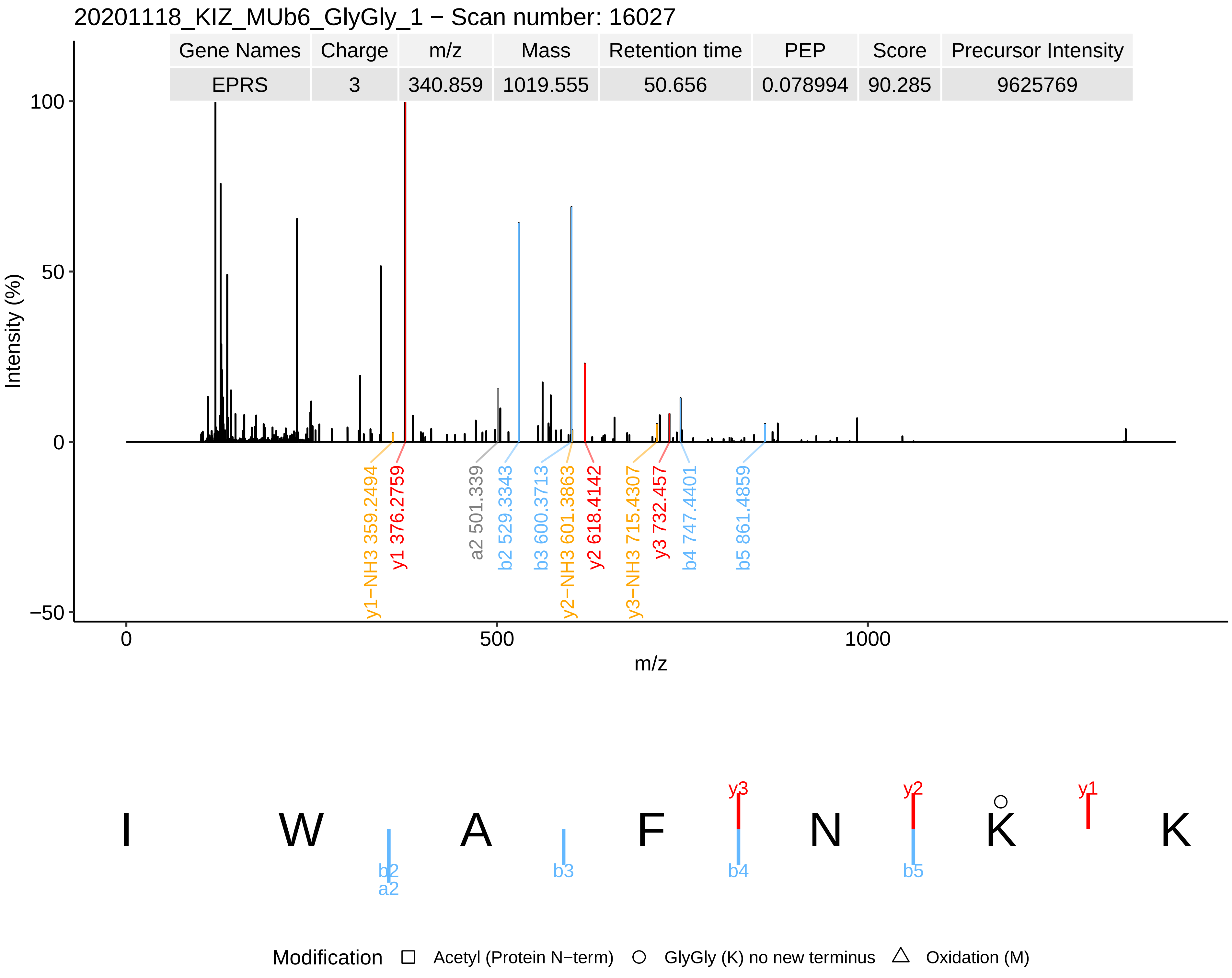

20201118_KIZ_MUb6_GlyGly_1 − Scan number: 16027
Gene Names | Charge | m/z | Mass | Retention time | PEP | Score | Precursor Intensity
EPRS | 3 | 340.859 | 1019.555 | 50.656 | 0.078994 | 90.285 | 9625769
Intensity (%)
100
50
0
−50
0
500
1000
m/z
y1−NH3 359.2494
y1 376.2759
a2 501.339
b2 529.3343
b3 600.3713
y2−NH3 601.3863
y2 618.4142
y3−NH3 715.4307
y3 732.457
b4 747.4401
b5 861.4859
I W A F N K K
y3 y2 y1
b2 a2 b3 b4 b5
Modification
Acetyl (Protein N−term)
GlyGly (K) no new terminus
Oxidation (M)

20200921_KIZ_MUb4_GlyGly_10plex − Scan number: 5911

Gene Names	Charge	m/z	Mass	Retention time	PEP	Score	Precursor Intensity
PHB2	3	340.1891	1017.545	19.388	0.044163	77.192	5229710

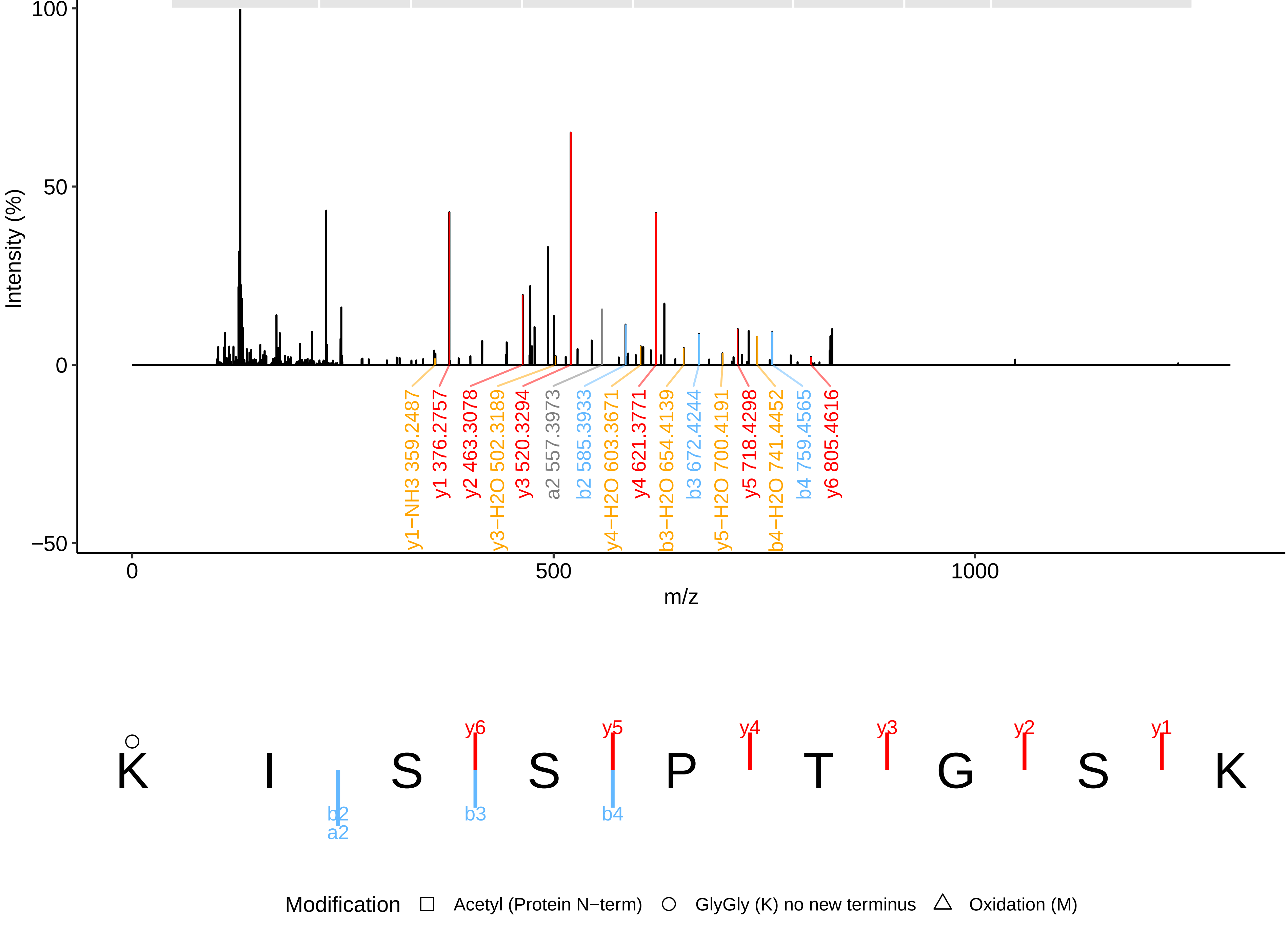

20200921_KIZ_MUb4_GlyGly_10plex − Scan number: 7230

Gene Names	Charge	m/z	Mass	Retention time	PEP	Score	Precursor Intensity
SCAMP3	3	338.5257	1012.555	24.189	0.024394	93.649	5311484

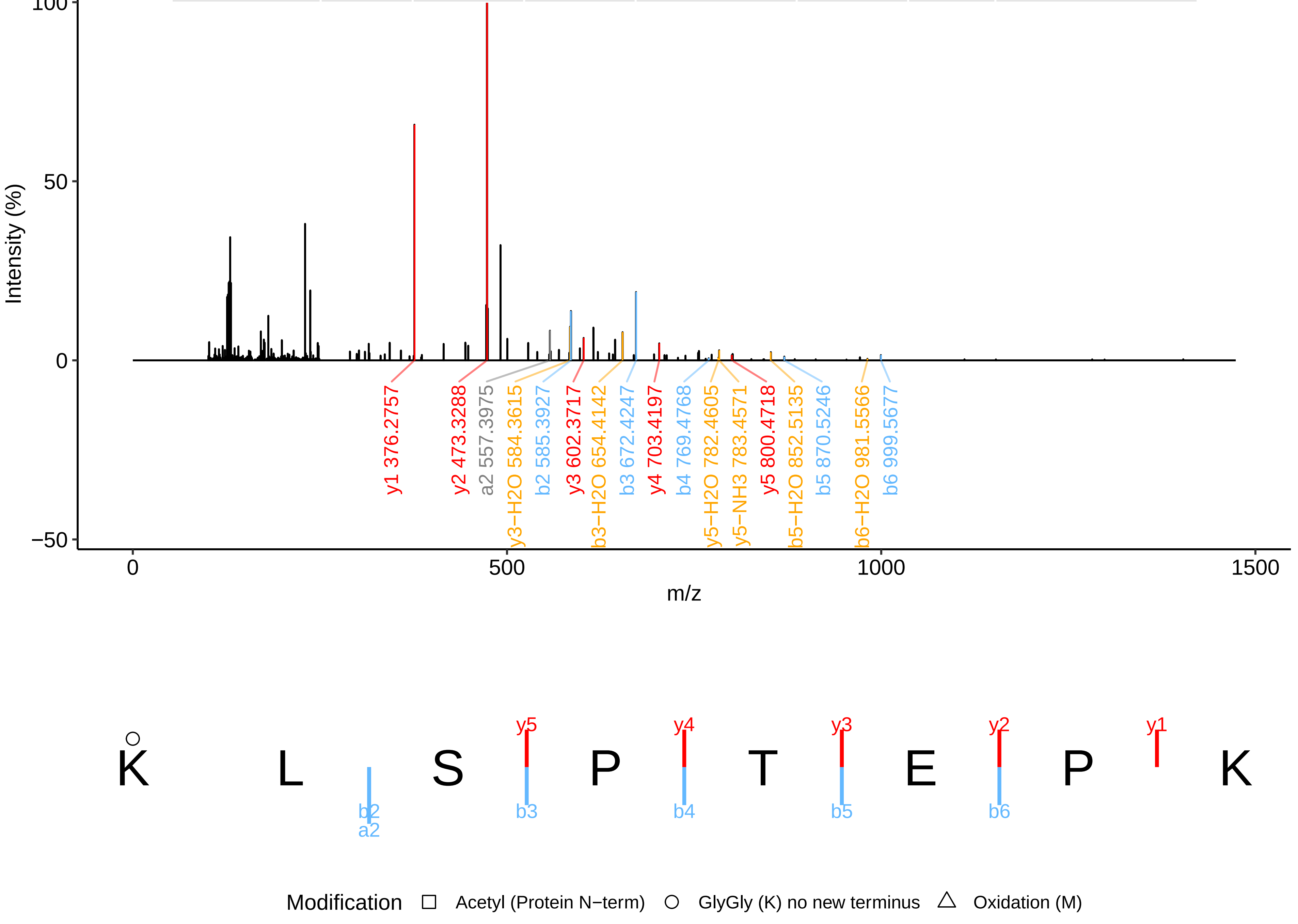

20200924_KIZ_MUb5_GlyGly_1 − Scan number: 6383

Gene Names	Charge	m/z	Mass	Retention time	PEP	Score	Precursor Intensity
SCAMP3	3	338.5257	1012.555	20.058	0.048791	78.516	3887682

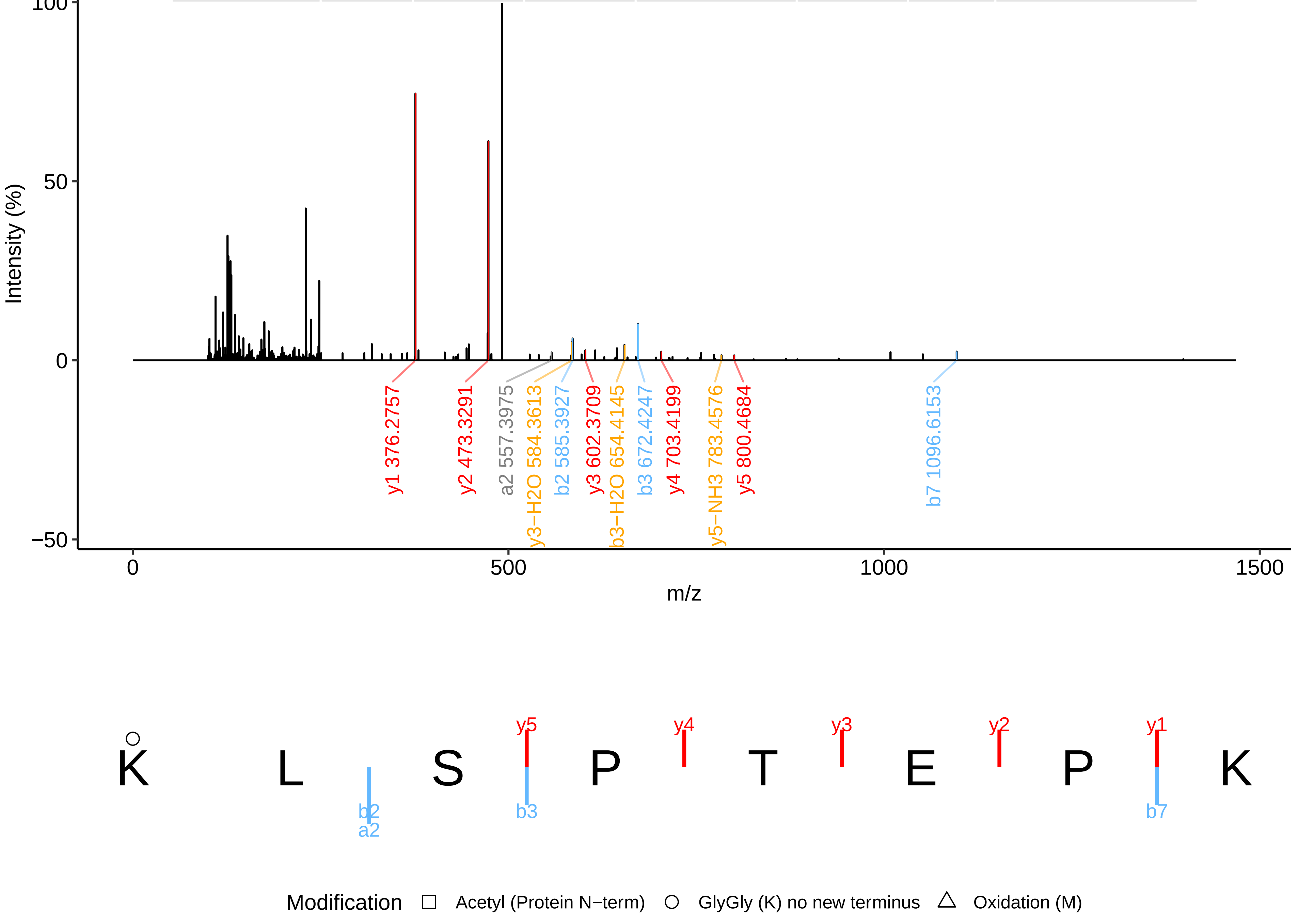

20200921_KIZ_MUb4_GlyGly_10plex − Scan number: 5293

Gene Names	Charge	m/z	Mass	Retention time	PEP	Score	Precursor Intensity
BCAP31	3	335.5172	1003.53	17.311	0.051089	78.098	NA

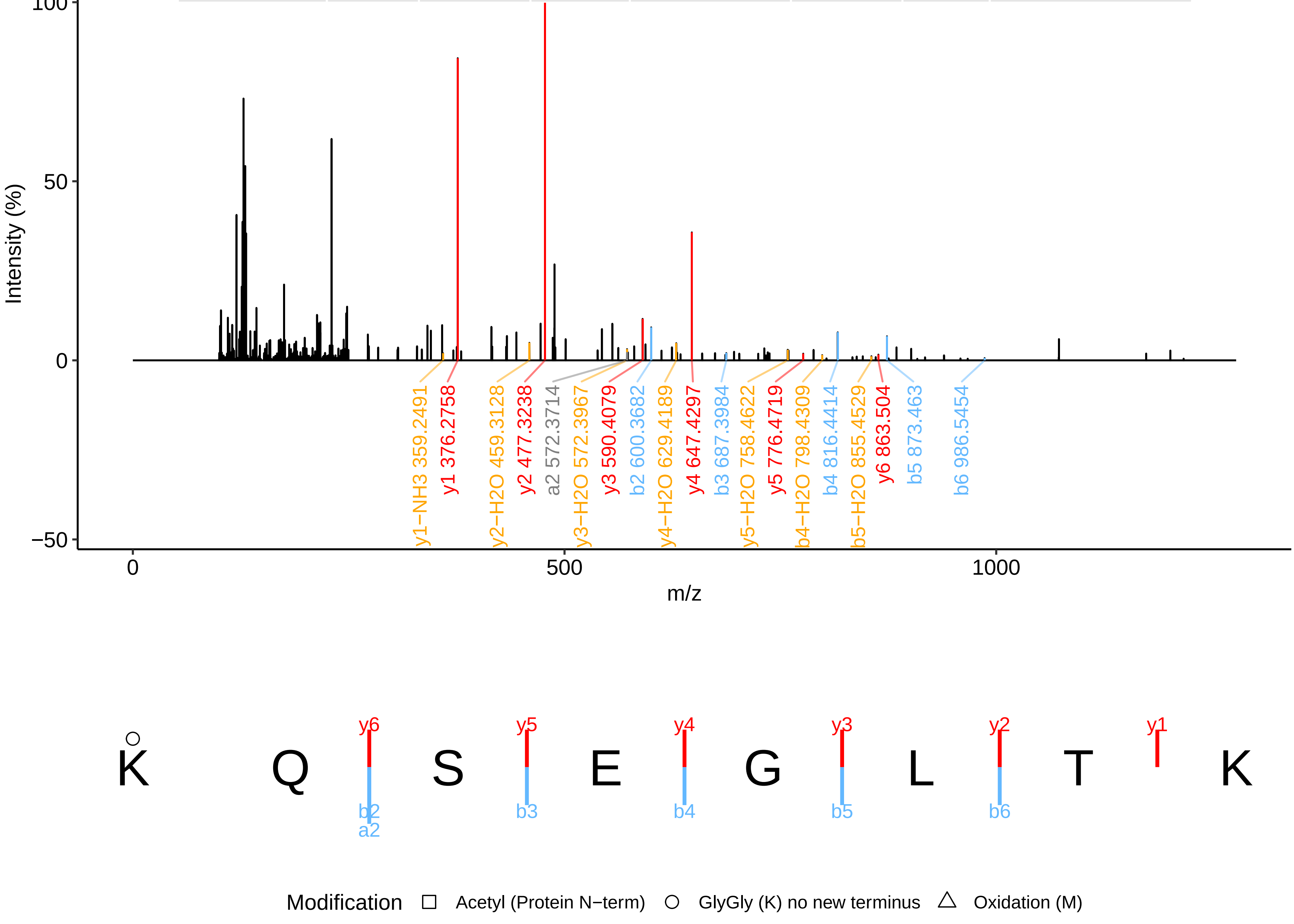

20200921_KIZ_MUb4_GlyGly_10plex − Scan number: 16380

Gene Names	Charge	m/z	Mass	Retention time	PEP	Score	Precursor Intensity
RRM1	3	409.9188	1226.735	58.807	2.6374e−05	109.44	5774593

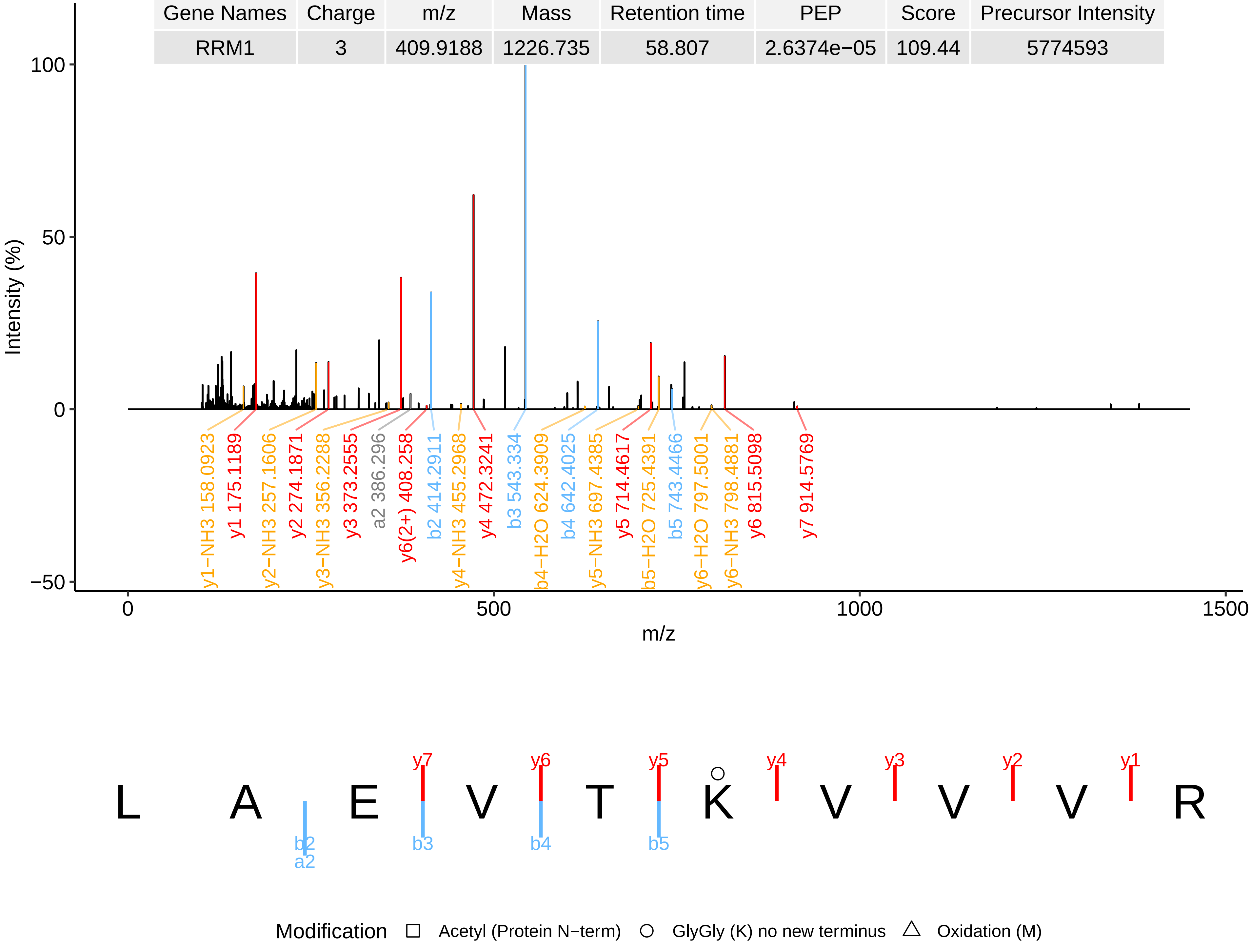

20200921_KIZ_MUb4_GlyGly_10plex_2 − Scan number: 15202

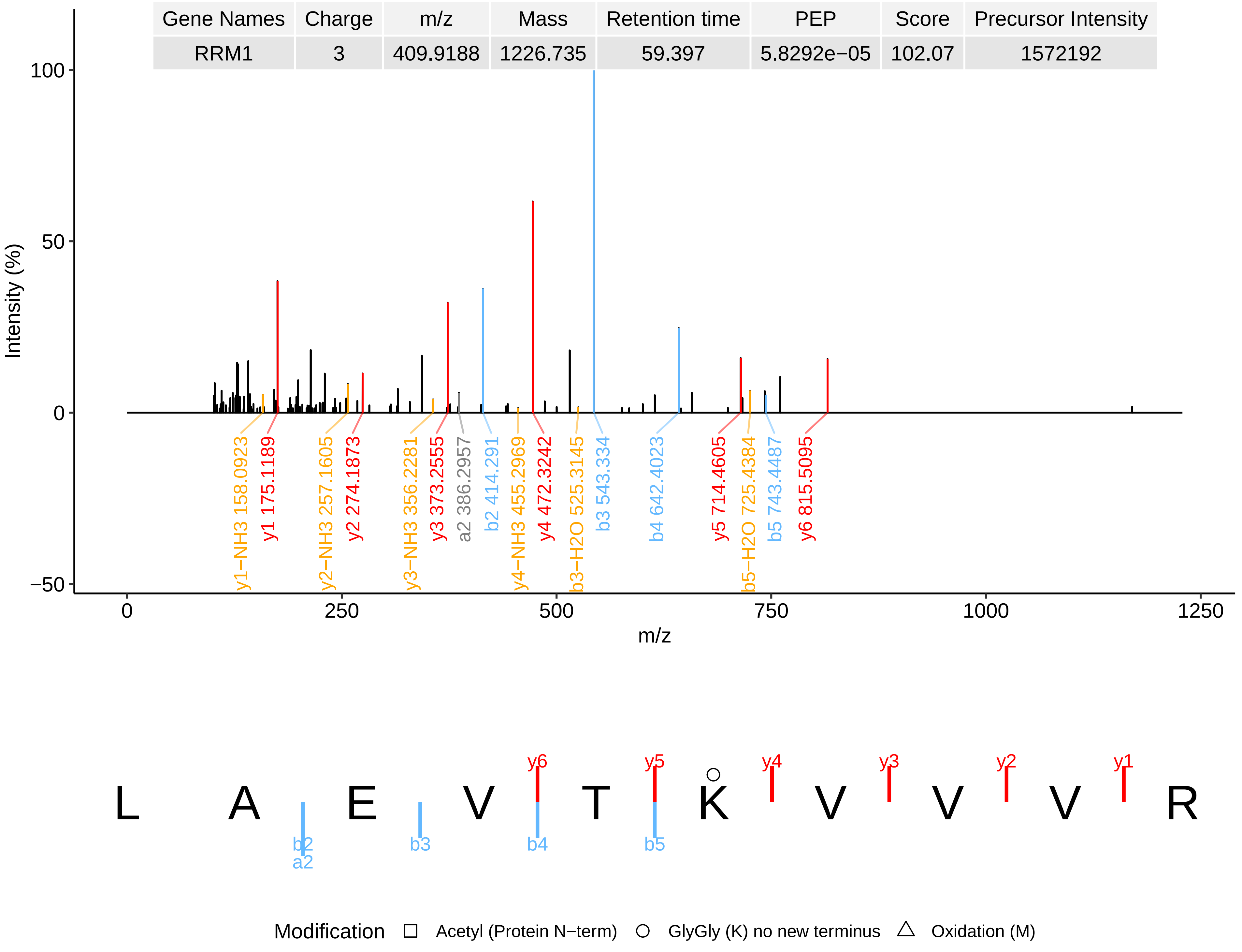

20200921_KIZ_MUb4_GlyGly_10plex_2 − Scan number: 10402

Gene Names	Charge	m/z	Mass	Retention time	PEP	Score	Precursor Intensity
EIF3E	3	335.1855	1002.535	39.79	0.052185	57.96	820970

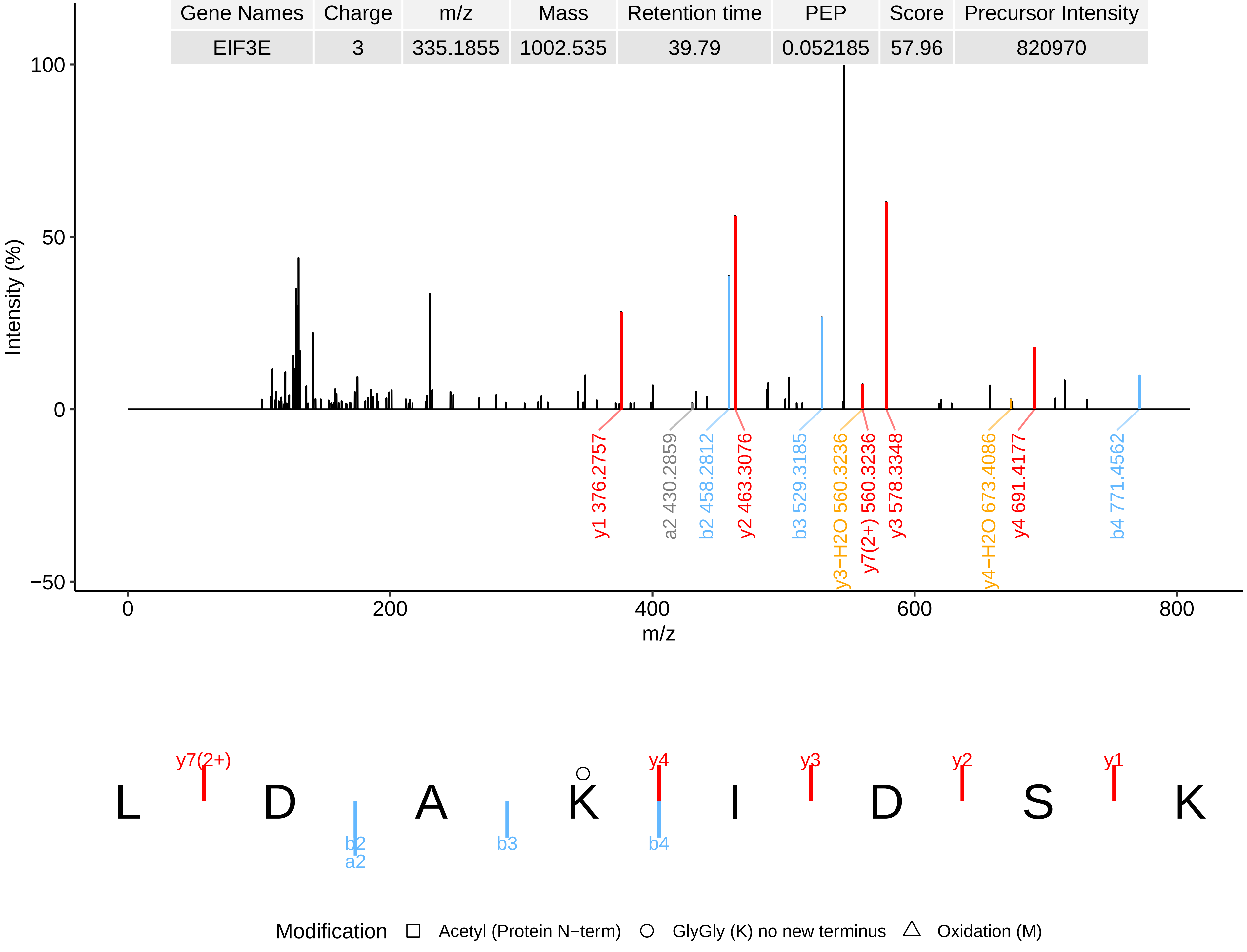

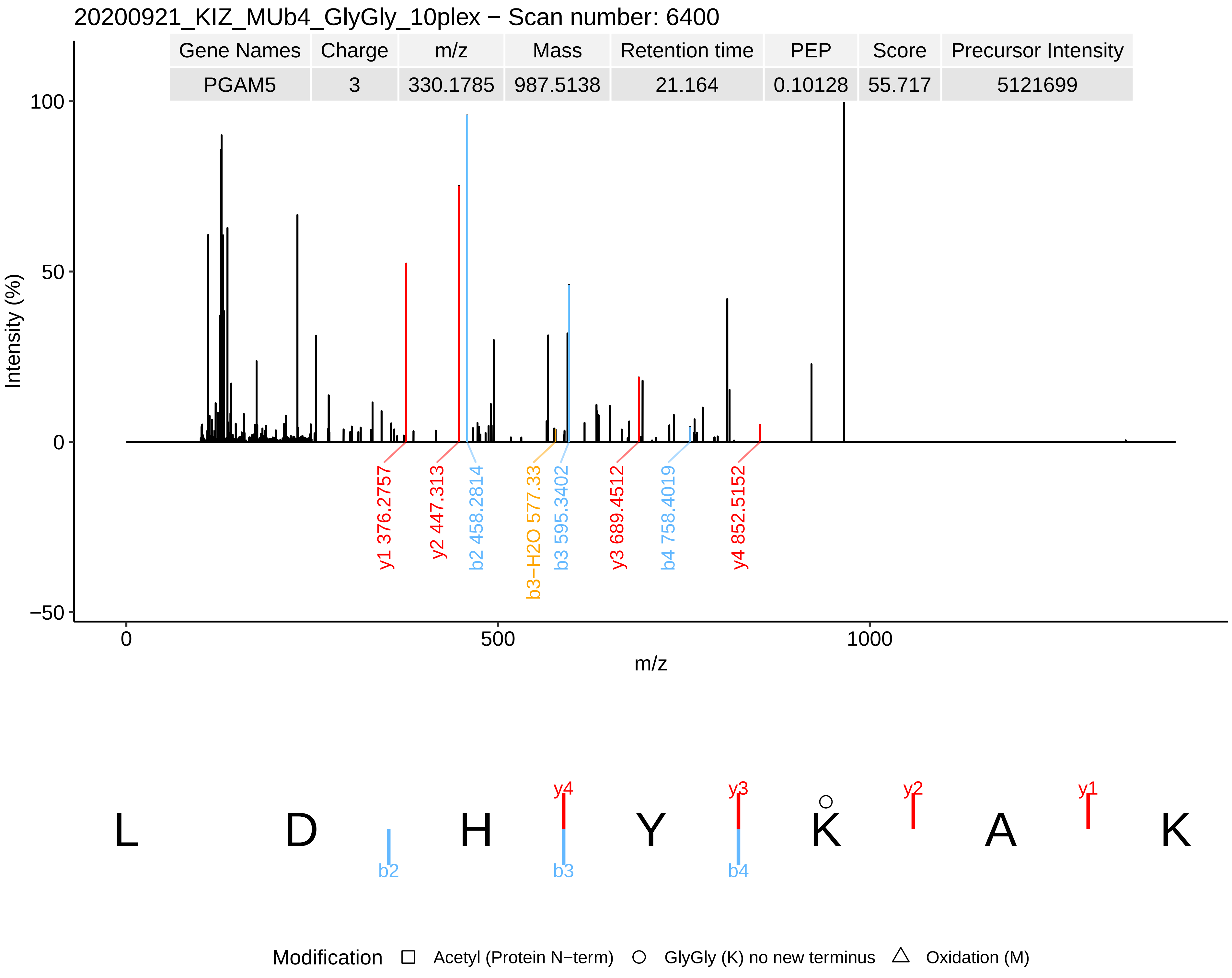

20200921_KIZ_MUb4_GlyGly_10plex − Scan number: 6400
Gene Names | Charge | m/z | Mass | Retention time | PEP | Score | Precursor Intensity
PGAM5 | 3 | 330.1785 | 987.5138 | 21.164 | 0.10128 | 55.717 | 5121699
Intensity (%)
100
50
0
−50
0
500
1000
m/z
y1 376.2757
y2 447.313
b2 458.2814
b3−H2O 577.33
b3 595.3402
y3 689.4512
b4 758.4019
y4 852.5152
L
D
H
Y
K
A
K
y4
y3
y2
y1
b2
b3
b4
Modification
Acetyl (Protein N−term)
GlyGly (K) no new terminus
Oxidation (M)

20200924_KIZ_MUb5_GlyGly_1 − Scan number: 8911

Gene Names	Charge	m/z	Mass	Retention time	PEP	Score	Precursor Intensity
HSP90AB1;HSP90AB3P;HSP90AB2P	3	335.1855	1002.535	28.584	0.020761	71.223	16504527

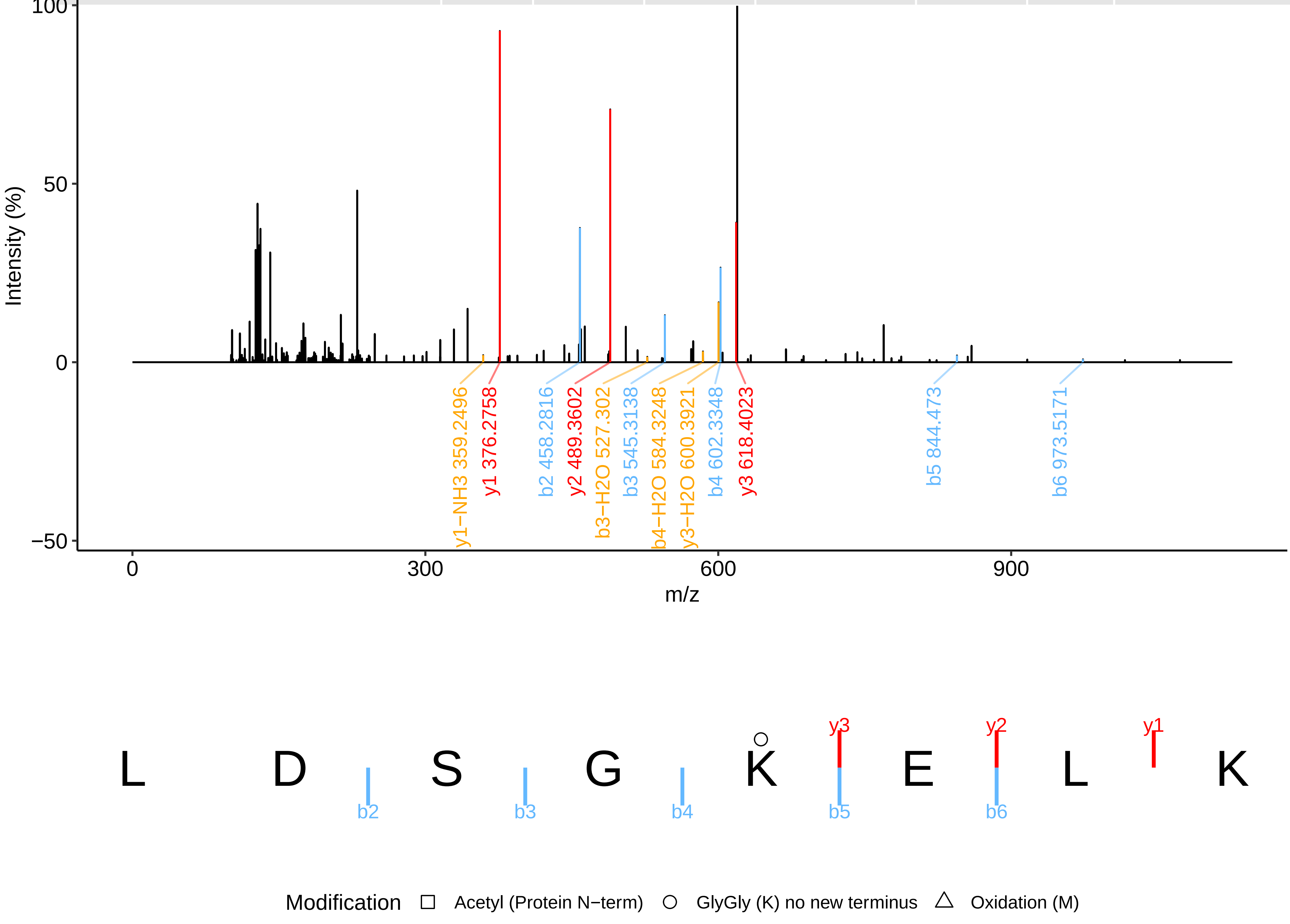

20200921_KIZ_MUb4_GlyGly_10plex_2 − Scan number: 15343

Gene Names	Charge	m/z	Mass	Retention time	PEP	Score	Precursor Intensity
GNAI3	3	411.8911	1232.651	59.982	0.010483	72.848	1392414

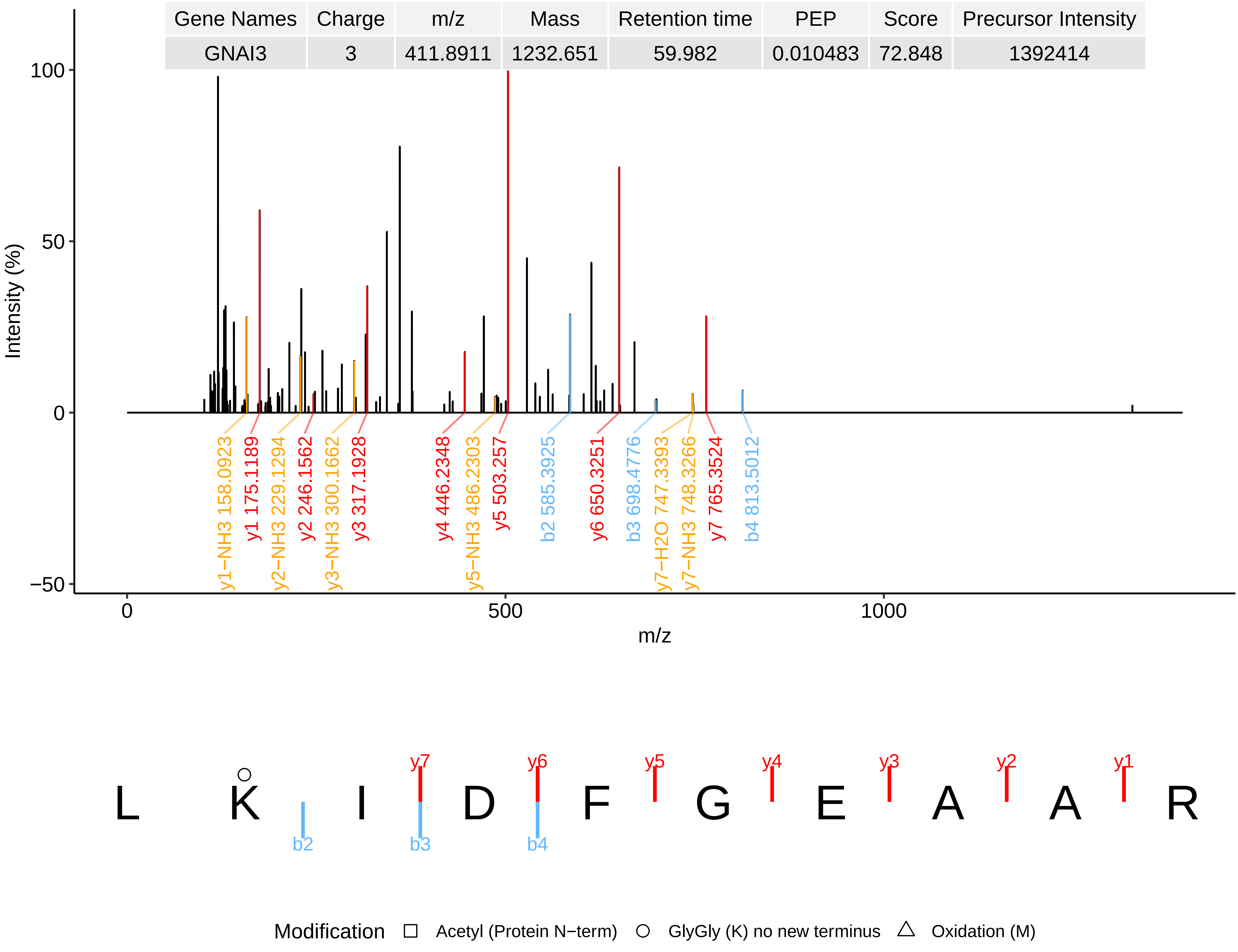

20201118_KIZ_MUb6_GlyGly_2 − Scan number: 17853

Gene Names	Charge	m/z	Mass	Retention time	PEP	Score	Precursor Intensity
GNAI3	3	411.8911	1232.651	60.374	0.022885	63.32	5309298

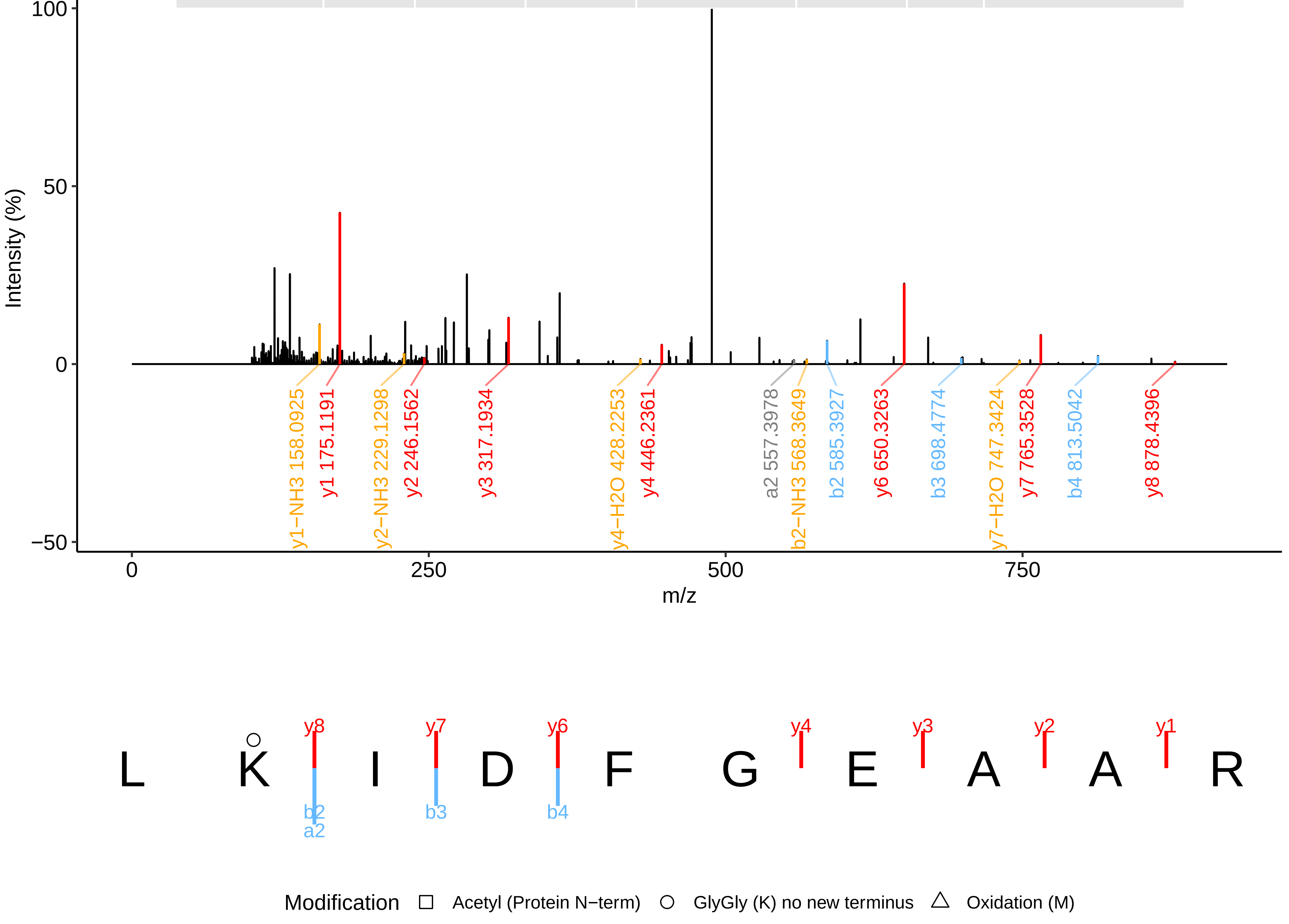

20200921_KIZ_MUb4_GlyGly_10plex − Scan number: 6680

Gene Names	Charge	m/z	Mass	Retention time	PEP	Score	Precursor Intensity
UBA1	3	406.2263	1215.657	22.2	0.013827	65.879	7749798

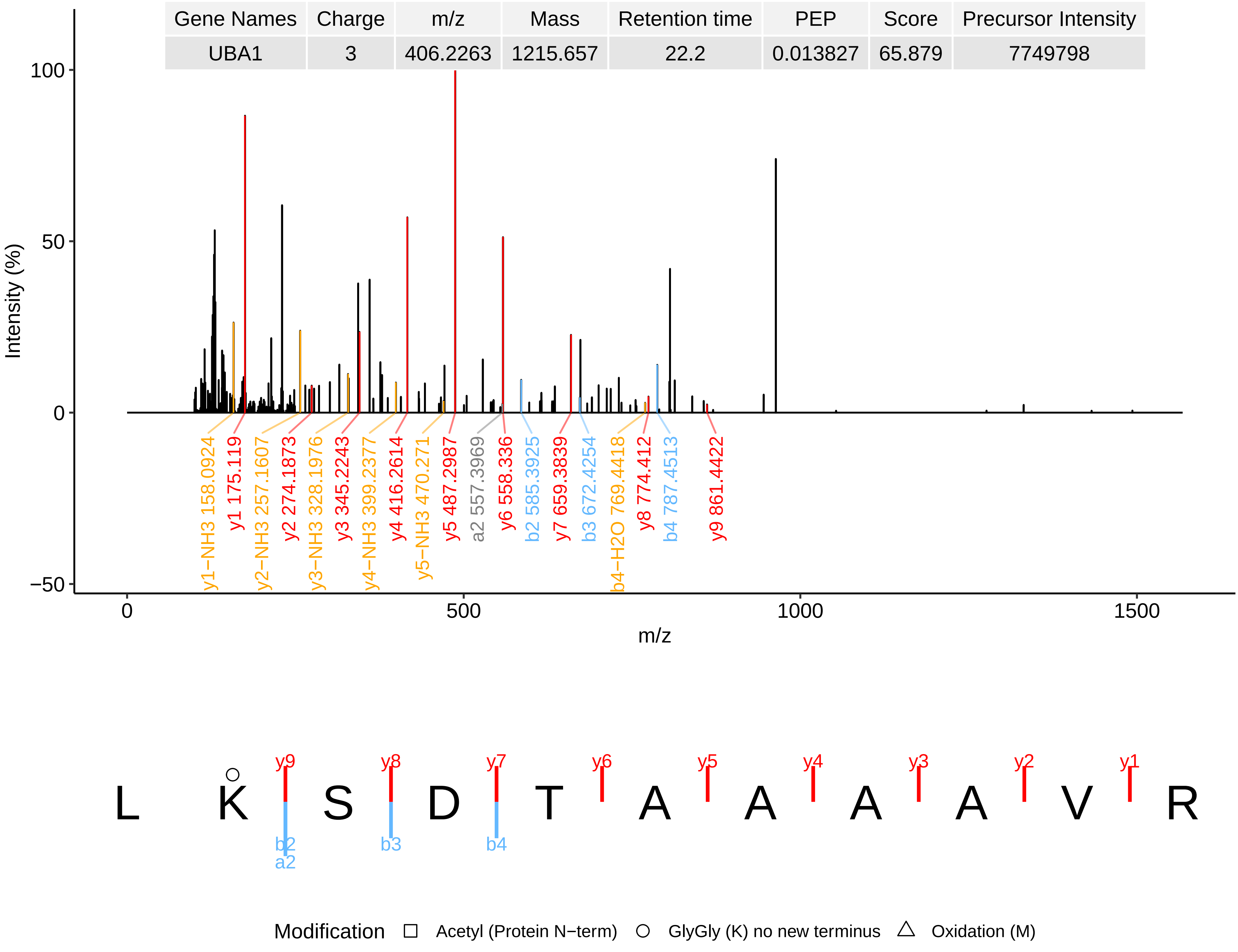

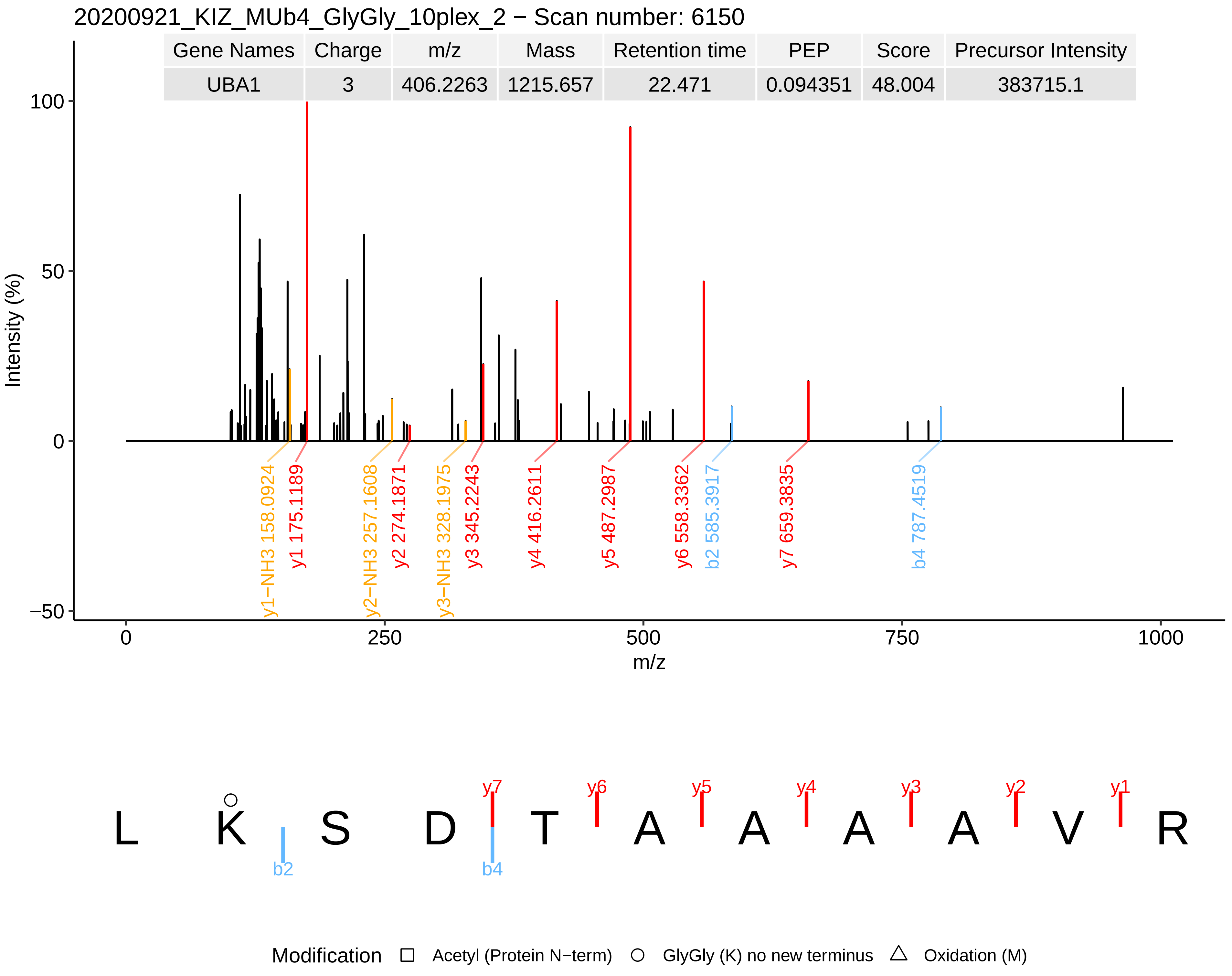
20200921_KIZ_MUb4_GlyGly_10plex_2 − Scan number: 6150
Gene Names
Charge
m/z
Mass
Retention time
PEP
Score
Precursor Intensity
UBA1
3
406.2263
1215.657
22.471
0.094351
48.004
383715.1
Intensity (%)
100
50
0
−50
0
250
500
750
1000
m/z
y1−NH3 158.0924
y1 175.1189
y2−NH3 257.1608
y2 274.1871
y3−NH3 328.1975
y3 345.2243
y4 416.2611
y5 487.2987
y6 558.3362
b2 585.3917
y7 659.3835
b4 787.4519
L K S D T A A A A V R
y7
y6
y5
y4
y3
y2
y1
b2
b4
Modification
Acetyl (Protein N−term)
GlyGly (K) no new terminus
Oxidation (M)

20200921_KIZ_MUb4_GlyGly_10plex − Scan number: 10721

Gene Names	Charge	m/z	Mass	Retention time	PEP	Score	Precursor Intensity
PTRF	4	250.6632	998.6237	37.186	0.042647	99.919	4298299

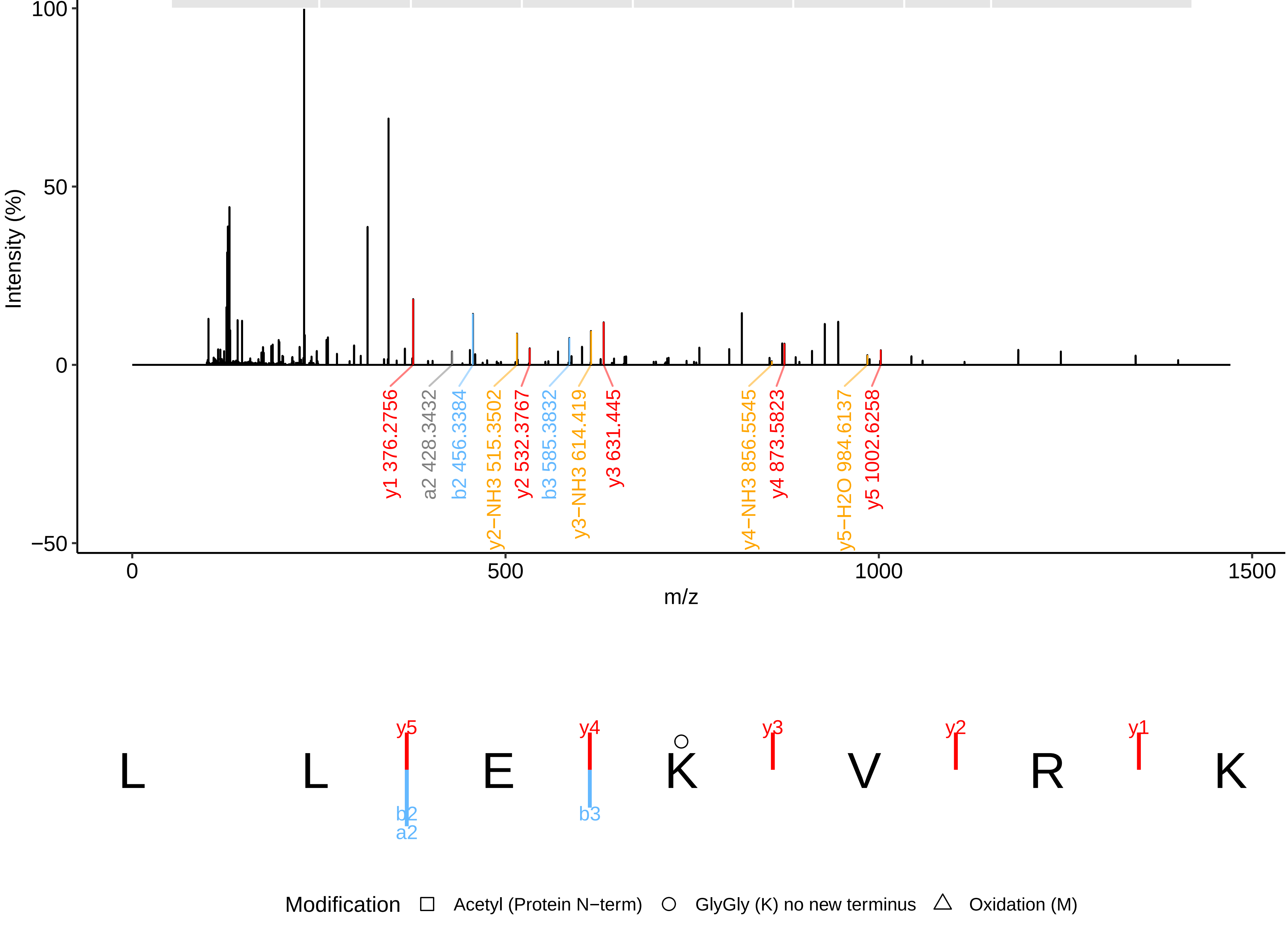

20200921_KIZ_MUb4_GlyGly_10plex − Scan number: 10739

Gene Names	Charge	m/z	Mass	Retention time	PEP	Score	Precursor Intensity
PTRF	3	333.8818	998.6237	37.254	0.026698	84.244	23891690

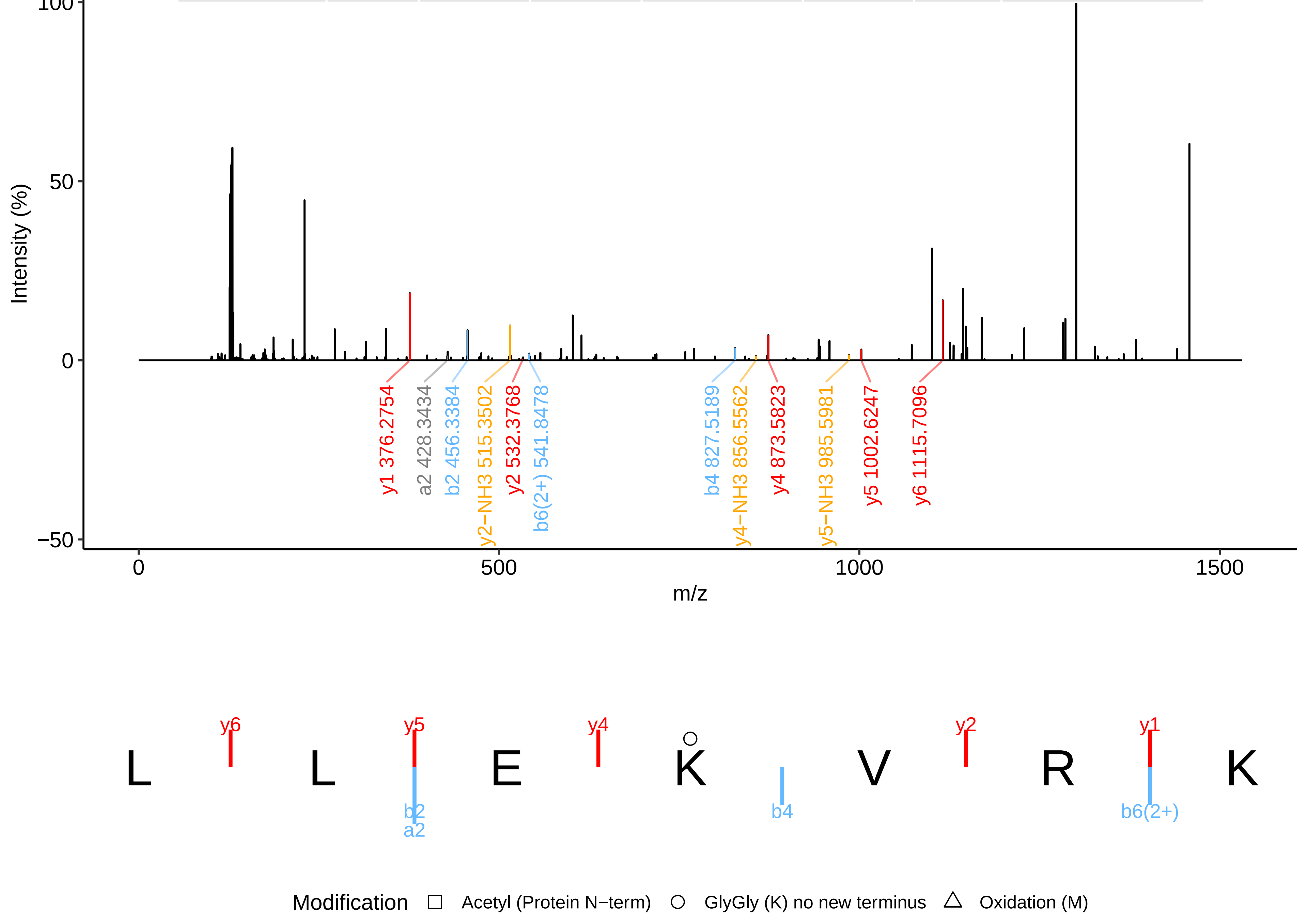

20200921_KIZ_MUb4_GlyGly_10plex_2 − Scan number: 10002

Gene Names	Charge	m/z	Mass	Retention time	PEP	Score	Precursor Intensity
PTRF	4	250.6632	998.6237	38.147	0.085172	89.673	1194499

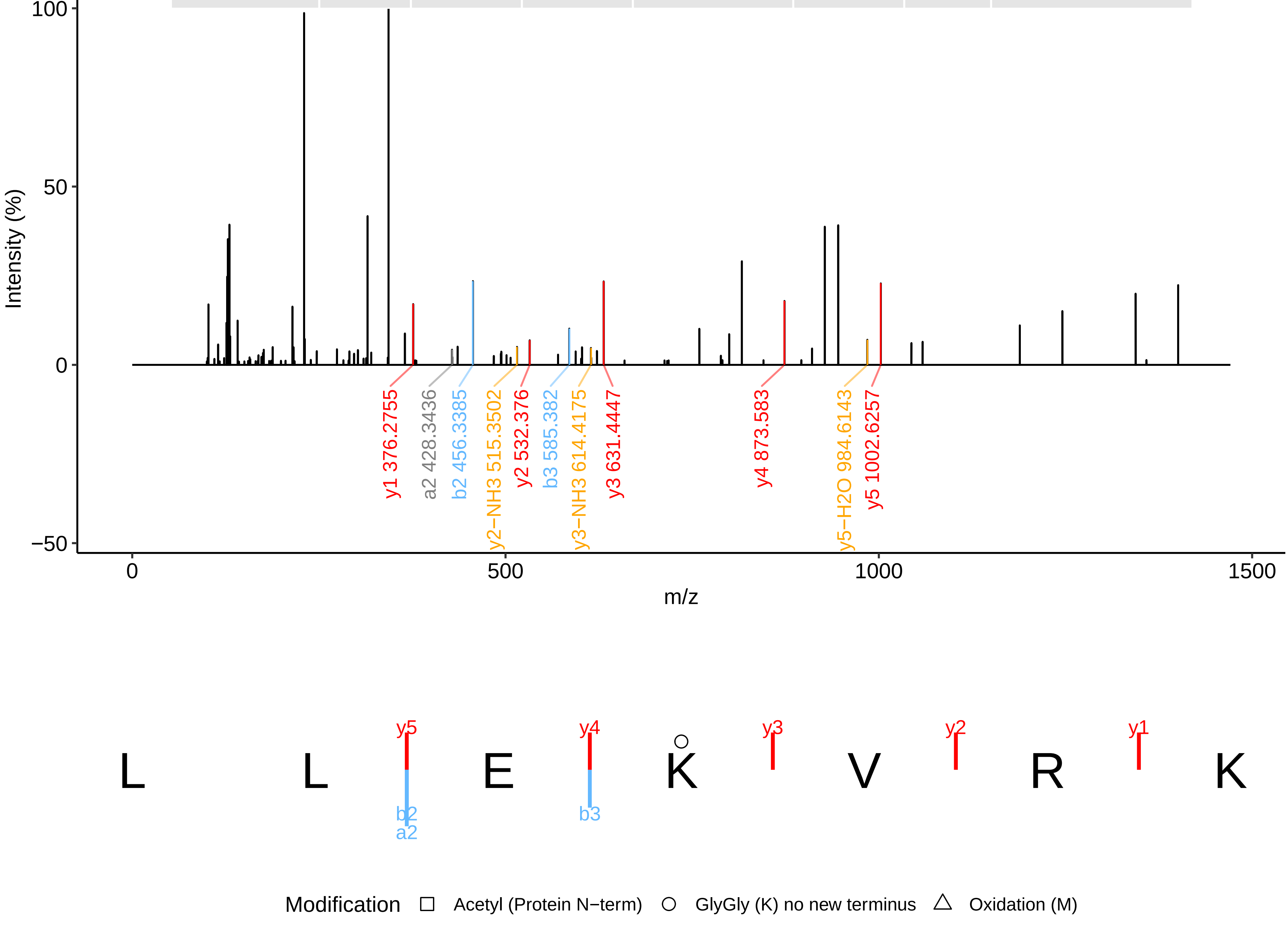

20200921_KIZ_MUb4_GlyGly_10plex_2 − Scan number: 10031

Gene Names	Charge	m/z	Mass	Retention time	PEP	Score	Precursor Intensity
PTRF	3	333.8818	998.6237	38.269	0.091933	65.252	7723958

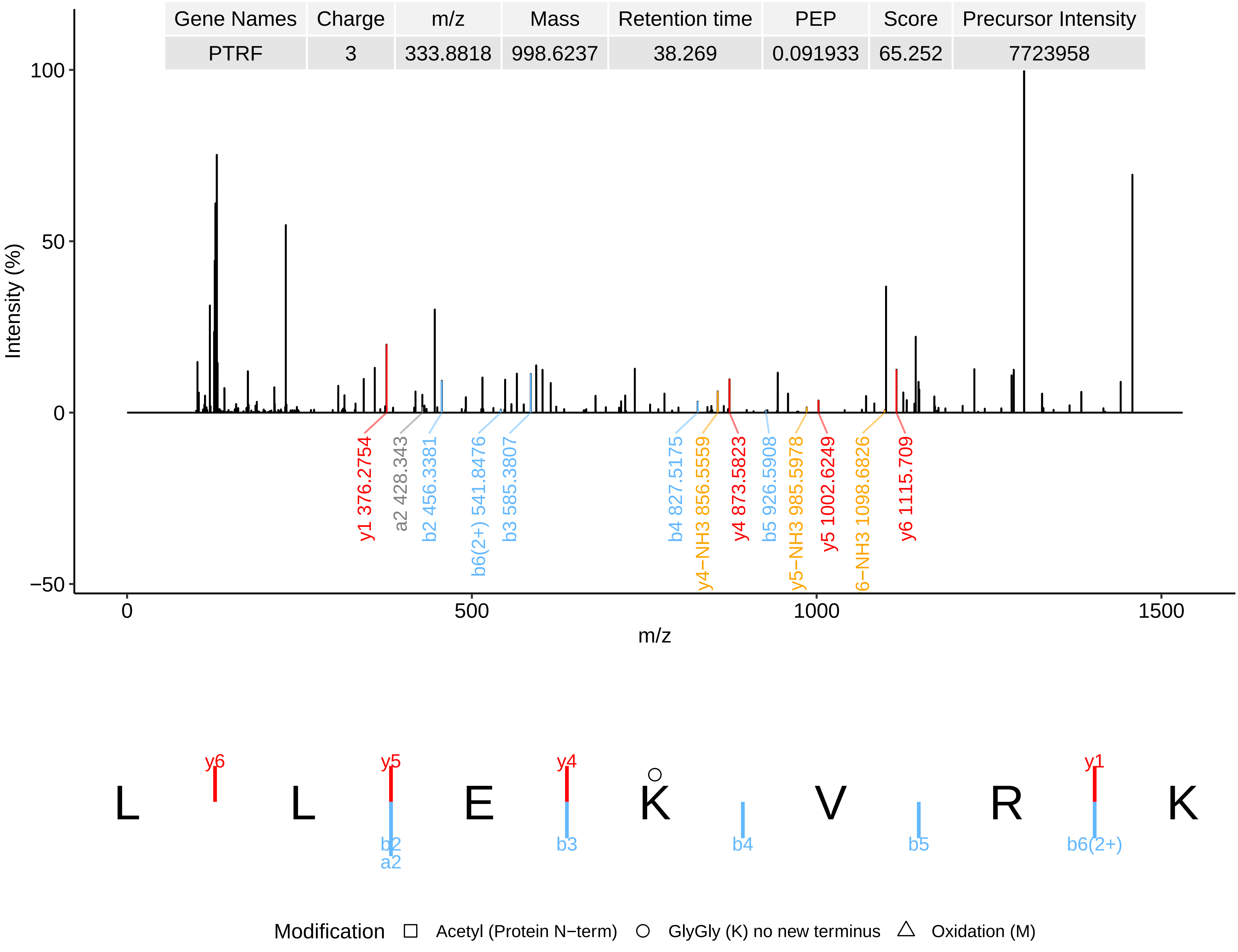

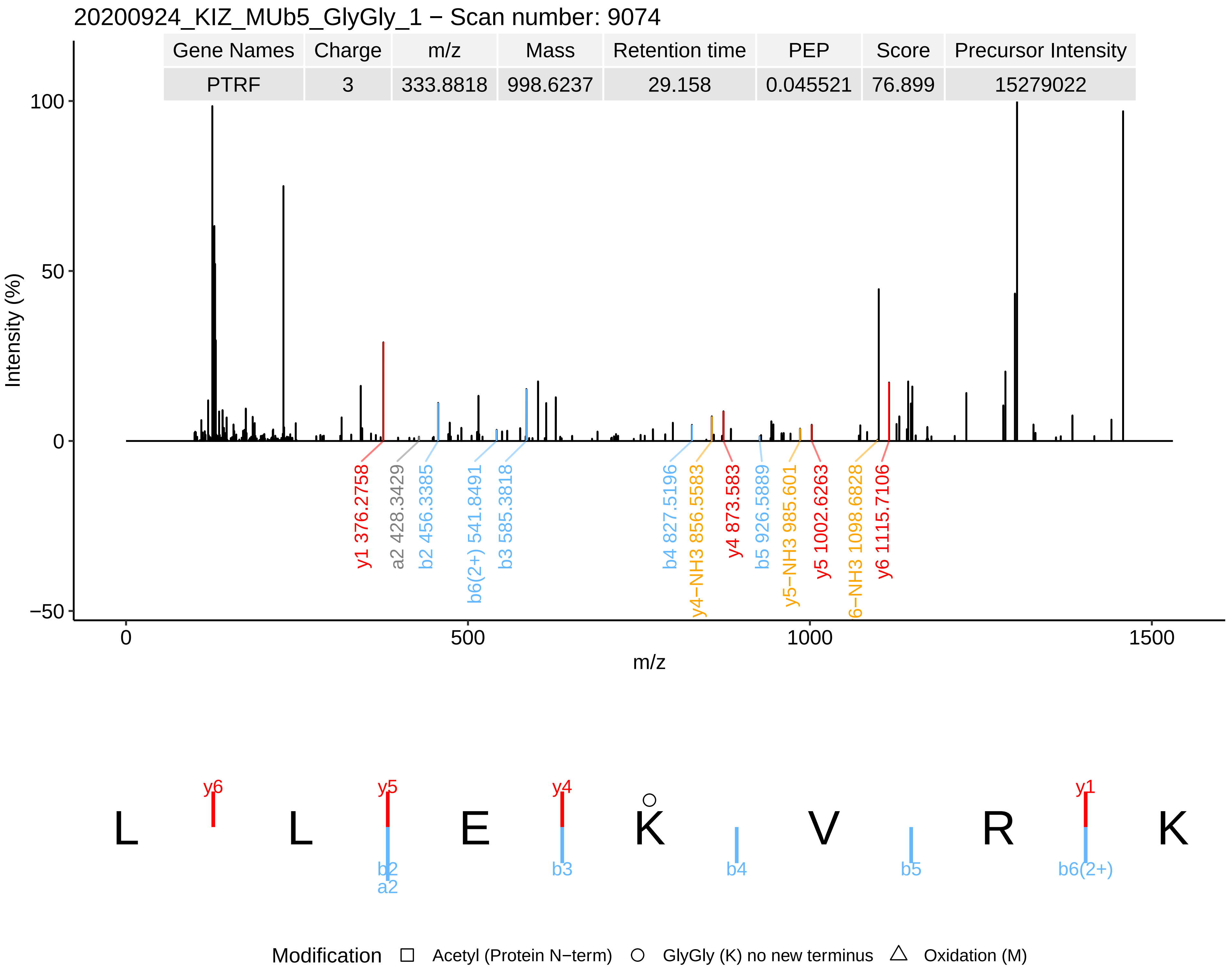
20200924_KIZ_MUb5_GlyGly_1 − Scan number: 9074
Gene Names | Charge | m/z | Mass | Retention time | PEP | Score | Precursor Intensity
PTRF | 3 | 333.8818 | 998.6237 | 29.158 | 0.045521 | 76.899 | 15279022
Intensity (%)
m/z
y1 376.2758
a2 428.3429
b2 456.3385
b6(2+) 541.8491
b3 585.3818
b4 827.5196
y4−NH3 856.5583
y4 873.583
b5 926.5889
y5−NH3 985.601
y5 1002.6263
6−NH3 1098.6828
y6 1115.7106
L L E K V R K
y6 y5 y4 y1
b2 a2 b3 b4 b5 b6(2+)
Modification
Acetyl (Protein N−term)
GlyGly (K) no new terminus
Oxidation (M)

20200924_KIZ_MUb5_GlyGly_1 − Scan number: 9229

Gene Names	Charge	m/z	Mass	Retention time	PEP	Score	Precursor Intensity
PTRF	3	333.8818	998.6237	29.657	0.023637	86.953	48410592

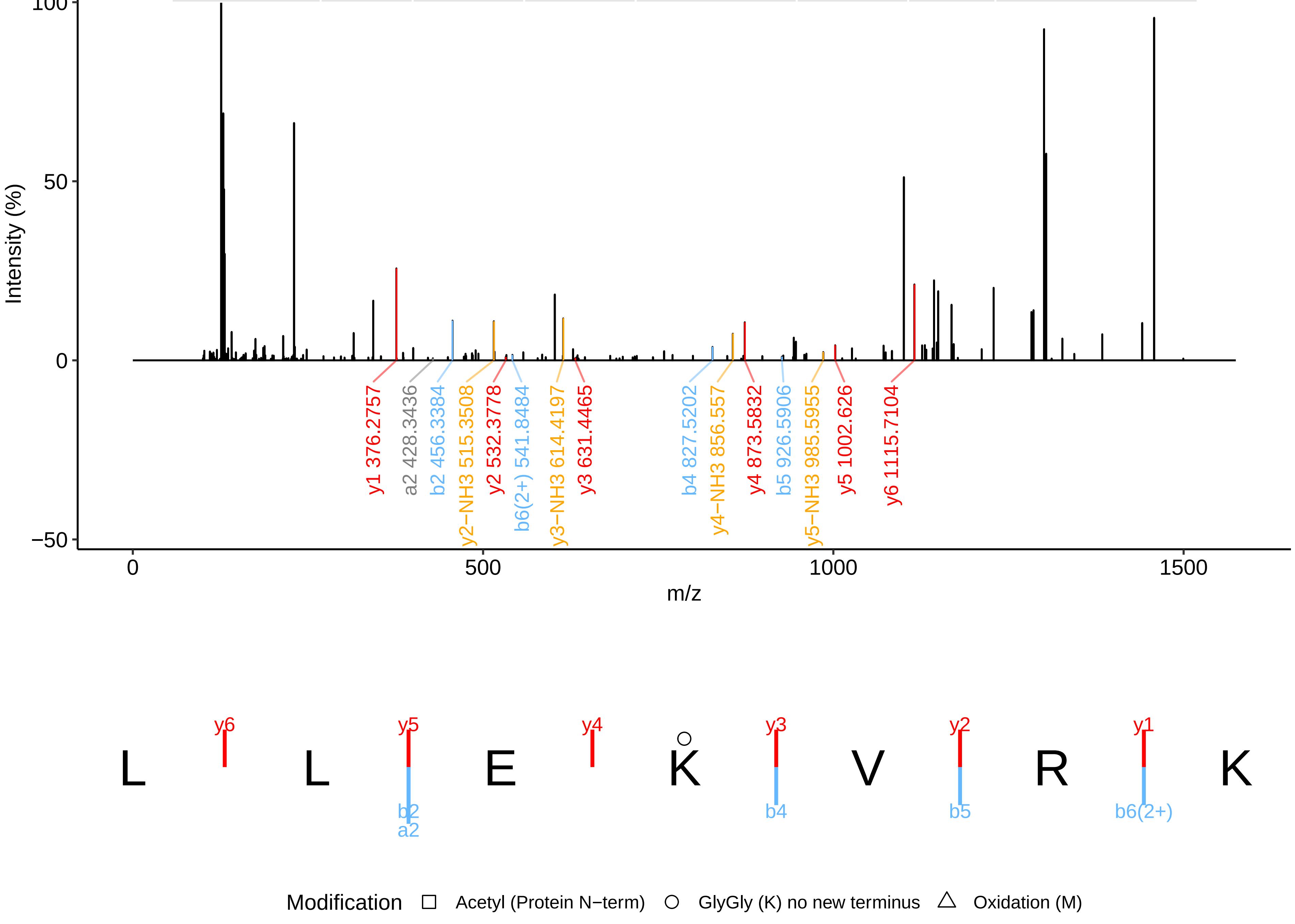

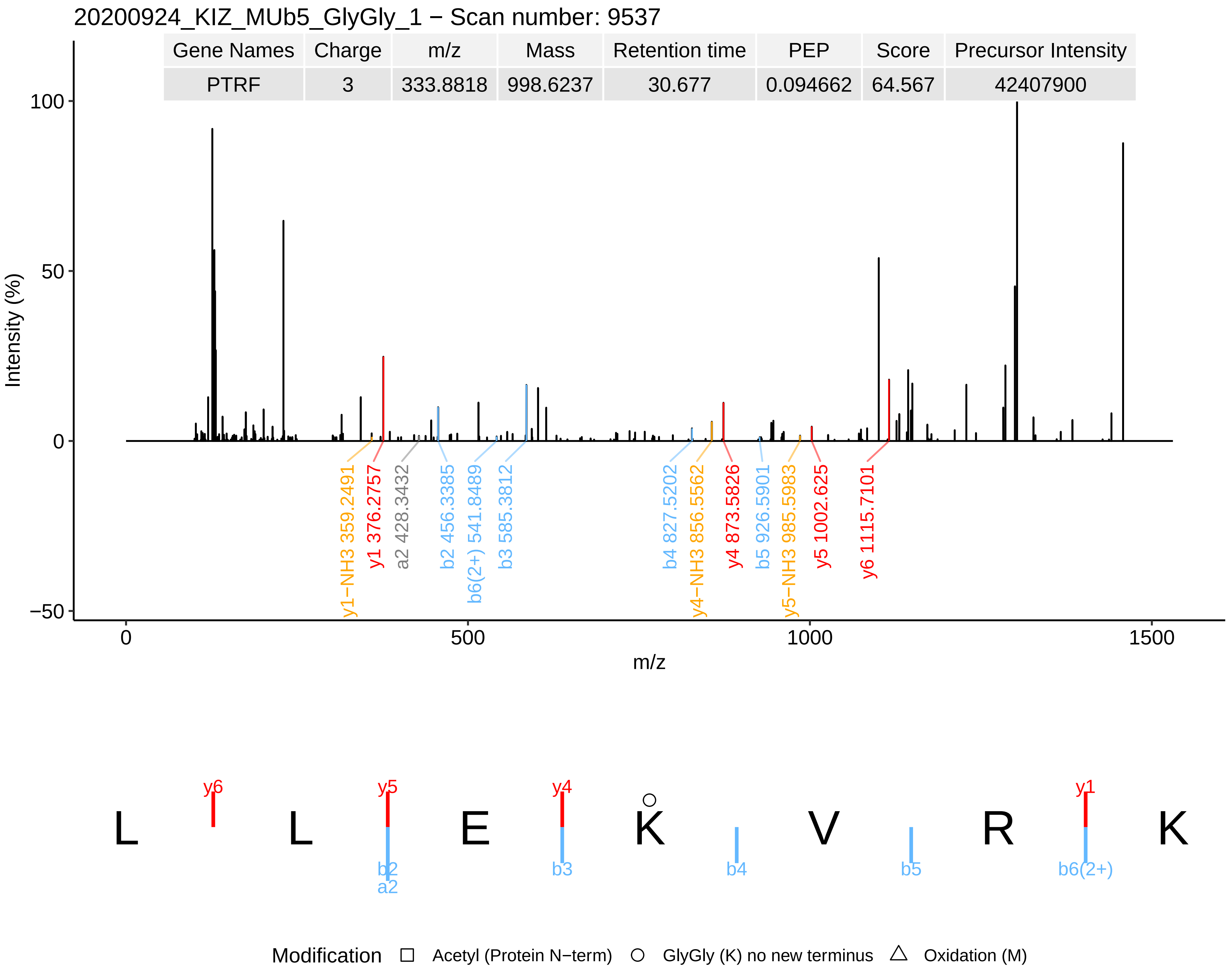
20200924_KIZ_MUb5_GlyGly_1 − Scan number: 9537
Gene Names
Charge
m/z
Mass
Retention time
PEP
Score
Precursor Intensity
PTRF
3
333.8818
998.6237
30.677
0.094662
64.567
42407900
Intensity (%)
100
50
0
−50
0
500
1000
1500
m/z
y1−NH3 359.2491
y1 376.2757
a2 428.3432
b2 456.3385
b6(2+) 541.8489
b3 585.3812
b4 827.5202
y4−NH3 856.5562
y4 873.5826
b5 926.5901
y5−NH3 985.5983
y5 1002.625
y6 1115.7101
L
L
E
K
V
R
K
y6
y5
y4
y1
b2
a2
b3
b4
b5
b6(2+)
Modification
Acetyl (Protein N−term)
GlyGly (K) no new terminus
Oxidation (M)

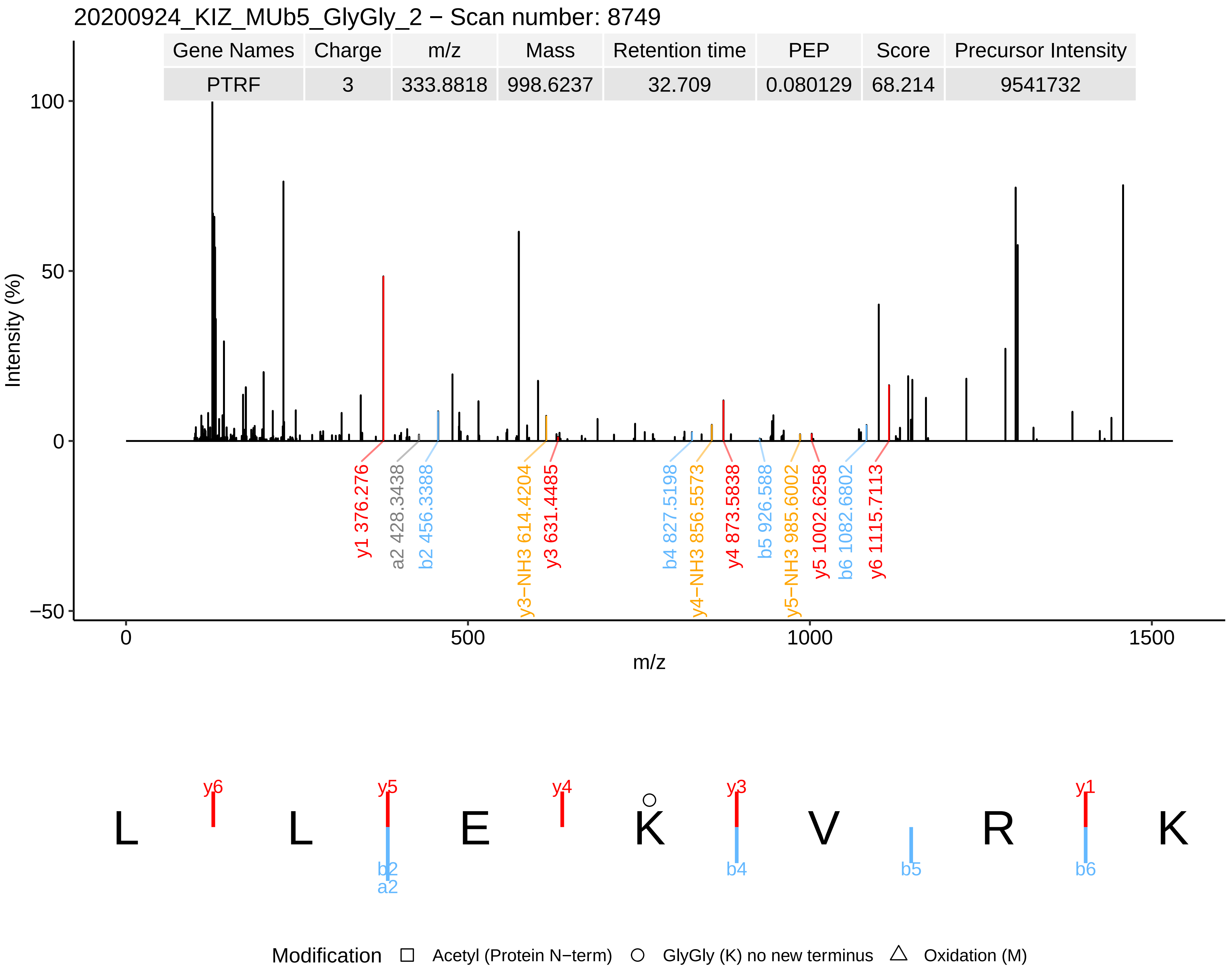
20200924_KIZ_MUb5_GlyGly_2 − Scan number: 8749
Gene Names | Charge | m/z | Mass | Retention time | PEP | Score | Precursor Intensity
PTRF | 3 | 333.8818 | 998.6237 | 32.709 | 0.080129 | 68.214 | 9541732
Intensity (%)
100
50
0
−50
y1 376.276
a2 428.3438
b2 456.3388
y3−NH3 614.4204
y3 631.4485
b4 827.5198
y4−NH3 856.5573
y4 873.5838
b5 926.588
y5−NH3 985.6002
y5 1002.6258
b6 1082.6802
y6 1115.7113
0
500
1000
1500
m/z
L L E K V R K
y6 y5 y4 y3 y1
b2 a2 b4 b5 b6
Modification
Acetyl (Protein N−term)
GlyGly (K) no new terminus
Oxidation (M)

20200924_KIZ_MUb5_GlyGly_2 − Scan number: 8884

Gene Names	Charge	m/z	Mass	Retention time	PEP	Score	Precursor Intensity
PTRF	3	333.8818	998.6237	33.213	0.089841	65.777	8660436

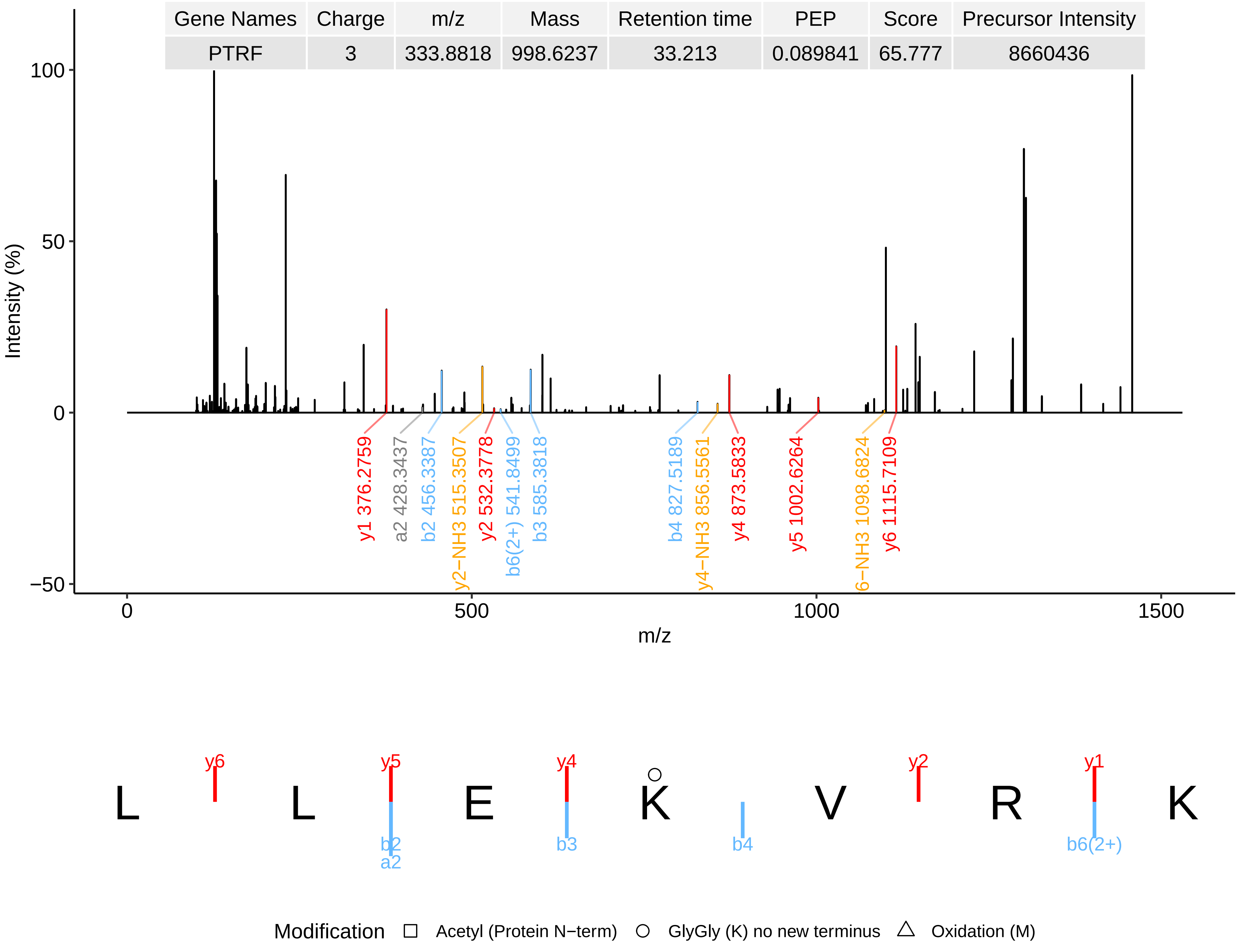

20200924_KIZ_MUb5_GlyGly_2 − Scan number: 9022

Gene Names	Charge	m/z	Mass	Retention time	PEP	Score	Precursor Intensity
PTRF	3	333.8818	998.6237	33.72	0.026698	89.046	11409369

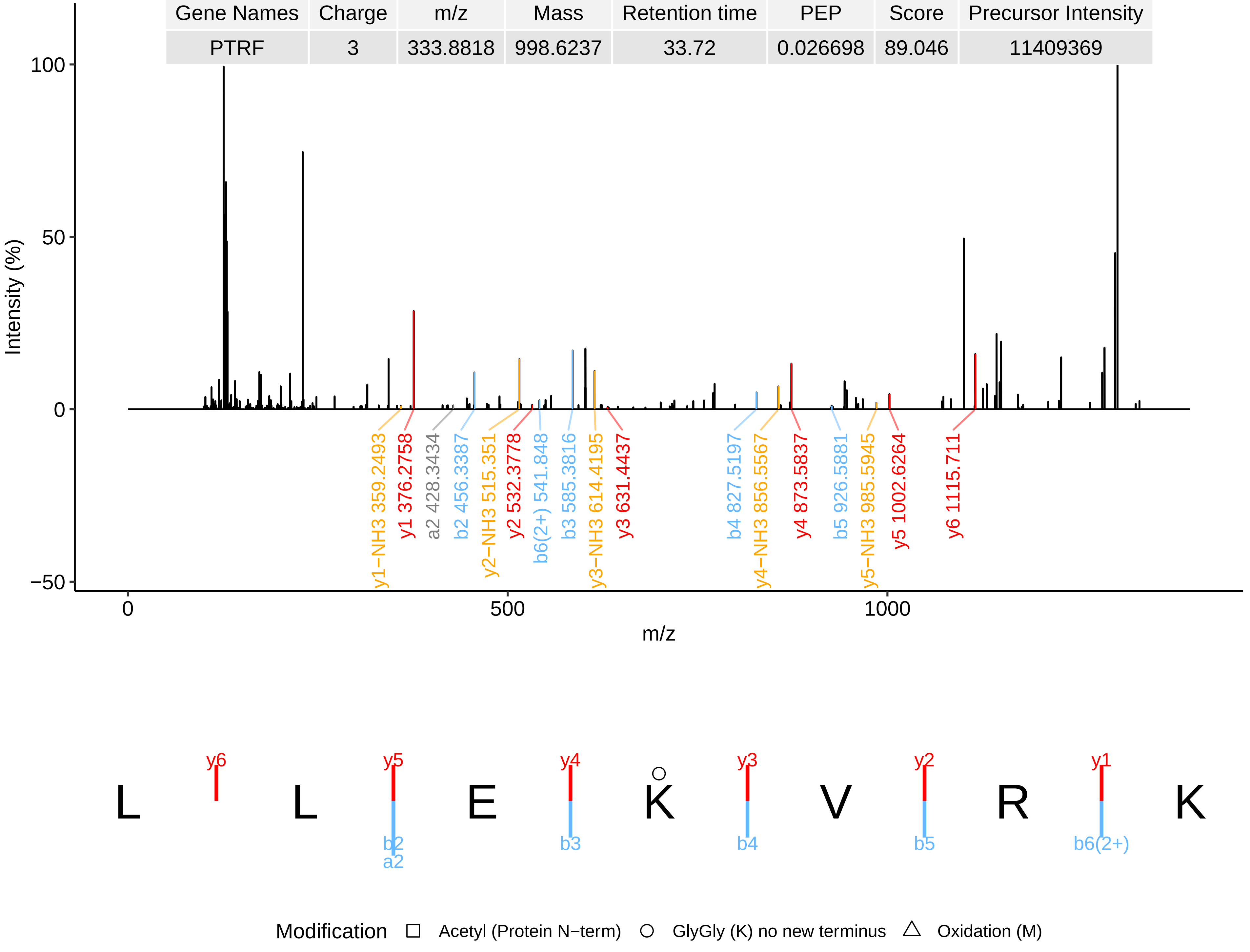

20201118_KIZ_MUb6_GlyGly_1 − Scan number: 9124

Gene Names	Charge	m/z	Mass	Retention time	PEP	Score	Precursor Intensity
PTRF	3	333.8818	998.6237	28.879	0.049684	75.854	36983024

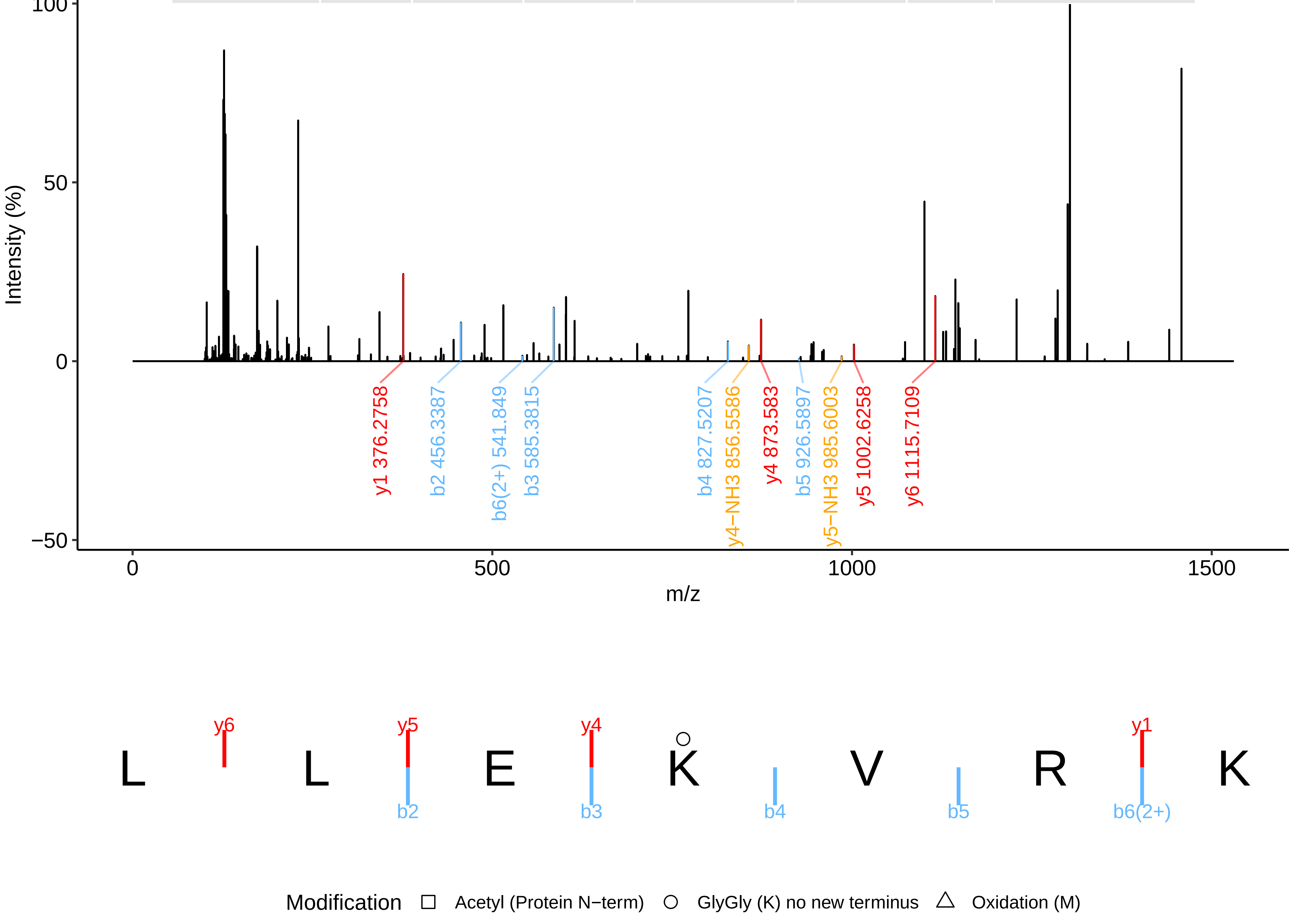

20201118_KIZ_MUb6_GlyGly_2 − Scan number: 12189

Gene Names	Charge	m/z	Mass	Retention time	PEP	Score	Precursor Intensity
PTRF	3	333.8818	998.6237	41.014	0.0095216	101.64	8912523

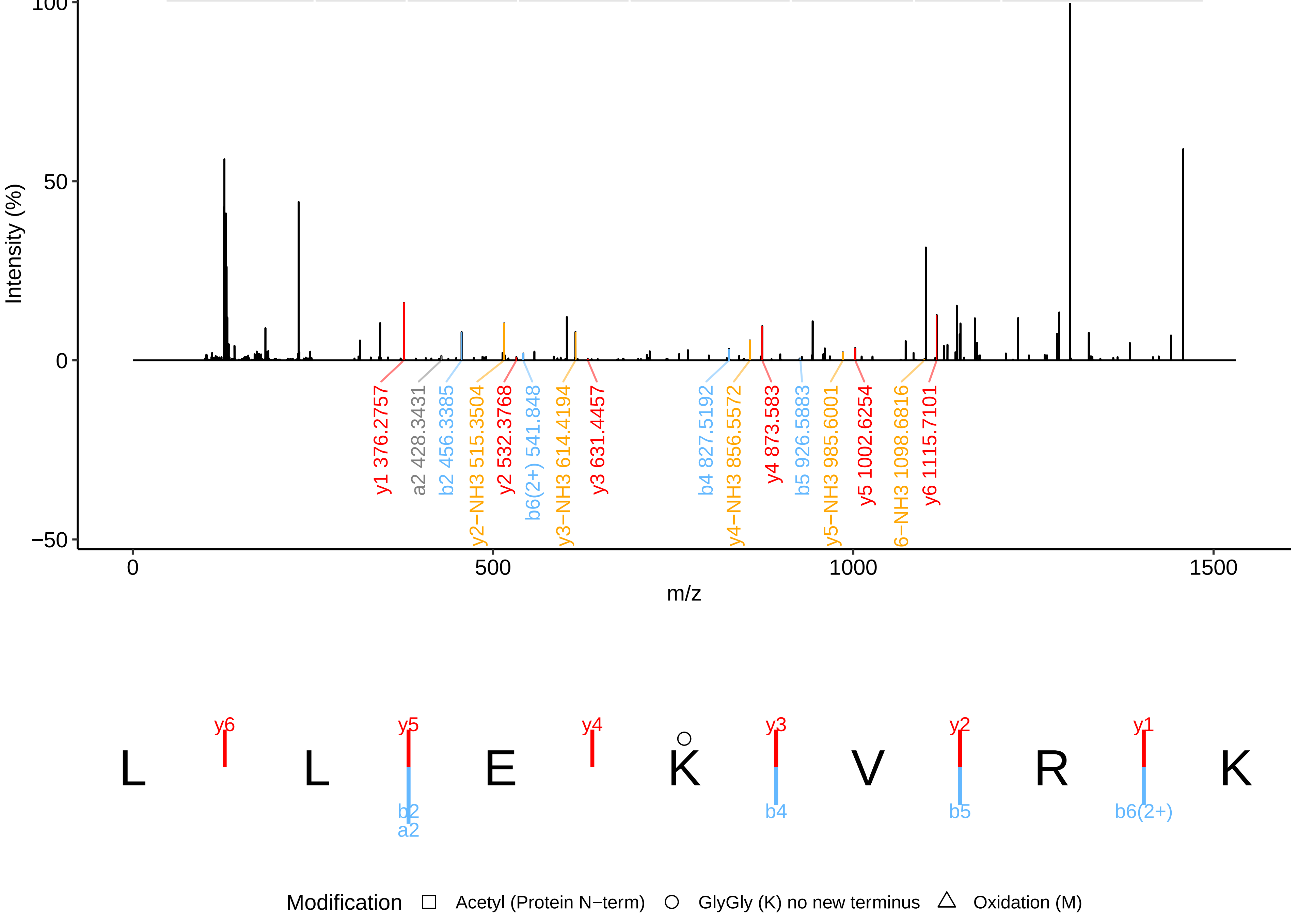

20200921_KIZ_MUb4_GlyGly_10plex − Scan number: 15655

Gene Names	Charge	m/z	Mass	Retention time	PEP	Score	Precursor Intensity
PRKDC	3	407.9153	1220.724	55.983	5.1071e−05	113.62	5412765

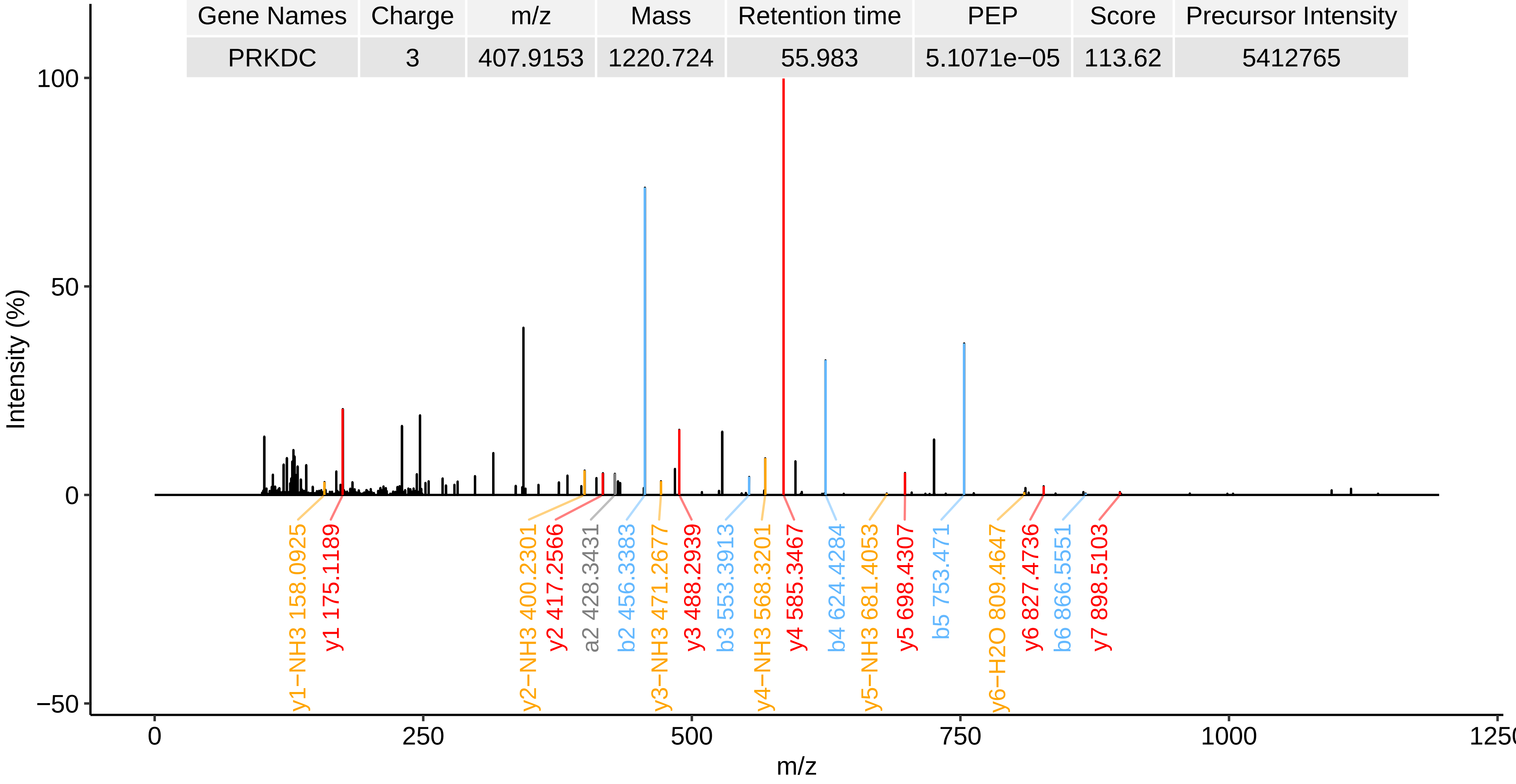

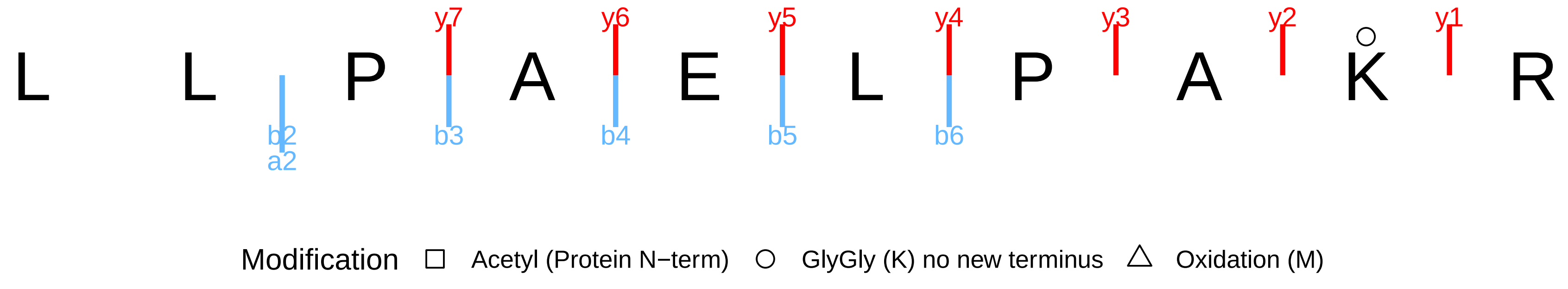

20200921_KIZ_MUb4_GlyGly_10plex − Scan number: 9717

Gene Names	Charge	m/z	Mass	Retention time	PEP	Score	Precursor Intensity
RPL4	3	403.2133	1206.618	33.37	7.968e−06	126.31	8314618

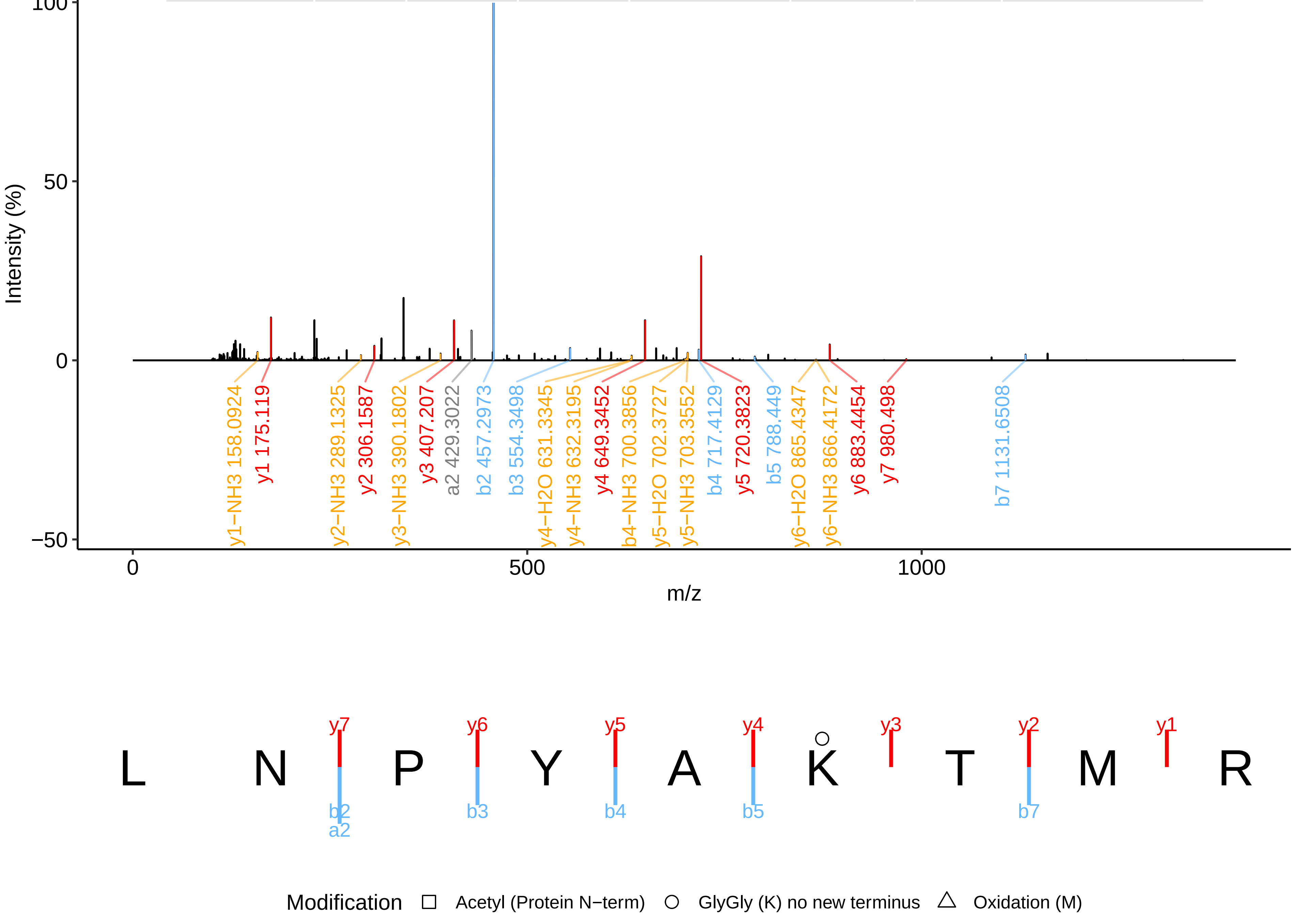

20200921_KIZ_MUb4_GlyGly_10plex_2 − Scan number: 8973

Gene Names	Charge	m/z	Mass	Retention time	PEP	Score	Precursor Intensity
RPL4	3	403.2133	1206.618	33.978	0.015983	68.893	1524397

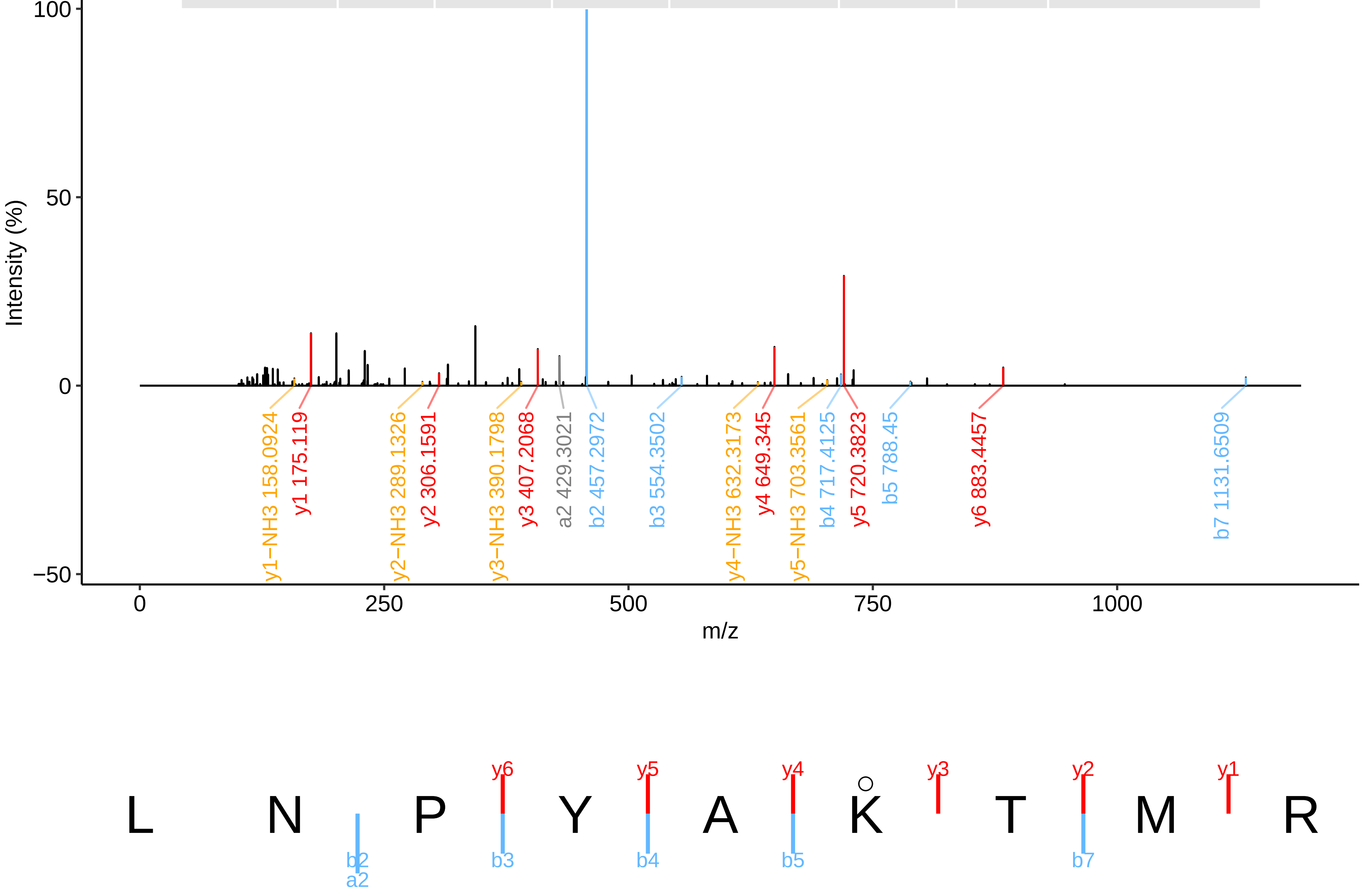

Modification □ Acetyl (Protein N−term) ○ GlyGly (K) no new terminus △ Oxidation (M)

20200924_KIZ_MUb5_GlyGly_1 − Scan number: 8523

Gene Names	Charge	m/z	Mass	Retention time	PEP	Score	Precursor Intensity
RPL4	3	403.2133	1206.618	27.258	0.026423	62.271	9799600

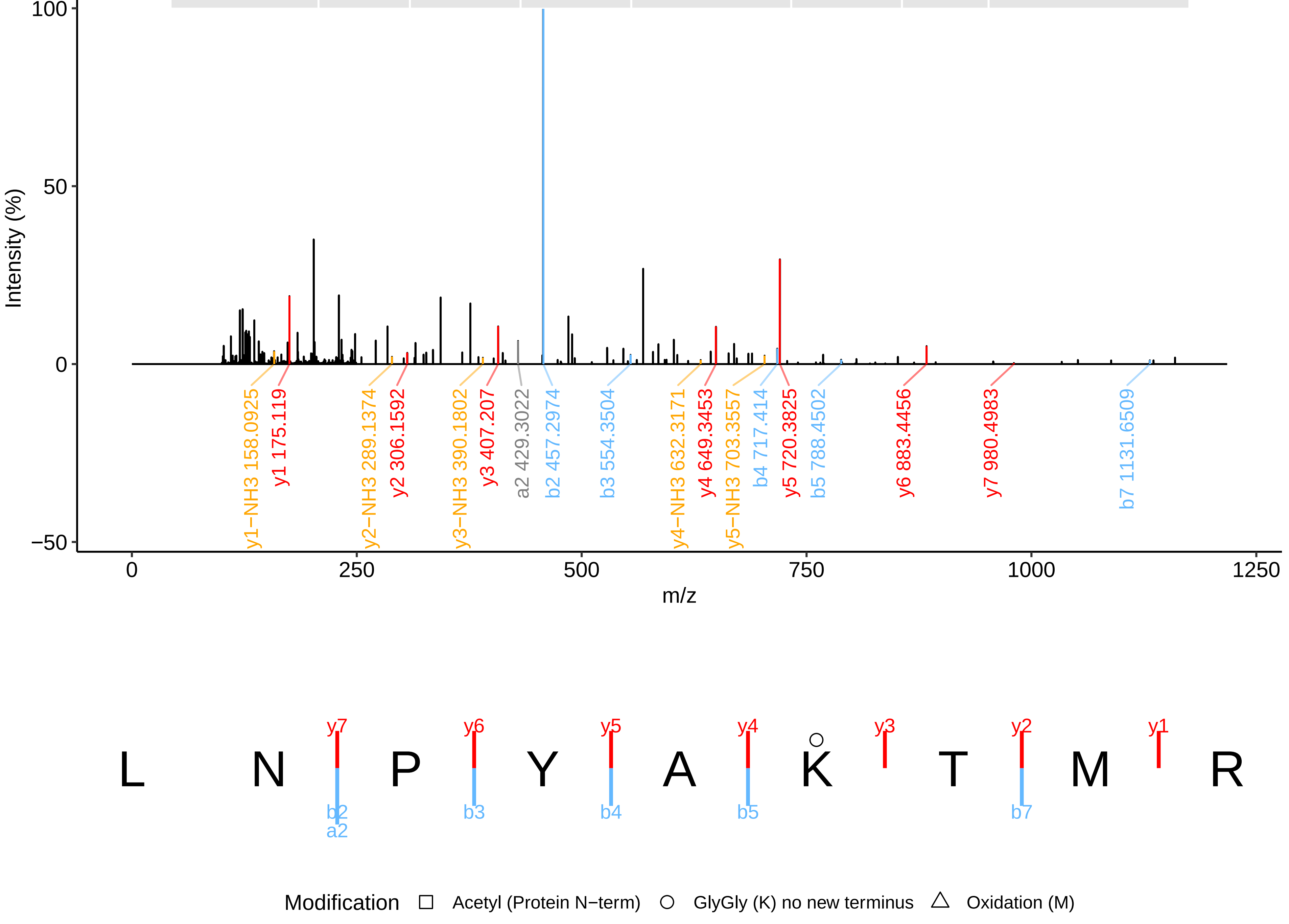

20200921_KIZ_MUb4_GlyGly_10plex − Scan number: 14529

Gene Names	Charge	m/z	Mass	Retention time	PEP	Score	Precursor Intensity
PTRF	3	330.5378	988.5917	51.671	0.0056631	89.296	6754729

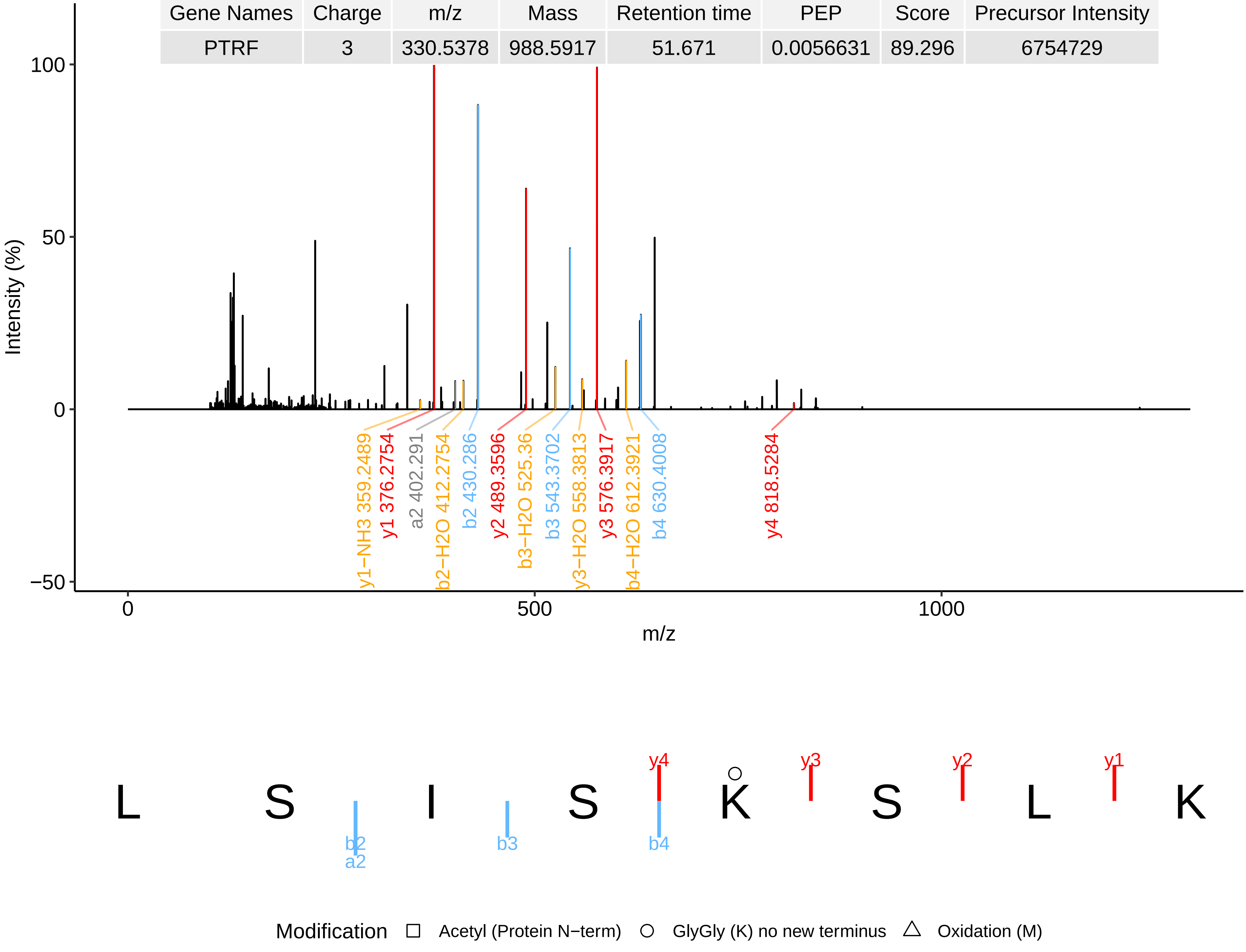

20200921_KIZ_MUb4_GlyGly_10plex_2 − Scan number: 13429

Gene Names	Charge	m/z	Mass	Retention time	PEP	Score	Precursor Intensity
PTRF	3	330.5378	988.5917	52.139	0.029052	64.567	457647.7

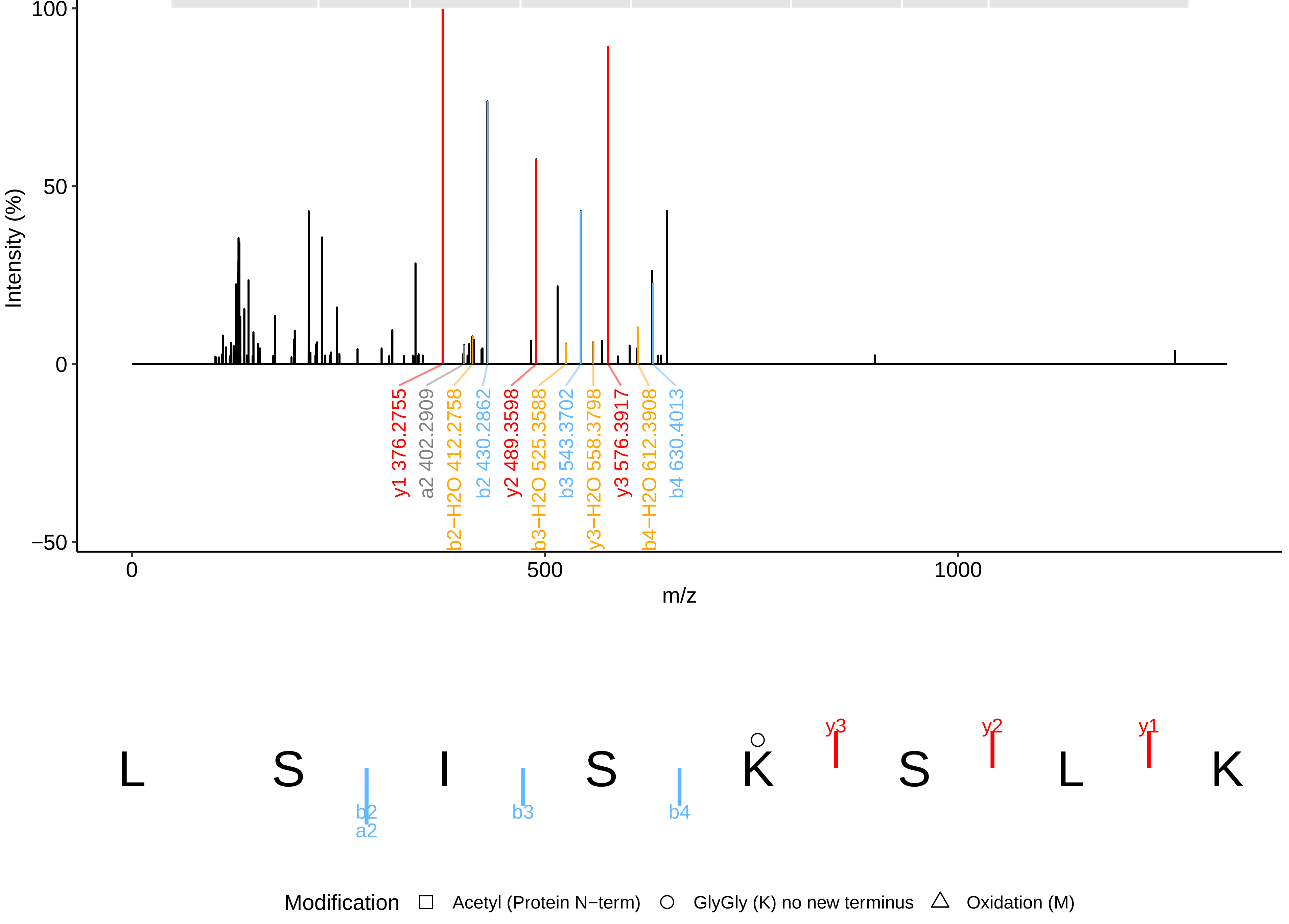

20200924_KIZ_MUb5_GlyGly_2 − Scan number: 12557

Gene Names	Charge	m/z	Mass	Retention time	PEP	Score	Precursor Intensity
PTRF	3	330.5378	988.5917	46.942	0.030361	68.214	3616172

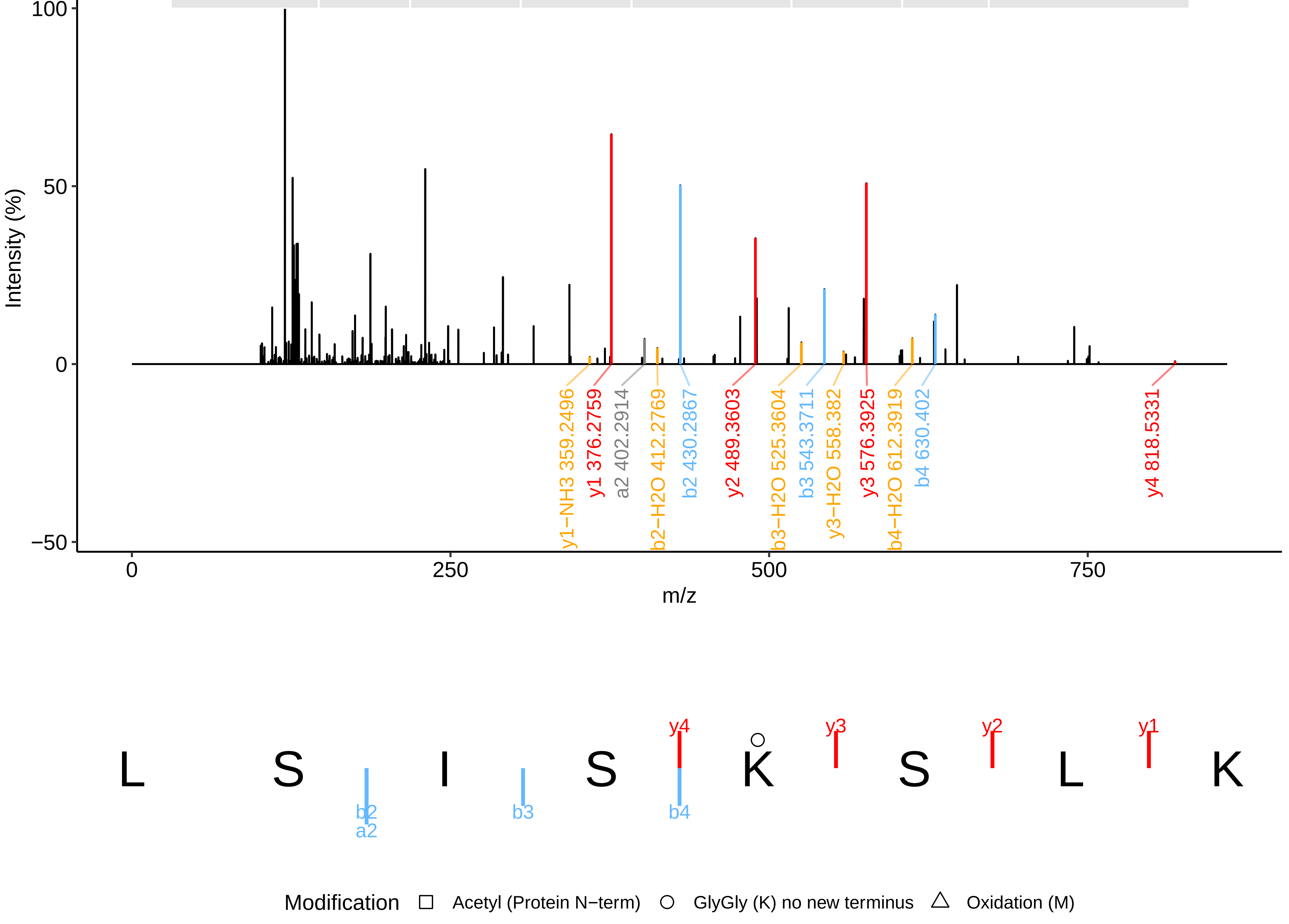

20201118_KIZ_MUb6_GlyGly_2 − Scan number: 15865

Gene Names	Charge	m/z	Mass	Retention time	PEP	Score	Precursor Intensity
PTRF	3	330.5378	988.5917	53.497	0.024509	68.214	5646864

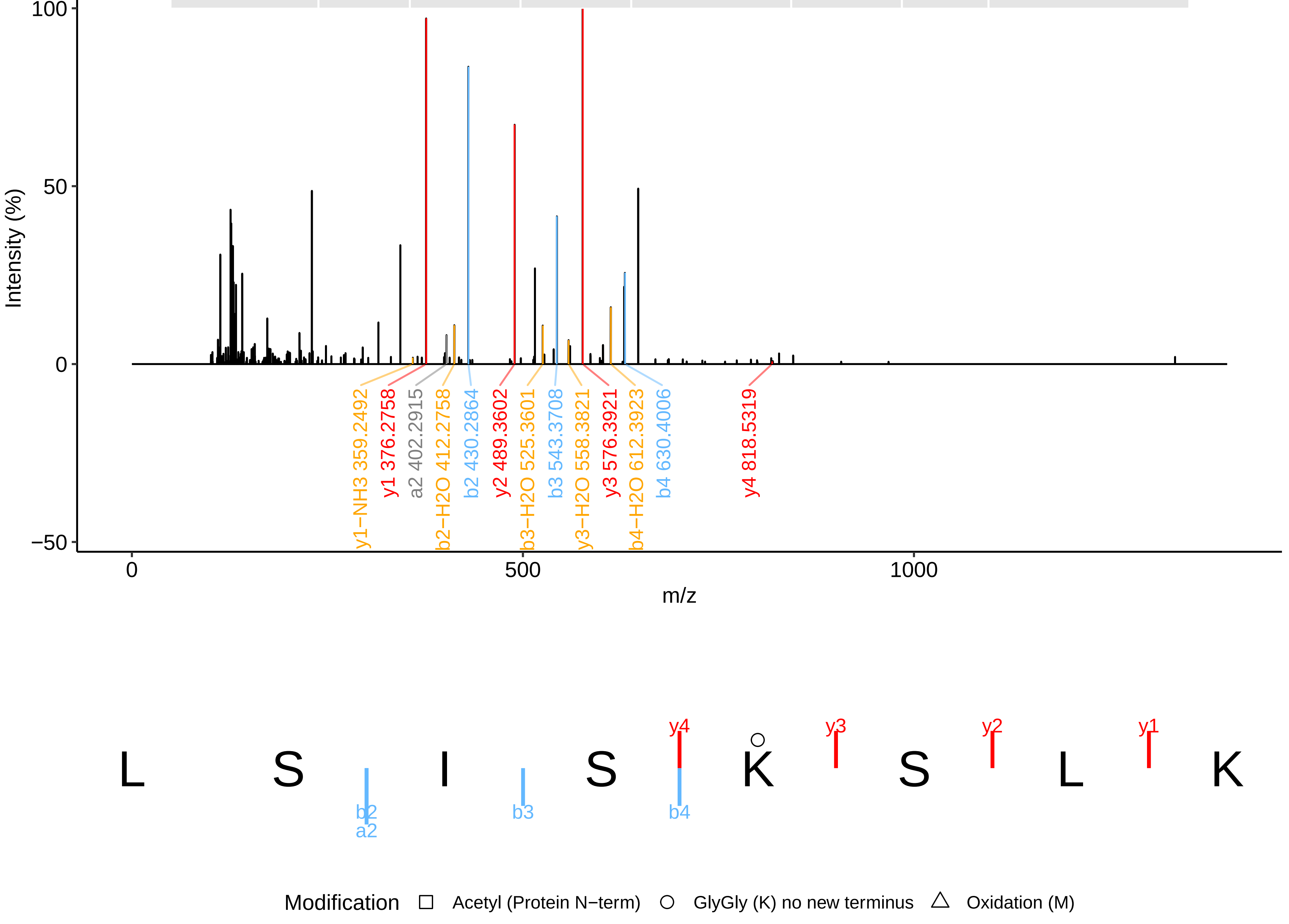

20200921_KIZ_MUb4_GlyGly_10plex − Scan number: 11473

Gene Names	Charge	m/z	Mass	Retention time	PEP	Score	Precursor Intensity
TUBA1C;TUBA1A;TUBA1B;TUBA4A	3	341.8538	1022.54	39.988	0.0041169	111.82	3795744

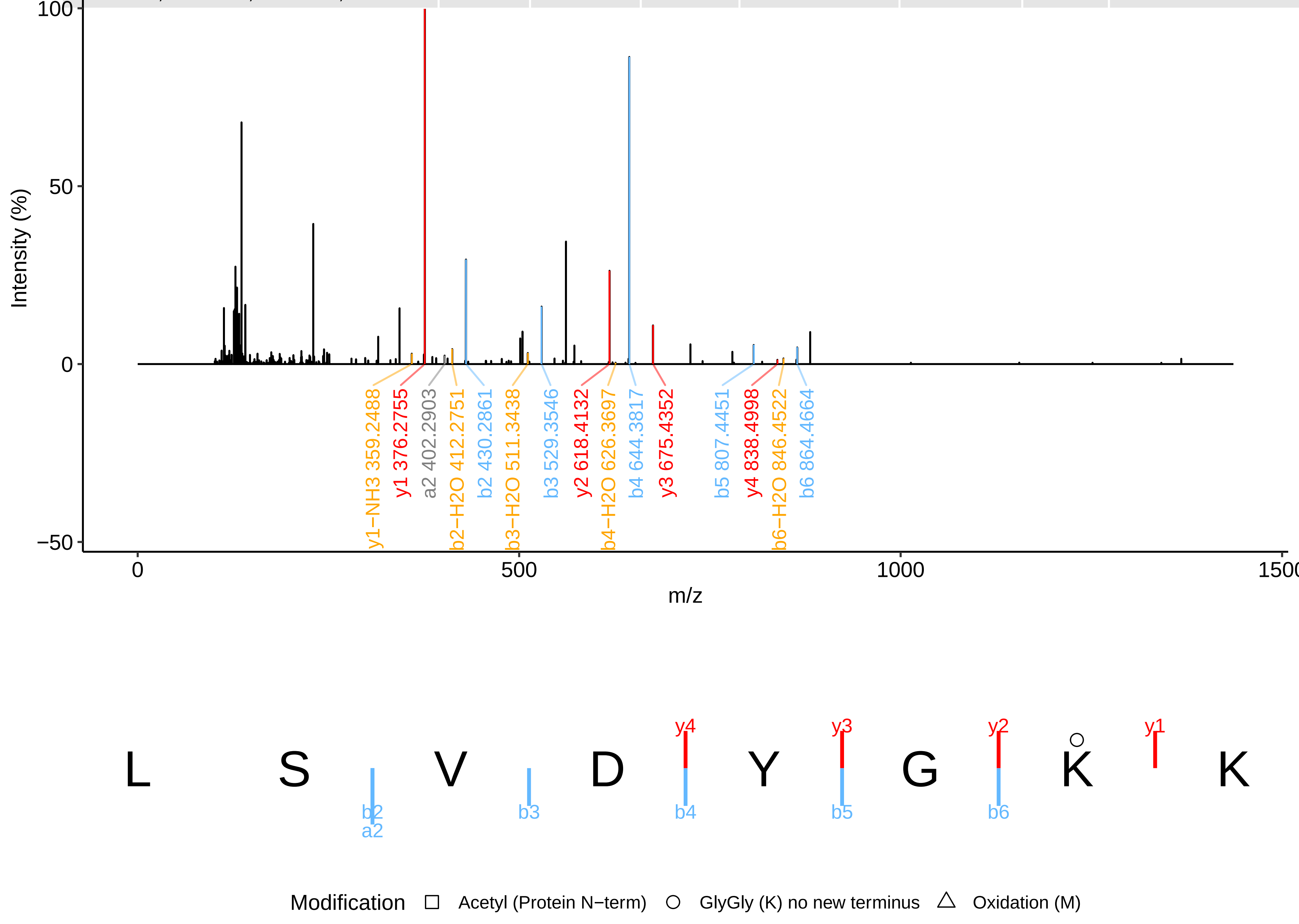

20200921_KIZ_MUb4_GlyGly_10plex − Scan number: 11546

Gene Names	Charge	m/z	Mass	Retention time	PEP	Score	Precursor Intensity
TUBA1C;TUBA1A;TUBA1B;TUBA4A	2	512.2771	1022.54	40.267	0.0061856	131.06	NA

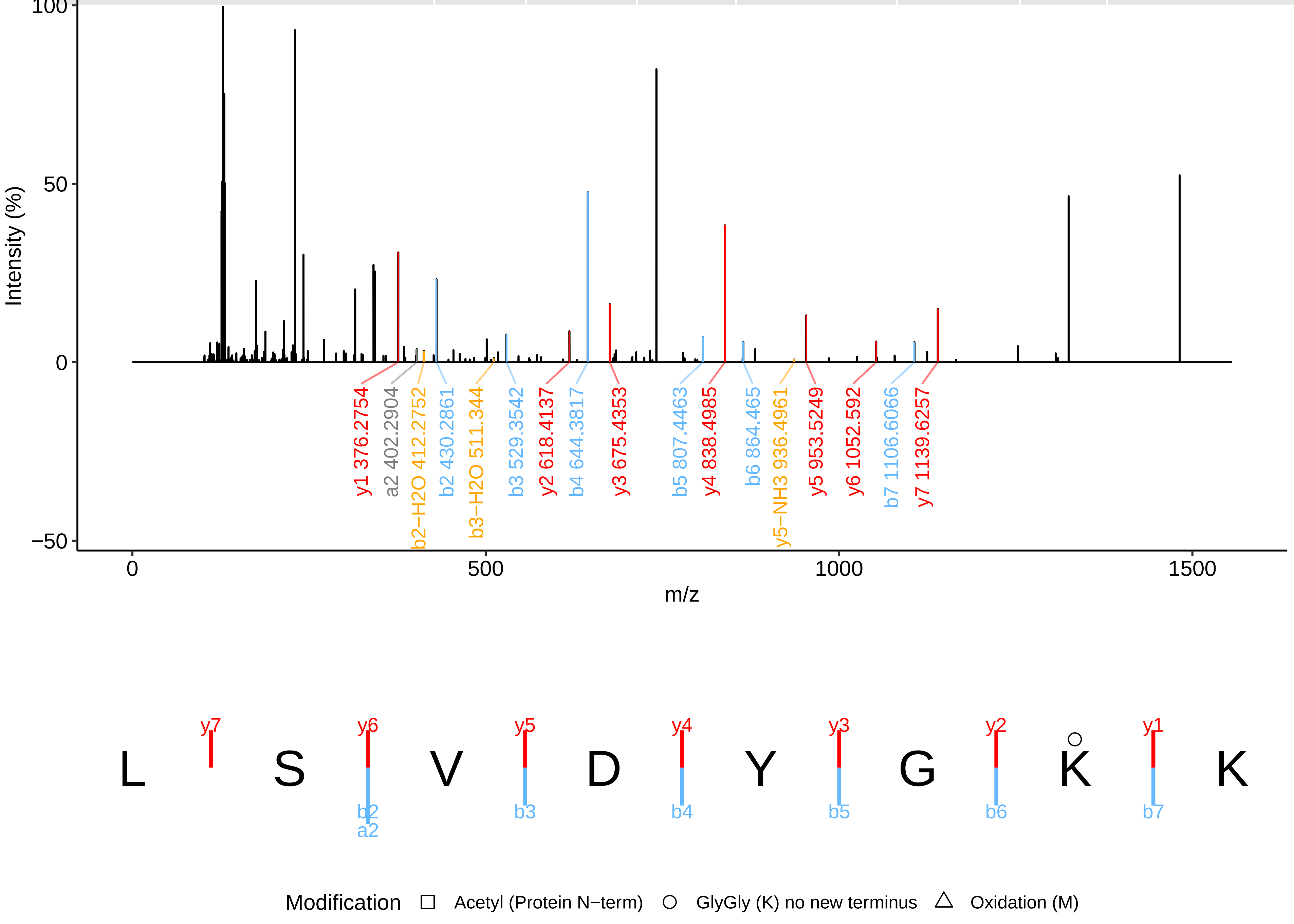

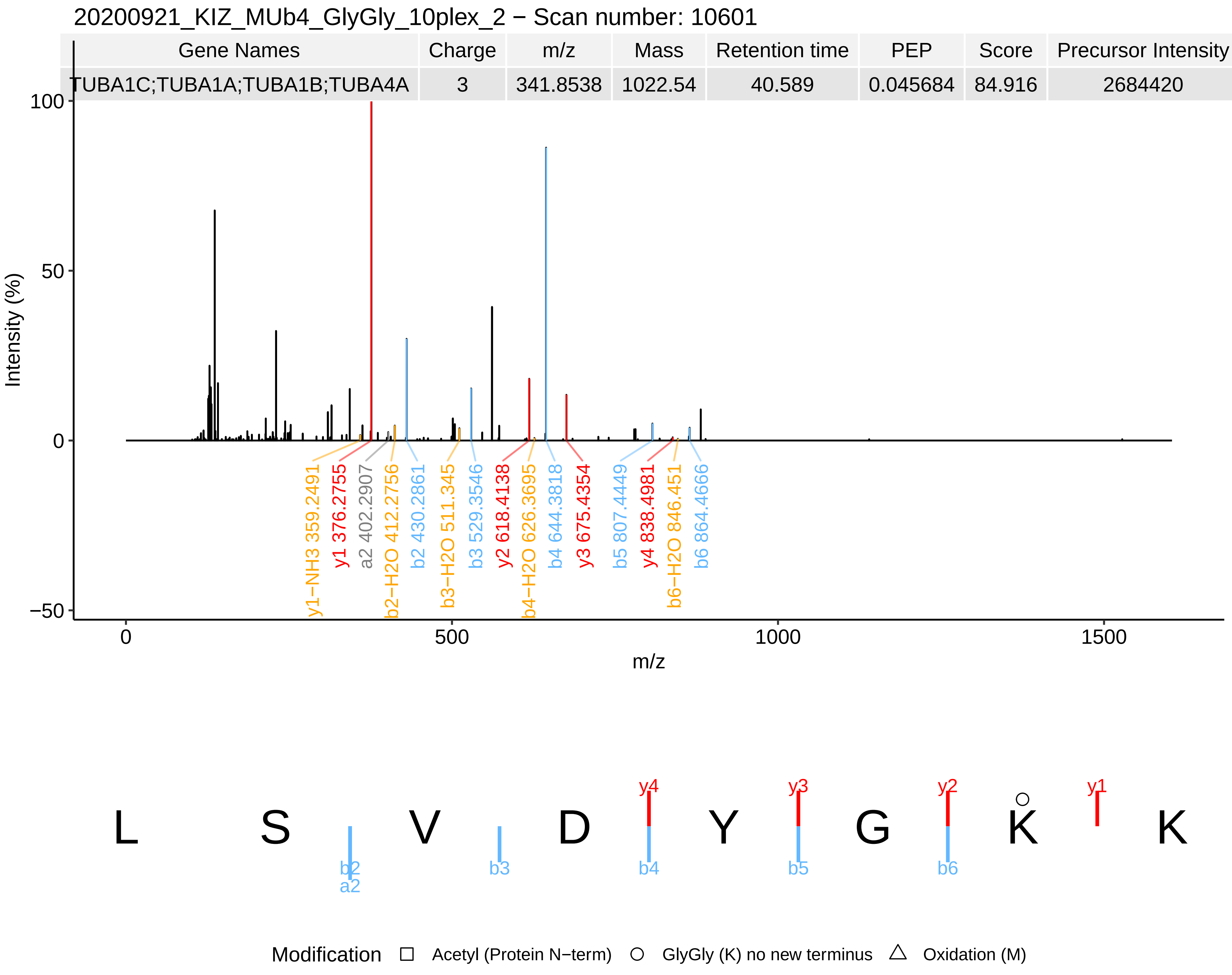
20200921_KIZ_MUb4_GlyGly_10plex_2 − Scan number: 10601
Gene Names
Charge
m/z
Mass
Retention time
PEP
Score
Precursor Intensity
TUBA1C;TUBA1A;TUBA1B;TUBA4A
3
341.8538
1022.54
40.589
0.045684
84.916
2684420
Intensity (%)
m/z
y1−NH3 359.2491
y1 376.2755
a2 402.2907
b2−H2O 412.2756
b2 430.2861
b3−H2O 511.345
b3 529.3546
y2 618.4138
b4−H2O 626.3695
b4 644.3818
y3 675.4354
b5 807.4449
y4 838.4981
b6−H2O 846.451
b6 864.4666
L S V D Y G K K
y4 y3 y2 y1
b2 a2 b3 b4 b5 b6
Modification
Acetyl (Protein N−term)
GlyGly (K) no new terminus
Oxidation (M)

20200924_KIZ_MUb5_GlyGly_1 − Scan number: 10301

Gene Names	Charge	m/z	Mass	Retention time	PEP	Score	Precursor Intensity
TUBA1C;TUBA1A;TUBA1B;TUBA4A	3	341.8538	1022.54	33.225	0.083297	77.072	7304244

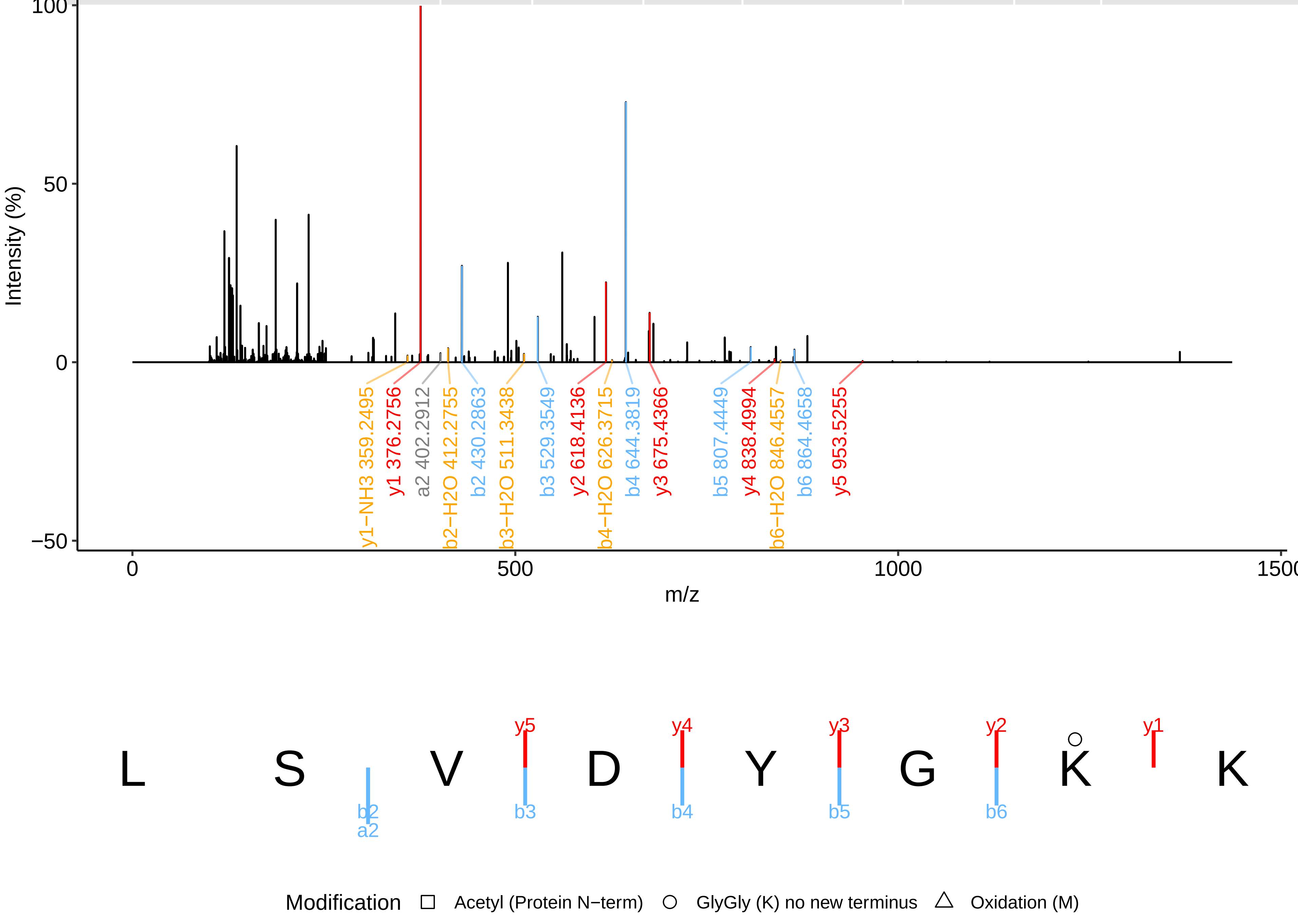

20200924_KIZ_MUb5_GlyGly_1 − Scan number: 10447

Gene Names	Charge	m/z	Mass	Retention time	PEP	Score	Precursor Intensity
TUBA1C;TUBA1A;TUBA1B;TUBA4A	3	341.8538	1022.54	33.723	0.041105	86.794	88627200

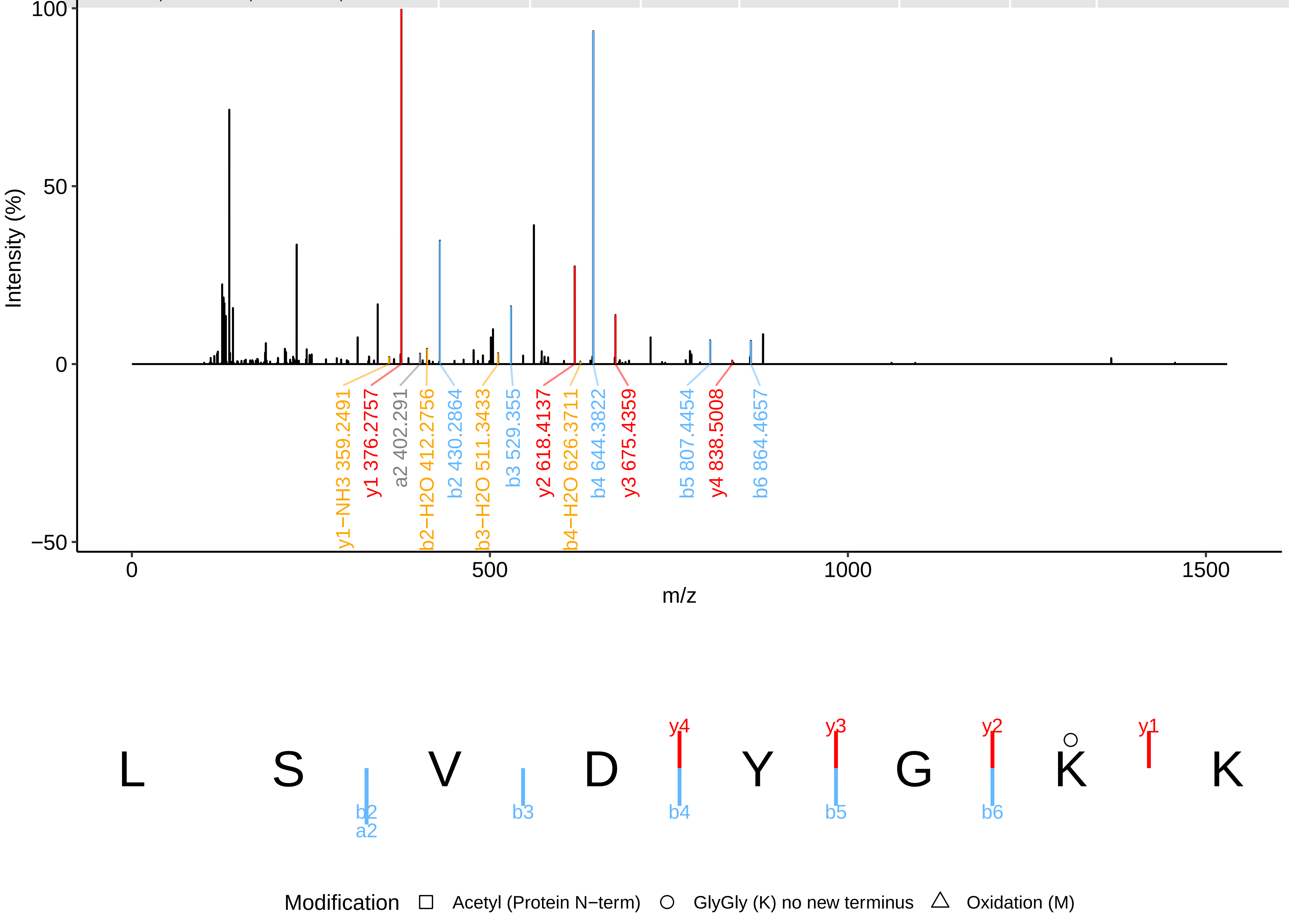

20200924_KIZ_MUb5_GlyGly_1 − Scan number: 10598

Gene Names	Charge	m/z	Mass	Retention time	PEP	Score	Precursor Intensity
TUBA1C;TUBA1A;TUBA1B;TUBA4A	3	341.8538	1022.54	34.237	0.023558	93.992	43011060

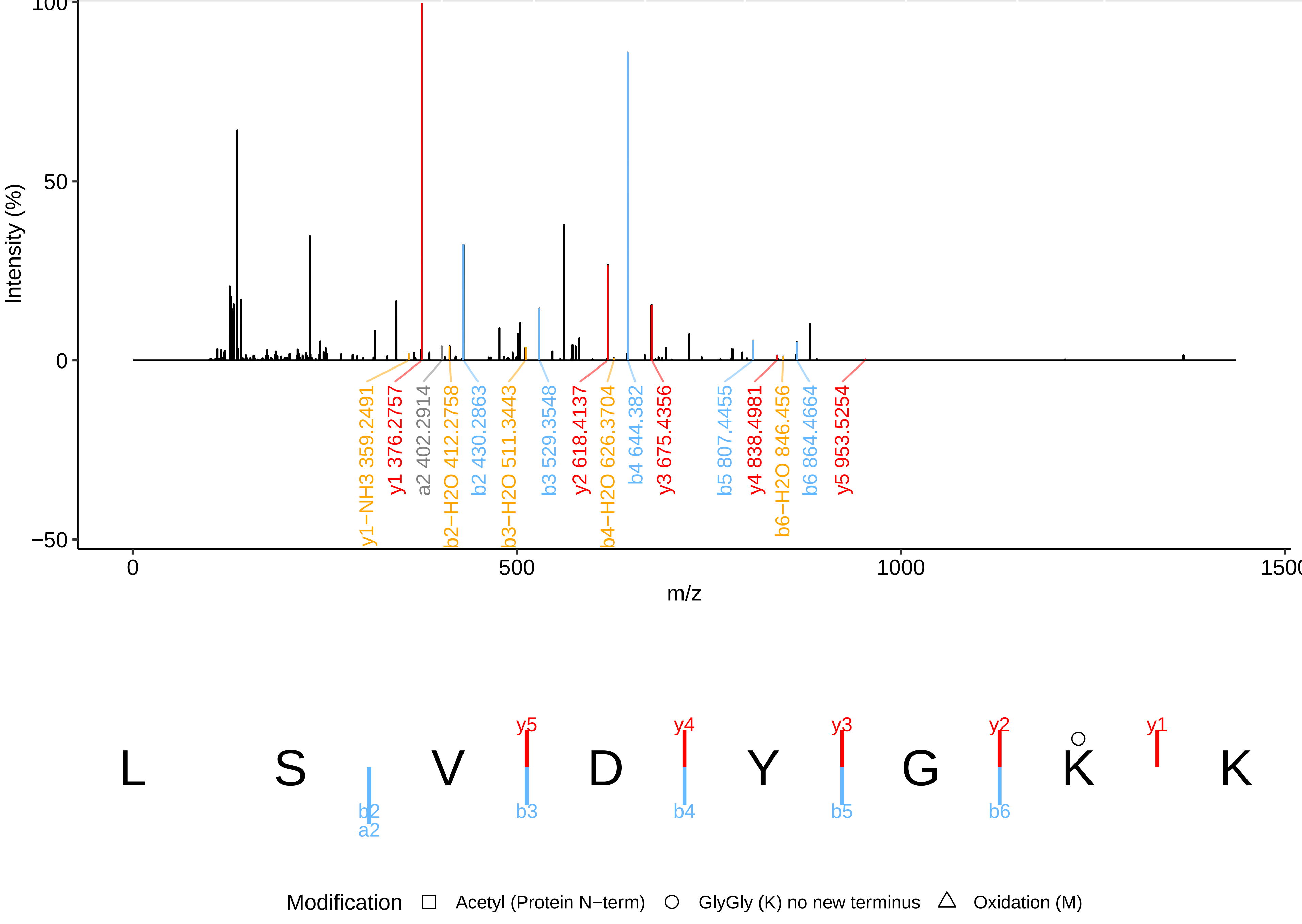

20200924_KIZ_MUb5_GlyGly_2 − Scan number: 9756

Gene Names	Charge	m/z	Mass	Retention time	PEP	Score	Precursor Intensity
TUBA1C;TUBA1A;TUBA1B;TUBA4A	3	341.8538	1022.54	36.455	0.035614	89.046	24000504

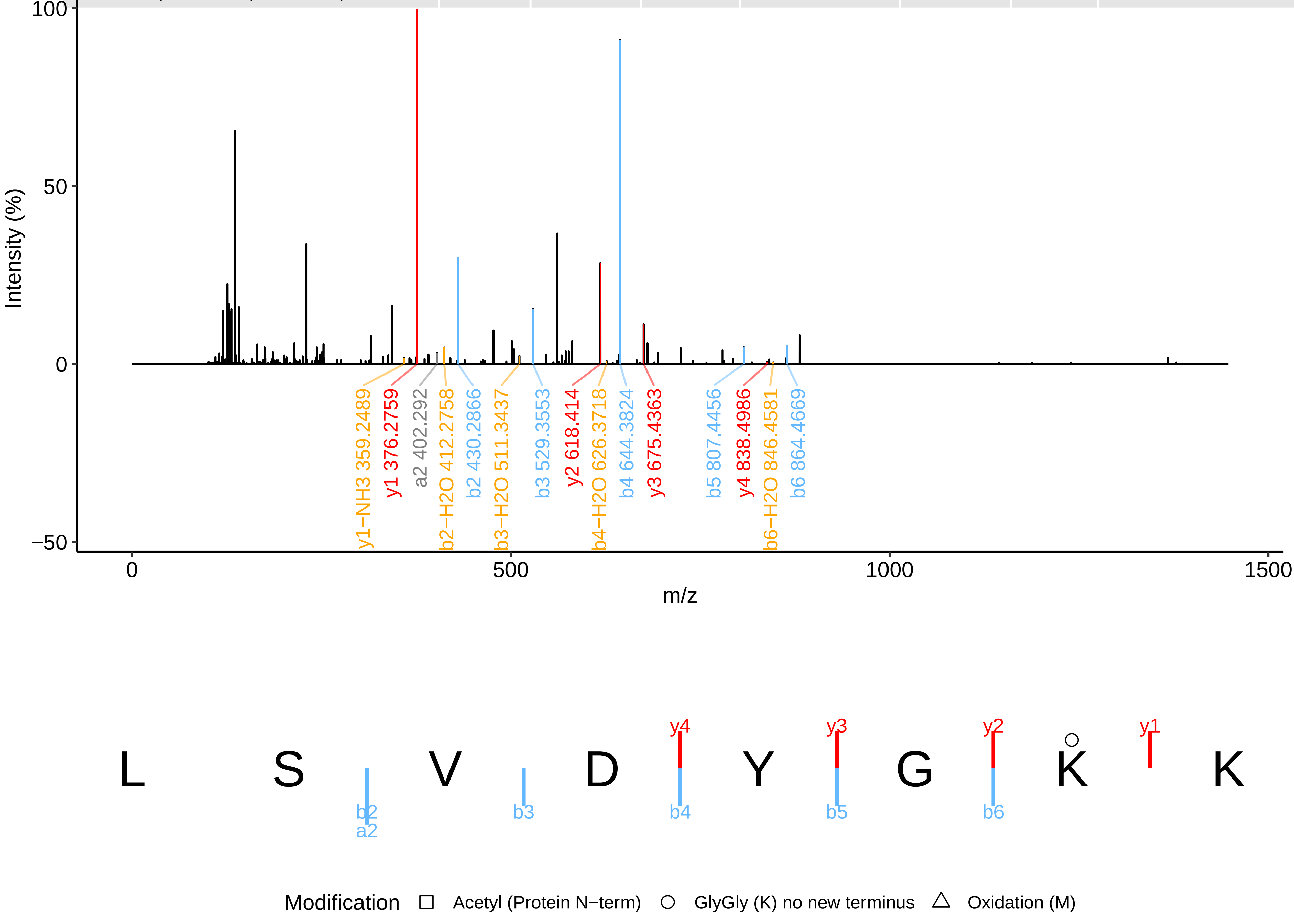

20200924_KIZ_MUb5_GlyGly_2 − Scan number: 9892

Gene Names	Charge	m/z	Mass	Retention time	PEP	Score	Precursor Intensity
TUBA1C;TUBA1A;TUBA1B;TUBA4A	3	341.8538	1022.54	36.973	0.035614	89.046	17070224

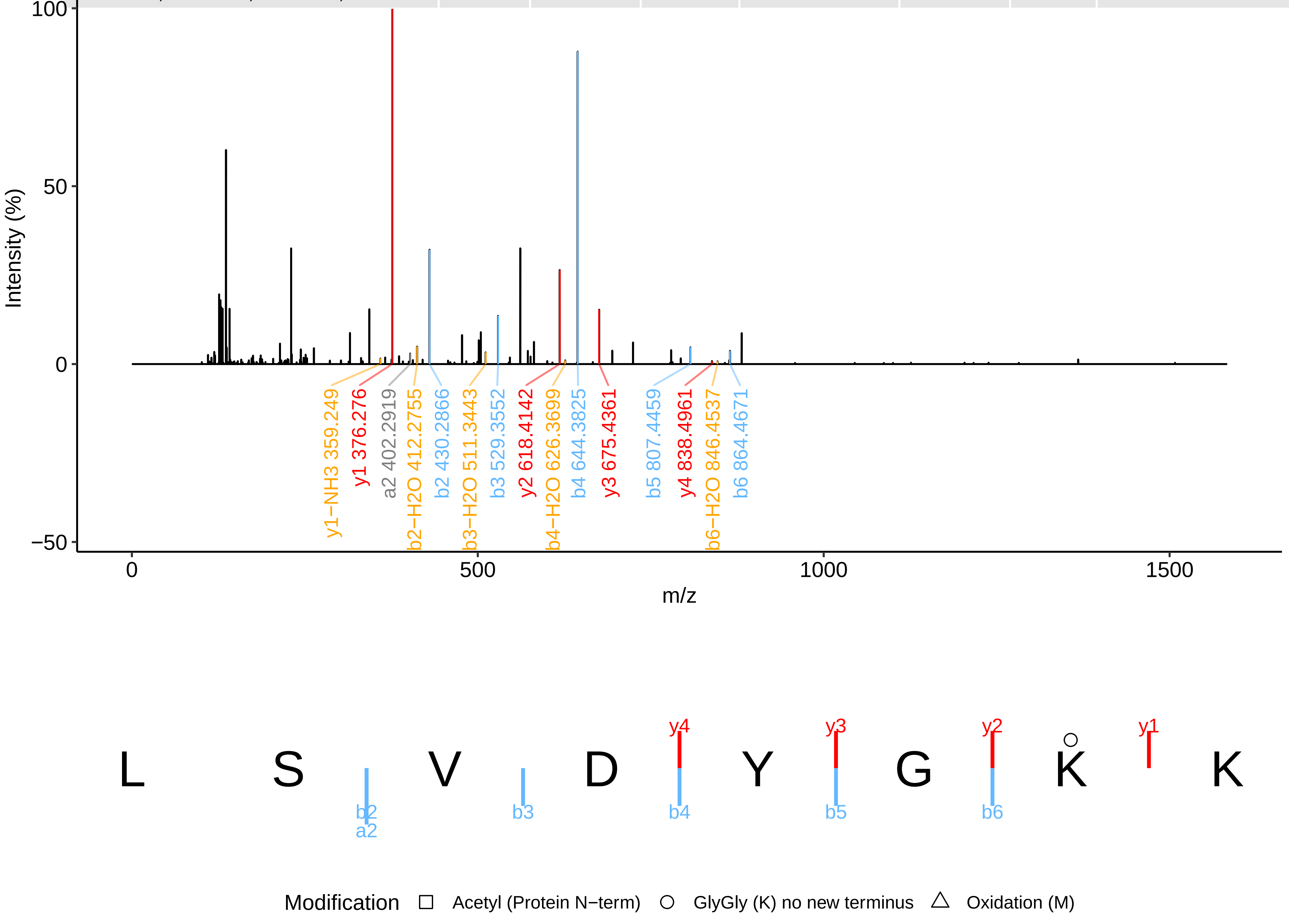

20201118_KIZ_MUb6_GlyGly_1 − Scan number: 10177

Gene Names	Charge	m/z	Mass	Retention time	PEP	Score	Precursor Intensity
TUBA1C;TUBA1A;TUBA1B;TUBA4A	3	341.8538	1022.54	32.126	0.027267	92.47	15249043

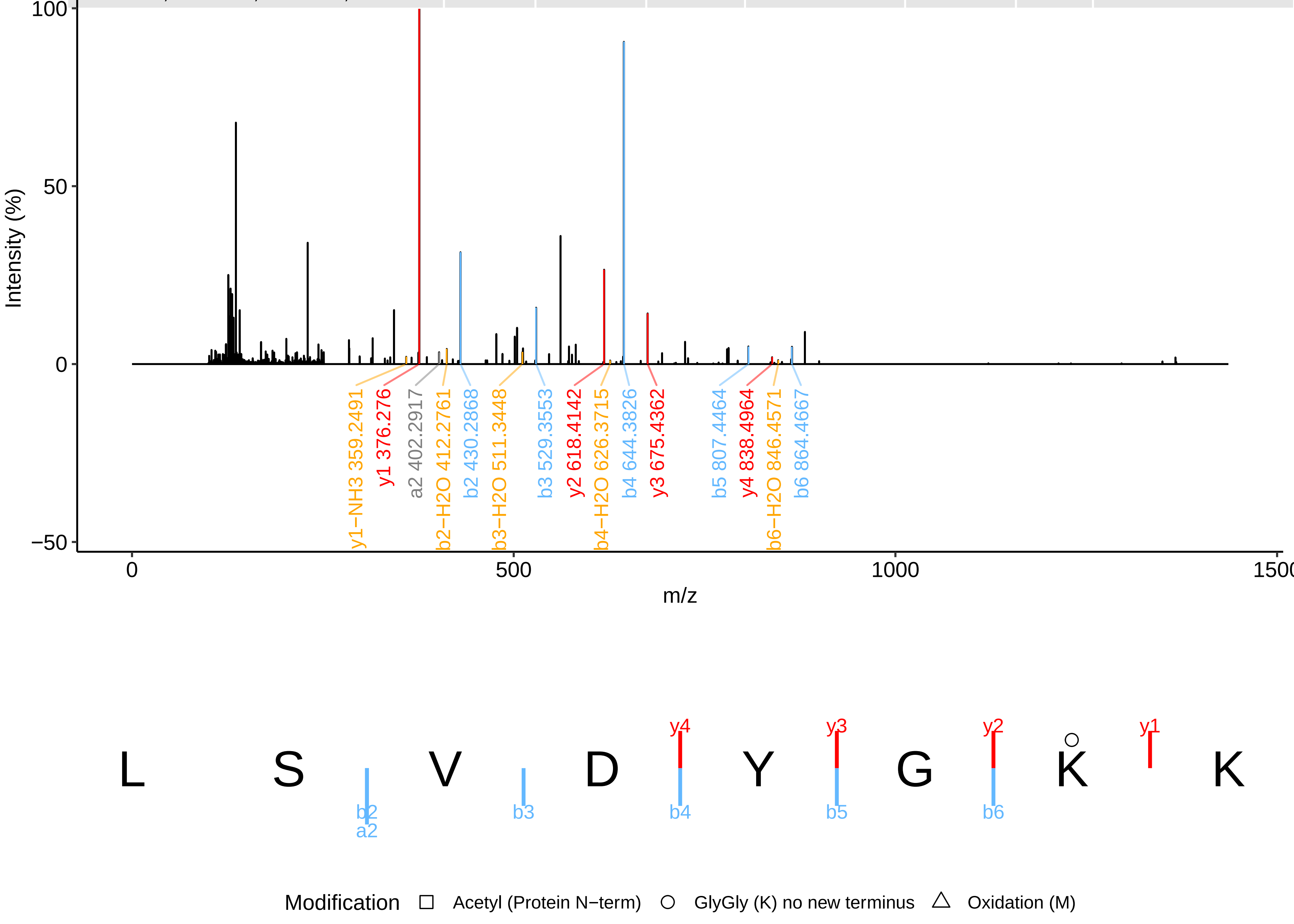

20201118_KIZ_MUb6_GlyGly_2 − Scan number: 12183

Gene Names	Charge	m/z	Mass	Retention time	PEP	Score	Precursor Intensity
TUBA1C;TUBA1A;TUBA1B;TUBA4A	3	341.8538	1022.54	40.99	0.0041169	111.82	7878309

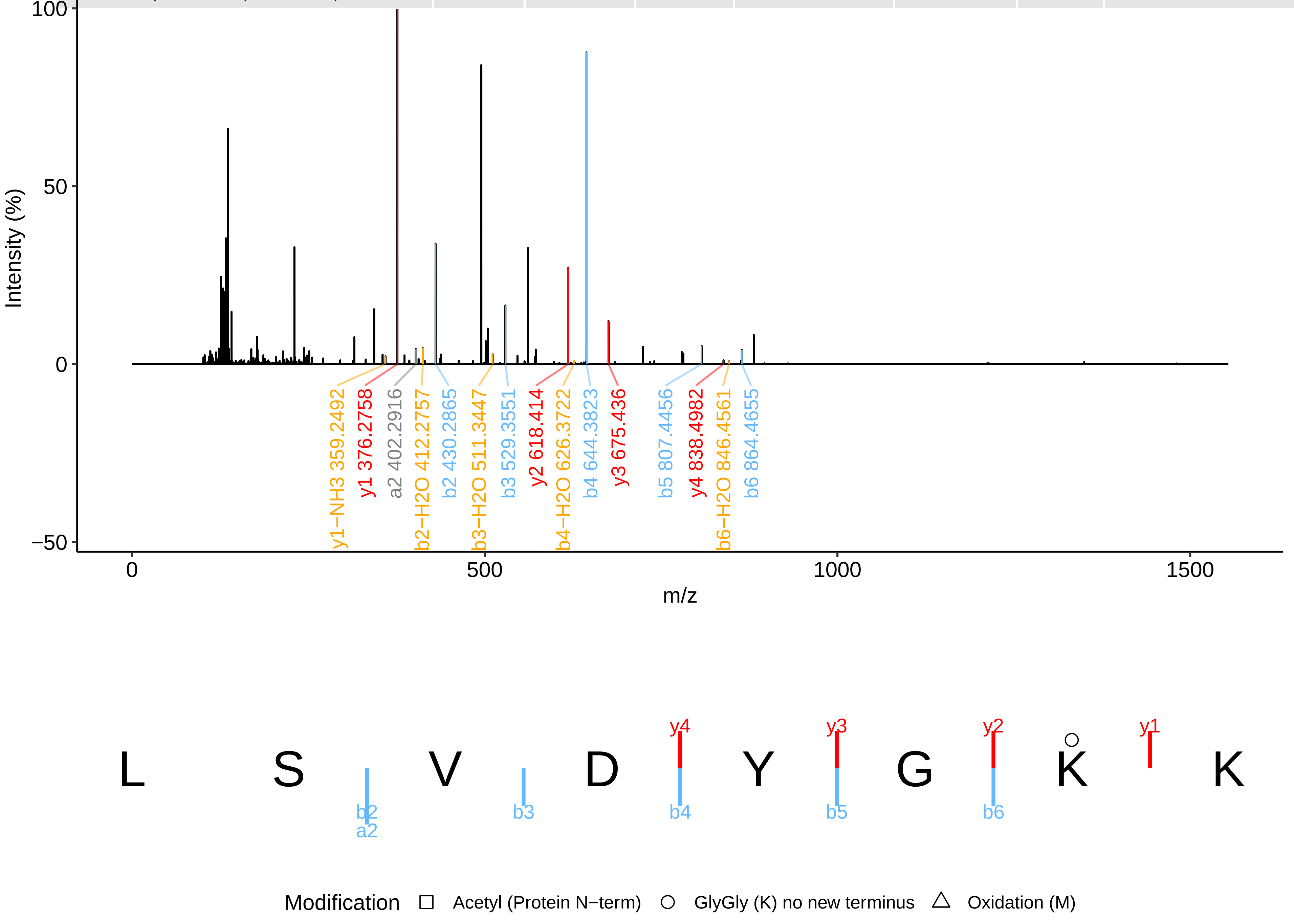

20200921_KIZ_MUb4_GlyGly_10plex − Scan number: 14445

Gene Names	Charge	m/z	Mass	Retention time	PEP	Score	Precursor Intensity
SPG20	3	343.2097	1026.607	51.339	0.058984	80.229	8646838

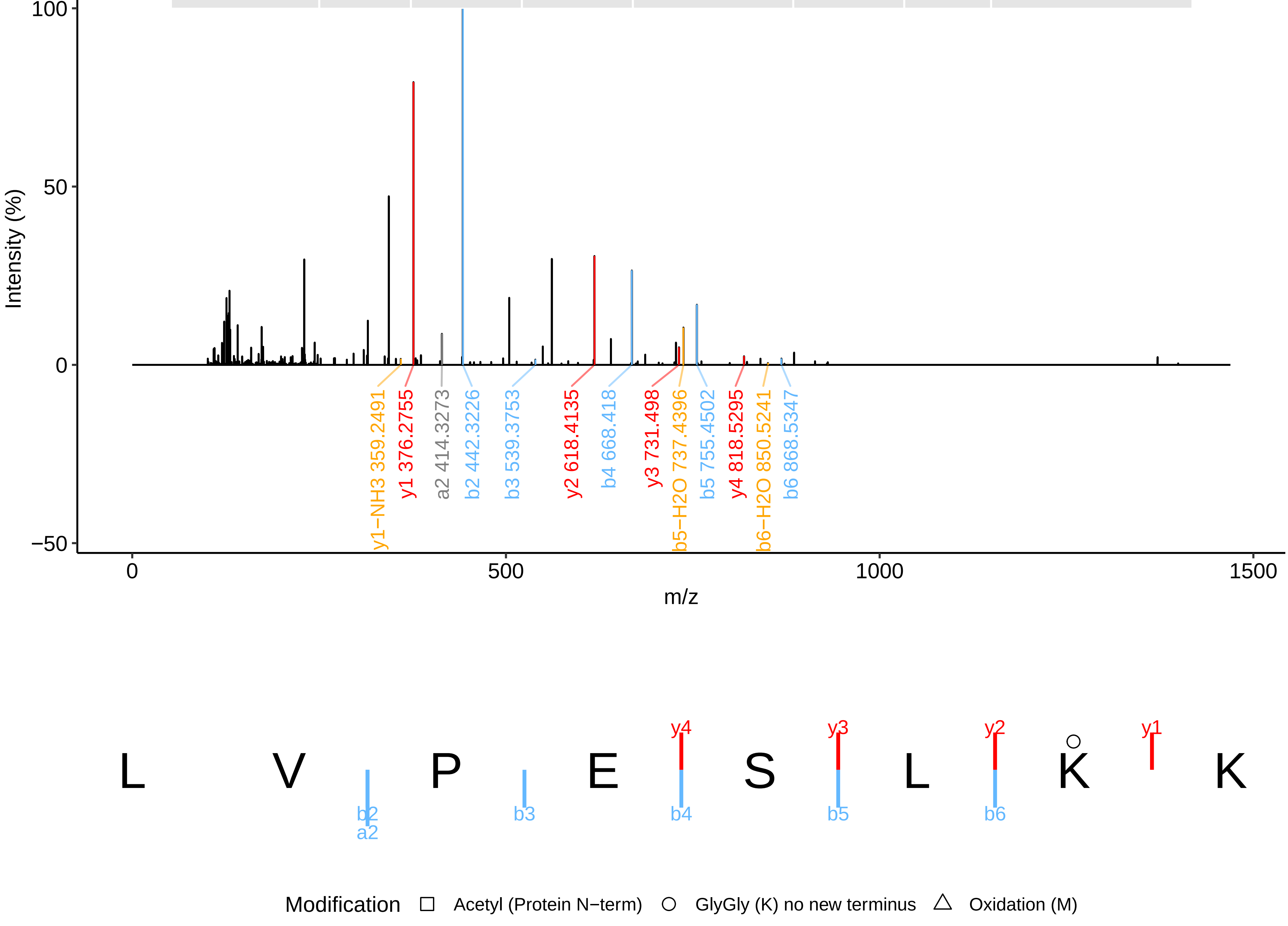

20200924_KIZ_MUb5_GlyGly_1 − Scan number: 13408

Gene Names	Charge	m/z	Mass	Retention time	PEP	Score	Precursor Intensity
SPG20	3	343.2097	1026.607	43.71	0.077726	73.248	4907954

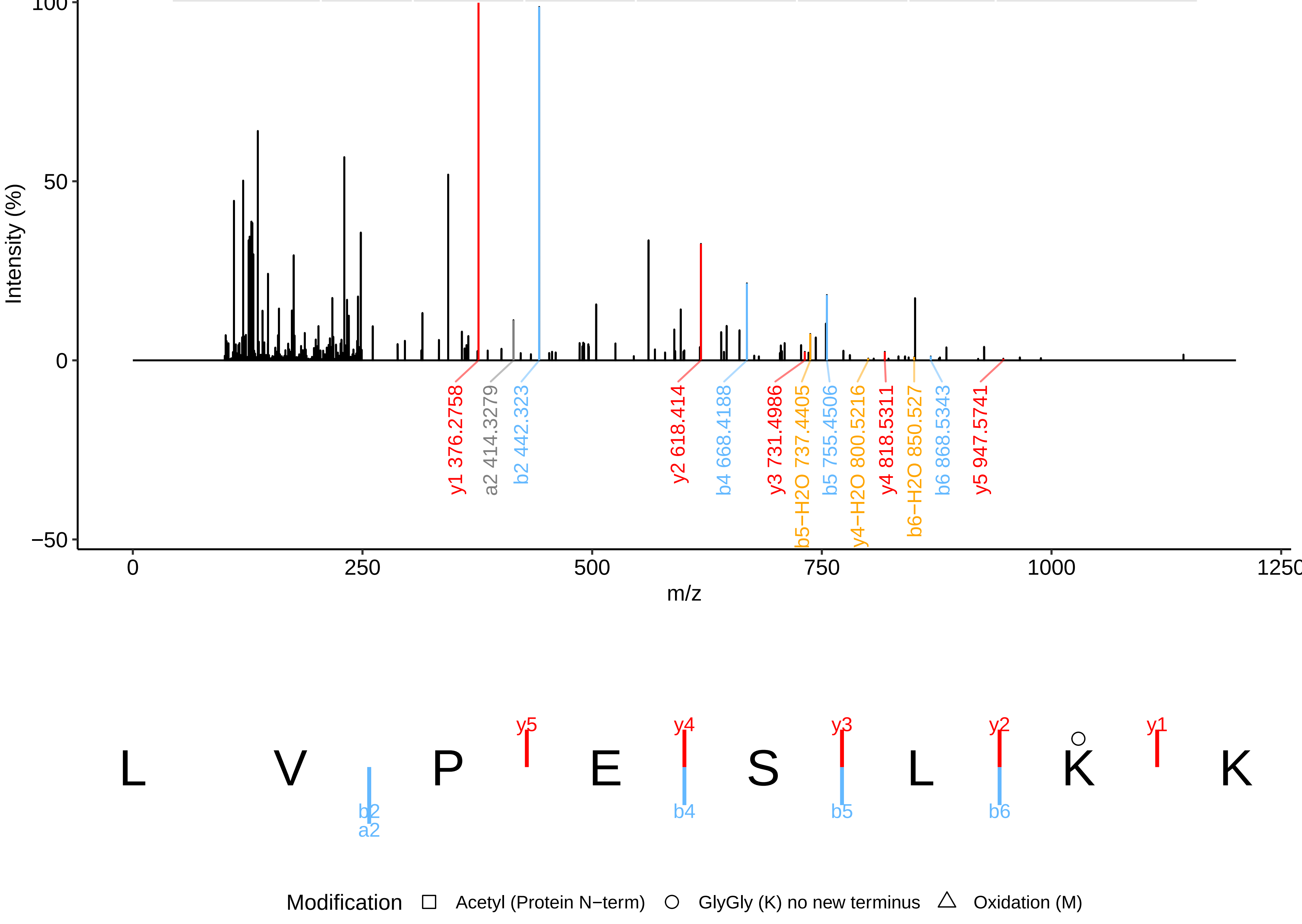

20200921_KIZ_MUb4_GlyGly_10plex − Scan number: 5140

Gene Names	Charge	m/z	Mass	Retention time	PEP	Score	Precursor Intensity
PSMA3	3	403.8668	1208.579	16.78	1.1954e−06	141.2	10067903

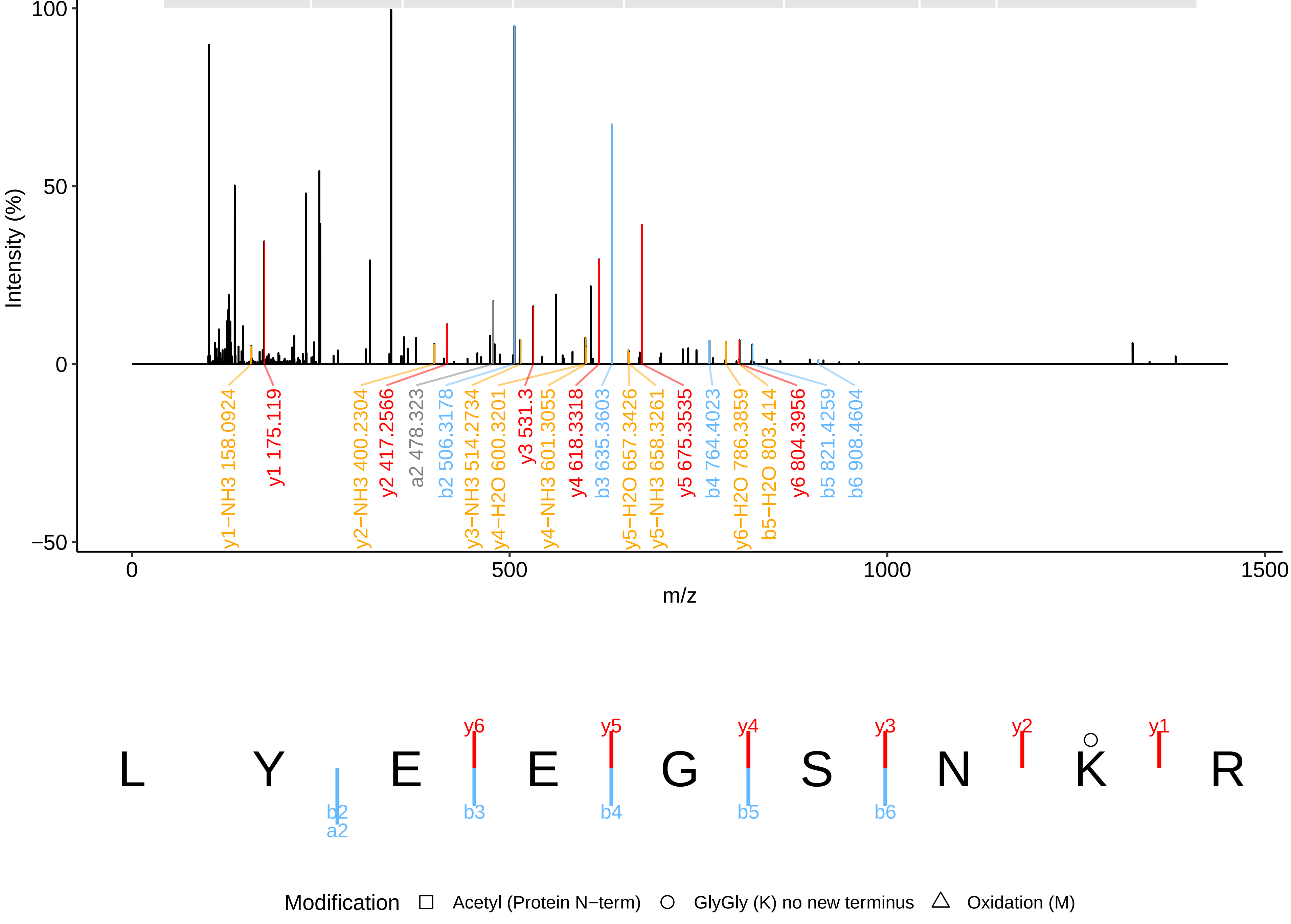

20200921_KIZ_MUb4_GlyGly_10plex_2 − Scan number: 5619

Gene Names	Charge	m/z	Mass	Retention time	PEP	Score	Precursor Intensity
TRAF2	3	404.5328	1210.576	20.292	0.0026651	85.676	790721.5

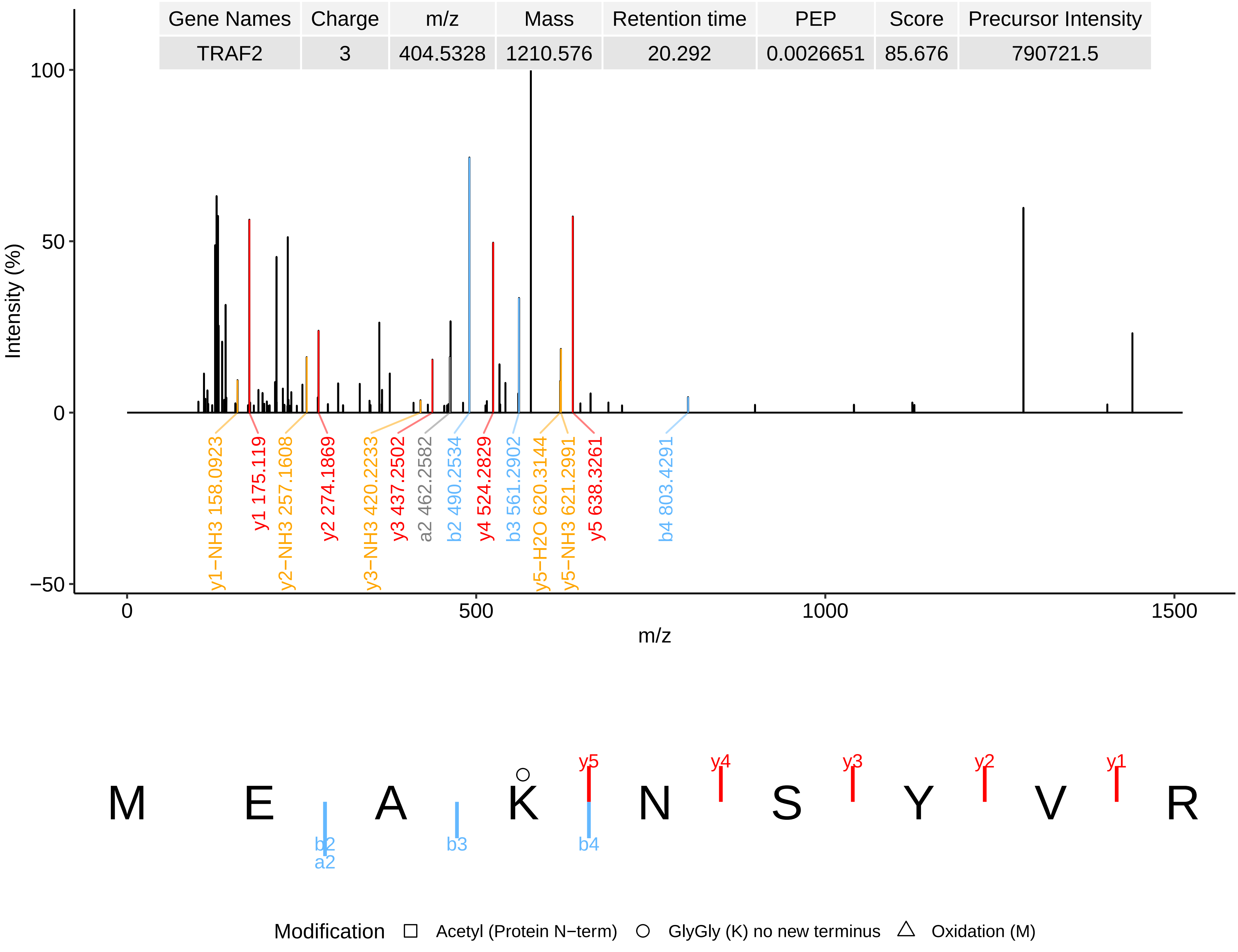

20200921_KIZ_MUb4_GlyGly_10plex − Scan number: 5983

Gene Names	Charge	m/z	Mass	Retention time	PEP	Score	Precursor Intensity
SLC46A1	2	721.3406	1440.667	19.647	6.4988e−06	79.148	4398838

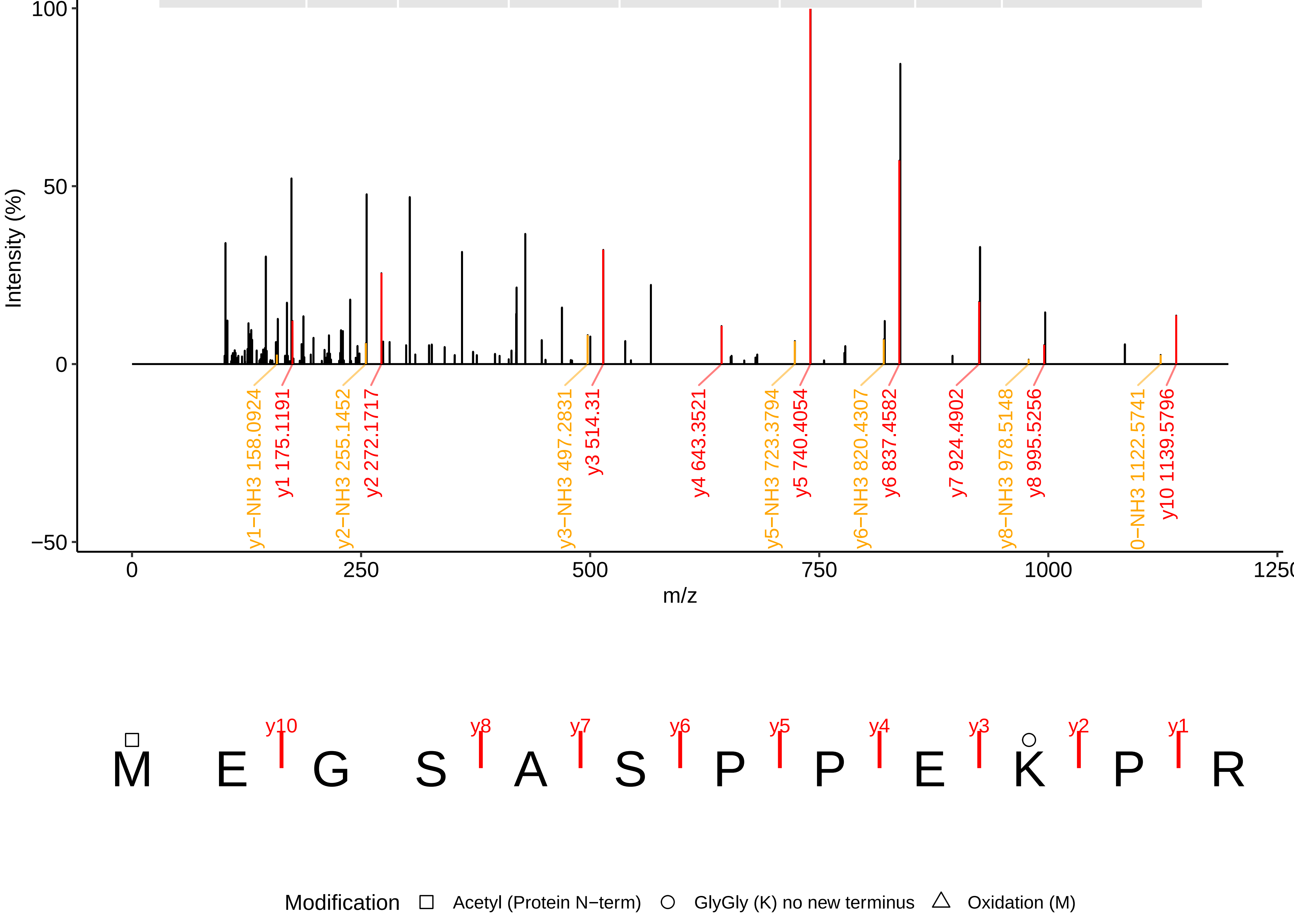

20200921_KIZ_MUb4_GlyGly_10plex_2 − Scan number: 5374

Gene Names	Charge	m/z	Mass	Retention time	PEP	Score	Precursor Intensity
SLC46A1	2	721.3406	1440.667	19.289	0.00017718	76.228	397501.8

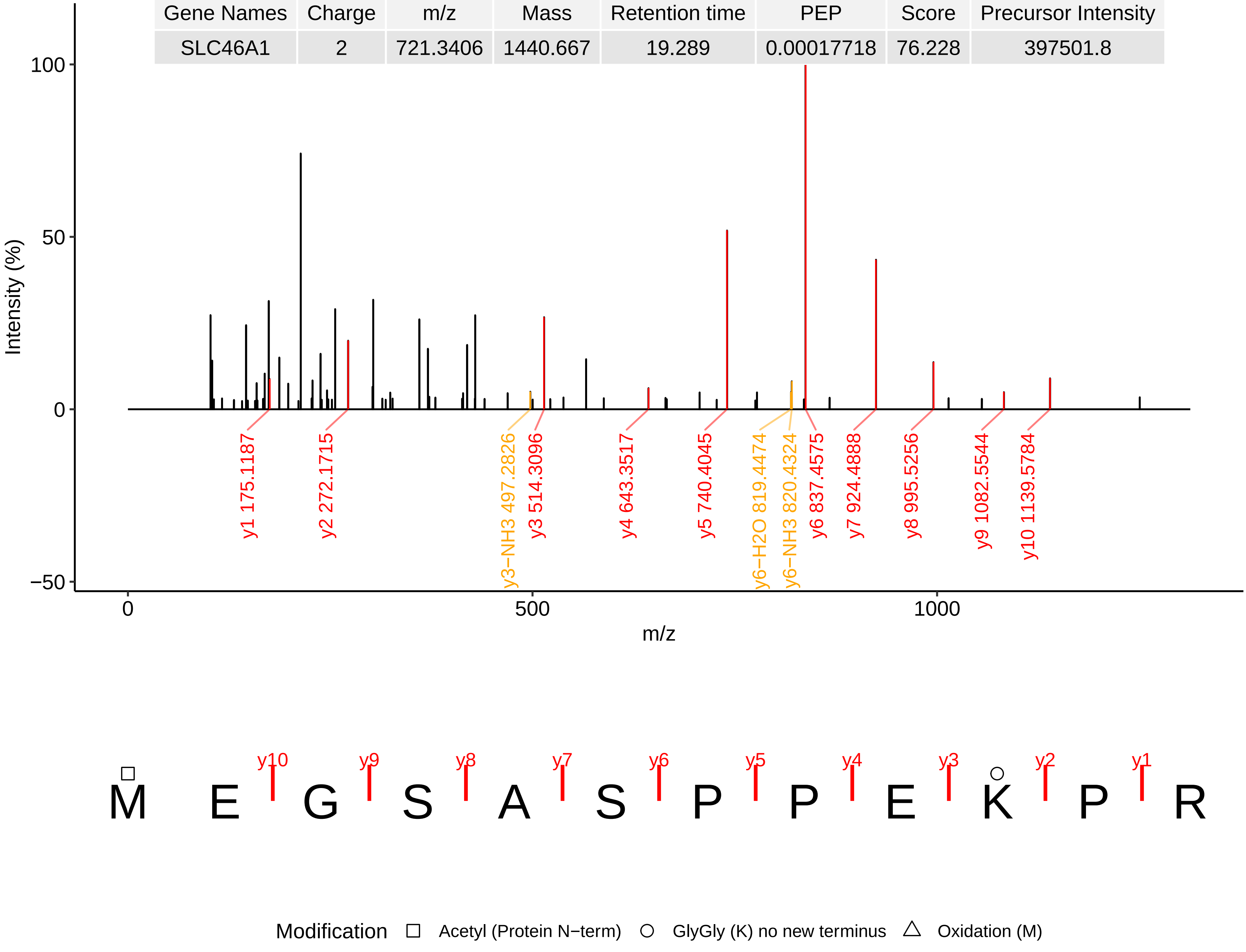

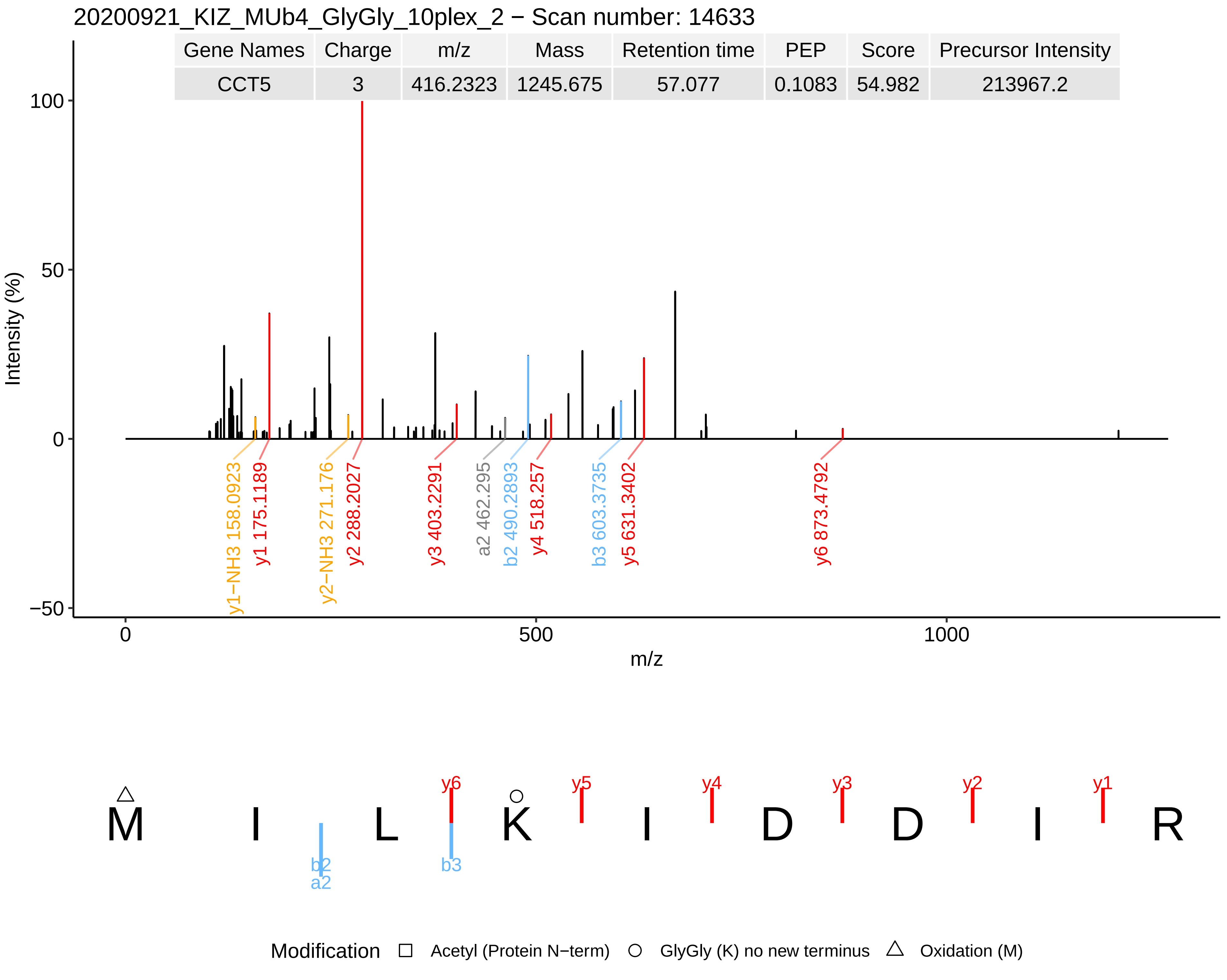

Gene Names	Charge	m/z	Mass	Retention time	PEP	Score	Precursor Intensity
CCT5	3	416.2323	1245.675	57.077	0.1083	54.982	213967.2

20200921_KIZ_MUb4_GlyGly_10plex_2 − Scan number: 18067

Gene Names	Charge	m/z	Mass	Retention time	PEP	Score	Precursor Intensity
CCT5	3	410.9007	1229.68	70.708	0.06573	57.59	322122.9

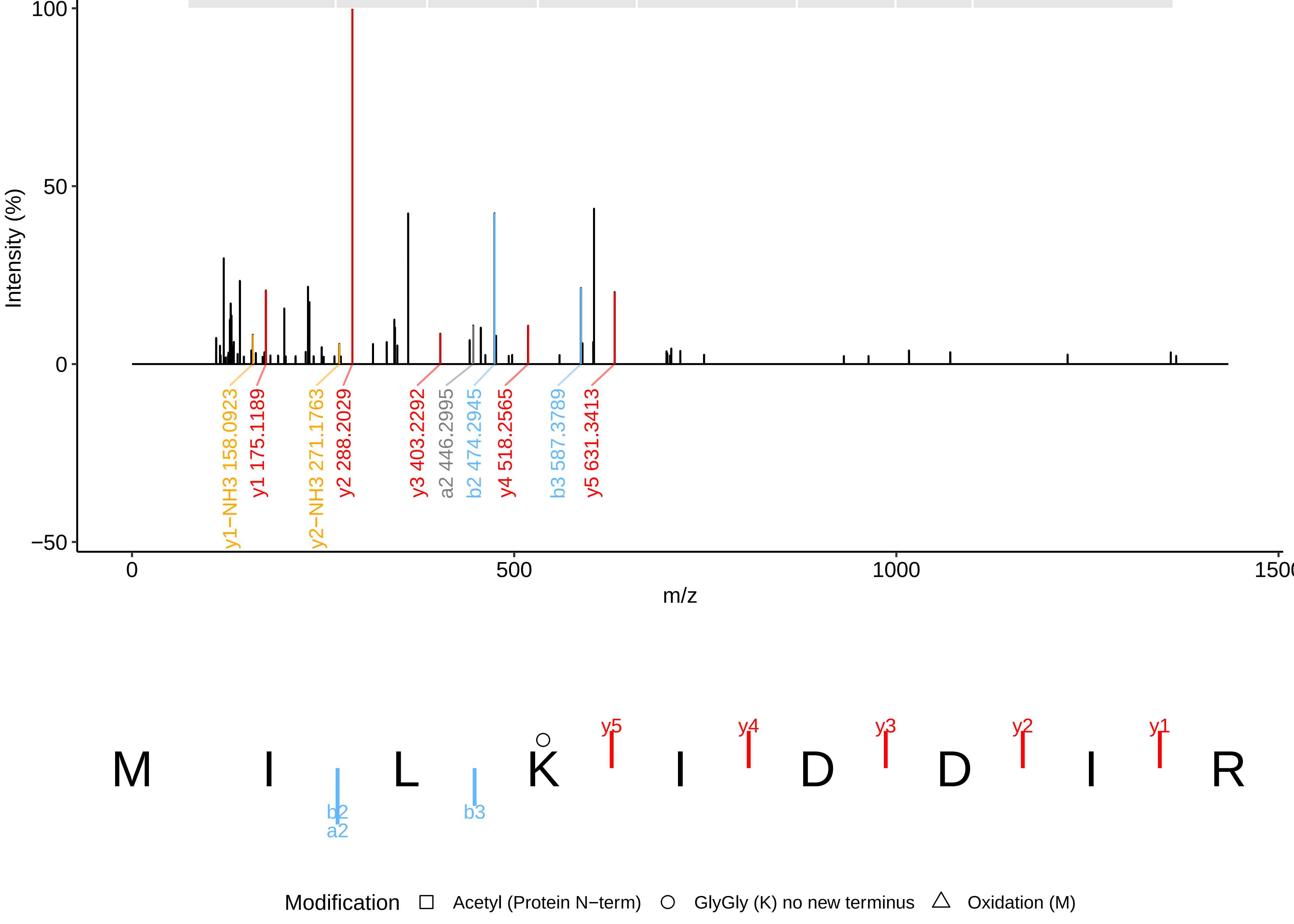

20200921_KIZ_MUb4_GlyGly_10plex − Scan number: 12086

Gene Names	Charge	m/z	Mass	Retention time	PEP	Score	Precursor Intensity
LAPTM4B	3	417.2108	1248.611	42.297	0.058458	52.579	5582400

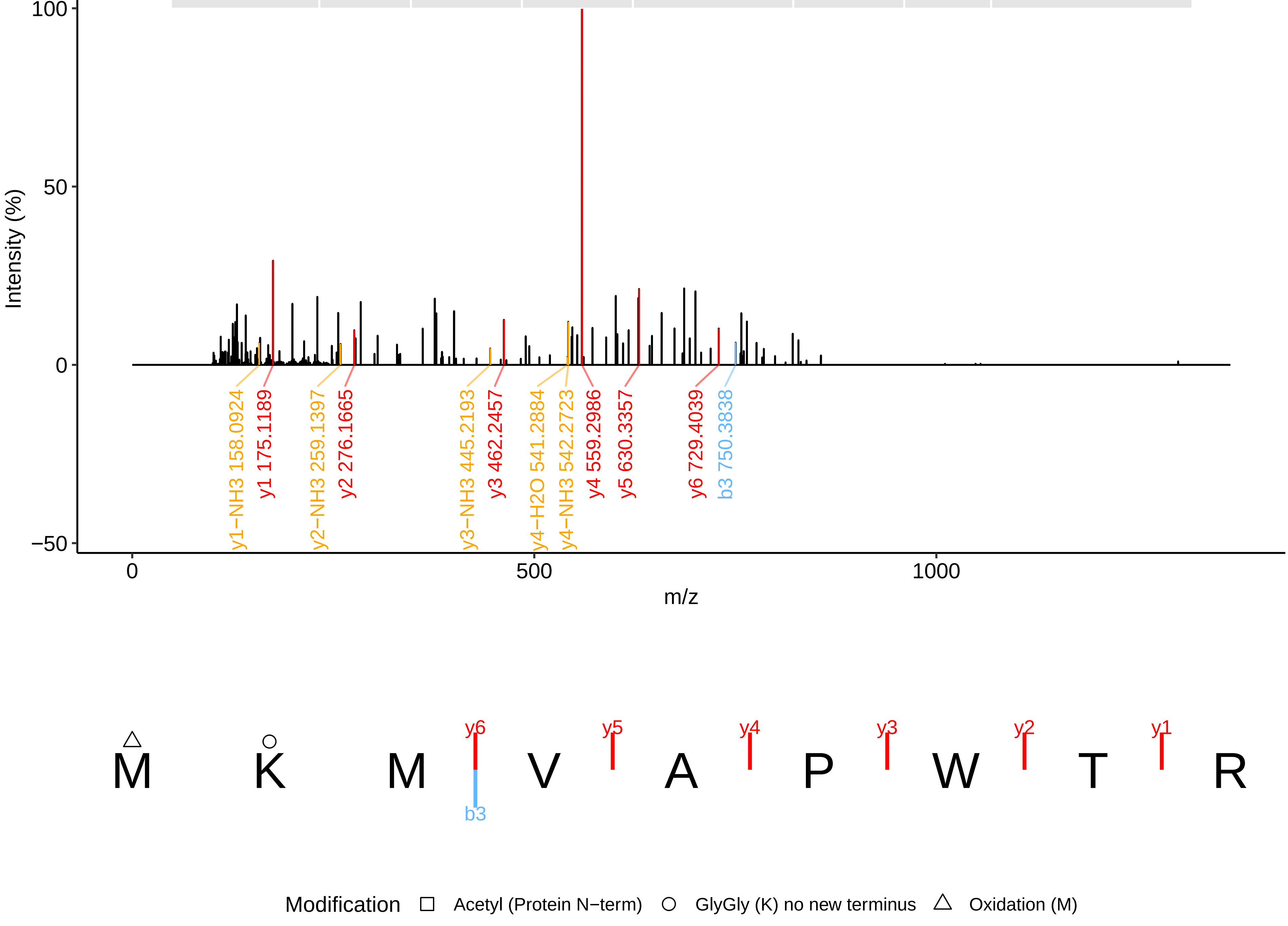

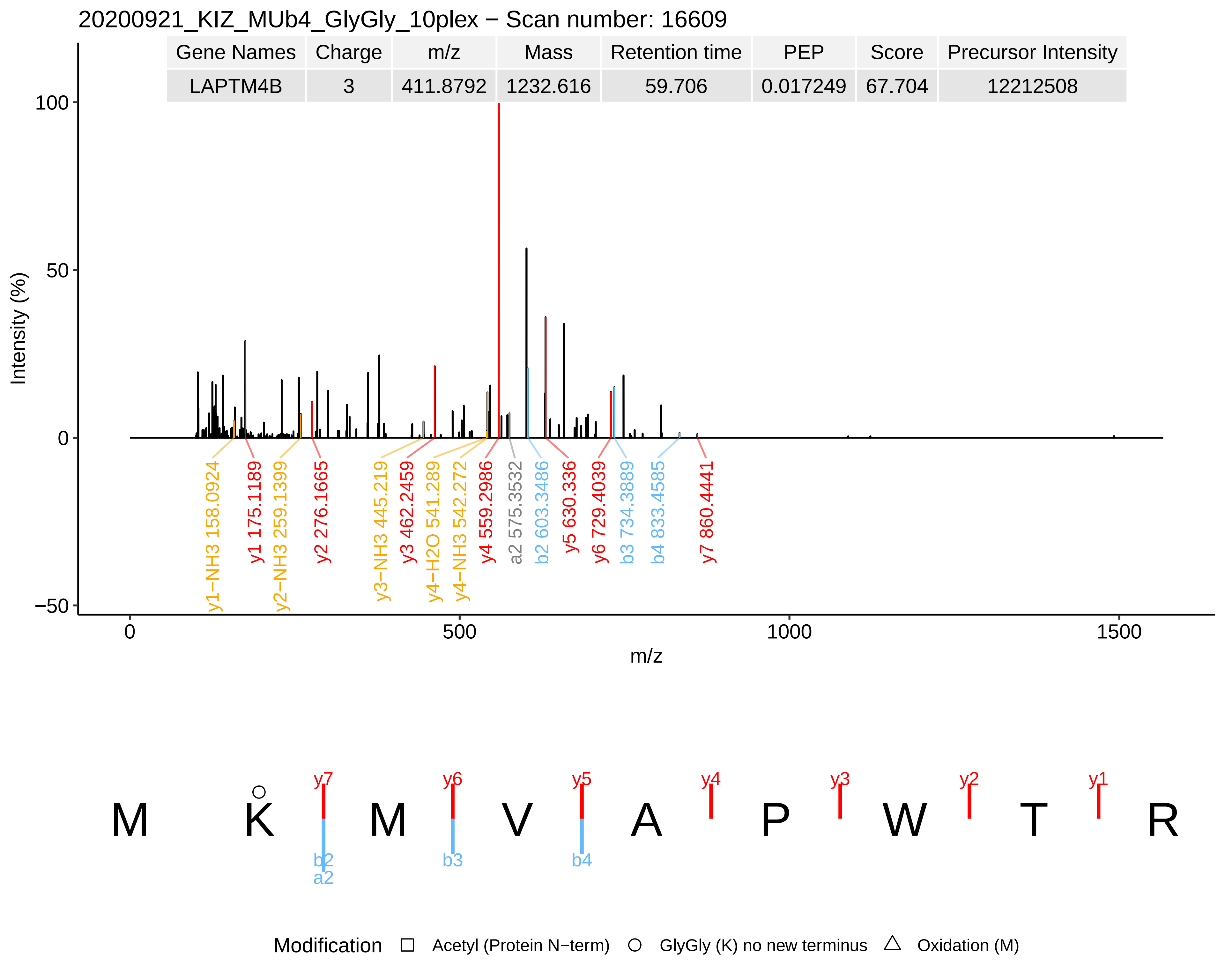
20200921_KIZ_MUb4_GlyGly_10plex − Scan number: 16609
Gene Names | Charge | m/z | Mass | Retention time | PEP | Score | Precursor Intensity
LAPTM4B | 3 | 411.8792 | 1232.616 | 59.706 | 0.017249 | 67.704 | 12212508
Intensity (%)
100
50
0
−50
0
500
1000
1500
m/z
y1−NH3 158.0924
y1 175.1189
y2−NH3 259.1399
y2 276.1665
y3−NH3 445.219
y3 462.2459
y4−H2O 541.289
y4−NH3 542.272
y4 559.2986
a2 575.3532
b2 603.3486
y5 630.336
y6 729.4039
b3 734.3889
b4 833.4585
y7 860.4441
M K M V A P W T R
y7 y6 y5 y4 y3 y2 y1
b2 a2 b3 b4
Modification
Acetyl (Protein N−term)
GlyGly (K) no new terminus
Oxidation (M)

20200921_KIZ_MUb4_GlyGly_10plex_2 − Scan number: 15434

Gene Names	Charge	m/z	Mass	Retention time	PEP	Score	Precursor Intensity
LAPTM4B	3	411.8792	1232.616	60.351	0.03707	60.064	1229044

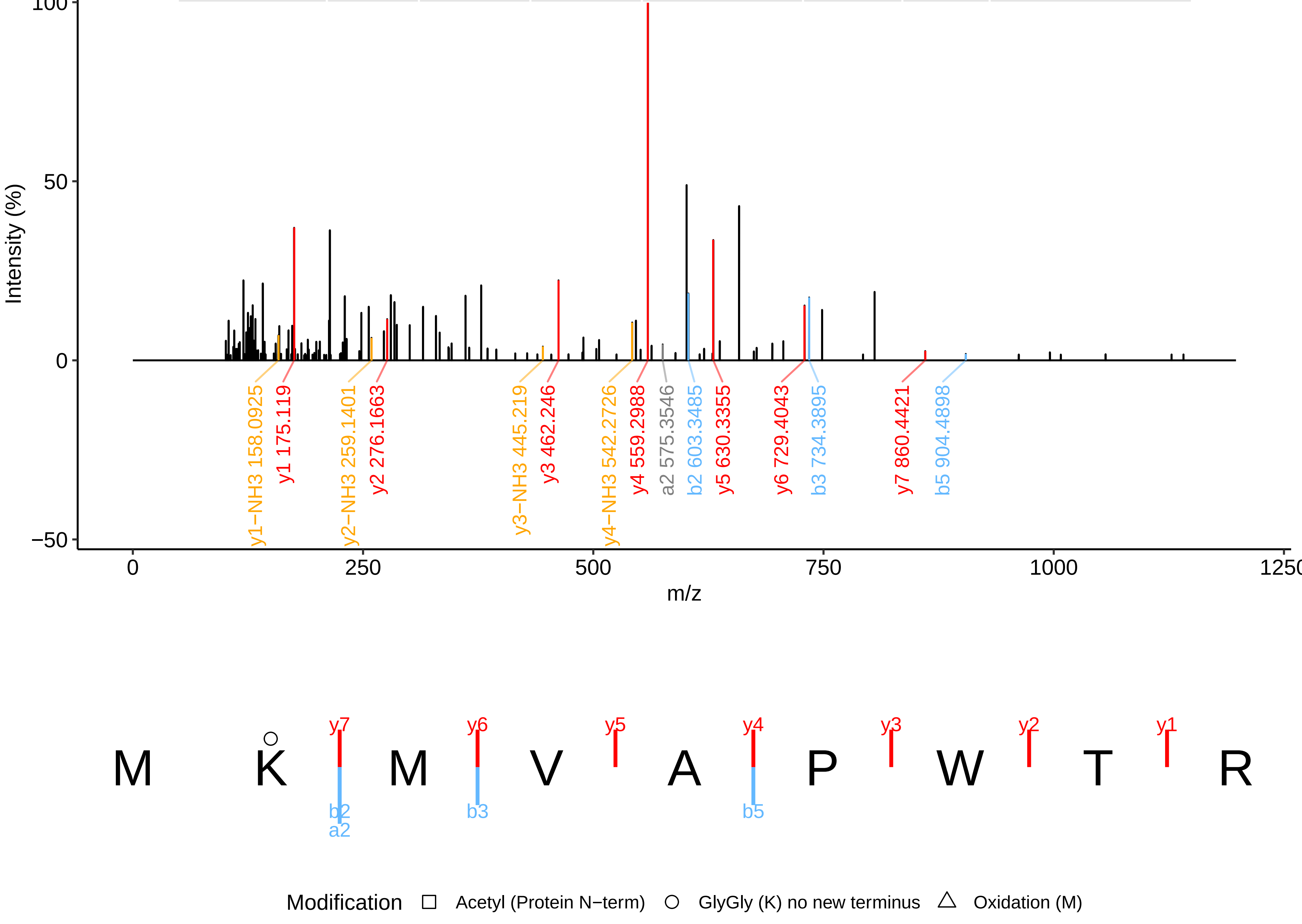

20200924_KIZ_MUb5_GlyGly_1 − Scan number: 15391

Gene Names	Charge	m/z	Mass	Retention time	PEP	Score	Precursor Intensity
LAPTM4B	3	411.8792	1232.616	50.245	0.085101	50.108	5787380

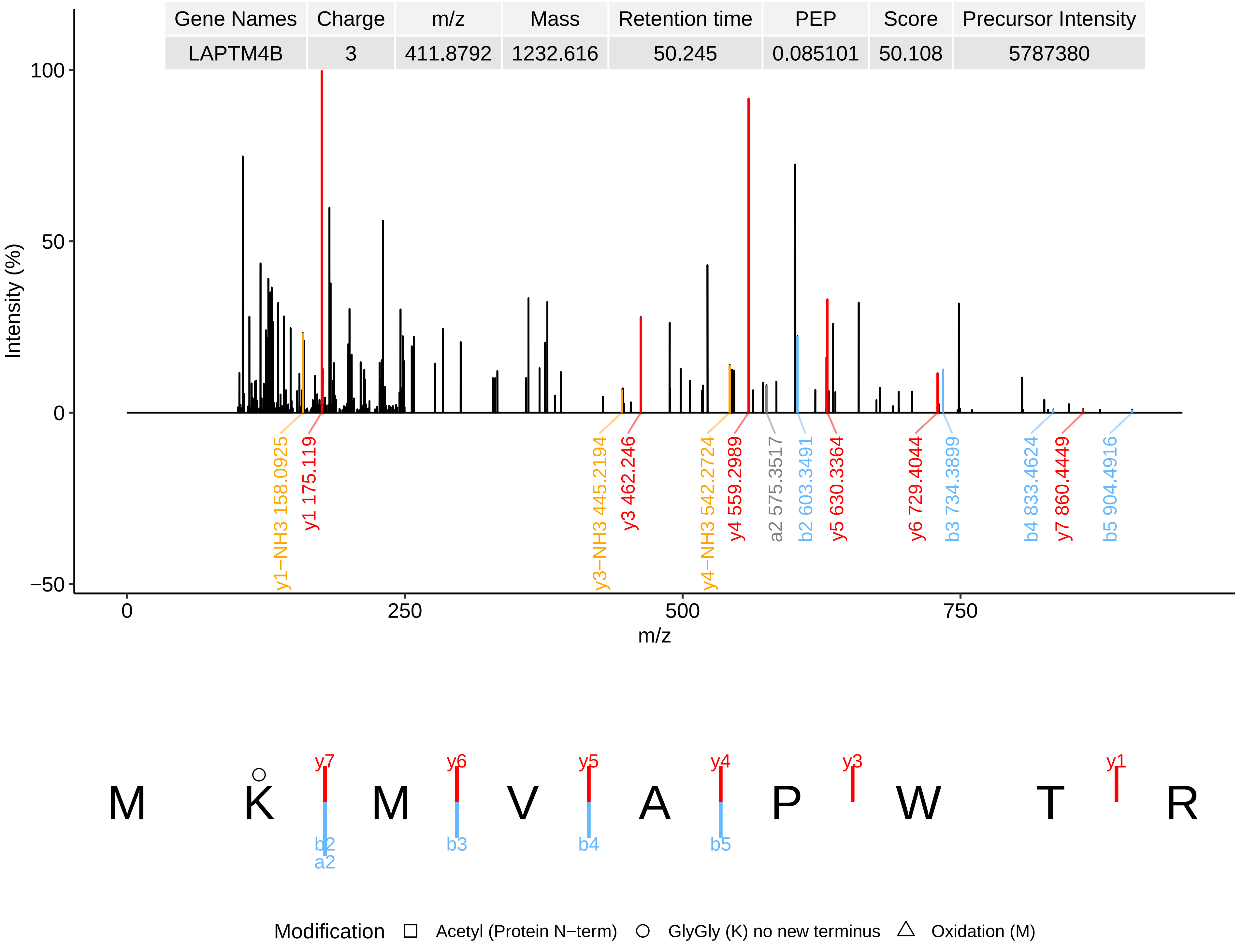

20200924_KIZ_MUb5_GlyGly_1 − Scan number: 15549

Gene Names	Charge	m/z	Mass	Retention time	PEP	Score	Precursor Intensity
LAPTM4B	3	411.8792	1232.616	50.765	0.045242	58.37	19650464

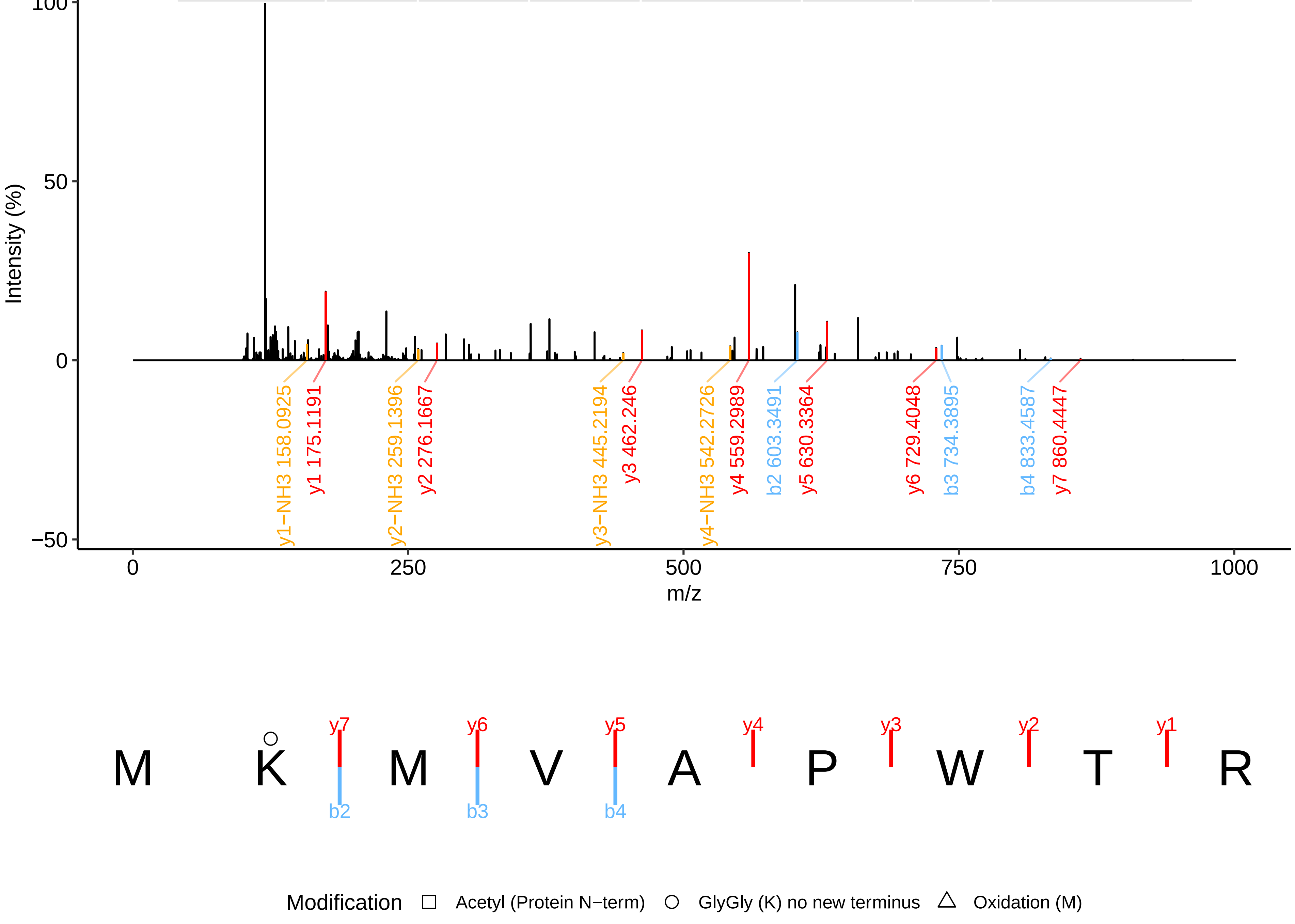

20201118_KIZ_MUb6_GlyGly_2 − Scan number: 18022

Gene Names	Charge	m/z	Mass	Retention time	PEP	Score	Precursor Intensity
LAPTM4B	3	411.8792	1232.616	60.963	0.10536	45.908	8519964

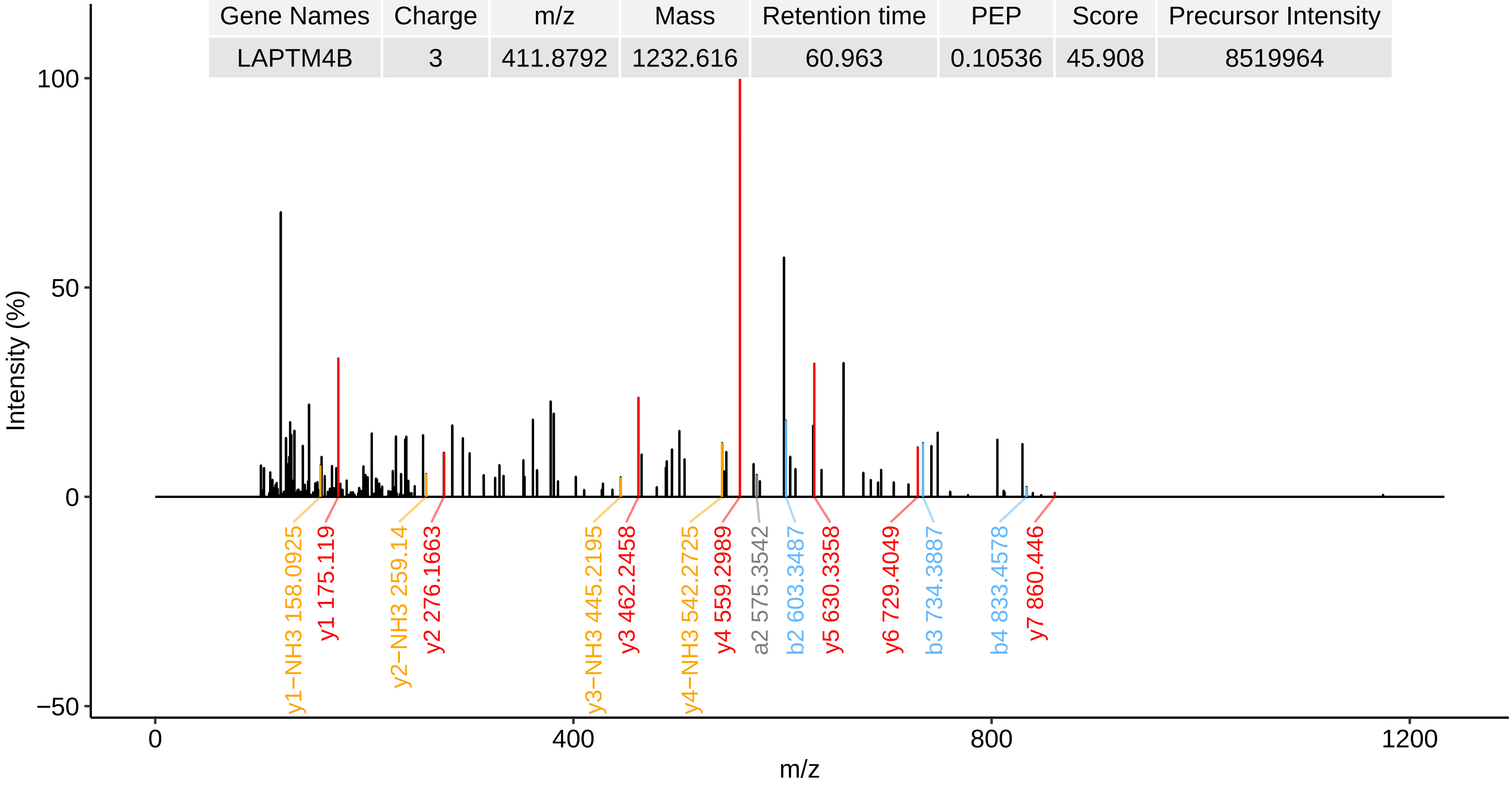

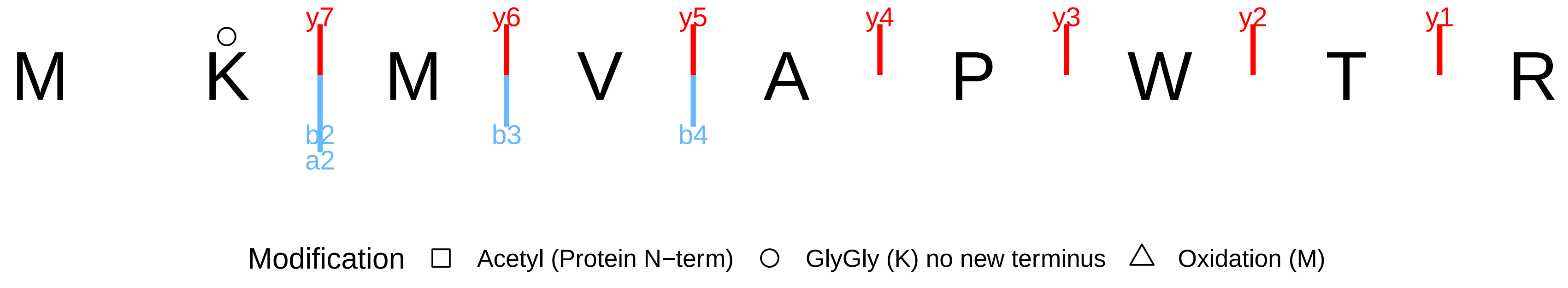

20200921_KIZ_MUb4_GlyGly_10plex − Scan number: 3883

Gene Names	Charge	m/z	Mass	Retention time	PEP	Score	Precursor Intensity
CPD	3	333.8248	998.4525	12.542	0.056866	49.833	2563820

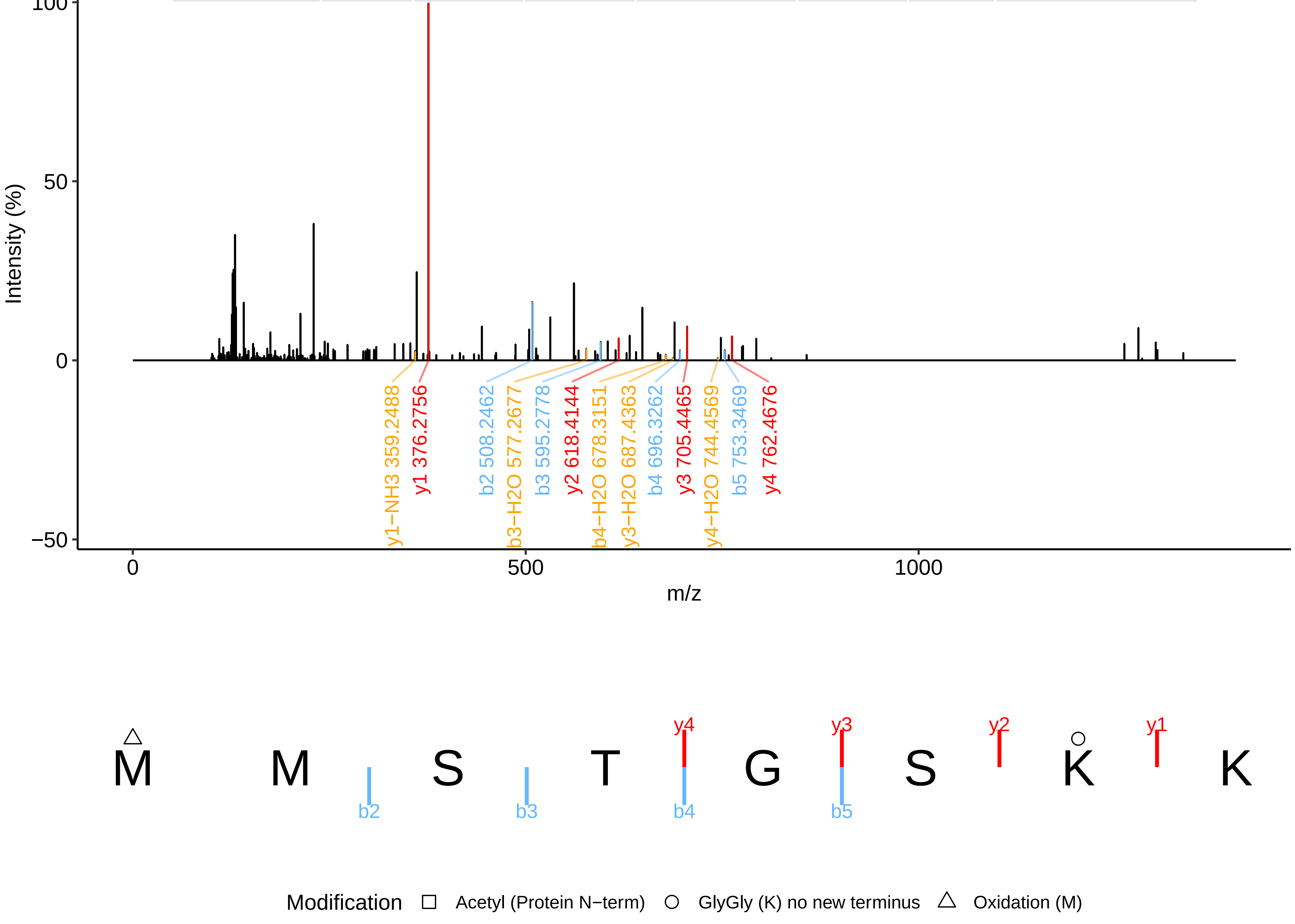

20200921_KIZ_MUb4_GlyGly_10plex − Scan number: 6007

Gene Names	Charge	m/z	Mass	Retention time	PEP	Score	Precursor Intensity
CPD	3	328.4931	982.4576	19.733	0.0090659	104.75	18476810

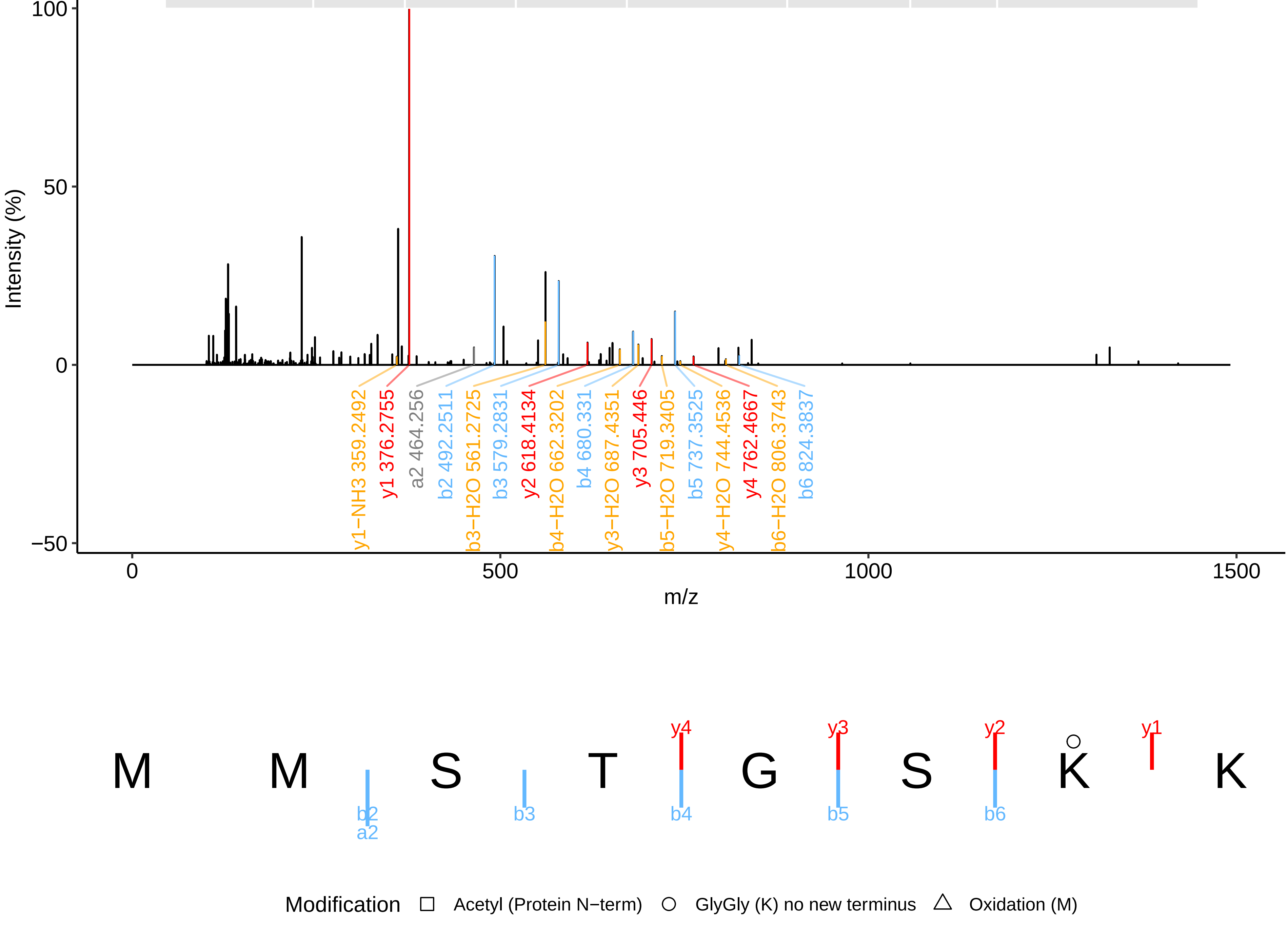

20200921_KIZ_MUb4_GlyGly_10plex_2 − Scan number: 5501

Gene Names	Charge	m/z	Mass	Retention time	PEP	Score	Precursor Intensity
CPD	3	328.4931	982.4576	19.806	0.0029383	113.5	2237483

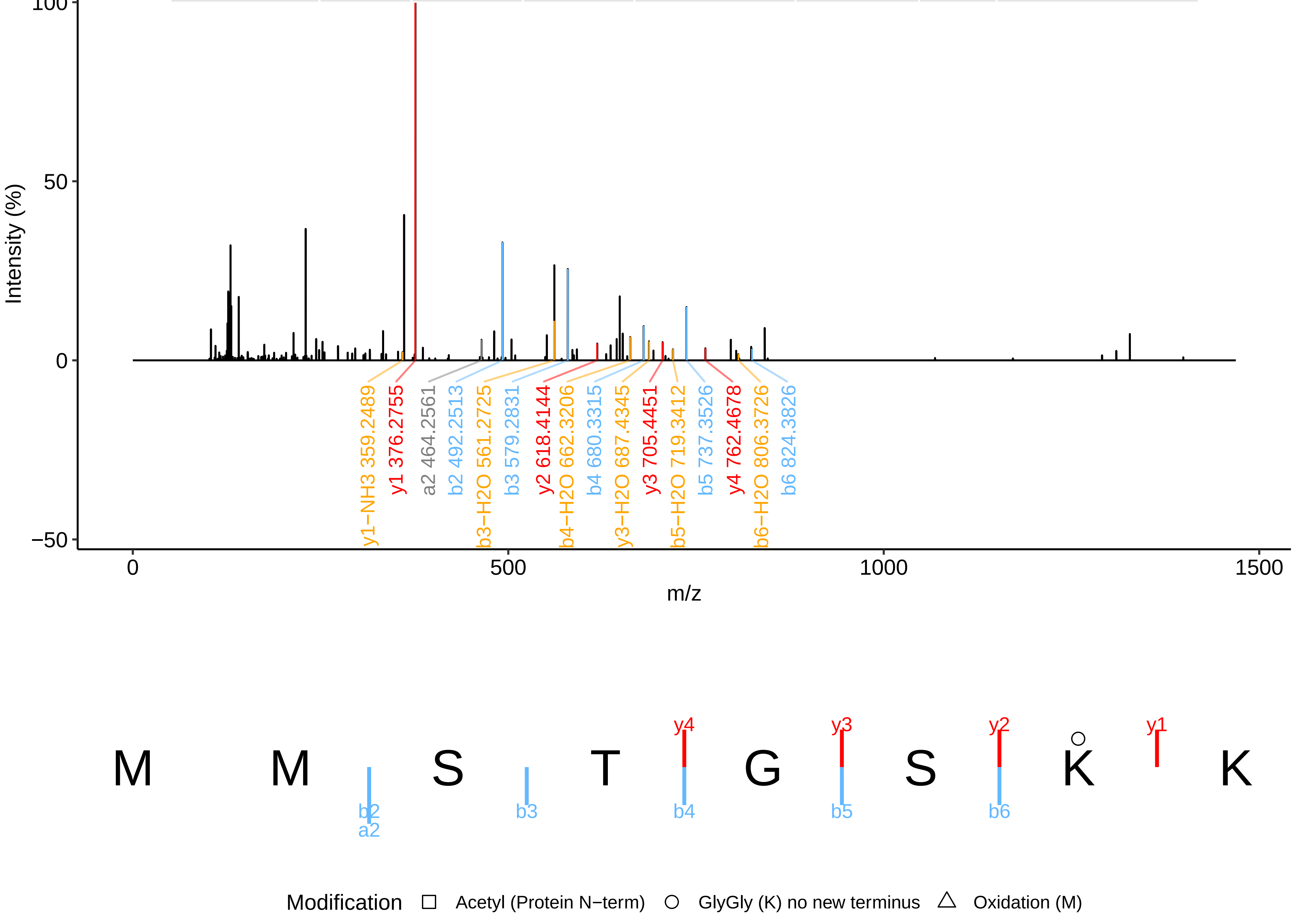

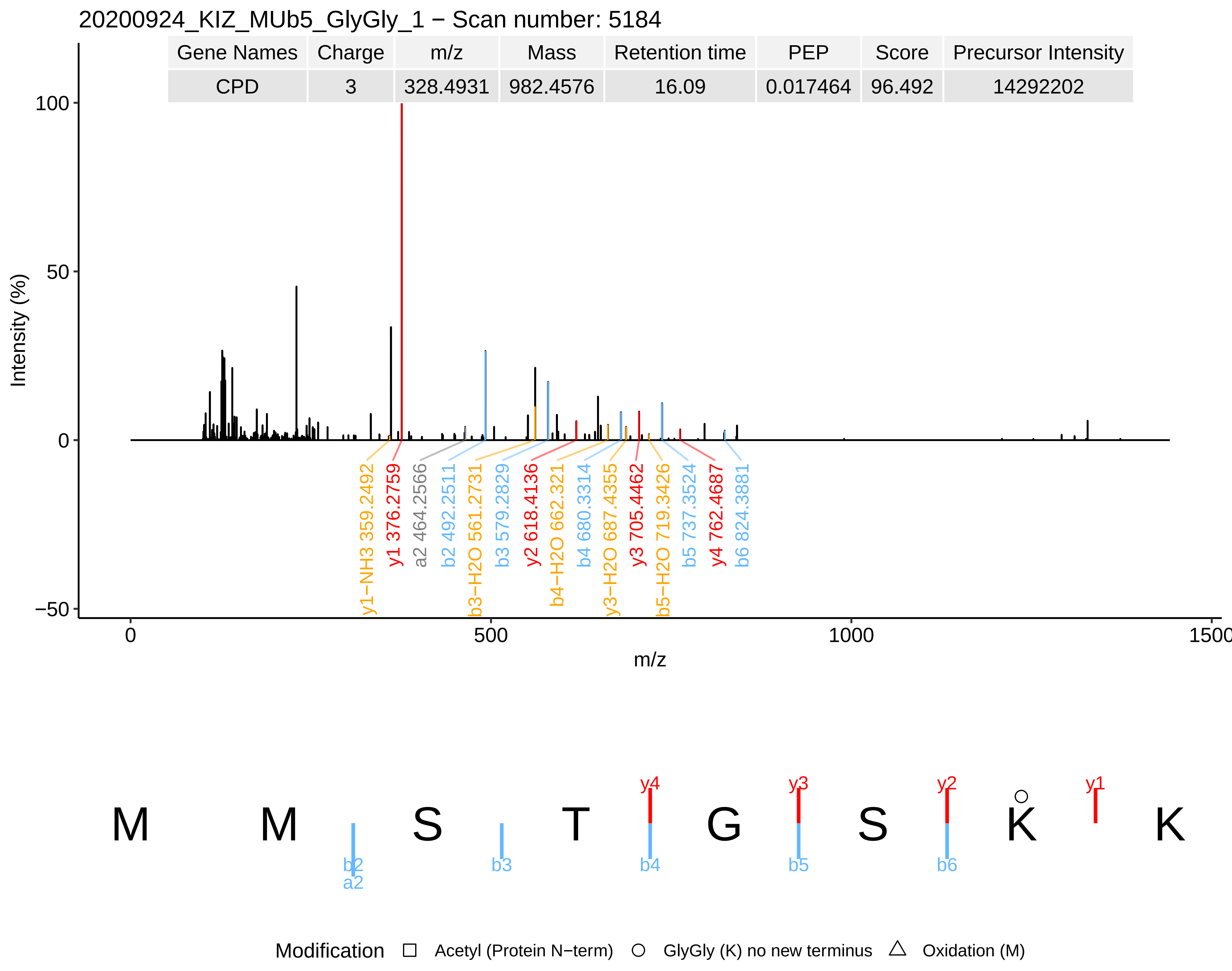

20200924_KIZ_MUb5_GlyGly_1 − Scan number: 5184
Gene Names | Charge | m/z | Mass | Retention time | PEP | Score | Precursor Intensity
CPD | 3 | 328.4931 | 982.4576 | 16.09 | 0.017464 | 96.492 | 14292202
Intensity (%)
m/z
y1−NH3 359.2492
y1 376.2759
a2 464.2566
b2 492.2511
b3−H2O 561.2731
b3 579.2829
y2 618.4136
b4−H2O 662.321
b4 680.3314
y3−H2O 687.4355
y3 705.4462
b5−H2O 719.3426
b5 737.3524
y4 762.4687
b6 824.3881
M M S T G S K K
y4 y3 y2 y1
b2 a2 b3 b4 b5 b6
Modification
Acetyl (Protein N−term)
GlyGly (K) no new terminus
Oxidation (M)

20200921_KIZ_MUb4_GlyGly_10plex − Scan number: 10857

Gene Names	Charge	m/z	Mass	Retention time	PEP	Score	Precursor Intensity
PPAP2B	2	623.2821	1244.55	37.72	0.0018623	76.899	5248456

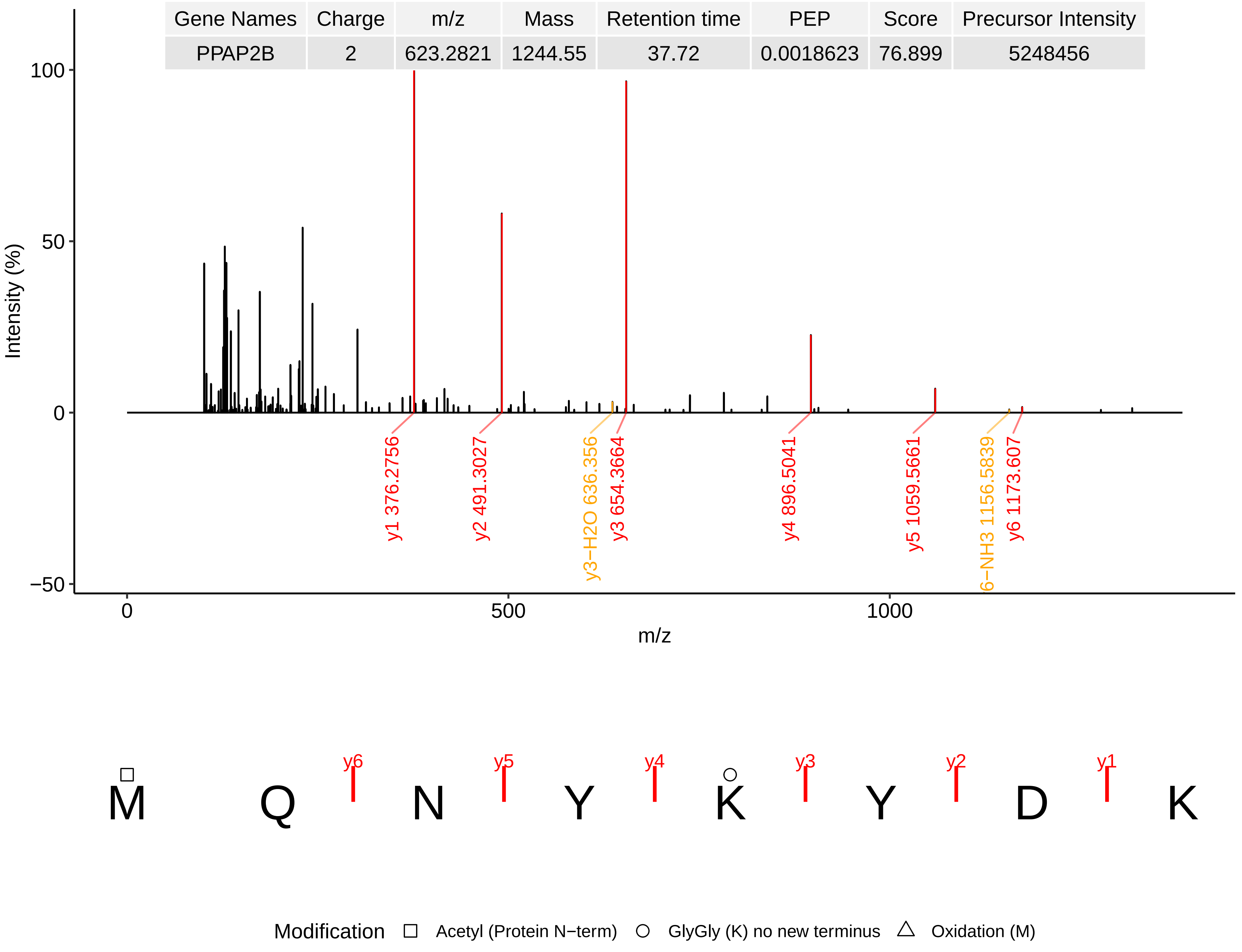

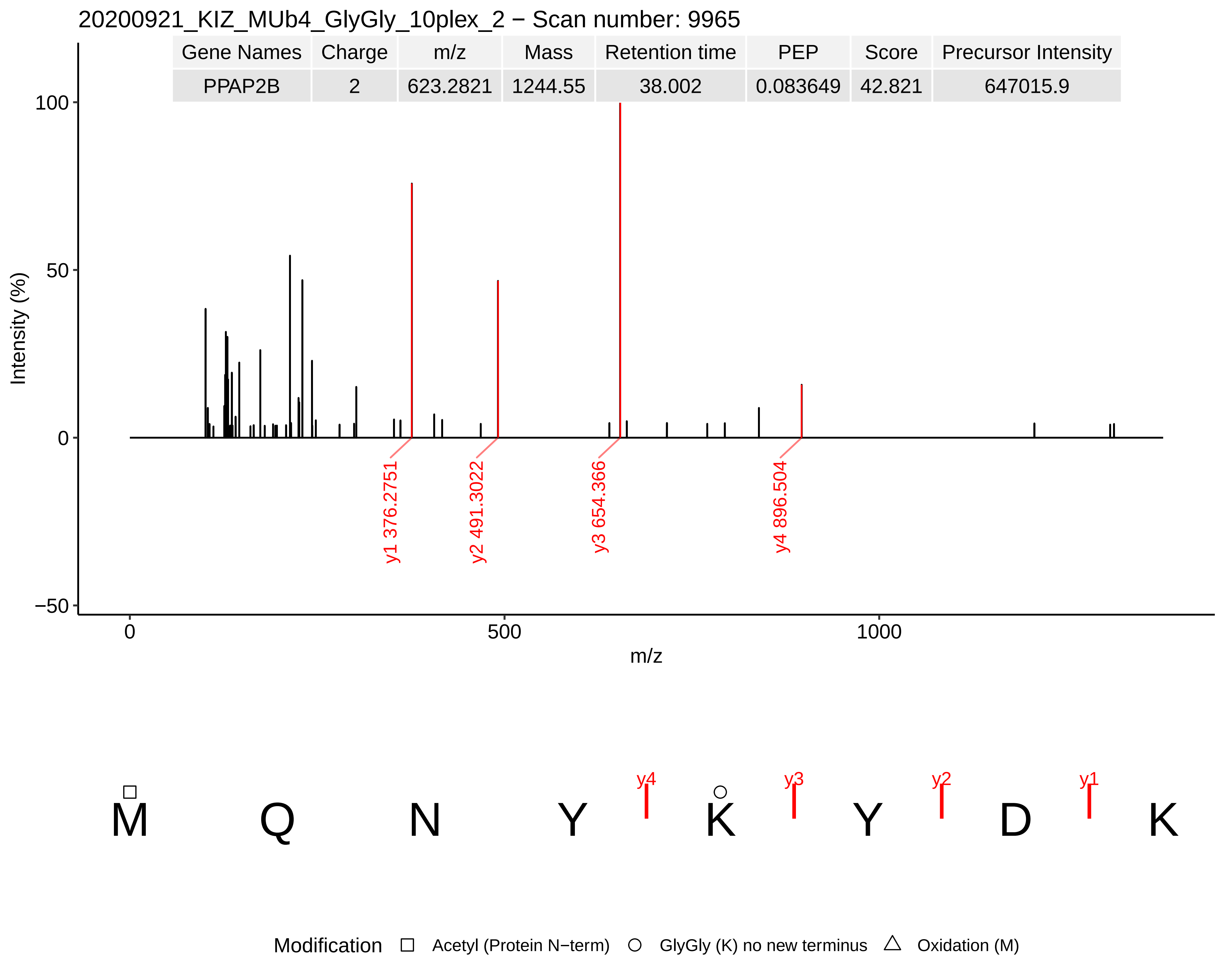
20200921_KIZ_MUb4_GlyGly_10plex_2 − Scan number: 9965
Gene Names | Charge | m/z | Mass | Retention time | PEP | Score | Precursor Intensity
PPAP2B | 2 | 623.2821 | 1244.55 | 38.002 | 0.083649 | 42.821 | 647015.9
Intensity (%)
100
50
0
−50
0
500
1000
m/z
y1 376.2751
y2 491.3022
y3 654.366
y4 896.504
M Q N Y K Y D K
y4
y3
y2
y1
Modification
Acetyl (Protein N−term)
GlyGly (K) no new terminus
Oxidation (M)

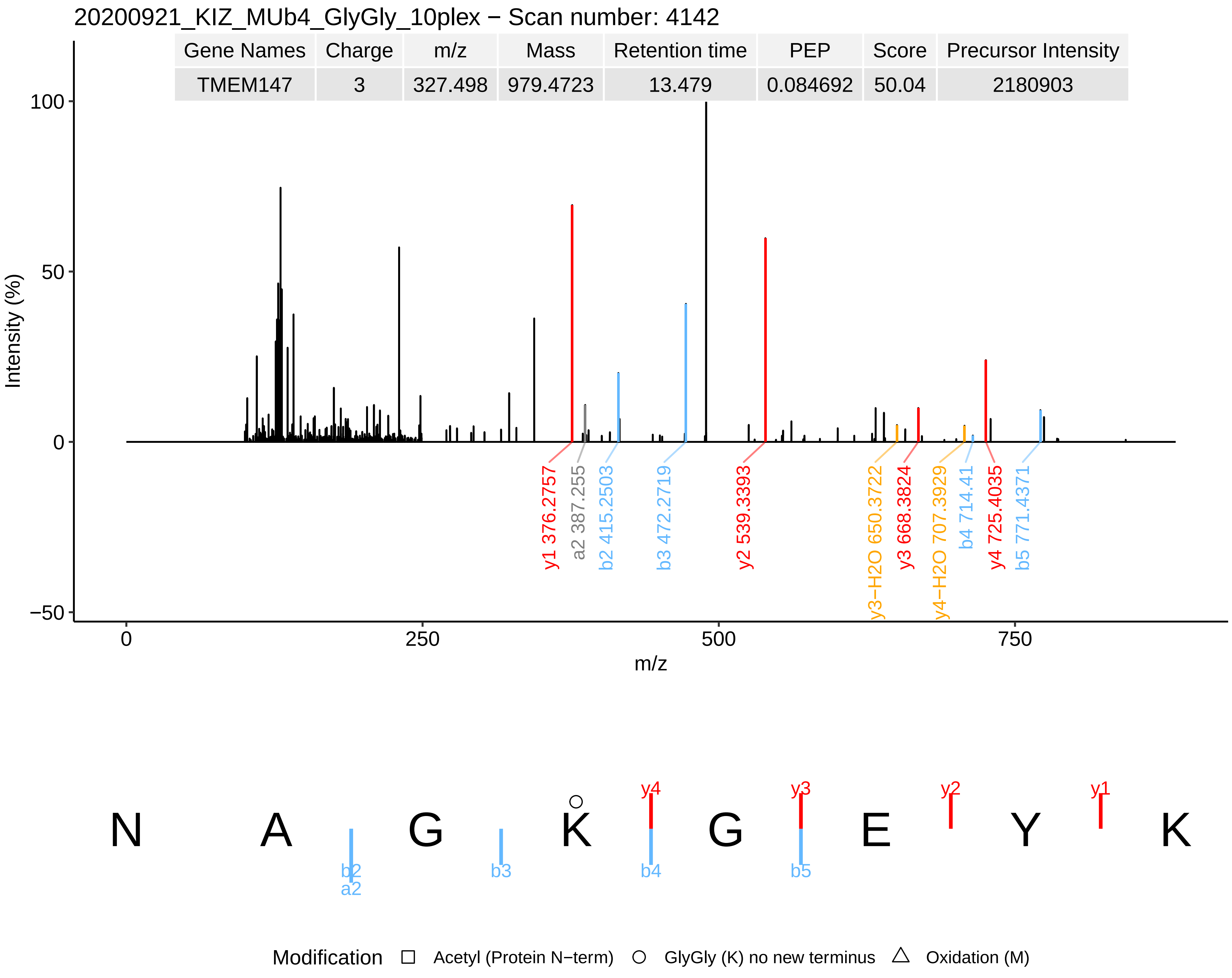

20200921_KIZ_MUb4_GlyGly_10plex − Scan number: 4142
Gene Names | Charge | m/z | Mass | Retention time | PEP | Score | Precursor Intensity
TMEM147 | 3 | 327.498 | 979.4723 | 13.479 | 0.084692 | 50.04 | 2180903
Intensity (%)
100
50
0
−50
0
250
500
750
m/z
y1 376.2757
a2 387.255
b2 415.2503
b3 472.2719
y2 539.3393
y3−H2O 650.3722
y3 668.3824
y4−H2O 707.3929
b4 714.41
y4 725.4035
b5 771.4371
N A G K G E Y K
y4
y3
y2
y1
b2
a2
b3
b4
b5
Modification
Acetyl (Protein N−term)
GlyGly (K) no new terminus
Oxidation (M)

20200921_KIZ_MUb4_GlyGly_10plex − Scan number: 15365

Gene Names	Charge	m/z	Mass	Retention time	PEP	Score	Precursor Intensity
RPL9	3	418.5594	1252.656	54.868	0.0020369	96.331	NA

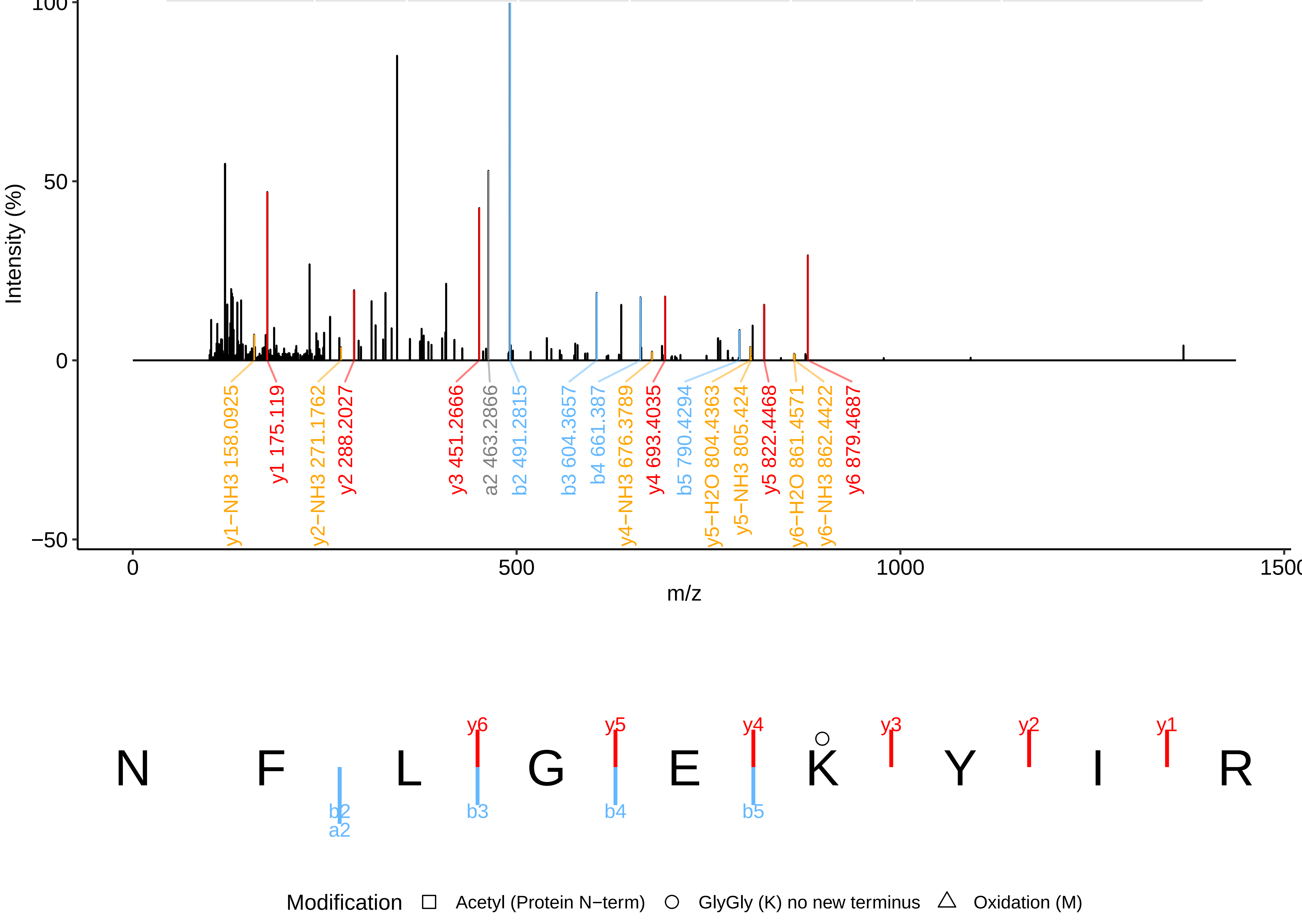

20200921_KIZ_MUb4_GlyGly_10plex − Scan number: 5306

Gene Names	Charge	m/z	Mass	Retention time	PEP	Score	Precursor Intensity
S100A11	3	419.2178	1254.632	17.356	0.002259	82.914	8922127

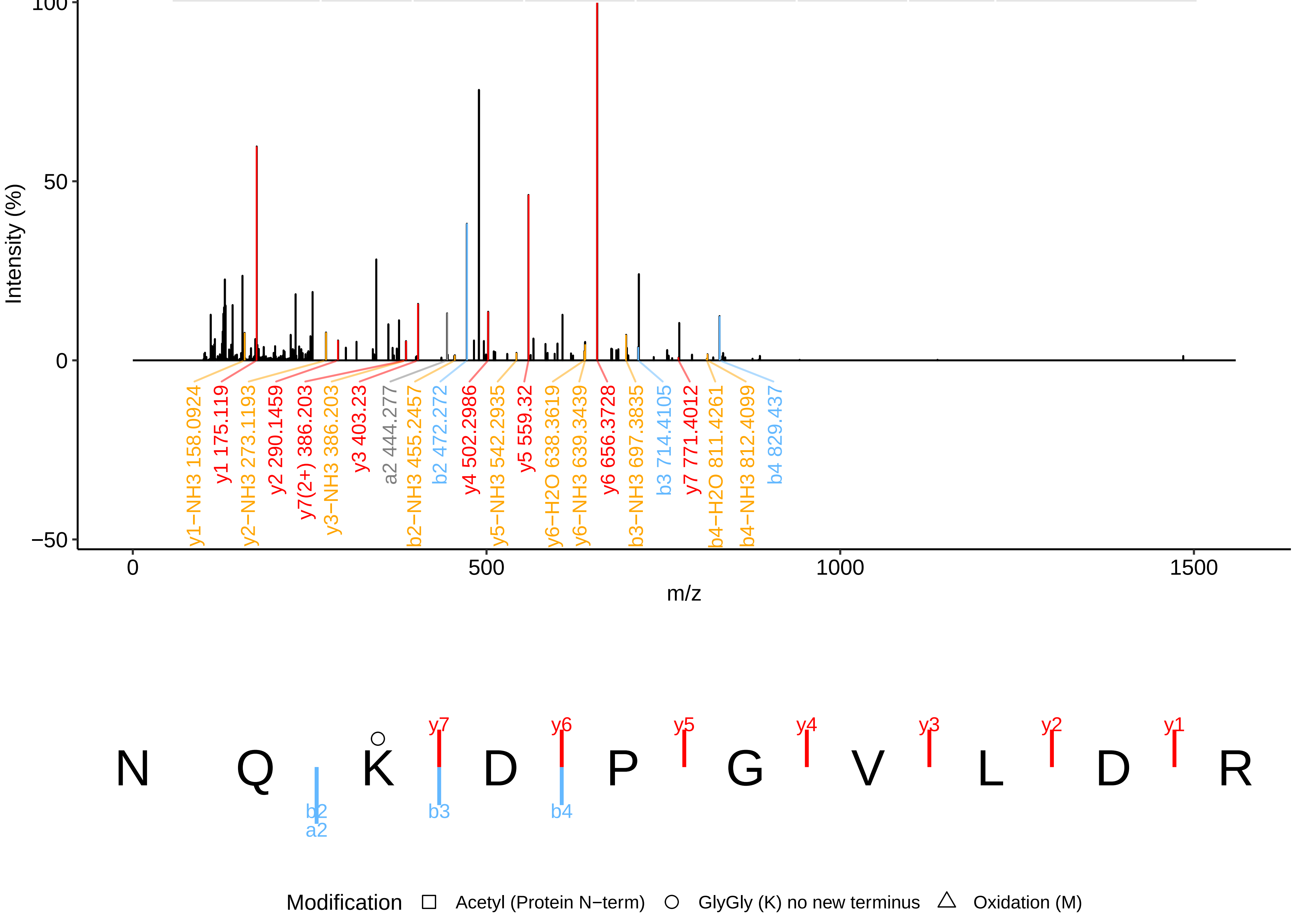

20200921_KIZ_MUb4_GlyGly_10plex_2 − Scan number: 4861

Gene Names	Charge	m/z	Mass	Retention time	PEP	Score	Precursor Intensity
S100A11	3	419.2178	1254.632	17.233	0.0015036	88.134	3120971

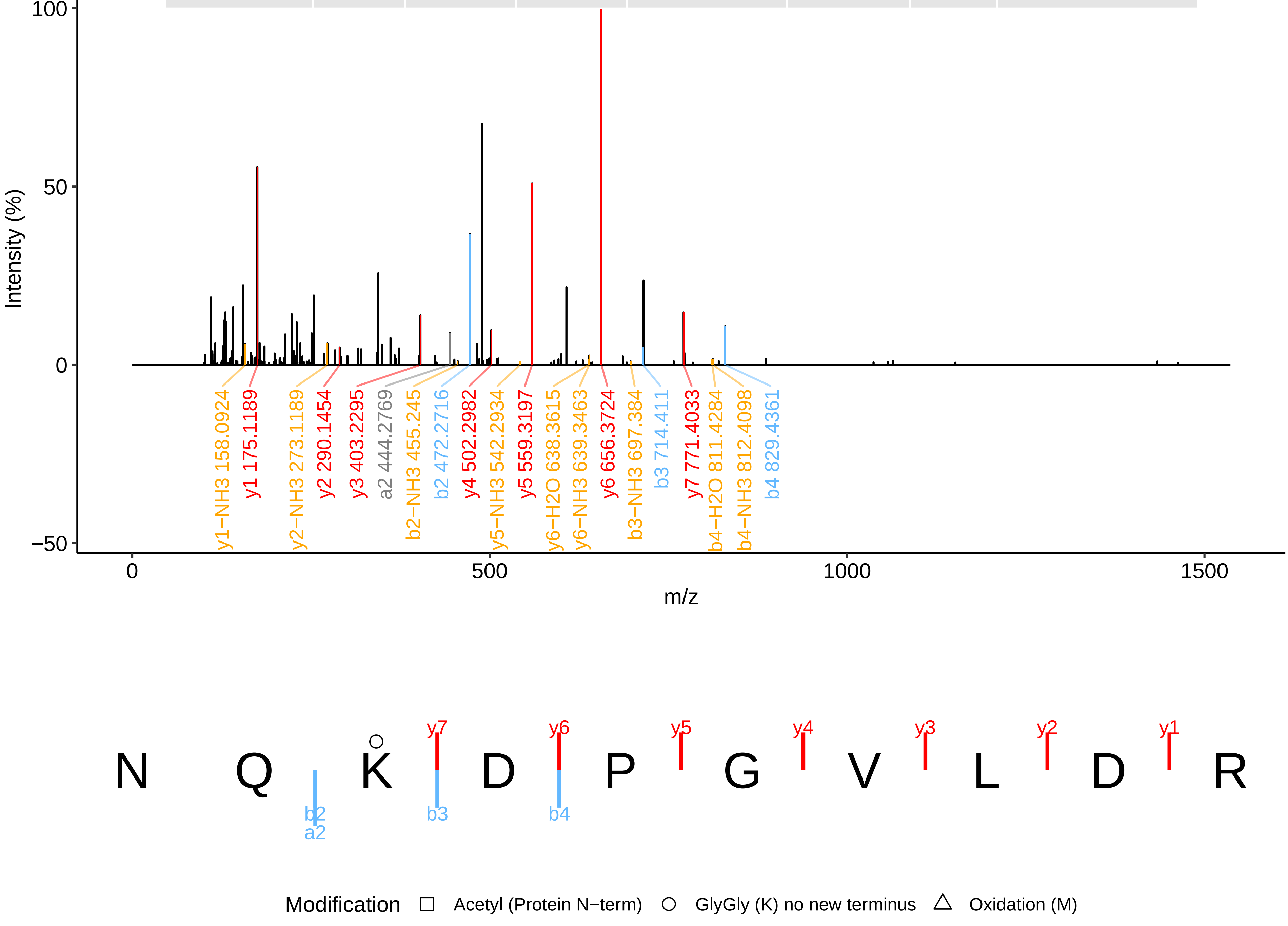

20200921_KIZ_MUb4_GlyGly_10plex − Scan number: 6939

Gene Names	Charge	m/z	Mass	Retention time	PEP	Score	Precursor Intensity
HSPA9	3	339.5294	1015.566	23.142	0.071944	74.301	NA

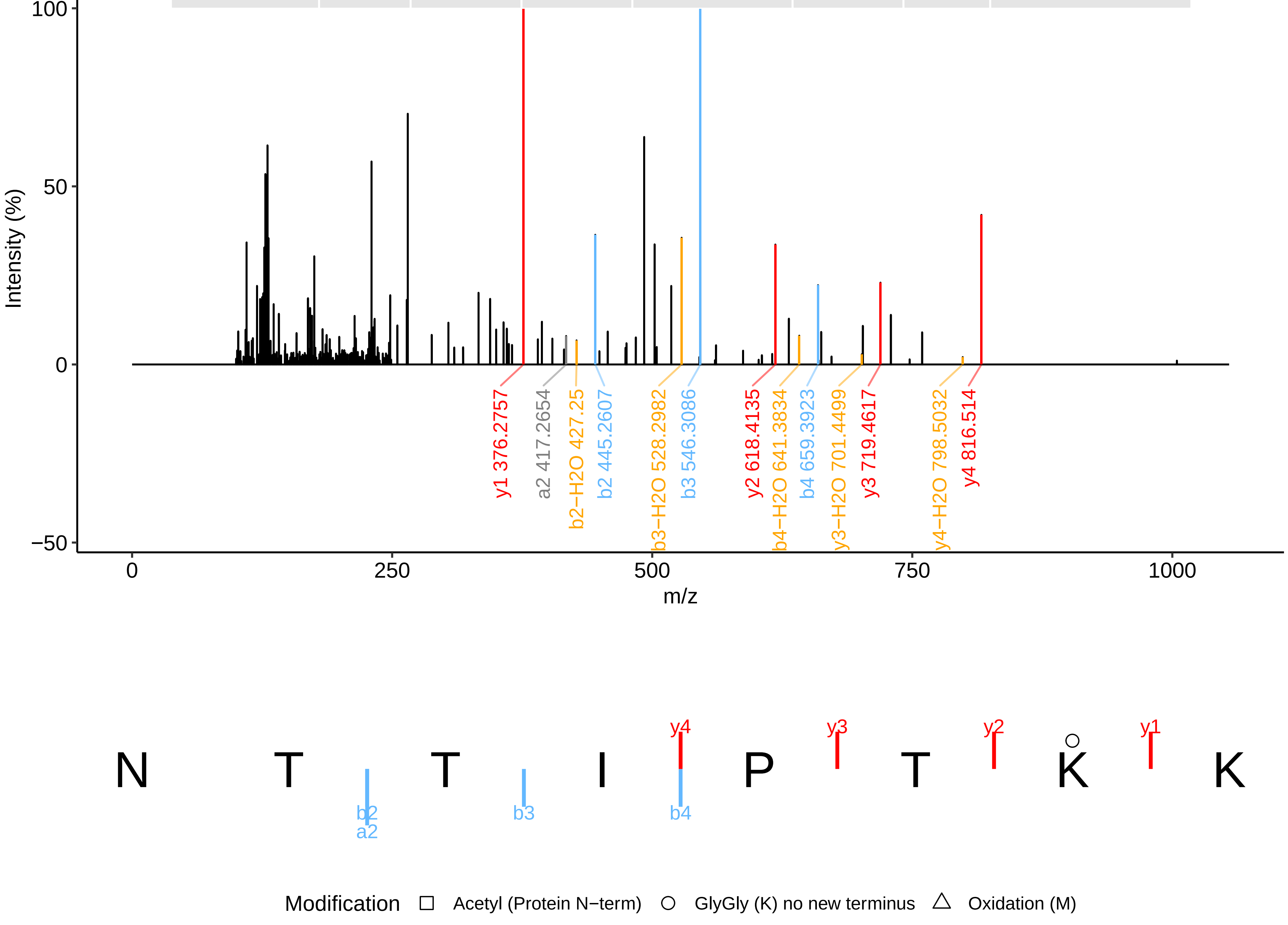

20200924_KIZ_MUb5_GlyGly_1 − Scan number: 13329

Gene Names	Charge	m/z	Mass	Retention time	PEP	Score	Precursor Intensity
CMAS	2	605.3442	1208.674	43.44	0.038193	87.754	32894888

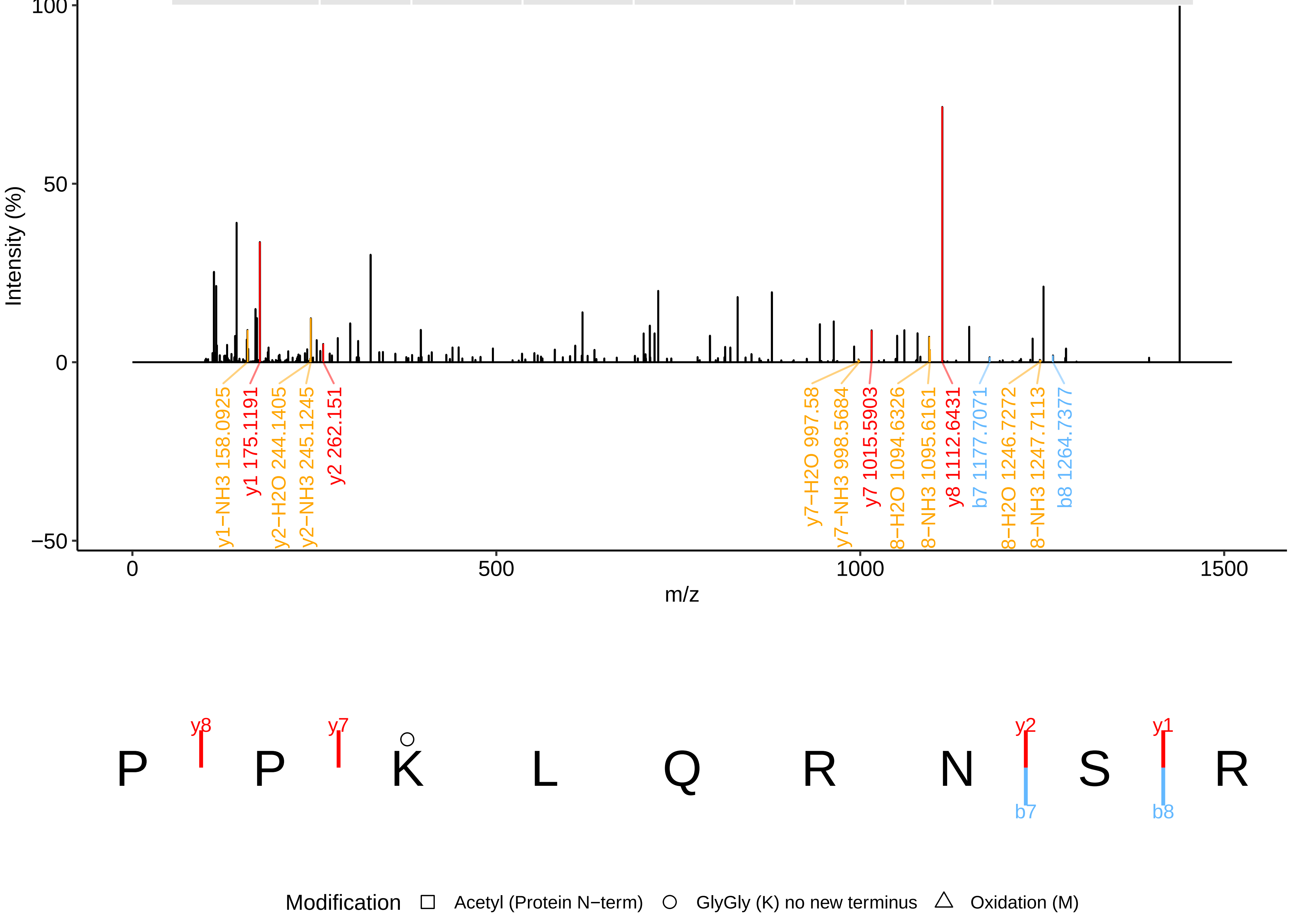

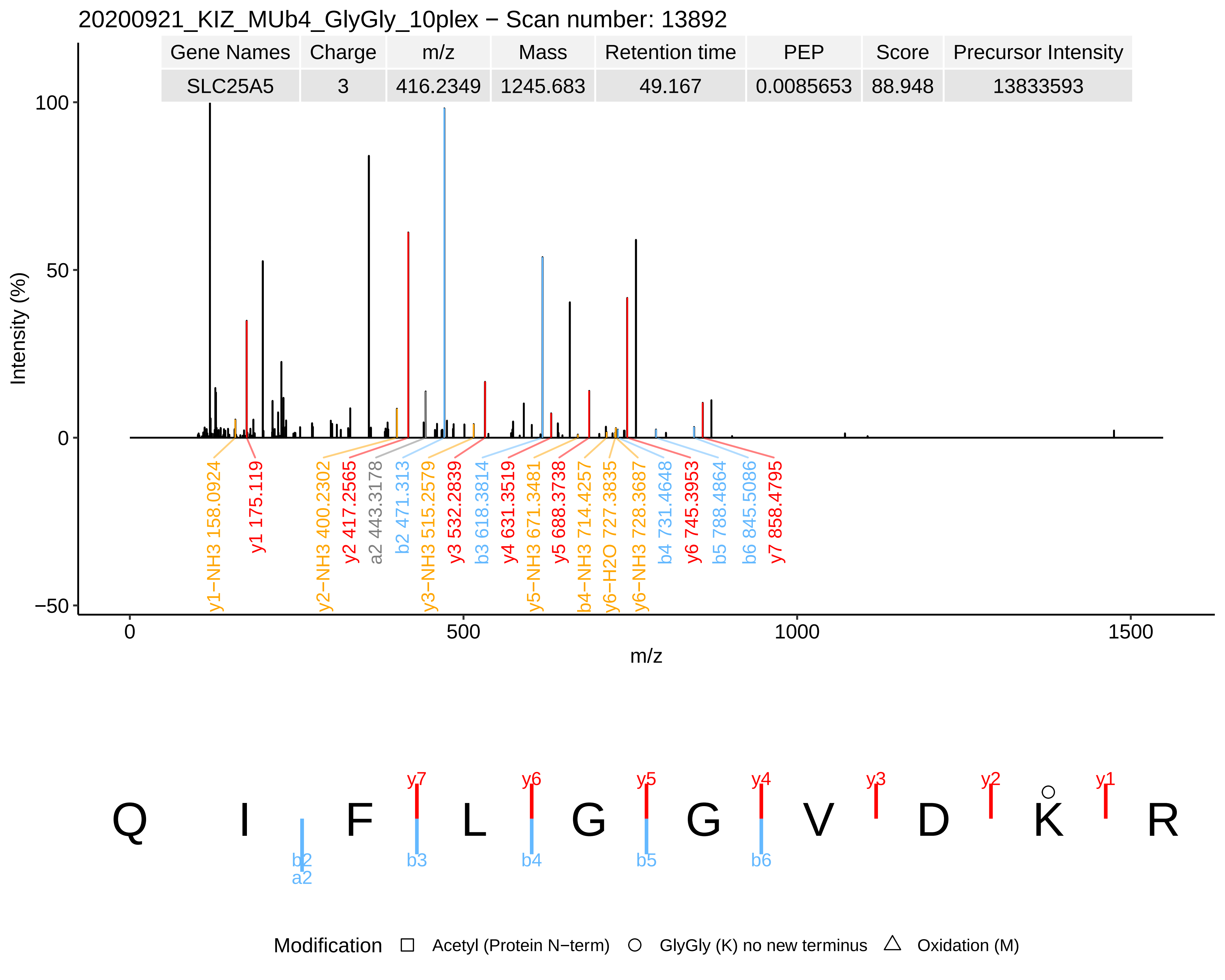
20200921_KIZ_MUb4_GlyGly_10plex − Scan number: 13892
Gene Names
Charge
m/z
Mass
Retention time
PEP
Score
Precursor Intensity
SLC25A5
3
416.2349
1245.683
49.167
0.0085653
88.948
13833593
Intensity (%)
100
50
0
−50
0
500
1000
1500
m/z
y1−NH3 158.0924
y1 175.119
y2−NH3 400.2302
y2 417.2565
a2 443.3178
b2 471.313
y3−NH3 515.2579
y3 532.2839
b3 618.3814
y4 631.3519
y5−NH3 671.3481
y5 688.3738
b4−NH3 714.4257
y6−H2O 727.3835
y6−NH3 728.3687
b4 731.4648
y6 745.3953
b5 788.4864
b6 845.5086
y7 858.4795
Q I F L G G V D K R
y7 y6 y5 y4 y3 y2 y1
b2 a2 b3 b4 b5 b6
Modification
Acetyl (Protein N−term)
GlyGly (K) no new terminus
Oxidation (M)

20200921_KIZ_MUb4_GlyGly_10plex_2 − Scan number: 12796

Gene Names	Charge	m/z	Mass	Retention time	PEP	Score	Precursor Intensity
SLC25A5	3	416.2349	1245.683	49.589	0.0001378	110.38	3945389

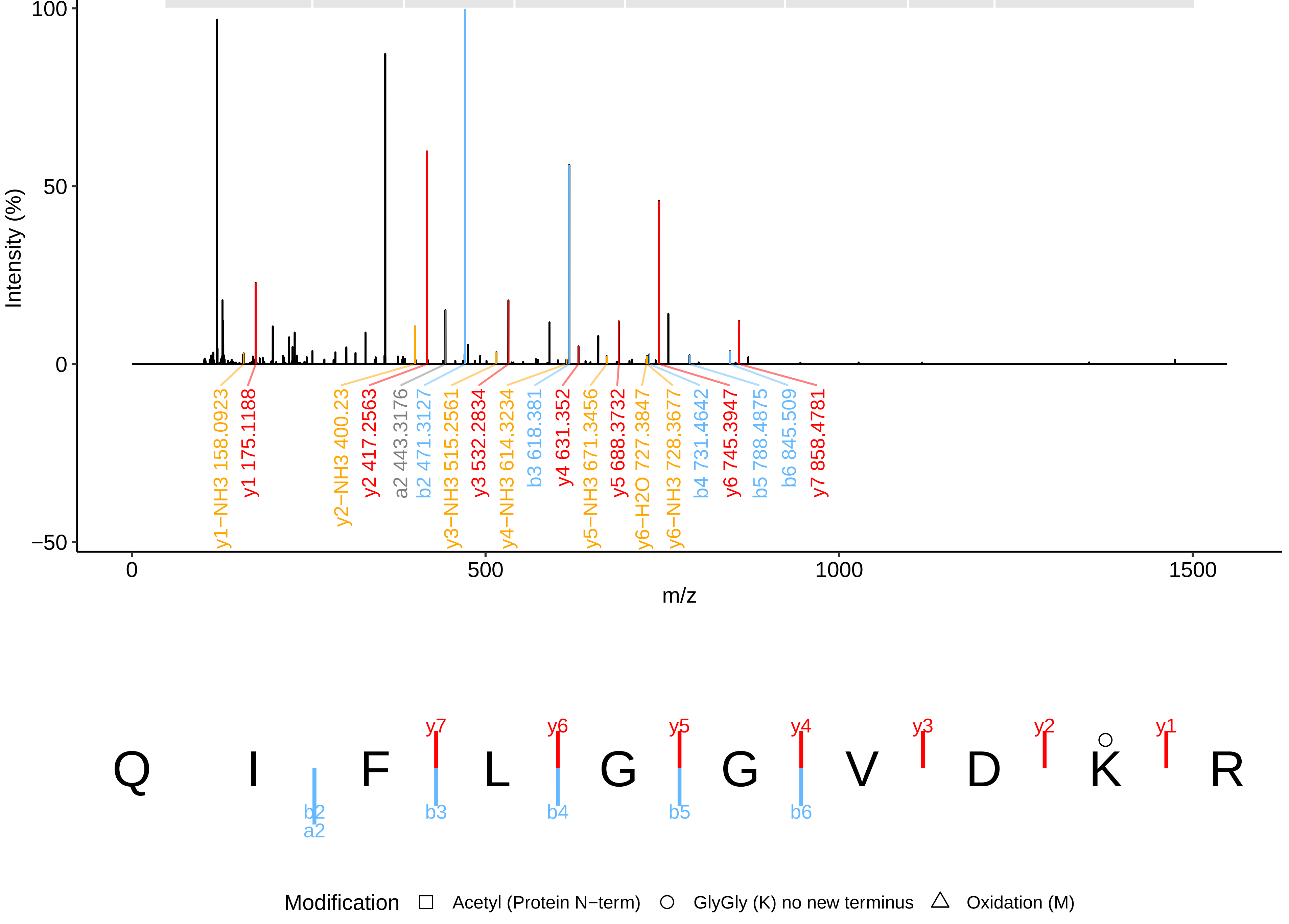

20200924_KIZ_MUb5_GlyGly_1 − Scan number: 12693

Gene Names	Charge	m/z	Mass	Retention time	PEP	Score	Precursor Intensity
SLC25A5	3	416.2349	1245.683	41.335	0.0022887	112.15	9525324

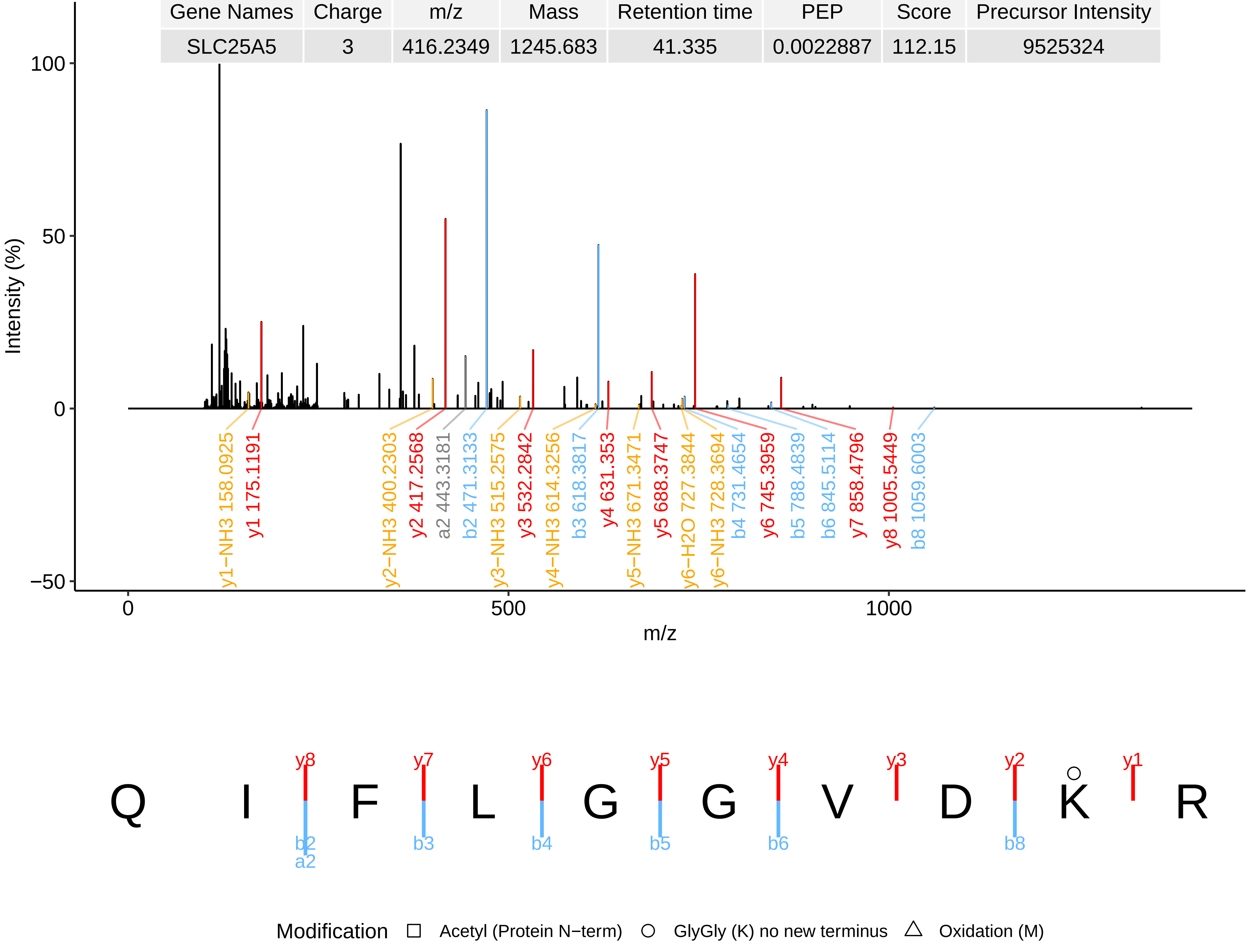

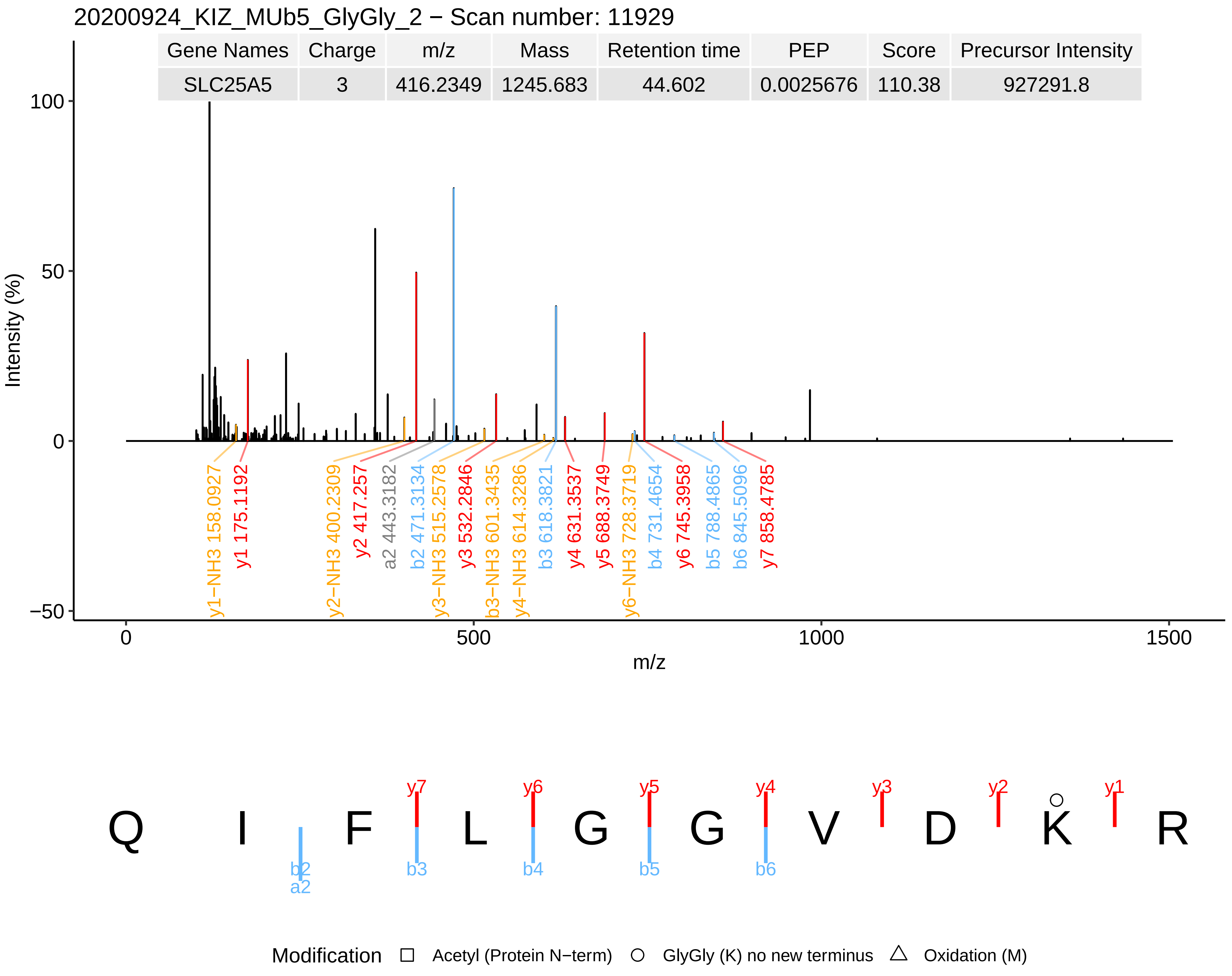

20200924_KIZ_MUb5_GlyGly_2 − Scan number: 11929
Gene Names | Charge | m/z | Mass | Retention time | PEP | Score | Precursor Intensity
SLC25A5 | 3 | 416.2349 | 1245.683 | 44.602 | 0.0025676 | 110.38 | 927291.8
Intensity (%)
m/z
y1−NH3 158.0927
y1 175.1192
y2−NH3 400.2309
y2 417.257
a2 443.3182
b2 471.3134
y3−NH3 515.2578
y3 532.2846
b3−NH3 601.3435
y4−NH3 614.3286
b3 618.3821
y4 631.3537
y5 688.3749
y6−NH3 728.3719
b4 731.4654
y6 745.3958
b5 788.4865
b6 845.5096
y7 858.4785
Q I F L G G V D K R
Modification
Acetyl (Protein N−term)
GlyGly (K) no new terminus
Oxidation (M)

20200921_KIZ_MUb4_GlyGly_10plex_2 − Scan number: 8361

Gene Names	Charge	m/z	Mass	Retention time	PEP	Score	Precursor Intensity
ATAD1	3	333.8697	998.5873	31.476	0.044373	61.999	826731.6

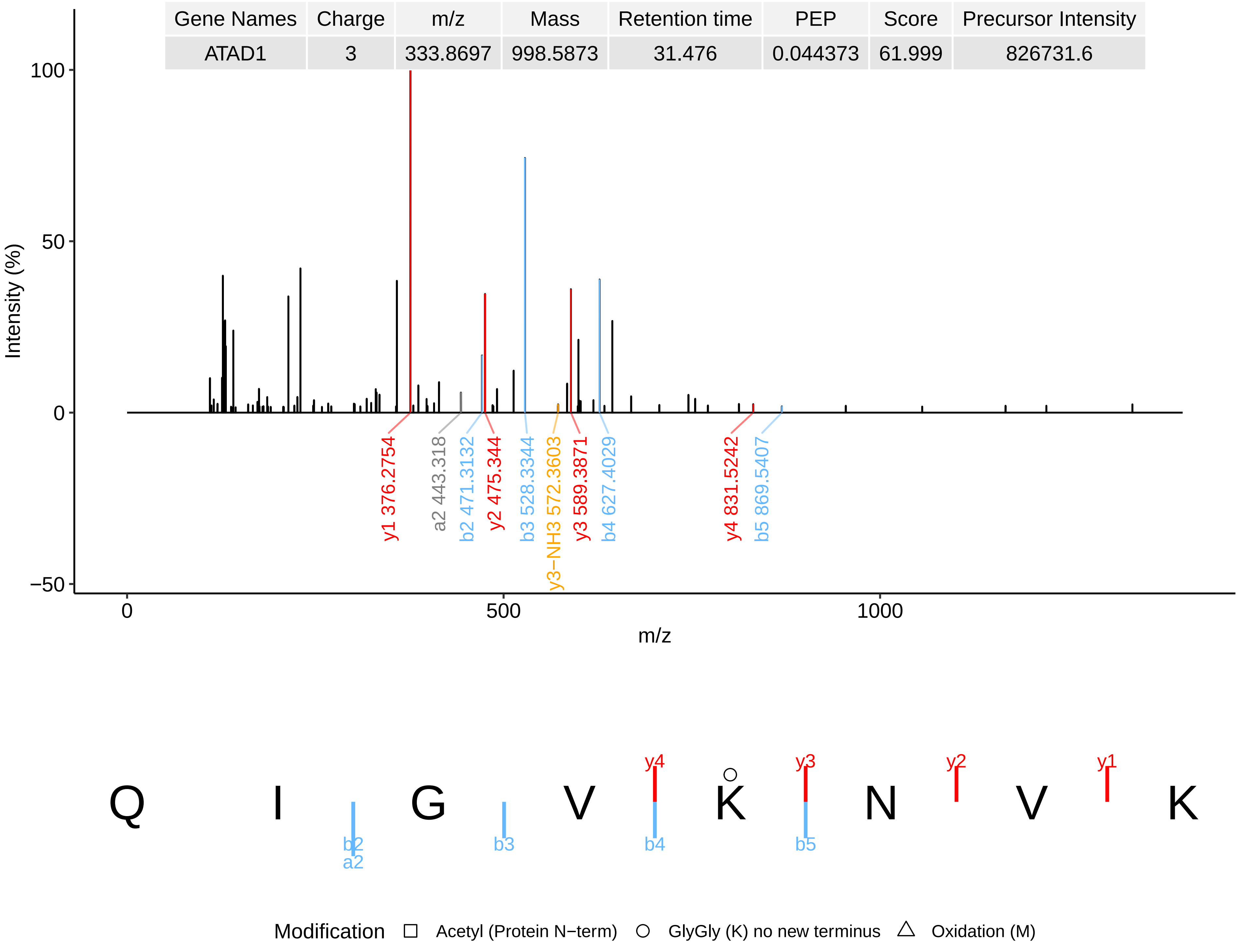

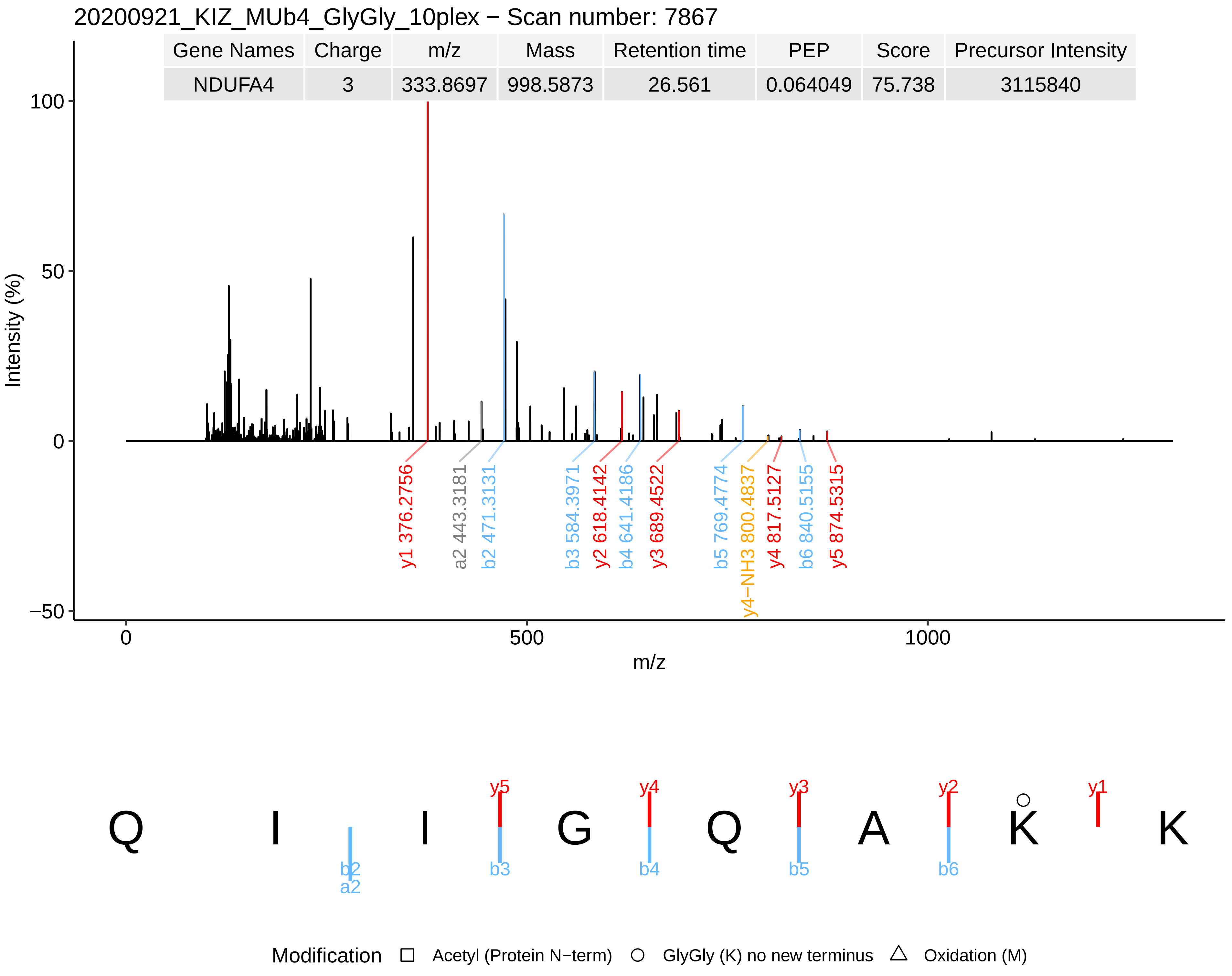
20200921_KIZ_MUb4_GlyGly_10plex − Scan number: 7867
Gene Names
Charge
m/z
Mass
Retention time
PEP
Score
Precursor Intensity
NDUFA4
3
333.8697
998.5873
26.561
0.064049
75.738
3115840
Intensity (%)
100
50
0
−50
0
500
1000
m/z
y1 376.2756
a2 443.3181
b2 471.3131
b3 584.3971
y2 618.4142
b4 641.4186
y3 689.4522
b5 769.4774
y4−NH3 800.4837
y4 817.5127
b6 840.5155
y5 874.5315
Q
I
I
G
Q
A
K
K
y5
y4
y3
y2
y1
b2
a2
b3
b4
b5
b6
Modification
Acetyl (Protein N−term)
GlyGly (K) no new terminus
Oxidation (M)

20200921_KIZ_MUb4_GlyGly_10plex − Scan number: 6277

Gene Names	Charge	m/z	Mass	Retention time	PEP	Score	Precursor Intensity
ABCC4	3	329.5137	985.5193	20.709	0.068954	78.934	5895587

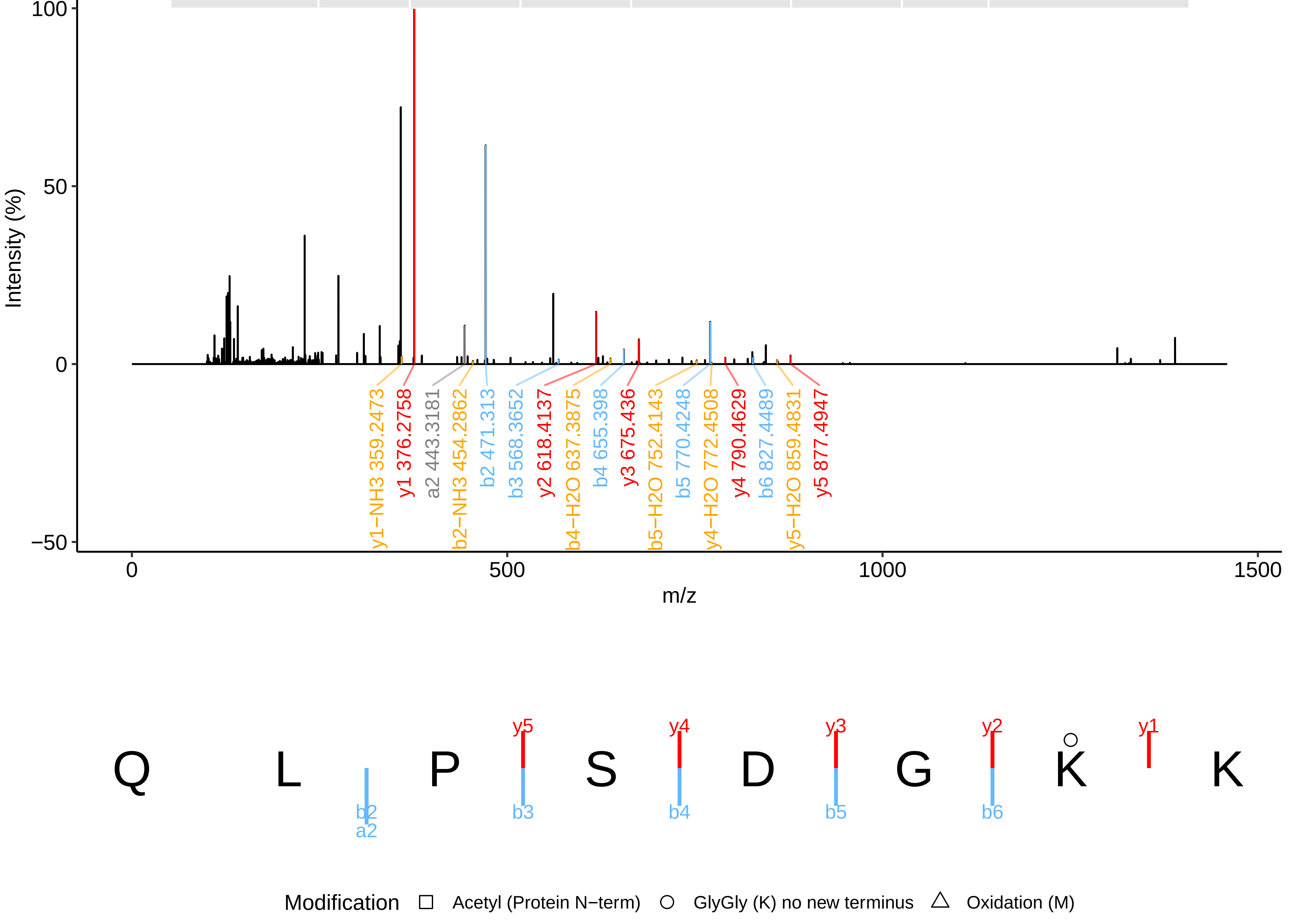

20200924_KIZ_MUb5_GlyGly_1 − Scan number: 5419

Gene Names	Charge	m/z	Mass	Retention time	PEP	Score	Precursor Intensity
ABCC4	3	329.5137	985.5193	16.851	0.028346	87.308	12067764

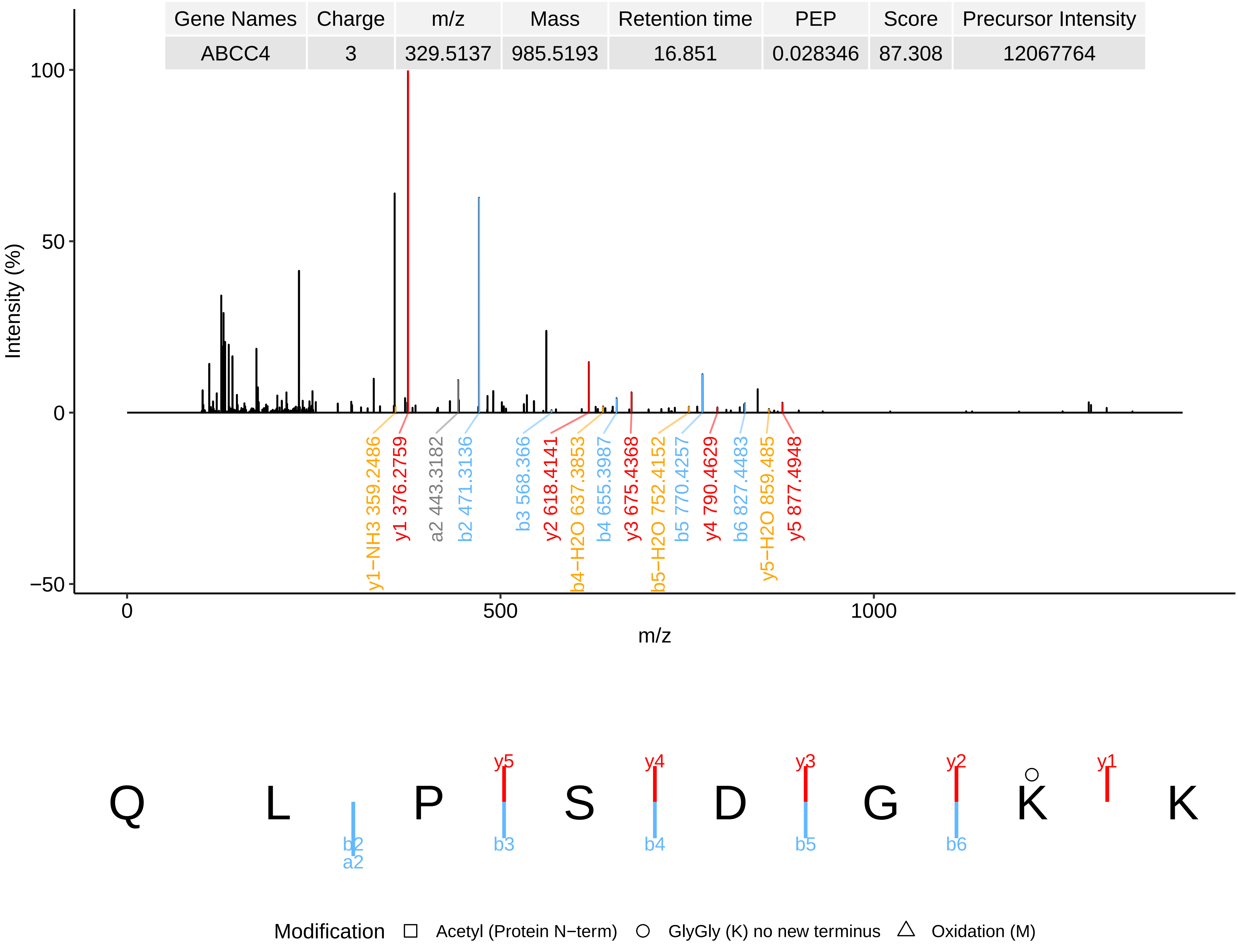

20200924_KIZ_MUb5_GlyGly_2 − Scan number: 5059

Gene Names	Charge	m/z	Mass	Retention time	PEP	Score	Precursor Intensity
ABCC4	3	329.5137	985.5193	18.718	0.10452	68.371	1480400

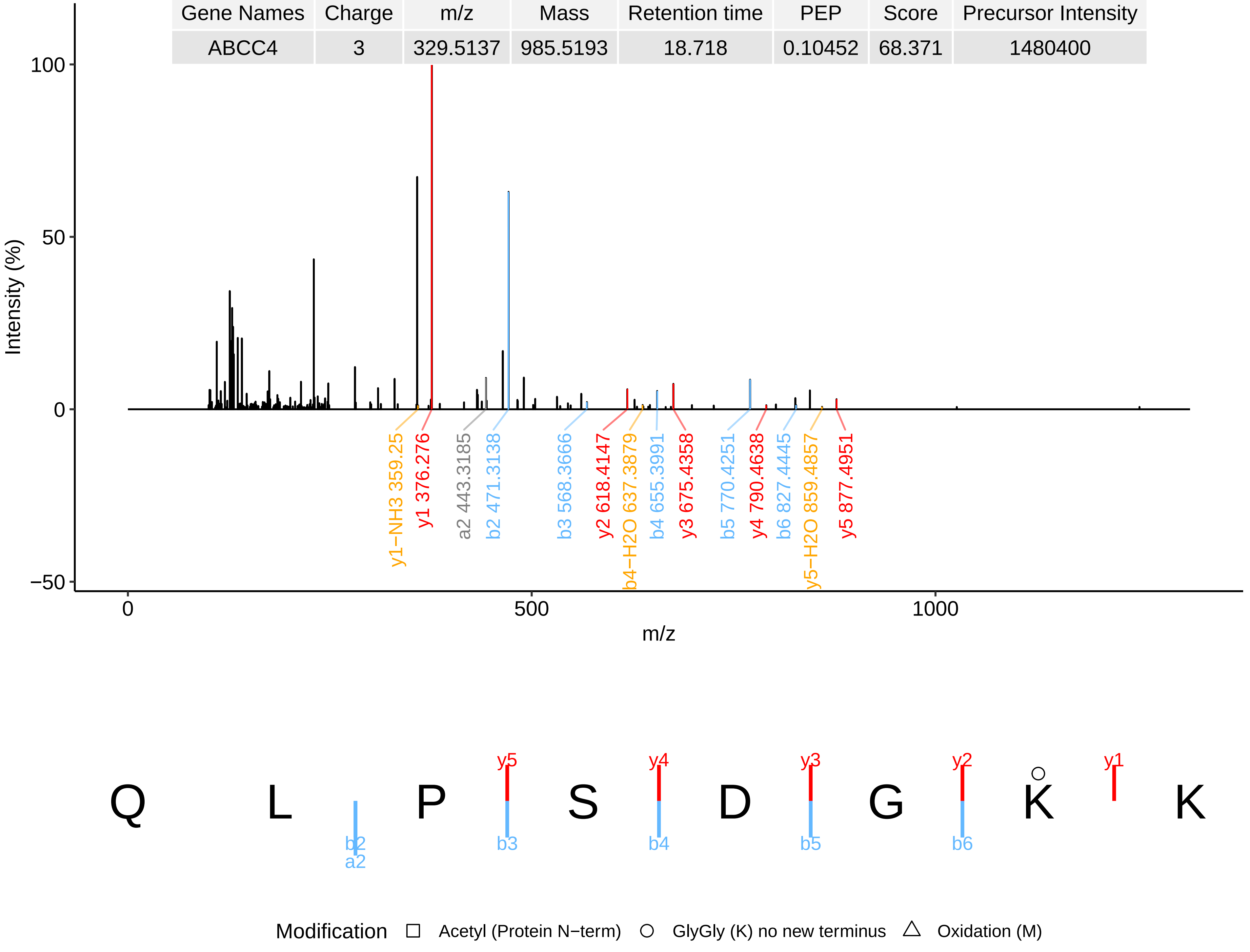

20200924_KIZ_MUb5_GlyGly_1 − Scan number: 22968

Gene Names	Charge	m/z	Mass	Retention time	PEP	Score	Precursor Intensity
OR4F21	3	335.8746	1004.602	73.867	0.097726	86.113	12665667

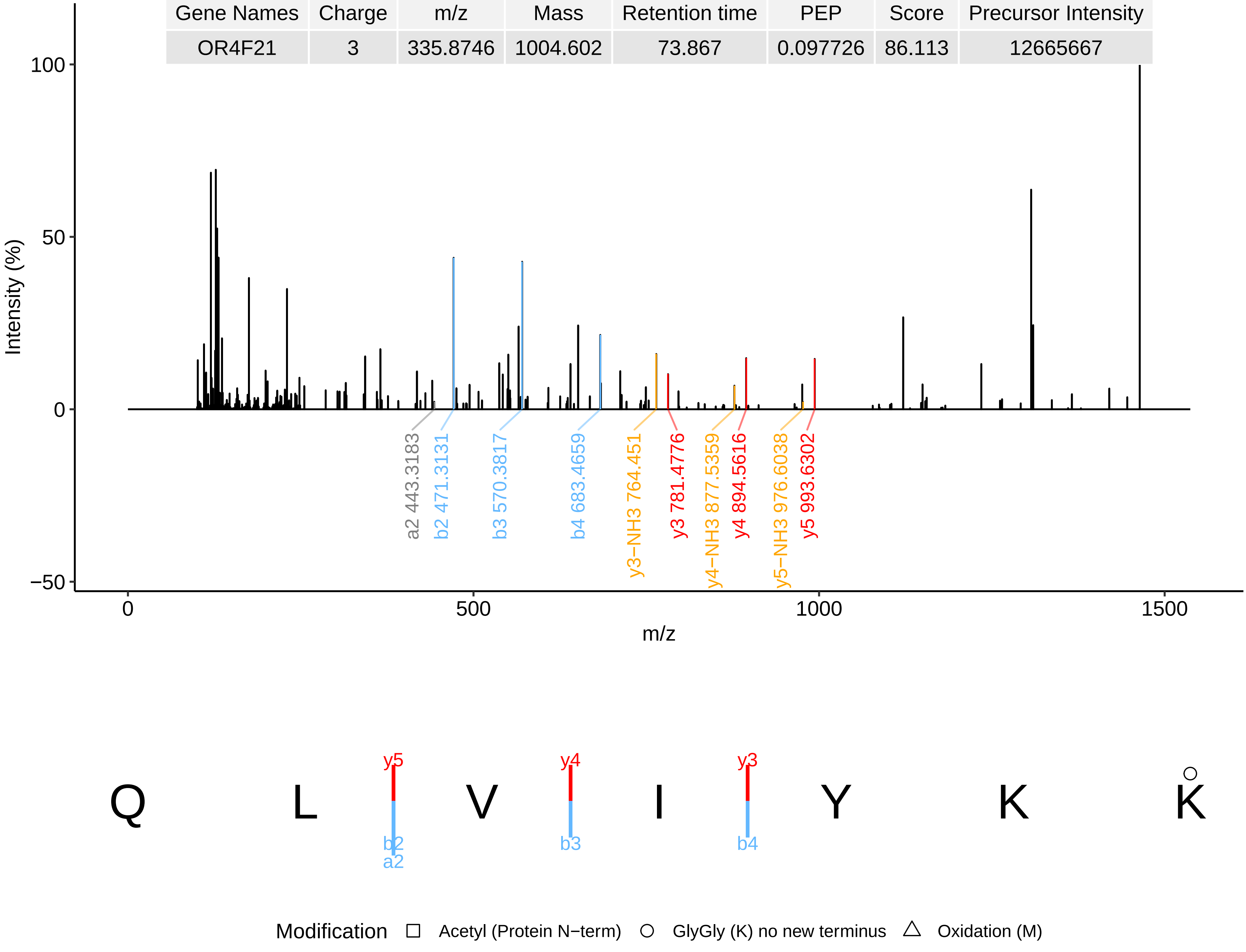

20200924_KIZ_MUb5_GlyGly_1 − Scan number: 23289

Gene Names	Charge	m/z	Mass	Retention time	PEP	Score	Precursor Intensity
OR4F21	3	335.8746	1004.602	74.873	0.038062	101.52	15048075

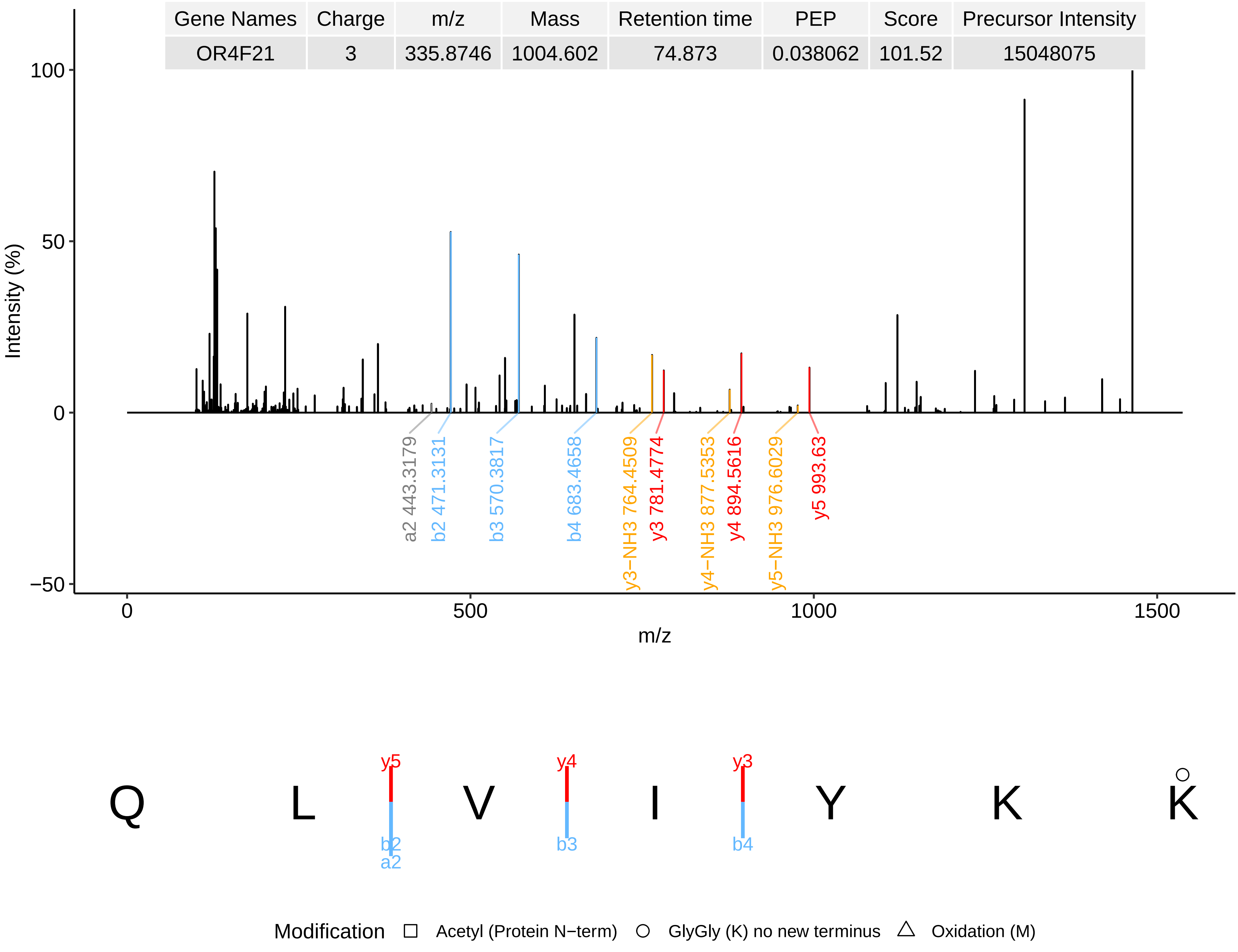

20200924_KIZ_MUb5_GlyGly_1 − Scan number: 23467

Gene Names	Charge	m/z	Mass	Retention time	PEP	Score	Precursor Intensity
OR4F21	3	335.8746	1004.602	75.431	0.109	76.847	12089657

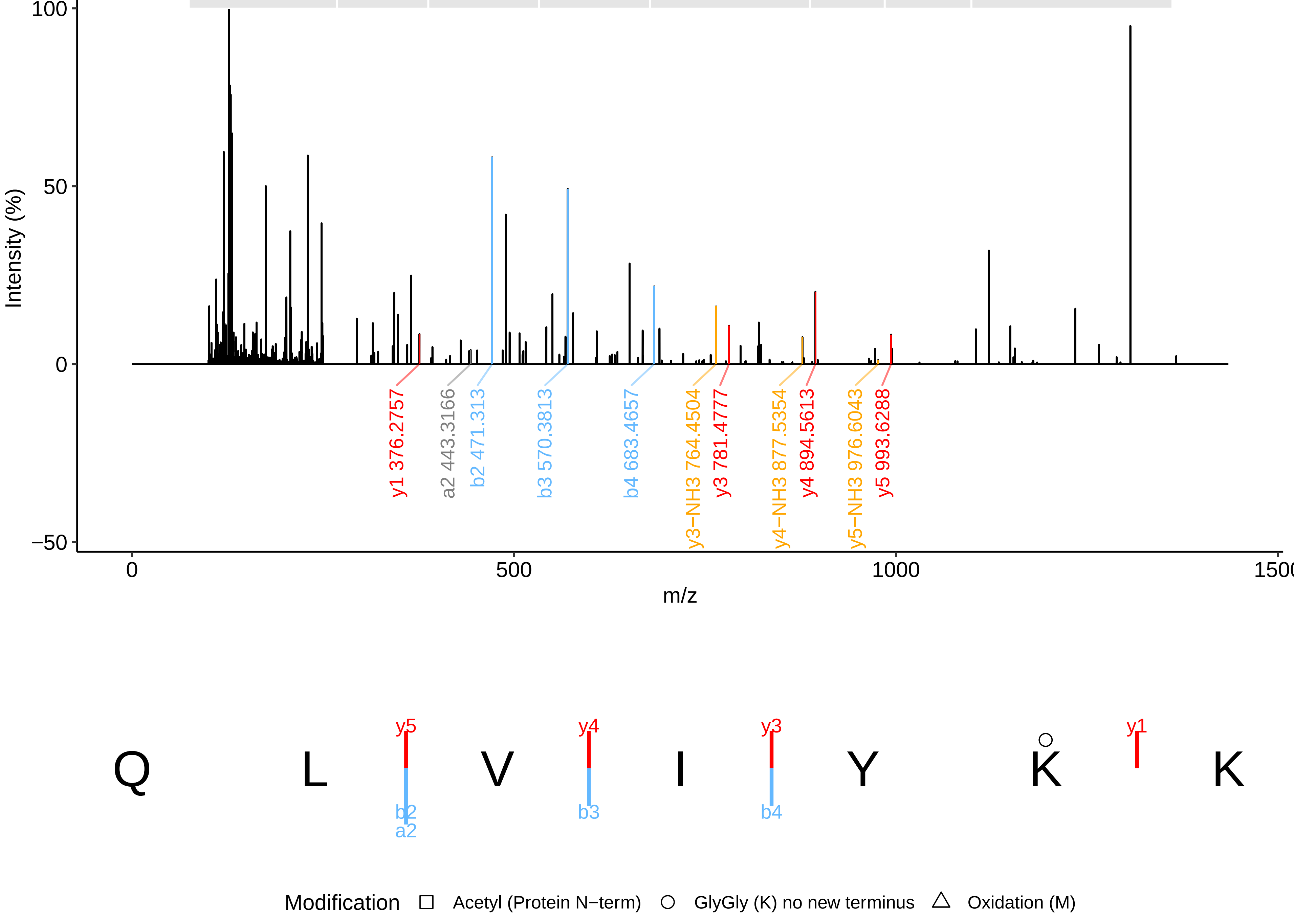

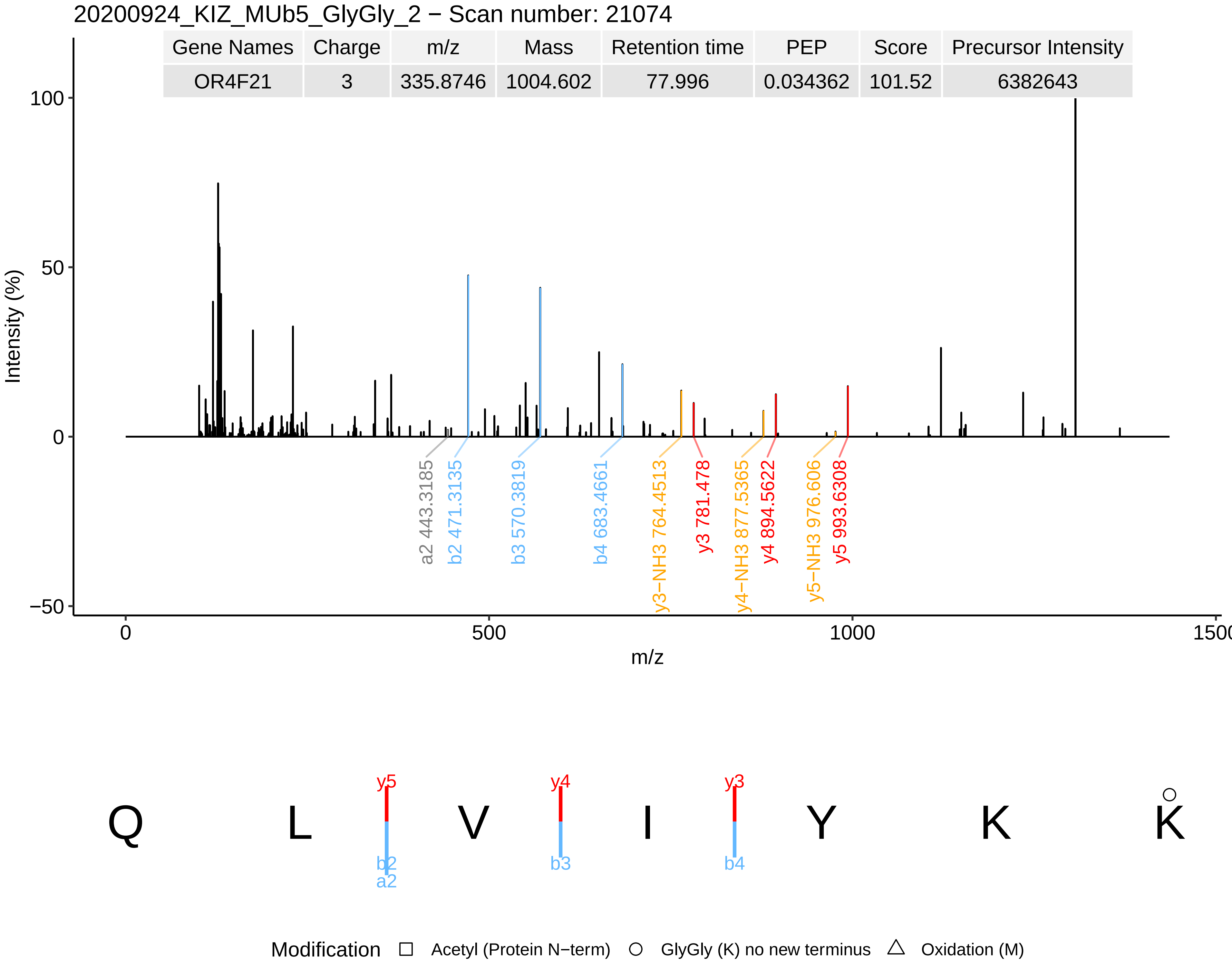
20200924_KIZ_MUb5_GlyGly_2 − Scan number: 21074
Gene Names | Charge | m/z | Mass | Retention time | PEP | Score | Precursor Intensity
OR4F21 | 3 | 335.8746 | 1004.602 | 77.996 | 0.034362 | 101.52 | 6382643
Intensity (%)
100
50
0
−50
0
500
1000
1500
m/z
a2 443.3185
b2 471.3135
b3 570.3819
b4 683.4661
y3−NH3 764.4513
y3 781.478
y4−NH3 877.5365
y4 894.5622
y5−NH3 976.606
y5 993.6308
Q L V I Y K K
y5
y4
y3
b2
a2
b3
b4
Modification
Acetyl (Protein N−term)
GlyGly (K) no new terminus
Oxidation (M)

20201118_KIZ_MUb6_GlyGly_2 − Scan number: 26255

Gene Names	Charge	m/z	Mass	Retention time	PEP	Score	Precursor Intensity
OR4F21	3	335.8746	1004.602	87.603	0.038062	101.52	13001797

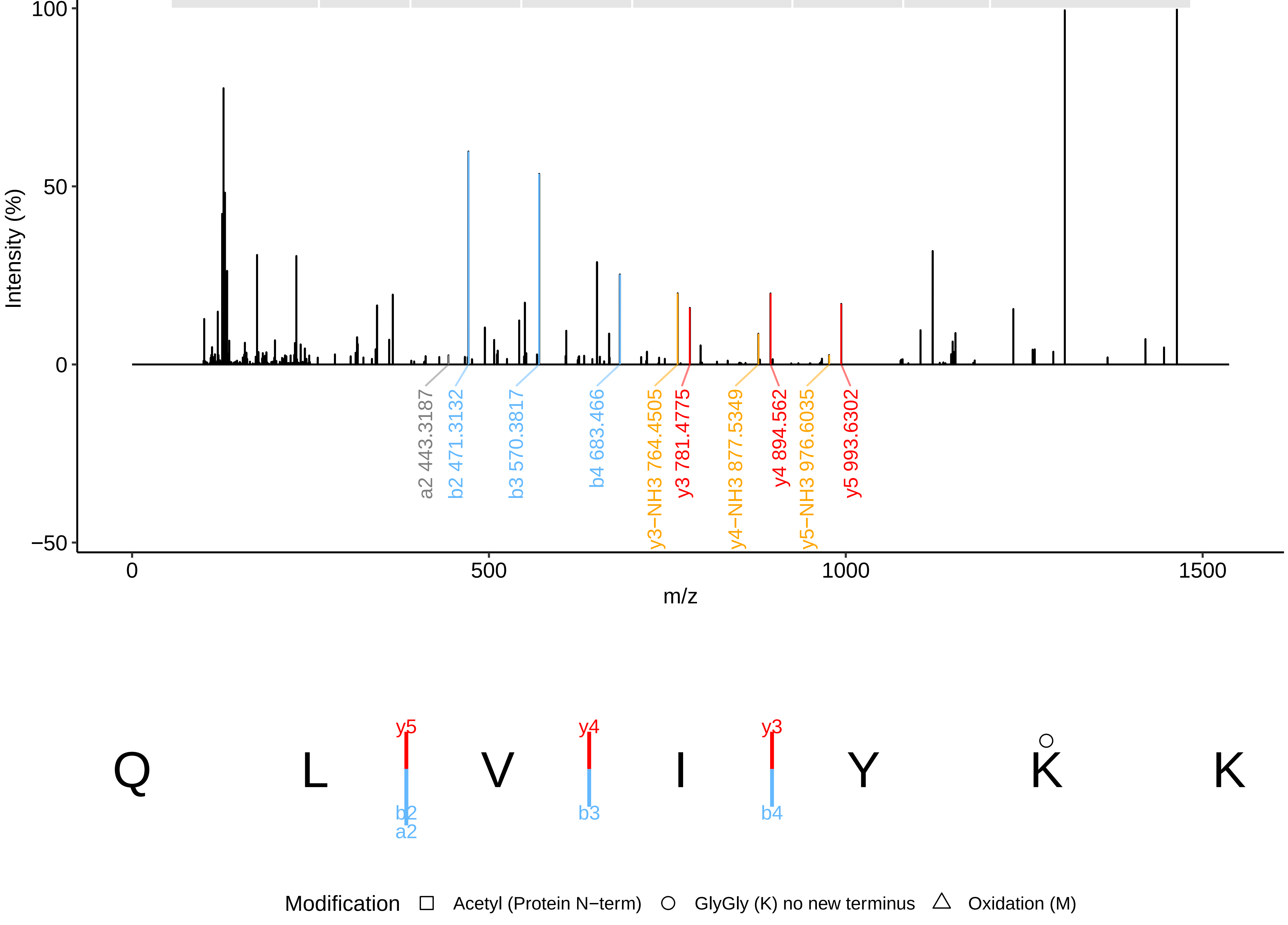

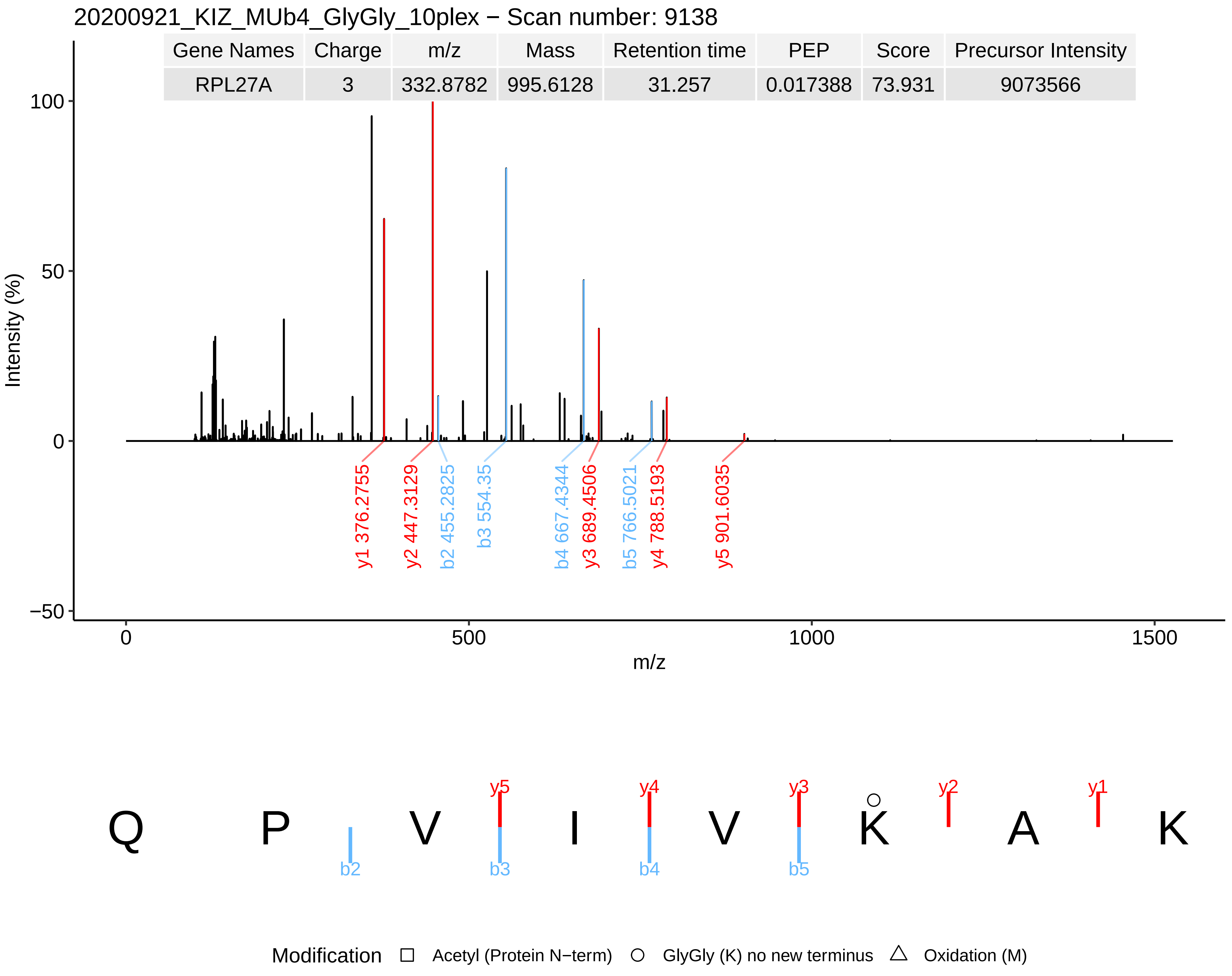
20200921_KIZ_MUb4_GlyGly_10plex − Scan number: 9138
Gene Names
Charge
m/z
Mass
Retention time
PEP
Score
Precursor Intensity
RPL27A
3
332.8782
995.6128
31.257
0.017388
73.931
9073566
Intensity (%)
100
50
0
−50
0
500
1000
1500
m/z
y1 376.2755
y2 447.3129
b2 455.2825
b3 554.35
b4 667.4344
y3 689.4506
b5 766.5021
y4 788.5193
y5 901.6035
Q
P
V
I
V
K
A
K
y5
y4
y3
y2
y1
b2
b3
b4
b5
Modification
Acetyl (Protein N−term)
GlyGly (K) no new terminus
Oxidation (M)

20200921_KIZ_MUb4_GlyGly_10plex_2 − Scan number: 8468

Gene Names	Charge	m/z	Mass	Retention time	PEP	Score	Precursor Intensity
RPL27A	3	332.8782	995.6128	31.91	0.0063255	87.616	1439374

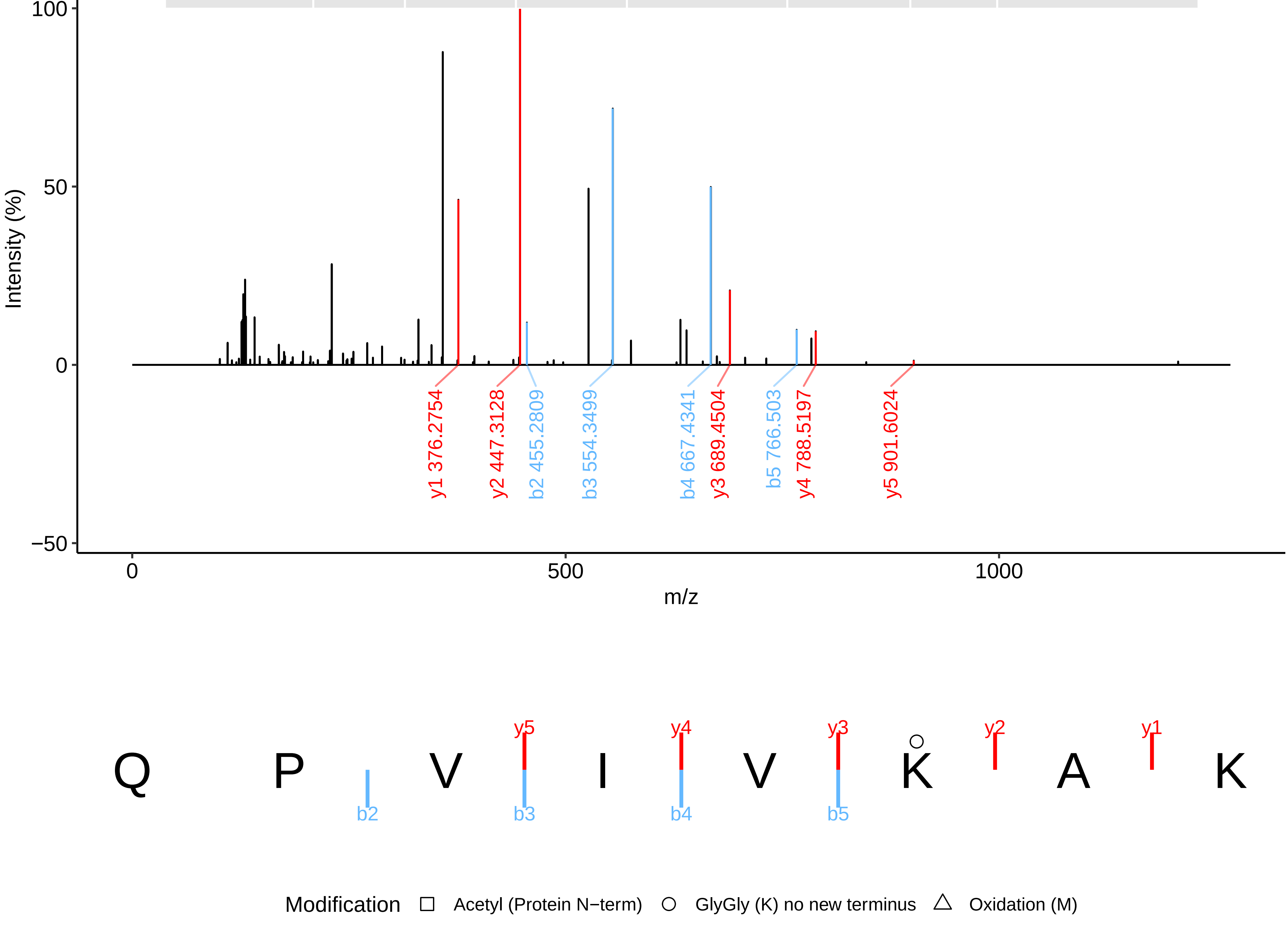

20200924_KIZ_MUb5_GlyGly_2 − Scan number: 7549

Gene Names	Charge	m/z	Mass	Retention time	PEP	Score	Precursor Intensity
RPL27A	3	332.8782	995.6128	28.188	0.021314	73.931	1745998

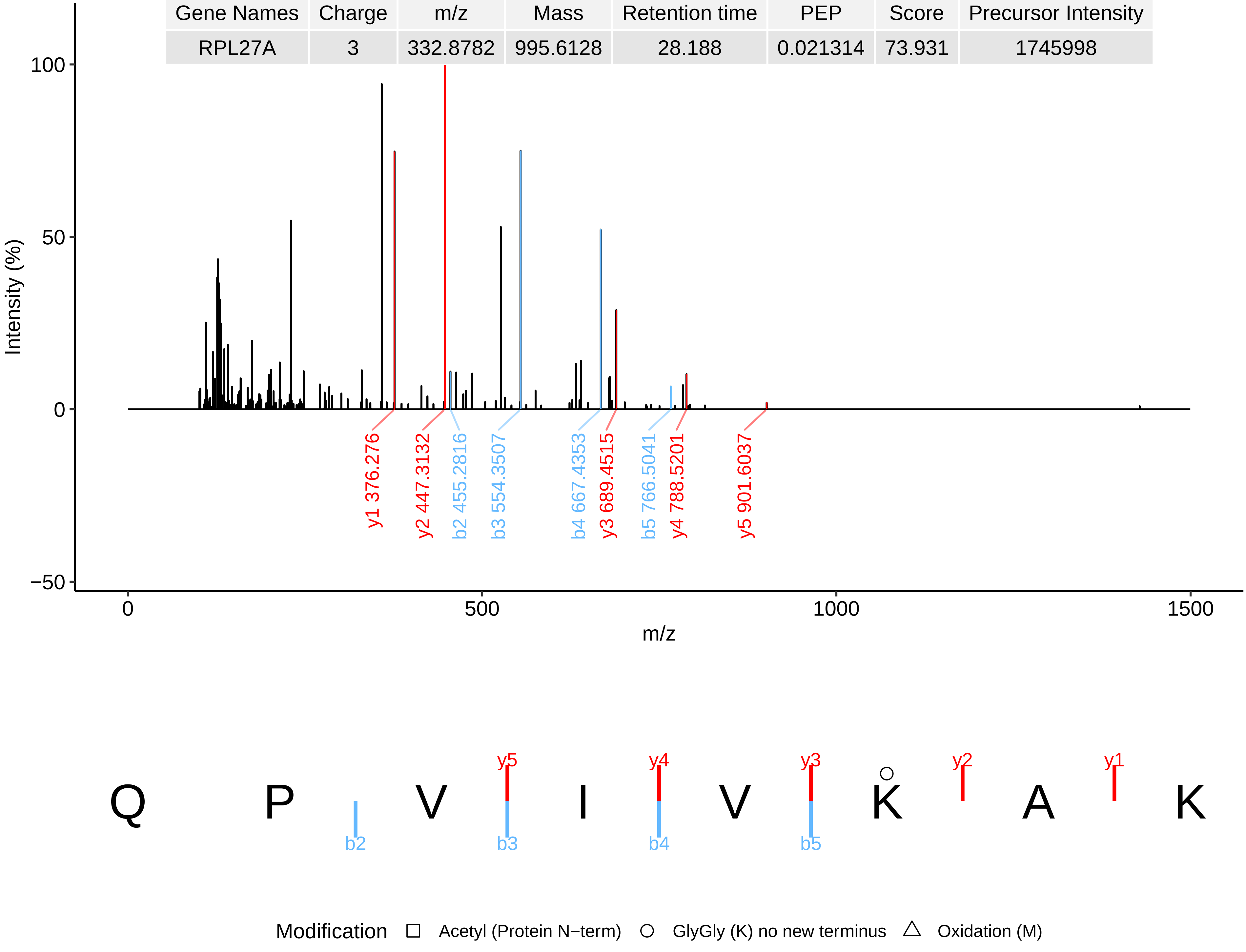

20200921_KIZ_MUb4_GlyGly_10plex_2 − Scan number: 3270

Gene Names	Charge	m/z	Mass	Retention time	PEP	Score	Precursor Intensity
PRKCD;PRKCQ	3	329.2016	984.5829	10.902	0.06312	62.659	1212440

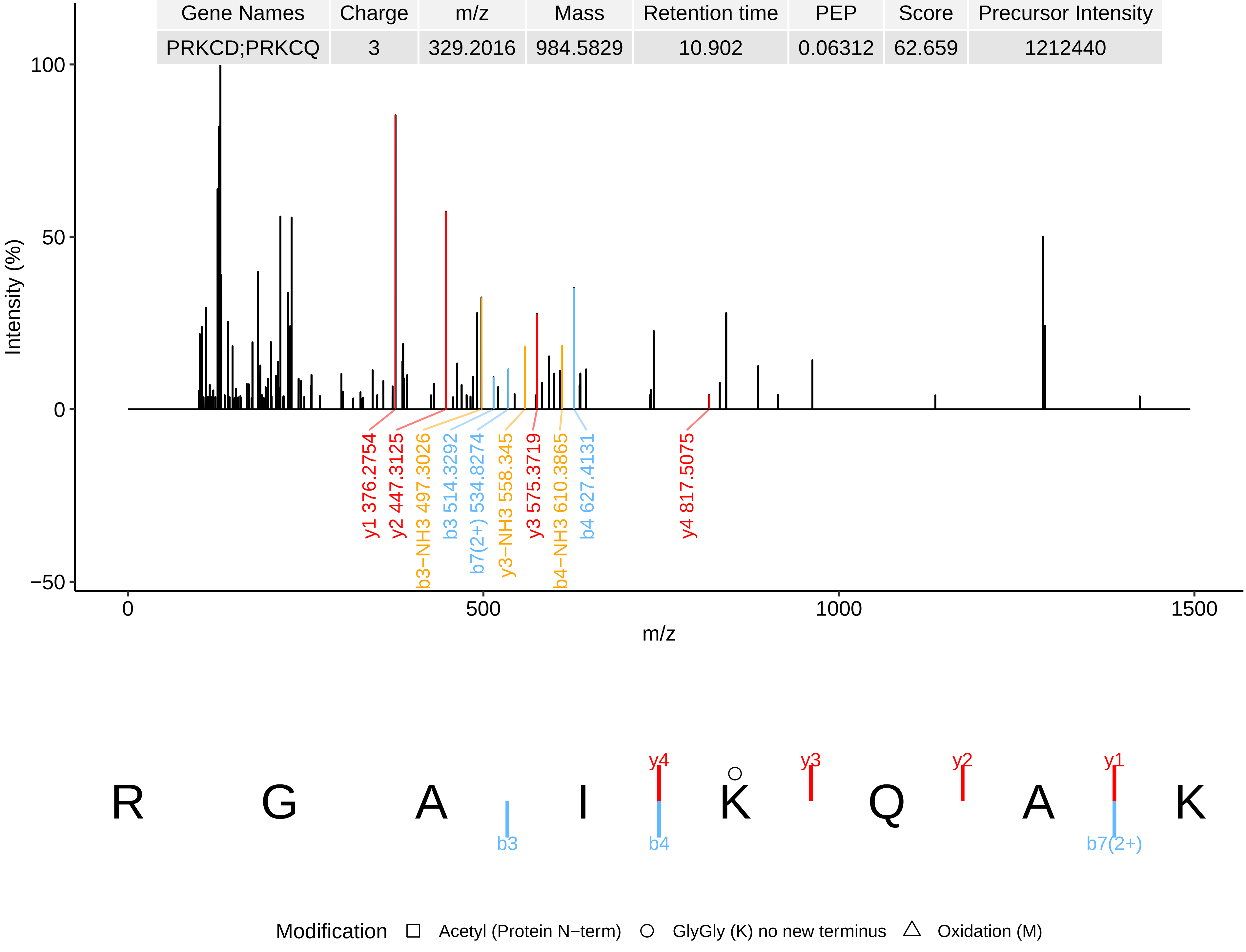

20200921_KIZ_MUb4_GlyGly_10plex − Scan number: 2735

Gene Names	Charge	m/z	Mass	Retention time	PEP	Score	Precursor Intensity
ATAD3B;ATAD3A	4	309.1691	1232.647	8.6004	0.08428	71.614	8602855

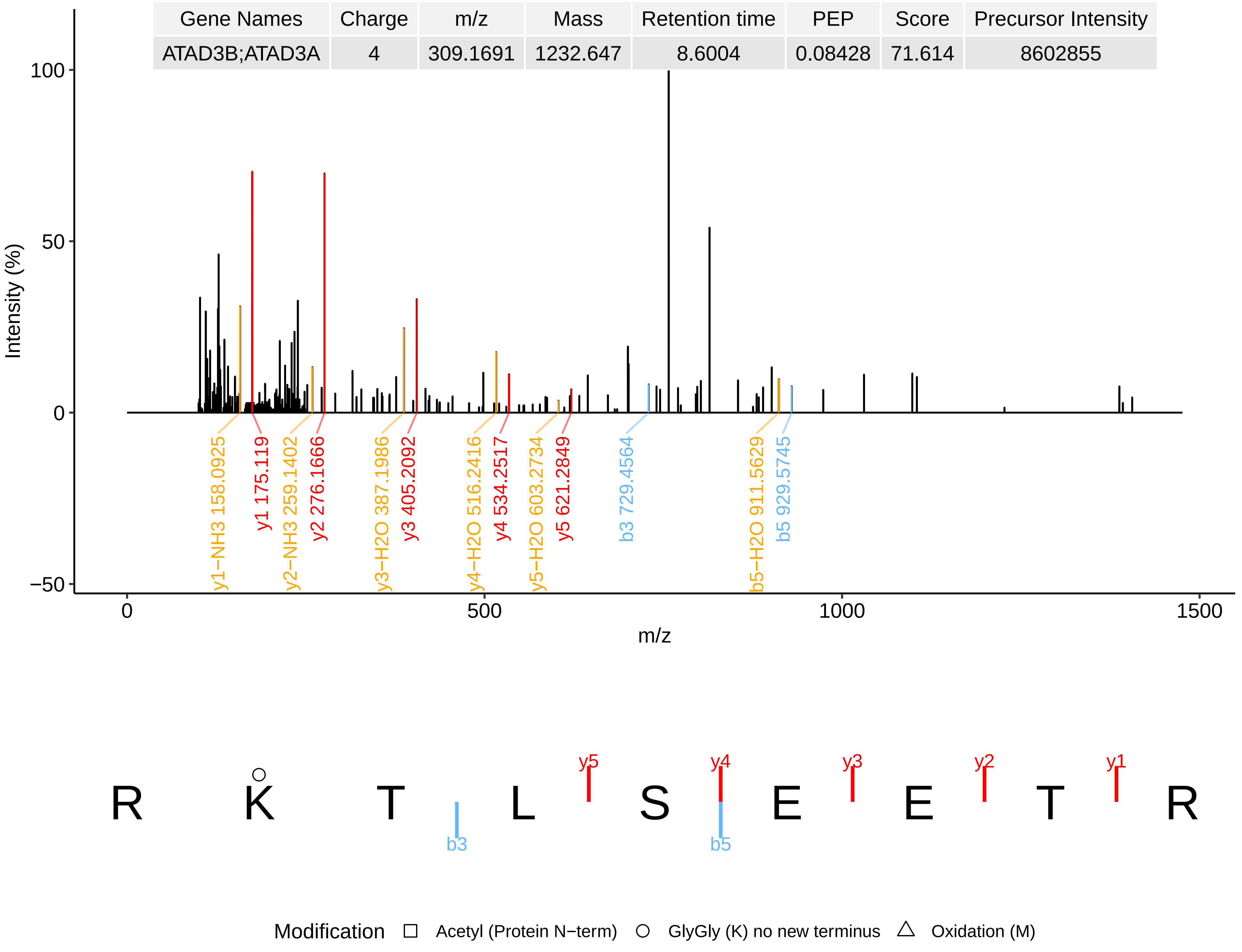

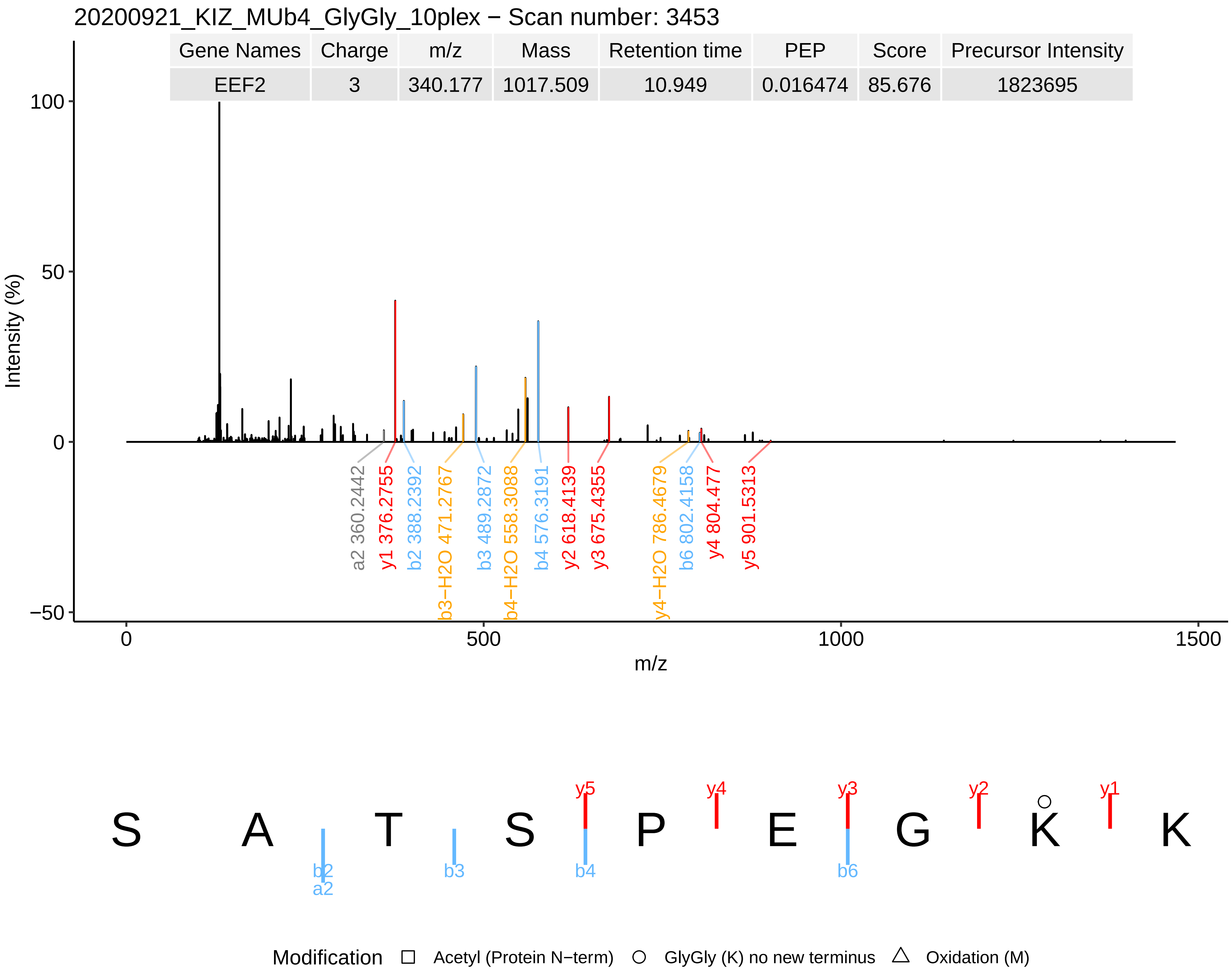
20200921_KIZ_MUb4_GlyGly_10plex − Scan number: 3453
Gene Names | Charge | m/z | Mass | Retention time | PEP | Score | Precursor Intensity
EEF2 | 3 | 340.177 | 1017.509 | 10.949 | 0.016474 | 85.676 | 1823695
Intensity (%)
100
50
0
−50
0
500
1000
1500
m/z
a2 360.2442
y1 376.2755
b2 388.2392
b3−H2O 471.2767
b3 489.2872
b4−H2O 558.3088
b4 576.3191
y2 618.4139
y3 675.4355
y4−H2O 786.4679
b6 802.4158
y4 804.477
y5 901.5313
S A T S P E G K K
y5 y4 y3 y2 y1
b2 a2 b3 b4 b6
Modification
Acetyl (Protein N−term)
GlyGly (K) no new terminus
Oxidation (M)

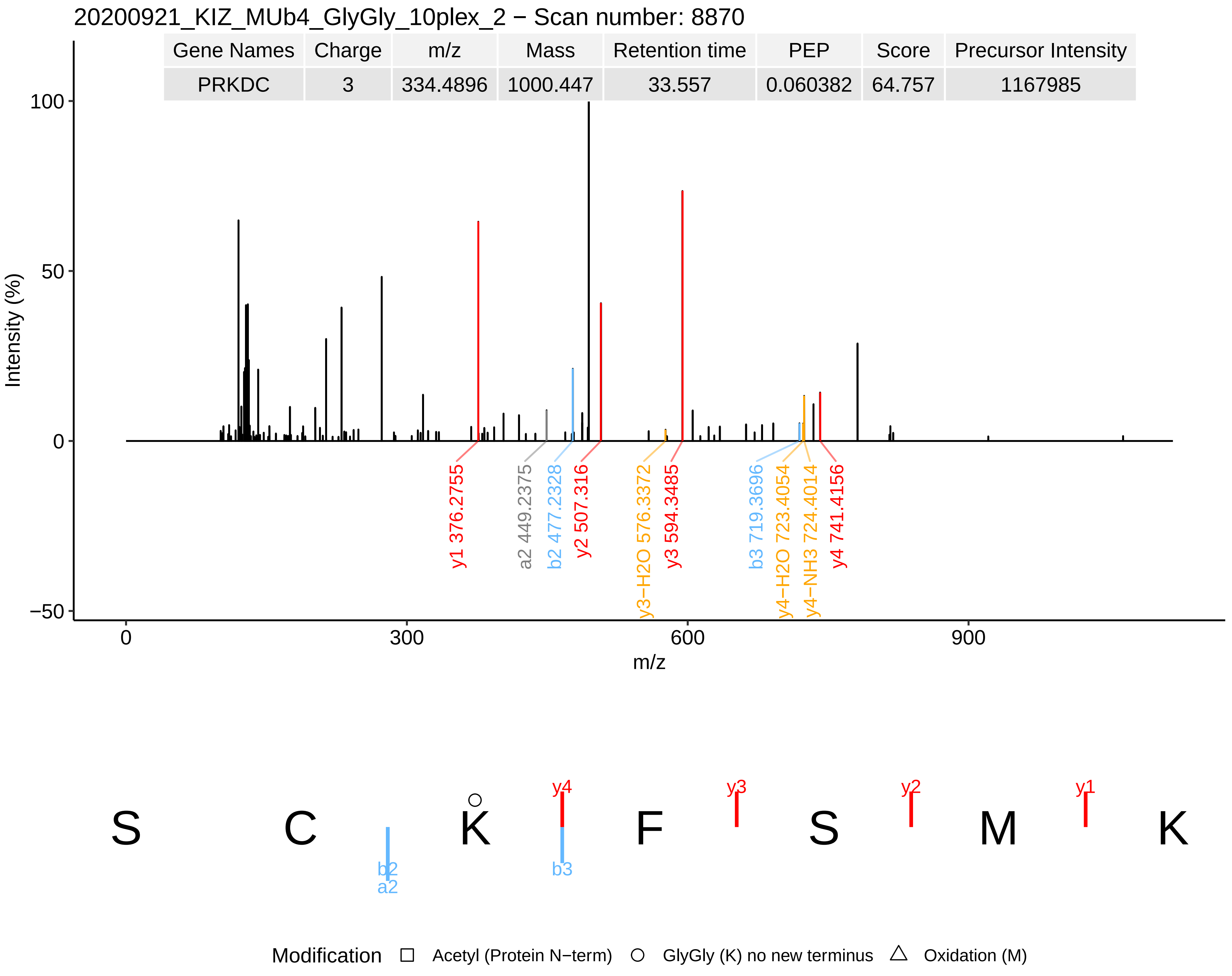
20200921_KIZ_MUb4_GlyGly_10plex_2 − Scan number: 8870
Gene Names | Charge | m/z | Mass | Retention time | PEP | Score | Precursor Intensity
PRKDC | 3 | 334.4896 | 1000.447 | 33.557 | 0.060382 | 64.757 | 1167985
Intensity (%)
100
50
0
−50
0
300
600
900
m/z
y1 376.2755
a2 449.2375
b2 477.2328
y2 507.316
y3−H2O 576.3372
y3 594.3485
b3 719.3696
y4−H2O 723.4054
y4−NH3 724.4014
y4 741.4156
S C K F S M K
y4 y3 y2 y1
b2 a2 b3
Modification
Acetyl (Protein N−term)
GlyGly (K) no new terminus
Oxidation (M)

20200921_KIZ_MUb4_GlyGly_10plex − Scan number: 8346

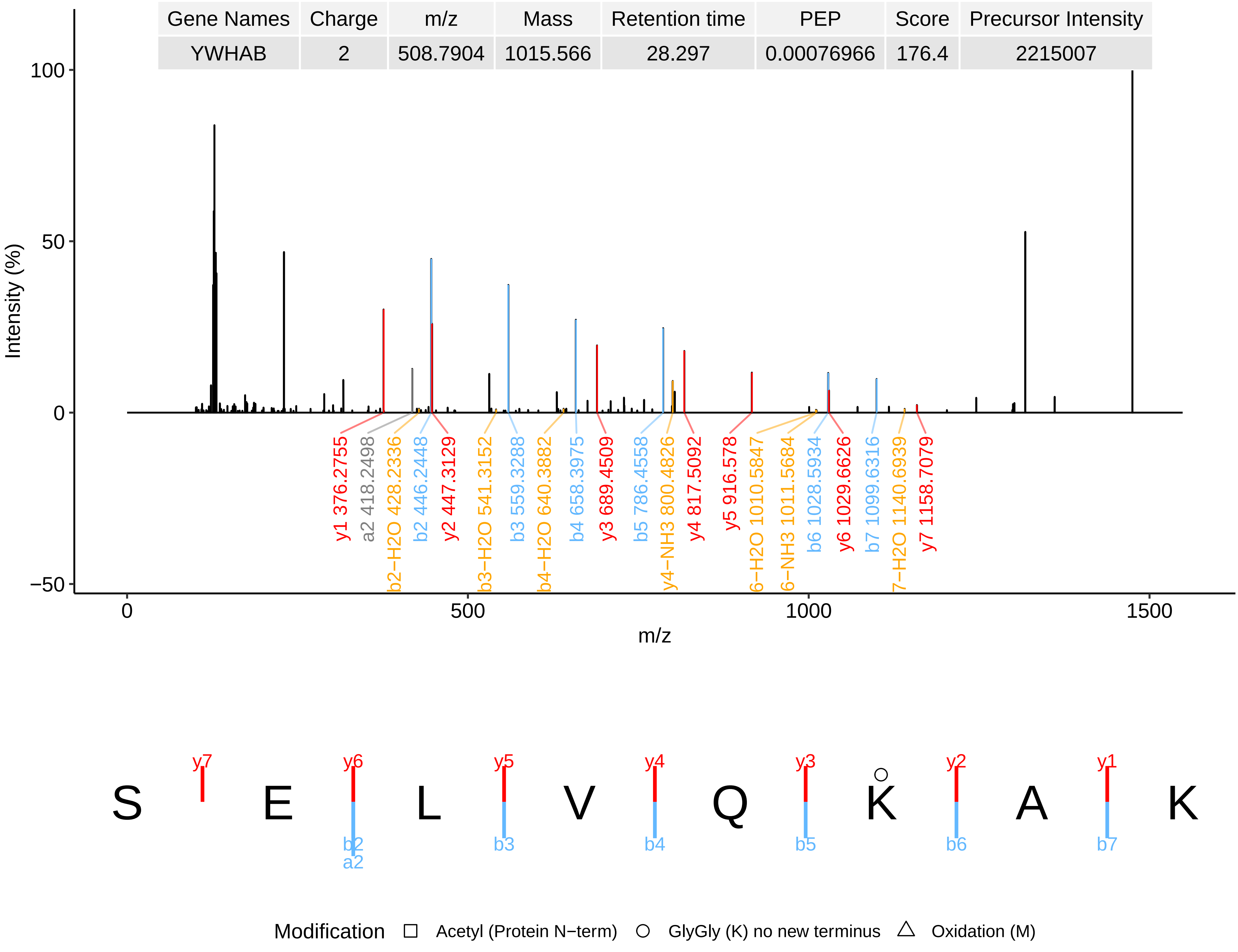

20200921_KIZ_MUb4_GlyGly_10plex_2 − Scan number: 7715

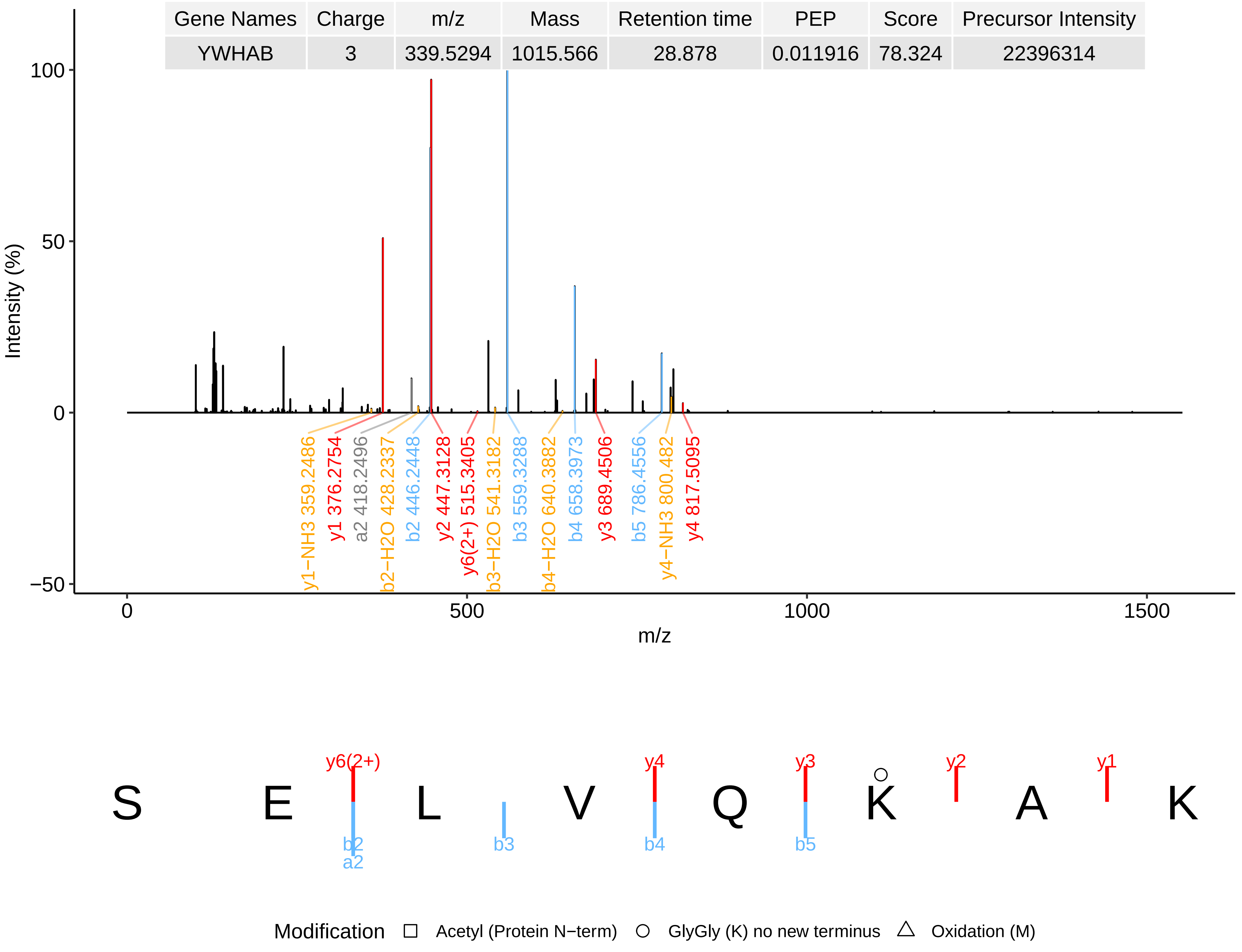

20200921_KIZ_MUb4_GlyGly_10plex_2 − Scan number: 7725

Gene Names	Charge	m/z	Mass	Retention time	PEP	Score	Precursor Intensity
YWHAB	2	508.7904	1015.566	28.915	0.00016639	145.63	606615.8

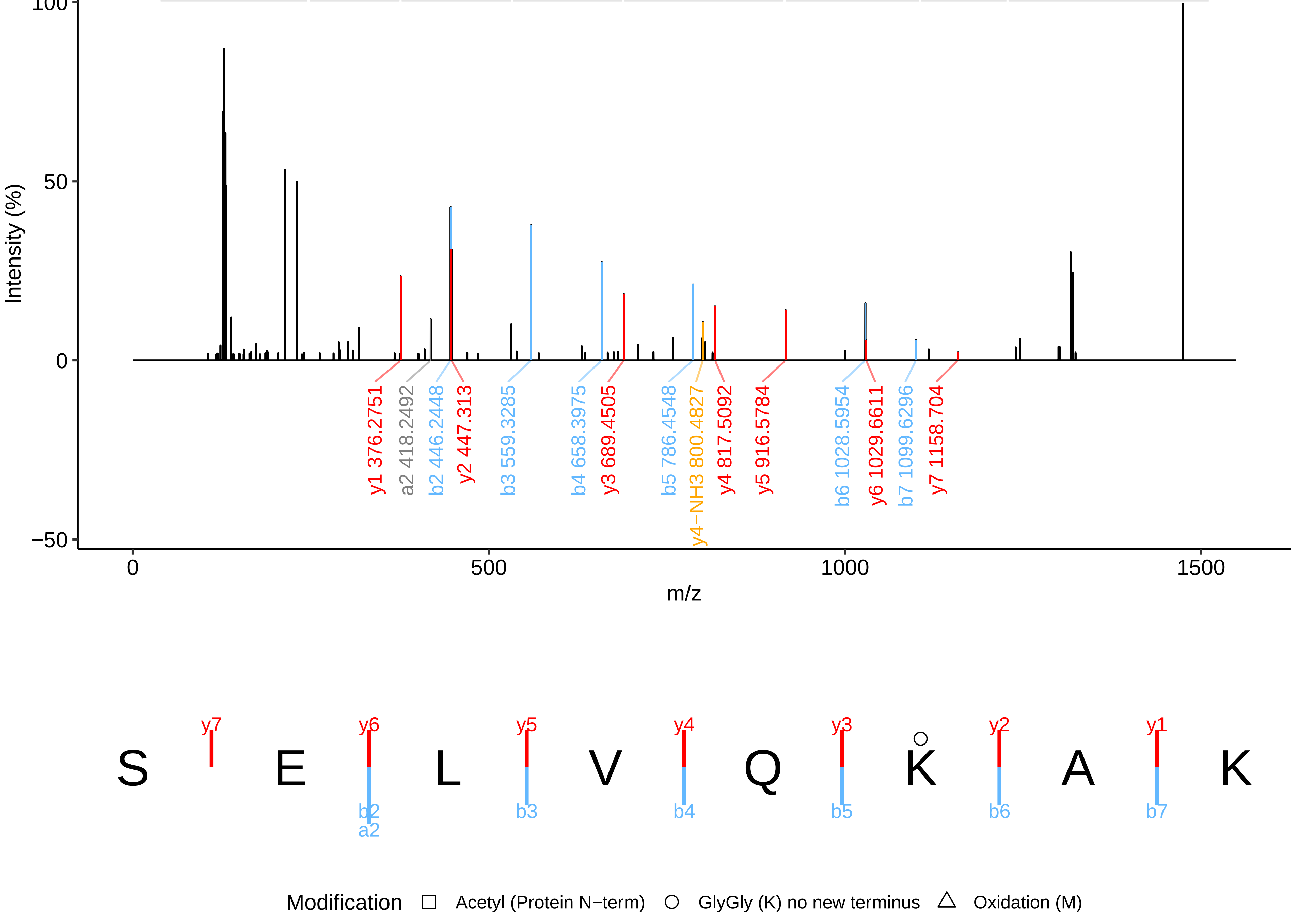

20200924_KIZ_MUb5_GlyGly_1 − Scan number: 7317

Gene Names	Charge	m/z	Mass	Retention time	PEP	Score	Precursor Intensity
YWHAB	3	339.5294	1015.566	23.207	0.011916	78.324	63994752

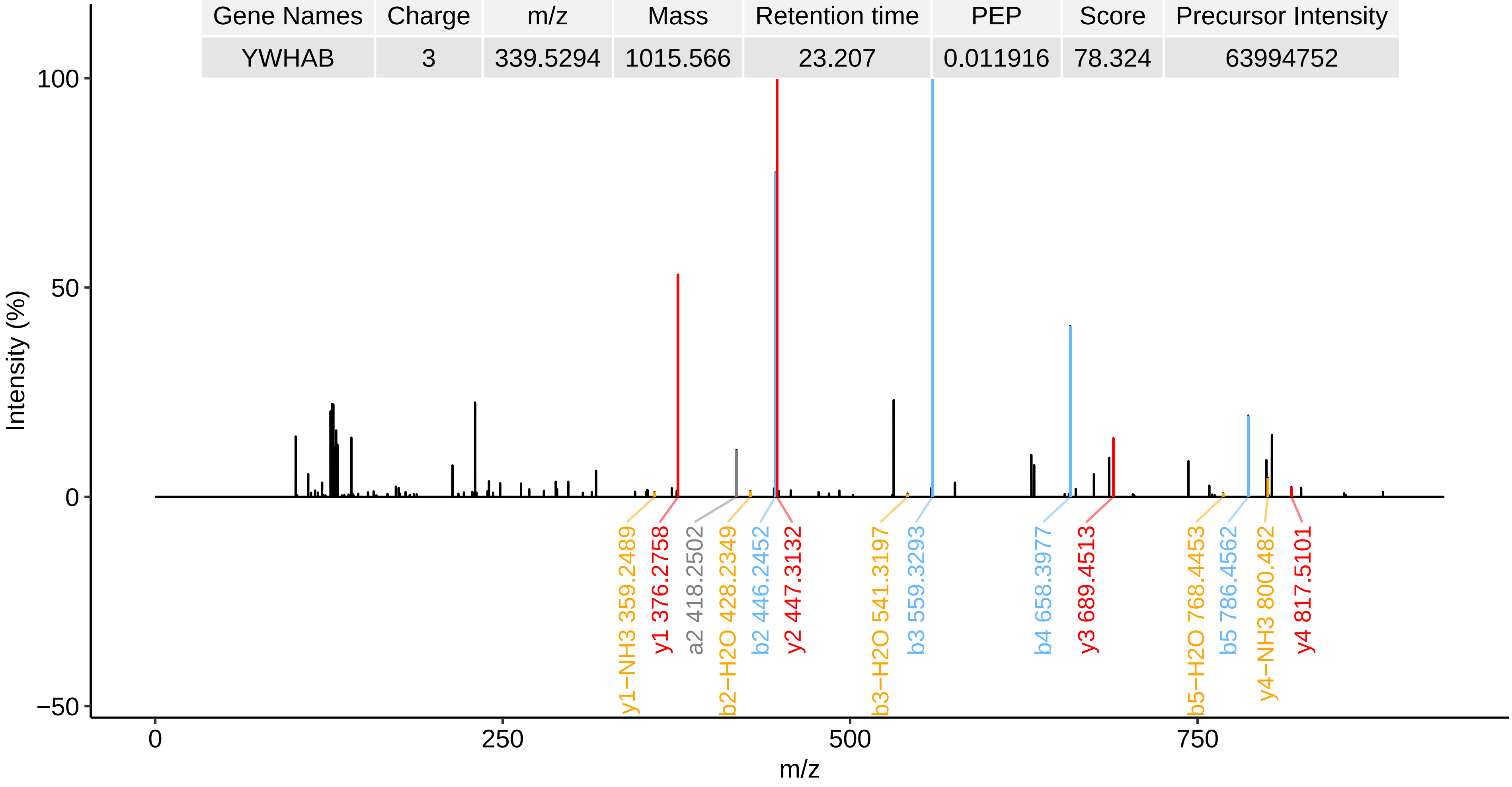

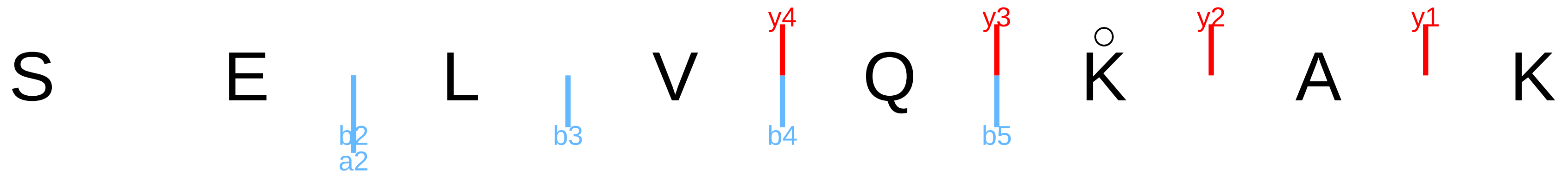

Modification □ Acetyl (Protein N−term) ○ GlyGly (K) no new terminus △ Oxidation (M)

20200924_KIZ_MUb5_GlyGly_1 − Scan number: 7467

Gene Names	Charge	m/z	Mass	Retention time	PEP	Score	Precursor Intensity
YWHAB	3	339.5294	1015.566	23.712	0.015791	75.213	39611700

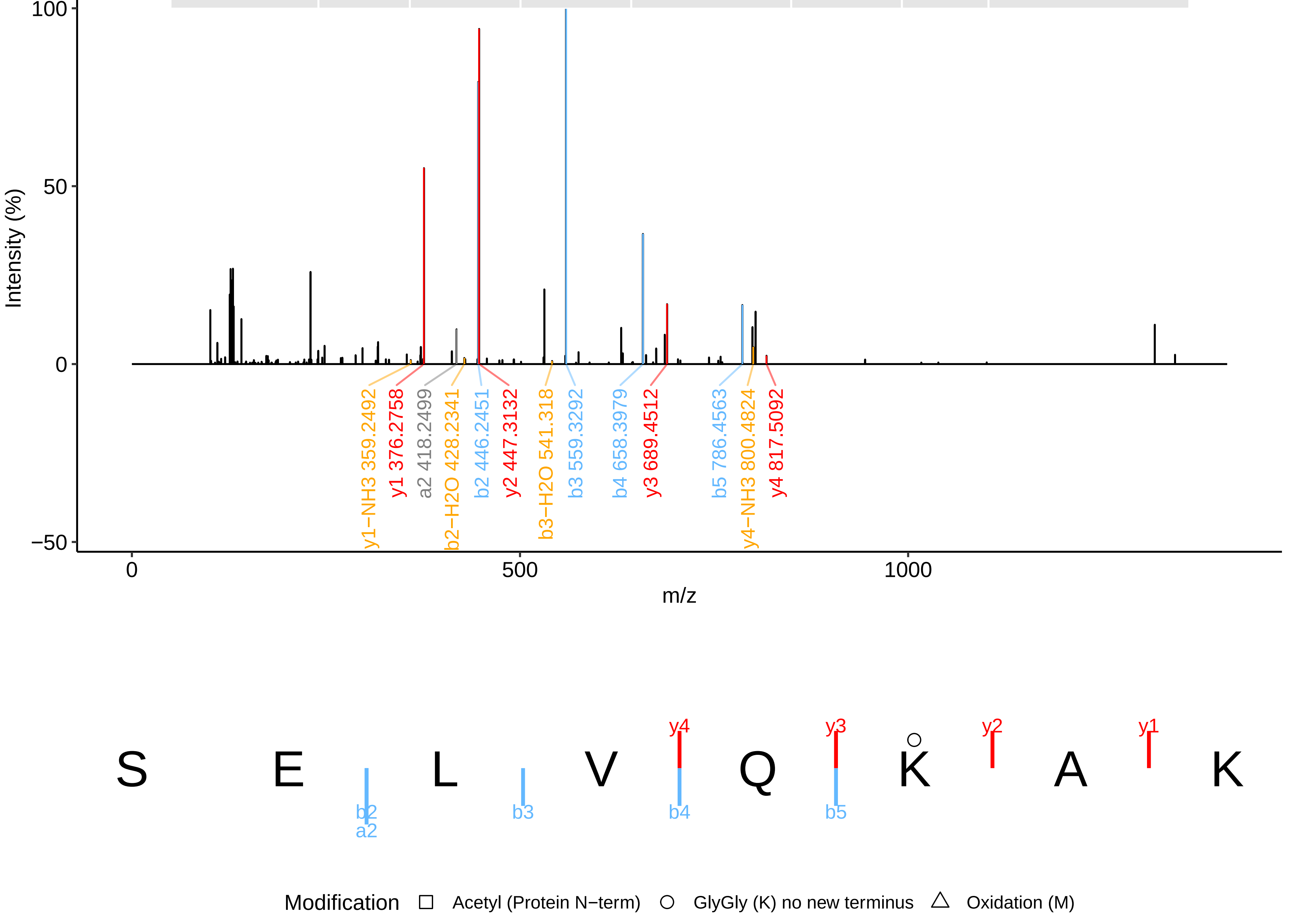

20200924_KIZ_MUb5_GlyGly_1 − Scan number: 7327

Gene Names	Charge	m/z	Mass	Retention time	PEP	Score	Precursor Intensity
YWHAB	2	508.7904	1015.566	23.241	0.014066	153.78	12977547

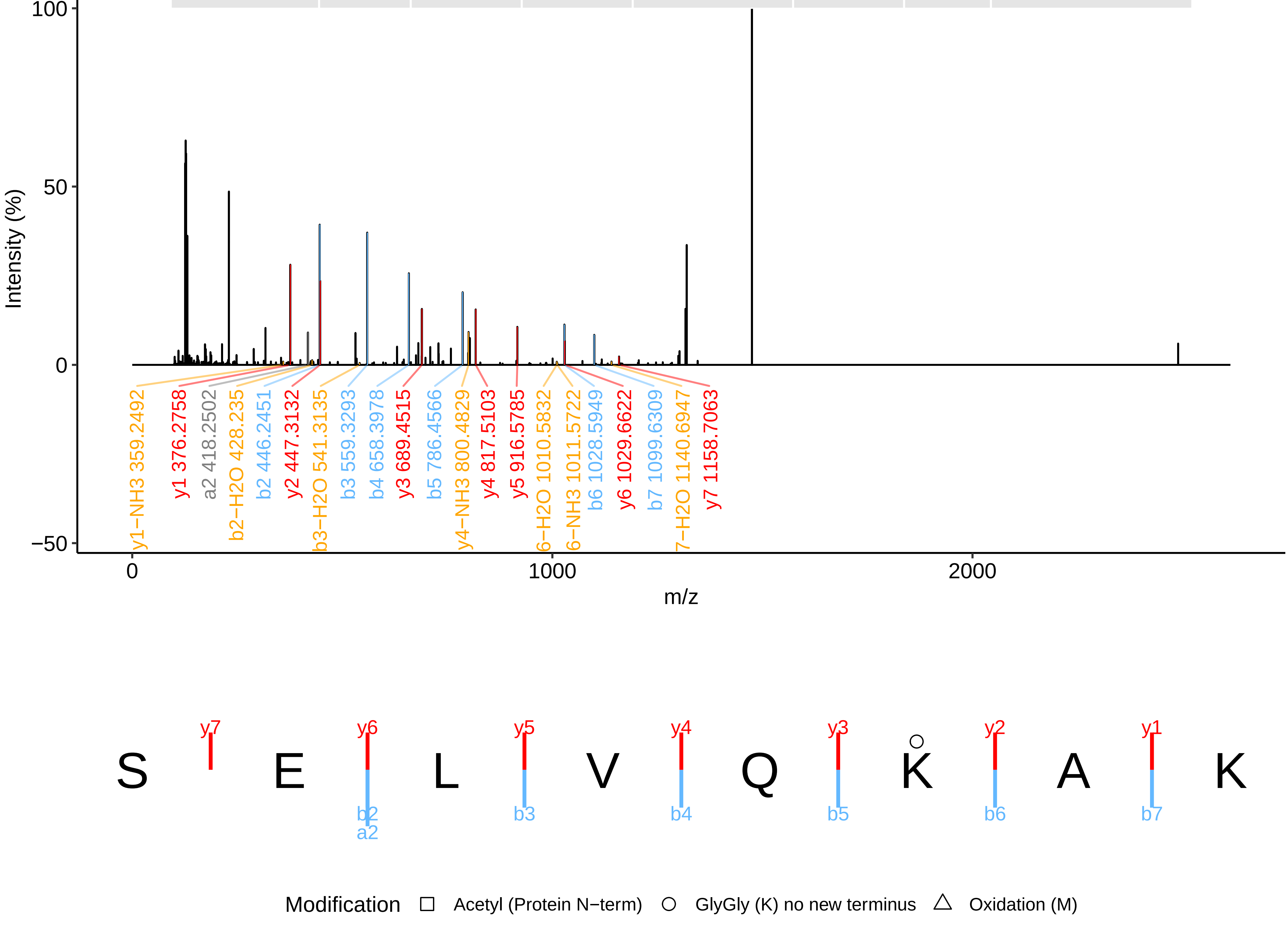

20200924_KIZ_MUb5_GlyGly_2 − Scan number: 6824

Gene Names	Charge	m/z	Mass	Retention time	PEP	Score	Precursor Intensity
YWHAB	3	339.5294	1015.566	25.452	0.0057615	89.046	9240778

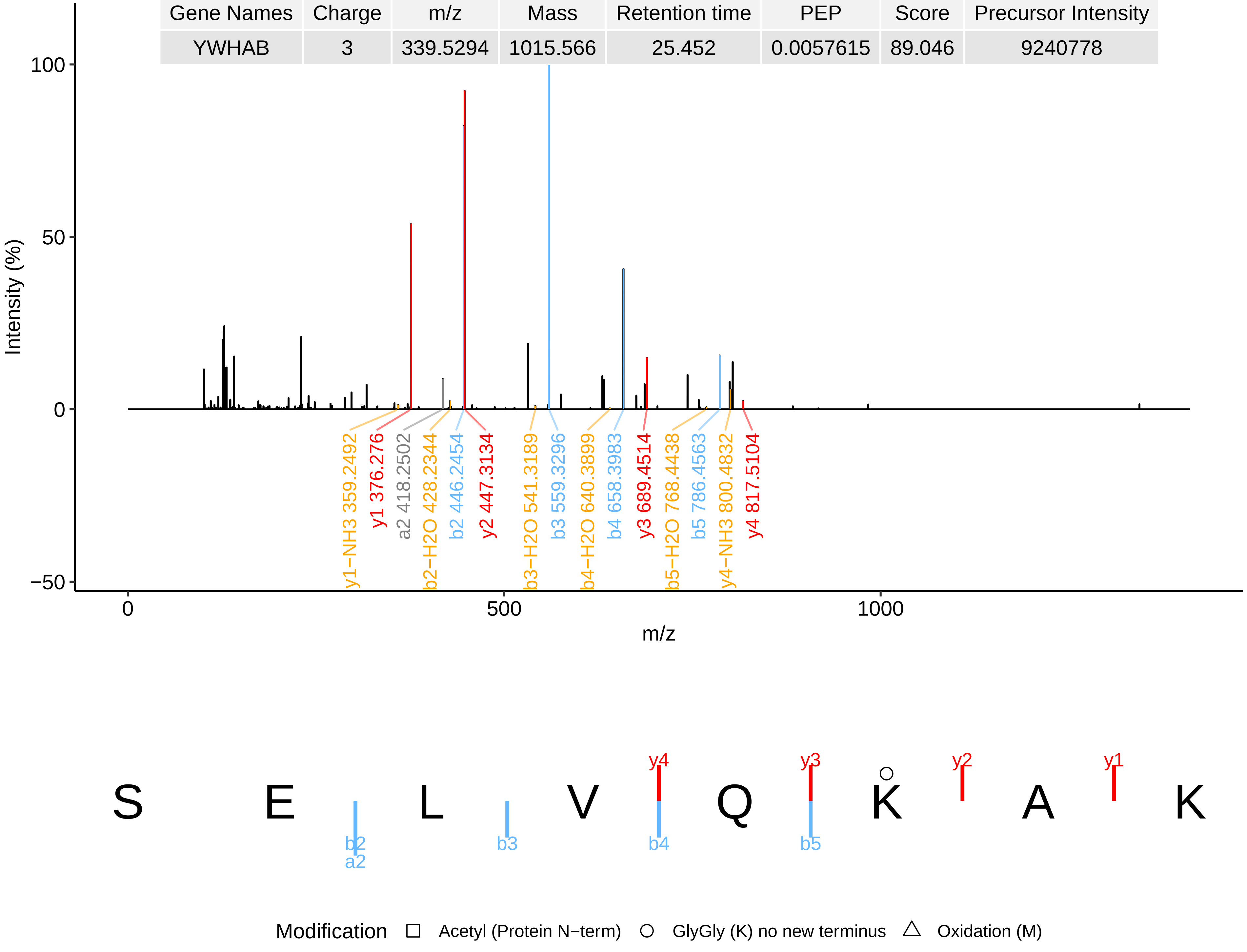

20201118_KIZ_MUb6_GlyGly_1 − Scan number: 7039

Gene Names	Charge	m/z	Mass	Retention time	PEP	Score	Precursor Intensity
YWHAB	3	339.5294	1015.566	22.311	0.01918	72.492	22828522

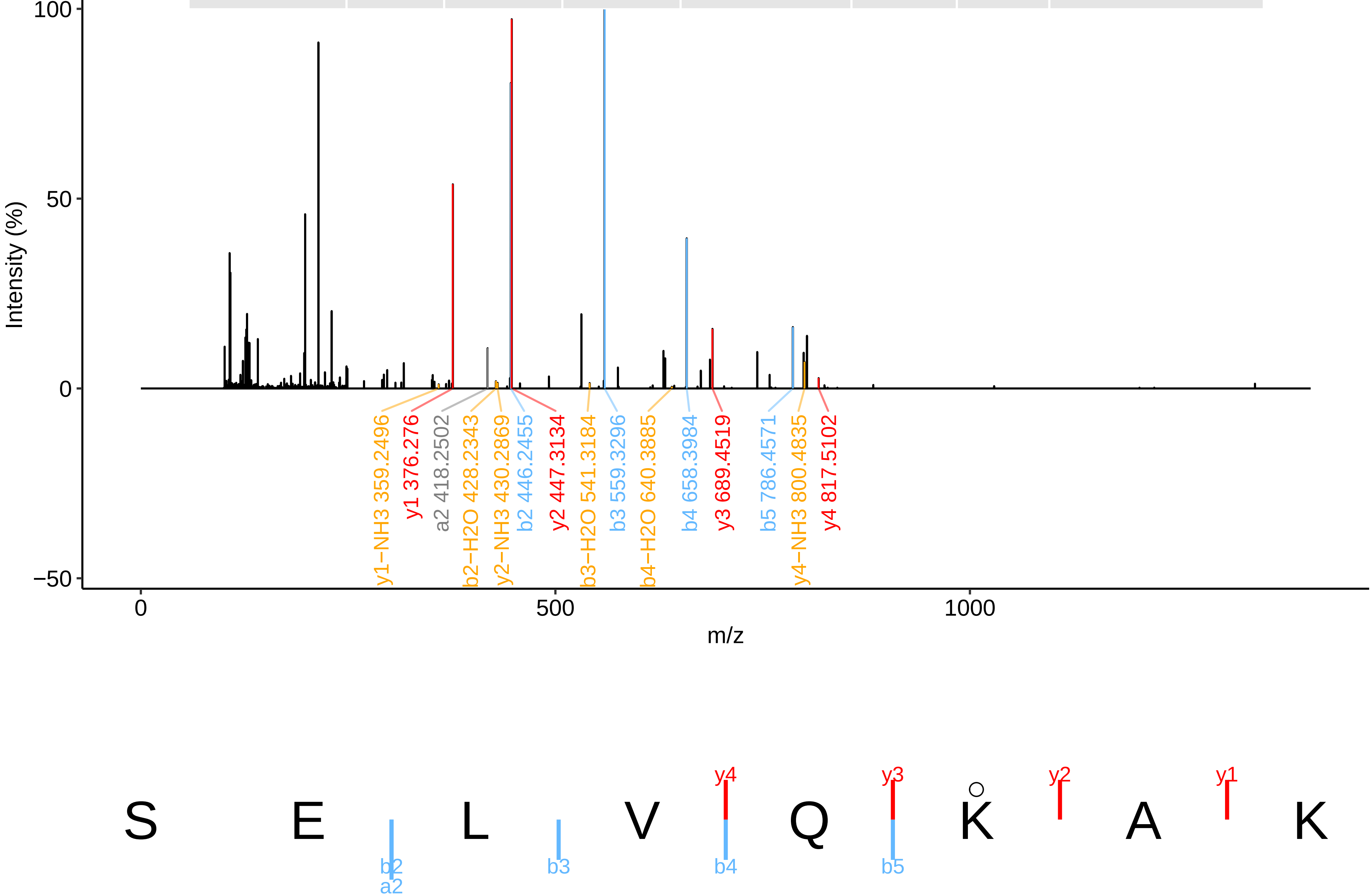

Modification □ Acetyl (Protein N−term) ○ GlyGly (K) no new terminus △ Oxidation (M)

20200921_KIZ_MUb4_GlyGly_10plex − Scan number: 7740

Gene Names	Charge	m/z	Mass	Retention time	PEP	Score	Precursor Intensity
GNG5	2	604.8006	1207.587	26.09	1.5412e−08	69.954	4946419

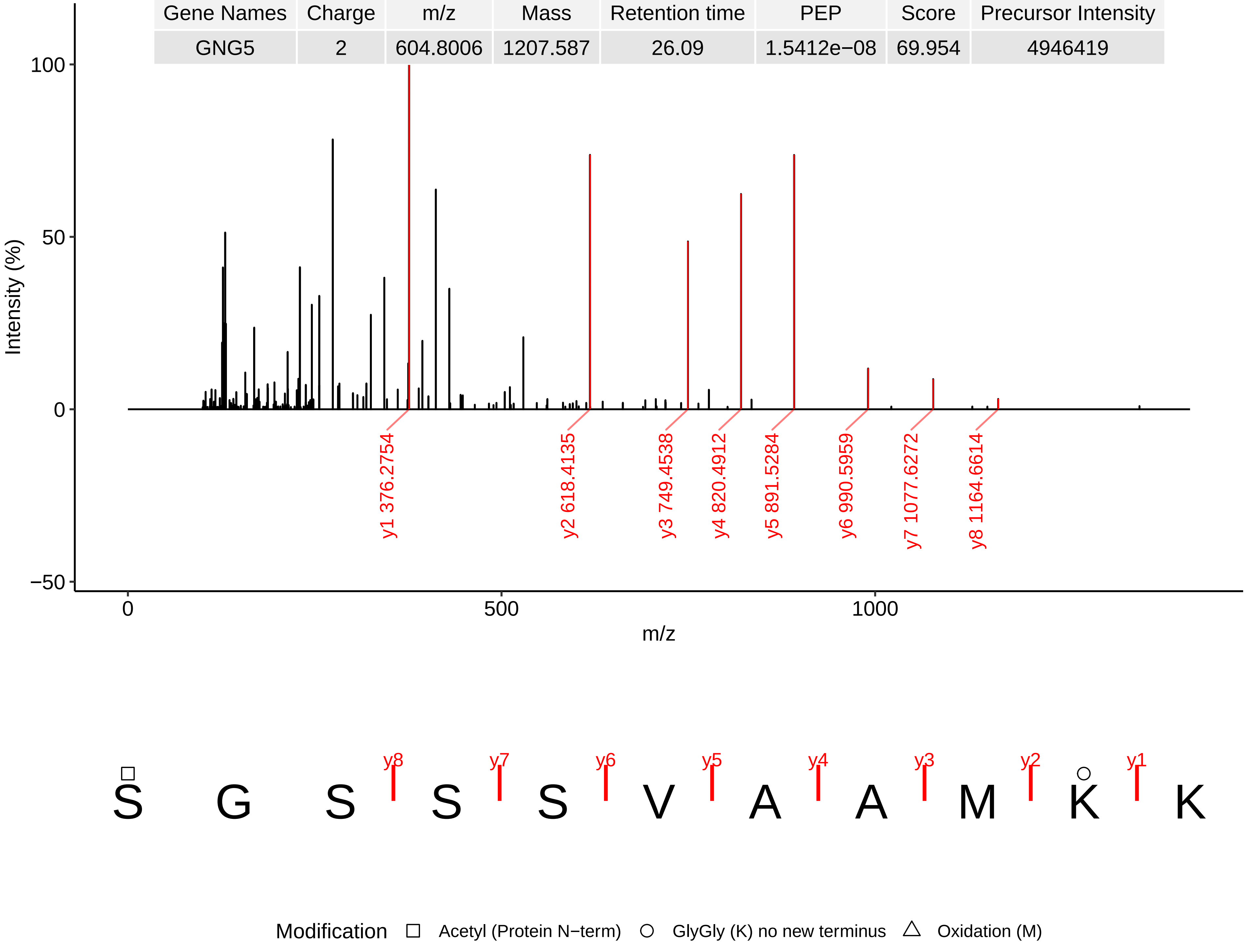

20200924_KIZ_MUb5_GlyGly_1 − Scan number: 6862

Gene Names	Charge	m/z	Mass	Retention time	PEP	Score	Precursor Intensity
GNG5	2	604.8006	1207.587	21.693	6.8896e−05	69.954	NA

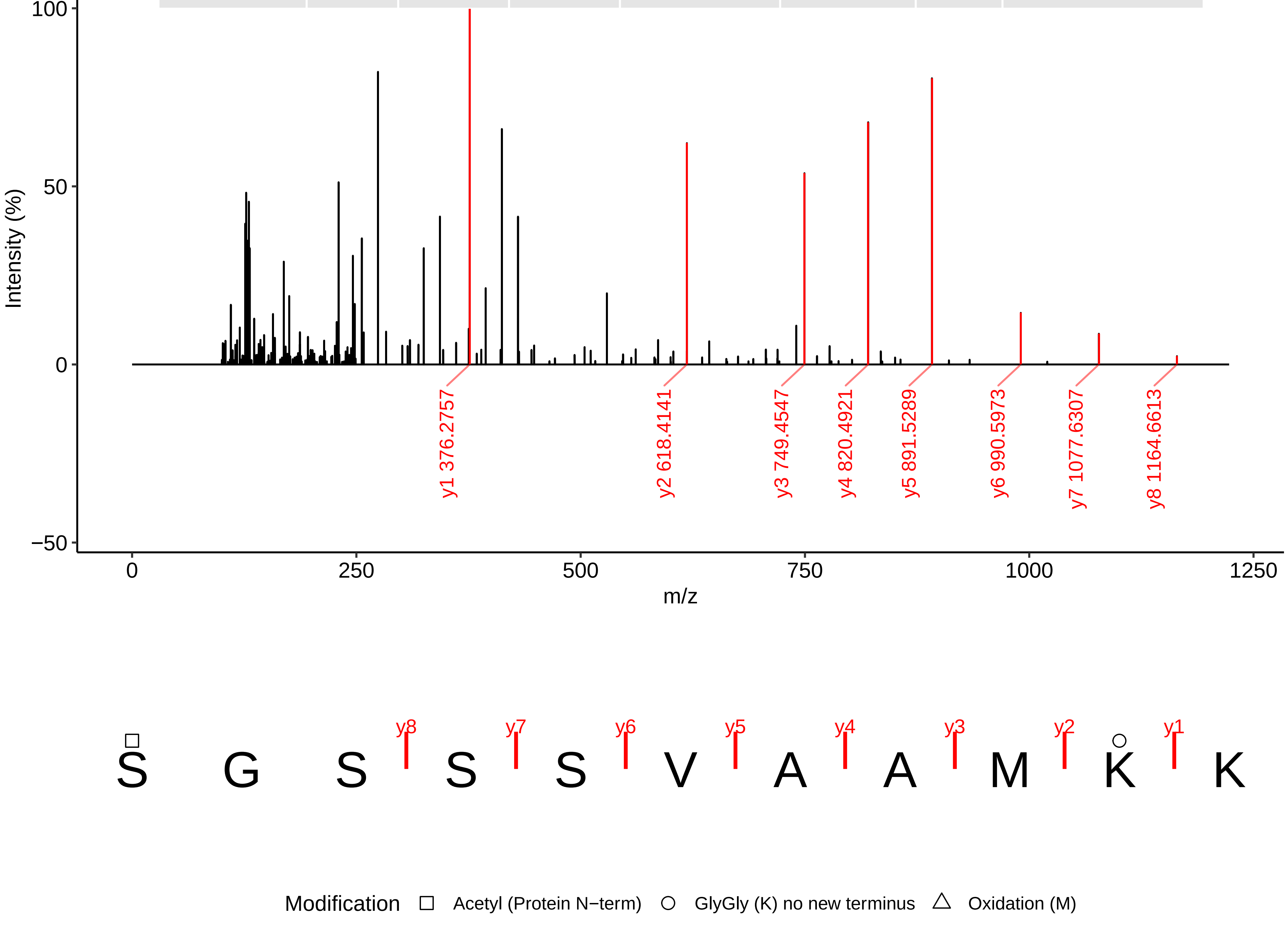

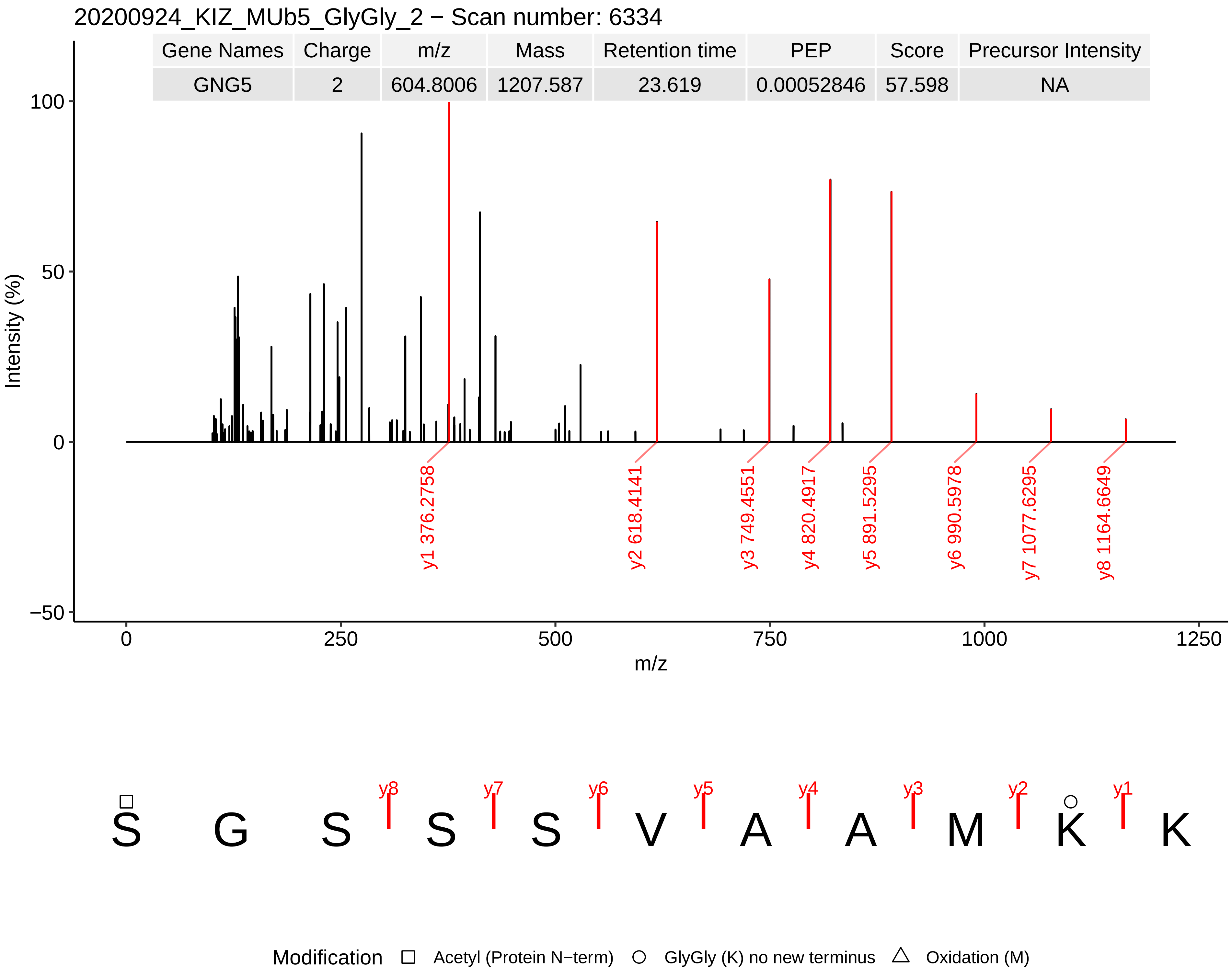
20200924_KIZ_MUb5_GlyGly_2 − Scan number: 6334
Gene Names
Charge
m/z
Mass
Retention time
PEP
Score
Precursor Intensity
GNG5
2
604.8006
1207.587
23.619
0.00052846
57.598
NA
Intensity (%)
100
50
0
−50
0
250
500
750
1000
1250
m/z
y1 376.2758
y2 618.4141
y3 749.4551
y4 820.4917
y5 891.5295
y6 990.5978
y7 1077.6295
y8 1164.6649
S G S S S V A A M K K
y8
y7
y6
y5
y4
y3
y2
y1
Modification
Acetyl (Protein N−term)
GlyGly (K) no new terminus
Oxidation (M)

20201118_KIZ_MUb6_GlyGly_2 − Scan number: 7680

Gene Names	Charge	m/z	Mass	Retention time	PEP	Score	Precursor Intensity
GNG5	2	604.8006	1207.587	26.209	6.9143e−05	72.547	NA

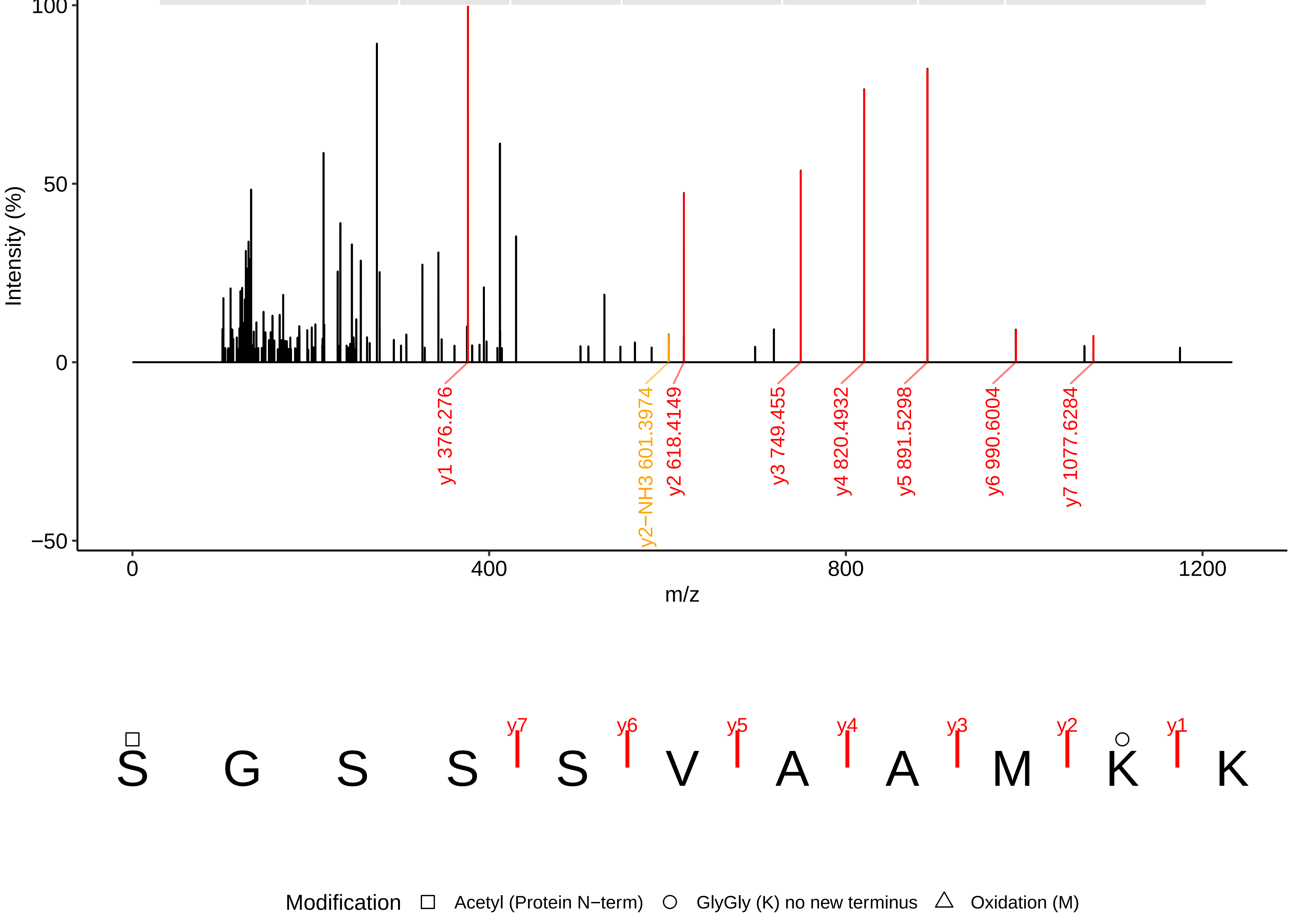

20200921_KIZ_MUb4_GlyGly_10plex − Scan number: 8688

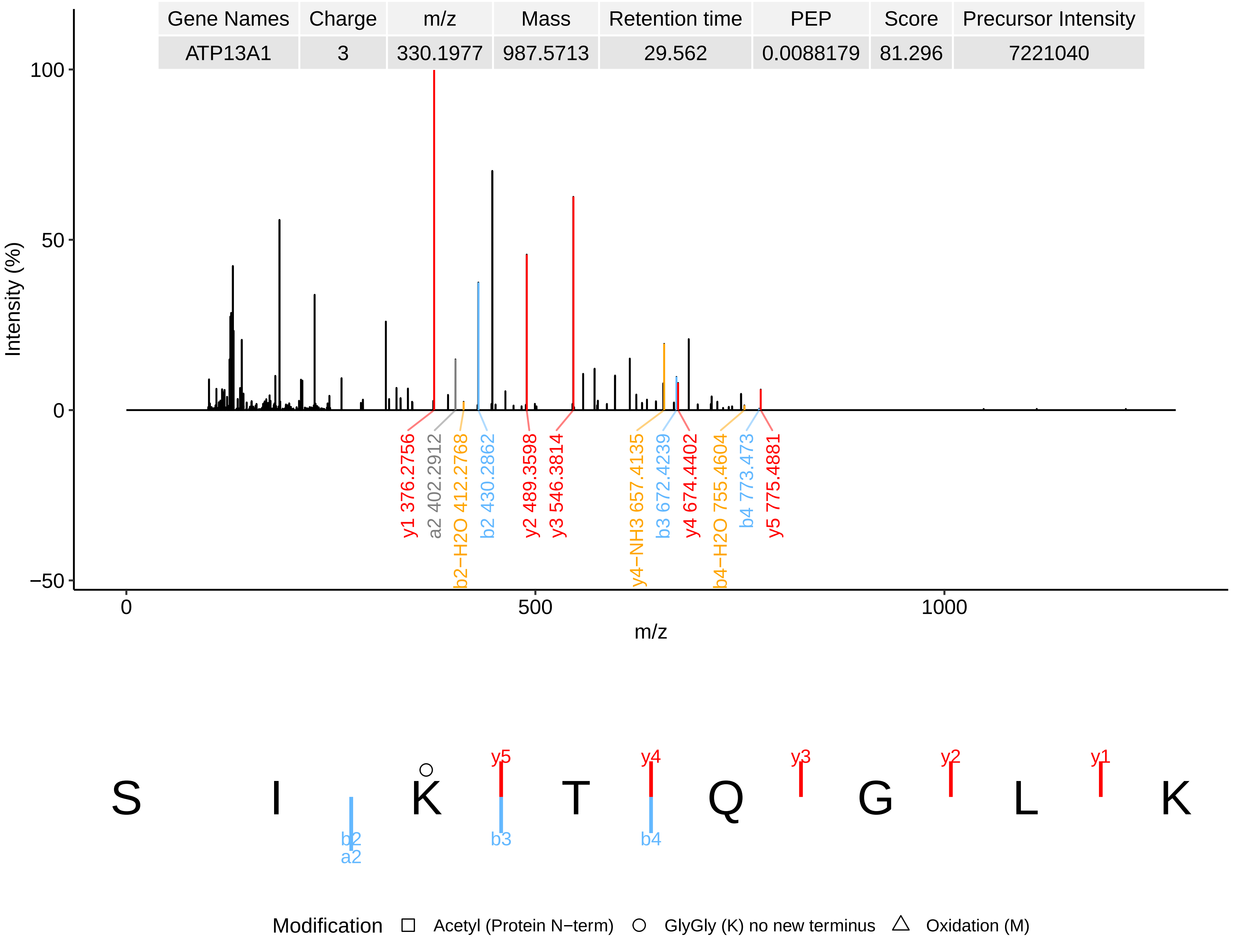

20200921_KIZ_MUb4_GlyGly_10plex_2 − Scan number: 8033

Gene Names	Charge	m/z	Mass	Retention time	PEP	Score	Precursor Intensity
ATP13A1	3	330.1977	987.5713	30.178	0.0083619	82.452	2995538

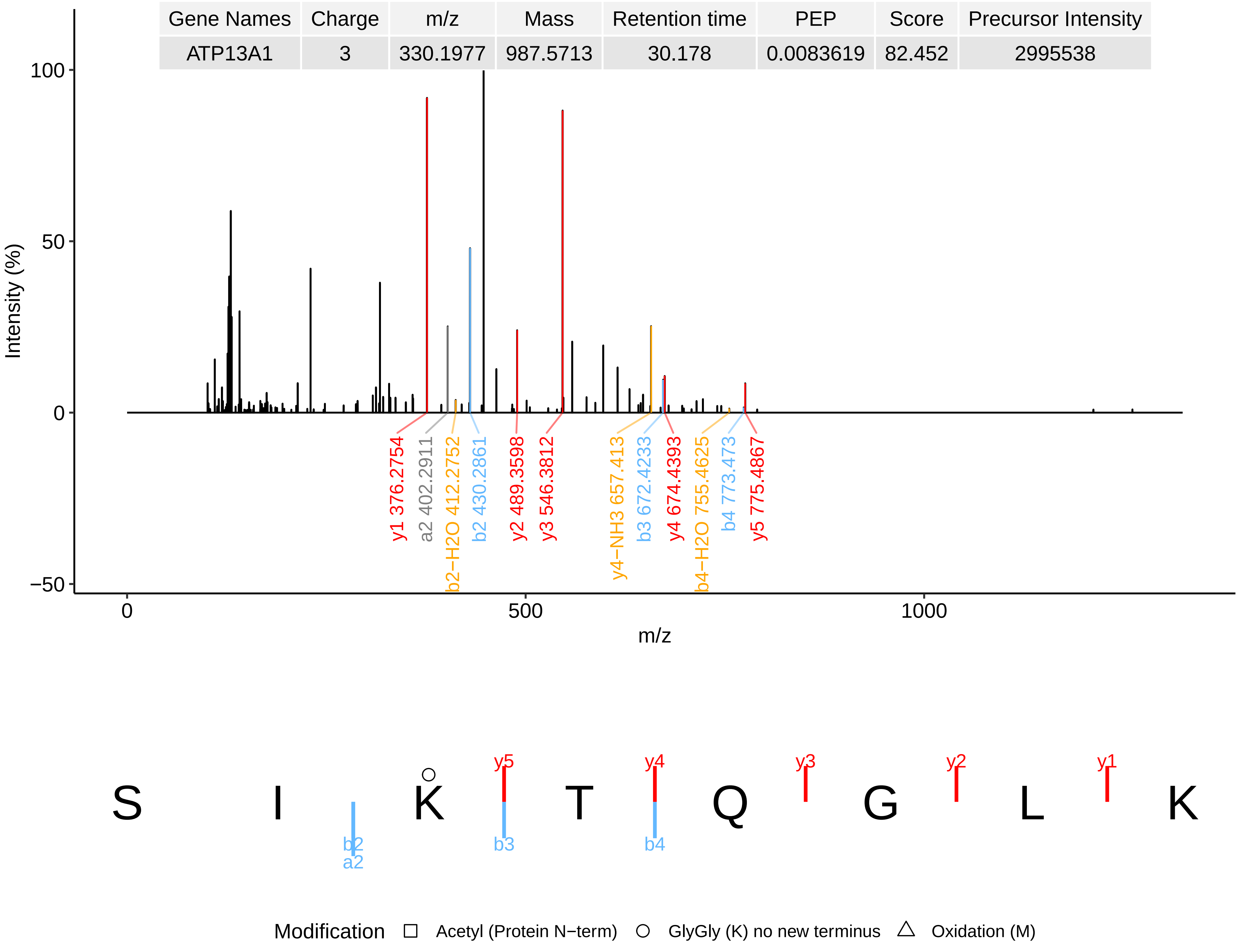

20200921_KIZ_MUb4_GlyGly_10plex_2 − Scan number: 5060

Gene Names	Charge	m/z	Mass	Retention time	PEP	Score	Precursor Intensity
WWP1	3	418.8739	1253.6	18.018	4.9251e−08	117.25	2530198

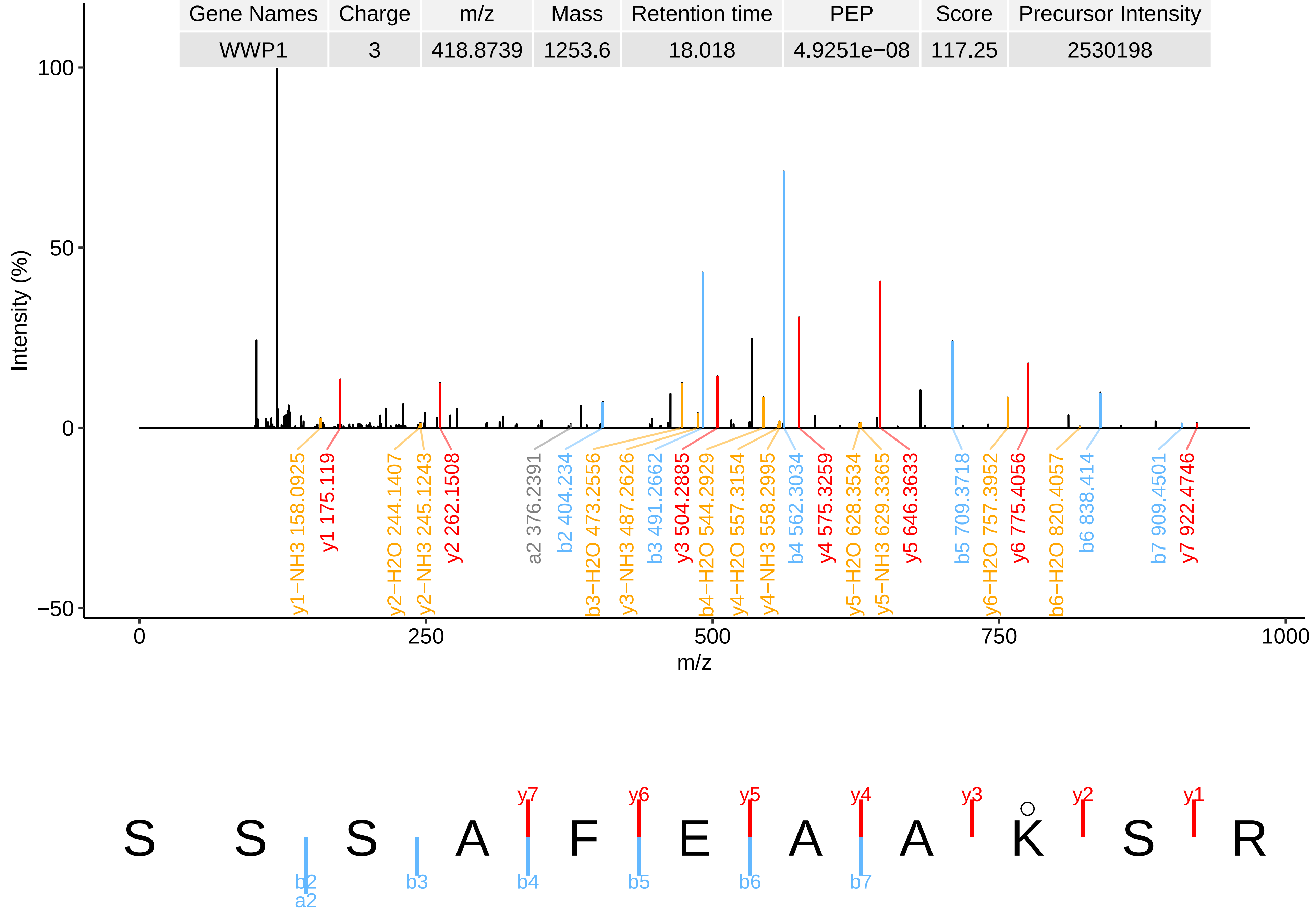

Modification □ Acetyl (Protein N−term) ○ GlyGly (K) no new terminus △ Oxidation (M)

20200924_KIZ_MUb5_GlyGly_2 − Scan number: 4440

Gene Names	Charge	m/z	Mass	Retention time	PEP	Score	Precursor Intensity
WWP1	3	418.8739	1253.6	16.287	0.017313	67.385	1339126

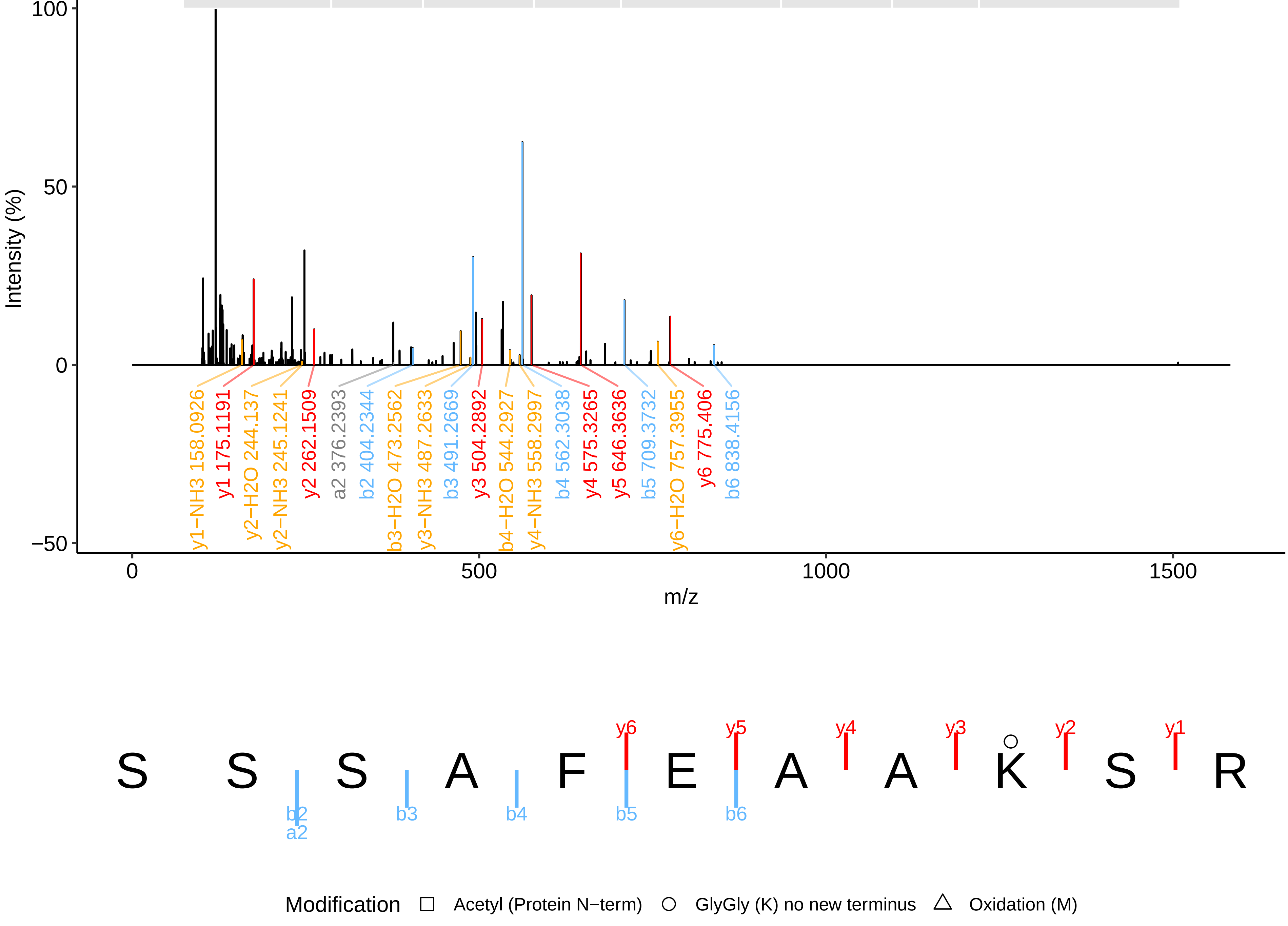

20201118_KIZ_MUb6_GlyGly_1 − Scan number: 4598

Gene Names	Charge	m/z	Mass	Retention time	PEP	Score	Precursor Intensity
WWP1	3	418.8739	1253.6	14.437	0.0031708	78.83	6488025

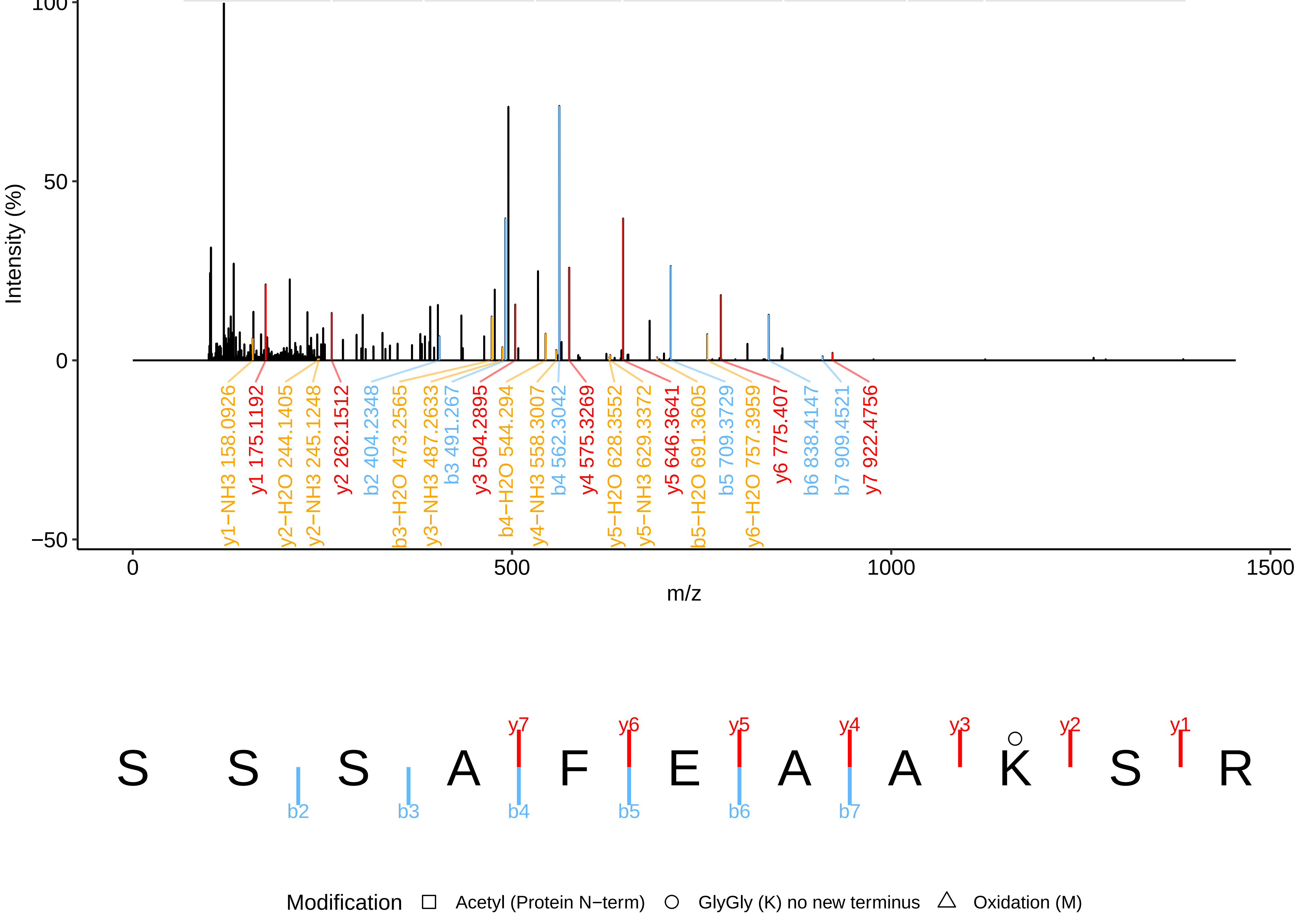

20201118_KIZ_MUb6_GlyGly_1 − Scan number: 32300

Gene Names	Charge	m/z	Mass	Retention time	PEP	Score	Precursor Intensity
LRMP	2	617.8306	1233.647	95.669	0.019404	90.657	NA

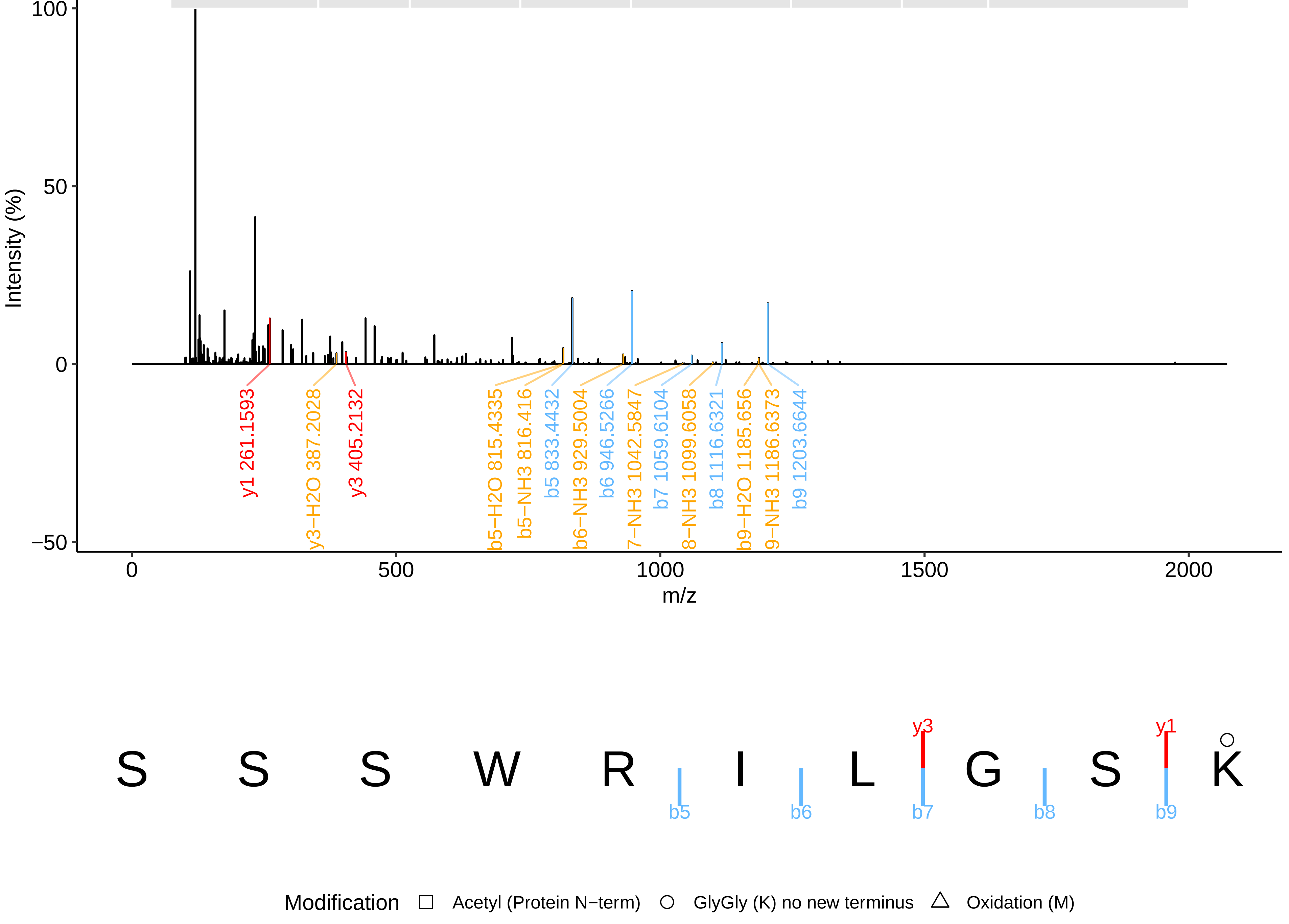

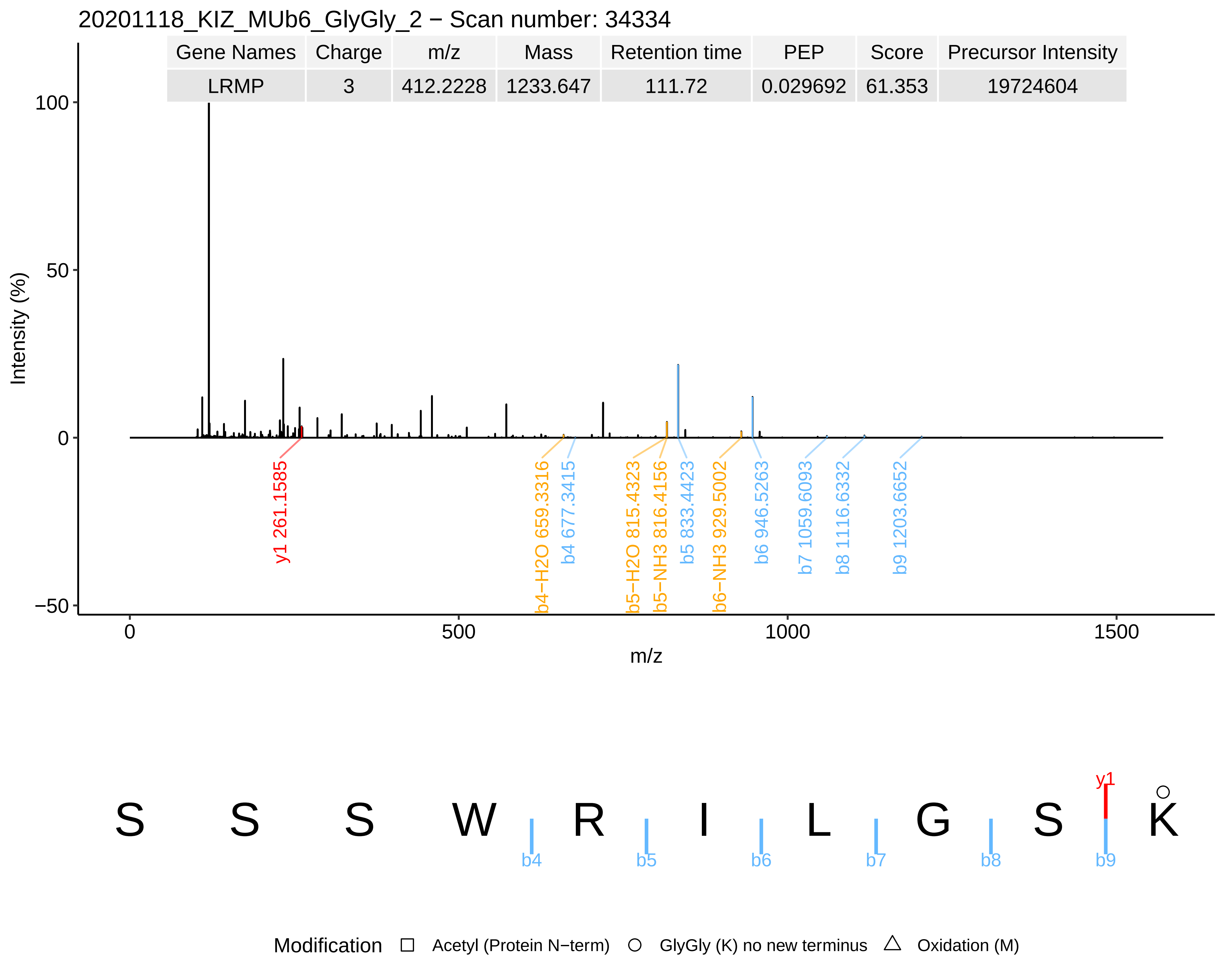
20201118_KIZ_MUb6_GlyGly_2 − Scan number: 34334
Gene Names | Charge | m/z | Mass | Retention time | PEP | Score | Precursor Intensity
LRMP | 3 | 412.2228 | 1233.647 | 111.72 | 0.029692 | 61.353 | 19724604
Intensity (%)
m/z
y1 261.1585
b4−H2O 659.3316
b4 677.3415
b5−H2O 815.4323
b5−NH3 816.4156
b5 833.4423
b6−NH3 929.5002
b6 946.5263
b7 1059.6093
b8 1116.6332
b9 1203.6652
S S S W R I L G S K
y1
b4 b5 b6 b7 b8 b9
Modification
Acetyl (Protein N−term)
GlyGly (K) no new terminus
Oxidation (M)

20200921_KIZ_MUb4_GlyGly_10plex − Scan number: 3043

Gene Names	Charge	m/z	Mass	Retention time	PEP	Score	Precursor Intensity
KIDINS220	3	340.8367	1019.488	9.543	0.04101	86.833	9065679

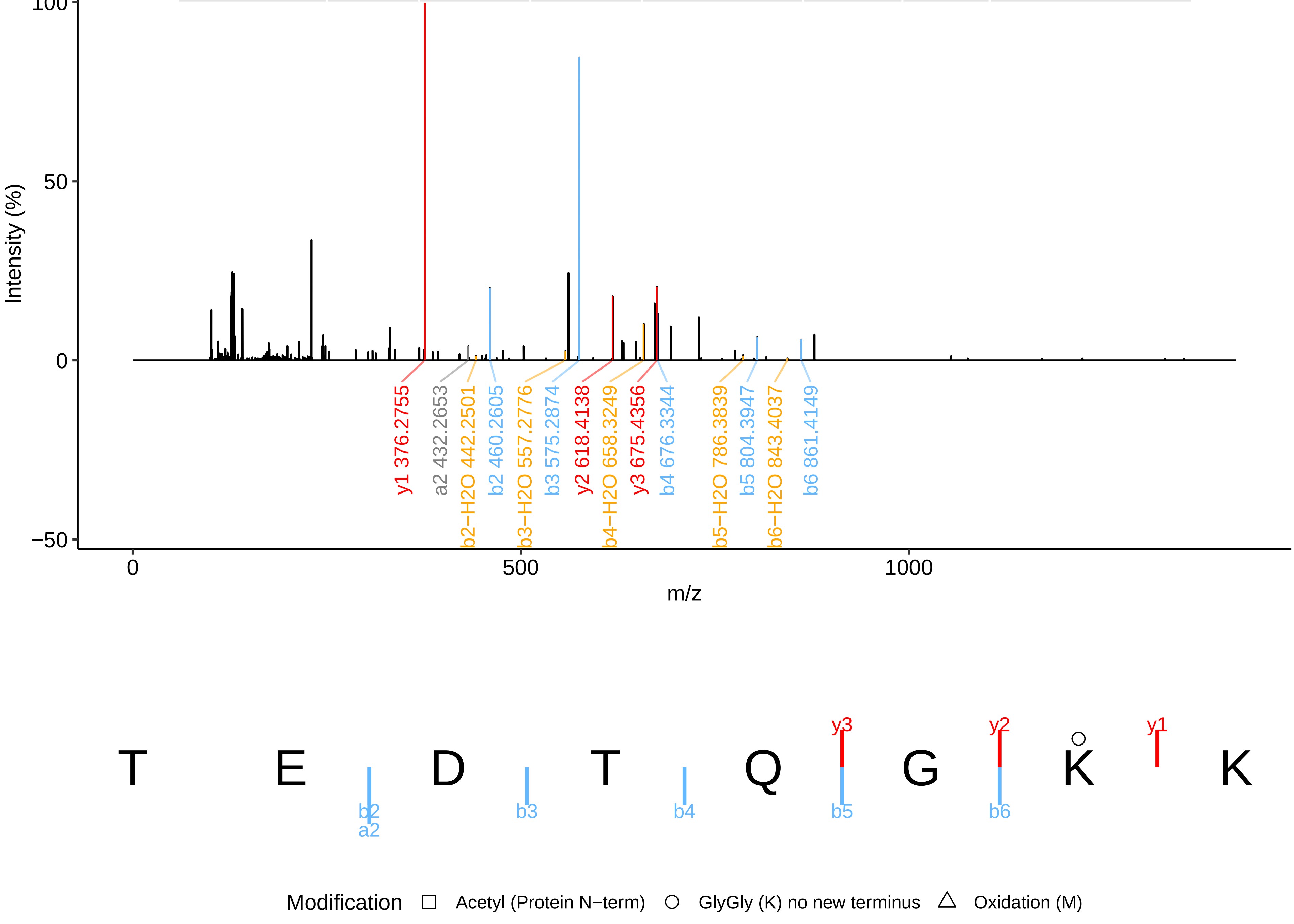

20200921_KIZ_MUb4_GlyGly_10plex_2 − Scan number: 2951

Gene Names	Charge	m/z	Mass	Retention time	PEP	Score	Precursor Intensity
KIDINS220	3	340.8367	1019.488	9.6307	0.093565	75.738	1187685

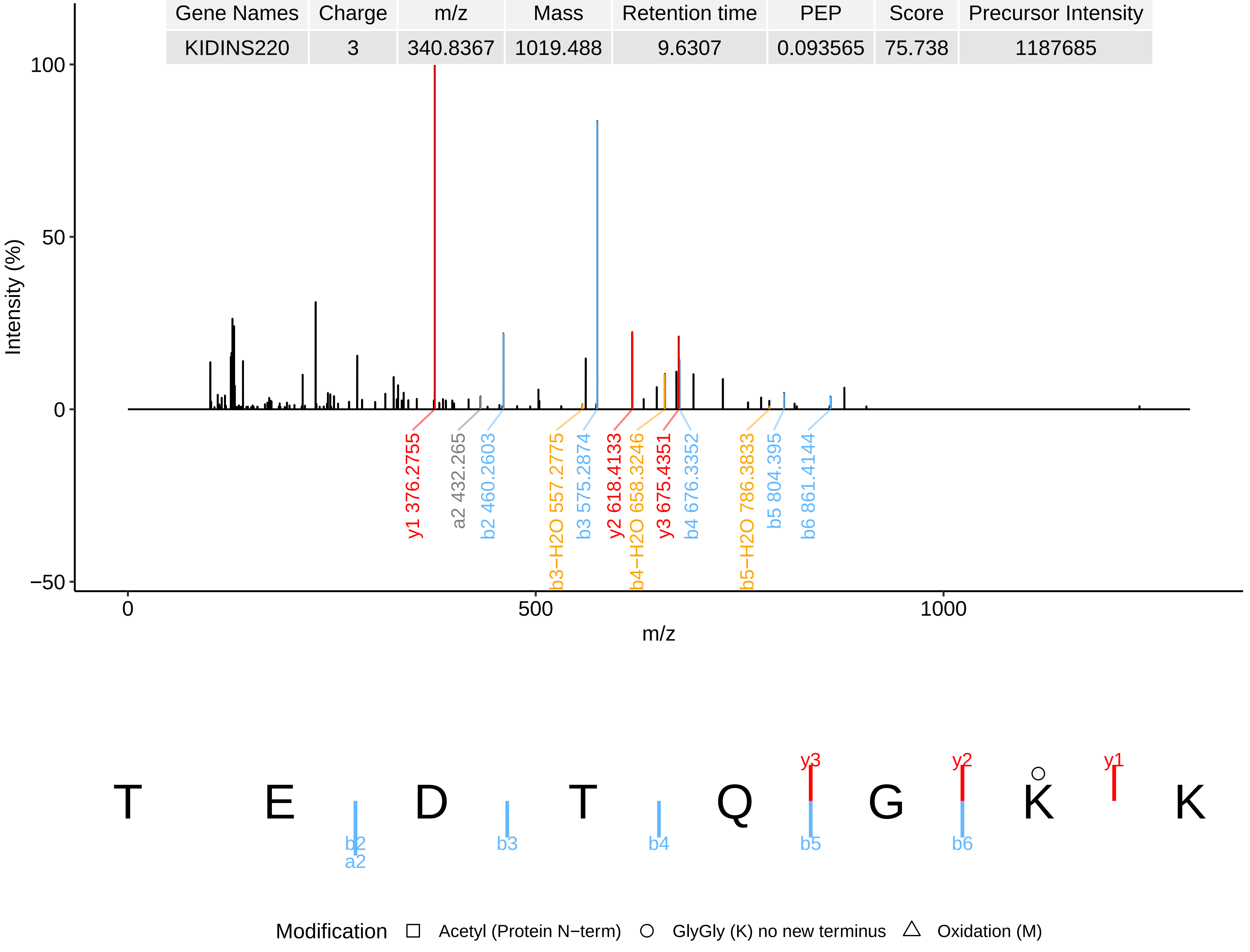

20200921_KIZ_MUb4_GlyGly_10plex − Scan number: 6110

Gene Names	Charge	m/z	Mass	Retention time	PEP	Score	Precursor Intensity
SNAP23	3	342.1735	1023.499	20.108	0.065474	54.722	2103332

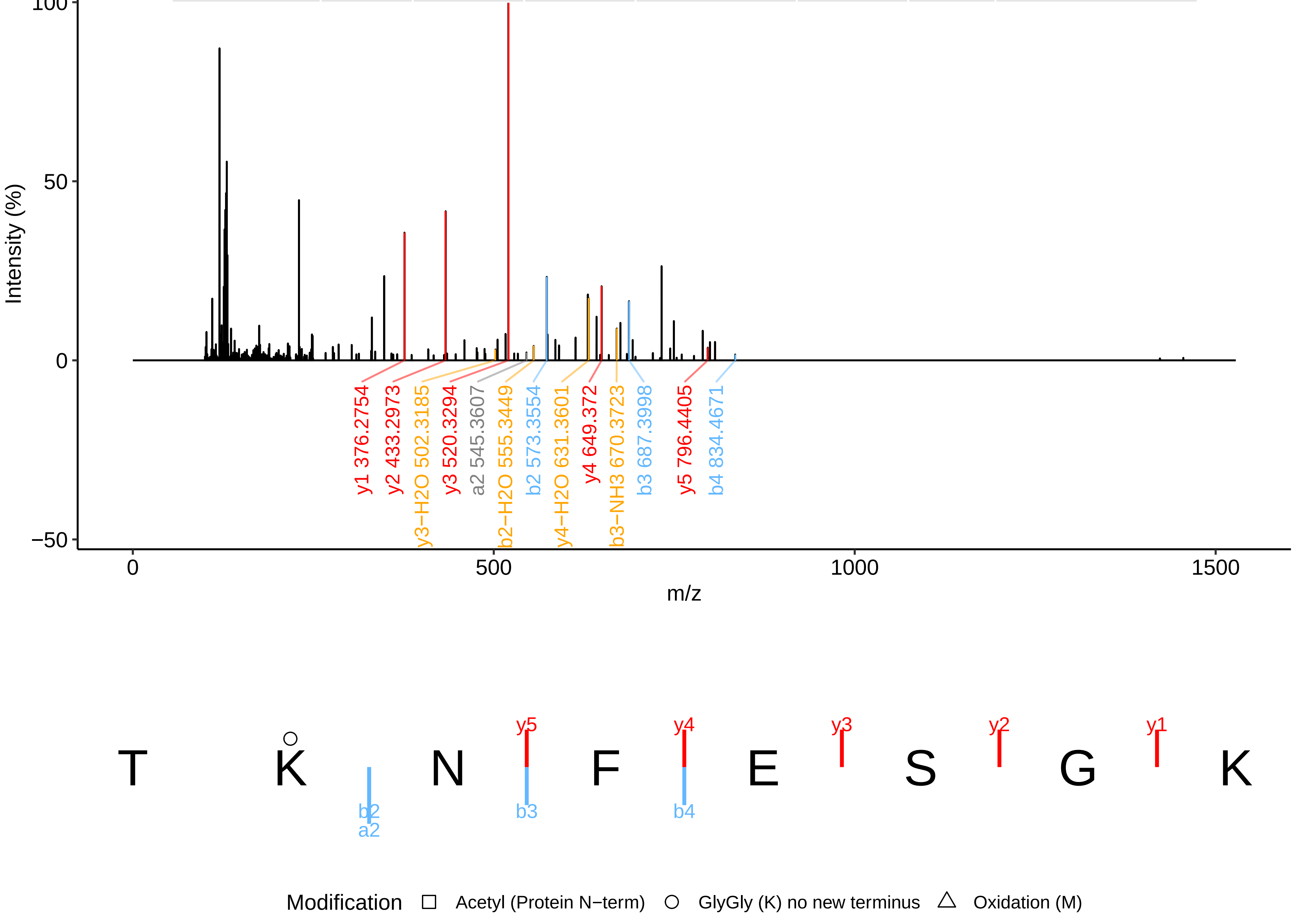

20200921_KIZ_MUb4_GlyGly_10plex_2 − Scan number: 5600

Gene Names	Charge	m/z	Mass	Retention time	PEP	Score	Precursor Intensity
SNAP23	3	342.1735	1023.499	20.214	0.084692	50.04	1115584

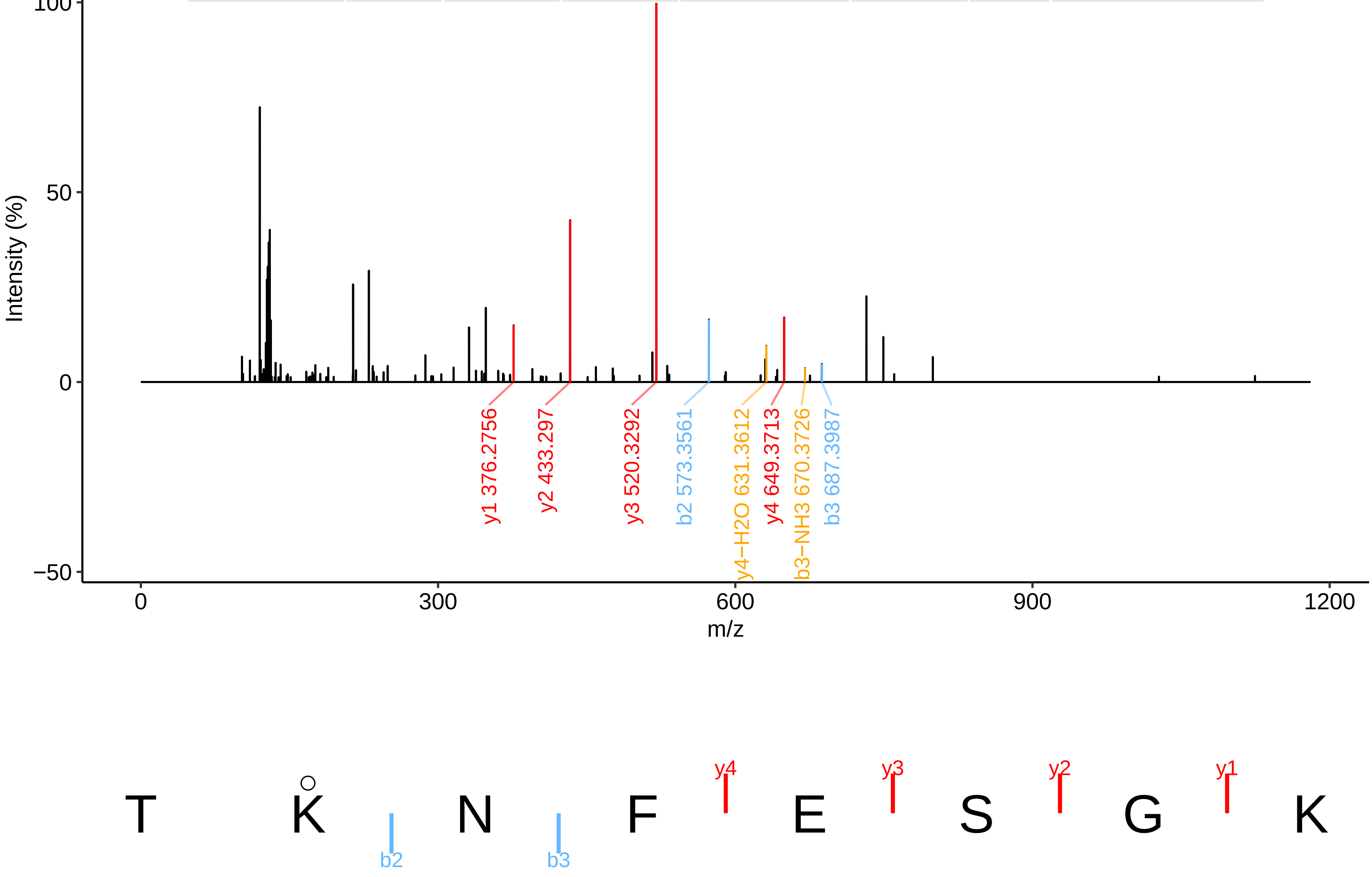

Modification □ Acetyl (Protein N−term) ○ GlyGly (K) no new terminus △ Oxidation (M)

20200924_KIZ_MUb5_GlyGly_1 − Scan number: 5279

Gene Names	Charge	m/z	Mass	Retention time	PEP	Score	Precursor Intensity
SNAP23	3	342.1735	1023.499	16.383	0.014457	78.264	6667103

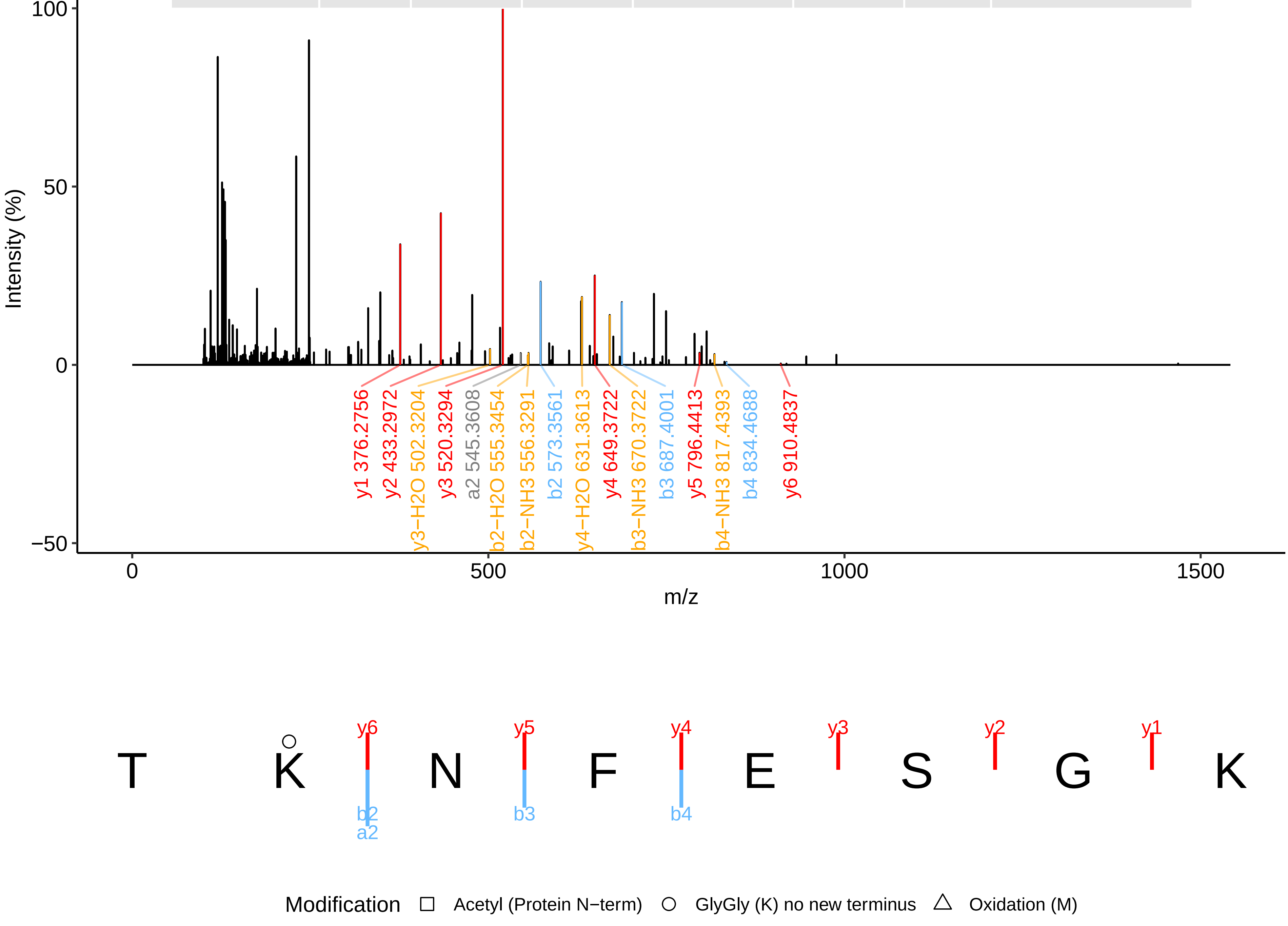

20200924_KIZ_MUb5_GlyGly_2 − Scan number: 4976

Gene Names	Charge	m/z	Mass	Retention time	PEP	Score	Precursor Intensity
SNAP23	3	342.1735	1023.499	18.383	0.036365	64.42	2235641

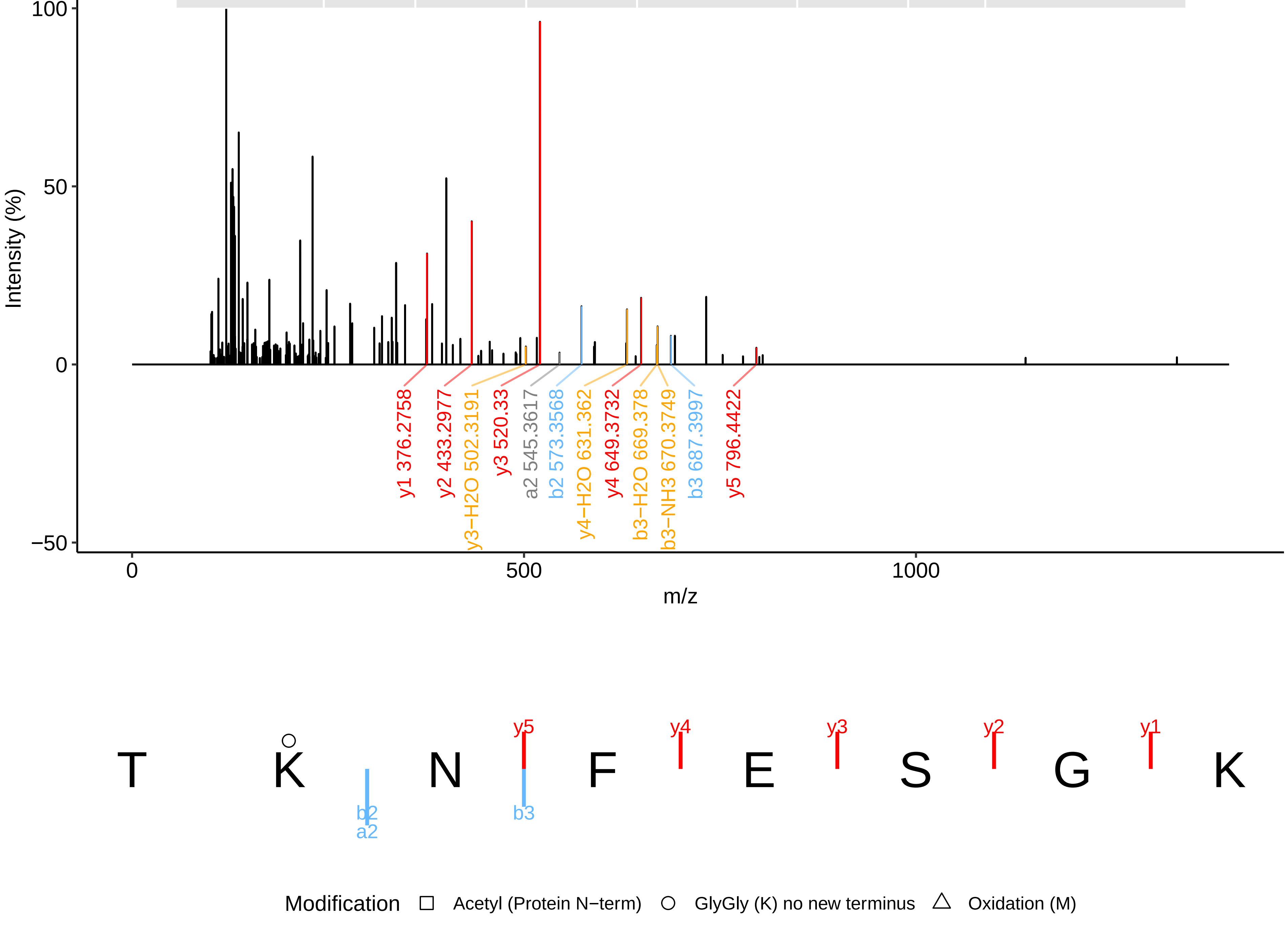

20201118_KIZ_MUb6_GlyGly_1 − Scan number: 5063

Gene Names	Charge	m/z	Mass	Retention time	PEP	Score	Precursor Intensity
SNAP23	3	342.1735	1023.499	16.009	0.012133	78.149	25955726

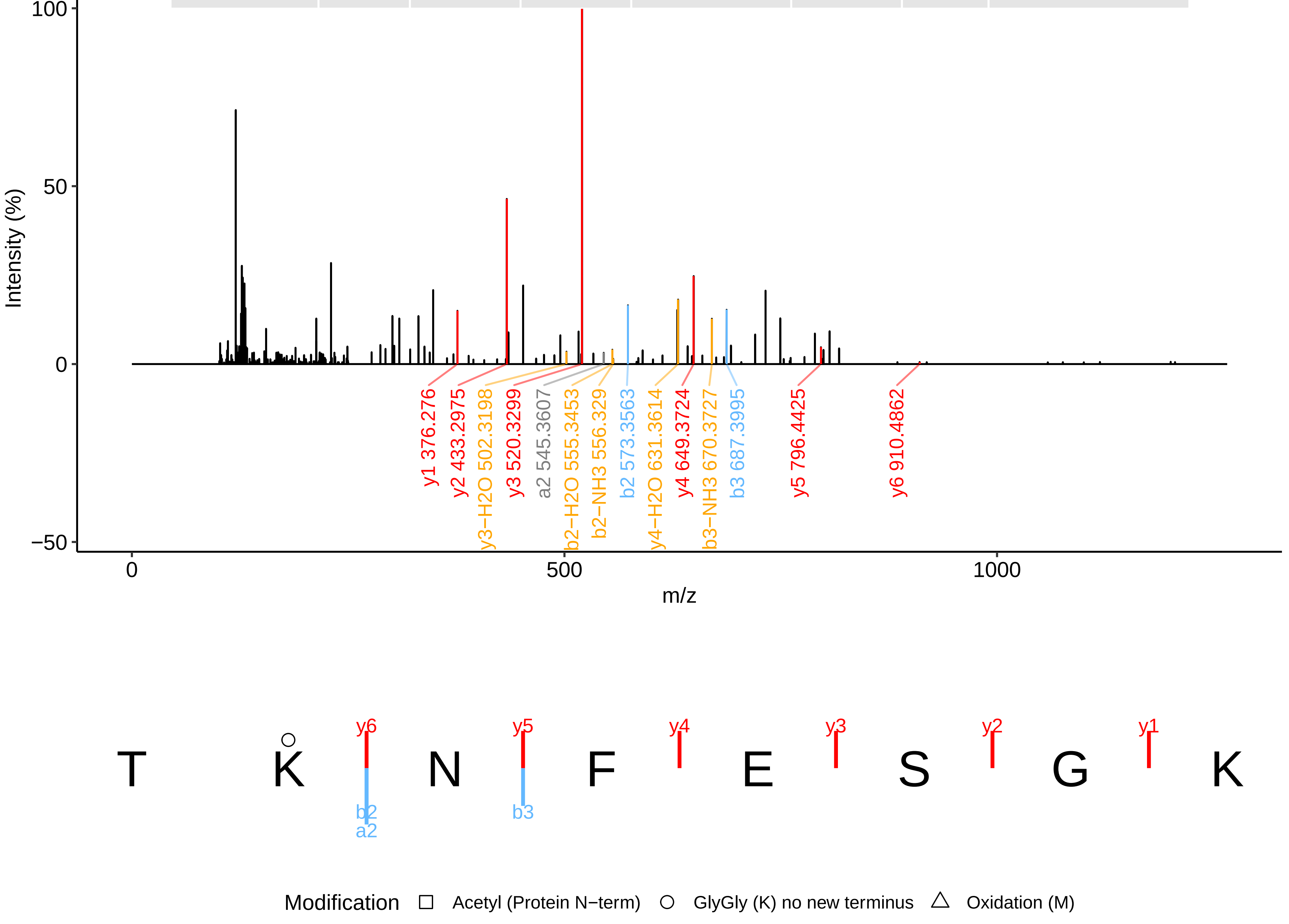

20200921_KIZ_MUb4_GlyGly_10plex − Scan number: 12357

Gene Names	Charge	m/z	Mass	Retention time	PEP	Score	Precursor Intensity
	3	411.2141	1230.62	43.291	0.10959	41.029	365992384

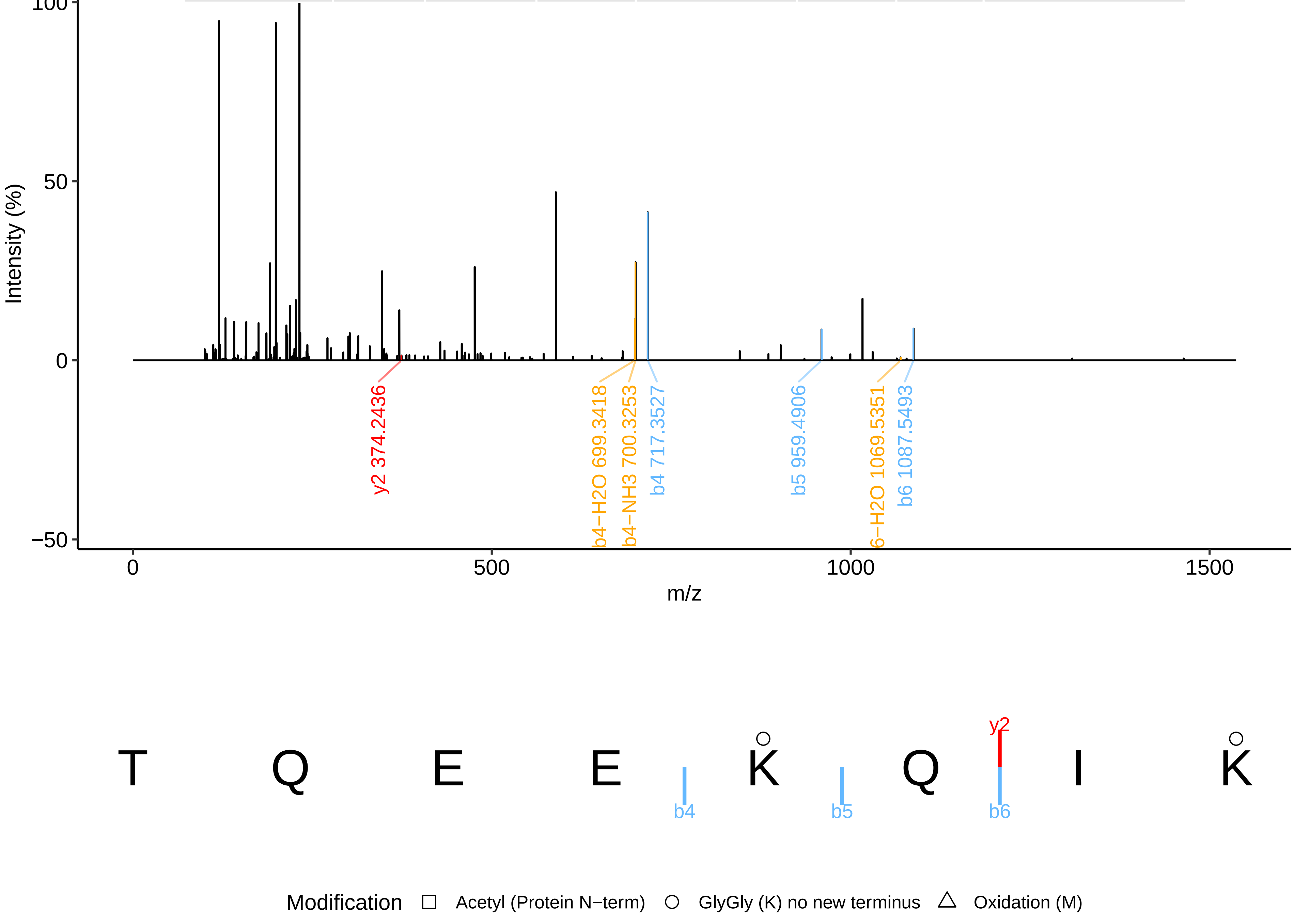

20200924_KIZ_MUb5_GlyGly_1 − Scan number: 11054

Gene Names	Charge	m/z	Mass	Retention time	PEP	Score	Precursor Intensity
	3	411.2141	1230.62	35.805	0.10959	41.029	314596384

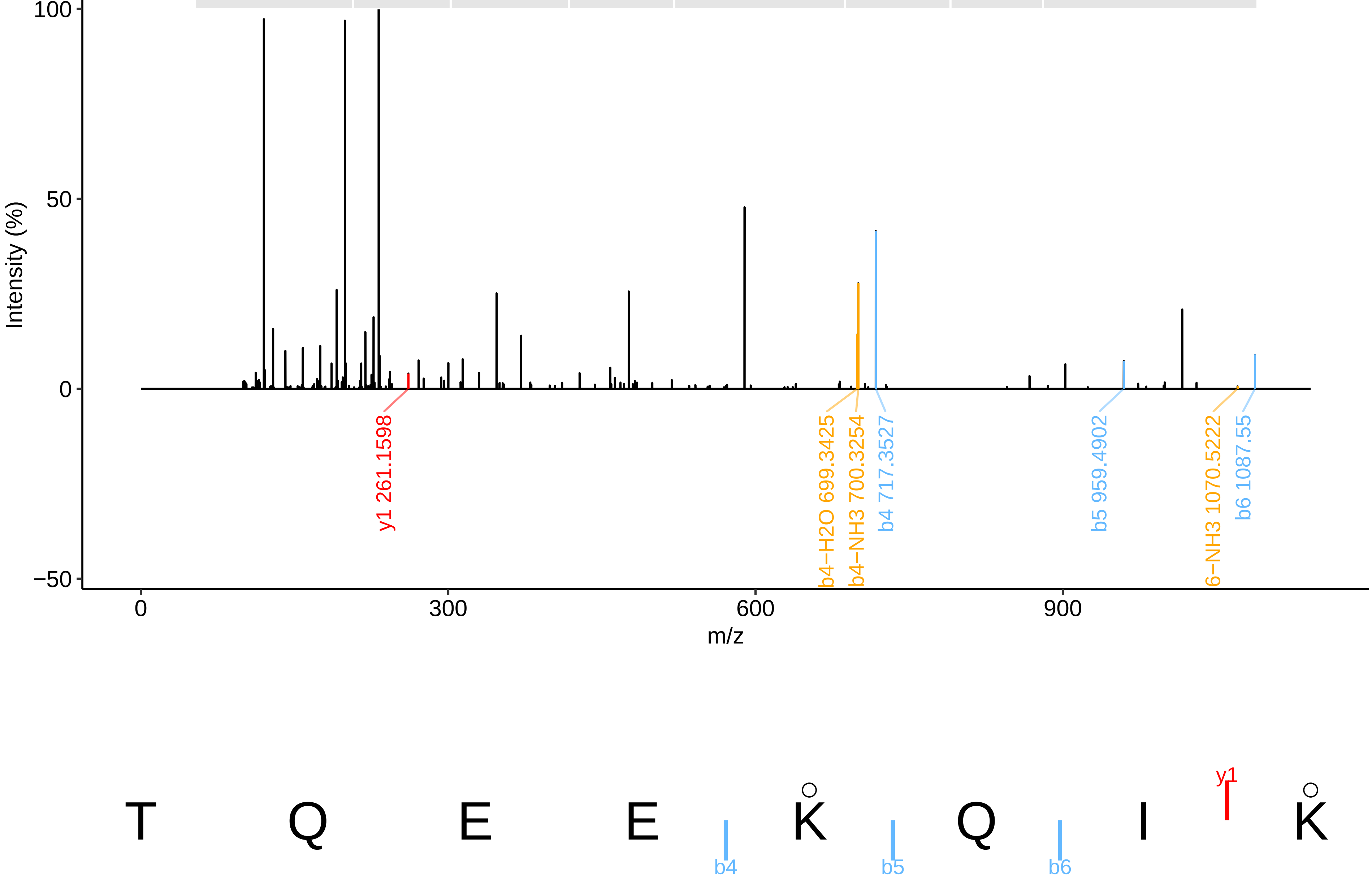

Modification □ Acetyl (Protein N−term) ○ GlyGly (K) no new terminus △ Oxidation (M)

20200924_KIZ_MUb5_GlyGly_2 − Scan number: 10320

Gene Names	Charge	m/z	Mass	Retention time	PEP	Score	Precursor Intensity
	3	411.2141	1230.62	38.531	0.10573	42.109	11721668

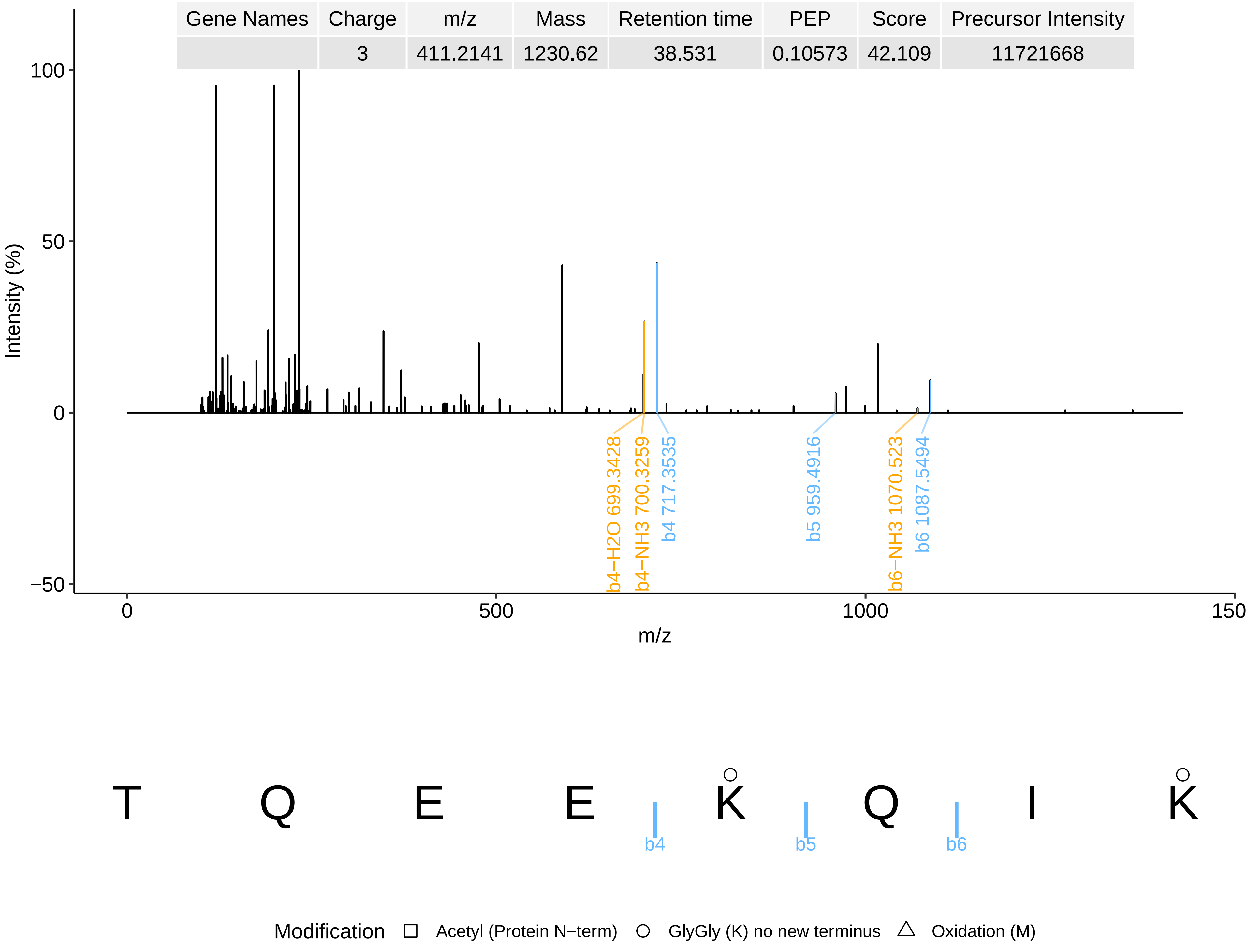

20201118_KIZ_MUb6_GlyGly_1 − Scan number: 10902

Gene Names	Charge	m/z	Mass	Retention time	PEP	Score	Precursor Intensity
	3	411.2141	1230.62	34.413	0.065133	53.166	115801640

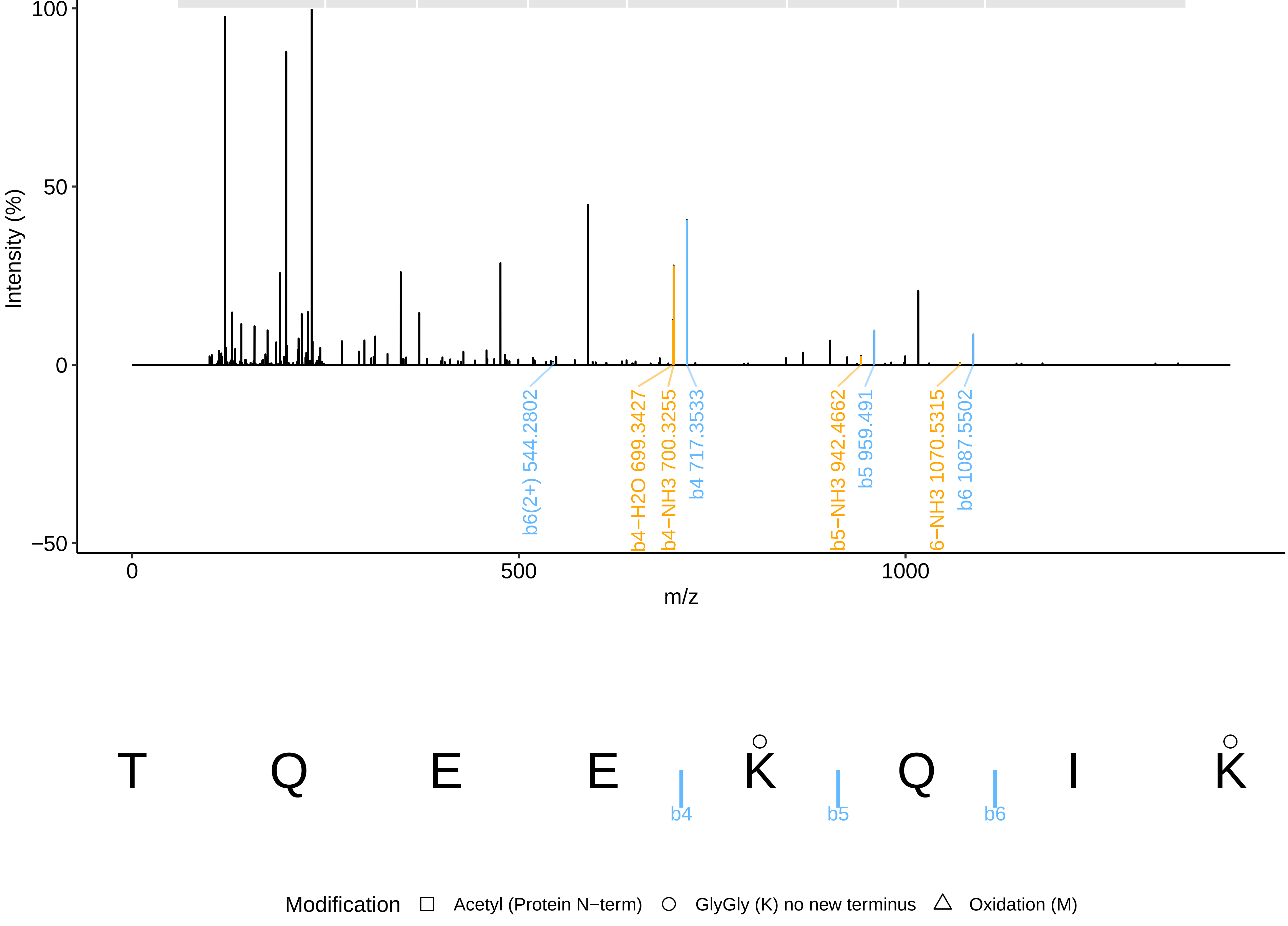

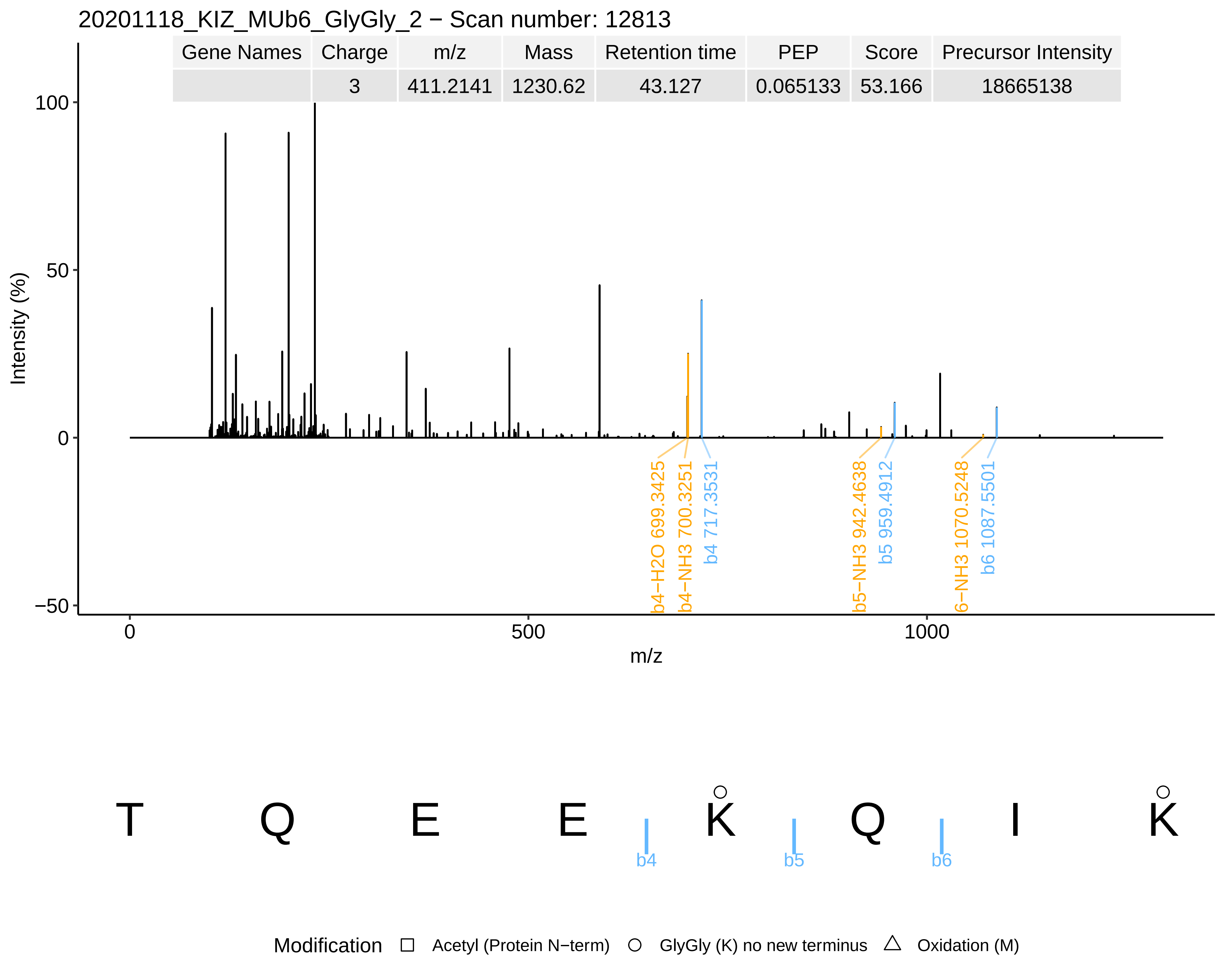

20201118_KIZ_MUb6_GlyGly_2 − Scan number: 12813
Gene Names
Charge
m/z
Mass
Retention time
PEP
Score
Precursor Intensity
3
411.2141
1230.62
43.127
0.065133
53.166
18665138
Intensity (%)
100
50
0
−50
0
500
1000
m/z
b4−H2O 699.3425
b4−NH3 700.3251
b4 717.3531
b5−NH3 942.4638
b5 959.4912
6−NH3 1070.5248
b6 1087.5501
T Q E E K Q I K
b4
b5
b6
Modification
Acetyl (Protein N−term)
GlyGly (K) no new terminus
Oxidation (M)

20200921_KIZ_MUb4_GlyGly_10plex − Scan number: 5585

Gene Names	Charge	m/z	Mass	Retention time	PEP	Score	Precursor Intensity
RPS3	3	405.8862	1214.637	18.321	0.0010576	91.215	4339193

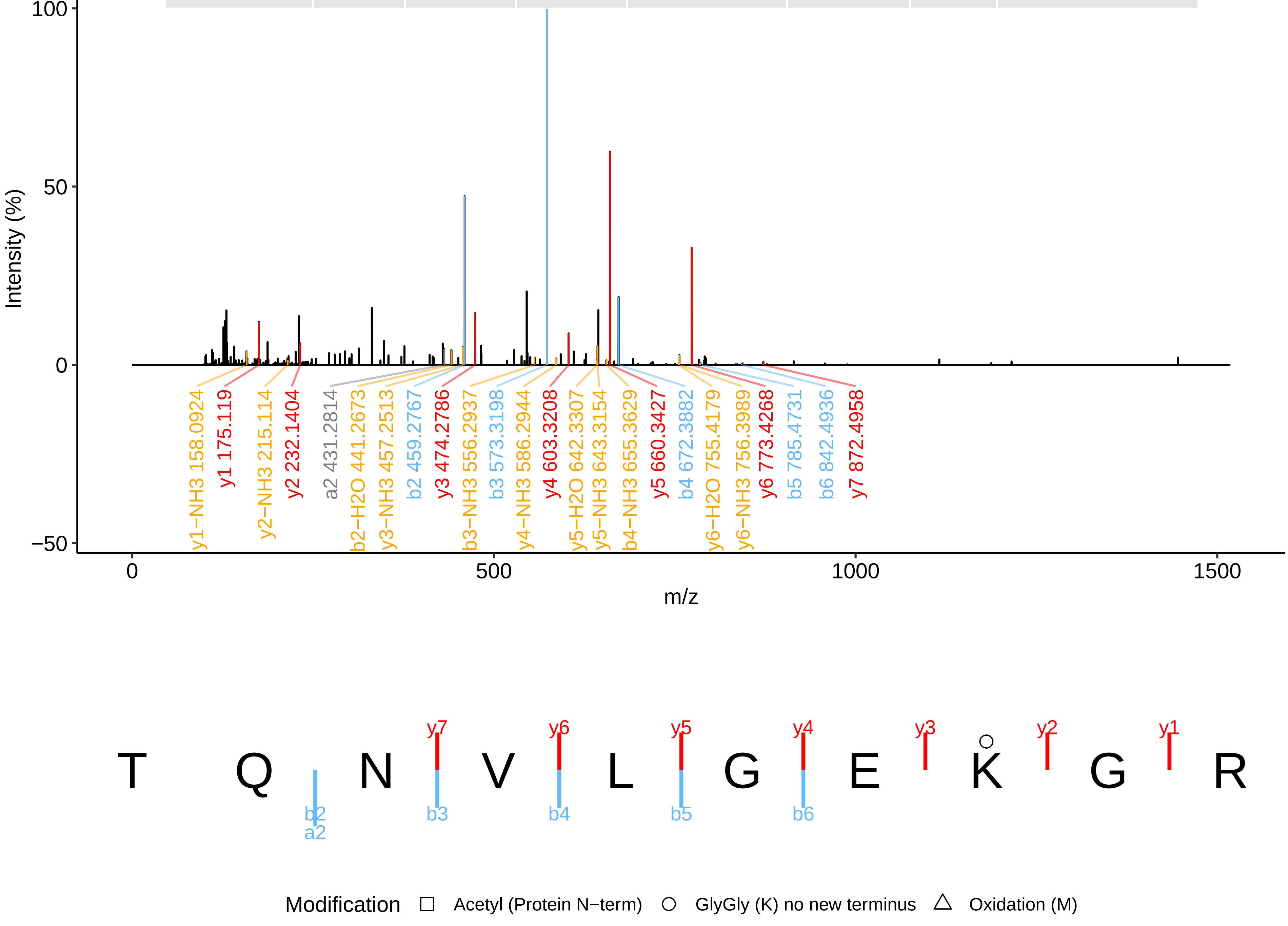

20200924_KIZ_MUb5_GlyGly_1 − Scan number: 4874

Gene Names	Charge	m/z	Mass	Retention time	PEP	Score	Precursor Intensity
RPS3	3	405.8862	1214.637	15.089	0.002674	89.9	7050249

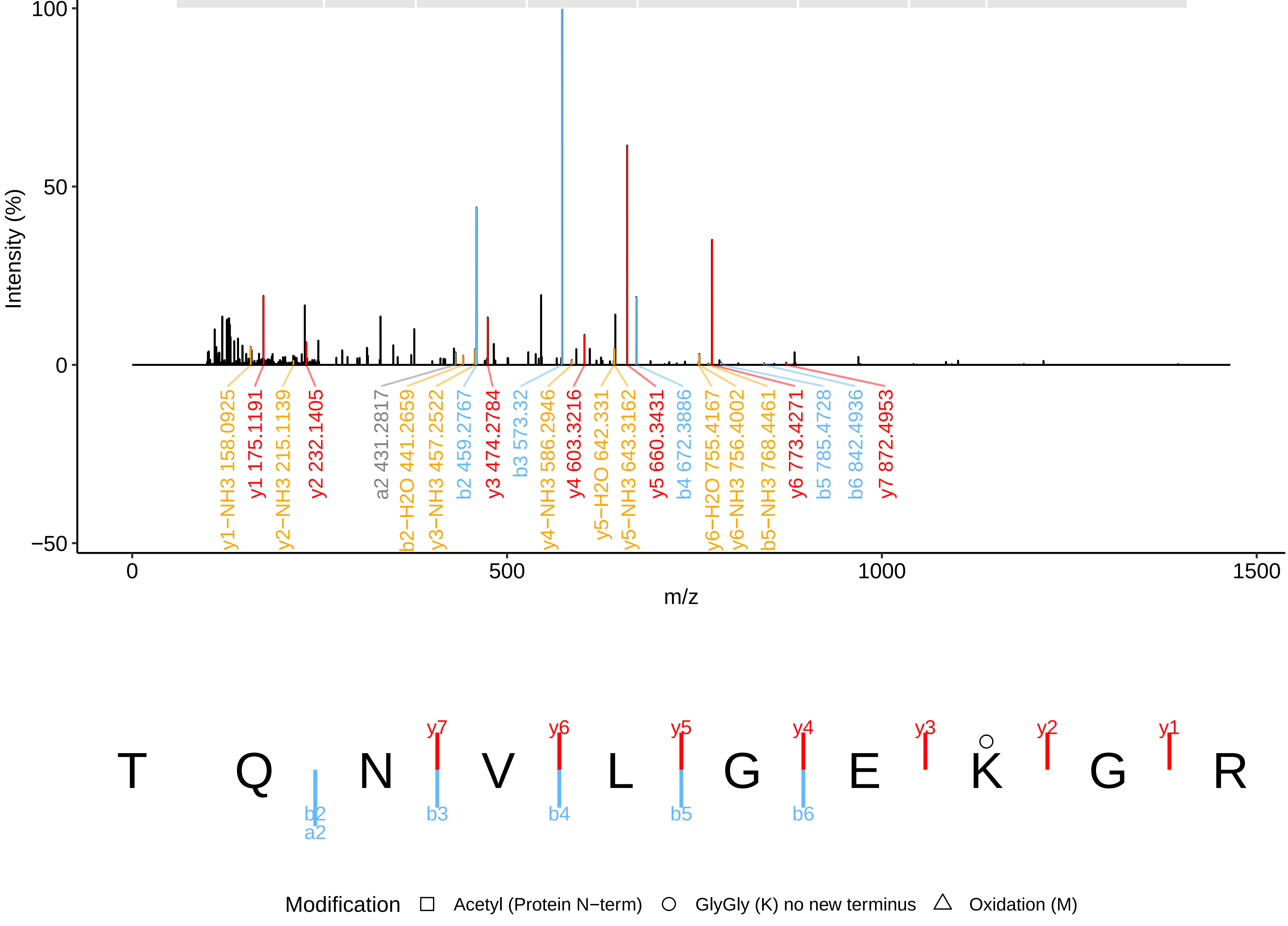

20200924_KIZ_MUb5_GlyGly_2 − Scan number: 4466

Gene Names	Charge	m/z	Mass	Retention time	PEP	Score	Precursor Intensity
RPS3	3	405.8862	1214.637	16.391	3.2813e−05	107.95	1606149

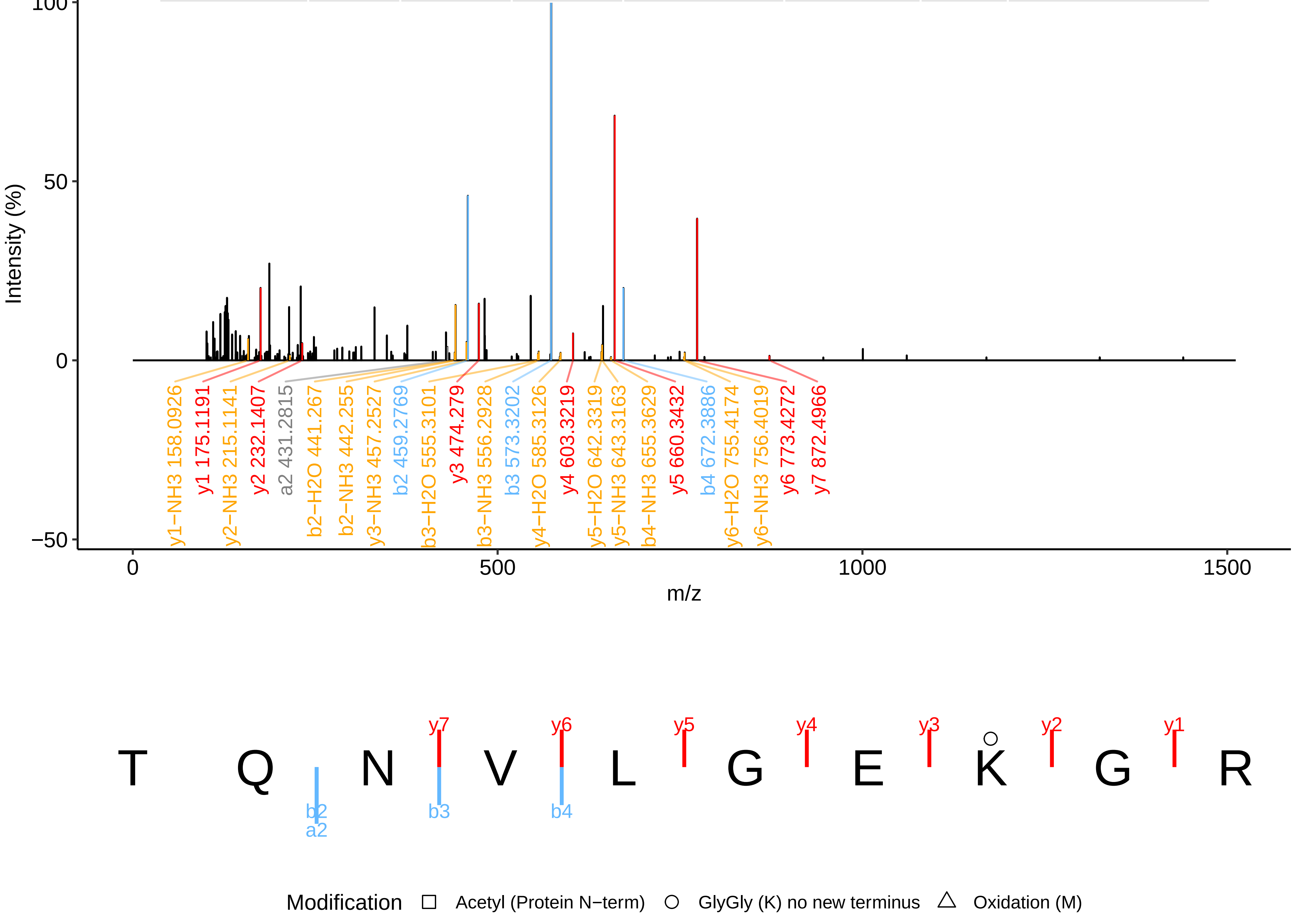

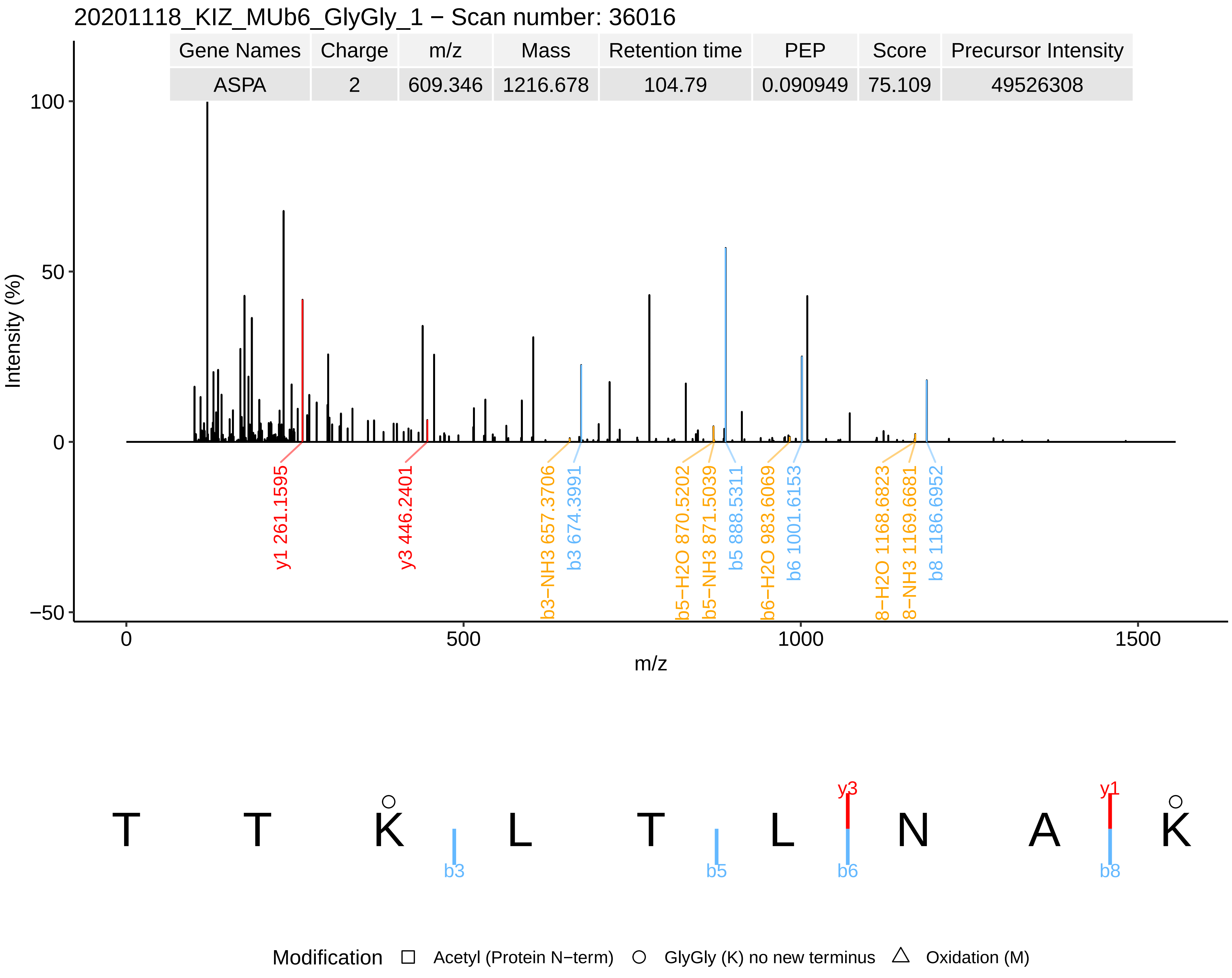
20201118_KIZ_MUb6_GlyGly_1 − Scan number: 36016
Gene Names | Charge | m/z | Mass | Retention time | PEP | Score | Precursor Intensity
ASPA | 2 | 609.346 | 1216.678 | 104.79 | 0.090949 | 75.109 | 49526308
Intensity (%)
100
50
0
−50
0
500
1000
1500
m/z
y1 261.1595
y3 446.2401
b3−NH3 657.3706
b3 674.3991
b5−H2O 870.5202
b5−NH3 871.5039
b5 888.5311
b6−H2O 983.6069
b6 1001.6153
8−H2O 1168.6823
8−NH3 1169.6681
b8 1186.6952
T T K L T L N A K
y3
y1
b3
b5
b6
b8
Modification
Acetyl (Protein N−term)
GlyGly (K) no new terminus
Oxidation (M)

20201118_KIZ_MUb6_GlyGly_2 − Scan number: 36673

Gene Names	Charge	m/z	Mass	Retention time	PEP	Score	Precursor Intensity
ASPA	2	609.346	1216.678	118.35	0.023171	87.323	18032456

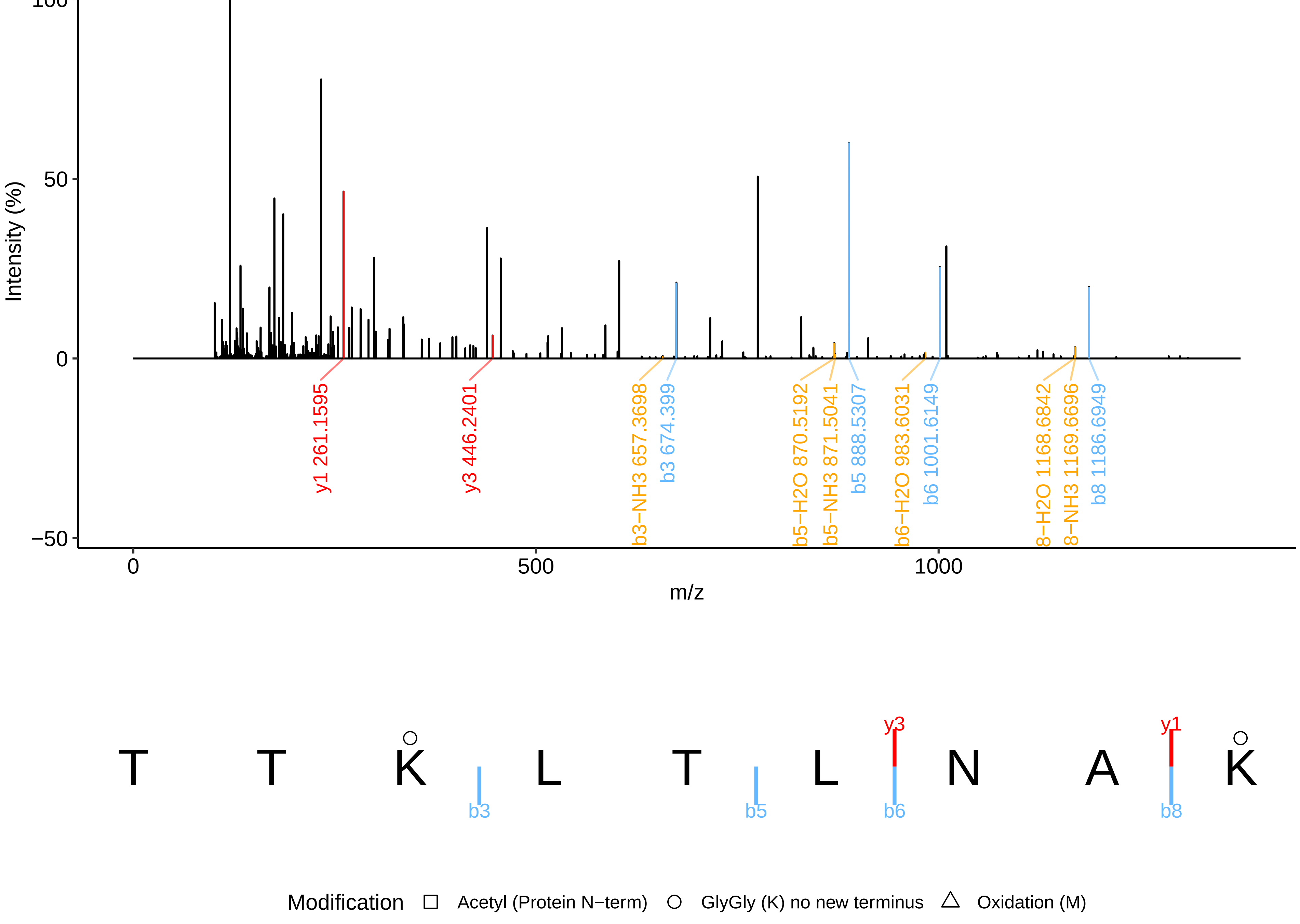

20200921_KIZ_MUb4_GlyGly_10plex_2 − Scan number: 5772

Gene Names	Charge	m/z	Mass	Retention time	PEP	Score	Precursor Intensity
LMNA	3	418.2142	1251.621	20.91	0.086498	50.297	899596.6

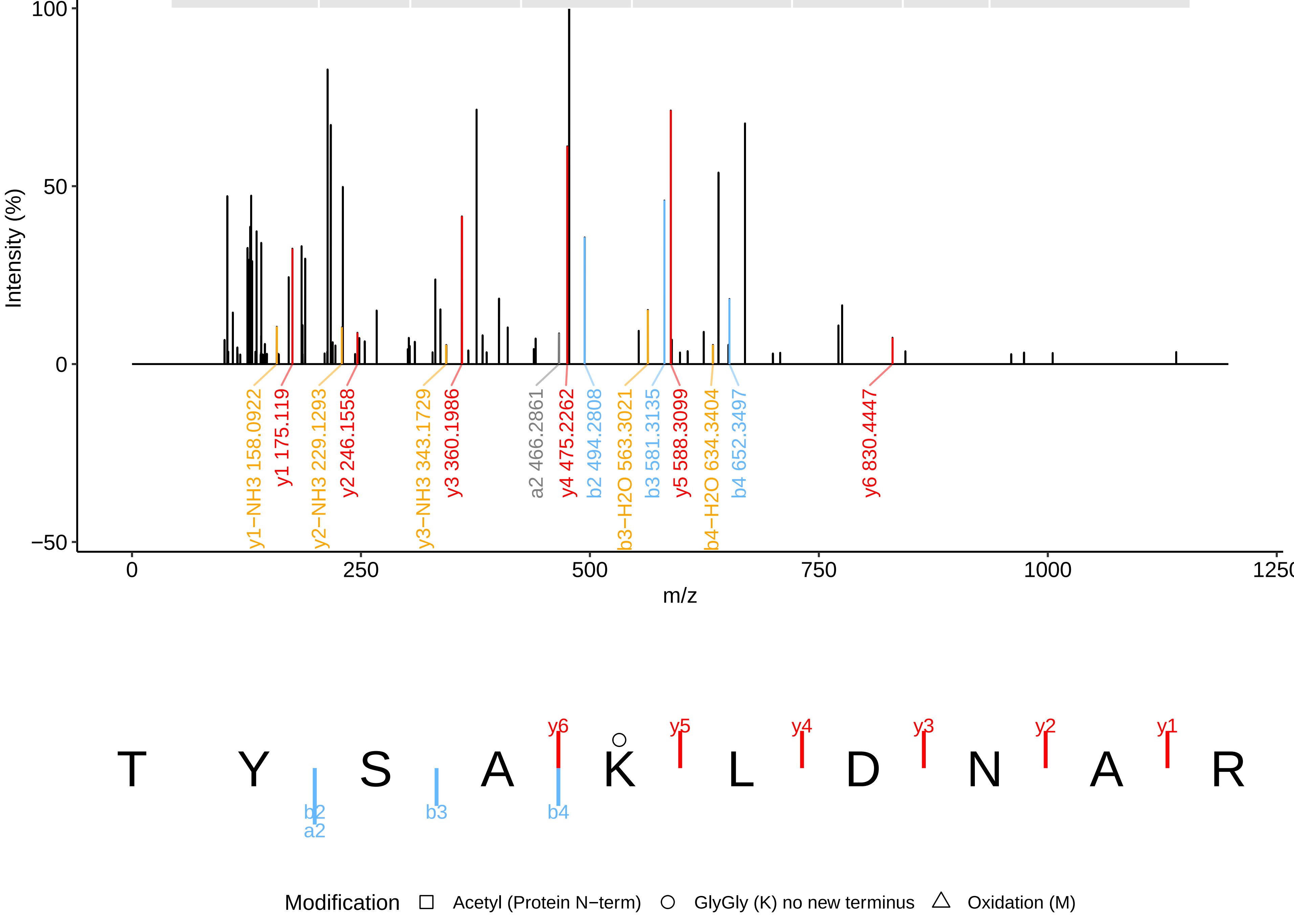

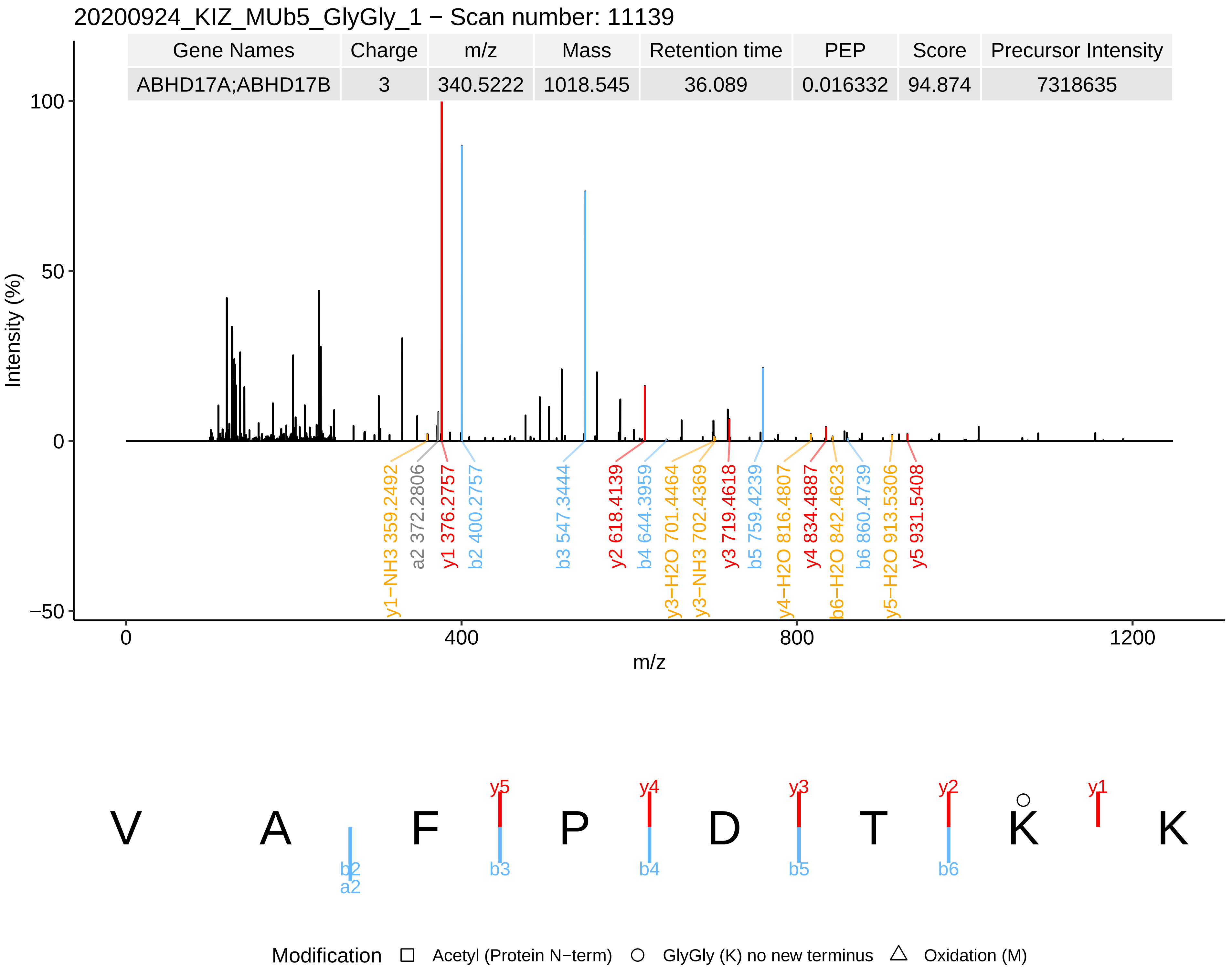
20200924_KIZ_MUb5_GlyGly_1 − Scan number: 11139
Gene Names | Charge | m/z | Mass | Retention time | PEP | Score | Precursor Intensity
ABHD17A;ABHD17B | 3 | 340.5222 | 1018.545 | 36.089 | 0.016332 | 94.874 | 7318635
Intensity (%)
m/z
y1−NH3 359.2492
a2 372.2806
y1 376.2757
b2 400.2757
b3 547.3444
y2 618.4139
b4 644.3959
y3−H2O 701.4464
y3−NH3 702.4369
y3 719.4618
b5 759.4239
y4−H2O 816.4807
y4 834.4887
b6−H2O 842.4623
b6 860.4739
y5−H2O 913.5306
y5 931.5408
V A F P D T K K
y5 y4 y3 y2 y1
b2 a2 b3 b4 b5 b6
Modification □ Acetyl (Protein N−term) ○ GlyGly (K) no new terminus △ Oxidation (M)

20200921_KIZ_MUb4_GlyGly_10plex − Scan number: 6476

Gene Names	Charge	m/z	Mass	Retention time	PEP	Score	Precursor Intensity
RAN	3	329.8576	986.5509	21.444	0.091956	65.246	6091699

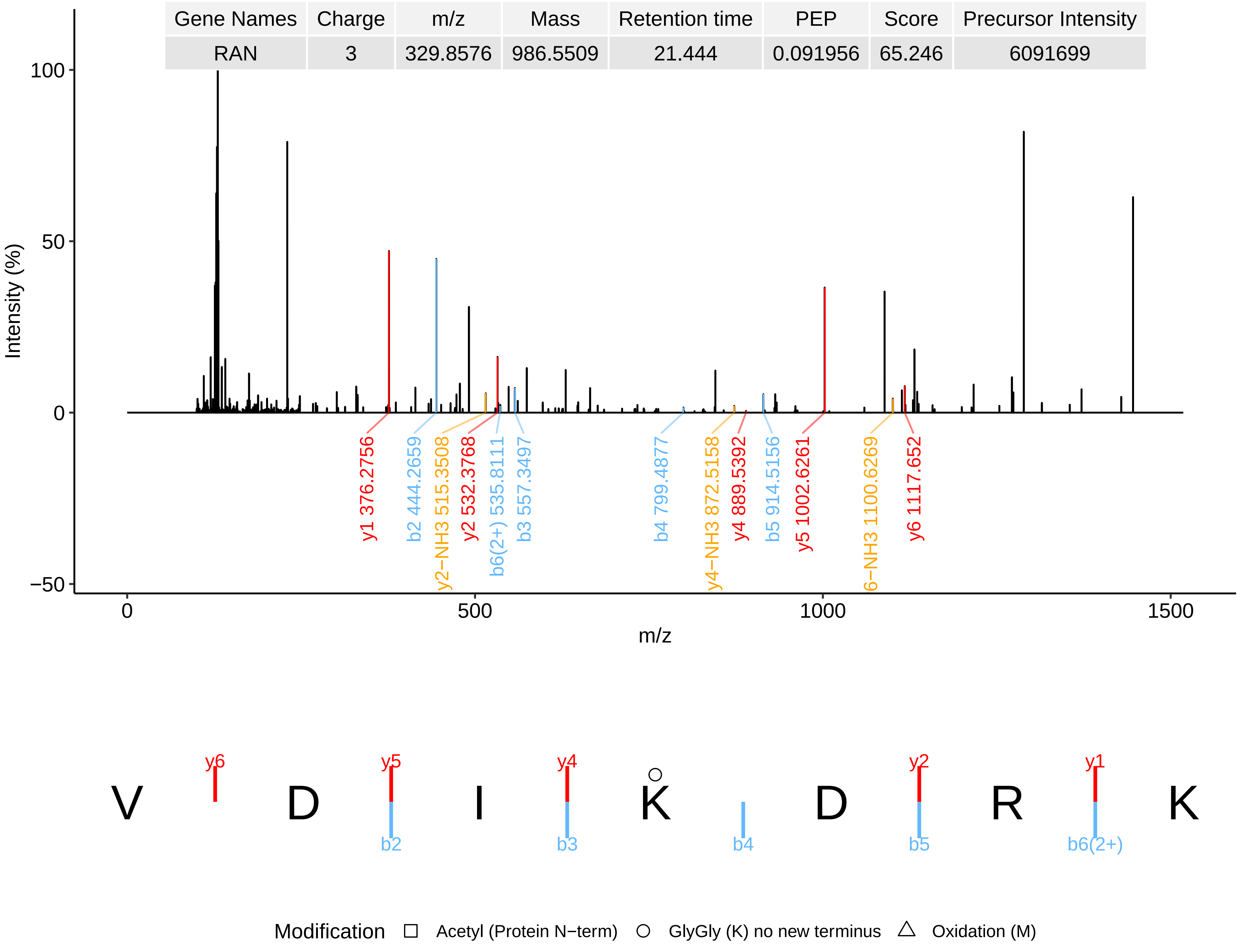

20200921_KIZ_MUb4_GlyGly_10plex − Scan number: 5816

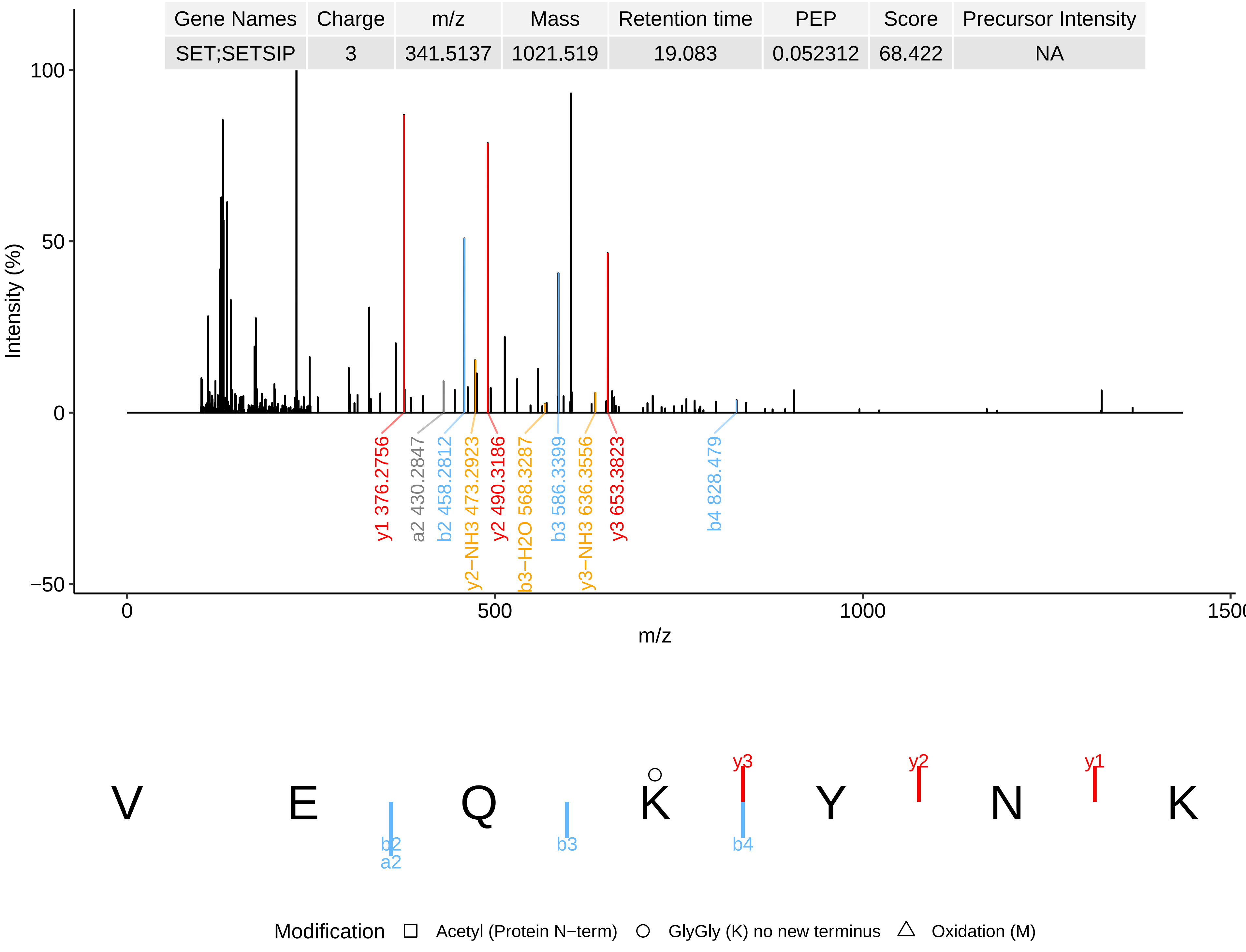

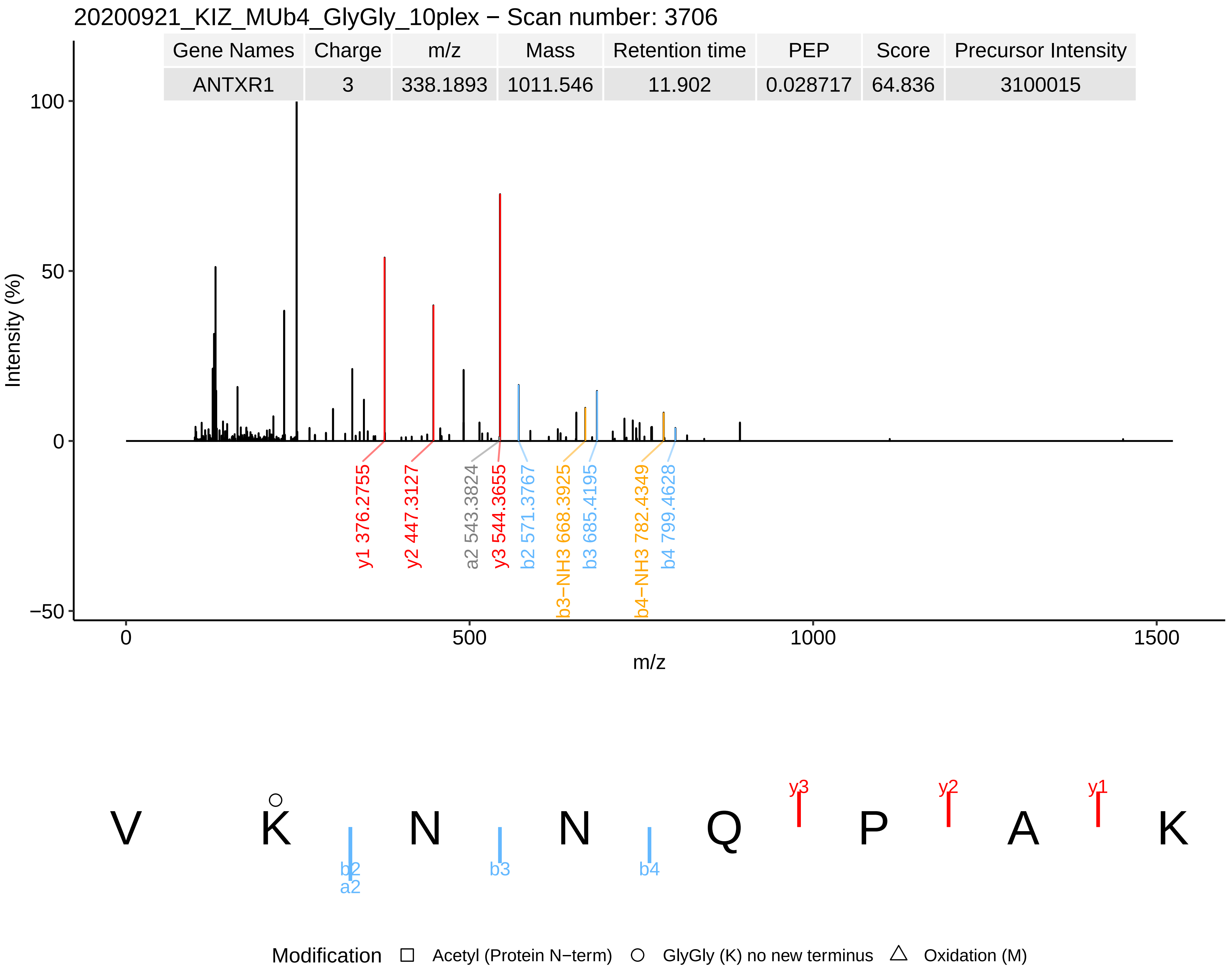
20200921_KIZ_MUb4_GlyGly_10plex − Scan number: 3706
Gene Names | Charge | m/z | Mass | Retention time | PEP | Score | Precursor Intensity
ANTXR1 | 3 | 338.1893 | 1011.546 | 11.902 | 0.028717 | 64.836 | 3100015
Intensity (%)
100
50
0
−50
0
500
1000
1500
m/z
y1 376.2755
y2 447.3127
a2 543.3824
y3 544.3655
b2 571.3767
b3−NH3 668.3925
b3 685.4195
b4−NH3 782.4349
b4 799.4628
V K N N Q P A K
y3
y2
y1
b2
a2
b3
b4
Modification
Acetyl (Protein N−term)
GlyGly (K) no new terminus
Oxidation (M)

20201118_KIZ_MUb6_GlyGly_1 − Scan number: 3238

Gene Names	Charge	m/z	Mass	Retention time	PEP	Score	Precursor Intensity
ANTXR1	3	338.1893	1011.546	9.9628	0.0064412	87.323	36211788

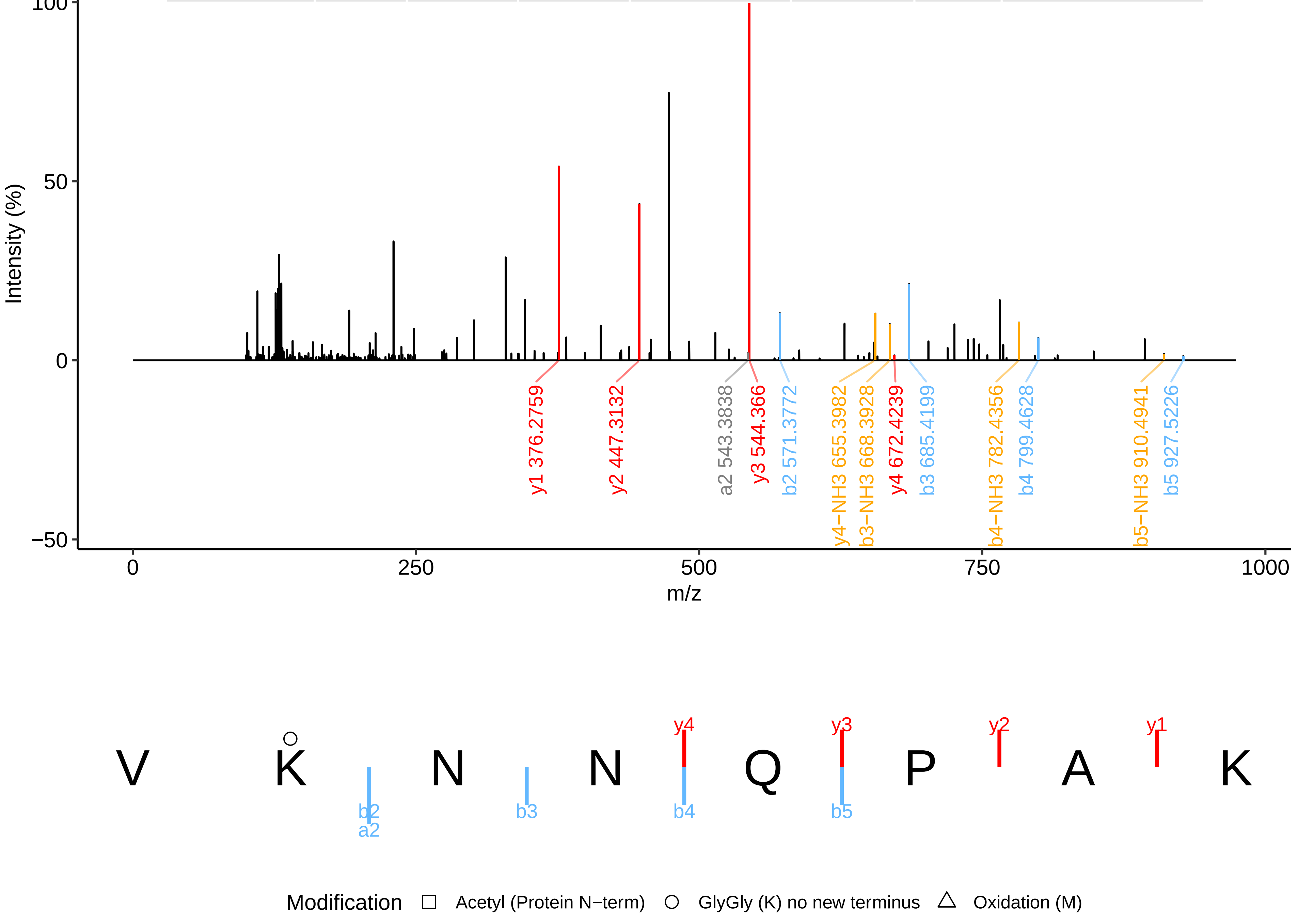

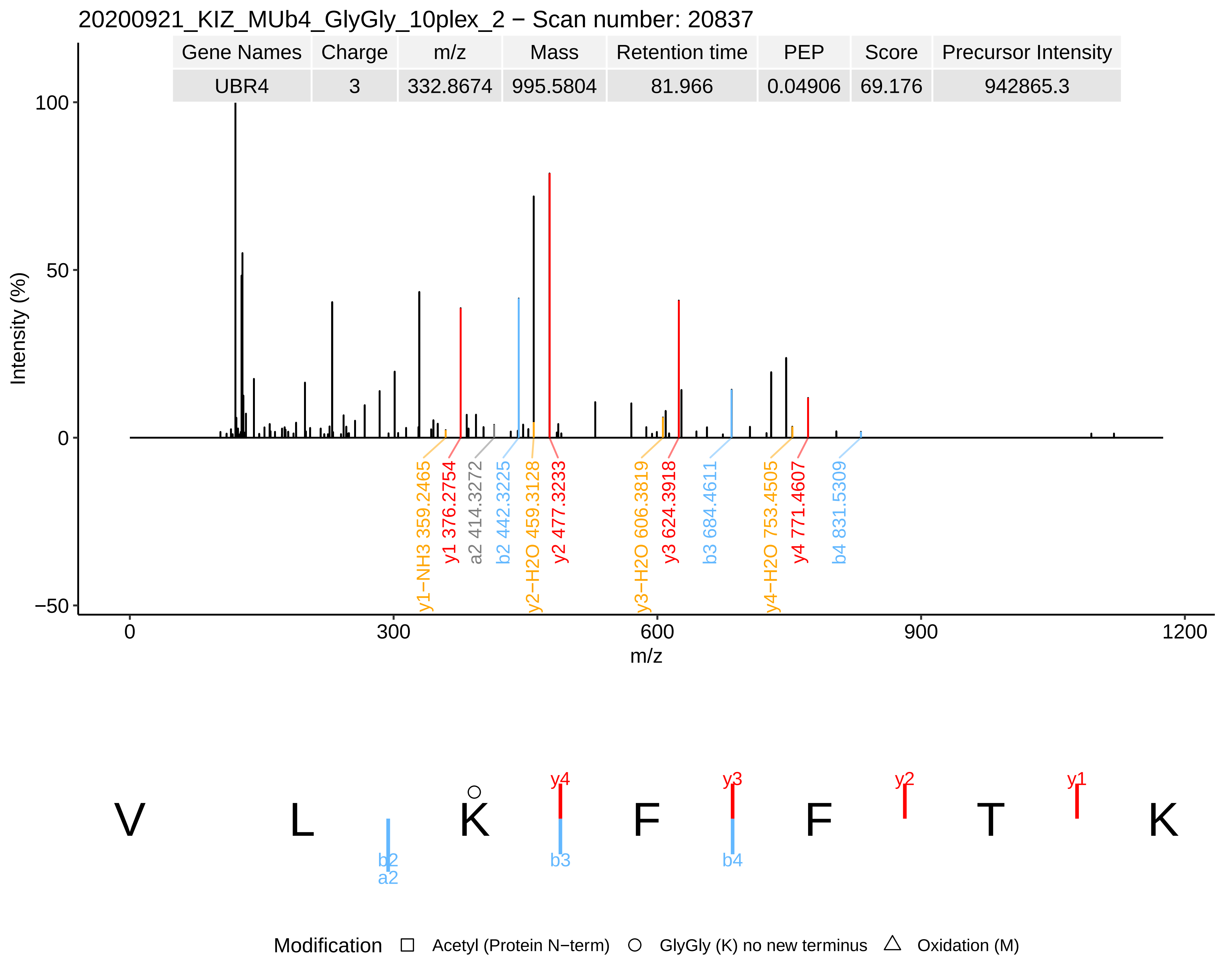

20200921_KIZ_MUb4_GlyGly_10plex_2 − Scan number: 20837
Gene Names | Charge | m/z | Mass | Retention time | PEP | Score | Precursor Intensity
UBR4 | 3 | 332.8674 | 995.5804 | 81.966 | 0.04906 | 69.176 | 942865.3
Intensity (%)
m/z
y1−NH3 359.2465
y1 376.2754
a2 414.3272
b2 442.3225
y2−H2O 459.3128
y2 477.3233
y3−H2O 606.3819
y3 624.3918
b3 684.4611
y4−H2O 753.4505
y4 771.4607
b4 831.5309
V L K F F T K
y4 y3 y2 y1
b2 a2 b3 b4
Modification □ Acetyl (Protein N−term) ○ GlyGly (K) no new terminus △ Oxidation (M)

20200924_KIZ_MUb5_GlyGly_2 − Scan number: 20417

Gene Names	Charge	m/z	Mass	Retention time	PEP	Score	Precursor Intensity
UBR4	3	332.8674	995.5804	75.647	0.052583	68.312	NA

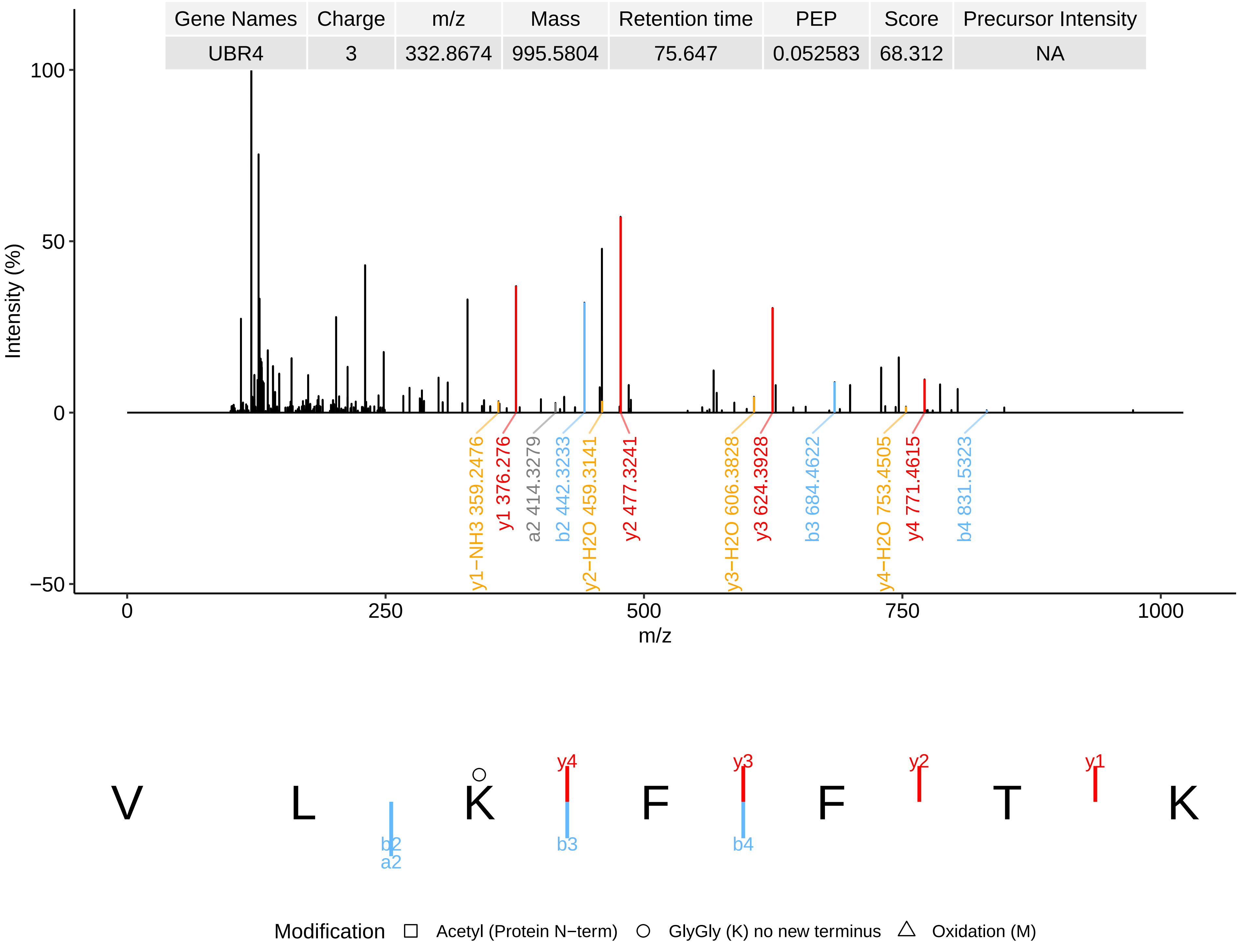

20200921_KIZ_MUb4_GlyGly_10plex_2 − Scan number: 9211

Gene Names	Charge	m/z	Mass	Retention time	PEP	Score	Precursor Intensity
ACTR2	3	334.5257	1000.555	34.957	0.053705	57.59	1364493

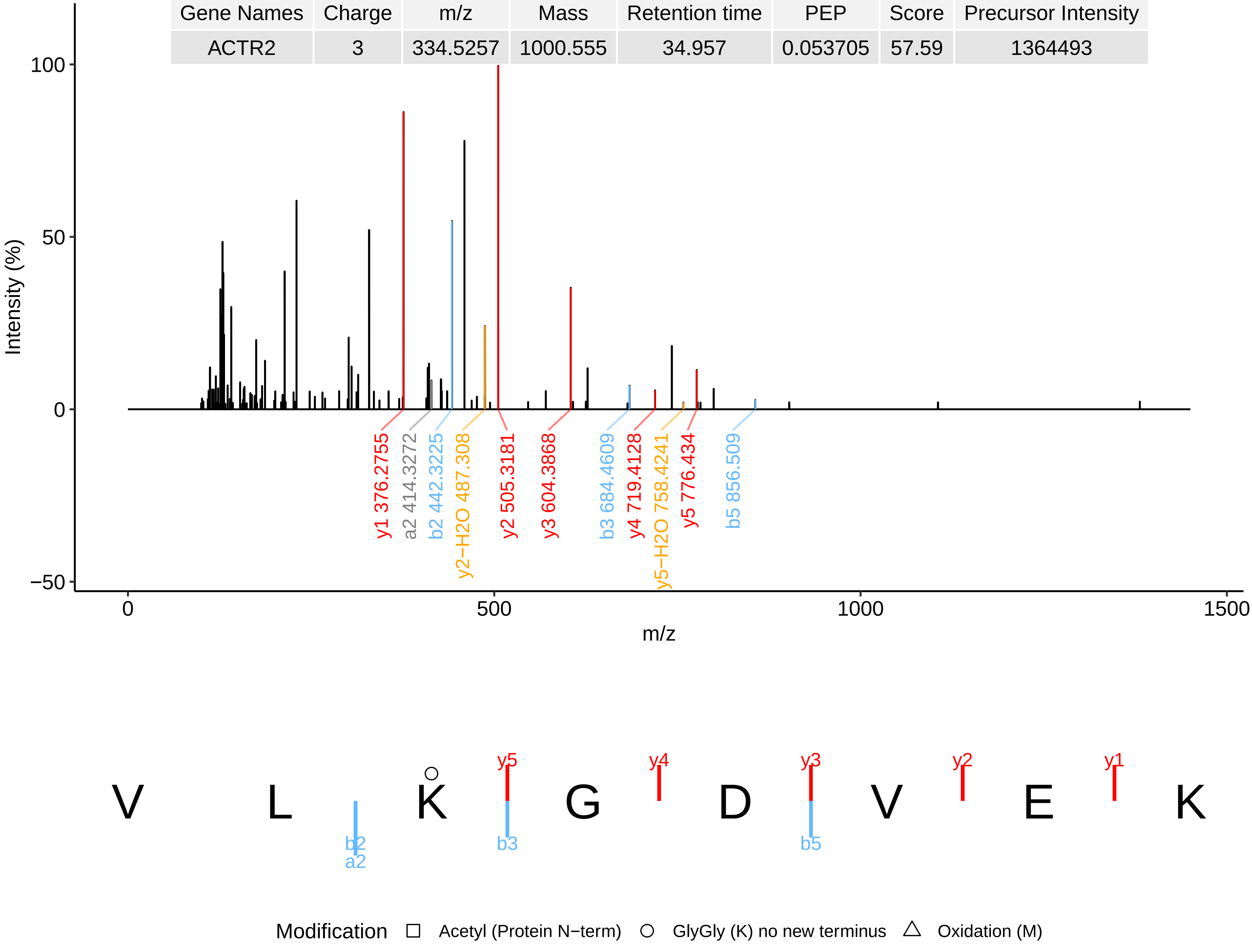

20200924_KIZ_MUb5_GlyGly_2 − Scan number: 8507

Gene Names	Charge	m/z	Mass	Retention time	PEP	Score	Precursor Intensity
ACTR2	3	334.5257	1000.555	31.785	0.14	48.423	1824938

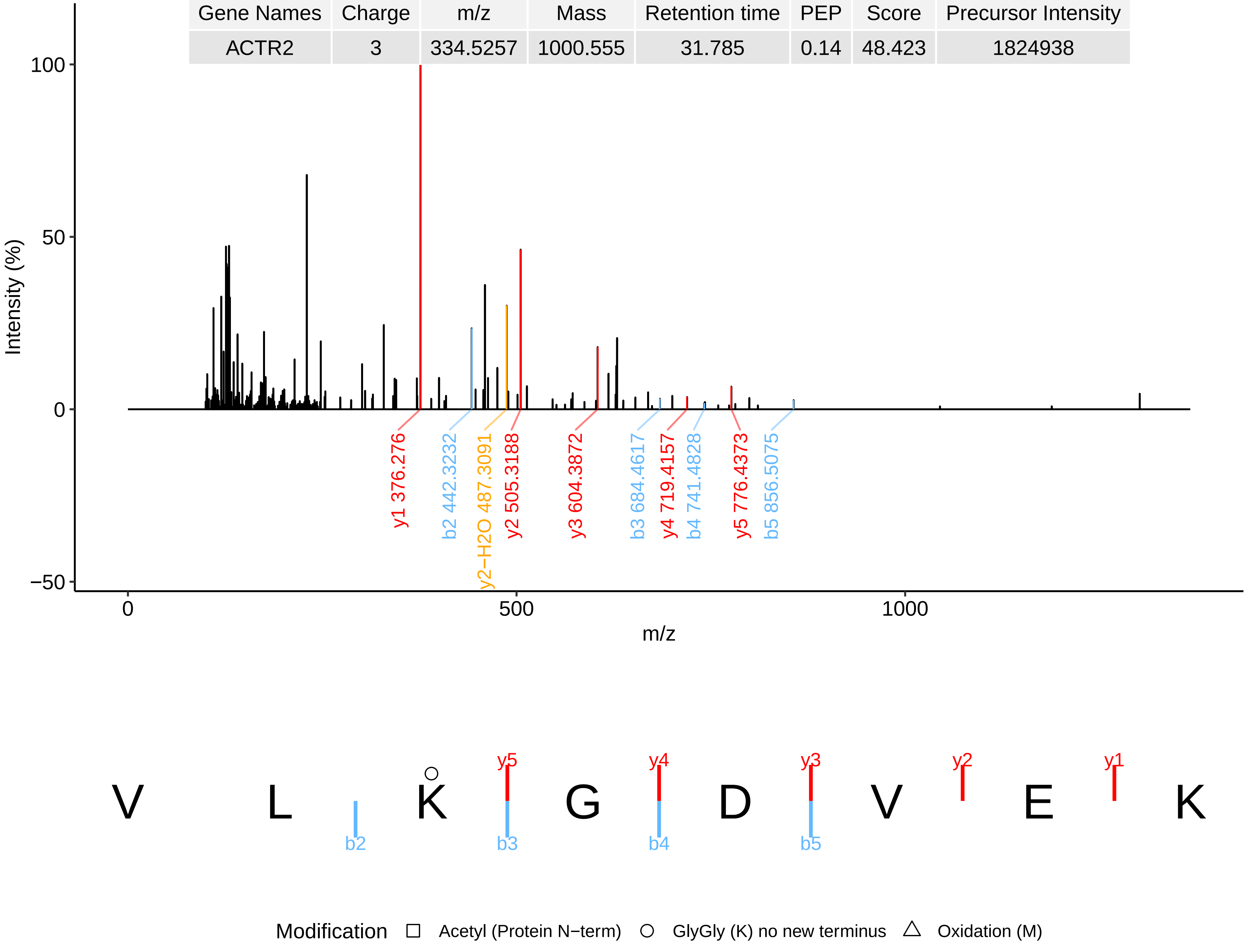

20200921_KIZ_MUb4_GlyGly_10plex − Scan number: 7918

Gene Names	Charge	m/z	Mass	Retention time	PEP	Score	Precursor Intensity
MCM3	4	313.4386	1249.726	26.739	0.0010851	84.817	12673864

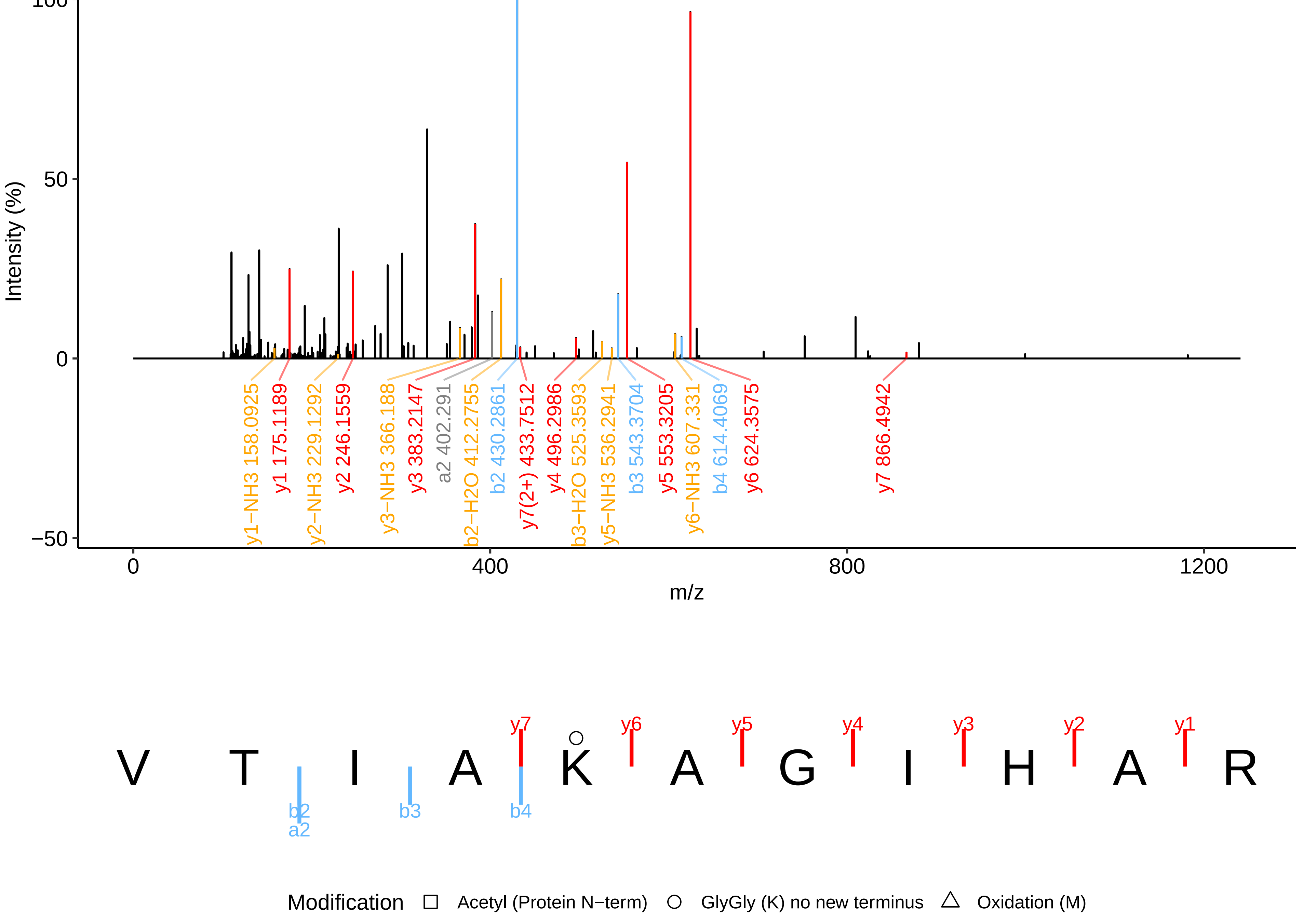

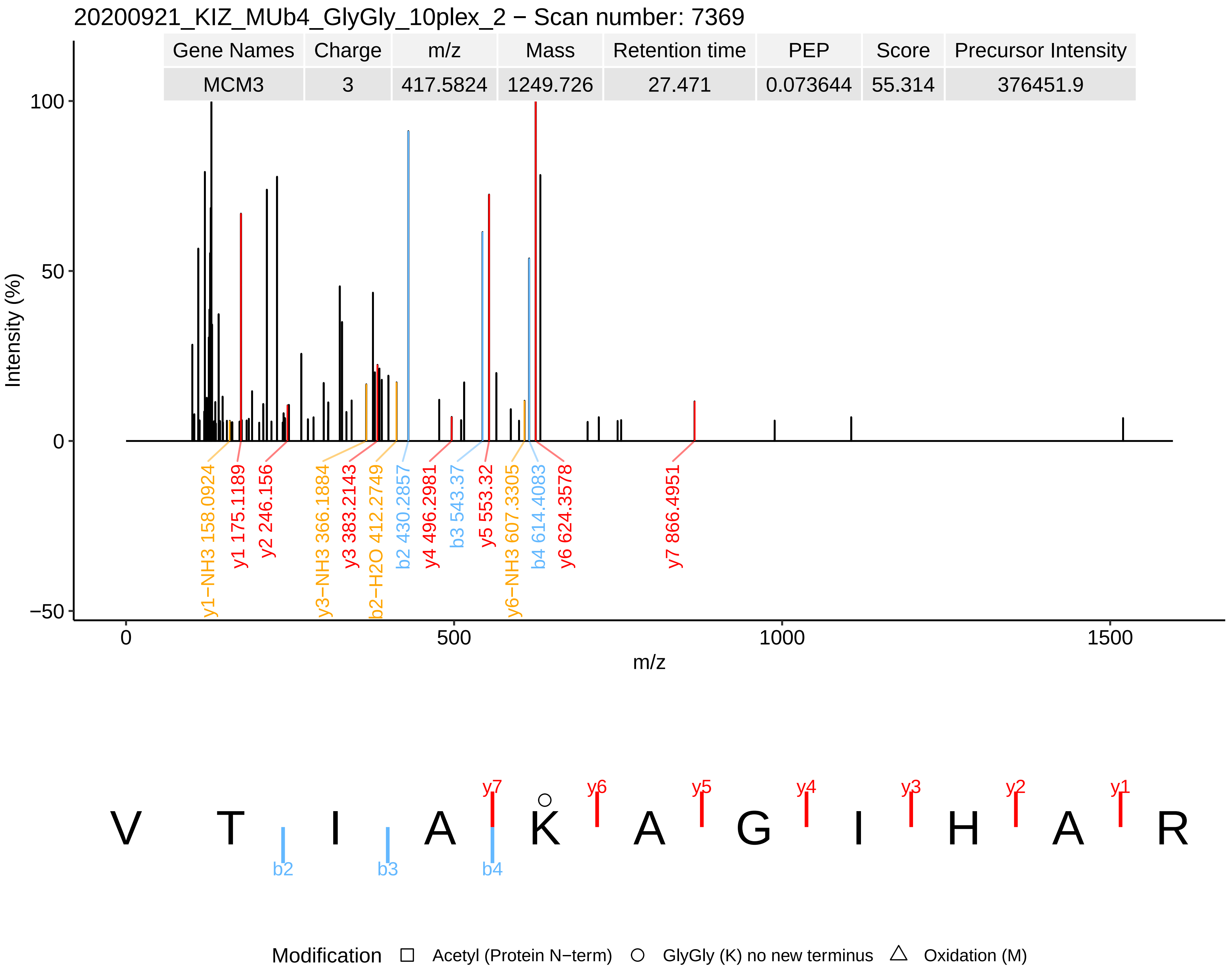

20200921_KIZ_MUb4_GlyGly_10plex_2 − Scan number: 7369
Gene Names | Charge | m/z | Mass | Retention time | PEP | Score | Precursor Intensity
MCM3 | 3 | 417.5824 | 1249.726 | 27.471 | 0.073644 | 55.314 | 376451.9
Intensity (%)
100
50
0
−50
0
500
1000
1500
m/z
y1−NH3 158.0924
y1 175.1189
y2 246.156
y3−NH3 366.1884
y3 383.2143
b2−H2O 412.2749
b2 430.2857
y4 496.2981
b3 543.37
y5 553.32
y6−NH3 607.3305
b4 614.4083
y6 624.3578
y7 866.4951
V T I A K A G I H A R
y7 y6 y5 y4 y3 y2 y1
b2 b3 b4
Modification
Acetyl (Protein N−term)
GlyGly (K) no new terminus
Oxidation (M)

20200921_KIZ_MUb4_GlyGly_10plex_2 − Scan number: 9349

Gene Names	Charge	m/z	Mass	Retention time	PEP	Score	Precursor Intensity
LAGE3	3	415.5701	1243.688	35.512	0.0080687	75.229	925567.1

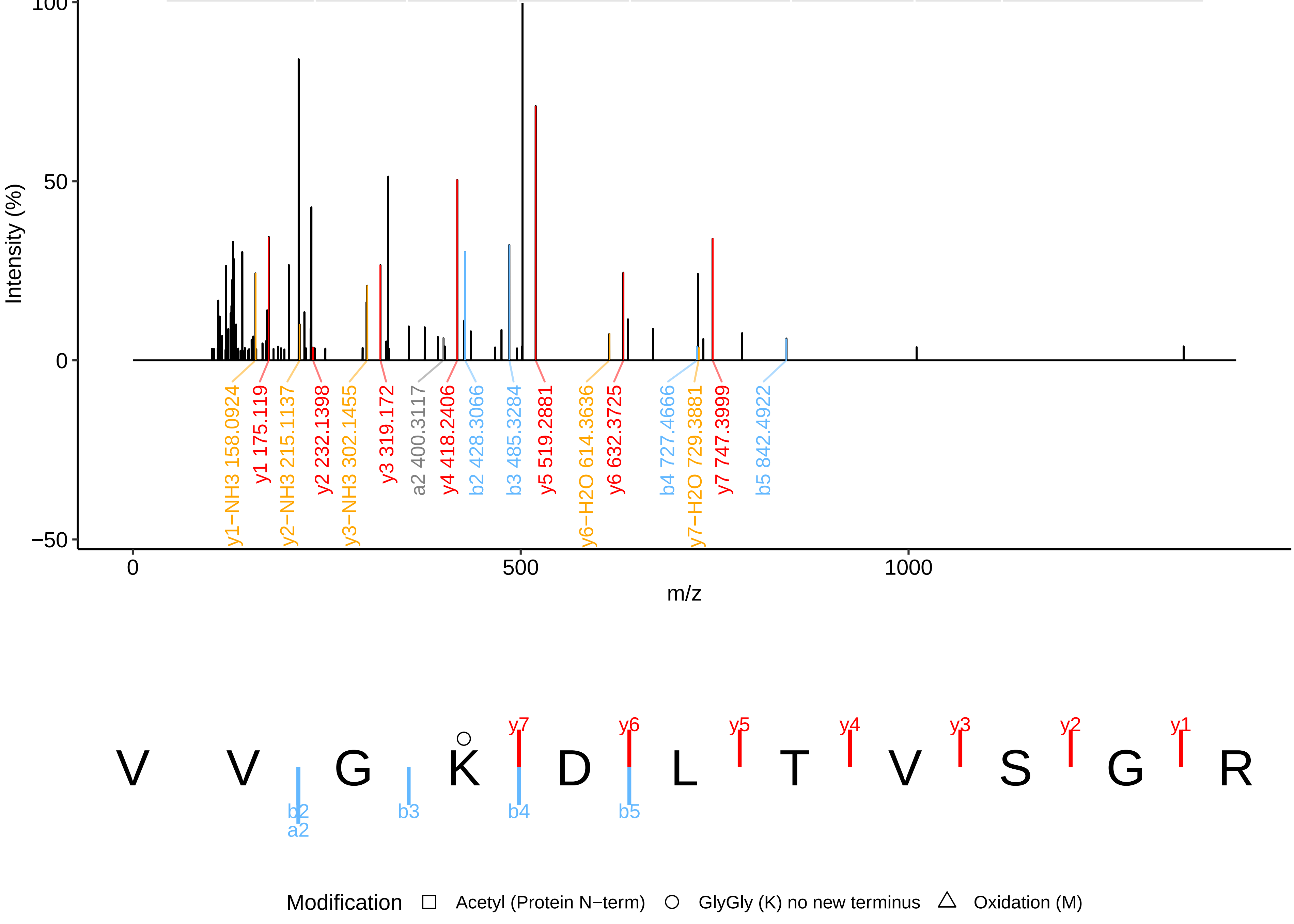

20200921_KIZ_MUb4_GlyGly_10plex − Scan number: 5523

Gene Names	Charge	m/z	Mass	Retention time	PEP	Score	Precursor Intensity
TXNIP	3	412.8826	1235.626	18.097	3.9127e−08	120.45	9990351

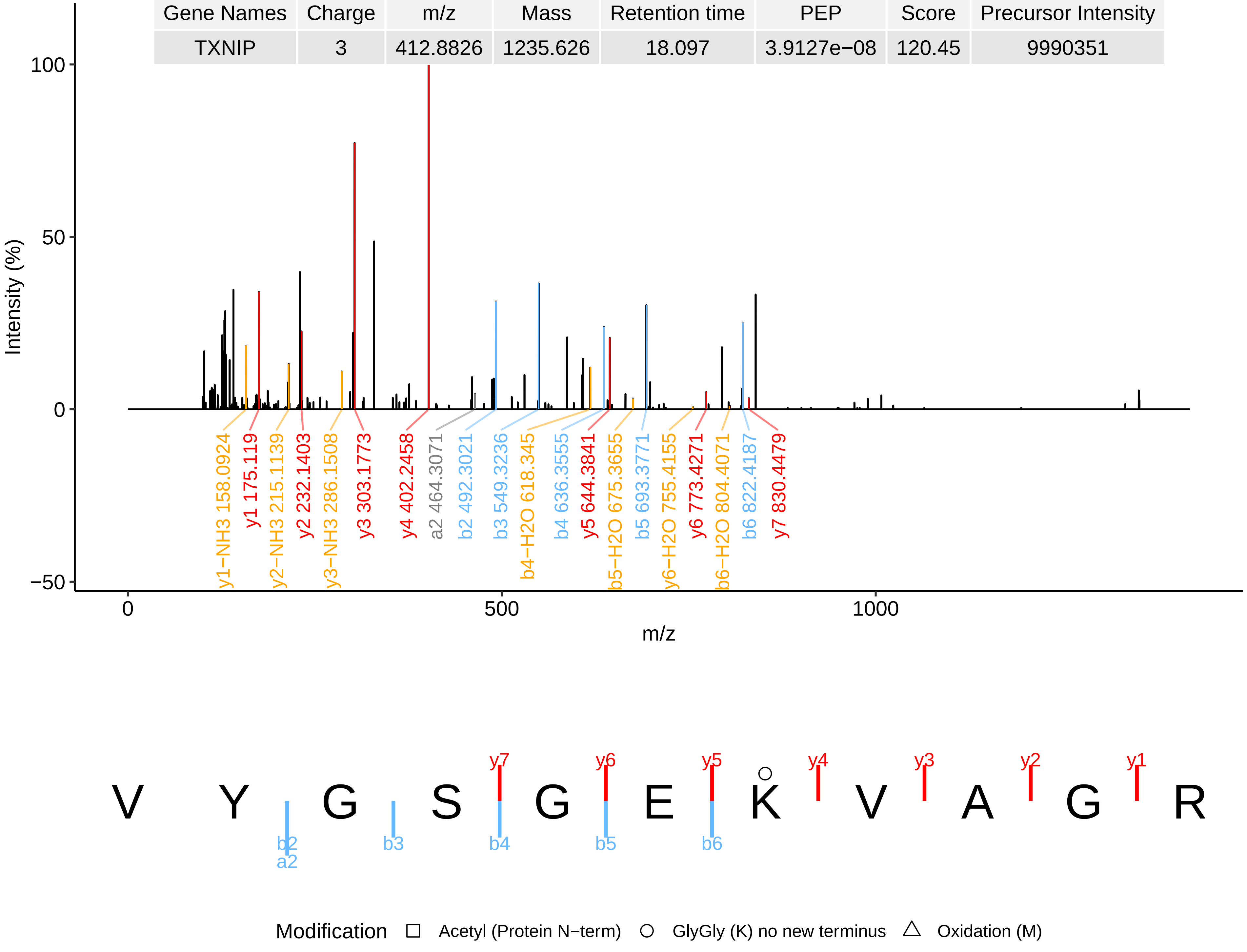

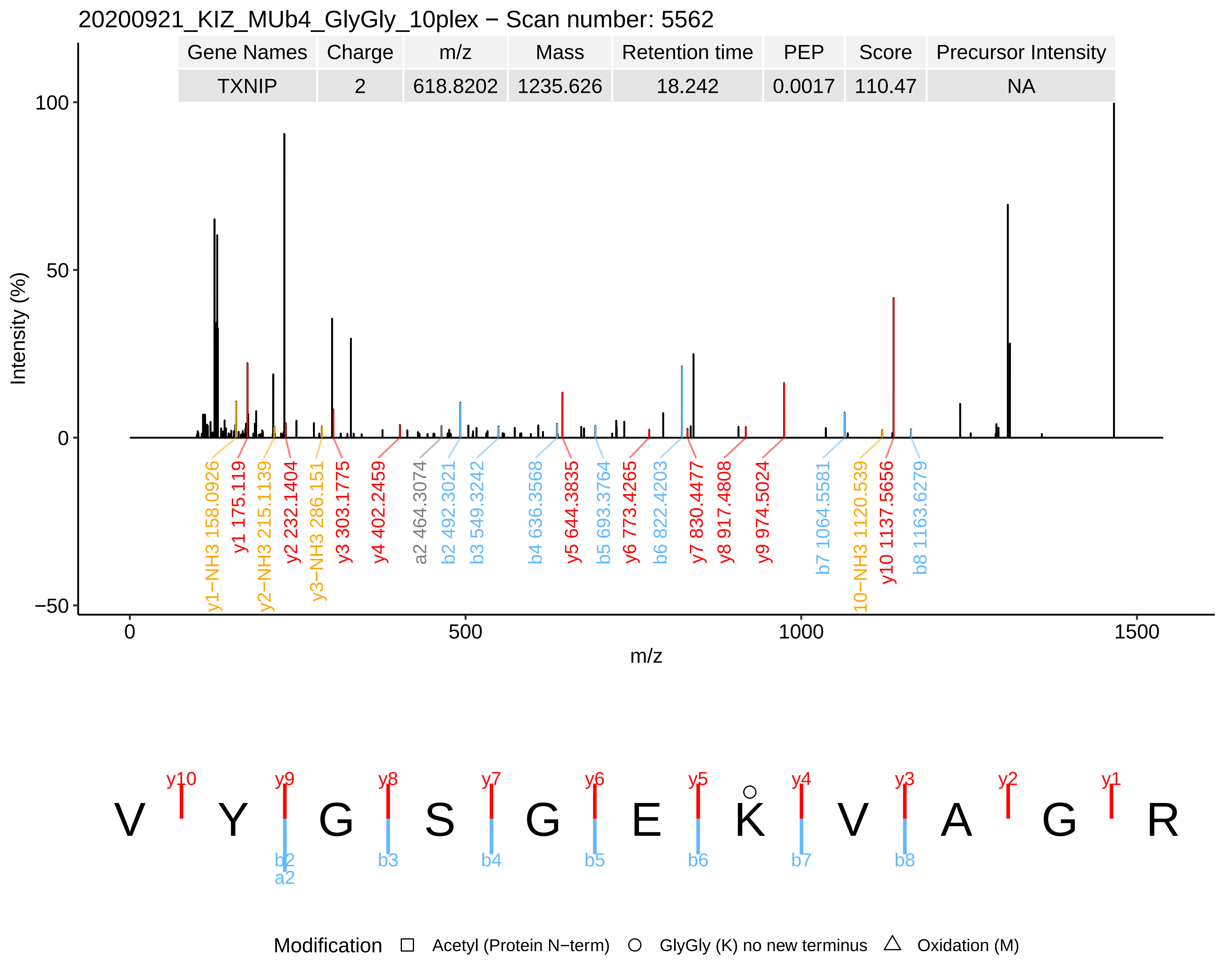

20200921_KIZ_MUb4_GlyGly_10plex − Scan number: 5562
Gene Names | Charge | m/z | Mass | Retention time | PEP | Score | Precursor Intensity
TXNIP | 2 | 618.8202 | 1235.626 | 18.242 | 0.0017 | 110.47 | NA
Intensity (%)
m/z
y1−NH3 158.0926
y1 175.119
y2−NH3 215.1139
y2 232.1404
y3−NH3 286.151
y3 303.1775
y4 402.2459
a2 464.3074
b2 492.3021
b3 549.3242
b4 636.3568
y5 644.3835
b5 693.3764
y6 773.4265
b6 822.4203
y7 830.4477
y8 917.4808
y9 974.5024
b7 1064.5581
10−NH3 1120.539
y10 1137.5656
b8 1163.6279
V Y G S G E K V A G R
y10 y9 y8 y7 y6 y5 y4 y3 y2 y1
b2 a2 b3 b4 b5 b6 b7 b8
Modification
Acetyl (Protein N−term)
GlyGly (K) no new terminus
Oxidation (M)

20200921_KIZ_MUb4_GlyGly_10plex_2 − Scan number: 5053

Gene Names	Charge	m/z	Mass	Retention time	PEP	Score	Precursor Intensity
TXNIP	3	412.8826	1235.626	17.992	5.1824e−07	115.23	1032404

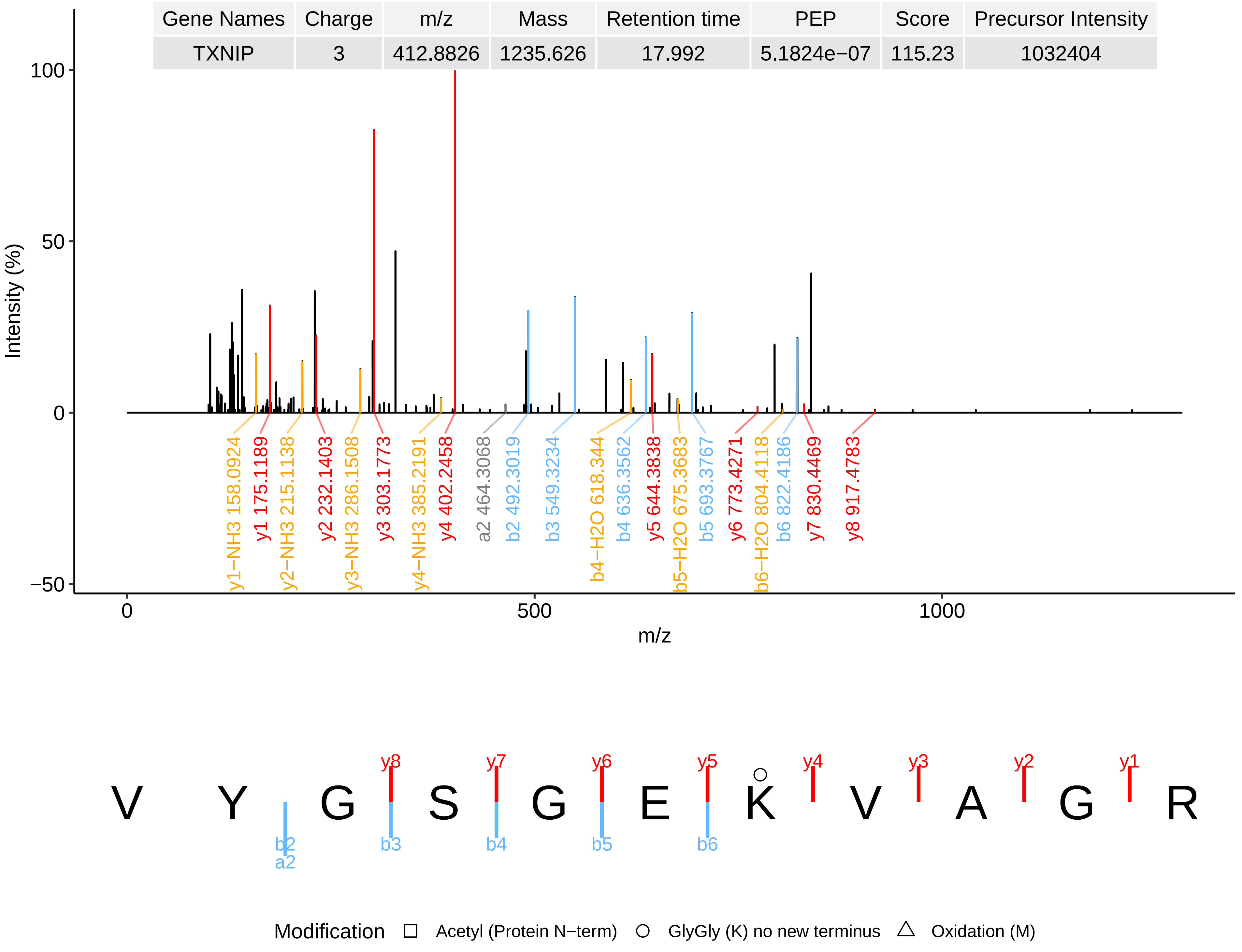

20200924_KIZ_MUb5_GlyGly_1 − Scan number: 4824

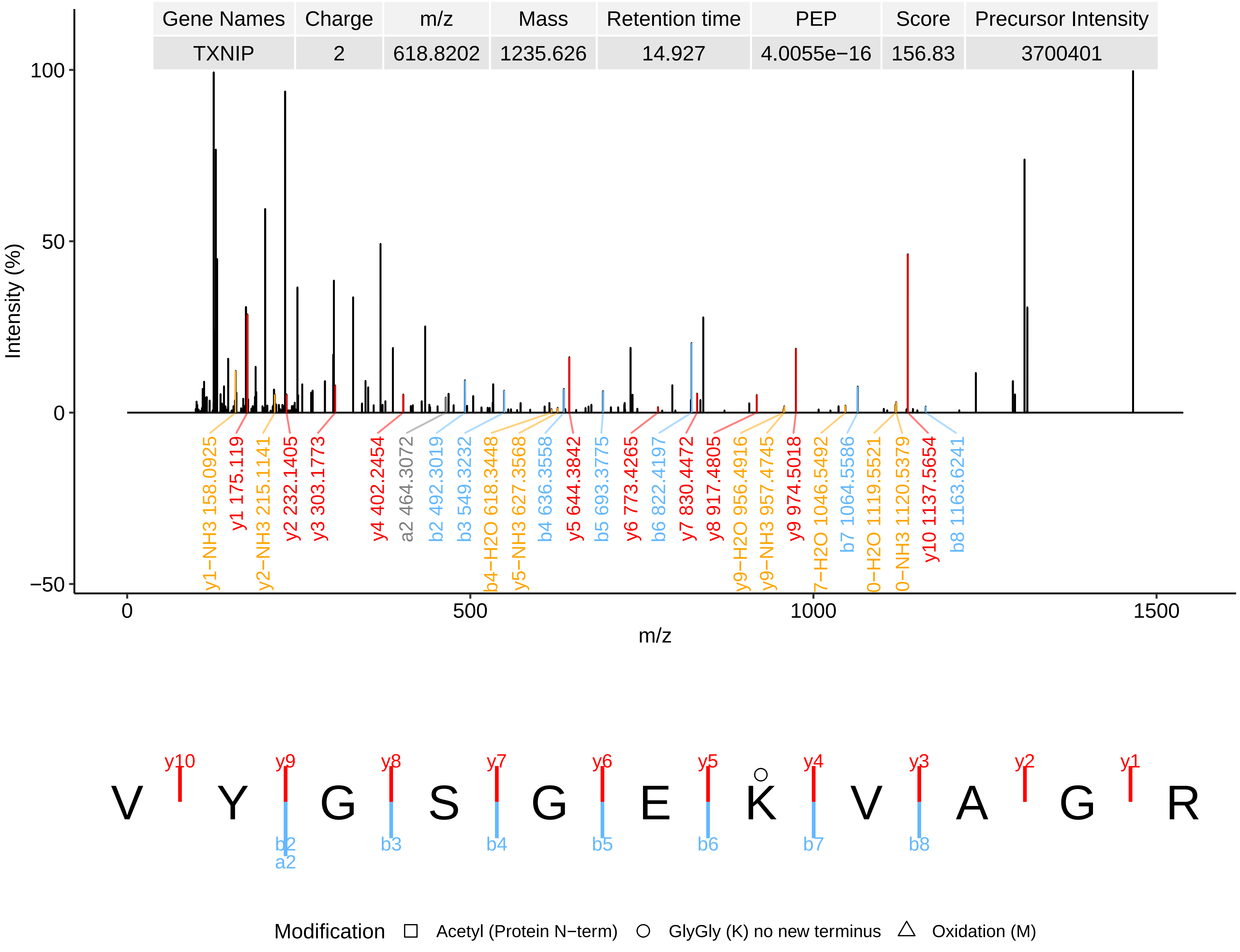

20200924_KIZ_MUb5_GlyGly_1 − Scan number: 4861

Gene Names	Charge	m/z	Mass	Retention time	PEP	Score	Precursor Intensity
TXNIP	3	412.8826	1235.626	15.048	3.8144e−08	120.76	42695300

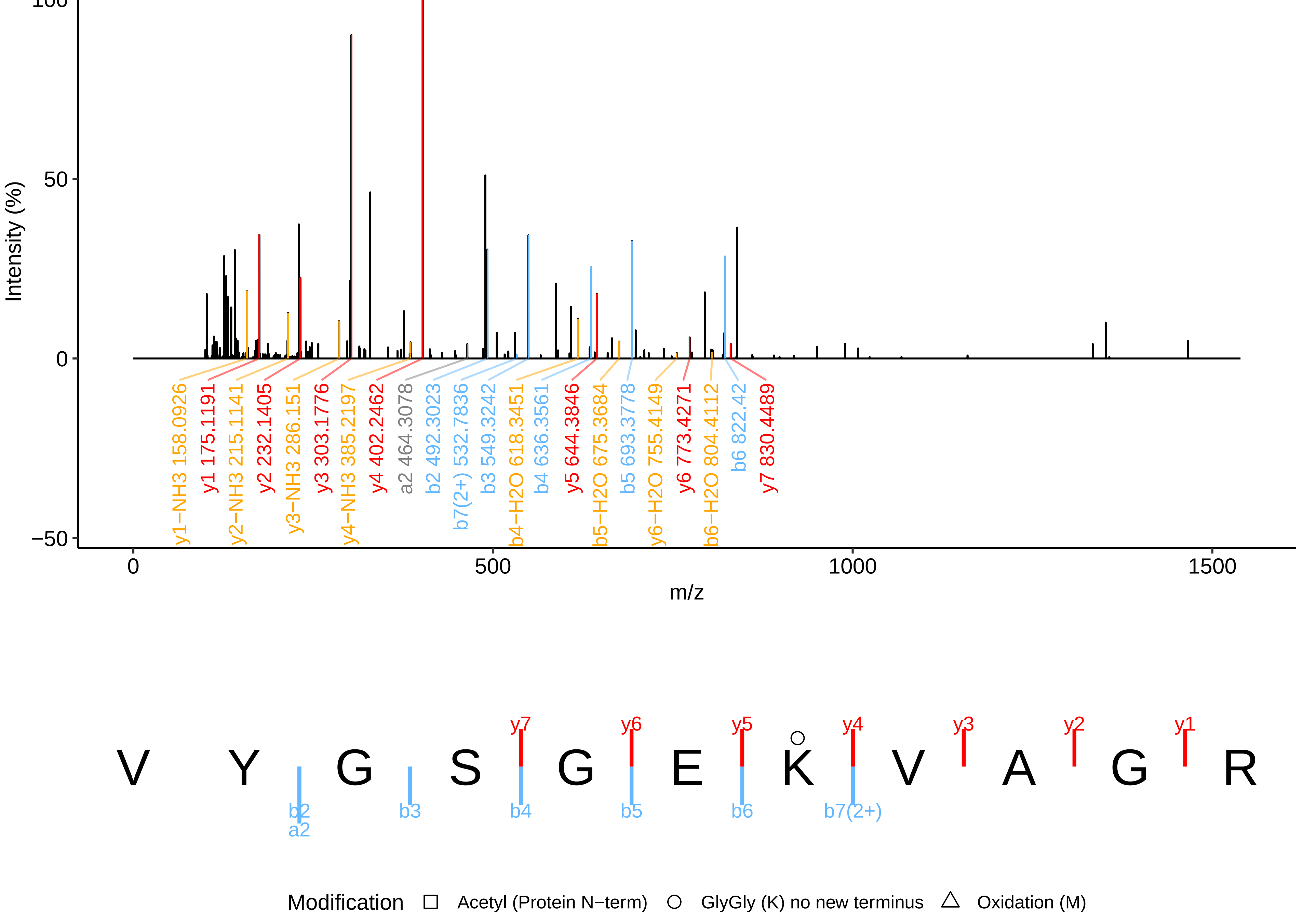

20200924_KIZ_MUb5_GlyGly_2 − Scan number: 4473

Gene Names	Charge	m/z	Mass	Retention time	PEP	Score	Precursor Intensity
TXNIP	3	412.8826	1235.626	16.417	1.2627e−05	110.53	10550699

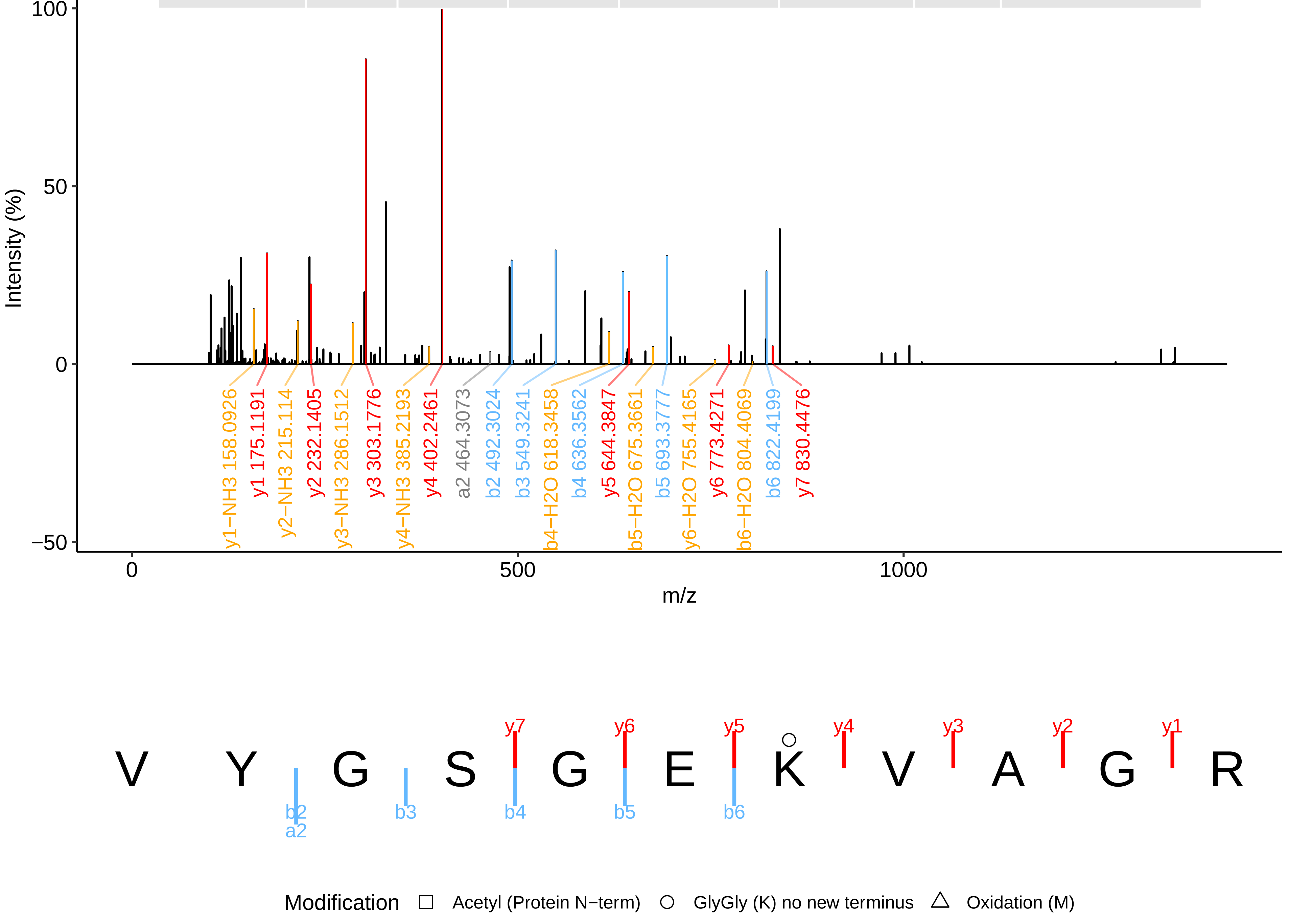

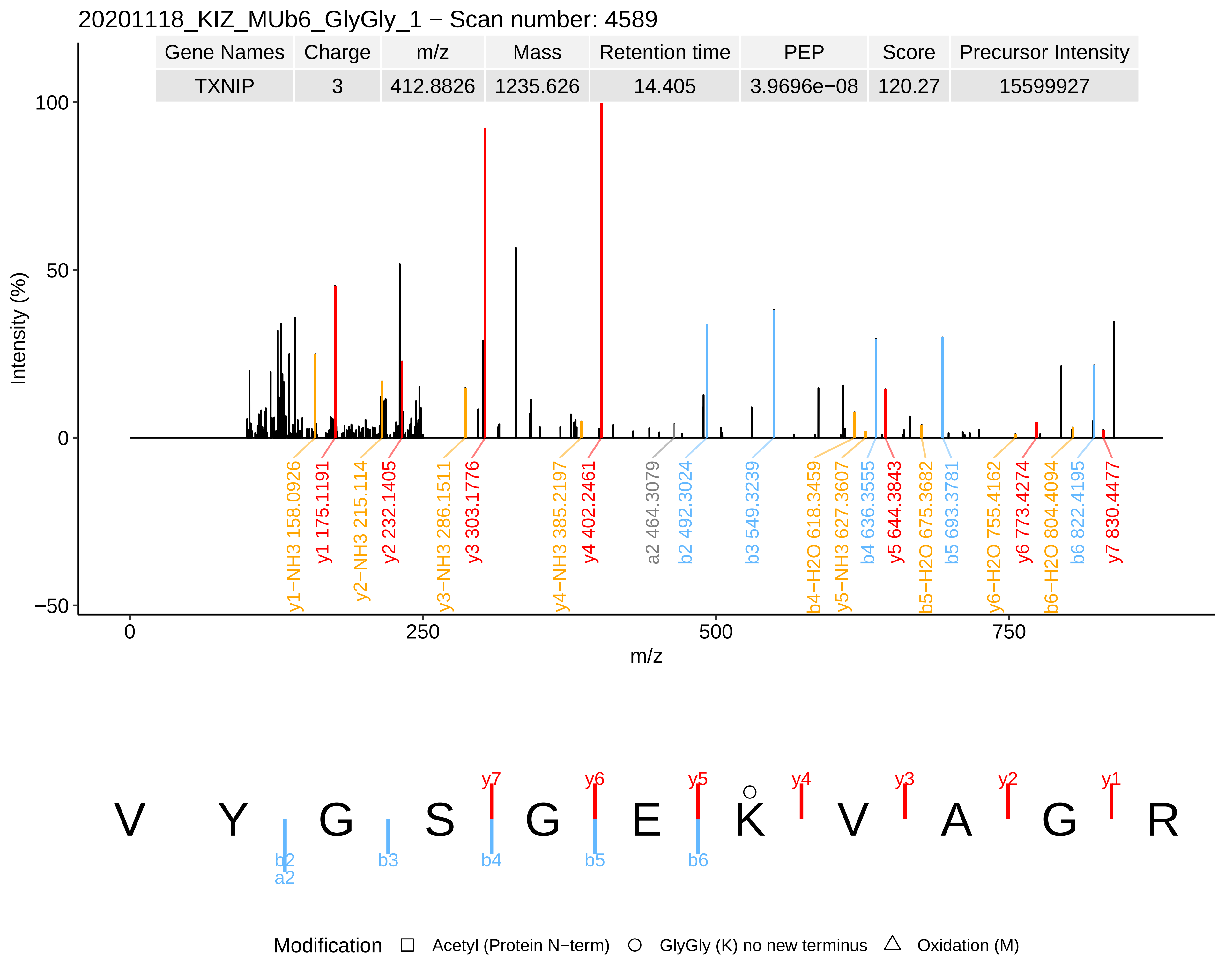

Gene Names	Charge	m/z	Mass	Retention time	PEP	Score	Precursor Intensity
TXNIP	3	412.8826	1235.626	14.405	3.9696e−08	120.27	15599927

20201118_KIZ_MUb6_GlyGly_1 − Scan number: 4594

Gene Names	Charge	m/z	Mass	Retention time	PEP	Score	Precursor Intensity
TXNIP	3	412.8826	1235.626	14.422	1.7692e−12	139.43	16834716

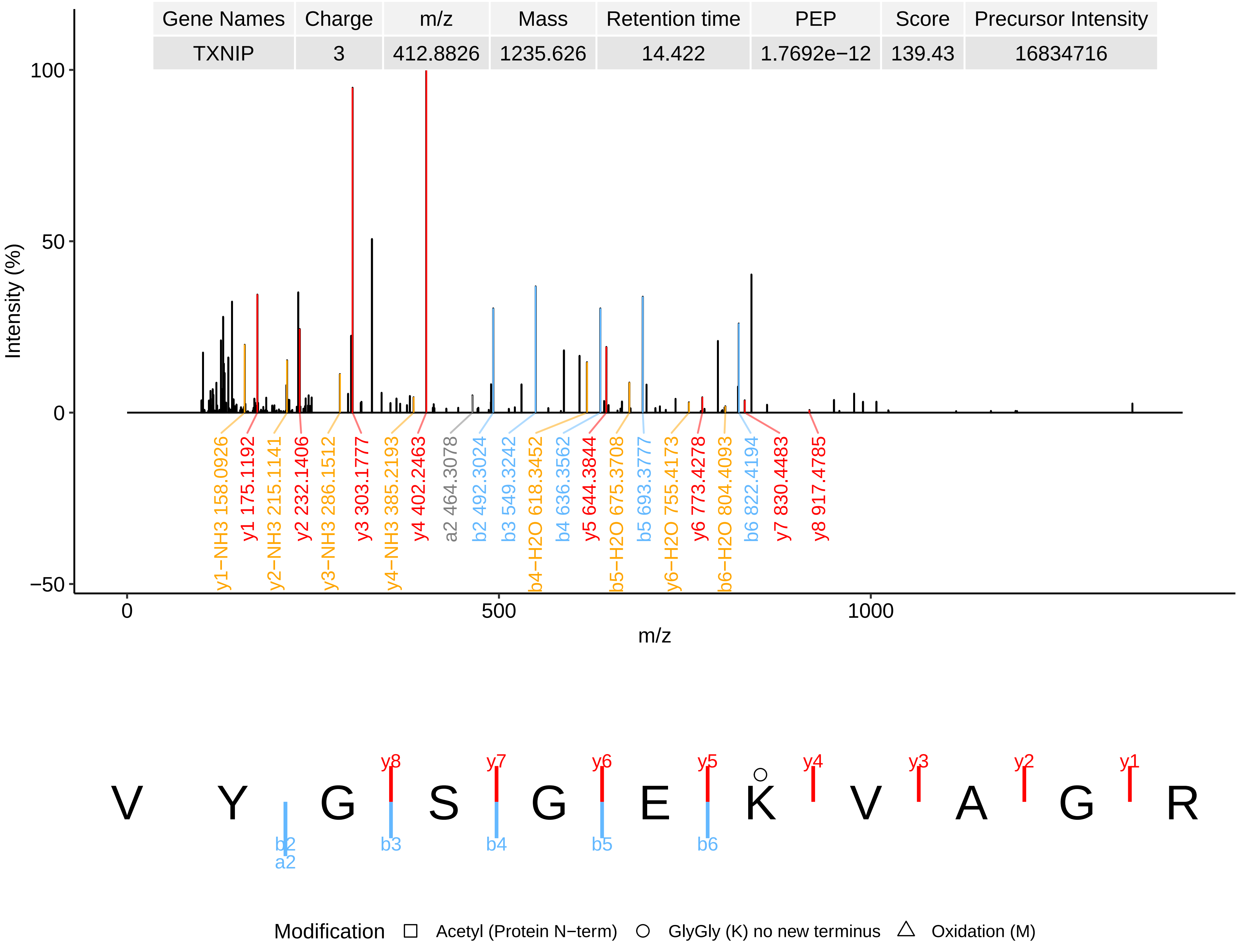

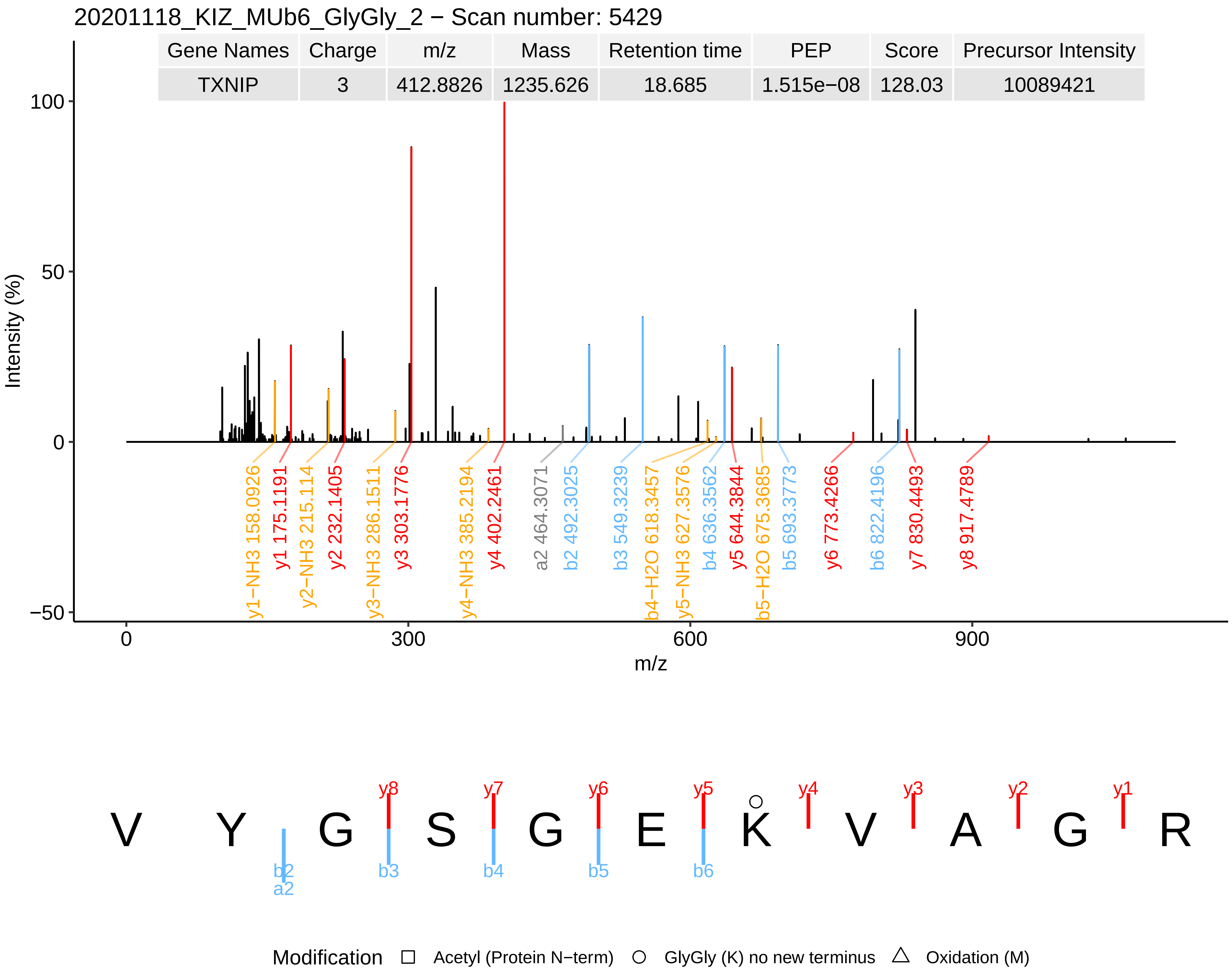
20201118_KIZ_MUb6_GlyGly_2 − Scan number: 5429
Gene Names | Charge | m/z | Mass | Retention time | PEP | Score | Precursor Intensity
TXNIP | 3 | 412.8826 | 1235.626 | 18.685 | 1.515e−08 | 128.03 | 10089421
Intensity (%)
100
50
0
−50
0
300
600
900
m/z
y1−NH3 158.0926
y1 175.1191
y2−NH3 215.114
y2 232.1405
y3−NH3 286.1511
y3 303.1776
y4−NH3 385.2194
y4 402.2461
a2 464.3071
b2 492.3025
b3 549.3239
b4−H2O 618.3457
y5−NH3 627.3576
b4 636.3562
y5 644.3844
b5−H2O 675.3685
b5 693.3773
y6 773.4266
b6 822.4196
y7 830.4493
y8 917.4789
V Y G S G E K V A G R
y8 y7 y6 y5 y4 y3 y2 y1
b2 a2 b3 b4 b5 b6
Modification
Acetyl (Protein N−term)
GlyGly (K) no new terminus
Oxidation (M)

20200924_KIZ_MUb5_GlyGly_1 − Scan number: 17399

Gene Names	Charge	m/z	Mass	Retention time	PEP	Score	Precursor Intensity
ATG16L2	2	628.8598	1255.705	56.169	0.081851	45.882	17665604

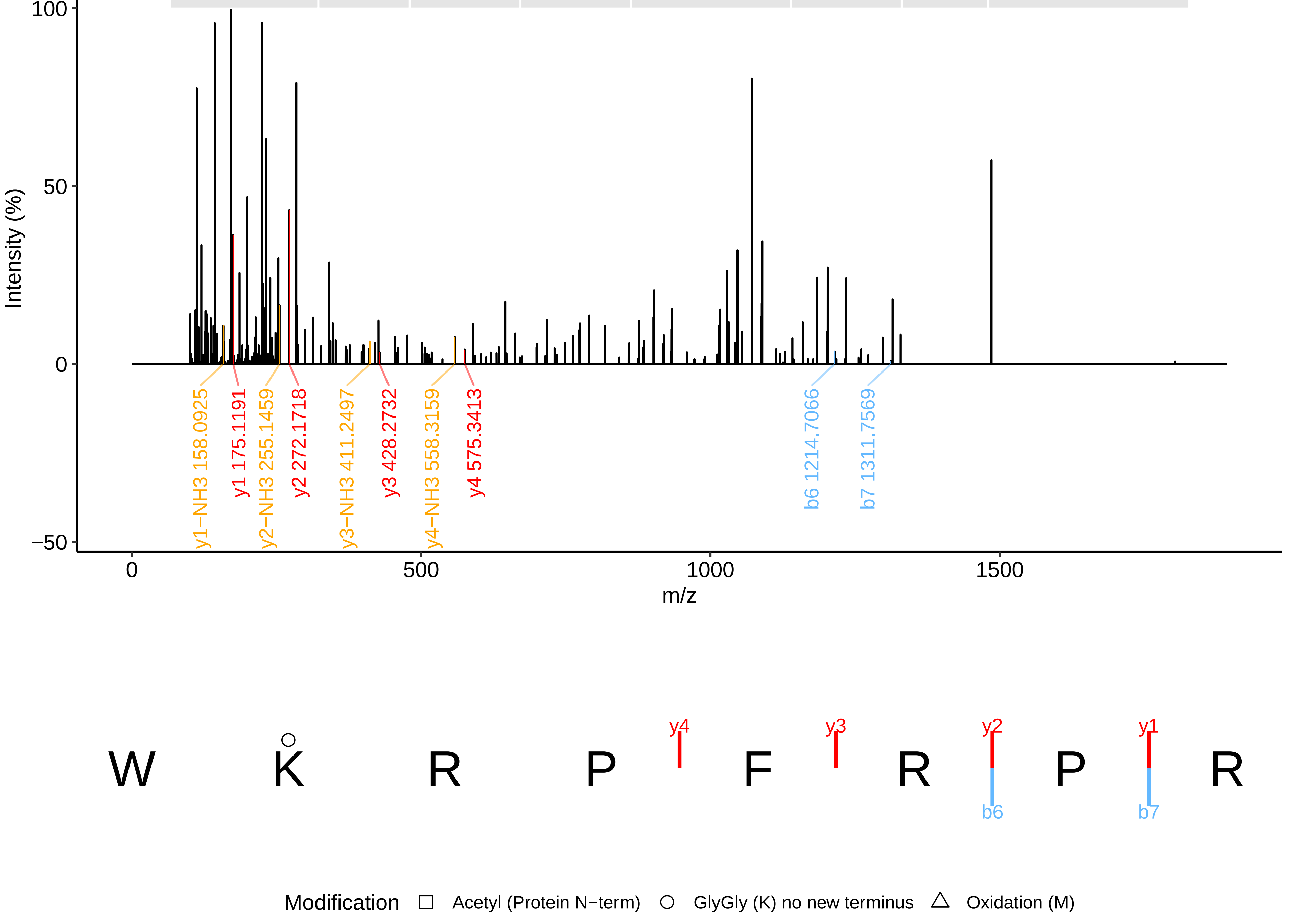

20200921_KIZ_MUb4_GlyGly_10plex − Scan number: 4989

Gene Names	Charge	m/z	Mass	Retention time	PEP	Score	Precursor Intensity
HSPA8	3	332.8259	995.456	16.262	0.061645	64.265	6665705

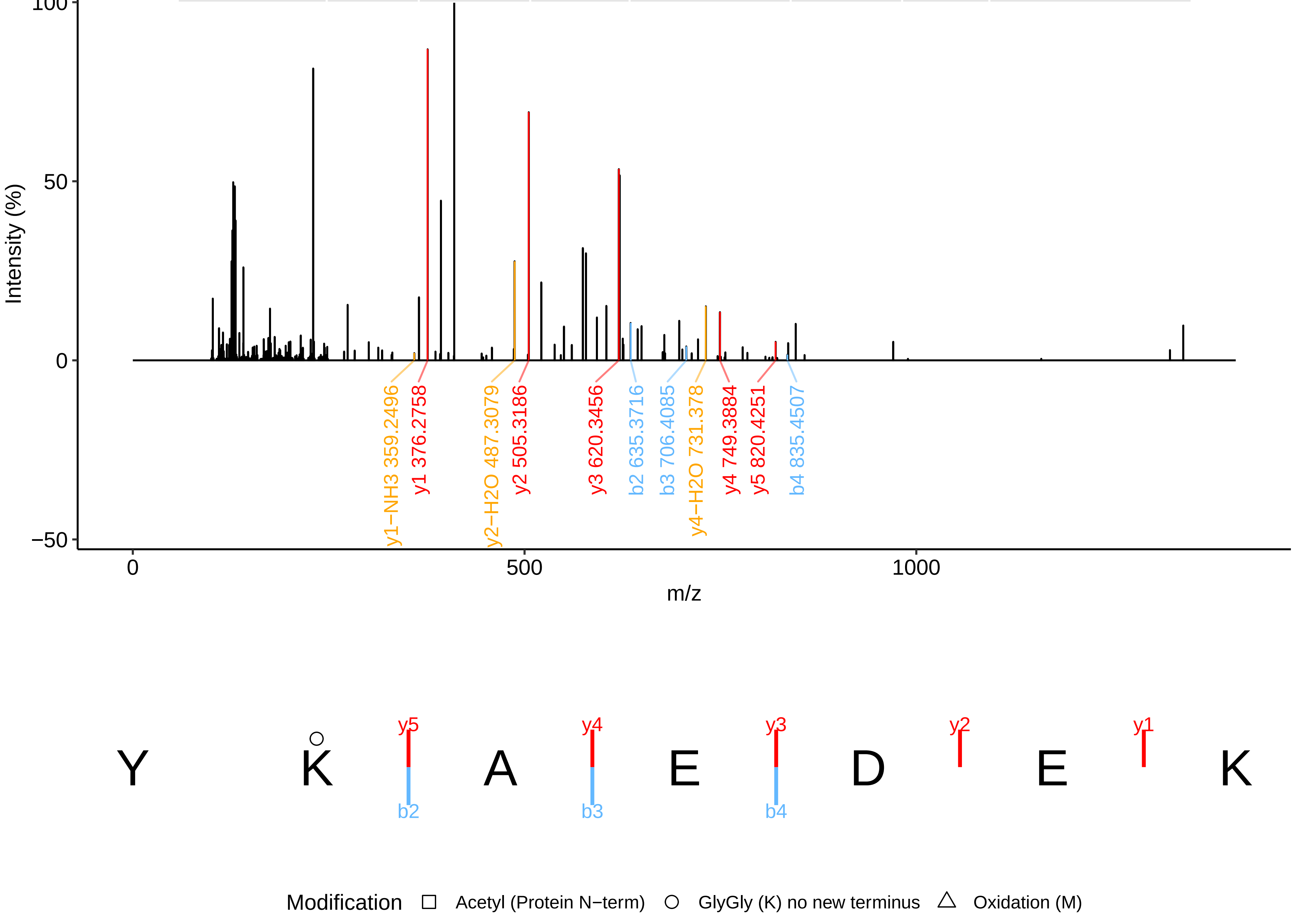

20200921_KIZ_MUb4_GlyGly_10plex_2 − Scan number: 4610

Gene Names	Charge	m/z	Mass	Retention time	PEP	Score	Precursor Intensity
HSPA8	3	332.8259	995.456	16.233	0.076749	60.682	1193147

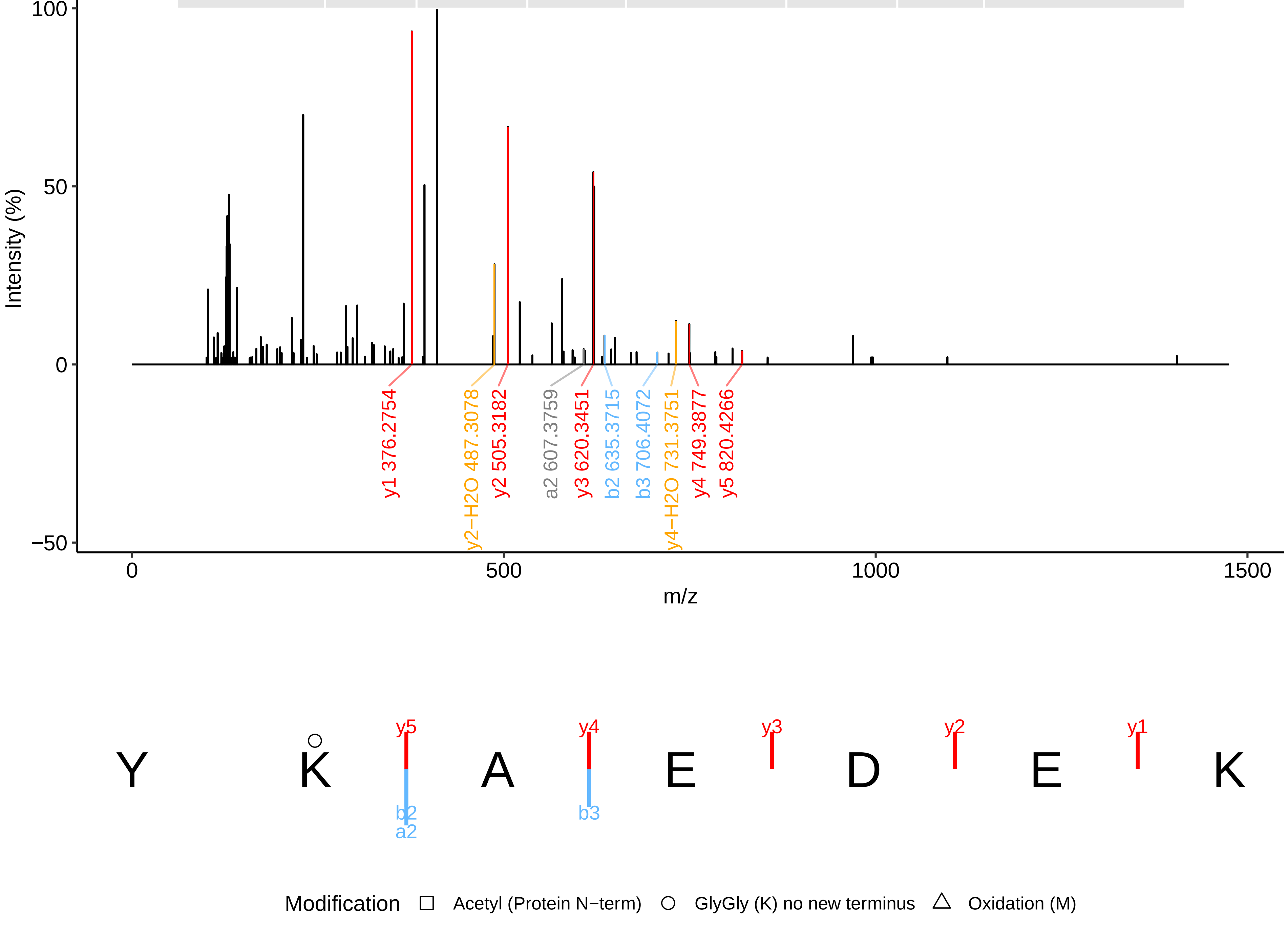

20200924_KIZ_MUb5_GlyGly_2 − Scan number: 4099

Gene Names	Charge	m/z	Mass	Retention time	PEP	Score	Precursor Intensity
HSPA8	3	332.8259	995.456	14.994	0.10992	53.968	1498879

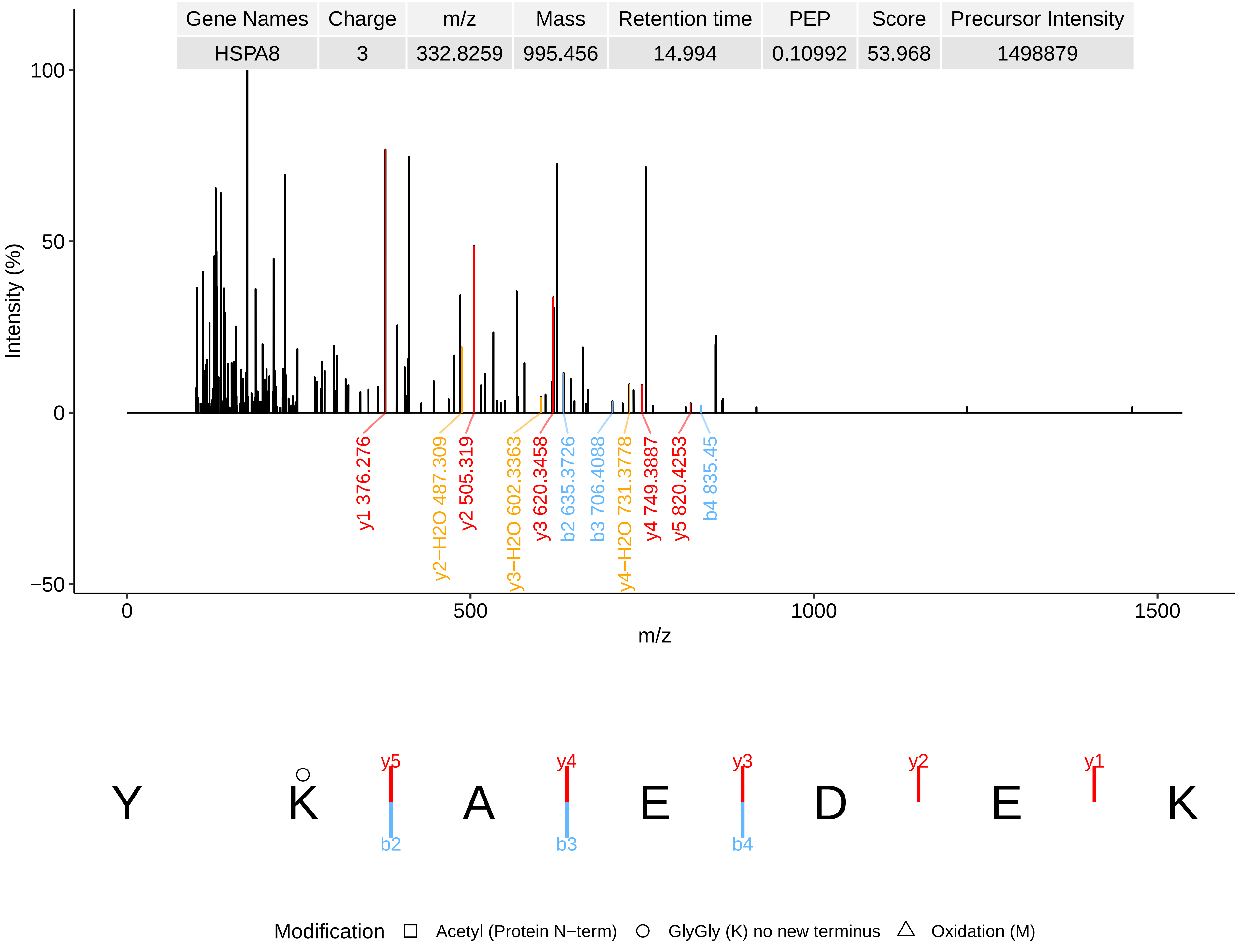

20200921_KIZ_MUb4_GlyGly_10plex − Scan number: 4631

Gene Names	Charge	m/z	Mass	Retention time	PEP	Score	Precursor Intensity
HSPA1B;HSPA1A;HSPA1L	3	417.8703	1250.589	15.094	0.0089366	75.509	3630555

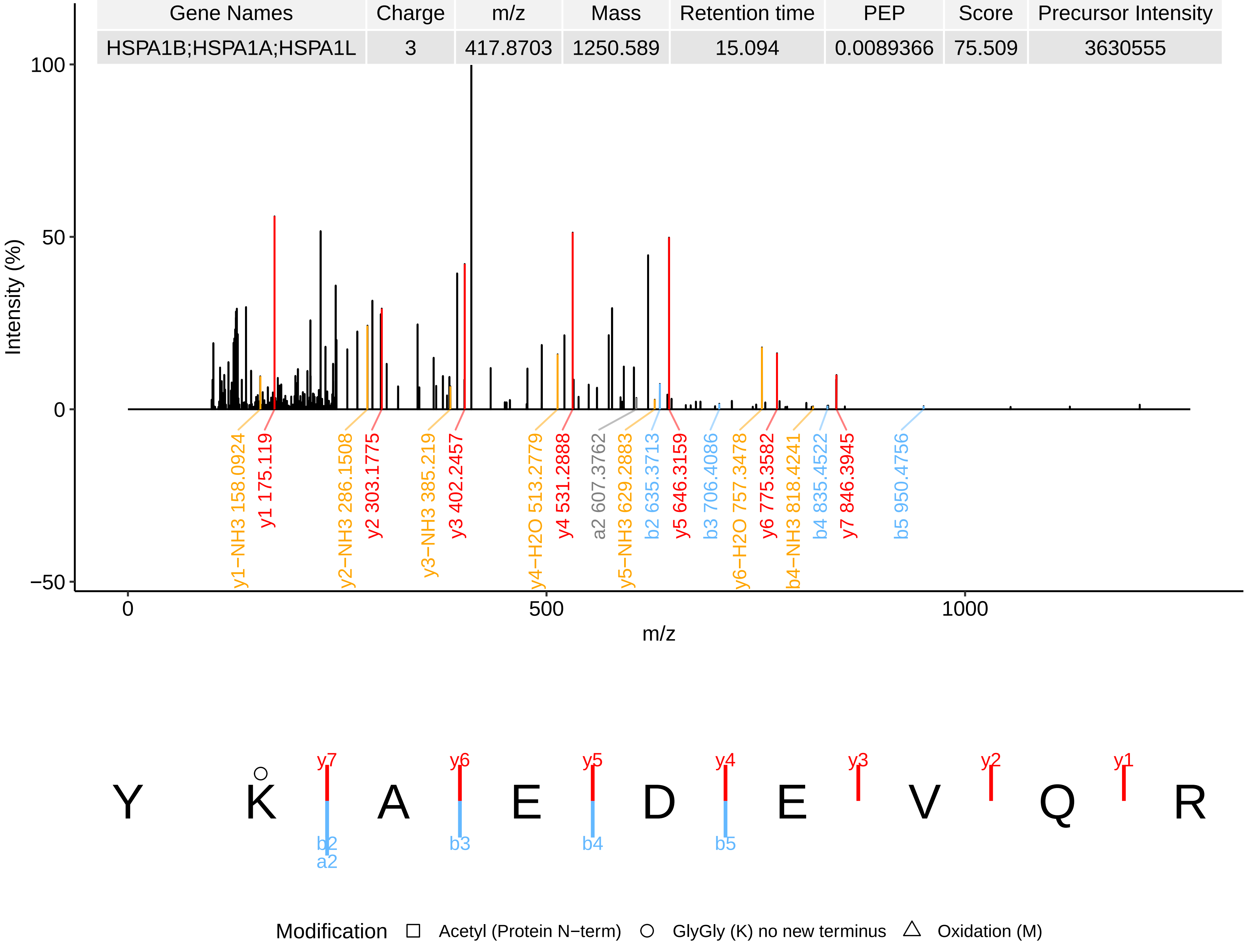

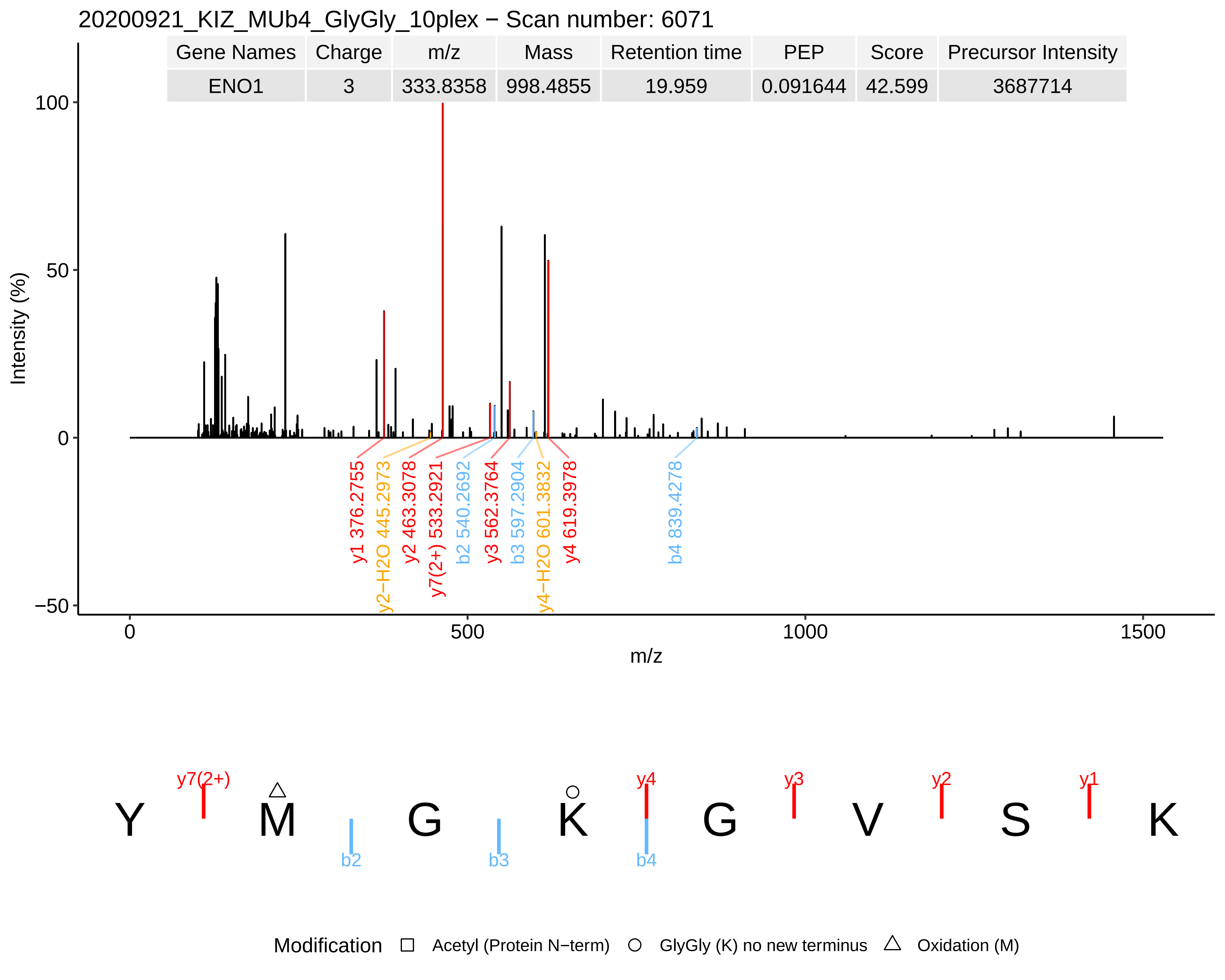

20200921_KIZ_MUb4_GlyGly_10plex − Scan number: 6071
Gene Names | Charge | m/z | Mass | Retention time | PEP | Score | Precursor Intensity
ENO1 | 3 | 333.8358 | 998.4855 | 19.959 | 0.091644 | 42.599 | 3687714
Intensity (%)
m/z
y1 376.2755
y2−H2O 445.2973
y2 463.3078
y7(2+) 533.2921
b2 540.2692
y3 562.3764
b3 597.2904
y4−H2O 601.3832
y4 619.3978
b4 839.4278
Y M G K G V S K
y7(2+) y4 y3 y2 y1
b2 b3 b4
Modification
Acetyl (Protein N−term)
GlyGly (K) no new terminus
Oxidation (M)

20200921_KIZ_MUb4_GlyGly_10plex − Scan number: 8228

Gene Names	Charge	m/z	Mass	Retention time	PEP	Score	Precursor Intensity
ENO1	3	328.5041	982.4906	27.866	0.073423	52.786	11424234

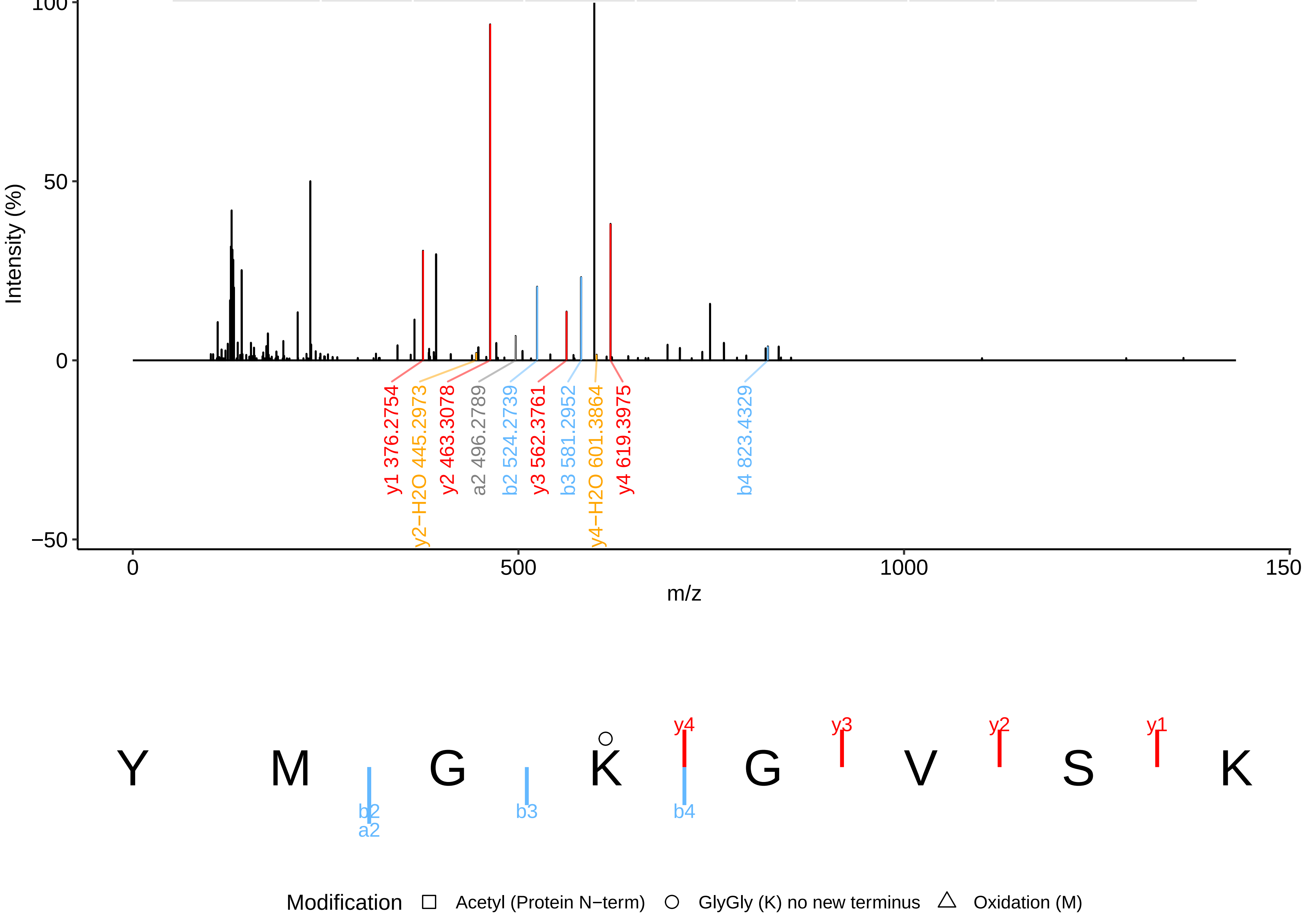

20200921_KIZ_MUb4_GlyGly_10plex − Scan number: 4279

Gene Names	Charge	m/z	Mass	Retention time	PEP	Score	Precursor Intensity
S100A4	3	333.1577	996.4512	13.94	0.053162	57.722	16252891

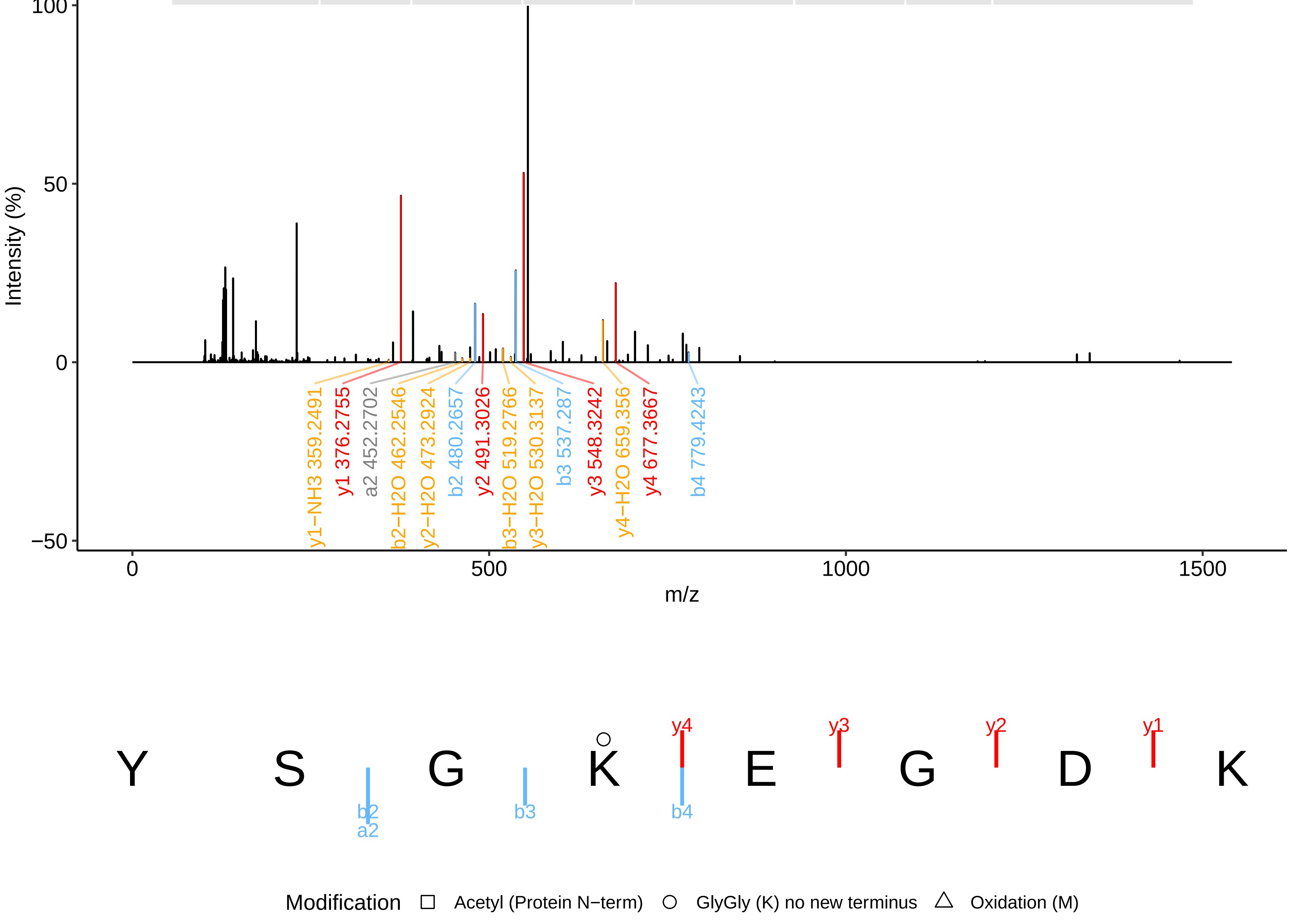

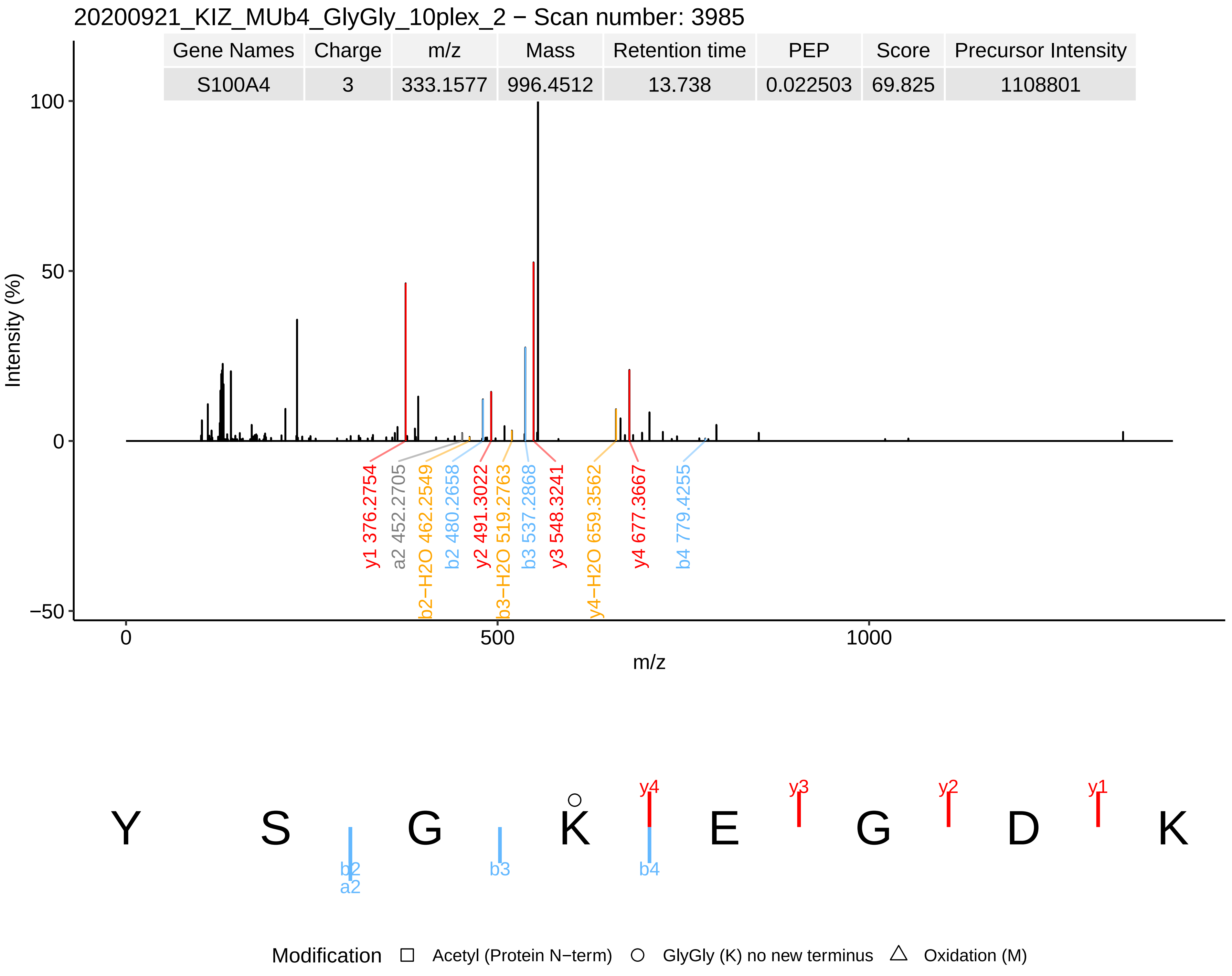
20200921_KIZ_MUb4_GlyGly_10plex_2 − Scan number: 3985
Gene Names | Charge | m/z | Mass | Retention time | PEP | Score | Precursor Intensity
S100A4 | 3 | 333.1577 | 996.4512 | 13.738 | 0.022503 | 69.825 | 1108801
Intensity (%)
100
50
0
−50
0
500
1000
m/z
y1 376.2754
a2 452.2705
b2−H2O 462.2549
b2 480.2658
y2 491.3022
b3−H2O 519.2763
b3 537.2868
y3 548.3241
y4−H2O 659.3562
y4 677.3667
b4 779.4255
Y S G K E G D K
y4
y3
y2
y1
b2
a2
b3
b4
Modification
Acetyl (Protein N−term)
GlyGly (K) no new terminus
Oxidation (M)

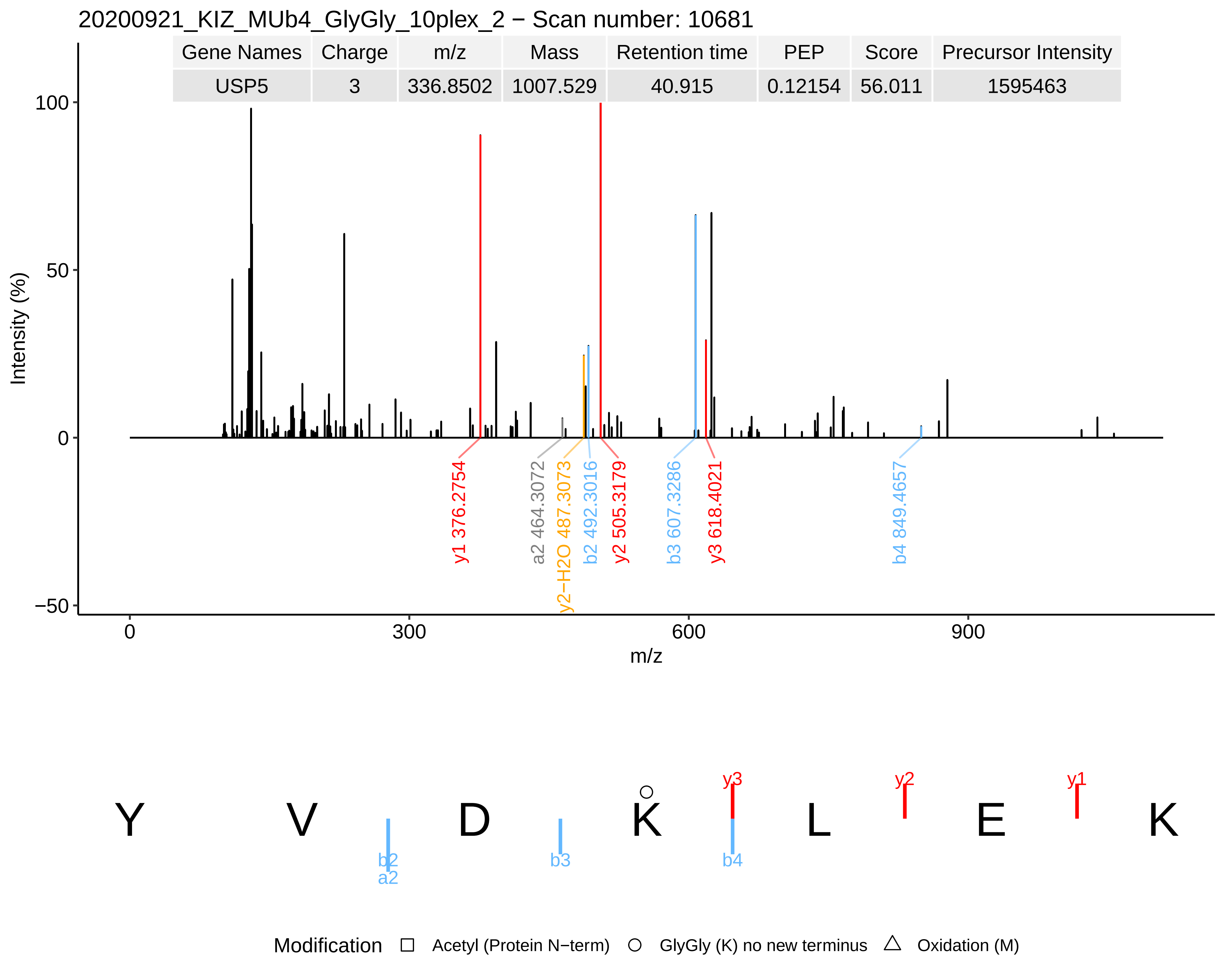
20200921_KIZ_MUb4_GlyGly_10plex_2 − Scan number: 10681
Gene Names | Charge | m/z | Mass | Retention time | PEP | Score | Precursor Intensity
USP5 | 3 | 336.8502 | 1007.529 | 40.915 | 0.12154 | 56.011 | 1595463
Intensity (%)
100
50
0
−50
0
300
600
900
m/z
y1 376.2754
a2 464.3072
y2−H2O 487.3073
b2 492.3016
y2 505.3179
b3 607.3286
y3 618.4021
b4 849.4657
Y V D K L E K
y3
y2
y1
b2
a2
b3
b4
Modification
Acetyl (Protein N−term)
GlyGly (K) no new terminus
Oxidation (M)

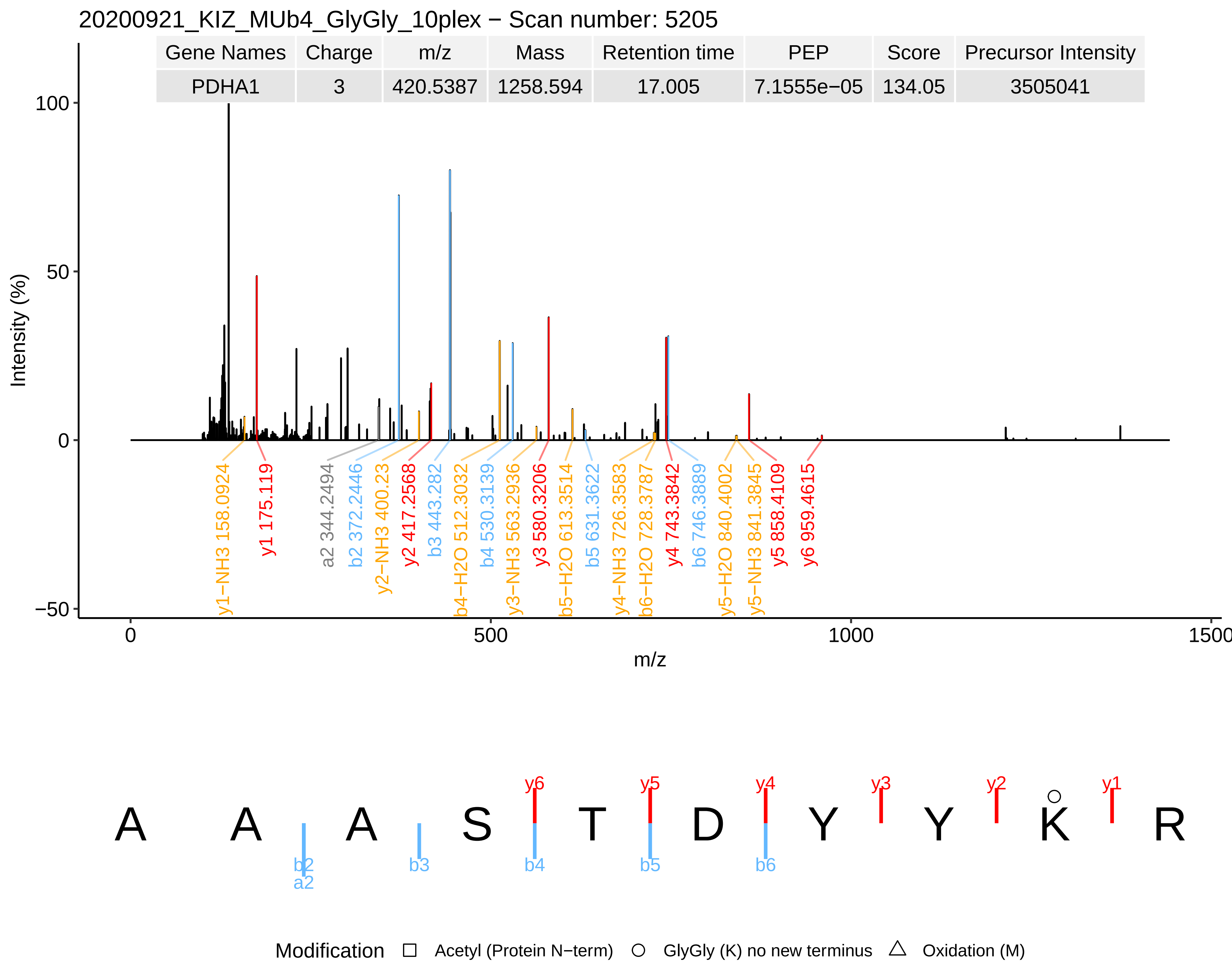
20200921_KIZ_MUb4_GlyGly_10plex − Scan number: 5205
Gene Names | Charge | m/z | Mass | Retention time | PEP | Score | Precursor Intensity
PDHA1 | 3 | 420.5387 | 1258.594 | 17.005 | 7.1555e−05 | 134.05 | 3505041
Intensity (%)
100
50
0
−50
0
500
1000
1500
m/z
y1−NH3 158.0924
y1 175.119
a2 344.2494
b2 372.2446
y2−NH3 400.23
y2 417.2568
b3 443.282
b4−H2O 512.3032
b4 530.3139
y3−NH3 563.2936
y3 580.3206
b5−H2O 613.3514
b5 631.3622
y4−NH3 726.3583
b6−H2O 728.3787
y4 743.3842
b6 746.3889
y5−H2O 840.4002
y5−NH3 841.3845
y5 858.4109
y6 959.4615
A A A S T D Y Y K R
y6 y5 y4 y3 y2 y1
b2 a2 b3 b4 b5 b6
Modification
Acetyl (Protein N−term)
GlyGly (K) no new terminus
Oxidation (M)

20200921_KIZ_MUb4_GlyGly_10plex_2 − Scan number: 4806

Gene Names	Charge	m/z	Mass	Retention time	PEP	Score	Precursor Intensity
PDHA1	3	420.5387	1258.594	17.019	0.0033353	105.52	1488138

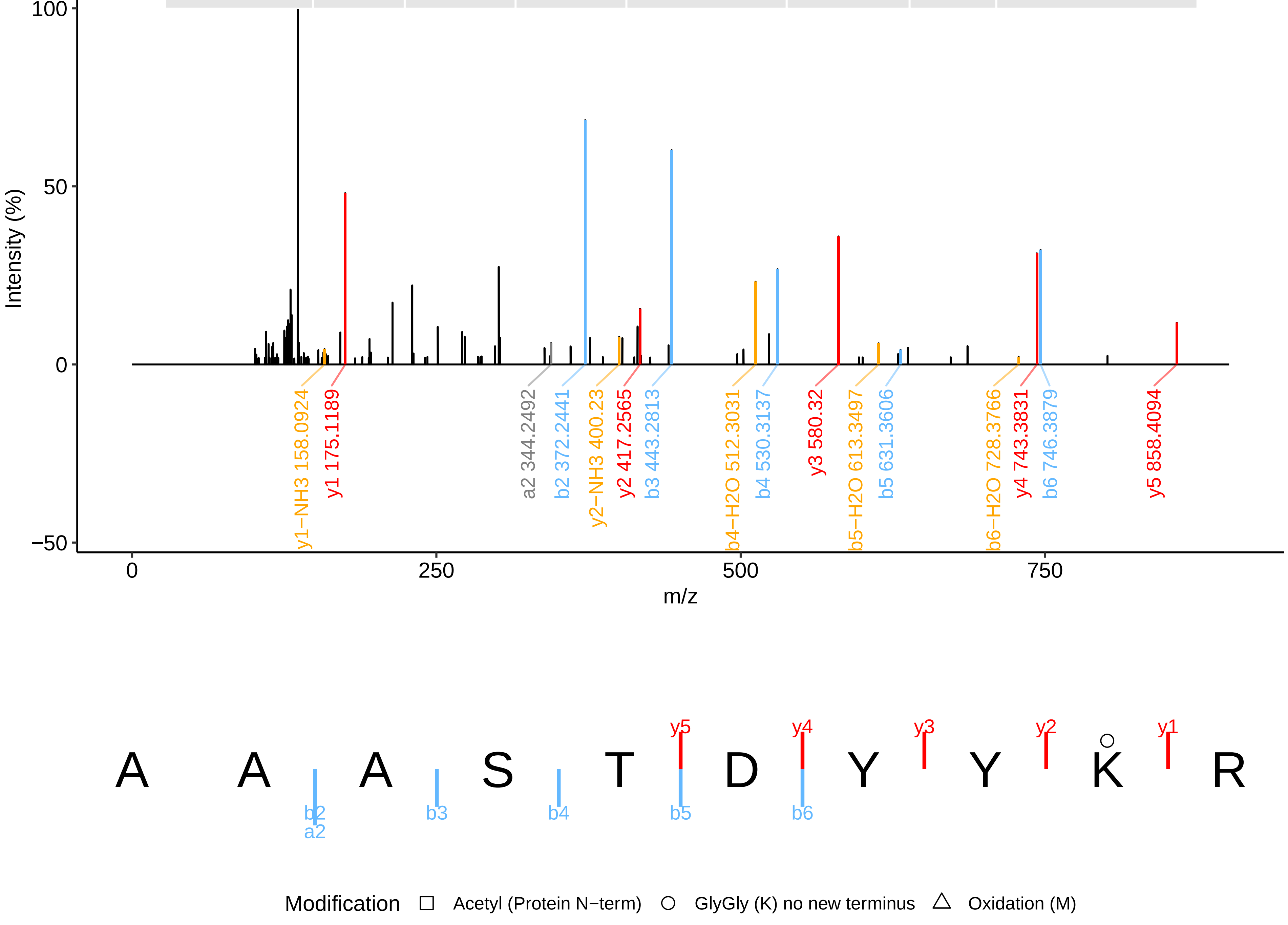

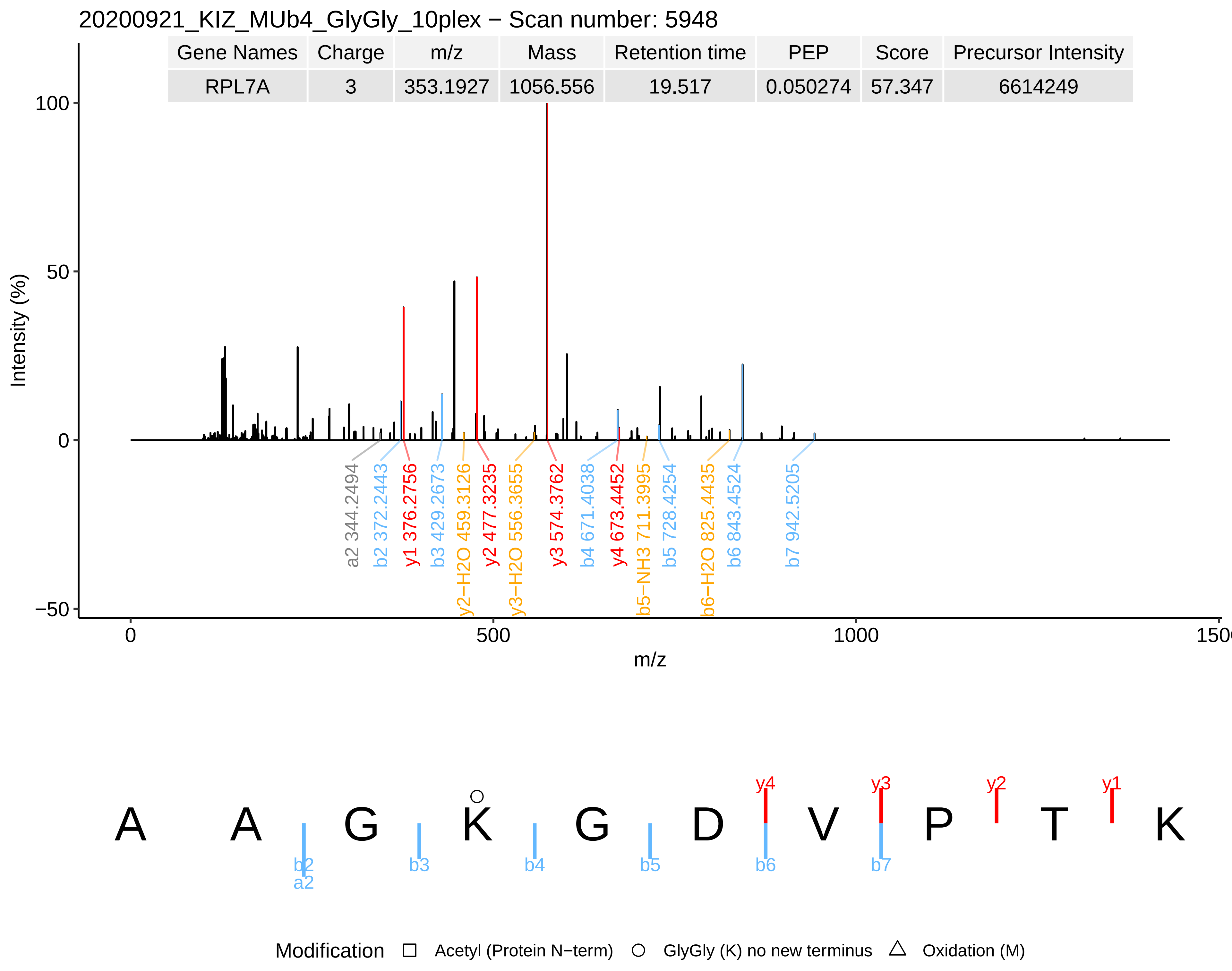
20200921_KIZ_MUb4_GlyGly_10plex − Scan number: 5948
Gene Names
Charge
m/z
Mass
Retention time
PEP
Score
Precursor Intensity
RPL7A
3
353.1927
1056.556
19.517
0.050274
57.347
6614249
Intensity (%)
100
50
0
−50
0
500
1000
150
m/z
a2 344.2494
b2 372.2443
y1 376.2756
b3 429.2673
y2−H2O 459.3126
y2 477.3235
y3−H2O 556.3655
y3 574.3762
b4 671.4038
y4 673.4452
b5−NH3 711.3995
b5 728.4254
b6−H2O 825.4435
b6 843.4524
b7 942.5205
A
A
G
K
G
D
V
P
T
K
y4
y3
y2
y1
b2
a2
b3
b4
b5
b6
b7
Modification
Acetyl (Protein N−term)
GlyGly (K) no new terminus
Oxidation (M)

20200921_KIZ_MUb4_GlyGly_10plex_2 − Scan number: 5411

Gene Names	Charge	m/z	Mass	Retention time	PEP	Score	Precursor Intensity
RPL7A	3	353.1927	1056.556	19.439	0.036119	60.102	928350.7

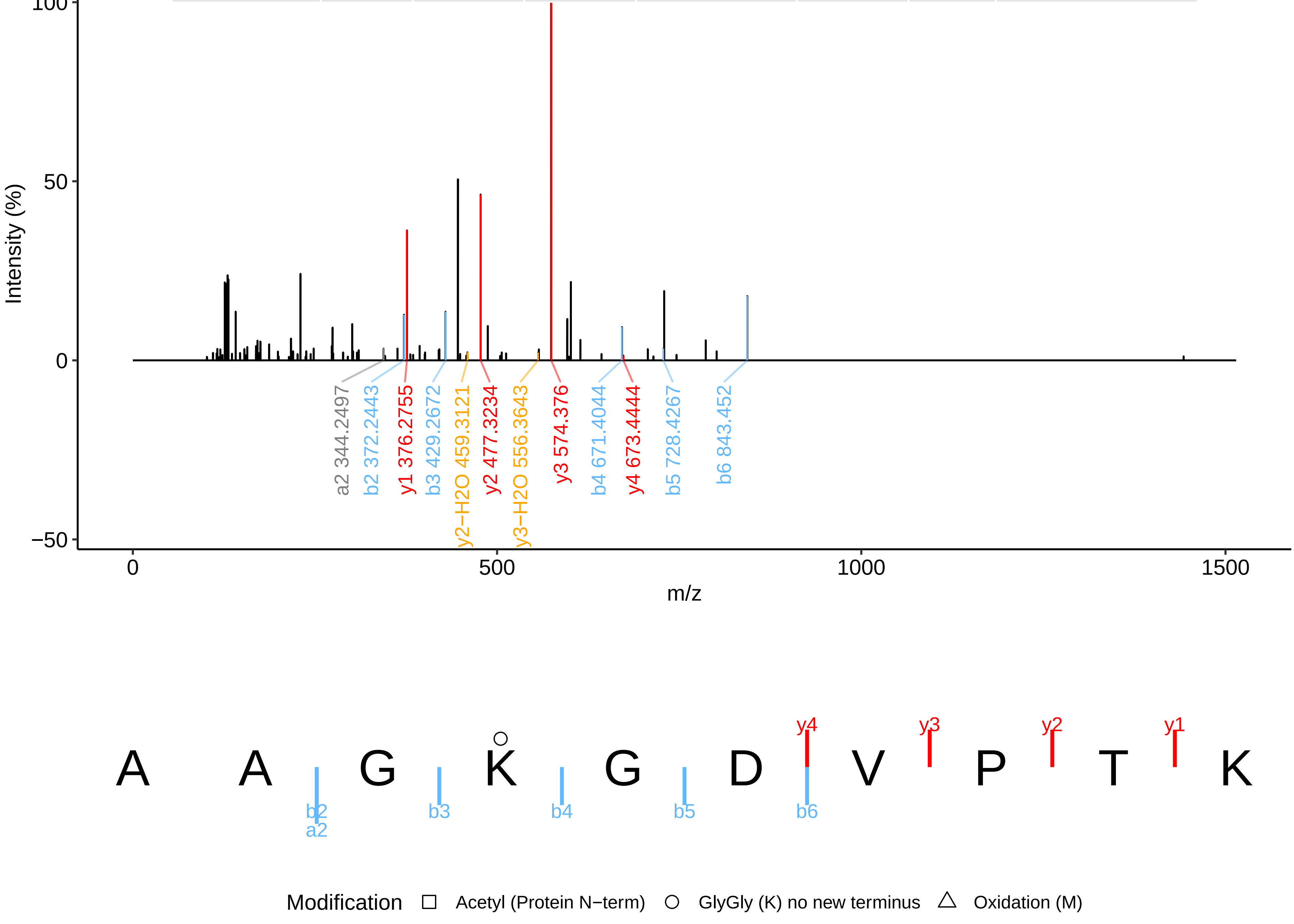

20200924_KIZ_MUb5_GlyGly_1 − Scan number: 5171

Gene Names	Charge	m/z	Mass	Retention time	PEP	Score	Precursor Intensity
RPL7A	3	353.1927	1056.556	16.051	0.082558	51.064	7333398

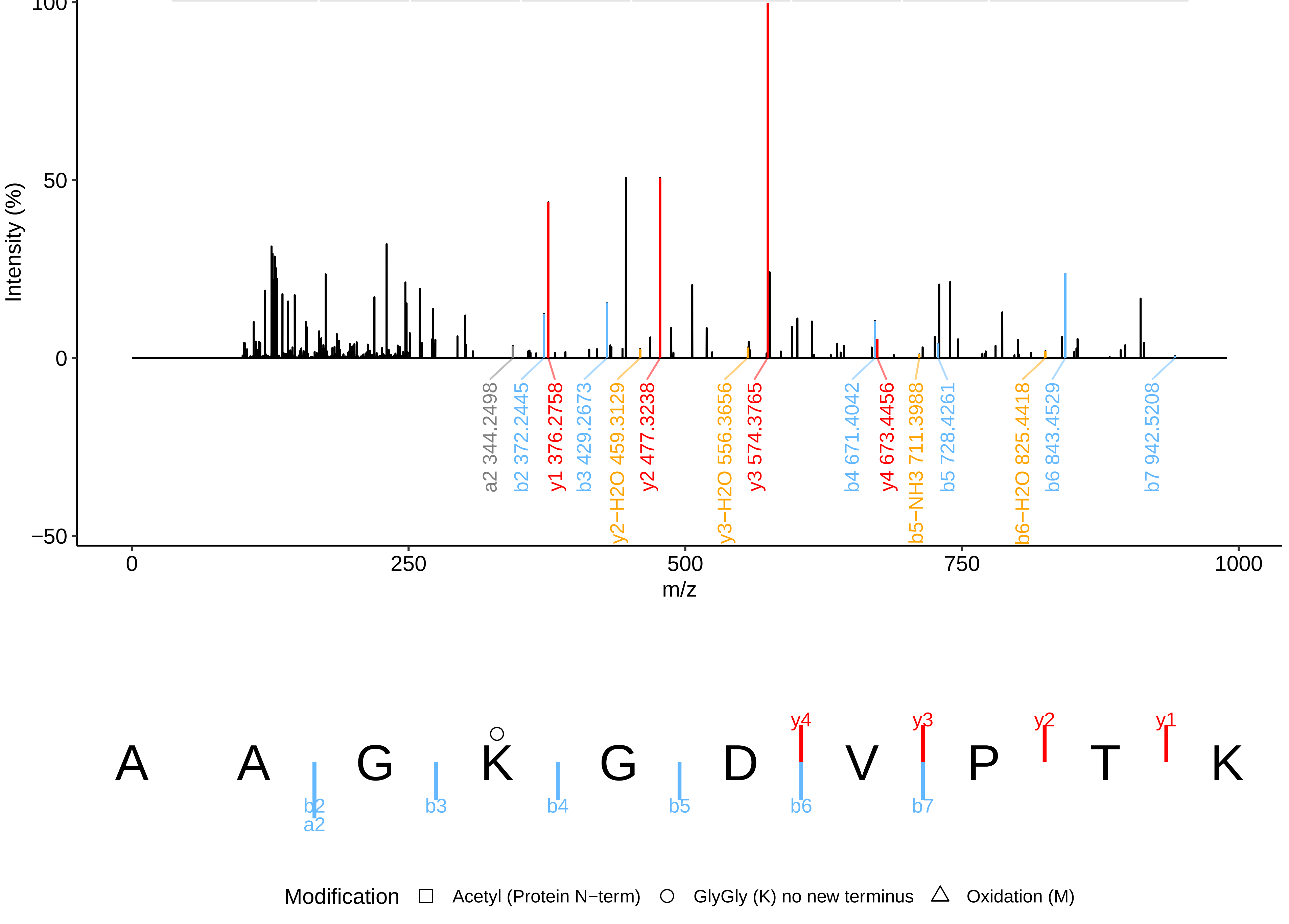

20200921_KIZ_MUb4_GlyGly_10plex − Scan number: 3316

Gene Names	Charge	m/z	Mass	Retention time	PEP	Score	Precursor Intensity
BASP1	3	345.1647	1032.472	10.412	0.040075	60.91	3360084

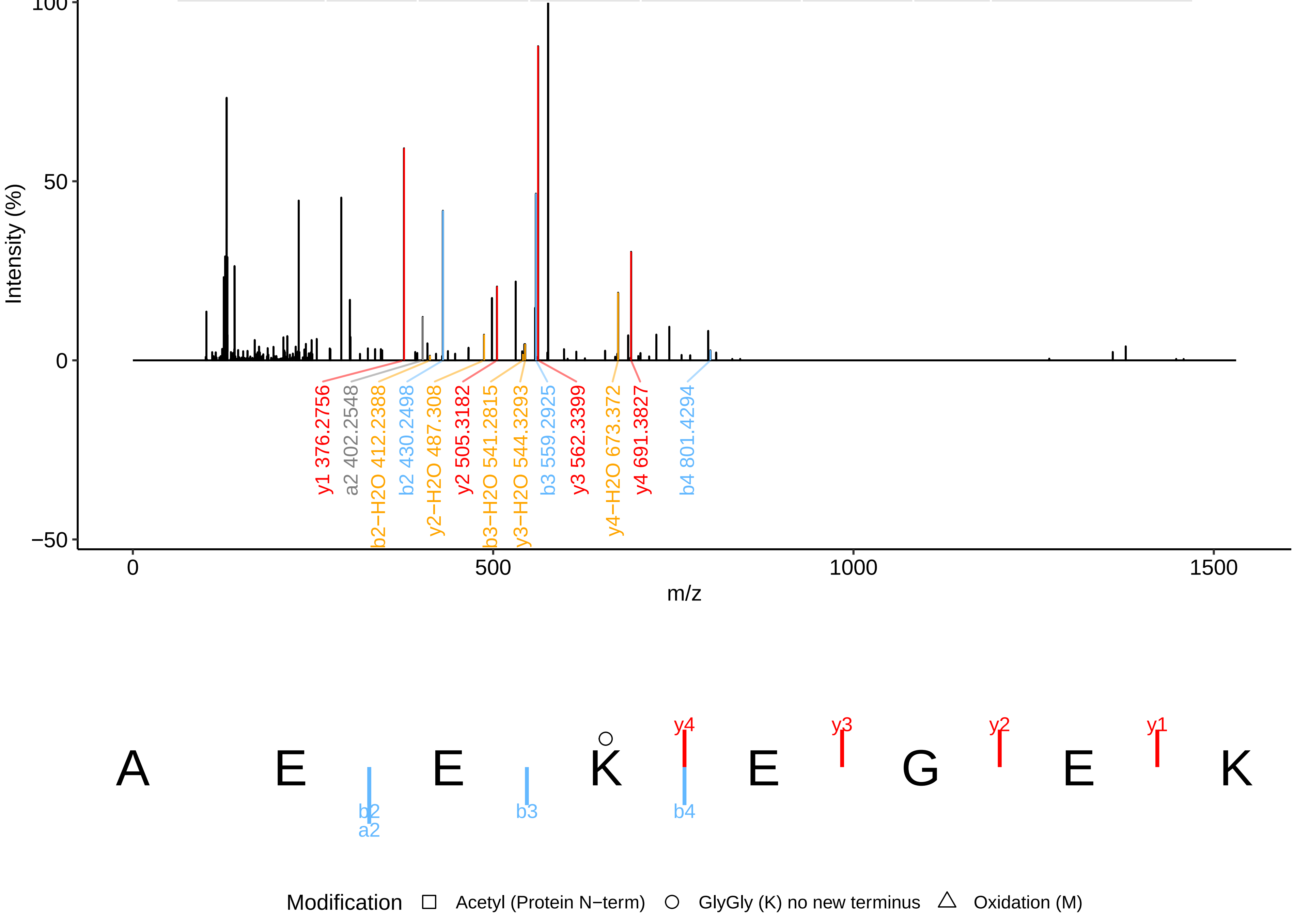

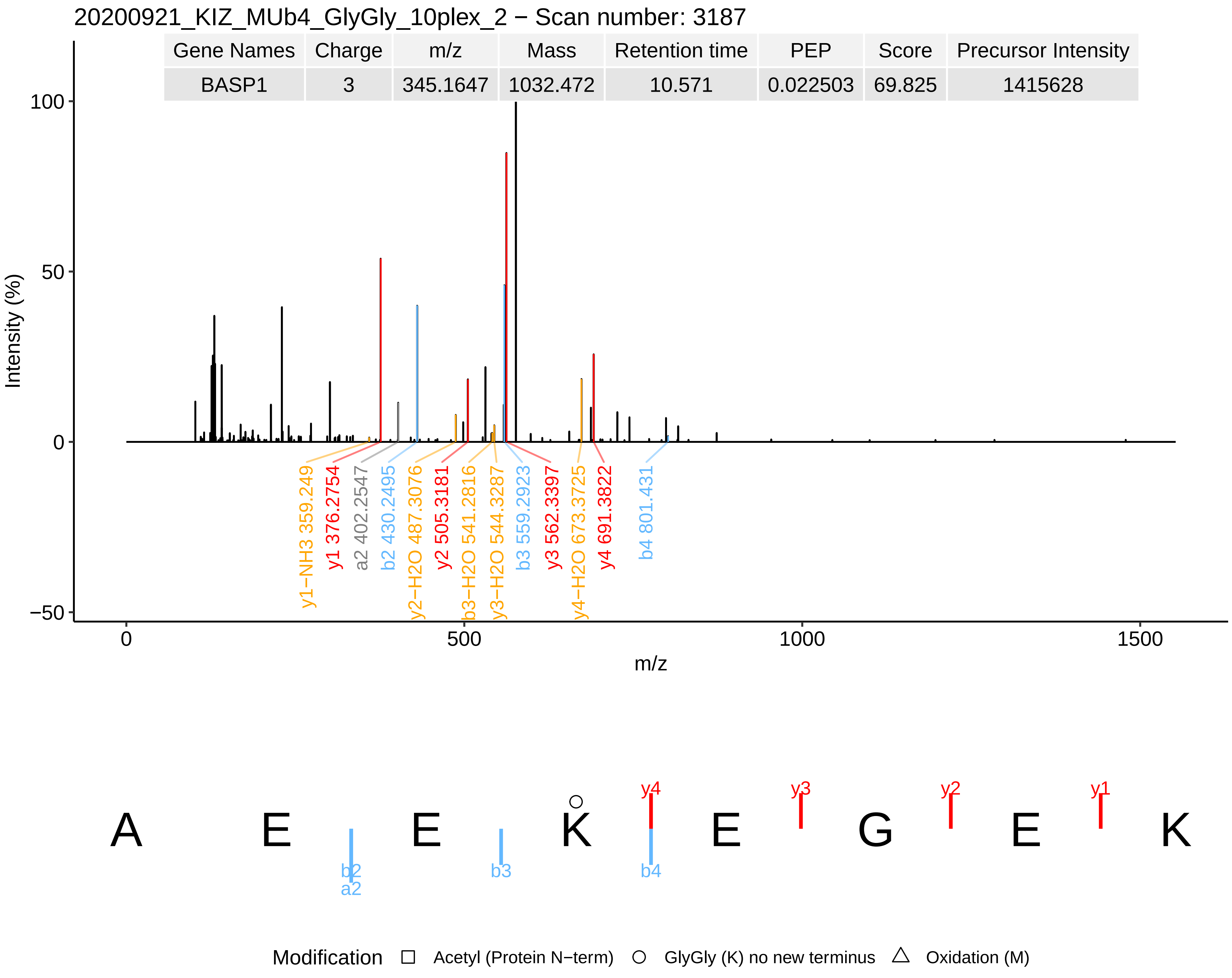
20200921_KIZ_MUb4_GlyGly_10plex_2 − Scan number: 3187
Gene Names | Charge | m/z | Mass | Retention time | PEP | Score | Precursor Intensity
BASP1 | 3 | 345.1647 | 1032.472 | 10.571 | 0.022503 | 69.825 | 1415628
Intensity (%)
100
50
0
−50
y1−NH3 359.249
y1 376.2754
a2 402.2547
b2 430.2495
y2−H2O 487.3076
y2 505.3181
b3−H2O 541.2816
y3−H2O 544.3287
b3 559.2923
y3 562.3397
y4−H2O 673.3725
y4 691.3822
b4 801.431
0
500
1000
1500
m/z
A E E K E G E K
y4 y3 y2 y1
b2 a2 b3 b4
Modification
Acetyl (Protein N−term)
GlyGly (K) no new terminus
Oxidation (M)

20200924_KIZ_MUb5_GlyGly_1 − Scan number: 3101

Gene Names	Charge	m/z	Mass	Retention time	PEP	Score	Precursor Intensity
BASP1	3	345.1647	1032.472	9.5629	0.040075	60.91	11544538

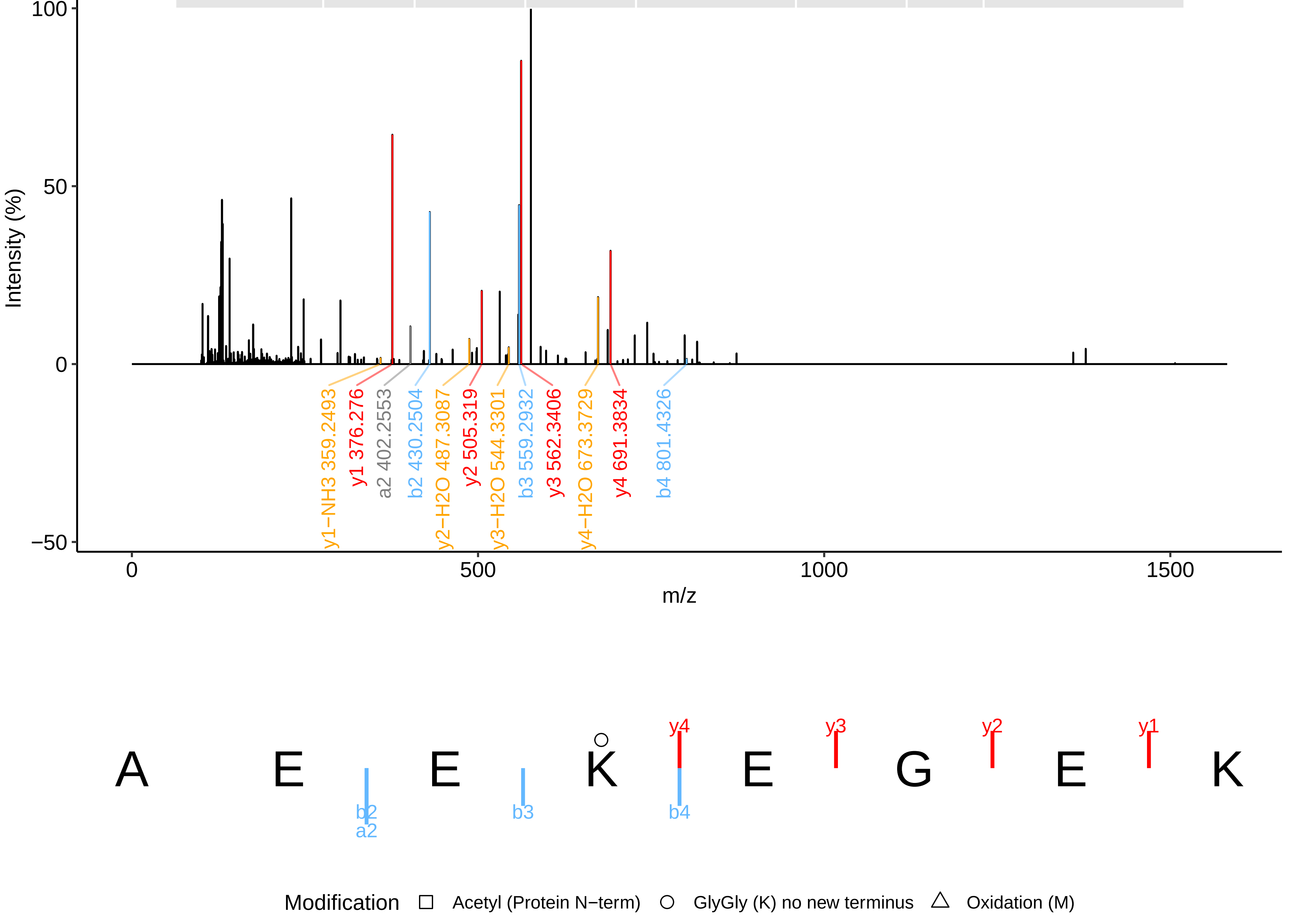

20200924_KIZ_MUb5_GlyGly_2 − Scan number: 2858

Gene Names	Charge	m/z	Mass	Retention time	PEP	Score	Precursor Intensity
BASP1	3	345.1647	1032.472	10.245	0.1023	53.775	1130255

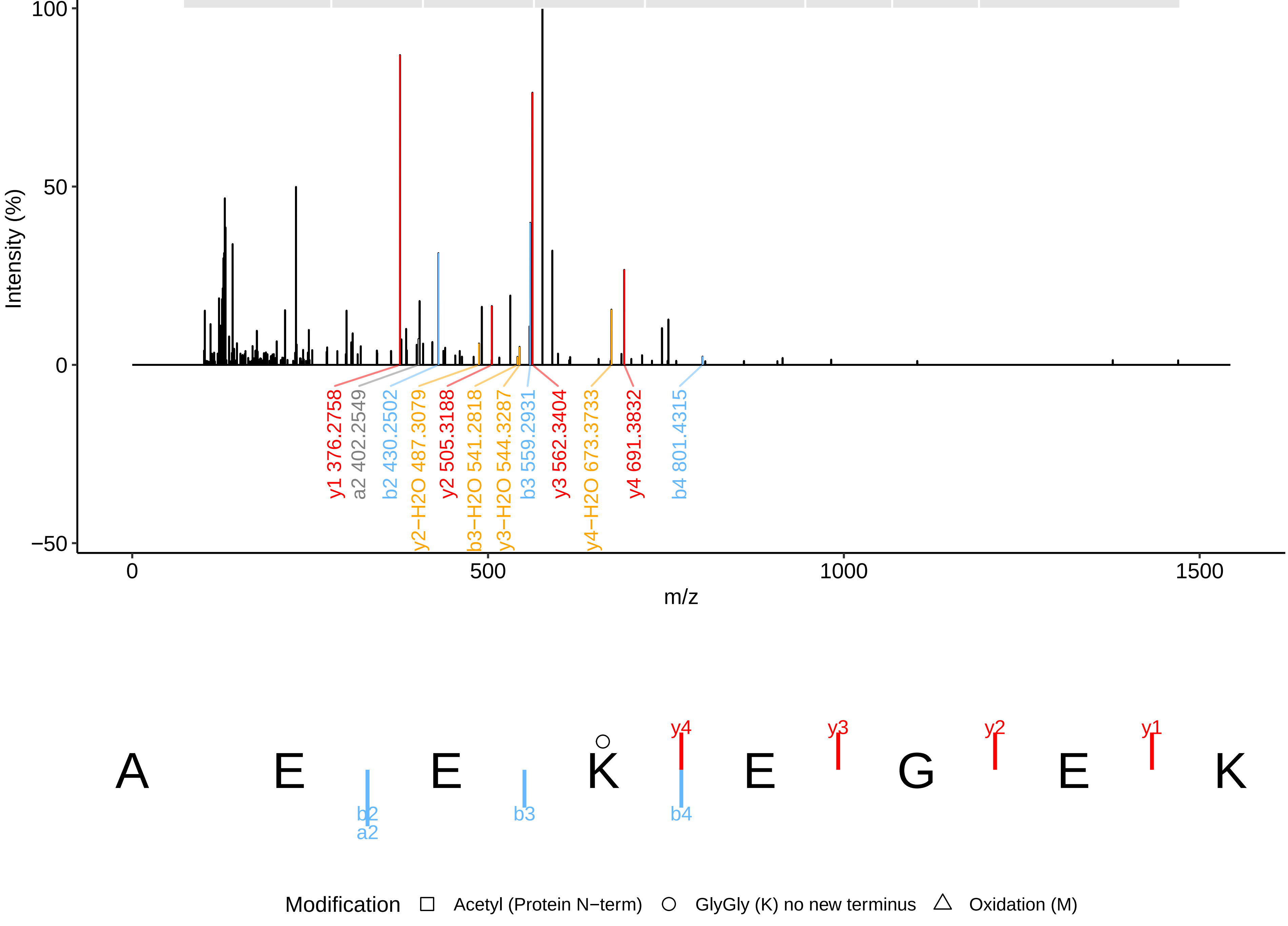

20201118_KIZ_MUb6_GlyGly_1 − Scan number: 3067

Gene Names	Charge	m/z	Mass	Retention time	PEP	Score	Precursor Intensity
BASP1	3	345.1647	1032.472	9.4432	0.024904	67.897	9256302

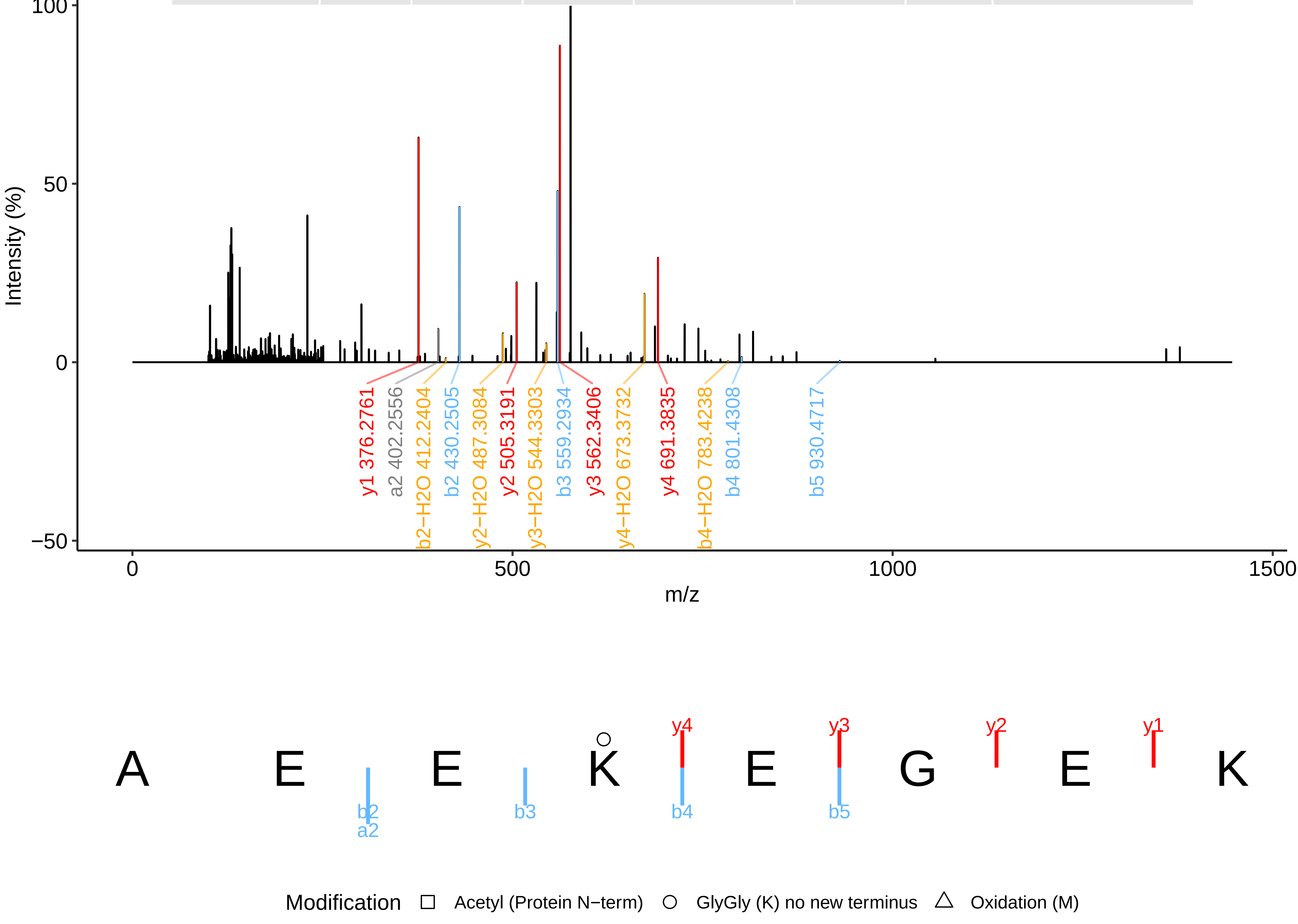

20201118_KIZ_MUb6_GlyGly_2 − Scan number: 3308

Gene Names	Charge	m/z	Mass	Retention time	PEP	Score	Precursor Intensity
BASP1	3	345.1647	1032.472	10.818	0.040075	60.91	2160783

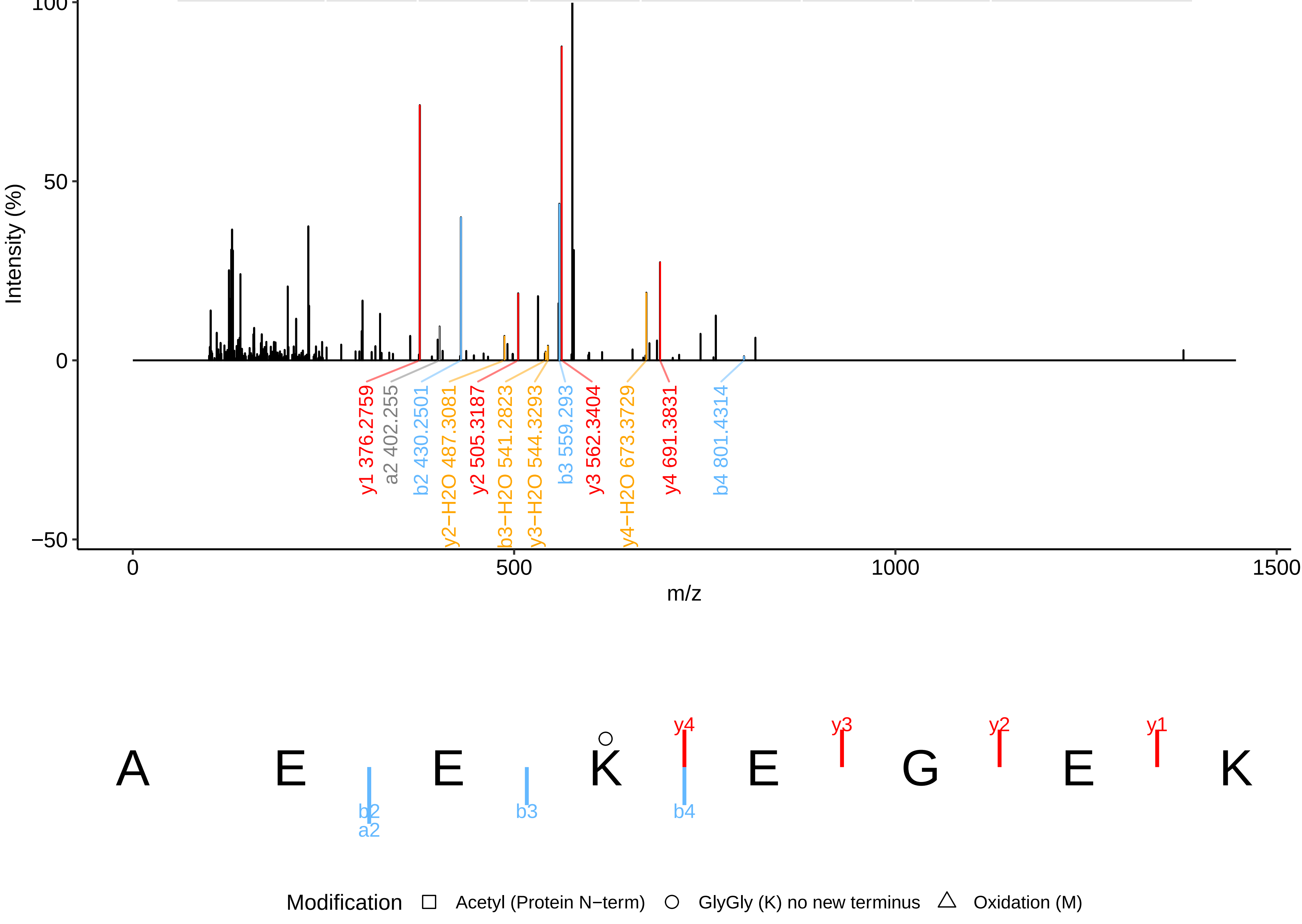

20200921_KIZ_MUb4_GlyGly_10plex_2 − Scan number: 3319

Gene Names	Charge	m/z	Mass	Retention time	PEP	Score	Precursor Intensity
TMPO	3	421.2055	1260.595	11.096	0.0010093	91.549	1026608

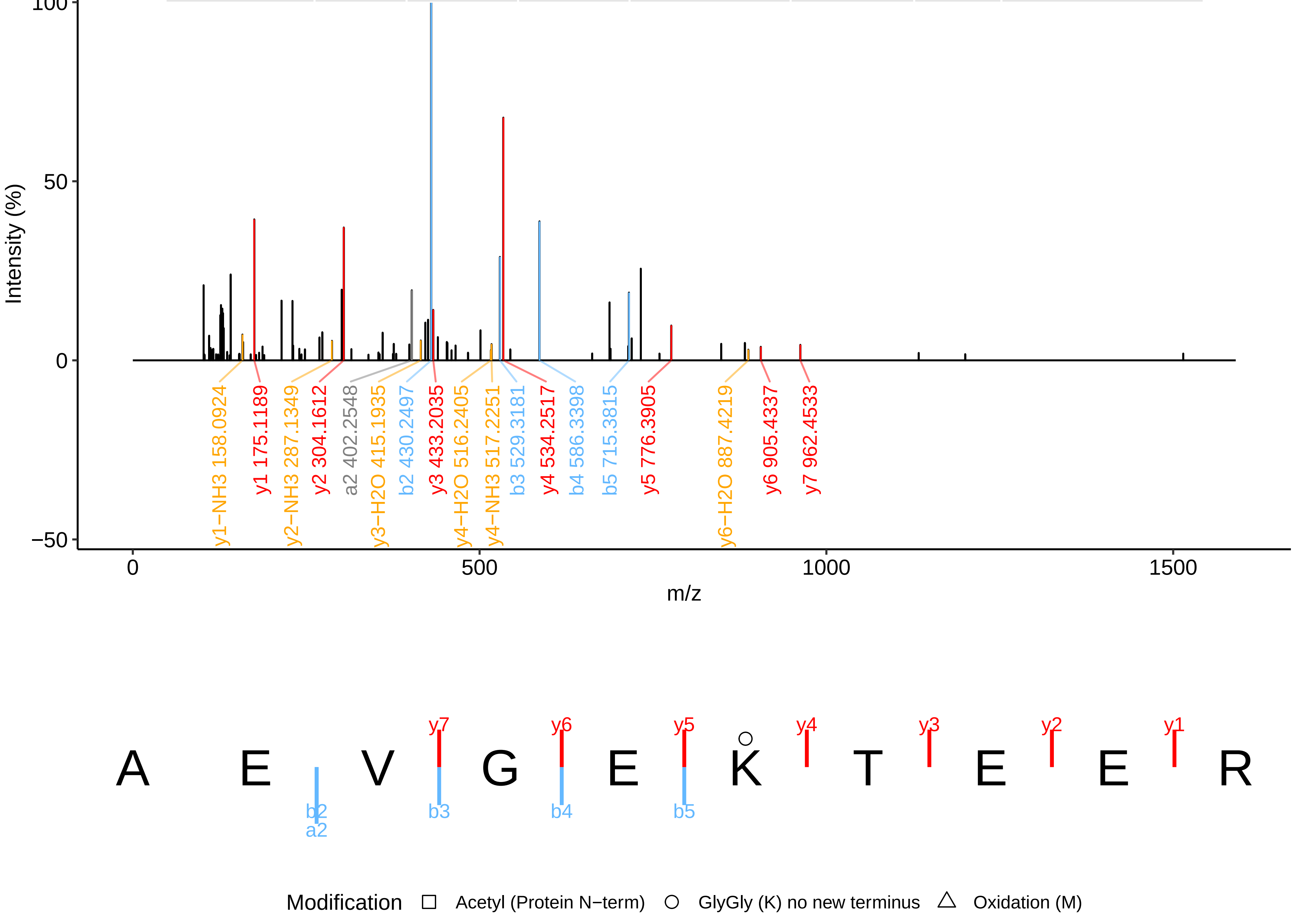

20200924_KIZ_MUb5_GlyGly_2 − Scan number: 2961

Gene Names	Charge	m/z	Mass	Retention time	PEP	Score	Precursor Intensity
TMPO	3	421.2055	1260.595	10.622	0.011404	73.781	NA

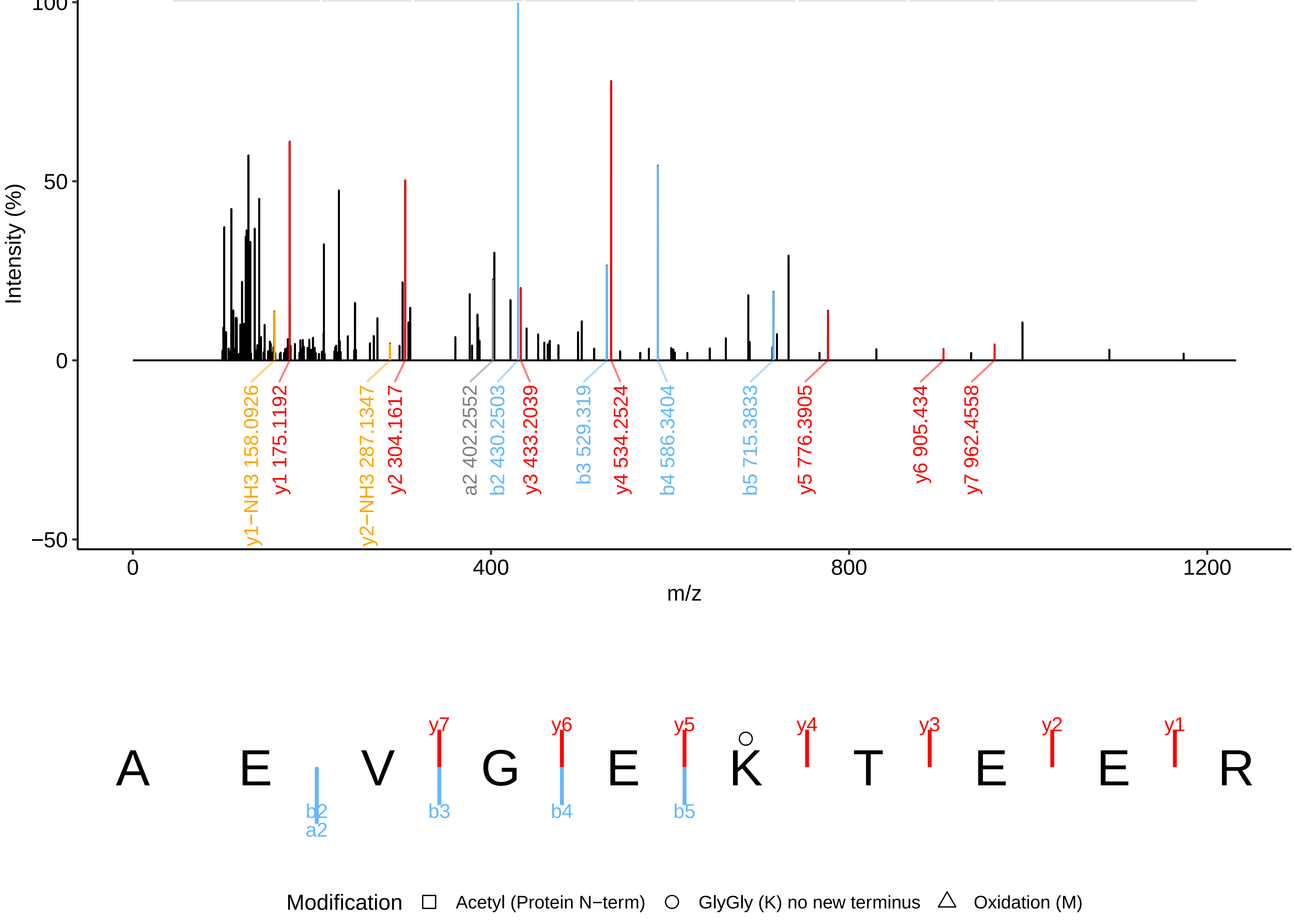

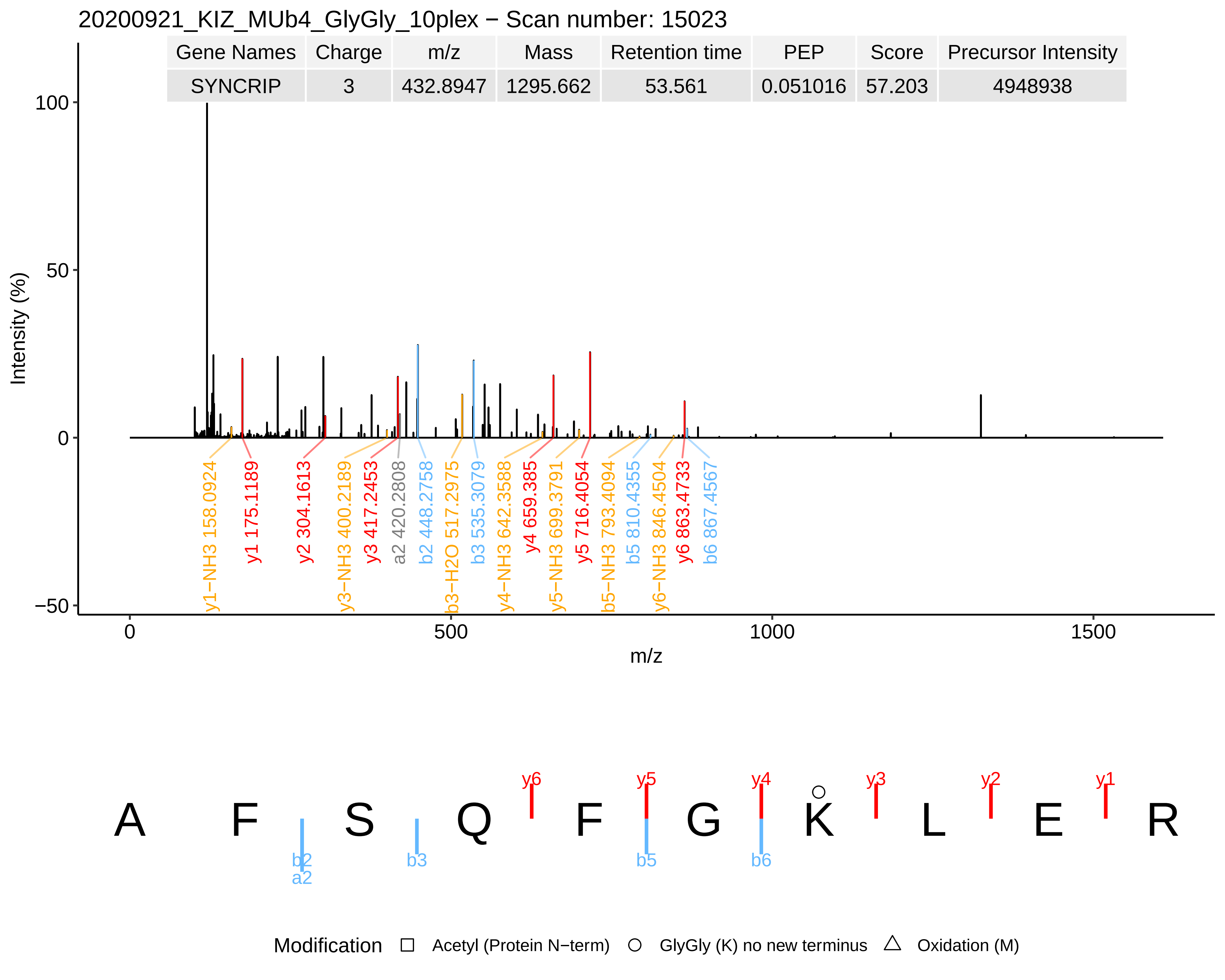

Gene Names	Charge	m/z	Mass	Retention time	PEP	Score	Precursor Intensity
SYNCRIP	3	432.8947	1295.662	53.561	0.051016	57.203	4948938

20200921_KIZ_MUb4_GlyGly_10plex_2 − Scan number: 13912

Gene Names	Charge	m/z	Mass	Retention time	PEP	Score	Precursor Intensity
SYNCRIP	3	432.8947	1295.662	54.12	0.058629	55.721	938074.3

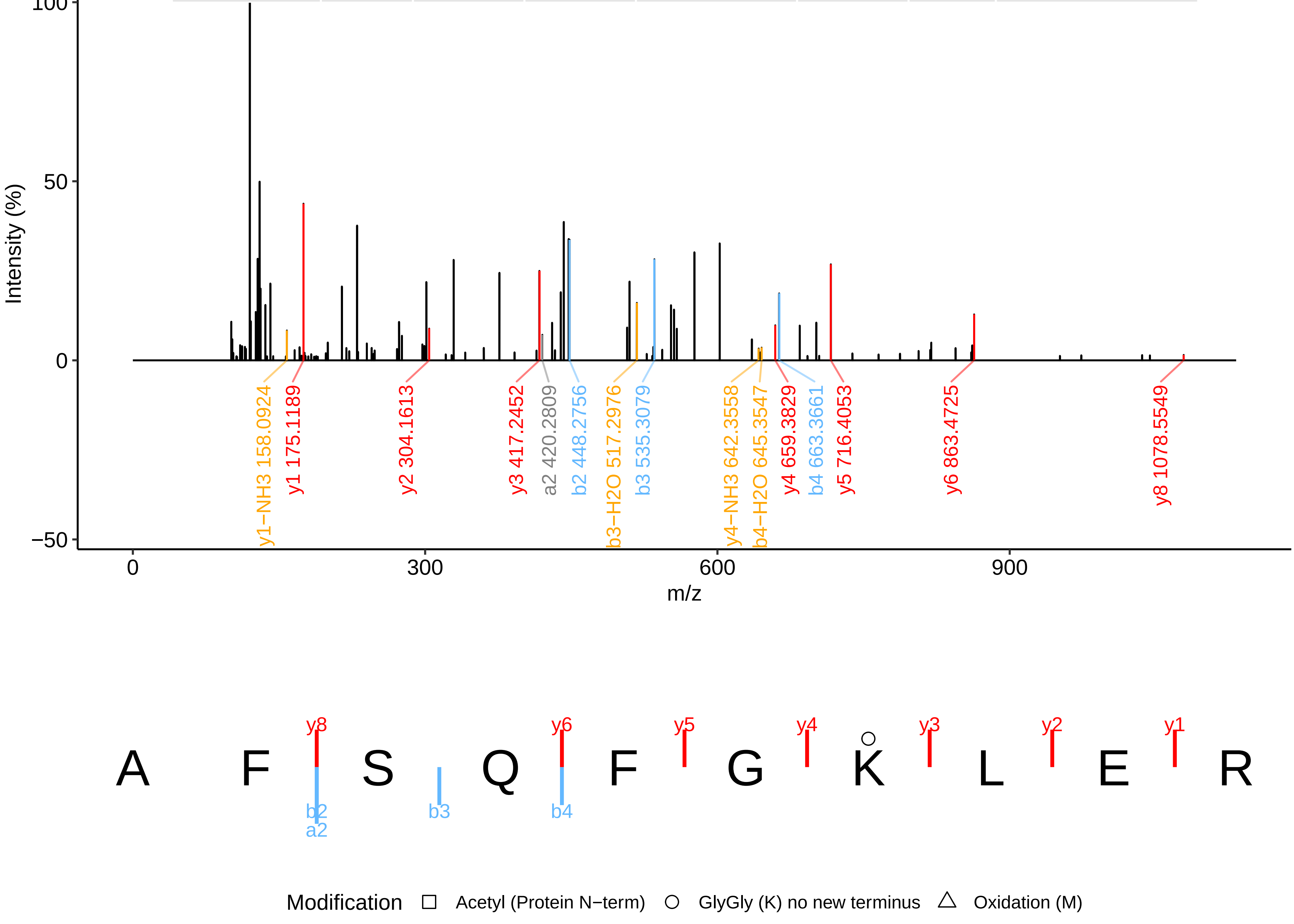

20200921_KIZ_MUb4_GlyGly_10plex − Scan number: 9817

Gene Names	Charge	m/z	Mass	Retention time	PEP	Score	Precursor Intensity
ABCC5	3	426.5702	1276.689	33.754	1.5371e−06	166.19	5561579

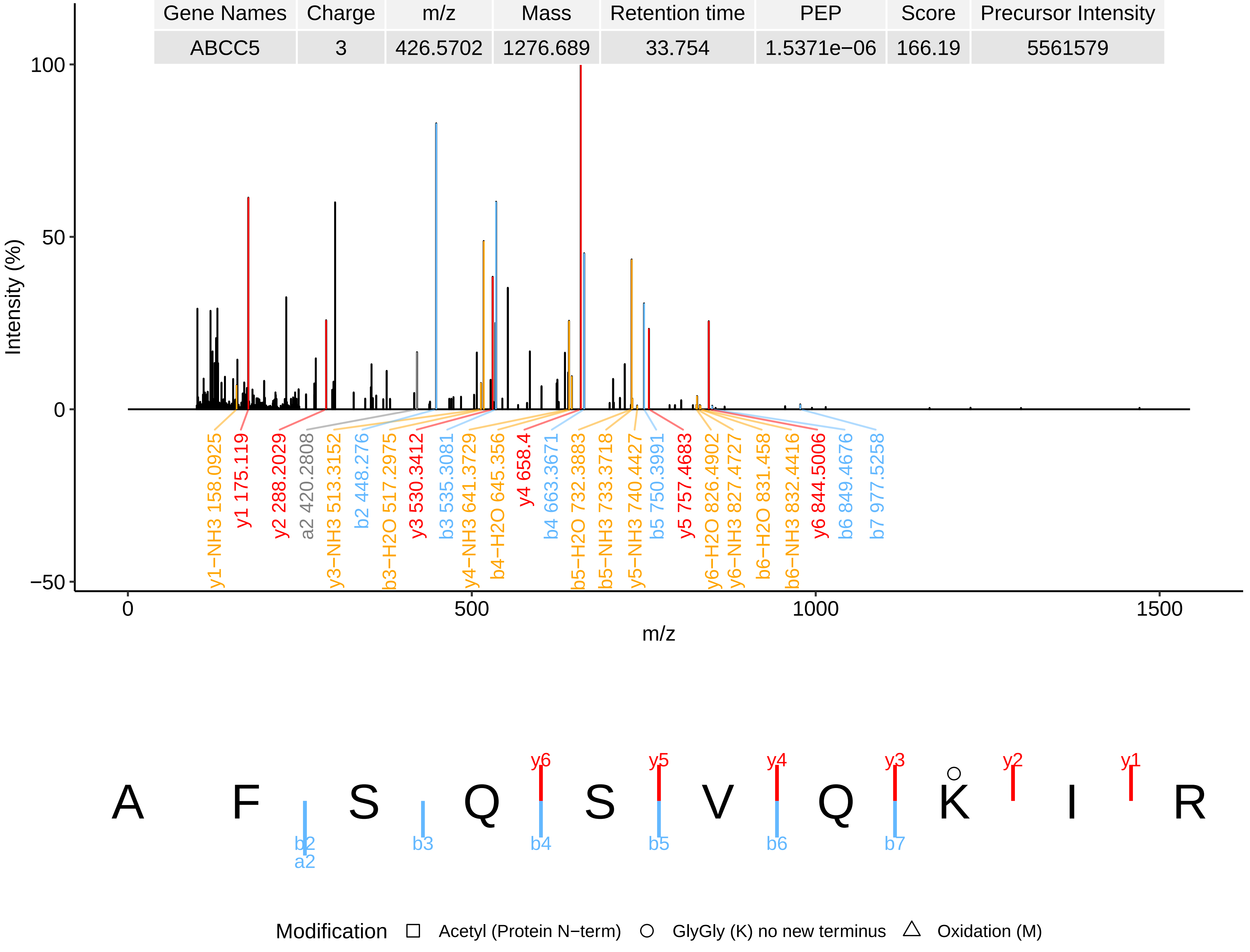

20200921_KIZ_MUb4_GlyGly_10plex_2 − Scan number: 9064

Gene Names	Charge	m/z	Mass	Retention time	PEP	Score	Precursor Intensity
ABCC5	3	426.5702	1276.689	34.35	7.5711e−05	98.044	1343891

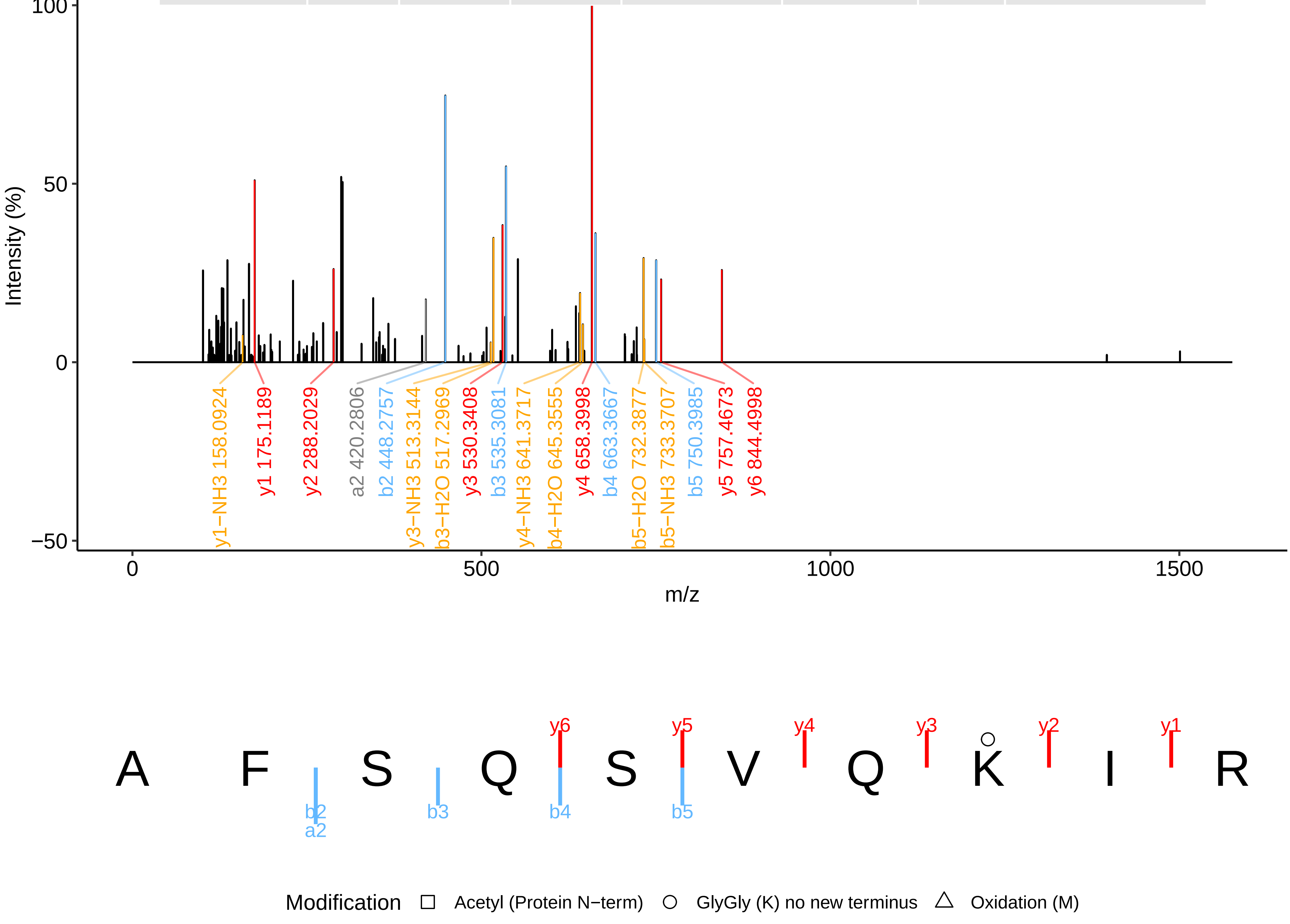

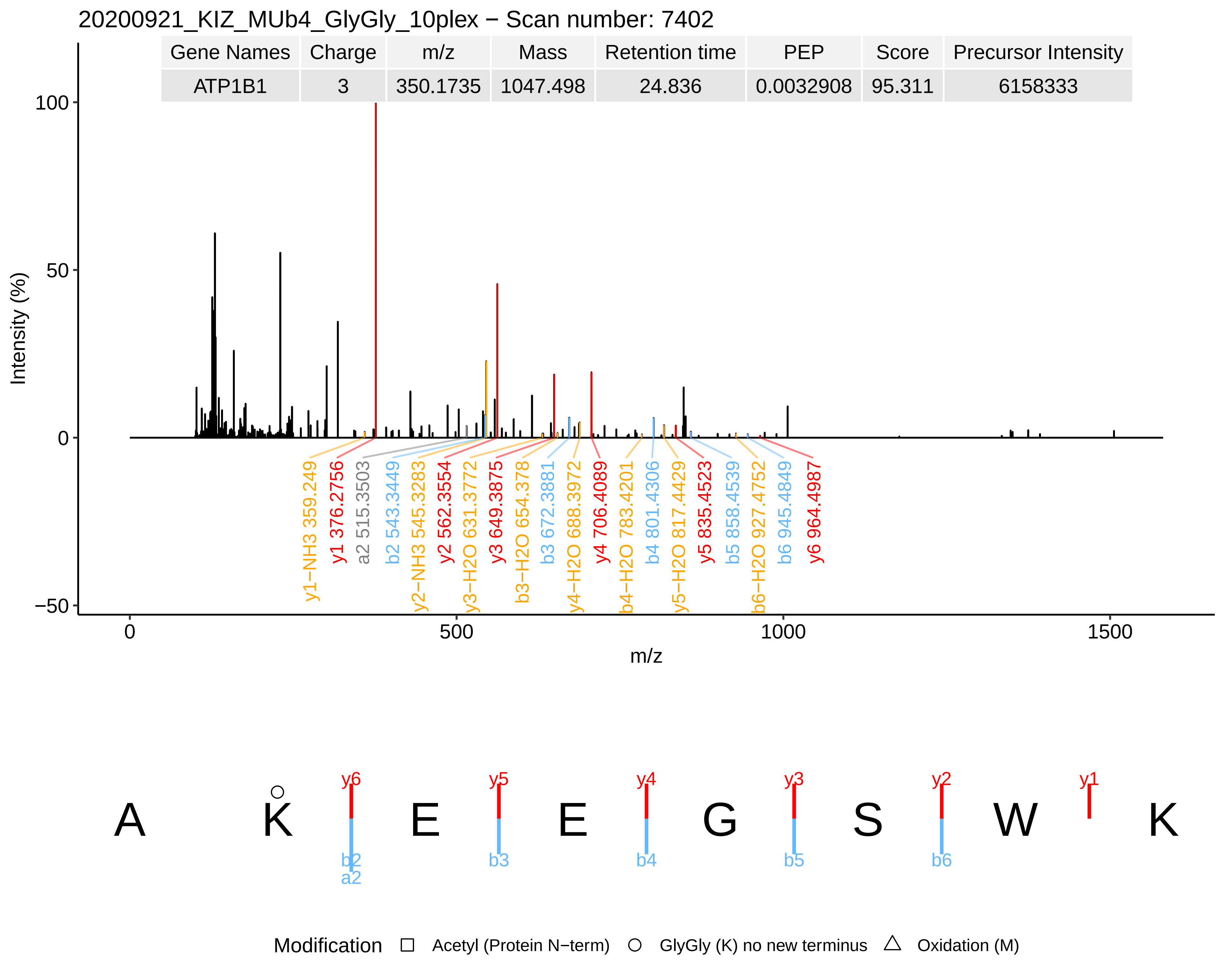
20200921_KIZ_MUb4_GlyGly_10plex − Scan number: 7402
Gene Names | Charge | m/z | Mass | Retention time | PEP | Score | Precursor Intensity
ATP1B1 | 3 | 350.1735 | 1047.498 | 24.836 | 0.0032908 | 95.311 | 6158333
Intensity (%)
100
50
0
−50
0
500
1000
1500
m/z
y1−NH3 359.249
y1 376.2756
a2 515.3503
b2 543.3449
y2−NH3 545.3283
y2 562.3554
y3−H2O 631.3772
y3 649.3875
b3−H2O 654.378
b3 672.3881
y4−H2O 688.3972
y4 706.4089
b4−H2O 783.4201
b4 801.4306
y5−H2O 817.4429
y5 835.4523
b5 858.4539
b6−H2O 927.4752
b6 945.4849
y6 964.4987
A K E E G S W K
y6 y5 y4 y3 y2 y1
b2 a2 b3 b4 b5 b6
Modification
Acetyl (Protein N−term)
GlyGly (K) no new terminus
Oxidation (M)

20200924_KIZ_MUb5_GlyGly_1 − Scan number: 6481

Gene Names	Charge	m/z	Mass	Retention time	PEP	Score	Precursor Intensity
ATP1B1	3	350.1735	1047.498	20.384	0.014599	76.17	5100050

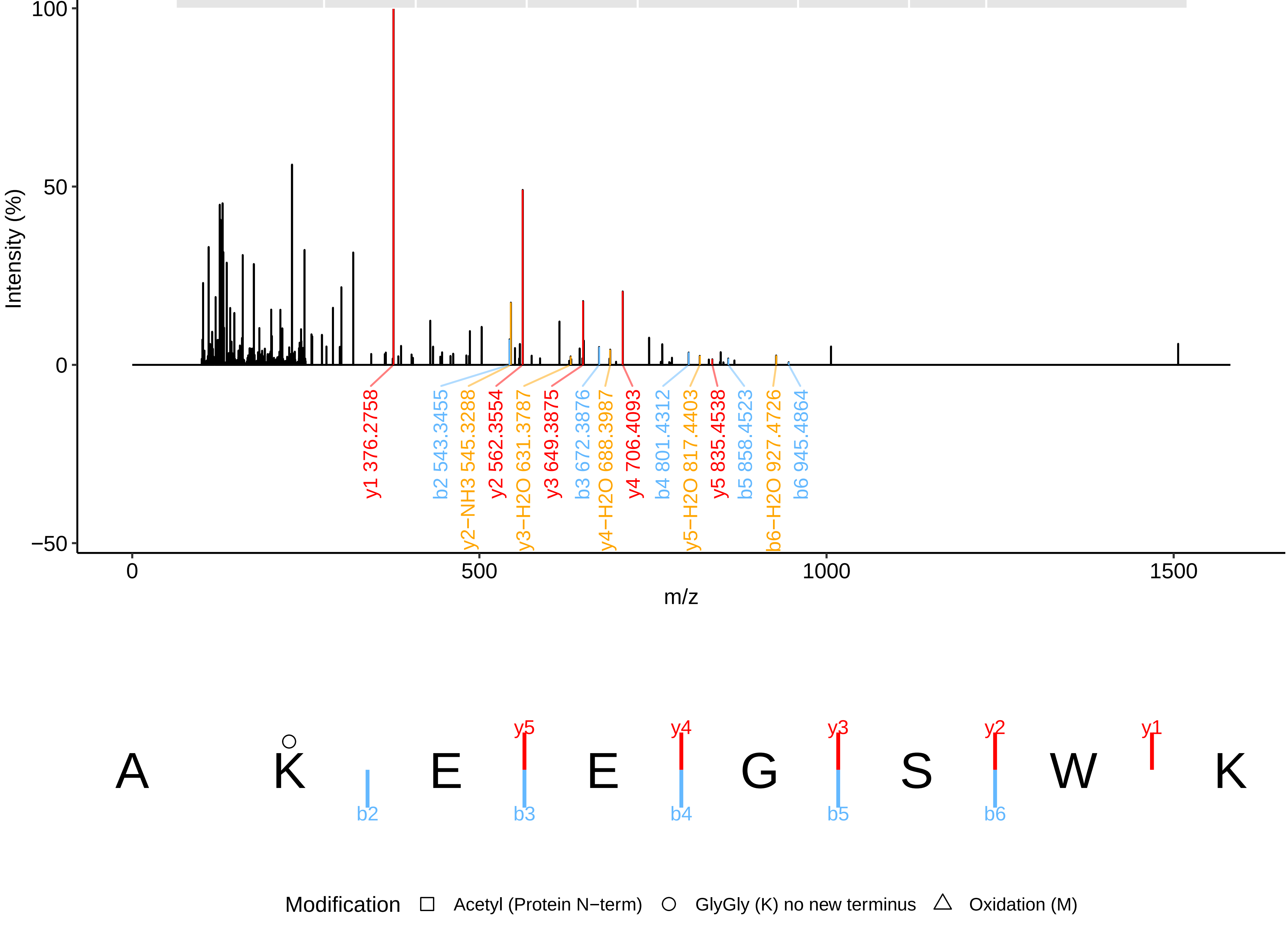

20200921_KIZ_MUb4_GlyGly_10plex − Scan number: 28566

Gene Names	Charge	m/z	Mass	Retention time	PEP	Score	Precursor Intensity
ATP6V0D1	2	654.3533	1306.692	104.55	0.092298	61.999	5392906

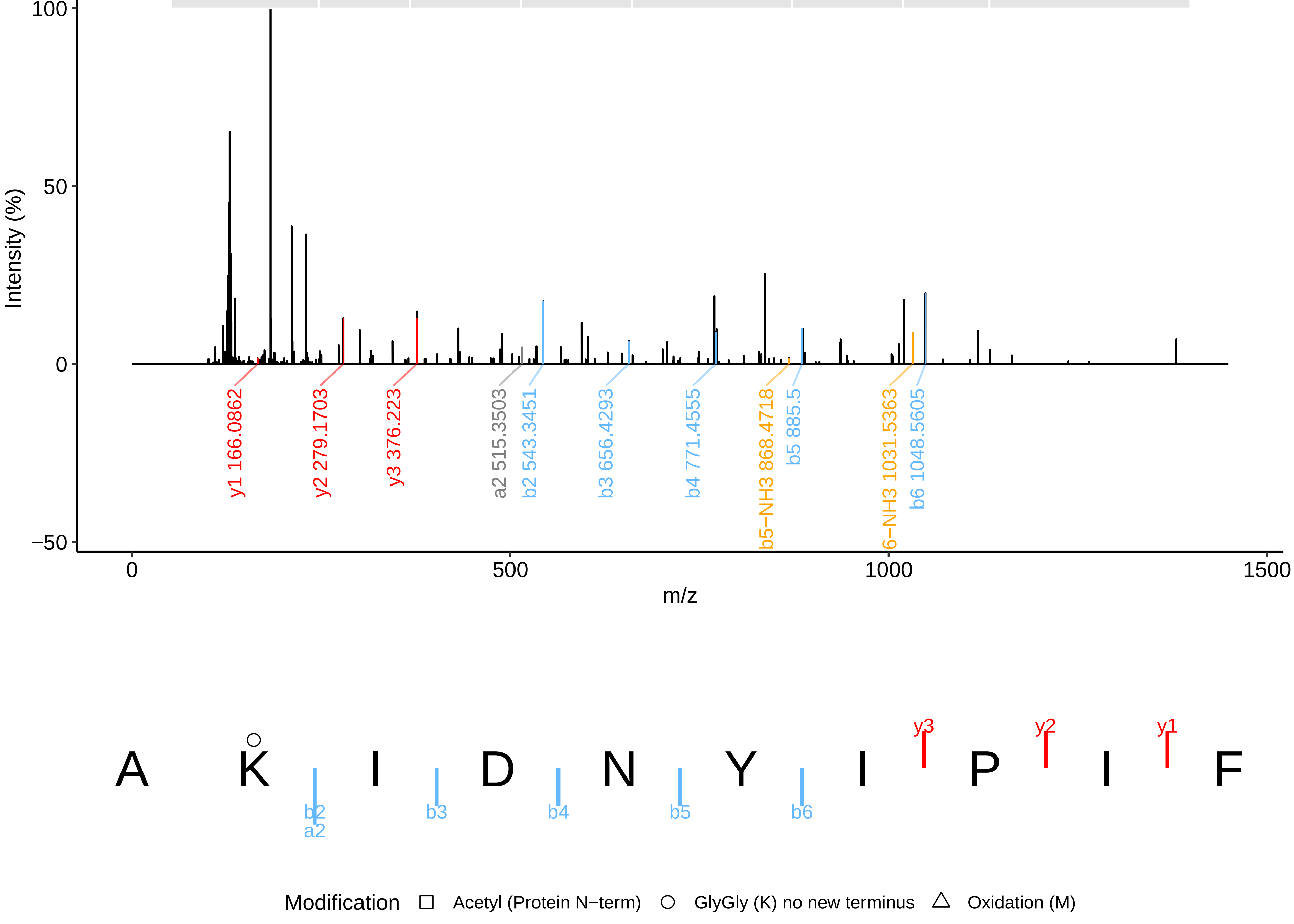

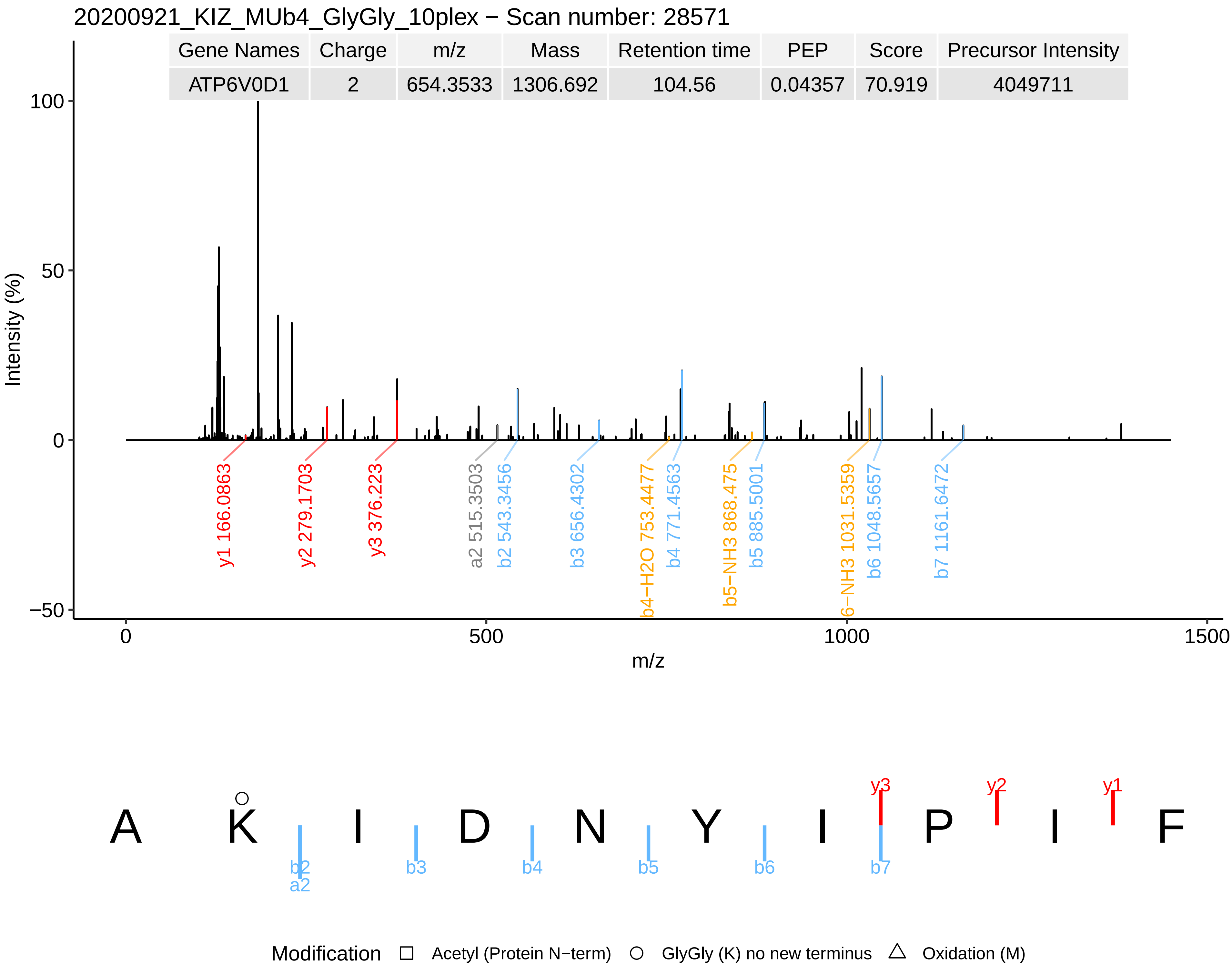

20200921_KIZ_MUb4_GlyGly_10plex − Scan number: 28571
Gene Names | Charge | m/z | Mass | Retention time | PEP | Score | Precursor Intensity
ATP6V0D1 | 2 | 654.3533 | 1306.692 | 104.56 | 0.04357 | 70.919 | 4049711
Intensity (%)
m/z
y1 166.0863
y2 279.1703
y3 376.223
a2 515.3503
b2 543.3456
b3 656.4302
b4−H2O 753.4477
b4 771.4563
b5−NH3 868.475
b5 885.5001
b6−NH3 1031.5359
b6 1048.5657
b7 1161.6472
A K I D N Y I P I F
Modification
Acetyl (Protein N−term)
GlyGly (K) no new terminus
Oxidation (M)

20200924_KIZ_MUb5_GlyGly_1 − Scan number: 29167

Gene Names	Charge	m/z	Mass	Retention time	PEP	Score	Precursor Intensity
ATP6V0D1	2	654.3533	1306.692	93.09	0.0077281	83.265	23151376

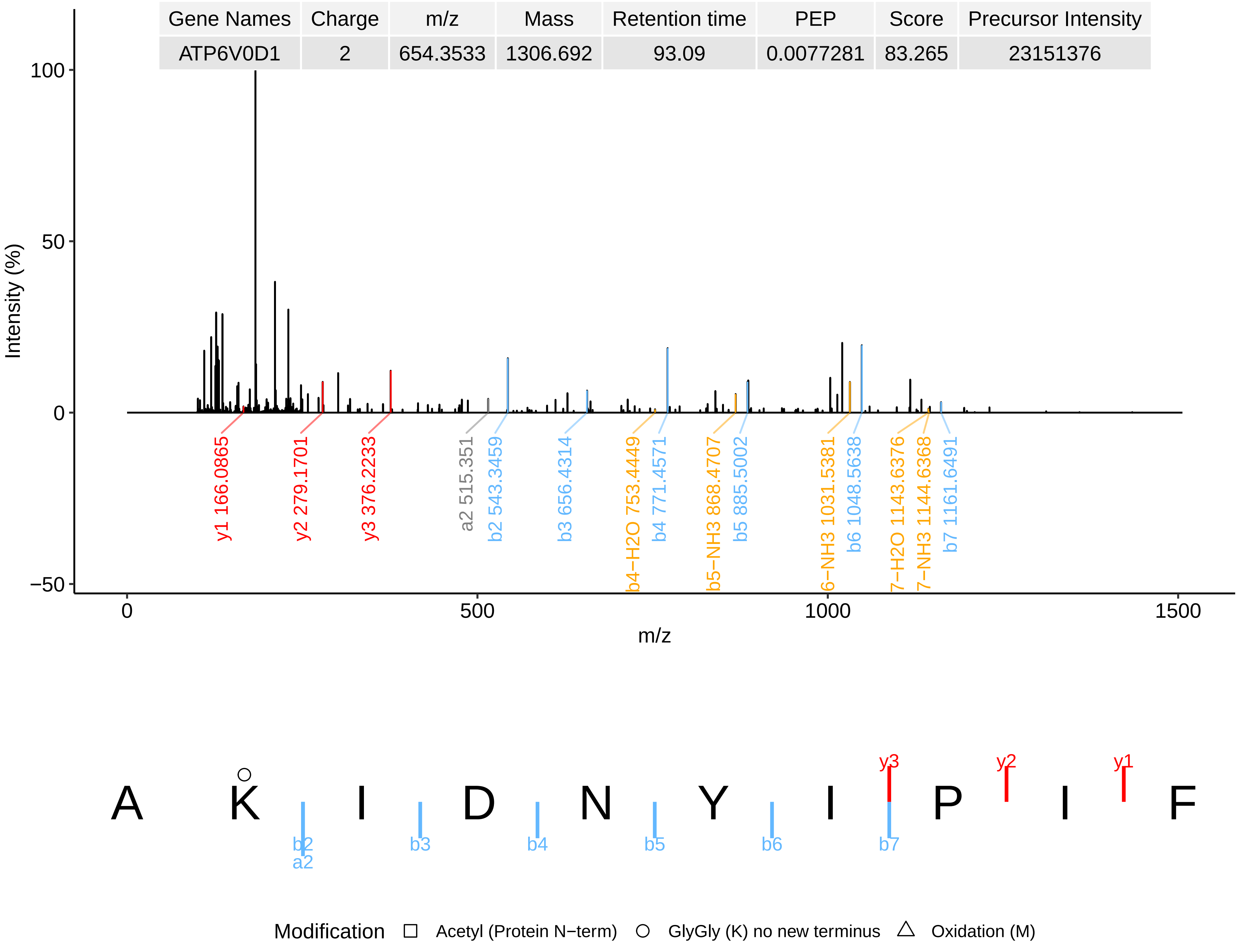

20201118_KIZ_MUb6_GlyGly_2 − Scan number: 31237

Gene Names	Charge	m/z	Mass	Retention time	PEP	Score	Precursor Intensity
ATP6V0D1	2	654.3533	1306.692	102.8	0.024539	86.833	5073967

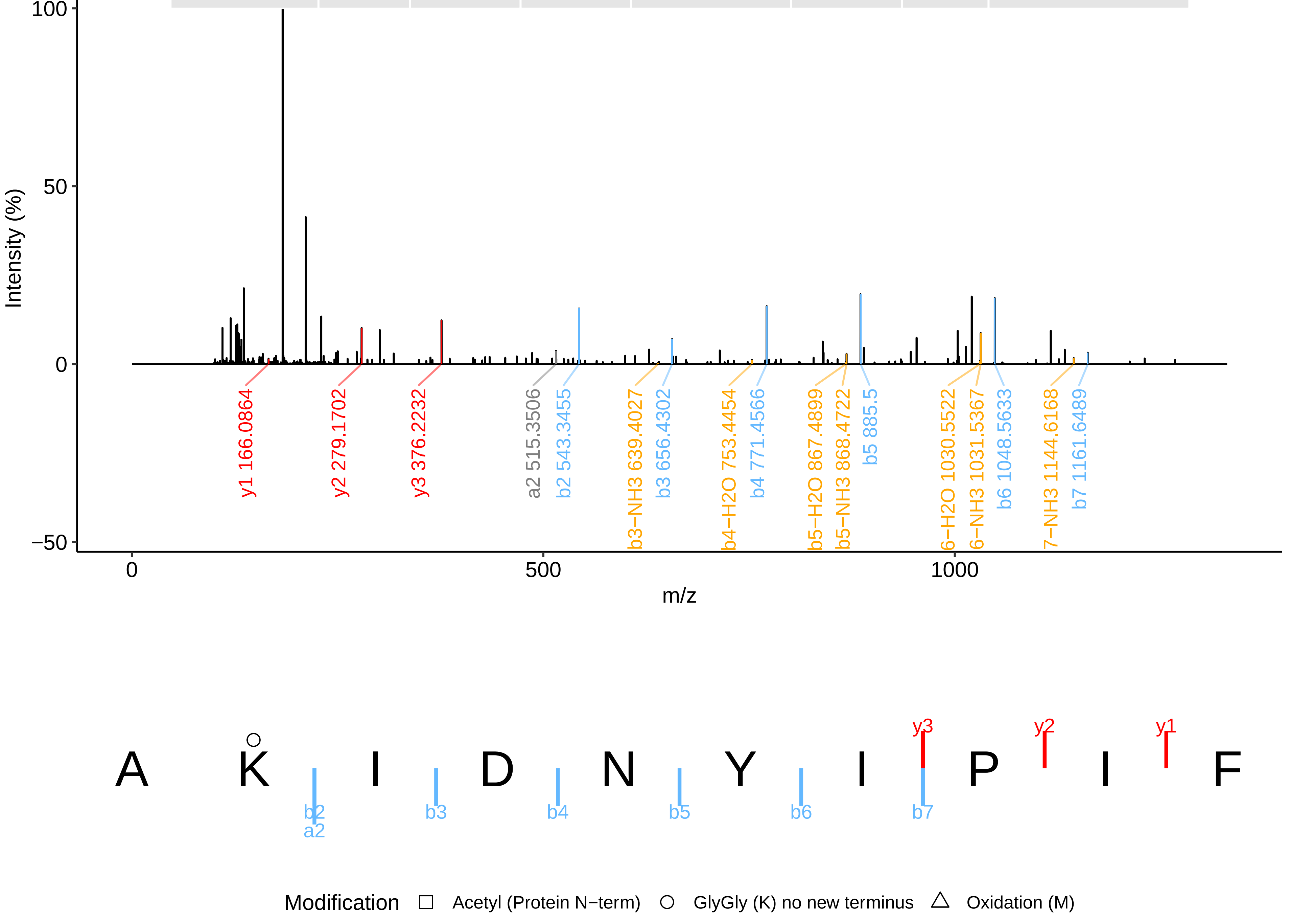

20200921_KIZ_MUb4_GlyGly_10plex − Scan number: 4103

Gene Names	Charge	m/z	Mass	Retention time	PEP	Score	Precursor Intensity
MP68	3	424.2265	1269.658	13.354	0.015669	62.633	5660331

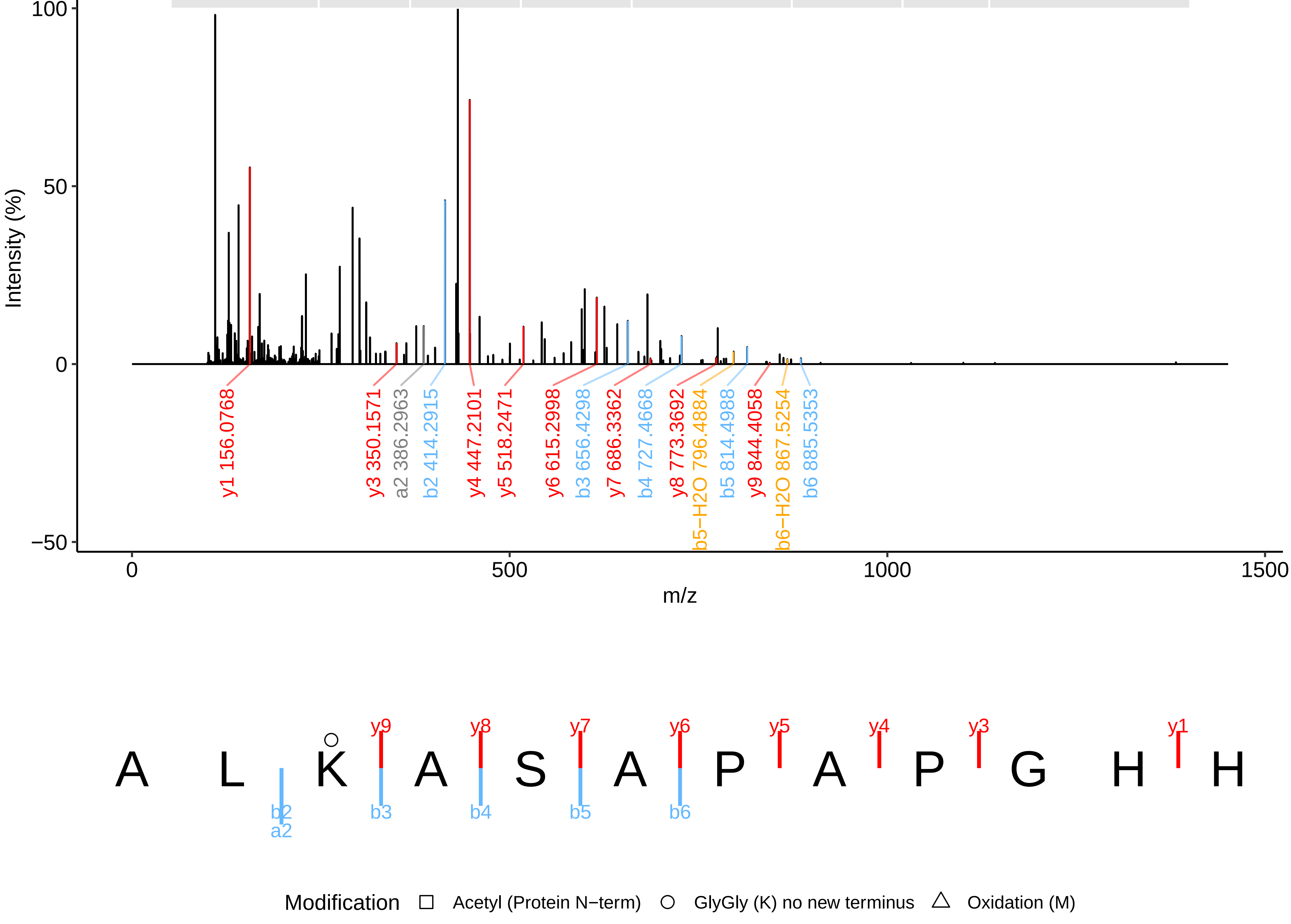

20200921_KIZ_MUb4_GlyGly_10plex − Scan number: 4130

Gene Names	Charge	m/z	Mass	Retention time	PEP	Score	Precursor Intensity
MP68	4	318.4217	1269.658	13.434	0.091514	43.958	22620294

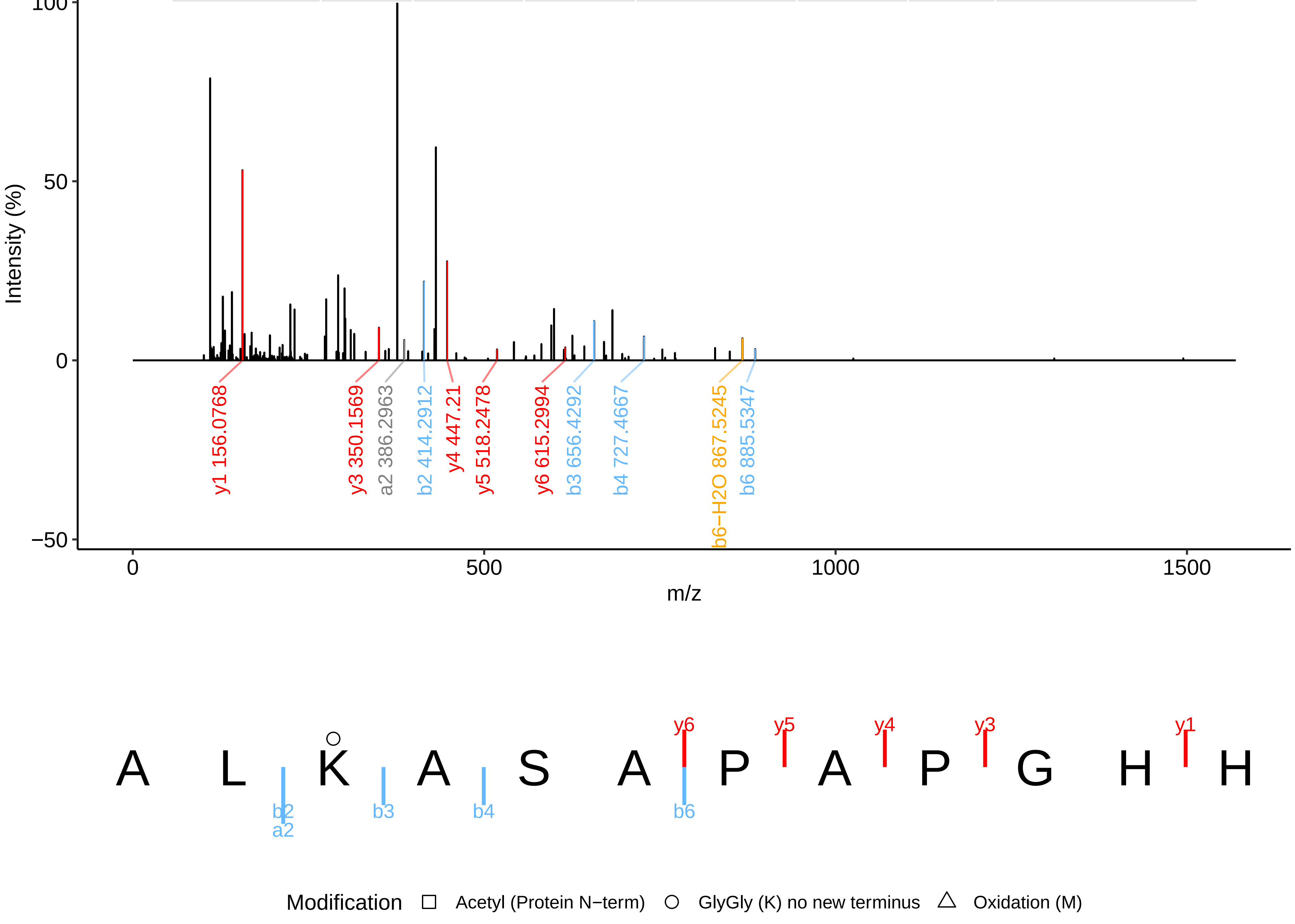

20200921_KIZ_MUb4_GlyGly_10plex_2 − Scan number: 3842

Gene Names	Charge	m/z	Mass	Retention time	PEP	Score	Precursor Intensity
MP68	4	318.4217	1269.658	13.168	0.022704	60.342	3443297

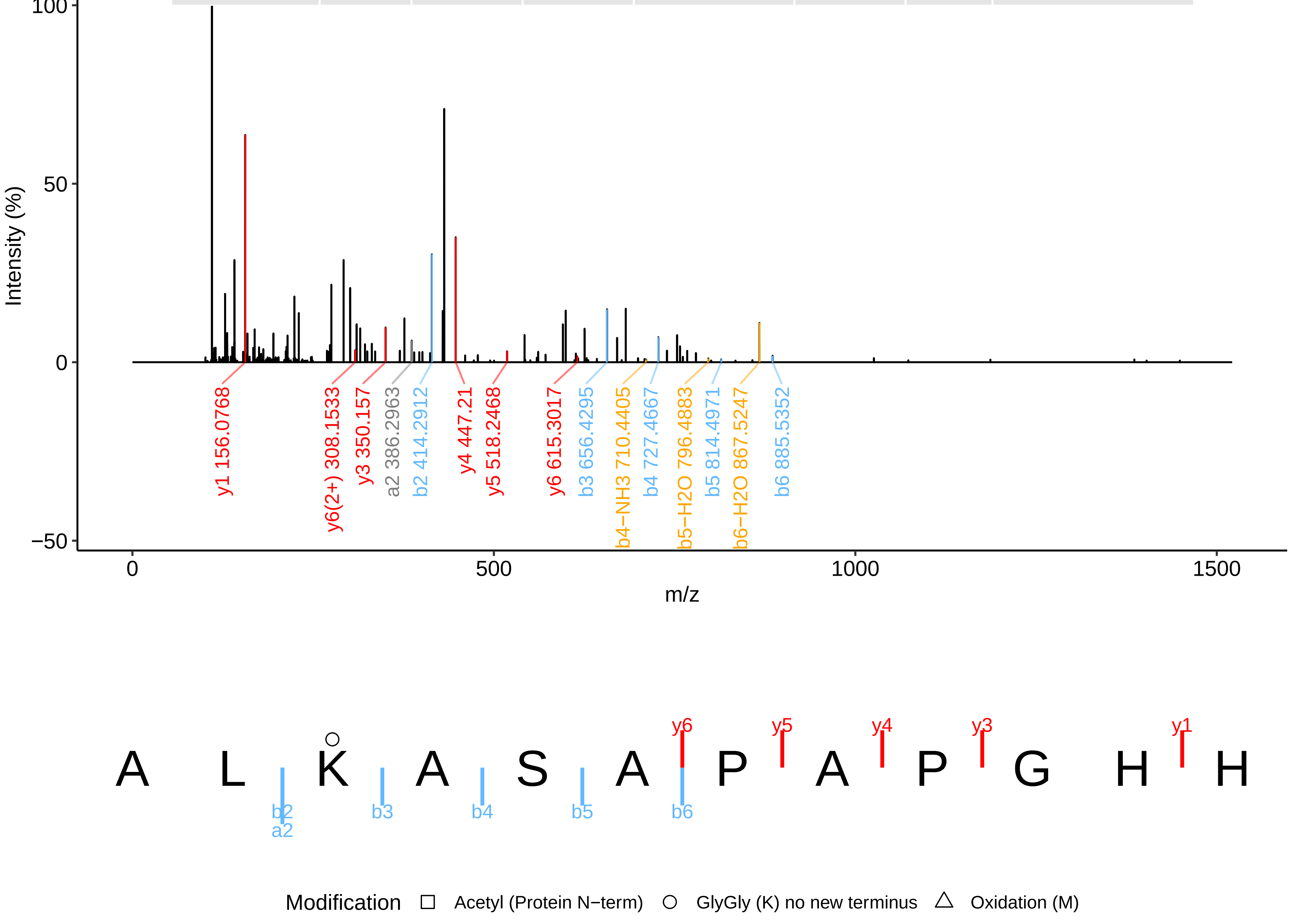

20200921_KIZ_MUb4_GlyGly_10plex_2 − Scan number: 3848

Gene Names	Charge	m/z	Mass	Retention time	PEP	Score	Precursor Intensity
MP68	3	424.2265	1269.658	13.192	0.010575	66.595	3064359

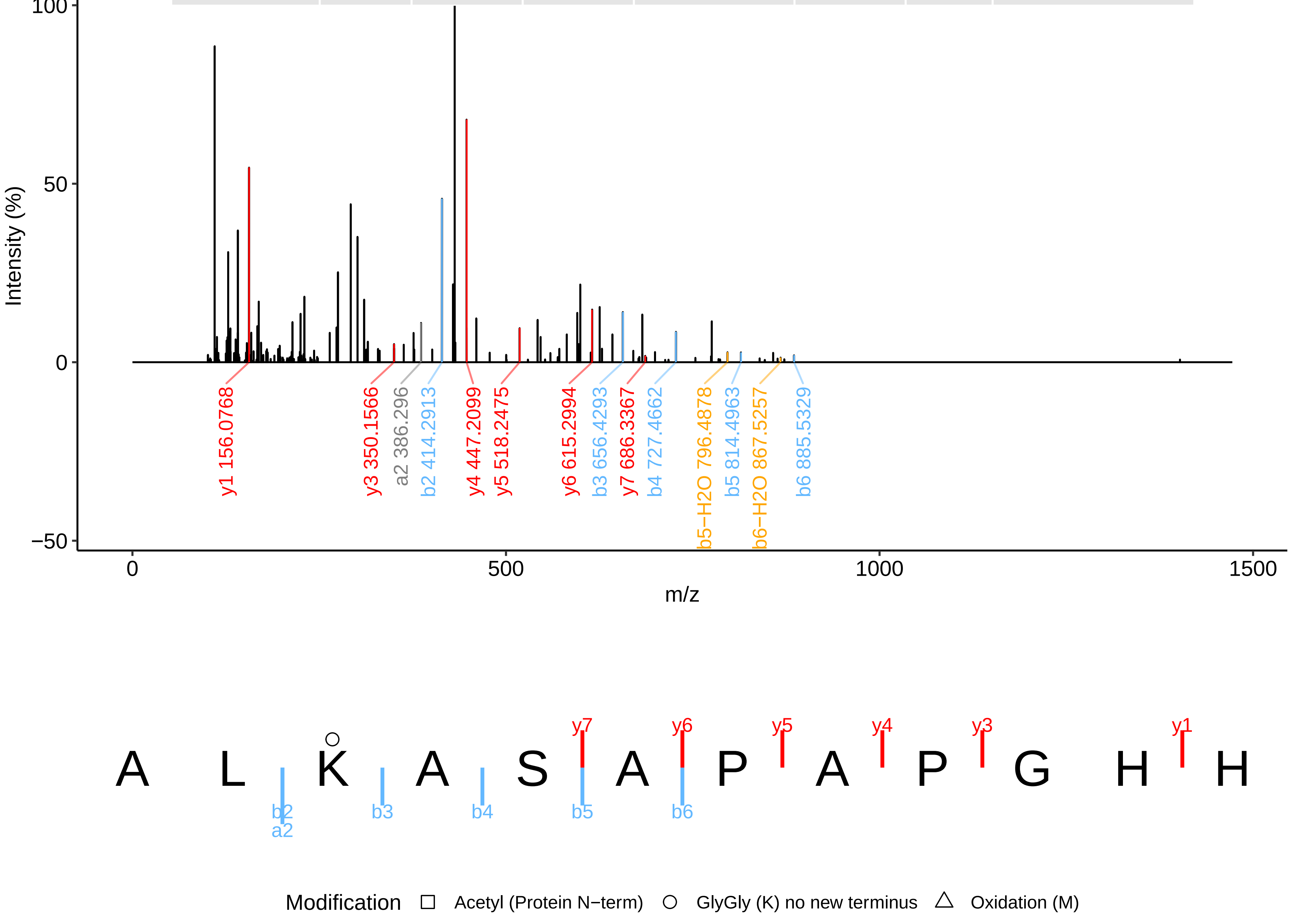

20200924_KIZ_MUb5_GlyGly_1 − Scan number: 3547

Gene Names	Charge	m/z	Mass	Retention time	PEP	Score	Precursor Intensity
MP68	3	424.2265	1269.658	10.851	0.056503	57.174	9312634

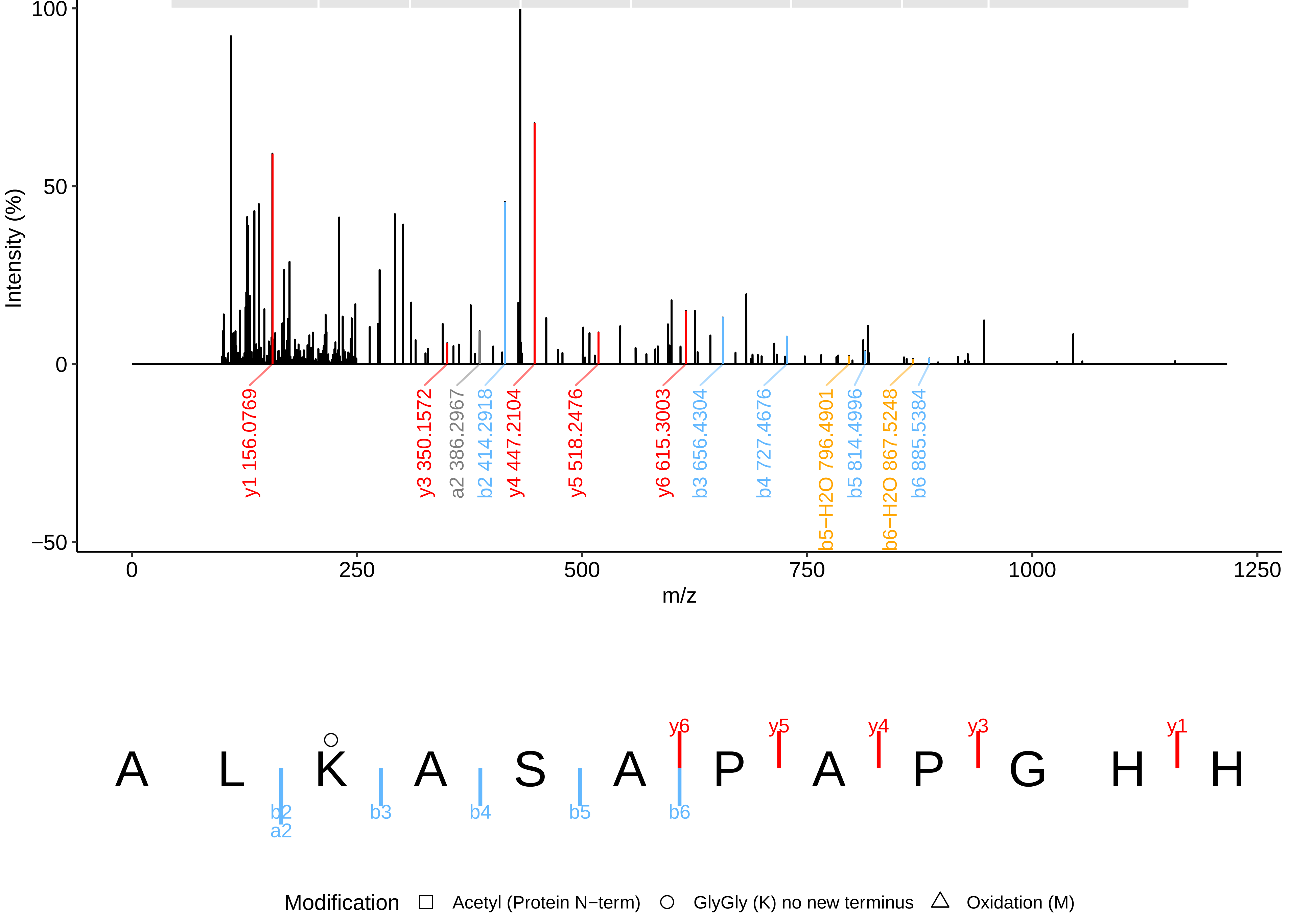

20201118_KIZ_MUb6_GlyGly_1 − Scan number: 3385

Gene Names	Charge	m/z	Mass	Retention time	PEP	Score	Precursor Intensity
MP68	4	318.4217	1269.658	10.396	0.091514	43.958	21207784

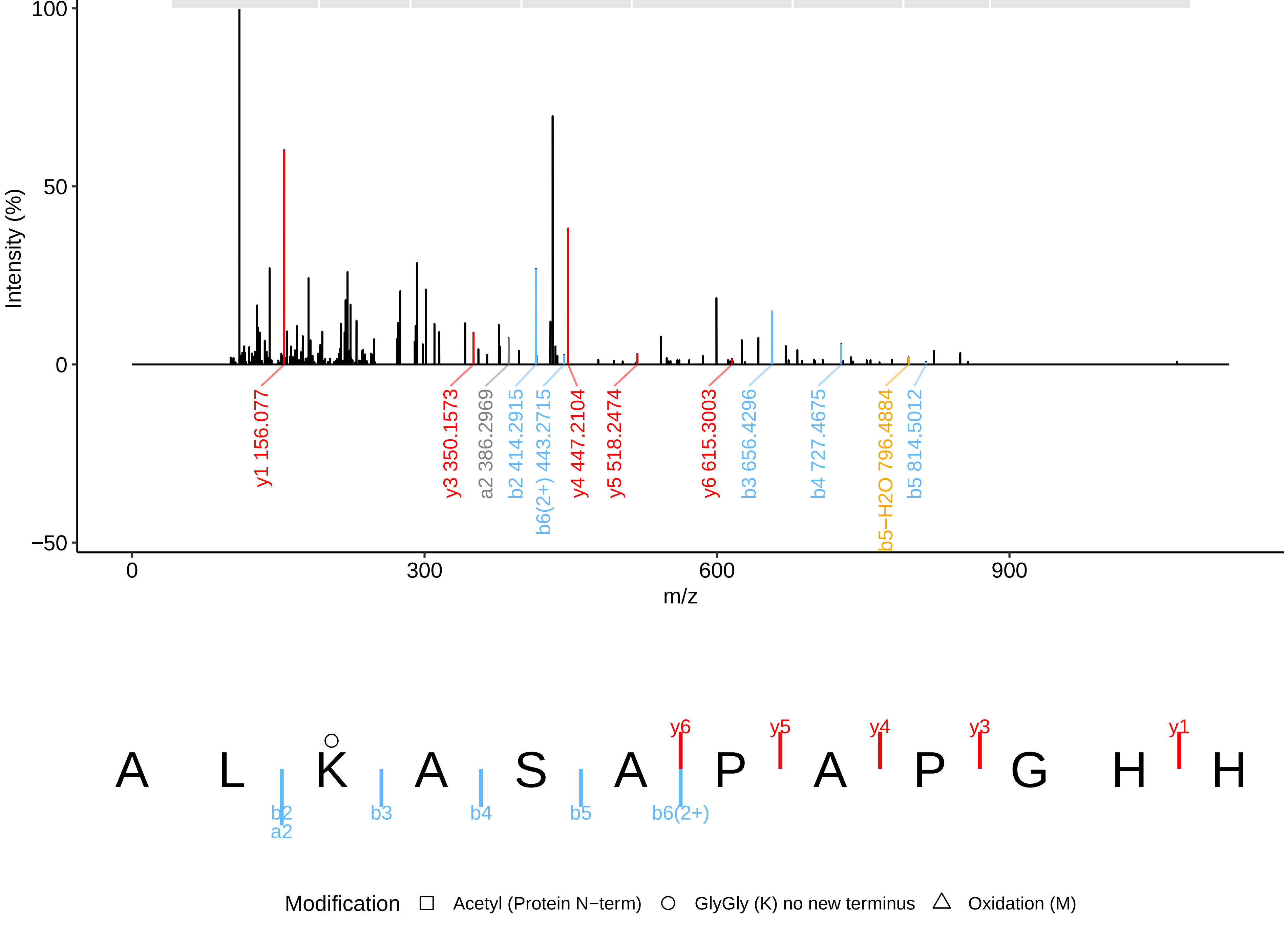

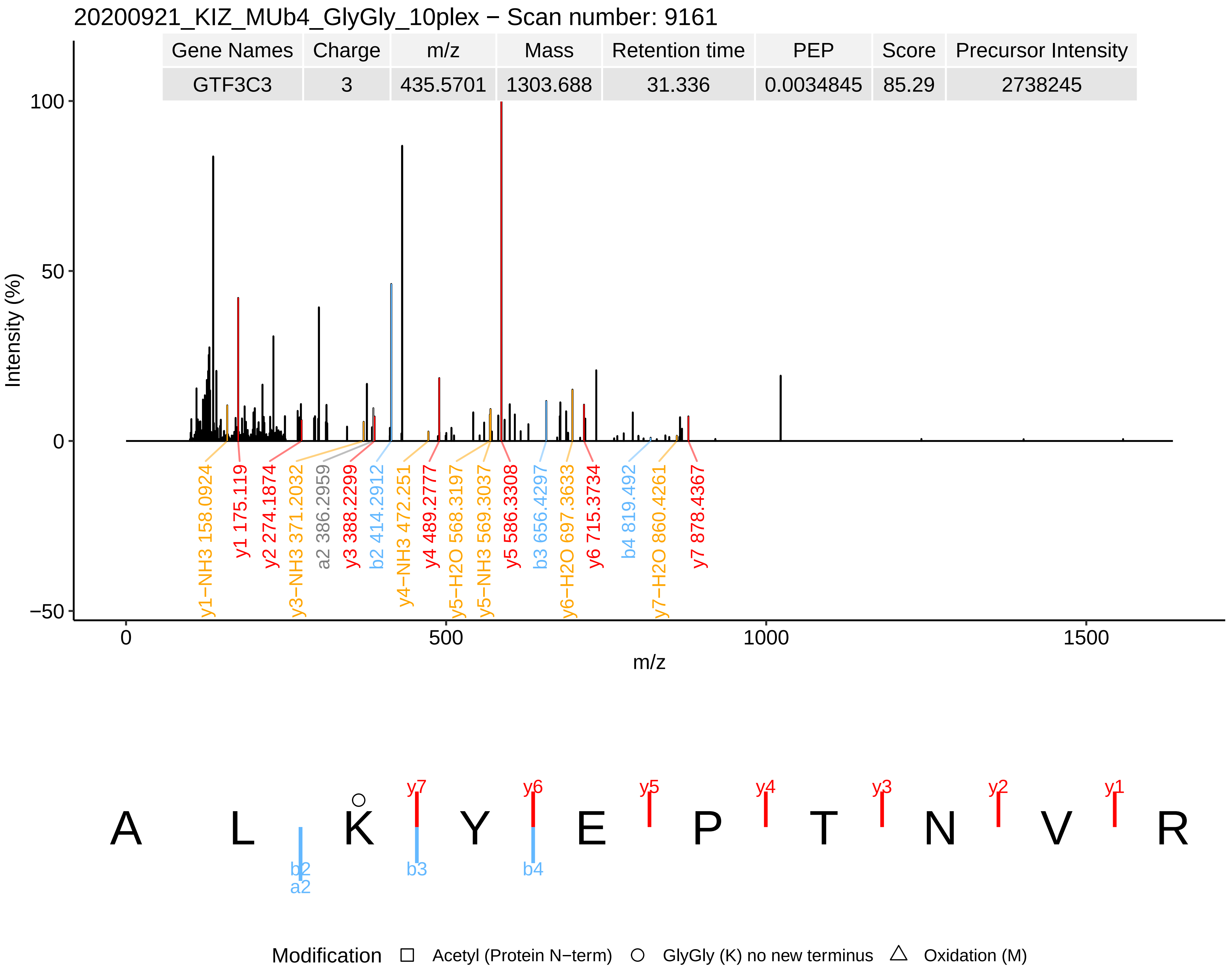
20200921_KIZ_MUb4_GlyGly_10plex − Scan number: 9161
Gene Names
Charge
m/z
Mass
Retention time
PEP
Score
Precursor Intensity
GTF3C3
3
435.5701
1303.688
31.336
0.0034845
85.29
2738245
Intensity (%)
100
50
0
−50
0
500
1000
1500
m/z
y1−NH3 158.0924
y1 175.119
y2 274.1874
y3−NH3 371.2032
a2 386.2959
y3 388.2299
b2 414.2912
y4−NH3 472.251
y4 489.2777
y5−H2O 568.3197
y5−NH3 569.3037
y5 586.3308
b3 656.4297
y6−H2O 697.3633
y6 715.3734
b4 819.492
y7−H2O 860.4261
y7 878.4367
A L K Y E P T N V R
y7 y6 y5 y4 y3 y2 y1
b2 a2 b3 b4
Modification
Acetyl (Protein N−term)
GlyGly (K) no new terminus
Oxidation (M)

20200921_KIZ_MUb4_GlyGly_10plex_2 − Scan number: 16523

Gene Names	Charge	m/z	Mass	Retention time	PEP	Score	Precursor Intensity
ATXN10	3	430.9103	1289.709	64.507	0.0012117	90.15	960662.9

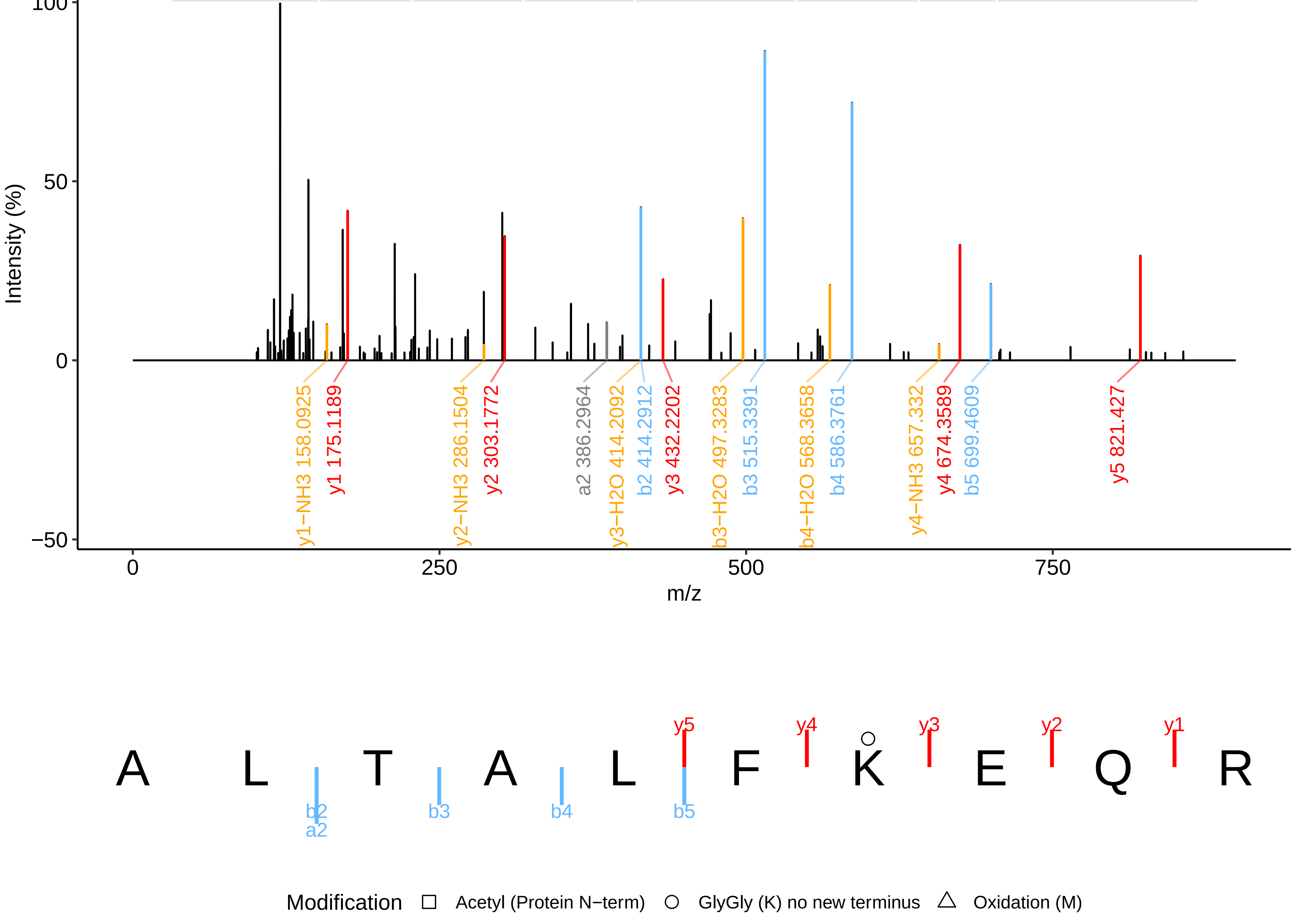

20200921_KIZ_MUb4_GlyGly_10plex − Scan number: 13237

Gene Names	Charge	m/z	Mass	Retention time	PEP	Score	Precursor Intensity
FAM129A	3	424.9139	1271.72	46.633	0.00027481	95.618	4963838

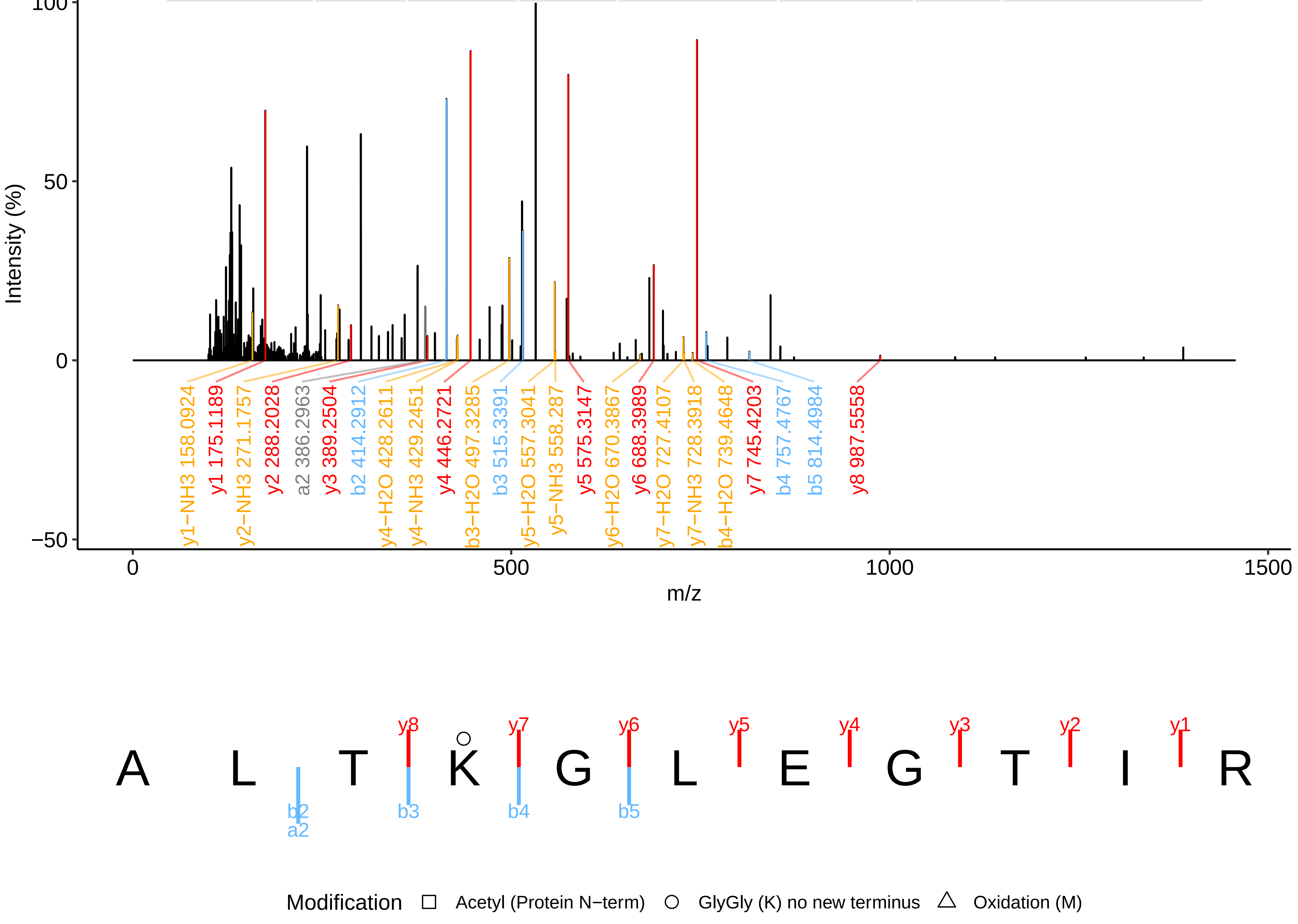

20200921_KIZ_MUb4_GlyGly_10plex − Scan number: 14925

Gene Names	Charge	m/z	Mass	Retention time	PEP	Score	Precursor Intensity
SLC35B2	2	633.8563	1265.698	53.17	0.00066885	98.458	10968003

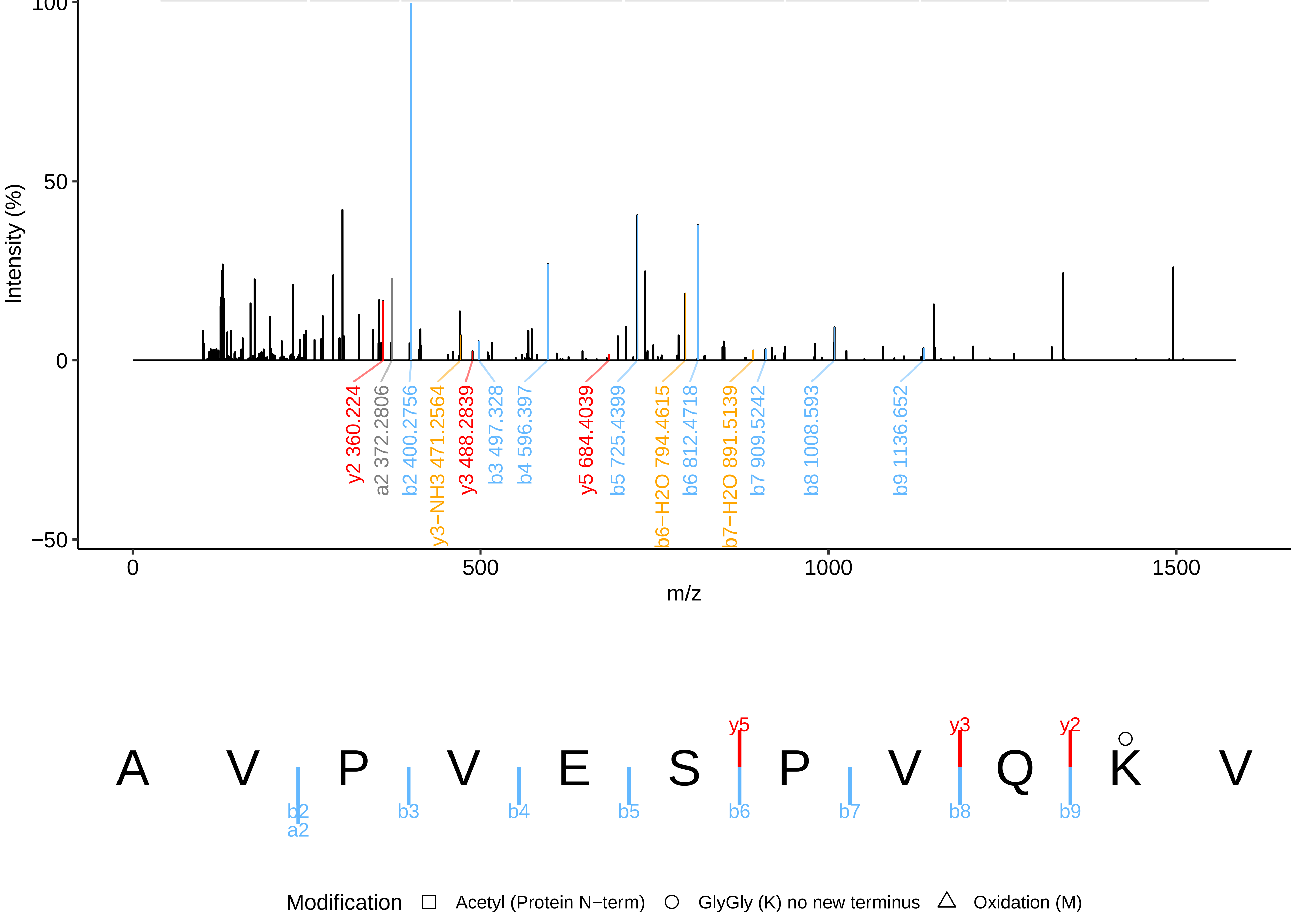

20200921_KIZ_MUb4_GlyGly_10plex_2 − Scan number: 13701

Gene Names	Charge	m/z	Mass	Retention time	PEP	Score	Precursor Intensity
SLC35B2	2	633.8563	1265.698	53.249	0.019566	85.212	1489630

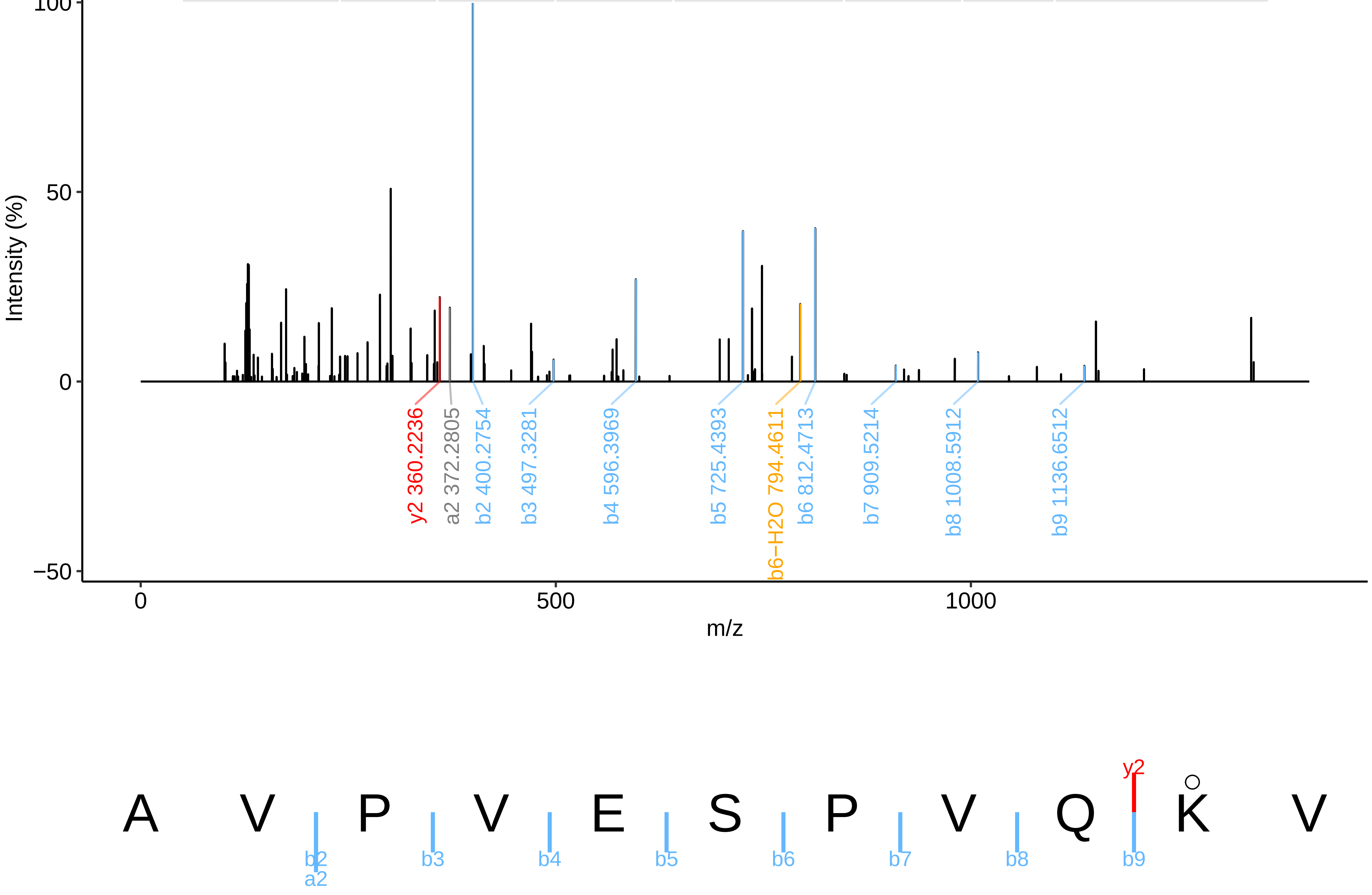

Modification □ Acetyl (Protein N−term) ○ GlyGly (K) no new terminus △ Oxidation (M)

20200924_KIZ_MUb5_GlyGly_1 − Scan number: 14262

Gene Names	Charge	m/z	Mass	Retention time	PEP	Score	Precursor Intensity
SLC35B2	2	633.8563	1265.698	46.559	0.00037004	106.04	10792902

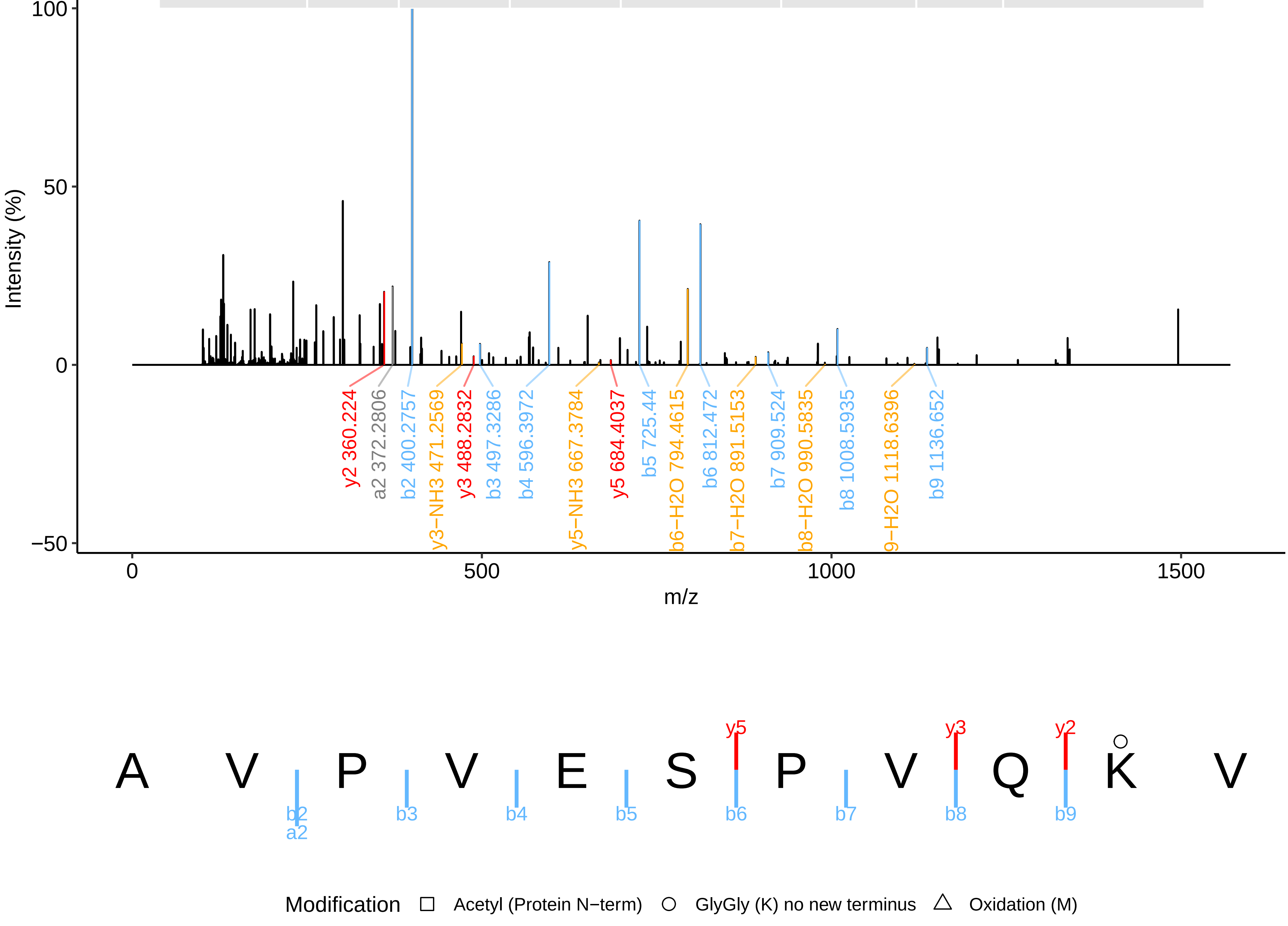

20200924_KIZ_MUb5_GlyGly_2 − Scan number: 13199

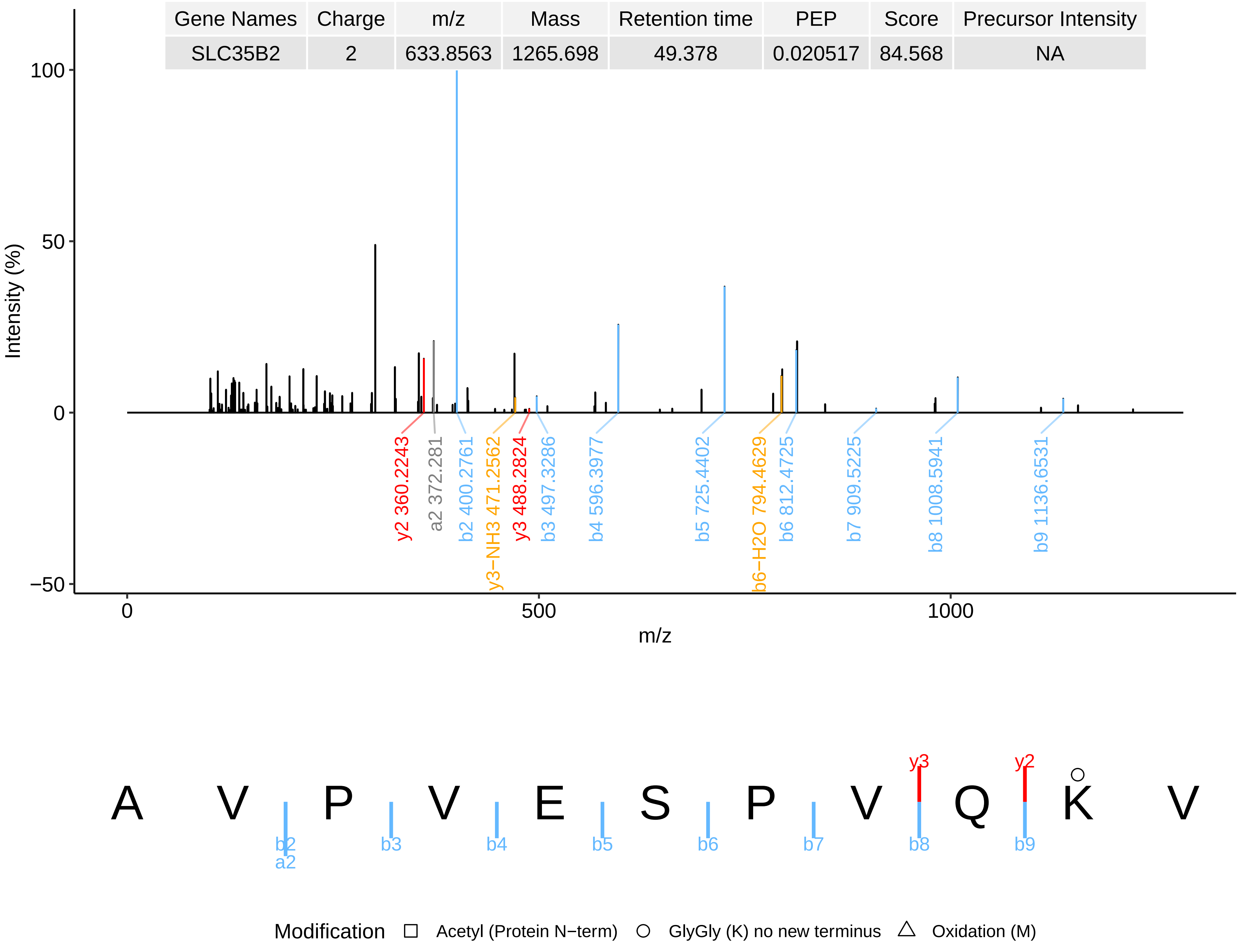

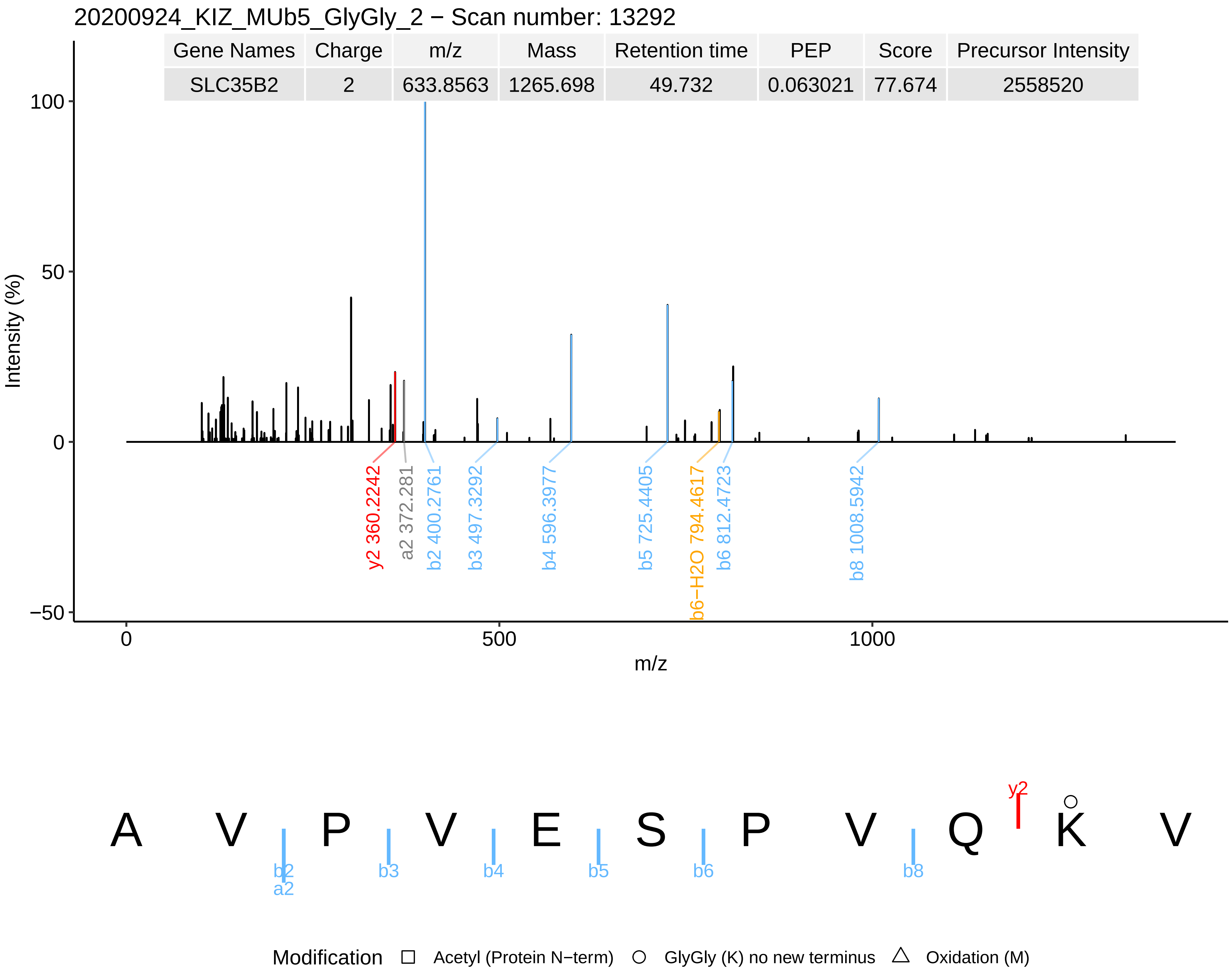
20200924_KIZ_MUb5_GlyGly_2 − Scan number: 13292
Gene Names | Charge | m/z | Mass | Retention time | PEP | Score | Precursor Intensity
SLC35B2 | 2 | 633.8563 | 1265.698 | 49.732 | 0.063021 | 77.674 | 2558520
Intensity (%)
100
50
0
−50
y2 360.2242
a2 372.281
b2 400.2761
b3 497.3292
b4 596.3977
b5 725.4405
b6−H2O 794.4617
b6 812.4723
b8 1008.5942
0
500
1000
m/z
A V P V E S P V Q K V
y2
b2
a2
b3
b4
b5
b6
b8
Modification
Acetyl (Protein N−term)
GlyGly (K) no new terminus
Oxidation (M)

20201118_KIZ_MUb6_GlyGly_2 − Scan number: 15802

Gene Names	Charge	m/z	Mass	Retention time	PEP	Score	Precursor Intensity
SLC35B2	2	633.8563	1265.698	53.283	0.00079426	116.25	9640161

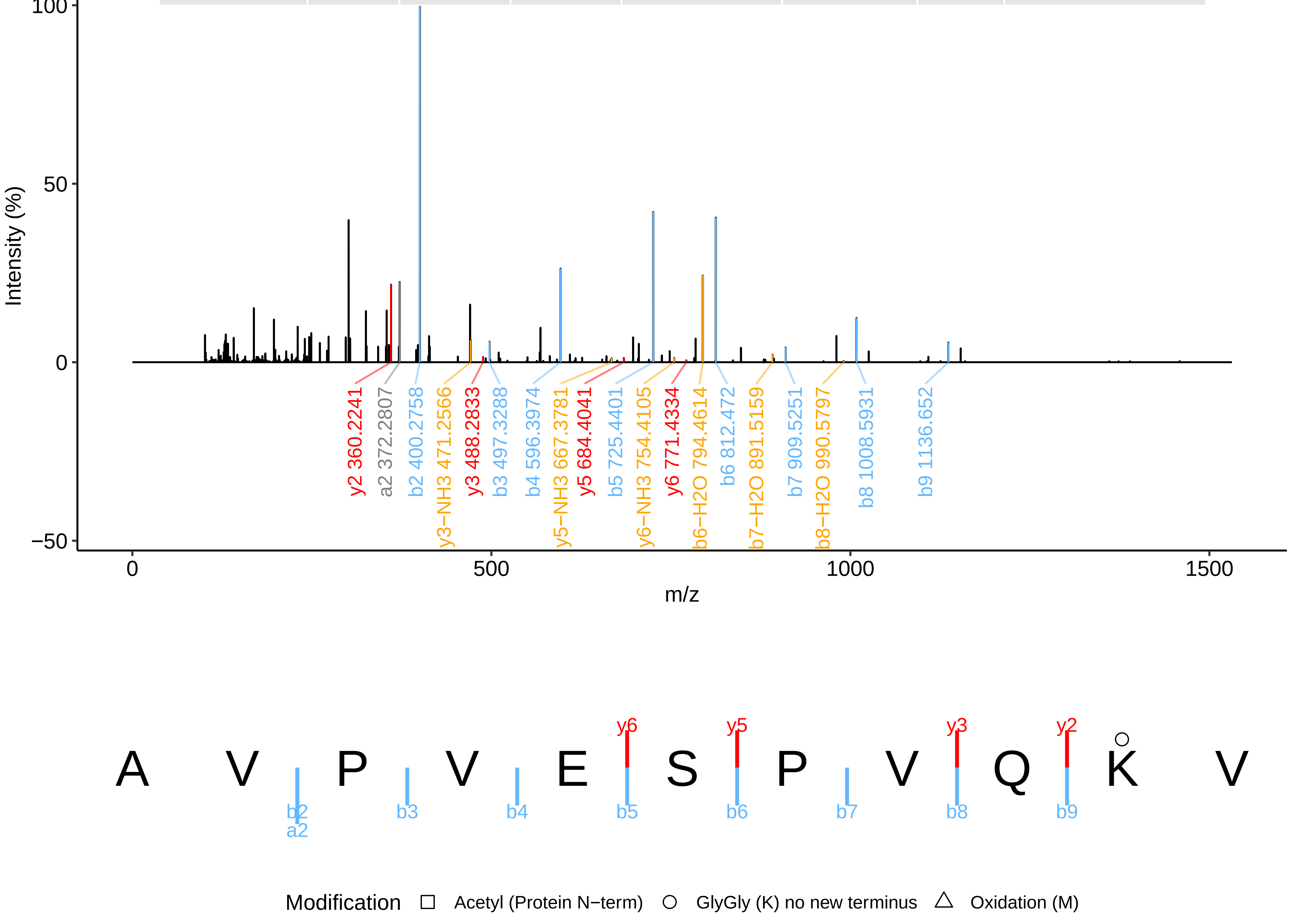

20200921_KIZ_MUb4_GlyGly_10plex − Scan number: 7636

Gene Names	Charge	m/z	Mass	Retention time	PEP	Score	Precursor Intensity
H2BFS;HIST1H2BK;HIST1H2BJ	3	361.5329	1081.577	25.69	0.0082329	76.17	11511504

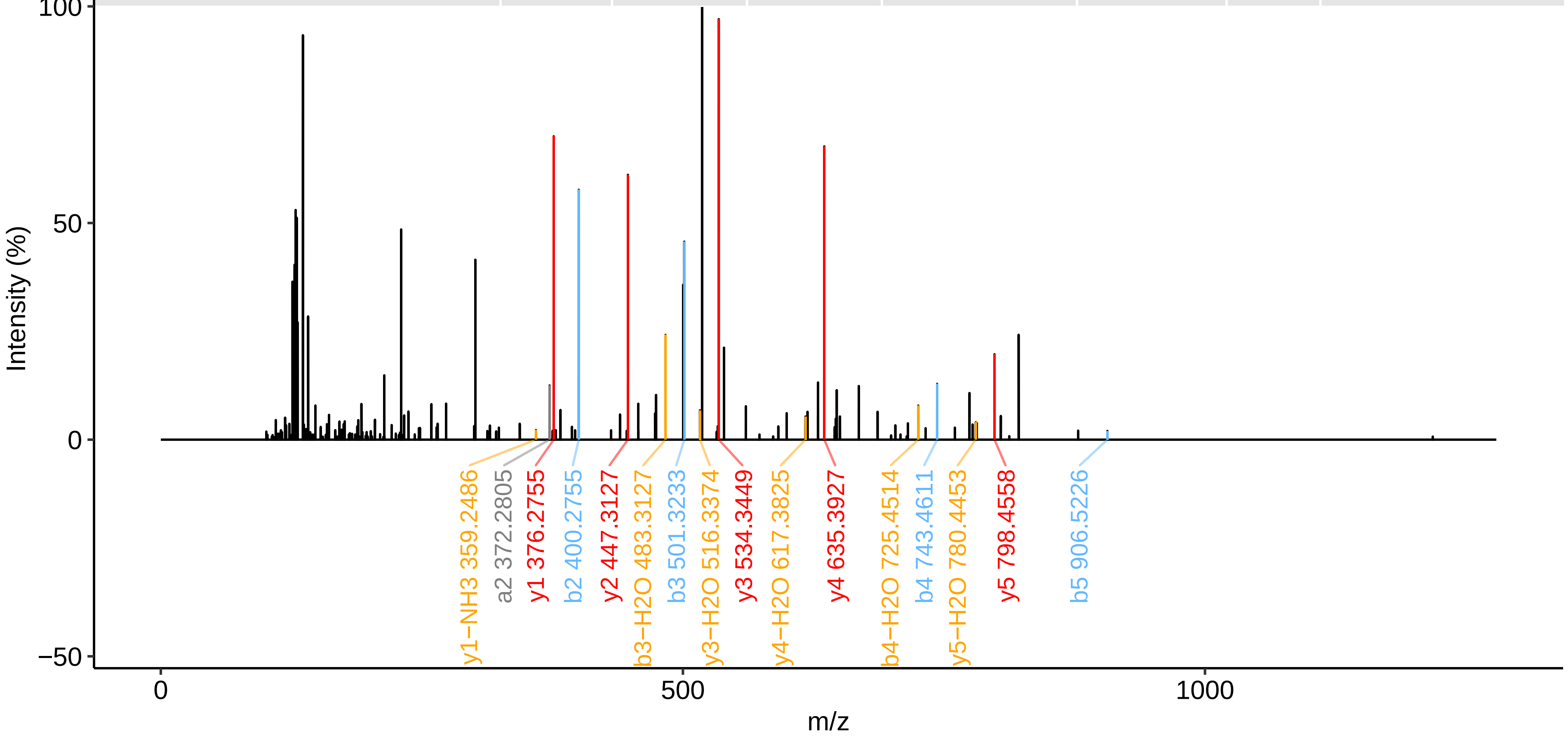

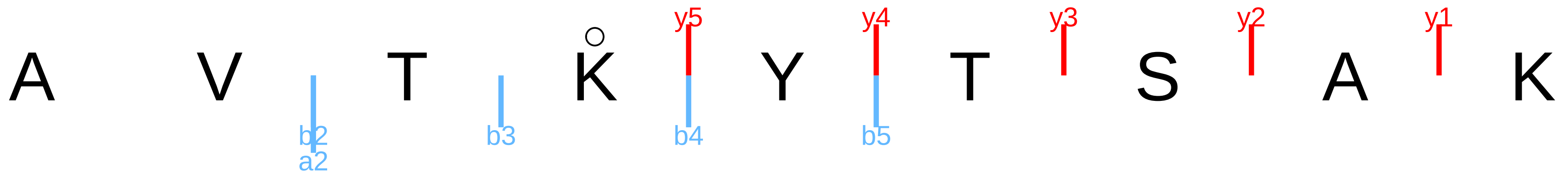

Modification □ Acetyl (Protein N−term) ○ GlyGly (K) no new terminus △ Oxidation (M)

20200921_KIZ_MUb4_GlyGly_10plex_2 − Scan number: 7054

Gene Names	Charge	m/z	Mass	Retention time	PEP	Score	Precursor Intensity
H2BFS;HIST1H2BK;HIST1H2BJ	3	361.5329	1081.577	26.188	0.00066749	97.431	936785.6

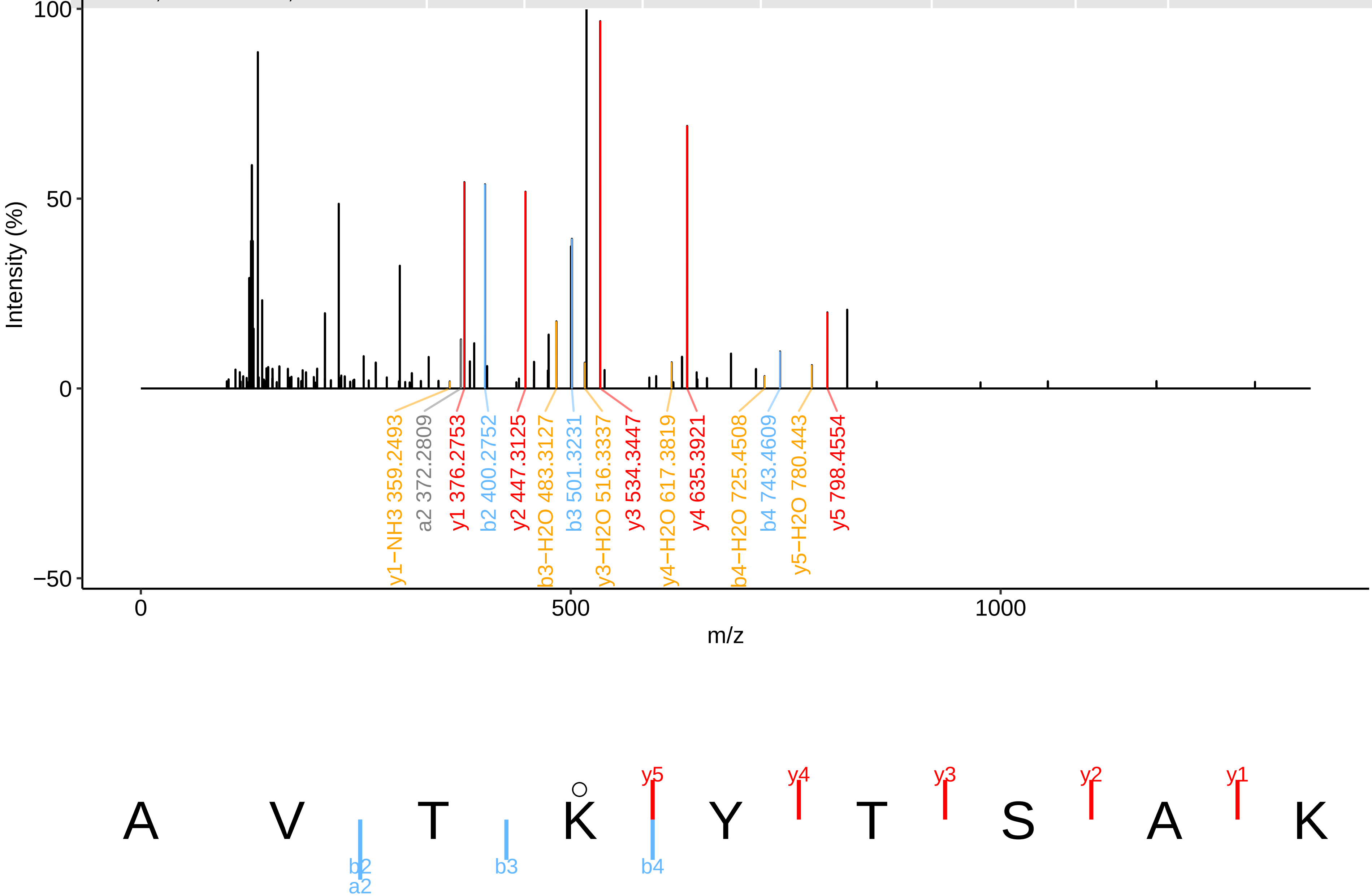

Modification □ Acetyl (Protein N−term) ○ GlyGly (K) no new terminus △ Oxidation (M)

20200921_KIZ_MUb4_GlyGly_10plex_2 − Scan number: 7087

Gene Names	Charge	m/z	Mass	Retention time	PEP	Score	Precursor Intensity
H2BFS;HIST1H2BK;HIST1H2BJ	2	541.7957	1081.577	26.319	6.0768e−07	142.43	1075833

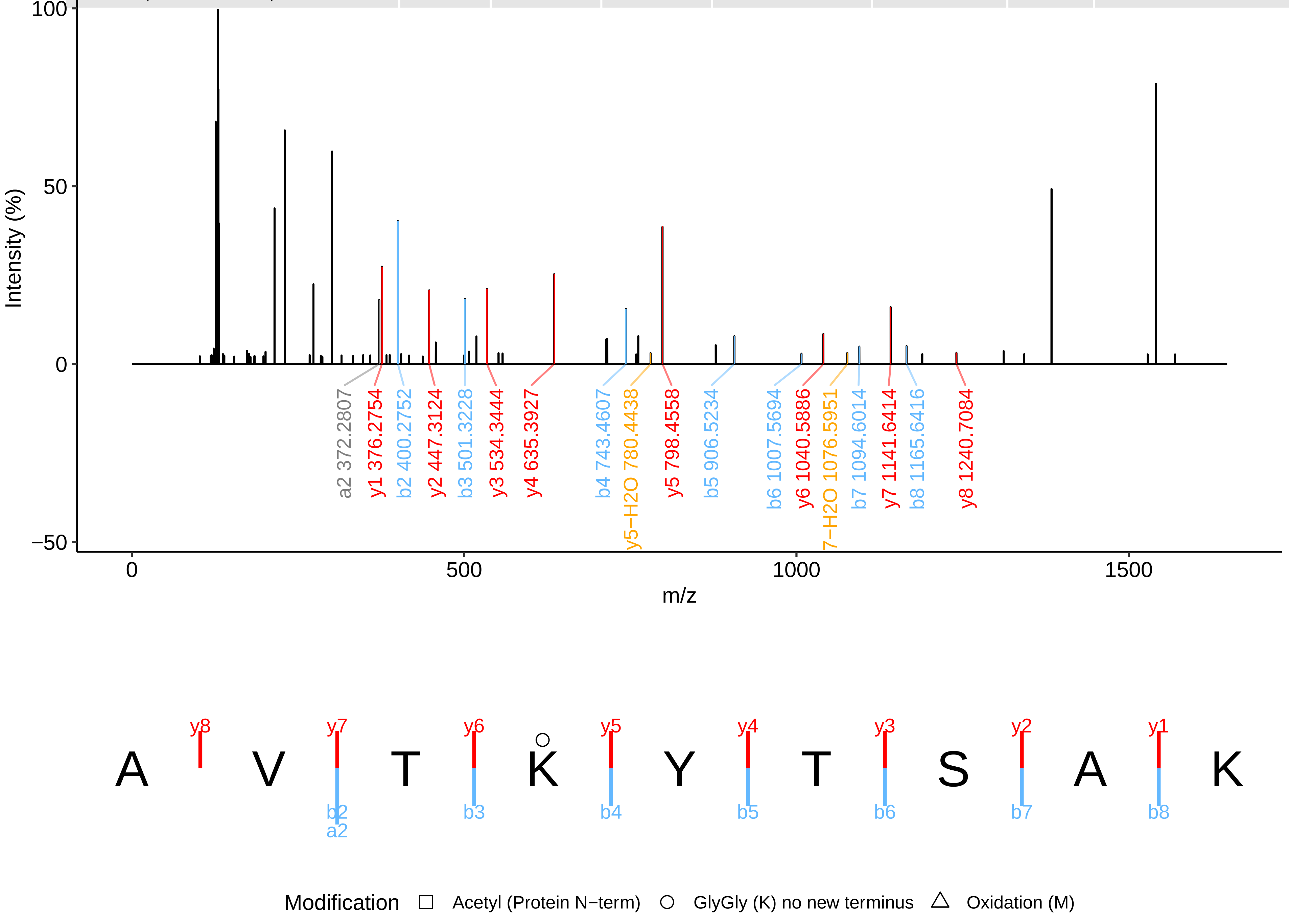

20200924_KIZ_MUb5_GlyGly_1 − Scan number: 6513

Gene Names	Charge	m/z	Mass	Retention time	PEP	Score	Precursor Intensity
H2BFS;HIST1H2BK;HIST1H2BJ	3	361.5329	1081.577	20.486	0.016539	68.371	11487831

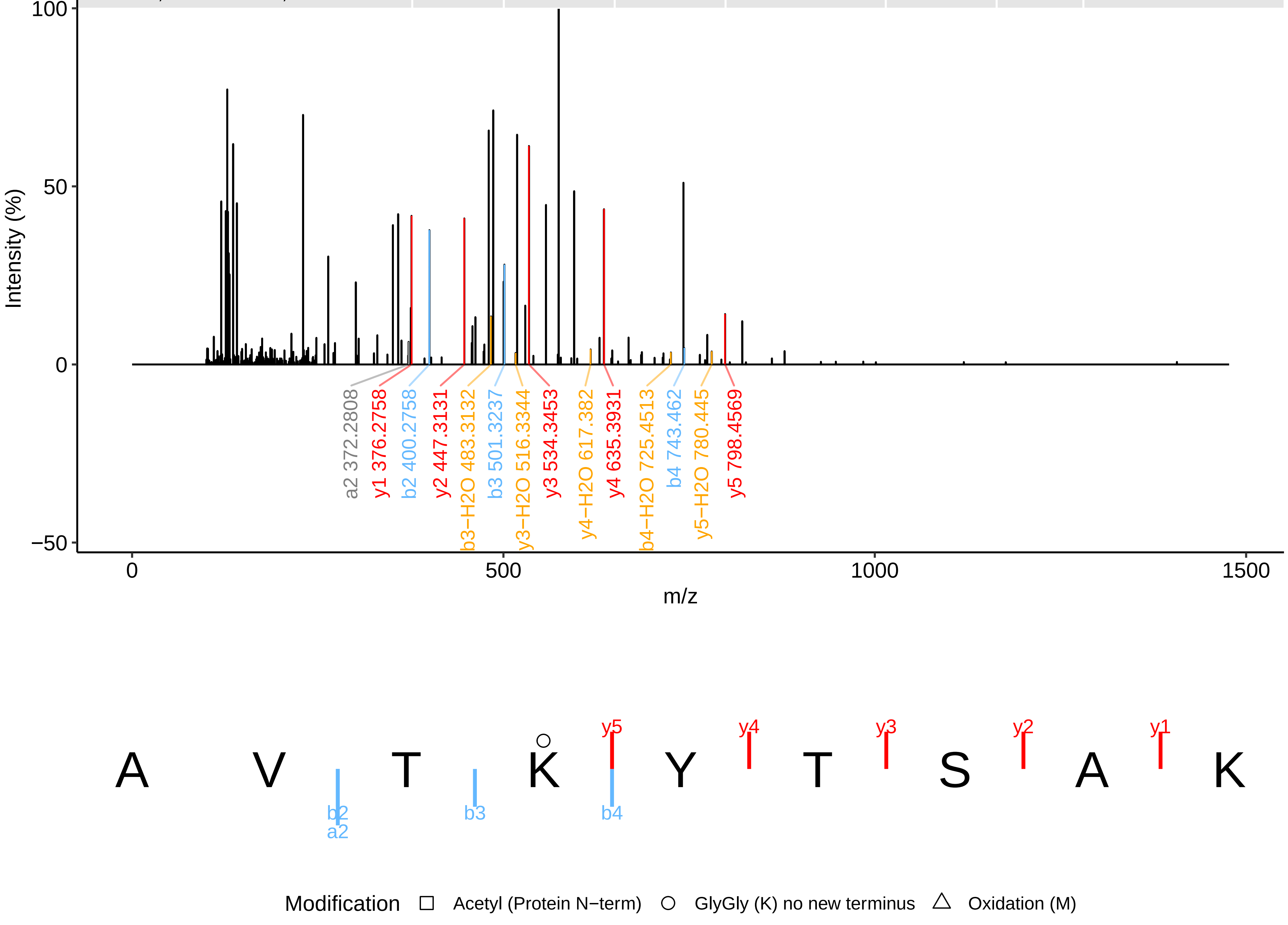

20200924_KIZ_MUb5_GlyGly_1 − Scan number: 6659

Gene Names	Charge	m/z	Mass	Retention time	PEP	Score	Precursor Intensity
H2BFS;HIST1H2BK;HIST1H2BJ	3	361.5329	1081.577	21	0.013934	70.816	130230352

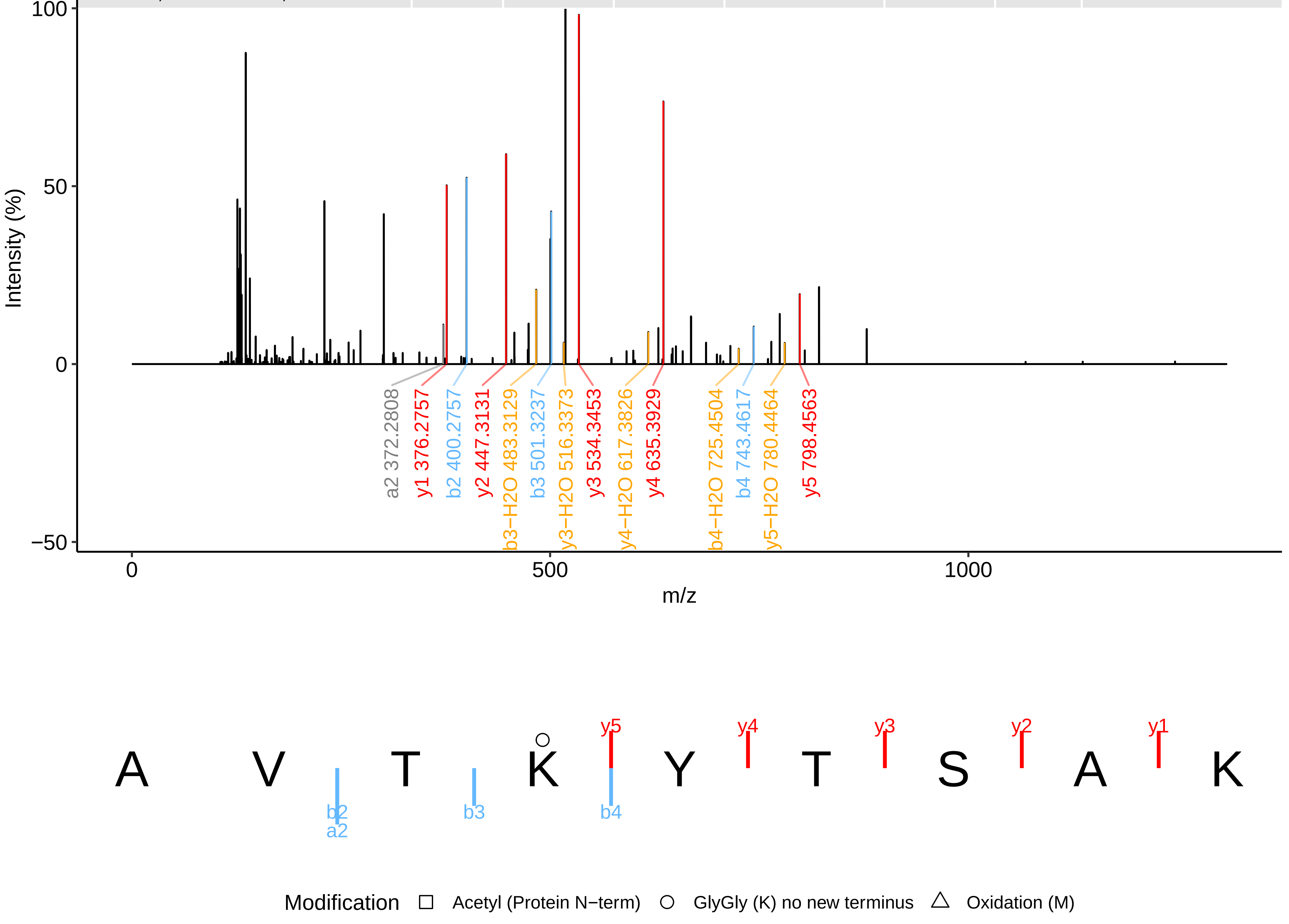

20200924_KIZ_MUb5_GlyGly_1 − Scan number: 6722

Gene Names	Charge	m/z	Mass	Retention time	PEP	Score	Precursor Intensity
H2BFS;HIST1H2BK;HIST1H2BJ	3	361.5329	1081.577	21.224	0.01036	74.173	9031199

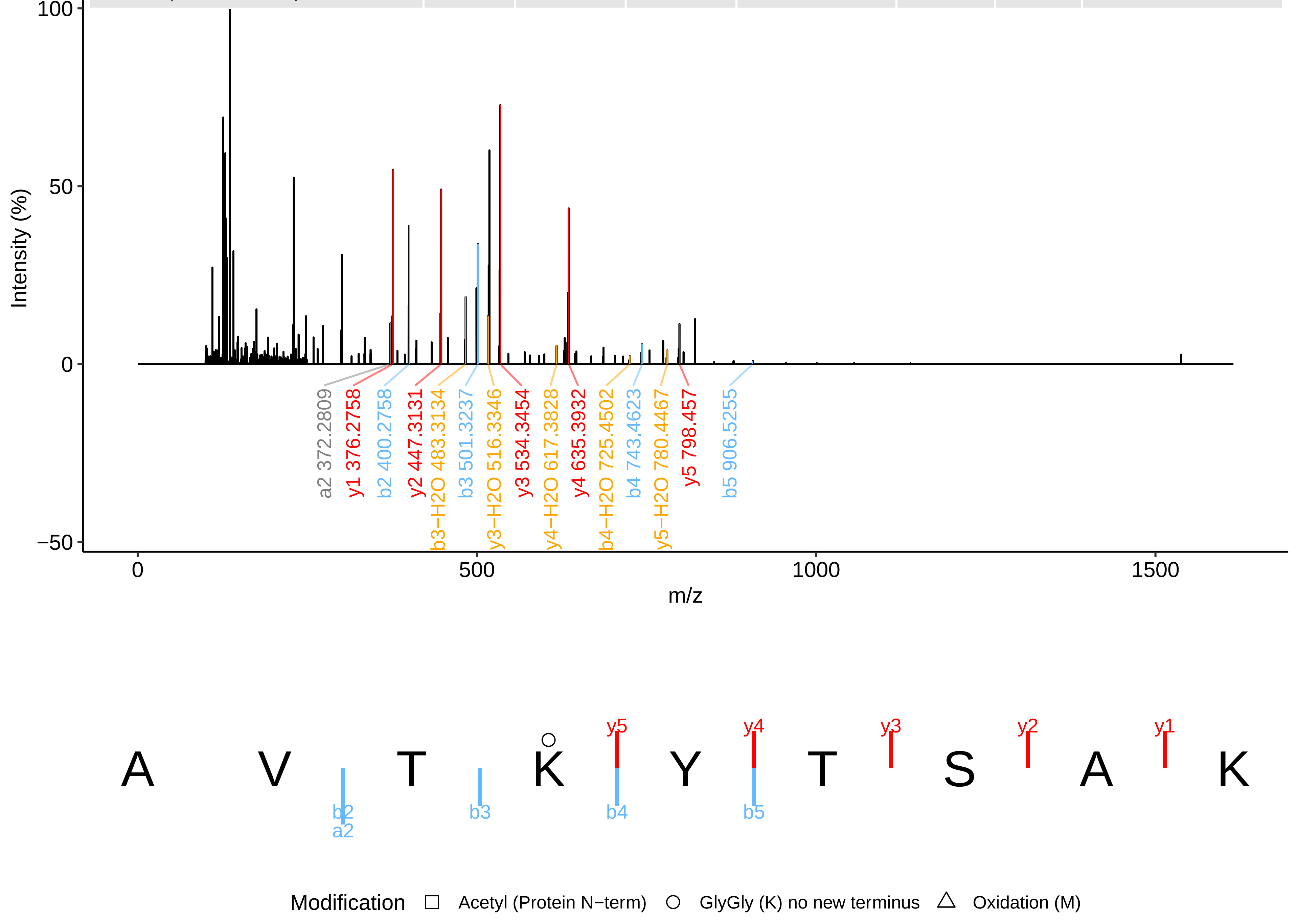

20200924_KIZ_MUb5_GlyGly_1 − Scan number: 6873

Gene Names	Charge	m/z	Mass	Retention time	PEP	Score	Precursor Intensity
H2BFS;HIST1H2BK;HIST1H2BJ	3	361.5329	1081.577	21.732	0.0061636	78.113	74921336

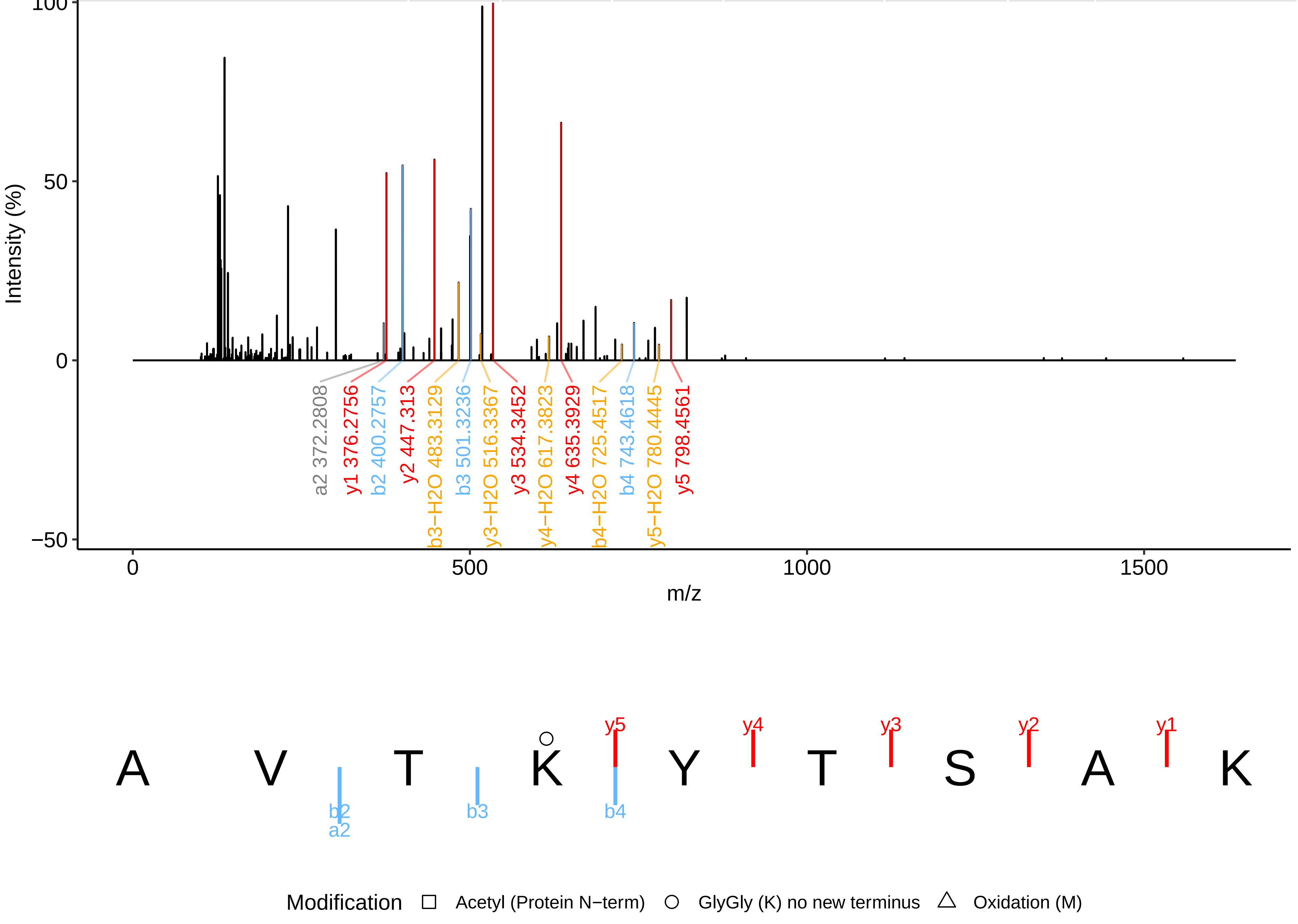

20200924_KIZ_MUb5_GlyGly_2 − Scan number: 6300

Gene Names	Charge	m/z	Mass	Retention time	PEP	Score	Precursor Intensity
H2BFS;HIST1H2BK;HIST1H2BJ	3	361.5329	1081.577	23.49	0.0029137	84.213	45956892

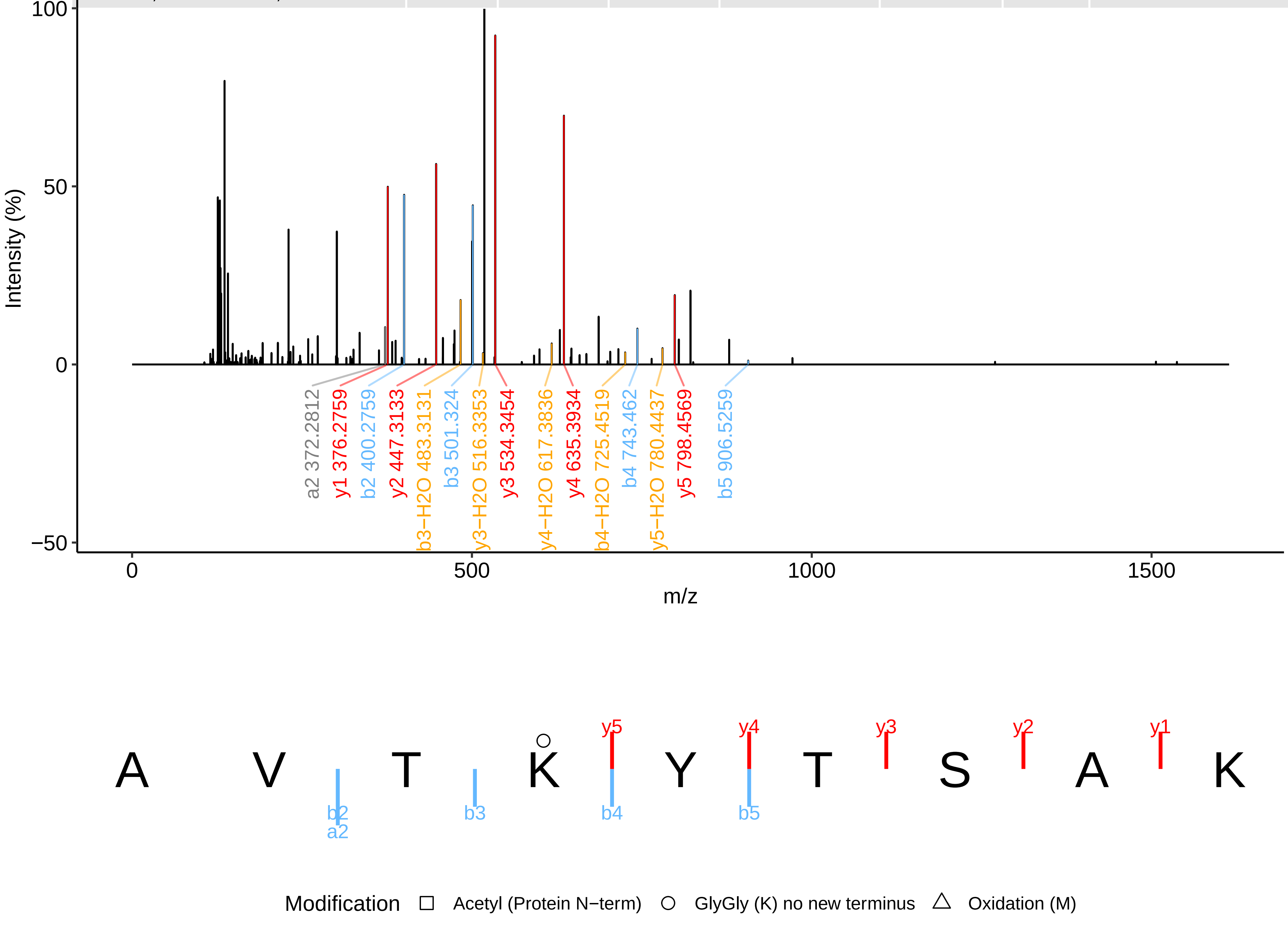

20200924_KIZ_MUb5_GlyGly_2 − Scan number: 6435

Gene Names	Charge	m/z	Mass	Retention time	PEP	Score	Precursor Intensity
H2BFS;HIST1H2BK;HIST1H2BJ	3	361.5329	1081.577	24	0.0013902	93.178	9339978

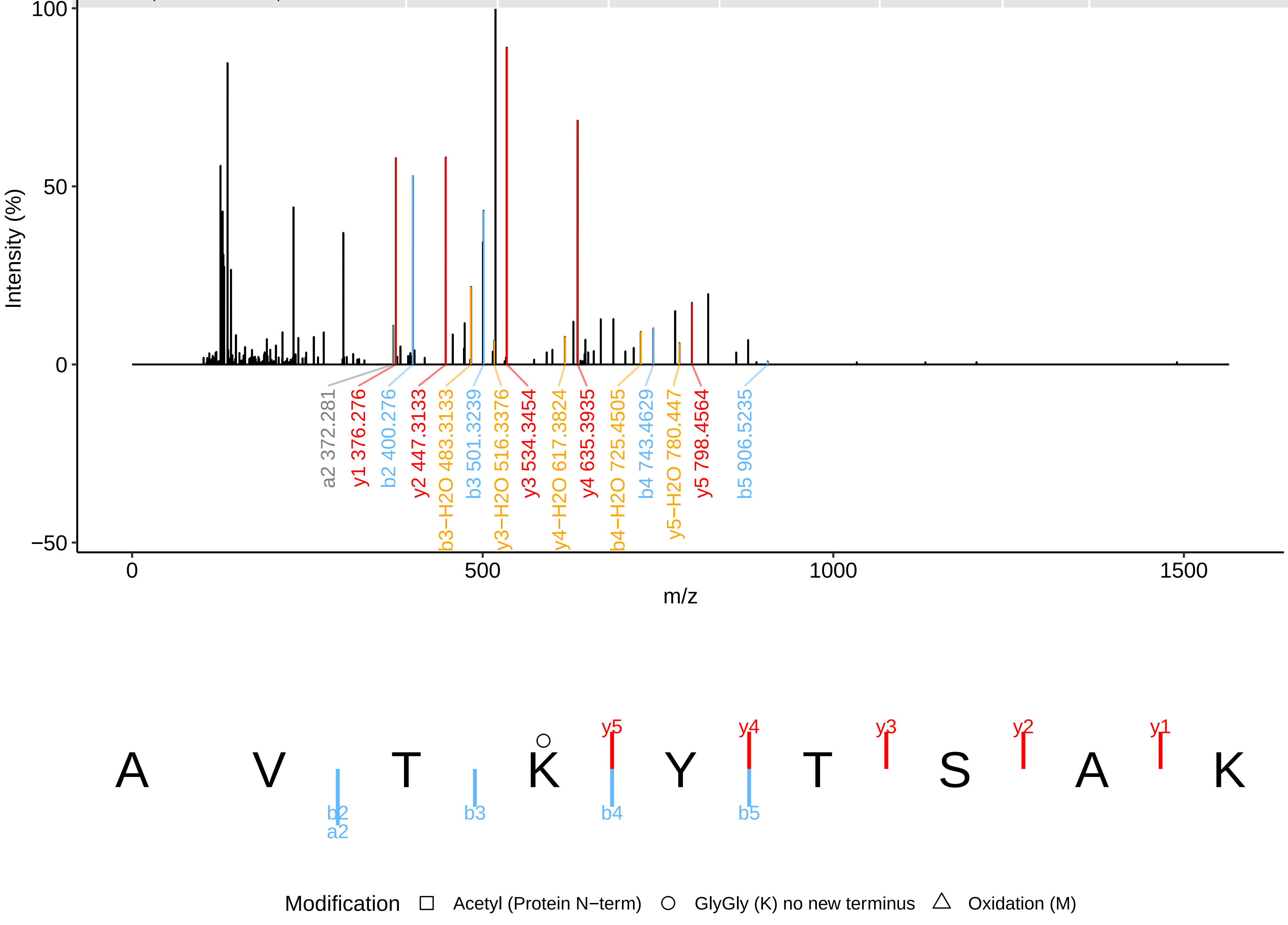

20200924_KIZ_MUb5_GlyGly_2 − Scan number: 6034

Gene Names	Charge	m/z	Mass	Retention time	PEP	Score	Precursor Intensity
H2BFS;HIST1H2BK;HIST1H2BJ	3	361.5329	1081.577	22.466	0.018568	70.816	6571097

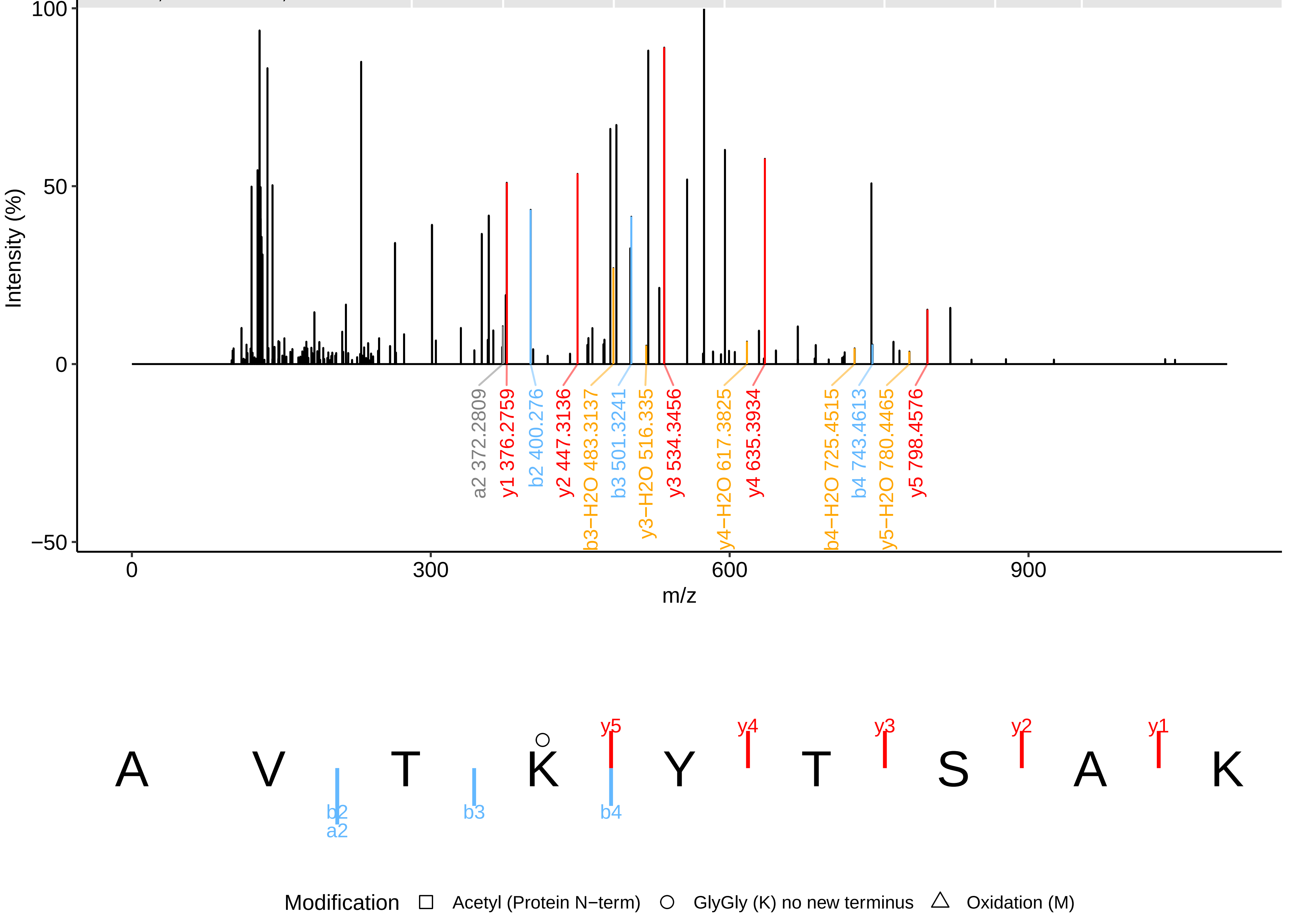

20200924_KIZ_MUb5_GlyGly_2 − Scan number: 6167

Gene Names	Charge	m/z	Mass	Retention time	PEP	Score	Precursor Intensity
H2BFS;HIST1H2BK;HIST1H2BJ	3	361.5329	1081.577	22.976	0.0030405	92.838	23635692

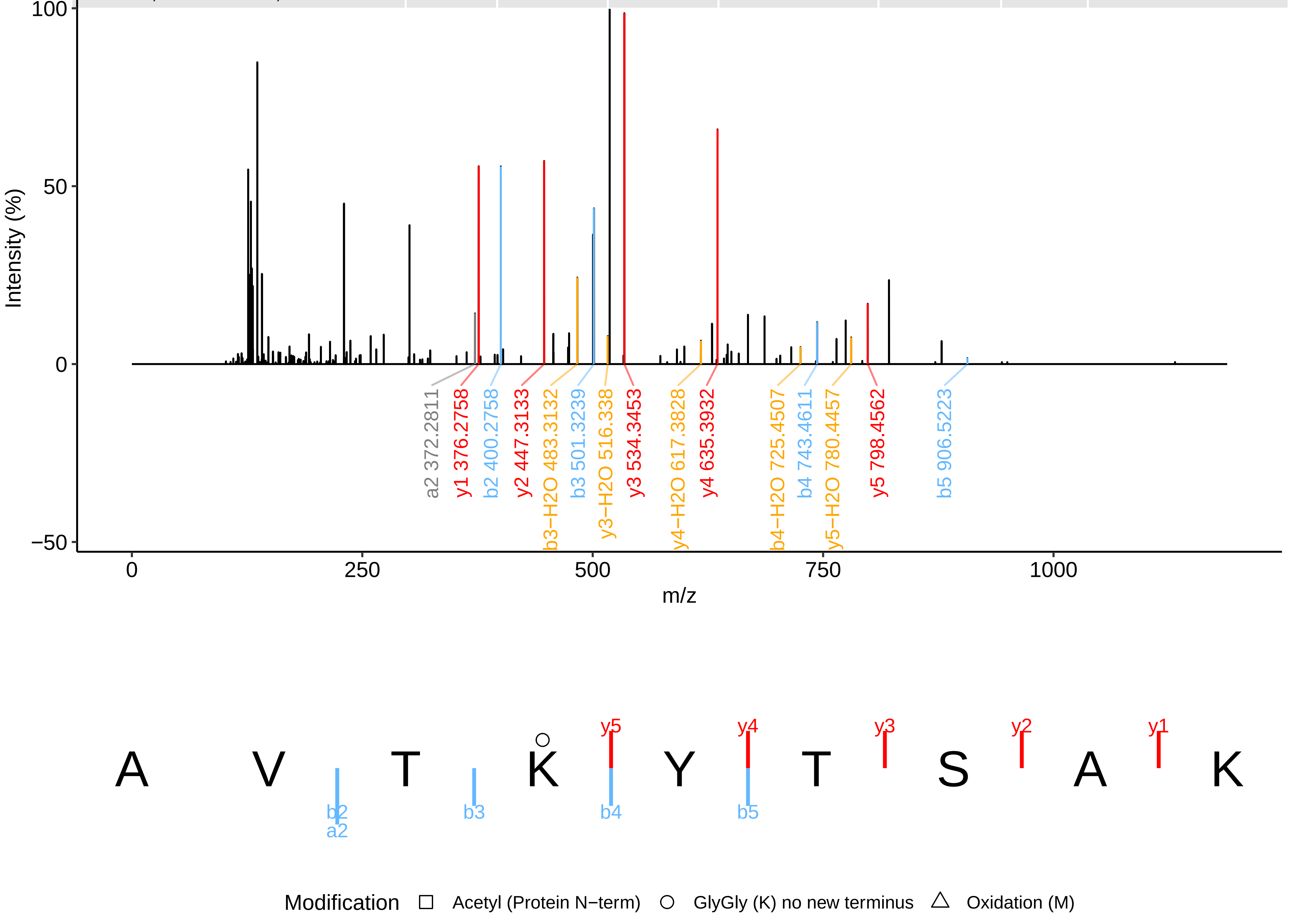

20201118_KIZ_MUb6_GlyGly_1 − Scan number: 6263

Gene Names	Charge	m/z	Mass	Retention time	PEP	Score	Precursor Intensity
H2BFS;HIST1H2BK;HIST1H2BJ	3	361.5329	1081.577	19.794	0.004169	79.986	23429538

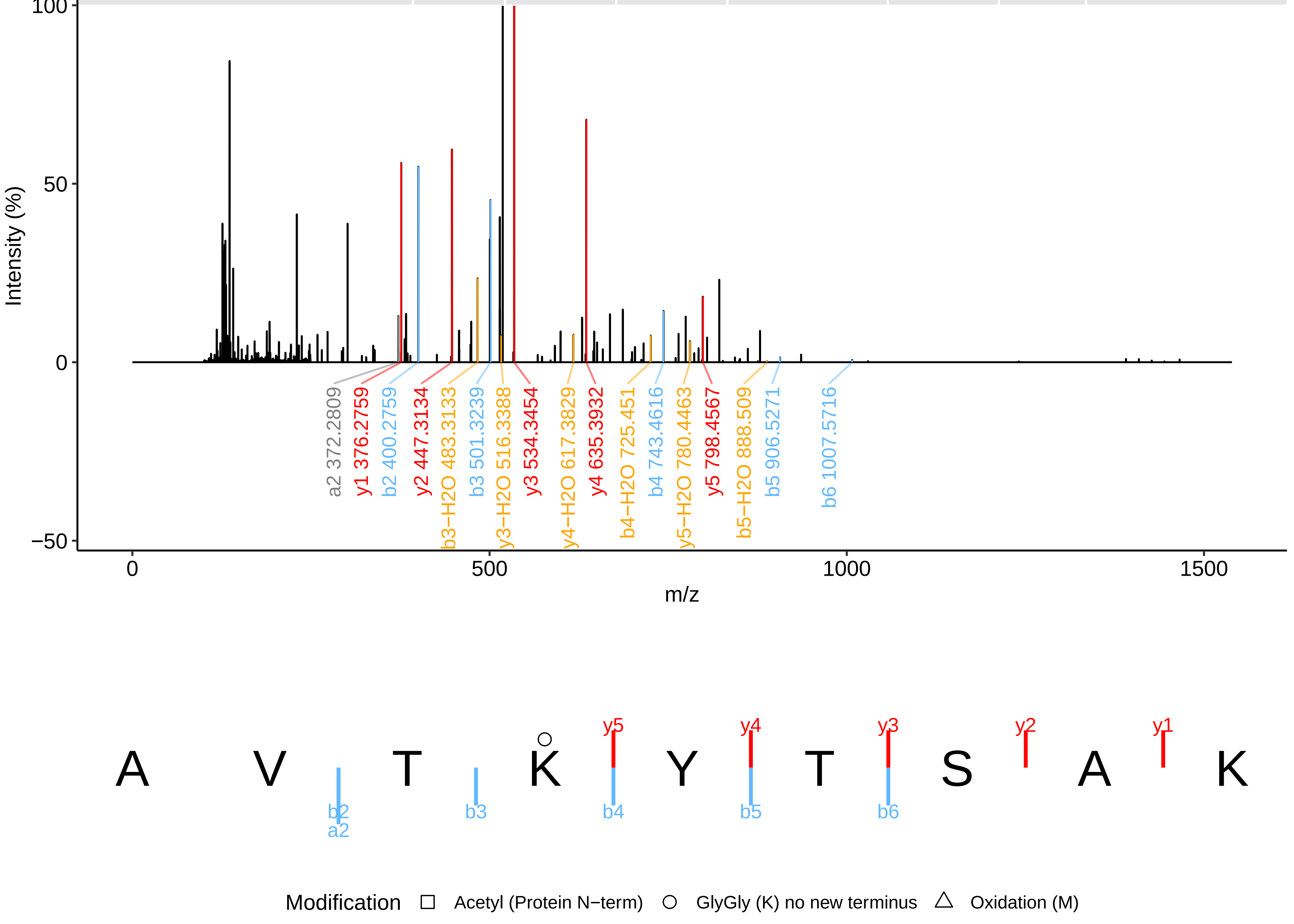

20201118_KIZ_MUb6_GlyGly_2 − Scan number: 8055

Gene Names	Charge	m/z	Mass	Retention time	PEP	Score	Precursor Intensity
H2BFS;HIST1H2BK;HIST1H2BJ	3	361.5329	1081.577	27.445	0.009564	74.92	17232192

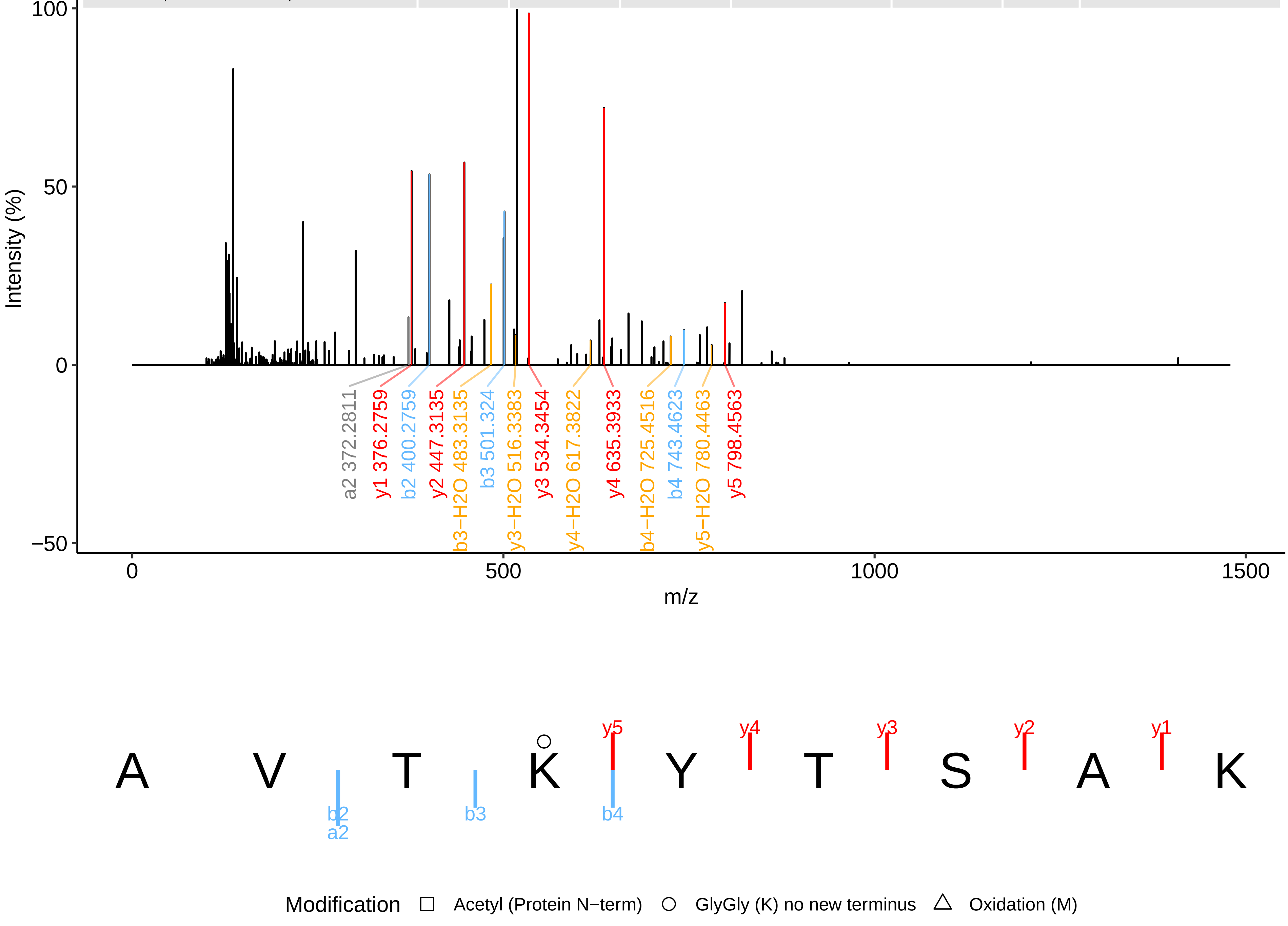

20200921_KIZ_MUb4_GlyGly_10plex − Scan number: 12069

Gene Names	Charge	m/z	Mass	Retention time	PEP	Score	Precursor Intensity
XRCC6	3	350.8696	1049.587	42.232	0.053913	59.245	3062478

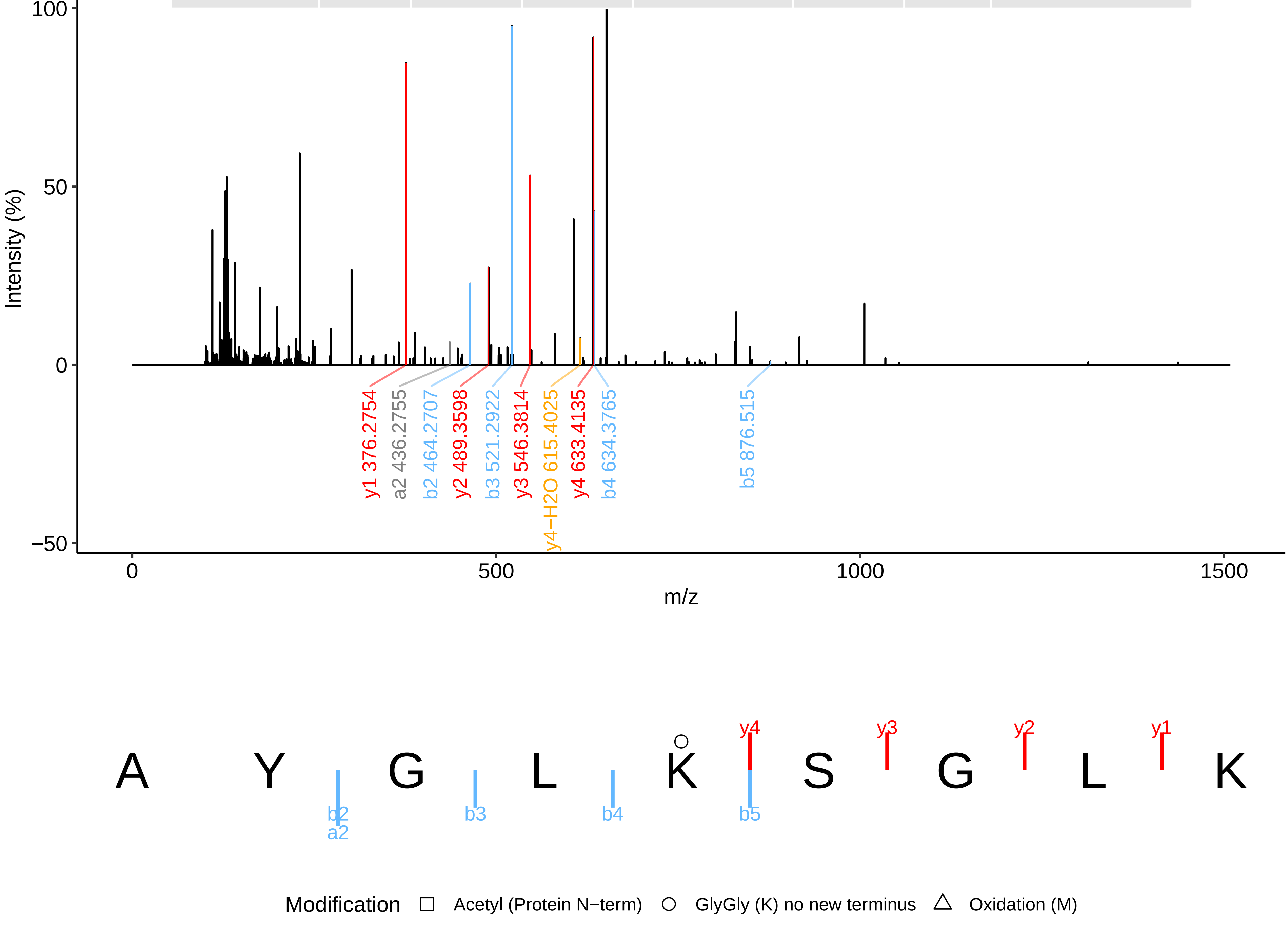

20200921_KIZ_MUb4_GlyGly_10plex_2 − Scan number: 11184

Gene Names	Charge	m/z	Mass	Retention time	PEP	Score	Precursor Intensity
XRCC6	3	350.8696	1049.587	42.982	0.079128	51.346	505830.9

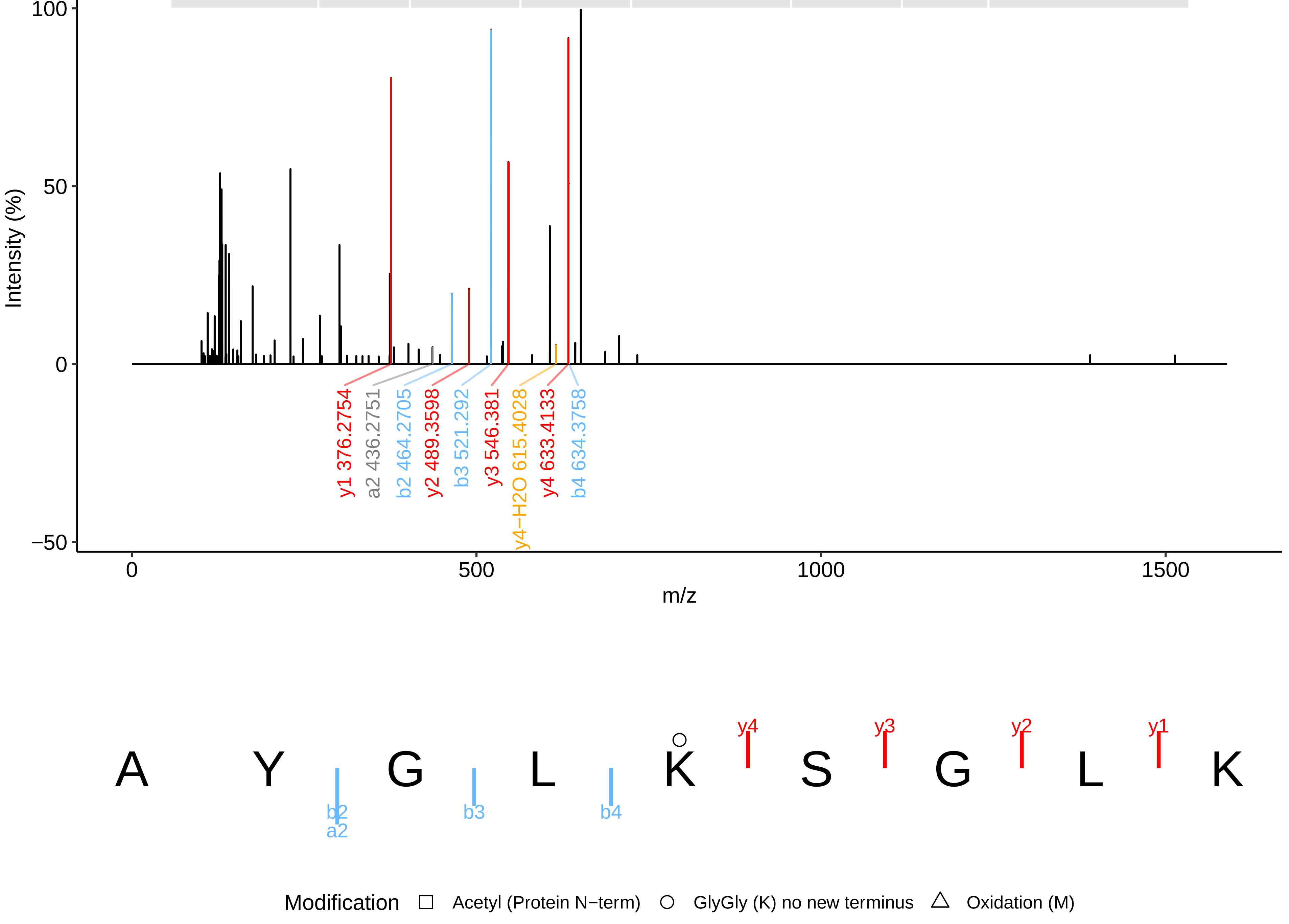

20200921_KIZ_MUb4_GlyGly_10plex_2 − Scan number: 6316

Gene Names	Charge	m/z	Mass	Retention time	PEP	Score	Precursor Intensity
	3	355.2013	1062.582	23.156	0.079128	51.346	1076744

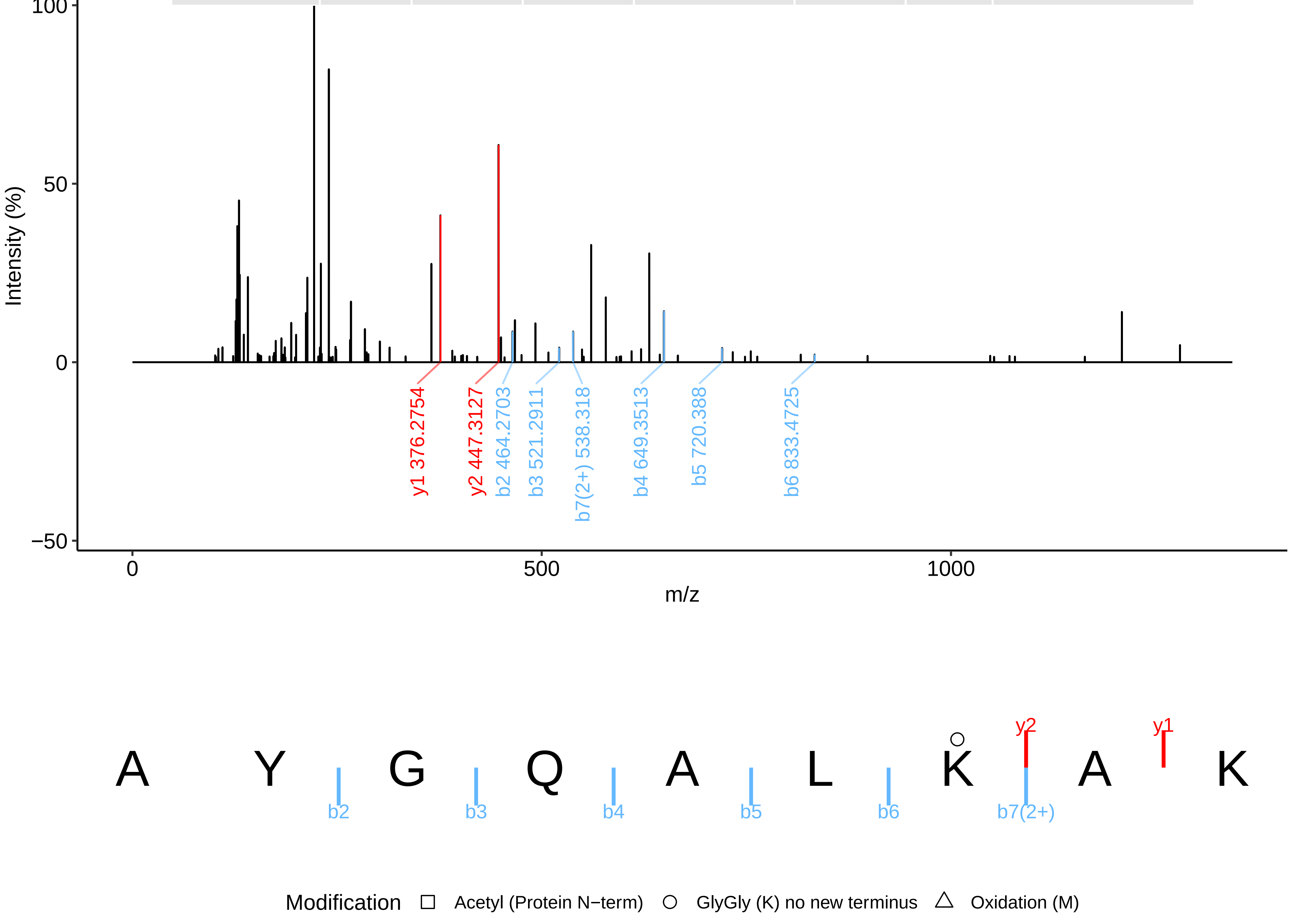

20200921_KIZ_MUb4_GlyGly_10plex_2 − Scan number: 7771

Gene Names	Charge	m/z	Mass	Retention time	PEP	Score	Precursor Intensity
SLC25A3	3	346.5077	1036.501	29.108	0.076668	51.995	983354.8

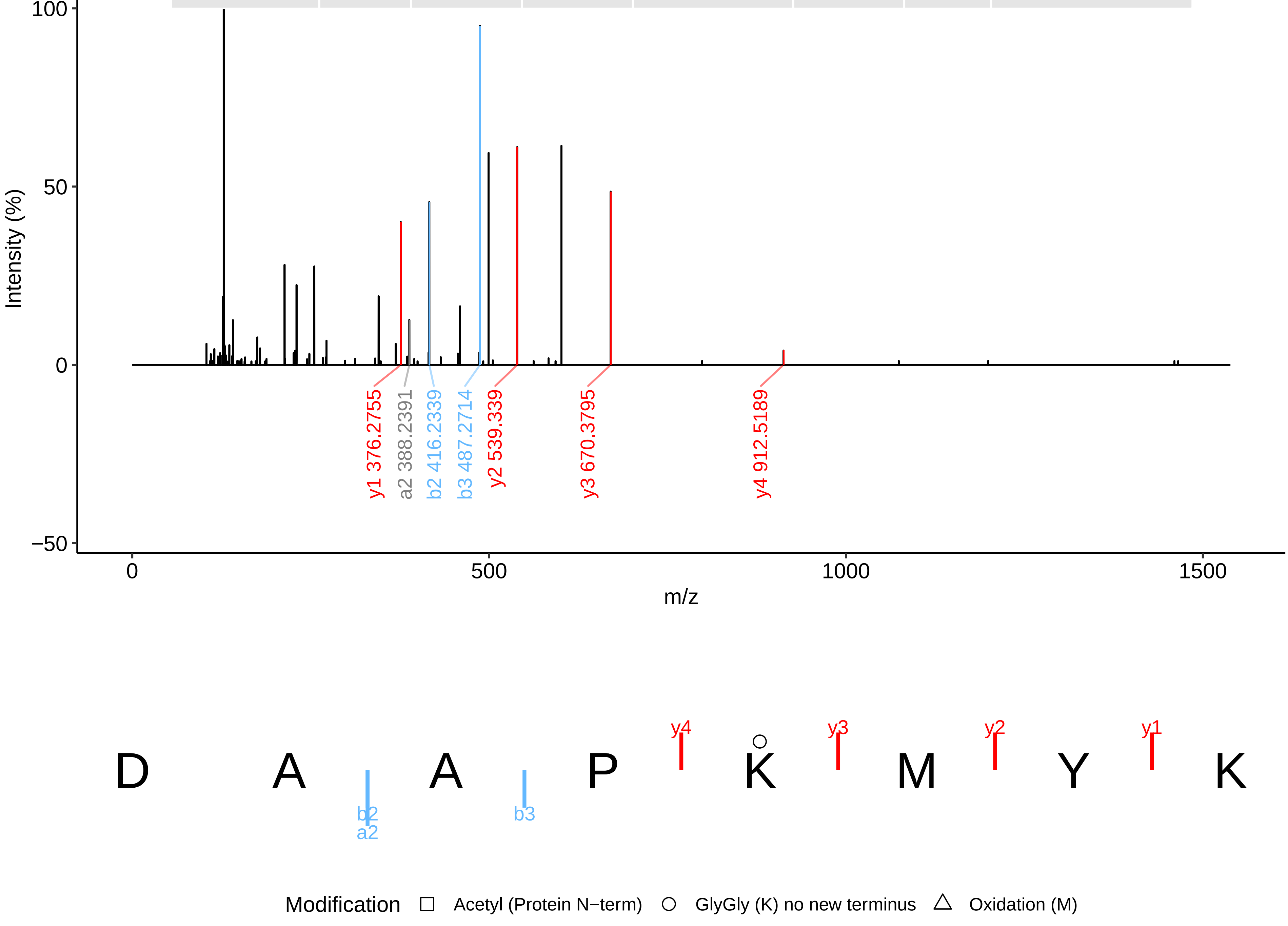

20200921_KIZ_MUb4_GlyGly_10plex − Scan number: 12715

Gene Names	Charge	m/z	Mass	Retention time	PEP	Score	Precursor Intensity
ATP5EP2;ATP5E	3	355.8573	1064.55	44.68	0.10976	52.716	NA

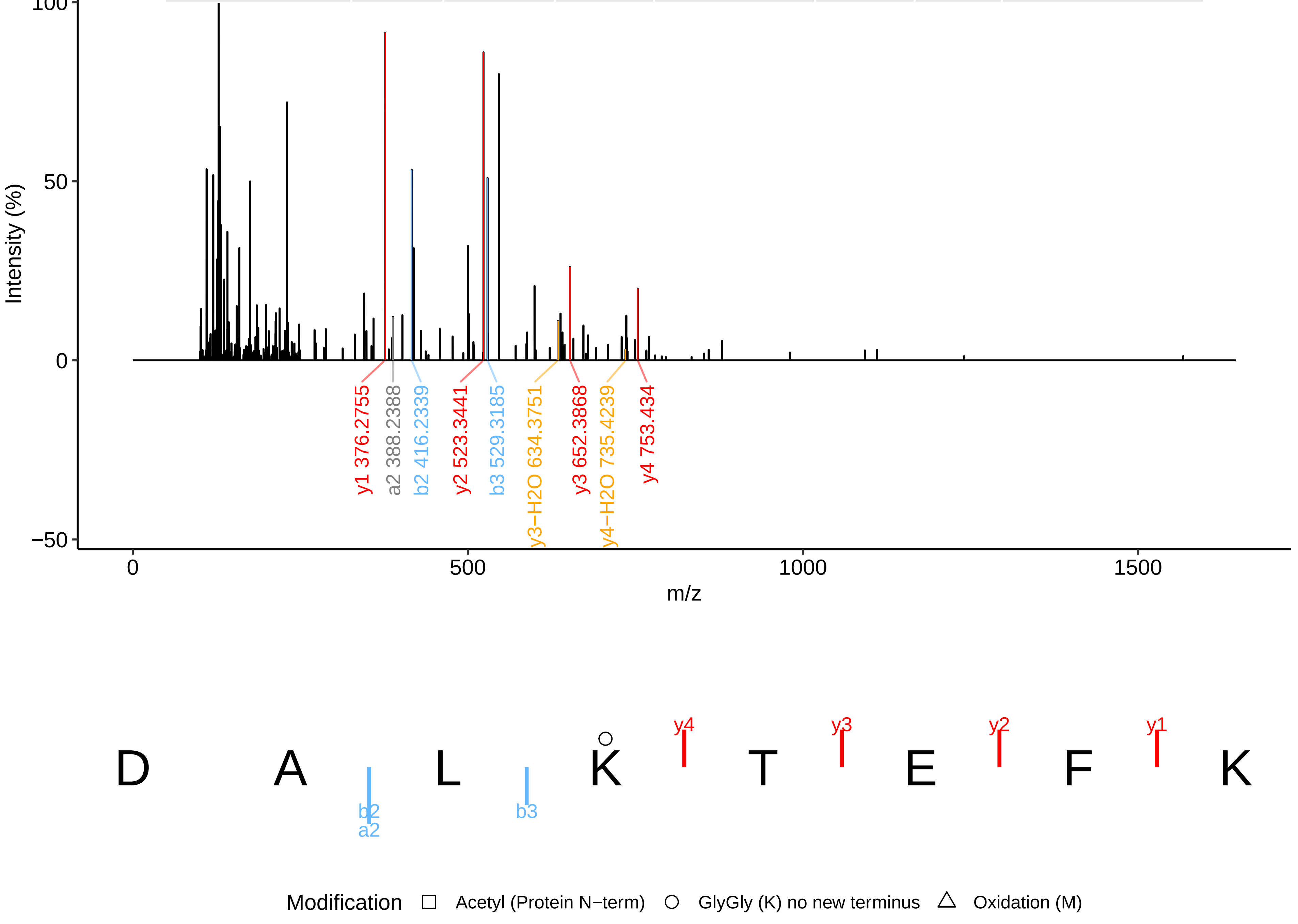

20200921_KIZ_MUb4_GlyGly_10plex − Scan number: 2578

Gene Names	Charge	m/z	Mass	Retention time	PEP	Score	Precursor Intensity
TP53	3	429.541	1285.601	8.1084	0.00034056	93.348	11114519

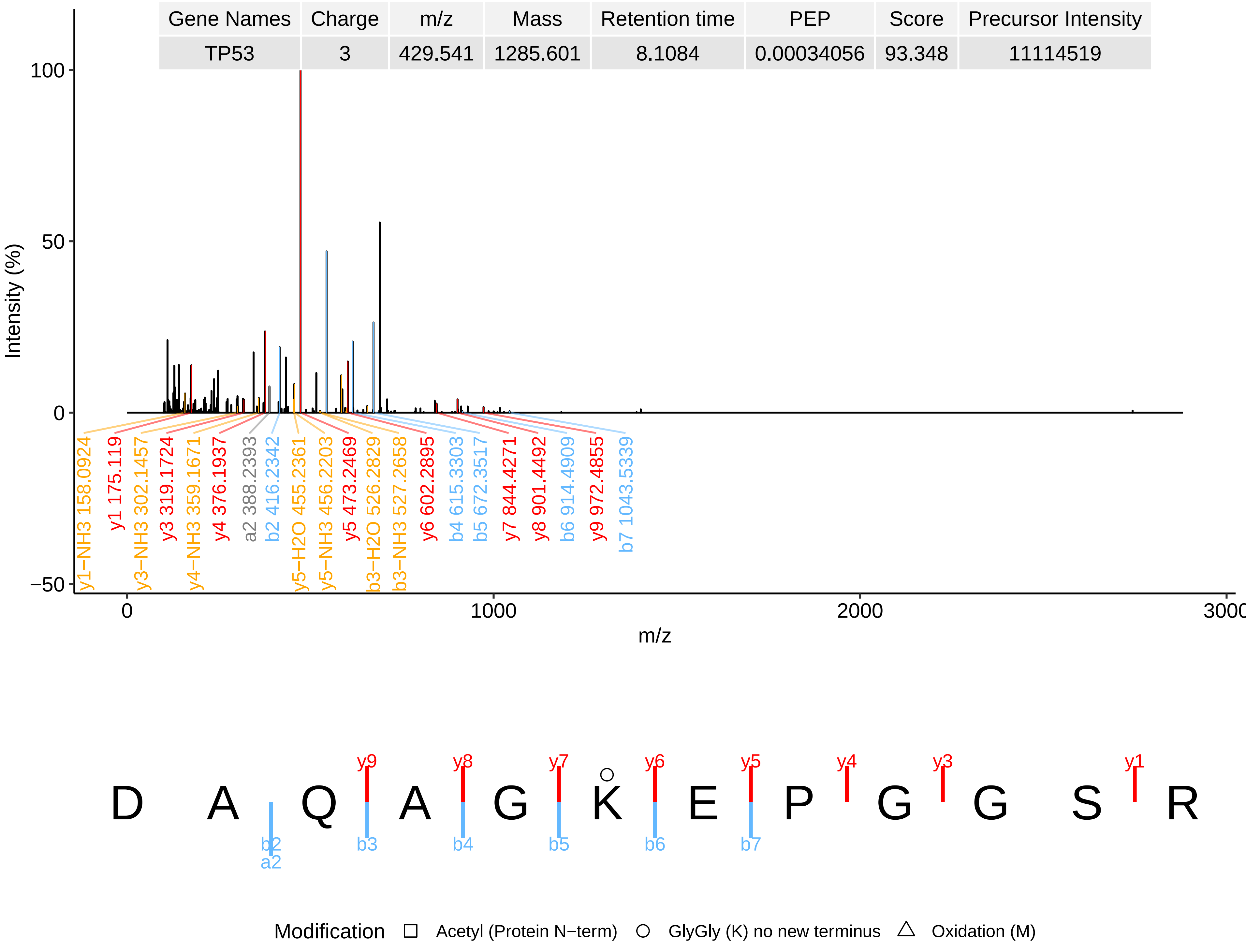

20200921_KIZ_MUb4_GlyGly_10plex_2 − Scan number: 2545

Gene Names	Charge	m/z	Mass	Retention time	PEP	Score	Precursor Intensity
TP53	3	429.541	1285.601	8.0534	1.6558e−05	101.53	4209644

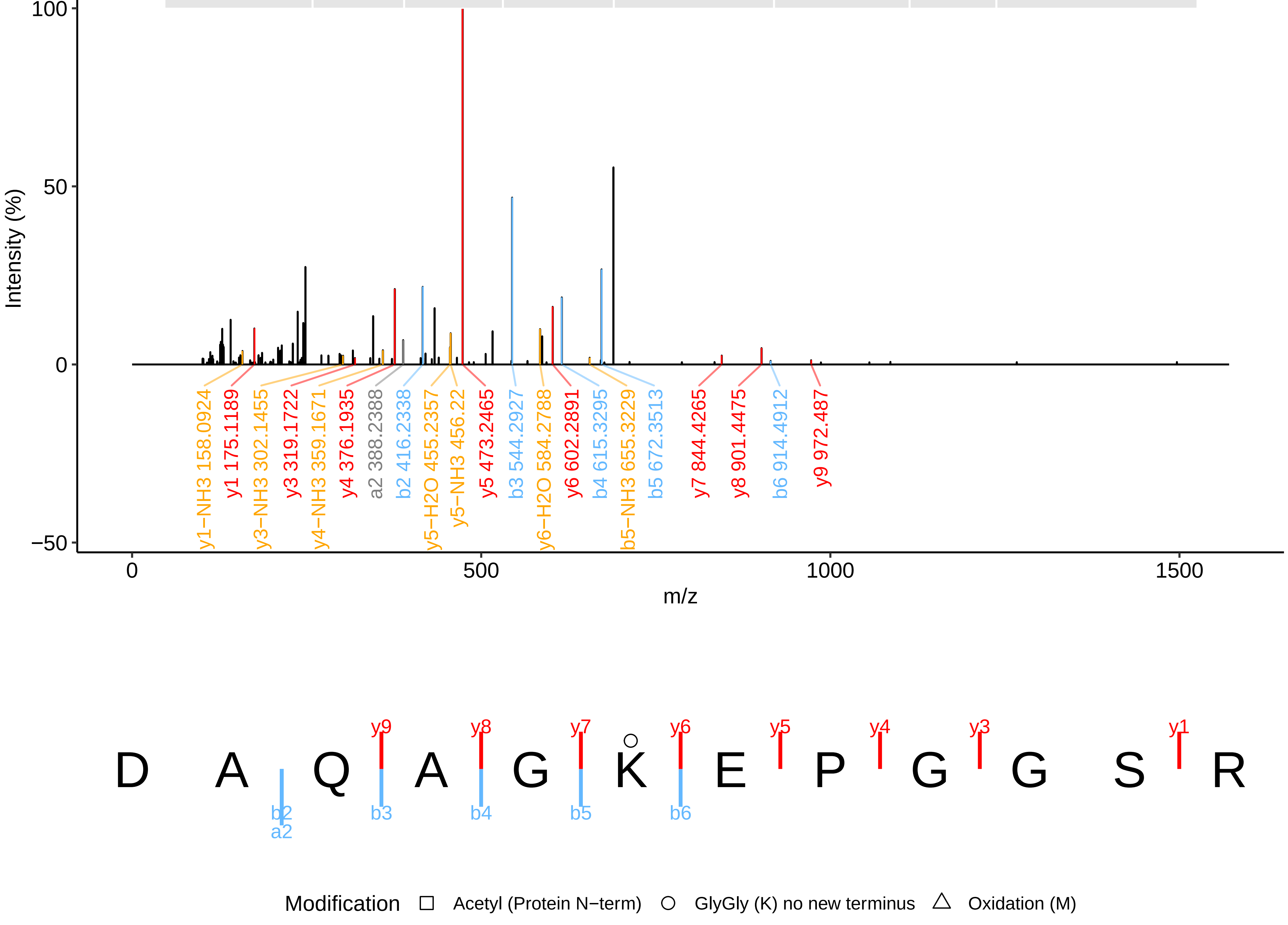

20201118_KIZ_MUb6_GlyGly_2 − Scan number: 2711

Gene Names	Charge	m/z	Mass	Retention time	PEP	Score	Precursor Intensity
TP53	3	429.541	1285.601	8.574	0.00059395	89.663	3908796

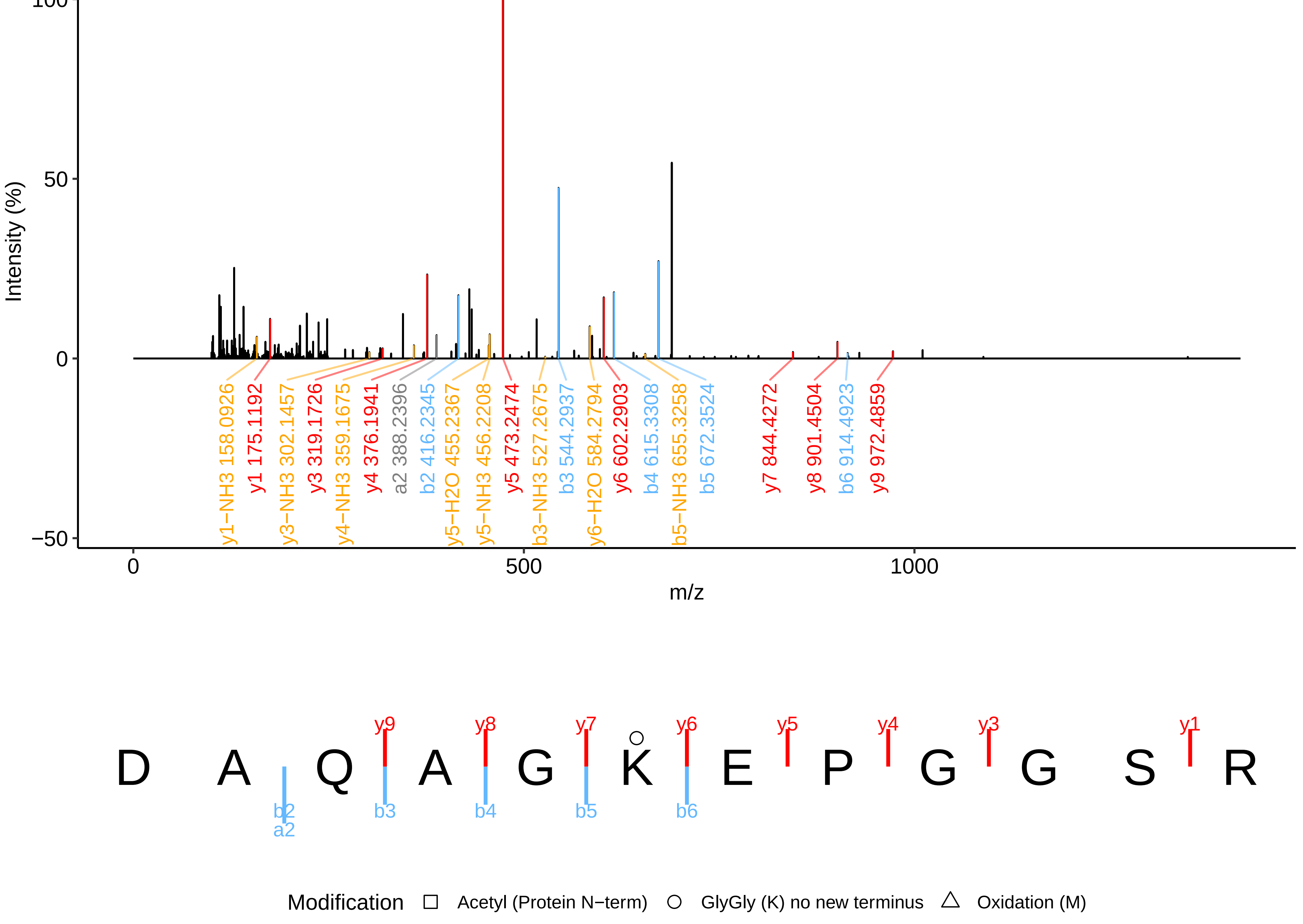

20200921_KIZ_MUb4_GlyGly_10plex − Scan number: 6120

Gene Names	Charge	m/z	Mass	Retention time	PEP	Score	Precursor Intensity
MCM6	3	431.8776	1292.611	20.146	0.005251	76.302	4590523

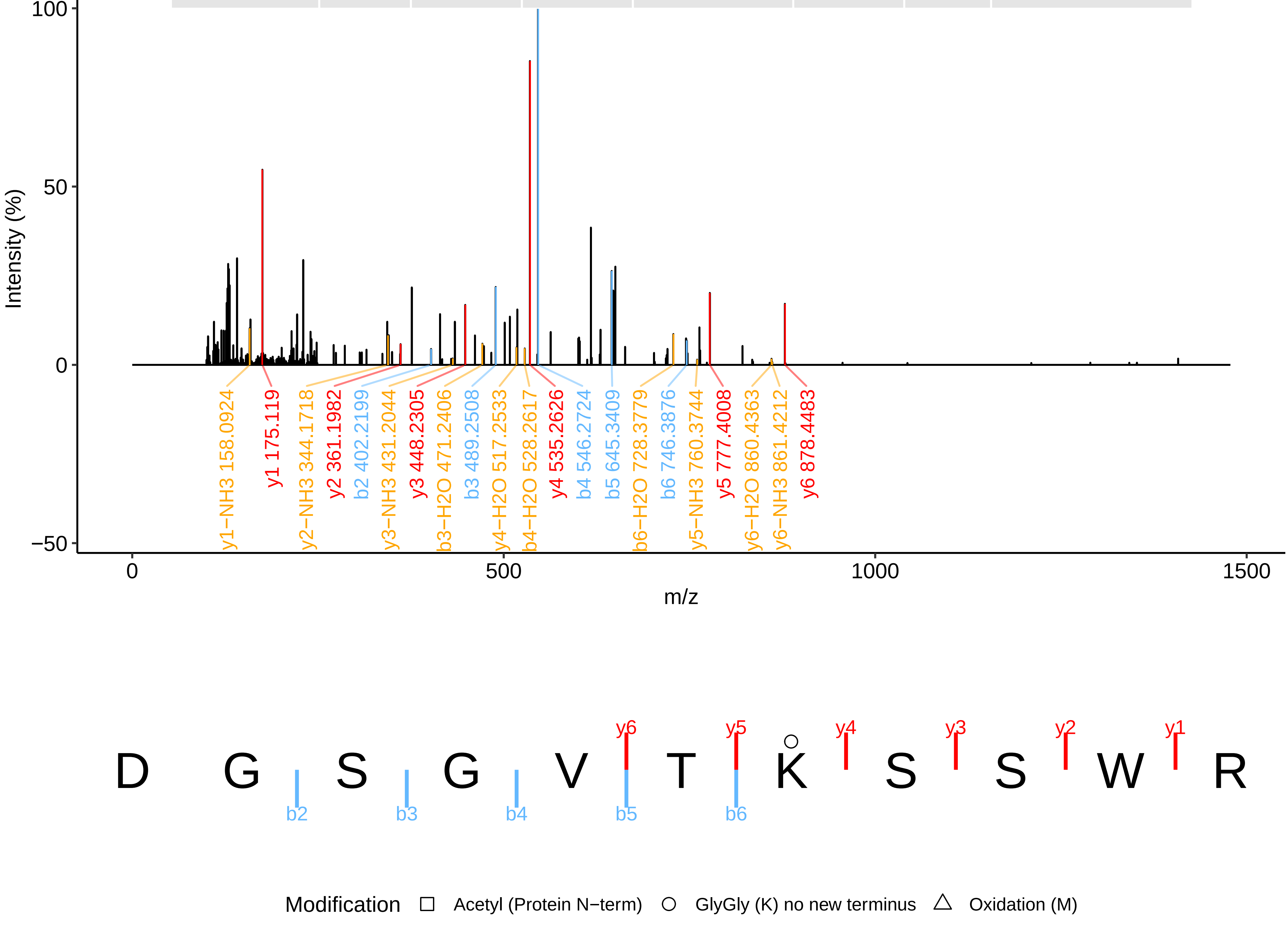

20200921_KIZ_MUb4_GlyGly_10plex − Scan number: 10707

Gene Names	Charge	m/z	Mass	Retention time	PEP	Score	Precursor Intensity
SLC6A6	3	356.8731	1067.598	37.133	0.021151	64.04	4544002

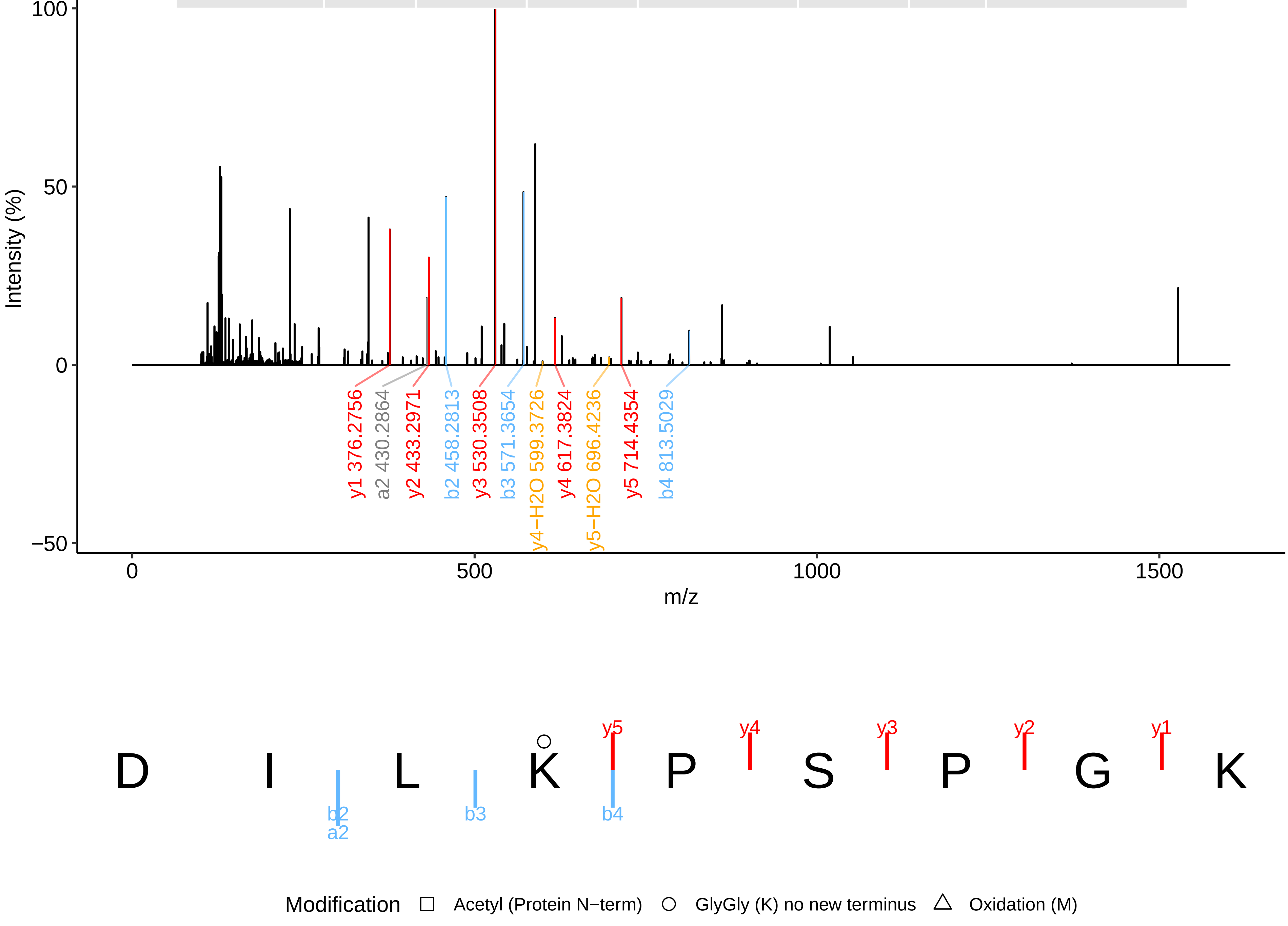

20200921_KIZ_MUb4_GlyGly_10plex − Scan number: 5535

Gene Names	Charge	m/z	Mass	Retention time	PEP	Score	Precursor Intensity
STAM	3	348.5208	1042.541	18.141	0.013644	88.37	4428633

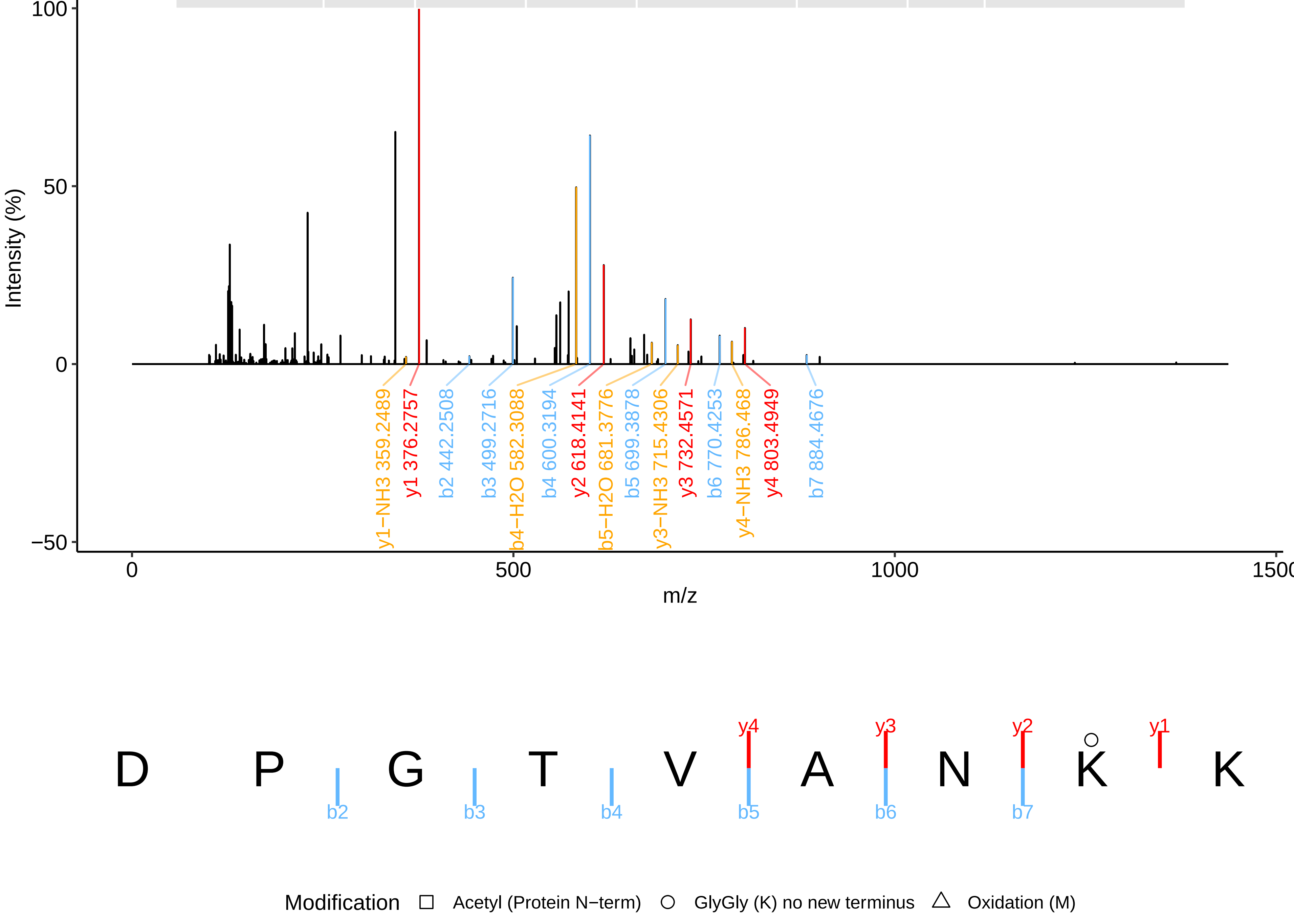

20200924_KIZ_MUb5_GlyGly_1 − Scan number: 4855

Gene Names	Charge	m/z	Mass	Retention time	PEP	Score	Precursor Intensity
STAM	3	348.5208	1042.541	15.029	0.0034154	98.582	15086378

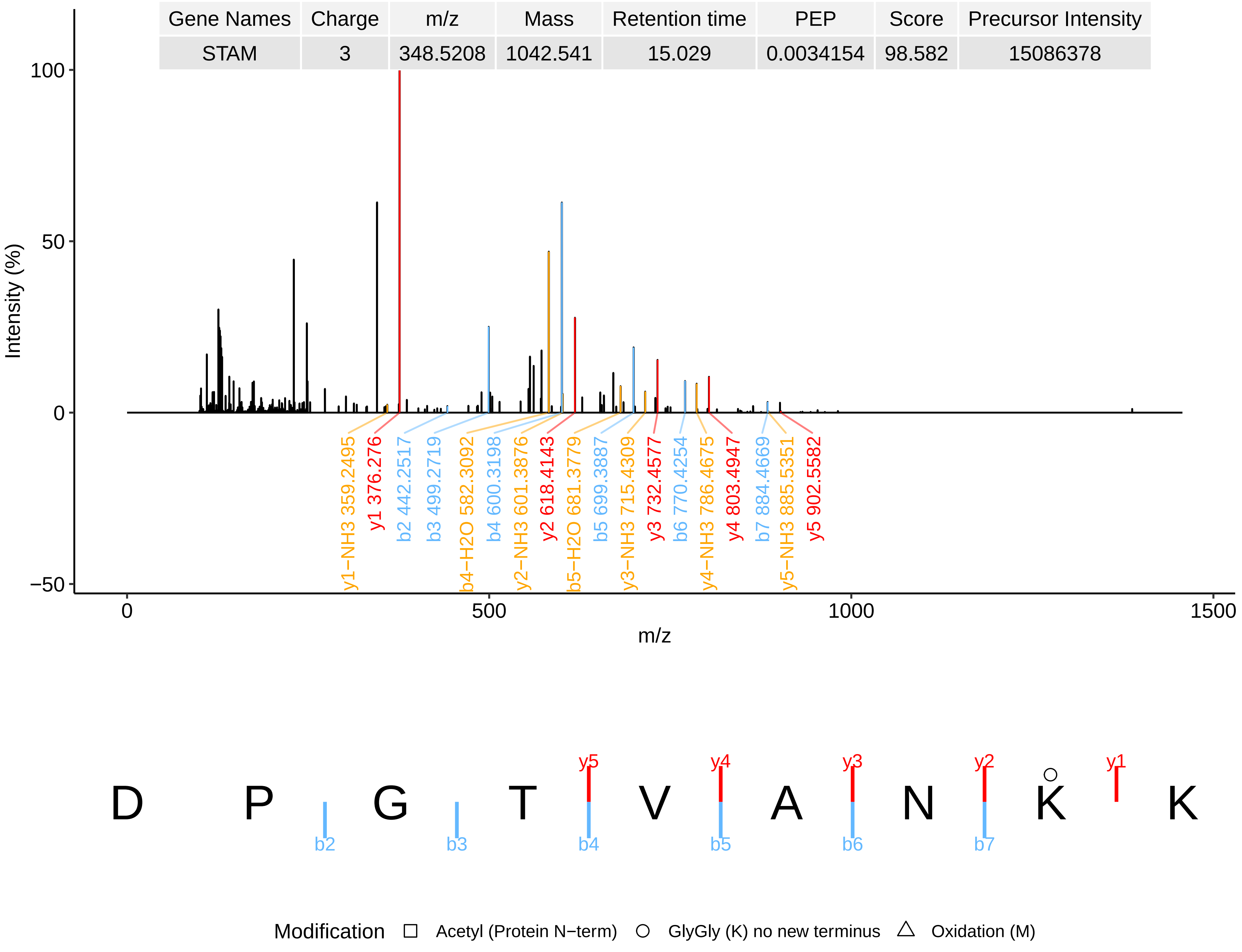

20200924_KIZ_MUb5_GlyGly_2 − Scan number: 4478

Gene Names	Charge	m/z	Mass	Retention time	PEP	Score	Precursor Intensity
STAM	3	348.5208	1042.541	16.436	0.0046333	96.948	1718547

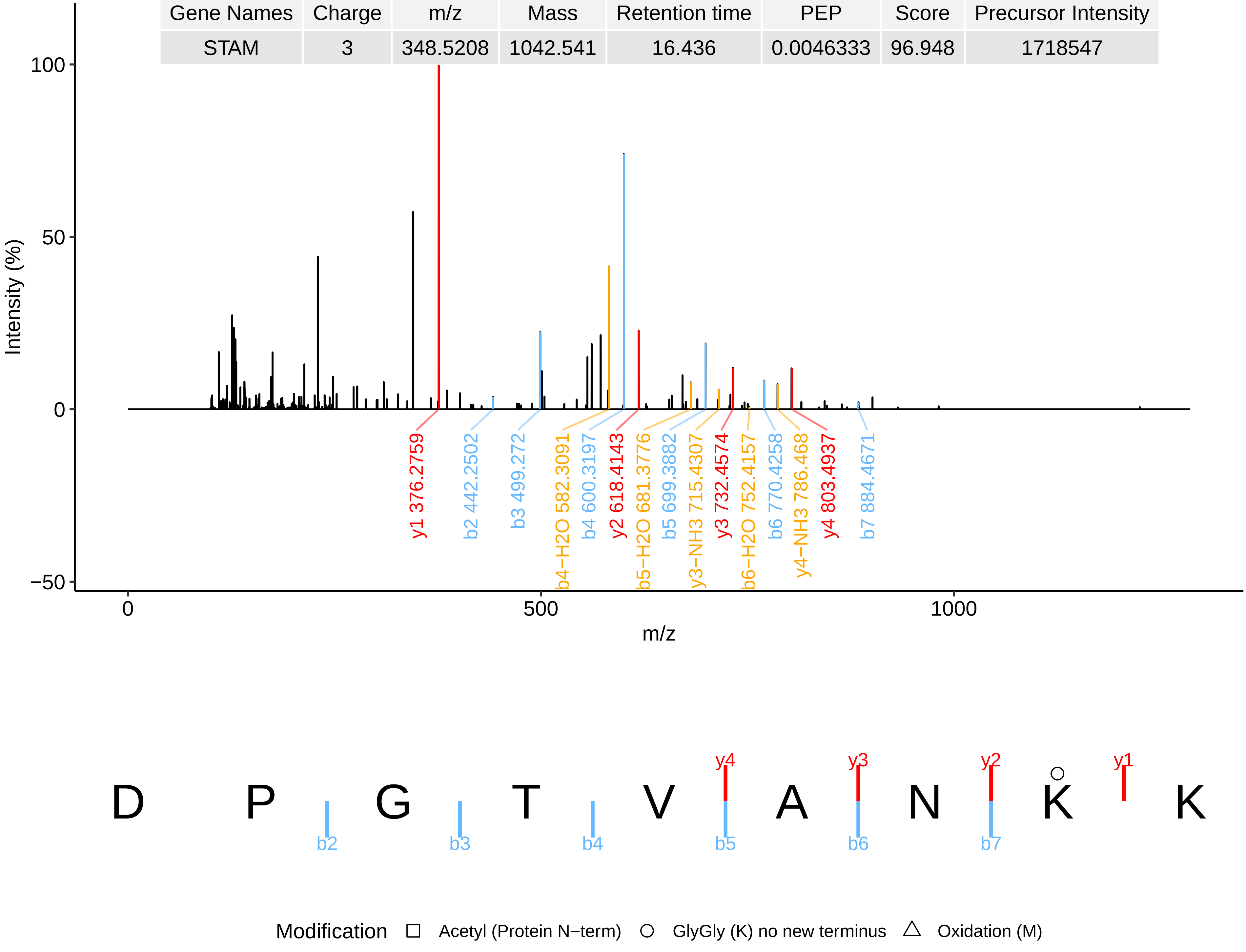

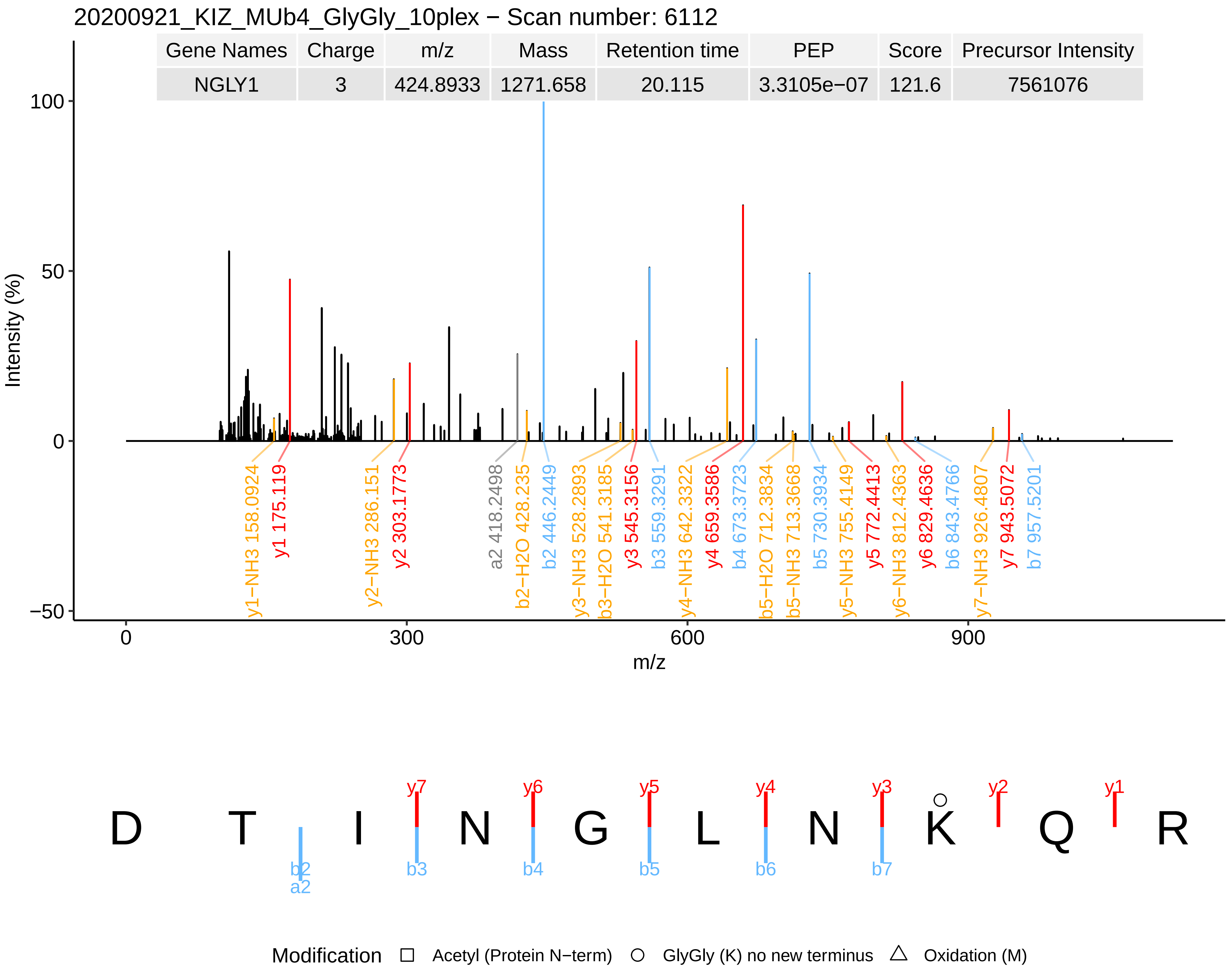
20200921_KIZ_MUb4_GlyGly_10plex − Scan number: 6112
Gene Names | Charge | m/z | Mass | Retention time | PEP | Score | Precursor Intensity
NGLY1 | 3 | 424.8933 | 1271.658 | 20.115 | 3.3105e−07 | 121.6 | 7561076
Intensity (%)
100
50
0
−50
0
300
600
900
m/z
y1−NH3 158.0924
y1 175.119
y2−NH3 286.151
y2 303.1773
a2 418.2498
b2−H2O 428.235
b2 446.2449
y3−NH3 528.2893
b3−H2O 541.3185
y3 545.3156
b3 559.3291
y4−NH3 642.3322
y4 659.3586
b4 673.3723
b5−H2O 712.3834
b5−NH3 713.3668
b5 730.3934
y5−NH3 755.4149
y5 772.4413
y6−NH3 812.4363
y6 829.4636
b6 843.4766
y7−NH3 926.4807
y7 943.5072
b7 957.5201
D T I N G L N K Q R
y7 y6 y5 y4 y3 y2 y1
b2 a2 b3 b4 b5 b6 b7
Modification
Acetyl (Protein N−term)
GlyGly (K) no new terminus
Oxidation (M)

20200921_KIZ_MUb4_GlyGly_10plex_2 − Scan number: 5570

Gene Names	Charge	m/z	Mass	Retention time	PEP	Score	Precursor Intensity
NGLY1	3	424.8933	1271.658	20.091	2.5401e−05	109.66	869704.5

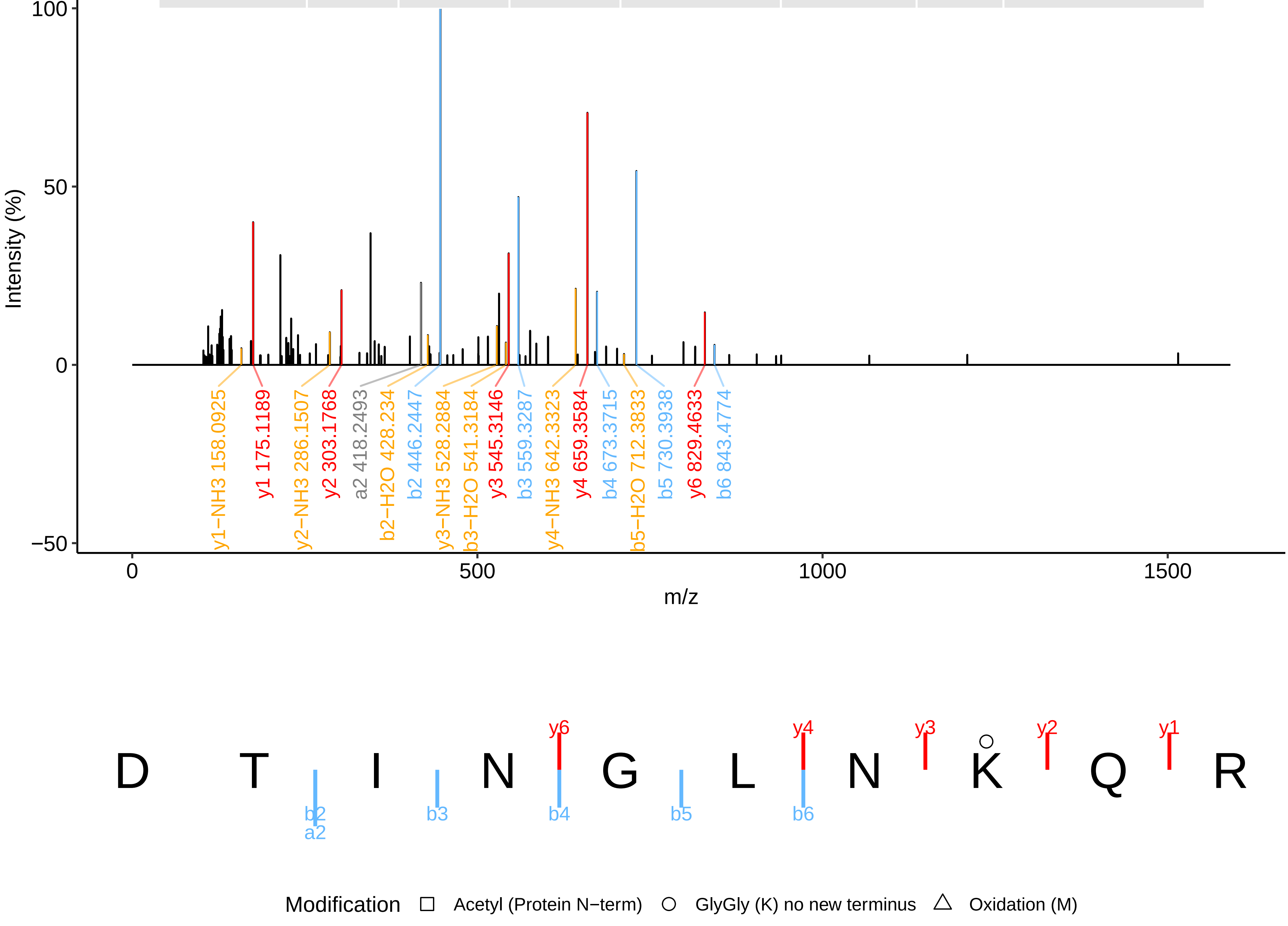

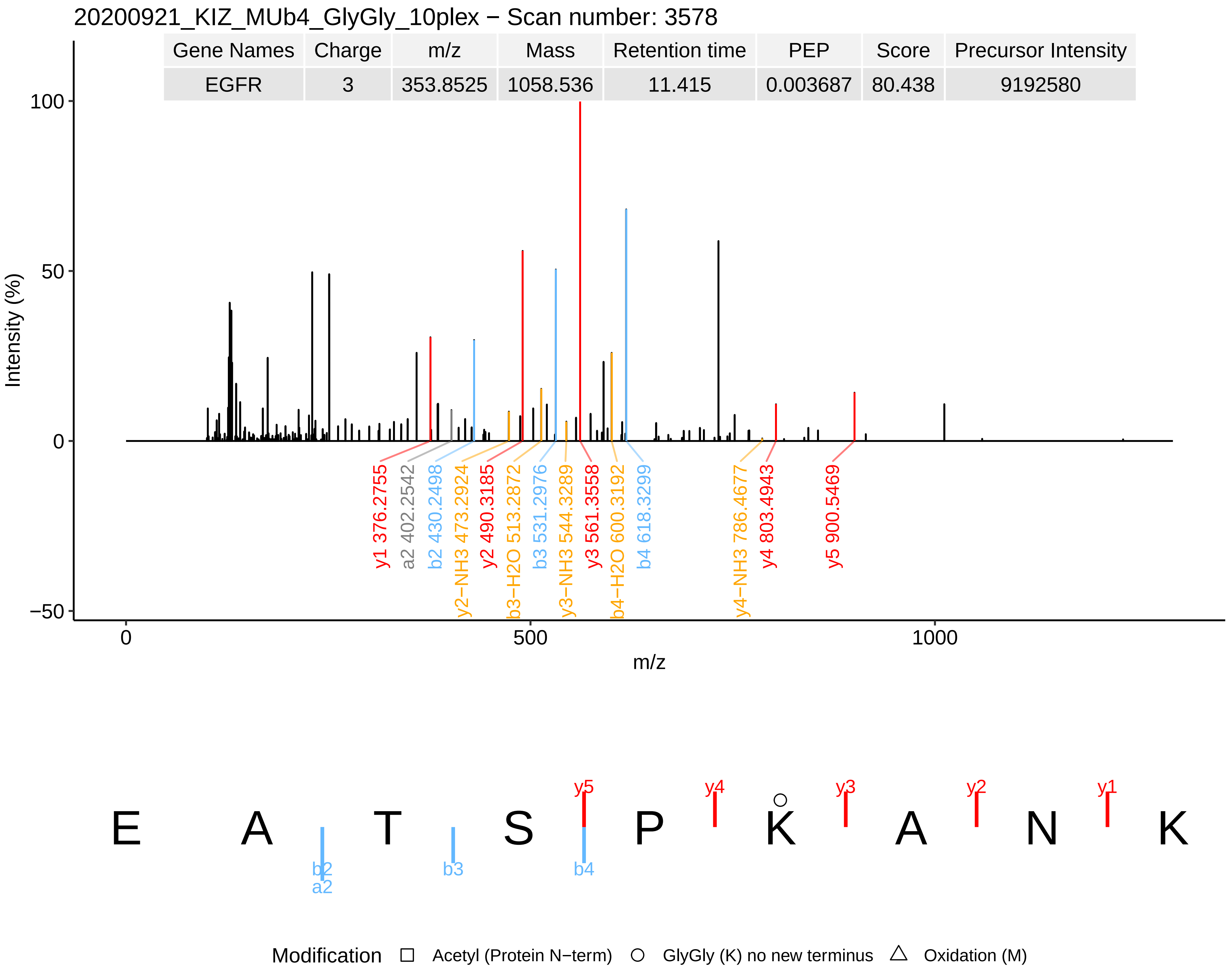

20200921_KIZ_MUb4_GlyGly_10plex − Scan number: 3578
Gene Names | Charge | m/z | Mass | Retention time | PEP | Score | Precursor Intensity
EGFR | 3 | 353.8525 | 1058.536 | 11.415 | 0.003687 | 80.438 | 9192580
Intensity (%)
100
50
0
−50
0
500
1000
m/z
y1 376.2755
a2 402.2542
b2 430.2498
y2−NH3 473.2924
y2 490.3185
b3−H2O 513.2872
b3 531.2976
y3−NH3 544.3289
y3 561.3558
b4−H2O 600.3192
b4 618.3299
y4−NH3 786.4677
y4 803.4943
y5 900.5469
E A T S P K A N K
y5 y4 y3 y2 y1
b2 a2 b3 b4
Modification
Acetyl (Protein N−term)
GlyGly (K) no new terminus
Oxidation (M)

20200921_KIZ_MUb4_GlyGly_10plex_2 − Scan number: 3408

Gene Names	Charge	m/z	Mass	Retention time	PEP	Score	Precursor Intensity
EGFR	3	353.8525	1058.536	11.447	0.02928	61.679	2176546

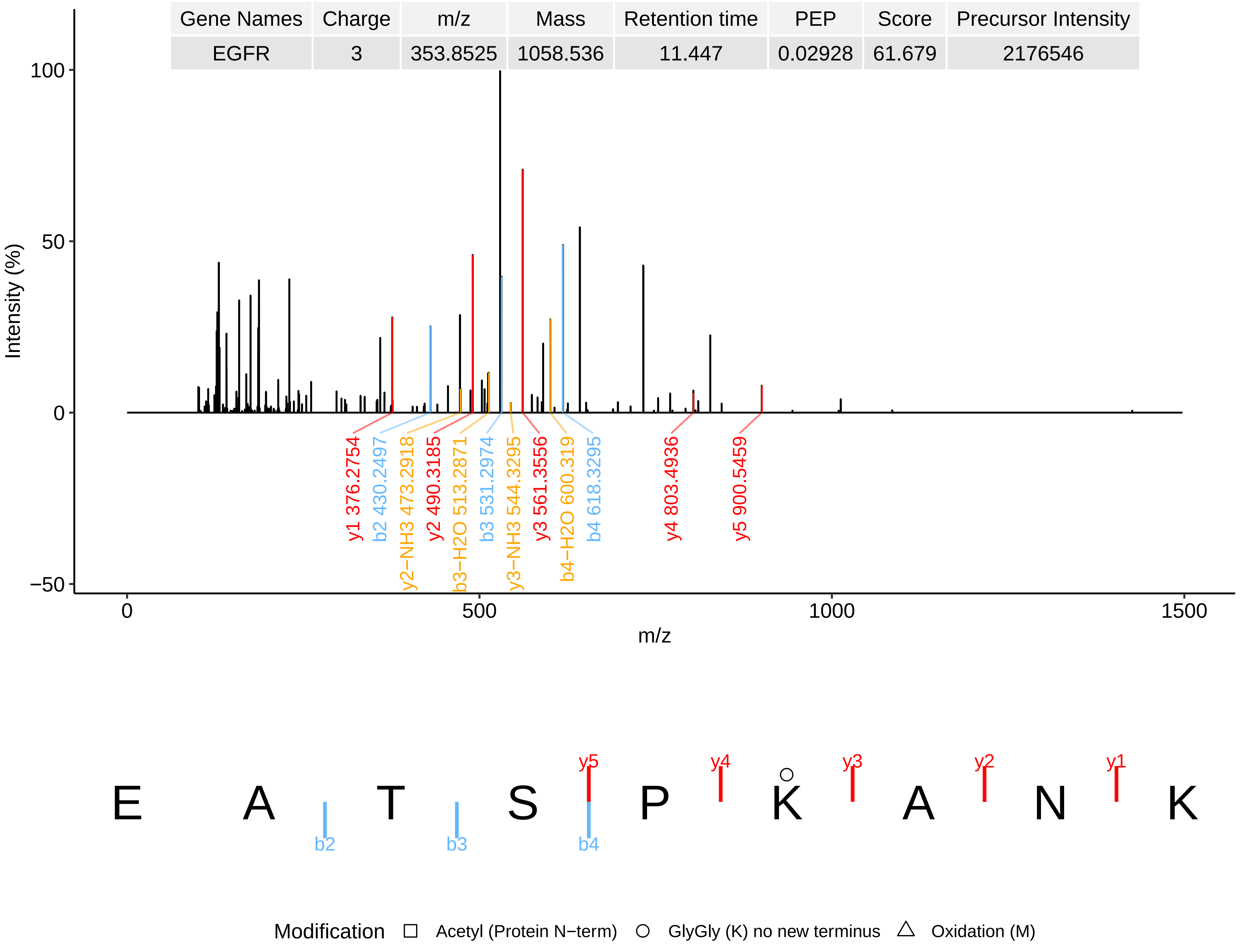

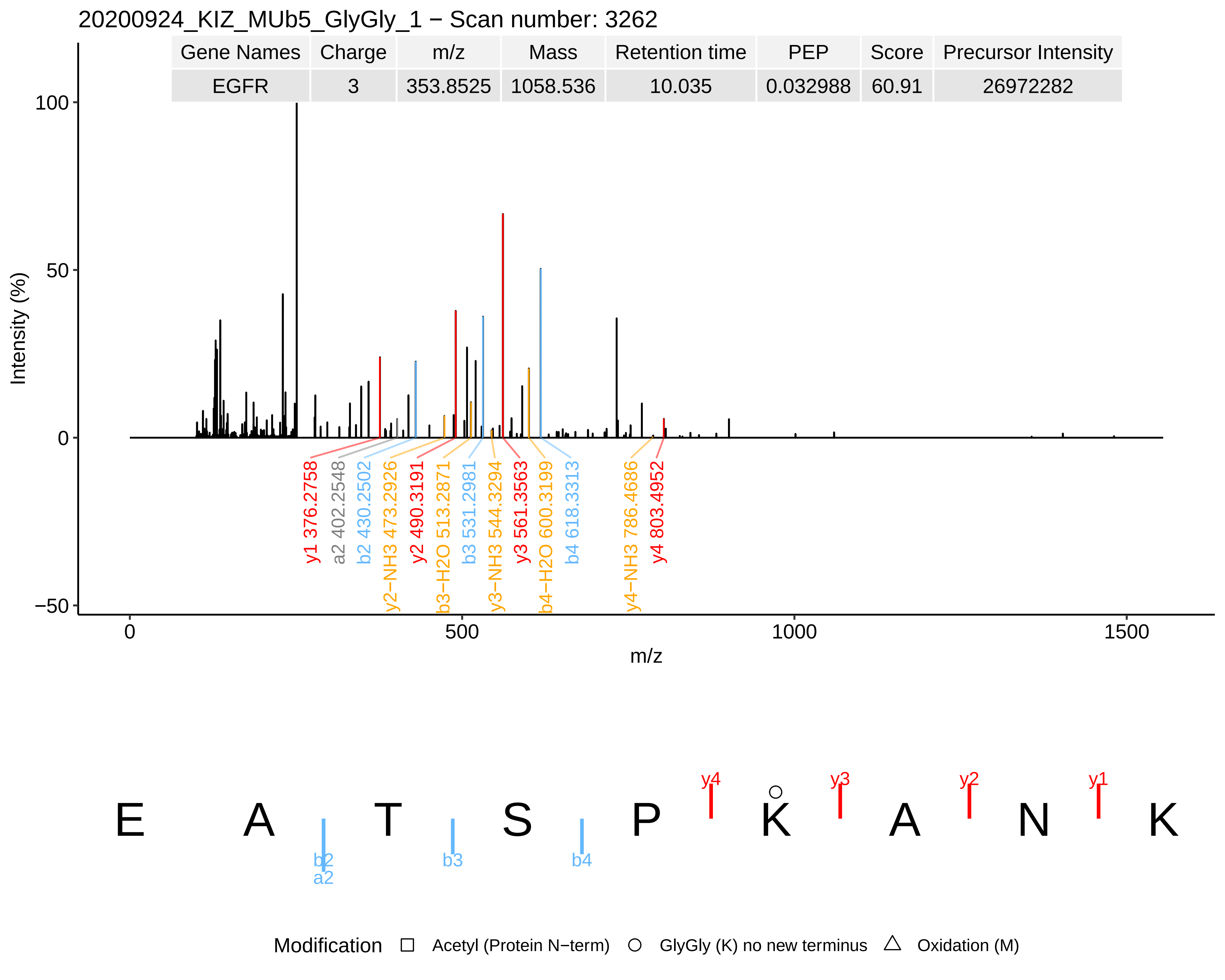
20200924_KIZ_MUb5_GlyGly_1 − Scan number: 3262
Gene Names | Charge | m/z | Mass | Retention time | PEP | Score | Precursor Intensity
EGFR | 3 | 353.8525 | 1058.536 | 10.035 | 0.032988 | 60.91 | 26972282
Intensity (%)
100
50
0
−50
m/z
0
500
1000
1500
y1 376.2758
a2 402.2548
b2 430.2502
y2−NH3 473.2926
y2 490.3191
b3−H2O 513.2871
b3 531.2981
y3−NH3 544.3294
y3 561.3563
b4−H2O 600.3199
b4 618.3313
y4−NH3 786.4686
y4 803.4952
E A T S P K A N K
y4 y3 y2 y1
b2 a2 b3 b4
Modification
Acetyl (Protein N−term)
GlyGly (K) no new terminus
Oxidation (M)

20200924_KIZ_MUb5_GlyGly_2 − Scan number: 3065

Gene Names	Charge	m/z	Mass	Retention time	PEP	Score	Precursor Intensity
EGFR	3	353.8525	1058.536	11.003	0.020115	69.598	2129336

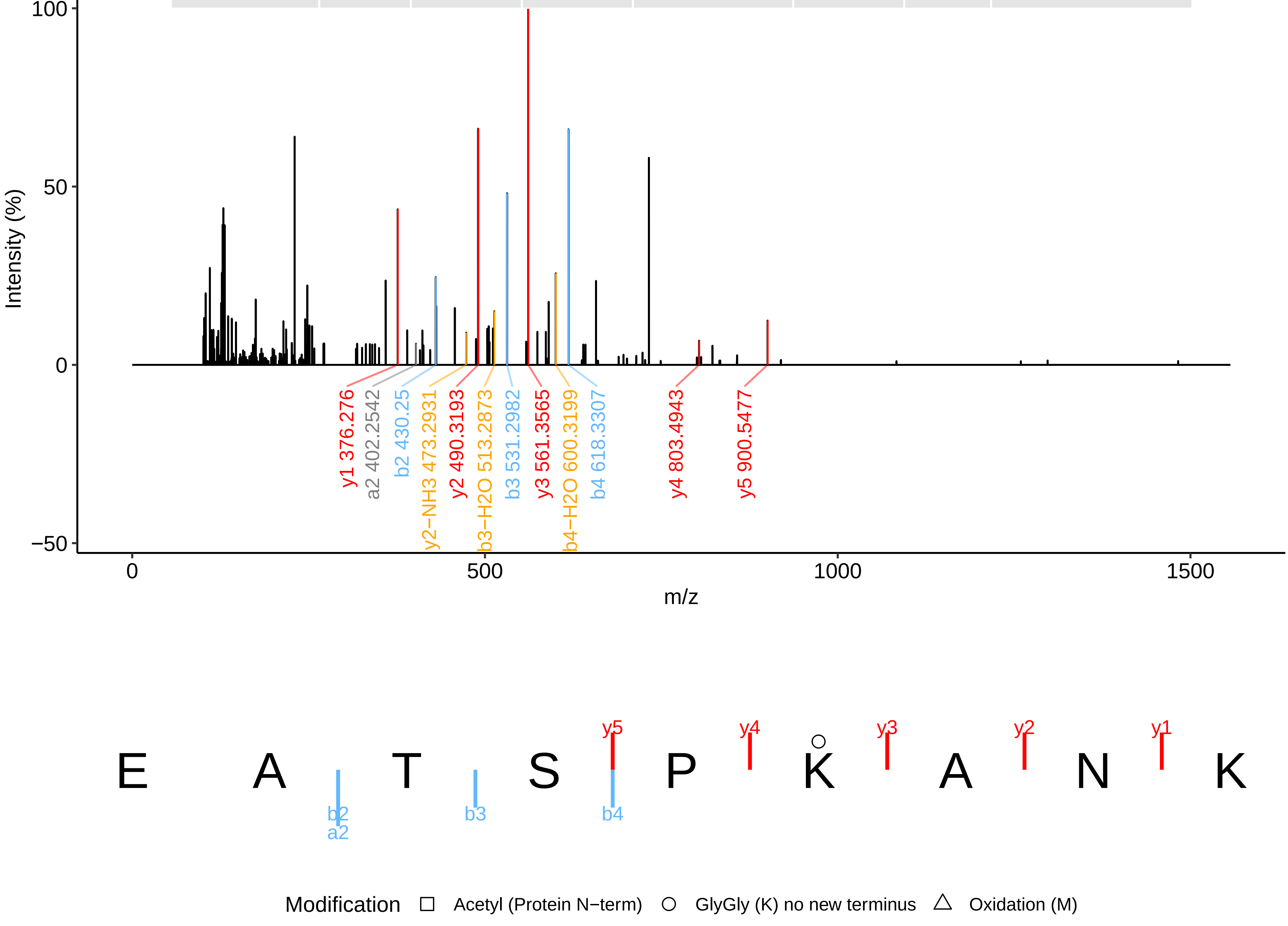

20201118_KIZ_MUb6_GlyGly_1 − Scan number: 3181

Gene Names	Charge	m/z	Mass	Retention time	PEP	Score	Precursor Intensity
EGFR	3	353.8525	1058.536	9.8027	0.0017062	91.318	8024294

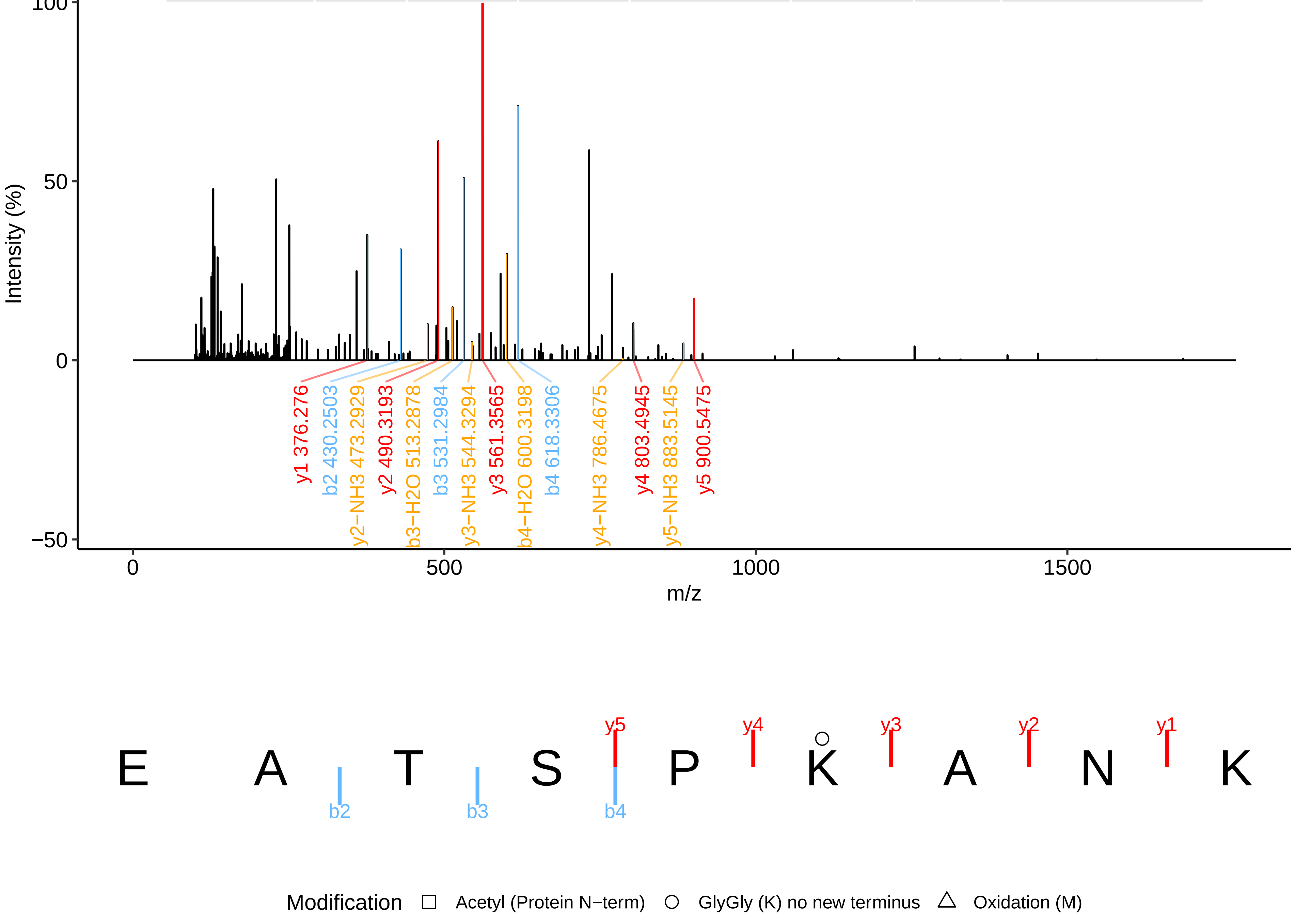

20201118_KIZ_MUb6_GlyGly_2 − Scan number: 3533

Gene Names	Charge	m/z	Mass	Retention time	PEP	Score	Precursor Intensity
EGFR	3	353.8525	1058.536	11.719	0.003687	80.438	2951193

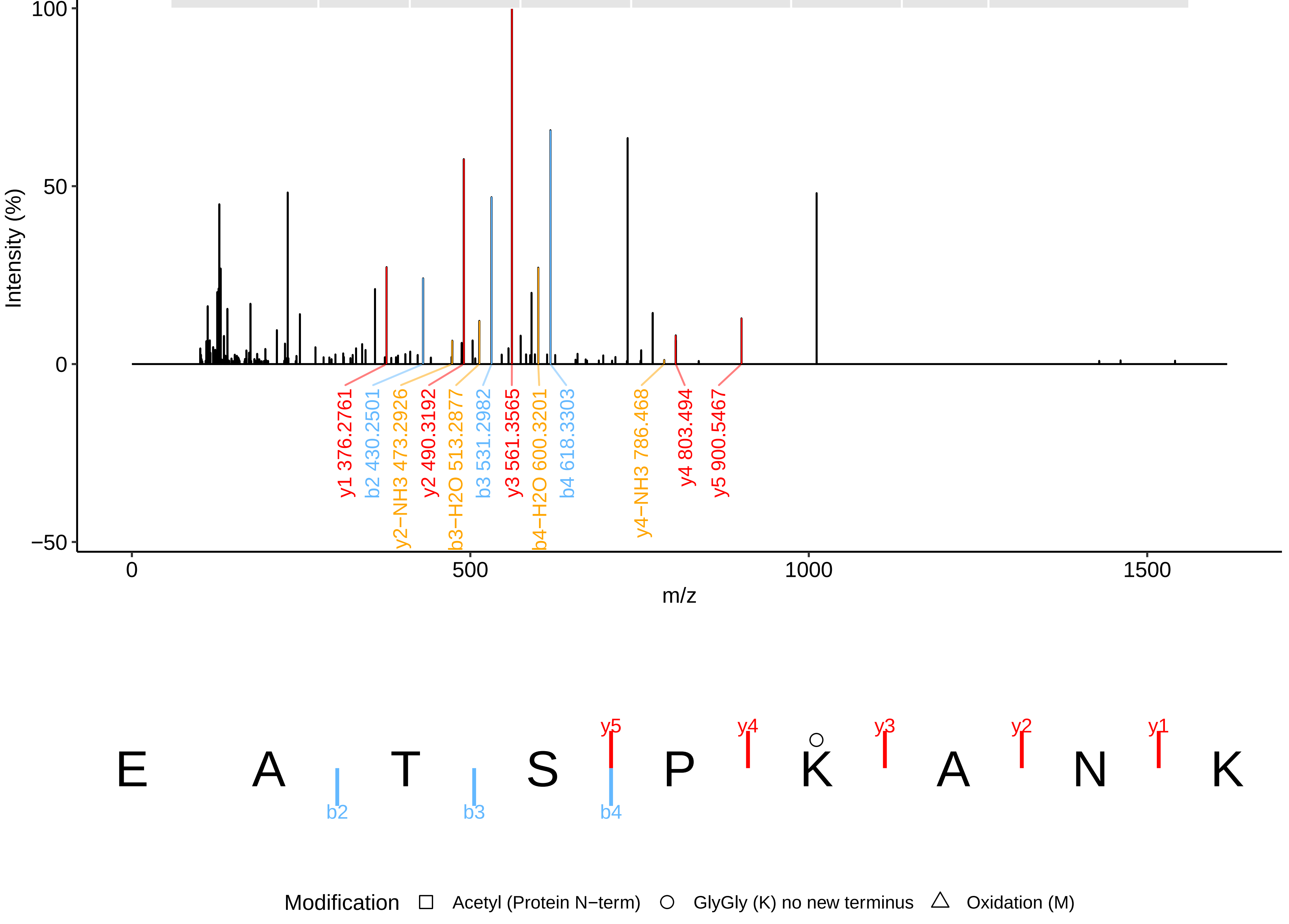

20200921_KIZ_MUb4_GlyGly_10plex_2 − Scan number: 12744

Gene Names	Charge	m/z	Mass	Retention time	PEP	Score	Precursor Intensity
IFNGR1	3	347.8816	1040.623	49.37	0.06778	58.676	319789.6

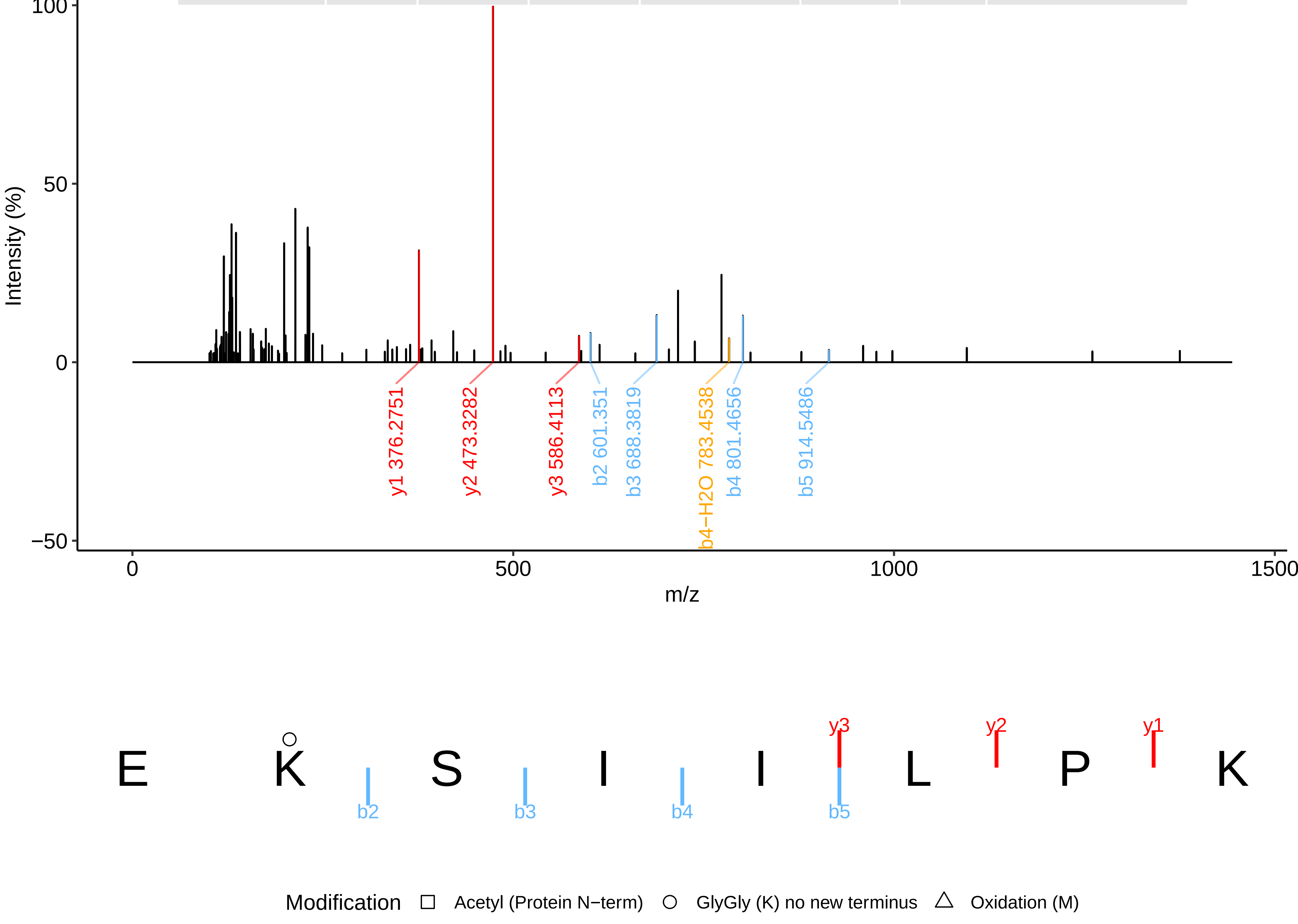

20200921_KIZ_MUb4_GlyGly_10plex_2 − Scan number: 5258

Gene Names	Charge	m/z	Mass	Retention time	PEP	Score	Precursor Intensity
DCAF6	3	425.2176	1272.631	18.827	0.089171	49.777	827083.4

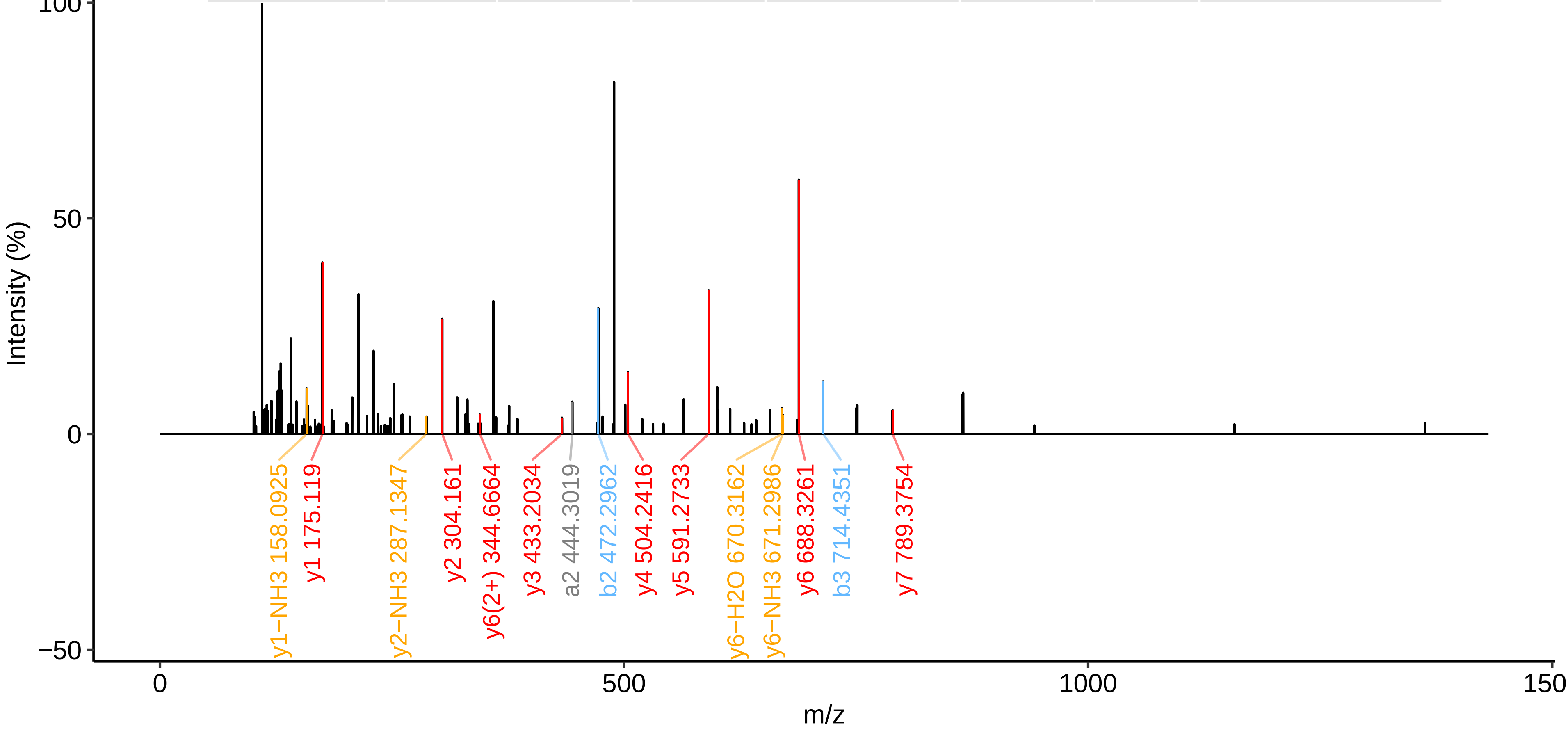

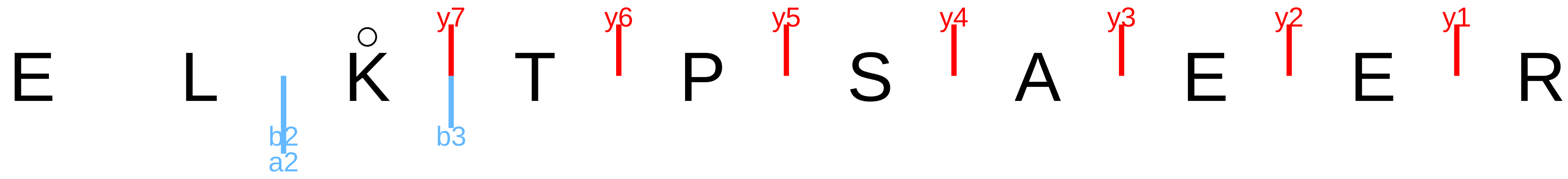

Modification □ Acetyl (Protein N−term) ○ GlyGly (K) no new terminus △ Oxidation (M)

20200924_KIZ_MUb5_GlyGly_2 − Scan number: 4642

Gene Names	Charge	m/z	Mass	Retention time	PEP	Score	Precursor Intensity
DCAF6	3	425.2176	1272.631	17.078	0.025673	62.466	NA

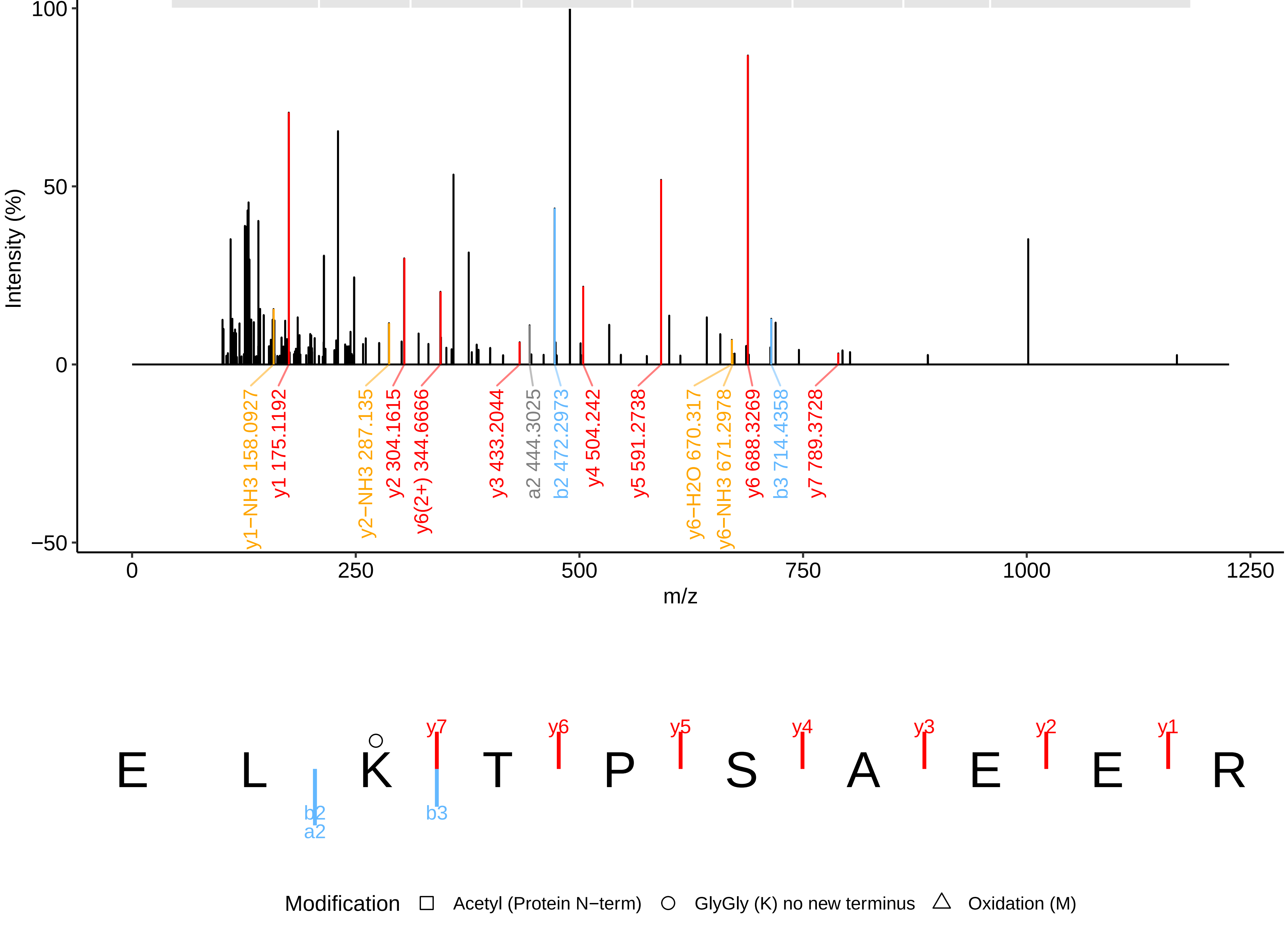

20200921_KIZ_MUb4_GlyGly_10plex_2 − Scan number: 8077

Gene Names	Charge	m/z	Mass	Retention time	PEP	Score	Precursor Intensity
CKS2	3	348.2012	1041.582	30.361	0.029429	64.265	663757.9

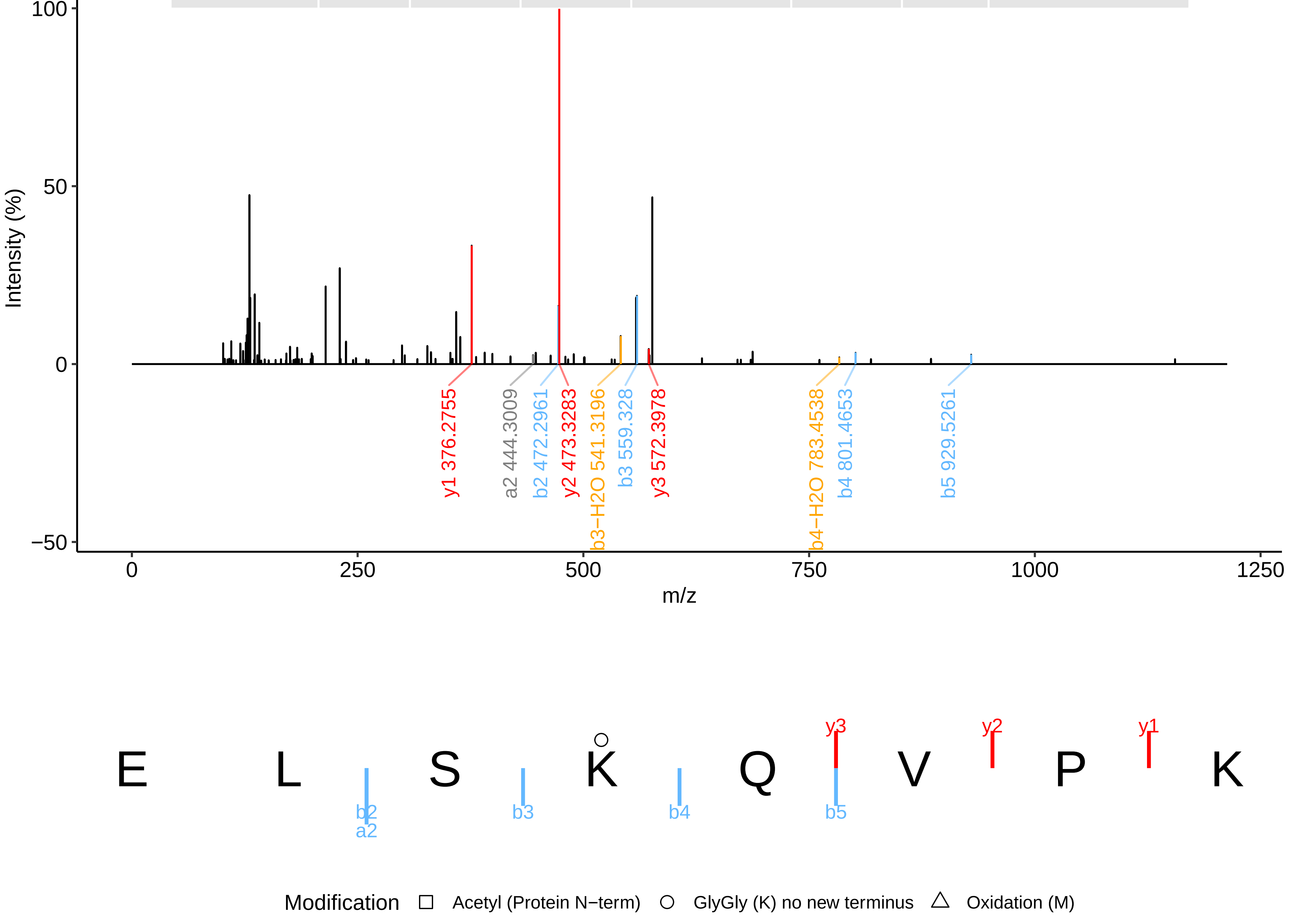

20200921_KIZ_MUb4_GlyGly_10plex − Scan number: 16776

Gene Names	Charge	m/z	Mass	Retention time	PEP	Score	Precursor Intensity
EZR;RDX	3	431.563	1291.667	60.373	7.4639e−05	113.71	9030942

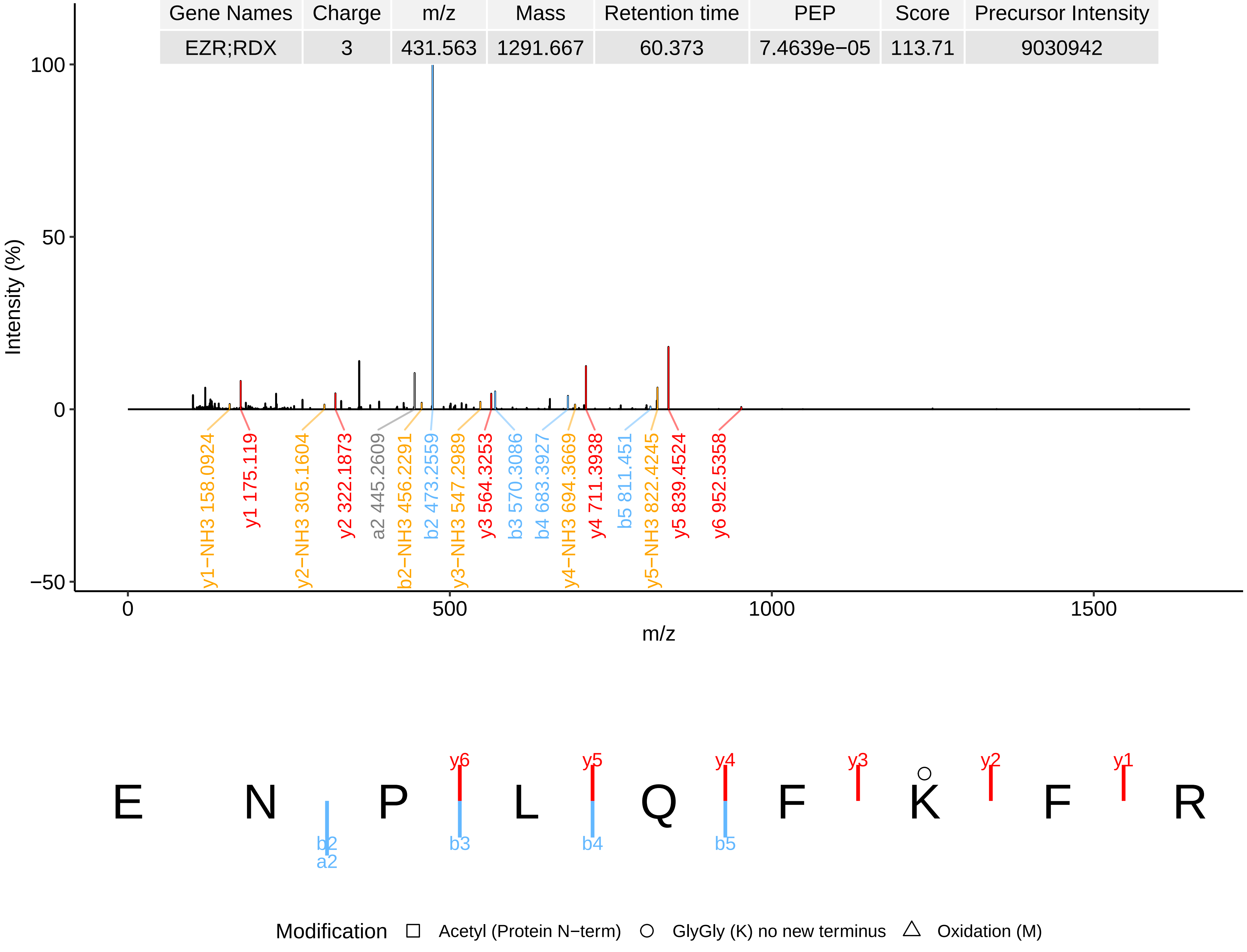

20200921_KIZ_MUb4_GlyGly_10plex_2 − Scan number: 15626

Gene Names	Charge	m/z	Mass	Retention time	PEP	Score	Precursor Intensity
EZR;RDX	3	431.563	1291.667	61.147	0.0020582	89.247	1361632

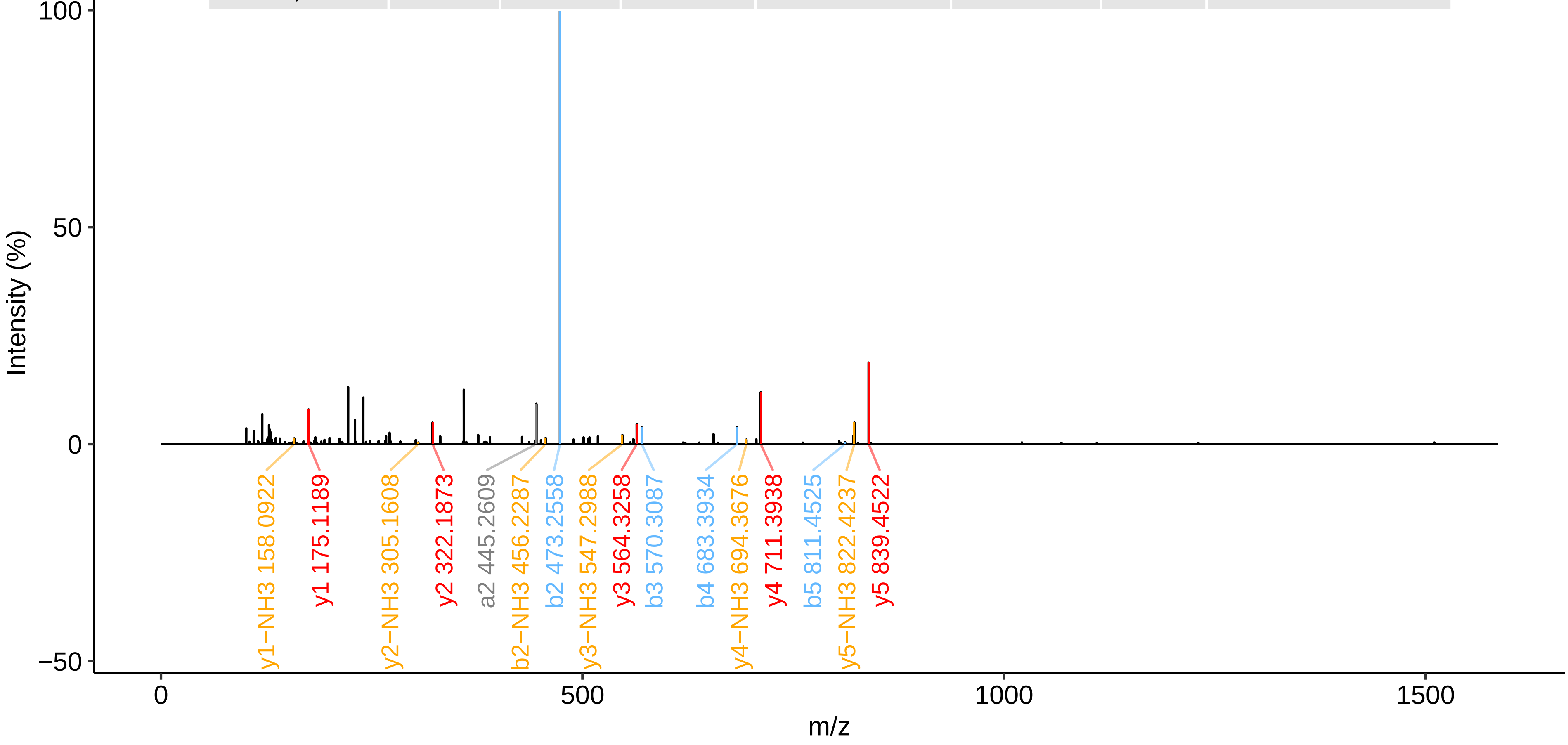

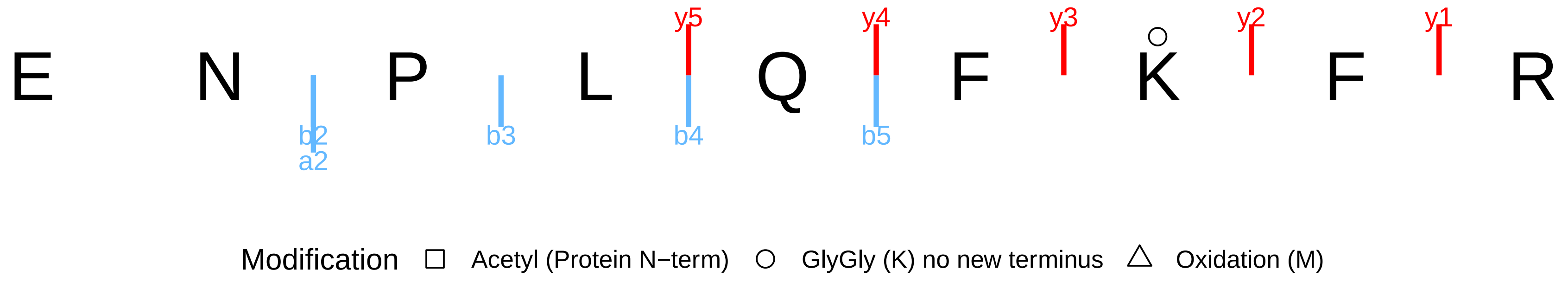

20200924_KIZ_MUb5_GlyGly_1 − Scan number: 15644

Gene Names	Charge	m/z	Mass	Retention time	PEP	Score	Precursor Intensity
EZR;RDX	3	431.563	1291.667	51.046	2.0404e−05	115.71	23782174

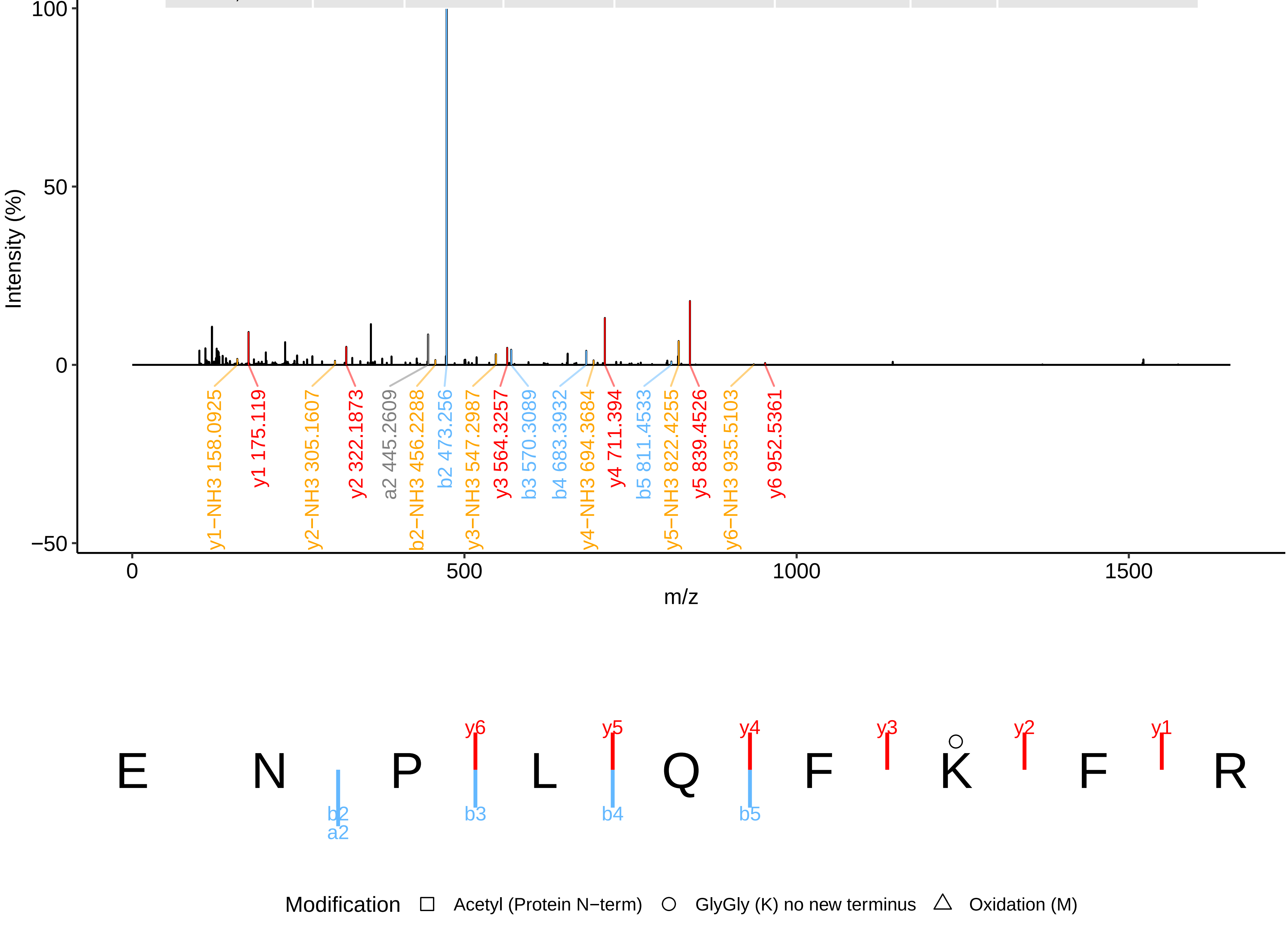

20200924_KIZ_MUb5_GlyGly_2 − Scan number: 14970

Gene Names	Charge	m/z	Mass	Retention time	PEP	Score	Precursor Intensity
EZR;RDX	3	431.563	1291.667	55.947	7.4639e−05	113.71	8216695

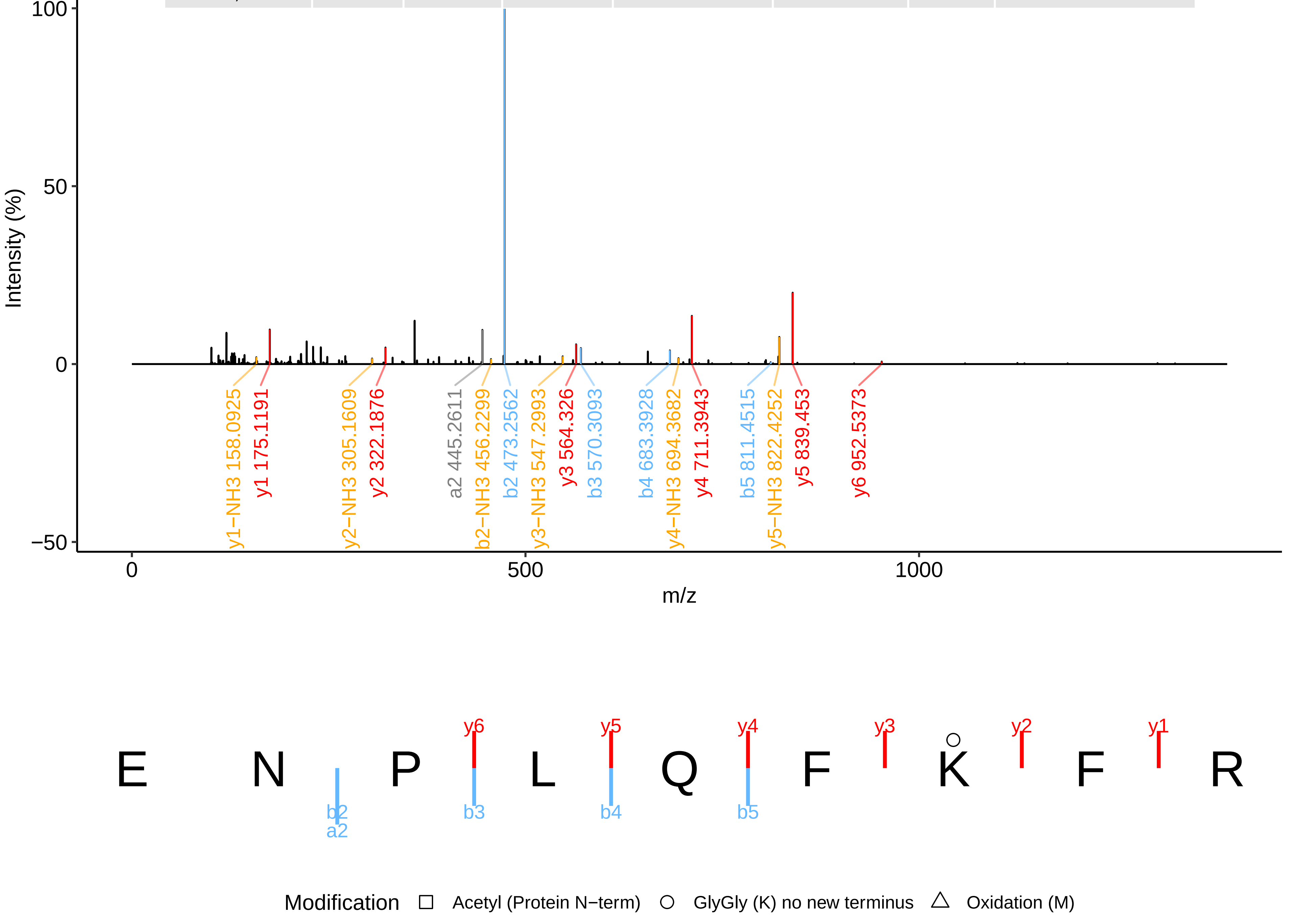

20201118_KIZ_MUb6_GlyGly_1 − Scan number: 15862

Gene Names	Charge	m/z	Mass	Retention time	PEP	Score	Precursor Intensity
EZR;RDX	3	431.563	1291.667	50.17	2.0404e−05	115.71	26012684

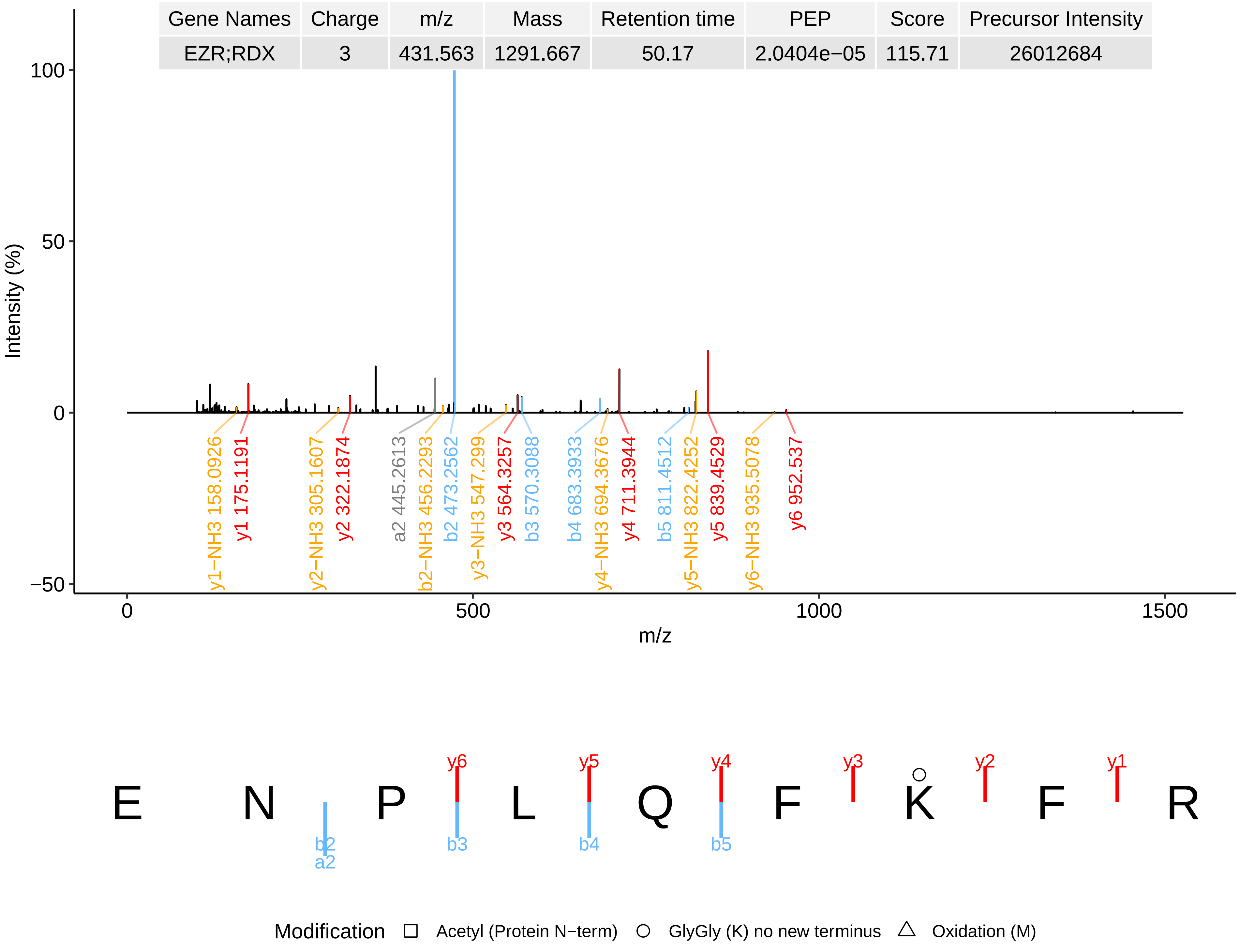

20201118_KIZ_MUb6_GlyGly_2 − Scan number: 18213

Gene Names	Charge	m/z	Mass	Retention time	PEP	Score	Precursor Intensity
EZR;RDX	3	431.563	1291.667	61.599	2.0404e−05	115.71	10827655

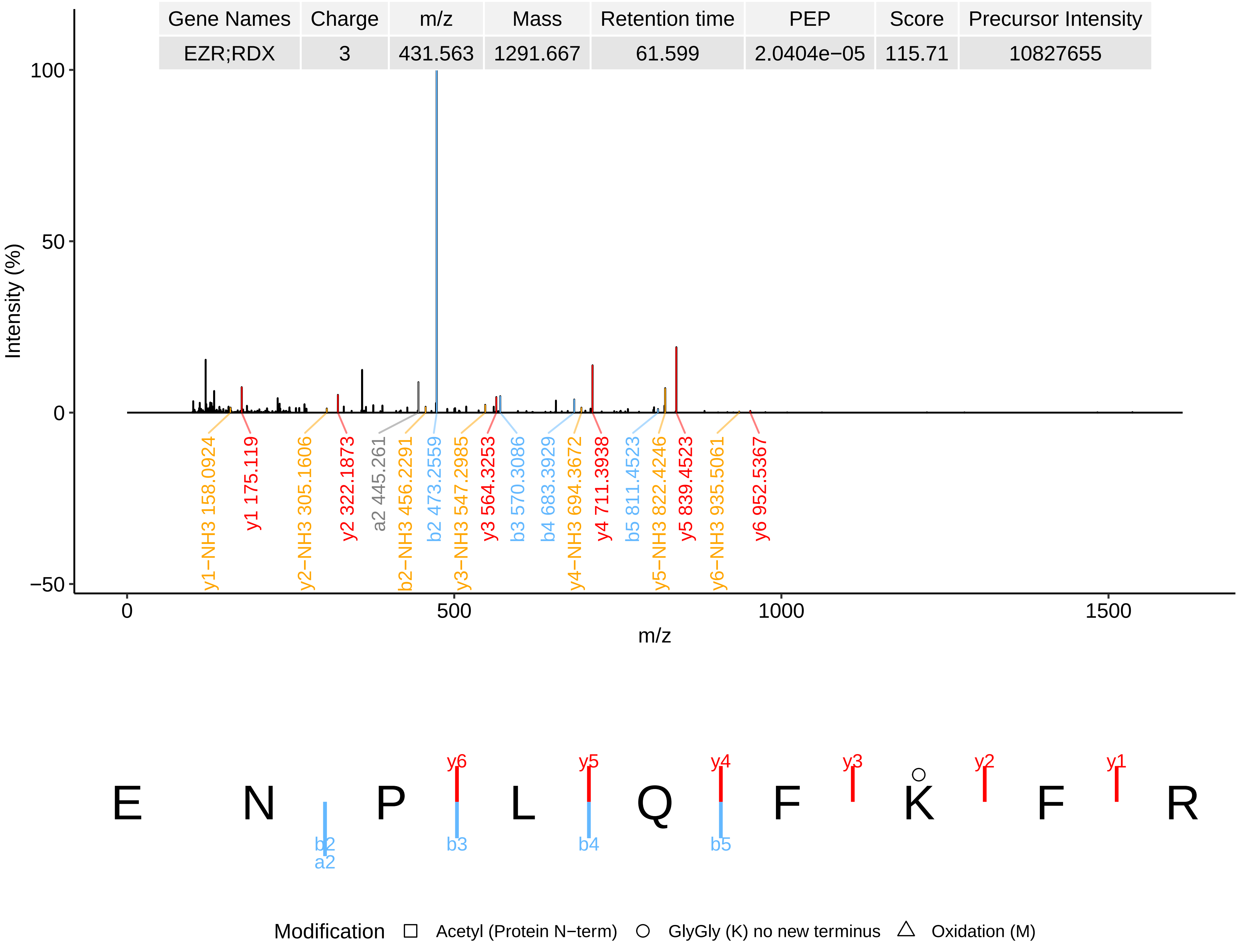

20200921_KIZ_MUb4_GlyGly_10plex_2 − Scan number: 5125

Gene Names	Charge	m/z	Mass	Retention time	PEP	Score	Precursor Intensity
RWDD4	3	349.1927	1044.556	18.284	0.094814	47.574	718819.2

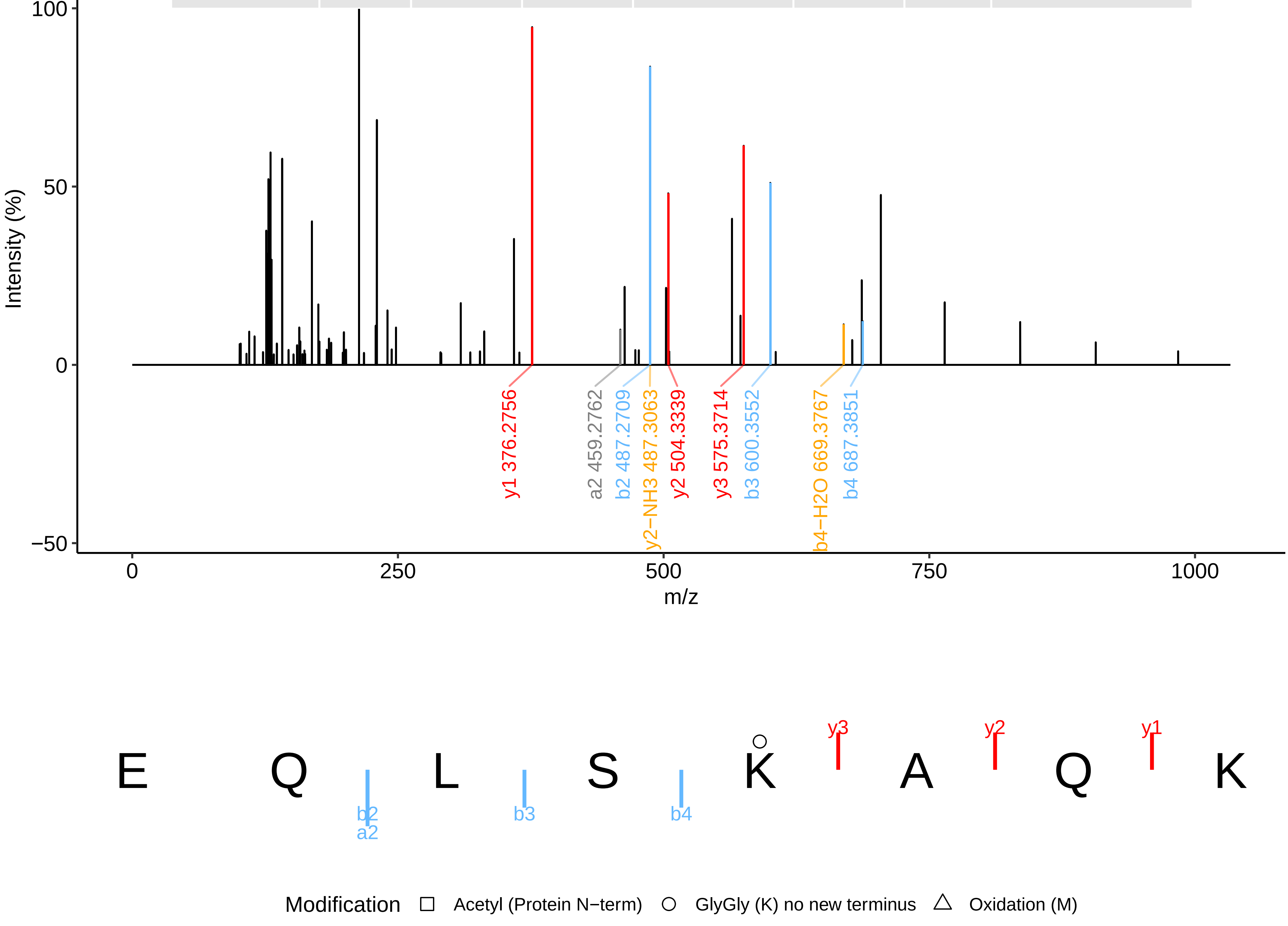

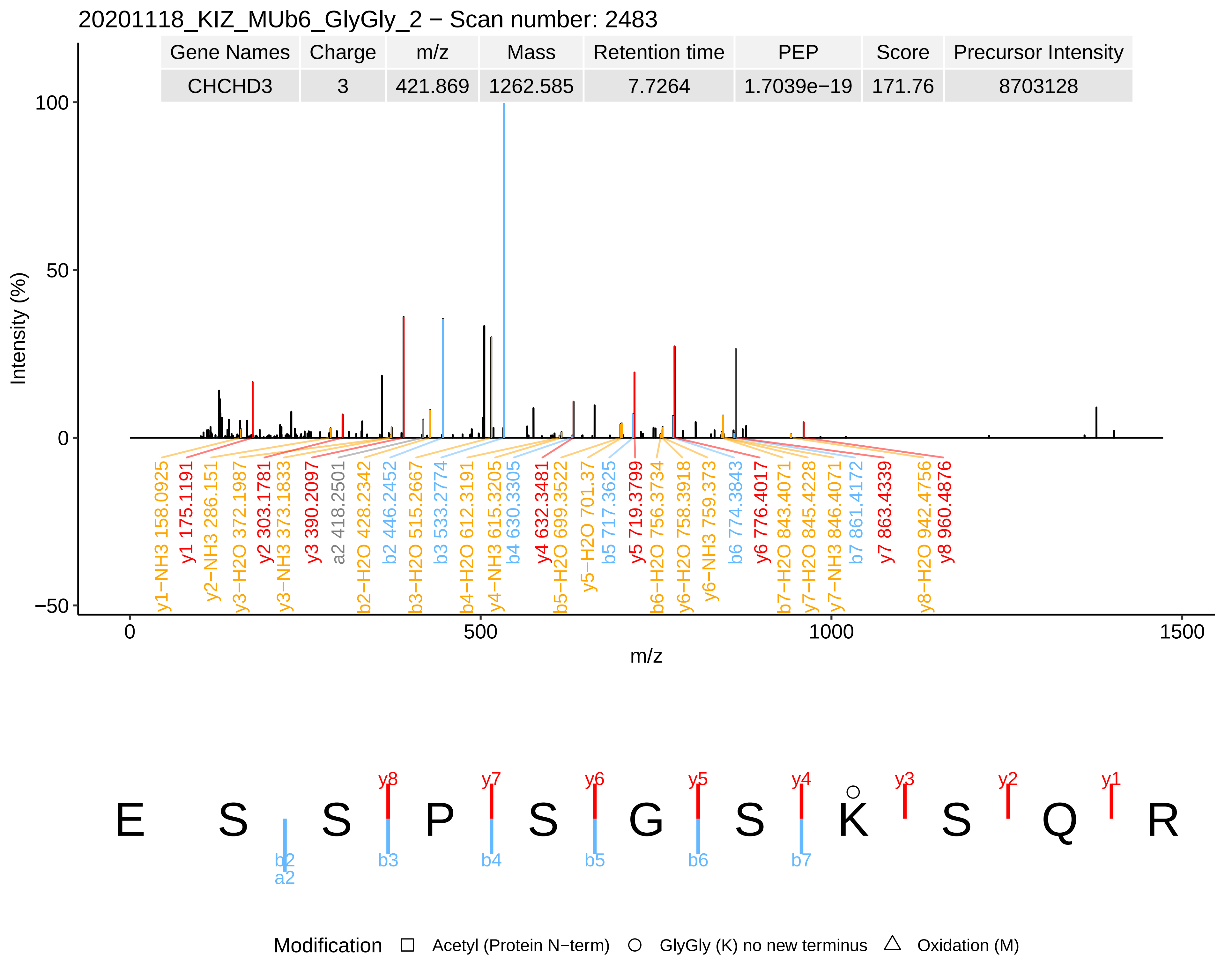
20201118_KIZ_MUb6_GlyGly_2 − Scan number: 2483
Gene Names | Charge | m/z | Mass | Retention time | PEP | Score | Precursor Intensity
CHCHD3 | 3 | 421.869 | 1262.585 | 7.7264 | 1.7039e−19 | 171.76 | 8703128
Intensity (%)
100
50
0
−50
0
500
1000
1500
m/z
y1−NH3 158.0925
y1 175.1191
y2−NH3 286.151
y3−H2O 372.1987
y2 303.1781
y3−NH3 373.1833
y3 390.2097
a2 418.2501
b2−H2O 428.2342
b2 446.2452
b3−H2O 515.2667
b3 533.2774
b4−H2O 612.3191
y4−NH3 615.3205
b4 630.3305
y4 632.3481
b5−H2O 699.3522
y5−H2O 701.37
b5 717.3625
y5 719.3799
b6−H2O 756.3734
y6−H2O 758.3918
y6−NH3 759.373
b6 774.3843
y6 776.4017
b7−H2O 843.4071
y7−H2O 845.4228
y7−NH3 846.4071
b7 861.4172
y7 863.4339
y8−H2O 942.4756
y8 960.4876
E S S P S G S K S Q R
y8 y7 y6 y5 y4 y3 y2 y1
b2 a2 b3 b4 b5 b6 b7
Modification
Acetyl (Protein N−term)
GlyGly (K) no new terminus
Oxidation (M)

20200921_KIZ_MUb4_GlyGly_10plex − Scan number: 3715

Gene Names	Charge	m/z	Mass	Retention time	PEP	Score	Precursor Intensity
VTI1A	3	431.2128	1290.616	11.936	2.4083e−17	165.39	3770122

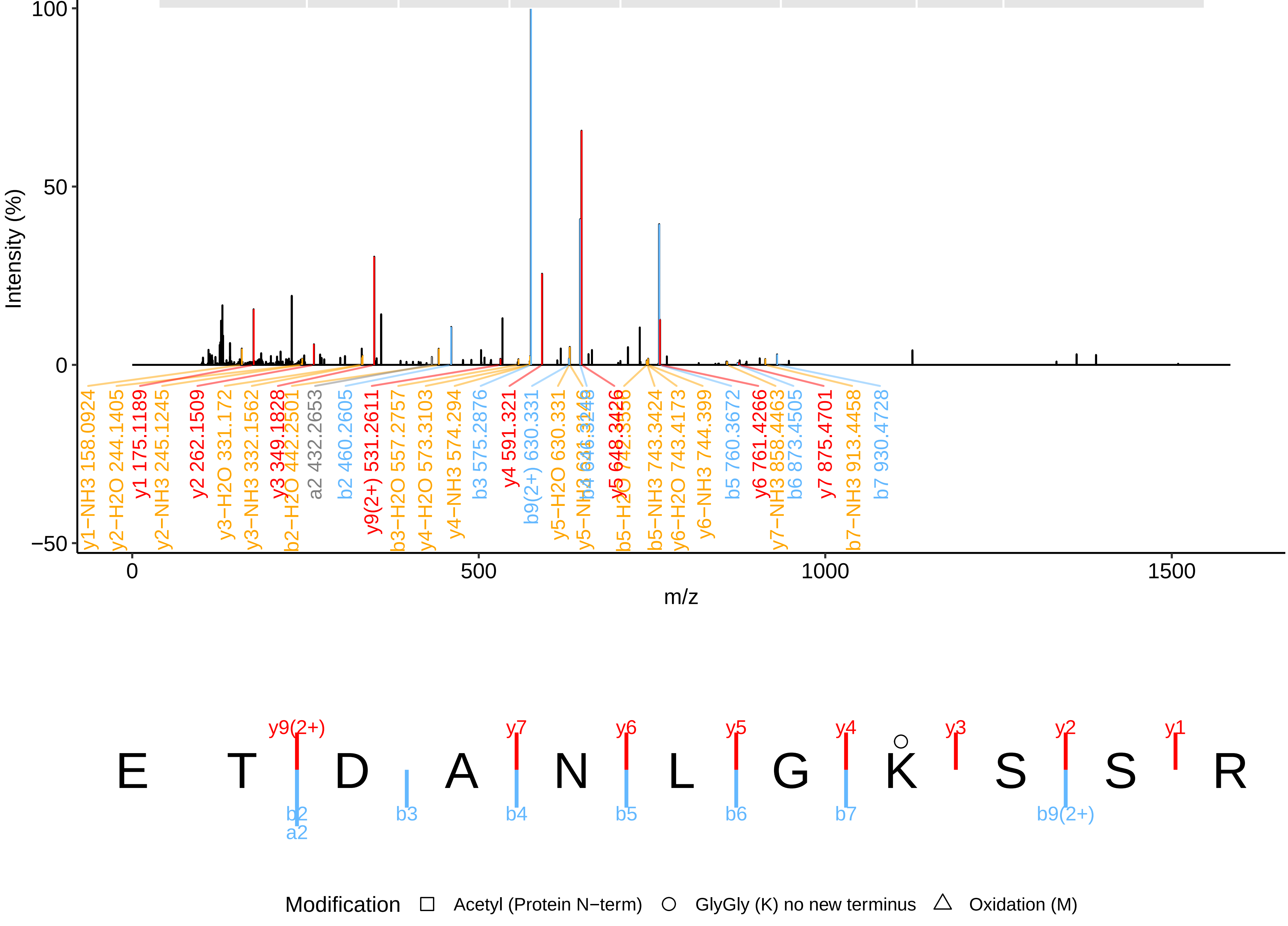

20200921_KIZ_MUb4_GlyGly_10plex_2 − Scan number: 3522

Gene Names	Charge	m/z	Mass	Retention time	PEP	Score	Precursor Intensity
VTI1A	3	431.2128	1290.616	11.878	1.5971e−05	109.24	1185617

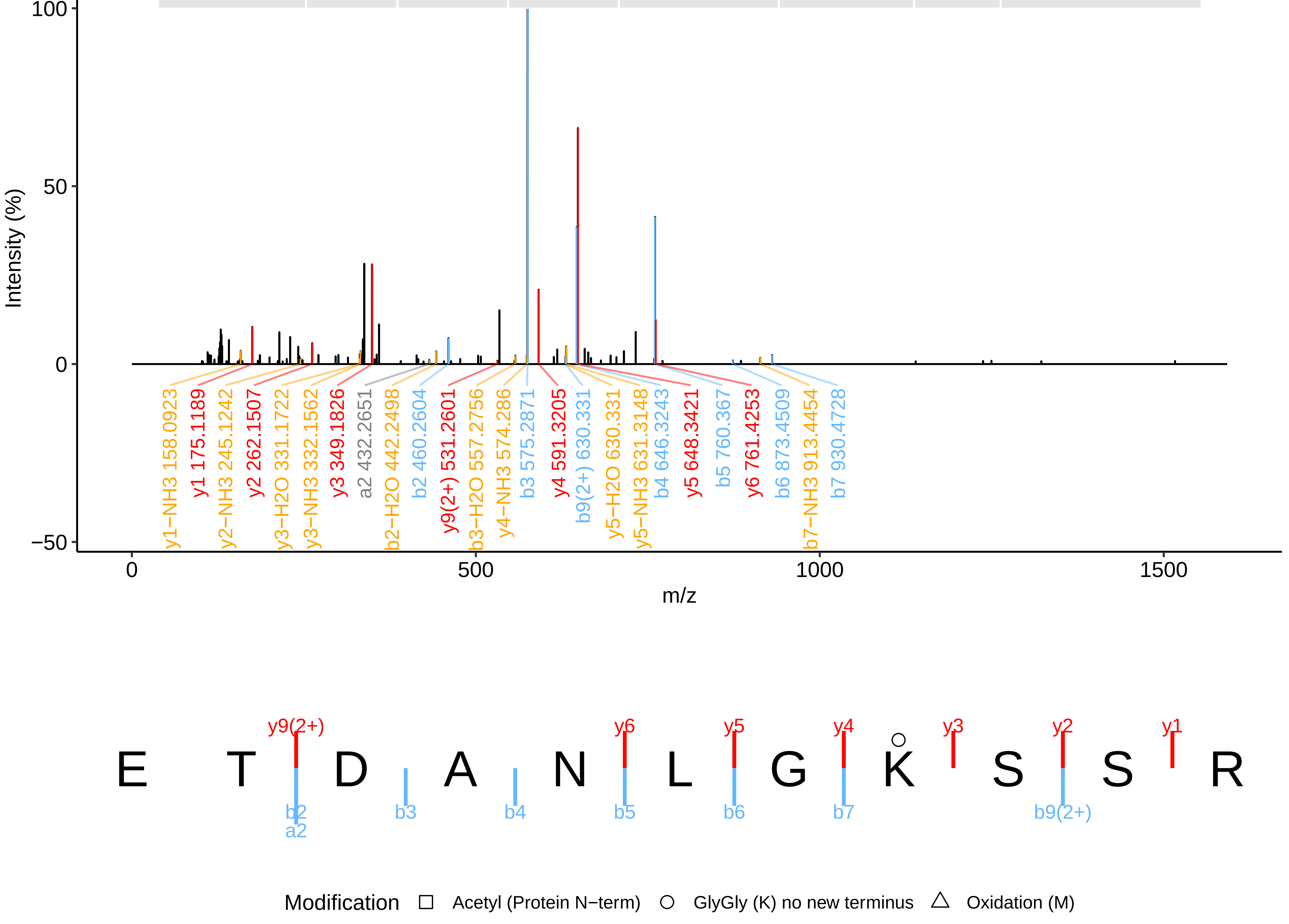

20200924_KIZ_MUb5_GlyGly_2 − Scan number: 3095

Gene Names	Charge	m/z	Mass	Retention time	PEP	Score	Precursor Intensity
VTI1A	3	431.2128	1290.616	11.118	6.9003e−05	97.749	2212326

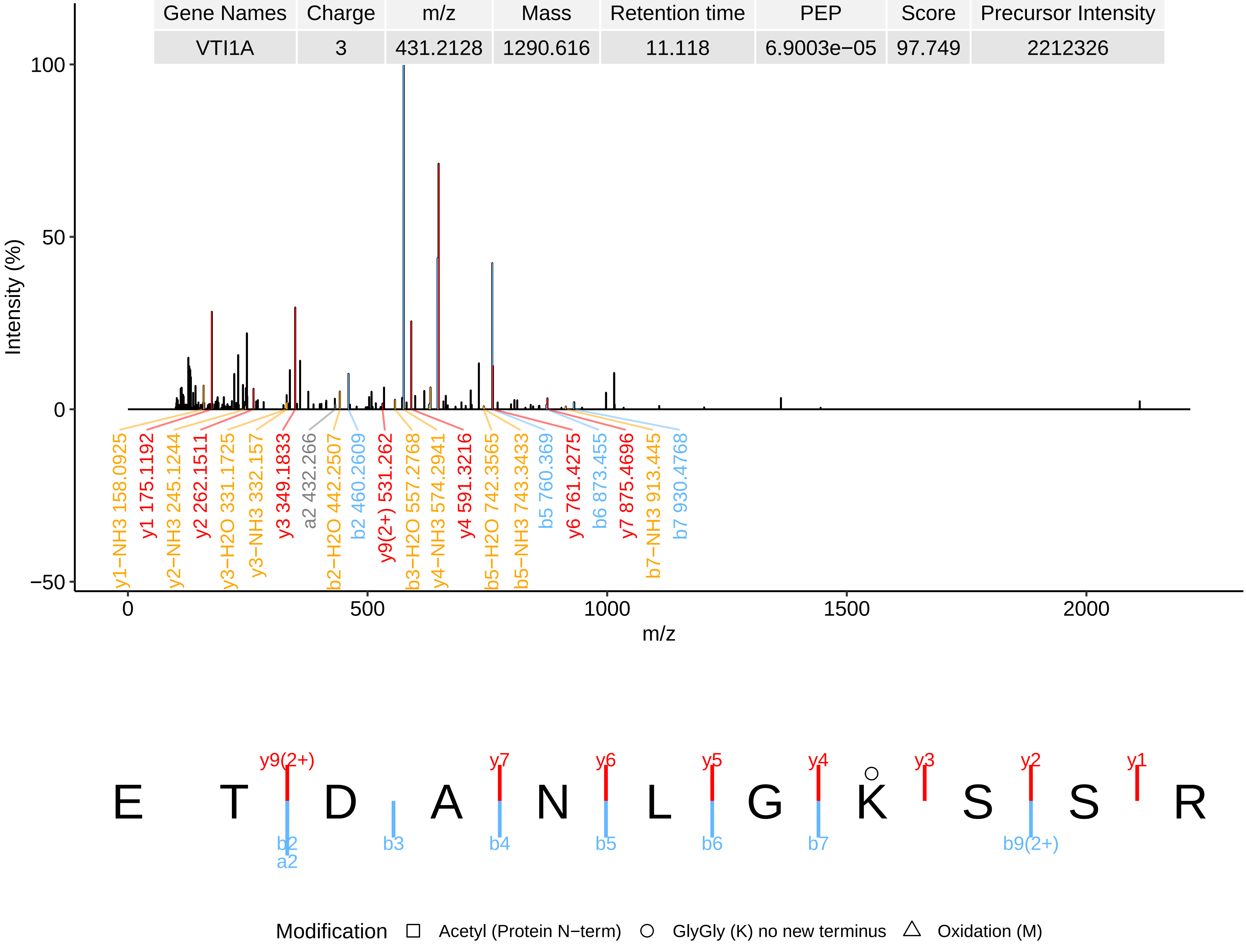

20201118_KIZ_MUb6_GlyGly_1 − Scan number: 3231

Gene Names	Charge	m/z	Mass	Retention time	PEP	Score	Precursor Intensity
VTI1A	3	431.2128	1290.616	9.9444	2.7329e−08	124.18	12489922

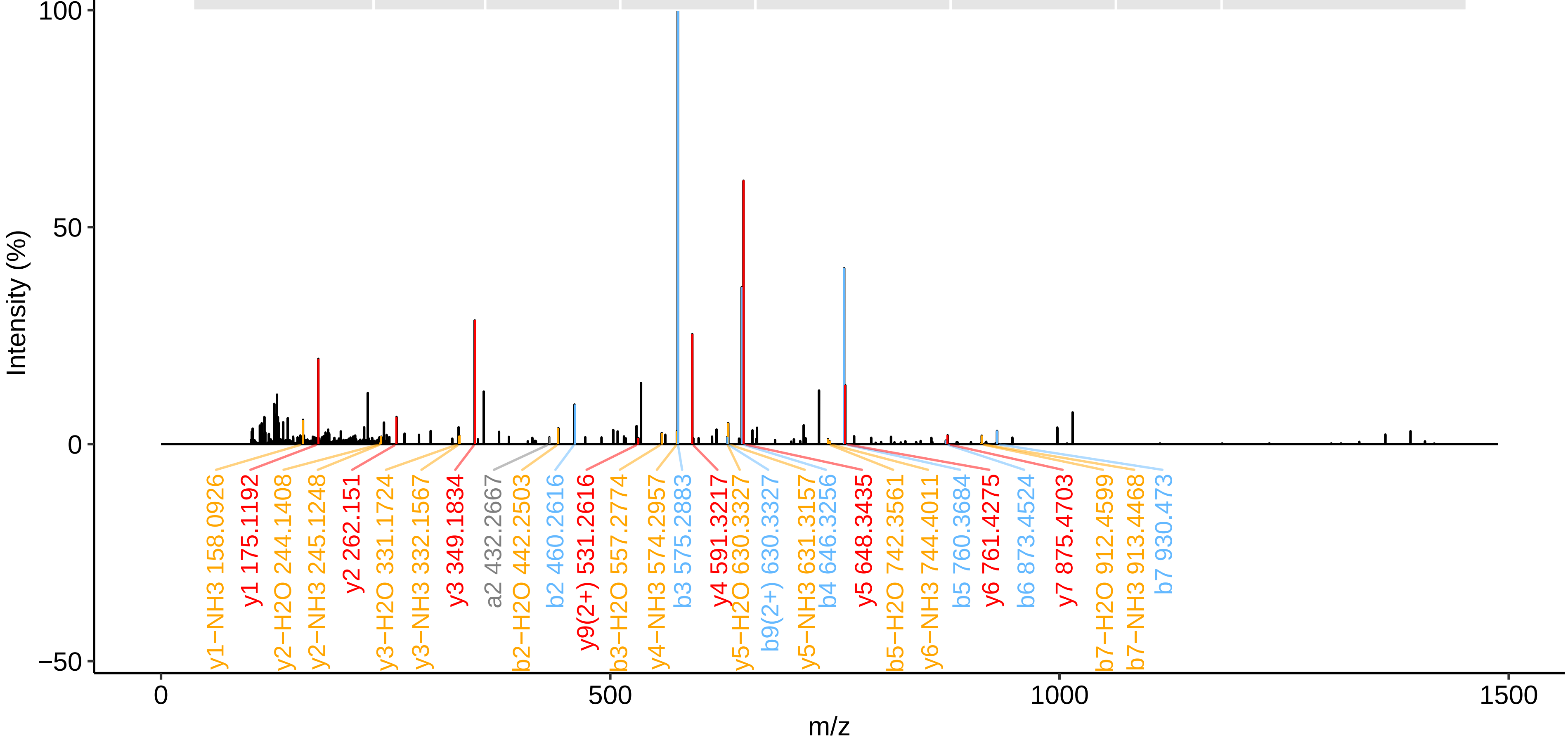

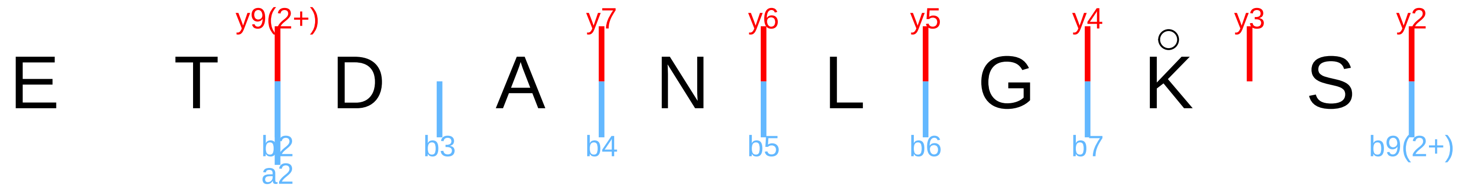

Modification □ Acetyl (Protein N−term) ○ GlyGly (K) no new terminus △ Oxidation (M)

20201118_KIZ_MUb6_GlyGly_2 − Scan number: 3550

Gene Names	Charge	m/z	Mass	Retention time	PEP	Score	Precursor Intensity
VTI1A	3	431.2128	1290.616	11.785	0.010153	70.345	5097970

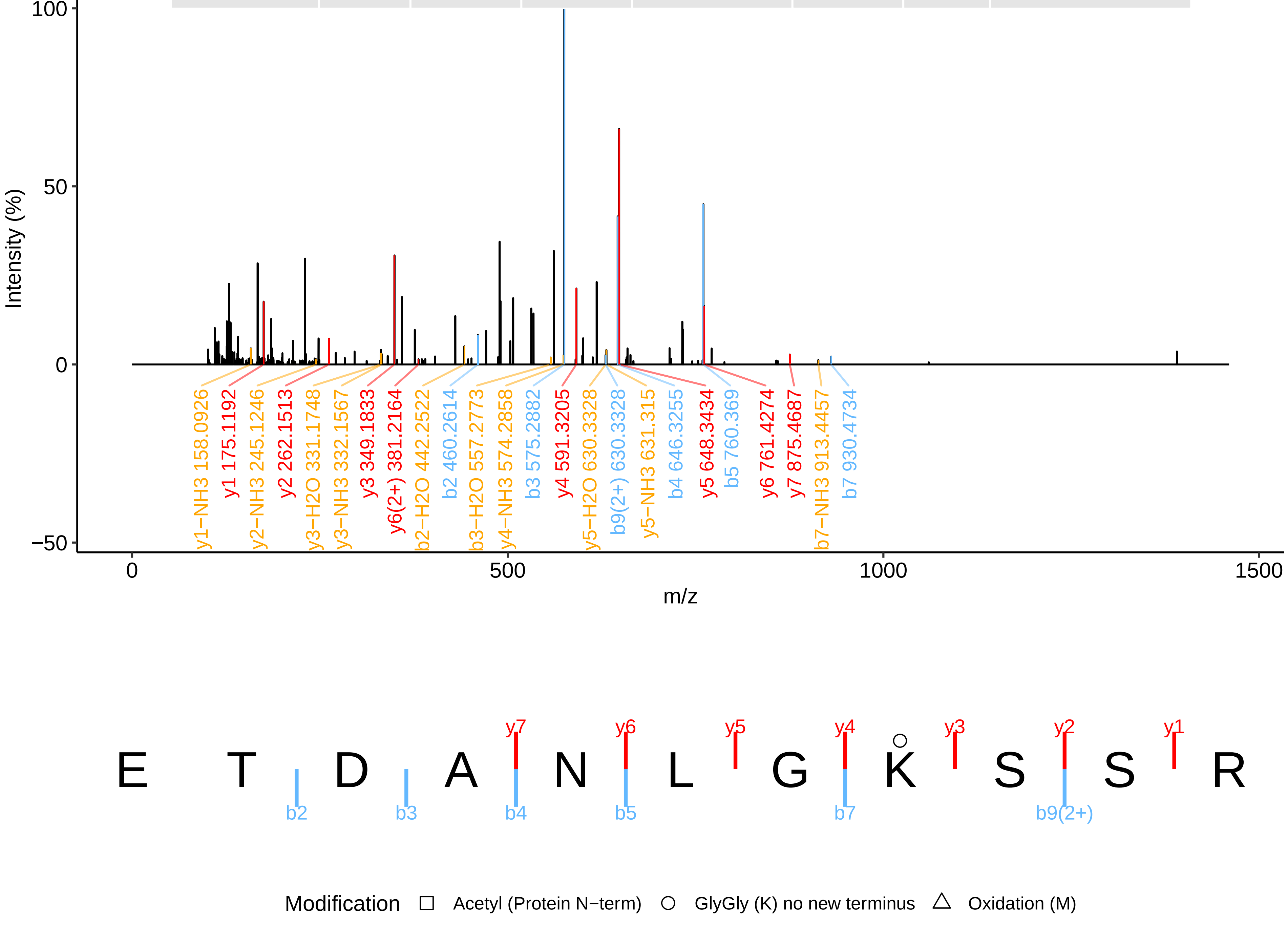

20200921_KIZ_MUb4_GlyGly_10plex_2 − Scan number: 6151

Gene Names	Charge	m/z	Mass	Retention time	PEP	Score	Precursor Intensity
NCAPD2	3	426.2153	1275.624	22.475	0.10491	46.001	391931.2

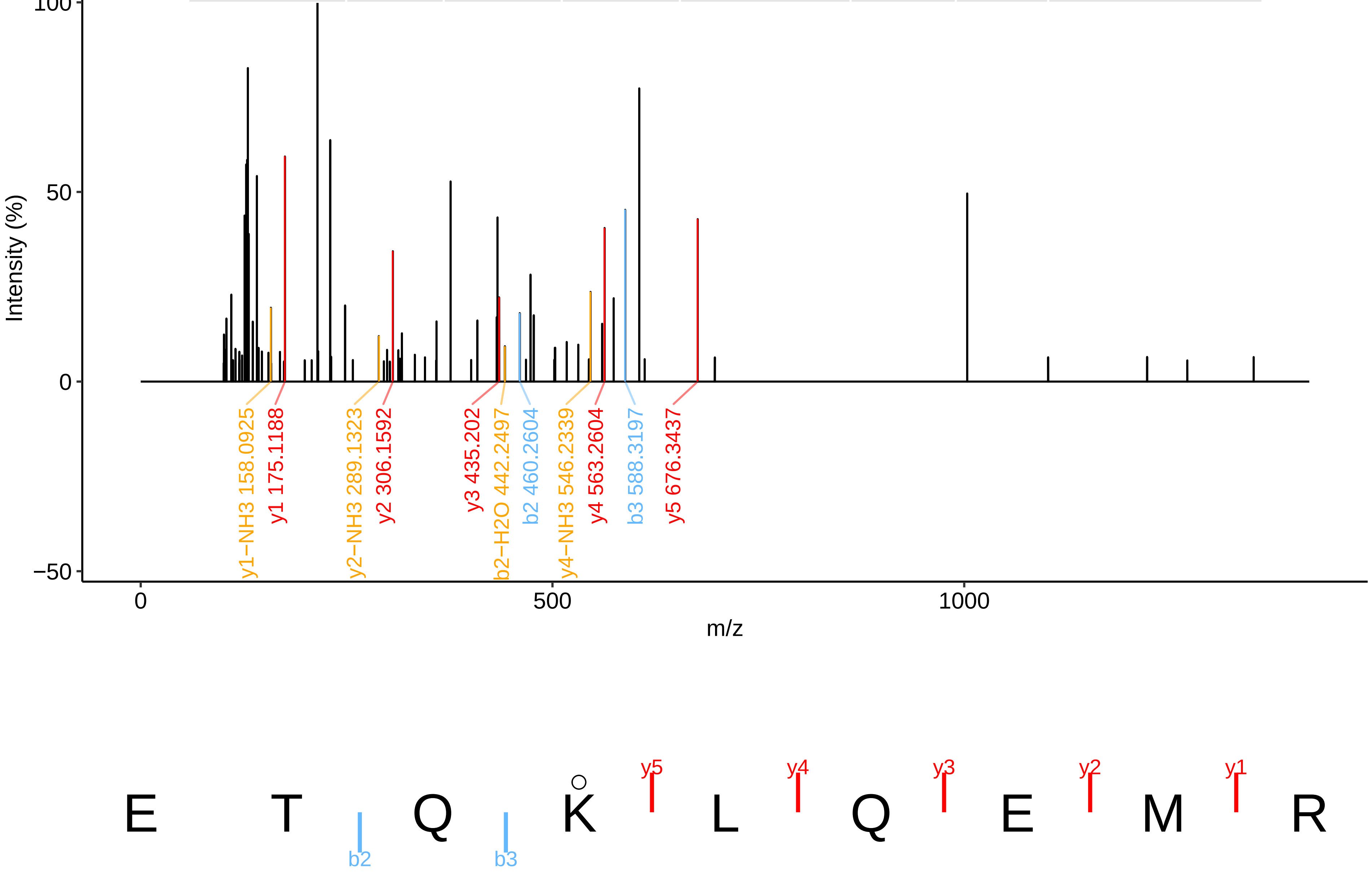

Modification □ Acetyl (Protein N−term) ○ GlyGly (K) no new terminus △ Oxidation (M)

20200921_KIZ_MUb4_GlyGly_10plex_2 − Scan number: 11850

Gene Names	Charge	m/z	Mass	Retention time	PEP	Score	Precursor Intensity
PCBP1;PCBP2;PCBP4;PCBP3	3	348.8731	1043.598	45.714	0.040606	77.732	759582.7

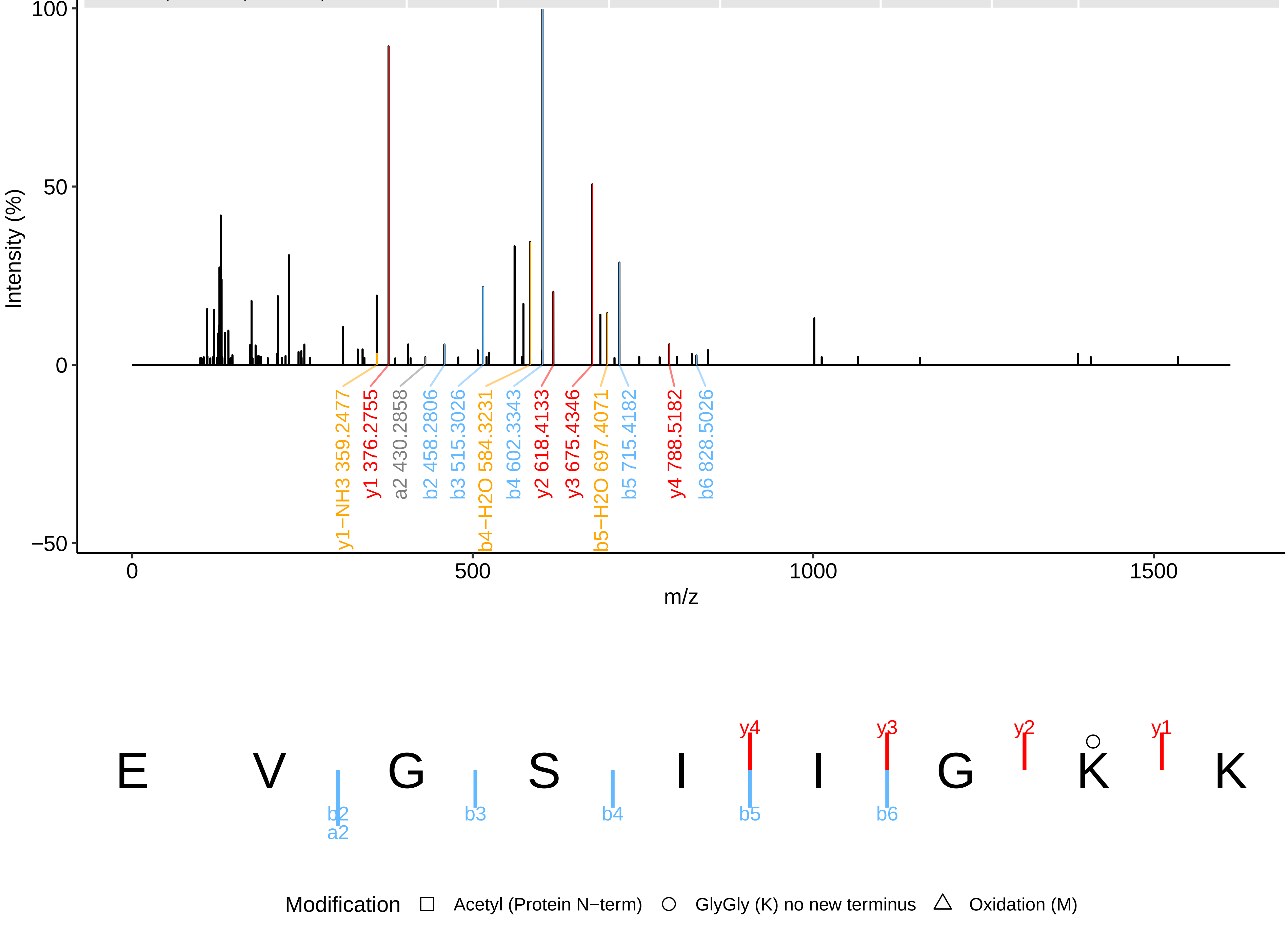

20200921_KIZ_MUb4_GlyGly_10plex_2 − Scan number: 11664

Gene Names	Charge	m/z	Mass	Retention time	PEP	Score	Precursor Intensity
ACOT13	3	345.1952	1032.564	44.94	0.023003	69.423	265102.1

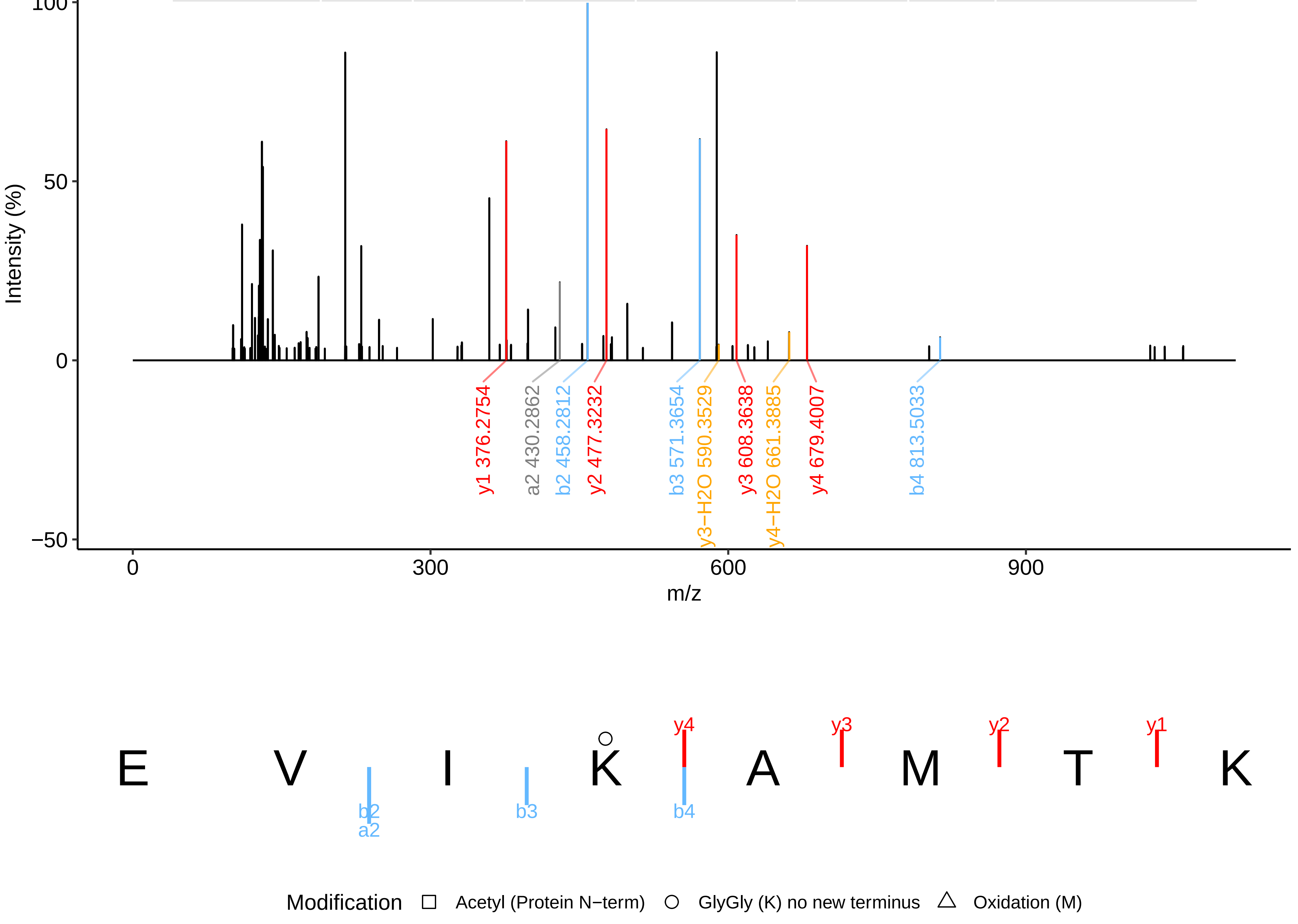

20200921_KIZ_MUb4_GlyGly_10plex − Scan number: 11492

Gene Names	Charge	m/z	Mass	Retention time	PEP	Score	Precursor Intensity
EEF1A1;EEF1A1P5	3	361.2011	1080.582	40.057	0.013091	98.997	1775109

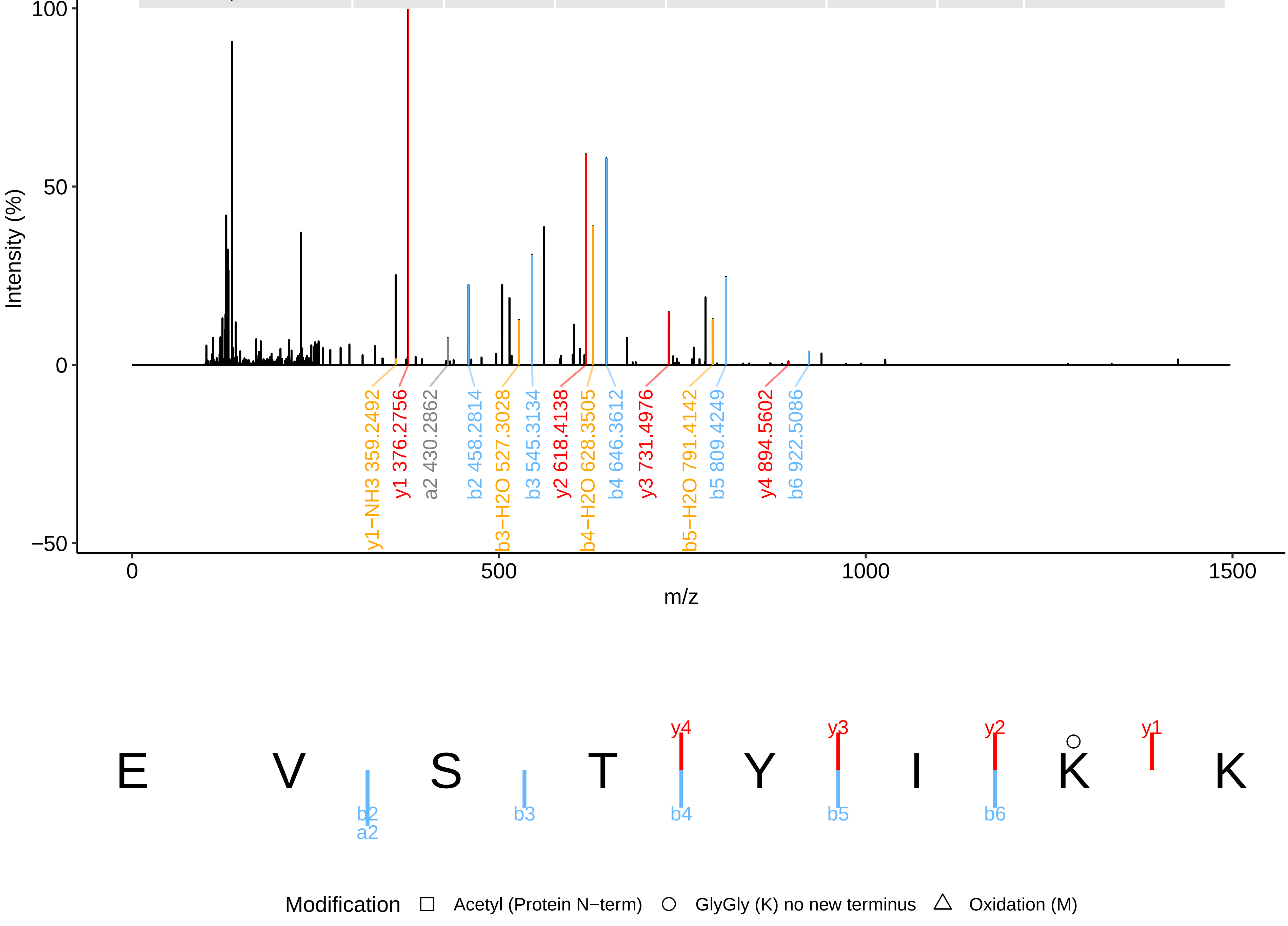

20200921_KIZ_MUb4_GlyGly_10plex_2 − Scan number: 10605

Gene Names	Charge	m/z	Mass	Retention time	PEP	Score	Precursor Intensity
EEF1A1;EEF1A1P5	3	361.2011	1080.582	40.606	0.012073	100.45	1227155

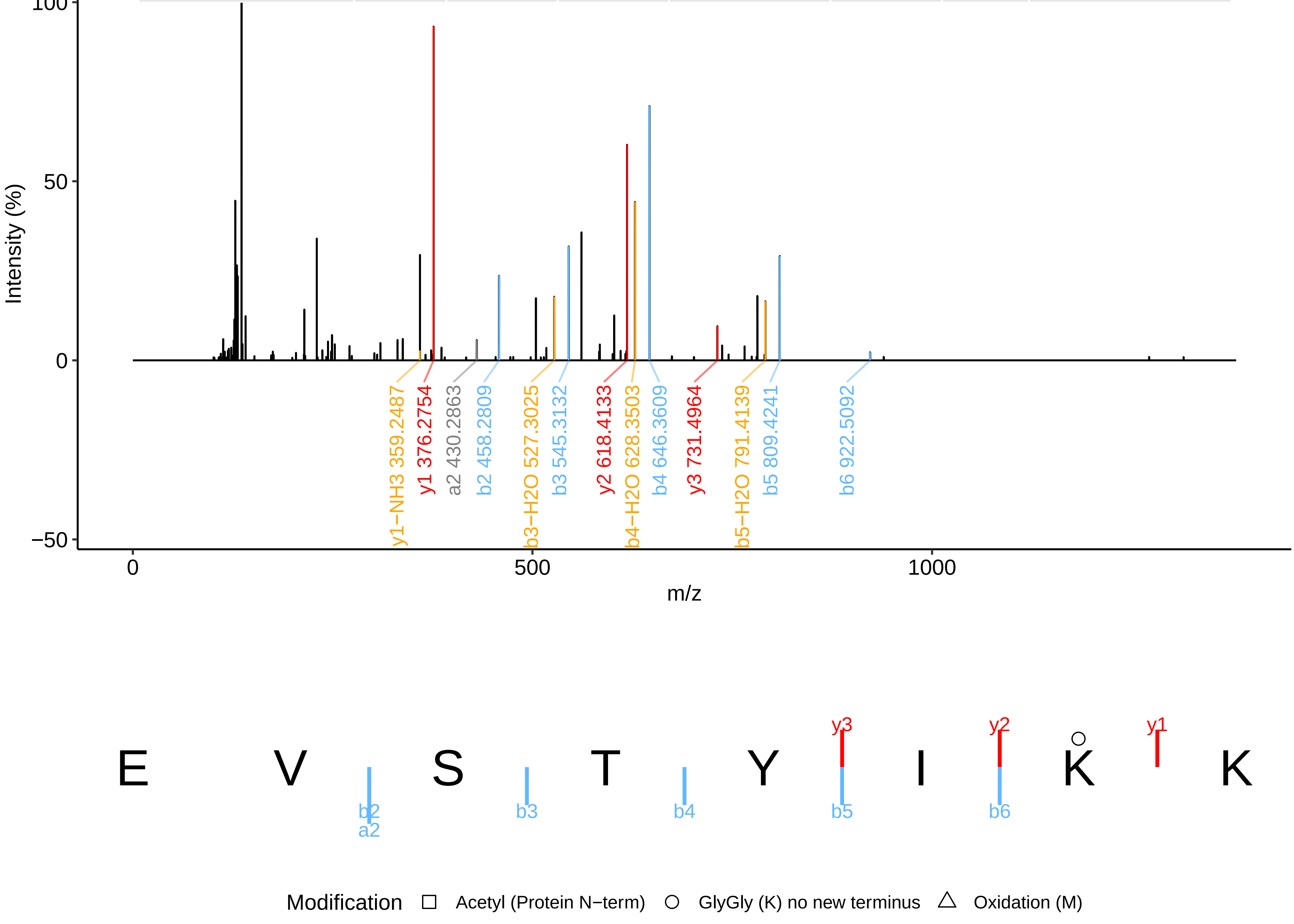

20200924_KIZ_MUb5_GlyGly_1 − Scan number: 10546

Gene Names	Charge	m/z	Mass	Retention time	PEP	Score	Precursor Intensity
EEF1A1;EEF1A1P5	3	361.2011	1080.582	34.062	0.011915	97.798	18114052

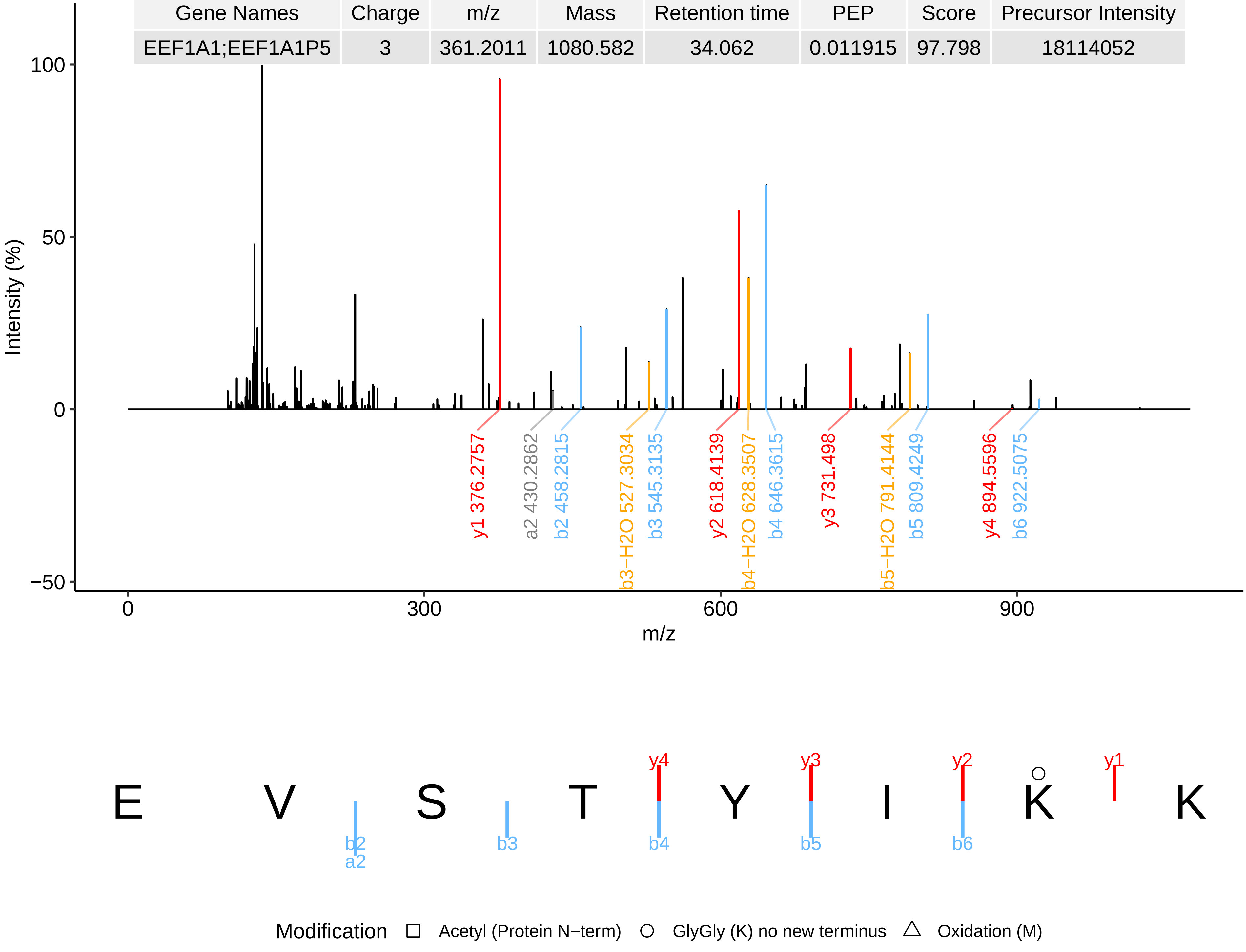

20200924_KIZ_MUb5_GlyGly_2 − Scan number: 9686

Gene Names	Charge	m/z	Mass	Retention time	PEP	Score	Precursor Intensity
EEF1A1;EEF1A1P5	3	361.2011	1080.582	36.178	0.025433	89.142	3432641

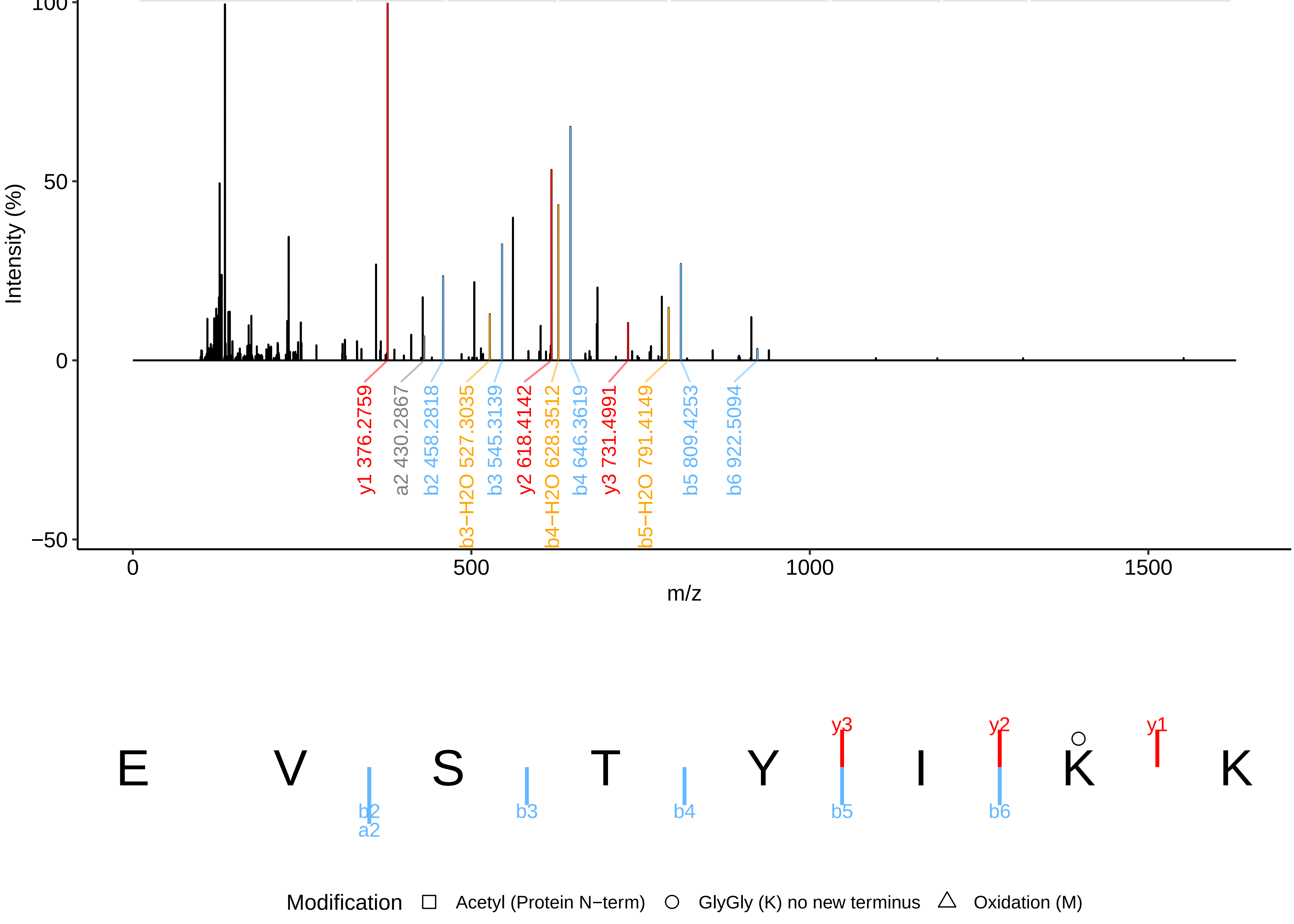

20200924_KIZ_MUb5_GlyGly_2 − Scan number: 9818

Gene Names	Charge	m/z	Mass	Retention time	PEP	Score	Precursor Intensity
EEF1A1;EEF1A1P5	3	361.2011	1080.582	36.692	0.005309	111.01	4354474

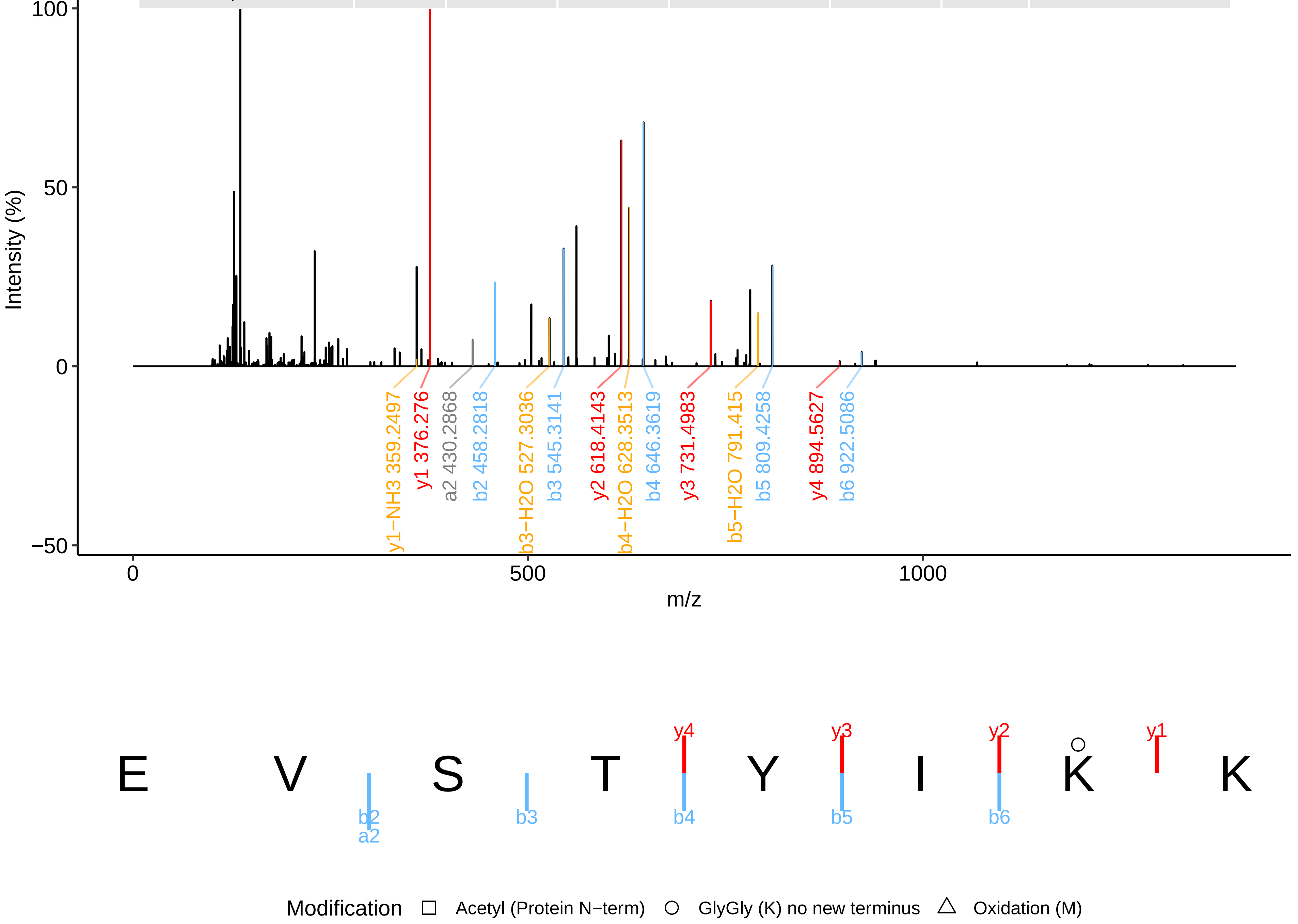

20200921_KIZ_MUb4_GlyGly_10plex − Scan number: 8828

Gene Names	Charge	m/z	Mass	Retention time	PEP	Score	Precursor Intensity
PRDX1	3	348.5101	1042.508	30.086	0.04461	70.912	8289051

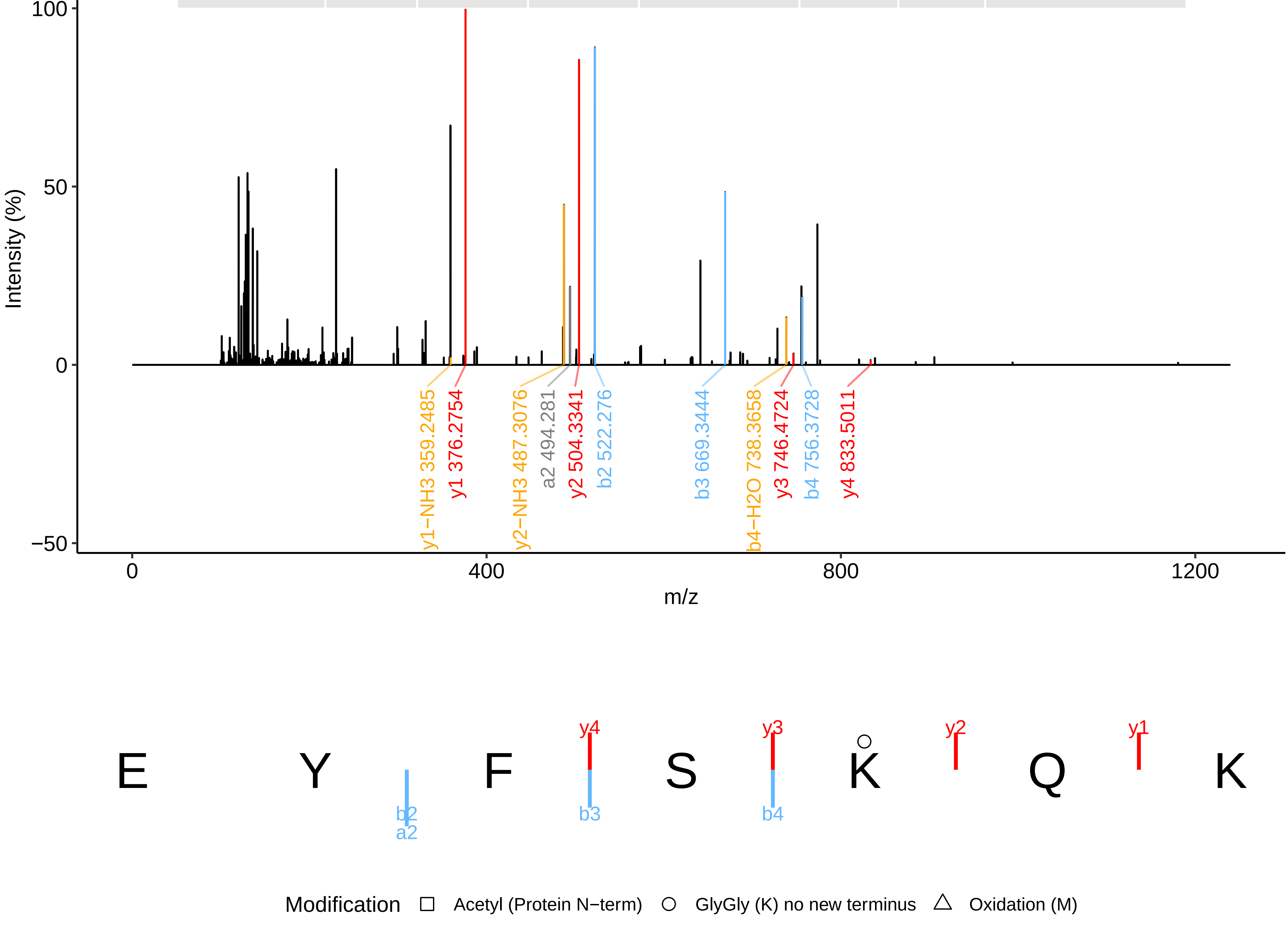

20200921_KIZ_MUb4_GlyGly_10plex − Scan number: 8975

Gene Names	Charge	m/z	Mass	Retention time	PEP	Score	Precursor Intensity
RAD23B	3	346.8416	1037.503	30.638	0.10148	55.676	16577445

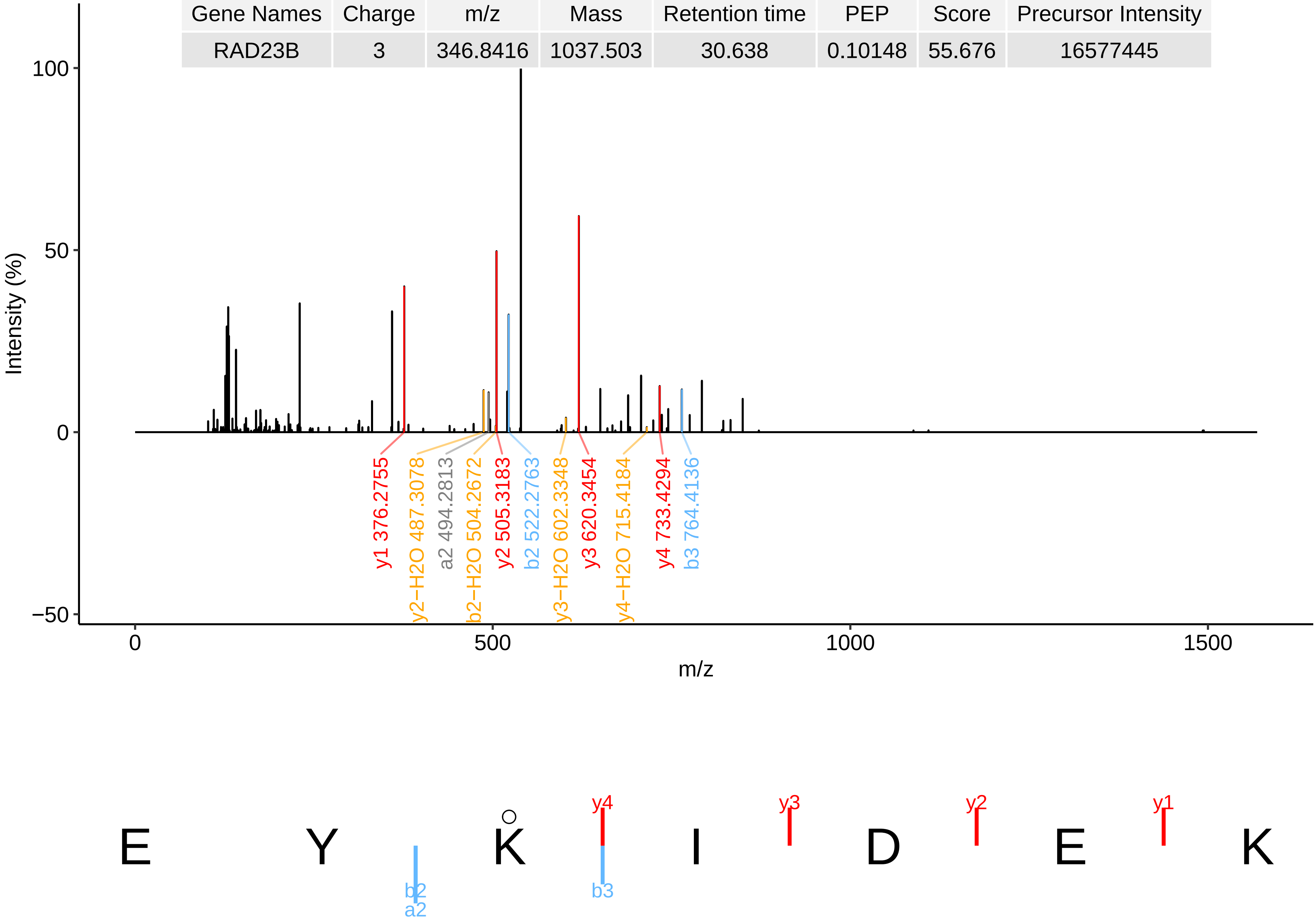

Modification □ Acetyl (Protein N−term) ○ GlyGly (K) no new terminus △ Oxidation (M)

20200921_KIZ_MUb4_GlyGly_10plex_2 − Scan number: 8288

Gene Names	Charge	m/z	Mass	Retention time	PEP	Score	Precursor Intensity
RAD23B	3	346.8416	1037.503	31.187	0.036372	74.127	1977247

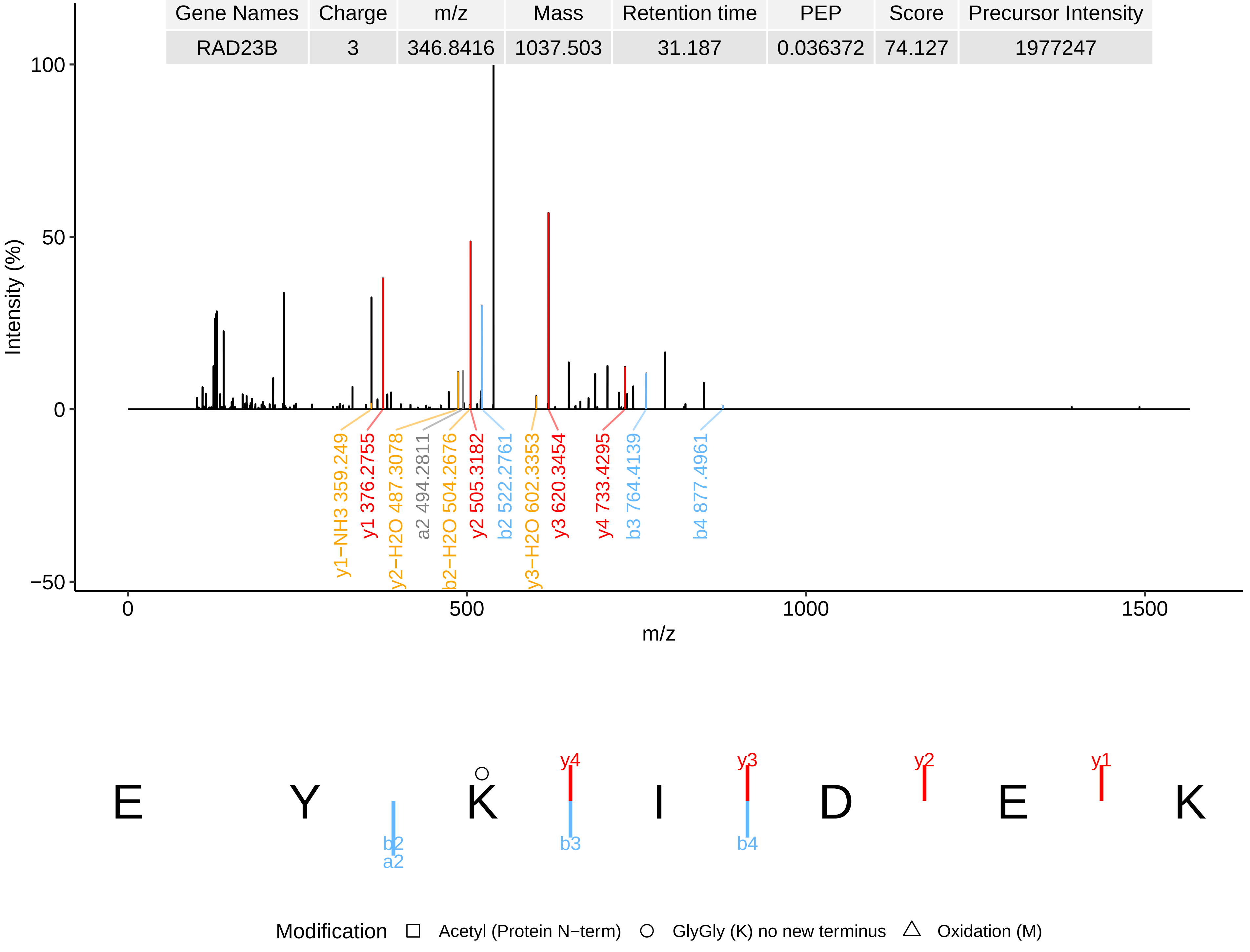

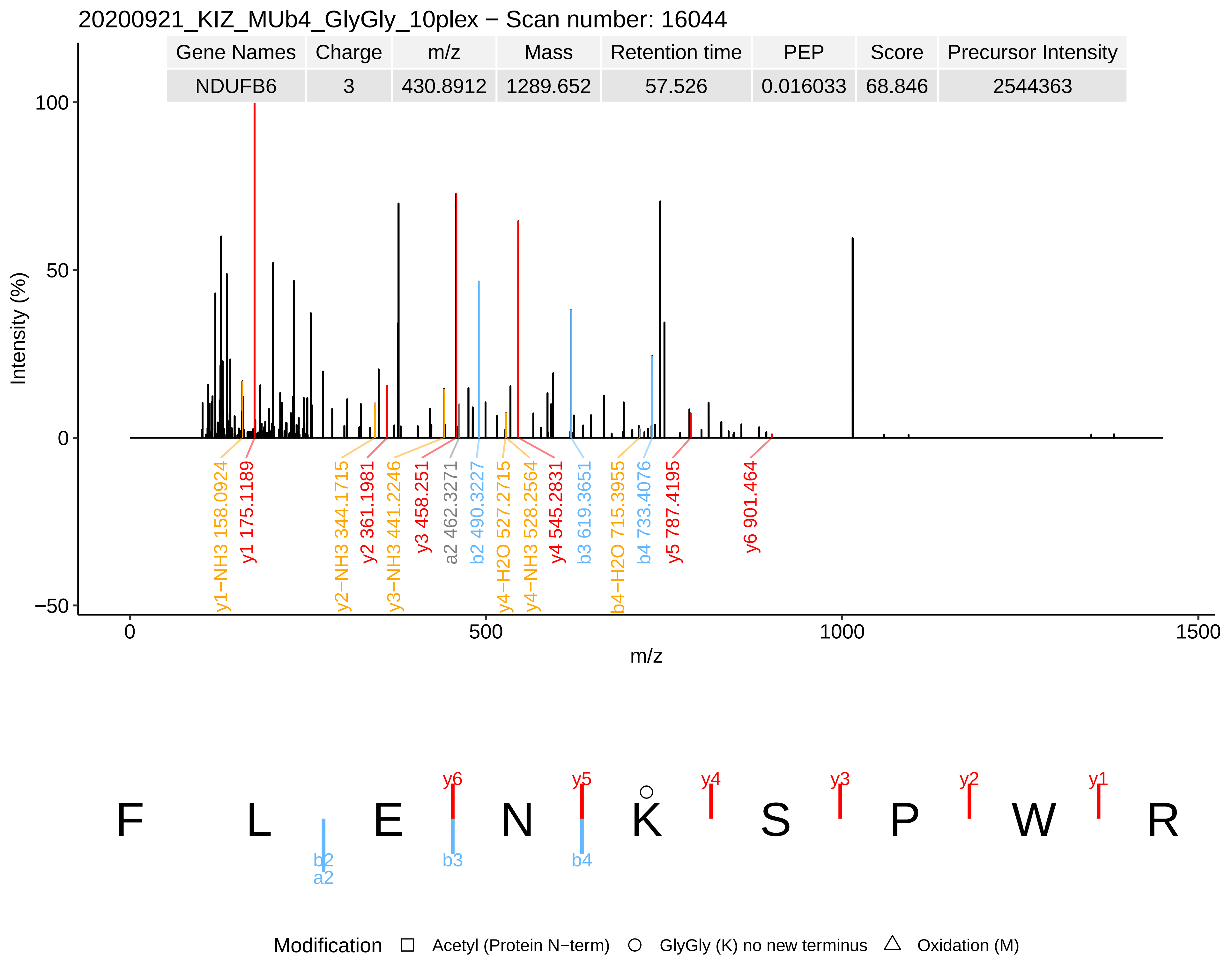
20200921_KIZ_MUb4_GlyGly_10plex − Scan number: 16044
Gene Names | Charge | m/z | Mass | Retention time | PEP | Score | Precursor Intensity
NDUFB6 | 3 | 430.8912 | 1289.652 | 57.526 | 0.016033 | 68.846 | 2544363
Intensity (%)
m/z
y1−NH3 158.0924
y1 175.1189
y2−NH3 344.1715
y2 361.1981
y3−NH3 441.2246
y3 458.251
a2 462.3271
b2 490.3227
y4−H2O 527.2715
y4−NH3 528.2564
y4 545.2831
b3 619.3651
b4−H2O 715.3955
b4 733.4076
y5 787.4195
y6 901.464
F L E N K S P W R
y6 y5 y4 y3 y2 y1
b2 a2 b3 b4
Modification
Acetyl (Protein N−term)
GlyGly (K) no new terminus
Oxidation (M)

20200921_KIZ_MUb4_GlyGly_10plex − Scan number: 10616

Gene Names	Charge	m/z	Mass	Retention time	PEP	Score	Precursor Intensity
IMMT	3	431.23	1290.668	36.787	0.00078547	93.096	3161462

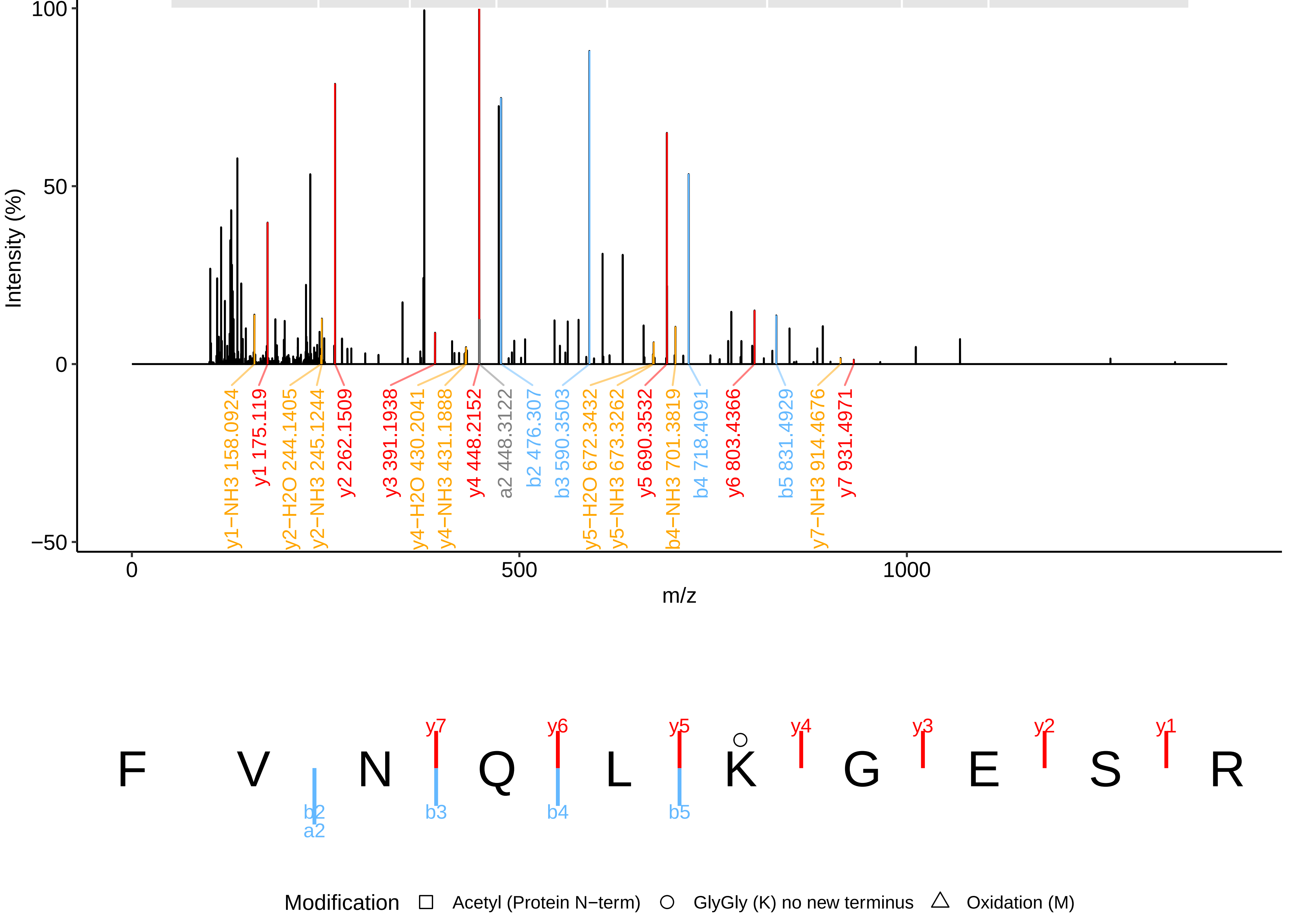

20200921_KIZ_MUb4_GlyGly_10plex_2 − Scan number: 9820

Gene Names	Charge	m/z	Mass	Retention time	PEP	Score	Precursor Intensity
IMMT	3	431.23	1290.668	37.453	0.0076246	75.652	1975795

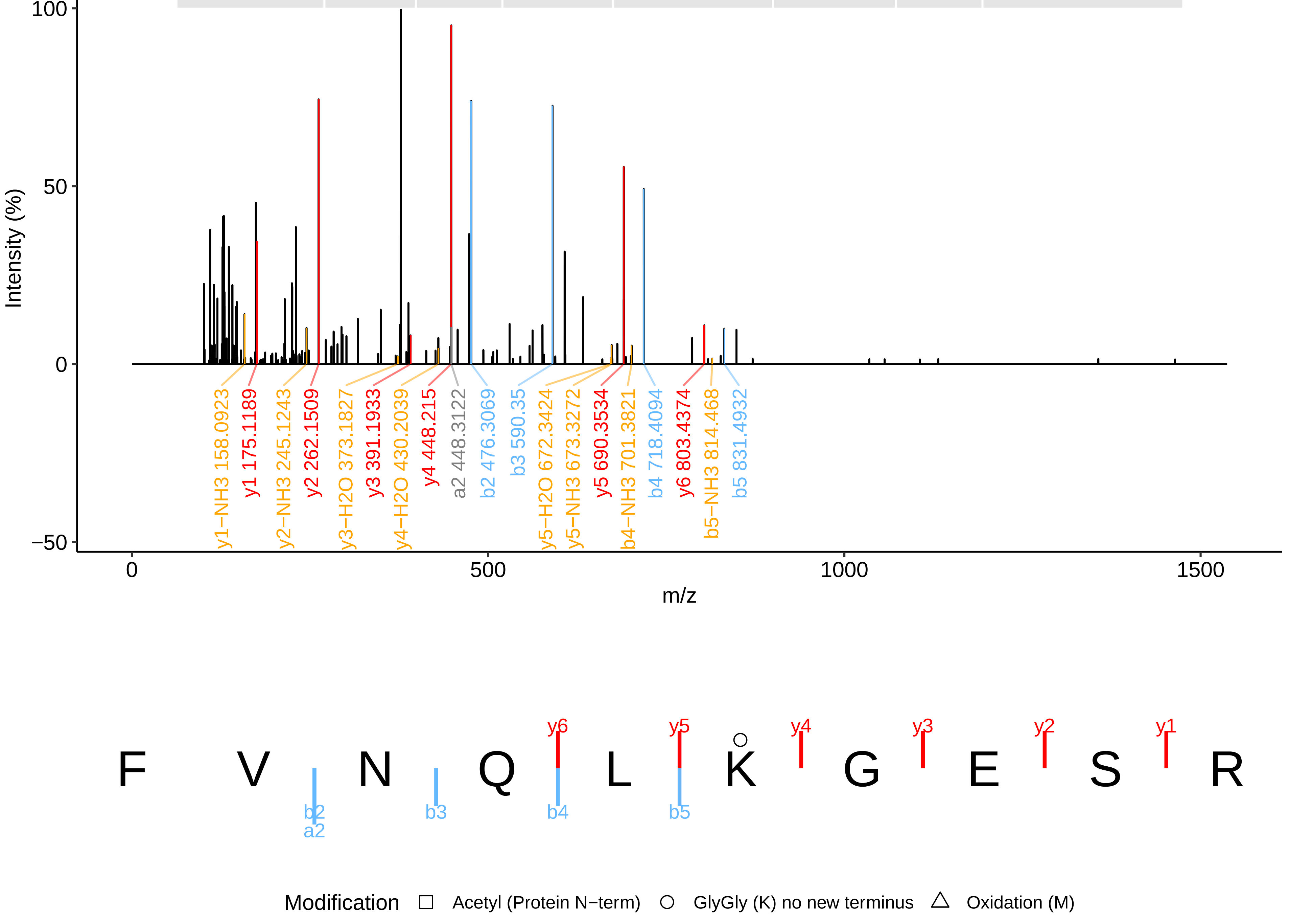

20201118_KIZ_MUb6_GlyGly_1 − Scan number: 8958

Gene Names	Charge	m/z	Mass	Retention time	PEP	Score	Precursor Intensity
IMMT	3	431.23	1290.668	28.355	0.014481	70.977	NA

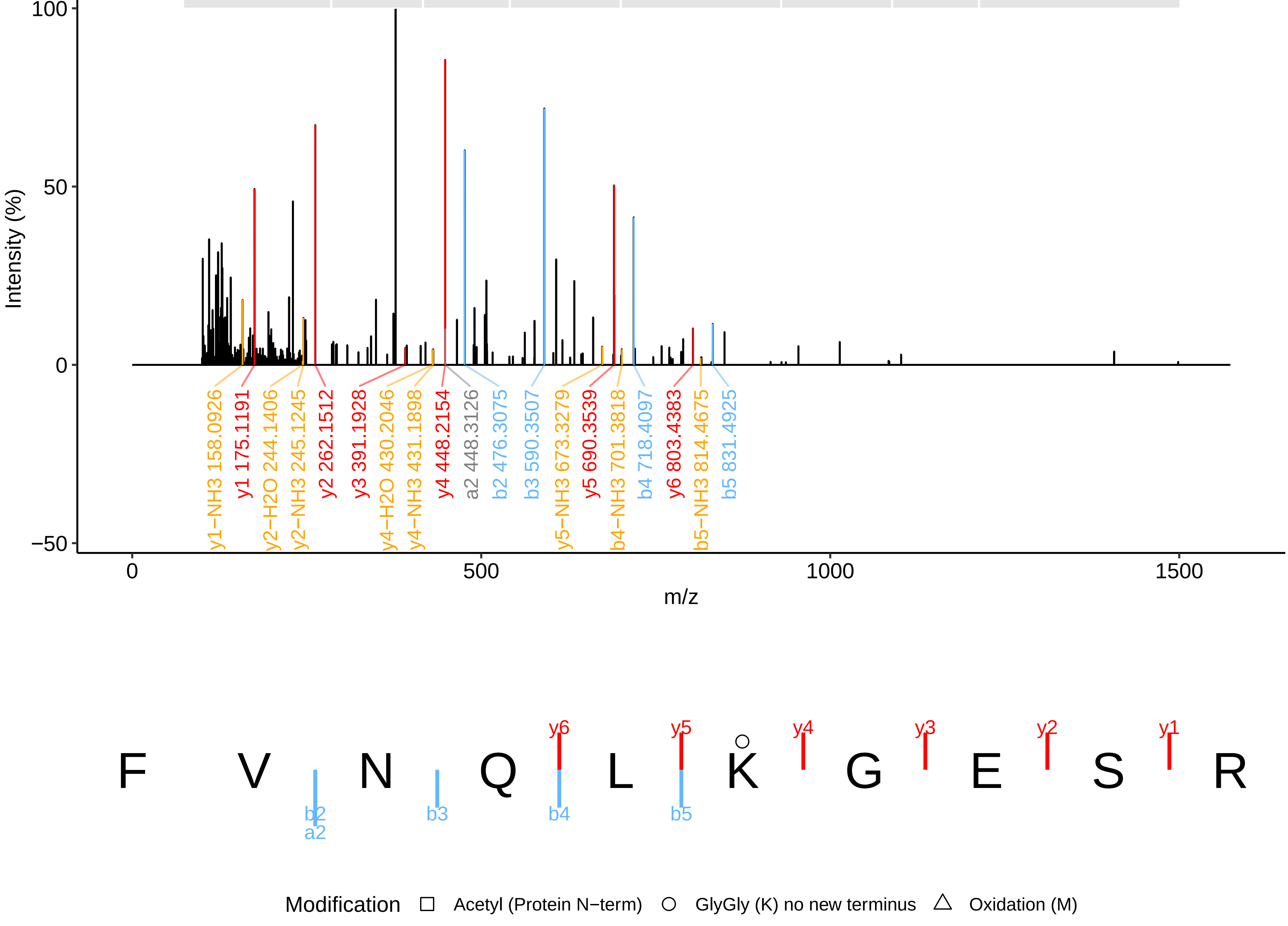

20200921_KIZ_MUb4_GlyGly_10plex − Scan number: 6599

Gene Names	Charge	m/z	Mass	Retention time	PEP	Score	Precursor Intensity
RPL10;RPL10L	3	434.9019	1301.684	21.895	1.5367e−05	102.53	6527241

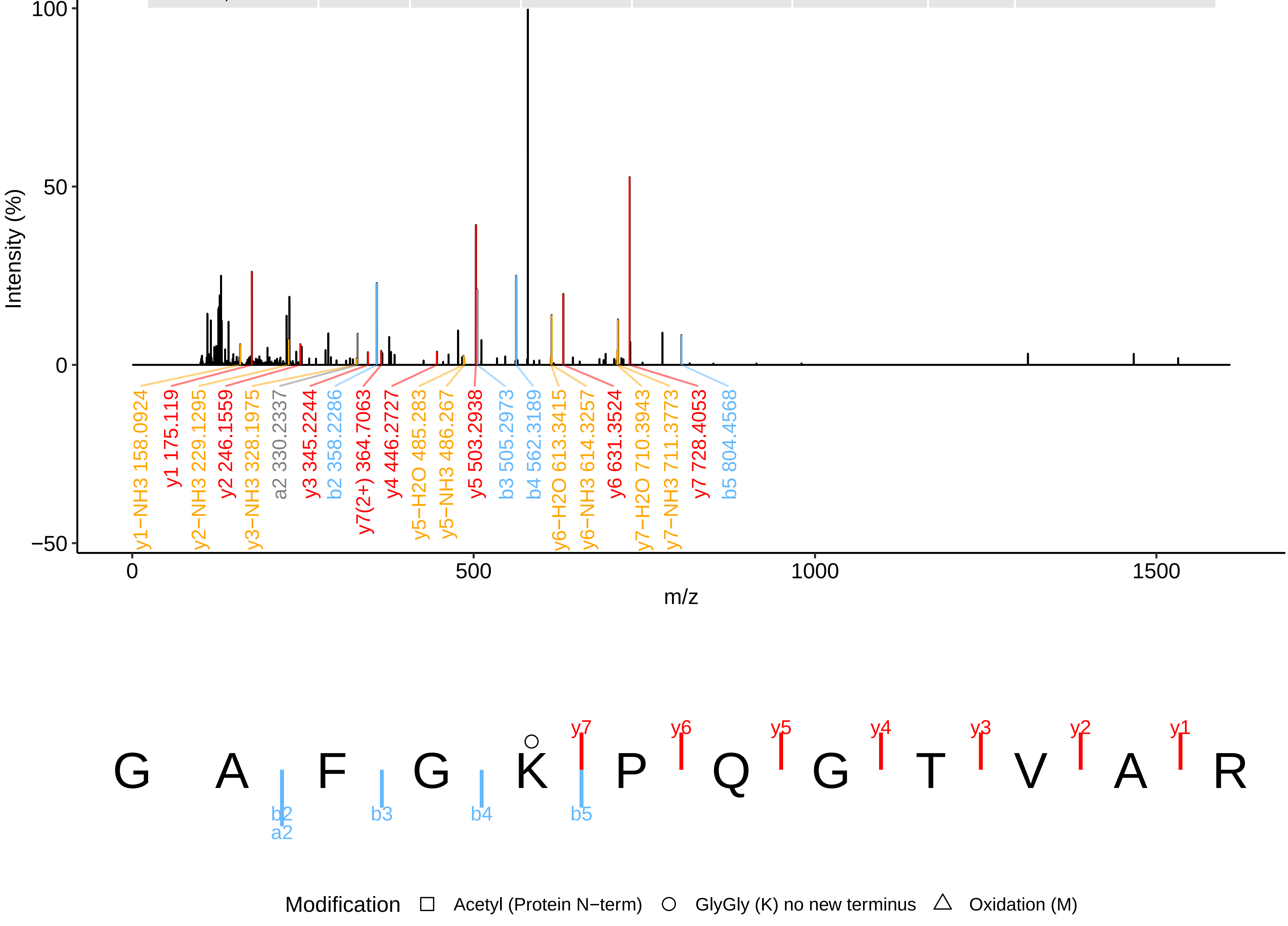

20200921_KIZ_MUb4_GlyGly_10plex_2 − Scan number: 6107

Gene Names	Charge	m/z	Mass	Retention time	PEP	Score	Precursor Intensity
RPL10;RPL10L	3	434.9019	1301.684	22.295	0.10631	45.077	352904.4

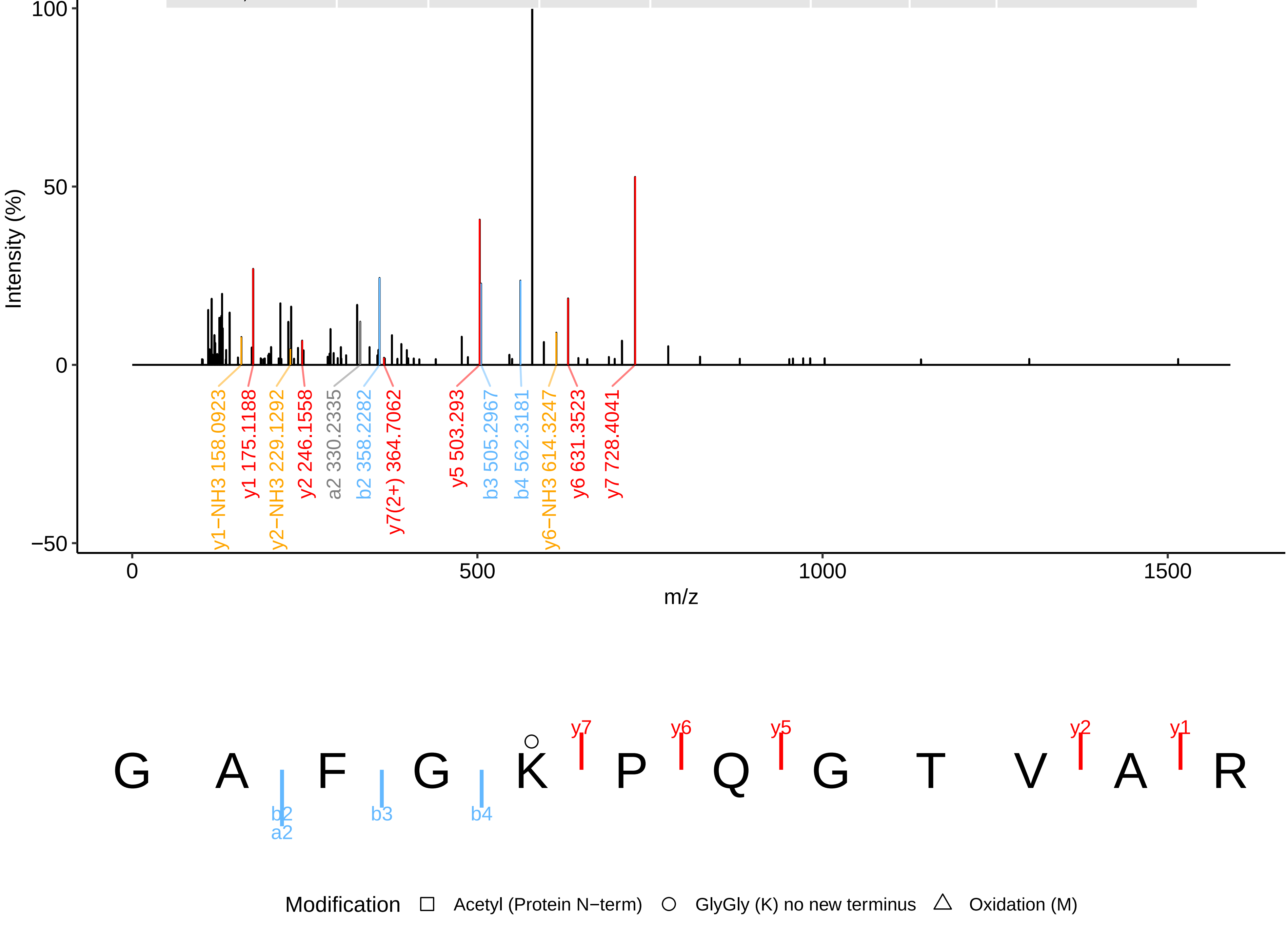

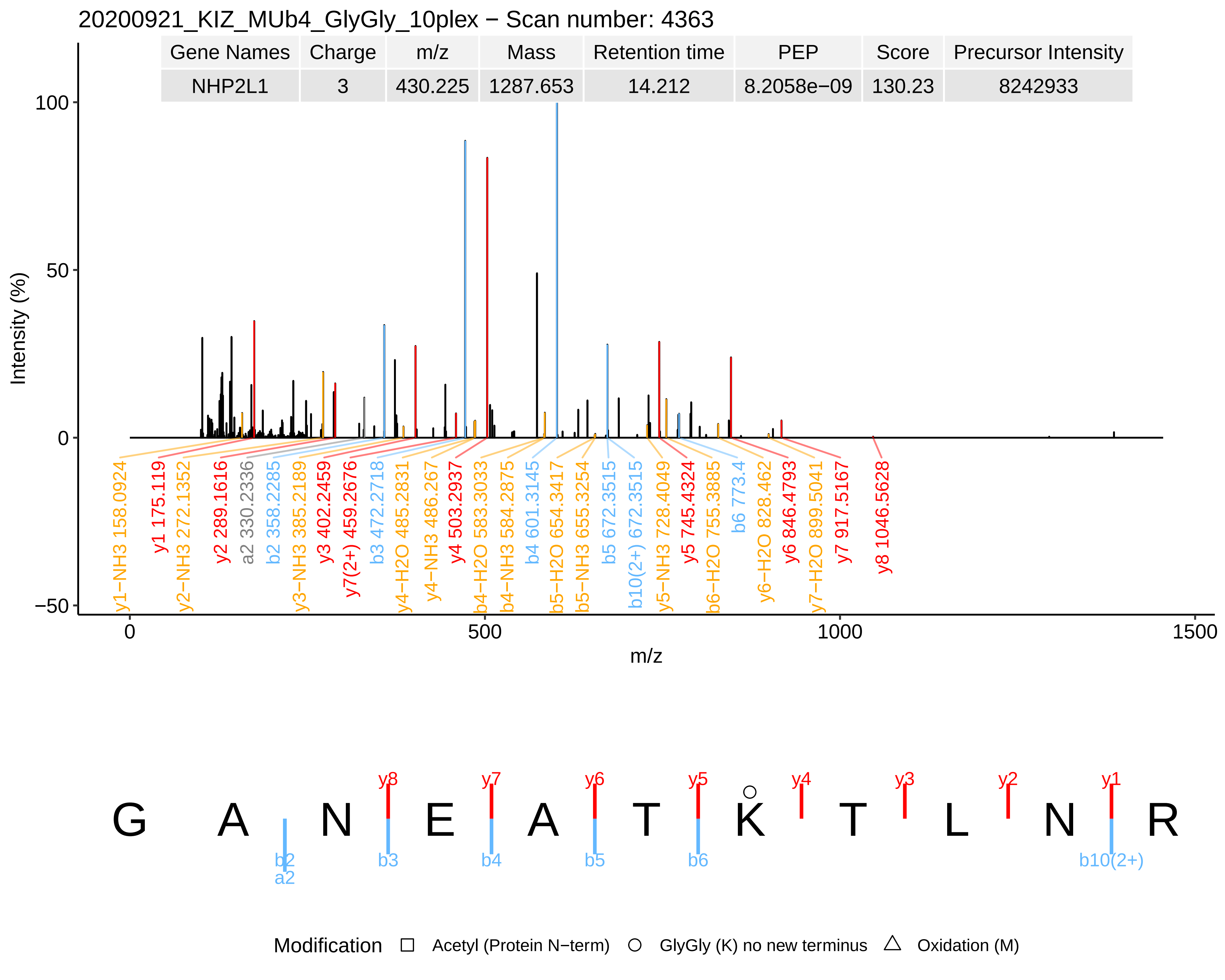
20200921_KIZ_MUb4_GlyGly_10plex − Scan number: 4363
Gene Names
Charge
m/z
Mass
Retention time
PEP
Score
Precursor Intensity
NHP2L1
3
430.225
1287.653
14.212
8.2058e−09
130.23
8242933
Intensity (%)
100
50
0
−50
m/z
0
500
1000
1500
y1−NH3 158.0924
y1 175.119
y2−NH3 272.1352
y2 289.1616
a2 330.2336
b2 358.2285
y3−NH3 385.2189
y3 402.2459
y7(2+) 459.2676
b3 472.2718
y4−H2O 485.2831
y4−NH3 486.267
y4 503.2937
b4−H2O 583.3033
b4−NH3 584.2875
b4 601.3145
b5−H2O 654.3417
b5−NH3 655.3254
b5 672.3515
b10(2+) 672.3515
y5−NH3 728.4049
y5 745.4324
b6−H2O 755.3885
b6 773.4
y6−H2O 828.462
y6 846.4793
y7−H2O 899.5041
y7 917.5167
y8 1046.5628
G A N E A T K T L N R
y8
y7
y6
y5
y4
y3
y2
y1
b2
a2
b3
b4
b5
b6
b10(2+)
Modification
Acetyl (Protein N−term)
GlyGly (K) no new terminus
Oxidation (M)

20200921_KIZ_MUb4_GlyGly_10plex_2 − Scan number: 4048

Gene Names	Charge	m/z	Mass	Retention time	PEP	Score	Precursor Intensity
NHP2L1	3	430.225	1287.653	13.991	3.7662e−05	100.82	1751629

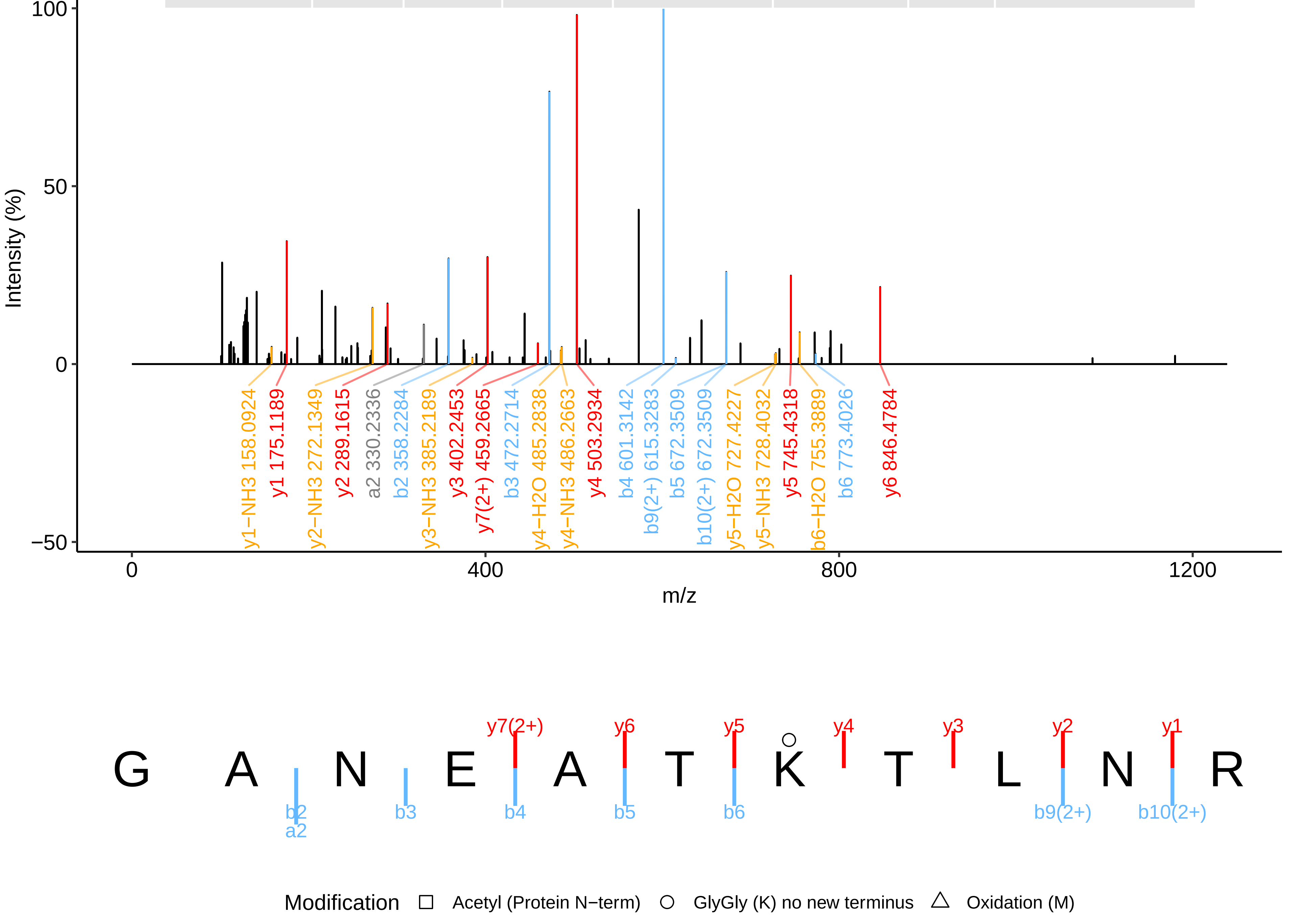

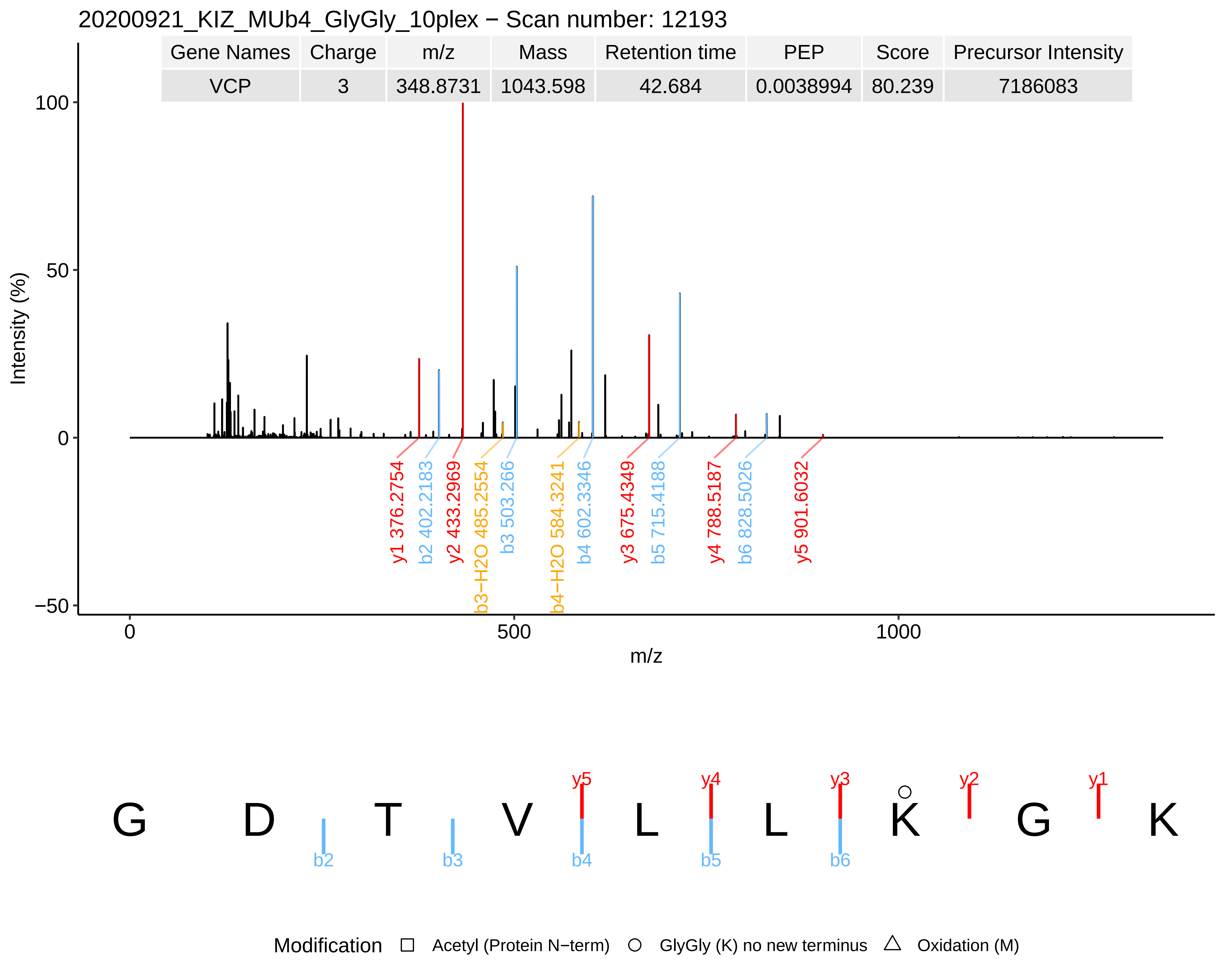
20200921_KIZ_MUb4_GlyGly_10plex − Scan number: 12193
Gene Names | Charge | m/z | Mass | Retention time | PEP | Score | Precursor Intensity
VCP | 3 | 348.8731 | 1043.598 | 42.684 | 0.0038994 | 80.239 | 7186083
Intensity (%)
100
50
0
−50
0
500
1000
m/z
y1 376.2754
b2 402.2183
y2 433.2969
b3−H2O 485.2554
b3 503.266
b4−H2O 584.3241
b4 602.3346
y3 675.4349
b5 715.4188
y4 788.5187
b6 828.5026
y5 901.6032
G D T V L L K G K
y5 y4 y3 y2 y1
b2 b3 b4 b5 b6
Modification
Acetyl (Protein N−term)
GlyGly (K) no new terminus
Oxidation (M)

20200921_KIZ_MUb4_GlyGly_10plex_2 − Scan number: 11257

Gene Names	Charge	m/z	Mass	Retention time	PEP	Score	Precursor Intensity
VCP	3	348.8731	1043.598	43.28	0.0038994	80.239	996898.1

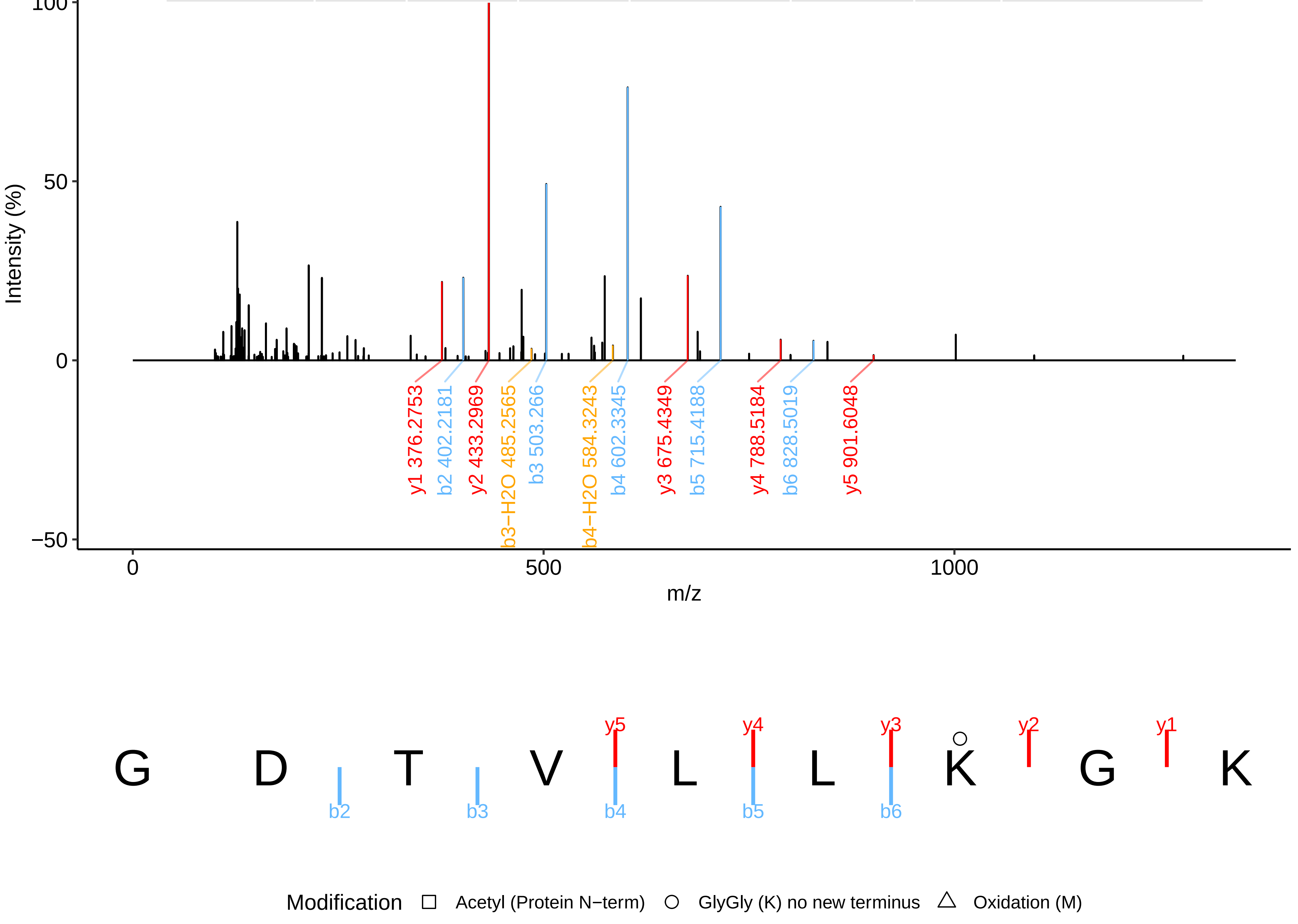

20200921_KIZ_MUb4_GlyGly_10plex − Scan number: 14778

Gene Names	Charge	m/z	Mass	Retention time	PEP	Score	Precursor Intensity
TOMM70A	3	355.1976	1062.571	52.598	0.10597	74.127	3153637

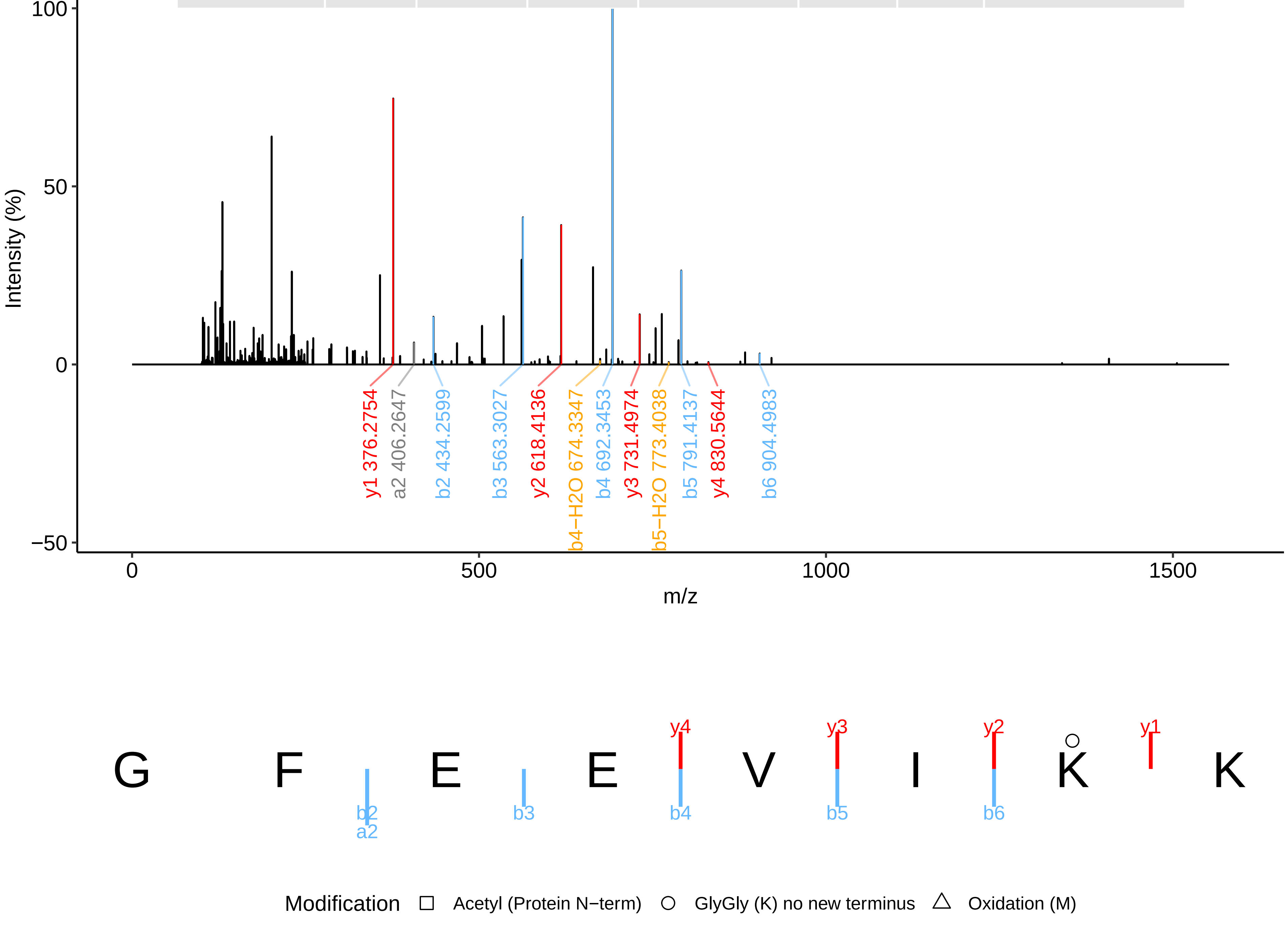

20200921_KIZ_MUb4_GlyGly_10plex − Scan number: 3413

Gene Names	Charge	m/z	Mass	Retention time	PEP	Score	Precursor Intensity
EIF3H	3	359.5159	1075.526	10.796	0.010034	70.488	2122905

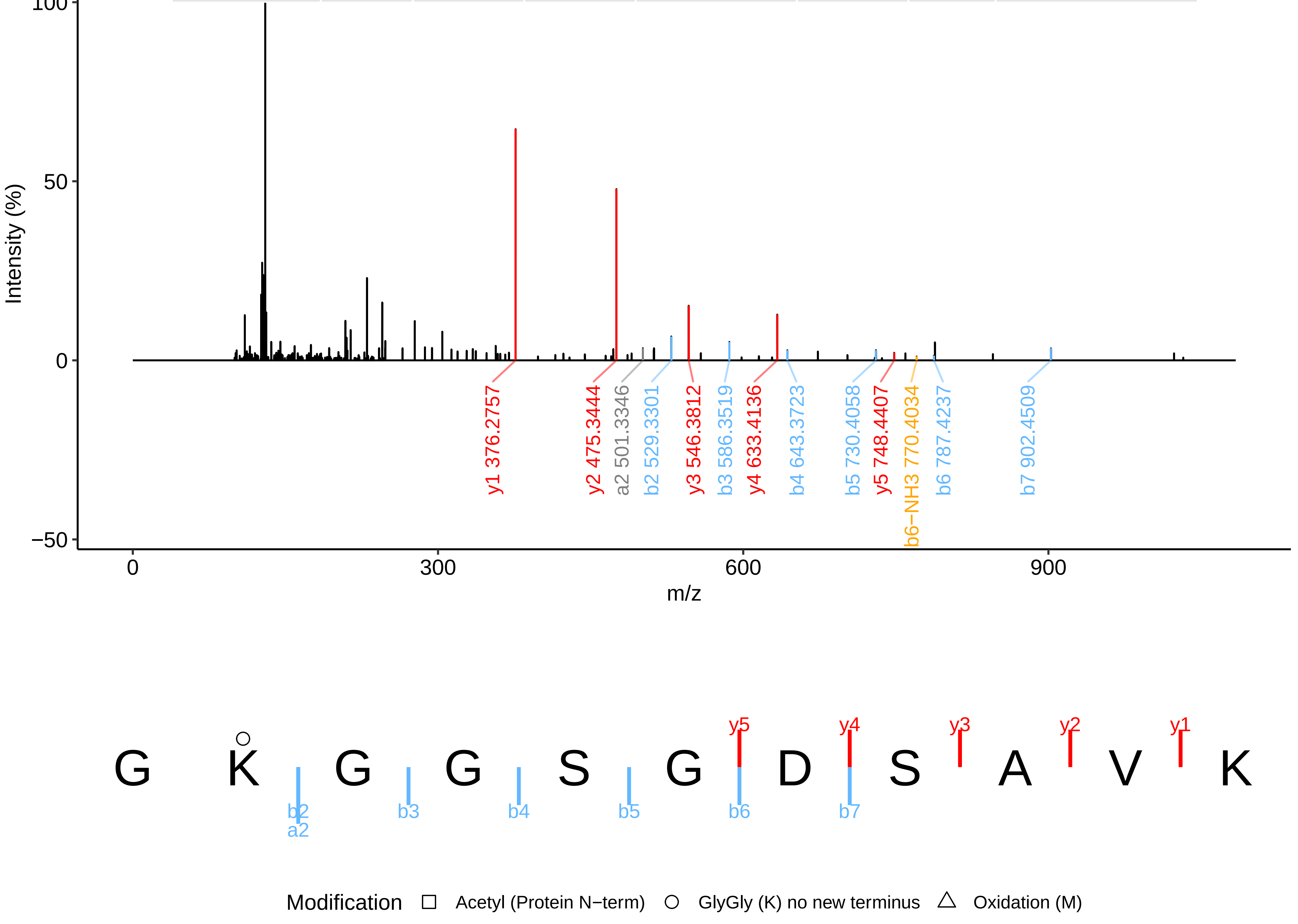

20200921_KIZ_MUb4_GlyGly_10plex_2 − Scan number: 4917

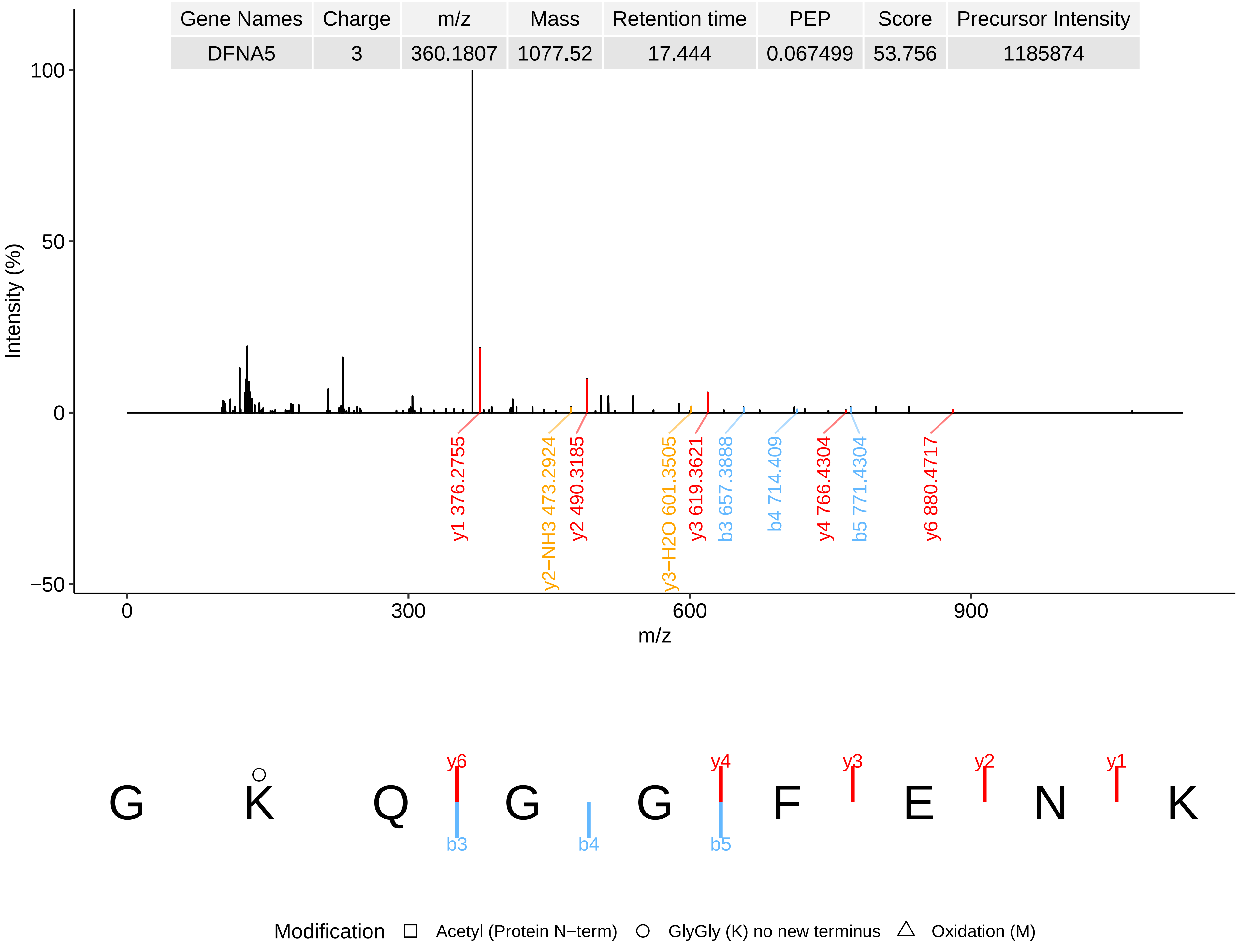

20200921_KIZ_MUb4_GlyGly_10plex − Scan number: 8451

Gene Names	Charge	m/z	Mass	Retention time	PEP	Score	Precursor Intensity
PCNX;PCNXL3	3	433.9091	1298.706	28.676	3.8346e−05	100.55	9222159

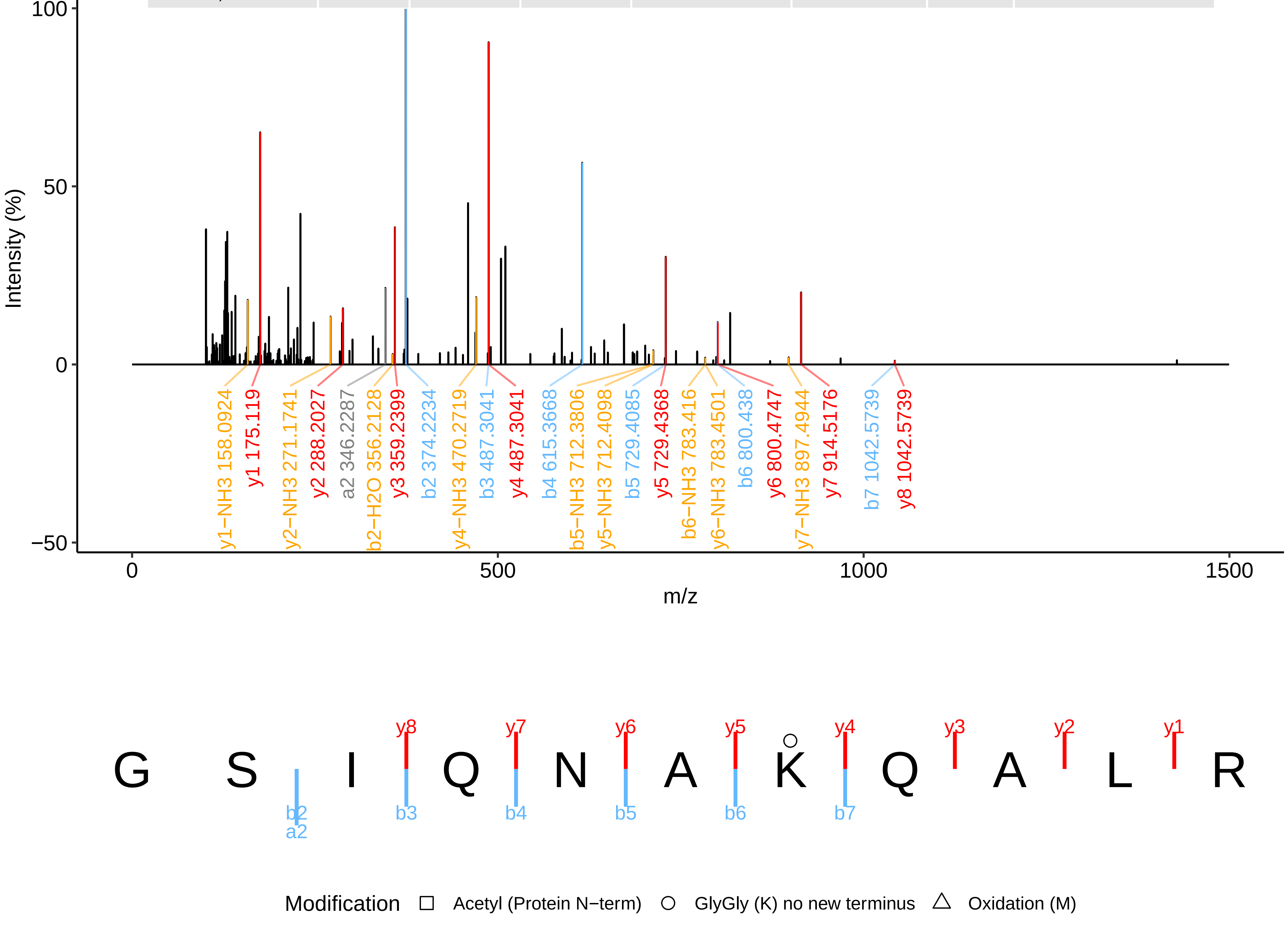

20200921_KIZ_MUb4_GlyGly_10plex_2 − Scan number: 7835

Gene Names	Charge	m/z	Mass	Retention time	PEP	Score	Precursor Intensity
PCNX;PCNXL3	3	433.9091	1298.706	29.365	4.1622e−05	99.283	1353288

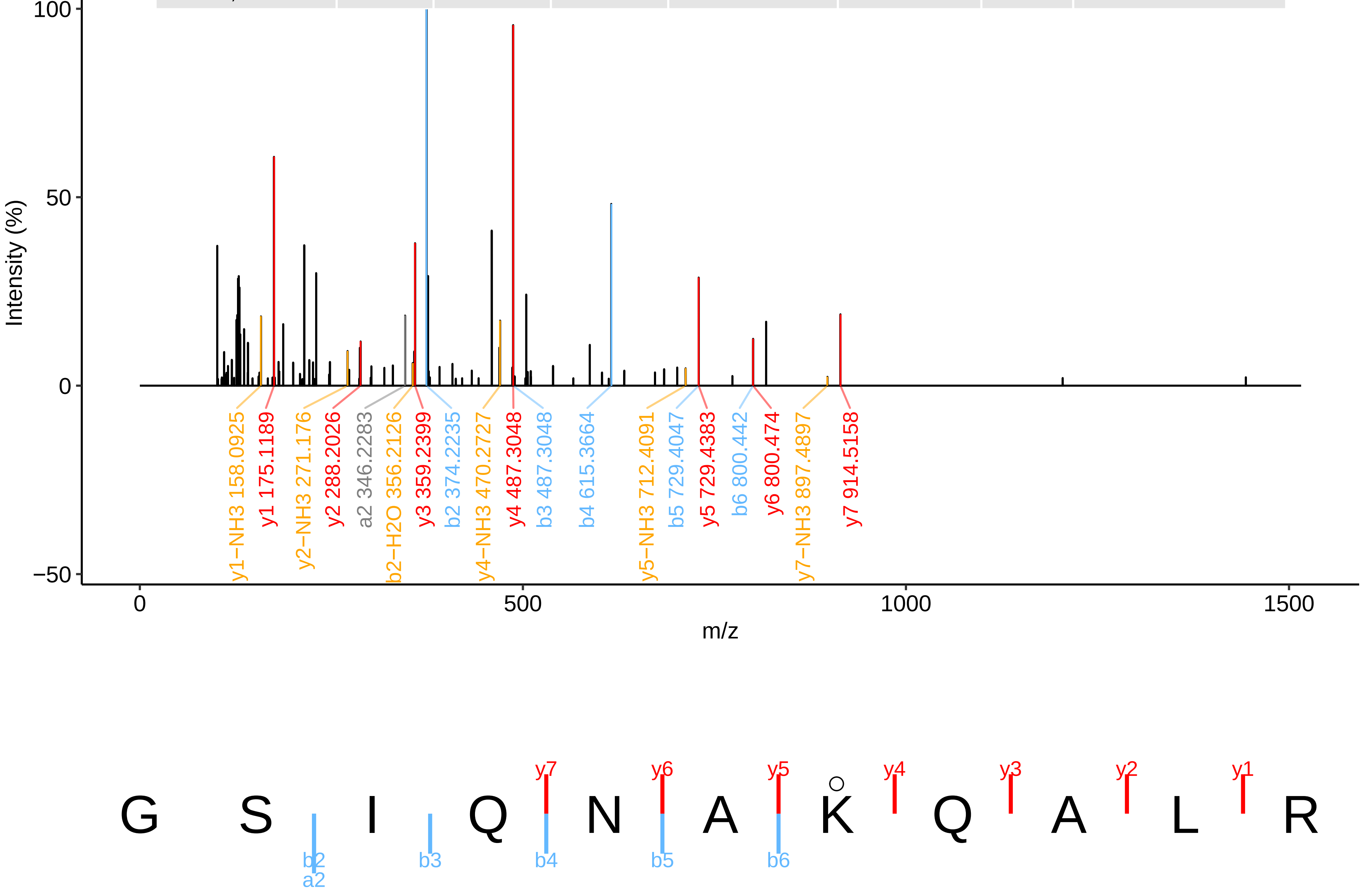

Modification □ Acetyl (Protein N−term) ○ GlyGly (K) no new terminus △ Oxidation (M)

20200921_KIZ_MUb4_GlyGly_10plex − Scan number: 14447

Gene Names	Charge	m/z	Mass	Retention time	PEP	Score	Precursor Intensity
CXCR4	3	349.545	1045.613	51.346	0.0017455	91.087	3145964

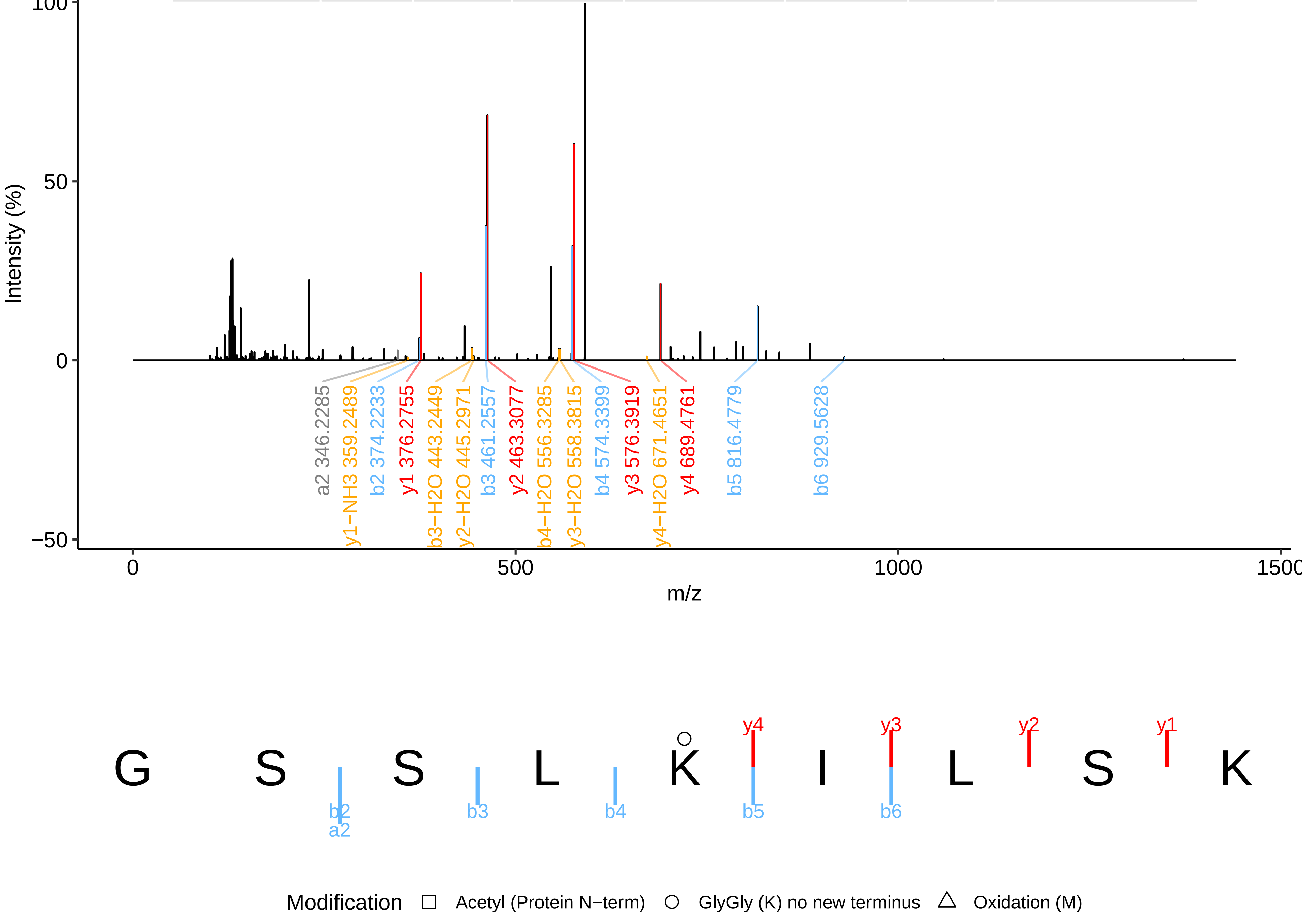

20200921_KIZ_MUb4_GlyGly_10plex − Scan number: 14496

Gene Names	Charge	m/z	Mass	Retention time	PEP	Score	Precursor Intensity
CXCR4	2	523.8139	1045.613	51.54	0.0061884	115.78	4500454

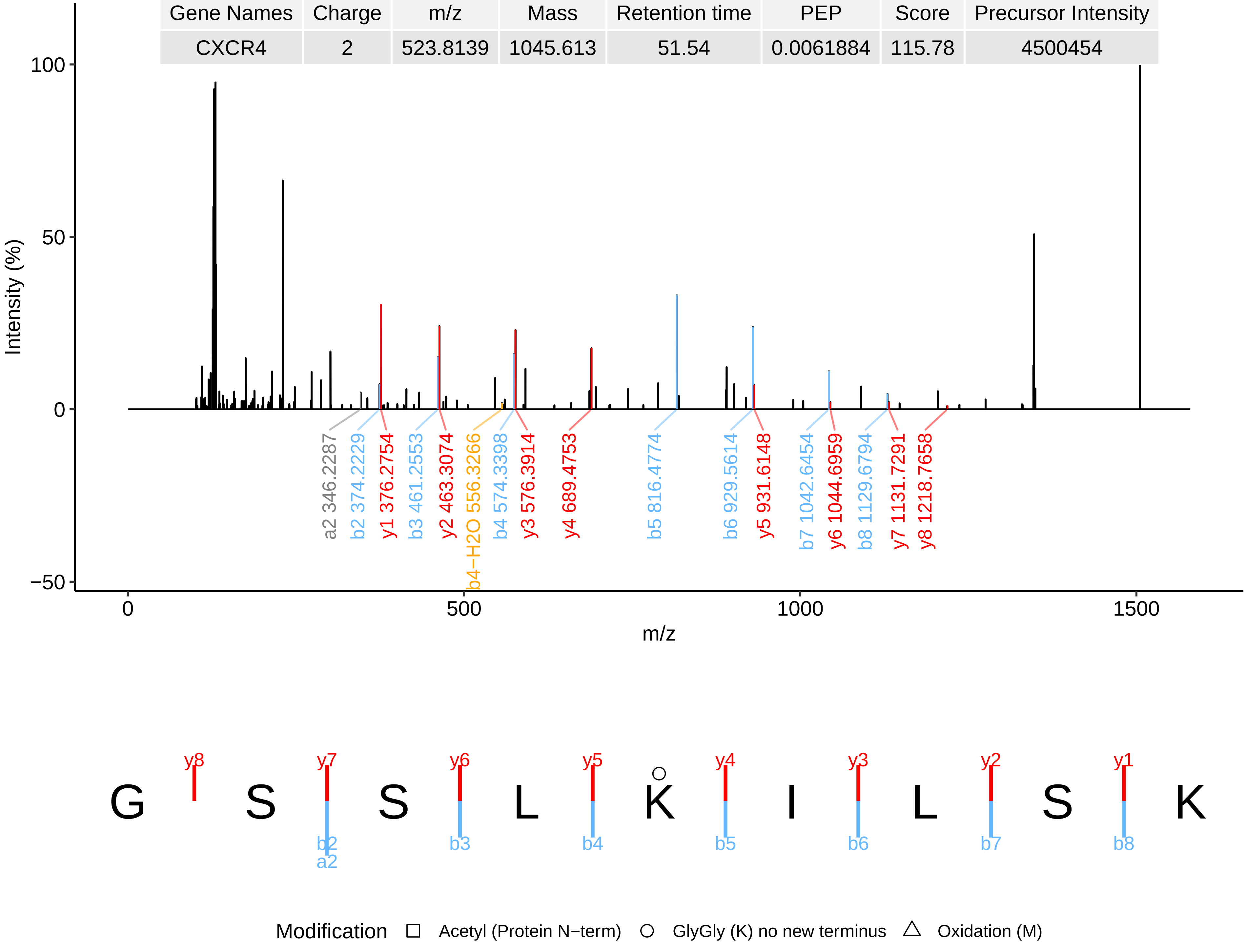

20200921_KIZ_MUb4_GlyGly_10plex_2 − Scan number: 13367

Gene Names	Charge	m/z	Mass	Retention time	PEP	Score	Precursor Intensity
CXCR4	3	349.545	1045.613	51.883	0.037713	59.931	1485736

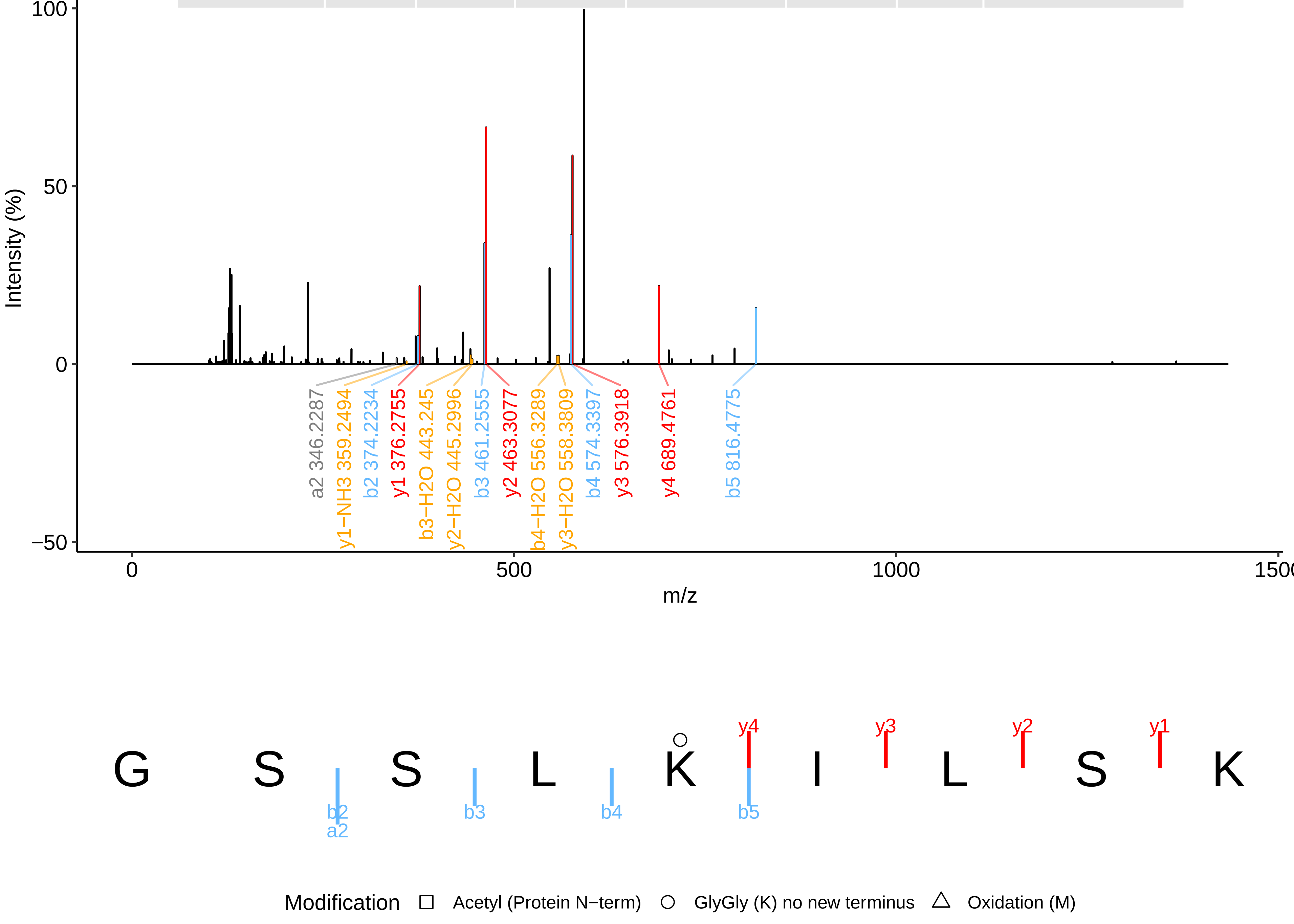

20200924_KIZ_MUb5_GlyGly_1 − Scan number: 13346

Gene Names	Charge	m/z	Mass	Retention time	PEP	Score	Precursor Intensity
CXCR4	3	349.545	1045.613	43.499	0.0061684	81.95	13562301

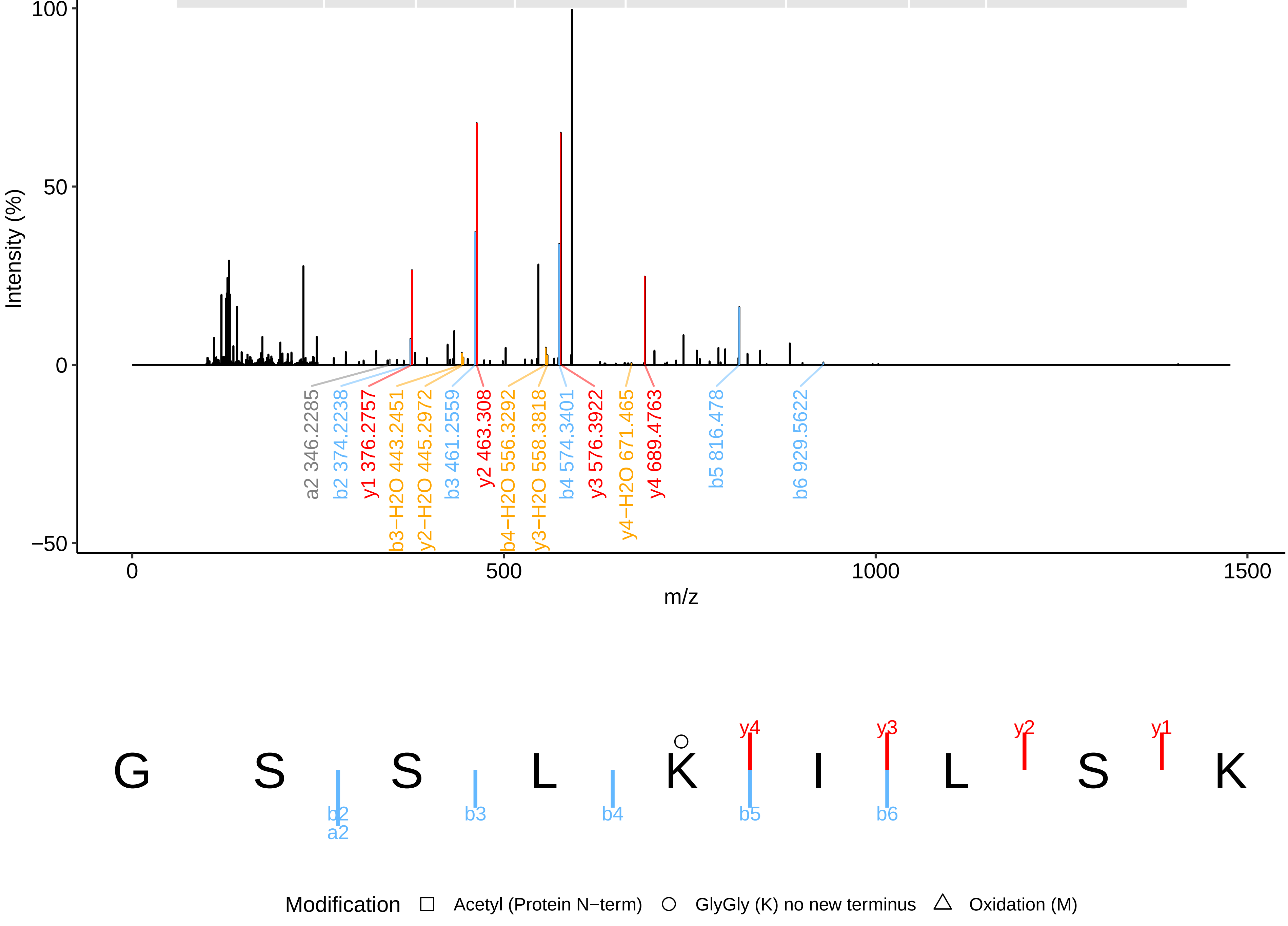

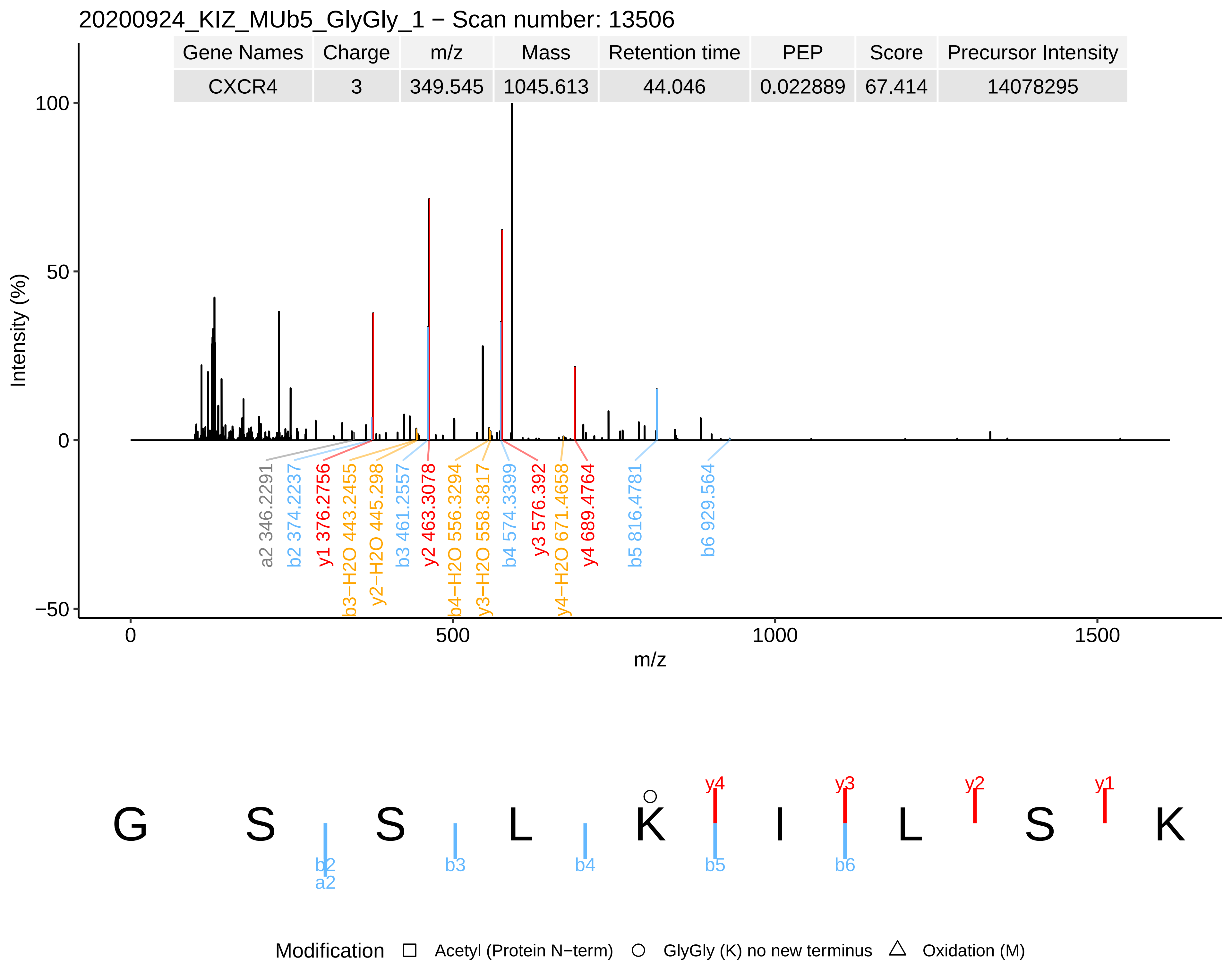
20200924_KIZ_MUb5_GlyGly_1 − Scan number: 13506
Gene Names	Charge	m/z	Mass	Retention time	PEP	Score	Precursor Intensity
CXCR4	3	349.545	1045.613	44.046	0.022889	67.414	14078295
Intensity (%)
100
50
0
−50
0
500
1000
1500
m/z
a2 346.2291
b2 374.2237
y1 376.2756
b3−H2O 443.2455
y2−H2O 445.298
b3 461.2557
y2 463.3078
b4−H2O 556.3294
y3−H2O 558.3817
b4 574.3399
y3 576.392
y4−H2O 671.4658
y4 689.4764
b5 816.4781
b6 929.564
G S S L K I L S K
y4 y3 y2 y1
b2 a2 b3 b4 b5 b6
Modification
Acetyl (Protein N−term)
GlyGly (K) no new terminus
Oxidation (M)

20200924_KIZ_MUb5_GlyGly_2 − Scan number: 12453

Gene Names	Charge	m/z	Mass	Retention time	PEP	Score	Precursor Intensity
CXCR4	3	349.545	1045.613	46.534	0.08421	50.292	1472332

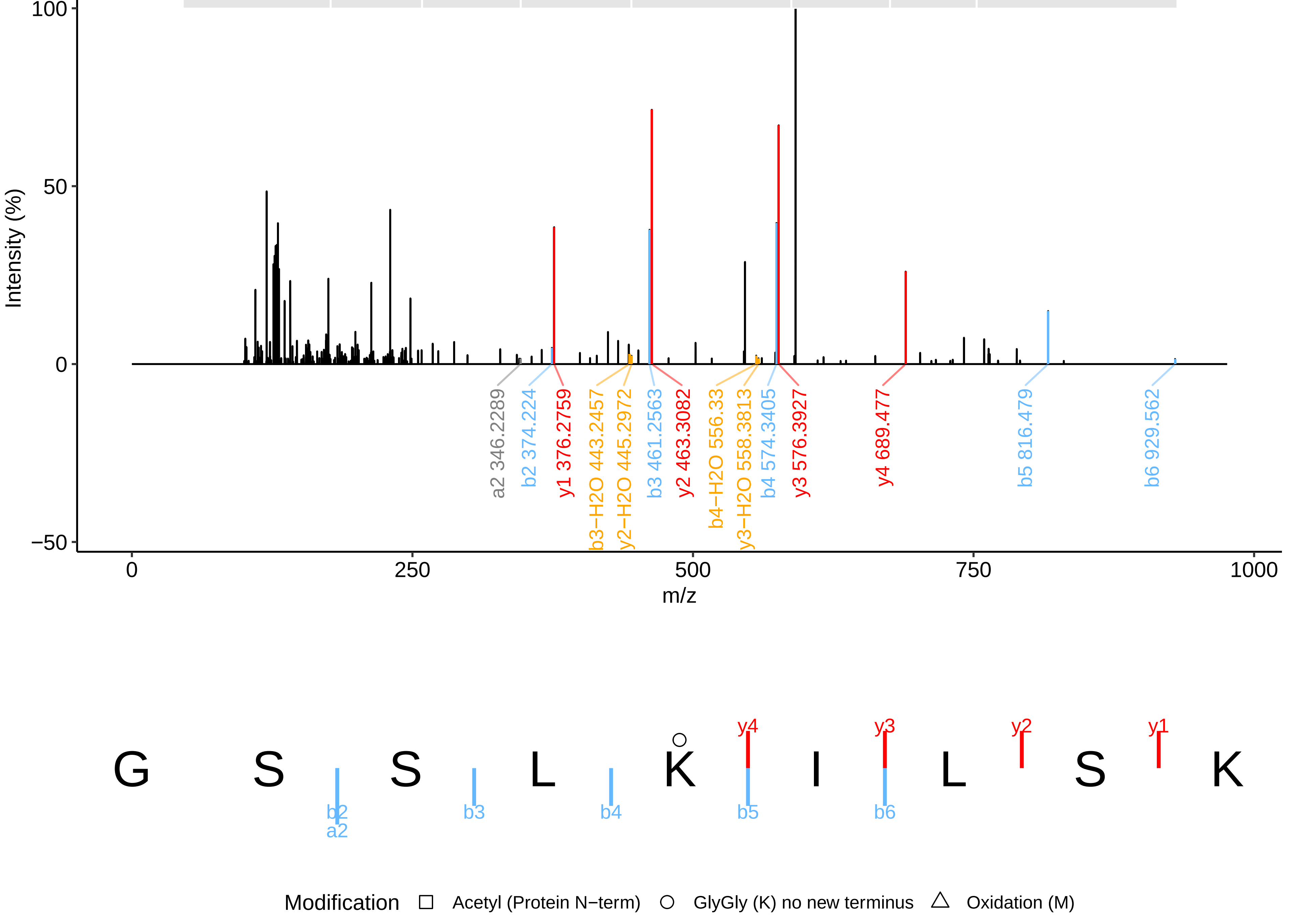

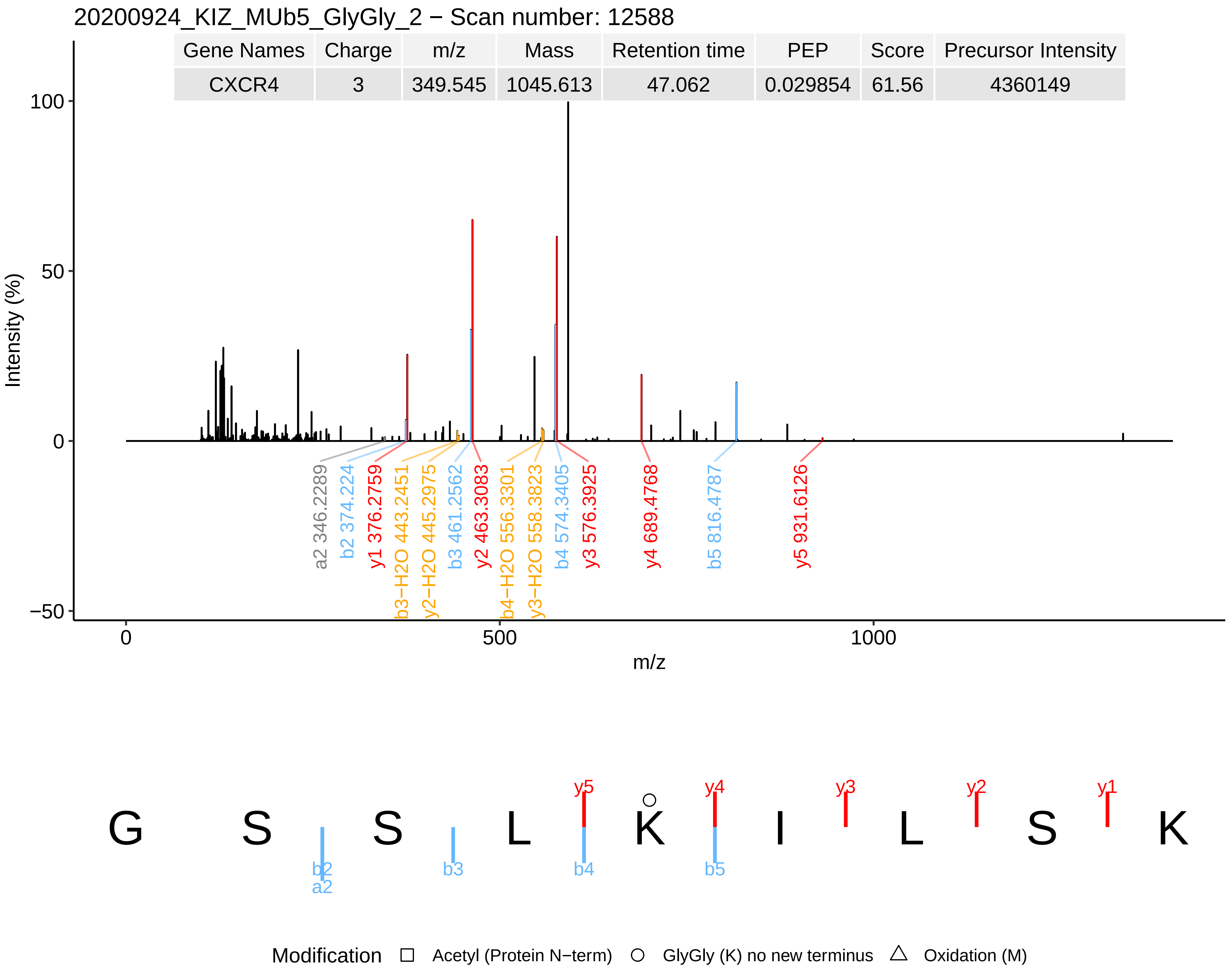

20200924_KIZ_MUb5_GlyGly_2 − Scan number: 12588
Gene Names
Charge
m/z
Mass
Retention time
PEP
Score
Precursor Intensity
CXCR4
3
349.545
1045.613
47.062
0.029854
61.56
4360149
Intensity (%)
m/z
a2 346.2289
b2 374.224
y1 376.2759
b3−H2O 443.2451
y2−H2O 445.2975
b3 461.2562
y2 463.3083
b4−H2O 556.3301
y3−H2O 558.3823
b4 574.3405
y3 576.3925
y4 689.4768
b5 816.4787
y5 931.6126
G S S L K I L S K
y5 y4 y3 y2 y1
b2 a2 b3 b4 b5
Modification
Acetyl (Protein N−term)
GlyGly (K) no new terminus
Oxidation (M)

20200921_KIZ_MUb4_GlyGly_10plex − Scan number: 14793

Gene Names	Charge	m/z	Mass	Retention time	PEP	Score	Precursor Intensity
HSPA8	3	438.2455	1311.715	52.657	1.0741e−05	111.27	5365357

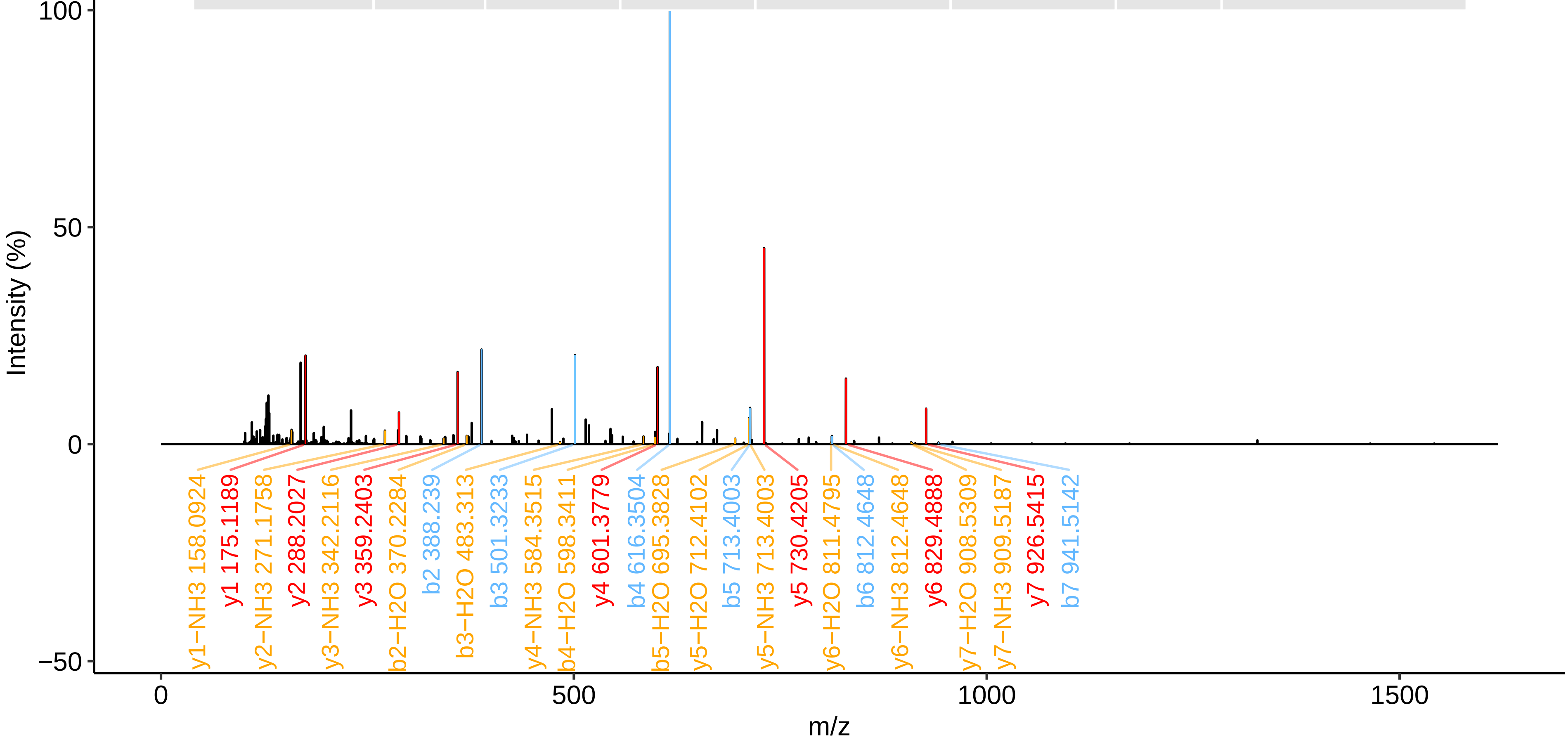

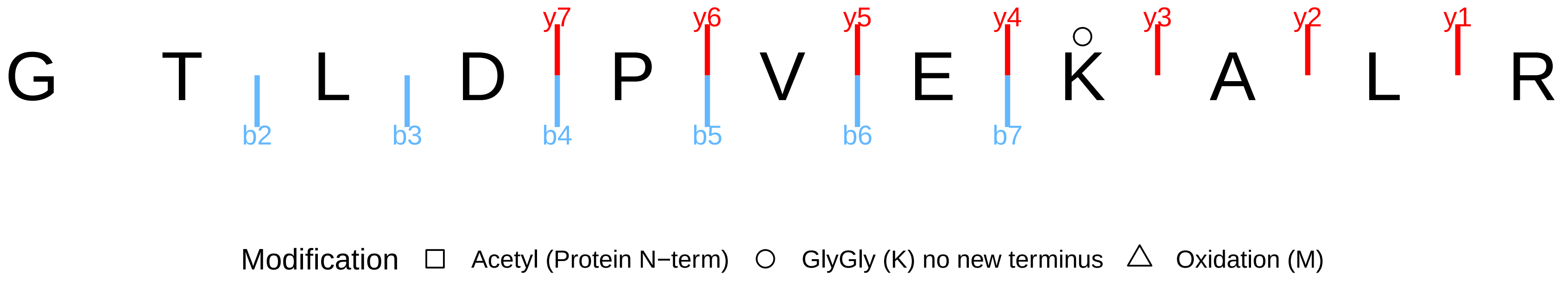

20200921_KIZ_MUb4_GlyGly_10plex_2 − Scan number: 13623

Gene Names	Charge	m/z	Mass	Retention time	PEP	Score	Precursor Intensity
HSPA8	3	438.2455	1311.715	52.938	0.001455	83.397	1471928

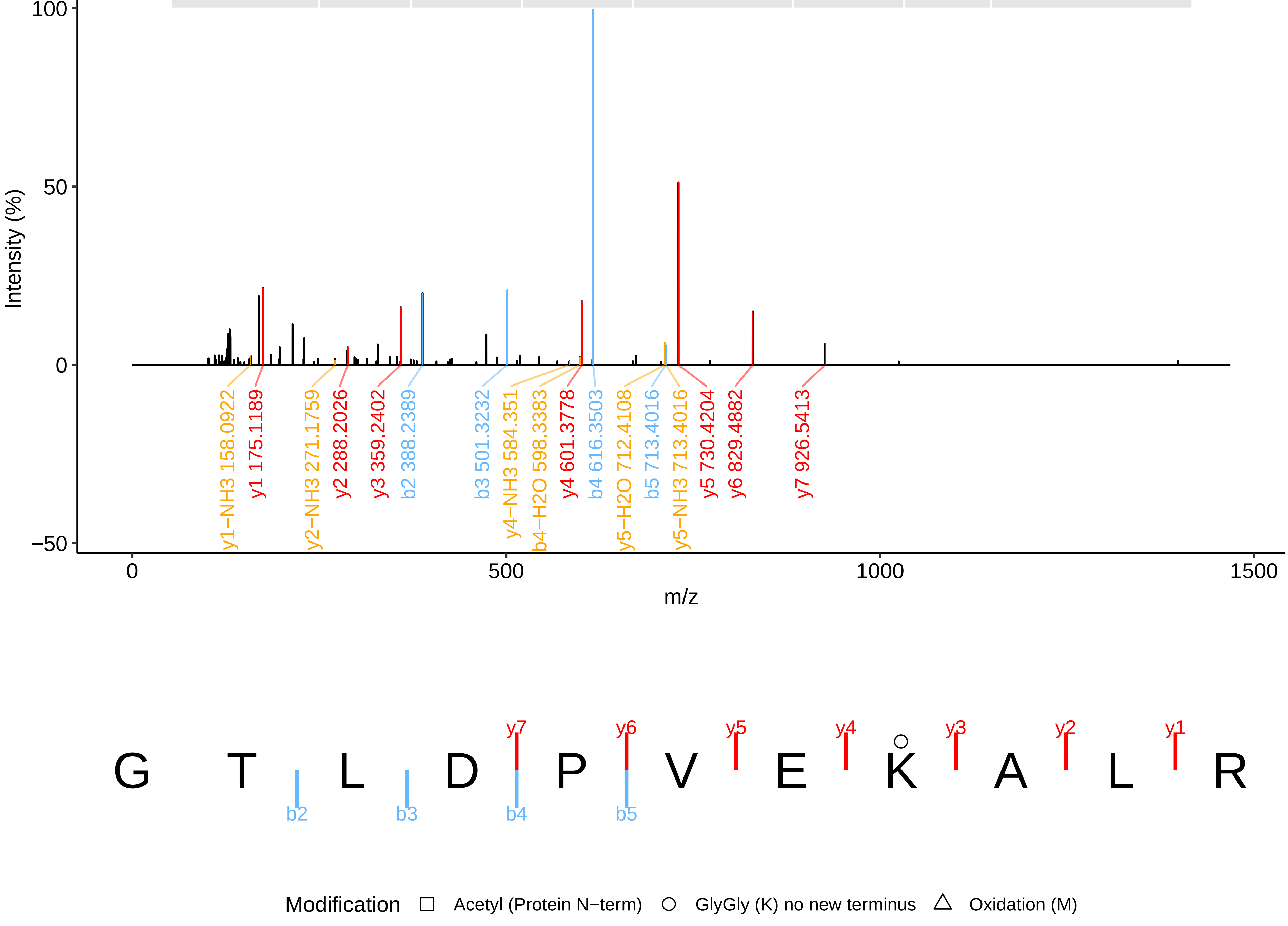

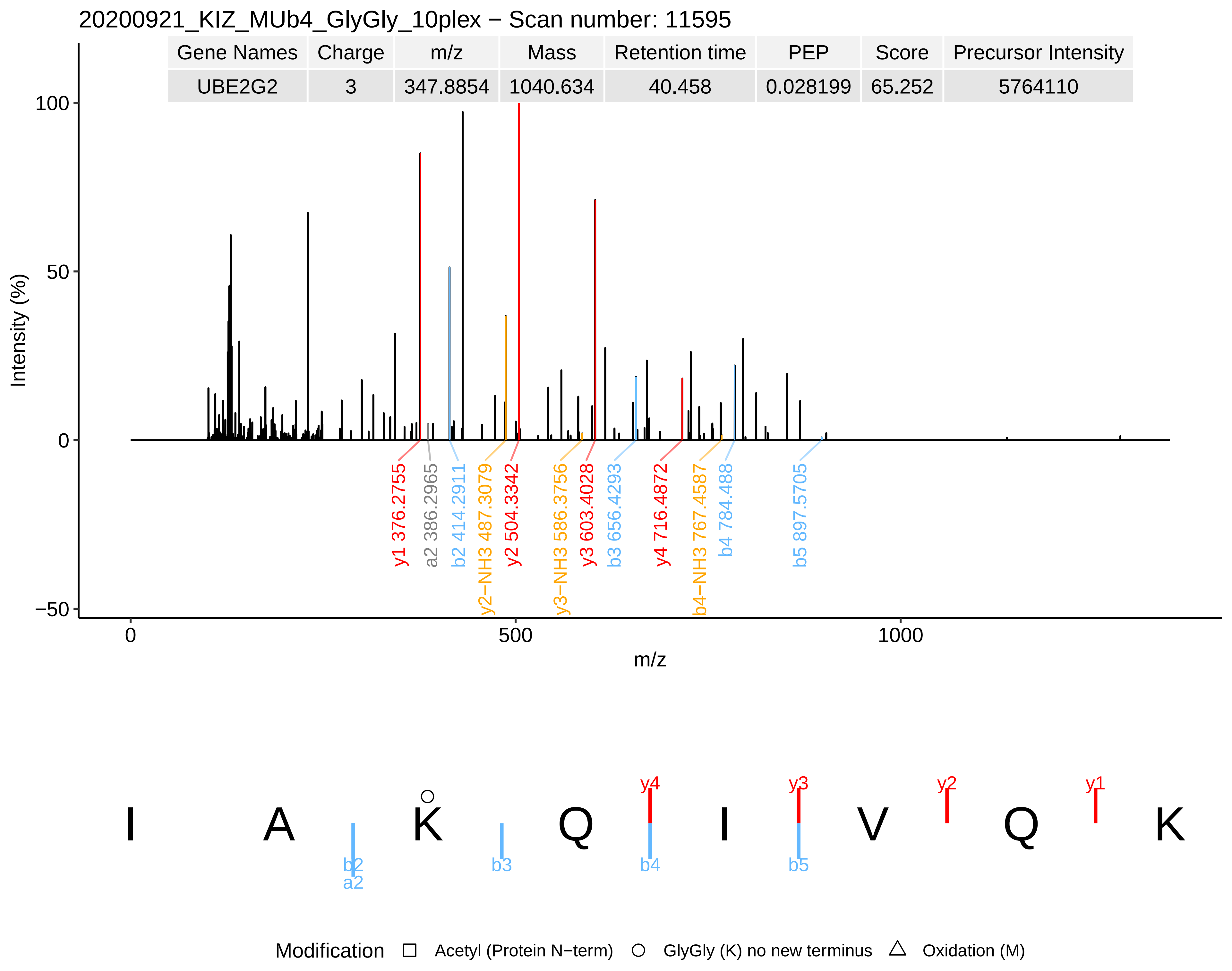

20200921_KIZ_MUb4_GlyGly_10plex − Scan number: 11595
Gene Names | Charge | m/z | Mass | Retention time | PEP | Score | Precursor Intensity
UBE2G2 | 3 | 347.8854 | 1040.634 | 40.458 | 0.028199 | 65.252 | 5764110
Intensity (%)
m/z
y1 376.2755
a2 386.2965
b2 414.2911
y2−NH3 487.3079
y2 504.3342
y3−NH3 586.3756
y3 603.4028
b3 656.4293
y4 716.4872
b4−NH3 767.4587
b4 784.488
b5 897.5705
I A K Q I V Q K
Modification
Acetyl (Protein N−term)
GlyGly (K) no new terminus
Oxidation (M)

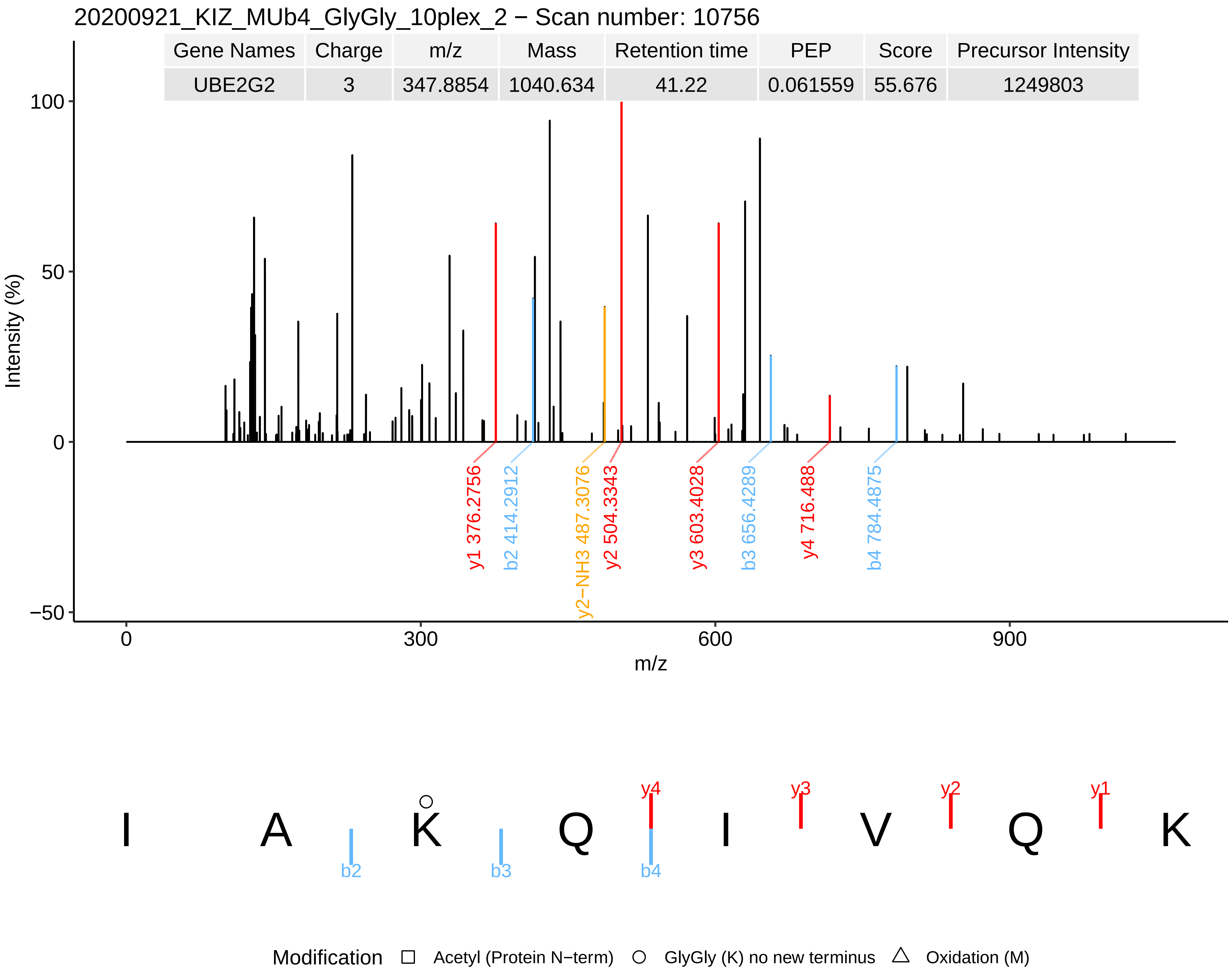
20200921_KIZ_MUb4_GlyGly_10plex_2 − Scan number: 10756
Gene Names | Charge | m/z | Mass | Retention time | PEP | Score | Precursor Intensity
UBE2G2 | 3 | 347.8854 | 1040.634 | 41.22 | 0.061559 | 55.676 | 1249803
Intensity (%)
100
50
0
−50
0
300
600
900
m/z
y1 376.2756
b2 414.2912
y2−NH3 487.3076
y2 504.3343
y3 603.4028
b3 656.4289
y4 716.488
b4 784.4875
I A K Q I V Q K
y4 y3 y2 y1
b2 b3 b4
Modification
Acetyl (Protein N−term)
GlyGly (K) no new terminus
Oxidation (M)

20200921_KIZ_MUb4_GlyGly_10plex − Scan number: 9666

Gene Names	Charge	m/z	Mass	Retention time	PEP	Score	Precursor Intensity
NUP85	3	421.2176	1260.631	33.172	0.015577	69.979	4196339

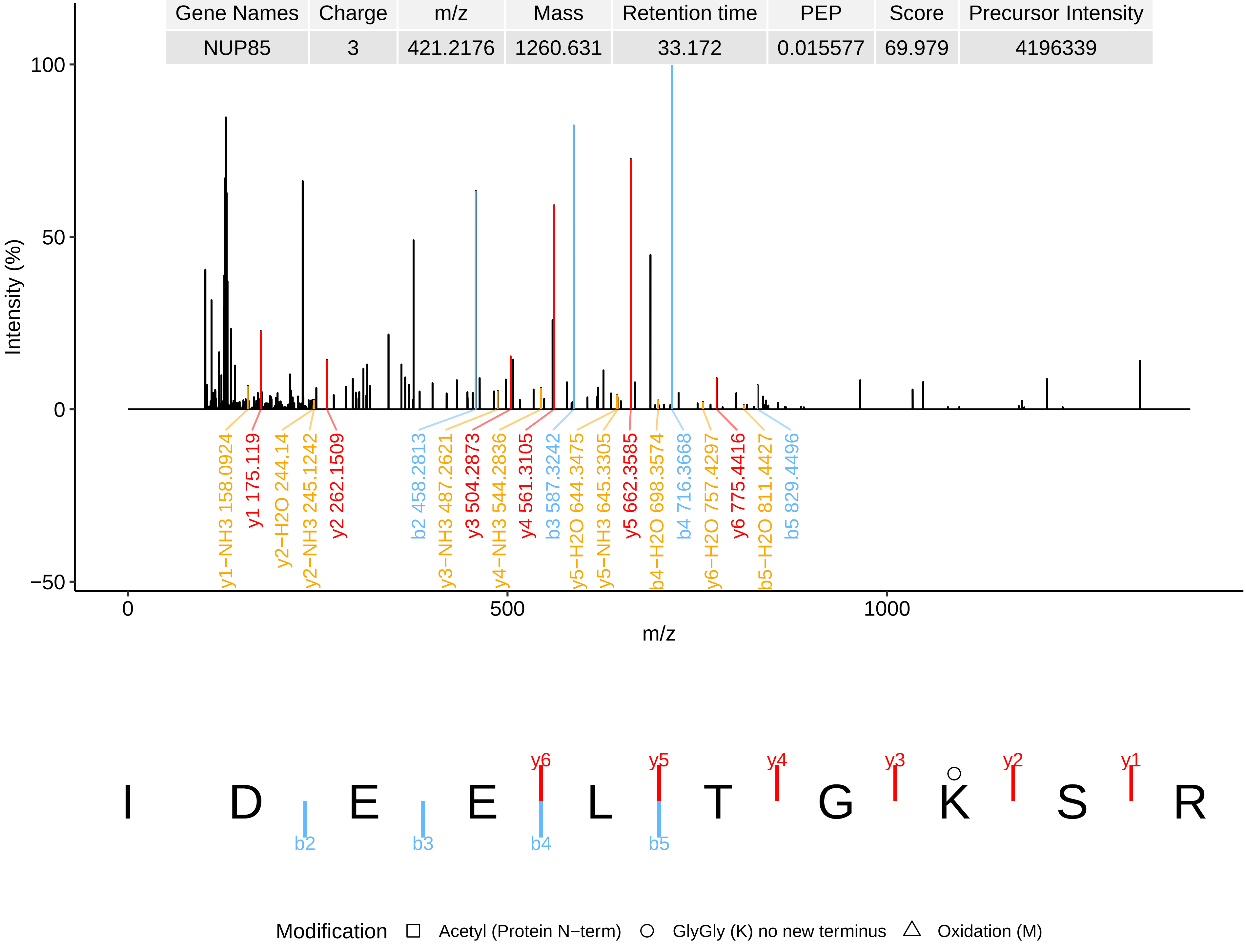

20200921_KIZ_MUb4_GlyGly_10plex − Scan number: 5274

Gene Names	Charge	m/z	Mass	Retention time	PEP	Score	Precursor Intensity
MFSD1	4	267.4056	1065.593	17.244	0.016156	72.138	9115645

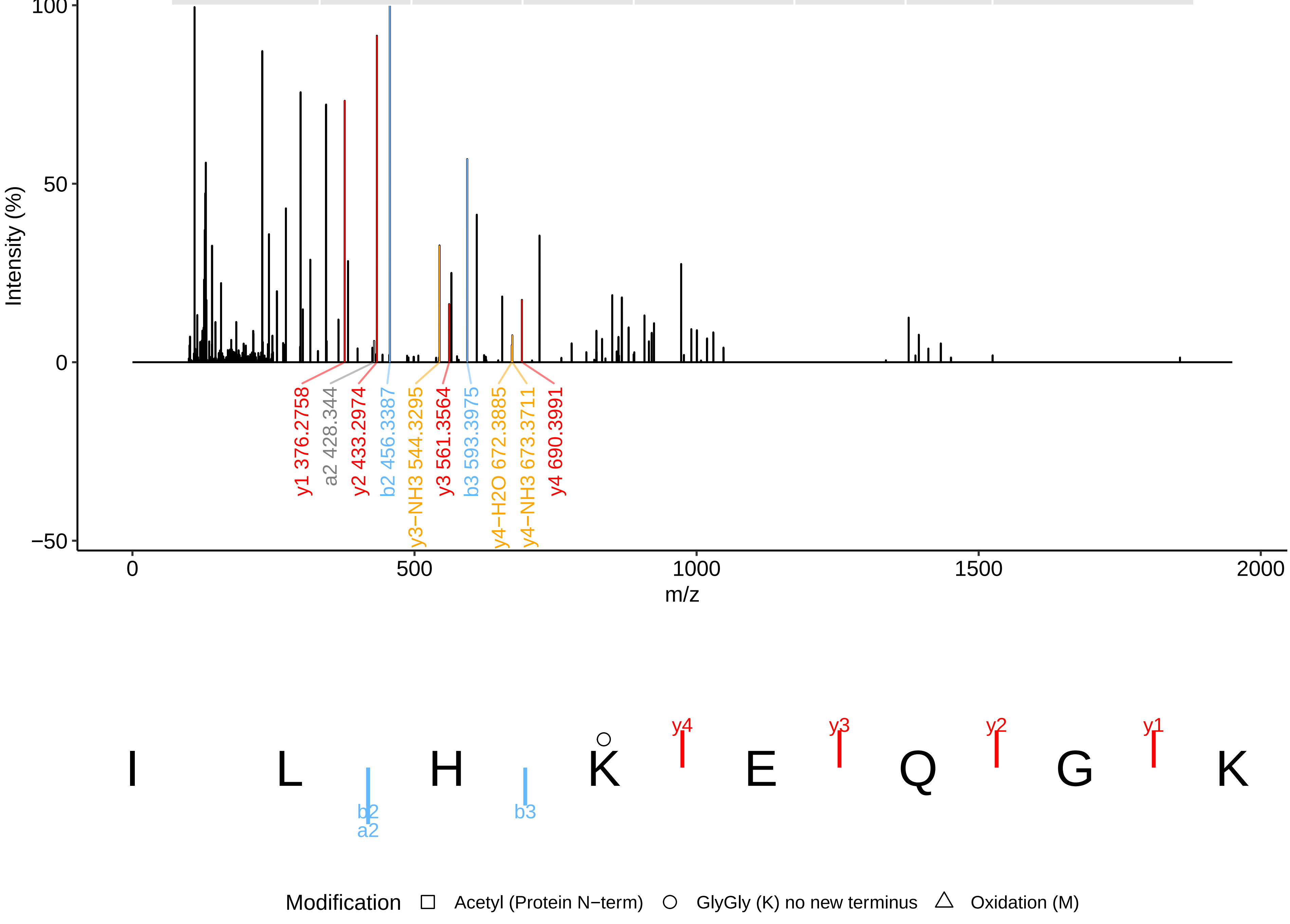

20200924_KIZ_MUb5_GlyGly_1 − Scan number: 4592

Gene Names	Charge	m/z	Mass	Retention time	PEP	Score	Precursor Intensity
MFSD1	3	356.205	1065.593	14.196	0.0087602	86.011	2186642

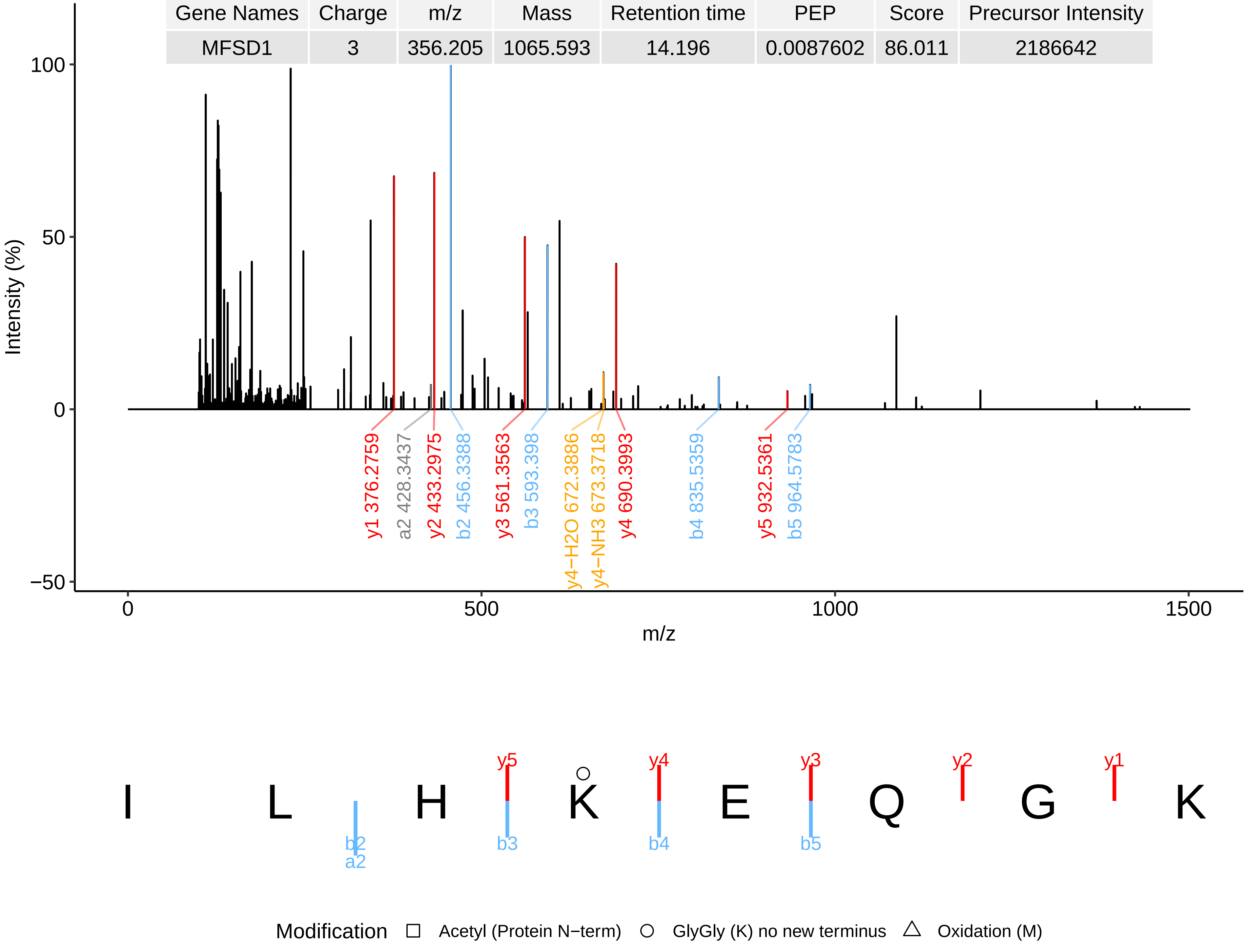

20200921_KIZ_MUb4_GlyGly_10plex_2 − Scan number: 7253

Gene Names	Charge	m/z	Mass	Retention time	PEP	Score	Precursor Intensity
COPA	3	358.8623	1073.565	26.996	0.096848	54.549	672052.6

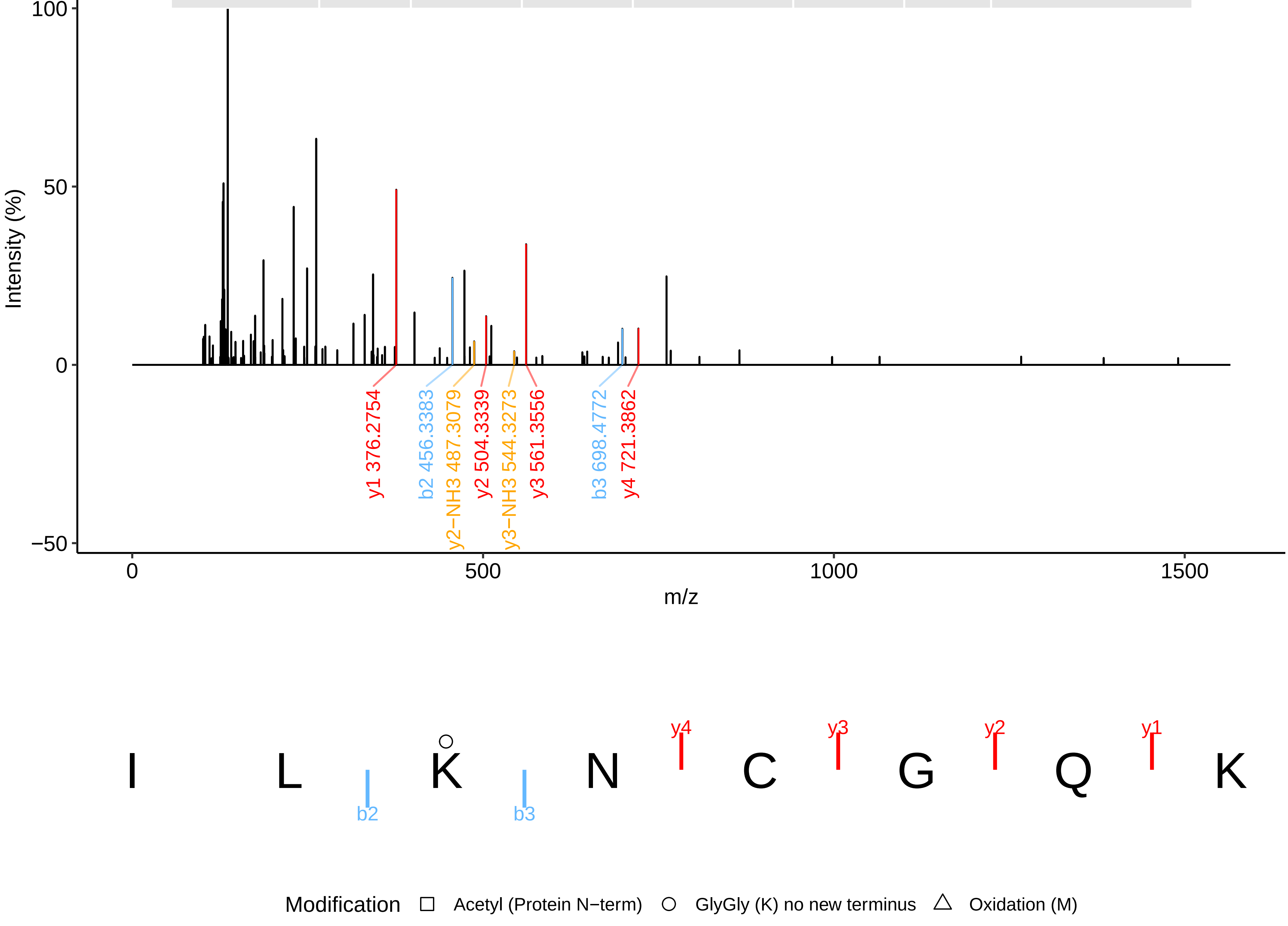

20201118_KIZ_MUb6_GlyGly_2 − Scan number: 8522

Gene Names	Charge	m/z	Mass	Retention time	PEP	Score	Precursor Intensity
COPA	3	358.8623	1073.565	28.949	0.013108	79.116	NA

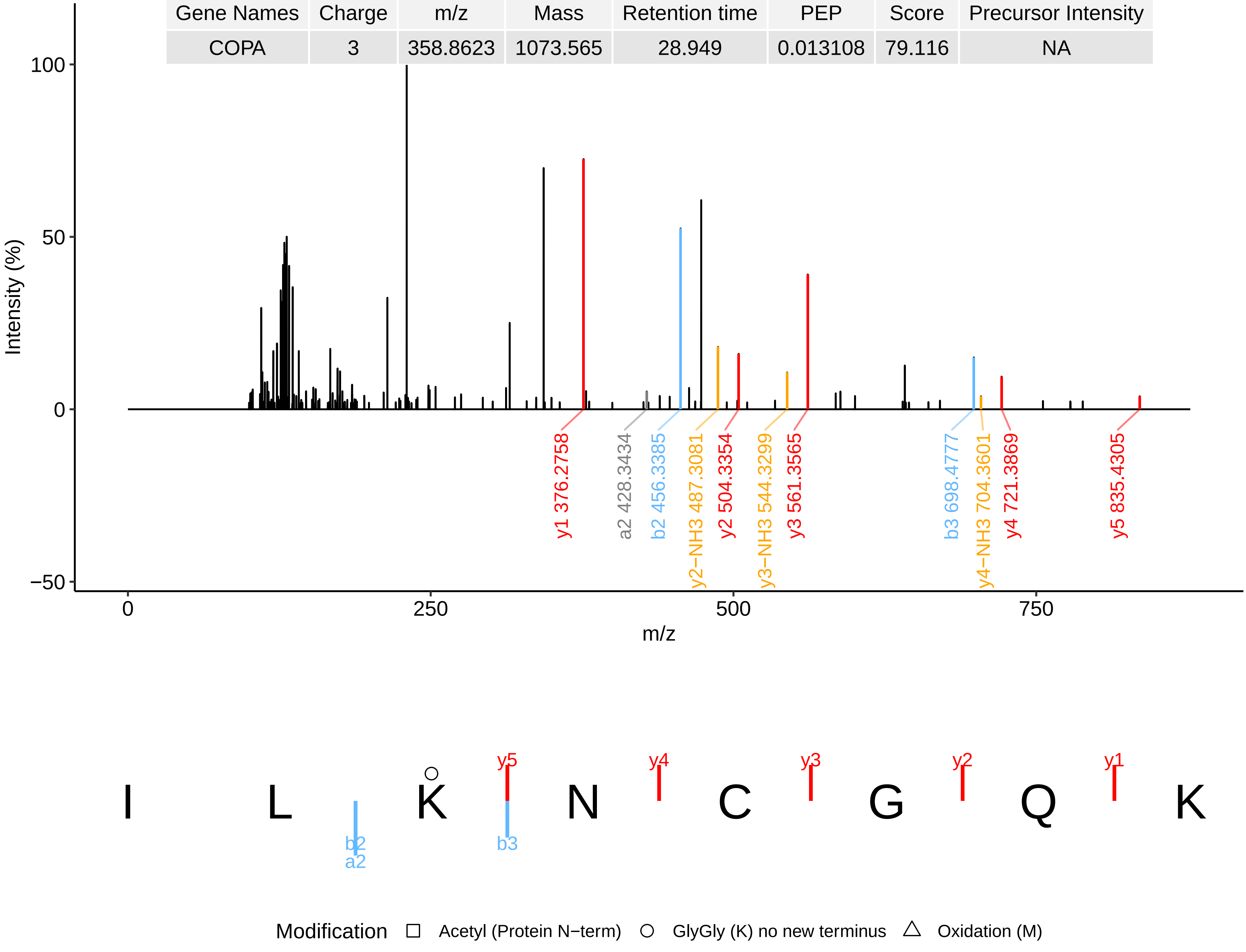

20200921_KIZ_MUb4_GlyGly_10plex − Scan number: 10506

Gene Names	Charge	m/z	Mass	Retention time	PEP	Score	Precursor Intensity
AP2M1	3	343.5415	1027.603	36.381	0.013493	77.058	NA

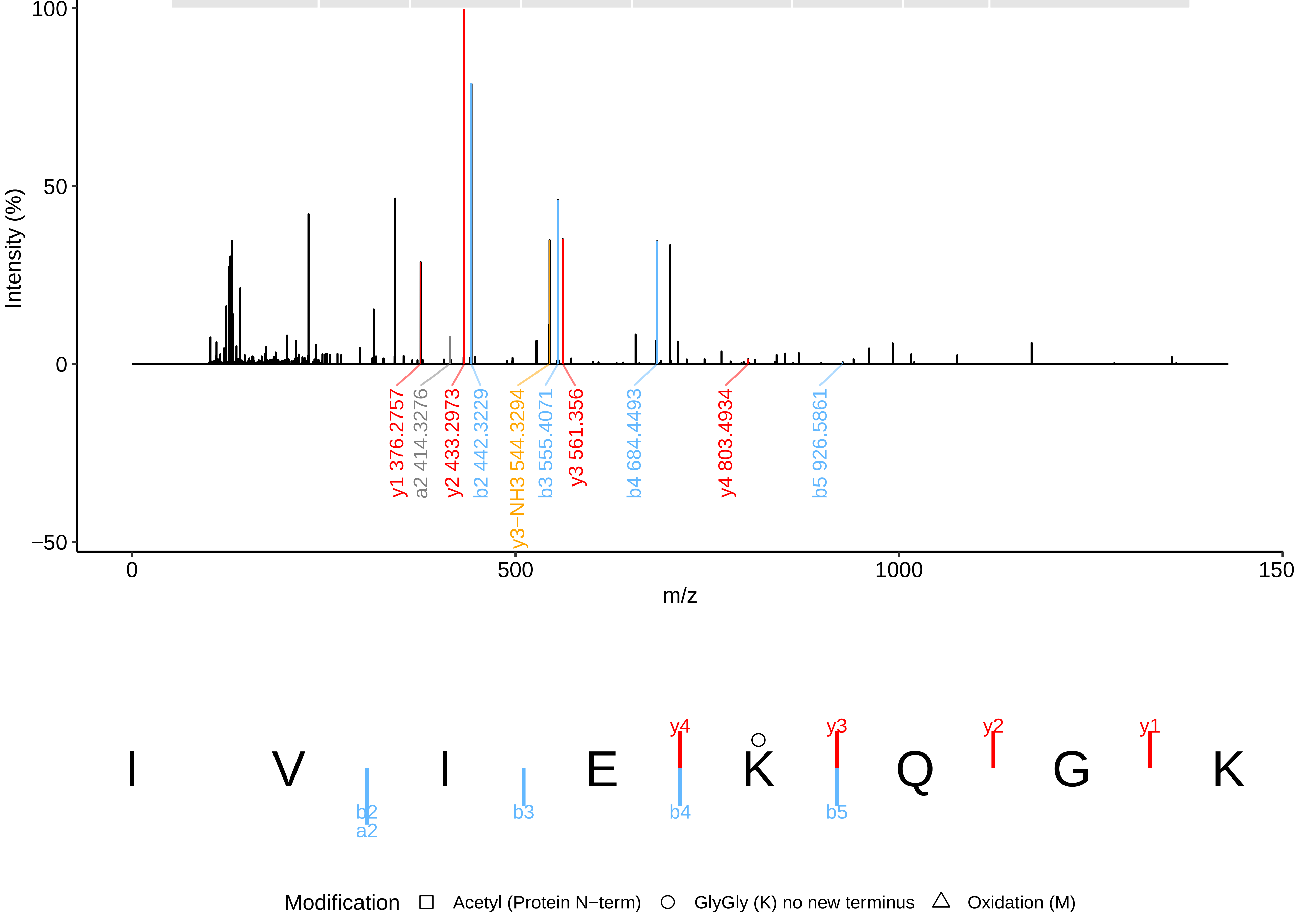

20200921_KIZ_MUb4_GlyGly_10plex_2 − Scan number: 9734

Gene Names	Charge	m/z	Mass	Retention time	PEP	Score	Precursor Intensity
AP2M1	3	343.5415	1027.603	37.097	0.01033	79.597	2014508

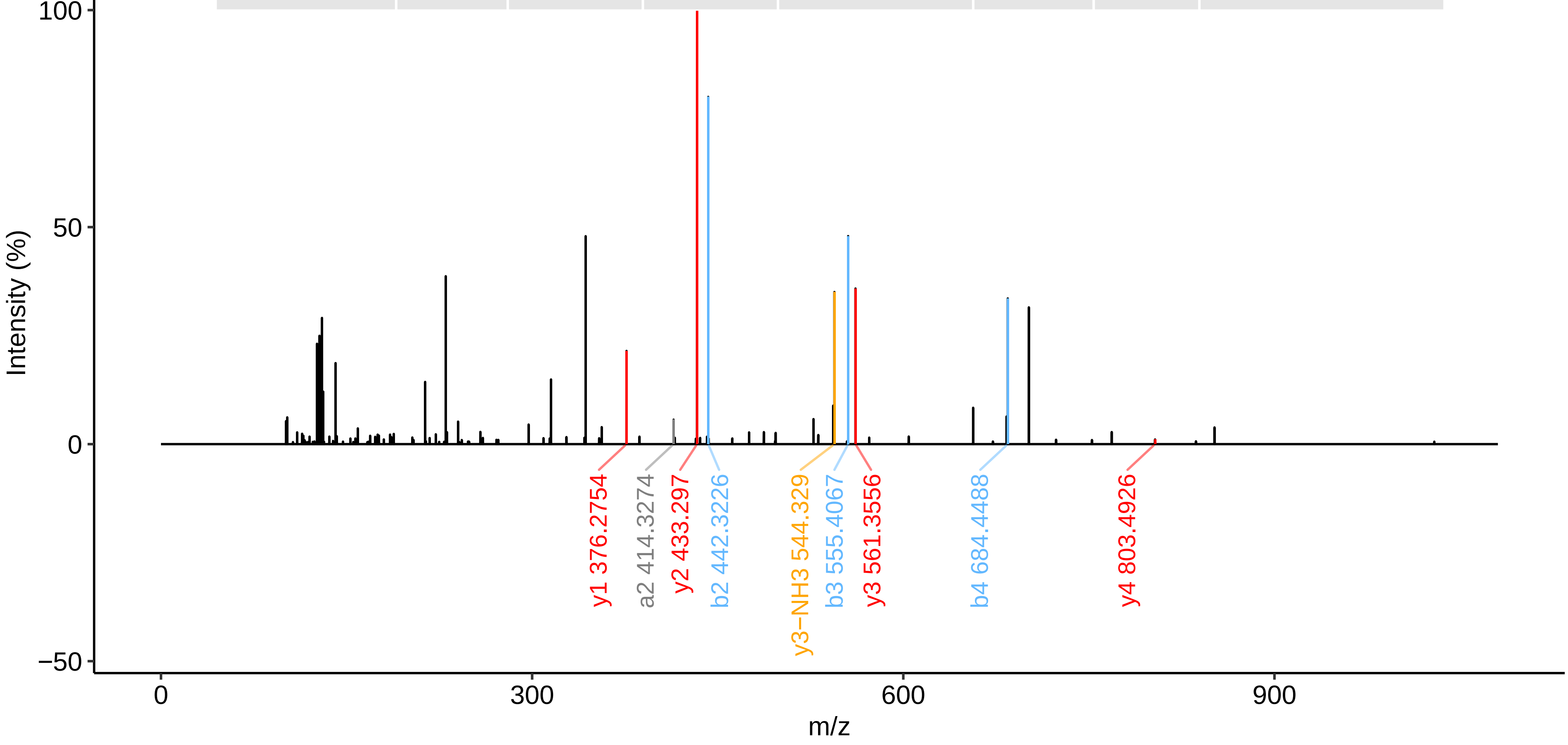

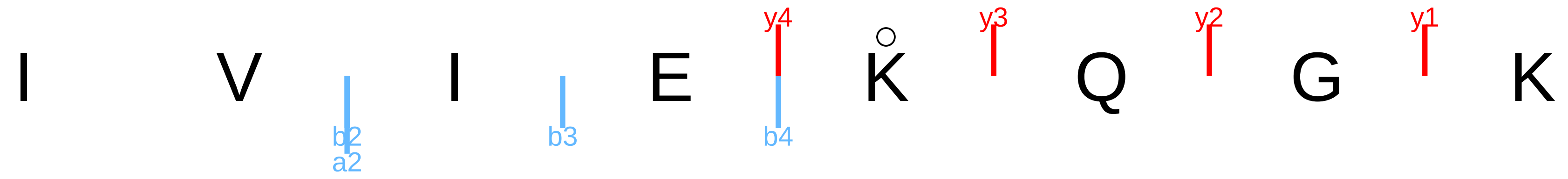

Modification □ Acetyl (Protein N−term) ○ GlyGly (K) no new terminus △ Oxidation (M)

20200924_KIZ_MUb5_GlyGly_1 − Scan number: 9560

Gene Names	Charge	m/z	Mass	Retention time	PEP	Score	Precursor Intensity
AP2M1	3	343.5415	1027.603	30.757	0.012415	77.923	11685292

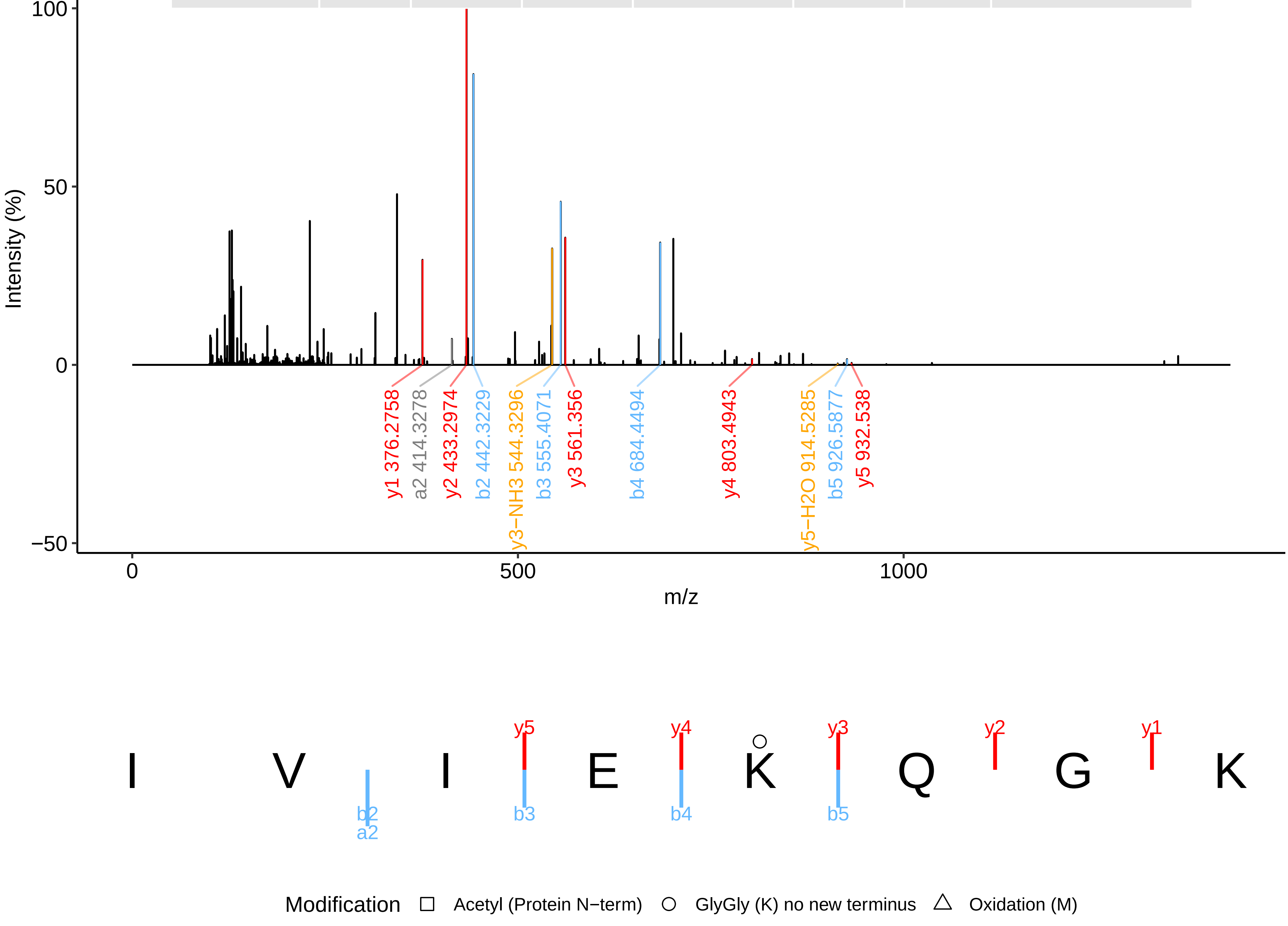

20200924_KIZ_MUb5_GlyGly_2 − Scan number: 8828

Gene Names	Charge	m/z	Mass	Retention time	PEP	Score	Precursor Intensity
AP2M1	3	343.5415	1027.603	33.002	0.054972	57.281	4113931

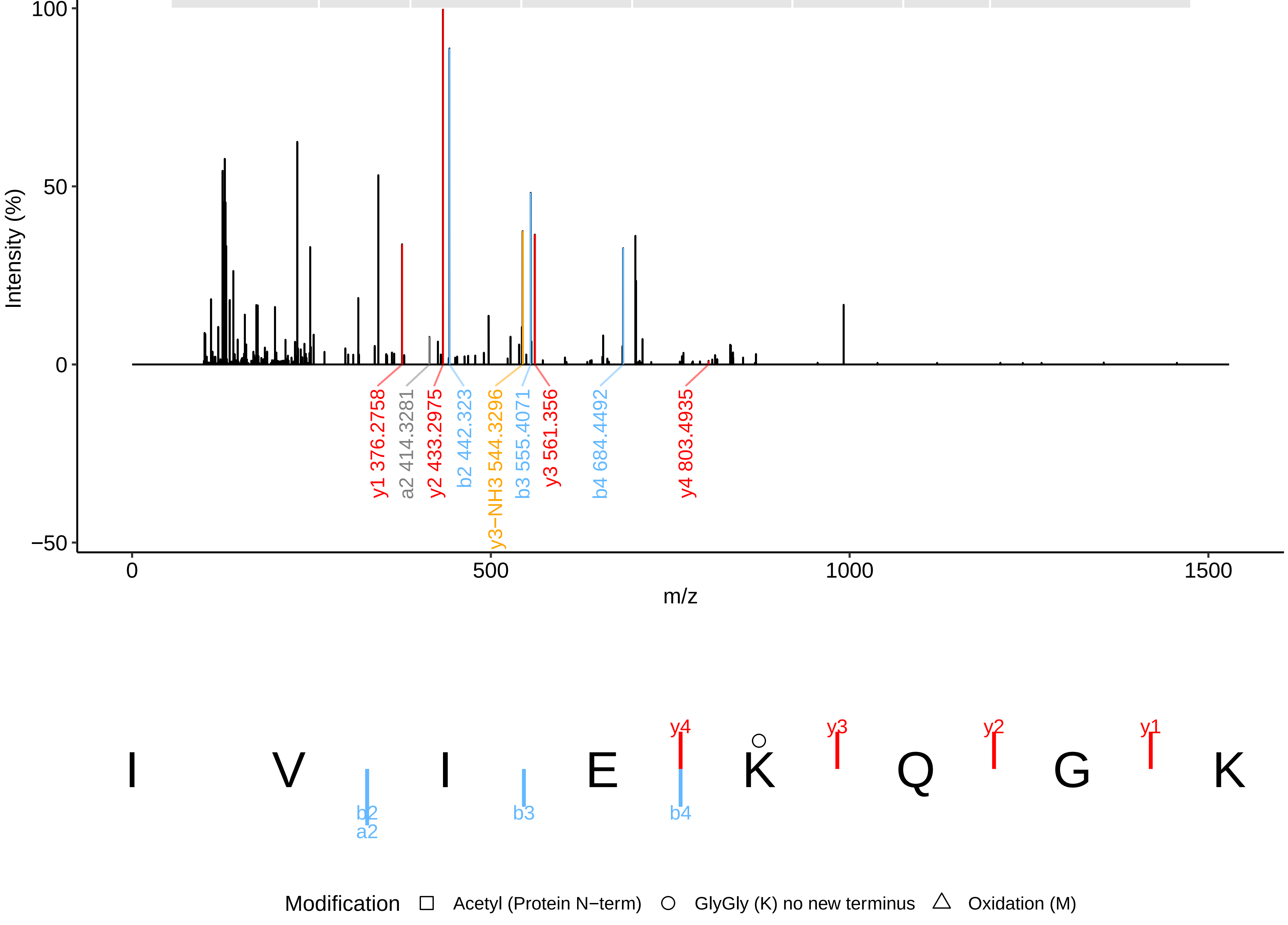

20200921_KIZ_MUb4_GlyGly_10plex_2 − Scan number: 5676

Gene Names	Charge	m/z	Mass	Retention time	PEP	Score	Precursor Intensity
RAB11A	3	435.8908	1304.651	20.518	0.00070282	93.667	307934.8

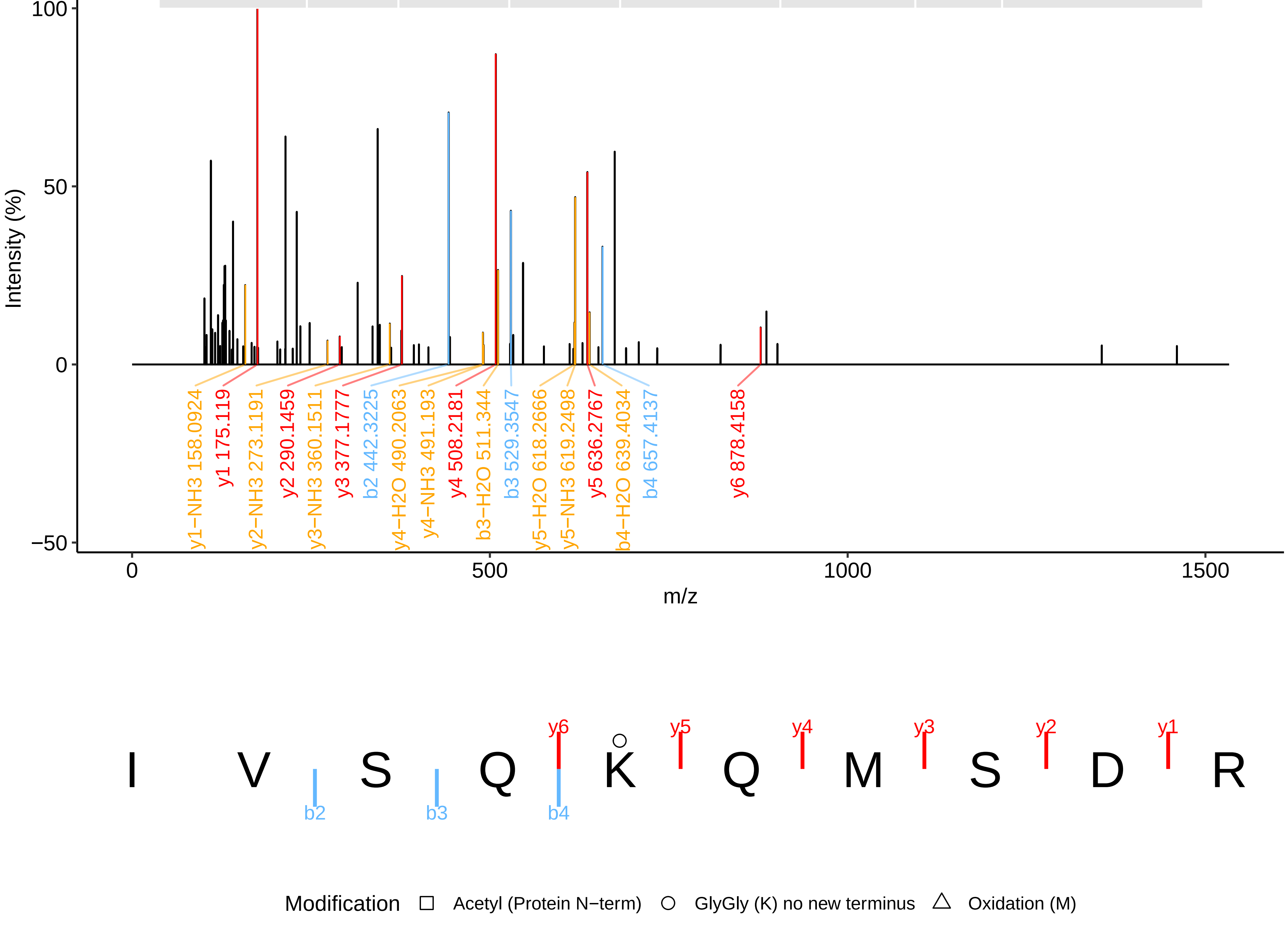

20200921_KIZ_MUb4_GlyGly_10plex − Scan number: 7992

Gene Names	Charge	m/z	Mass	Retention time	PEP	Score	Precursor Intensity
VARS	4	261.6554	1042.592	26.997	0.047357	62.52	11766893

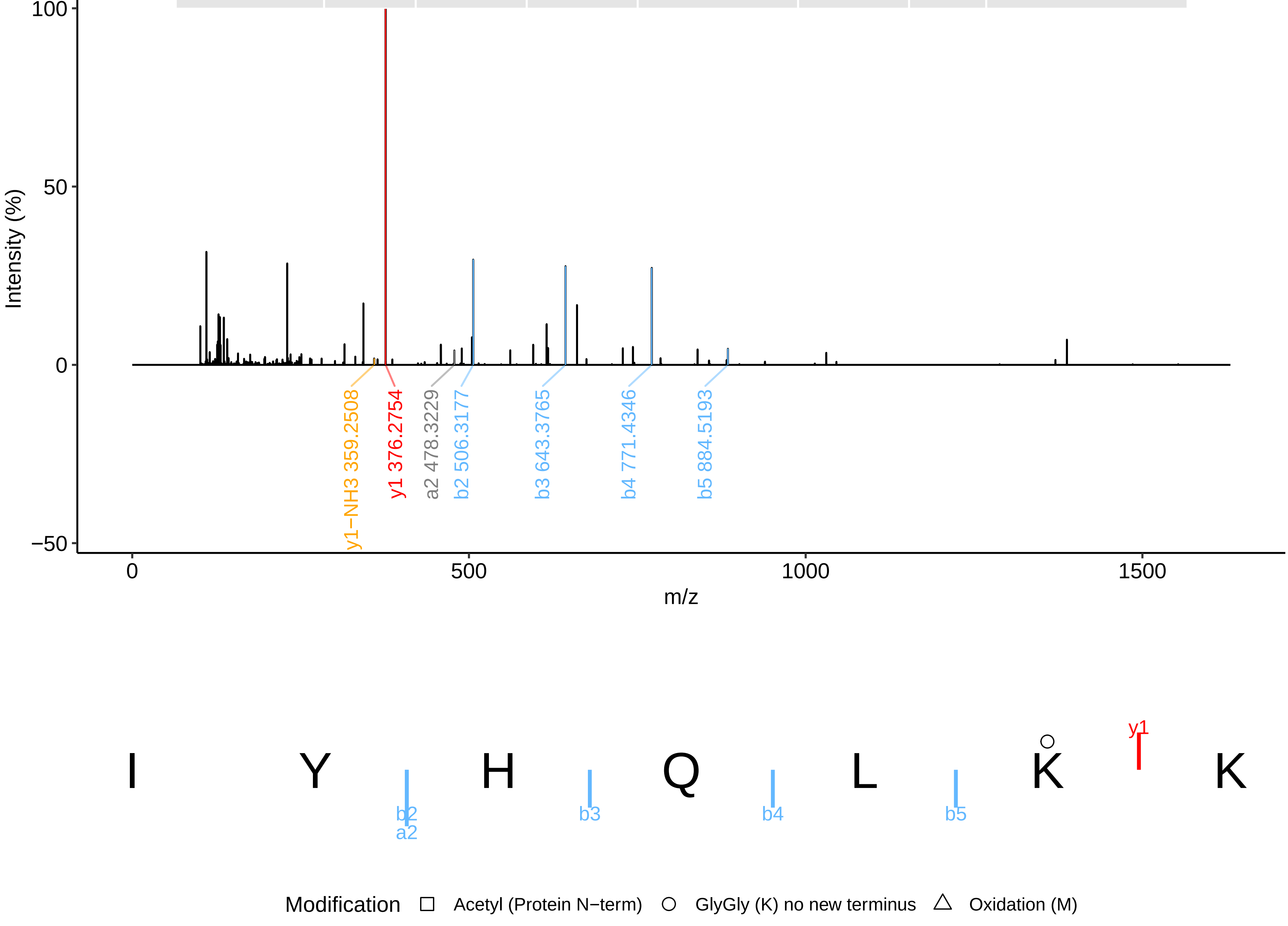

20200921_KIZ_MUb4_GlyGly_10plex_2 − Scan number: 7499

Gene Names	Charge	m/z	Mass	Retention time	PEP	Score	Precursor Intensity
VARS	4	261.6554	1042.592	28.004	0.047357	62.52	1754666

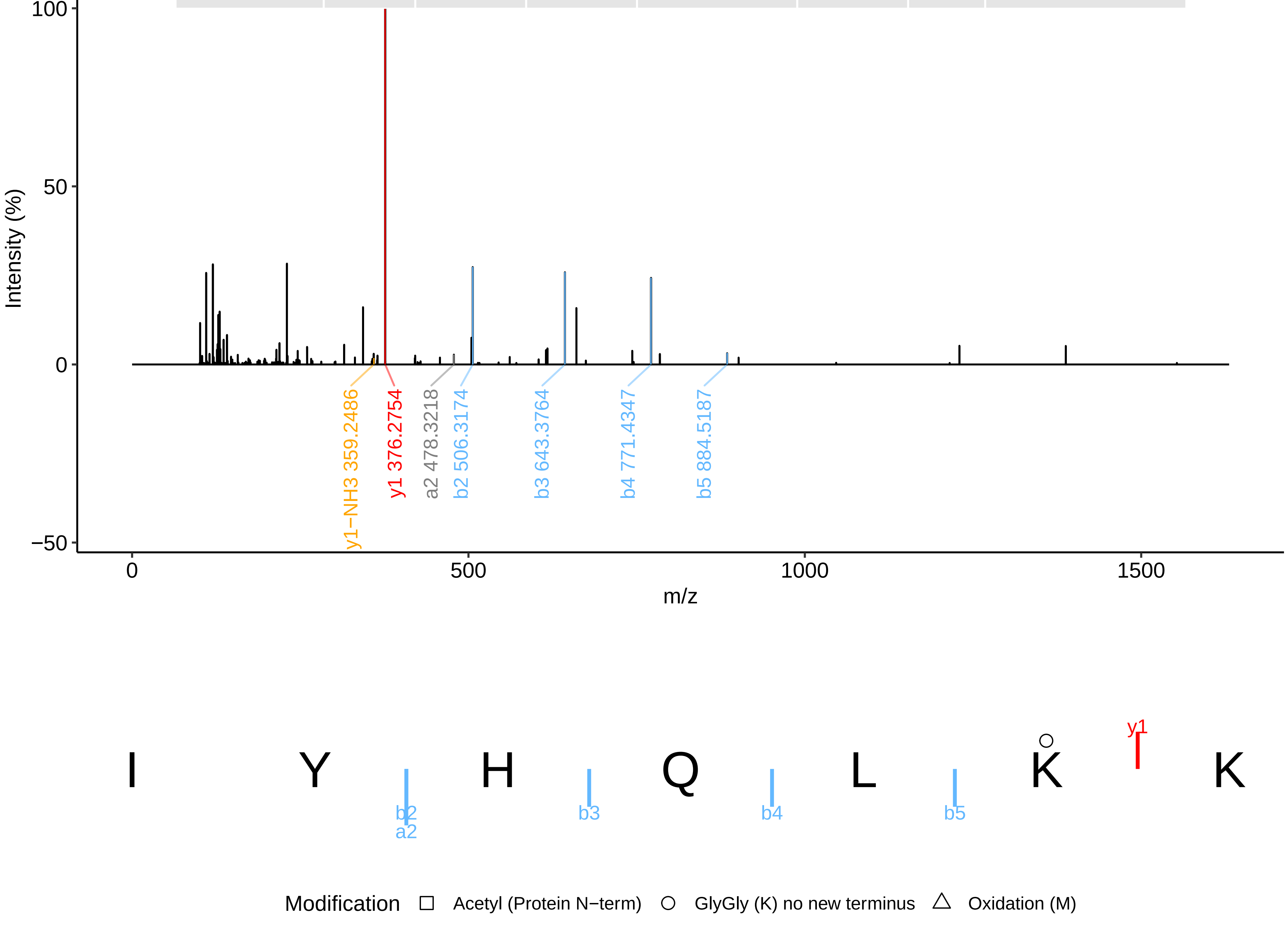

20200921_KIZ_MUb4_GlyGly_10plex − Scan number: 10458

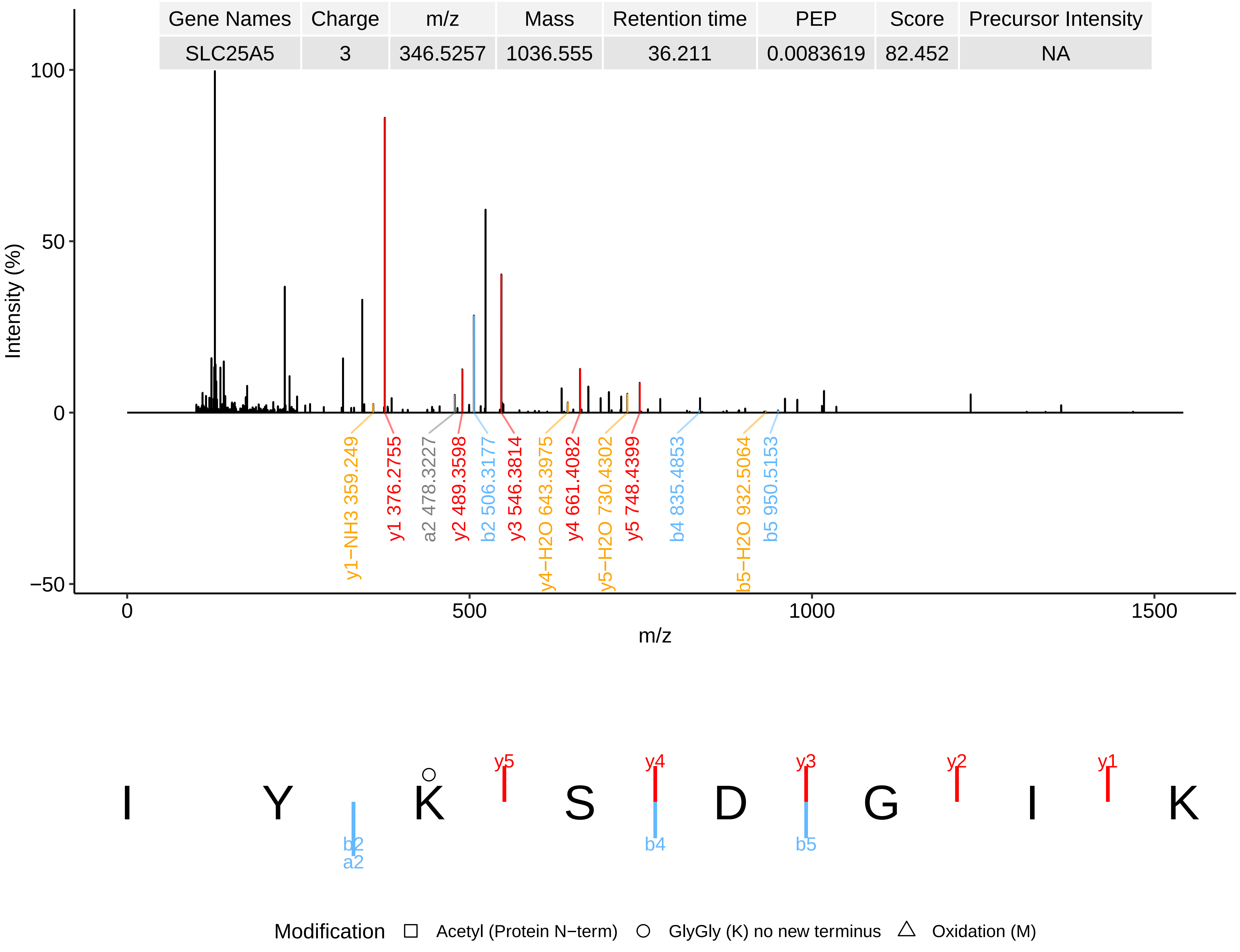

20200921_KIZ_MUb4_GlyGly_10plex_2 − Scan number: 9677

Gene Names	Charge	m/z	Mass	Retention time	PEP	Score	Precursor Intensity
SLC25A5	3	346.5257	1036.555	36.865	0.067794	54.157	621834.5

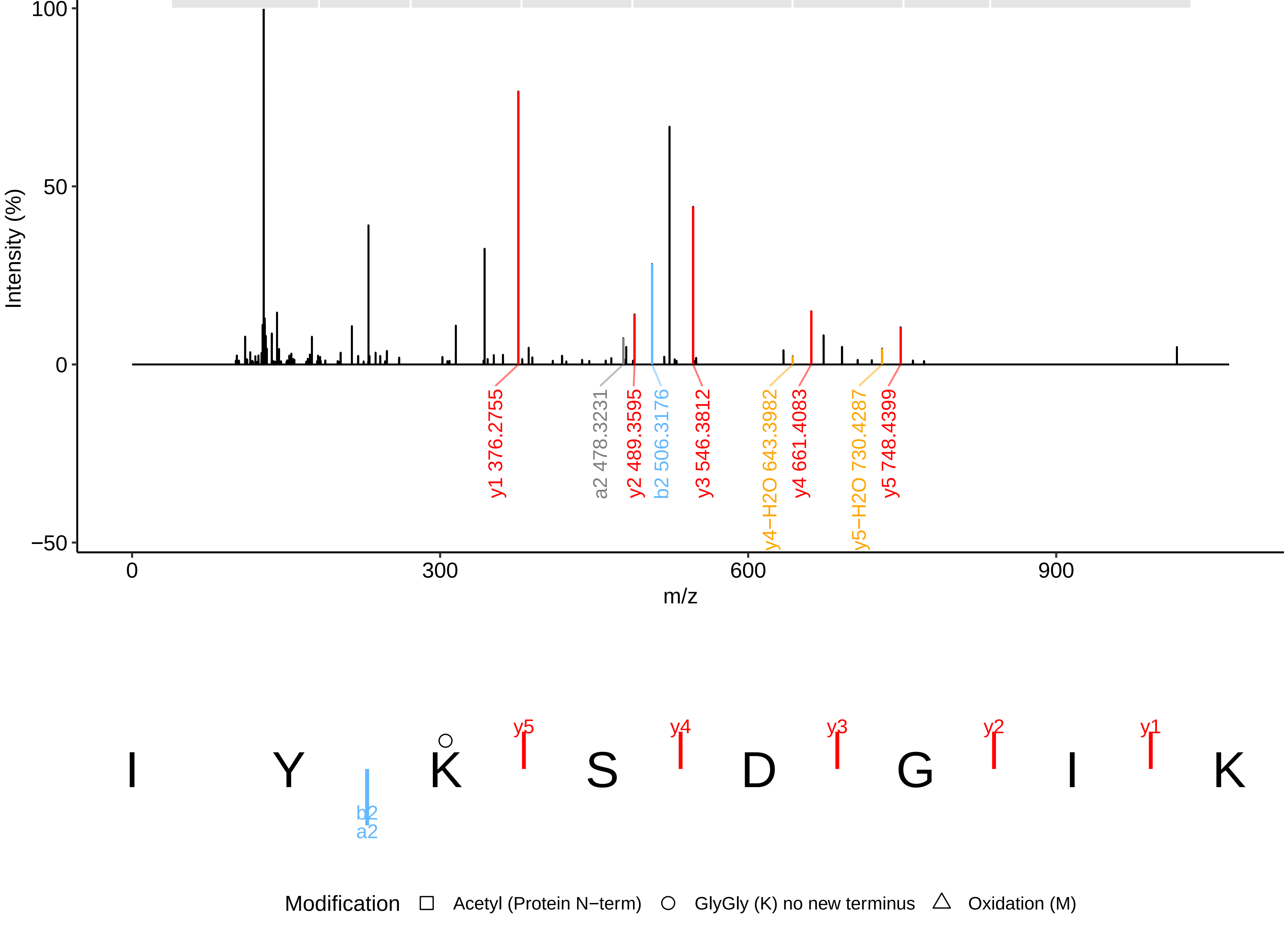

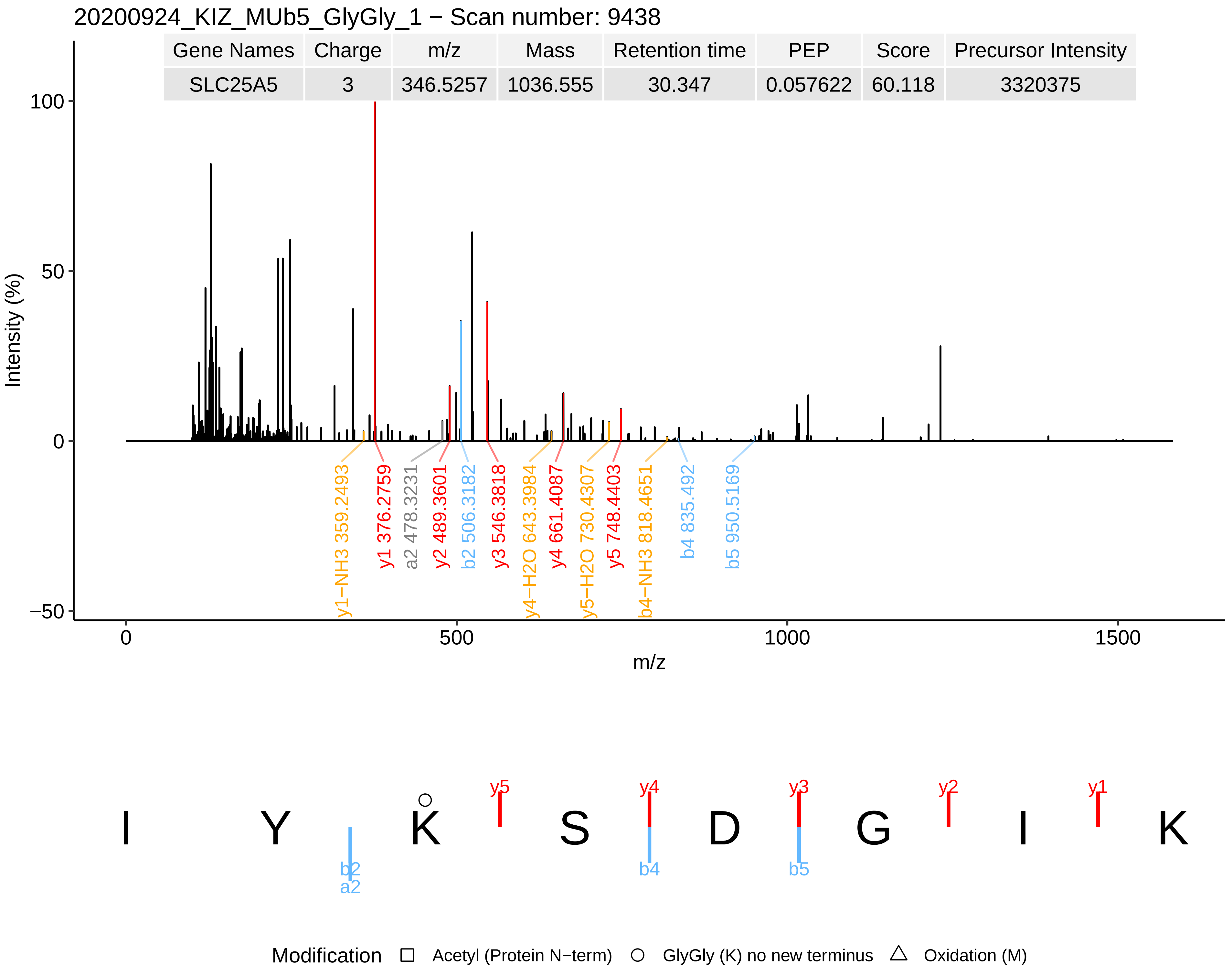
20200924_KIZ_MUb5_GlyGly_1 − Scan number: 9438
Gene Names
Charge
m/z
Mass
Retention time
PEP
Score
Precursor Intensity
SLC25A5
3
346.5257
1036.555
30.347
0.057622
60.118
3320375
Intensity (%)
m/z
y1−NH3 359.2493
y1 376.2759
a2 478.3231
y2 489.3601
b2 506.3182
y3 546.3818
y4−H2O 643.3984
y4 661.4087
y5−H2O 730.4307
y5 748.4403
b4−NH3 818.4651
b4 835.492
b5 950.5169
I Y K S D G I K
y5 y4 y3 y2 y1
b2 a2 b4 b5
Modification
Acetyl (Protein N−term)
GlyGly (K) no new terminus
Oxidation (M)

20200921_KIZ_MUb4_GlyGly_10plex − Scan number: 6700

Gene Names	Charge	m/z	Mass	Retention time	PEP	Score	Precursor Intensity
TKT	4	266.6528	1062.582	22.268	0.060884	49.715	2624074

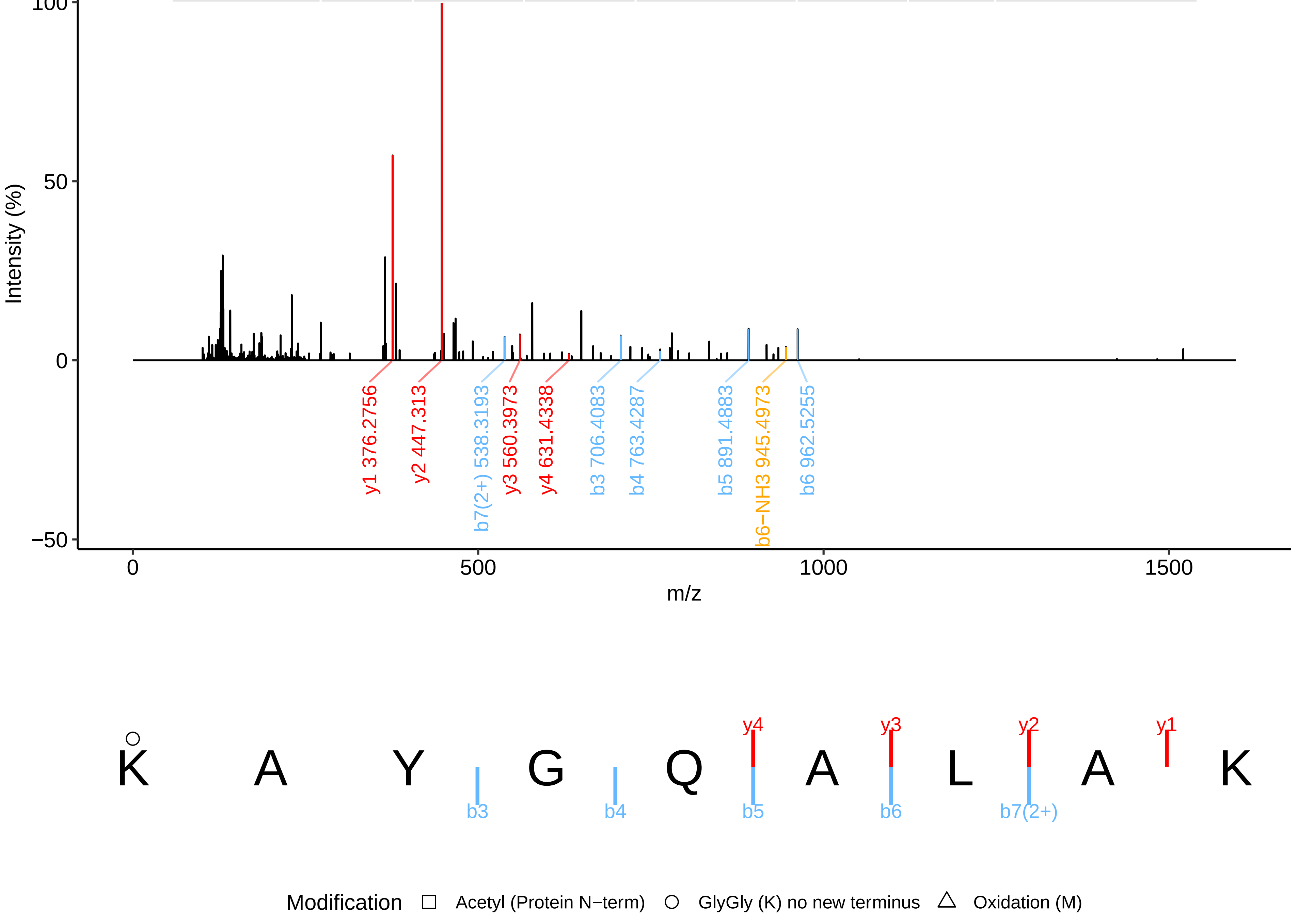

20200921_KIZ_MUb4_GlyGly_10plex − Scan number: 8668

Gene Names	Charge	m/z	Mass	Retention time	PEP	Score	Precursor Intensity
TKT	3	355.2013	1062.582	29.492	0.11329	66.692	10755018

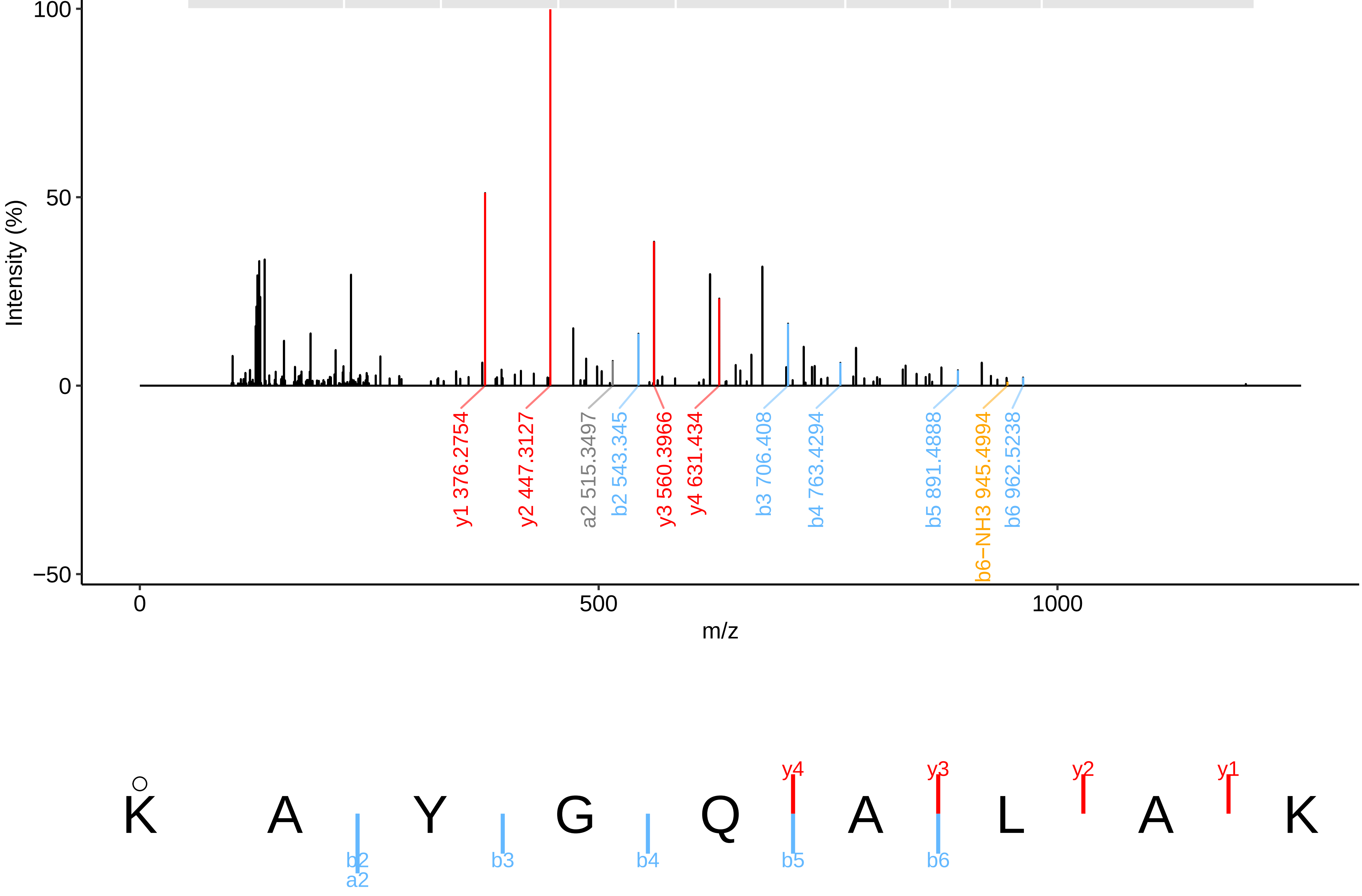

Modification □ Acetyl (Protein N−term) ○ GlyGly (K) no new terminus △ Oxidation (M)

20200924_KIZ_MUb5_GlyGly_2 − Scan number: 7195

Gene Names	Charge	m/z	Mass	Retention time	PEP	Score	Precursor Intensity
TKT	3	355.2013	1062.582	26.838	0.10755	67.563	4690397

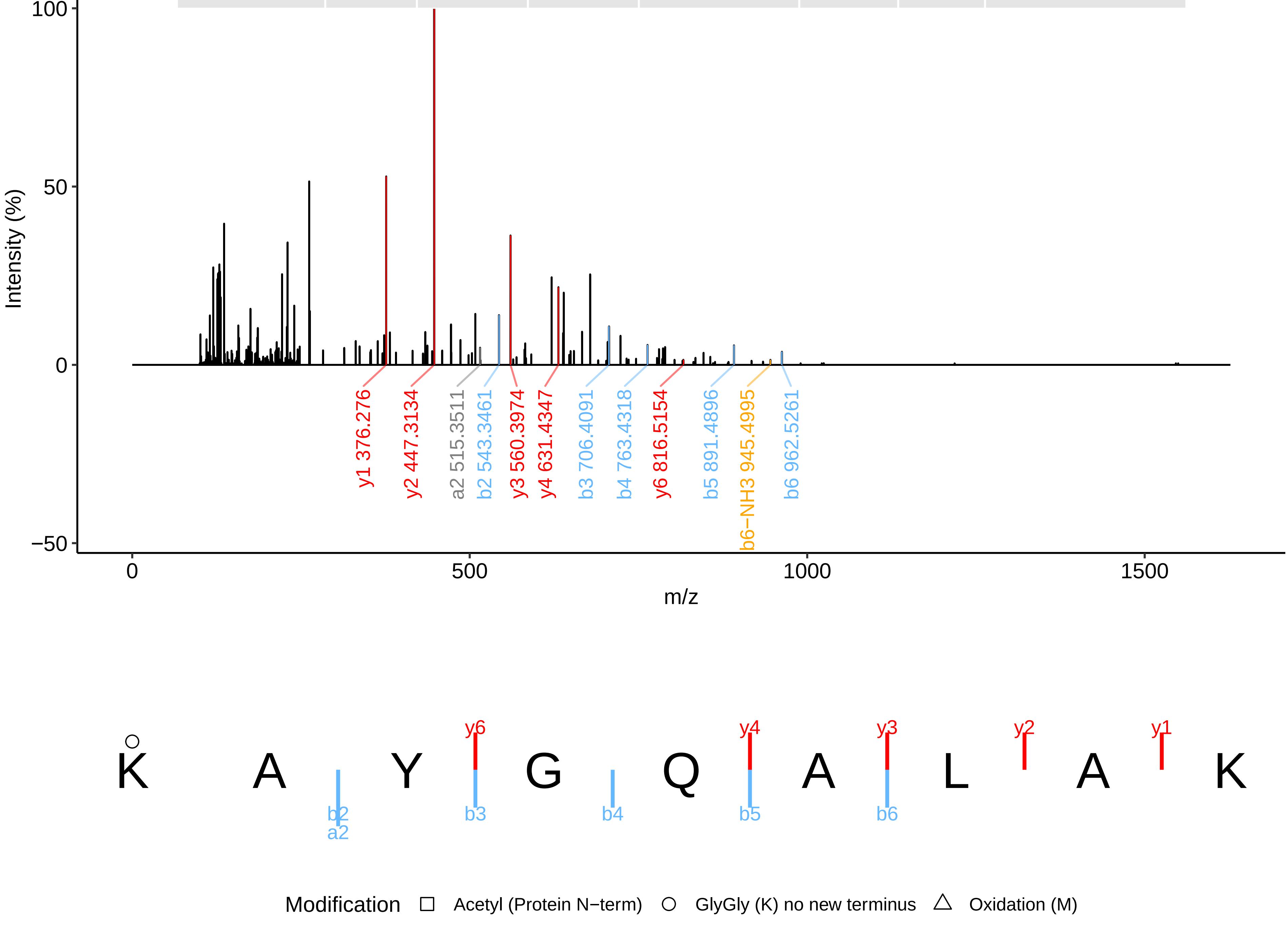

20201118_KIZ_MUb6_GlyGly_1 − Scan number: 7272

Gene Names	Charge	m/z	Mass	Retention time	PEP	Score	Precursor Intensity
TKT	3	355.2013	1062.582	23.04	0.11329	66.692	21498232

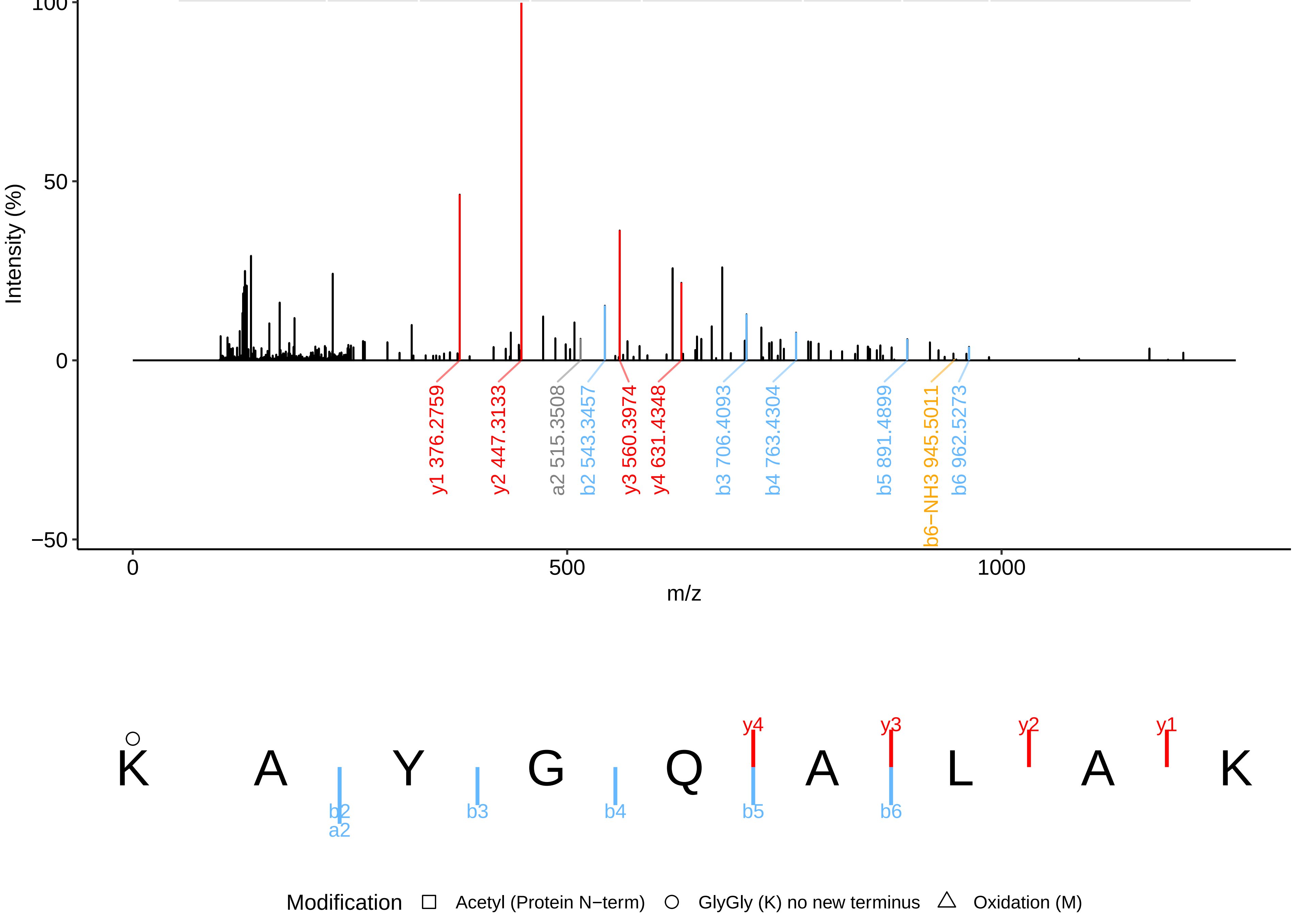

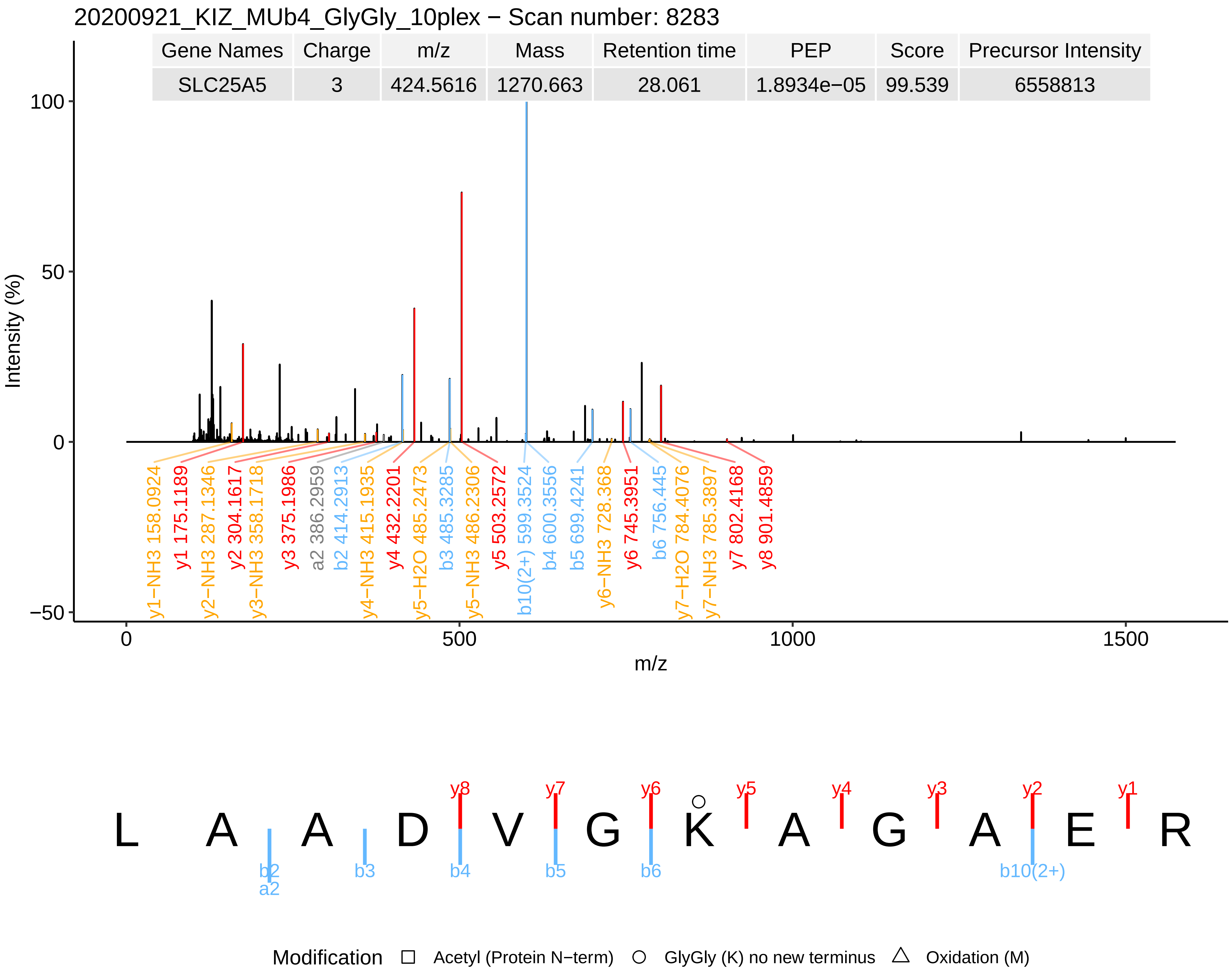

20200921_KIZ_MUb4_GlyGly_10plex − Scan number: 8283
Gene Names | Charge | m/z | Mass | Retention time | PEP | Score | Precursor Intensity
SLC25A5 | 3 | 424.5616 | 1270.663 | 28.061 | 1.8934e−05 | 99.539 | 6558813
Intensity (%)
100
50
0
−50
0
500
1000
1500
m/z
y1−NH3 158.0924
y1 175.1189
y2−NH3 287.1346
y2 304.1617
y3−NH3 358.1718
y3 375.1986
a2 386.2959
b2 414.2913
y4−NH3 415.1935
y4 432.2201
y5−H2O 485.2473
b3 485.3285
y5−NH3 486.2306
y5 503.2572
b10(2+) 599.3524
b4 600.3556
b5 699.4241
y6−NH3 728.368
y6 745.3951
b6 756.445
y7−H2O 784.4076
y7−NH3 785.3897
y7 802.4168
y8 901.4859
y8 y7 y6 y5 y4 y3 y2 y1
L A A D V G K A G A E R
b2 a2 b3 b4 b5 b6 b10(2+)
Modification
Acetyl (Protein N−term)
GlyGly (K) no new terminus
Oxidation (M)

20200921_KIZ_MUb4_GlyGly_10plex_2 − Scan number: 7654

Gene Names	Charge	m/z	Mass	Retention time	PEP	Score	Precursor Intensity
SLC25A5	3	424.5616	1270.663	28.636	0.0035335	77.124	1387192

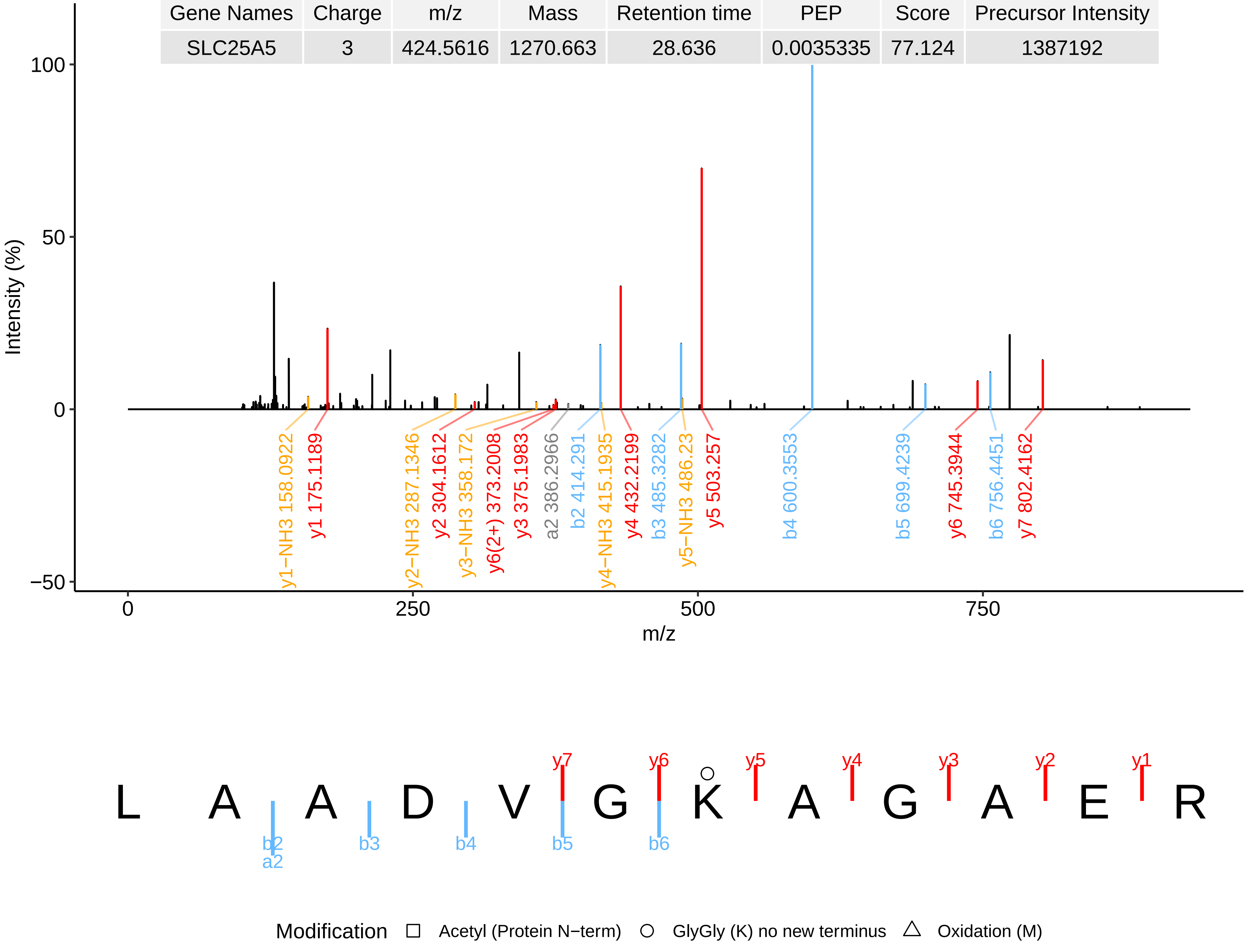

20200924_KIZ_MUb5_GlyGly_1 − Scan number: 7205

Gene Names	Charge	m/z	Mass	Retention time	PEP	Score	Precursor Intensity
SLC25A5	3	424.5616	1270.663	22.84	0.038122	58.313	17721346

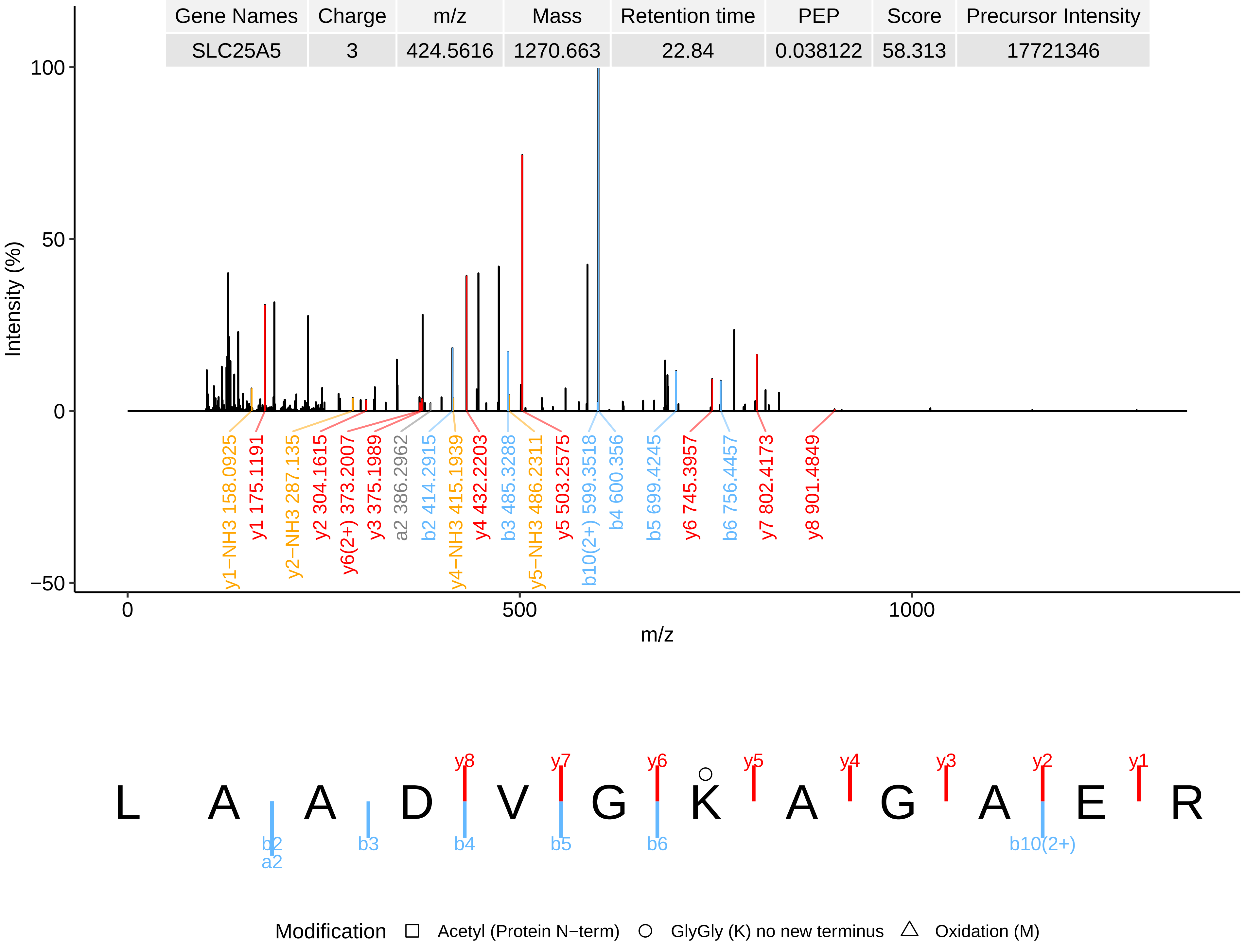

20200921_KIZ_MUb4_GlyGly_10plex − Scan number: 16562

Gene Names	Charge	m/z	Mass	Retention time	PEP	Score	Precursor Intensity
SLC25A24	3	345.866	1034.576	59.52	0.01592	75.109	4244741

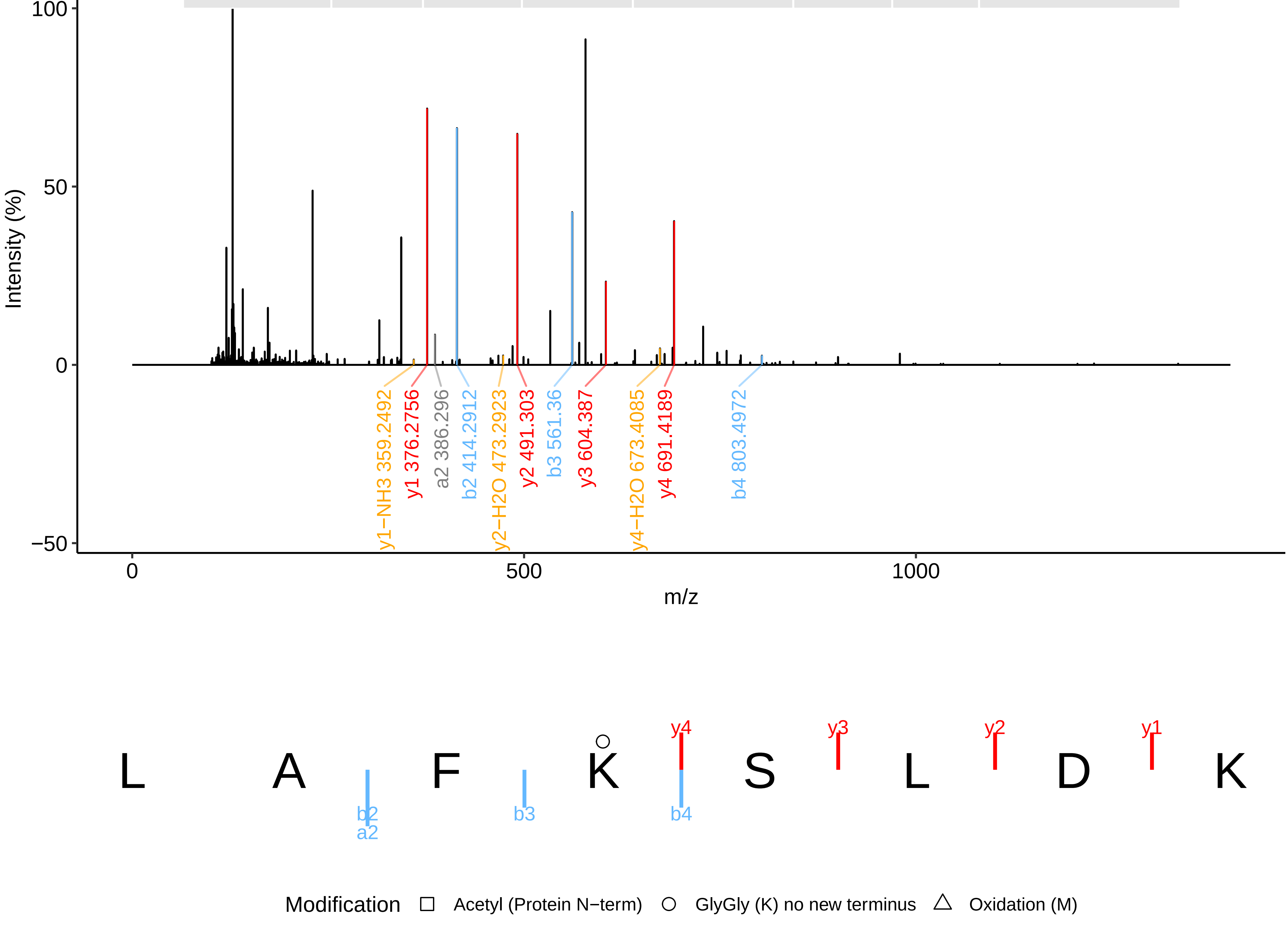

20200921_KIZ_MUb4_GlyGly_10plex_2 − Scan number: 15387

Gene Names	Charge	m/z	Mass	Retention time	PEP	Score	Precursor Intensity
SLC25A24	3	345.866	1034.576	60.159	0.073423	52.786	1058813

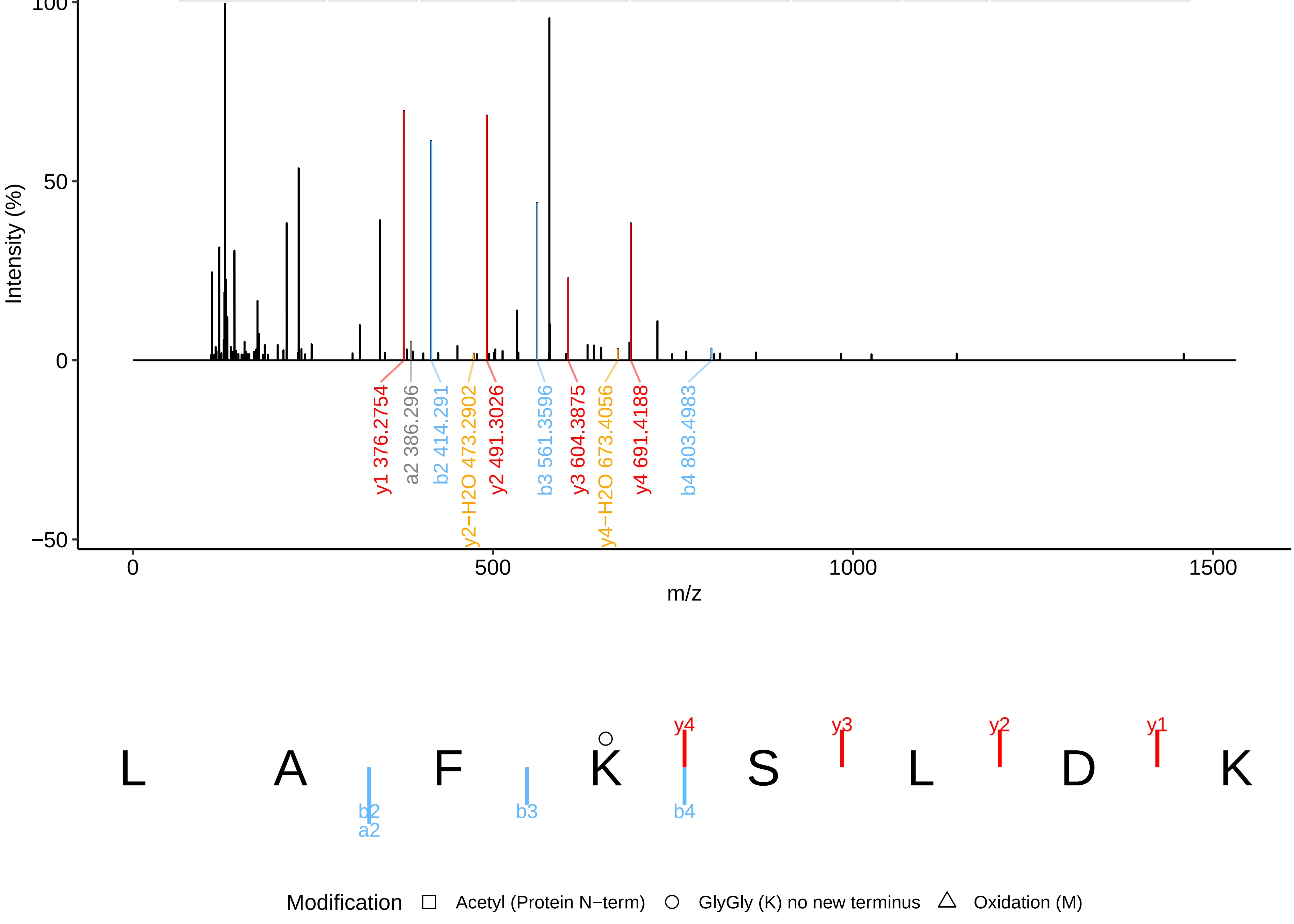

20200921_KIZ_MUb4_GlyGly_10plex − Scan number: 5268

Gene Names	Charge	m/z	Mass	Retention time	PEP	Score	Precursor Intensity
TIMM8A	3	359.1963	1074.567	17.228	0.012133	78.149	3841896

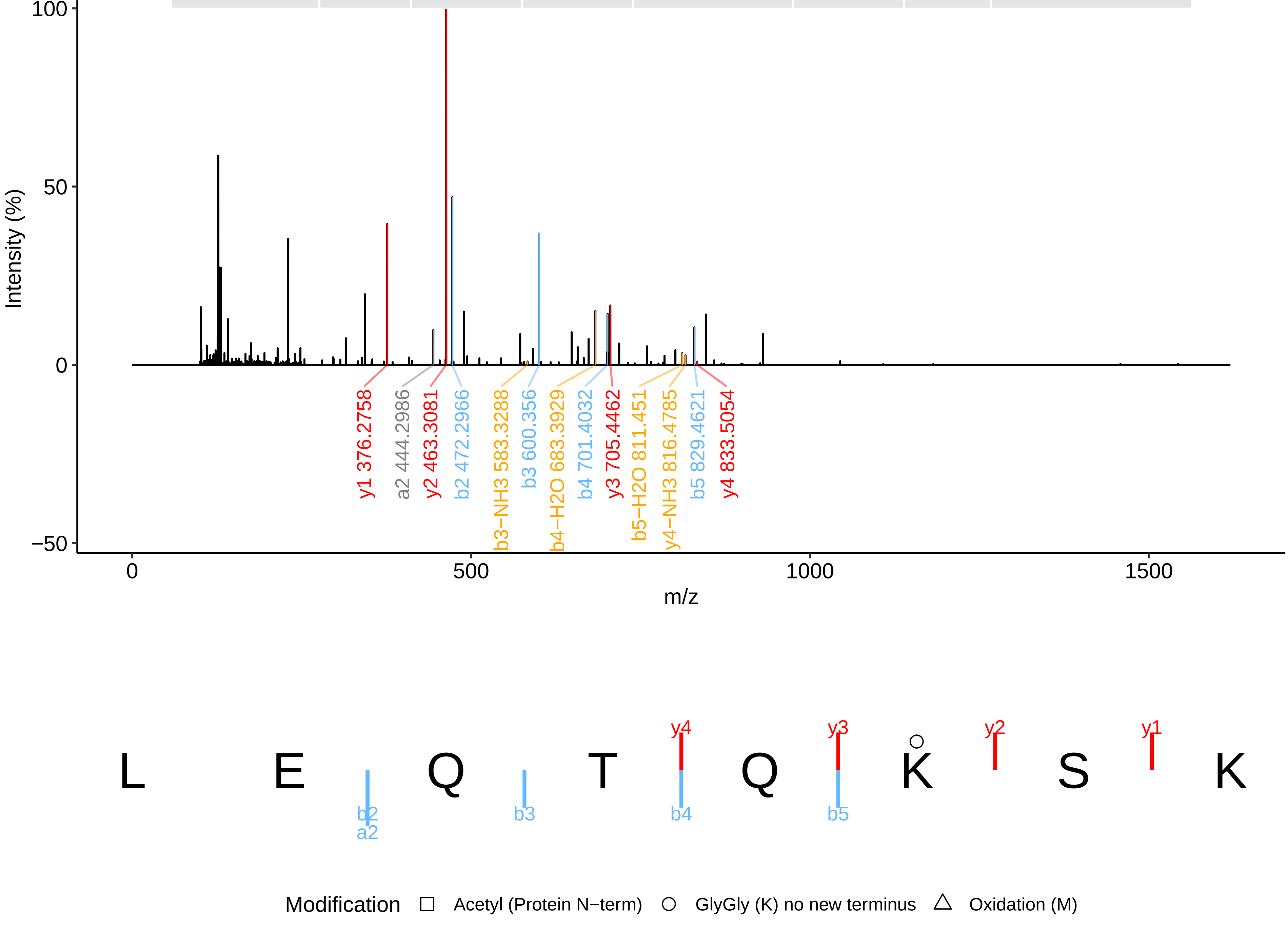

20200921_KIZ_MUb4_GlyGly_10plex_2 − Scan number: 4856

Gene Names	Charge	m/z	Mass	Retention time	PEP	Score	Precursor Intensity
TIMM8A	3	359.1963	1074.567	17.215	0.018698	72.879	1979031

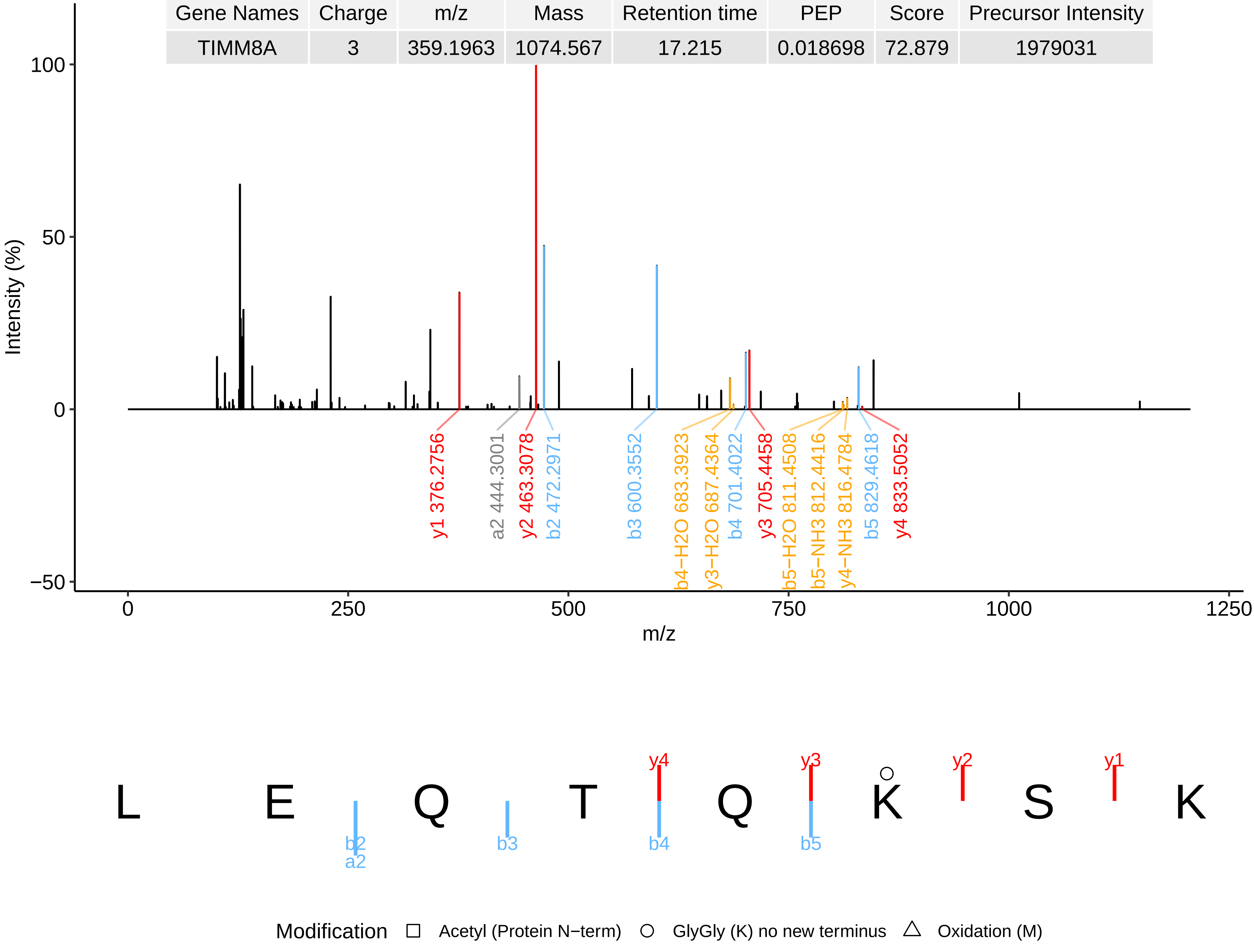

20200924_KIZ_MUb5_GlyGly_1 − Scan number: 4594

Gene Names	Charge	m/z	Mass	Retention time	PEP	Score	Precursor Intensity
TIMM8A	3	359.1963	1074.567	14.202	0.024756	68.016	7496749

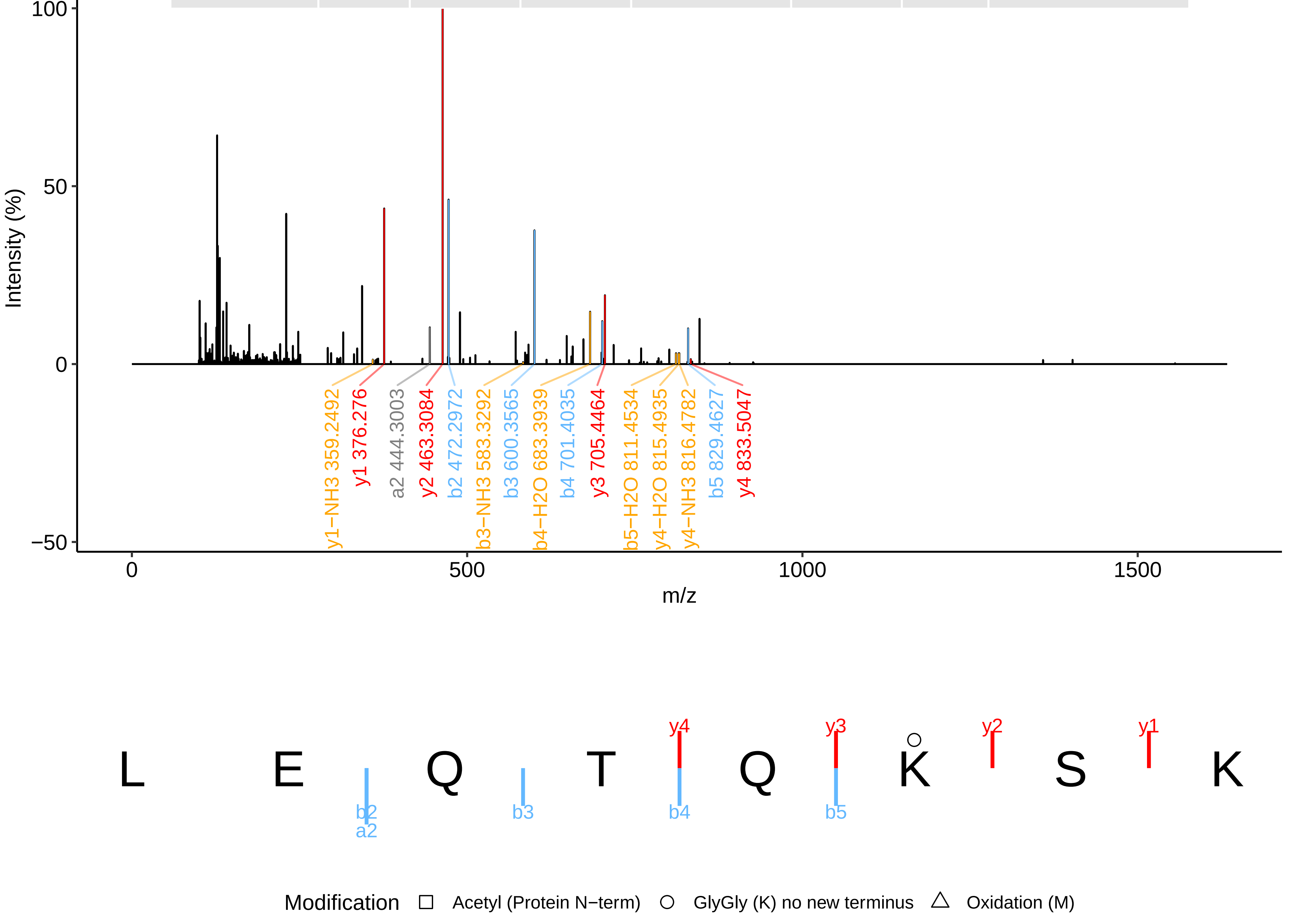

20200924_KIZ_MUb5_GlyGly_2 − Scan number: 4209

Gene Names	Charge	m/z	Mass	Retention time	PEP	Score	Precursor Intensity
TIMM8A	3	359.1963	1074.567	15.416	0.024785	67.993	1231109

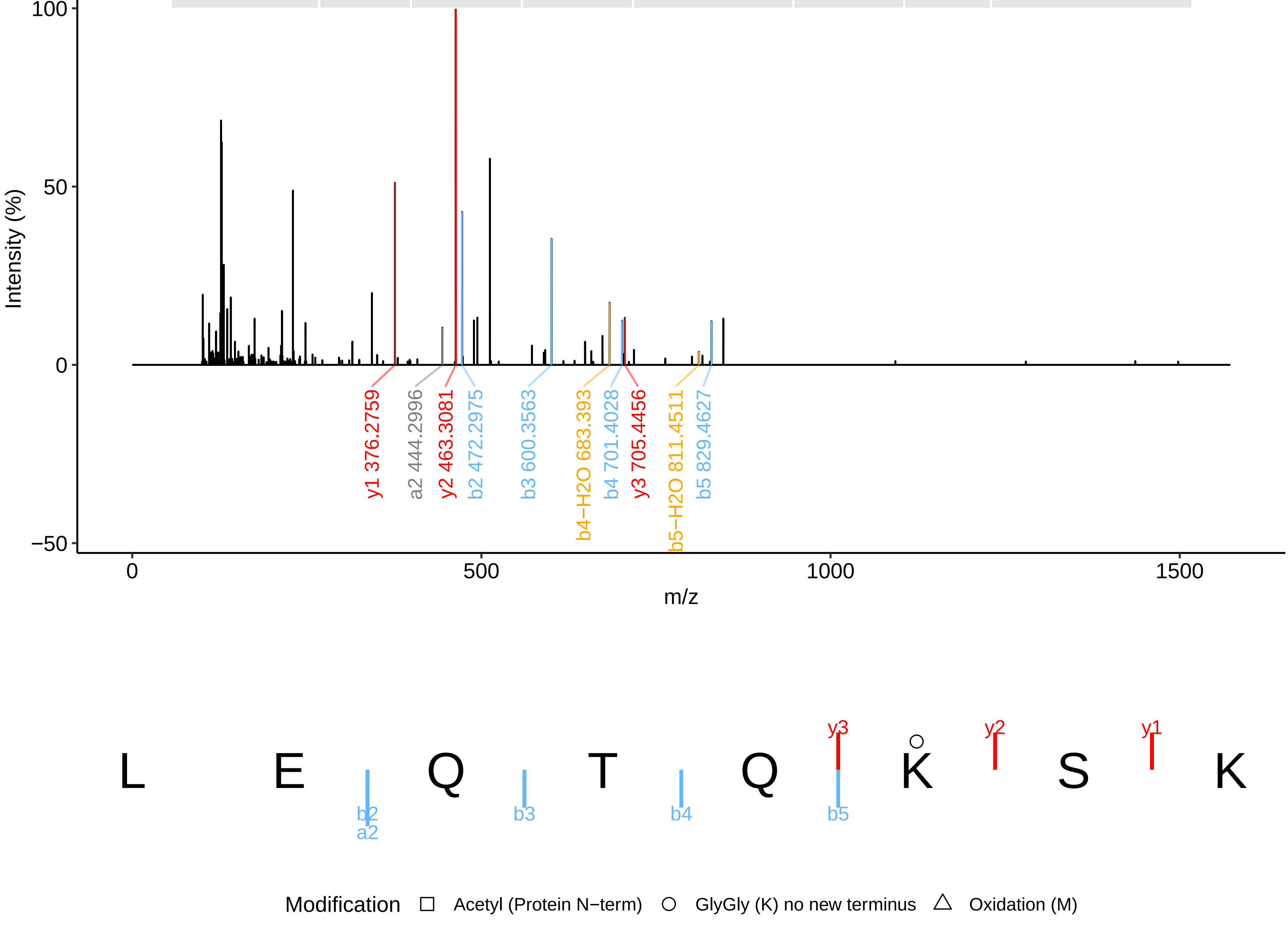

20200924_KIZ_MUb5_GlyGly_2 − Scan number: 4348

Gene Names	Charge	m/z	Mass	Retention time	PEP	Score	Precursor Intensity
TIMM8A	3	359.1963	1074.567	15.942	0.024498	68.224	1563878

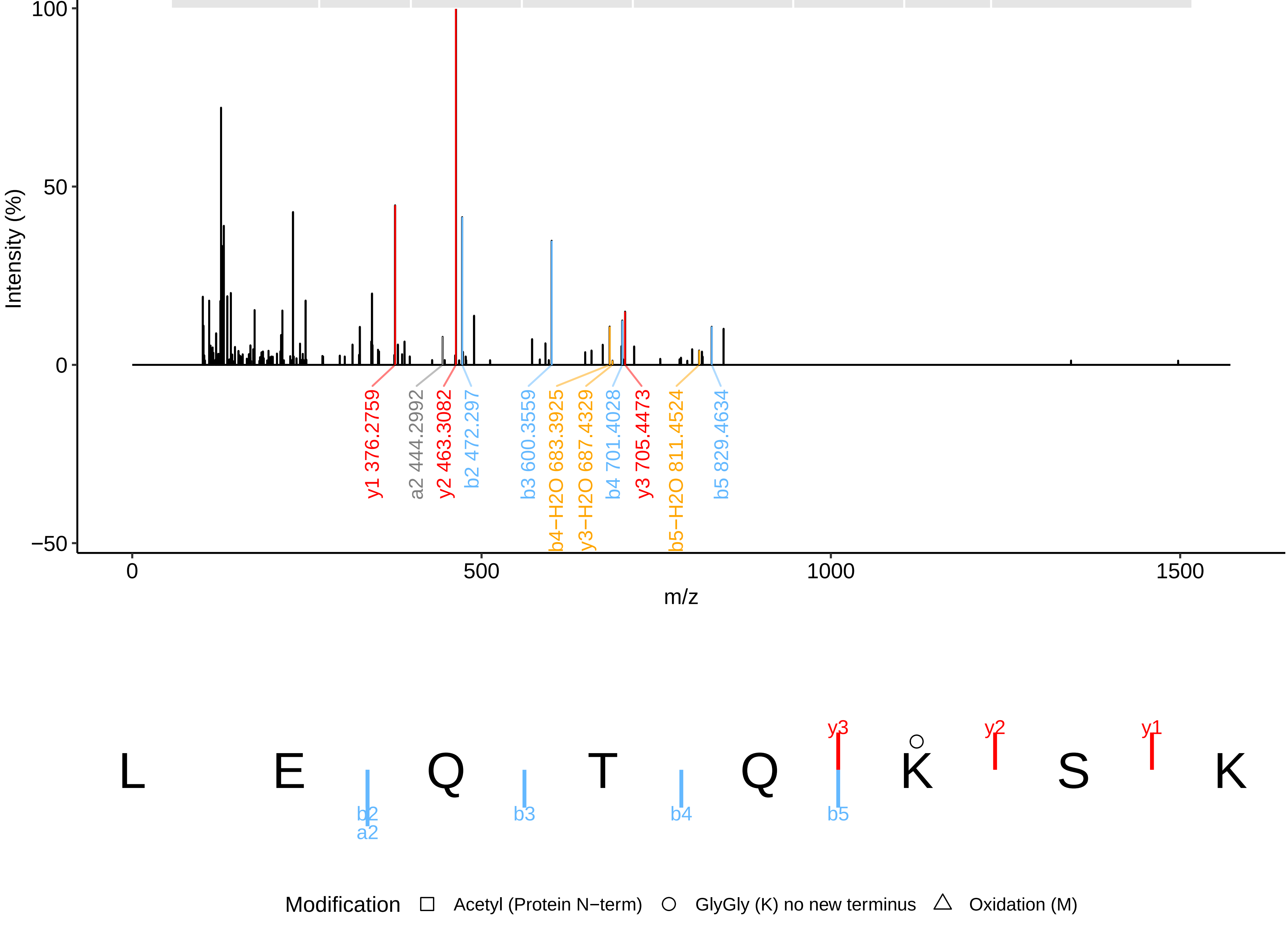

20201118_KIZ_MUb6_GlyGly_1 − Scan number: 4455

Gene Names	Charge	m/z	Mass	Retention time	PEP	Score	Precursor Intensity
TIMM8A	3	359.1963	1074.567	13.945	0.013965	76.679	17613472

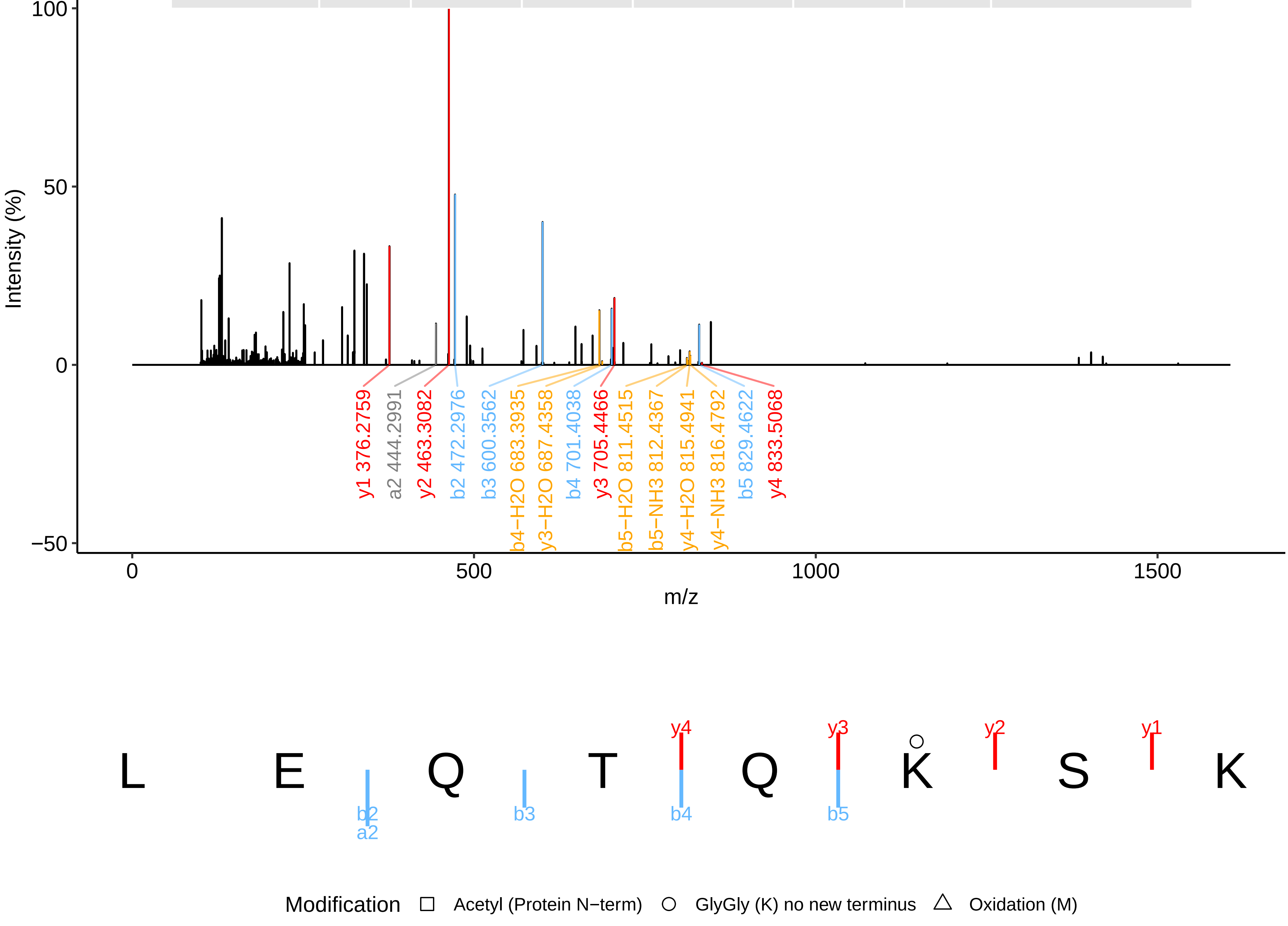

20200921_KIZ_MUb4_GlyGly_10plex − Scan number: 10207

Gene Names	Charge	m/z	Mass	Retention time	PEP	Score	Precursor Intensity
EMC7	3	352.1891	1053.545	35.241	0.010143	74.376	5527998

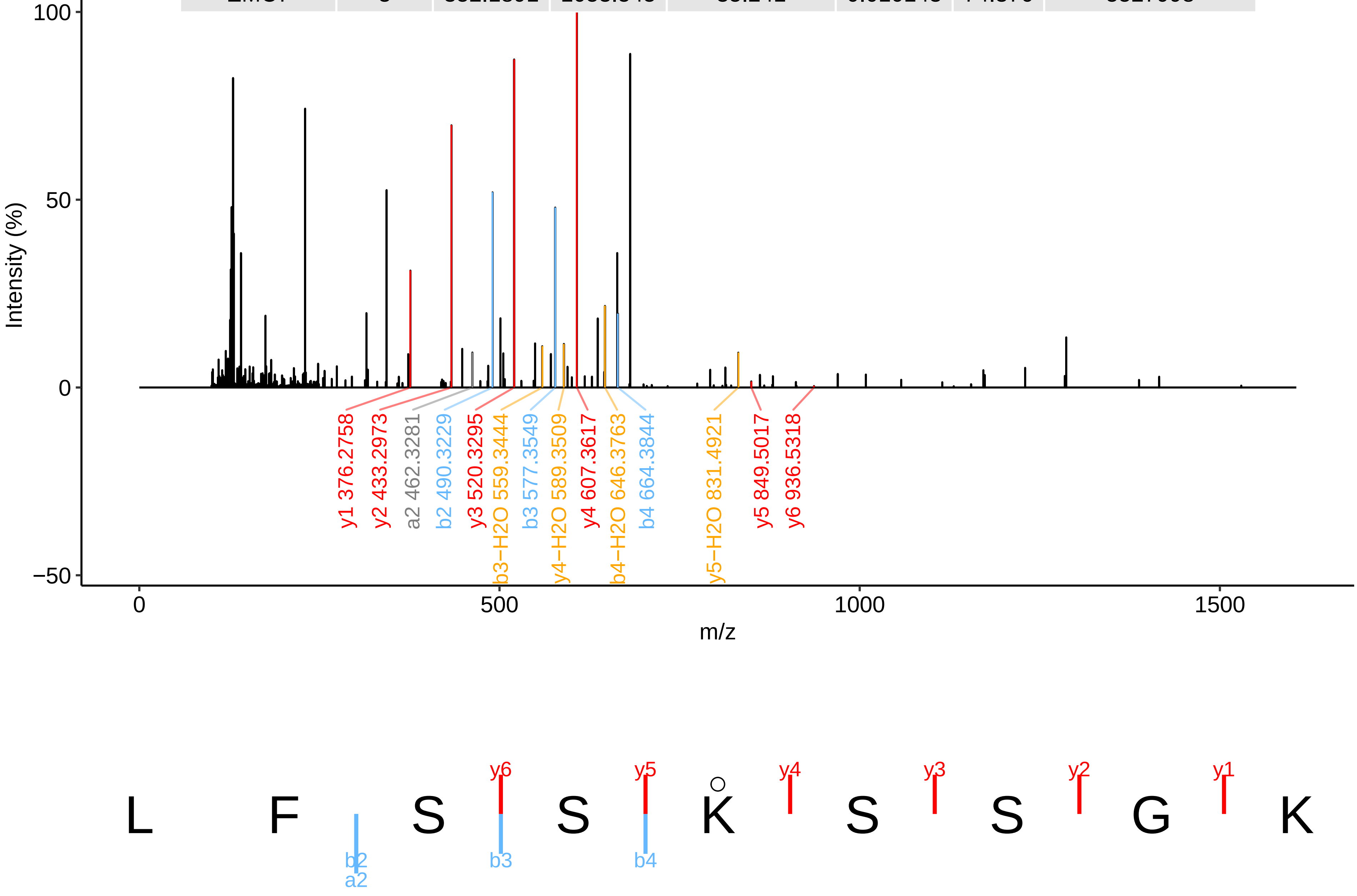

Modification □ Acetyl (Protein N−term) ○ GlyGly (K) no new terminus △ Oxidation (M)

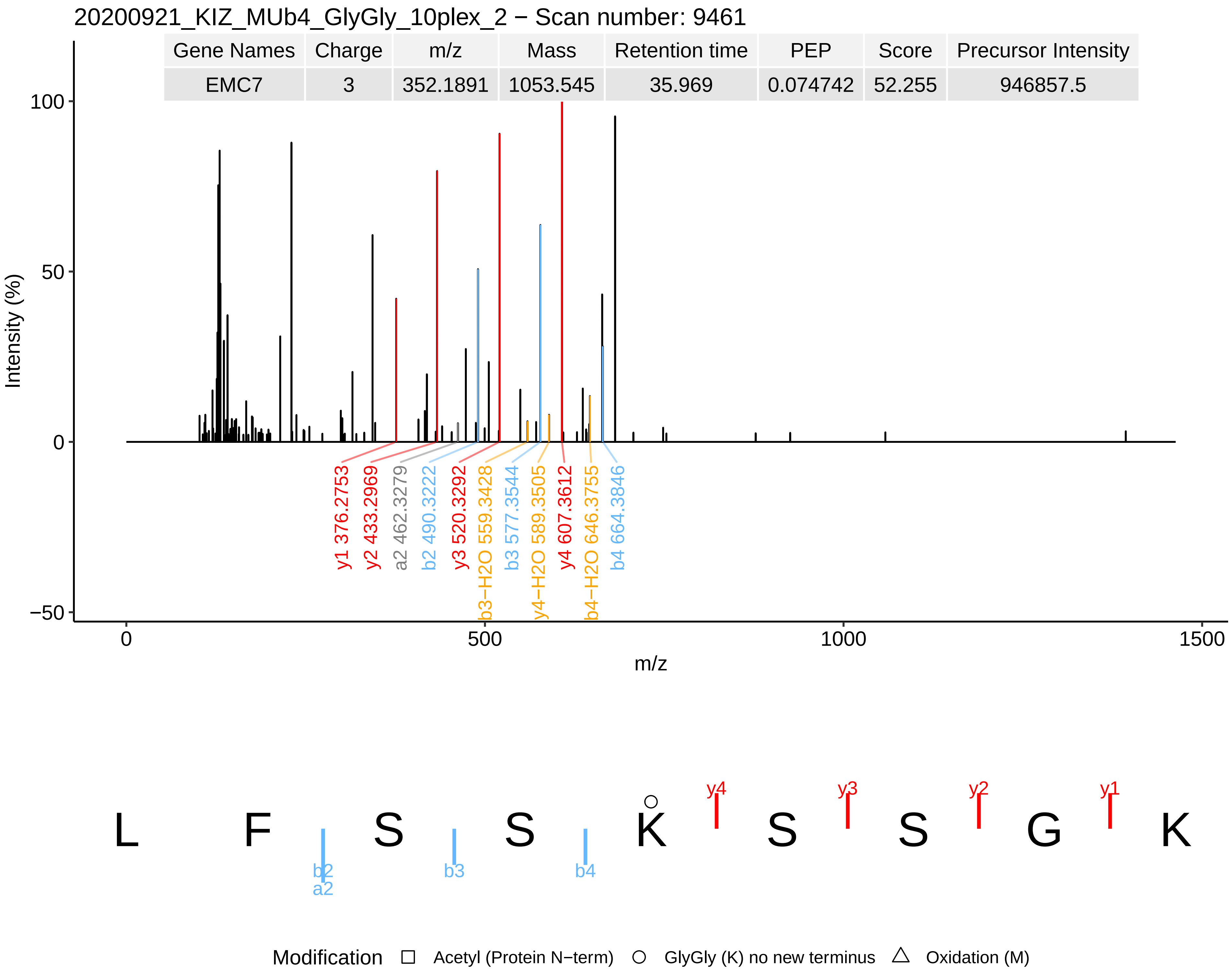
20200921_KIZ_MUb4_GlyGly_10plex_2 − Scan number: 9461
Gene Names | Charge | m/z | Mass | Retention time | PEP | Score | Precursor Intensity
EMC7 | 3 | 352.1891 | 1053.545 | 35.969 | 0.074742 | 52.255 | 946857.5
Intensity (%)
m/z
y1 376.2753
y2 433.2969
a2 462.3279
b2 490.3222
y3 520.3292
b3−H2O 559.3428
b3 577.3544
y4−H2O 589.3505
y4 607.3612
b4−H2O 646.3755
b4 664.3846
L F S S K S S G K
y4 y3 y2 y1
b2 a2 b3 b4
Modification
Acetyl (Protein N−term)
GlyGly (K) no new terminus
Oxidation (M)

20200924_KIZ_MUb5_GlyGly_1 − Scan number: 8929

Gene Names	Charge	m/z	Mass	Retention time	PEP	Score	Precursor Intensity
EMC7	3	352.1891	1053.545	28.644	0.0066659	80.219	5774453

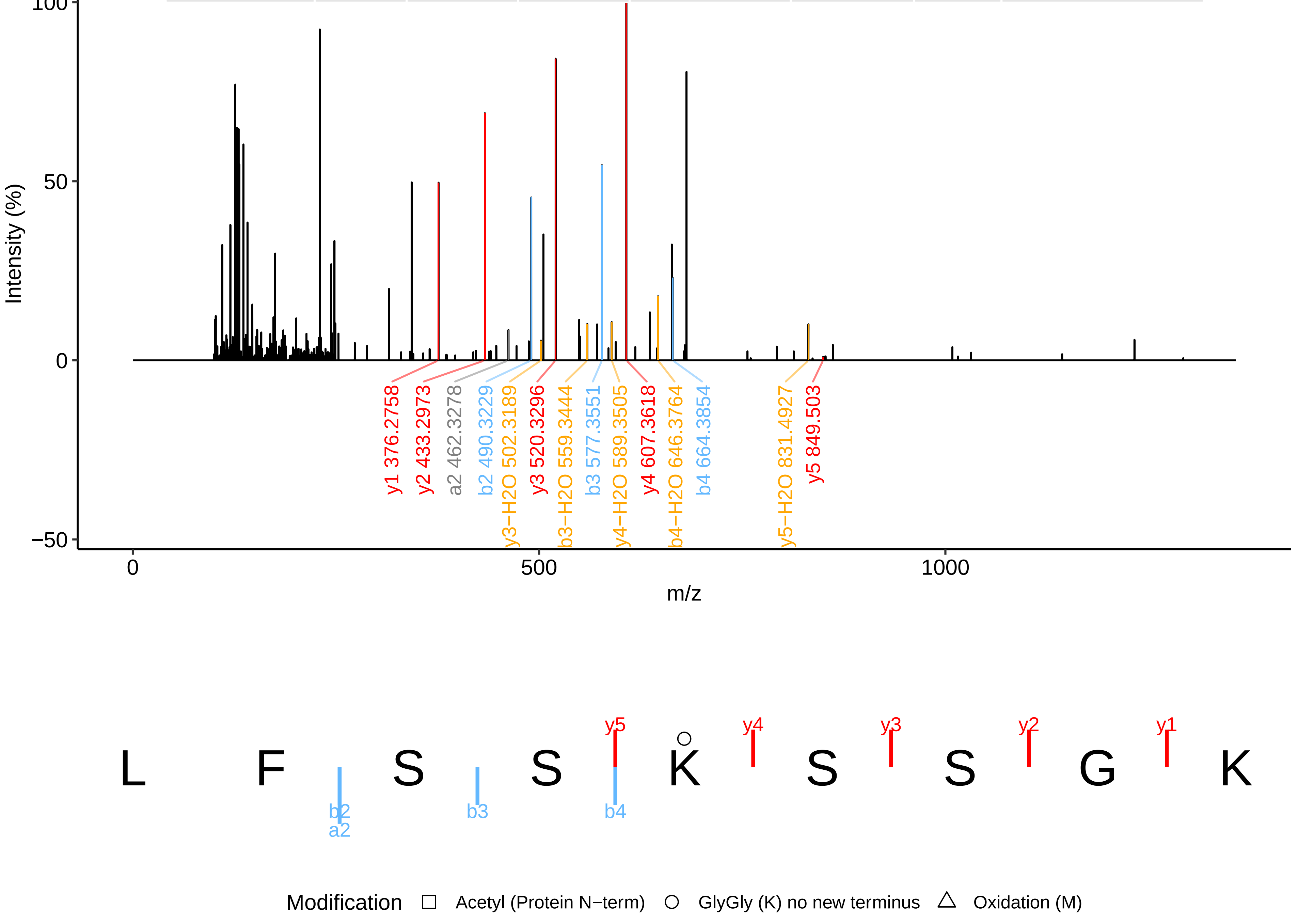

20200921_KIZ_MUb4_GlyGly_10plex − Scan number: 5738

Gene Names	Charge	m/z	Mass	Retention time	PEP	Score	Precursor Intensity
ANXA1	4	328.6792	1310.688	18.82	0.077691	48.004	9678241

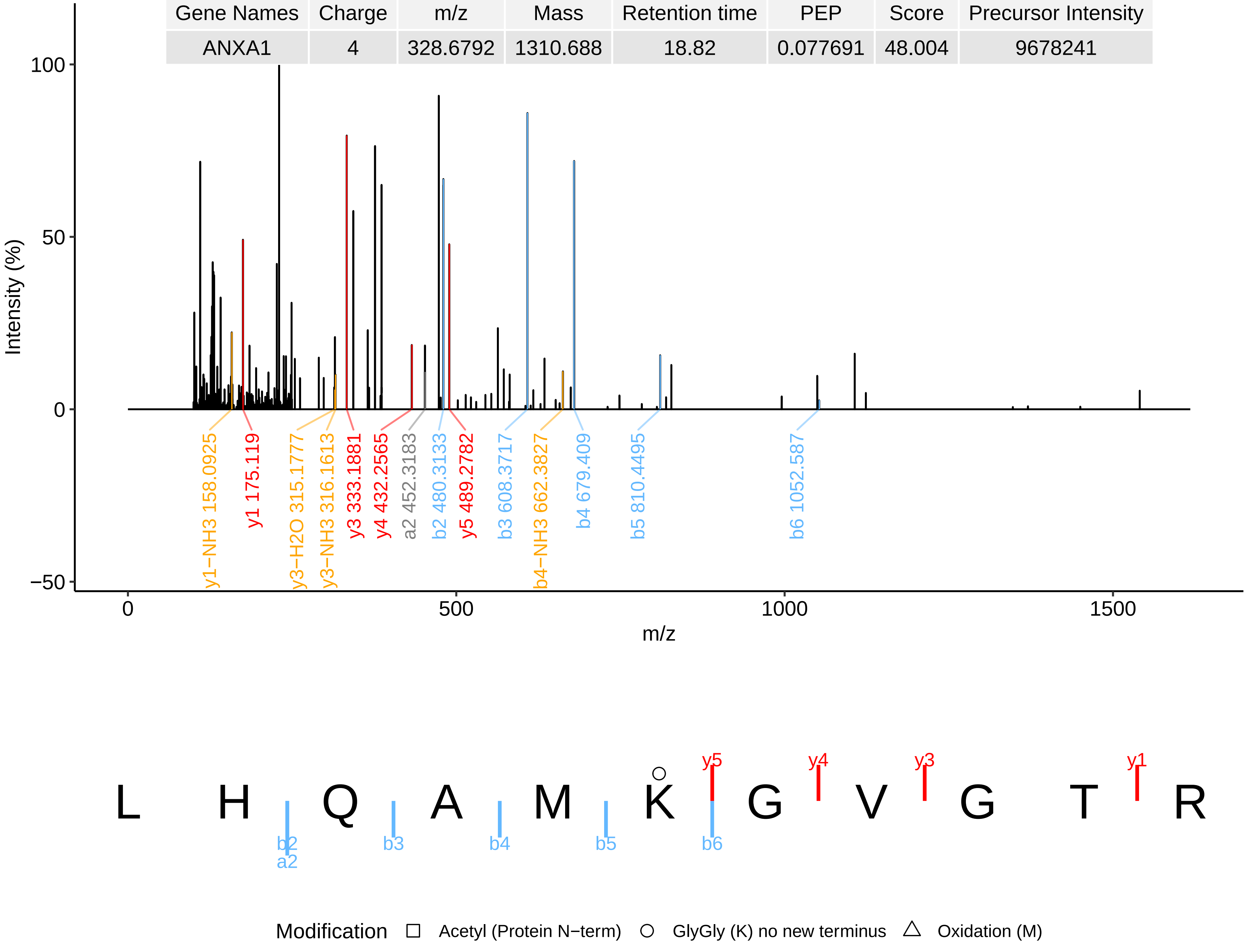

20200921_KIZ_MUb4_GlyGly_10plex_2 − Scan number: 5314

Gene Names	Charge	m/z	Mass	Retention time	PEP	Score	Precursor Intensity
ANXA1	4	328.6792	1310.688	19.05	0.074558	48.741	1398879

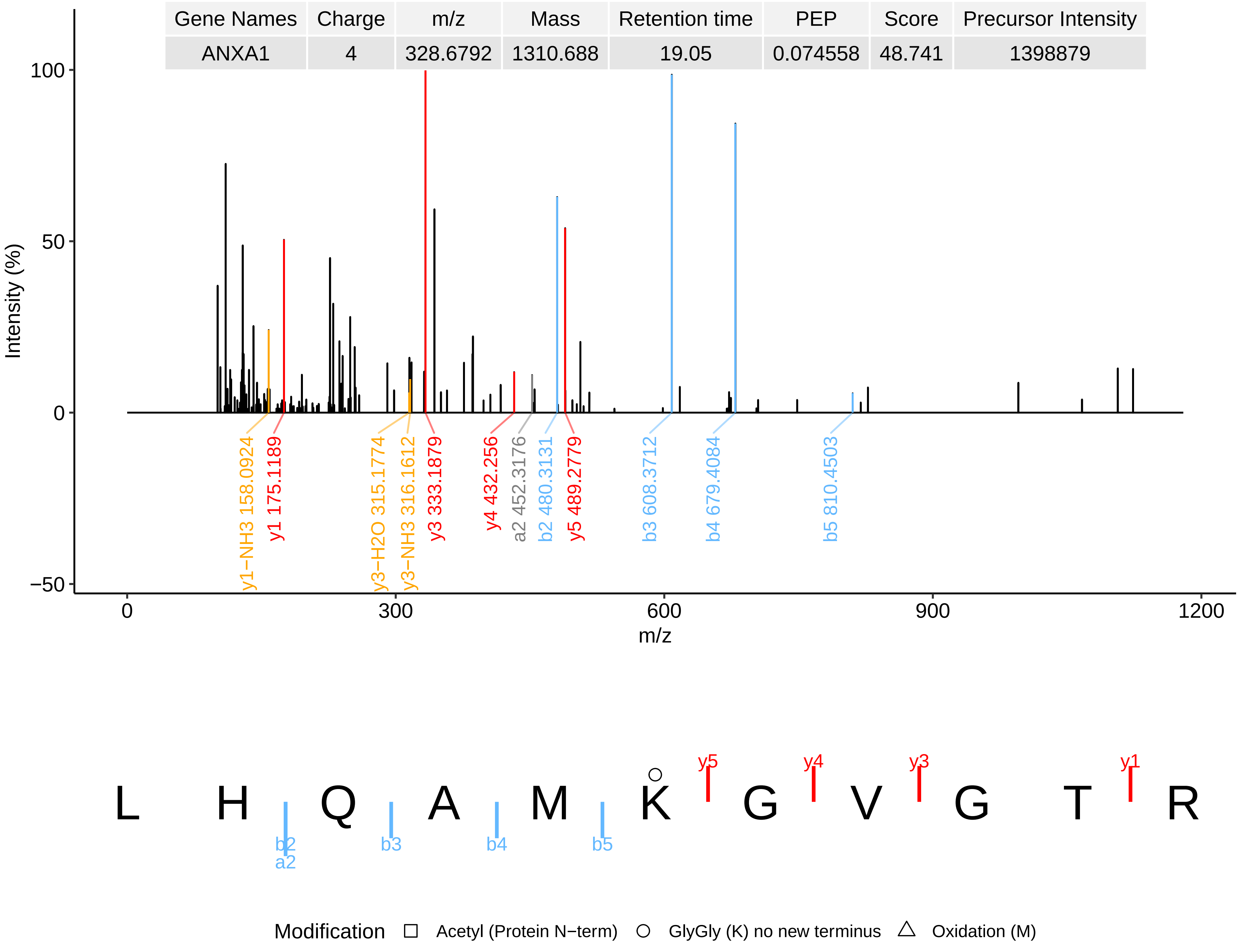

20200921_KIZ_MUb4_GlyGly_10plex − Scan number: 13736

Gene Names	Charge	m/z	Mass	Retention time	PEP	Score	Precursor Intensity
CCDC50	3	359.1987	1074.574	48.56	0.099973	69.198	7619161

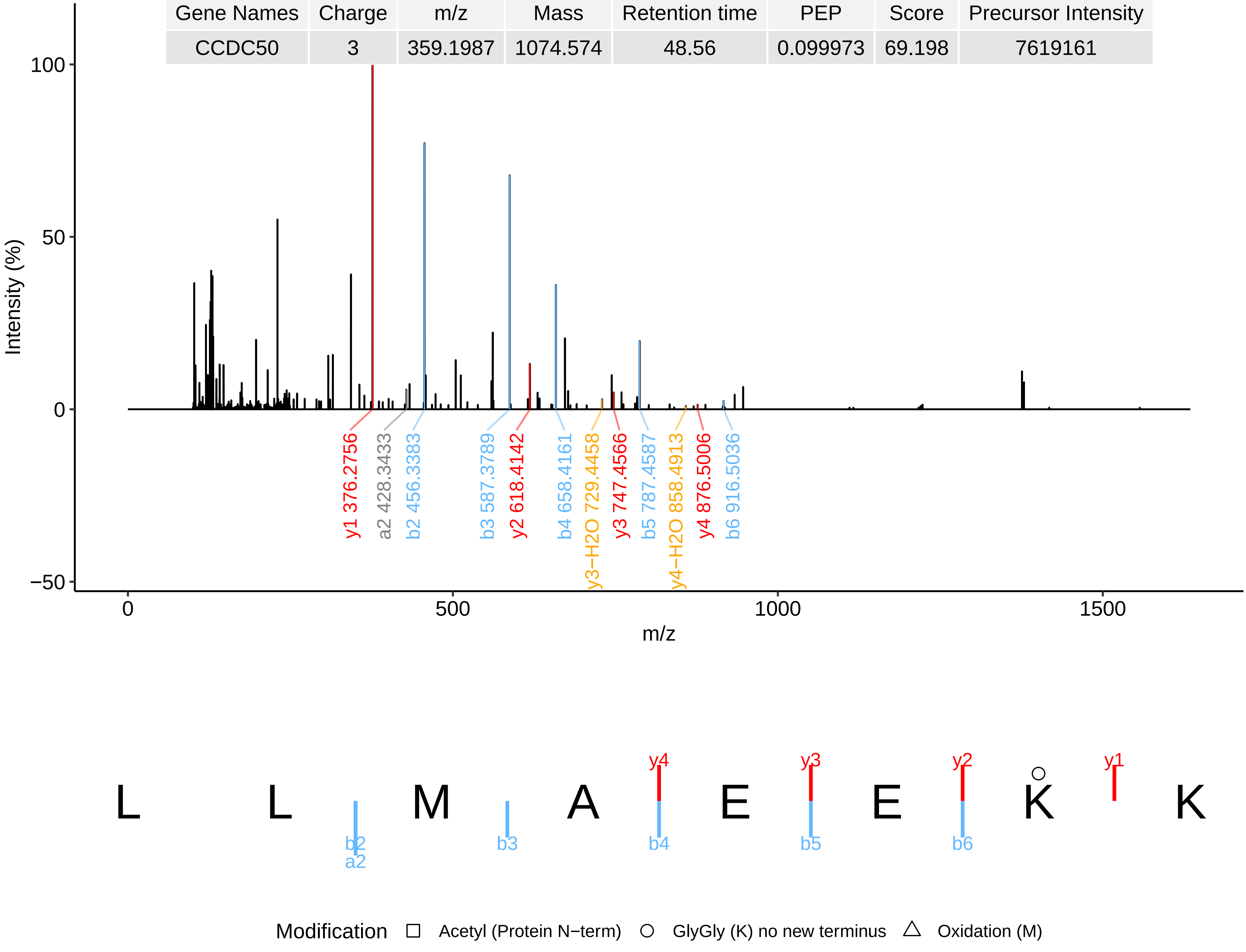

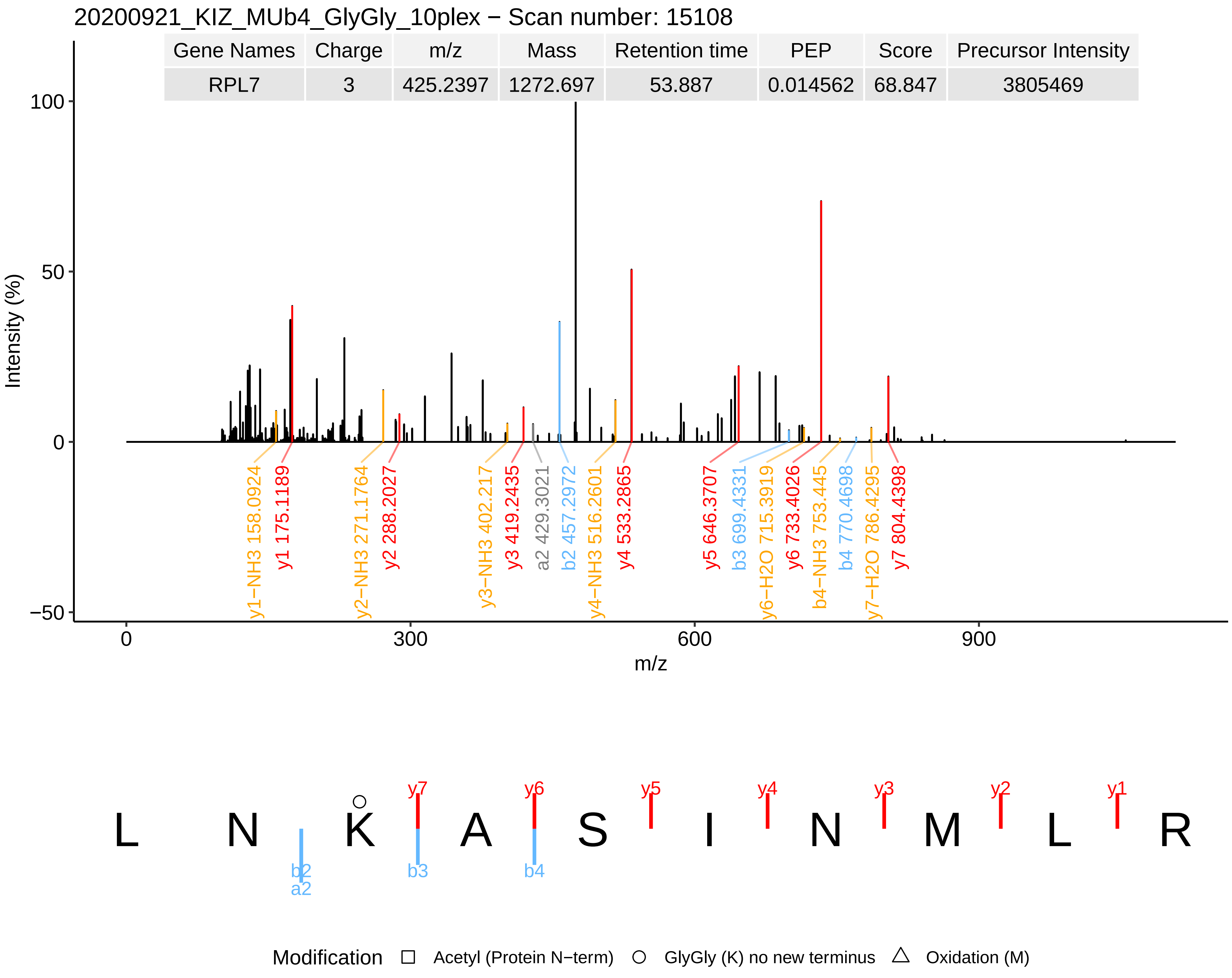

20200921_KIZ_MUb4_GlyGly_10plex − Scan number: 15108
Gene Names | Charge | m/z | Mass | Retention time | PEP | Score | Precursor Intensity
RPL7 | 3 | 425.2397 | 1272.697 | 53.887 | 0.014562 | 68.847 | 3805469
Intensity (%)
m/z
y1−NH3 158.0924
y1 175.1189
y2−NH3 271.1764
y2 288.2027
y3−NH3 402.217
y3 419.2435
a2 429.3021
b2 457.2972
y4−NH3 516.2601
y4 533.2865
y5 646.3707
b3 699.4331
y6−H2O 715.3919
y6 733.4026
b4−NH3 753.445
b4 770.4698
y7−H2O 786.4295
y7 804.4398
L N K A S I N M L R
y7 y6 y5 y4 y3 y2 y1
b2 a2 b3 b4
Modification
Acetyl (Protein N−term)
GlyGly (K) no new terminus
Oxidation (M)

20200921_KIZ_MUb4_GlyGly_10plex − Scan number: 16822

Gene Names	Charge	m/z	Mass	Retention time	PEP	Score	Precursor Intensity
PTRF	3	357.5571	1069.649	60.552	0.021151	64.04	6674800

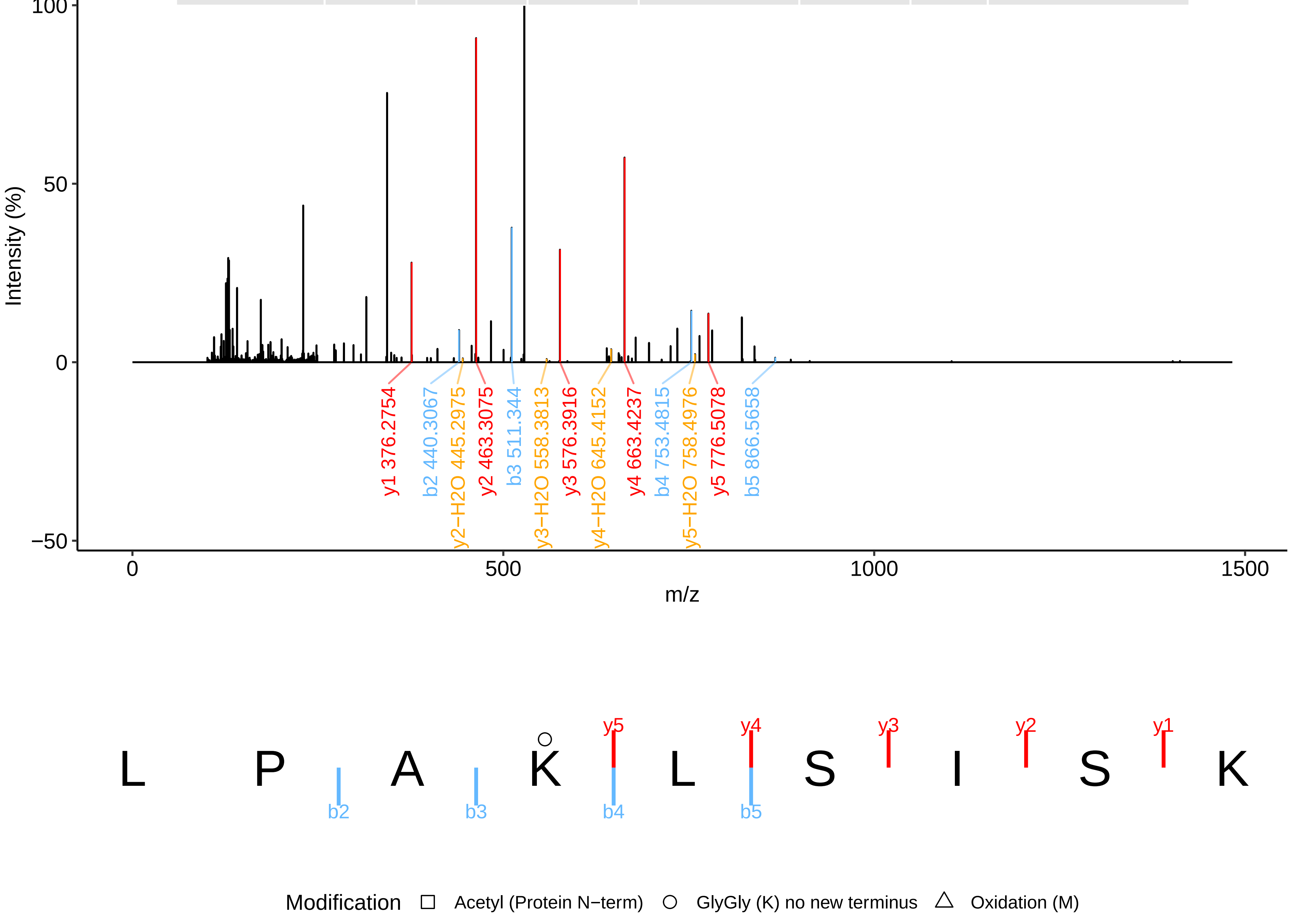

20200921_KIZ_MUb4_GlyGly_10plex_2 − Scan number: 15645

Gene Names	Charge	m/z	Mass	Retention time	PEP	Score	Precursor Intensity
PTRF	3	357.5571	1069.649	61.228	0.061588	54.982	707328.2

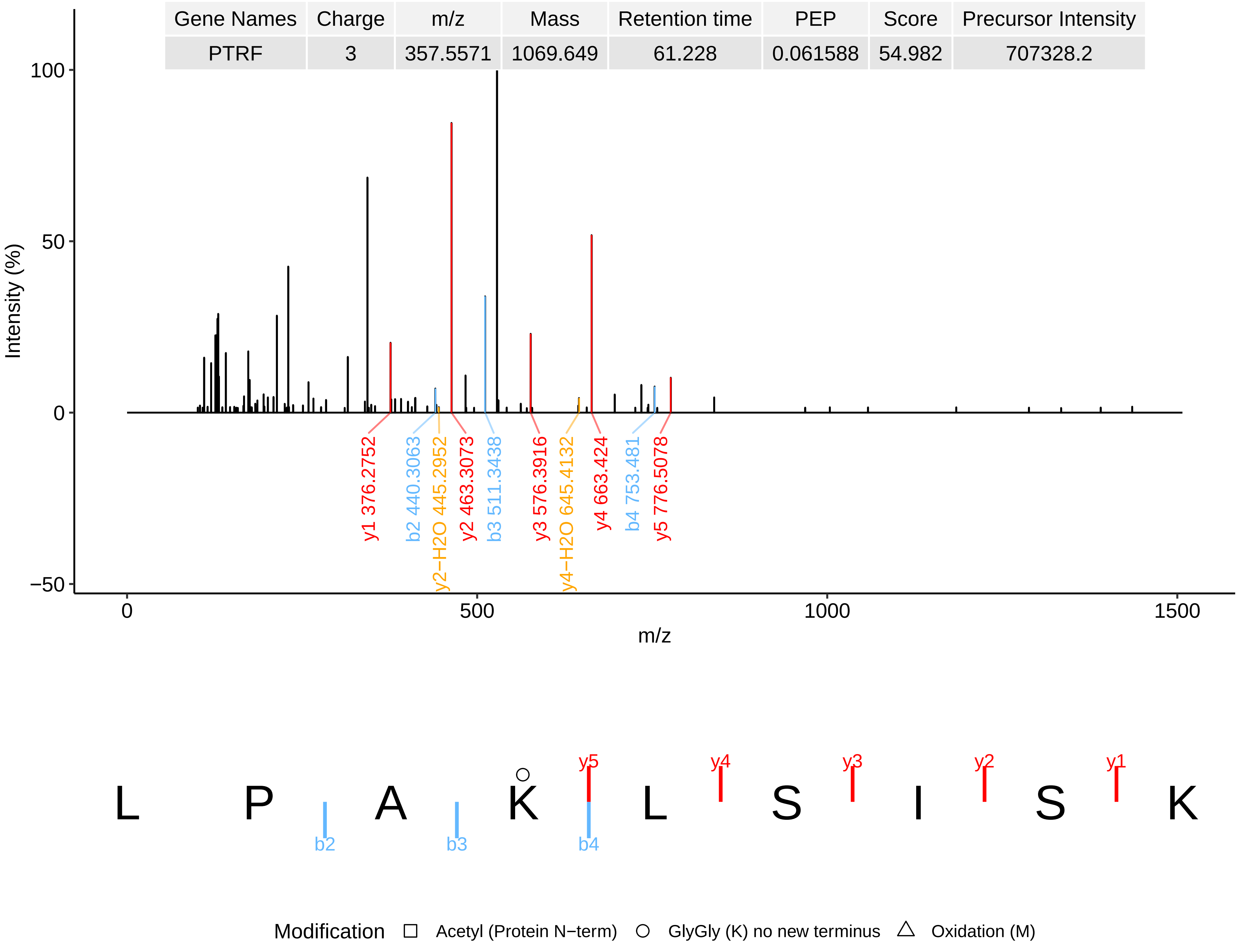

20200924_KIZ_MUb5_GlyGly_1 − Scan number: 15733

Gene Names	Charge	m/z	Mass	Retention time	PEP	Score	Precursor Intensity
PTRF	3	357.5571	1069.649	51.285	0.063391	54.608	39473452

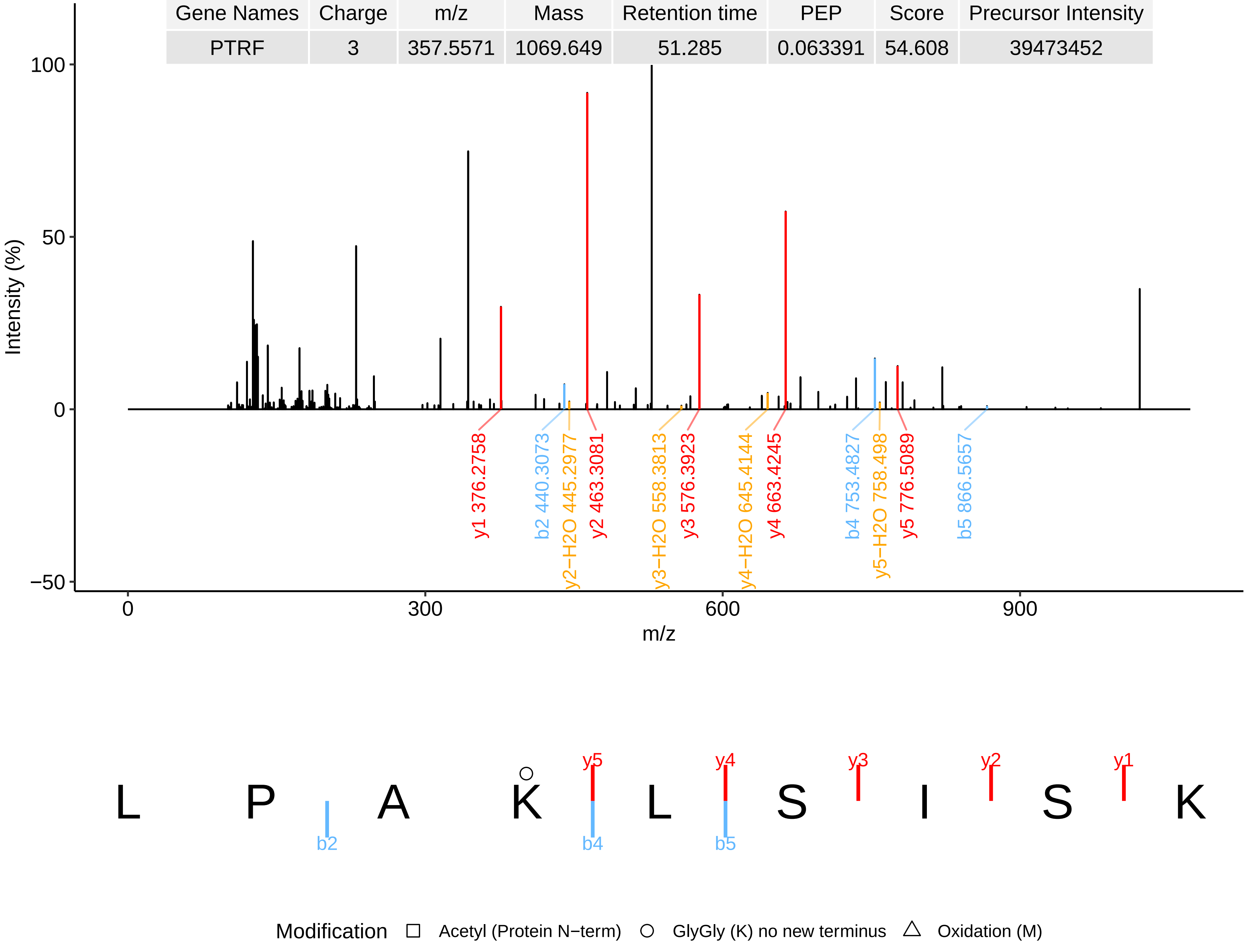

20200924_KIZ_MUb5_GlyGly_2 − Scan number: 15061

Gene Names	Charge	m/z	Mass	Retention time	PEP	Score	Precursor Intensity
PTRF	3	357.5571	1069.649	56.286	0.012773	75.378	3777366

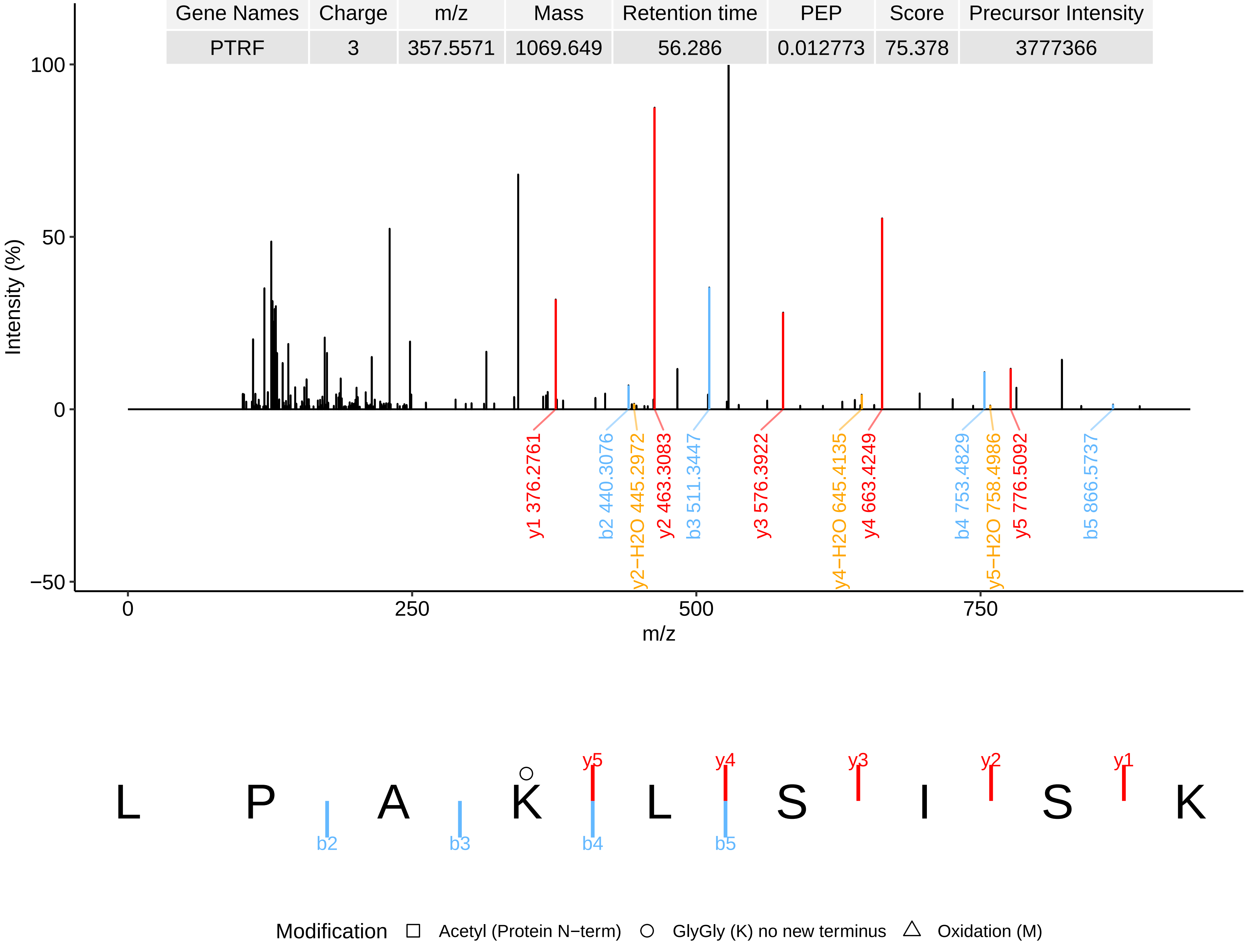

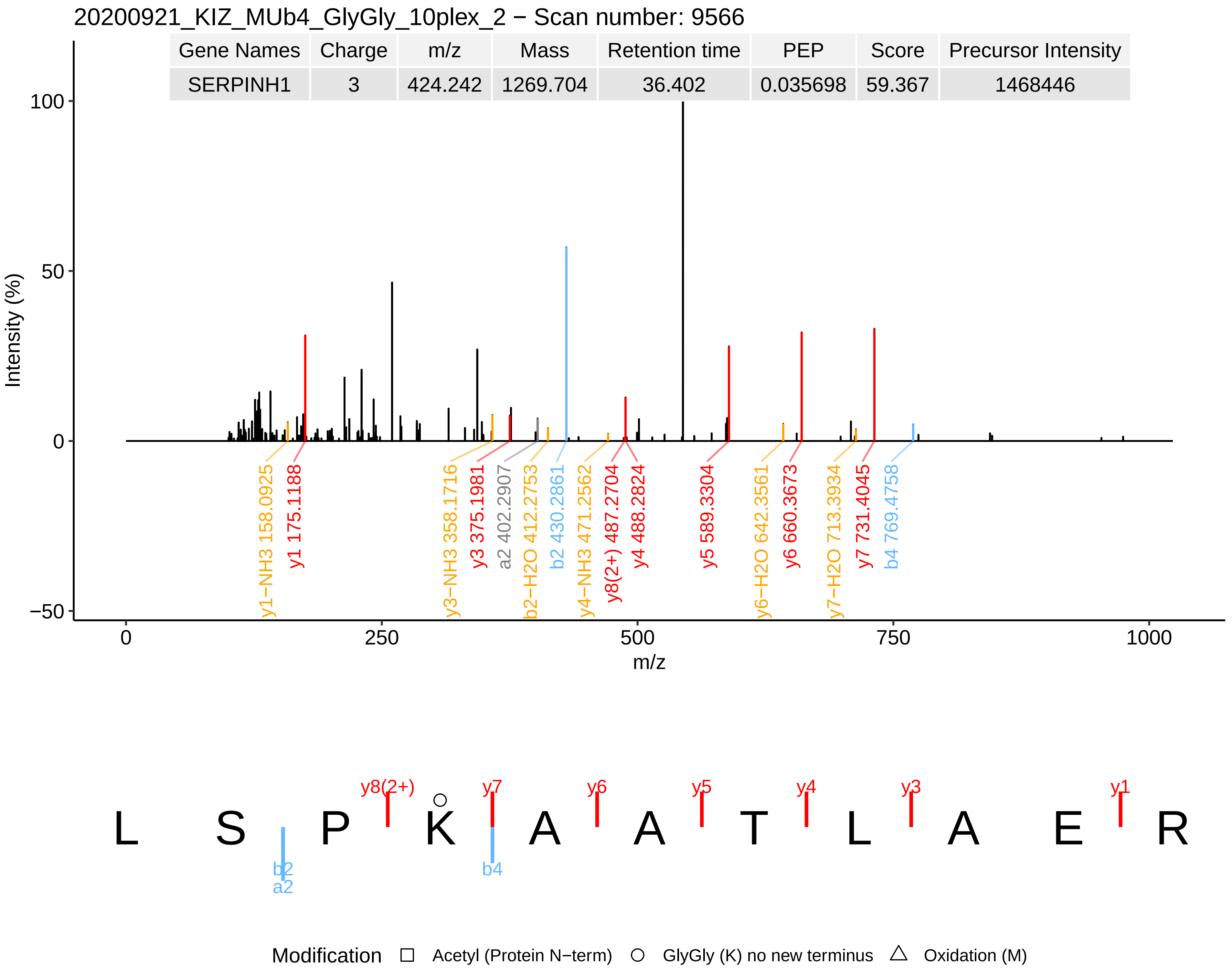
20200921_KIZ_MUb4_GlyGly_10plex_2 − Scan number: 9566
Gene Names | Charge | m/z | Mass | Retention time | PEP | Score | Precursor Intensity
SERPINH1 | 3 | 424.242 | 1269.704 | 36.402 | 0.035698 | 59.367 | 1468446
Intensity (%)
m/z
y1−NH3 158.0925
y1 175.1188
y3−NH3 358.1716
y3 375.1981
a2 402.2907
b2−H2O 412.2753
b2 430.2861
y4−NH3 471.2562
y8(2+) 487.2704
y4 488.2824
y5 589.3304
y6−H2O 642.3561
y6 660.3673
y7−H2O 713.3934
y7 731.4045
b4 769.4758
L S P K A A T L A E R
y8(2+) y7 y6 y5 y4 y3 y1
b2 a2 b4
Modification
Acetyl (Protein N−term)
GlyGly (K) no new terminus
Oxidation (M)

20200921_KIZ_MUb4_GlyGly_10plex − Scan number: 15228

Gene Names	Charge	m/z	Mass	Retention time	PEP	Score	Precursor Intensity
CAD	3	424.5629	1270.667	54.353	1.0936e−09	133.99	5922853

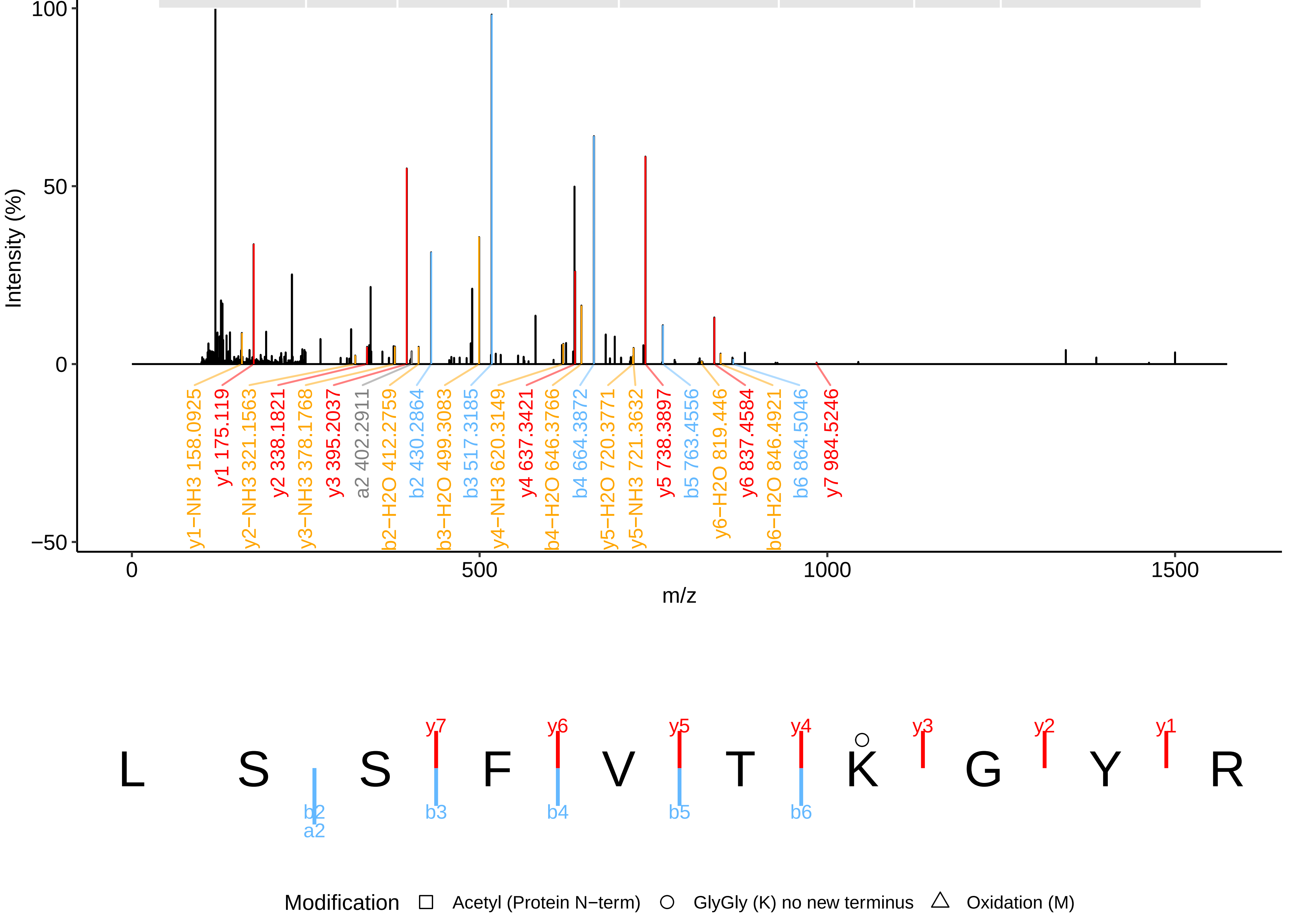

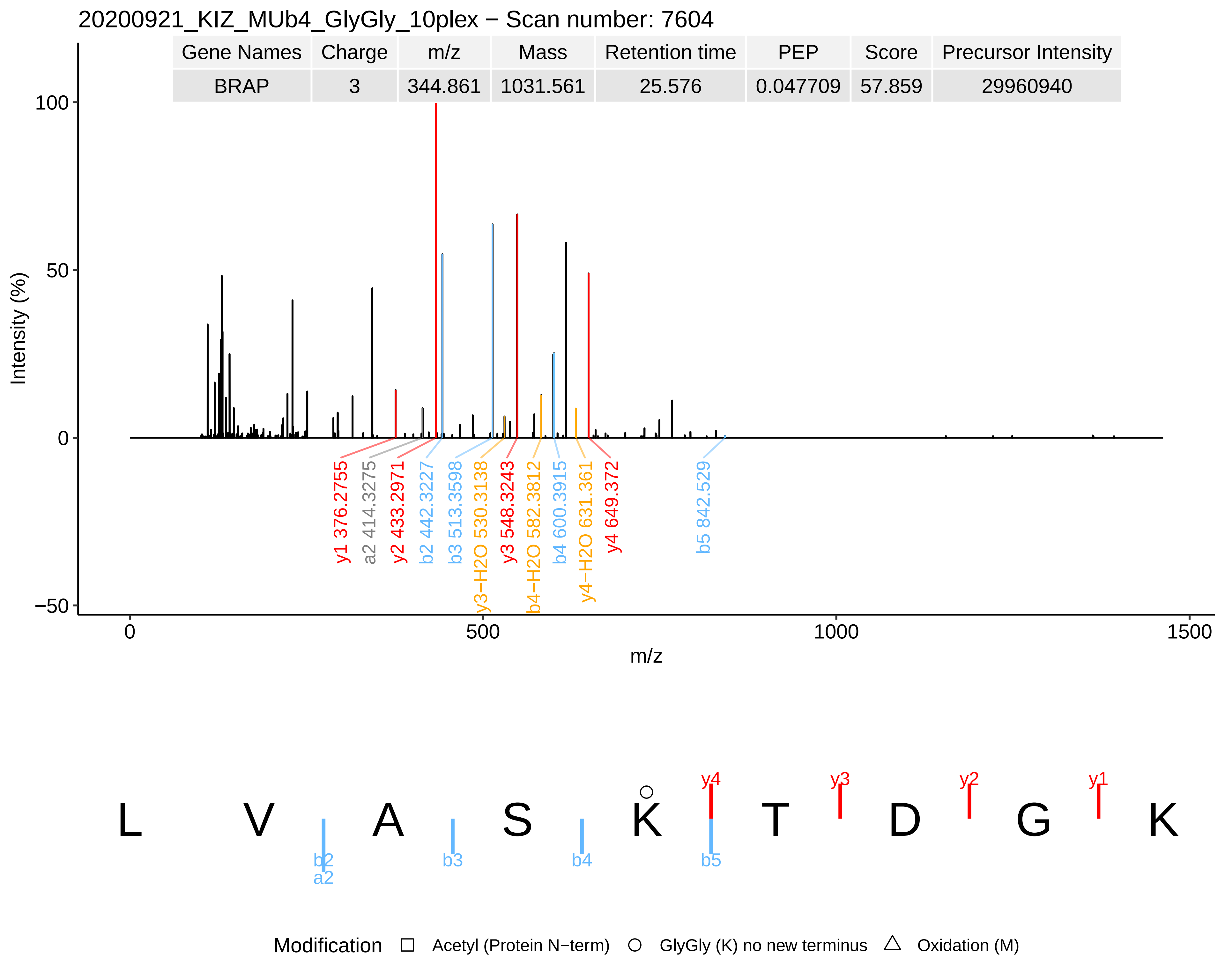

20200921_KIZ_MUb4_GlyGly_10plex − Scan number: 7604
Gene Names | Charge | m/z | Mass | Retention time | PEP | Score | Precursor Intensity
BRAP | 3 | 344.861 | 1031.561 | 25.576 | 0.047709 | 57.859 | 29960940
Intensity (%)
m/z
y1 376.2755
a2 414.3275
y2 433.2971
b2 442.3227
b3 513.3598
y3−H2O 530.3138
y3 548.3243
b4−H2O 582.3812
b4 600.3915
y4−H2O 631.361
y4 649.372
b5 842.529
L V A S K T D G K
y4 y3 y2 y1
b2 a2 b3 b4 b5
Modification
Acetyl (Protein N−term)
GlyGly (K) no new terminus
Oxidation (M)

20200921_KIZ_MUb4_GlyGly_10plex_2 − Scan number: 7005

Gene Names	Charge	m/z	Mass	Retention time	PEP	Score	Precursor Intensity
BRAP	3	344.861	1031.561	25.988	0.042296	58.981	3068524

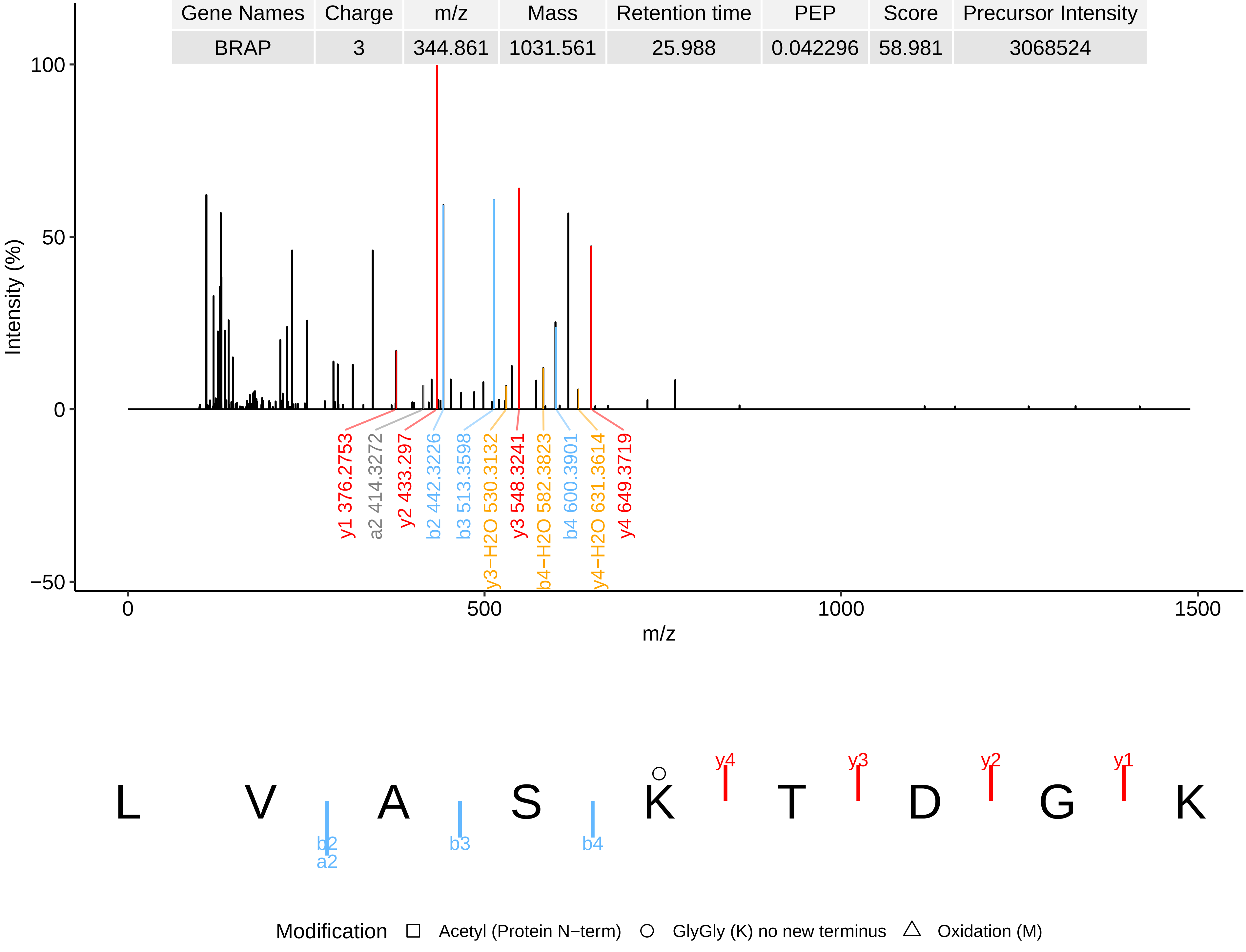

20200924_KIZ_MUb5_GlyGly_1 − Scan number: 6594

Gene Names	Charge	m/z	Mass	Retention time	PEP	Score	Precursor Intensity
BRAP	3	344.861	1031.561	20.77	0.04261	58.916	15404578

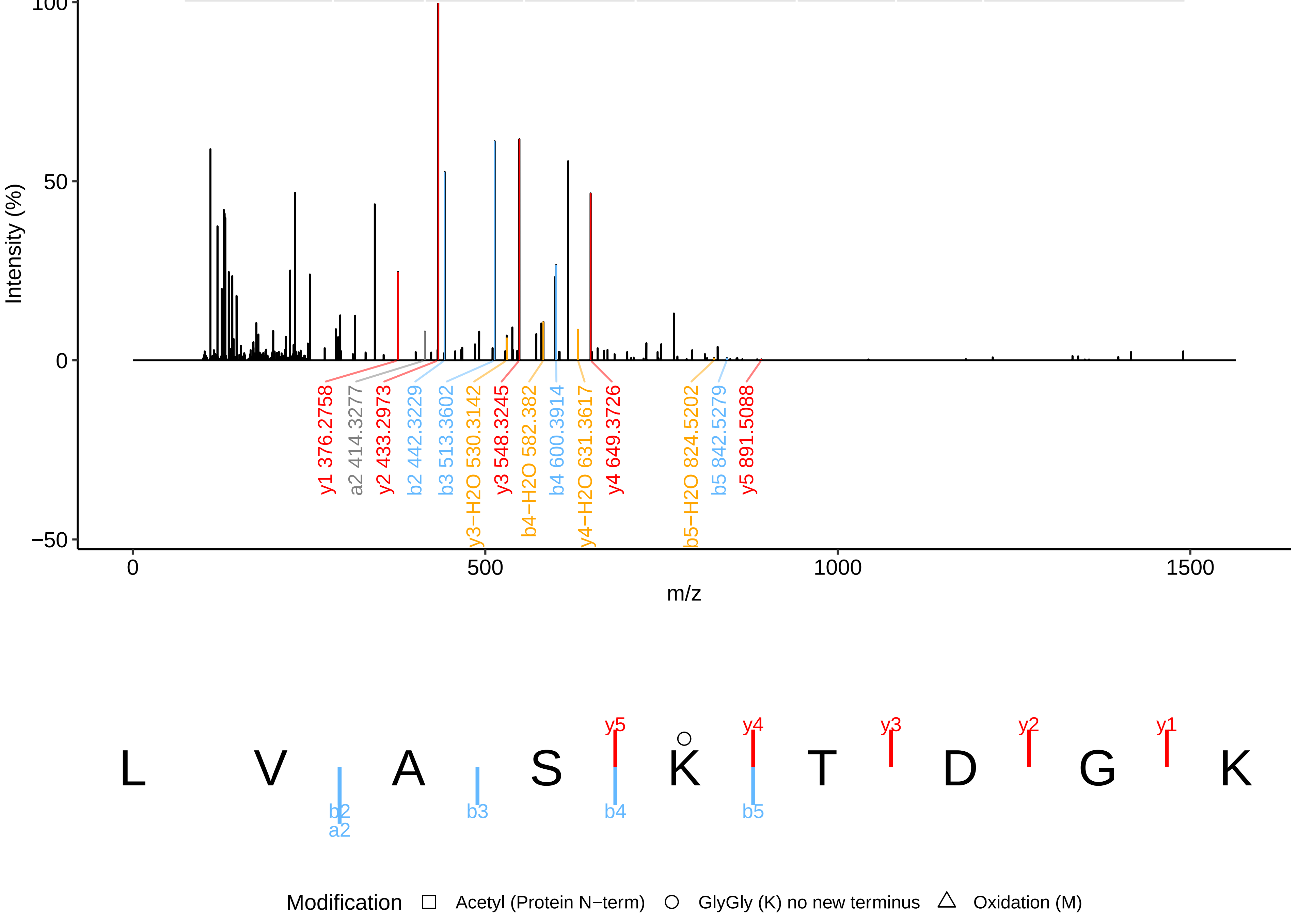

20200924_KIZ_MUb5_GlyGly_1 − Scan number: 6741

Gene Names	Charge	m/z	Mass	Retention time	PEP	Score	Precursor Intensity
BRAP	3	344.861	1031.561	21.281	0.037287	60.019	17797756

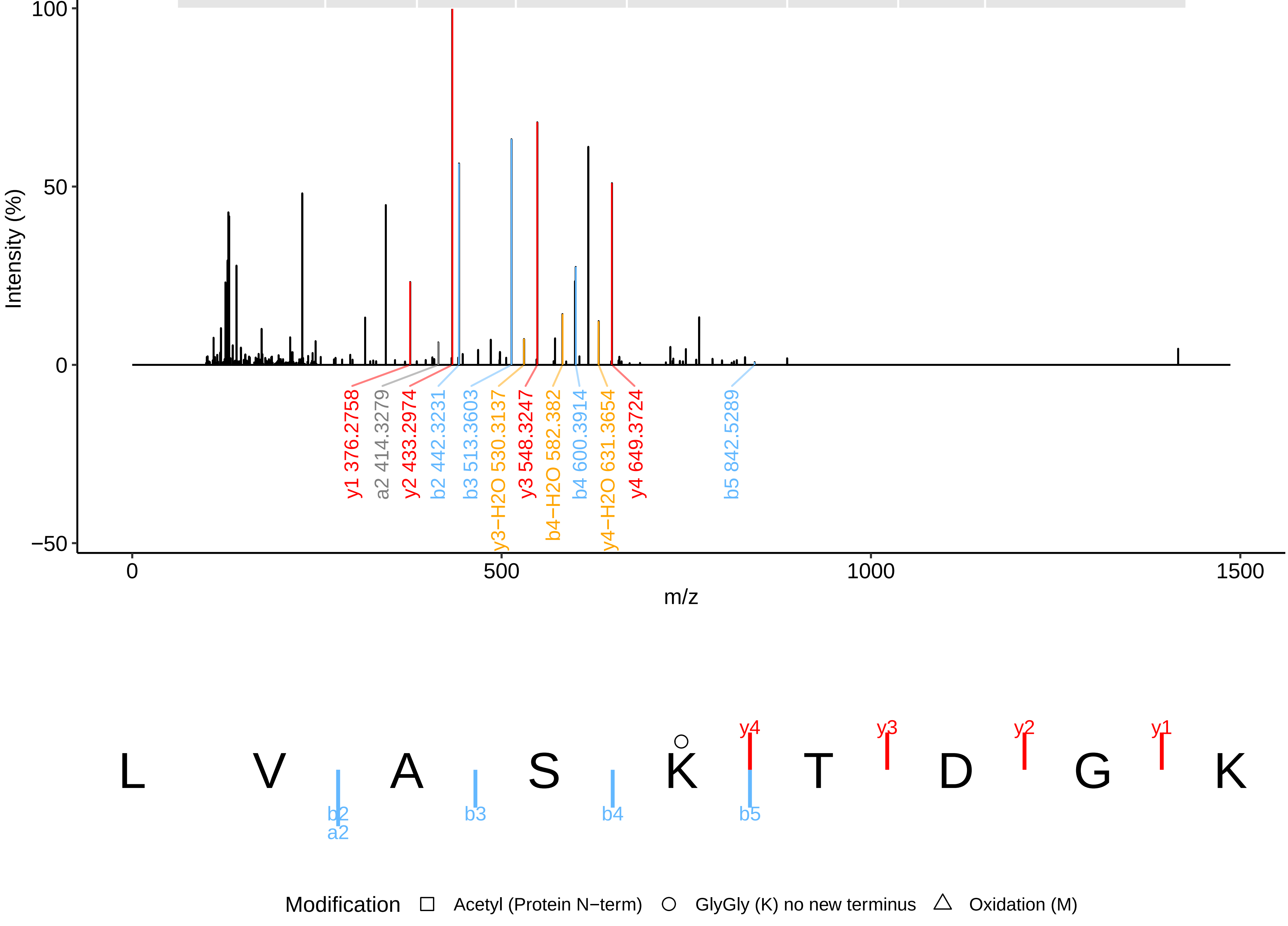

20200924_KIZ_MUb5_GlyGly_2 − Scan number: 6161

Gene Names	Charge	m/z	Mass	Retention time	PEP	Score	Precursor Intensity
BRAP	3	344.861	1031.561	22.956	0.002666	85.67	4293918

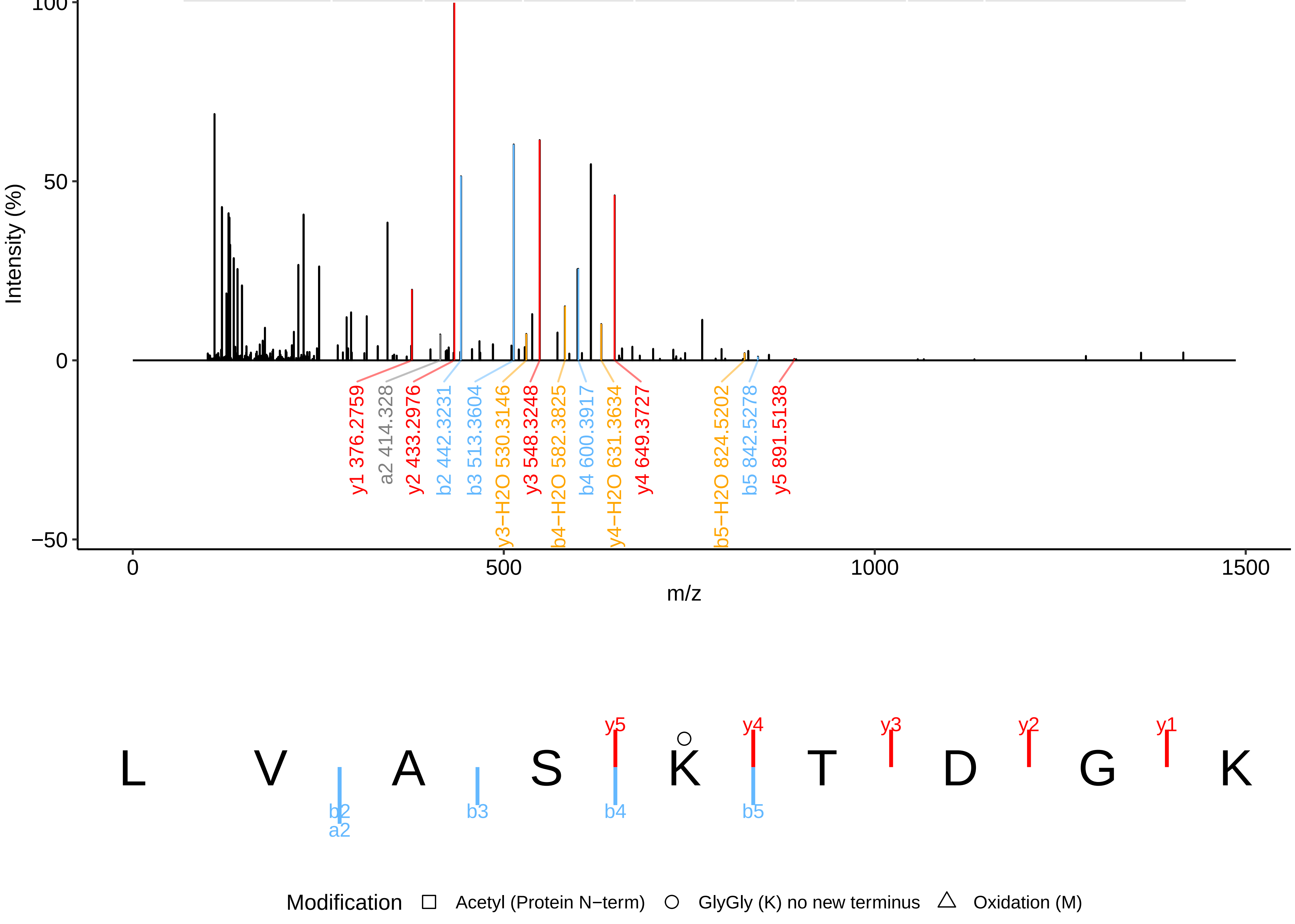

20200924_KIZ_MUb5_GlyGly_2 − Scan number: 6294

Gene Names	Charge	m/z	Mass	Retention time	PEP	Score	Precursor Intensity
BRAP	3	344.861	1031.561	23.471	0.071294	56.81	2654933

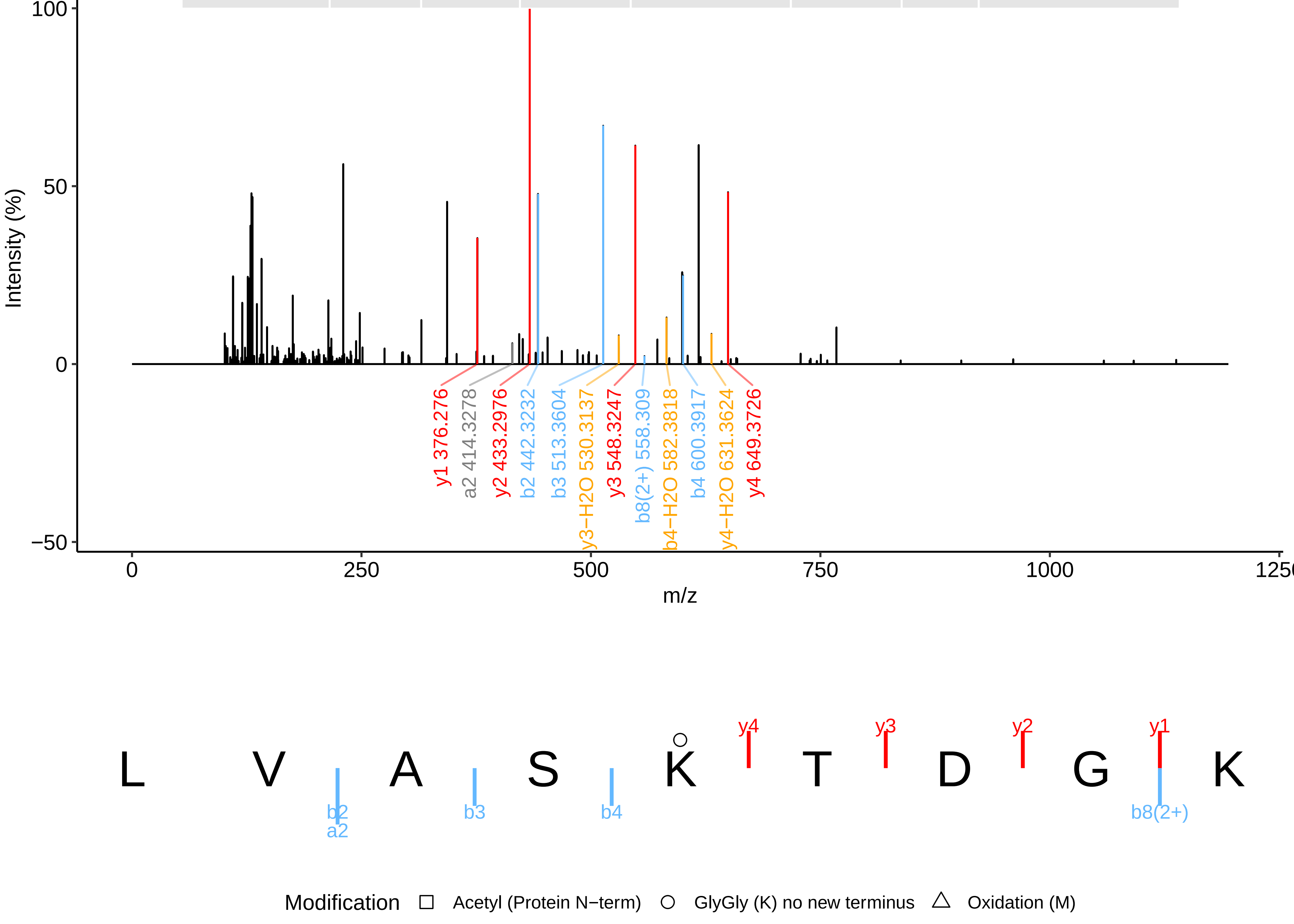

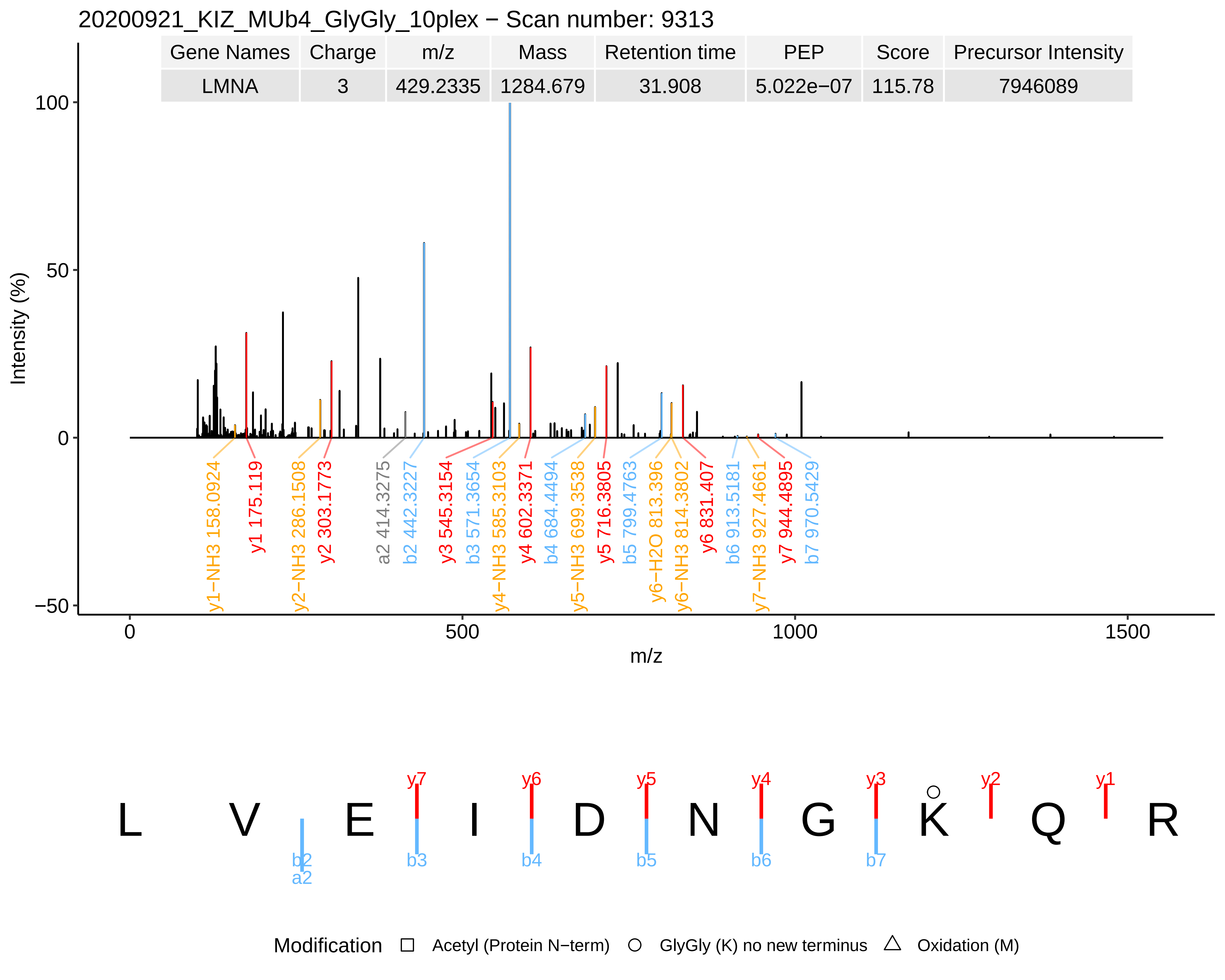

20200921_KIZ_MUb4_GlyGly_10plex − Scan number: 9313
Gene Names | Charge | m/z | Mass | Retention time | PEP | Score | Precursor Intensity
LMNA | 3 | 429.2335 | 1284.679 | 31.908 | 5.022e−07 | 115.78 | 7946089
Intensity (%)
100
50
0
−50
y1−NH3 158.0924
y1 175.119
y2−NH3 286.1508
y2 303.1773
a2 414.3275
b2 442.3227
y3 545.3154
b3 571.3654
y4−NH3 585.3103
y4 602.3371
b4 684.4494
y5−NH3 699.3538
y5 716.3805
b5 799.4763
y6−H2O 813.396
y6−NH3 814.3802
y6 831.407
b6 913.5181
y7−NH3 927.4661
y7 944.4895
b7 970.5429
0
500
1000
1500
m/z
L V E I D N G K Q R
y7 y6 y5 y4 y3 y2 y1
b2 a2 b3 b4 b5 b6 b7
Modification
Acetyl (Protein N−term)
GlyGly (K) no new terminus
Oxidation (M)

20200921_KIZ_MUb4_GlyGly_10plex_2 − Scan number: 8600

Gene Names	Charge	m/z	Mass	Retention time	PEP	Score	Precursor Intensity
LMNA	3	429.2335	1284.679	32.45	0.00055448	94.692	802136

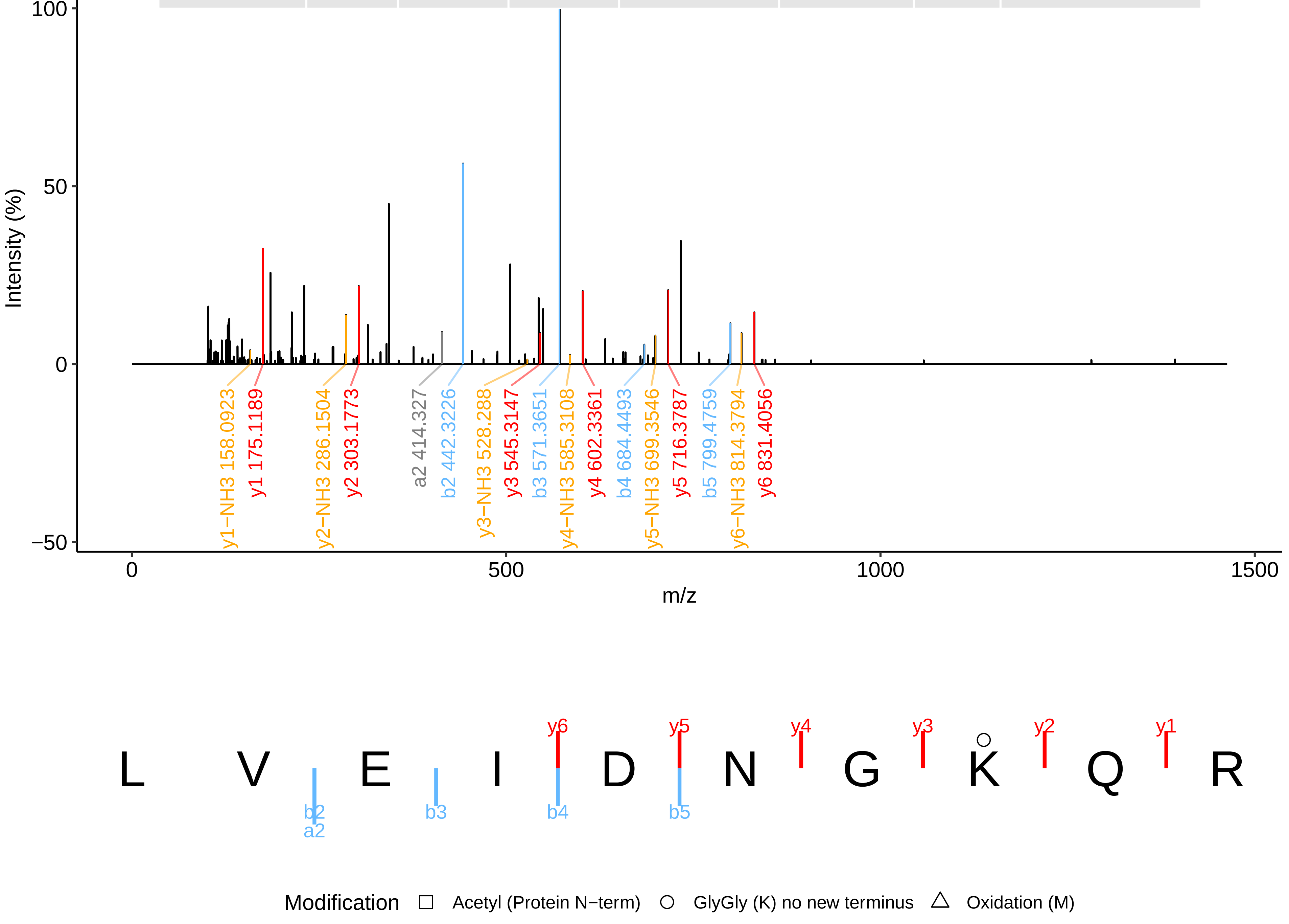

20200924_KIZ_MUb5_GlyGly_1 − Scan number: 8276

Gene Names	Charge	m/z	Mass	Retention time	PEP	Score	Precursor Intensity
LMNA	3	429.2335	1284.679	26.429	0.0012119	90.149	12269269

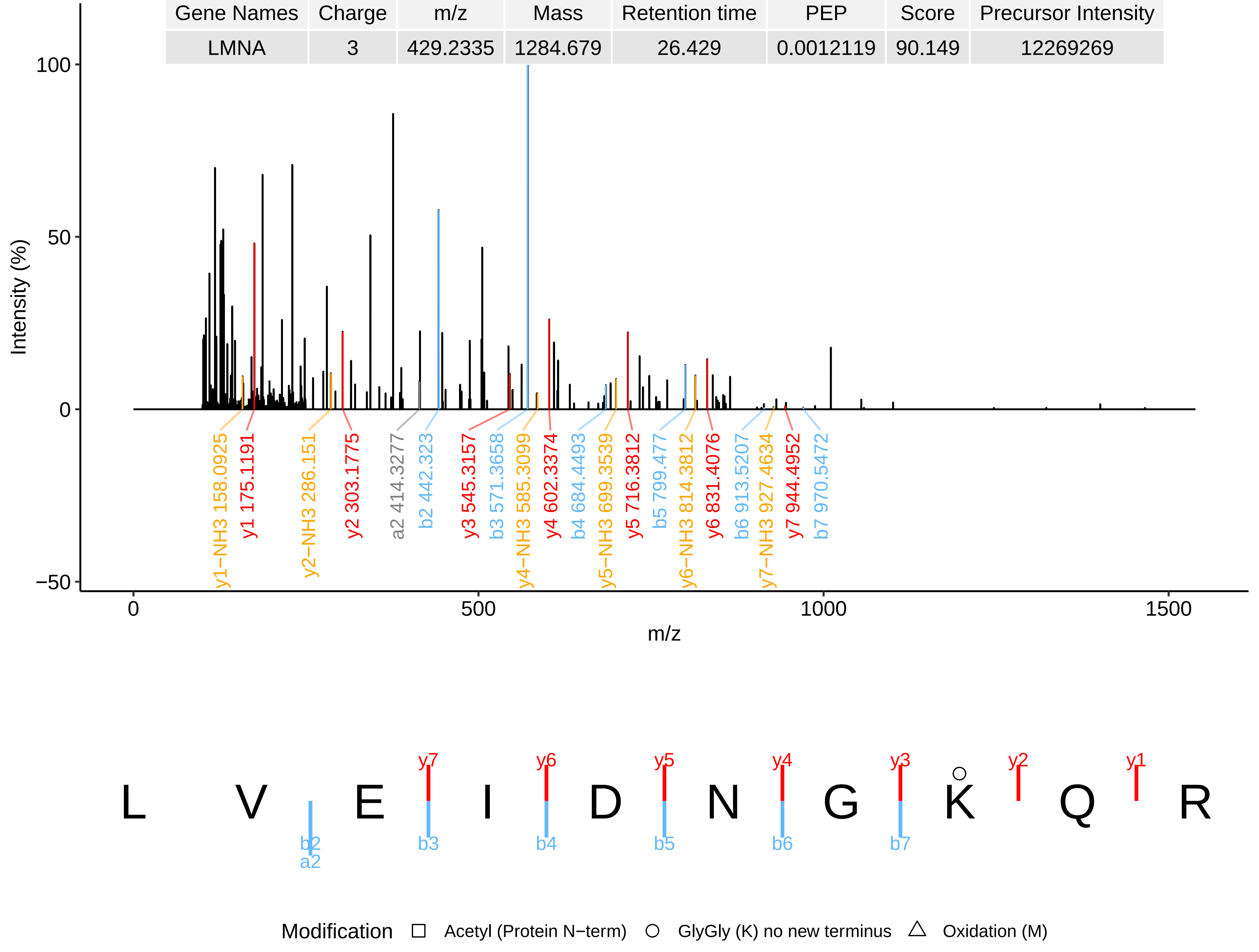

20200924_KIZ_MUb5_GlyGly_2 − Scan number: 7638

Gene Names	Charge	m/z	Mass	Retention time	PEP	Score	Precursor Intensity
LMNA	3	429.2335	1284.679	28.523	0.029041	61.48	3183202

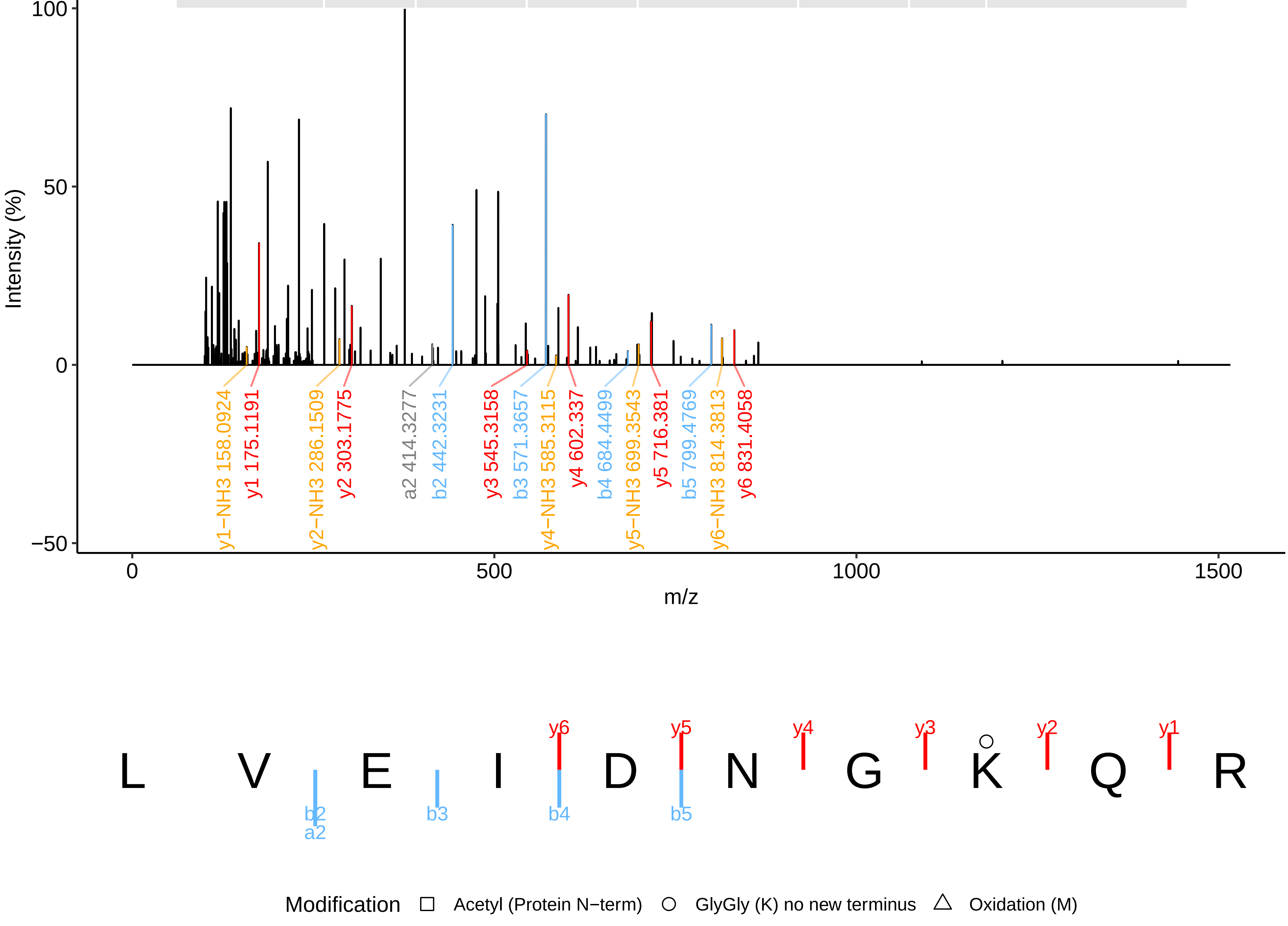

20200924_KIZ_MUb5_GlyGly_2 − Scan number: 14162

Gene Names	Charge	m/z	Mass	Retention time	PEP	Score	Precursor Intensity
CDC2;CDK1	2	656.8081	1311.602	52.943	0.01217	51.727	4364766

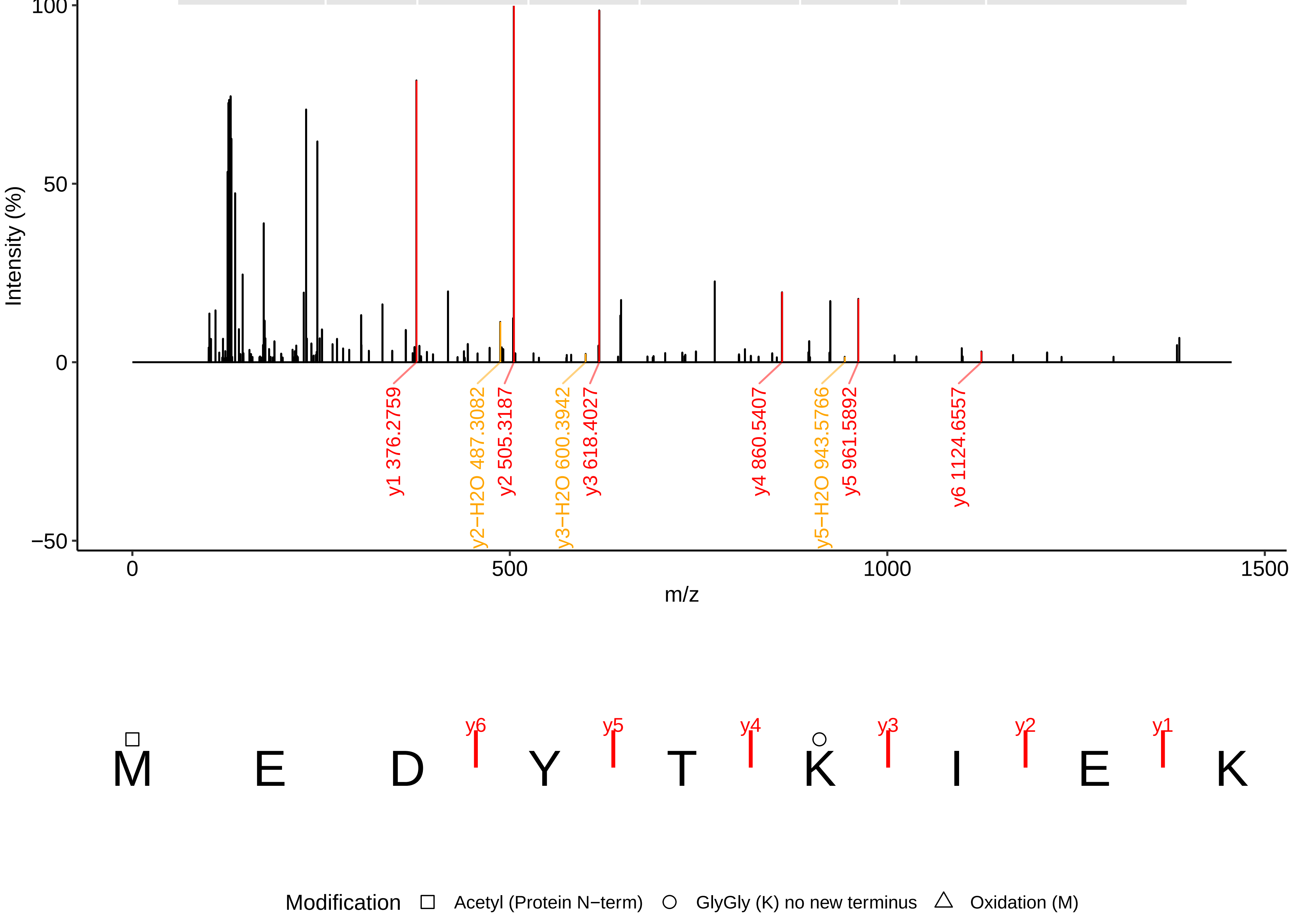

20200921_KIZ_MUb4_GlyGly_10plex − Scan number: 2479

Gene Names	Charge	m/z	Mass	Retention time	PEP	Score	Precursor Intensity
HIP1	4	327.1556	1304.593	7.8015	0.076582	49.418	3852217

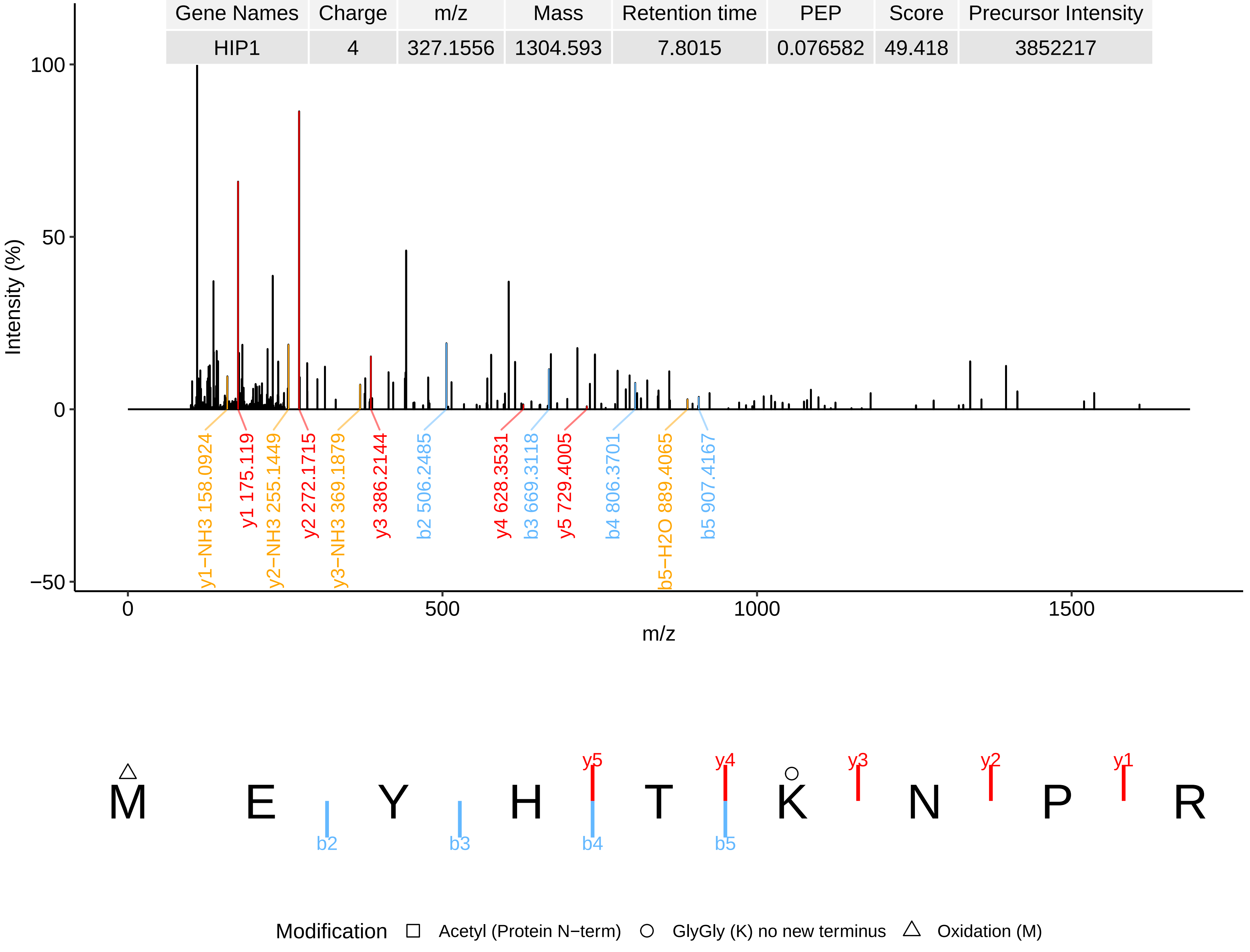

20200921_KIZ_MUb4_GlyGly_10plex − Scan number: 3402

Gene Names	Charge	m/z	Mass	Retention time	PEP	Score	Precursor Intensity
HIP1	4	323.1568	1288.598	10.753	0.00044048	99.139	4498828

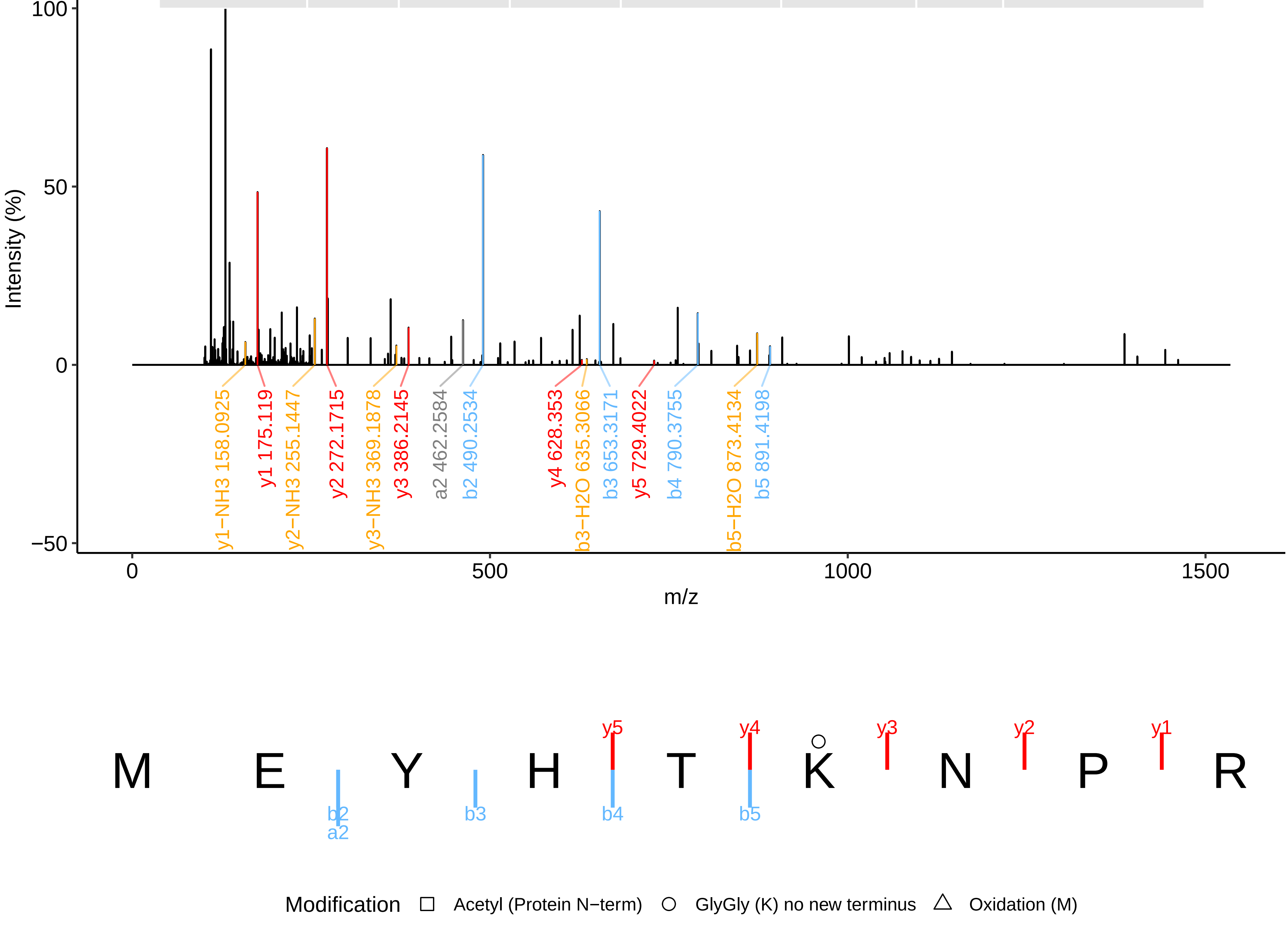

20200921_KIZ_MUb4_GlyGly_10plex − Scan number: 3411

Gene Names	Charge	m/z	Mass	Retention time	PEP	Score	Precursor Intensity
HIP1	3	430.54	1288.598	10.79	2.7339e−07	136.21	3634511

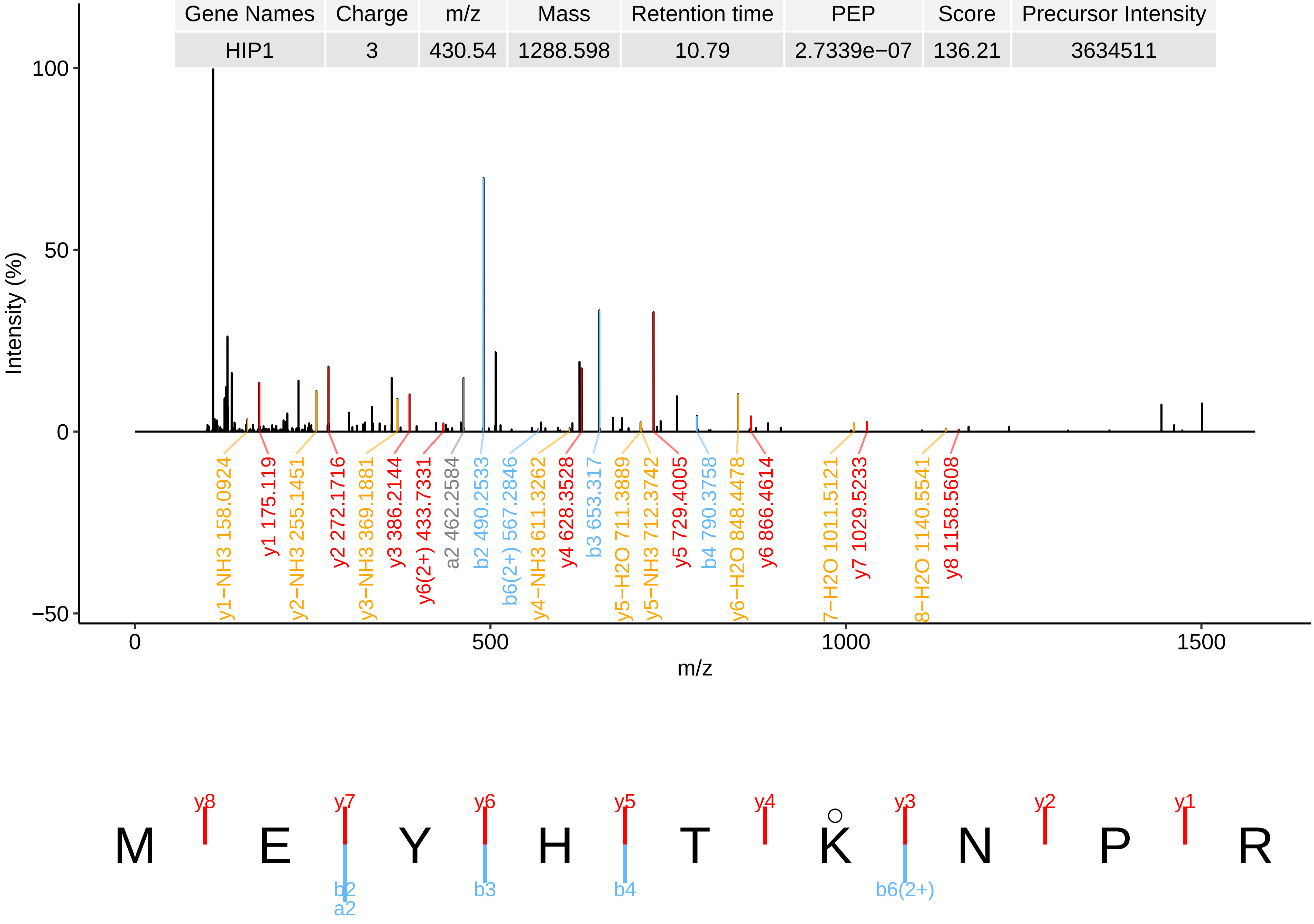

Modification □ Acetyl (Protein N−term) ○ GlyGly (K) no new terminus △ Oxidation (M)

20200921_KIZ_MUb4_GlyGly_10plex_2 − Scan number: 3283

Gene Names	Charge	m/z	Mass	Retention time	PEP	Score	Precursor Intensity
HIP1	4	323.1568	1288.598	10.954	0.0026455	82.452	2012734

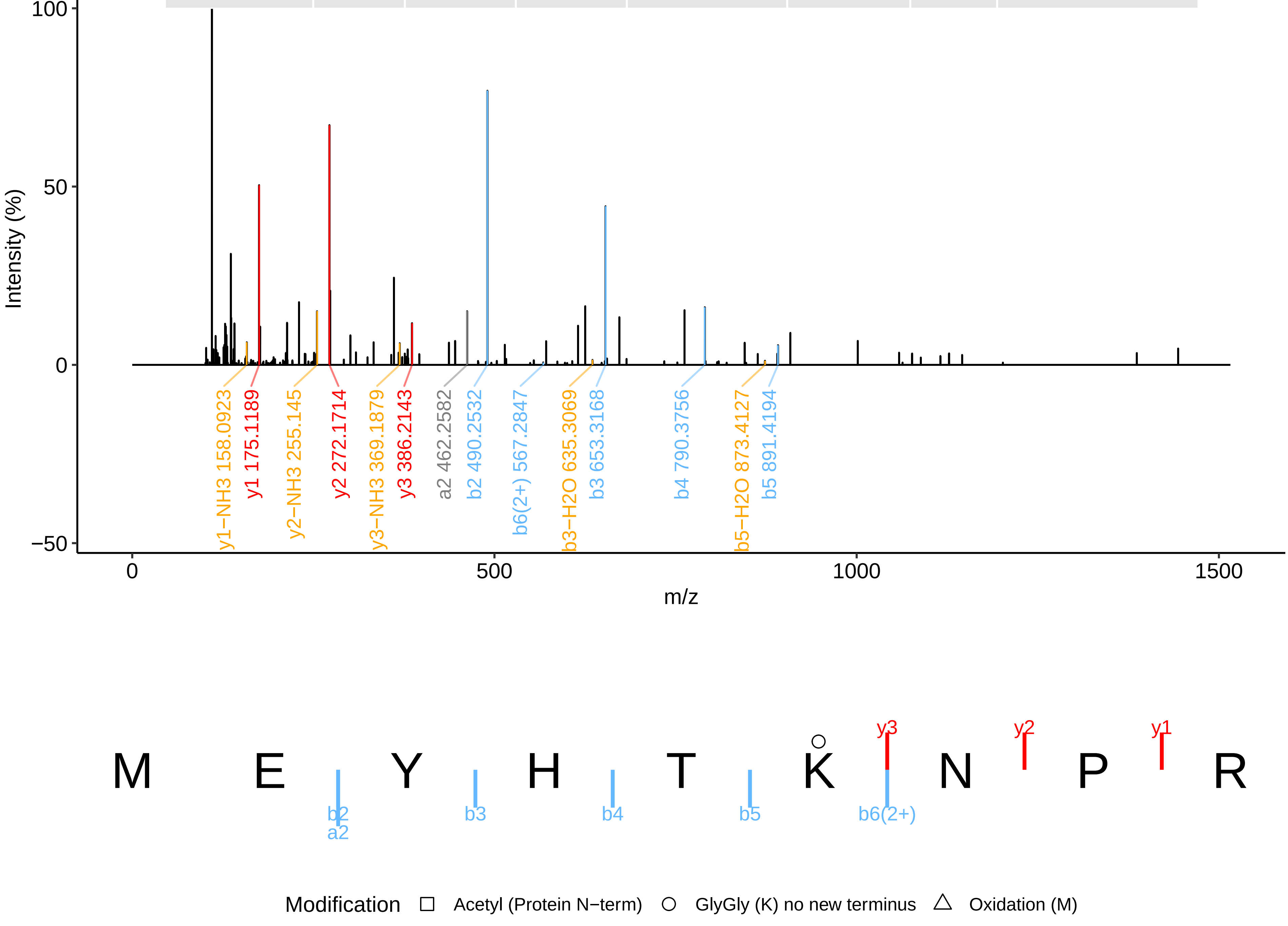

20200921_KIZ_MUb4_GlyGly_10plex_2 − Scan number: 3288

Gene Names	Charge	m/z	Mass	Retention time	PEP	Score	Precursor Intensity
HIP1	3	430.54	1288.598	10.974	5.8466e−06	128.12	1391411

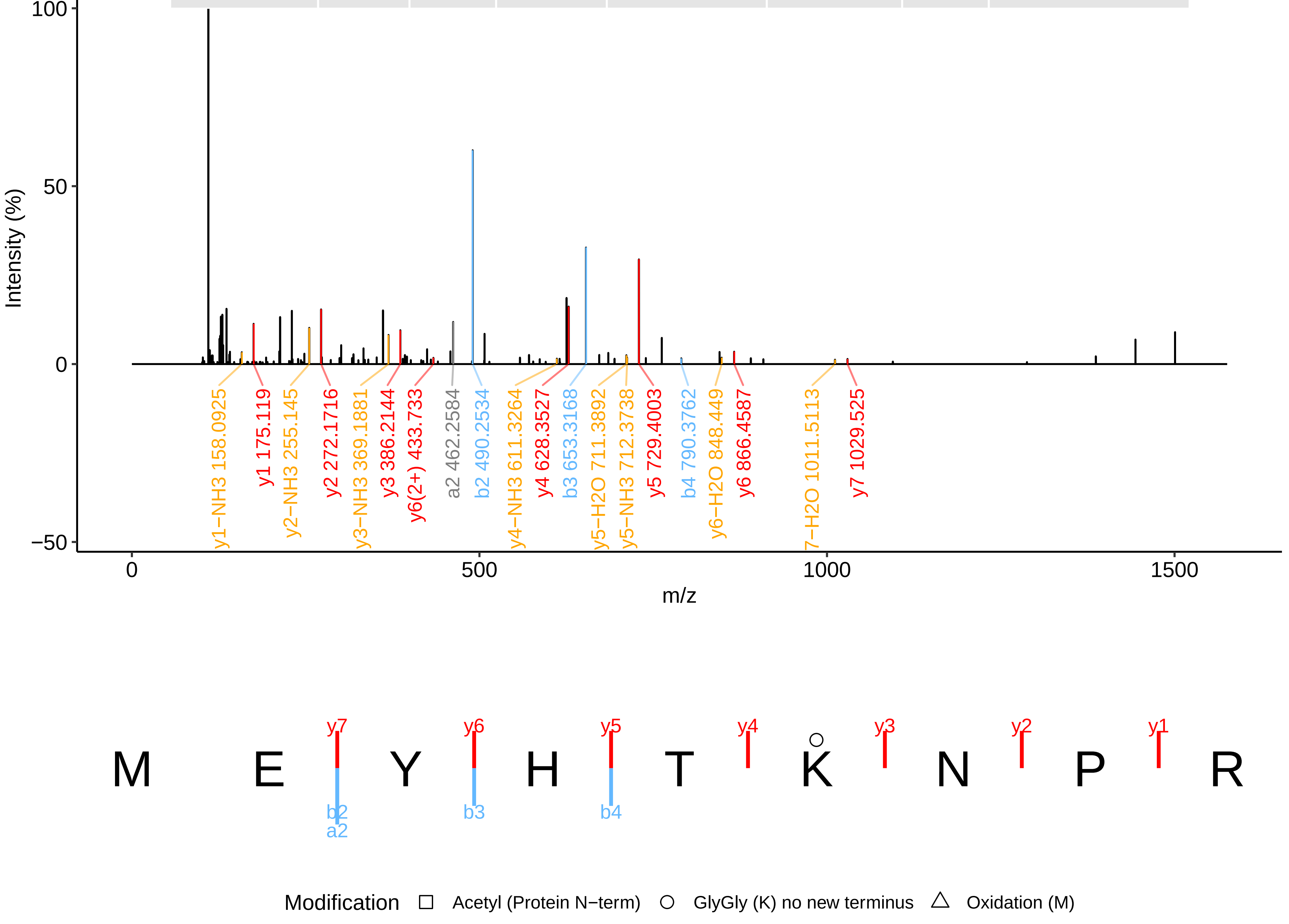

20200924_KIZ_MUb5_GlyGly_1 − Scan number: 3145

Gene Names	Charge	m/z	Mass	Retention time	PEP	Score	Precursor Intensity
HIP1	4	323.1568	1288.598	9.7038	0.015461	66.27	18713922

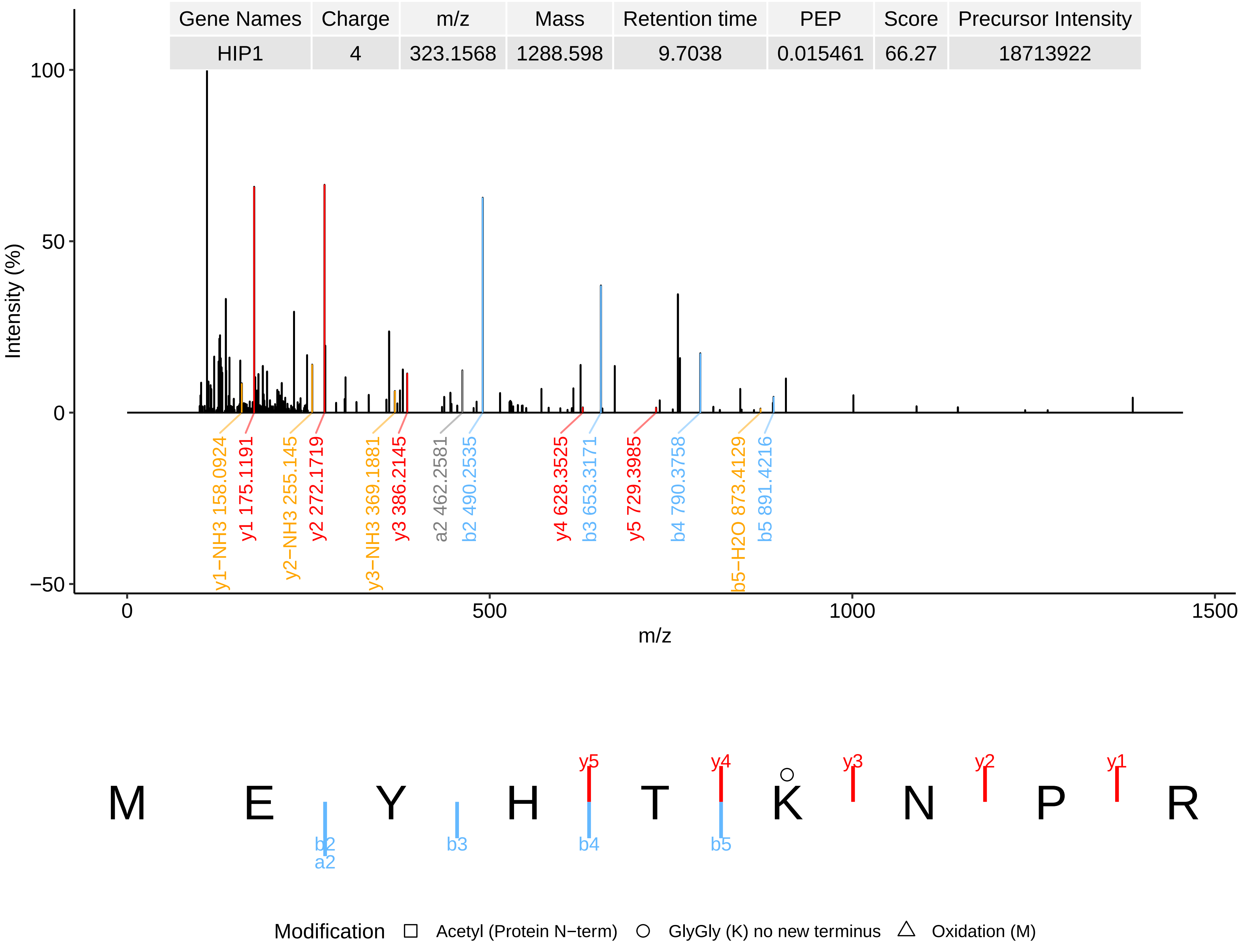

20200924_KIZ_MUb5_GlyGly_1 − Scan number: 3174

Gene Names	Charge	m/z	Mass	Retention time	PEP	Score	Precursor Intensity
HIP1	3	430.54	1288.598	9.7935	0.00045571	101.65	12311933

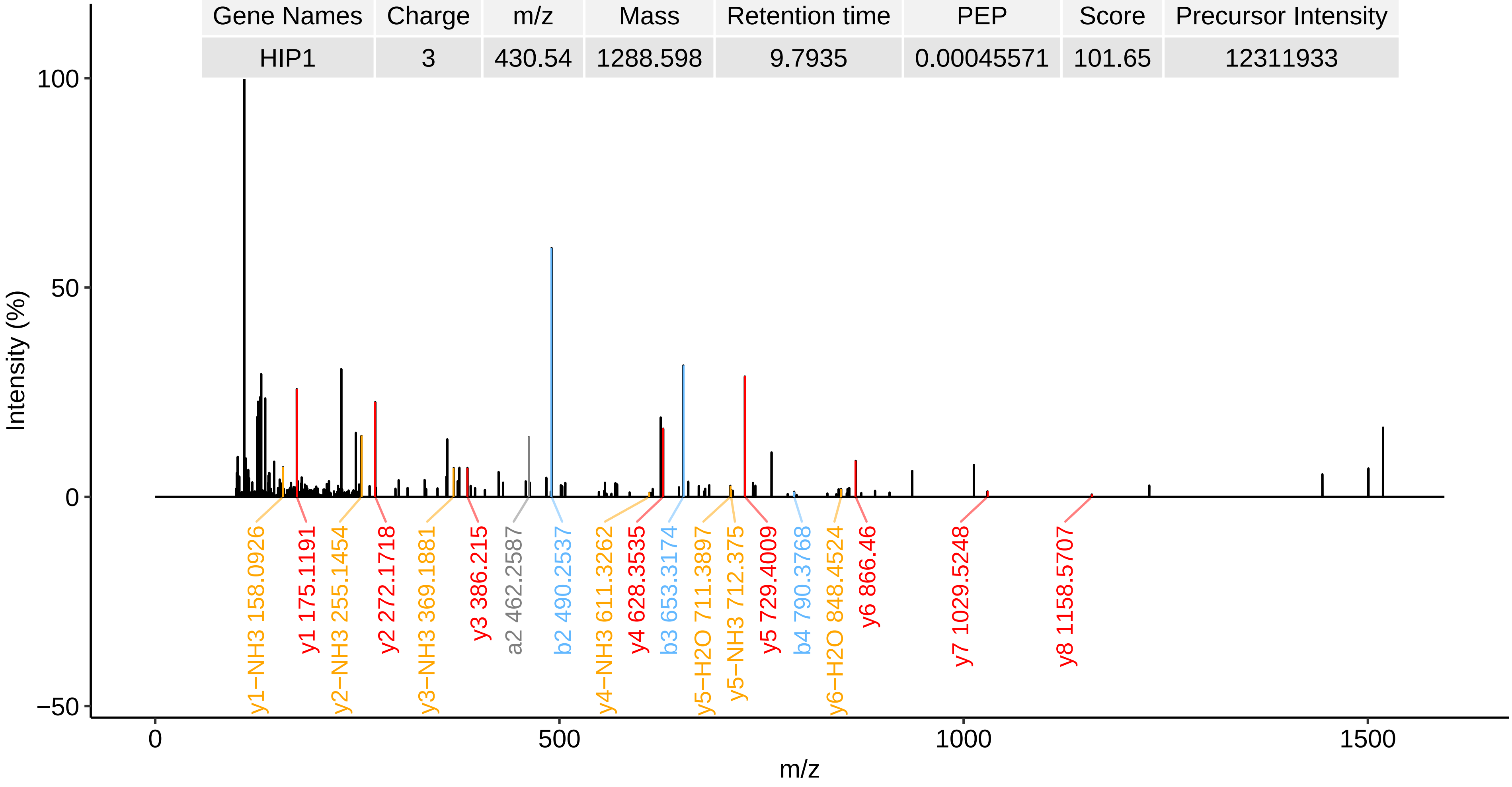

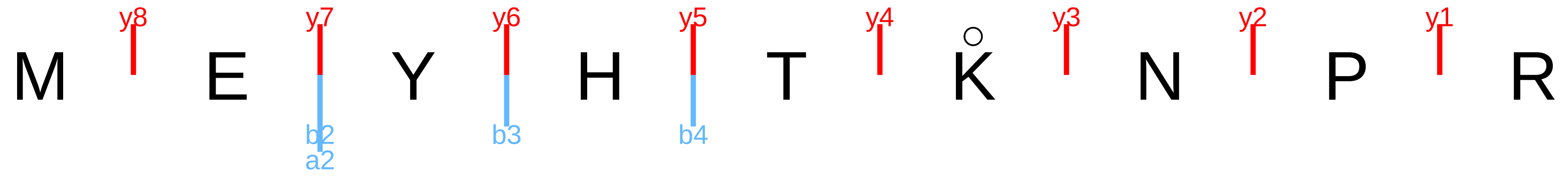

Modification □ Acetyl (Protein N−term) ○ GlyGly (K) no new terminus △ Oxidation (M)

20201118_KIZ_MUb6_GlyGly_2 − Scan number: 3436

Gene Names	Charge	m/z	Mass	Retention time	PEP	Score	Precursor Intensity
HIP1	4	323.1568	1288.598	11.335	0.031574	59.8	7244076

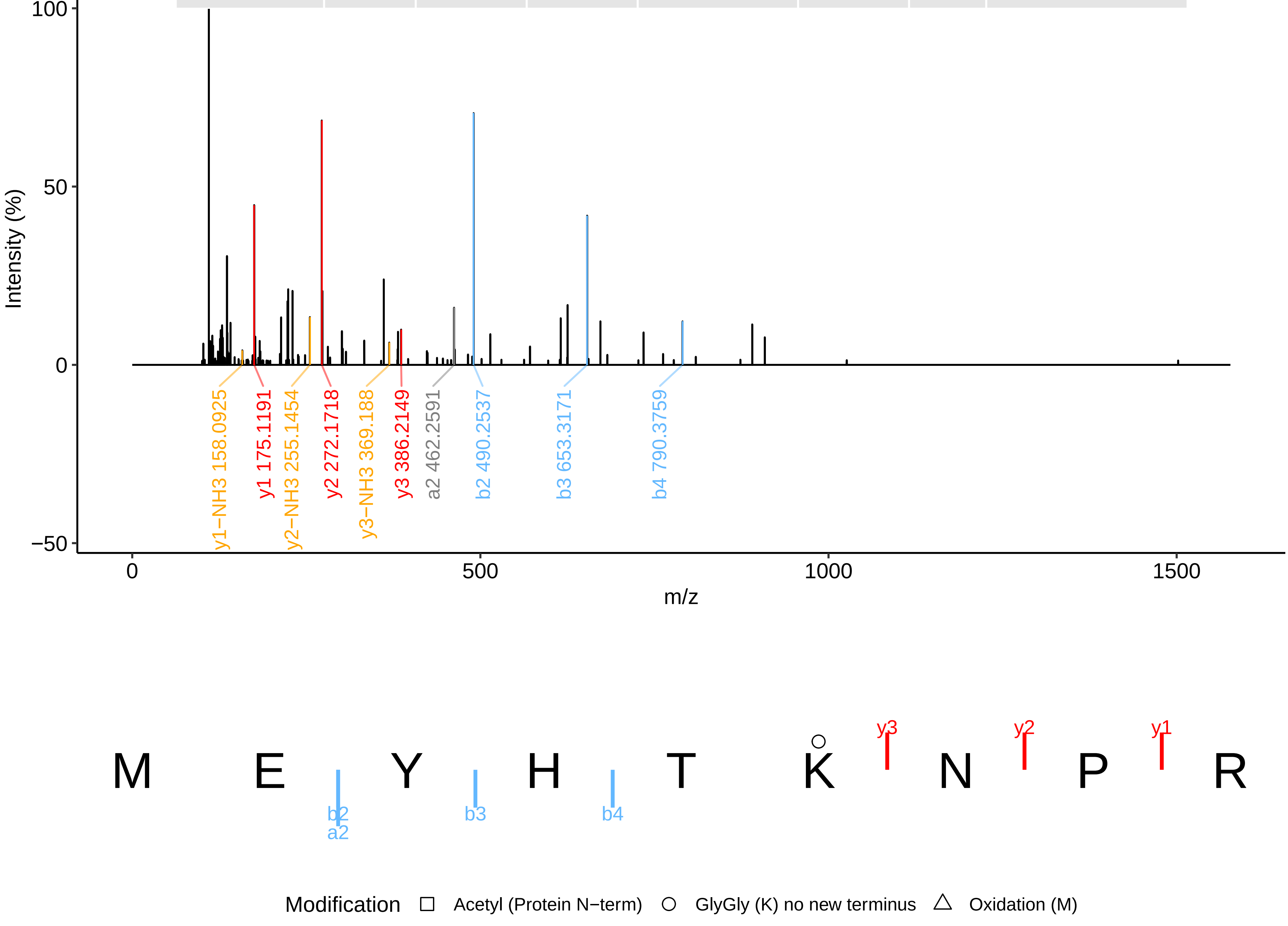

20200921_KIZ_MUb4_GlyGly_10plex_2 − Scan number: 5740

Gene Names	Charge	m/z	Mass	Retention time	PEP	Score	Precursor Intensity
SLC25A11	3	431.219	1290.635	20.782	0.0035873	78.324	737269.9

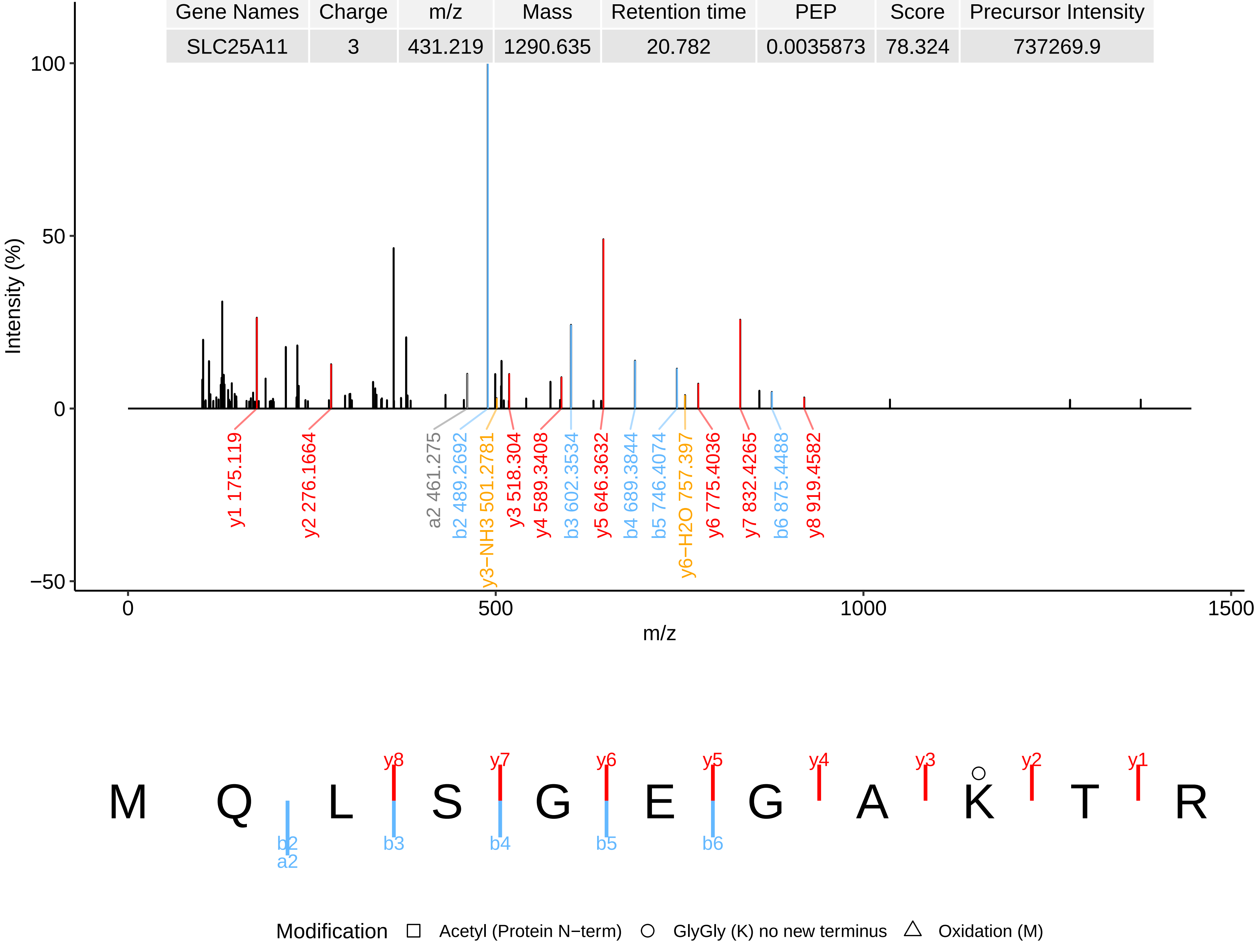

20200924_KIZ_MUb5_GlyGly_1 − Scan number: 4991

Gene Names	Charge	m/z	Mass	Retention time	PEP	Score	Precursor Intensity
SLC1A3	2	638.7955	1275.576	15.486	0.00068005	103.83	NA

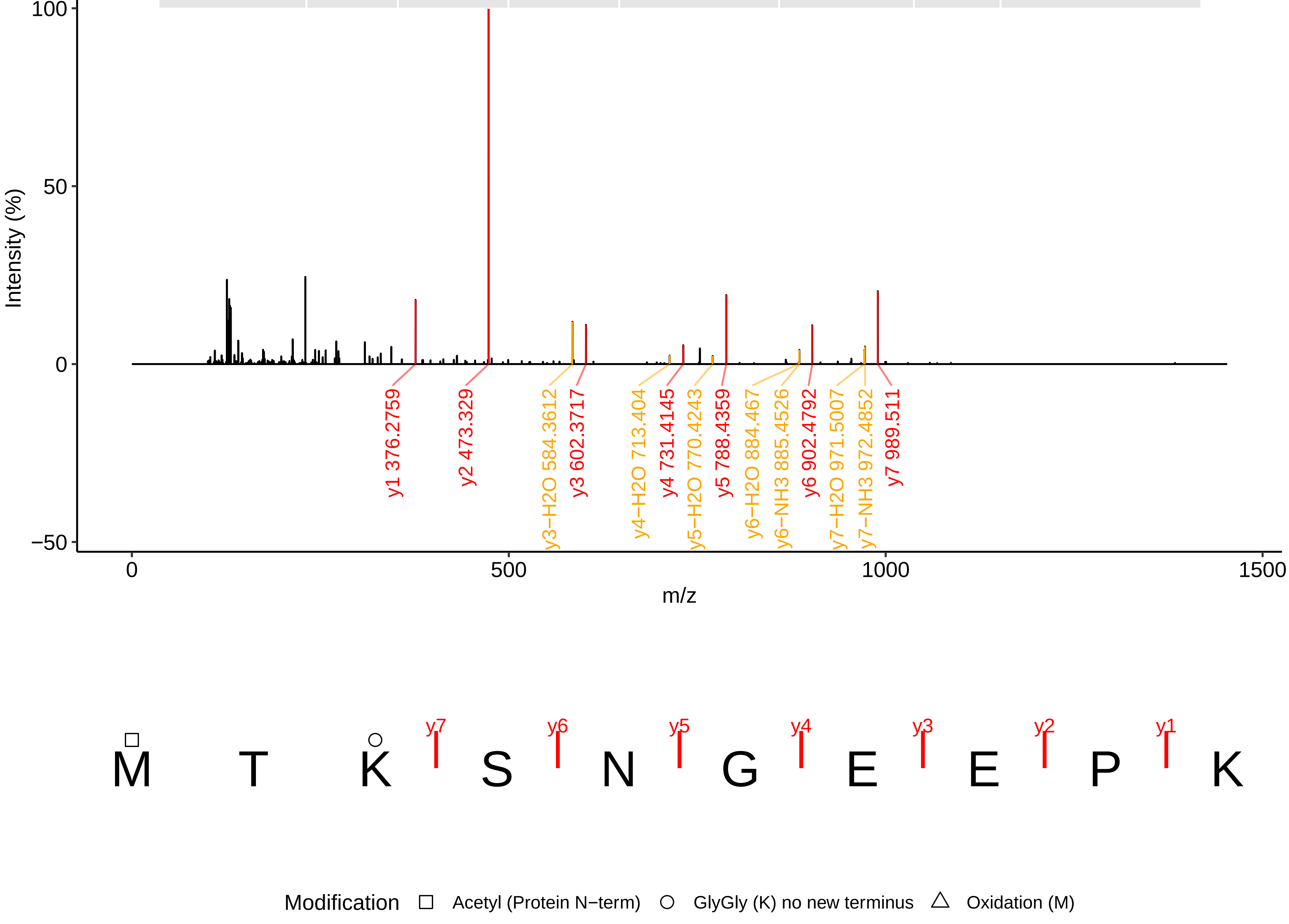

20200924_KIZ_MUb5_GlyGly_2 − Scan number: 4570

Gene Names	Charge	m/z	Mass	Retention time	PEP	Score	Precursor Intensity
SLC1A3	2	638.7955	1275.576	16.79	0.0083784	80.438	NA

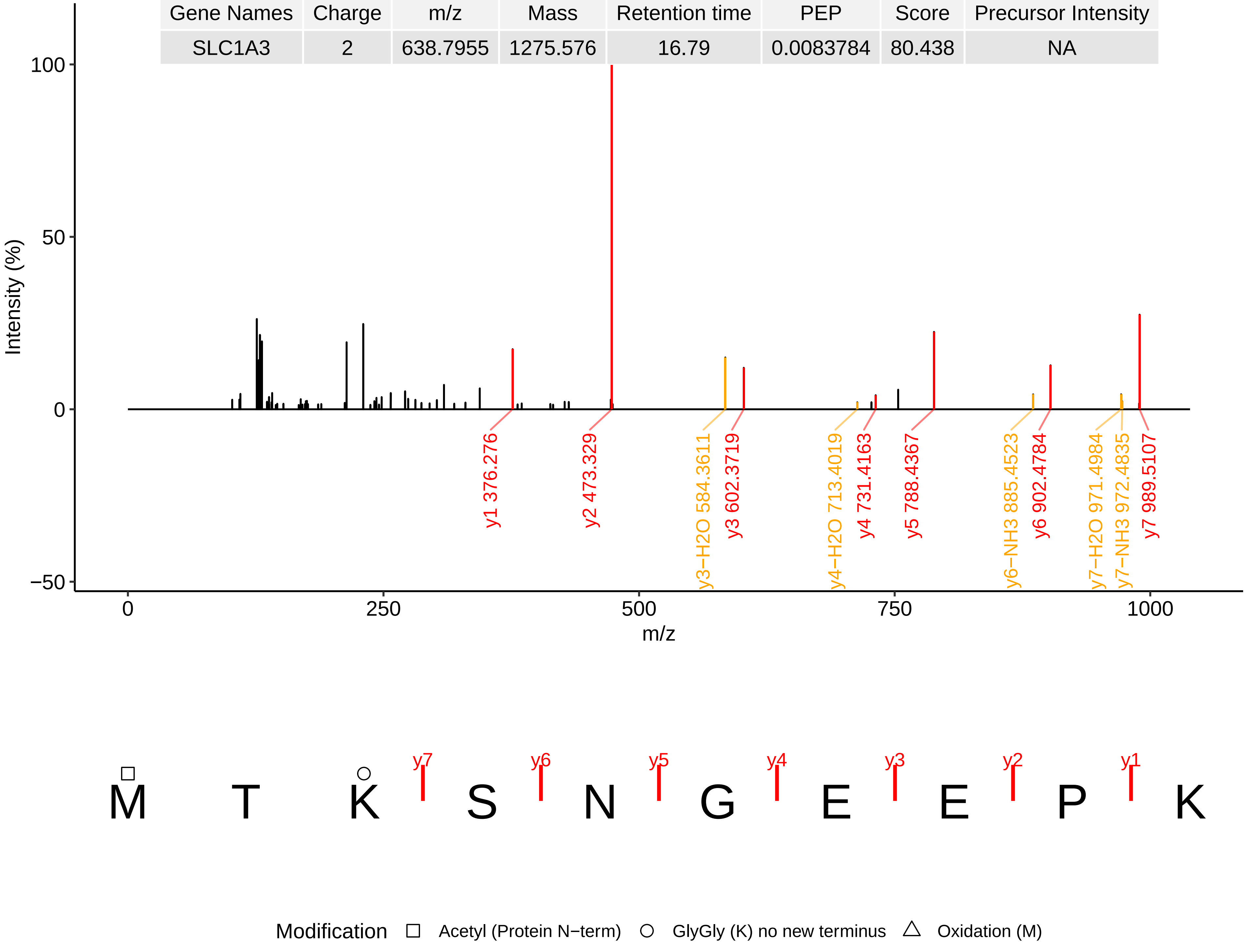

20200921_KIZ_MUb4_GlyGly_10plex − Scan number: 19259

Gene Names	Charge	m/z	Mass	Retention time	PEP	Score	Precursor Intensity
RPL13A;RPL13a;RPL13AP3	3	432.9322	1295.775	69.391	1.2814e−06	139.96	2494148

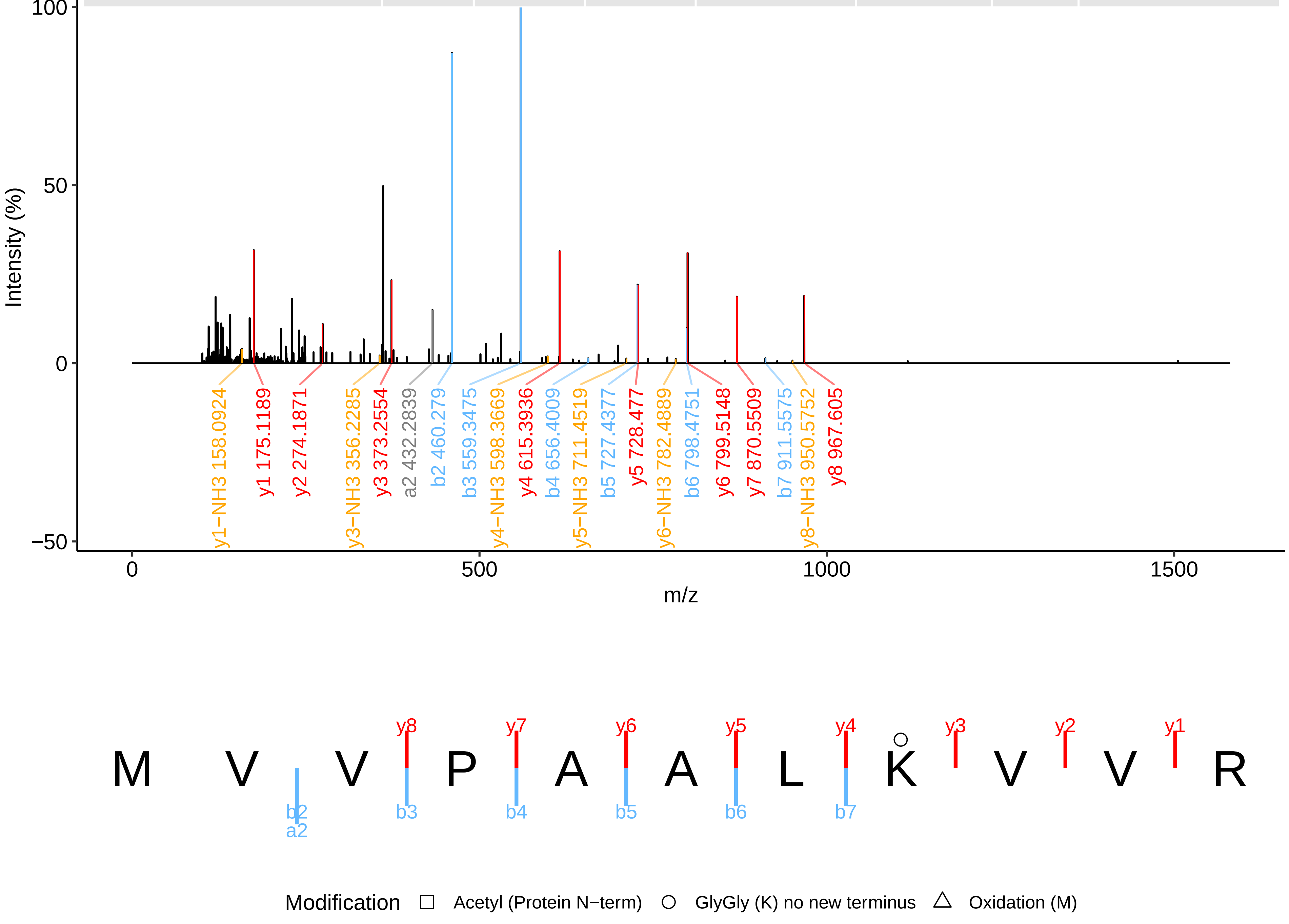

20200921_KIZ_MUb4_GlyGly_10plex_2 − Scan number: 17787

Gene Names	Charge	m/z	Mass	Retention time	PEP	Score	Precursor Intensity
RPL13A;RPL13a;RPL13AP3	3	432.9322	1295.775	69.588	4.025e−05	99.815	905648.2

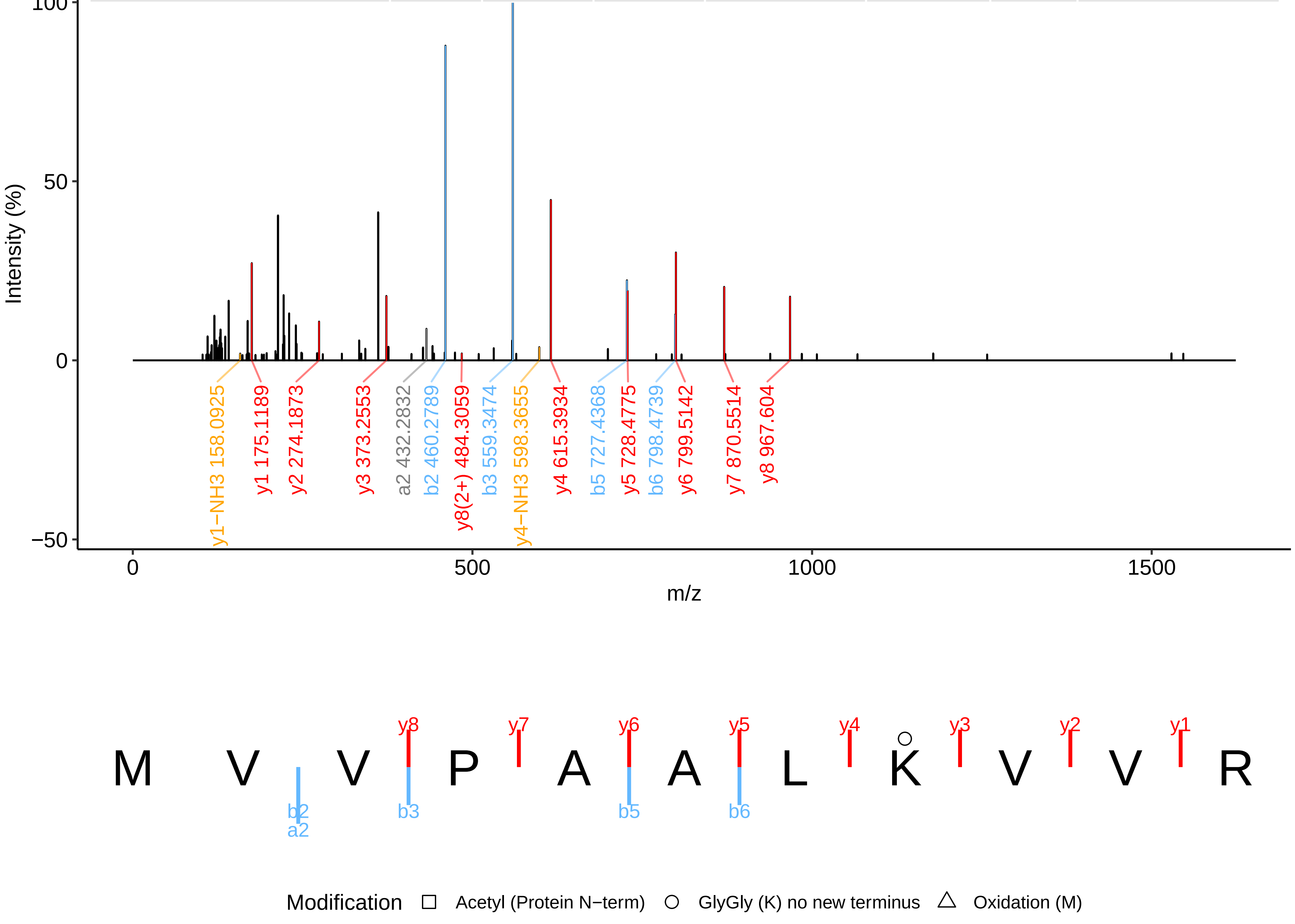

20200921_KIZ_MUb4_GlyGly_10plex_2 − Scan number: 14311

Gene Names	Charge	m/z	Mass	Retention time	PEP	Score	Precursor Intensity
RPL13A;RPL13a;RPL13AP3	3	438.2638	1311.77	55.776	0.064873	58.699	928371.8

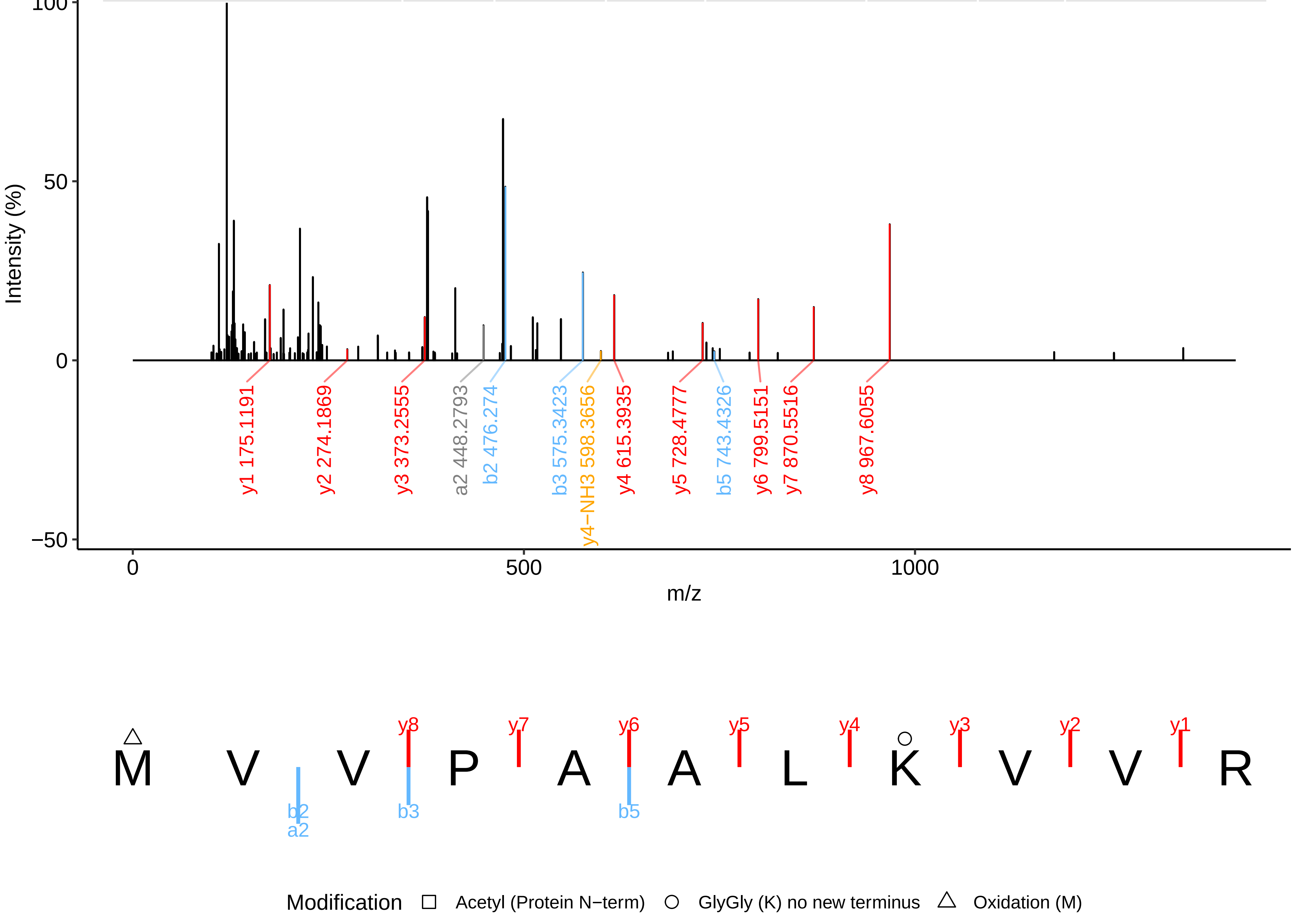

20200921_KIZ_MUb4_GlyGly_10plex − Scan number: 6086

Gene Names	Charge	m/z	Mass	Retention time	PEP	Score	Precursor Intensity
MCL1	3	426.2091	1275.605	20.017	7.2636e−05	98.754	6216507

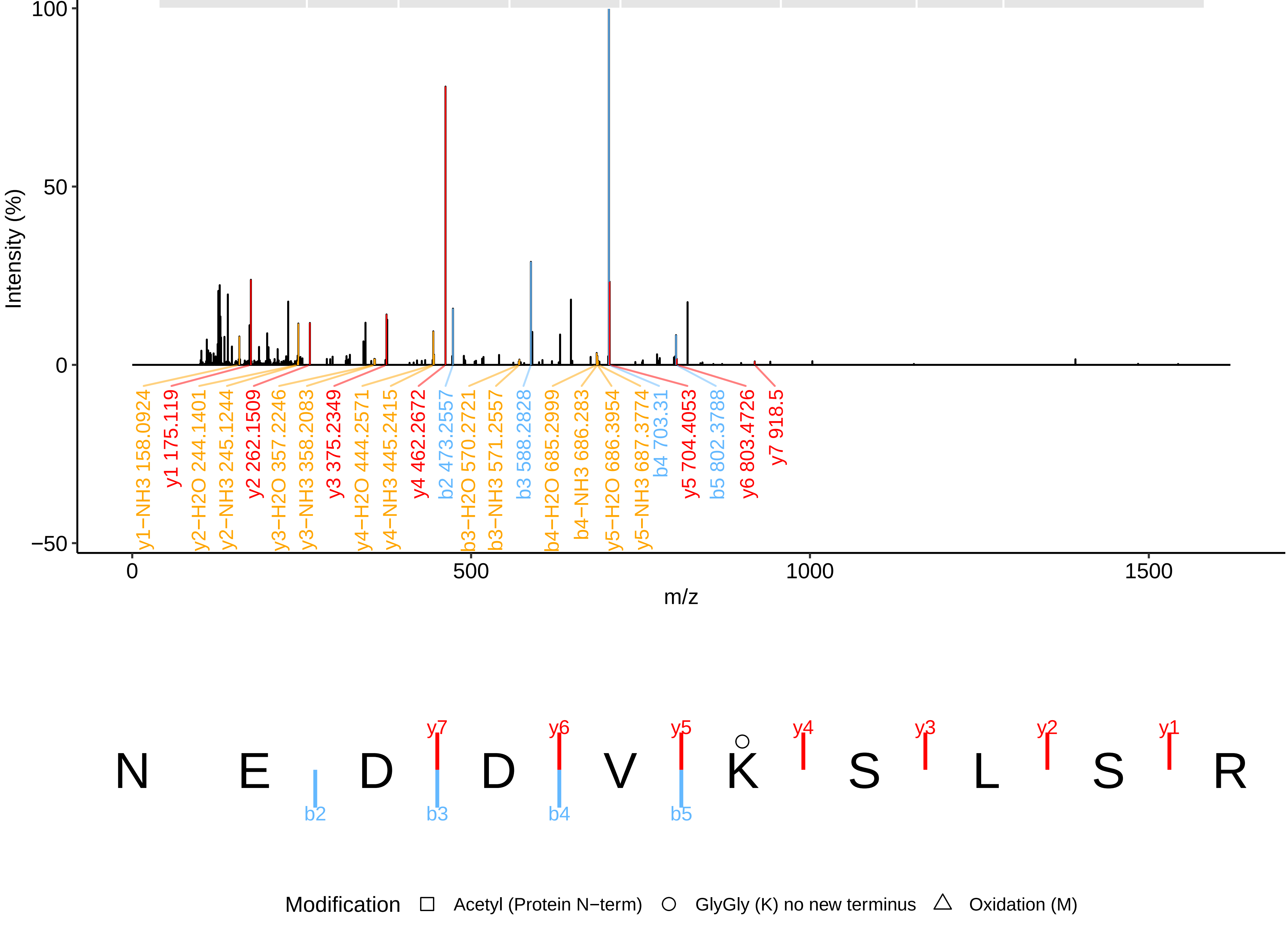

20200921_KIZ_MUb4_GlyGly_10plex_2 − Scan number: 5511

Gene Names	Charge	m/z	Mass	Retention time	PEP	Score	Precursor Intensity
MCL1	3	426.2091	1275.605	19.85	0.014879	68.536	1394917

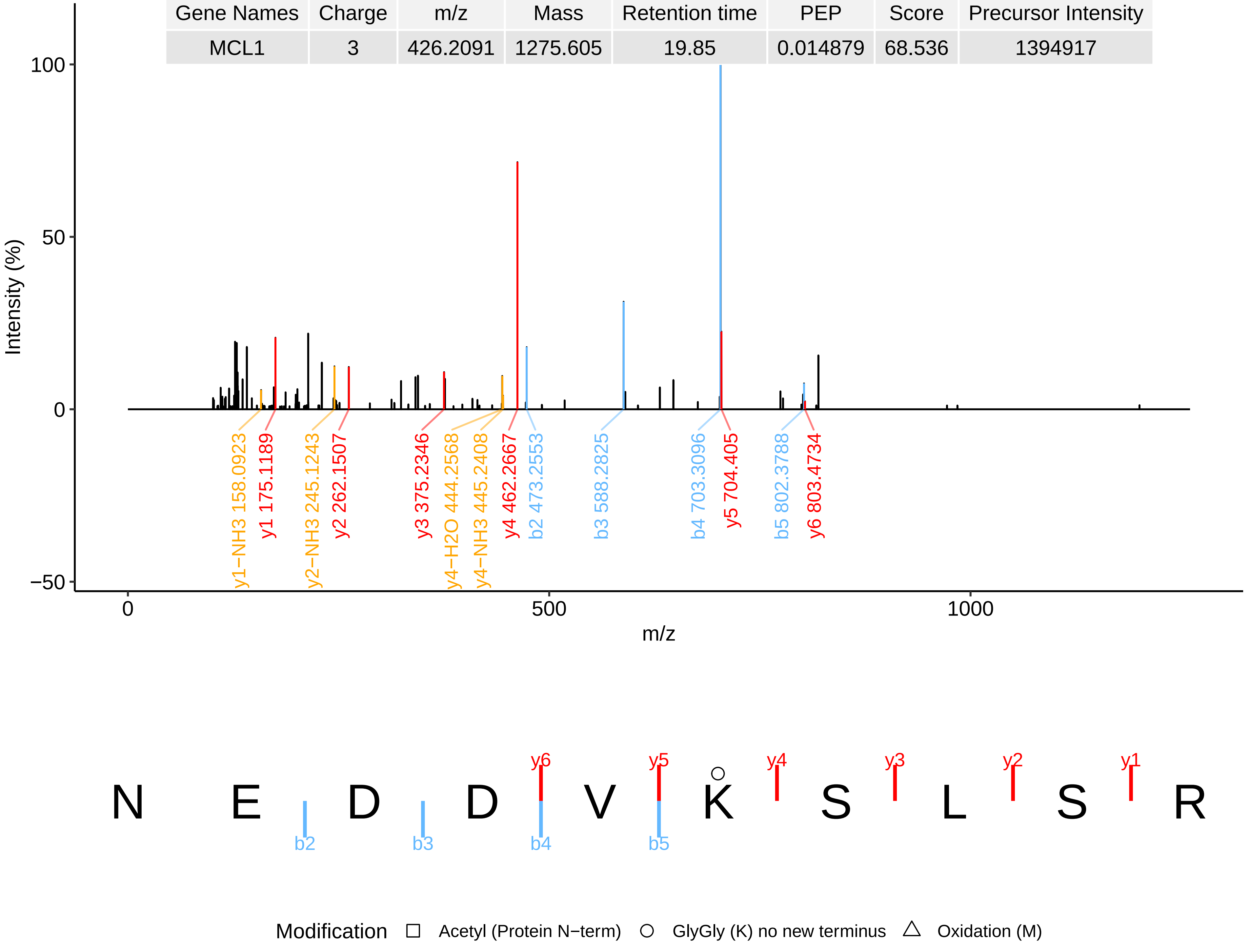

20201118_KIZ_MUb6_GlyGly_1 − Scan number: 5060

Gene Names	Charge	m/z	Mass	Retention time	PEP	Score	Precursor Intensity
MCL1	3	426.2091	1275.605	15.999	0.018801	64.688	8208697

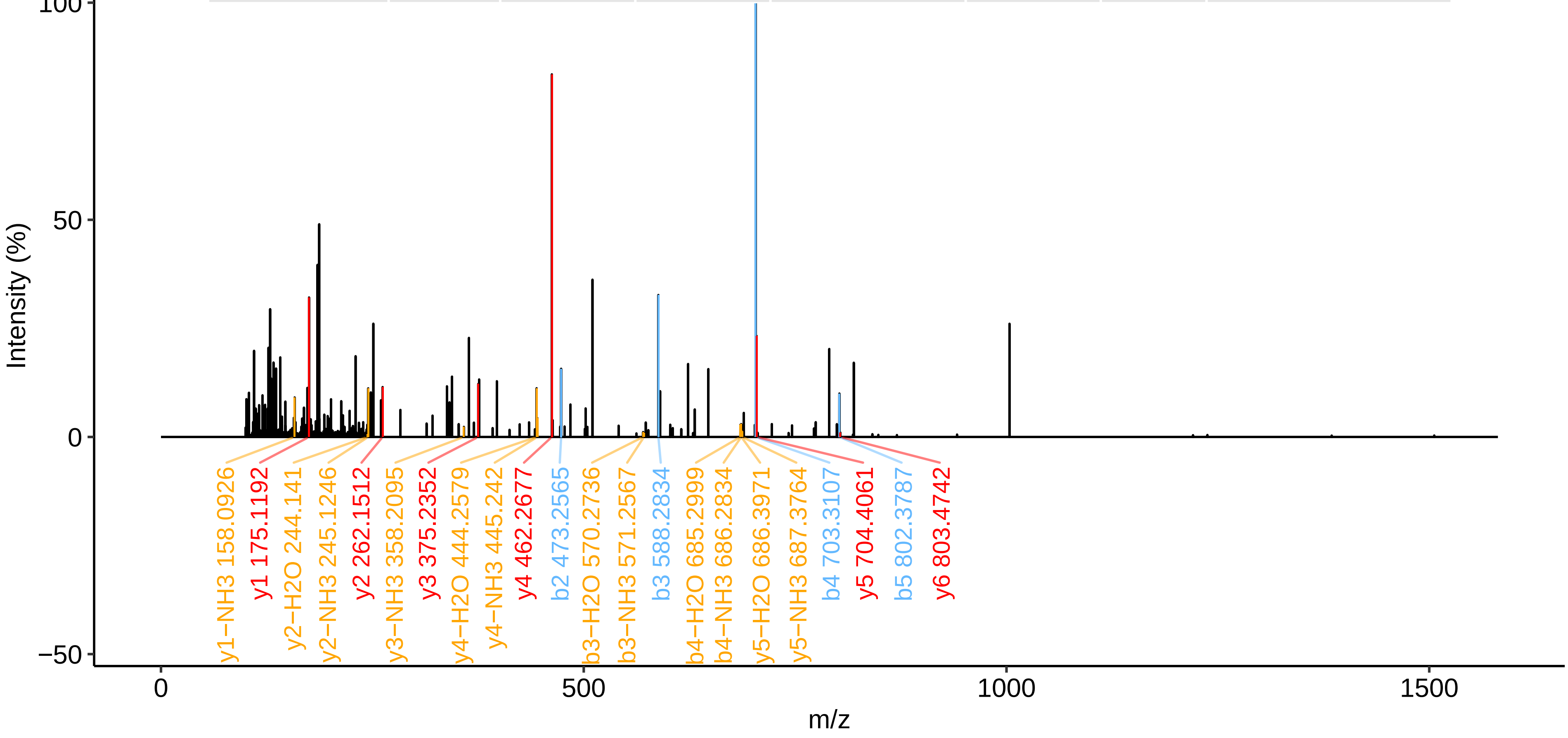

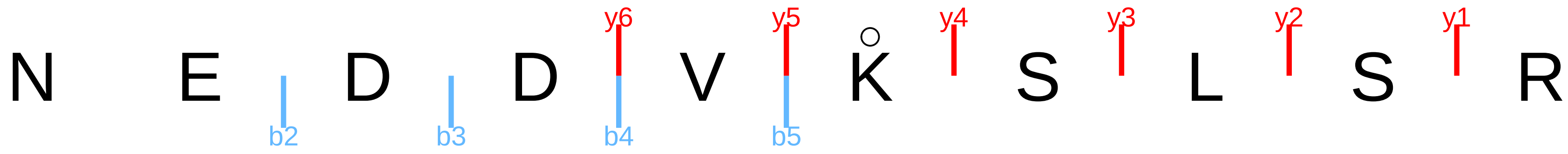

Modification □ Acetyl (Protein N−term) ○ GlyGly (K) no new terminus △ Oxidation (M)

20200921_KIZ_MUb4_GlyGly_10plex − Scan number: 8032

Gene Names	Charge	m/z	Mass	Retention time	PEP	Score	Precursor Intensity
YWHAZ	3	348.533	1042.577	27.147	0.053669	57.598	23098686

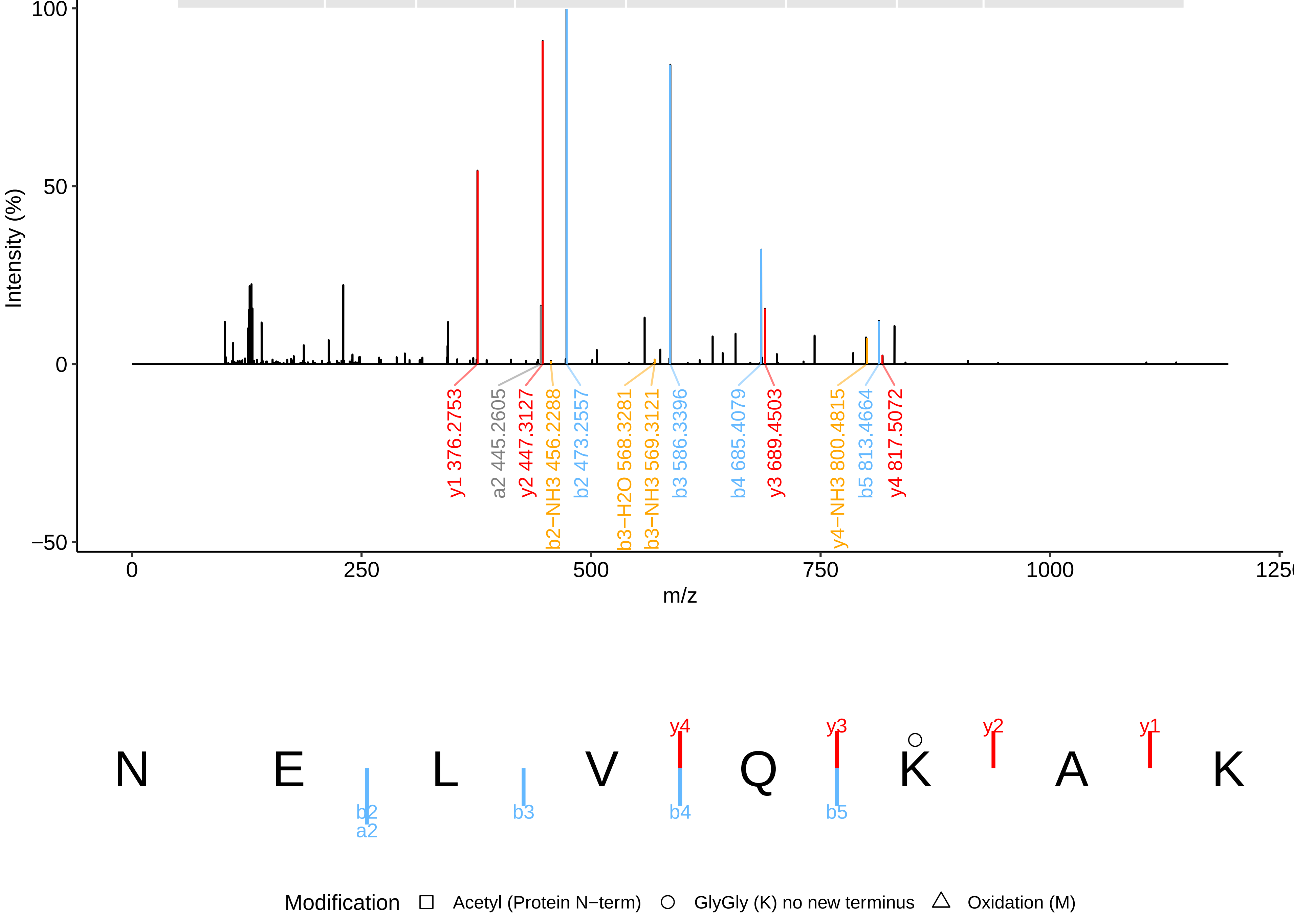

20200921_KIZ_MUb4_GlyGly_10plex − Scan number: 8077

Gene Names	Charge	m/z	Mass	Retention time	PEP	Score	Precursor Intensity
YWHAZ	2	522.2958	1042.577	27.323	0.00035832	138.1	2100485

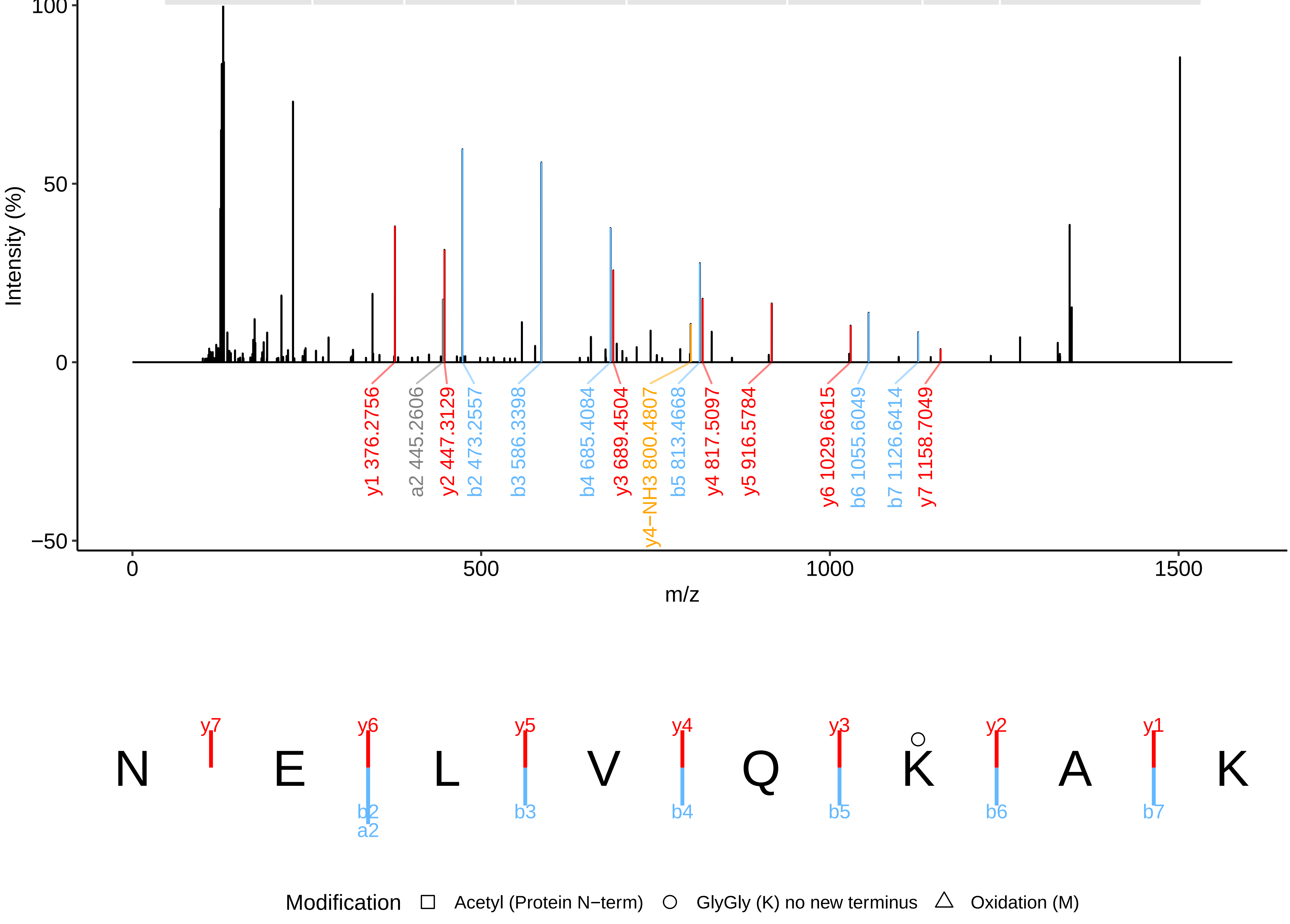

20200921_KIZ_MUb4_GlyGly_10plex_2 − Scan number: 7416

Gene Names	Charge	m/z	Mass	Retention time	PEP	Score	Precursor Intensity
YWHAZ	3	348.533	1042.577	27.663	0.04648	59.35	1709891

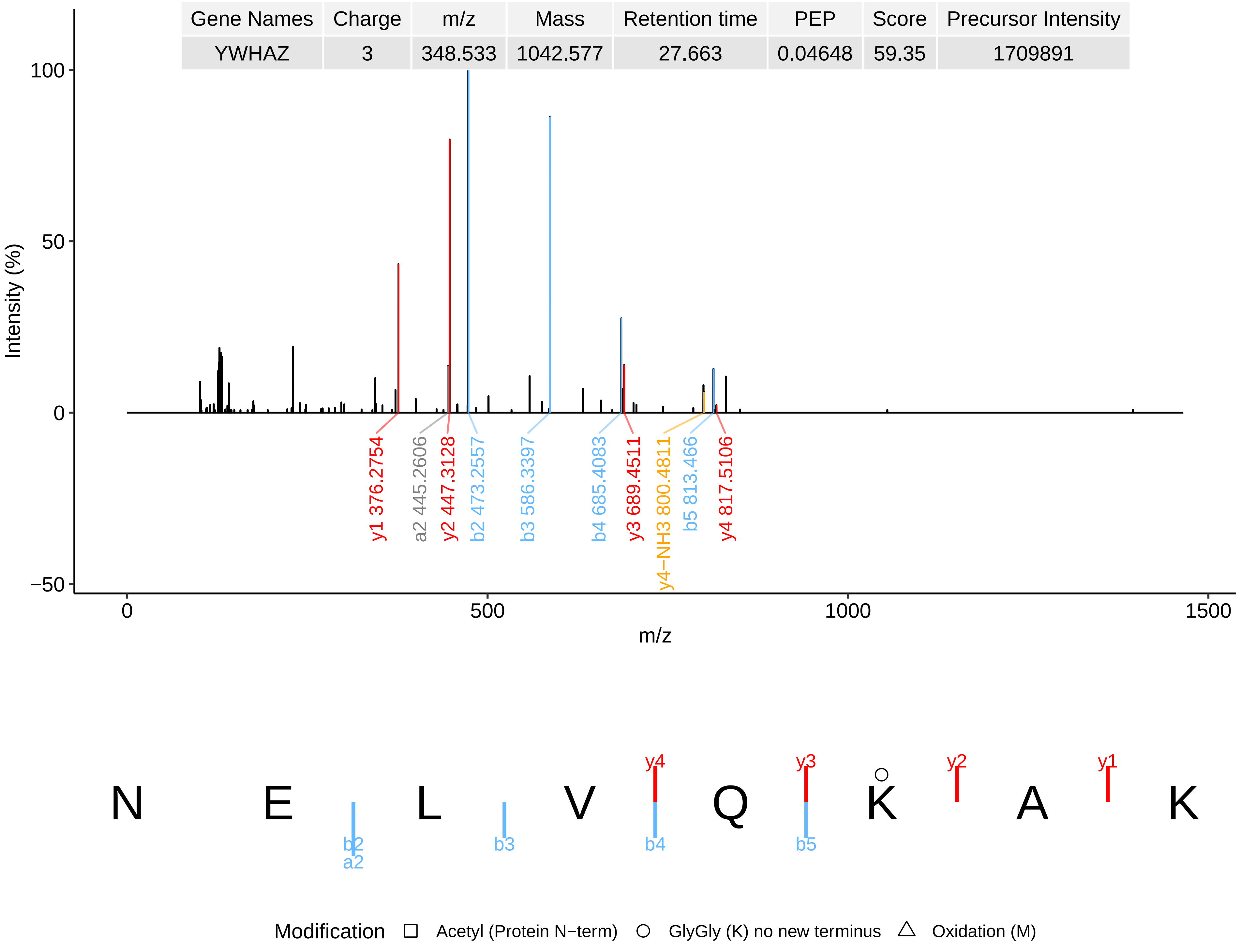

20200924_KIZ_MUb5_GlyGly_1 − Scan number: 6964

Gene Names	Charge	m/z	Mass	Retention time	PEP	Score	Precursor Intensity
YWHAZ	3	348.533	1042.577	22.034	0.037318	61.582	26600024

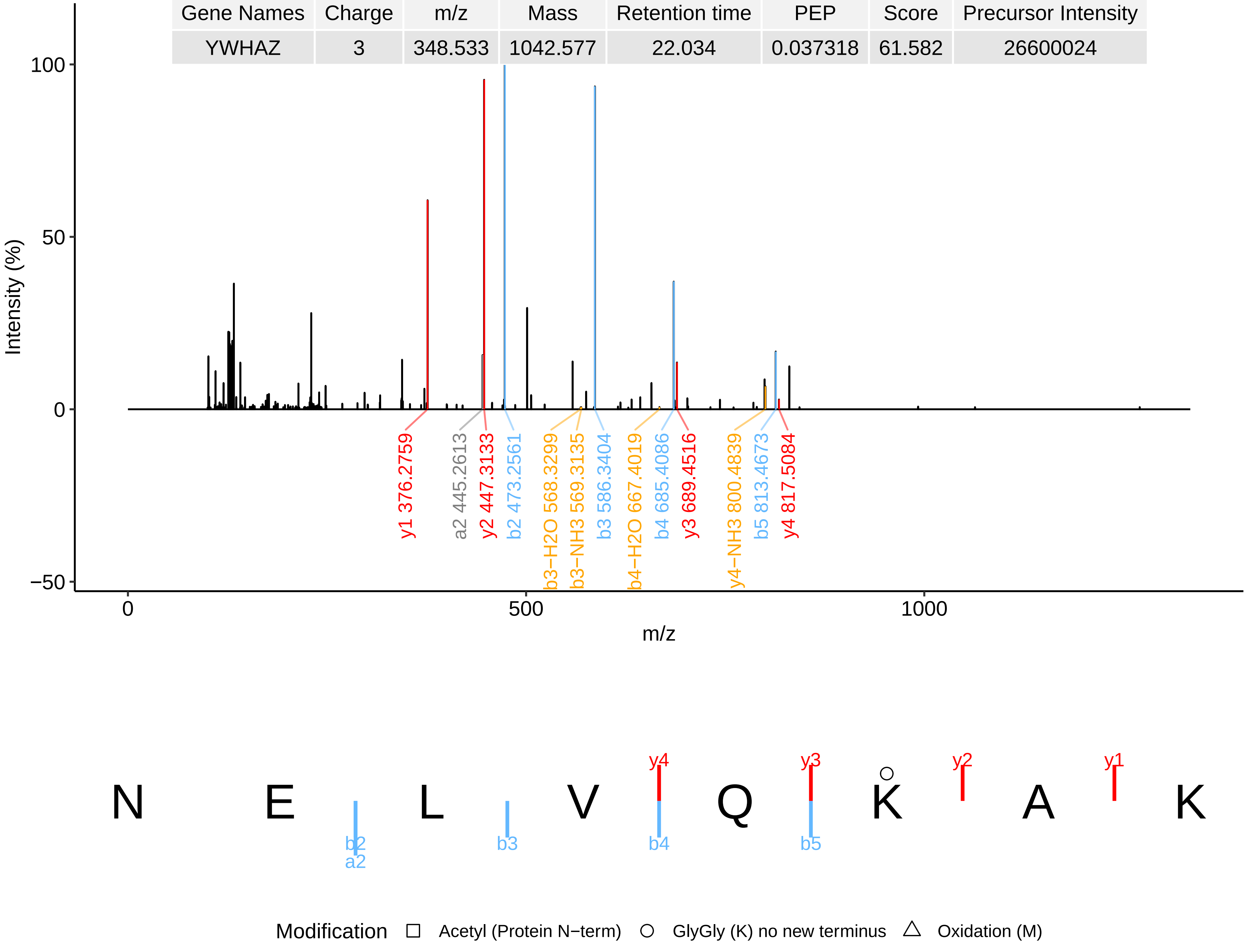

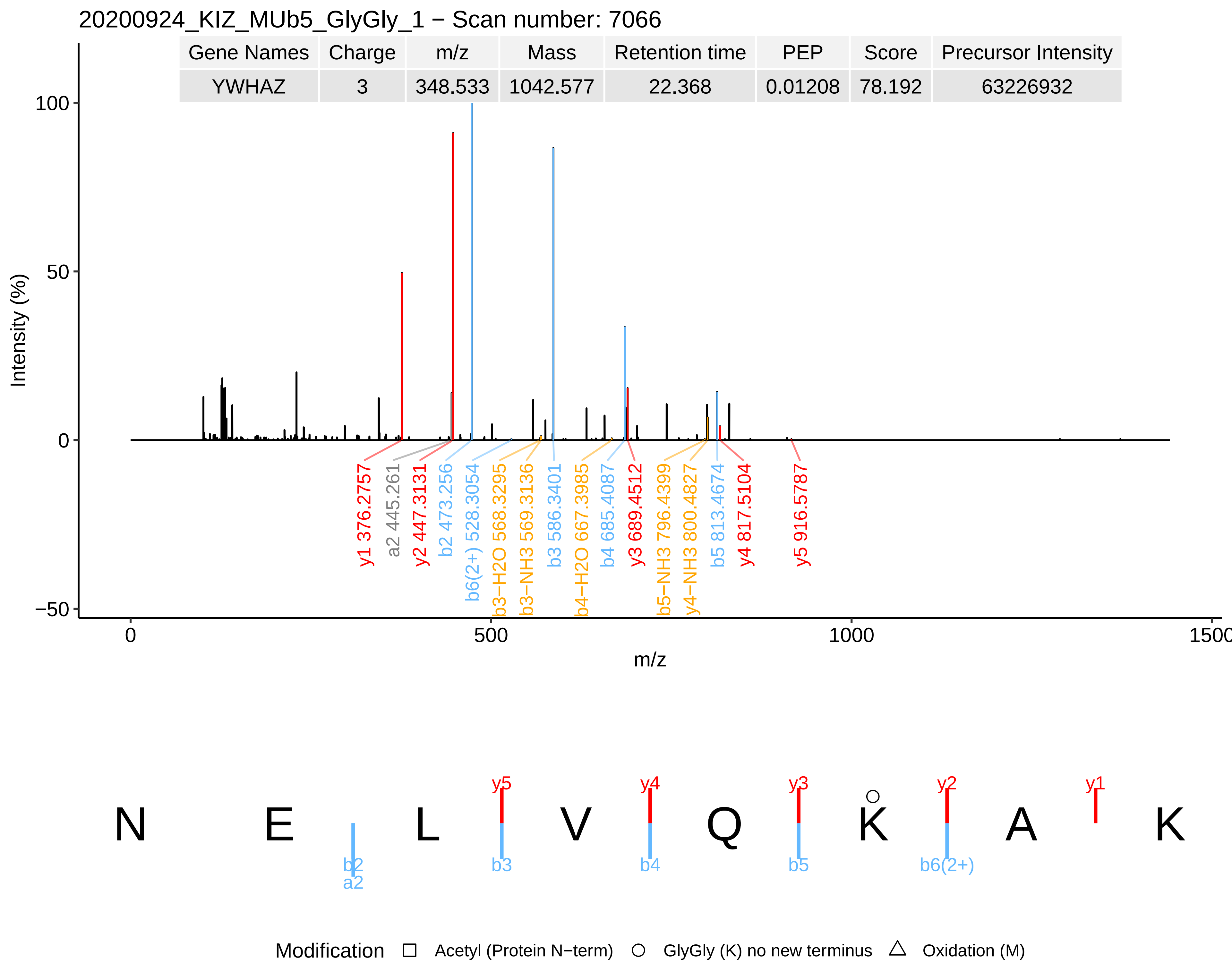
20200924_KIZ_MUb5_GlyGly_1 − Scan number: 7066
Gene Names | Charge | m/z | Mass | Retention time | PEP | Score | Precursor Intensity
YWHAZ | 3 | 348.533 | 1042.577 | 22.368 | 0.01208 | 78.192 | 63226932
Intensity (%)
100
50
0
−50
0
500
1000
1500
m/z
y1 376.2757
a2 445.261
y2 447.3131
b2 473.256
b6(2+) 528.3054
b3−H2O 568.3295
b3−NH3 569.3136
b3 586.3401
b4−H2O 667.3985
b4 685.4087
y3 689.4512
b5−NH3 796.4399
y4−NH3 800.4827
b5 813.4674
y4 817.5104
y5 916.5787
N E L V Q K A K
y5 y4 y3 y2 y1
b2 a2 b3 b4 b5 b6(2+)
Modification
Acetyl (Protein N−term)
GlyGly (K) no new terminus
Oxidation (M)

20200924_KIZ_MUb5_GlyGly_1 − Scan number: 7220

Gene Names	Charge	m/z	Mass	Retention time	PEP	Score	Precursor Intensity
YWHAZ	3	348.533	1042.577	22.886	0.09938	46.462	13763200

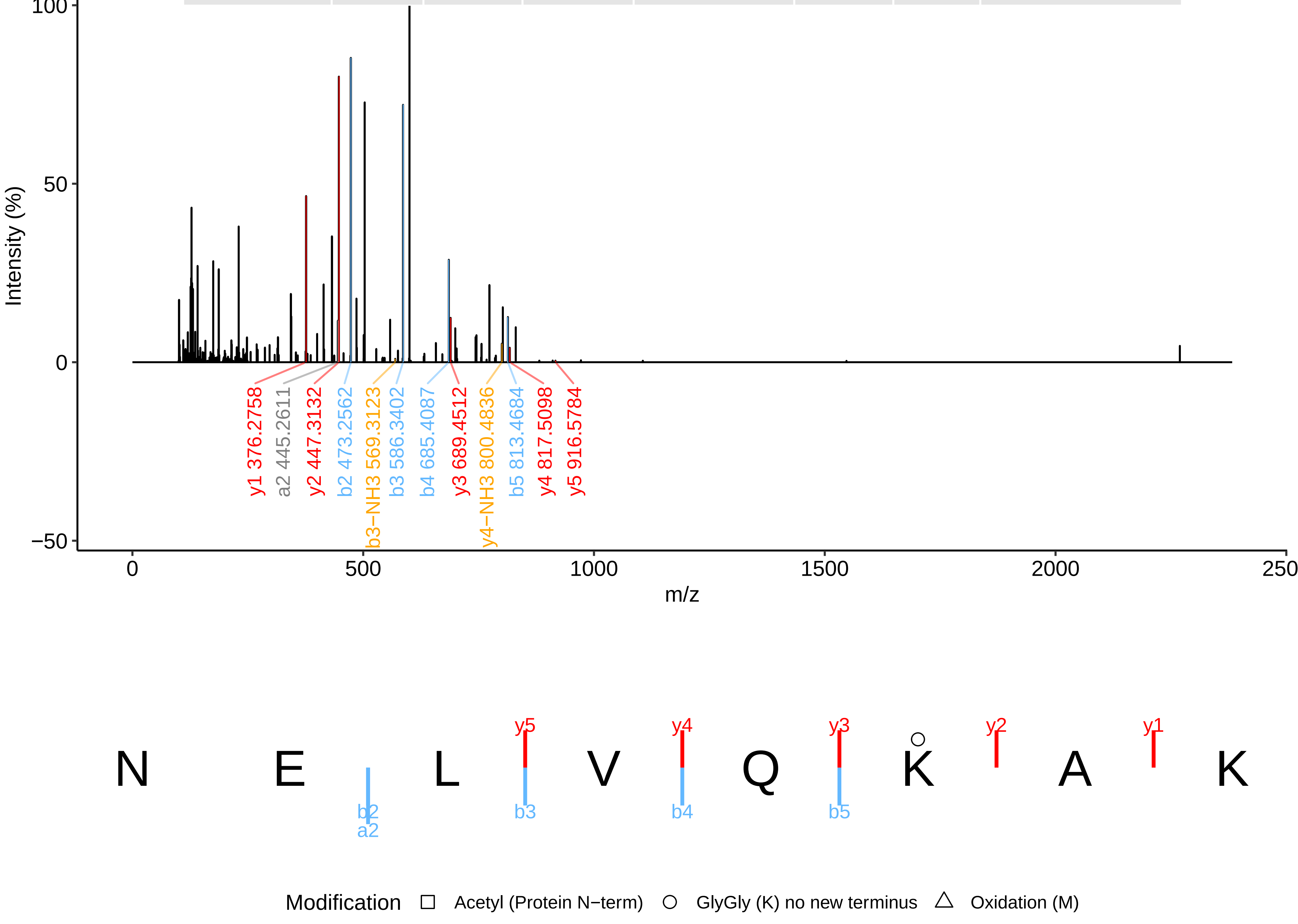

20200924_KIZ_MUb5_GlyGly_2 − Scan number: 6415

Gene Names	Charge	m/z	Mass	Retention time	PEP	Score	Precursor Intensity
YWHAZ	3	348.533	1042.577	23.922	0.049245	58.676	2925799

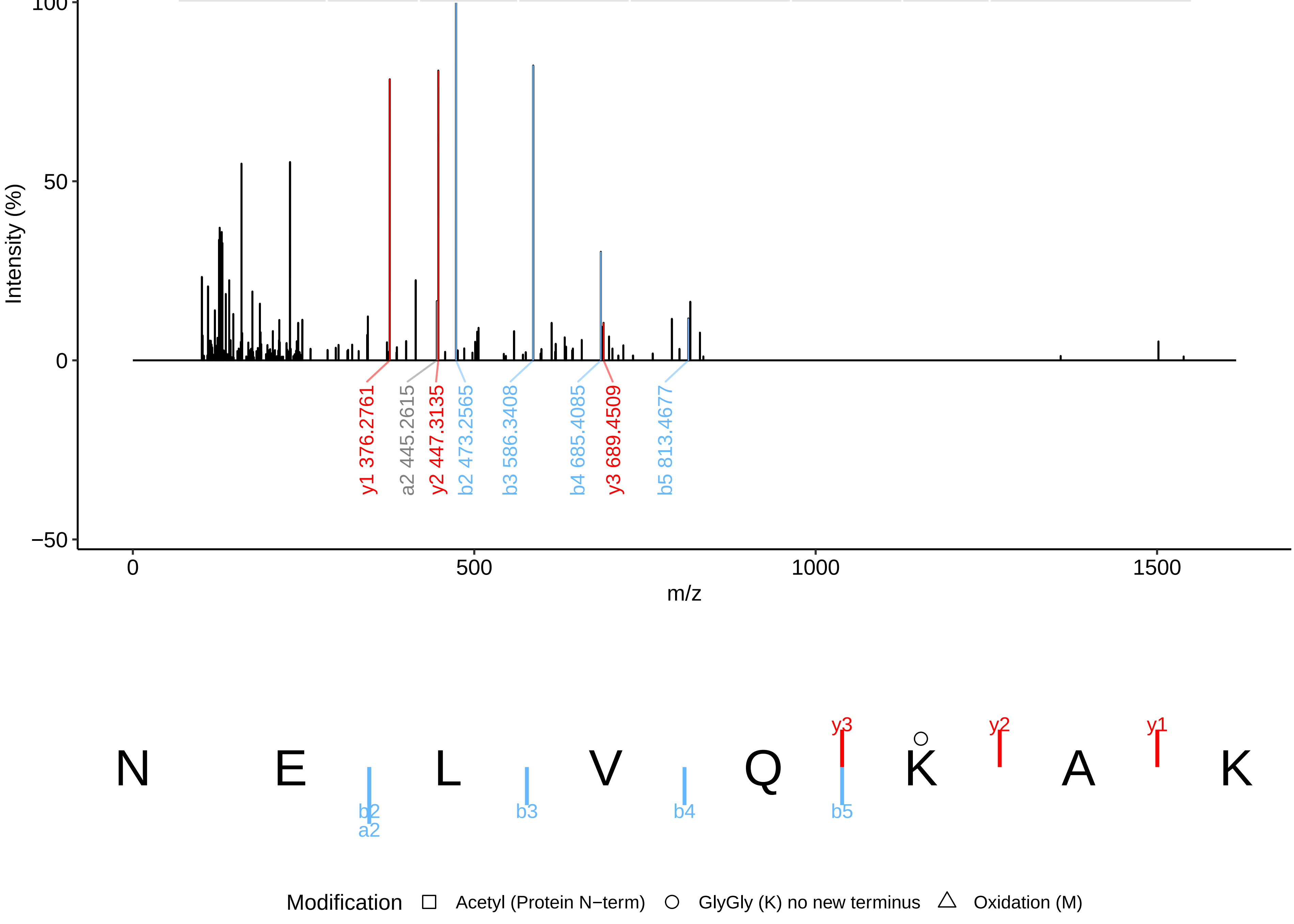

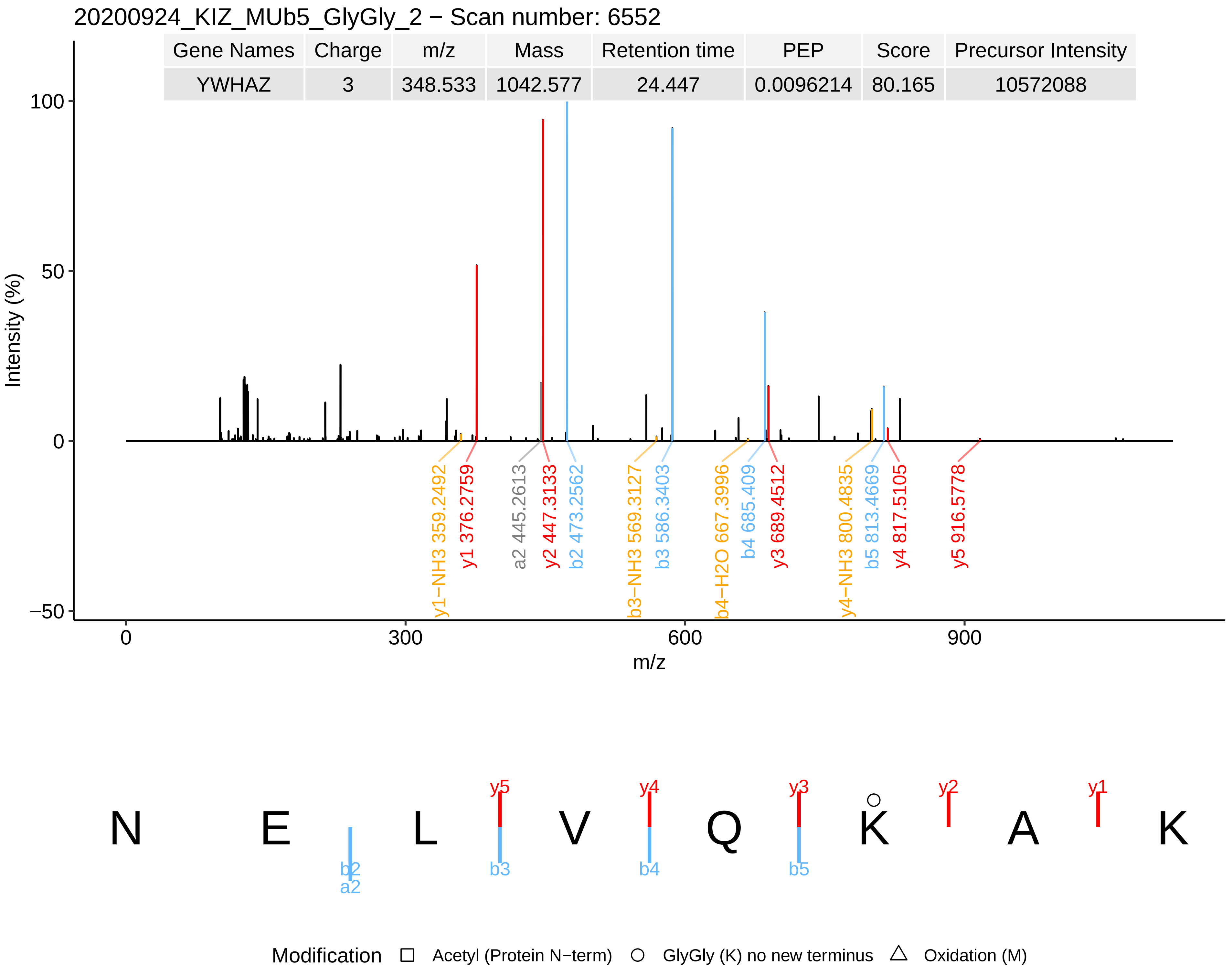
20200924_KIZ_MUb5_GlyGly_2 − Scan number: 6552
Gene Names | Charge | m/z | Mass | Retention time | PEP | Score | Precursor Intensity
YWHAZ | 3 | 348.533 | 1042.577 | 24.447 | 0.0096214 | 80.165 | 10572088
Intensity (%)
100
50
0
−50
0
300
600
900
m/z
y1−NH3 359.2492
y1 376.2759
a2 445.2613
y2 447.3133
b2 473.2562
b3−NH3 569.3127
b3 586.3403
b4−H2O 667.3996
b4 685.409
y3 689.4512
y4−NH3 800.4835
b5 813.4669
y4 817.5105
y5 916.5778
N E L V Q K A K
y5 y4 y3 y2 y1
b2 a2 b3 b4 b5
Modification
Acetyl (Protein N−term)
GlyGly (K) no new terminus
Oxidation (M)

20200924_KIZ_MUb5_GlyGly_2 − Scan number: 6686

Gene Names	Charge	m/z	Mass	Retention time	PEP	Score	Precursor Intensity
YWHAZ	3	348.533	1042.577	24.951	0.023593	68.95	10765971

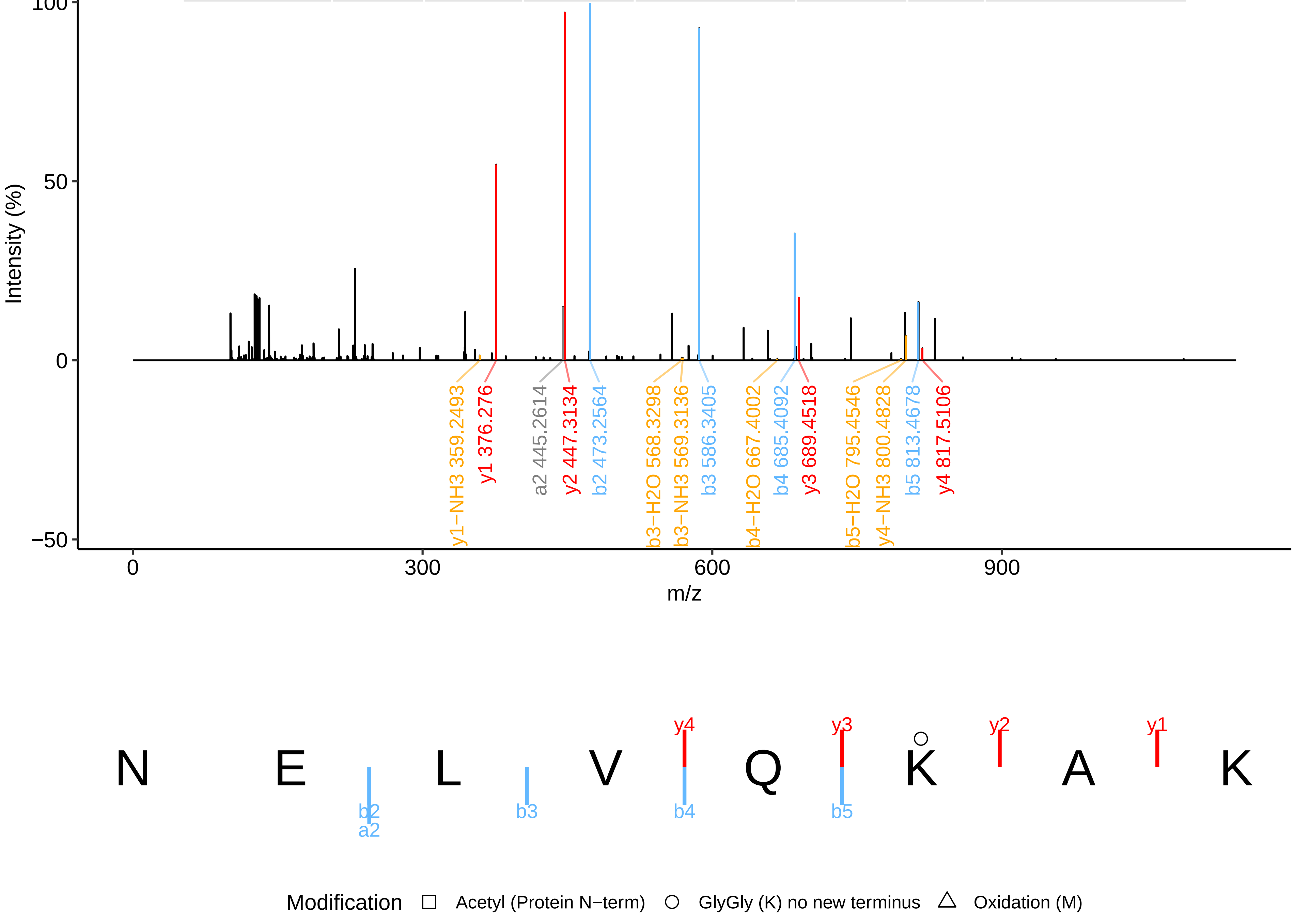

20201118_KIZ_MUb6_GlyGly_2 − Scan number: 8534

Gene Names	Charge	m/z	Mass	Retention time	PEP	Score	Precursor Intensity
YWHAZ	3	348.533	1042.577	28.994	0.053669	57.598	4457944

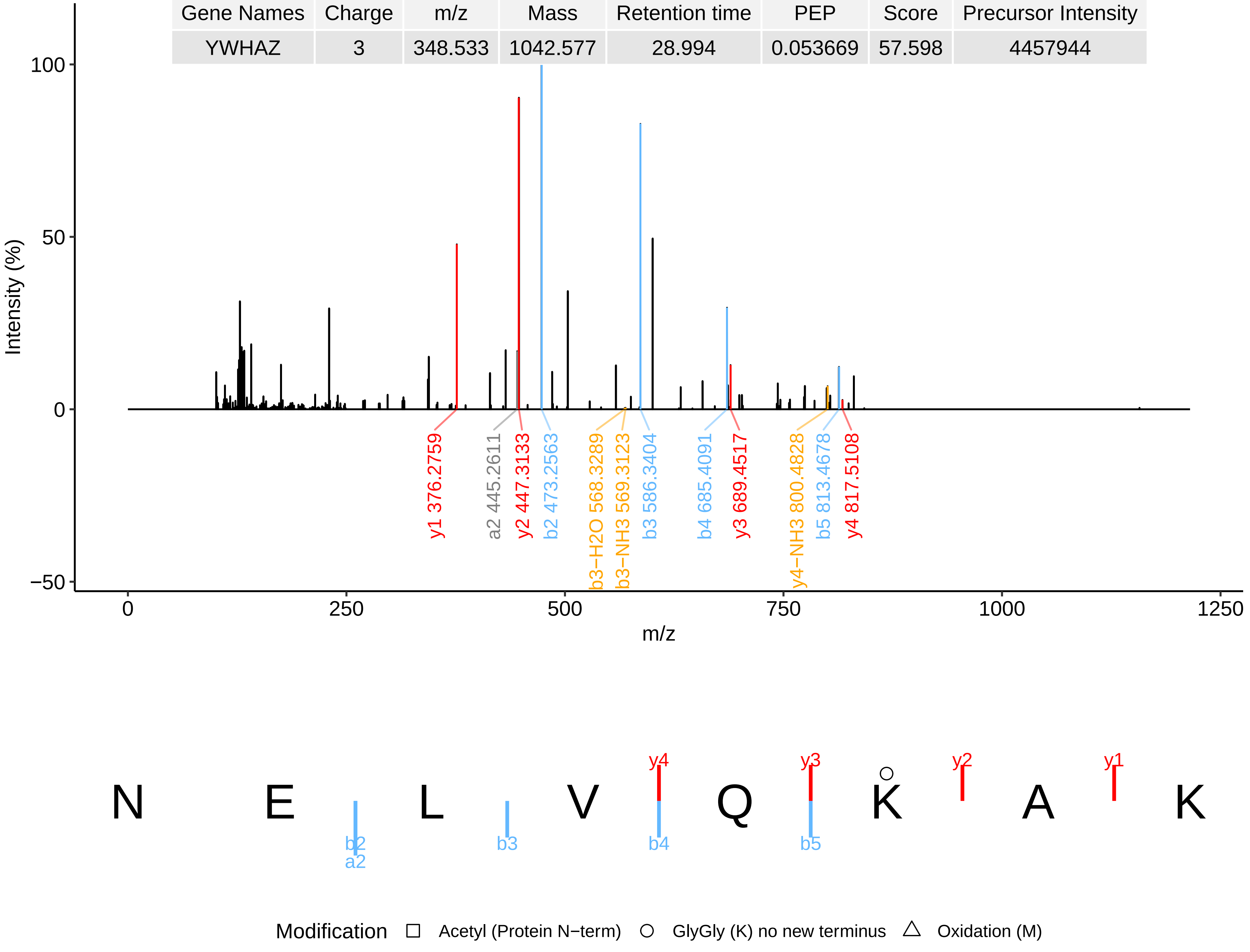

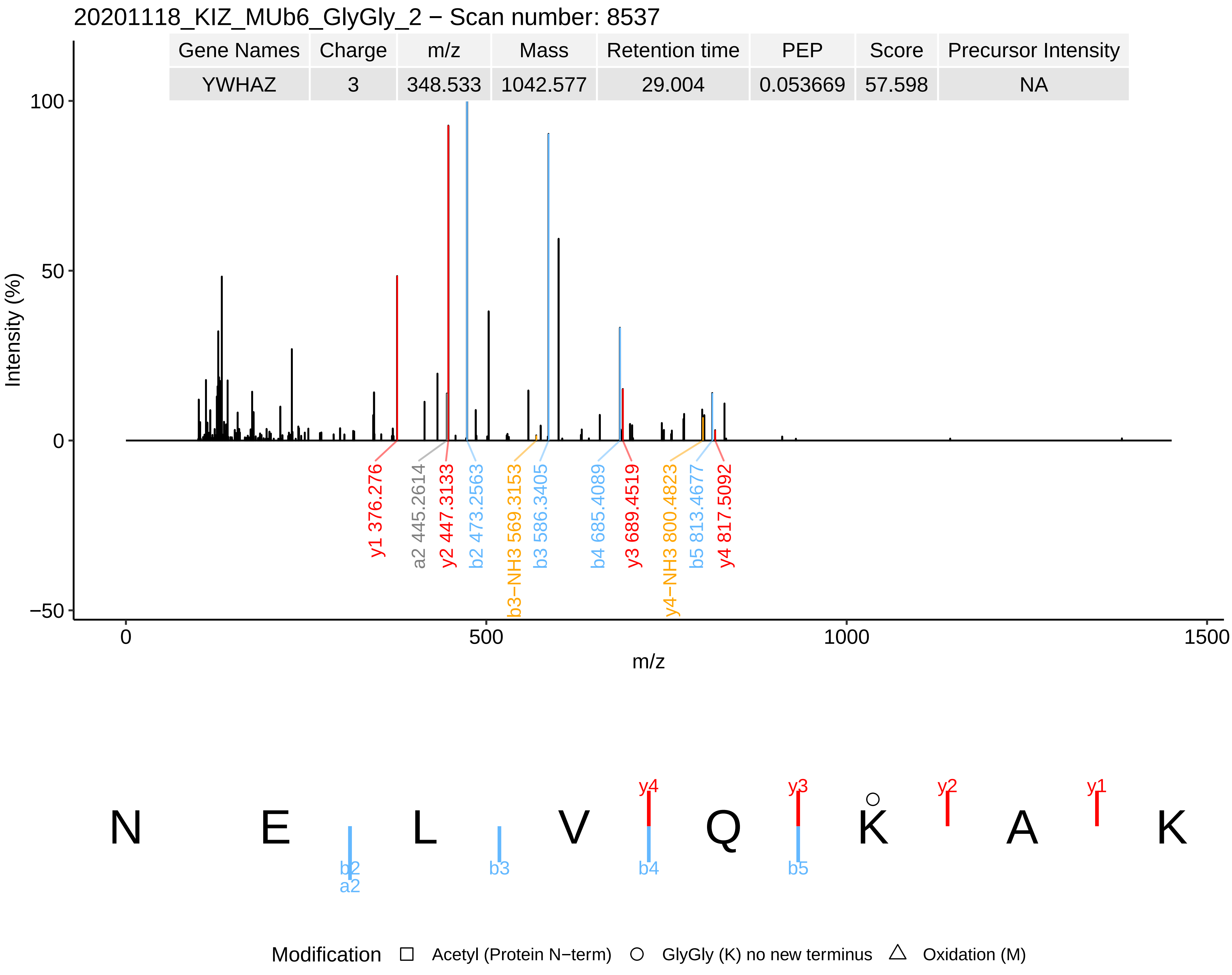
20201118_KIZ_MUb6_GlyGly_2 − Scan number: 8537
Gene Names | Charge | m/z | Mass | Retention time | PEP | Score | Precursor Intensity
YWHAZ | 3 | 348.533 | 1042.577 | 29.004 | 0.053669 | 57.598 | NA
Intensity (%)
100
50
0
−50
y1 376.276
a2 445.2614
y2 447.3133
b2 473.2563
b3−NH3 569.3153
b3 586.3405
b4 685.4089
y3 689.4519
y4−NH3 800.4823
b5 813.4677
y4 817.5092
0
500
1000
1500
m/z
N E L V Q K A K
y4 y3 y2 y1
b2 a2 b3 b4 b5
Modification
Acetyl (Protein N−term)
GlyGly (K) no new terminus
Oxidation (M)

20200921_KIZ_MUb4_GlyGly_10plex − Scan number: 16770

Gene Names	Charge	m/z	Mass	Retention time	PEP	Score	Precursor Intensity
RPL7	3	359.5415	1075.603	60.35	0.04691	59.245	4195917

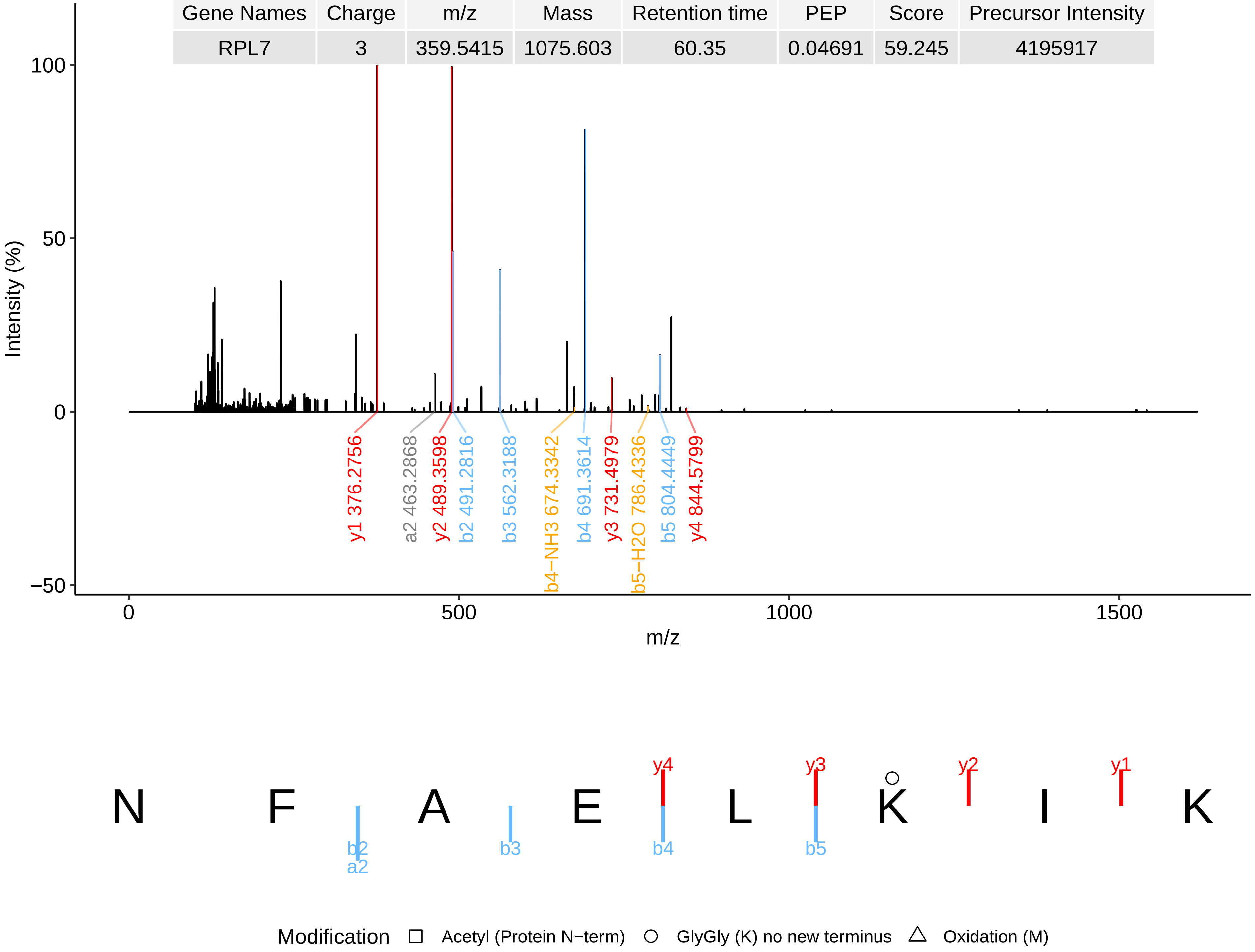

20200921_KIZ_MUb4_GlyGly_10plex − Scan number: 7774

Gene Names	Charge	m/z	Mass	Retention time	PEP	Score	Precursor Intensity
ACSL4	4	326.1704	1300.652	26.211	0.050713	54.982	3089805

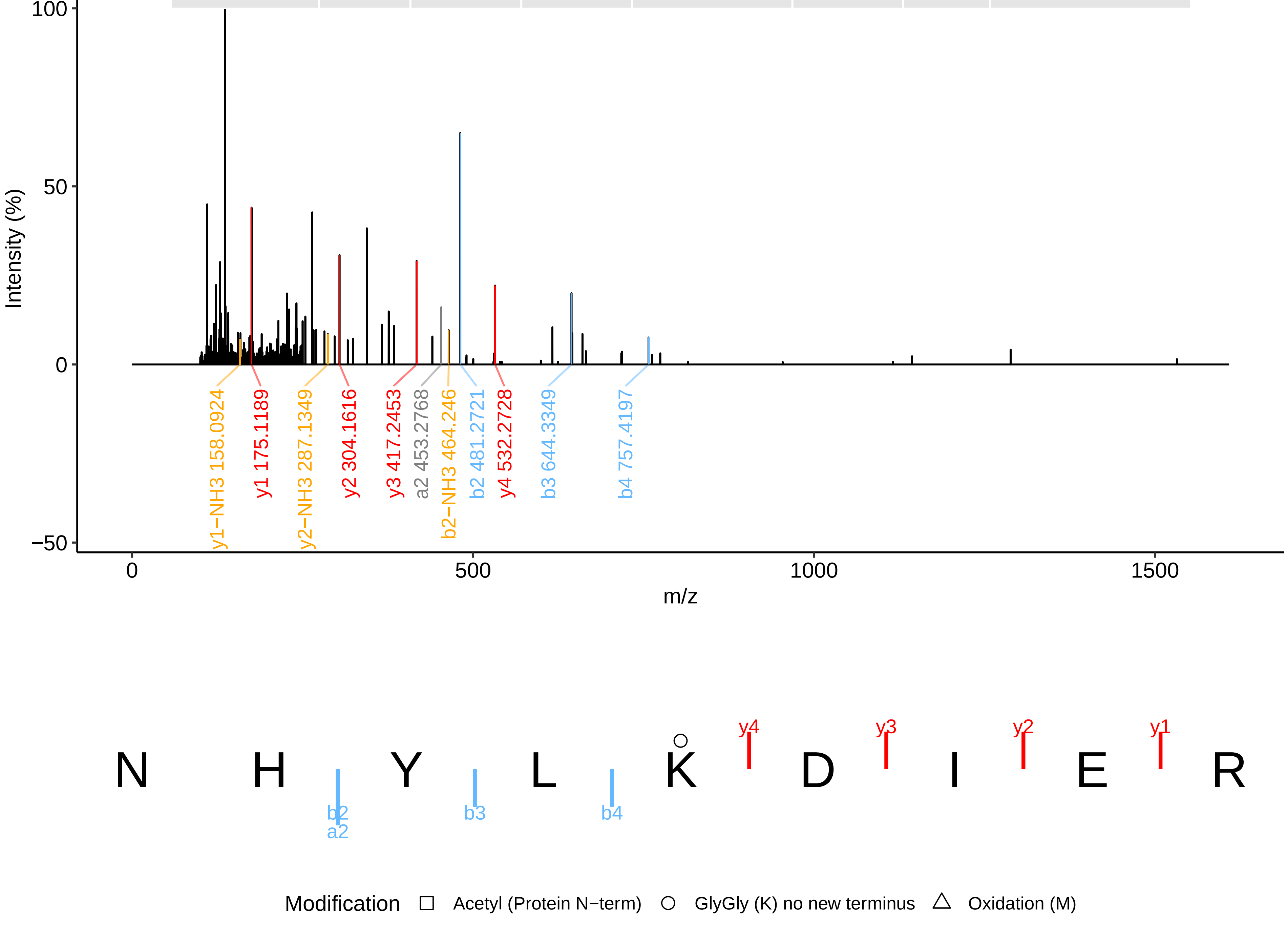

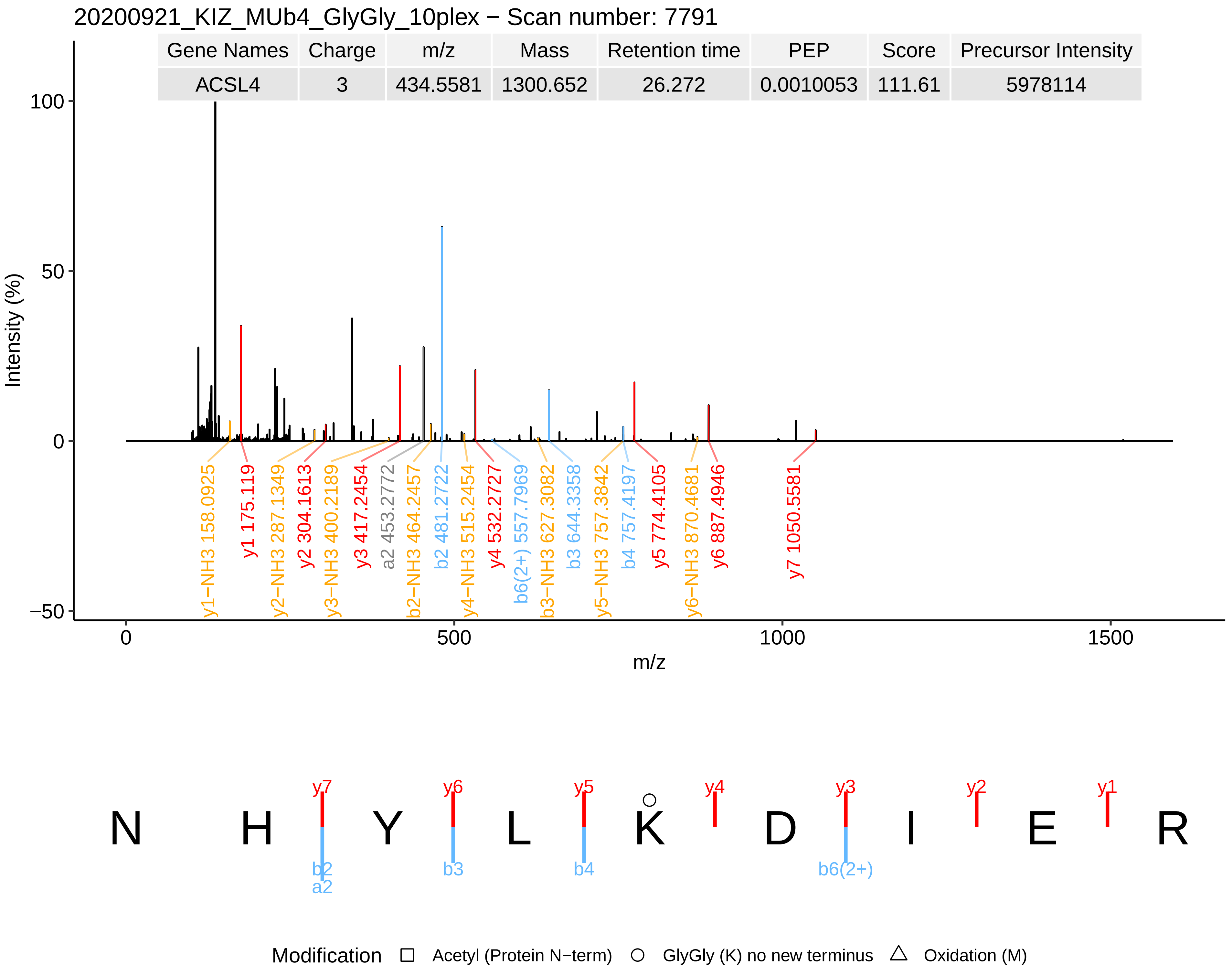

20200921_KIZ_MUb4_GlyGly_10plex − Scan number: 7791
Gene Names | Charge | m/z | Mass | Retention time | PEP | Score | Precursor Intensity
ACSL4 | 3 | 434.5581 | 1300.652 | 26.272 | 0.0010053 | 111.61 | 5978114
Intensity (%)
m/z
y1−NH3 158.0925
y1 175.119
y2−NH3 287.1349
y2 304.1613
y3−NH3 400.2189
y3 417.2454
a2 453.2772
b2−NH3 464.2457
b2 481.2722
y4−NH3 515.2454
y4 532.2727
b6(2+) 557.7969
b3−NH3 627.3082
b3 644.3358
y5−NH3 757.3842
b4 757.4197
y5 774.4105
y6−NH3 870.4681
y6 887.4946
y7 1050.5581
N H Y L K D I E R
y7 y6 y5 y4 y3 y2 y1
b2 a2 b3 b4 b6(2+)
Modification
Acetyl (Protein N−term)
GlyGly (K) no new terminus
Oxidation (M)

20200921_KIZ_MUb4_GlyGly_10plex − Scan number: 3148

Gene Names	Charge	m/z	Mass	Retention time	PEP	Score	Precursor Intensity
CPNE3	3	343.521	1027.541	9.8626	0.0090595	80.684	5591594

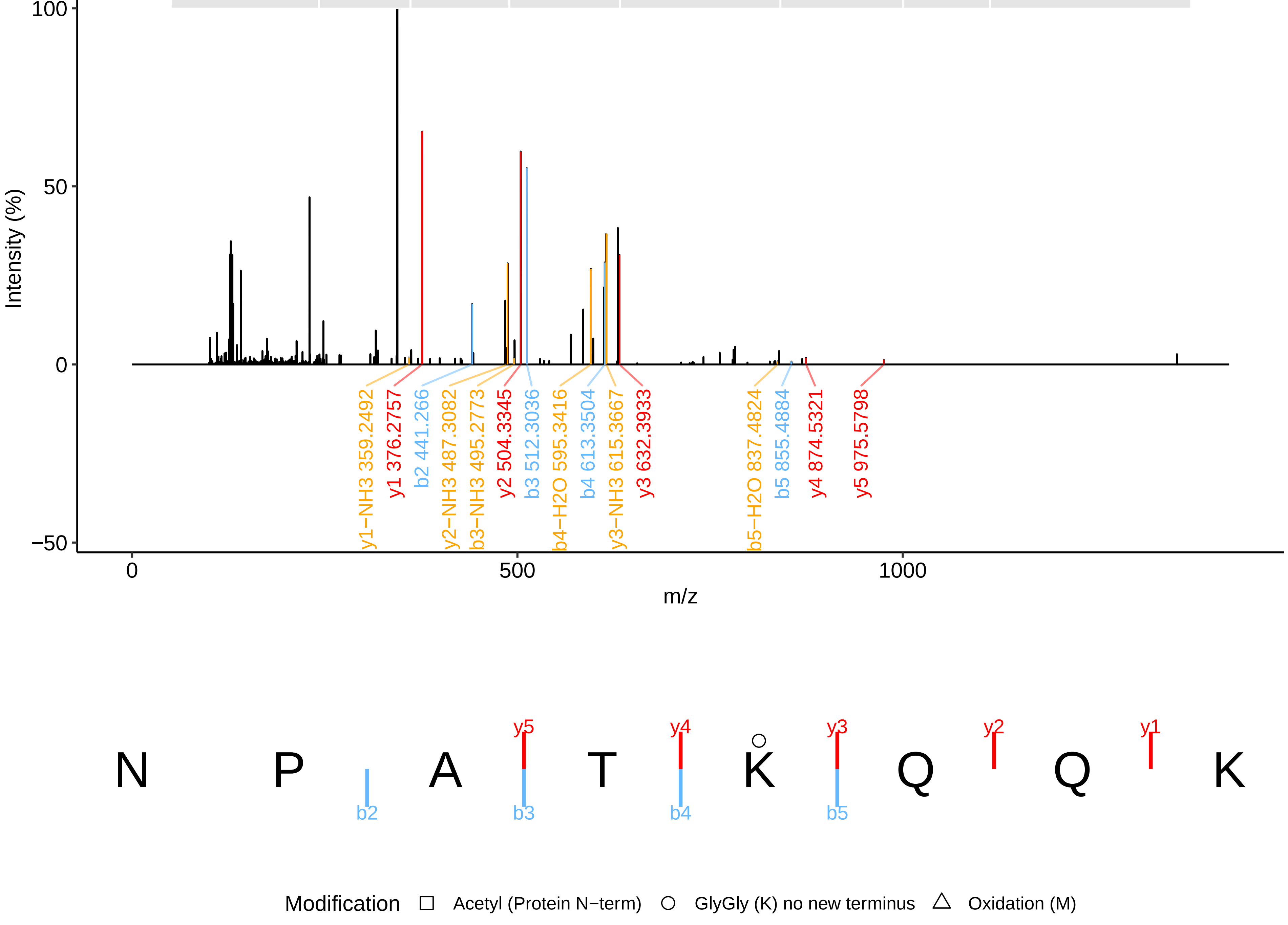

20200921_KIZ_MUb4_GlyGly_10plex_2 − Scan number: 3060

Gene Names	Charge	m/z	Mass	Retention time	PEP	Score	Precursor Intensity
CPNE3	3	343.521	1027.541	10.064	0.055397	57.177	4286773

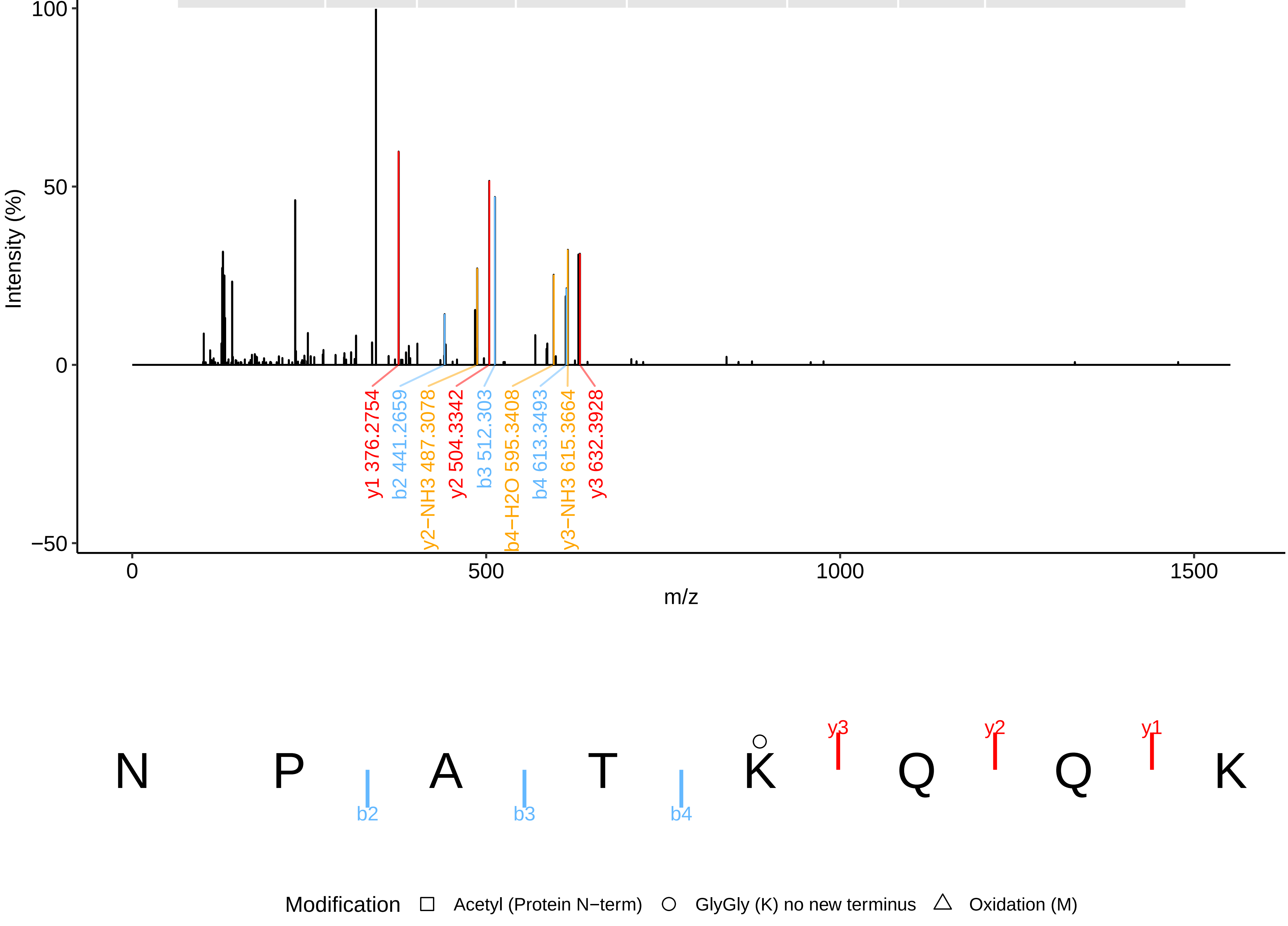

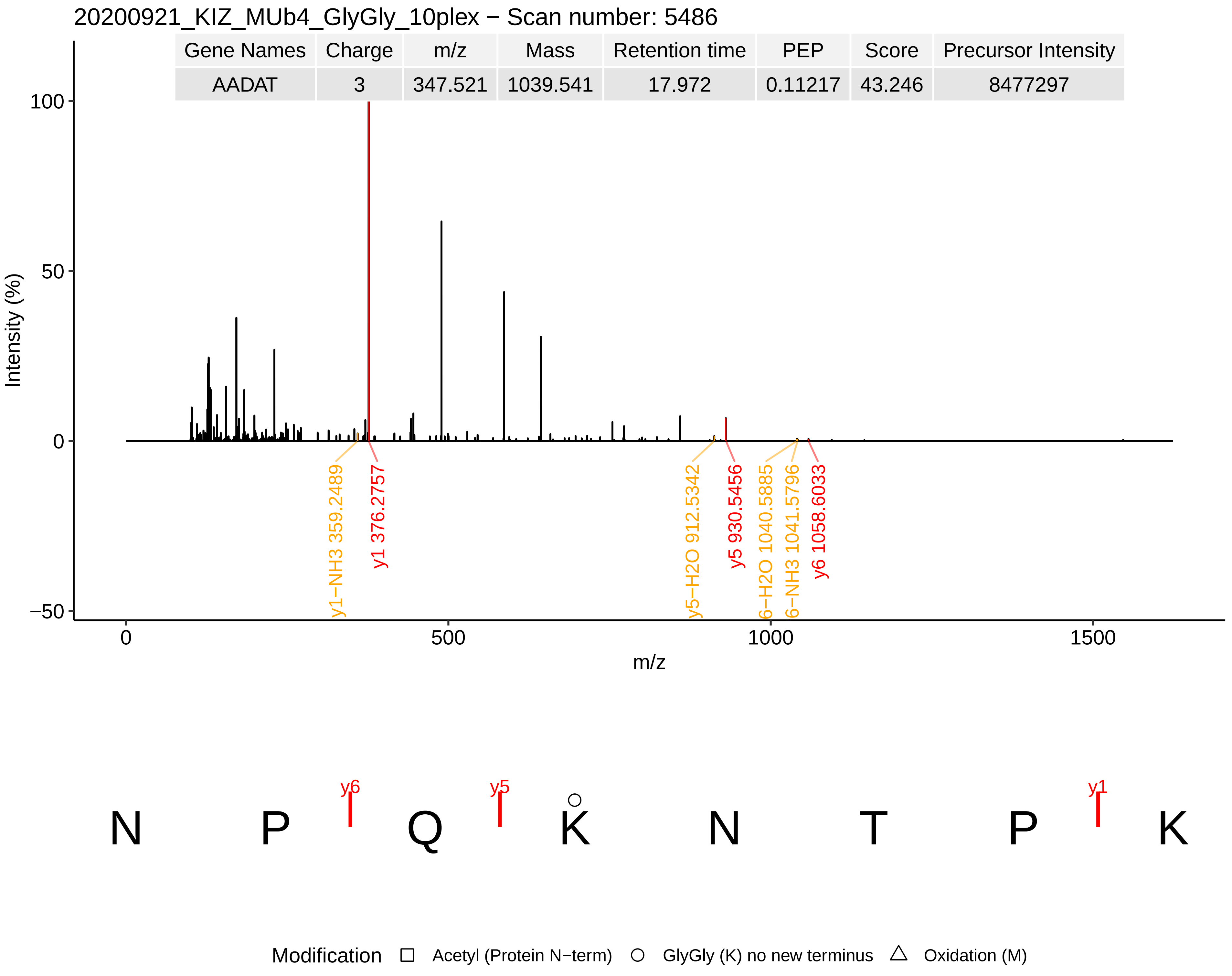

20200921_KIZ_MUb4_GlyGly_10plex − Scan number: 5486
Gene Names | Charge | m/z | Mass | Retention time | PEP | Score | Precursor Intensity
AADAT | 3 | 347.521 | 1039.541 | 17.972 | 0.11217 | 43.246 | 8477297
Intensity (%)
100
50
0
−50
0
500
1000
1500
m/z
y1−NH3 359.2489
y1 376.2757
y5−H2O 912.5342
y5 930.5456
6−H2O 1040.5885
6−NH3 1041.5796
y6 1058.6033
N P Q K N T P K
y6
y5
y1
Modification
Acetyl (Protein N−term)
GlyGly (K) no new terminus
Oxidation (M)

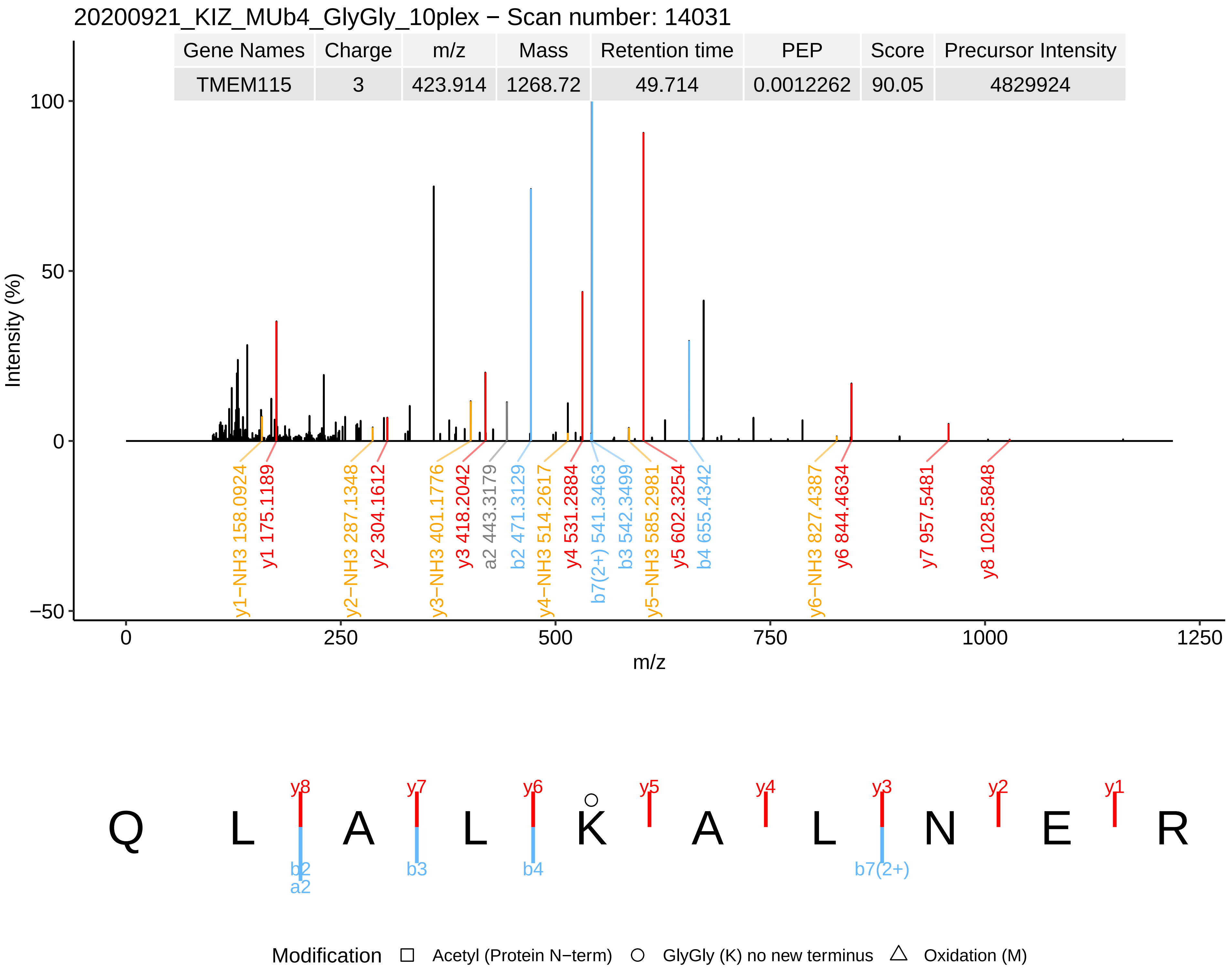
20200921_KIZ_MUb4_GlyGly_10plex − Scan number: 14031
Gene Names
Charge
m/z
Mass
Retention time
PEP
Score
Precursor Intensity
TMEM115
3
423.914
1268.72
49.714
0.0012262
90.05
4829924
Intensity (%)
100
50
0
−50
0
250
500
750
1000
1250
m/z
y1−NH3 158.0924
y1 175.1189
y2−NH3 287.1348
y2 304.1612
y3−NH3 401.1776
y3 418.2042
a2 443.3179
b2 471.3129
y4−NH3 514.2617
y4 531.2884
b7(2+) 541.3463
b3 542.3499
y5−NH3 585.2981
y5 602.3254
b4 655.4342
y6−NH3 827.4387
y6 844.4634
y7 957.5481
y8 1028.5848
Q L A L K A L N E R
y8
y7
y6
y5
y4
y3
y2
y1
b2
a2
b3
b4
b7(2+)
Modification
Acetyl (Protein N−term)
GlyGly (K) no new terminus
Oxidation (M)

20200921_KIZ_MUb4_GlyGly_10plex_2 − Scan number: 6383

Gene Names	Charge	m/z	Mass	Retention time	PEP	Score	Precursor Intensity
MORC3	3	421.5434	1261.609	23.432	0.034878	60.518	3261987

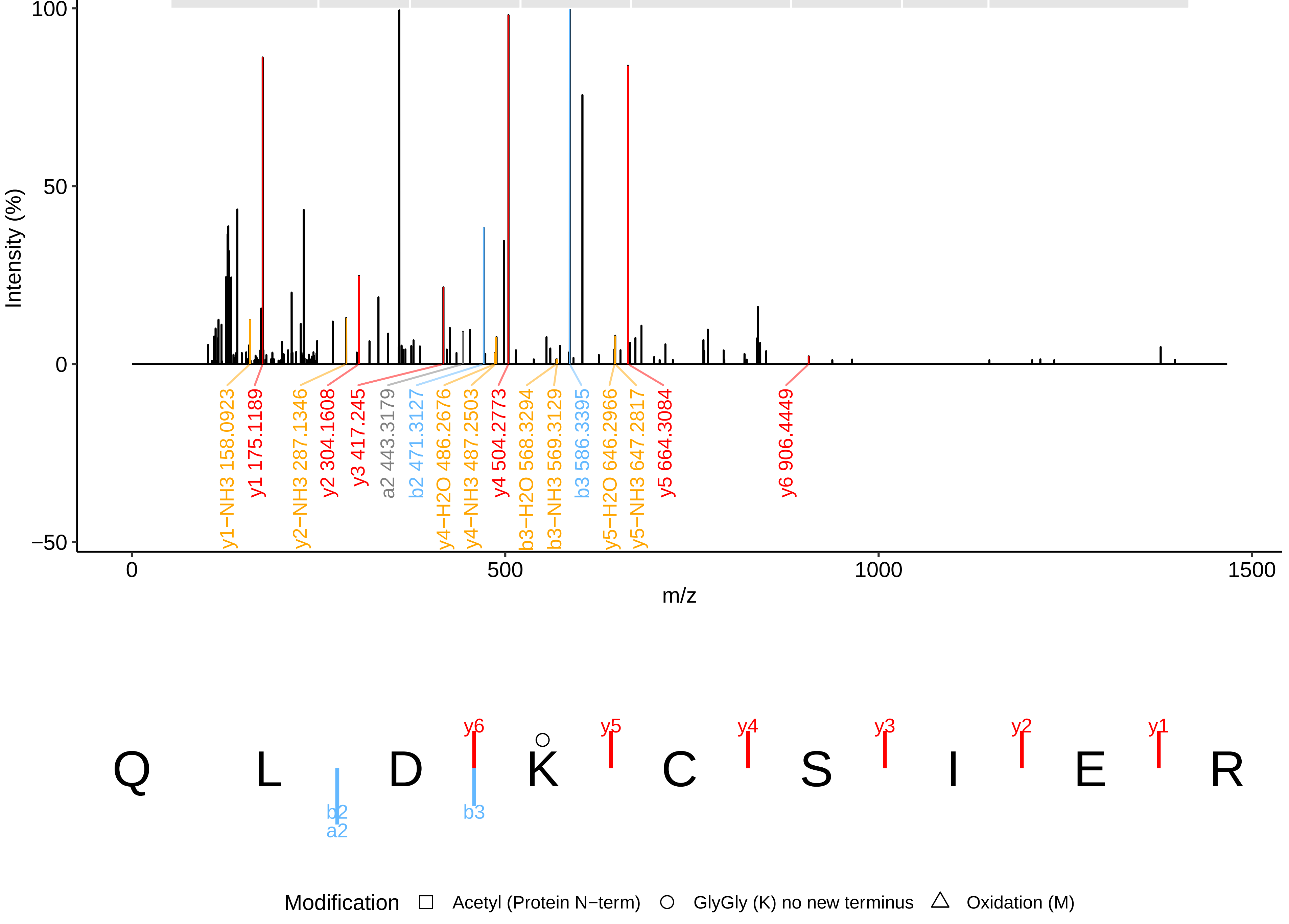

20201118_KIZ_MUb6_GlyGly_1 − Scan number: 5533

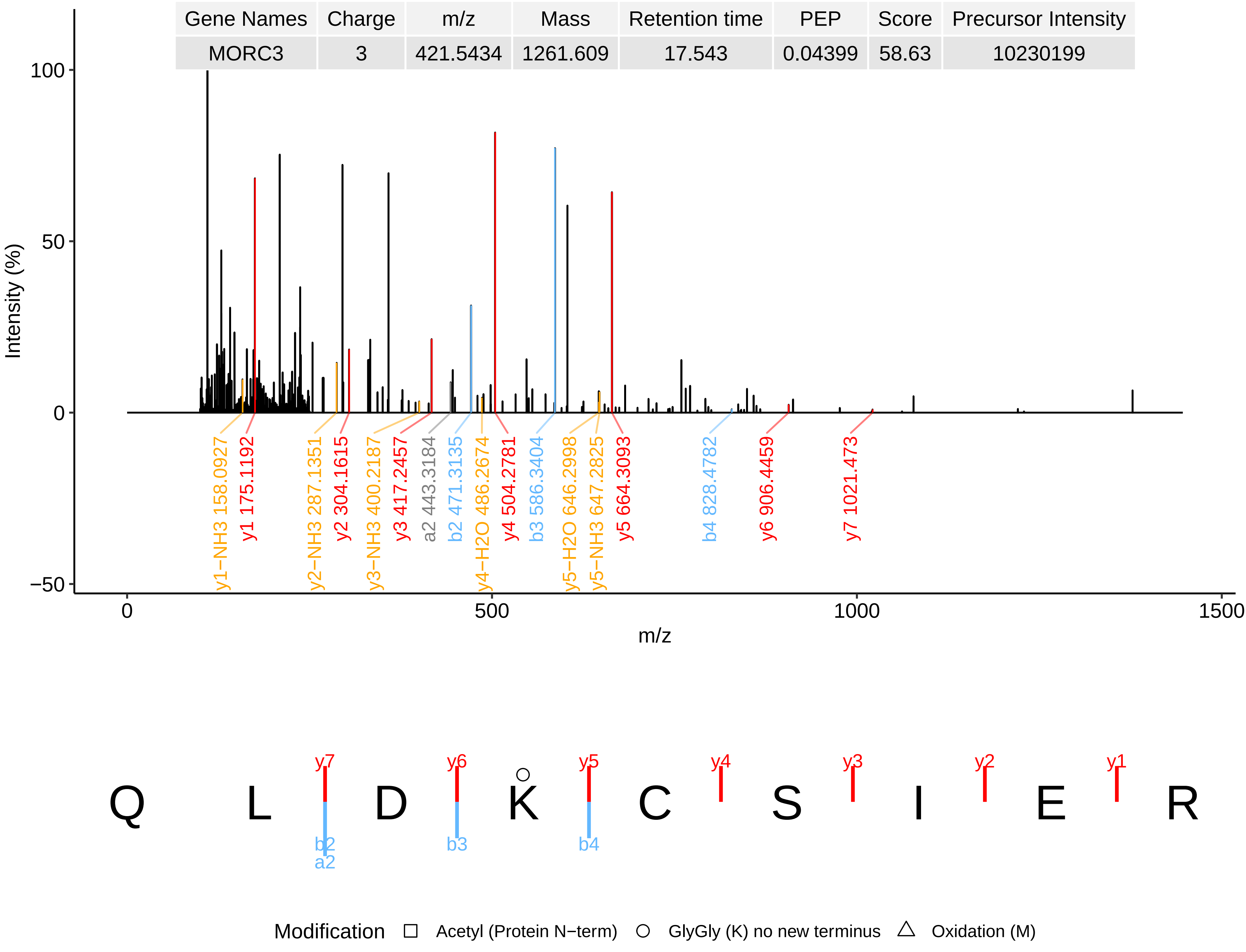

20200921_KIZ_MUb4_GlyGly_10plex − Scan number: 4070

Gene Names	Charge	m/z	Mass	Retention time	PEP	Score	Precursor Intensity
SPG20	3	429.5531	1285.638	13.236	8.7824e−08	129.88	14624458

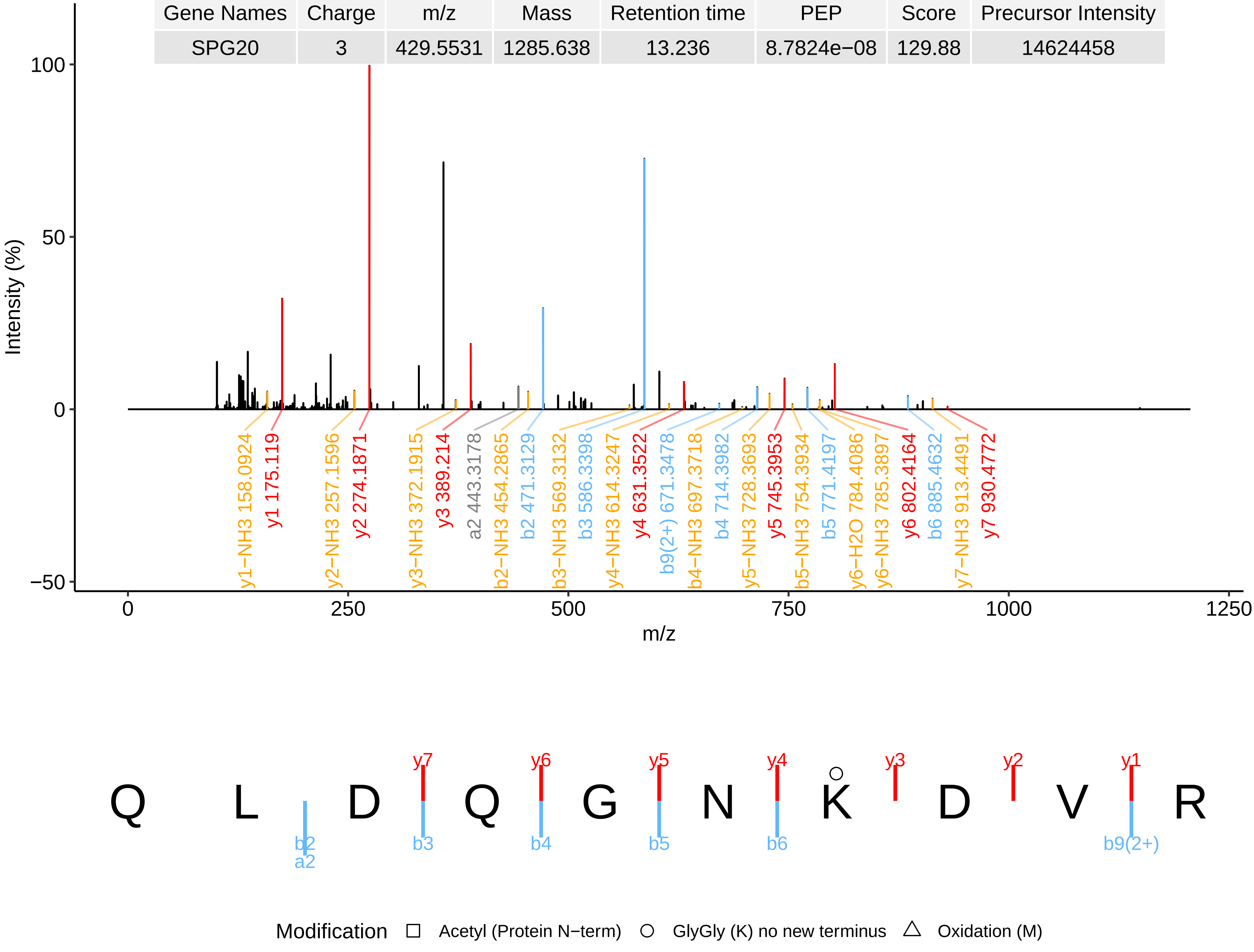

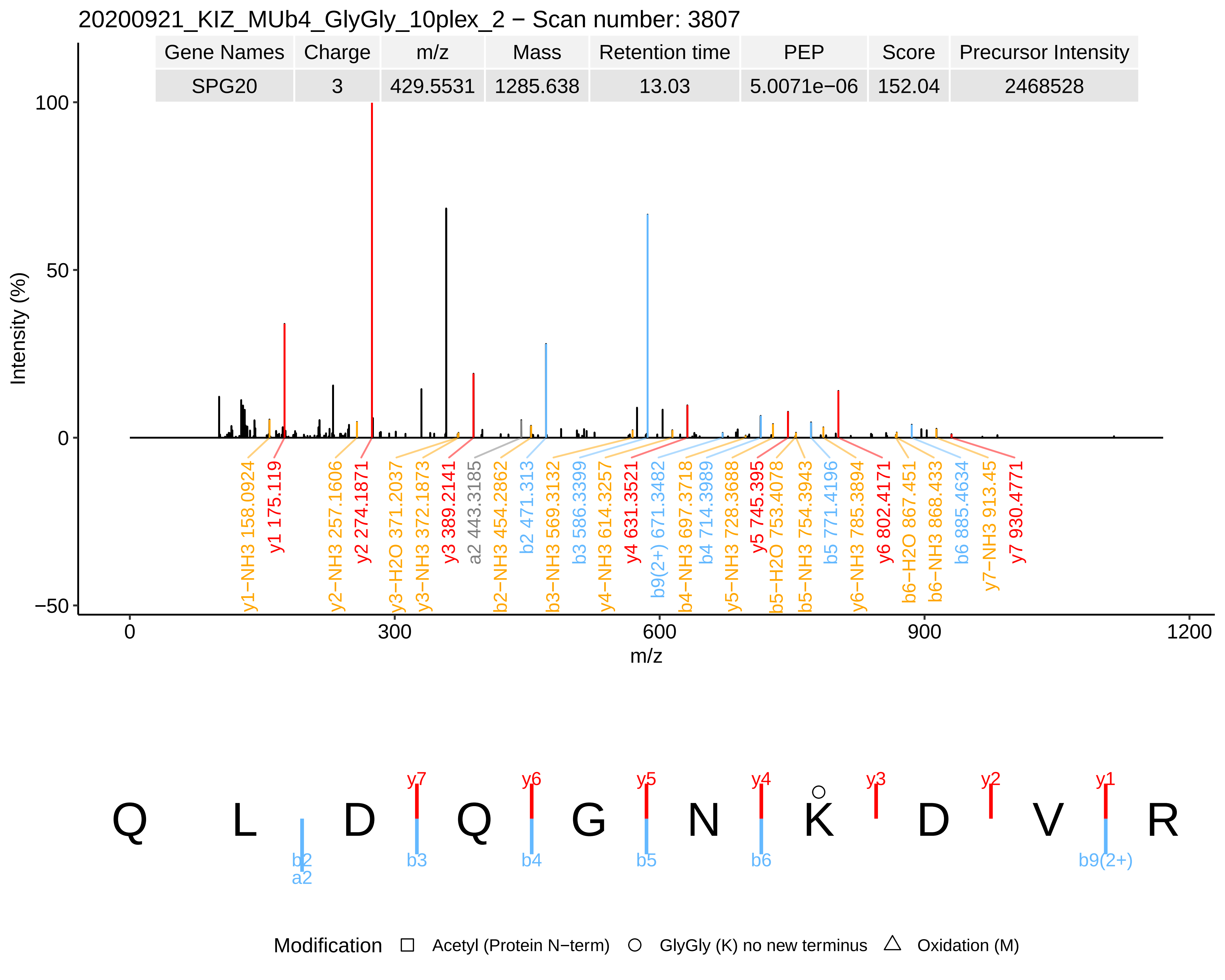
20200921_KIZ_MUb4_GlyGly_10plex_2 − Scan number: 3807
Gene Names | Charge | m/z | Mass | Retention time | PEP | Score | Precursor Intensity
SPG20 | 3 | 429.5531 | 1285.638 | 13.03 | 5.0071e−06 | 152.04 | 2468528
Intensity (%)
m/z
y1−NH3 158.0924
y1 175.119
y2−NH3 257.1606
y2 274.1871
y3−H2O 371.2037
y3−NH3 372.1873
y3 389.2141
a2 443.3185
b2−NH3 454.2862
b2 471.313
b3−NH3 569.3132
b3 586.3399
y4−NH3 614.3257
y4 631.3521
b9(2+) 671.3482
b4−NH3 697.3718
b4 714.3989
y5−NH3 728.3688
y5 745.395
b5−H2O 753.4078
b5−NH3 754.3943
b5 771.4196
y6−NH3 785.3894
y6 802.4171
b6−H2O 867.451
b6−NH3 868.433
b6 885.4634
y7−NH3 913.45
y7 930.4771
Q L D Q G N K D V R
y7 y6 y5 y4 y3 y2 y1
b2 a2 b3 b4 b5 b6 b9(2+)
Modification
Acetyl (Protein N−term)
GlyGly (K) no new terminus
Oxidation (M)

20200924_KIZ_MUb5_GlyGly_1 − Scan number: 3515

Gene Names	Charge	m/z	Mass	Retention time	PEP	Score	Precursor Intensity
SPG20	3	429.5531	1285.638	10.756	4.1938e−07	118.6	16959134

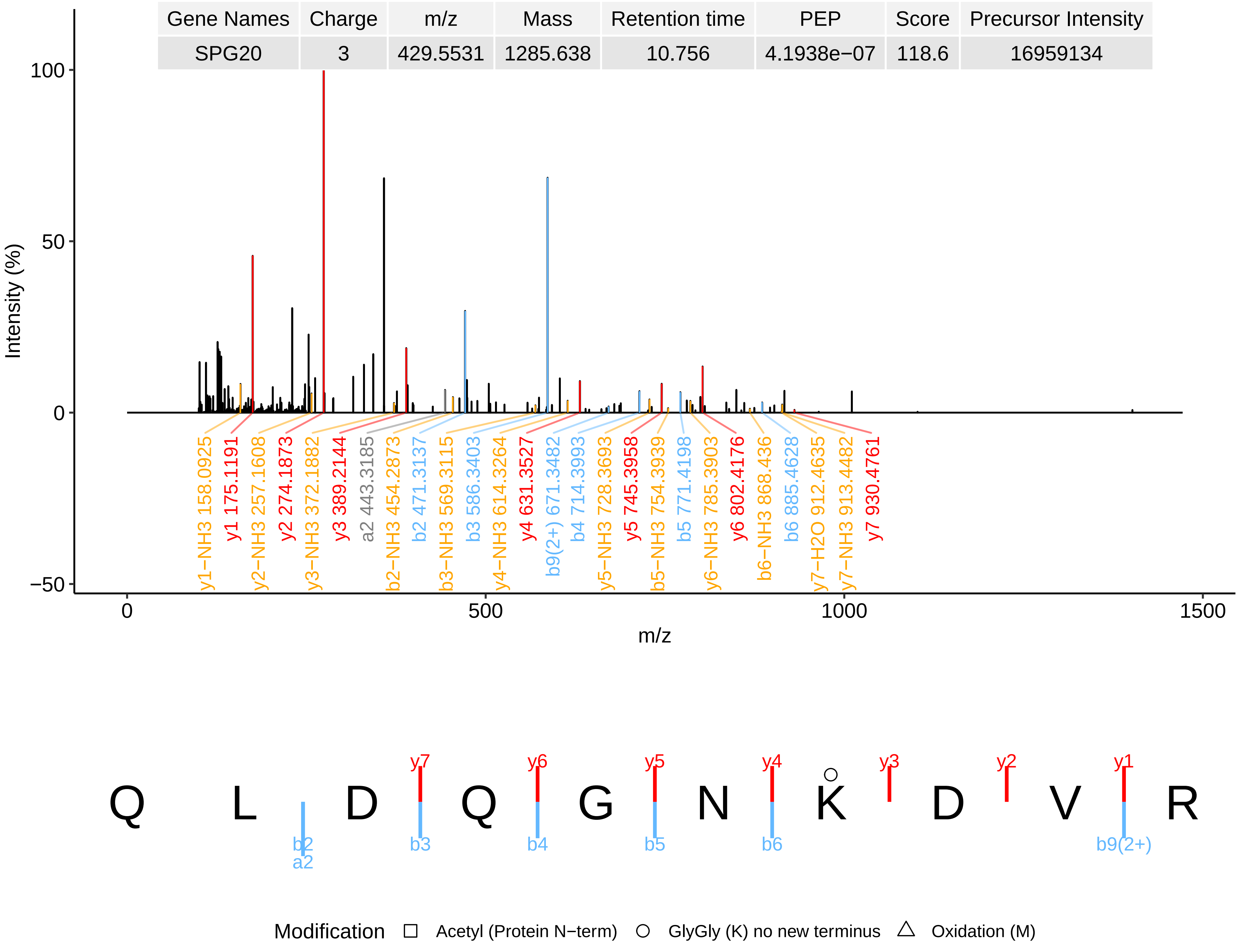

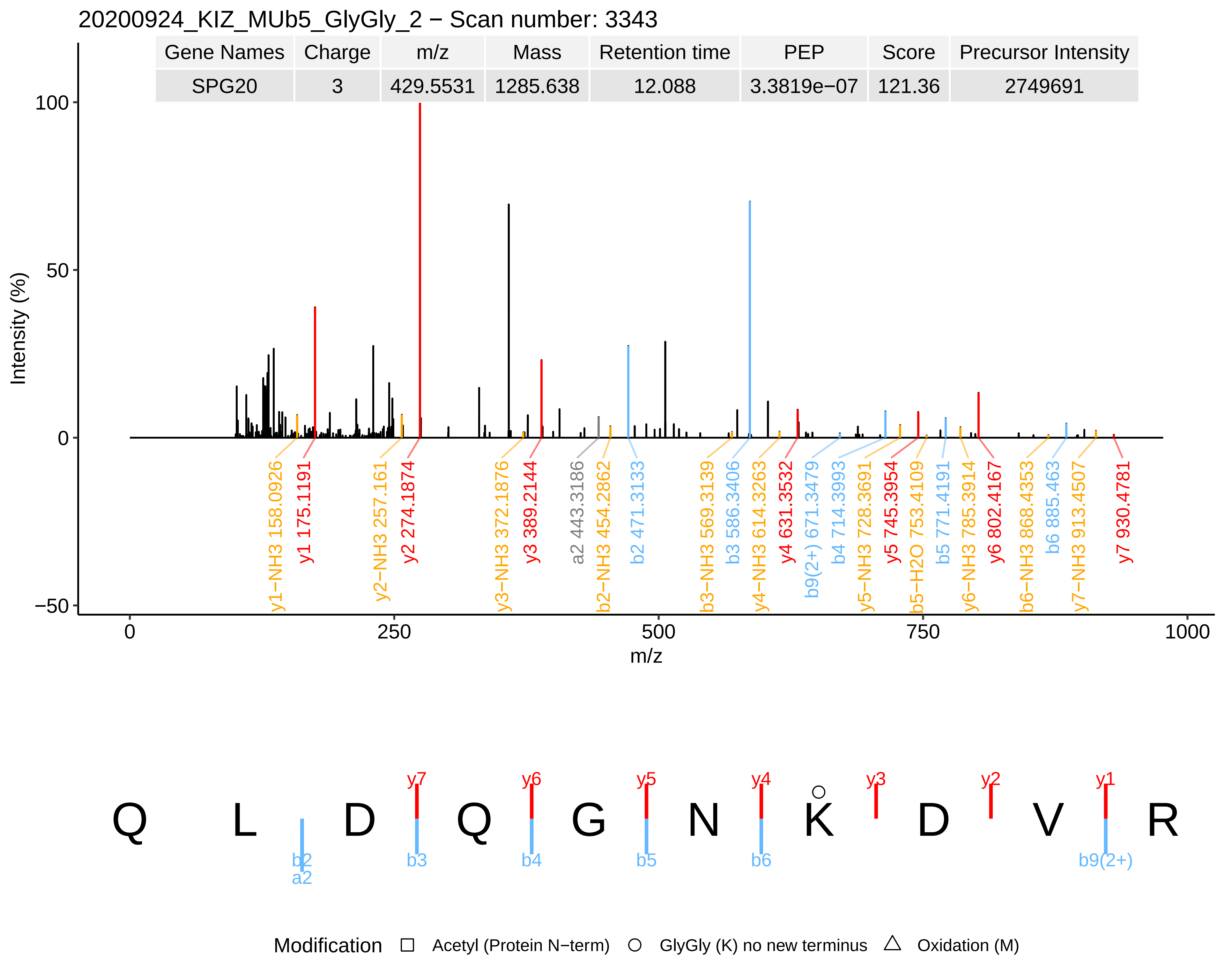

20200924_KIZ_MUb5_GlyGly_2 − Scan number: 3343
Gene Names | Charge | m/z | Mass | Retention time | PEP | Score | Precursor Intensity
SPG20 | 3 | 429.5531 | 1285.638 | 12.088 | 3.3819e−07 | 121.36 | 2749691
Intensity (%)
m/z
y1−NH3 158.0926
y1 175.1191
y2−NH3 257.161
y2 274.1874
y3−NH3 372.1876
y3 389.2144
a2 443.3186
b2−NH3 454.2862
b2 471.3133
b3−NH3 569.3139
b3 586.3406
y4−NH3 614.3263
y4 631.3532
b9(2+) 671.3479
b4 714.3993
y5−NH3 728.3691
y5 745.3954
b5−H2O 753.4109
b5 771.4191
y6−NH3 785.3914
y6 802.4167
b6−NH3 868.4353
b6 885.463
y7−NH3 913.4507
y7 930.4781
Q L D Q G N K D V R
y7 y6 y5 y4 y3 y2 y1
b2 a2 b3 b4 b5 b6 b9(2+)
Modification
Acetyl (Protein N−term)
GlyGly (K) no new terminus
Oxidation (M)

20200921_KIZ_MUb4_GlyGly_10plex − Scan number: 3348

Gene Names	Charge	m/z	Mass	Retention time	PEP	Score	Precursor Intensity
FAF2	3	429.8811	1286.621	10.544	0.0012181	107.15	NA

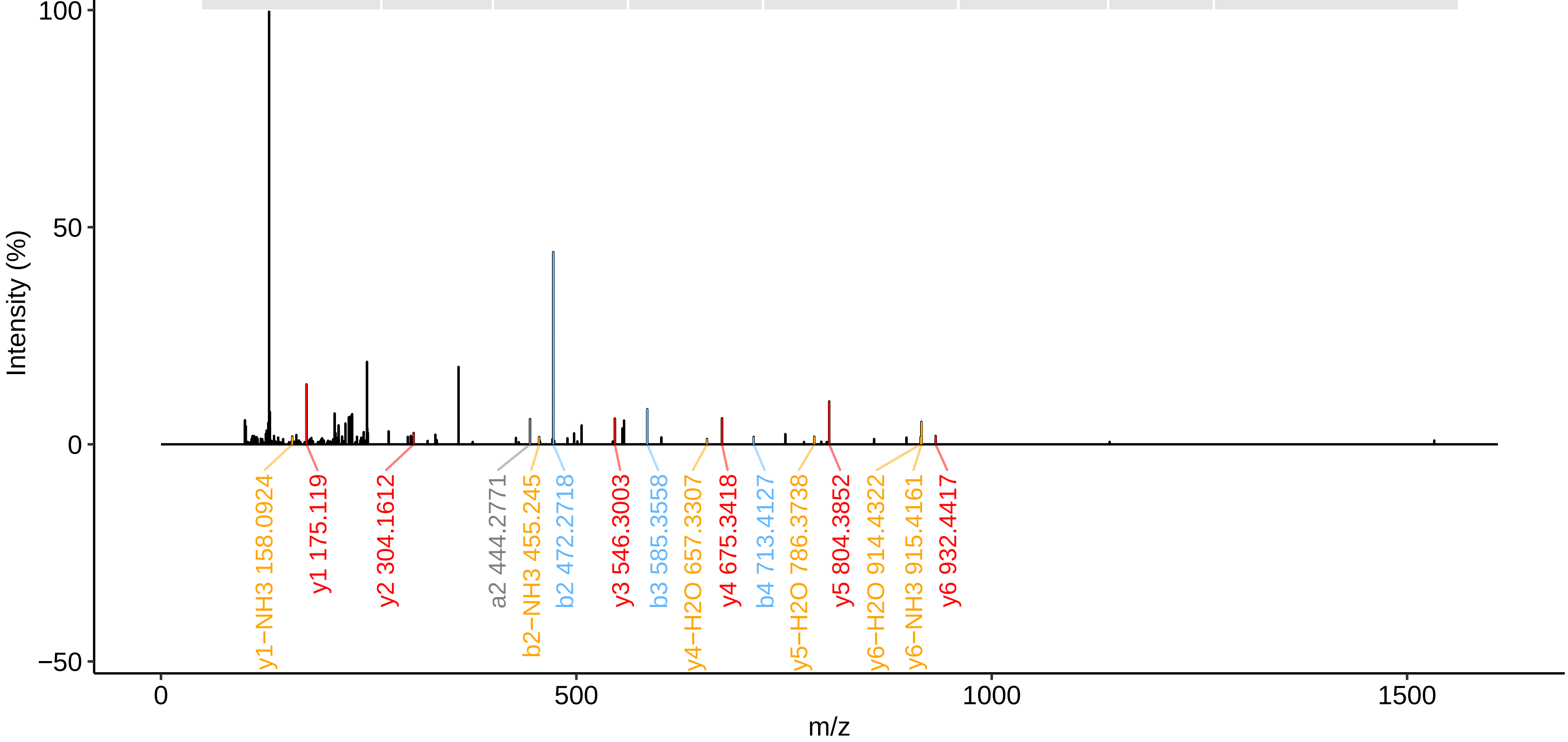

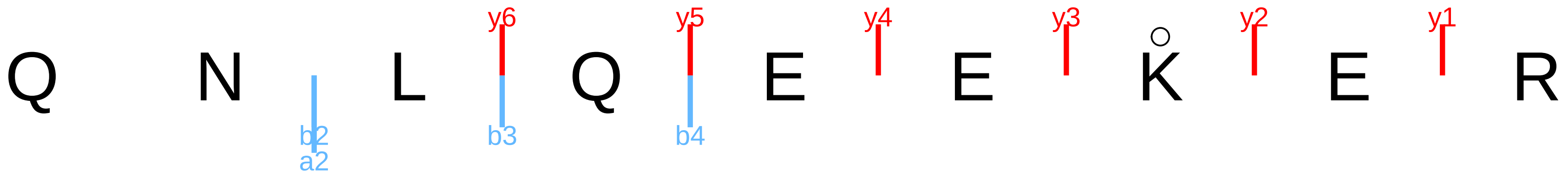

Modification □ Acetyl (Protein N−term) ○ GlyGly (K) no new terminus △ Oxidation (M)

20200921_KIZ_MUb4_GlyGly_10plex_2 − Scan number: 3213

Gene Names	Charge	m/z	Mass	Retention time	PEP	Score	Precursor Intensity
FAF2	3	429.8811	1286.621	10.674	9.0034e−05	113.22	156634

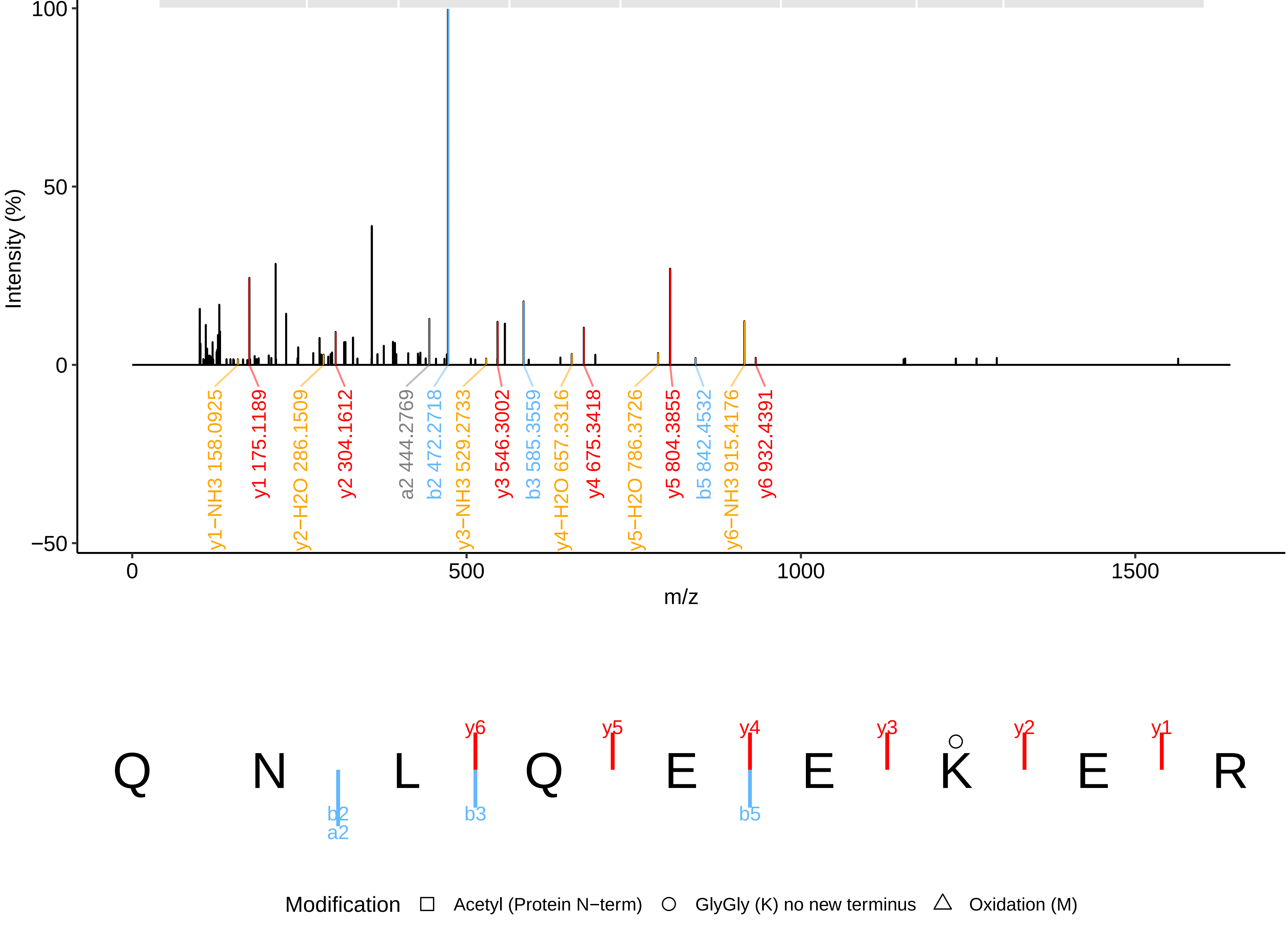

20200921_KIZ_MUb4_GlyGly_10plex − Scan number: 4858

Gene Names	Charge	m/z	Mass	Retention time	PEP	Score	Precursor Intensity
H3F3B;H3F3A	3	349.1927	1044.556	15.828	0.0018605	114.86	13607665

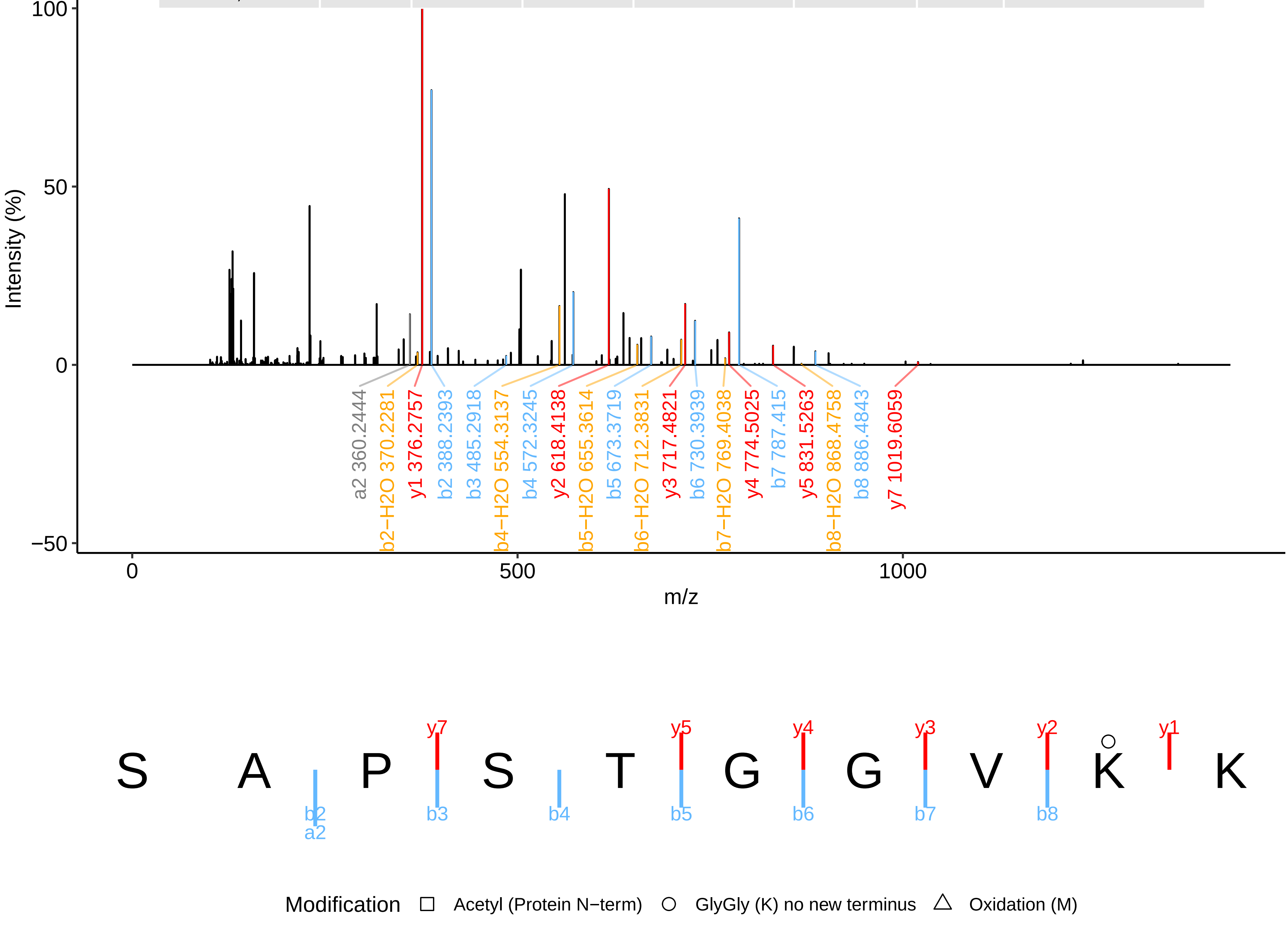

20200921_KIZ_MUb4_GlyGly_10plex_2 − Scan number: 4478

Gene Names	Charge	m/z	Mass	Retention time	PEP	Score	Precursor Intensity
H3F3B;H3F3A	3	349.1927	1044.556	15.736	0.00020722	107.79	1705447

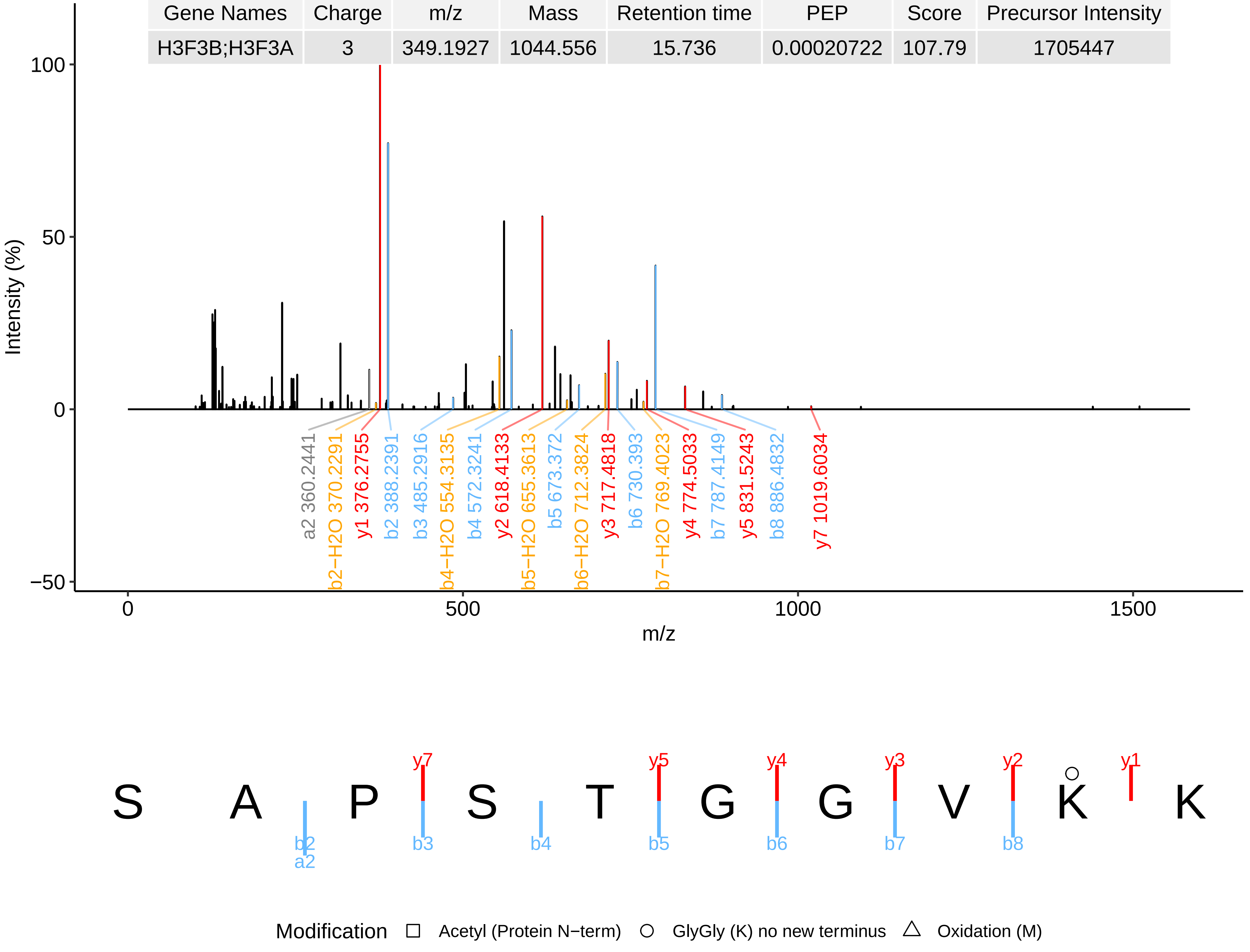

20200924_KIZ_MUb5_GlyGly_1 − Scan number: 4261

Gene Names	Charge	m/z	Mass	Retention time	PEP	Score	Precursor Intensity
H3F3B;H3F3A	3	349.1927	1044.556	13.13	0.023136	78.098	12966730

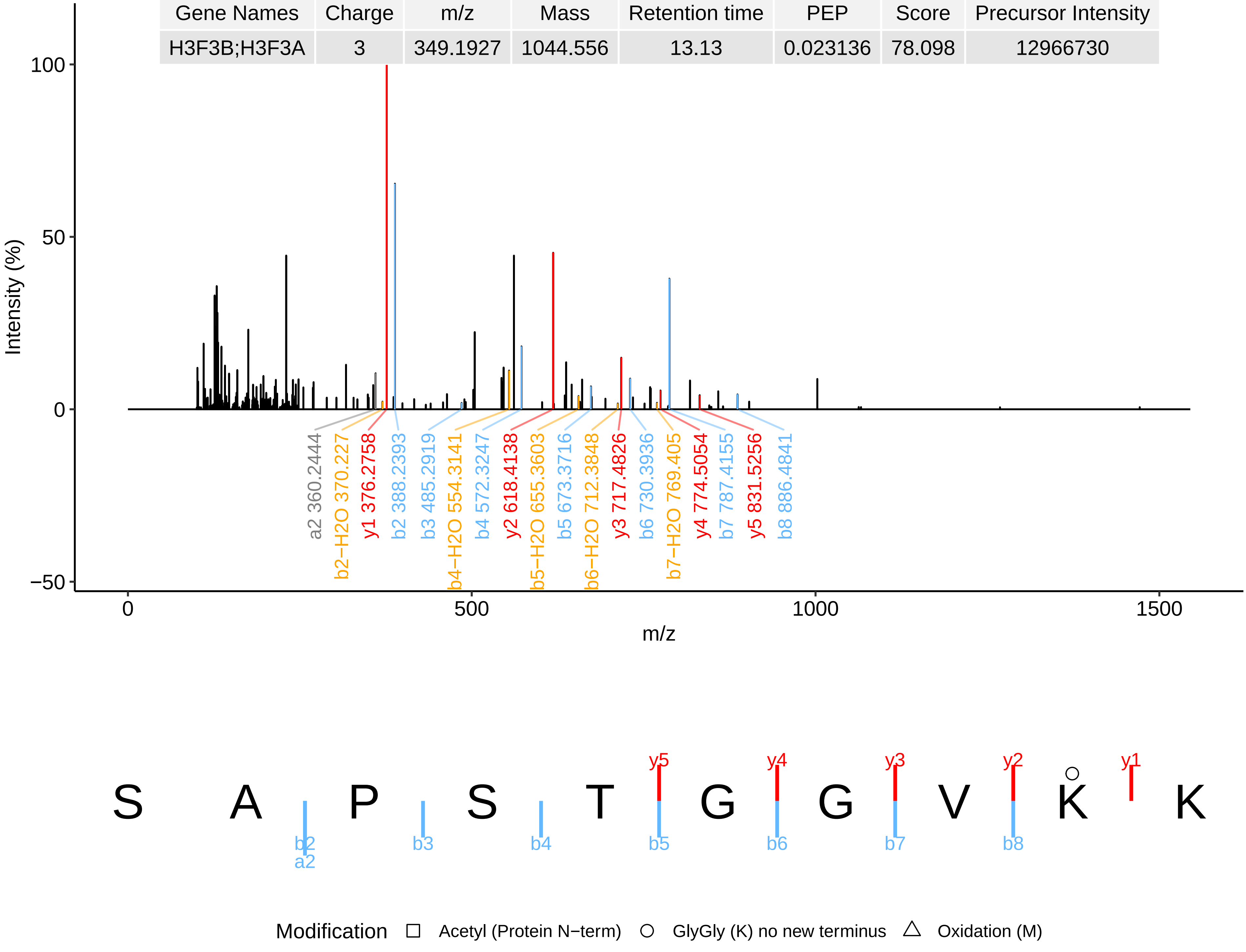

20200924_KIZ_MUb5_GlyGly_2 − Scan number: 3936

Gene Names	Charge	m/z	Mass	Retention time	PEP	Score	Precursor Intensity
H3F3B;H3F3A	3	349.1927	1044.556	14.39	0.0045286	97.965	NA

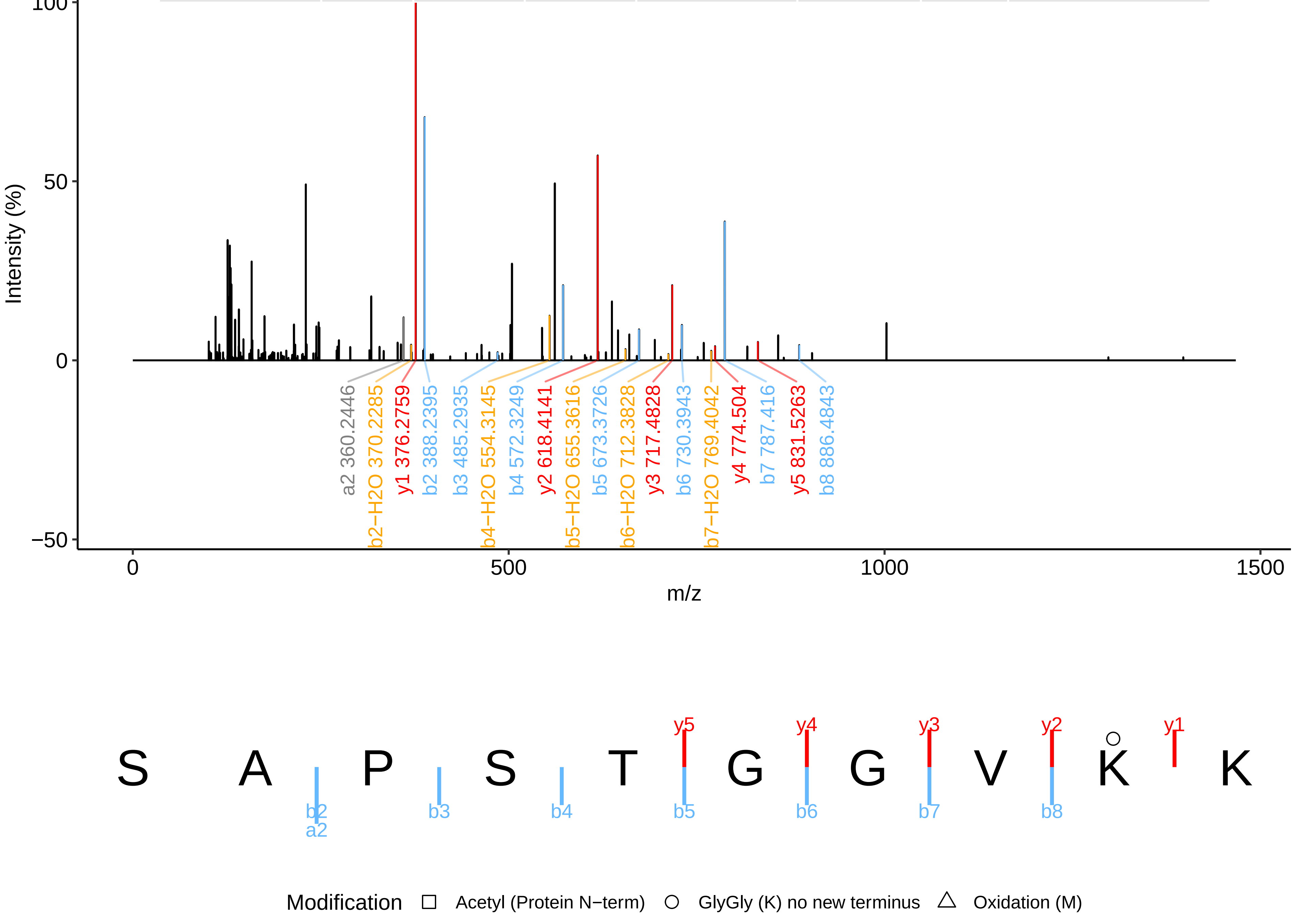

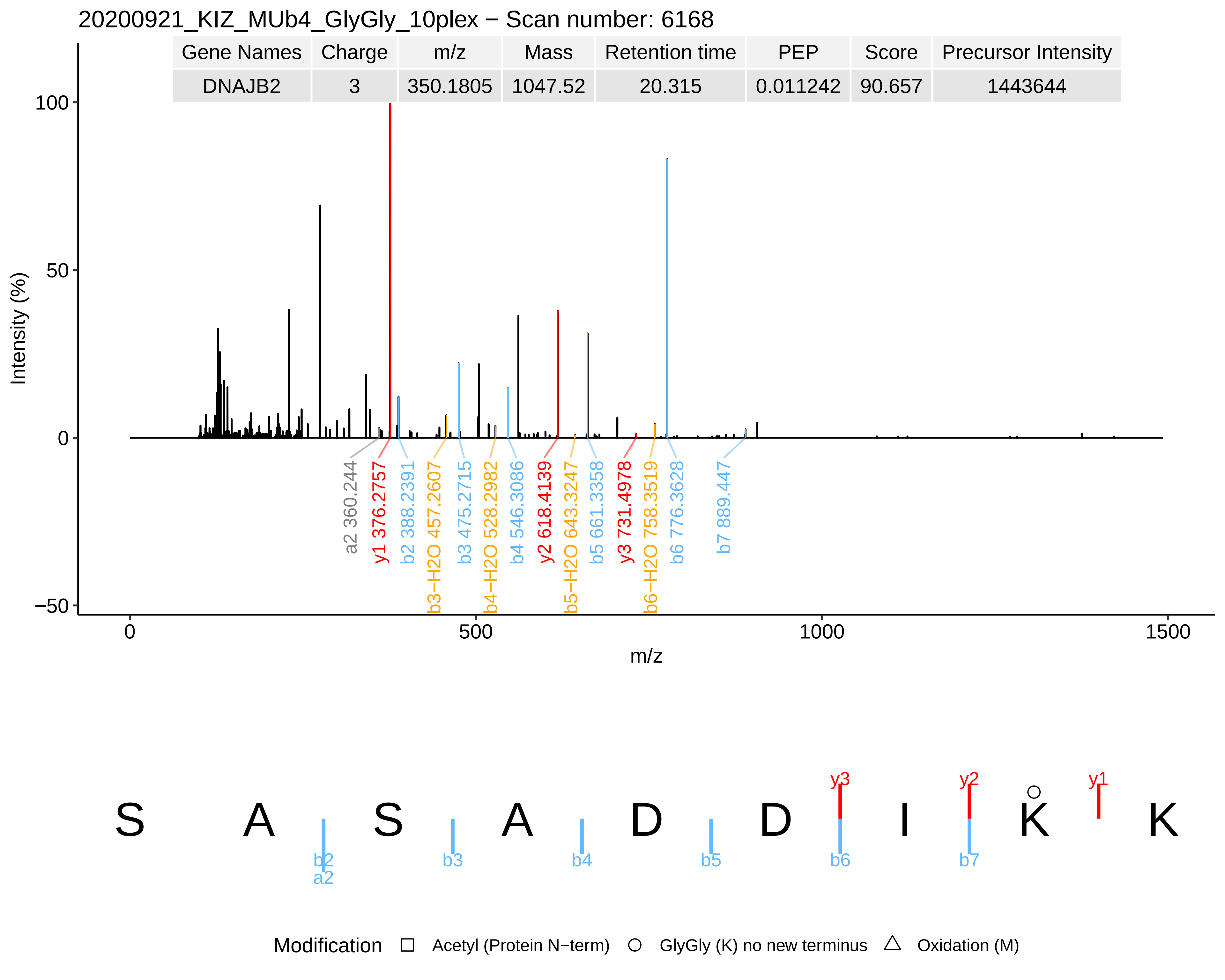
20200921_KIZ_MUb4_GlyGly_10plex − Scan number: 6168
Gene Names | Charge | m/z | Mass | Retention time | PEP | Score | Precursor Intensity
DNAJB2 | 3 | 350.1805 | 1047.52 | 20.315 | 0.011242 | 90.657 | 1443644
Intensity (%)
100
50
0
−50
a2 360.244
y1 376.2757
b2 388.2391
b3−H2O 457.2607
b3 475.2715
b4−H2O 528.2982
b4 546.3086
y2 618.4139
b5−H2O 643.3247
b5 661.3358
y3 731.4978
b6−H2O 758.3519
b6 776.3628
b7 889.447
0
500
1000
1500
m/z
S A S A D D I K K
y3 y2 y1
b2 a2 b3 b4 b5 b6 b7
Modification
Acetyl (Protein N−term)
GlyGly (K) no new terminus
Oxidation (M)

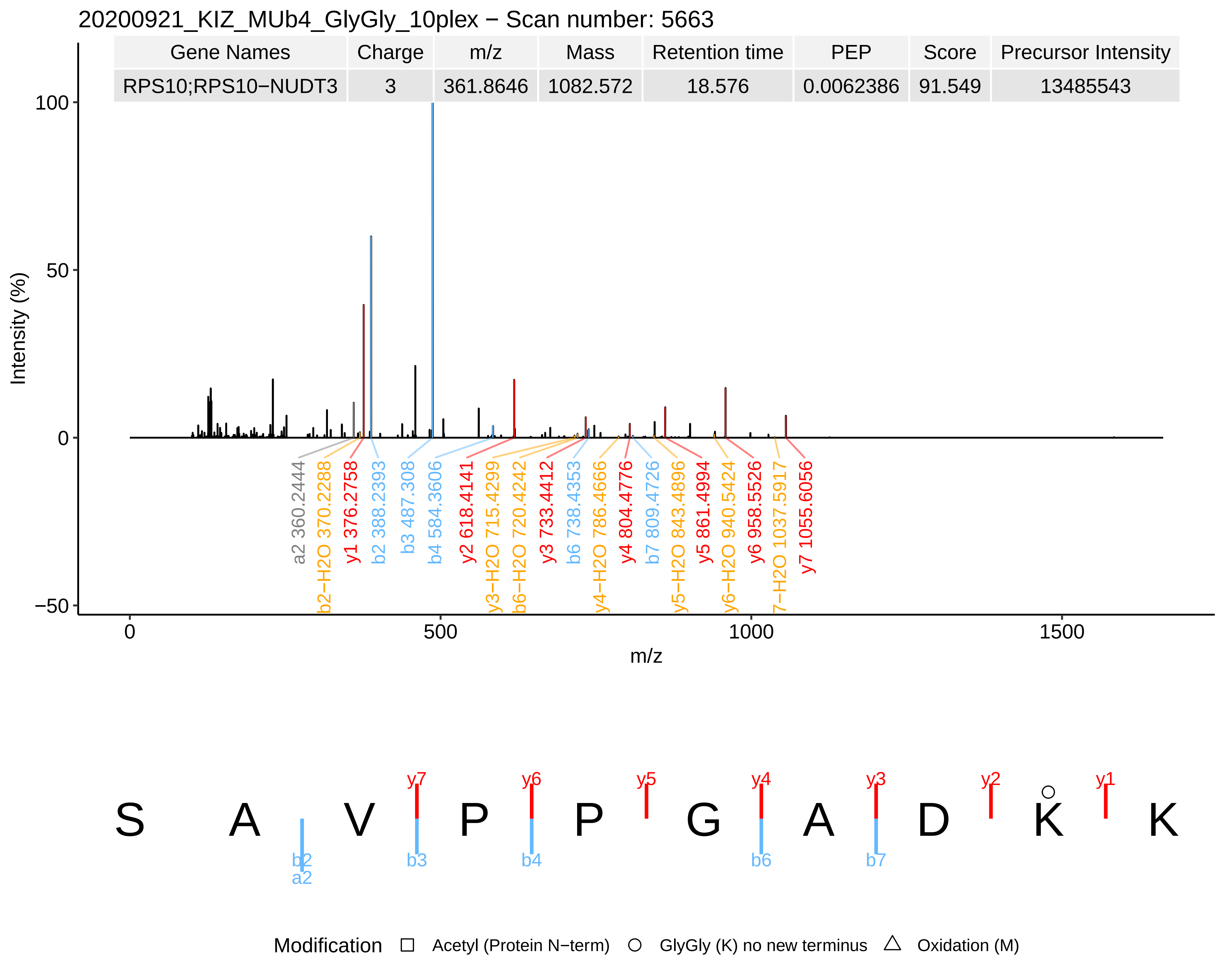
20200921_KIZ_MUb4_GlyGly_10plex − Scan number: 5663
Gene Names | Charge | m/z | Mass | Retention time | PEP | Score | Precursor Intensity
RPS10;RPS10−NUDT3 | 3 | 361.8646 | 1082.572 | 18.576 | 0.0062386 | 91.549 | 13485543
Intensity (%)
100
50
0
−50
m/z
0
500
1000
1500
a2 360.2444
b2−H2O 370.2288
y1 376.2758
b2 388.2393
b3 487.308
b4 584.3606
y2 618.4141
y3−H2O 715.4299
b6−H2O 720.4242
y3 733.4412
b6 738.4353
y4−H2O 786.4666
y4 804.4776
b7 809.4726
y5−H2O 843.4896
y5 861.4994
y6−H2O 940.5424
y6 958.5526
y7−H2O 1037.5917
y7 1055.6056
S A V P P G A D K K
y7 y6 y5 y4 y3 y2 y1
b2 a2 b3 b4 b6 b7
Modification
Acetyl (Protein N−term)
GlyGly (K) no new terminus
Oxidation (M)

20200921_KIZ_MUb4_GlyGly_10plex − Scan number: 5818

Gene Names	Charge	m/z	Mass	Retention time	PEP	Score	Precursor Intensity
RPS10;RPS10−NUDT3	3	361.8646	1082.572	19.089	0.0047104	93.258	28573634

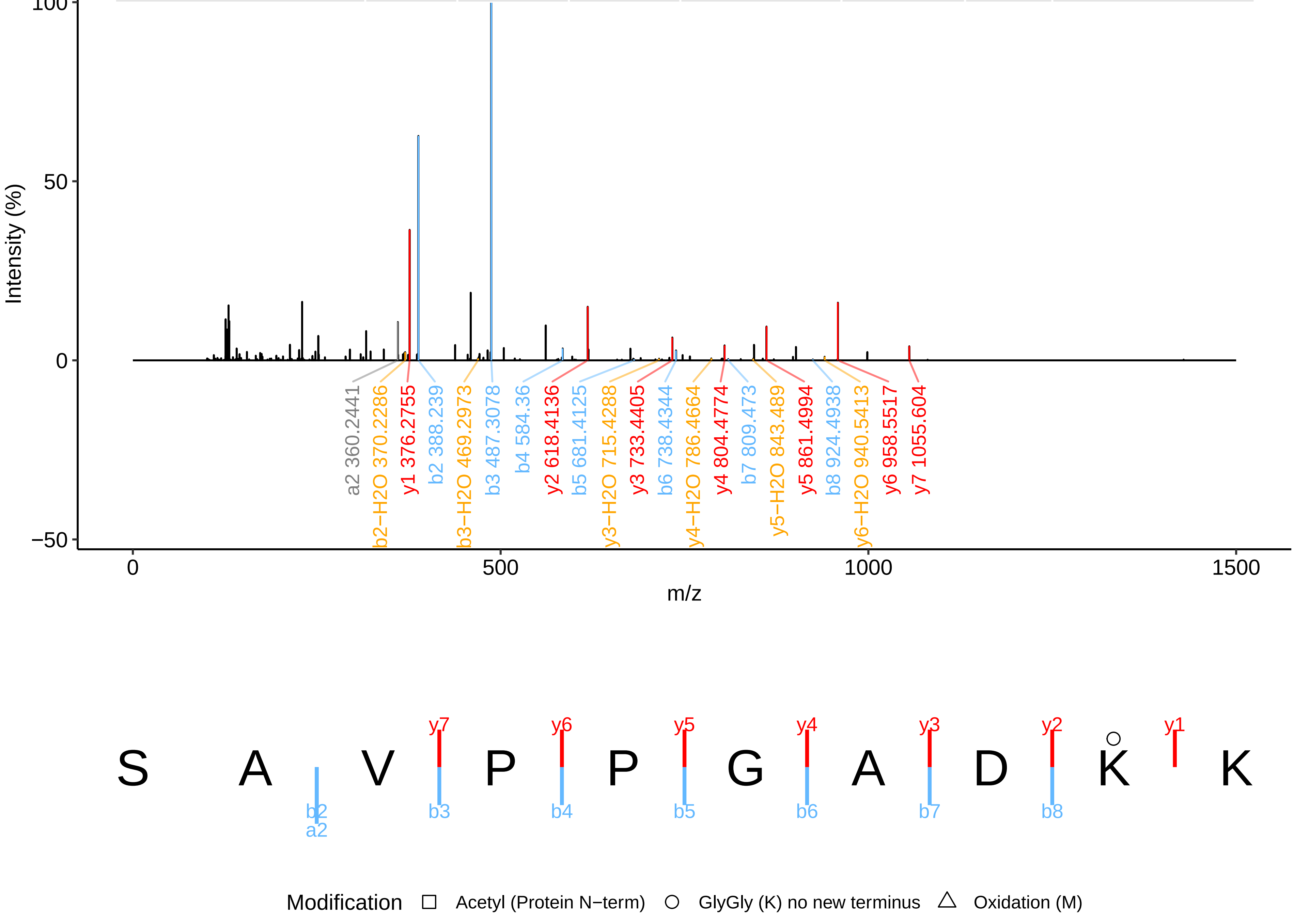

20200921_KIZ_MUb4_GlyGly_10plex_2 − Scan number: 5154

Gene Names	Charge	m/z	Mass	Retention time	PEP	Score	Precursor Intensity
RPS10;RPS10−NUDT3	3	361.8646	1082.572	18.4	0.00095434	97.456	1609329

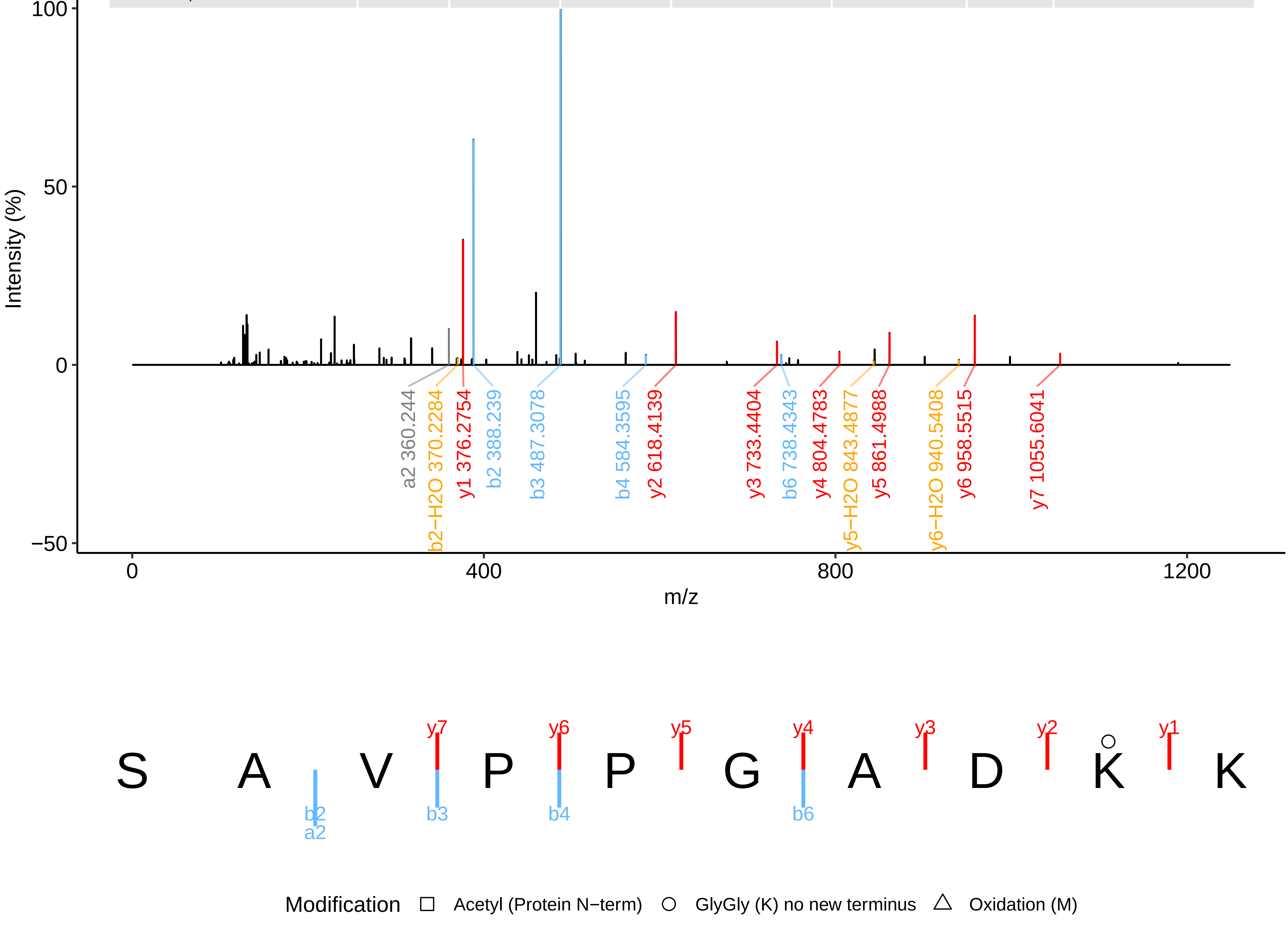

20200921_KIZ_MUb4_GlyGly_10plex_2 − Scan number: 5284

Gene Names	Charge	m/z	Mass	Retention time	PEP	Score	Precursor Intensity
RPS10;RPS10−NUDT3	3	361.8646	1082.572	18.93	0.051592	74.944	2917859

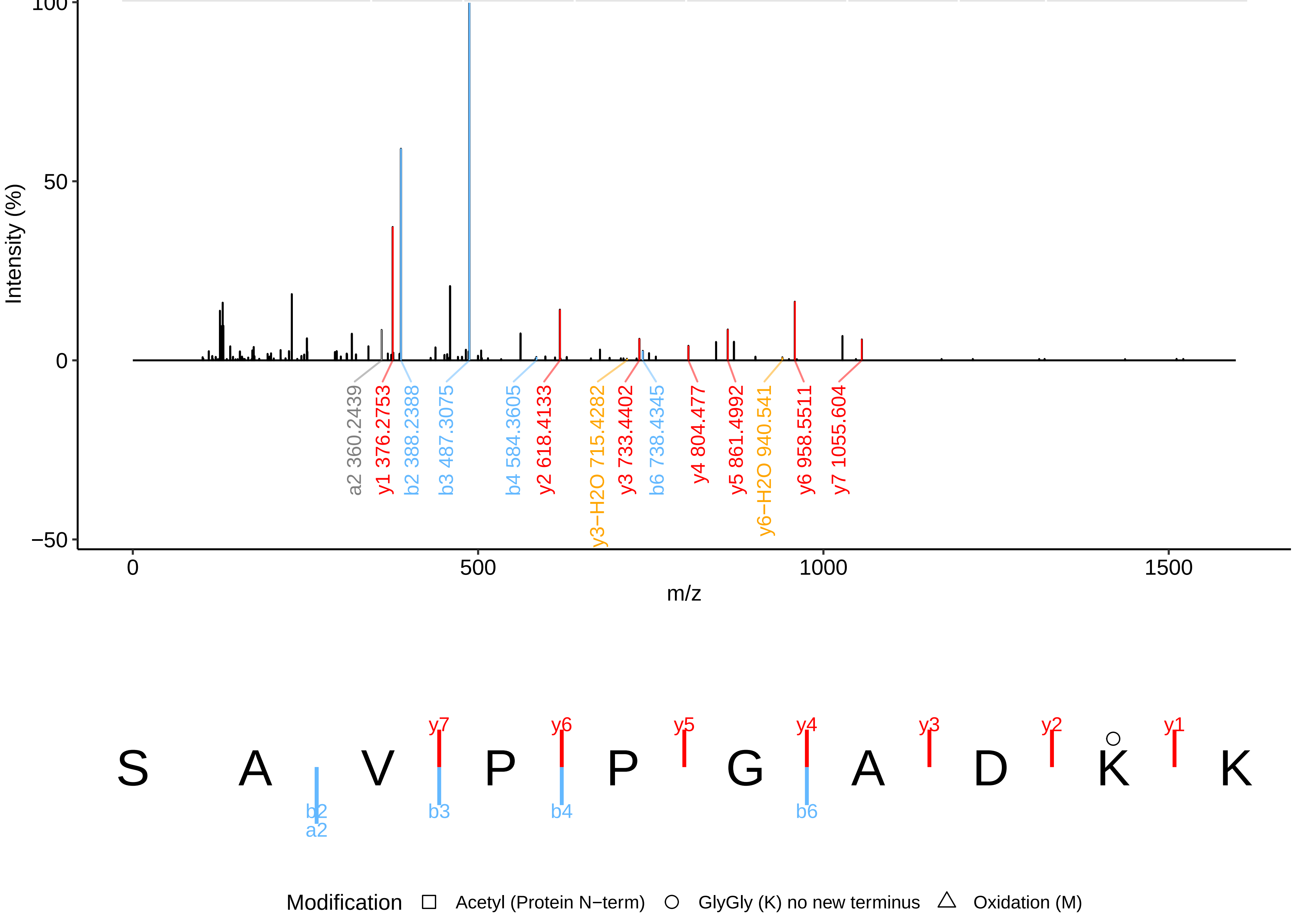

20200924_KIZ_MUb5_GlyGly_1 − Scan number: 4931

Gene Names	Charge	m/z	Mass	Retention time	PEP	Score	Precursor Intensity
RPS10;RPS10−NUDT3	3	361.8646	1082.572	15.286	0.05892	73.781	7791635

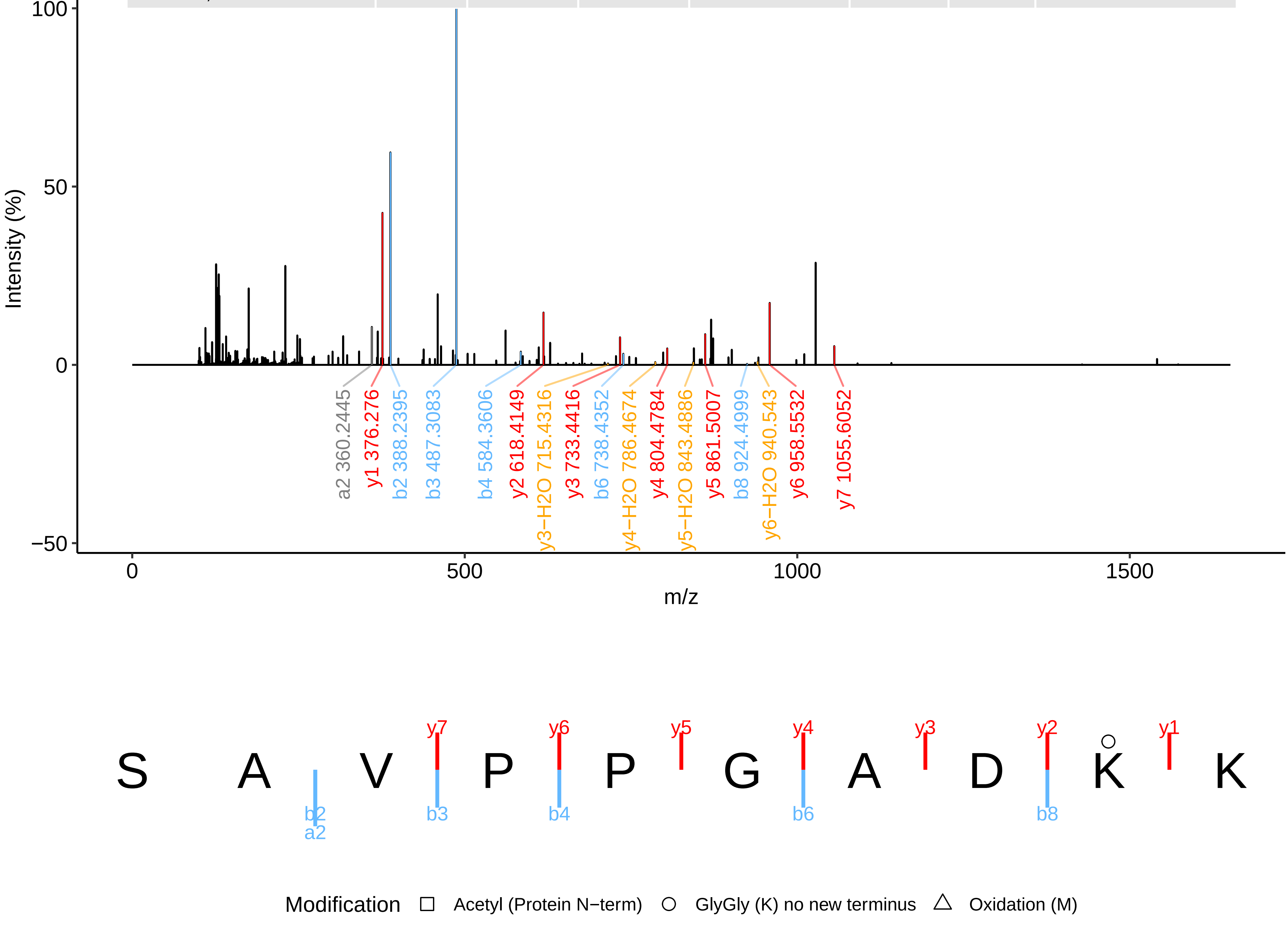

20200924_KIZ_MUb5_GlyGly_1 − Scan number: 5087

Gene Names	Charge	m/z	Mass	Retention time	PEP	Score	Precursor Intensity
RPS10;RPS10−NUDT3	3	361.8646	1082.572	15.8	0.00024437	106.4	34175208

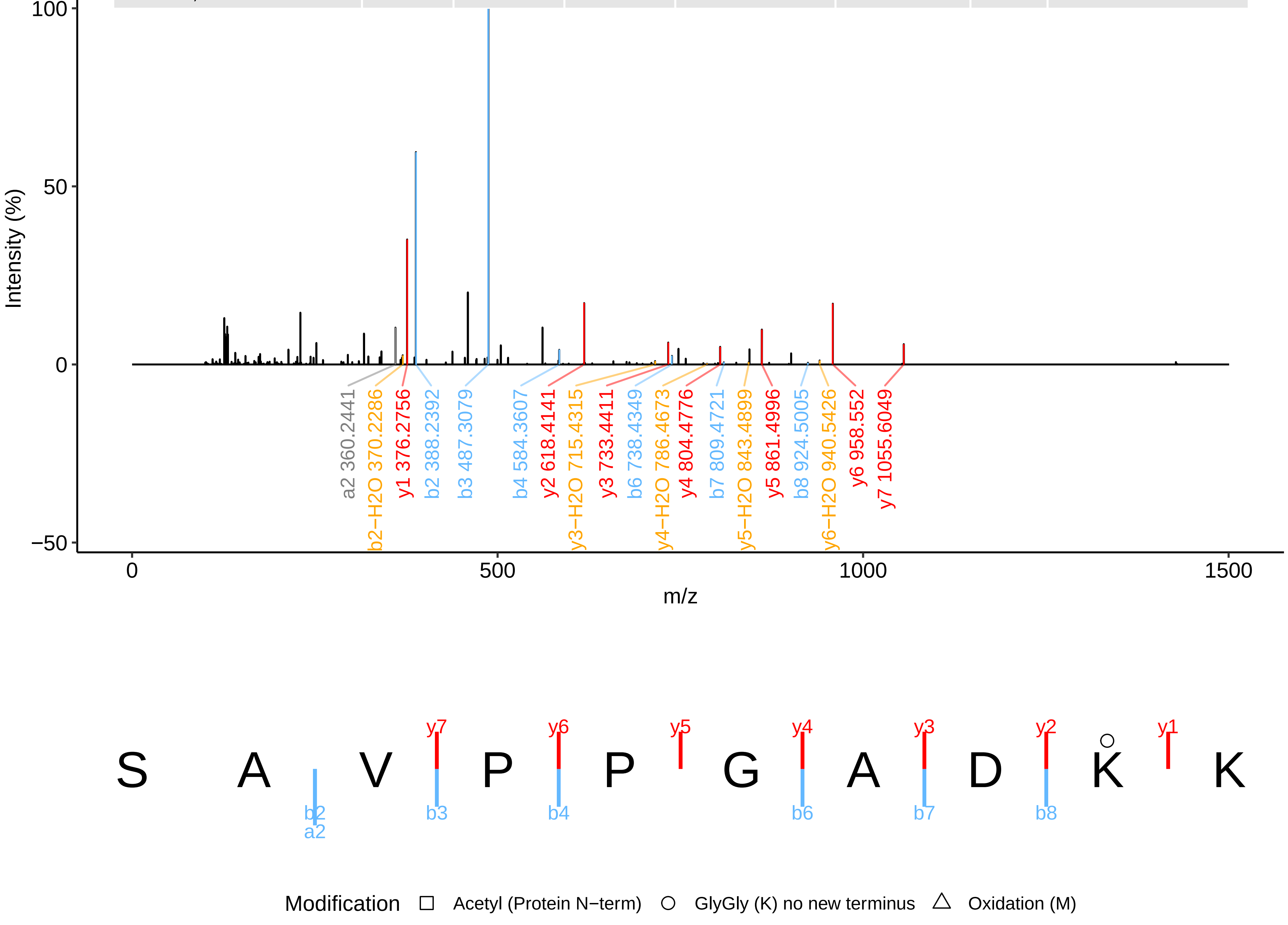

20200924_KIZ_MUb5_GlyGly_1 − Scan number: 5256

Gene Names	Charge	m/z	Mass	Retention time	PEP	Score	Precursor Intensity
RPS10;RPS10−NUDT3	3	361.8646	1082.572	16.307	0.05892	73.781	17534398

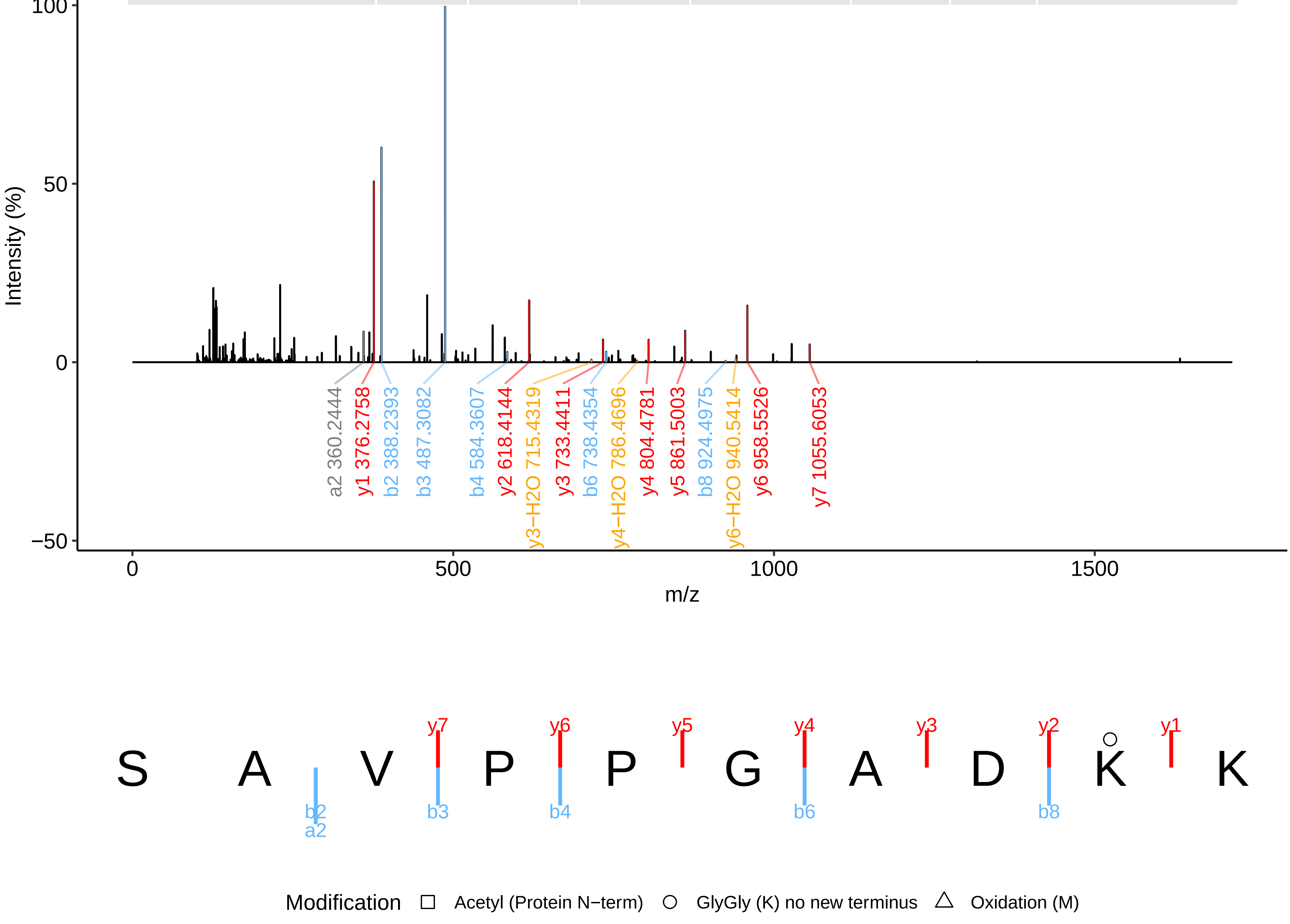

20200924_KIZ_MUb5_GlyGly_1 − Scan number: 5268

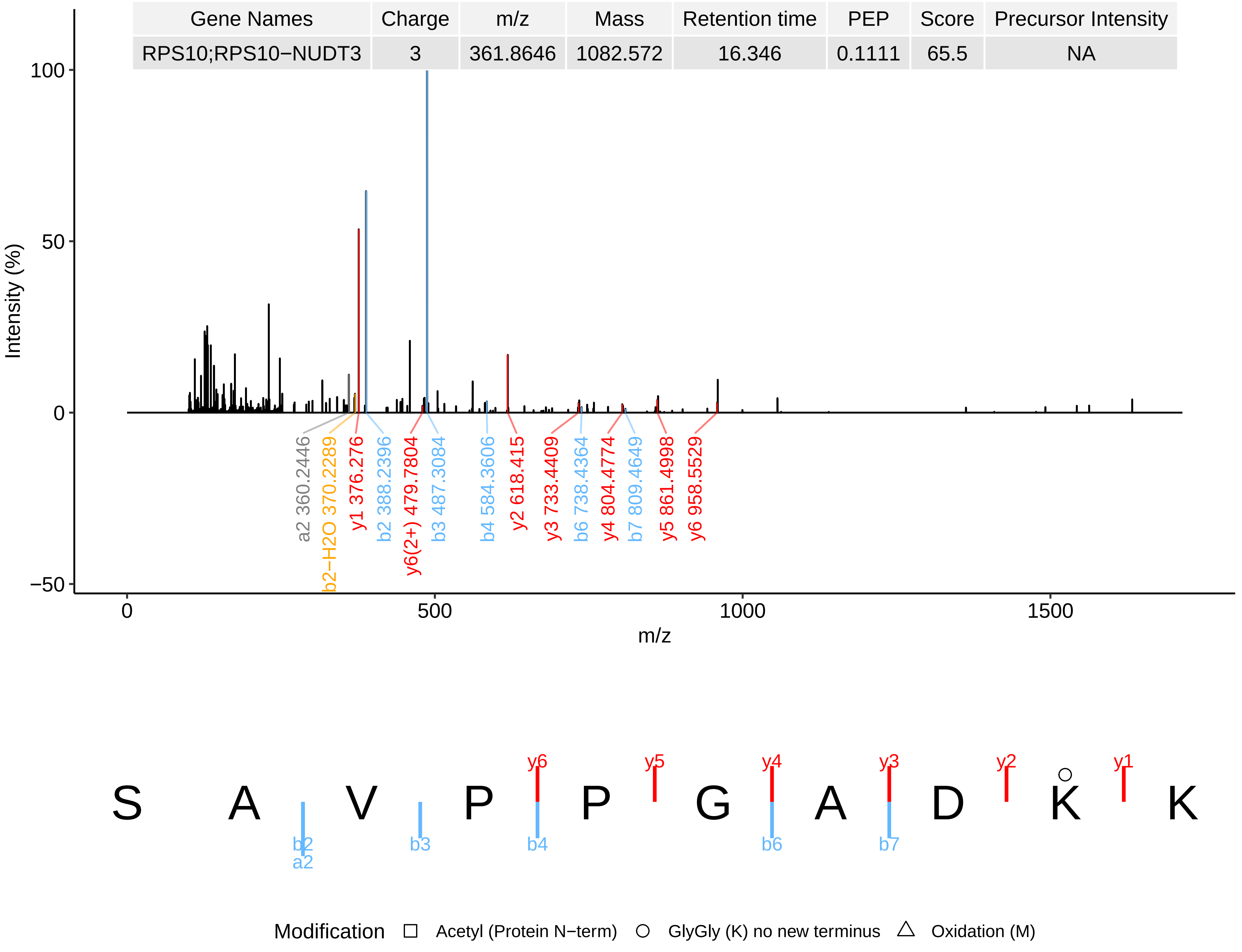

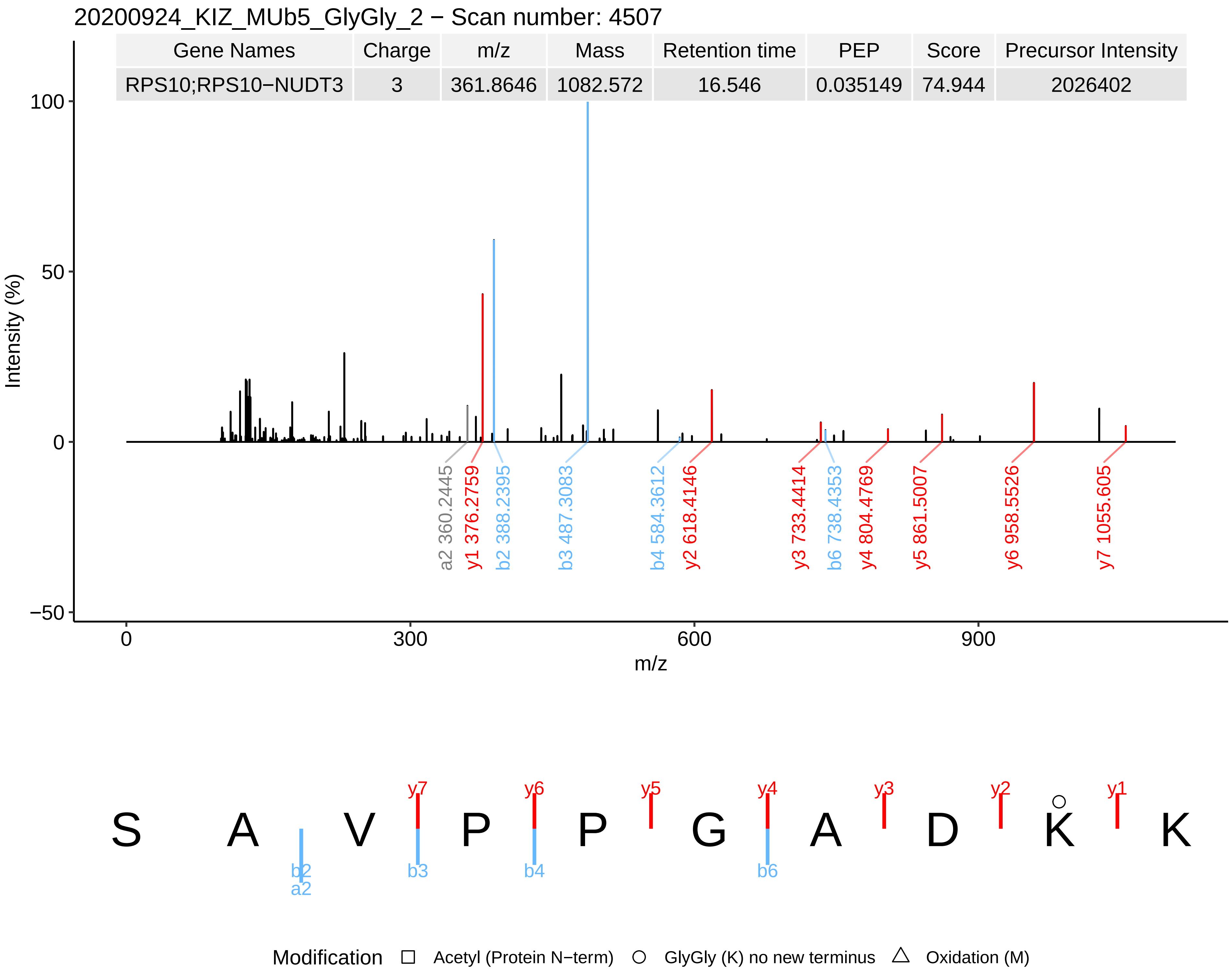
20200924_KIZ_MUb5_GlyGly_2 − Scan number: 4507
Gene Names
RPS10;RPS10−NUDT3
Charge
3
m/z
361.8646
Mass
1082.572
Retention time
16.546
PEP
0.035149
Score
74.944
Precursor Intensity
2026402
Intensity (%)
100
50
0
−50
0
300
600
900
m/z
a2 360.2445
y1 376.2759
b2 388.2395
b3 487.3083
b4 584.3612
y2 618.4146
y3 733.4414
b6 738.4353
y4 804.4769
y5 861.5007
y6 958.5526
y7 1055.605
S A V P P G A D K K
y7 y6 y5 y4 y3 y2 y1
b2 a2 b3 b4 b6
Modification
Acetyl (Protein N−term)
GlyGly (K) no new terminus
Oxidation (M)

20200924_KIZ_MUb5_GlyGly_2 − Scan number: 4638

Gene Names	Charge	m/z	Mass	Retention time	PEP	Score	Precursor Intensity
RPS10;RPS10−NUDT3	3	361.8646	1082.572	17.063	0.010406	88.056	7679794

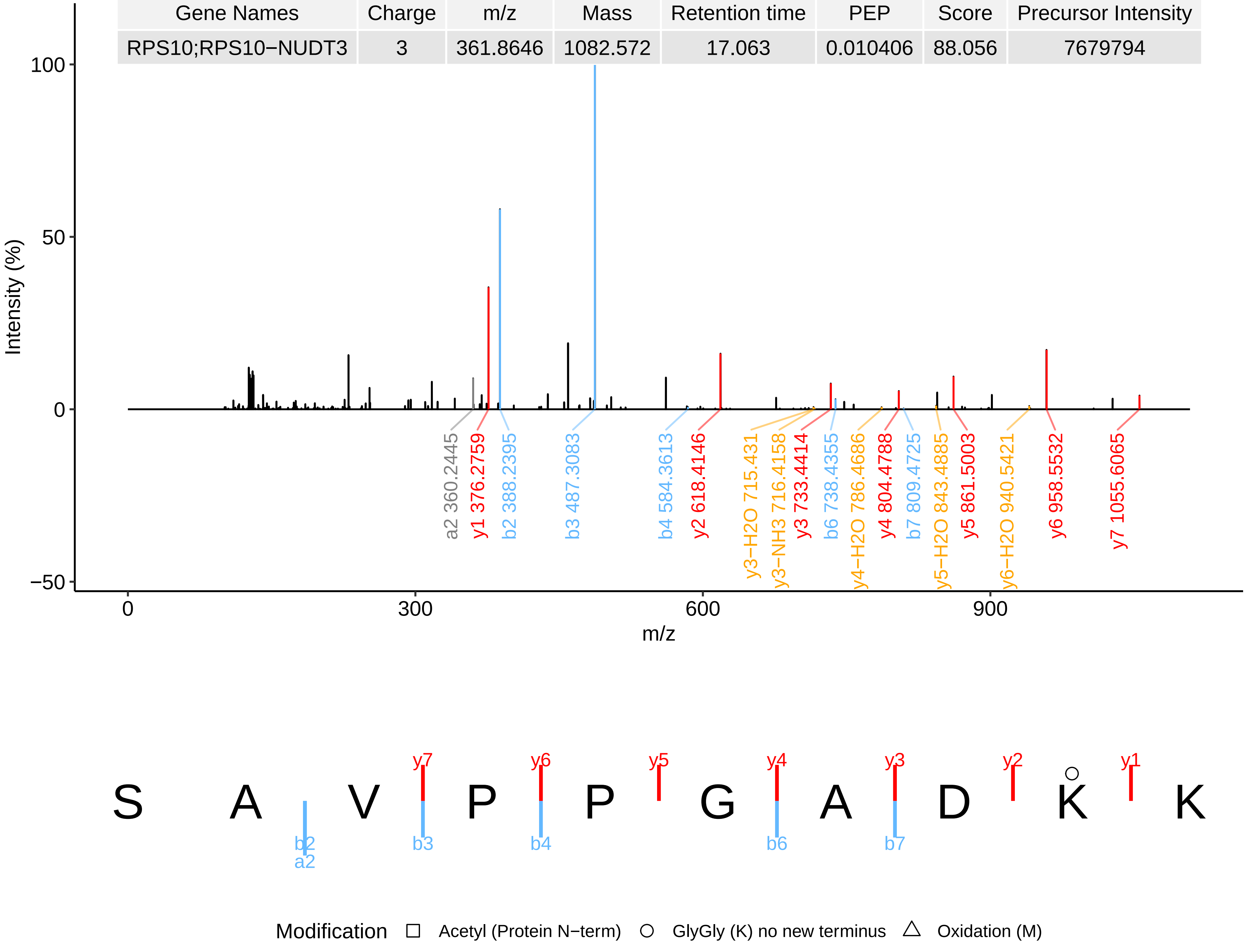

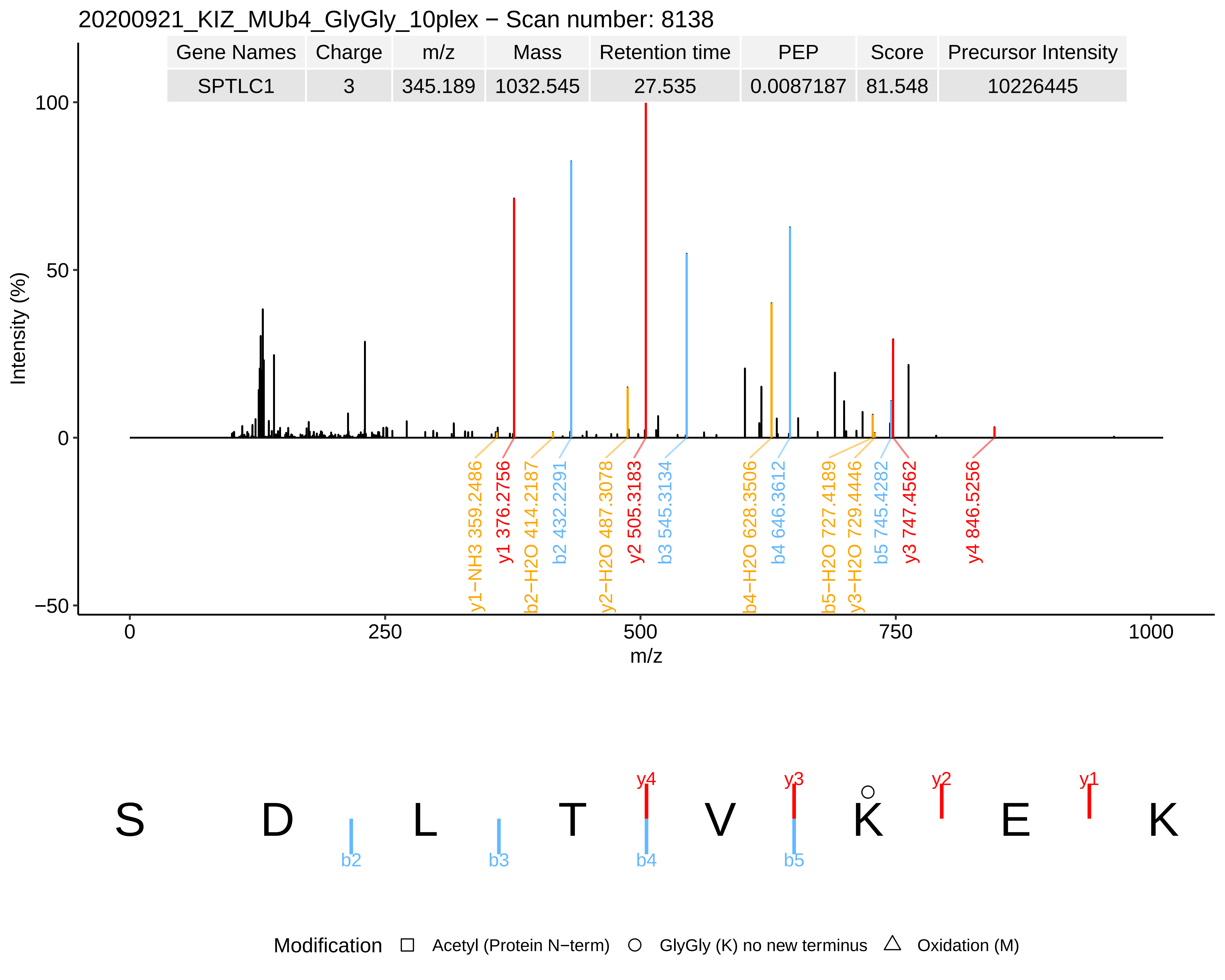
20200921_KIZ_MUb4_GlyGly_10plex − Scan number: 8138
Gene Names
SPTLC1
Charge
3
m/z
345.189
Mass
1032.545
Retention time
27.535
PEP
0.0087187
Score
81.548
Precursor Intensity
10226445
Intensity (%)
100
50
0
−50
0
250
500
750
1000
m/z
y1−NH3 359.2486
y1 376.2756
b2−H2O 414.2187
b2 432.2291
y2−H2O 487.3078
y2 505.3183
b3 545.3134
b4−H2O 628.3506
b4 646.3612
b5−H2O 727.4189
y3−H2O 729.4446
b5 745.4282
y3 747.4562
y4 846.5256
S D L T V K E K
y4 y3 y2 y1
b2 b3 b4 b5
Modification
Acetyl (Protein N−term)
GlyGly (K) no new terminus
Oxidation (M)

20200921_KIZ_MUb4_GlyGly_10plex_2 − Scan number: 7494

Gene Names	Charge	m/z	Mass	Retention time	PEP	Score	Precursor Intensity
SPTLC1	3	345.189	1032.545	27.985	0.007332	85.064	1569243

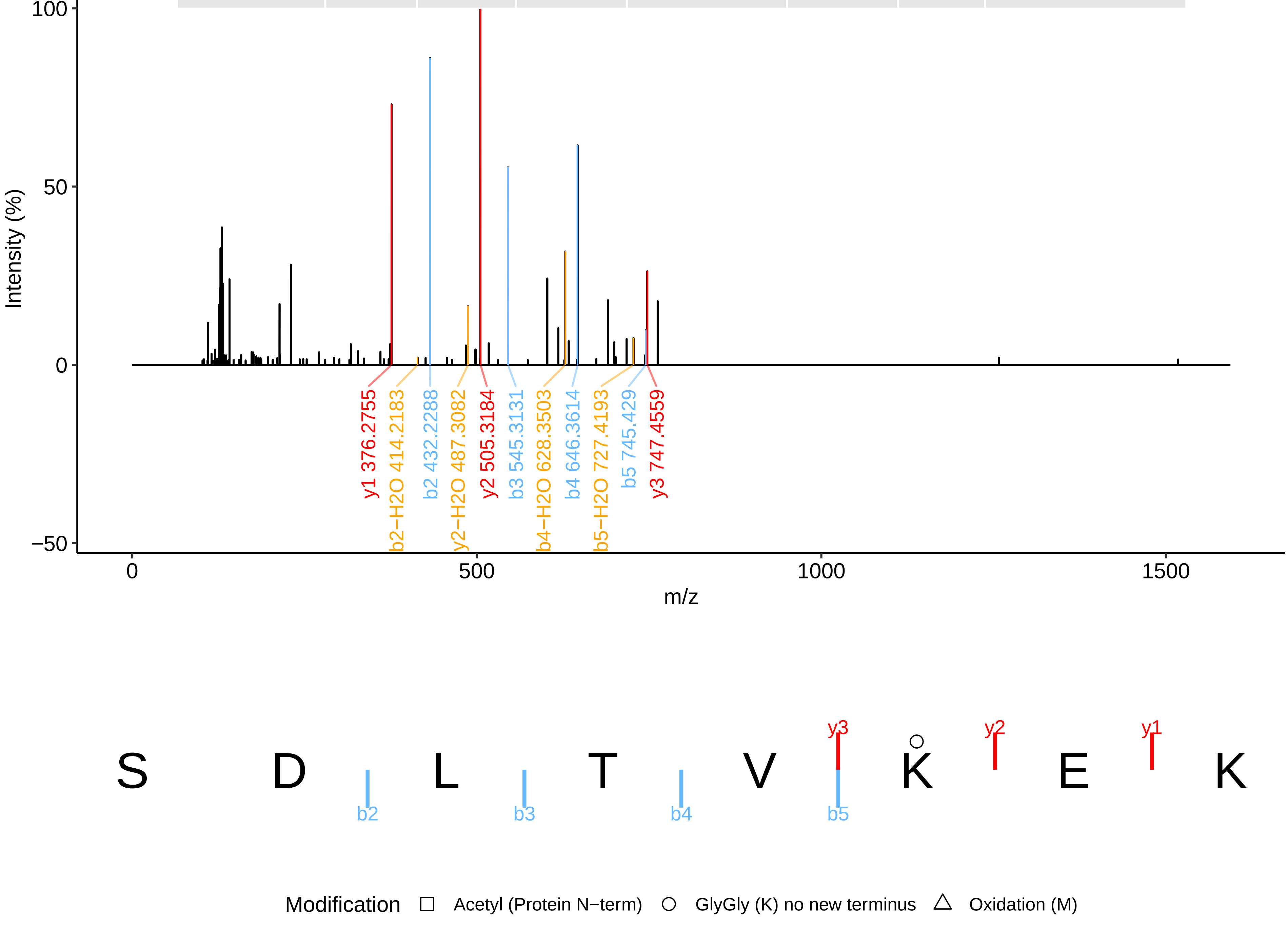

20200924_KIZ_MUb5_GlyGly_1 − Scan number: 7150

Gene Names	Charge	m/z	Mass	Retention time	PEP	Score	Precursor Intensity
SPTLC1	3	345.189	1032.545	22.65	0.0087187	81.548	11768829

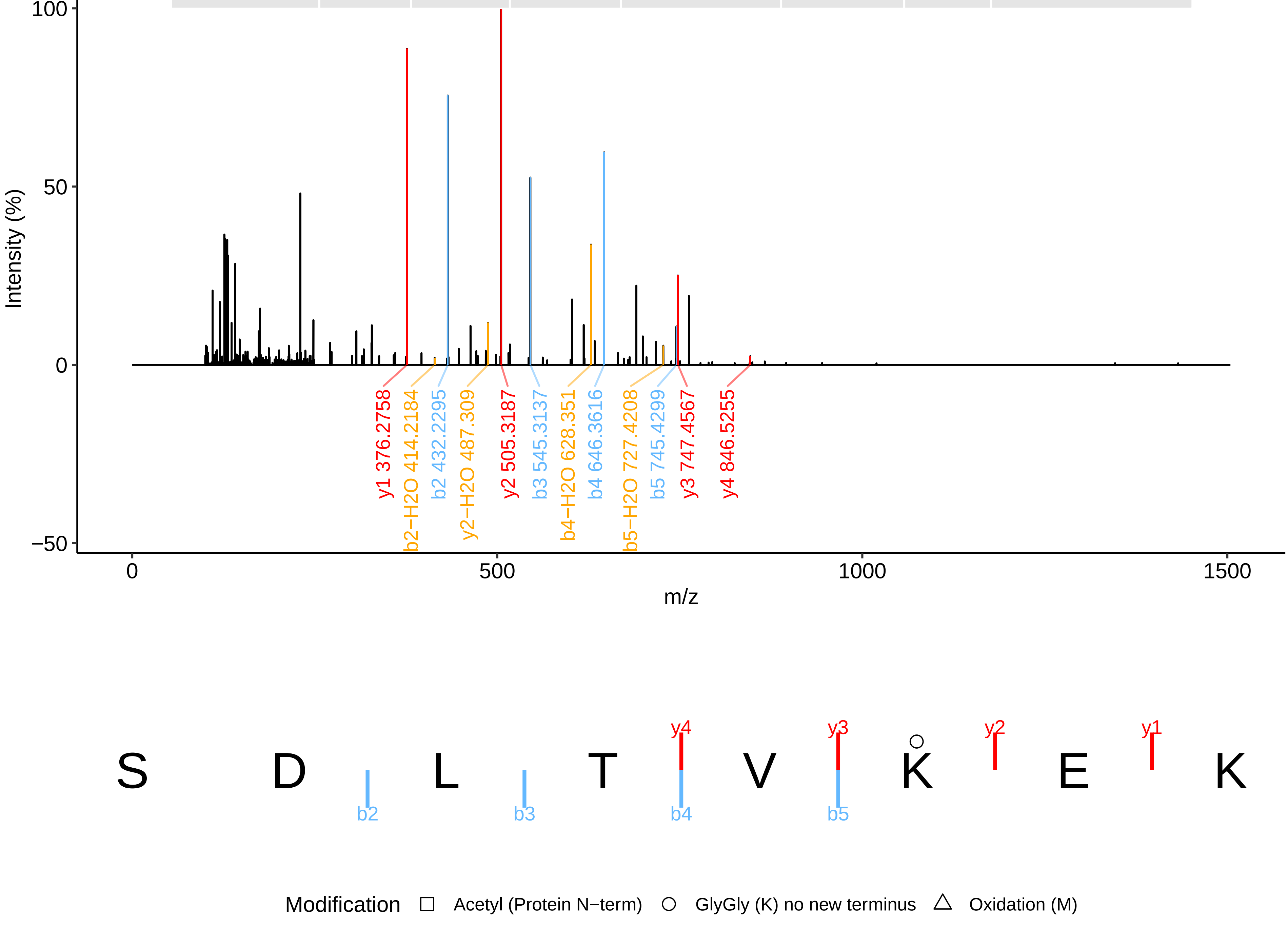

20200924_KIZ_MUb5_GlyGly_2 − Scan number: 6654

Gene Names	Charge	m/z	Mass	Retention time	PEP	Score	Precursor Intensity
SPTLC1	3	345.189	1032.545	24.835	0.025352	71.379	3055568

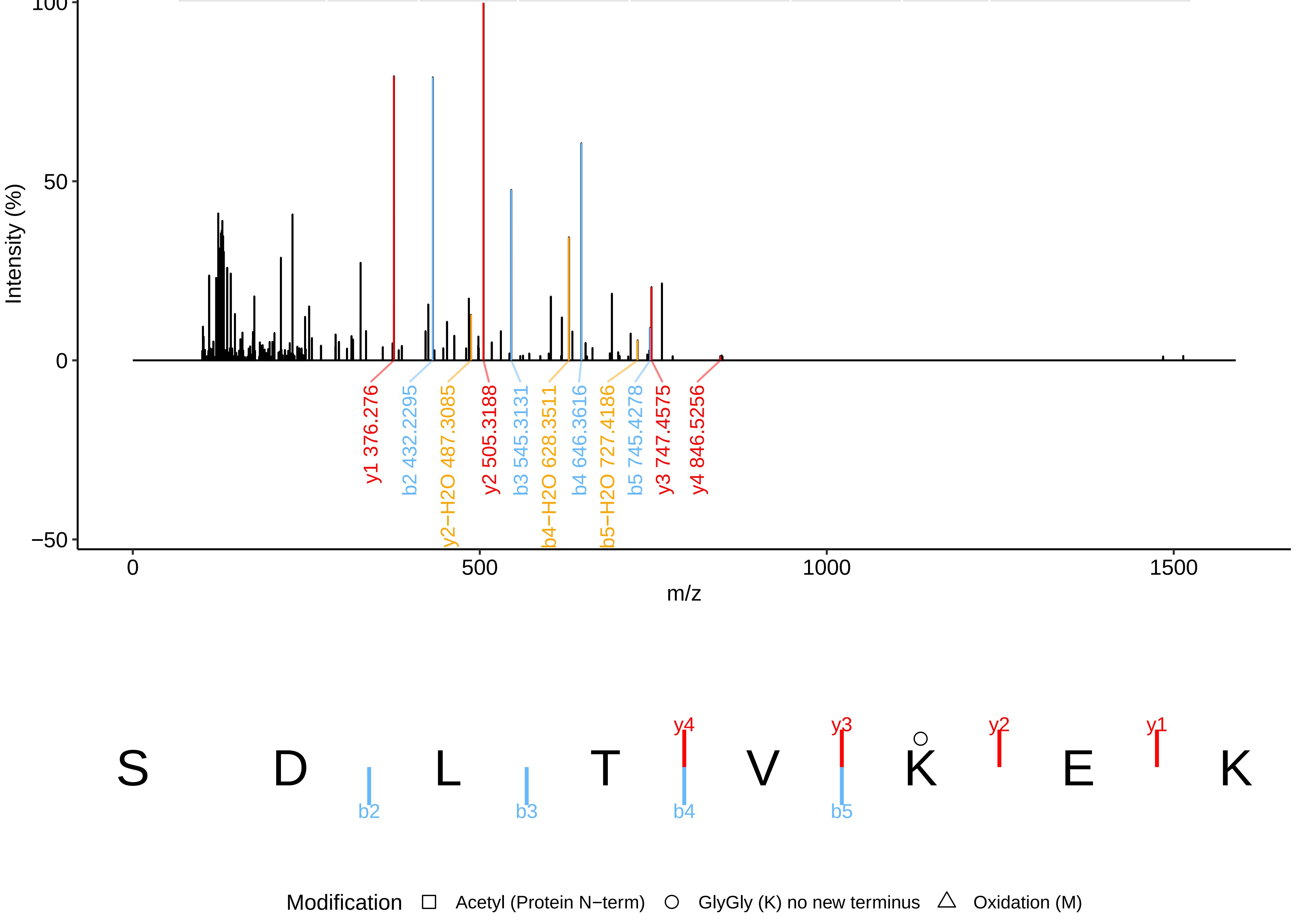

20200921_KIZ_MUb4_GlyGly_10plex − Scan number: 10163

Gene Names	Charge	m/z	Mass	Retention time	PEP	Score	Precursor Intensity
EPHA2	3	352.8731	1055.598	35.08	0.022503	69.825	3635800

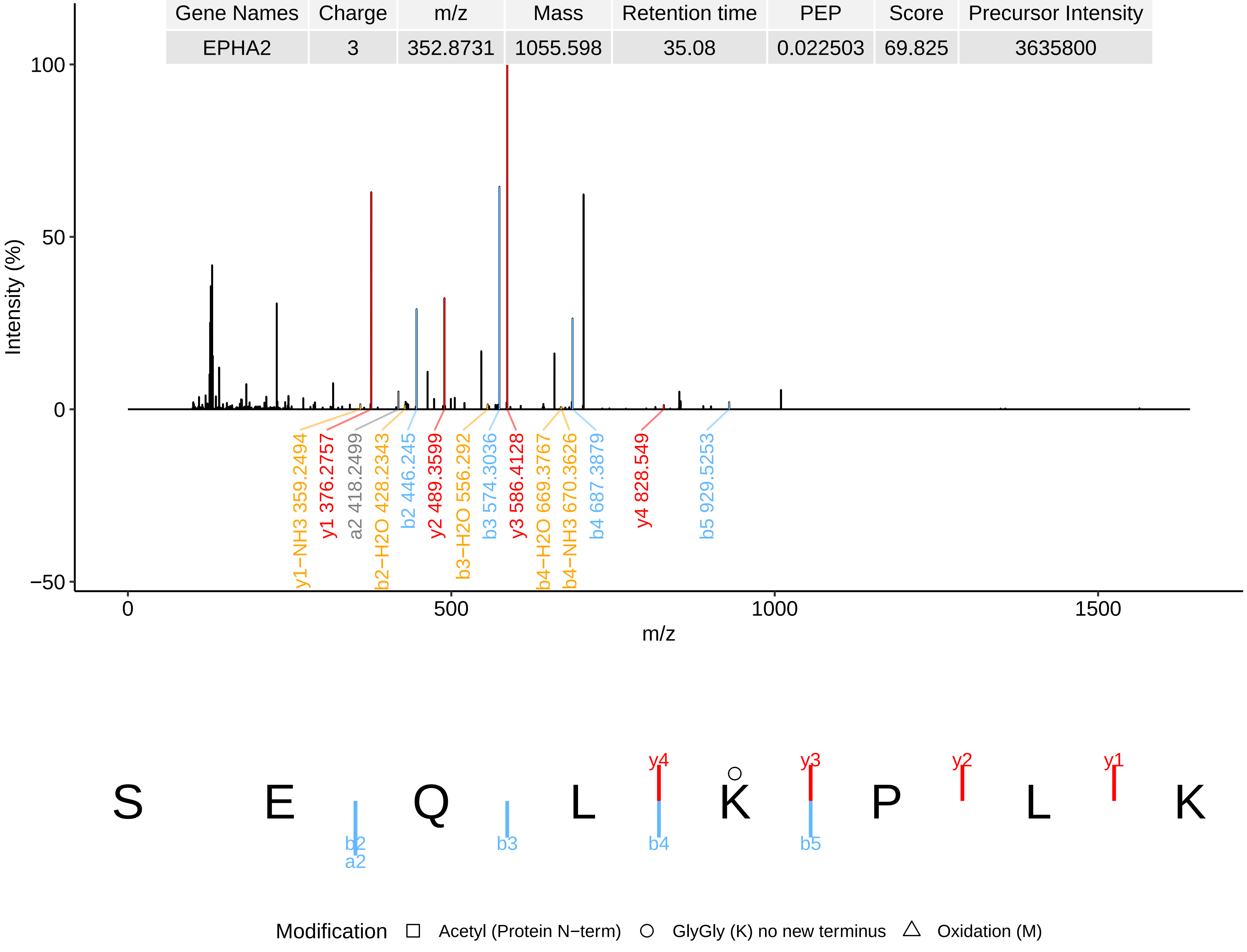

20200921_KIZ_MUb4_GlyGly_10plex_2 − Scan number: 9380

Gene Names	Charge	m/z	Mass	Retention time	PEP	Score	Precursor Intensity
EPHA2	3	352.8731	1055.598	35.639	0.10996	43.808	953860.7

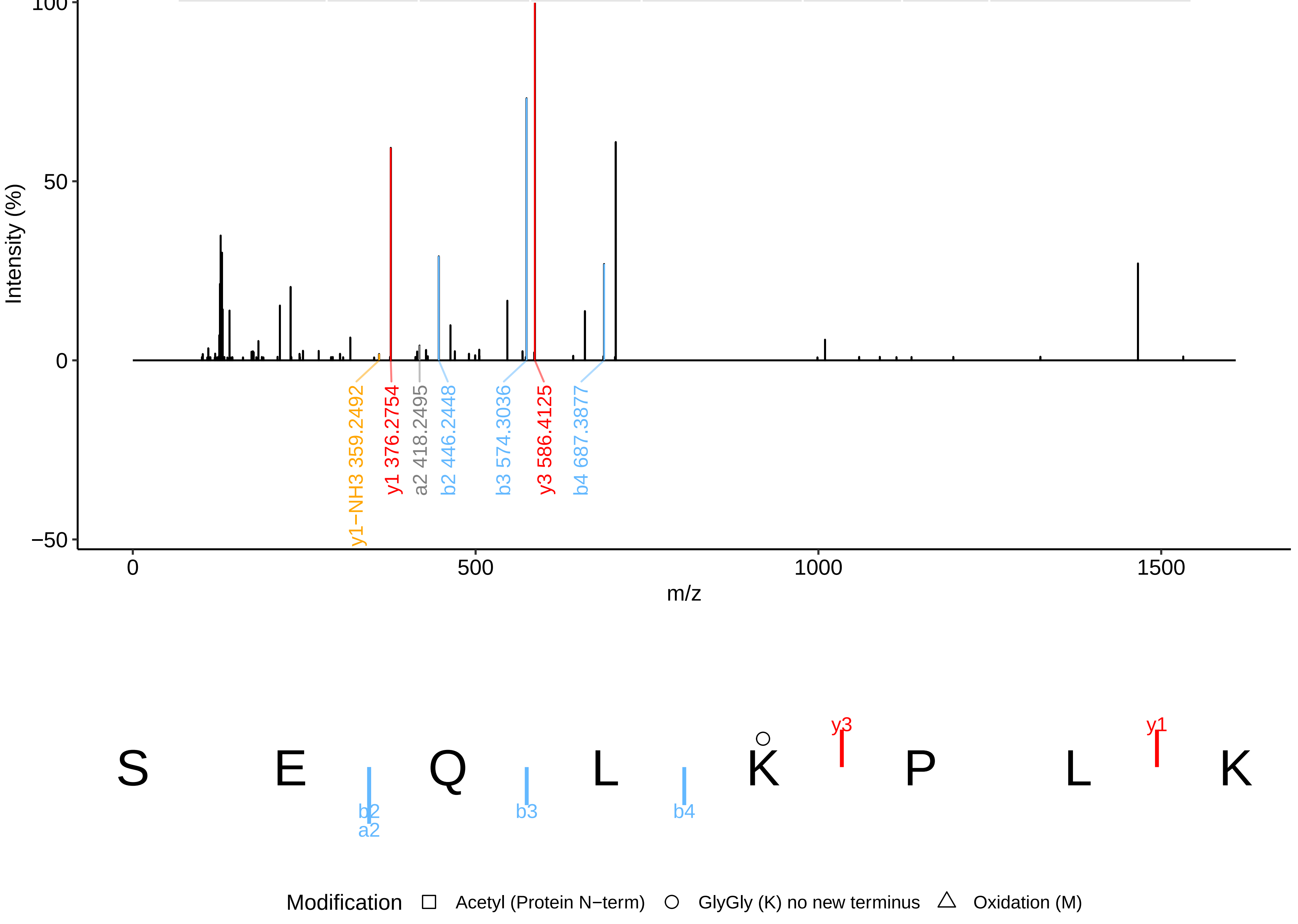

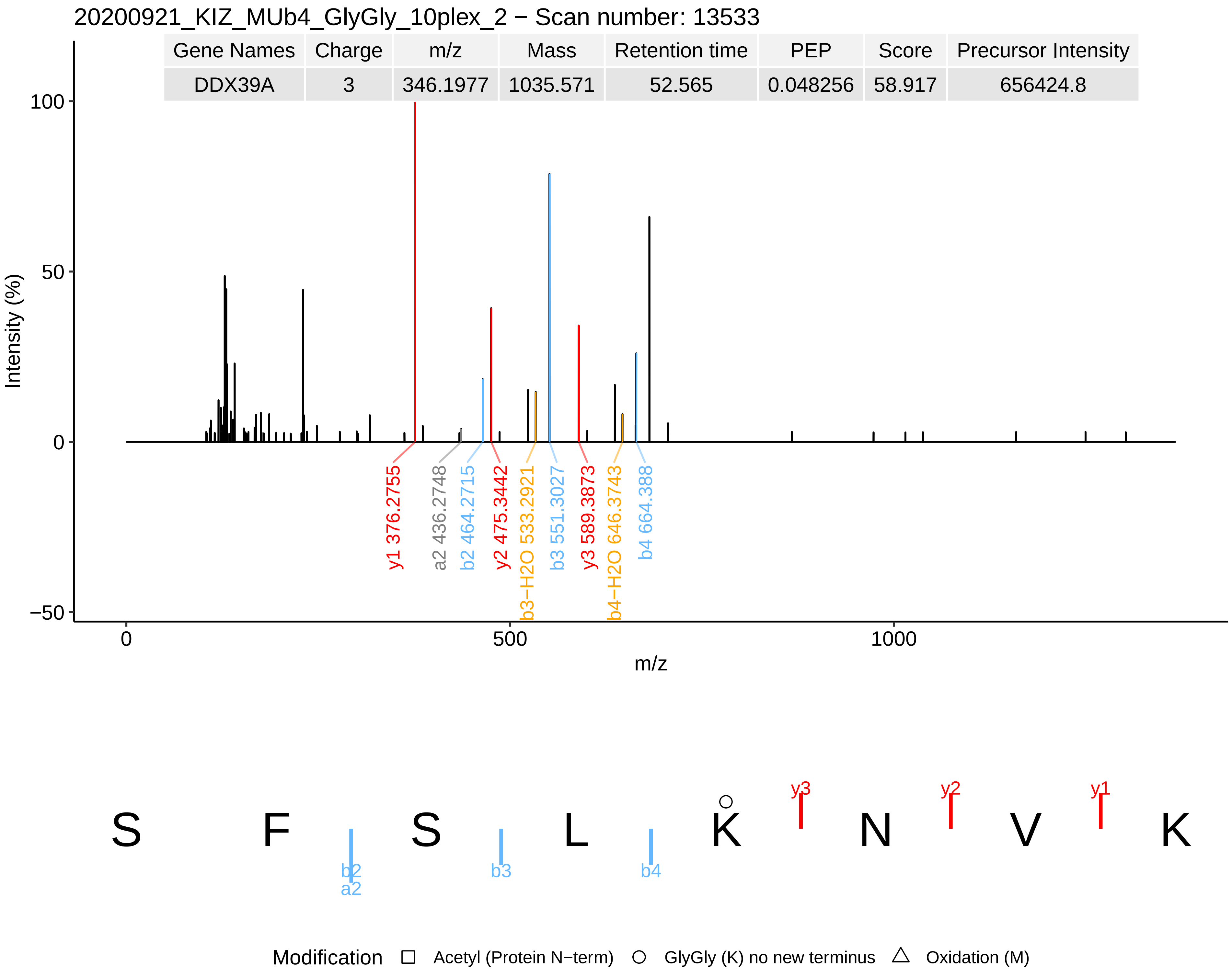
20200921_KIZ_MUb4_GlyGly_10plex_2 − Scan number: 13533
Gene Names | Charge | m/z | Mass | Retention time | PEP | Score | Precursor Intensity
DDX39A | 3 | 346.1977 | 1035.571 | 52.565 | 0.048256 | 58.917 | 656424.8
Intensity (%)
100
50
0
−50
0
500
1000
m/z
y1 376.2755
a2 436.2748
b2 464.2715
y2 475.3442
b3−H2O 533.2921
b3 551.3027
y3 589.3873
b4−H2O 646.3743
b4 664.388
S F S L K N V K
y3
y2
y1
b2
a2
b3
b4
Modification
Acetyl (Protein N−term)
GlyGly (K) no new terminus
Oxidation (M)

20200921_KIZ_MUb4_GlyGly_10plex − Scan number: 11297

Gene Names	Charge	m/z	Mass	Retention time	PEP	Score	Precursor Intensity
TKT	3	352.8731	1055.598	39.297	0.015983	68.893	6299471

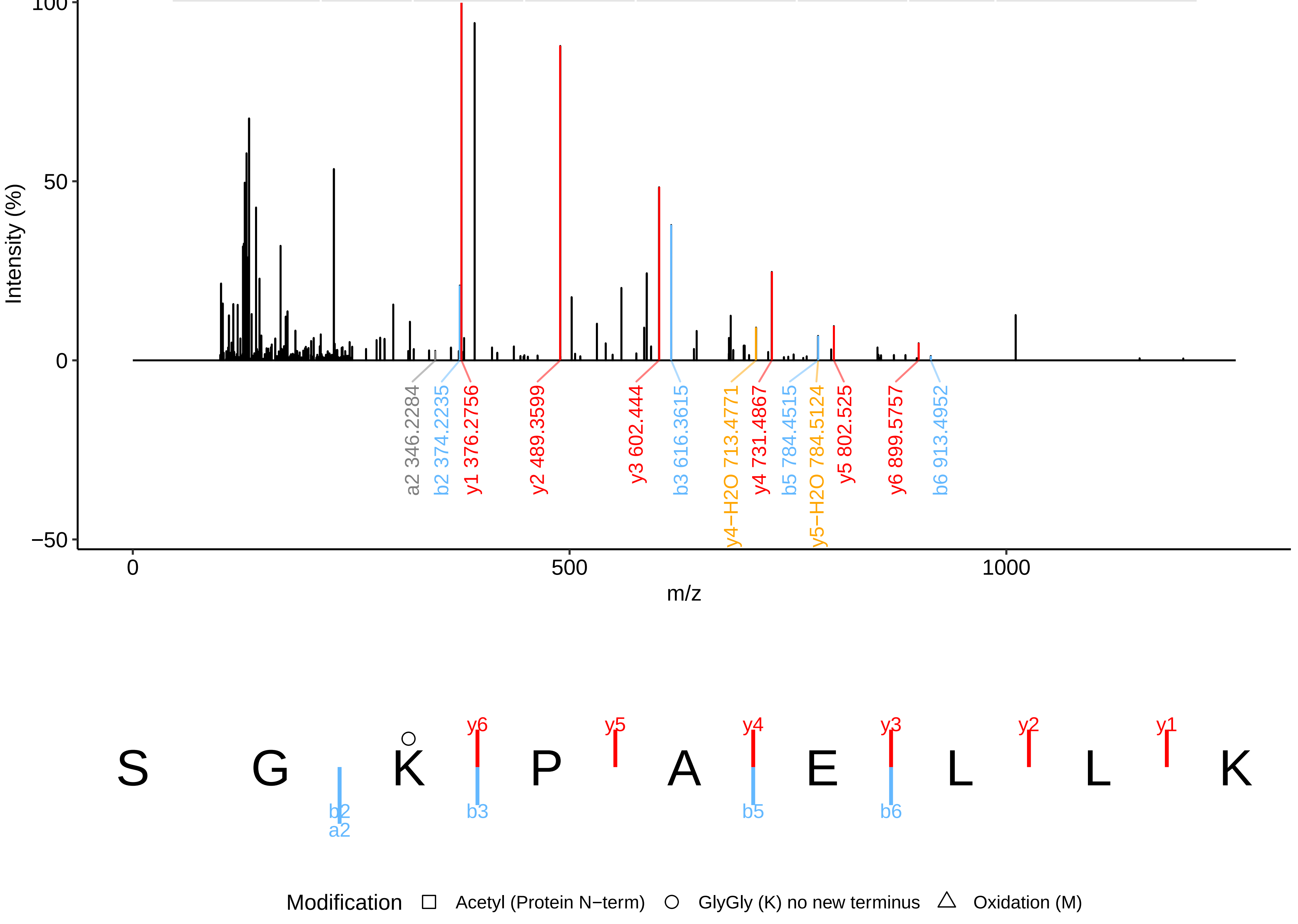

20200921_KIZ_MUb4_GlyGly_10plex_2 − Scan number: 10435

Gene Names	Charge	m/z	Mass	Retention time	PEP	Score	Precursor Intensity
TKT	3	352.8731	1055.598	39.922	0.10777	45.368	880981.4

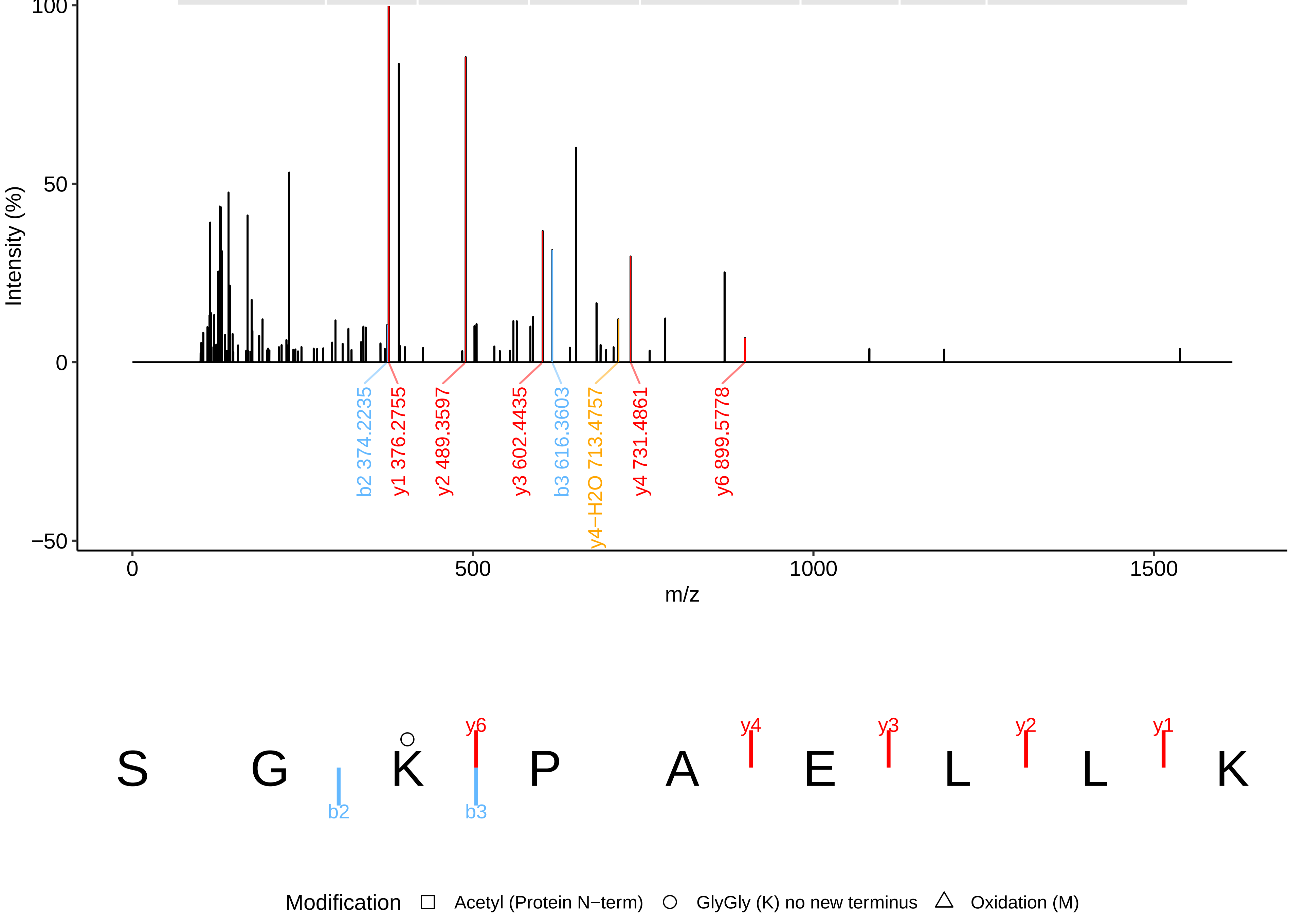

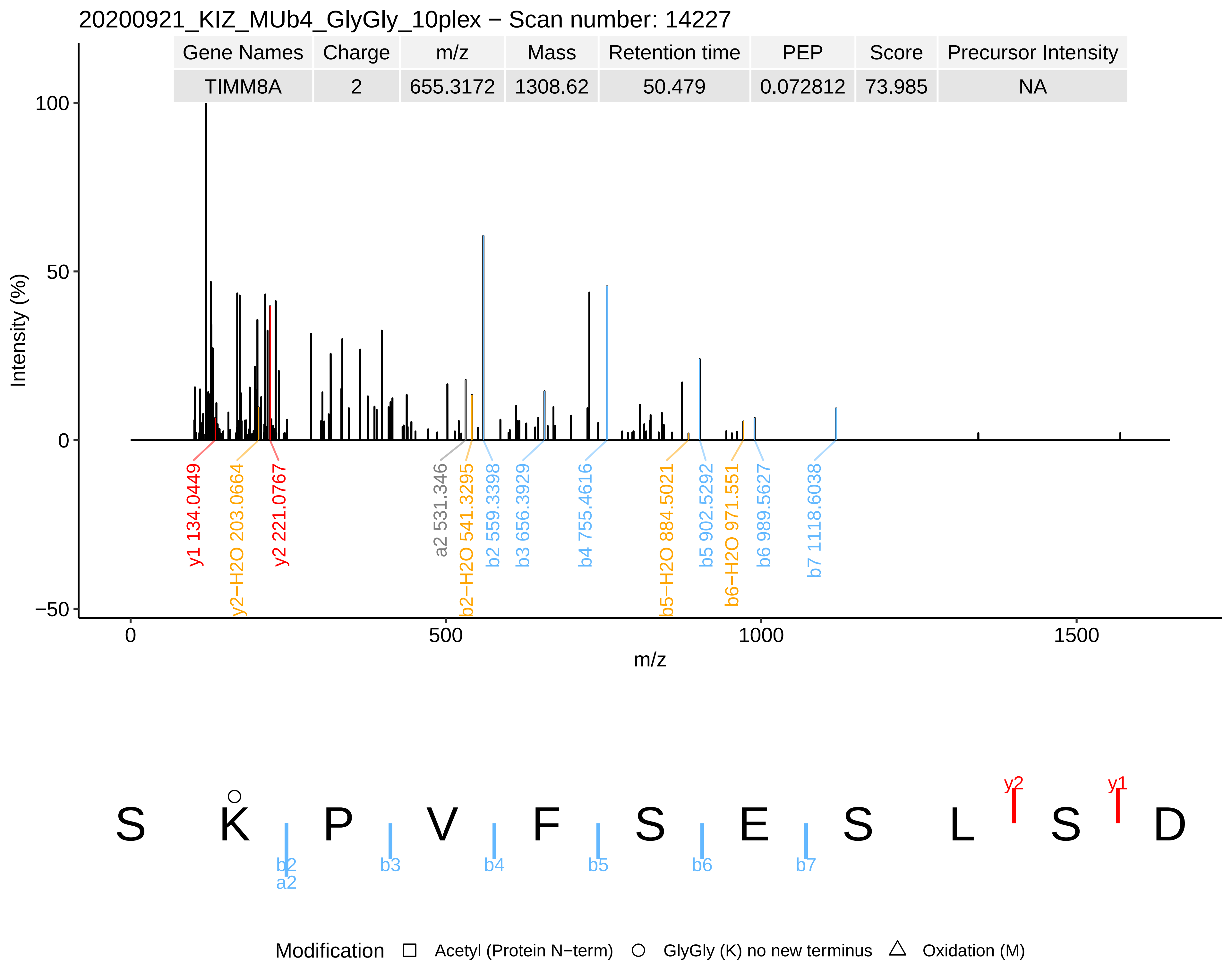
20200921_KIZ_MUb4_GlyGly_10plex − Scan number: 14227
Gene Names | Charge | m/z | Mass | Retention time | PEP | Score | Precursor Intensity
TIMM8A | 2 | 655.3172 | 1308.62 | 50.479 | 0.072812 | 73.985 | NA
Intensity (%)
m/z
y1 134.0449
y2−H2O 203.0664
y2 221.0767
a2 531.346
b2−H2O 541.3295
b2 559.3398
b3 656.3929
b4 755.4616
b5−H2O 884.5021
b5 902.5292
b6−H2O 971.551
b6 989.5627
b7 1118.6038
S K P V F S E S L S D
b2
a2
b3
b4
b5
b6
b7
y2
y1
Modification
Acetyl (Protein N−term)
GlyGly (K) no new terminus
Oxidation (M)

20200921_KIZ_MUb4_GlyGly_10plex − Scan number: 3265

Gene Names	Charge	m/z	Mass	Retention time	PEP	Score	Precursor Intensity
SLC1A3	3	425.8631	1274.567	10.217	0.0011187	86.879	3033009

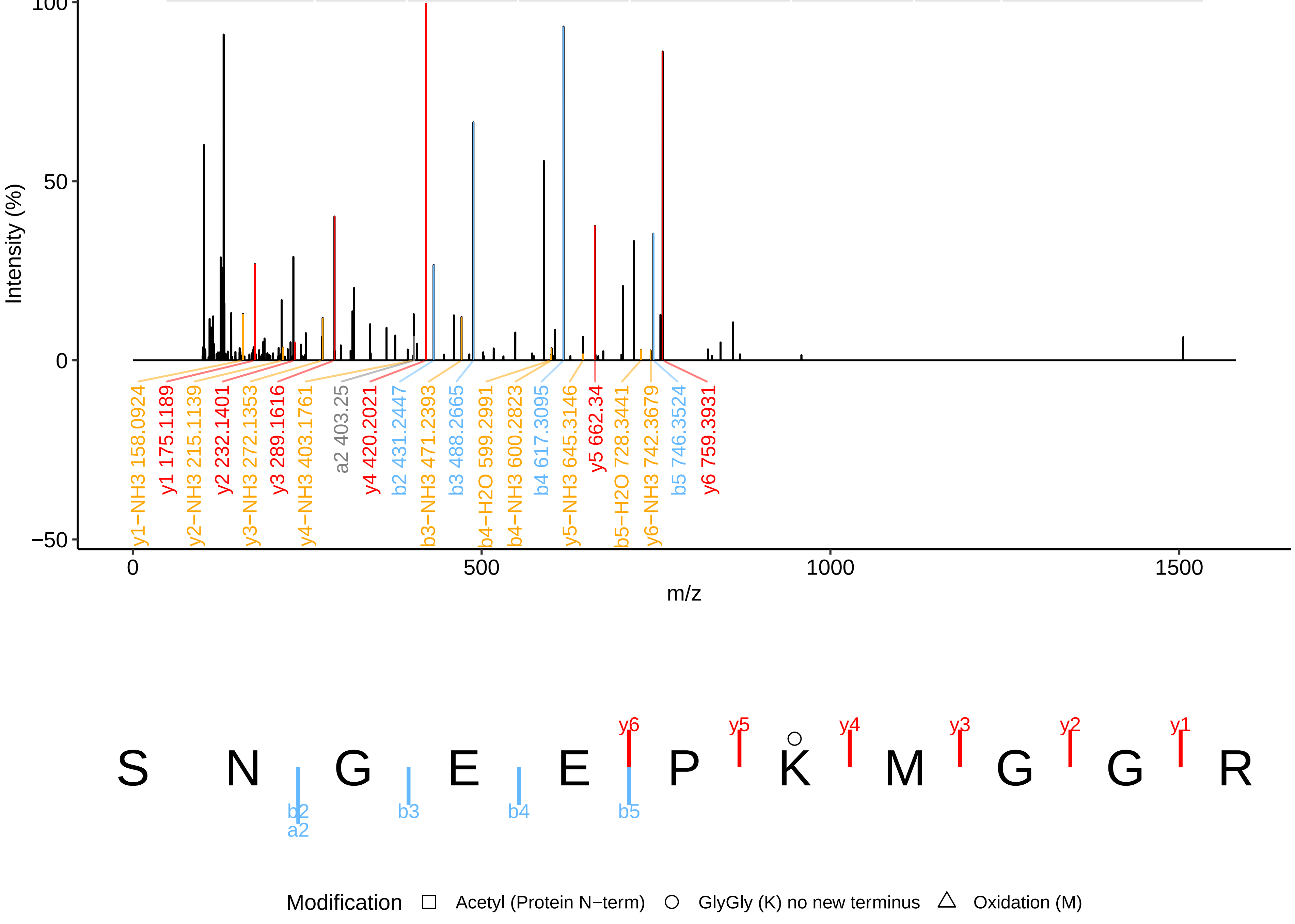

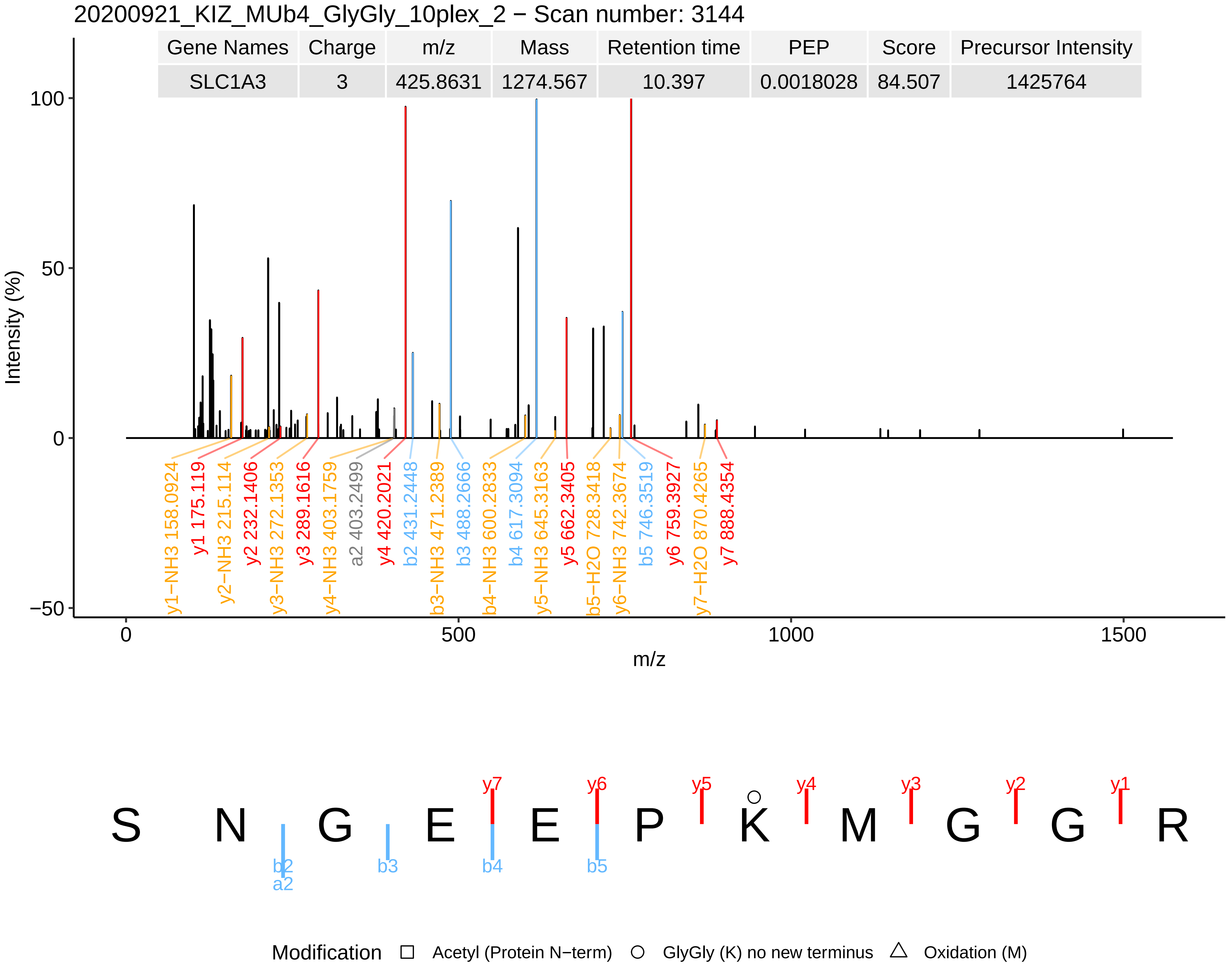

20200921_KIZ_MUb4_GlyGly_10plex_2 − Scan number: 3144
Gene Names | Charge | m/z | Mass | Retention time | PEP | Score | Precursor Intensity
SLC1A3 | 3 | 425.8631 | 1274.567 | 10.397 | 0.0018028 | 84.507 | 1425764
Intensity (%)
100
50
0
−50
m/z
0
500
1000
1500
y1−NH3 158.0924
y1 175.119
y2−NH3 215.114
y2 232.1406
y3−NH3 272.1353
y3 289.1616
y4−NH3 403.1759
a2 403.2499
y4 420.2021
b2 431.2448
b3−NH3 471.2389
b3 488.2666
b4−NH3 600.2833
b4 617.3094
y5−NH3 645.3163
y5 662.3405
b5−H2O 728.3418
y6−NH3 742.3674
b5 746.3519
y6 759.3927
y7−H2O 870.4265
y7 888.4354
S N G E E P K M G G R
y7 y6 y5 y4 y3 y2 y1
b2 a2 b3 b4 b5
Modification
Acetyl (Protein N−term)
GlyGly (K) no new terminus
Oxidation (M)

20200921_KIZ_MUb4_GlyGly_10plex − Scan number: 18026

Gene Names	Charge	m/z	Mass	Retention time	PEP	Score	Precursor Intensity
HUWE1	3	425.254	1272.74	64.741	0.00027134	123.86	NA

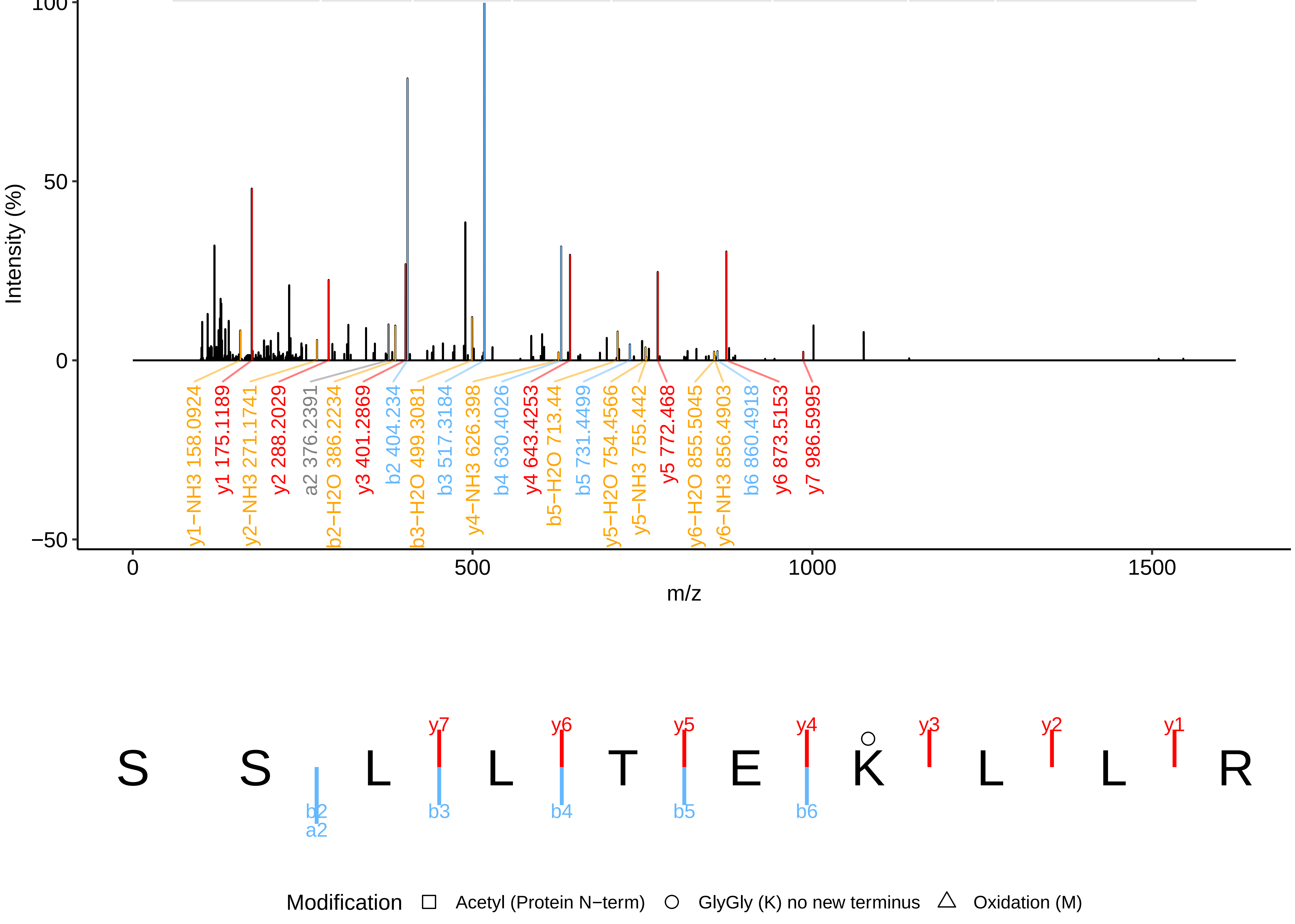

20200921_KIZ_MUb4_GlyGly_10plex − Scan number: 18245

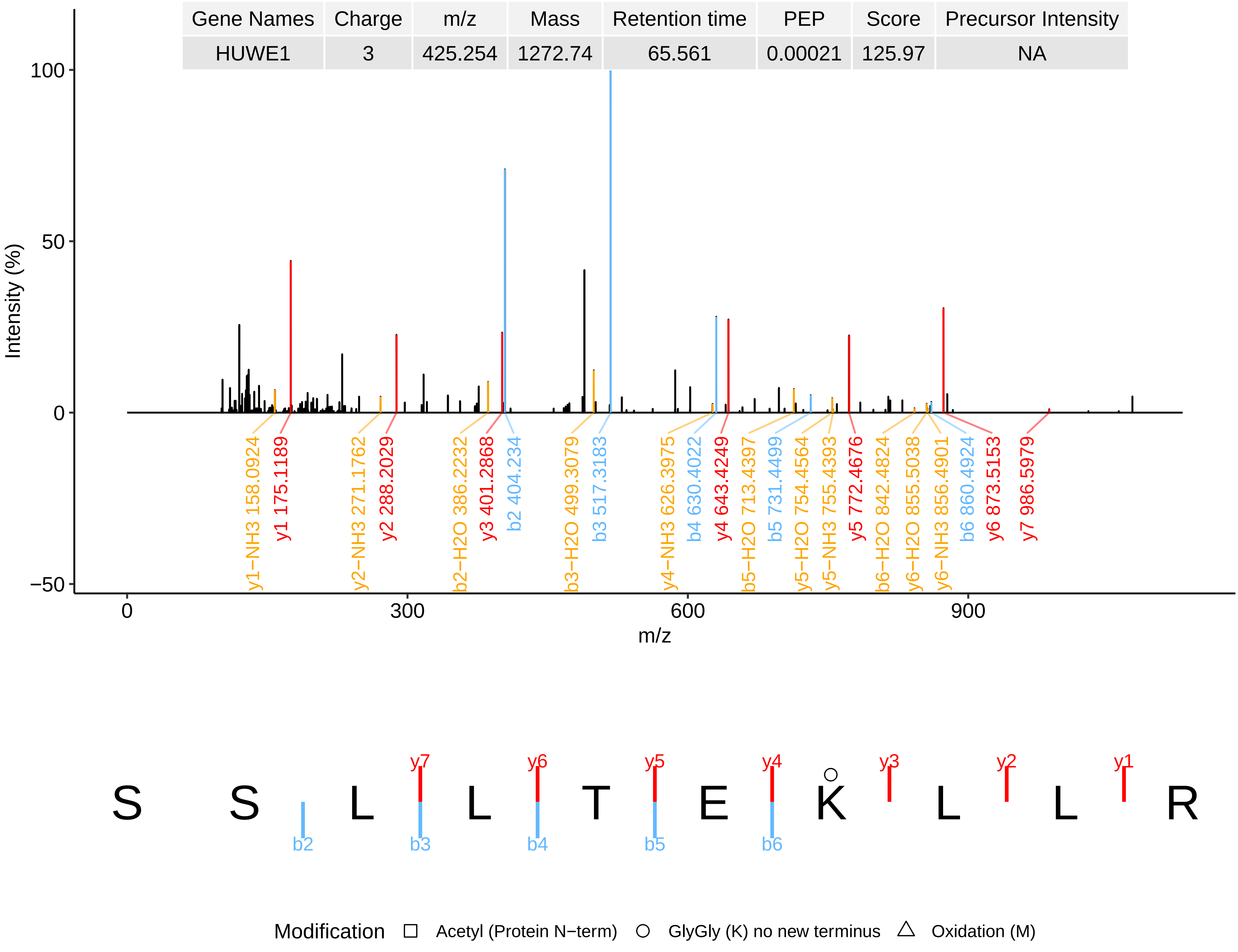

20200921_KIZ_MUb4_GlyGly_10plex_2 − Scan number: 16790

Gene Names	Charge	m/z	Mass	Retention time	PEP	Score	Precursor Intensity
HUWE1	3	425.254	1272.74	65.584	8.2818e−10	137.95	1872358

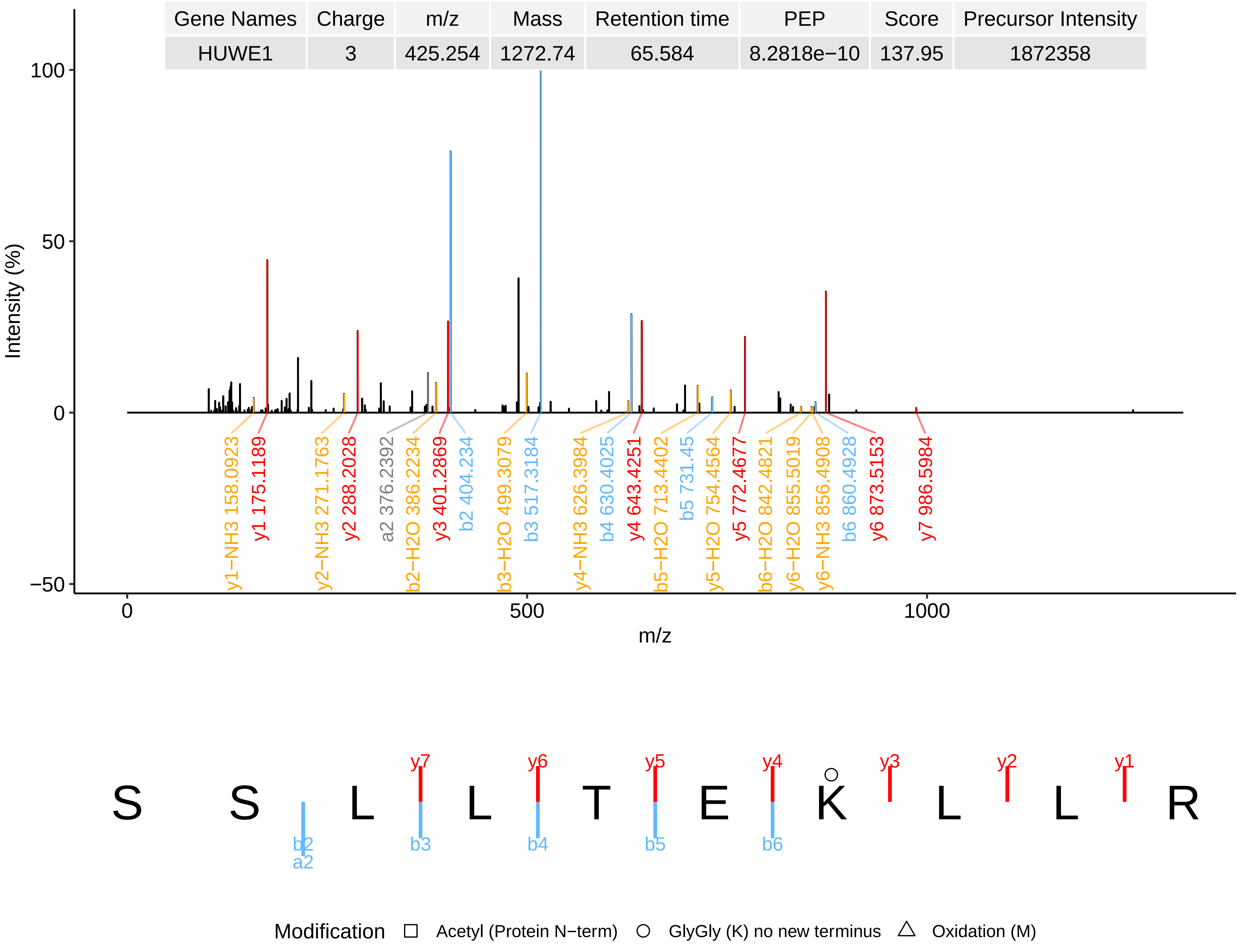

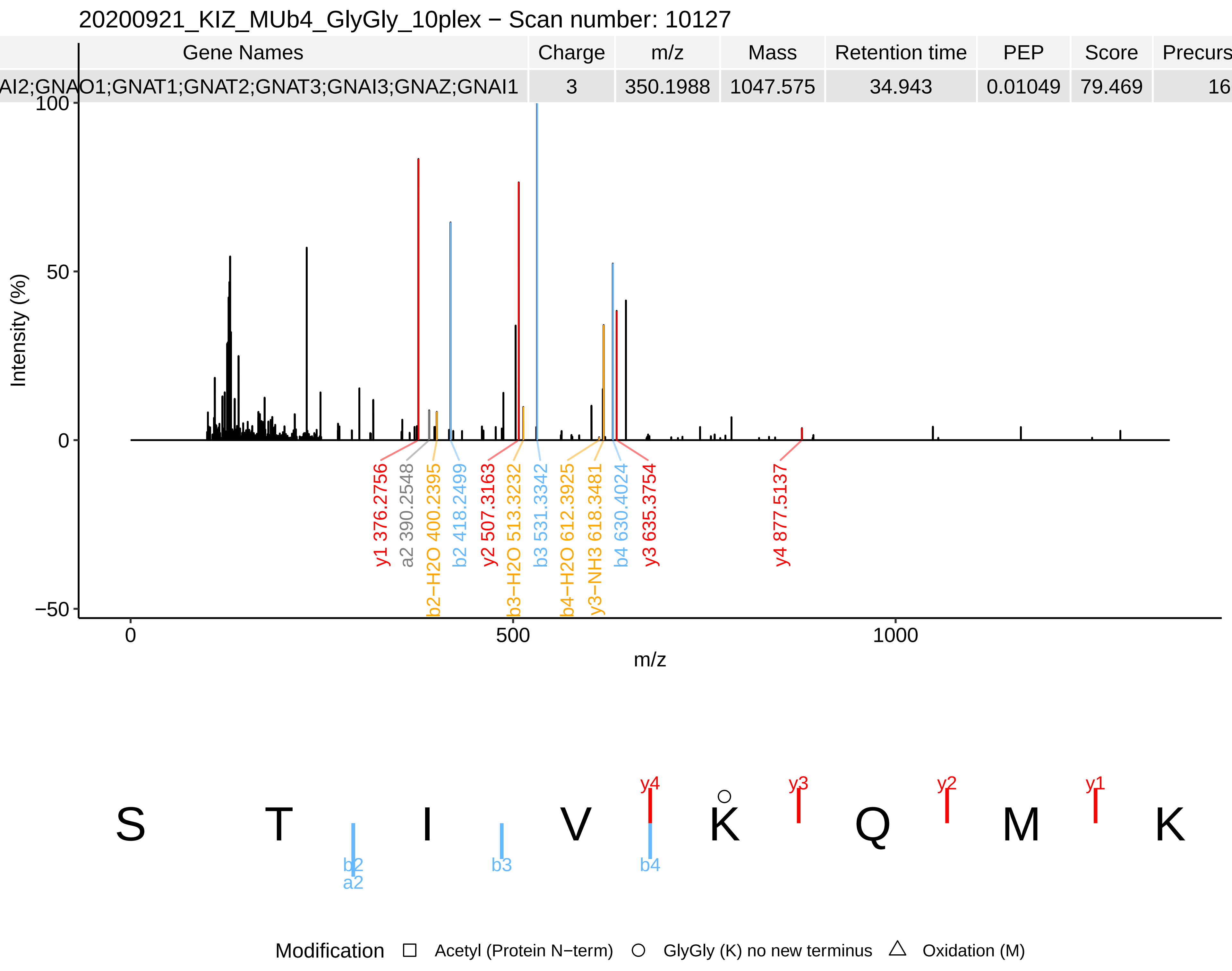

20200921_KIZ_MUb4_GlyGly_10plex − Scan number: 10127
Gene Names
Charge
m/z
Mass
Retention time
PEP
Score
Precurs
AI2;GNAO1;GNAT1;GNAT2;GNAT3;GNAI3;GNAZ;GNAI1
3
350.1988
1047.575
34.943
0.01049
79.469
16
Intensity (%)
100
50
0
−50
0
500
1000
m/z
y1 376.2756
a2 390.2548
b2−H2O 400.2395
b2 418.2499
y2 507.3163
b3−H2O 513.3232
b3 531.3342
b4−H2O 612.3925
y3−NH3 618.3481
b4 630.4024
y3 635.3754
y4 877.5137
S T I V K Q M K
y4 y3 y2 y1
b2 a2 b3 b4
Modification
Acetyl (Protein N−term)
GlyGly (K) no new terminus
Oxidation (M)

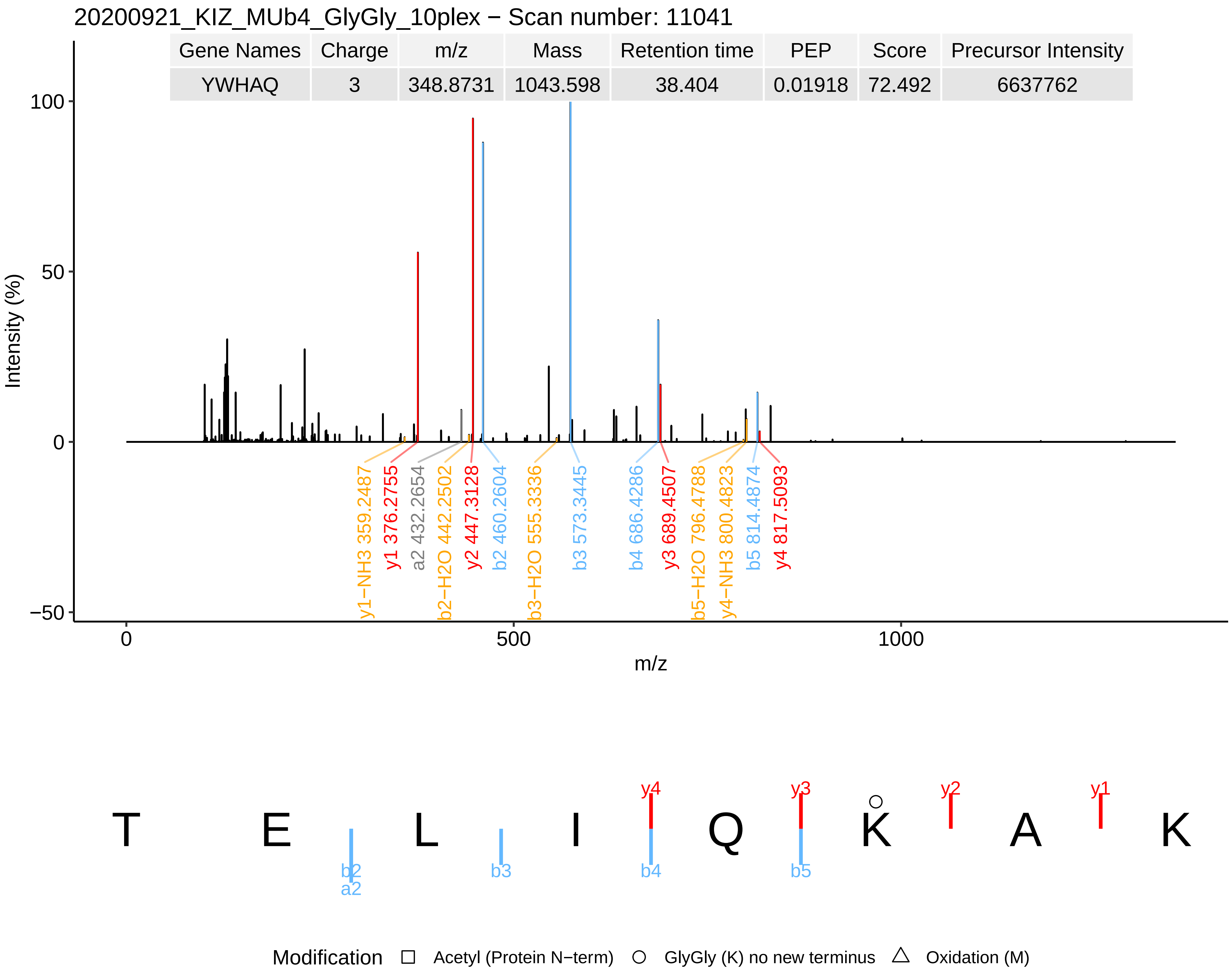

20200921_KIZ_MUb4_GlyGly_10plex − Scan number: 11041
Gene Names | Charge | m/z | Mass | Retention time | PEP | Score | Precursor Intensity
YWHAQ | 3 | 348.8731 | 1043.598 | 38.404 | 0.01918 | 72.492 | 6637762
Intensity (%)
100
50
0
−50
y1−NH3 359.2487
y1 376.2755
a2 432.2654
b2−H2O 442.2502
y2 447.3128
b2 460.2604
b3−H2O 555.3336
b3 573.3445
b4 686.4286
y3 689.4507
b5−H2O 796.4788
y4−NH3 800.4823
b5 814.4874
y4 817.5093
0
500
1000
m/z
T E L I Q K A K
y4 y3 y2 y1
b2 a2 b3 b4 b5
Modification
Acetyl (Protein N−term)
GlyGly (K) no new terminus
Oxidation (M)

20200921_KIZ_MUb4_GlyGly_10plex_2 − Scan number: 10204

Gene Names	Charge	m/z	Mass	Retention time	PEP	Score	Precursor Intensity
YWHAQ	3	348.8731	1043.598	38.979	0.034781	62.2	944456.6

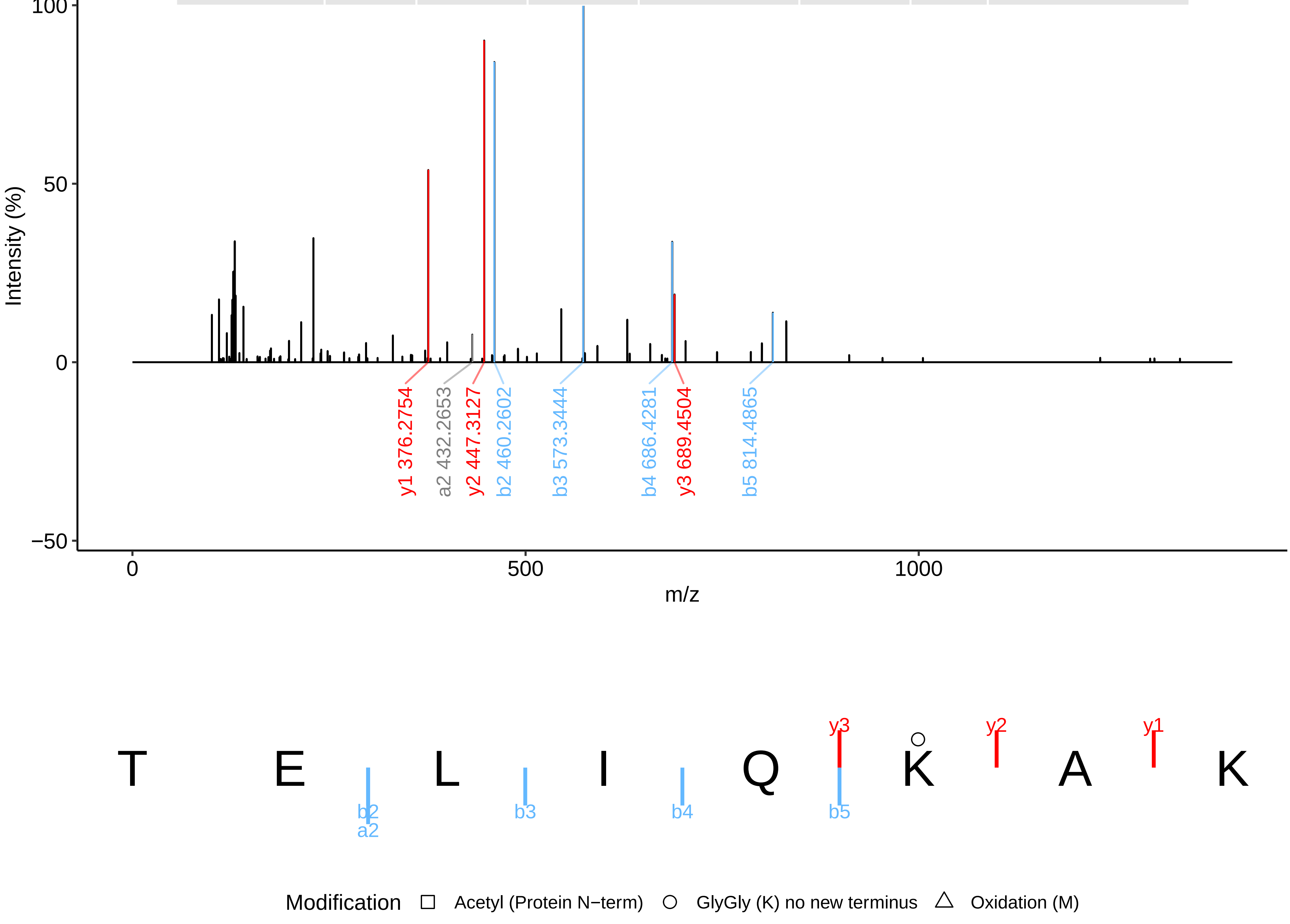

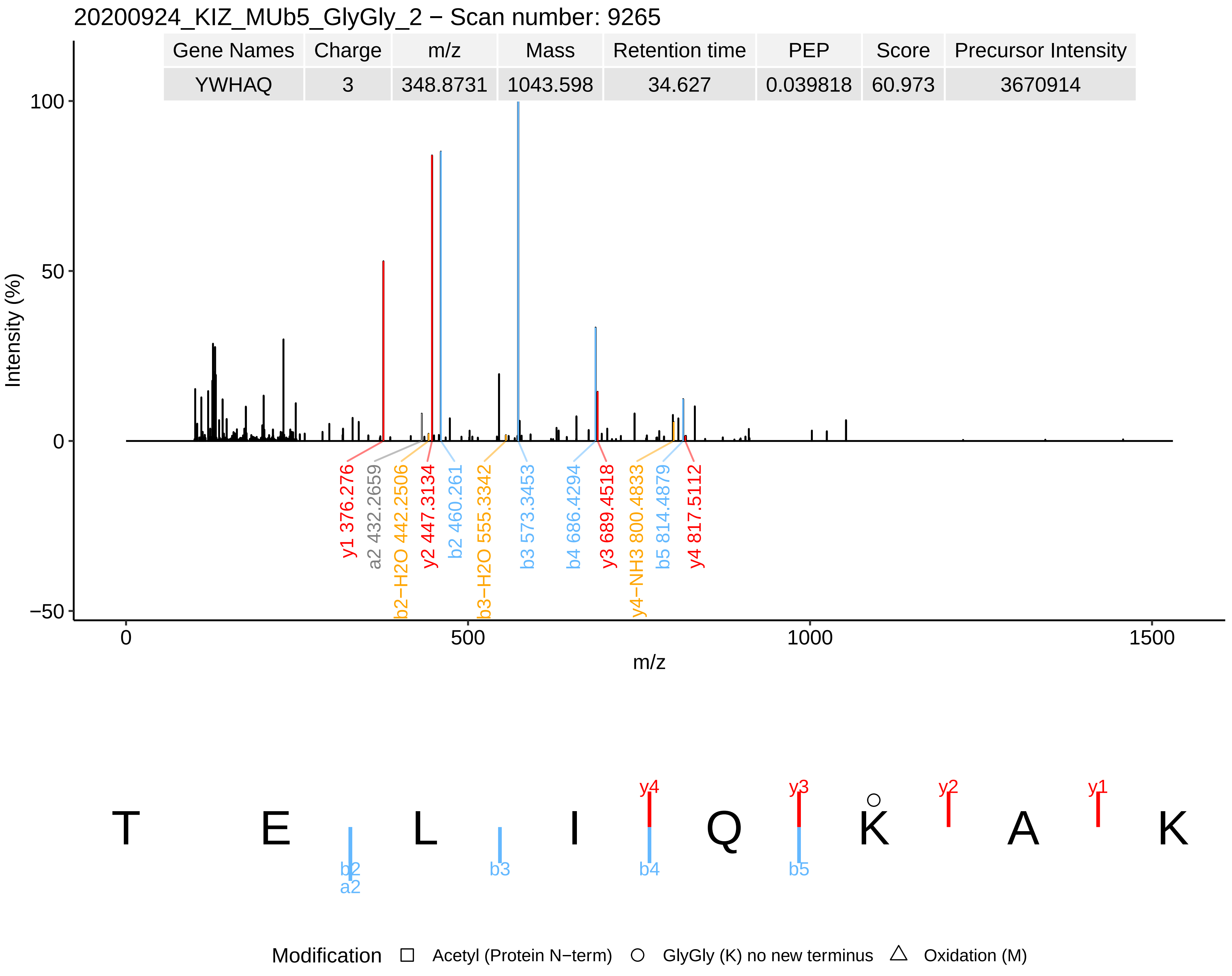
20200924_KIZ_MUb5_GlyGly_2 − Scan number: 9265
Gene Names | Charge | m/z | Mass | Retention time | PEP | Score | Precursor Intensity
YWHAQ | 3 | 348.8731 | 1043.598 | 34.627 | 0.039818 | 60.973 | 3670914
Intensity (%)
m/z
y1 376.276
a2 432.2659
b2−H2O 442.2506
y2 447.3134
b2 460.261
b3−H2O 555.3342
b3 573.3453
b4 686.4294
y3 689.4518
y4−NH3 800.4833
b5 814.4879
y4 817.5112
T E L I Q K A K
y4 y3 y2 y1
b2 a2 b3 b4 b5
Modification □ Acetyl (Protein N−term) ○ GlyGly (K) no new terminus △ Oxidation (M)

20200924_KIZ_MUb5_GlyGly_2 − Scan number: 9399

Gene Names	Charge	m/z	Mass	Retention time	PEP	Score	Precursor Intensity
YWHAQ	3	348.8731	1043.598	35.128	0.039818	60.973	6097308

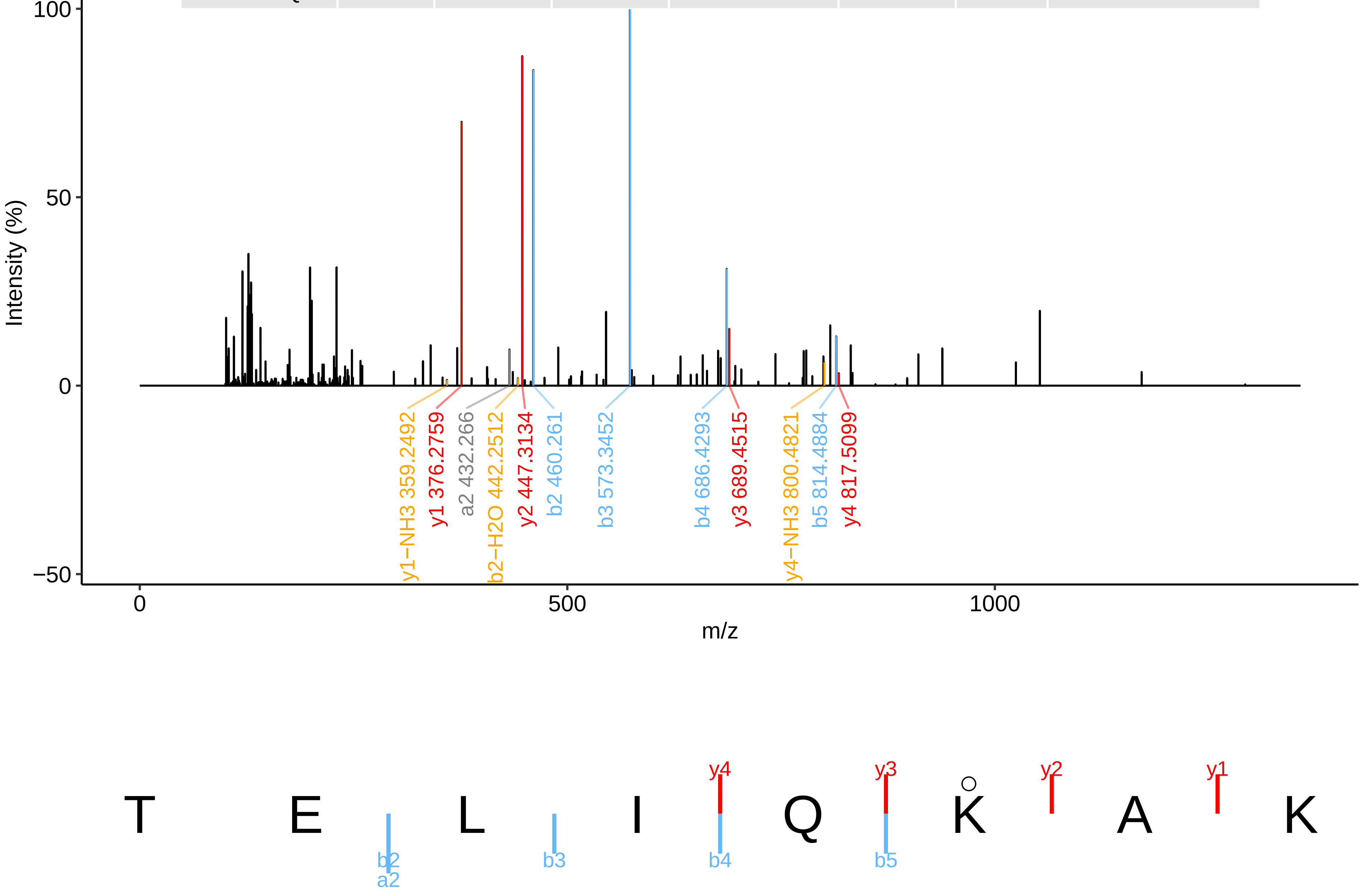

Modification □ Acetyl (Protein N−term) ○ GlyGly (K) no new terminus △ Oxidation (M)

20201118_KIZ_MUb6_GlyGly_2 − Scan number: 11885

Gene Names	Charge	m/z	Mass	Retention time	PEP	Score	Precursor Intensity
YWHAQ	3	348.8731	1043.598	40.071	0.0066497	86.794	12216633

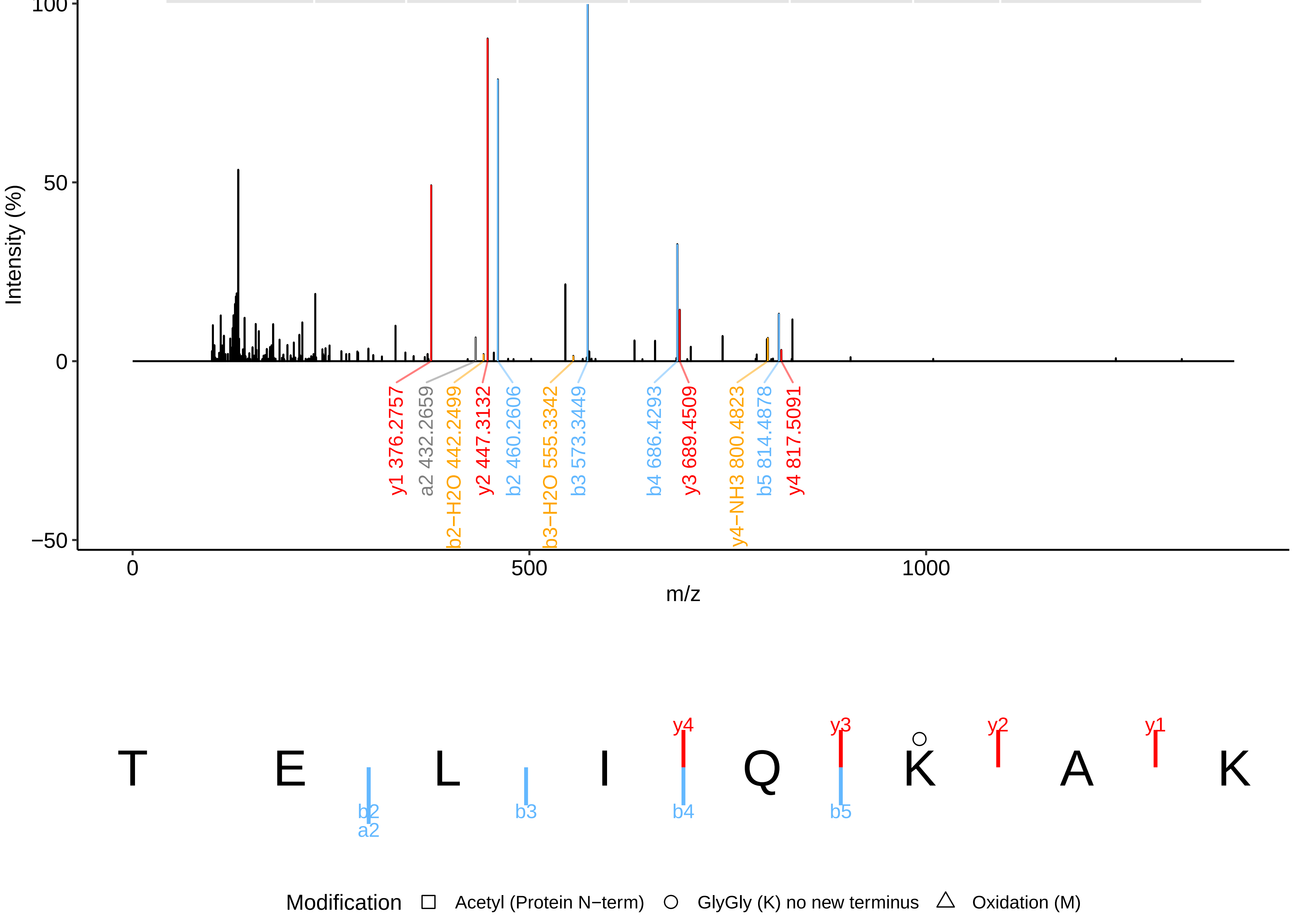

20200921_KIZ_MUb4_GlyGly_10plex − Scan number: 6504

Gene Names	Charge	m/z	Mass	Retention time	PEP	Score	Precursor Intensity
SLC7A1	2	631.2775	1260.541	21.542	0.011345	93.551	5509532

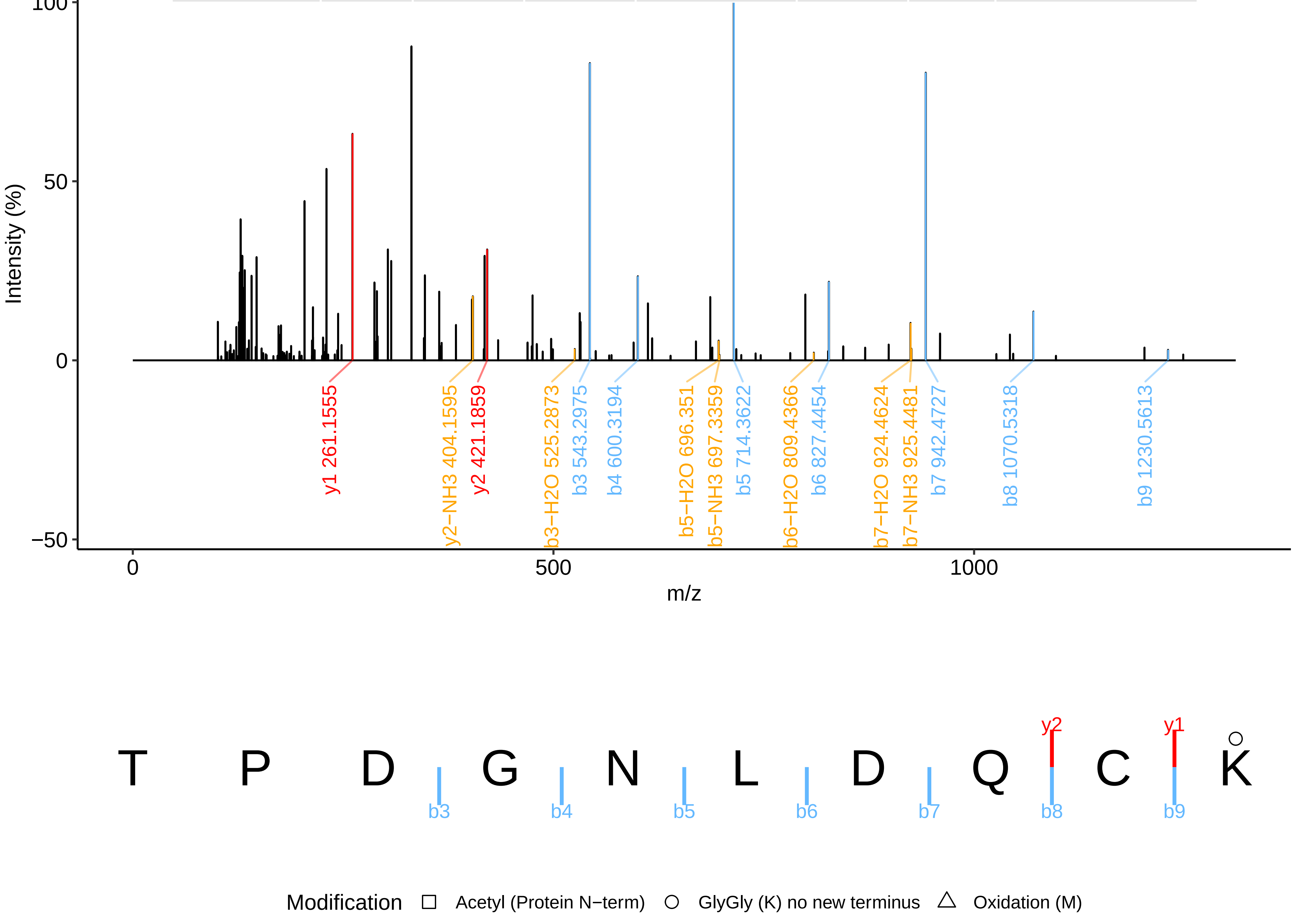

20200921_KIZ_MUb4_GlyGly_10plex − Scan number: 2939

Gene Names	Charge	m/z	Mass	Retention time	PEP	Score	Precursor Intensity
YWHAG	3	355.172	1062.494	9.2089	0.072231	72.928	5311497

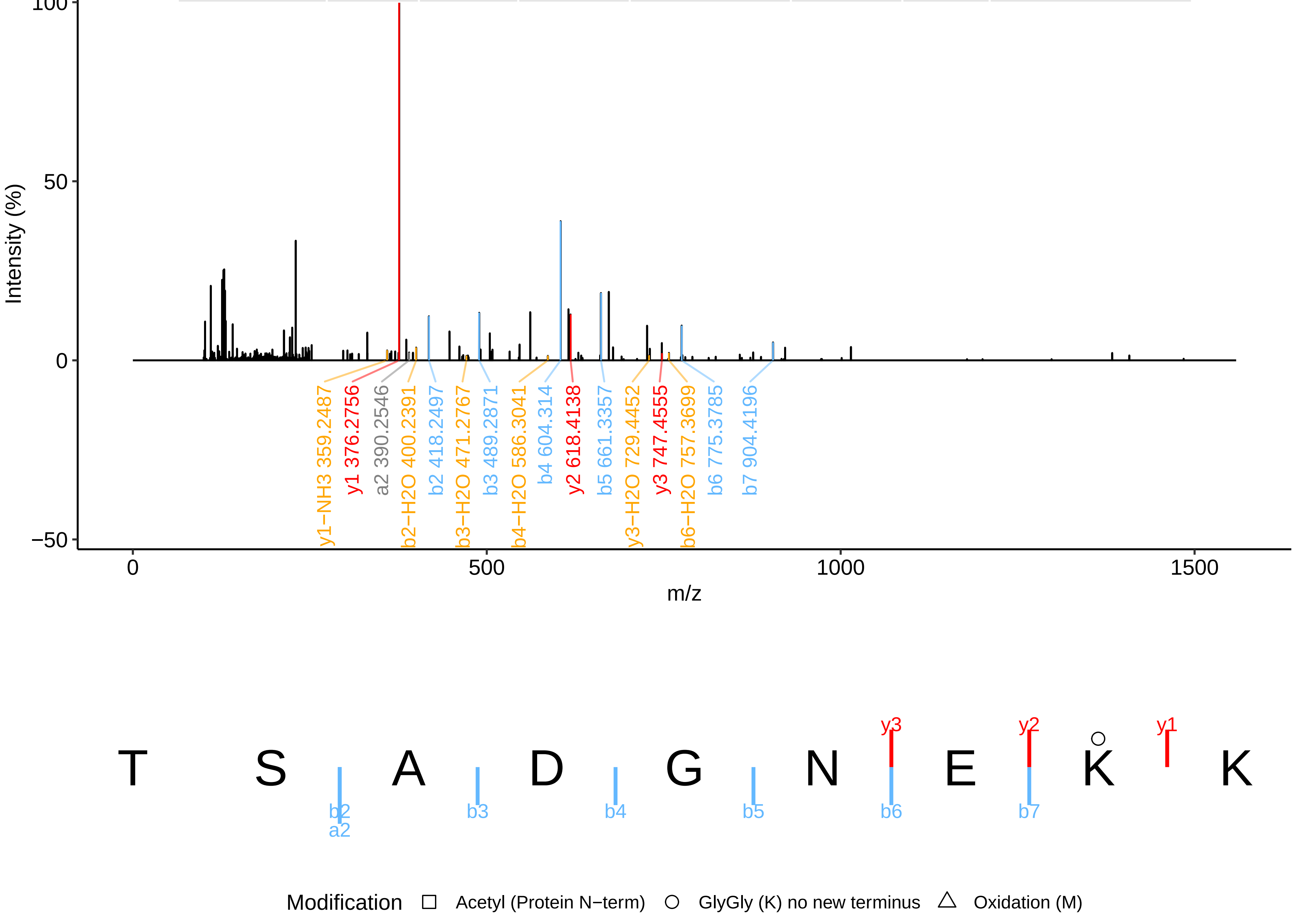

20200921_KIZ_MUb4_GlyGly_10plex_2 − Scan number: 11995

Gene Names	Charge	m/z	Mass	Retention time	PEP	Score	Precursor Intensity
SKP1	3	359.2025	1074.586	46.312	0.063391	54.608	745211.1

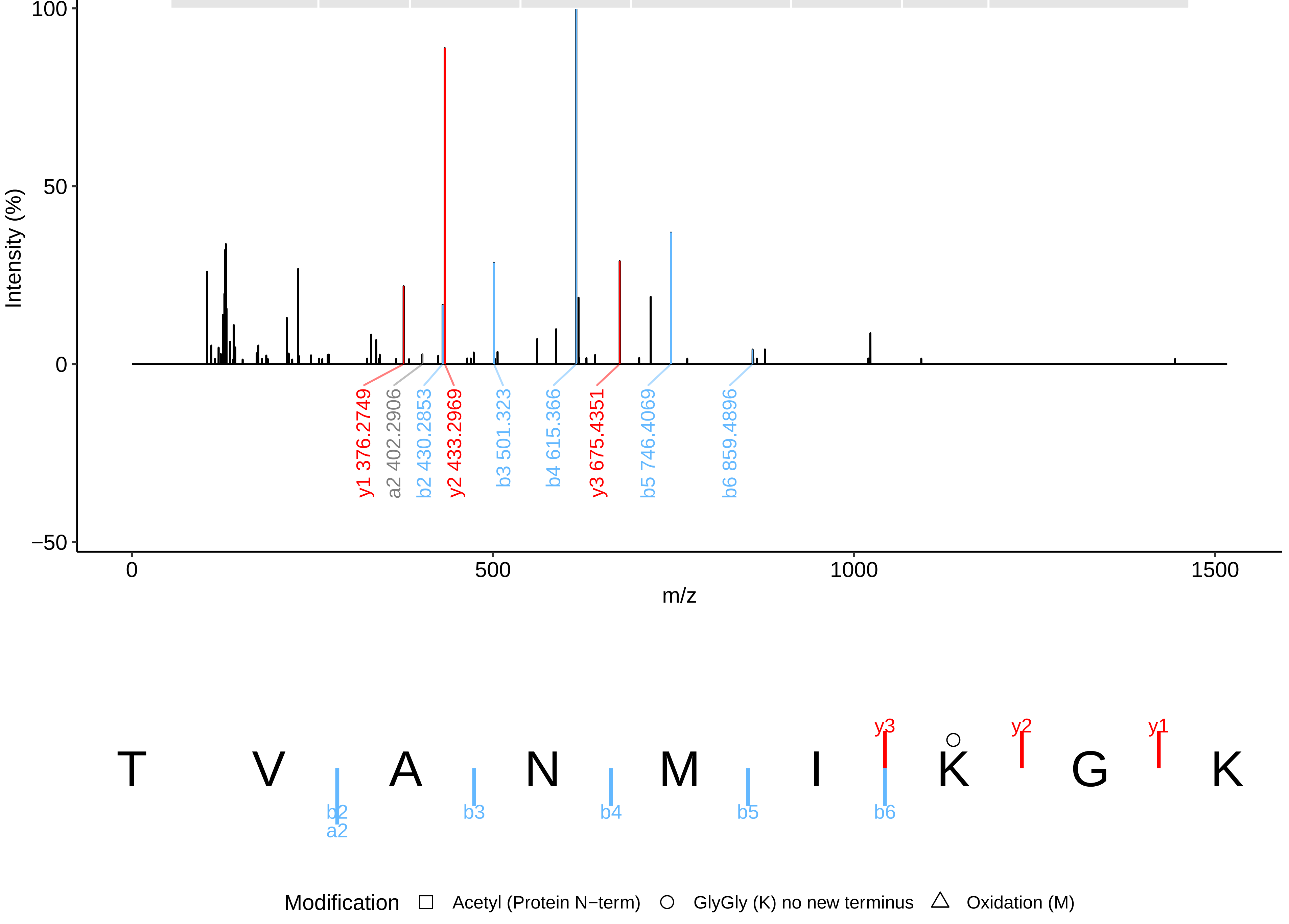

20200924_KIZ_MUb5_GlyGly_1 − Scan number: 11820

Gene Names	Charge	m/z	Mass	Retention time	PEP	Score	Precursor Intensity
SKP1	3	359.2025	1074.586	38.359	0.023761	62.823	5670227

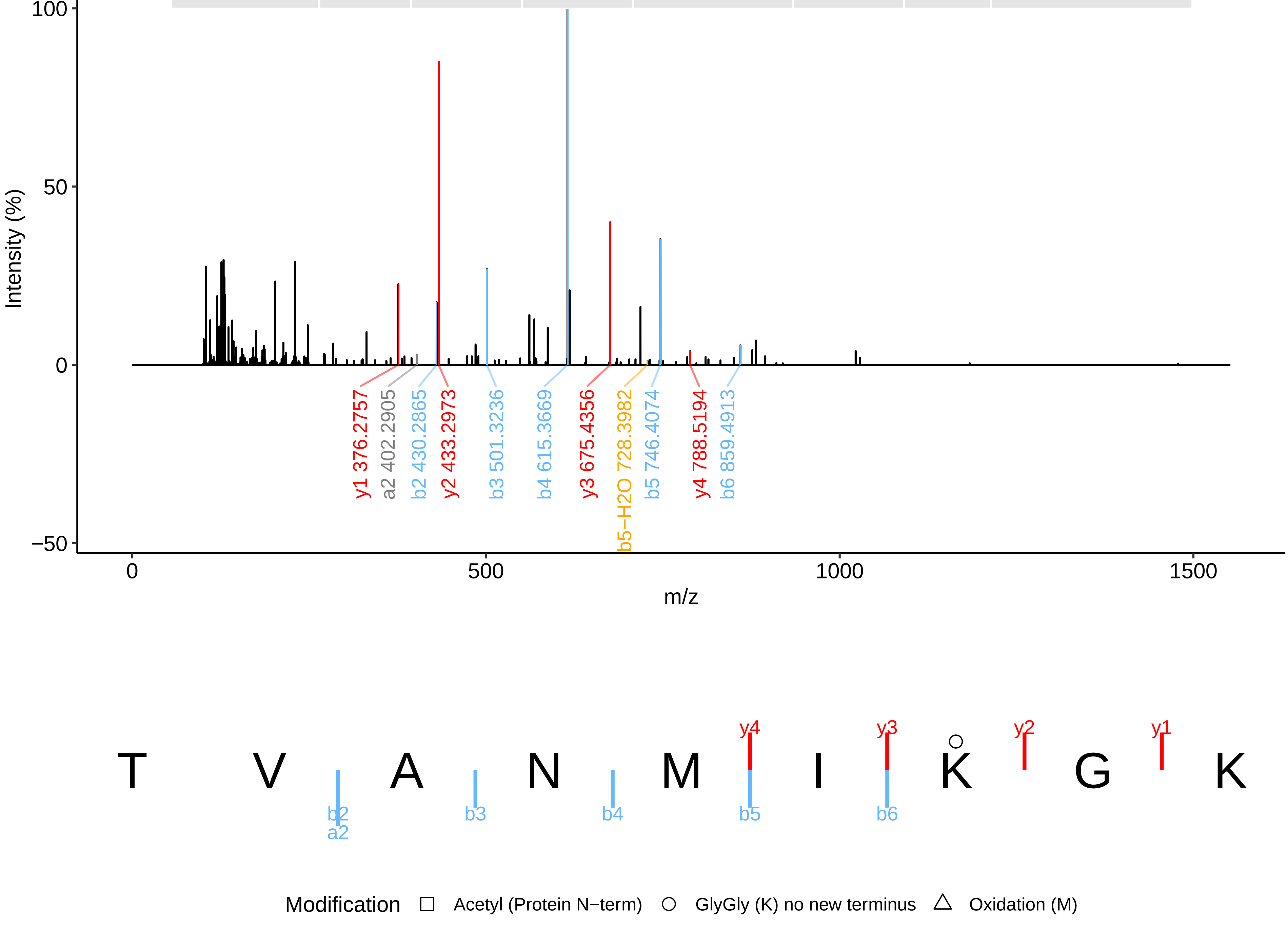

20201118_KIZ_MUb6_GlyGly_2 − Scan number: 14124

Gene Names	Charge	m/z	Mass	Retention time	PEP	Score	Precursor Intensity
SKP1	3	359.2025	1074.586	47.574	0.02872	62.823	5056811

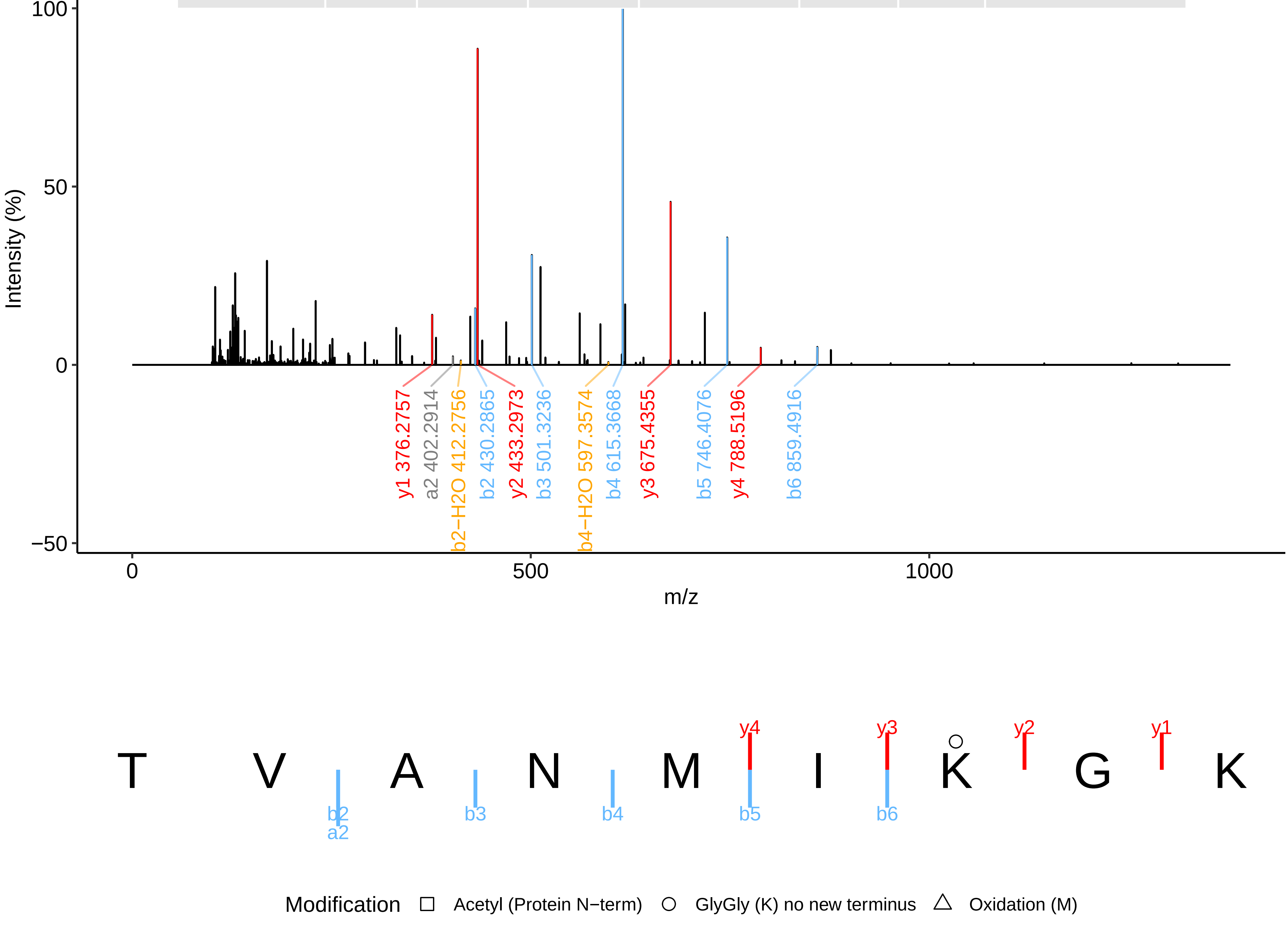

20200921_KIZ_MUb4_GlyGly_10plex − Scan number: 7406

Gene Names	Charge	m/z	Mass	Retention time	PEP	Score	Precursor Intensity
YWHAG	3	499.9426	1496.806	24.85	1.0952e−08	93.258	6982657

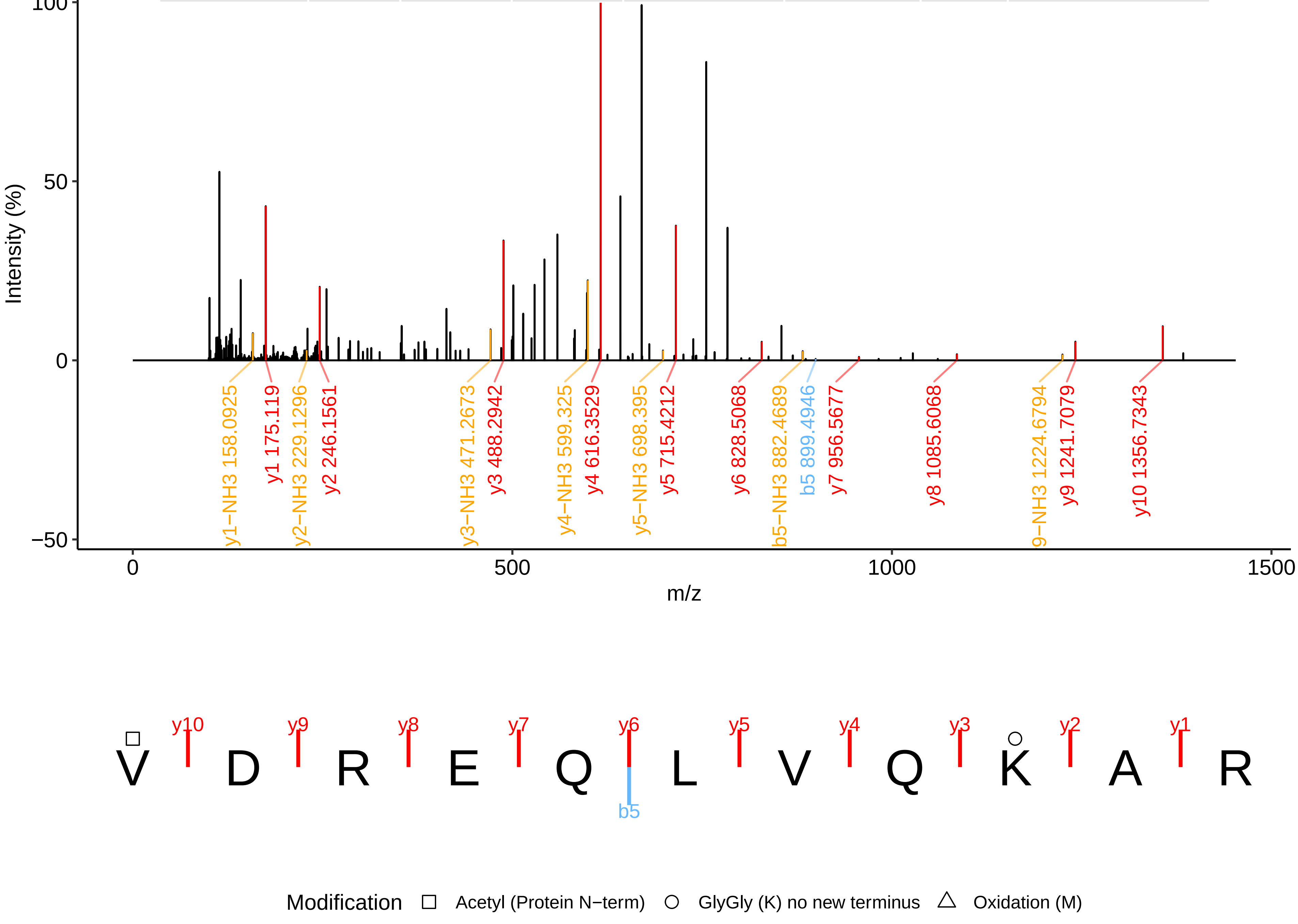

20200921_KIZ_MUb4_GlyGly_10plex_2 − Scan number: 6795

Gene Names	Charge	m/z	Mass	Retention time	PEP	Score	Precursor Intensity
YWHAG	3	499.9426	1496.806	25.128	0.0043058	47.302	1514451

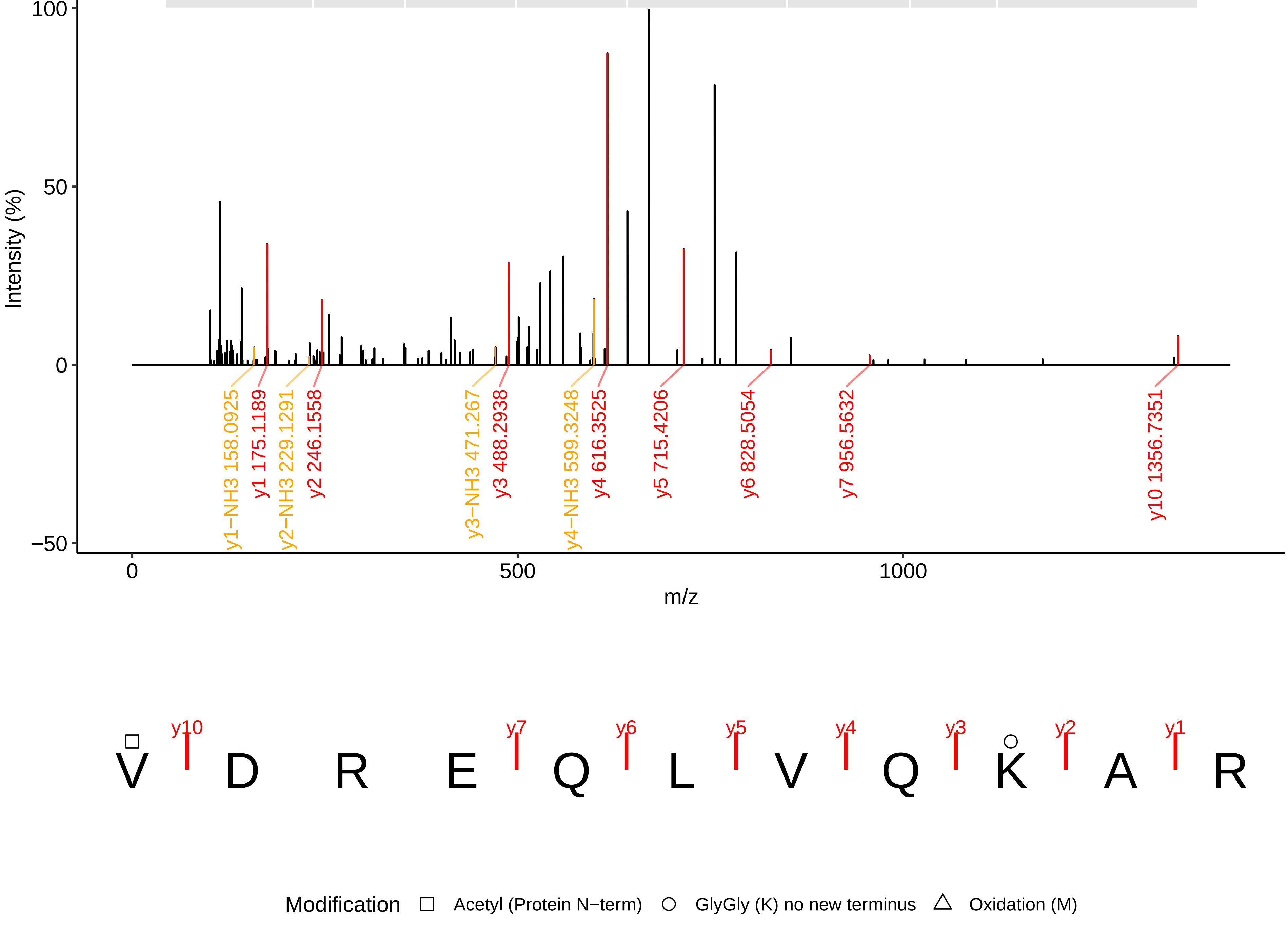

20200924_KIZ_MUb5_GlyGly_1 − Scan number: 6508

Gene Names	Charge	m/z	Mass	Retention time	PEP	Score	Precursor Intensity
YWHAG	3	499.9426	1496.806	20.47	5.8299e−07	74.944	21235176

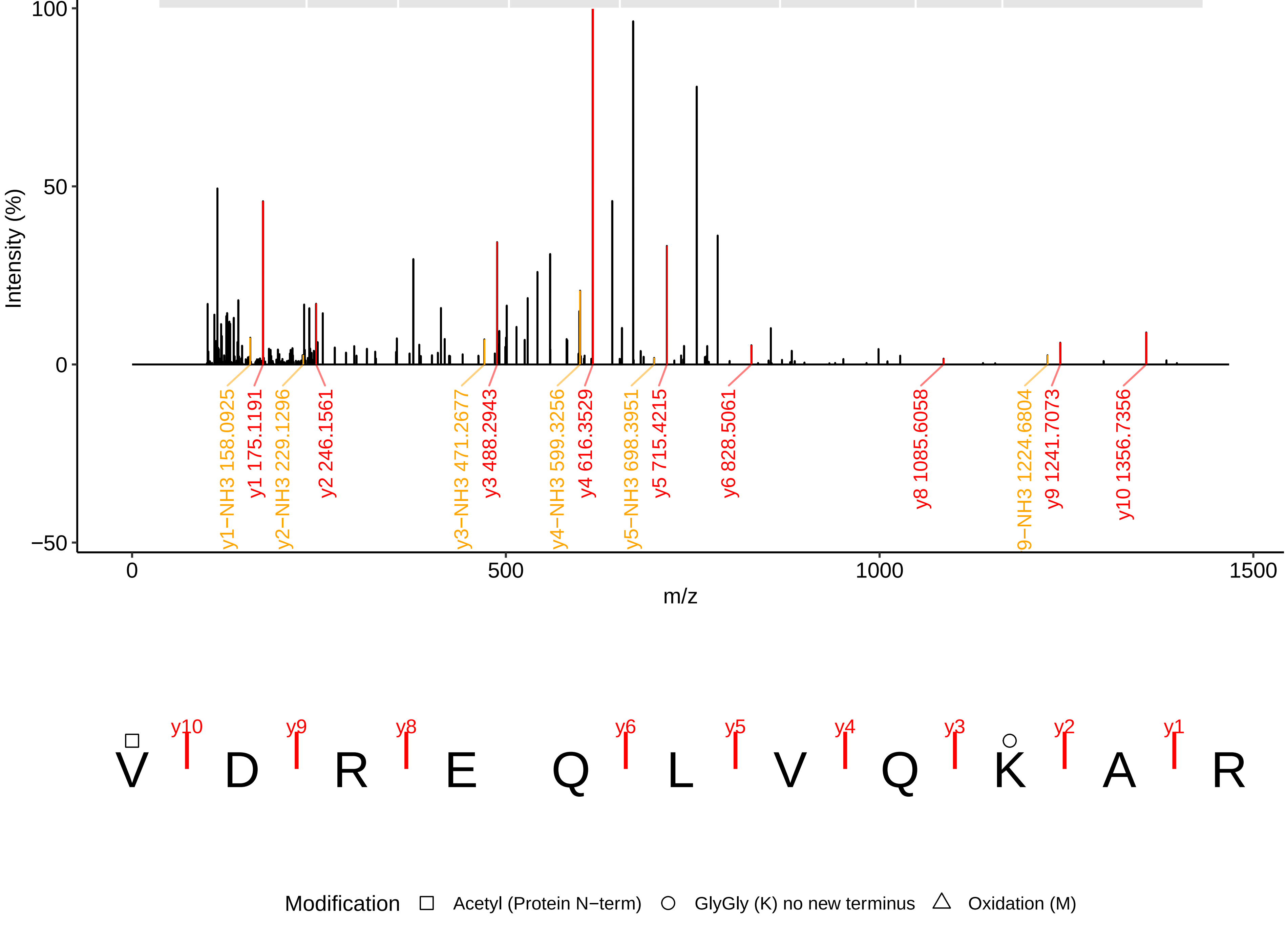

20200924_KIZ_MUb5_GlyGly_2 − Scan number: 6028

Gene Names	Charge	m/z	Mass	Retention time	PEP	Score	Precursor Intensity
YWHAG	3	499.9426	1496.806	22.443	0.00027214	57.532	4871927

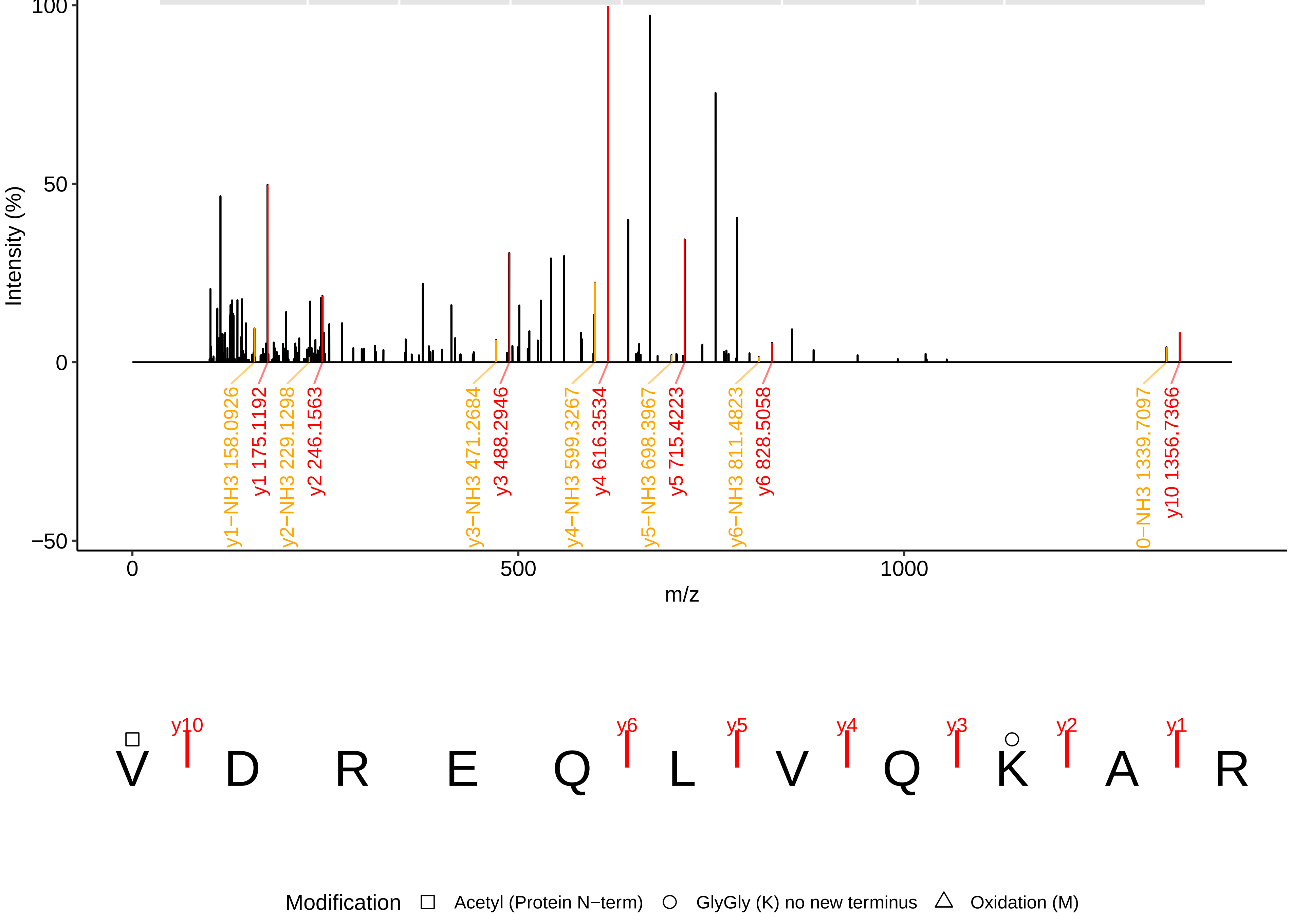

20201118_KIZ_MUb6_GlyGly_2 − Scan number: 7491

Gene Names	Charge	m/z	Mass	Retention time	PEP	Score	Precursor Intensity
YWHAG	3	499.9426	1496.806	25.554	0.00066009	74.944	6928127

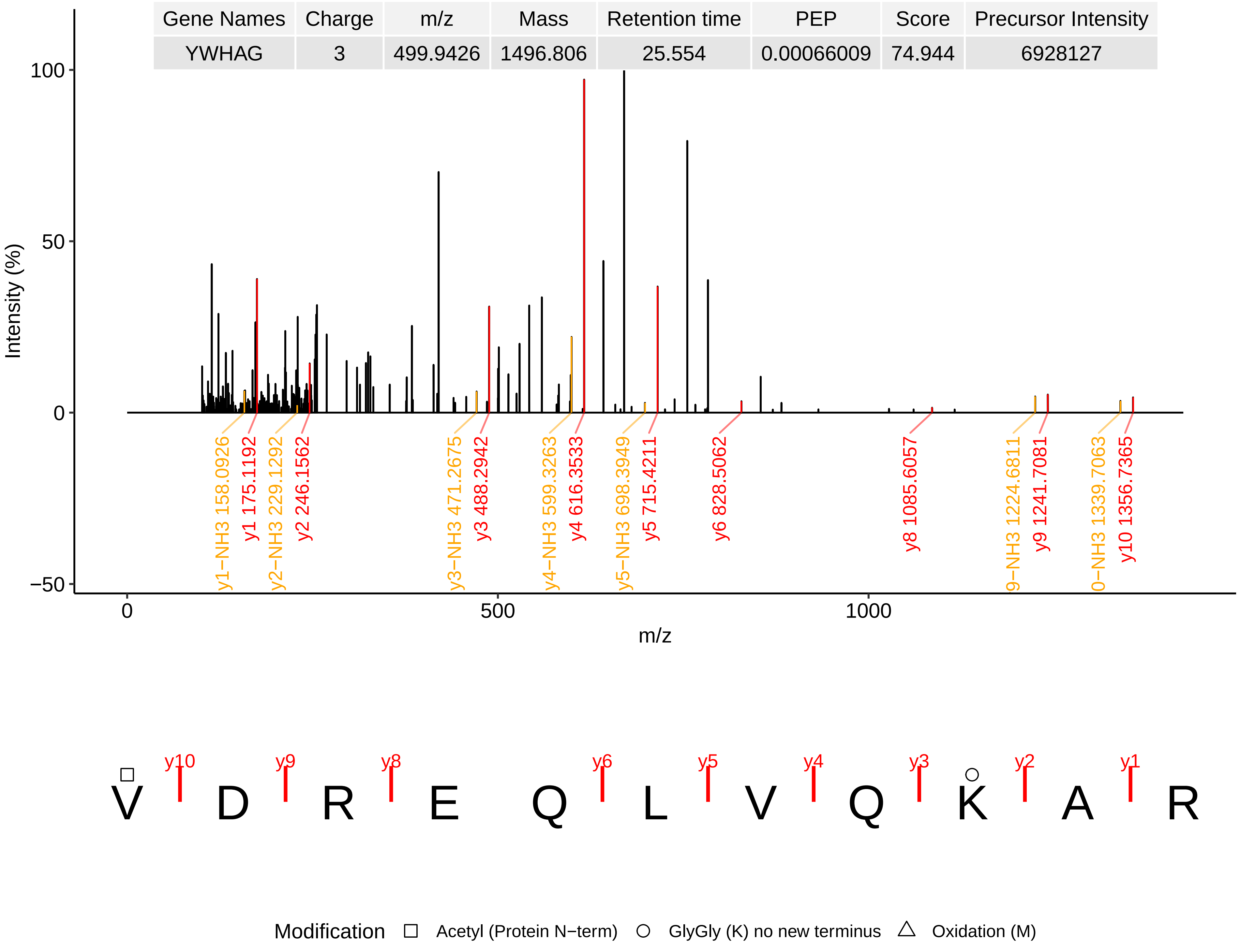

20200921_KIZ_MUb4_GlyGly_10plex − Scan number: 7937

Gene Names	Charge	m/z	Mass	Retention time	PEP	Score	Precursor Intensity
RPL18A	3	426.2153	1275.624	26.805	0.0011364	101.66	6819486

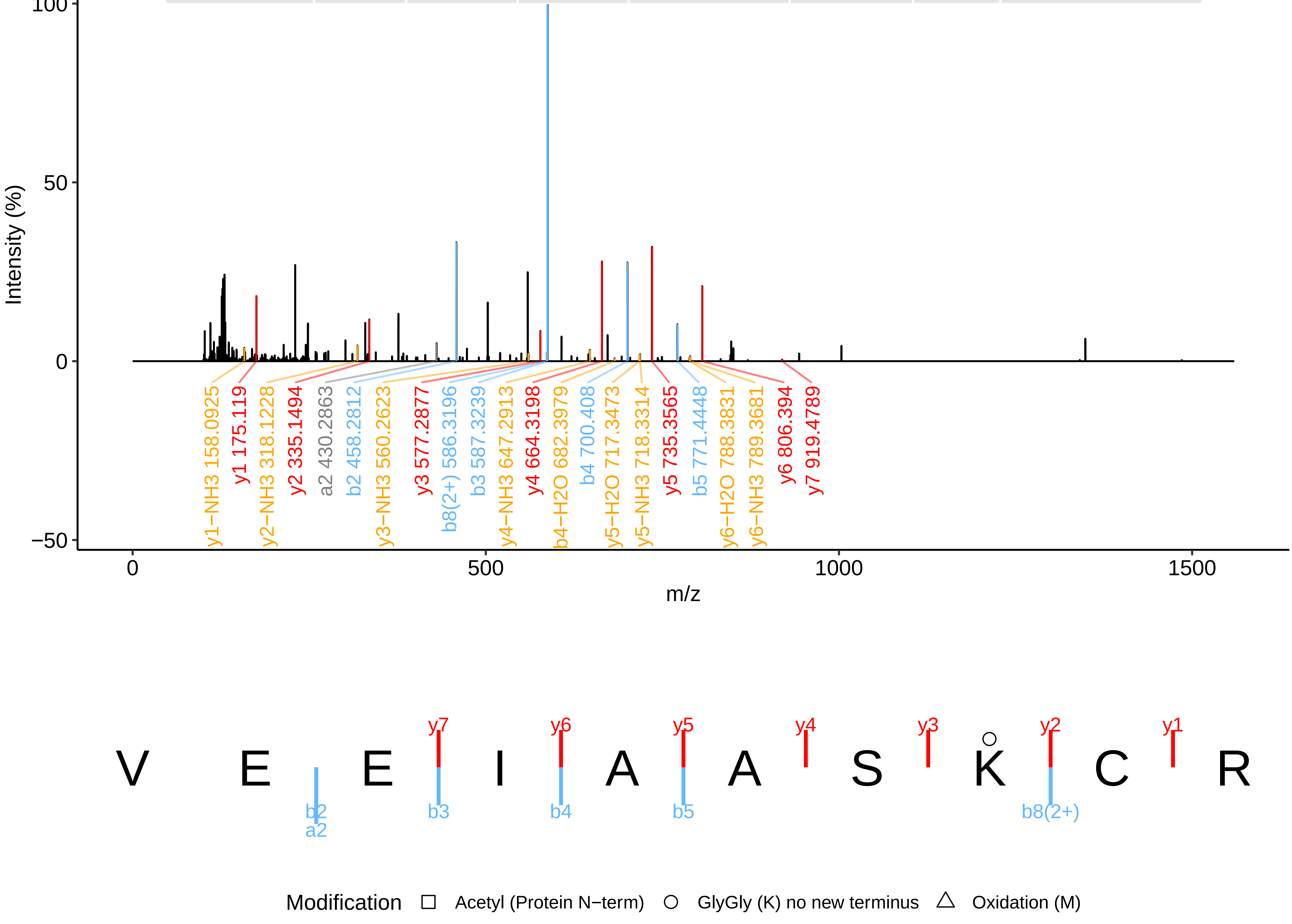

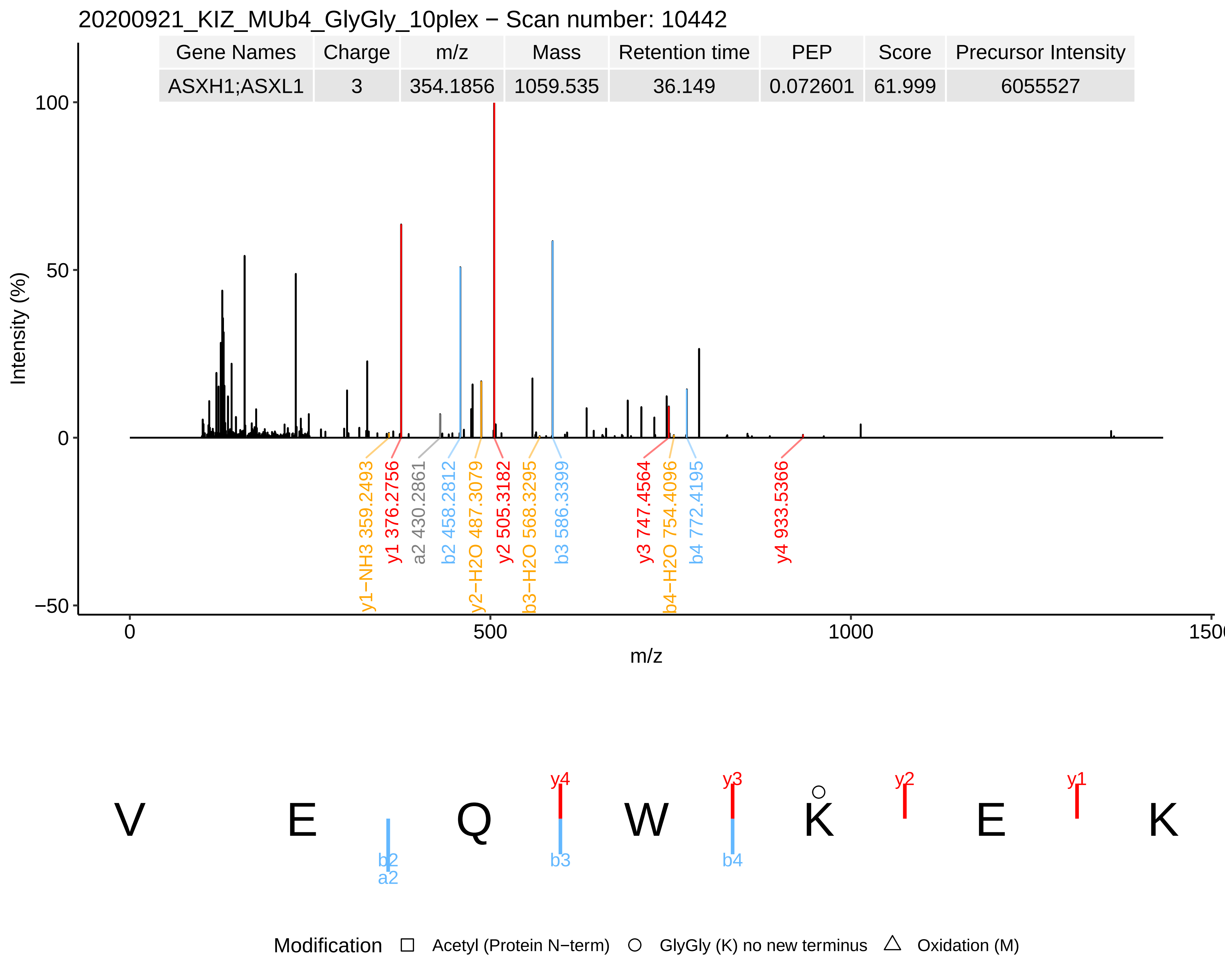
20200921_KIZ_MUb4_GlyGly_10plex − Scan number: 10442
Gene Names | Charge | m/z | Mass | Retention time | PEP | Score | Precursor Intensity
ASXH1;ASXL1 | 3 | 354.1856 | 1059.535 | 36.149 | 0.072601 | 61.999 | 6055527
Intensity (%)
100
50
0
−50
0
500
1000
m/z
y1−NH3 359.2493
y1 376.2756
a2 430.2861
b2 458.2812
y2−H2O 487.3079
y2 505.3182
b3−H2O 568.3295
b3 586.3399
y3 747.4564
b4−H2O 754.4096
b4 772.4195
y4 933.5366
V E Q W K E K
y4 y3 y2 y1
b2 a2 b3 b4
Modification
Acetyl (Protein N−term)
GlyGly (K) no new terminus
Oxidation (M)

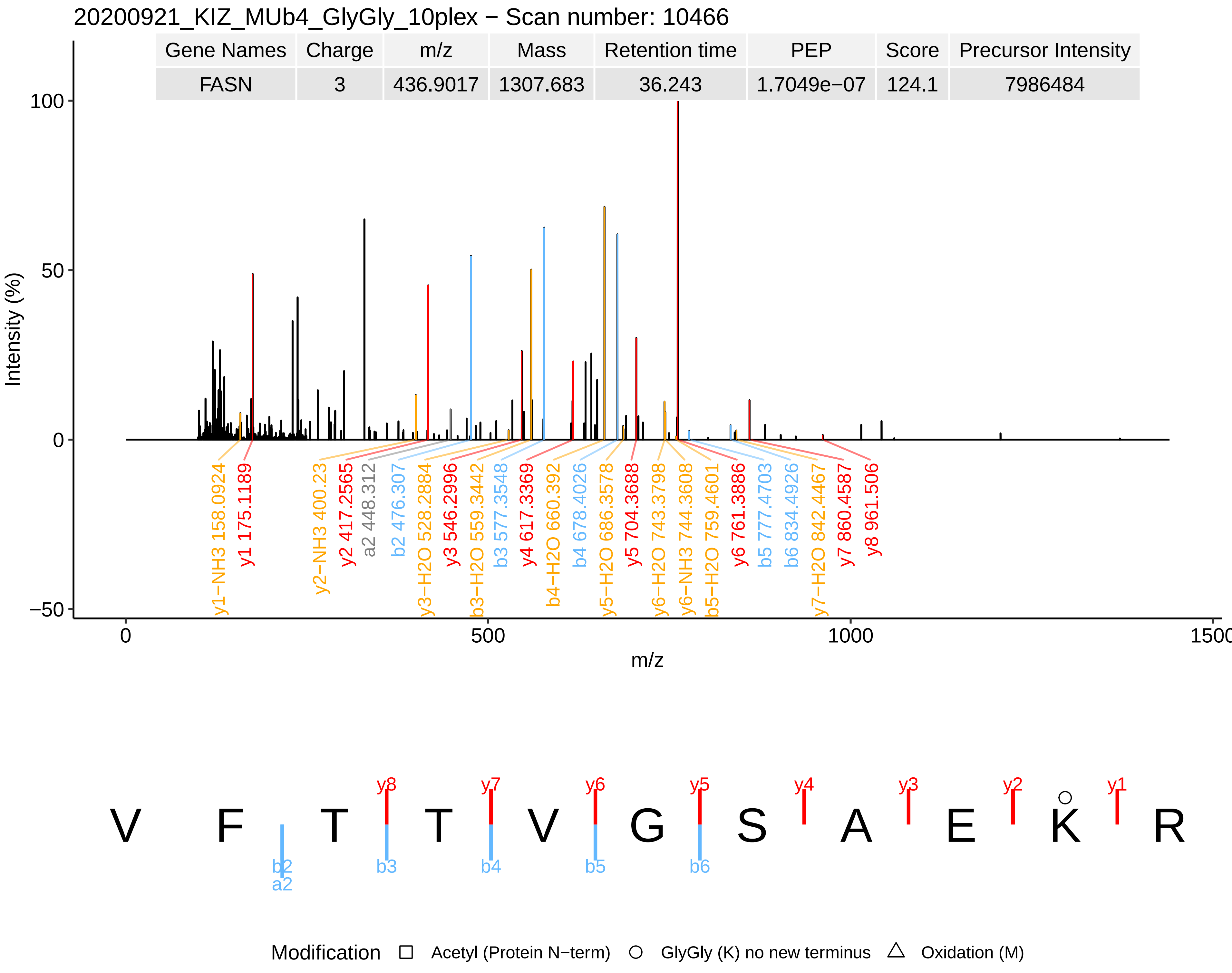

20200921_KIZ_MUb4_GlyGly_10plex − Scan number: 10466
Gene Names | Charge | m/z | Mass | Retention time | PEP | Score | Precursor Intensity
FASN | 3 | 436.9017 | 1307.683 | 36.243 | 1.7049e−07 | 124.1 | 7986484
Intensity (%)
100
50
0
−50
0
500
1000
1500
m/z
y1−NH3 158.0924
y1 175.1189
y2−NH3 400.23
y2 417.2565
a2 448.312
b2 476.307
y3−H2O 528.2884
y3 546.2996
b3−H2O 559.3442
b3 577.3548
y4 617.3369
b4−H2O 660.392
b4 678.4026
y5−H2O 686.3578
y5 704.3688
y6−H2O 743.3798
y6−NH3 744.3608
b5−H2O 759.4601
y6 761.3886
b5 777.4703
b6 834.4926
y7−H2O 842.4467
y7 860.4587
y8 961.506
V F T T V G S A E K R
y8 y7 y6 y5 y4 y3 y2 y1
b2 a2 b3 b4 b5 b6
Modification
Acetyl (Protein N−term)
GlyGly (K) no new terminus
Oxidation (M)

20200921_KIZ_MUb4_GlyGly_10plex_2 − Scan number: 18158

Gene Names	Charge	m/z	Mass	Retention time	PEP	Score	Precursor Int[illegible]
YWHAG;YWHAQ;YWHAB;SFN;YWHAZ;YWHAH	3	348.1966	1041.568	71.073	0.060104	64.866	250883

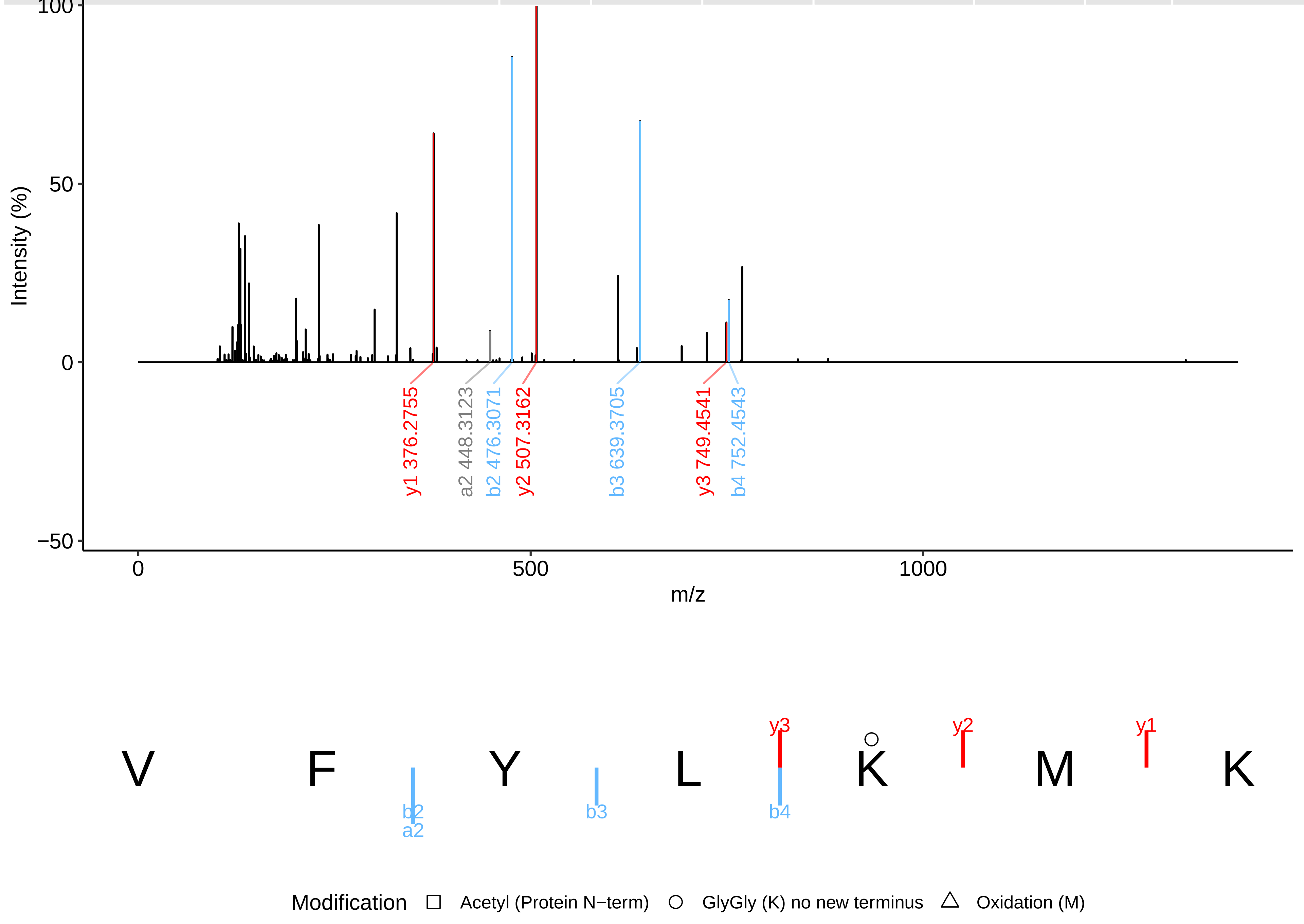

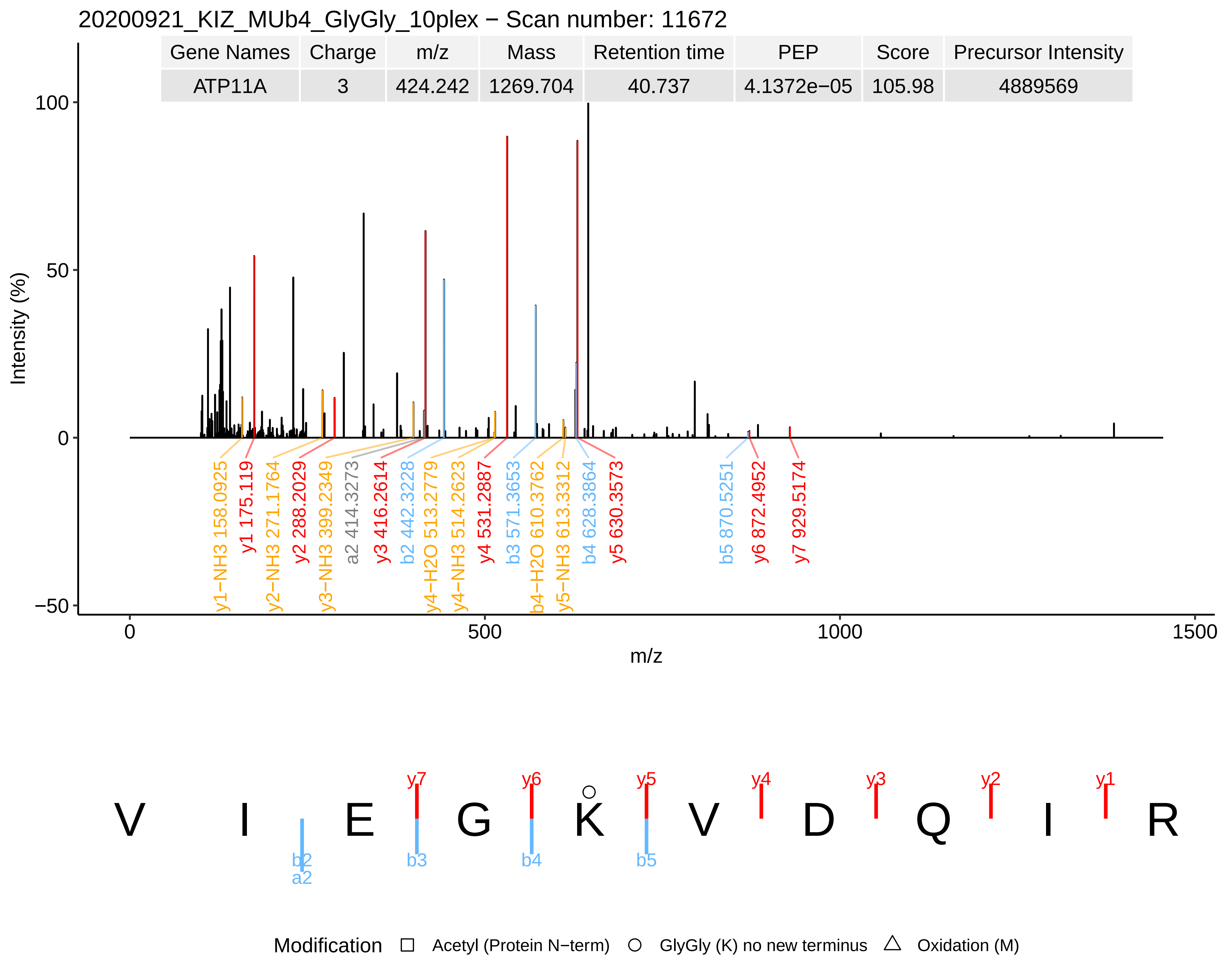
20200921_KIZ_MUb4_GlyGly_10plex − Scan number: 11672
Gene Names | Charge | m/z | Mass | Retention time | PEP | Score | Precursor Intensity
ATP11A | 3 | 424.242 | 1269.704 | 40.737 | 4.1372e−05 | 105.98 | 4889569
Intensity (%)
100
50
0
−50
0
500
1000
1500
m/z
y1−NH3 158.0925
y1 175.119
y2−NH3 271.1764
y2 288.2029
y3−NH3 399.2349
a2 414.3273
y3 416.2614
b2 442.3228
y4−H2O 513.2779
y4−NH3 514.2623
y4 531.2887
b3 571.3653
b4−H2O 610.3762
y5−NH3 613.3312
b4 628.3864
y5 630.3573
b5 870.5251
y6 872.4952
y7 929.5174
V I E G K V D Q I R
y7 y6 y5 y4 y3 y2 y1
b2 a2 b3 b4 b5
Modification
Acetyl (Protein N−term)
GlyGly (K) no new terminus
Oxidation (M)

20200921_KIZ_MUb4_GlyGly_10plex_2 − Scan number: 10775

Gene Names	Charge	m/z	Mass	Retention time	PEP	Score	Precursor Intensity
ATP11A	3	424.242	1269.704	41.298	0.045655	58.246	732075.3

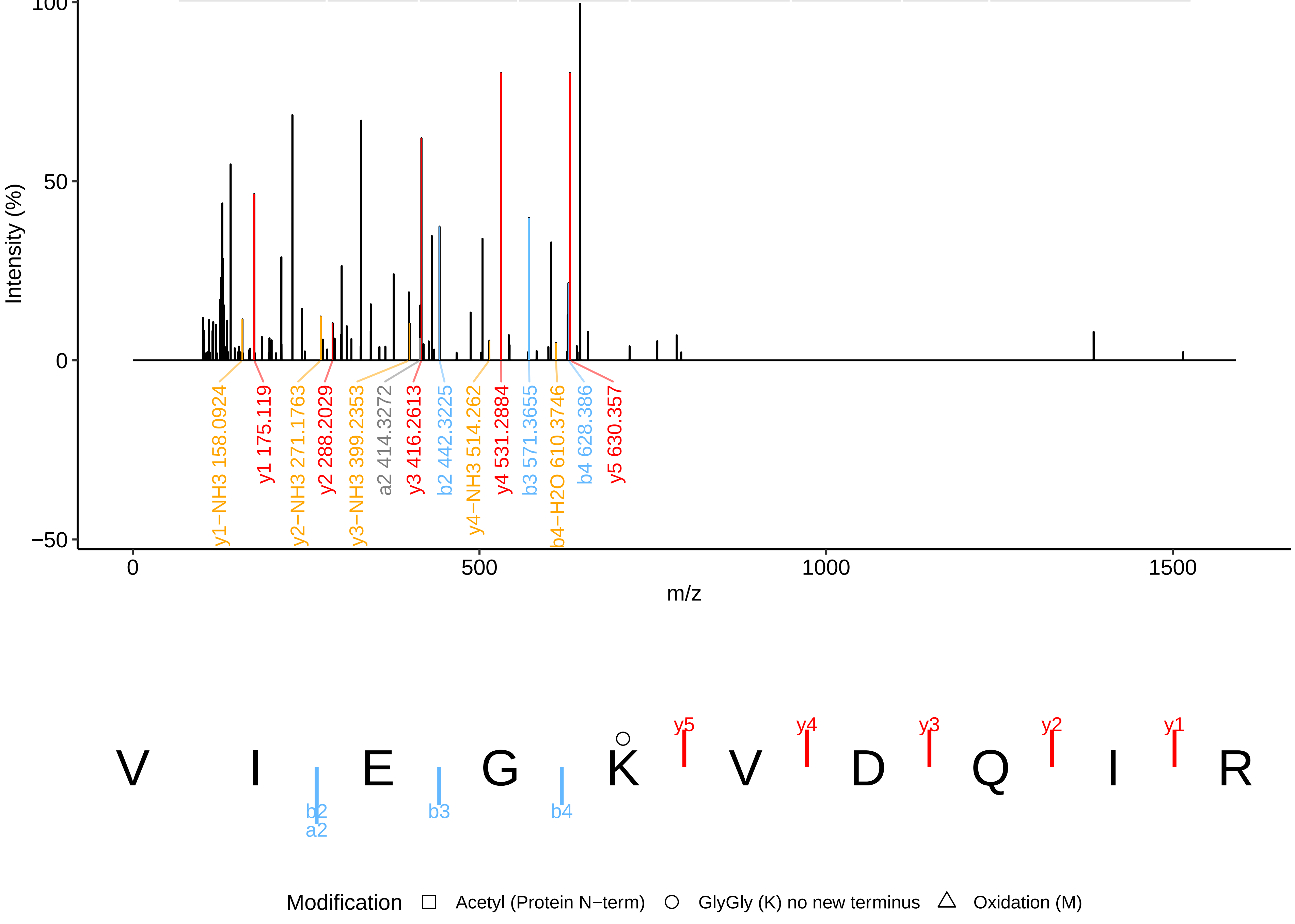

20200921_KIZ_MUb4_GlyGly_10plex_2 − Scan number: 13907

Gene Names	Charge	m/z	Mass	Retention time	PEP	Score	Precursor Intensity
RPS17	3	433.575	1297.703	54.1	0.0026558	85.731	2171701

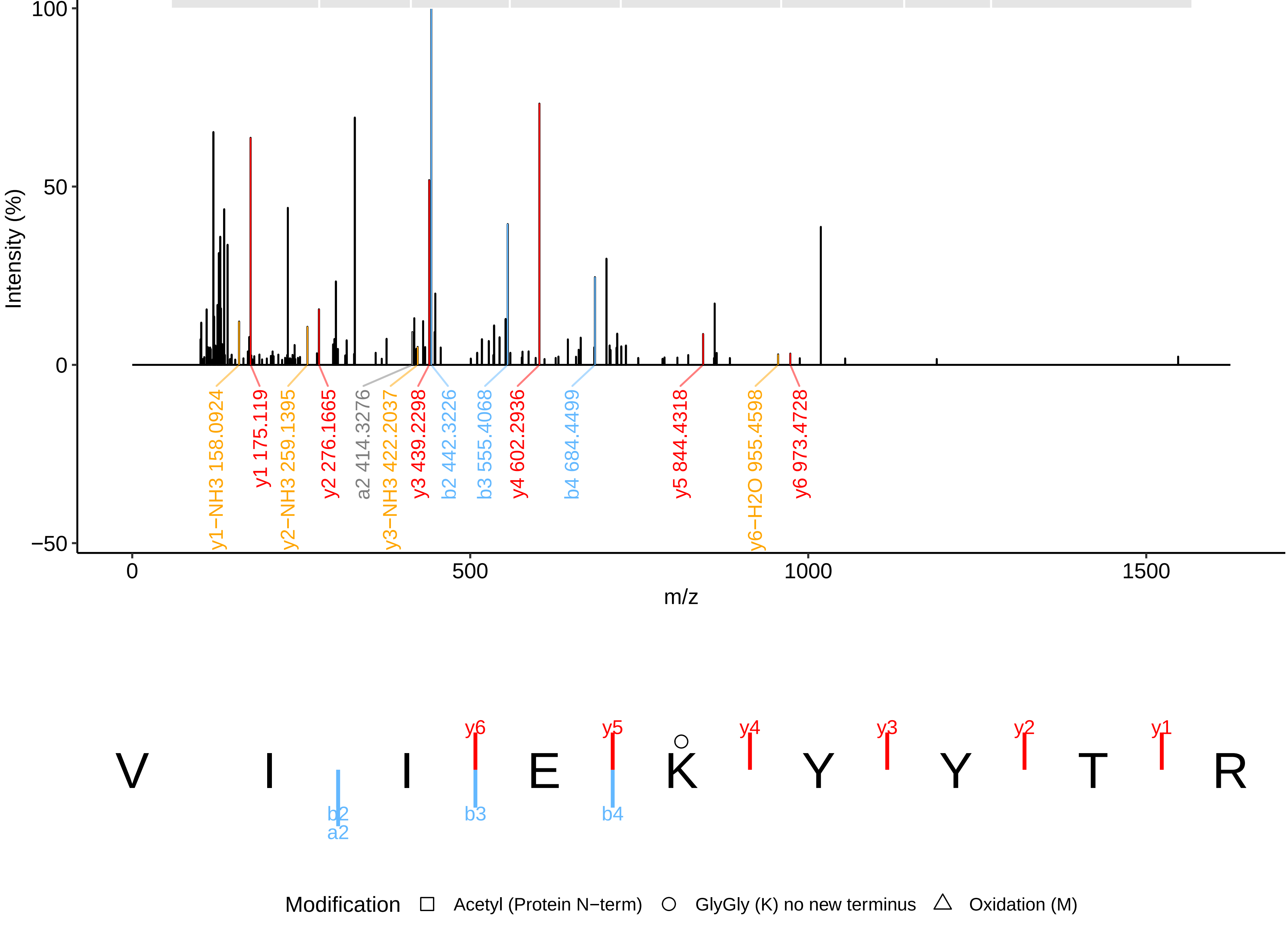

20200921_KIZ_MUb4_GlyGly_10plex − Scan number: 7684

Gene Names	Charge	m/z	Mass	Retention time	PEP	Score	Precursor Intensity
UFD1L	3	354.5147	1060.522	25.869	0.11941	51.346	NA

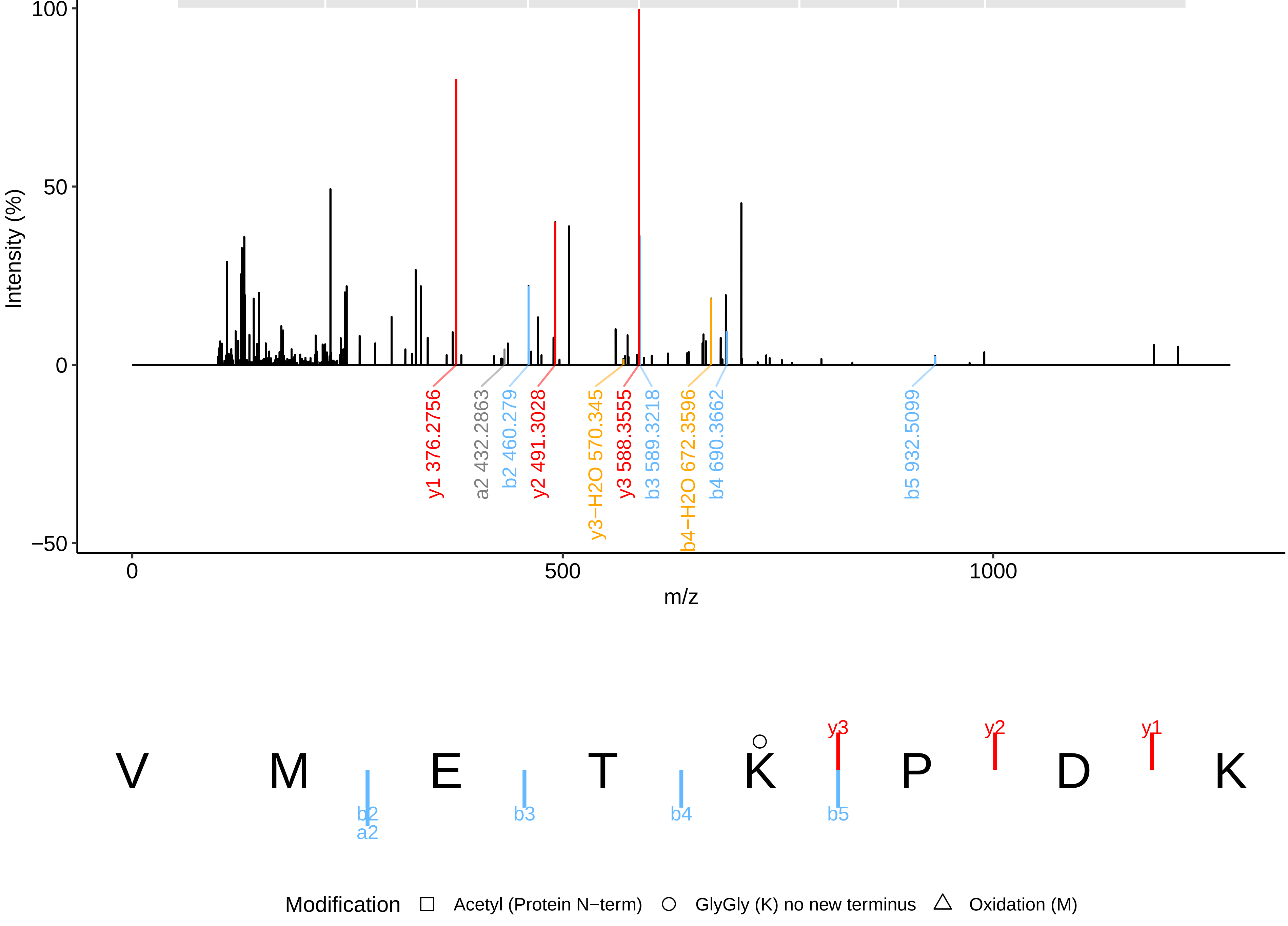

20200921_KIZ_MUb4_GlyGly_10plex − Scan number: 7558

Gene Names	Charge	m/z	Mass	Retention time	PEP	Score	Precursor Intensity
GK;GK3P	3	348.861	1043.561	25.409	0.011676	78.516	5734139

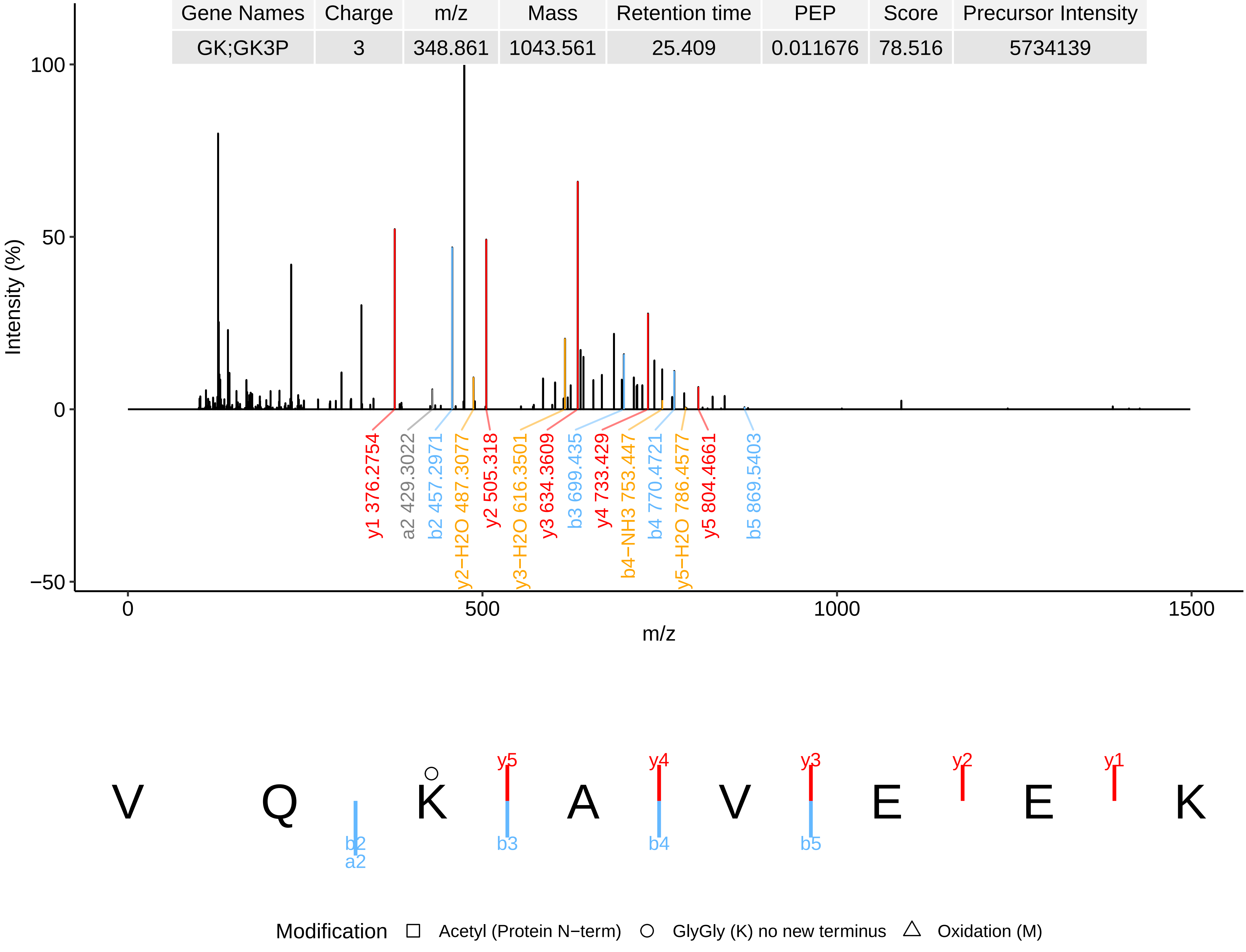

20200924_KIZ_MUb5_GlyGly_1 − Scan number: 6577

Gene Names	Charge	m/z	Mass	Retention time	PEP	Score	Precursor Intensity
GK;GK3P	3	348.861	1043.561	20.703	0.075602	52.255	13713219

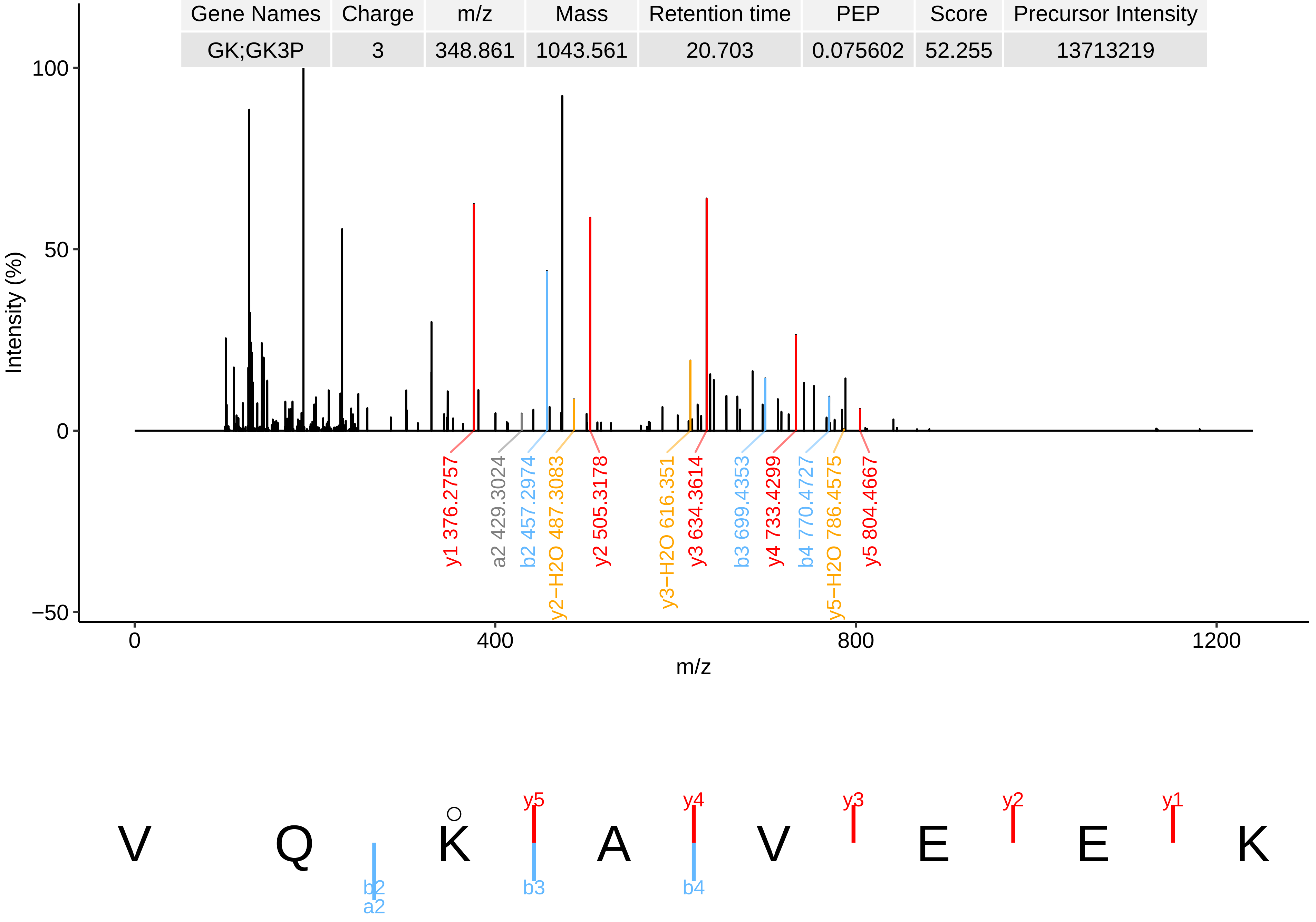

Modification □ Acetyl (Protein N−term) ○ GlyGly (K) no new terminus △ Oxidation (M)

20200924_KIZ_MUb5_GlyGly_2 − Scan number: 6157

Gene Names	Charge	m/z	Mass	Retention time	PEP	Score	Precursor Intensity
GK;GK3P	3	348.861	1043.561	22.942	0.024498	68.224	2350628

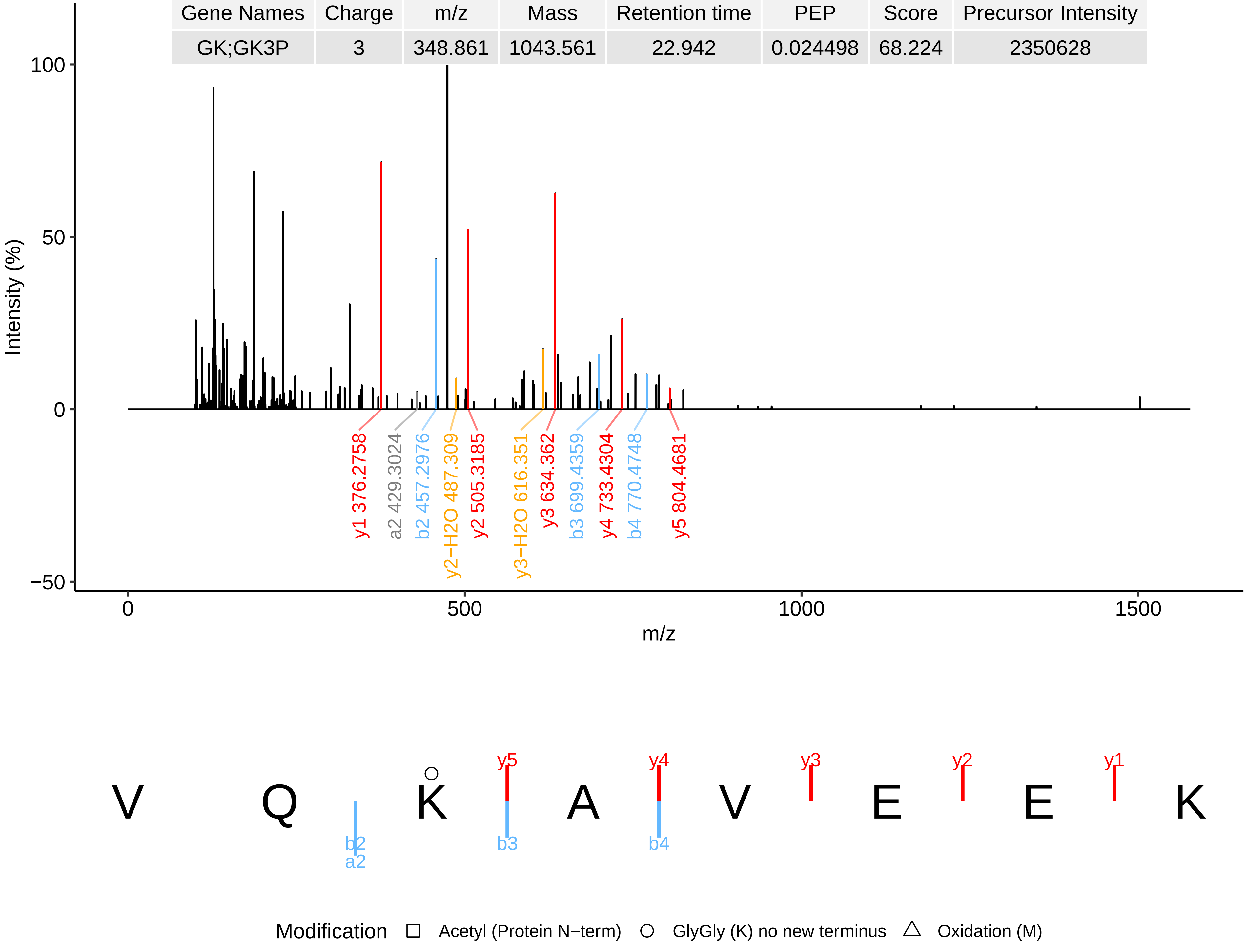

20201118_KIZ_MUb6_GlyGly_2 − Scan number: 7961

Gene Names	Charge	m/z	Mass	Retention time	PEP	Score	Precursor Intensity
GK;GK3P	3	348.861	1043.561	27.12	0.022785	69.598	6813584

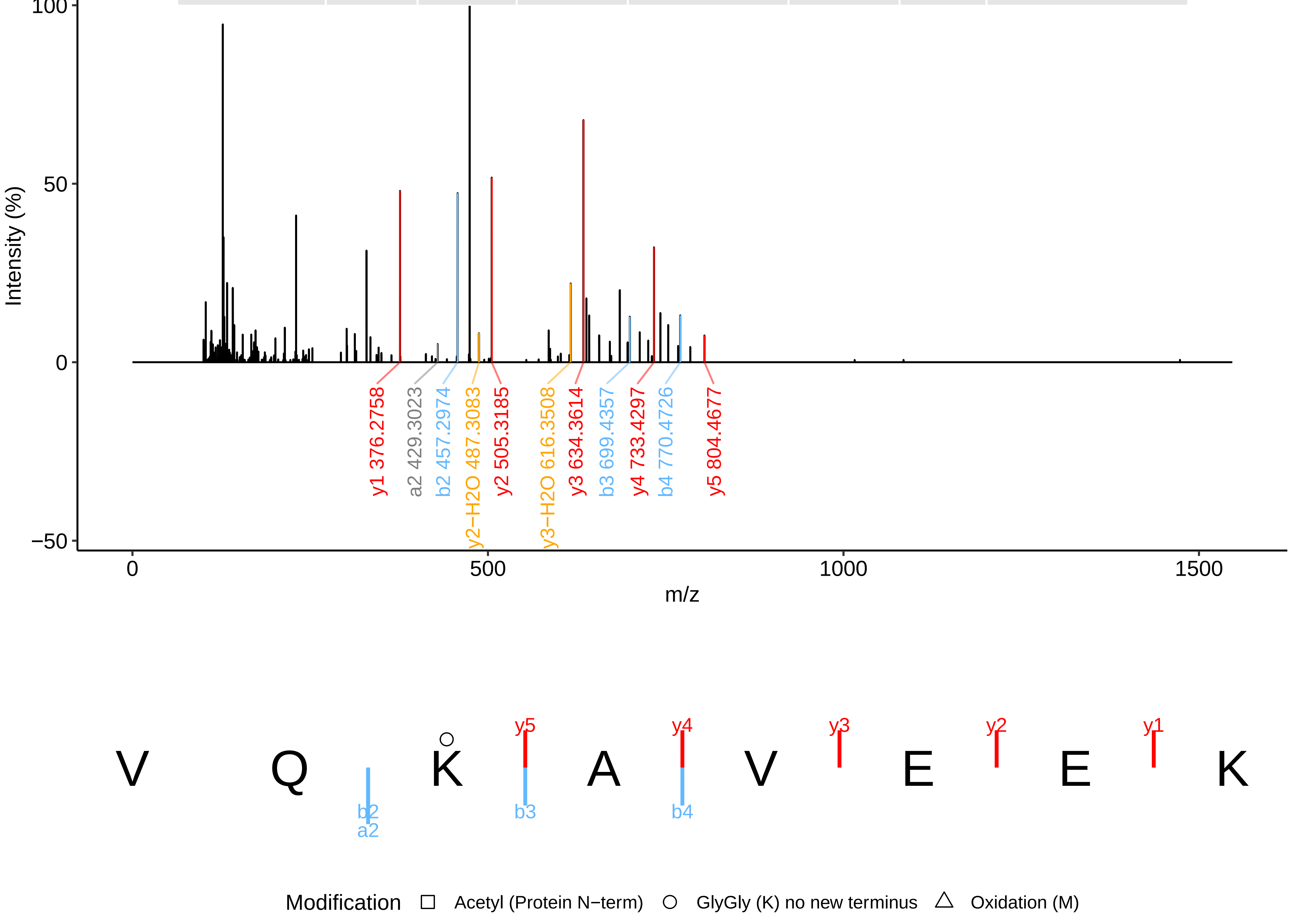

20200921_KIZ_MUb4_GlyGly_10plex − Scan number: 4119

Gene Names	Charge	m/z	Mass	Retention time	PEP	Score	Precursor Intensity
RAB13	3	353.8525	1058.536	13.403	0.030862	67.897	2576730

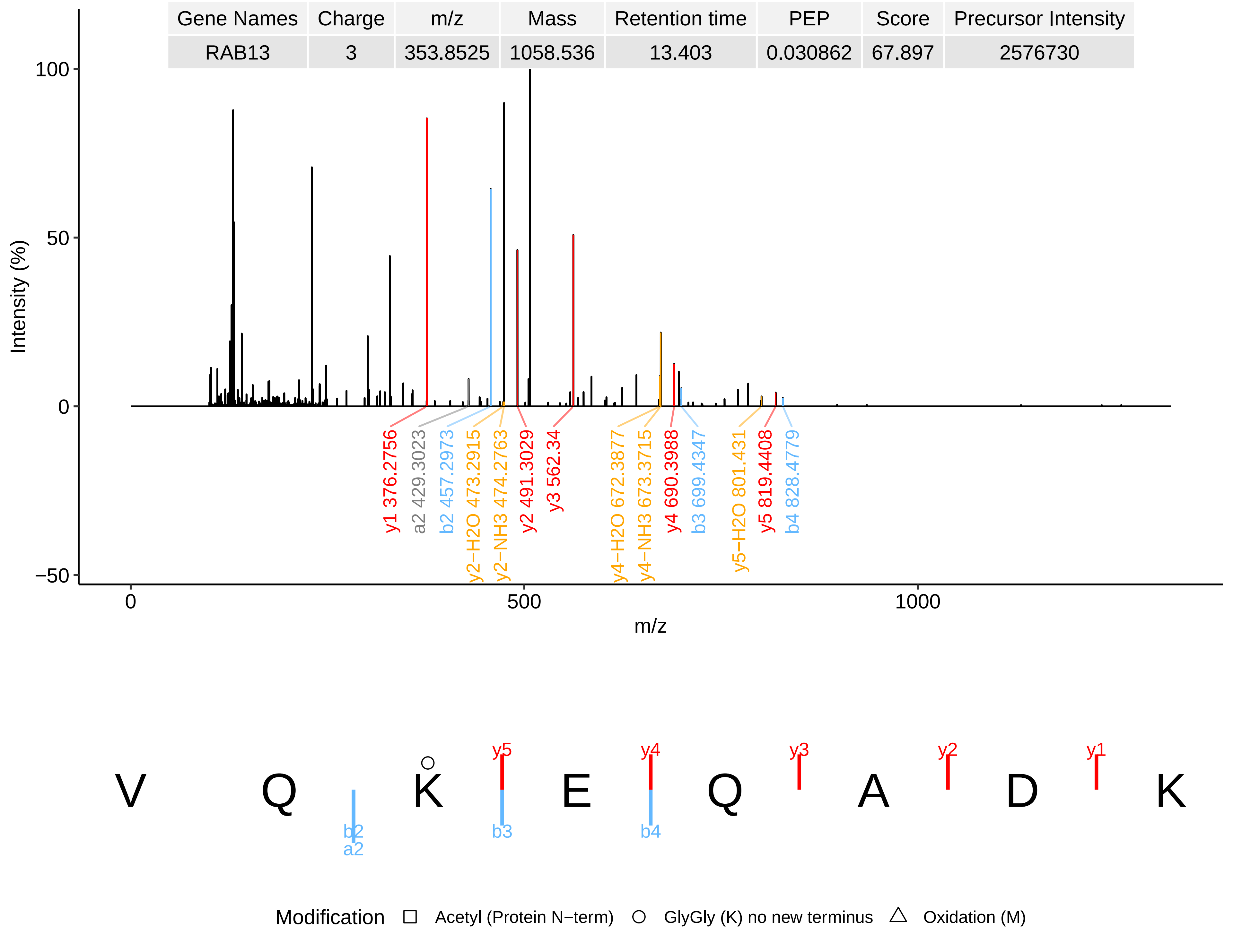

20200921_KIZ_MUb4_GlyGly_10plex − Scan number: 12487

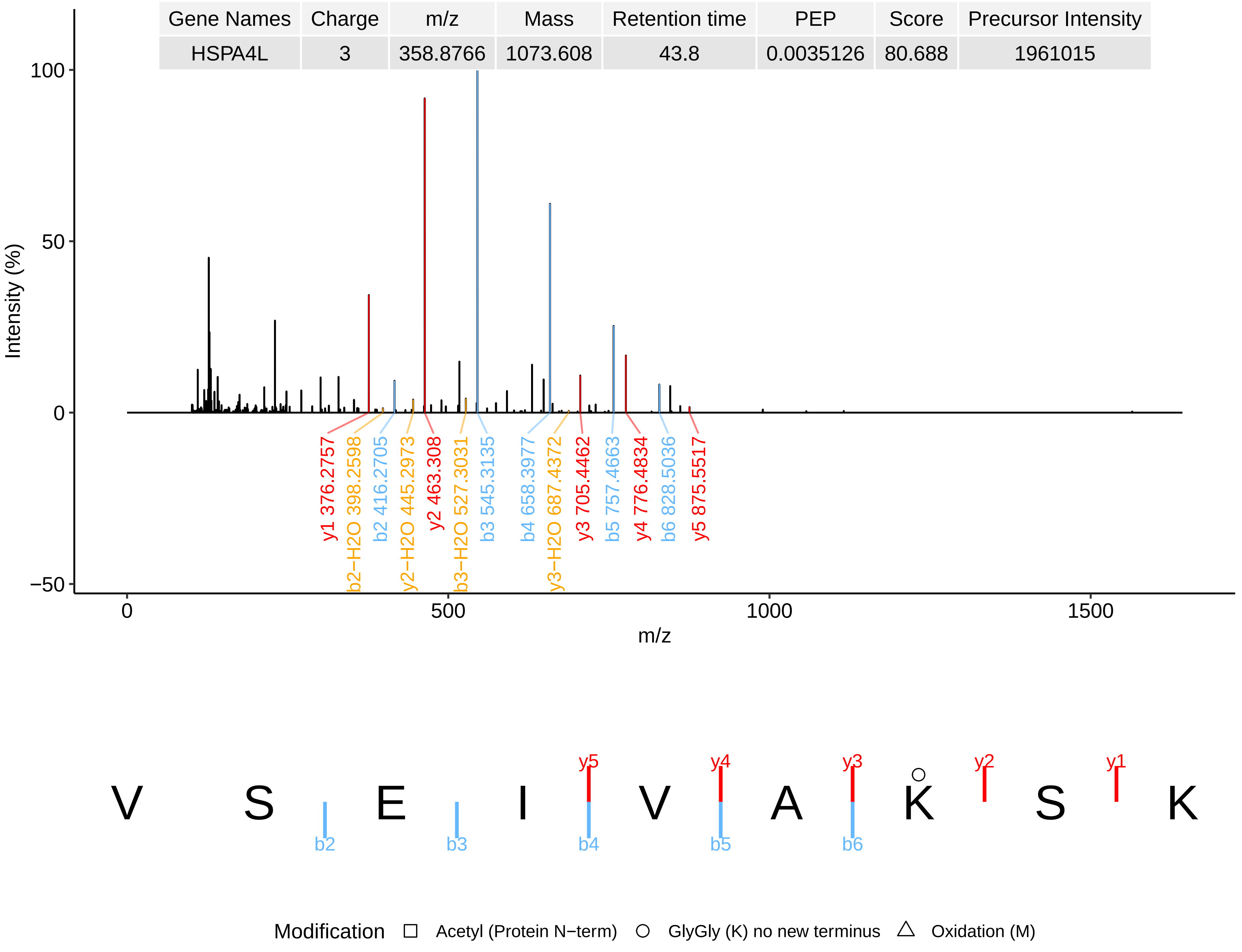

20200921_KIZ_MUb4_GlyGly_10plex − Scan number: 9158

Gene Names	Charge	m/z	Mass	Retention time	PEP	Score	Precursor Intensity
VDAC1	3	423.2262	1266.657	31.325	1.3509e−07	128.27	14856213

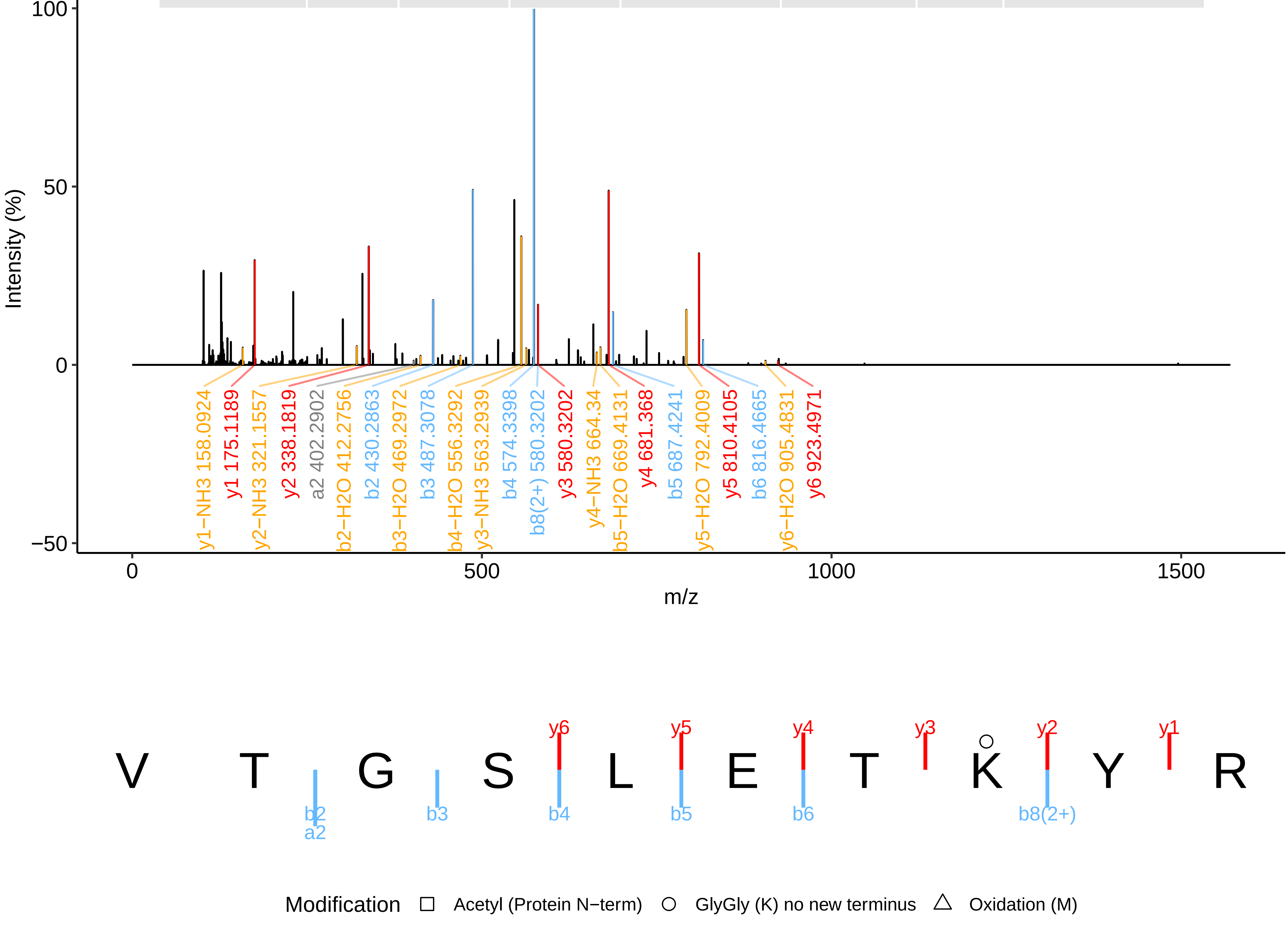

20200921_KIZ_MUb4_GlyGly_10plex − Scan number: 9188

Gene Names	Charge	m/z	Mass	Retention time	PEP	Score	Precursor Intensity
VDAC1	2	634.3357	1266.657	31.436	5.3505e−35	258.47	9133284

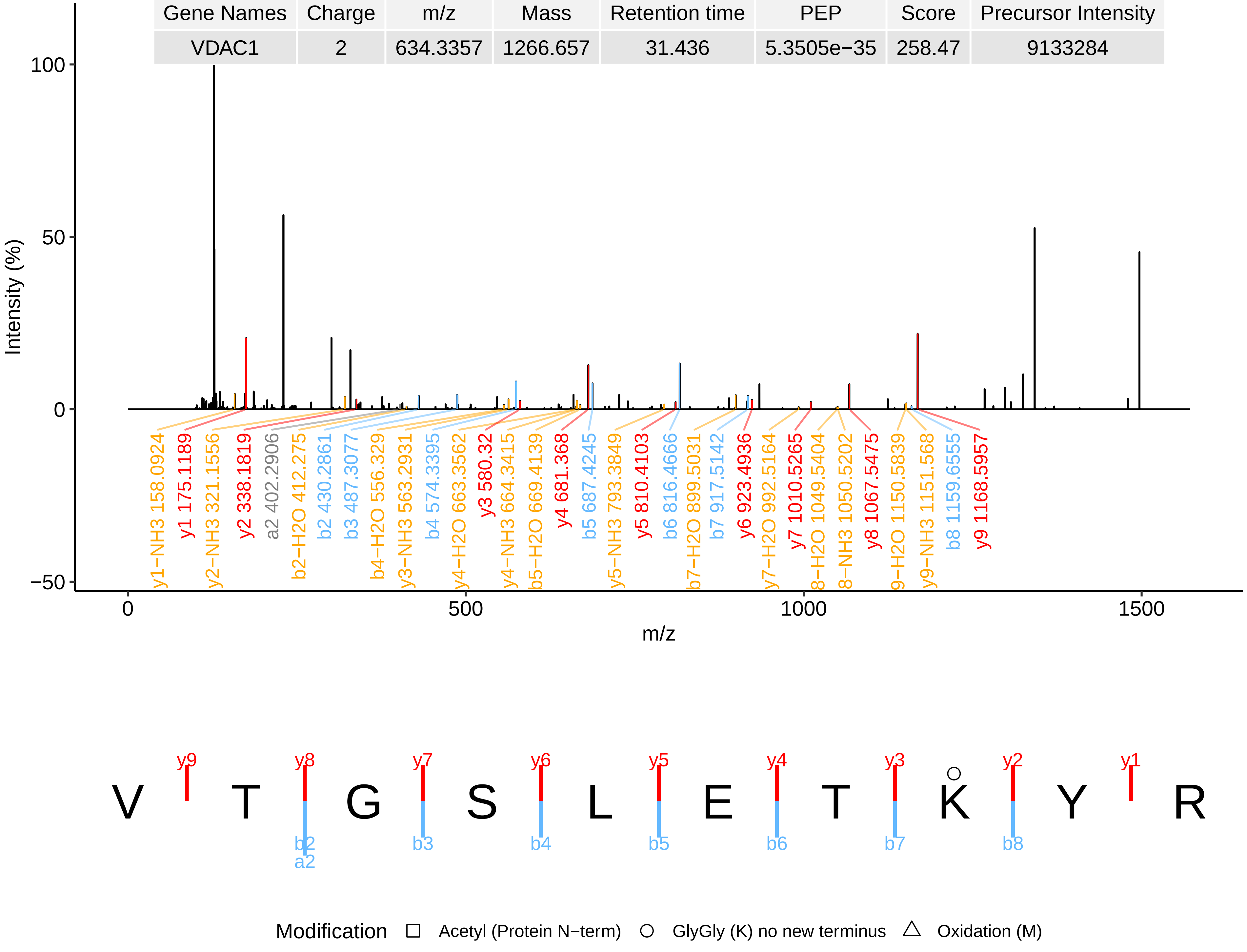

20200921_KIZ_MUb4_GlyGly_10plex_2 − Scan number: 8463

Gene Names	Charge	m/z	Mass	Retention time	PEP	Score	Precursor Intensity
VDAC1	3	423.2262	1266.657	31.89	3.3523e−05	107.79	1228366

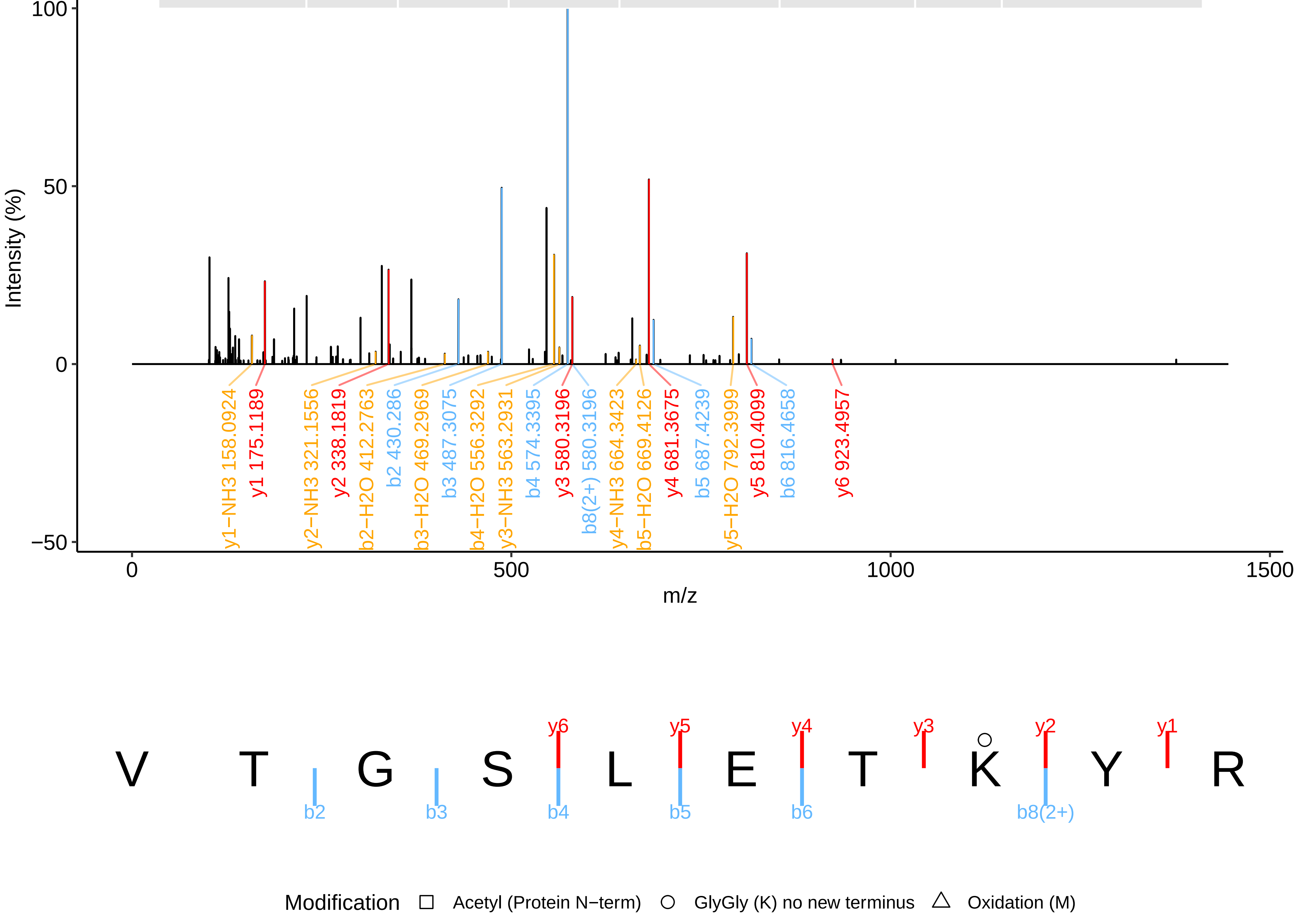

20200924_KIZ_MUb5_GlyGly_1 − Scan number: 8087

Gene Names	Charge	m/z	Mass	Retention time	PEP	Score	Precursor Intensity
VDAC1	3	423.2262	1266.657	25.776	0.0012461	89.913	23655294

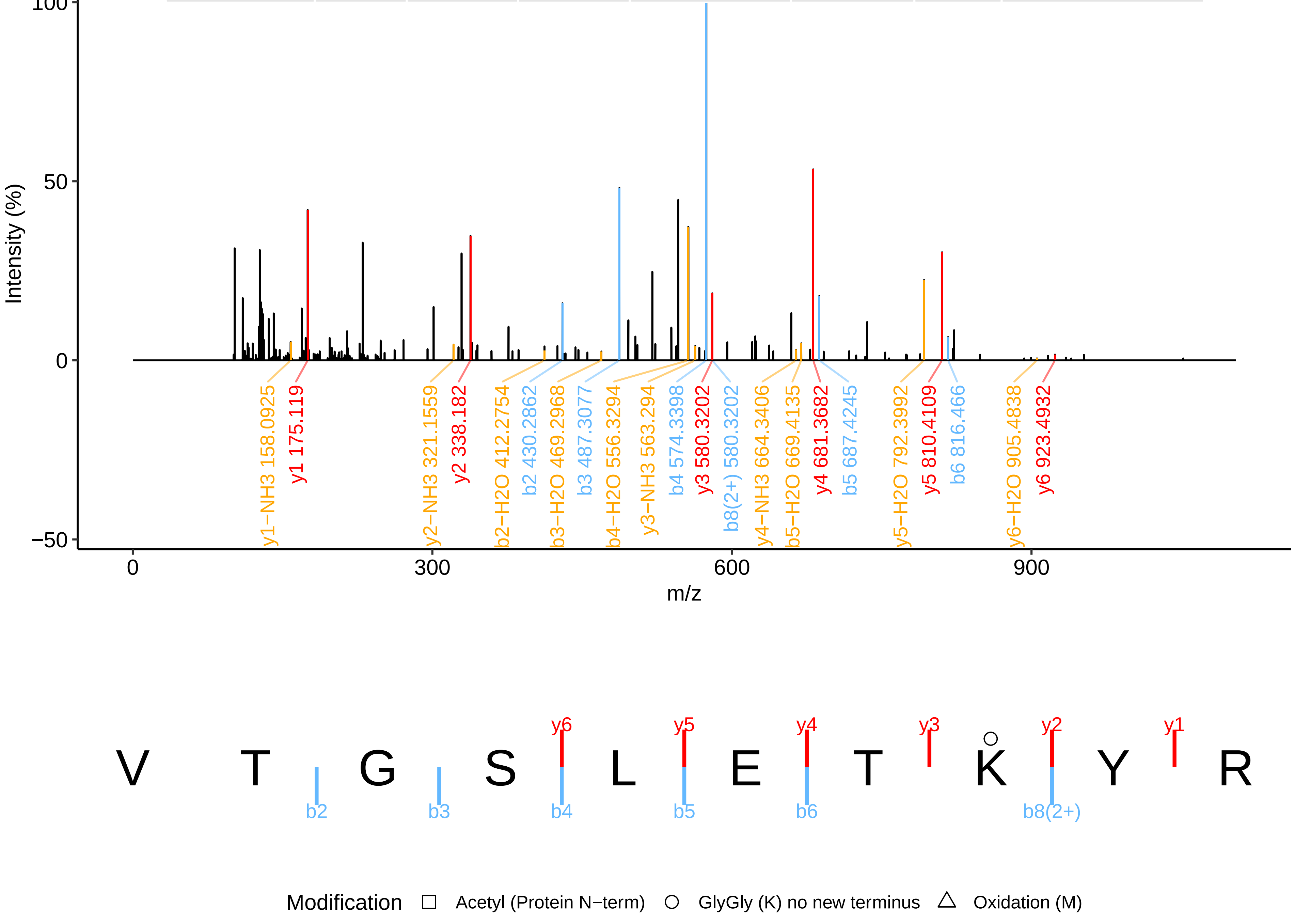

20200924_KIZ_MUb5_GlyGly_1 − Scan number: 8238

Gene Names	Charge	m/z	Mass	Retention time	PEP	Score	Precursor Intensity
VDAC1	3	423.2262	1266.657	26.293	4.919e−06	114.39	2674615

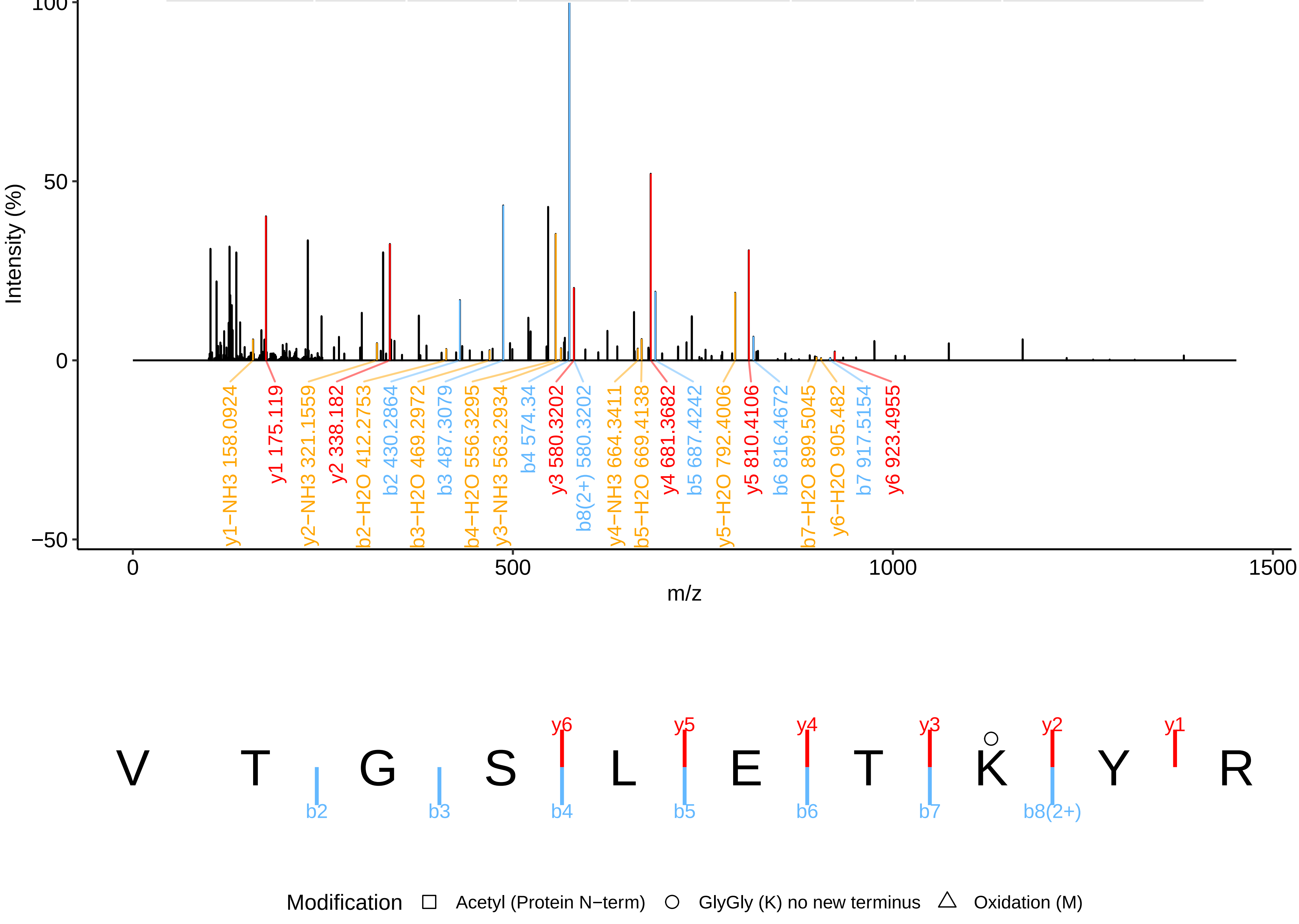

20200924_KIZ_MUb5_GlyGly_2 − Scan number: 7683

Gene Names	Charge	m/z	Mass	Retention time	PEP	Score	Precursor Intensity
VDAC1	3	423.2262	1266.657	28.697	2.5117e−08	132.01	10174964

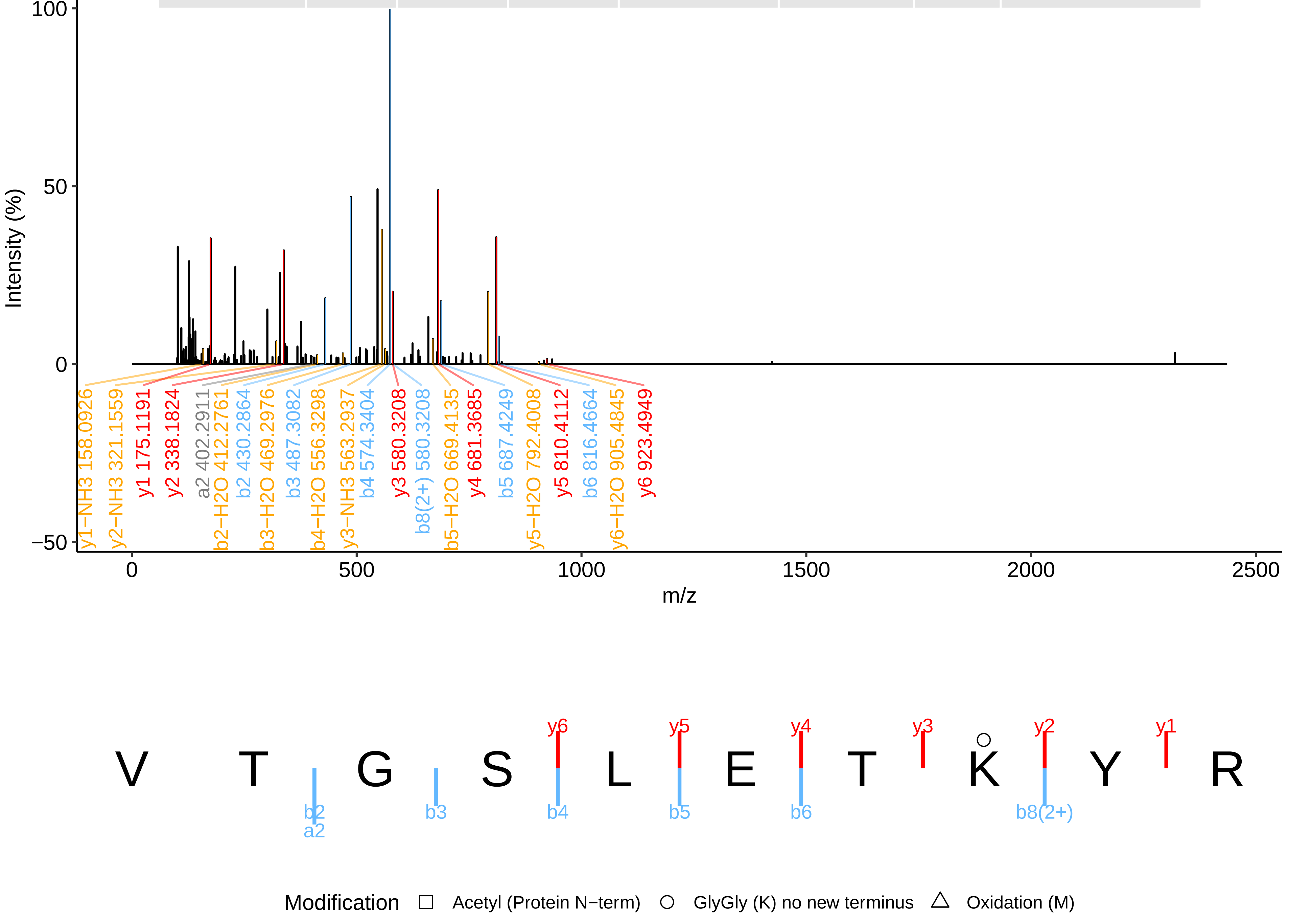

20200924_KIZ_MUb5_GlyGly_2 − Scan number: 7818

Gene Names	Charge	m/z	Mass	Retention time	PEP	Score	Precursor Intensity
VDAC1	3	423.2262	1266.657	29.211	0.00060984	94.309	3386652

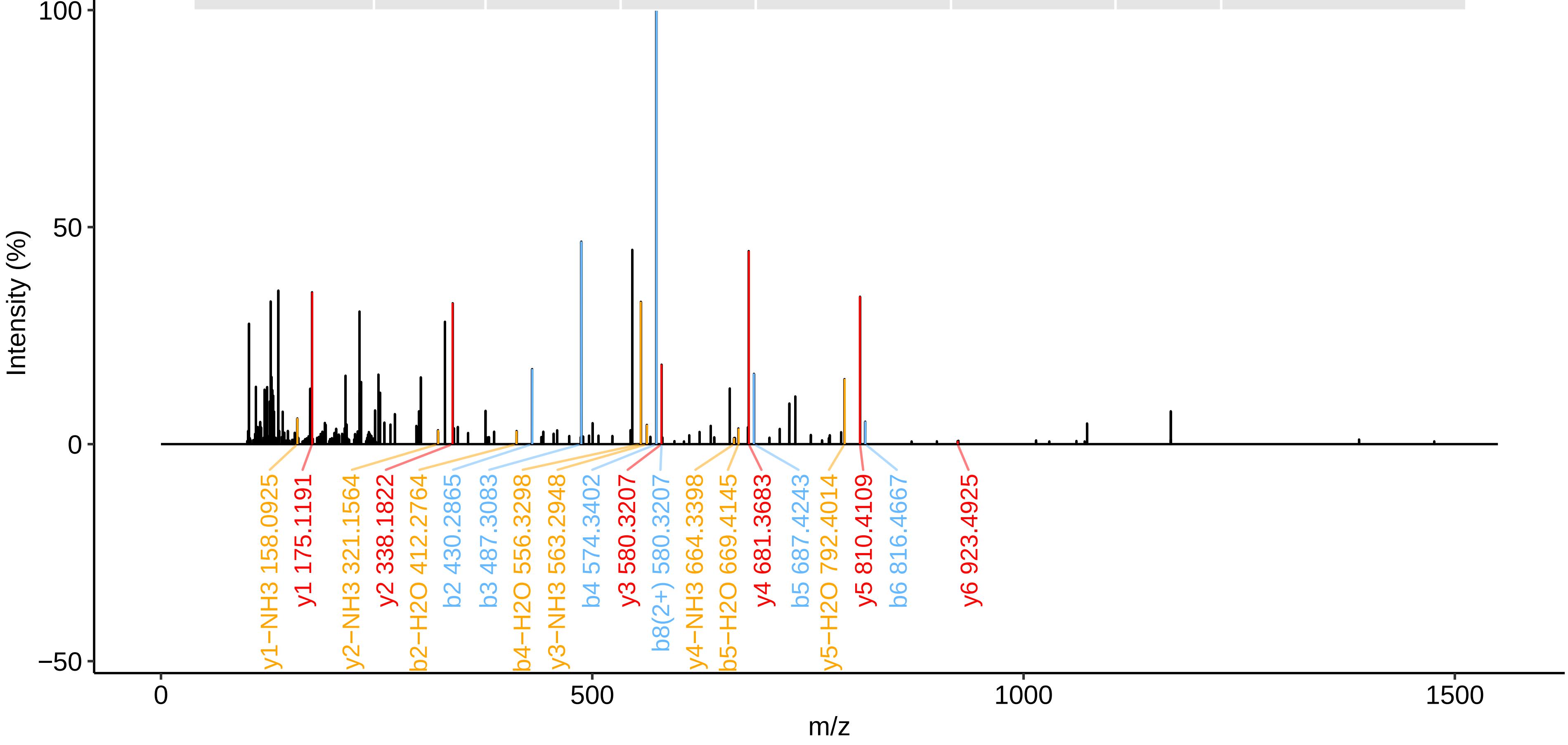

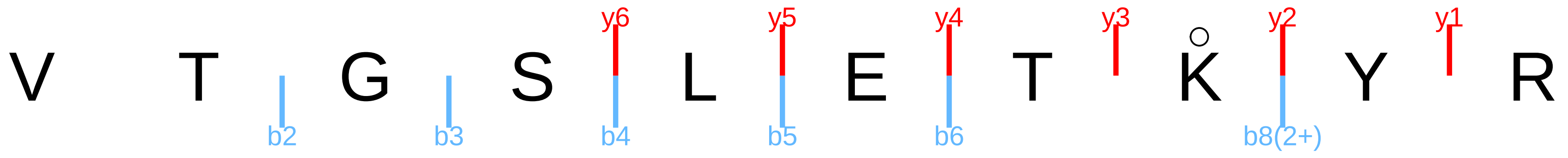

20201118_KIZ_MUb6_GlyGly_2 − Scan number: 9511

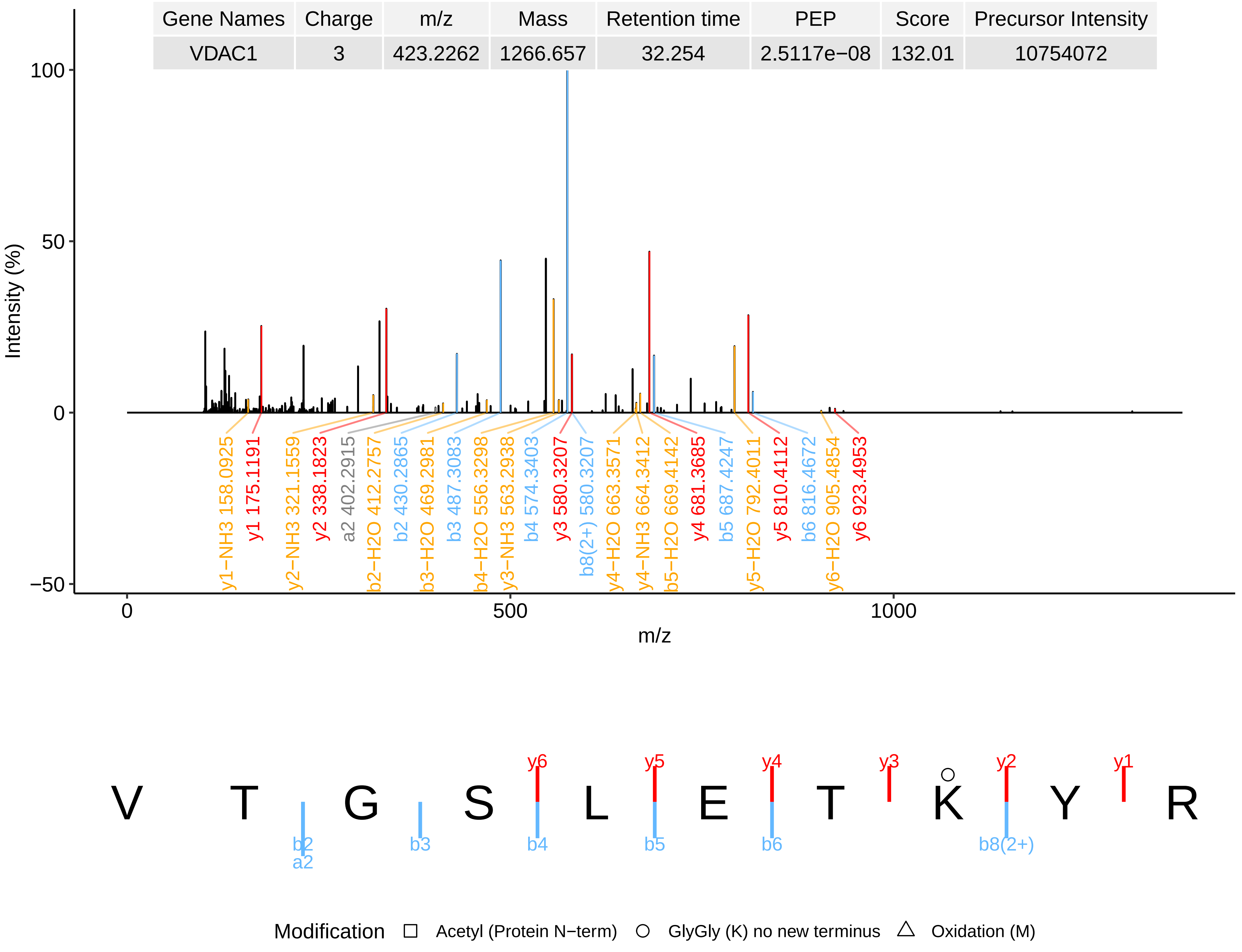

20200921_KIZ_MUb4_GlyGly_10plex − Scan number: 6301

Gene Names	Charge	m/z	Mass	Retention time	PEP	Score	Precursor Intensity
CPS1	3	359.8611	1076.562	20.795	0.005403	84.615	5114472

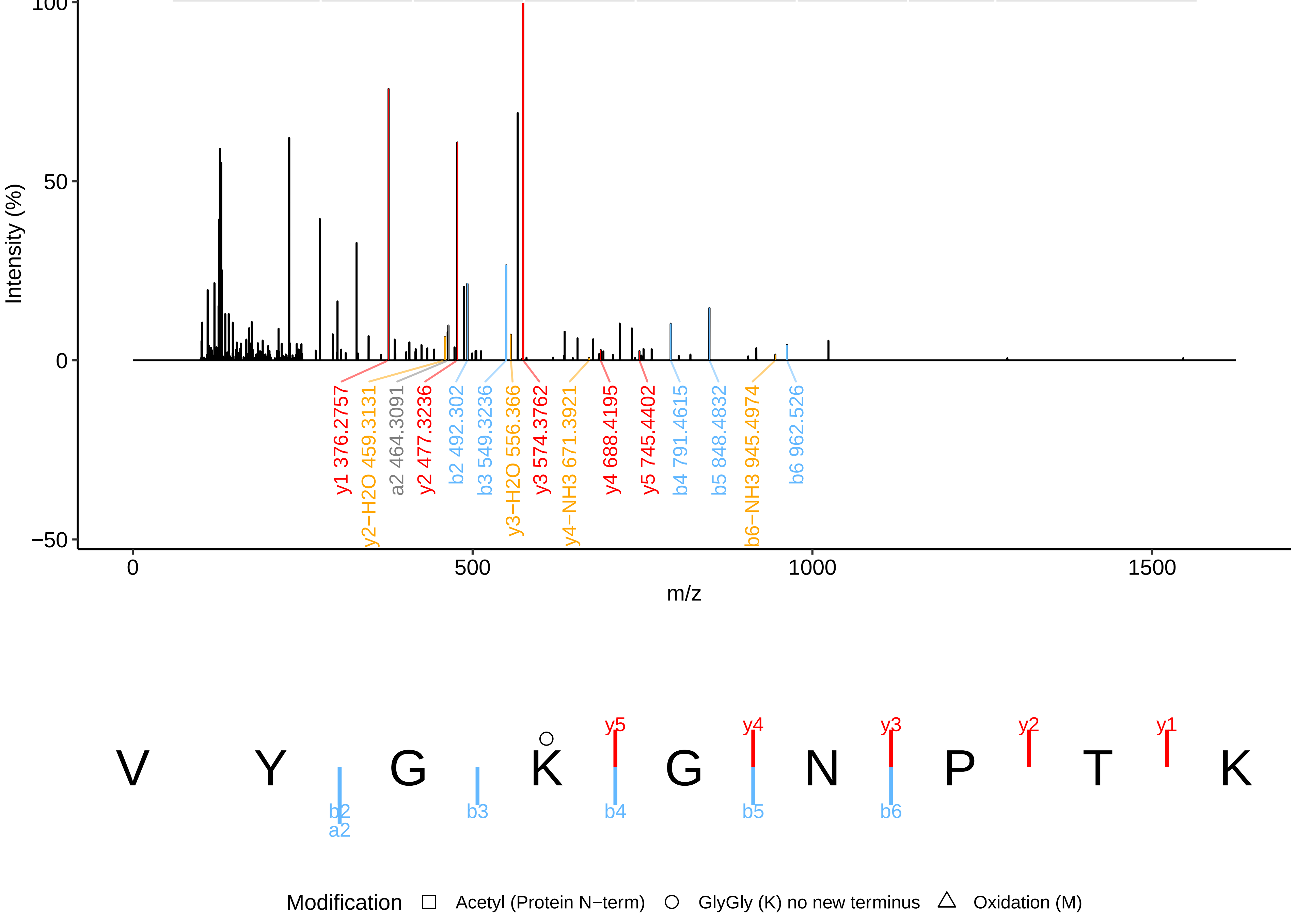

20200921_KIZ_MUb4_GlyGly_10plex − Scan number: 11783

Gene Names	Charge	m/z	Mass	Retention time	PEP	Score	Precursor Intensity
ANXA2;ANXA2P2	3	358.8478	1073.522	41.156	0.035635	74.415	3642469

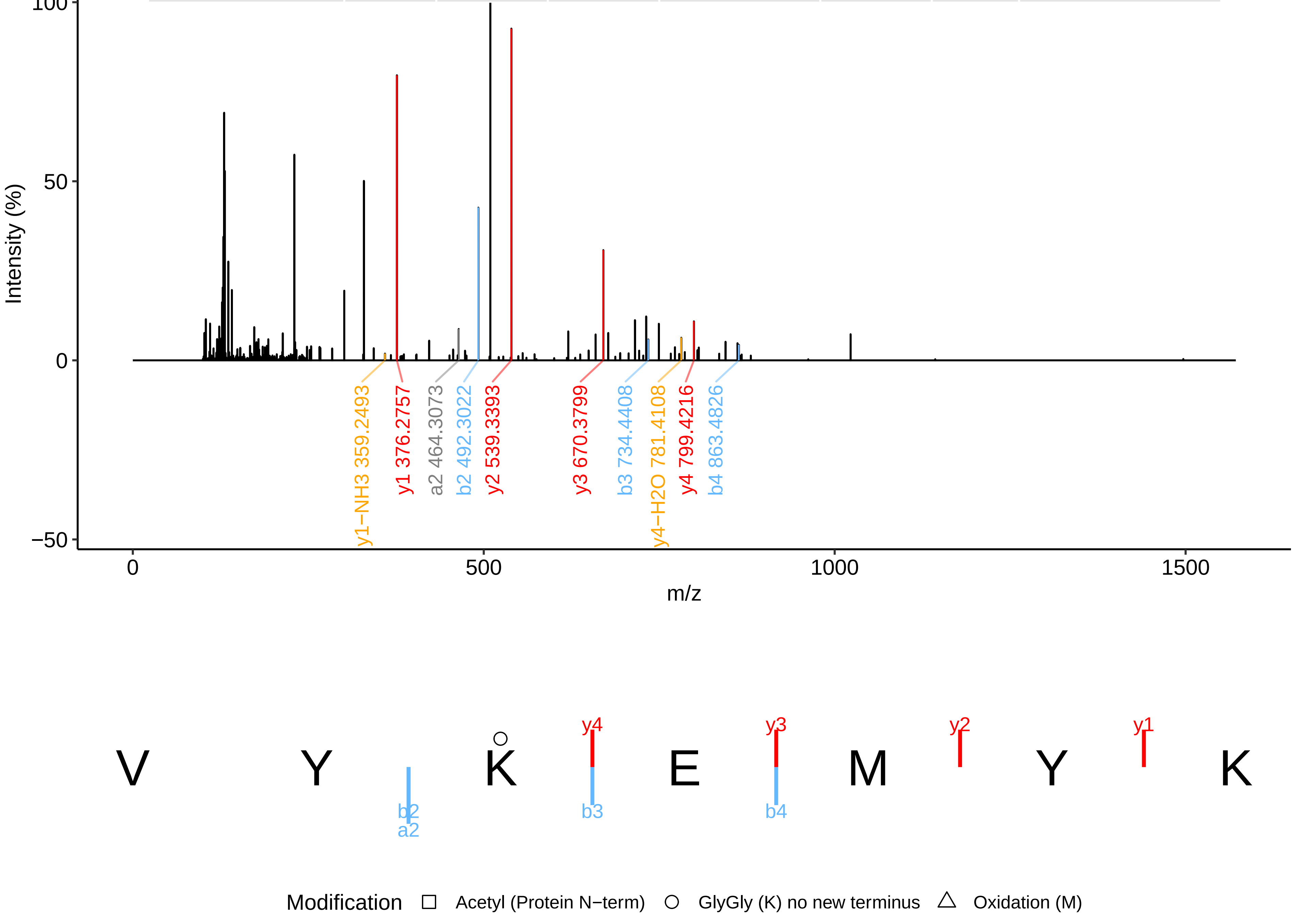

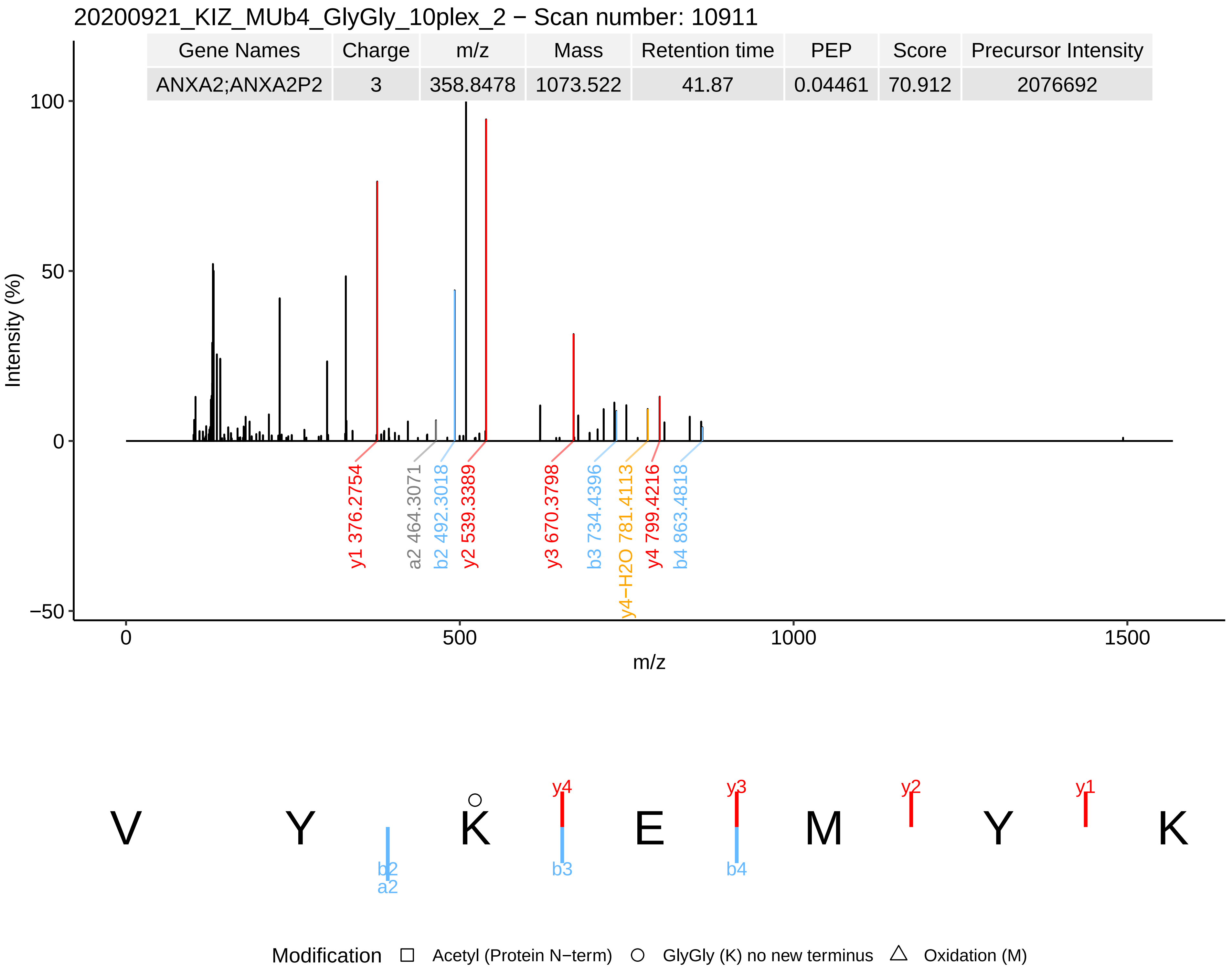

20200921_KIZ_MUb4_GlyGly_10plex_2 − Scan number: 10911

Gene Names	Charge	m/z	Mass	Retention time	PEP	Score	Precursor Intensity
ANXA2;ANXA2P2	3	358.8478	1073.522	41.87	0.04461	70.912	2076692

Intensity (%)
100
50
0
−50
0
500
1000
1500
m/z
y1 376.2754
a2 464.3071
b2 492.3018
y2 539.3389
y3 670.3798
b3 734.4396
y4−H2O 781.4113
y4 799.4216
b4 863.4818
V Y K E M Y K
y4 y3 y2 y1
b2 a2 b3 b4
Modification
Acetyl (Protein N−term)
GlyGly (K) no new terminus
Oxidation (M)

20200924_KIZ_MUb5_GlyGly_1 − Scan number: 10547

Gene Names	Charge	m/z	Mass	Retention time	PEP	Score	Precursor Intensity
ANXA2;ANXA2P2	3	358.8478	1073.522	34.064	0.09928	56.122	19089952

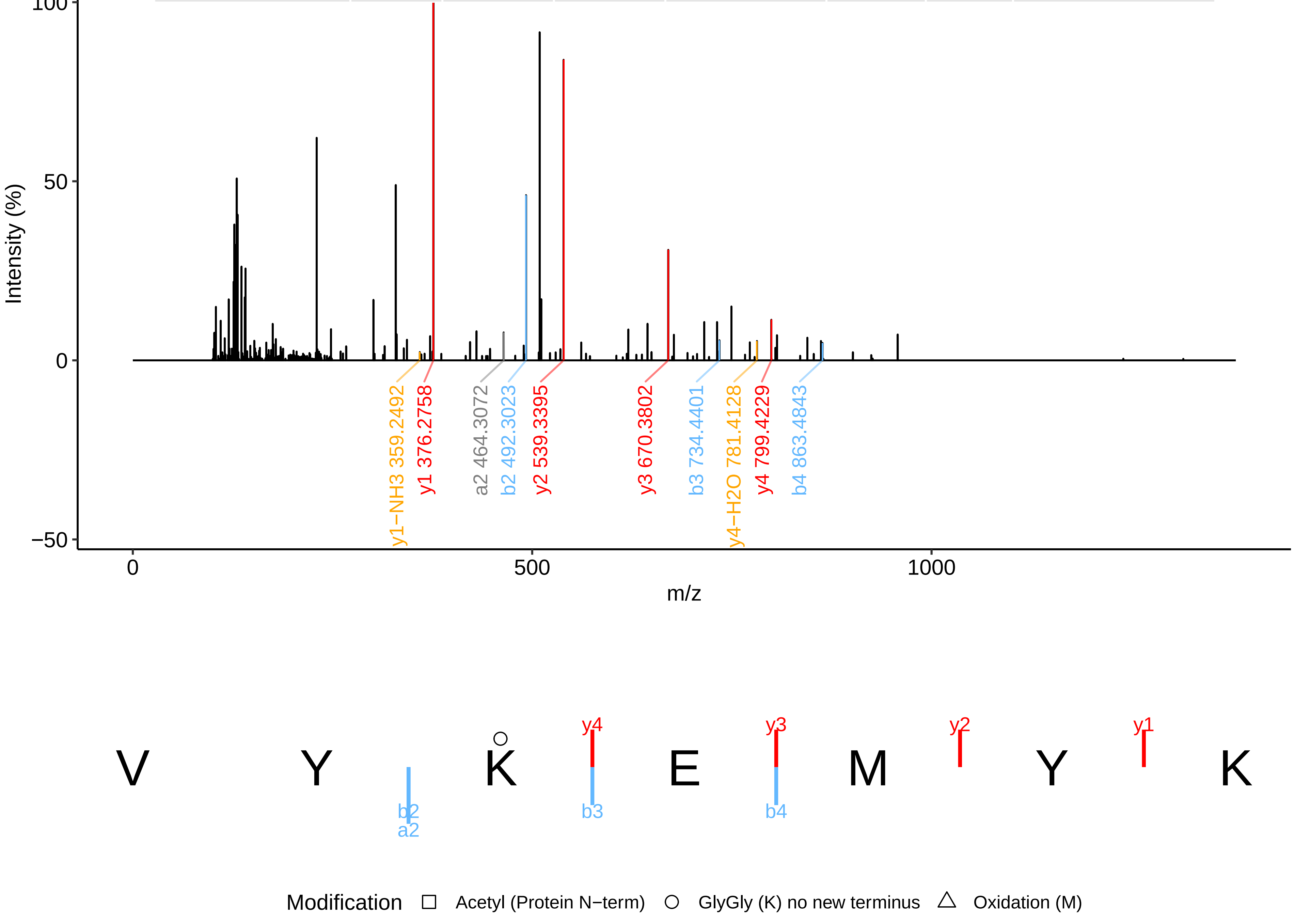

20200924_KIZ_MUb5_GlyGly_2 − Scan number: 10049

Gene Names	Charge	m/z	Mass	Retention time	PEP	Score	Precursor Intensity
ANXA2;ANXA2P2	3	358.8478	1073.522	37.527	0.05396	67.757	2918055

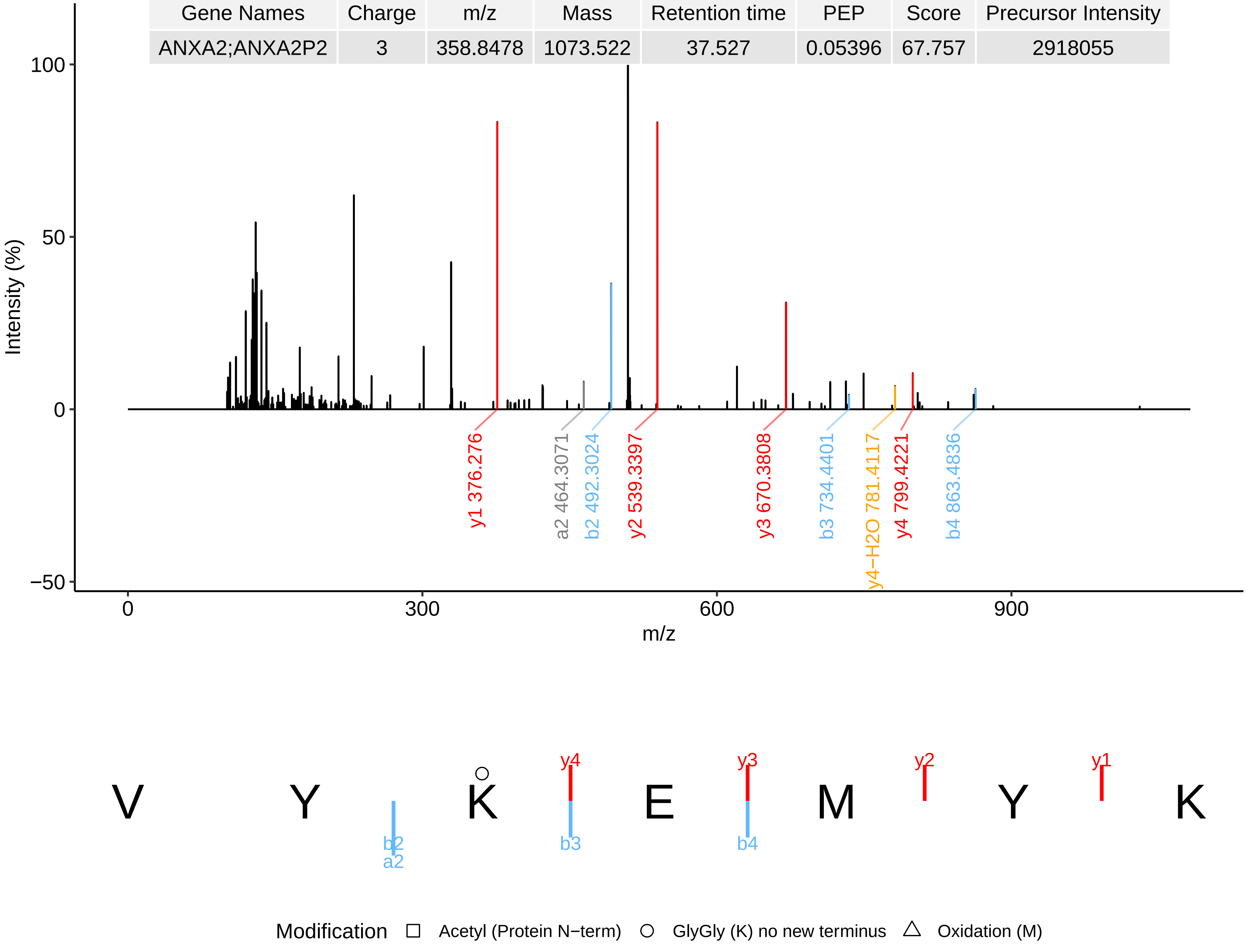

20200921_KIZ_MUb4_GlyGly_10plex − Scan number: 11370

Gene Names	Charge	m/z	Mass	Retention time	PEP	Score	Precursor Intensity
SDHA	3	435.5701	1303.688	39.58	0.0015734	82.171	2782550

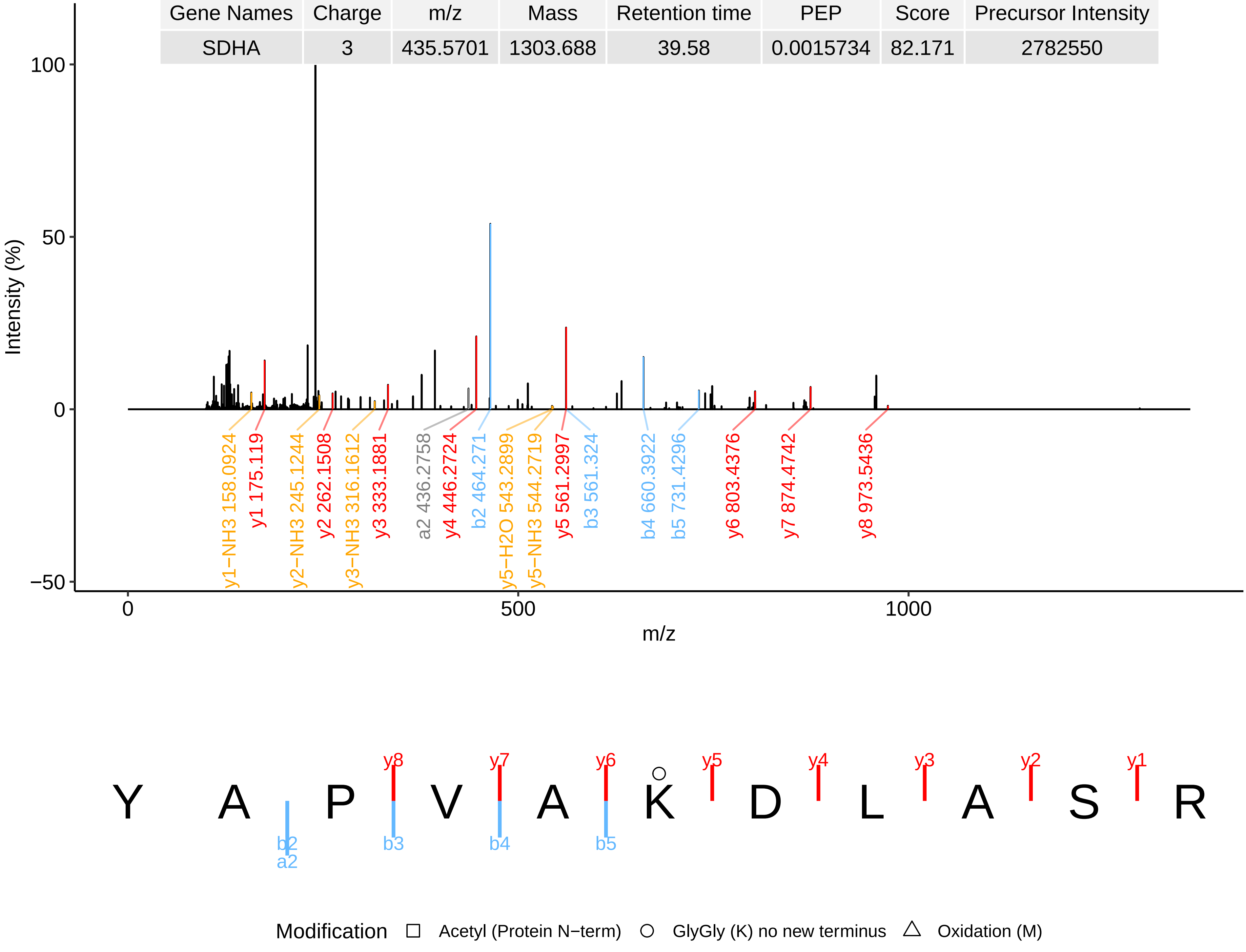

20200921_KIZ_MUb4_GlyGly_10plex − Scan number: 7879

Gene Names	Charge	m/z	Mass	Retention time	PEP	Score	Precursor Intensity
SEC31A	3	358.1856	1071.535	26.603	0.0057236	89.142	5176090

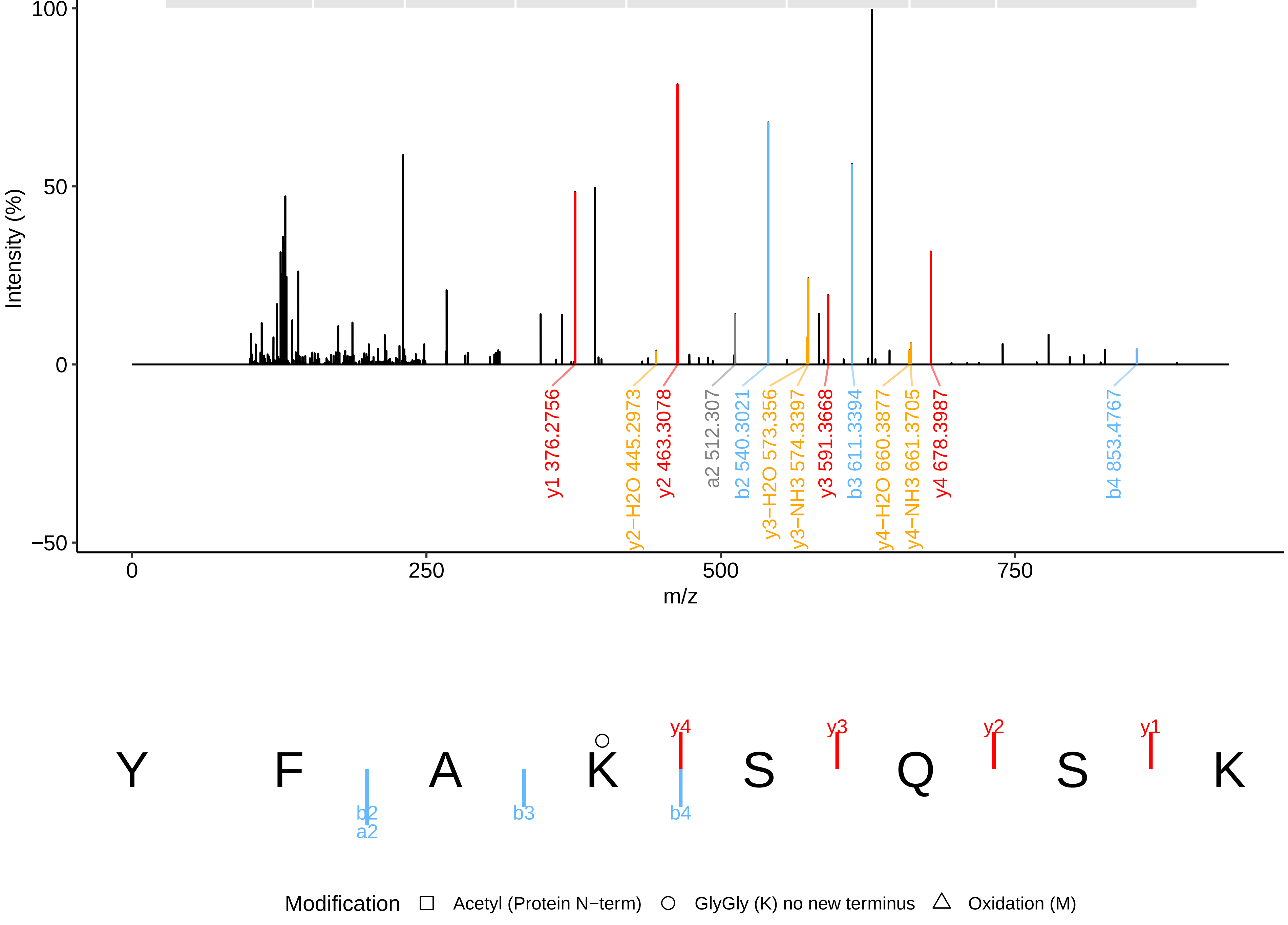

20200921_KIZ_MUb4_GlyGly_10plex − Scan number: 13929

Gene Names	Charge	m/z	Mass	Retention time	PEP	Score	Precursor Intensity
S100P	3	357.1941	1068.56	49.316	0.077246	51.854	4480929

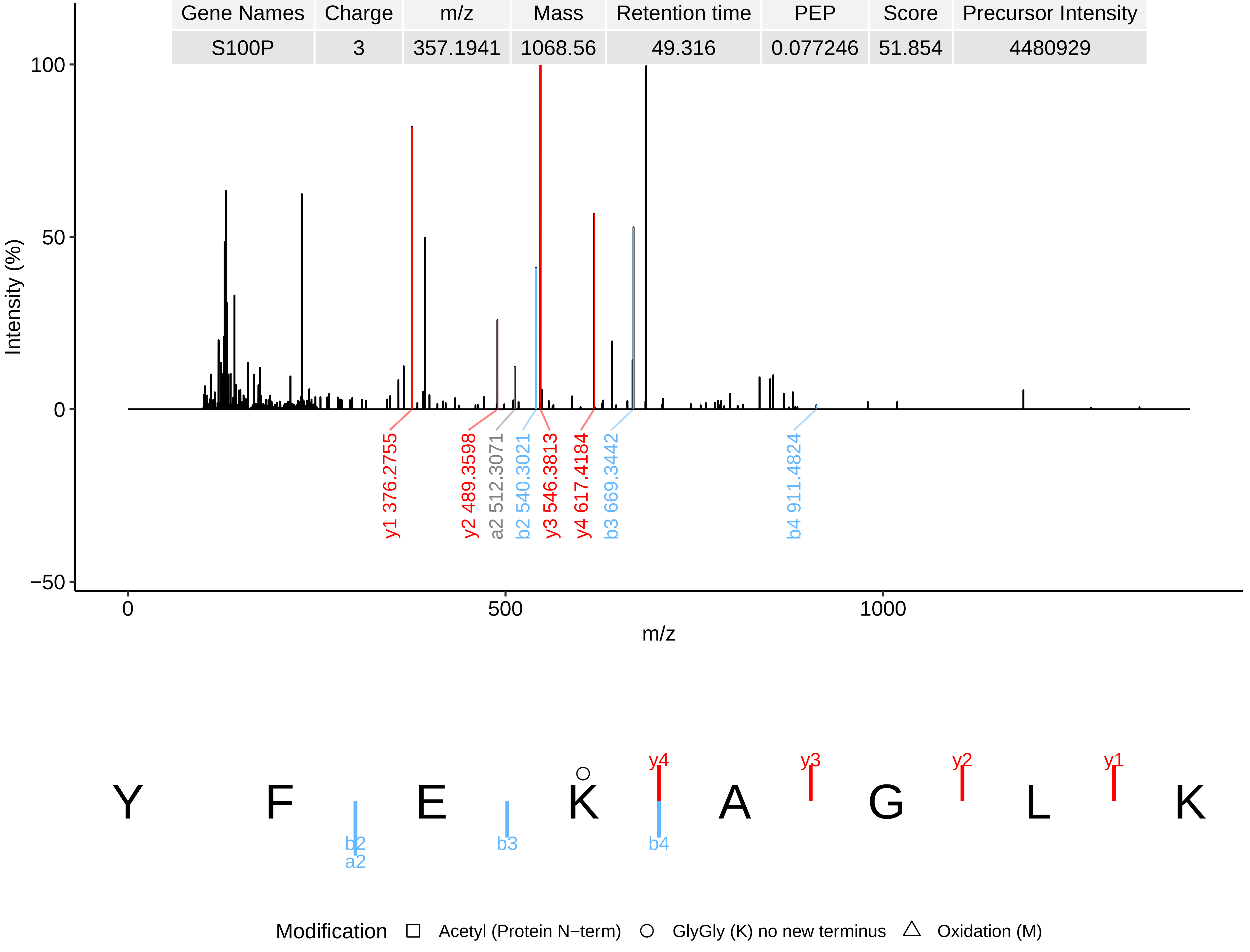

20200921_KIZ_MUb4_GlyGly_10plex − Scan number: 3275

Gene Names	Charge	m/z	Mass	Retention time	PEP	Score	Precursor Intensity
SCCPDH	3	359.1771	1074.509	10.256	0.022343	69.954	1854273

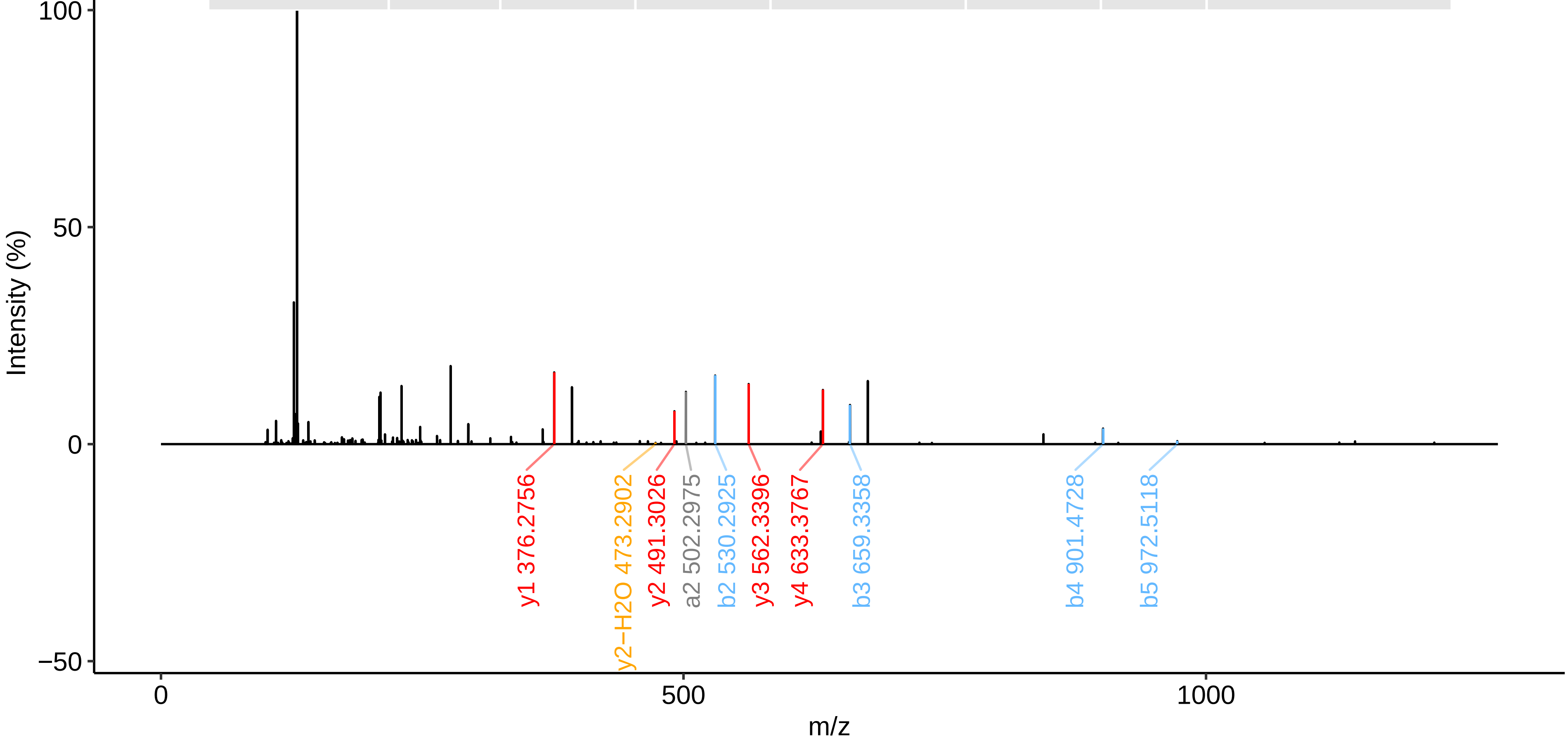

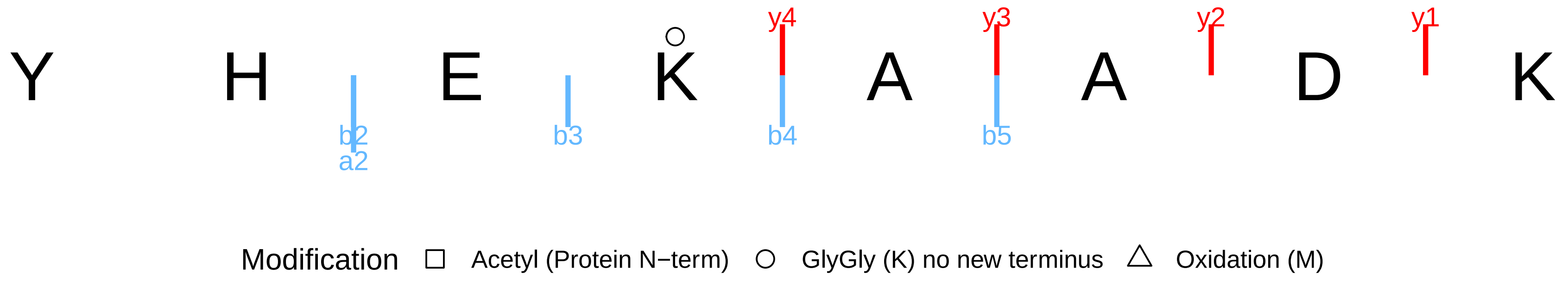

20200921_KIZ_MUb4_GlyGly_10plex − Scan number: 3282

Gene Names	Charge	m/z	Mass	Retention time	PEP	Score	Precursor Intensity
SCCPDH	4	269.6346	1074.509	10.282	0.067823	50.607	8143529

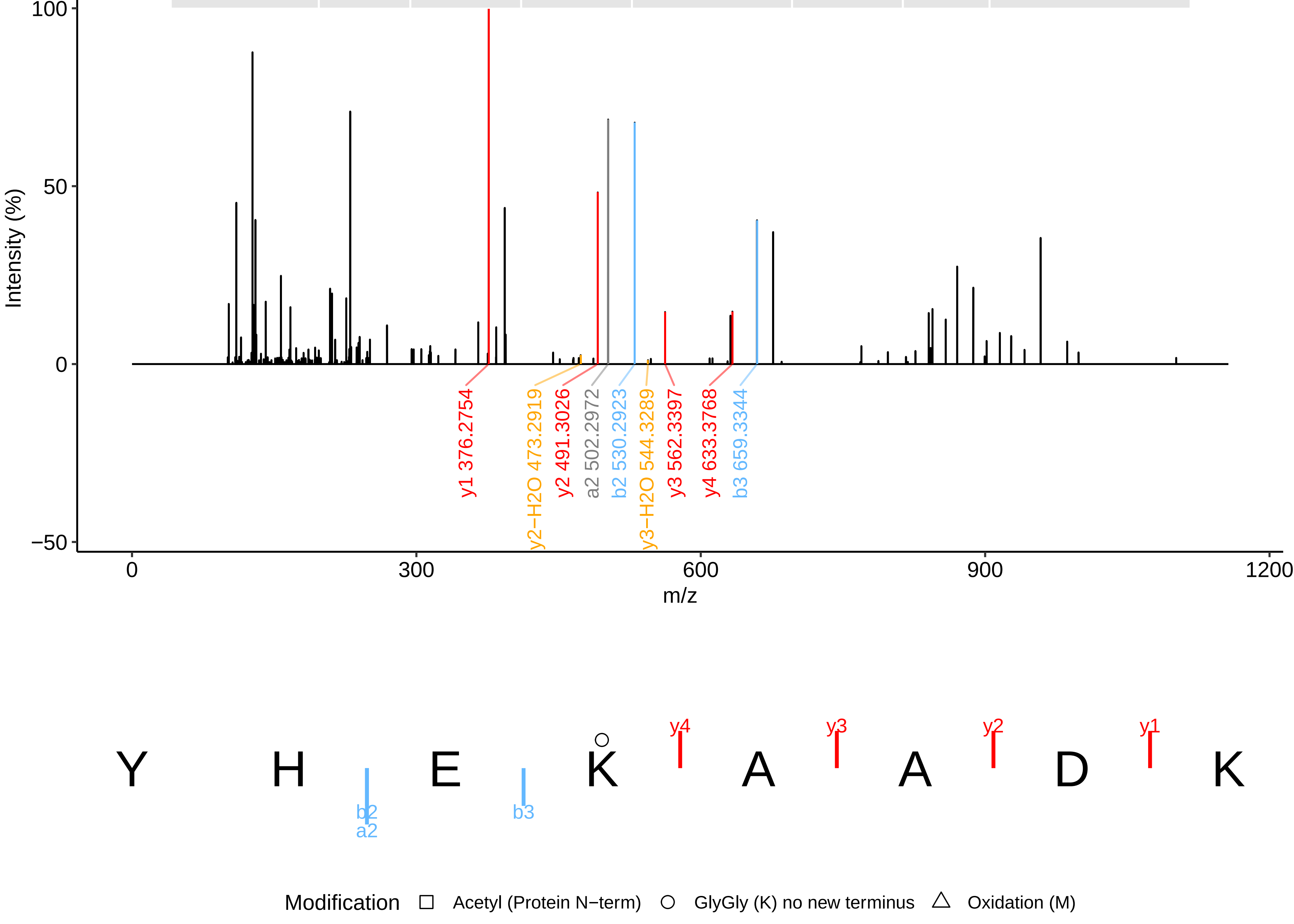

20200921_KIZ_MUb4_GlyGly_10plex_2 − Scan number: 3191

Gene Names	Charge	m/z	Mass	Retention time	PEP	Score	Precursor Intensity
SCCPDH	3	359.1771	1074.509	10.586	0.082367	50.607	300554.7

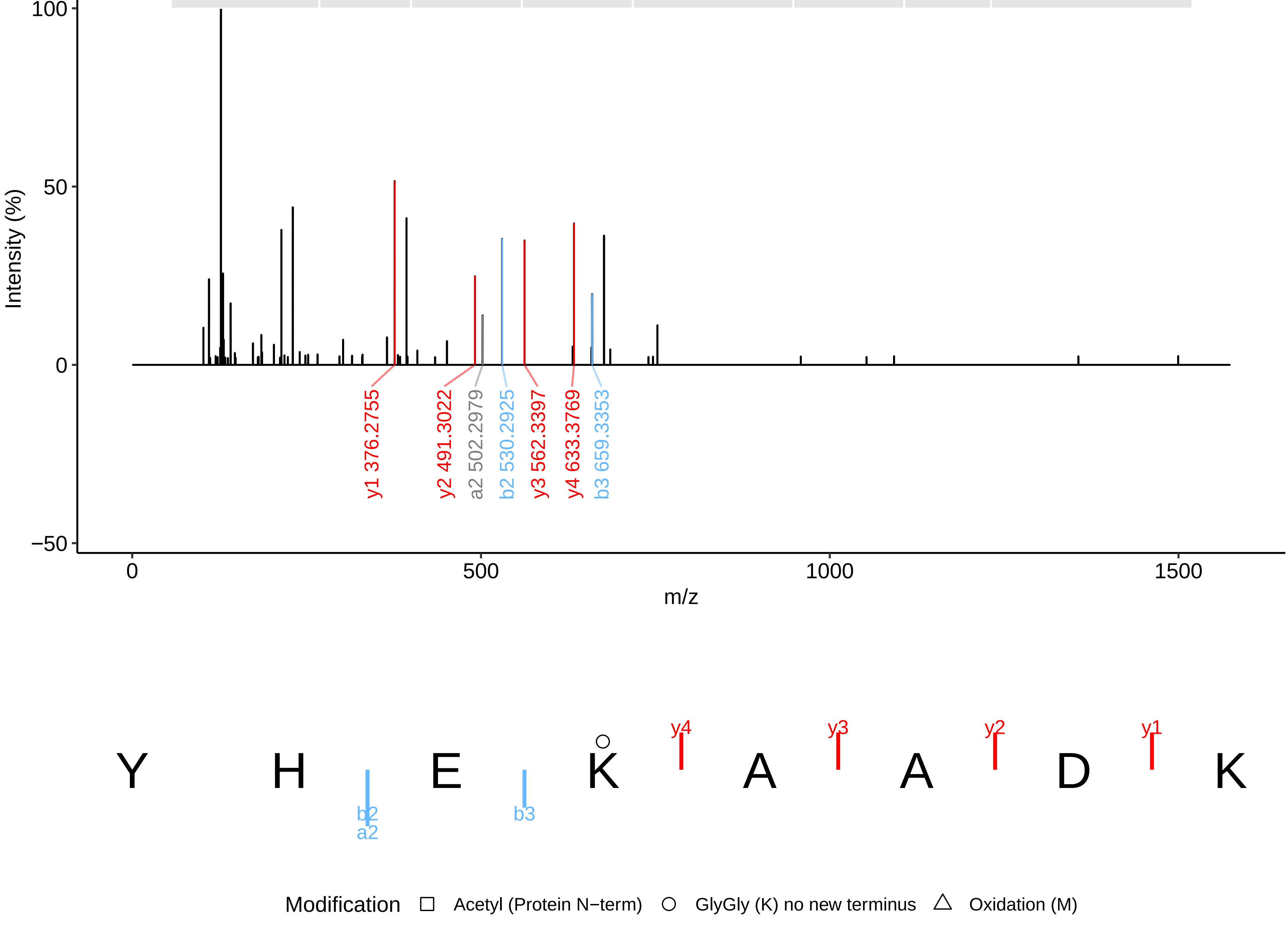

20200924_KIZ_MUb5_GlyGly_2 − Scan number: 2795

Gene Names	Charge	m/z	Mass	Retention time	PEP	Score	Precursor Intensity
SCCPDH	4	269.6346	1074.509	9.9865	0.067823	50.607	2019595

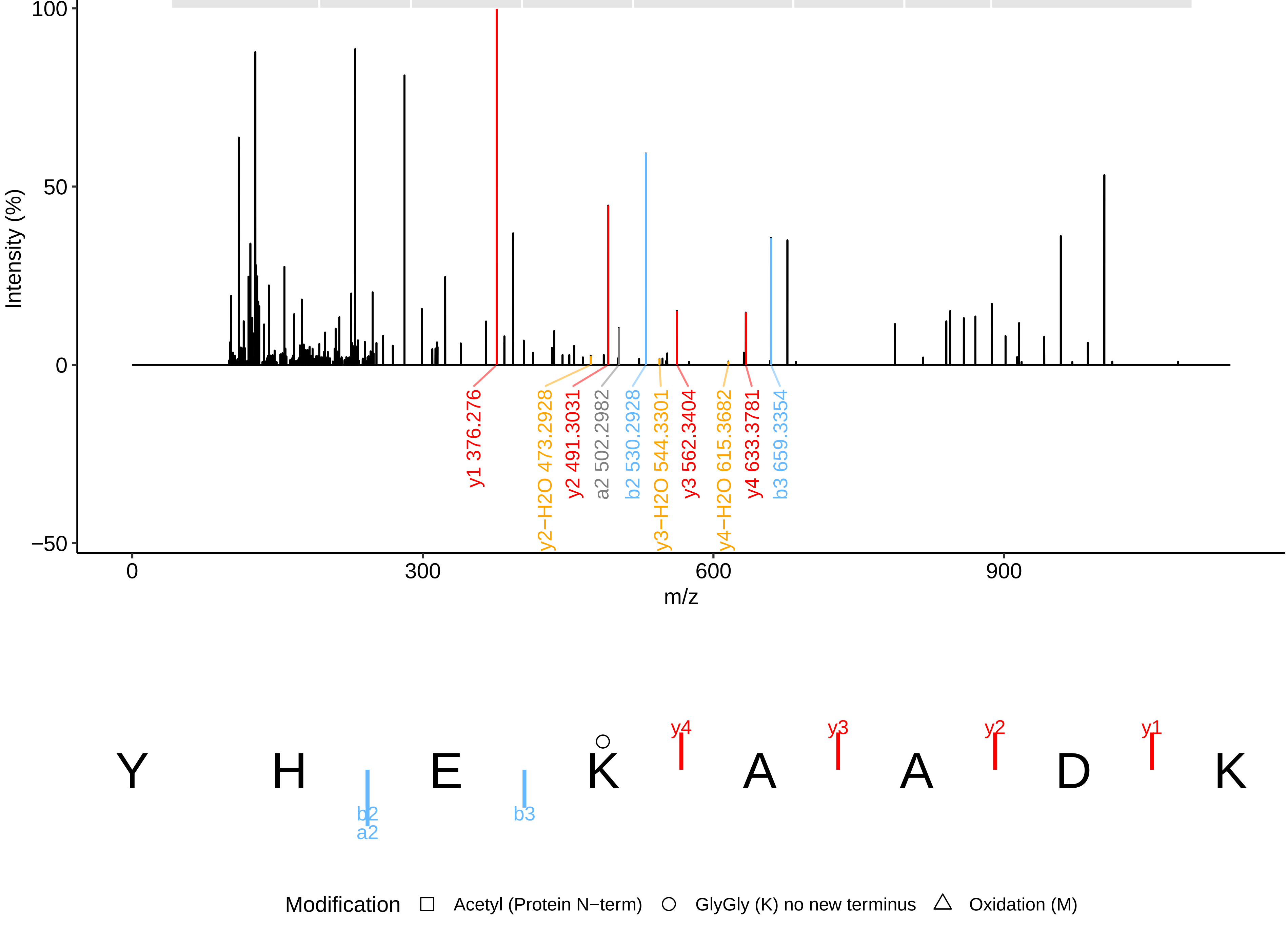

20200921_KIZ_MUb4_GlyGly_10plex − Scan number: 10754

Gene Names	Charge	m/z	Mass	Retention time	PEP	Score	Precursor Intensity
RPL37	3	361.1857	1080.535	37.316	0.028171	69.598	5372954

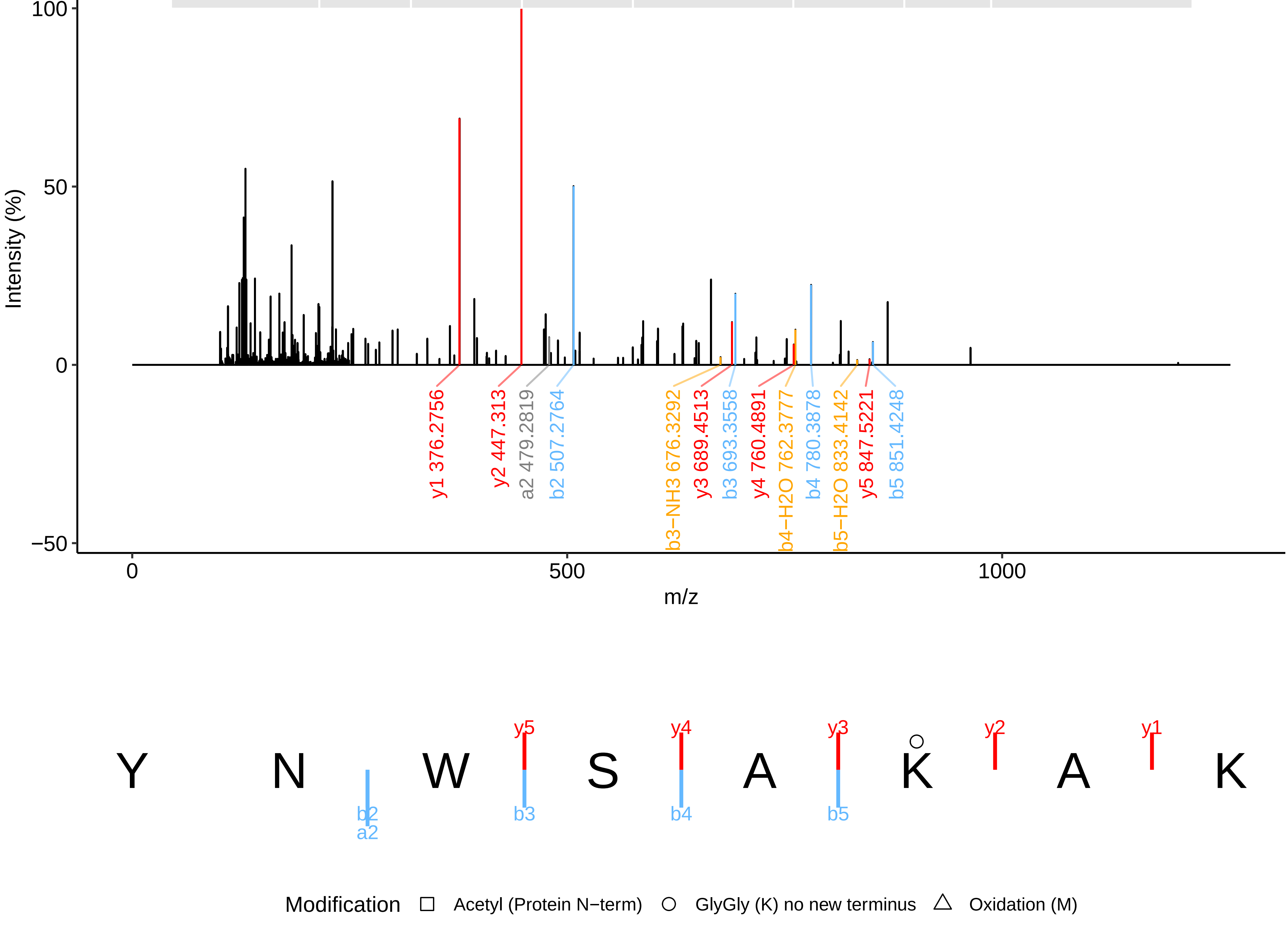

20200921_KIZ_MUb4_GlyGly_10plex − Scan number: 6116

Gene Names	Charge	m/z	Mass	Retention time	PEP	Score	Precursor Intensity
SLC25A11	2	778.3948	1554.775	20.129	2.5658e−87	164.04	3526656

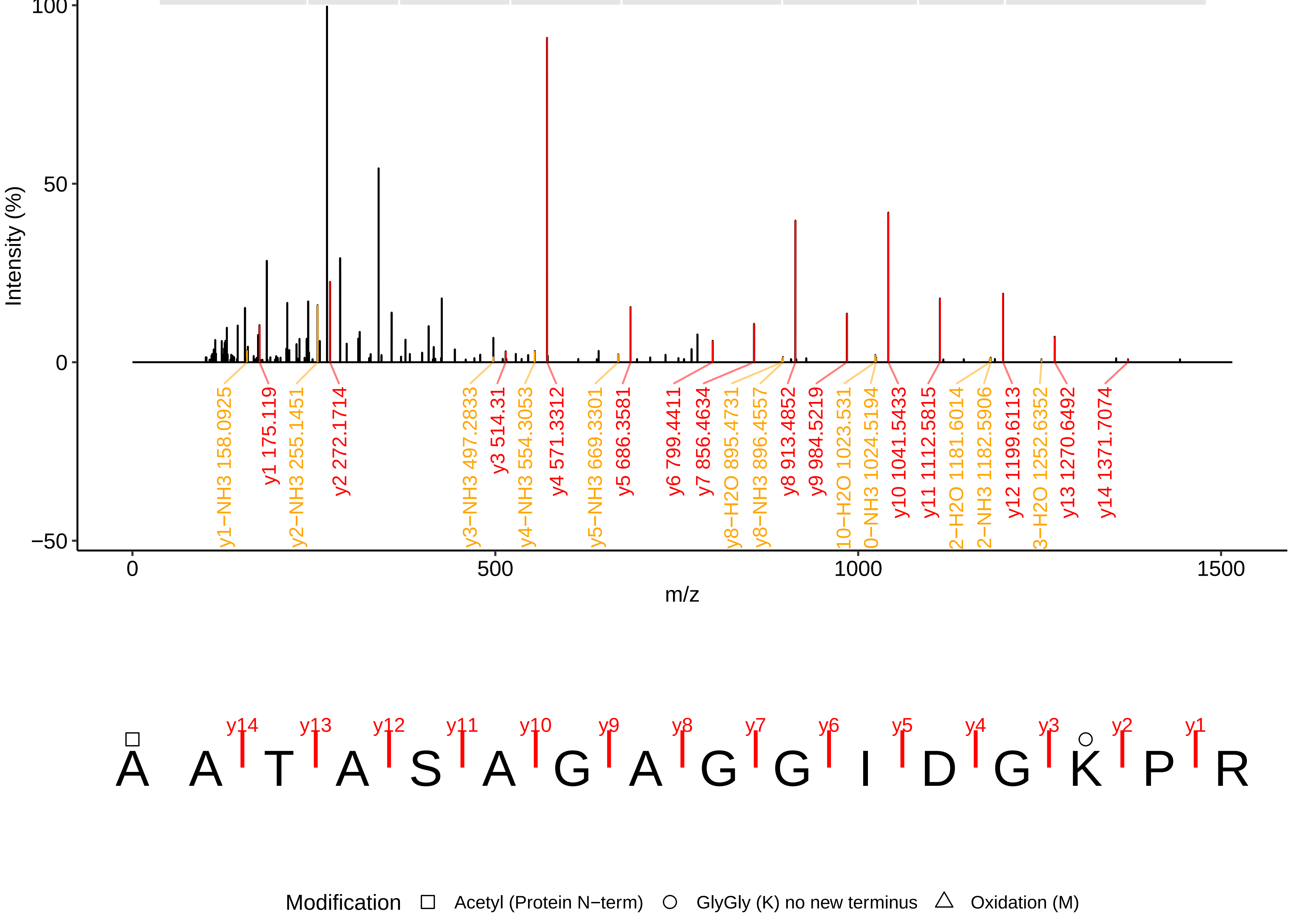

20200921_KIZ_MUb4_GlyGly_10plex_2 − Scan number: 5517

Gene Names	Charge	m/z	Mass	Retention time	PEP	Score	Precursor Intensity
SLC25A11	2	778.3948	1554.775	19.874	0.0042662	42.809	NA

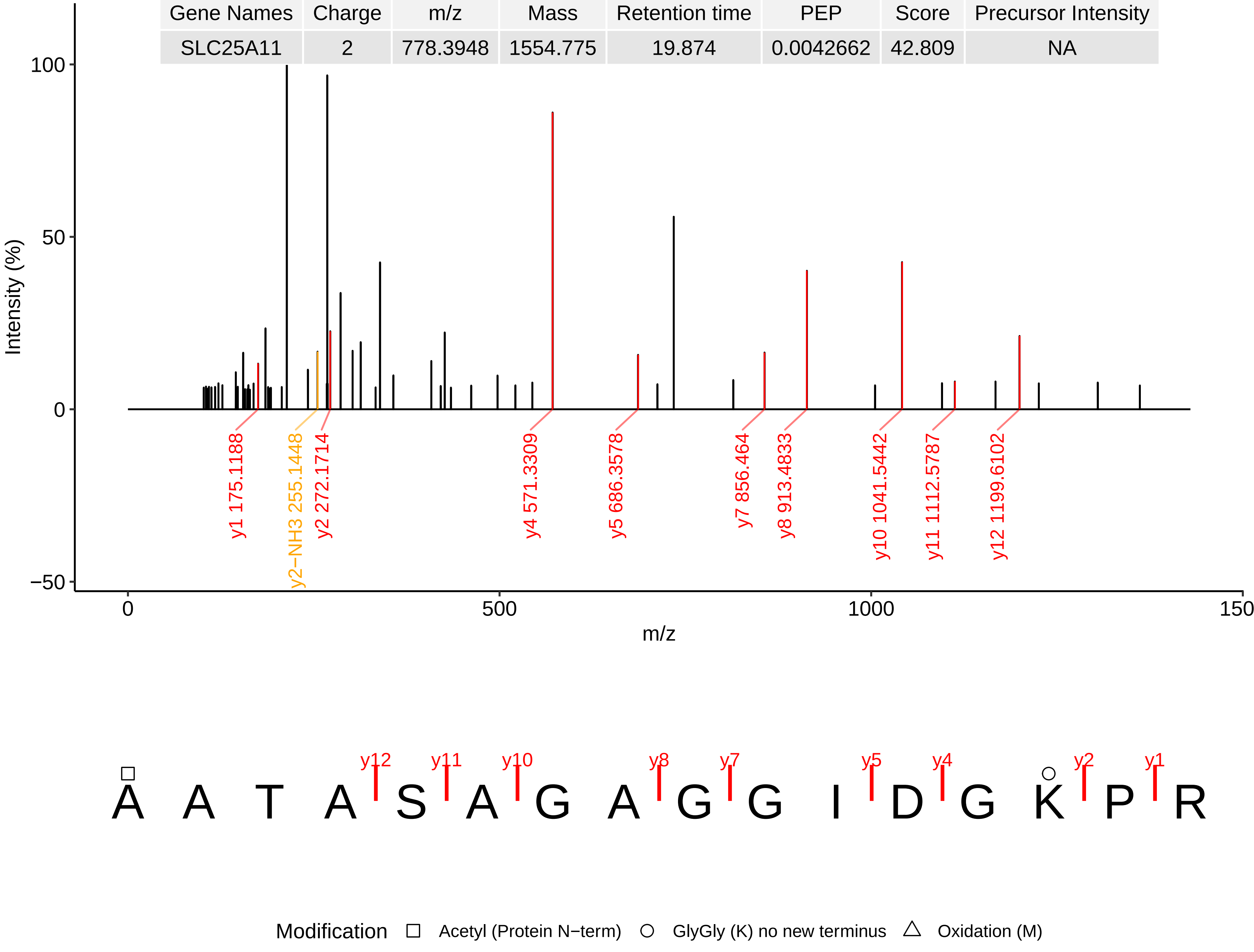

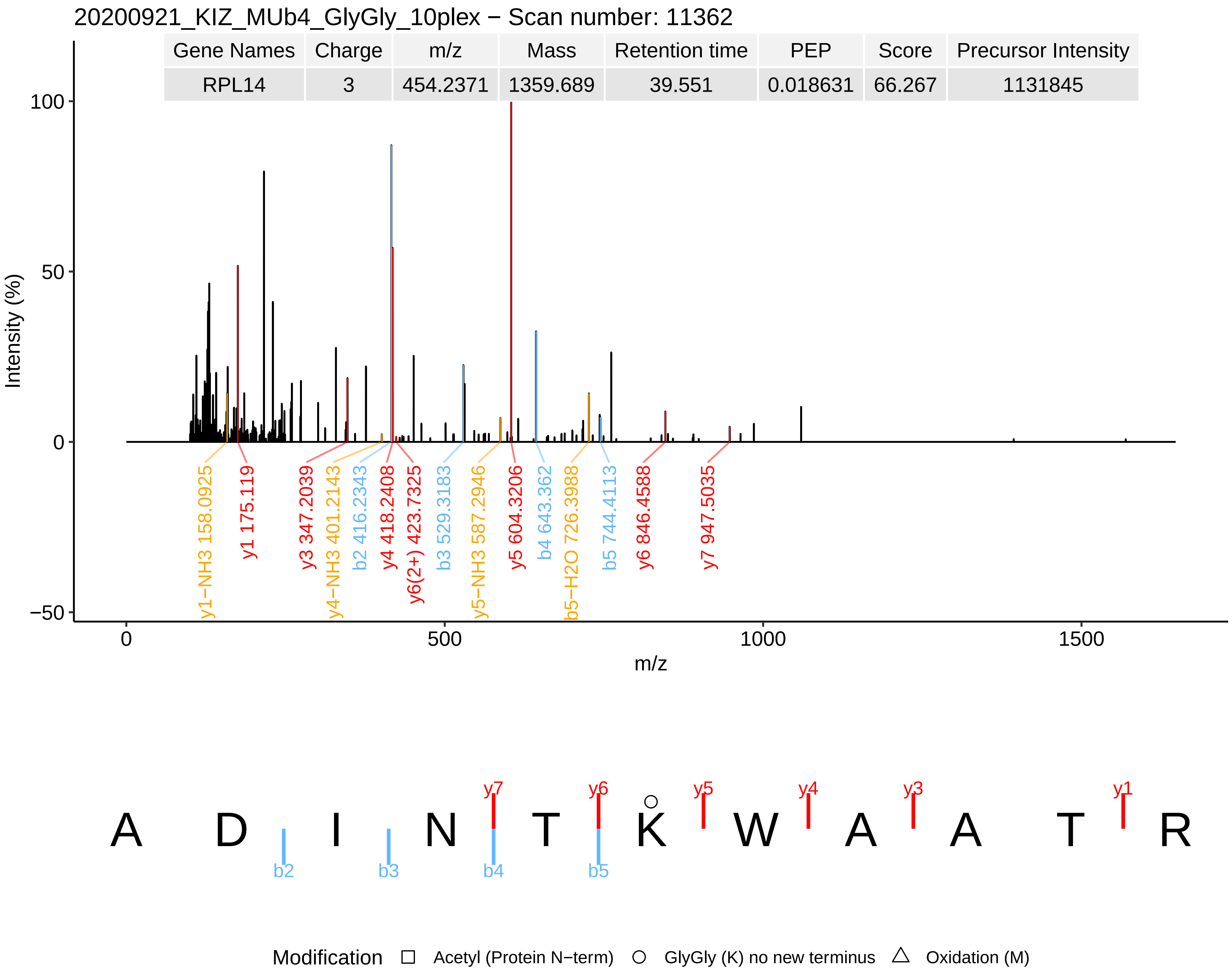

20200921_KIZ_MUb4_GlyGly_10plex − Scan number: 11362
Gene Names | Charge | m/z | Mass | Retention time | PEP | Score | Precursor Intensity
RPL14 | 3 | 454.2371 | 1359.689 | 39.551 | 0.018631 | 66.267 | 1131845
Intensity (%)
100
50
0
−50
0
500
1000
1500
m/z
y1−NH3 158.0925
y1 175.119
y3 347.2039
y4−NH3 401.2143
b2 416.2343
y4 418.2408
y6(2+) 423.7325
b3 529.3183
y5−NH3 587.2946
y5 604.3206
b4 643.362
b5−H2O 726.3988
b5 744.4113
y6 846.4588
y7 947.5035
A D I N T K W A A T R
y7 y6 y5 y4 y3 y1
b2 b3 b4 b5
Modification
Acetyl (Protein N−term)
GlyGly (K) no new terminus
Oxidation (M)

20200924_KIZ_MUb5_GlyGly_2 − Scan number: 8285

Gene Names	Charge	m/z	Mass	Retention time	PEP	Score	Precursor Intensity
DHX16	3	363.5439	1087.61	30.947	0.091662	55.531	1347280

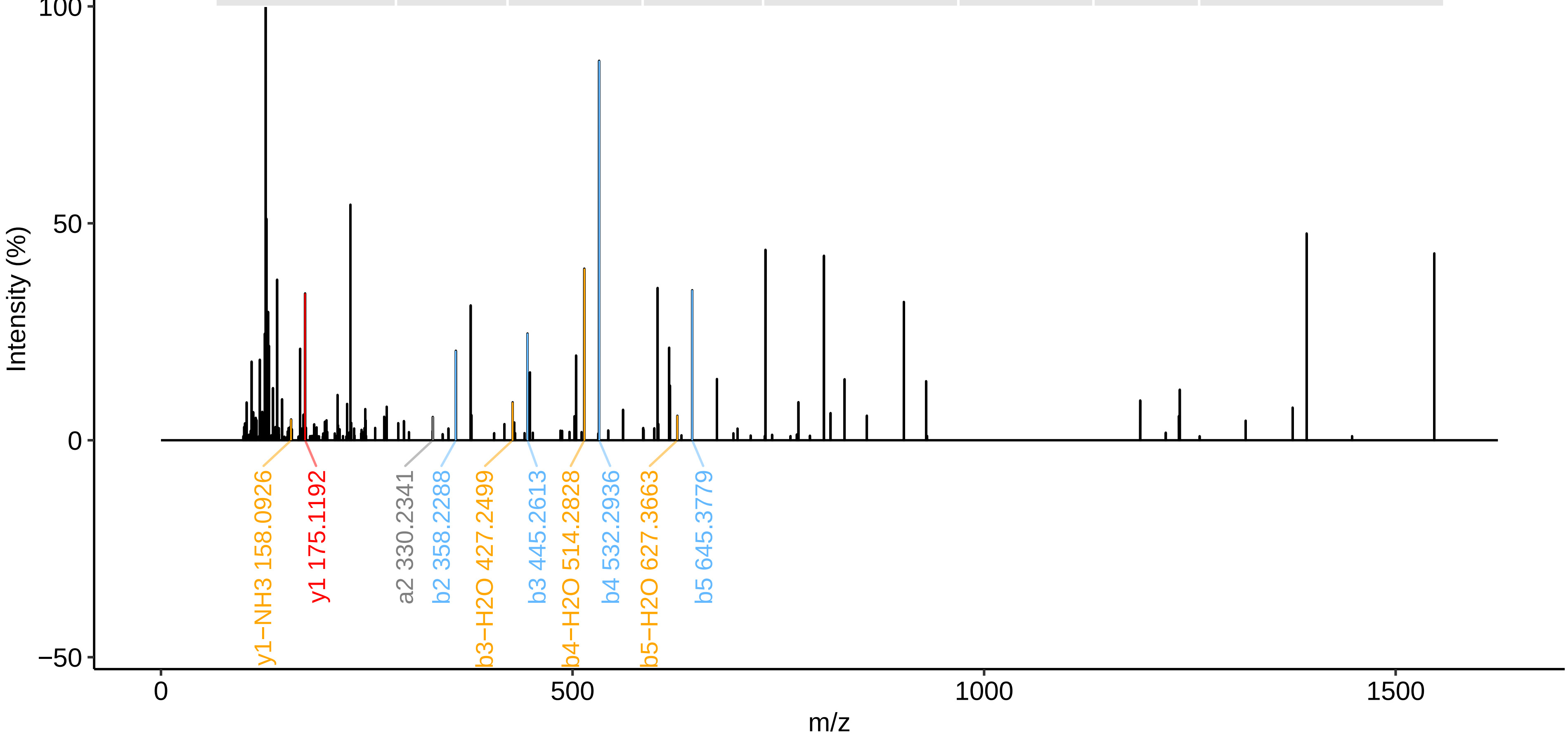

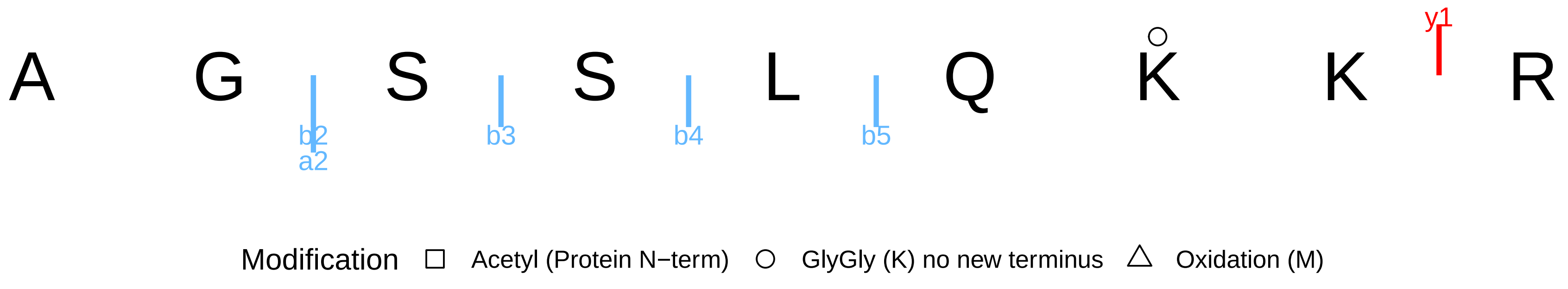

20200921_KIZ_MUb4_GlyGly_10plex − Scan number: 18172

Gene Names	Charge	m/z	Mass	Retention time	PEP	Score	Precursor Intensity
RPL36	3	378.218	1131.632	65.283	0.017399	67.563	NA

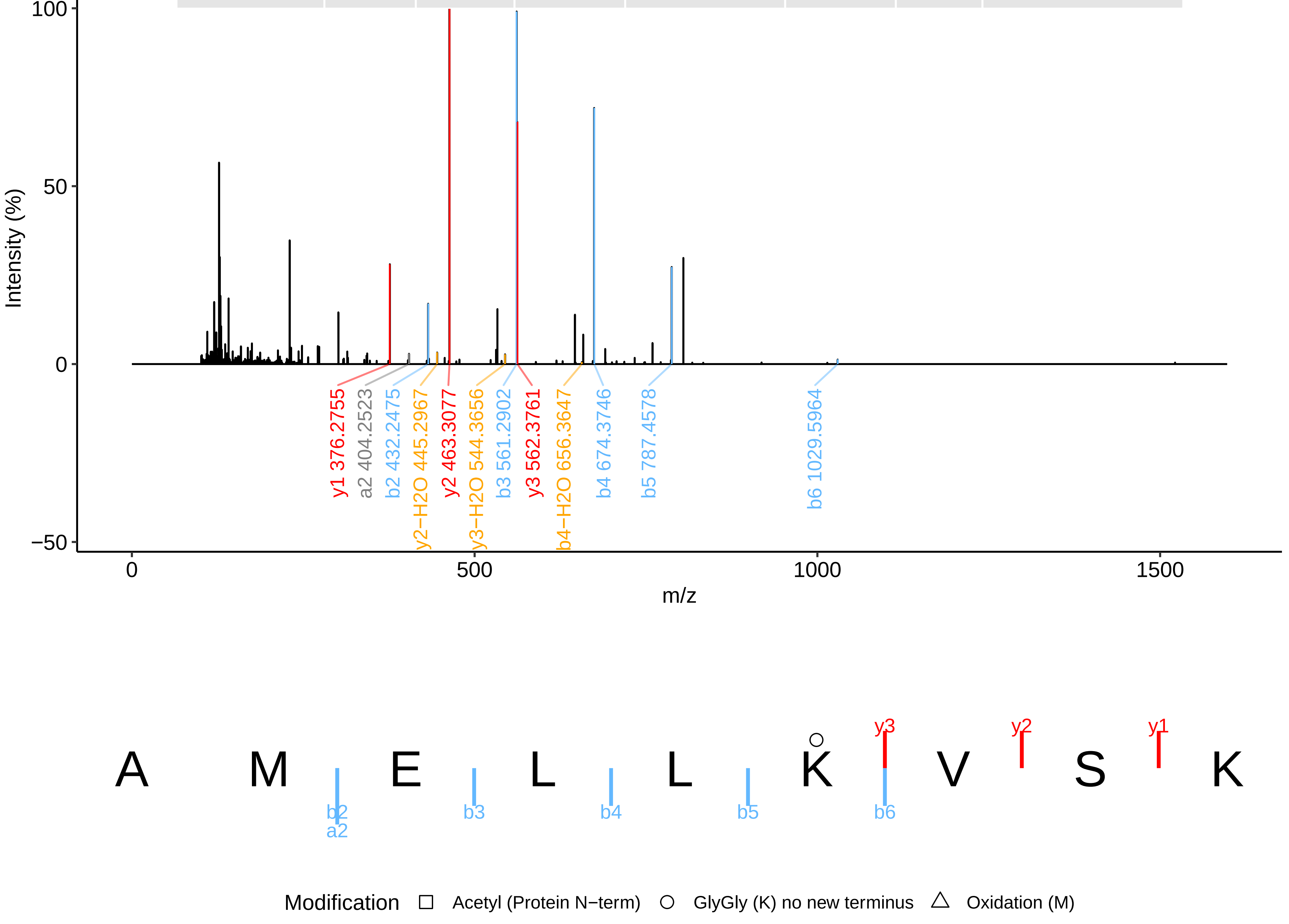

20200921_KIZ_MUb4_GlyGly_10plex − Scan number: 18190

Gene Names	Charge	m/z	Mass	Retention time	PEP	Score	Precursor Intensity
RPL36	3	378.218	1131.632	65.354	0.058764	55.567	2124959

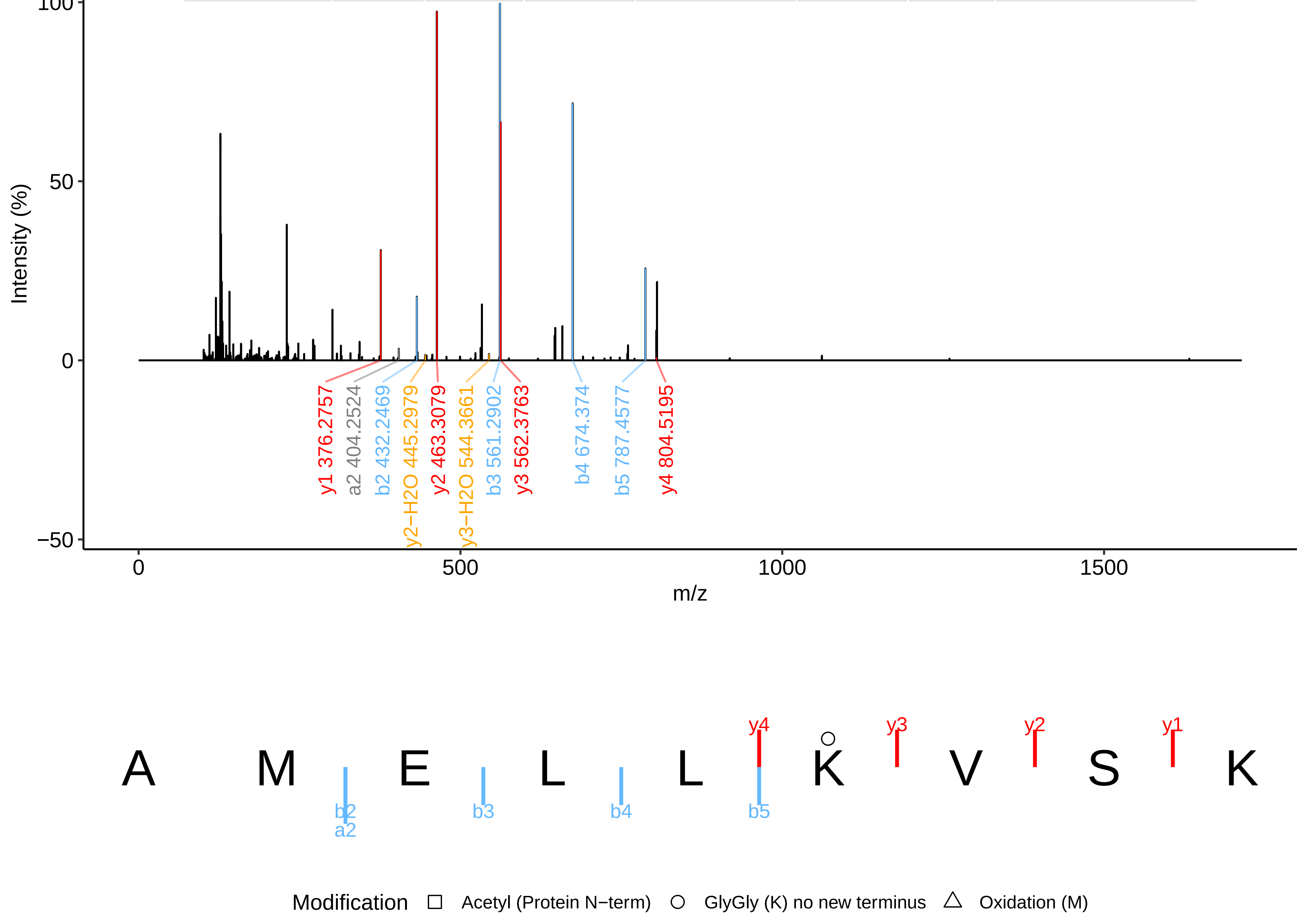

20200921_KIZ_MUb4_GlyGly_10plex_2 − Scan number: 16892

Gene Names	Charge	m/z	Mass	Retention time	PEP	Score	Precursor Intensity
RPL36	3	378.218	1131.632	65.992	0.048964	57.598	882710.4

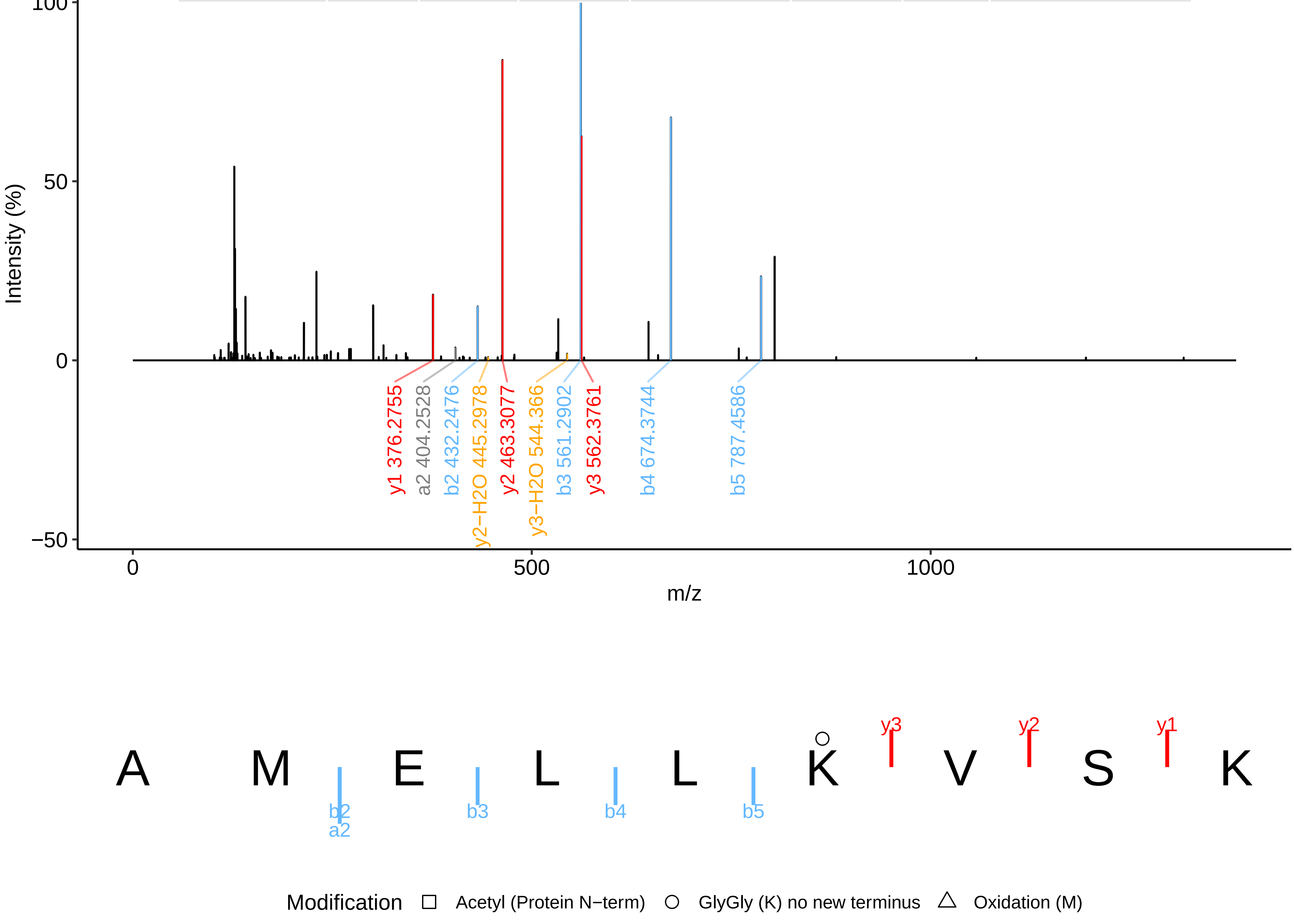

20200924_KIZ_MUb5_GlyGly_1 − Scan number: 17513

Gene Names	Charge	m/z	Mass	Retention time	PEP	Score	Precursor Intensity
RPL36	3	378.218	1131.632	56.545	0.065667	57.598	12958896

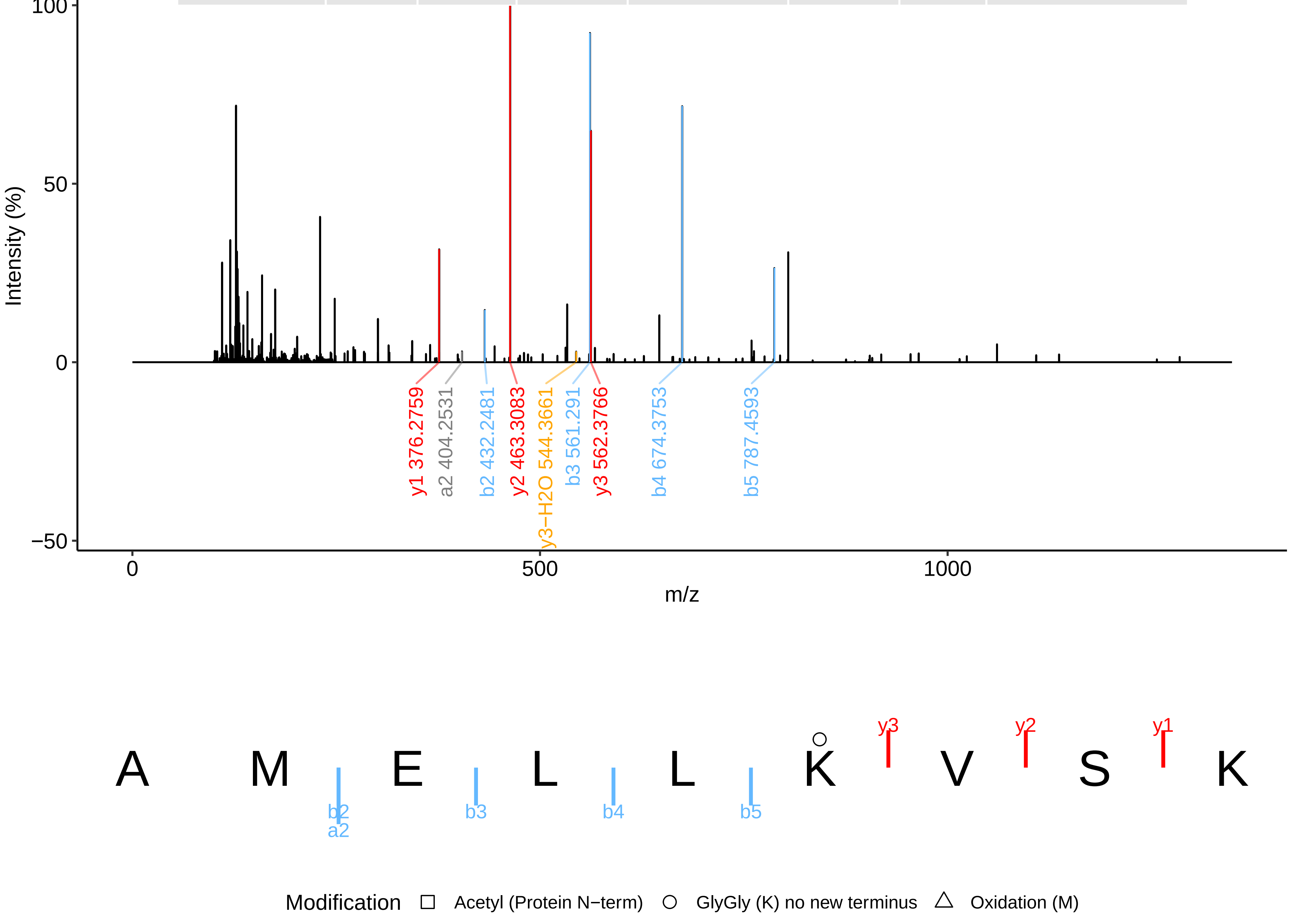

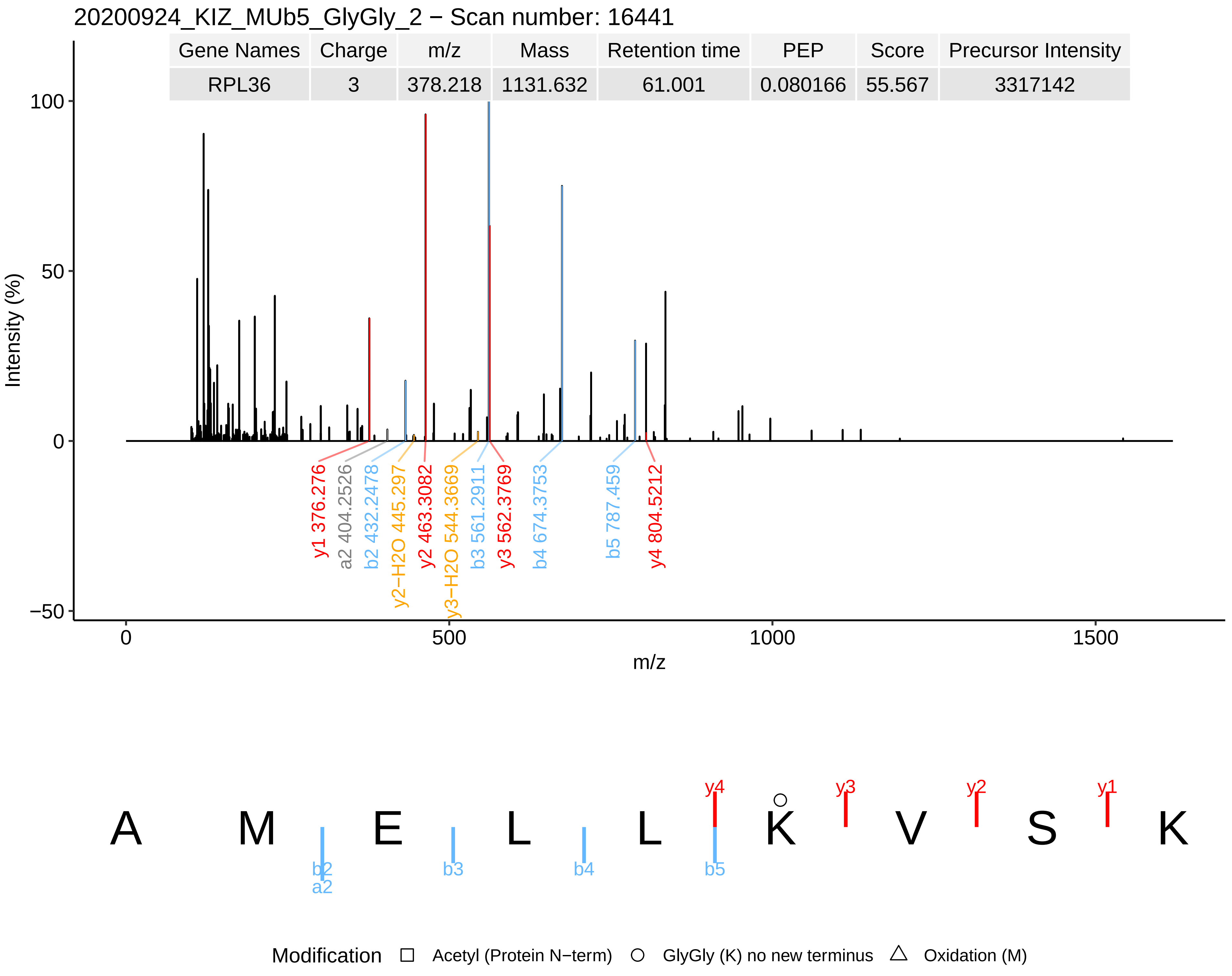
20200924_KIZ_MUb5_GlyGly_2 − Scan number: 16441
Gene Names
Charge
m/z
Mass
Retention time
PEP
Score
Precursor Intensity
RPL36
3
378.218
1131.632
61.001
0.080166
55.567
3317142
Intensity (%)
100
50
0
−50
y1 376.276
a2 404.2526
b2 432.2478
y2−H2O 445.297
y2 463.3082
y3−H2O 544.3669
b3 561.2911
y3 562.3769
b4 674.3753
b5 787.459
y4 804.5212
0
500
1000
1500
m/z
A M E L L K V S K
y4 y3 y2 y1
b2 a2 b3 b4 b5
Modification
Acetyl (Protein N−term)
GlyGly (K) no new terminus
Oxidation (M)

20200921_KIZ_MUb4_GlyGly_10plex − Scan number: 6683

Gene Names	Charge	m/z	Mass	Retention time	PEP	Score	Precursor Intensity
HSPA1B;HSPA1A;HSPA1L;HSPA6	3	454.898	1361.672	22.21	0.00071684	89.171	3456006

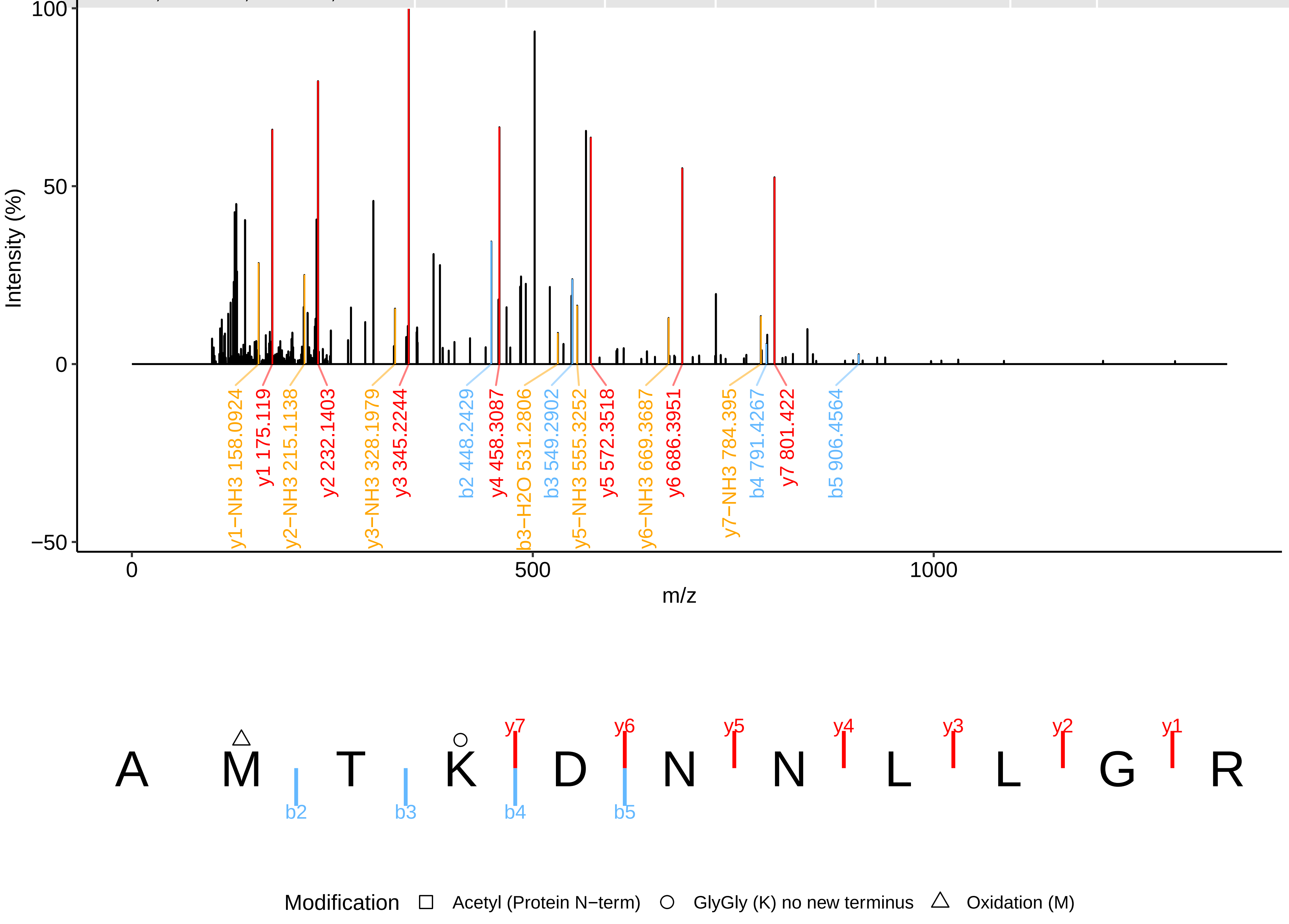

20200921_KIZ_MUb4_GlyGly_10plex_2 − Scan number: 6156

Gene Names	Charge	m/z	Mass	Retention time	PEP	Score	Precursor Intensity
HSPA1B;HSPA1A;HSPA1L;HSPA6	3	454.898	1361.672	22.497	0.0042077	76.282	618150

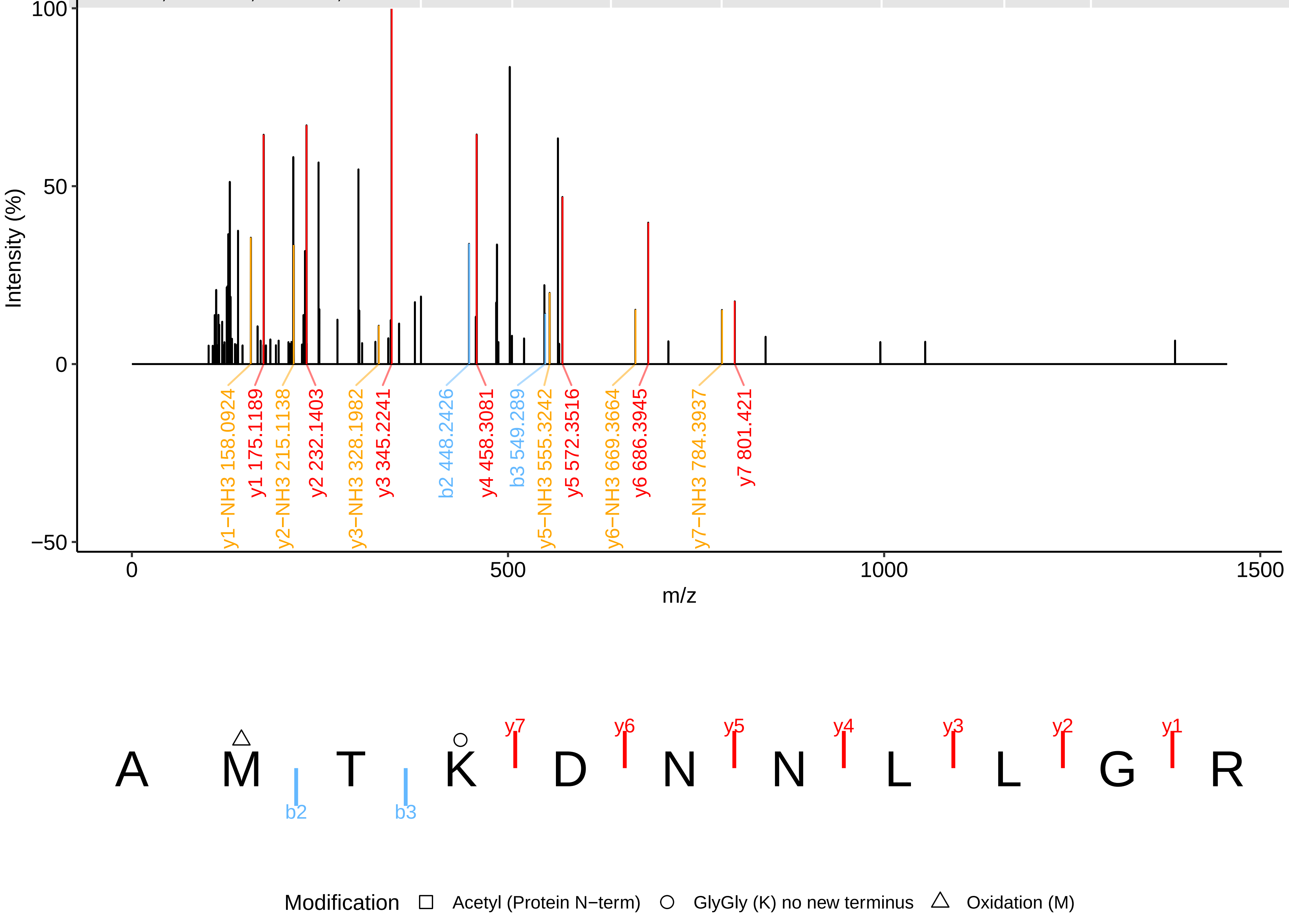

20200921_KIZ_MUb4_GlyGly_10plex − Scan number: 9860

Gene Names	Charge	m/z	Mass	Retention time	PEP	Score	Precursor Intensity
HSPA1B;HSPA1A;HSPA1L;HSPA6	3	449.5663	1345.677	33.918	2.2417e−08	125.74	22115358

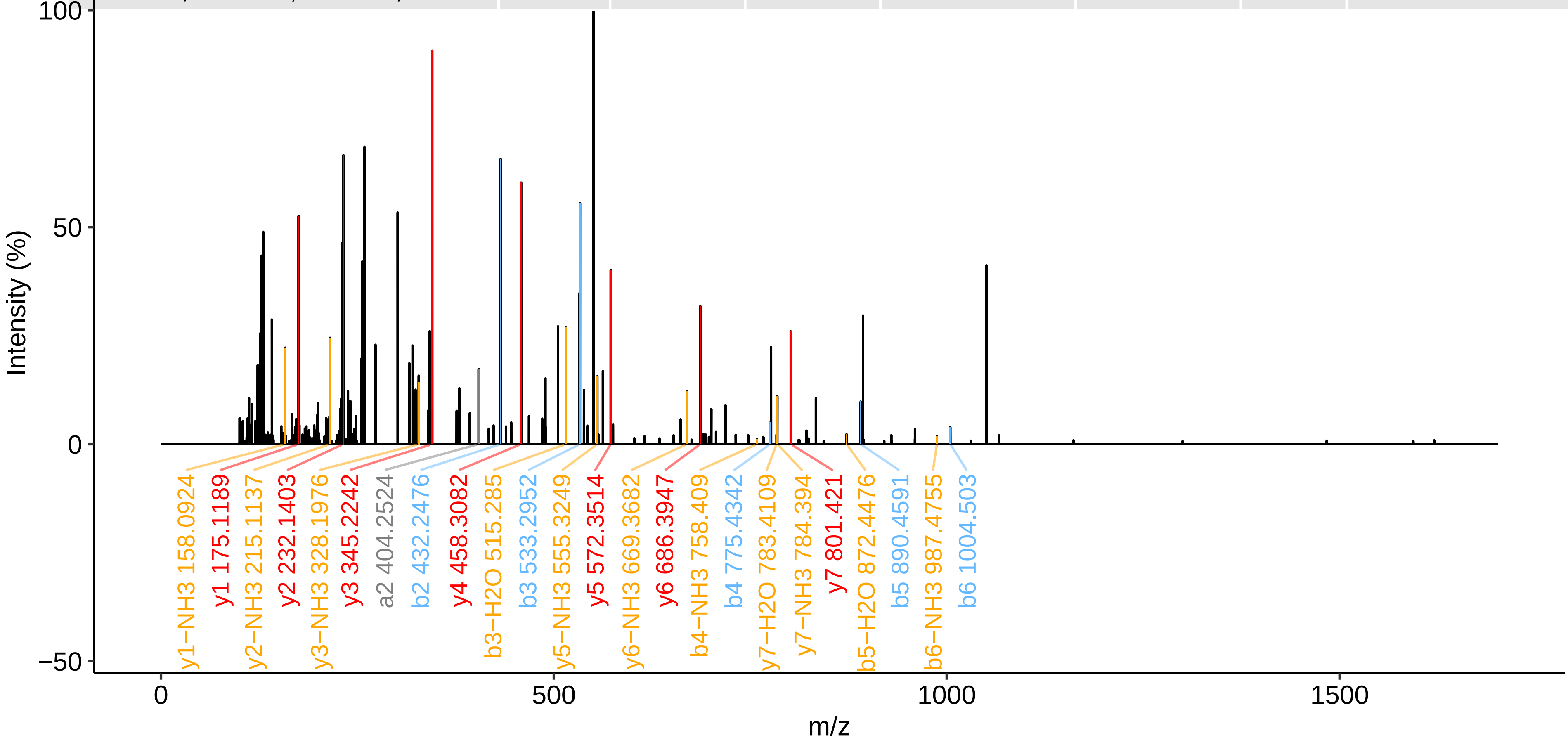

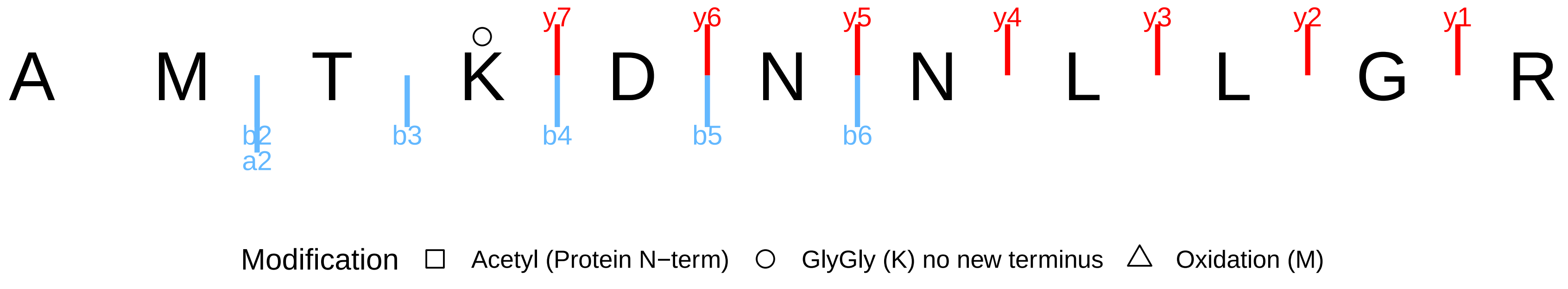

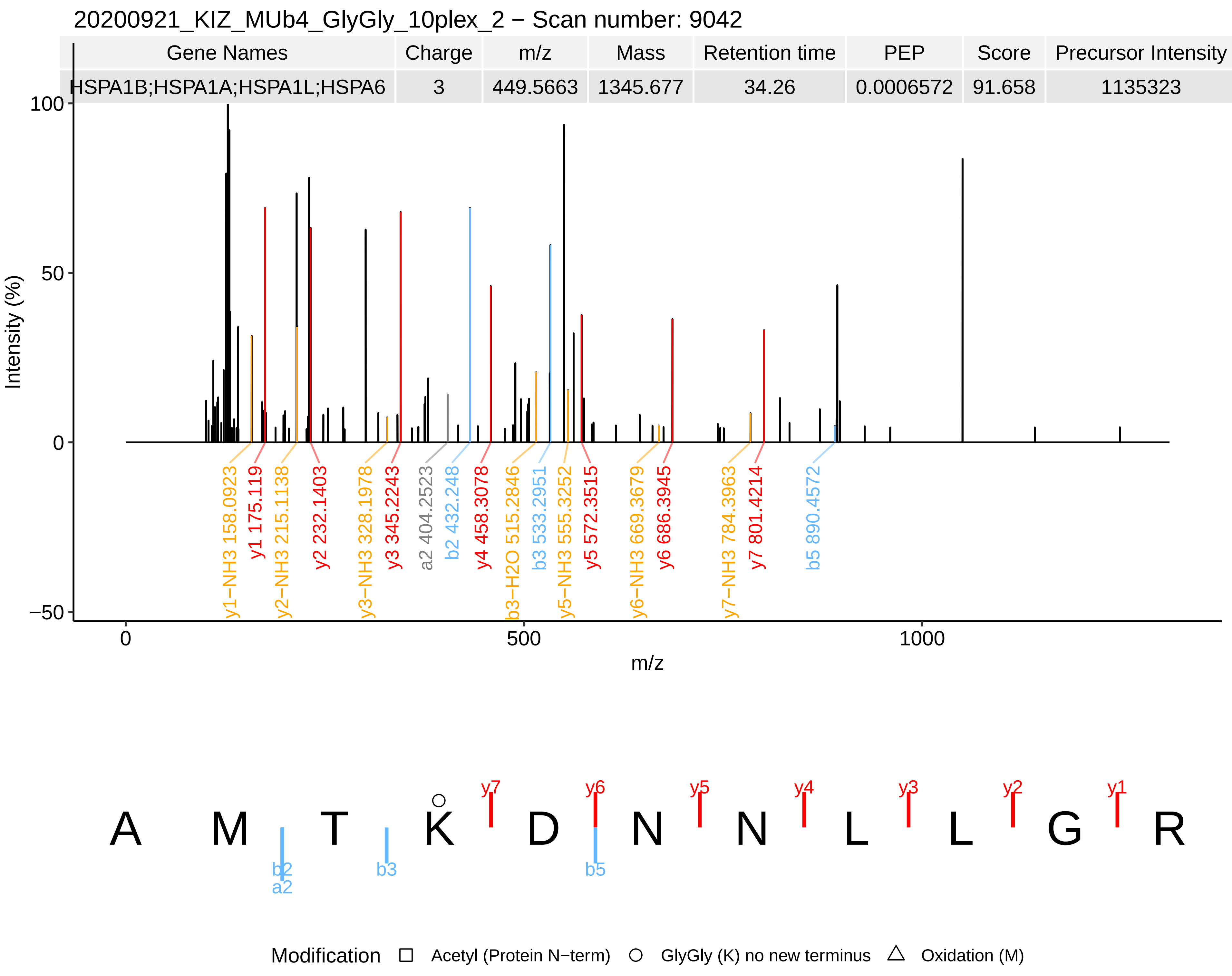

20200921_KIZ_MUb4_GlyGly_10plex_2 − Scan number: 9042
Gene Names
HSPA1B;HSPA1A;HSPA1L;HSPA6
Charge
3
m/z
449.5663
Mass
1345.677
Retention time
34.26
PEP
0.0006572
Score
91.658
Precursor Intensity
1135323
Intensity (%)
m/z
y1−NH3 158.0923
y1 175.119
y2−NH3 215.1138
y2 232.1403
y3−NH3 328.1978
y3 345.2243
a2 404.2523
b2 432.248
y4 458.3078
b3−H2O 515.2846
b3 533.2951
y5−NH3 555.3252
y5 572.3515
y6−NH3 669.3679
y6 686.3945
y7−NH3 784.3963
y7 801.4214
b5 890.4572
A M T K D N N L L G R
y7 y6 y5 y4 y3 y2 y1
b2 a2 b3 b5
Modification
Acetyl (Protein N−term)
GlyGly (K) no new terminus
Oxidation (M)

20200924_KIZ_MUb5_GlyGly_1 − Scan number: 8724

Gene Names	Charge	m/z	Mass	Retention time	PEP	Score	Precursor Intensity
HSPA1B;HSPA1A;HSPA1L;HSPA6	3	449.5663	1345.677	27.945	1.3898e−06	114.89	18000908

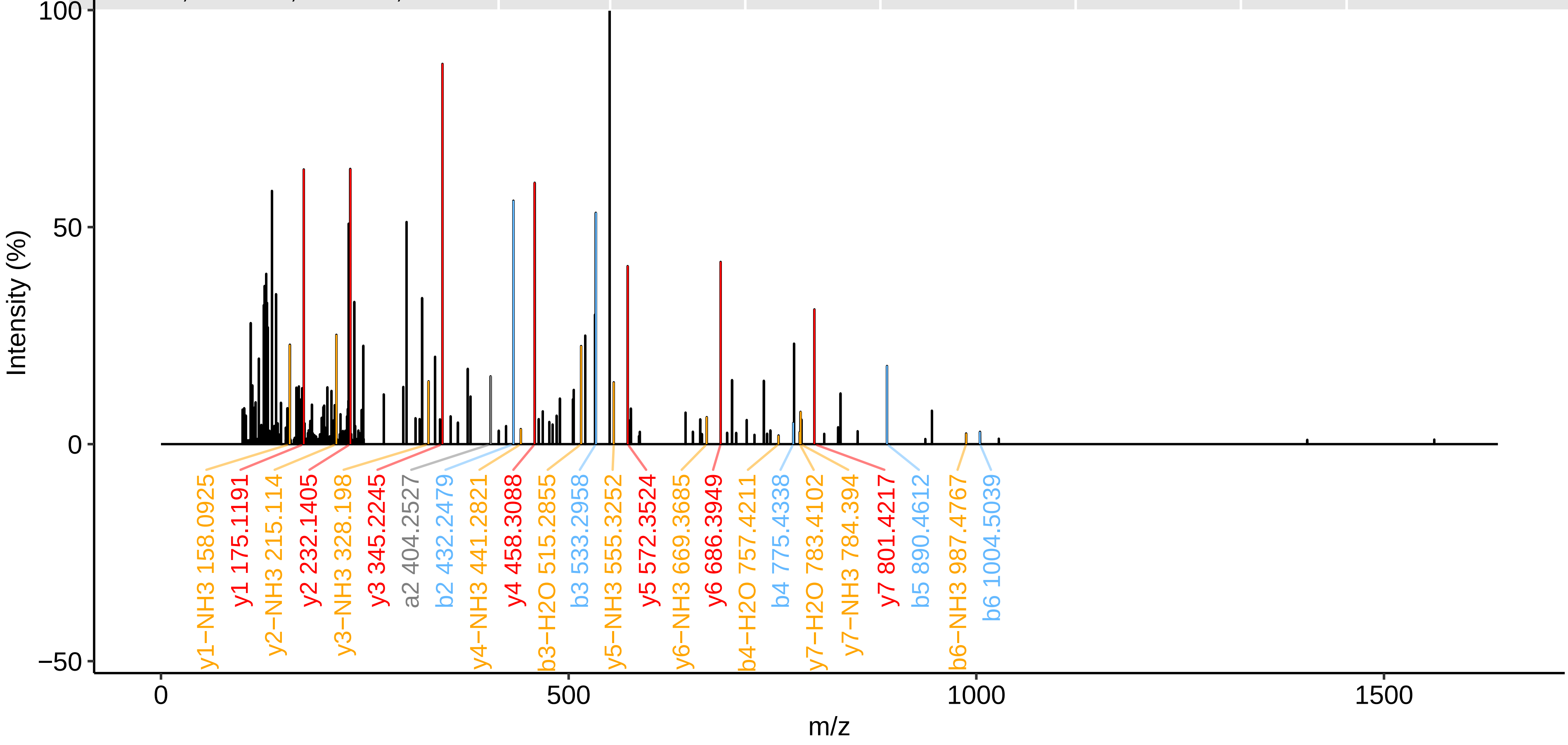

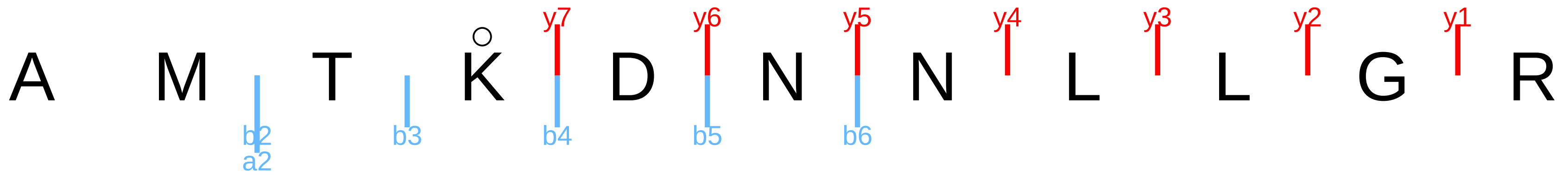

Modification □ Acetyl (Protein N−term) ○ GlyGly (K) no new terminus △ Oxidation (M)

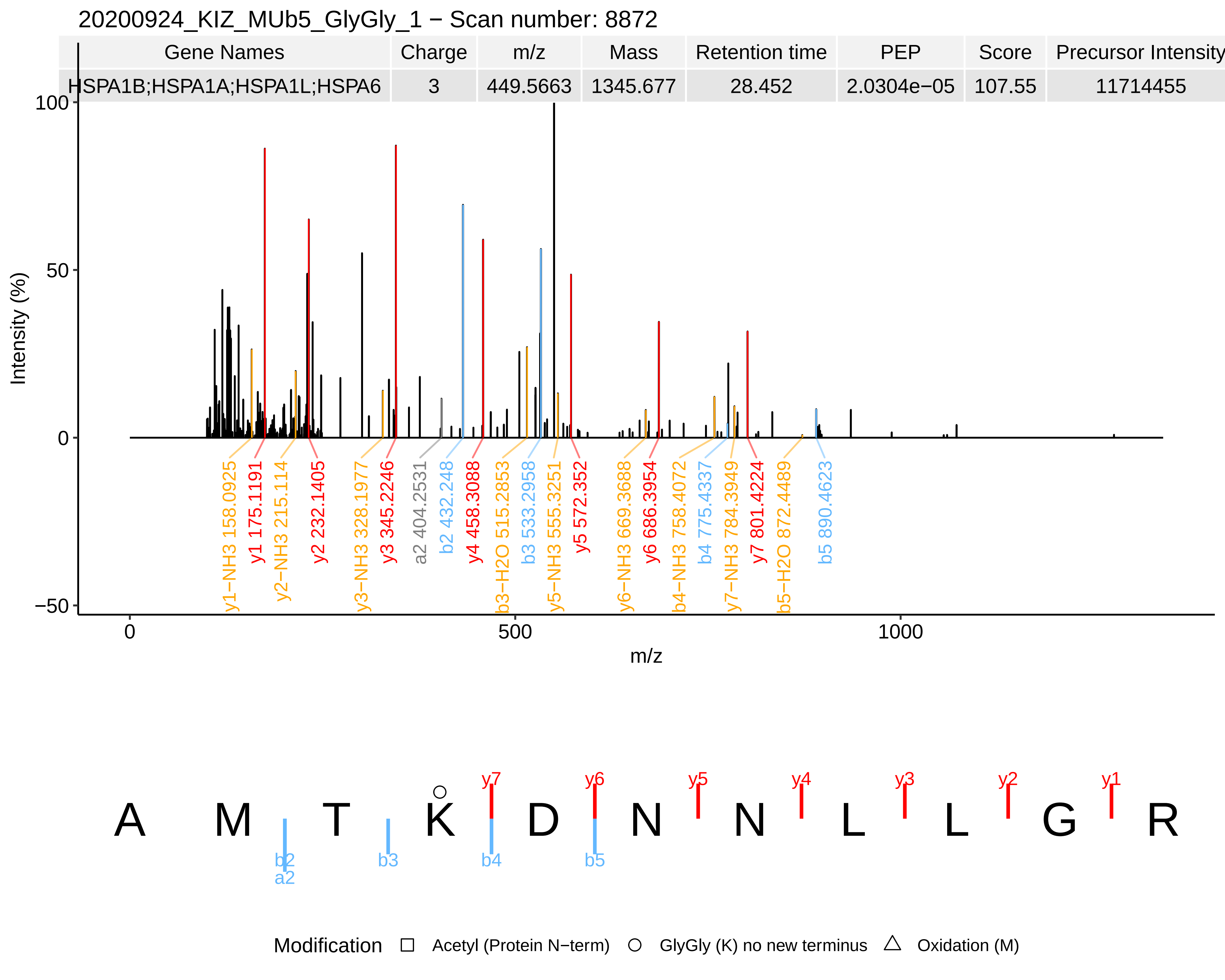

20200924_KIZ_MUb5_GlyGly_1 − Scan number: 8872
Gene Names | Charge | m/z | Mass | Retention time | PEP | Score | Precursor Intensity
HSPA1B;HSPA1A;HSPA1L;HSPA6 | 3 | 449.5663 | 1345.677 | 28.452 | 2.0304e−05 | 107.55 | 11714455
Intensity (%)
m/z
y1−NH3 158.0925
y1 175.1191
y2−NH3 215.114
y2 232.1405
y3−NH3 328.1977
y3 345.2246
a2 404.2531
b2 432.248
y4 458.3088
b3−H2O 515.2853
b3 533.2958
y5−NH3 555.3251
y5 572.352
y6−NH3 669.3688
y6 686.3954
b4−NH3 758.4072
b4 775.4337
y7−NH3 784.3949
y7 801.4224
b5−H2O 872.4489
b5 890.4623
A M T K D N N L L G R
y7 y6 y5 y4 y3 y2 y1
b2 a2 b3 b4 b5
Modification
Acetyl (Protein N−term)
GlyGly (K) no new terminus
Oxidation (M)

20200924_KIZ_MUb5_GlyGly_2 − Scan number: 8258

Gene Names	Charge	m/z	Mass	Retention time	PEP	Score	Precursor Intensity
HSPA1B;HSPA1A;HSPA1L;HSPA6	3	449.5663	1345.677	30.837	0.0021776	80.037	1898326

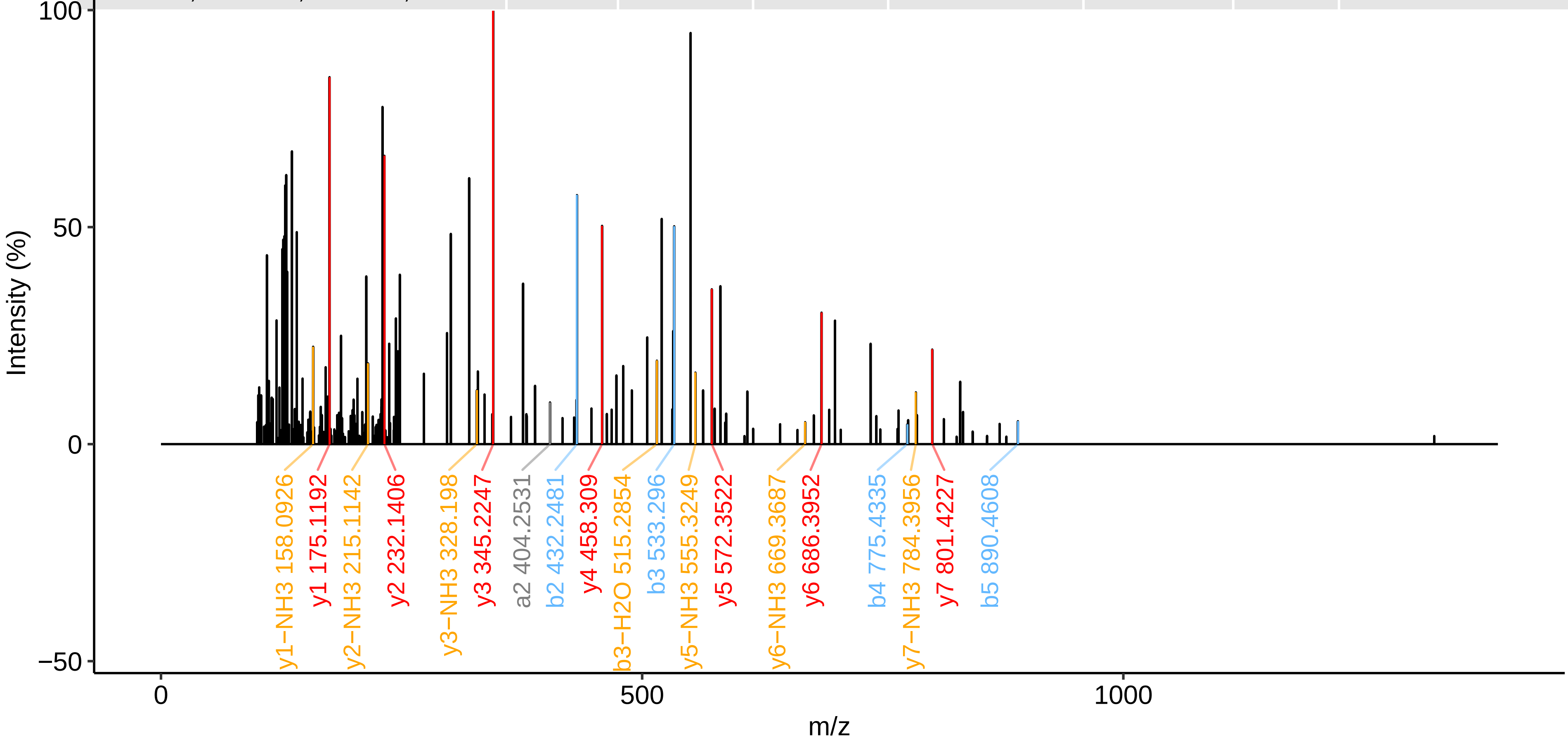

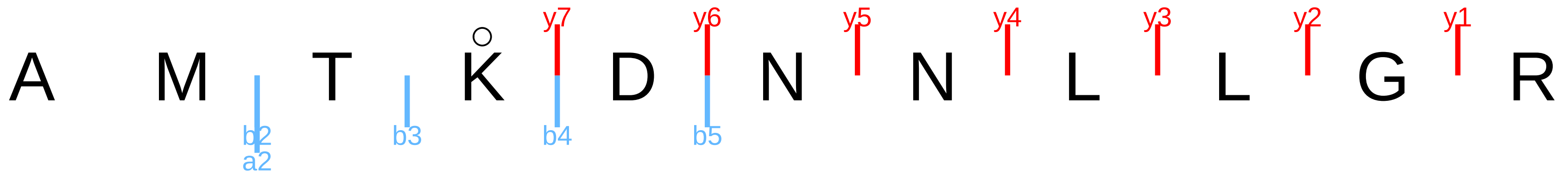

Modification □ Acetyl (Protein N−term) ○ GlyGly (K) no new terminus △ Oxidation (M)

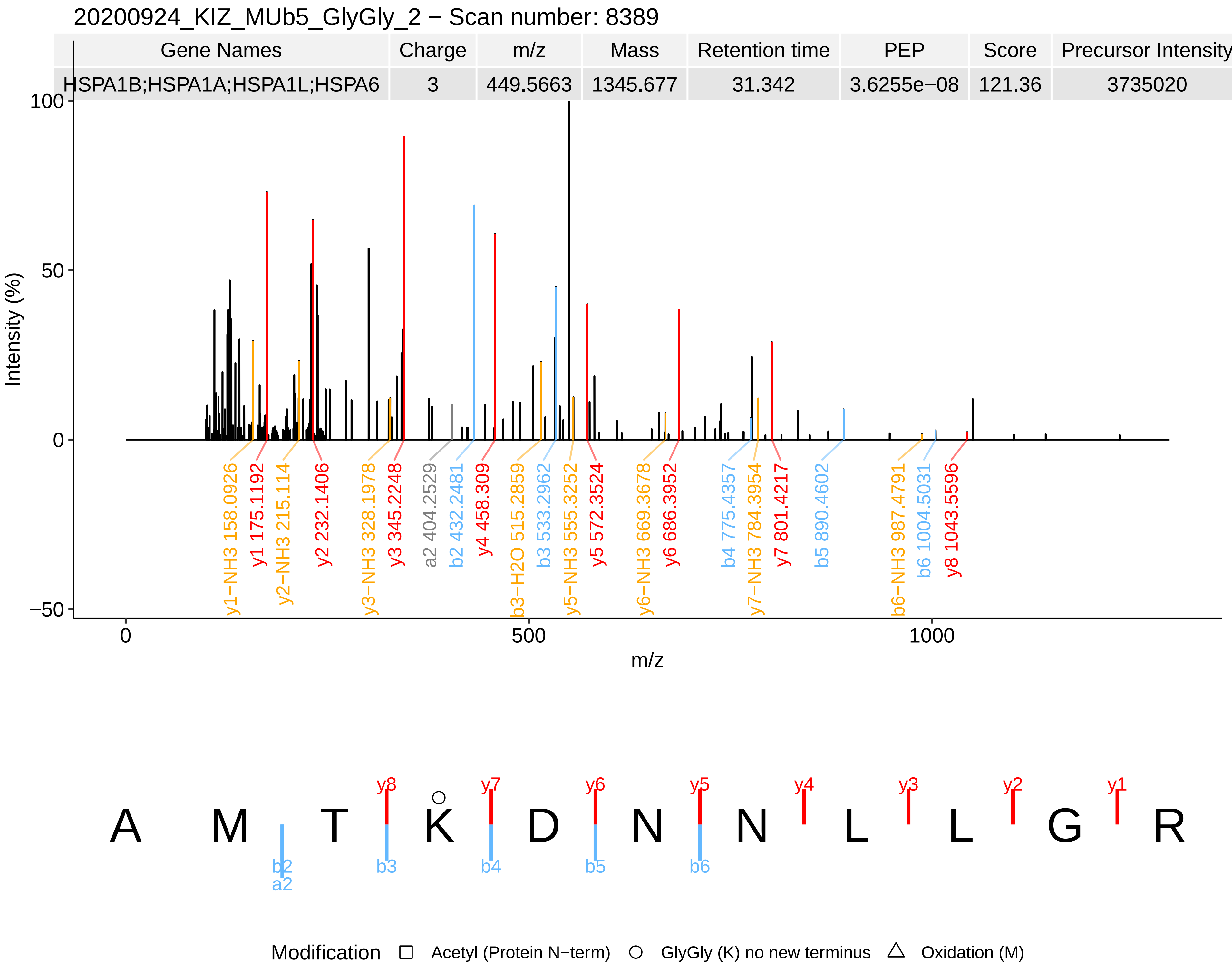
20200924_KIZ_MUb5_GlyGly_2 − Scan number: 8389
Gene Names | Charge | m/z | Mass | Retention time | PEP | Score | Precursor Intensity
HSPA1B;HSPA1A;HSPA1L;HSPA6 | 3 | 449.5663 | 1345.677 | 31.342 | 3.6255e−08 | 121.36 | 3735020
Intensity (%)
100
50
0
−50
0
500
1000
m/z
y1−NH3 158.0926
y1 175.1192
y2−NH3 215.114
y2 232.1406
y3−NH3 328.1978
y3 345.2248
a2 404.2529
b2 432.2481
y4 458.309
b3−H2O 515.2859
b3 533.2962
y5−NH3 555.3252
y5 572.3524
y6−NH3 669.3678
y6 686.3952
b4 775.4357
y7−NH3 784.3954
y7 801.4217
b5 890.4602
b6−NH3 987.4791
b6 1004.5031
y8 1043.5596
A M T K D N N L L G R
y8 y7 y6 y5 y4 y3 y2 y1
b2 a2 b3 b4 b5 b6
Modification
Acetyl (Protein N−term)
GlyGly (K) no new terminus
Oxidation (M)

20201118_KIZ_MUb6_GlyGly_2 − Scan number: 10279

Gene Names	Charge	m/z	Mass	Retention time	PEP	Score	Precursor Intensity
HSPA1B;HSPA1A;HSPA1L;HSPA6	3	449.5663	1345.677	34.686	1.9591e−05	107.83	14963774

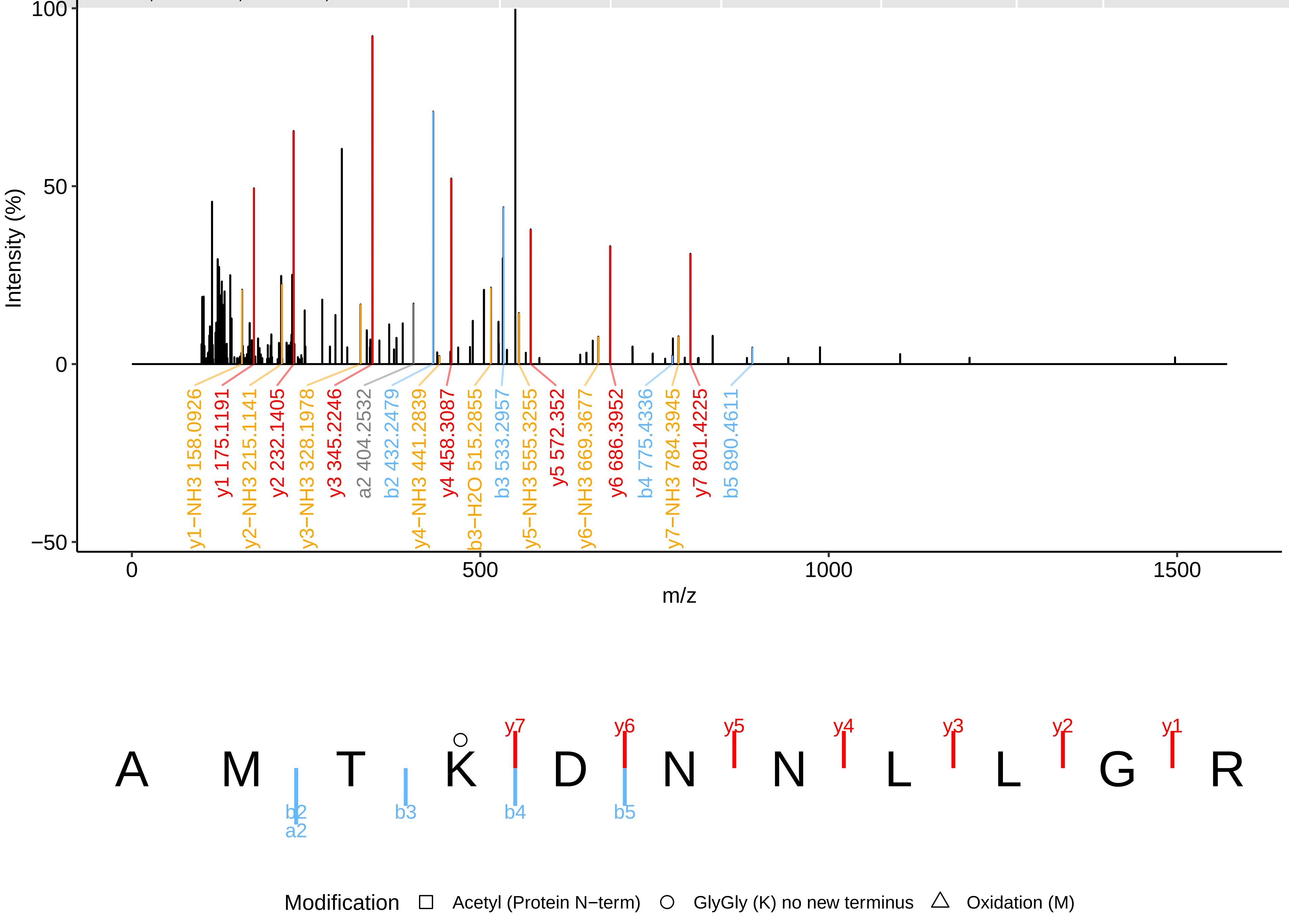

20200921_KIZ_MUb4_GlyGly_10plex_2 − Scan number: 3023

Gene Names	Charge	m/z	Mass	Retention time	PEP	Score	Precursor Intensity
PRKDC	4	285.1585	1136.605	9.912	0.077322	48.283	2978364

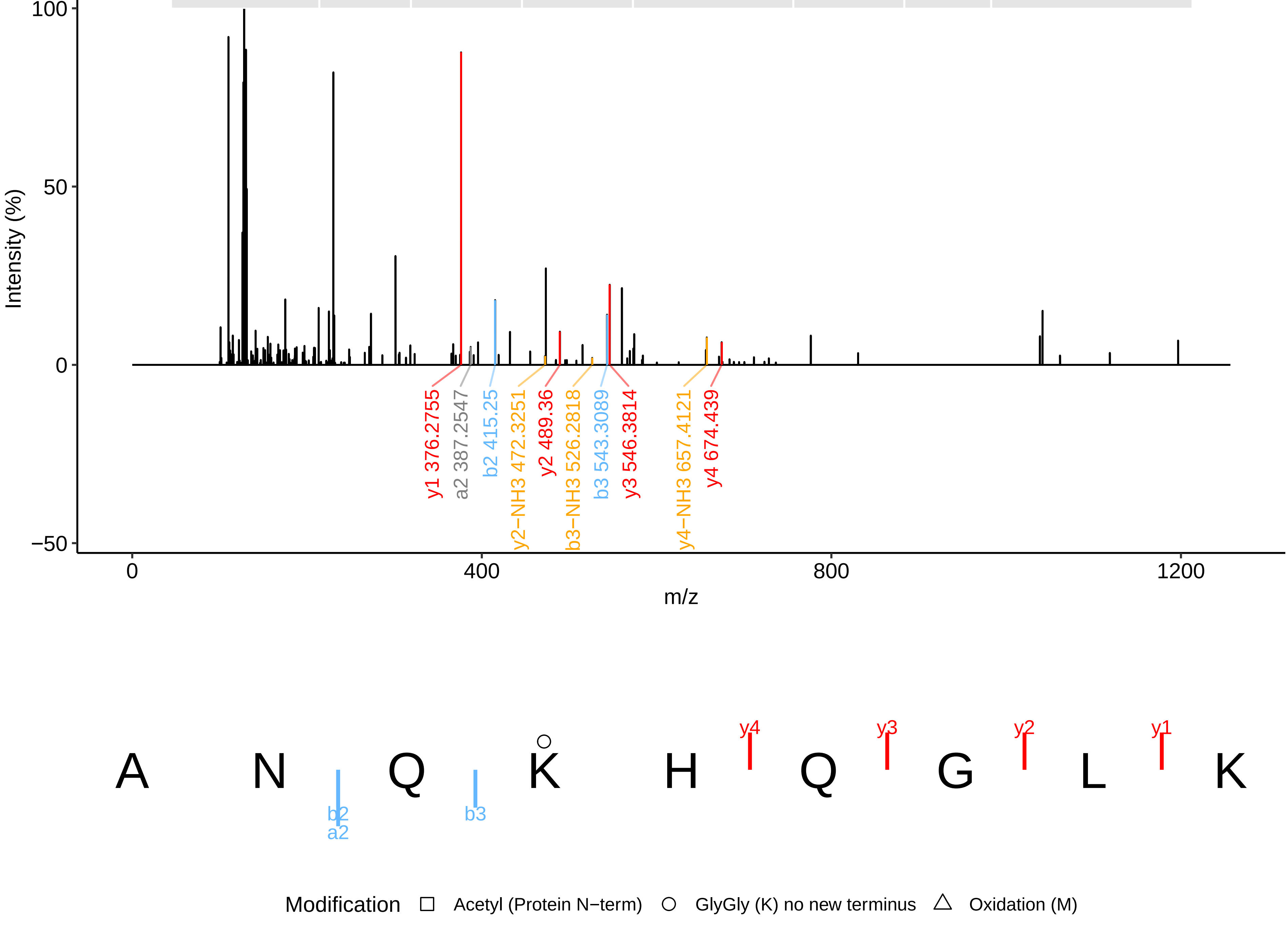

20200921_KIZ_MUb4_GlyGly_10plex − Scan number: 8088

Gene Names	Charge	m/z	Mass	Retention time	PEP	Score	Precursor Intensity
ABCF3	3	368.212	1101.614	27.364	0.00059215	120.99	2700985

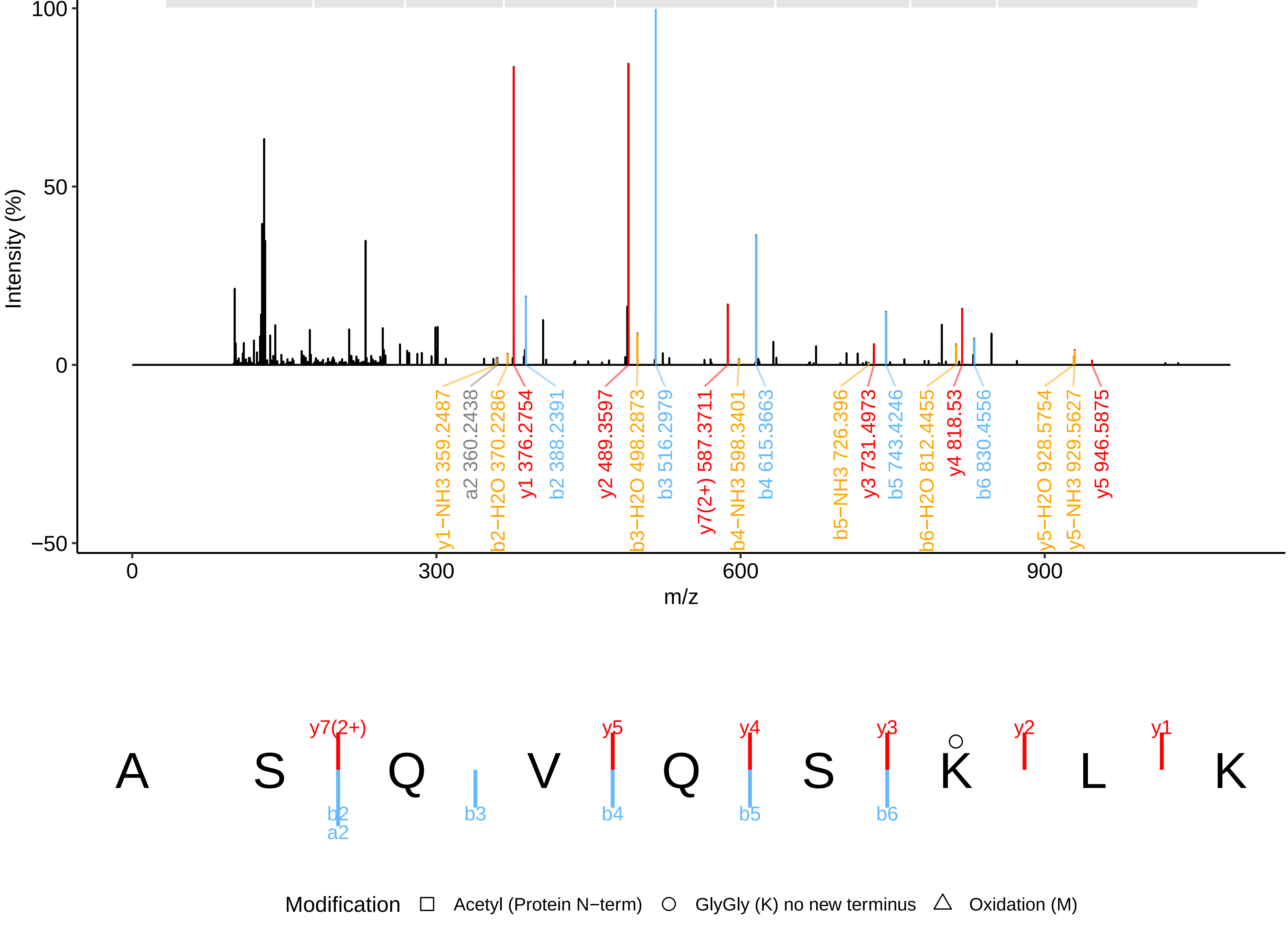

20200921_KIZ_MUb4_GlyGly_10plex_2 − Scan number: 7462

Gene Names	Charge	m/z	Mass	Retention time	PEP	Score	Precursor Intensity
ABCF3	3	368.212	1101.614	27.853	0.0031624	82.749	731586

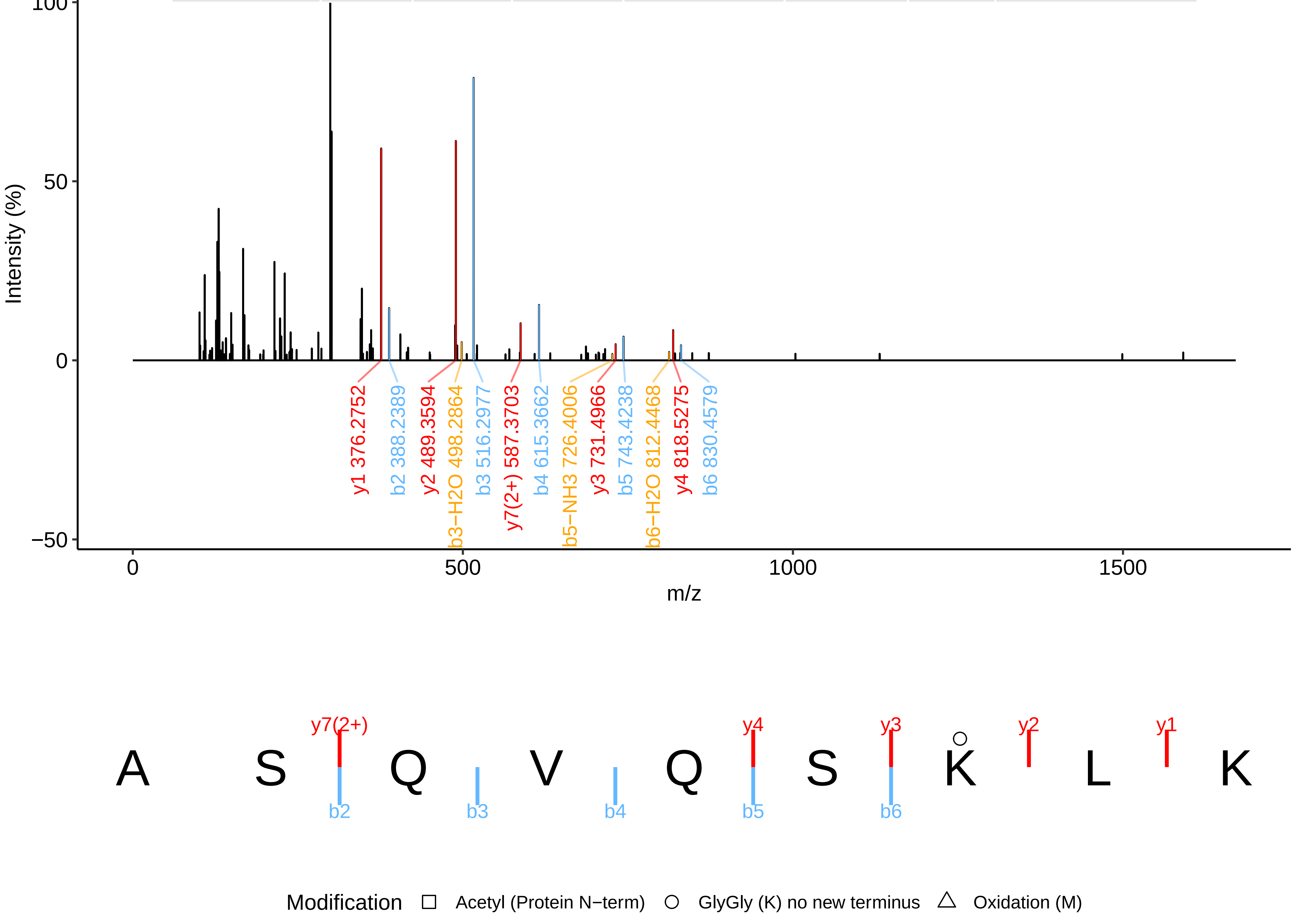

20200924_KIZ_MUb5_GlyGly_1 − Scan number: 7072

Gene Names	Charge	m/z	Mass	Retention time	PEP	Score	Precursor Intensity
ABCF3	3	368.212	1101.614	22.39	0.045685	60.398	NA

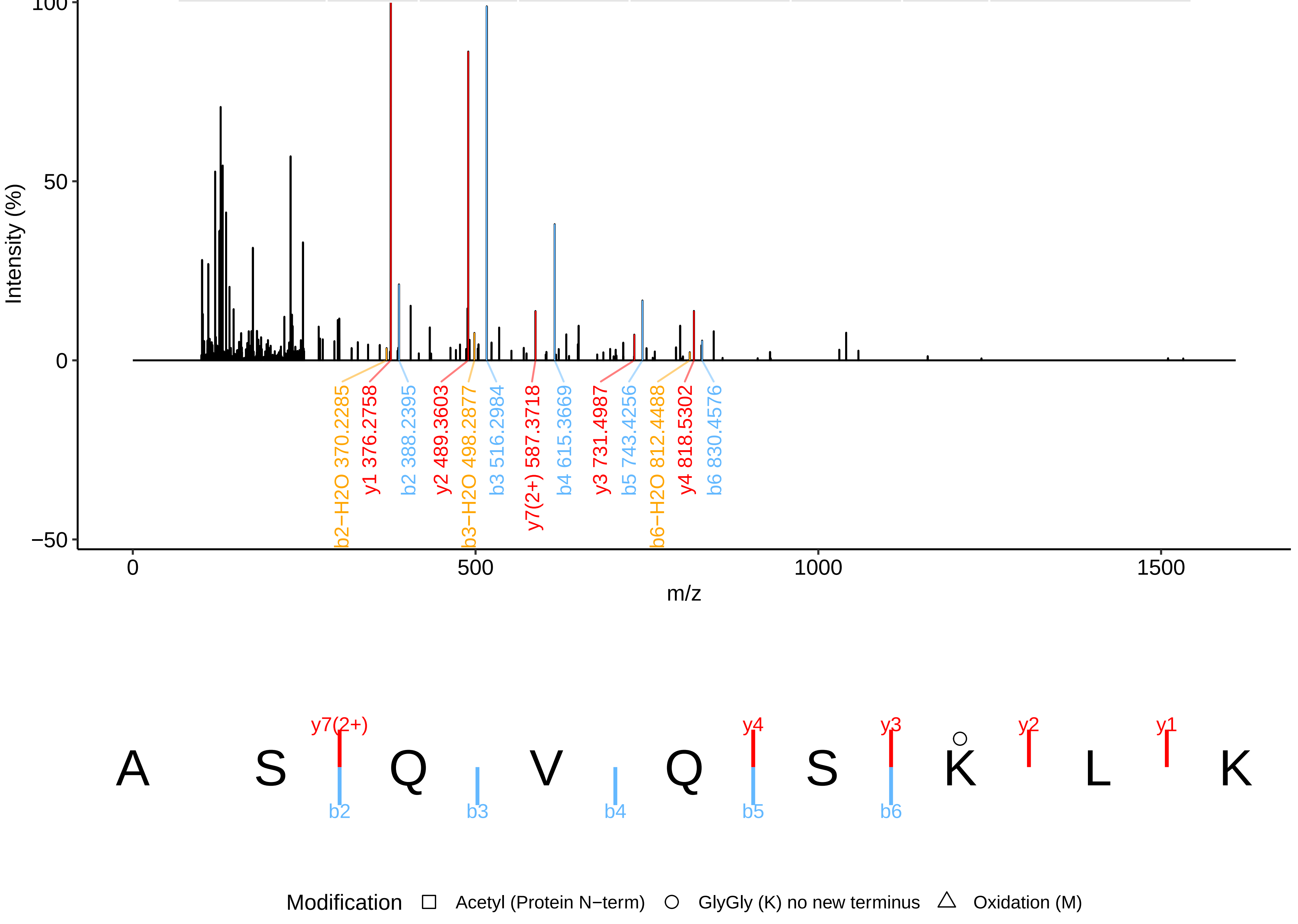

20200921_KIZ_MUb4_GlyGly_10plex_2 − Scan number: 3235

Gene Names	Charge	m/z	Mass	Retention time	PEP	Score	Precursor Intensity
RPS3A	3	449.8881	1346.643	10.762	0.0010097	83.617	984582.9

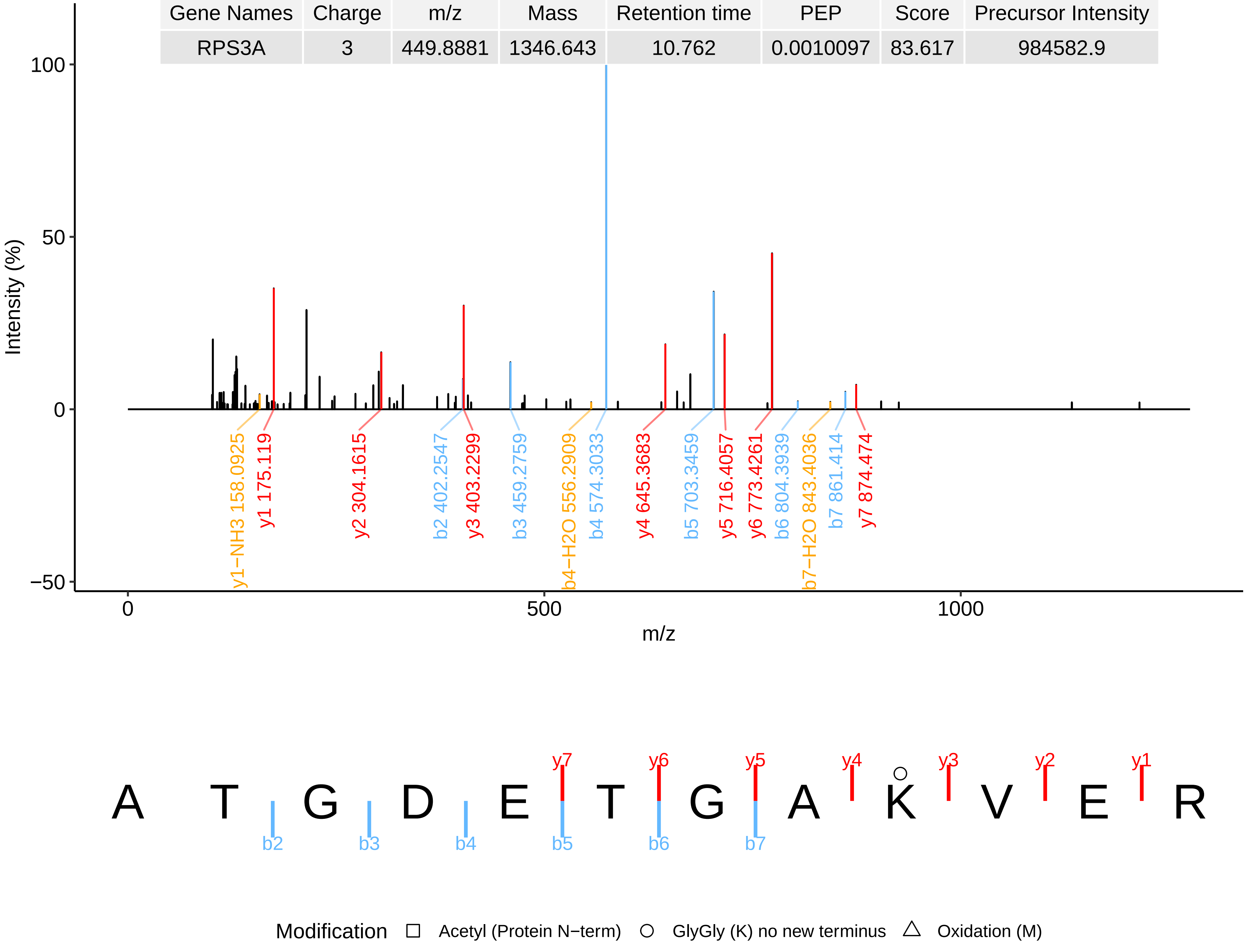

20200924_KIZ_MUb5_GlyGly_1 − Scan number: 3134

Gene Names	Charge	m/z	Mass	Retention time	PEP	Score	Precursor Intensity
RPS3A	3	449.8881	1346.643	9.6736	0.0034928	77.185	8257569

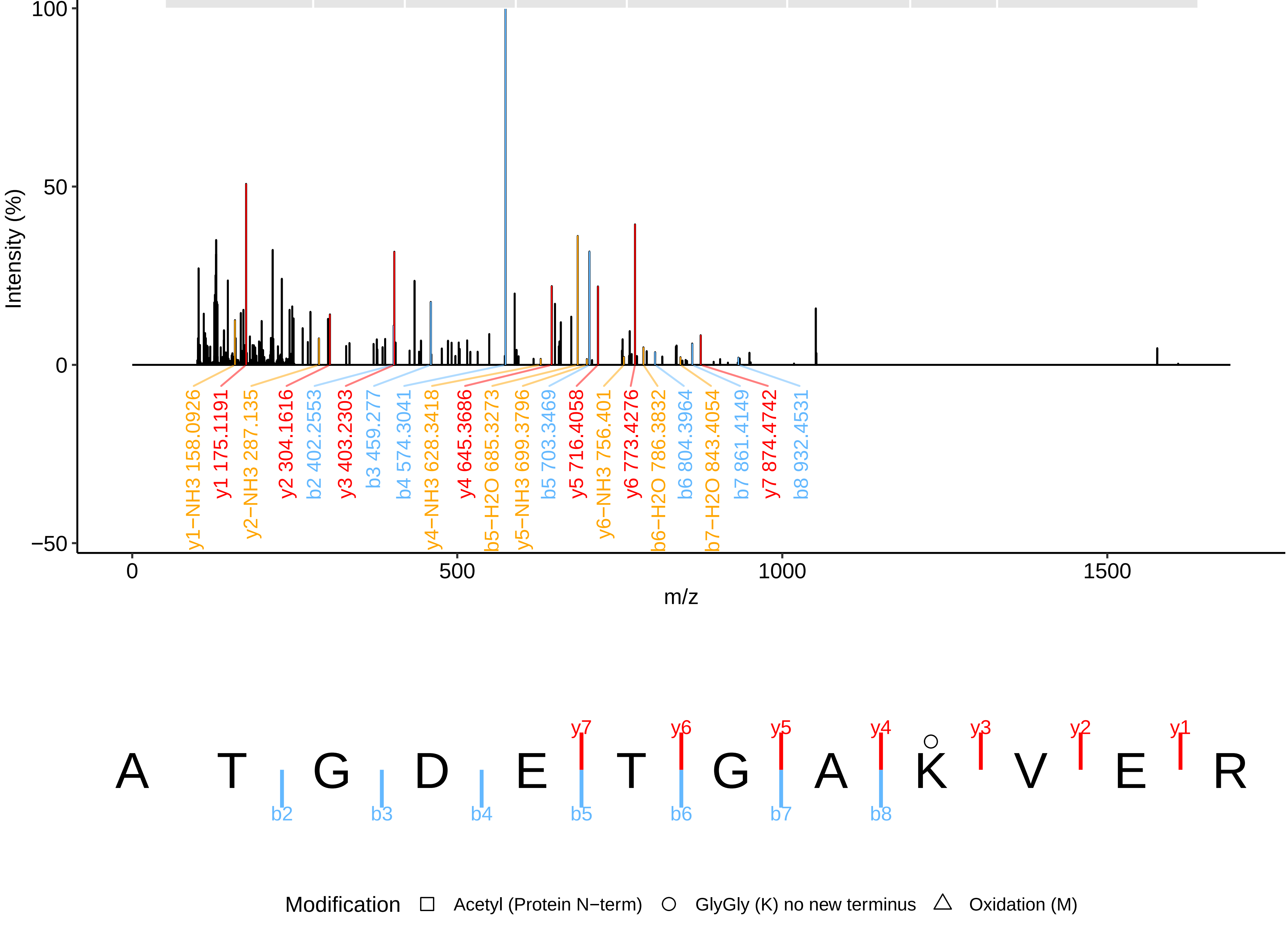

20200921_KIZ_MUb4_GlyGly_10plex − Scan number: 8768

Gene Names	Charge	m/z	Mass	Retention time	PEP	Score	Precursor Intensity
RNF114	3	438.5772	1312.71	29.858	0.00072917	90.913	4321020

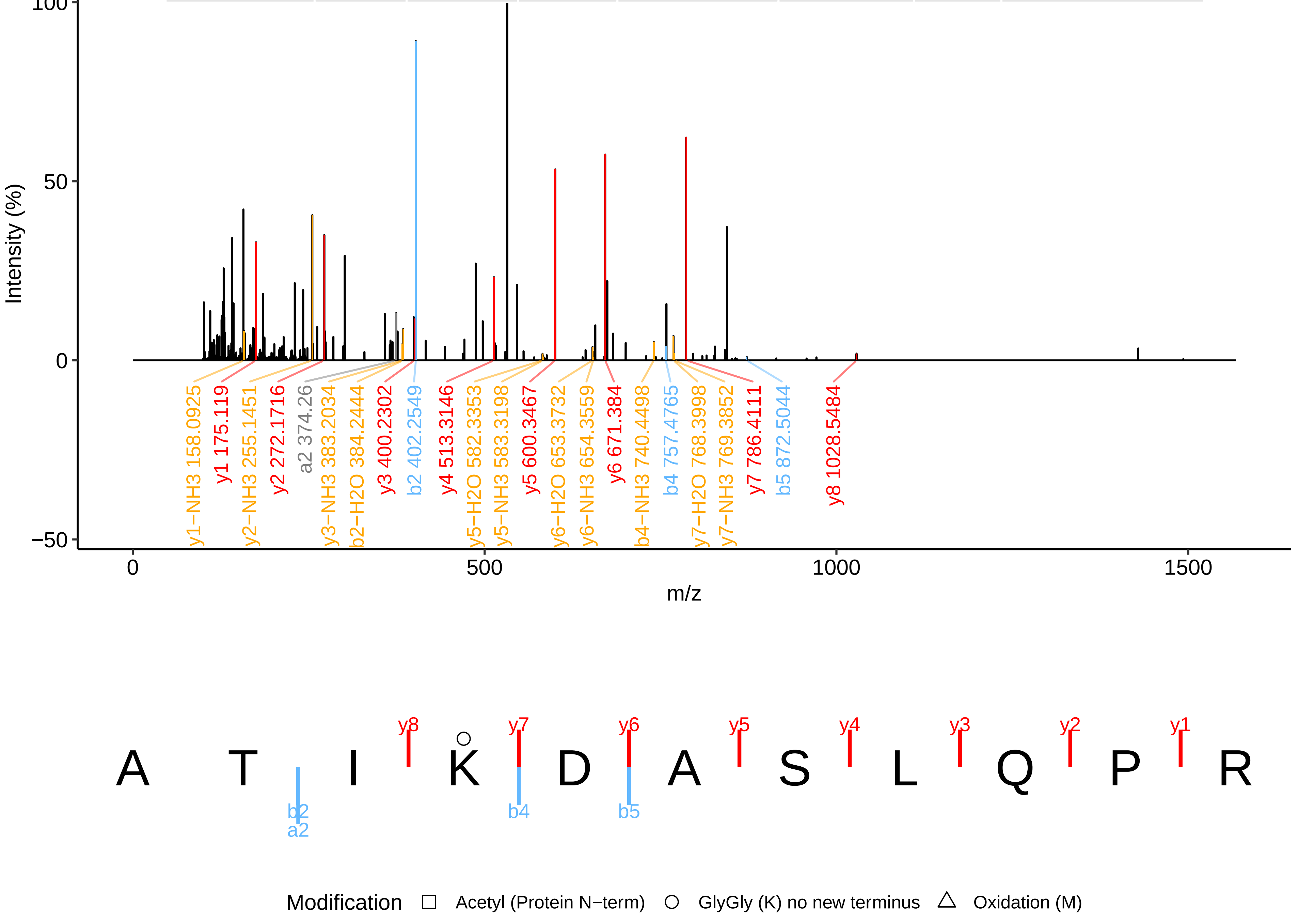

20200921_KIZ_MUb4_GlyGly_10plex_2 − Scan number: 8067

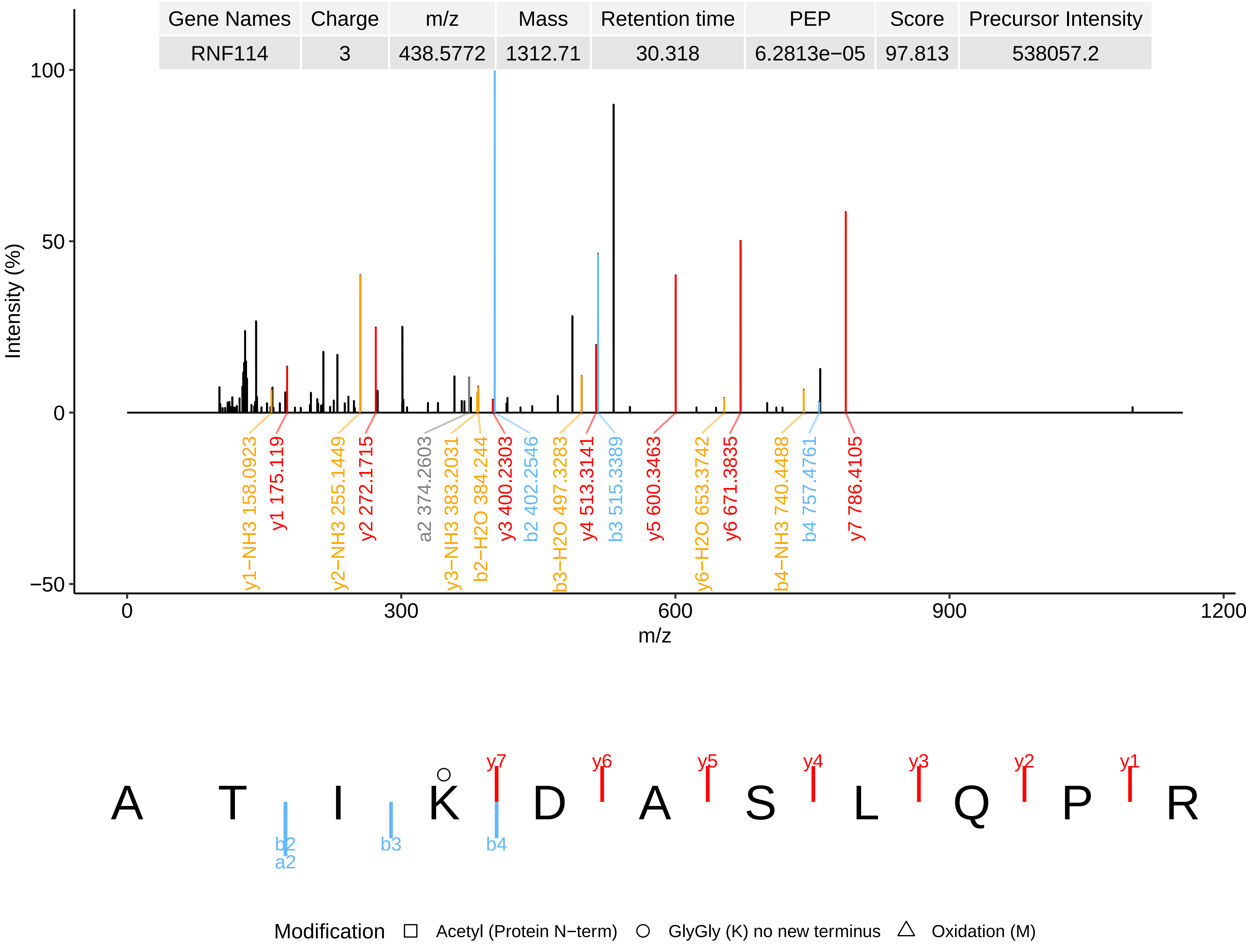

Gene Names	Charge	m/z	Mass	Retention time	PEP	Score	Precursor Intensity
RNF114	3	438.5772	1312.71	30.318	6.2813e−05	97.813	538057.2

20200924_KIZ_MUb5_GlyGly_1 − Scan number: 7812

Gene Names	Charge	m/z	Mass	Retention time	PEP	Score	Precursor Intensity
RNF114	3	438.5772	1312.71	24.885	3.2049e−08	122.69	23299184

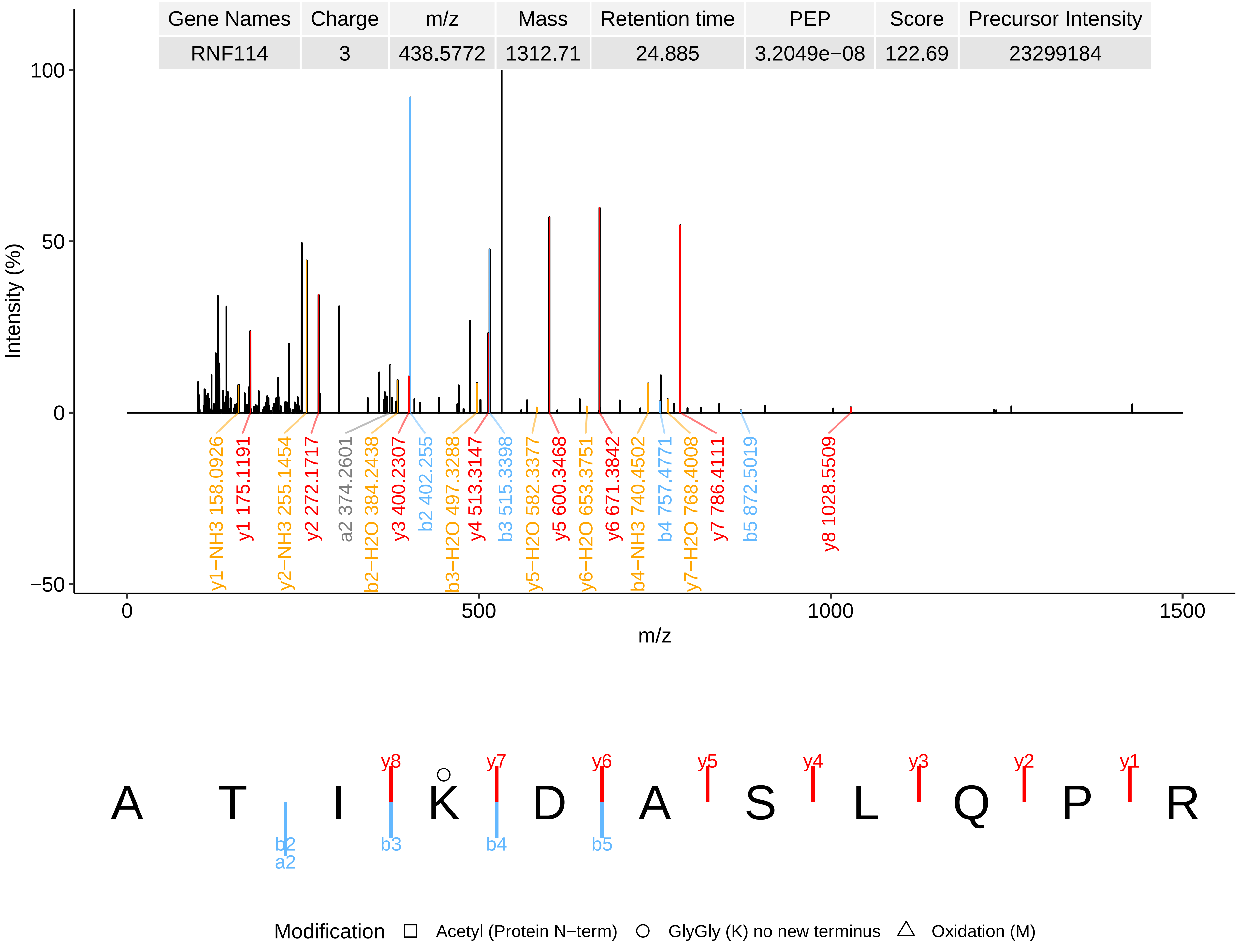

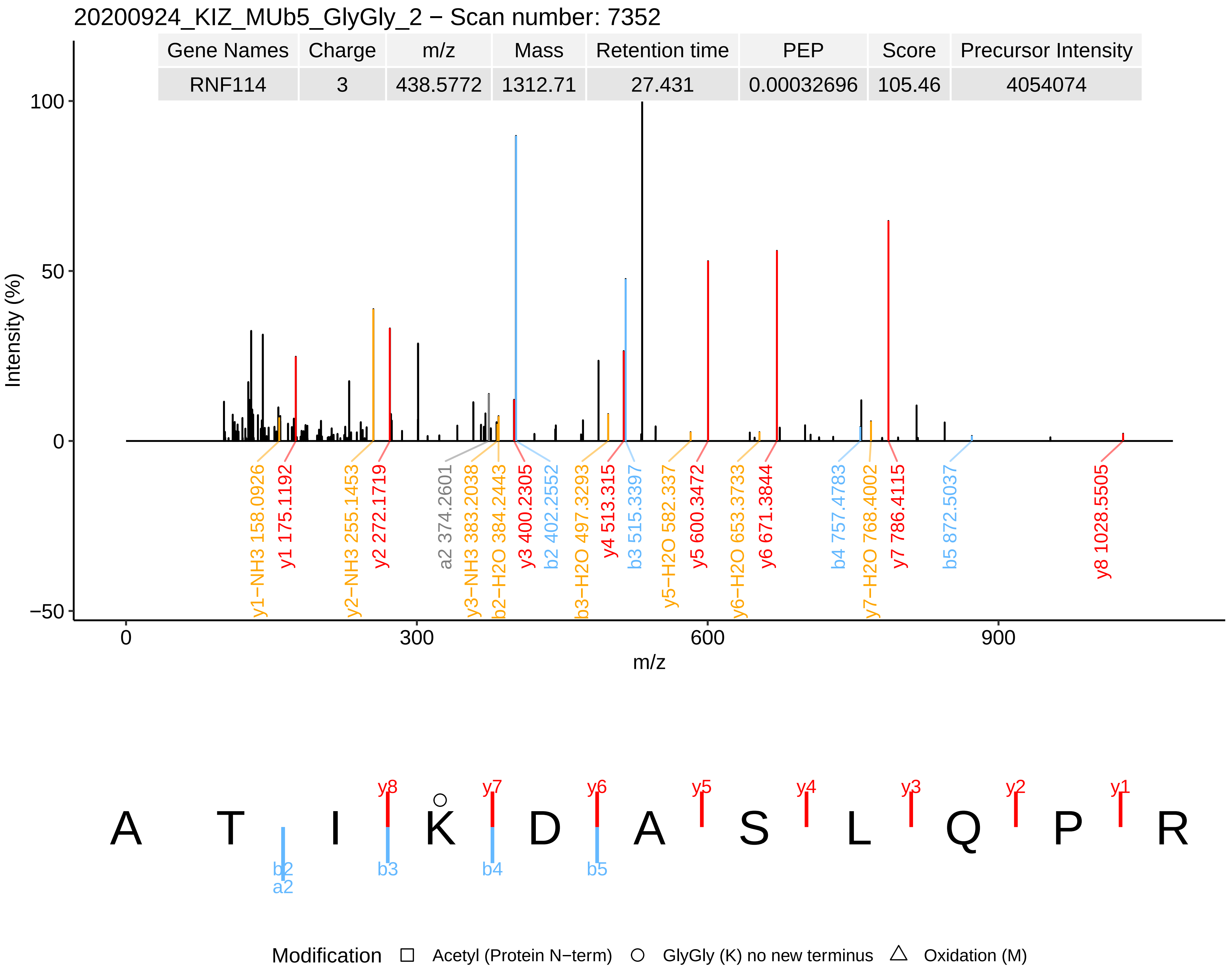

20200924_KIZ_MUb5_GlyGly_2 − Scan number: 7352
Gene Names | Charge | m/z | Mass | Retention time | PEP | Score | Precursor Intensity
RNF114 | 3 | 438.5772 | 1312.71 | 27.431 | 0.00032696 | 105.46 | 4054074
Intensity (%)
100
50
0
−50
m/z
0
300
600
900
y1−NH3 158.0926
y1 175.1192
y2−NH3 255.1453
y2 272.1719
a2 374.2601
y3−NH3 383.2038
b2−H2O 384.2443
y3 400.2305
b2 402.2552
b3−H2O 497.3293
y4 513.315
b3 515.3397
y5−H2O 582.337
y5 600.3472
y6−H2O 653.3733
y6 671.3844
b4 757.4783
y7−H2O 768.4002
y7 786.4115
b5 872.5037
y8 1028.5505
A T I K D A S L Q P R
y8 y7 y6 y5 y4 y3 y2 y1
b2 a2 b3 b4 b5
Modification
Acetyl (Protein N−term)
GlyGly (K) no new terminus
Oxidation (M)

20200921_KIZ_MUb4_GlyGly_10plex − Scan number: 13664

Gene Names	Charge	m/z	Mass	Retention time	PEP	Score	Precursor Intensity
RUVBL2	2	771.9279	1541.841	48.288	3.3561e−13	85.958	NA

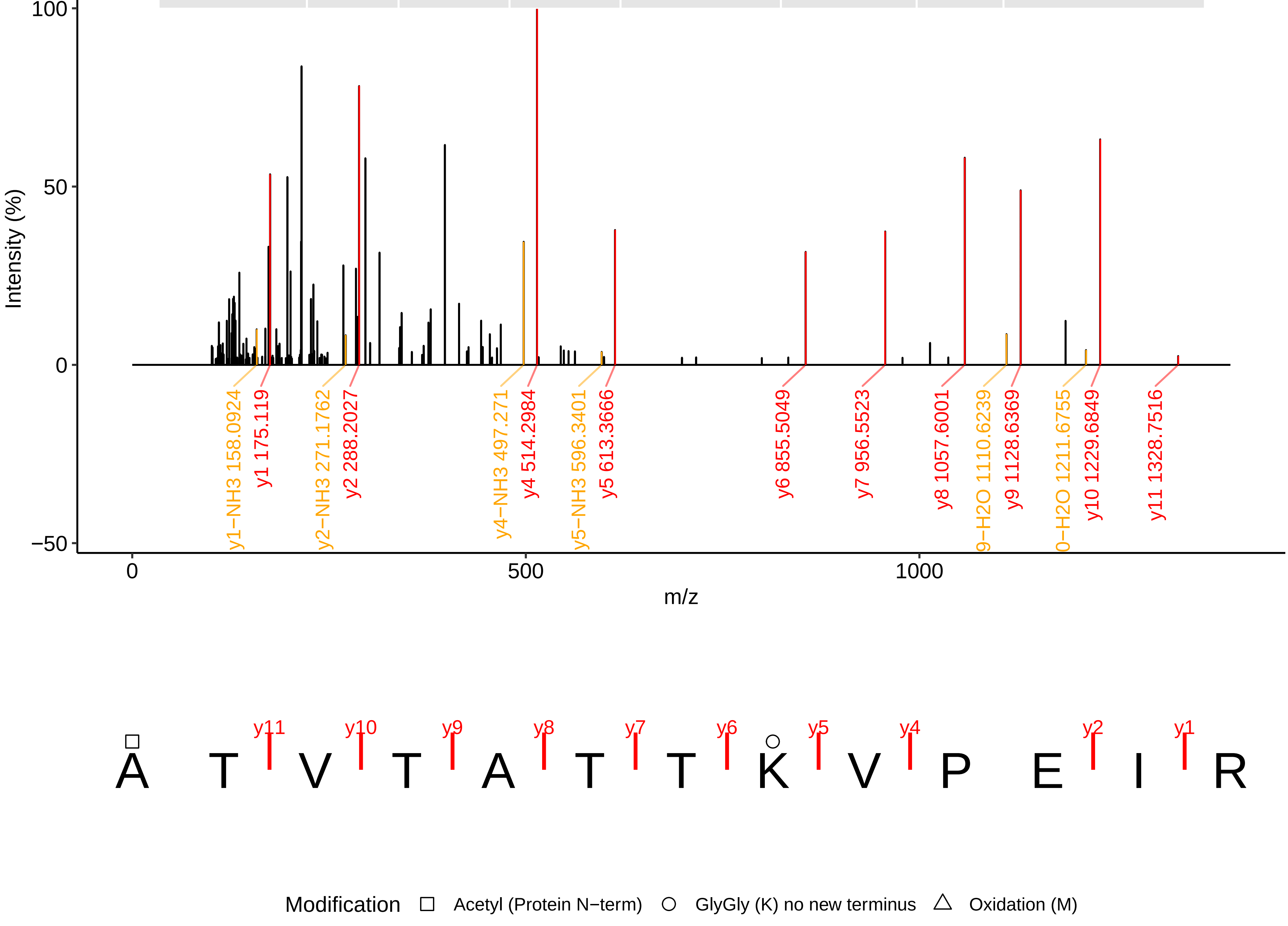

20200921_KIZ_MUb4_GlyGly_10plex − Scan number: 7099

Gene Names	Charge	m/z	Mass
HIST1H2BL;HIST1H2BM;HIST2H2BF;HIST2H2BE;HIST1H2BO;HIST1H2BB;HIST3H2BB;HIST1H2BA	3	366.8645	1097.572

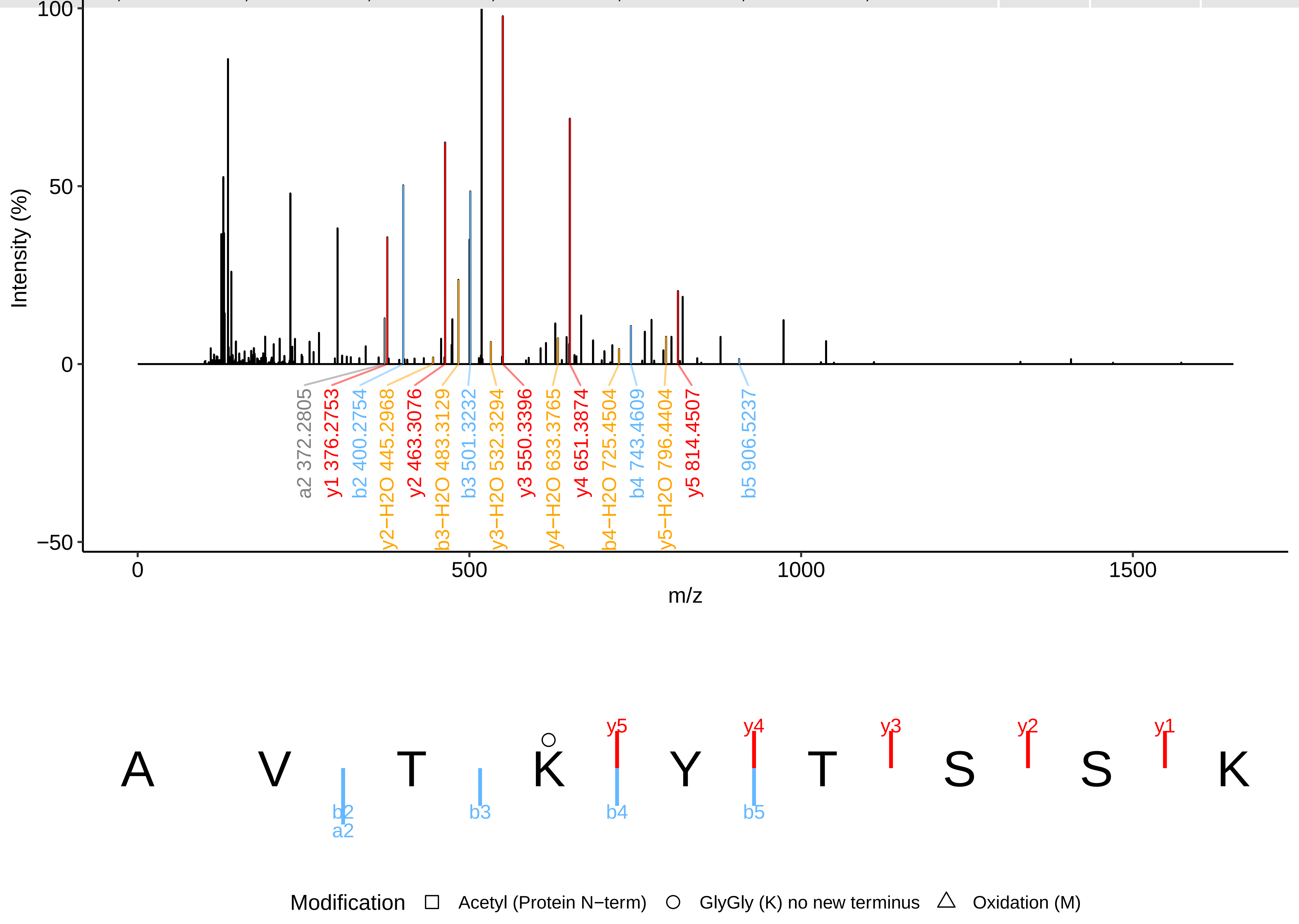

20200921_KIZ_MUb4_GlyGly_10plex − Scan number: 7148

Gene Names	Charge	m/z	Mass
HIST1H2BL;HIST1H2BM;HIST2H2BF;HIST2H2BE;HIST1H2BO;HIST1H2BB;HIST3H2BB;HIST1H2BA	3	366.8645	1097.572

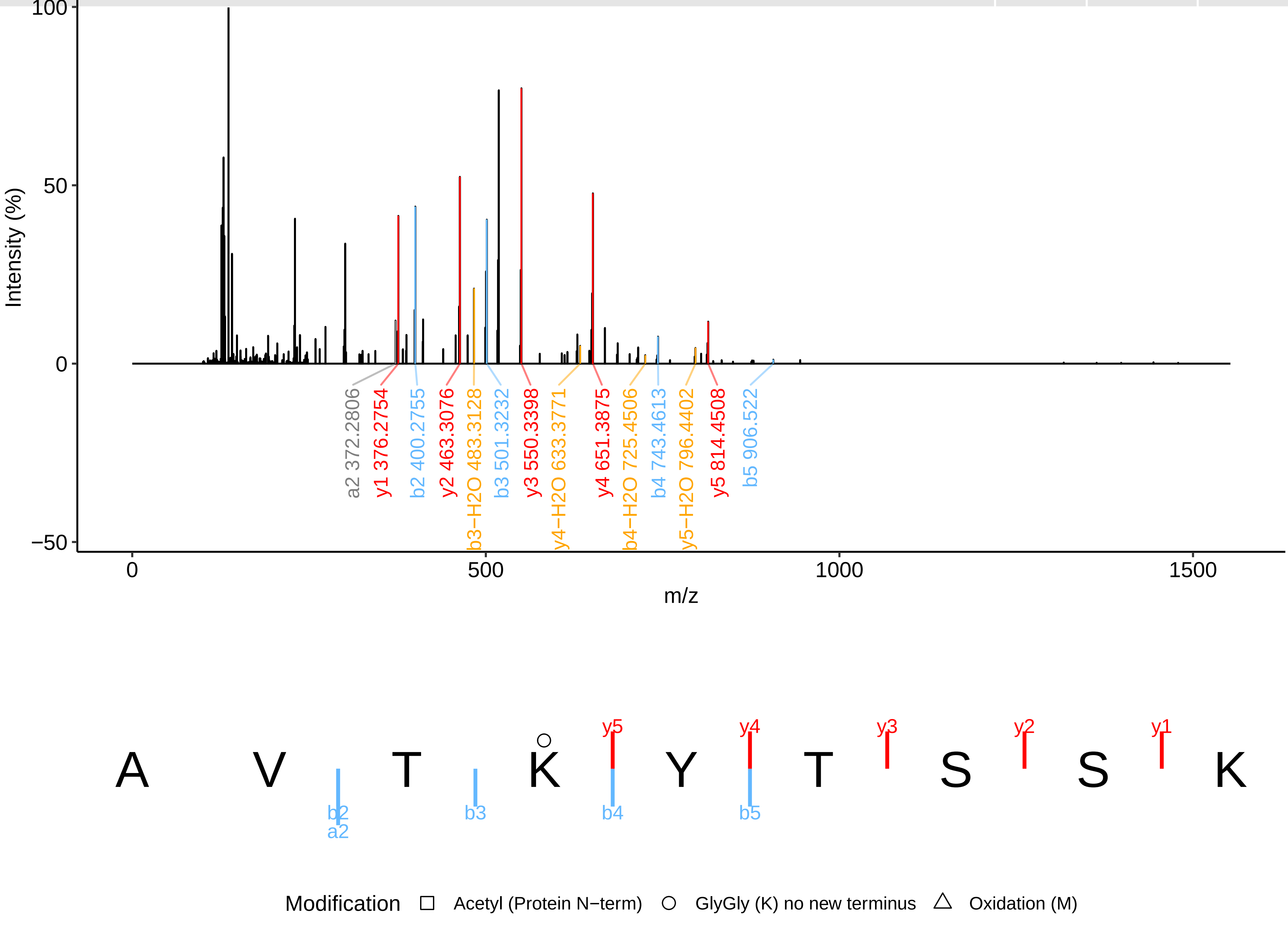

20200921_KIZ_MUb4_GlyGly_10plex − Scan number: 7124

Gene Names	Charge	m/z	Mass
HIST1H2BL;HIST1H2BM;HIST2H2BF;HIST2H2BE;HIST1H2BO;HIST1H2BB;HIST3H2BB;HIST1H2BA	2	549.7931	1097.572

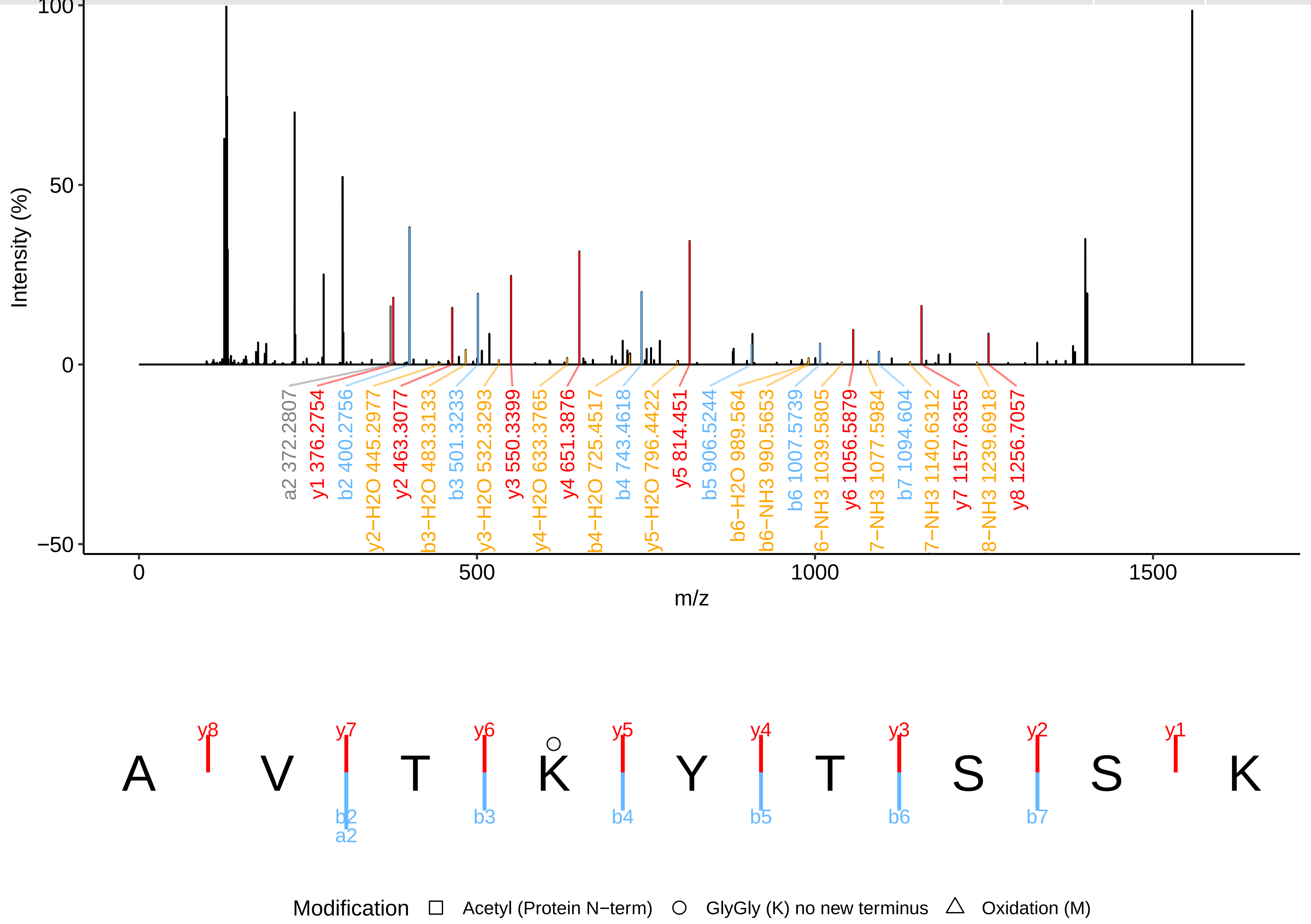

20200921_KIZ_MUb4_GlyGly_10plex_2 − Scan number: 6587

Gene Names	Charge	m/z	Mass
HIST1H2BL;HIST1H2BM;HIST2H2BF;HIST2H2BE;HIST1H2BO;HIST1H2BB;HIST3H2BB;HIST1H2BA	3	366.8645	1097.572

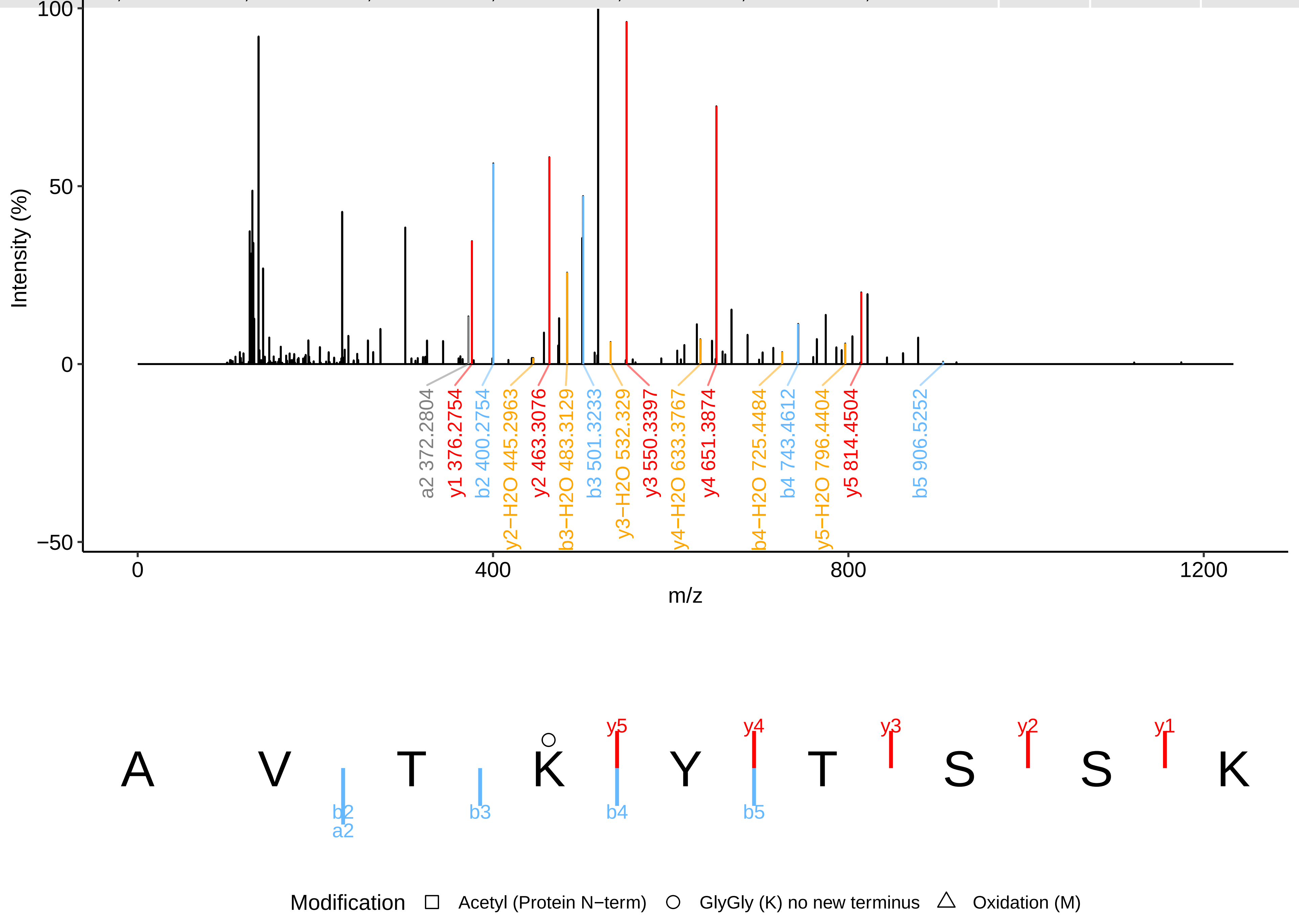

20200921_KIZ_MUb4_GlyGly_10plex_2 − Scan number: 6606

Gene Names	Charge	m/z	Mass
HIST1H2BL;HIST1H2BM;HIST2H2BF;HIST2H2BE;HIST1H2BO;HIST1H2BB;HIST3H2BB;HIST1H2BA	2	549.7931	1097.572

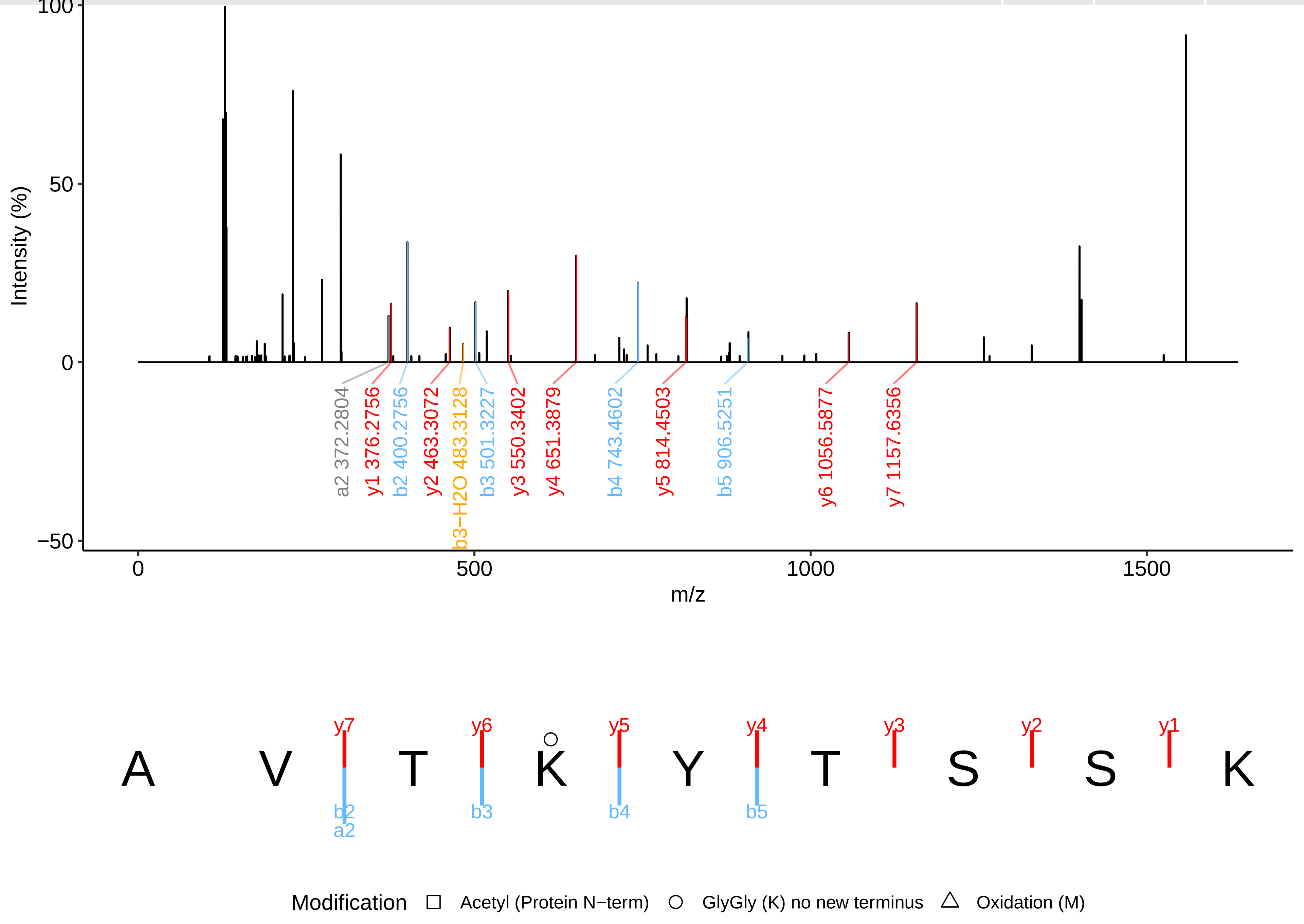

20200924_KIZ_MUb5_GlyGly_1 − Scan number: 5967

Gene Names	Charge	m/z	Mass
;HIST1H2BL;HIST1H2BM;HIST2H2BF;HIST2H2BE;HIST1H2BO;HIST1H2BB;HIST3H2BB;HIST1H2BA	3	366.8645	1097.57

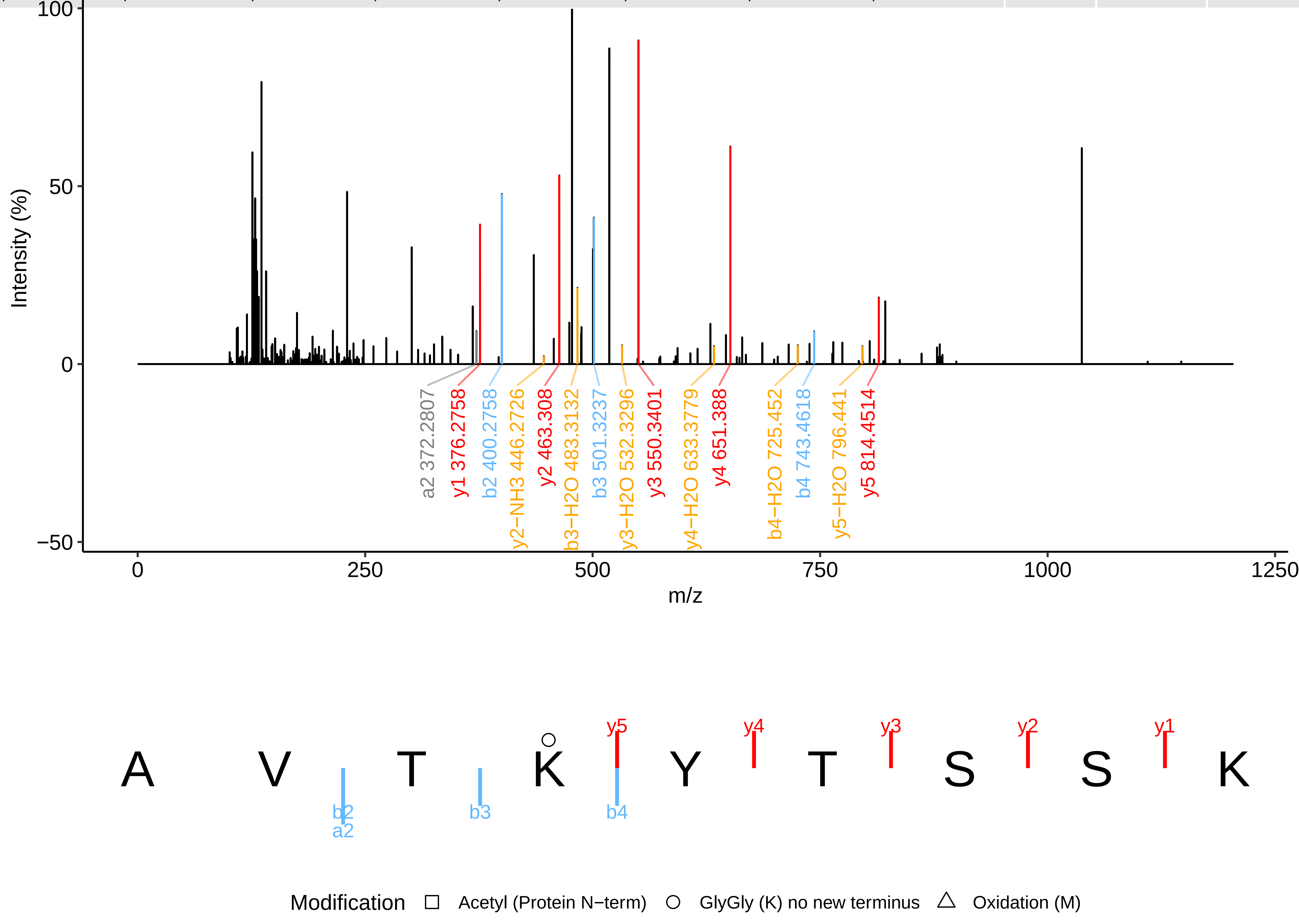

20200924_KIZ_MUb5_GlyGly_1 − Scan number: 6117

Gene Names	Charge	m/z	Mass
;HIST1H2BL;HIST1H2BM;HIST2H2BF;HIST2H2BE;HIST1H2BO;HIST1H2BB;HIST3H2BB;HIST1H2BA	3	366.8645	1097.572

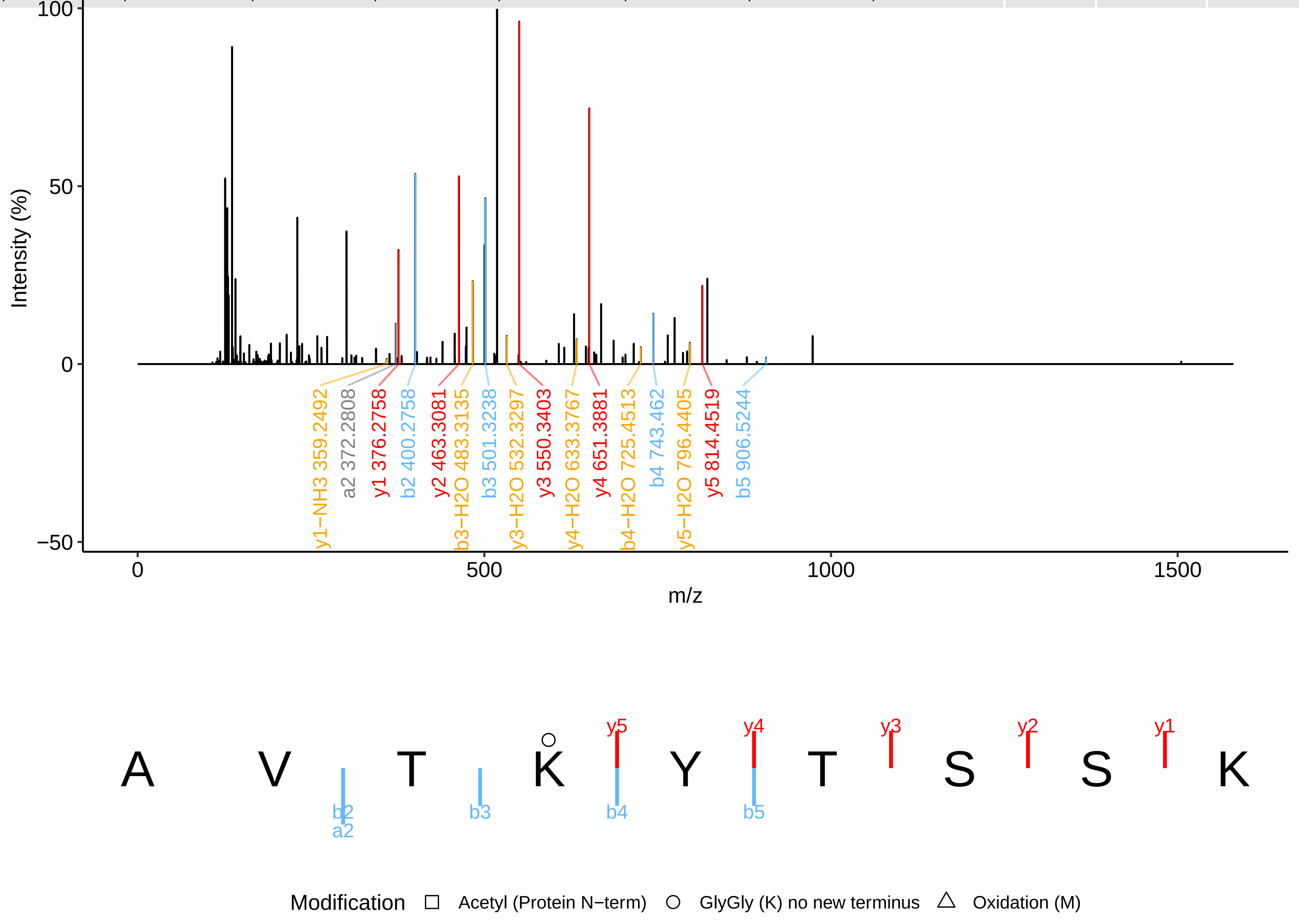

20200924_KIZ_MUb5_GlyGly_1 − Scan number: 6346

Gene Names	Charge	m/z	Mass
HIST1H2BL;HIST1H2BM;HIST2H2BF;HIST2H2BE;HIST1H2BO;HIST1H2BB;HIST3H2BB;HIST1H2BA	3	366.8645	1097.572

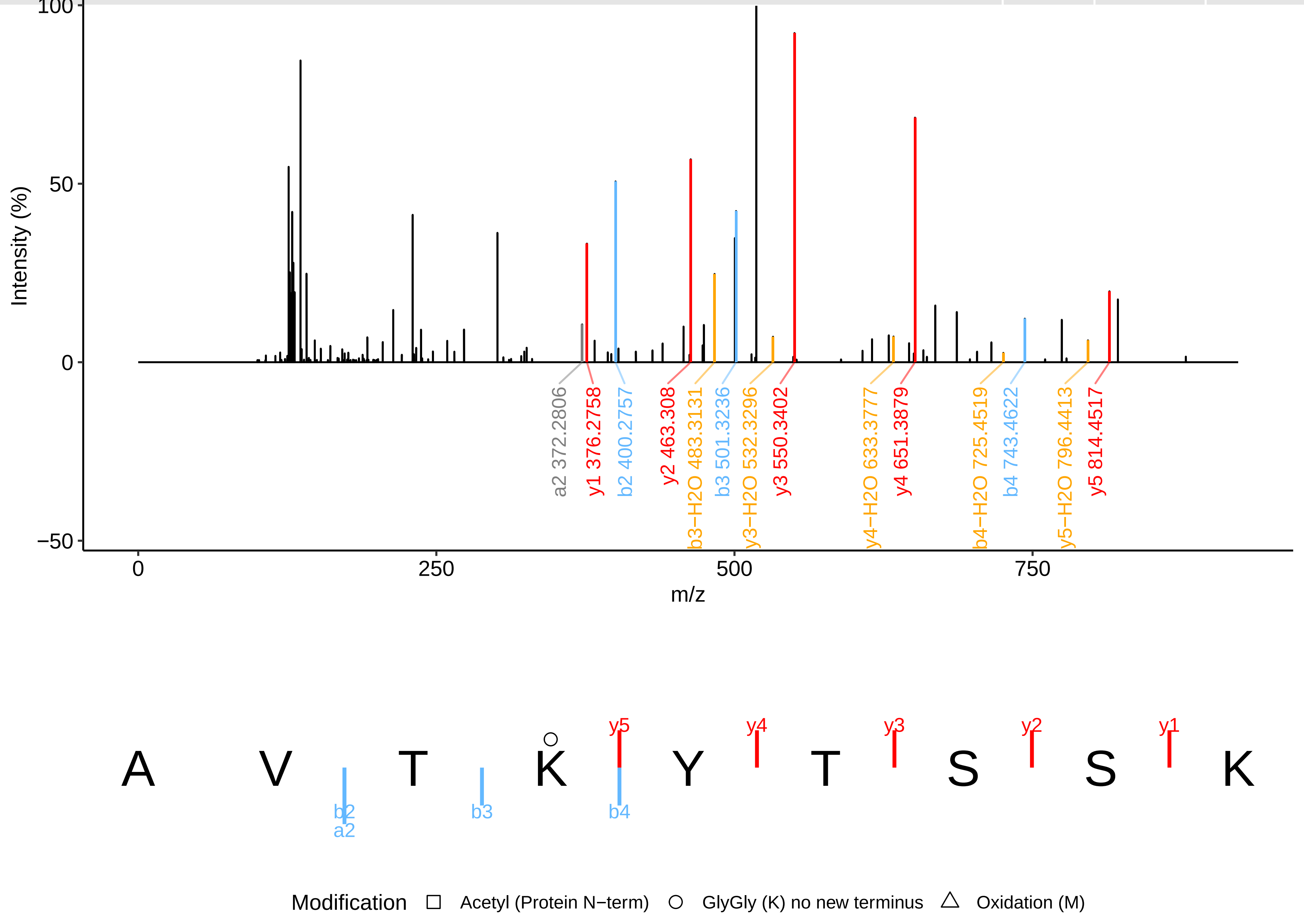

20200924_KIZ_MUb5_GlyGly_1 − Scan number: 6501

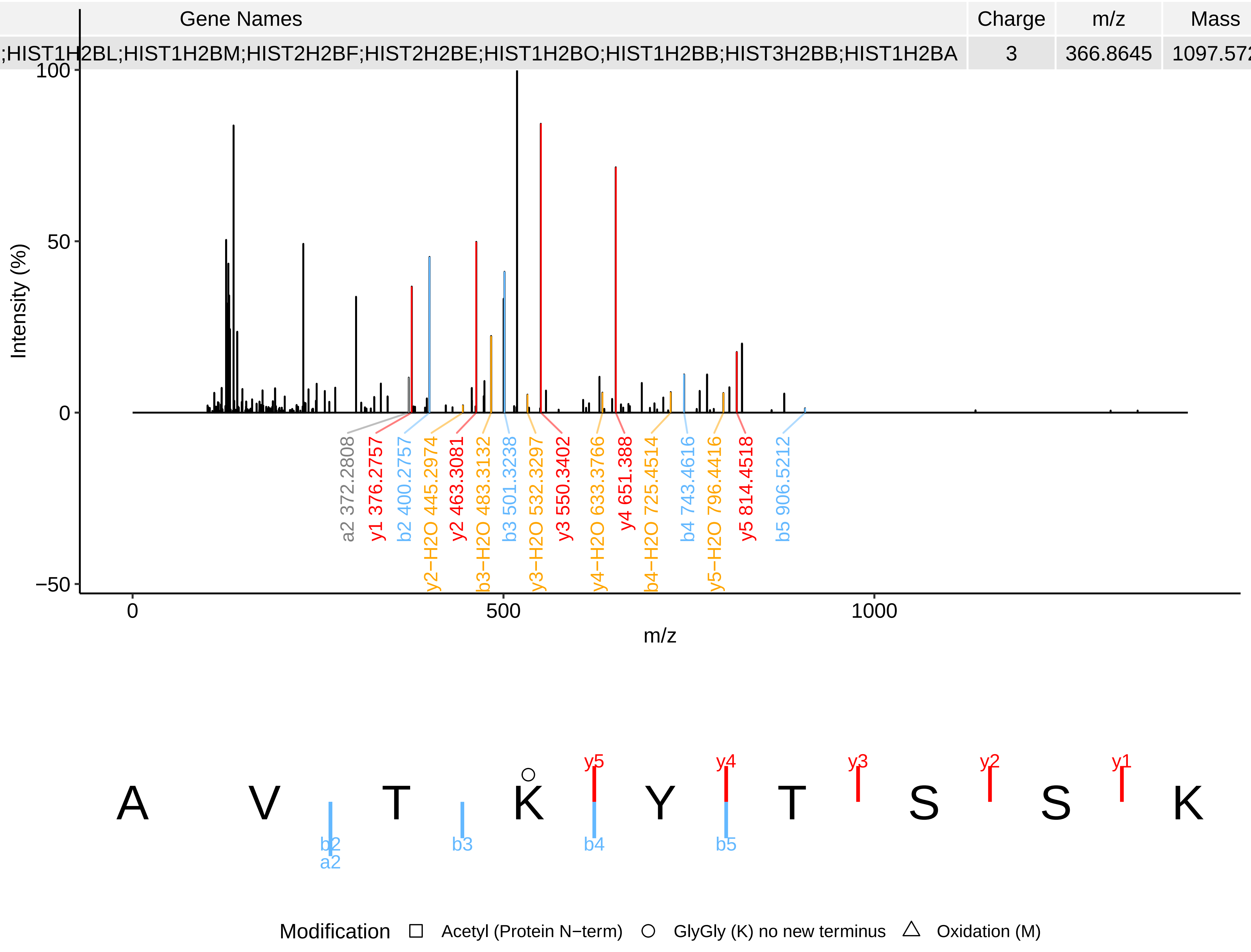

20200924_KIZ_MUb5_GlyGly_1 − Scan number: 6185

Gene Names	Charge	m/z	Mass
HIST1H2BL;HIST1H2BM;HIST2H2BF;HIST2H2BE;HIST1H2BO;HIST1H2BB;HIST3H2BB;HIST1H2BA	2	549.7931	1097.572

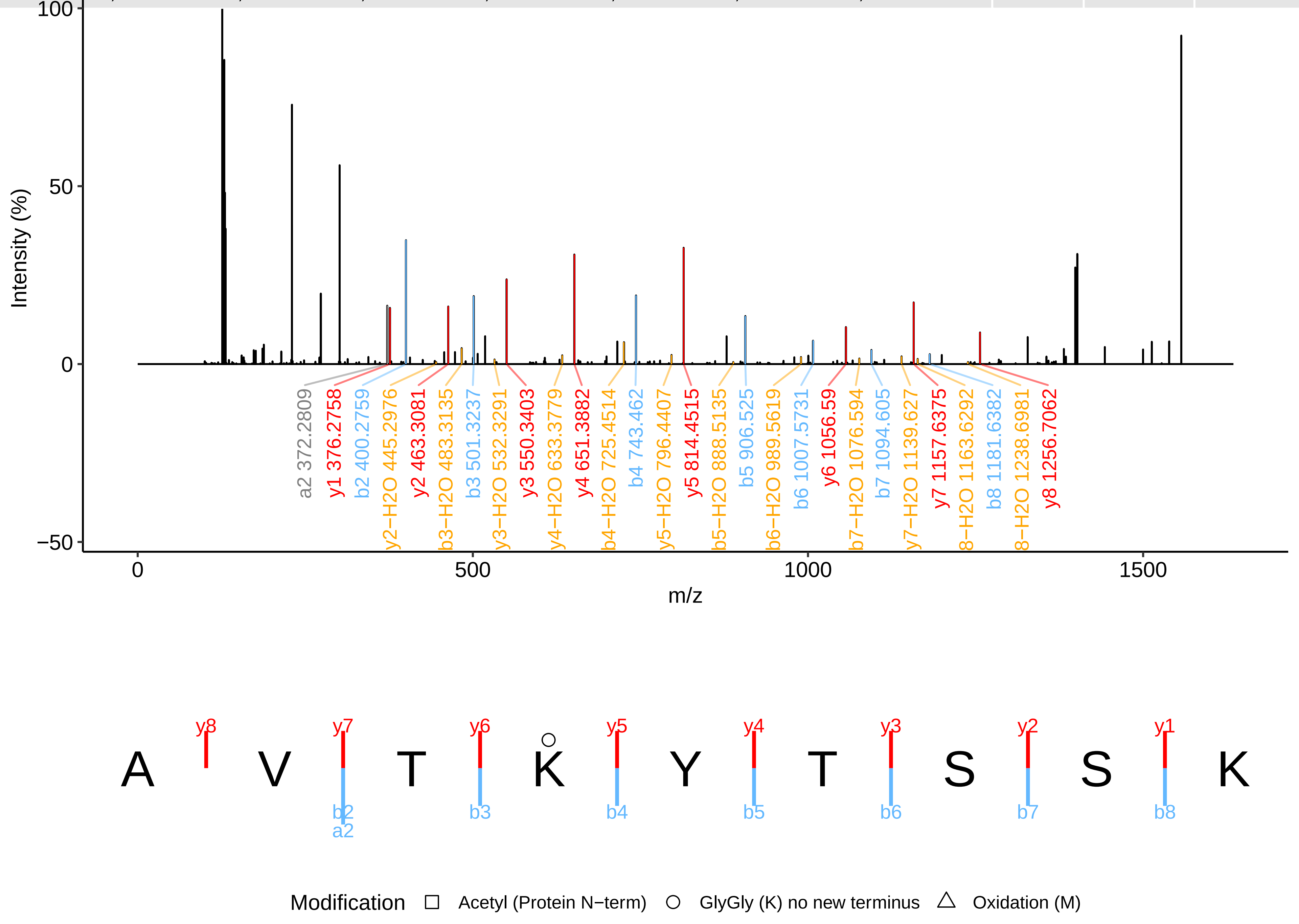

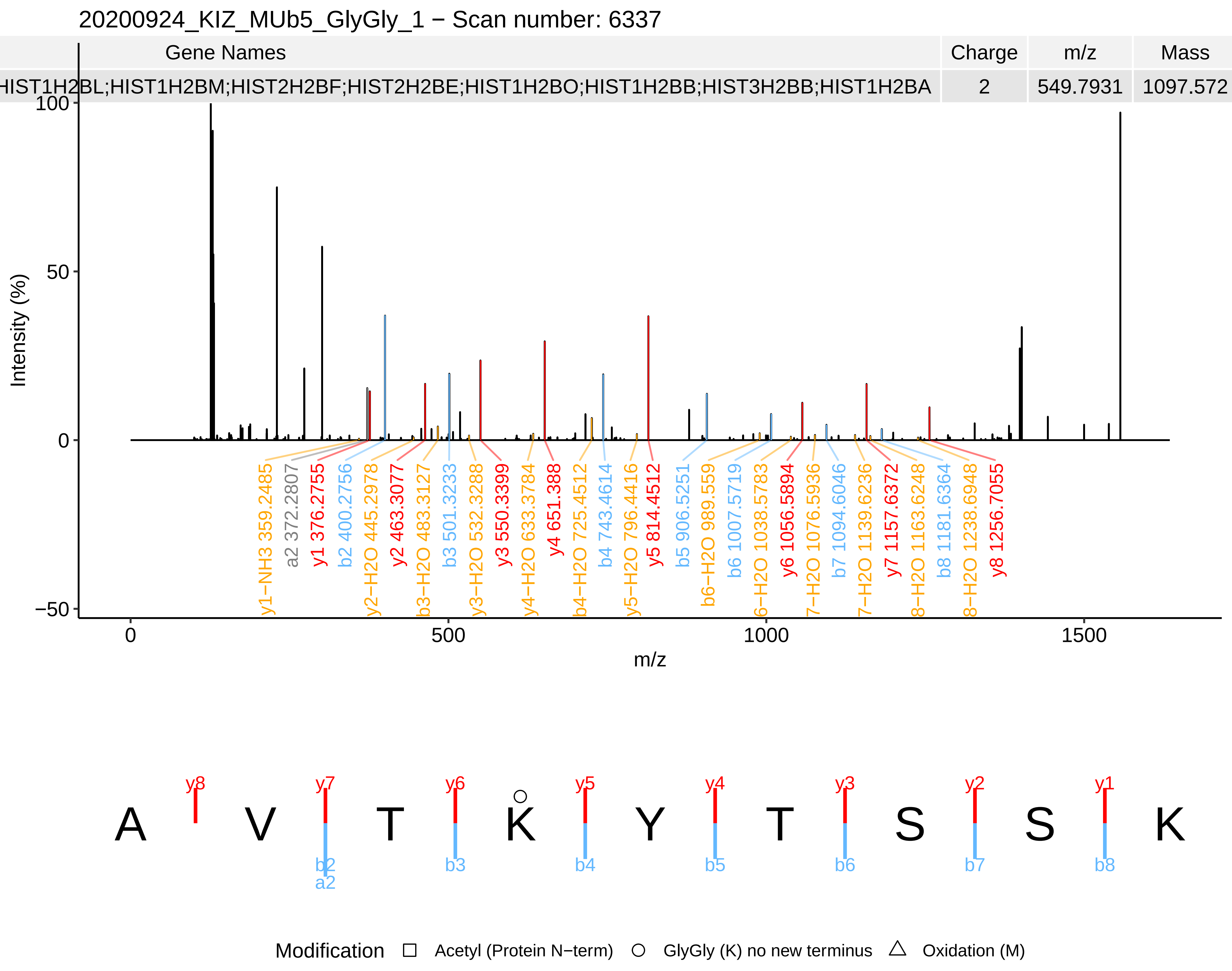

20200924_KIZ_MUb5_GlyGly_1 − Scan number: 6337
Gene Names
HIST1H2BL;HIST1H2BM;HIST2H2BF;HIST2H2BE;HIST1H2BO;HIST1H2BB;HIST3H2BB;HIST1H2BA
Charge
2
m/z
549.7931
Mass
1097.572
Intensity (%)
m/z
y1−NH3 359.2485
a2 372.2807
y1 376.2755
b2 400.2756
y2−H2O 445.2978
y2 463.3077
b3−H2O 483.3127
b3 501.3233
y3−H2O 532.3288
y3 550.3399
y4−H2O 633.3784
y4 651.388
b4−H2O 725.4512
b4 743.4614
y5−H2O 796.4416
y5 814.4512
b5 906.5251
b6−H2O 989.559
b6 1007.5719
y6−H2O 1038.5783
y6 1056.5894
b7−H2O 1076.5936
b7 1094.6046
y7−H2O 1139.6236
y7 1157.6372
b8−H2O 1163.6248
b8 1181.6364
y8−H2O 1238.6948
y8 1256.7055
A V T K Y T S S K
Modification
Acetyl (Protein N−term)
GlyGly (K) no new terminus
Oxidation (M)

20200924_KIZ_MUb5_GlyGly_1 − Scan number: 6648

Gene Names	Charge	m/z	Mass
;HIST1H2BL;HIST1H2BM;HIST2H2BF;HIST2H2BE;HIST1H2BO;HIST1H2BB;HIST3H2BB;HIST1H2BA	3	366.8645	1097.572

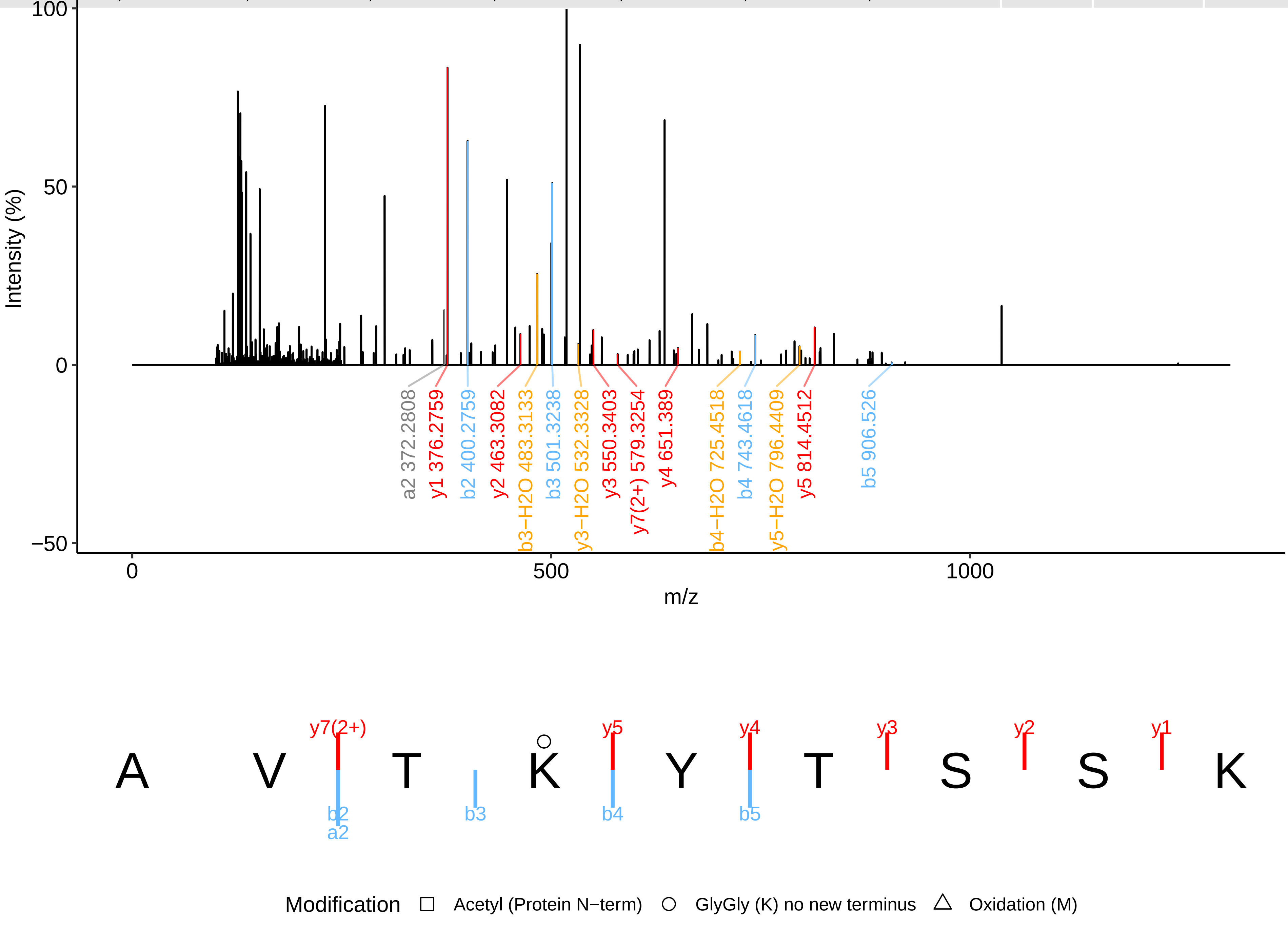

20200924_KIZ_MUb5_GlyGly_2 − Scan number: 5573

Gene Names	Charge	m/z	Mass
;HIST1H2BL;HIST1H2BM;HIST2H2BF;HIST2H2BE;HIST1H2BO;HIST1H2BB;HIST3H2BB;HIST1H2BA	3	366.8645	1097.57

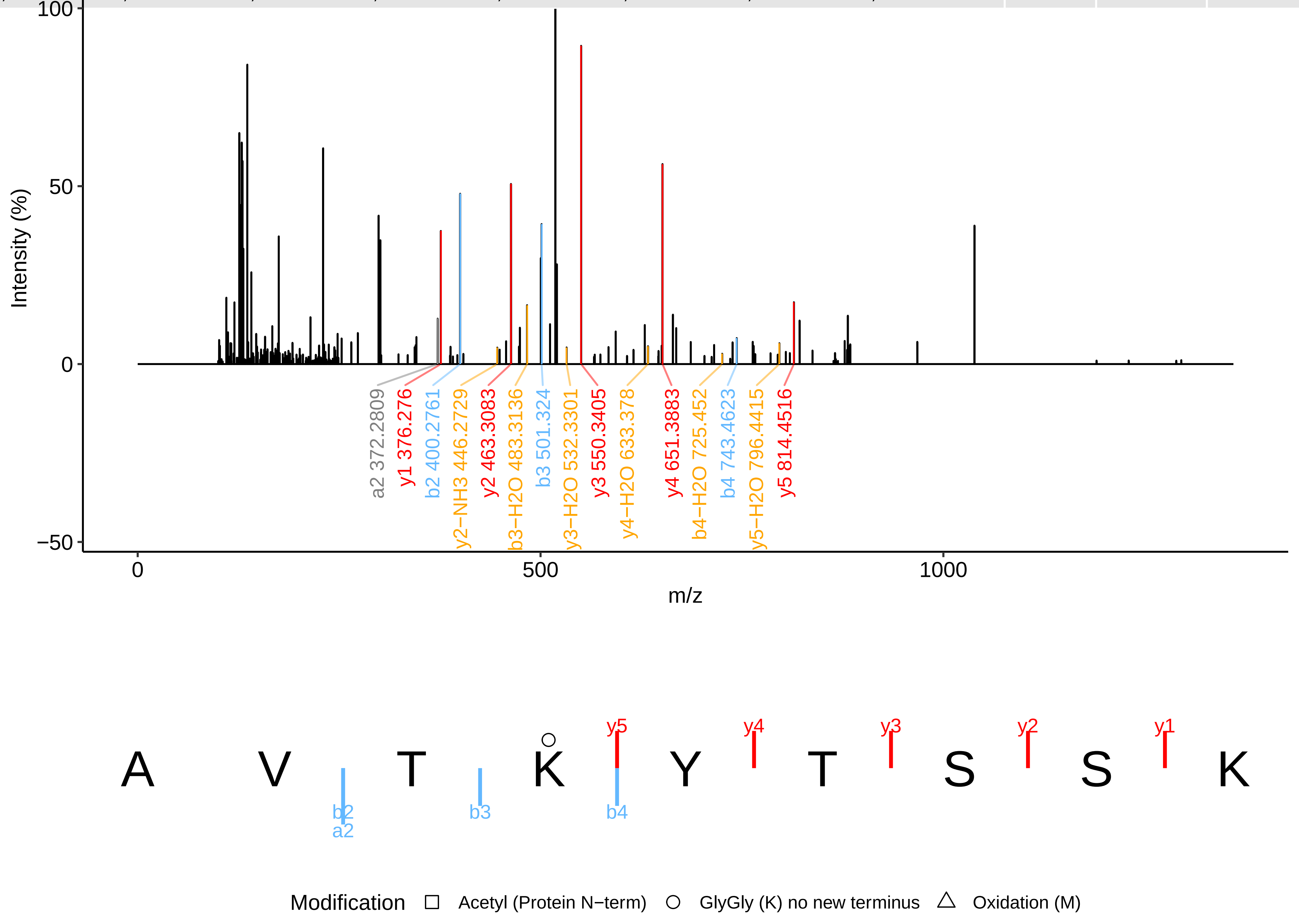

20200924_KIZ_MUb5_GlyGly_2 − Scan number: 5704

Gene Names	Charge	m/z	Mass
HIST1H2BL;HIST1H2BM;HIST2H2BF;HIST2H2BE;HIST1H2BO;HIST1H2BB;HIST3H2BB;HIST1H2BA	3	366.8645	1097.572

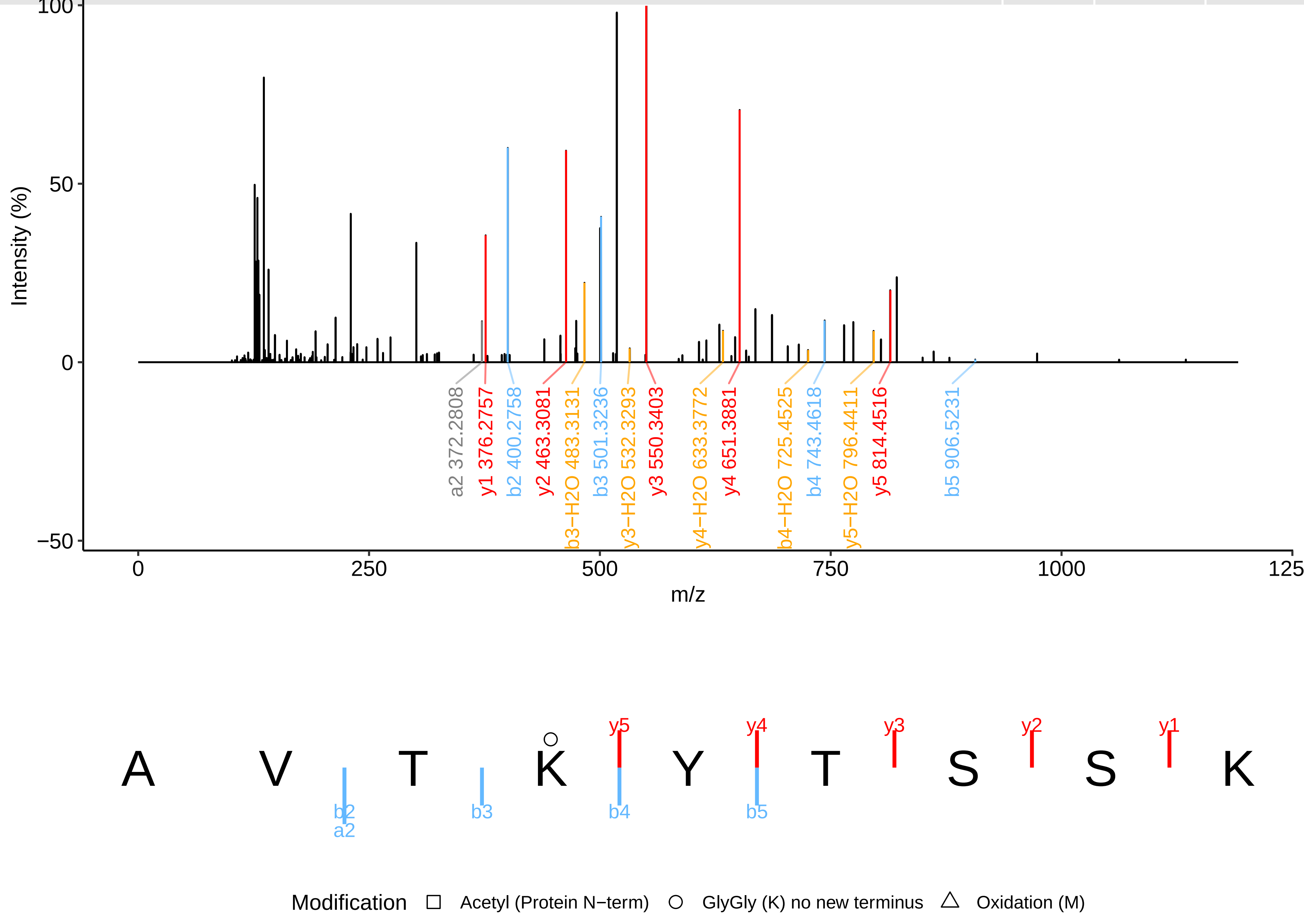

20200924_KIZ_MUb5_GlyGly_2 − Scan number: 5838

Gene Names	Charge	m/z	Mass
HIST1H2BL;HIST1H2BM;HIST2H2BF;HIST2H2BE;HIST1H2BO;HIST1H2BB;HIST3H2BB;HIST1H2BA	3	366.8645	1097.572

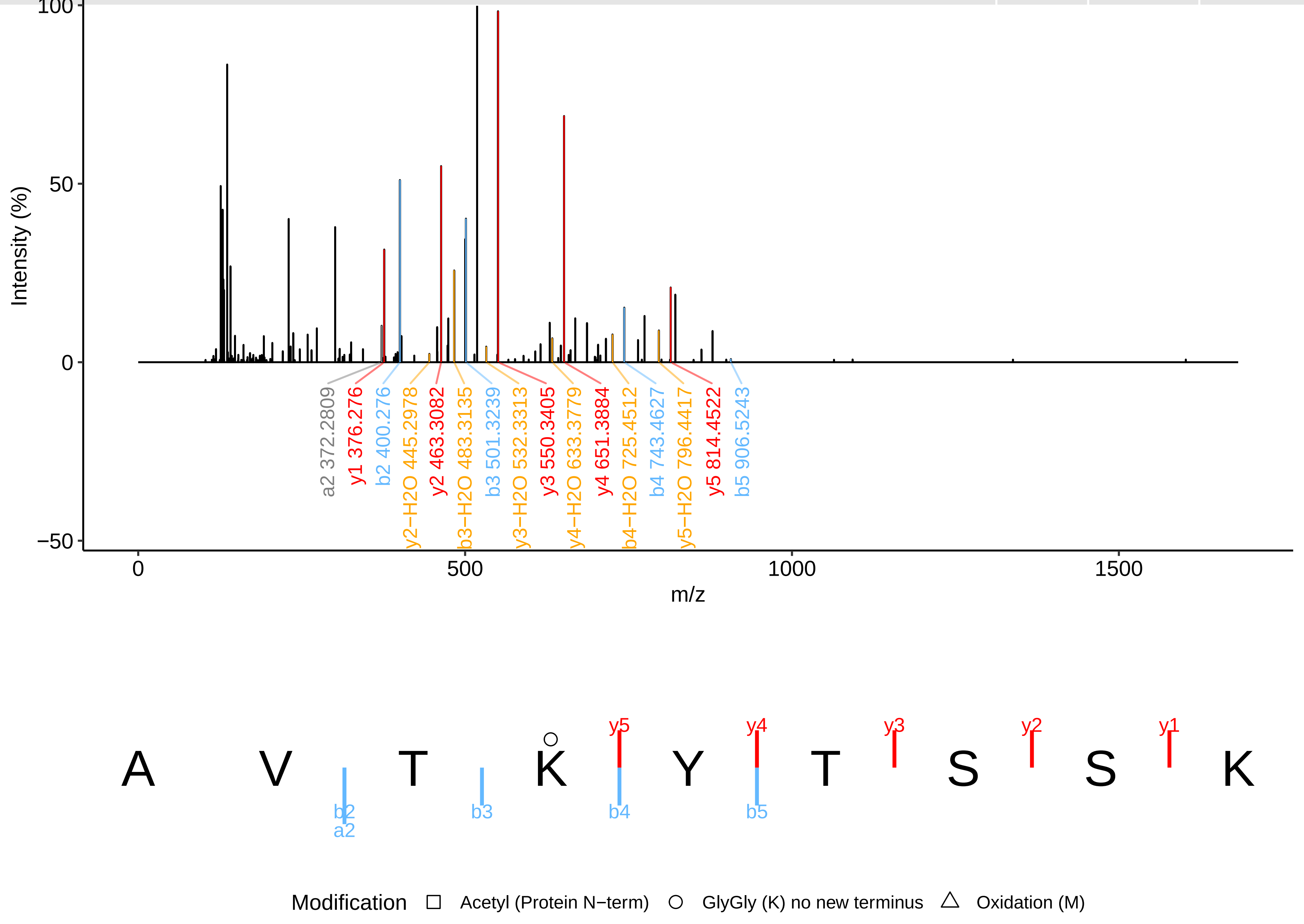

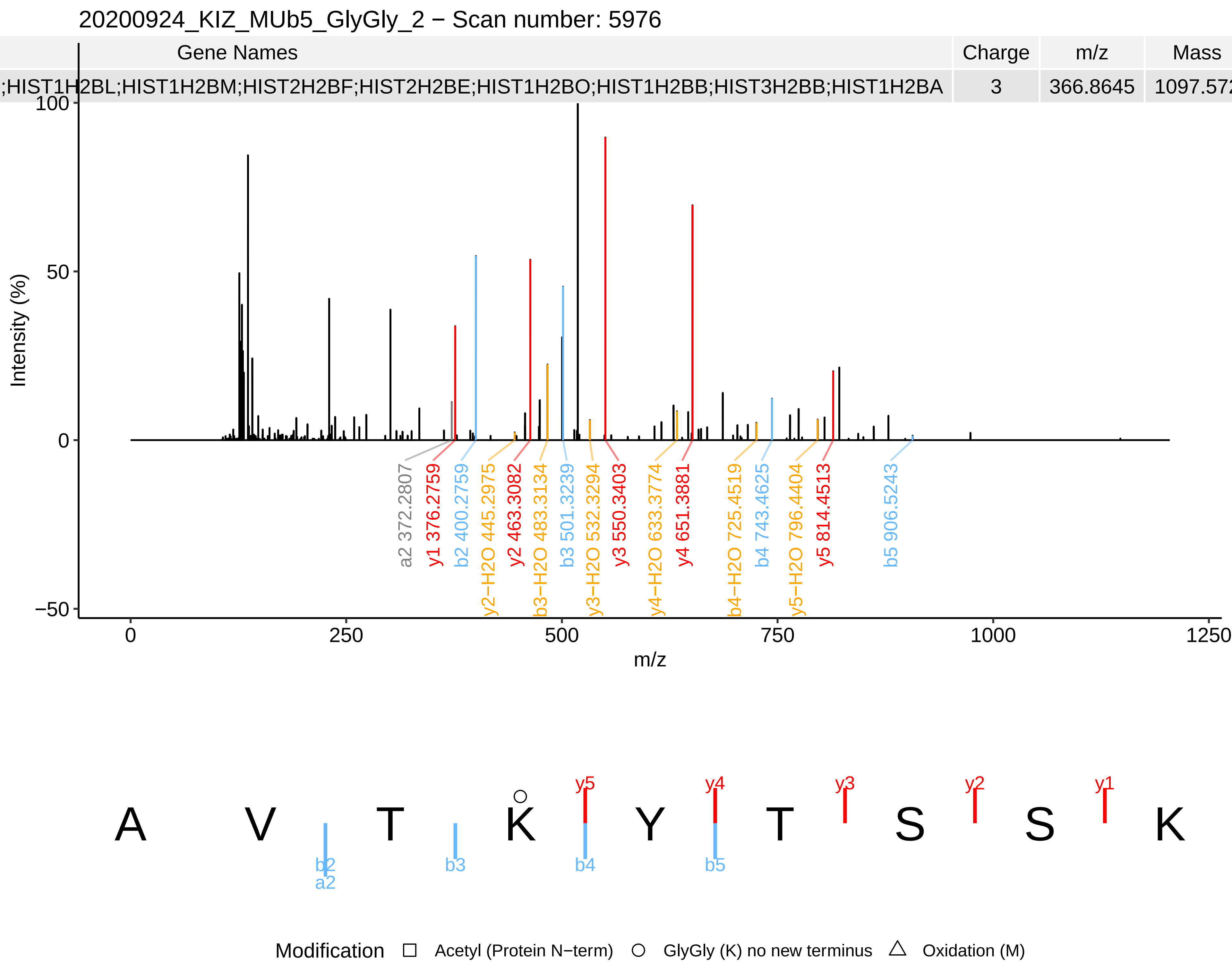

20200924_KIZ_MUb5_GlyGly_2 − Scan number: 5976
Gene Names
Charge
m/z
Mass
;HIST1H2BL;HIST1H2BM;HIST2H2BF;HIST2H2BE;HIST1H2BO;HIST1H2BB;HIST3H2BB;HIST1H2BA
3
366.8645
1097.57
Intensity (%)
100
50
0
−50
0
250
500
750
1000
1250
m/z
a2 372.2807
y1 376.2759
b2 400.2759
y2−H2O 445.2975
y2 463.3082
b3−H2O 483.3134
b3 501.3239
y3−H2O 532.3294
y3 550.3403
y4−H2O 633.3774
y4 651.3881
b4−H2O 725.4519
b4 743.4625
y5−H2O 796.4404
y5 814.4513
b5 906.5243
A V T K Y T S S K
y5 y4 y3 y2 y1
b2 a2 b3 b4 b5
Modification
Acetyl (Protein N−term)
GlyGly (K) no new terminus
Oxidation (M)

20200924_KIZ_MUb5_GlyGly_2 − Scan number: 5663

Gene Names	Charge	m/z	Mass
HIST1H2BL;HIST1H2BM;HIST2H2BF;HIST2H2BE;HIST1H2BO;HIST1H2BB;HIST3H2BB;HIST1H2BA	2	549.7931	1097.572

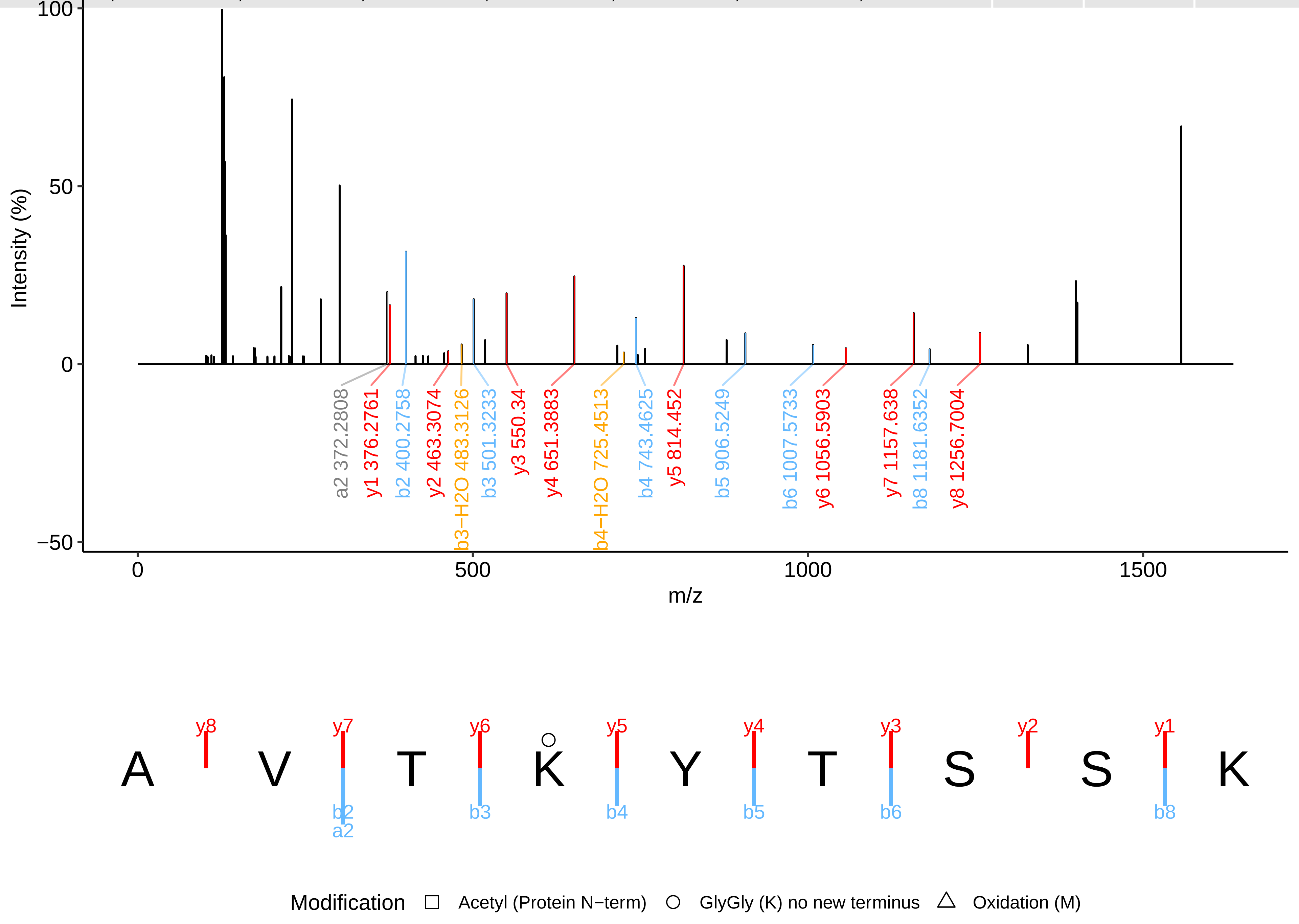

20200924_KIZ_MUb5_GlyGly_2 − Scan number: 5796

Gene Names	Charge	m/z	Mass
HIST1H2BL;HIST1H2BM;HIST2H2BF;HIST2H2BE;HIST1H2BO;HIST1H2BB;HIST3H2BB;HIST1H2BA	2	549.7931	1097.572

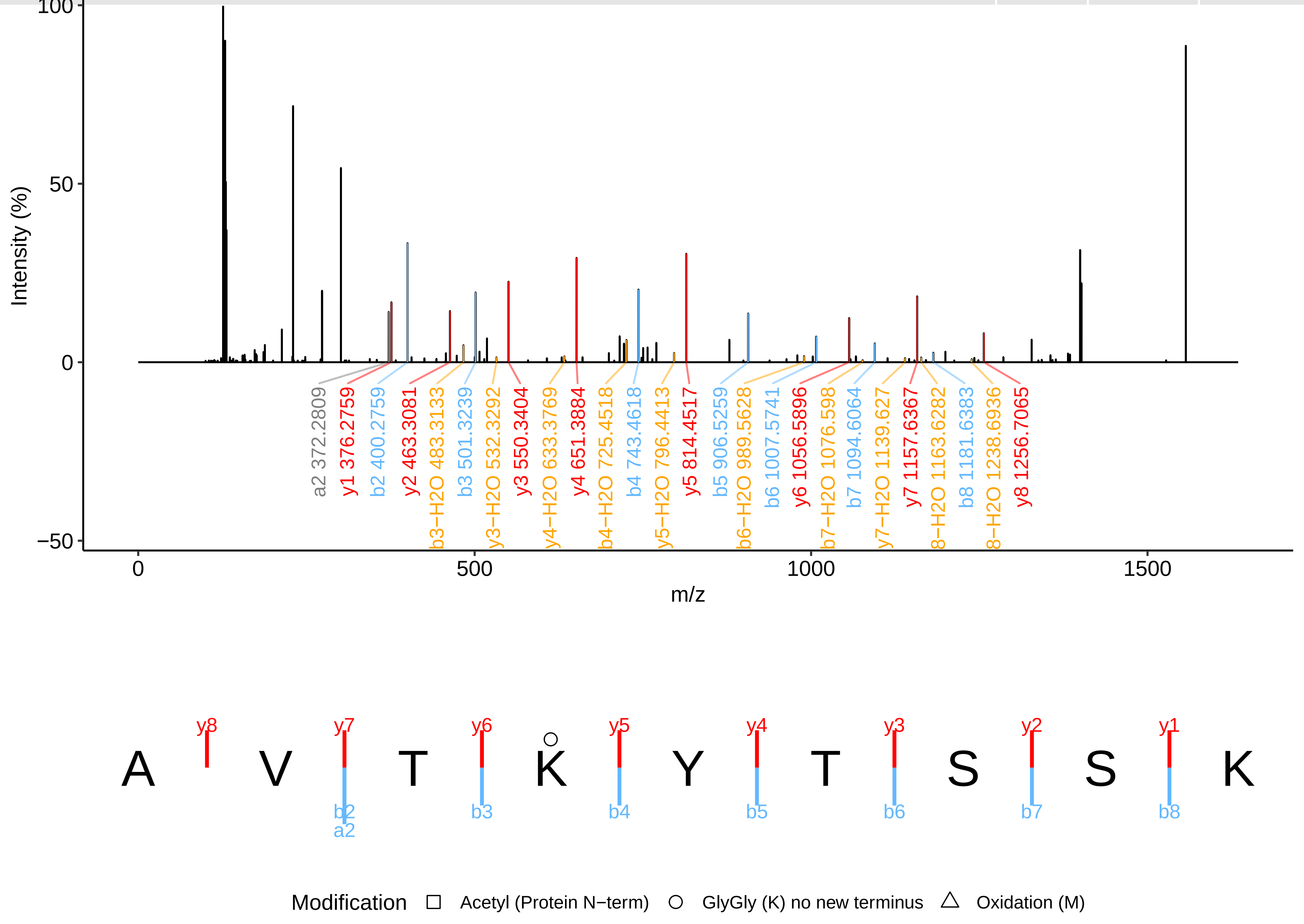

20200924_KIZ_MUb5_GlyGly_2 − Scan number: 6249

Gene Names	Charge	m/z	Mass
;HIST1H2BL;HIST1H2BM;HIST2H2BF;HIST2H2BE;HIST1H2BO;HIST1H2BB;HIST3H2BB;HIST1H2BA	3	366.8645	1097.572

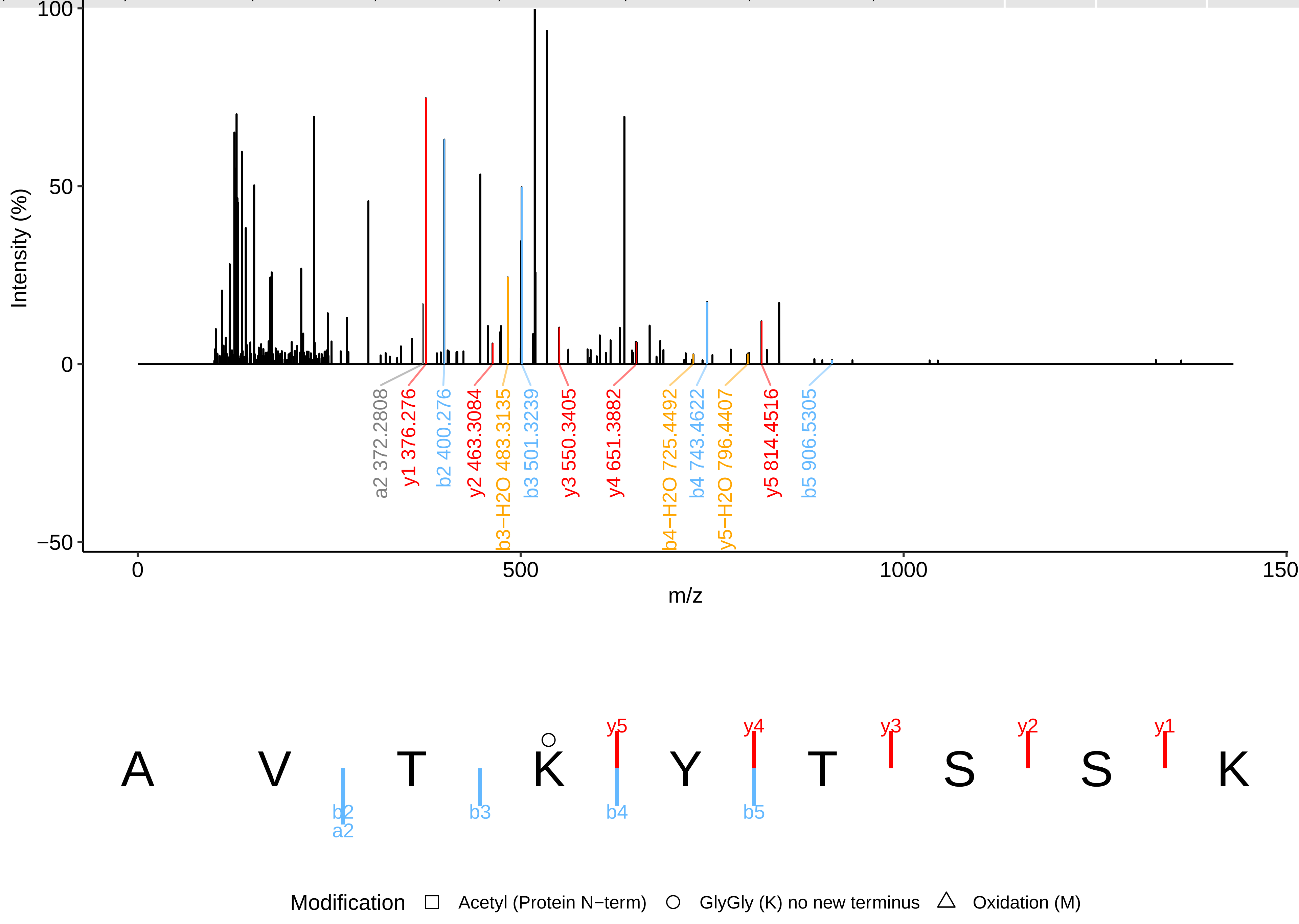

20200924_KIZ_MUb5_GlyGly_2 − Scan number: 5933

Gene Names	Charge	m/z	Mass
HIST1H2BL;HIST1H2BM;HIST2H2BF;HIST2H2BE;HIST1H2BO;HIST1H2BB;HIST3H2BB;HIST1H2BA	2	549.7931	1097.572

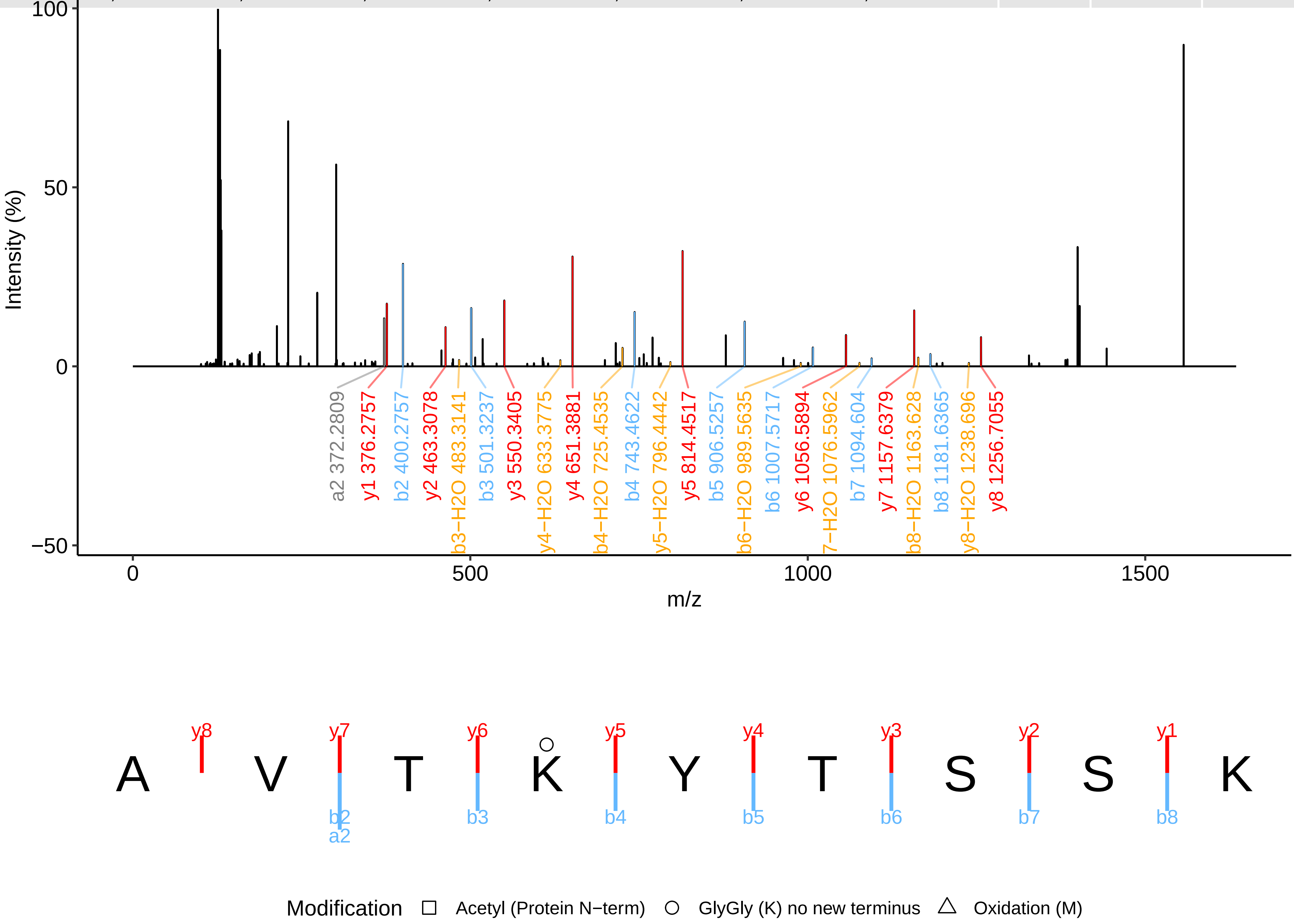

20200924_KIZ_MUb5_GlyGly_2 − Scan number: 6112

Gene Names	Charge	m/z	Mass
[illegible];HIST1H2BL;HIST1H2BM;HIST2H2BF;HIST2H2BE;HIST1H2BO;HIST1H2BB;HIST3H2BB;HIST1H2BA	3	366.8645	1097.57[illegible]

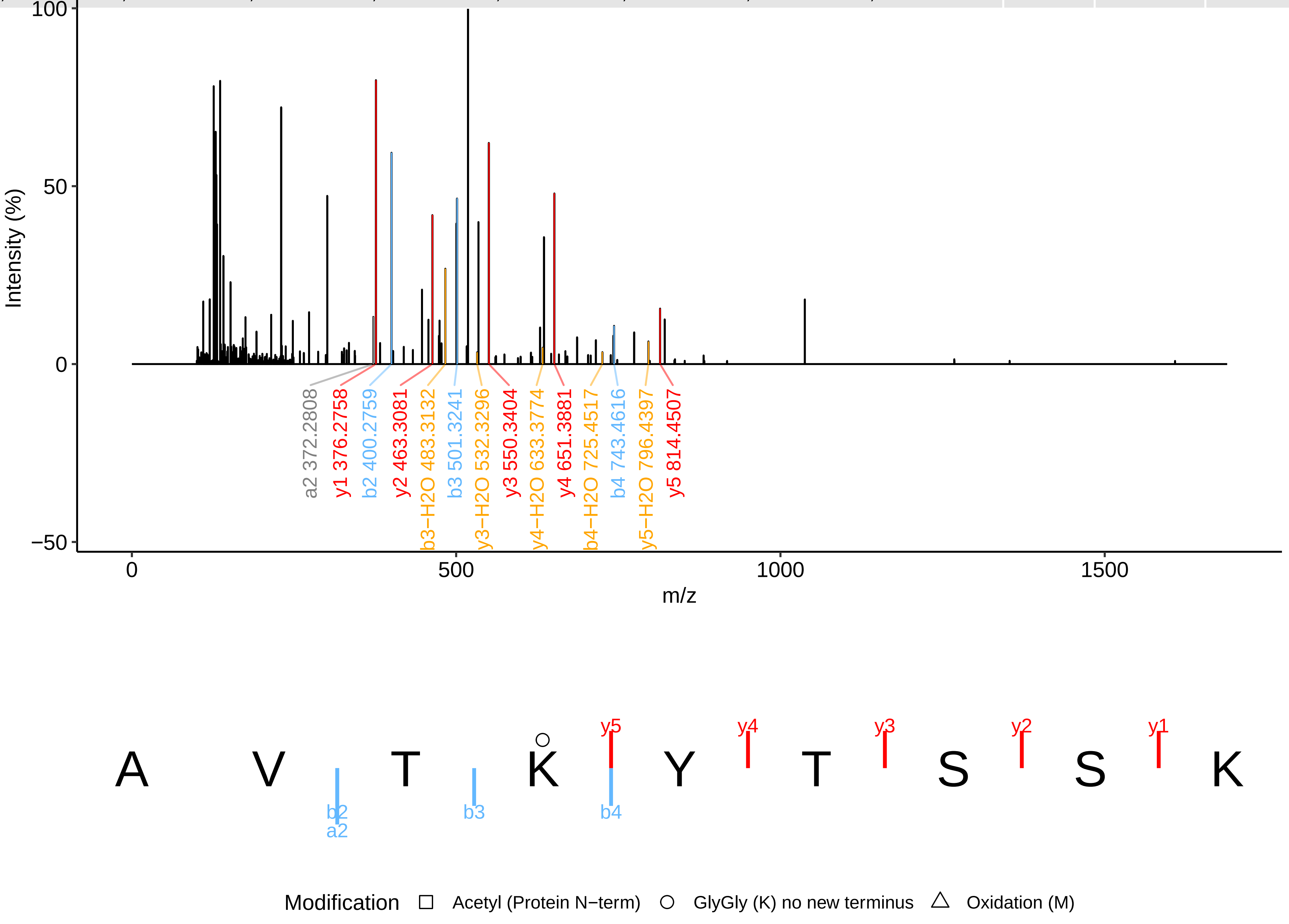

20201118_KIZ_MUb6_GlyGly_1 − Scan number: 5723

Gene Names	Charge	m/z	Mass
;HIST1H2BL;HIST1H2BM;HIST2H2BF;HIST2H2BE;HIST1H2BO;HIST1H2BB;HIST3H2BB;HIST1H2BA	3	366.8645	1097.572

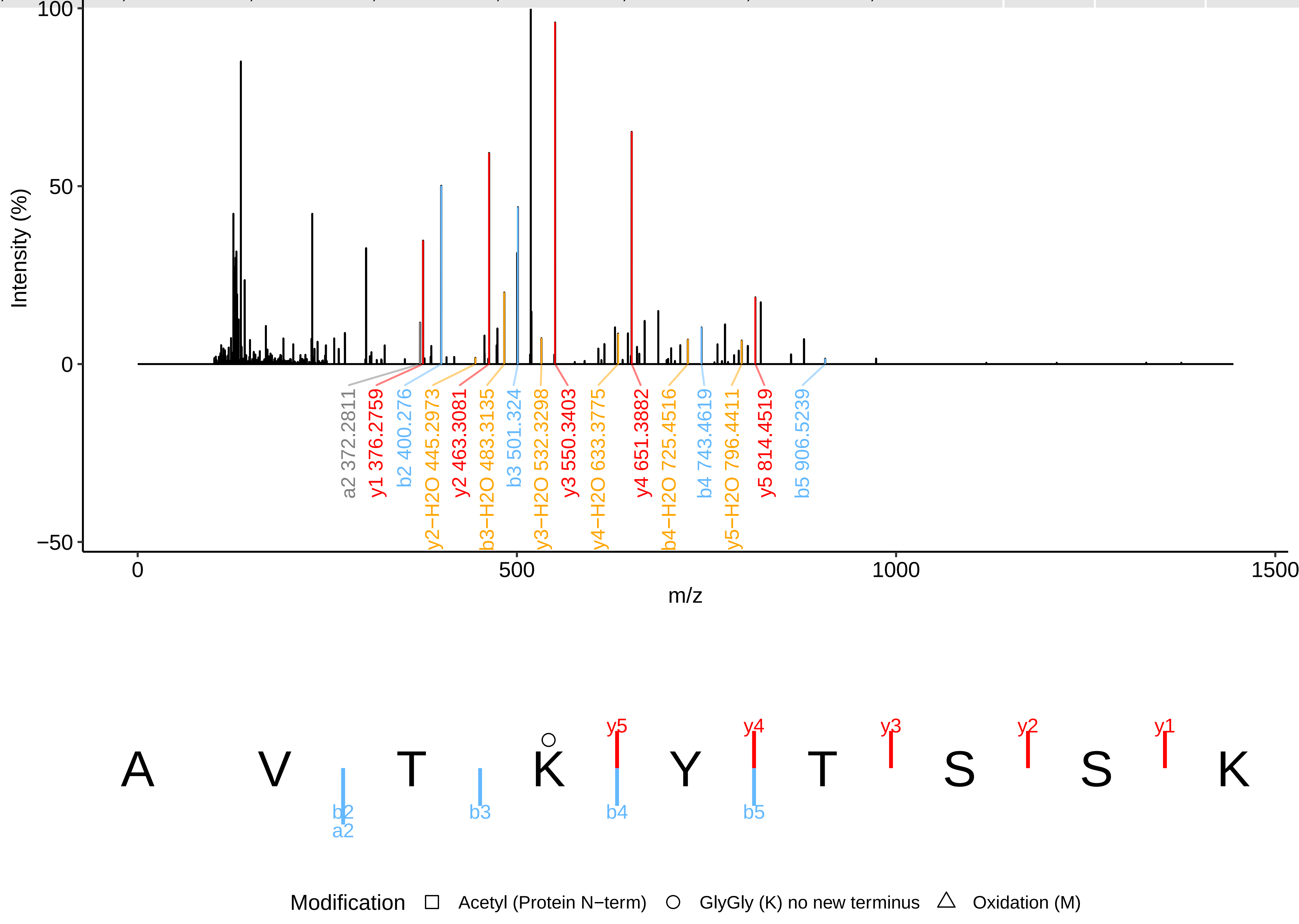

20201118_KIZ_MUb6_GlyGly_1 − Scan number: 5751

Gene Names	Charge	m/z	Mass
;HIST1H2BL;HIST1H2BM;HIST2H2BF;HIST2H2BE;HIST1H2BO;HIST1H2BB;HIST3H2BB;HIST1H2BA	3	366.8645	1097.572

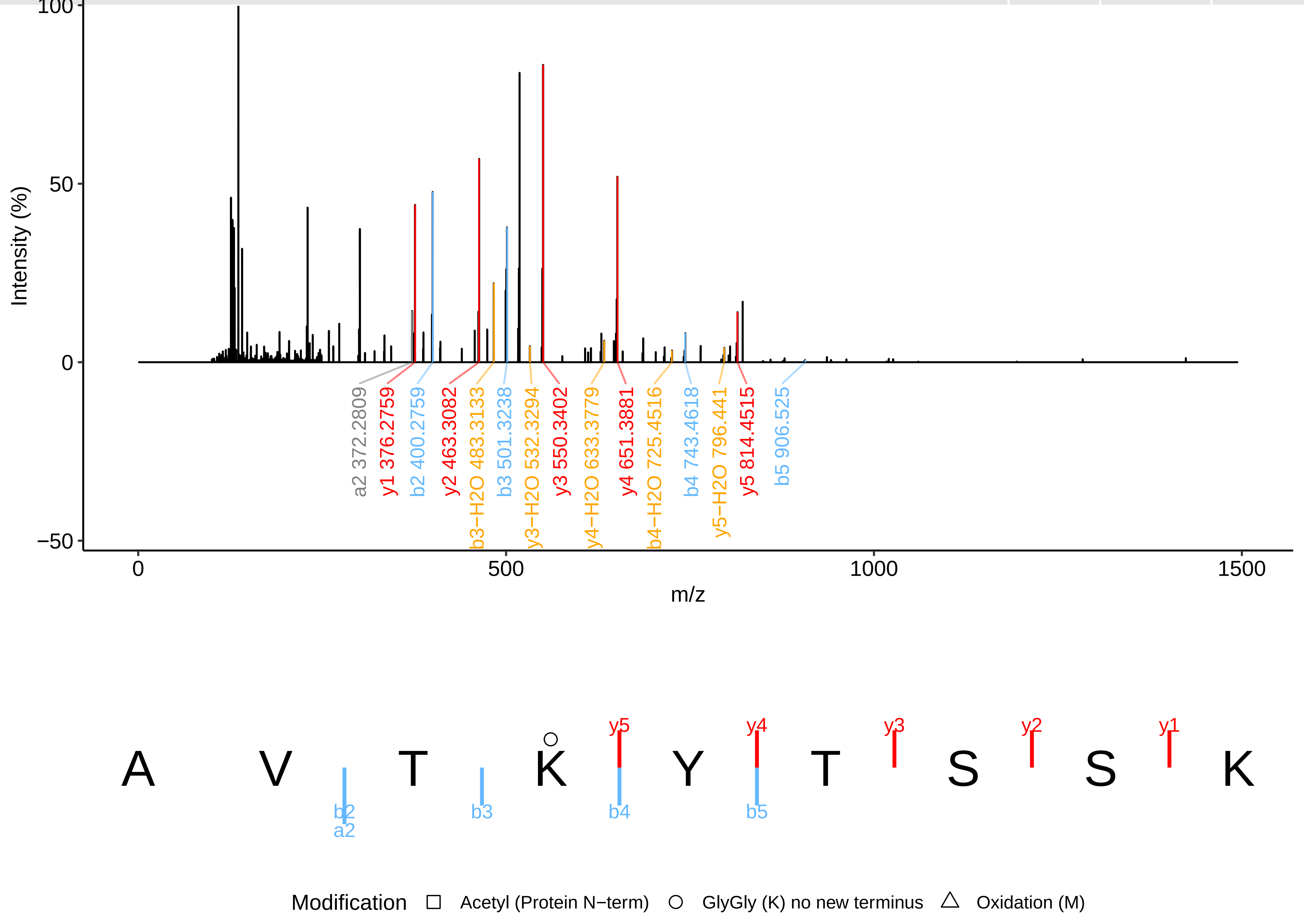

20201118_KIZ_MUb6_GlyGly_1 − Scan number: 5736

Gene Names	Charge	m/z	Mass
HIST1H2BL;HIST1H2BM;HIST2H2BF;HIST2H2BE;HIST1H2BO;HIST1H2BB;HIST3H2BB;HIST1H2BA	2	549.7931	1097.572

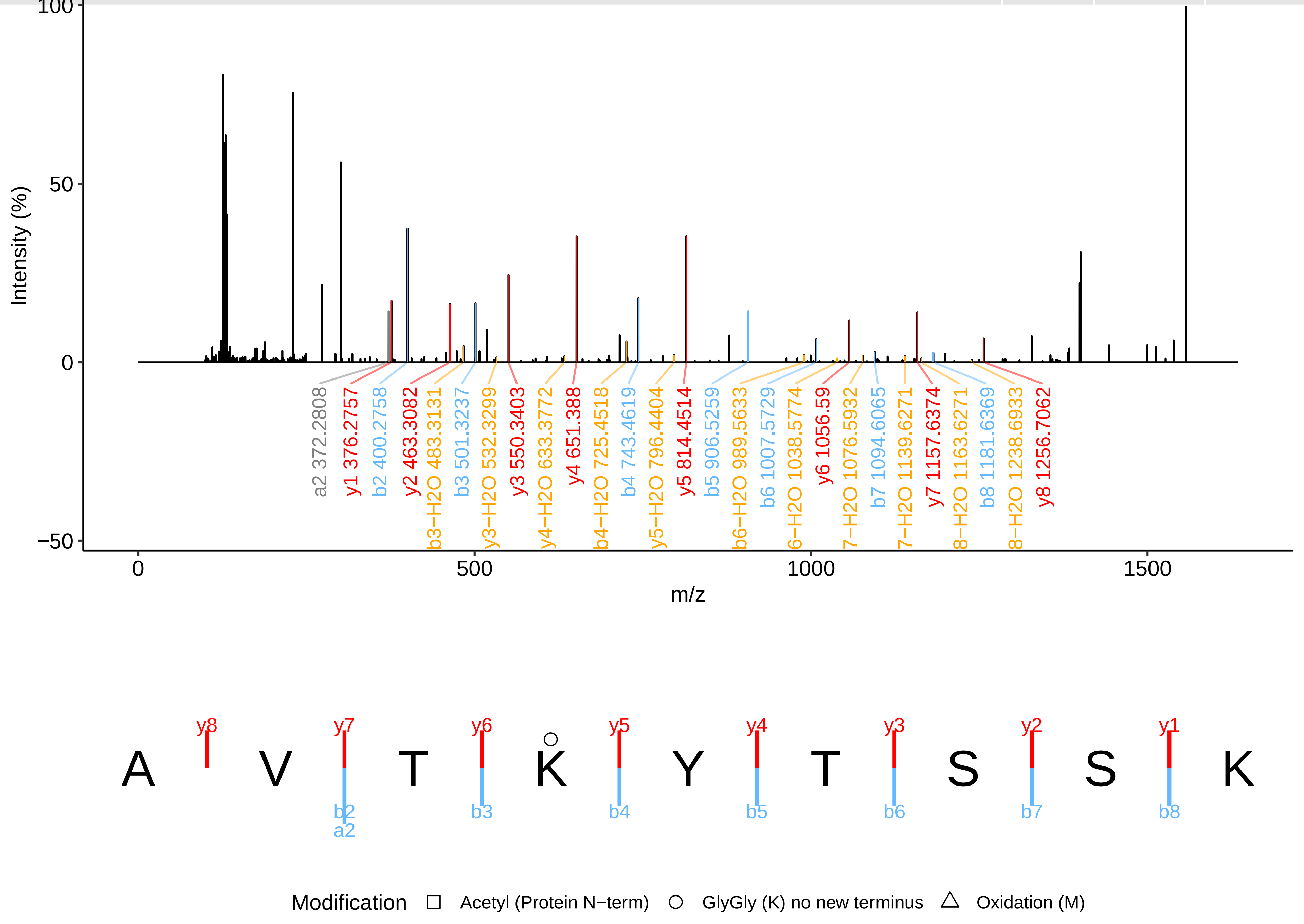

20201118_KIZ_MUb6_GlyGly_2 − Scan number: 7448

Gene Names	Charge	m/z	Mass
HIST1H2BL;HIST1H2BM;HIST2H2BF;HIST2H2BE;HIST1H2BO;HIST1H2BB;HIST3H2BB;HIST1H2BA	3	366.8645	1097.572

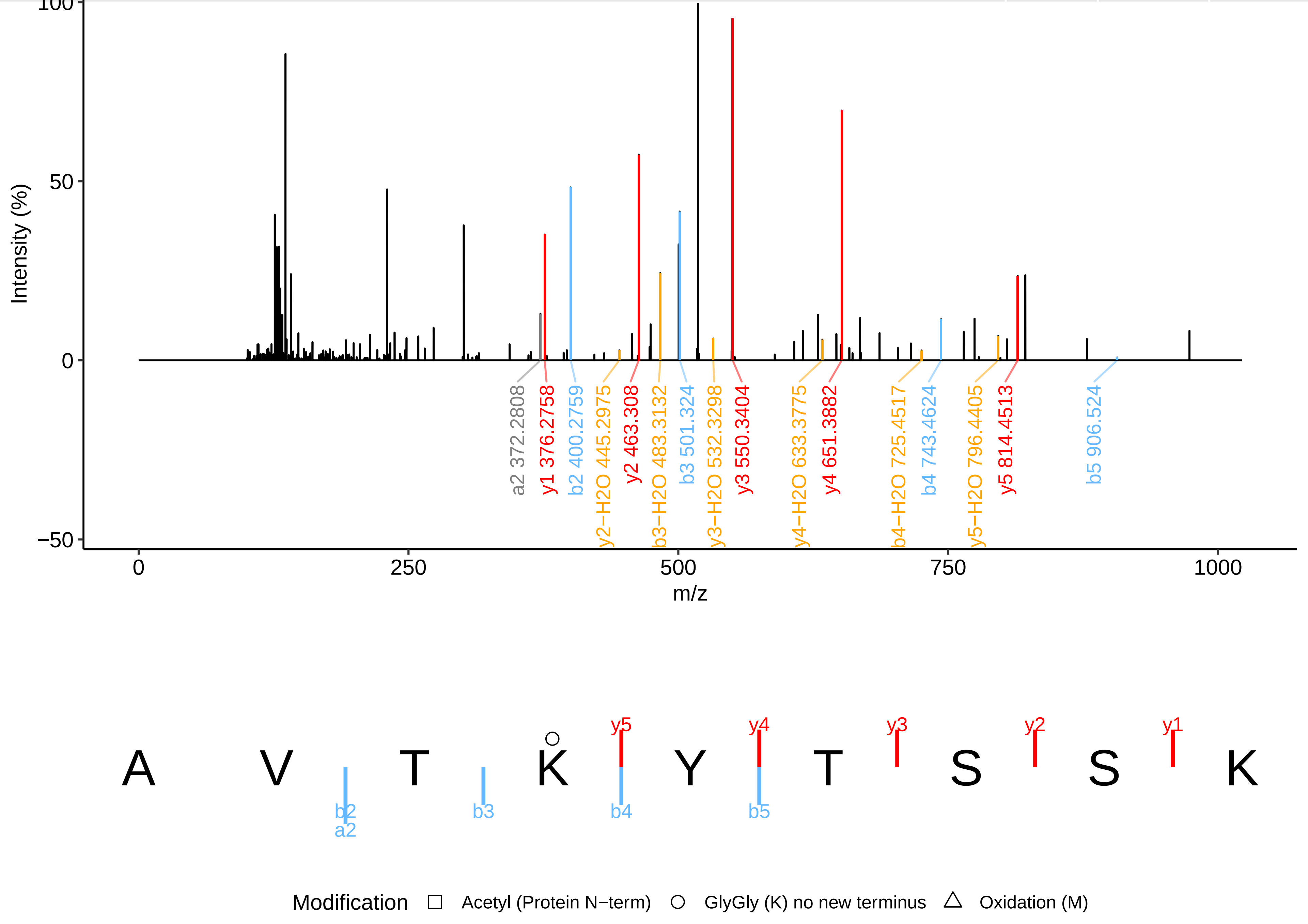

20201118_KIZ_MUb6_GlyGly_2 − Scan number: 7479

Gene Names	Charge	m/z	Mass
HIST1H2BL;HIST1H2BM;HIST2H2BF;HIST2H2BE;HIST1H2BO;HIST1H2BB;HIST3H2BB;HIST1H2BA	2	549.7931	1097.572

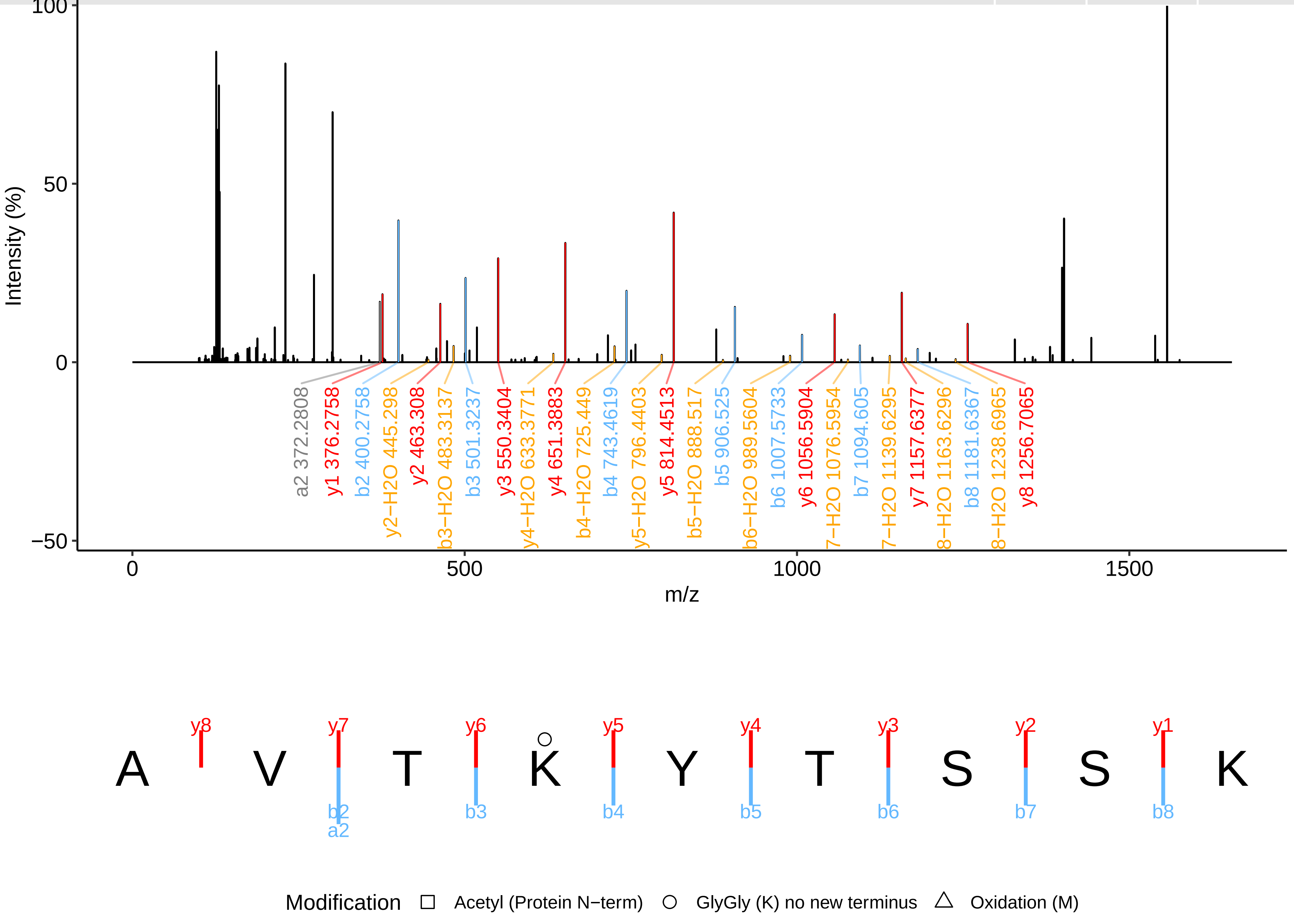

20200921_KIZ_MUb4_GlyGly_10plex − Scan number: 11593

Gene Names	Charge	m/z	Mass	Retention time	PEP	Score	Precursor Intensity
SQSTM1	3	440.9017	1319.683	40.451	4.6754e−08	118.04	3480463

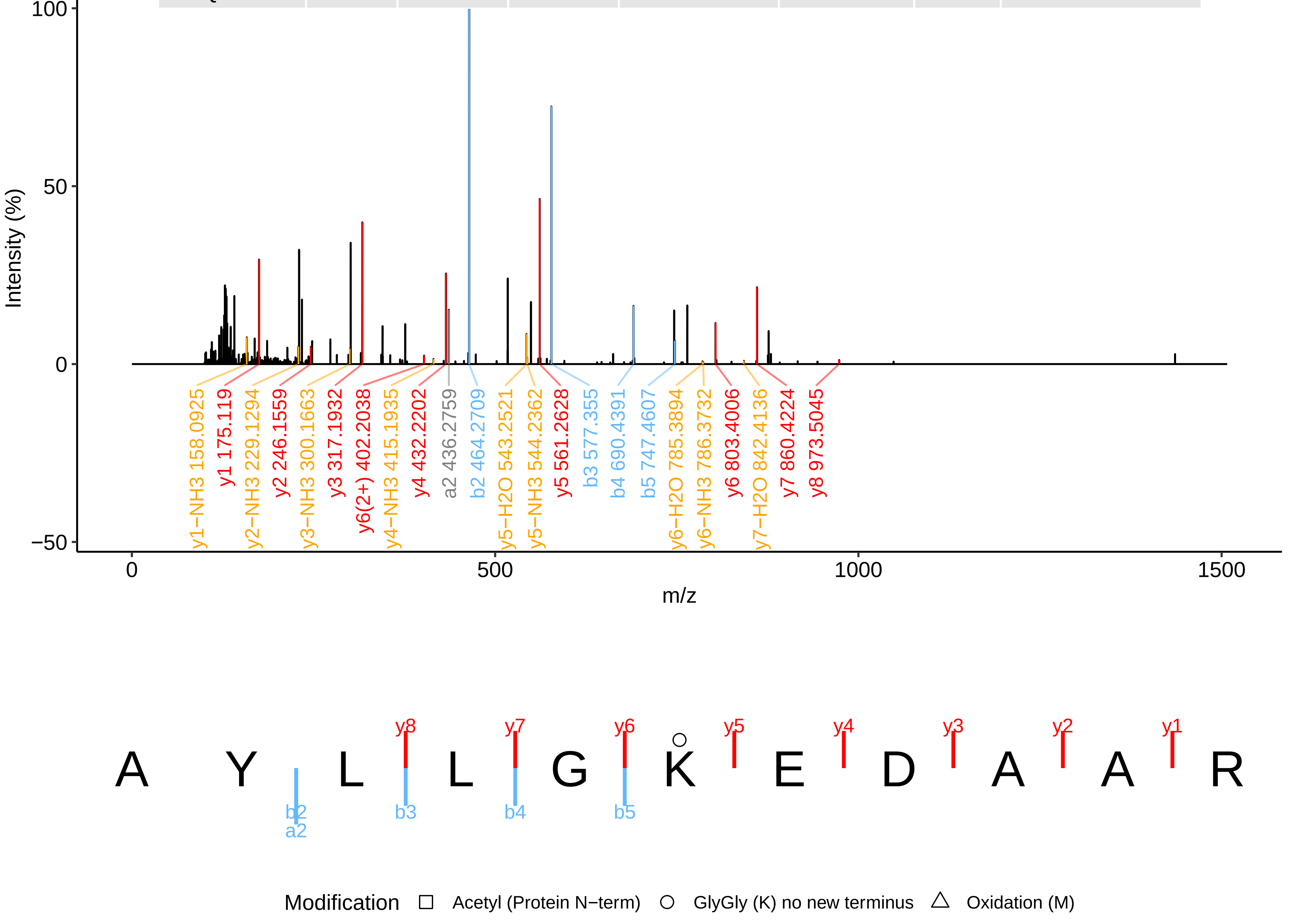

20200921_KIZ_MUb4_GlyGly_10plex_2 − Scan number: 10736

Gene Names	Charge	m/z	Mass	Retention time	PEP	Score	Precursor Intensity
SQSTM1	3	440.9017	1319.683	41.138	1.6722e−16	151.83	2631041

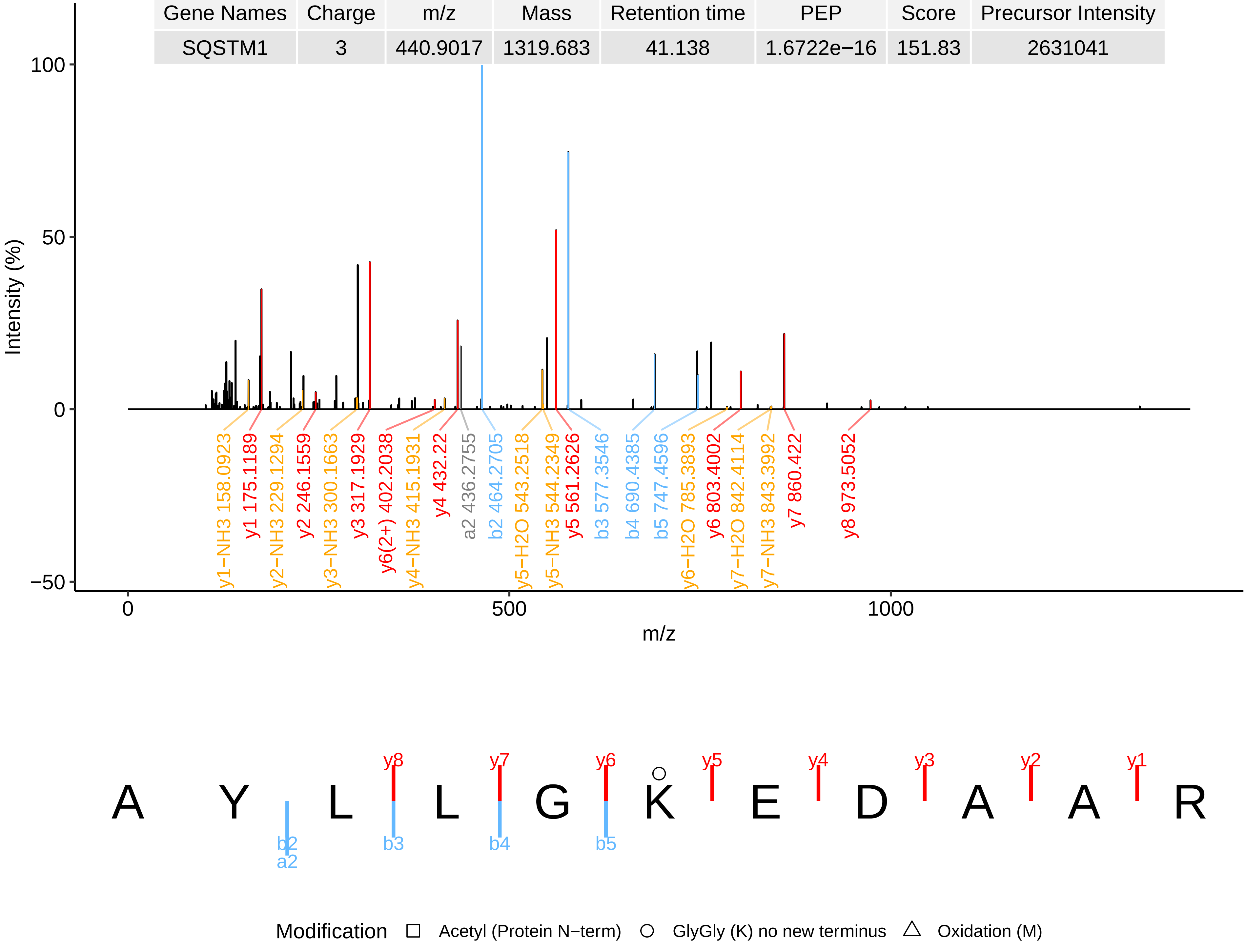

20200924_KIZ_MUb5_GlyGly_1 − Scan number: 10527

Gene Names	Charge	m/z	Mass	Retention time	PEP	Score	Precursor Intensity
SQSTM1	3	440.9017	1319.683	34	4.4272e−05	98.254	5221304

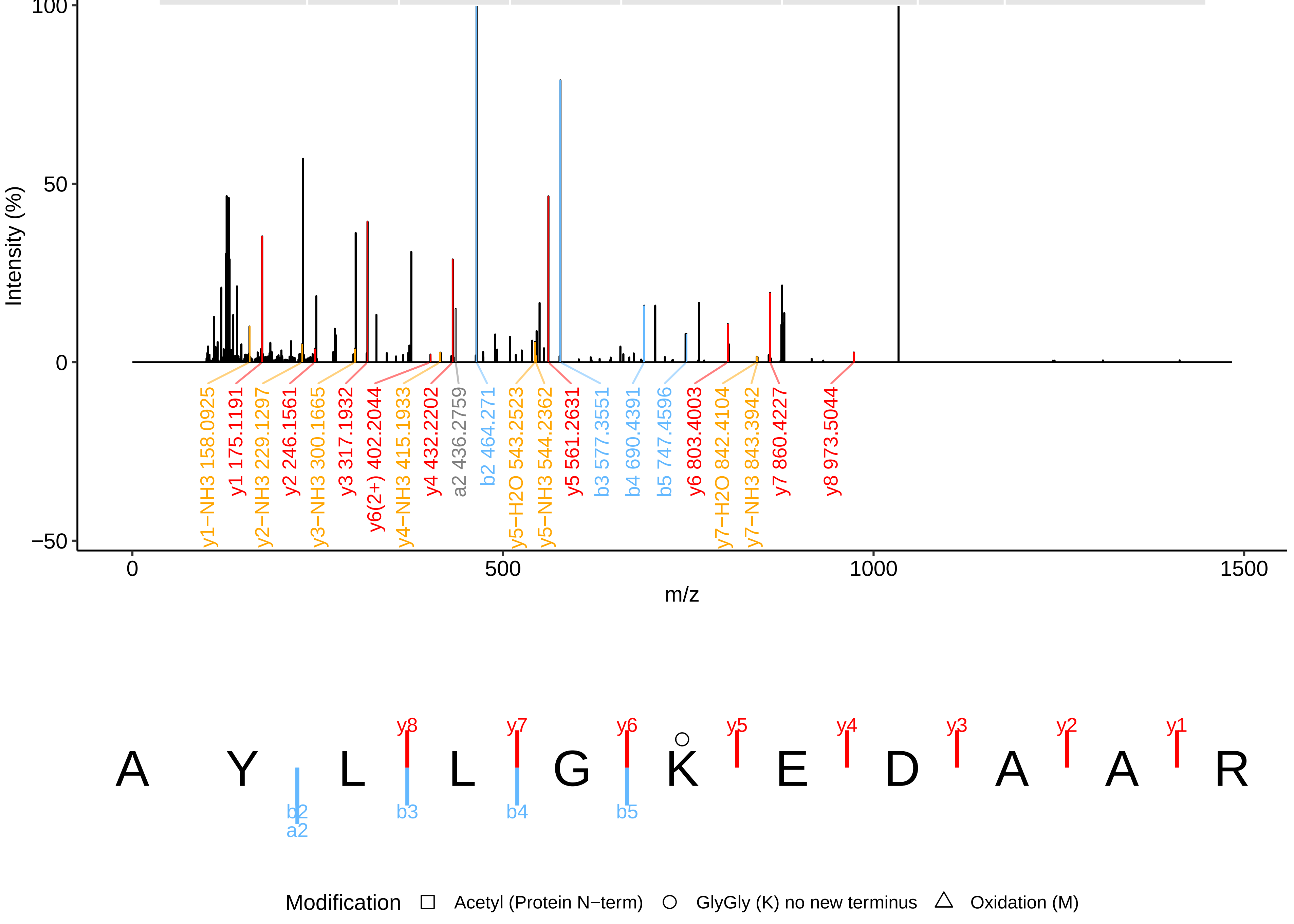

20200924_KIZ_MUb5_GlyGly_1 − Scan number: 10383

Gene Names	Charge	m/z	Mass	Retention time	PEP	Score	Precursor Intensity
SQSTM1	3	440.9017	1319.683	33.495	0.0013574	88.948	6972192

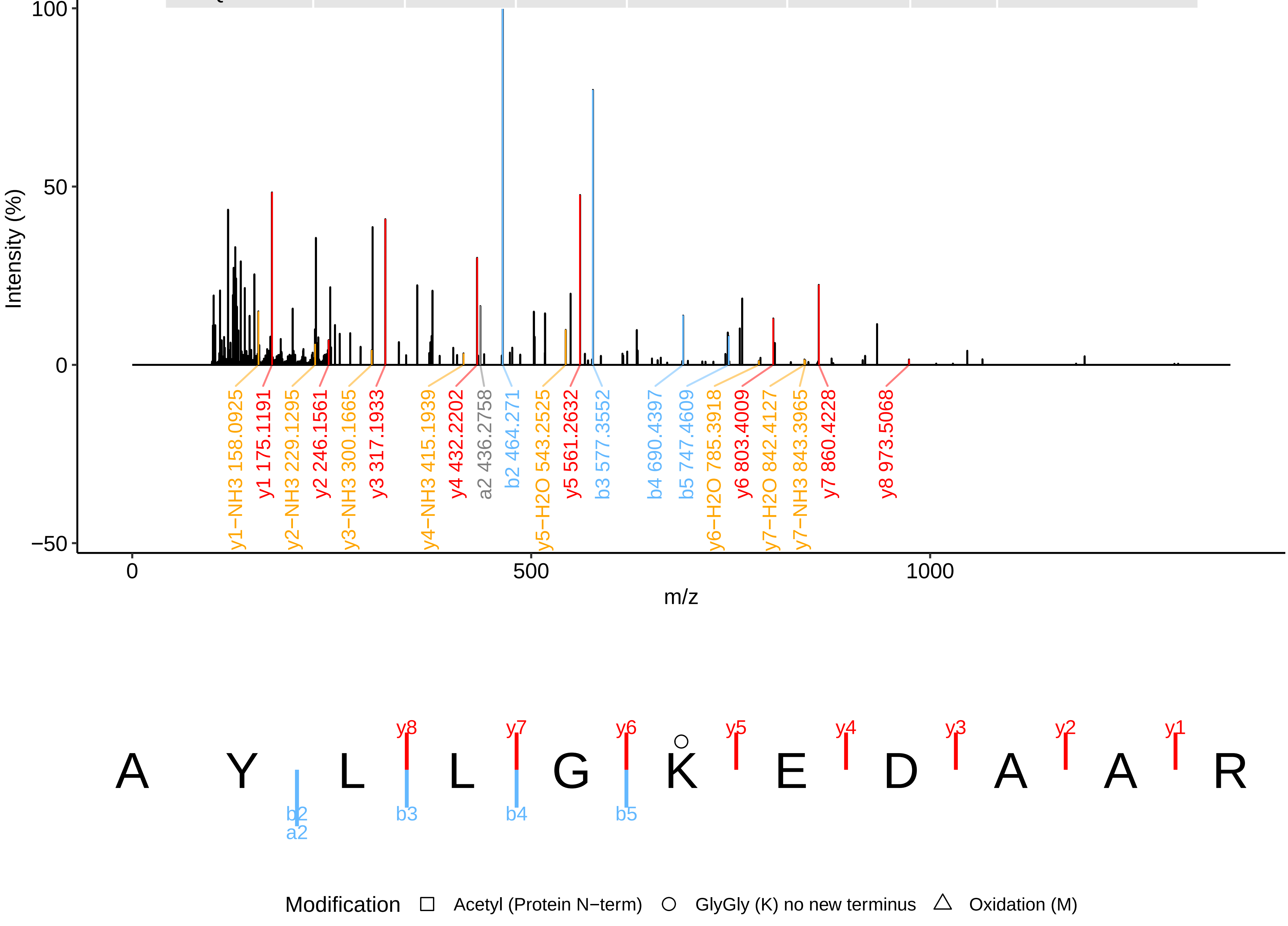

20200924_KIZ_MUb5_GlyGly_2 − Scan number: 9799

Gene Names	Charge	m/z	Mass	Retention time	PEP	Score	Precursor Intensity
SQSTM1	3	440.9017	1319.683	36.621	0.014139	65.5	2789246

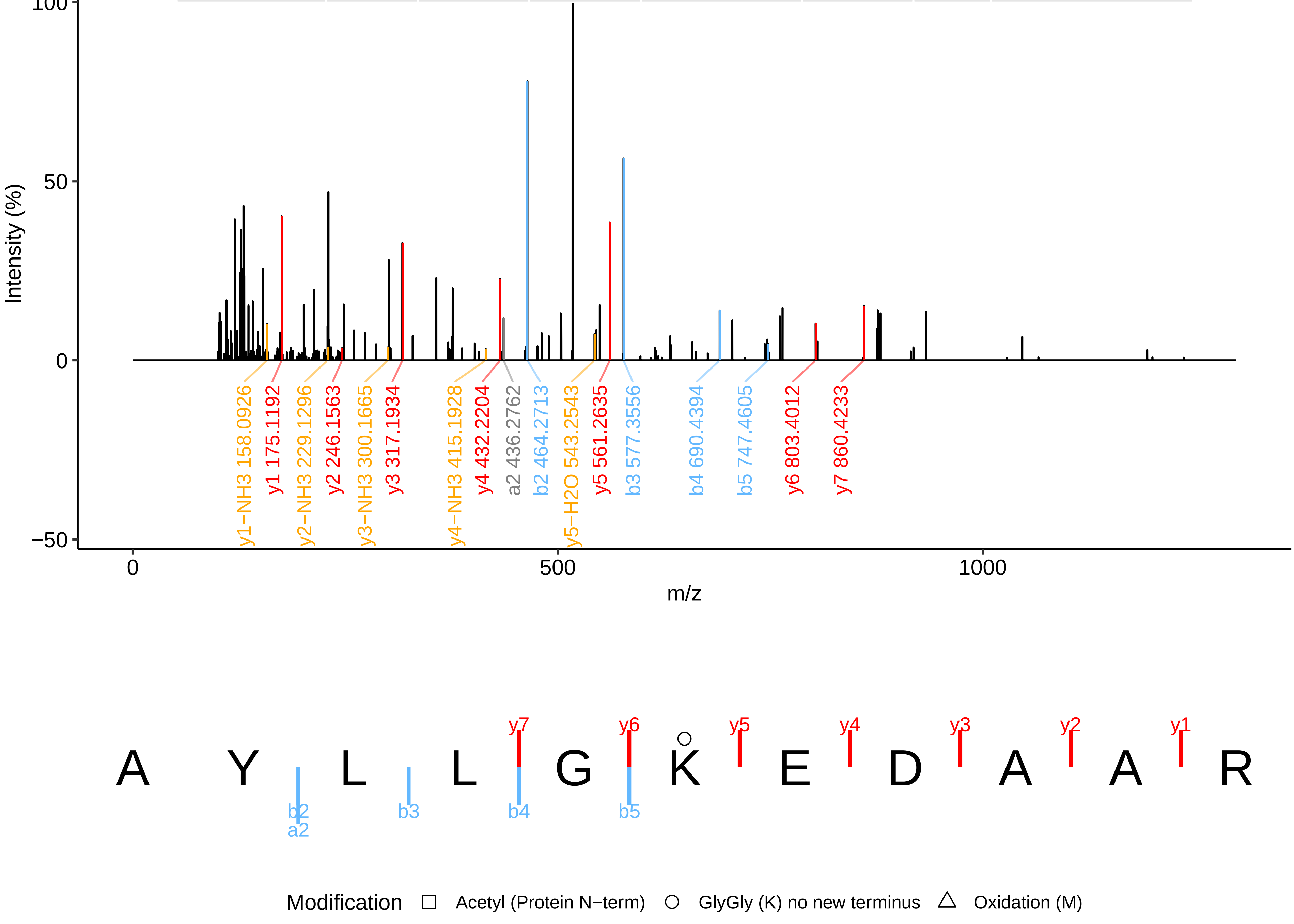

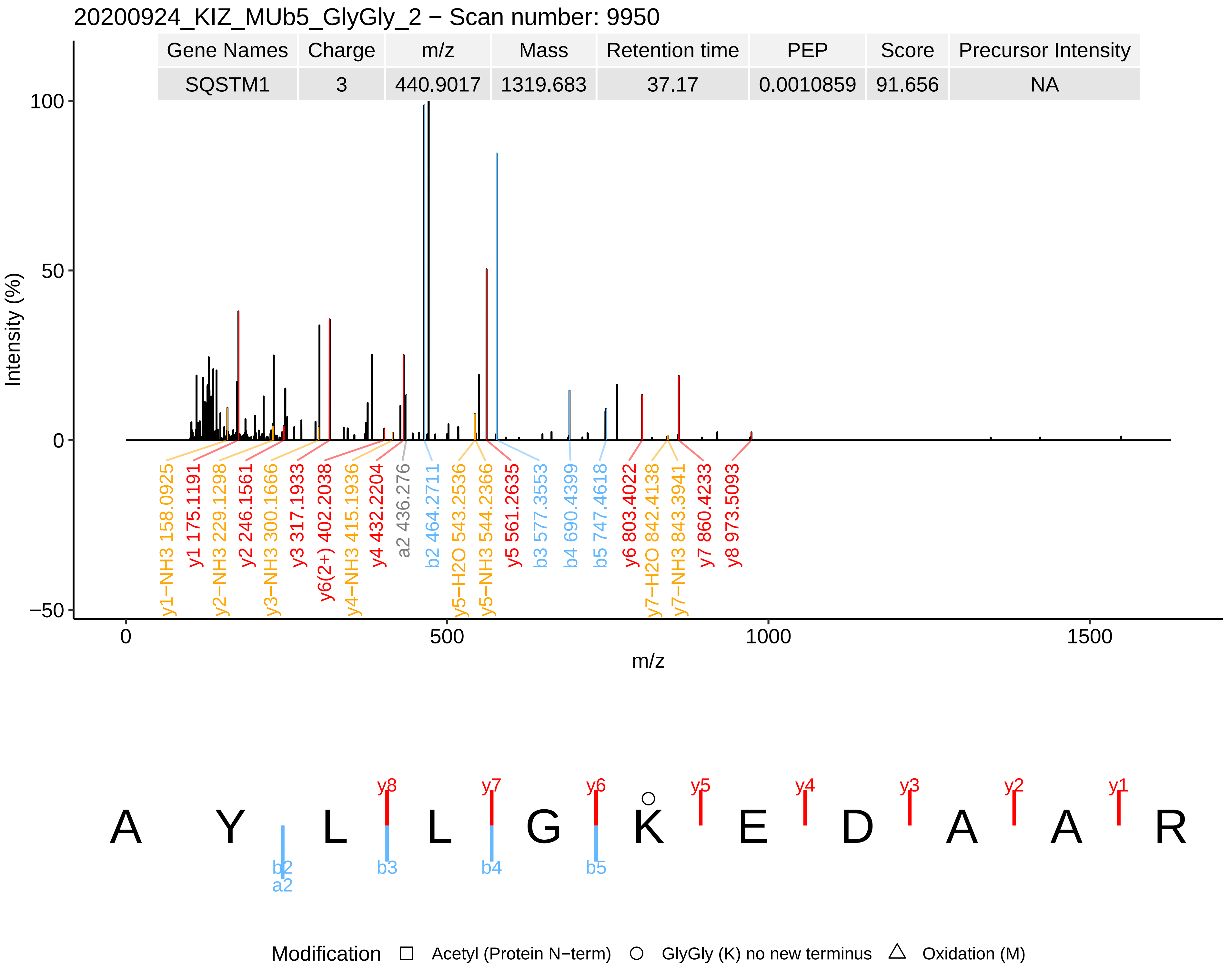
20200924_KIZ_MUb5_GlyGly_2 − Scan number: 9950
Gene Names | Charge | m/z | Mass | Retention time | PEP | Score | Precursor Intensity
SQSTM1 | 3 | 440.9017 | 1319.683 | 37.17 | 0.0010859 | 91.656 | NA
Intensity (%)
m/z
y1−NH3 158.0925
y1 175.1191
y2−NH3 229.1298
y2 246.1561
y3−NH3 300.1666
y3 317.1933
y6(2+) 402.2038
y4−NH3 415.1936
y4 432.2204
a2 436.276
b2 464.2711
y5−H2O 543.2536
y5−NH3 544.2366
y5 561.2635
b3 577.3553
b4 690.4399
b5 747.4618
y6 803.4022
y7−H2O 842.4138
y7−NH3 843.3941
y7 860.4233
y8 973.5093
A Y L L G K E D A A R
y8 y7 y6 y5 y4 y3 y2 y1
b2 a2 b3 b4 b5
Modification
Acetyl (Protein N−term)
GlyGly (K) no new terminus
Oxidation (M)

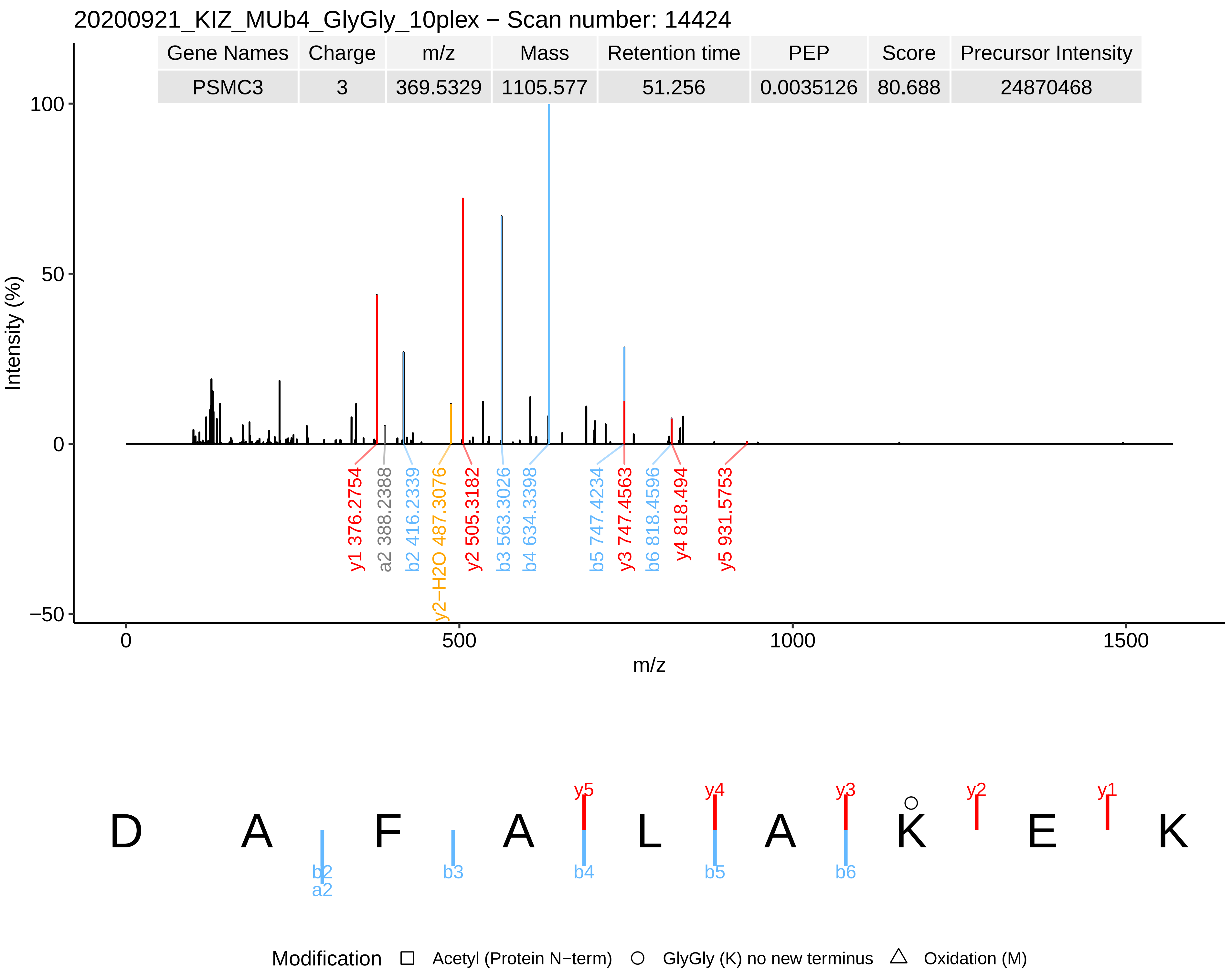

20200921_KIZ_MUb4_GlyGly_10plex − Scan number: 14424
Gene Names | Charge | m/z | Mass | Retention time | PEP | Score | Precursor Intensity
PSMC3 | 3 | 369.5329 | 1105.577 | 51.256 | 0.0035126 | 80.688 | 24870468
Intensity (%)
100
50
0
−50
0
500
1000
1500
m/z
y1 376.2754
a2 388.2388
b2 416.2339
y2−H2O 487.3076
y2 505.3182
b3 563.3026
b4 634.3398
b5 747.4234
y3 747.4563
b6 818.4596
y4 818.494
y5 931.5753
D A F A L A K E K
y5 y4 y3 y2 y1
b2 a2 b3 b4 b5 b6
Modification
Acetyl (Protein N−term)
GlyGly (K) no new terminus
Oxidation (M)

20200921_KIZ_MUb4_GlyGly_10plex_2 − Scan number: 13300

Gene Names	Charge	m/z	Mass	Retention time	PEP	Score	Precursor Intensity
PSMC3	3	369.5329	1105.577	51.606	0.022029	63.216	3162742

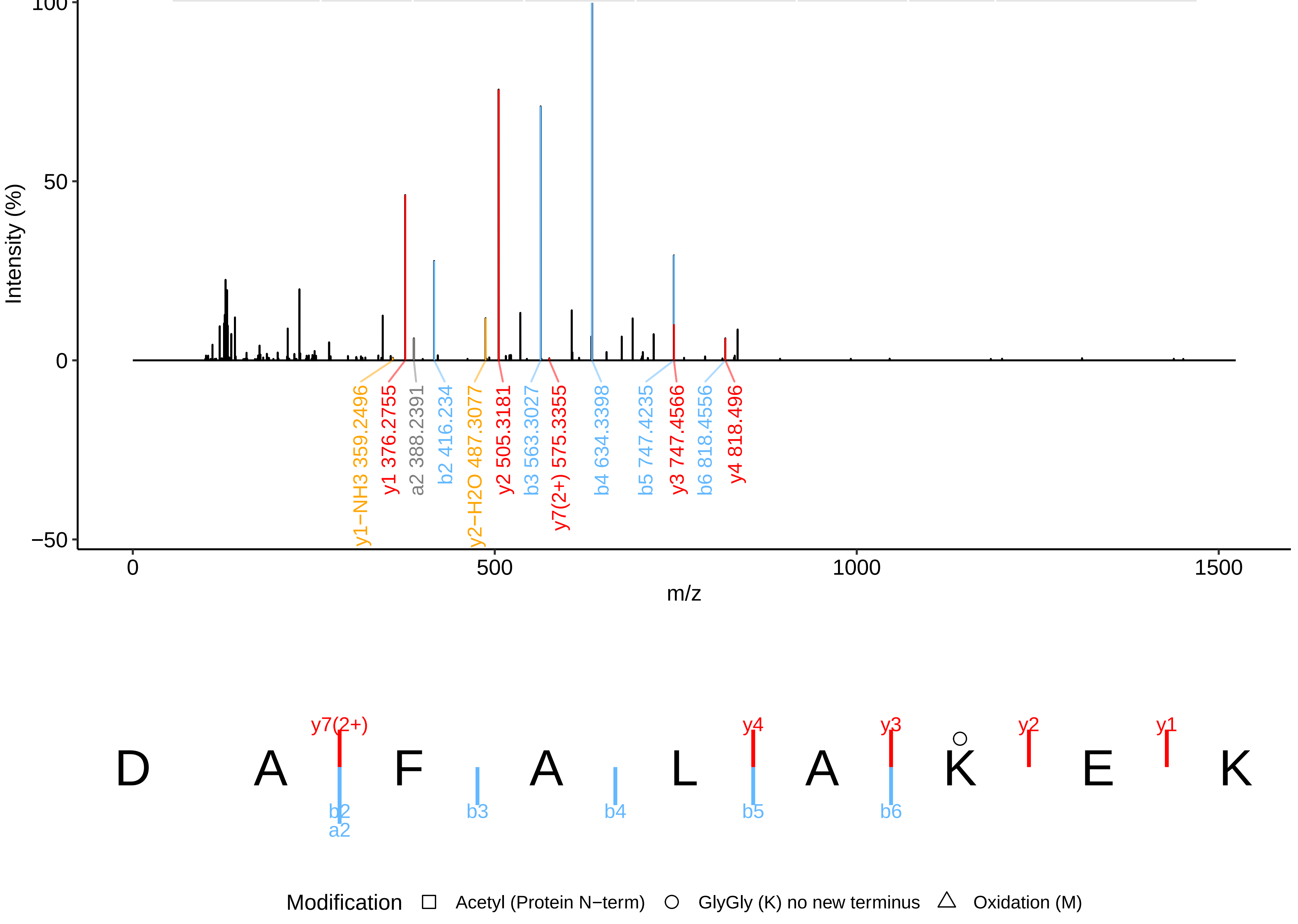

20200924_KIZ_MUb5_GlyGly_1 − Scan number: 13324

Gene Names	Charge	m/z	Mass	Retention time	PEP	Score	Precursor Intensity
PSMC3	3	369.5329	1105.577	43.423	0.029854	61.56	10561615

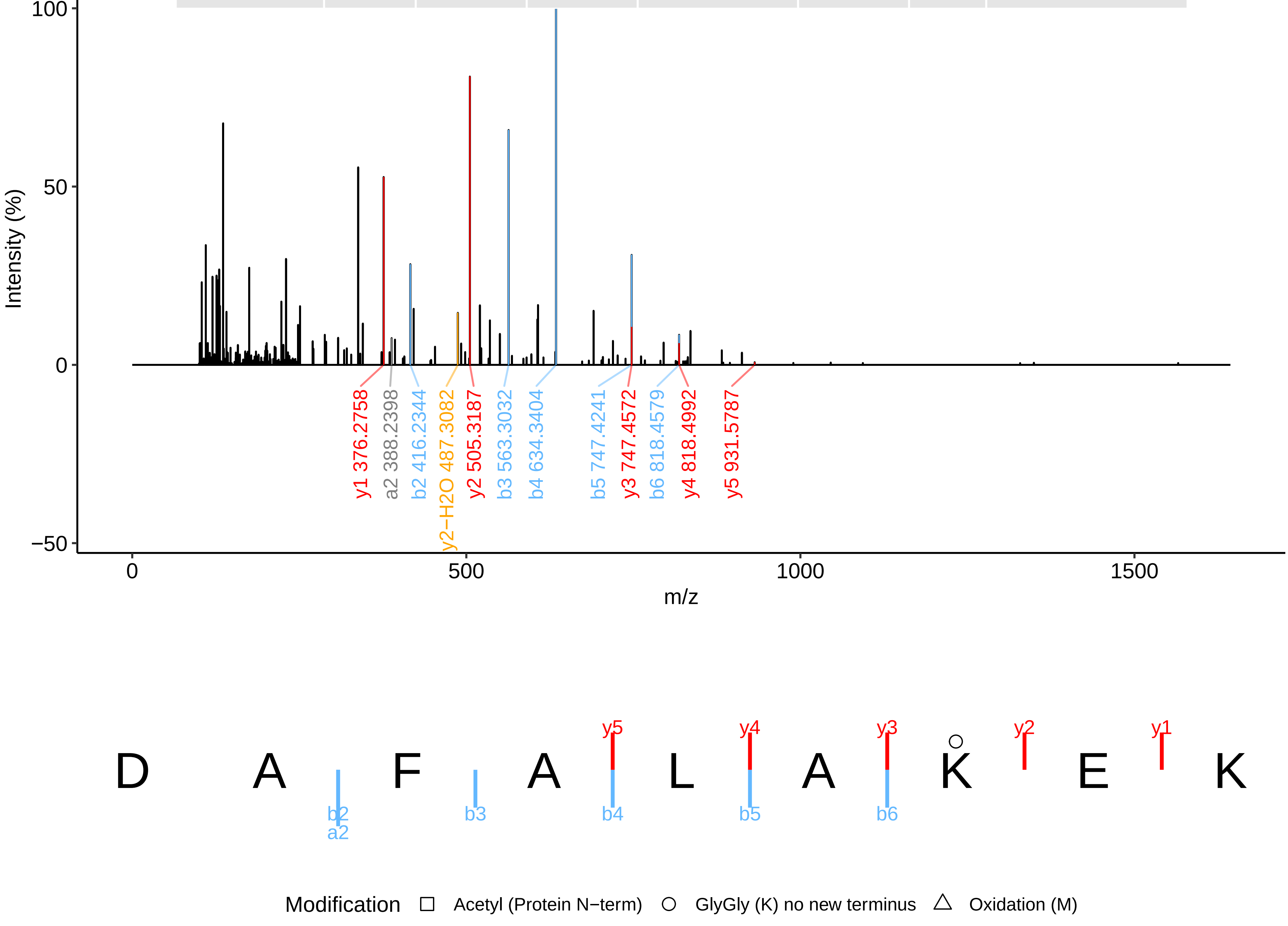

20200921_KIZ_MUb4_GlyGly_10plex − Scan number: 12781

Gene Names	Charge	m/z	Mass	Retention time	PEP	Score	Precursor Intensity
SLC25A6;SLC25A5	3	371.5172	1111.53	44.934	0.021151	64.04	NA

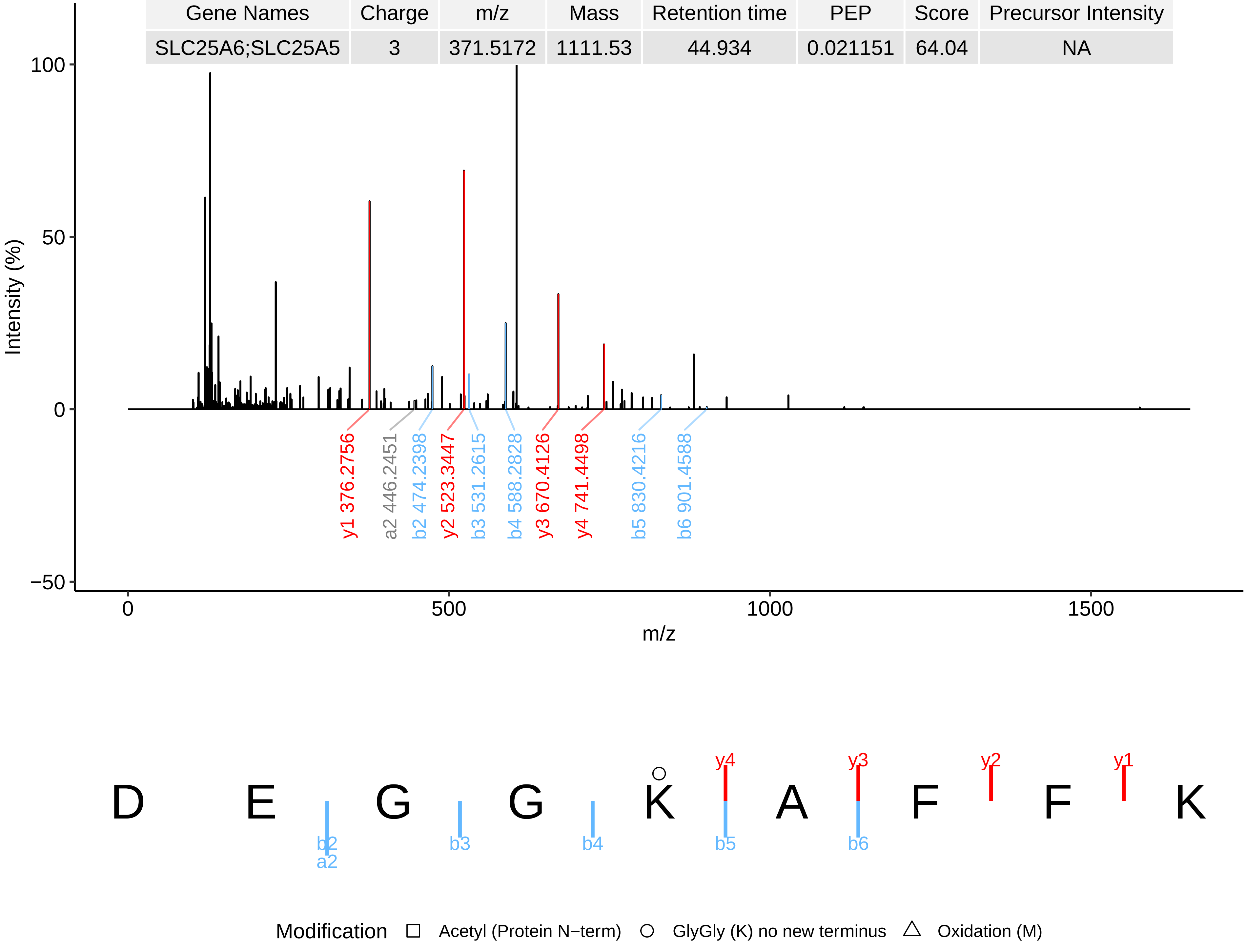

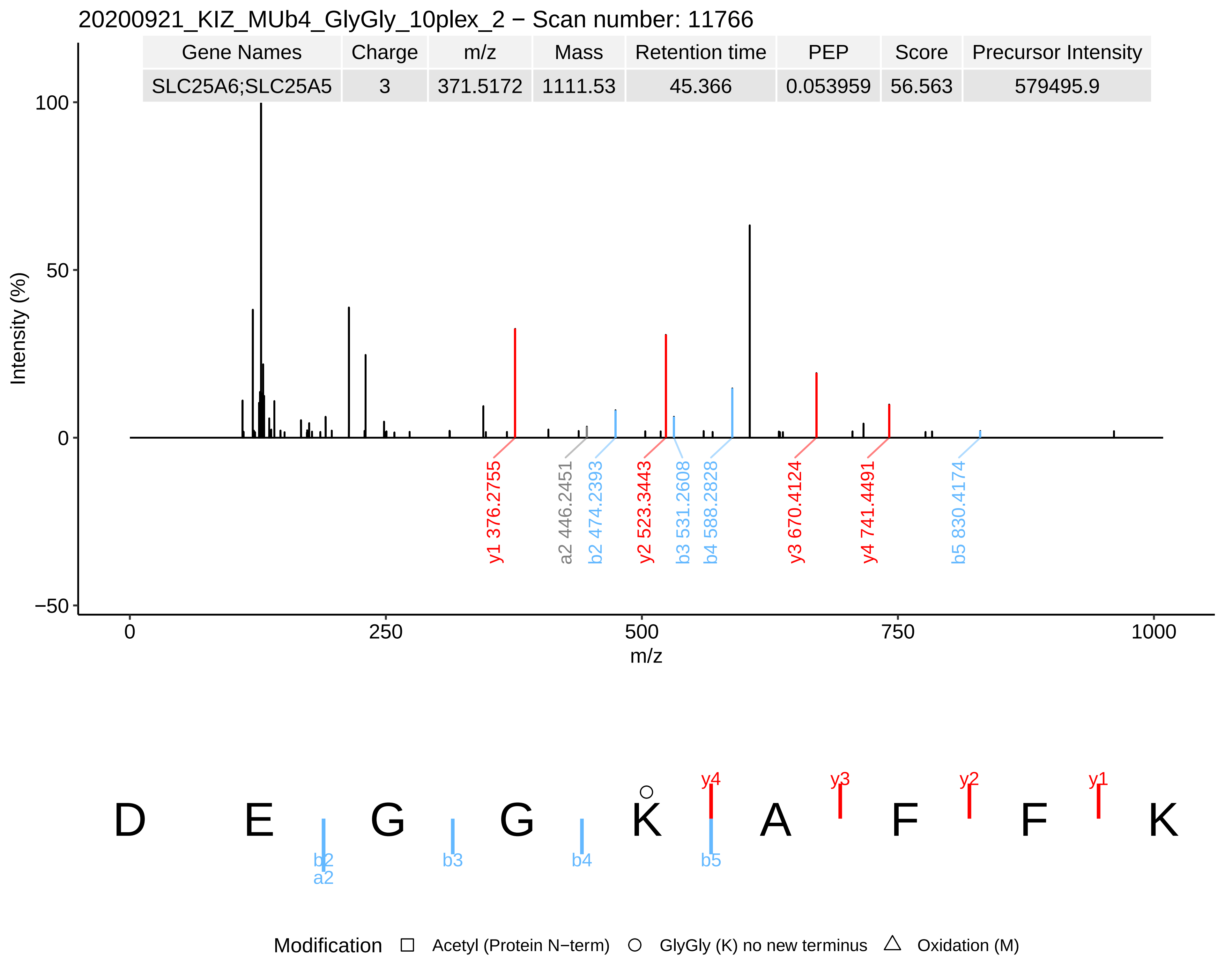

20200921_KIZ_MUb4_GlyGly_10plex_2 − Scan number: 11766
Gene Names
Charge
m/z
Mass
Retention time
PEP
Score
Precursor Intensity
SLC25A6;SLC25A5
3
371.5172
1111.53
45.366
0.053959
56.563
579495.9
Intensity (%)
m/z
y1 376.2755
a2 446.2451
b2 474.2393
y2 523.3443
b3 531.2608
b4 588.2828
y3 670.4124
y4 741.4491
b5 830.4174
D E G G K A F F K
Modification
Acetyl (Protein N−term)
GlyGly (K) no new terminus
Oxidation (M)

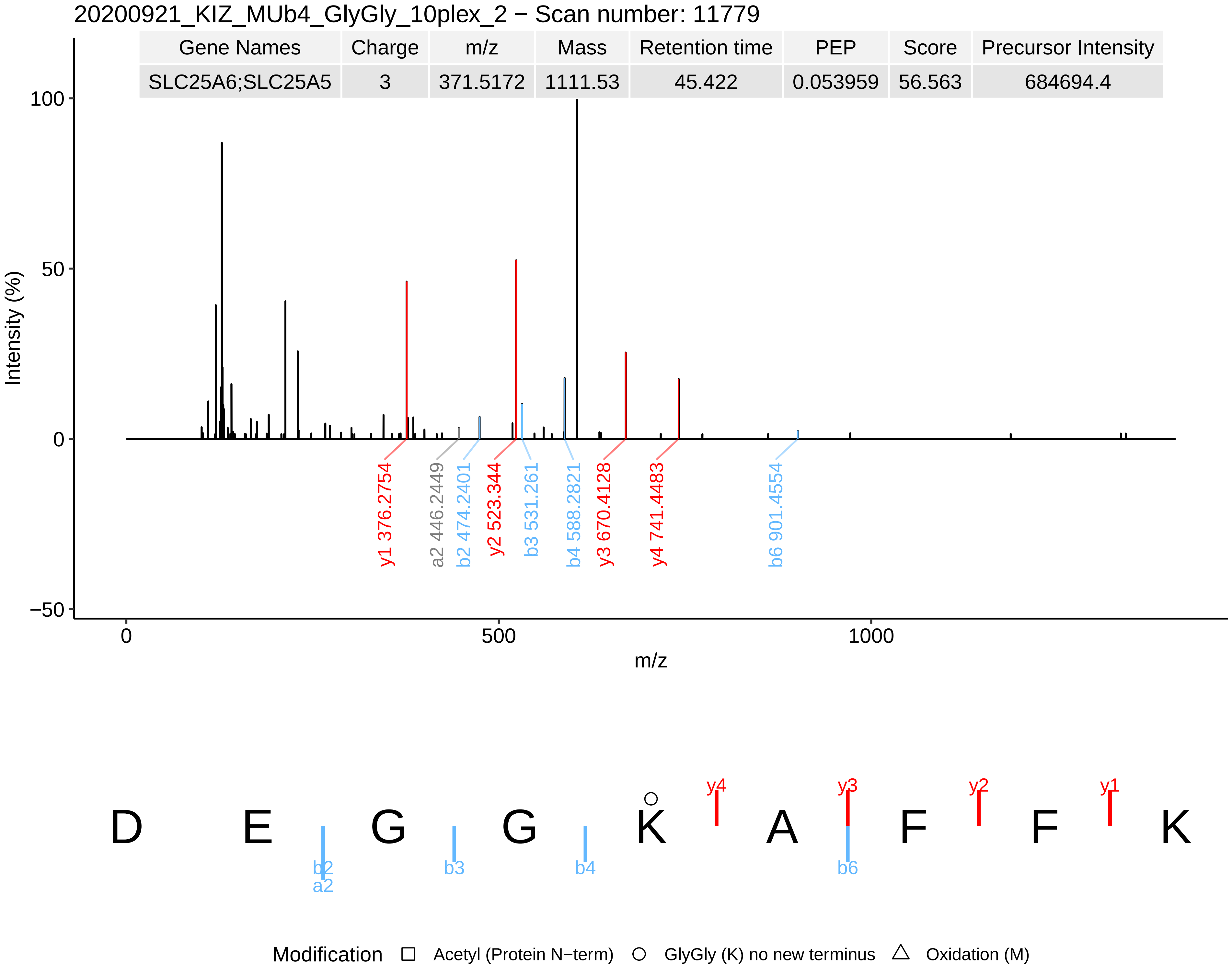

20200921_KIZ_MUb4_GlyGly_10plex_2 − Scan number: 11779
Gene Names | Charge | m/z | Mass | Retention time | PEP | Score | Precursor Intensity
SLC25A6;SLC25A5 | 3 | 371.5172 | 1111.53 | 45.422 | 0.053959 | 56.563 | 684694.4
Intensity (%)
m/z
y1 376.2754
a2 446.2449
b2 474.2401
y2 523.344
b3 531.261
b4 588.2821
y3 670.4128
y4 741.4483
b6 901.4554
D E G G K A F F K
y4 y3 y2 y1
b2 a2 b3 b4 b6
Modification
Acetyl (Protein N−term)
GlyGly (K) no new terminus
Oxidation (M)

20200921_KIZ_MUb4_GlyGly_10plex − Scan number: 10460

Gene Names	Charge	m/z	Mass	Retention time	PEP	Score	Precursor Intensity
WRNIP1	3	372.1891	1113.545	36.219	0.005734	78.516	10851176

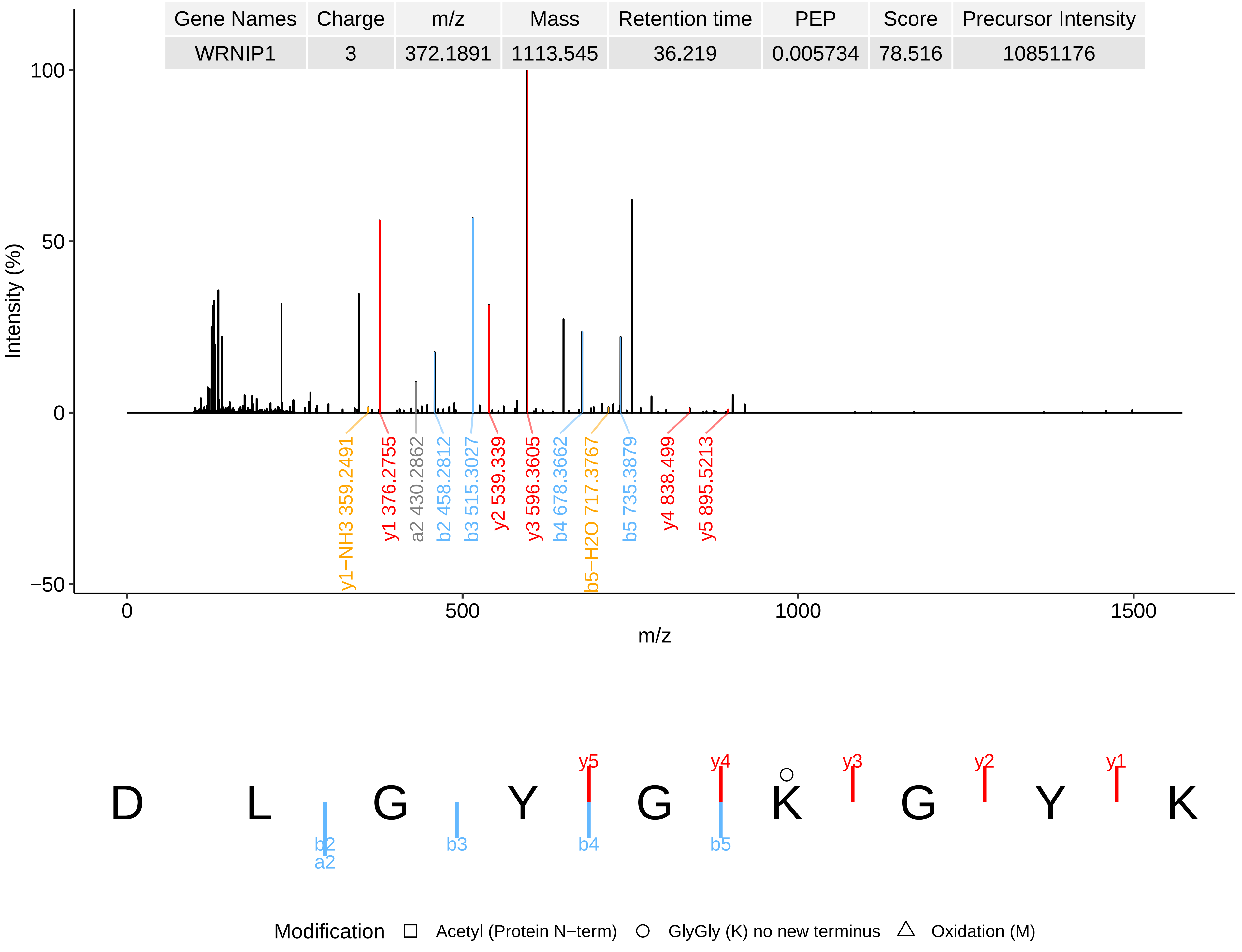

20200921_KIZ_MUb4_GlyGly_10plex_2 − Scan number: 9650

Gene Names	Charge	m/z	Mass	Retention time	PEP	Score	Precursor Intensity
WRNIP1	3	372.1891	1113.545	36.754	0.049007	57.59	2284243

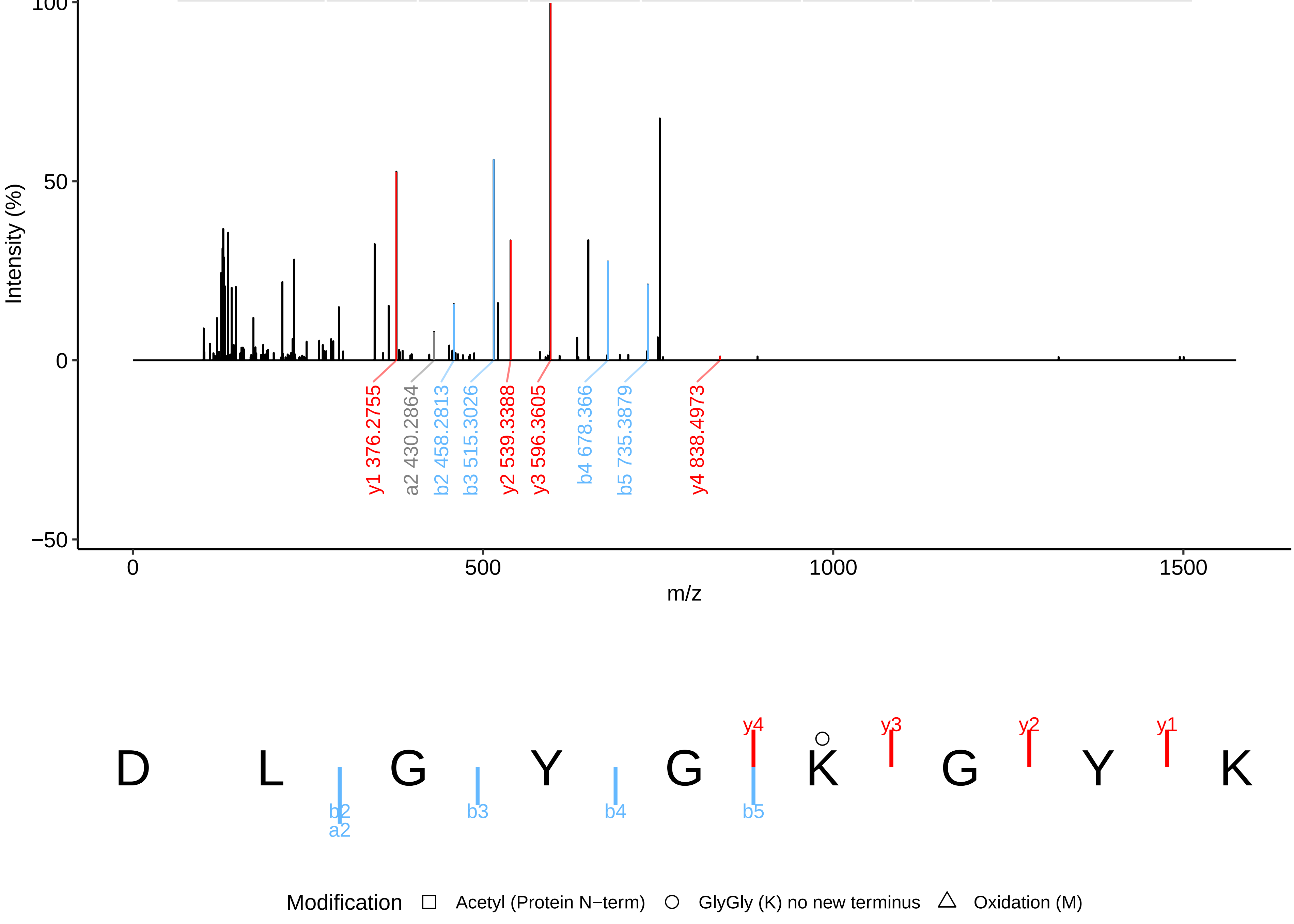

20200924_KIZ_MUb5_GlyGly_1 − Scan number: 9465

Gene Names	Charge	m/z	Mass	Retention time	PEP	Score	Precursor Intensity
WRNIP1	3	372.1891	1113.545	30.424	0.047709	57.859	6640488

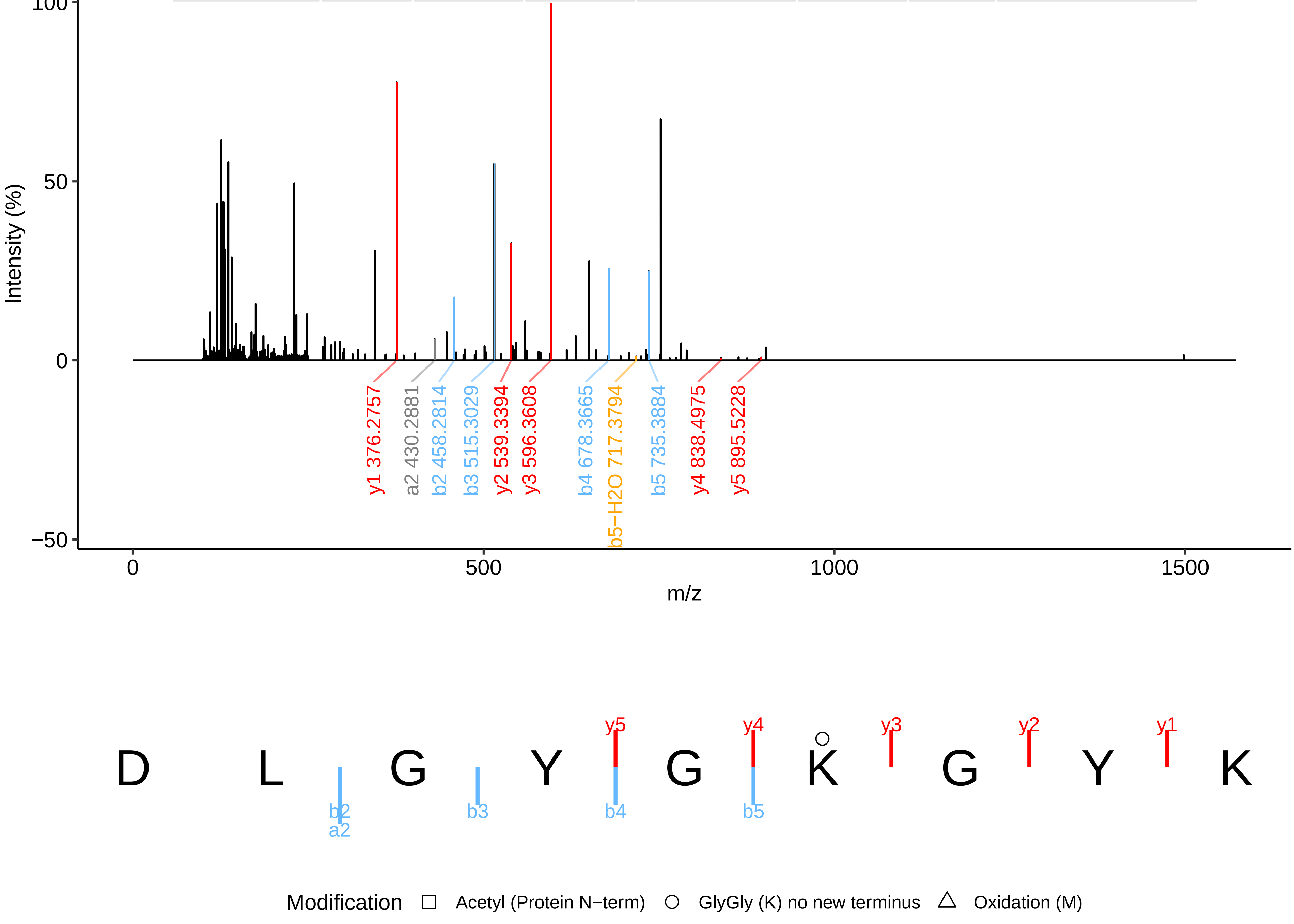

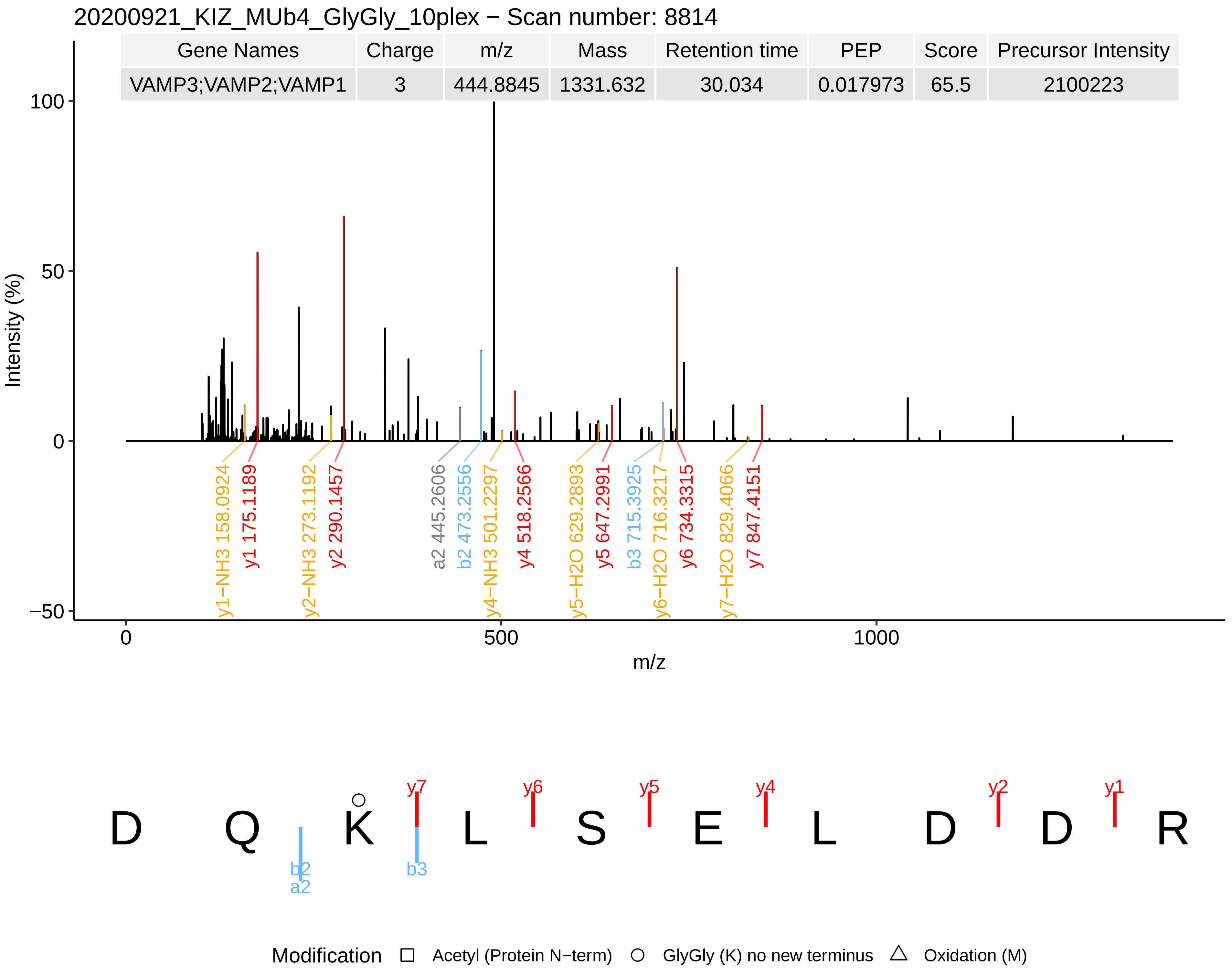
20200921_KIZ_MUb4_GlyGly_10plex − Scan number: 8814
Gene Names | Charge | m/z | Mass | Retention time | PEP | Score | Precursor Intensity
VAMP3;VAMP2;VAMP1 | 3 | 444.8845 | 1331.632 | 30.034 | 0.017973 | 65.5 | 2100223
Intensity (%)
100
50
0
−50
0
500
1000
m/z
y1−NH3 158.0924
y1 175.1189
y2−NH3 273.1192
y2 290.1457
a2 445.2606
b2 473.2556
y4−NH3 501.2297
y4 518.2566
y5−H2O 629.2893
y5 647.2991
b3 715.3925
y6−H2O 716.3217
y6 734.3315
y7−H2O 829.4066
y7 847.4151
D Q K L S E L D D R
y7 y6 y5 y4 y2 y1
b2 a2 b3
Modification
Acetyl (Protein N−term)
GlyGly (K) no new terminus
Oxidation (M)

20200921_KIZ_MUb4_GlyGly_10plex_2 − Scan number: 8113

Gene Names	Charge	m/z	Mass	Retention time	PEP	Score	Precursor Intensity
VAMP3;VAMP2;VAMP1	3	444.8845	1331.632	30.511	0.076479	52.247	772660.6

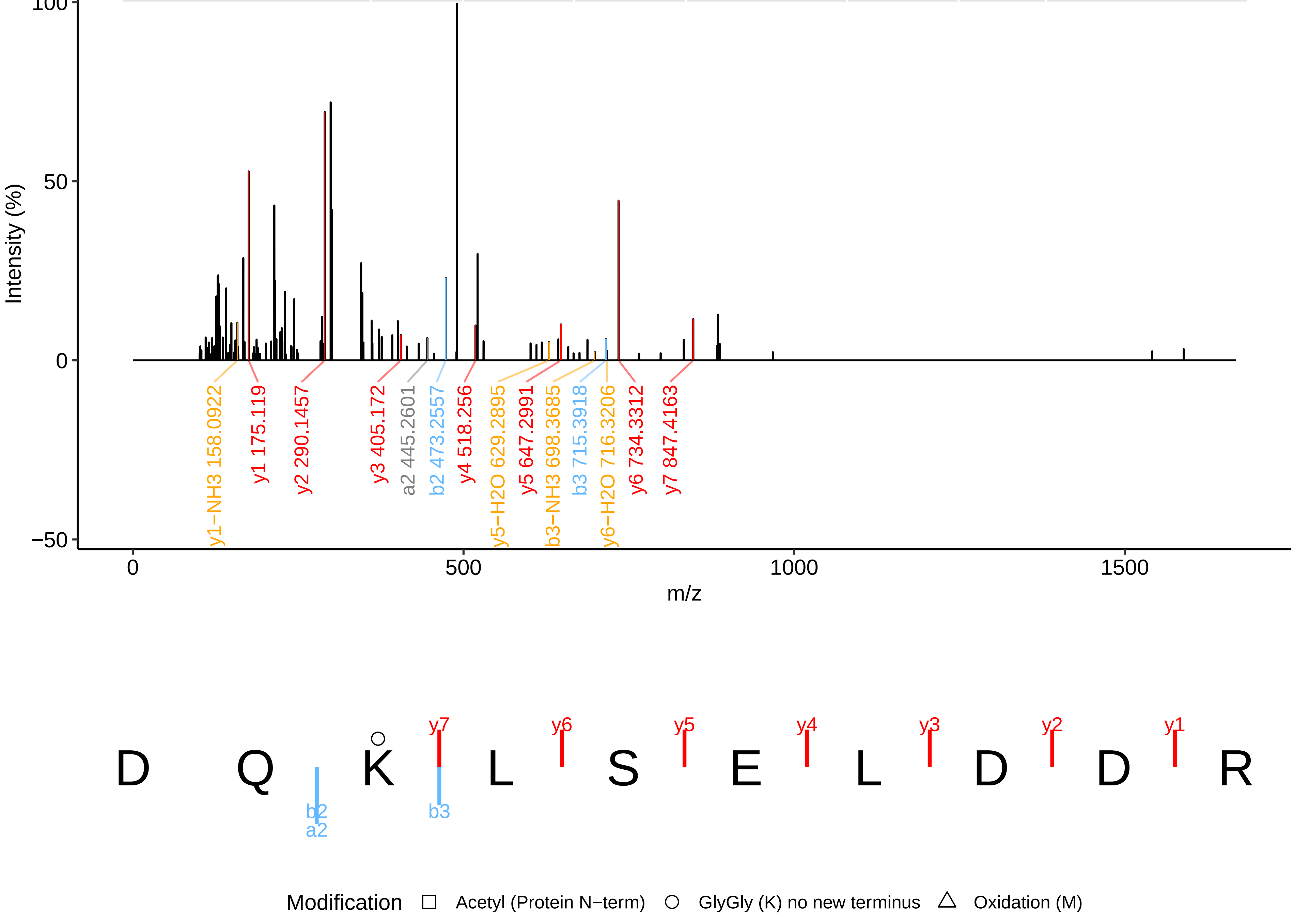

20200921_KIZ_MUb4_GlyGly_10plex − Scan number: 7929

Gene Names	Charge	m/z	Mass	Retention time	PEP	Score	Precursor Intensity
EHMT1	3	378.5314	1132.572	26.779	0.00046815	101.25	8752183

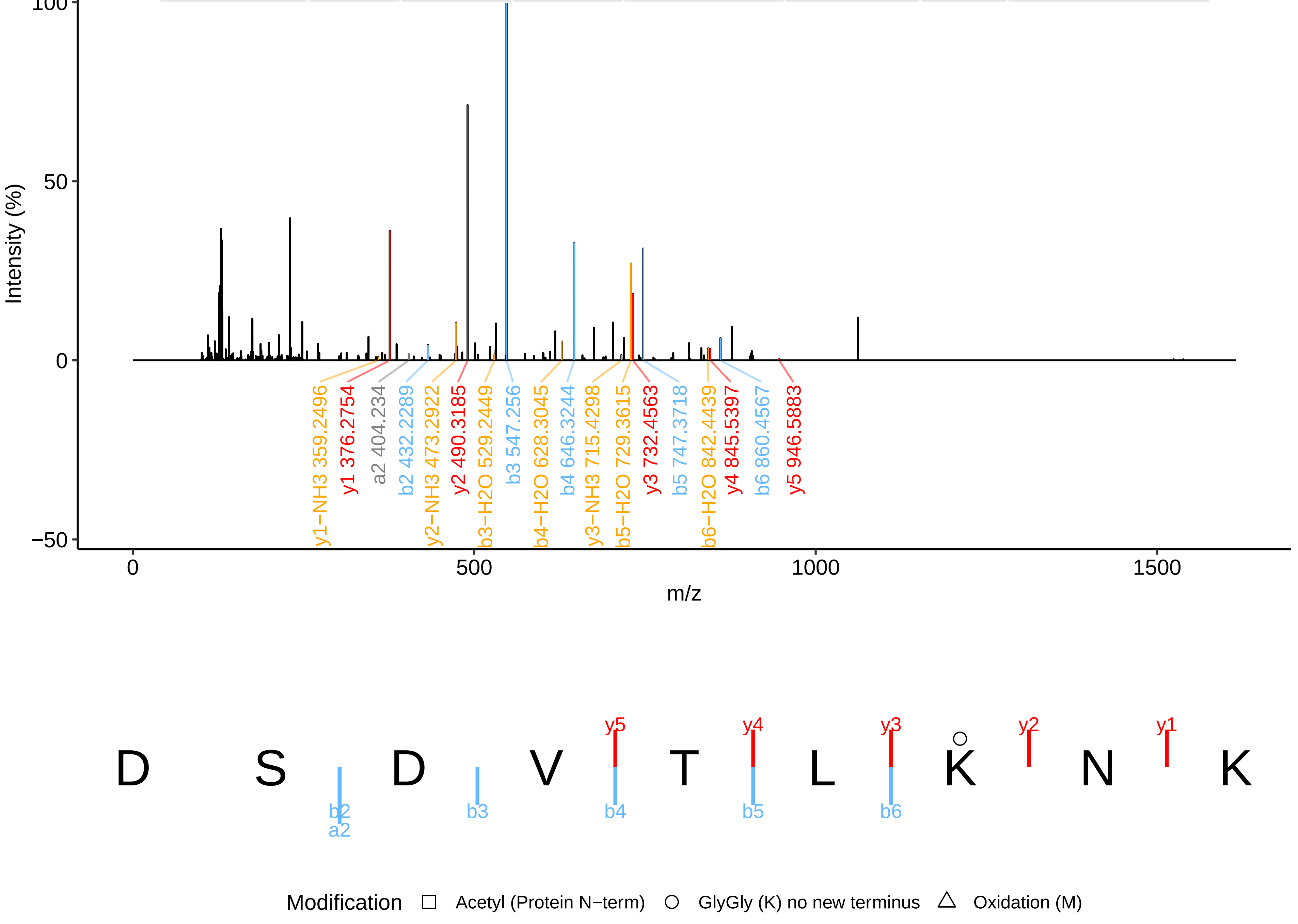

20200924_KIZ_MUb5_GlyGly_1 − Scan number: 6996

Gene Names	Charge	m/z	Mass	Retention time	PEP	Score	Precursor Intensity
EHMT1	3	378.5314	1132.572	22.131	0.0032633	92.062	7951946

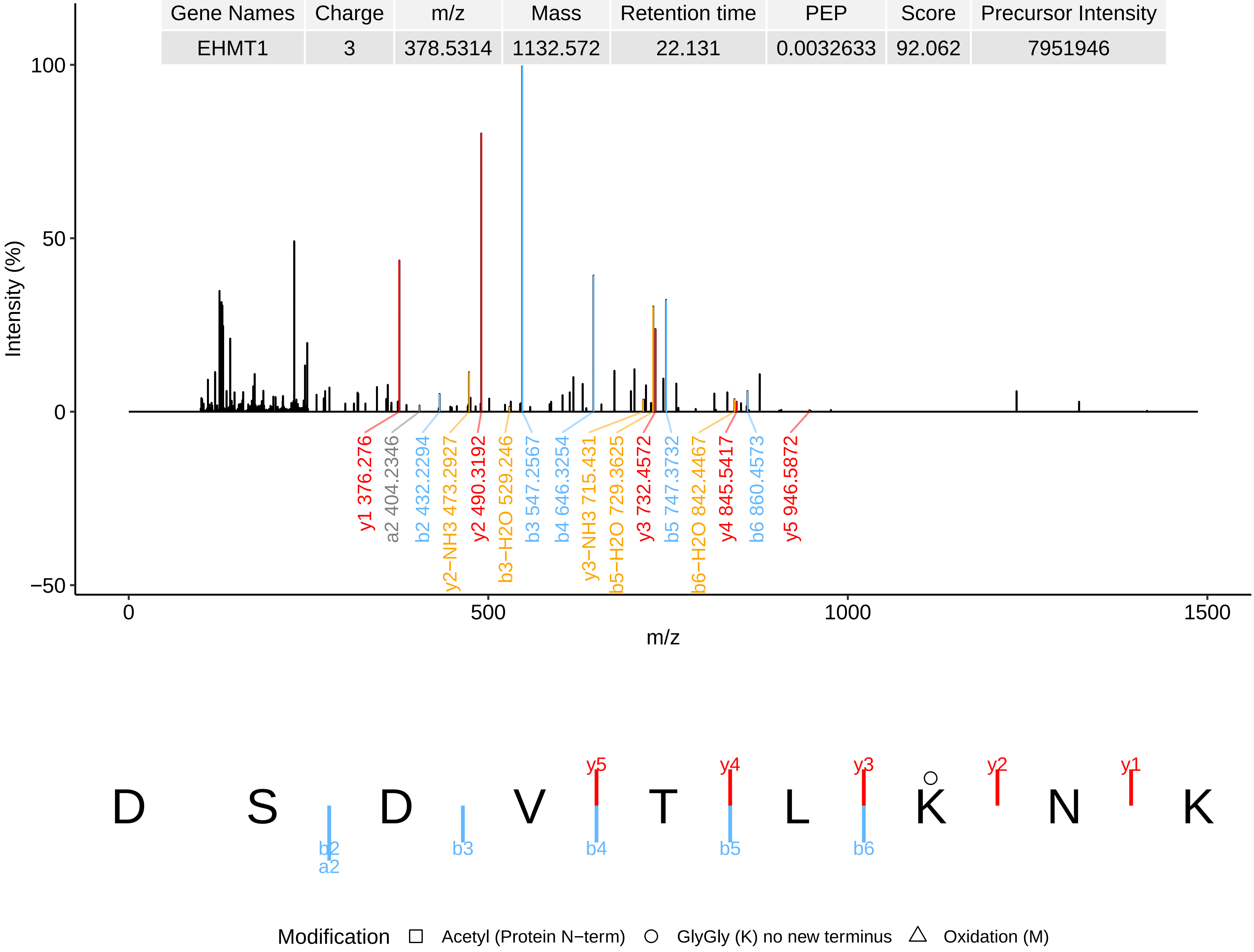

20200924_KIZ_MUb5_GlyGly_2 − Scan number: 6476

Gene Names	Charge	m/z	Mass	Retention time	PEP	Score	Precursor Intensity
EHMT1	3	378.5314	1132.572	24.153	0.0087877	78.516	2697728

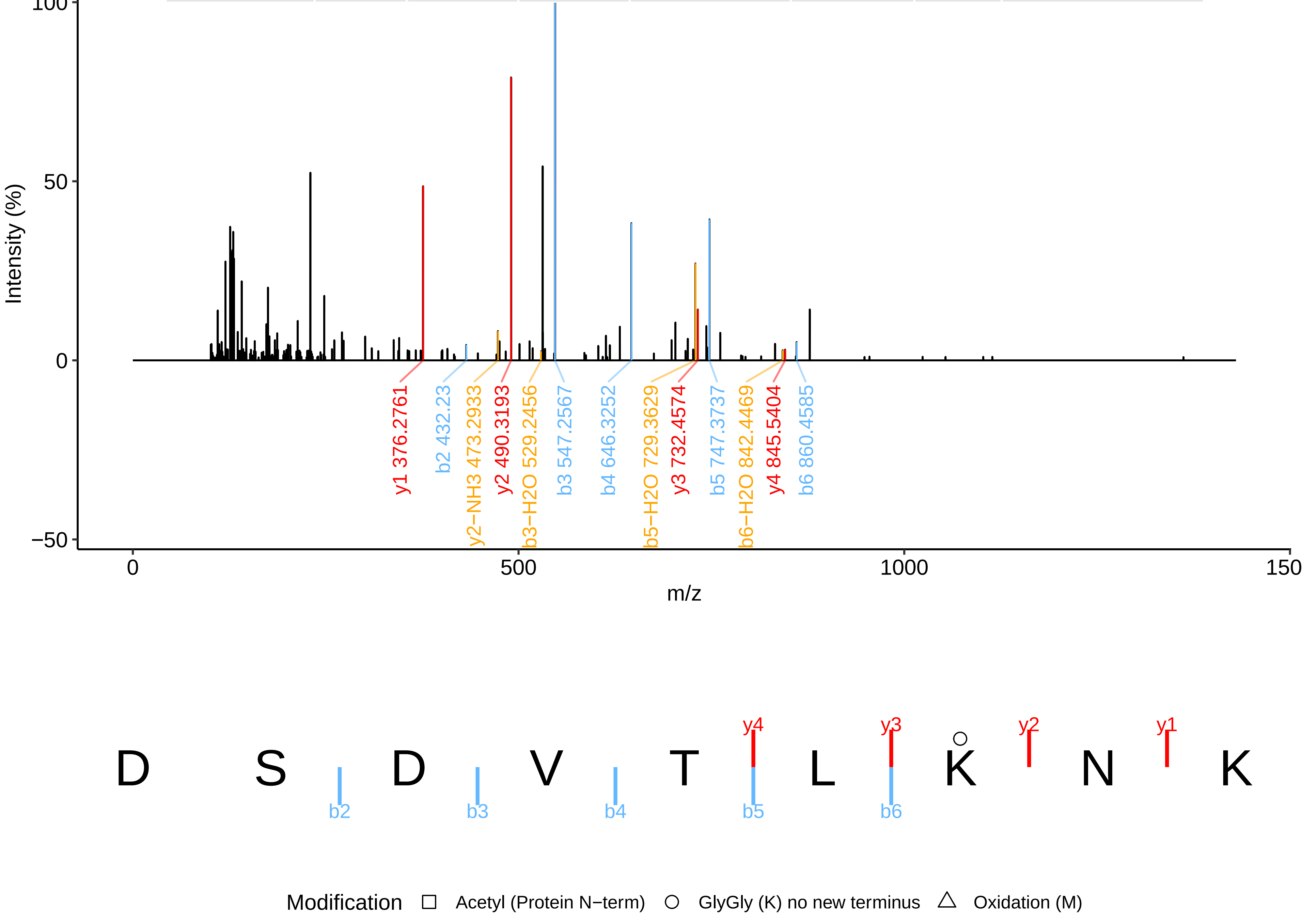

20200921_KIZ_MUb4_GlyGly_10plex_2 − Scan number: 3443

Gene Names	Charge	m/z	Mass	Retention time	PEP	Score	Precursor Intensity
POLD1	3	452.2164	1353.627	11.581	0.00023087	110.08	1293097

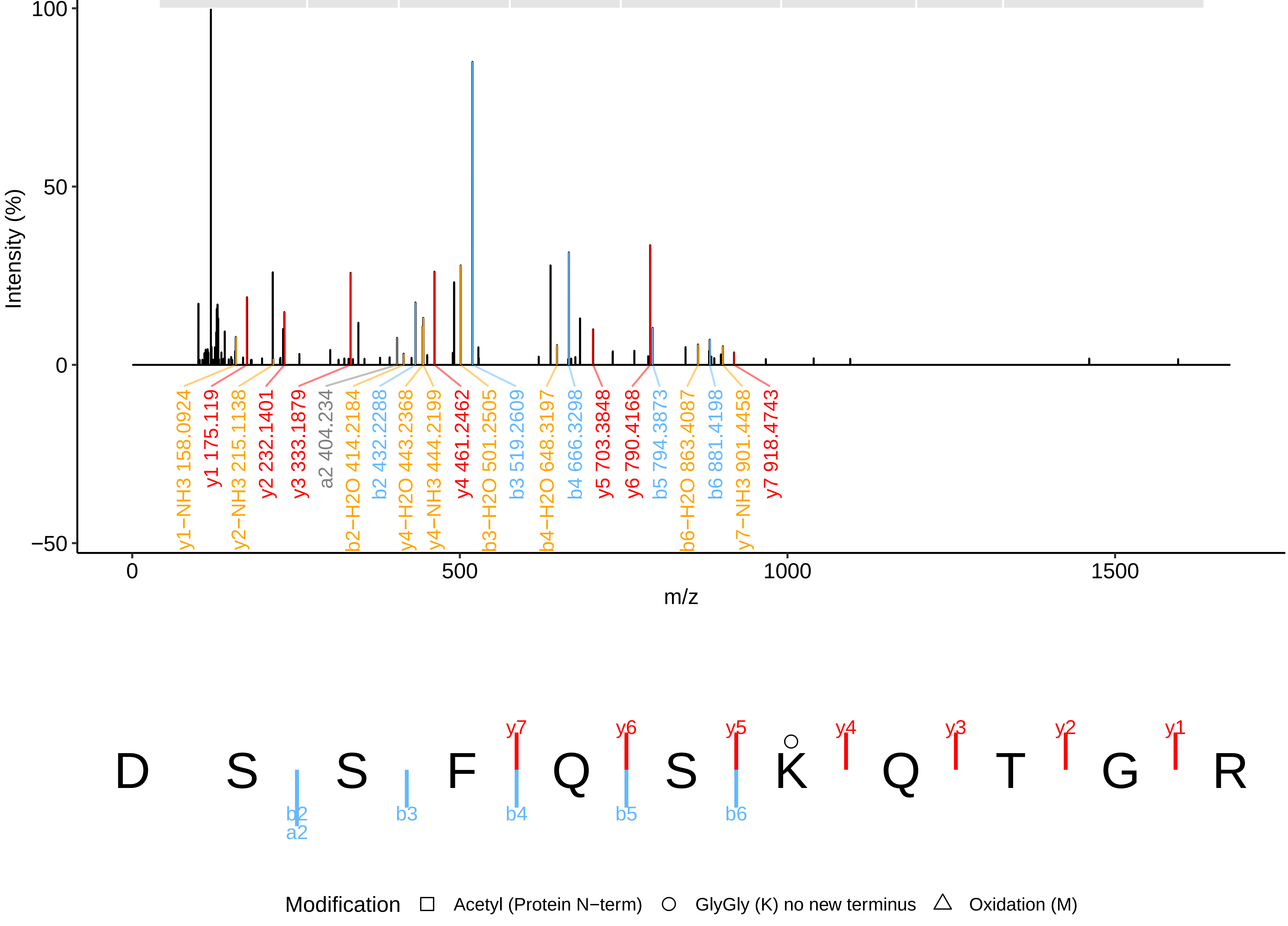

20200924_KIZ_MUb5_GlyGly_1 − Scan number: 3293

Gene Names	Charge	m/z	Mass	Retention time	PEP	Score	Precursor Intensity
POLD1	3	452.2164	1353.627	10.123	0.011654	72.187	NA

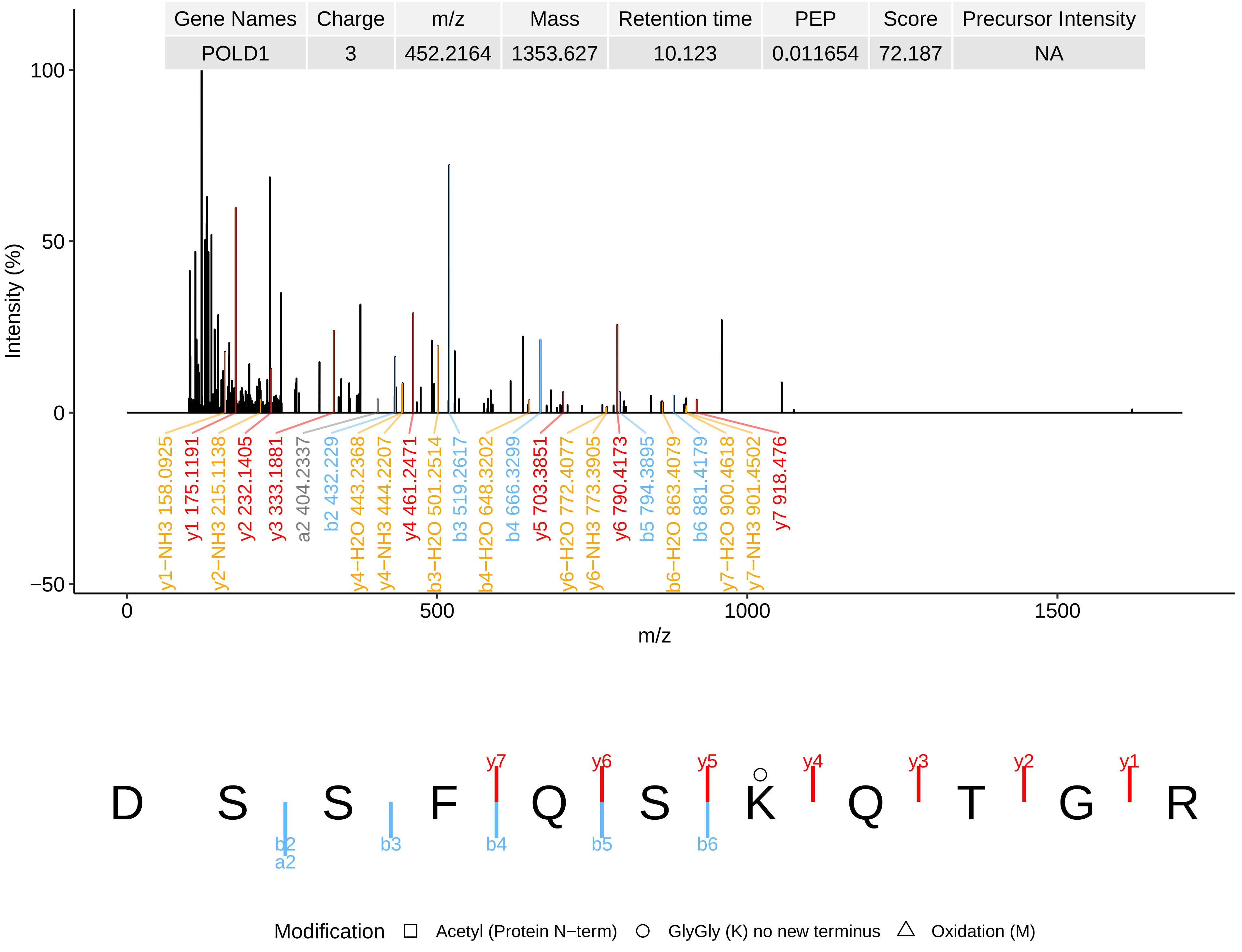

20200921_KIZ_MUb4_GlyGly_10plex − Scan number: 26719

Gene Names	Charge	m/z	Mass	Retention time	PEP	Score	Precursor Intensity
UBFD1	2	685.8406	1369.667	97.386	0.026377	75.738	7475614

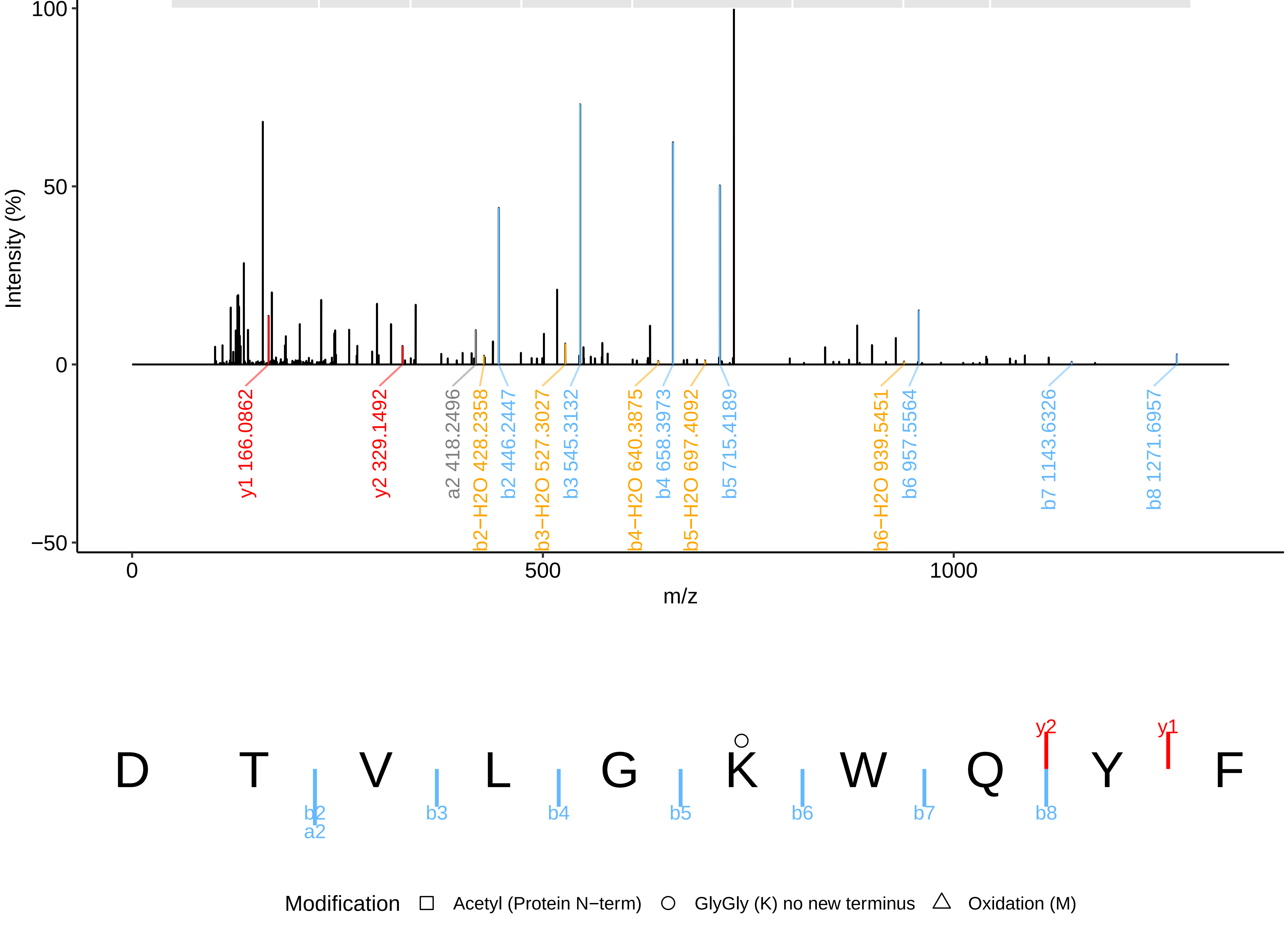

20200921_KIZ_MUb4_GlyGly_10plex − Scan number: 26857

Gene Names	Charge	m/z	Mass	Retention time	PEP	Score	Precursor Intensity
UBFD1	2	685.8406	1369.667	97.911	0.04015	71.877	11038040

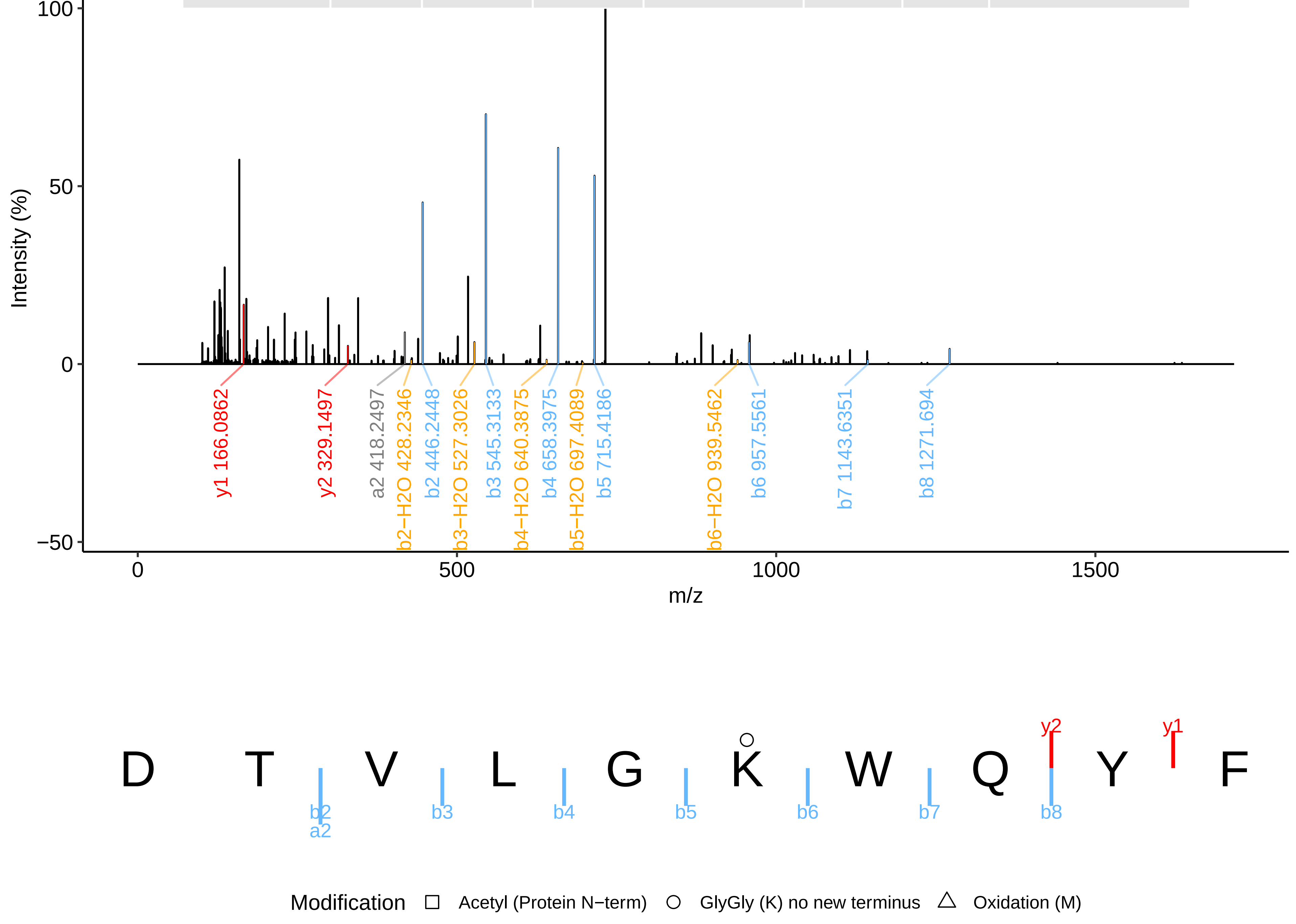

20200924_KIZ_MUb5_GlyGly_1 − Scan number: 27090

Gene Names	Charge	m/z	Mass	Retention time	PEP	Score	Precursor Intensity
UBFD1	2	685.8406	1369.667	86.627	0.041493	71.501	15553624

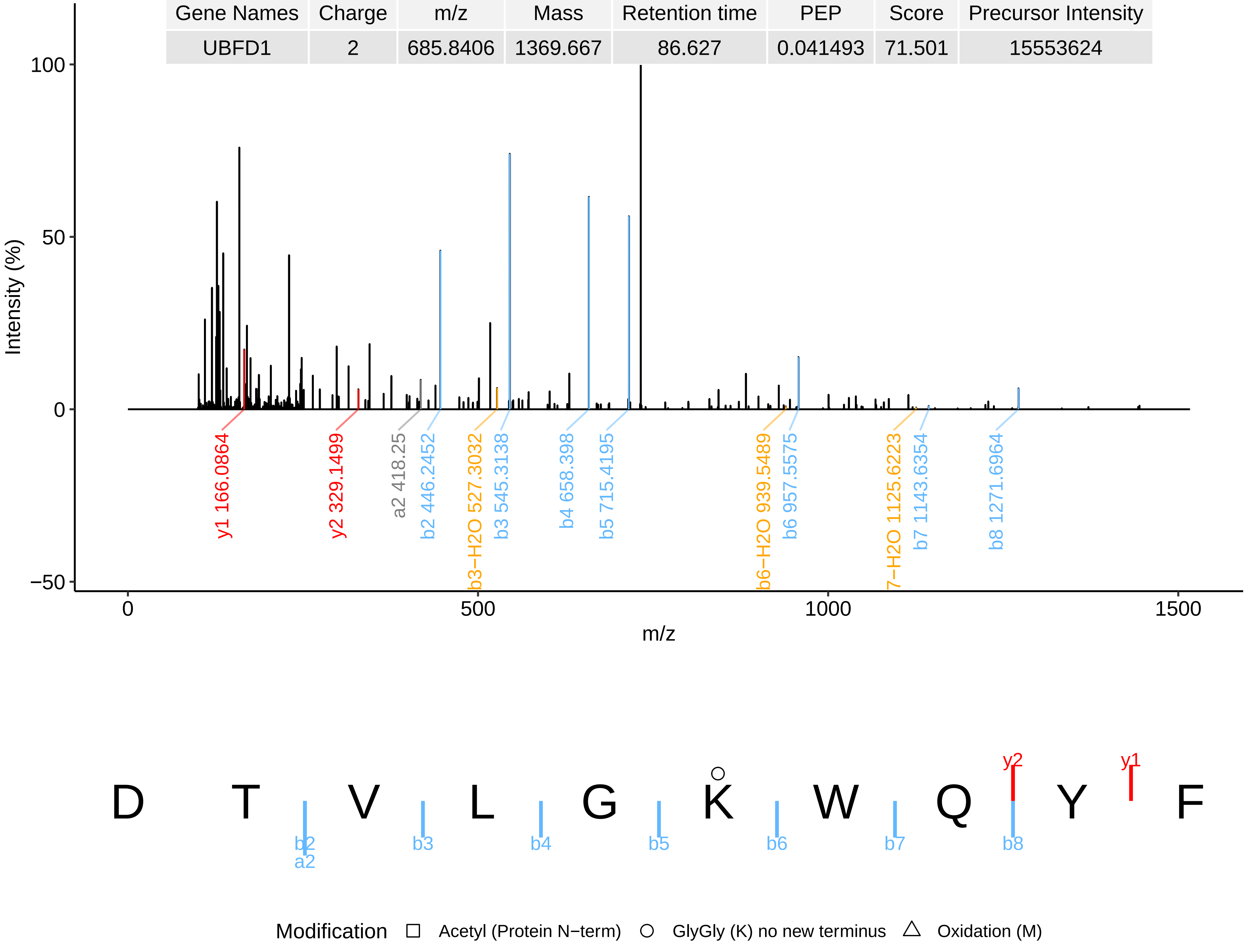

20200924_KIZ_MUb5_GlyGly_1 − Scan number: 27257

Gene Names	Charge	m/z	Mass	Retention time	PEP	Score	Precursor Intensity
UBFD1	2	685.8406	1369.667	87.149	0.0011498	96.253	25452646

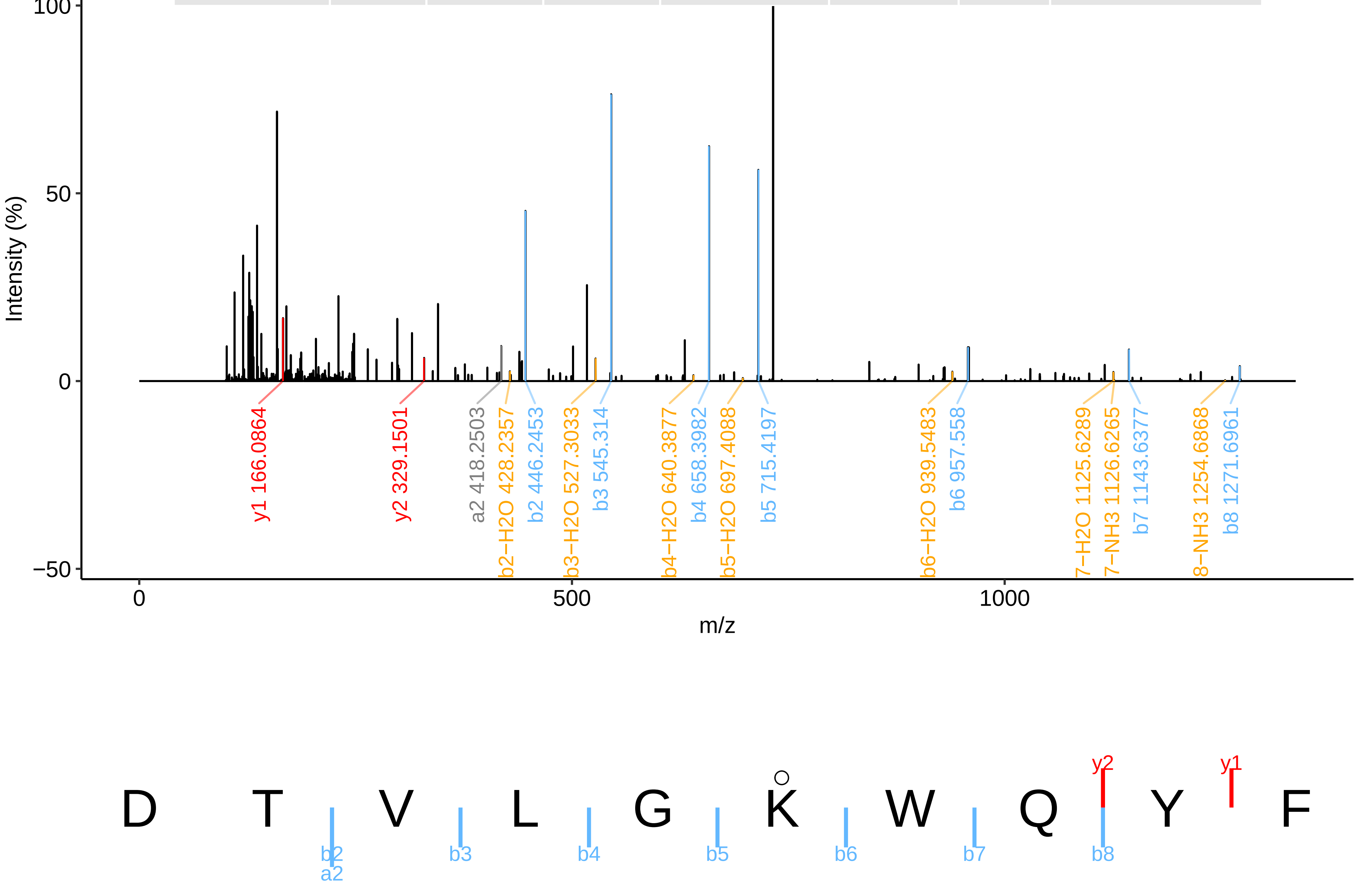

Modification □ Acetyl (Protein N−term) ○ GlyGly (K) no new terminus △ Oxidation (M)

20200924_KIZ_MUb5_GlyGly_2 − Scan number: 24265

Gene Names	Charge	m/z	Mass	Retention time	PEP	Score	Precursor Intensity
UBFD1	2	685.8406	1369.667	89.902	0.060857	66.073	5940522

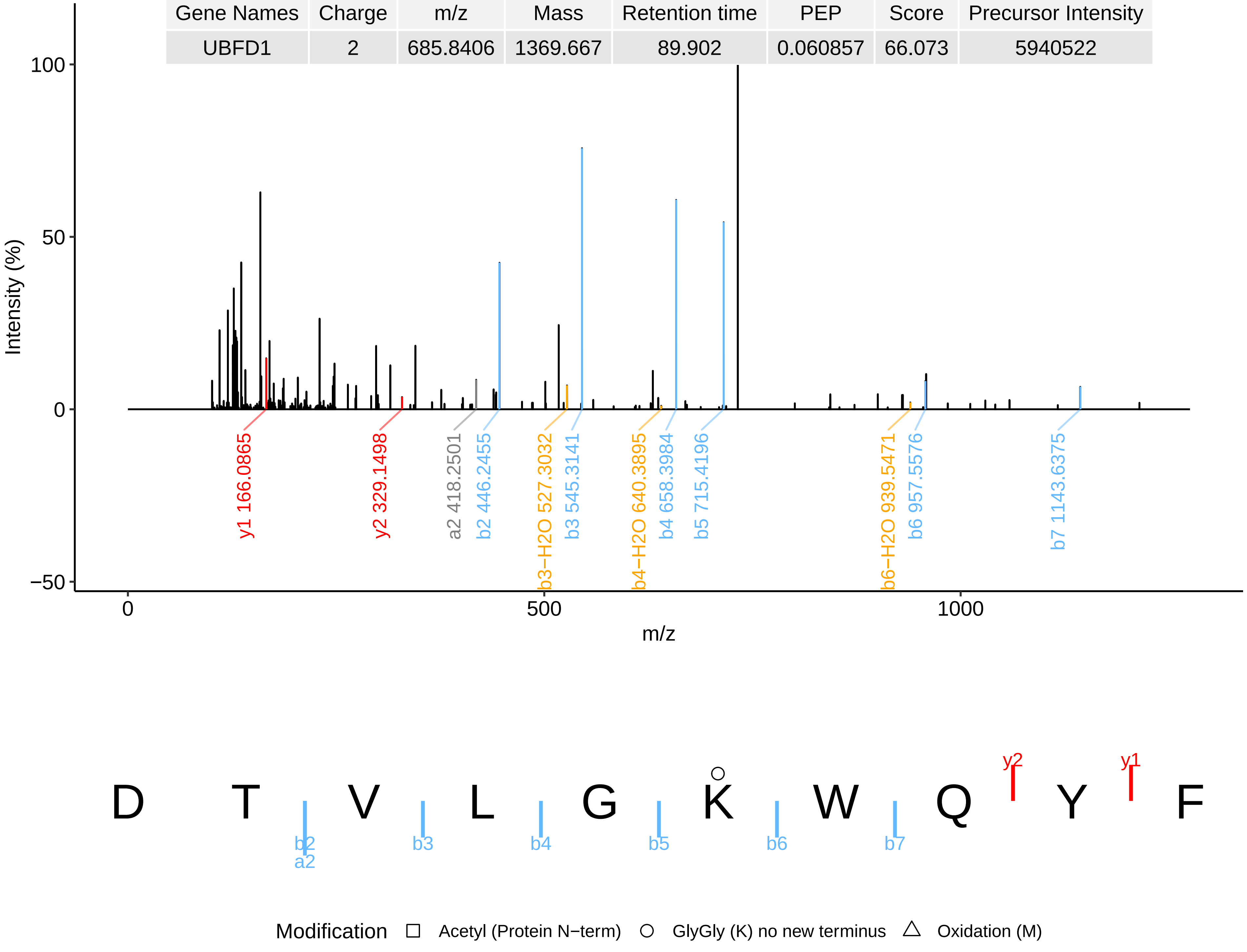

20200924_KIZ_MUb5_GlyGly_2 − Scan number: 24404

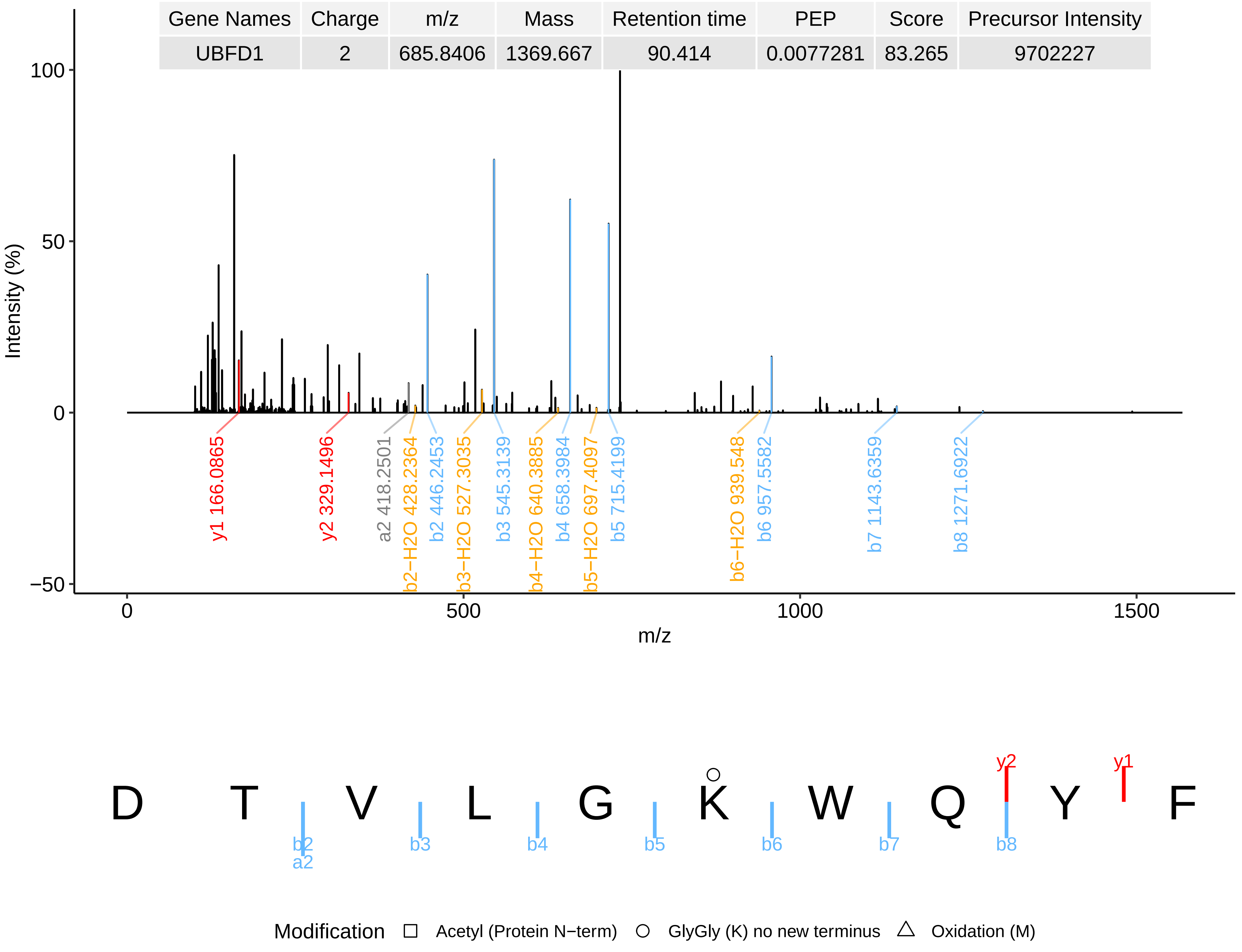

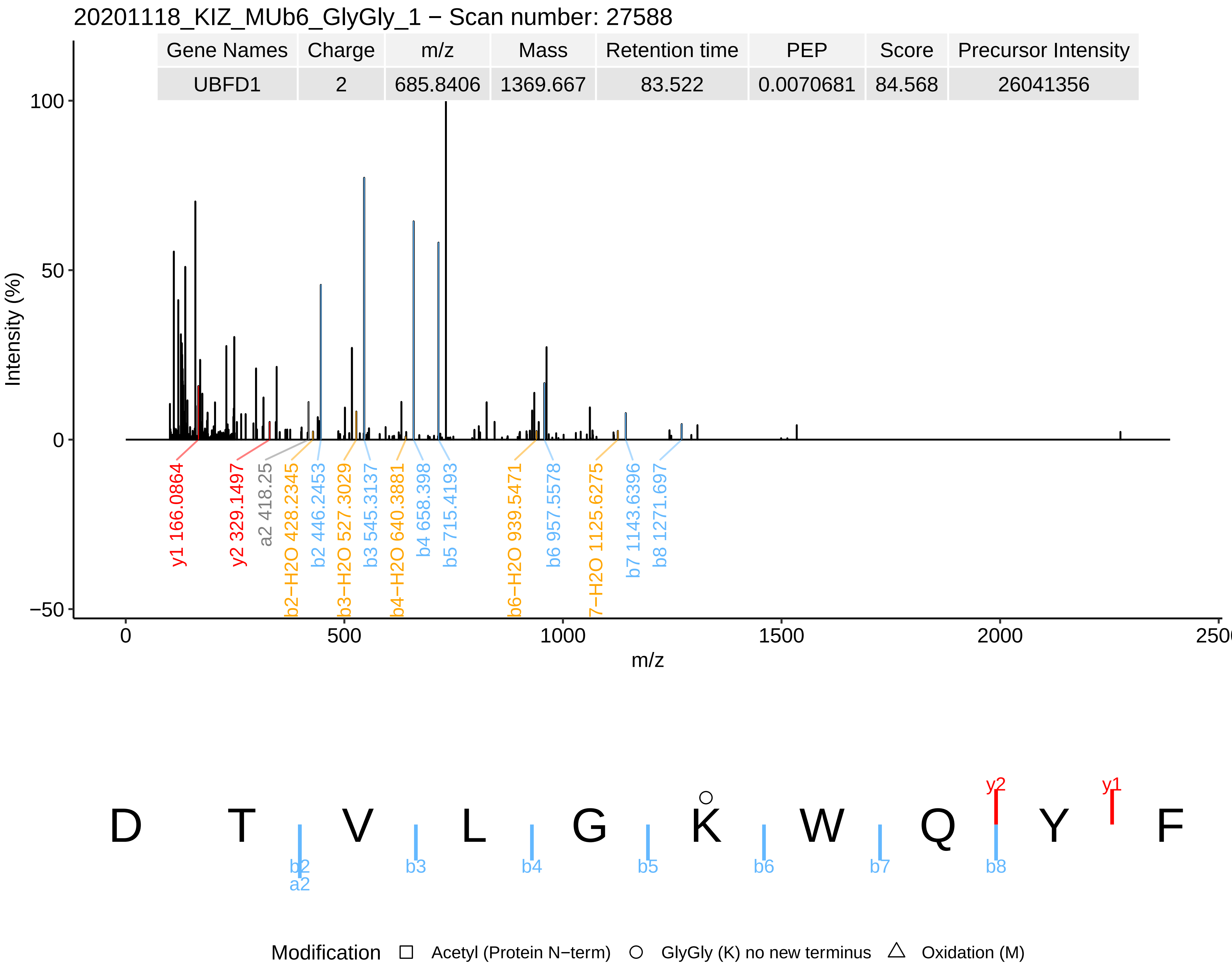
20201118_KIZ_MUb6_GlyGly_1 − Scan number: 27588
Gene Names | Charge | m/z | Mass | Retention time | PEP | Score | Precursor Intensity
UBFD1 | 2 | 685.8406 | 1369.667 | 83.522 | 0.0070681 | 84.568 | 26041356
Intensity (%)
m/z
y1 166.0864
y2 329.1497
a2 418.25
b2−H2O 428.2345
b2 446.2453
b3−H2O 527.3029
b3 545.3137
b4−H2O 640.3881
b4 658.398
b5 715.4193
b6−H2O 939.5471
b6 957.5578
7−H2O 1125.6275
b7 1143.6396
b8 1271.697
D T V L G K W Q Y F
y2 y1
b2 a2 b3 b4 b5 b6 b7 b8
Modification
Acetyl (Protein N−term)
GlyGly (K) no new terminus
Oxidation (M)

20200921_KIZ_MUb4_GlyGly_10plex_2 − Scan number: 8926

Gene Names	Charge	m/z	Mass	Retention time	PEP	Score	Precursor Intensity
MCM7	3	370.5206	1108.54	33.79	0.054301	60.59	271795.7

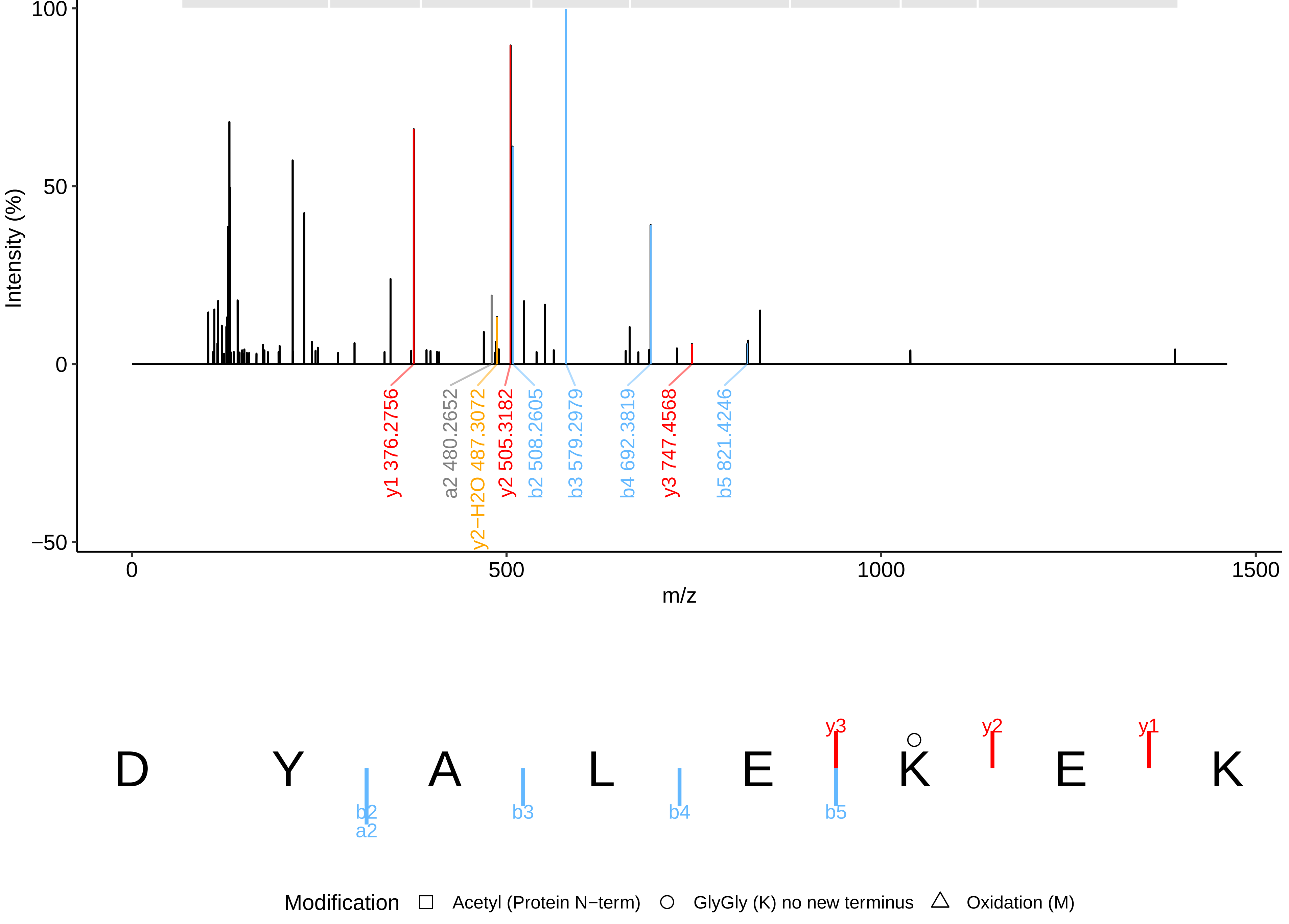

20200921_KIZ_MUb4_GlyGly_10plex − Scan number: 4752

Gene Names	Charge	m/z	Mass	Retention time	PEP	Score	Precursor Intensity
RPS12	3	380.5158	1138.525	15.494	0.0029501	83.998	7327560

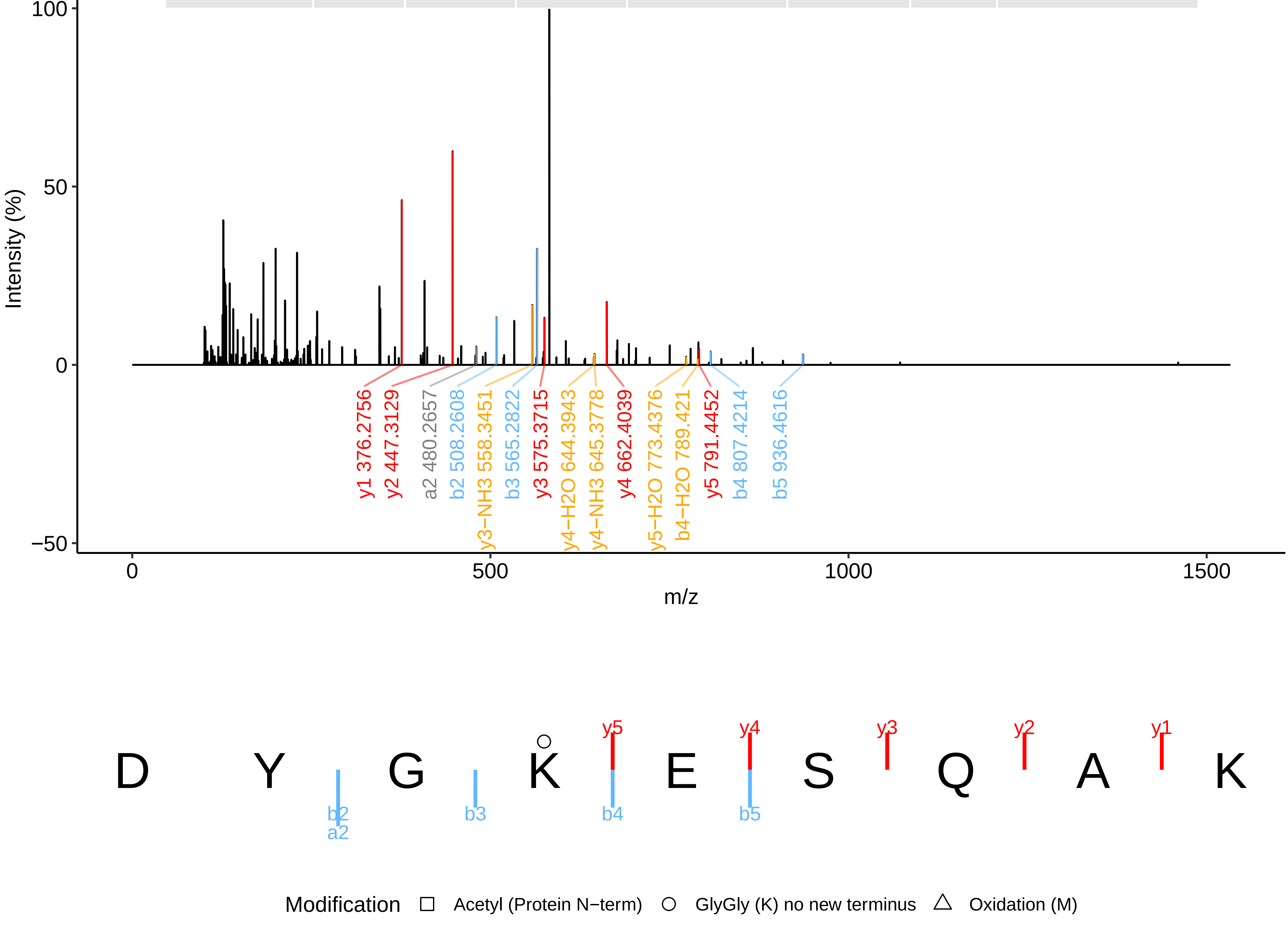

20200921_KIZ_MUb4_GlyGly_10plex − Scan number: 2945

Gene Names	Charge	m/z	Mass	Retention time	PEP	Score	Precursor Intensity
YWHAE	3	373.8353	1118.484	9.2284	0.008784	75.652	5417030

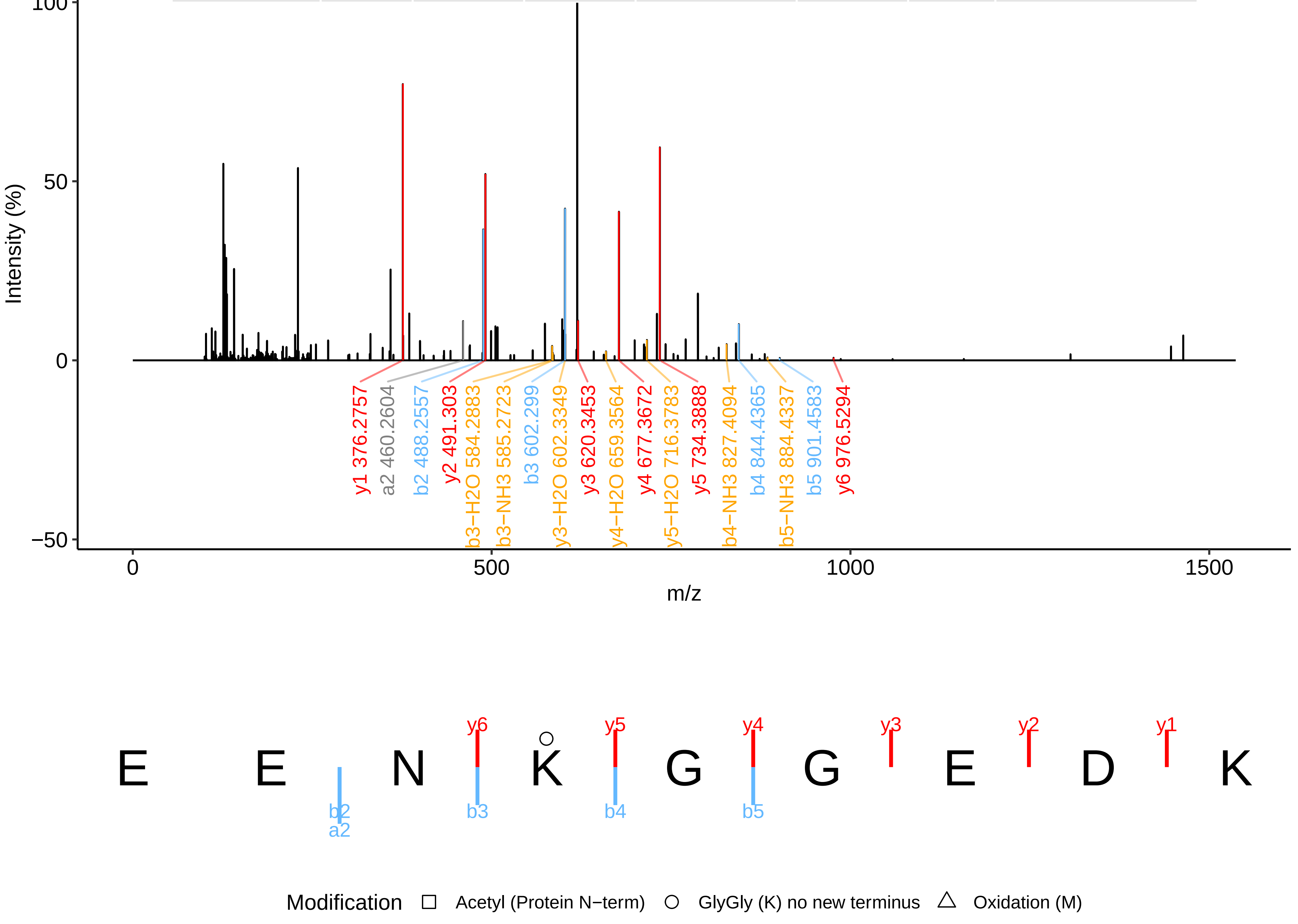

20200921_KIZ_MUb4_GlyGly_10plex_2 − Scan number: 2885

Gene Names	Charge	m/z	Mass	Retention time	PEP	Score	Precursor Intensity
YWHAE	3	373.8353	1118.484	9.3693	0.022063	63.184	1072856

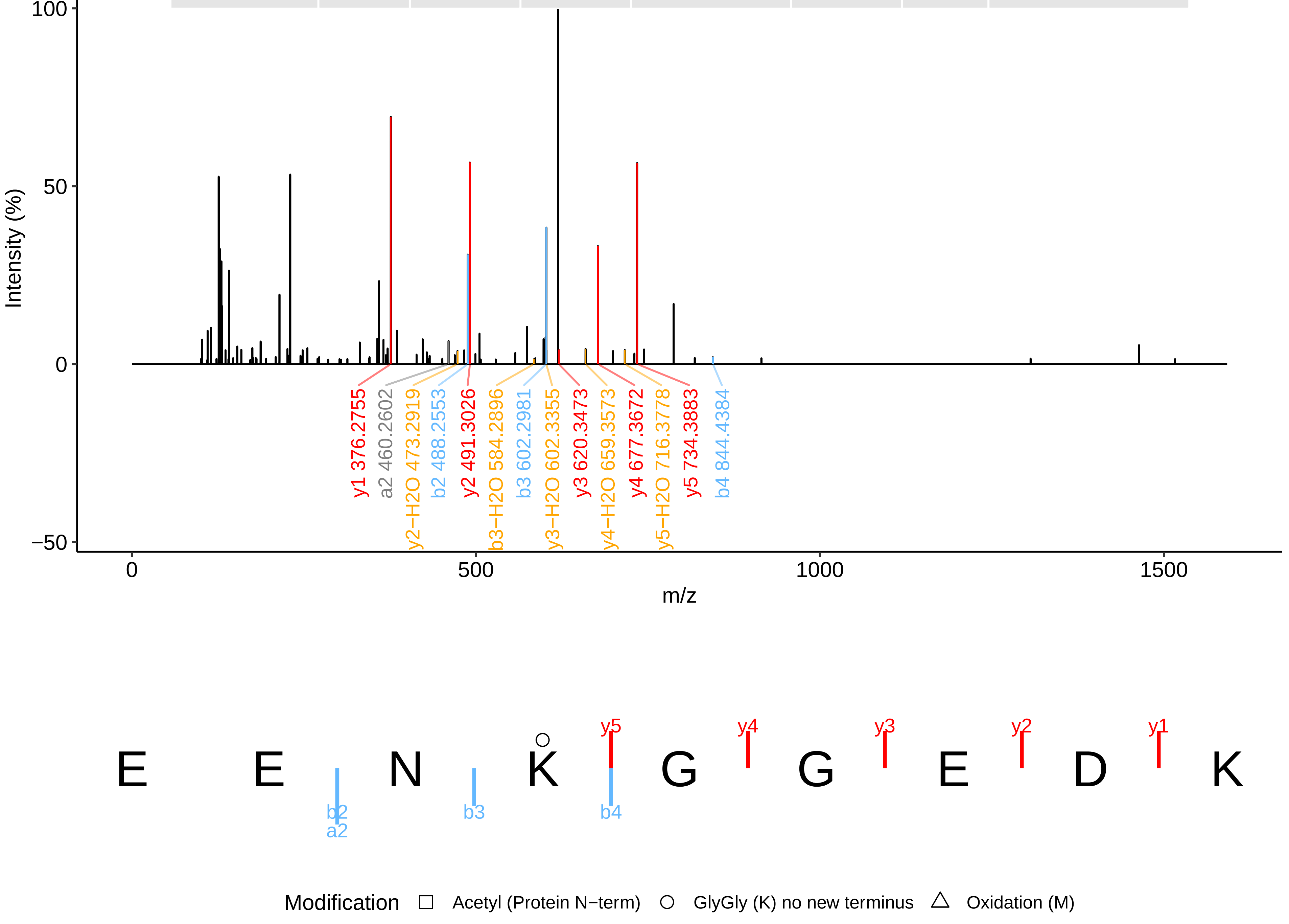

20200924_KIZ_MUb5_GlyGly_1 − Scan number: 12430

Gene Names	Charge	m/z	Mass	Retention time	PEP	Score	Precursor Intensity
DDB1	3	373.8576	1118.551	40.439	0.13739	48.794	10946787

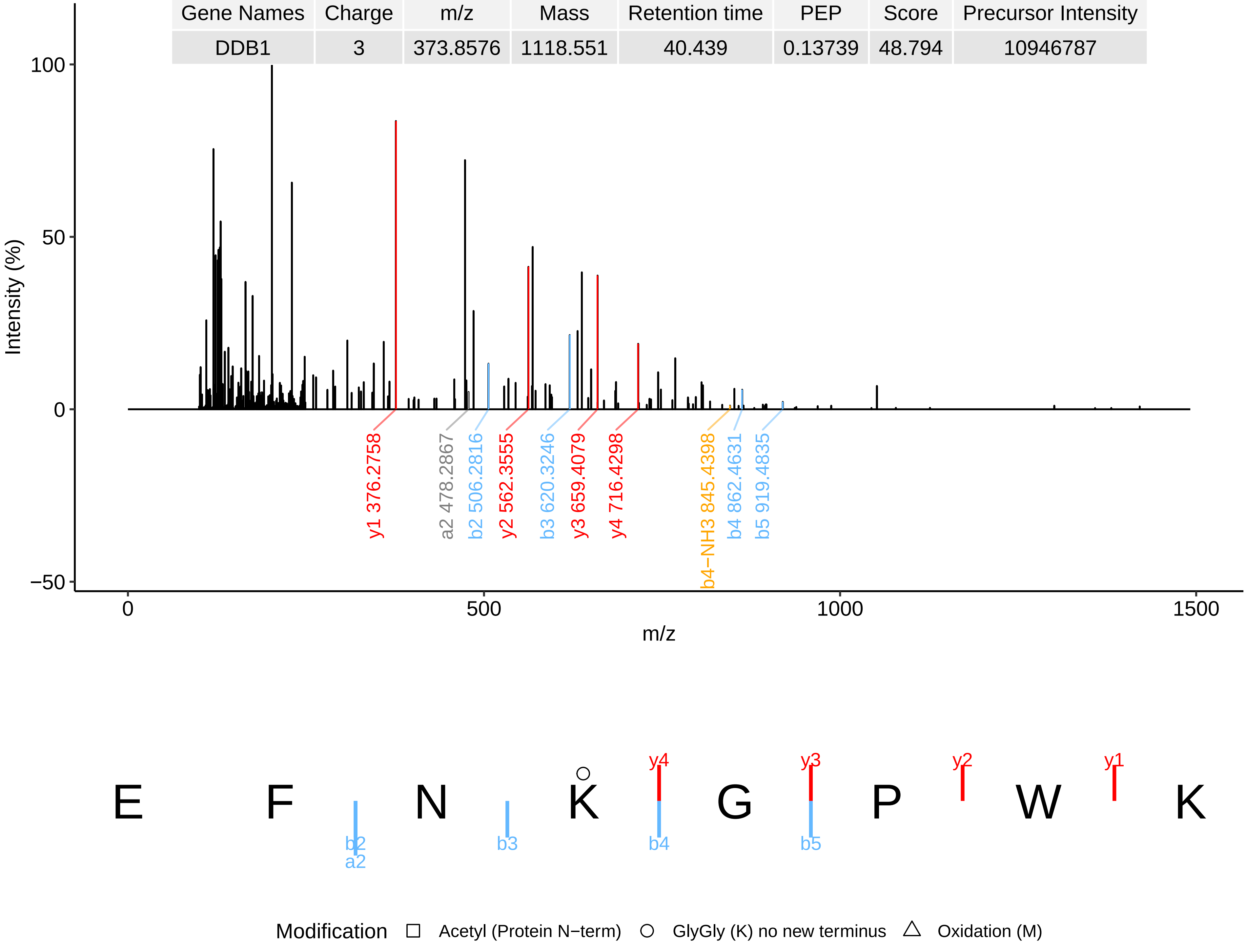

20200921_KIZ_MUb4_GlyGly_10plex − Scan number: 4167

Gene Names	Charge	m/z	Mass	Retention time	PEP	Score	Precursor Intensity
BASP1	3	368.5158	1102.525	13.564	0.019397	64.103	5325633

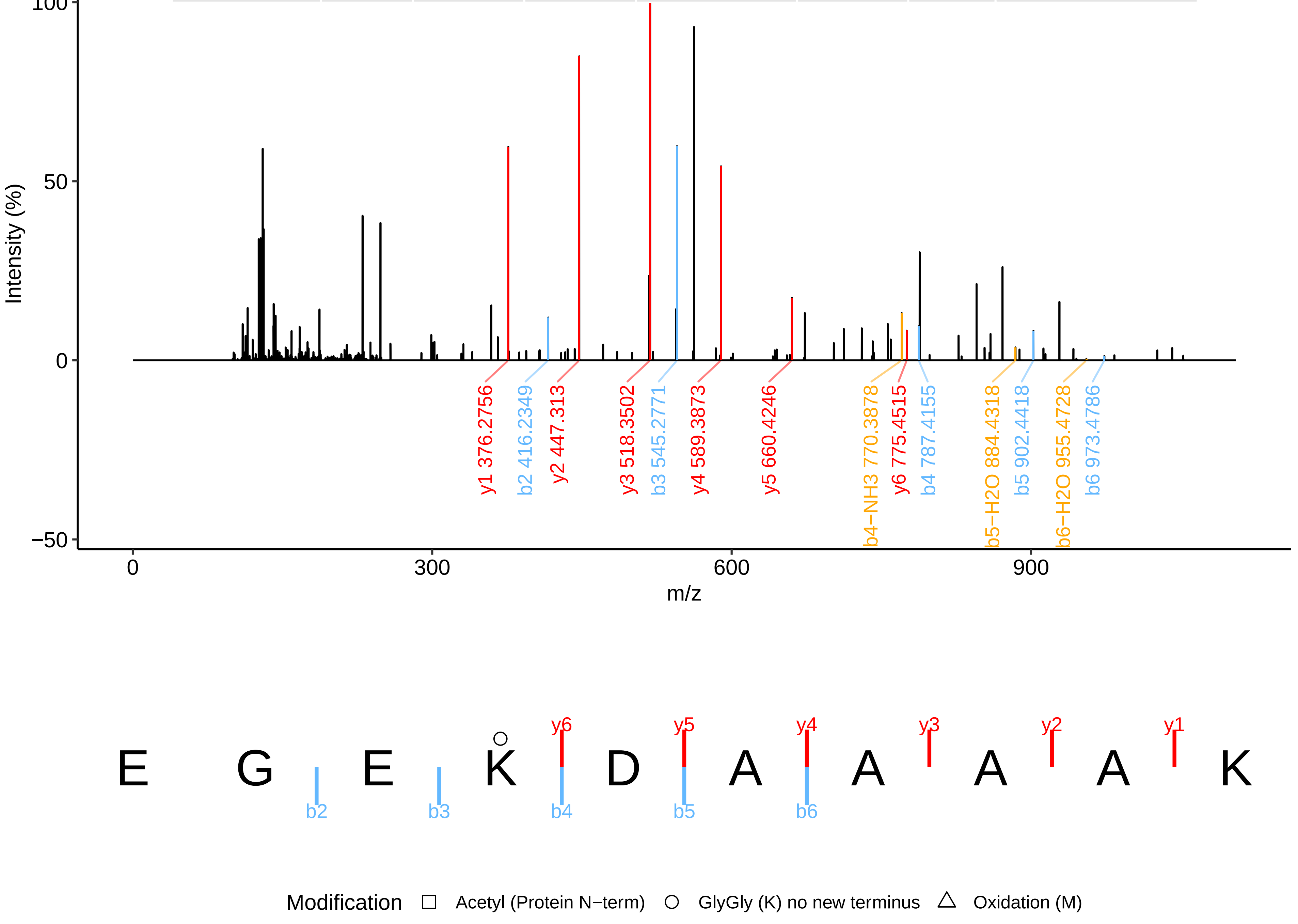

20200921_KIZ_MUb4_GlyGly_10plex_2 − Scan number: 3884

Gene Names	Charge	m/z	Mass	Retention time	PEP	Score	Precursor Intensity
BASP1	3	368.5158	1102.525	13.333	0.017973	65.5	3603177

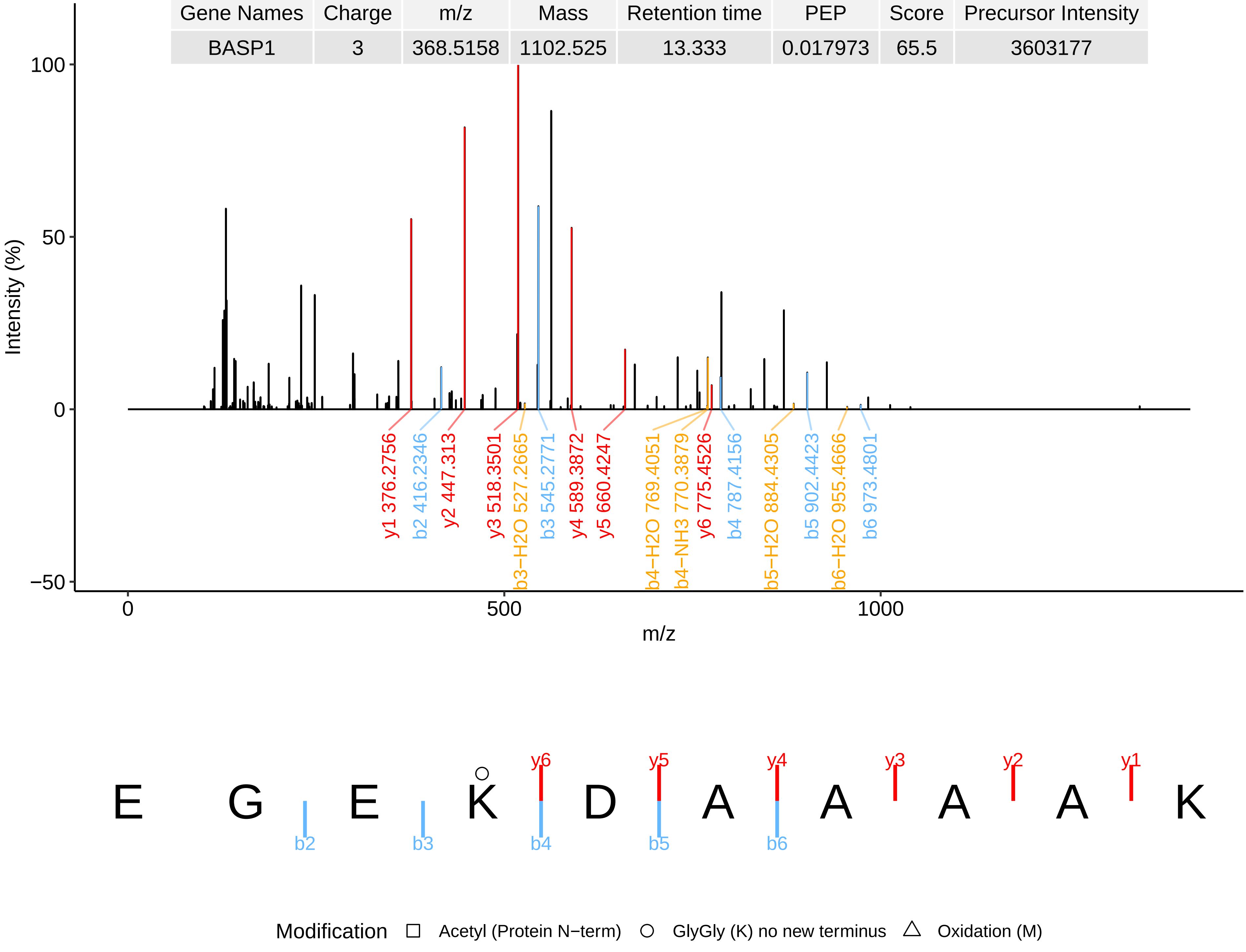

20200924_KIZ_MUb5_GlyGly_1 − Scan number: 3568

Gene Names	Charge	m/z	Mass	Retention time	PEP	Score	Precursor Intensity
BASP1	3	368.5158	1102.525	10.918	0.019885	63.624	13154980

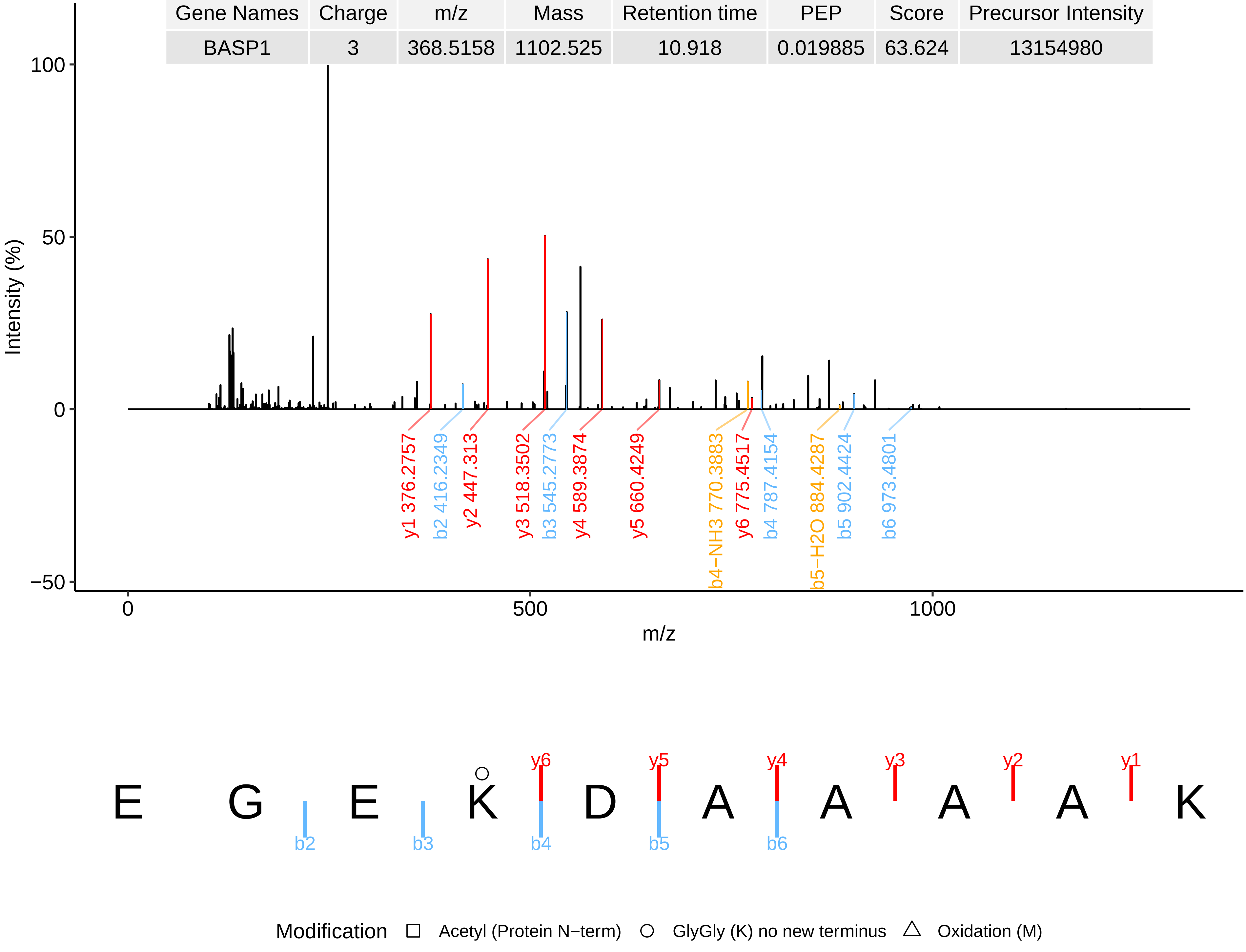

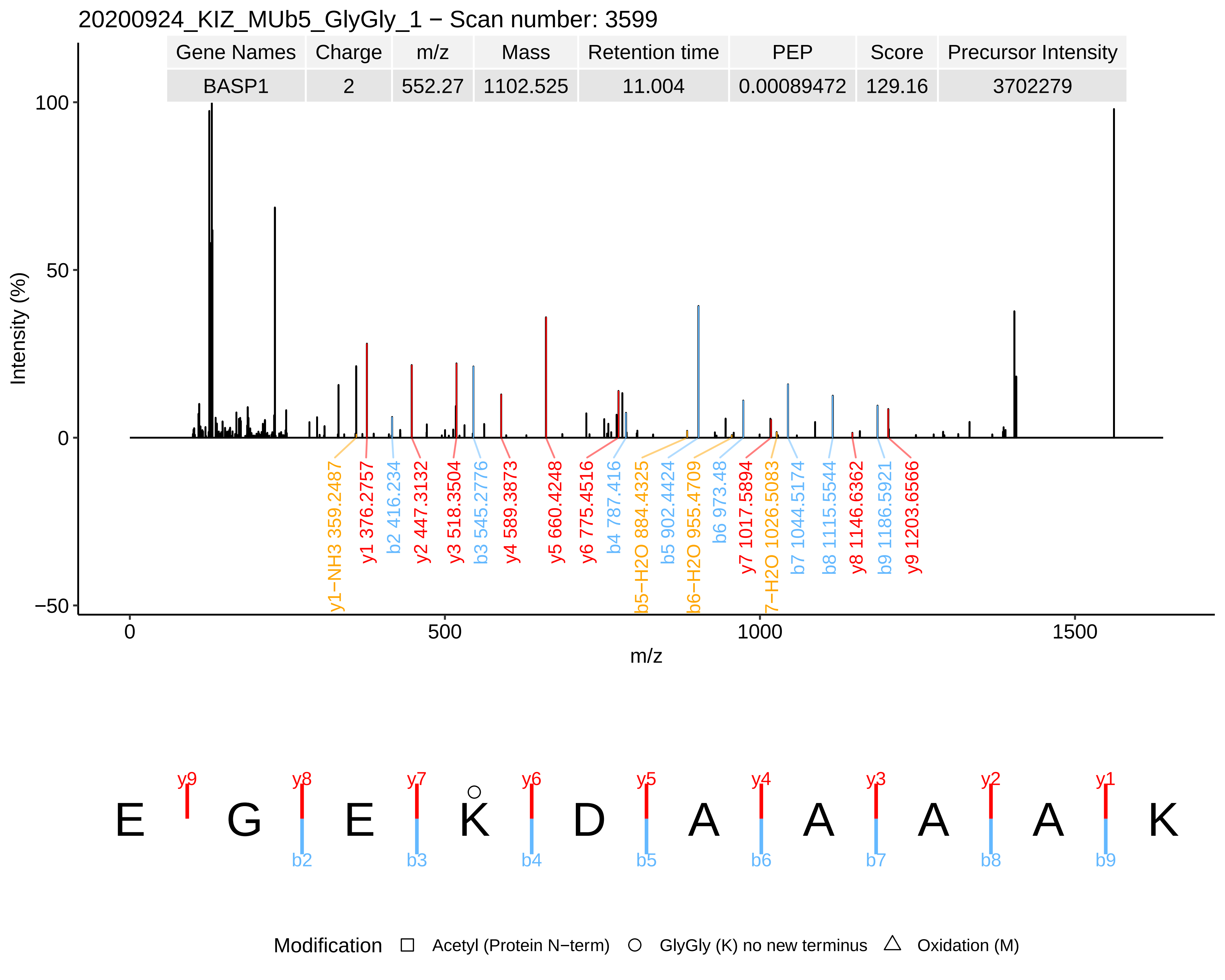

20200924_KIZ_MUb5_GlyGly_1 − Scan number: 3599
Gene Names | Charge | m/z | Mass | Retention time | PEP | Score | Precursor Intensity
BASP1 | 2 | 552.27 | 1102.525 | 11.004 | 0.00089472 | 129.16 | 3702279
Intensity (%)
100
50
0
−50
0
500
1000
1500
m/z
y1−NH3 359.2487
y1 376.2757
b2 416.234
y2 447.3132
y3 518.3504
b3 545.2776
y4 589.3873
y5 660.4248
y6 775.4516
b4 787.416
b5−H2O 884.4325
b5 902.4424
b6−H2O 955.4709
b6 973.48
y7 1017.5894
y7−H2O 1026.5083
b7 1044.5174
b8 1115.5544
y8 1146.6362
b9 1186.5921
y9 1203.6566
E G E K D A A A A K
y9 y8 y7 y6 y5 y4 y3 y2 y1
b2 b3 b4 b5 b6 b7 b8 b9
Modification
Acetyl (Protein N−term)
GlyGly (K) no new terminus
Oxidation (M)

20200924_KIZ_MUb5_GlyGly_2 − Scan number: 3435

Gene Names	Charge	m/z	Mass	Retention time	PEP	Score	Precursor Intensity
BASP1	3	368.5158	1102.525	12.448	0.01775	65.719	2654504

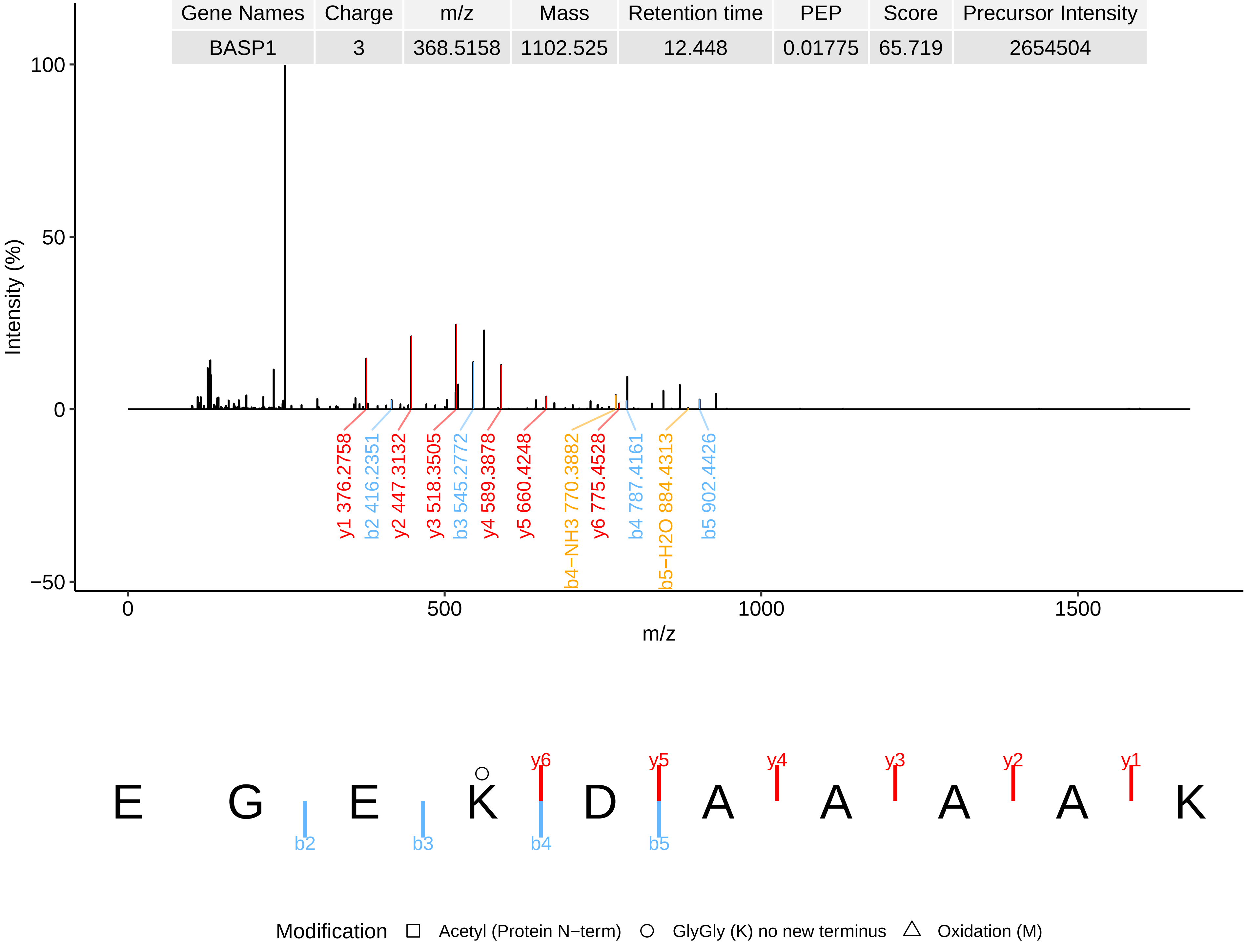

20201118_KIZ_MUb6_GlyGly_1 − Scan number: 3436

Gene Names	Charge	m/z	Mass	Retention time	PEP	Score	Precursor Intensity
BASP1	3	368.5158	1102.525	10.556	0.060276	55.401	18367554

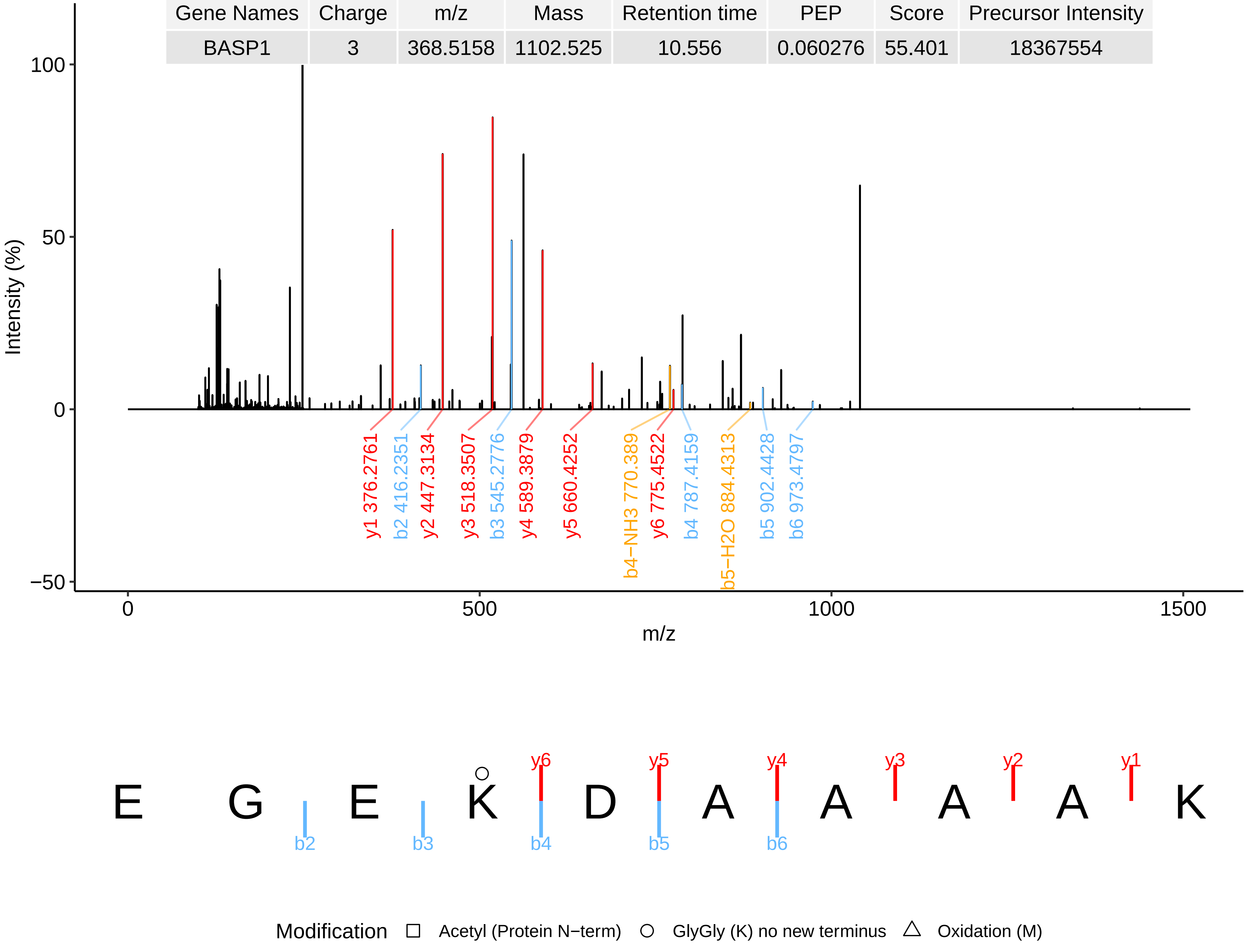

20201118_KIZ_MUb6_GlyGly_2 − Scan number: 4205

Gene Names	Charge	m/z	Mass	Retention time	PEP	Score	Precursor Intensity
BASP1	3	368.5158	1102.525	14.259	0.0021218	83.862	5409194

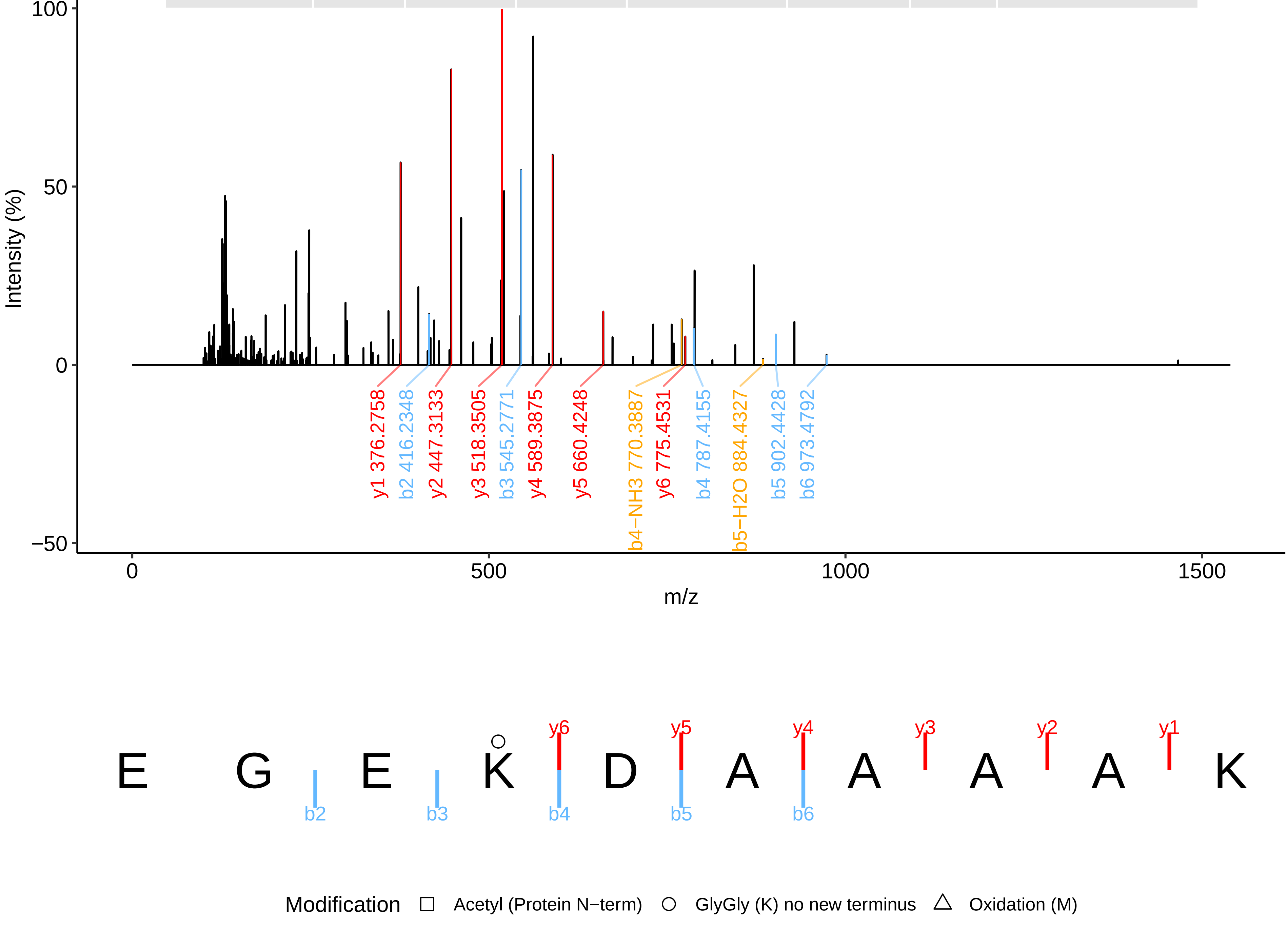

20200921_KIZ_MUb4_GlyGly_10plex − Scan number: 13324

Gene Names	Charge	m/z	Mass	Retention time	PEP	Score	Precursor Intensity
SUMO1	3	365.2011	1092.582	46.971	0.063773	59.245	4892214

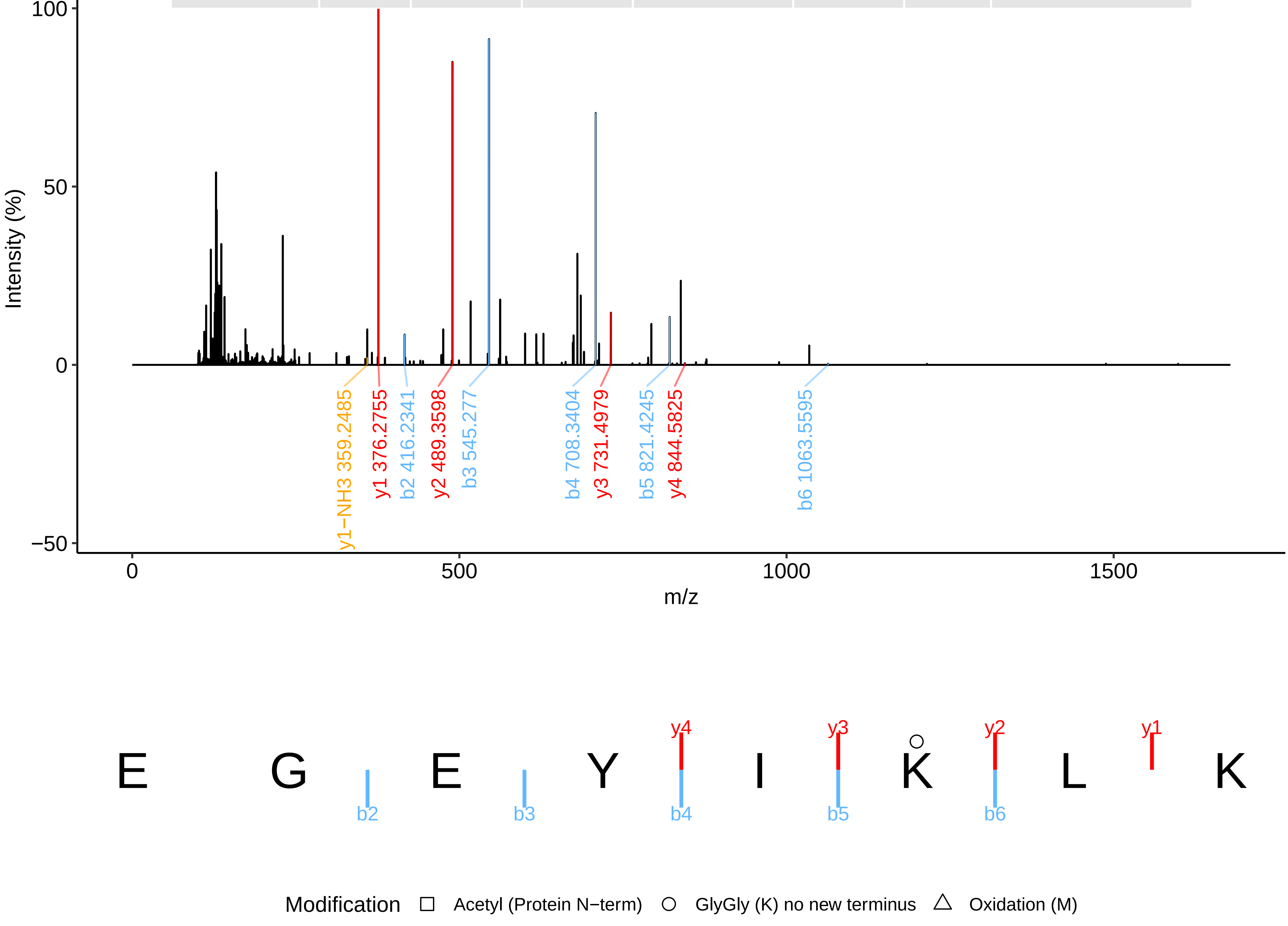

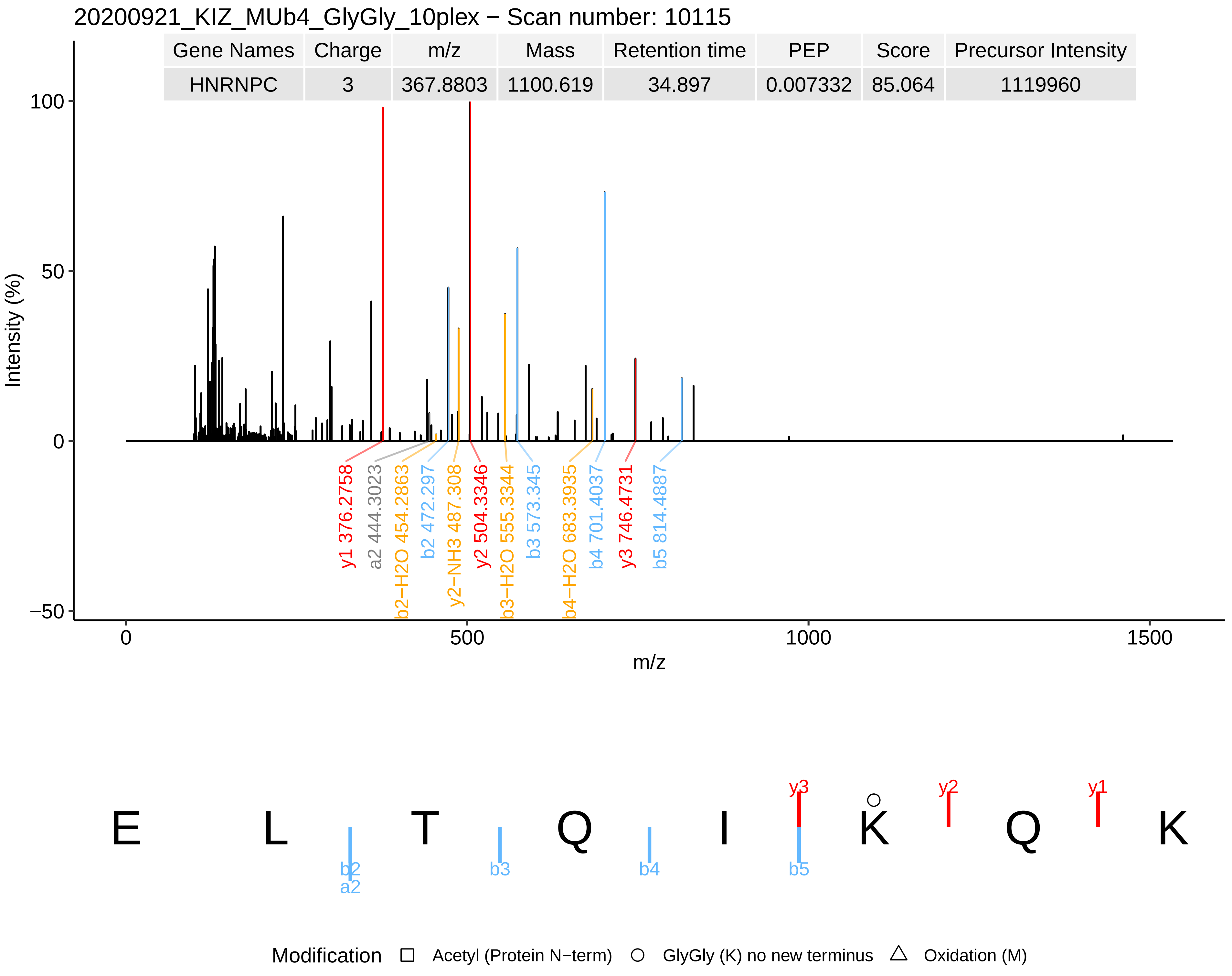

20200921_KIZ_MUb4_GlyGly_10plex − Scan number: 10115

Gene Names	Charge	m/z	Mass	Retention time	PEP	Score	Precursor Intensity
HNRNPC	3	367.8803	1100.619	34.897	0.007332	85.064	1119960

Intensity (%)
100
50
0
−50
0
500
1000
1500
m/z
y1 376.2758
a2 444.3023
b2−H2O 454.2863
b2 472.297
y2−NH3 487.308
y2 504.3346
b3−H2O 555.3344
b3 573.345
b4−H2O 683.3935
b4 701.4037
y3 746.4731
b5 814.4887
E L T Q I K Q K
y3
y2
y1
b2
a2
b3
b4
b5
Modification
Acetyl (Protein N−term)
GlyGly (K) no new terminus
Oxidation (M)

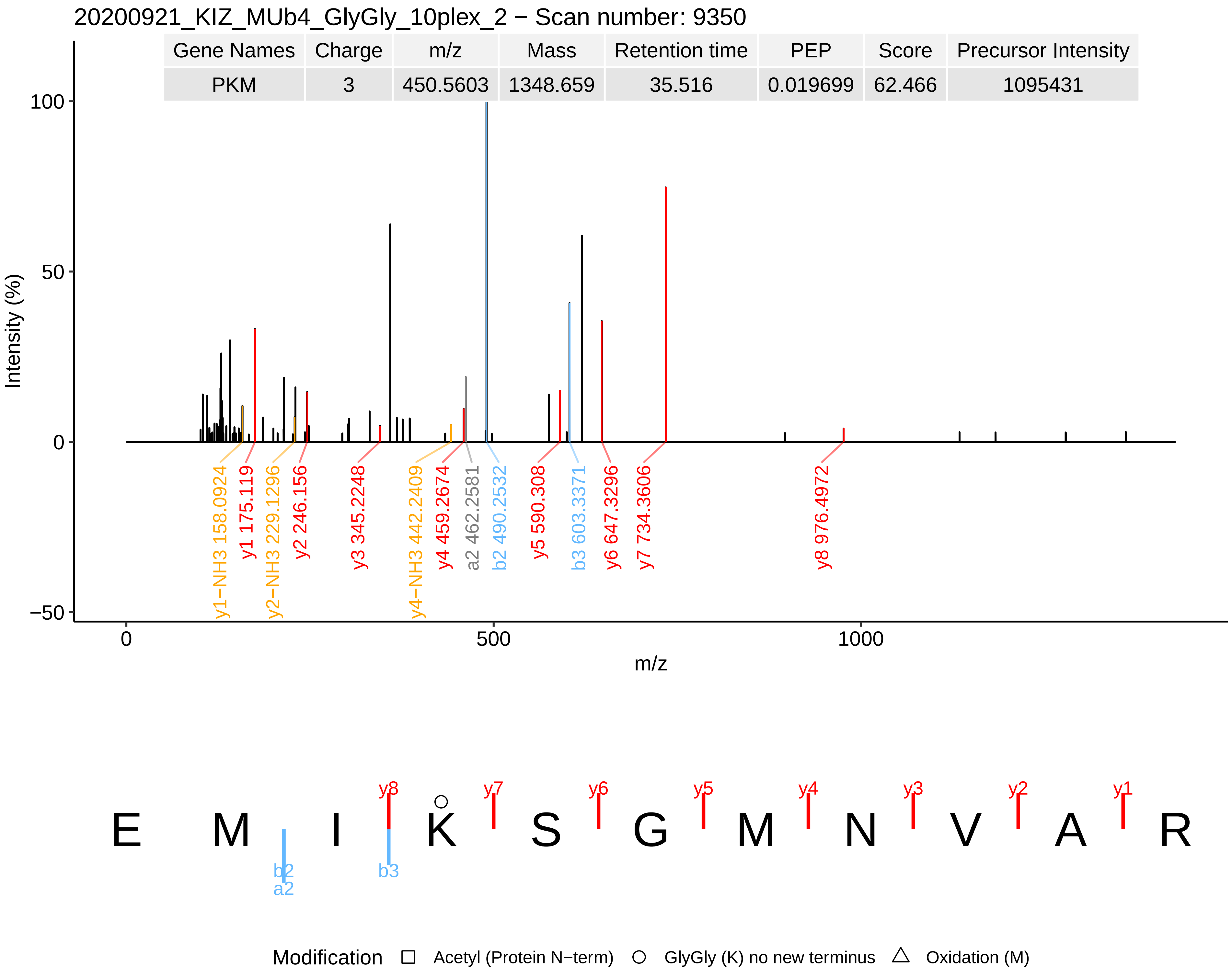
20200921_KIZ_MUb4_GlyGly_10plex_2 − Scan number: 9350
Gene Names | Charge | m/z | Mass | Retention time | PEP | Score | Precursor Intensity
PKM | 3 | 450.5603 | 1348.659 | 35.516 | 0.019699 | 62.466 | 1095431
Intensity (%)
100
50
0
−50
y1−NH3 158.0924
y1 175.119
y2−NH3 229.1296
y2 246.156
y3 345.2248
y4−NH3 442.2409
y4 459.2674
a2 462.2581
b2 490.2532
y5 590.308
b3 603.3371
y6 647.3296
y7 734.3606
y8 976.4972
0
500
1000
m/z
y8 y7 y6 y5 y4 y3 y2 y1
E M I K S G M N V A R
b2
a2
b3
Modification
Acetyl (Protein N−term)
GlyGly (K) no new terminus
Oxidation (M)

20200921_KIZ_MUb4_GlyGly_10plex − Scan number: 8910

Gene Names	Charge	m/z	Mass	Retention time	PEP	Score	Precursor Intensity
GMNN	3	439.8968	1316.668	30.39	0.0024654	95.401	5836760

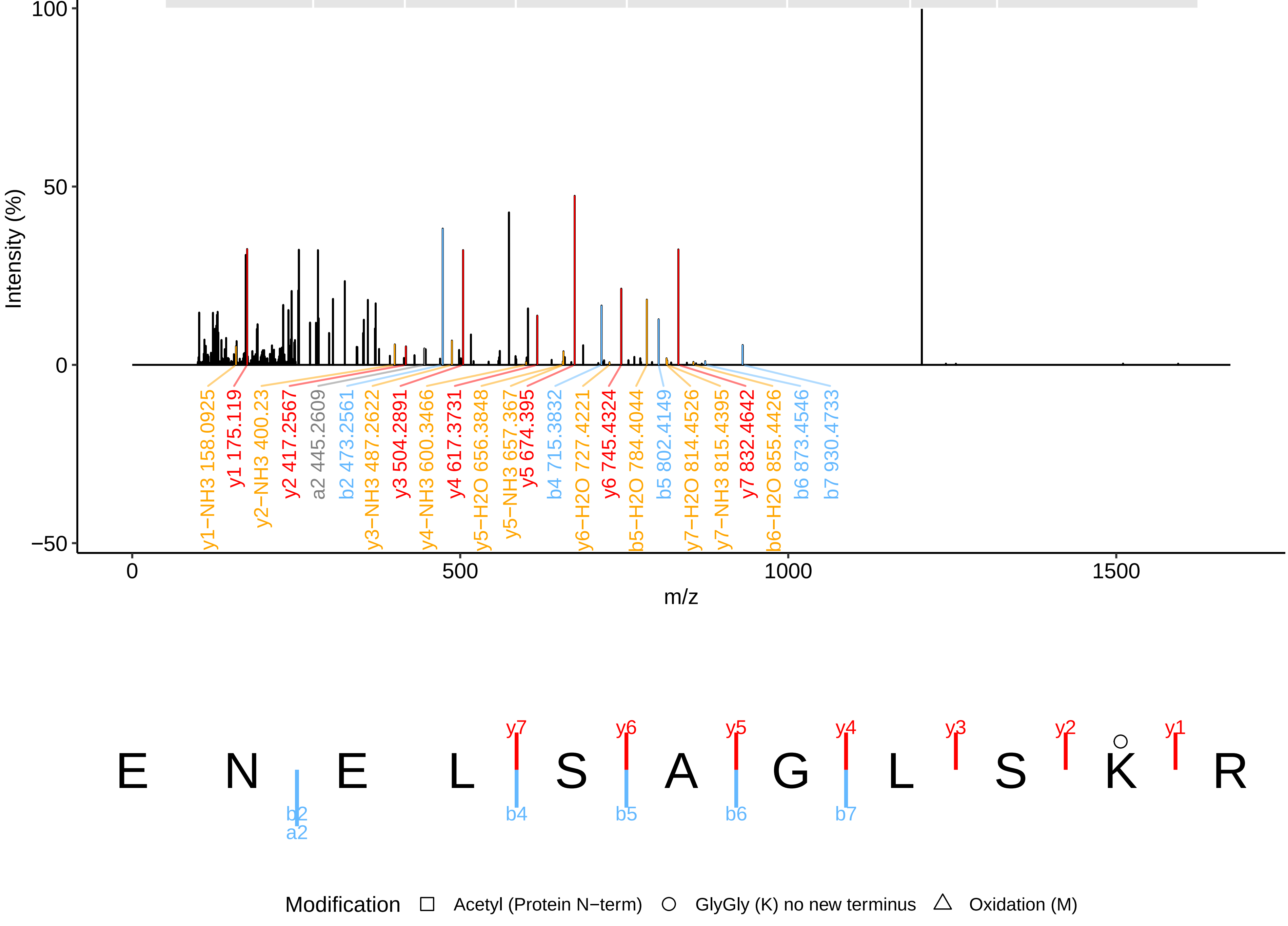

20200921_KIZ_MUb4_GlyGly_10plex − Scan number: 13875

Gene Names	Charge	m/z	Mass	Retention time	PEP	Score	Precursor Intensity
RPS13	3	369.545	1105.613	49.096	0.026406	66.692	4333095

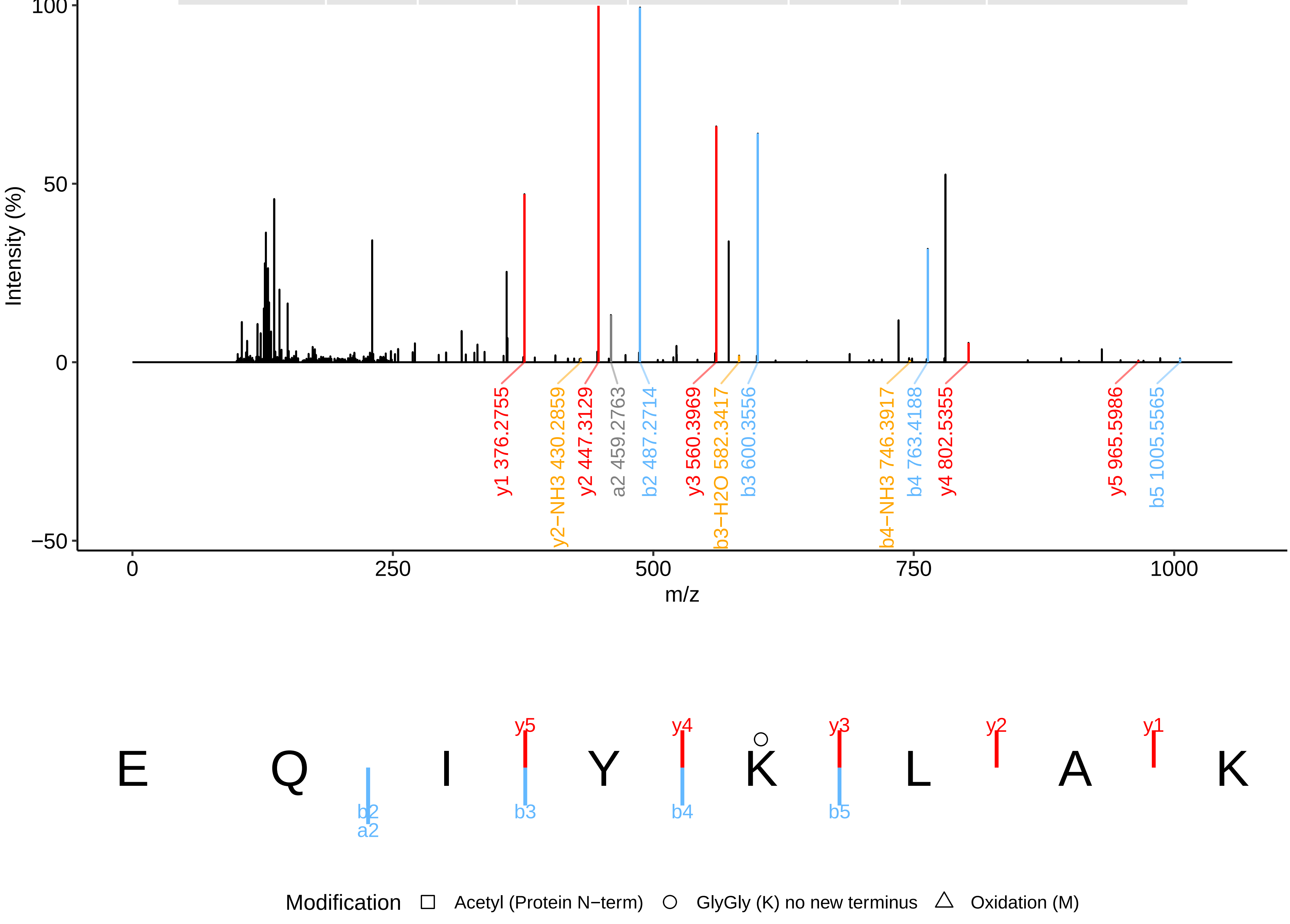

20200921_KIZ_MUb4_GlyGly_10plex_2 − Scan number: 12784

Gene Names	Charge	m/z	Mass	Retention time	PEP	Score	Precursor Intensity
RPS13	3	369.545	1105.613	49.539	0.084692	50.04	1037798

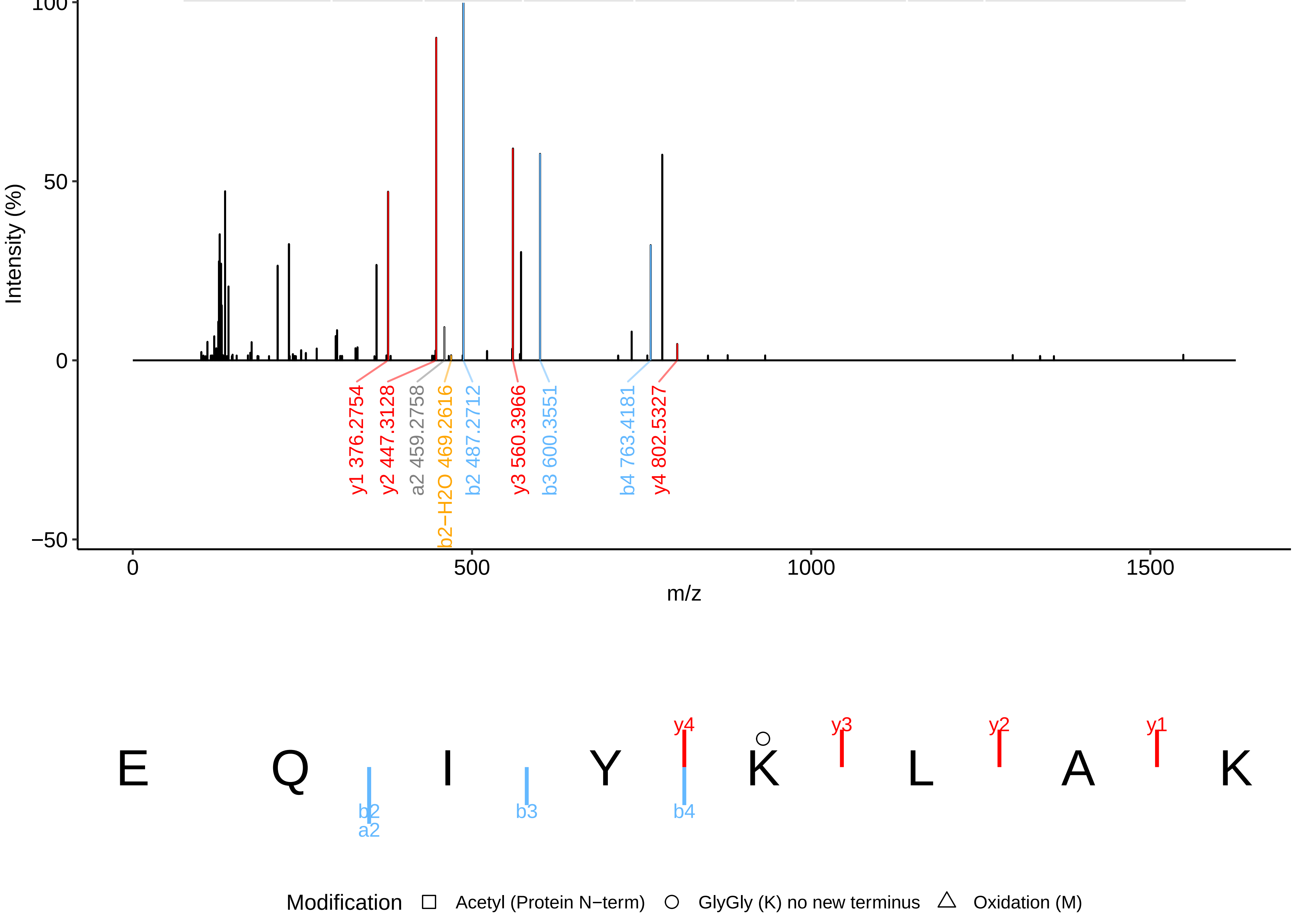

20200924_KIZ_MUb5_GlyGly_1 − Scan number: 12796

Gene Names	Charge	m/z	Mass	Retention time	PEP	Score	Precursor Intensity
RPS13	3	369.545	1105.613	41.689	0.059866	59.8	NA

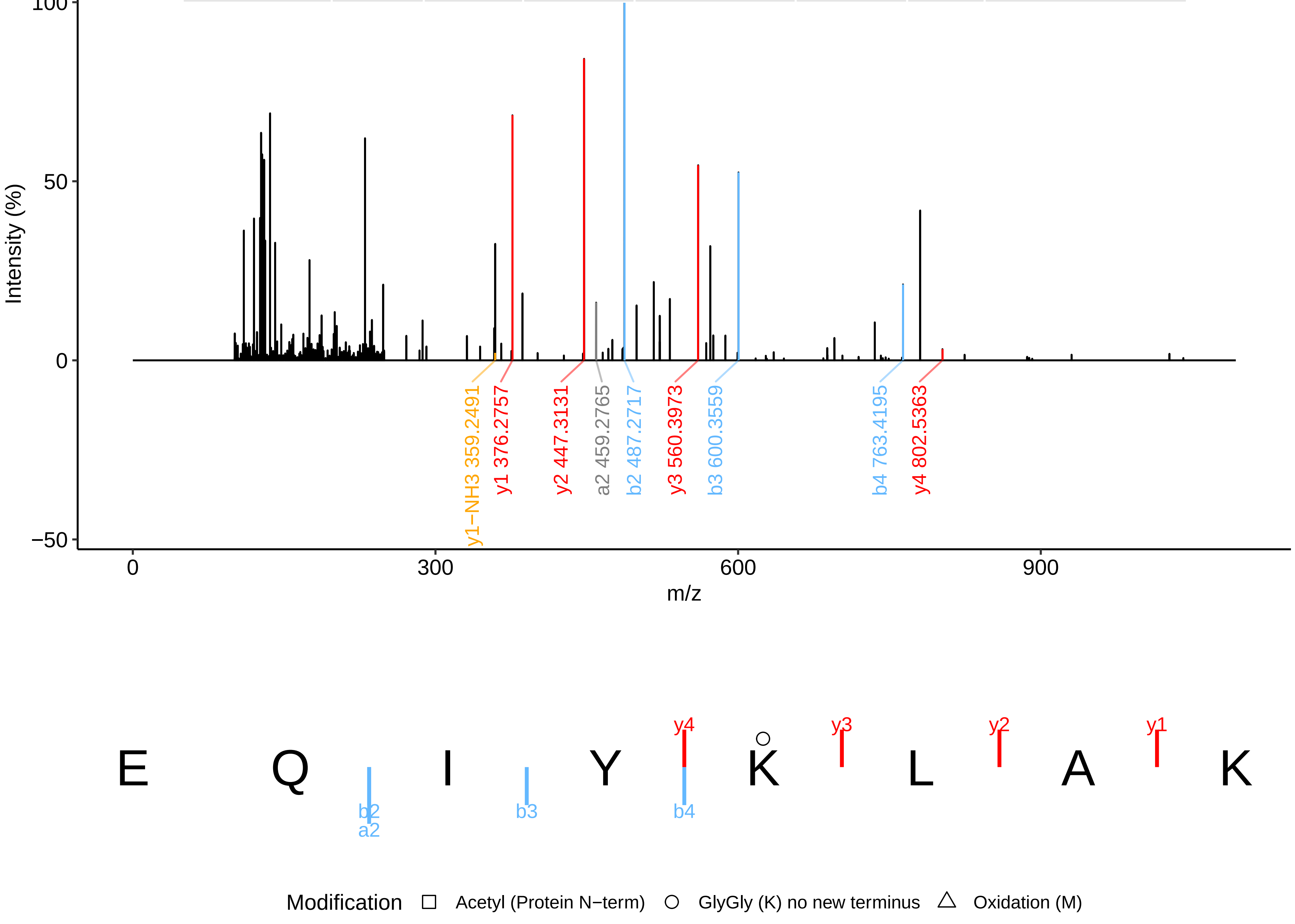

20200921_KIZ_MUb4_GlyGly_10plex_2 − Scan number: 2797

Gene Names	Charge	m/z	Mass	Retention time	PEP	Score	Precursor Intensity
SLC16A1	3	363.5159	1087.526	9.0132	0.028133	65.305	2500839

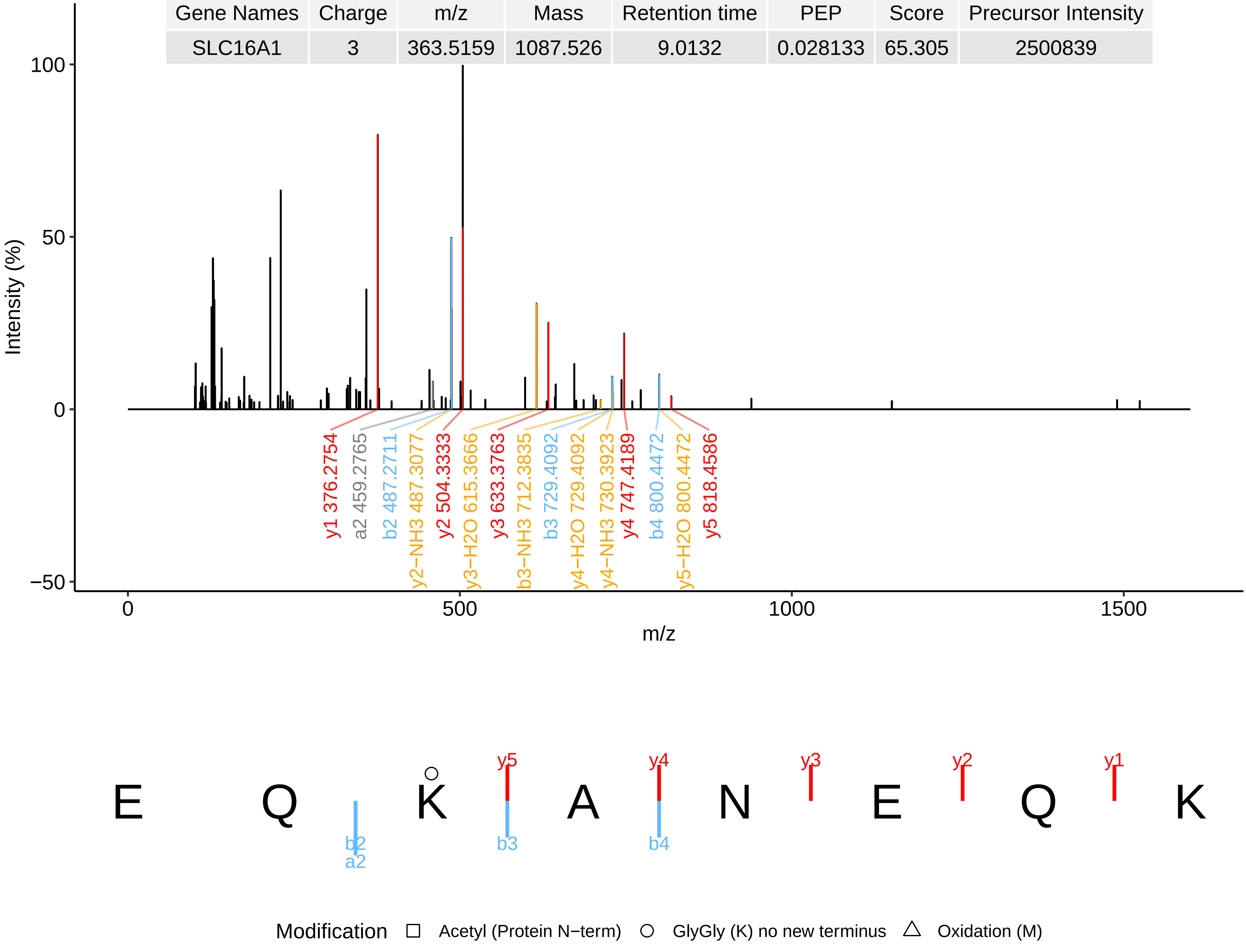

20200921_KIZ_MUb4_GlyGly_10plex − Scan number: 12402

Gene Names	Charge	m/z	Mass	Retention time	PEP	Score	Precursor Intensity
PSMC6	3	368.2083	1101.603	43.469	0.0085515	81.972	9756326

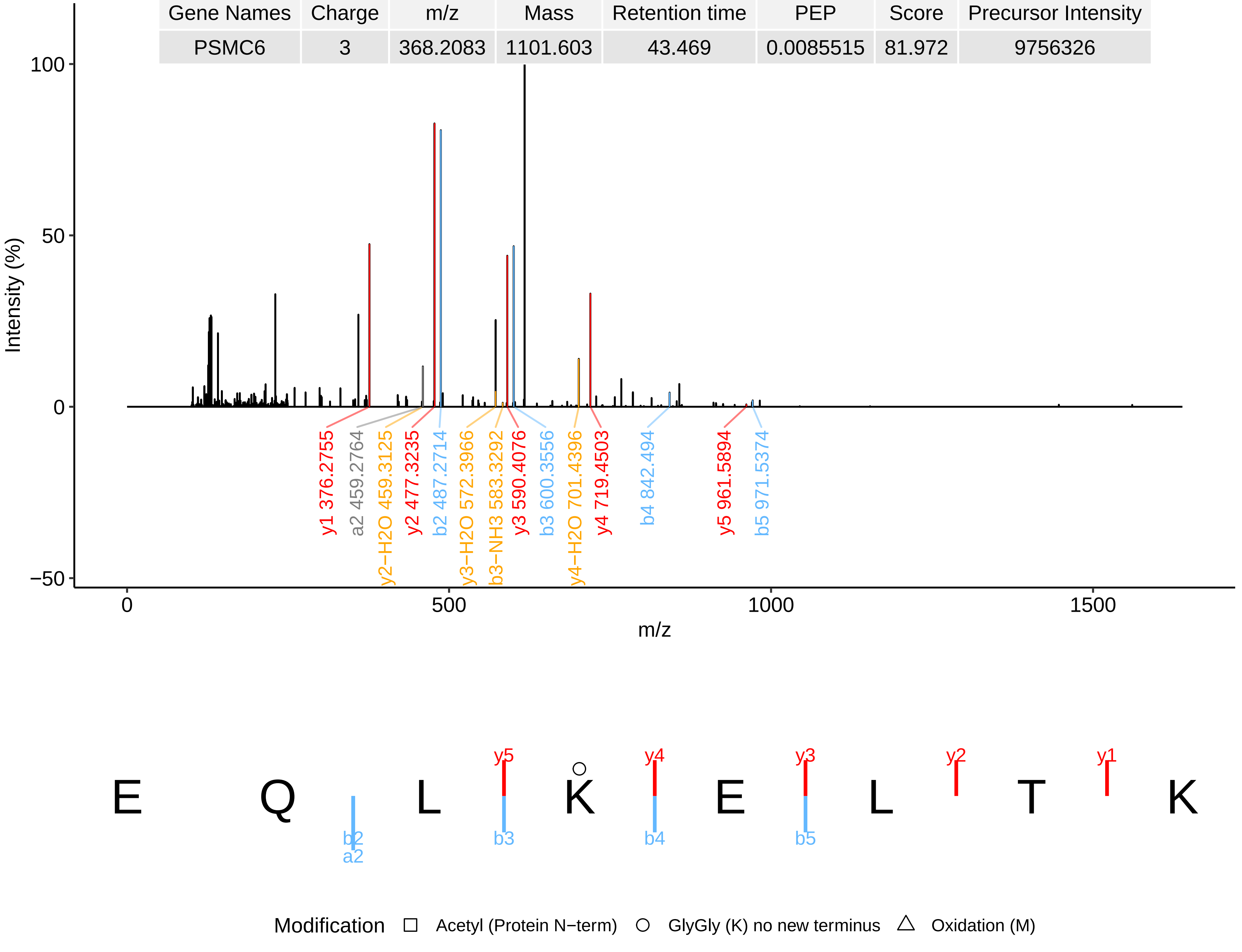

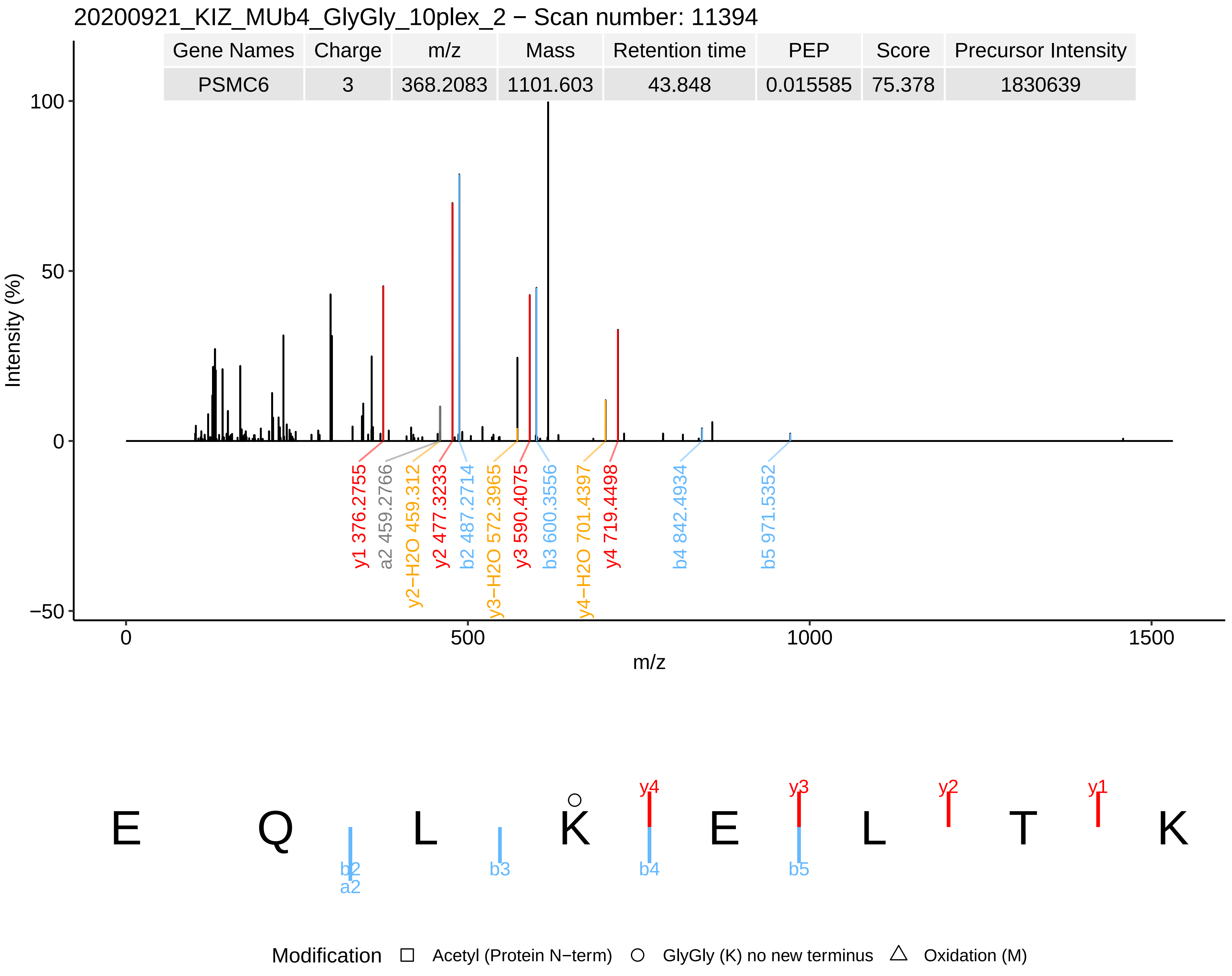

20200921_KIZ_MUb4_GlyGly_10plex_2 − Scan number: 11394
Gene Names | Charge | m/z | Mass | Retention time | PEP | Score | Precursor Intensity
PSMC6 | 3 | 368.2083 | 1101.603 | 43.848 | 0.015585 | 75.378 | 1830639
Intensity (%)
m/z
y1 376.2755
a2 459.2766
y2−H2O 459.312
y2 477.3233
b2 487.2714
y3−H2O 572.3965
y3 590.4075
b3 600.3556
y4−H2O 701.4397
y4 719.4498
b4 842.4934
b5 971.5352
E Q L K E L T K
y4 y3 y2 y1
b2 a2 b3 b4 b5
Modification
Acetyl (Protein N−term)
GlyGly (K) no new terminus
Oxidation (M)

20200921_KIZ_MUb4_GlyGly_10plex − Scan number: 8989

Gene Names	Charge	m/z	Mass	Retention time	PEP	Score	Precursor Intensity
NUP155	3	380.1961	1137.567	30.692	0.00111	107.9	6330530

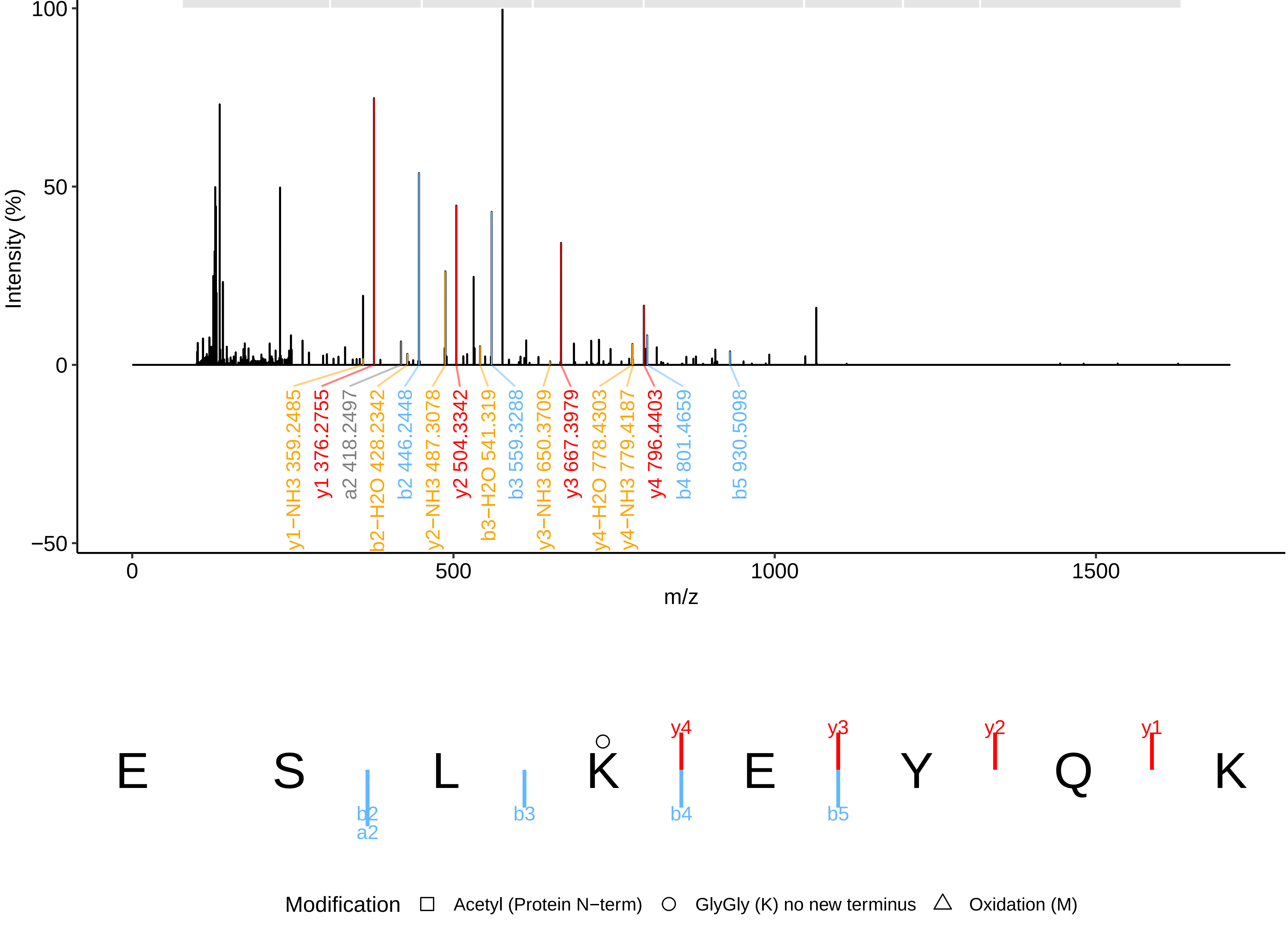

20200921_KIZ_MUb4_GlyGly_10plex_2 − Scan number: 8311

Gene Names	Charge	m/z	Mass	Retention time	PEP	Score	Precursor Intensity
NUP155	3	380.1961	1137.567	31.277	0.0088179	81.296	870015.8

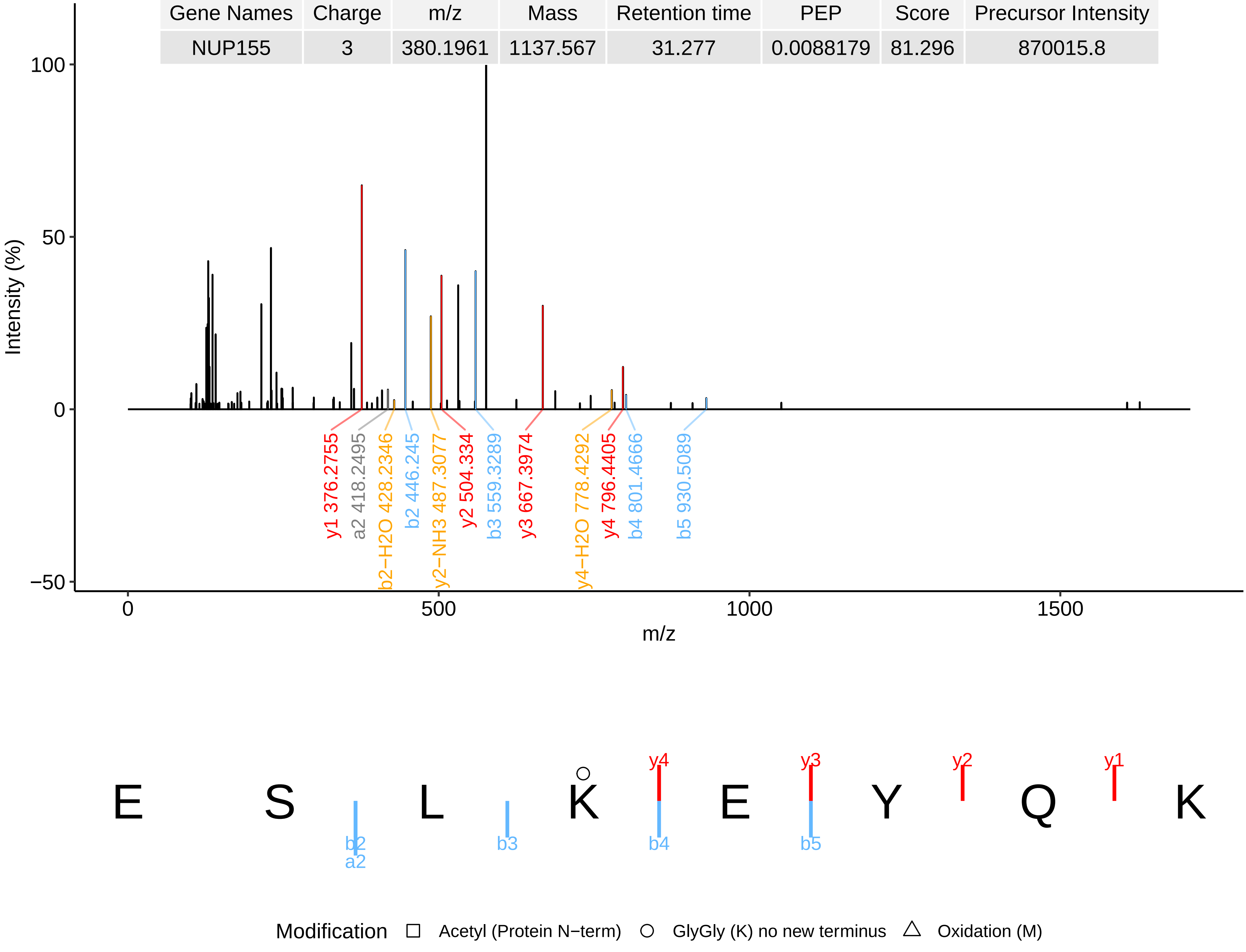

20200924_KIZ_MUb5_GlyGly_1 − Scan number: 7939

Gene Names	Charge	m/z	Mass	Retention time	PEP	Score	Precursor Intensity
NUP155	3	380.1961	1137.567	25.29	0.0087187	81.548	8104888

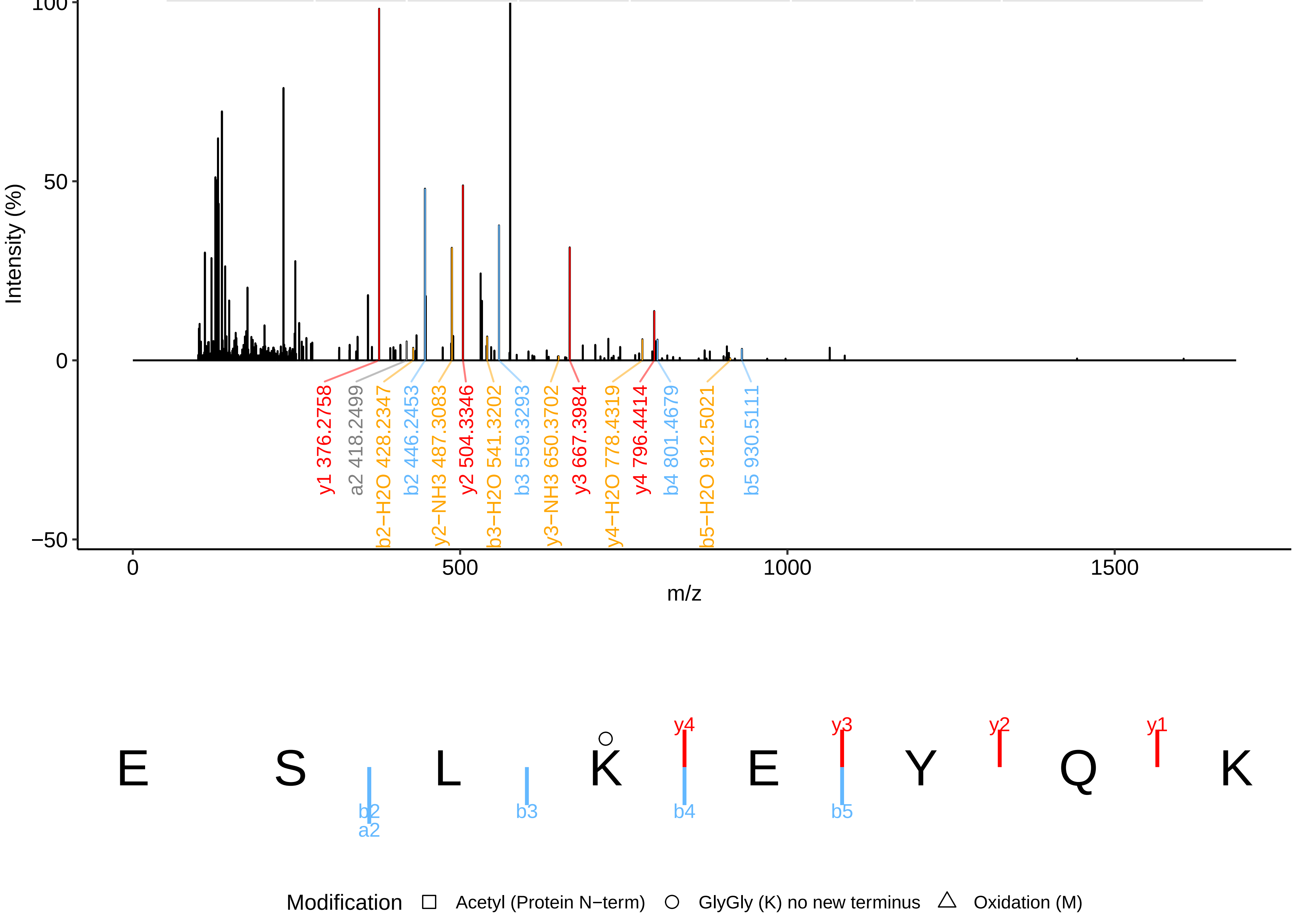

20200921_KIZ_MUb4_GlyGly_10plex − Scan number: 4325

Gene Names	Charge	m/z	Mass	Retention time	PEP	Score	Precursor Intensity
CCDC127	3	364.184	1089.53	14.094	0.028206	65.246	3862917

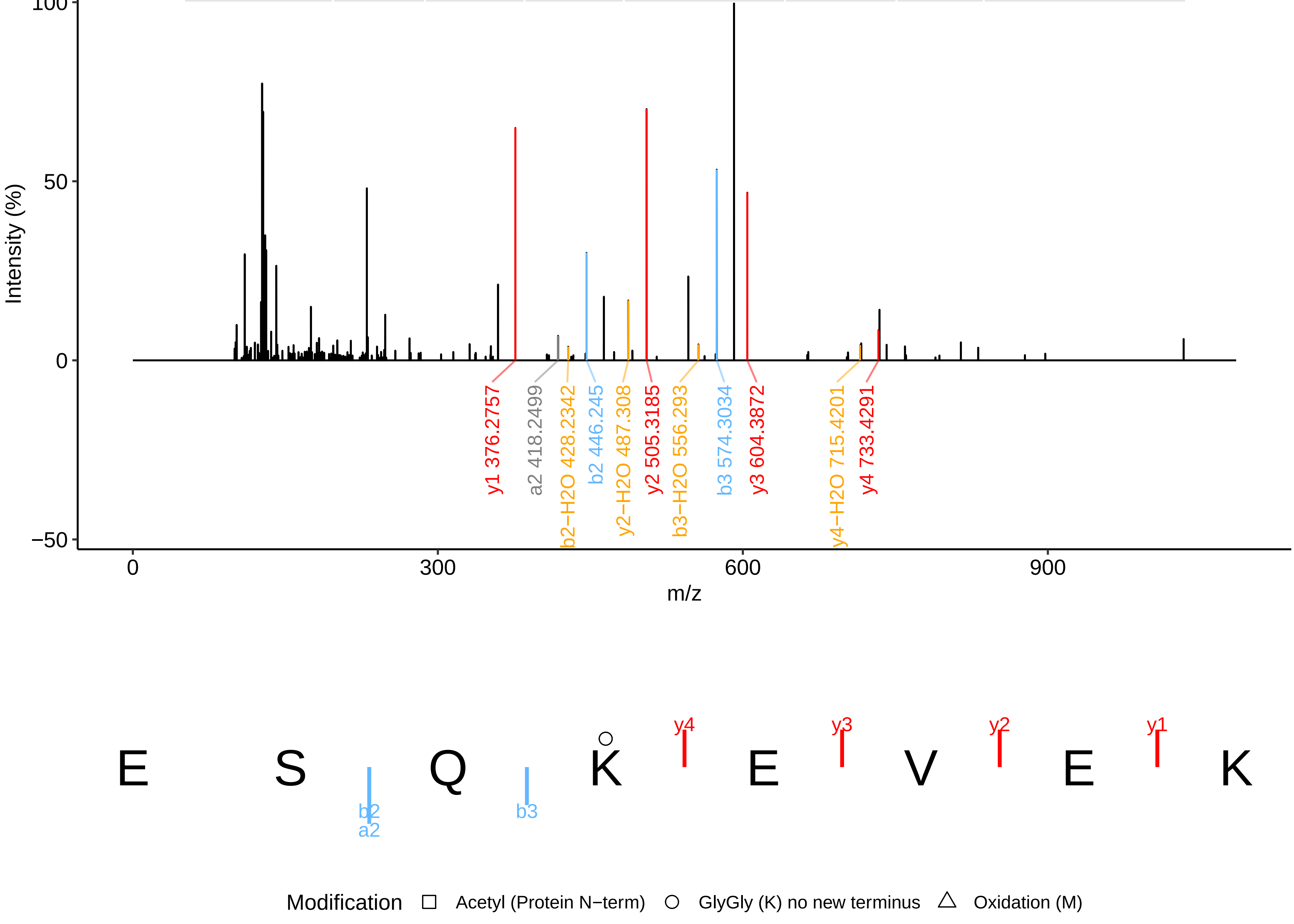

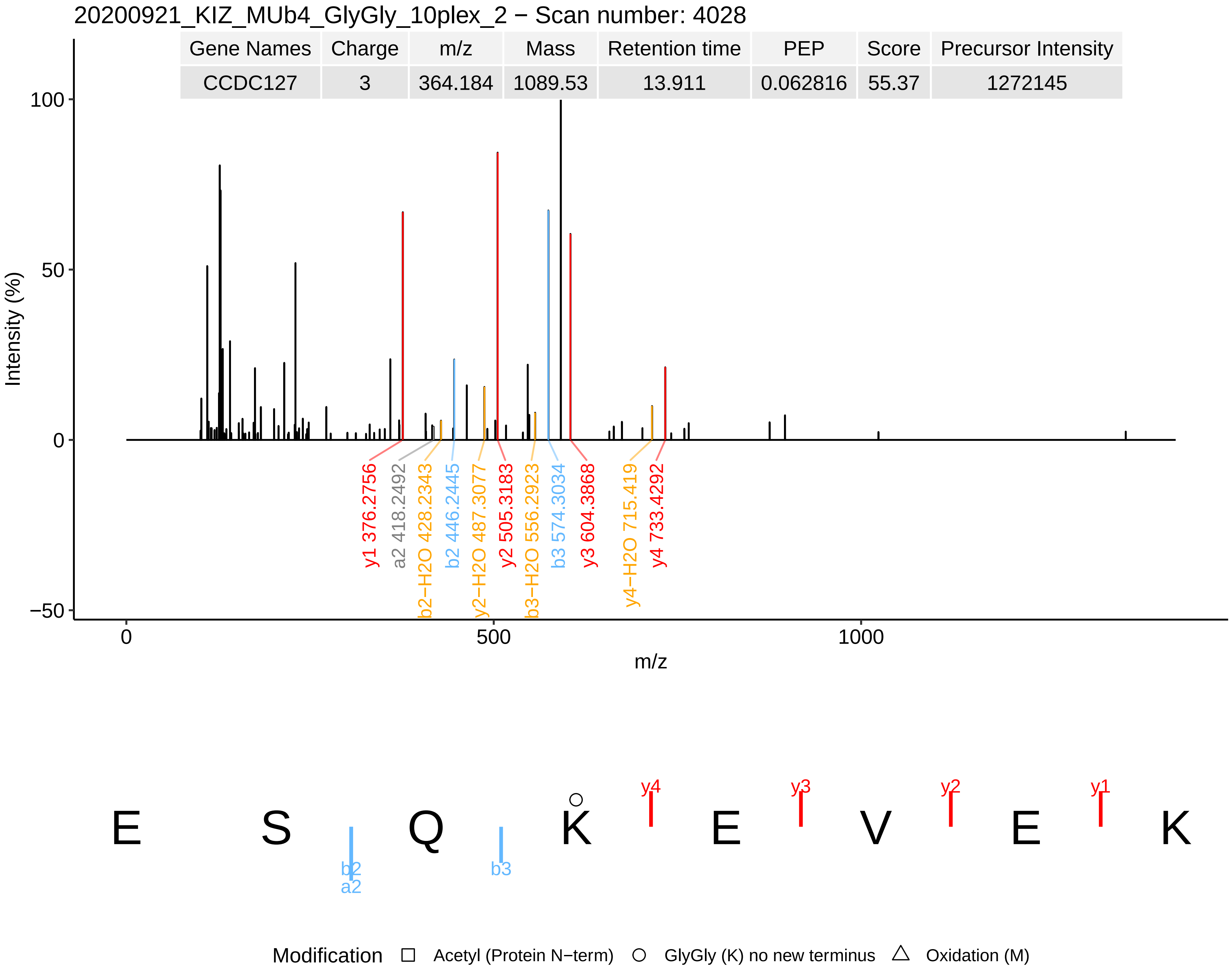
20200921_KIZ_MUb4_GlyGly_10plex_2 − Scan number: 4028
Gene Names | Charge | m/z | Mass | Retention time | PEP | Score | Precursor Intensity
CCDC127 | 3 | 364.184 | 1089.53 | 13.911 | 0.062816 | 55.37 | 1272145
Intensity (%)
100
50
0
−50
0
500
1000
m/z
y1 376.2756
a2 418.2492
b2−H2O 428.2343
b2 446.2445
y2−H2O 487.3077
y2 505.3183
b3−H2O 556.2923
b3 574.3034
y3 604.3868
y4−H2O 715.419
y4 733.4292
E S Q K E V E K
y4
y3
y2
y1
b2
a2
b3
Modification
Acetyl (Protein N−term)
GlyGly (K) no new terminus
Oxidation (M)

20200921_KIZ_MUb4_GlyGly_10plex − Scan number: 10252

Gene Names	Charge	m/z	Mass	Retention time	PEP	Score	Precursor Intensity
USP14	3	367.8803	1100.619	35.408	0.0031801	82.645	3190091

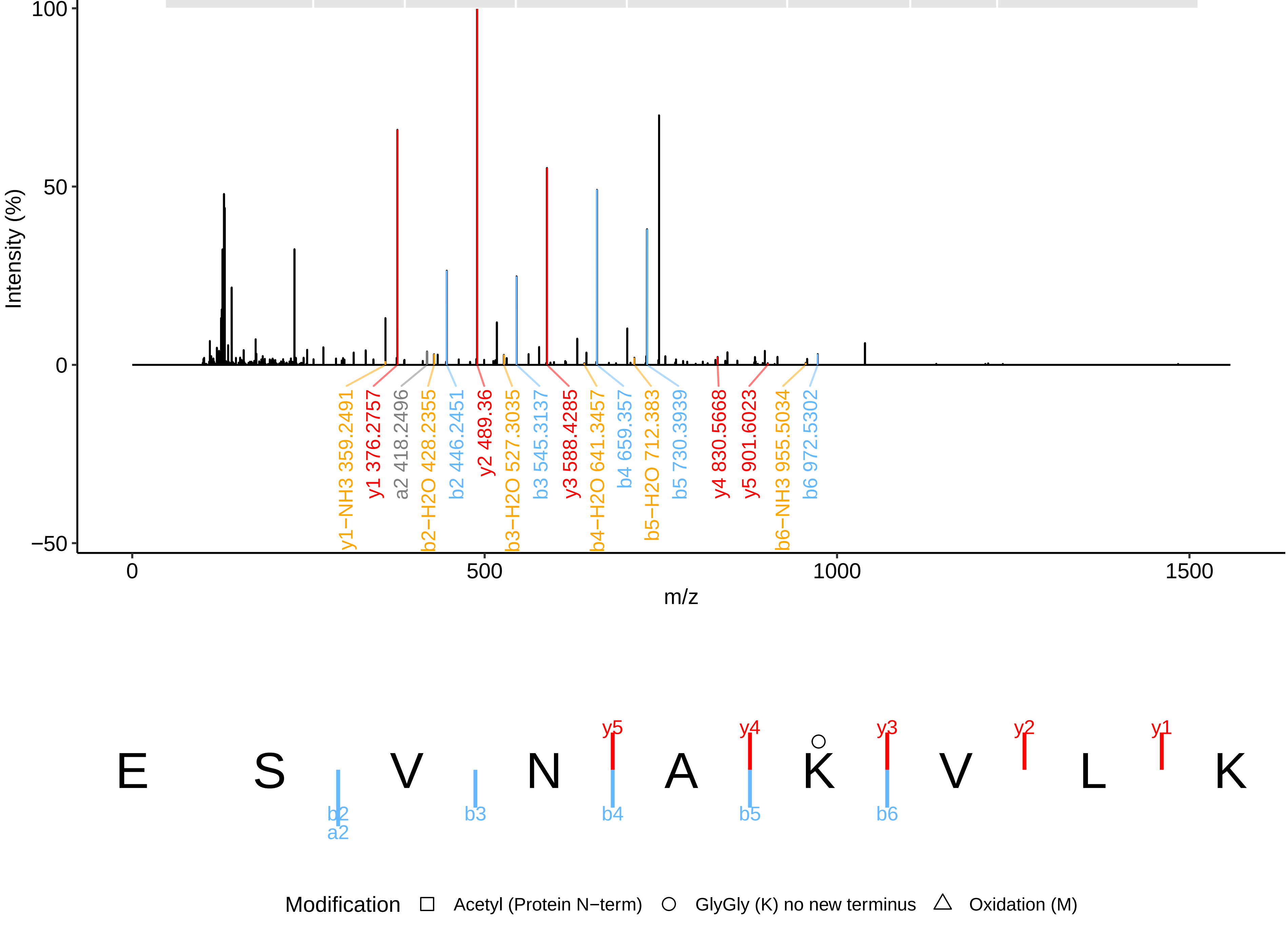

20200921_KIZ_MUb4_GlyGly_10plex_2 − Scan number: 9457

Gene Names	Charge	m/z	Mass	Retention time	PEP	Score	Precursor Intensity
USP14	3	367.8803	1100.619	35.952	0.10867	45.161	903632.9

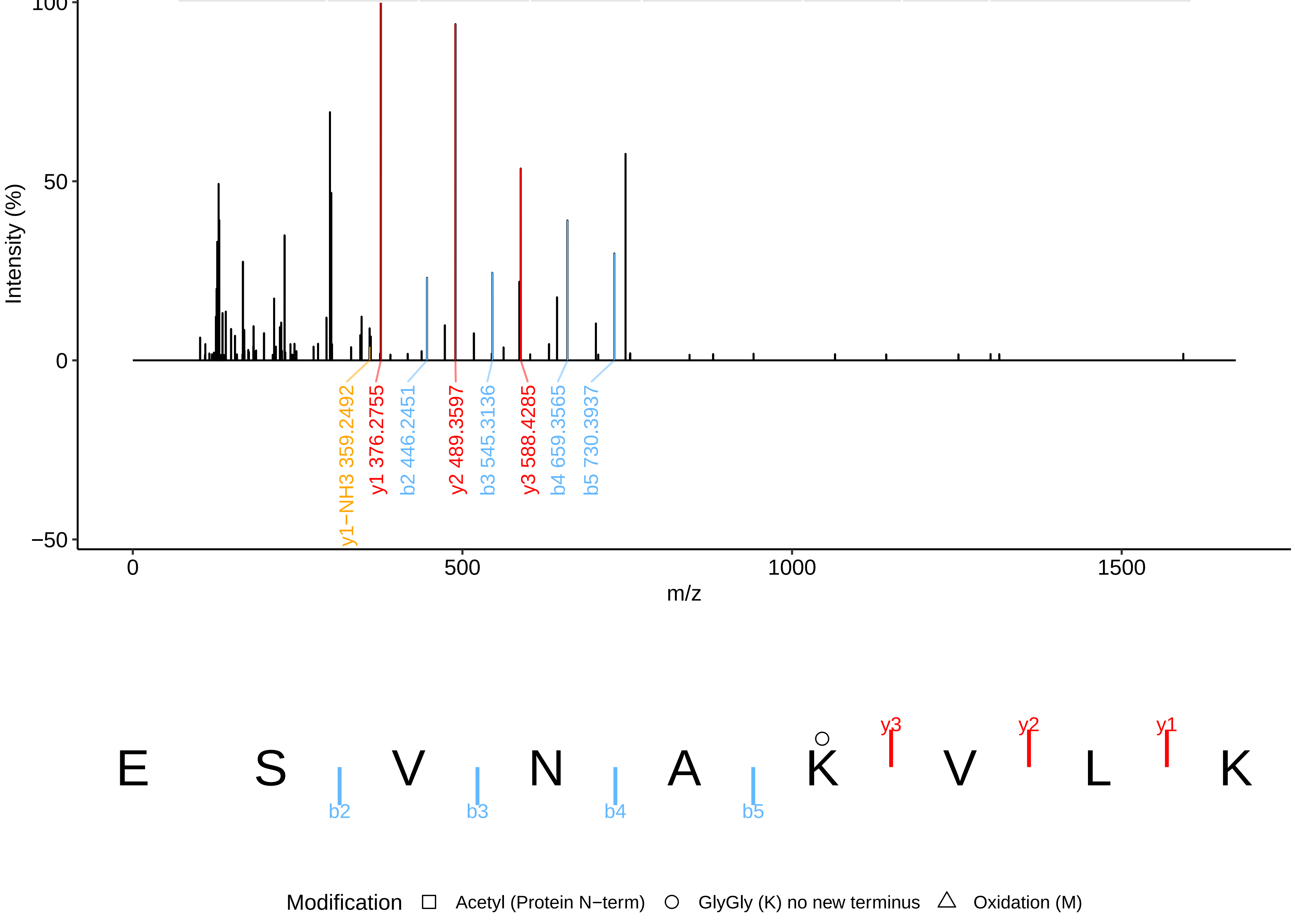

20200921_KIZ_MUb4_GlyGly_10plex − Scan number: 13830

Gene Names	Charge	m/z	Mass	Retention time	PEP	Score	Precursor Intensity
RPS2	3	449.5677	1345.681	48.917	0.0020144	81.099	8618164

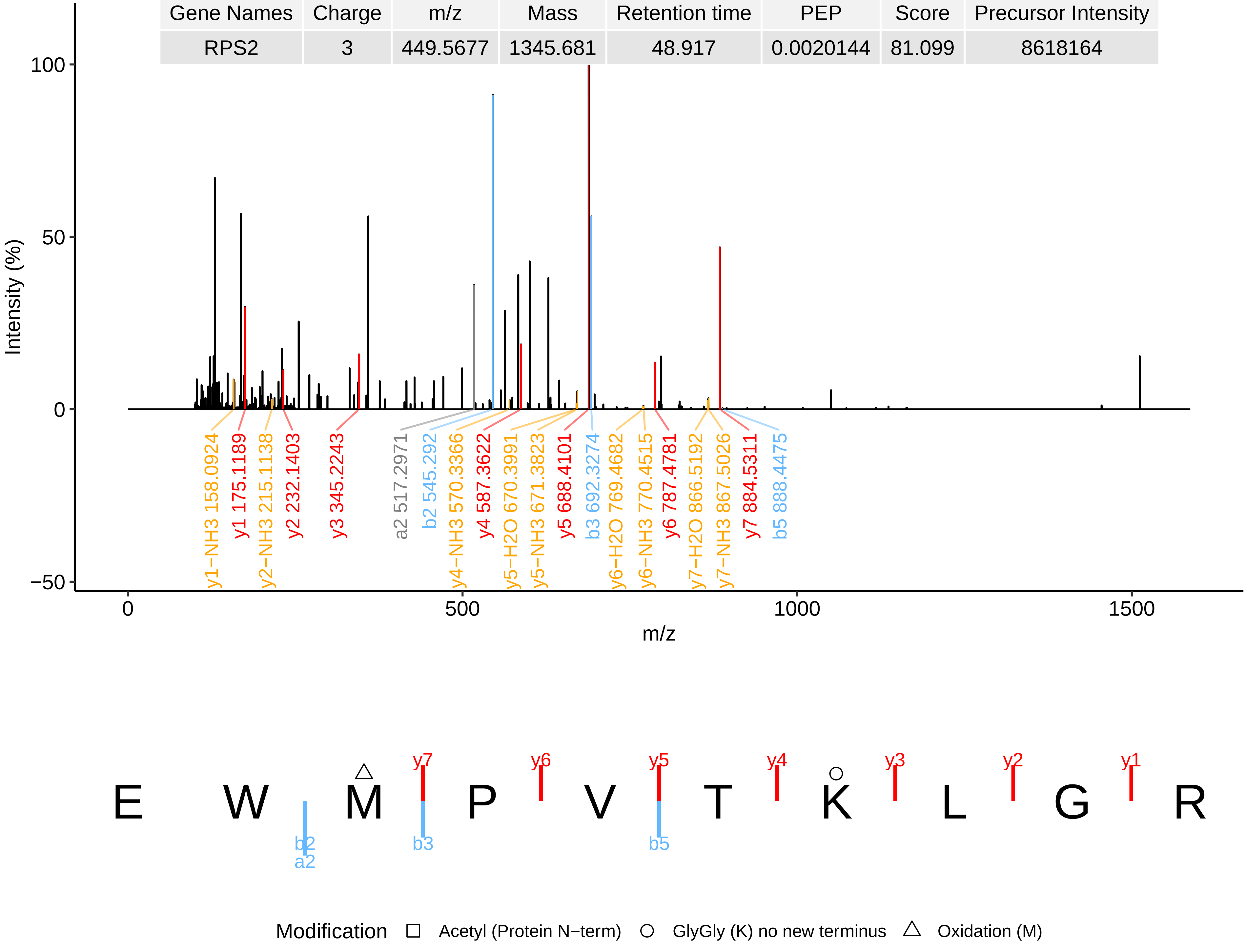

20200921_KIZ_MUb4_GlyGly_10plex_2 − Scan number: 12715

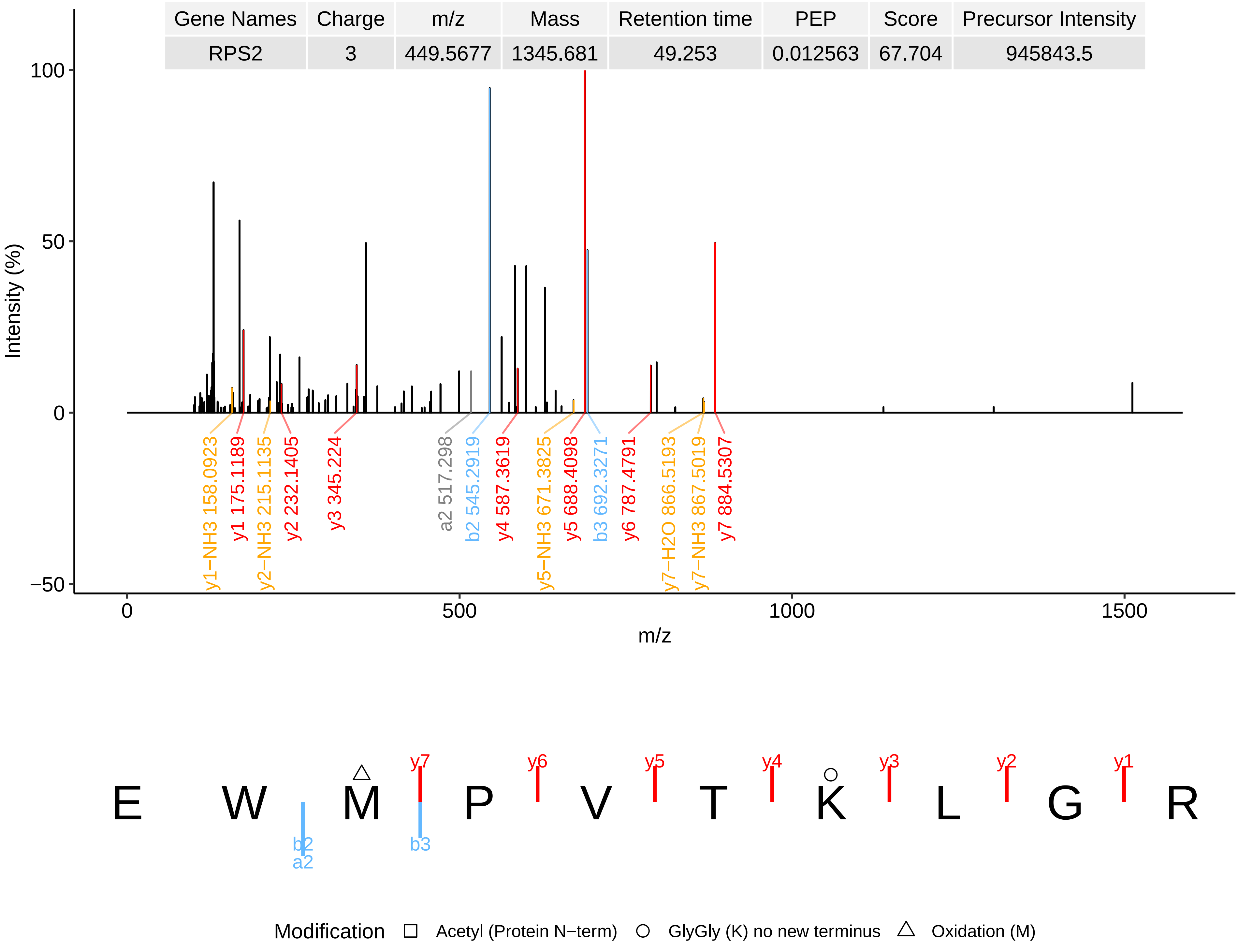

20200924_KIZ_MUb5_GlyGly_1 − Scan number: 12725

Gene Names	Charge	m/z	Mass	Retention time	PEP	Score	Precursor Intensity
RPS2	3	449.5677	1345.681	41.444	0.11617	53.597	3288116

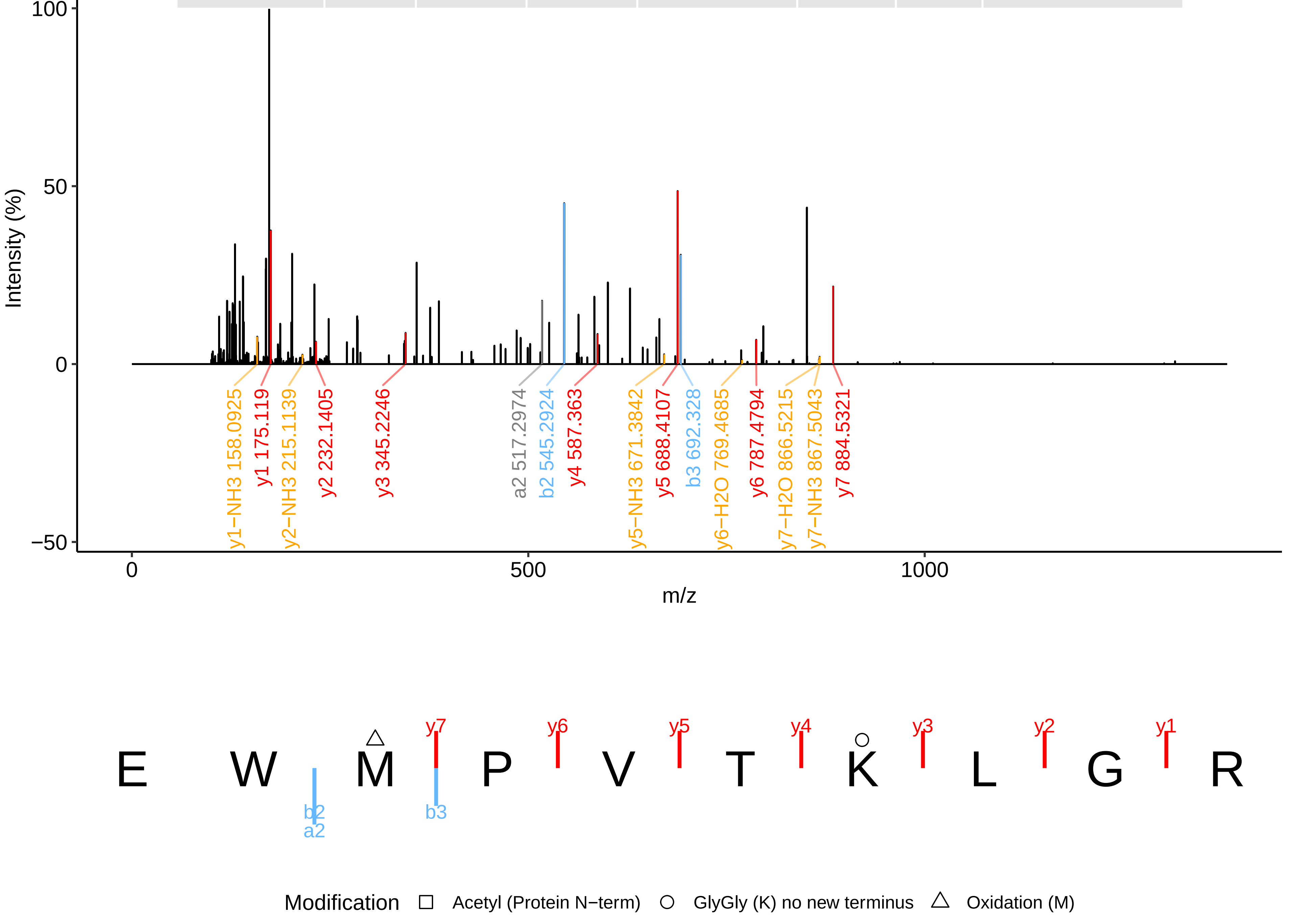

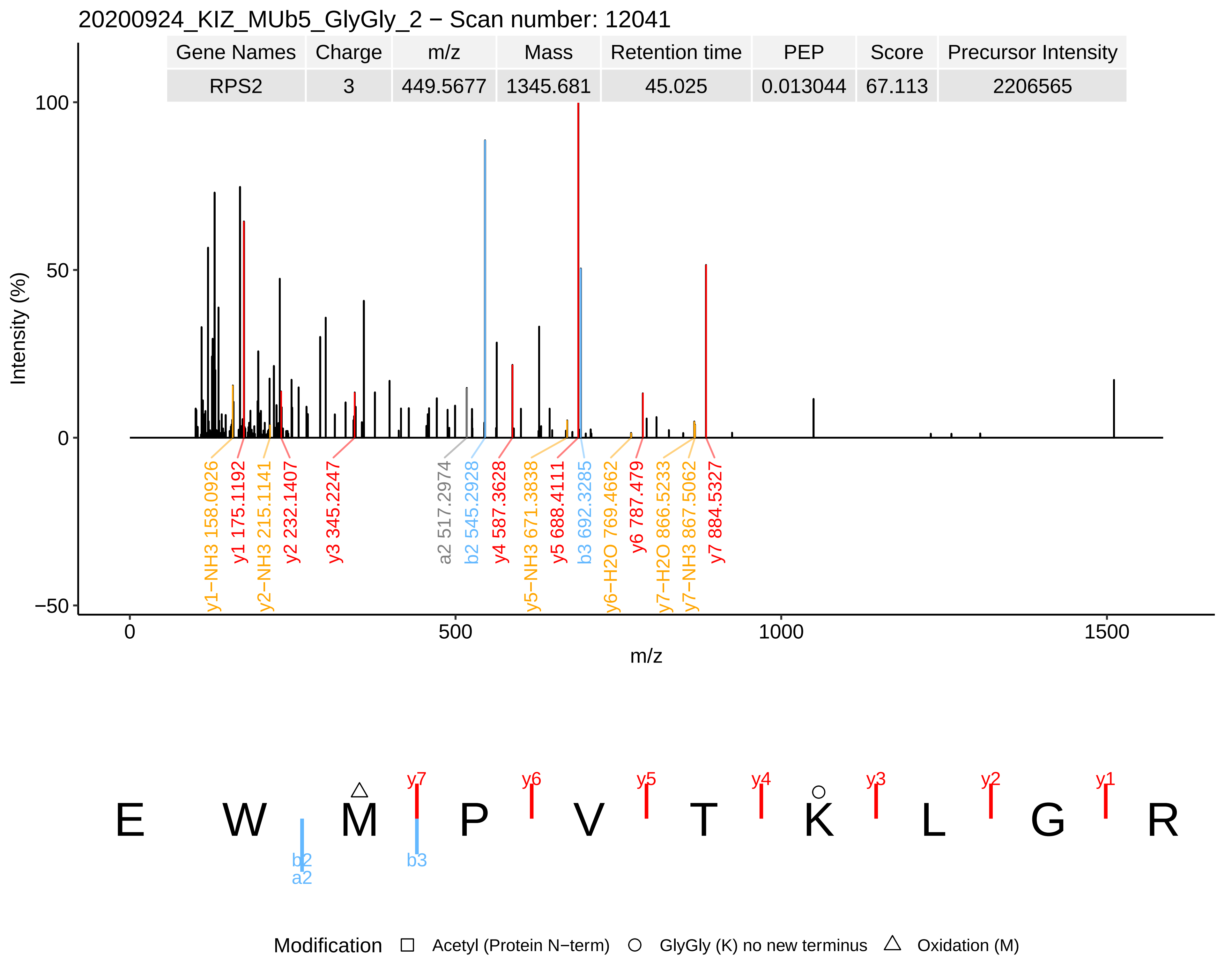
20200924_KIZ_MUb5_GlyGly_2 − Scan number: 12041
Gene Names | Charge | m/z | Mass | Retention time | PEP | Score | Precursor Intensity
RPS2 | 3 | 449.5677 | 1345.681 | 45.025 | 0.013044 | 67.113 | 2206565
Intensity (%)
100
50
0
−50
0
500
1000
1500
m/z
y1−NH3 158.0926
y1 175.1192
y2−NH3 215.1141
y2 232.1407
y3 345.2247
a2 517.2974
b2 545.2928
y4 587.3628
y5−NH3 671.3838
y5 688.4111
b3 692.3285
y6−H2O 769.4662
y6 787.479
y7−H2O 866.5233
y7−NH3 867.5062
y7 884.5327
E W M P V T K L G R
y7 y6 y5 y4 y3 y2 y1
b2
a2
b3
Modification
Acetyl (Protein N−term)
GlyGly (K) no new terminus
Oxidation (M)

20200924_KIZ_MUb5_GlyGly_2 − Scan number: 11971

Gene Names	Charge	m/z	Mass	Retention time	PEP	Score	Precursor Intensity
RPS2	3	449.5677	1345.681	44.756	0.021597	68.847	3365591

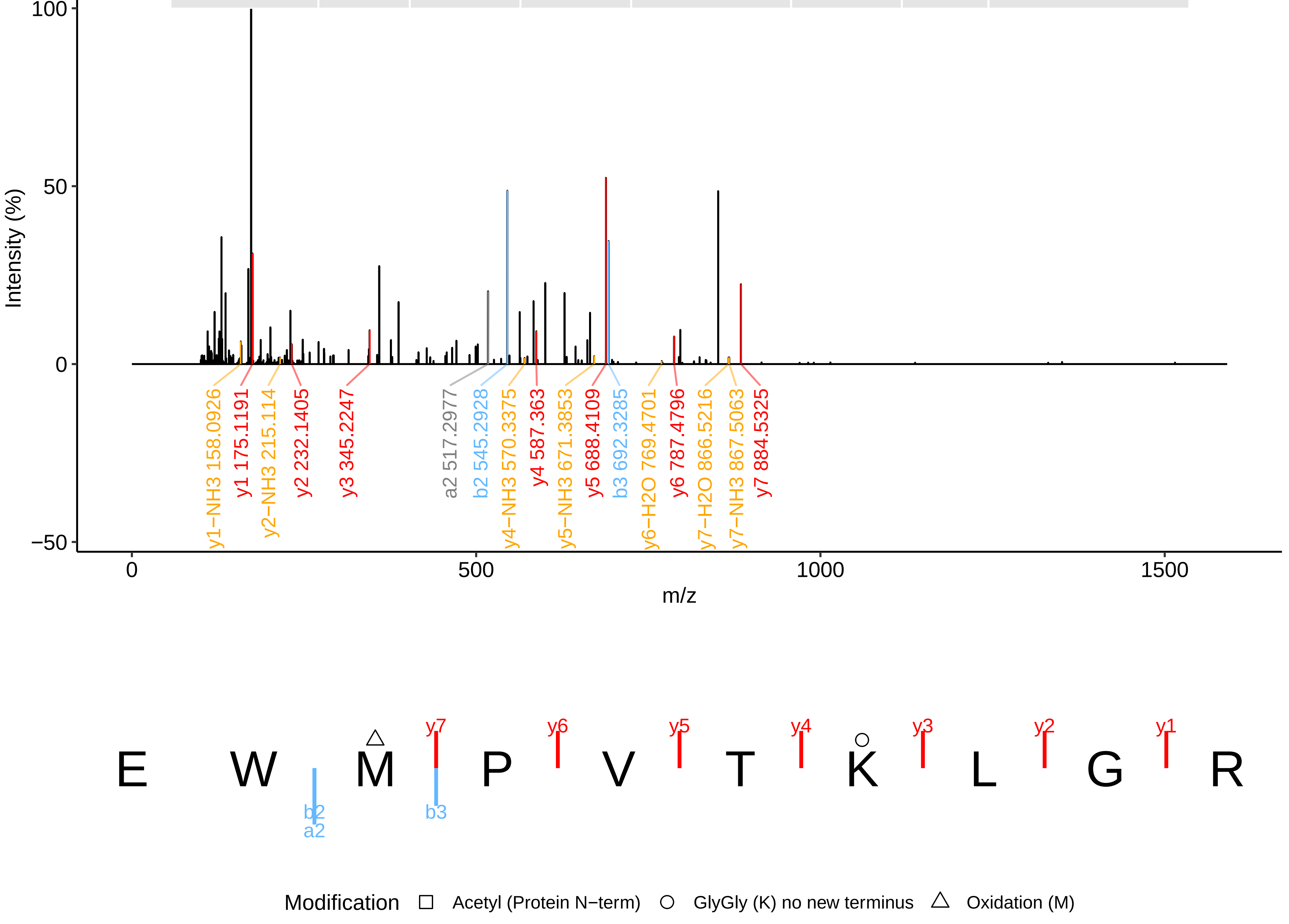

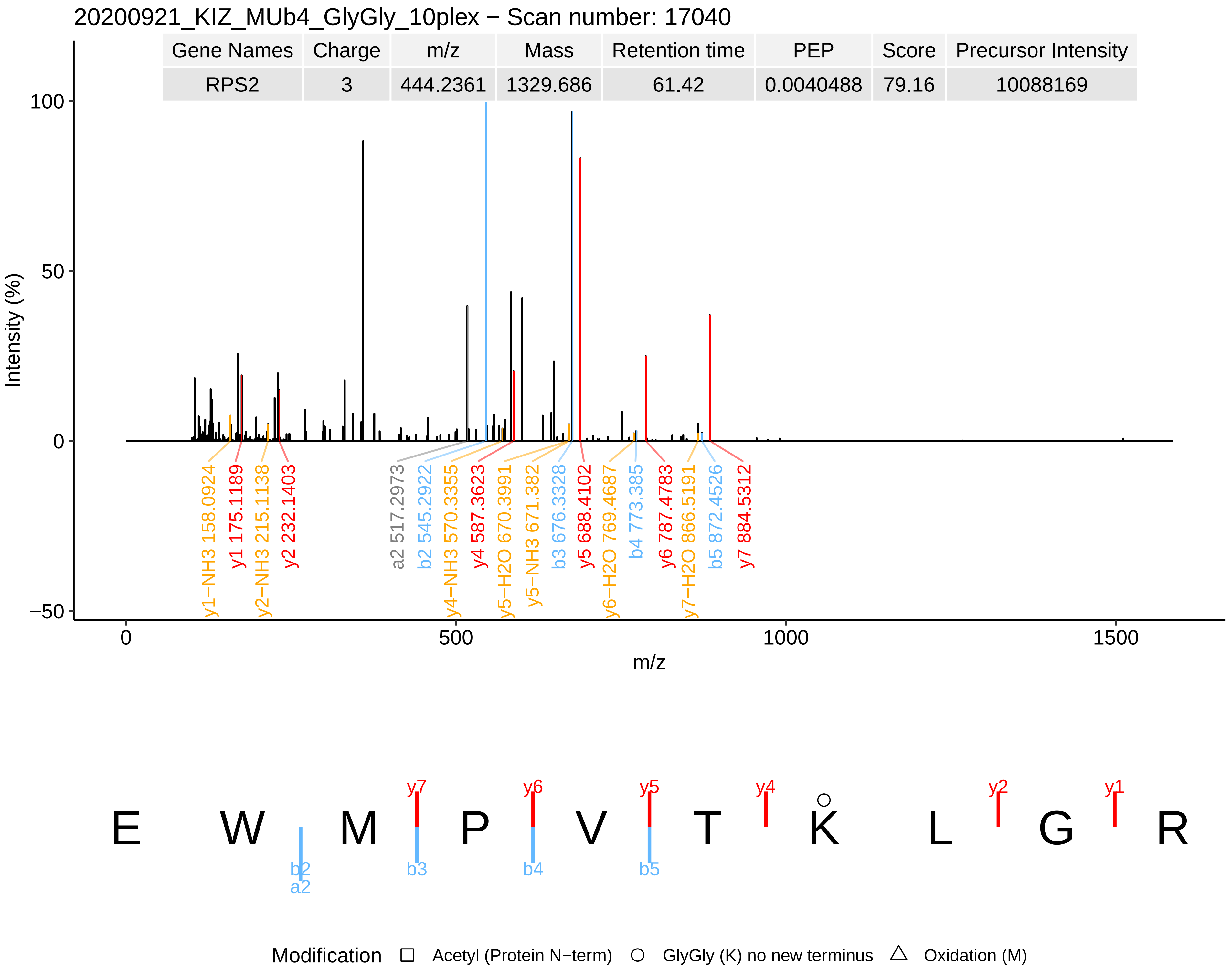
20200921_KIZ_MUb4_GlyGly_10plex − Scan number: 17040
Gene Names | Charge | m/z | Mass | Retention time | PEP | Score | Precursor Intensity
RPS2 | 3 | 444.2361 | 1329.686 | 61.42 | 0.0040488 | 79.16 | 10088169
Intensity (%)
100
50
0
−50
m/z
0
500
1000
1500
y1−NH3 158.0924
y1 175.1189
y2−NH3 215.1138
y2 232.1403
a2 517.2973
b2 545.2922
y4−NH3 570.3355
y4 587.3623
y5−H2O 670.3991
y5−NH3 671.382
b3 676.3328
y5 688.4102
y6−H2O 769.4687
b4 773.385
y6 787.4783
y7−H2O 866.5191
b5 872.4526
y7 884.5312
E W M P V T K L G R
y7 y6 y5 y4 y2 y1
b2 a2 b3 b4 b5
Modification
Acetyl (Protein N−term)
GlyGly (K) no new terminus
Oxidation (M)

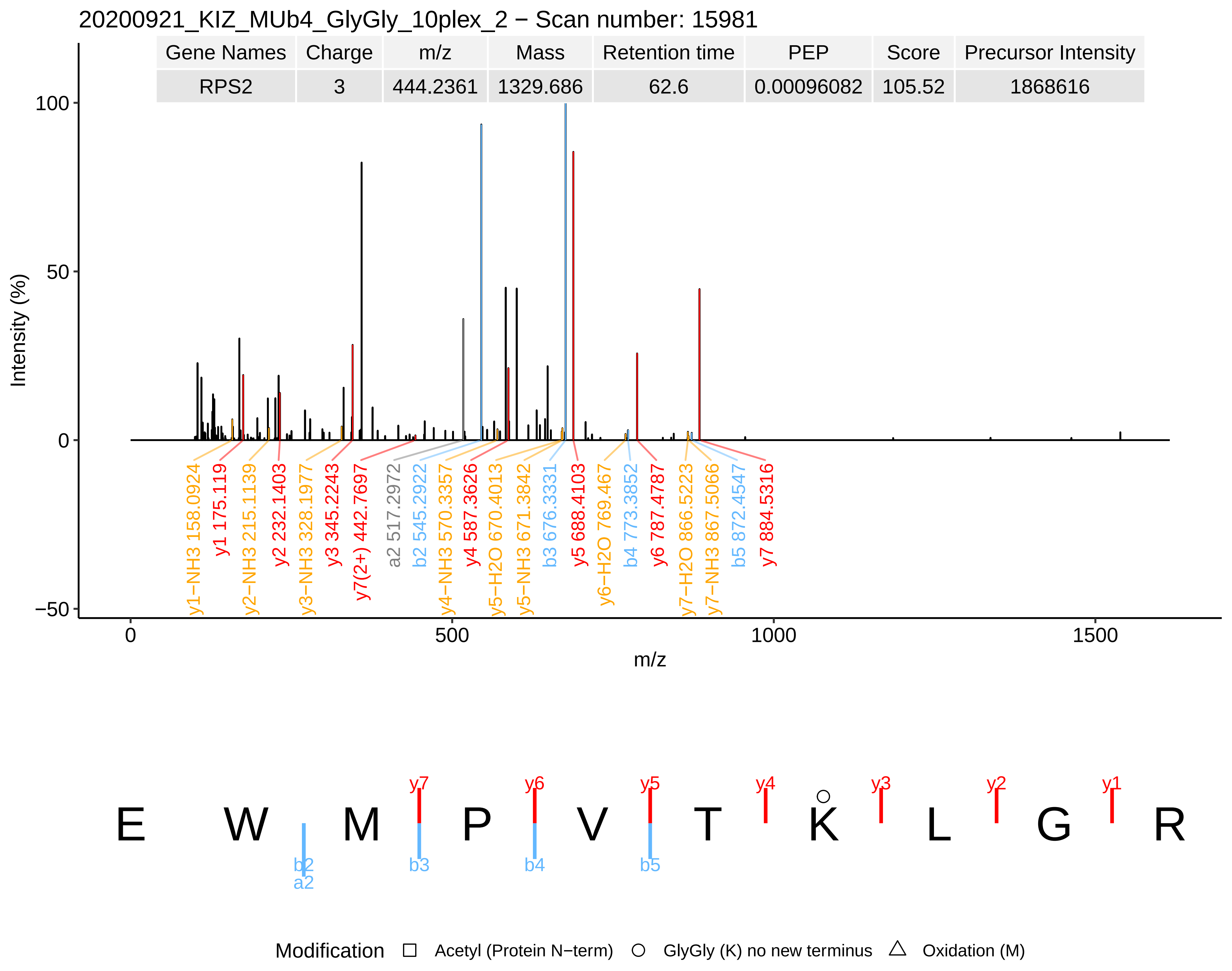
20200921_KIZ_MUb4_GlyGly_10plex_2 − Scan number: 15981
Gene Names | Charge | m/z | Mass | Retention time | PEP | Score | Precursor Intensity
RPS2 | 3 | 444.2361 | 1329.686 | 62.6 | 0.00096082 | 105.52 | 1868616
Intensity (%)
100
50
0
−50
0
500
1000
1500
m/z
y1−NH3 158.0924
y1 175.119
y2−NH3 215.1139
y2 232.1403
y3−NH3 328.1977
y3 345.2243
y7(2+) 442.7697
a2 517.2972
b2 545.2922
y4−NH3 570.3357
y4 587.3626
y5−H2O 670.4013
y5−NH3 671.3842
b3 676.3331
y5 688.4103
y6−H2O 769.467
b4 773.3852
y6 787.4787
y7−H2O 866.5223
y7−NH3 867.5066
b5 872.4547
y7 884.5316
E W M P V T K L G R
y7 y6 y5 y4 y3 y2 y1
b2 a2 b3 b4 b5
Modification
Acetyl (Protein N−term)
GlyGly (K) no new terminus
Oxidation (M)

20200924_KIZ_MUb5_GlyGly_2 − Scan number: 15286

Gene Names	Charge	m/z	Mass	Retention time	PEP	Score	Precursor Intensity
RPS2	3	444.2361	1329.686	57.129	0.0011419	101.54	20289852

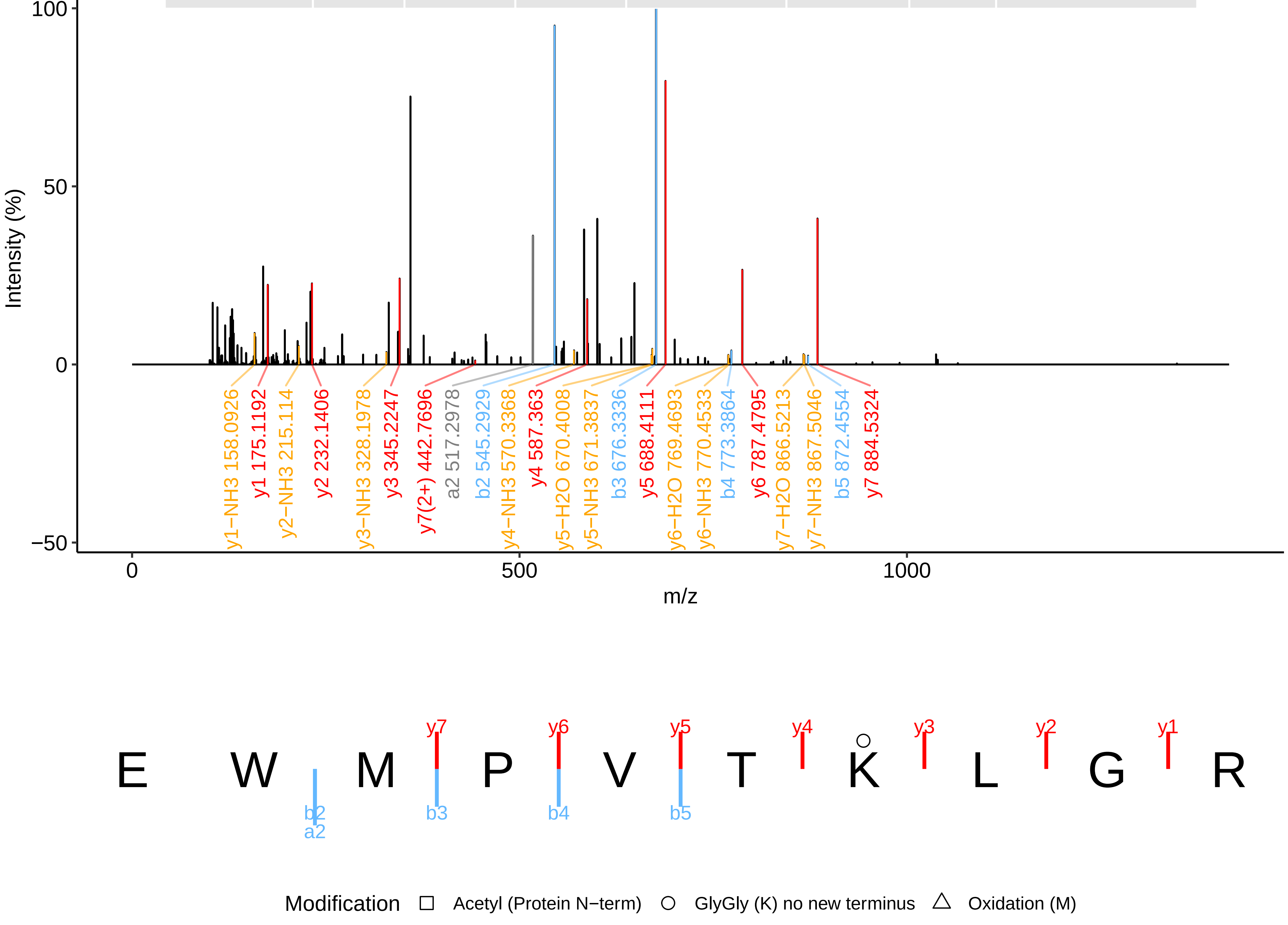

20201118_KIZ_MUb6_GlyGly_1 − Scan number: 16044

Gene Names	Charge	m/z	Mass	Retention time	PEP	Score	Precursor Intensity
RPS2	3	444.2361	1329.686	50.699	4.9238e−07	116.12	35362044

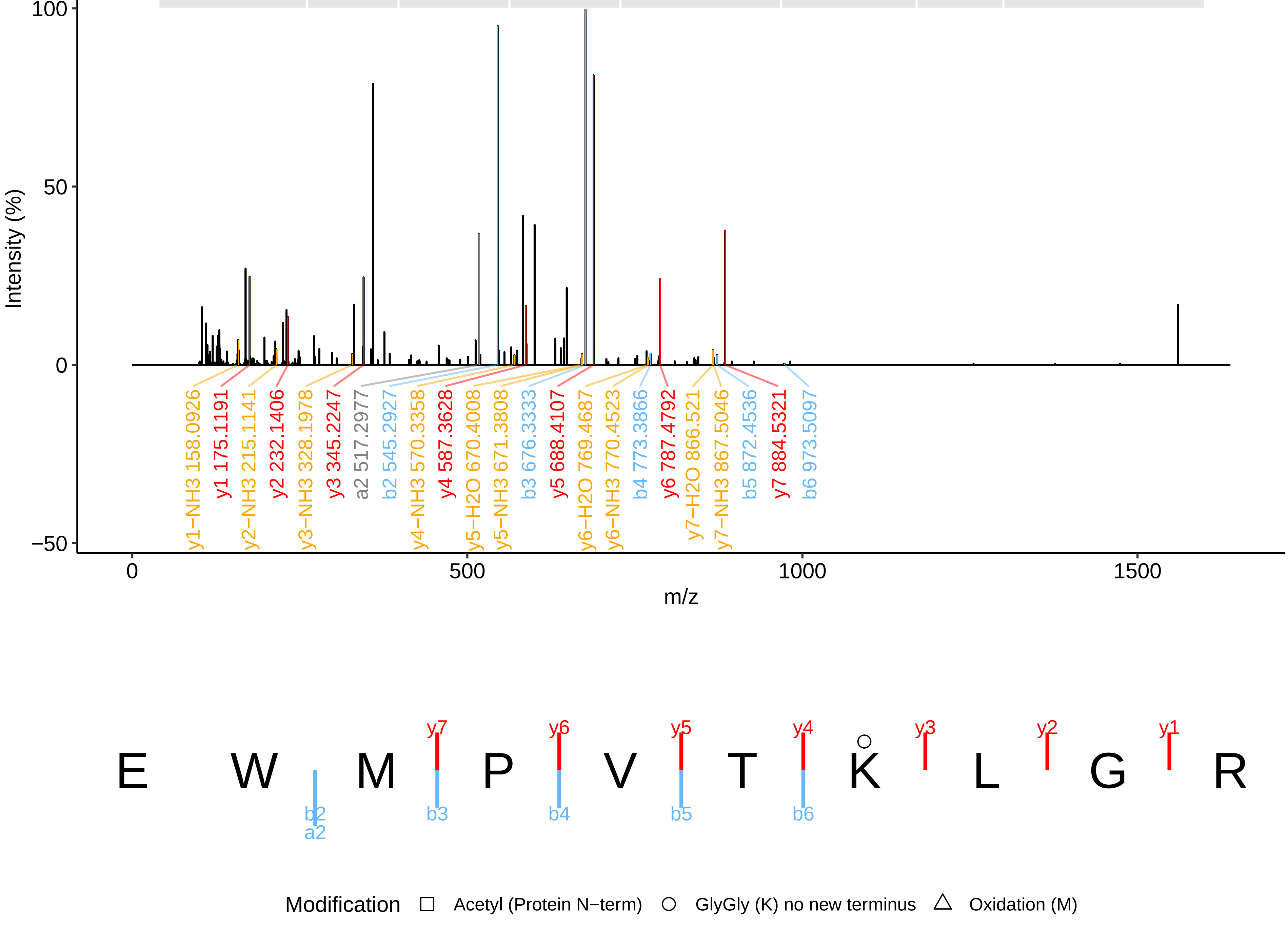

20201118_KIZ_MUb6_GlyGly_2 − Scan number: 18574

Gene Names	Charge	m/z	Mass	Retention time	PEP	Score	Precursor Intensity
RPS2	3	444.2361	1329.686	62.783	5.0092e−07	115.82	11788238

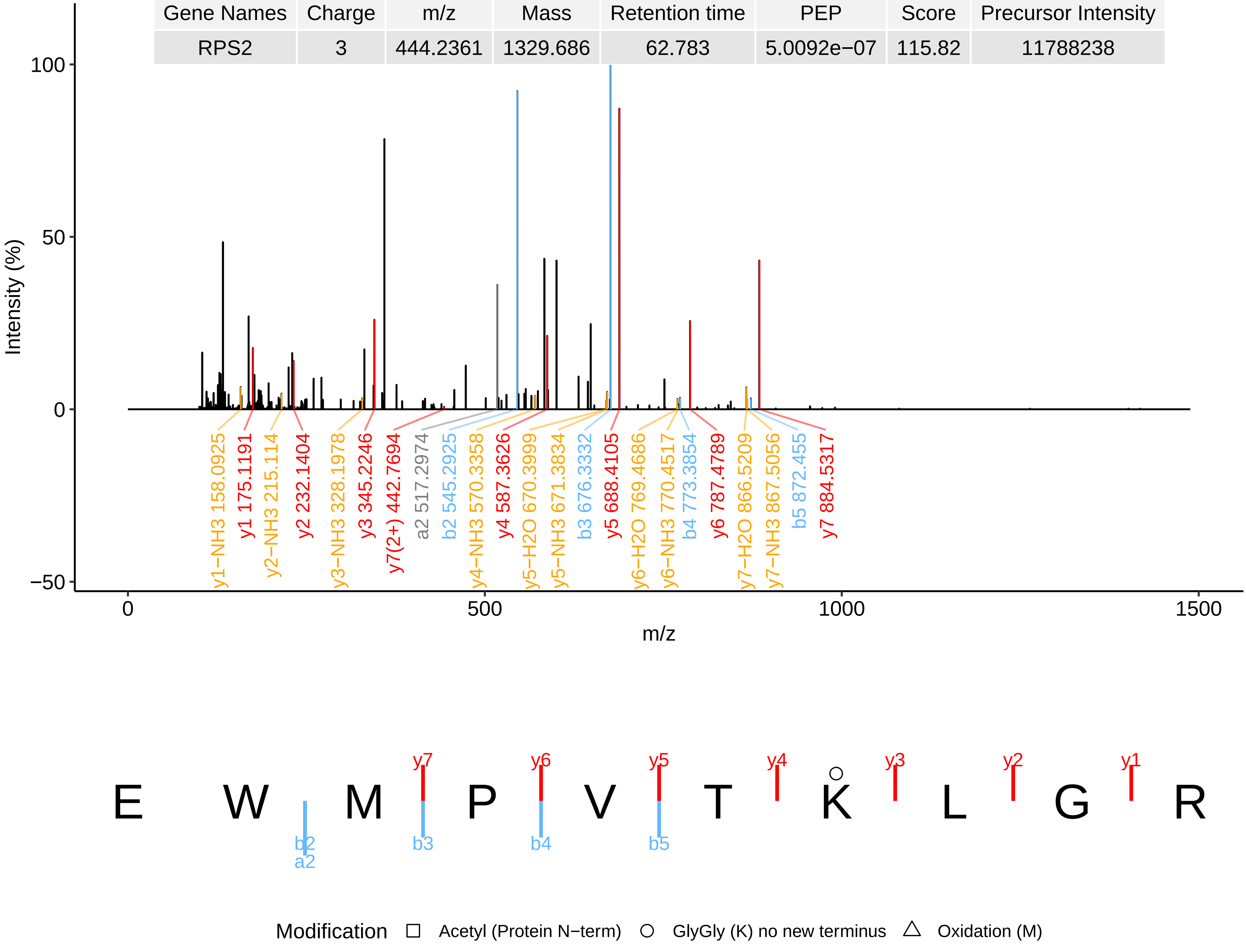

20200921_KIZ_MUb4_GlyGly_10plex − Scan number: 9593

Gene Names	Charge	m/z	Mass	Retention time	PEP	Score	Precursor Intensity
COX4I1	3	368.533	1102.577	32.886	0.020177	71.692	3724429

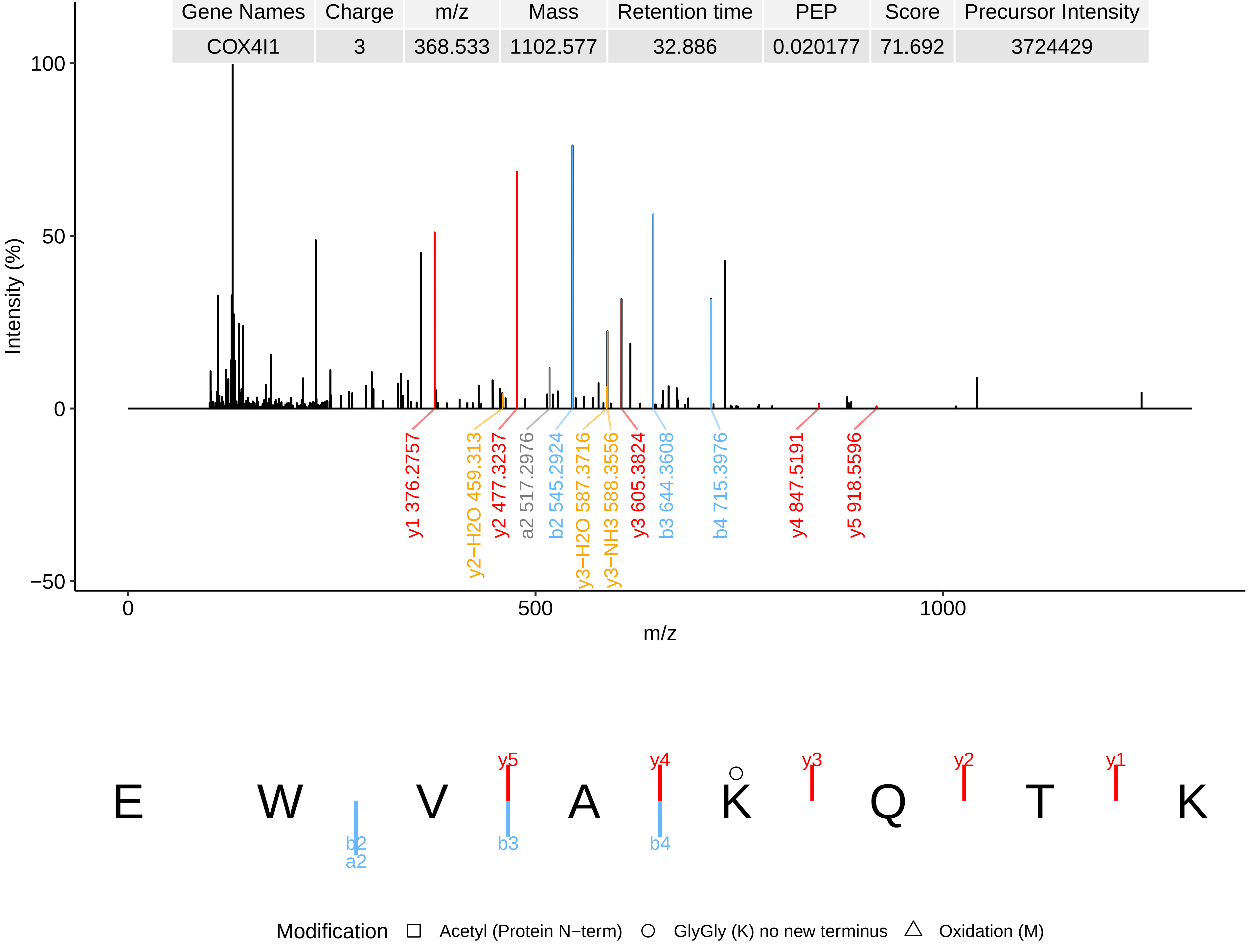

20200921_KIZ_MUb4_GlyGly_10plex_2 − Scan number: 8871

Gene Names	Charge	m/z	Mass	Retention time	PEP	Score	Precursor Intensity
COX4I1	3	368.533	1102.577	33.561	0.028206	65.246	655197.4

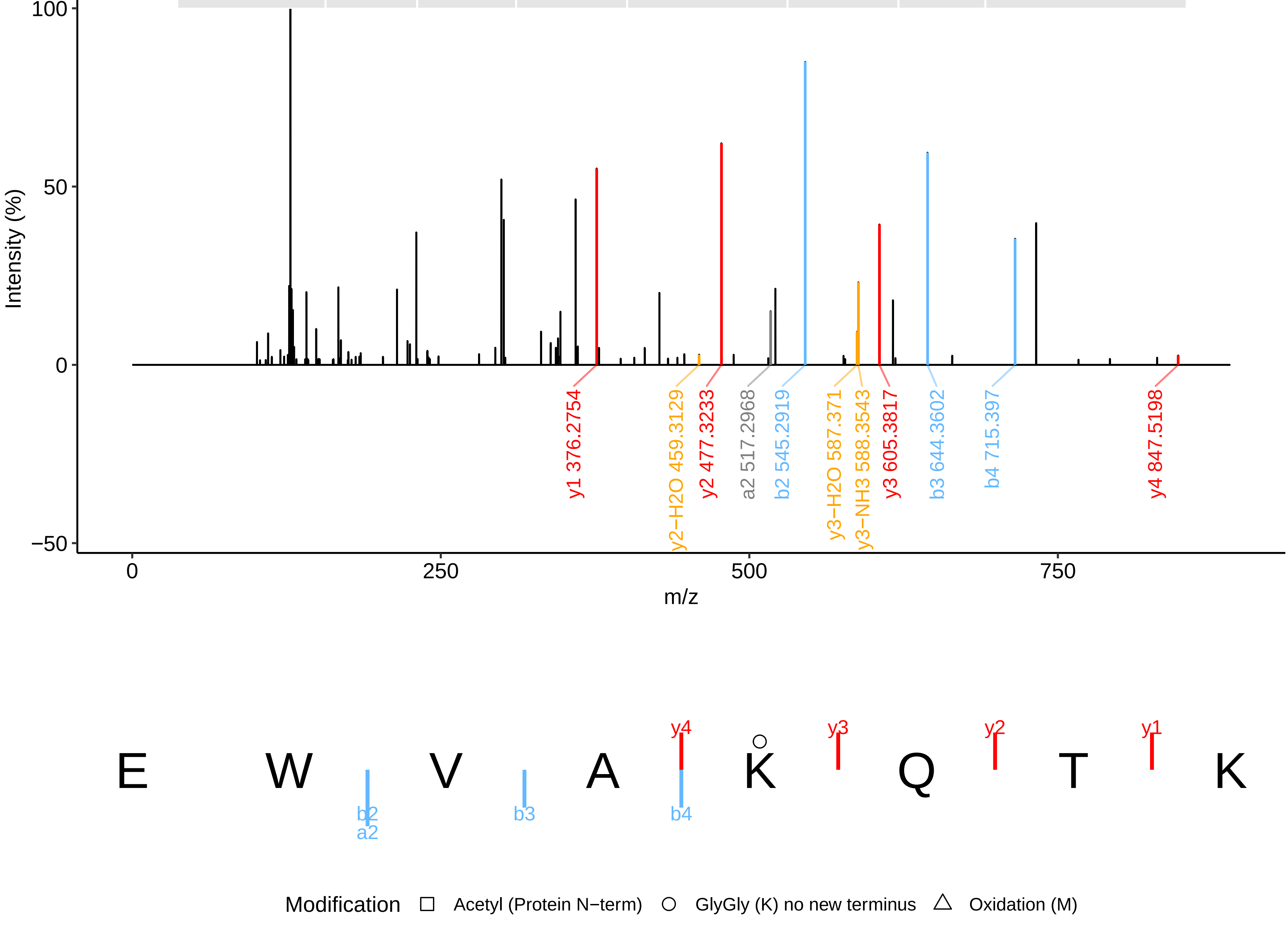

20200921_KIZ_MUb4_GlyGly_10plex_2 − Scan number: 10712

Gene Names	Charge	m/z	Mass	Retention time	PEP	Score	Precursor Intensity
LRRC59	3	374.8645	1121.572	41.04	0.10493	45.997	1135171

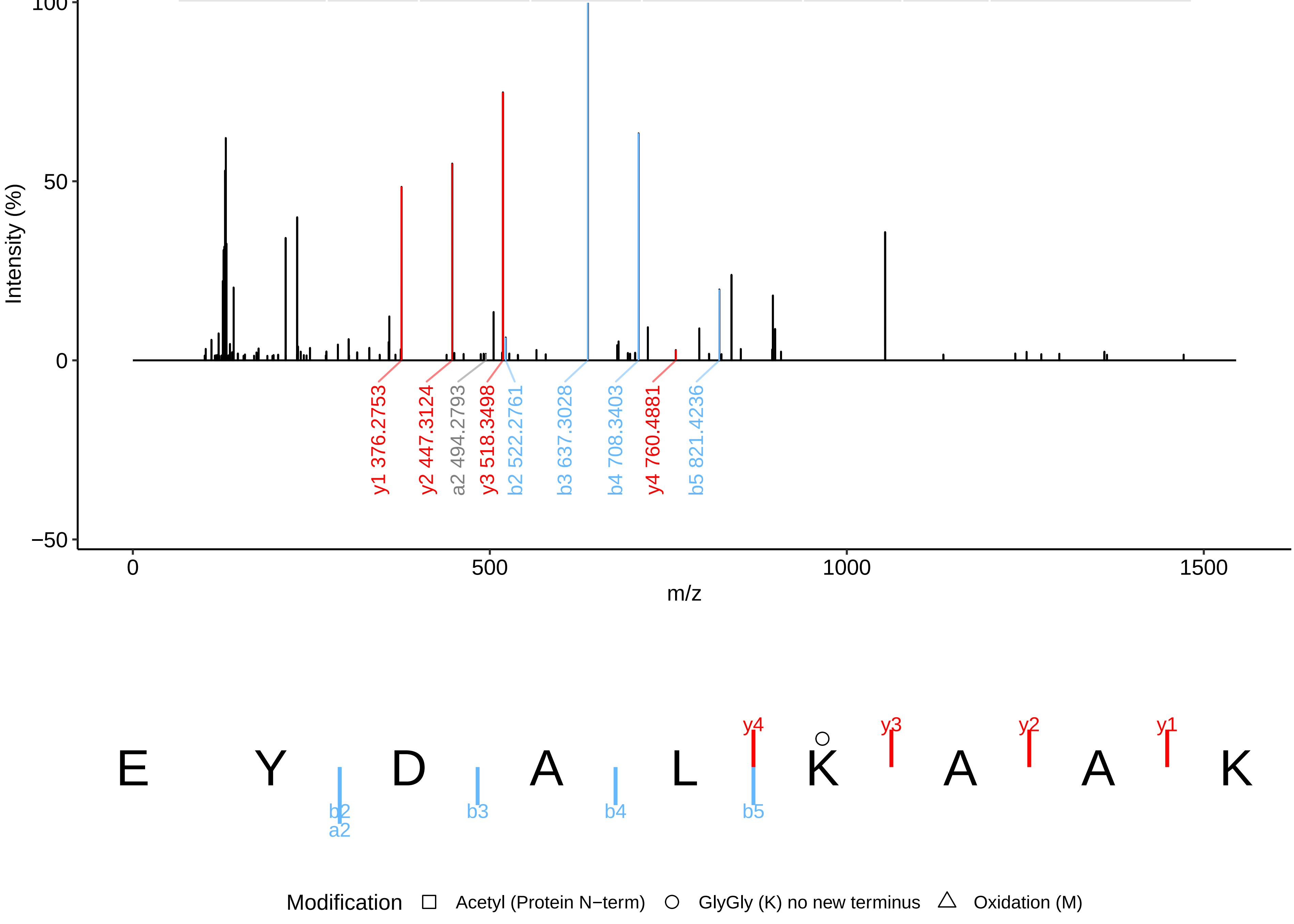

20200921_KIZ_MUb4_GlyGly_10plex − Scan number: 12330

Gene Names	Charge	m/z	Mass	Retention time	PEP	Score	Precursor Intensity
CLTC	3	370.5328	1108.576	43.192	0.0081439	83.005	6036251

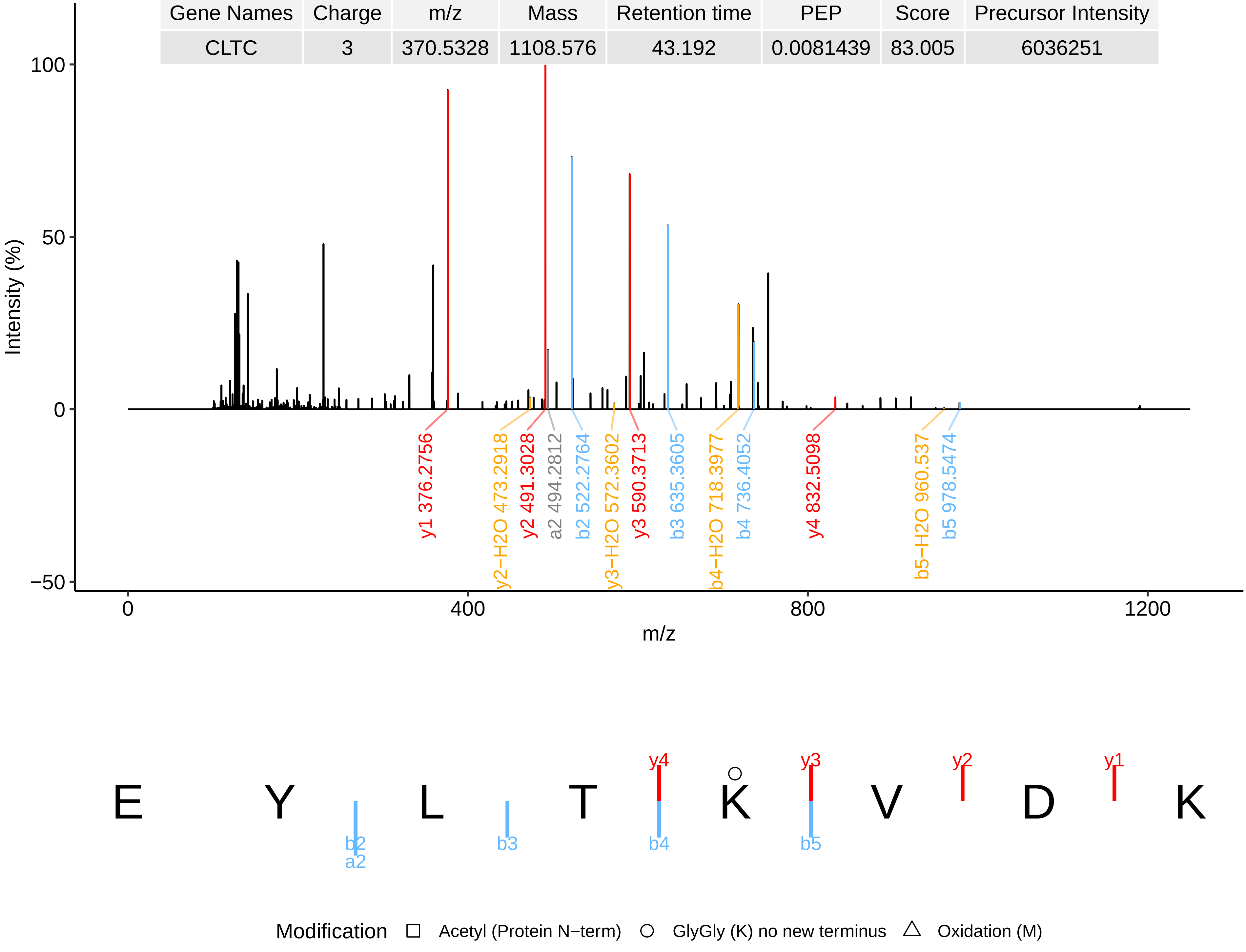

20200921_KIZ_MUb4_GlyGly_10plex_2 − Scan number: 11350

Gene Names	Charge	m/z	Mass	Retention time	PEP	Score	Precursor Intensity
CLTC	3	370.5328	1108.576	43.666	0.04202	60.436	4653682

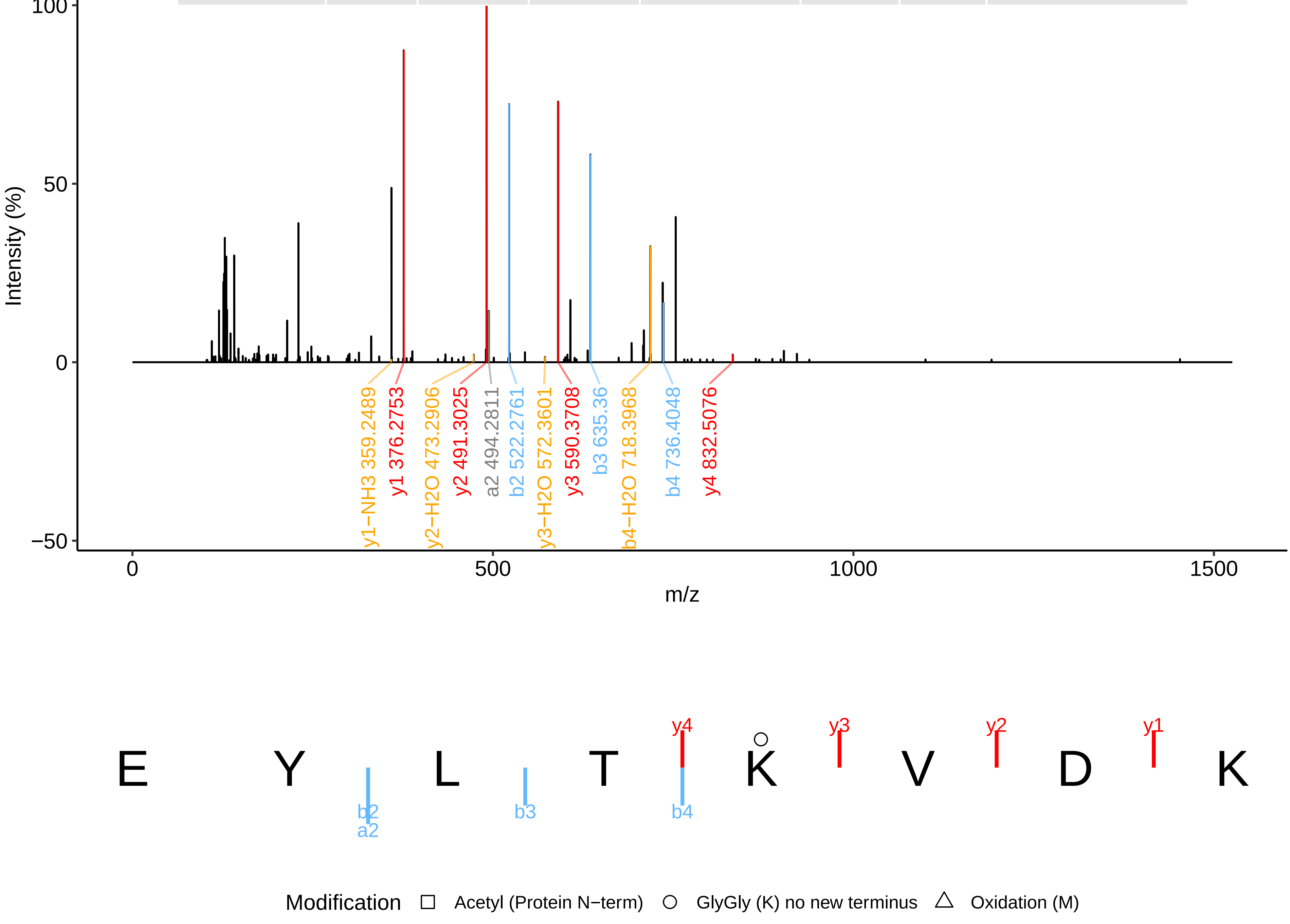

20200924_KIZ_MUb5_GlyGly_1 − Scan number: 11126

Gene Names	Charge	m/z	Mass	Retention time	PEP	Score	Precursor Intensity
CLTC	3	370.5328	1108.576	36.045	0.059866	59.8	15353129

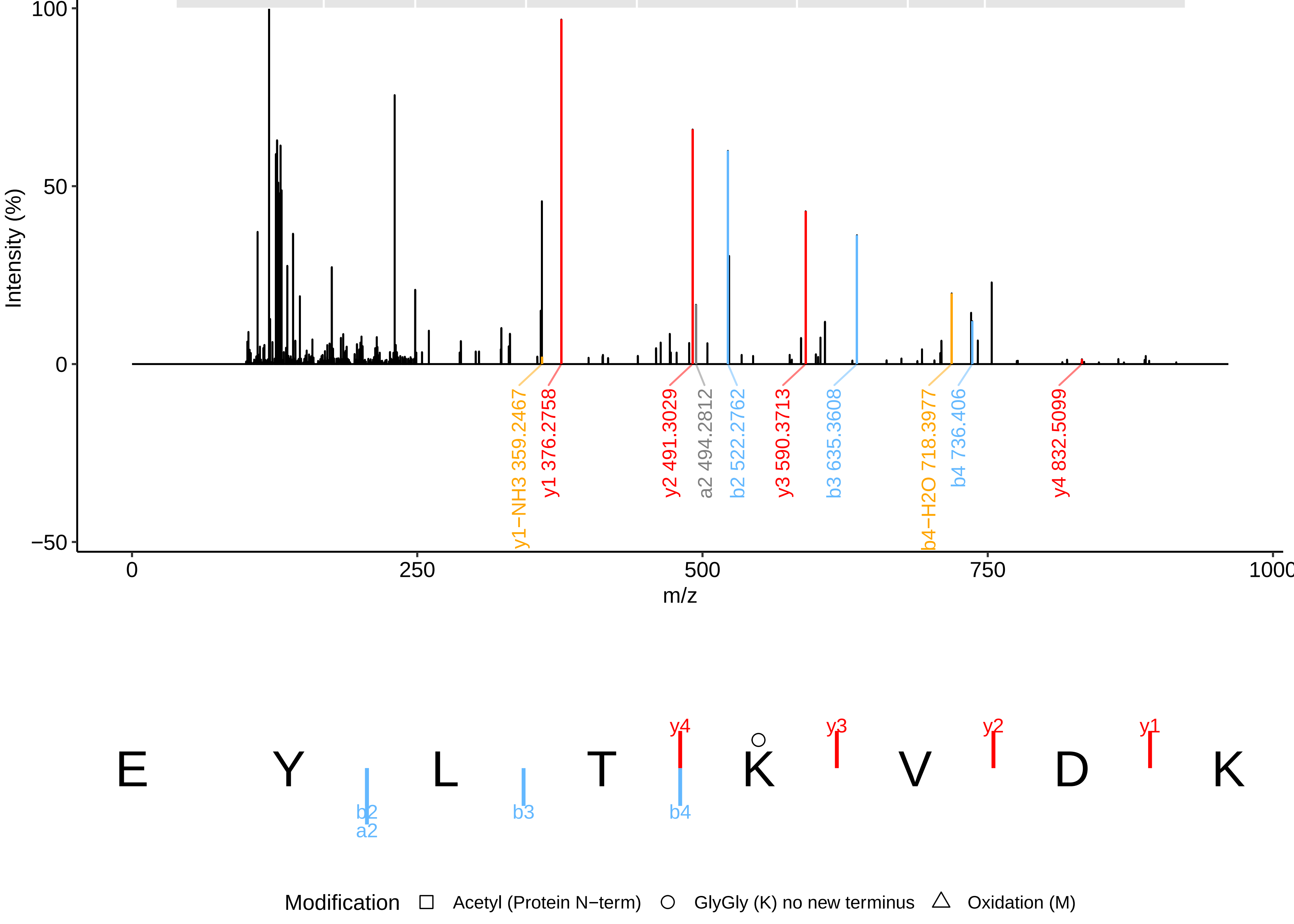

20200924_KIZ_MUb5_GlyGly_2 − Scan number: 10501

Gene Names	Charge	m/z	Mass	Retention time	PEP	Score	Precursor Intensity
CLTC	3	370.5328	1108.576	39.193	0.055382	60.436	4987781

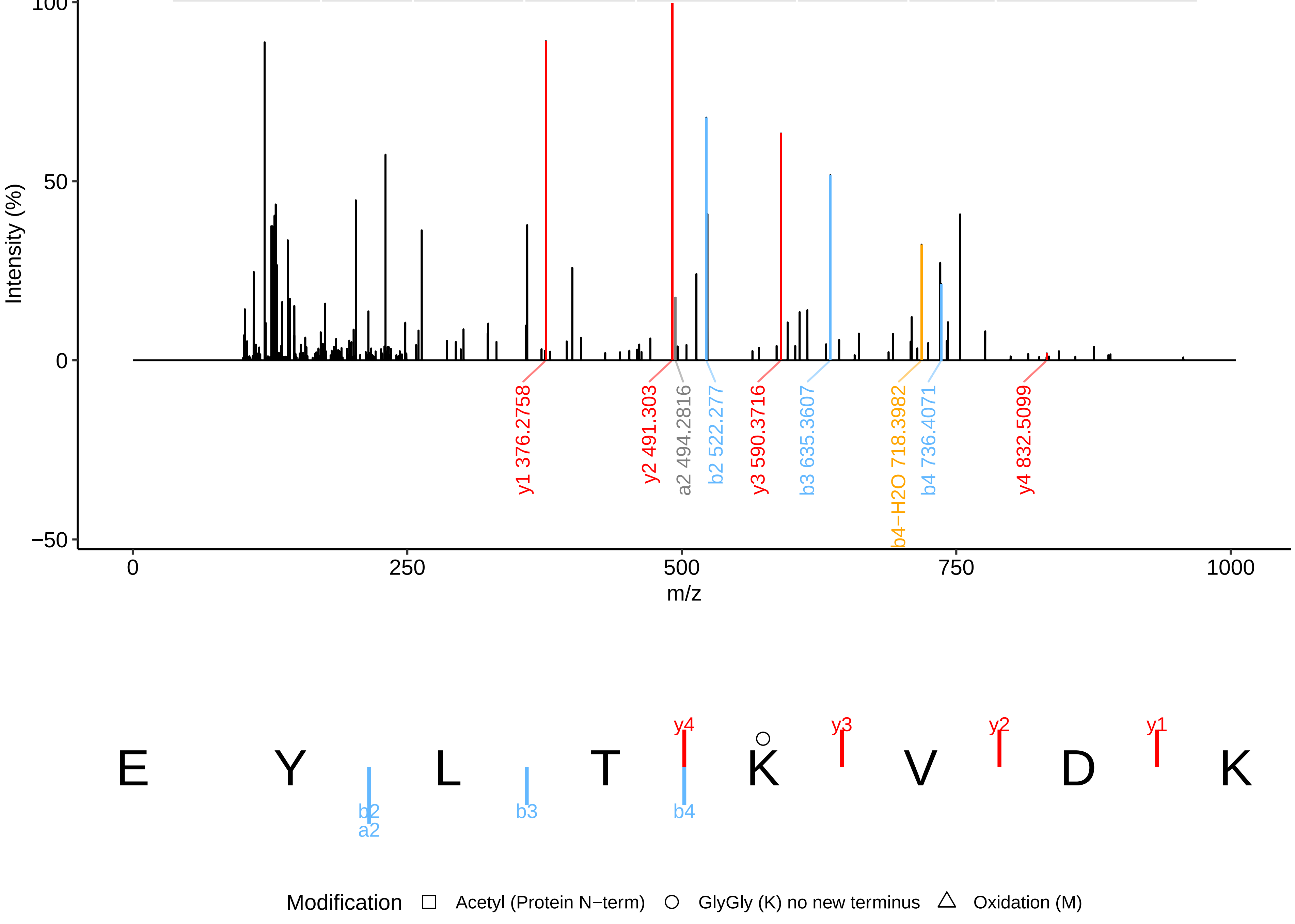

20200924_KIZ_MUb5_GlyGly_2 − Scan number: 10634

Gene Names	Charge	m/z	Mass	Retention time	PEP	Score	Precursor Intensity
CLTC	3	370.5328	1108.576	39.703	0.055382	60.436	3082703

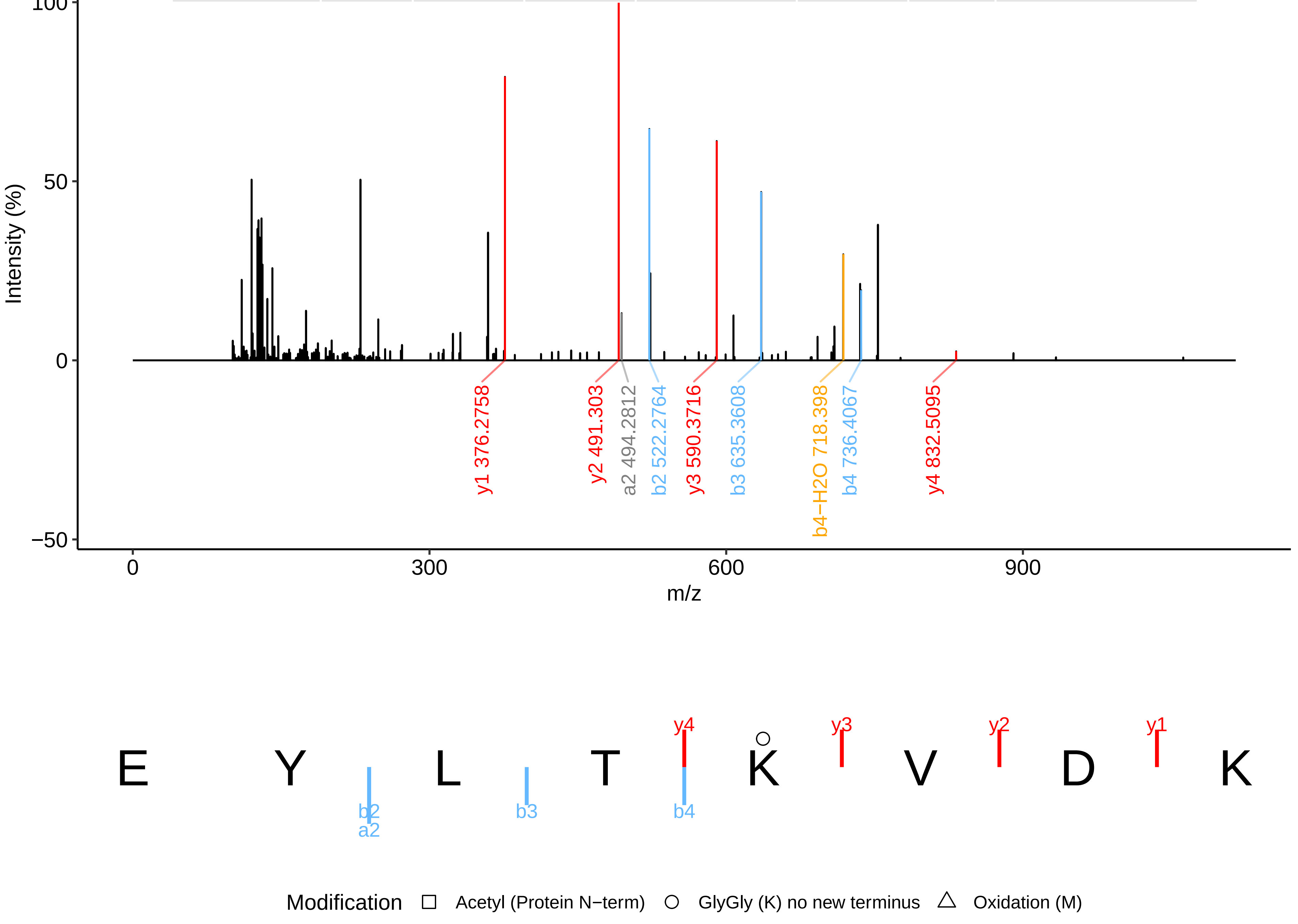

20201118_KIZ_MUb6_GlyGly_2 − Scan number: 13123

Gene Names	Charge	m/z	Mass	Retention time	PEP	Score	Precursor Intensity
CLTC	3	370.5328	1108.576	44.214	0.022343	69.954	4919915

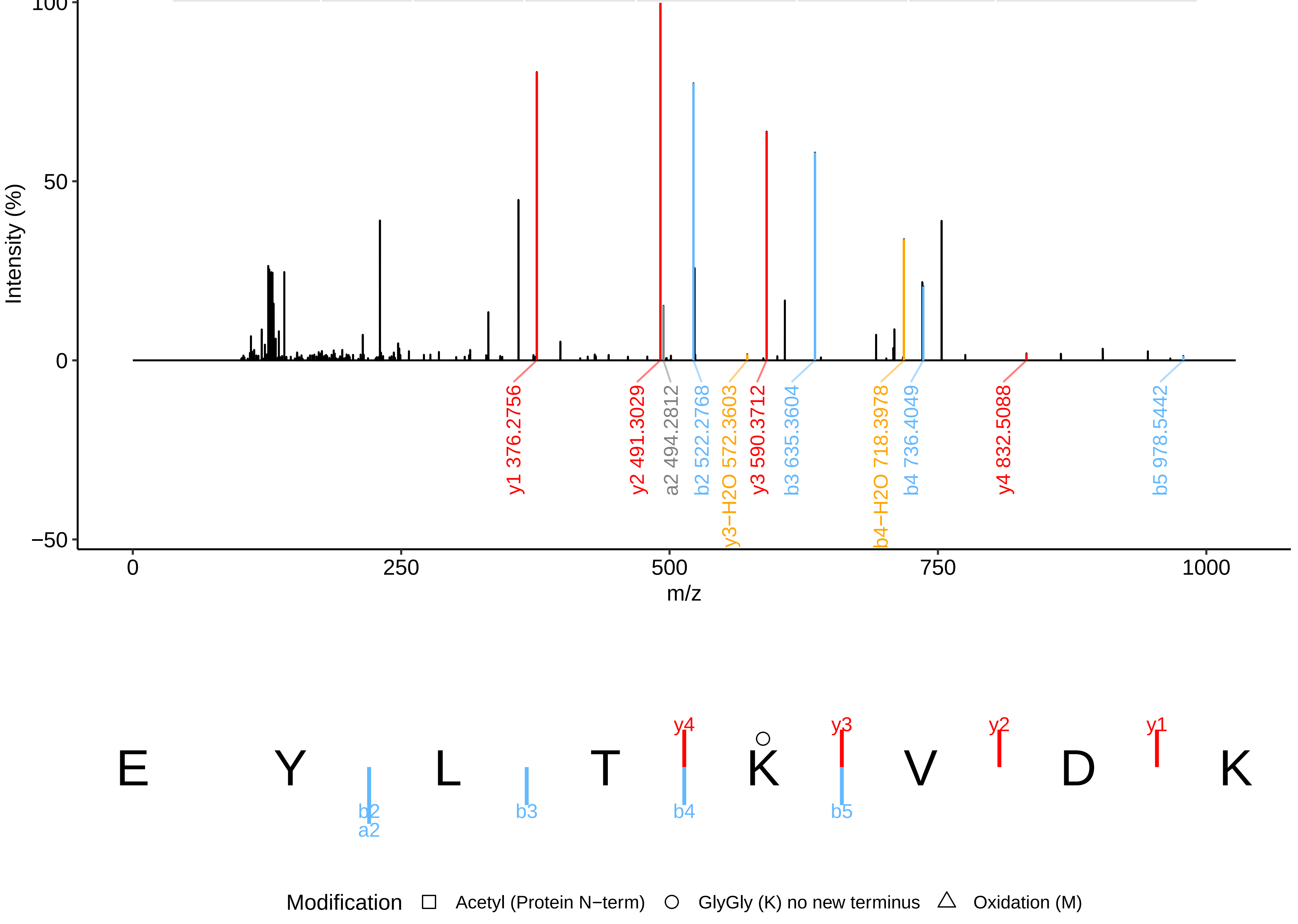

20200921_KIZ_MUb4_GlyGly_10plex − Scan number: 9998

Gene Names	Charge	m/z	Mass	Retention time	PEP	Score	Precursor Intensity
CYB5R3	3	363.5452	1087.614	34.444	0.0079724	90.657	8656833

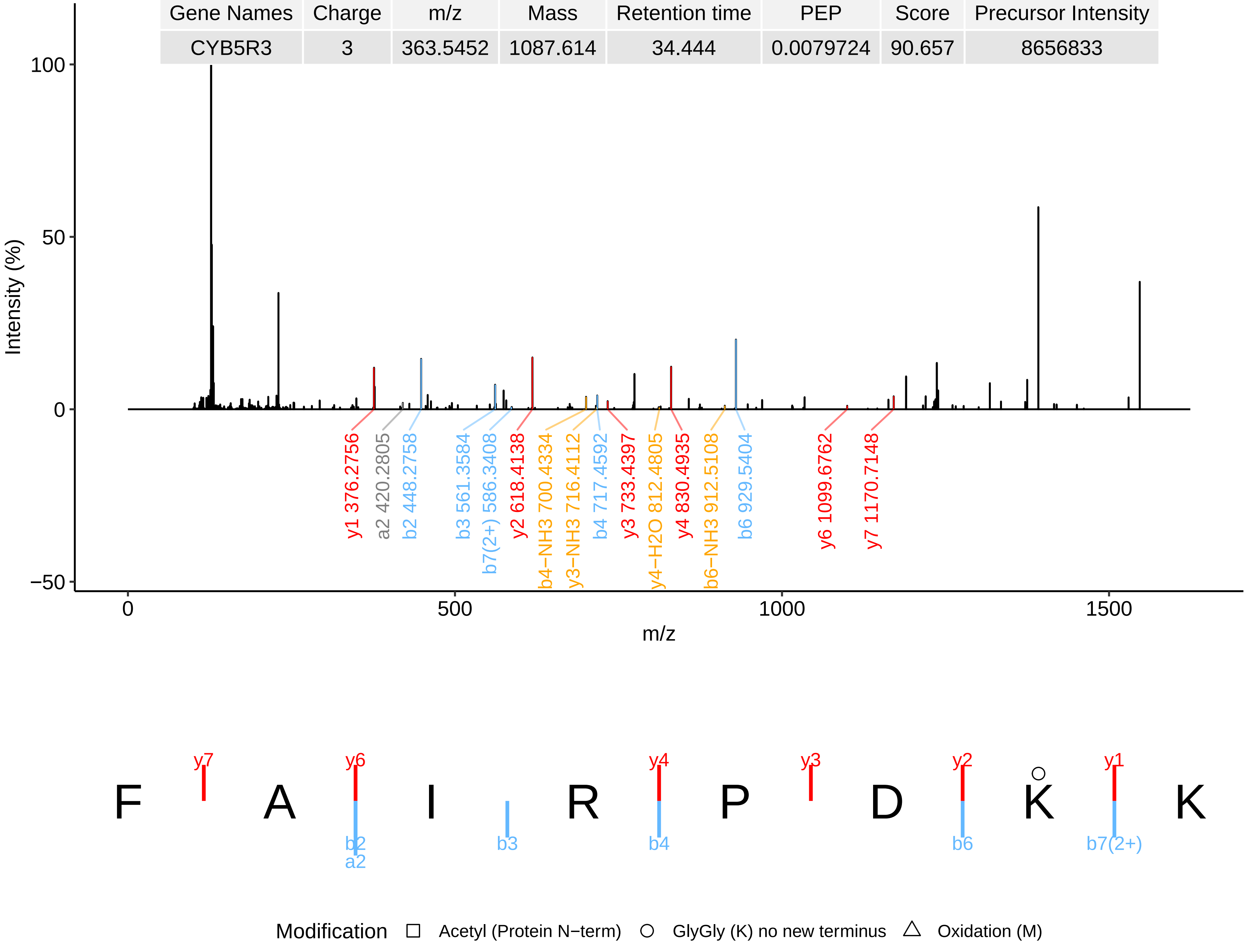

20200924_KIZ_MUb5_GlyGly_1 − Scan number: 8770

Gene Names	Charge	m/z	Mass	Retention time	PEP	Score	Precursor Intensity
CYB5R3	3	363.5452	1087.614	28.101	0.018787	78.192	6959963

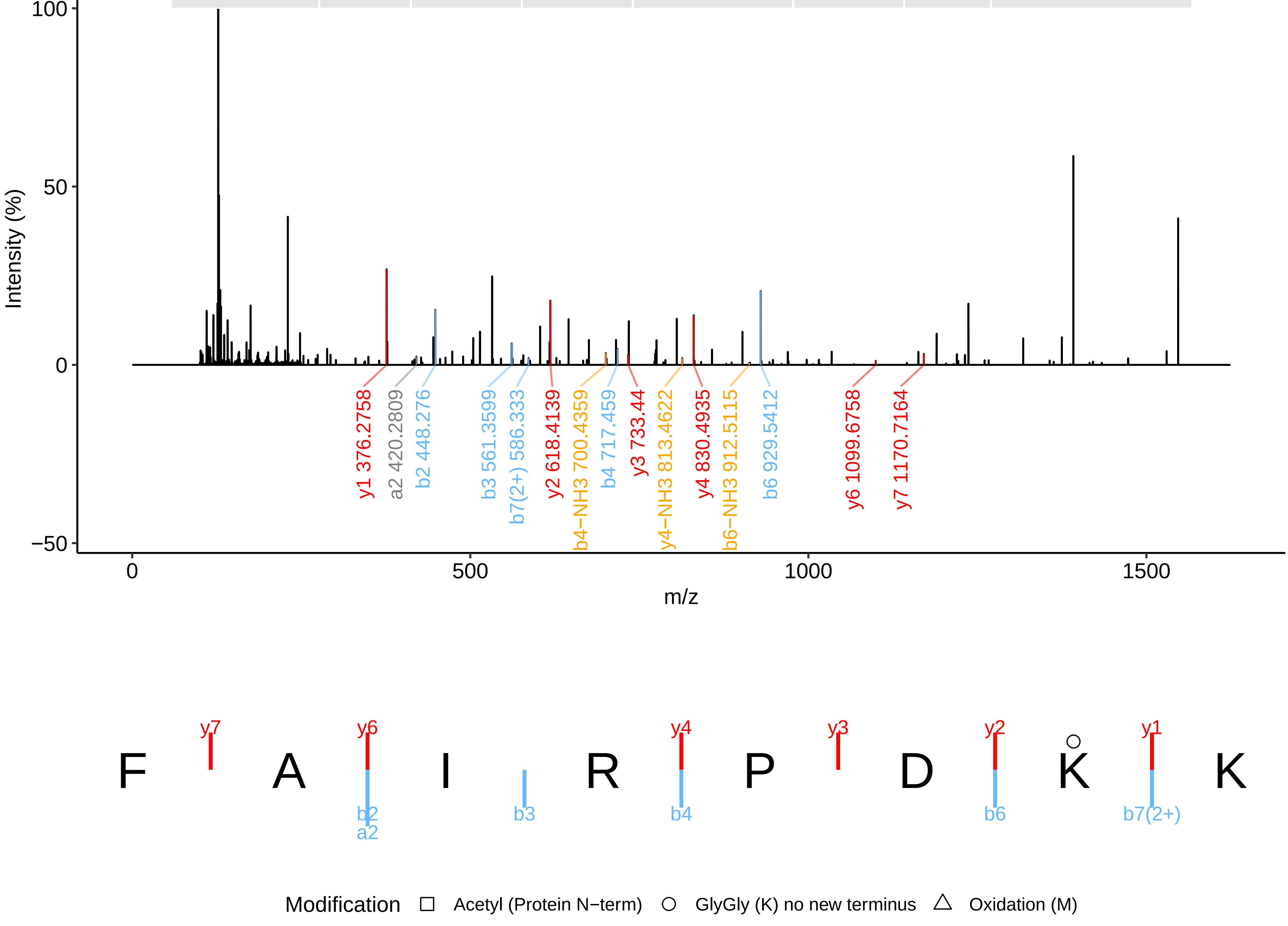

20200924_KIZ_MUb5_GlyGly_1 − Scan number: 8601

Gene Names	Charge	m/z	Mass	Retention time	PEP	Score	Precursor Intensity
CYB5R3	3	363.5452	1087.614	27.53	0.052919	66.351	12352633

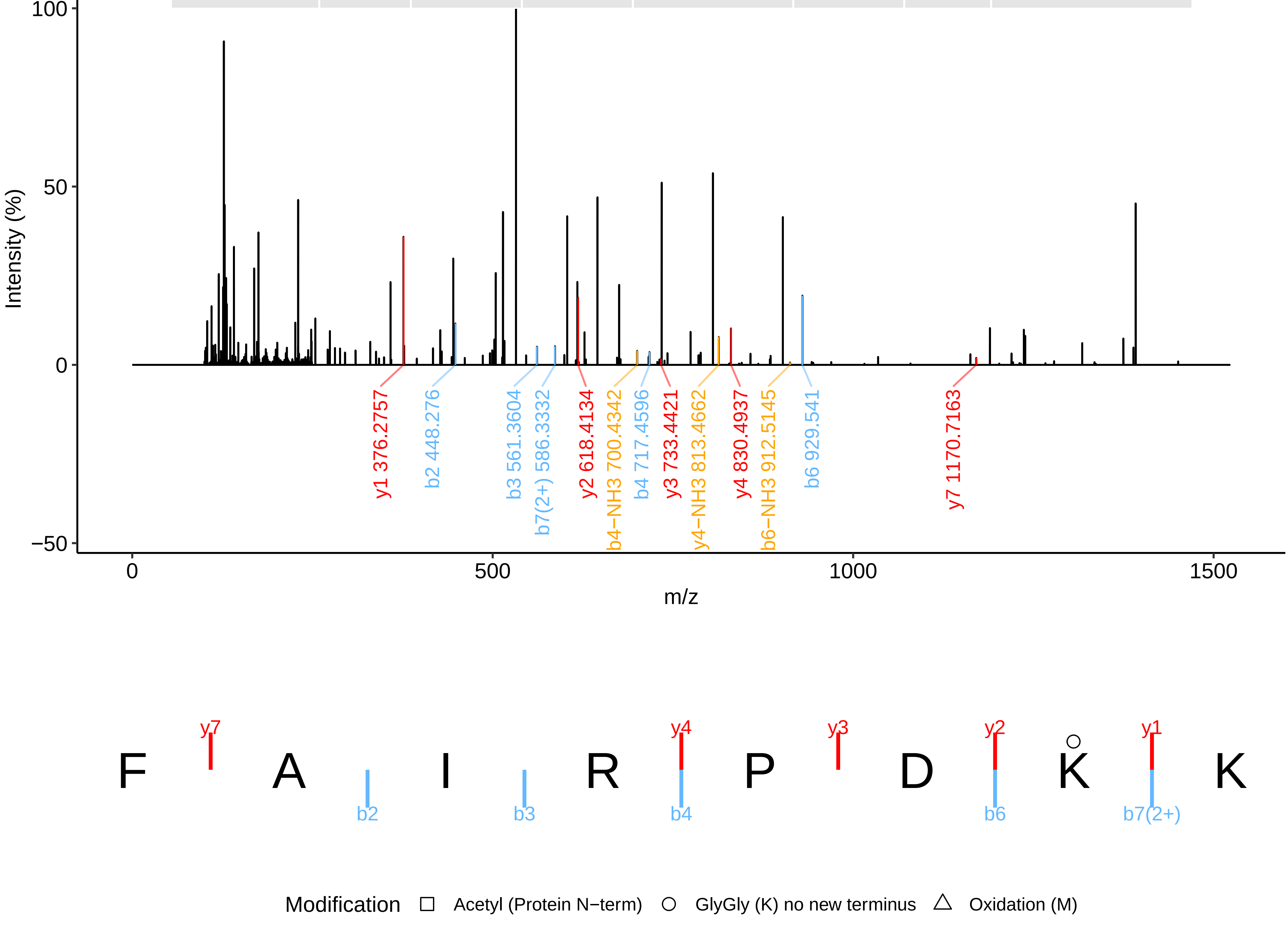

20200924_KIZ_MUb5_GlyGly_1 − Scan number: 8930

Gene Names	Charge	m/z	Mass	Retention time	PEP	Score	Precursor Intensity
CYB5R3	3	363.5452	1087.614	28.649	0.025965	77.072	4583612

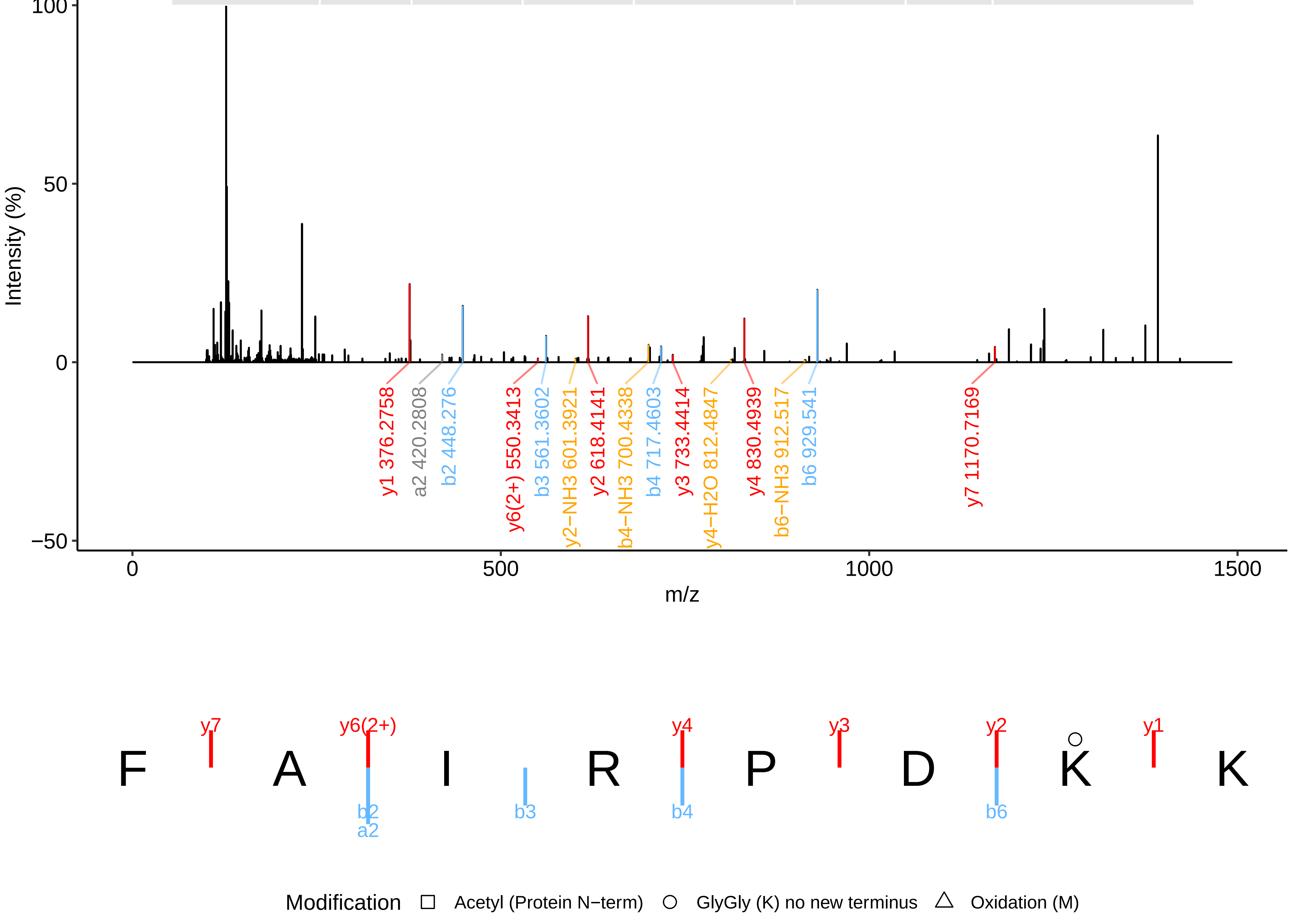

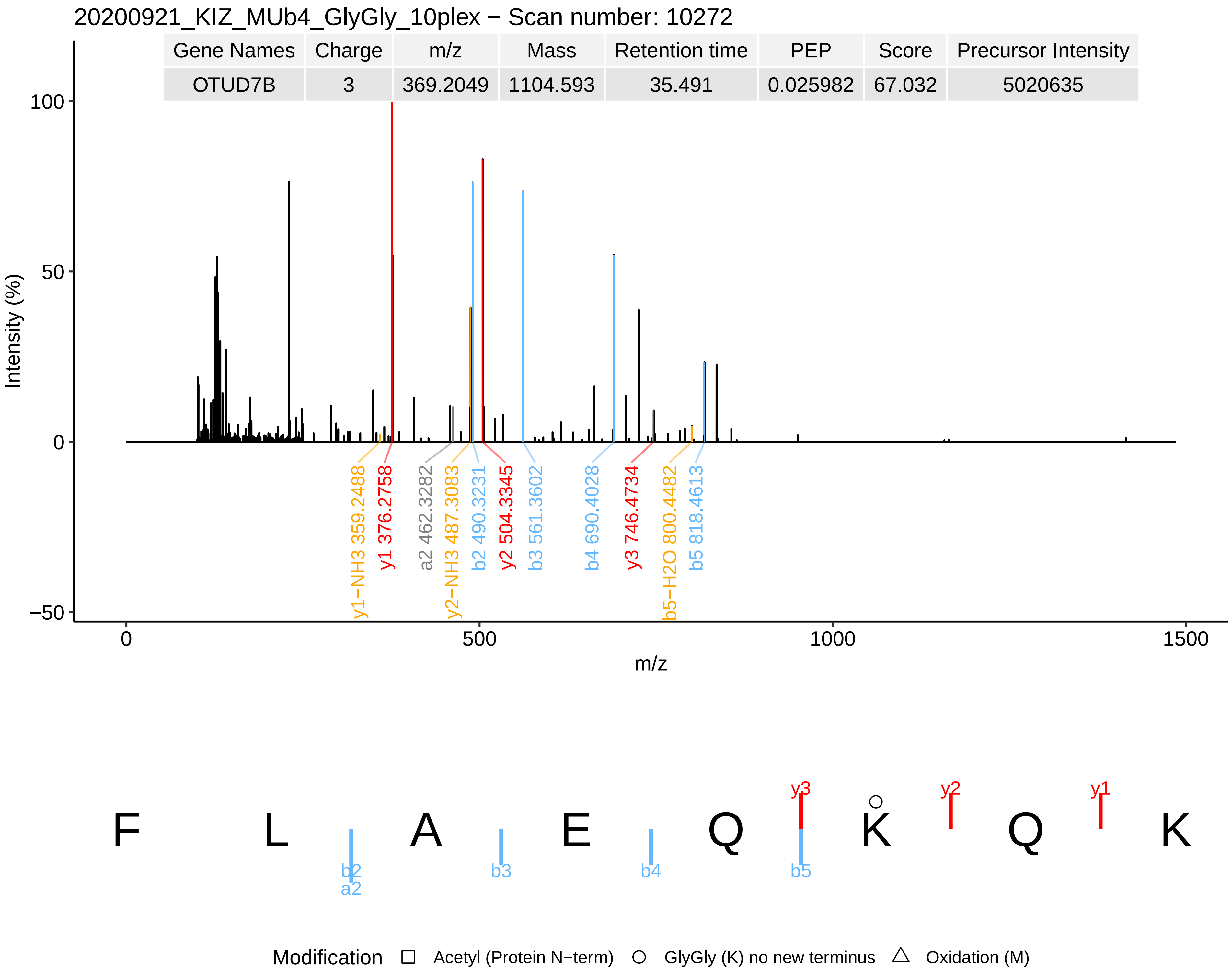

20200921_KIZ_MUb4_GlyGly_10plex − Scan number: 10272
Gene Names | Charge | m/z | Mass | Retention time | PEP | Score | Precursor Intensity
OTUD7B | 3 | 369.2049 | 1104.593 | 35.491 | 0.025982 | 67.032 | 5020635
Intensity (%)
100
50
0
−50
0
500
1000
1500
m/z
y1−NH3 359.2488
y1 376.2758
a2 462.3282
y2−NH3 487.3083
b2 490.3231
y2 504.3345
b3 561.3602
b4 690.4028
y3 746.4734
b5−H2O 800.4482
b5 818.4613
F L A E Q K Q K
y3
y2
y1
b2
a2
b3
b4
b5
Modification
Acetyl (Protein N−term)
GlyGly (K) no new terminus
Oxidation (M)

20200921_KIZ_MUb4_GlyGly_10plex_2 − Scan number: 9517

Gene Names	Charge	m/z	Mass	Retention time	PEP	Score	Precursor Intensity
OTUD7B	3	369.2049	1104.593	36.2	0.032786	66.682	766232.6

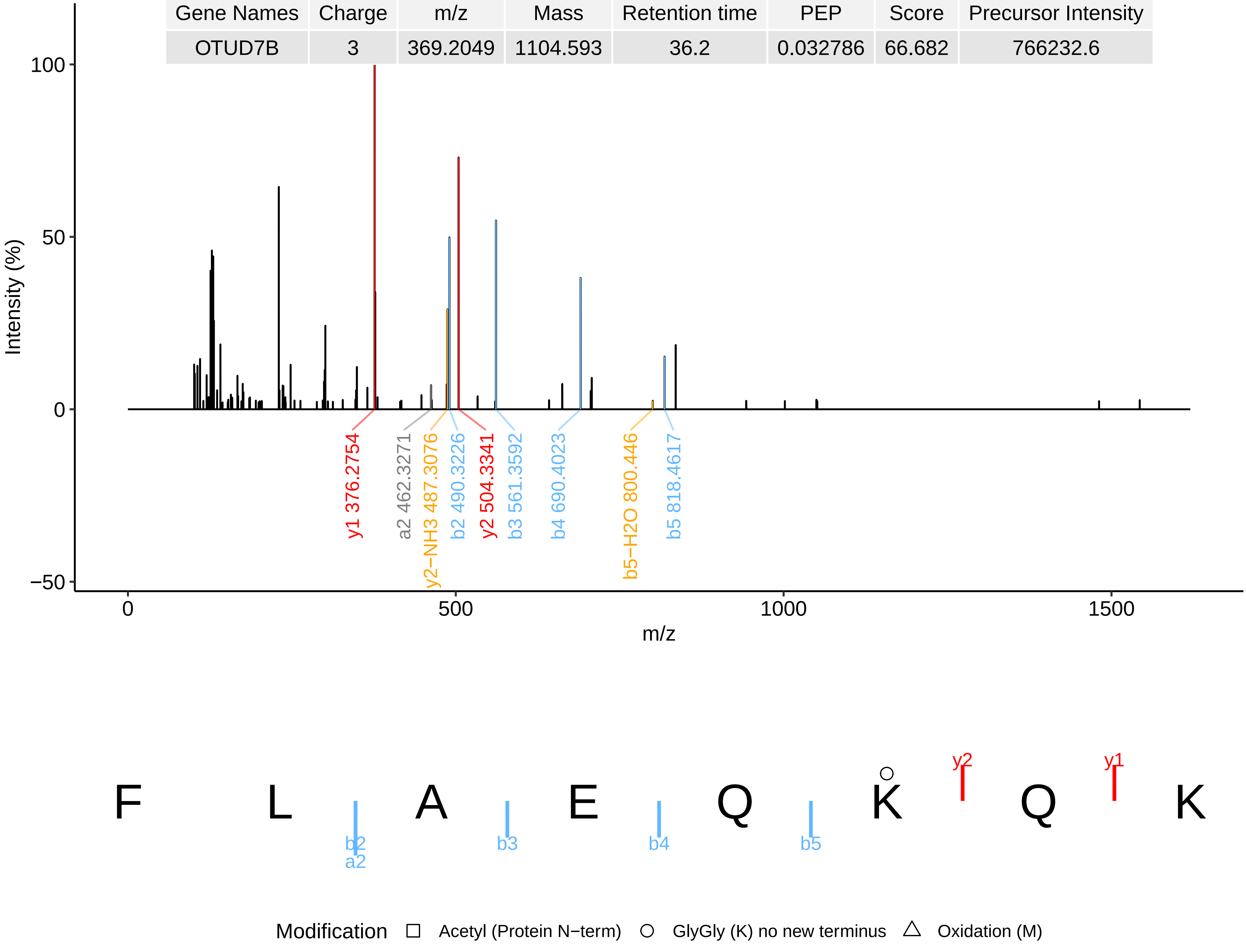

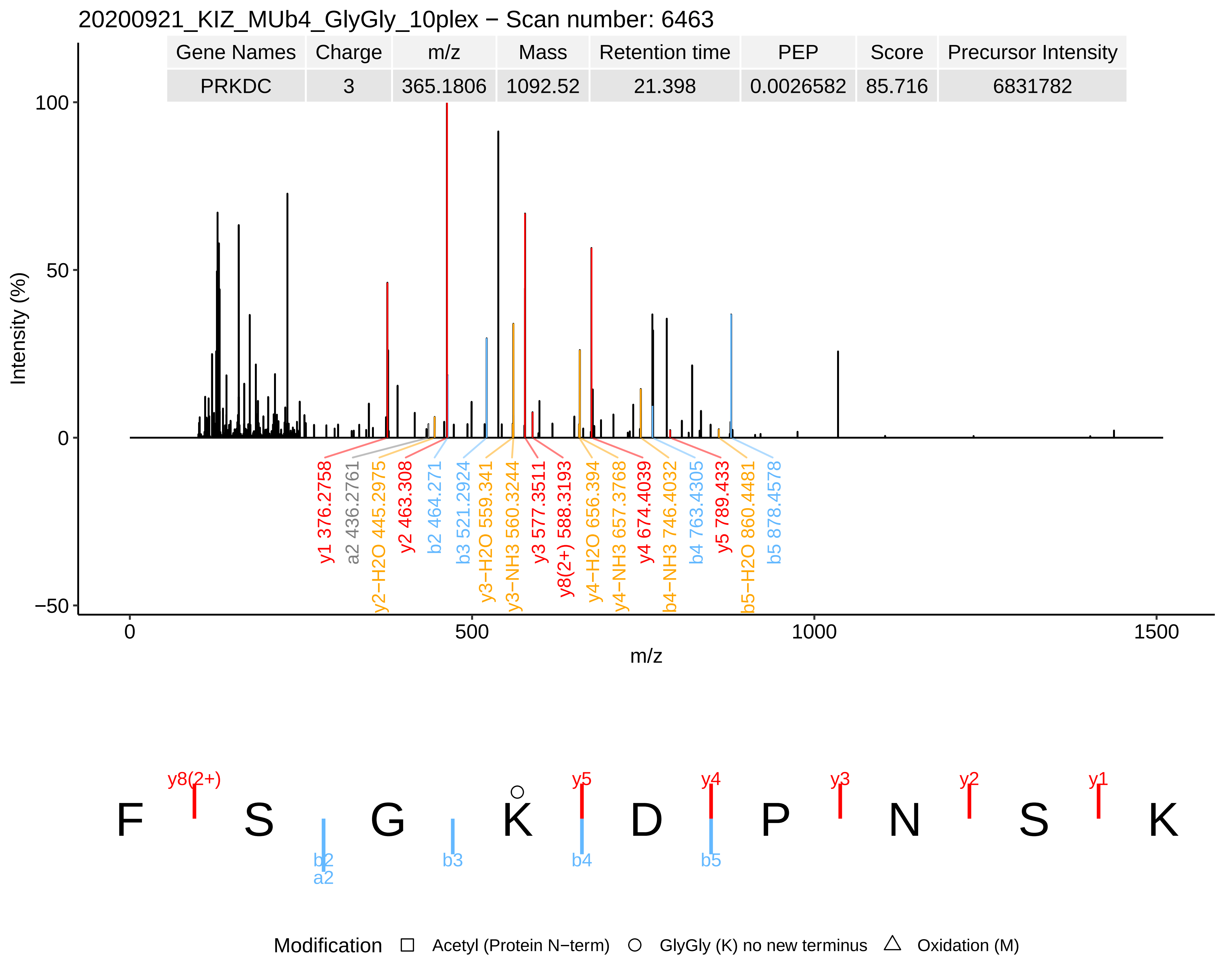
20200921_KIZ_MUb4_GlyGly_10plex − Scan number: 6463
Gene Names | Charge | m/z | Mass | Retention time | PEP | Score | Precursor Intensity
PRKDC | 3 | 365.1806 | 1092.52 | 21.398 | 0.0026582 | 85.716 | 6831782
Intensity (%)
100
50
0
−50
0
500
1000
1500
m/z
y1 376.2758
a2 436.2761
y2−H2O 445.2975
y2 463.308
b2 464.271
b3 521.2924
y3−H2O 559.341
y3−NH3 560.3244
y3 577.3511
y8(2+) 588.3193
y4−H2O 656.394
y4−NH3 657.3768
y4 674.4039
b4−NH3 746.4032
b4 763.4305
y5 789.433
b5−H2O 860.4481
b5 878.4578
F S G K D P N S K
y8(2+) y5 y4 y3 y2 y1
b2 a2 b3 b4 b5
Modification
Acetyl (Protein N−term)
GlyGly (K) no new terminus
Oxidation (M)

20200924_KIZ_MUb5_GlyGly_1 − Scan number: 5583

Gene Names	Charge	m/z	Mass	Retention time	PEP	Score	Precursor Intensity
PRKDC	3	365.1806	1092.52	17.399	0.002247	88.136	5123119

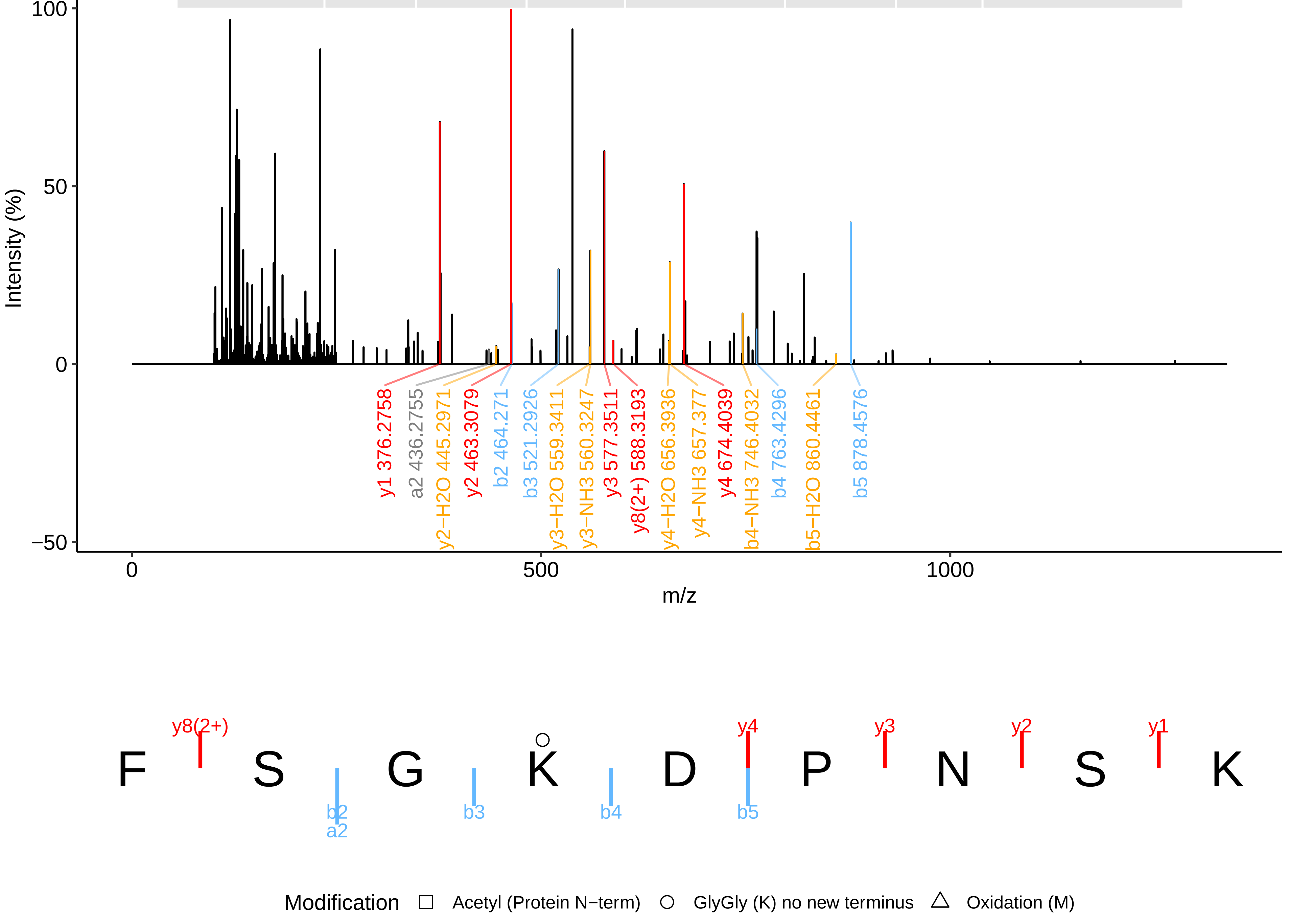

20200921_KIZ_MUb4_GlyGly_10plex − Scan number: 12870

Gene Names	Charge	m/z	Mass	Retention time	PEP	Score	Precursor Intensity
ALDOA	3	362.5486	1084.624	45.276	0.019461	64.04	11384365

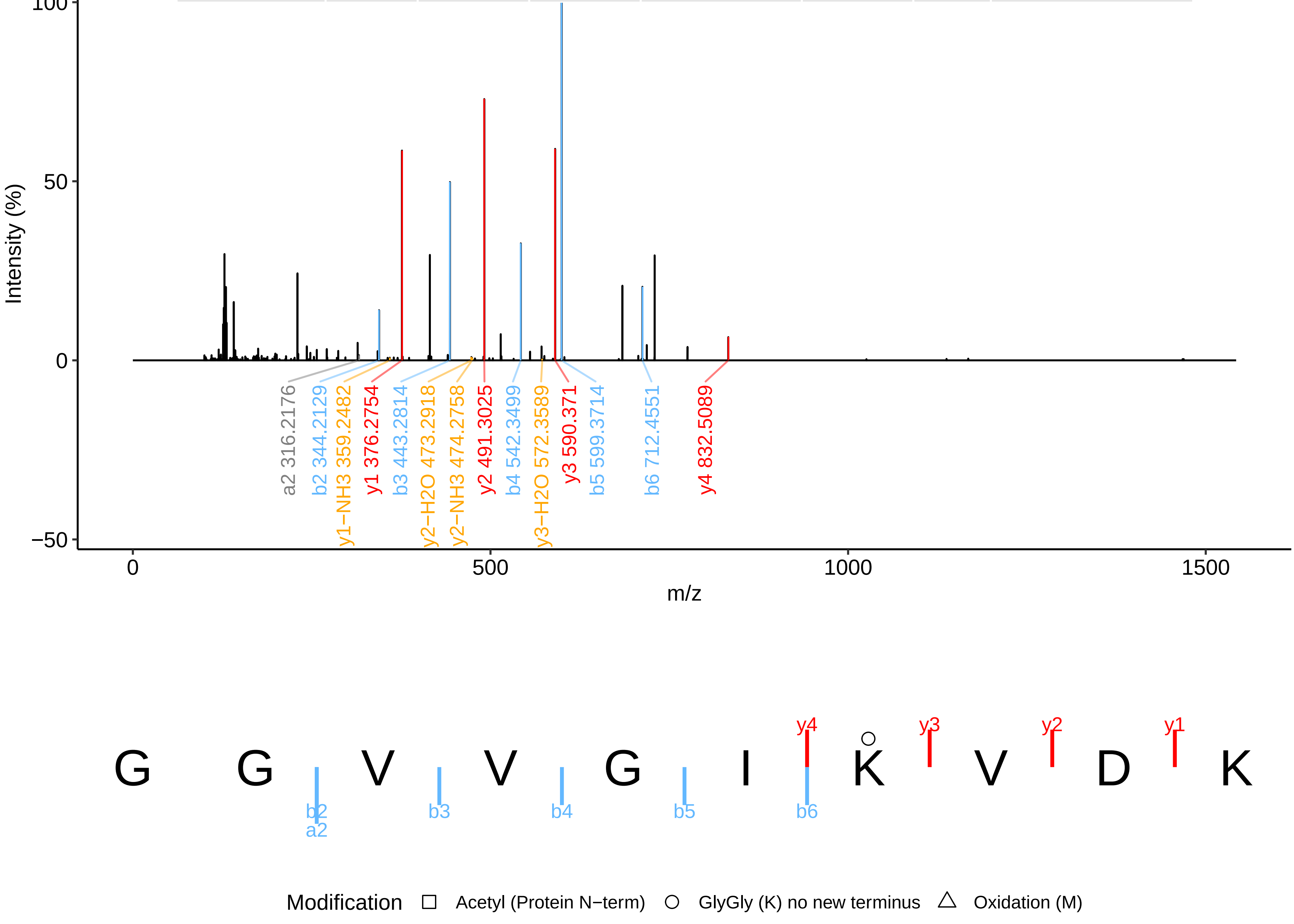

20200921_KIZ_MUb4_GlyGly_10plex_2 − Scan number: 11814

Gene Names	Charge	m/z	Mass	Retention time	PEP	Score	Precursor Intensity
ALDOA	3	362.5486	1084.624	45.563	0.019461	64.04	691228.3

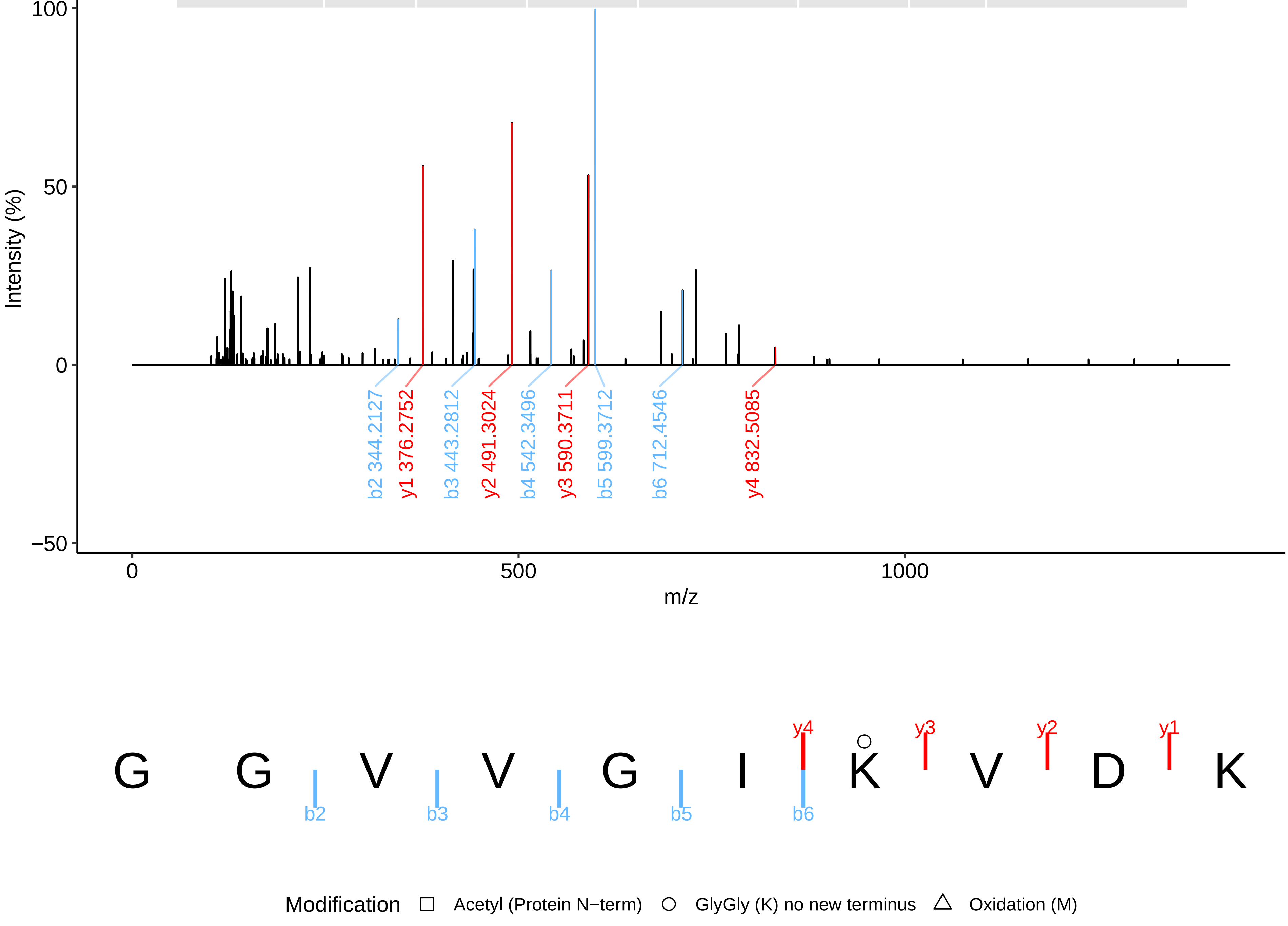

20200921_KIZ_MUb4_GlyGly_10plex − Scan number: 11517

Gene Names	Charge	m/z	Mass	Retention time	PEP	Score	Precursor Intensity
NONO	3	449.2456	1344.715	40.159	1.4554e−05	103.21	6573525

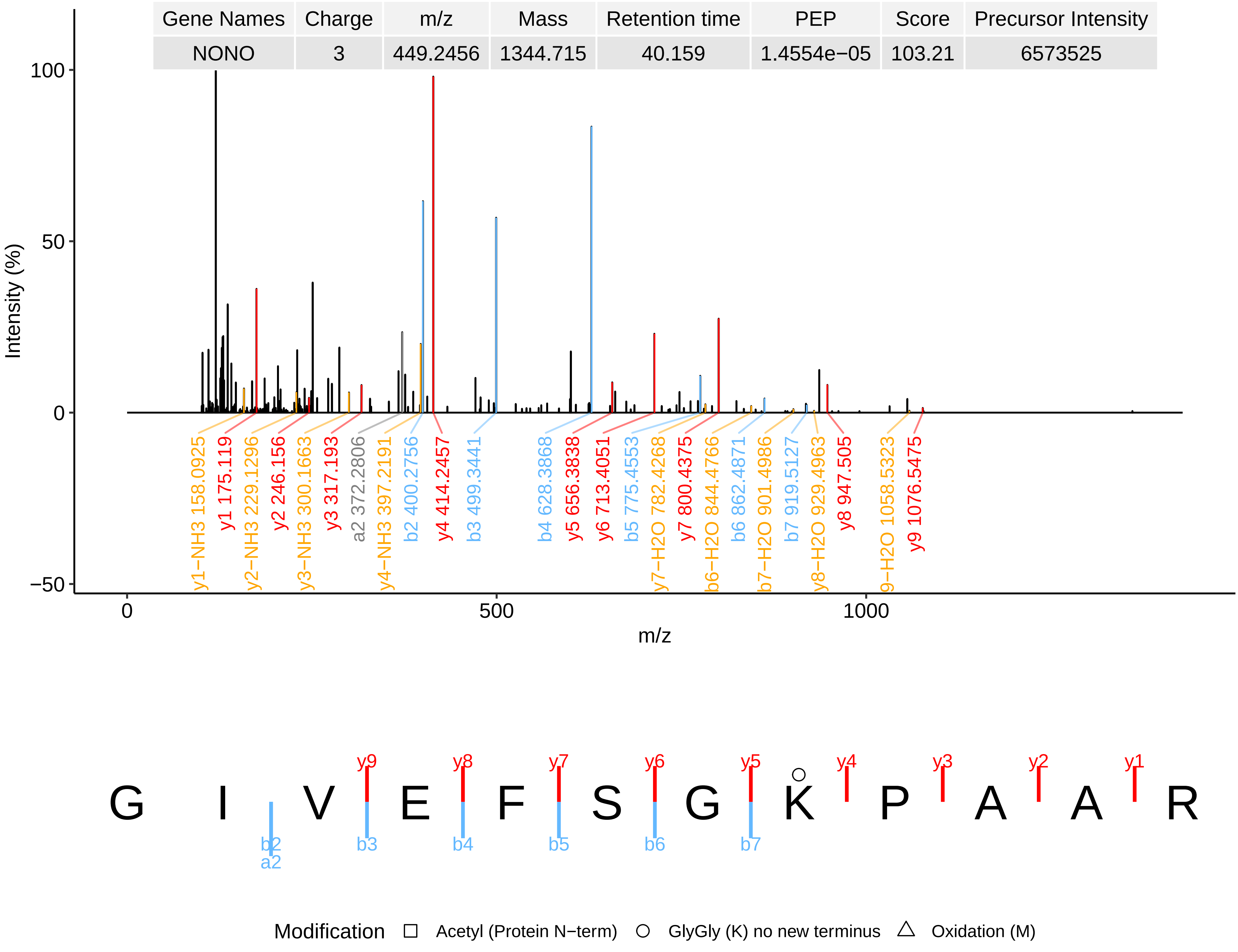

20200921_KIZ_MUb4_GlyGly_10plex_2 − Scan number: 10631

Gene Names	Charge	m/z	Mass	Retention time	PEP	Score	Precursor Intensity
NONO	3	449.2456	1344.715	40.708	7.7721e−06	108.9	1955884

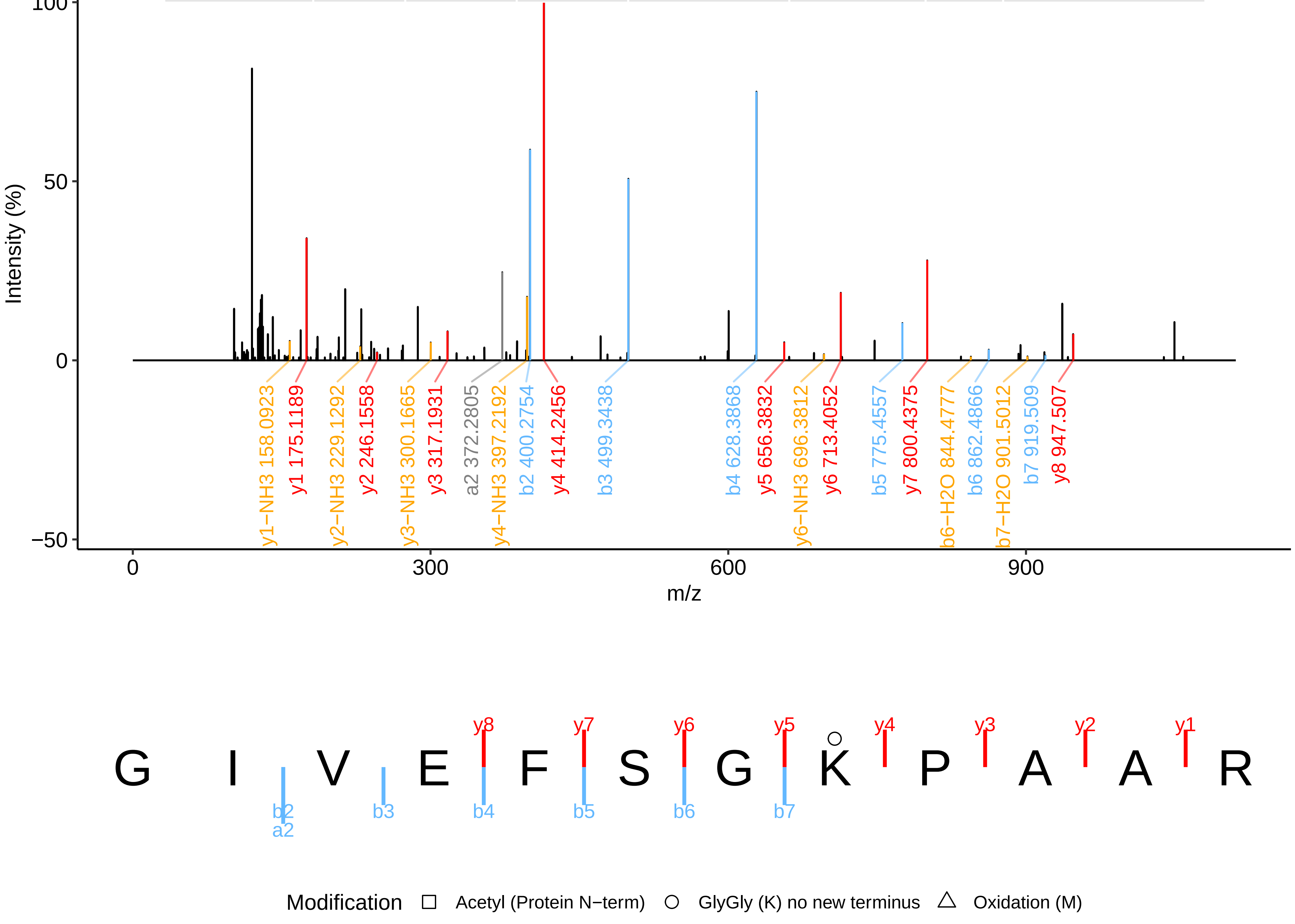

20200921_KIZ_MUb4_GlyGly_10plex − Scan number: 11004

Gene Names	Charge	m/z	Mass	Retention time	PEP	Score	Precursor Intensity
SAR1A	3	443.5809	1327.721	38.268	0.0020047	82.494	3909702

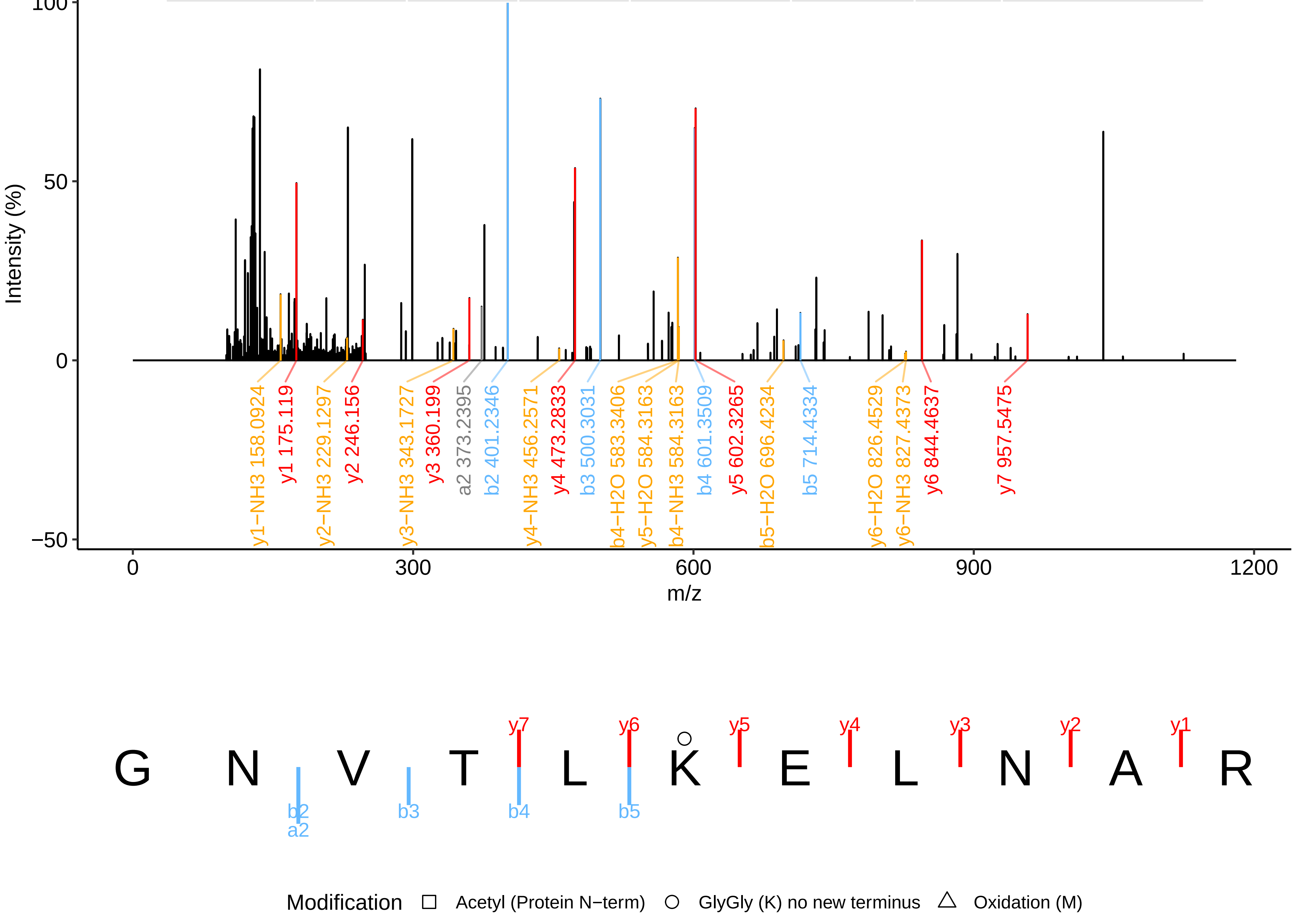

20200921_KIZ_MUb4_GlyGly_10plex − Scan number: 5287

Gene Names	Charge	m/z	Mass	Retention time	PEP	Score	Precursor Intensity
HSPA8	3	439.5688	1315.684	17.29	3.6721e−05	110.08	6066282

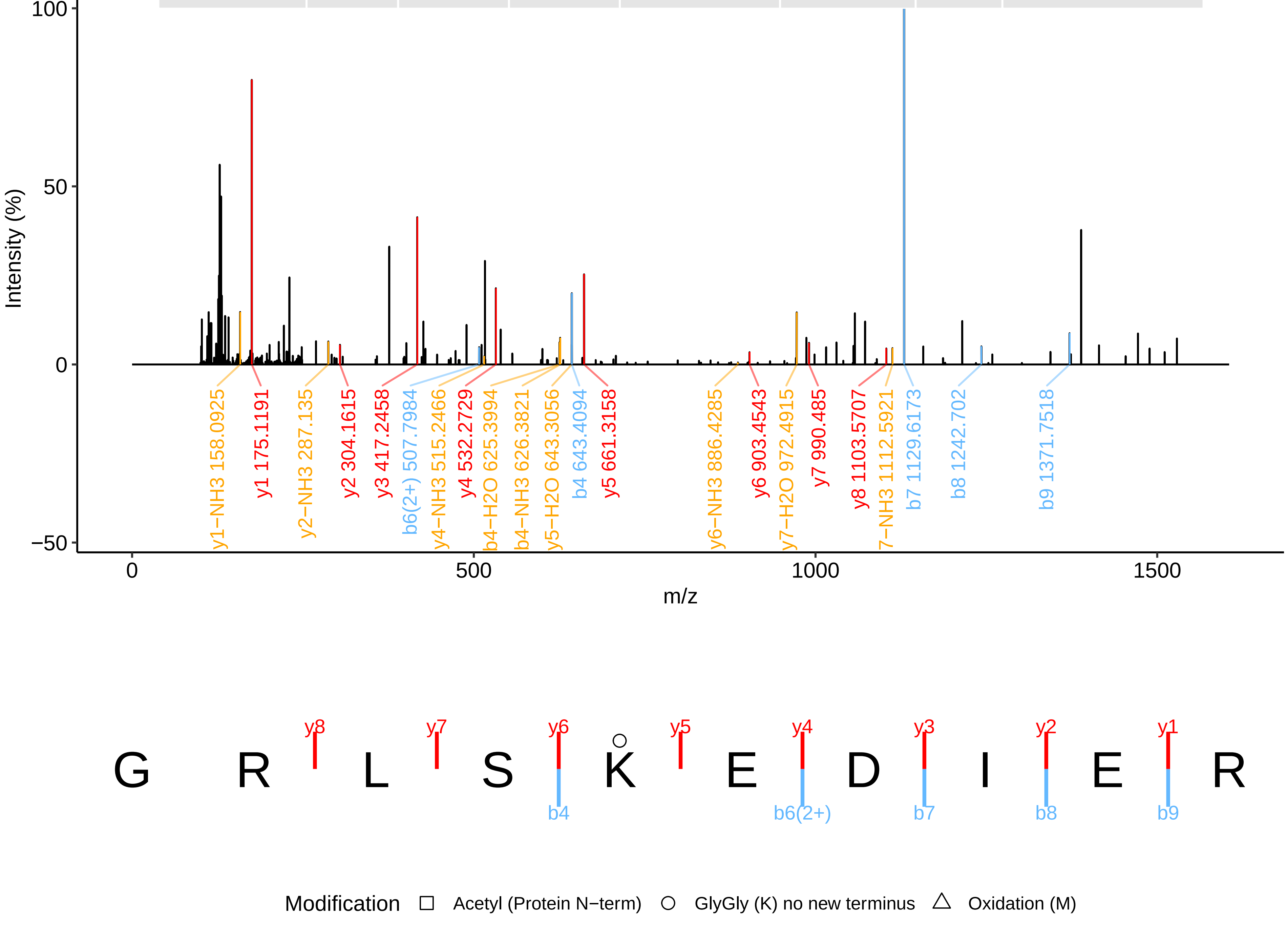

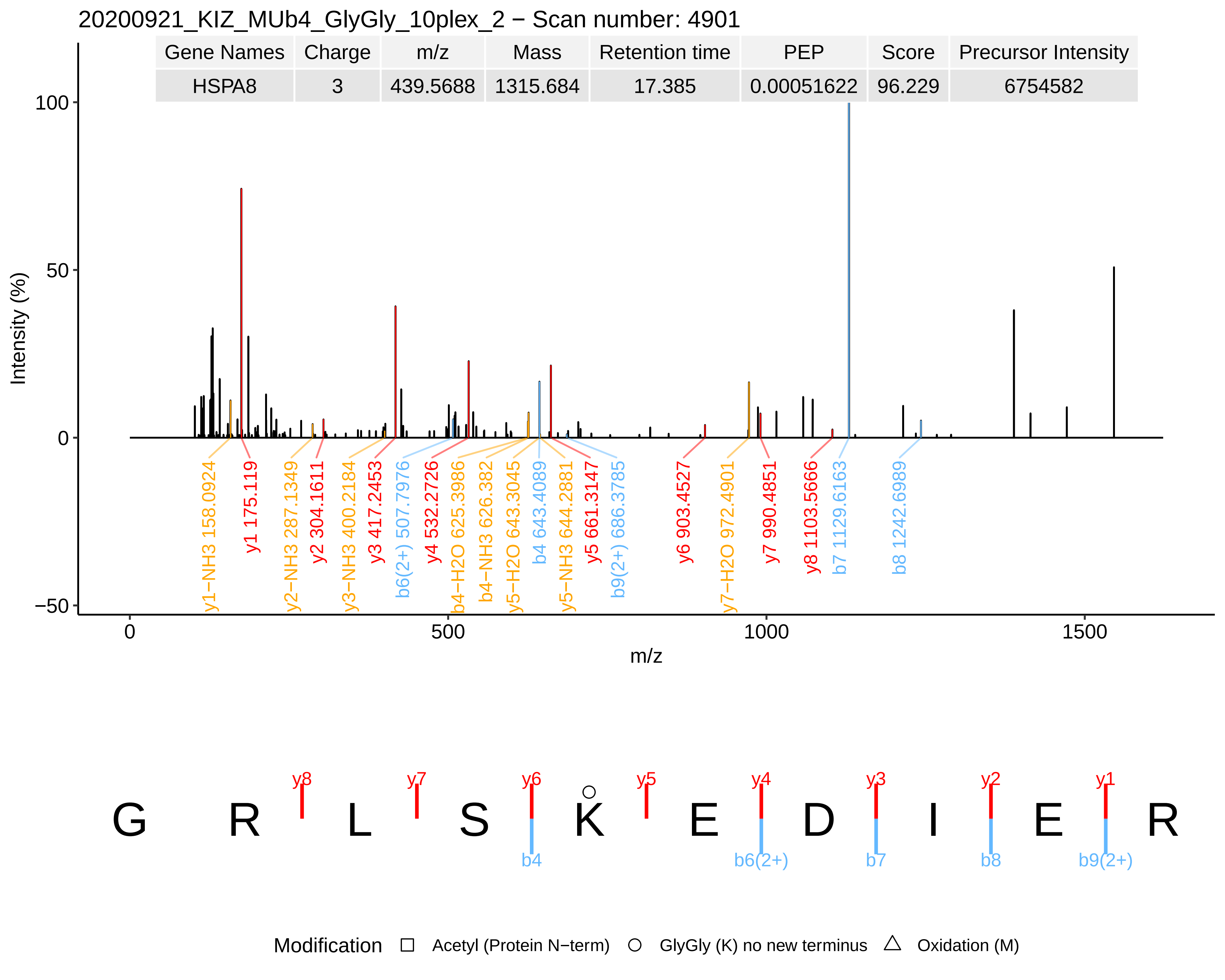
20200921_KIZ_MUb4_GlyGly_10plex_2 − Scan number: 4901
Gene Names | Charge | m/z | Mass | Retention time | PEP | Score | Precursor Intensity
HSPA8 | 3 | 439.5688 | 1315.684 | 17.385 | 0.00051622 | 96.229 | 6754582
Intensity (%)
100
50
0
−50
0
500
1000
1500
m/z
y1−NH3 158.0924
y1 175.119
y2−NH3 287.1349
y2 304.1611
y3−NH3 400.2184
y3 417.2453
b6(2+) 507.7976
y4 532.2726
b4−H2O 625.3986
b4−NH3 626.382
y5−H2O 643.3045
b4 643.4089
y5−NH3 644.2881
y5 661.3147
b9(2+) 686.3785
y6 903.4527
y7−H2O 972.4901
y7 990.4851
y8 1103.5666
b7 1129.6163
b8 1242.6989
G R L S K E D I E R
y8 y7 y6 y5 y4 y3 y2 y1
b4 b6(2+) b7 b8 b9(2+)
Modification
Acetyl (Protein N−term)
GlyGly (K) no new terminus
Oxidation (M)

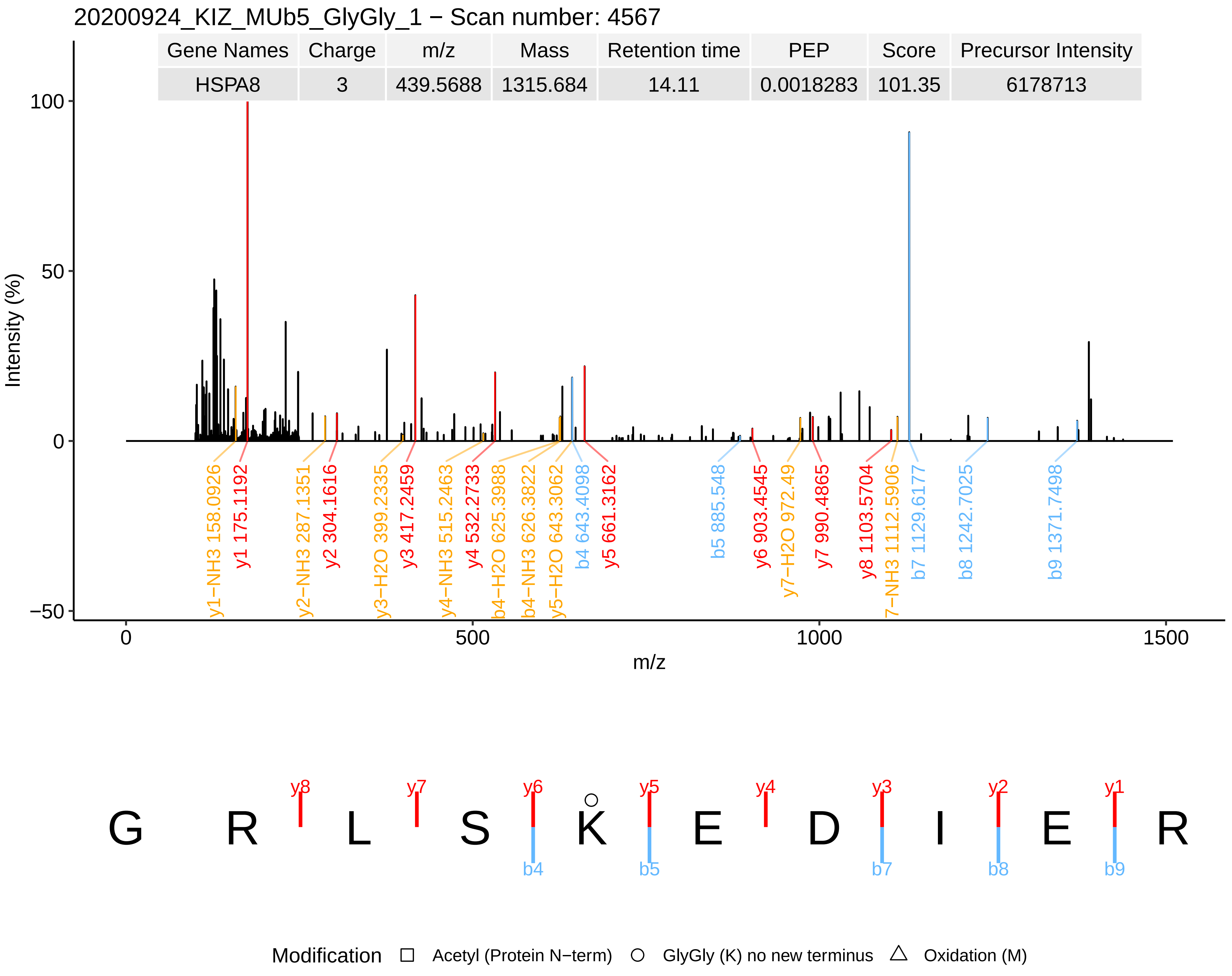

20200924_KIZ_MUb5_GlyGly_1 − Scan number: 4567
Gene Names | Charge | m/z | Mass | Retention time | PEP | Score | Precursor Intensity
HSPA8 | 3 | 439.5688 | 1315.684 | 14.11 | 0.0018283 | 101.35 | 6178713
Intensity (%)
100
50
0
−50
0
500
1000
1500
m/z
y1−NH3 158.0926
y1 175.1192
y2−NH3 287.1351
y2 304.1616
y3−H2O 399.2335
y3 417.2459
y4−NH3 515.2463
y4 532.2733
b4−H2O 625.3988
b4−NH3 626.3822
y5−H2O 643.3062
b4 643.4098
y5 661.3162
b5 885.548
y6 903.4545
y7−H2O 972.49
y7 990.4865
y8 1103.5704
7−NH3 1112.5906
b7 1129.6177
b8 1242.7025
b9 1371.7498
G R L S K E D I E R
y8 y7 y6 y5 y4 y3 y2 y1
b4 b5 b7 b8 b9
Modification
Acetyl (Protein N−term)
GlyGly (K) no new terminus
Oxidation (M)

20200924_KIZ_MUb5_GlyGly_2 − Scan number: 4230

Gene Names	Charge	m/z	Mass	Retention time	PEP	Score	Precursor Intensity
HSPA8	3	439.5688	1315.684	15.5	0.0025126	96.113	2680115

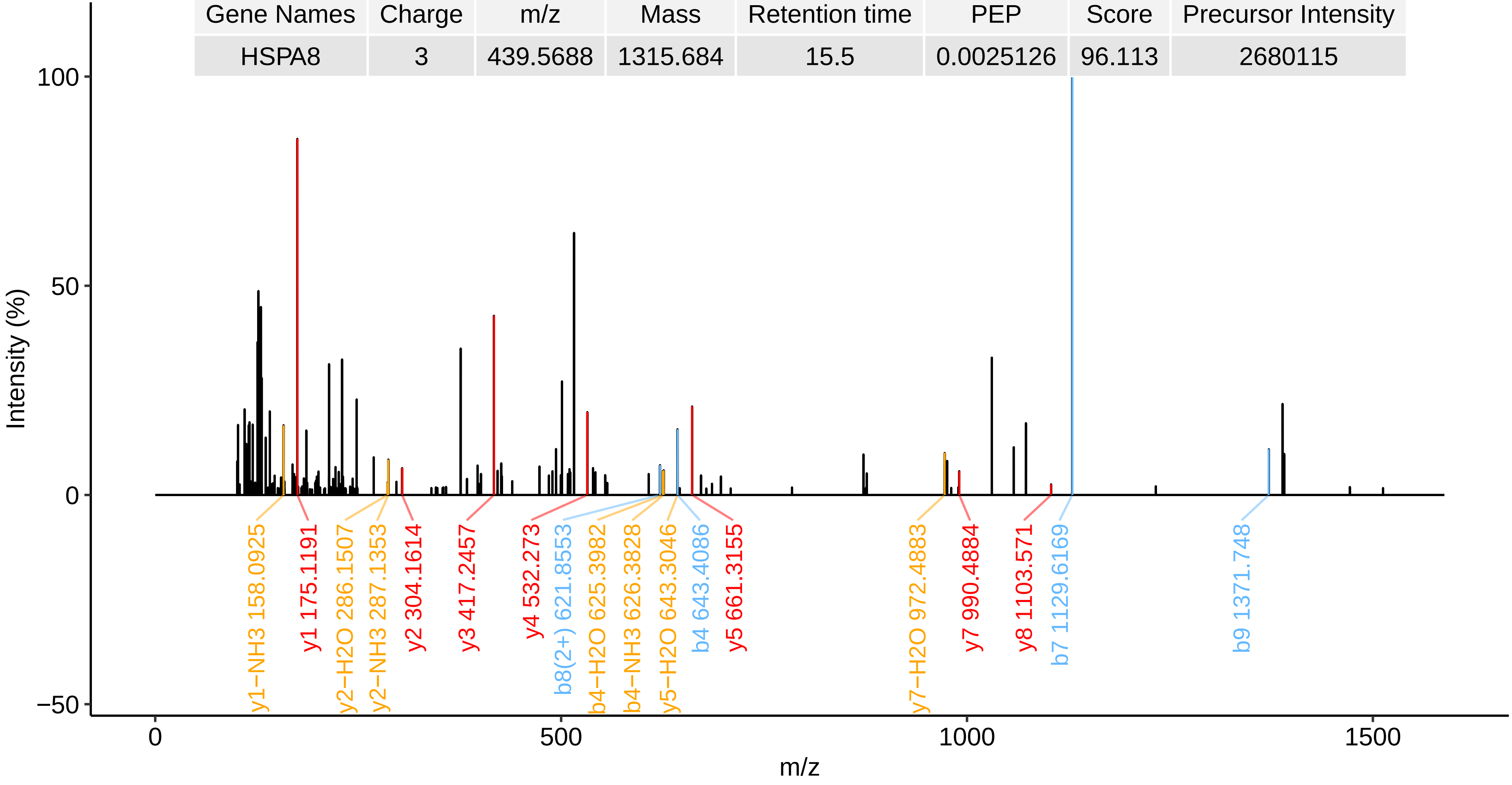

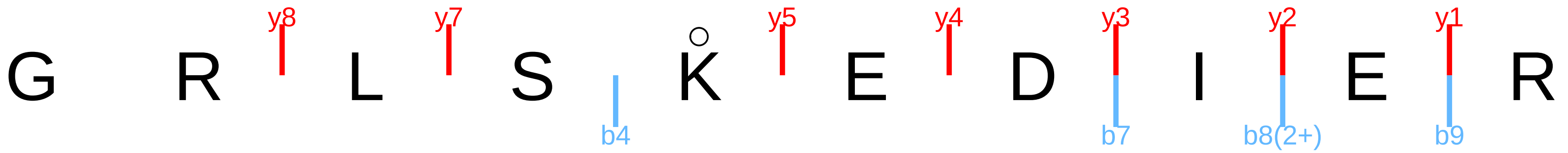

Modification □ Acetyl (Protein N−term) ○ GlyGly (K) no new terminus △ Oxidation (M)

20200921_KIZ_MUb4_GlyGly_10plex − Scan number: 5065

Gene Names	Charge	m/z	Mass	Retention time	PEP	Score	Precursor Intensity
HSPA1B;HSPA1A;HSPA1L	3	444.2406	1329.7	16.533	0.00042373	96.64	4438935

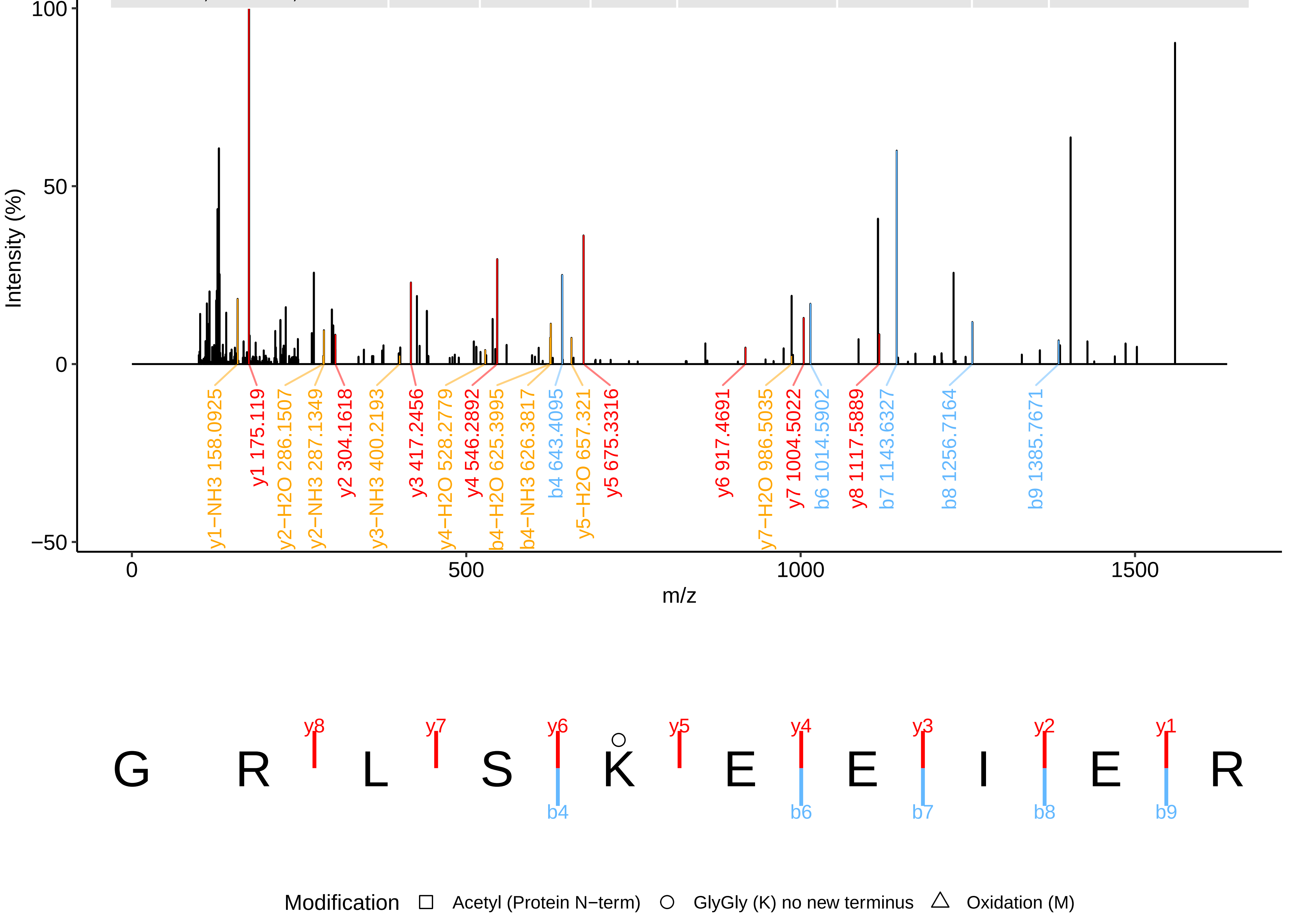

20200921_KIZ_MUb4_GlyGly_10plex_2 − Scan number: 4713

Gene Names	Charge	m/z	Mass	Retention time	PEP	Score	Precursor Intensity
HSPA1B;HSPA1A;HSPA1L	3	444.2406	1329.7	16.647	0.0023264	88.187	930280.8

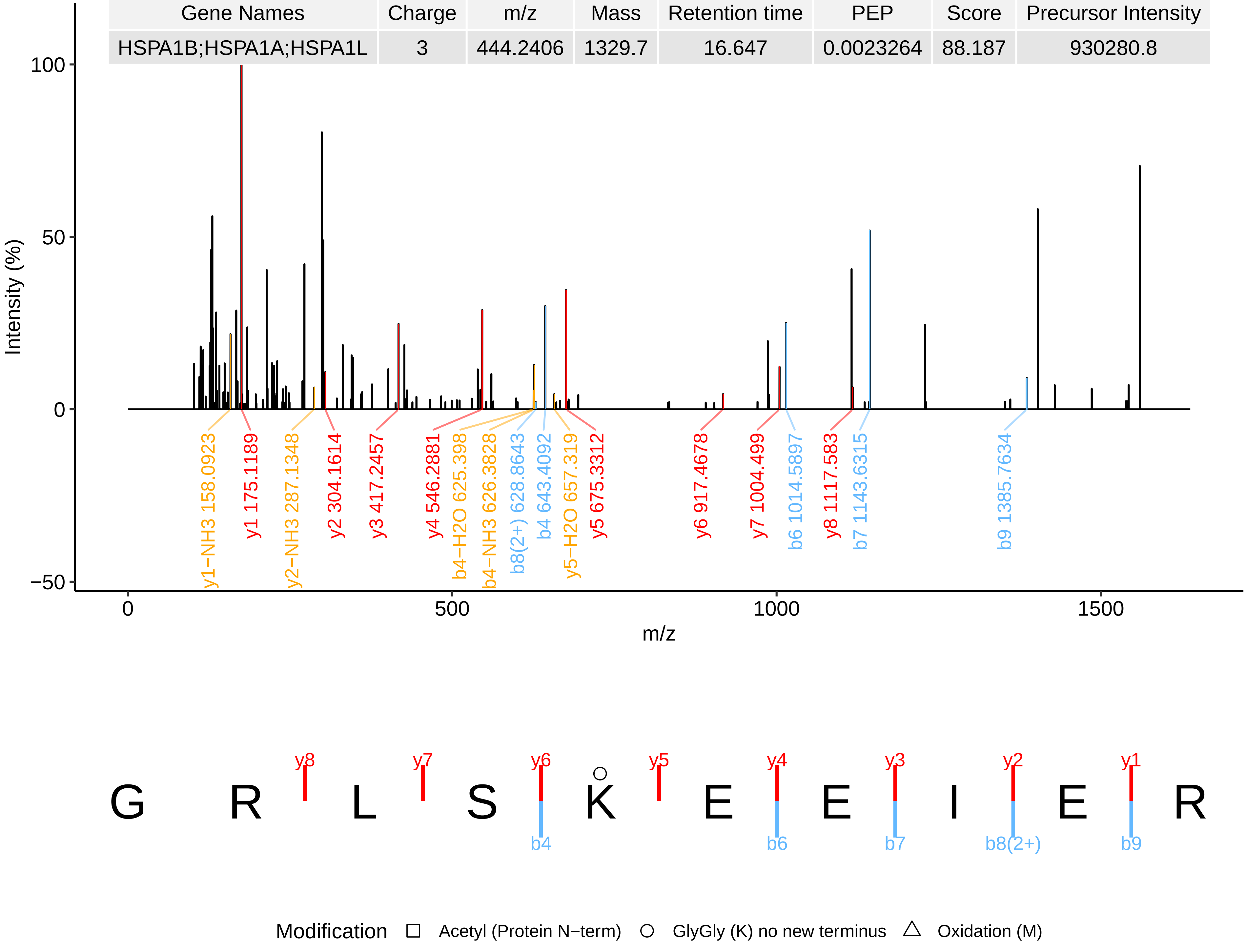

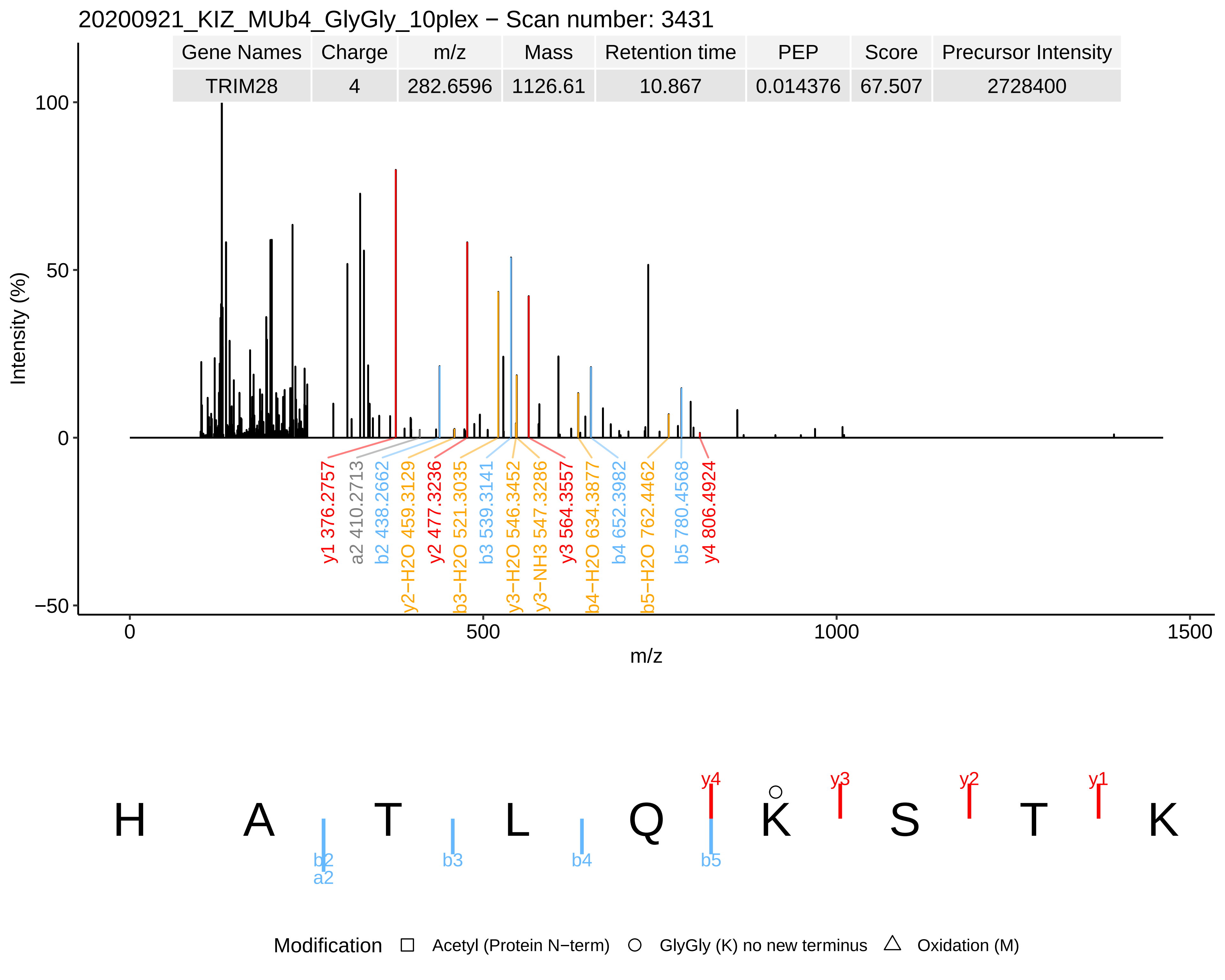
20200921_KIZ_MUb4_GlyGly_10plex − Scan number: 3431
Gene Names
Charge
m/z
Mass
Retention time
PEP
Score
Precursor Intensity
TRIM28
4
282.6596
1126.61
10.867
0.014376
67.507
2728400
Intensity (%)
m/z
y1 376.2757
a2 410.2713
b2 438.2662
y2−H2O 459.3129
y2 477.3236
b3−H2O 521.3035
b3 539.3141
y3−H2O 546.3452
y3−NH3 547.3286
y3 564.3557
b4−H2O 634.3877
b4 652.3982
b5−H2O 762.4462
b5 780.4568
y4 806.4924
H A T L Q K S T K
y4 y3 y2 y1
b2 a2 b3 b4 b5
Modification
Acetyl (Protein N−term)
GlyGly (K) no new terminus
Oxidation (M)

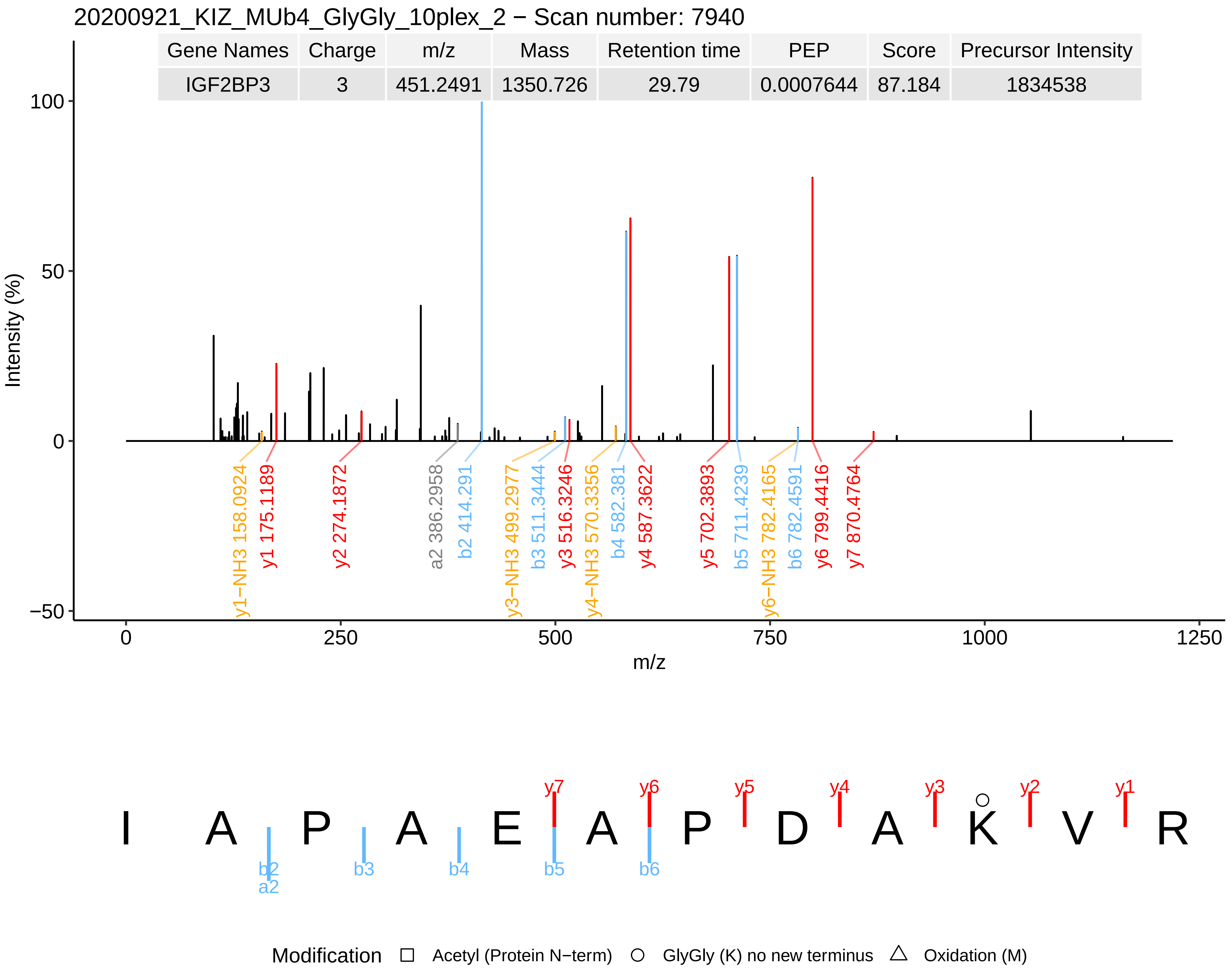

20200921_KIZ_MUb4_GlyGly_10plex_2 − Scan number: 7940
Gene Names | Charge | m/z | Mass | Retention time | PEP | Score | Precursor Intensity
IGF2BP3 | 3 | 451.2491 | 1350.726 | 29.79 | 0.0007644 | 87.184 | 1834538
Intensity (%)
100
50
0
−50
0
250
500
750
1000
1250
m/z
y1−NH3 158.0924
y1 175.1189
y2 274.1872
a2 386.2958
b2 414.291
y3−NH3 499.2977
b3 511.3444
y3 516.3246
y4−NH3 570.3356
b4 582.381
y4 587.3622
y5 702.3893
b5 711.4239
y6−NH3 782.4165
b6 782.4591
y6 799.4416
y7 870.4764
y7
y6
y5
y4
y3
y2
y1
I A P A E A P D A K V R
b2
a2
b3
b4
b5
b6
Modification
Acetyl (Protein N−term)
GlyGly (K) no new terminus
Oxidation (M)

20200921_KIZ_MUb4_GlyGly_10plex − Scan number: 19225

Gene Names	Charge	m/z	Mass	Retention time	PEP	Score	Precursor Intensity
FAF2	3	439.5822	1315.725	69.26	0.00041692	95.642	8234930

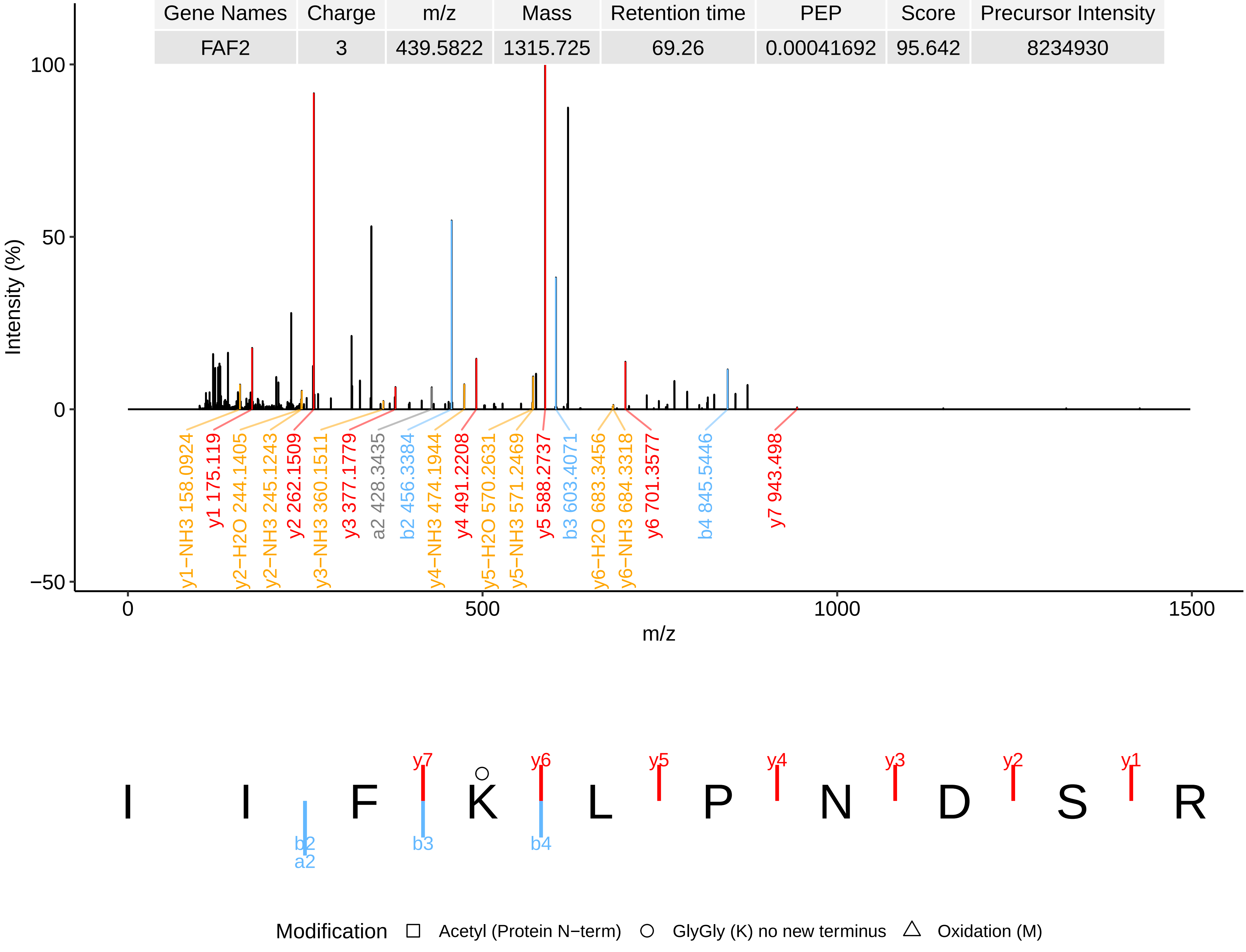

20200921_KIZ_MUb4_GlyGly_10plex − Scan number: 19356

Gene Names	Charge	m/z	Mass	Retention time	PEP	Score	Precursor Intensity
FAF2	3	439.5822	1315.725	69.775	0.0051318	78.098	7225854

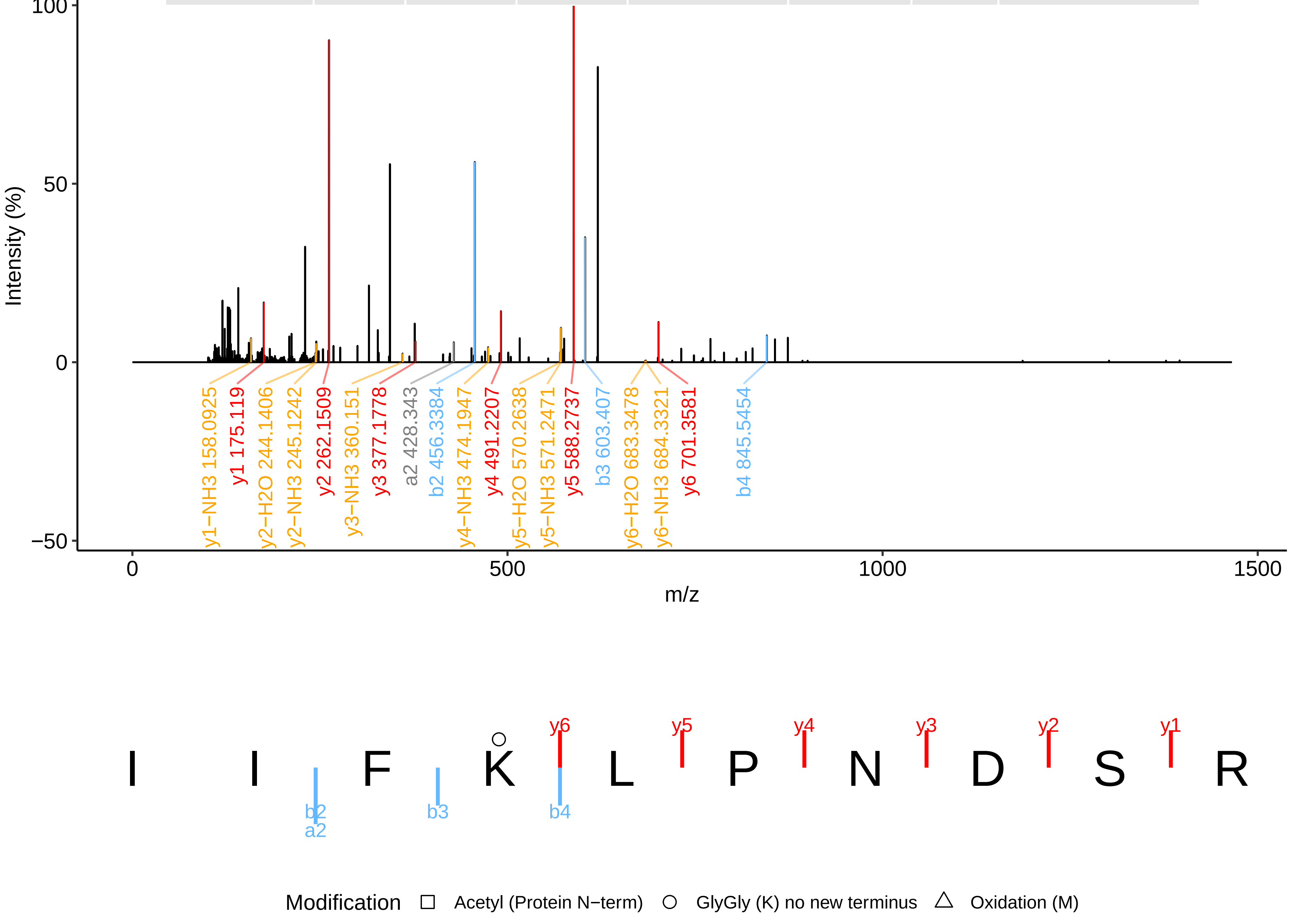

20200921_KIZ_MUb4_GlyGly_10plex_2 − Scan number: 17813

Gene Names	Charge	m/z	Mass	Retention time	PEP	Score	Precursor Intensity
FAF2	3	439.5822	1315.725	69.69	0.0055755	77.662	747135.3

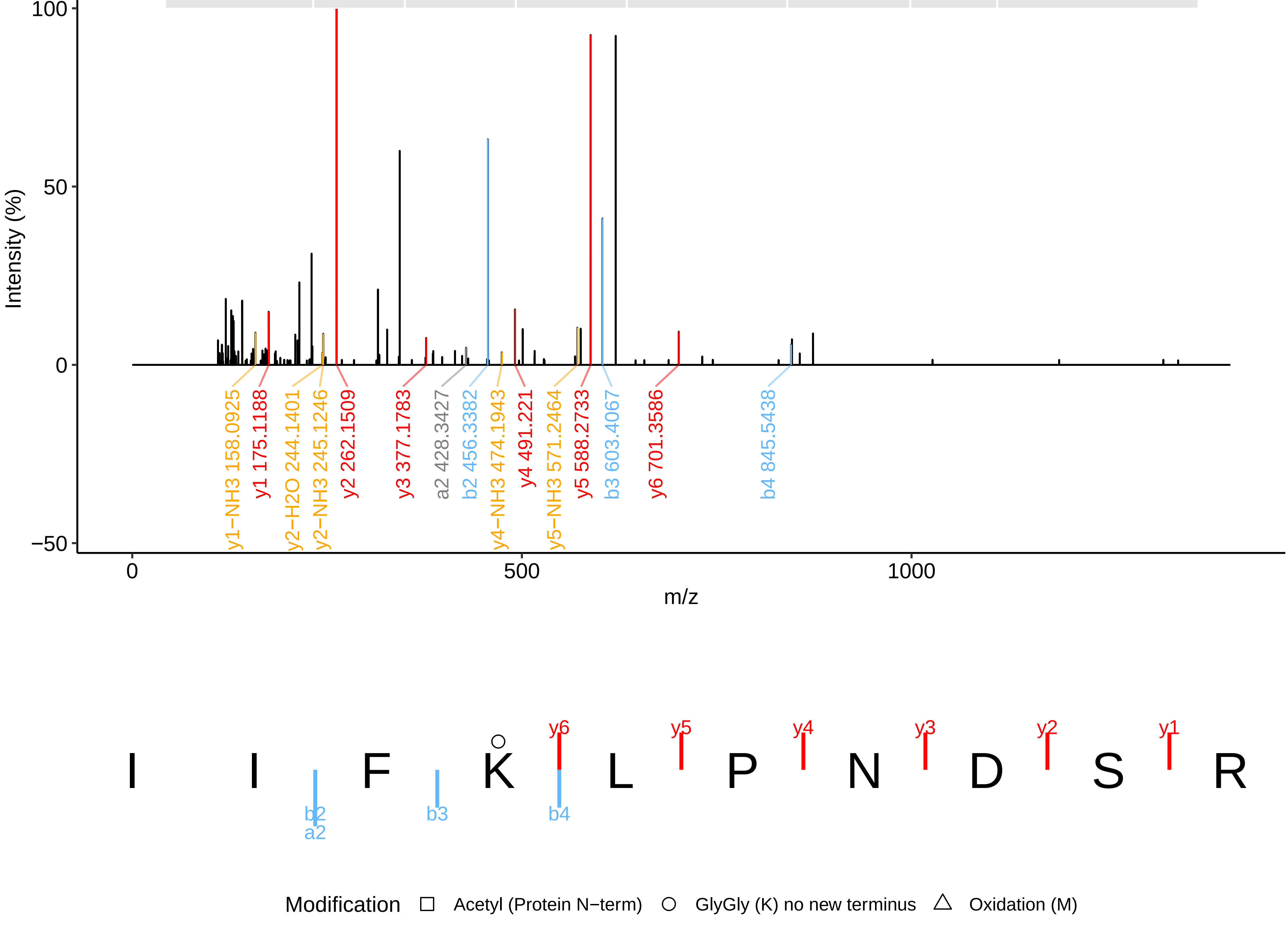

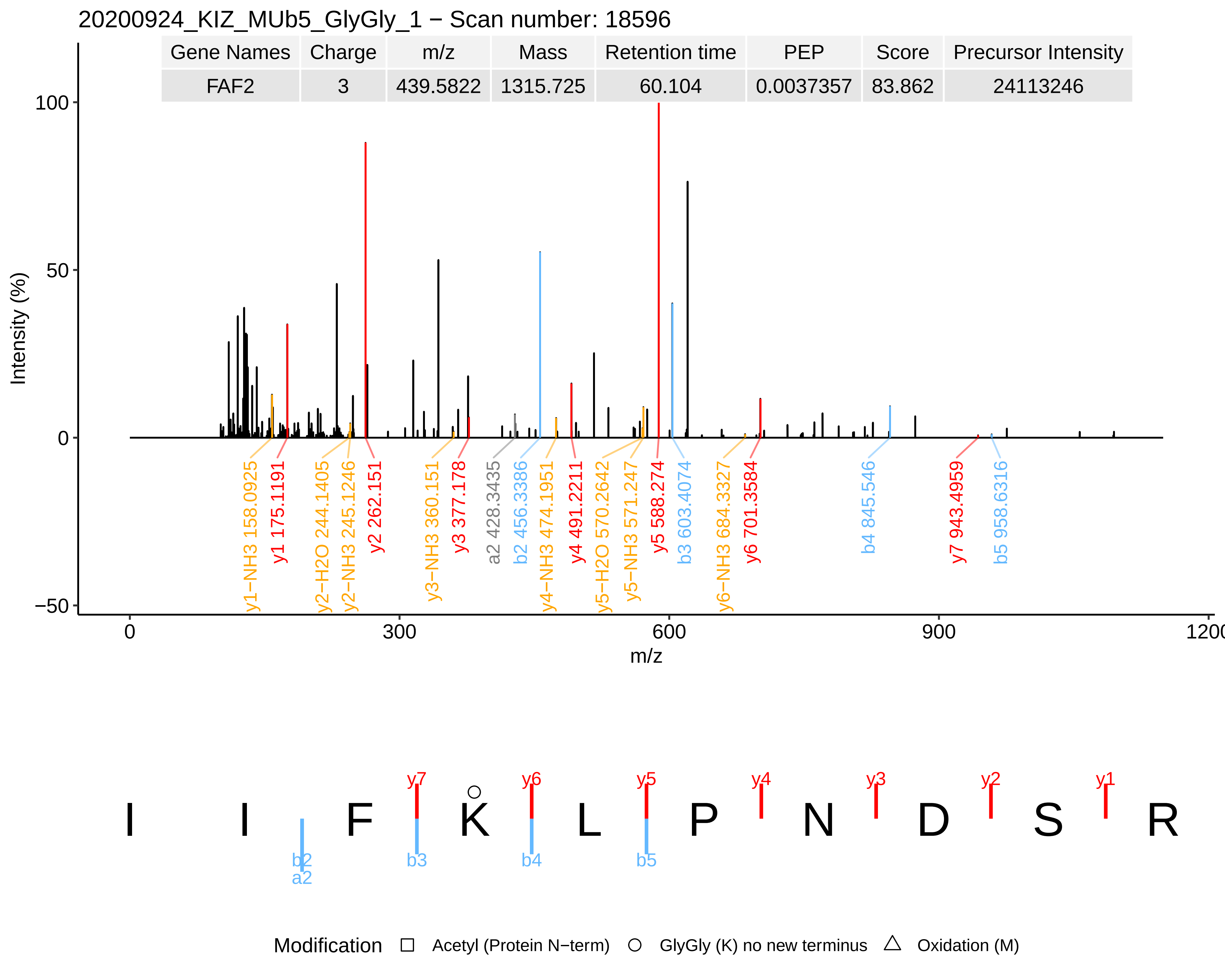

20200924_KIZ_MUb5_GlyGly_1 − Scan number: 18596
Gene Names | Charge | m/z | Mass | Retention time | PEP | Score | Precursor Intensity
FAF2 | 3 | 439.5822 | 1315.725 | 60.104 | 0.0037357 | 83.862 | 24113246
Intensity (%)
m/z
y1−NH3 158.0925
y1 175.1191
y2−H2O 244.1405
y2−NH3 245.1246
y2 262.151
y3−NH3 360.151
y3 377.178
a2 428.3435
b2 456.3386
y4−NH3 474.1951
y4 491.2211
y5−H2O 570.2642
y5−NH3 571.247
y5 588.274
b3 603.4074
y6−NH3 684.3327
y6 701.3584
b4 845.546
y7 943.4959
b5 958.6316
I I F K L P N D S R
Modification
Acetyl (Protein N−term)
GlyGly (K) no new terminus
Oxidation (M)

20200924_KIZ_MUb5_GlyGly_1 − Scan number: 18757

Gene Names	Charge	m/z	Mass	Retention time	PEP	Score	Precursor Intensity
FAF2	3	439.5822	1315.725	60.615	0.030798	61.765	15974222

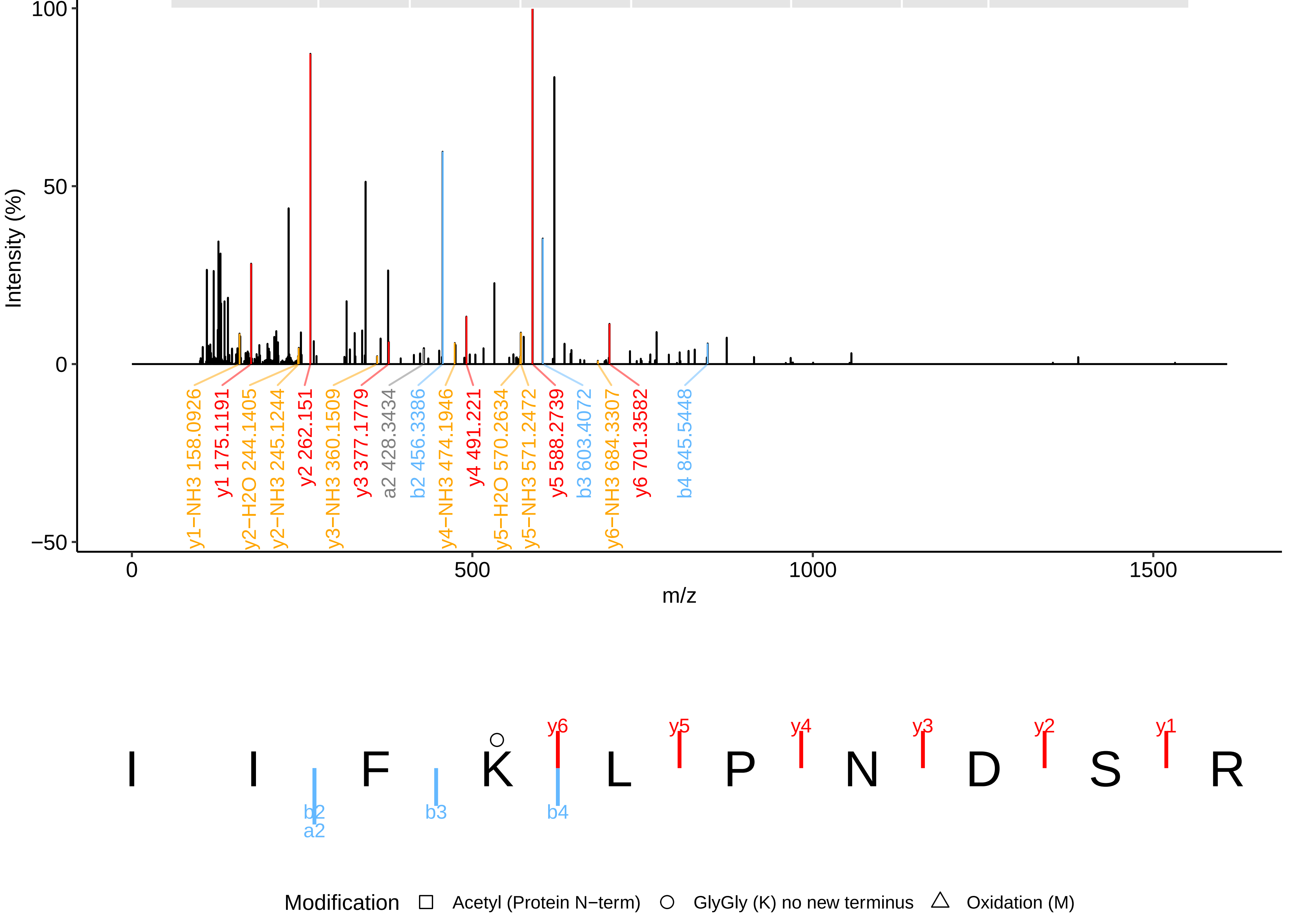

20200924_KIZ_MUb5_GlyGly_2 − Scan number: 17483

Gene Names	Charge	m/z	Mass	Retention time	PEP	Score	Precursor Intensity
FAF2	3	439.5822	1315.725	64.79	0.004058	82.029	6081016

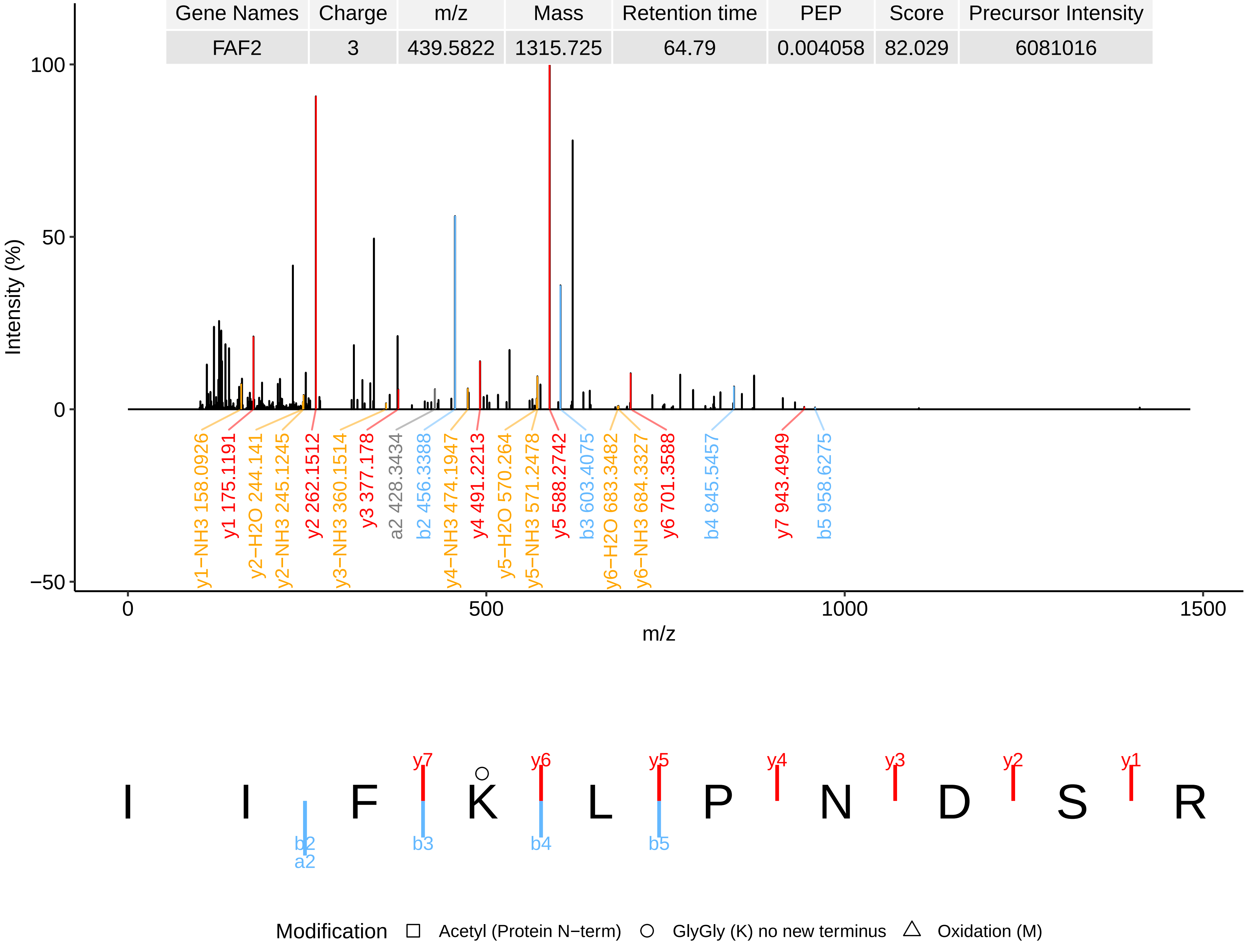

20201118_KIZ_MUb6_GlyGly_2 − Scan number: 21082

Gene Names	Charge	m/z	Mass	Retention time	PEP	Score	Precursor Intensity
FAF2	3	439.5822	1315.725	71.036	0.0023648	91.658	11838245

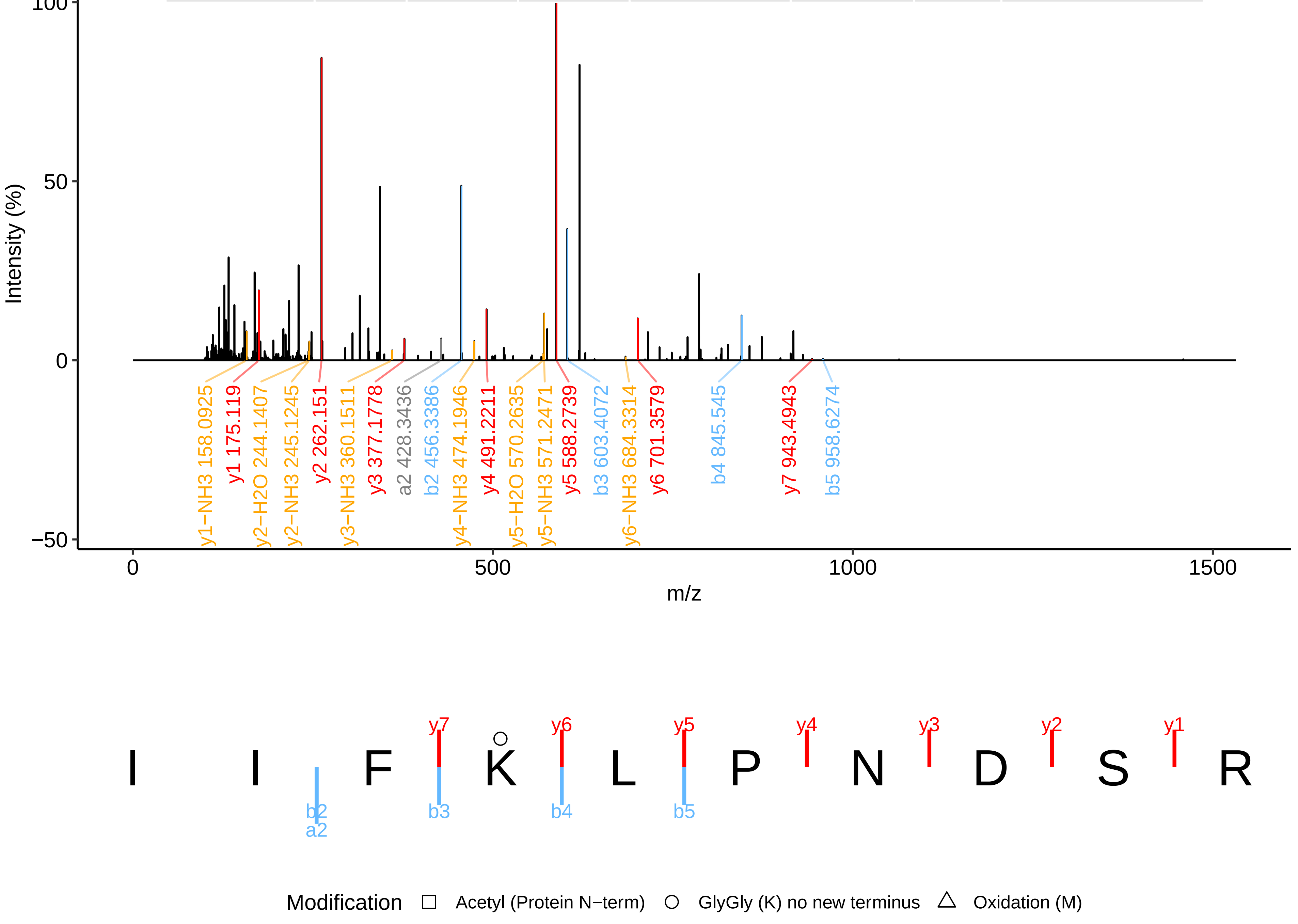

20200921_KIZ_MUb4_GlyGly_10plex − Scan number: 8123

Gene Names	Charge	m/z	Mass	Retention time	PEP	Score	Precursor Intensity
SYNJ2BP−COX16;SYNJ2BP	3	443.5688	1327.684	27.485	1.5605e−05	102.33	5826382

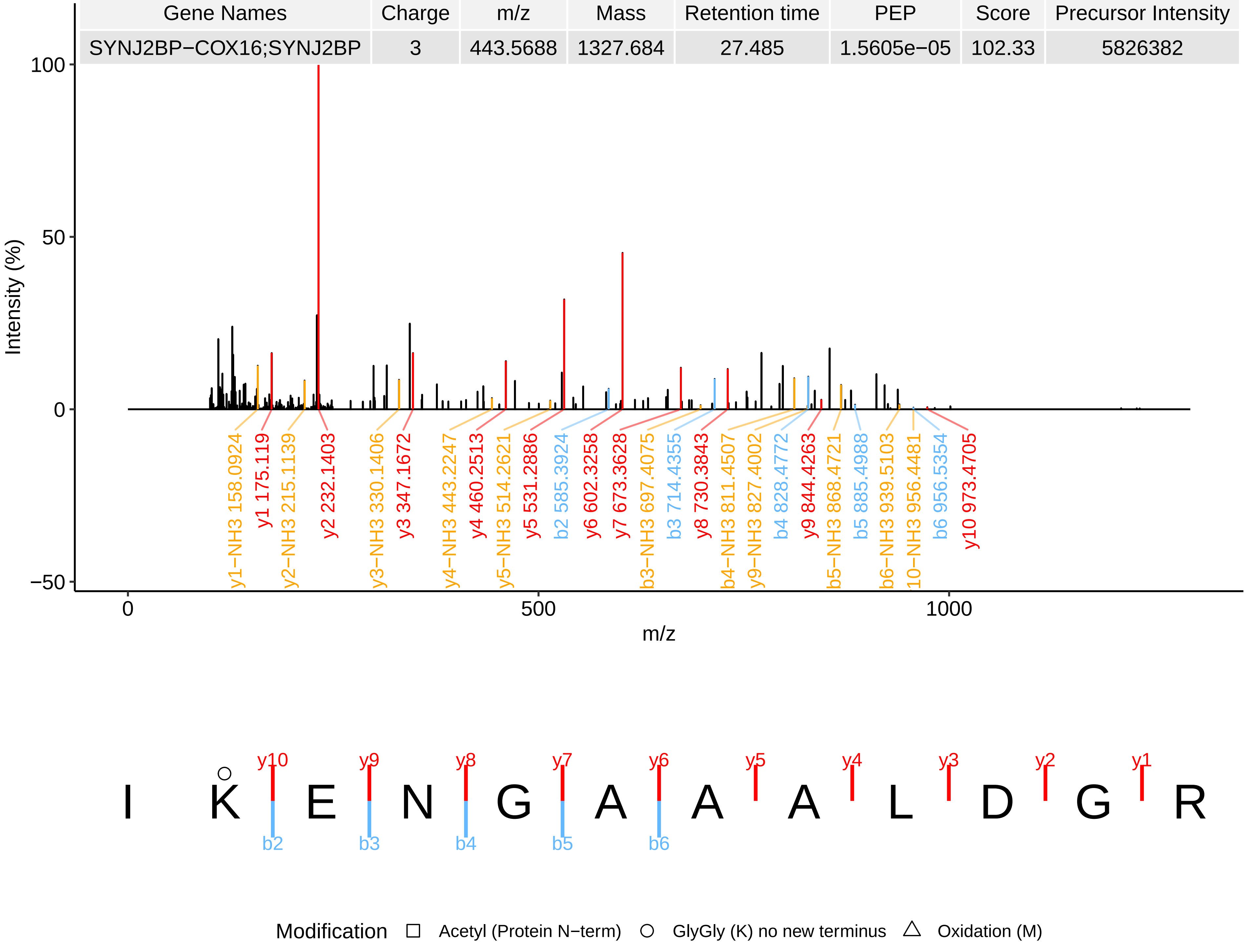

20200921_KIZ_MUb4_GlyGly_10plex_2 − Scan number: 7495

Gene Names	Charge	m/z	Mass	Retention time	PEP	Score	Precursor Intensity
SYNJ2BP−COX16;SYNJ2BP	3	443.5688	1327.684	27.989	0.021389	61.532	1365732

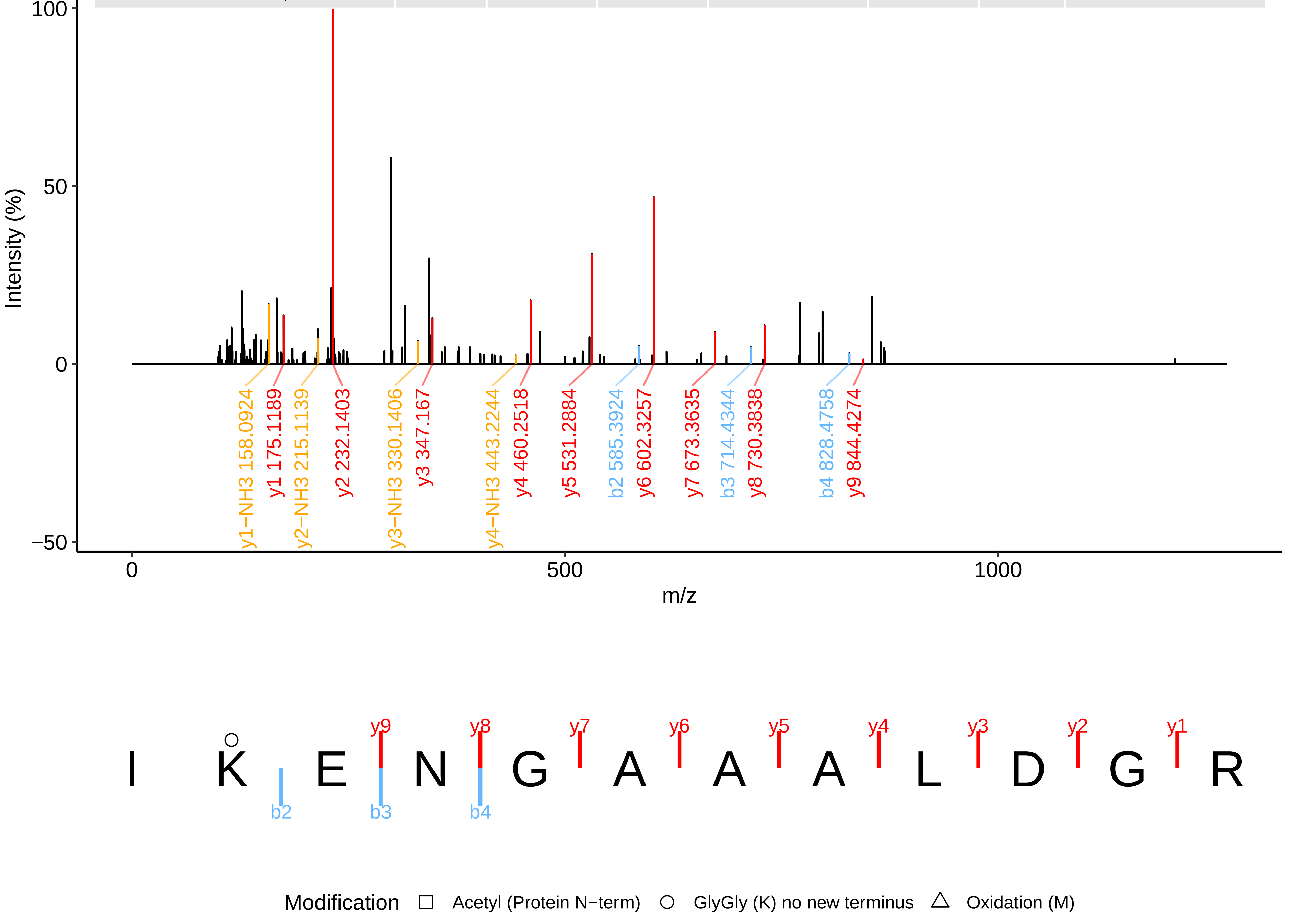

20200921_KIZ_MUb4_GlyGly_10plex − Scan number: 13426

Gene Names	Charge	m/z	Mass	Retention time	PEP	Score	Precursor Intensity
RNF213	3	448.5808	1342.72	47.363	0.00045332	114.89	9988302

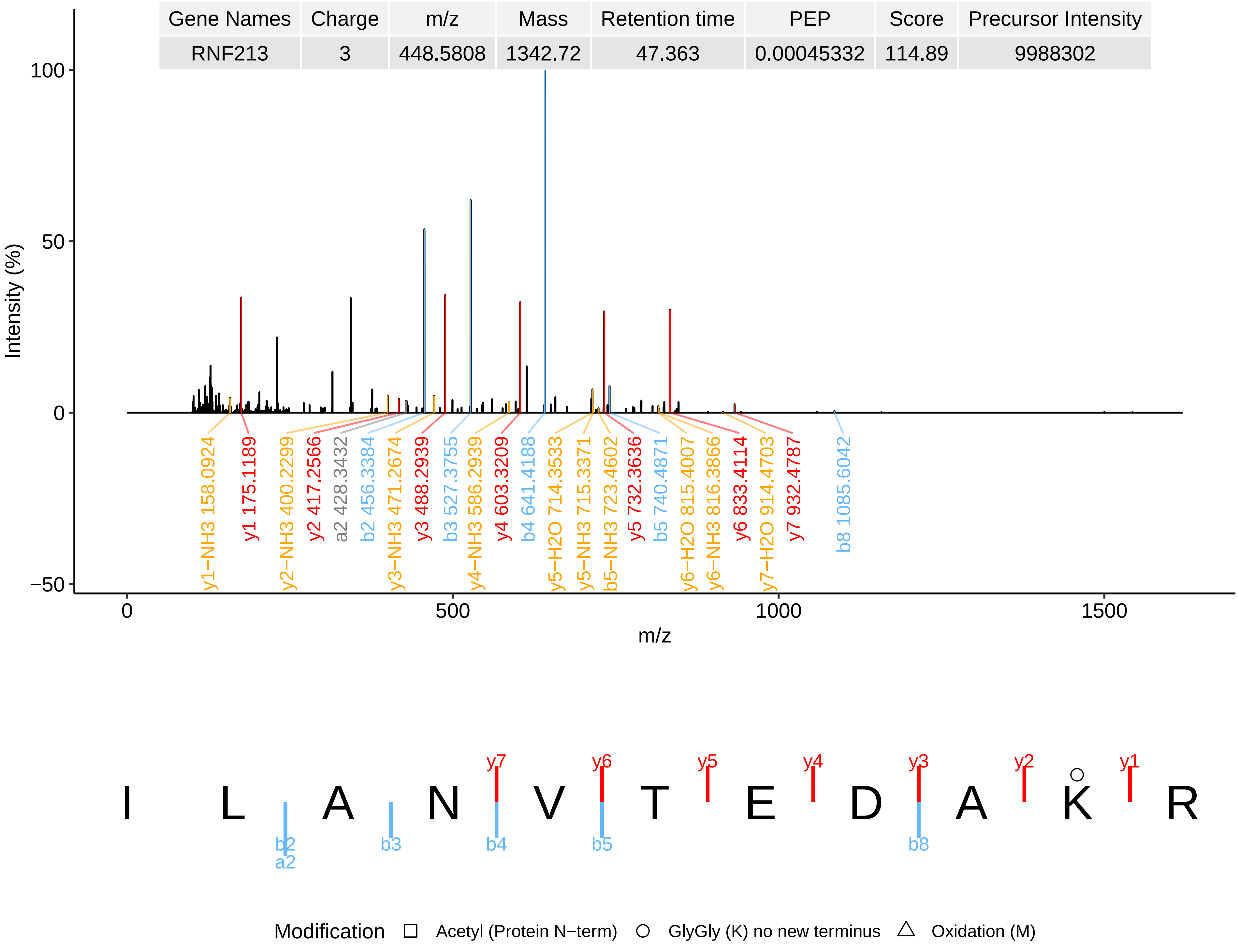

20200921_KIZ_MUb4_GlyGly_10plex_2 − Scan number: 12377

Gene Names	Charge	m/z	Mass	Retention time	PEP	Score	Precursor Intensity
RNF213	3	448.5808	1342.72	47.872	0.003537	92.538	1658554

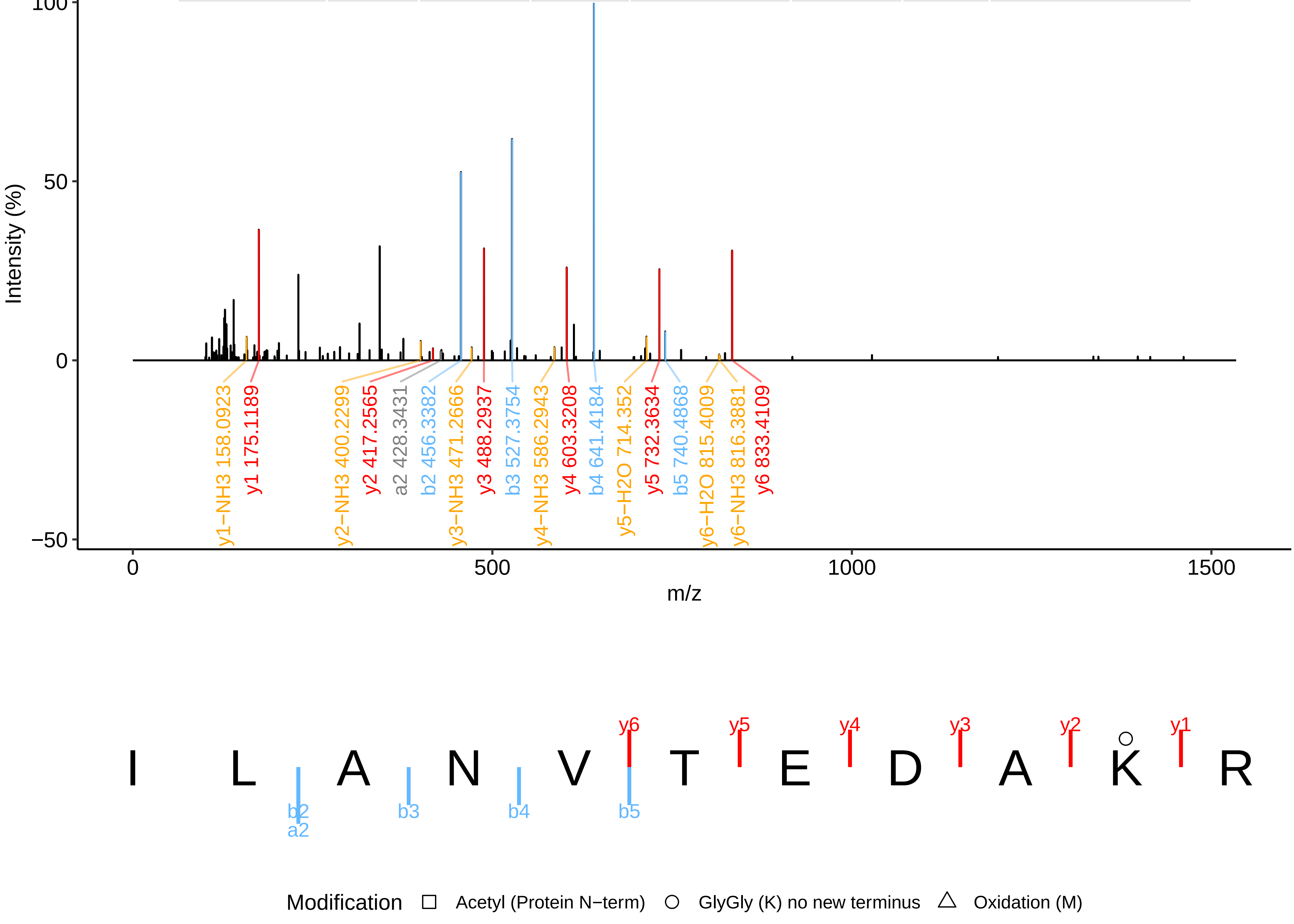

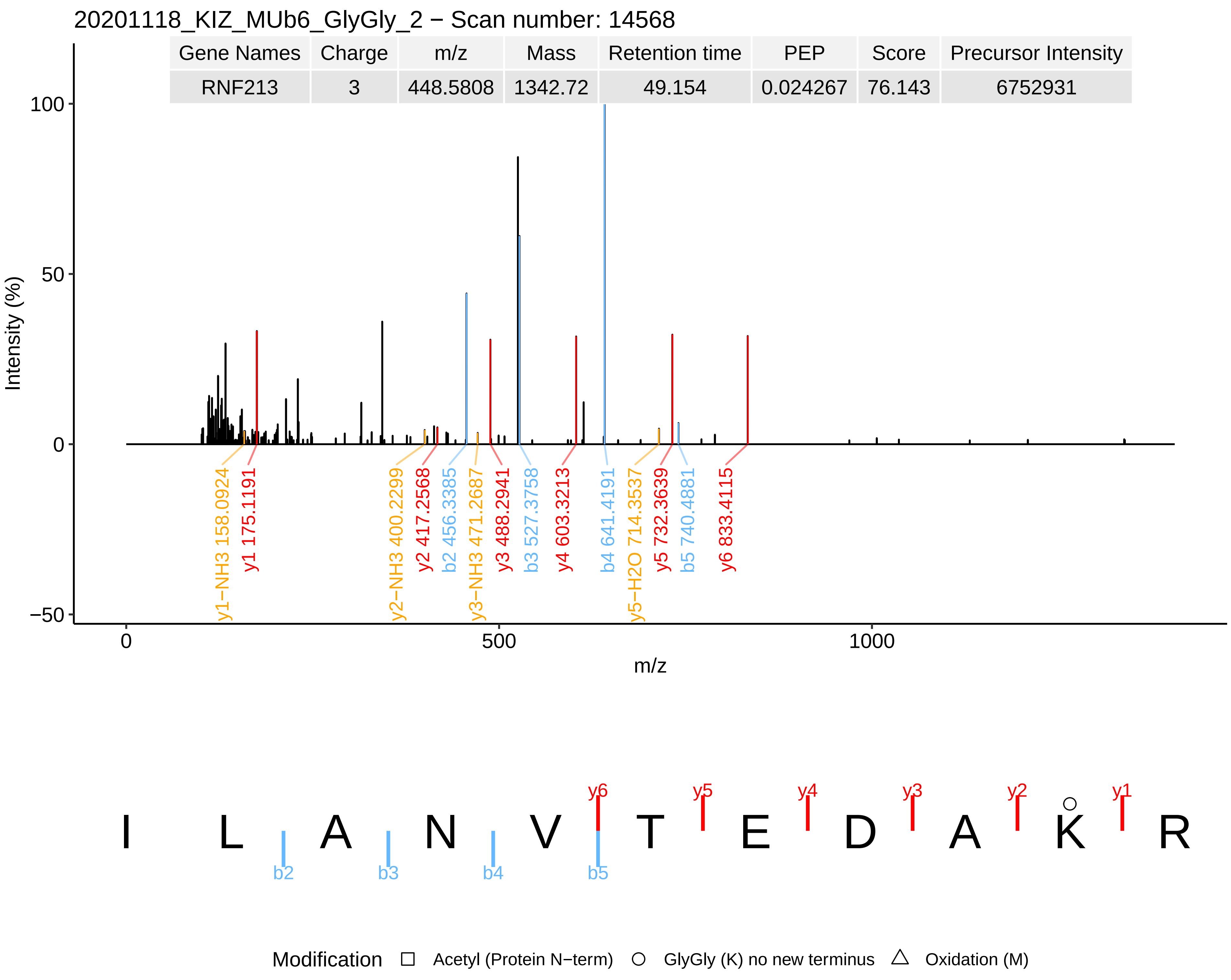

20201118_KIZ_MUb6_GlyGly_2 − Scan number: 14568
Gene Names | Charge | m/z | Mass | Retention time | PEP | Score | Precursor Intensity
RNF213 | 3 | 448.5808 | 1342.72 | 49.154 | 0.024267 | 76.143 | 6752931
Intensity (%)
m/z
y1−NH3 158.0924
y1 175.1191
y2−NH3 400.2299
y2 417.2568
b2 456.3385
y3−NH3 471.2687
y3 488.2941
b3 527.3758
y4 603.3213
b4 641.4191
y5−H2O 714.3537
y5 732.3639
b5 740.4881
y6 833.4115
I L A N V T E D A K R
y6 y5 y4 y3 y2 y1
b2 b3 b4 b5
Modification
Acetyl (Protein N−term)
GlyGly (K) no new terminus
Oxidation (M)

20200921_KIZ_MUb4_GlyGly_10plex − Scan number: 15863

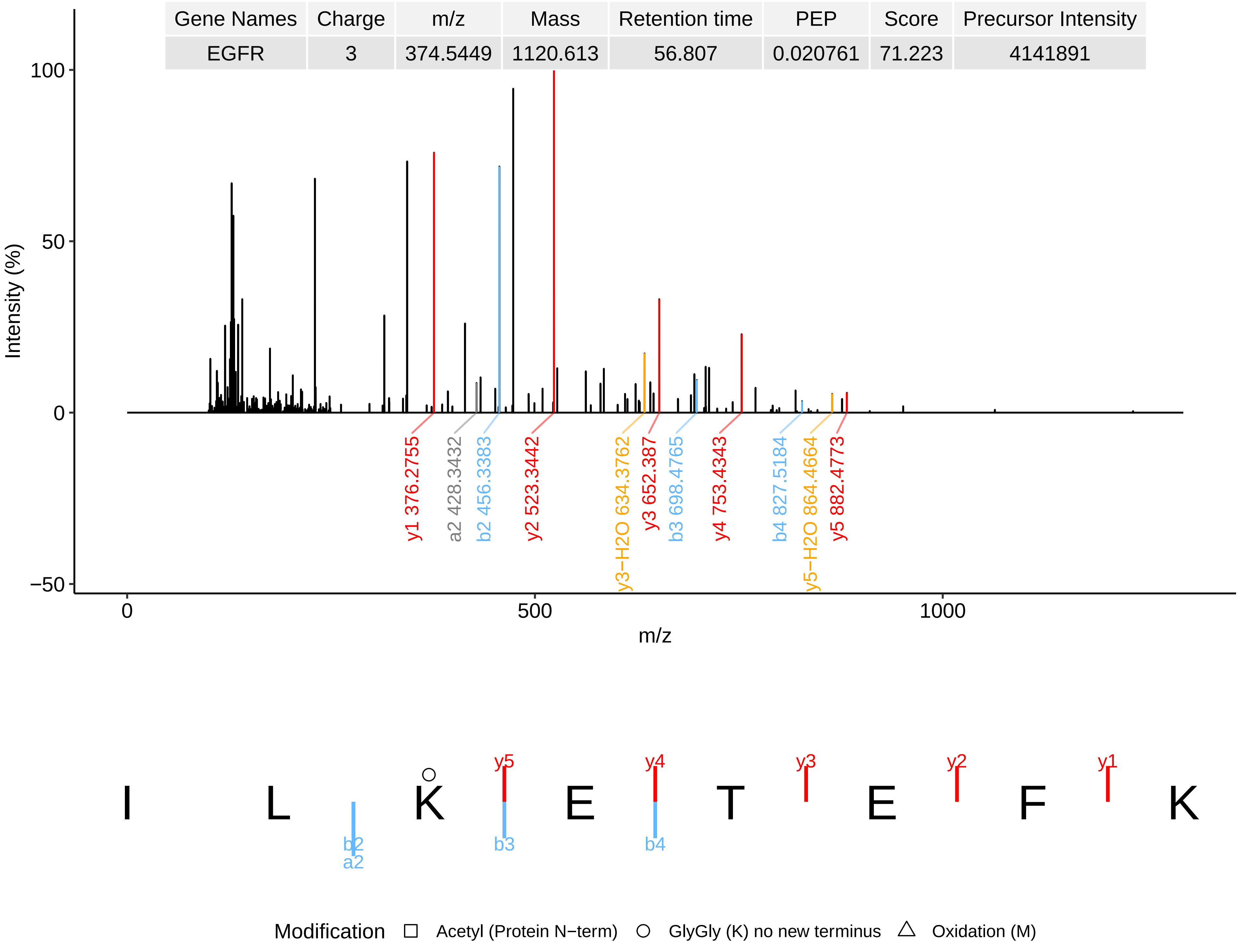

20200921_KIZ_MUb4_GlyGly_10plex_2 − Scan number: 14759

Gene Names	Charge	m/z	Mass	Retention time	PEP	Score	Precursor Intensity
EGFR	3	374.5449	1120.613	57.597	0.12271	50.878	NA

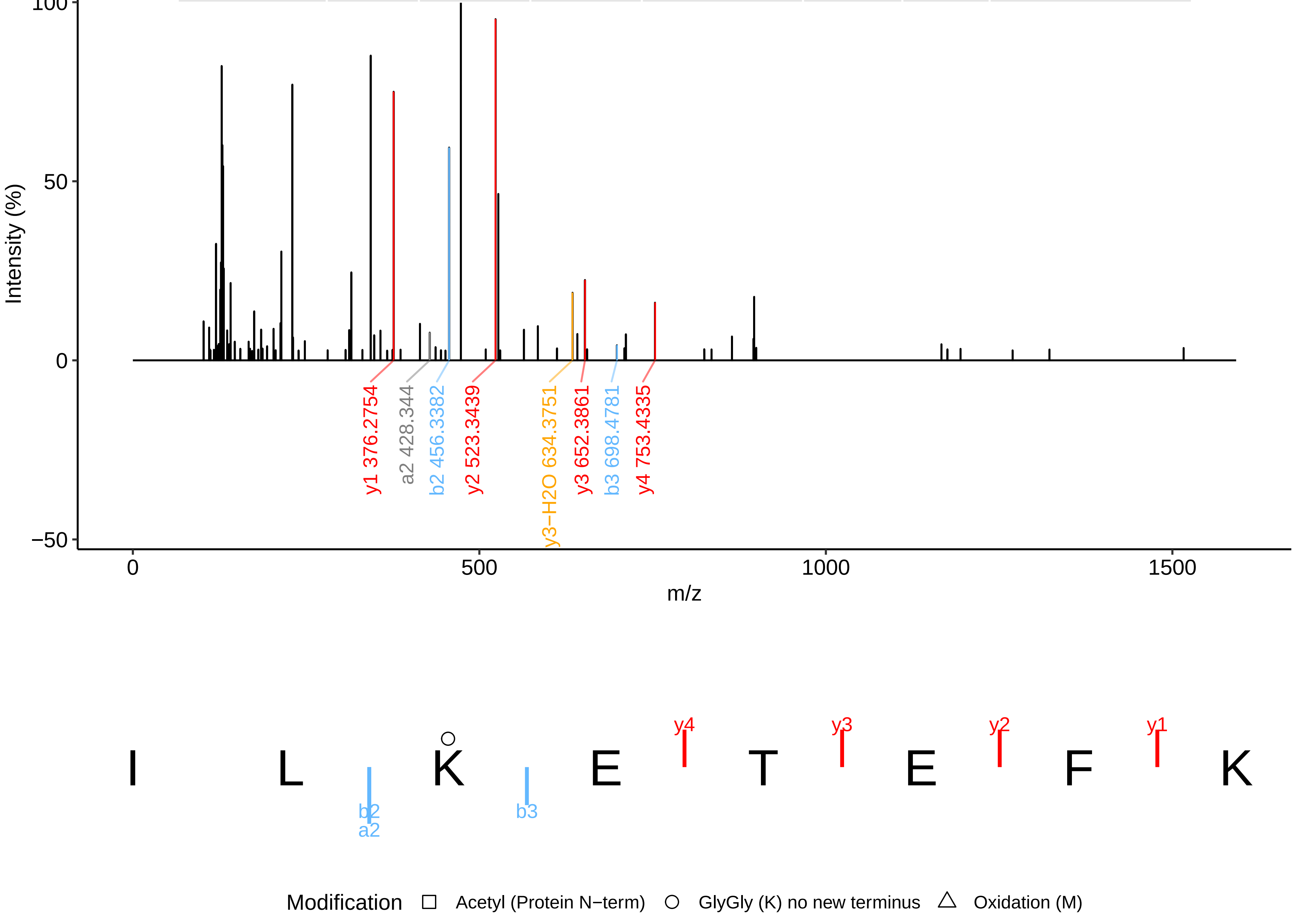

20200921_KIZ_MUb4_GlyGly_10plex − Scan number: 12731

Gene Names	Charge	m/z	Mass	Retention time	PEP	Score	Precursor Intensity
SLC43A2	3	366.8696	1097.587	44.738	0.044633	59.8	4282657

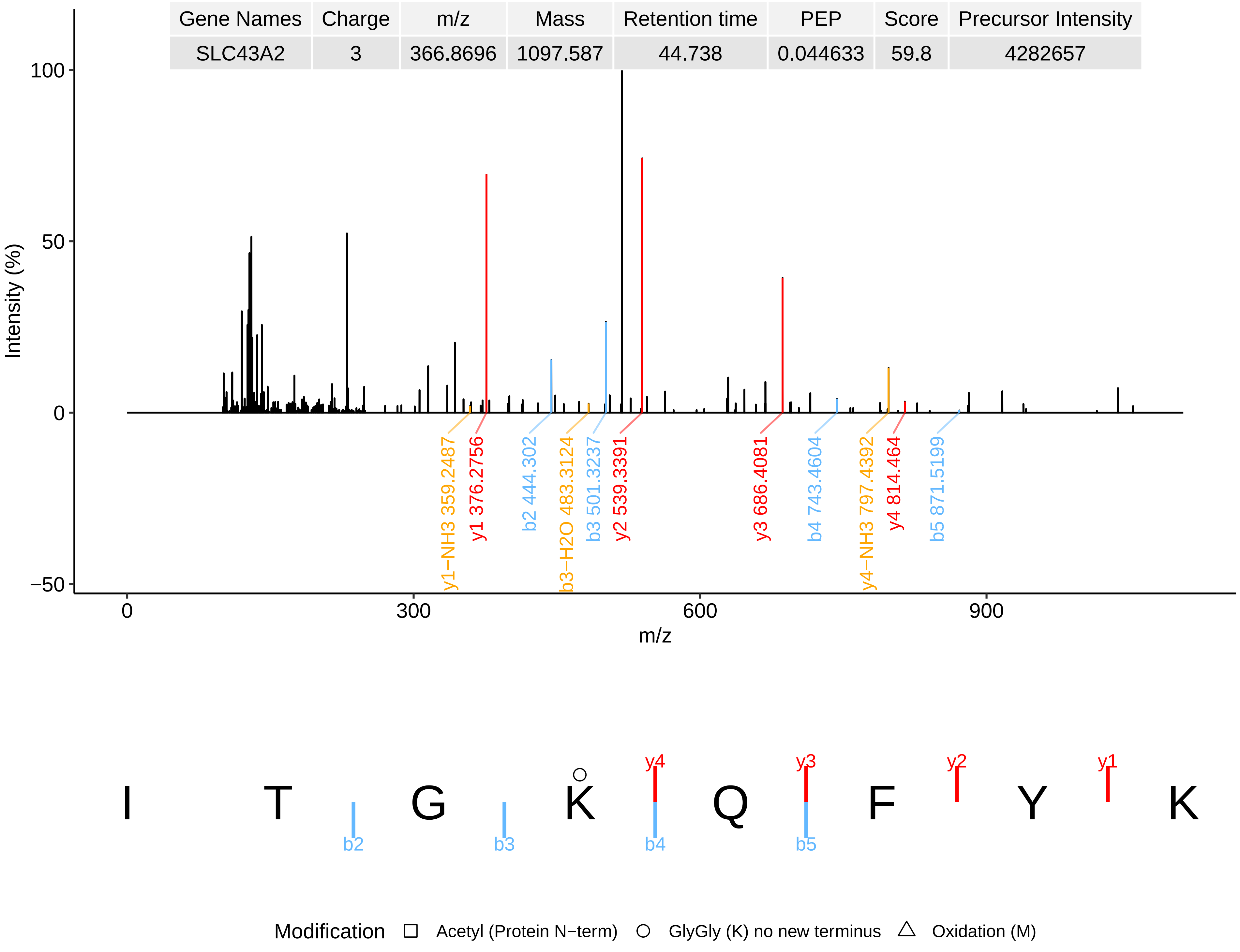

20200921_KIZ_MUb4_GlyGly_10plex − Scan number: 12784

Gene Names	Charge	m/z	Mass	Retention time	PEP	Score	Precursor Intensity
SLC43A2	3	366.8696	1097.587	44.945	0.03336	62.546	7880696

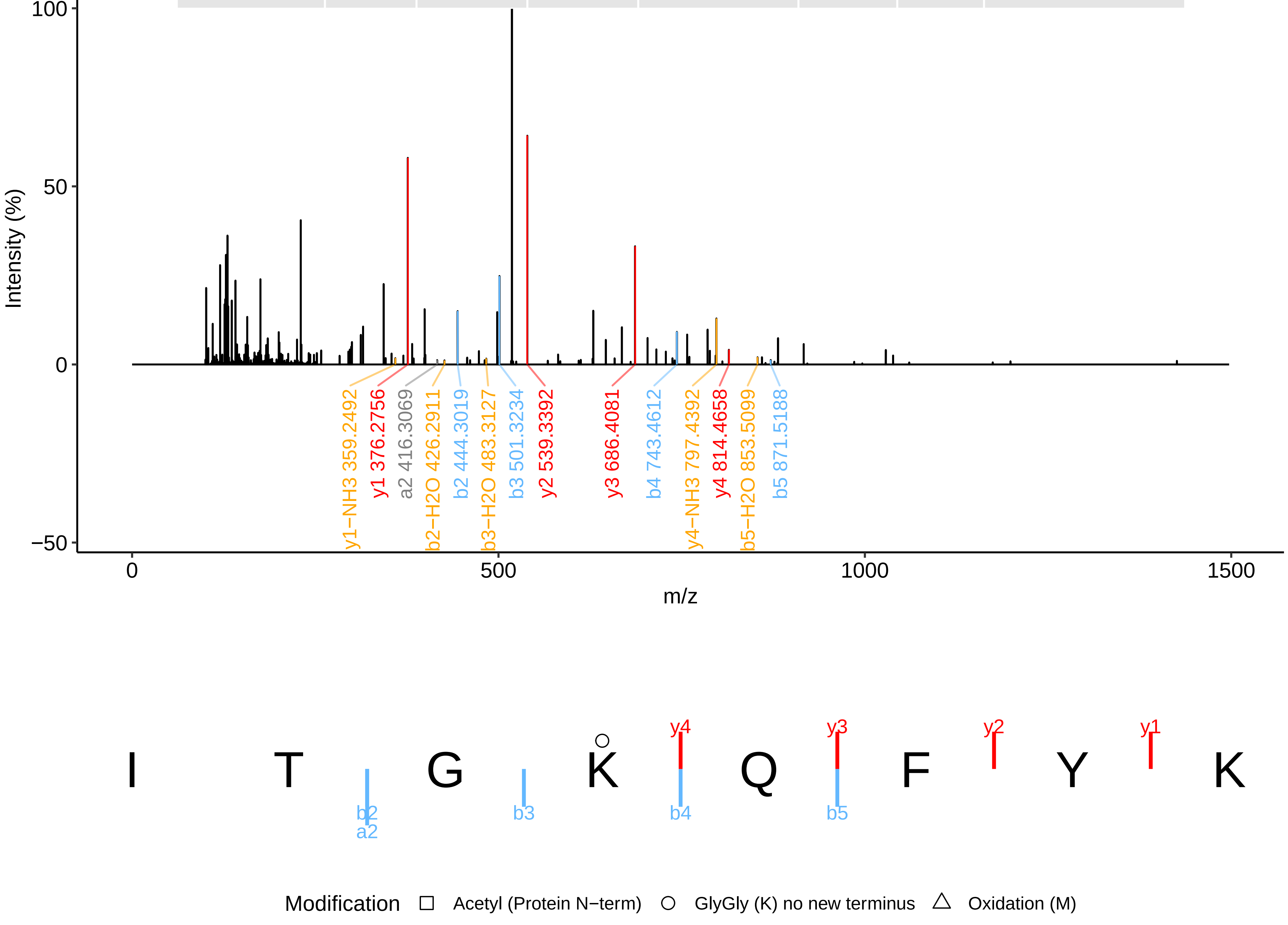

20200921_KIZ_MUb4_GlyGly_10plex_2 − Scan number: 11744

Gene Names	Charge	m/z	Mass	Retention time	PEP	Score	Precursor Intensity
SLC43A2	3	366.8696	1097.587	45.274	0.030301	63.565	1840511

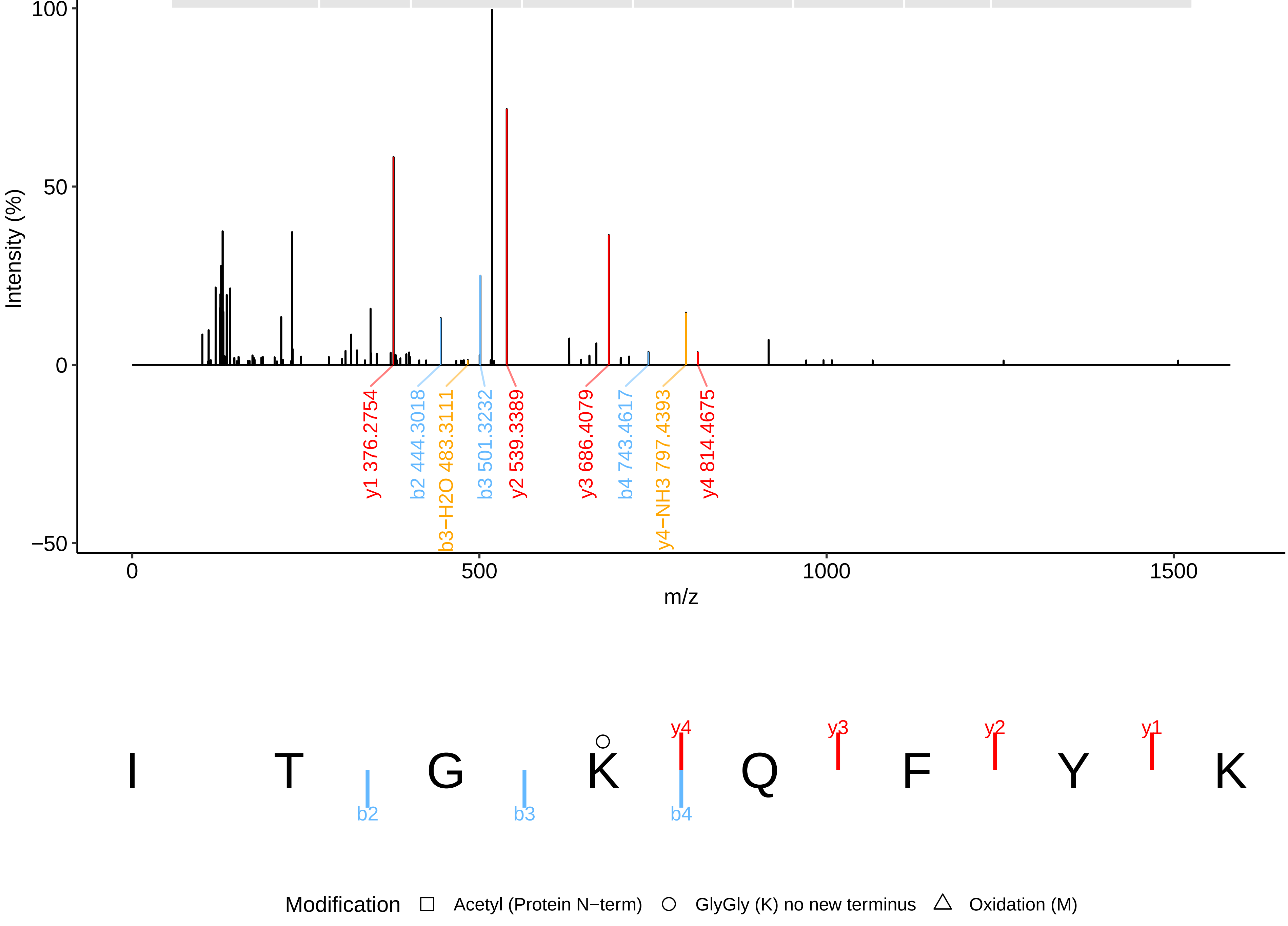

20200921_KIZ_MUb4_GlyGly_10plex − Scan number: 10534

Gene Names	Charge	m/z	Mass	Retention time	PEP	Score	Precursor Intensity
ALDH9A1	3	445.8873	1334.64	36.487	0.019105	64.39	7729467

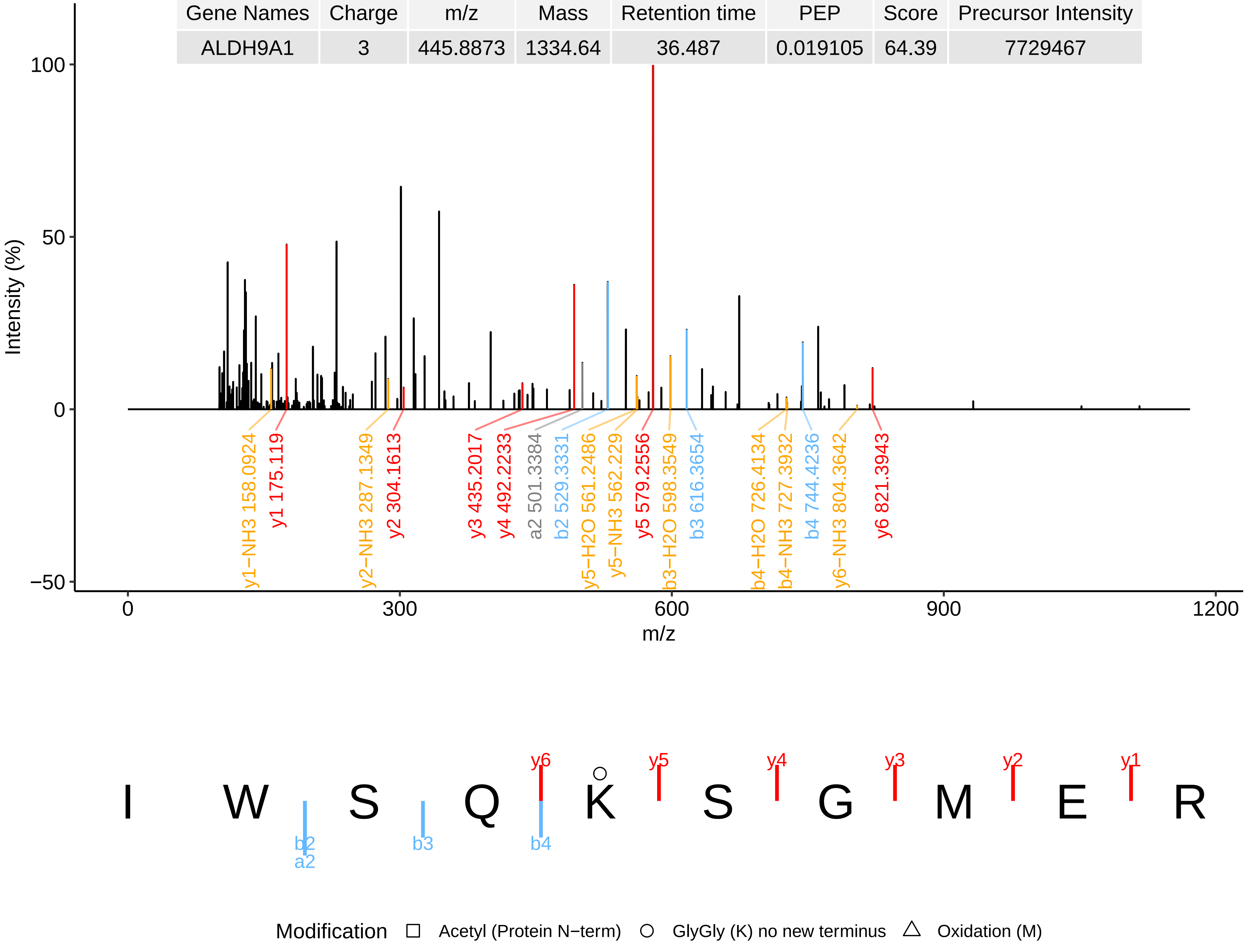

20200924_KIZ_MUb5_GlyGly_2 − Scan number: 8892

Gene Names	Charge	m/z	Mass	Retention time	PEP	Score	Precursor Intensity
ALDH9A1	3	445.8873	1334.64	33.237	0.067618	56.725	3042965

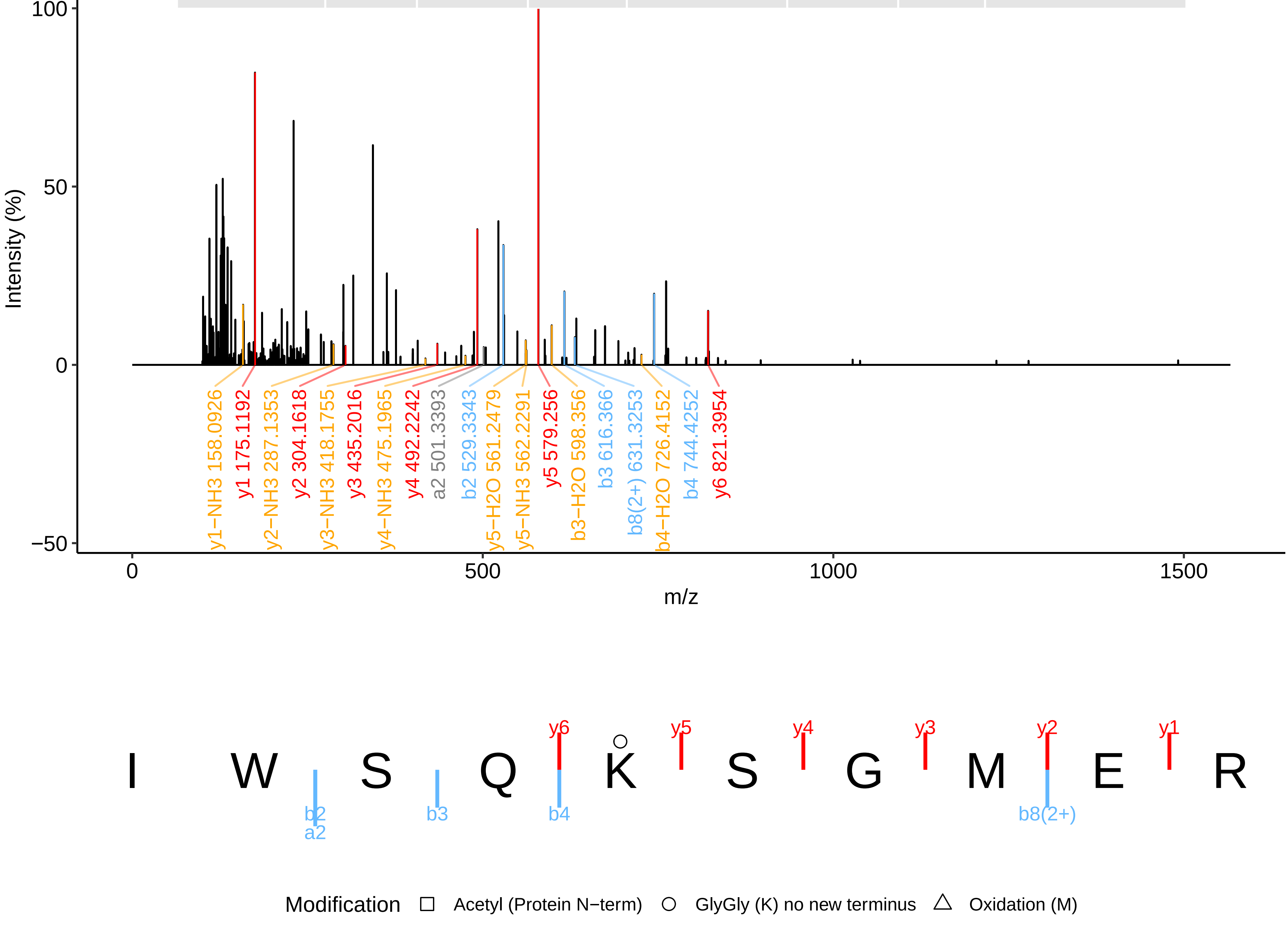

20201118_KIZ_MUb6_GlyGly_1 − Scan number: 8765

Gene Names	Charge	m/z	Mass	Retention time	PEP	Score	Precursor Intensity
ALDH9A1	3	445.8873	1334.64	27.699	0.04502	58.37	11668397

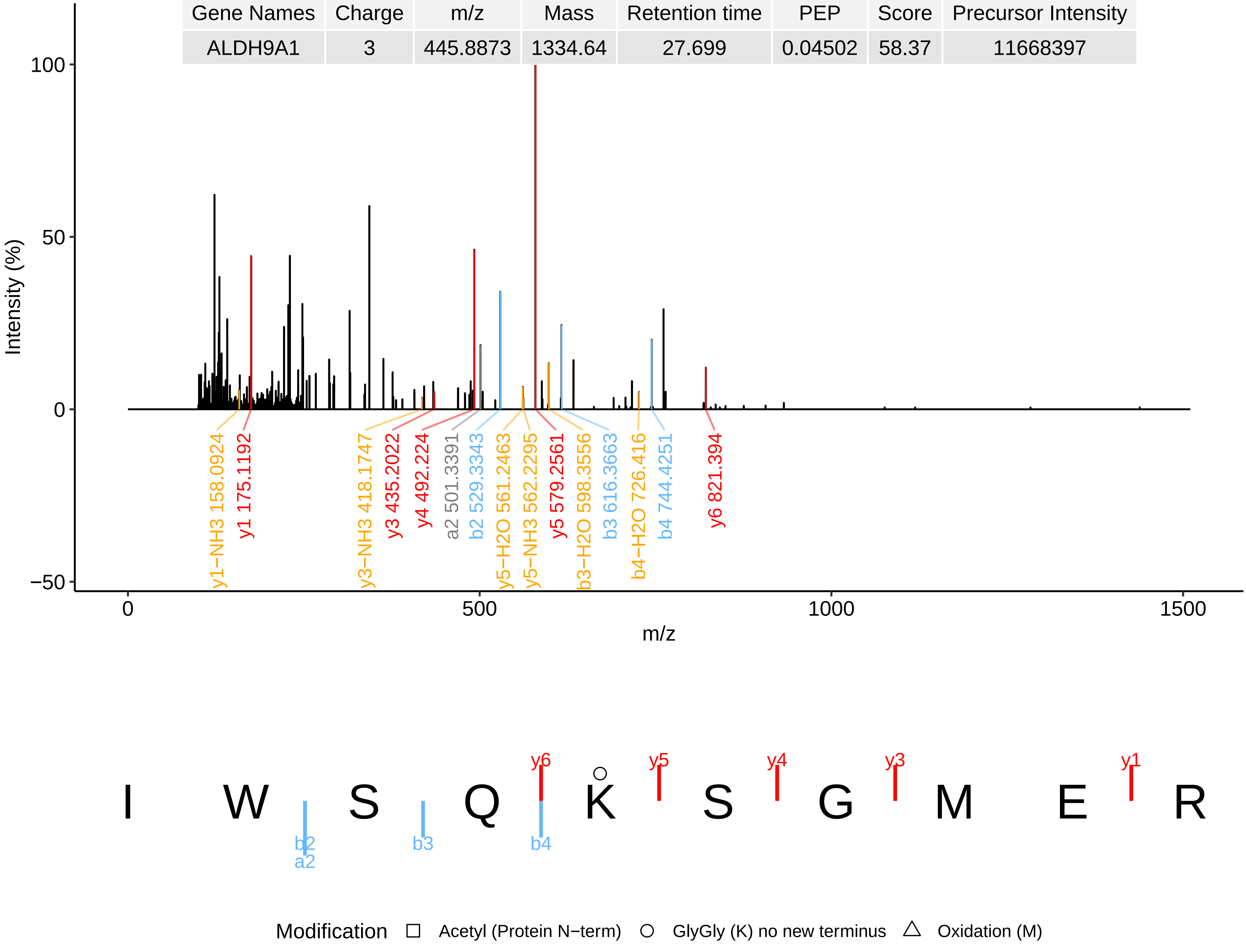

20200921_KIZ_MUb4_GlyGly_10plex − Scan number: 7655

Gene Names	Charge	m/z	Mass	Retention time	PEP	Score	Precursor Intensity
ALDH9A1	3	451.219	1350.635	25.759	0.13338	51.762	2497277

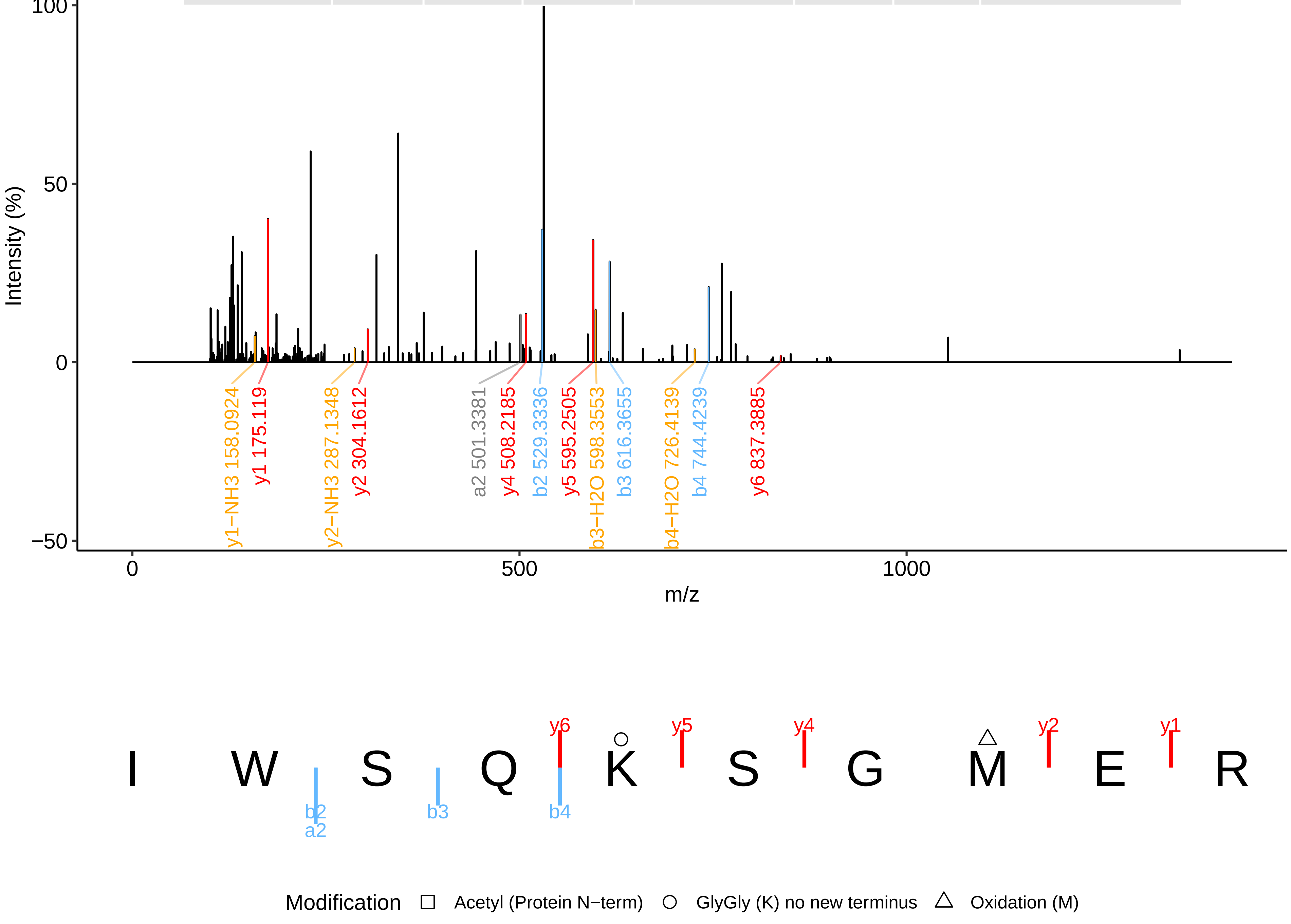

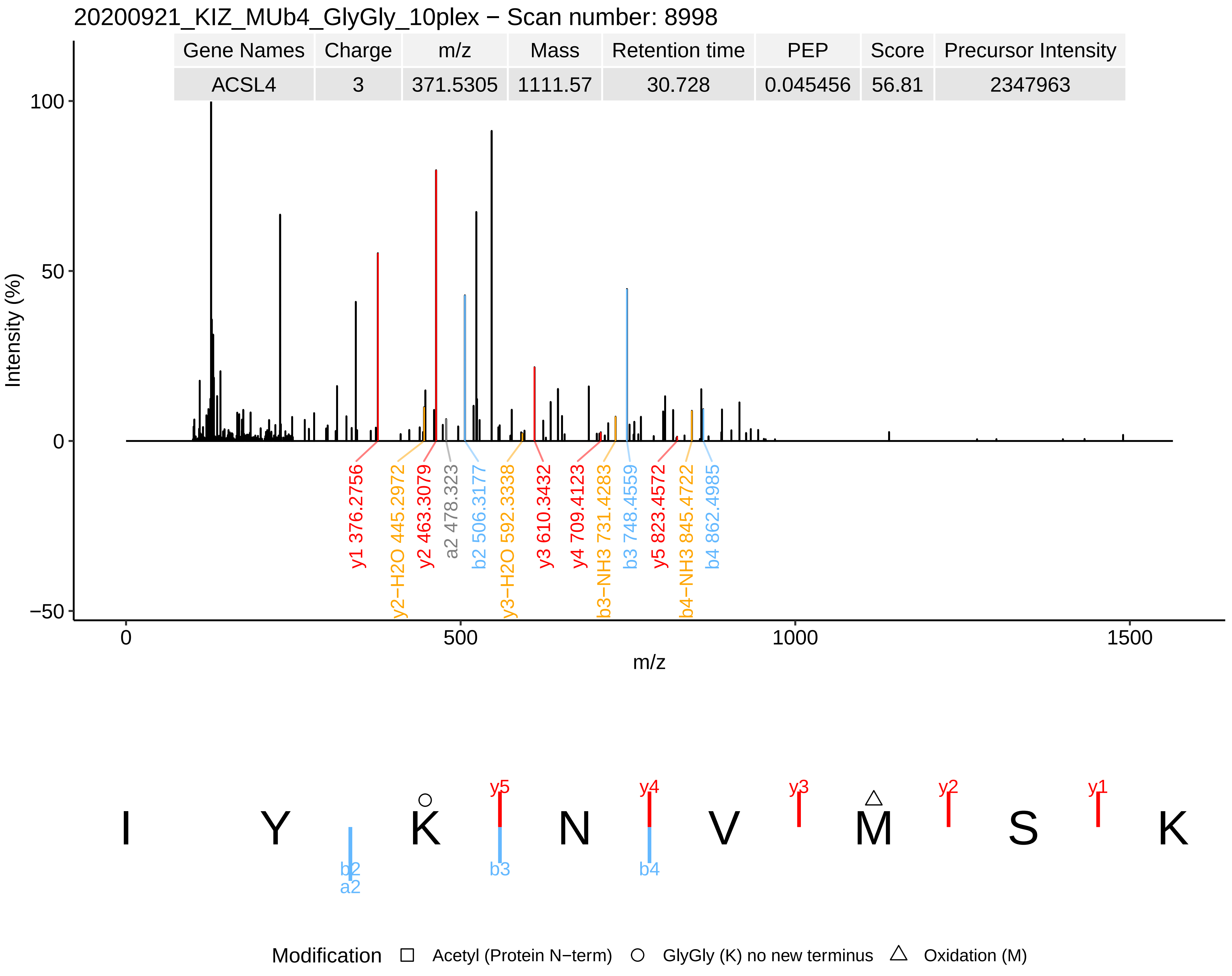
20200921_KIZ_MUb4_GlyGly_10plex − Scan number: 8998
Gene Names | Charge | m/z | Mass | Retention time | PEP | Score | Precursor Intensity
ACSL4 | 3 | 371.5305 | 1111.57 | 30.728 | 0.045456 | 56.81 | 2347963
Intensity (%)
m/z
y1 376.2756
y2−H2O 445.2972
y2 463.3079
a2 478.323
b2 506.3177
y3−H2O 592.3338
y3 610.3432
y4 709.4123
b3−NH3 731.4283
b3 748.4559
y5 823.4572
b4−NH3 845.4722
b4 862.4985
I Y K N V M S K
y5 y4 y3 y2 y1
b2 a2 b3 b4
Modification
Acetyl (Protein N−term)
GlyGly (K) no new terminus
Oxidation (M)

20200921_KIZ_MUb4_GlyGly_10plex − Scan number: 9025

Gene Names	Charge	m/z	Mass	Retention time	PEP	Score	Precursor Intensity
ACSL4	3	371.5305	1111.57	30.837	0.095327	41.429	4635503

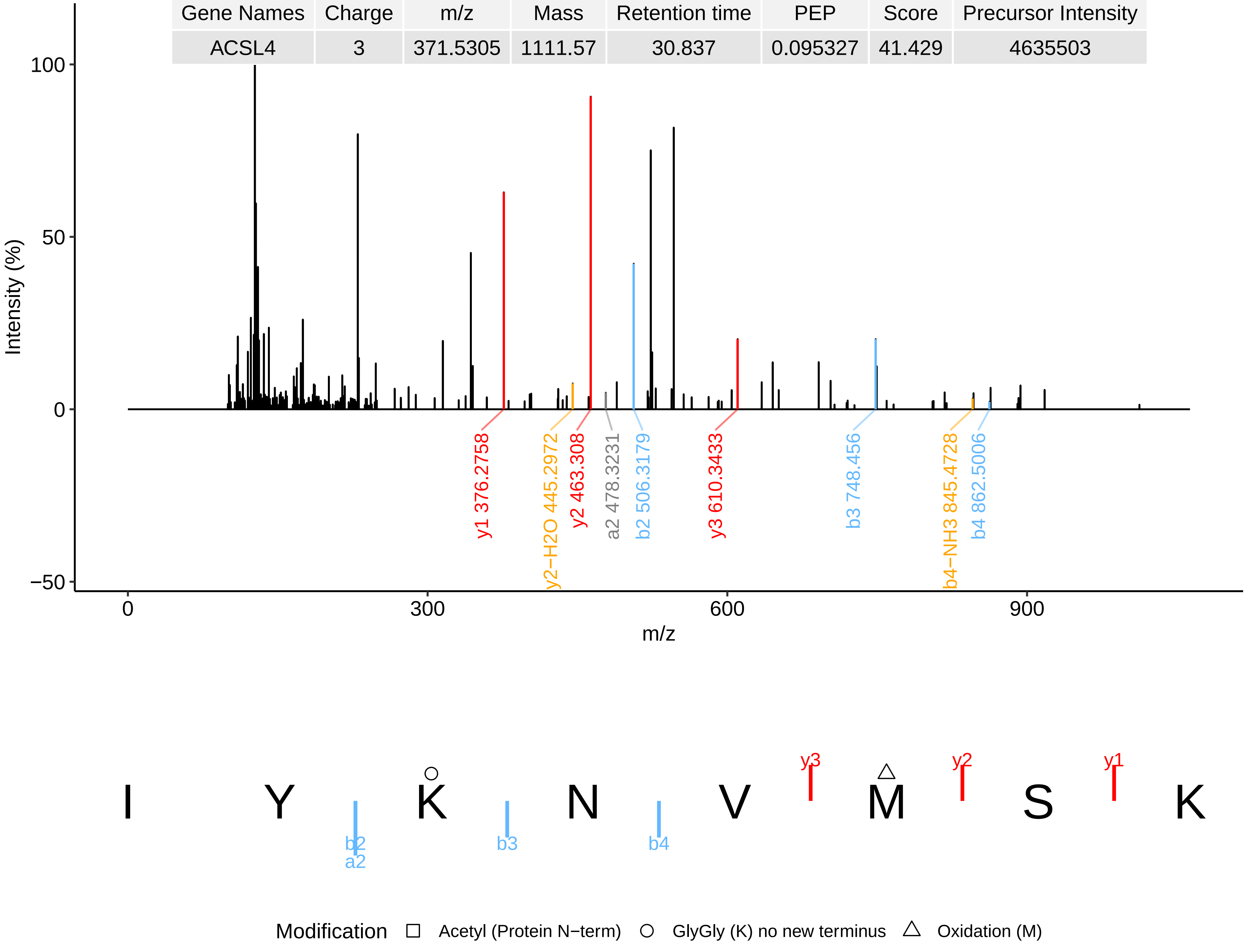

20200921_KIZ_MUb4_GlyGly_10plex_2 − Scan number: 8337

Gene Names	Charge	m/z	Mass	Retention time	PEP	Score	Precursor Intensity
ACSL4	3	371.5305	1111.57	31.378	0.096587	41.029	1291314

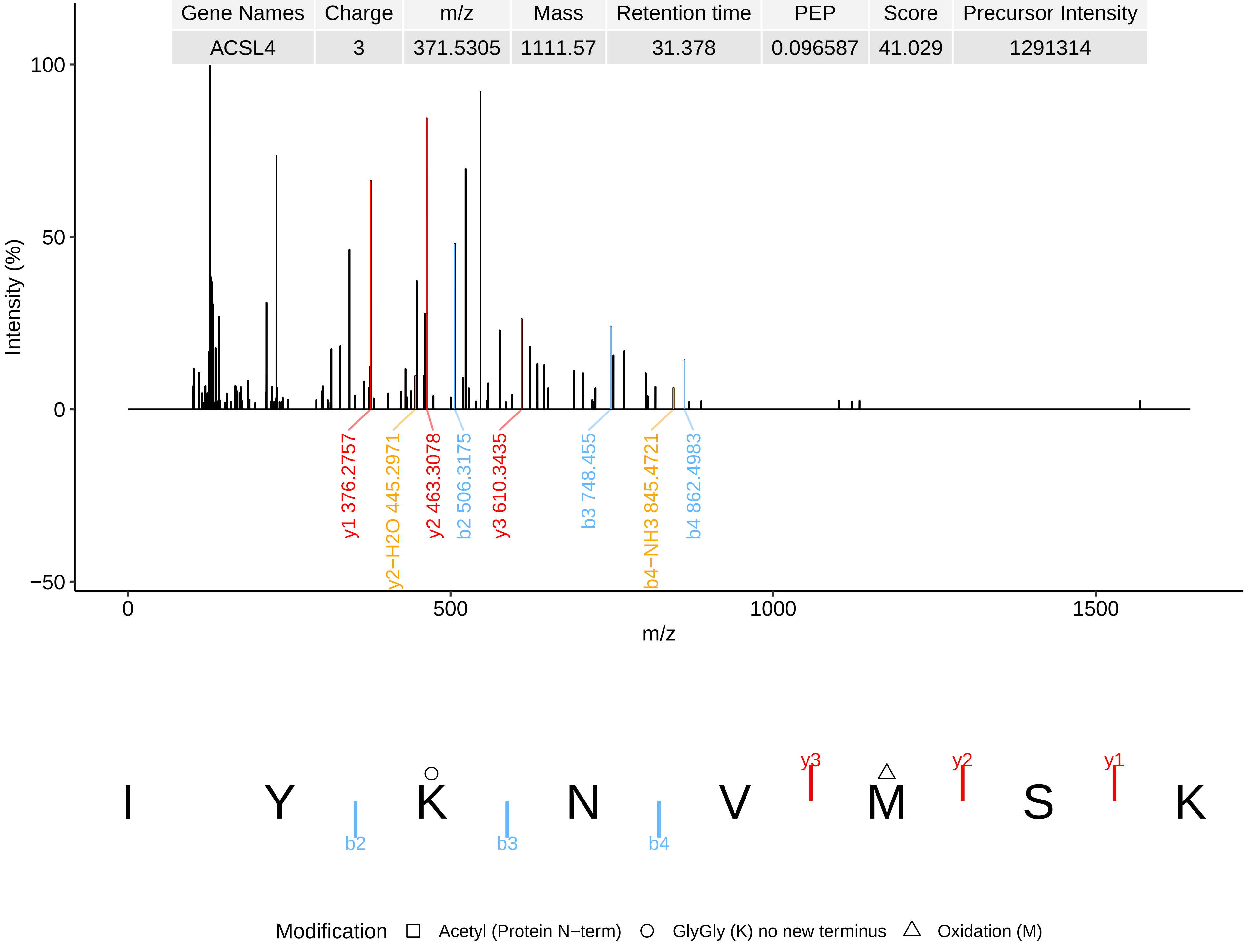

20200921_KIZ_MUb4_GlyGly_10plex − Scan number: 13493

Gene Names	Charge	m/z	Mass	Retention time	PEP	Score	Precursor Intensity
ACSL4	3	366.1988	1095.575	47.63	0.0074585	84.743	13810531

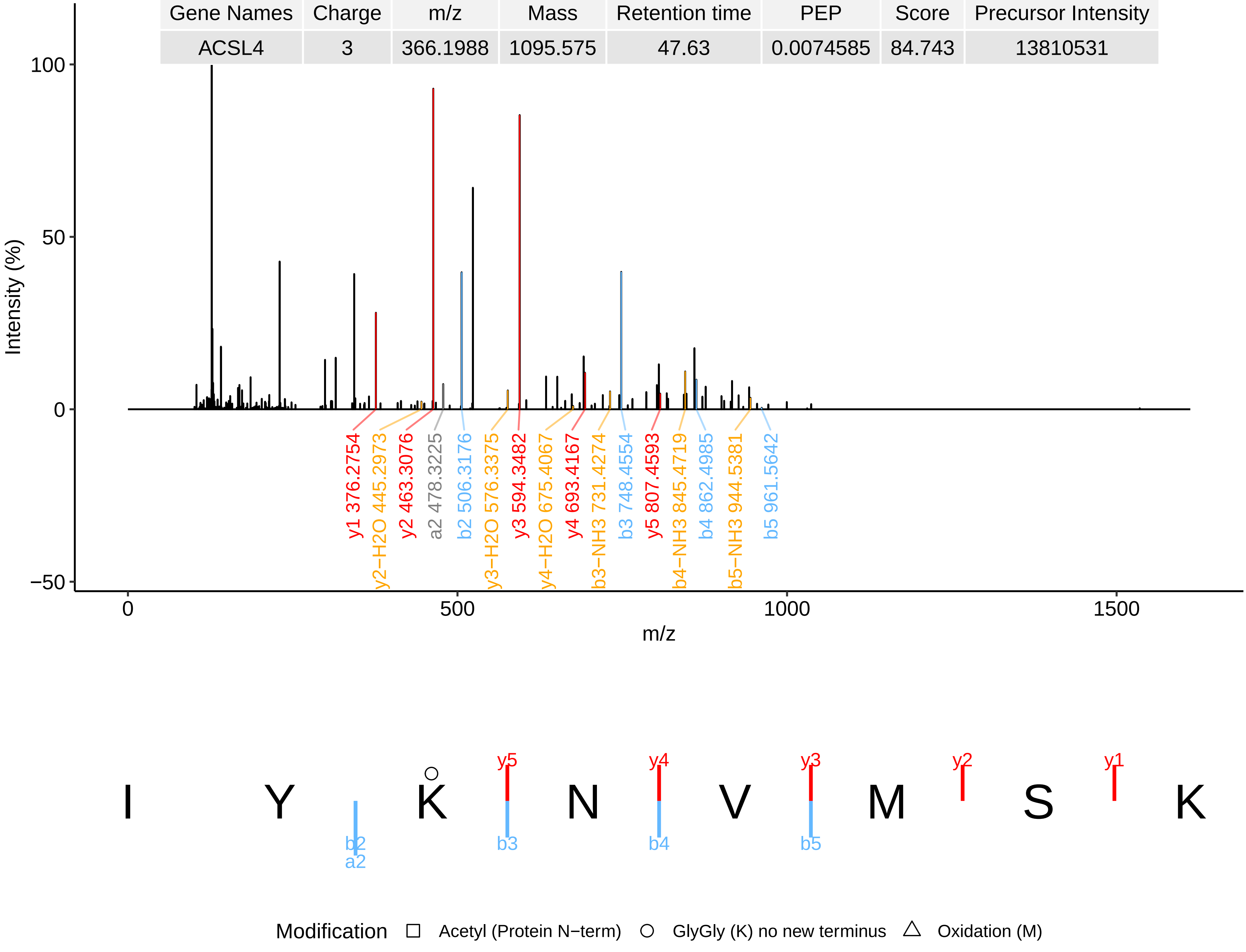

20200921_KIZ_MUb4_GlyGly_10plex_2 − Scan number: 12459

Gene Names	Charge	m/z	Mass	Retention time	PEP	Score	Precursor Intensity
ACSL4	3	366.1988	1095.575	48.204	0.049245	58.676	1169224

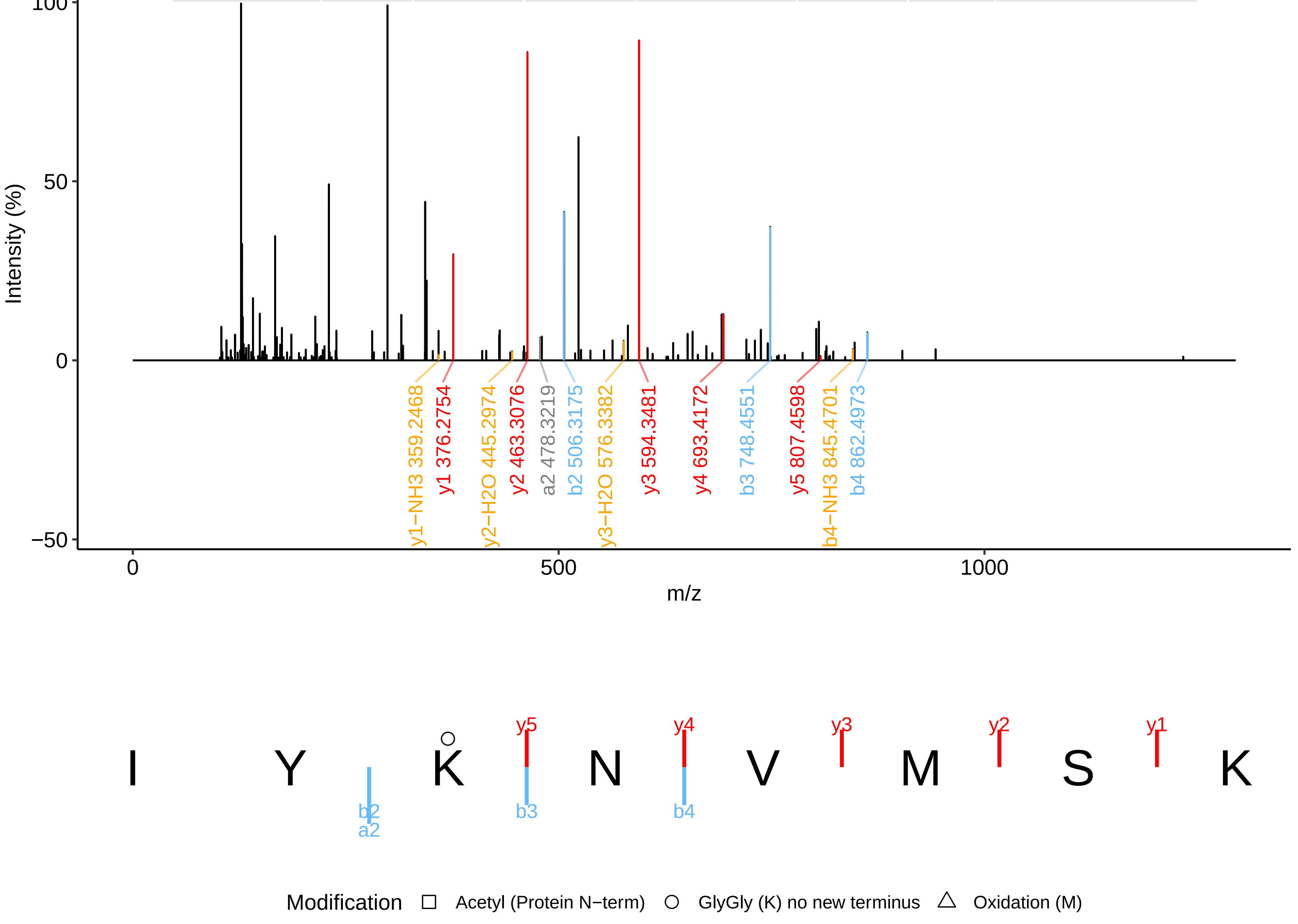

20200924_KIZ_MUb5_GlyGly_1 − Scan number: 12375

Gene Names	Charge	m/z	Mass	Retention time	PEP	Score	Precursor Intensity
ACSL4	3	366.1988	1095.575	40.257	0.0040548	93.374	20732134

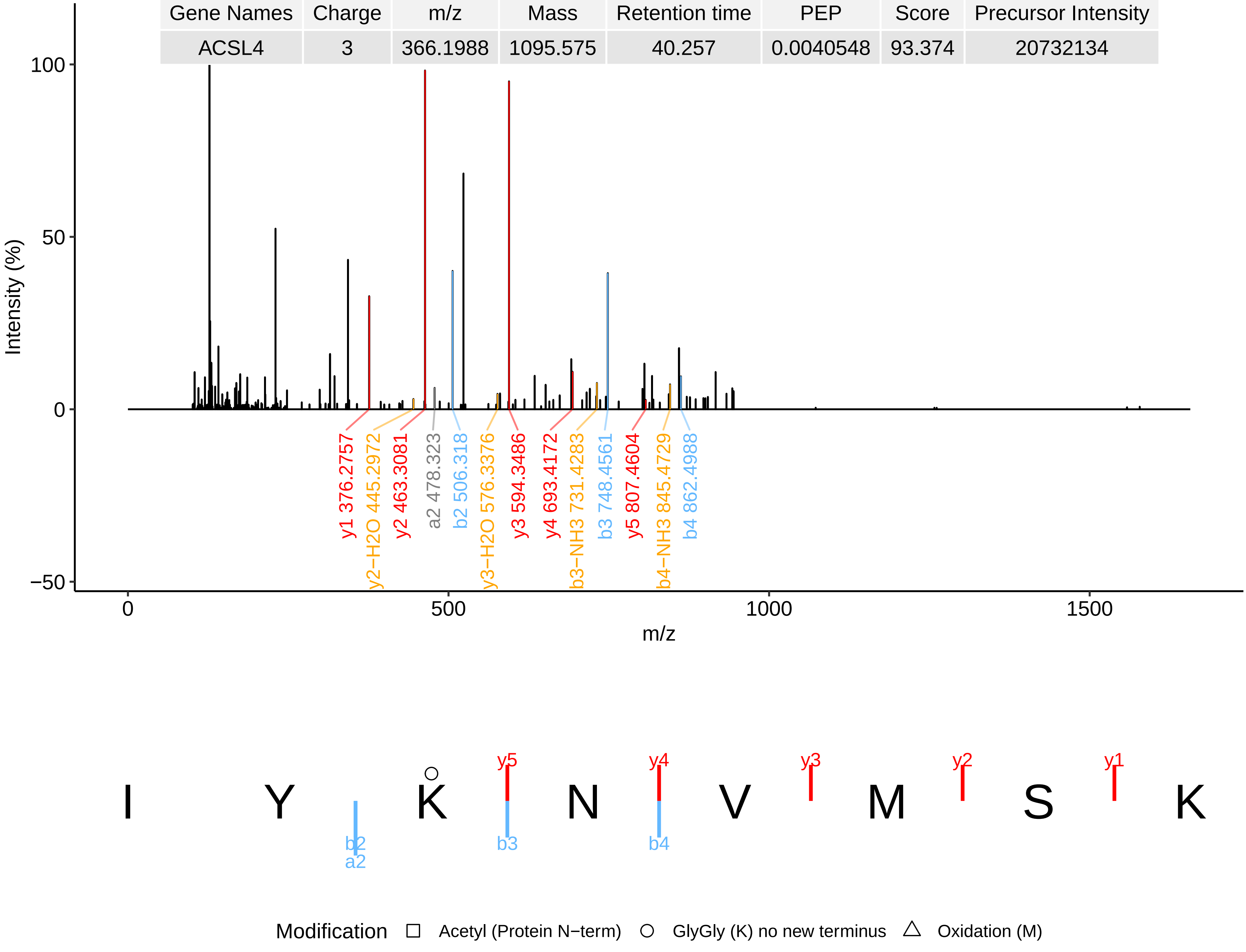

20200924_KIZ_MUb5_GlyGly_1 − Scan number: 12207

Gene Names	Charge	m/z	Mass	Retention time	PEP	Score	Precursor Intensity
ACSL4	3	366.1988	1095.575	39.677	0.028124	69.628	11042540

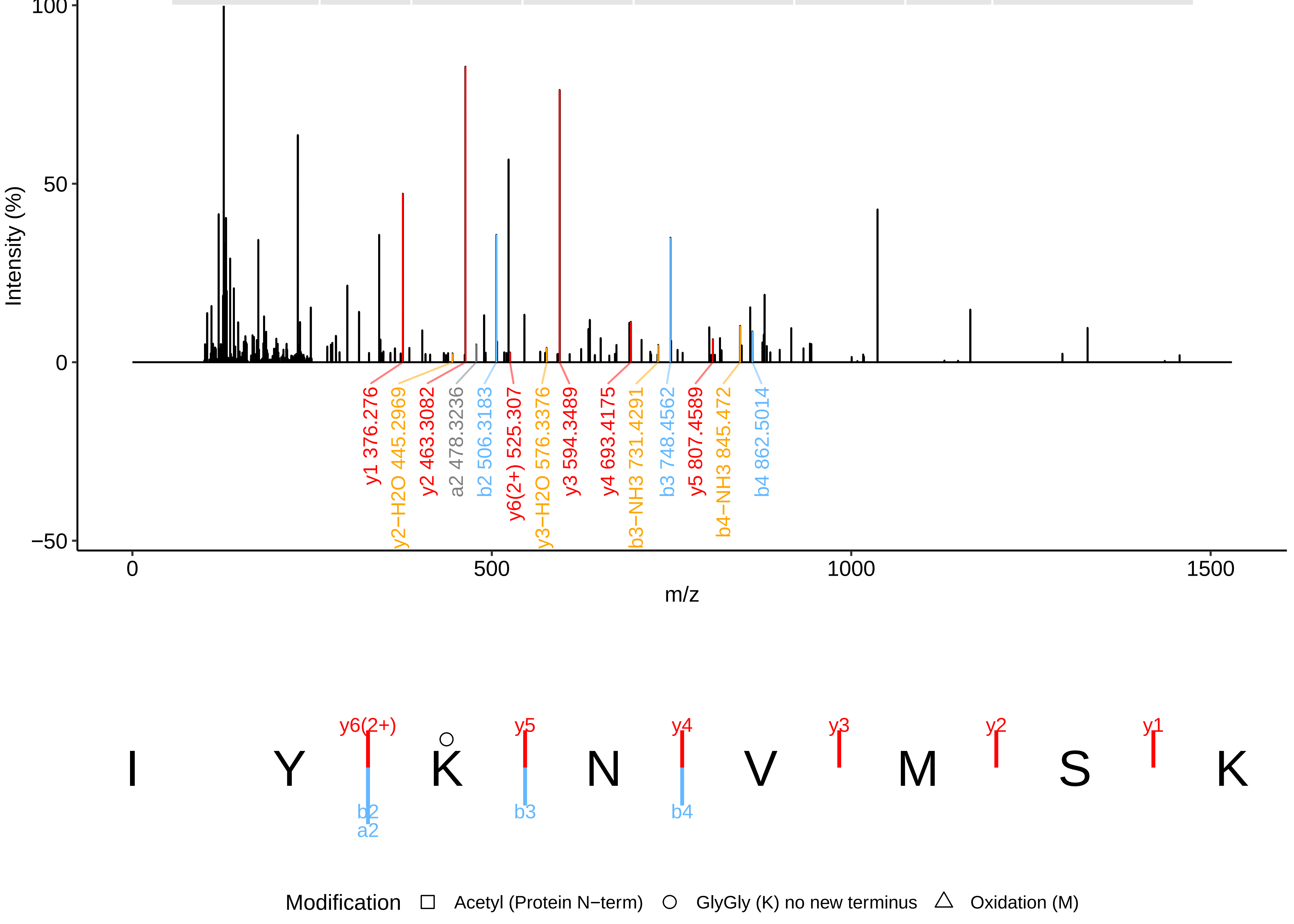

20200924_KIZ_MUb5_GlyGly_2 − Scan number: 11732

Gene Names	Charge	m/z	Mass	Retention time	PEP	Score	Precursor Intensity
ACSL4	3	366.1988	1095.575	43.853	0.021735	73.665	NA

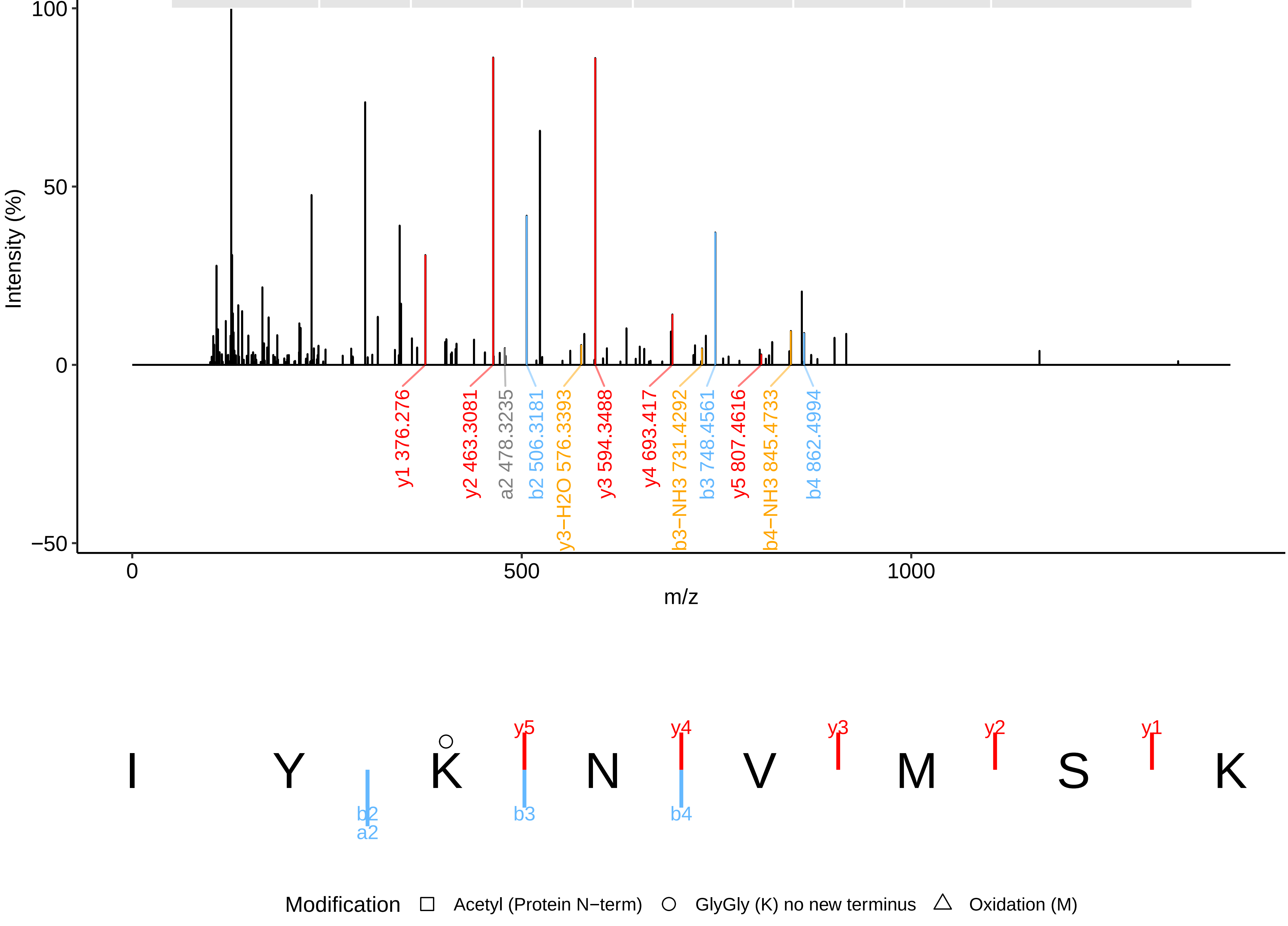

20200921_KIZ_MUb4_GlyGly_10plex_2 − Scan number: 9828

Gene Names	Charge	m/z	Mass	Retention time	PEP	Score	Precursor Intensity
FAM127A;FAM127B	3	450.2334	1347.678	37.488	0.013532	83.397	1505094

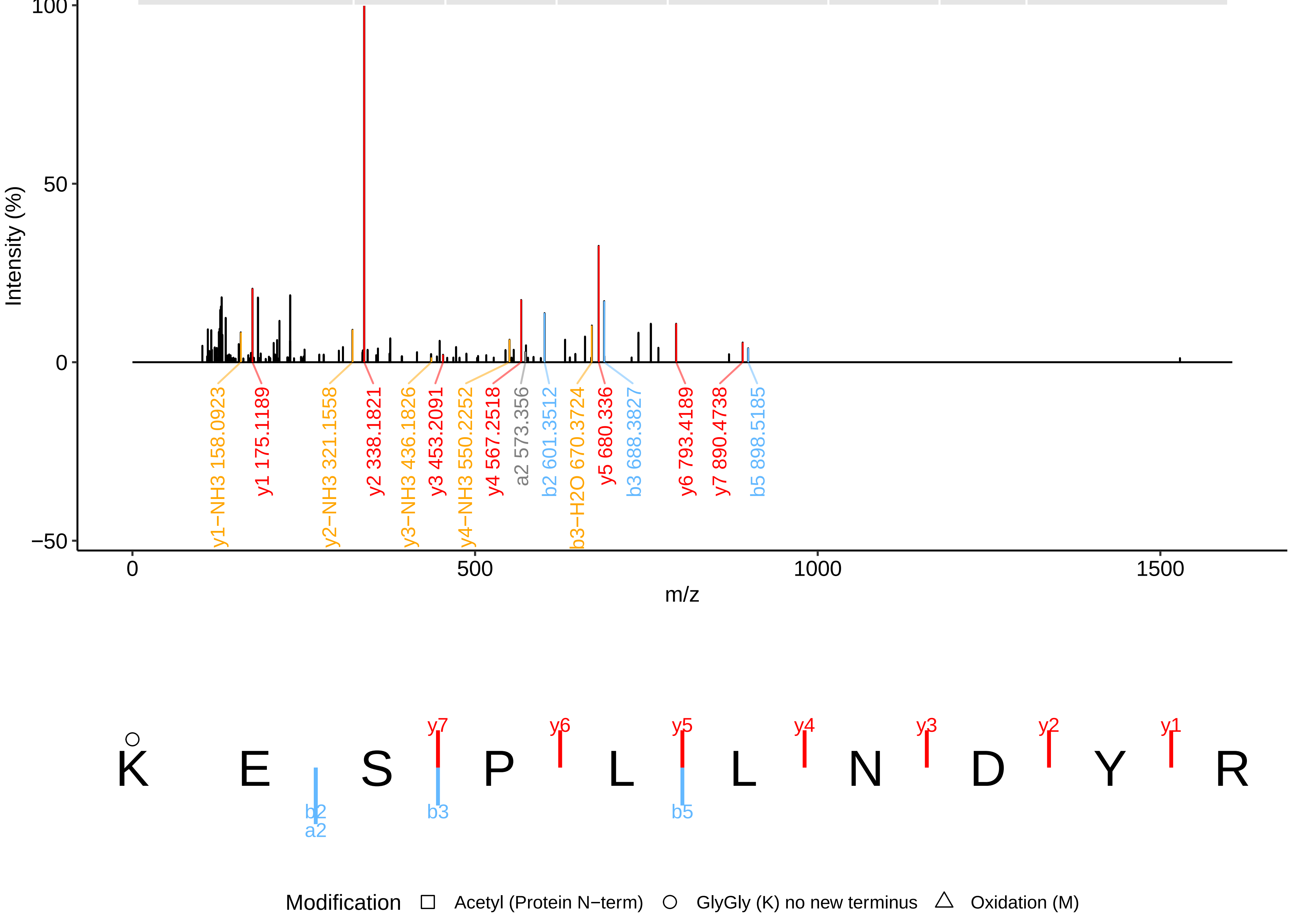

20200921_KIZ_MUb4_GlyGly_10plex − Scan number: 3533

Gene Names	Charge	m/z	Mass	Retention time	PEP	Score	Precursor Intensity
	4	279.9056	1115.593	11.249	0.11793	47.743	11218007

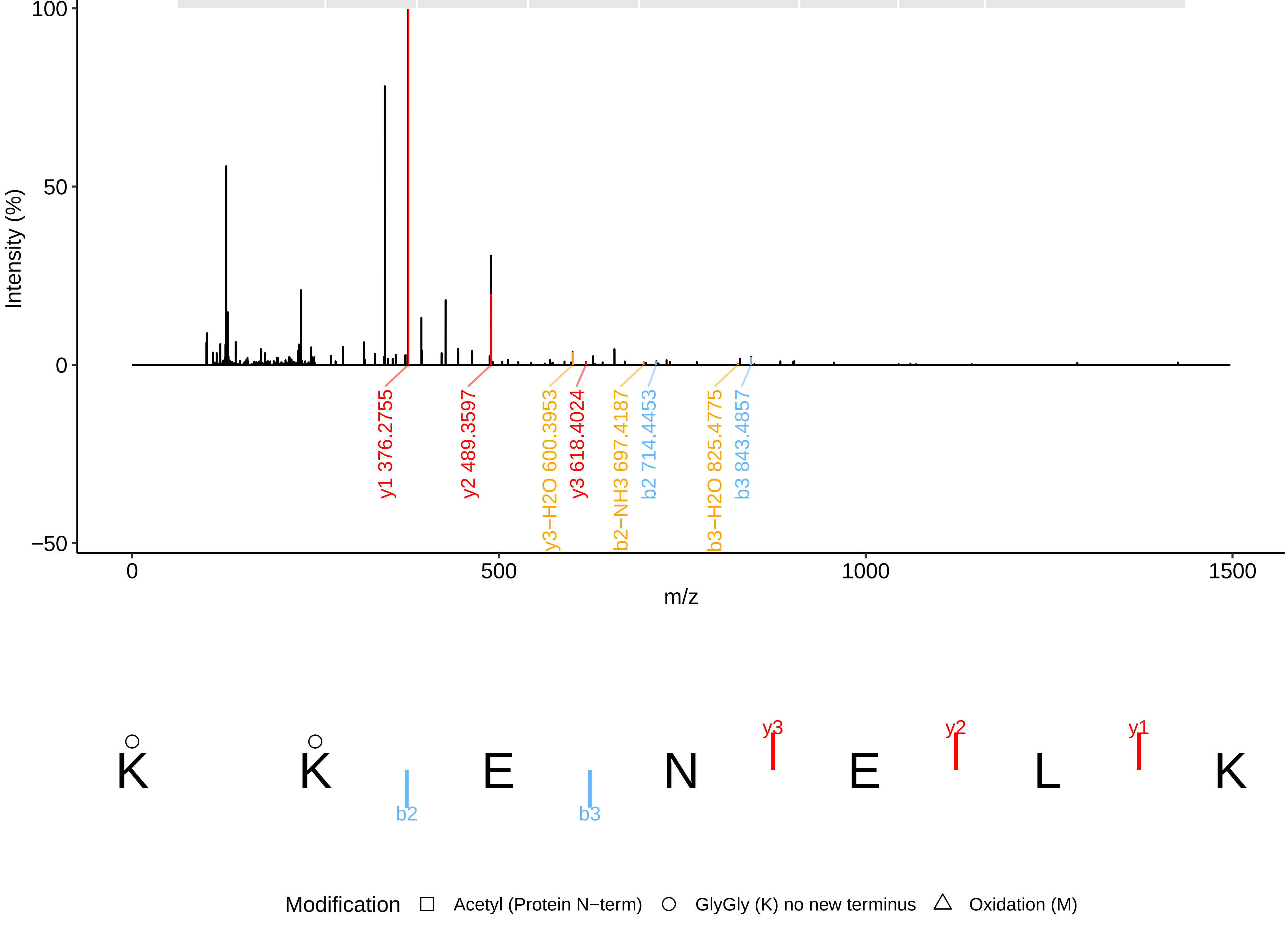

20200921_KIZ_MUb4_GlyGly_10plex_2 − Scan number: 2906

Gene Names	Charge	m/z	Mass	Retention time	PEP	Score	Precursor Intensity
FAM63B	3	439.5566	1315.648	9.4557	0.091388	55.567	1276088

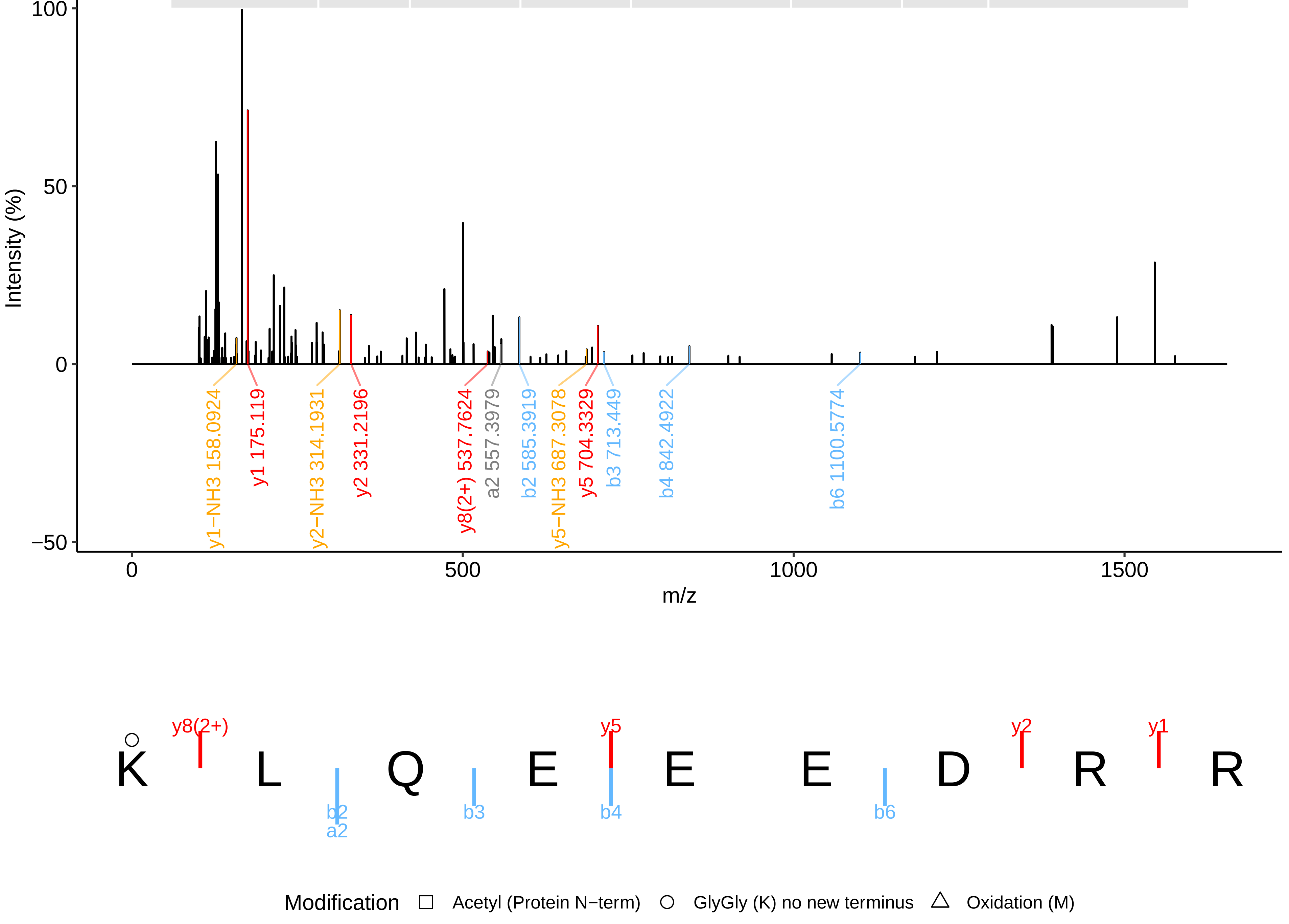

20200924_KIZ_MUb5_GlyGly_1 − Scan number: 2824

Gene Names	Charge	m/z	Mass	Retention time	PEP	Score	Precursor Intensity
FAM63B	3	439.5566	1315.648	8.7192	0.0016786	110.53	6388564

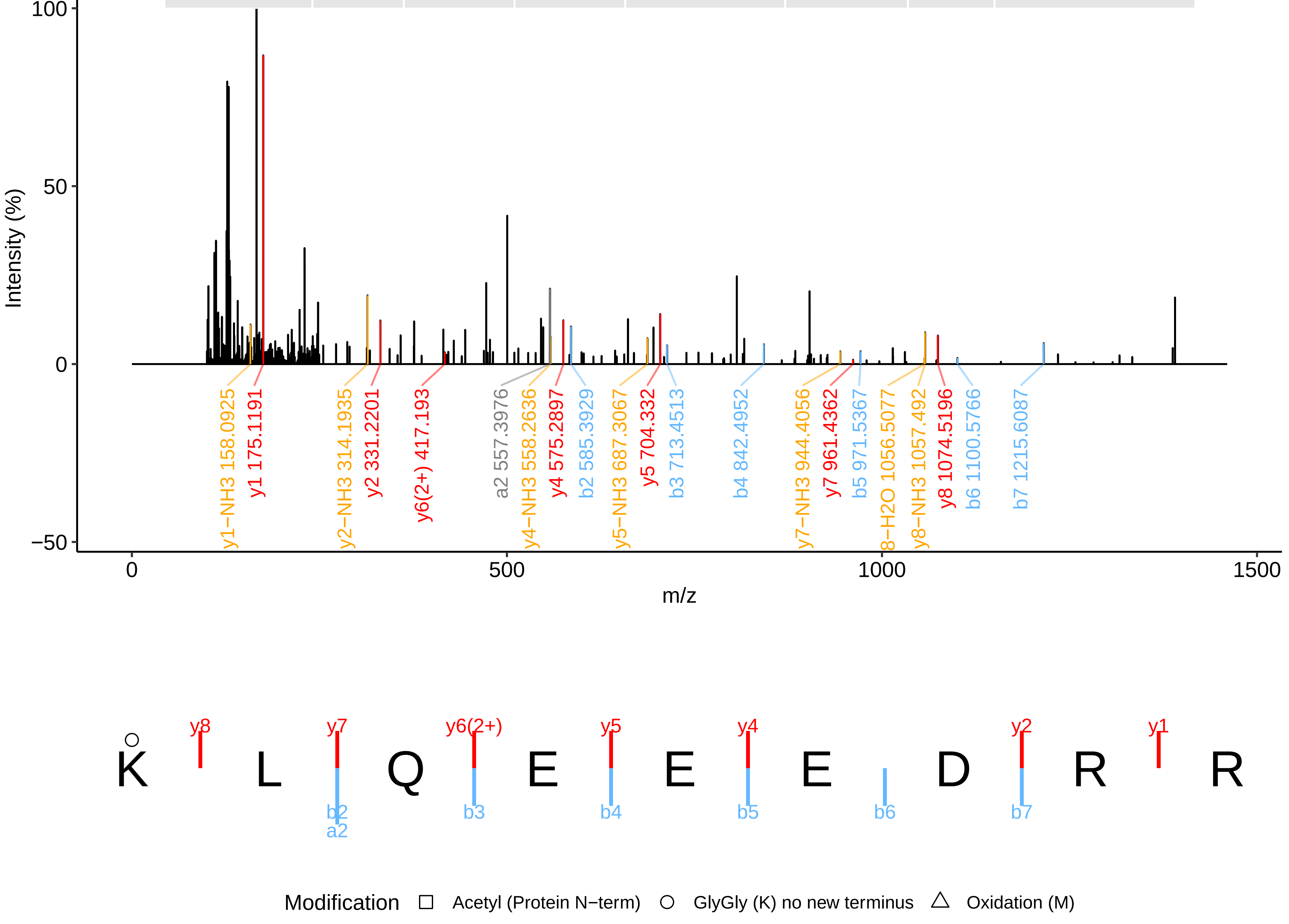

20201118_KIZ_MUb6_GlyGly_1 − Scan number: 2827

Gene Names	Charge	m/z	Mass	Retention time	PEP	Score	Precursor Intensity
FAM63B	3	439.5566	1315.648	8.652	0.0013258	96.342	9675477

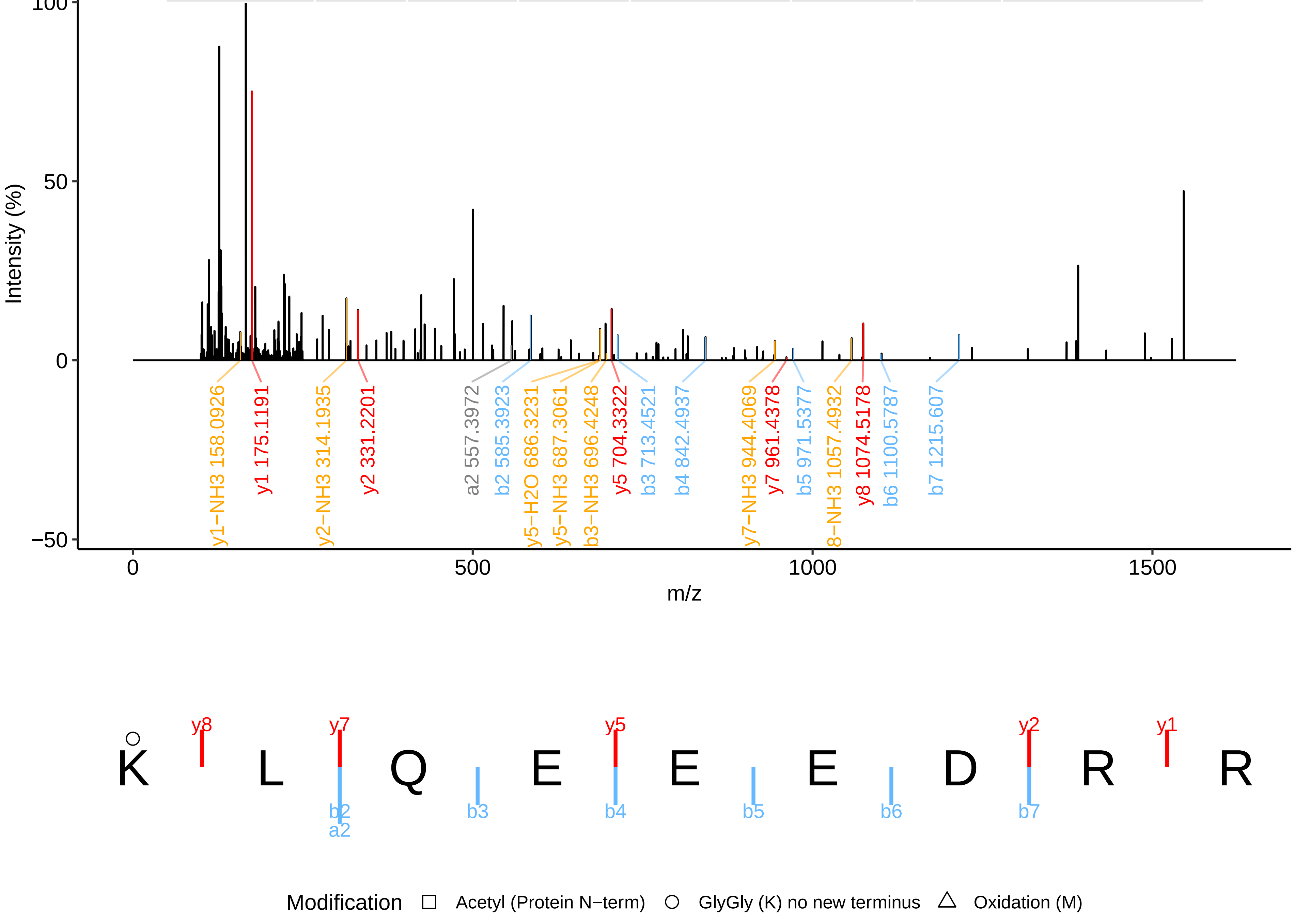

20201118_KIZ_MUb6_GlyGly_2 − Scan number: 3089

Gene Names	Charge	m/z	Mass	Retention time	PEP	Score	Precursor Intensity
FAM63B	4	329.9193	1315.648	10.01	0.0017287	107.57	3591934

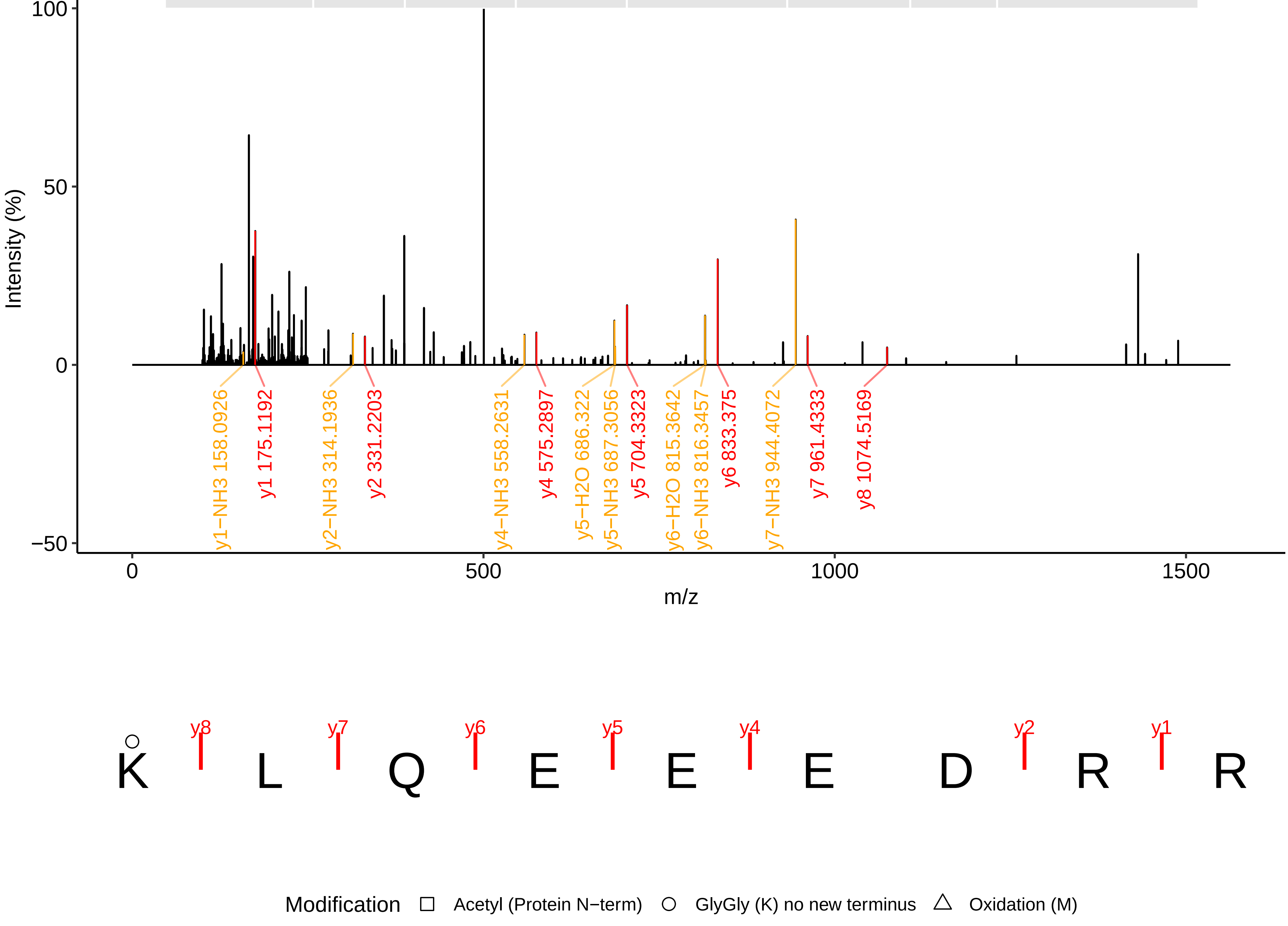

20200921_KIZ_MUb4_GlyGly_10plex − Scan number: 2873

Gene Names	Charge	m/z	Mass	Retention time	PEP	Score	Precursor Intensity
KRT18	4	282.3926	1125.542	8.9965	0.019903	65.06	11695543

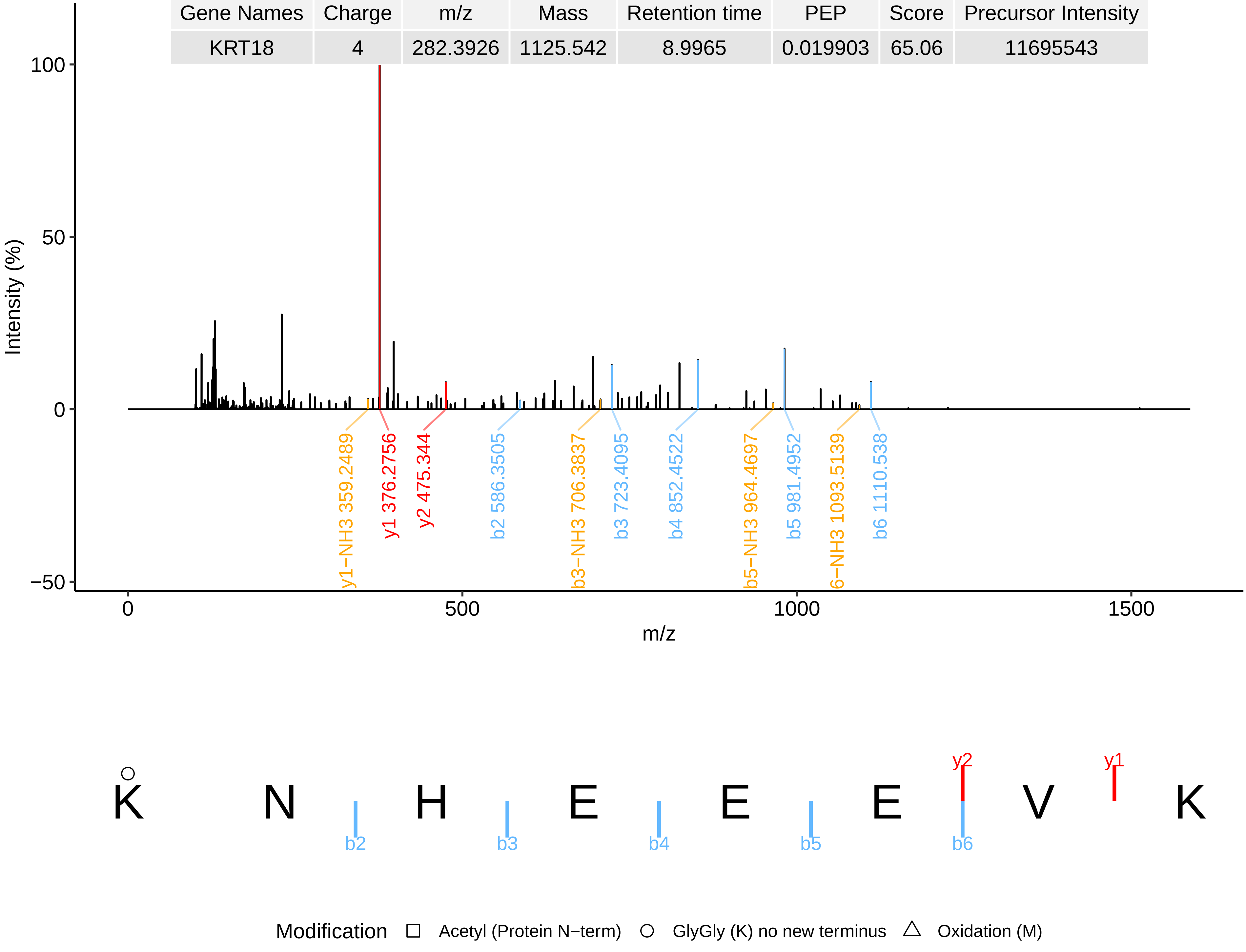

20200921_KIZ_MUb4_GlyGly_10plex_2 − Scan number: 2844

Gene Names	Charge	m/z	Mass	Retention time	PEP	Score	Precursor Intensity
KRT18	3	376.1878	1125.542	9.2038	0.052112	82.279	1071206

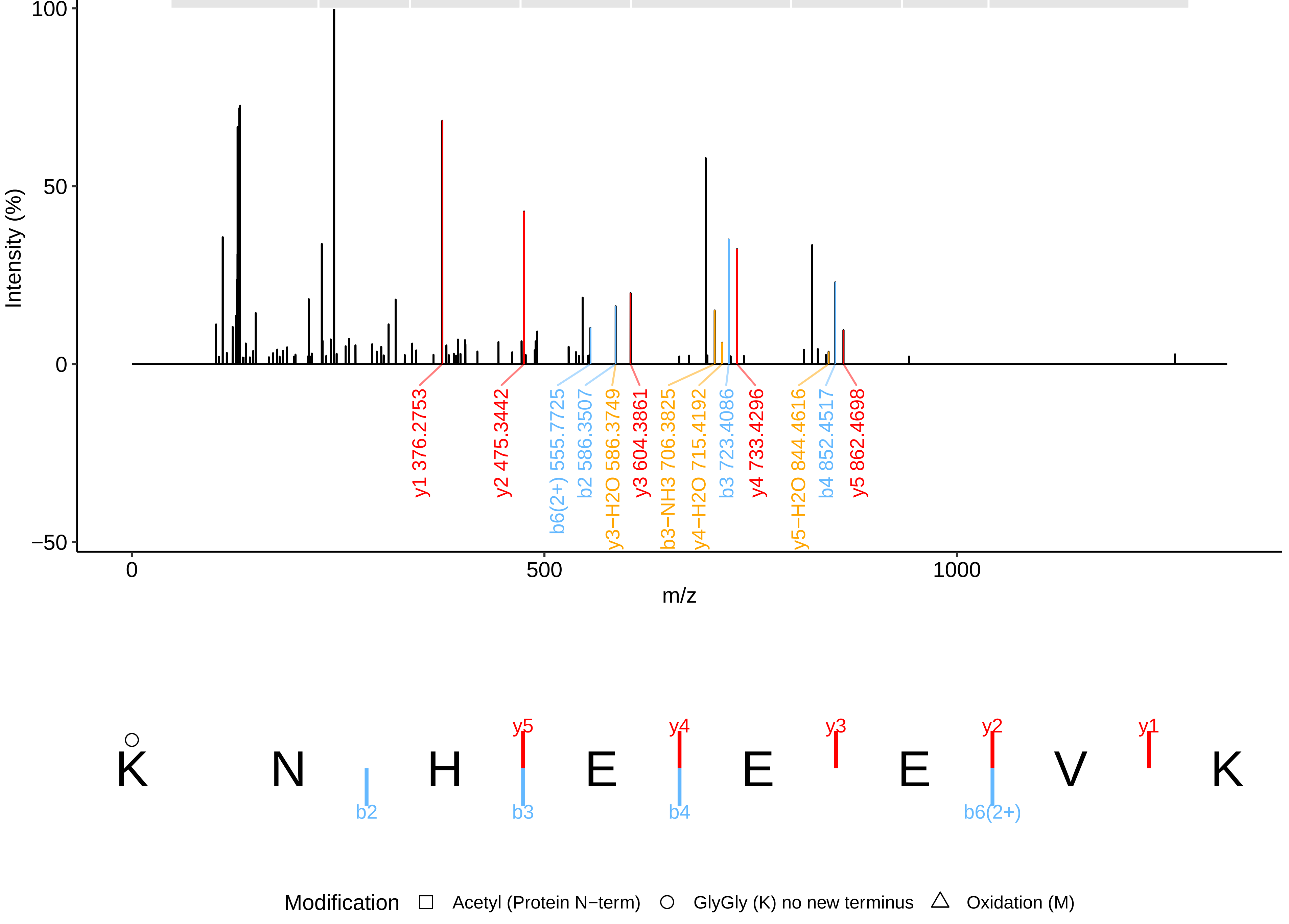

20200921_KIZ_MUb4_GlyGly_10plex − Scan number: 5406

Gene Names	Charge	m/z	Mass	Retention time	PEP	Score	Precursor Intensity
SDC2	3	365.1927	1092.556	17.704	0.057295	75.197	8642051

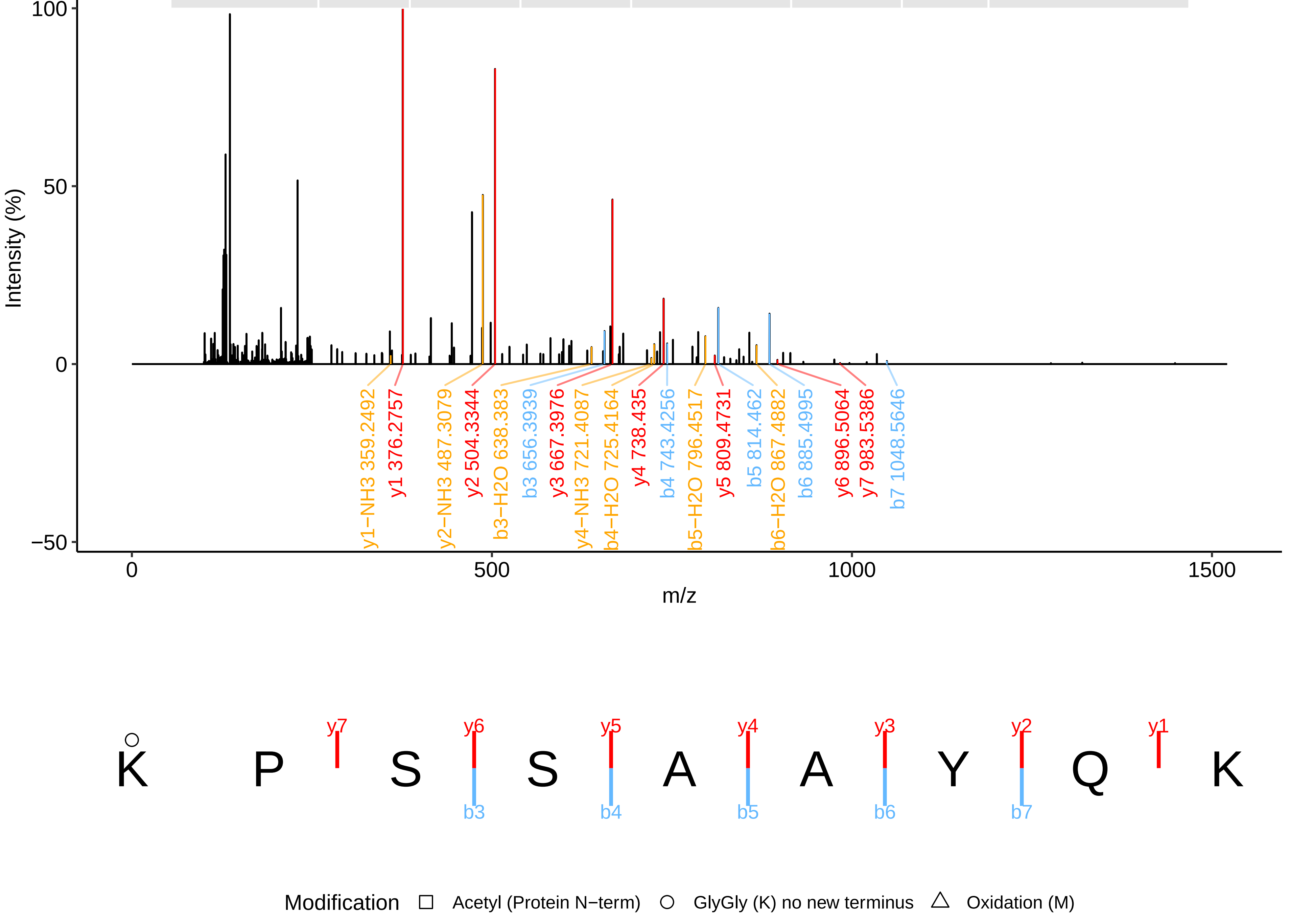

20200921_KIZ_MUb4_GlyGly_10plex_2 − Scan number: 4978

Gene Names	Charge	m/z	Mass	Retention time	PEP	Score	Precursor Intensity
SDC2	3	365.1927	1092.556	17.688	0.076532	72.275	4125463

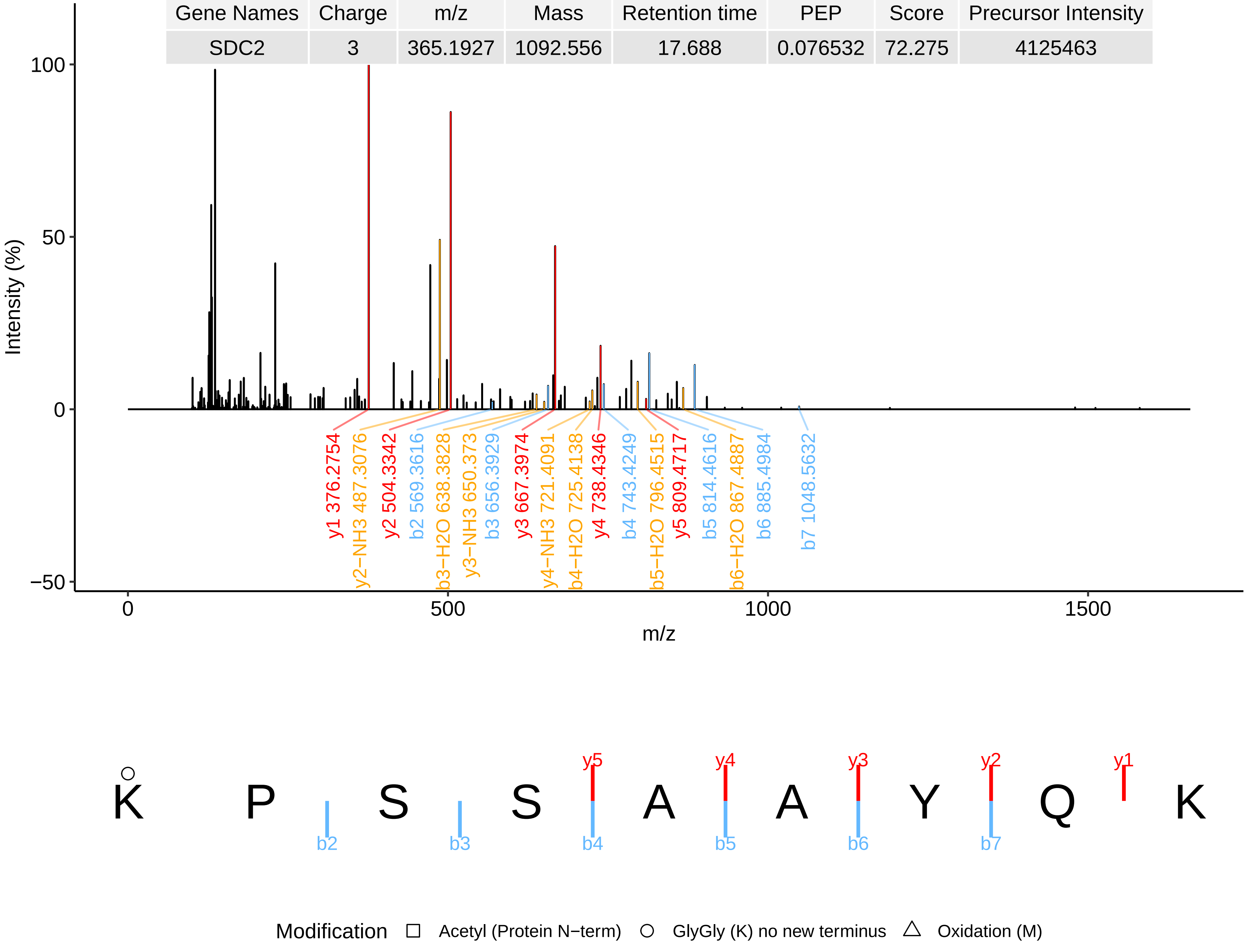

20200924_KIZ_MUb5_GlyGly_1 − Scan number: 4685

Gene Names	Charge	m/z	Mass	Retention time	PEP	Score	Precursor Intensity
SDC2	3	365.1927	1092.556	14.493	0.085394	70.929	NA

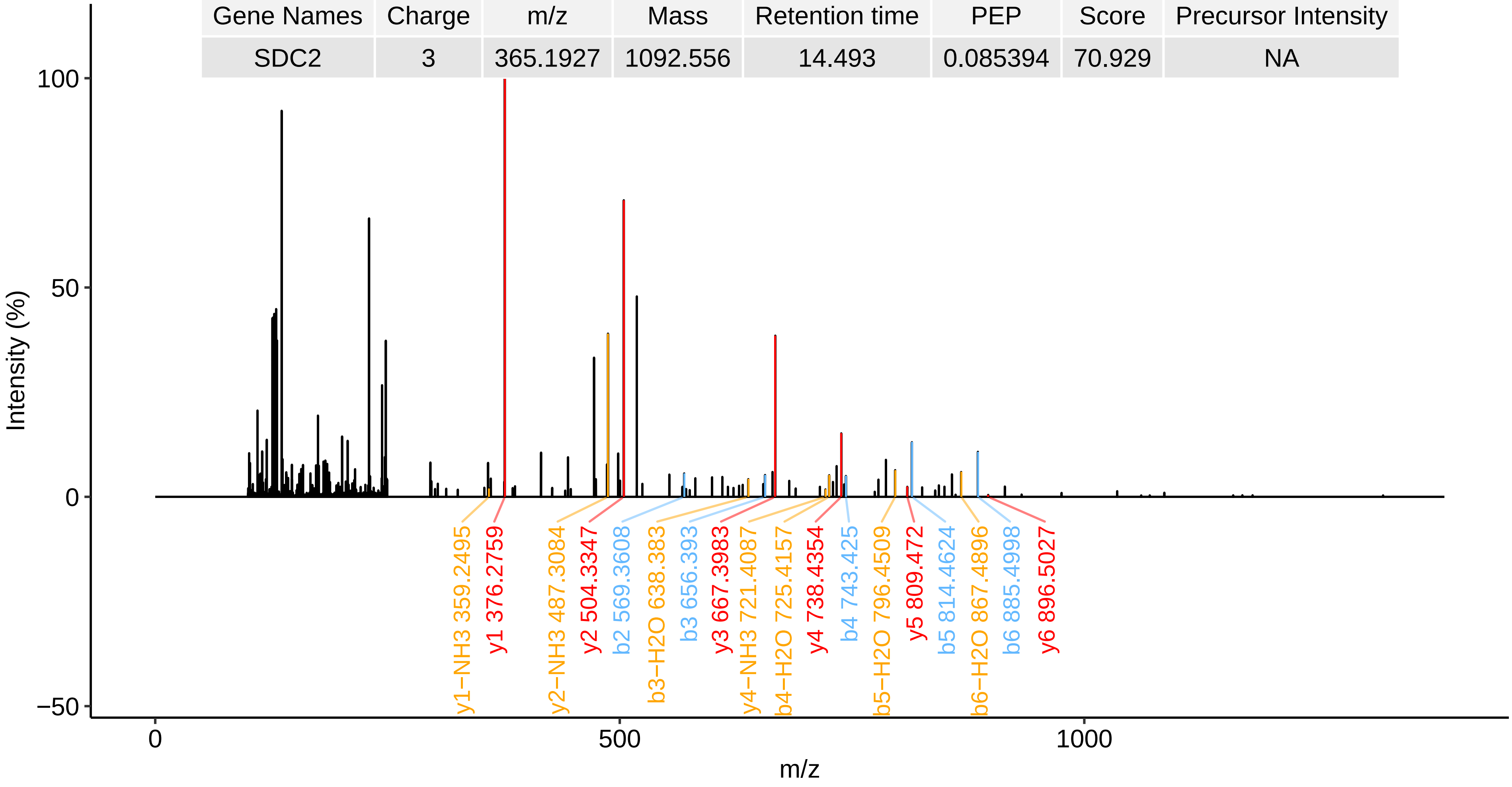

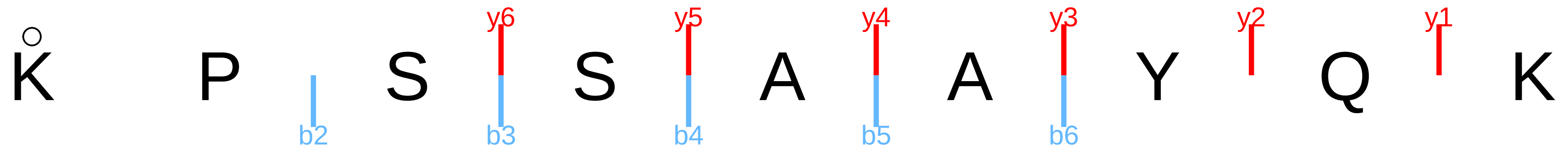

Modification □ Acetyl (Protein N−term) ○ GlyGly (K) no new terminus △ Oxidation (M)

20200924_KIZ_MUb5_GlyGly_2 − Scan number: 4306

Gene Names	Charge	m/z	Mass	Retention time	PEP	Score	Precursor Intensity
SDC2	3	365.1927	1092.556	15.785	0.016416	85.731	1717005

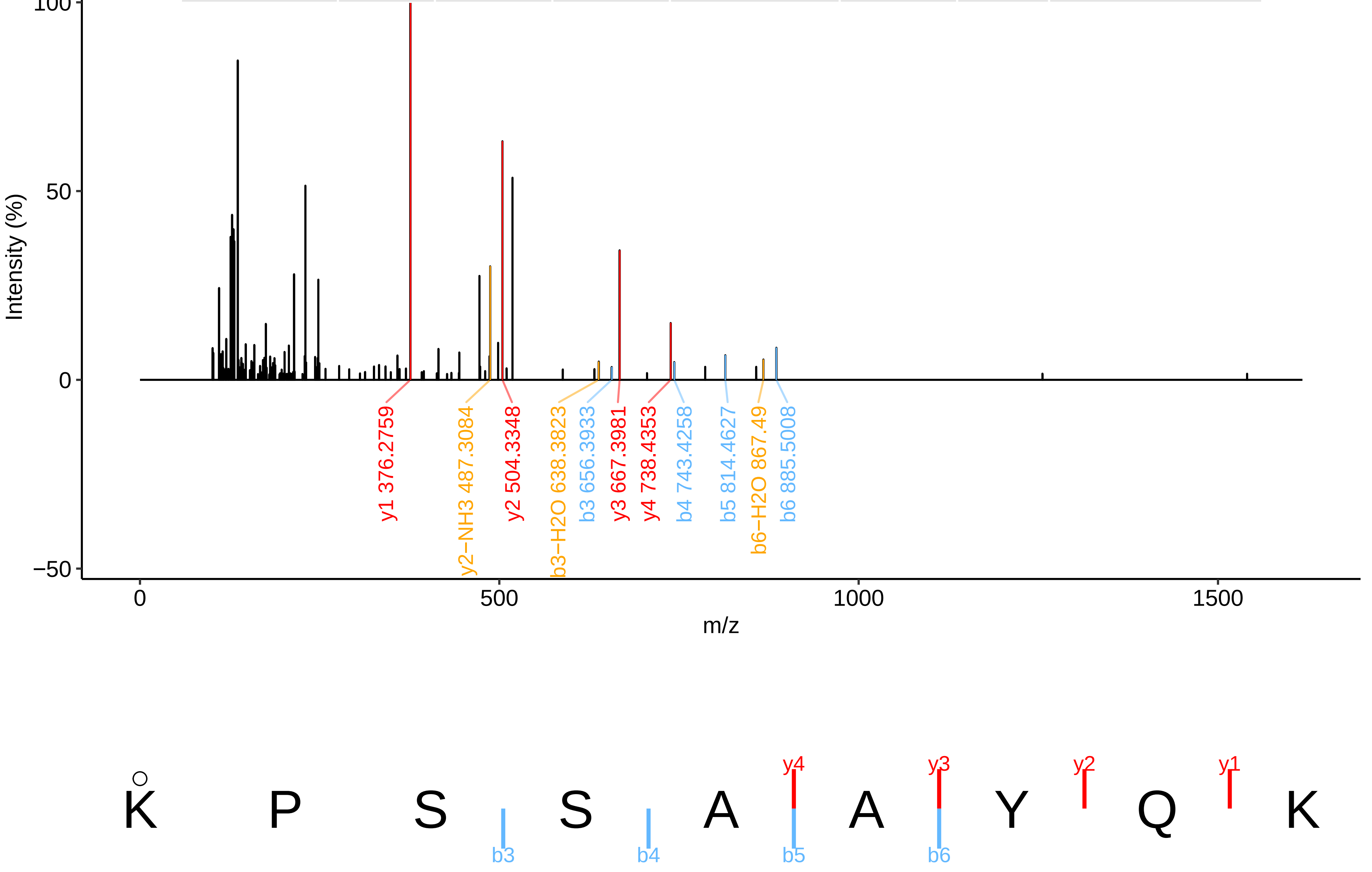

Modification □ Acetyl (Protein N−term) ○ GlyGly (K) no new terminus △ Oxidation (M)

20201118_KIZ_MUb6_GlyGly_1 − Scan number: 4602

Gene Names	Charge	m/z	Mass	Retention time	PEP	Score	Precursor Intensity
SDC2	3	365.1927	1092.556	14.452	0.074142	72.638	4486010

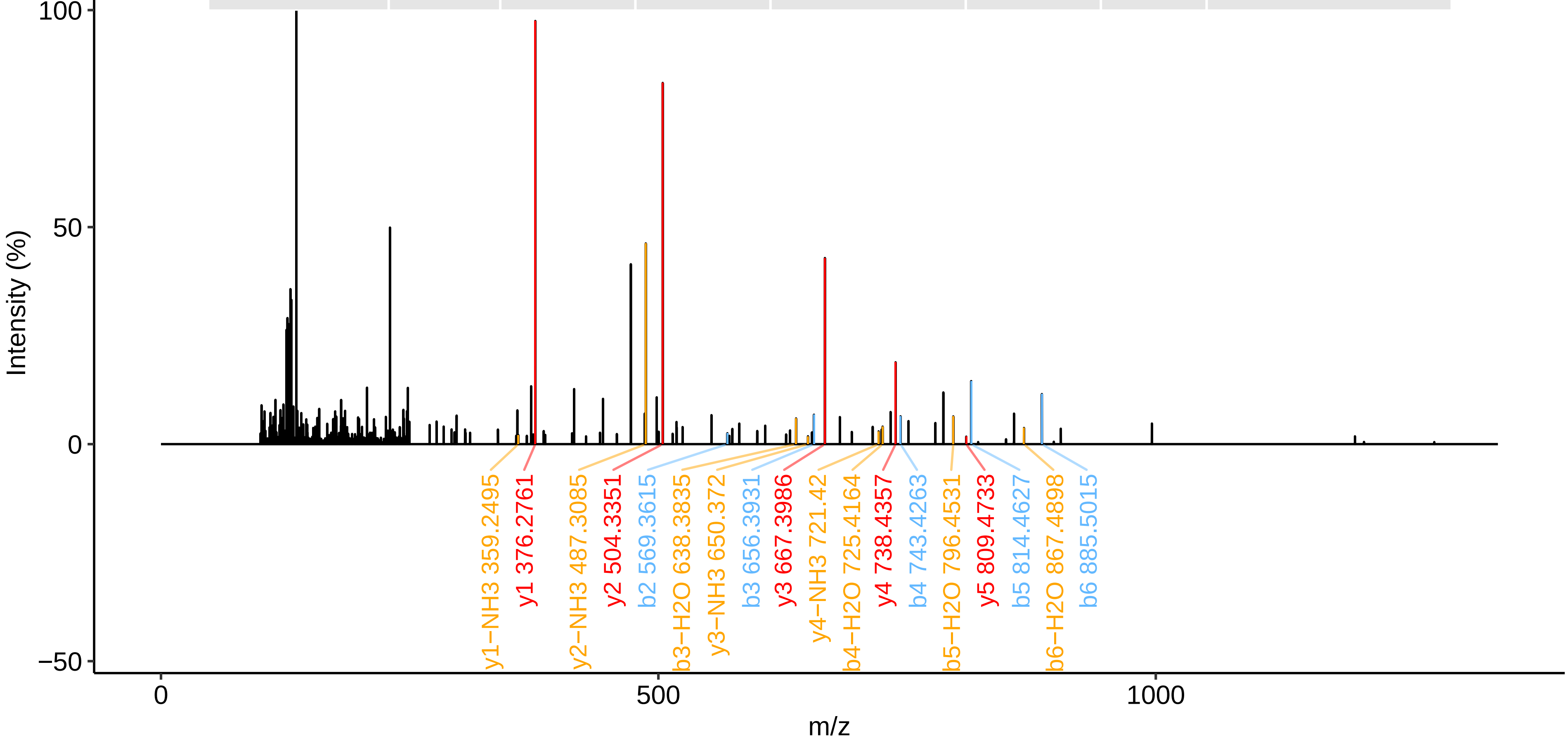

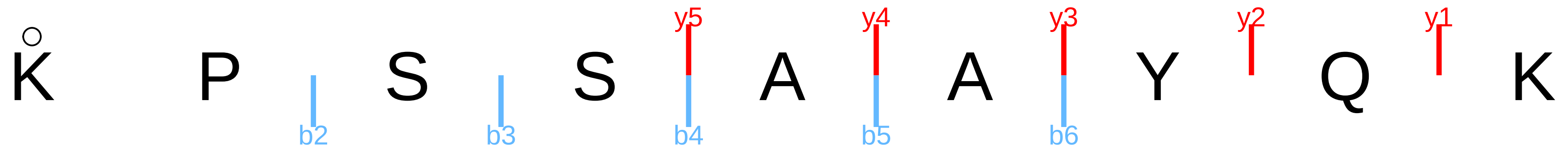

Modification □ Acetyl (Protein N−term) ○ GlyGly (K) no new terminus △ Oxidation (M)

20200921_KIZ_MUb4_GlyGly_10plex − Scan number: 6751

Gene Names	Charge	m/z	Mass	Retention time	PEP	Score	Precursor Intensity
SLC25A6	3	439.8968	1316.668	22.458	1.8565e−13	141.89	28941236

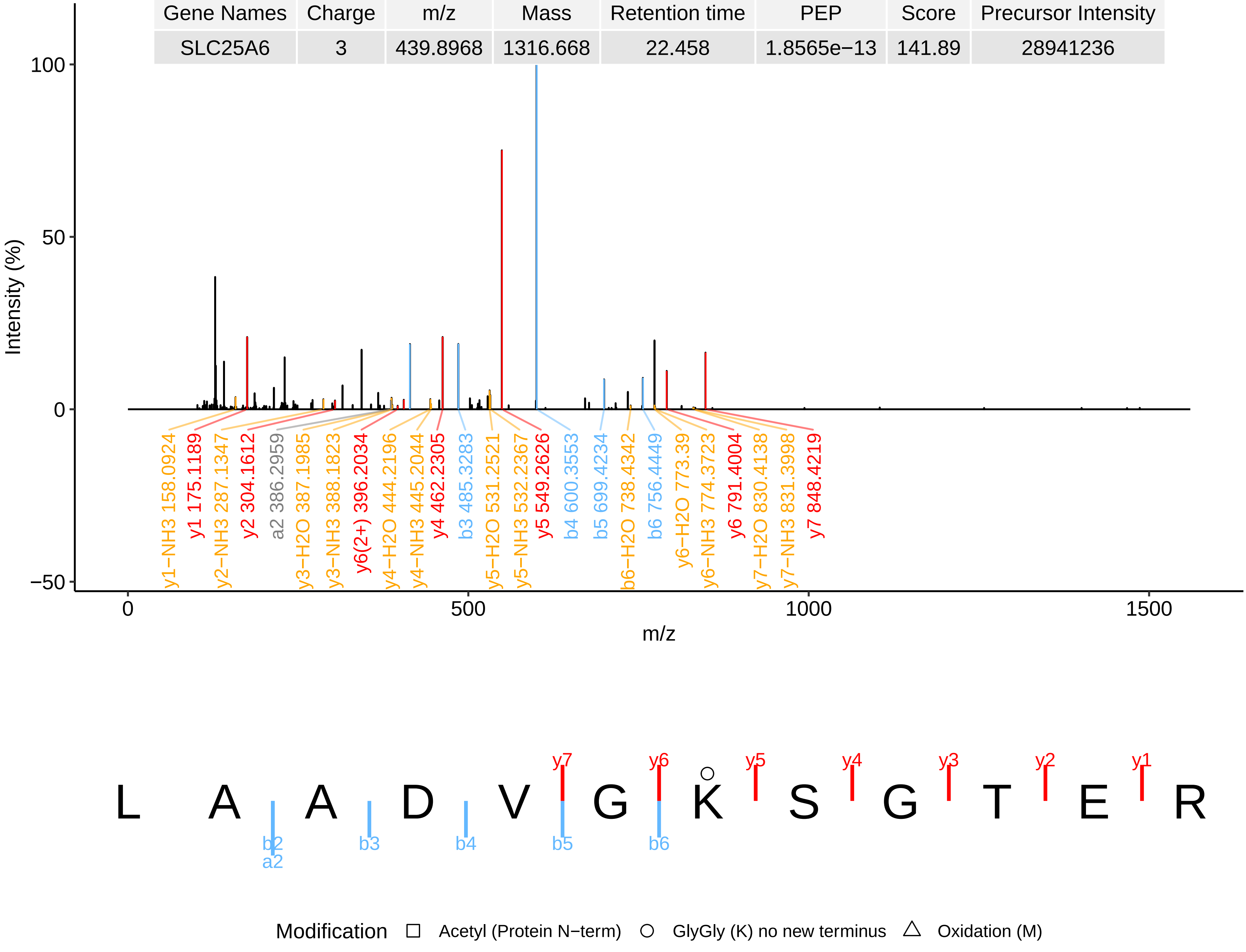

20200921_KIZ_MUb4_GlyGly_10plex_2 − Scan number: 6214

Gene Names	Charge	m/z	Mass	Retention time	PEP	Score	Precursor Intensity
SLC25A6	3	439.8968	1316.668	22.734	0.0003684	92.943	872967.1

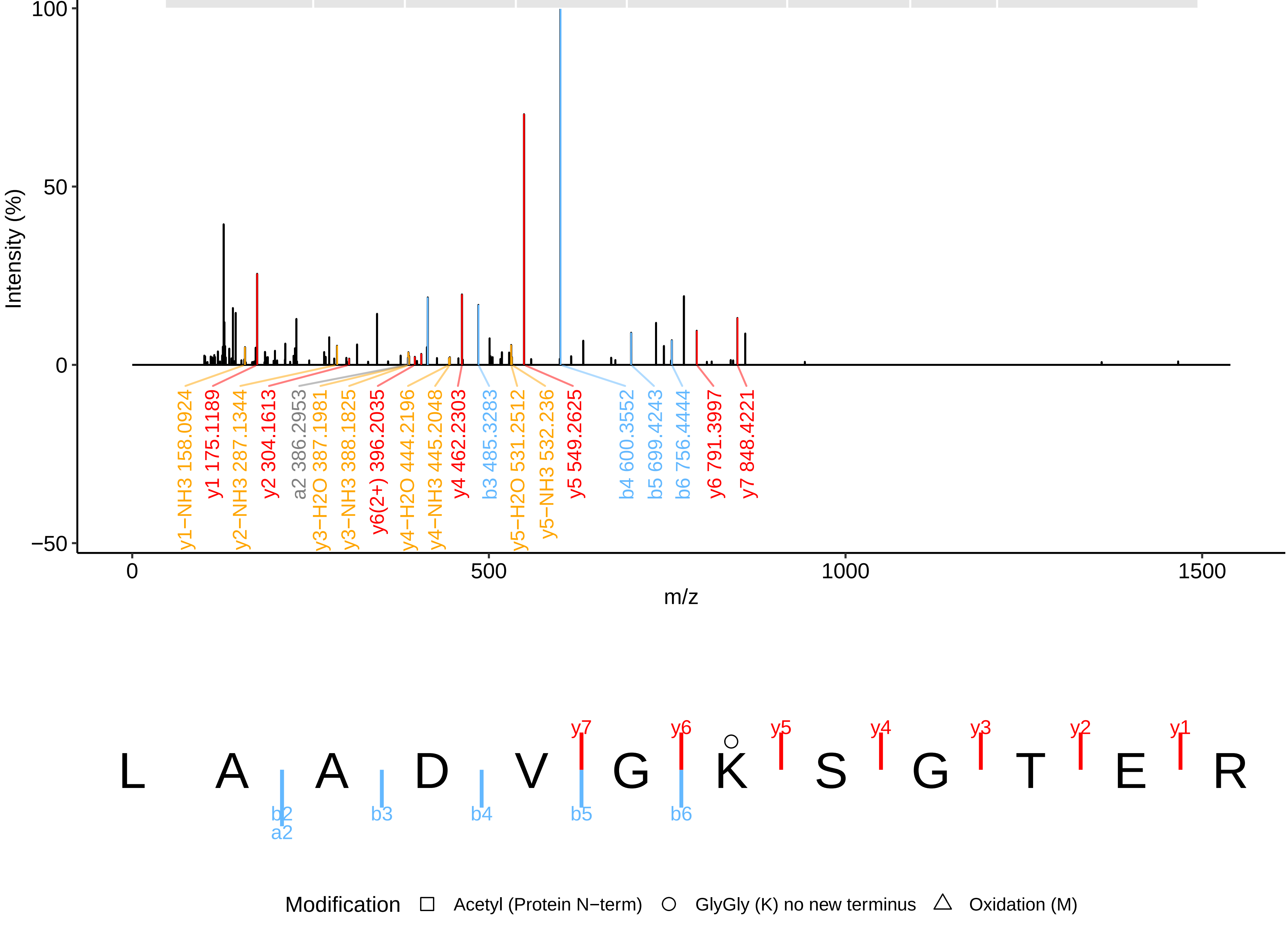

20200924_KIZ_MUb5_GlyGly_1 − Scan number: 5786

Gene Names	Charge	m/z	Mass	Retention time	PEP	Score	Precursor Intensity
SLC25A6	3	439.8968	1316.668	18.068	1.13e−05	105.94	14289459

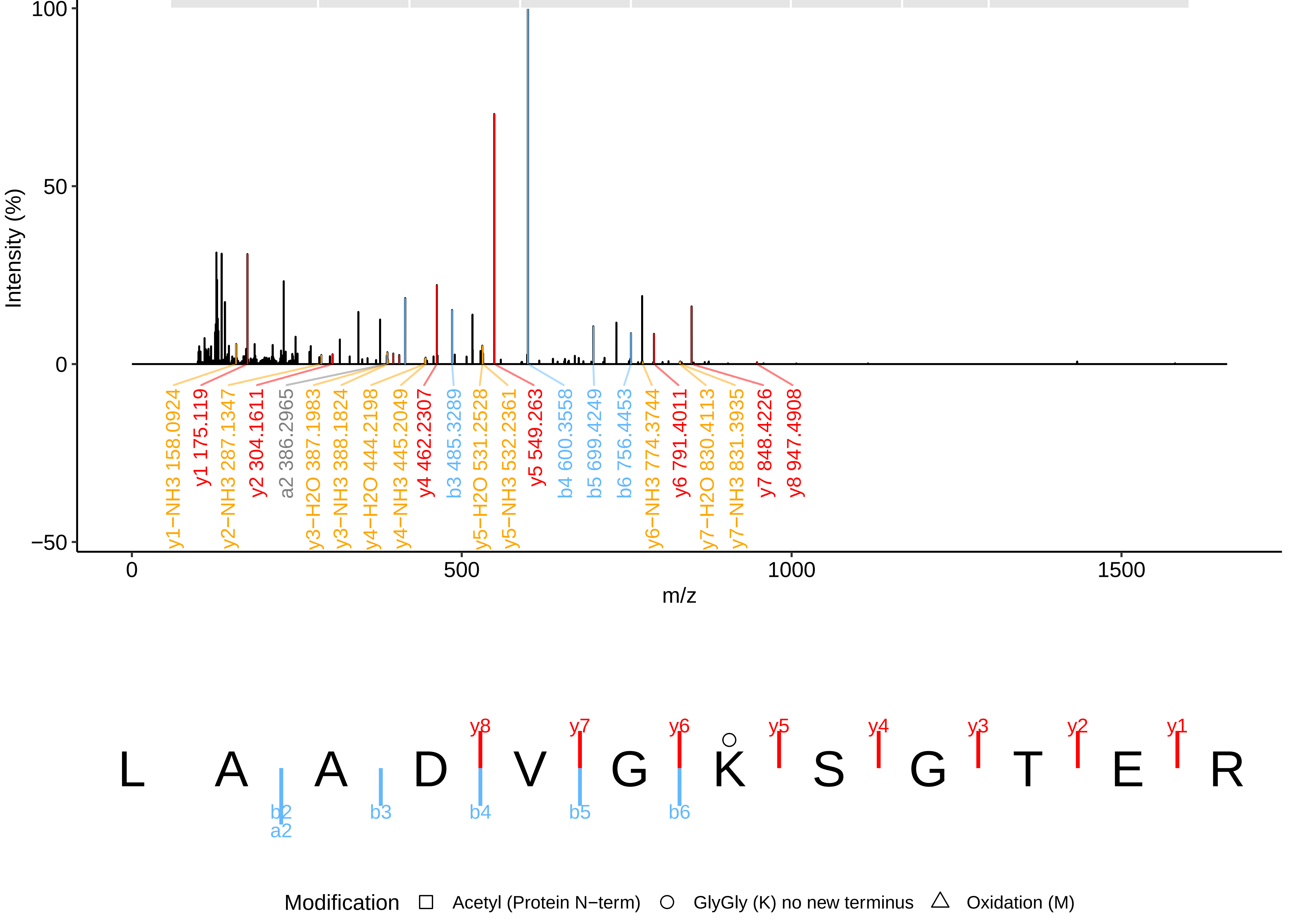

20201118_KIZ_MUb6_GlyGly_1 − Scan number: 5431

Gene Names	Charge	m/z	Mass	Retention time	PEP	Score	Precursor Intensity
SLC25A6	3	439.8968	1316.668	17.195	0.0023437	78.903	11518486

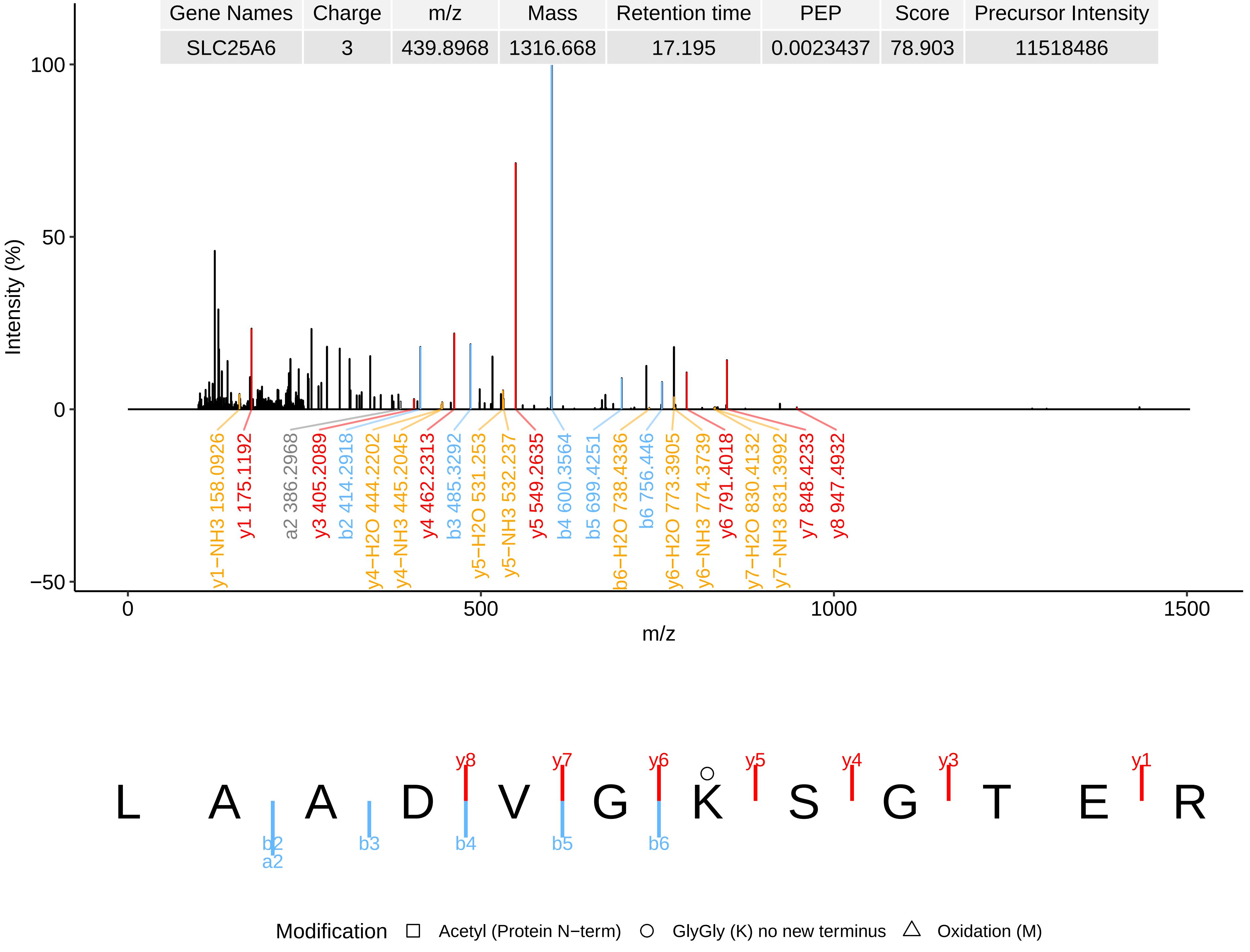

20200921_KIZ_MUb4_GlyGly_10plex − Scan number: 11132

Gene Names	Charge	m/z	Mass	Retention time	PEP	Score	Precursor Intensity
TIMM50	3	368.54	1102.598	38.74	0.0050981	85.676	NA

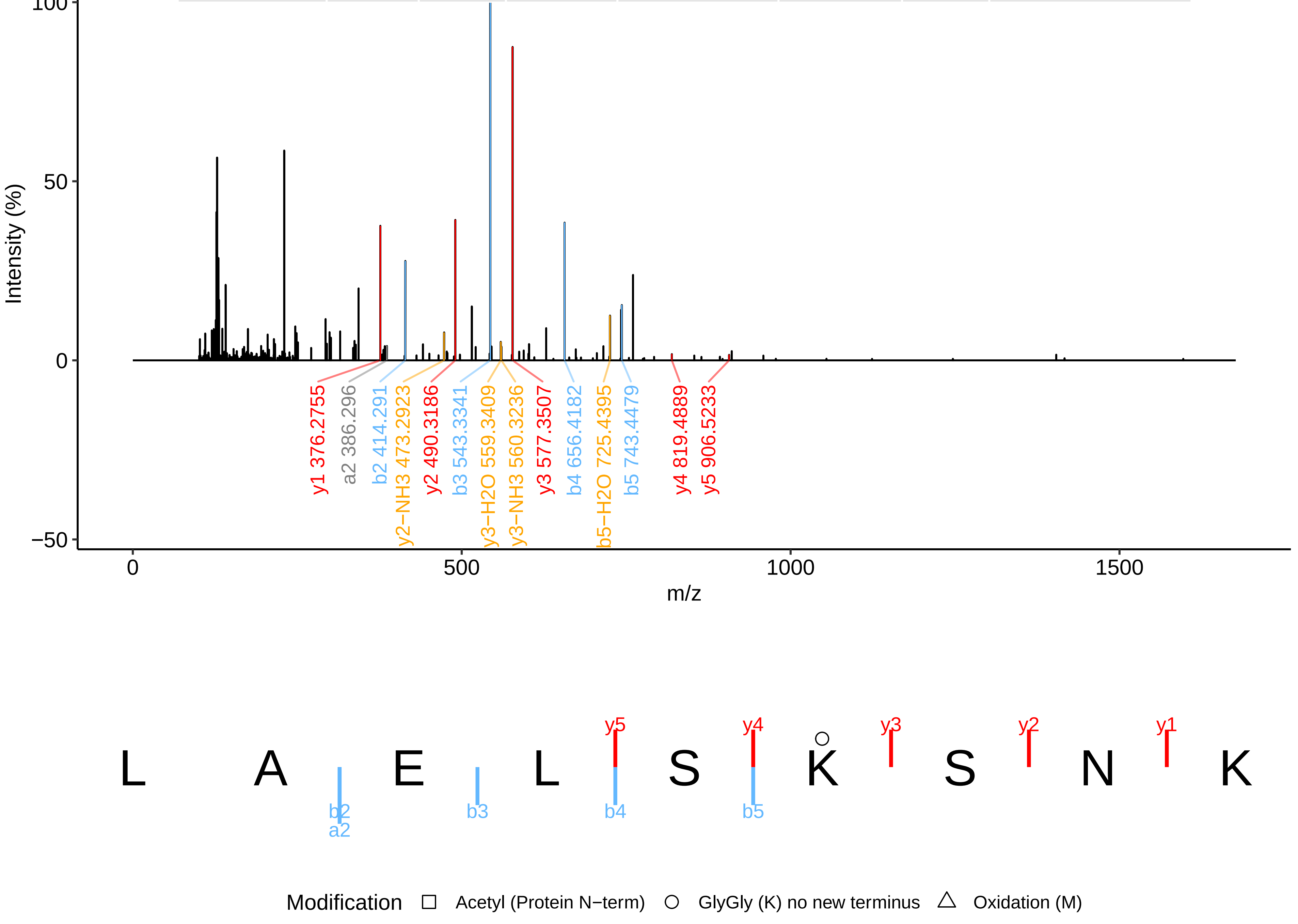

20200921_KIZ_MUb4_GlyGly_10plex − Scan number: 15852

Gene Names	Charge	m/z	Mass	Retention time	PEP	Score	Precursor Intensity
UBE2M	3	369.217	1104.629	56.765	0.013846	76.774	5707045

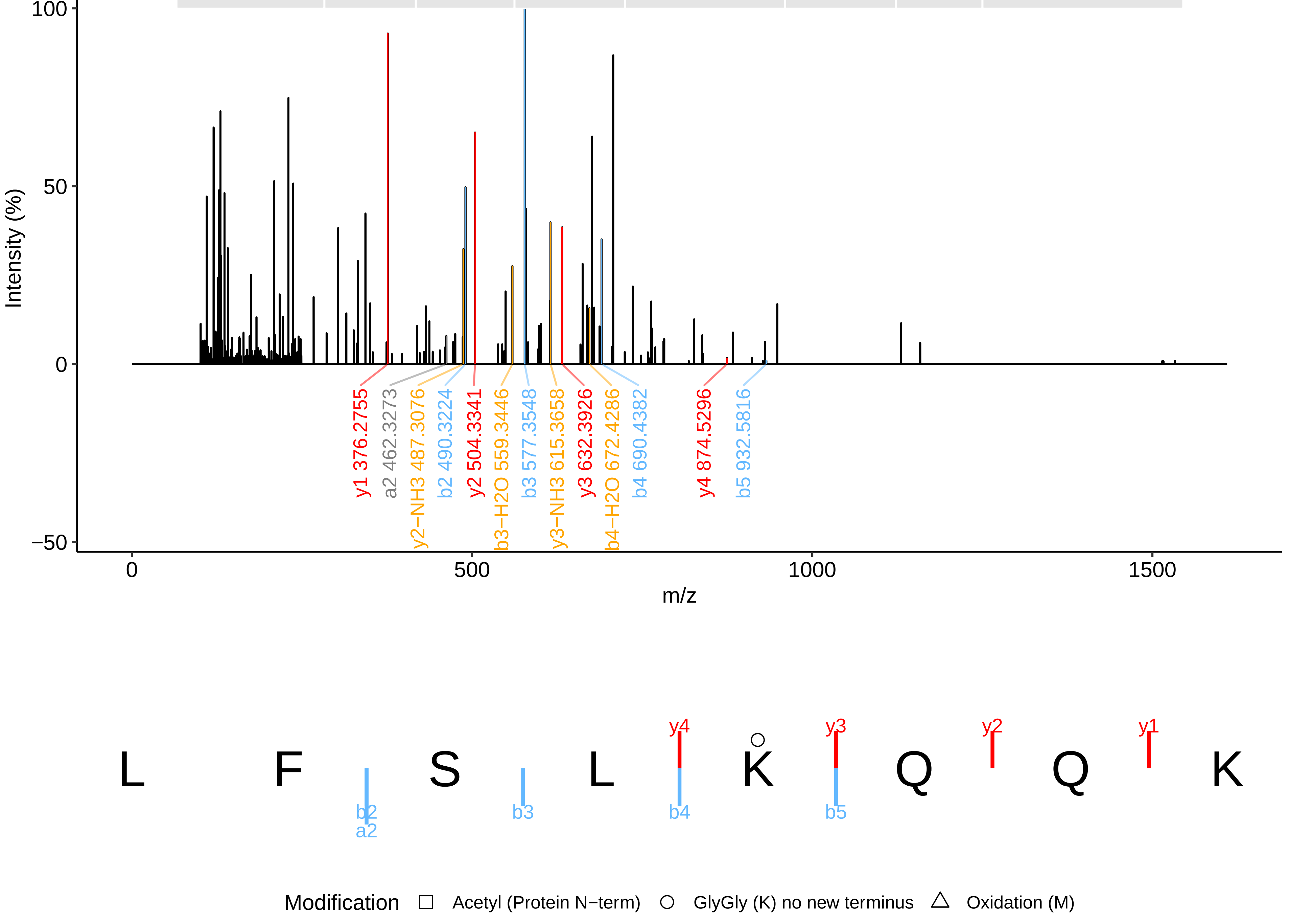

20200921_KIZ_MUb4_GlyGly_10plex_2 − Scan number: 14735

Gene Names	Charge	m/z	Mass	Retention time	PEP	Score	Precursor Intensity
UBE2M	3	369.217	1104.629	57.497	0.0016969	102.71	479038.3

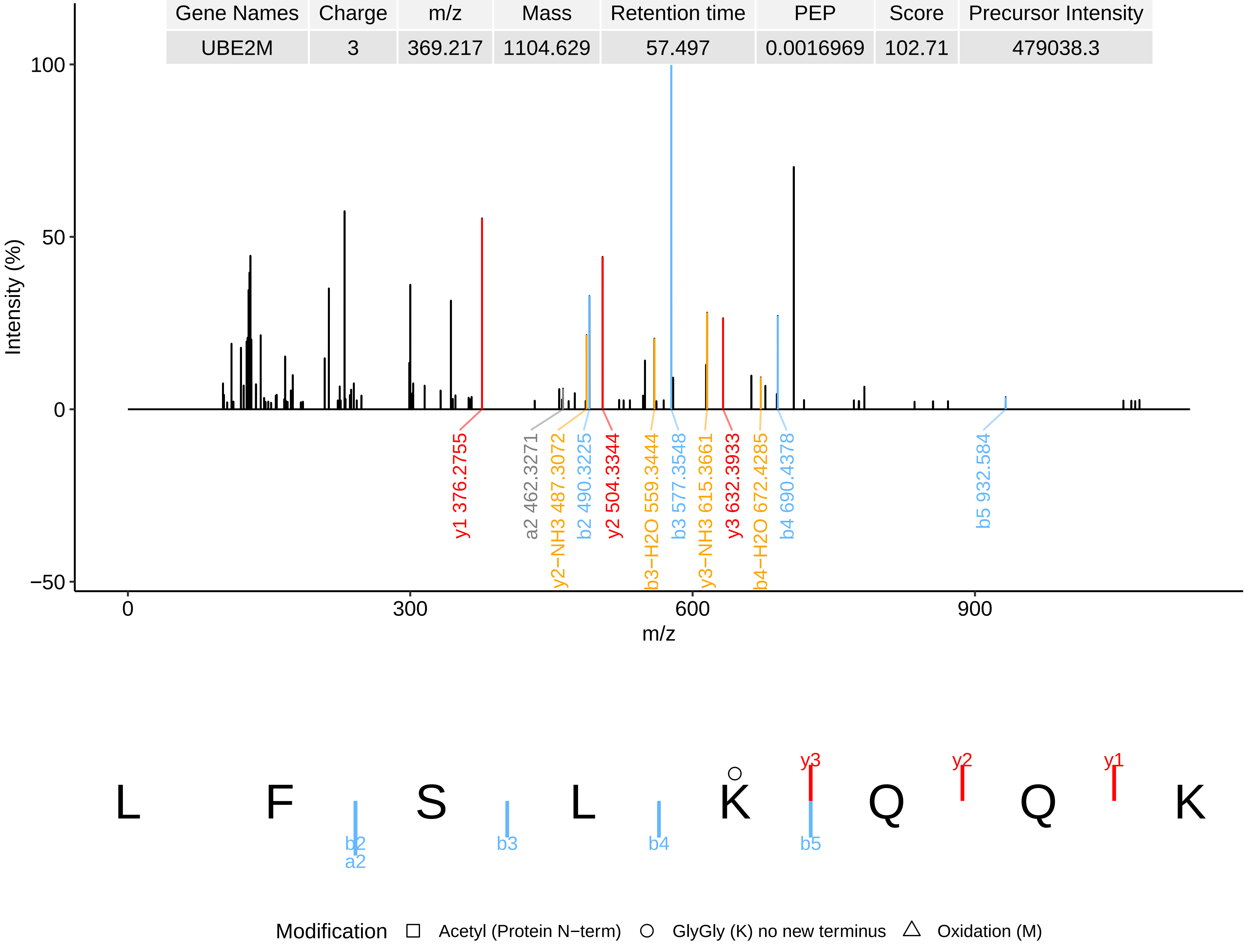

20200921_KIZ_MUb4_GlyGly_10plex − Scan number: 19860

Gene Names	Charge	m/z	Mass	Retention time	PEP	Score	Precursor Intensity
PSTPIP2	3	374.229	1119.665	71.695	0.093903	48.283	5910511

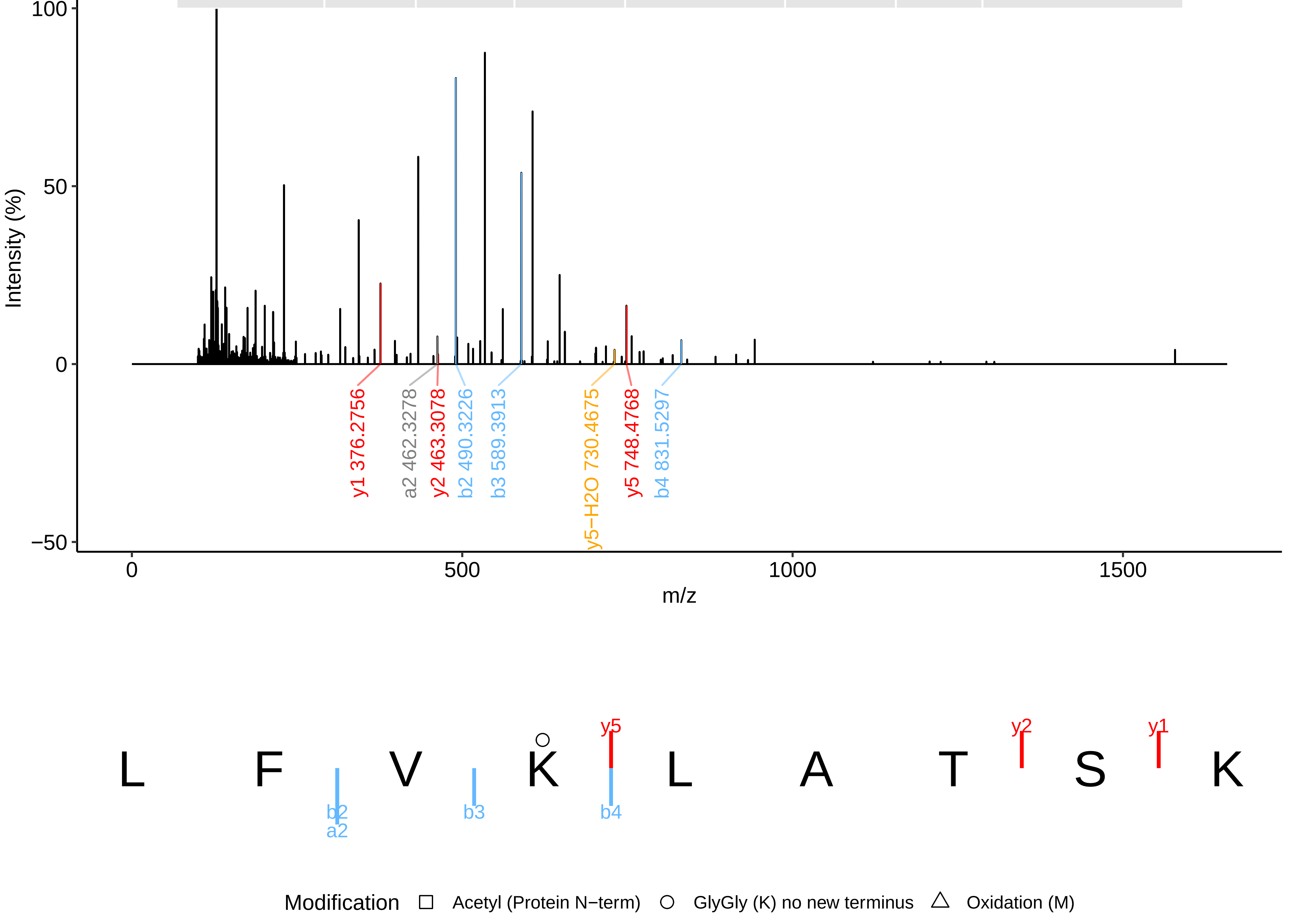

20200921_KIZ_MUb4_GlyGly_10plex_2 − Scan number: 17895

Gene Names	Charge	m/z	Mass	Retention time	PEP	Score	Precursor Intensity
PSTPIP2	3	374.229	1119.665	70.025	0.093903	48.283	1262409

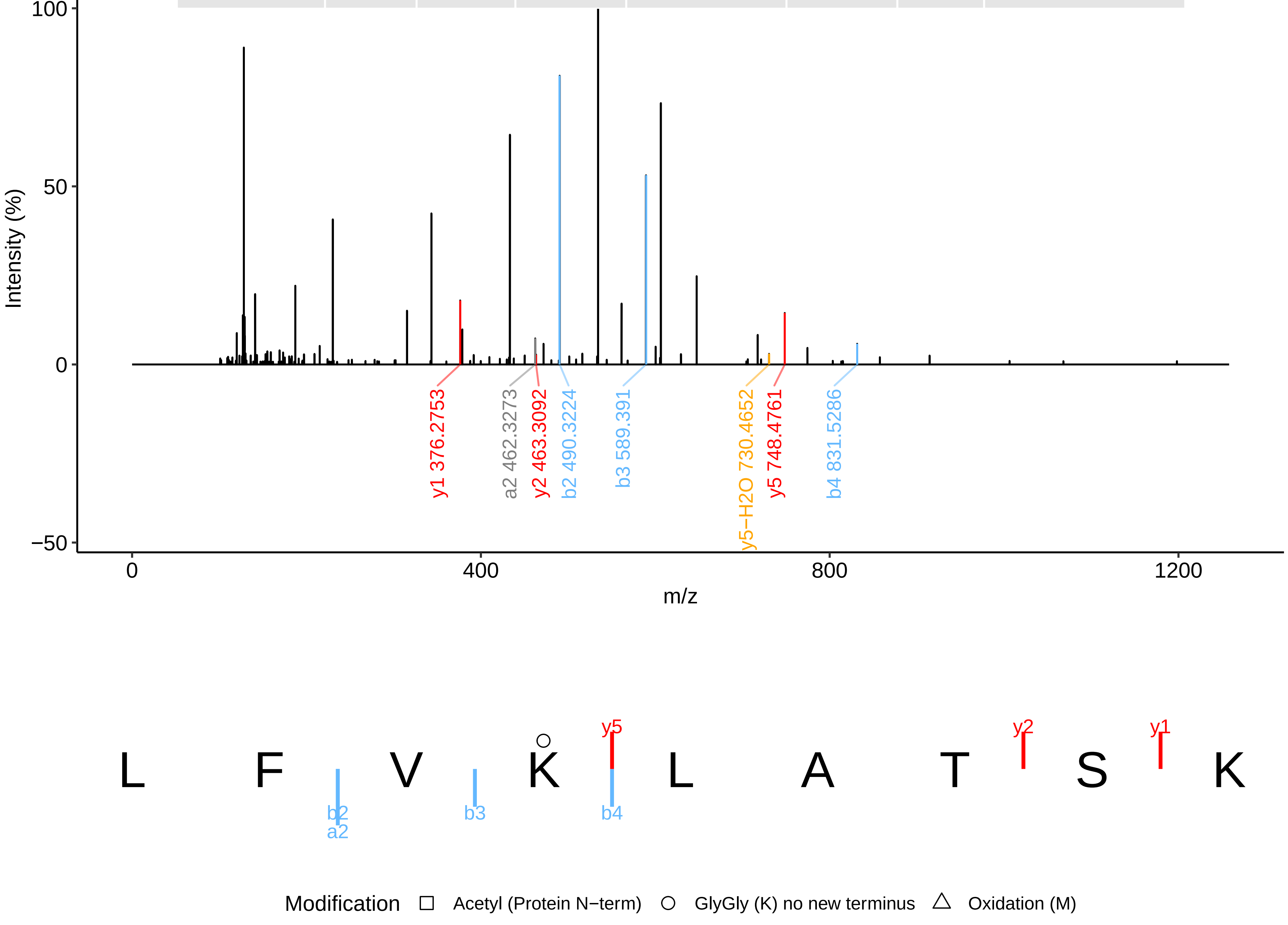

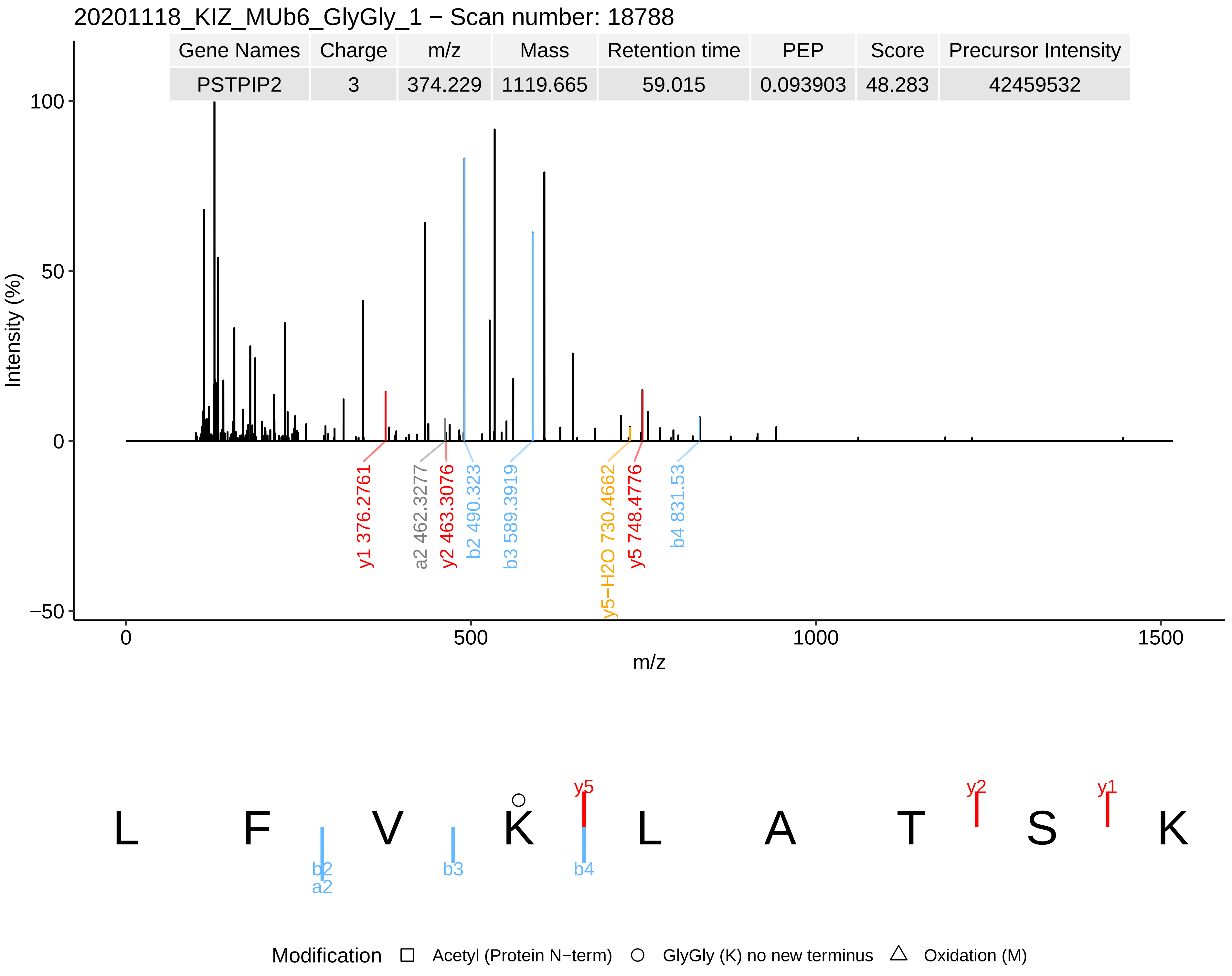

20201118_KIZ_MUb6_GlyGly_1 − Scan number: 18788
Gene Names | Charge | m/z | Mass | Retention time | PEP | Score | Precursor Intensity
PSTPIP2 | 3 | 374.229 | 1119.665 | 59.015 | 0.093903 | 48.283 | 42459532
Intensity (%)
m/z
y1 376.2761
a2 462.3277
y2 463.3076
b2 490.323
b3 589.3919
y5−H2O 730.4662
y5 748.4776
b4 831.53
L F V K L A T S K
y5 y2 y1
b2 a2 b3 b4
Modification
Acetyl (Protein N−term)
GlyGly (K) no new terminus
Oxidation (M)

20200921_KIZ_MUb4_GlyGly_10plex − Scan number: 11568

Gene Names	Charge	m/z	Mass	Retention time	PEP	Score	Precursor Intensity
ATAD1	3	443.2491	1326.726	40.35	3.8758e−07	119.68	12072268

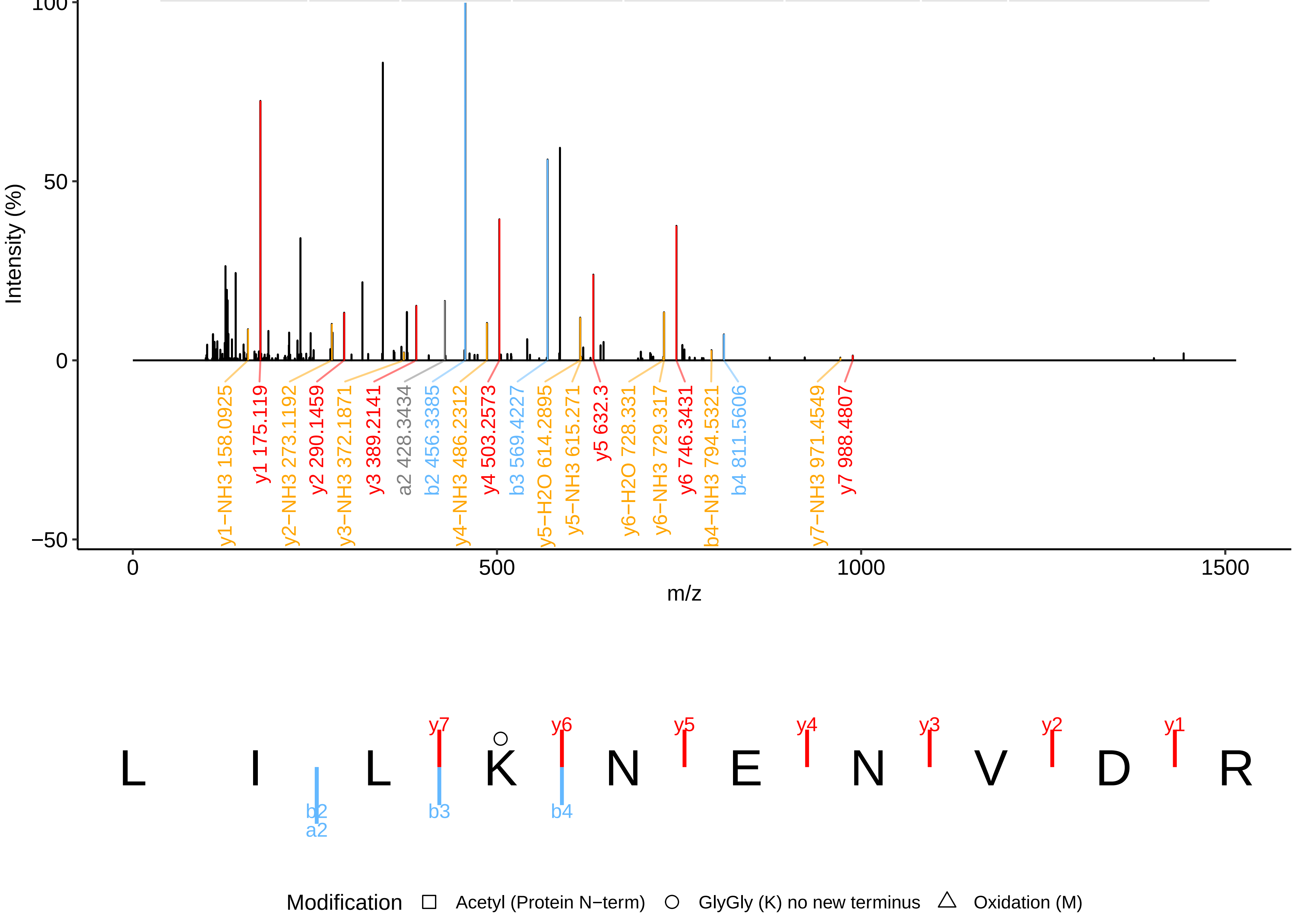

20200924_KIZ_MUb5_GlyGly_2 − Scan number: 9730

Gene Names	Charge	m/z	Mass	Retention time	PEP	Score	Precursor Intensity
ATAD1	3	443.2491	1326.726	36.354	0.00031567	96.342	1941166

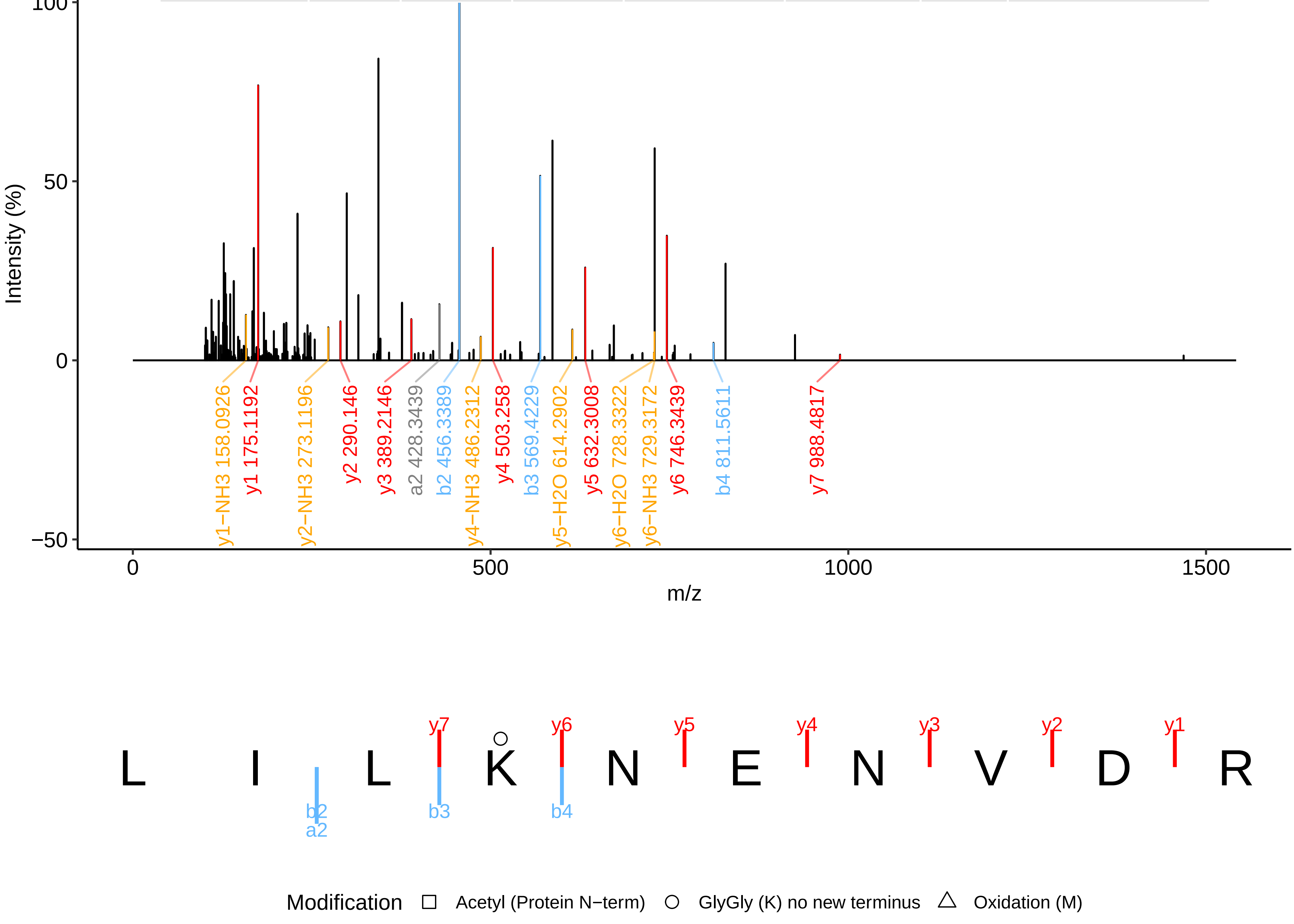

20200924_KIZ_MUb5_GlyGly_2 − Scan number: 9861

Gene Names	Charge	m/z	Mass	Retention time	PEP	Score	Precursor Intensity
ATAD1	3	443.2491	1326.726	36.858	0.00031567	96.342	2089404

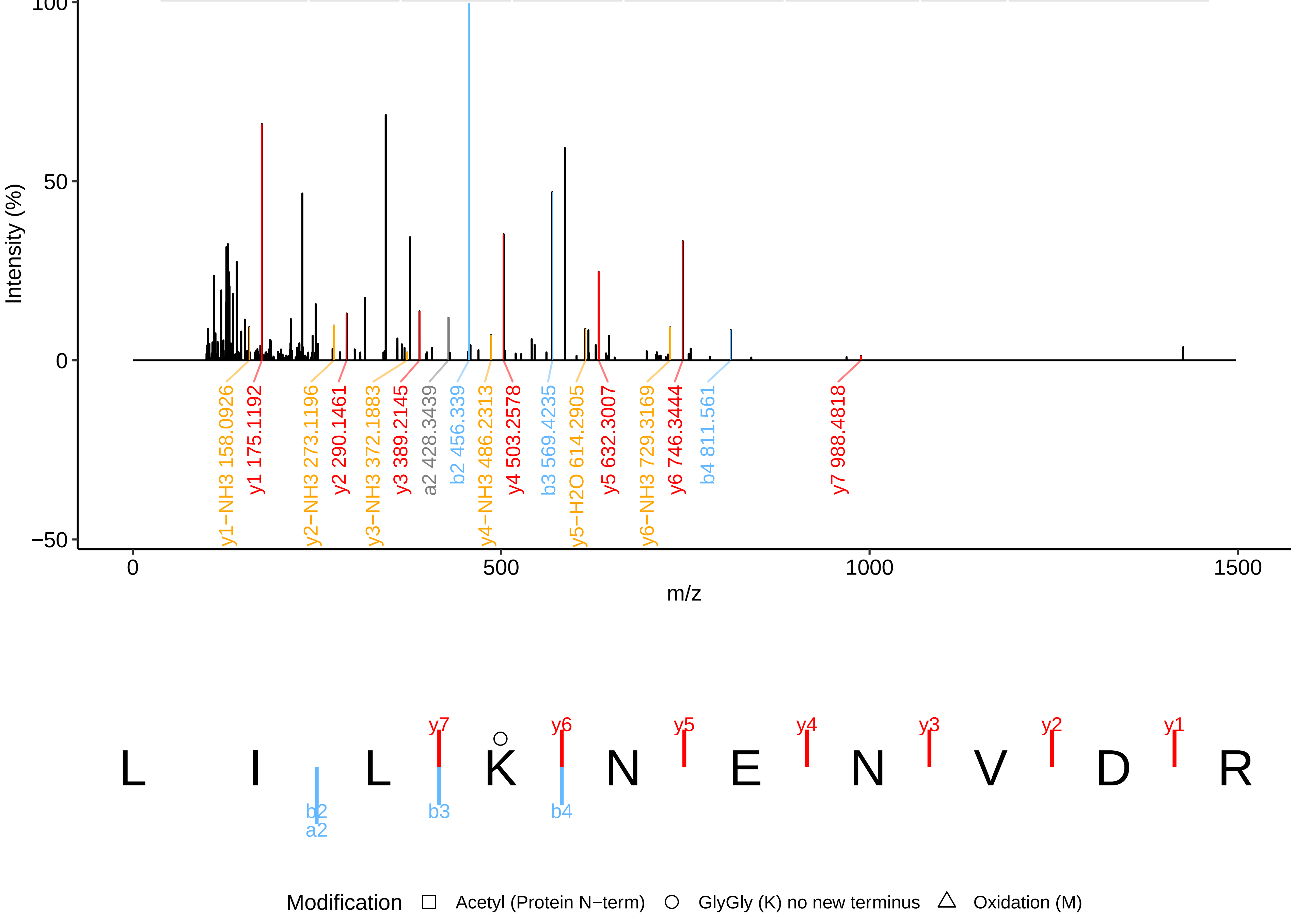

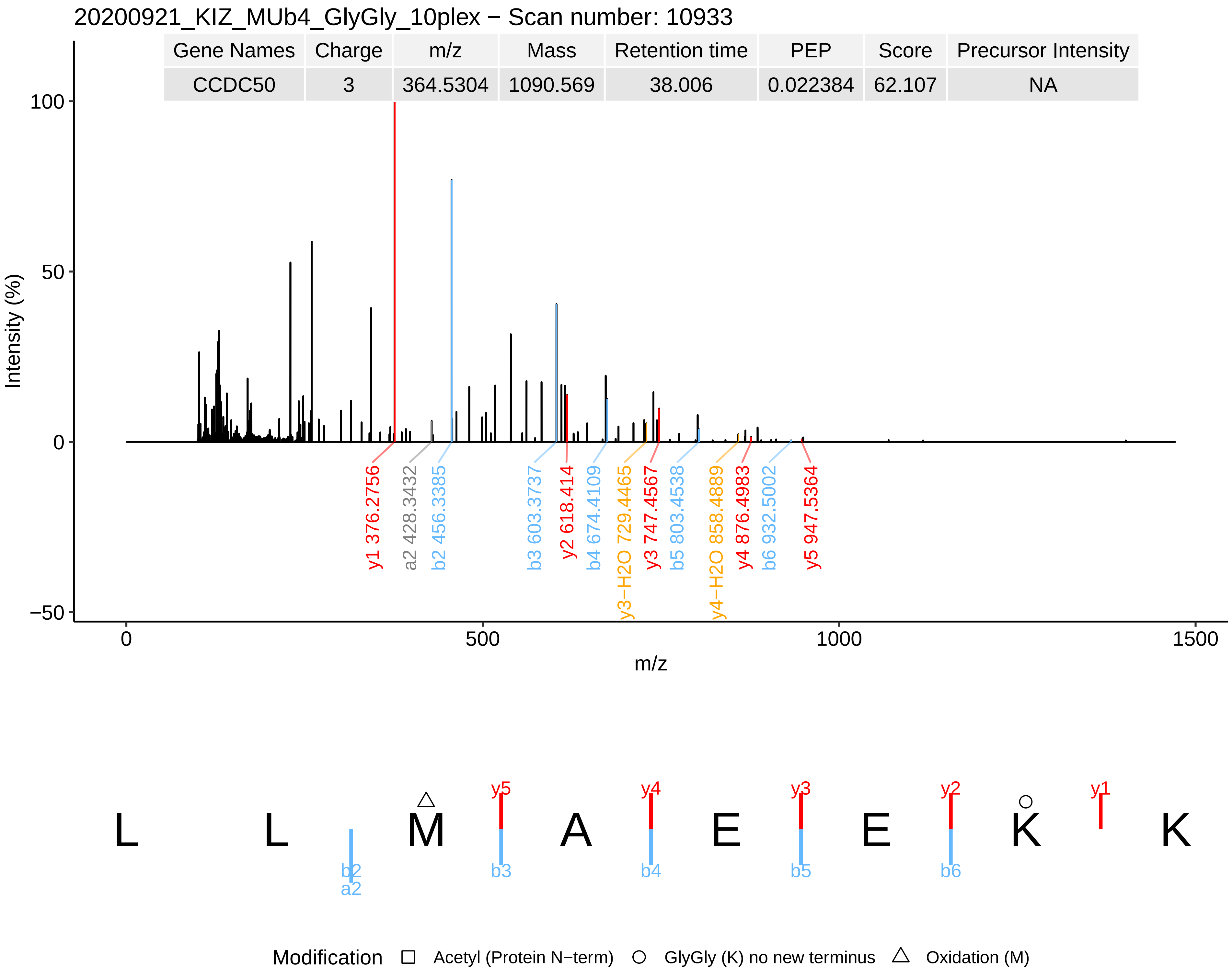

20200921_KIZ_MUb4_GlyGly_10plex − Scan number: 10933
Gene Names | Charge | m/z | Mass | Retention time | PEP | Score | Precursor Intensity
CCDC50 | 3 | 364.5304 | 1090.569 | 38.006 | 0.022384 | 62.107 | NA
Intensity (%)
100
50
0
−50
0
500
1000
1500
m/z
y1 376.2756
a2 428.3432
b2 456.3385
b3 603.3737
y2 618.414
b4 674.4109
y3−H2O 729.4465
y3 747.4567
b5 803.4538
y4−H2O 858.4889
y4 876.4983
b6 932.5002
y5 947.5364
L L M A E E K K
y5 y4 y3 y2 y1
b2 a2 b3 b4 b5 b6
Modification
Acetyl (Protein N−term)
GlyGly (K) no new terminus
Oxidation (M)

20200921_KIZ_MUb4_GlyGly_10plex_2 − Scan number: 10122

Gene Names	Charge	m/z	Mass	Retention time	PEP	Score	Precursor Intensity
CCDC50	3	364.5304	1090.569	38.648	0.065696	50.127	721313.1

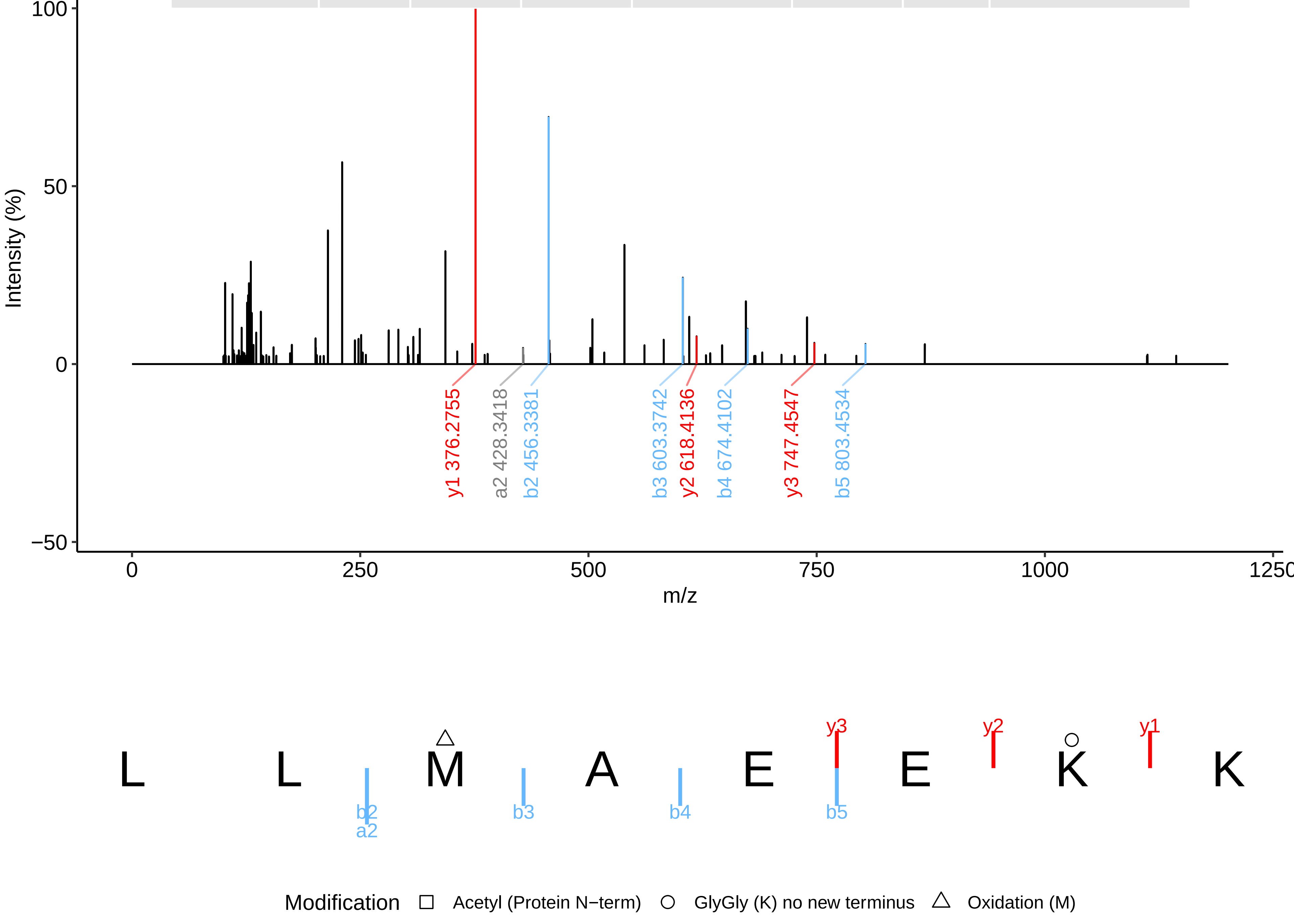

20200921_KIZ_MUb4_GlyGly_10plex − Scan number: 12730

Gene Names	Charge	m/z	Mass	Retention time	PEP	Score	Precursor Intensity
RPL30	3	378.5498	1132.627	44.734	0.079235	47.187	4290342

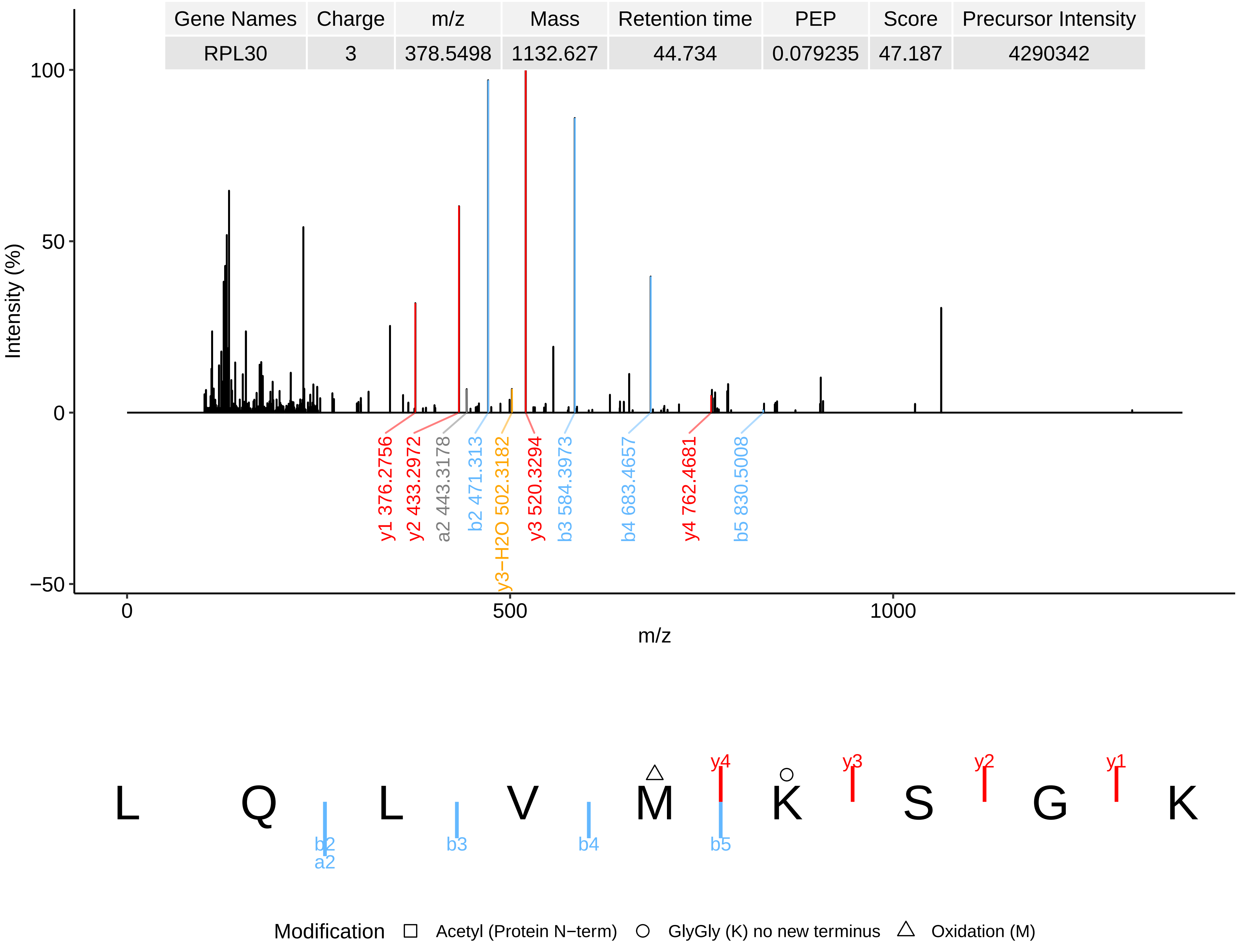

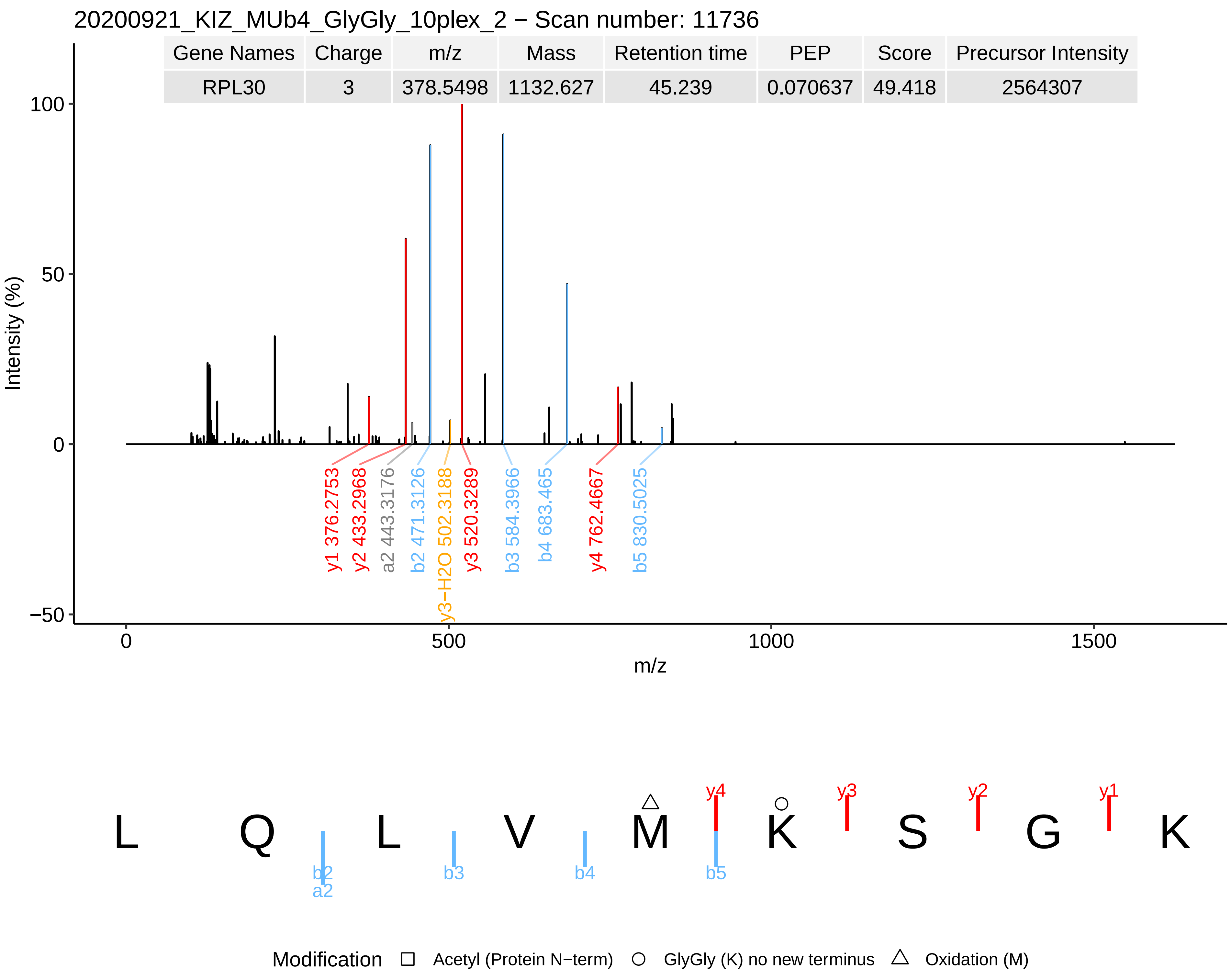

20200921_KIZ_MUb4_GlyGly_10plex_2 − Scan number: 11736
Gene Names | Charge | m/z | Mass | Retention time | PEP | Score | Precursor Intensity
RPL30 | 3 | 378.5498 | 1132.627 | 45.239 | 0.070637 | 49.418 | 2564307
Intensity (%)
m/z
y1 376.2753
y2 433.2968
a2 443.3176
b2 471.3126
y3−H2O 502.3188
y3 520.3289
b3 584.3966
b4 683.465
y4 762.4667
b5 830.5025
L Q L V M K S G K
Modification
Acetyl (Protein N−term)
GlyGly (K) no new terminus
Oxidation (M)

20200924_KIZ_MUb5_GlyGly_1 − Scan number: 11782

Gene Names	Charge	m/z	Mass	Retention time	PEP	Score	Precursor Intensity
RPL30	3	378.5498	1132.627	38.222	0.10294	55.567	NA

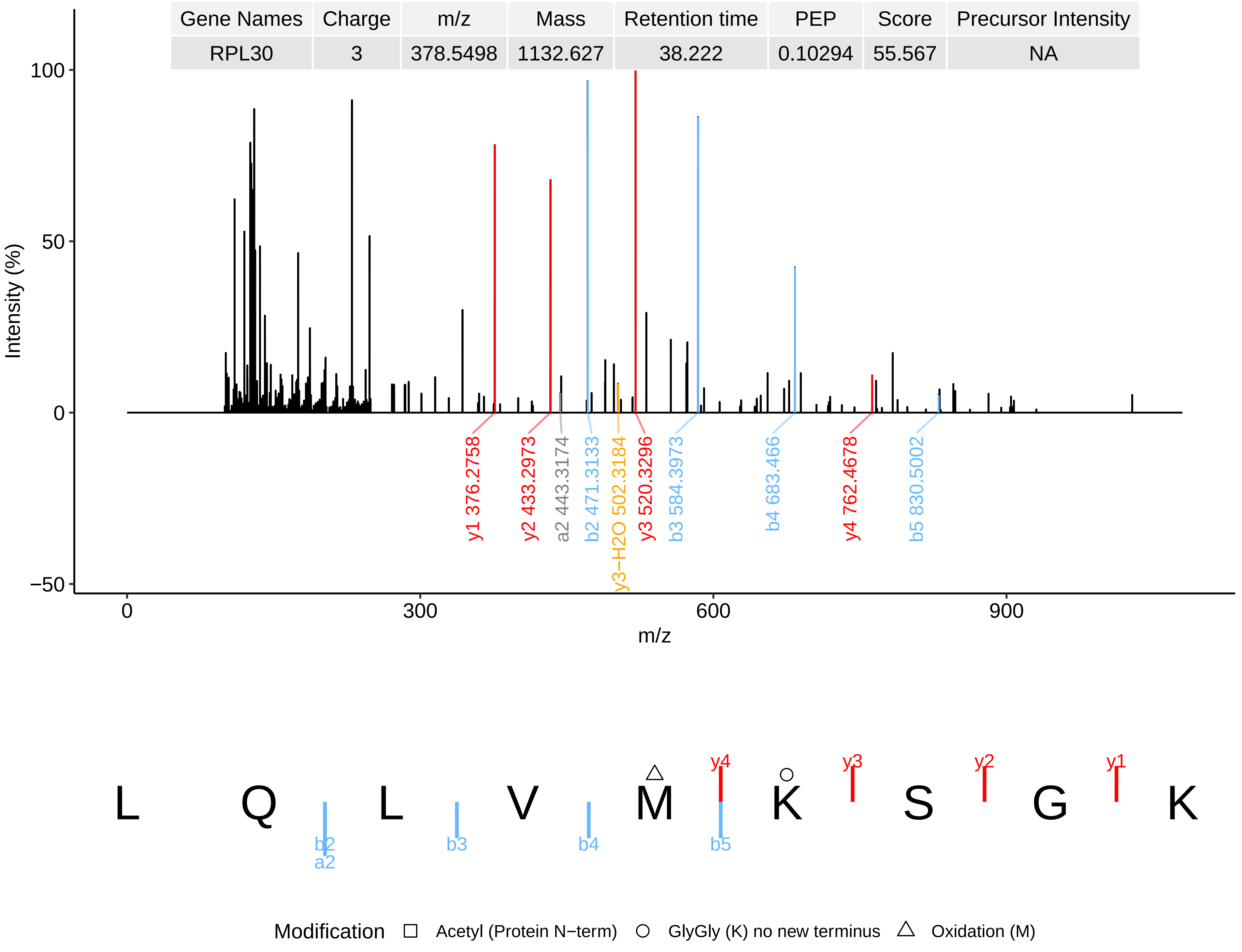

20200921_KIZ_MUb4_GlyGly_10plex − Scan number: 16309

Gene Names	Charge	m/z	Mass	Retention time	PEP	Score	Precursor Intensity
RPL30	3	373.2181	1116.632	58.541	0.01136	73.233	11566312

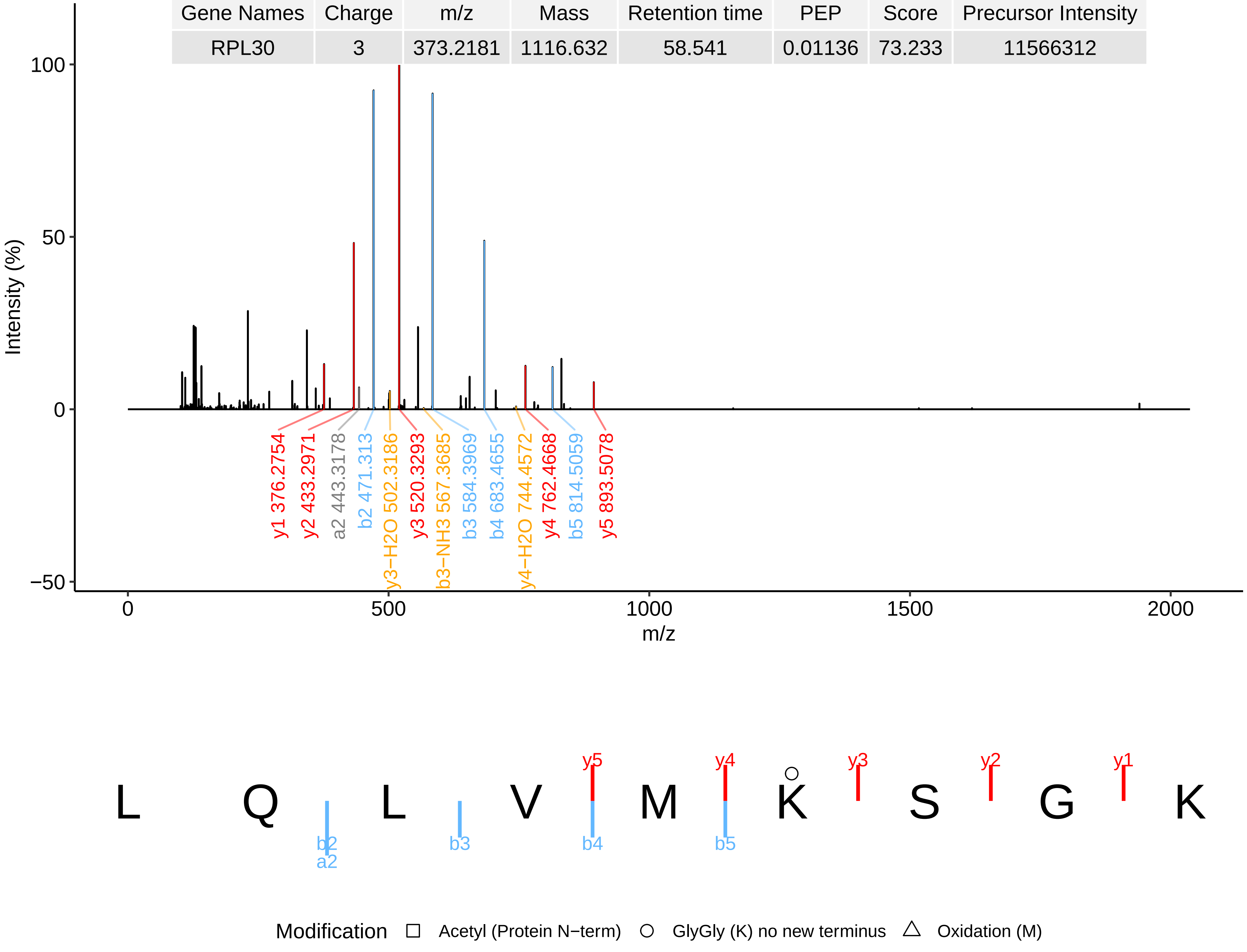

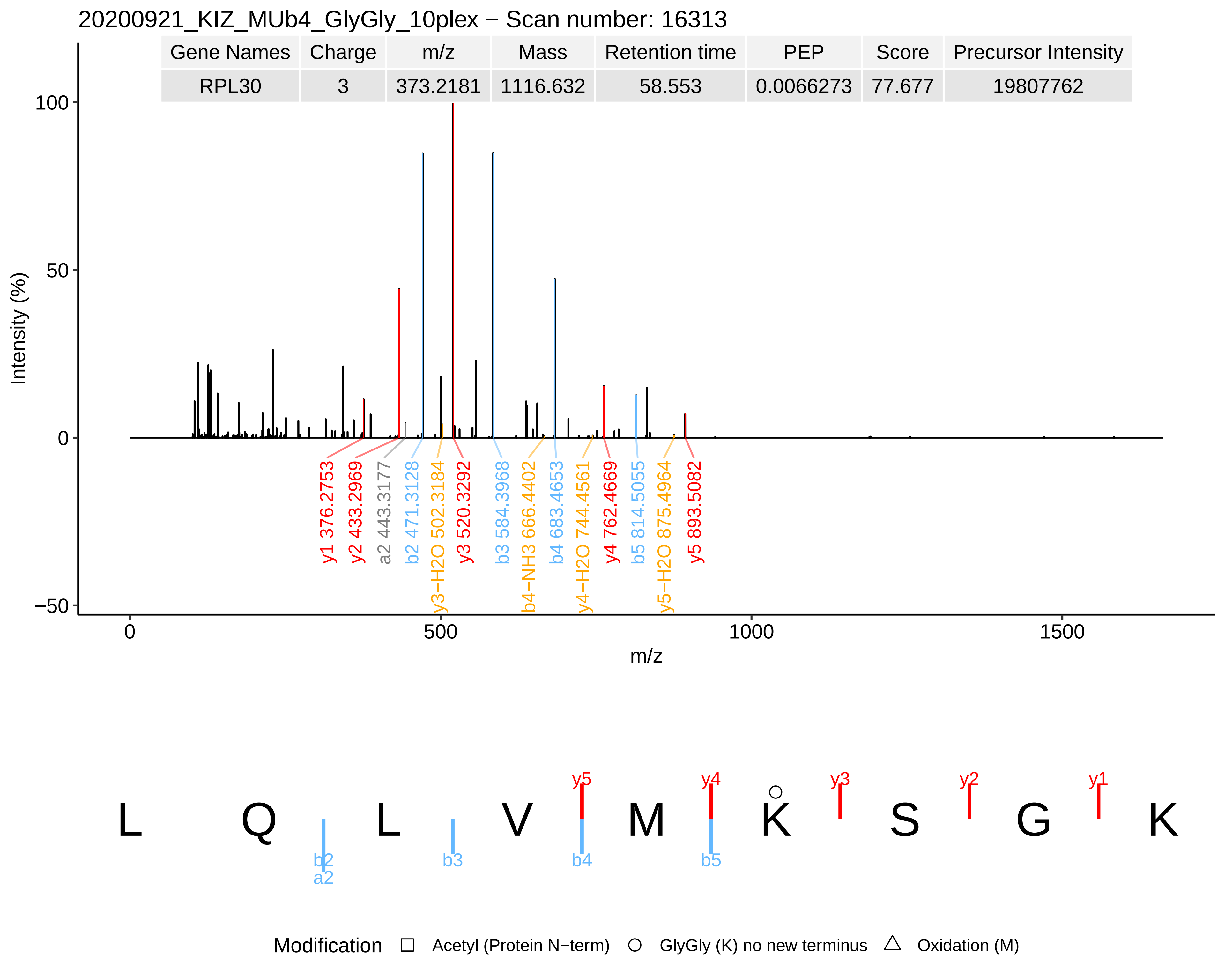

20200921_KIZ_MUb4_GlyGly_10plex − Scan number: 16313
Gene Names | Charge | m/z | Mass | Retention time | PEP | Score | Precursor Intensity
RPL30 | 3 | 373.2181 | 1116.632 | 58.553 | 0.0066273 | 77.677 | 19807762
Intensity (%)
100
50
0
−50
0
500
1000
1500
m/z
y1 376.2753
y2 433.2969
a2 443.3177
b2 471.3128
y3−H2O 502.3184
y3 520.3292
b3 584.3968
b4−NH3 666.4402
b4 683.4653
y4−H2O 744.4561
y4 762.4669
b5 814.5055
y5−H2O 875.4964
y5 893.5082
L Q L V M K S G K
y5 y4 y3 y2 y1
b2 a2 b3 b4 b5
Modification
Acetyl (Protein N−term)
GlyGly (K) no new terminus
Oxidation (M)

20200921_KIZ_MUb4_GlyGly_10plex_2 − Scan number: 15150

Gene Names	Charge	m/z	Mass	Retention time	PEP	Score	Precursor Intensity
RPL30	3	373.2181	1116.632	59.187	0.019759	65.347	788489.7

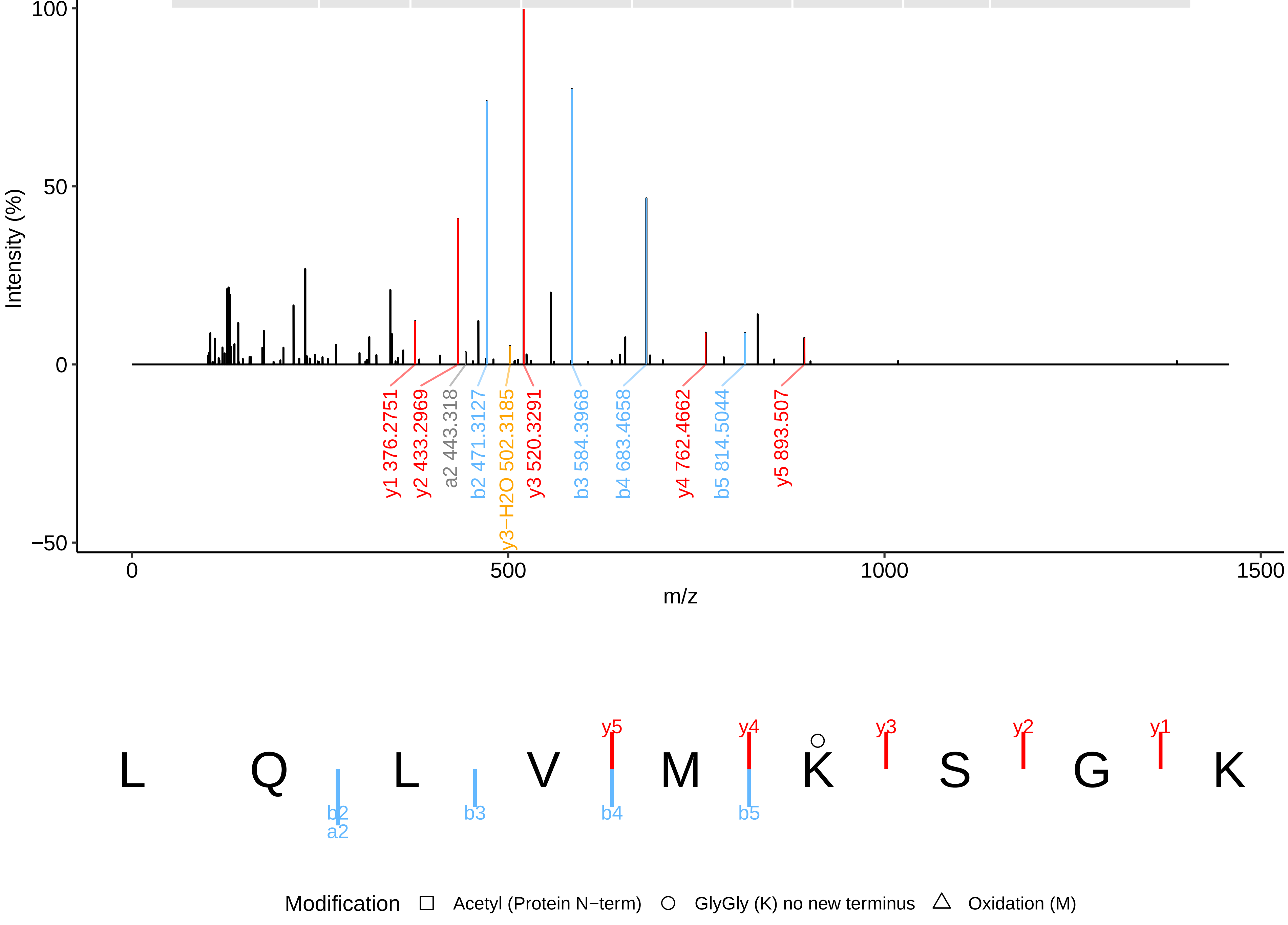

20200924_KIZ_MUb5_GlyGly_1 − Scan number: 15279

Gene Names	Charge	m/z	Mass	Retention time	PEP	Score	Precursor Intensity
RPL30	3	373.2181	1116.632	49.891	0.00099529	95.502	26353012

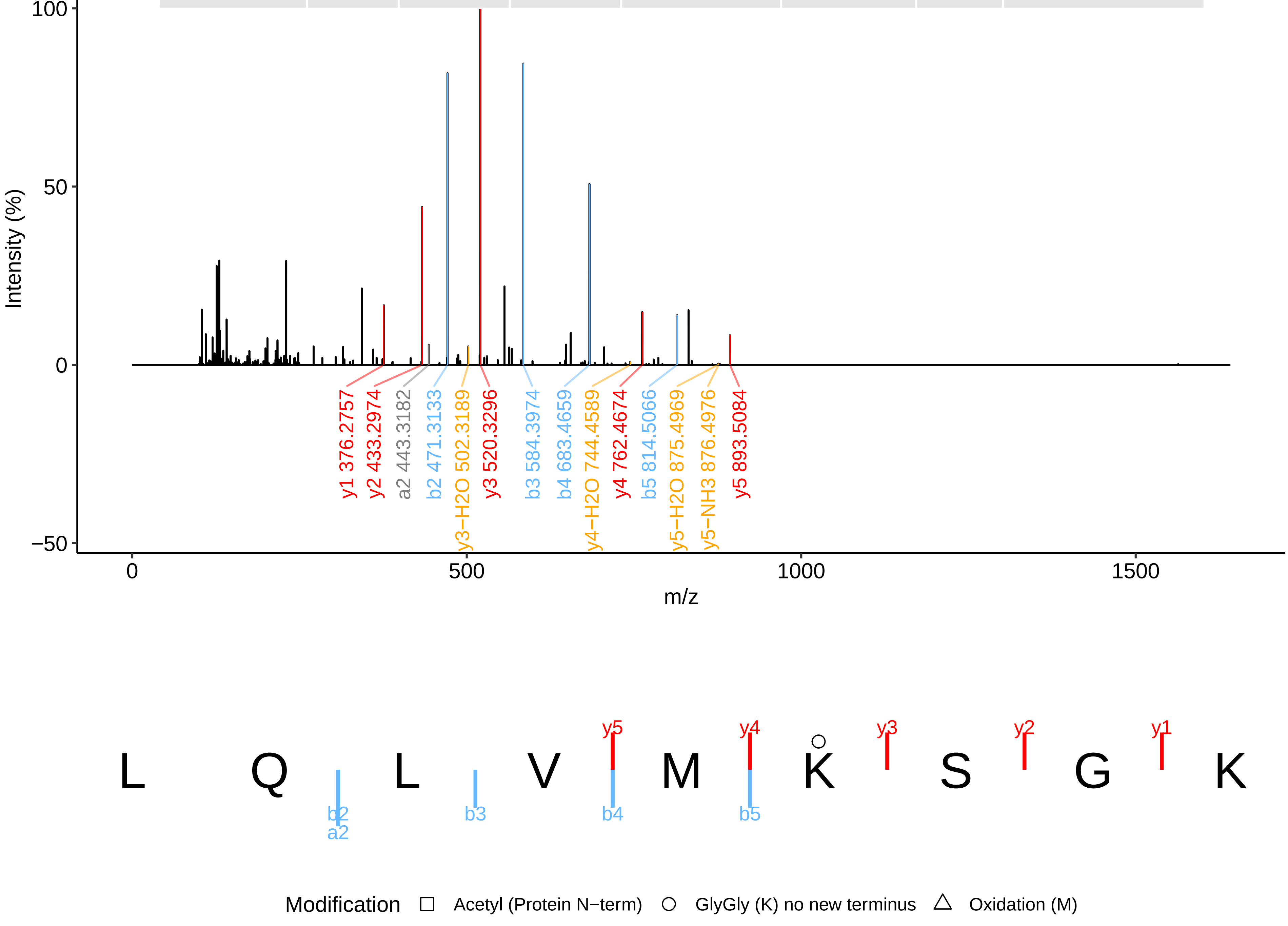

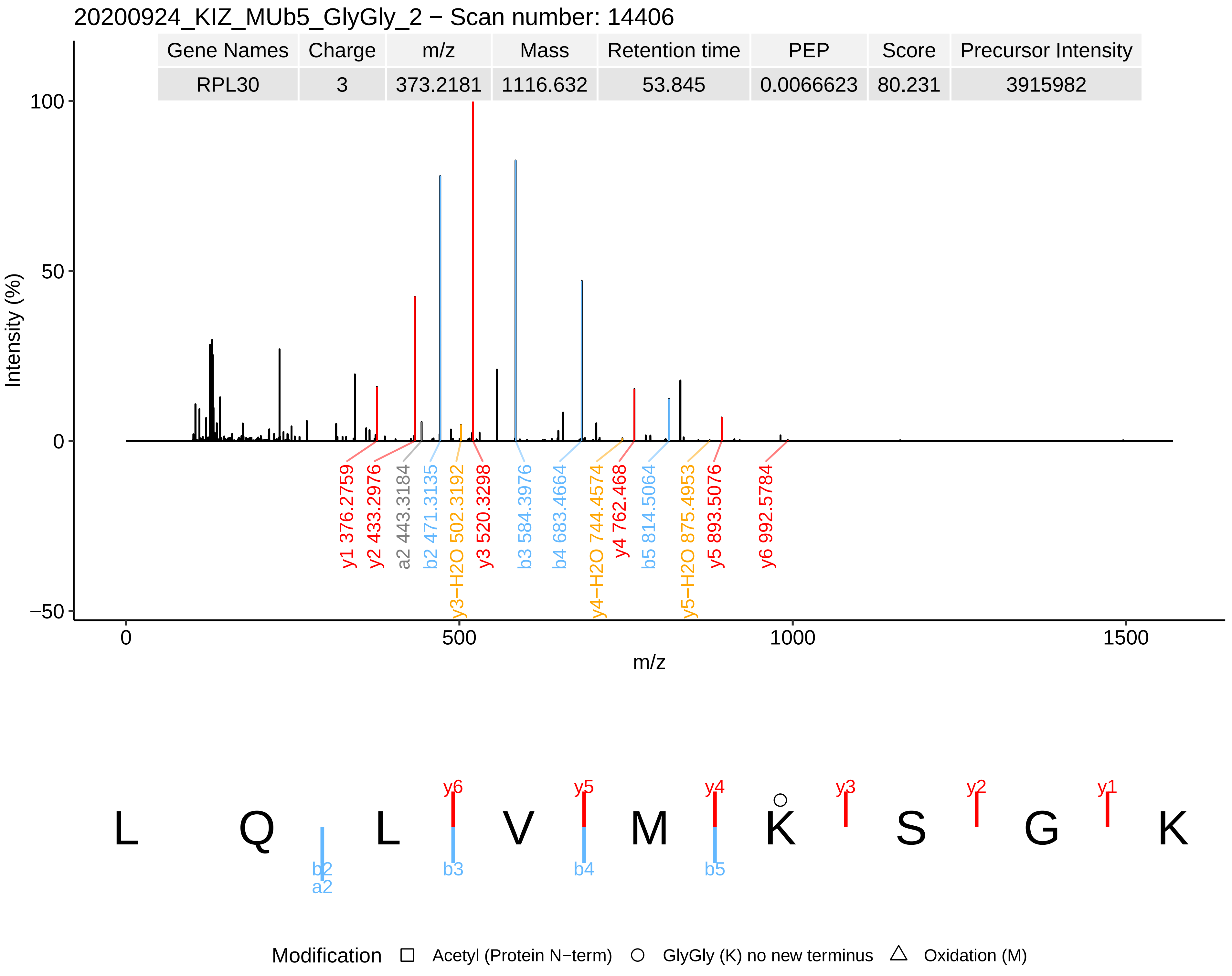
20200924_KIZ_MUb5_GlyGly_2 − Scan number: 14406
Gene Names | Charge | m/z | Mass | Retention time | PEP | Score | Precursor Intensity
RPL30 | 3 | 373.2181 | 1116.632 | 53.845 | 0.0066623 | 80.231 | 3915982
Intensity (%)
100
50
0
−50
y1 376.2759
y2 433.2976
a2 443.3184
b2 471.3135
y3−H2O 502.3192
y3 520.3298
b3 584.3976
b4 683.4664
y4−H2O 744.4574
y4 762.468
b5 814.5064
y5−H2O 875.4953
y5 893.5076
y6 992.5784
0
500
1000
1500
m/z
L Q L V M K S G K
y6 y5 y4 y3 y2 y1
b2 a2 b3 b4 b5
Modification
Acetyl (Protein N−term)
GlyGly (K) no new terminus
Oxidation (M)

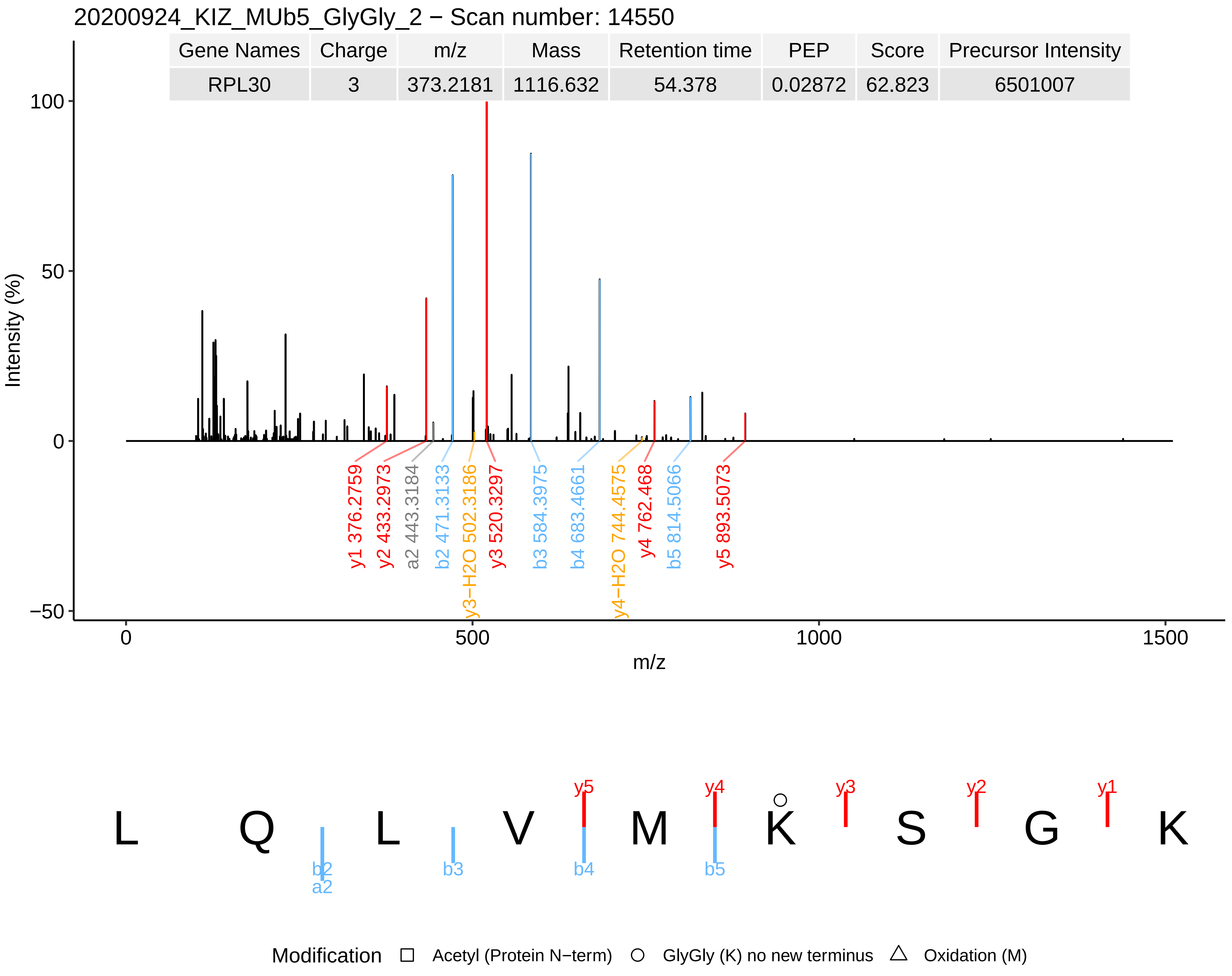

20200924_KIZ_MUb5_GlyGly_2 − Scan number: 14550
Gene Names | Charge | m/z | Mass | Retention time | PEP | Score | Precursor Intensity
RPL30 | 3 | 373.2181 | 1116.632 | 54.378 | 0.02872 | 62.823 | 6501007
Intensity (%)
100
50
0
−50
y1 376.2759
y2 433.2973
a2 443.3184
b2 471.3133
y3−H2O 502.3186
y3 520.3297
b3 584.3975
b4 683.4661
y4−H2O 744.4575
y4 762.468
b5 814.5066
y5 893.5073
0
500
1000
1500
m/z
L Q L V M K S G K
y5 y4 y3 y2 y1
b2 a2 b3 b4 b5
Modification
Acetyl (Protein N−term)
GlyGly (K) no new terminus
Oxidation (M)

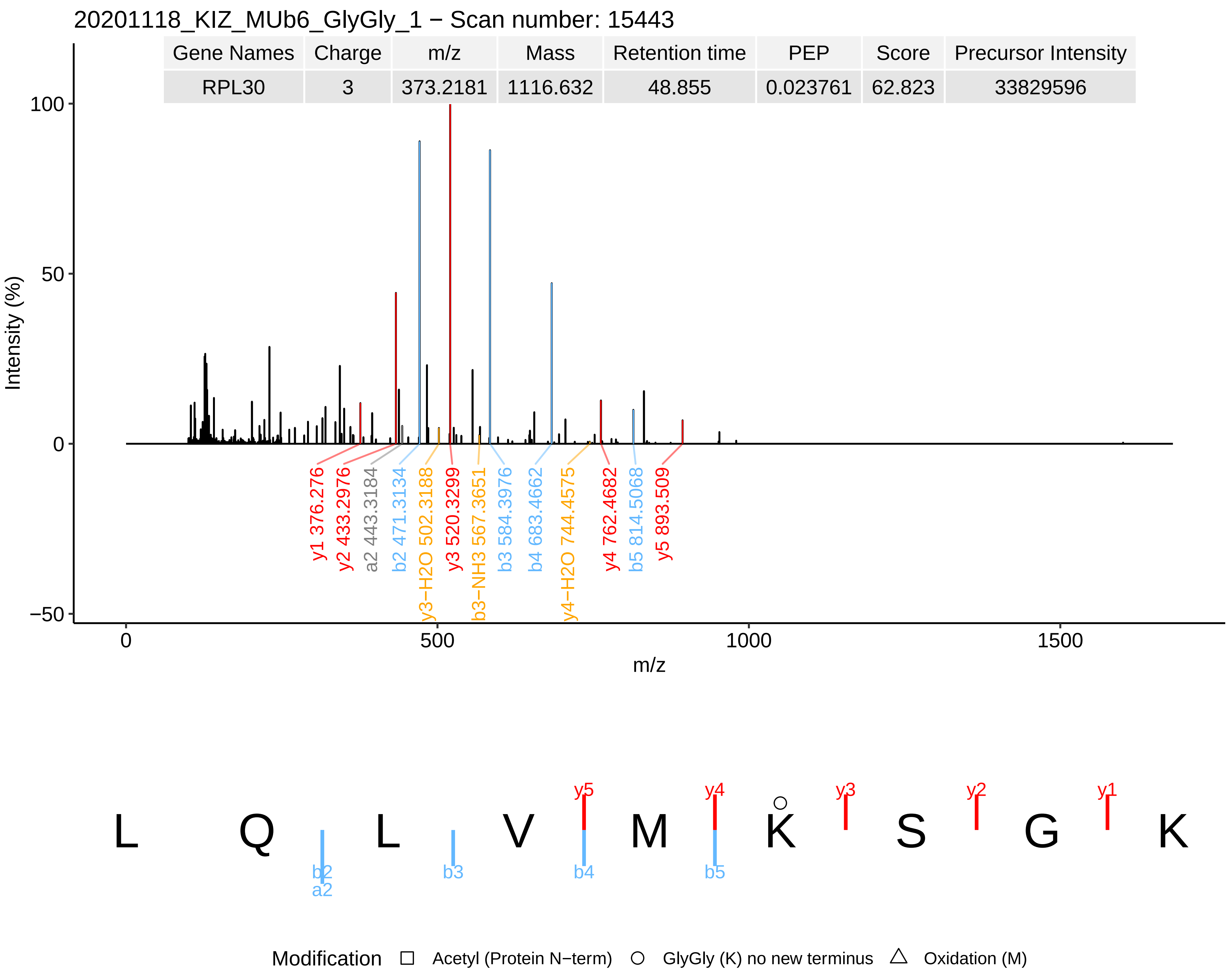
20201118_KIZ_MUb6_GlyGly_1 − Scan number: 15443
Gene Names
RPL30
Charge
3
m/z
373.2181
Mass
1116.632
Retention time
48.855
PEP
0.023761
Score
62.823
Precursor Intensity
33829596
Intensity (%)
m/z
y1 376.276
y2 433.2976
a2 443.3184
b2 471.3134
y3−H2O 502.3188
y3 520.3299
b3−NH3 567.3651
b3 584.3976
b4 683.4662
y4−H2O 744.4575
y4 762.4682
b5 814.5068
y5 893.509
L Q L V M K S G K
Modification
Acetyl (Protein N−term)
GlyGly (K) no new terminus
Oxidation (M)

20201118_KIZ_MUb6_GlyGly_2 − Scan number: 18044

Gene Names	Charge	m/z	Mass	Retention time	PEP	Score	Precursor Intensity
RPL30	3	373.2181	1116.632	61.038	0.0083074	76.1	17251888

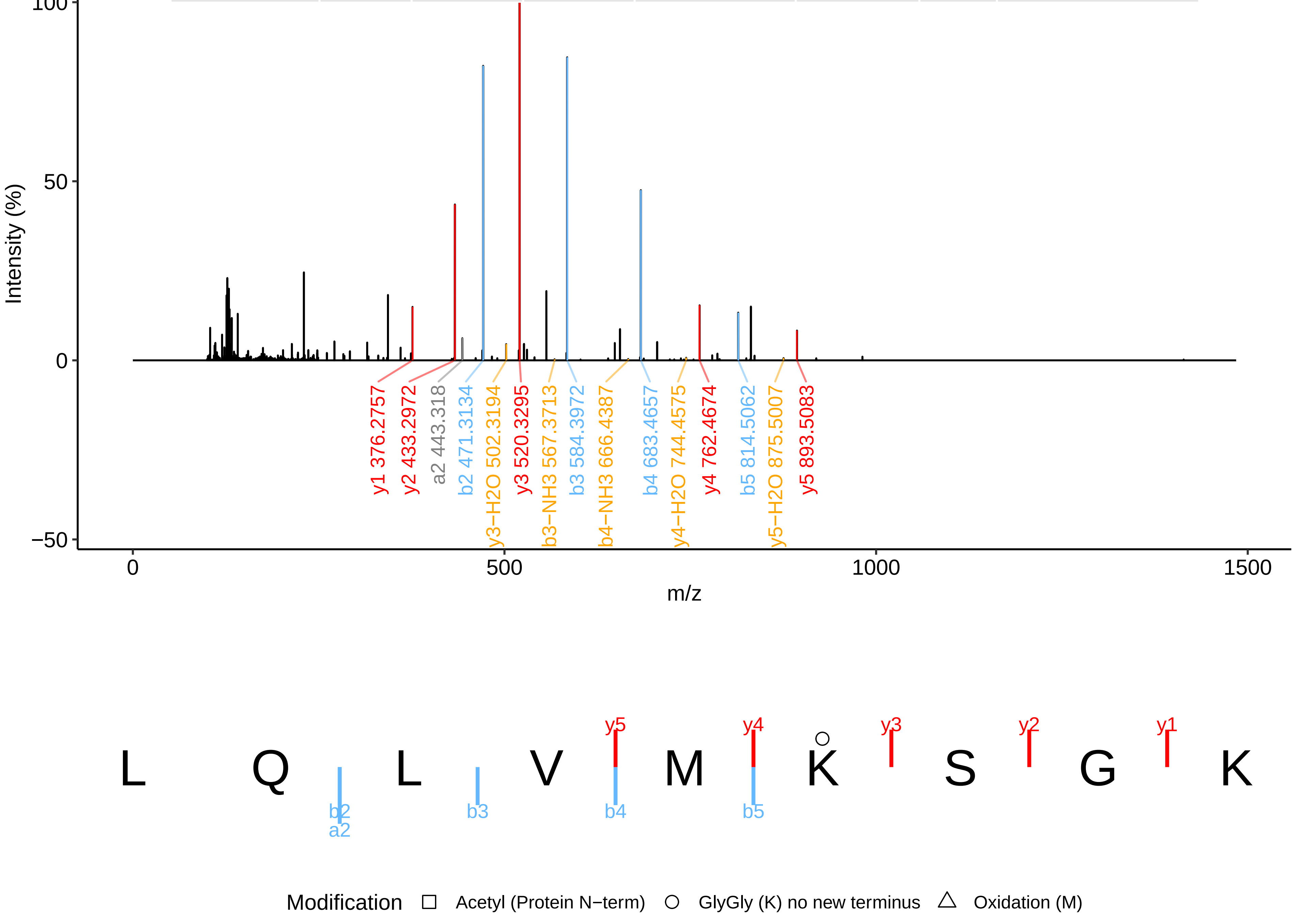

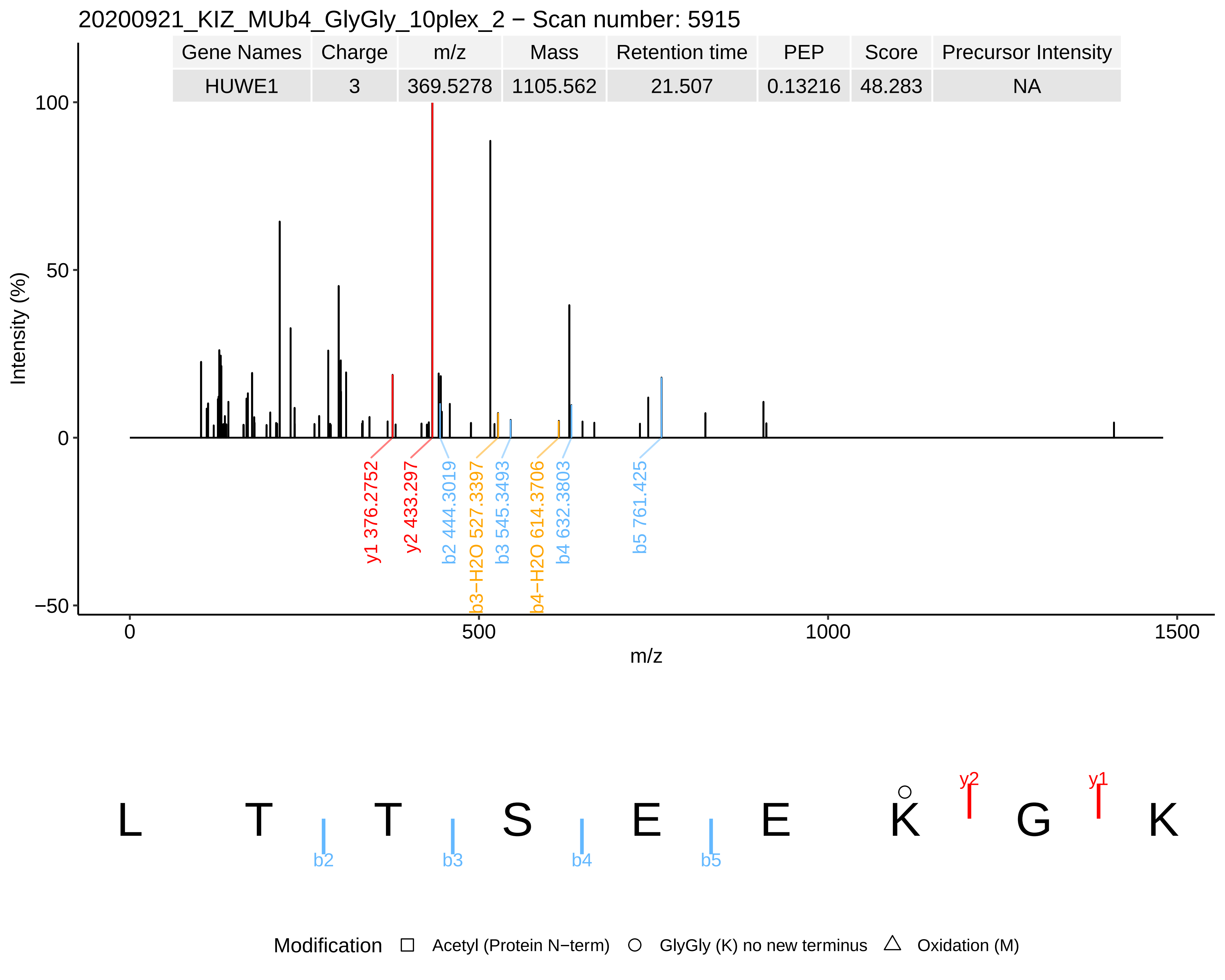
20200921_KIZ_MUb4_GlyGly_10plex_2 − Scan number: 5915
Gene Names | Charge | m/z | Mass | Retention time | PEP | Score | Precursor Intensity
HUWE1 | 3 | 369.5278 | 1105.562 | 21.507 | 0.13216 | 48.283 | NA
Intensity (%)
100
50
0
−50
0
500
1000
1500
m/z
y1 376.2752
y2 433.297
b2 444.3019
b3−H2O 527.3397
b3 545.3493
b4−H2O 614.3706
b4 632.3803
b5 761.425
L T T S E E K G K
b2
b3
b4
b5
y2
y1
Modification
Acetyl (Protein N−term)
GlyGly (K) no new terminus
Oxidation (M)

20200921_KIZ_MUb4_GlyGly_10plex − Scan number: 10392

Gene Names	Charge	m/z	Mass	Retention time	PEP	Score	Precursor Intensity
ARIH1	3	364.5451	1090.613	35.955	0.01539	77.674	10530055

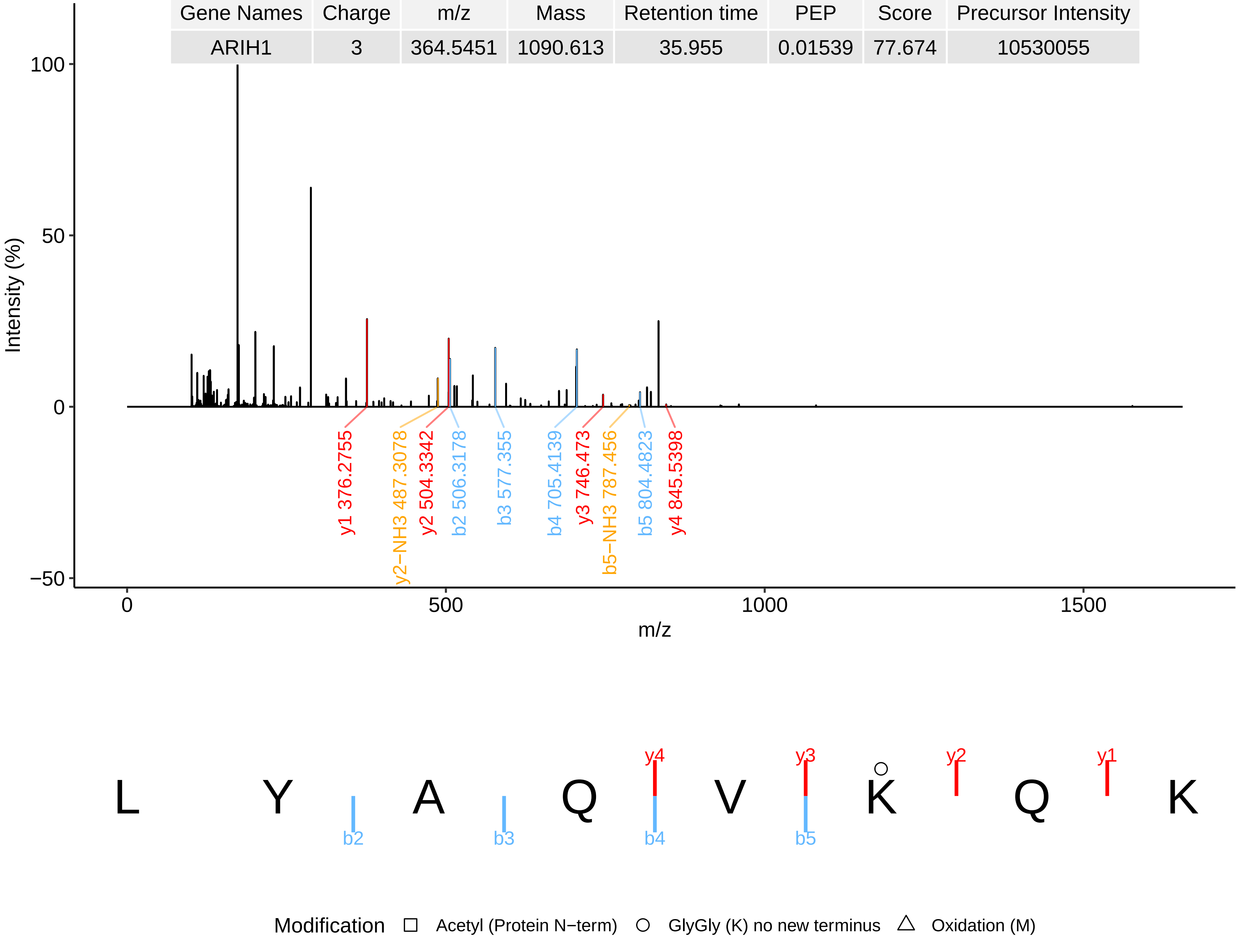

20200921_KIZ_MUb4_GlyGly_10plex − Scan number: 4782

Gene Names	Charge	m/z	Mass	Retention time	PEP	Score	Precursor Intensity
DCUN1D1	3	449.5505	1345.63	15.59	0.0009795	90.05	8974822

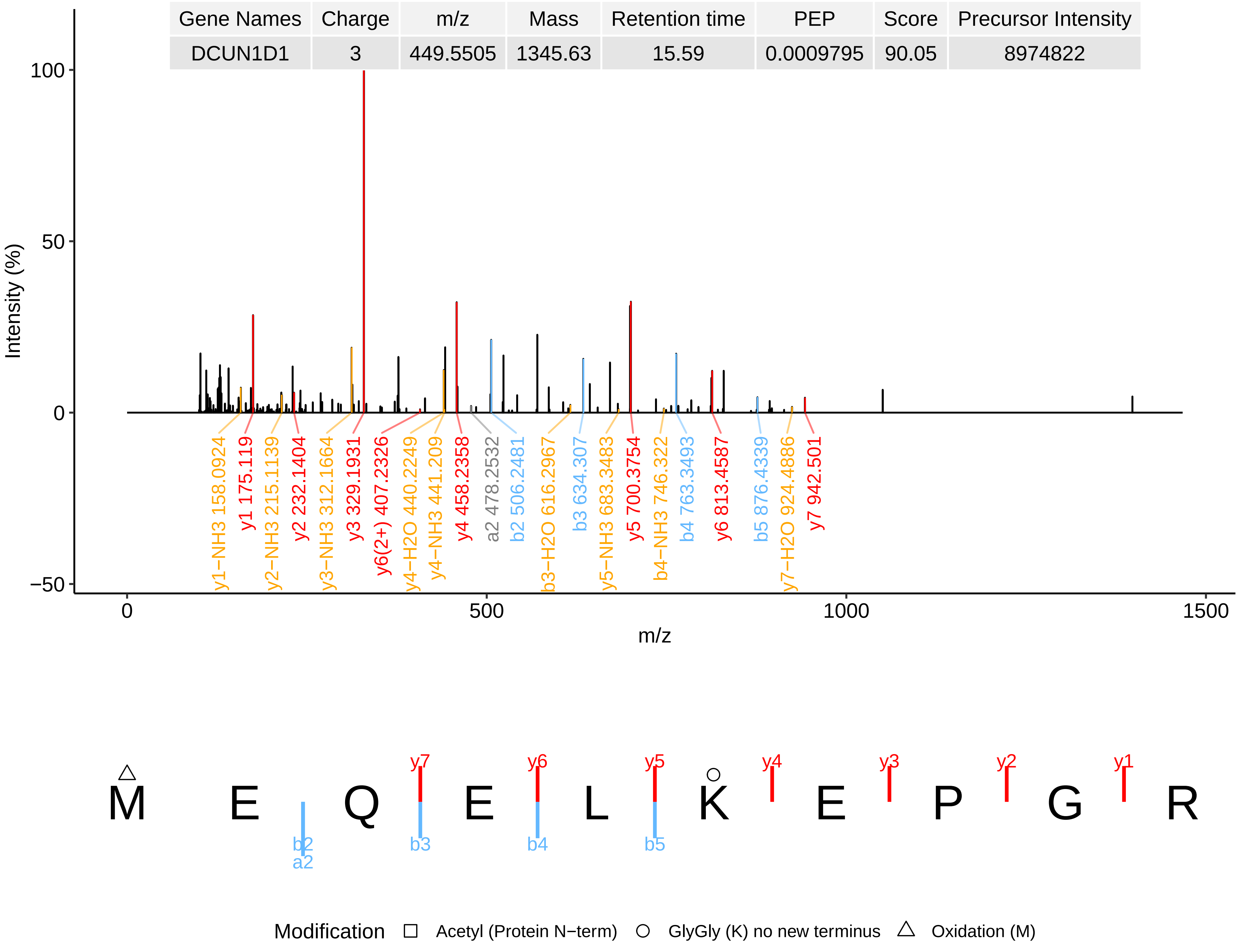

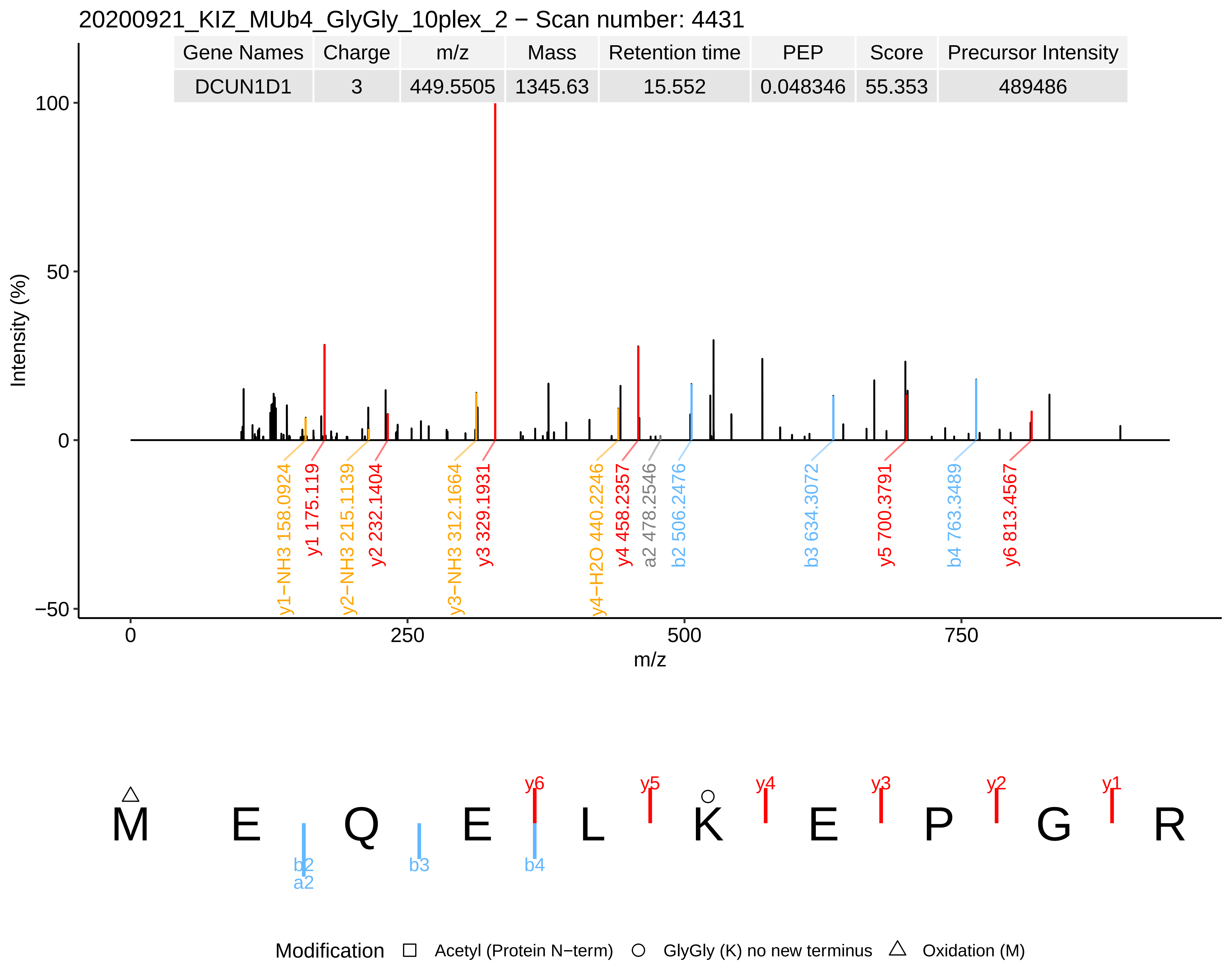

20200921_KIZ_MUb4_GlyGly_10plex_2 − Scan number: 4431
Gene Names | Charge | m/z | Mass | Retention time | PEP | Score | Precursor Intensity
DCUN1D1 | 3 | 449.5505 | 1345.63 | 15.552 | 0.048346 | 55.353 | 489486
Intensity (%)
m/z
y1−NH3 158.0924
y1 175.119
y2−NH3 215.1139
y2 232.1404
y3−NH3 312.1664
y3 329.1931
y4−H2O 440.2246
y4 458.2357
a2 478.2546
b2 506.2476
b3 634.3072
y5 700.3791
b4 763.3489
y6 813.4567
M E Q E L K E P G R
Modification
Acetyl (Protein N−term)
GlyGly (K) no new terminus
Oxidation (M)

20200921_KIZ_MUb4_GlyGly_10plex − Scan number: 8359

Gene Names	Charge	m/z	Mass	Retention time	PEP	Score	Precursor Intensity
DCUN1D1	3	444.2188	1329.635	28.347	9.2458e−10	136.52	7186669

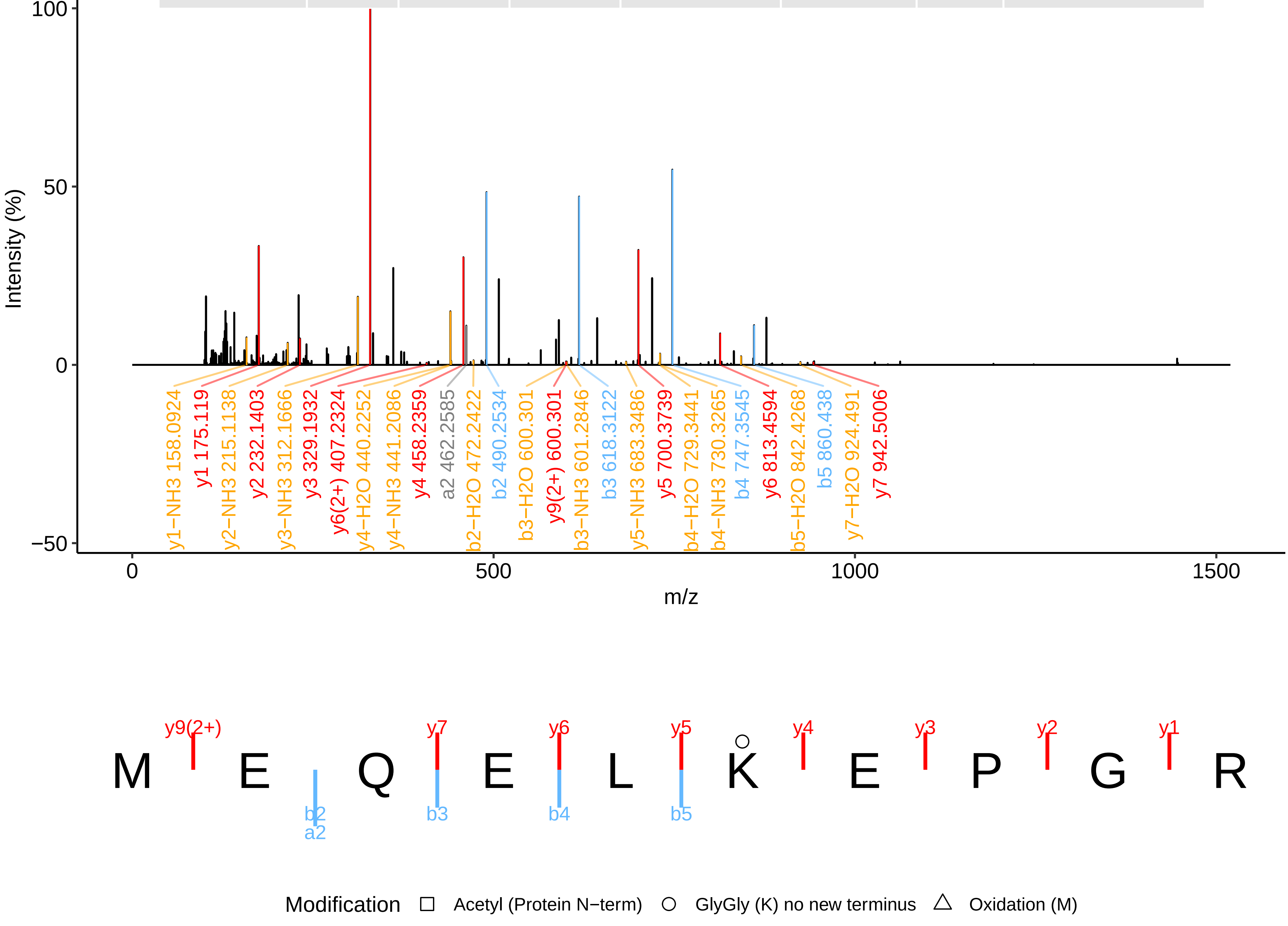

20200921_KIZ_MUb4_GlyGly_10plex_2 − Scan number: 7698

Gene Names	Charge	m/z	Mass	Retention time	PEP	Score	Precursor Intensity
DCUN1D1	3	444.2188	1329.635	28.811	6.3439e−05	100.88	2016107

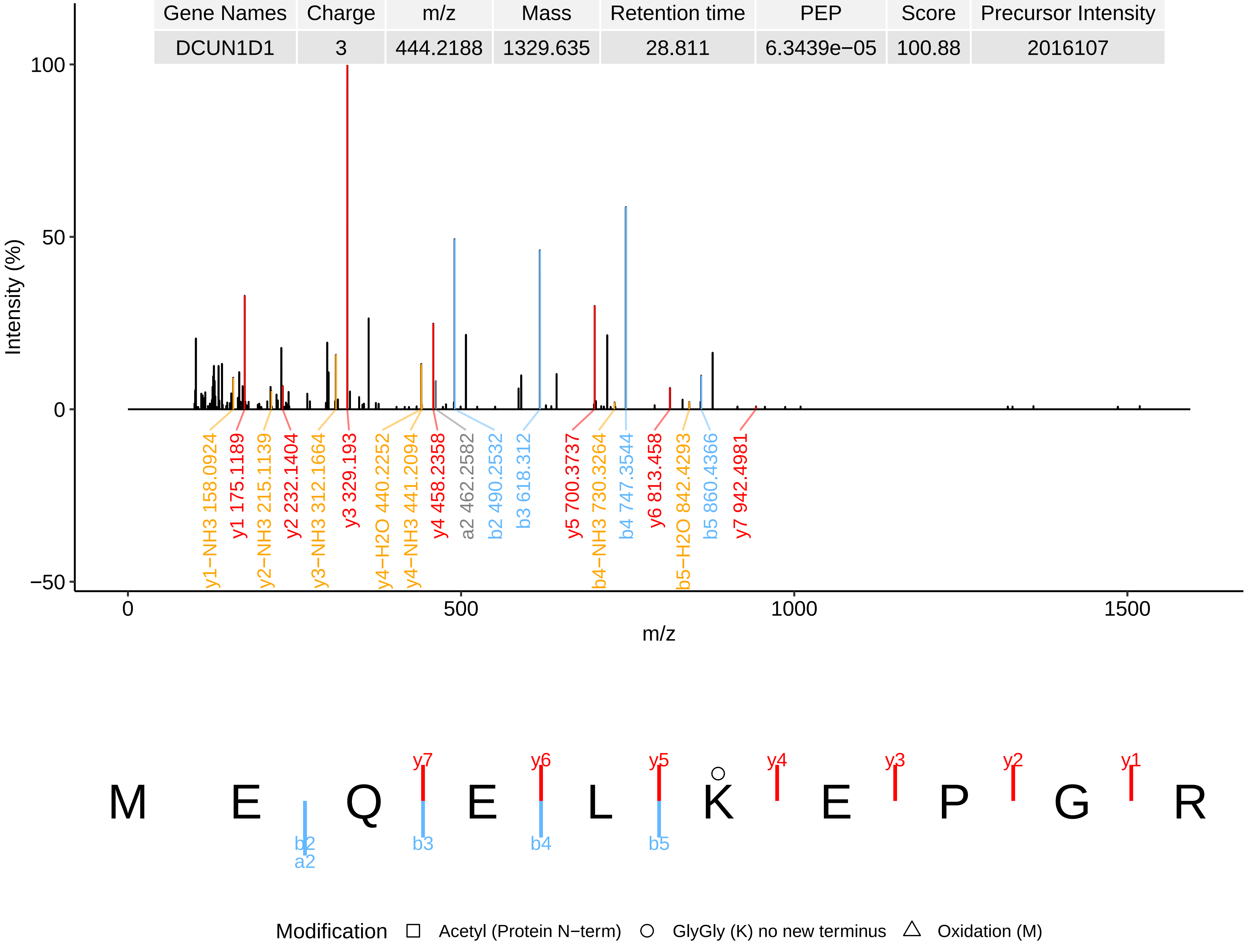

20200924_KIZ_MUb5_GlyGly_1 − Scan number: 7223

Gene Names	Charge	m/z	Mass	Retention time	PEP	Score	Precursor Intensity
DCUN1D1	3	444.2188	1329.635	22.896	3.057e−05	108.47	14459984

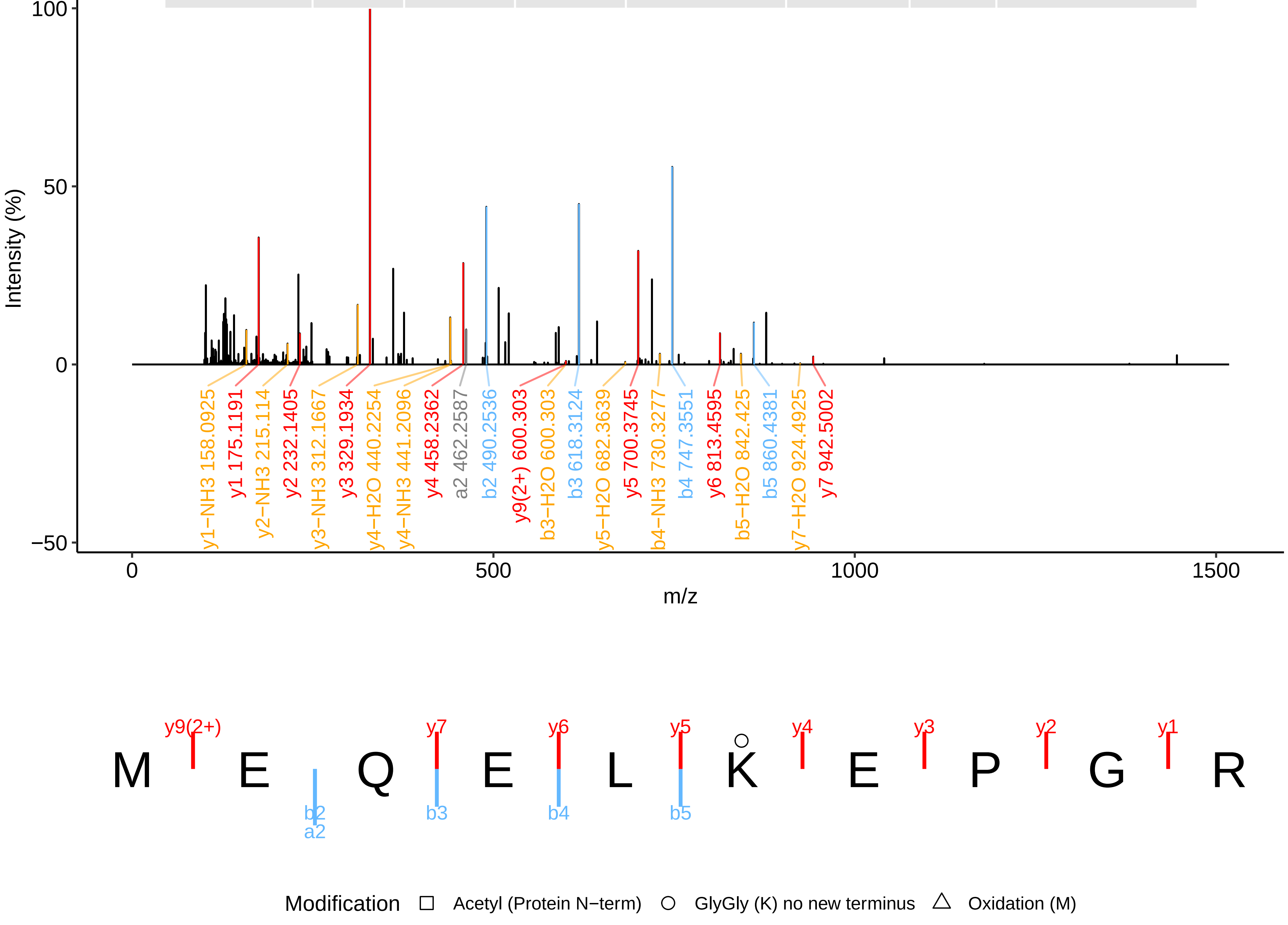

20200924_KIZ_MUb5_GlyGly_1 − Scan number: 7379

Gene Names	Charge	m/z	Mass	Retention time	PEP	Score	Precursor Intensity
DCUN1D1	3	444.2188	1329.635	23.409	1.7273e−10	147.73	42725028

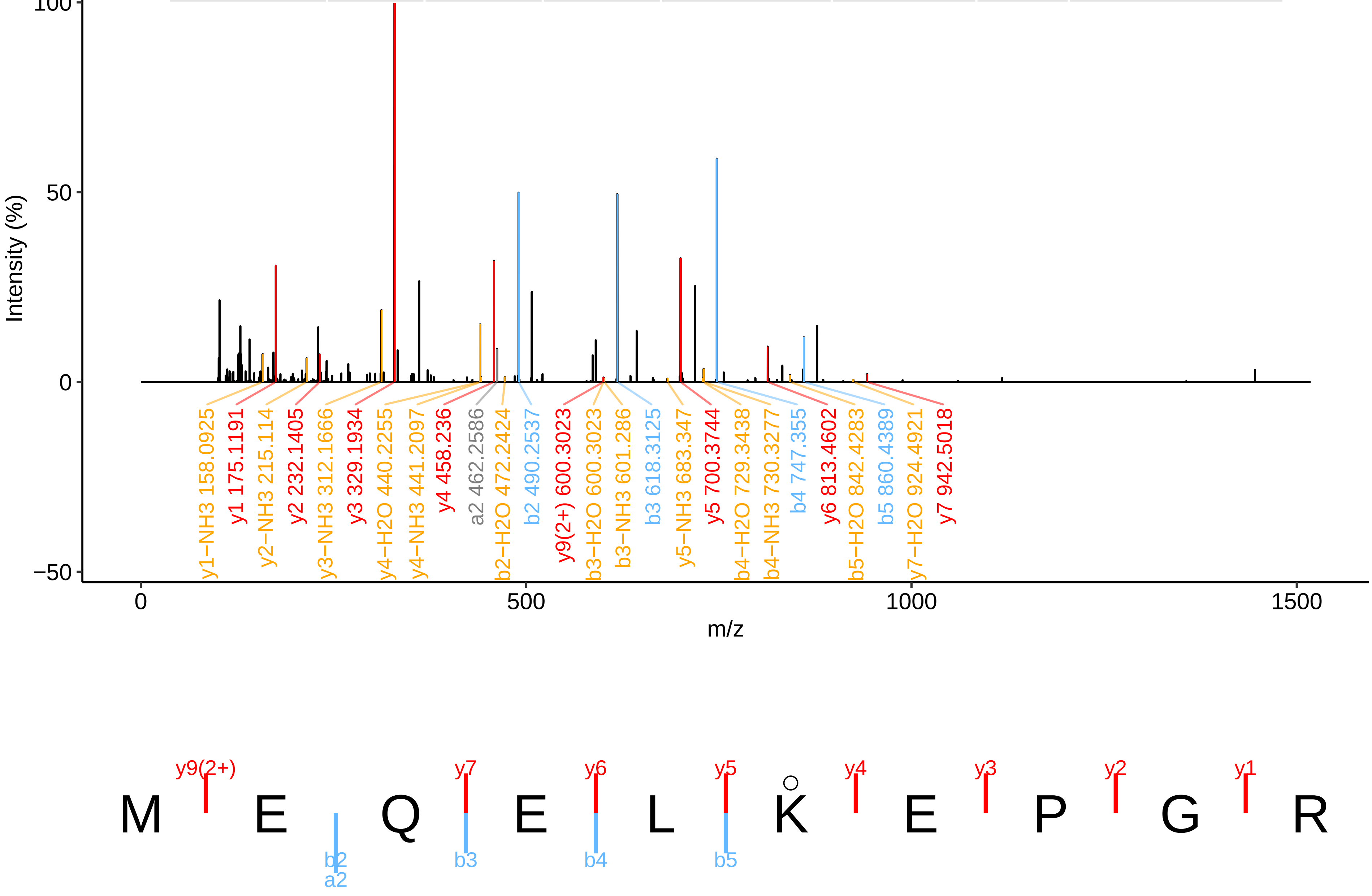

Modification □ Acetyl (Protein N−term) ○ GlyGly (K) no new terminus △ Oxidation (M)

20200924_KIZ_MUb5_GlyGly_2 − Scan number: 6989

Gene Names	Charge	m/z	Mass	Retention time	PEP	Score	Precursor Intensity
DCUN1D1	3	444.2188	1329.635	26.069	3.7017e−07	120.27	12626843

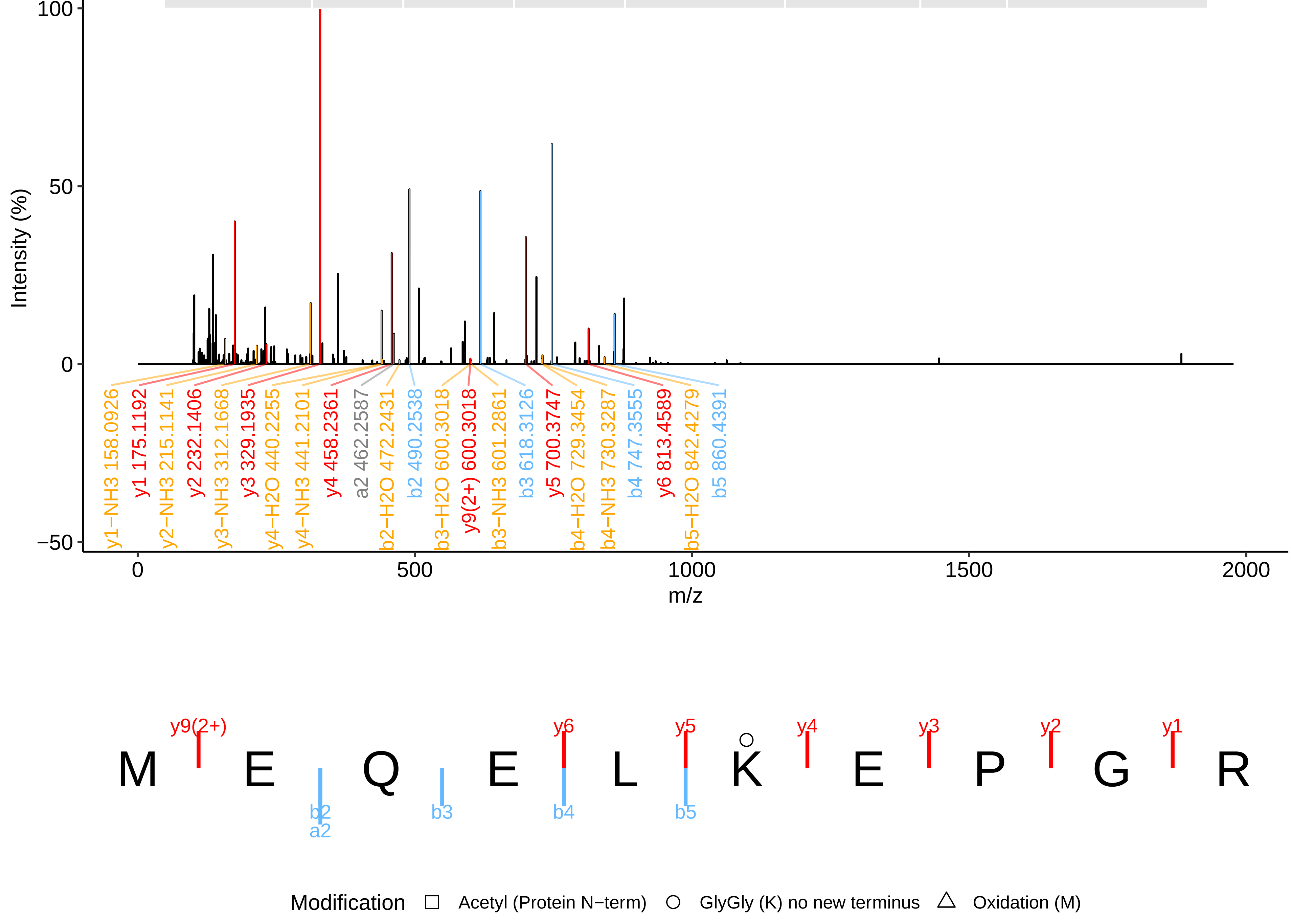

20201118_KIZ_MUb6_GlyGly_1 − Scan number: 6868

Gene Names	Charge	m/z	Mass	Retention time	PEP	Score	Precursor Intensity
DCUN1D1	3	444.2188	1329.635	21.734	3.9204e−07	119.53	9828748

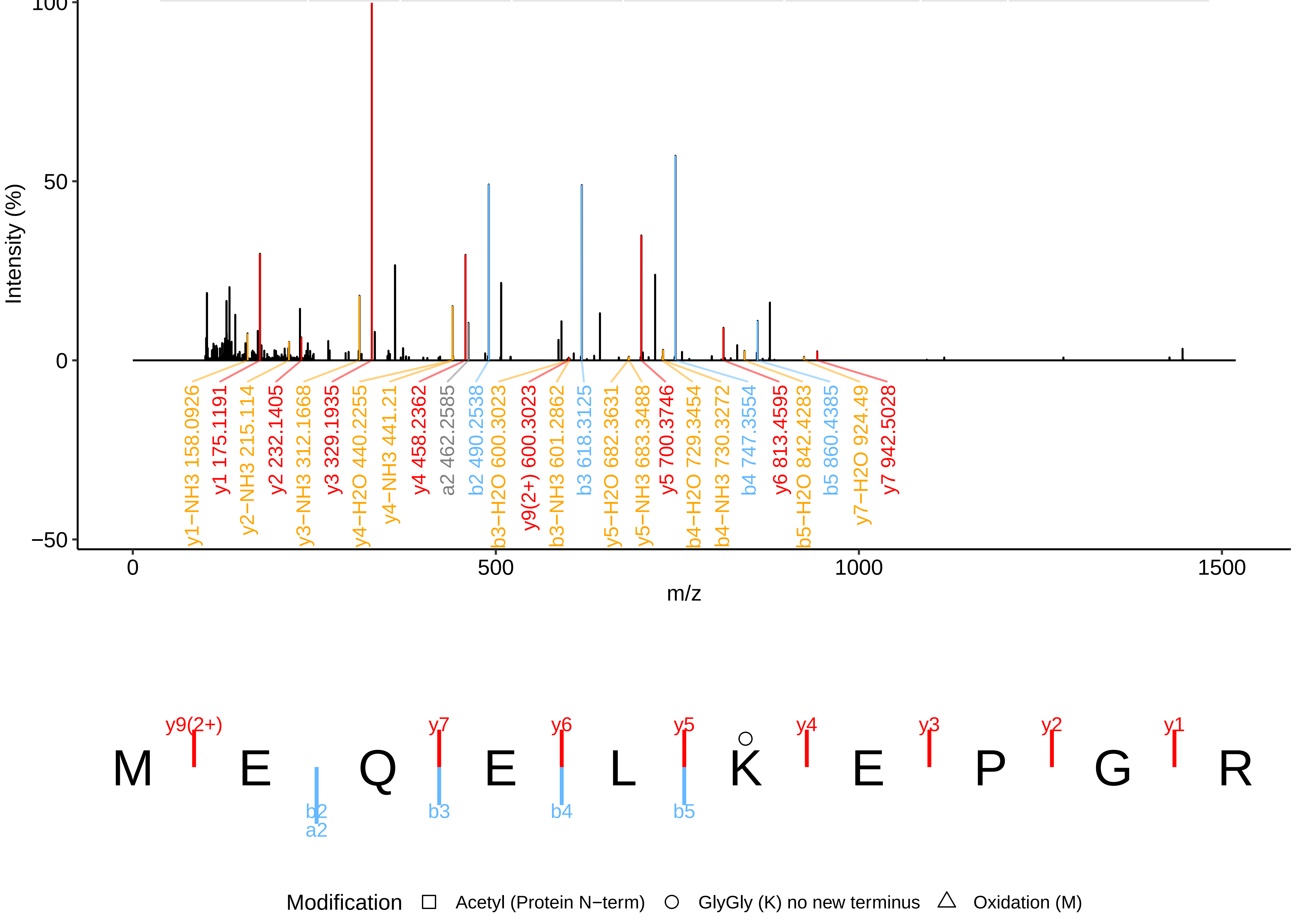

20201118_KIZ_MUb6_GlyGly_1 − Scan number: 6881

Gene Names	Charge	m/z	Mass	Retention time	PEP	Score	Precursor Intensity
DCUN1D1	3	444.2188	1329.635	21.771	4.333e−05	105.52	20648466

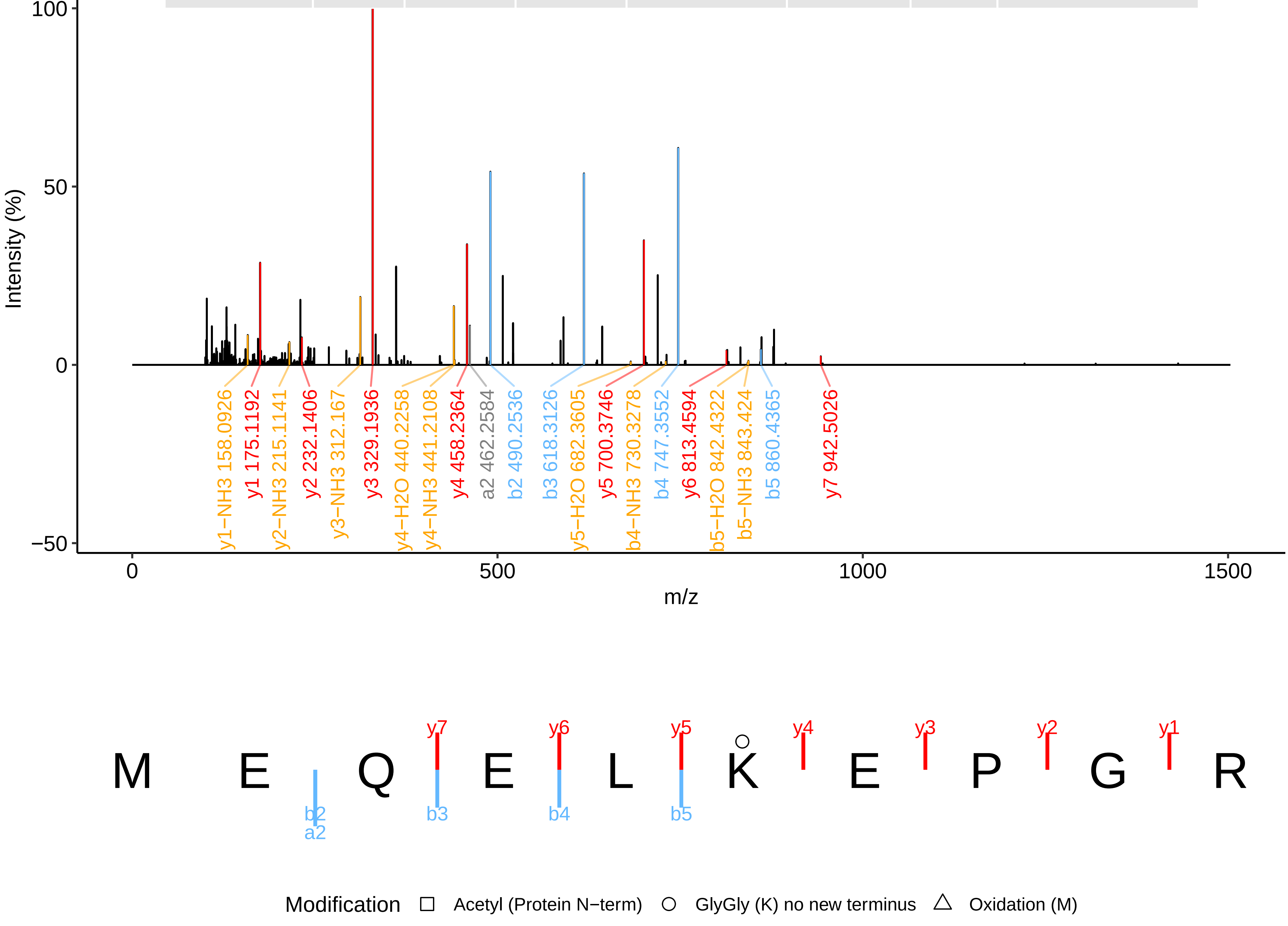

20201118_KIZ_MUb6_GlyGly_2 − Scan number: 8428

Gene Names	Charge	m/z	Mass	Retention time	PEP	Score	Precursor Intensity
DCUN1D1	3	444.2188	1329.635	28.625	6.3439e−05	100.88	9295205

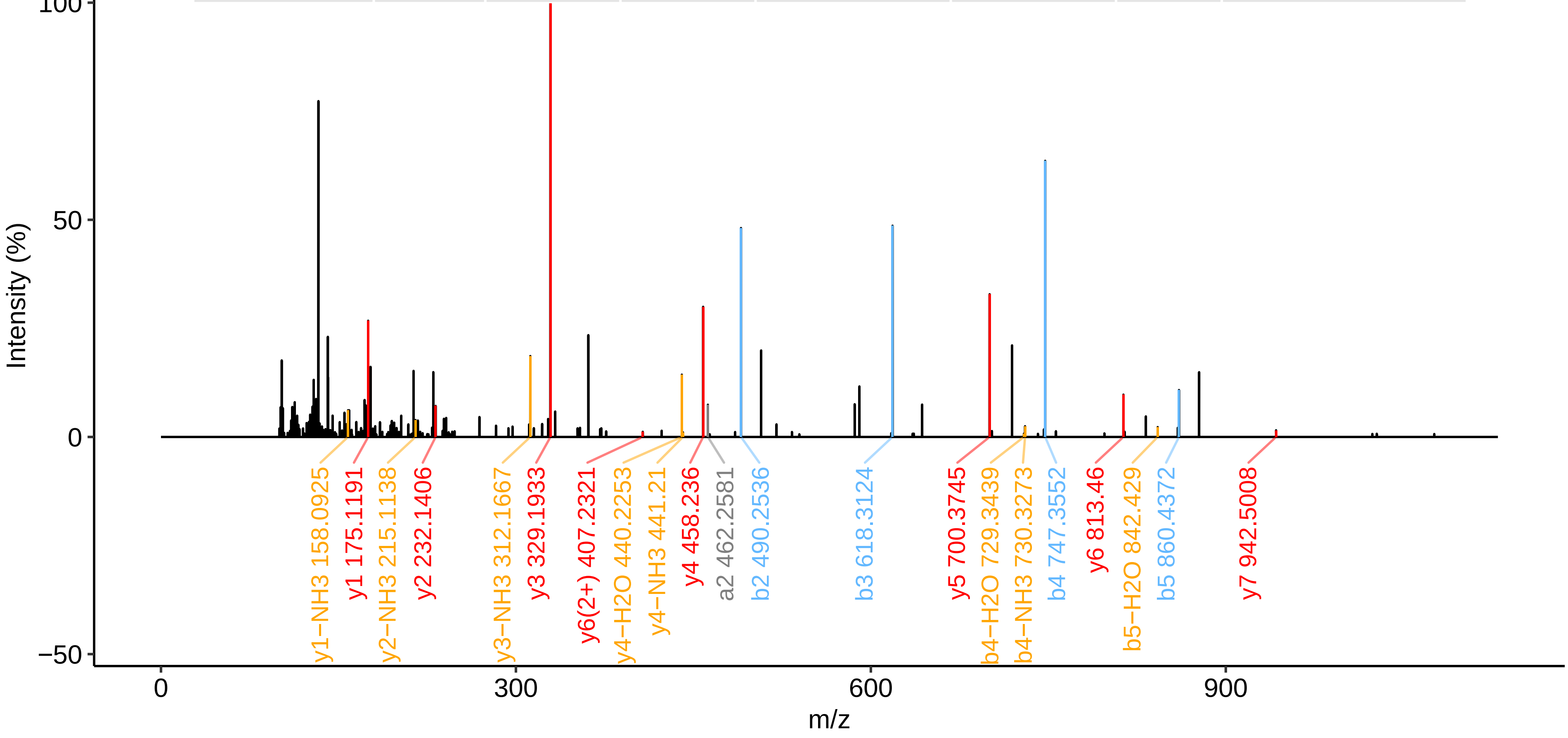

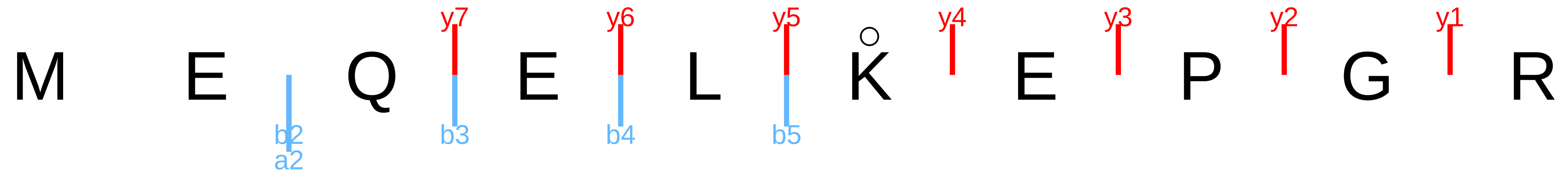

Modification □ Acetyl (Protein N−term) ○ GlyGly (K) no new terminus △ Oxidation (M)

20200921_KIZ_MUb4_GlyGly_10plex_2 − Scan number: 11427

Gene Names	Charge	m/z	Mass	Retention time	PEP	Score	Precursor Intensity
IMMT	3	378.5376	1132.591	43.984	0.084188	50.297	1952208

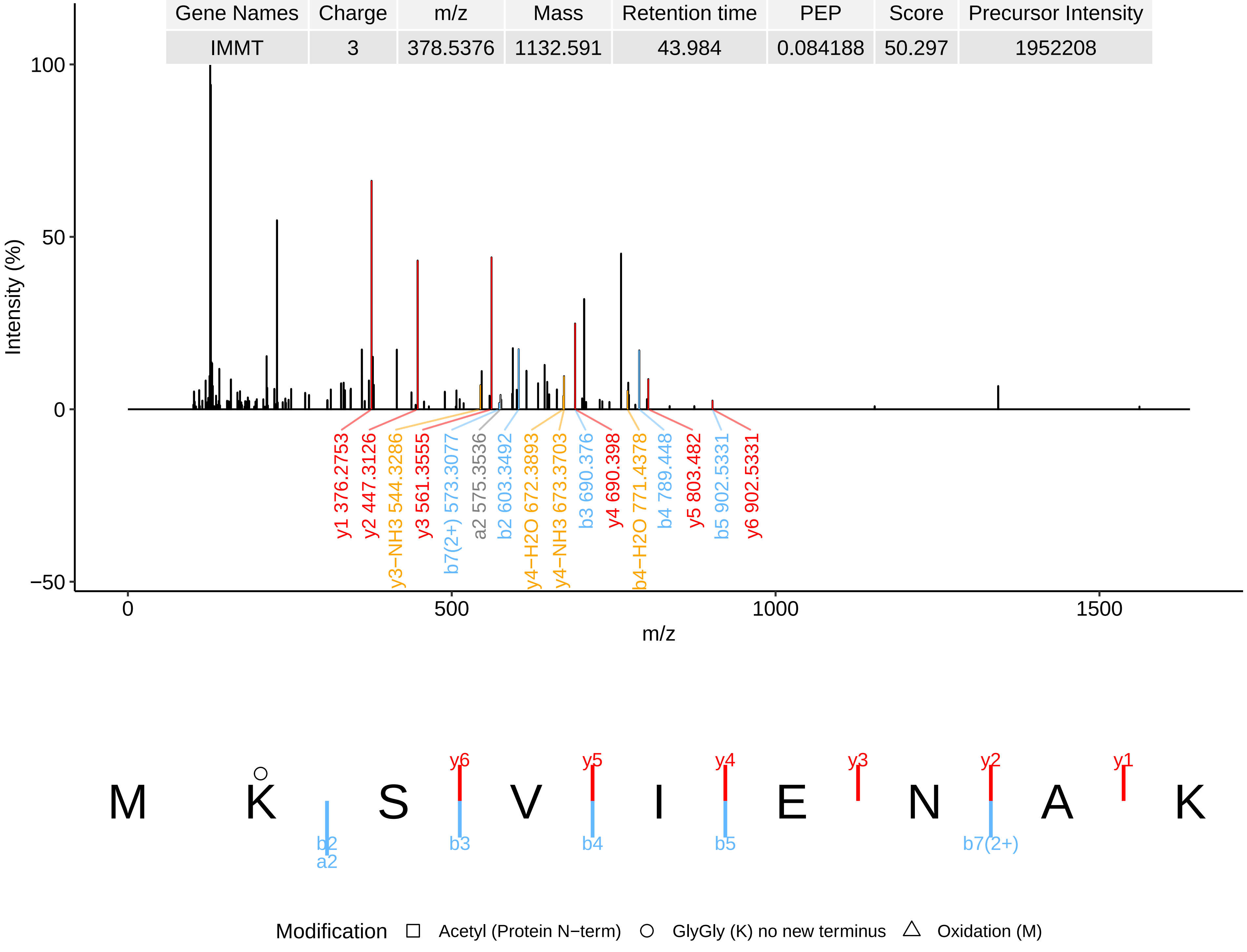

20200924_KIZ_MUb5_GlyGly_1 − Scan number: 11319

Gene Names	Charge	m/z	Mass	Retention time	PEP	Score	Precursor Intensity
IMMT	3	378.5376	1132.591	36.649	0.11185	44.425	NA

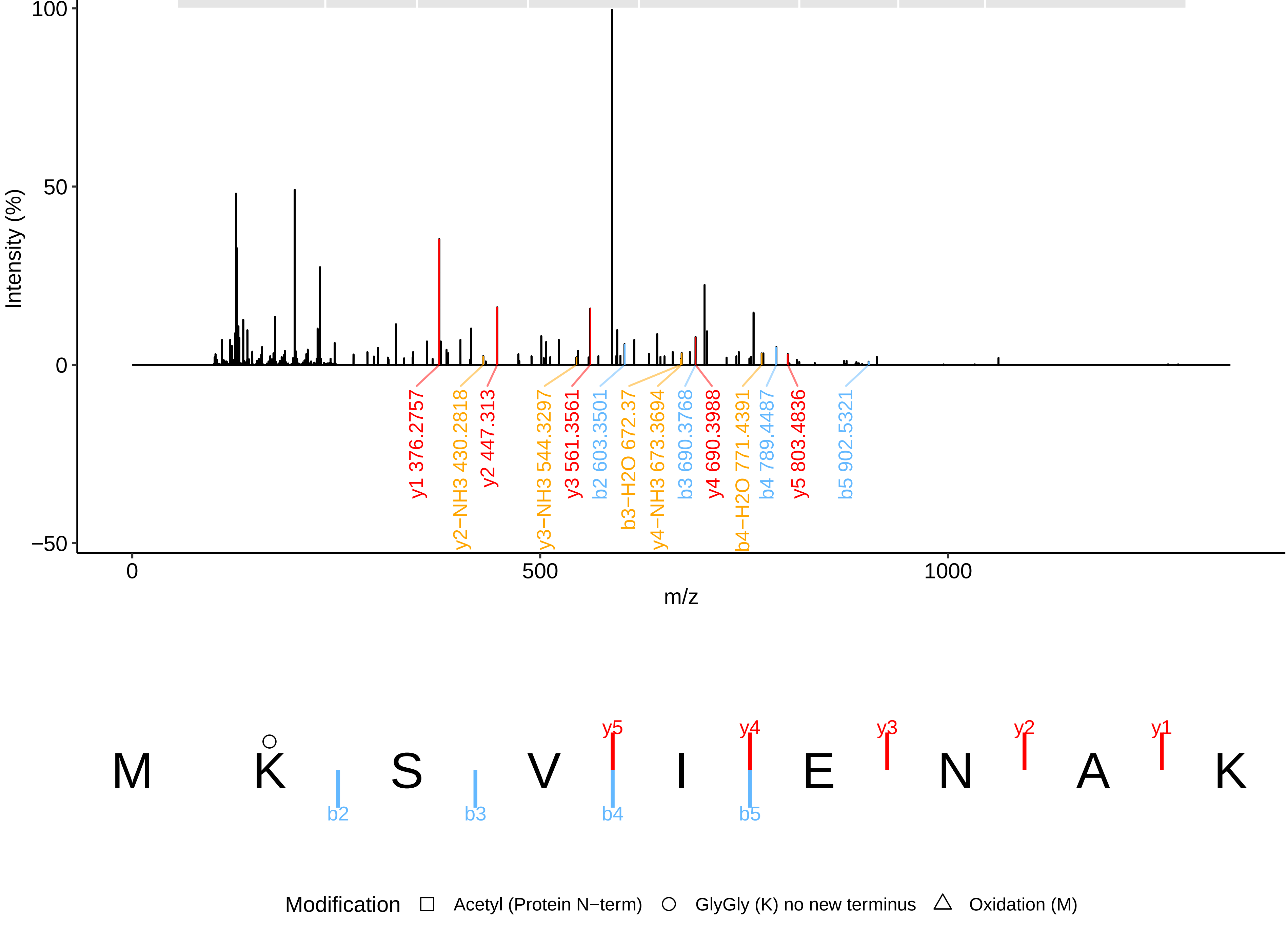

20200924_KIZ_MUb5_GlyGly_1 − Scan number: 11148

Gene Names	Charge	m/z	Mass	Retention time	PEP	Score	Precursor Intensity
IMMT	3	378.5376	1132.591	36.115	0.12569	49.189	16879388

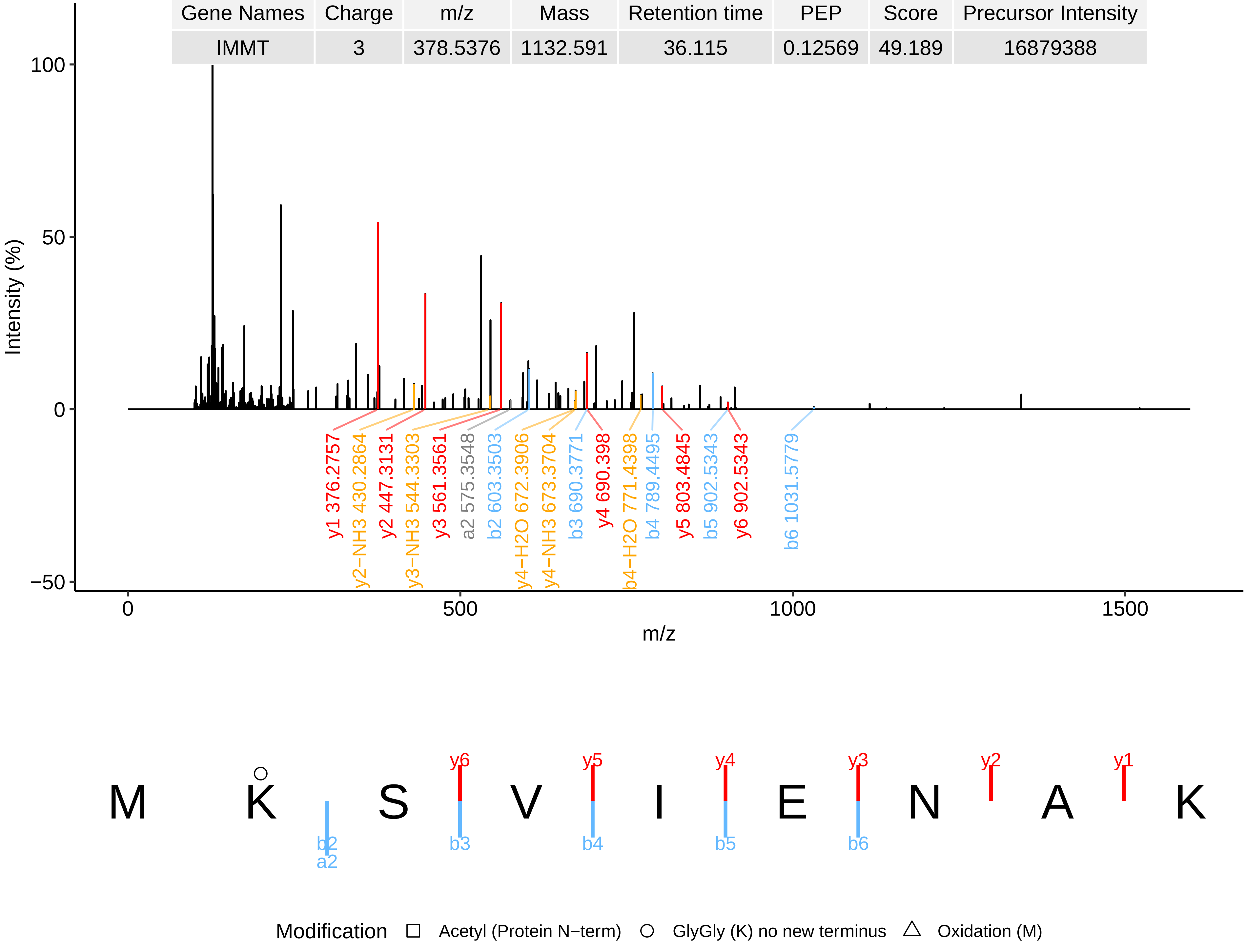

20200924_KIZ_MUb5_GlyGly_2 − Scan number: 10520

Gene Names	Charge	m/z	Mass	Retention time	PEP	Score	Precursor Intensity
IMMT	3	378.5376	1132.591	39.263	0.038865	61.353	NA

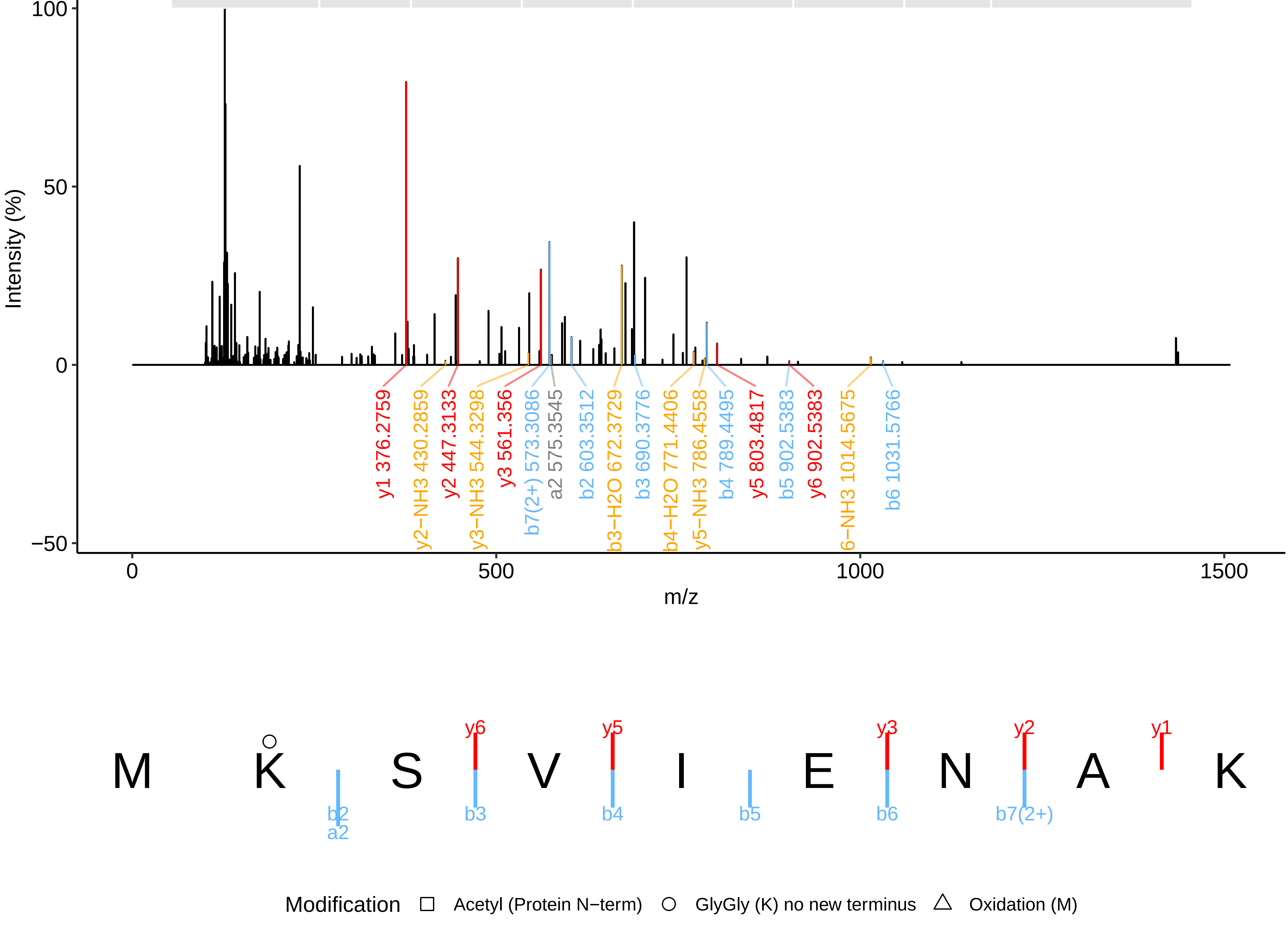

20200921_KIZ_MUb4_GlyGly_10plex − Scan number: 15547

Gene Names	Charge	m/z	Mass	Retention time	PEP	Score	Precursor Intensity
UBXN1	3	443.9151	1328.724	55.563	1.6358e−07	127.3	4861199

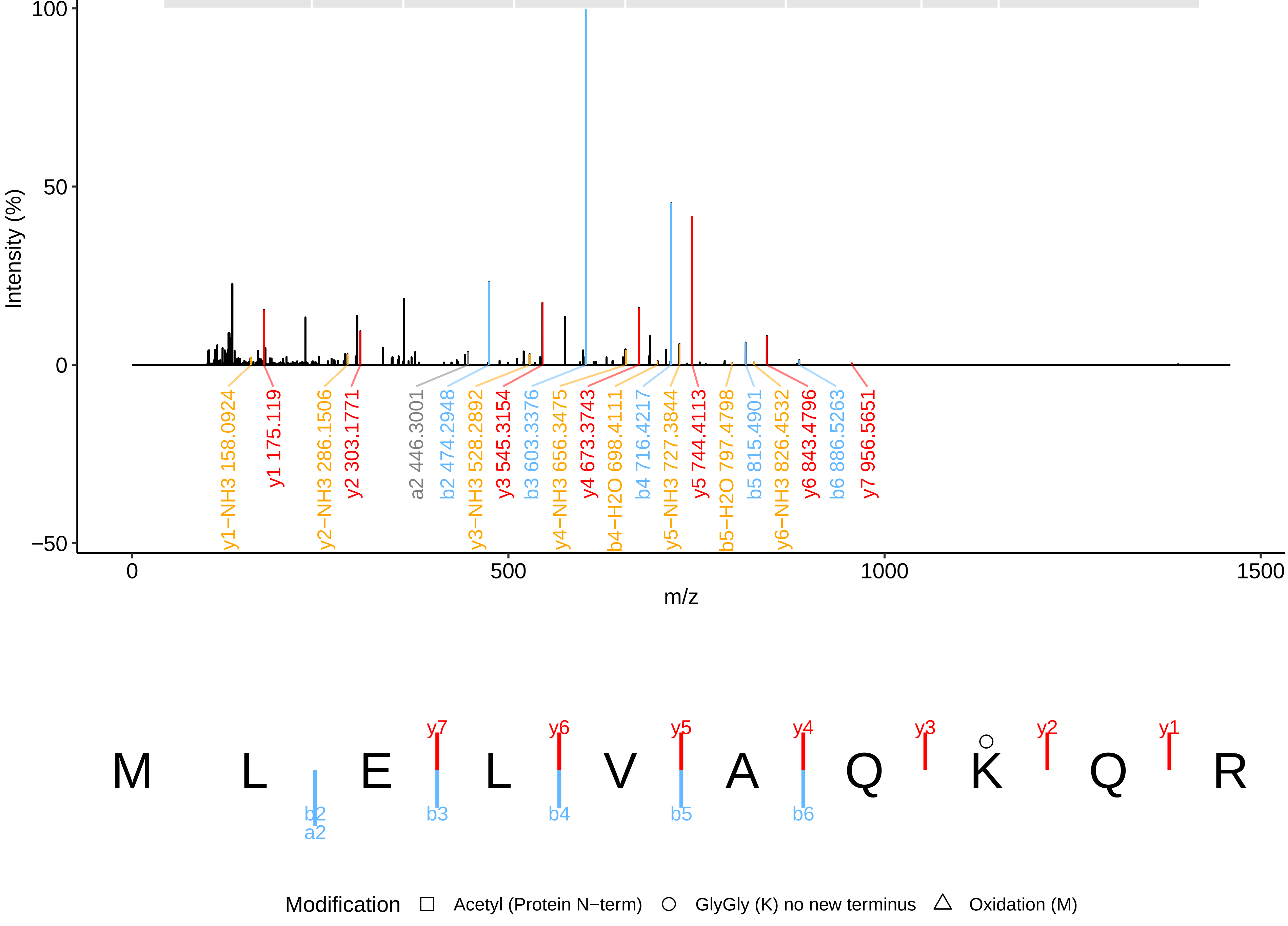

20200921_KIZ_MUb4_GlyGly_10plex_2 − Scan number: 14435

Gene Names	Charge	m/z	Mass	Retention time	PEP	Score	Precursor Intensity
UBXN1	3	443.9151	1328.724	56.285	1.1217e−09	133.57	1435123

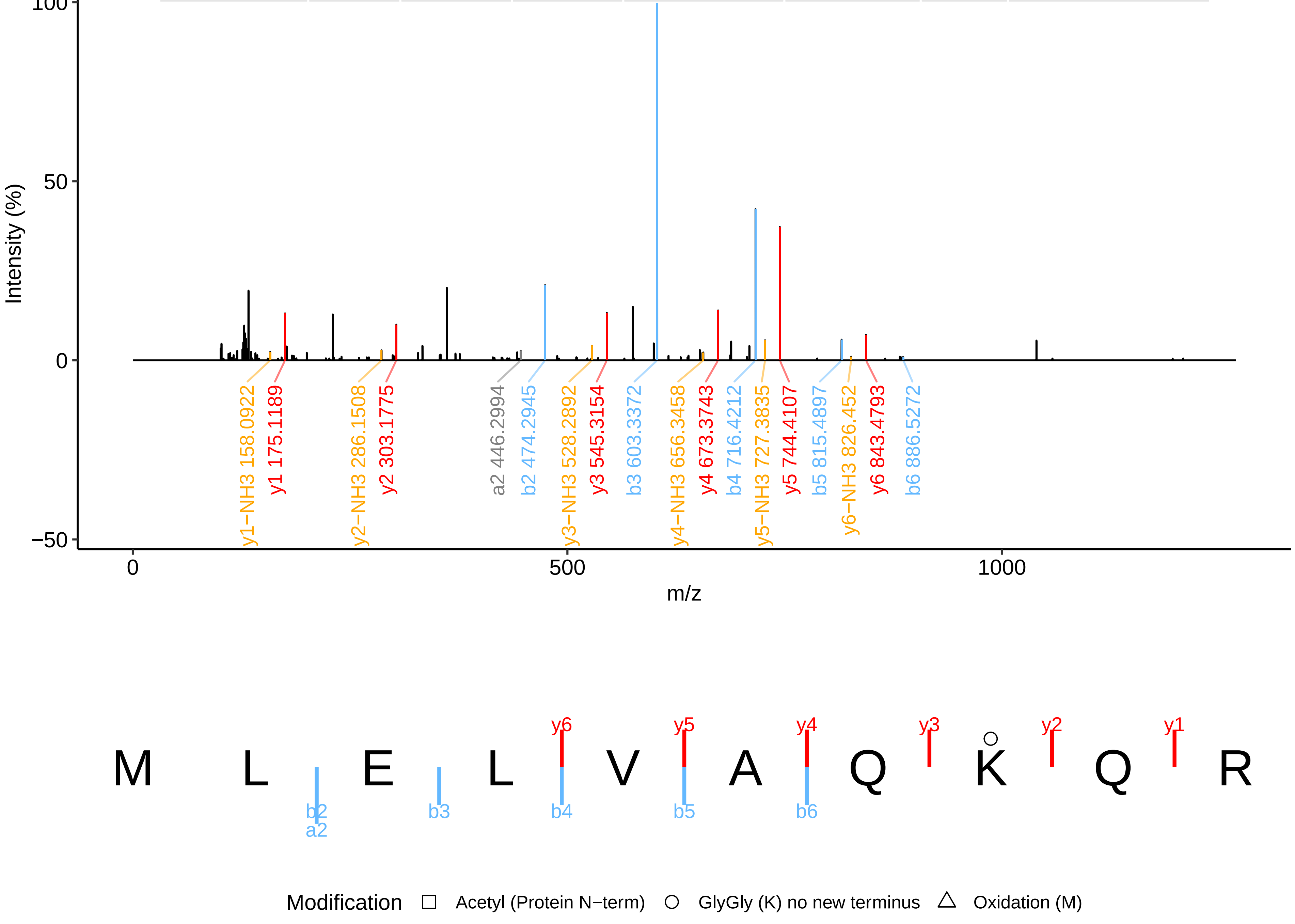

20200924_KIZ_MUb5_GlyGly_1 − Scan number: 14384

Gene Names	Charge	m/z	Mass	Retention time	PEP	Score	Precursor Intensity
UBXN1	3	443.9151	1328.724	46.968	0.00031744	122.28	15786516

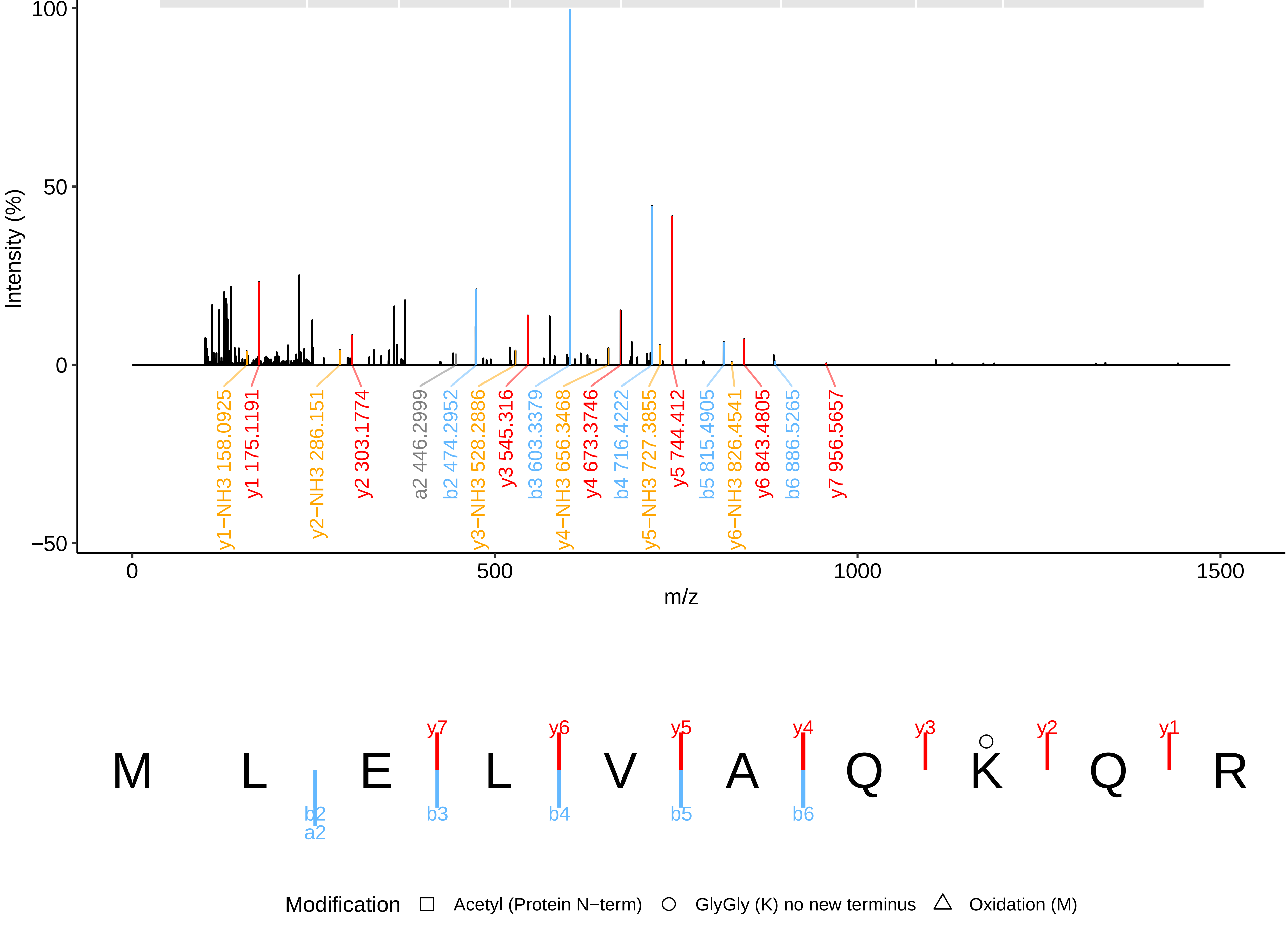

20200921_KIZ_MUb4_GlyGly_10plex − Scan number: 13709

Gene Names	Charge	m/z	Mass	Retention time	PEP	Score	Precursor Intensity
USP24	3	456.8958	1367.666	48.456	0.0031773	80.165	8513100

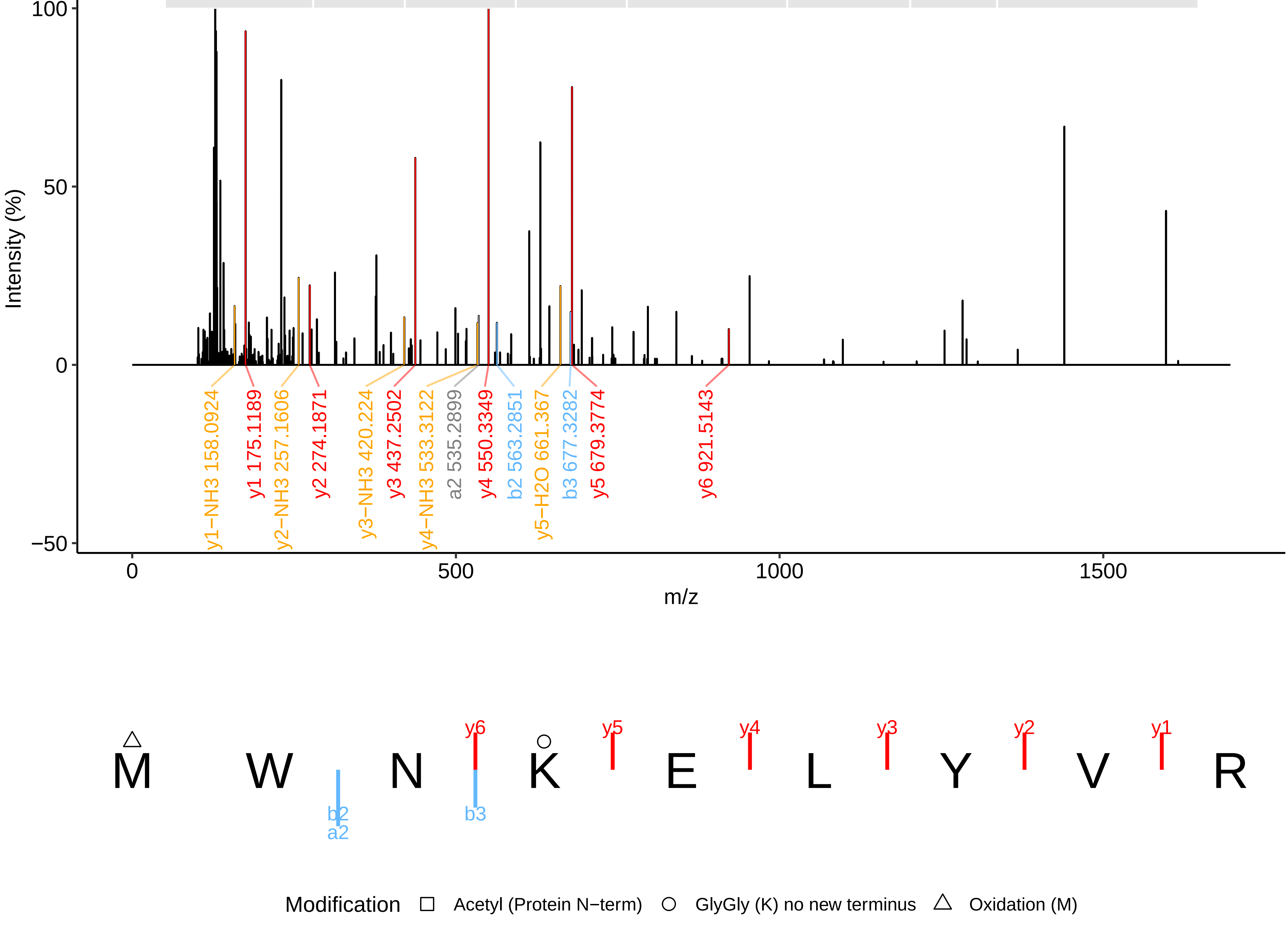

20200921_KIZ_MUb4_GlyGly_10plex_2 − Scan number: 12635

Gene Names	Charge	m/z	Mass	Retention time	PEP	Score	Precursor Intensity
USP24	3	456.8958	1367.666	48.925	0.011986	69.812	655309.9

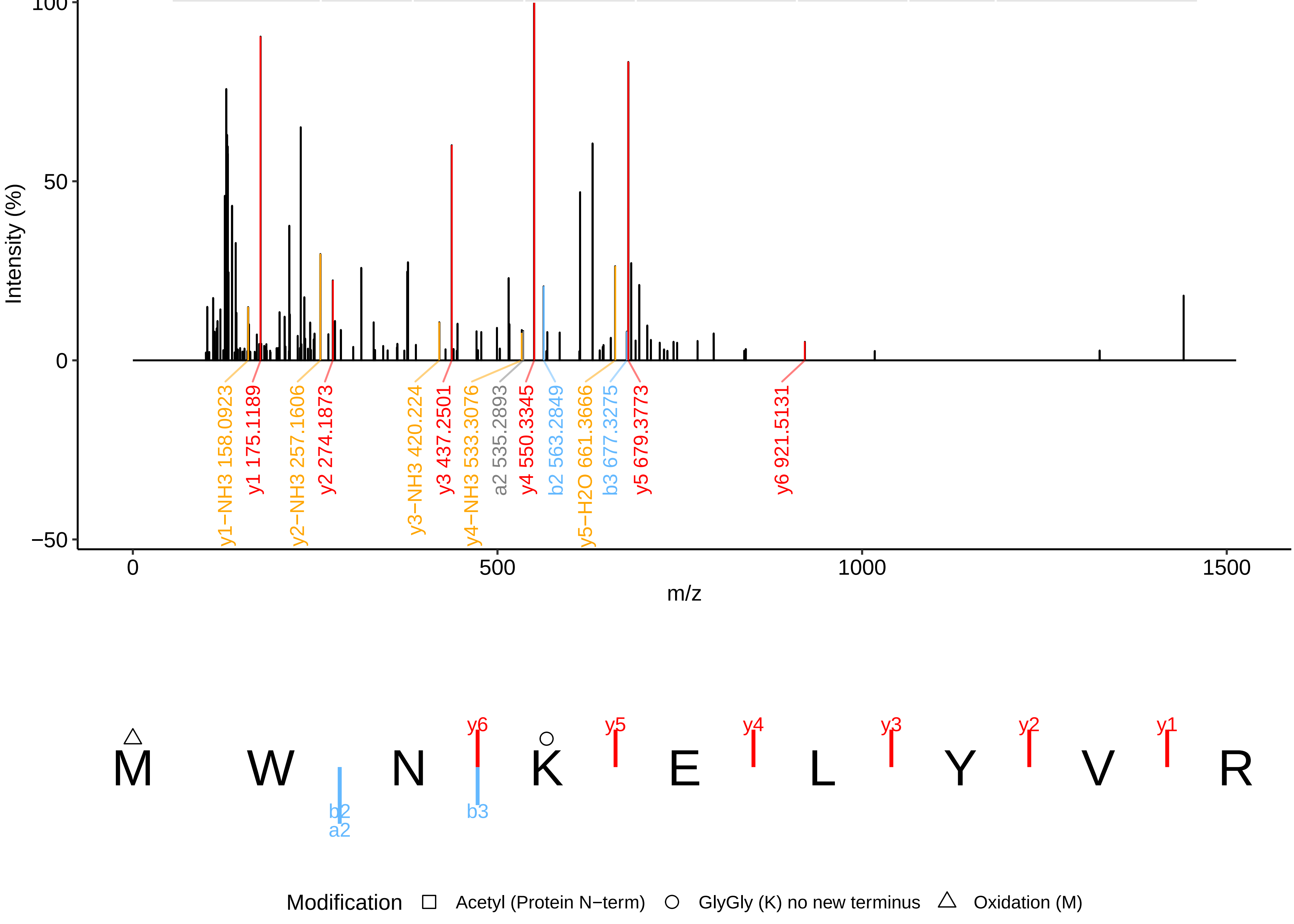

20200921_KIZ_MUb4_GlyGly_10plex − Scan number: 16118

Gene Names	Charge	m/z	Mass	Retention time	PEP	Score	Precursor Intensity
USP24	3	451.5642	1351.671	57.814	0.00099529	95.502	10669585

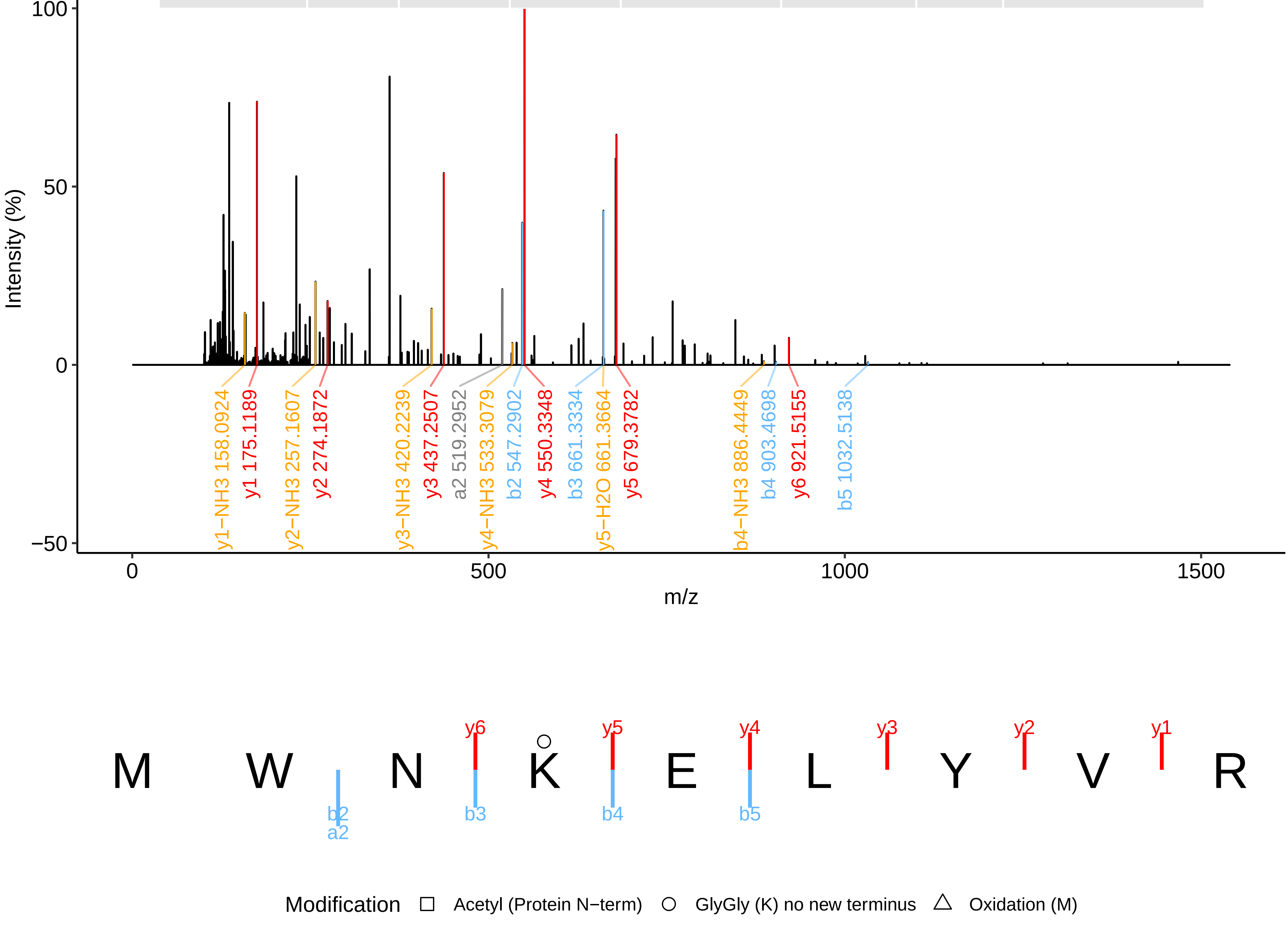

20200921_KIZ_MUb4_GlyGly_10plex_2 − Scan number: 15015

Gene Names	Charge	m/z	Mass	Retention time	PEP	Score	Precursor Intensity
USP24	3	451.5642	1351.671	58.634	0.00039514	103.56	1806877

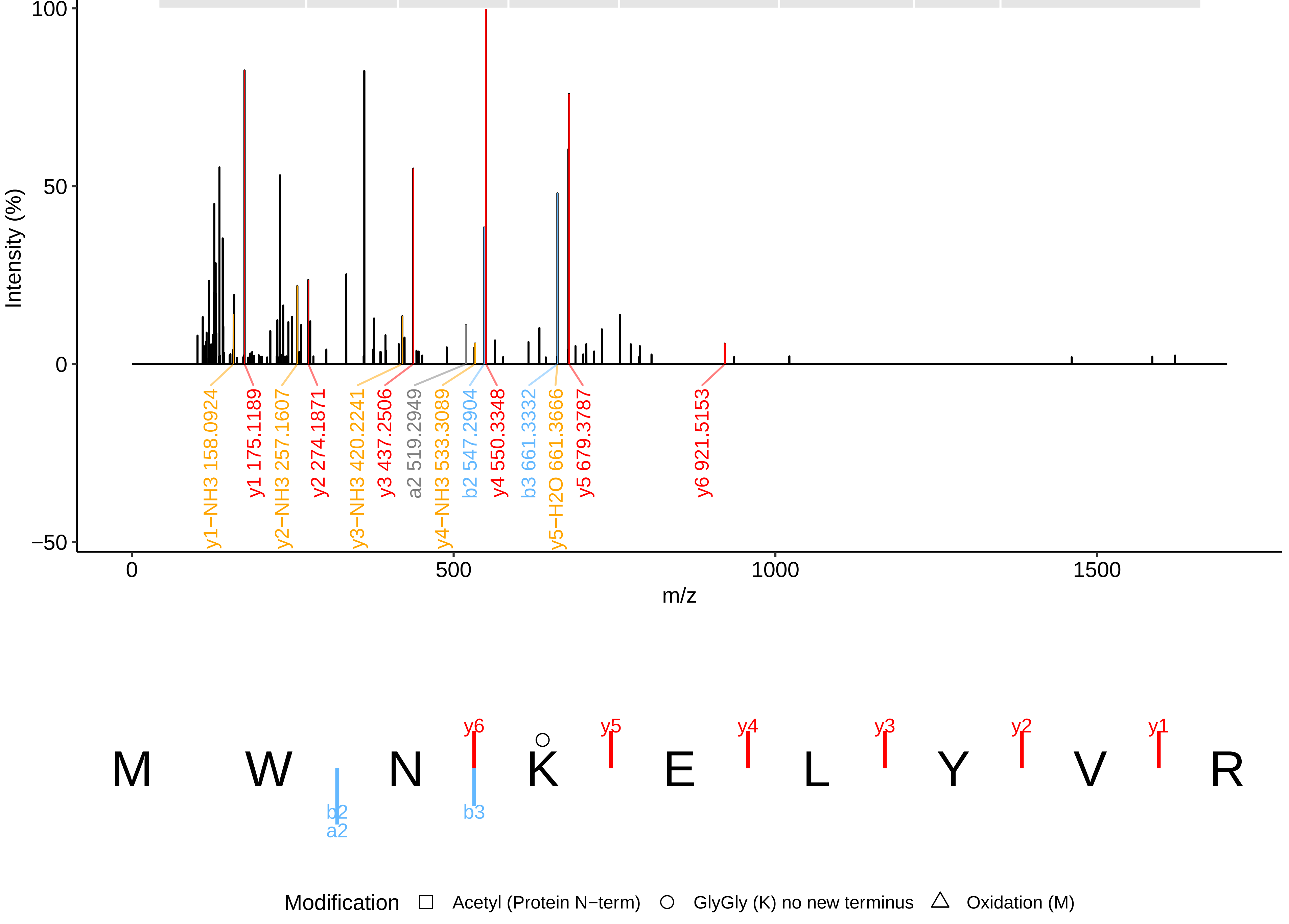

20200924_KIZ_MUb5_GlyGly_1 − Scan number: 14939

Gene Names	Charge	m/z	Mass	Retention time	PEP	Score	Precursor Intensity
USP24	3	451.5642	1351.671	48.802	0.0087877	78.516	7779953

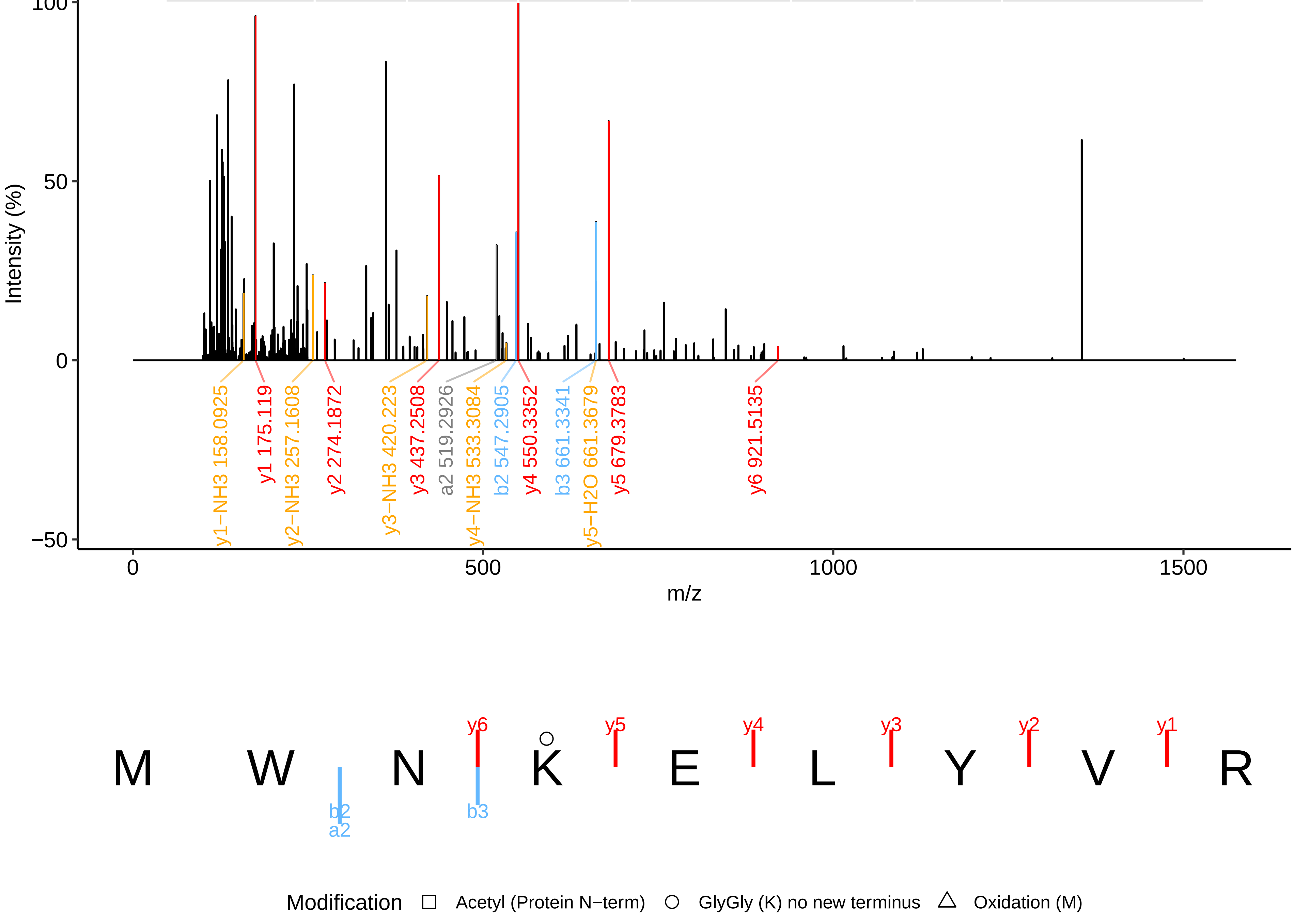

20200921_KIZ_MUb4_GlyGly_10plex − Scan number: 7976

Gene Names	Charge	m/z	Mass	Retention time	PEP	Score	Precursor Intensity
SLC25A3	3	376.5182	1126.533	26.94	0.049652	55.531	NA

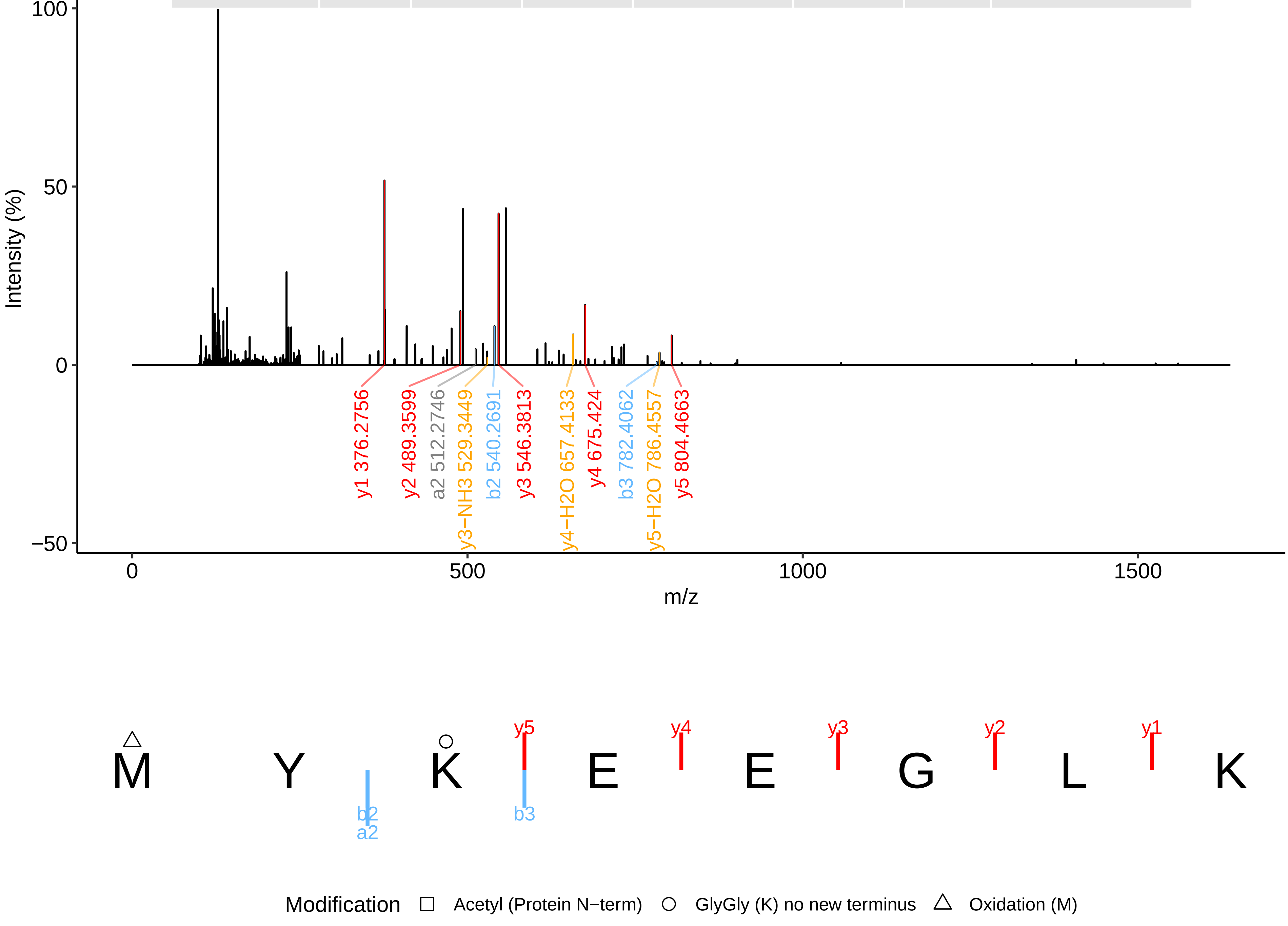

20201118_KIZ_MUb6_GlyGly_2 − Scan number: 11234

Gene Names	Charge	m/z	Mass	Retention time	PEP	Score	Precursor Intensity
SLC25A3	3	371.1866	1110.538	37.898	0.03336	62.546	4327660

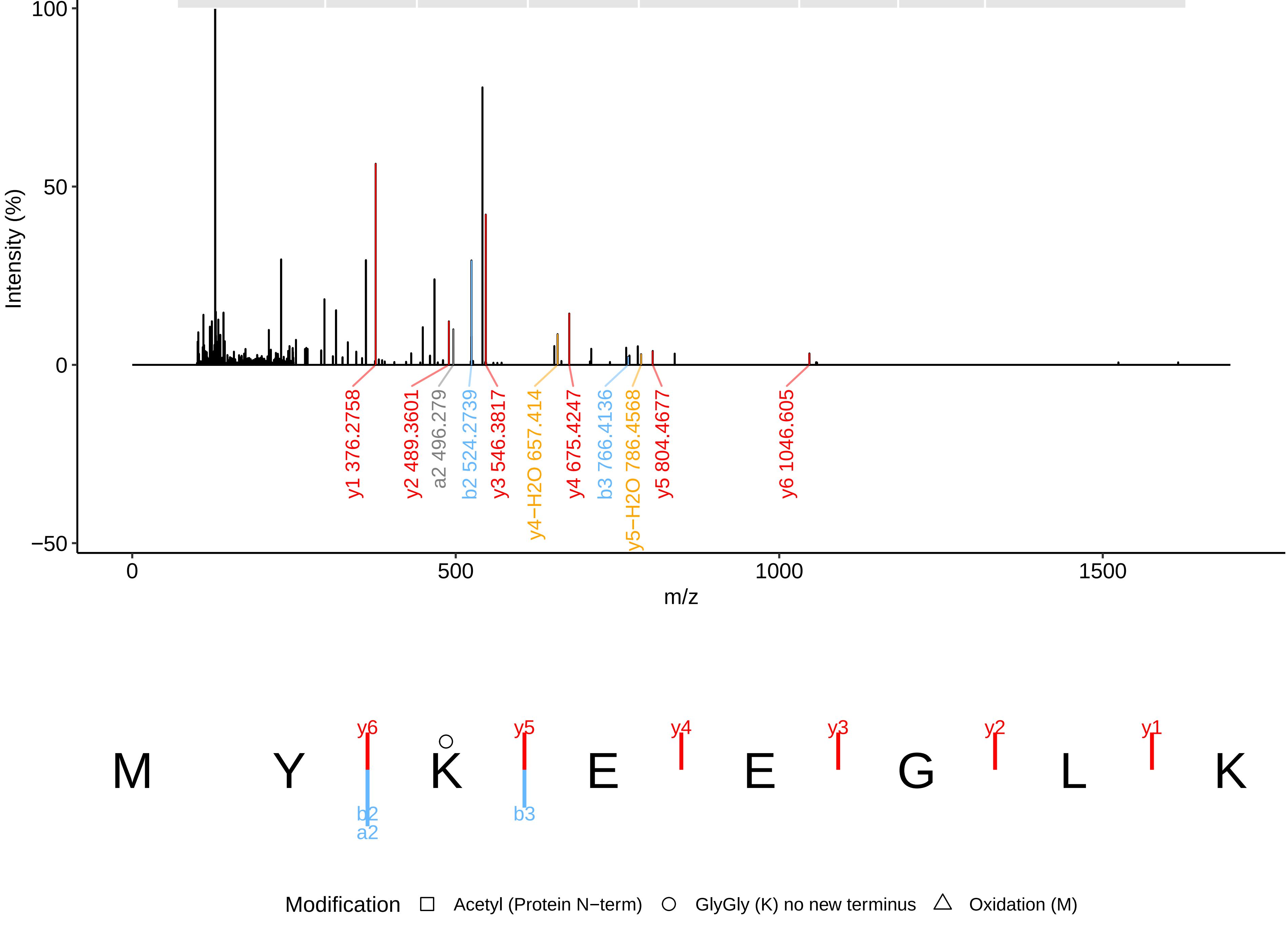

20200921_KIZ_MUb4_GlyGly_10plex_2 − Scan number: 8402

Gene Names	Charge	m/z	Mass	Retention time	PEP	Score	Precursor Intensity
EIF3B	3	379.856	1136.546	31.642	0.11669	43.308	526885.7

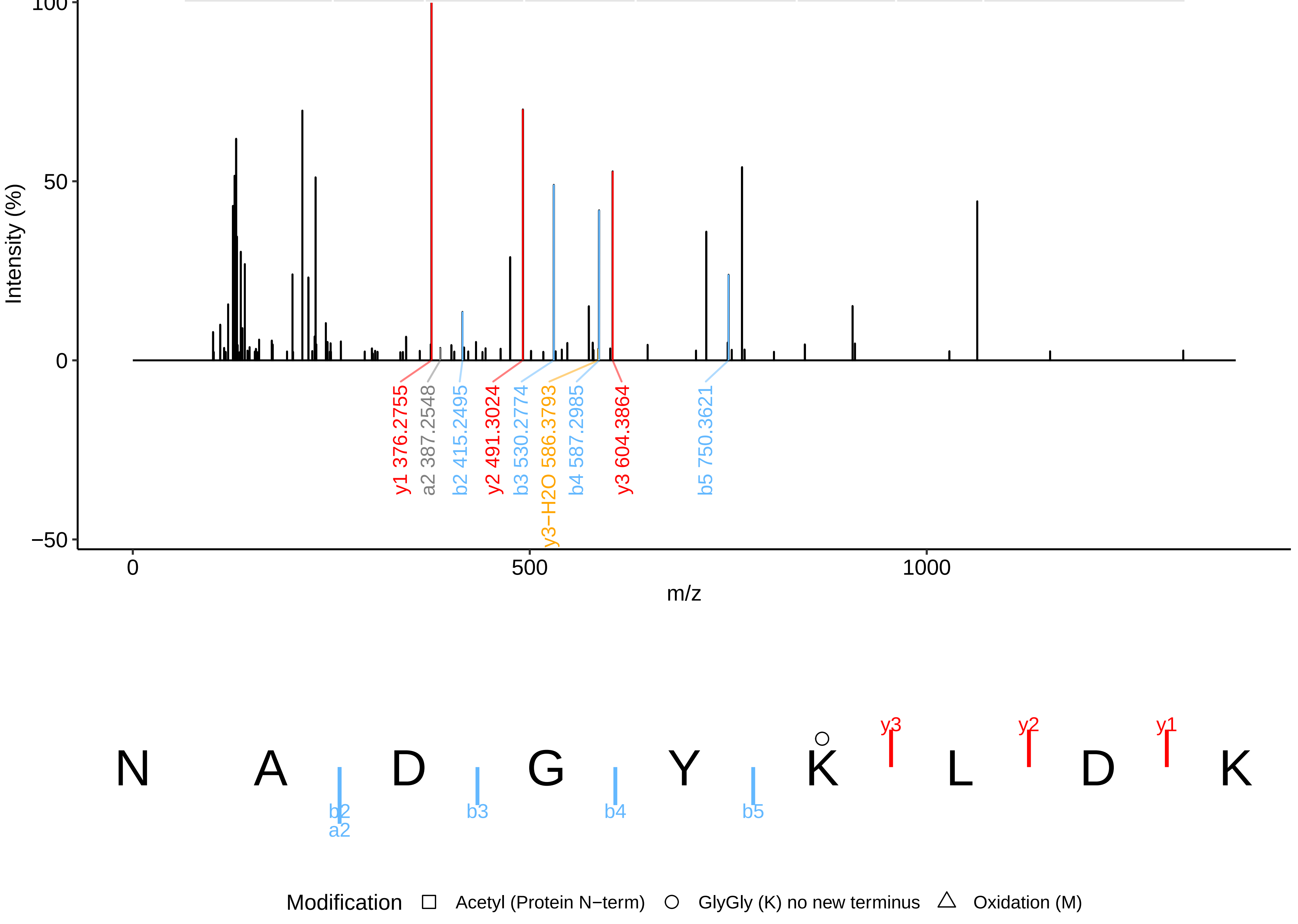

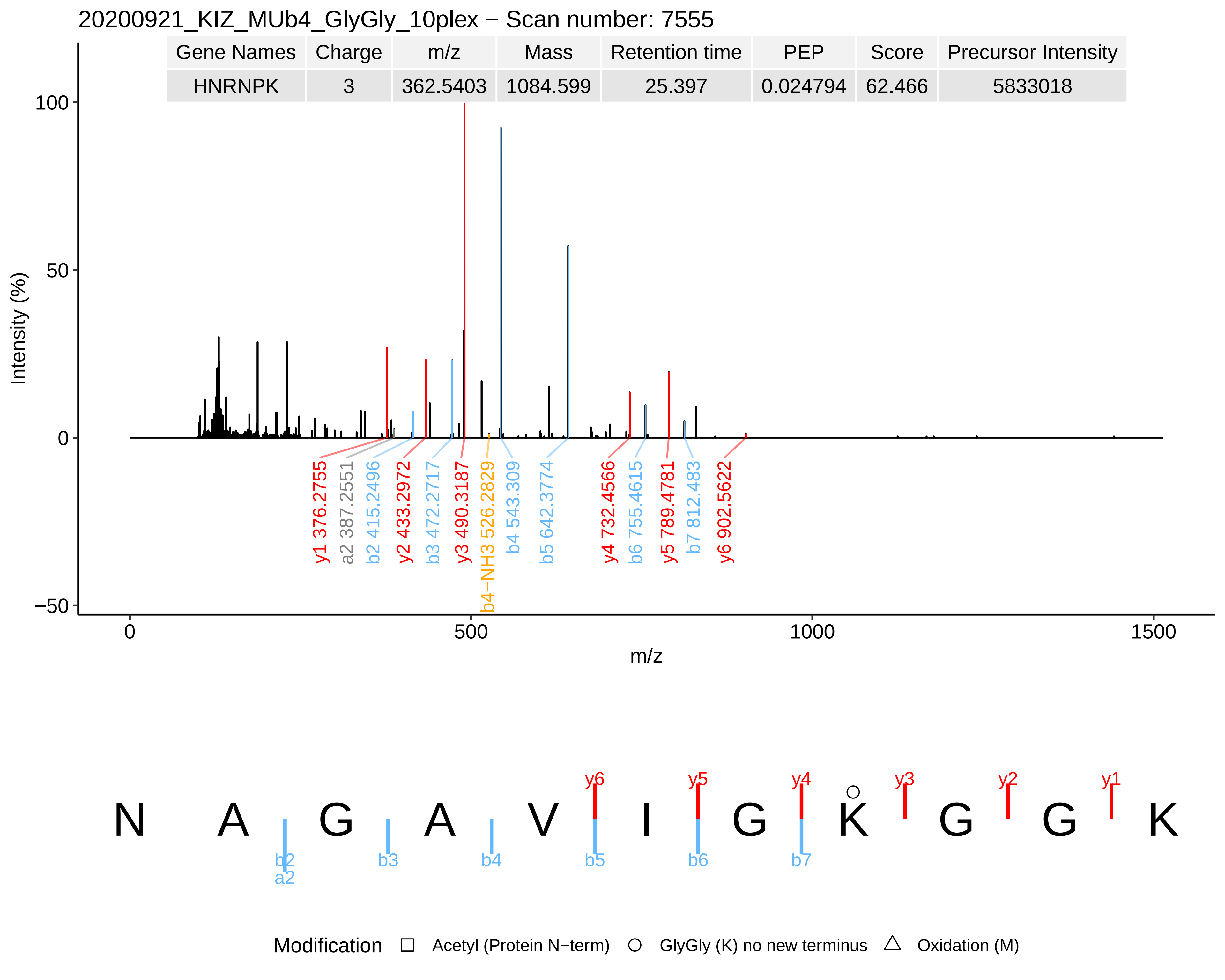
20200921_KIZ_MUb4_GlyGly_10plex − Scan number: 7555
Gene Names | Charge | m/z | Mass | Retention time | PEP | Score | Precursor Intensity
HNRNPK | 3 | 362.5403 | 1084.599 | 25.397 | 0.024794 | 62.466 | 5833018
Intensity (%)
m/z
y1 376.2755
a2 387.2551
b2 415.2496
y2 433.2972
b3 472.2717
y3 490.3187
b4−NH3 526.2829
b4 543.309
b5 642.3774
y4 732.4566
b6 755.4615
y5 789.4781
b7 812.483
y6 902.5622
N A G A V I G K G G K
y6 y5 y4 y3 y2 y1
b2 a2 b3 b4 b5 b6 b7
Modification
Acetyl (Protein N−term)
GlyGly (K) no new terminus
Oxidation (M)

20201118_KIZ_MUb6_GlyGly_1 − Scan number: 19085

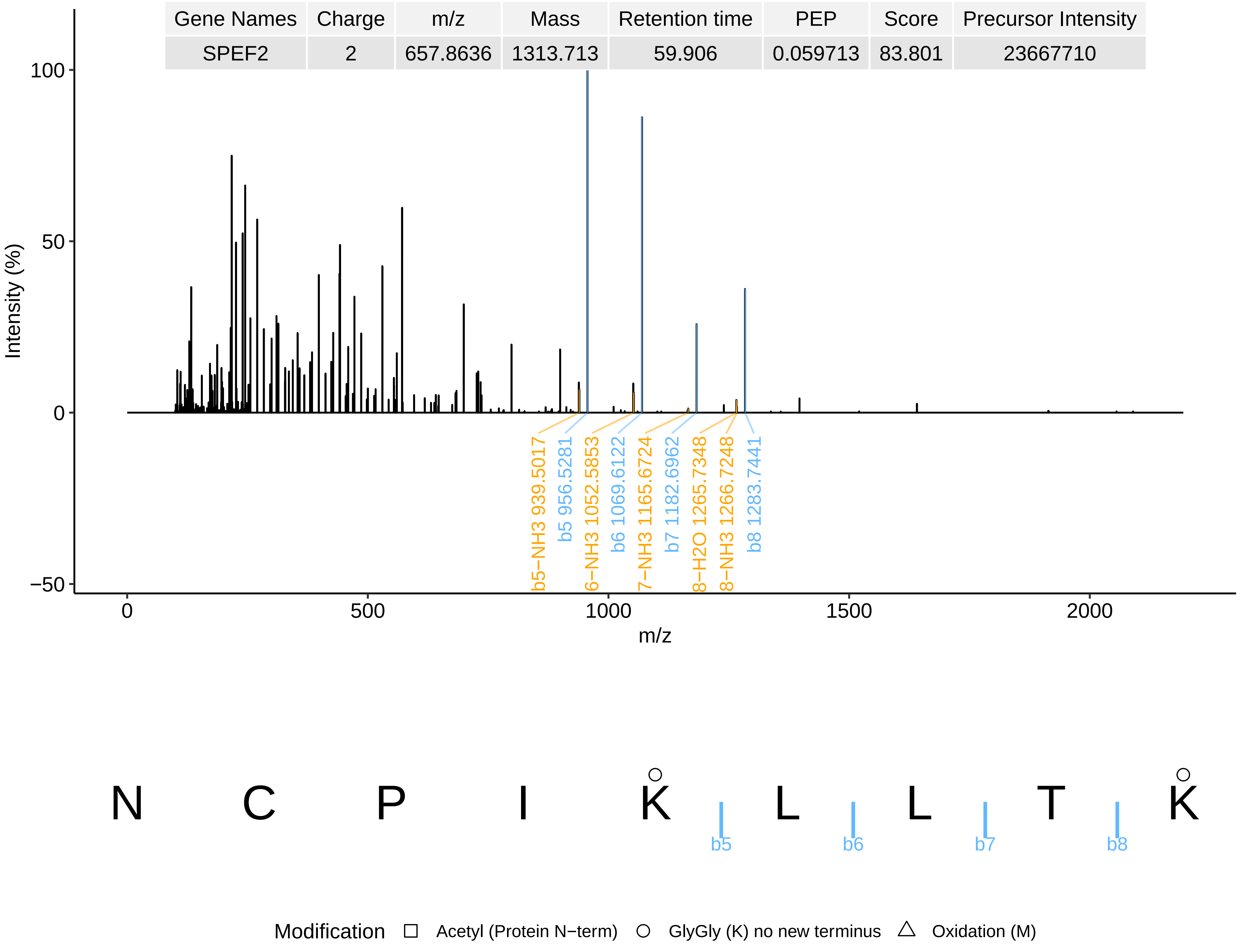

20200921_KIZ_MUb4_GlyGly_10plex − Scan number: 19334

Gene Names	Charge	m/z	Mass	Retention time	PEP	Score	Precursor Intensity
CD44	2	672.8506	1343.687	69.689	0.0042087	86.011	2367077

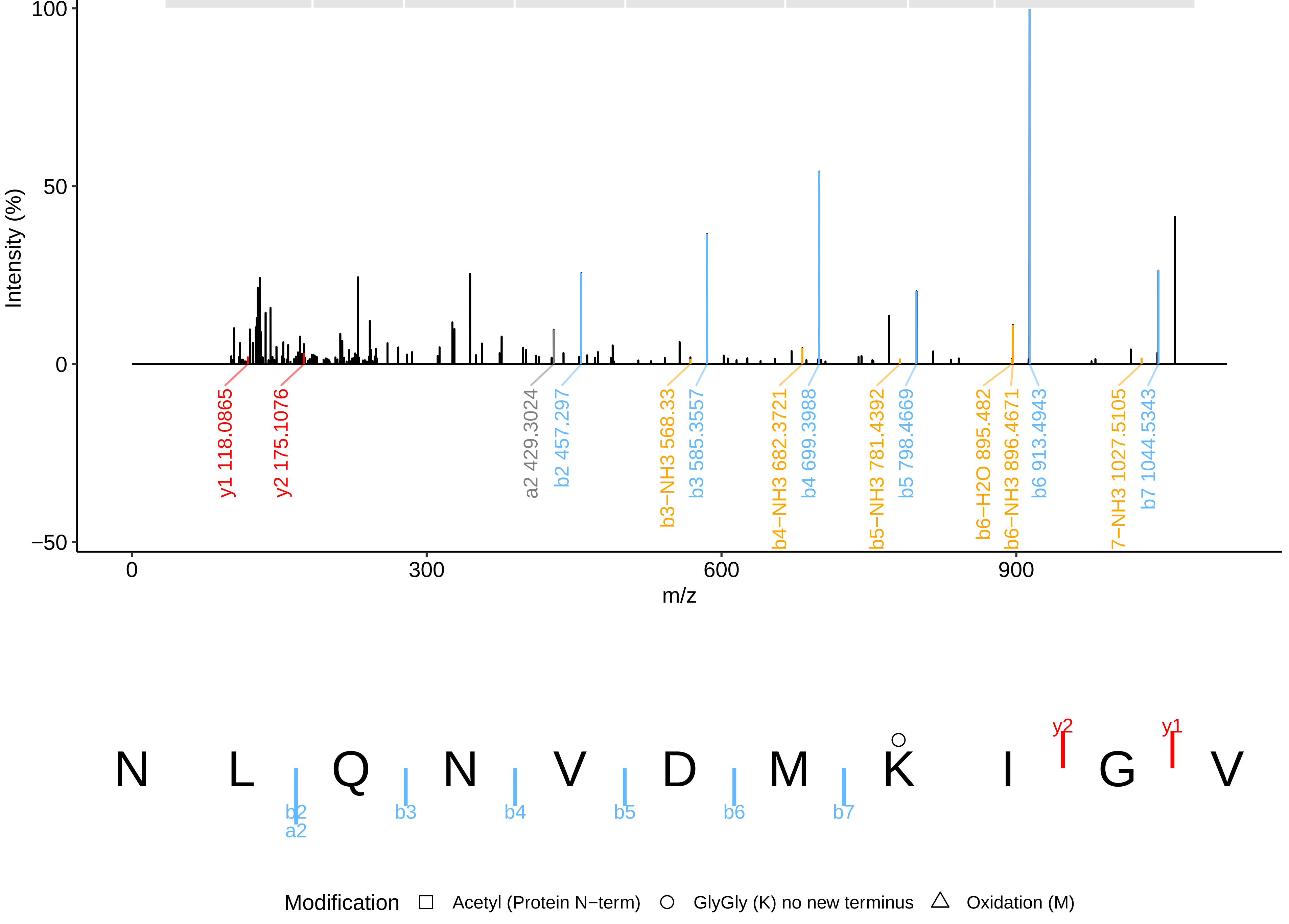

20200921_KIZ_MUb4_GlyGly_10plex_2 − Scan number: 17835

Gene Names	Charge	m/z	Mass	Retention time	PEP	Score	Precursor Intensity
CD44	2	672.8506	1343.687	69.78	0.029189	72.547	2144676

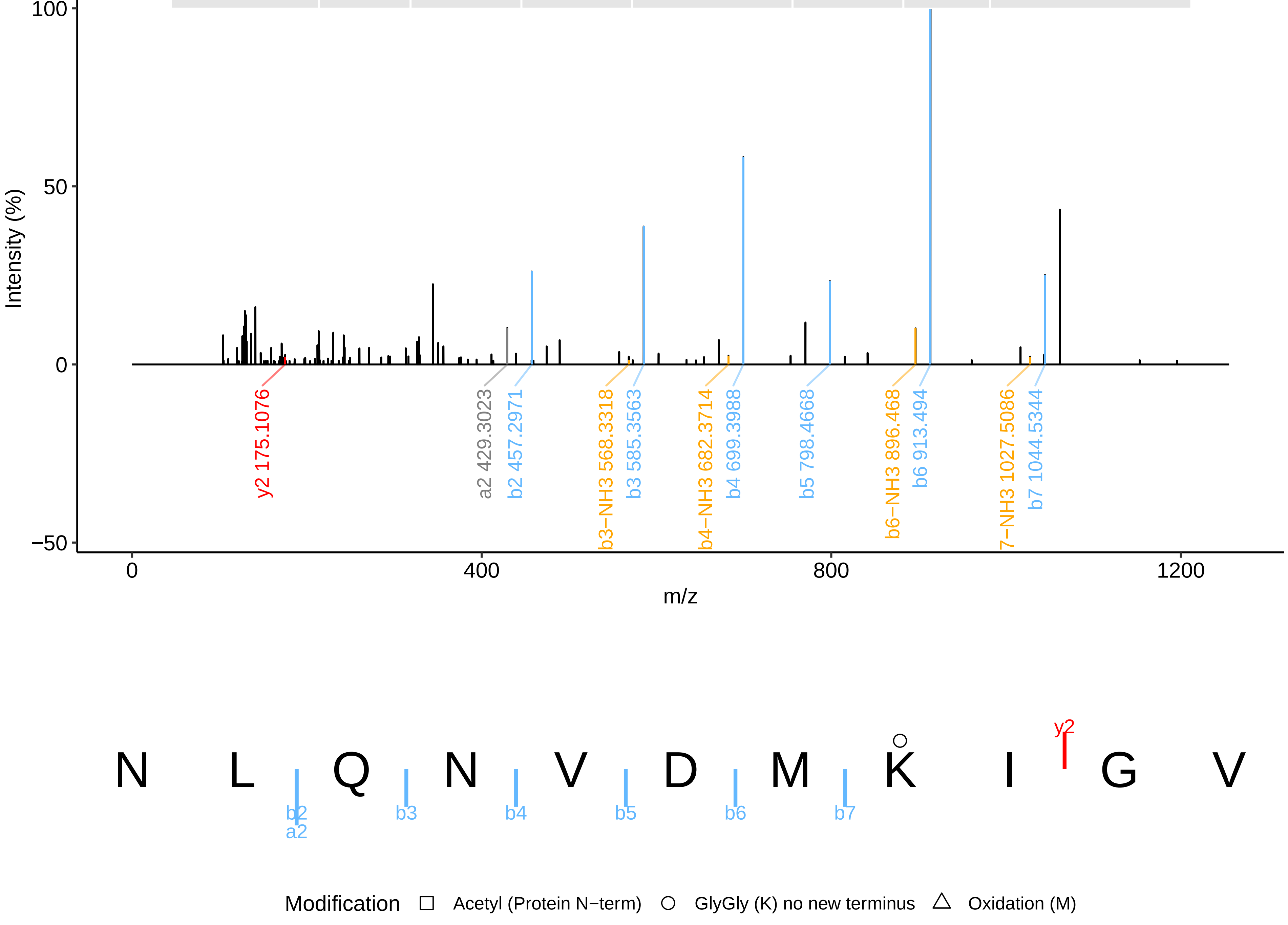

20200924_KIZ_MUb5_GlyGly_1 − Scan number: 19396

Gene Names	Charge	m/z	Mass	Retention time	PEP	Score	Precursor Intensity
CD44	2	672.8506	1343.687	62.701	0.014719	83.265	NA

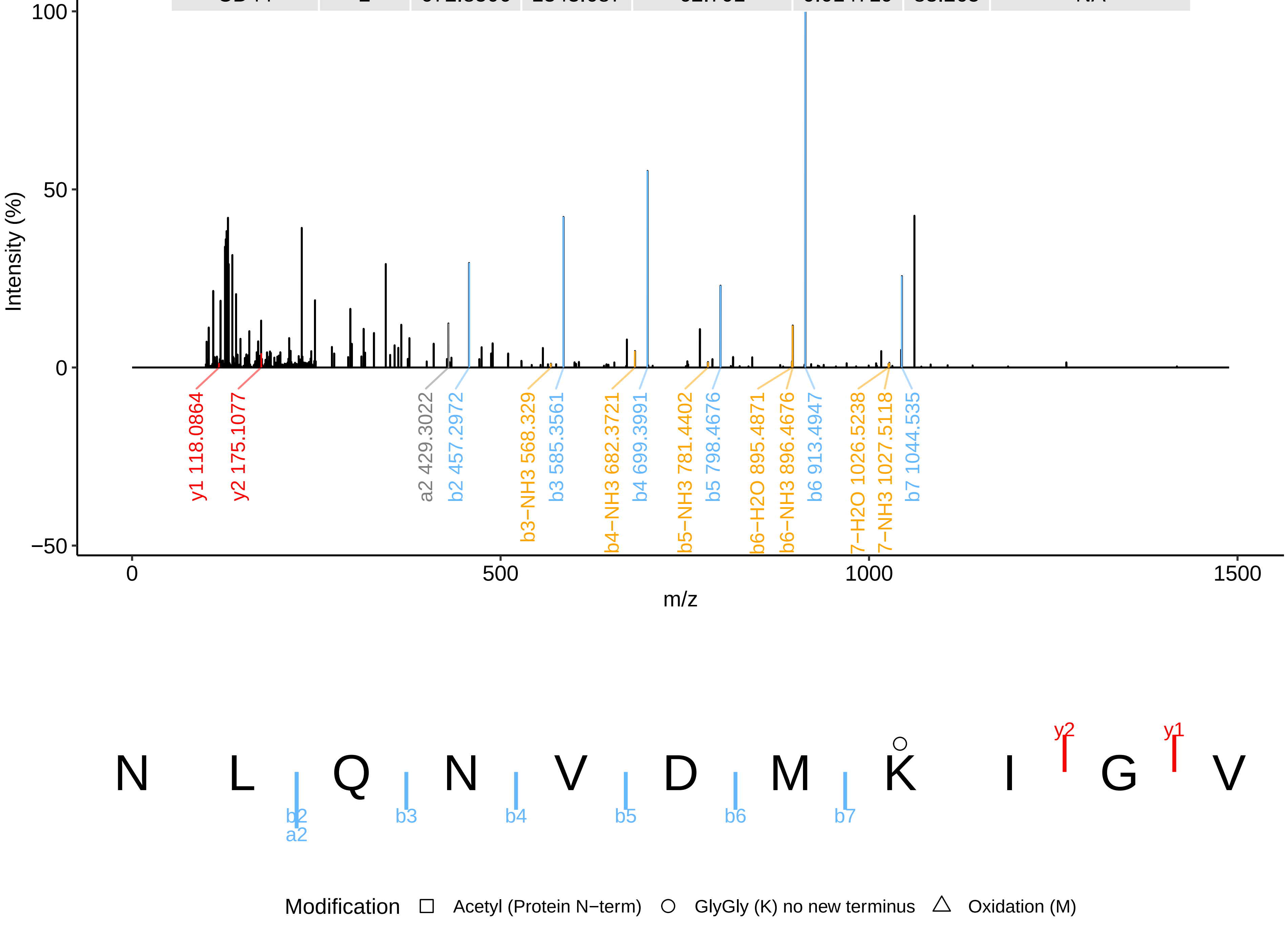

20201118_KIZ_MUb6_GlyGly_2 − Scan number: 20806

Gene Names	Charge	m/z	Mass	Retention time	PEP	Score	Precursor Intensity
CD44	2	672.8506	1343.687	70.112	0.00014881	98.933	10522706

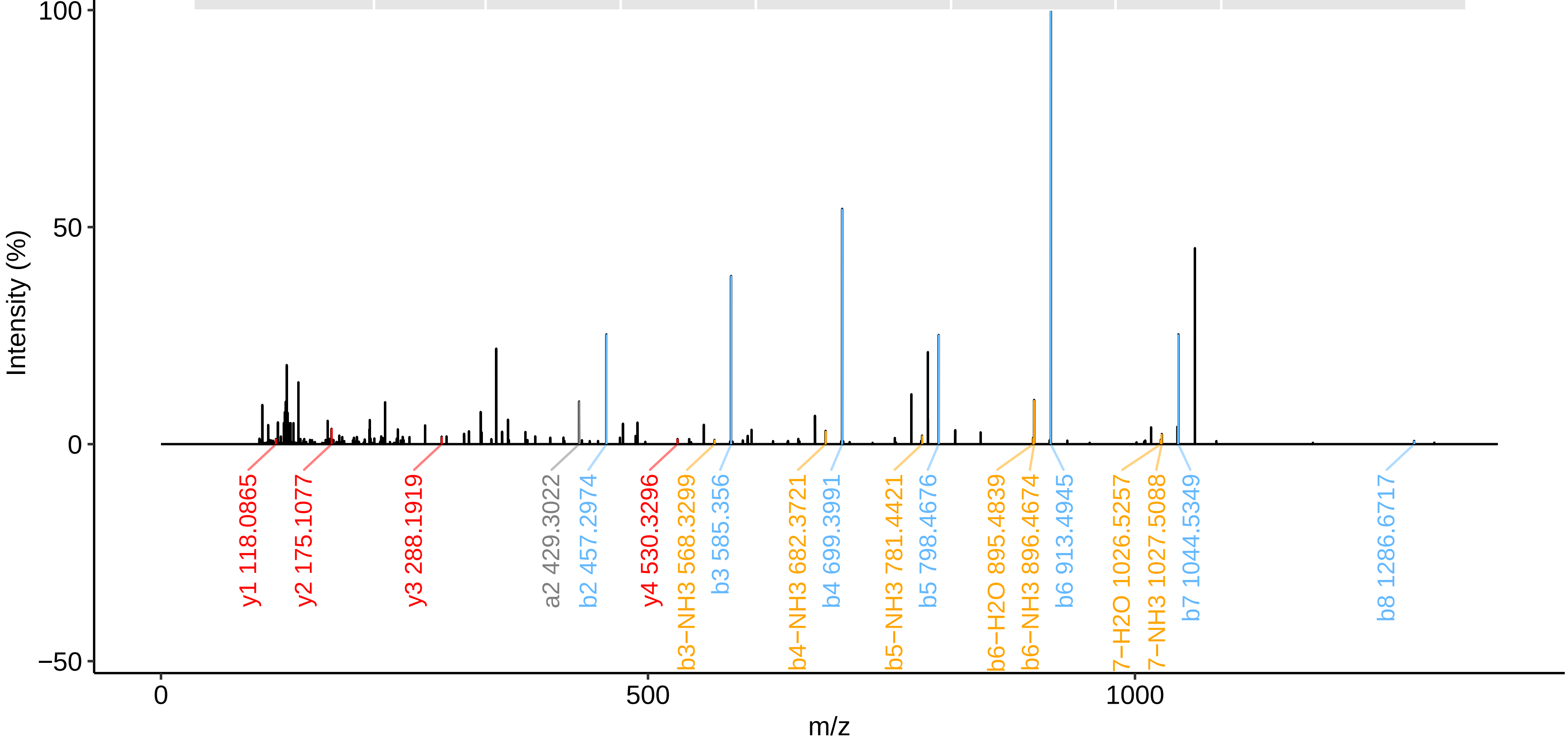

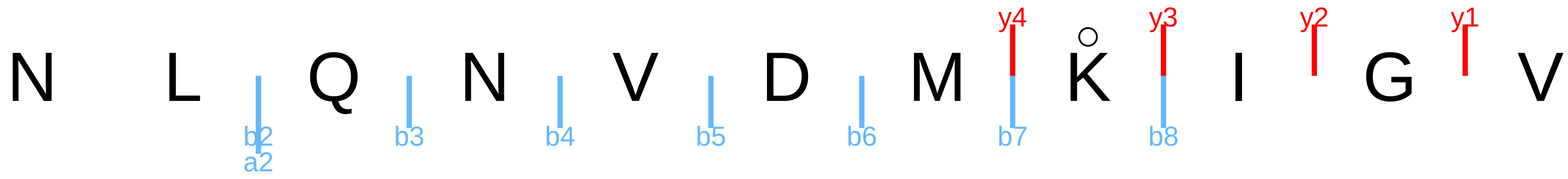

Modification □ Acetyl (Protein N−term) ○ GlyGly (K) no new terminus △ Oxidation (M)

20200921_KIZ_MUb4_GlyGly_10plex_2 − Scan number: 15038

Gene Names	Charge	m/z	Mass	Retention time	PEP	Score	Precursor Intensity
CD44	2	680.8481	1359.682	58.729	0.059697	59.8	770107.7

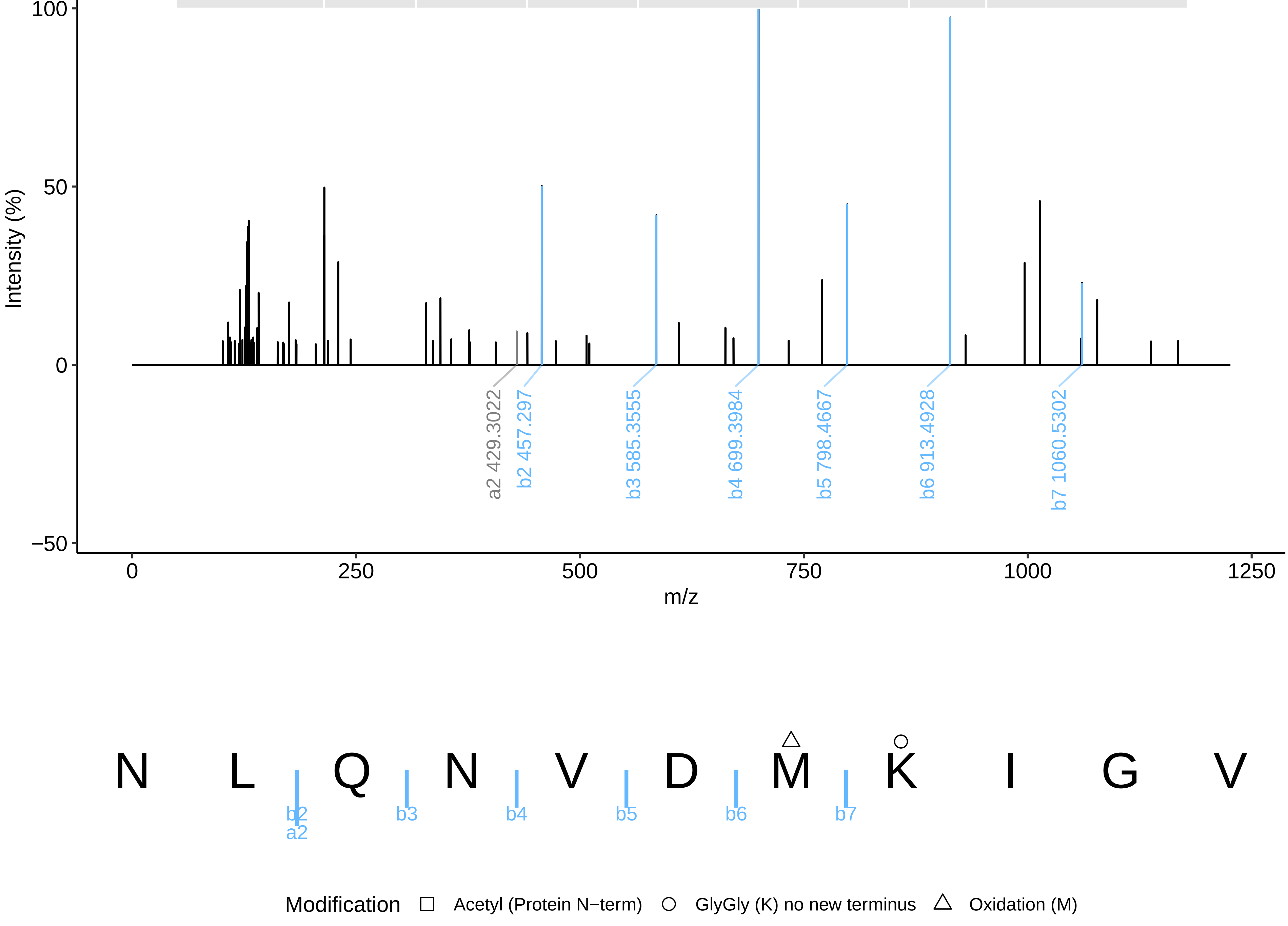

20200921_KIZ_MUb4_GlyGly_10plex_2 − Scan number: 2801

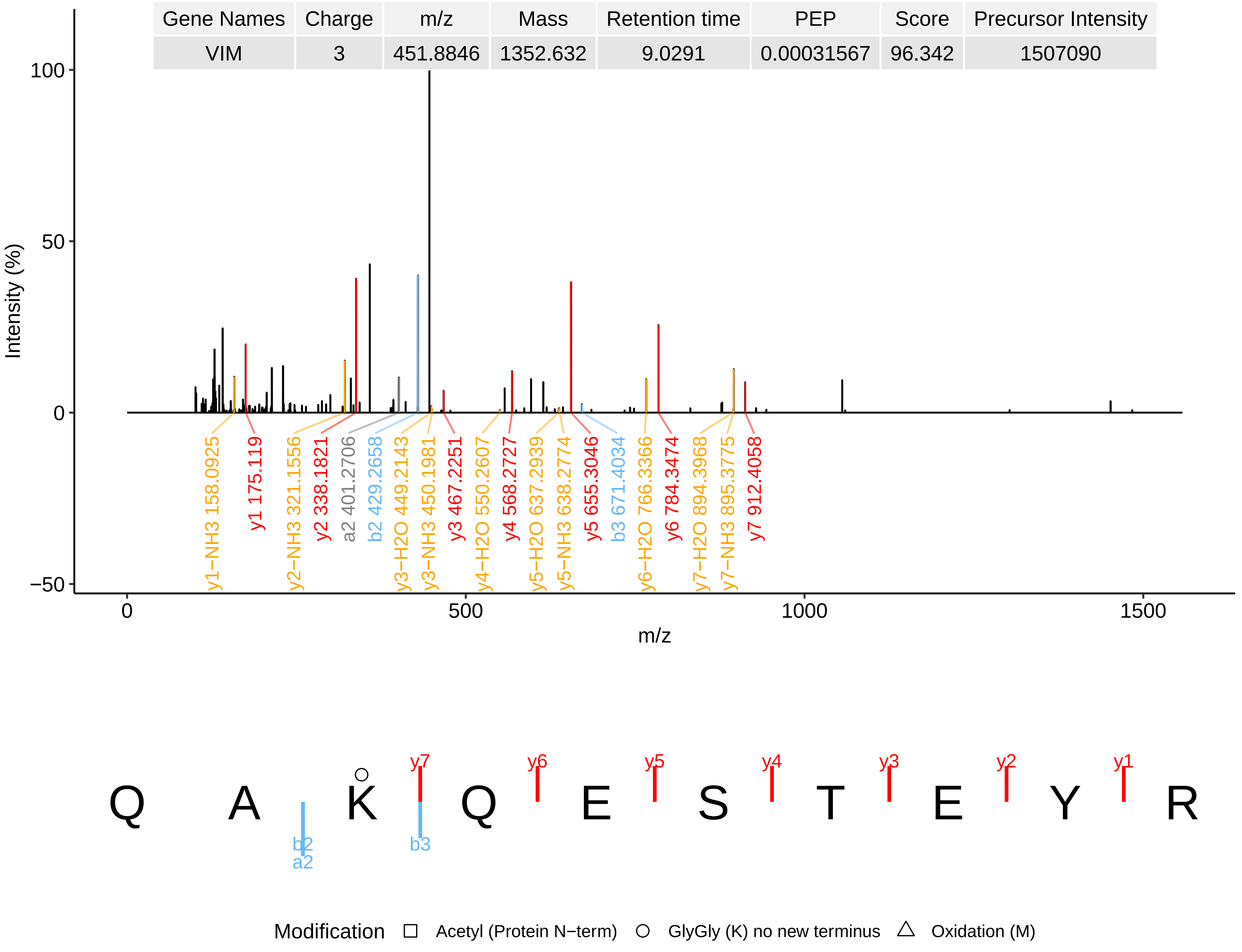

20200924_KIZ_MUb5_GlyGly_2 − Scan number: 2417

Gene Names	Charge	m/z	Mass	Retention time	PEP	Score	Precursor Intensity
VIM	3	451.8846	1352.632	8.5249	0.0031315	87.298	392233.8

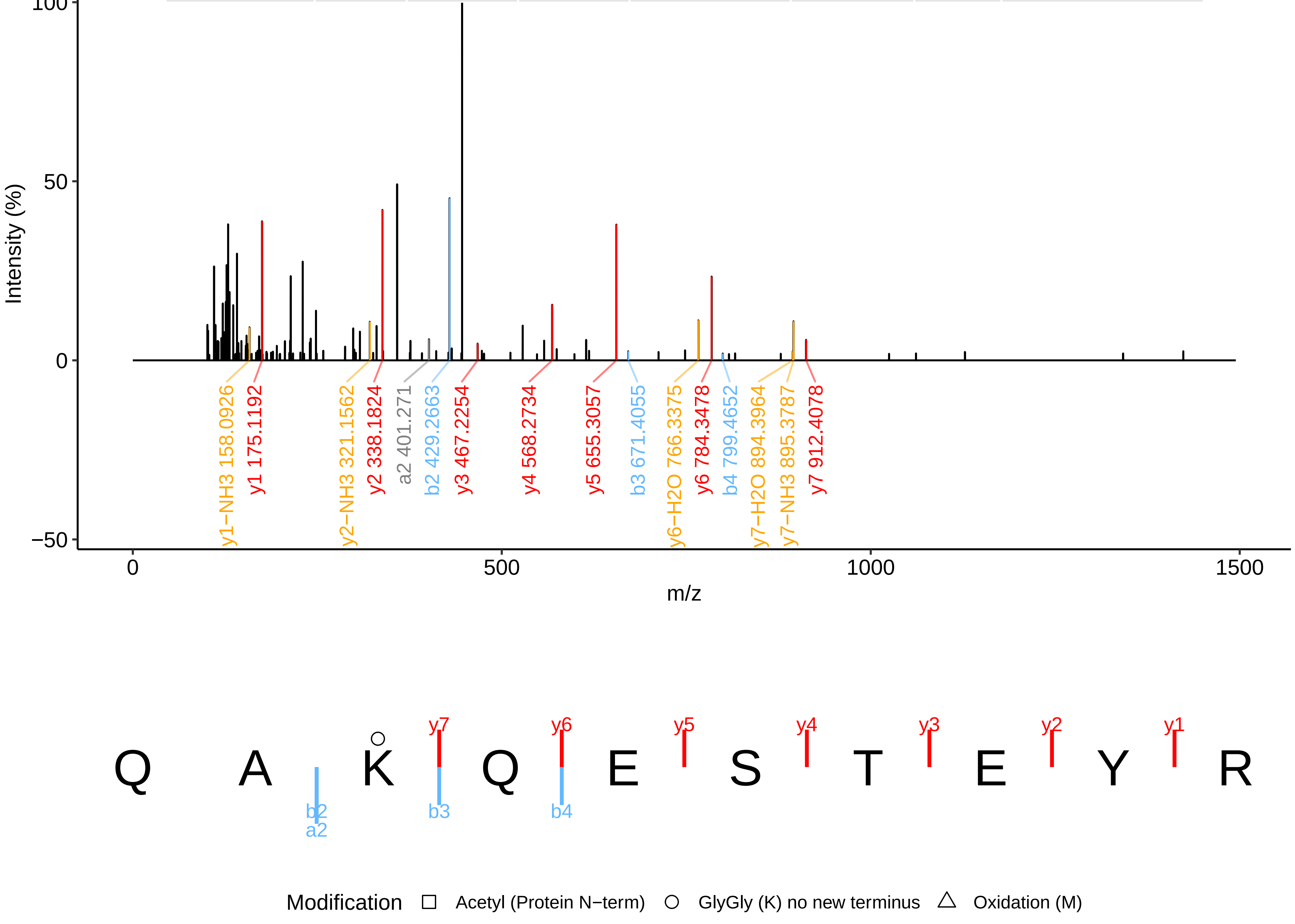

20201118_KIZ_MUb6_GlyGly_2 − Scan number: 3003

Gene Names	Charge	m/z	Mass	Retention time	PEP	Score	Precursor Intensity
VIM	3	451.8846	1352.632	9.6748	0.034417	60.434	2488112

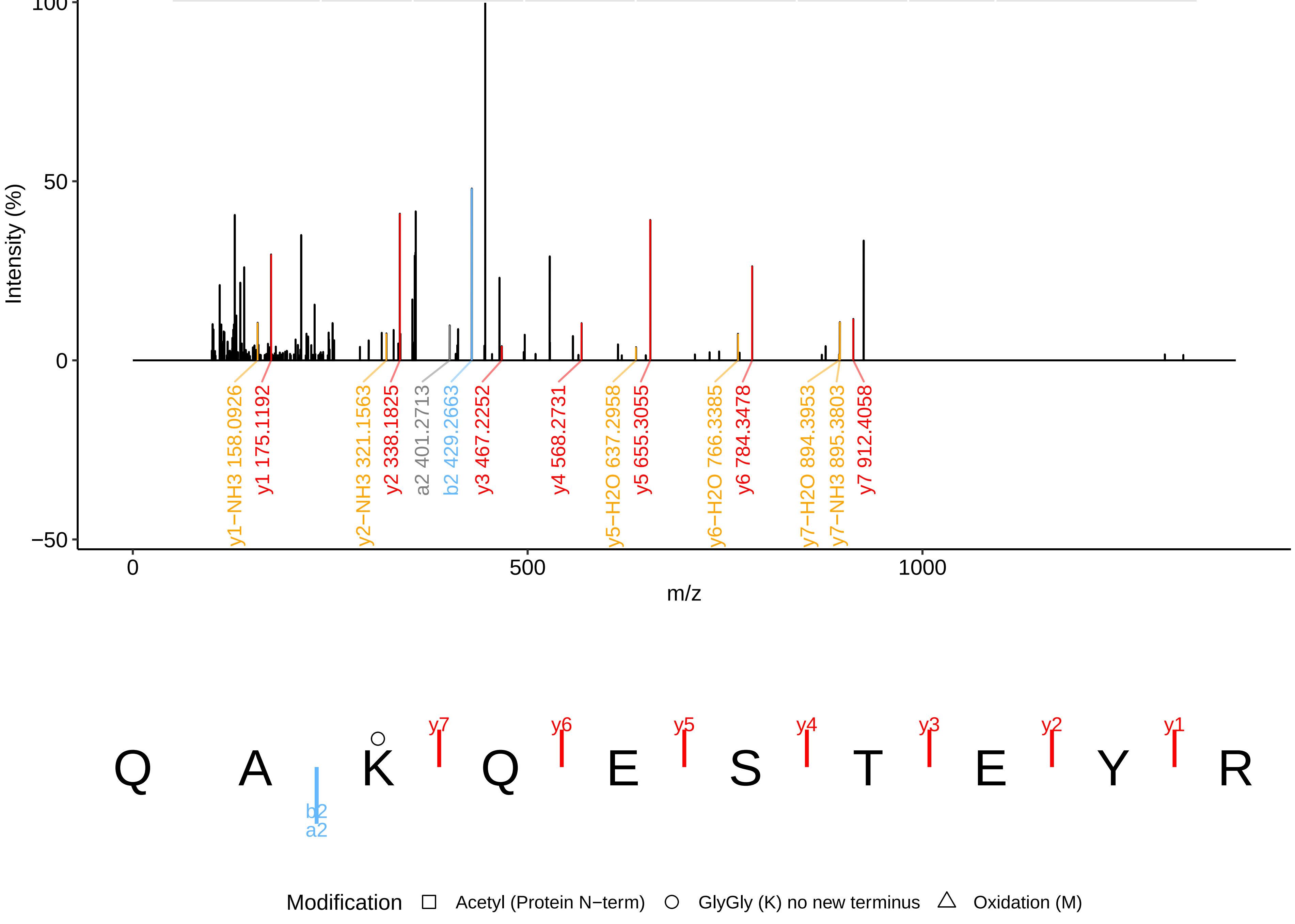

20200921_KIZ_MUb4_GlyGly_10plex − Scan number: 10597

Gene Names	Charge	m/z	Mass	Retention time	PEP	Score	Precursor Intensity
TMEM192	3	446.2334	1335.678	36.718	2.3213e−06	120.09	3701789

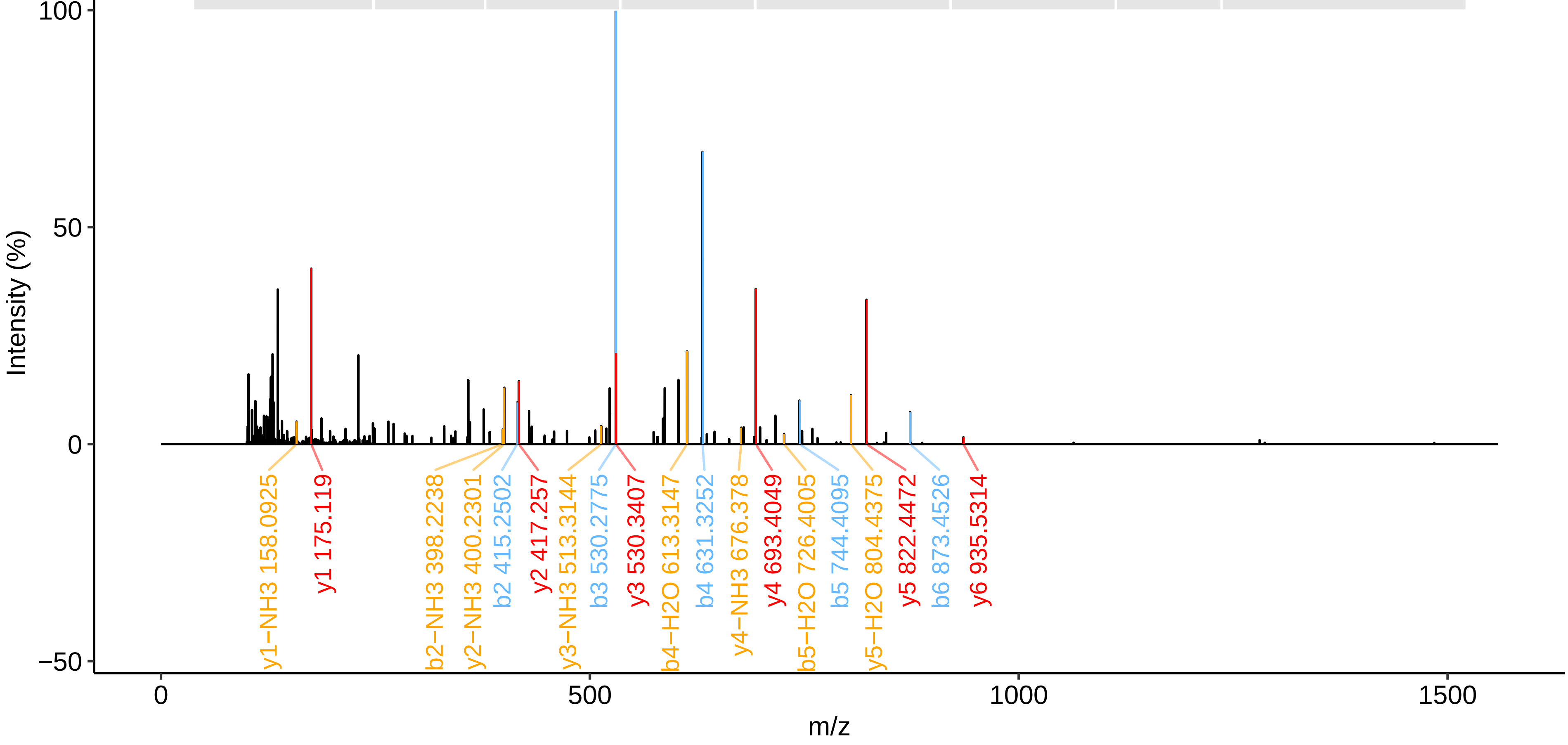

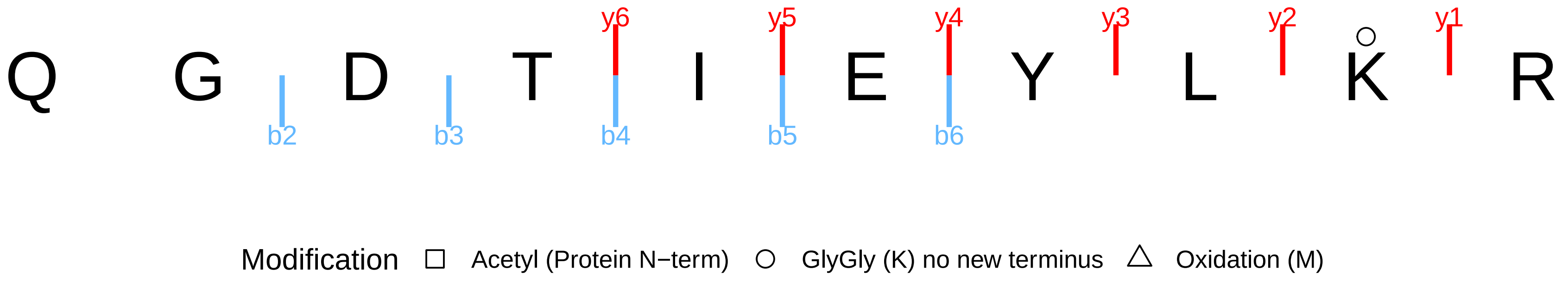

20200921_KIZ_MUb4_GlyGly_10plex − Scan number: 16674

Gene Names	Charge	m/z	Mass	Retention time	PEP	Score	Precursor Intensity
MBD3	3	364.8661	1091.576	59.968	0.085023	88.942	3683005

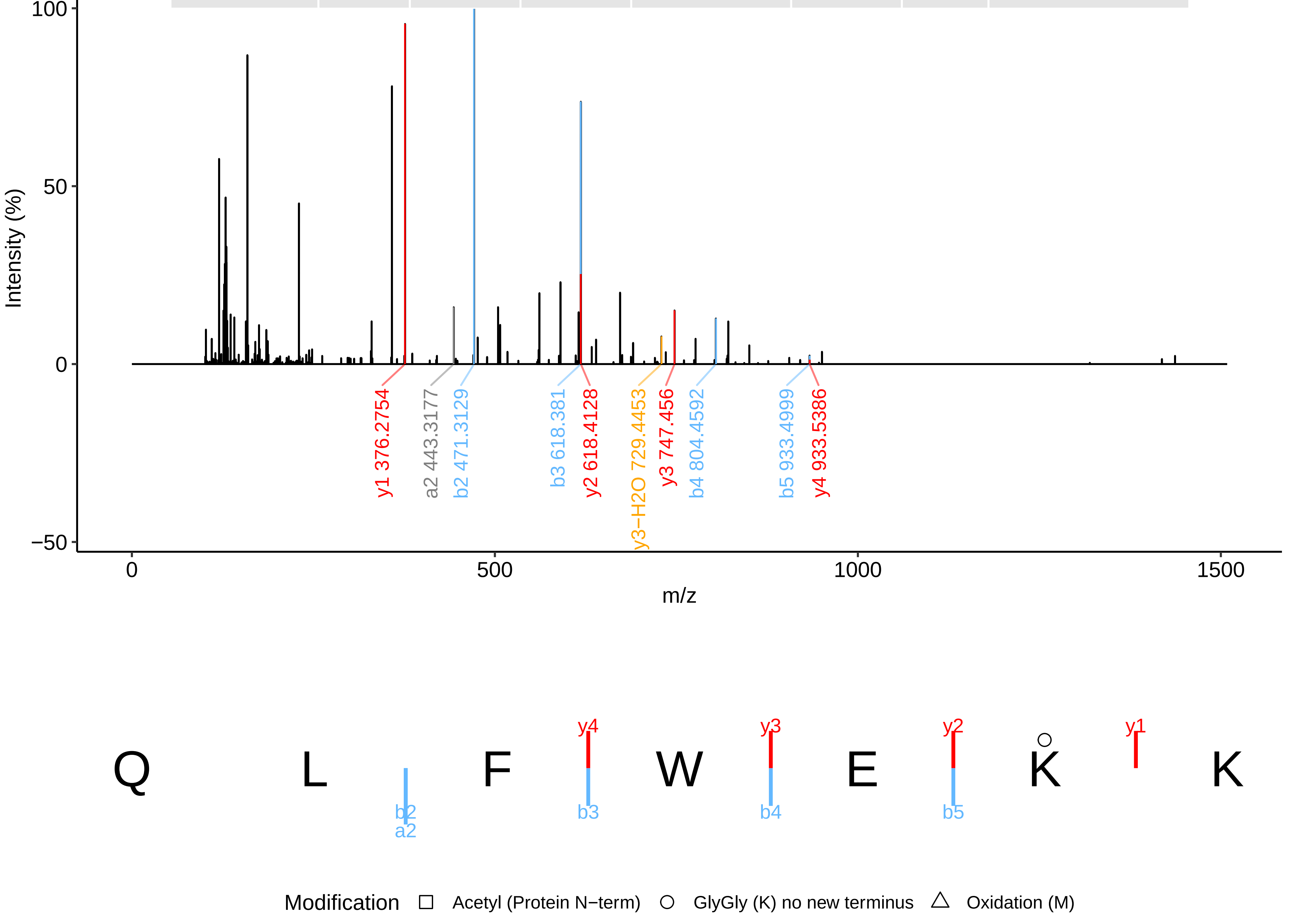

20200921_KIZ_MUb4_GlyGly_10plex − Scan number: 14945

Gene Names	Charge	m/z	Mass	Retention time	PEP	Score	Precursor Intensity
XPO1	3	448.2649	1341.773	53.246	1.9342e−08	126.71	4598278

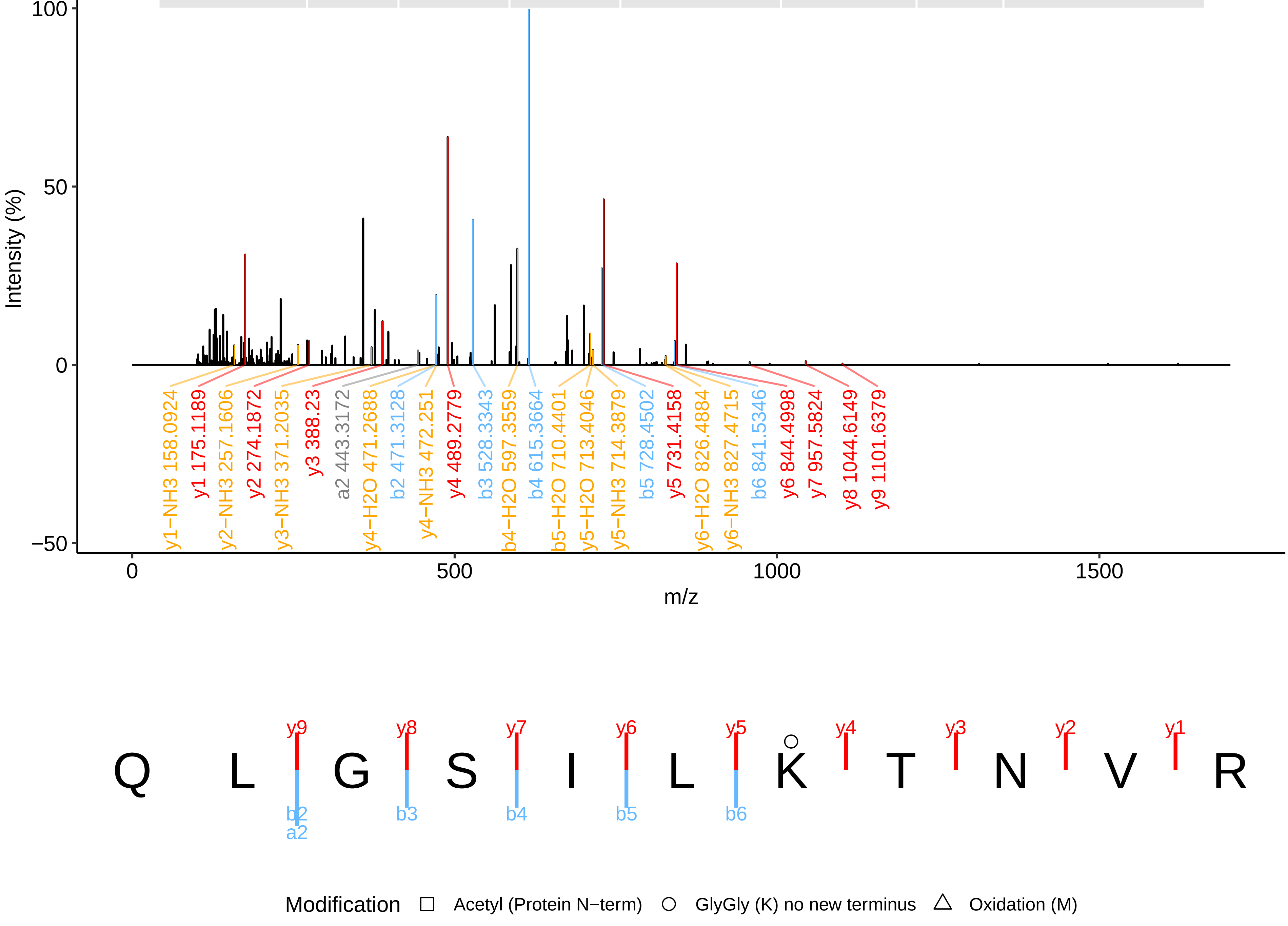

20200921_KIZ_MUb4_GlyGly_10plex_2 − Scan number: 13795

Gene Names	Charge	m/z	Mass	Retention time	PEP	Score	Precursor Intensity
XPO1	3	448.2649	1341.773	53.642	0.014618	64.918	1019666

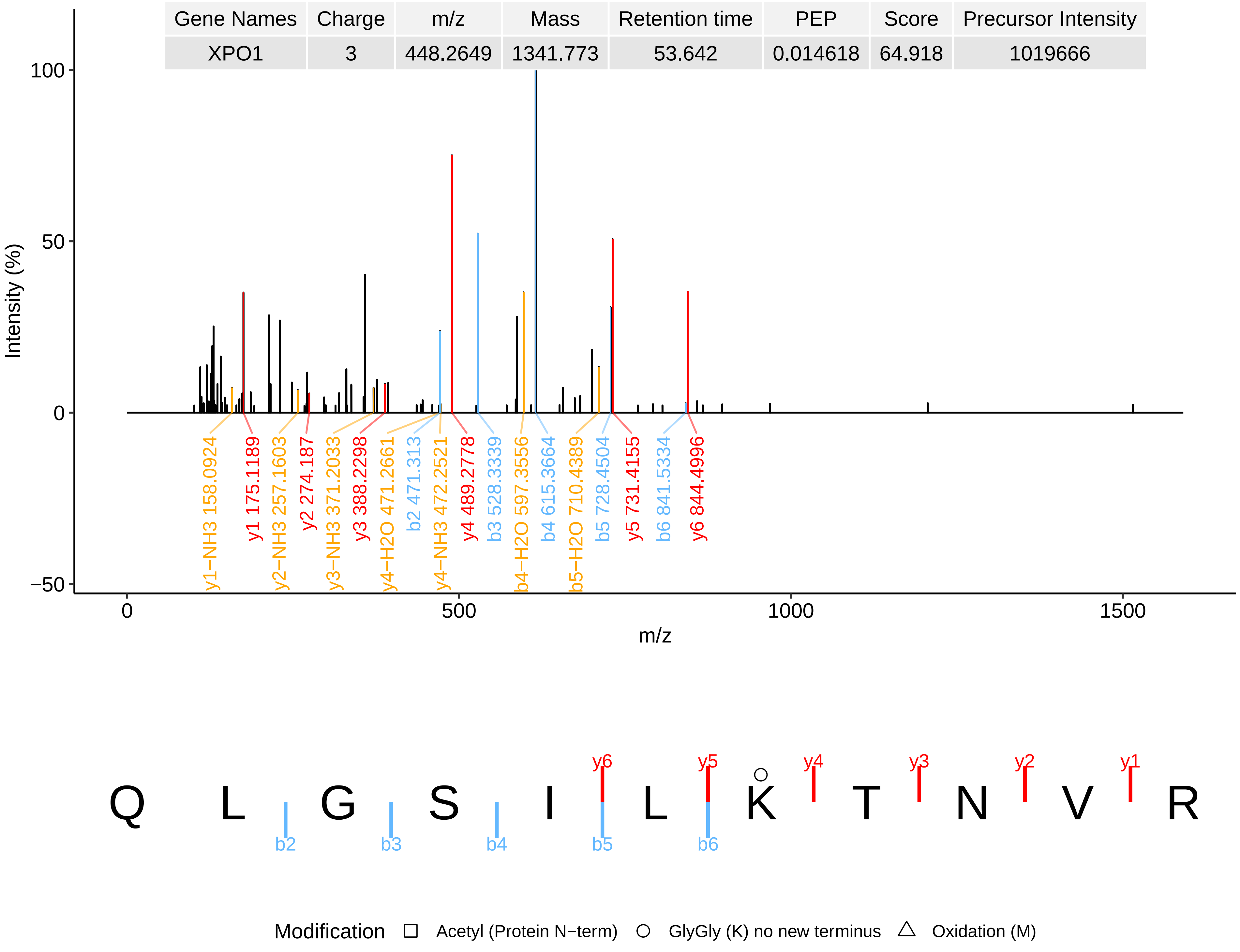

20200921_KIZ_MUb4_GlyGly_10plex − Scan number: 8176

Gene Names	Charge	m/z	Mass	Retention time	PEP	Score	Precursor Intensity
RECQL	3	376.8839	1127.63	27.679	0.039442	87.476	7225795

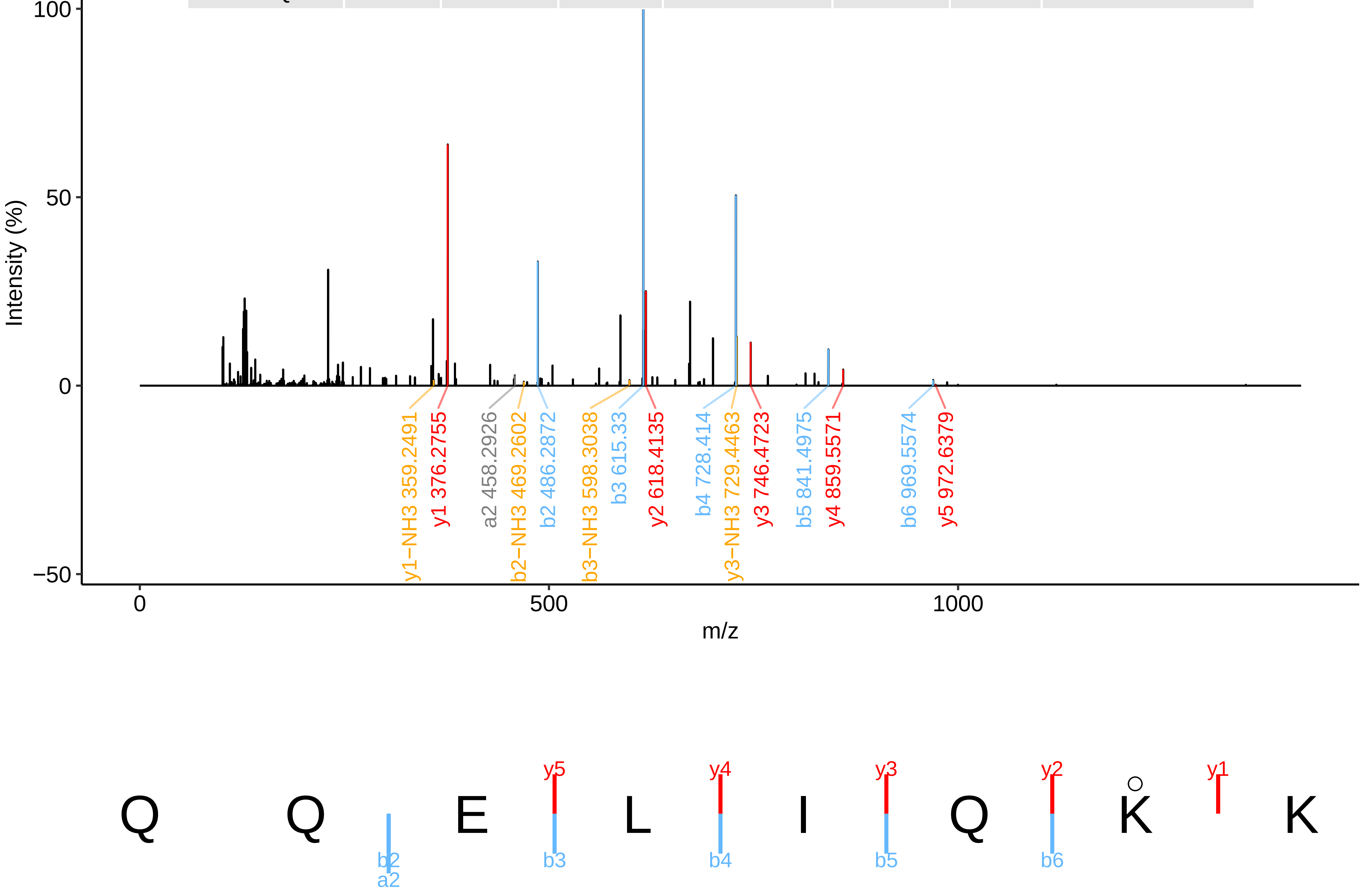

Modification □ Acetyl (Protein N−term) ○ GlyGly (K) no new terminus △ Oxidation (M)

20200921_KIZ_MUb4_GlyGly_10plex − Scan number: 8223

Gene Names	Charge	m/z	Mass	Retention time	PEP	Score	Precursor Intensity
RECQL	2	564.8222	1127.63	27.849	0.013321	152.47	3620949

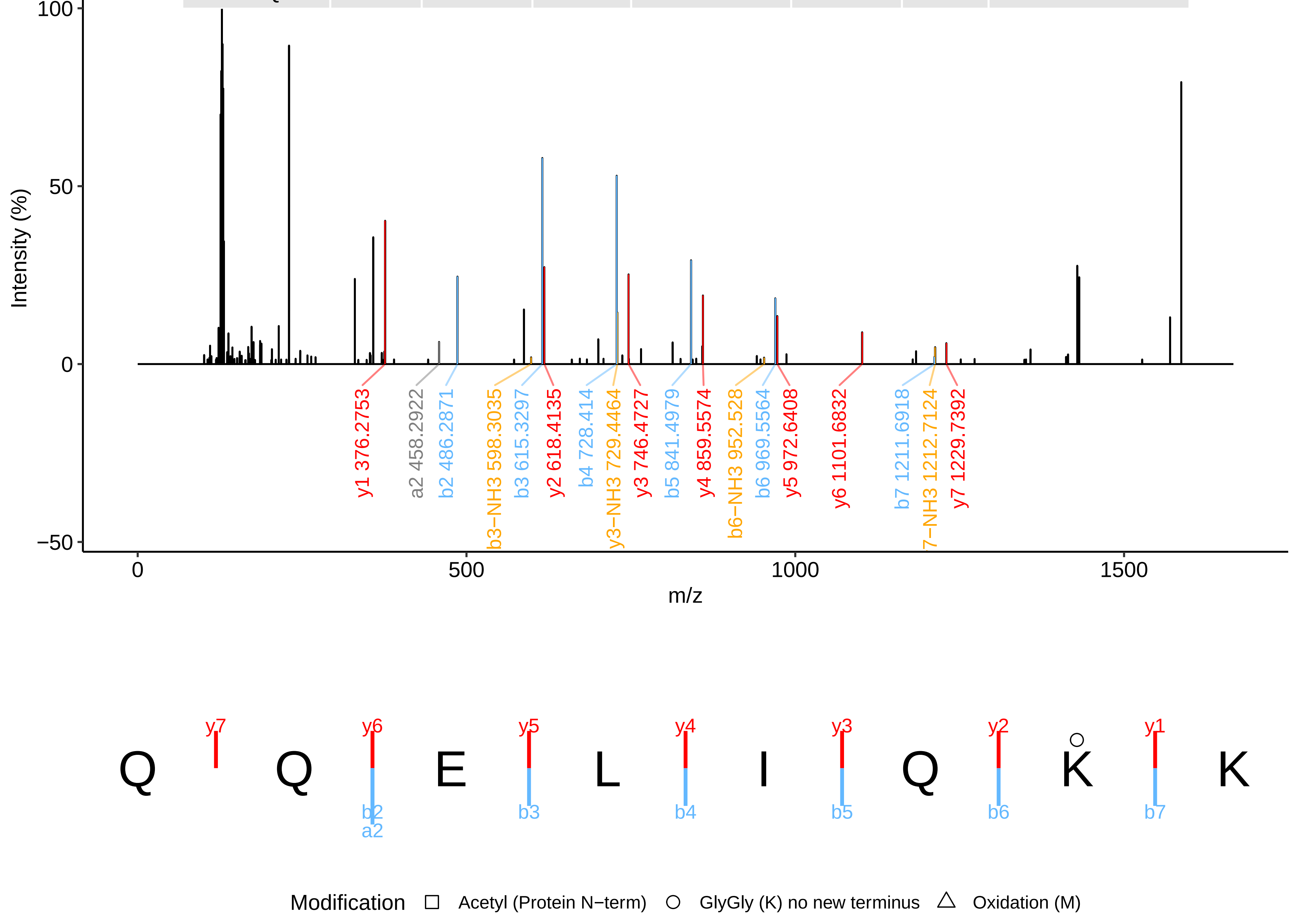

20200921_KIZ_MUb4_GlyGly_10plex_2 − Scan number: 7575

Gene Names	Charge	m/z	Mass	Retention time	PEP	Score	Precursor Intensity
RECQL	3	376.8839	1127.63	28.312	0.059474	80.165	2337694

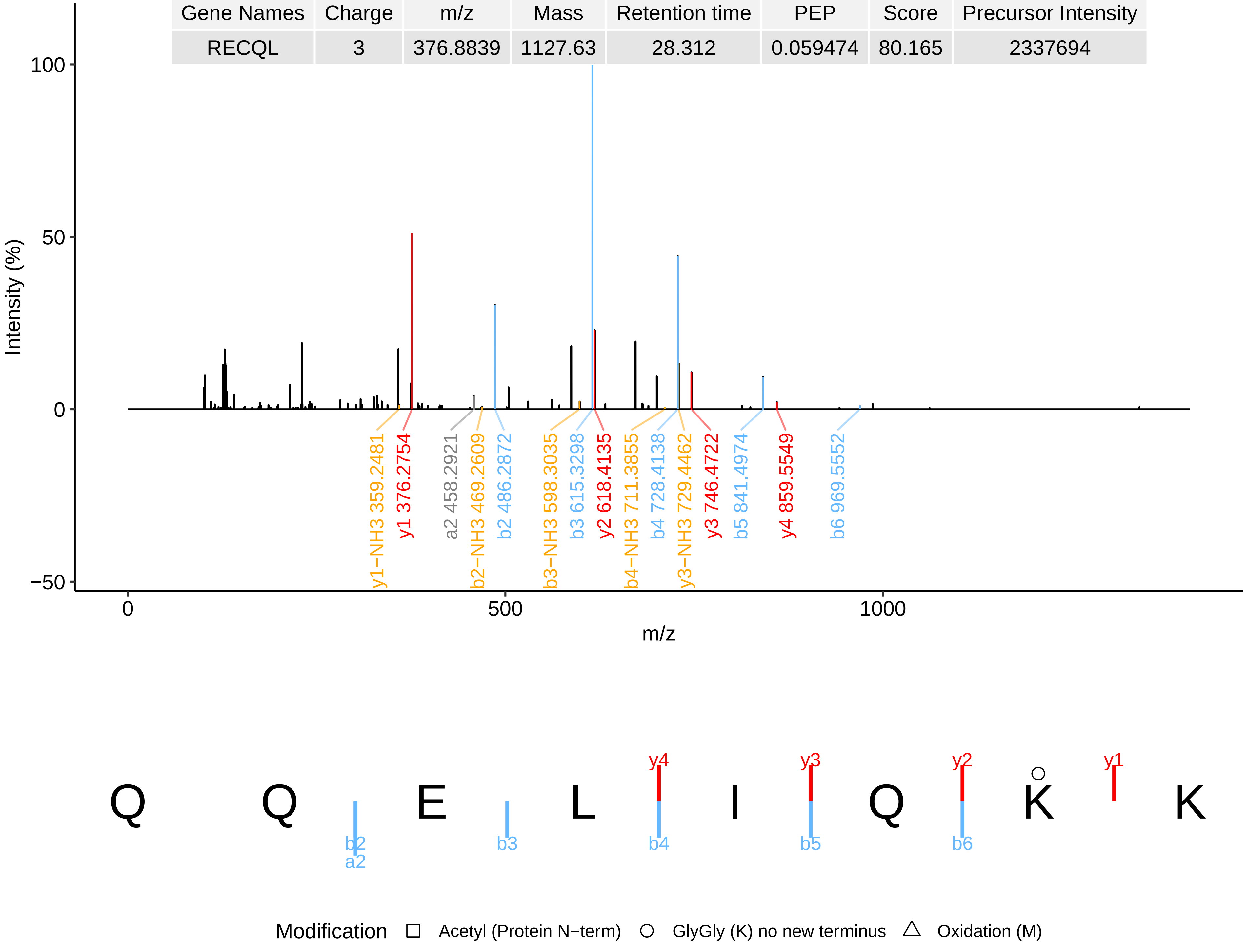

20200924_KIZ_MUb5_GlyGly_1 − Scan number: 7024

Gene Names	Charge	m/z	Mass	Retention time	PEP	Score	Precursor Intensity
RECQL	3	376.8839	1127.63	22.234	0.094	75.682	6686052

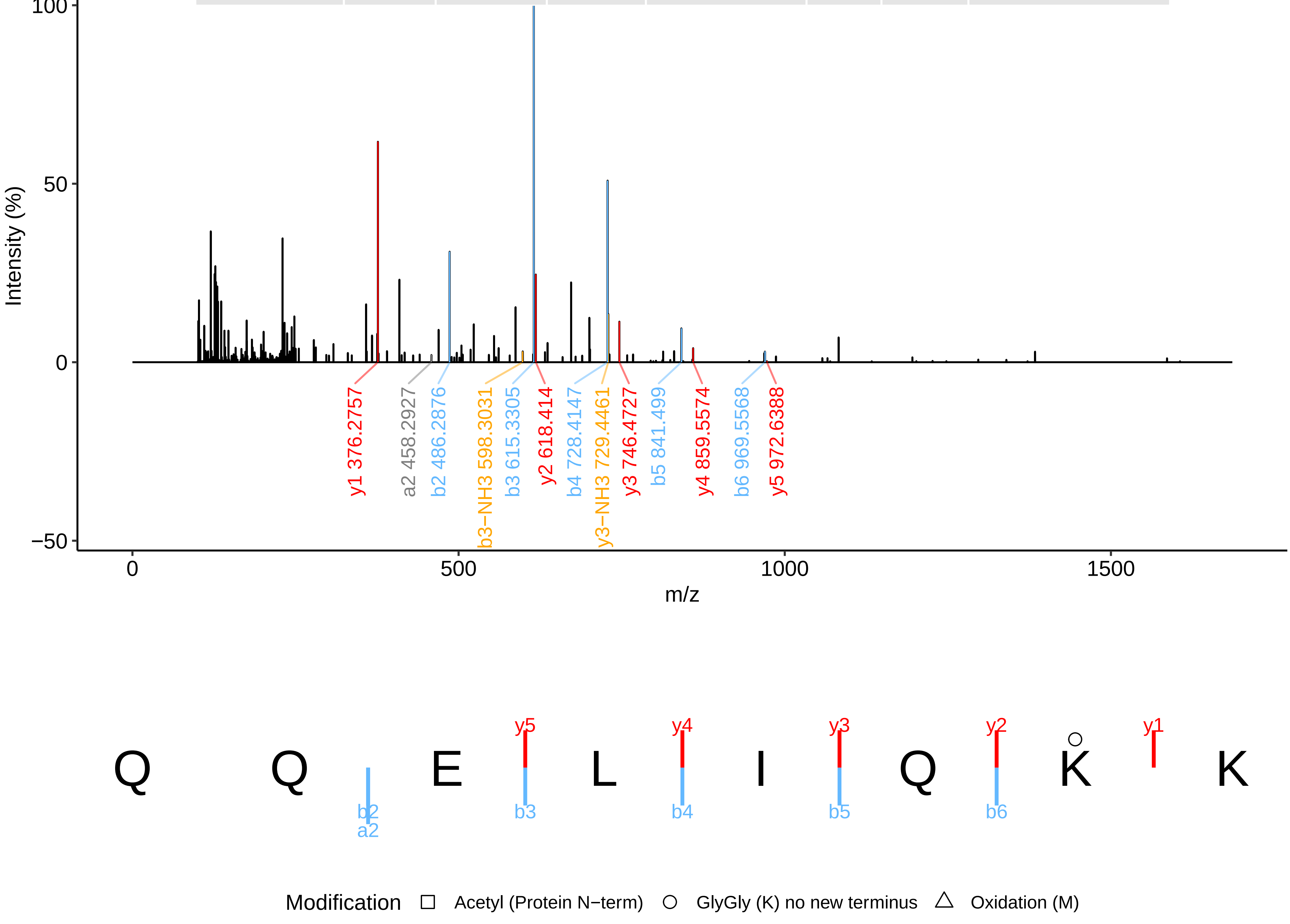

20200924_KIZ_MUb5_GlyGly_1 − Scan number: 7179

Gene Names	Charge	m/z	Mass	Retention time	PEP	Score	Precursor Intensity
RECQL	3	376.8839	1127.63	22.747	0.011387	101.43	89377880

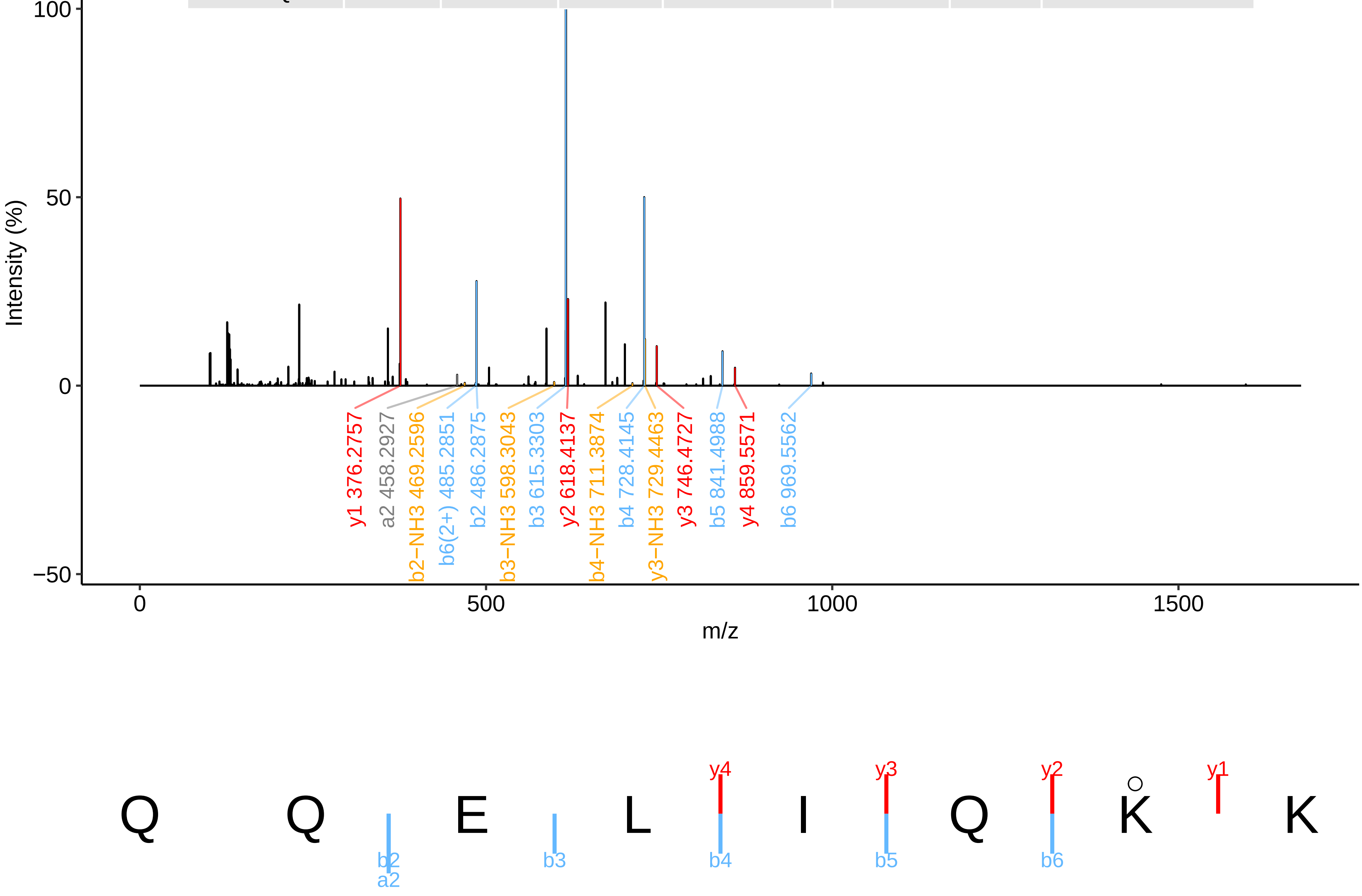

Modification □ Acetyl (Protein N−term) ○ GlyGly (K) no new terminus △ Oxidation (M)

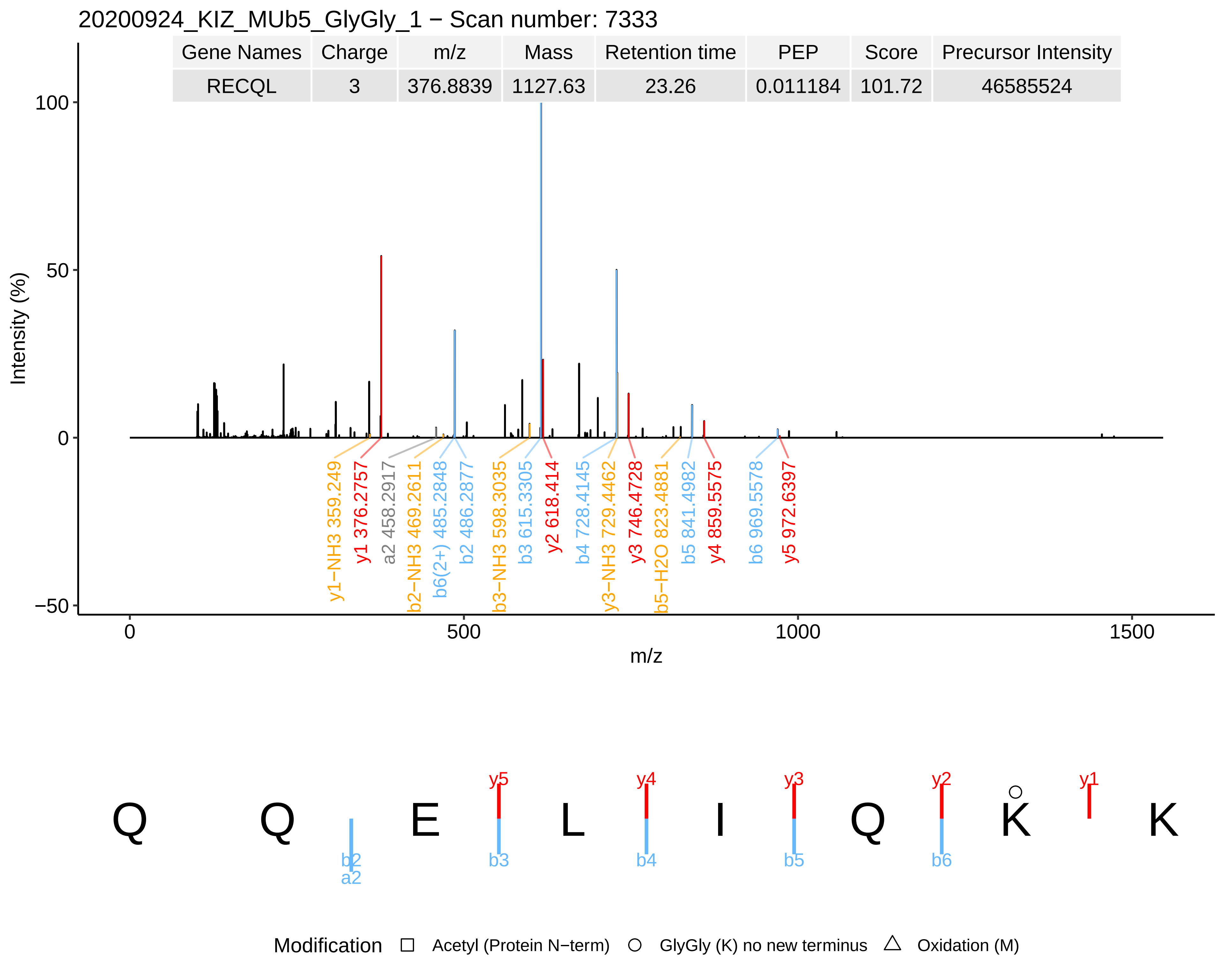
20200924_KIZ_MUb5_GlyGly_1 − Scan number: 7333
Gene Names
Charge
m/z
Mass
Retention time
PEP
Score
Precursor Intensity
RECQL
3
376.8839
1127.63
23.26
0.011184
101.72
46585524
Intensity (%)
m/z
y1−NH3 359.249
y1 376.2757
a2 458.2917
b2−NH3 469.2611
b6(2+) 485.2848
b2 486.2877
b3−NH3 598.3035
b3 615.3305
y2 618.414
b4 728.4145
y3−NH3 729.4462
y3 746.4728
b5−H2O 823.4881
b5 841.4982
y4 859.5575
b6 969.5578
y5 972.6397
Q Q E L I Q K K
Modification
Acetyl (Protein N−term)
GlyGly (K) no new terminus
Oxidation (M)

20200924_KIZ_MUb5_GlyGly_2 − Scan number: 6539

Gene Names	Charge	m/z	Mass	Retention time	PEP	Score	Precursor Intensity
RECQL	3	376.8839	1127.63	24.402	0.05378	81.594	NA

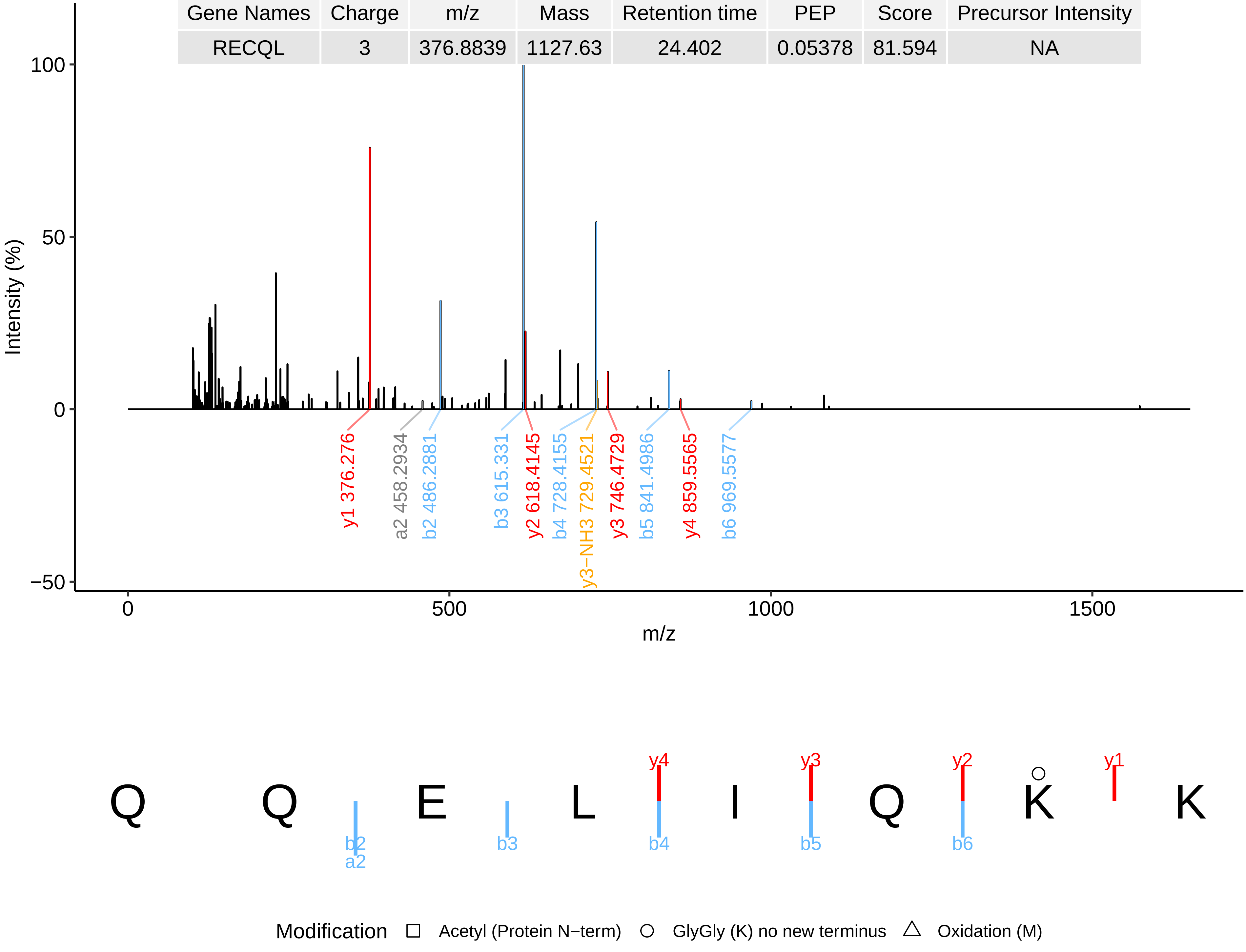

20200924_KIZ_MUb5_GlyGly_2 − Scan number: 6675

Gene Names	Charge	m/z	Mass	Retention time	PEP	Score	Precursor Intensity
RECQL	3	376.8839	1127.63	24.911	0.036168	88.819	14313901

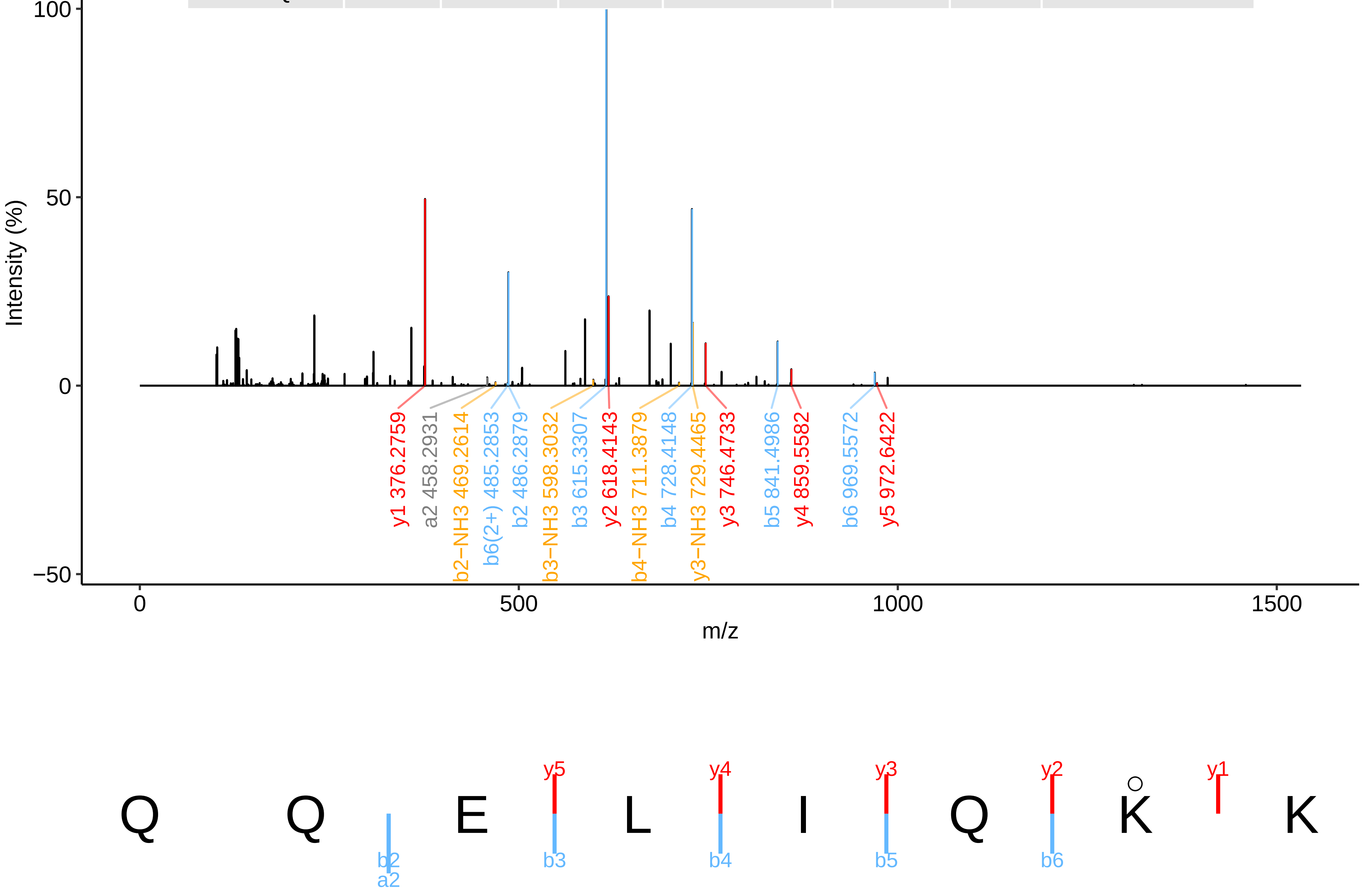

Modification □ Acetyl (Protein N−term) ○ GlyGly (K) no new terminus △ Oxidation (M)

20200924_KIZ_MUb5_GlyGly_2 − Scan number: 6812

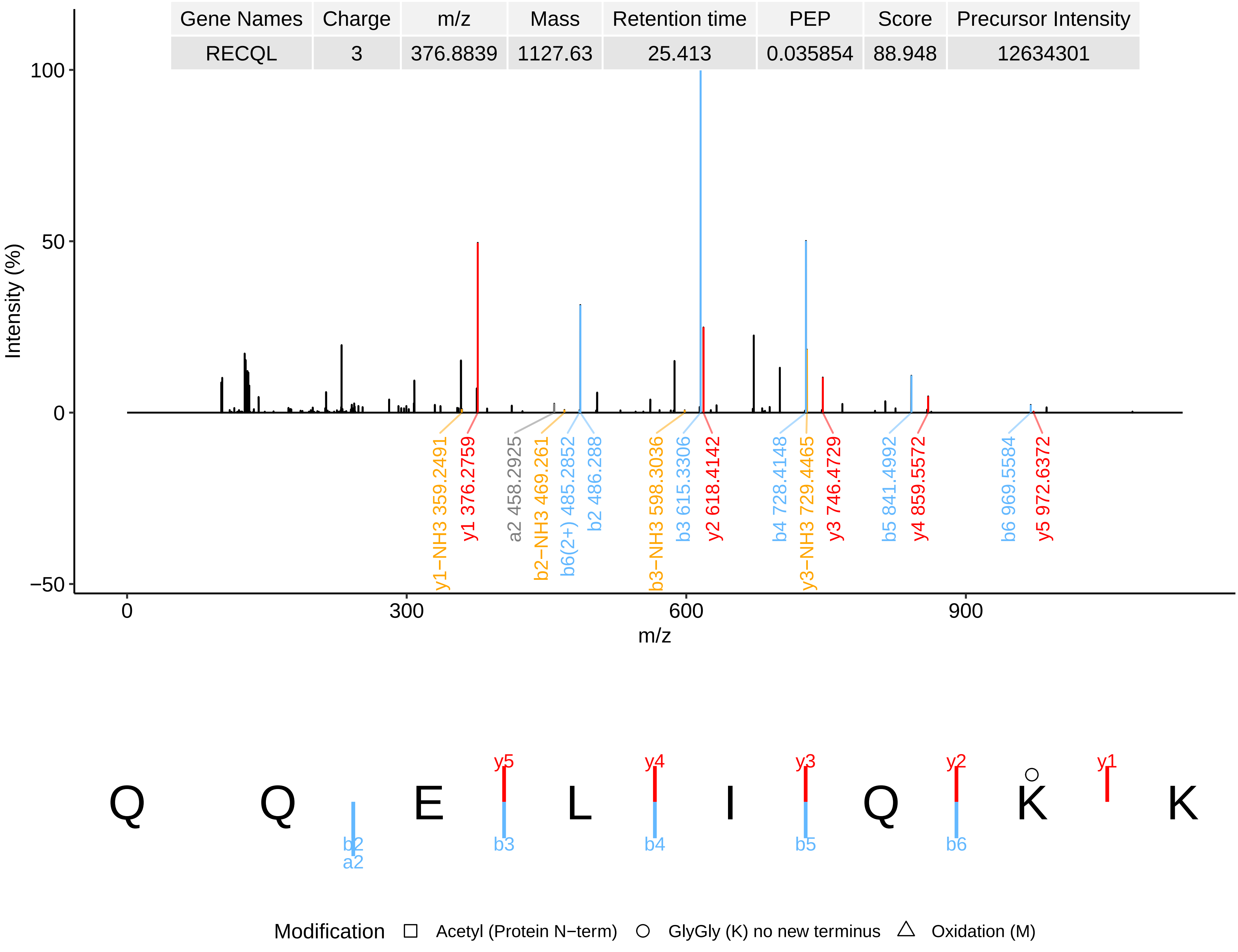

20200924_KIZ_MUb5_GlyGly_2 − Scan number: 6950

Gene Names	Charge	m/z	Mass	Retention time	PEP	Score	Precursor Intensity
RECQL	3	376.8839	1127.63	25.921	0.023028	90.657	2739293

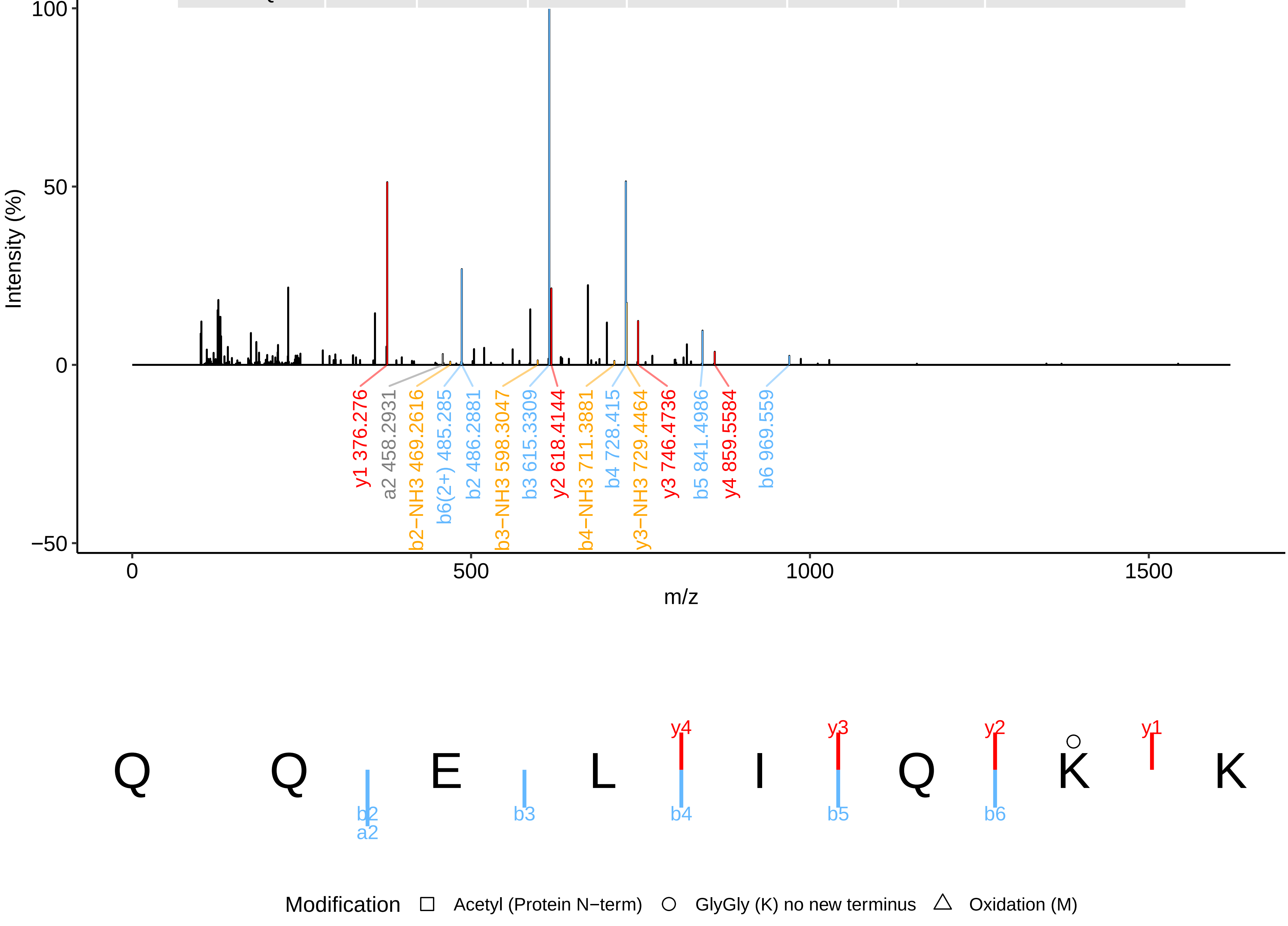

20201118_KIZ_MUb6_GlyGly_1 − Scan number: 6898

Gene Names	Charge	m/z	Mass	Retention time	PEP	Score	Precursor Intensity
RECQL	3	376.8839	1127.63	21.836	0.031688	90.657	22911280

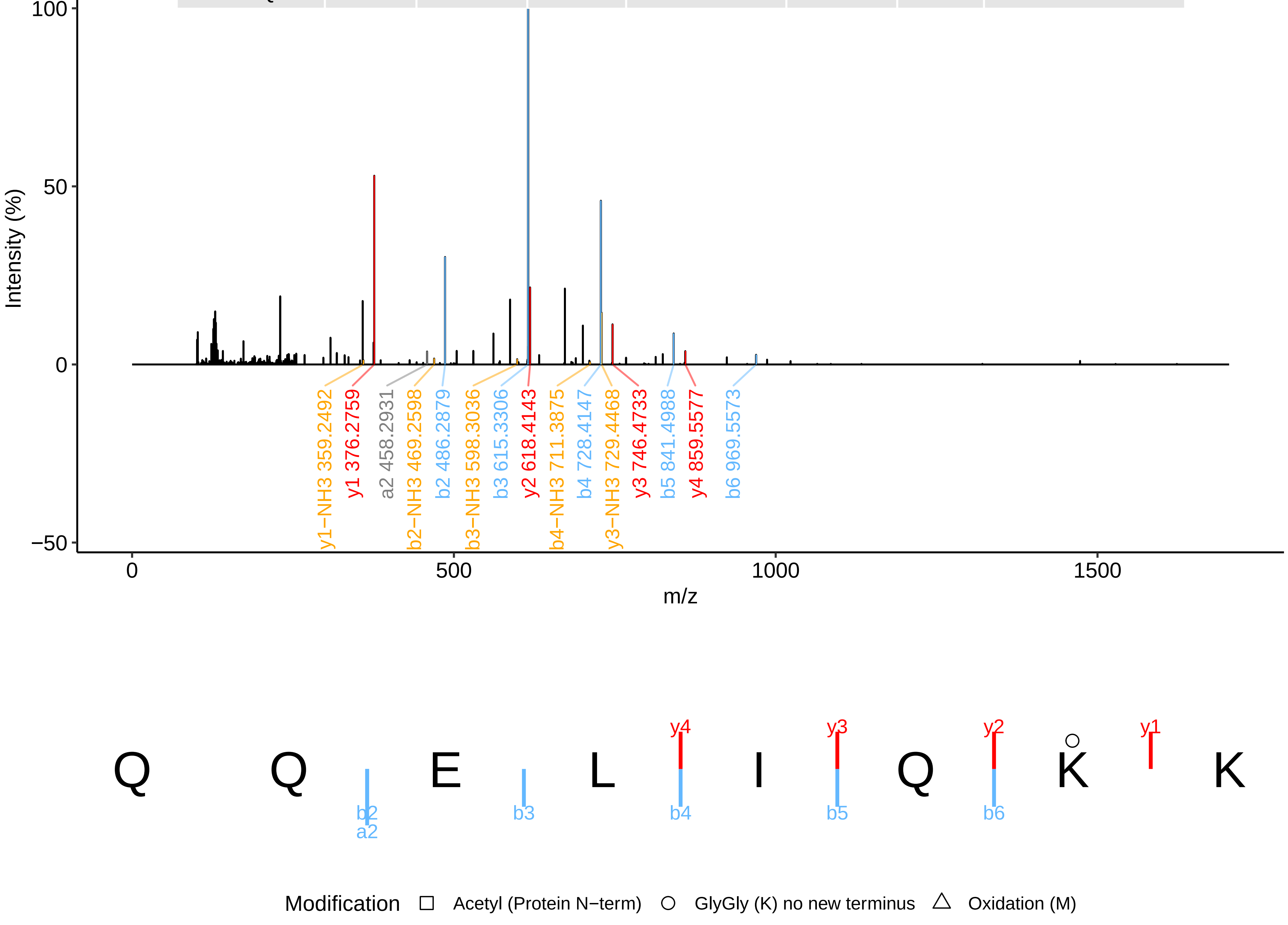

20201118_KIZ_MUb6_GlyGly_2 − Scan number: 8694

Gene Names	Charge	m/z	Mass	Retention time	PEP	Score	Precursor Intensity
RECQL	3	376.8839	1127.63	29.498	0.025626	93.143	15932923

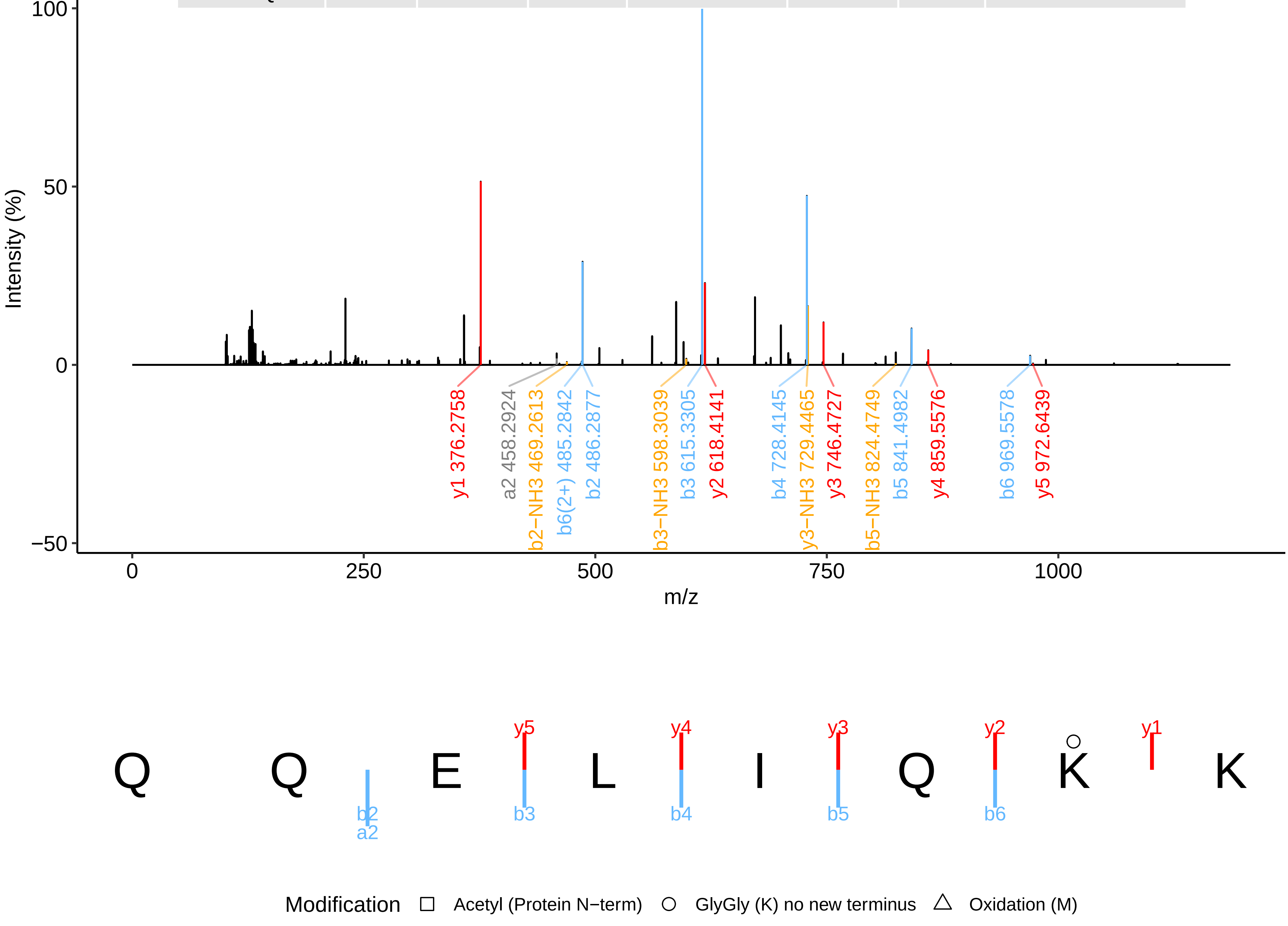

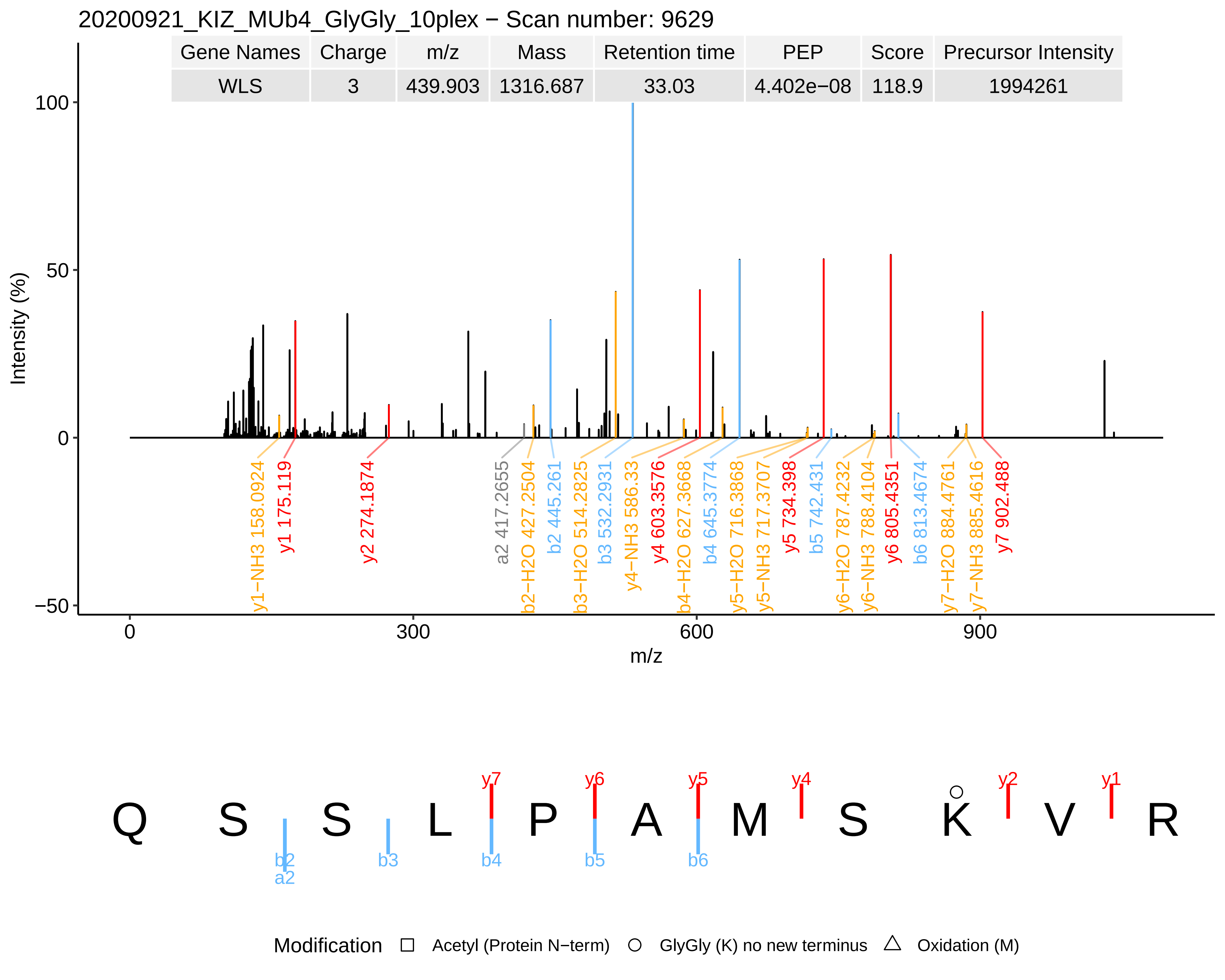

20200921_KIZ_MUb4_GlyGly_10plex − Scan number: 9629
Gene Names | Charge | m/z | Mass | Retention time | PEP | Score | Precursor Intensity
WLS | 3 | 439.903 | 1316.687 | 33.03 | 4.402e−08 | 118.9 | 1994261
Intensity (%)
100
50
0
−50
0
300
600
900
m/z
y1−NH3 158.0924
y1 175.119
y2 274.1874
a2 417.2655
b2−H2O 427.2504
b2 445.261
b3−H2O 514.2825
b3 532.2931
y4−NH3 586.33
y4 603.3576
b4−H2O 627.3668
b4 645.3774
y5−H2O 716.3868
y5−NH3 717.3707
y5 734.398
b5 742.431
y6−H2O 787.4232
y6−NH3 788.4104
y6 805.4351
b6 813.4674
y7−H2O 884.4761
y7−NH3 885.4616
y7 902.488
Q S S L P A M S K V R
y7 y6 y5 y4 y2 y1
b2 a2 b3 b4 b5 b6
Modification □ Acetyl (Protein N−term) ○ GlyGly (K) no new terminus △ Oxidation (M)

20200921_KIZ_MUb4_GlyGly_10plex − Scan number: 9761

Gene Names	Charge	m/z	Mass	Retention time	PEP	Score	Precursor Intensity
WLS	3	439.903	1316.687	33.533	2.6089e−12	134.3	11741627

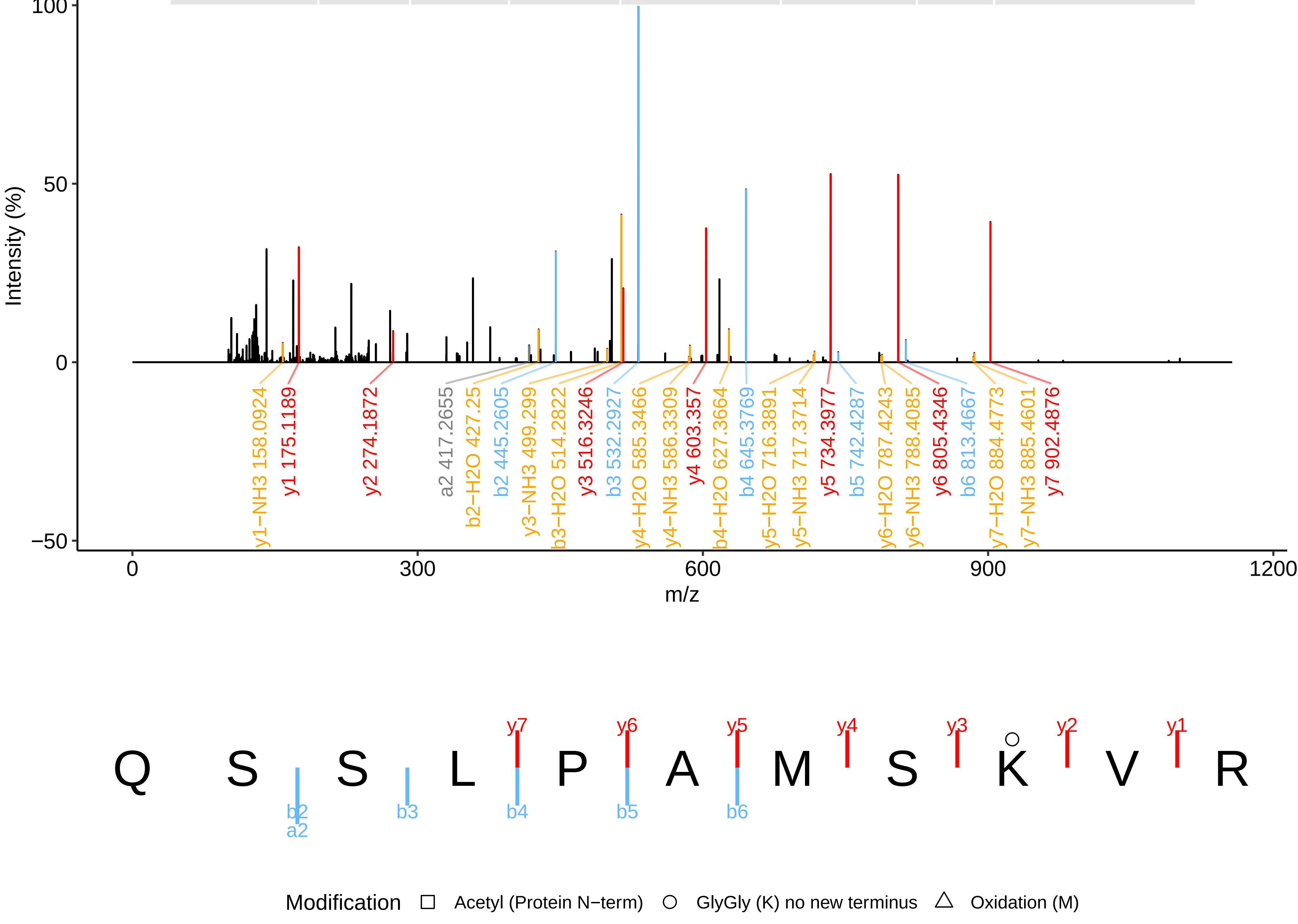

20200921_KIZ_MUb4_GlyGly_10plex_2 − Scan number: 8883

Gene Names	Charge	m/z	Mass	Retention time	PEP	Score	Precursor Intensity
WLS	3	439.903	1316.687	33.611	0.0014757	83.182	1067493

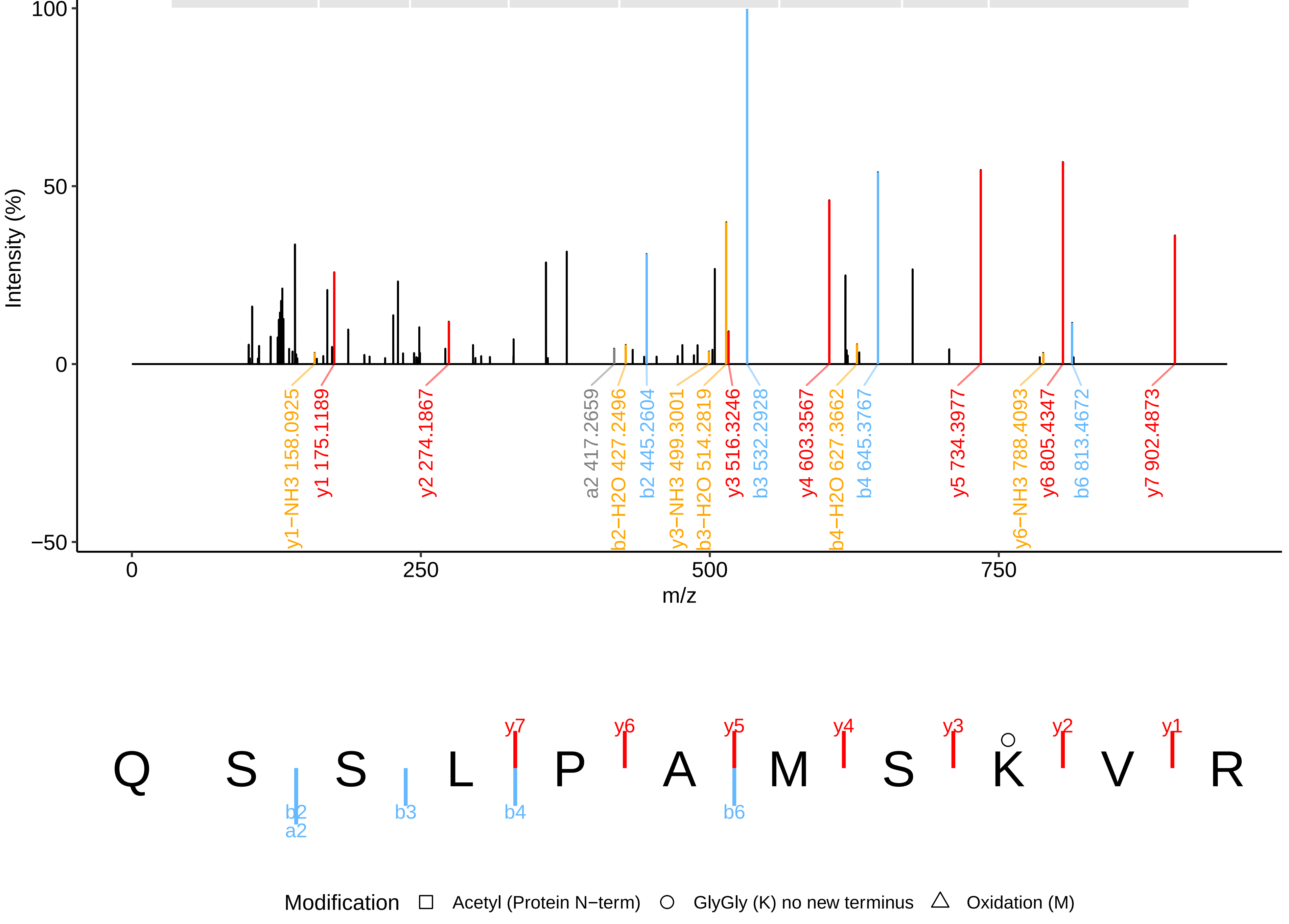

20200921_KIZ_MUb4_GlyGly_10plex_2 − Scan number: 8903

Gene Names	Charge	m/z	Mass	Retention time	PEP	Score	Precursor Intensity
WLS	3	439.903	1316.687	33.694	5.294e−06	113.38	1685912

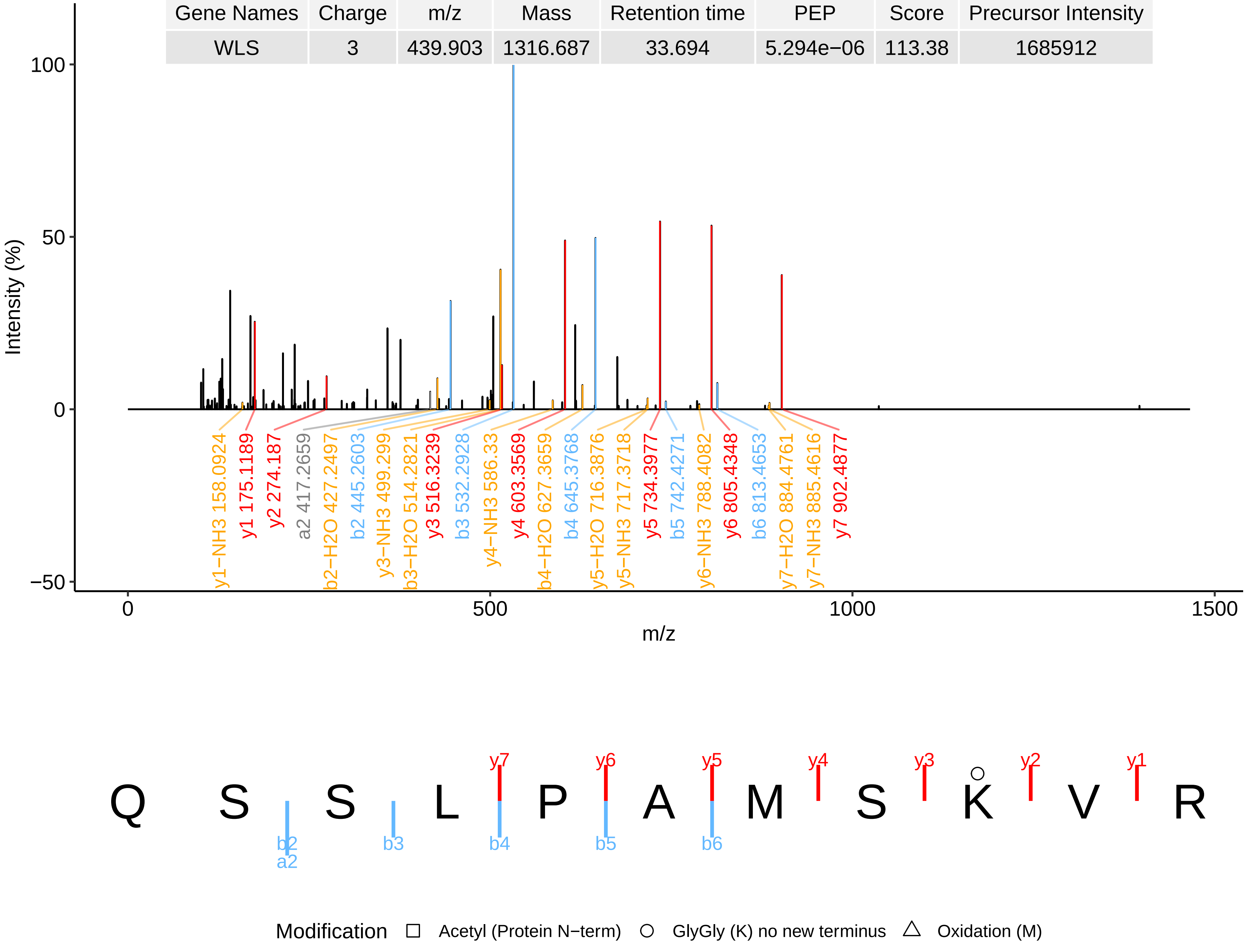

20200924_KIZ_MUb5_GlyGly_1 − Scan number: 8520

Gene Names	Charge	m/z	Mass	Retention time	PEP	Score	Precursor Intensity
WLS	3	439.903	1316.687	27.247	0.00044661	93.839	12586624

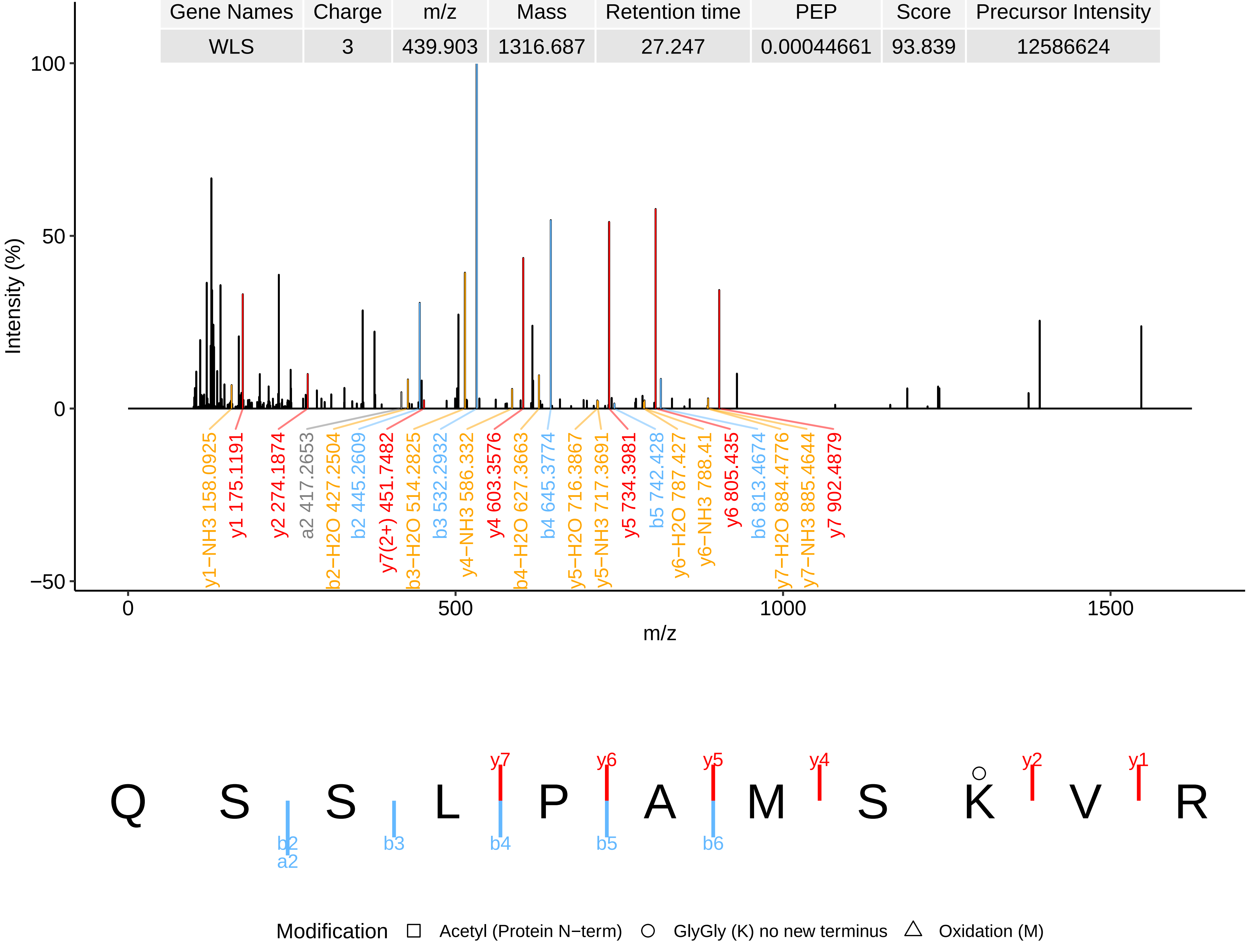

20200921_KIZ_MUb4_GlyGly_10plex_2 − Scan number: 3394

Gene Names	Charge	m/z	Mass	Retention time	PEP	Score	Precursor Intensity
NUP155	3	363.528	1087.562	11.389	0.082367	50.607	1441415

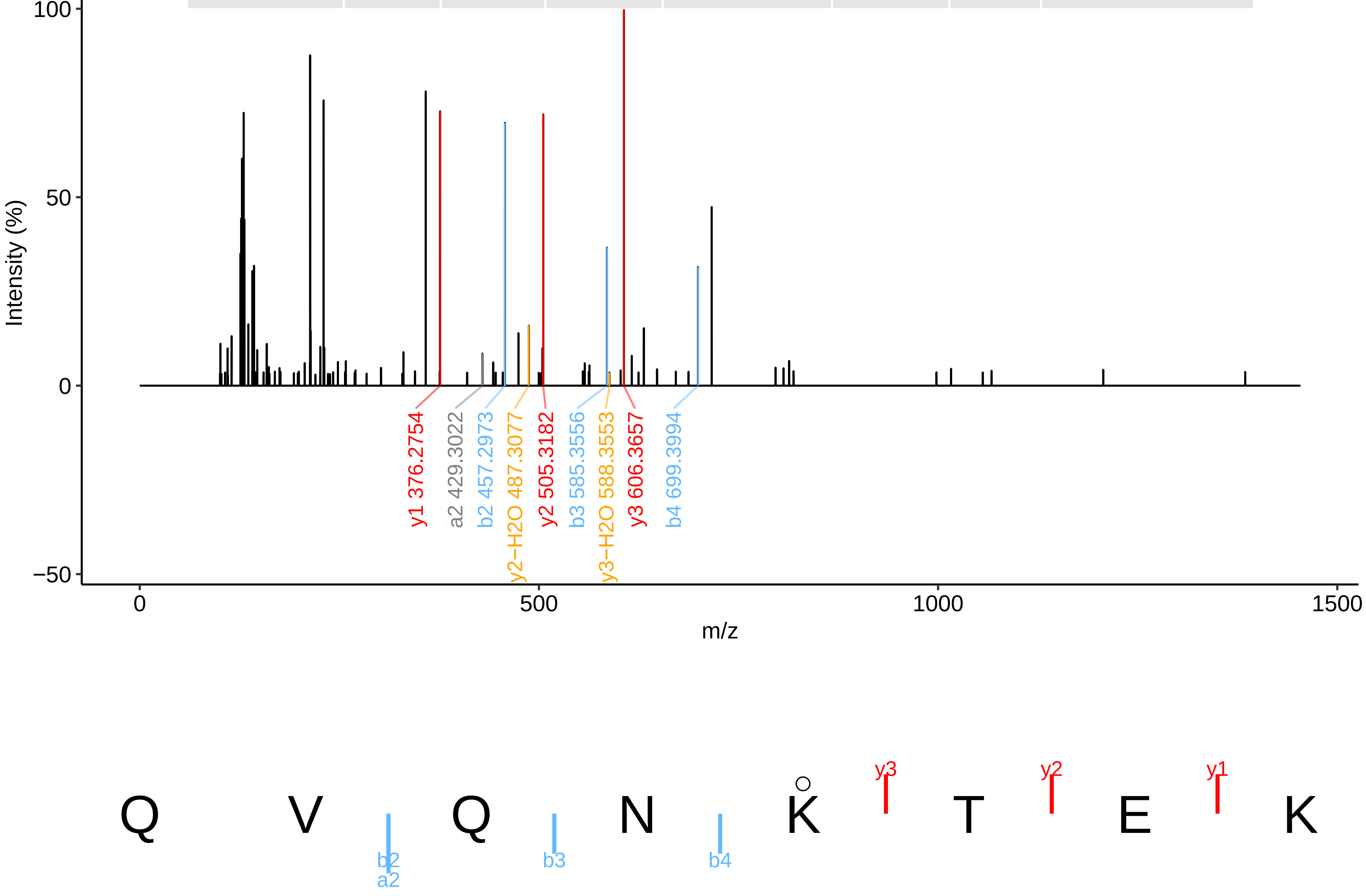

Modification □ Acetyl (Protein N−term) ○ GlyGly (K) no new terminus △ Oxidation (M)

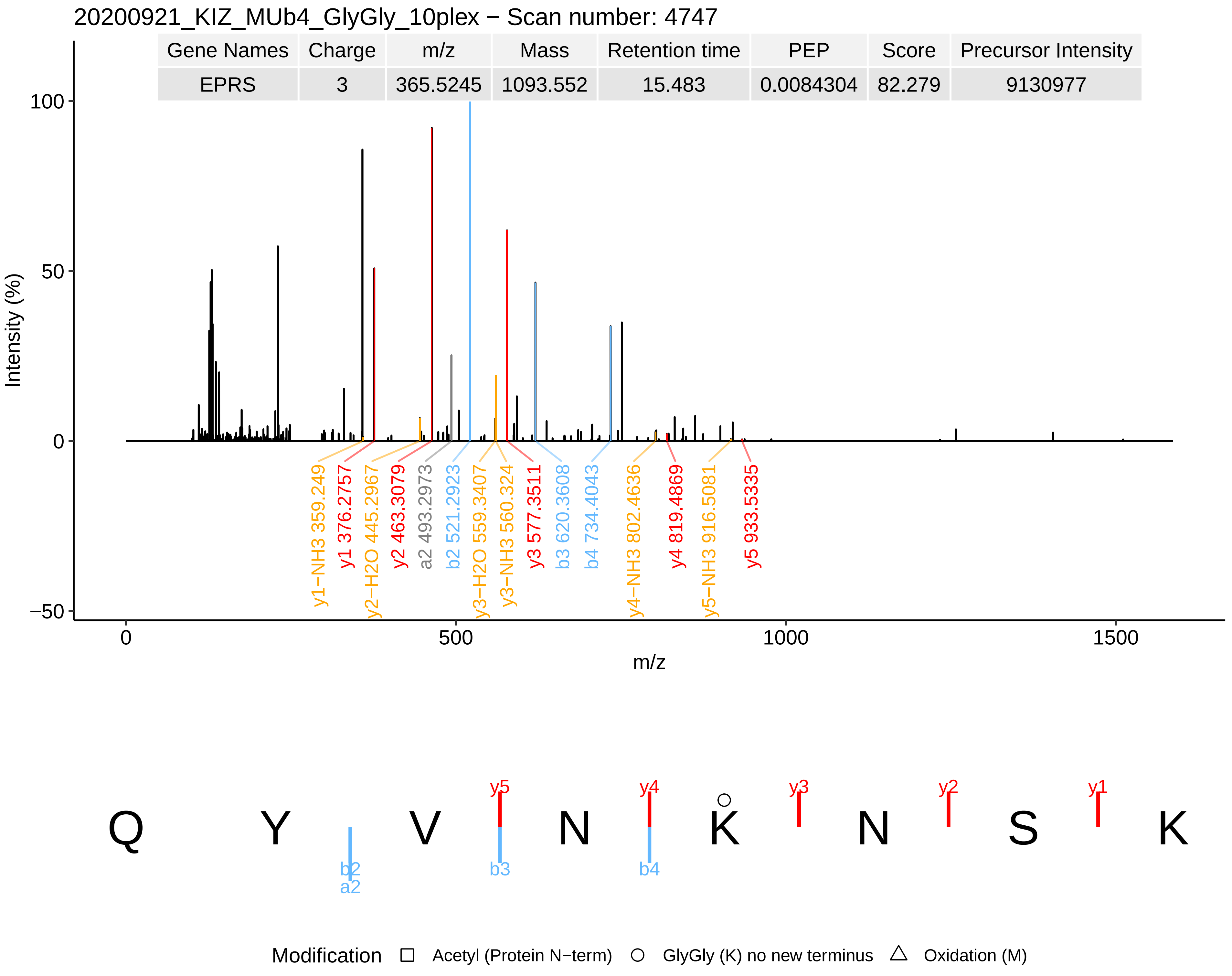
20200921_KIZ_MUb4_GlyGly_10plex − Scan number: 4747
Gene Names
Charge
m/z
Mass
Retention time
PEP
Score
Precursor Intensity
EPRS
3
365.5245
1093.552
15.483
0.0084304
82.279
9130977
Intensity (%)
100
50
0
−50
0
500
1000
1500
m/z
y1−NH3 359.249
y1 376.2757
y2−H2O 445.2967
y2 463.3079
a2 493.2973
b2 521.2923
y3−H2O 559.3407
y3−NH3 560.324
y3 577.3511
b3 620.3608
b4 734.4043
y4−NH3 802.4636
y4 819.4869
y5−NH3 916.5081
y5 933.5335
Q Y V N K N S K
y5
y4
y3
y2
y1
b2
a2
b3
b4
Modification
Acetyl (Protein N−term)
GlyGly (K) no new terminus
Oxidation (M)

20200921_KIZ_MUb4_GlyGly_10plex − Scan number: 5703

Gene Names	Charge	m/z	Mass	Retention time	PEP	Score	Precursor Intensity
RPL4	3	363.856	1088.546	18.709	0.055351	56.359	8273197

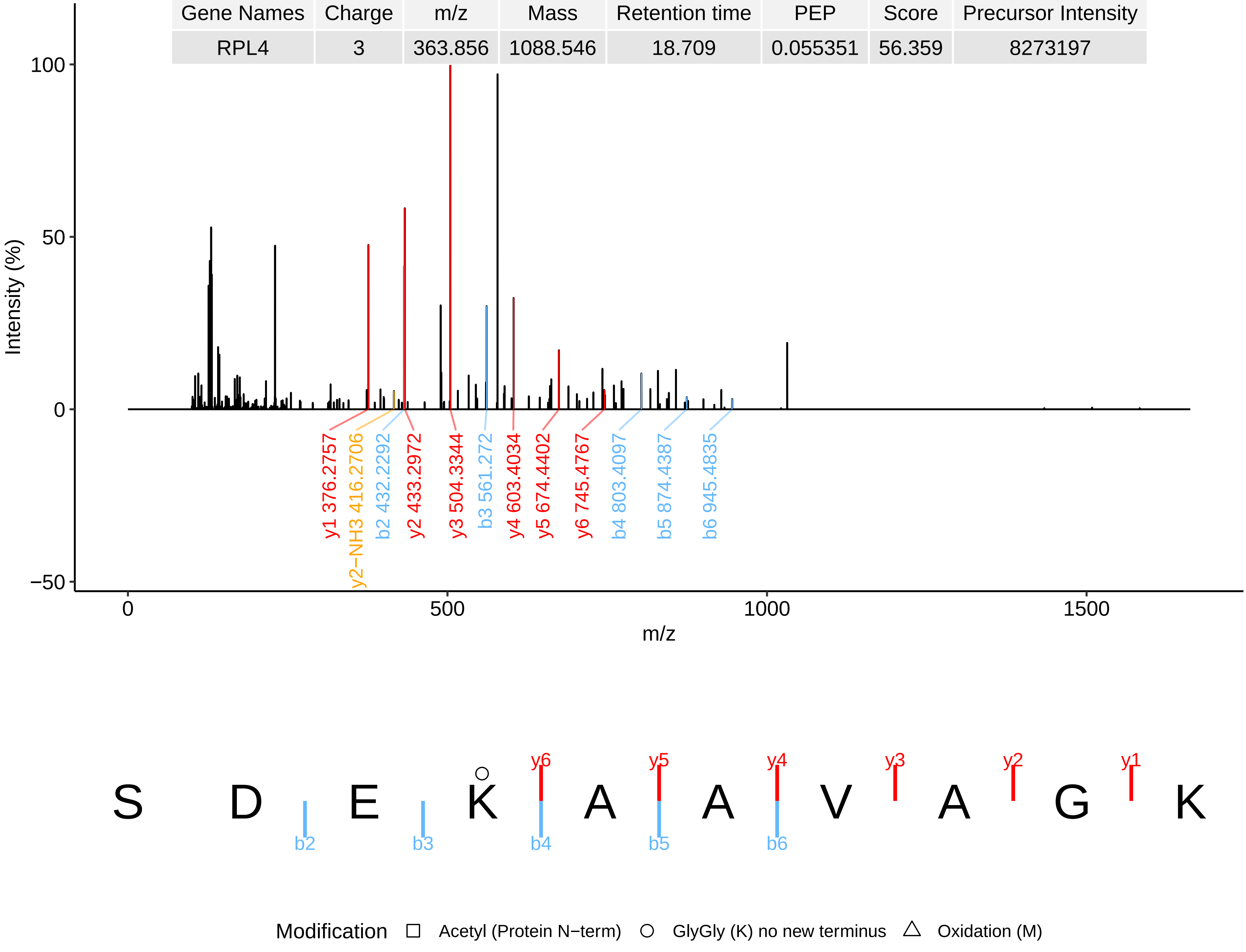

20201118_KIZ_MUb6_GlyGly_2 − Scan number: 2943

Gene Names	Charge	m/z	Mass	Retention time	PEP	Score	Precursor Intensity
DNAJA2	3	446.5398	1336.598	9.4545	7.0501e−05	97.734	3776347

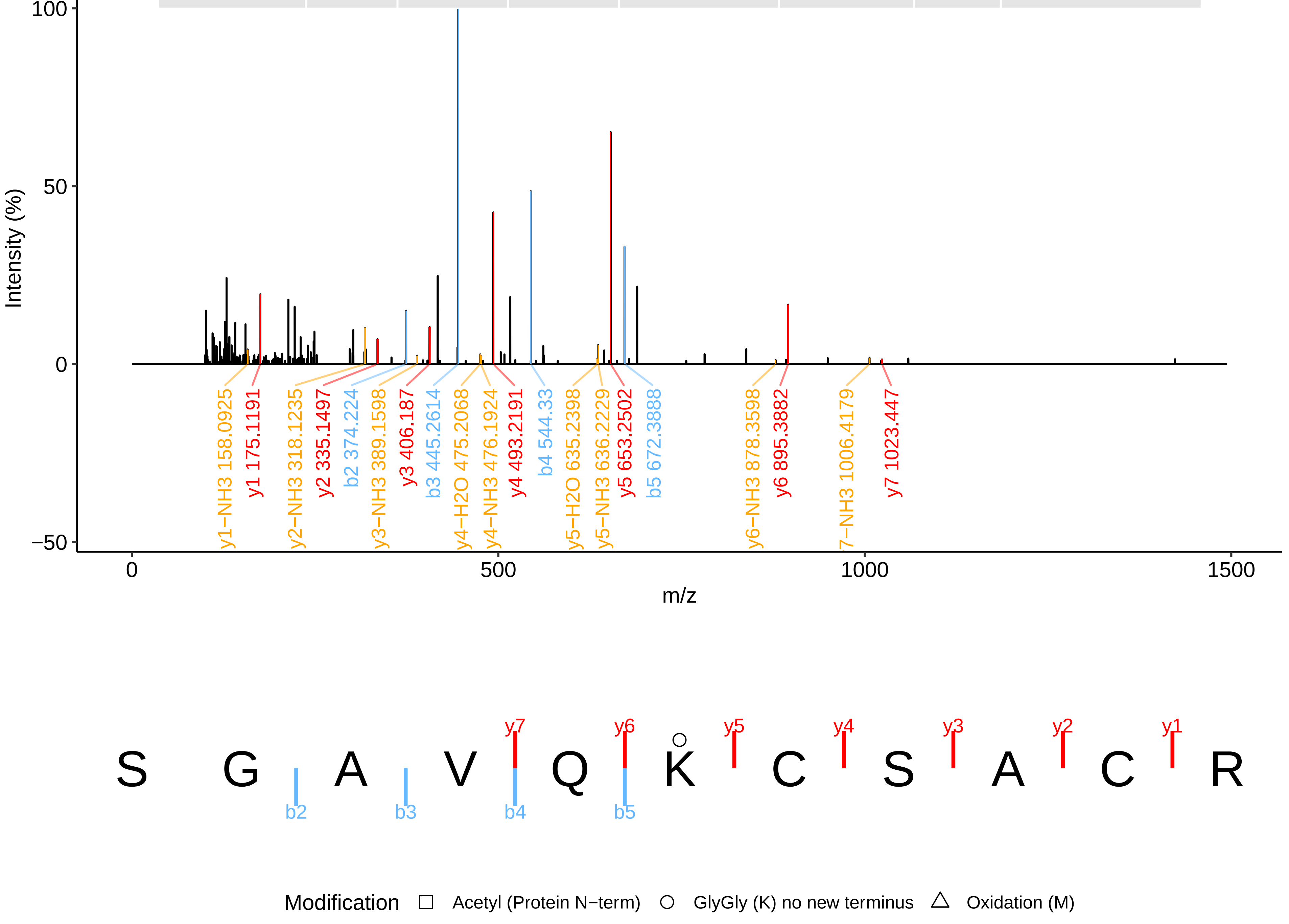

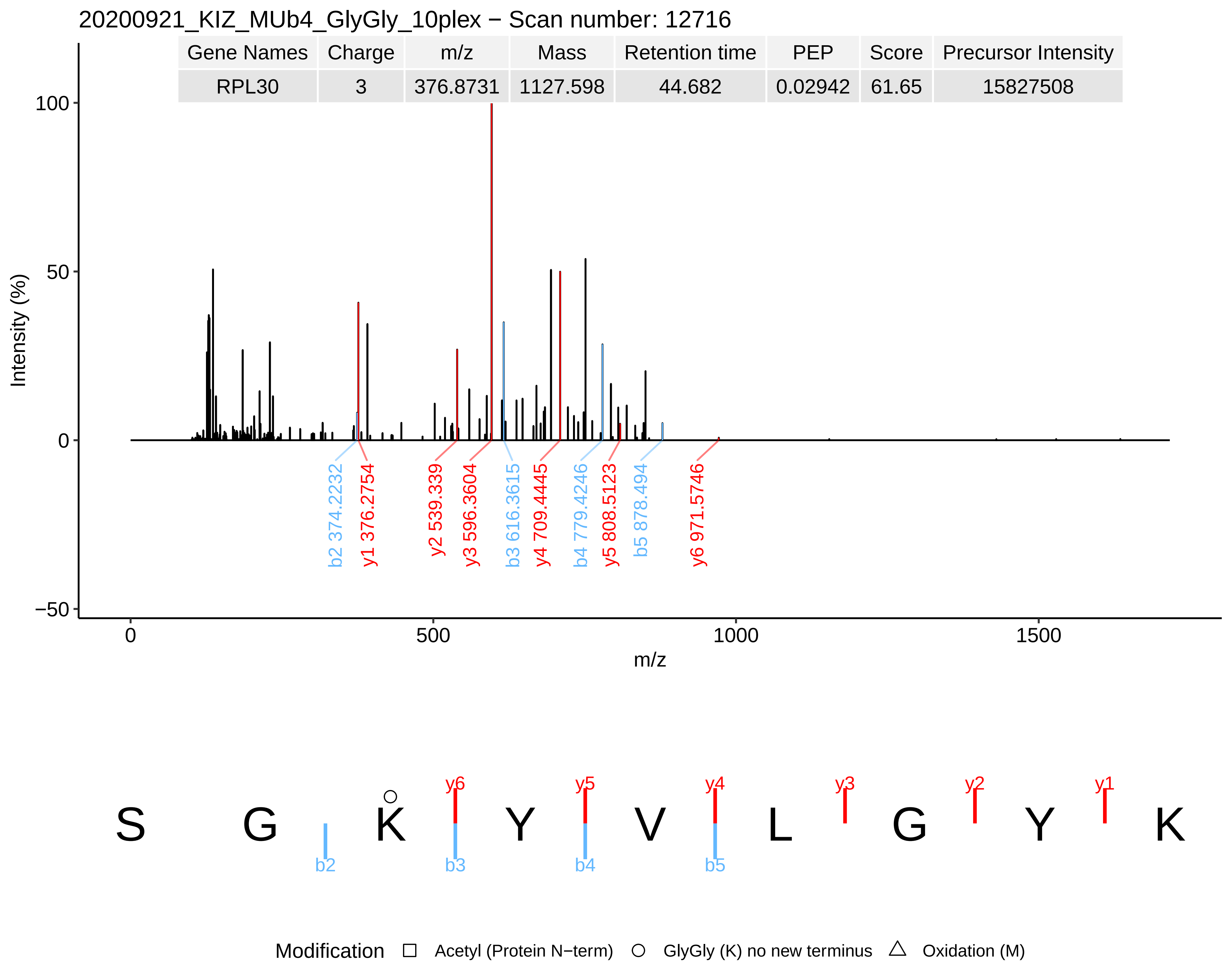

20200921_KIZ_MUb4_GlyGly_10plex − Scan number: 12716
Gene Names | Charge | m/z | Mass | Retention time | PEP | Score | Precursor Intensity
RPL30 | 3 | 376.8731 | 1127.598 | 44.682 | 0.02942 | 61.65 | 15827508
Intensity (%)
100
50
0
−50
0
500
1000
1500
m/z
b2 374.2232
y1 376.2754
y2 539.339
y3 596.3604
b3 616.3615
y4 709.4445
b4 779.4246
y5 808.5123
b5 878.494
y6 971.5746
S G K Y V L G Y K
y6 y5 y4 y3 y2 y1
b2 b3 b4 b5
Modification
Acetyl (Protein N−term)
GlyGly (K) no new terminus
Oxidation (M)

20200924_KIZ_MUb5_GlyGly_1 − Scan number: 11401

Gene Names	Charge	m/z	Mass	Retention time	PEP	Score	Precursor Intensity
RPL30	3	376.8731	1127.598	36.935	0.10364	52.278	3394592

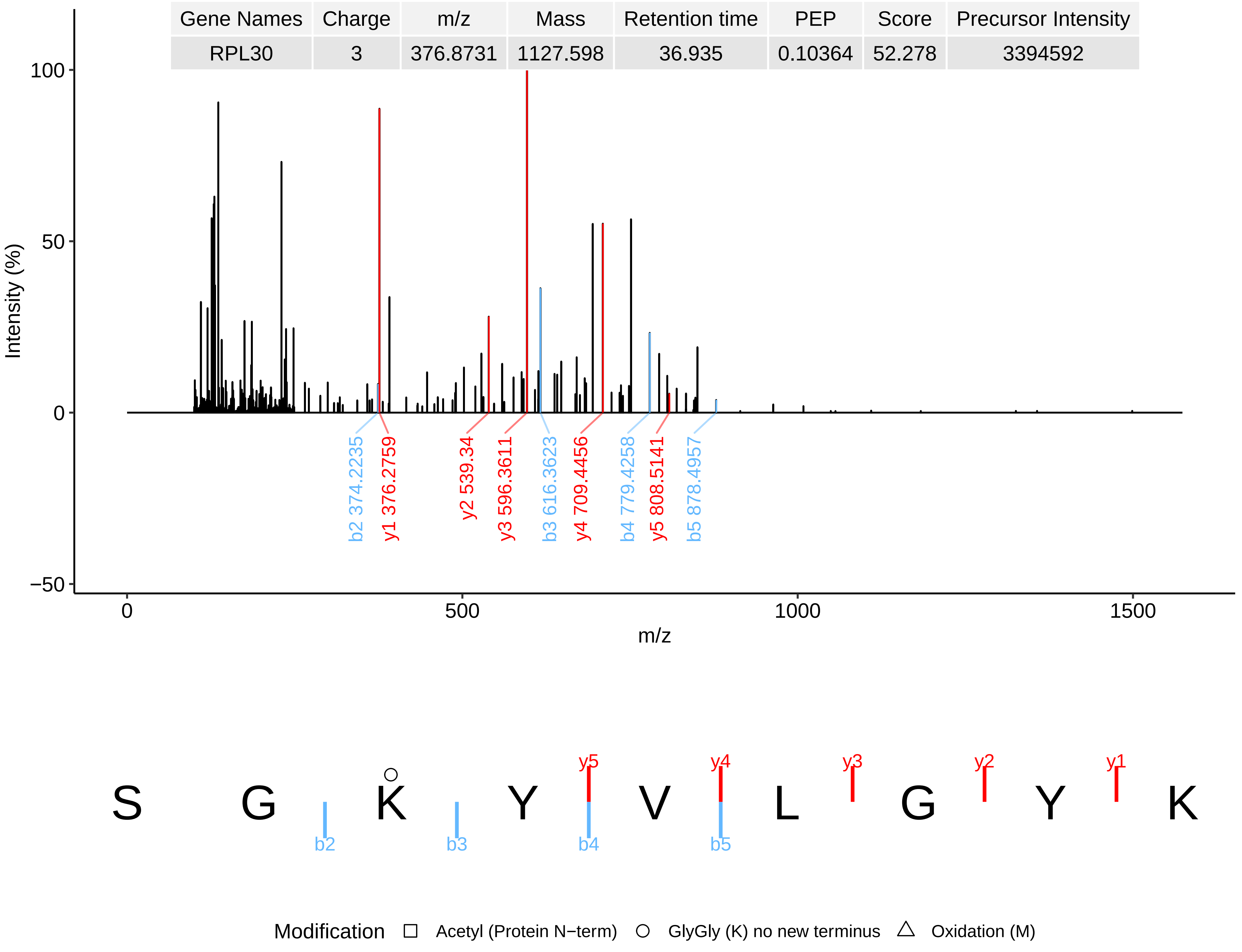

20201118_KIZ_MUb6_GlyGly_2 − Scan number: 13719

Gene Names	Charge	m/z	Mass	Retention time	PEP	Score	Precursor Intensity
RPL30	3	376.8731	1127.598	46.239	0.1168	43.282	15357844

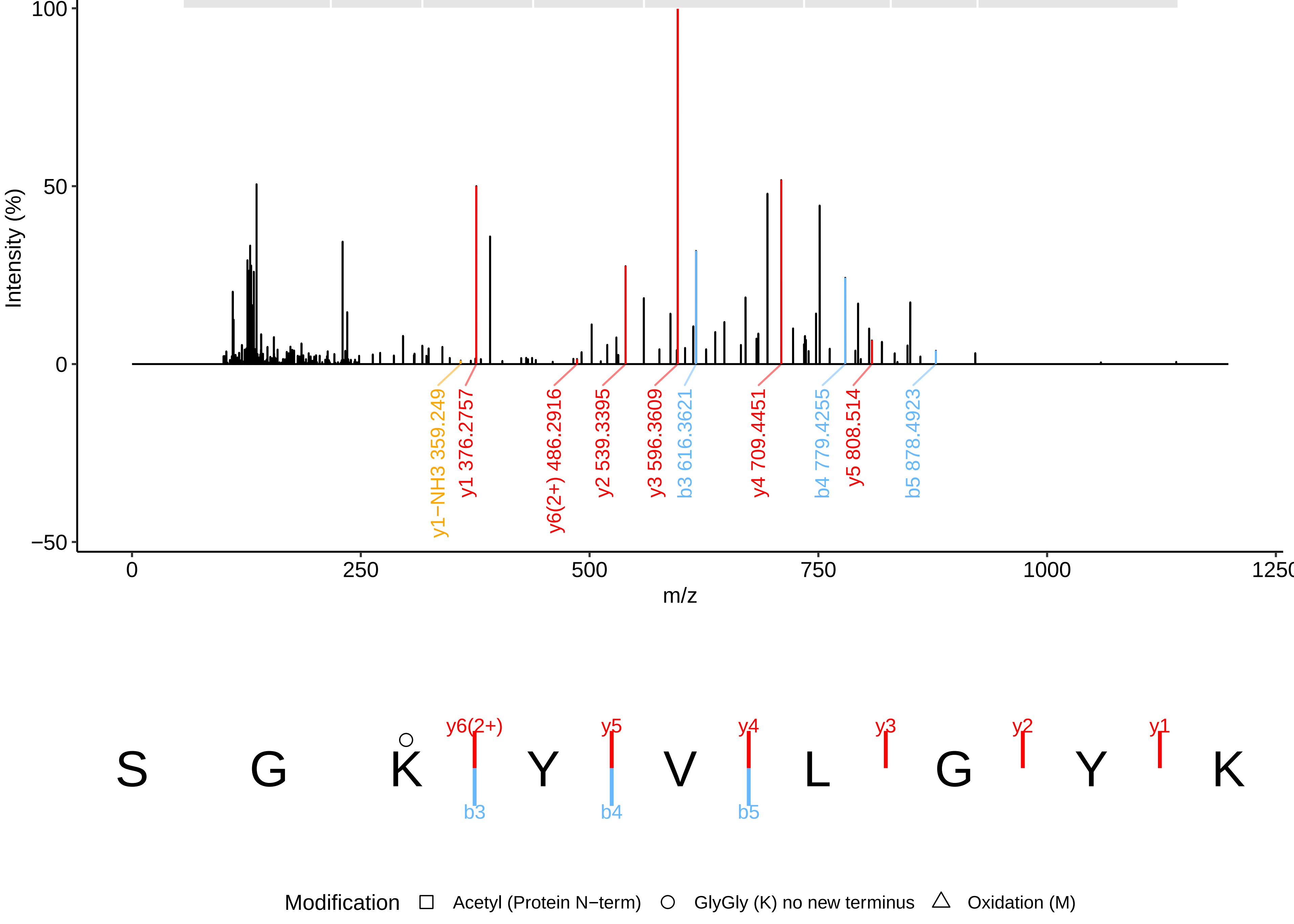

20200921_KIZ_MUb4_GlyGly_10plex_2 − Scan number: 10377

Gene Names	Charge	m/z	Mass	Retention time	PEP	Score	Precursor Intensity
HSPD1	3	374.8645	1121.572	39.687	0.099991	52.79	605616

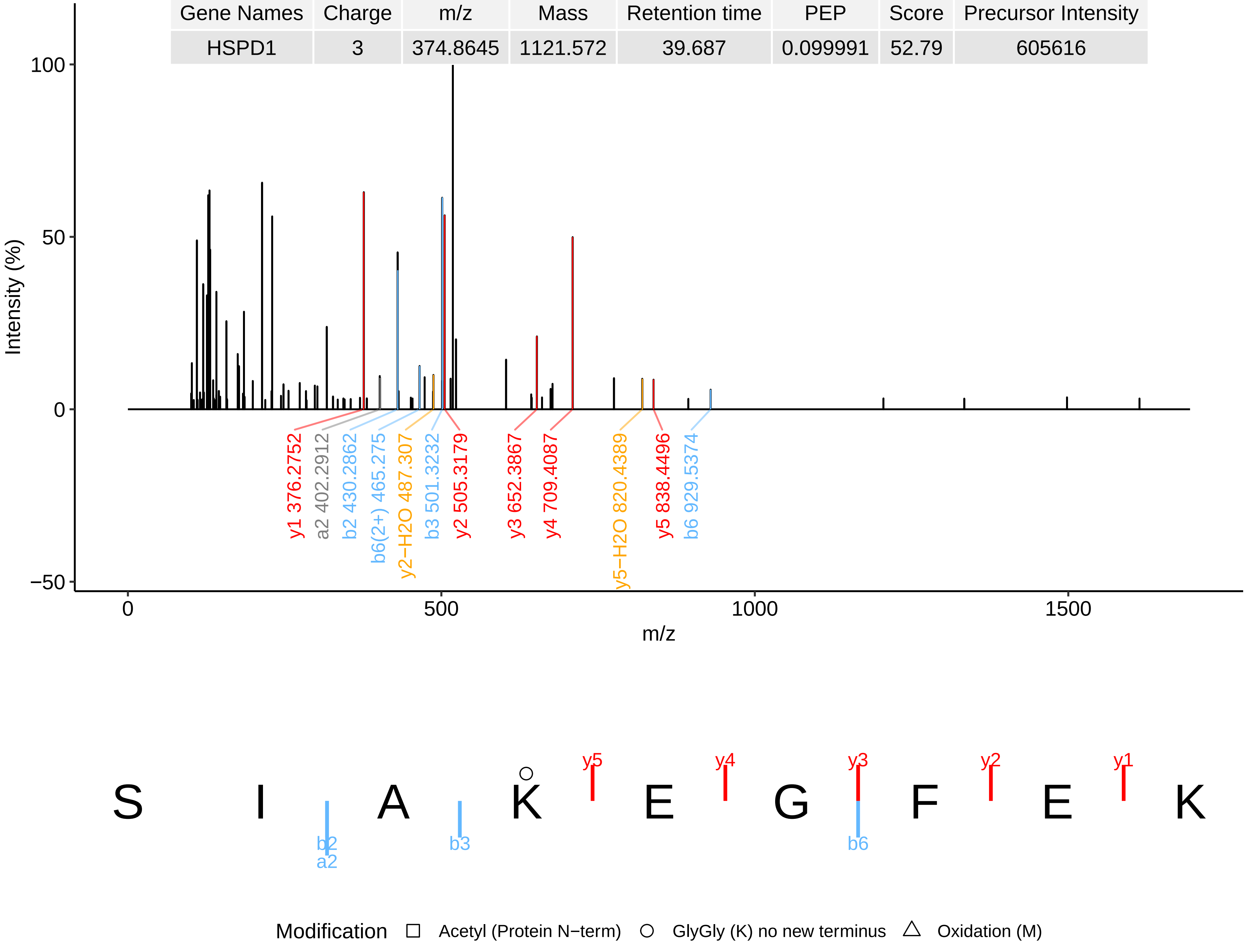

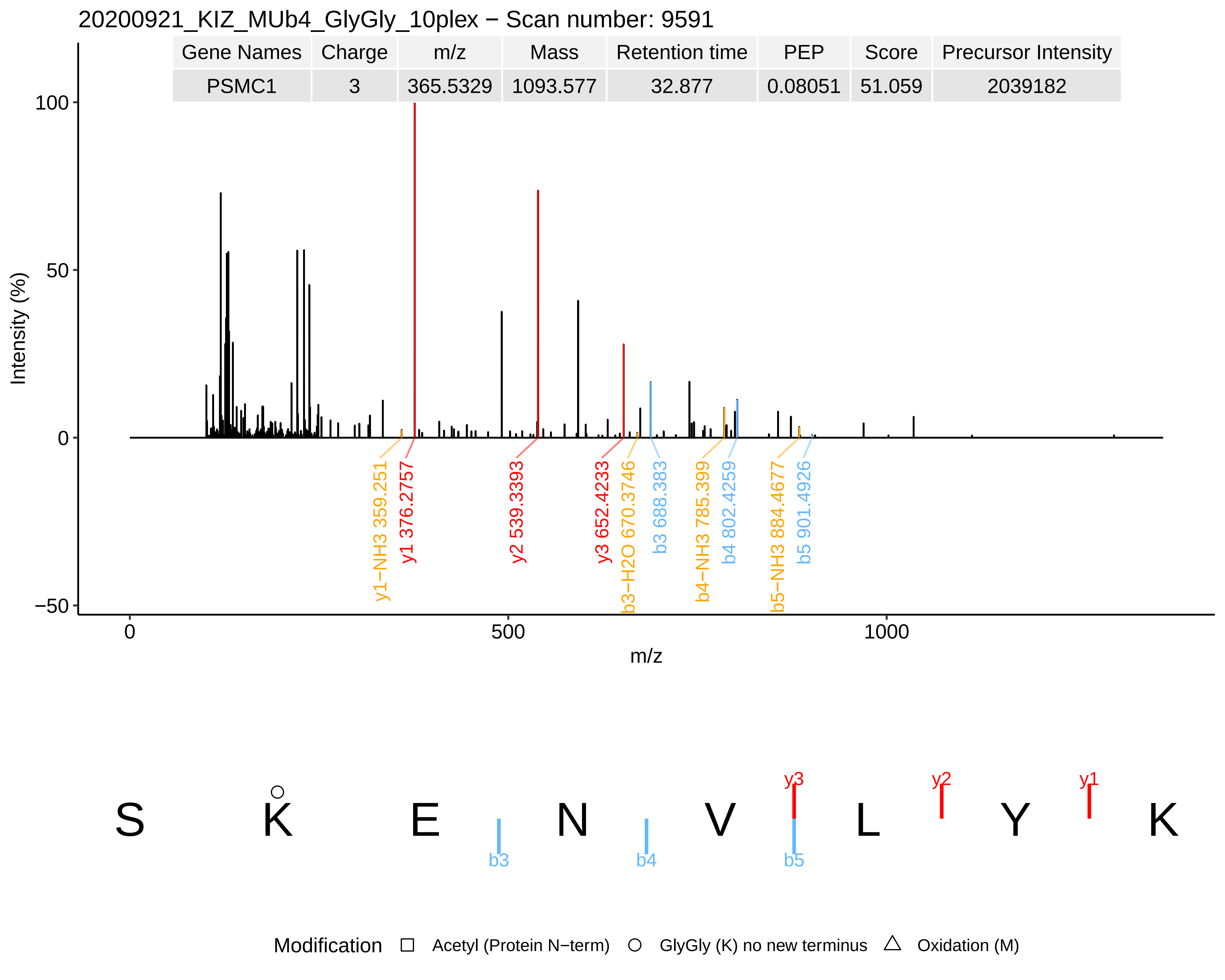
20200921_KIZ_MUb4_GlyGly_10plex − Scan number: 9591
Gene Names | Charge | m/z | Mass | Retention time | PEP | Score | Precursor Intensity
PSMC1 | 3 | 365.5329 | 1093.577 | 32.877 | 0.08051 | 51.059 | 2039182
Intensity (%)
100
50
0
−50
0
500
1000
m/z
y1−NH3 359.251
y1 376.2757
y2 539.3393
y3 652.4233
b3−H2O 670.3746
b3 688.383
b4−NH3 785.399
b4 802.4259
b5−NH3 884.4677
b5 901.4926
S K E N V L Y K
y3
y2
y1
b3
b4
b5
Modification
Acetyl (Protein N−term)
GlyGly (K) no new terminus
Oxidation (M)

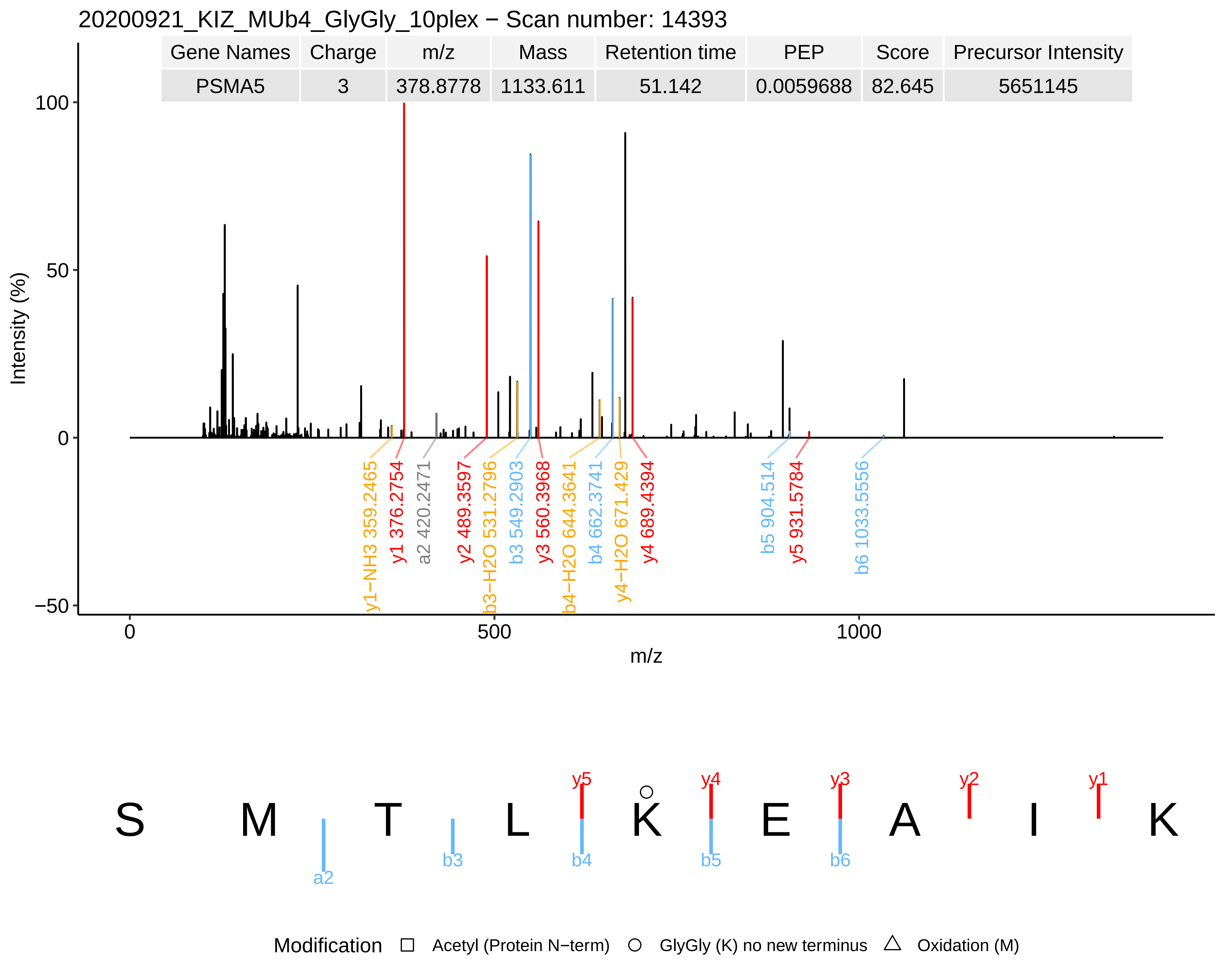

20200921_KIZ_MUb4_GlyGly_10plex − Scan number: 14393
Gene Names
Charge
m/z
Mass
Retention time
PEP
Score
Precursor Intensity
PSMA5
3
378.8778
1133.611
51.142
0.0059688
82.645
5651145
Intensity (%)
m/z
y1−NH3 359.2465
y1 376.2754
a2 420.2471
y2 489.3597
b3−H2O 531.2796
b3 549.2903
y3 560.3968
b4−H2O 644.3641
b4 662.3741
y4−H2O 671.429
y4 689.4394
b5 904.514
y5 931.5784
b6 1033.5556
S M T L K E A I K
Modification
Acetyl (Protein N−term)
GlyGly (K) no new terminus
Oxidation (M)

20200921_KIZ_MUb4_GlyGly_10plex − Scan number: 15130

Gene Names	Charge	m/z	Mass	Retention time	PEP	Score	Precursor Intensity
SGTA	3	377.224	1128.65	53.972	0.092366	48.602	5579215

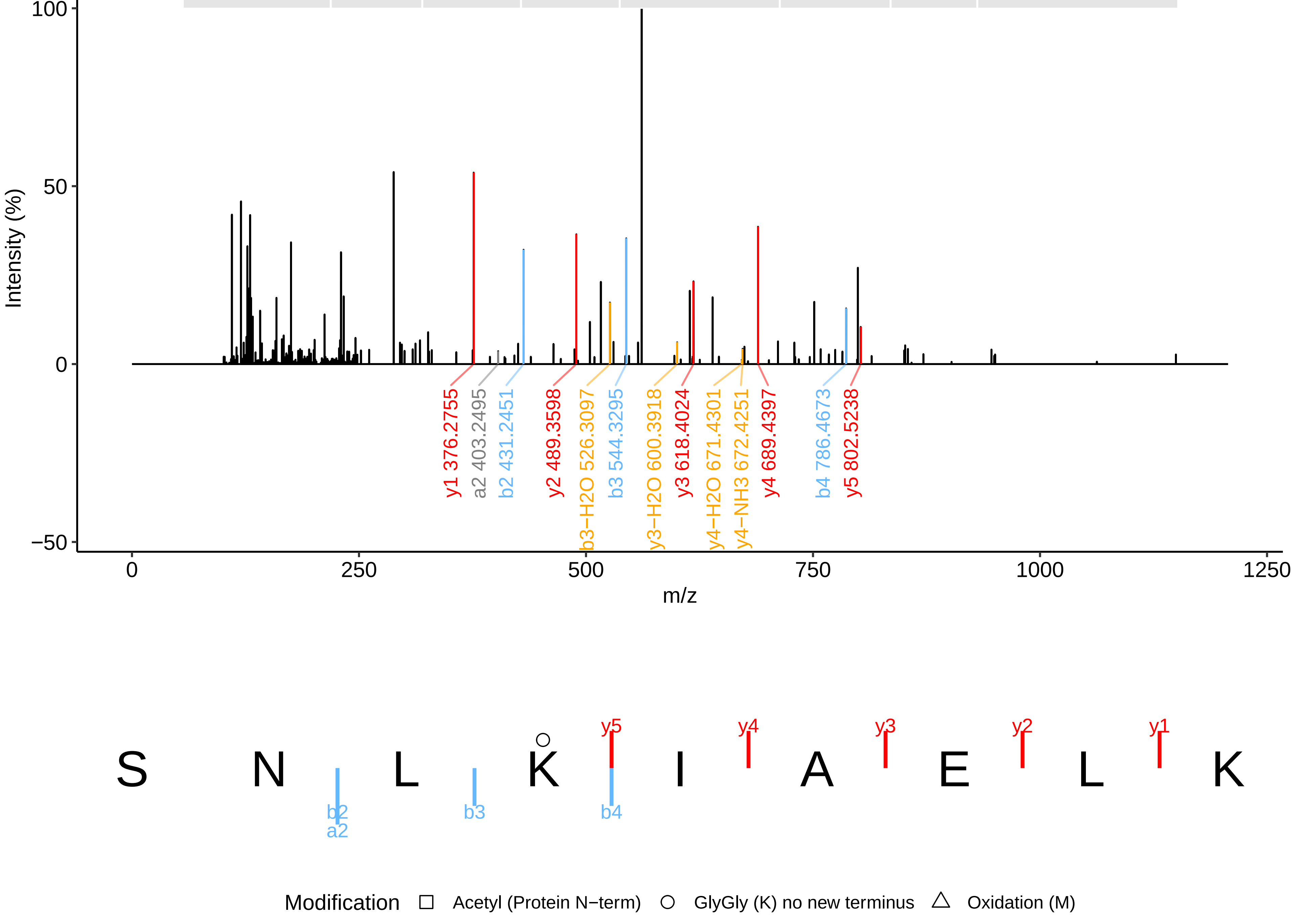

20200921_KIZ_MUb4_GlyGly_10plex_2 − Scan number: 14006

Gene Names	Charge	m/z	Mass	Retention time	PEP	Score	Precursor Intensity
SGTA	3	377.224	1128.65	54.508	0.083915	50.353	1318995

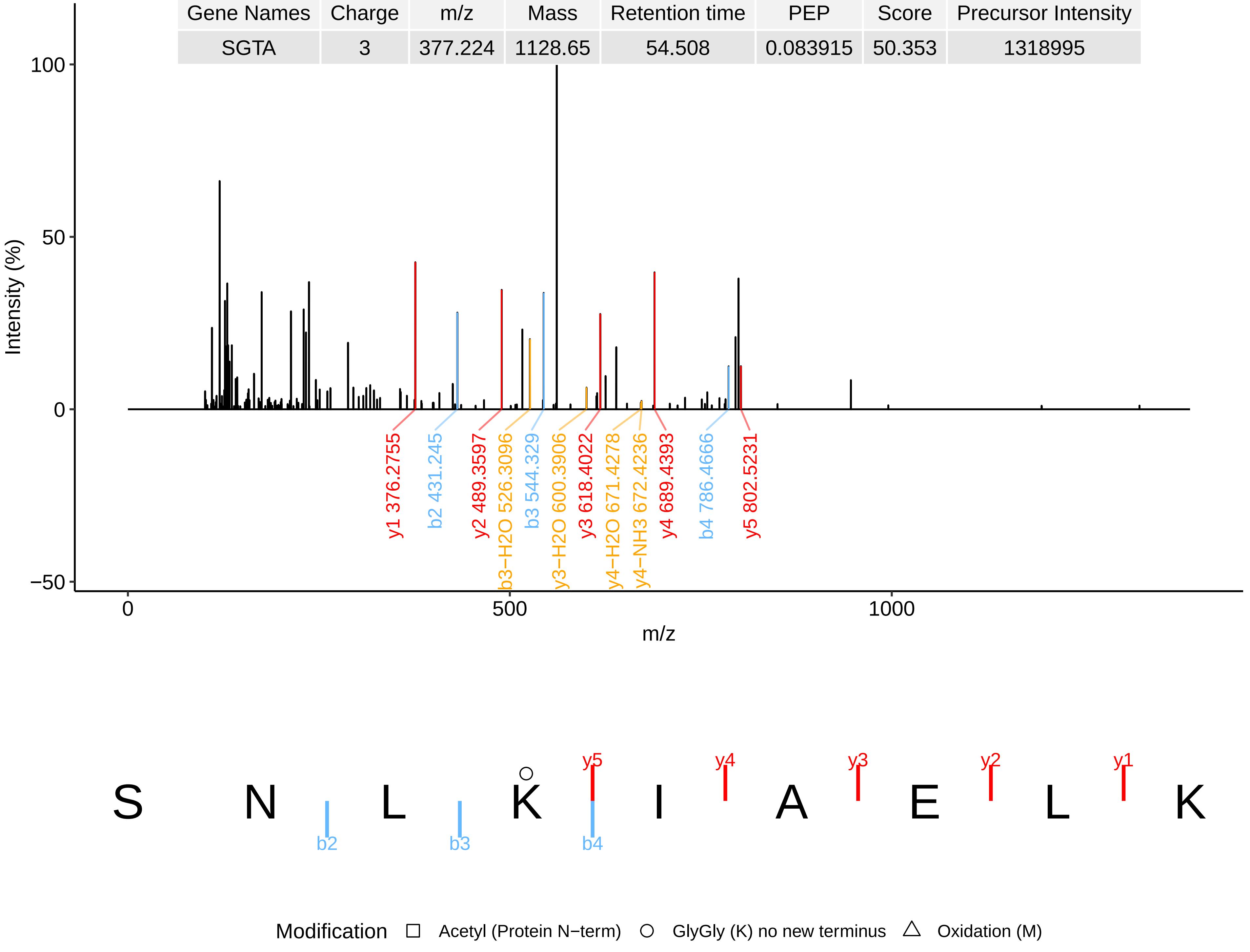

20201118_KIZ_MUb6_GlyGly_1 − Scan number: 14270

Gene Names	Charge	m/z	Mass	Retention time	PEP	Score	Precursor Intensity
SGTA	3	377.224	1128.65	45.134	0.014379	70.399	12574353

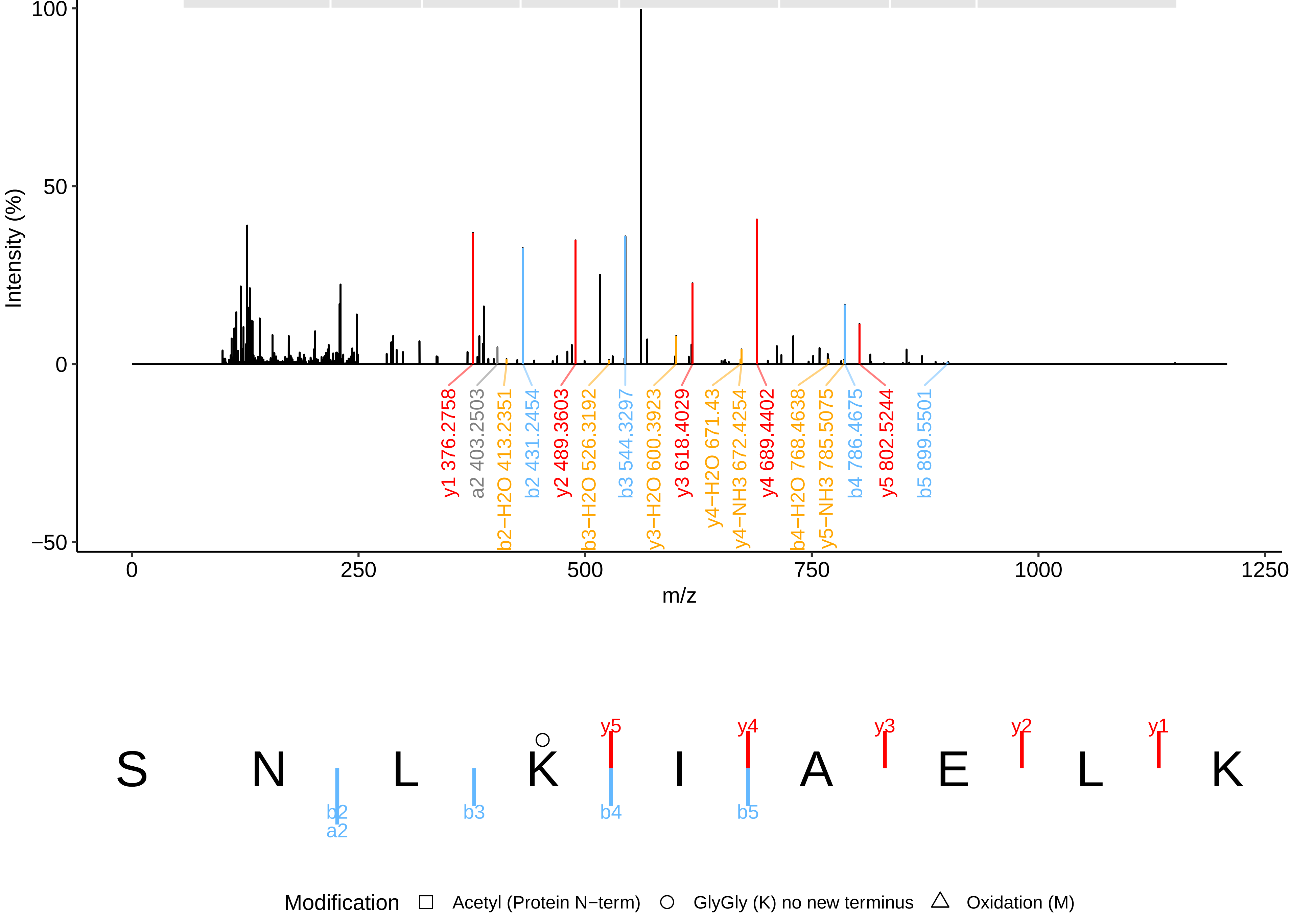

20201118_KIZ_MUb6_GlyGly_2 − Scan number: 16554

Gene Names	Charge	m/z	Mass	Retention time	PEP	Score	Precursor Intensity
SGTA	3	377.224	1128.65	55.922	0.016032	68.847	9596675

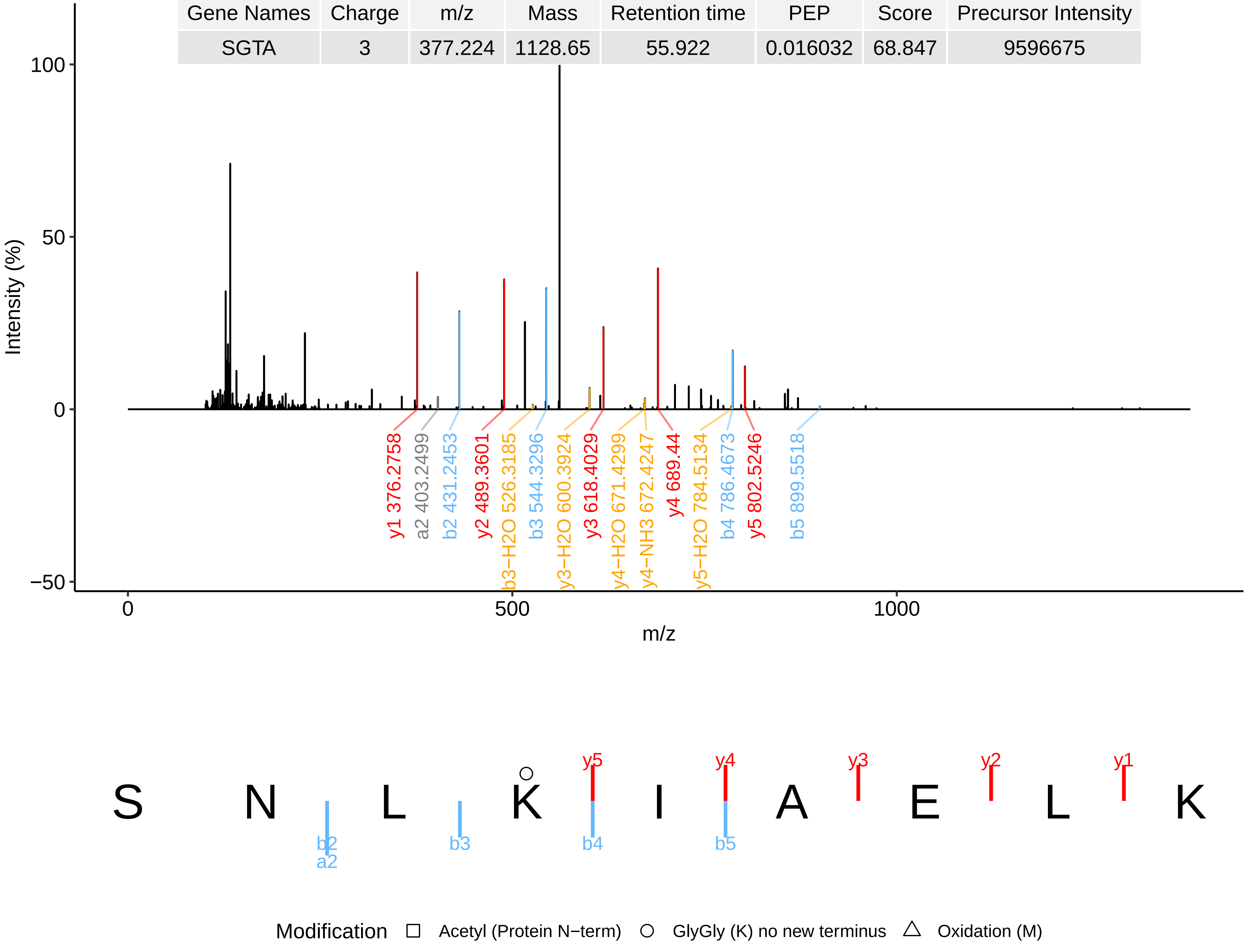

20200921_KIZ_MUb4_GlyGly_10plex_2 − Scan number: 11911

Gene Names	Charge	m/z	Mass	Retention time	PEP	Score	Precursor Intensity
ALDH3B1	3	443.903	1328.687	45.964	0.016565	63.073	1109579

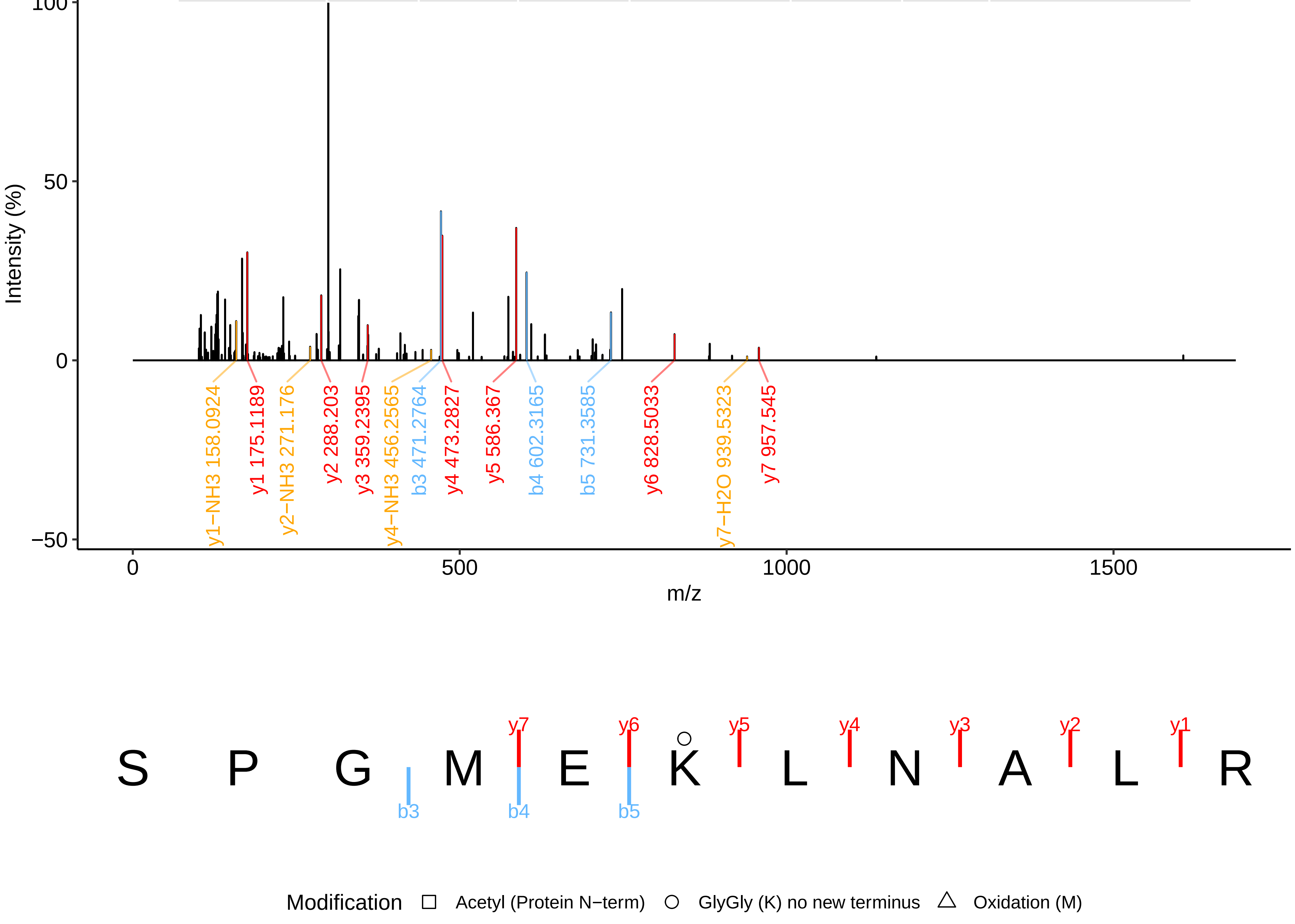

20200924_KIZ_MUb5_GlyGly_1 − Scan number: 11886

Gene Names	Charge	m/z	Mass	Retention time	PEP	Score	Precursor Intensity
ALDH3B1	3	443.903	1328.687	38.579	0.0018226	84.31	7894691

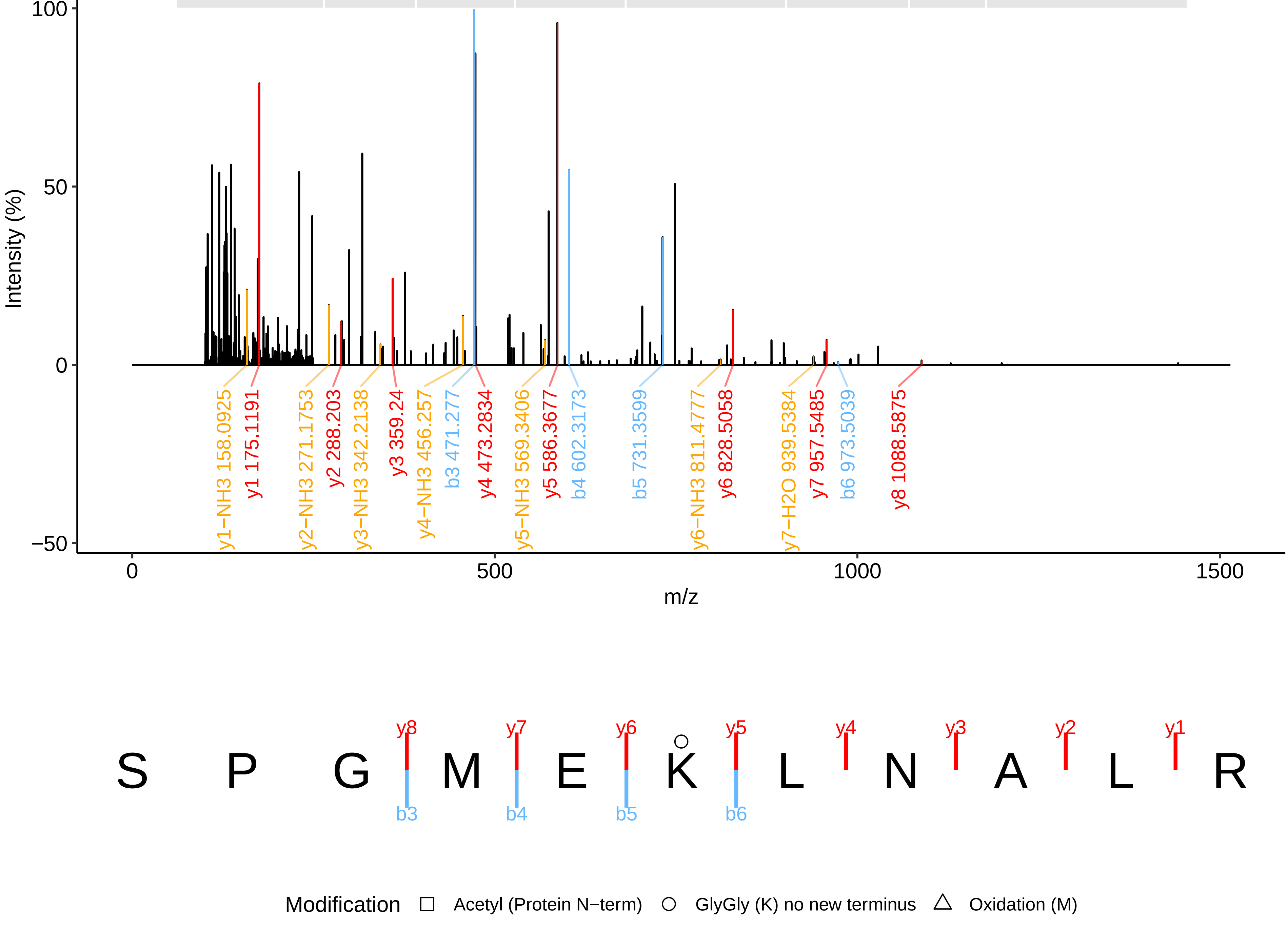

20200921_KIZ_MUb4_GlyGly_10plex − Scan number: 14370

Gene Names	Charge	m/z	Mass	Retention time	PEP	Score	Precursor Intensity
XRCC5	3	376.5643	1126.671	51.055	0.00031893	105.98	6387999

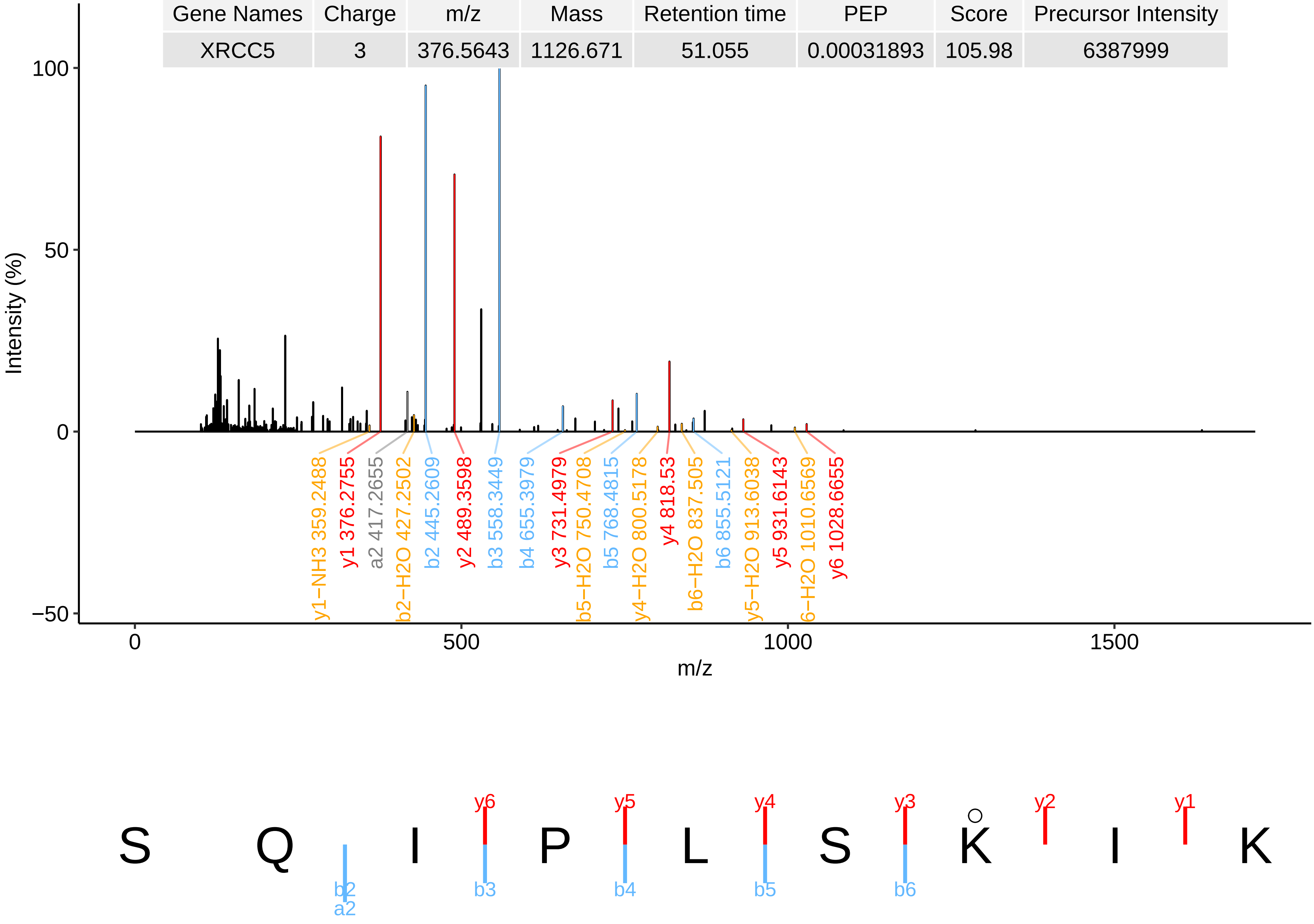

Modification □ Acetyl (Protein N−term) ○ GlyGly (K) no new terminus △ Oxidation (M)

20200921_KIZ_MUb4_GlyGly_10plex_2 − Scan number: 13525

Gene Names	Charge	m/z	Mass	Retention time	PEP	Score	Precursor Intensity
HSPA1B;HSPA1A;HSPA6;HSPA7	3	452.9209	1355.741	52.533	0.065626	53.569	272411.9

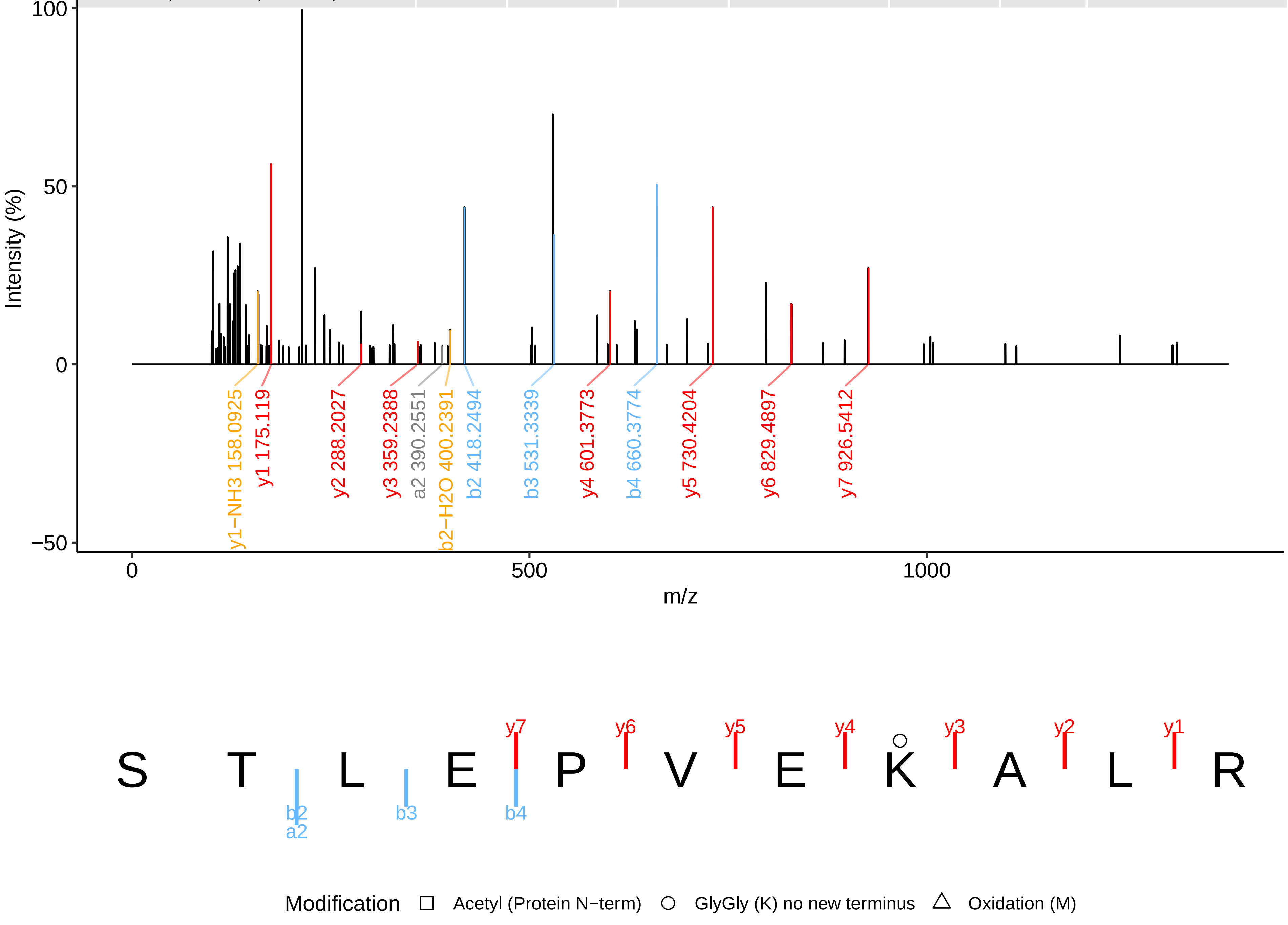

20201118_KIZ_MUb6_GlyGly_2 − Scan number: 4778

Gene Names	Charge	m/z	Mass	Retention time	PEP	Score	Precursor Intensity
RNF13	3	378.8513	1133.532	16.422	0.0081601	87.323	1468648

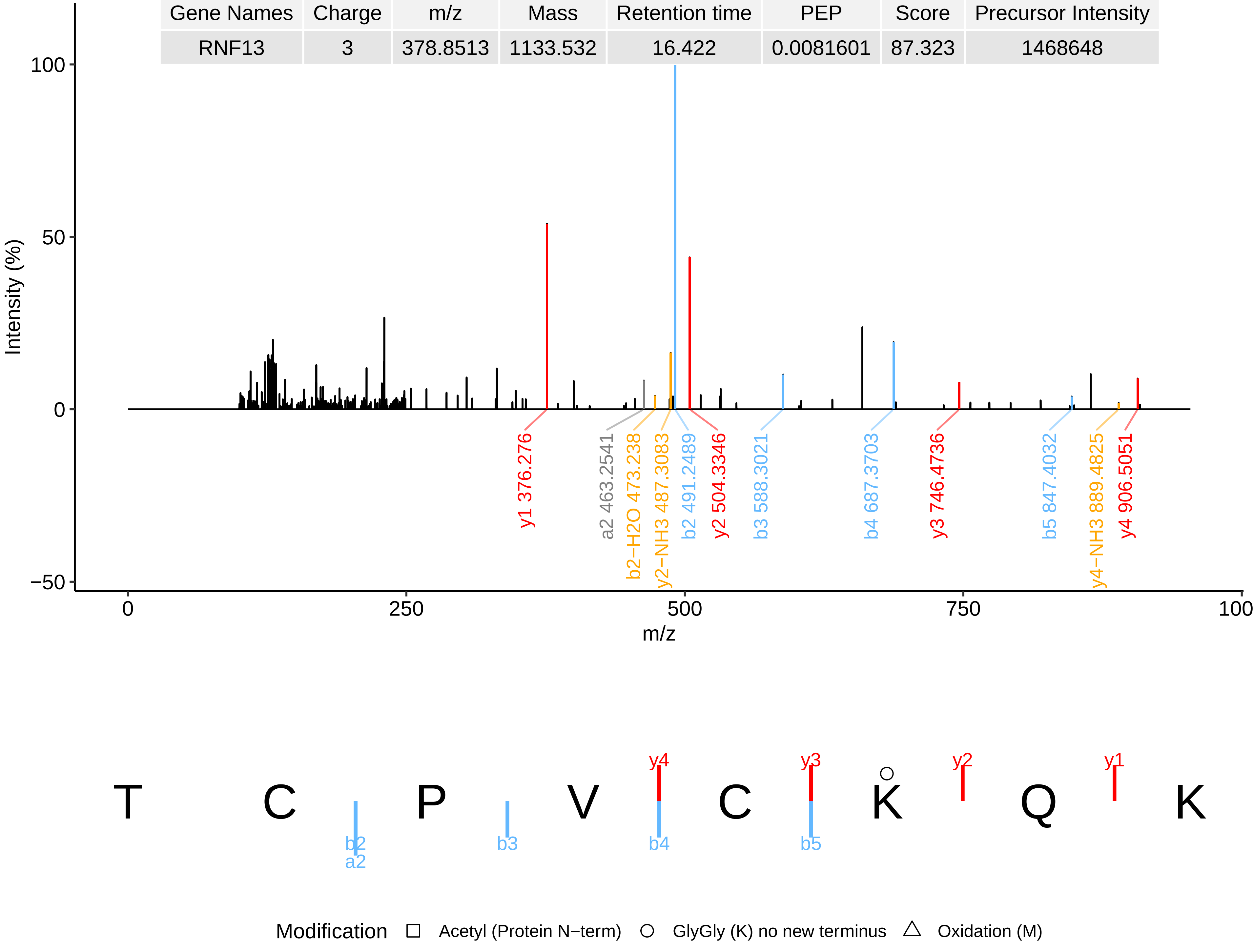

20200921_KIZ_MUb4_GlyGly_10plex − Scan number: 3099

Gene Names	Charge	m/z	Mass	Retention time	PEP	Score	Precursor Intensity
SLC1A3	3	368.5158	1102.525	9.7071	0.00051443	99.788	8413096

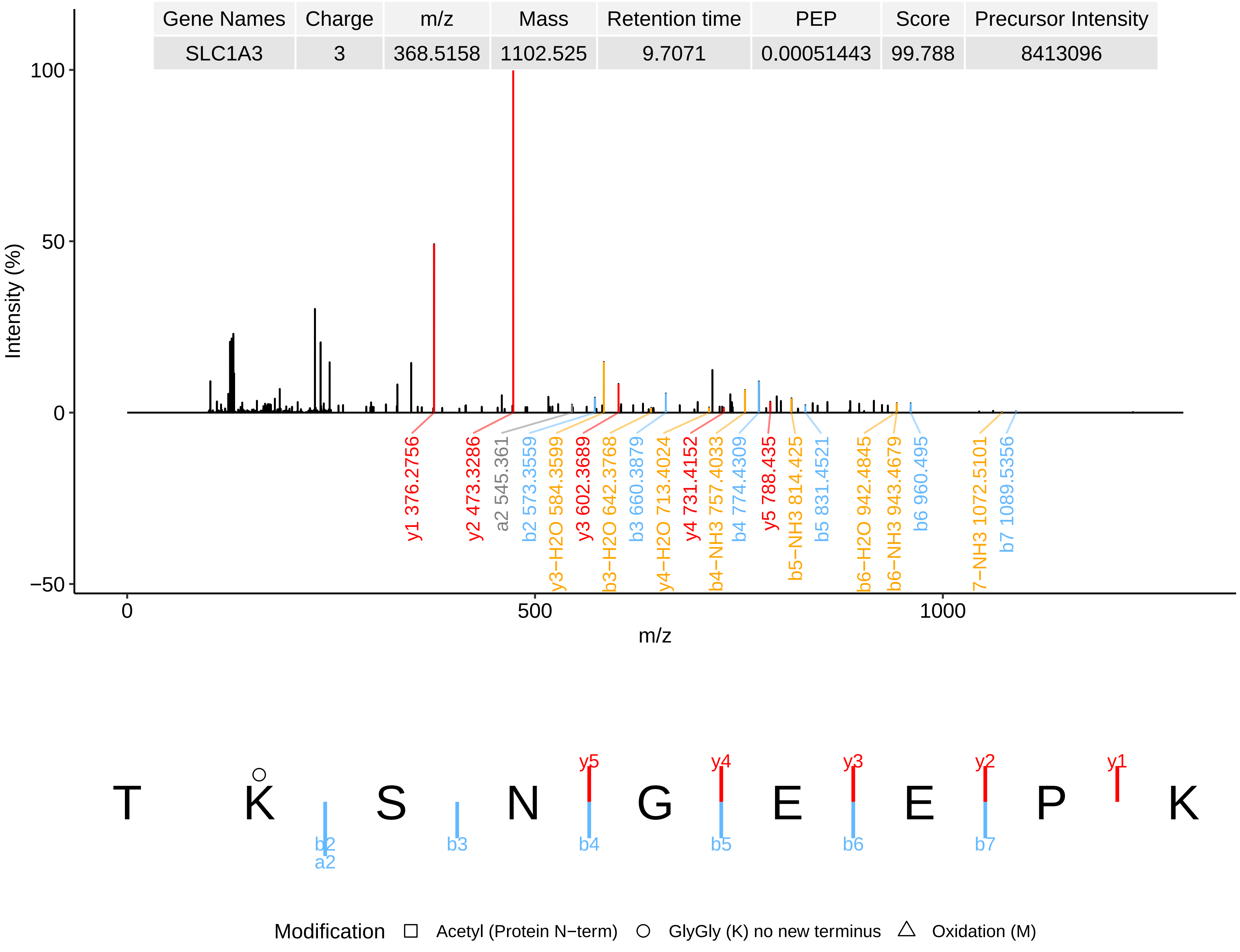

20200921_KIZ_MUb4_GlyGly_10plex_2 − Scan number: 2997

Gene Names	Charge	m/z	Mass	Retention time	PEP	Score	Precursor Intensity
SLC1A3	3	368.5158	1102.525	9.8124	0.029854	61.56	746134.2

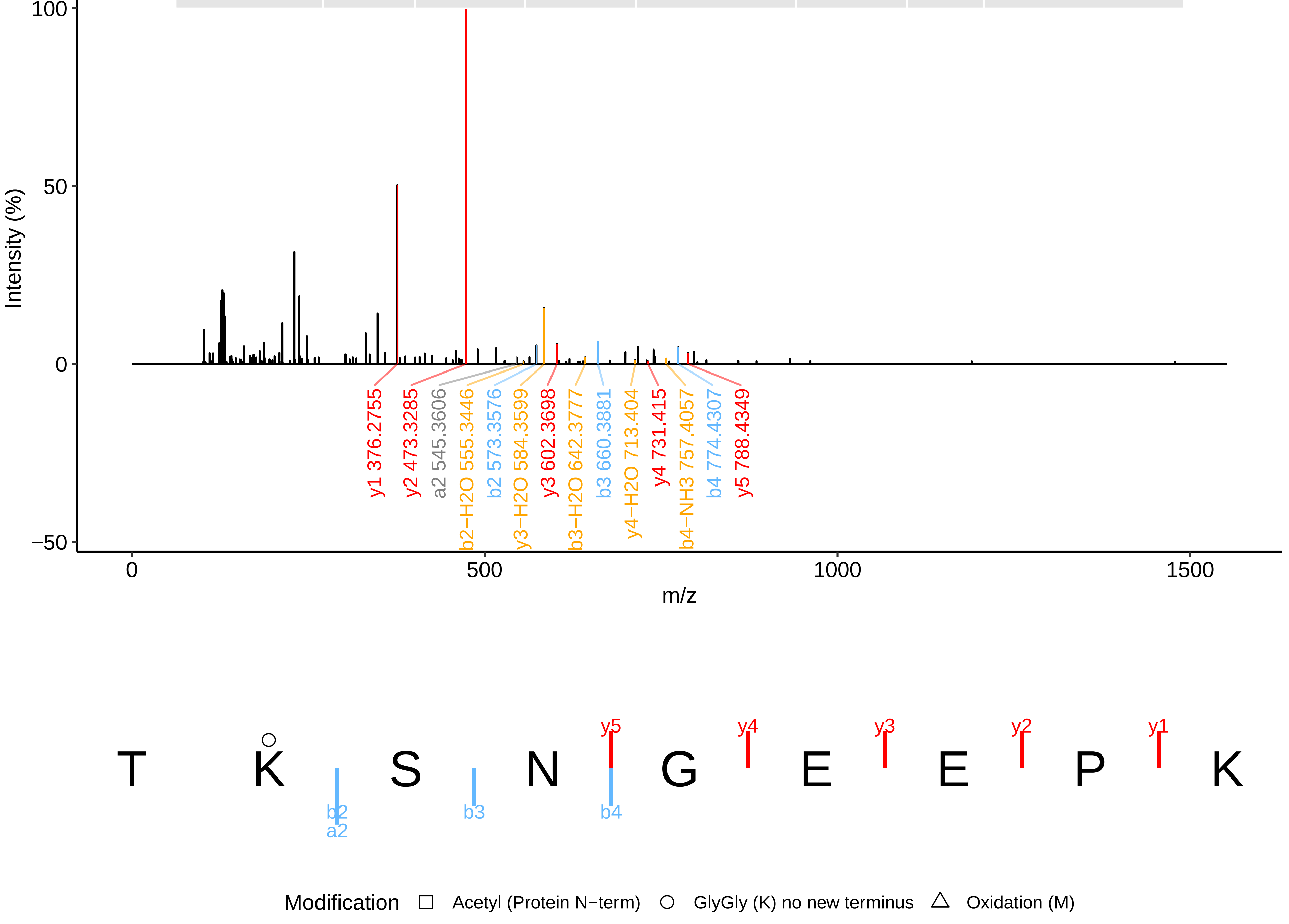

20201118_KIZ_MUb6_GlyGly_1 − Scan number: 2940

Gene Names	Charge	m/z	Mass	Retention time	PEP	Score	Precursor Intensity
SLC1A3	3	368.5158	1102.525	9.0153	0.0019597	89.827	20737092

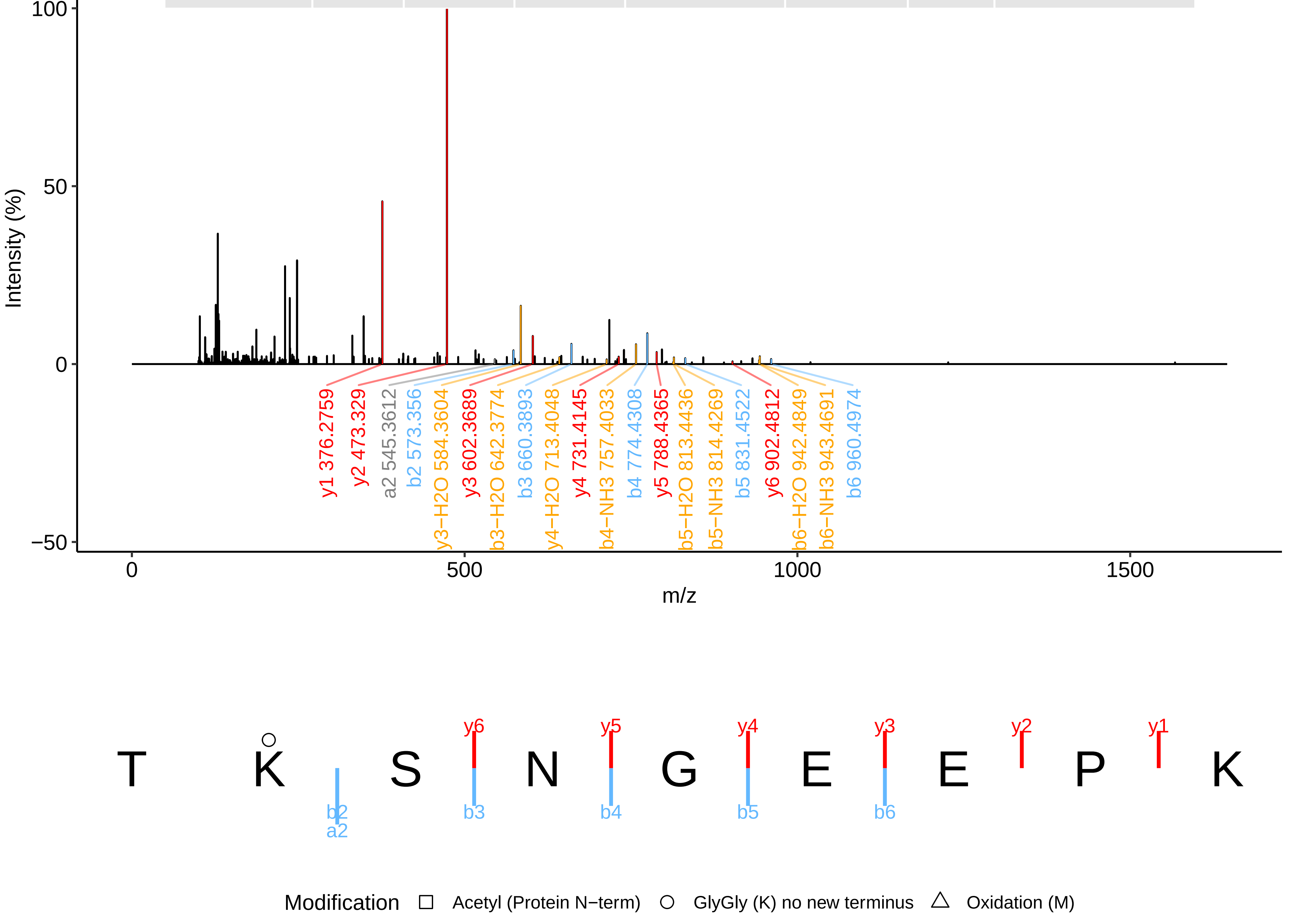

20201118_KIZ_MUb6_GlyGly_2 − Scan number: 3189

Gene Names	Charge	m/z	Mass	Retention time	PEP	Score	Precursor Intensity
SLC1A3	3	368.5158	1102.525	10.363	0.00033319	105.52	5266730

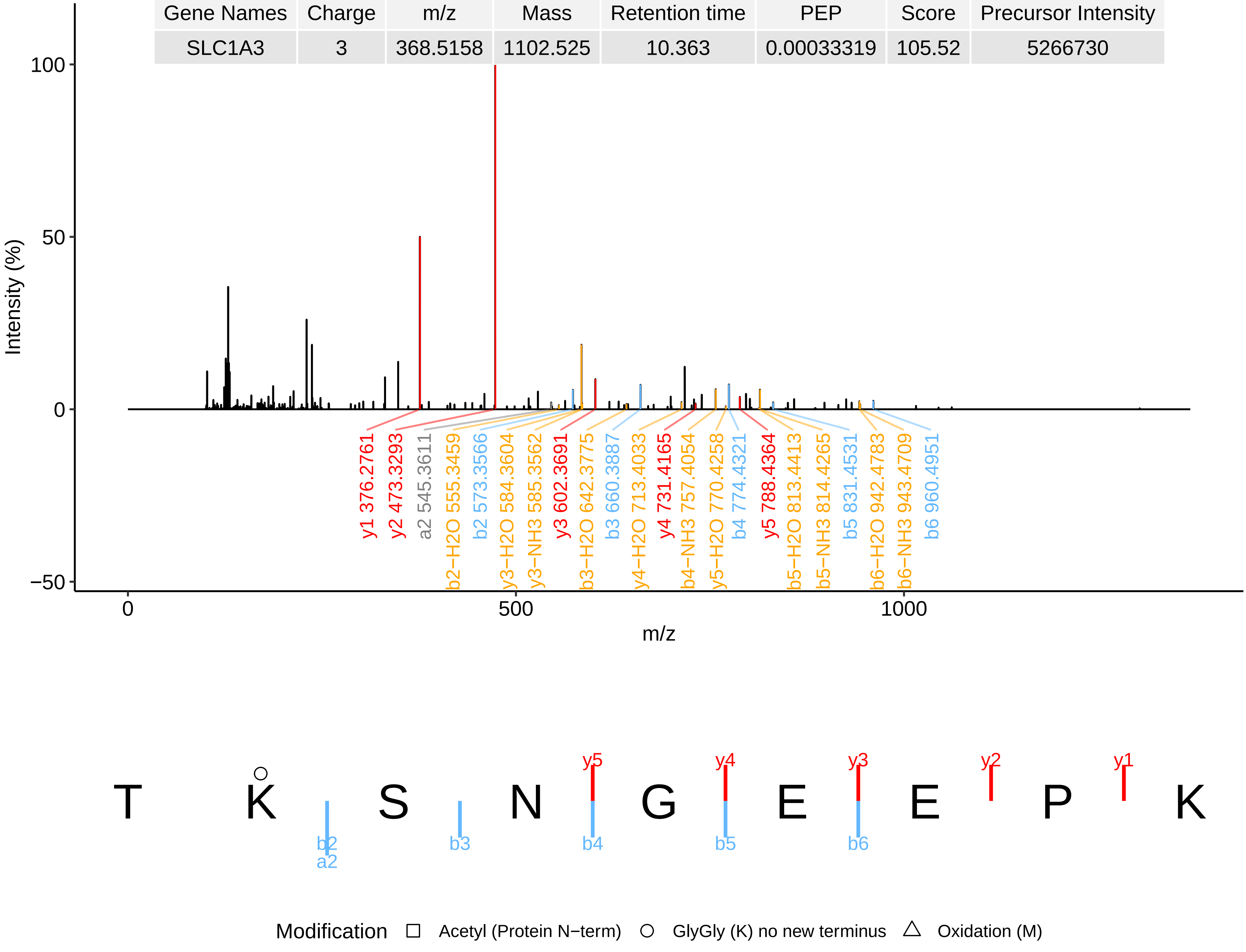

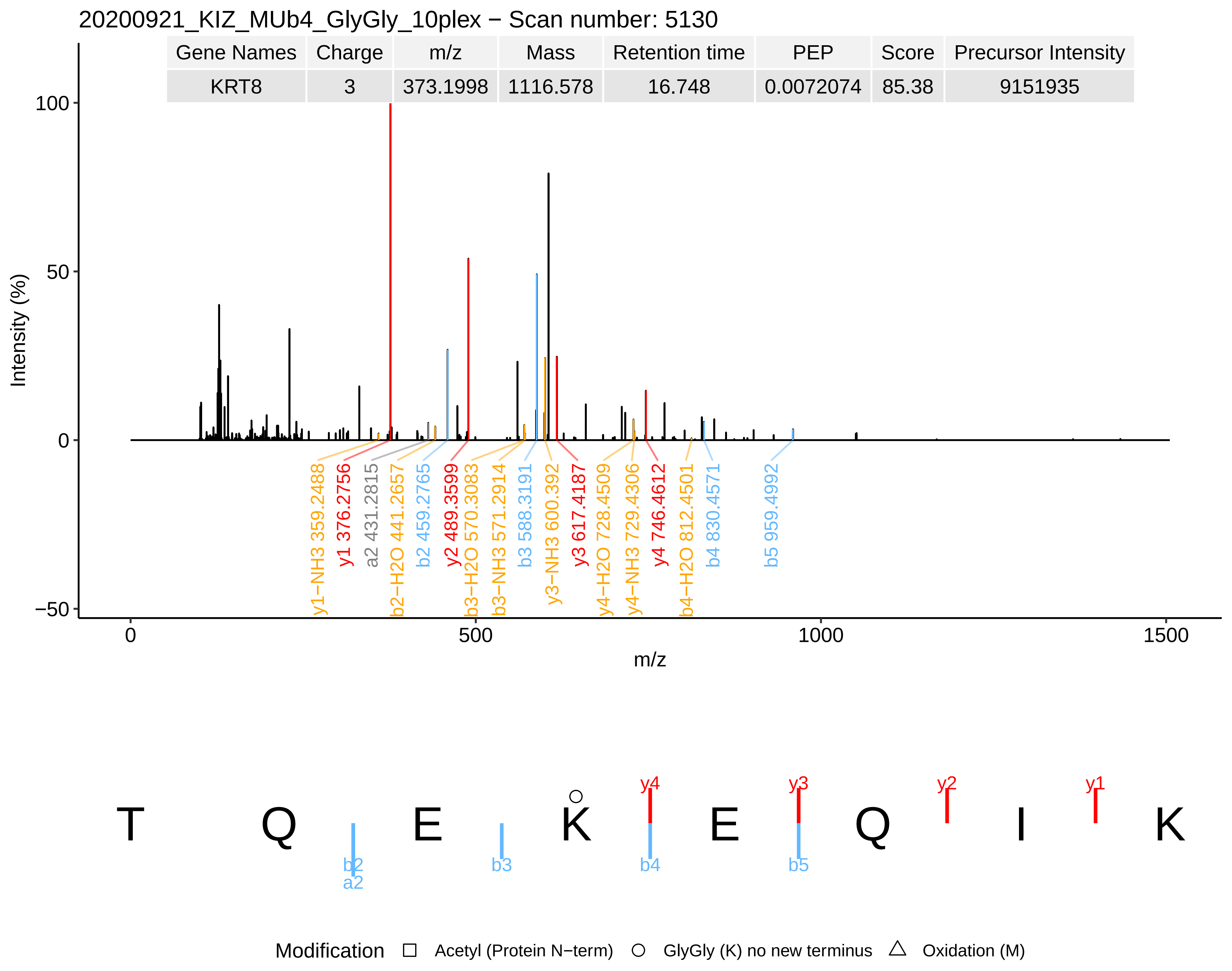
20200921_KIZ_MUb4_GlyGly_10plex − Scan number: 5130
Gene Names | Charge | m/z | Mass | Retention time | PEP | Score | Precursor Intensity
KRT8 | 3 | 373.1998 | 1116.578 | 16.748 | 0.0072074 | 85.38 | 9151935
Intensity (%)
m/z
y1−NH3 359.2488
y1 376.2756
a2 431.2815
b2−H2O 441.2657
b2 459.2765
y2 489.3599
b3−H2O 570.3083
b3−NH3 571.2914
b3 588.3191
y3−NH3 600.392
y3 617.4187
y4−H2O 728.4509
y4−NH3 729.4306
y4 746.4612
b4−H2O 812.4501
b4 830.4571
b5 959.4992
T Q E K E Q I K
y4 y3 y2 y1
b2 a2 b3 b4 b5
Modification
Acetyl (Protein N−term)
GlyGly (K) no new terminus
Oxidation (M)

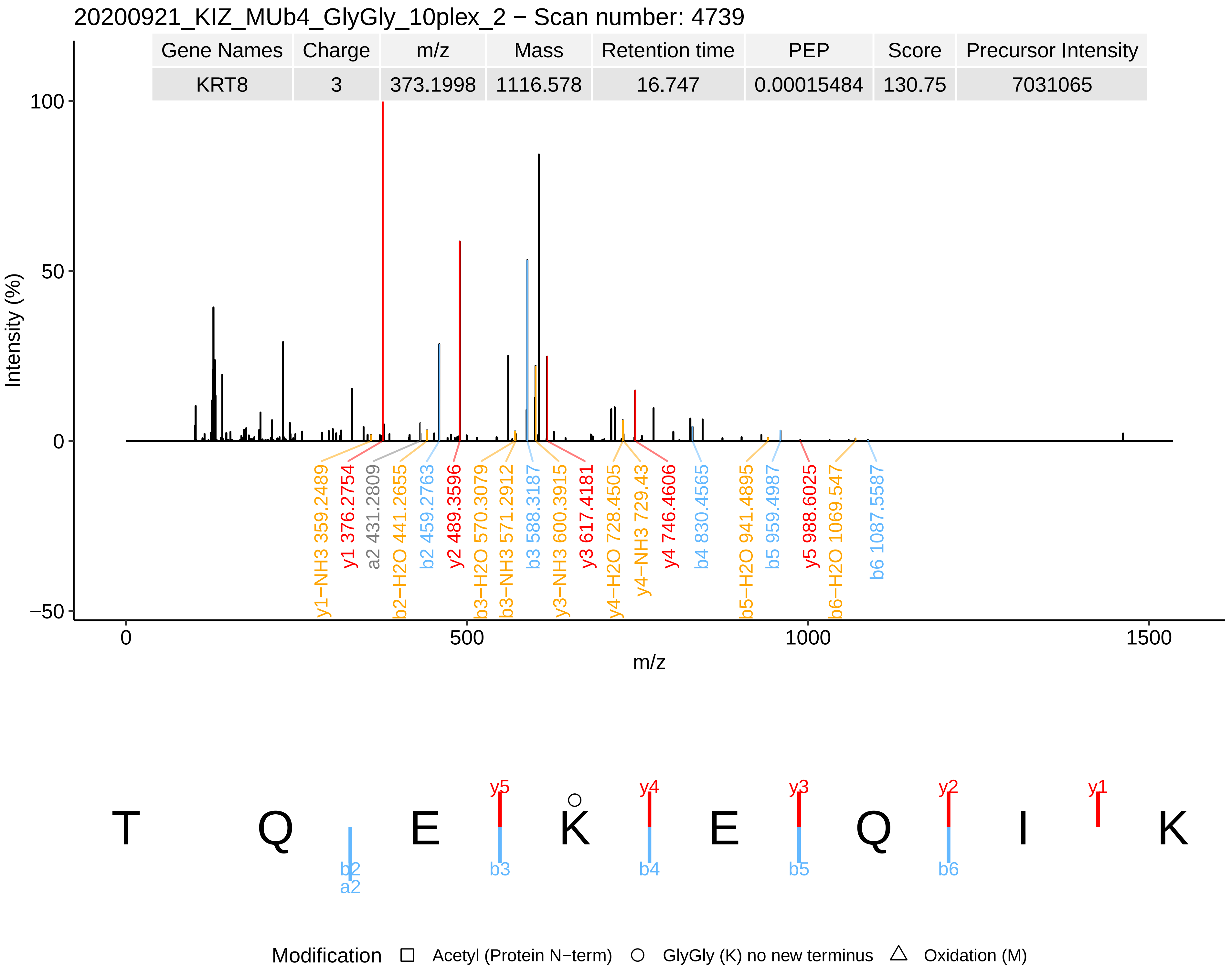

20200921_KIZ_MUb4_GlyGly_10plex_2 − Scan number: 4739
Gene Names | Charge | m/z | Mass | Retention time | PEP | Score | Precursor Intensity
KRT8 | 3 | 373.1998 | 1116.578 | 16.747 | 0.00015484 | 130.75 | 7031065
Intensity (%)
m/z
y1−NH3 359.2489
y1 376.2754
a2 431.2809
b2−H2O 441.2655
b2 459.2763
y2 489.3596
b3−H2O 570.3079
b3−NH3 571.2912
b3 588.3187
y3−NH3 600.3915
y3 617.4181
y4−H2O 728.4505
y4−NH3 729.43
y4 746.4606
b4 830.4565
b5−H2O 941.4895
b5 959.4987
y5 988.6025
b6−H2O 1069.547
b6 1087.5587
T Q E K E Q I K
y5 y4 y3 y2 y1
b2 a2 b3 b4 b5 b6
Modification
Acetyl (Protein N−term)
GlyGly (K) no new terminus
Oxidation (M)

20200924_KIZ_MUb5_GlyGly_1 − Scan number: 4481

Gene Names	Charge	m/z	Mass	Retention time	PEP	Score	Precursor Intensity
KRT8	3	373.1998	1116.578	13.83	0.099906	46.334	2985293

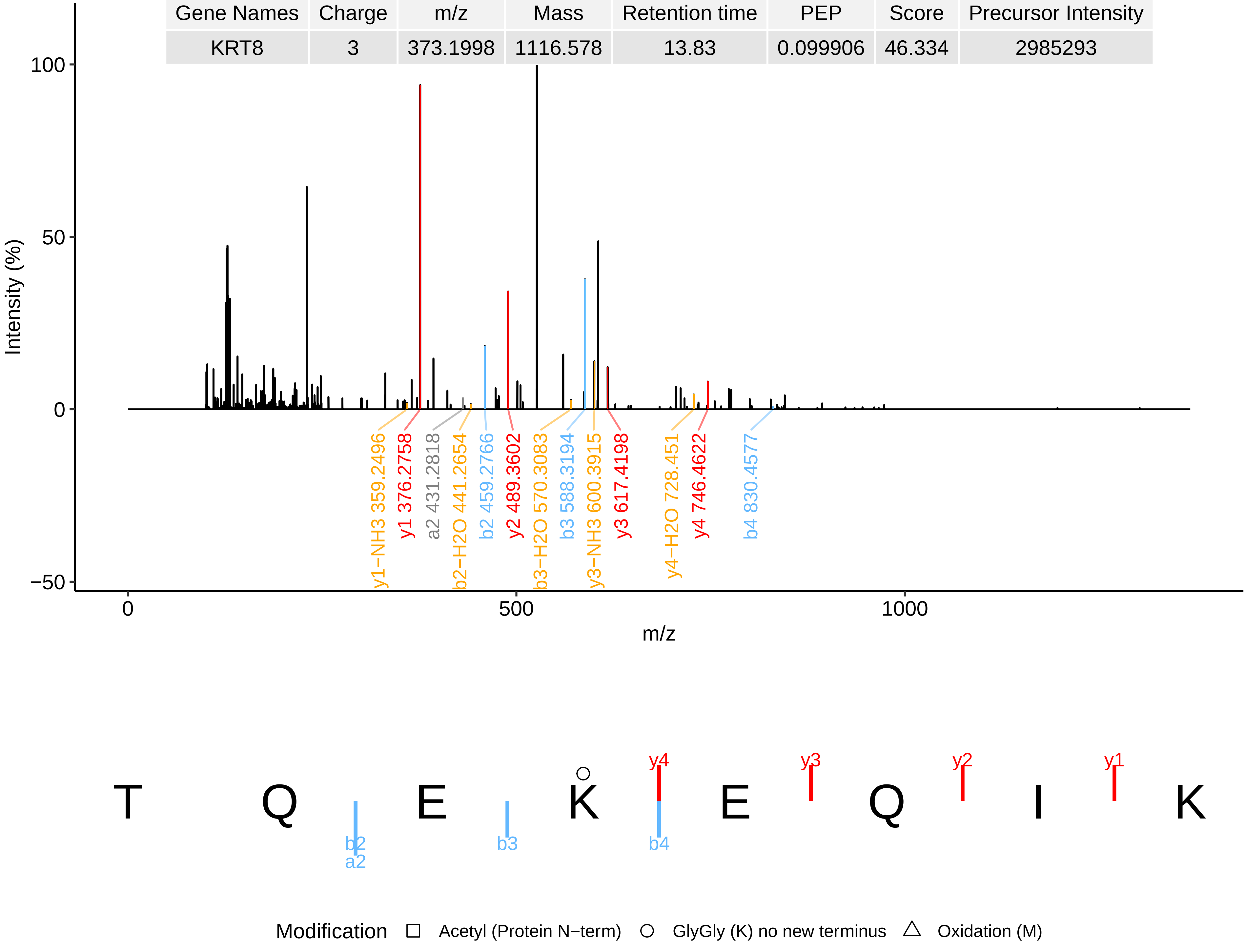

20200924_KIZ_MUb5_GlyGly_2 − Scan number: 4139

Gene Names	Charge	m/z	Mass	Retention time	PEP	Score	Precursor Intensity
KRT8	3	373.1998	1116.578	15.146	0.023997	68.625	3474277

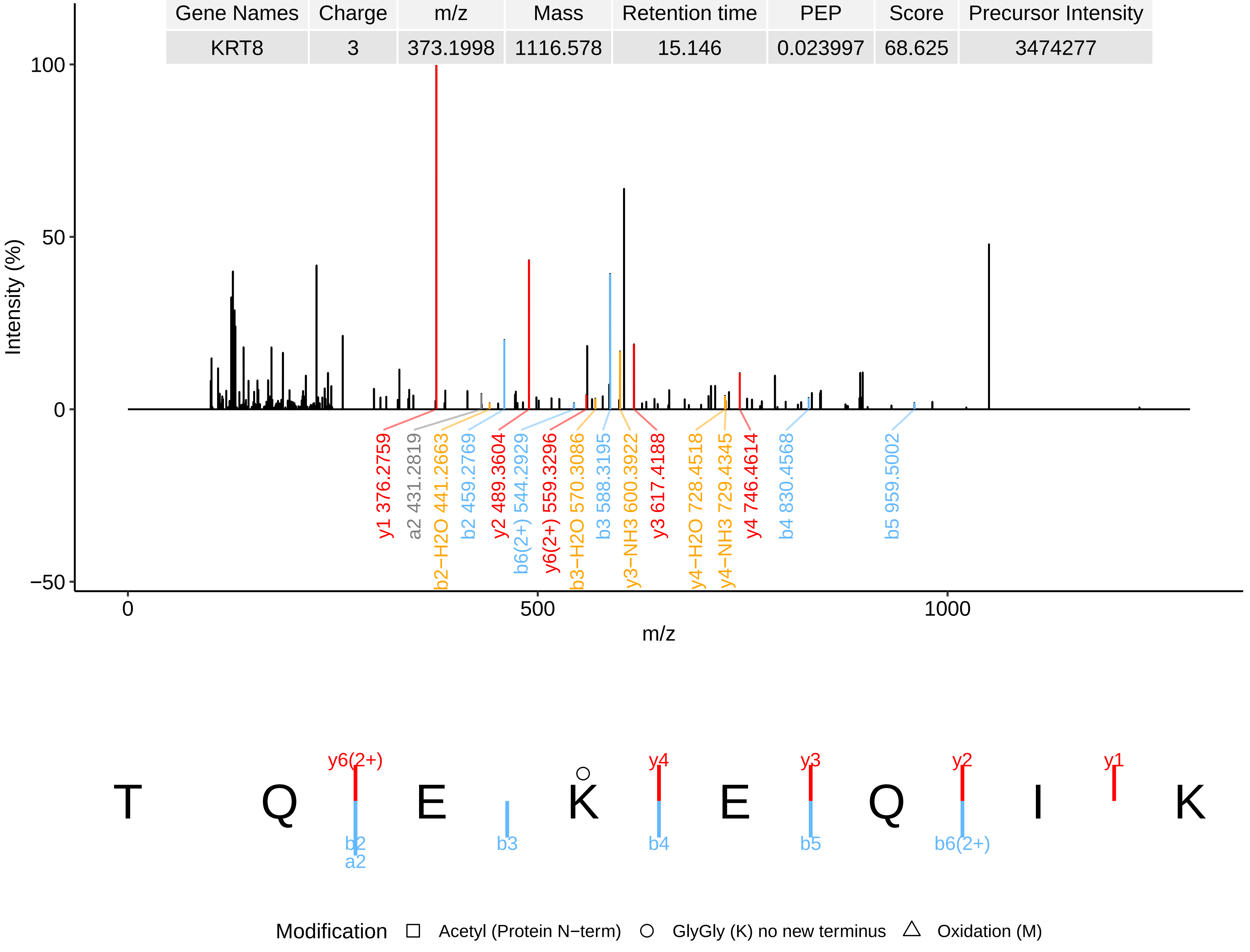

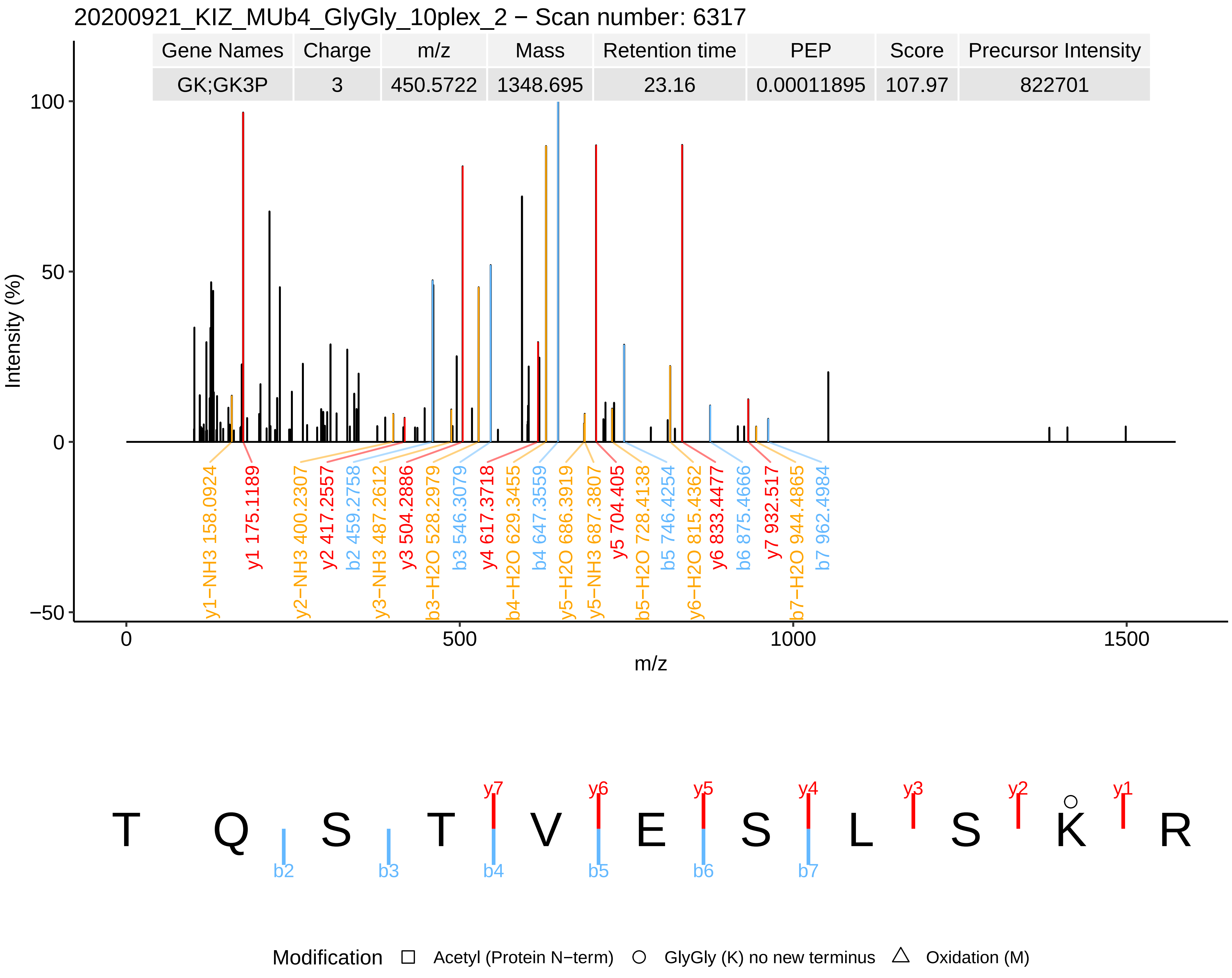

20200921_KIZ_MUb4_GlyGly_10plex_2 − Scan number: 6317
Gene Names
Charge
m/z
Mass
Retention time
PEP
Score
Precursor Intensity
GK;GK3P
3
450.5722
1348.695
23.16
0.00011895
107.97
822701
Intensity (%)
100
50
0
−50
0
500
1000
1500
m/z
y1−NH3 158.0924
y1 175.1189
y2−NH3 400.2307
y2 417.2557
b2 459.2758
y3−NH3 487.2612
y3 504.2886
b3−H2O 528.2979
b3 546.3079
y4 617.3718
b4−H2O 629.3455
b4 647.3559
y5−H2O 686.3919
y5−NH3 687.3807
y5 704.405
b5−H2O 728.4138
b5 746.4254
y6−H2O 815.4362
y6 833.4477
b6 875.4666
y7 932.517
b7−H2O 944.4865
b7 962.4984
T Q S T V E S L S K R
y7 y6 y5 y4 y3 y2 y1
b2 b3 b4 b5 b6 b7
Modification
Acetyl (Protein N−term)
GlyGly (K) no new terminus
Oxidation (M)

20200921_KIZ_MUb4_GlyGly_10plex − Scan number: 12354

Gene Names	Charge	m/z	Mass	Retention time	PEP	Score	Precursor Intensity
GAPDH	3	443.9018	1328.684	43.283	1.8151e−12	139.15	7902279

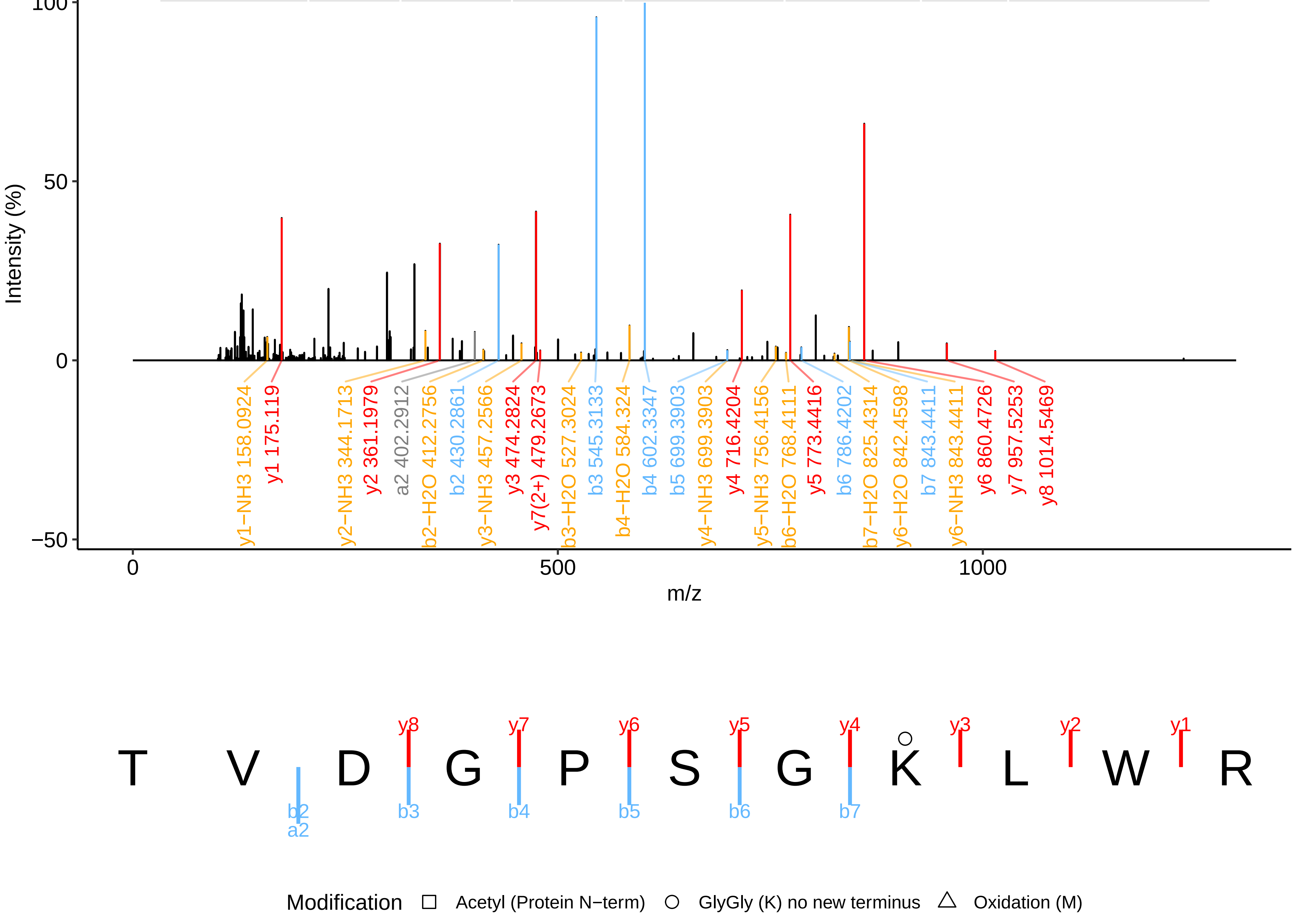

20200921_KIZ_MUb4_GlyGly_10plex_2 − Scan number: 11279

Gene Names	Charge	m/z	Mass	Retention time	PEP	Score	Precursor Intensity
GAPDH	3	443.9018	1328.684	43.373	2.2417e−08	125.74	1532676

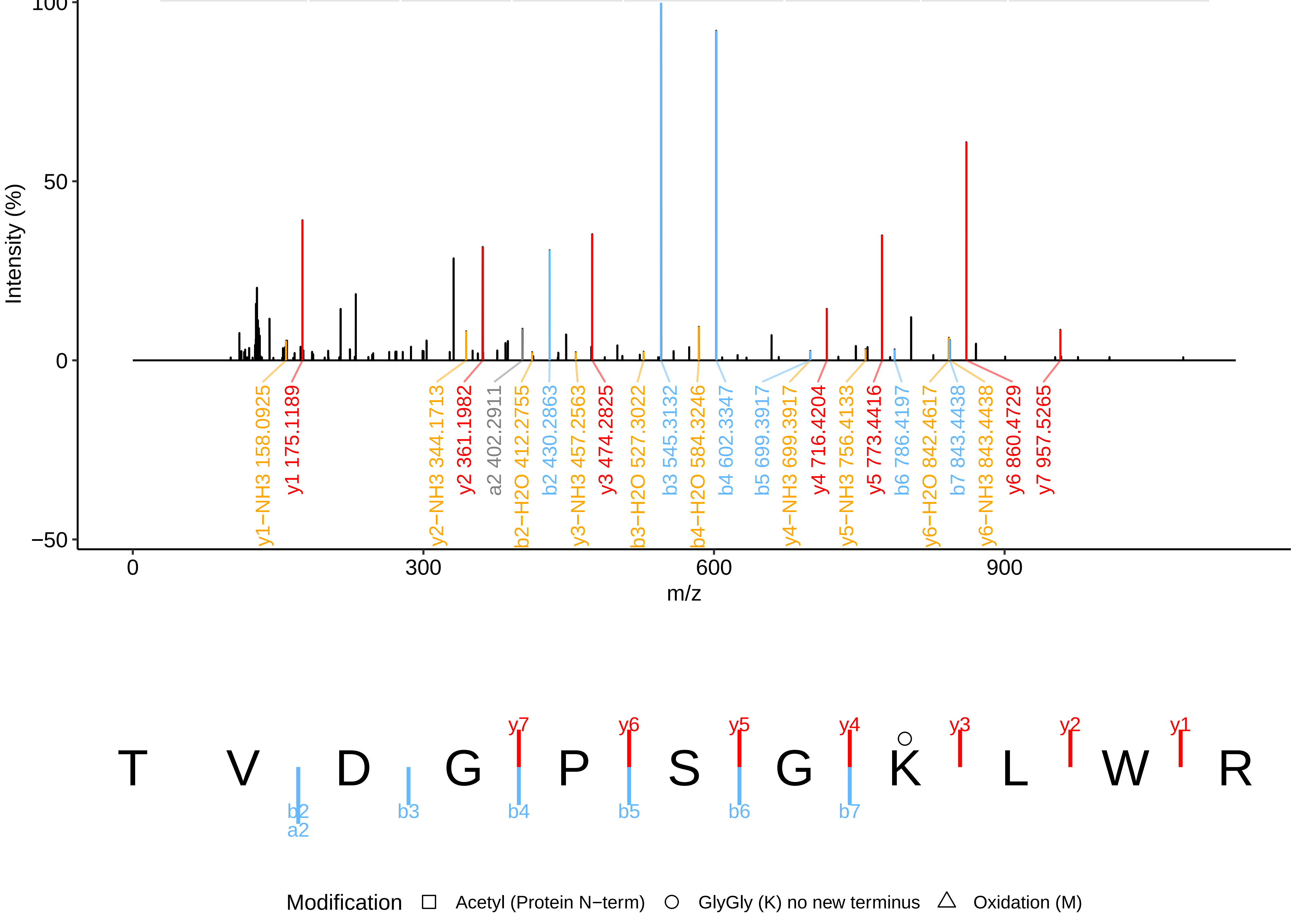

20200924_KIZ_MUb5_GlyGly_1 − Scan number: 10964

Gene Names	Charge	m/z	Mass	Retention time	PEP	Score	Precursor Intensity
GAPDH	3	443.9018	1328.684	35.501	3.74e−05	100.92	18038682

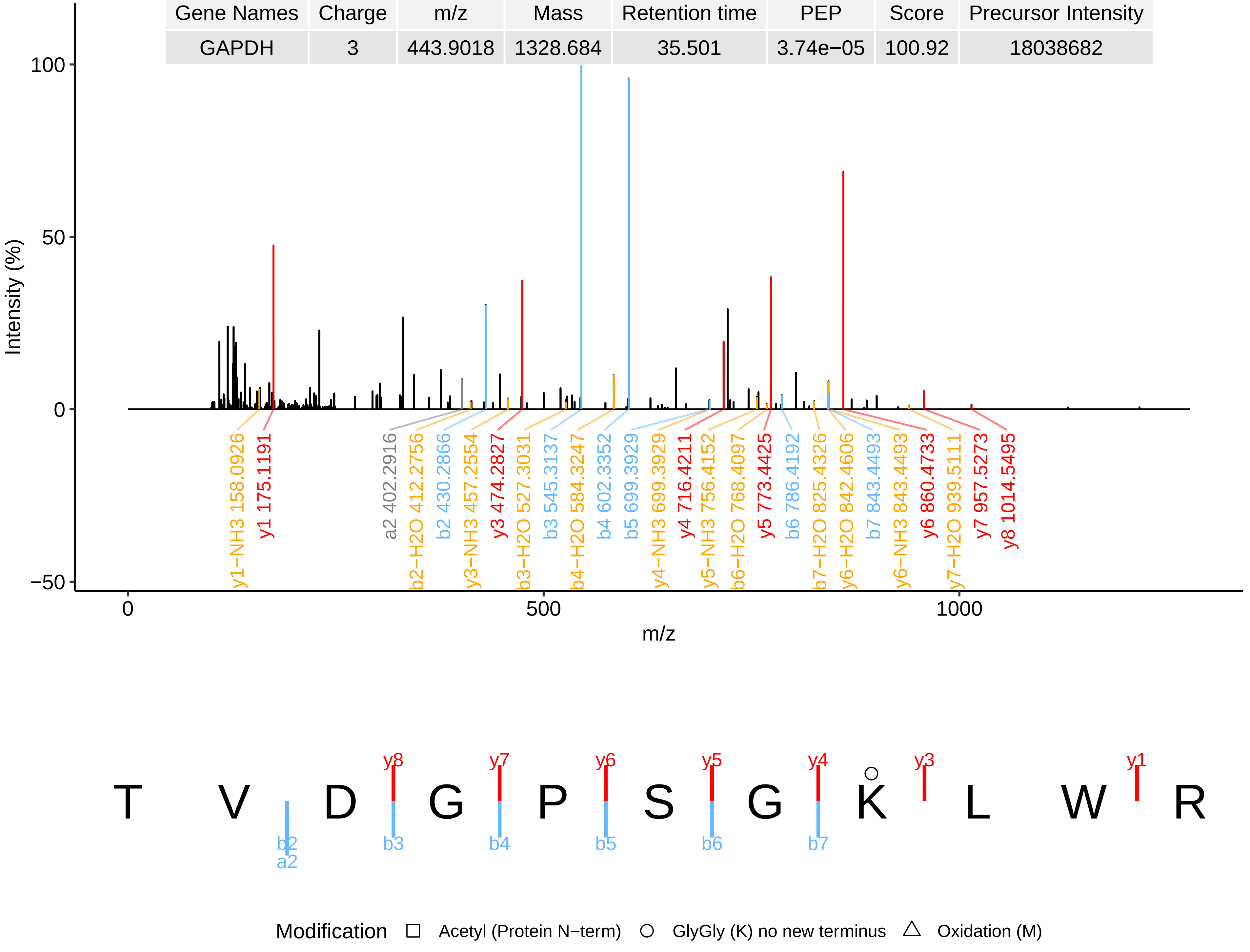

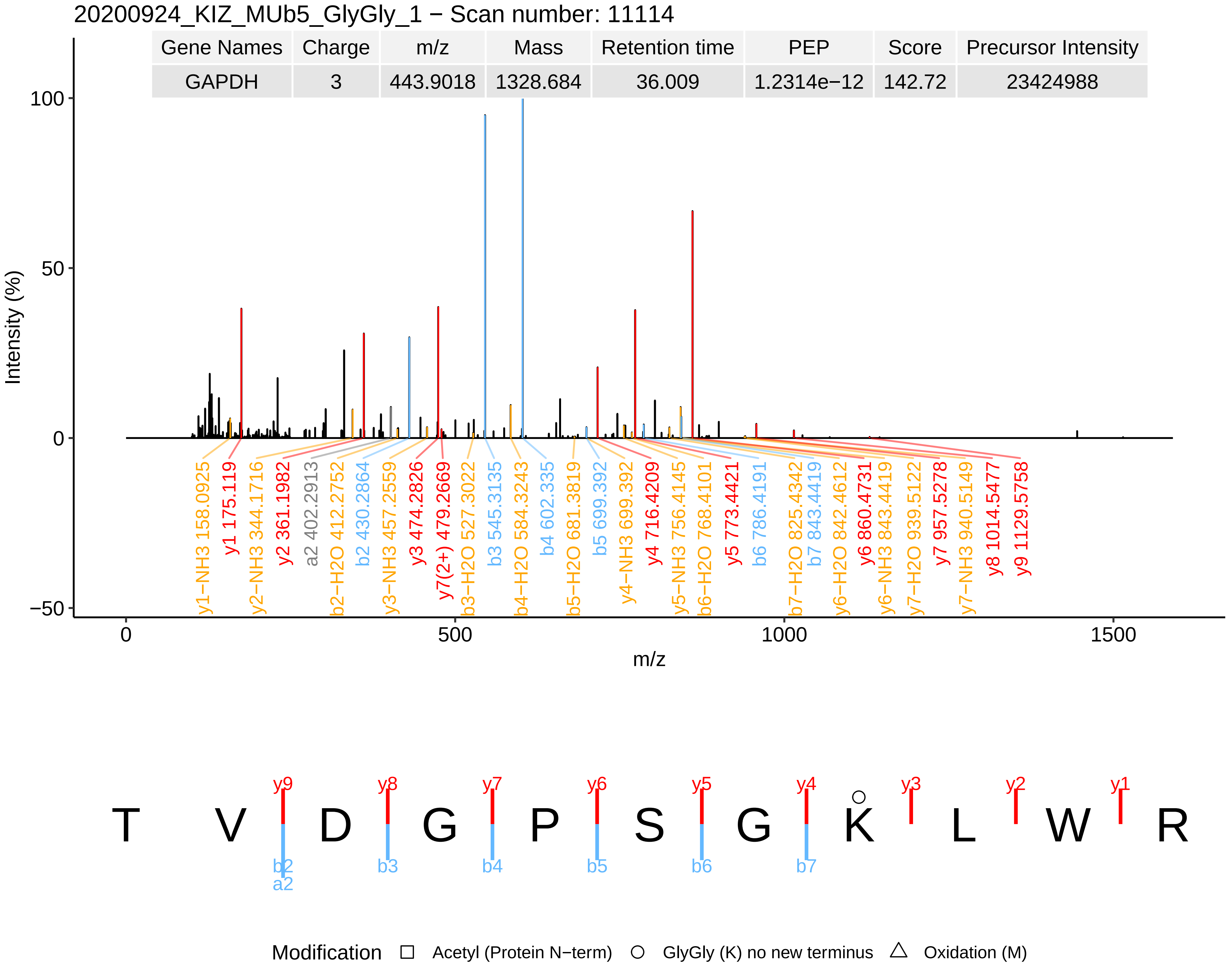
20200924_KIZ_MUb5_GlyGly_1 − Scan number: 11114
Gene Names | Charge | m/z | Mass | Retention time | PEP | Score | Precursor Intensity
GAPDH | 3 | 443.9018 | 1328.684 | 36.009 | 1.2314e−12 | 142.72 | 23424988
Intensity (%)
m/z
y1−NH3 158.0925
y1 175.119
y2−NH3 344.1716
y2 361.1982
a2 402.2913
b2−H2O 412.2752
b2 430.2864
y3−NH3 457.2559
y3 474.2826
y7(2+) 479.2669
b3−H2O 527.3022
b3 545.3135
b4−H2O 584.3243
b4 602.335
b5−H2O 681.3819
b5 699.392
y4−NH3 699.392
y4 716.4209
y5−NH3 756.4145
b6−H2O 768.4101
y5 773.4421
b6 786.4191
b7−H2O 825.4342
b7 843.4419
y6−H2O 842.4612
y6 860.4731
y6−NH3 843.4419
y7−H2O 939.5122
y7 957.5278
y7−NH3 940.5149
y8 1014.5477
y9 1129.5758
T V D G P S G K L W R
y9 y8 y7 y6 y5 y4 y3 y2 y1
b2 a2 b3 b4 b5 b6 b7
Modification □ Acetyl (Protein N−term) ○ GlyGly (K) no new terminus △ Oxidation (M)

20200924_KIZ_MUb5_GlyGly_1 − Scan number: 11280

Gene Names	Charge	m/z	Mass	Retention time	PEP	Score	Precursor Intensity
GAPDH	3	443.9018	1328.684	36.522	3.6255e−08	121.36	7458128

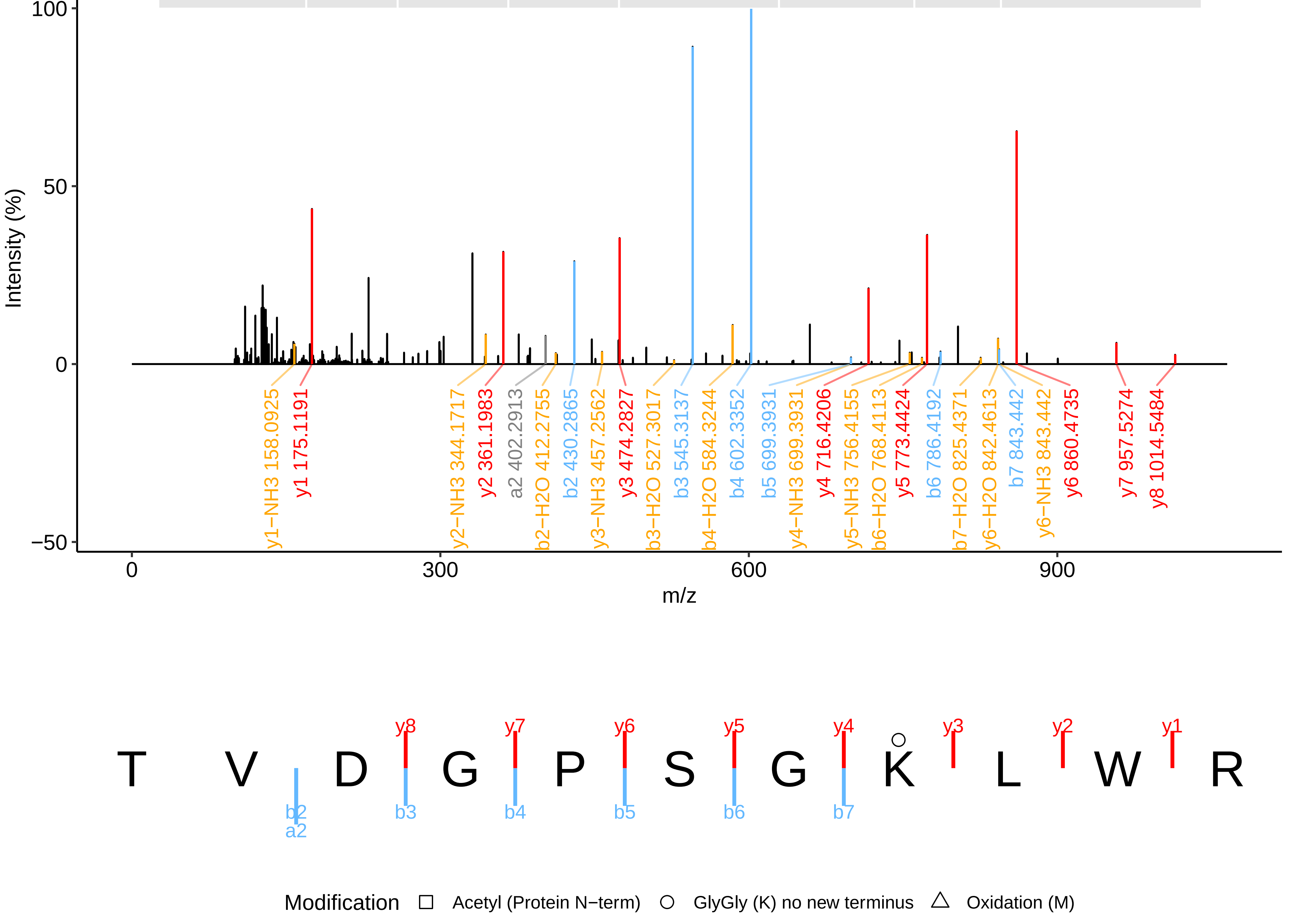

20200924_KIZ_MUb5_GlyGly_2 − Scan number: 10372

Gene Names	Charge	m/z	Mass	Retention time	PEP	Score	Precursor Intensity
GAPDH	3	443.9018	1328.684	38.725	3.5955e−05	101.48	3643854

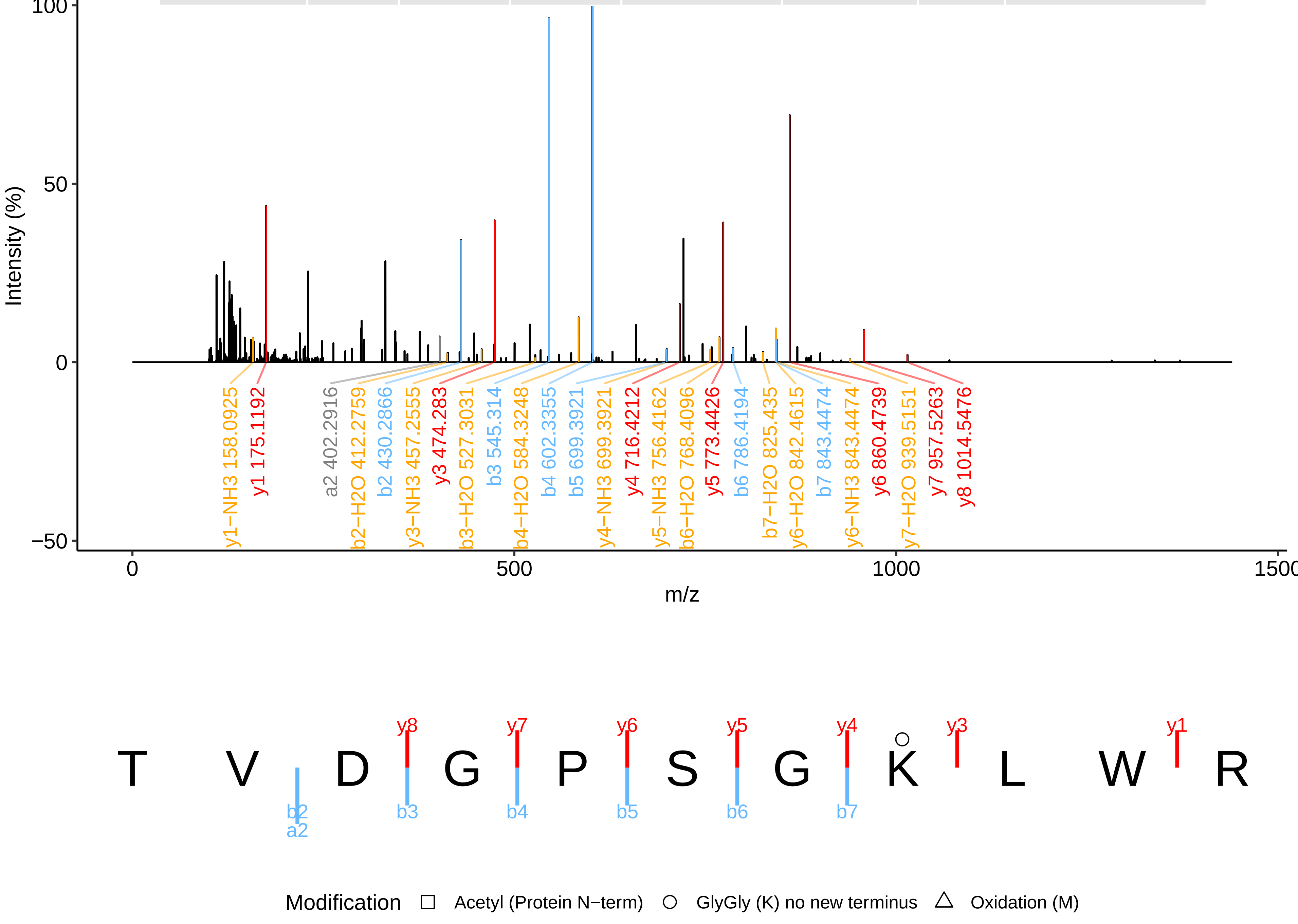

20200924_KIZ_MUb5_GlyGly_2 − Scan number: 10512

Gene Names	Charge	m/z	Mass	Retention time	PEP	Score	Precursor Intensity
GAPDH	3	443.9018	1328.684	39.231	3.6435e−05	101.3	10780731

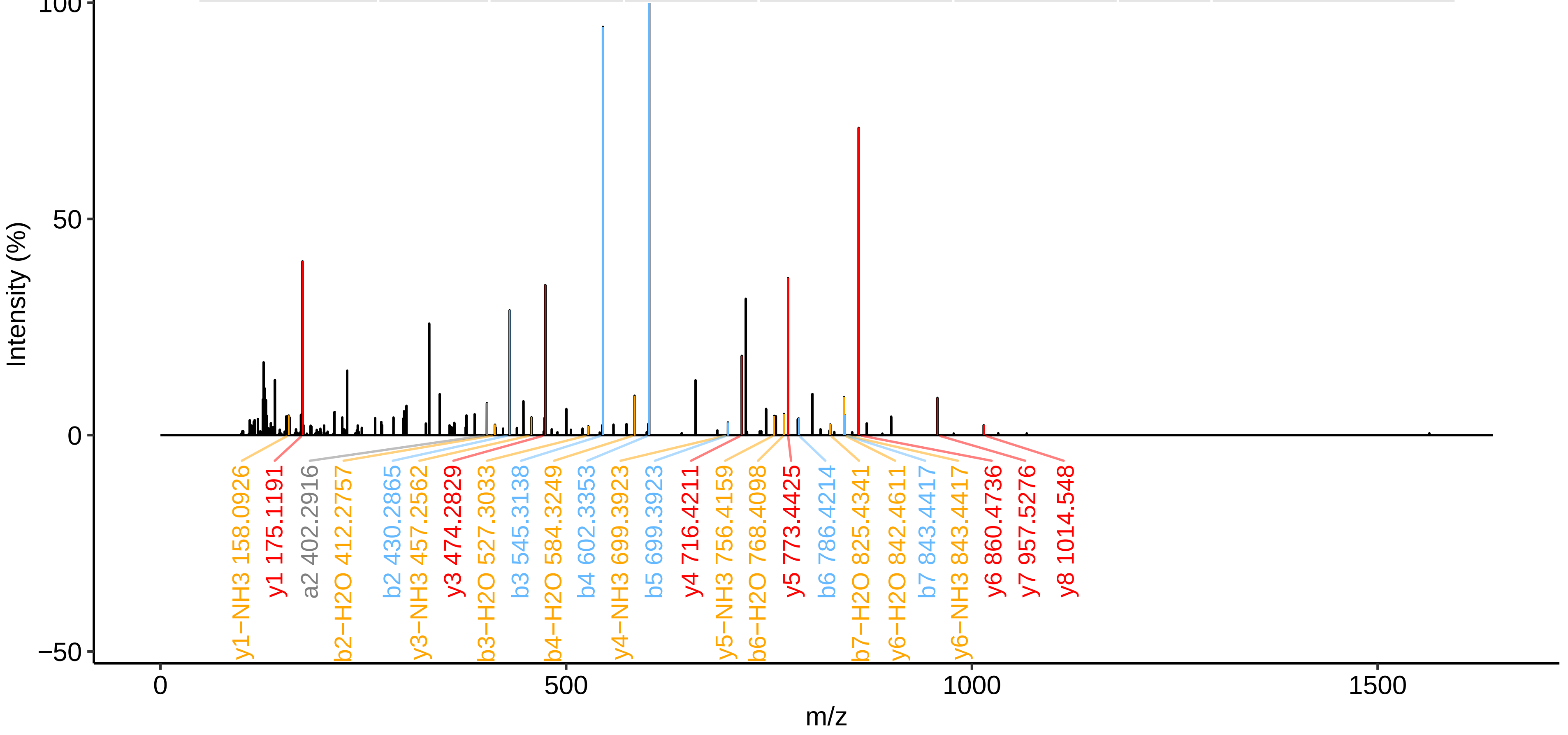

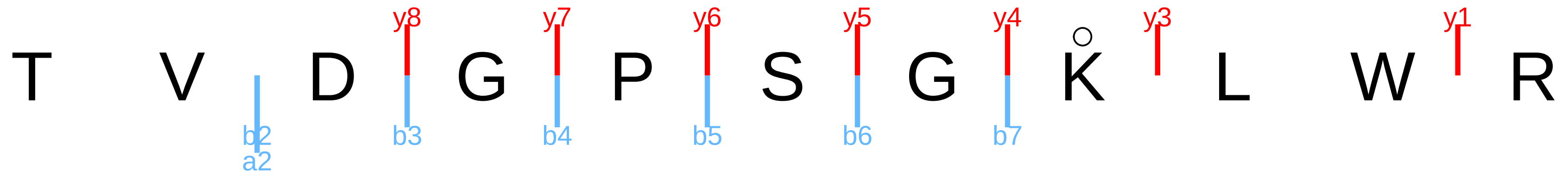

Modification □ Acetyl (Protein N−term) ○ GlyGly (K) no new terminus △ Oxidation (M)

20200924_KIZ_MUb5_GlyGly_2 − Scan number: 10648

Gene Names	Charge	m/z	Mass	Retention time	PEP	Score	Precursor Intensity
GAPDH	3	443.9018	1328.684	39.753	0.0083116	72.582	3274263

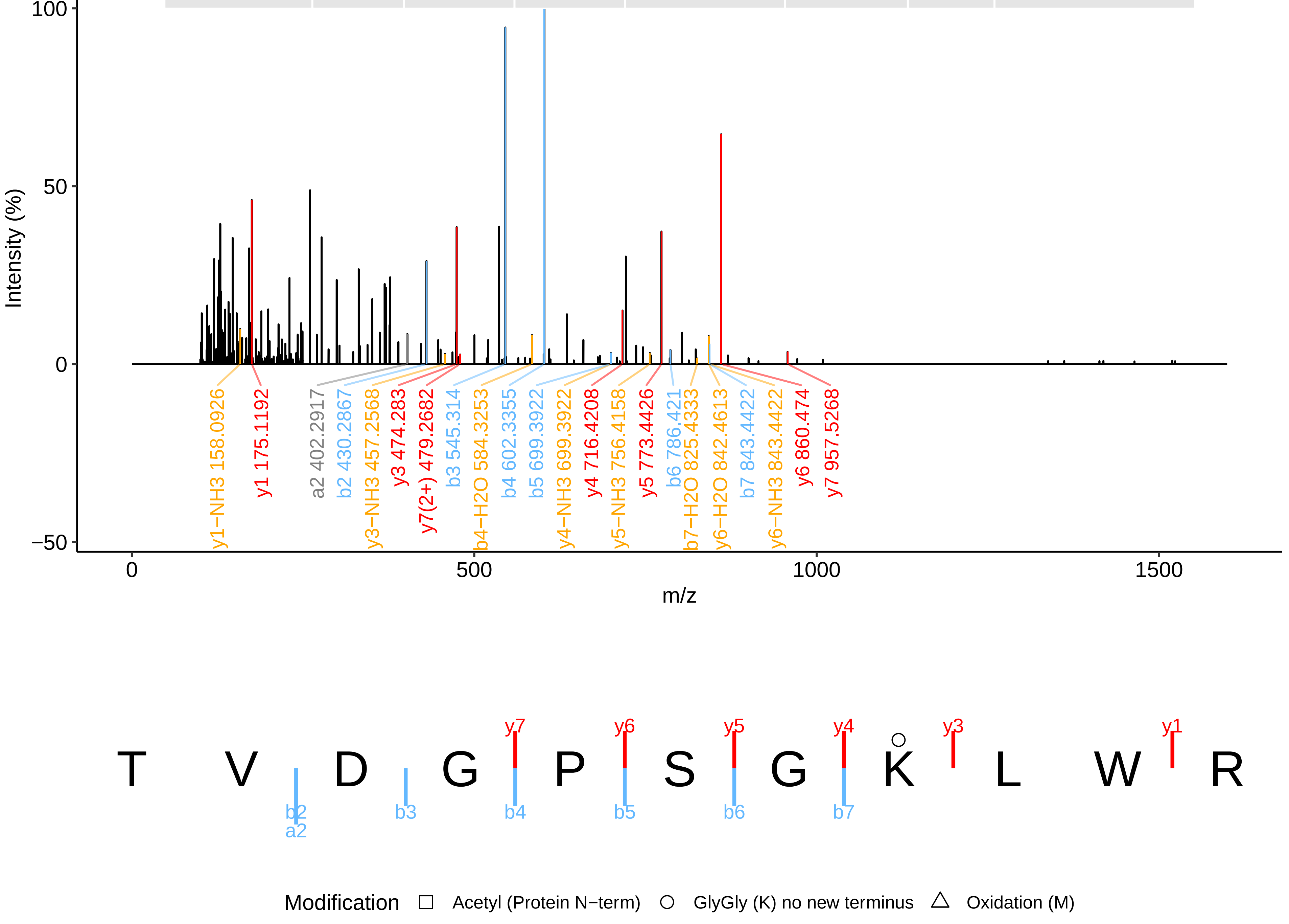

20201118_KIZ_MUb6_GlyGly_2 − Scan number: 12915

Gene Names	Charge	m/z	Mass	Retention time	PEP	Score	Precursor Intensity
GAPDH	3	443.9018	1328.684	43.47	2.5794e−08	124.67	7895386

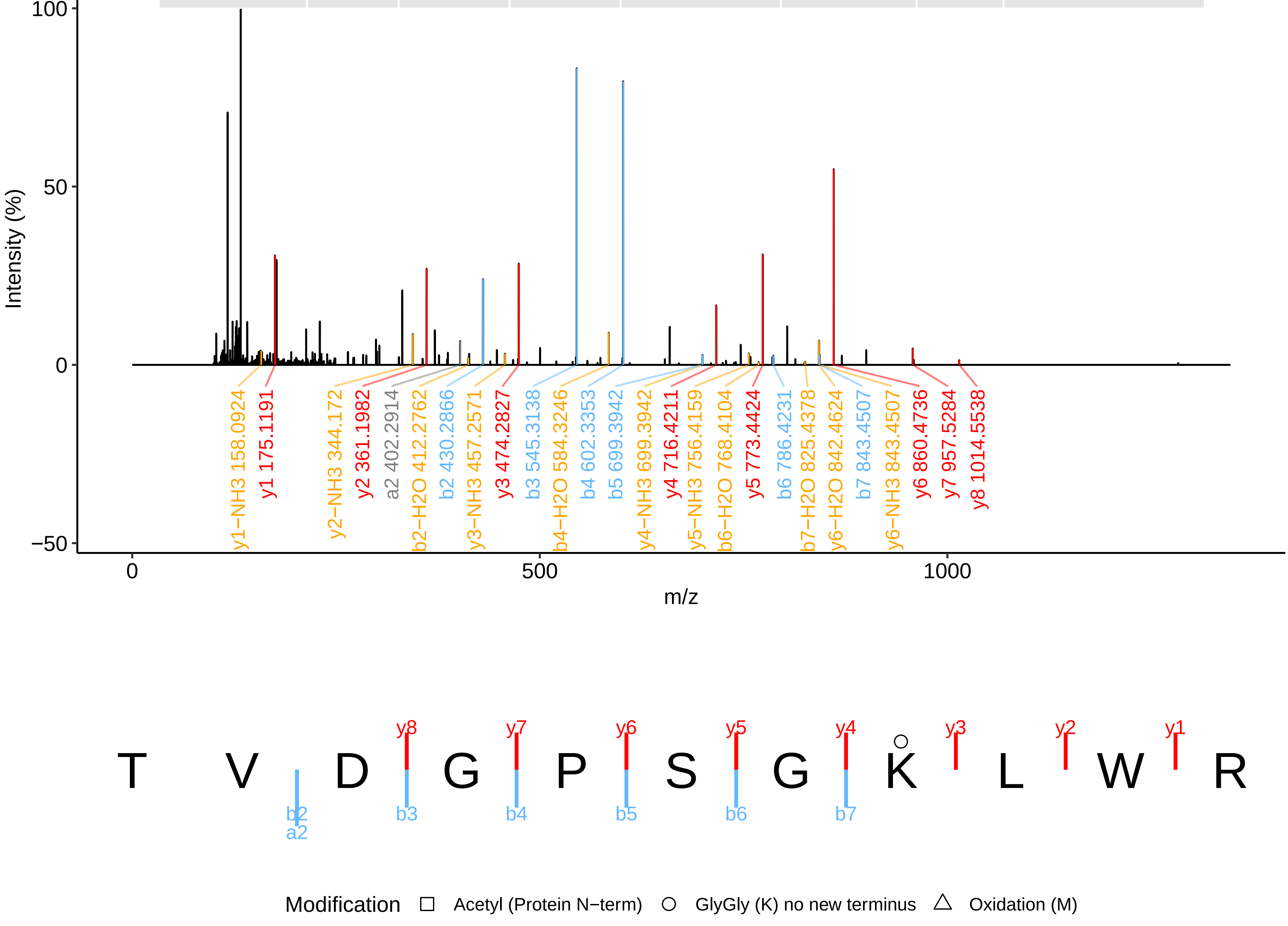

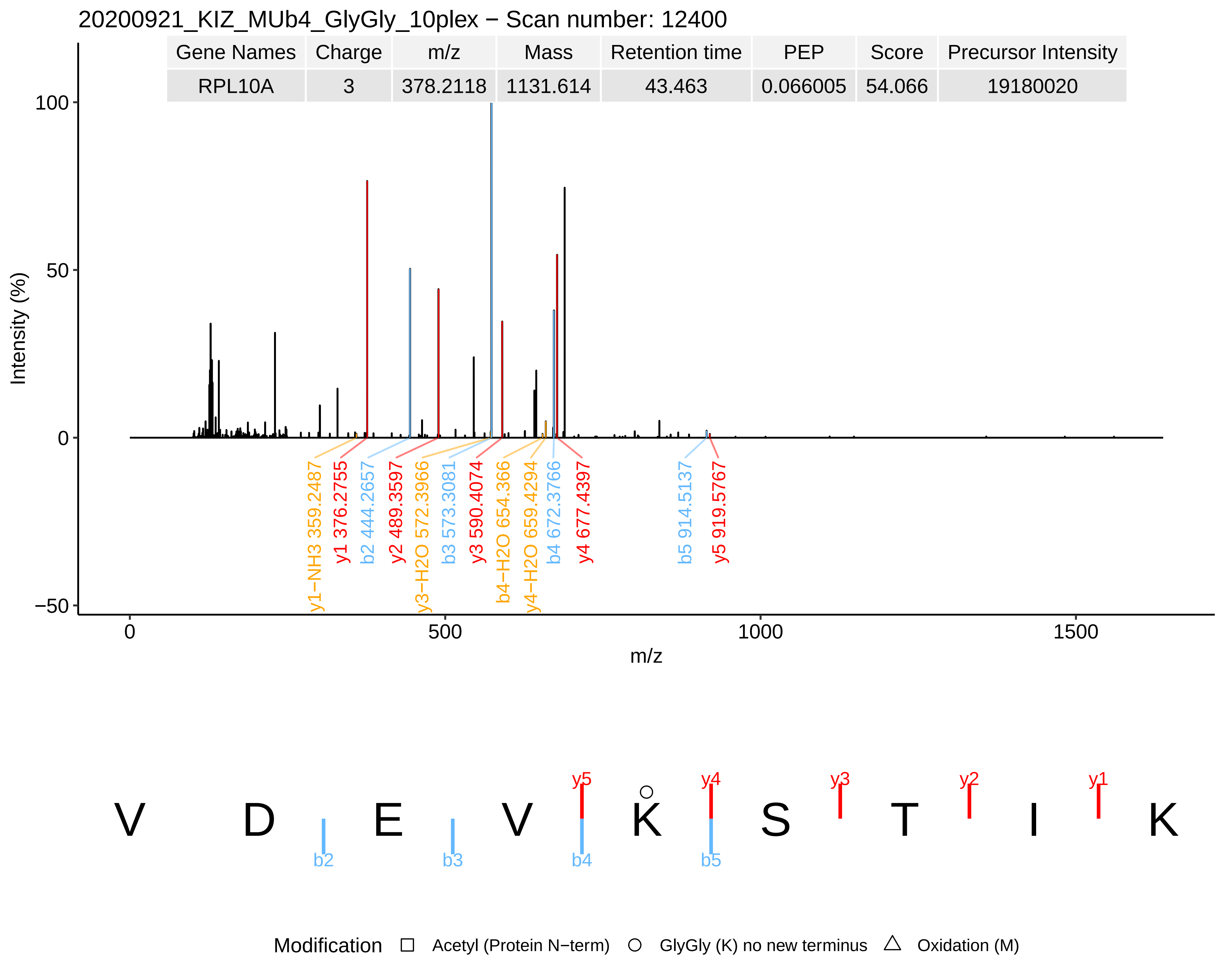
20200921_KIZ_MUb4_GlyGly_10plex − Scan number: 12400
Gene Names	Charge	m/z	Mass	Retention time	PEP	Score	Precursor Intensity
RPL10A	3	378.2118	1131.614	43.463	0.066005	54.066	19180020
Intensity (%)
100
50
0
−50
0
500
1000
1500
m/z
y1−NH3 359.2487
y1 376.2755
b2 444.2657
y2 489.3597
y3−H2O 572.3966
b3 573.3081
y3 590.4074
b4−H2O 654.366
y4−H2O 659.4294
b4 672.3766
y4 677.4397
b5 914.5137
y5 919.5767
V D E V K S T I K
y5 y4 y3 y2 y1
b2 b3 b4 b5
Modification
Acetyl (Protein N−term)
GlyGly (K) no new terminus
Oxidation (M)

20200921_KIZ_MUb4_GlyGly_10plex_2 − Scan number: 11360

Gene Names	Charge	m/z	Mass	Retention time	PEP	Score	Precursor Intensity
RPL10A	3	378.2118	1131.614	43.709	0.020877	64.297	3368034

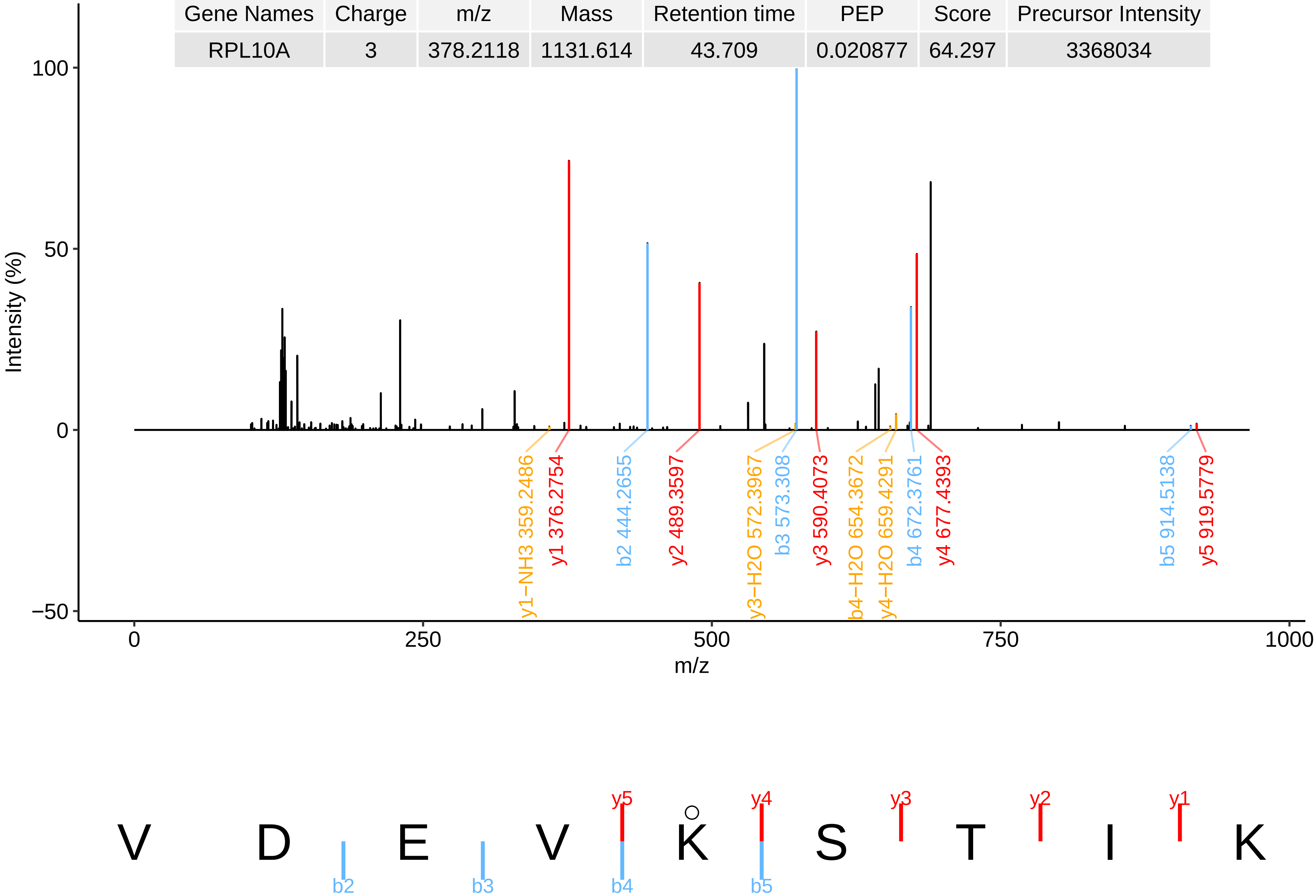

Modification □ Acetyl (Protein N−term) ○ GlyGly (K) no new terminus △ Oxidation (M)

20200924_KIZ_MUb5_GlyGly_1 − Scan number: 11493

Gene Names	Charge	m/z	Mass	Retention time	PEP	Score	Precursor Intensity
RPL10A	3	378.2118	1131.614	37.243	0.063391	54.608	13364722

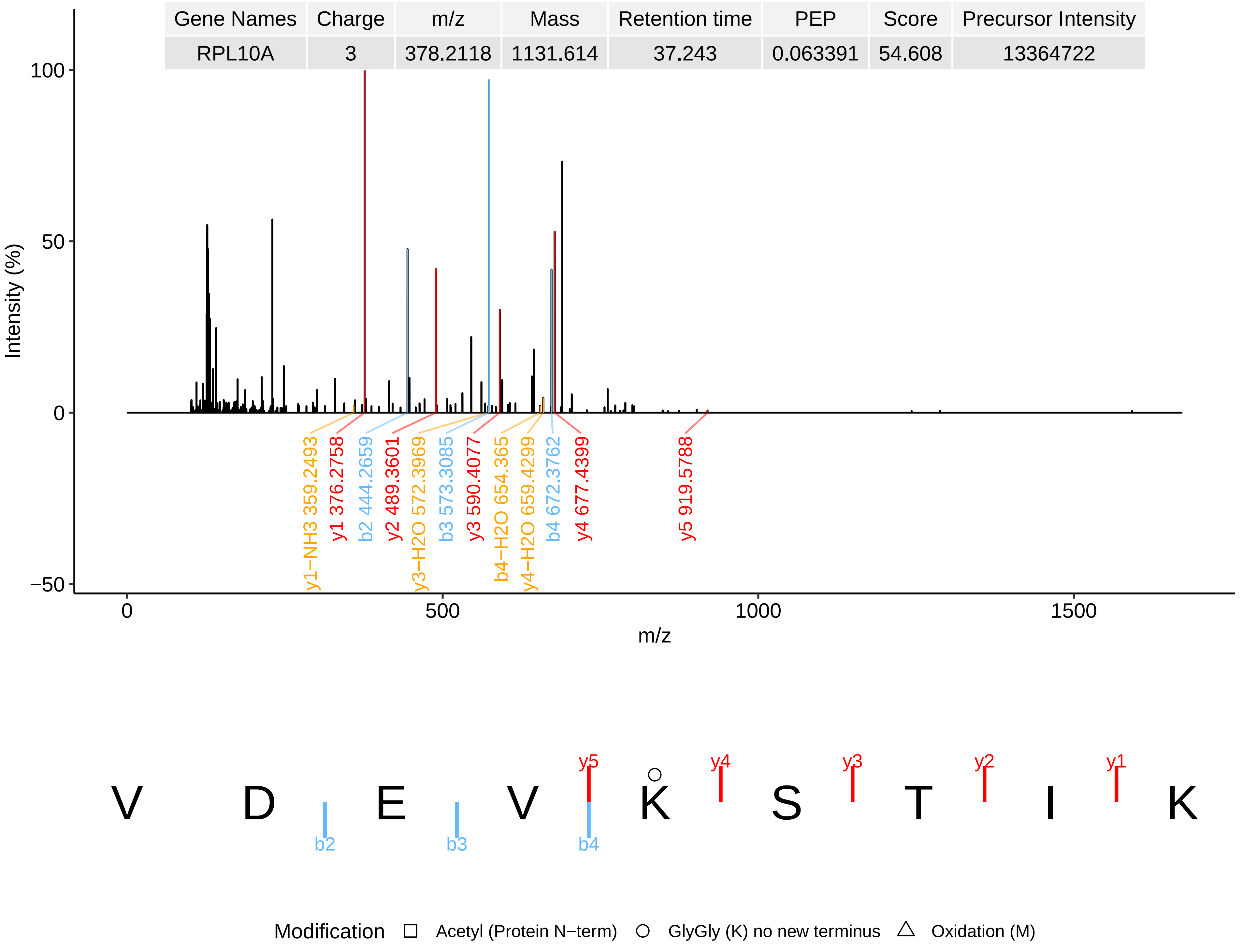

20201118_KIZ_MUb6_GlyGly_2 − Scan number: 13068

Gene Names	Charge	m/z	Mass	Retention time	PEP	Score	Precursor Intensity
RPL10A	3	378.2118	1131.614	44.018	0.020877	64.297	10265150

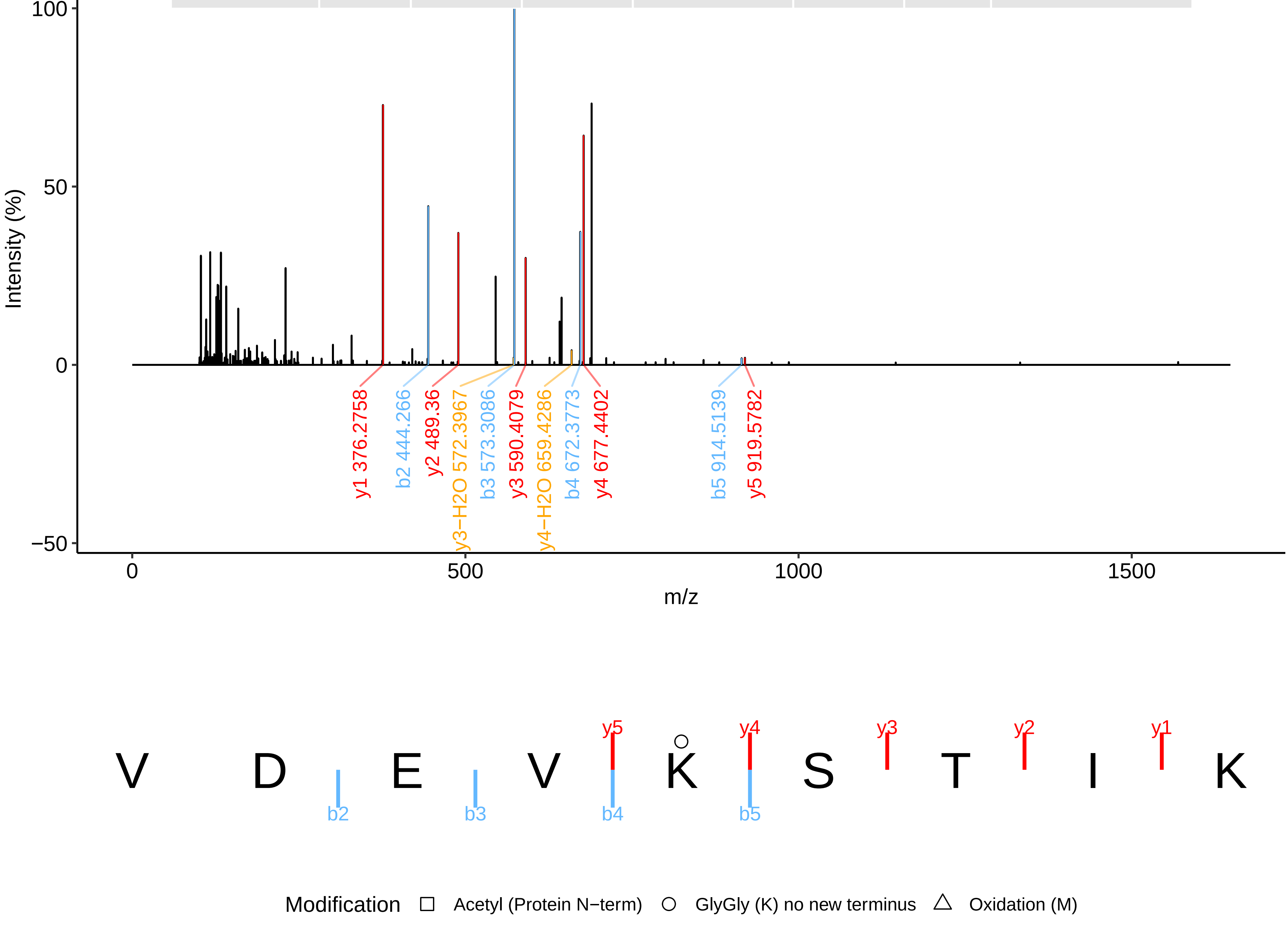

20200921_KIZ_MUb4_GlyGly_10plex − Scan number: 13059

Gene Names	Charge	m/z	Mass	Retention time	PEP	Score	Precursor Intensity
PGRMC2	3	365.5329	1093.577	45.932	0.011685	72.928	7768383

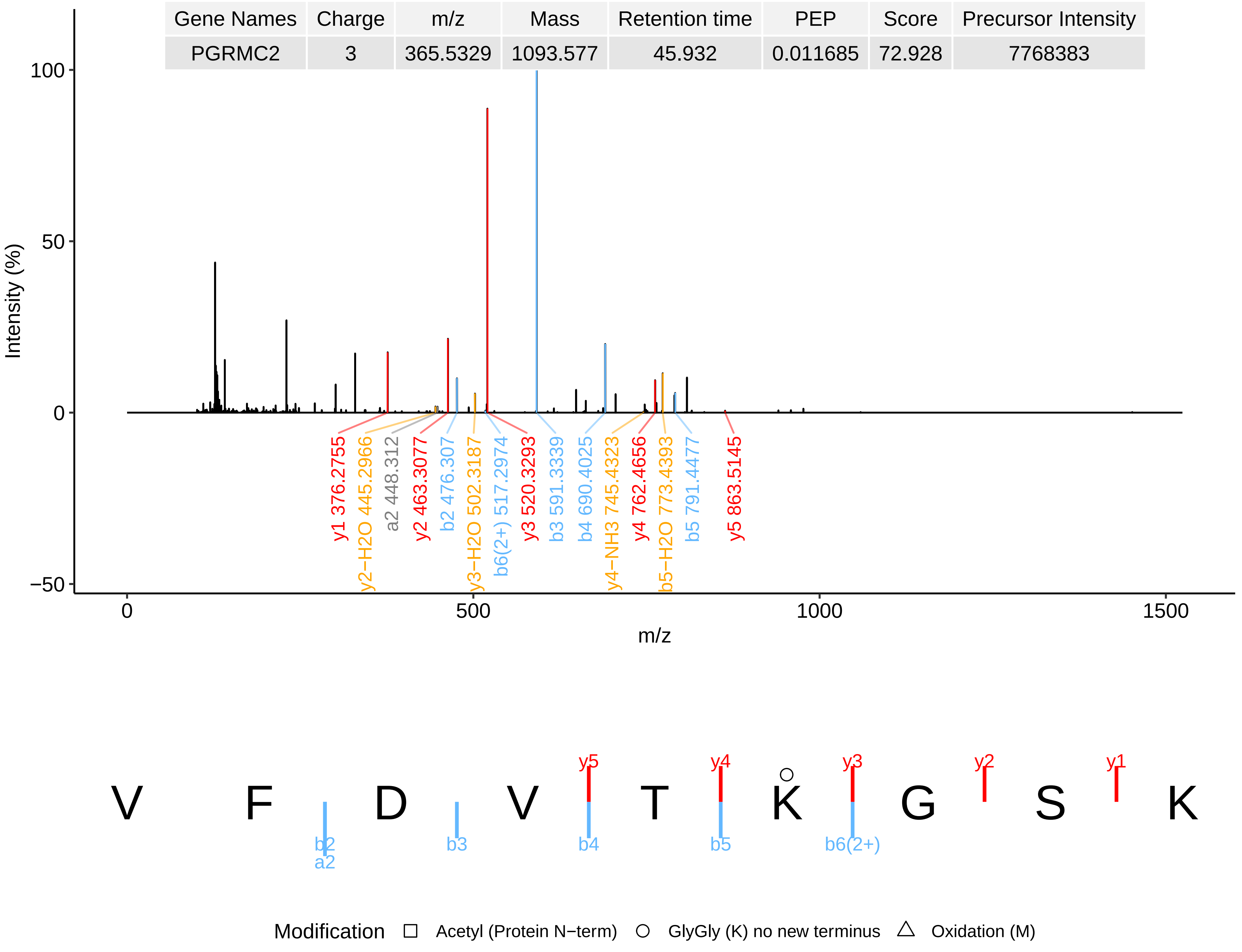

20200921_KIZ_MUb4_GlyGly_10plex_2 − Scan number: 12020

Gene Names	Charge	m/z	Mass	Retention time	PEP	Score	Precursor Intensity
PGRMC2	3	365.5329	1093.577	46.413	0.019804	65.305	1325113

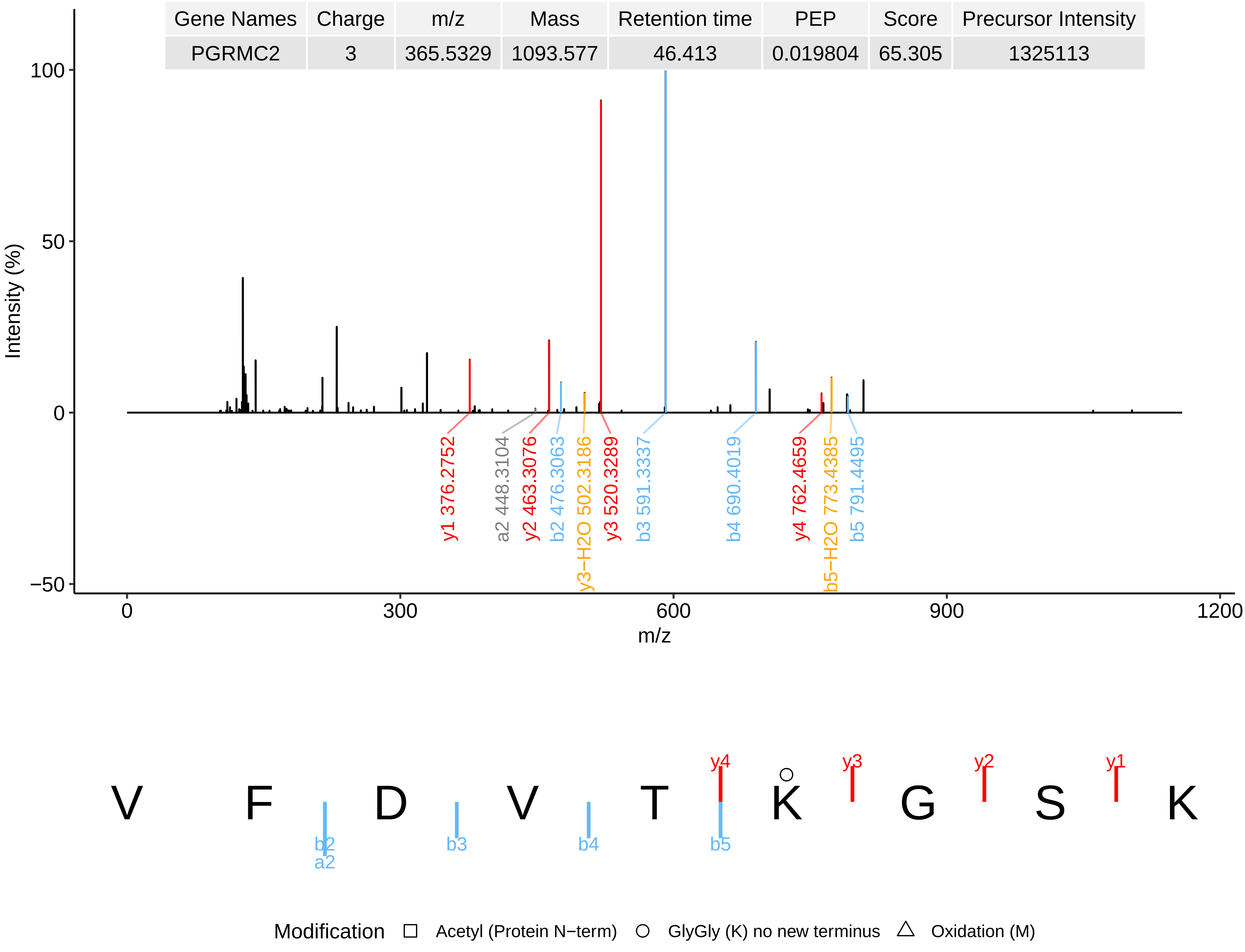

20200924_KIZ_MUb5_GlyGly_1 − Scan number: 11973

Gene Names	Charge	m/z	Mass	Retention time	PEP	Score	Precursor Intensity
PGRMC2	3	365.5329	1093.577	38.891	0.022029	63.216	8608752

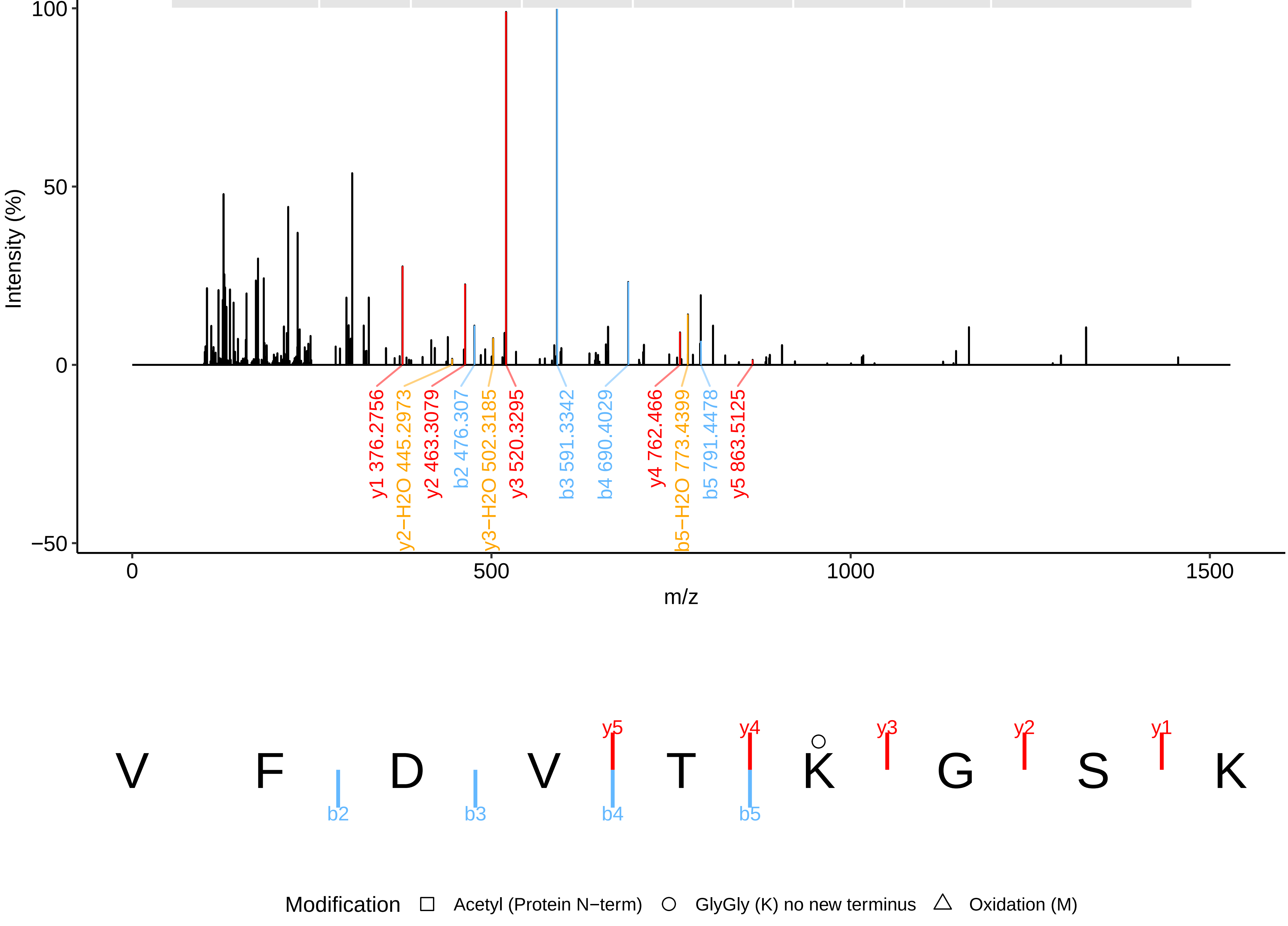

20200924_KIZ_MUb5_GlyGly_2 − Scan number: 11298

Gene Names	Charge	m/z	Mass	Retention time	PEP	Score	Precursor Intensity
PGRMC2	3	365.5329	1093.577	42.211	0.073996	56.432	NA

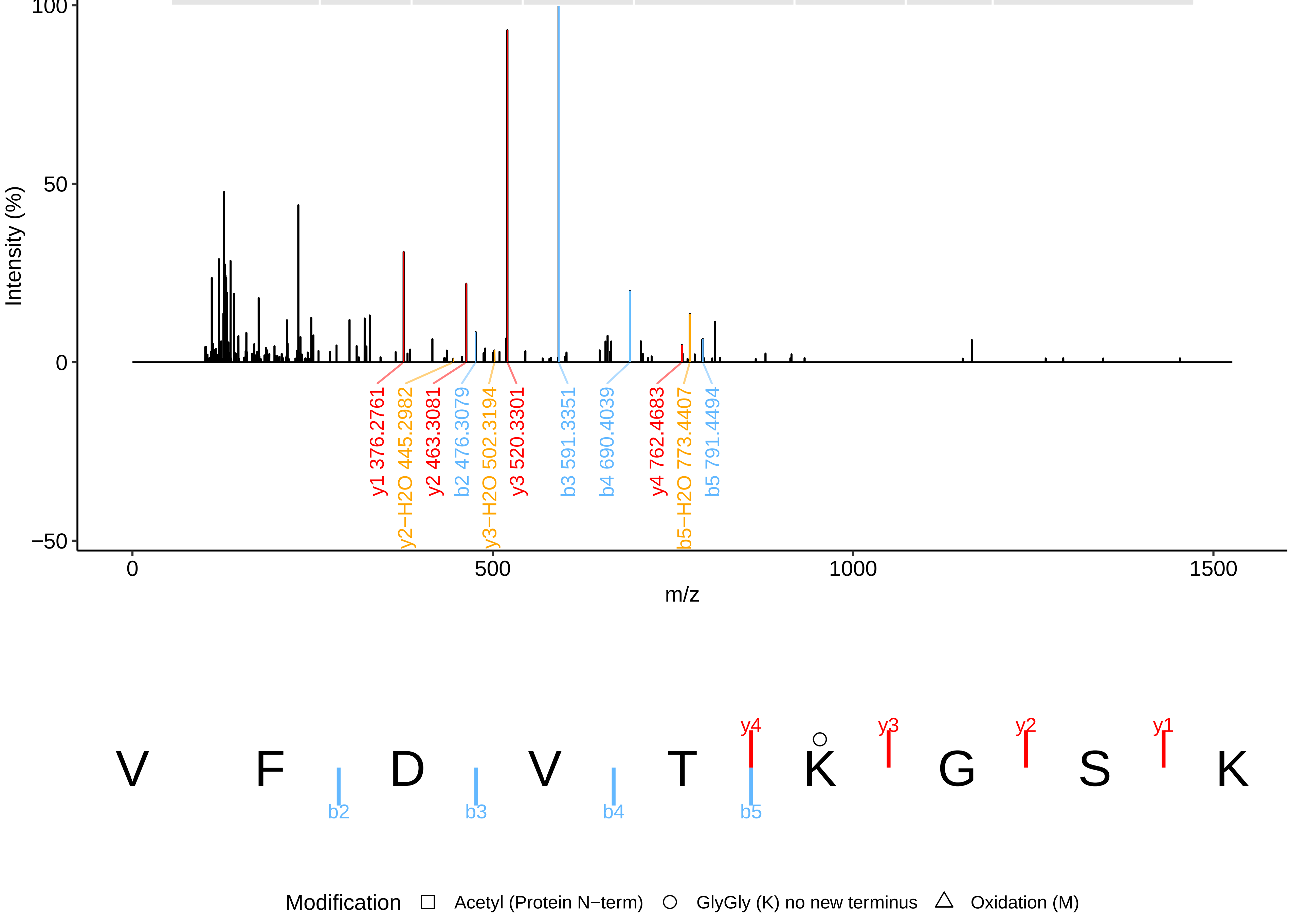

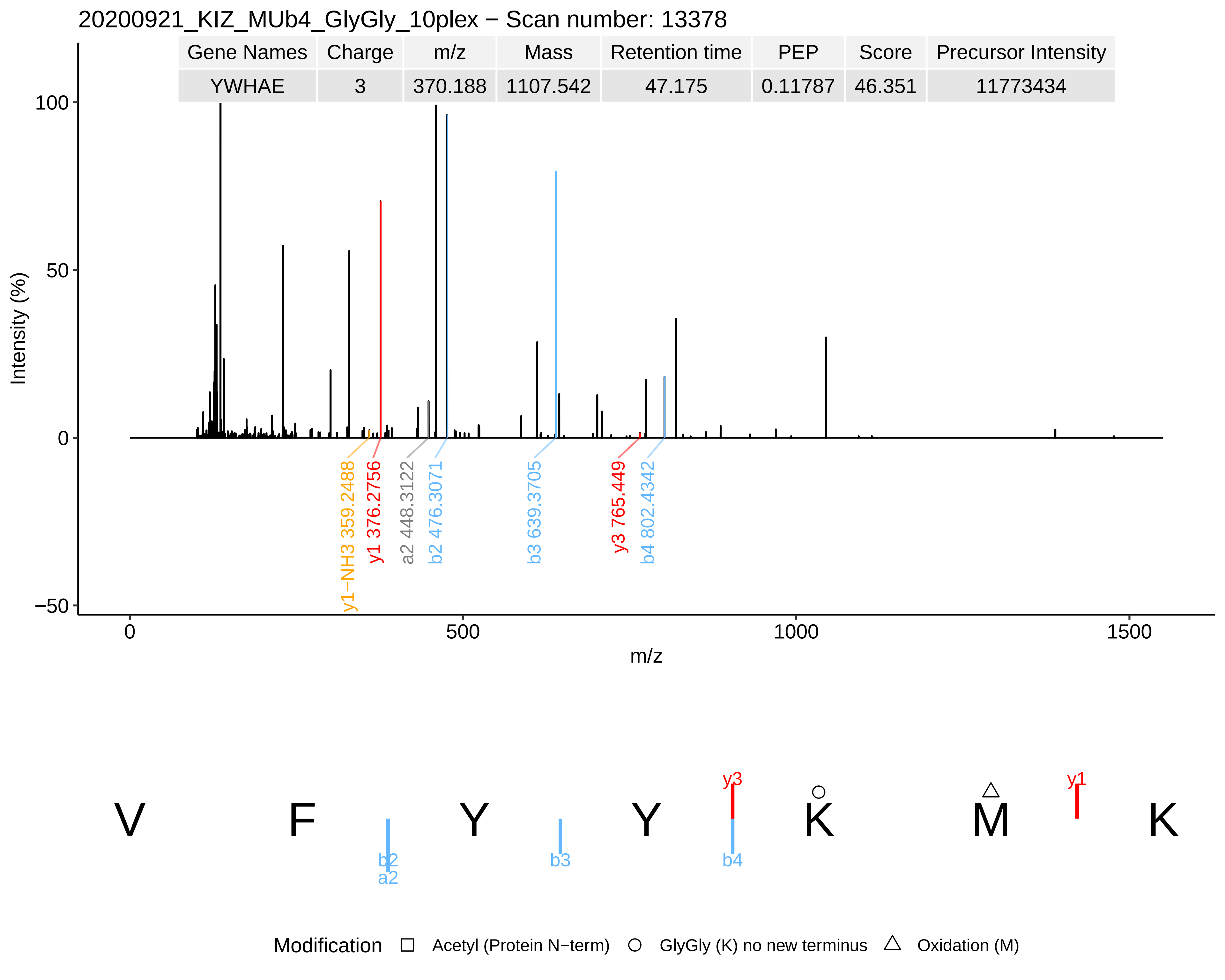

20200921_KIZ_MUb4_GlyGly_10plex − Scan number: 13378

Gene Names	Charge	m/z	Mass	Retention time	PEP	Score	Precursor Intensity
YWHAE	3	370.188	1107.542	47.175	0.11787	46.351	11773434

Intensity (%)
100
50
0
−50
0
500
1000
1500
m/z
y1−NH3 359.2488
y1 376.2756
a2 448.3122
b2 476.3071
b3 639.3705
y3 765.449
b4 802.4342
V F Y Y K M K
y3
y1
b2
a2
b3
b4
Modification
Acetyl (Protein N−term)
GlyGly (K) no new terminus
Oxidation (M)

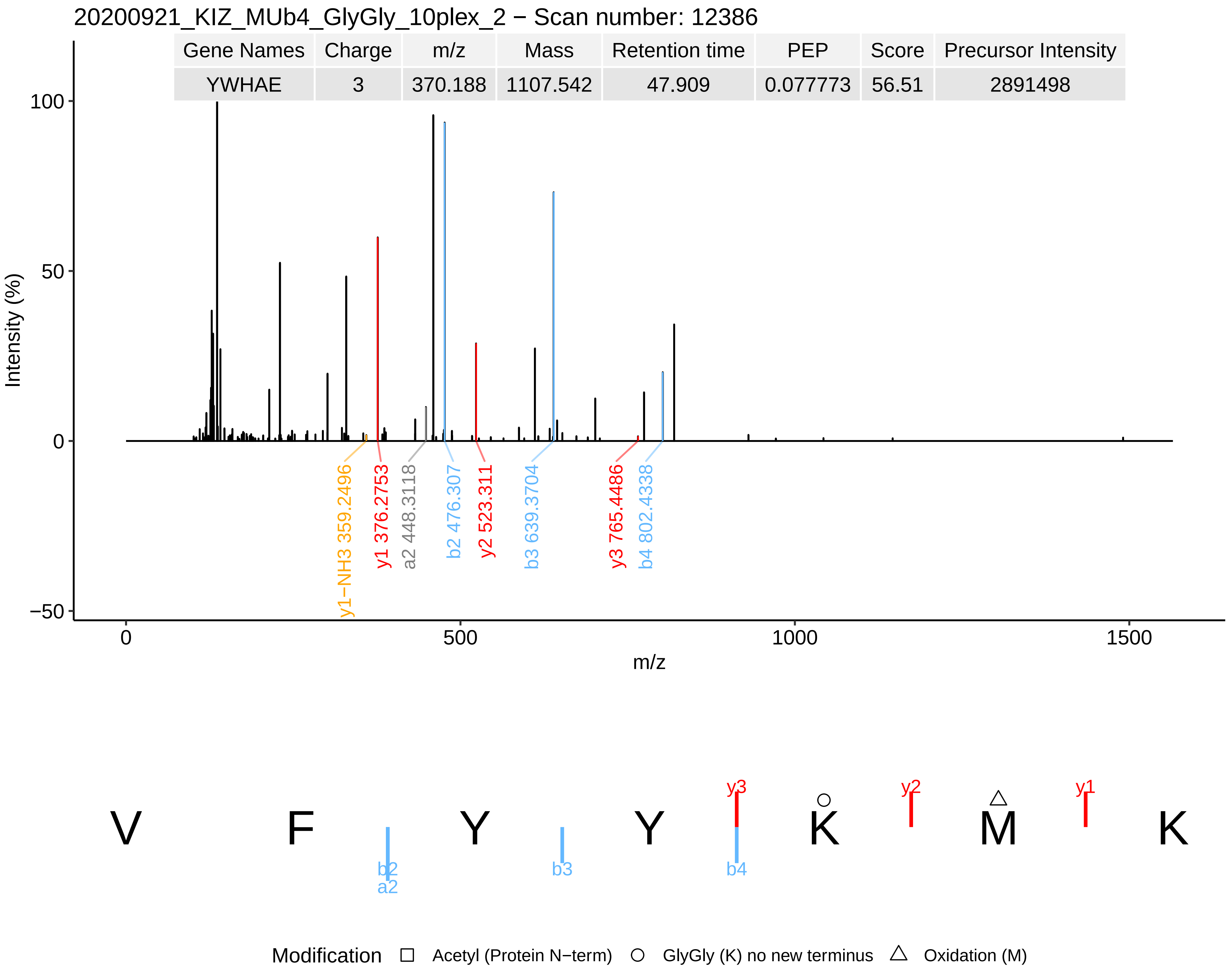
20200921_KIZ_MUb4_GlyGly_10plex_2 − Scan number: 12386
Gene Names | Charge | m/z | Mass | Retention time | PEP | Score | Precursor Intensity
YWHAE | 3 | 370.188 | 1107.542 | 47.909 | 0.077773 | 56.51 | 2891498
Intensity (%)
100
50
0
−50
0
500
1000
1500
m/z
y1−NH3 359.2496
y1 376.2753
a2 448.3118
b2 476.307
y2 523.311
b3 639.3704
y3 765.4486
b4 802.4338
V F Y Y K M K
y3
y2
y1
b2
a2
b3
b4
Modification
Acetyl (Protein N−term)
GlyGly (K) no new terminus
Oxidation (M)

20200921_KIZ_MUb4_GlyGly_10plex − Scan number: 16586

Gene Names	Charge	m/z	Mass	Retention time	PEP	Score	Precursor Intensity
YWHAE	3	364.8564	1091.547	59.613	0.030058	76.592	8120856

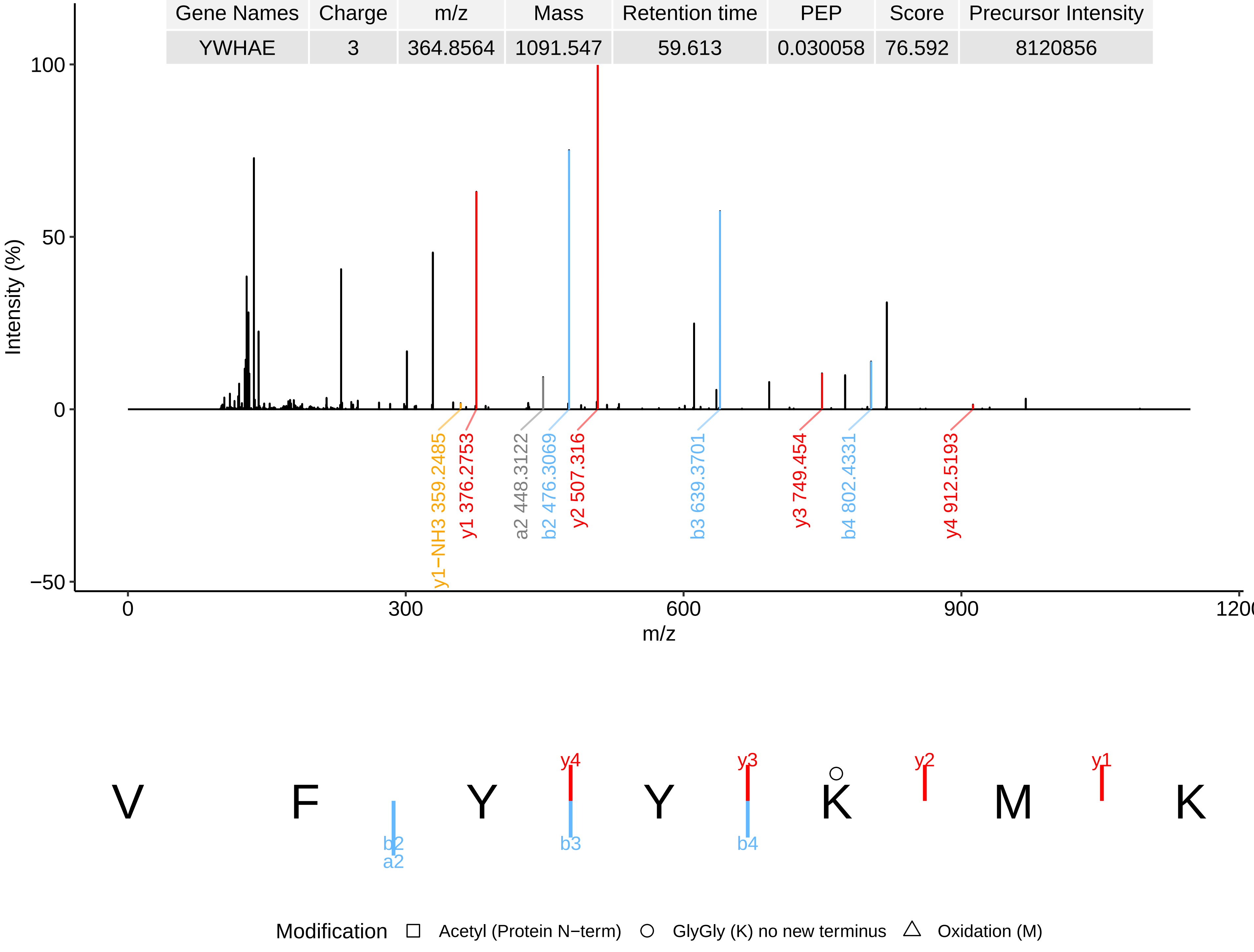

20200921_KIZ_MUb4_GlyGly_10plex_2 − Scan number: 15462

Gene Names	Charge	m/z	Mass	Retention time	PEP	Score	Precursor Intensity
YWHAE	3	364.8564	1091.547	60.464	0.097361	56.51	1544808

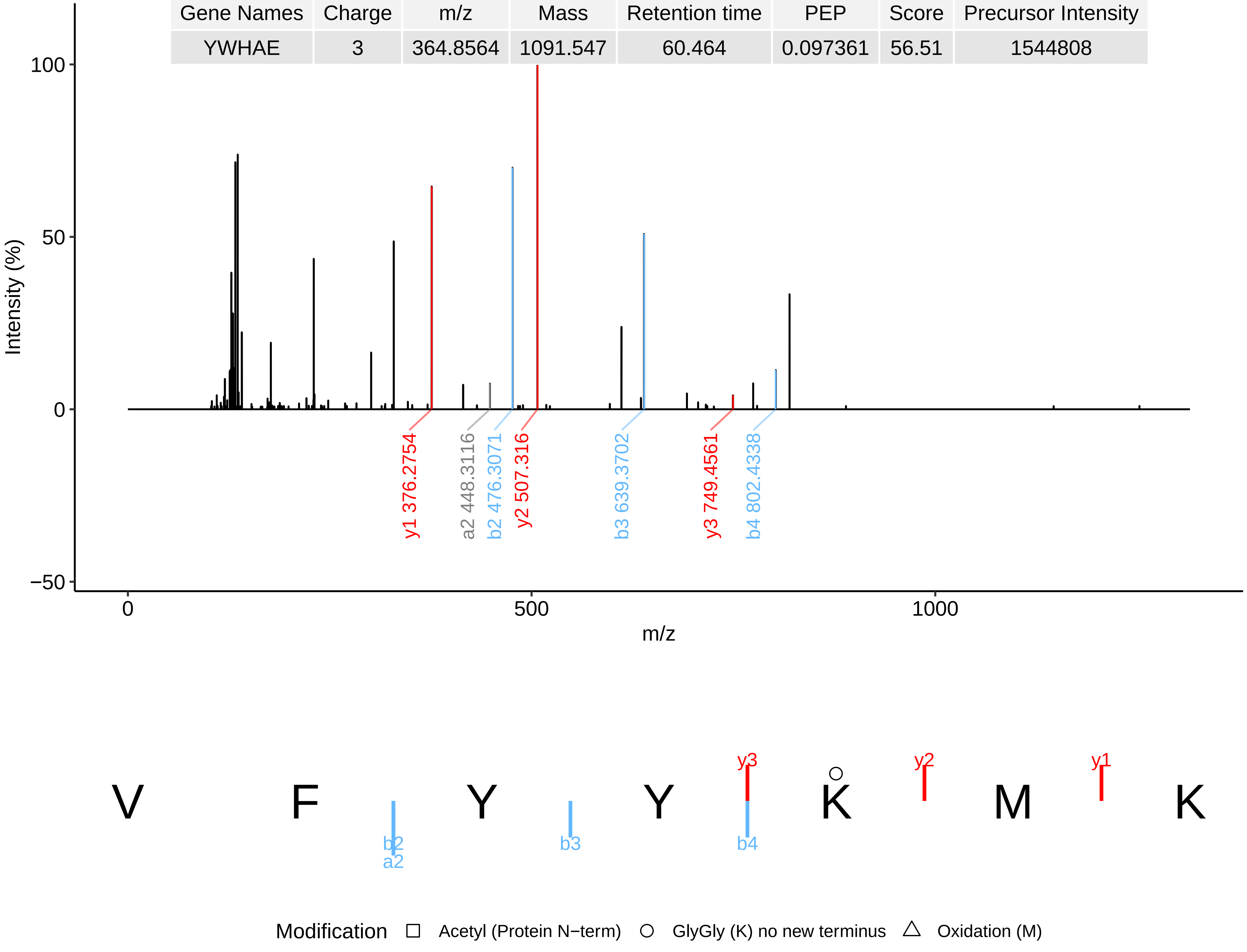

20200924_KIZ_MUb5_GlyGly_1 − Scan number: 15457

Gene Names	Charge	m/z	Mass	Retention time	PEP	Score	Precursor Intensity
YWHAE	3	364.8564	1091.547	50.469	0.054652	66.994	23329654

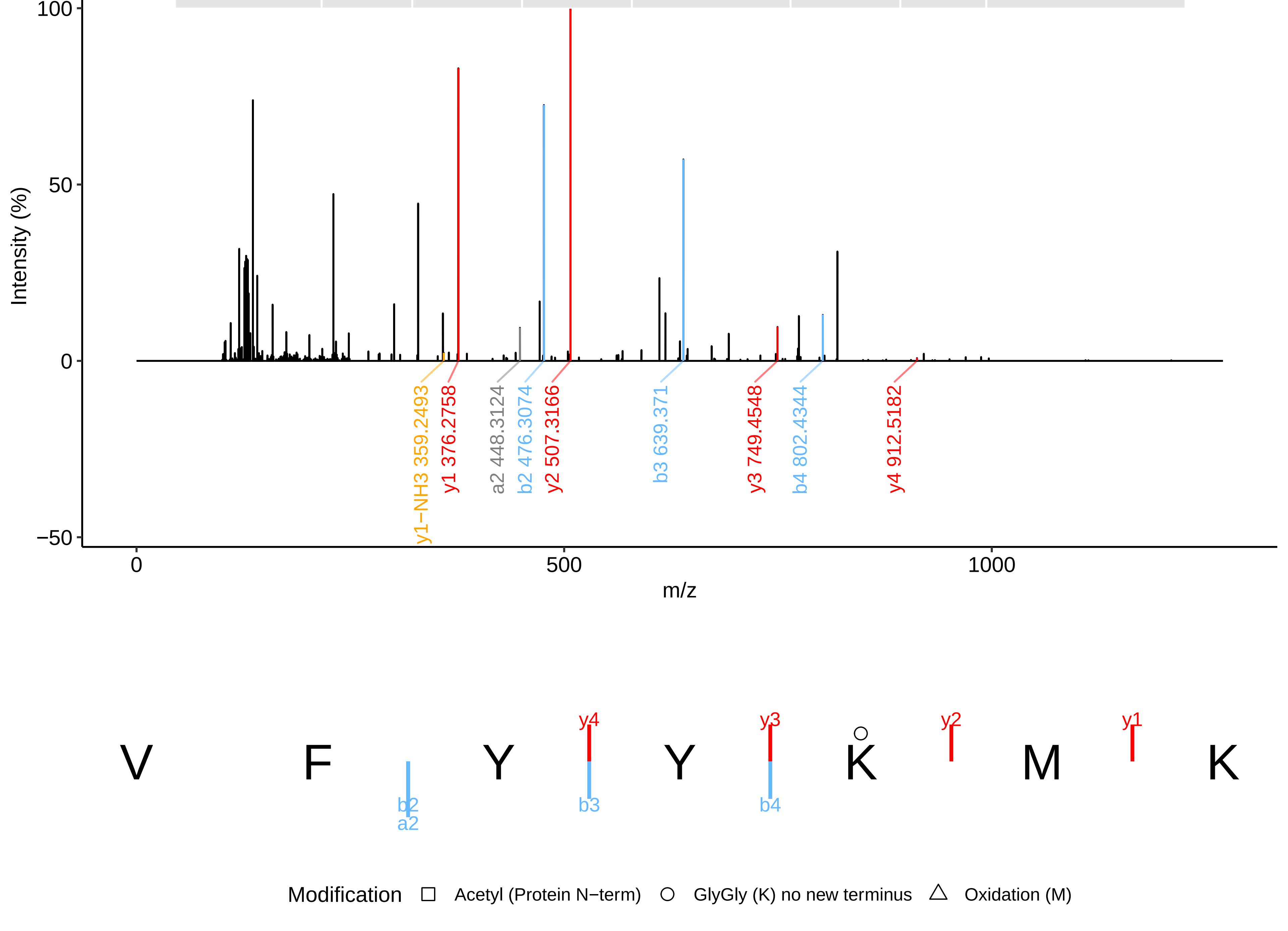

20201118_KIZ_MUb6_GlyGly_1 − Scan number: 15553

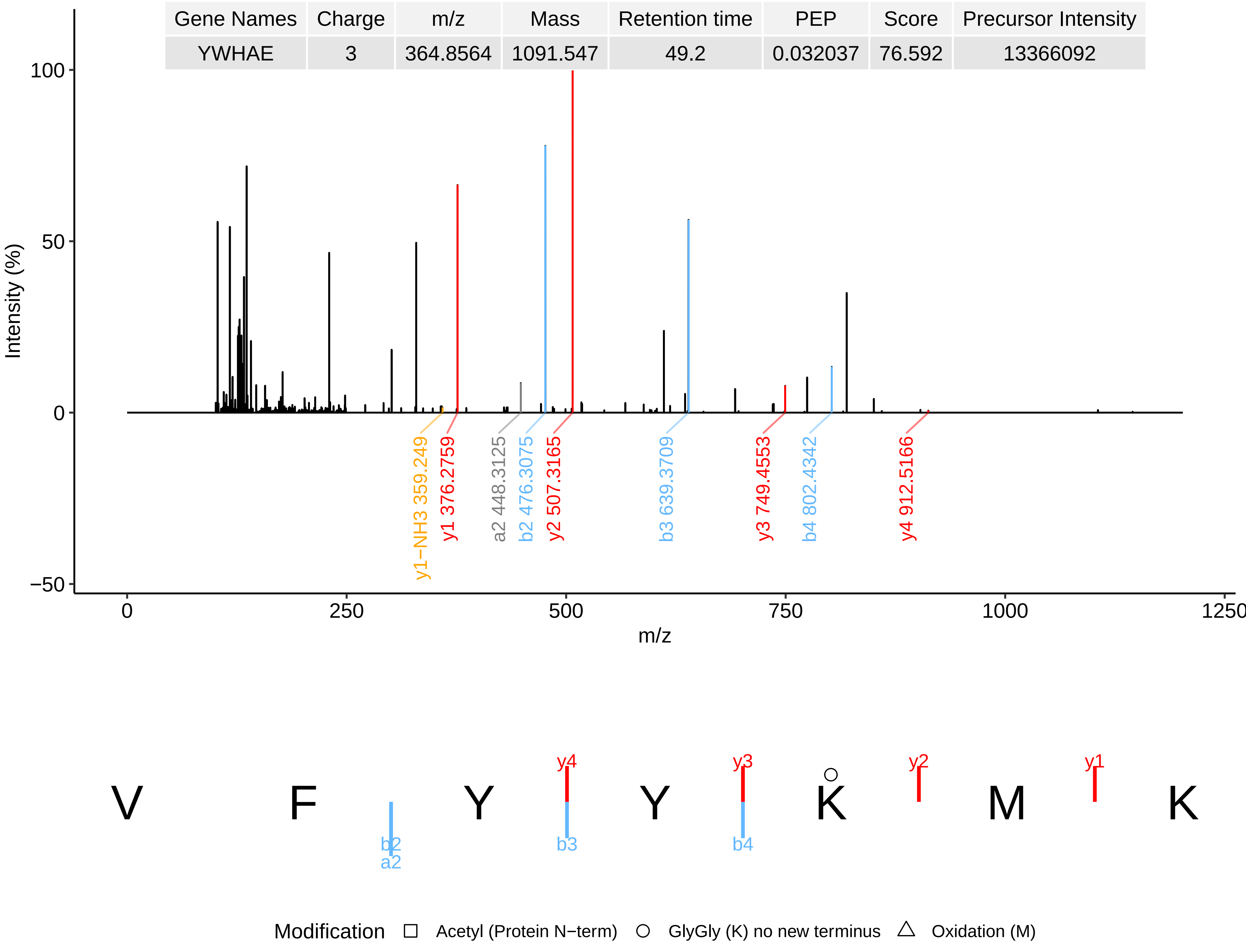

20201118_KIZ_MUb6_GlyGly_2 − Scan number: 18255

Gene Names	Charge	m/z	Mass	Retention time	PEP	Score	Precursor Intensity
YWHAE	3	364.8564	1091.547	61.738	0.034796	74.743	6451969

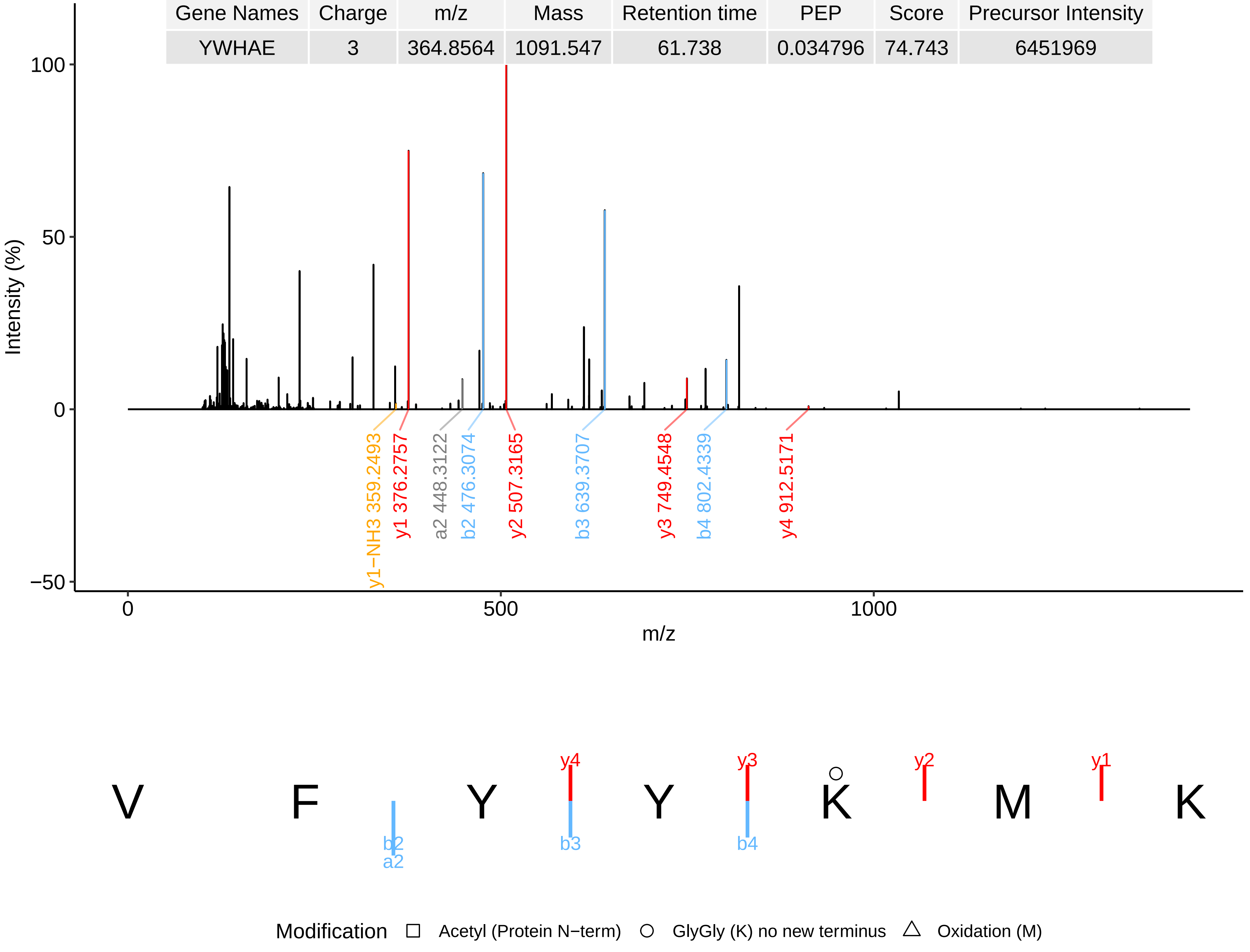

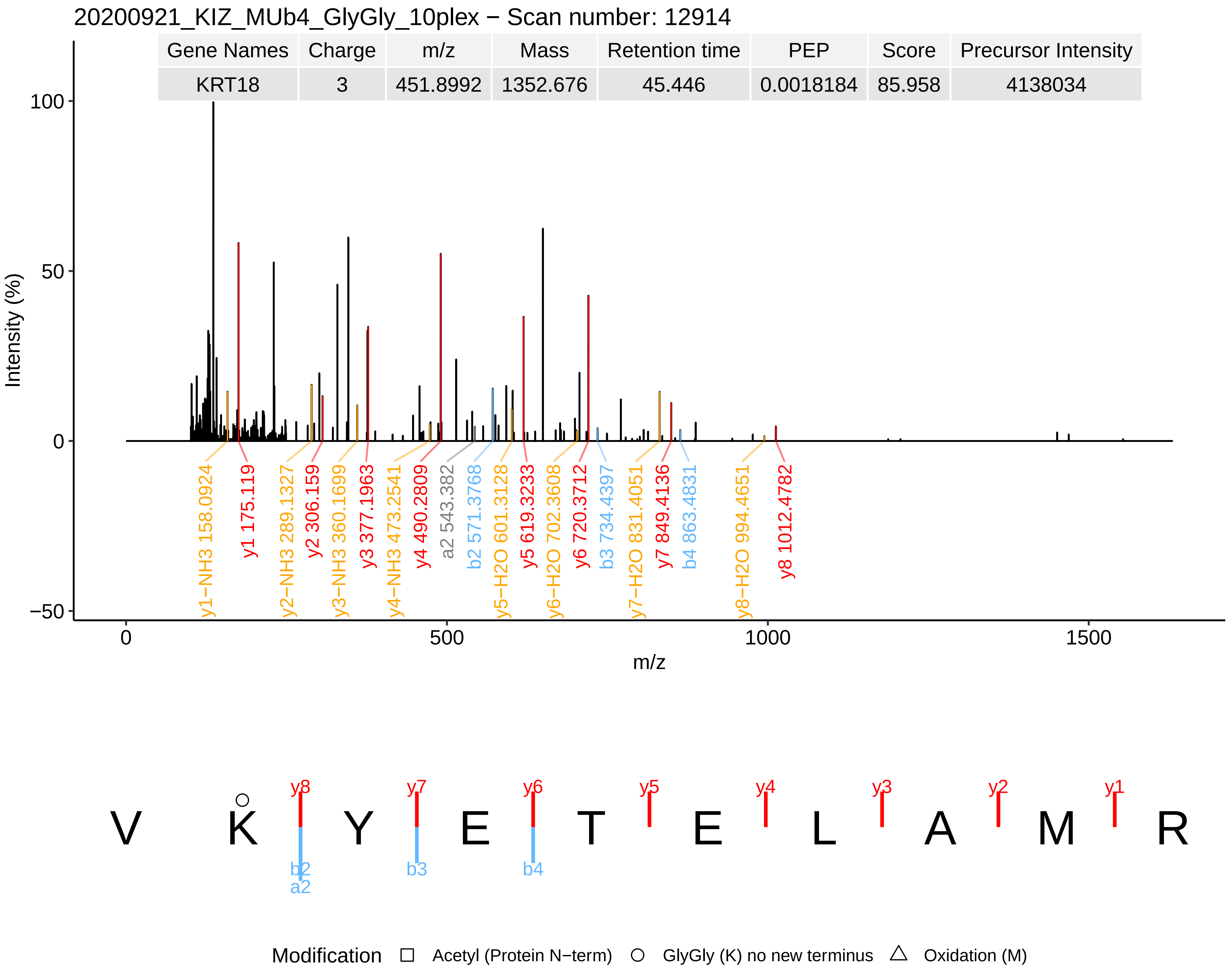

20200921_KIZ_MUb4_GlyGly_10plex − Scan number: 12914
Gene Names | Charge | m/z | Mass | Retention time | PEP | Score | Precursor Intensity
KRT18 | 3 | 451.8992 | 1352.676 | 45.446 | 0.0018184 | 85.958 | 4138034
Intensity (%)
m/z
y1−NH3 158.0924
y1 175.119
y2−NH3 289.1327
y2 306.159
y3−NH3 360.1699
y3 377.1963
y4−NH3 473.2541
y4 490.2809
a2 543.382
b2 571.3768
y5−H2O 601.3128
y5 619.3233
y6−H2O 702.3608
y6 720.3712
b3 734.4397
y7−H2O 831.4051
y7 849.4136
b4 863.4831
y8−H2O 994.4651
y8 1012.4782
V K Y E T E L A M R
y8 y7 y6 y5 y4 y3 y2 y1
b2 a2 b3 b4
Modification
Acetyl (Protein N−term)
GlyGly (K) no new terminus
Oxidation (M)

20200921_KIZ_MUb4_GlyGly_10plex_2 − Scan number: 11904

Gene Names	Charge	m/z	Mass	Retention time	PEP	Score	Precursor Intensity
KRT18	3	451.8992	1352.676	45.934	0.09981	47.706	1067987

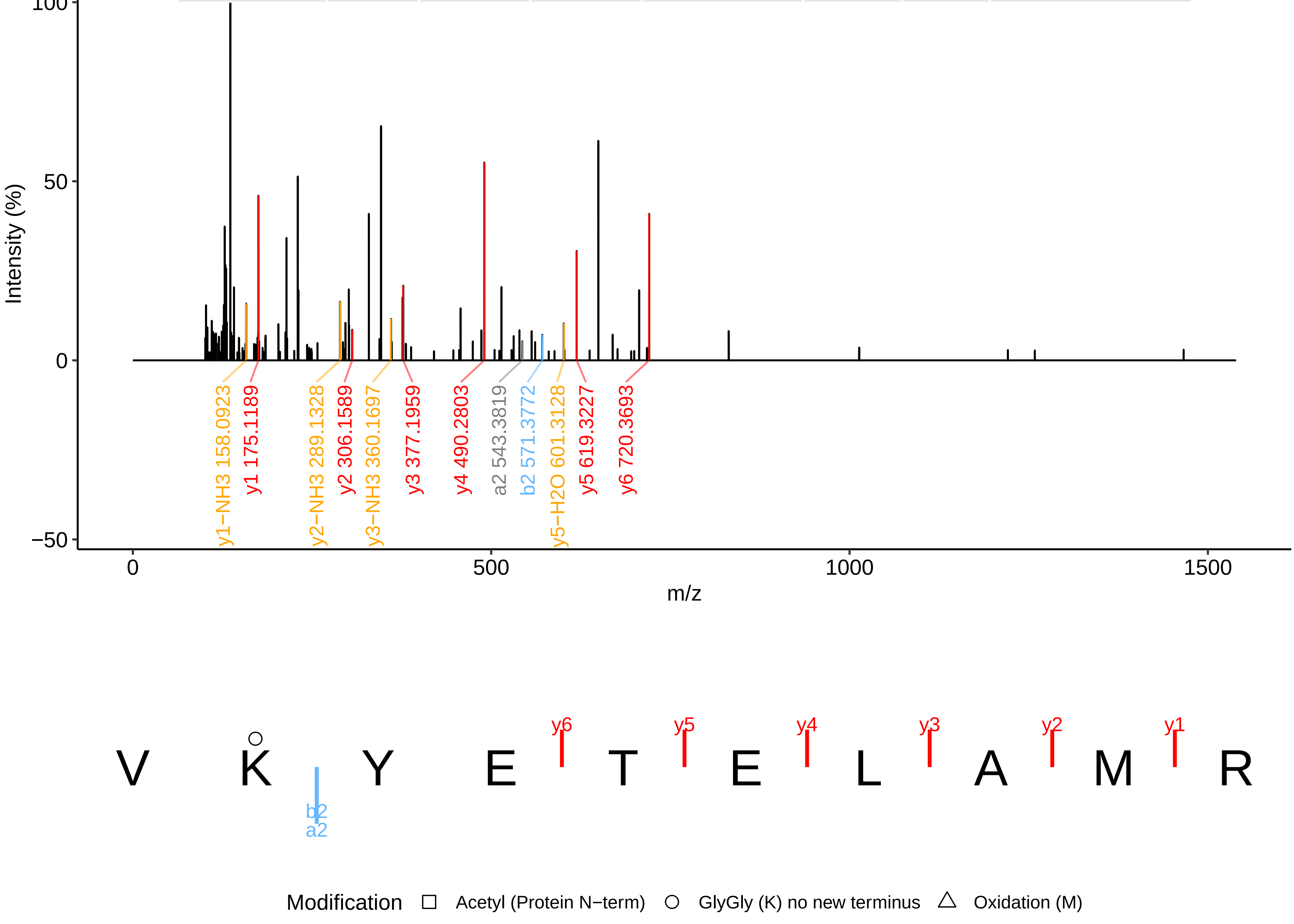

20200921_KIZ_MUb4_GlyGly_10plex_2 − Scan number: 6946

Gene Names	Charge	m/z	Mass	Retention time	PEP	Score	Precursor Intensity
UCK2	3	373.2119	1116.614	25.742	0.0015157	92.439	1460106

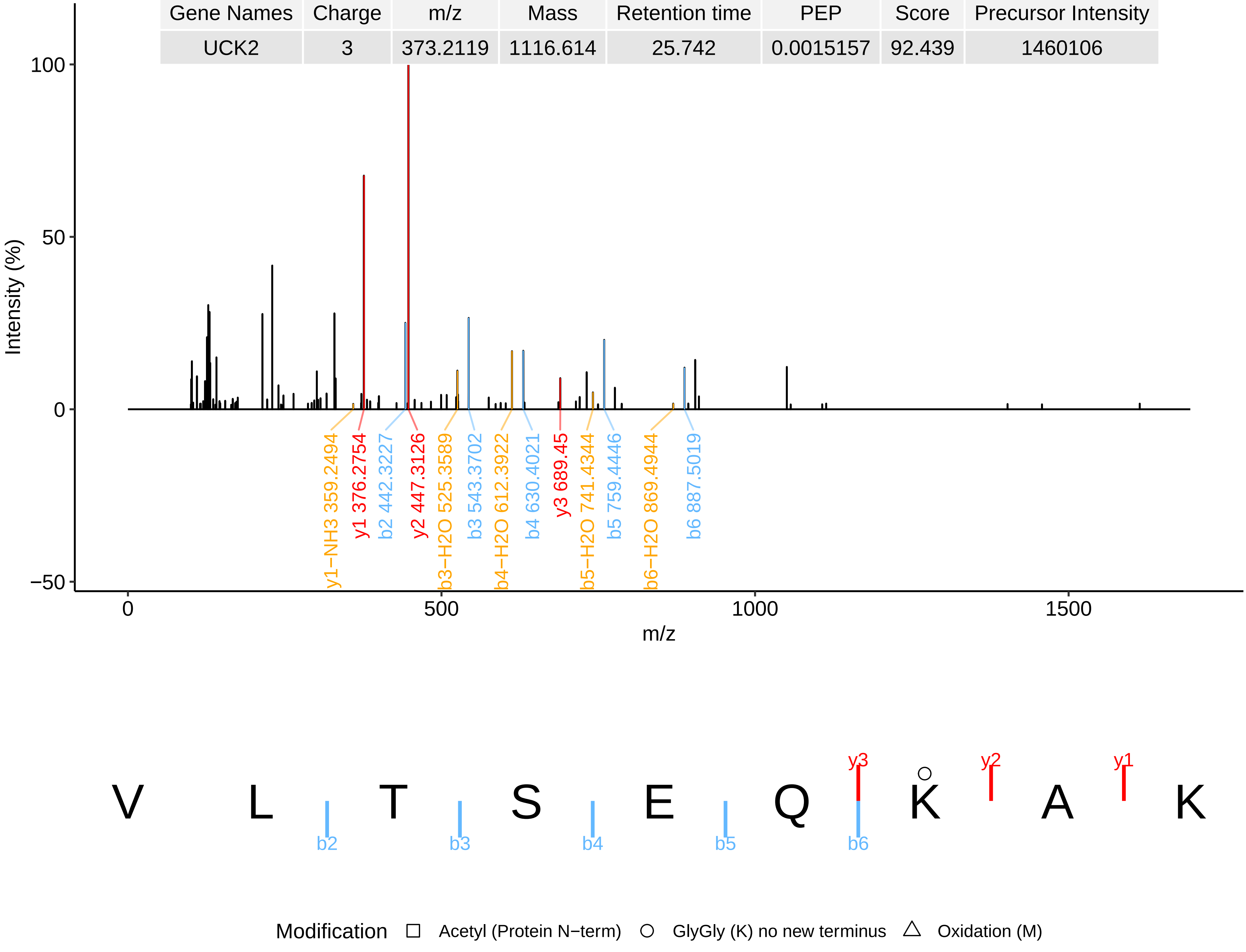

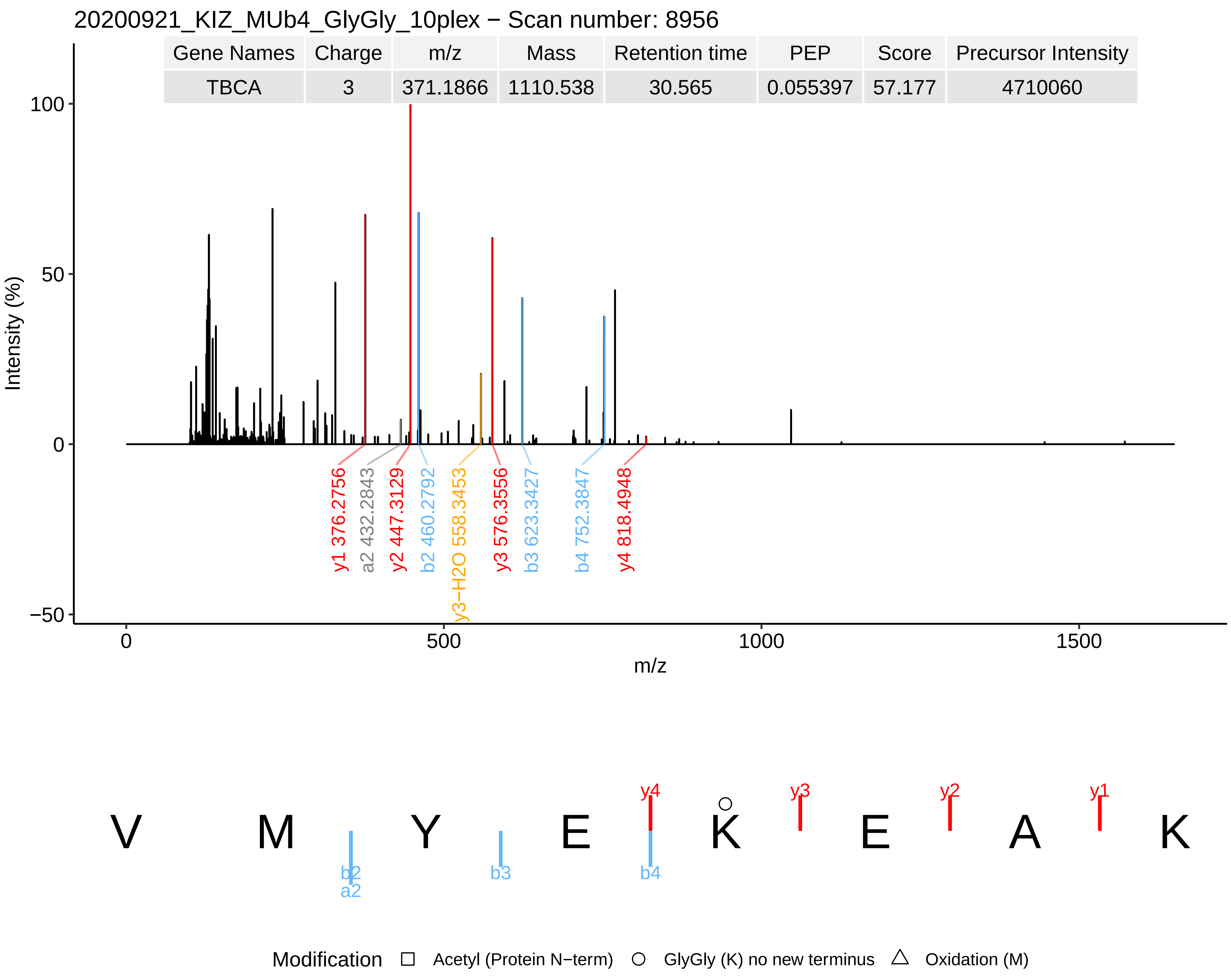
20200921_KIZ_MUb4_GlyGly_10plex − Scan number: 8956
Gene Names | Charge | m/z | Mass | Retention time | PEP | Score | Precursor Intensity
TBCA | 3 | 371.1866 | 1110.538 | 30.565 | 0.055397 | 57.177 | 4710060
Intensity (%)
100
50
0
−50
0
500
1000
1500
m/z
y1 376.2756
a2 432.2843
y2 447.3129
b2 460.2792
y3−H2O 558.3453
y3 576.3556
b3 623.3427
b4 752.3847
y4 818.4948
V M Y E K E A K
y4
y3
y2
y1
b2
a2
b3
b4
Modification
Acetyl (Protein N−term)
GlyGly (K) no new terminus
Oxidation (M)

20200921_KIZ_MUb4_GlyGly_10plex_2 − Scan number: 8314

Gene Names	Charge	m/z	Mass	Retention time	PEP	Score	Precursor Intensity
TBCA	3	371.1866	1110.538	31.288	0.071846	53.17	972505.6

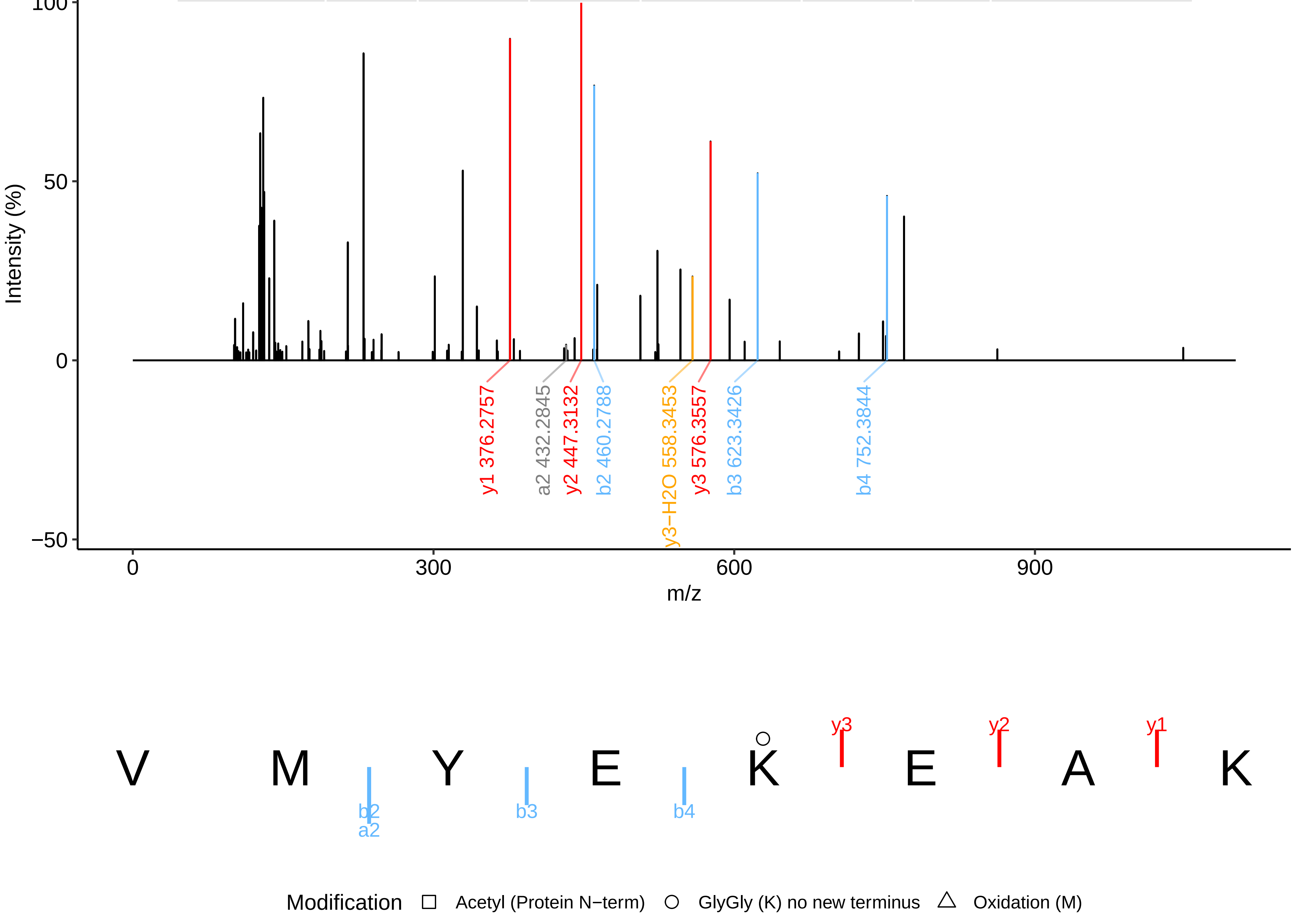

20200921_KIZ_MUb4_GlyGly_10plex − Scan number: 14302

Gene Names	Charge	m/z	Mass	Retention time	PEP	Score	Precursor Intensity
EIF2S3;EIF2S3L	3	367.8803	1100.619	50.773	0.00043995	95.483	7907206

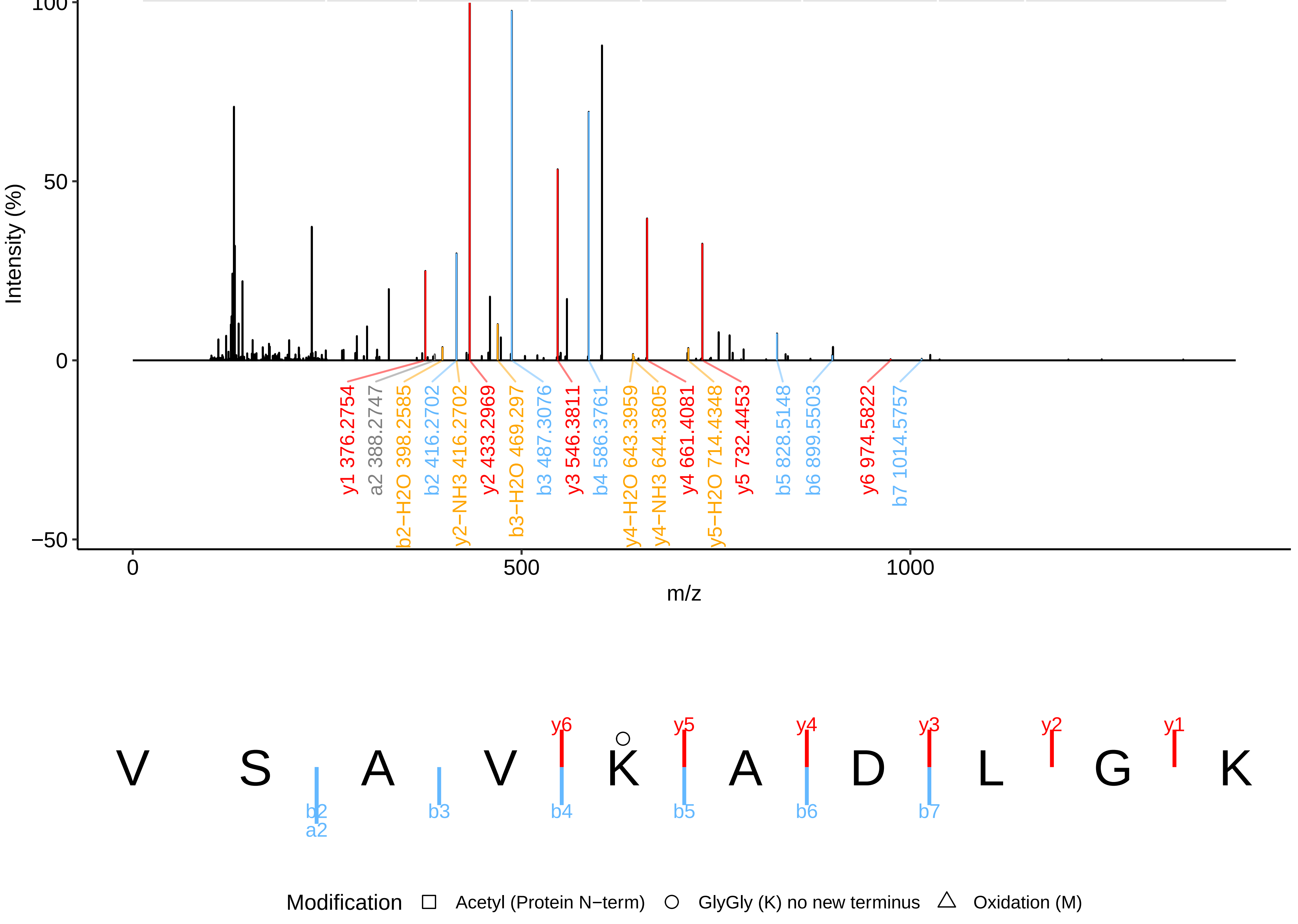

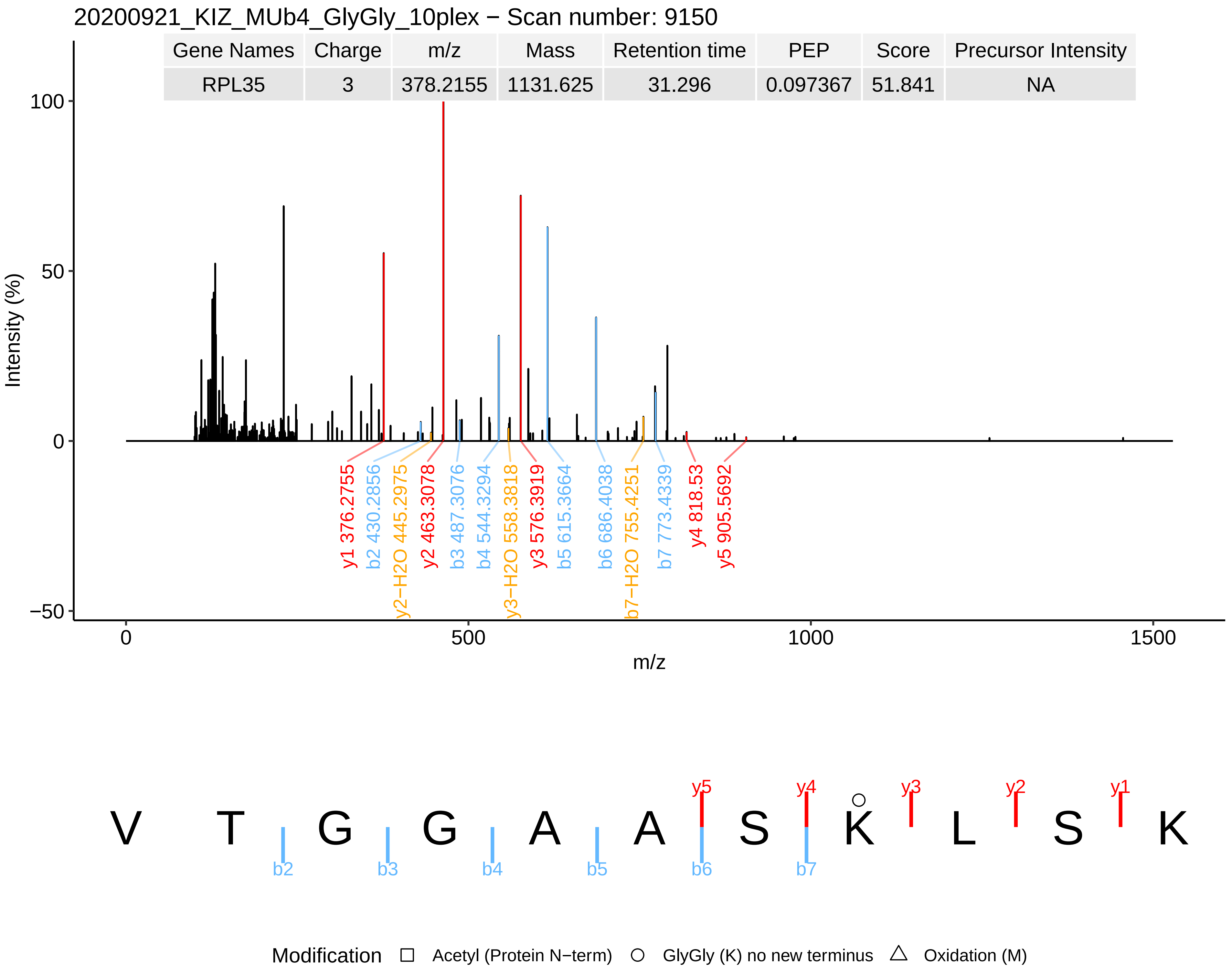

20200921_KIZ_MUb4_GlyGly_10plex − Scan number: 9150
Gene Names | Charge | m/z | Mass | Retention time | PEP | Score | Precursor Intensity
RPL35 | 3 | 378.2155 | 1131.625 | 31.296 | 0.097367 | 51.841 | NA
Intensity (%)
m/z
y1 376.2755
b2 430.2856
y2−H2O 445.2975
y2 463.3078
b3 487.3076
b4 544.3294
y3−H2O 558.3818
y3 576.3919
b5 615.3664
b6 686.4038
b7−H2O 755.4251
b7 773.4339
y4 818.53
y5 905.5692
V T G G A A S K L S K
y5 y4 y3 y2 y1
b2 b3 b4 b5 b6 b7
Modification
Acetyl (Protein N−term)
GlyGly (K) no new terminus
Oxidation (M)

20200921_KIZ_MUb4_GlyGly_10plex − Scan number: 9663

Gene Names	Charge	m/z	Mass	Retention time	PEP	Score	Precursor Intensity
PSMC1	3	365.5207	1093.54	33.159	0.001596	115.49	4190818

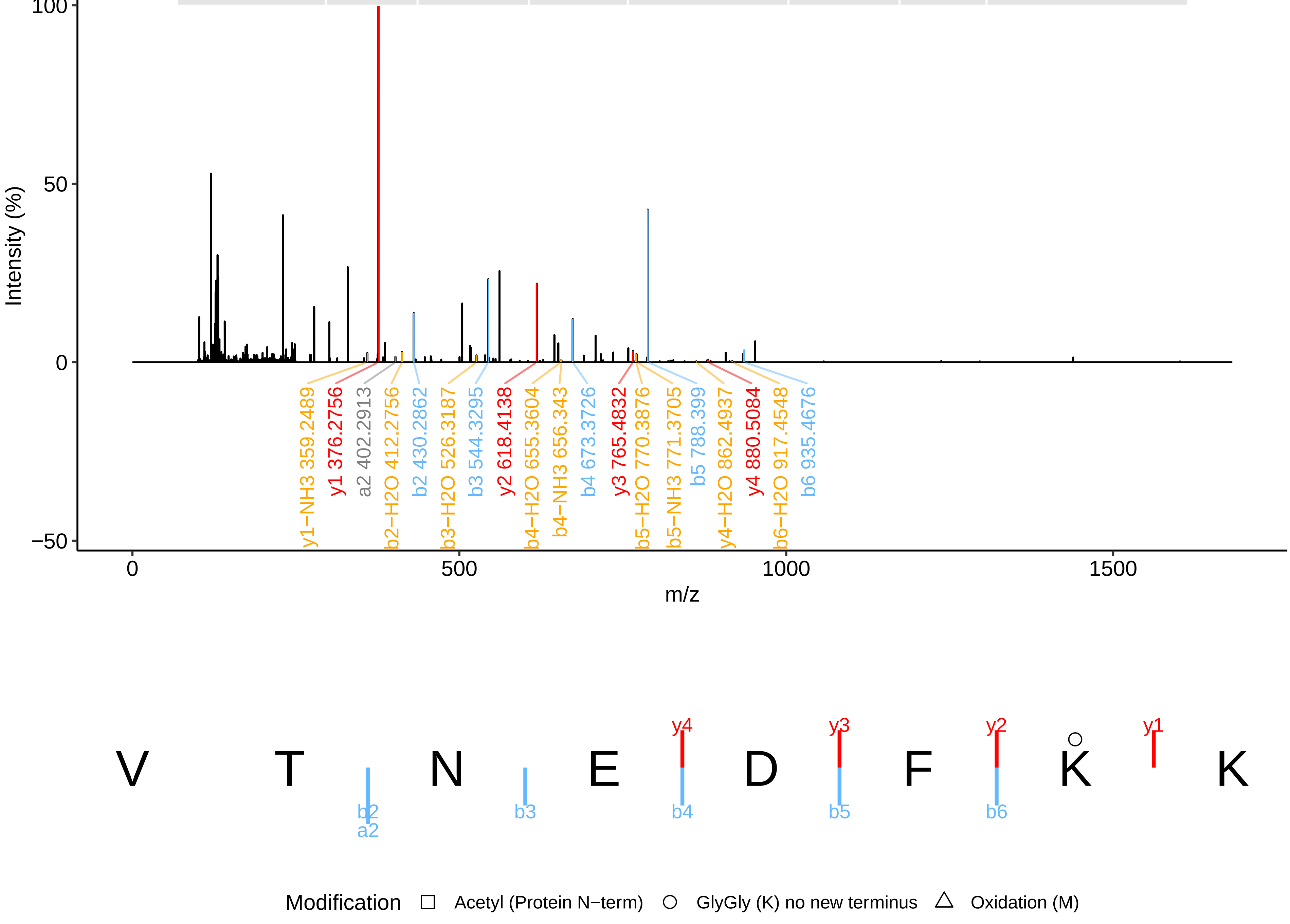

20200921_KIZ_MUb4_GlyGly_10plex − Scan number: 15444

Gene Names	Charge	m/z	Mass	Retention time	PEP	Score	Precursor Intensity
ANTXR1	3	364.8661	1091.576	55.165	0.0057236	89.142	7209970

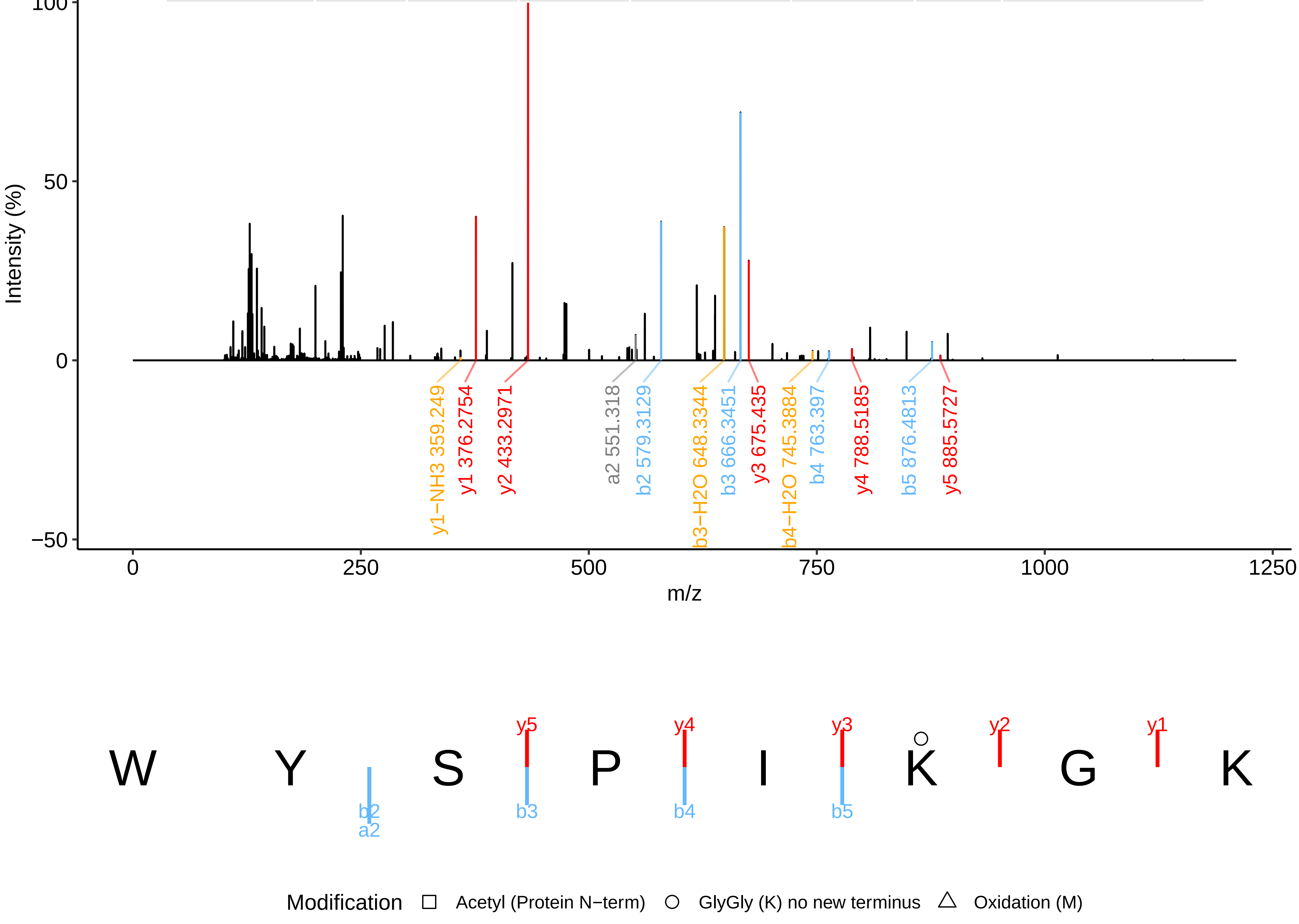

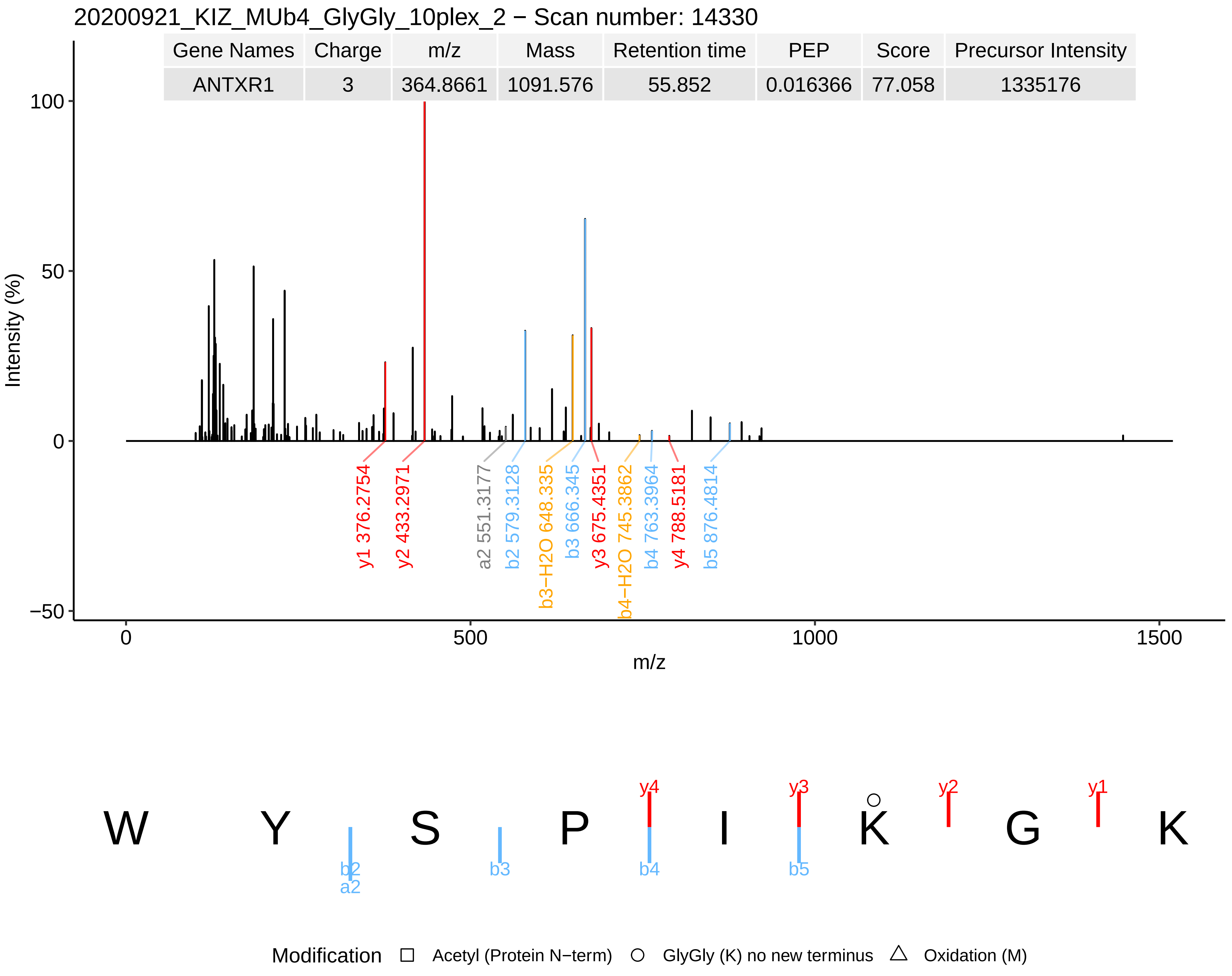
20200921_KIZ_MUb4_GlyGly_10plex_2 − Scan number: 14330
Gene Names
Charge
m/z
Mass
Retention time
PEP
Score
Precursor Intensity
ANTXR1
3
364.8661
1091.576
55.852
0.016366
77.058
1335176
Intensity (%)
100
50
0
−50
0
500
1000
1500
m/z
y1 376.2754
y2 433.2971
a2 551.3177
b2 579.3128
b3−H2O 648.335
b3 666.345
y3 675.4351
b4−H2O 745.3862
b4 763.3964
y4 788.5181
b5 876.4814
W
Y
S
P
I
K
G
K
y4
y3
y2
y1
b2
a2
b3
b4
b5
Modification
Acetyl (Protein N−term)
GlyGly (K) no new terminus
Oxidation (M)

20200921_KIZ_MUb4_GlyGly_10plex − Scan number: 15065

Gene Names	Charge	m/z	Mass	Retention time	PEP	Score	Precursor Intensity
EIF4A1	3	375.8816	1124.623	53.727	0.013691	76.899	6496647

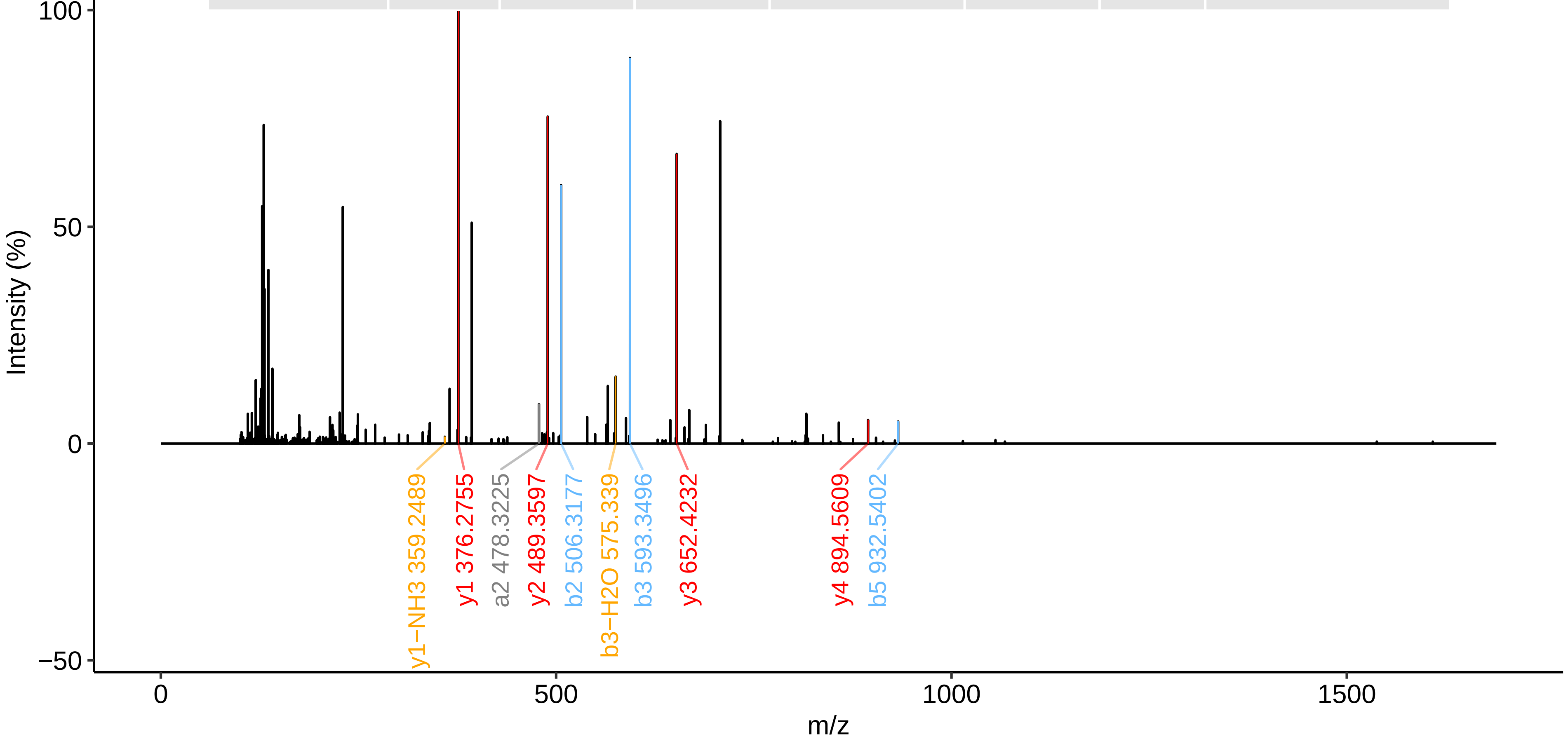

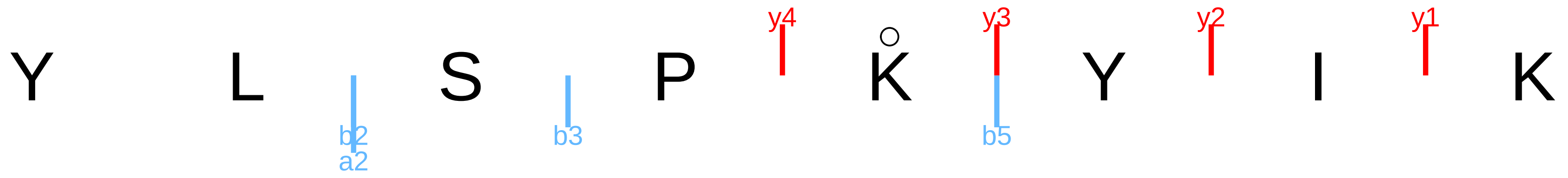

Modification □ Acetyl (Protein N−term) ○ GlyGly (K) no new terminus △ Oxidation (M)

20200921_KIZ_MUb4_GlyGly_10plex_2 − Scan number: 13978

Gene Names	Charge	m/z	Mass	Retention time	PEP	Score	Precursor Intensity
EIF4A1	3	375.8816	1124.623	54.394	0.014992	75.854	1289396

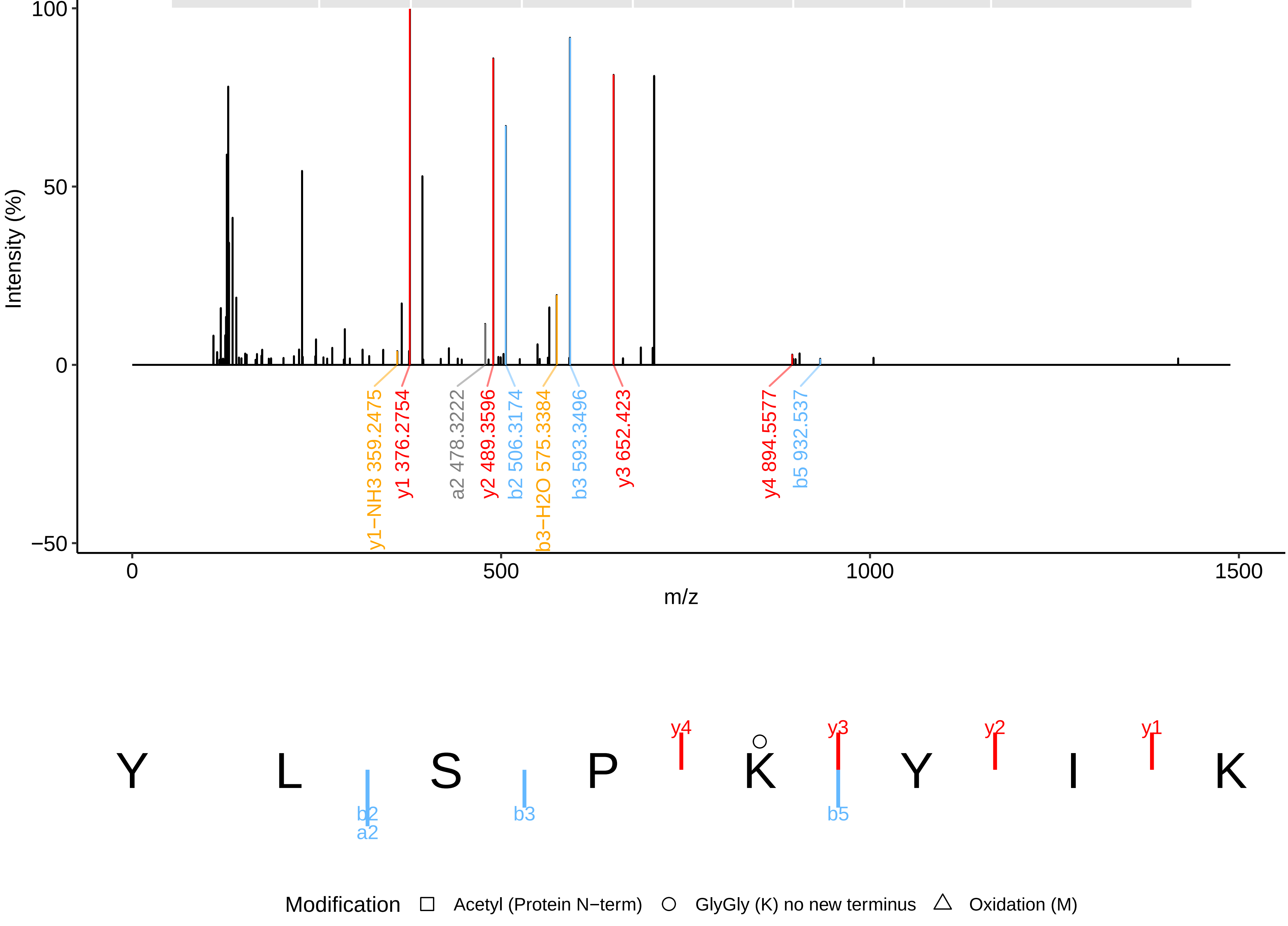

20200921_KIZ_MUb4_GlyGly_10plex − Scan number: 13262

Gene Names	Charge	m/z	Mass	Retention time	PEP	Score	Pr
3F3A;H3F3C;HIST1H3A;HIST2H3A;HIST3H3;HIST2H3PS2	3	455.5893	1363.746	46.735	0.0019835	84.817	

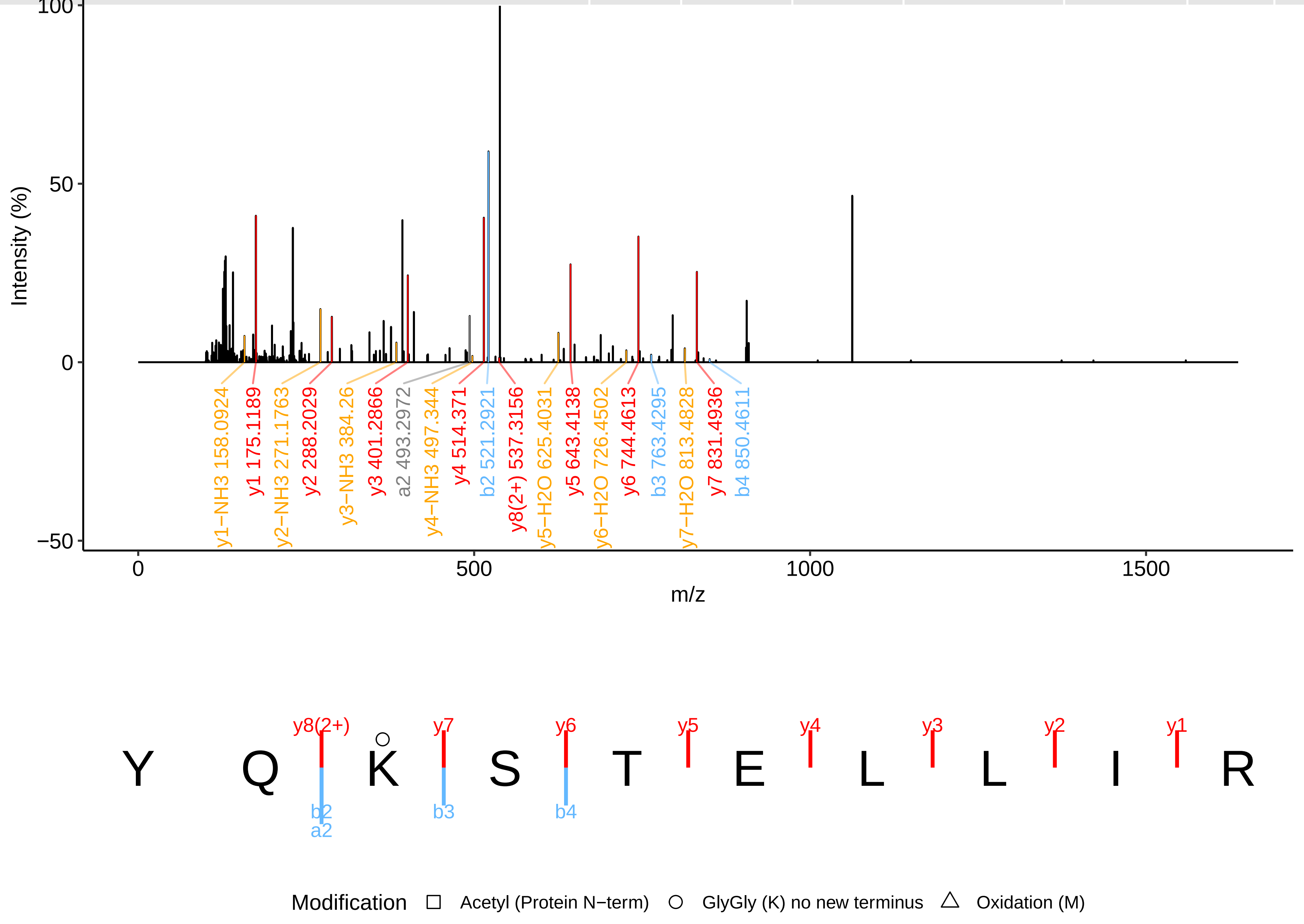

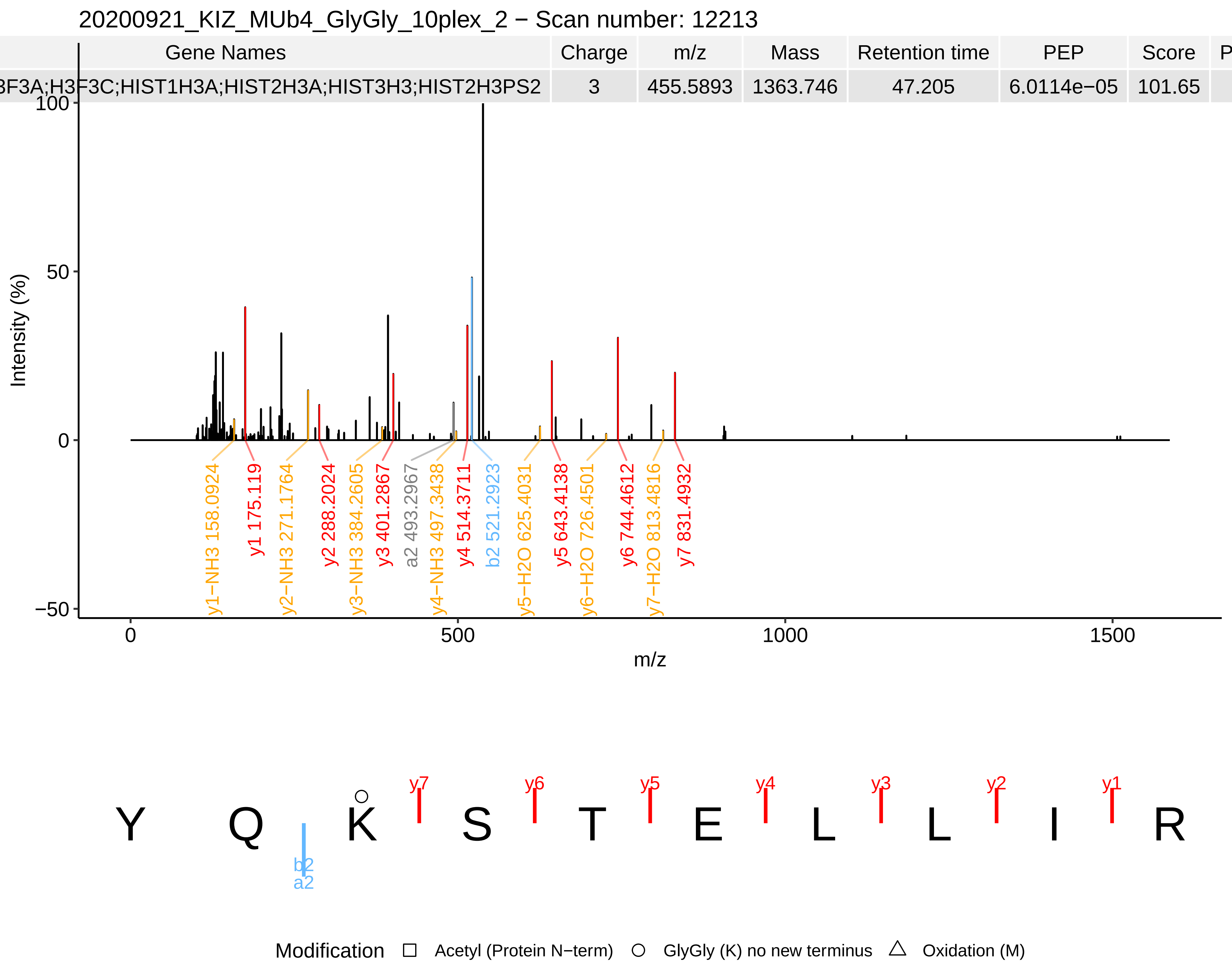
20200921_KIZ_MUb4_GlyGly_10plex_2 − Scan number: 12213
Gene Names
Charge
m/z
Mass
Retention time
PEP
Score
3F3A;H3F3C;HIST1H3A;HIST2H3A;HIST3H3;HIST2H3PS2
3
455.5893
1363.746
47.205
6.0114e−05
101.65
Intensity (%)
m/z
y1−NH3 158.0924
y1 175.119
y2−NH3 271.1764
y2 288.2024
y3−NH3 384.2605
y3 401.2867
a2 493.2967
y4−NH3 497.3438
y4 514.3711
b2 521.2923
y5−H2O 625.4031
y5 643.4138
y6−H2O 726.4501
y6 744.4612
y7−H2O 813.4816
y7 831.4932
Y Q K S T E L L I R
y7 y6 y5 y4 y3 y2 y1
b2
a2
Modification
Acetyl (Protein N−term)
GlyGly (K) no new terminus
Oxidation (M)

20200921_KIZ_MUb4_GlyGly_10plex_2 − Scan number: 14696

Gene Names	Charge	m/z	Mass	Retention time	PEP	Score	Precursor Intensity
ESYT1	3	374.2169	1119.629	57.333	0.053166	59.35	609535.6

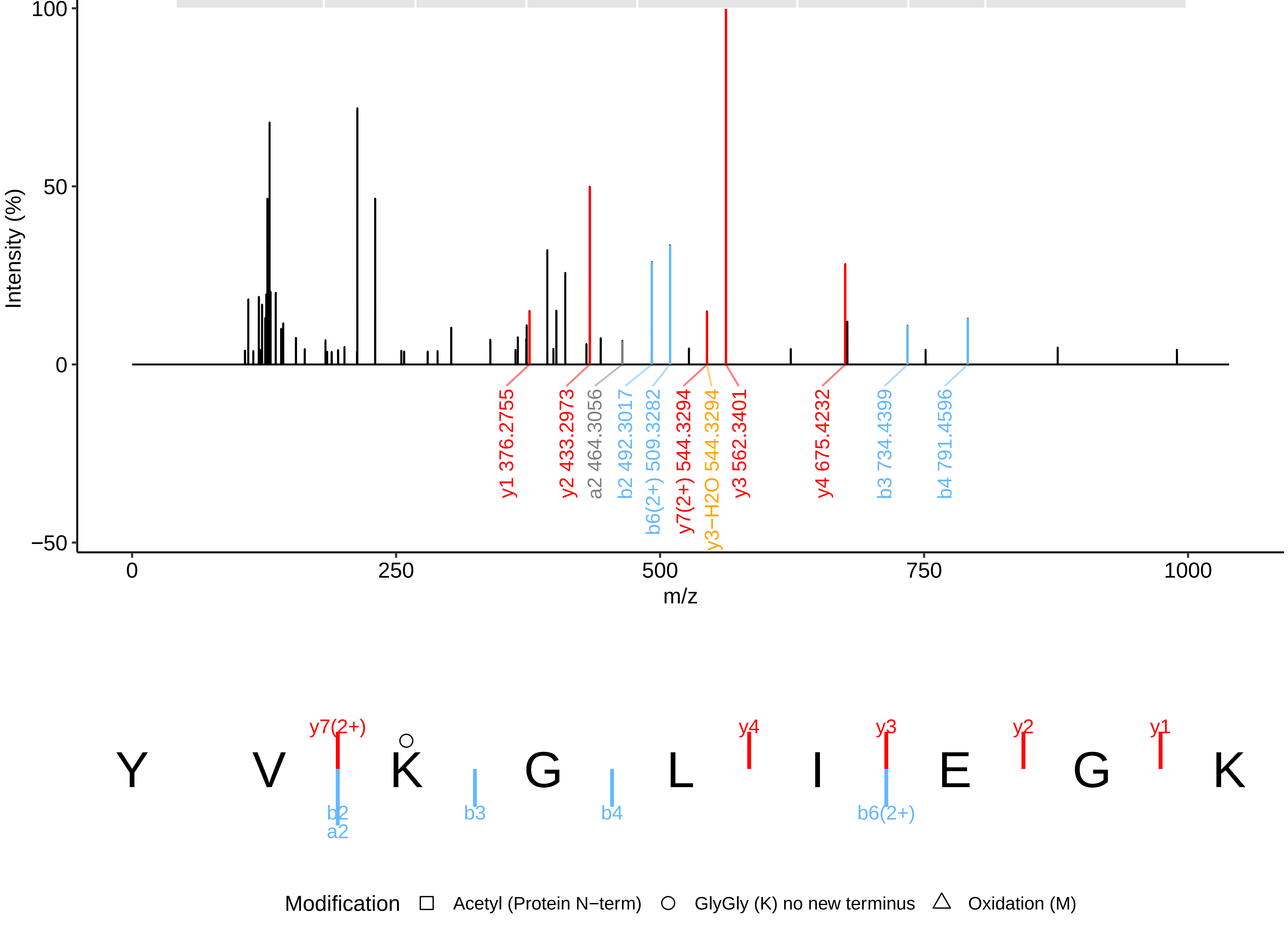

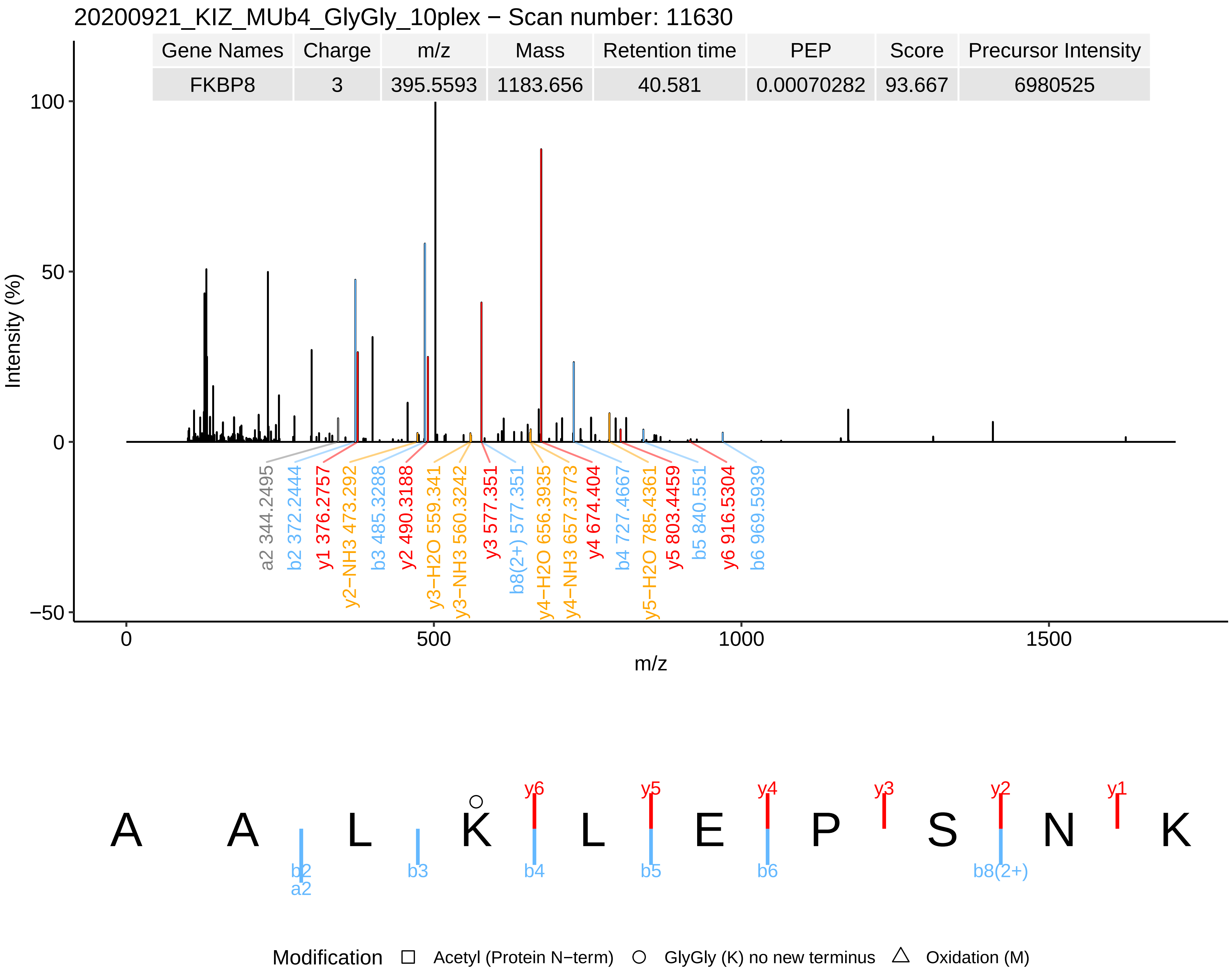

20200921_KIZ_MUb4_GlyGly_10plex − Scan number: 11630
Gene Names | Charge | m/z | Mass | Retention time | PEP | Score | Precursor Intensity
FKBP8 | 3 | 395.5593 | 1183.656 | 40.581 | 0.00070282 | 93.667 | 6980525
Intensity (%)
100
50
0
−50
m/z
0
500
1000
1500
a2 344.2495
b2 372.2444
y1 376.2757
y2−NH3 473.292
b3 485.3288
y2 490.3188
y3−H2O 559.341
y3−NH3 560.3242
y3 577.351
b8(2+) 577.351
y4−H2O 656.3935
y4−NH3 657.3773
y4 674.404
b4 727.4667
y5−H2O 785.4361
y5 803.4459
b5 840.551
y6 916.5304
b6 969.5939
A A L K L E P S N K
y6 y5 y4 y3 y2 y1
b2 a2 b3 b4 b5 b6 b8(2+)
Modification
Acetyl (Protein N−term)
GlyGly (K) no new terminus
Oxidation (M)

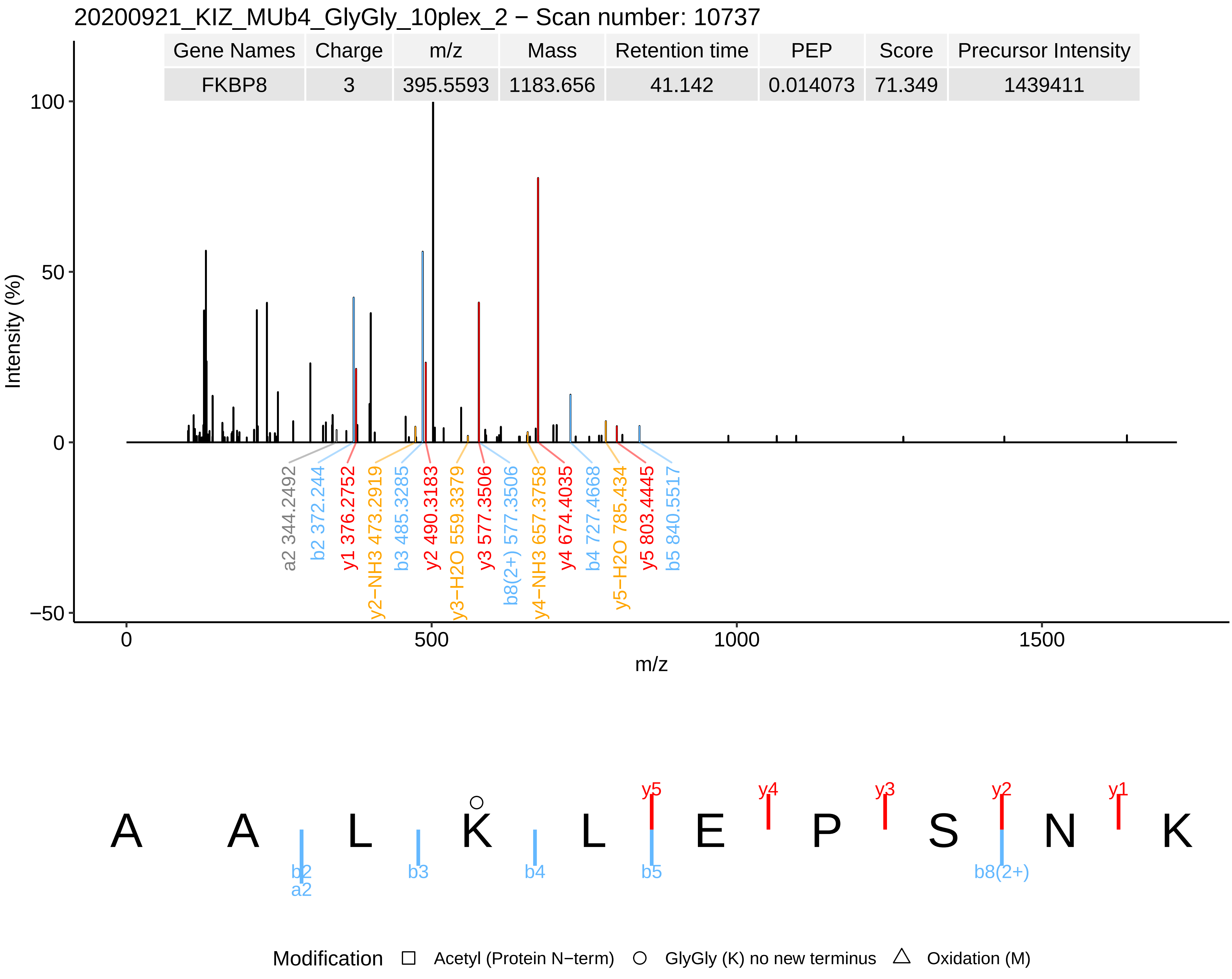

20200921_KIZ_MUb4_GlyGly_10plex_2 − Scan number: 10737
Gene Names | Charge | m/z | Mass | Retention time | PEP | Score | Precursor Intensity
FKBP8 | 3 | 395.5593 | 1183.656 | 41.142 | 0.014073 | 71.349 | 1439411
Intensity (%)
100
50
0
−50
0
500
1000
1500
m/z
a2 344.2492
b2 372.244
y1 376.2752
y2−NH3 473.2919
b3 485.3285
y2 490.3183
y3−H2O 559.3379
y3 577.3506
b8(2+) 577.3506
y4−NH3 657.3758
y4 674.4035
b4 727.4668
y5−H2O 785.434
y5 803.4445
b5 840.5517
A A L K L E P S N K
y5 y4 y3 y2 y1
b2 a2 b3 b4 b5 b8(2+)
Modification
Acetyl (Protein N−term)
GlyGly (K) no new terminus
Oxidation (M)

20200924_KIZ_MUb5_GlyGly_1 − Scan number: 10551

Gene Names	Charge	m/z	Mass	Retention time	PEP	Score	Precursor Intensity
FKBP8	3	395.5593	1183.656	34.077	0.13981	46.844	NA

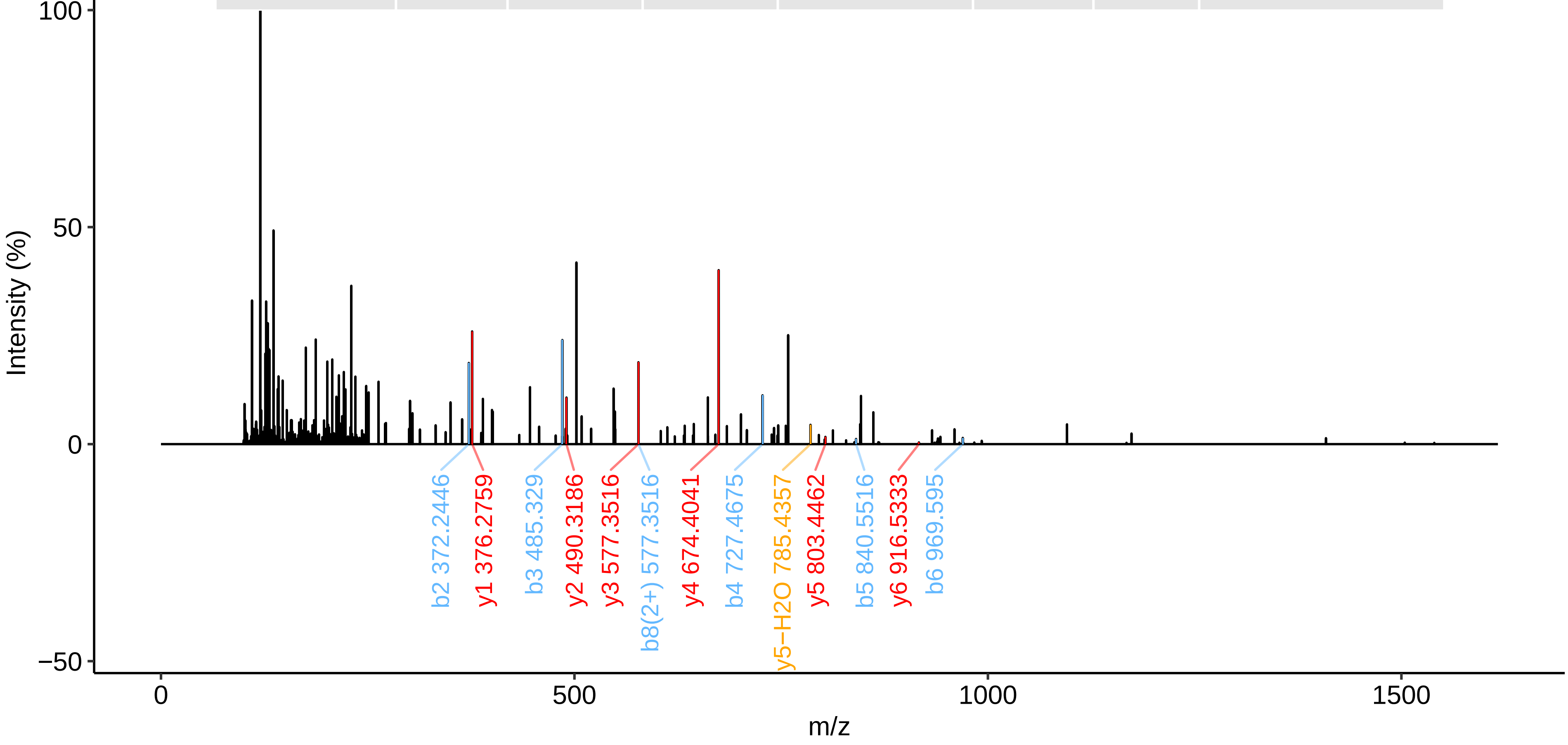

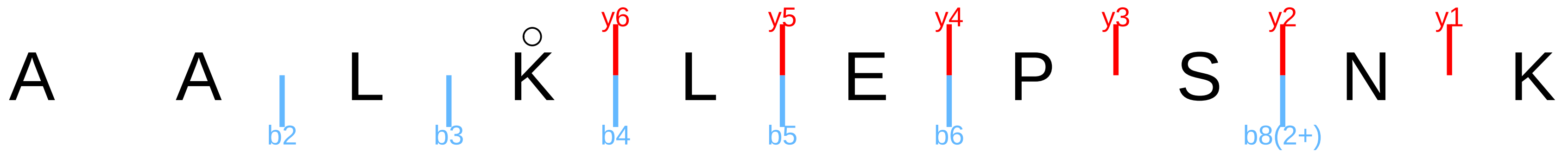

Modification □ Acetyl (Protein N−term) ○ GlyGly (K) no new terminus △ Oxidation (M)

20200921_KIZ_MUb4_GlyGly_10plex − Scan number: 6452

Gene Names	Charge	m/z	Mass	Retention time	PEP	Score	Precursor Intensity
TMEM104	4	353.4599	1409.81	21.358	0.054818	72.34	5362111

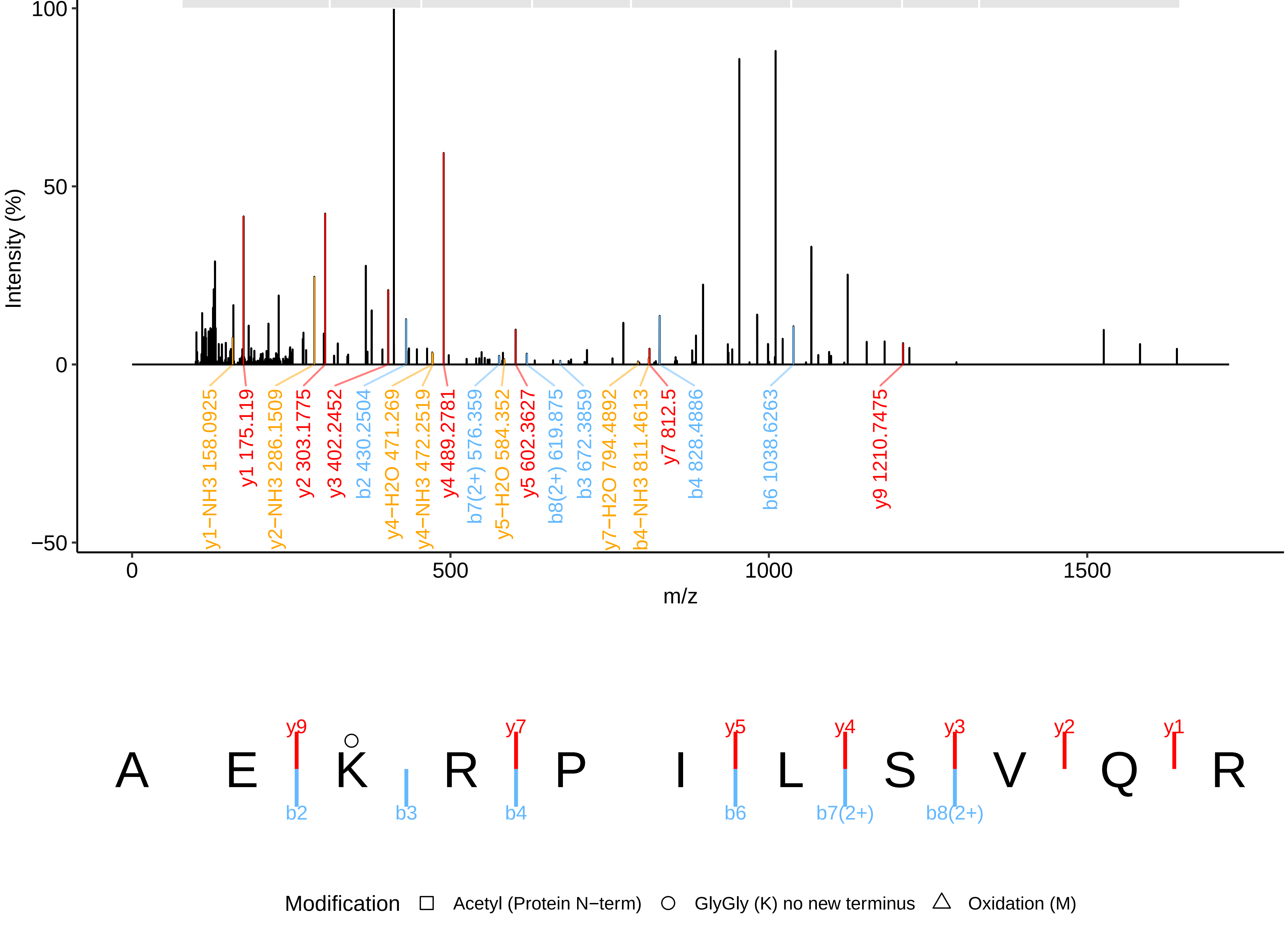

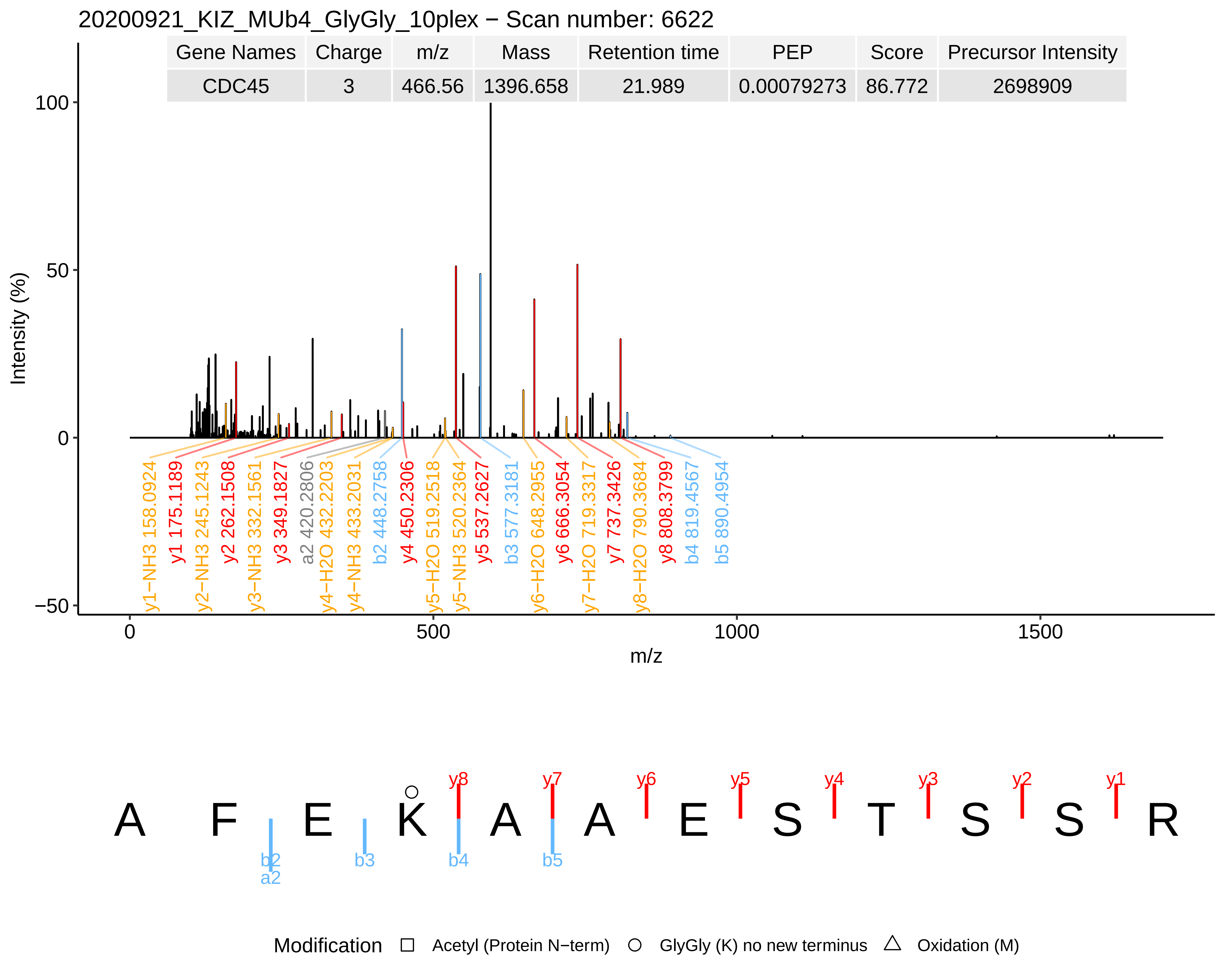
20200921_KIZ_MUb4_GlyGly_10plex − Scan number: 6622
Gene Names
Charge
m/z
Mass
Retention time
PEP
Score
Precursor Intensity
CDC45
3
466.56
1396.658
21.989
0.00079273
86.772
2698909
Intensity (%)
100
50
0
−50
0
500
1000
1500
m/z
y1−NH3 158.0924
y1 175.1189
y2−NH3 245.1243
y2 262.1508
y3−NH3 332.1561
y3 349.1827
a2 420.2806
y4−H2O 432.2203
y4−NH3 433.2031
b2 448.2758
y4 450.2306
y5−H2O 519.2518
y5−NH3 520.2364
y5 537.2627
b3 577.3181
y6−H2O 648.2955
y6 666.3054
y7−H2O 719.3317
y7 737.3426
y8−H2O 790.3684
y8 808.3799
b4 819.4567
b5 890.4954
A F E K A A E S T S S R
y8
y7
y6
y5
y4
y3
y2
y1
b2
a2
b3
b4
b5
Modification
Acetyl (Protein N−term)
GlyGly (K) no new terminus
Oxidation (M)

20200921_KIZ_MUb4_GlyGly_10plex_2 − Scan number: 6138

Gene Names	Charge	m/z	Mass	Retention time	PEP	Score	Precursor Intensity
CDC45	3	466.56	1396.658	22.421	0.013287	63.091	557724.1

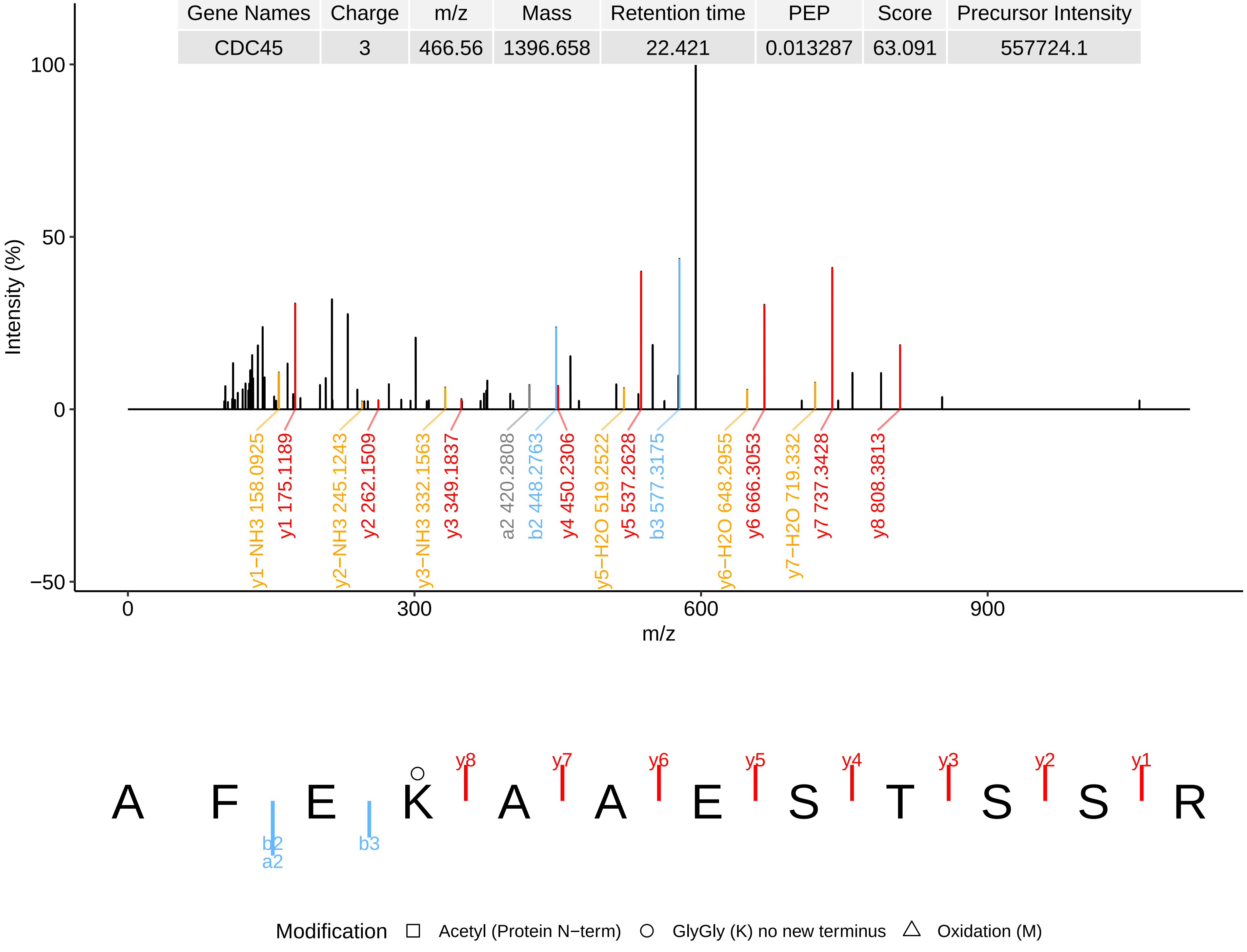

20200924_KIZ_MUb5_GlyGly_1 − Scan number: 5694

Gene Names	Charge	m/z	Mass	Retention time	PEP	Score	Precursor Intensity
CDC45	3	466.56	1396.658	17.764	0.0052747	76.522	NA

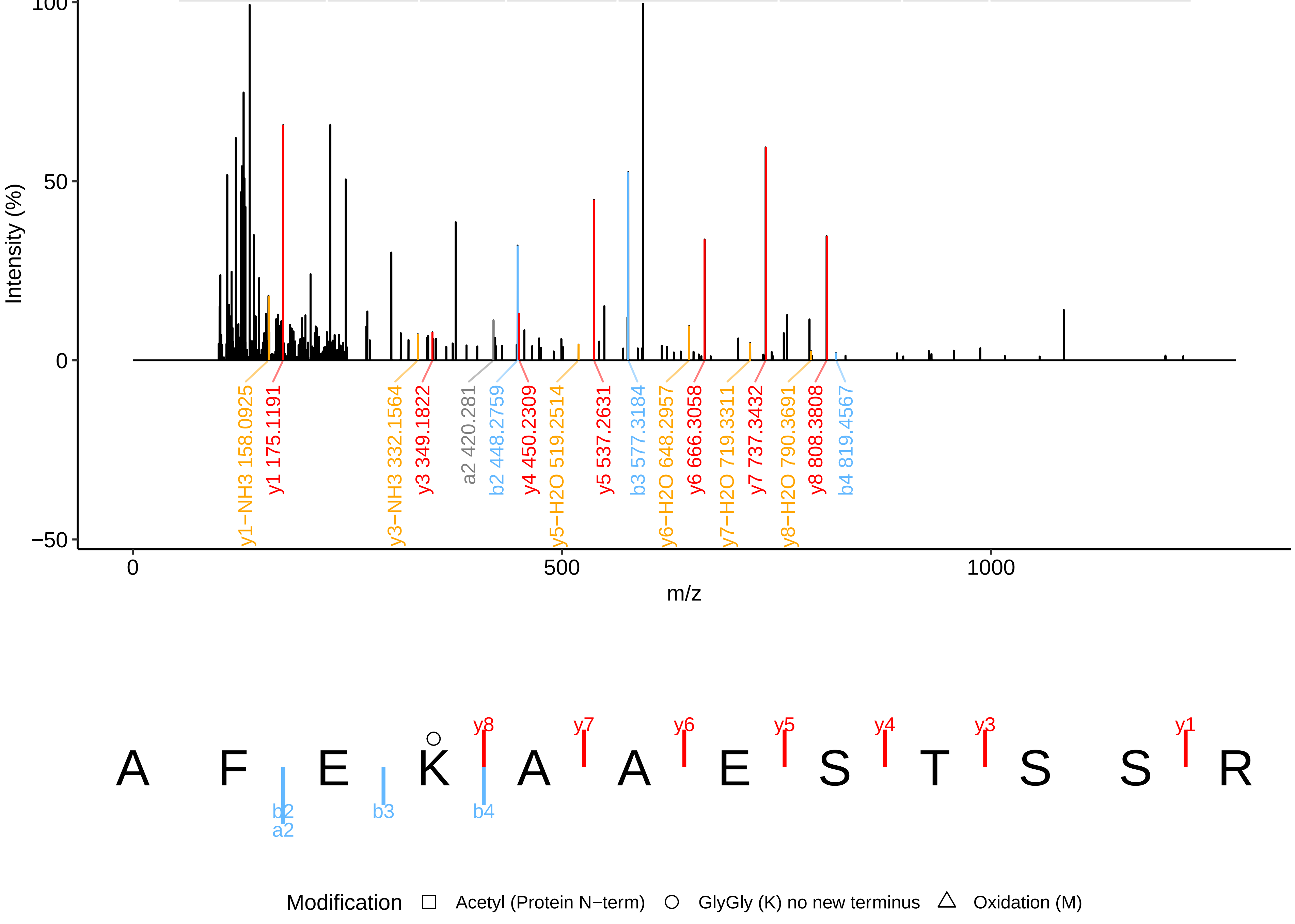

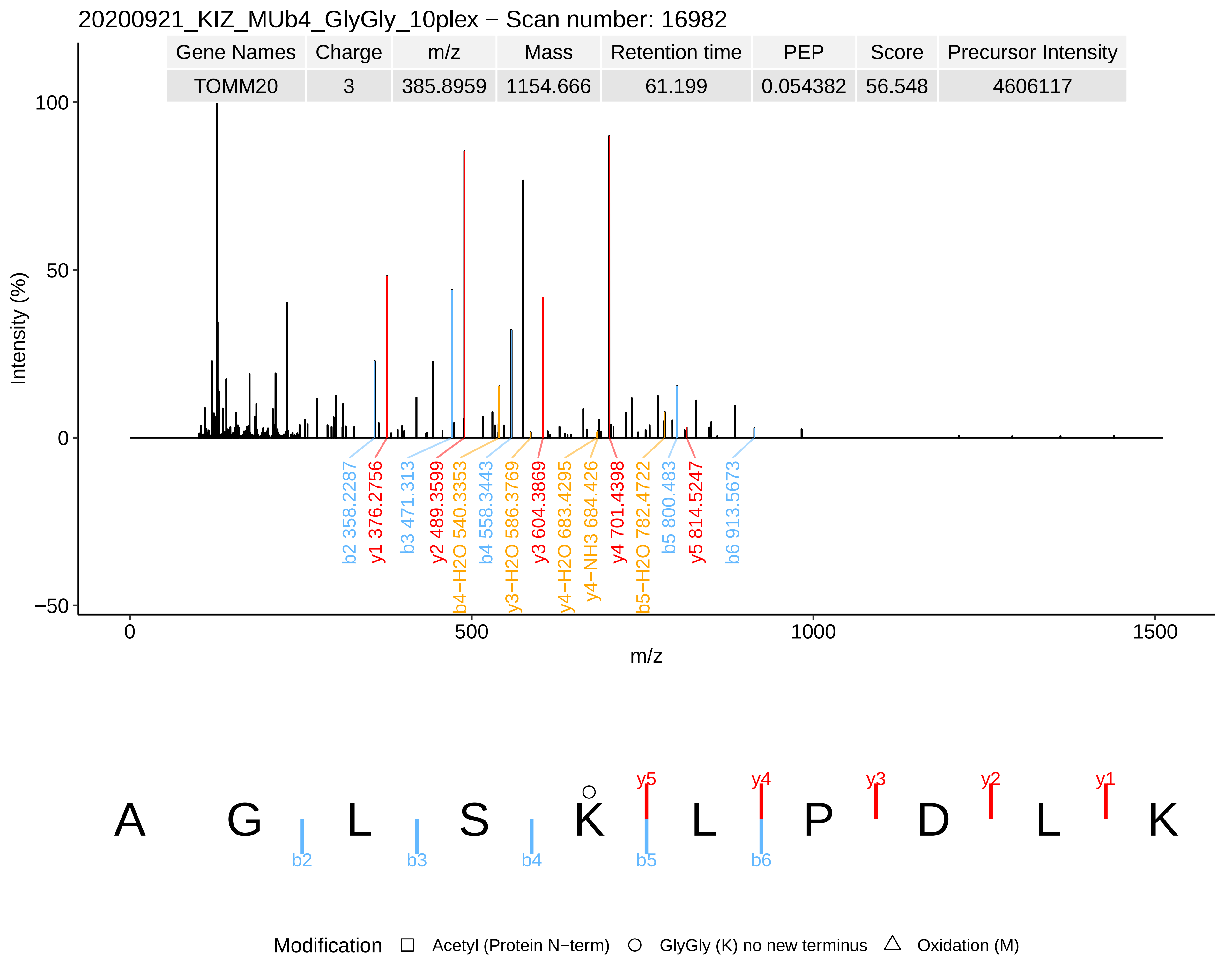

20200921_KIZ_MUb4_GlyGly_10plex − Scan number: 16982
Gene Names | Charge | m/z | Mass | Retention time | PEP | Score | Precursor Intensity
TOMM20 | 3 | 385.8959 | 1154.666 | 61.199 | 0.054382 | 56.548 | 4606117
Intensity (%)
100
50
0
−50
0
500
1000
1500
m/z
b2 358.2287
y1 376.2756
b3 471.313
y2 489.3599
b4−H2O 540.3353
b4 558.3443
y3−H2O 586.3769
y3 604.3869
y4−H2O 683.4295
y4−NH3 684.426
y4 701.4398
b5−H2O 782.4722
b5 800.483
y5 814.5247
b6 913.5673
A G L S K L P D L K
y5 y4 y3 y2 y1
b2 b3 b4 b5 b6
Modification
Acetyl (Protein N−term)
GlyGly (K) no new terminus
Oxidation (M)

20200921_KIZ_MUb4_GlyGly_10plex_2 − Scan number: 15883

Gene Names	Charge	m/z	Mass	Retention time	PEP	Score	Precursor Intensity
TOMM20	3	385.8959	1154.666	62.202	0.01775	65.719	1200195

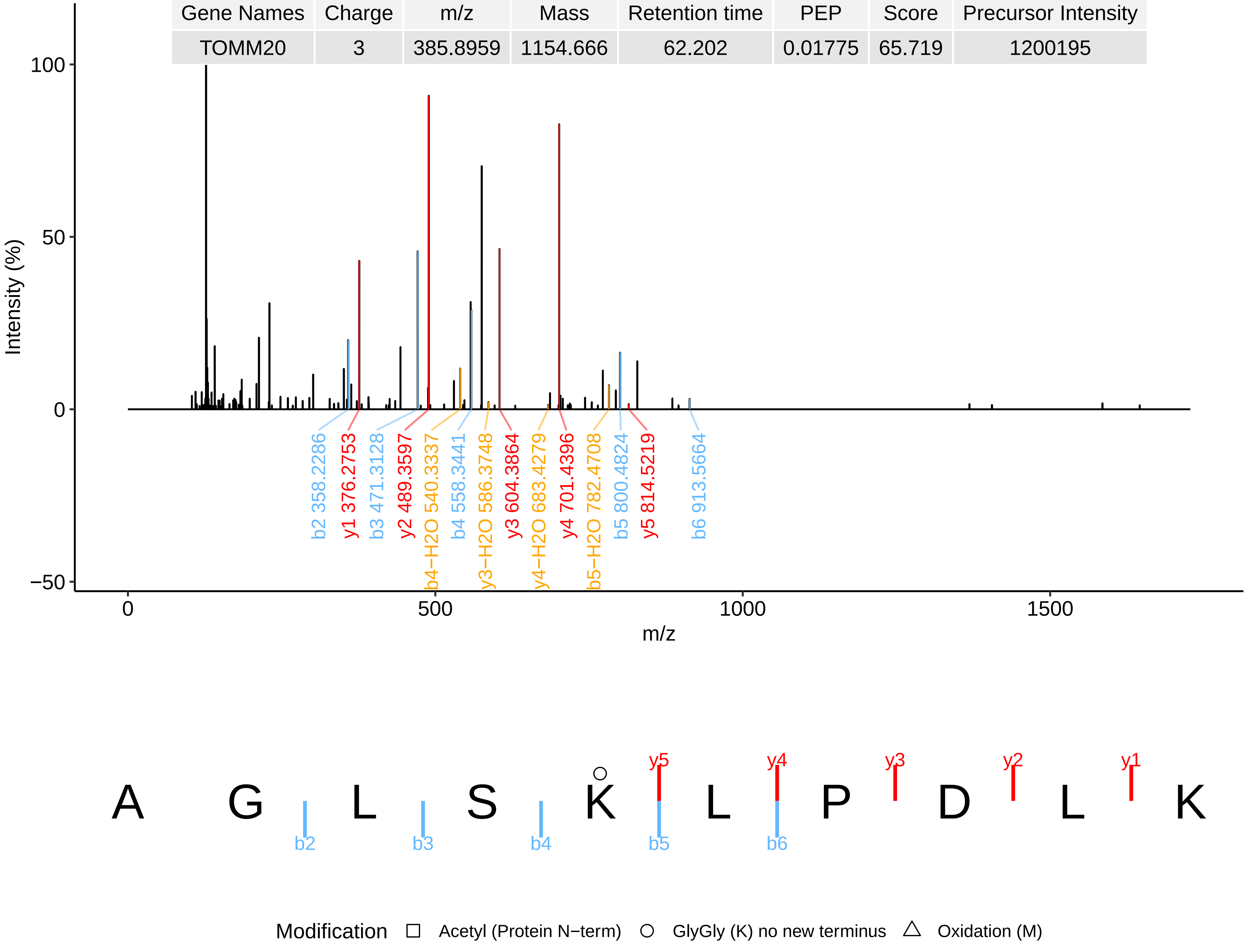

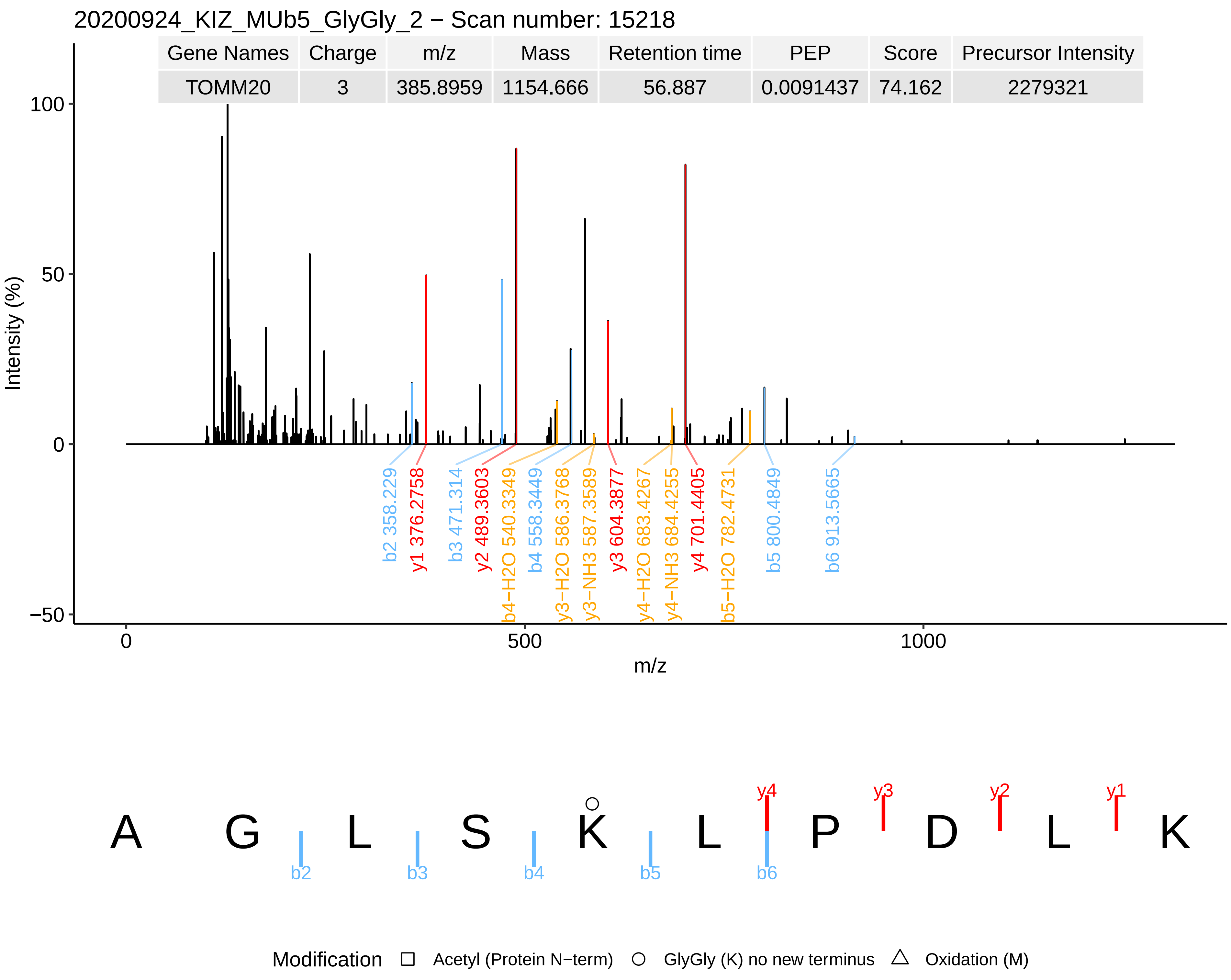

20200924_KIZ_MUb5_GlyGly_2 − Scan number: 15218
Gene Names
TOMM20
Charge
3
m/z
385.8959
Mass
1154.666
Retention time
56.887
PEP
0.0091437
Score
74.162
Precursor Intensity
2279321
Intensity (%)
m/z
b2 358.229
y1 376.2758
b3 471.314
y2 489.3603
b4−H2O 540.3349
b4 558.3449
y3−H2O 586.3768
y3−NH3 587.3589
y3 604.3877
y4−H2O 683.4267
y4−NH3 684.4255
y4 701.4405
b5−H2O 782.4731
b5 800.4849
b6 913.5665
A G L S K L P D L K
Modification
Acetyl (Protein N−term)
GlyGly (K) no new terminus
Oxidation (M)

20200921_KIZ_MUb4_GlyGly_10plex_2 − Scan number: 9225

Gene Names	Charge	m/z	Mass	Retention time	PEP	Score	Precursor Intensity
UBA6	3	465.5626	1393.666	35.011	0.025021	61.435	875035.4

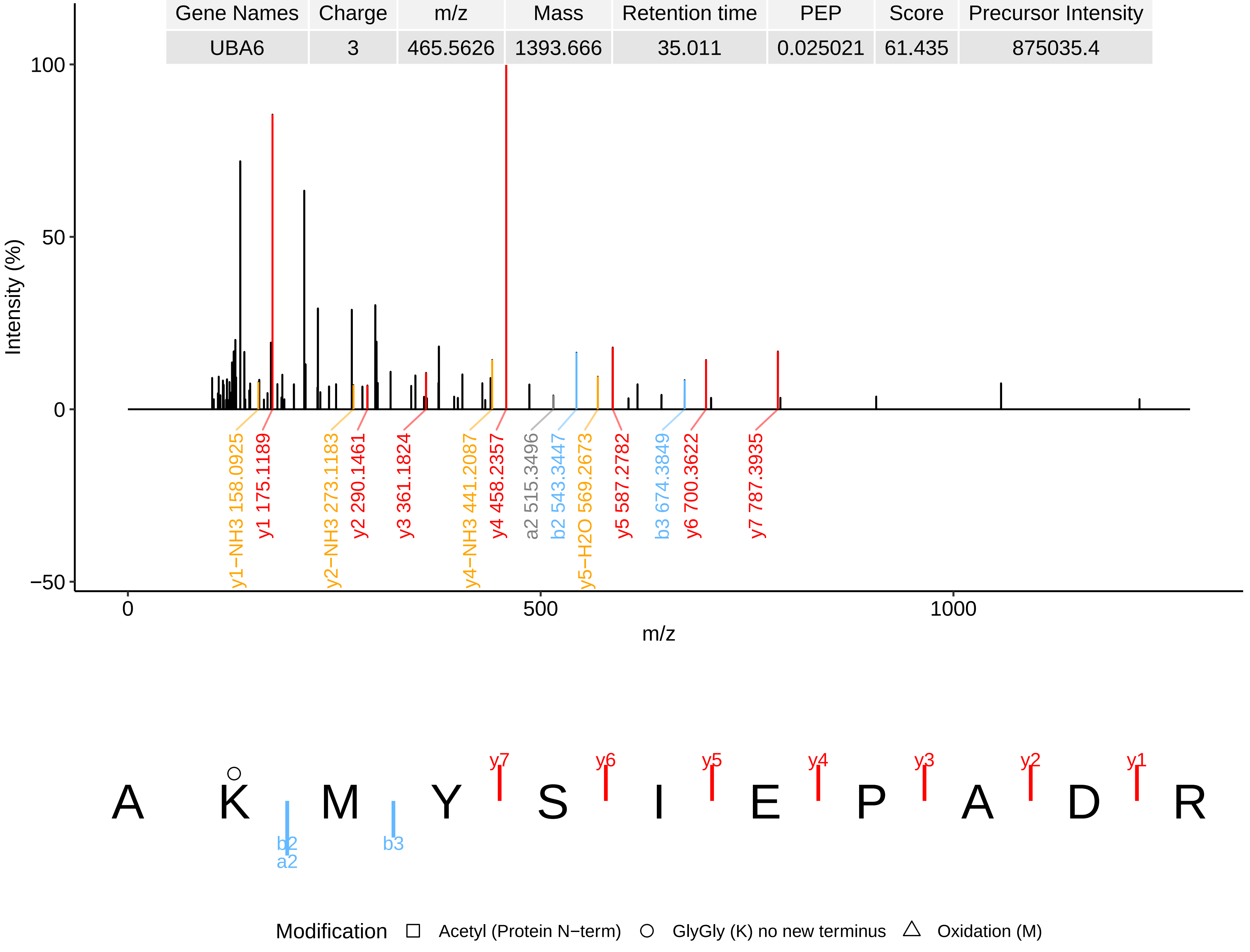

20200921_KIZ_MUb4_GlyGly_10plex − Scan number: 8139

Gene Names	Charge	m/z	Mass	Retention time	PEP	Score	Precursor Int
HSPA8;HSPA1B;HSPA1A;HSPA6;HSPA7;HSPA2	3	381.8875	1142.641	27.539	0.001733	94.801	664157

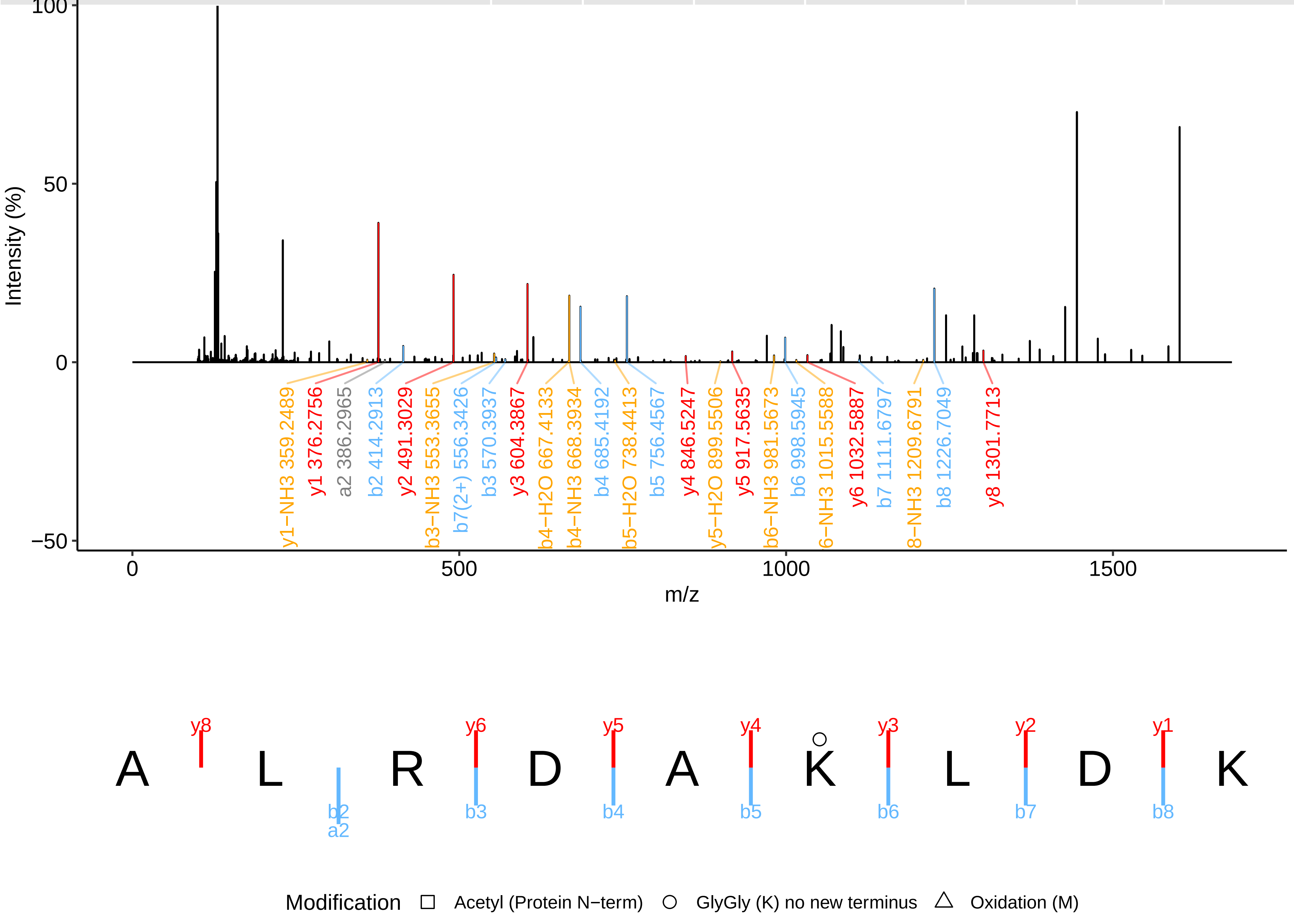

20200921_KIZ_MUb4_GlyGly_10plex_2 − Scan number: 7599

Gene Names	Charge	m/z	Mass	Retention time	PEP	Score	Precursor In
HSPA8;HSPA1B;HSPA1A;HSPA6;HSPA7;HSPA2	3	381.8875	1142.641	28.406	0.0046185	83.883	199859

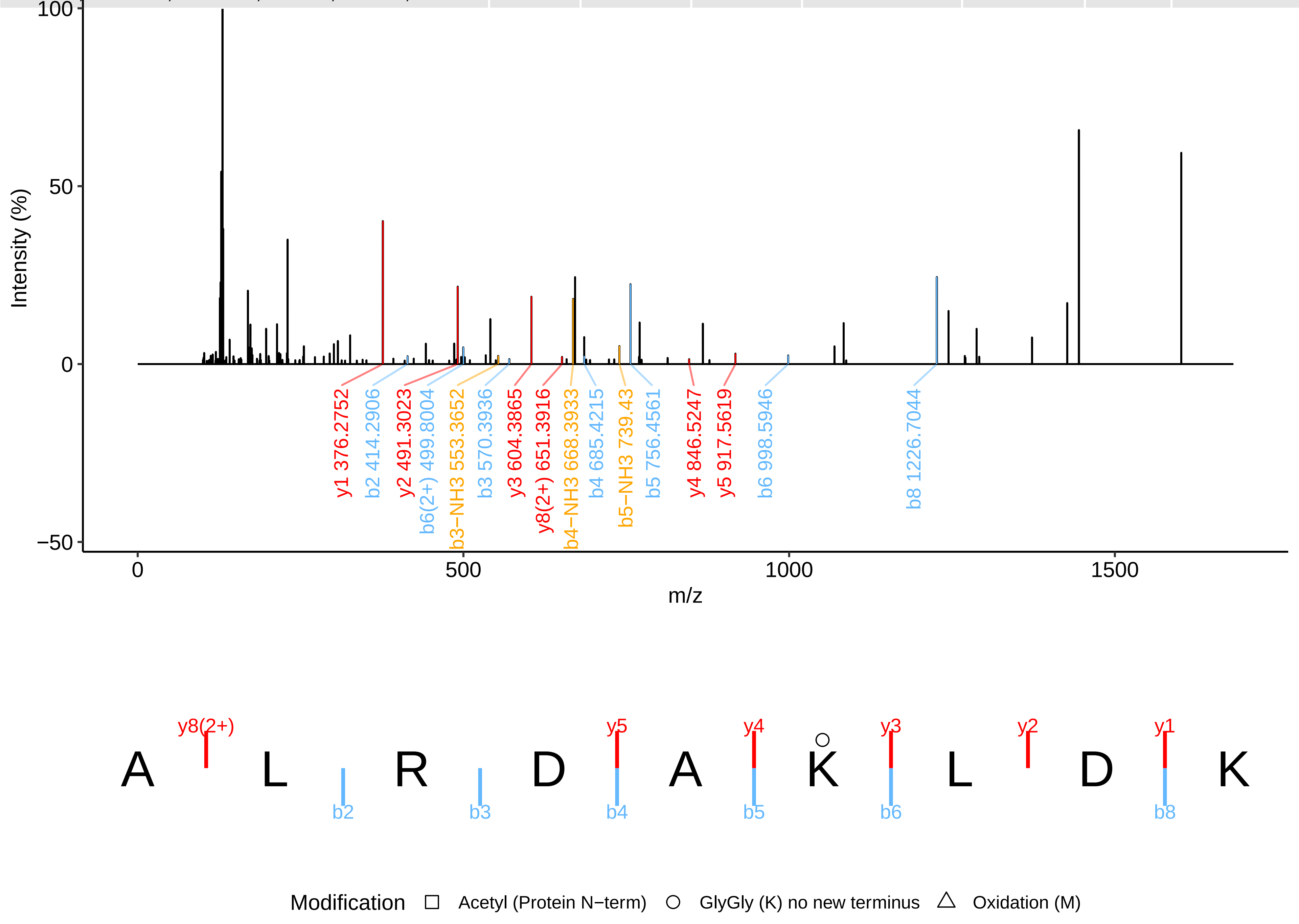

20200924_KIZ_MUb5_GlyGly_1 − Scan number: 7084

Gene Names	Charge	m/z	Mass	Retention time	PEP	Score	Precursor Int
HSPA8;HSPA1B;HSPA1A;HSPA6;HSPA7;HSPA2	3	381.8875	1142.641	22.426	0.005376	81.017	698227[illegible]

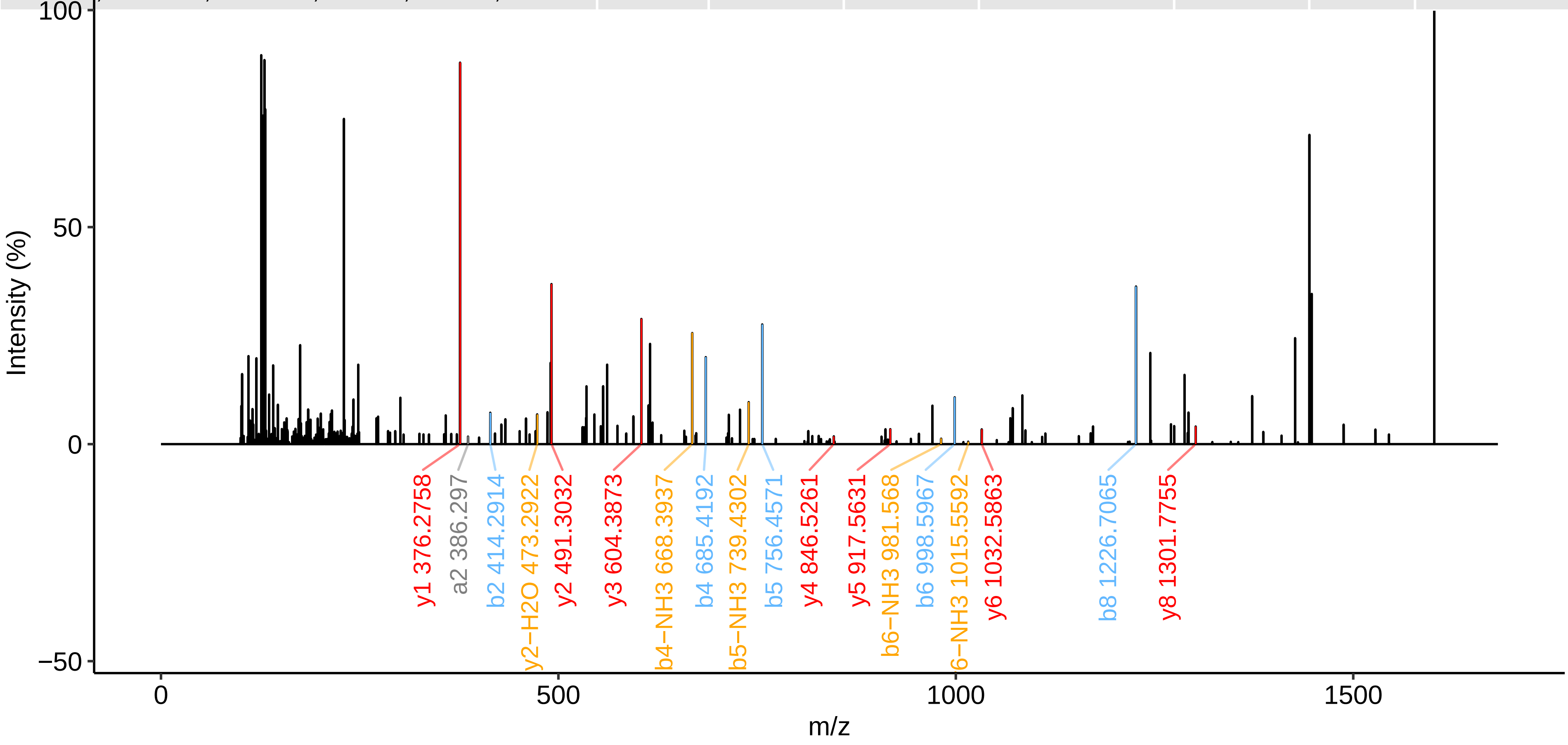

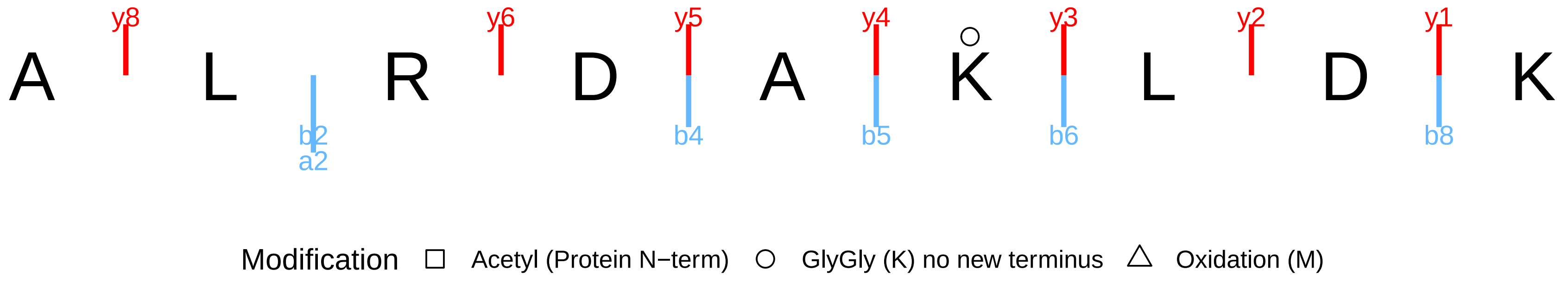

20200921_KIZ_MUb4_GlyGly_10plex − Scan number: 14310

Gene Names	Charge	m/z	Mass	Retention time	PEP	Score	Precursor Intensity
RPL36	3	383.5496	1147.627	50.807	0.058458	52.579	3158194

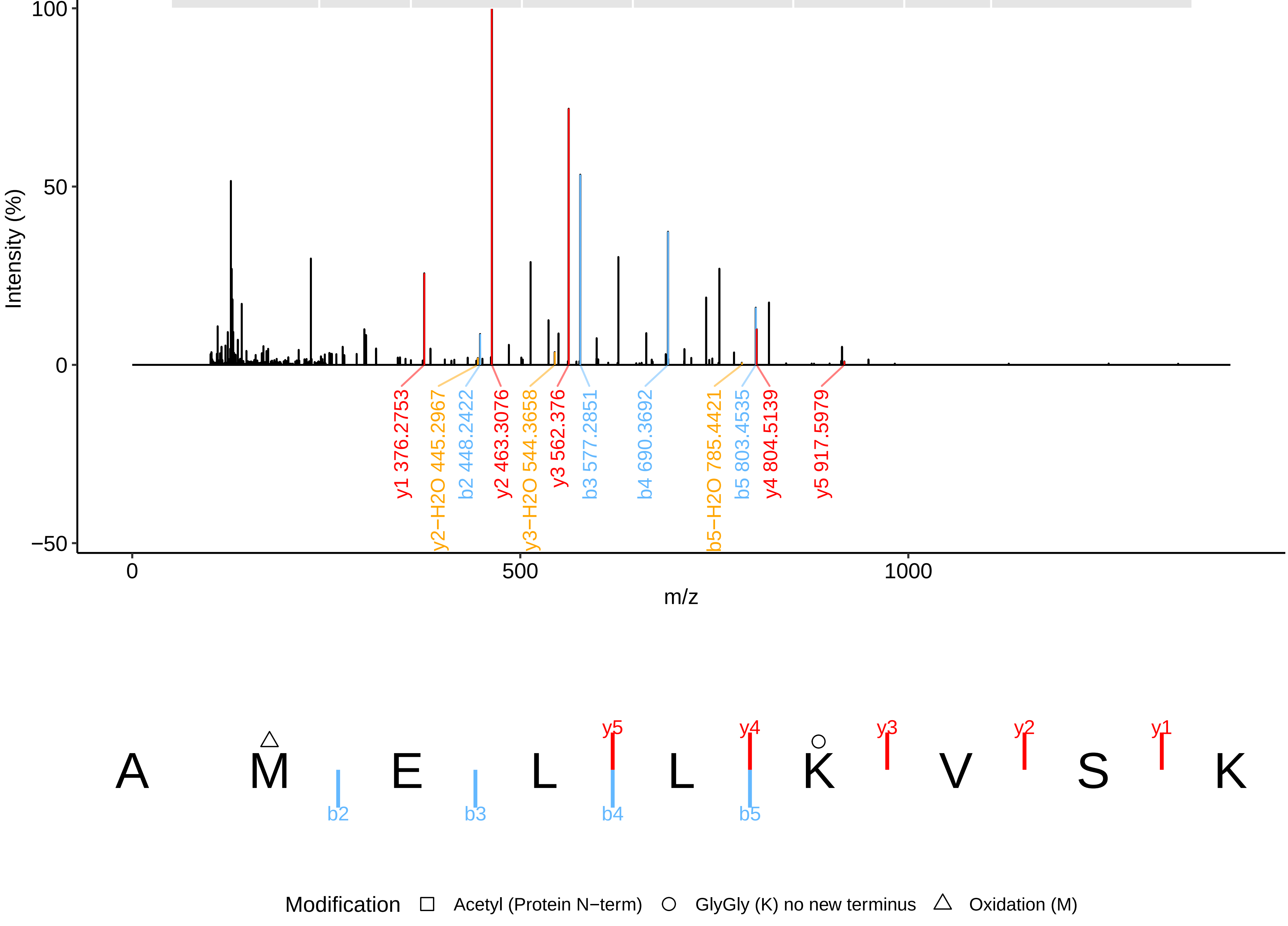

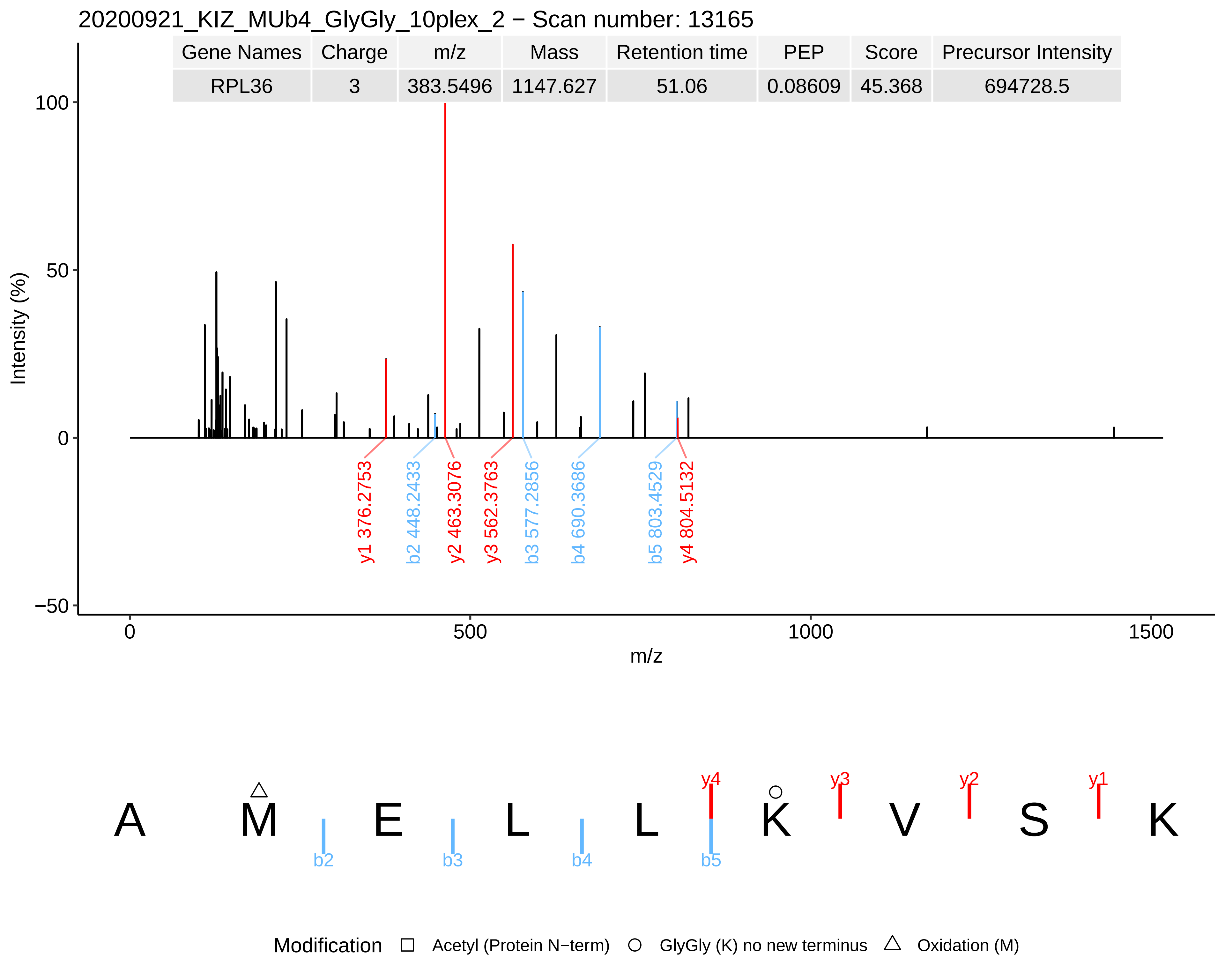

20200921_KIZ_MUb4_GlyGly_10plex_2 − Scan number: 13165
Gene Names | Charge | m/z | Mass | Retention time | PEP | Score | Precursor Intensity
RPL36 | 3 | 383.5496 | 1147.627 | 51.06 | 0.08609 | 45.368 | 694728.5
Intensity (%)
100
50
0
−50
0
500
1000
1500
m/z
y1 376.2753
b2 448.2433
y2 463.3076
y3 562.3763
b3 577.2856
b4 690.3686
b5 803.4529
y4 804.5132
A M E L L K V S K
y4 y3 y2 y1
b2 b3 b4 b5
Modification □ Acetyl (Protein N−term) ○ GlyGly (K) no new terminus △ Oxidation (M)

20200921_KIZ_MUb4_GlyGly_10plex − Scan number: 10152

Gene Names	Charge	m/z	Mass	Retention time	PEP	Score	Precursor Intensity
SEC22B	3	392.5508	1174.631	35.035	0.0070403	90.653	4619308

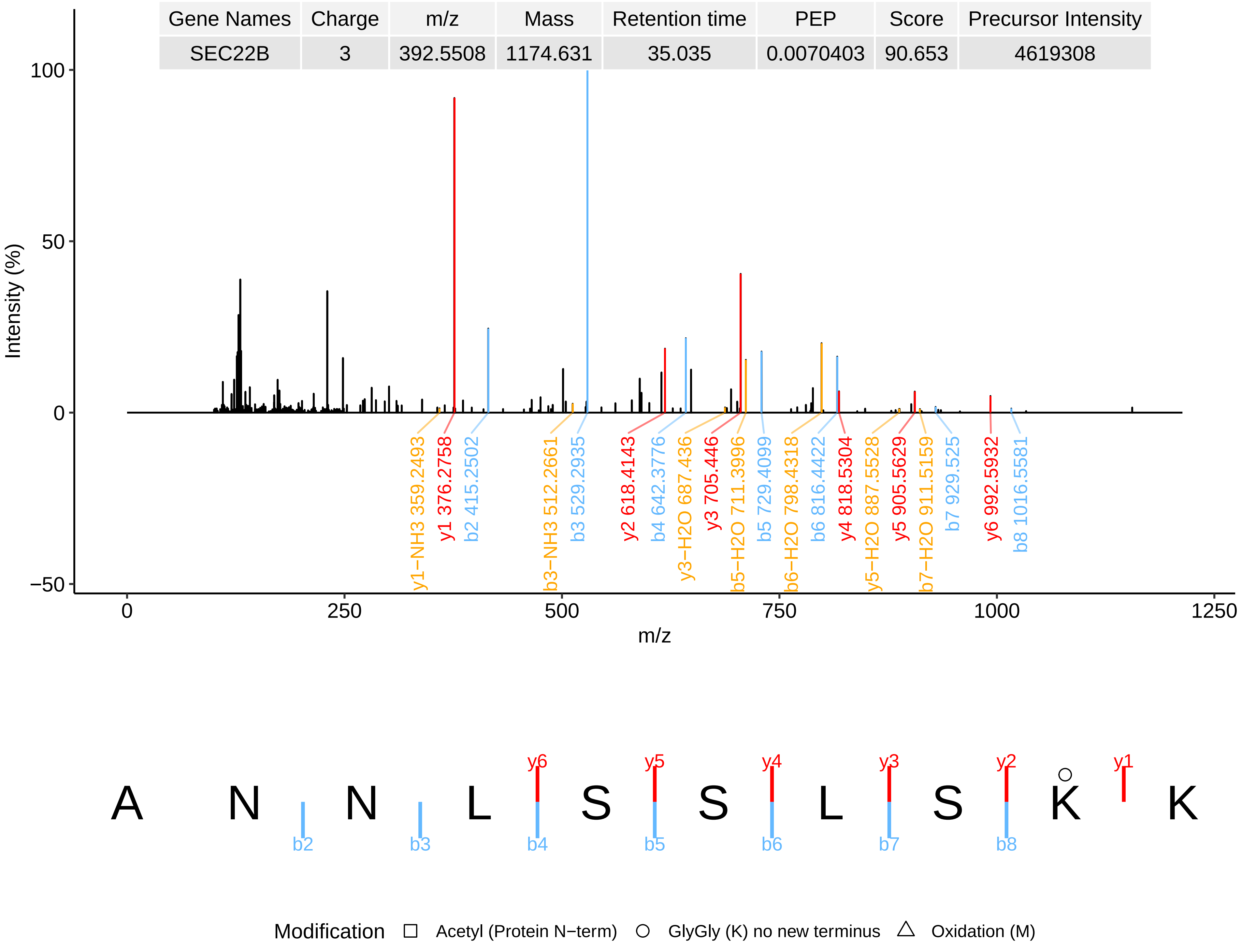

20200921_KIZ_MUb4_GlyGly_10plex_2 − Scan number: 9353

Gene Names	Charge	m/z	Mass	Retention time	PEP	Score	Precursor Intensity
SEC22B	3	392.5508	1174.631	35.529	0.013116	83.862	498121

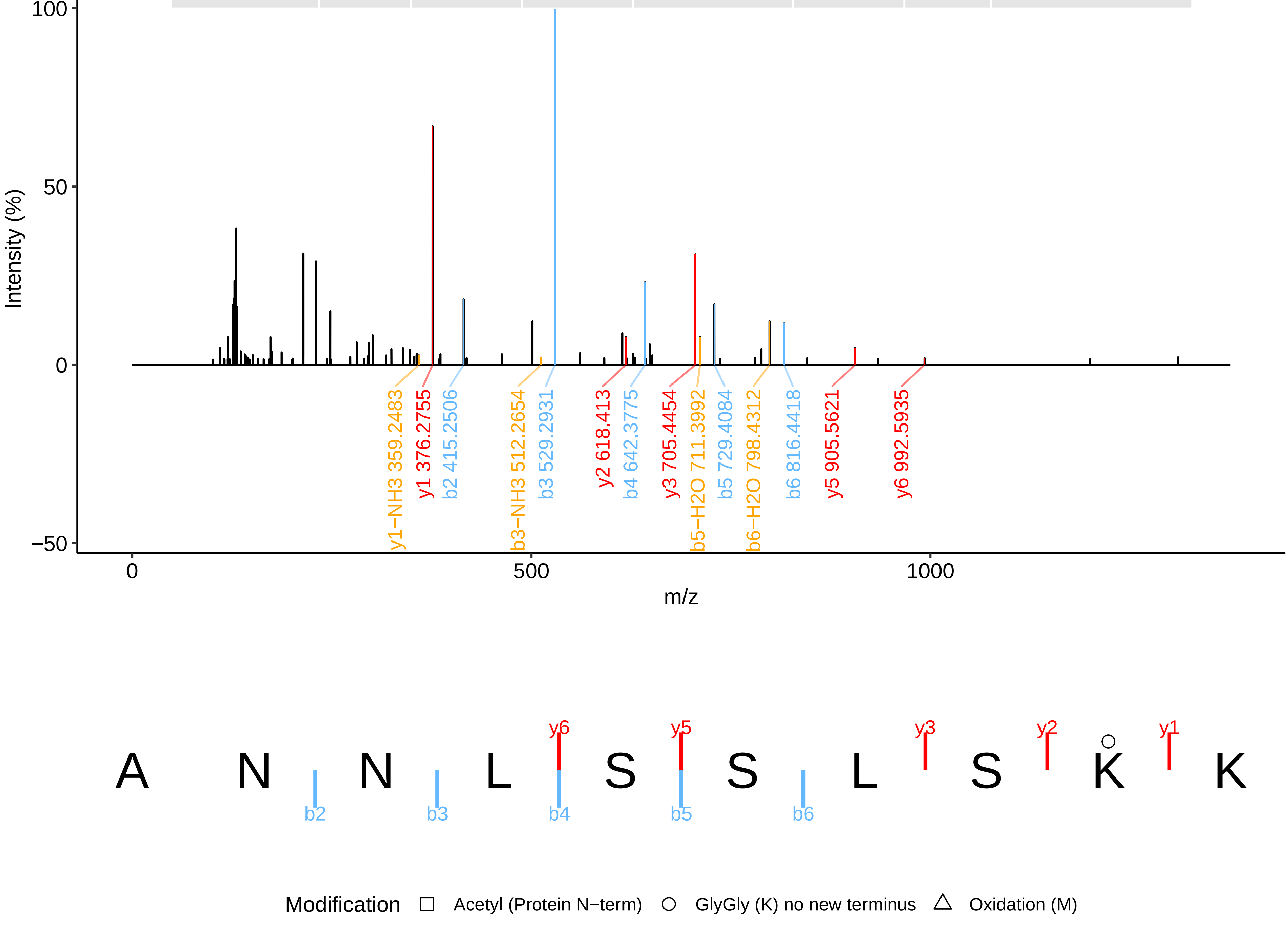

20200921_KIZ_MUb4_GlyGly_10plex − Scan number: 14939

Gene Names	Charge	m/z	Mass	Retention time	PEP	Score	Precursor Intensity
ARMC1	3	466.2492	1395.726	53.224	3.9397e−06	141.1	3531372

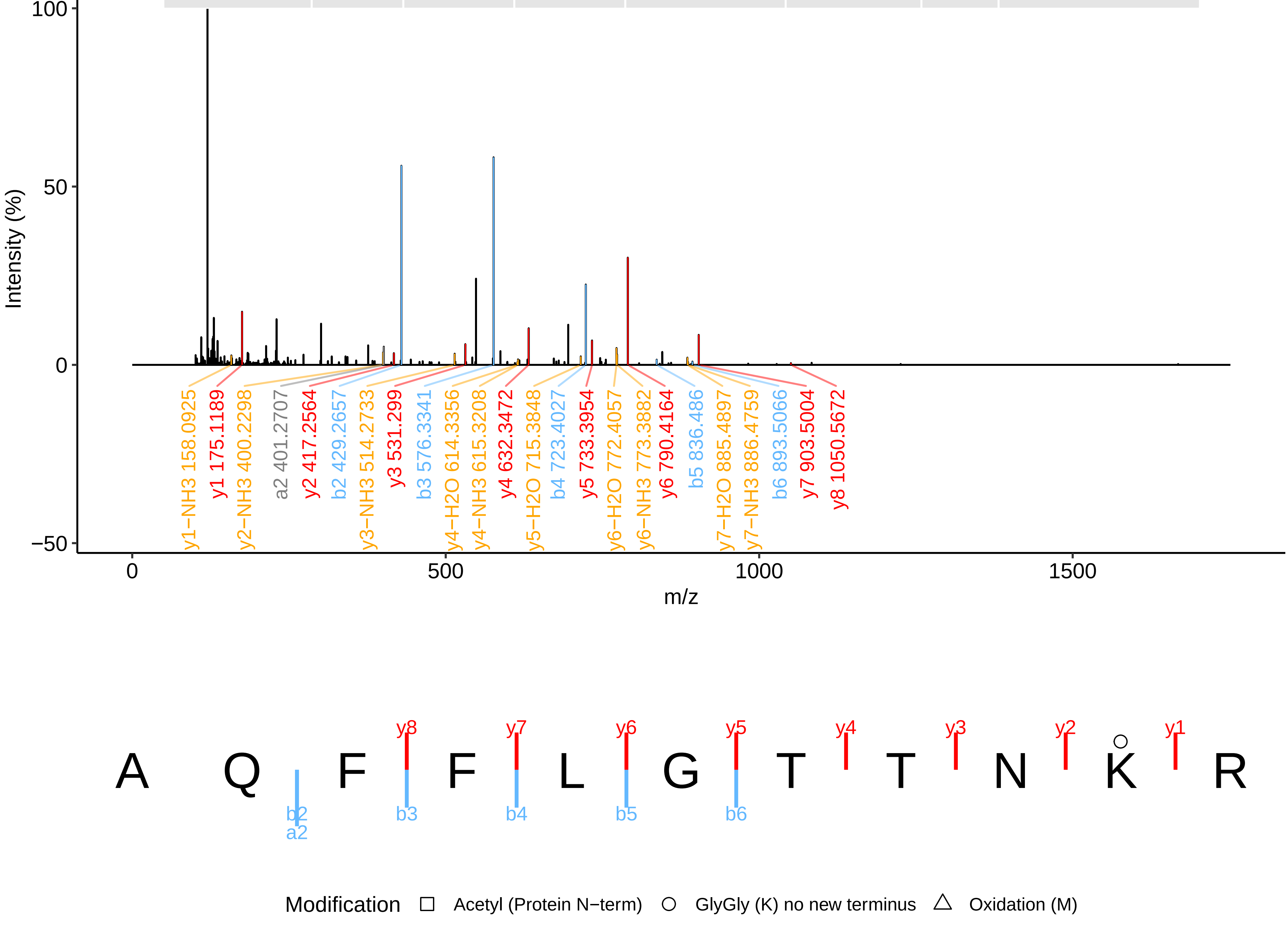

20200921_KIZ_MUb4_GlyGly_10plex − Scan number: 14846

Gene Names	Charge	m/z	Mass	Retention time	PEP	Score	Precursor Intensity
PHB2	3	383.2205	1146.64	52.866	0.011989	72.643	5158268

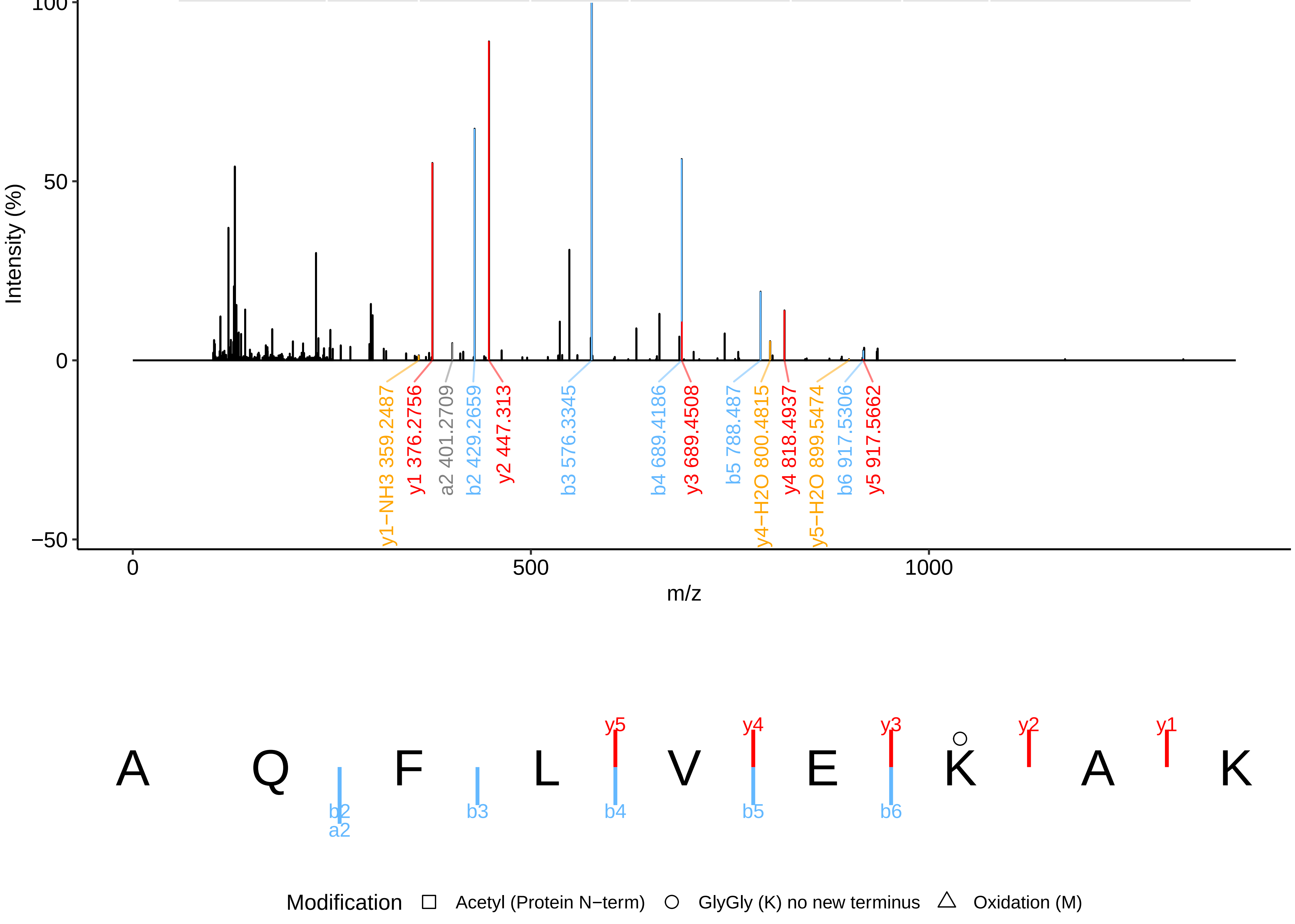

20200921_KIZ_MUb4_GlyGly_10plex − Scan number: 12450

Gene Names	Charge	m/z	Mass	Retention time	PEP	Score	Precursor Intensity
NCAPG	3	387.5434	1159.609	43.659	0.0029137	84.213	8378673

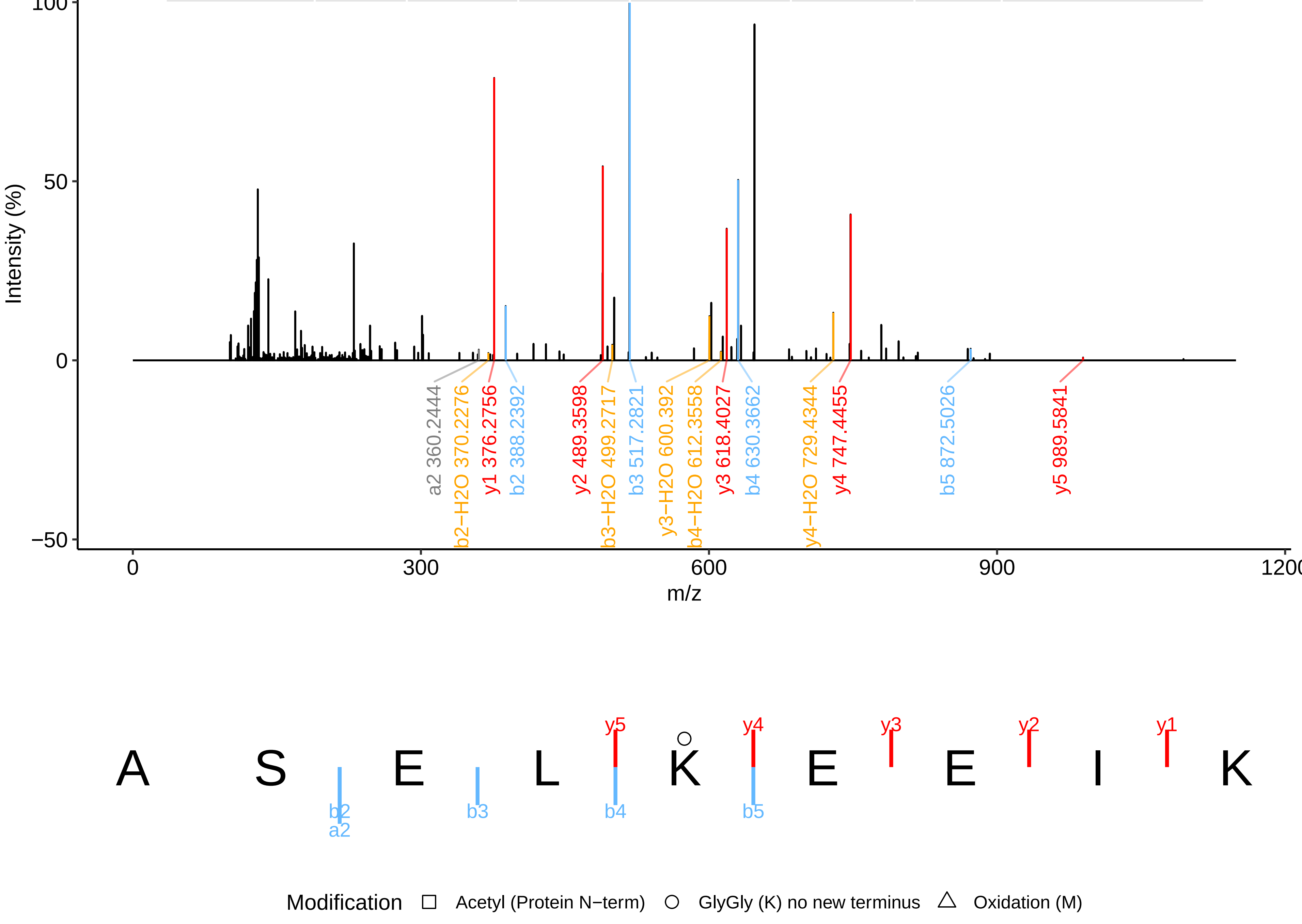

20200921_KIZ_MUb4_GlyGly_10plex_2 − Scan number: 11424

Gene Names	Charge	m/z	Mass	Retention time	PEP	Score	Precursor Intensity
NCAPG	3	387.5434	1159.609	43.971	0.0029137	84.213	2897695

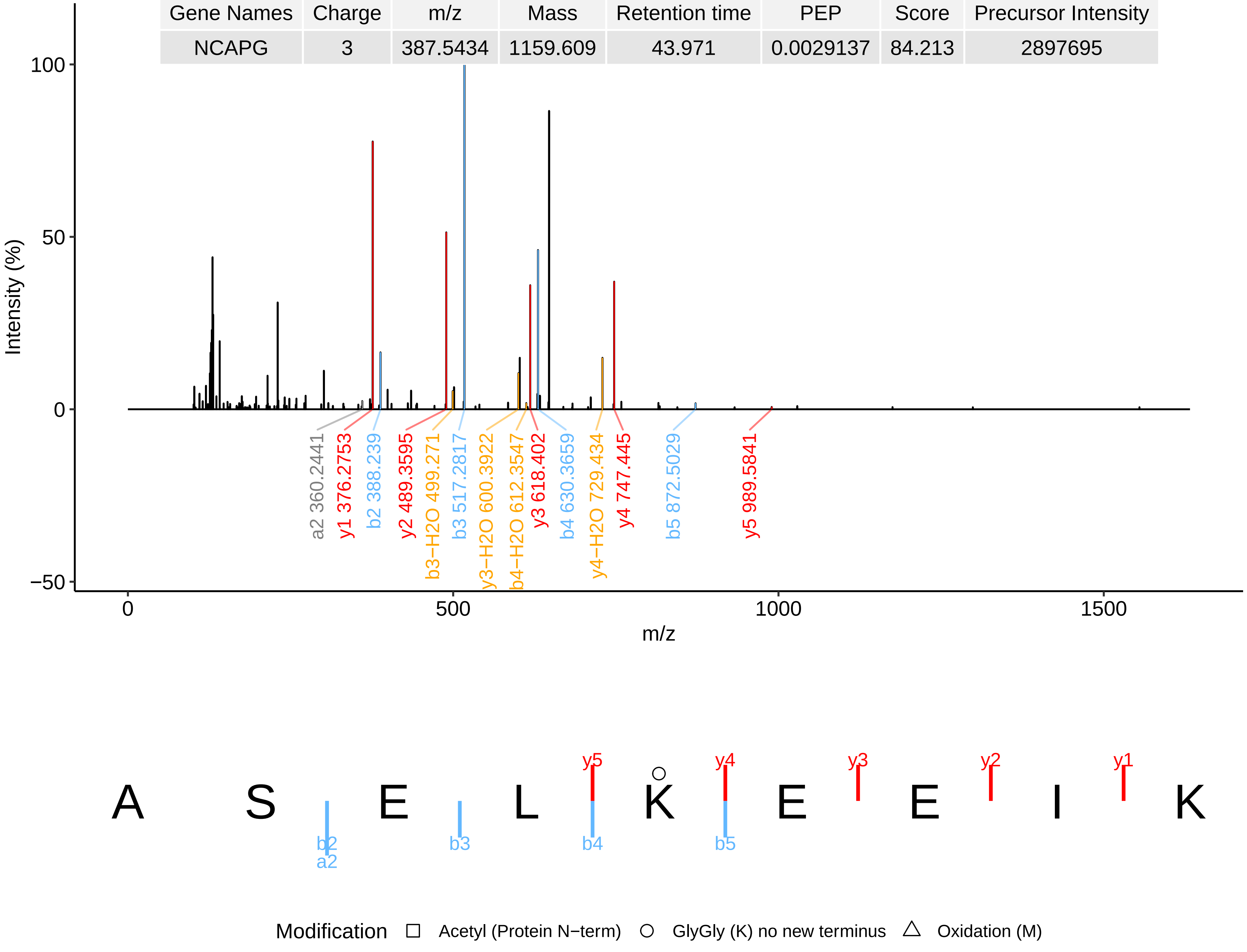

20200924_KIZ_MUb5_GlyGly_2 − Scan number: 10606

Gene Names	Charge	m/z	Mass	Retention time	PEP	Score	Precursor Intensity
NCAPG	3	387.5434	1159.609	39.591	0.080292	55.549	1502813

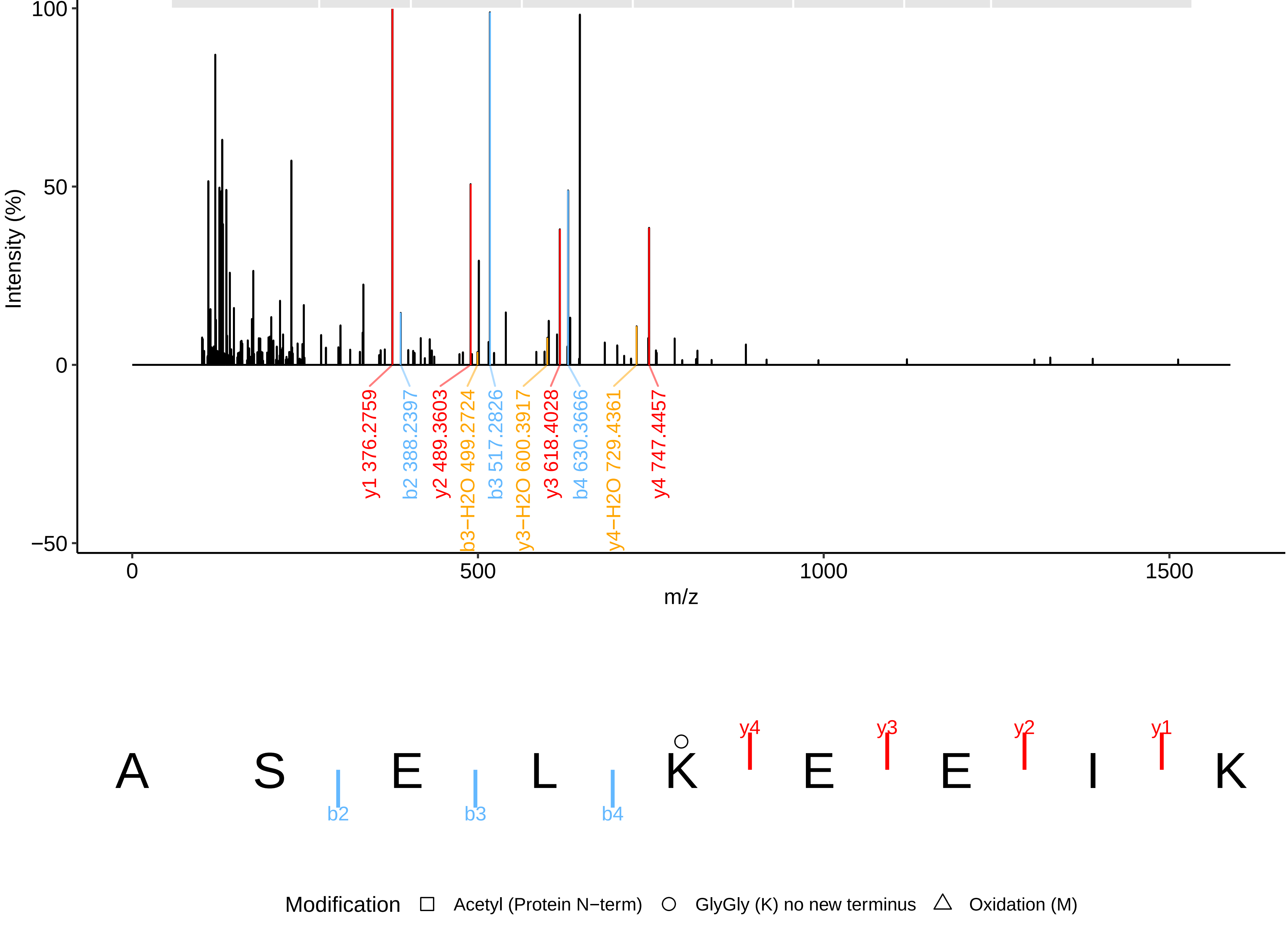

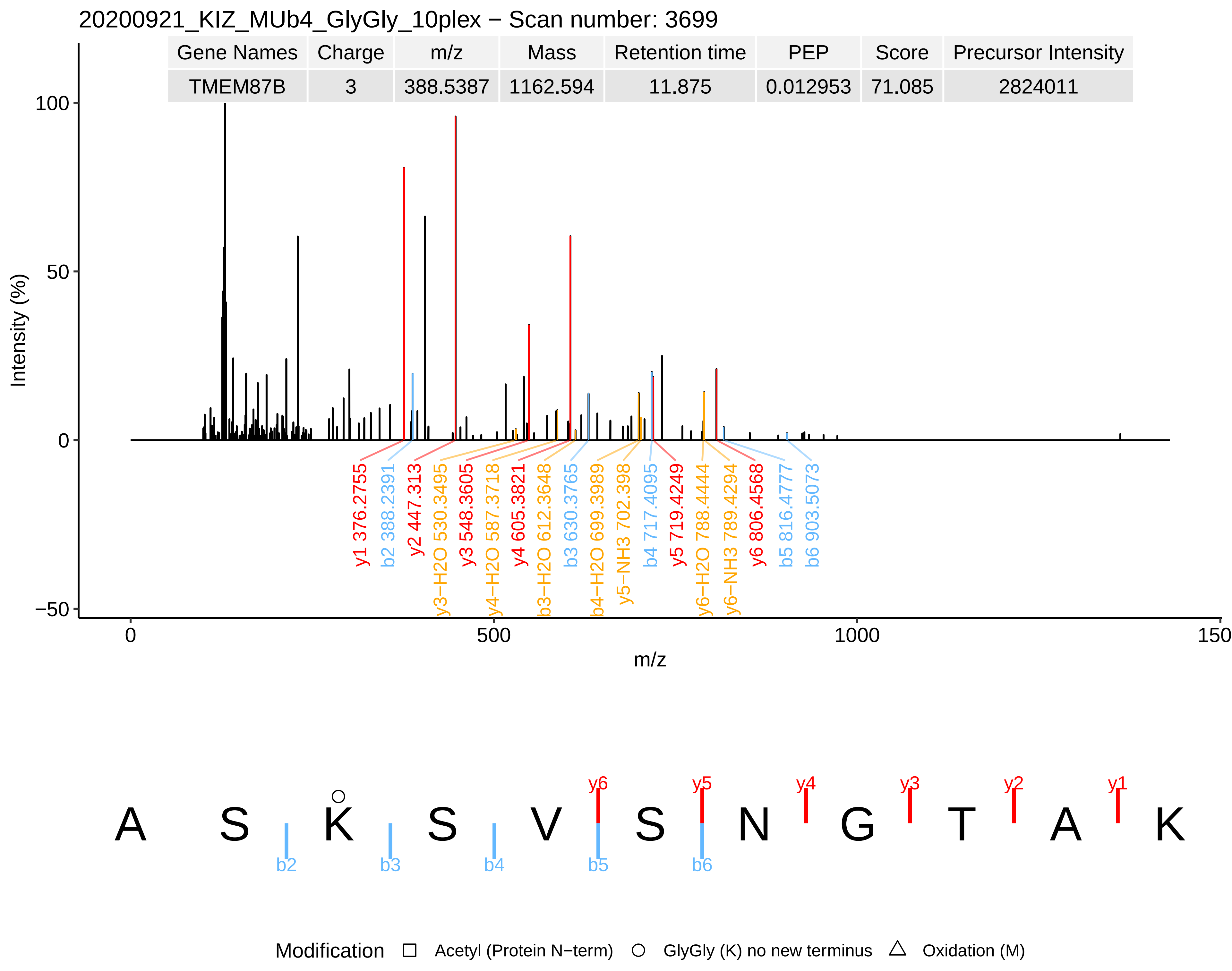
20200921_KIZ_MUb4_GlyGly_10plex − Scan number: 3699
Gene Names | Charge | m/z | Mass | Retention time | PEP | Score | Precursor Intensity
TMEM87B | 3 | 388.5387 | 1162.594 | 11.875 | 0.012953 | 71.085 | 2824011
Intensity (%)
100
50
0
−50
0
500
1000
150
m/z
y1 376.2755
b2 388.2391
y2 447.313
y3−H2O 530.3495
y3 548.3605
y4−H2O 587.3718
y4 605.3821
b3−H2O 612.3648
b3 630.3765
b4−H2O 699.3989
y5−NH3 702.398
b4 717.4095
y5 719.4249
y6−H2O 788.4444
y6−NH3 789.4294
y6 806.4568
b5 816.4777
b6 903.5073
A S K S V S N G T A K
y6 y5 y4 y3 y2 y1
b2 b3 b4 b5 b6
Modification
Acetyl (Protein N−term)
GlyGly (K) no new terminus
Oxidation (M)

20200921_KIZ_MUb4_GlyGly_10plex − Scan number: 19792

Gene Names	Charge	m/z	Mass	Retention time	PEP	Score	Precursor Intensity
PSME3	2	693.3854	1384.756	71.431	5.4553e−09	116.52	NA

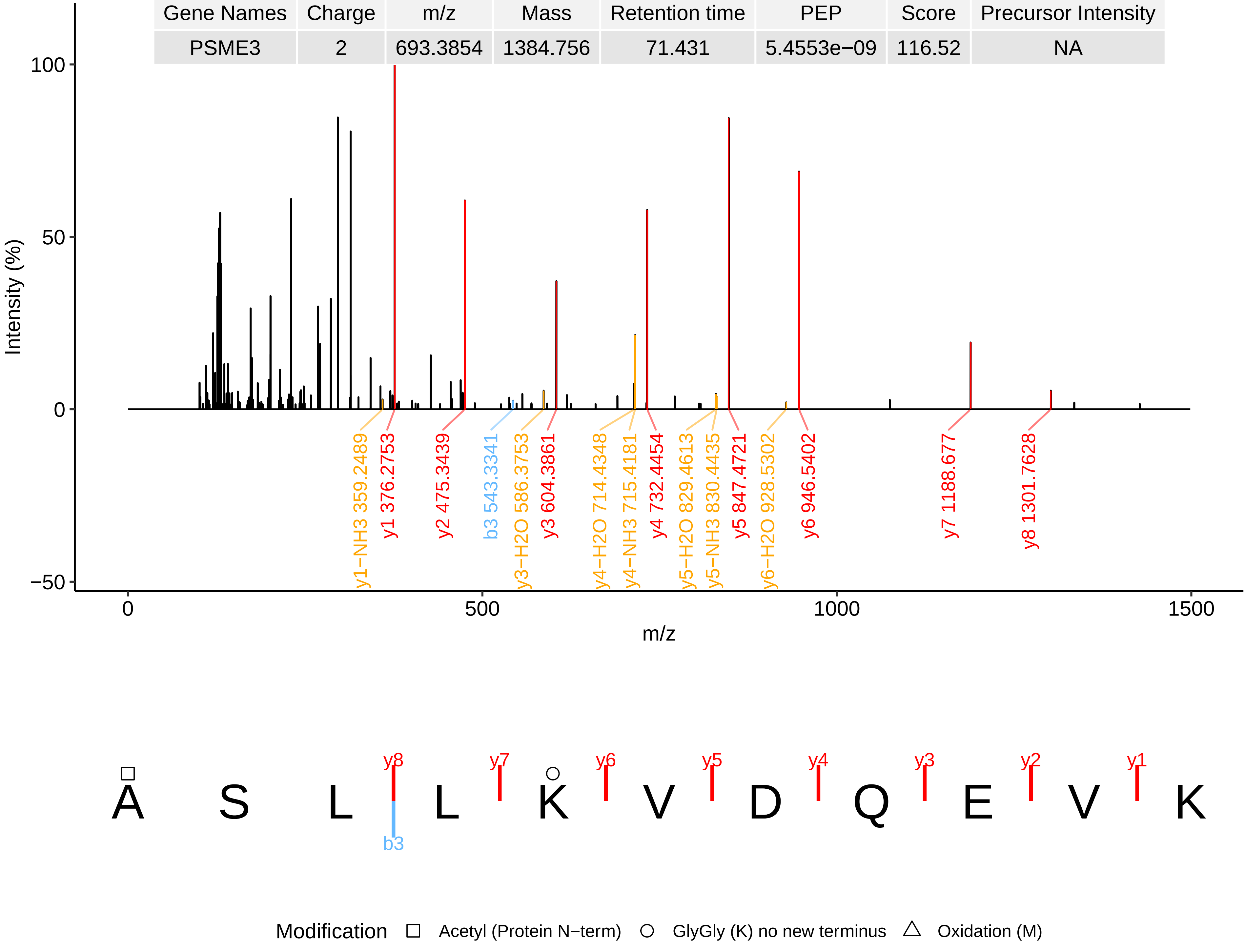

20200921_KIZ_MUb4_GlyGly_10plex − Scan number: 5517

Gene Names	Charge	m/z	Mass	Retention time	PEP	Score	Precursor Intensity
UGCG	3	384.5279	1150.562	18.083	0.036748	61.65	3156694

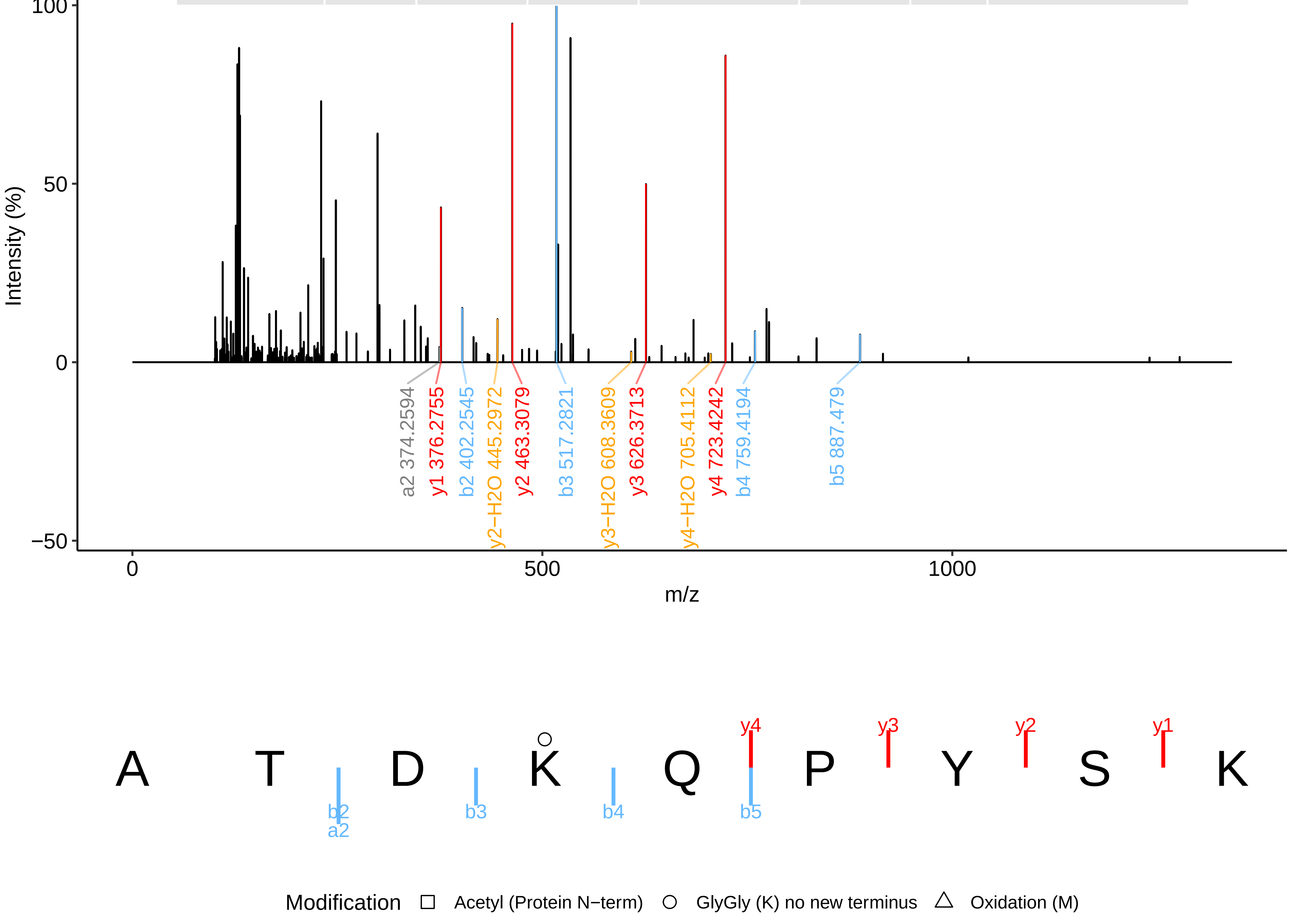

20200921_KIZ_MUb4_GlyGly_10plex − Scan number: 13411

Gene Names	Charge	m/z	Mass	Retention time	PEP	Score	Precursor Intensity
PRPF8	3	391.2084	1170.603	47.307	0.016032	68.847	2890945

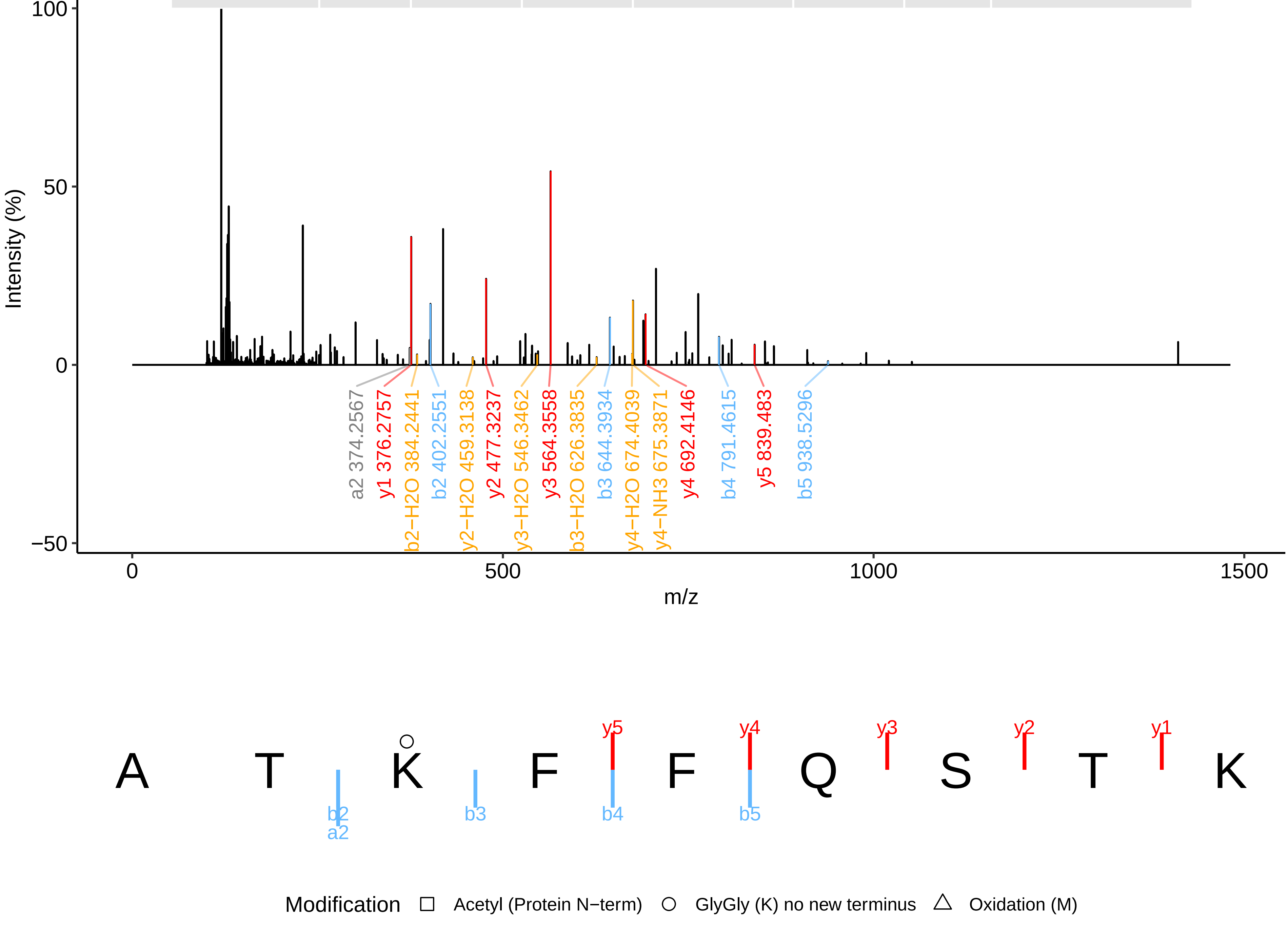

20200921_KIZ_MUb4_GlyGly_10plex − Scan number: 15279

Gene Names	Charge	m/z	Mass	Retention time	PEP	Score	Precursor Intensity
CLTC	3	390.5486	1168.624	54.549	0.0025869	86.136	21272288

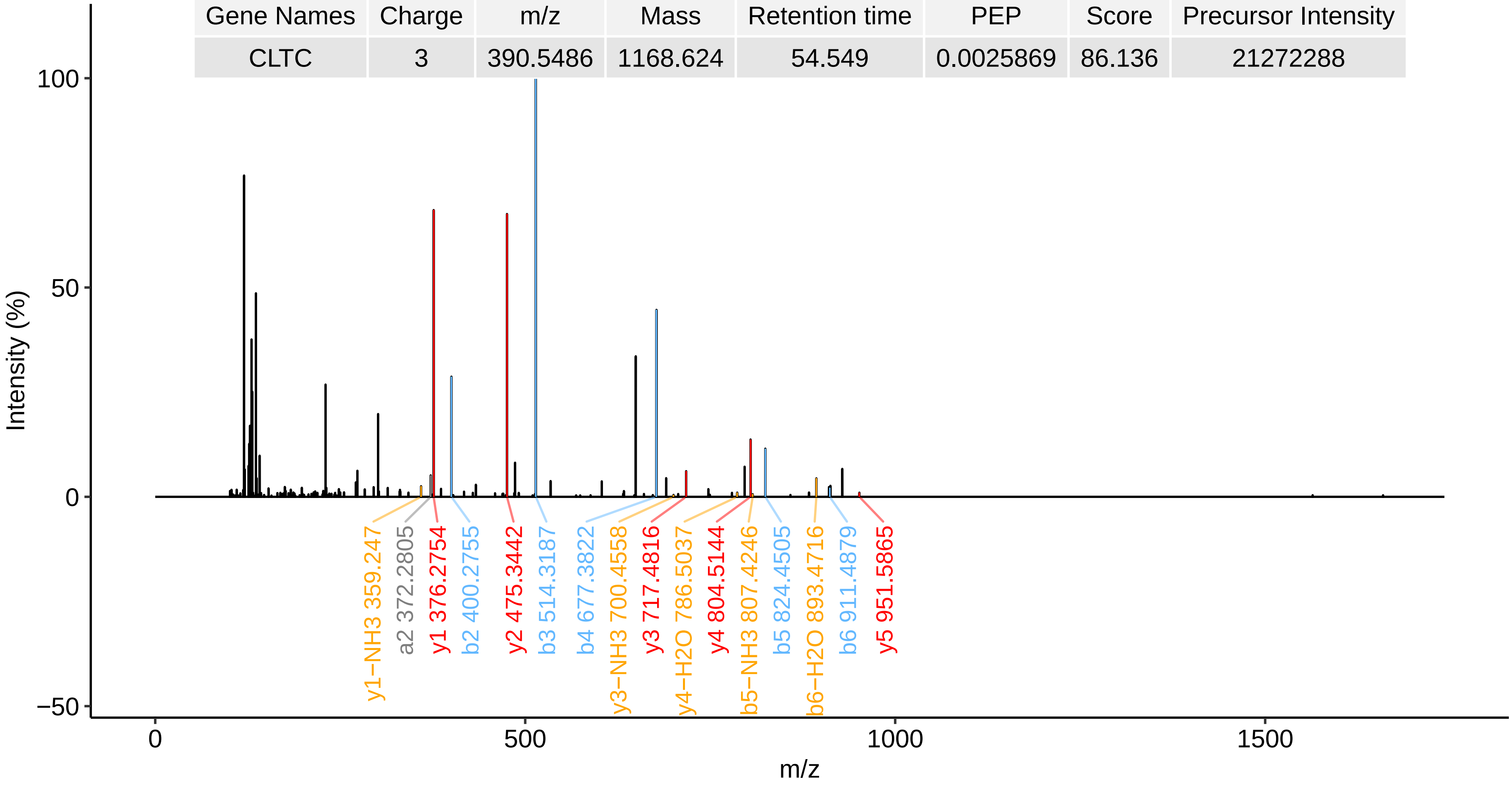

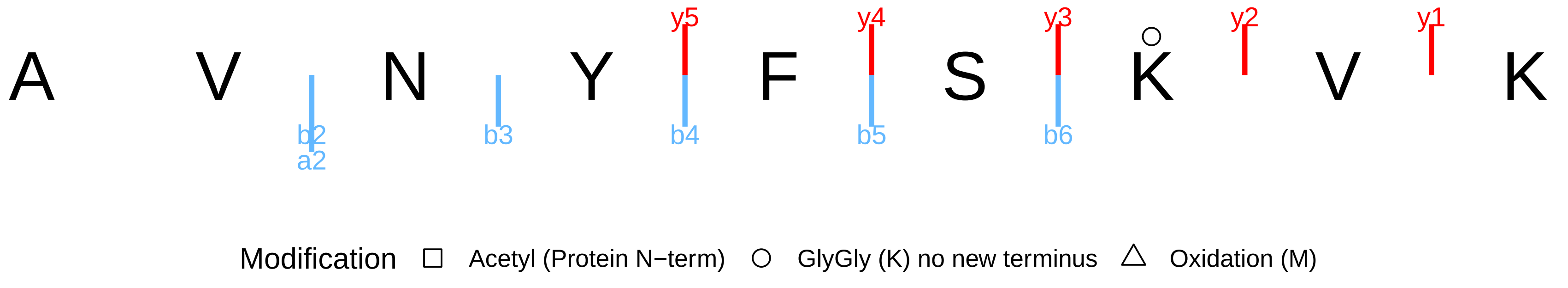

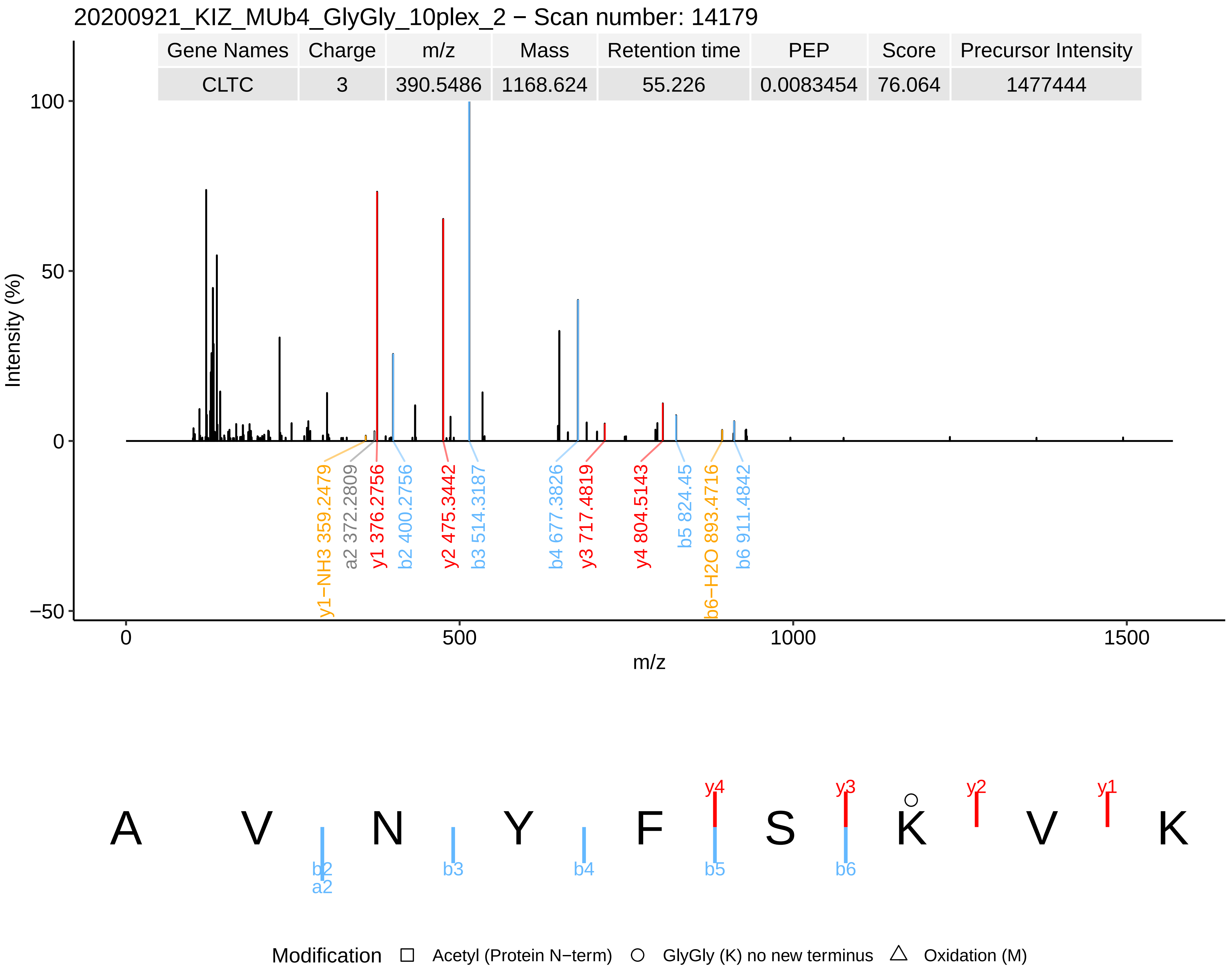

20200921_KIZ_MUb4_GlyGly_10plex_2 − Scan number: 14179
Gene Names | Charge | m/z | Mass | Retention time | PEP | Score | Precursor Intensity
CLTC | 3 | 390.5486 | 1168.624 | 55.226 | 0.0083454 | 76.064 | 1477444
Intensity (%)
m/z
y1−NH3 359.2479
a2 372.2809
y1 376.2756
b2 400.2756
y2 475.3442
b3 514.3187
b4 677.3826
y3 717.4819
y4 804.5143
b5 824.45
b6−H2O 893.4716
b6 911.4842
A V N Y F S K V K
y4 y3 y2 y1
b2 a2 b3 b4 b5 b6
Modification
Acetyl (Protein N−term)
GlyGly (K) no new terminus
Oxidation (M)

20200921_KIZ_MUb4_GlyGly_10plex − Scan number: 14281

Gene Names	Charge	m/z	Mass	Retention time	PEP	Score	Precursor Intensity
VDAC1	2	801.4177	1600.821	50.688	8.6398e−05	101.39	NA

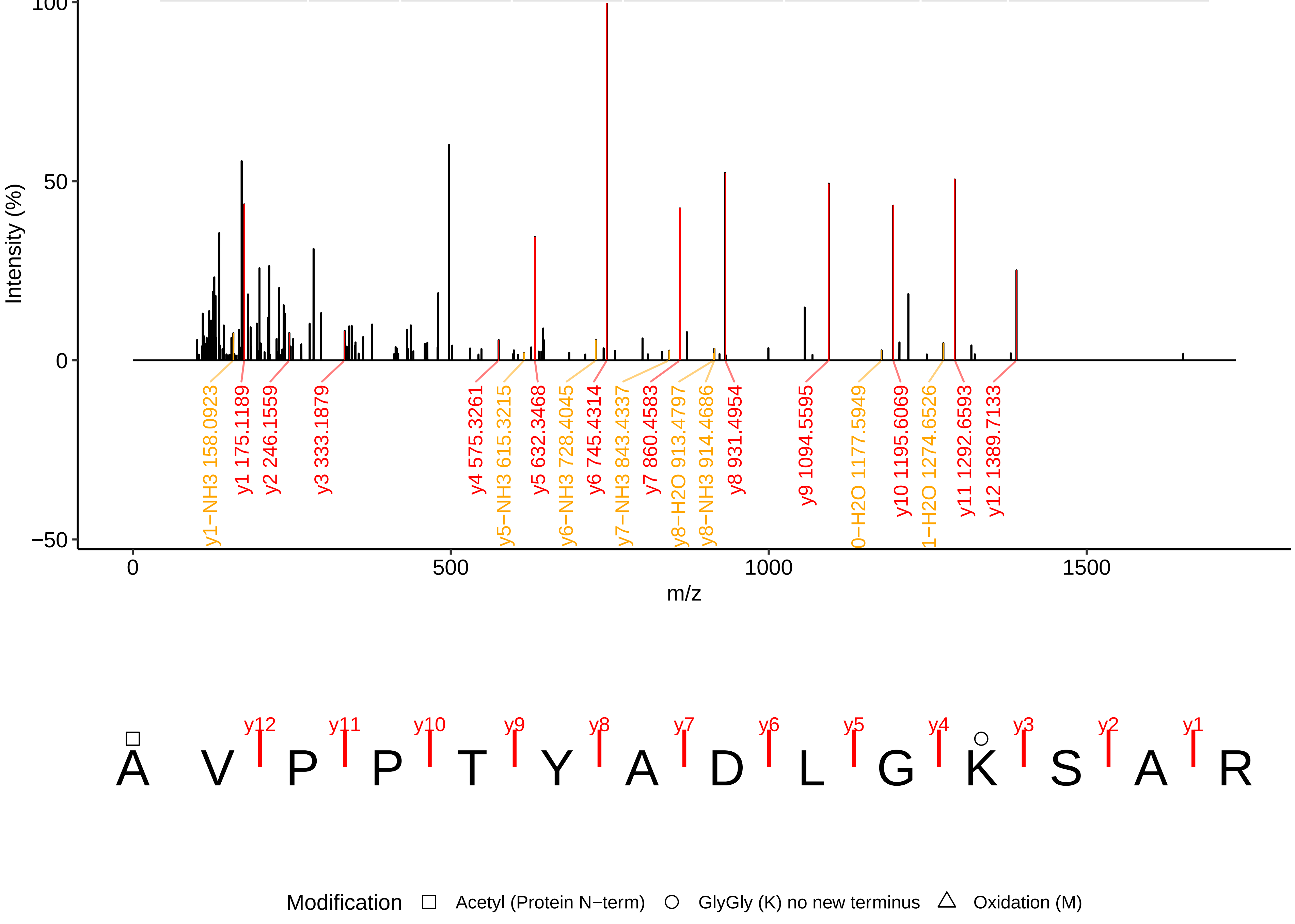

20200921_KIZ_MUb4_GlyGly_10plex − Scan number: 19232

Gene Names	Charge	m/z	Mass	Retention time	PEP	Score	Precursor Intensity
AP2B1	3	399.5765	1195.708	69.288	0.0043727	80.239	1638596

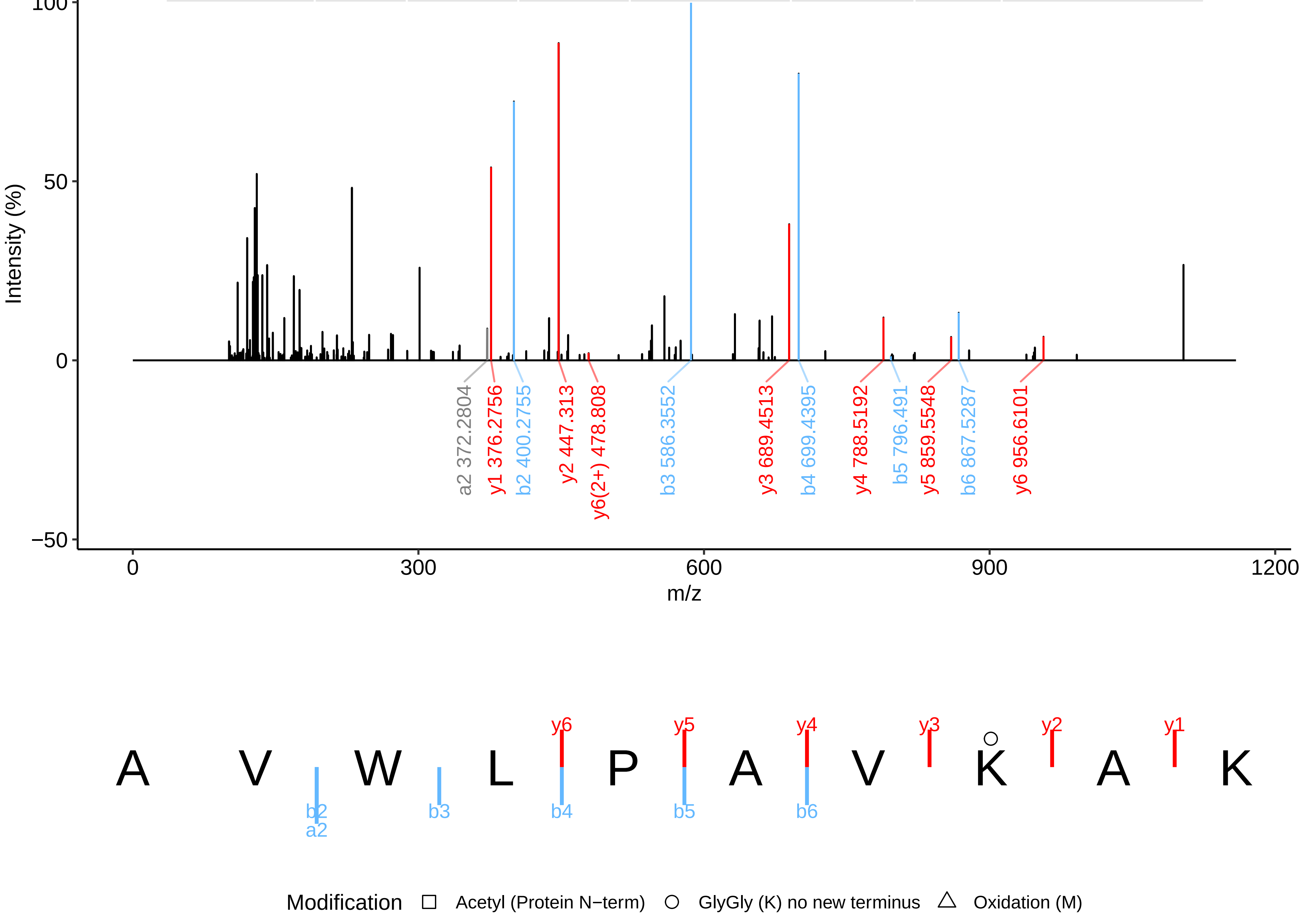

20200921_KIZ_MUb4_GlyGly_10plex − Scan number: 3315

Gene Names	Charge	m/z	Mass	Retention time	PEP	Score	Precursor Intensity
	3	474.8942	1421.661	10.408	0.11039	45.598	9024512

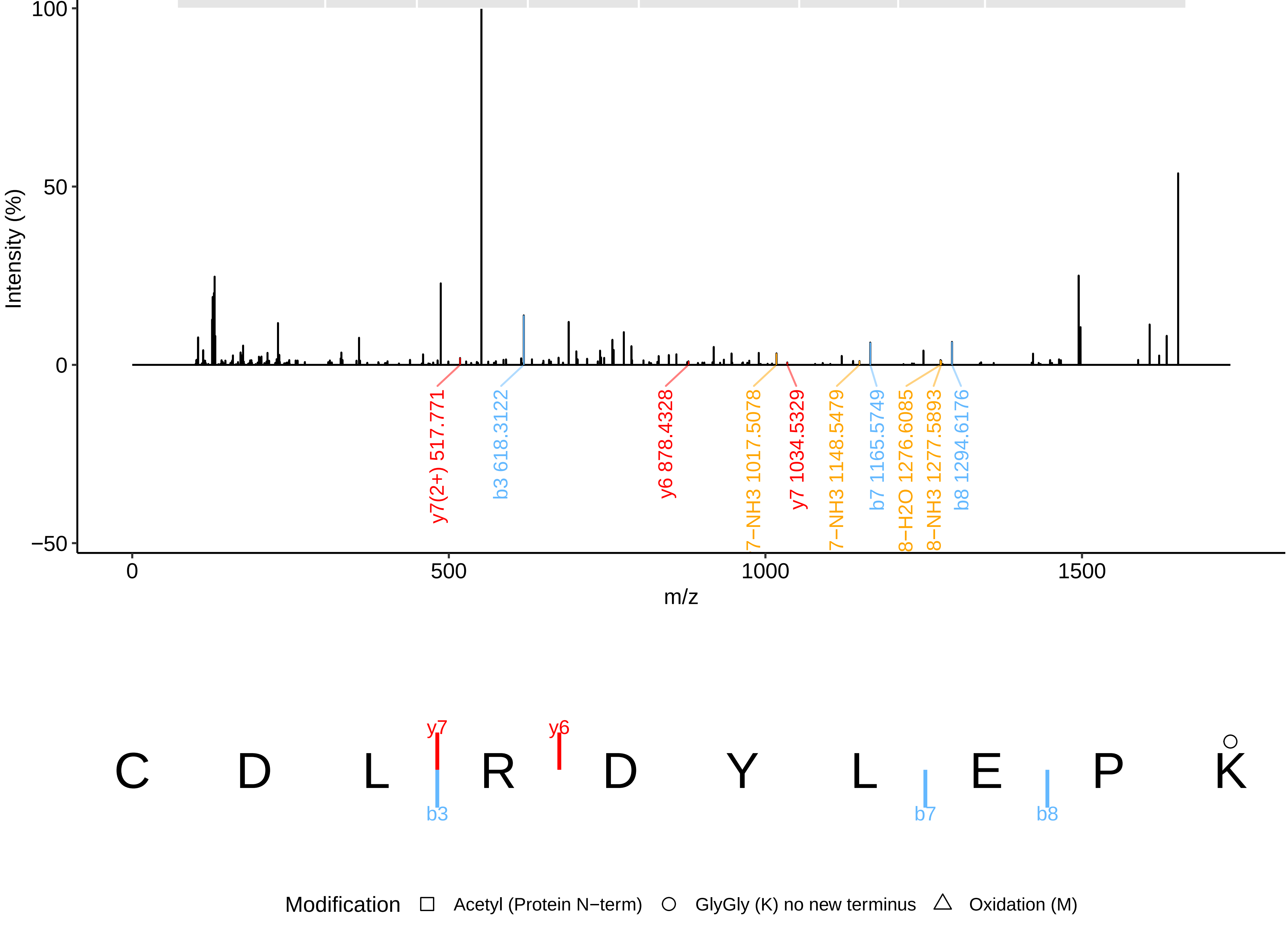

20200921_KIZ_MUb4_GlyGly_10plex_2 − Scan number: 3179

Gene Names	Charge	m/z	Mass	Retention time	PEP	Score	Precursor Intensity
	3	474.8942	1421.661	10.54	0.080597	51.445	2900046

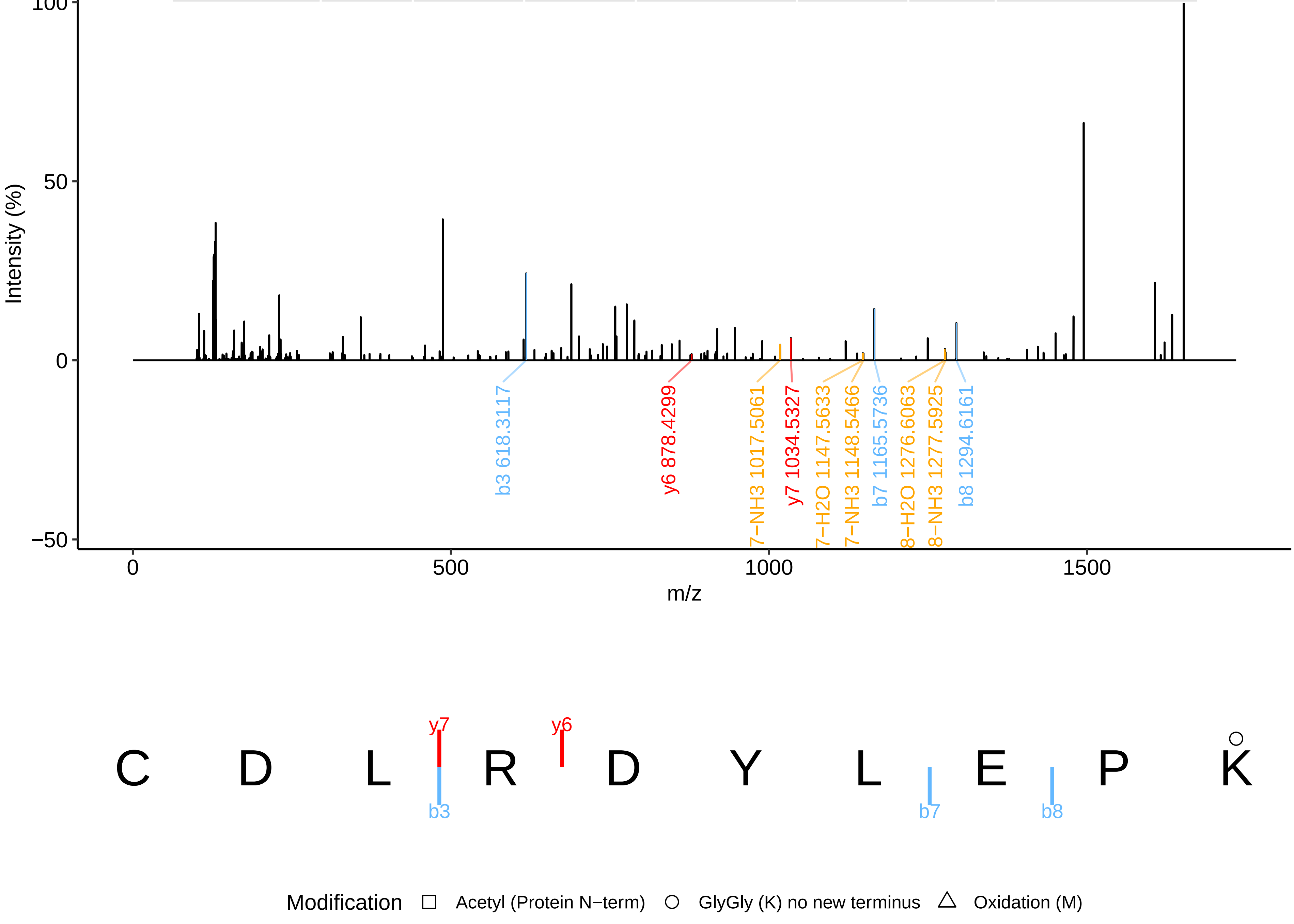

20200921_KIZ_MUb4_GlyGly_10plex − Scan number: 12880

Gene Names	Charge	m/z	Mass	Retention time	PEP	Score	Precursor Intensity
HNRNPK	3	462.588	1384.742	45.315	1.1306e−05	106.67	9176290

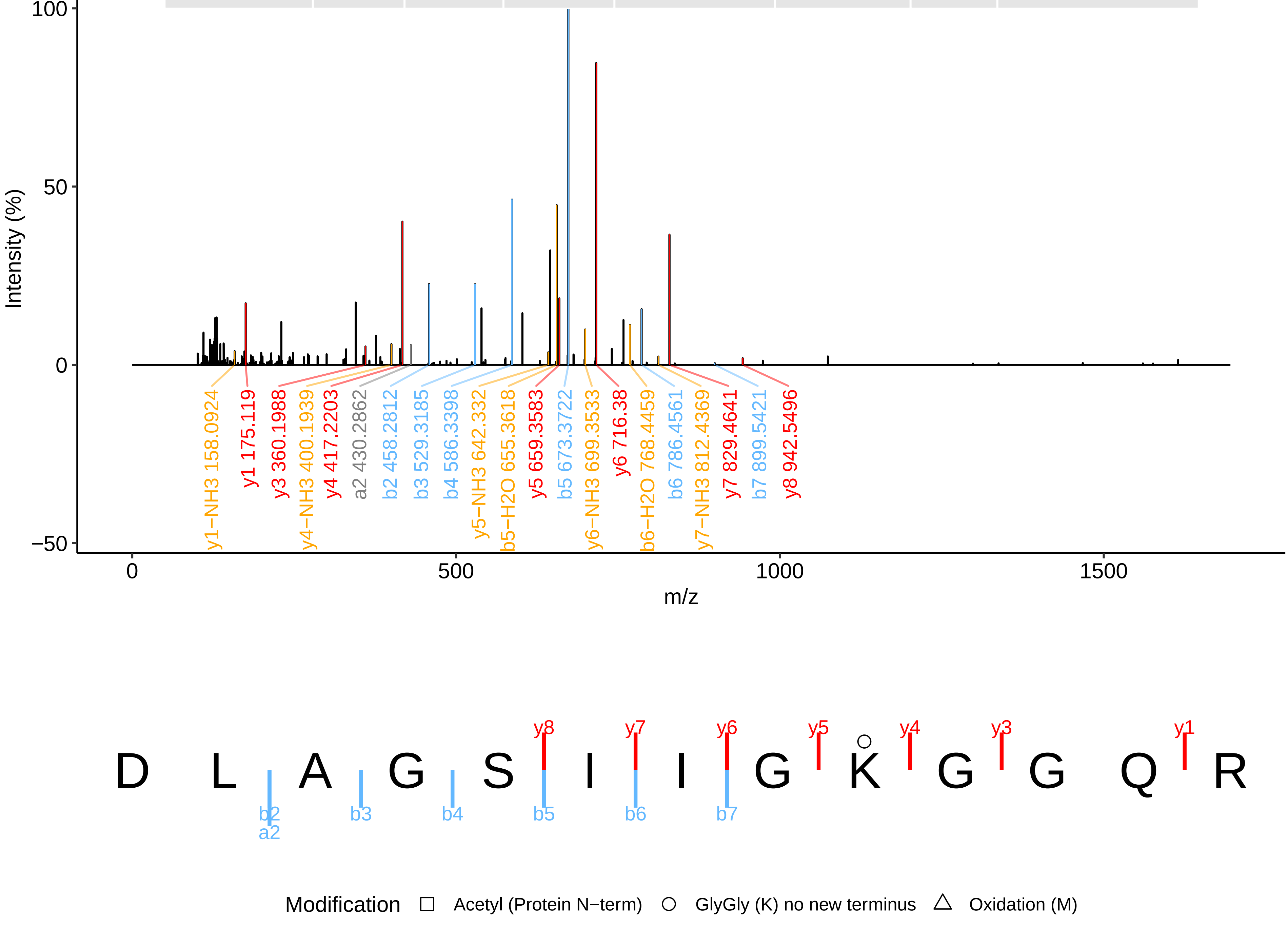

20200921_KIZ_MUb4_GlyGly_10plex_2 − Scan number: 11813

Gene Names	Charge	m/z	Mass	Retention time	PEP	Score	Precursor Intensity
HNRNPK	3	462.588	1384.742	45.559	0.0062989	71.085	777925.9

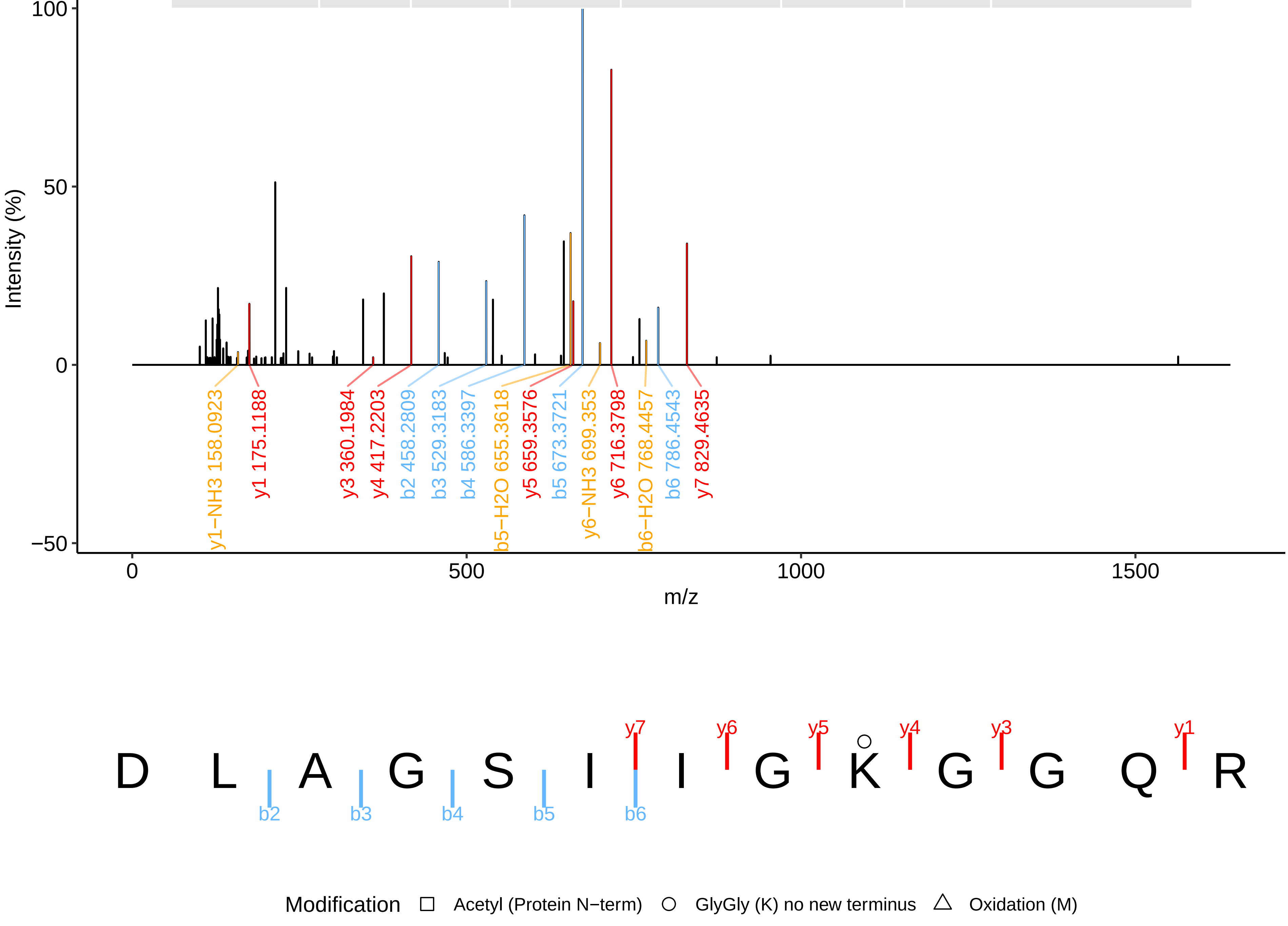

20200921_KIZ_MUb4_GlyGly_10plex − Scan number: 15778

Gene Names	Charge	m/z	Mass	Retention time	PEP	Score	Precursor Intensity
CLUH	3	457.9124	1370.715	56.467	0.0012789	89.731	NA

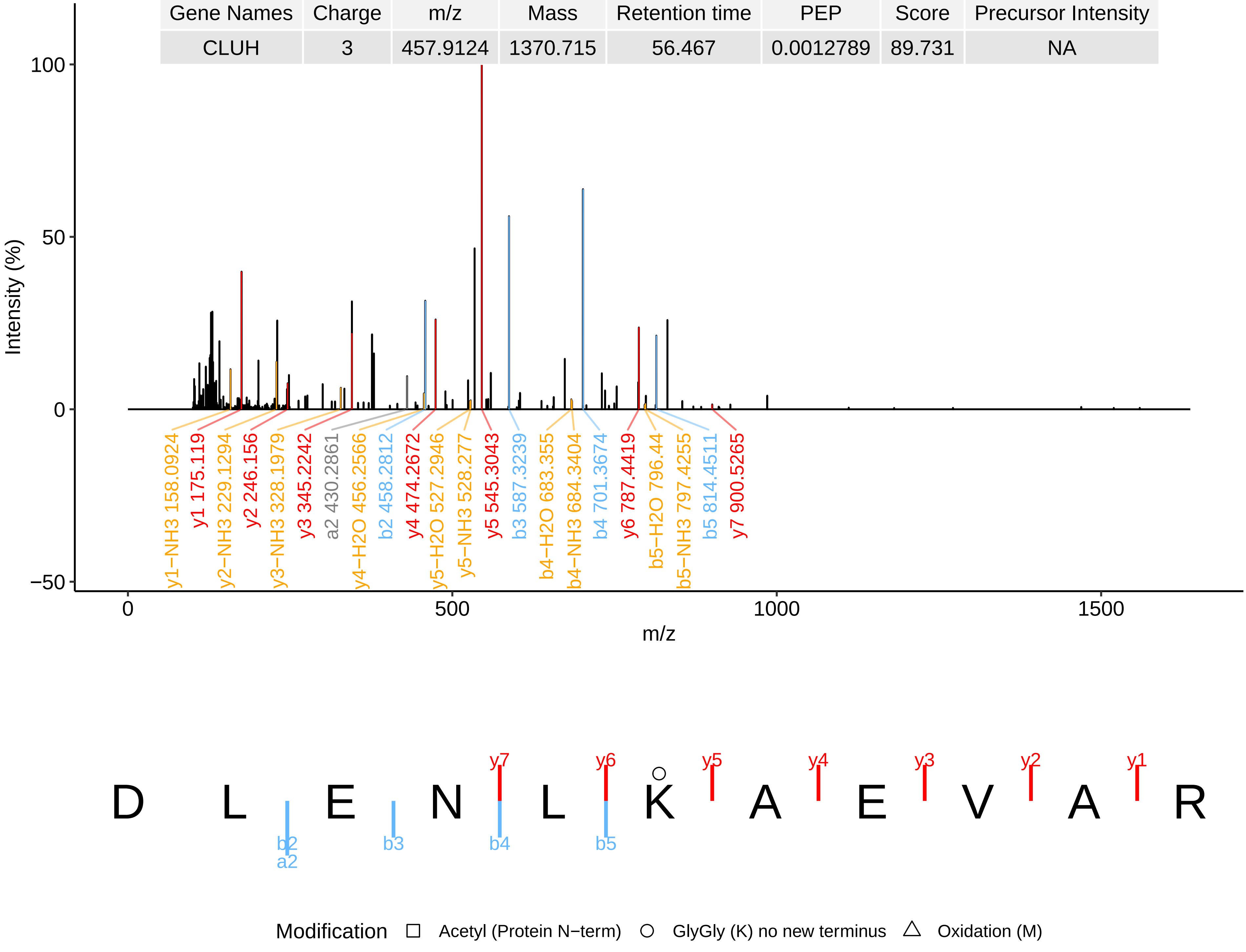

20200921_KIZ_MUb4_GlyGly_10plex_2 − Scan number: 14608

Gene Names	Charge	m/z	Mass	Retention time	PEP	Score	Precursor Intensity
CLUH	3	457.9124	1370.715	56.978	0.001489	83.045	413820.1

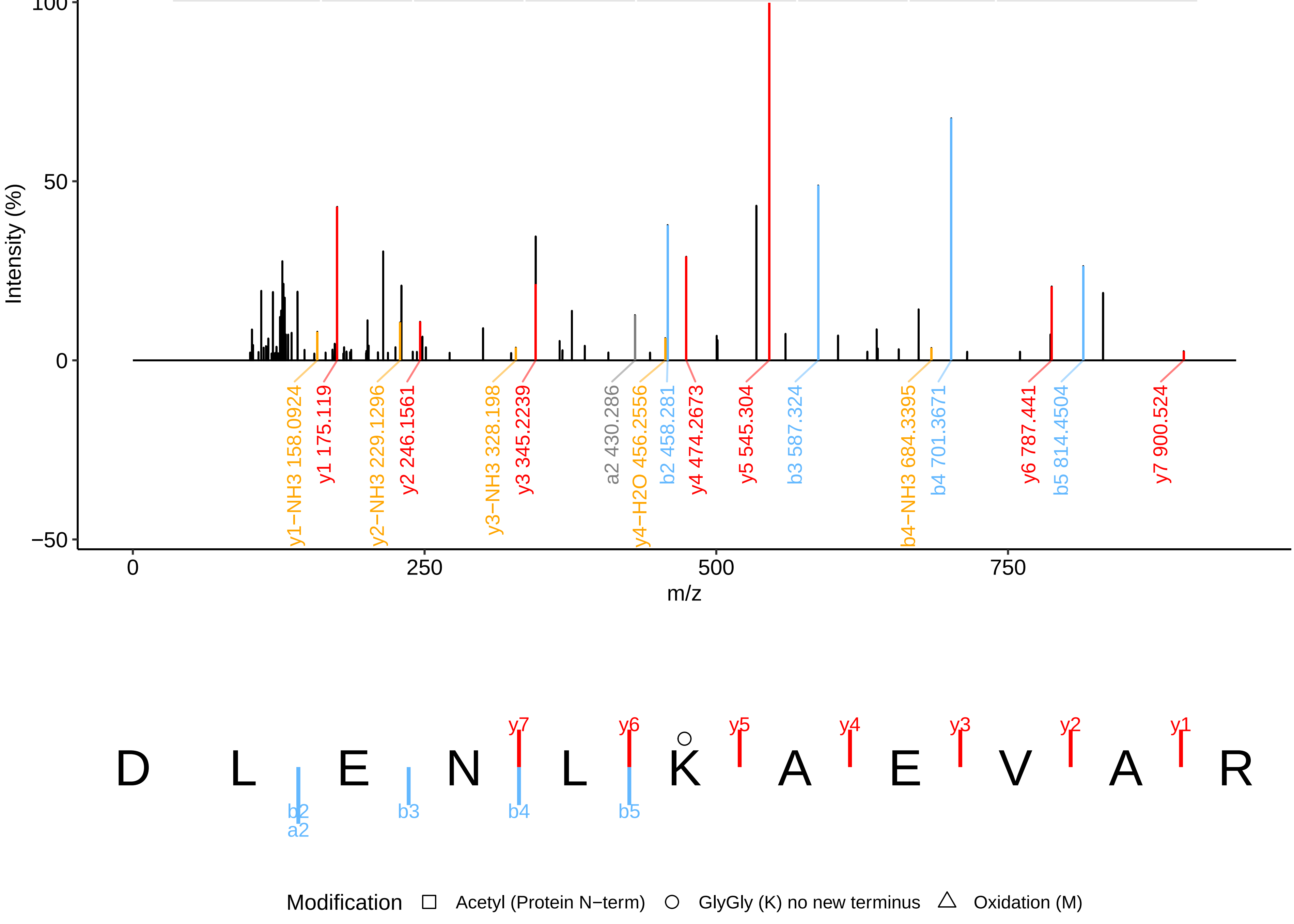

20200921_KIZ_MUb4_GlyGly_10plex − Scan number: 13601

Gene Names	Charge	m/z	Mass	Retention time	PEP	Score	Precursor Intensity
GORASP2	3	381.8838	1142.63	48.038	0.078693	51.436	5961273

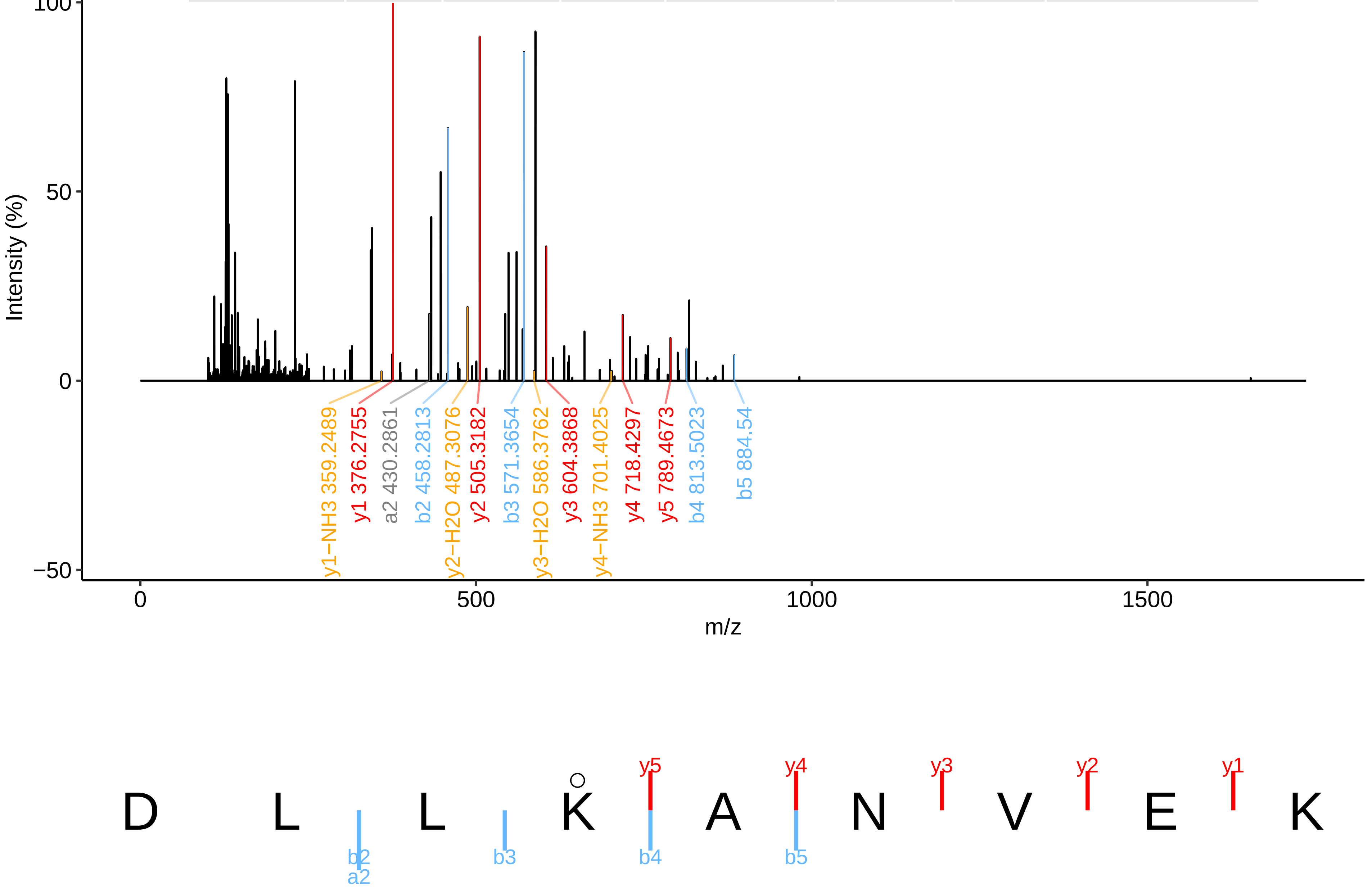

Modification □ Acetyl (Protein N−term) ○ GlyGly (K) no new terminus △ Oxidation (M)

20200921_KIZ_MUb4_GlyGly_10plex_2 − Scan number: 12519

Gene Names	Charge	m/z	Mass	Retention time	PEP	Score	Precursor Intensity
GORASP2	3	381.8838	1142.63	48.452	0.080353	51.092	1673130

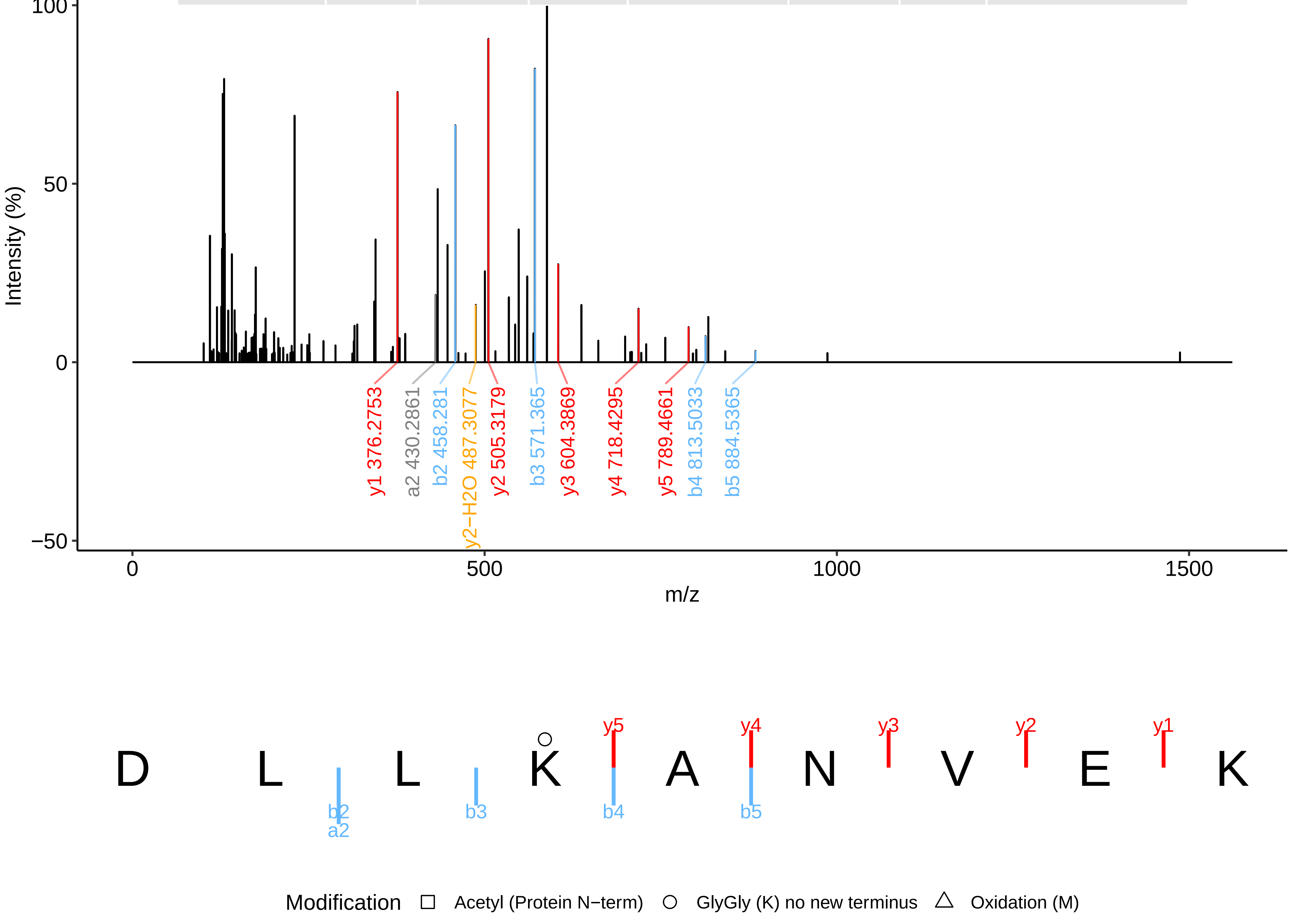

20200921_KIZ_MUb4_GlyGly_10plex − Scan number: 8041

Gene Names	Charge	m/z	Mass	Retention time	PEP	Score	Precursor Intensity
RTN3	3	387.8752	1160.604	27.179	0.054724	56.404	5050970

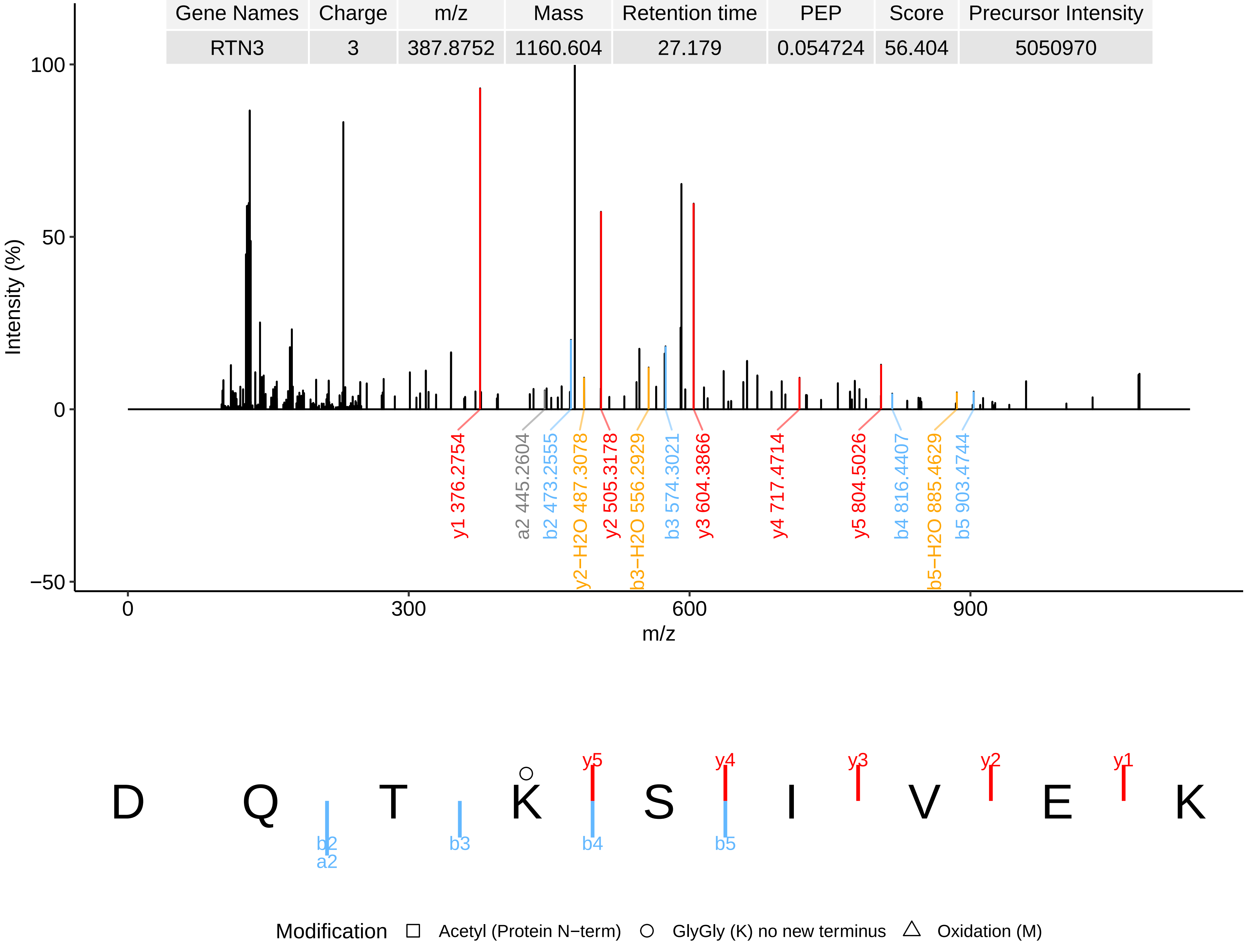

20200921_KIZ_MUb4_GlyGly_10plex − Scan number: 7790

Gene Names	Charge	m/z	Mass	Retention time	PEP	Score	Precursor Intensity
PSMC5	3	389.1888	1164.544	26.268	0.019712	69.915	6928788

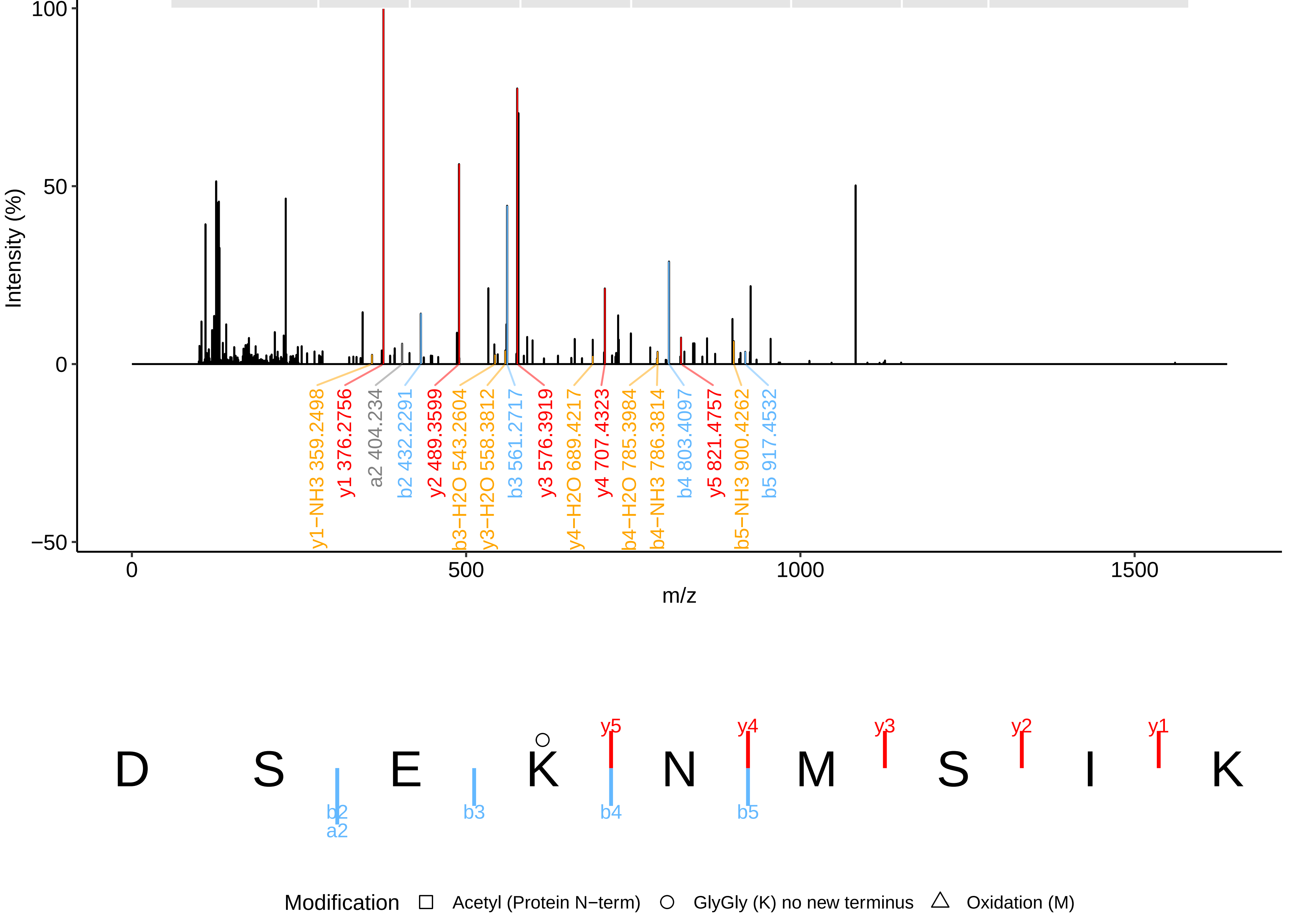

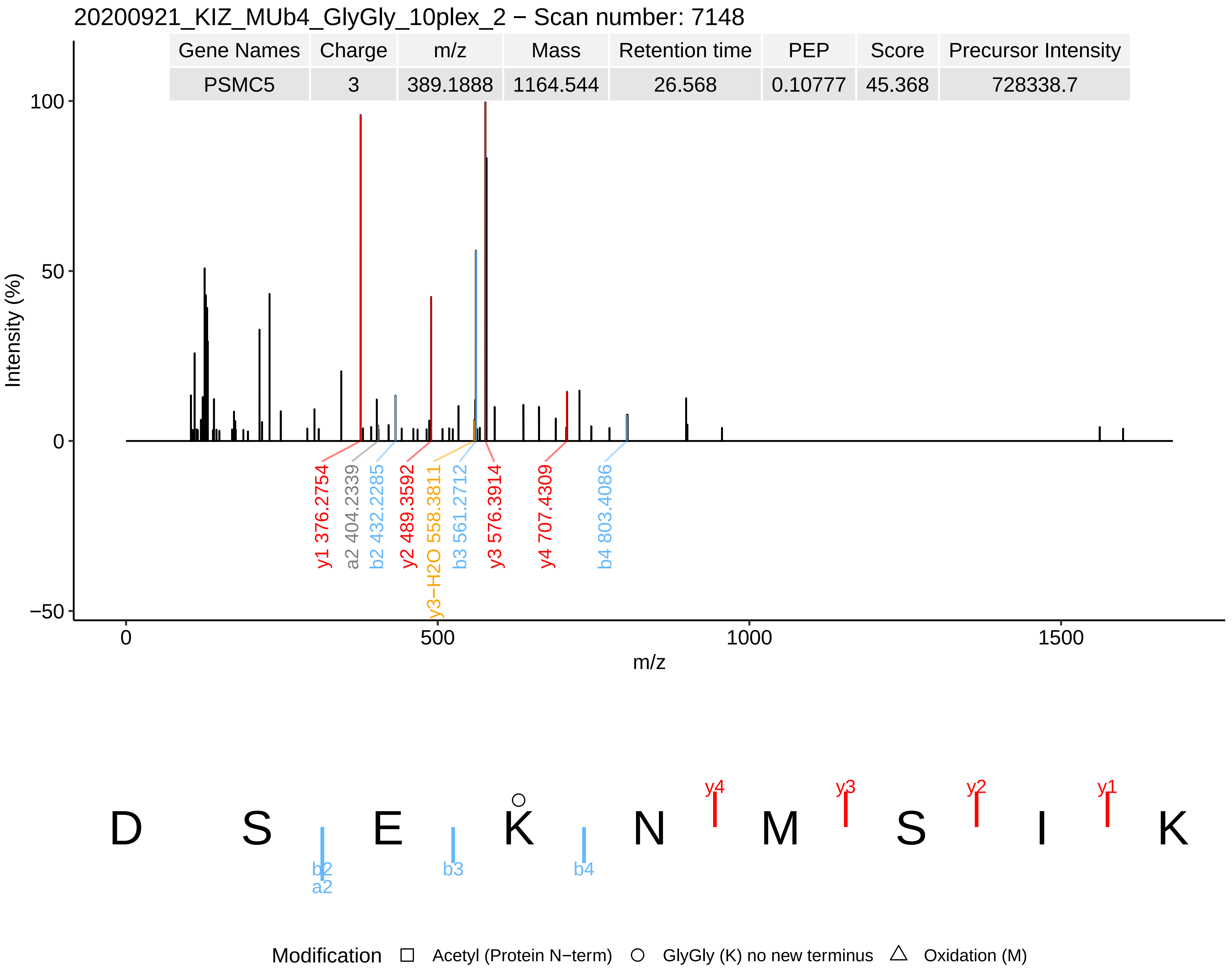

20200921_KIZ_MUb4_GlyGly_10plex_2 − Scan number: 7148
Gene Names | Charge | m/z | Mass | Retention time | PEP | Score | Precursor Intensity
PSMC5 | 3 | 389.1888 | 1164.544 | 26.568 | 0.10777 | 45.368 | 728338.7
Intensity (%)
100
50
0
−50
y1 376.2754
a2 404.2339
b2 432.2285
y2 489.3592
y3−H2O 558.3811
b3 561.2712
y3 576.3914
y4 707.4309
b4 803.4086
0
500
1000
1500
m/z
D S E K N M S I K
y4 y3 y2 y1
b2 a2 b3 b4
Modification
Acetyl (Protein N−term)
GlyGly (K) no new terminus
Oxidation (M)

20200924_KIZ_MUb5_GlyGly_1 − Scan number: 6813

Gene Names	Charge	m/z	Mass	Retention time	PEP	Score	Precursor Intensity
PSMC5	3	389.1888	1164.544	21.521	0.022889	67.414	8850509

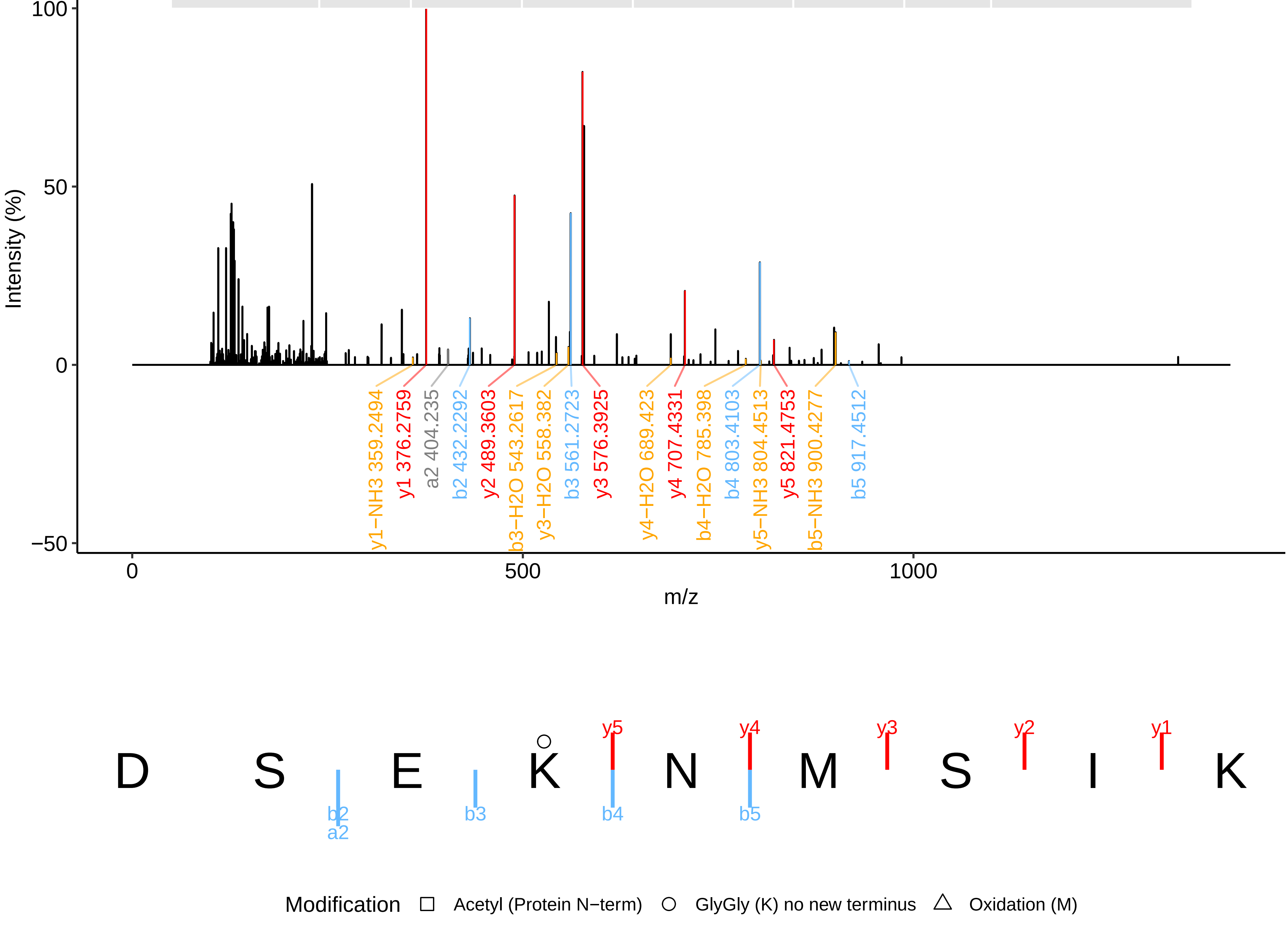

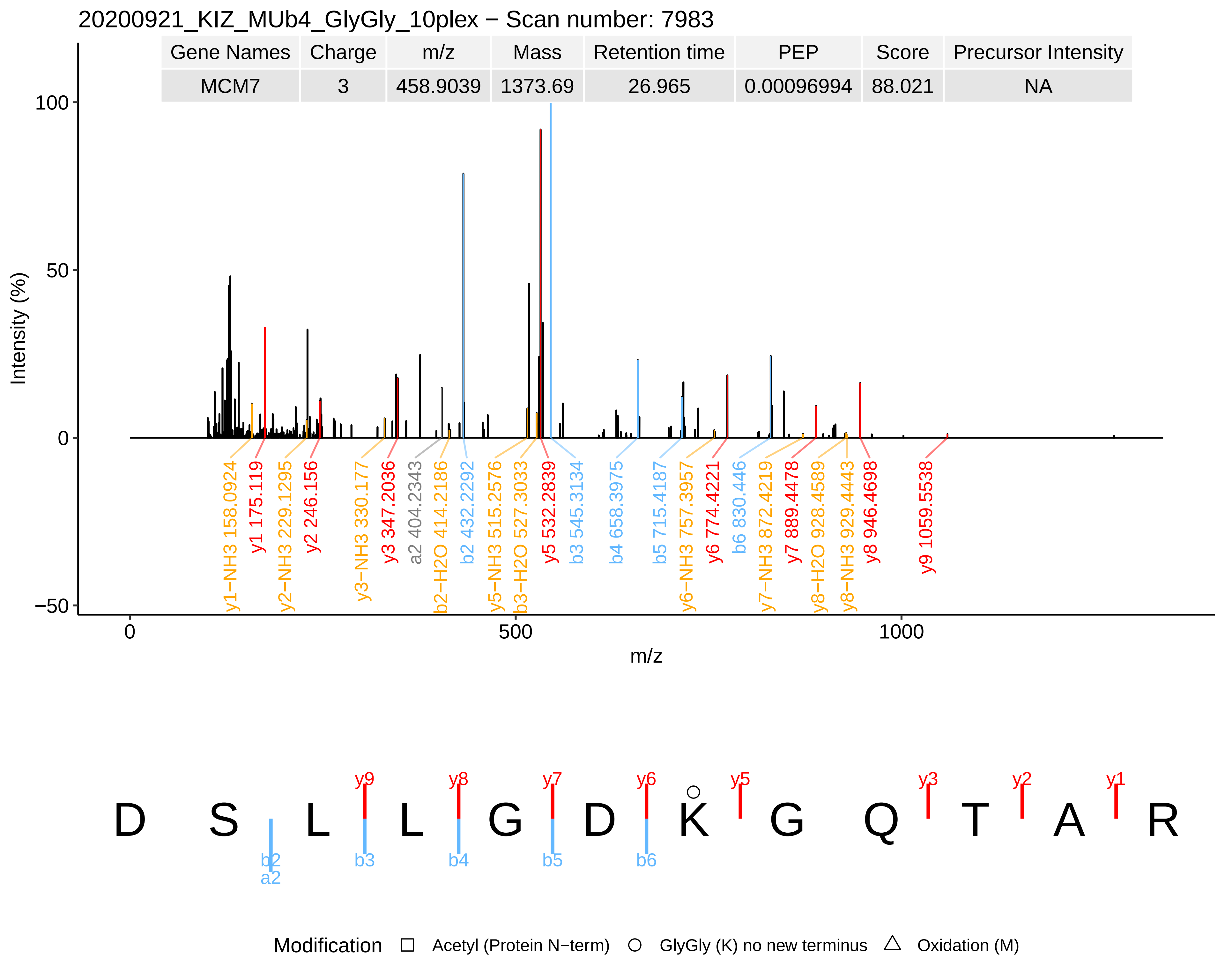
20200921_KIZ_MUb4_GlyGly_10plex − Scan number: 7983
Gene Names | Charge | m/z | Mass | Retention time | PEP | Score | Precursor Intensity
MCM7 | 3 | 458.9039 | 1373.69 | 26.965 | 0.00096994 | 88.021 | NA
Intensity (%)
100
50
0
−50
0
500
1000
m/z
y1−NH3 158.0924
y1 175.119
y2−NH3 229.1295
y2 246.156
y3−NH3 330.177
y3 347.2036
a2 404.2343
b2−H2O 414.2186
b2 432.2292
y5−NH3 515.2576
b3−H2O 527.3033
y5 532.2839
b3 545.3134
b4 658.3975
b5 715.4187
y6−NH3 757.3957
y6 774.4221
b6 830.446
y7−NH3 872.4219
y7 889.4478
y8−H2O 928.4589
y8−NH3 929.4443
y8 946.4698
y9 1059.5538
D S L L G D K G Q T A R
y9 y8 y7 y6 y5 y3 y2 y1
b2 a2 b3 b4 b5 b6
Modification
Acetyl (Protein N−term)
GlyGly (K) no new terminus
Oxidation (M)

20200921_KIZ_MUb4_GlyGly_10plex_2 − Scan number: 7332

Gene Names	Charge	m/z	Mass	Retention time	PEP	Score	Precursor Intensity
MCM7	3	458.9039	1373.69	27.321	0.0011409	81.709	663846.1

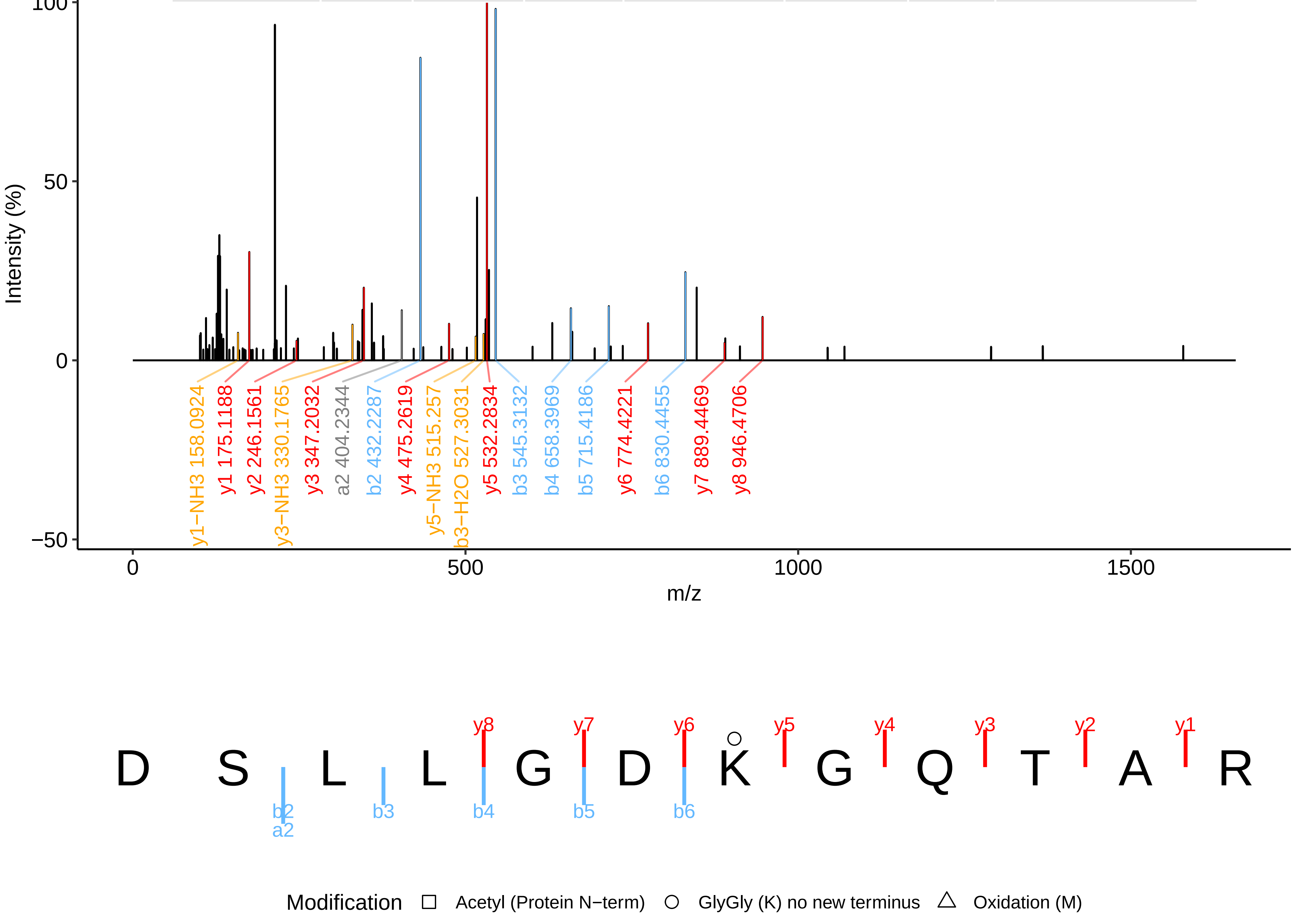

20200921_KIZ_MUb4_GlyGly_10plex − Scan number: 16962

Gene Names	Charge	m/z	Mass	Retention time	PEP	Score	Precursor Intensity
RPL19	3	392.2274	1173.66	61.124	0.067854	53.683	4616427

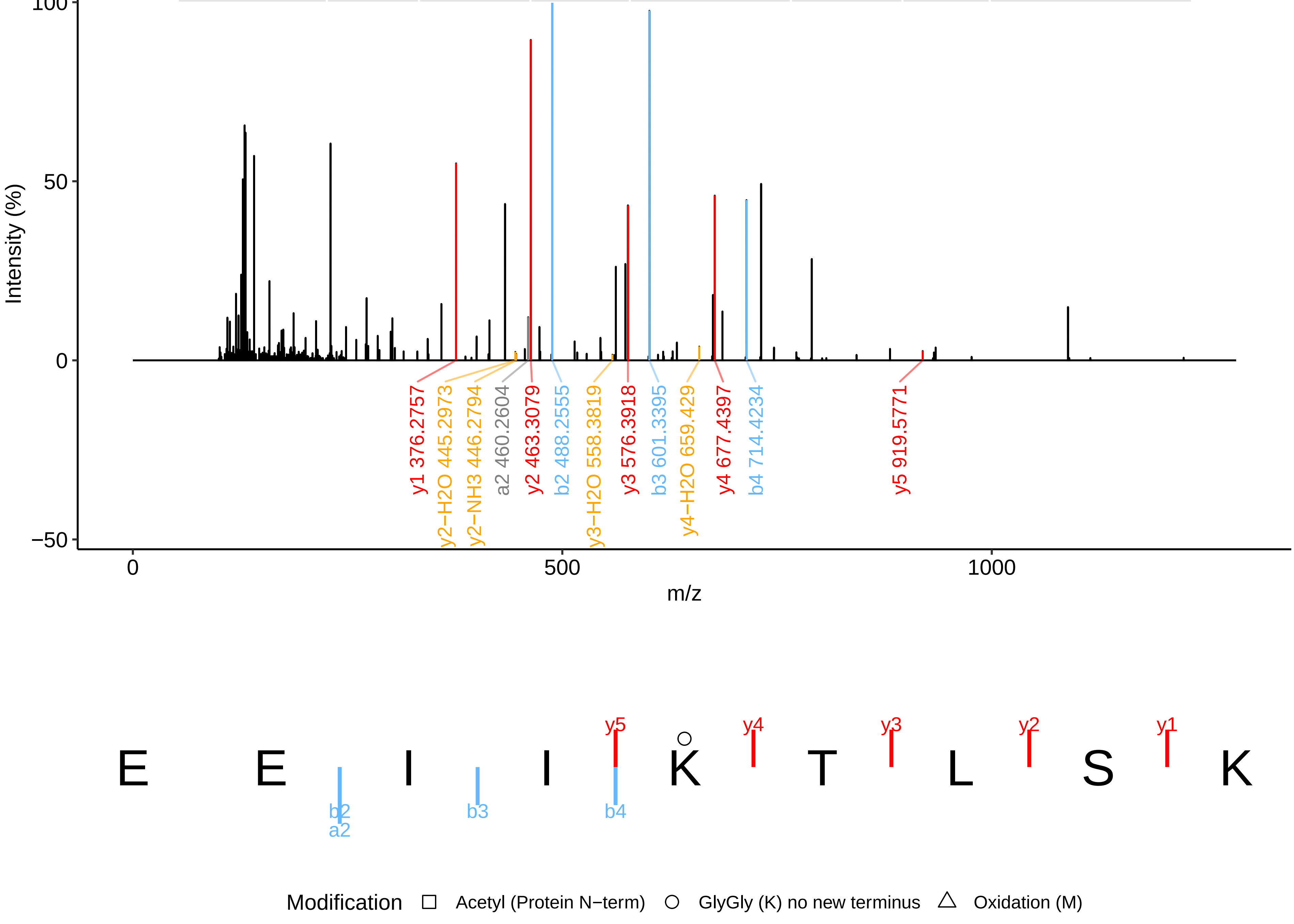

20200924_KIZ_MUb5_GlyGly_1 − Scan number: 4754

Gene Names	Charge	m/z	Mass	Retention time	PEP	Score	Precursor Intensity
UIMC1	3	387.5351	1159.583	14.708	0.014379	70.399	9884805

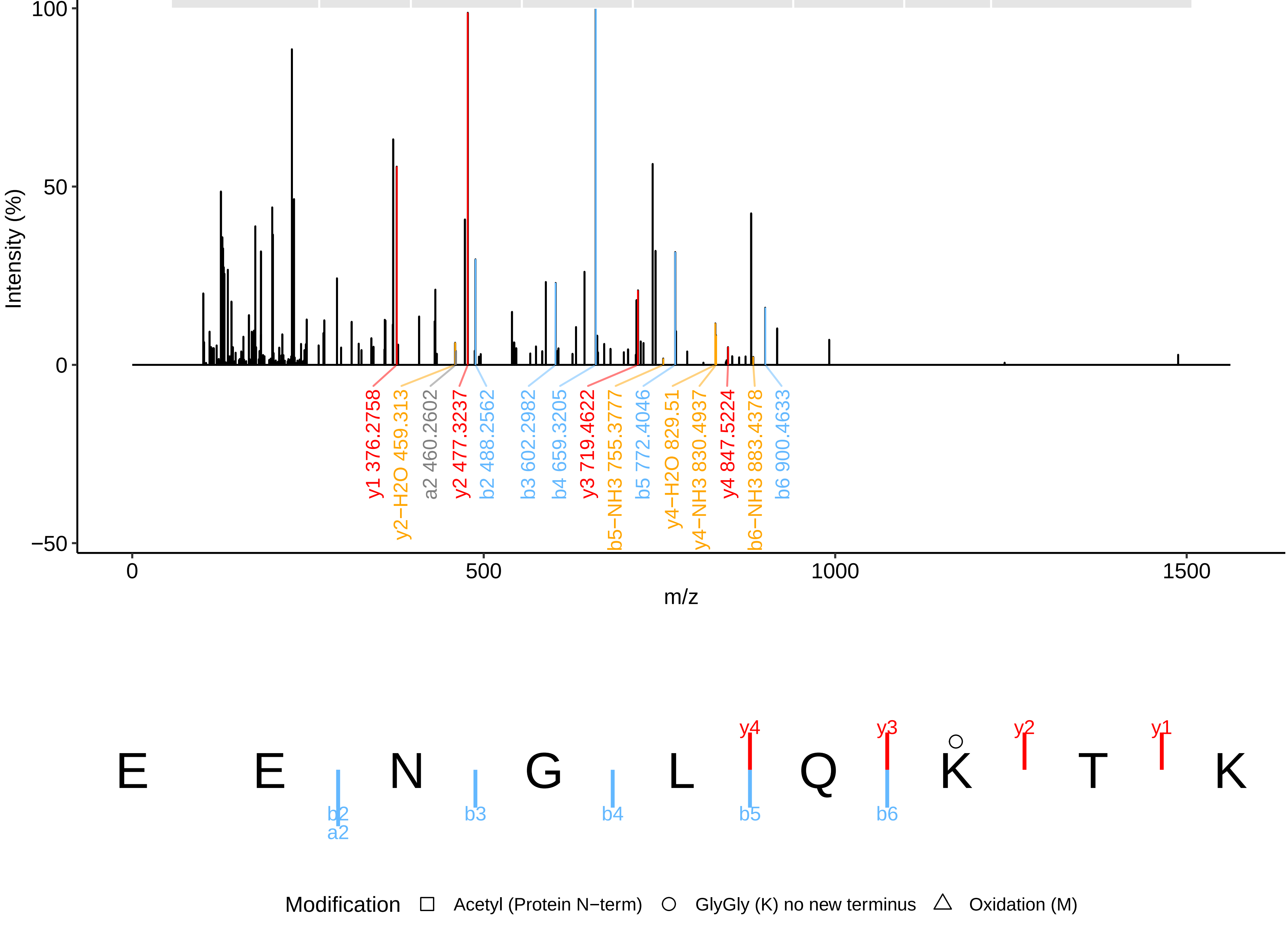

20200924_KIZ_MUb5_GlyGly_2 − Scan number: 4413

Gene Names	Charge	m/z	Mass	Retention time	PEP	Score	Precursor Intensity
UIMC1	3	387.5351	1159.583	16.182	0.023271	67.113	1840987

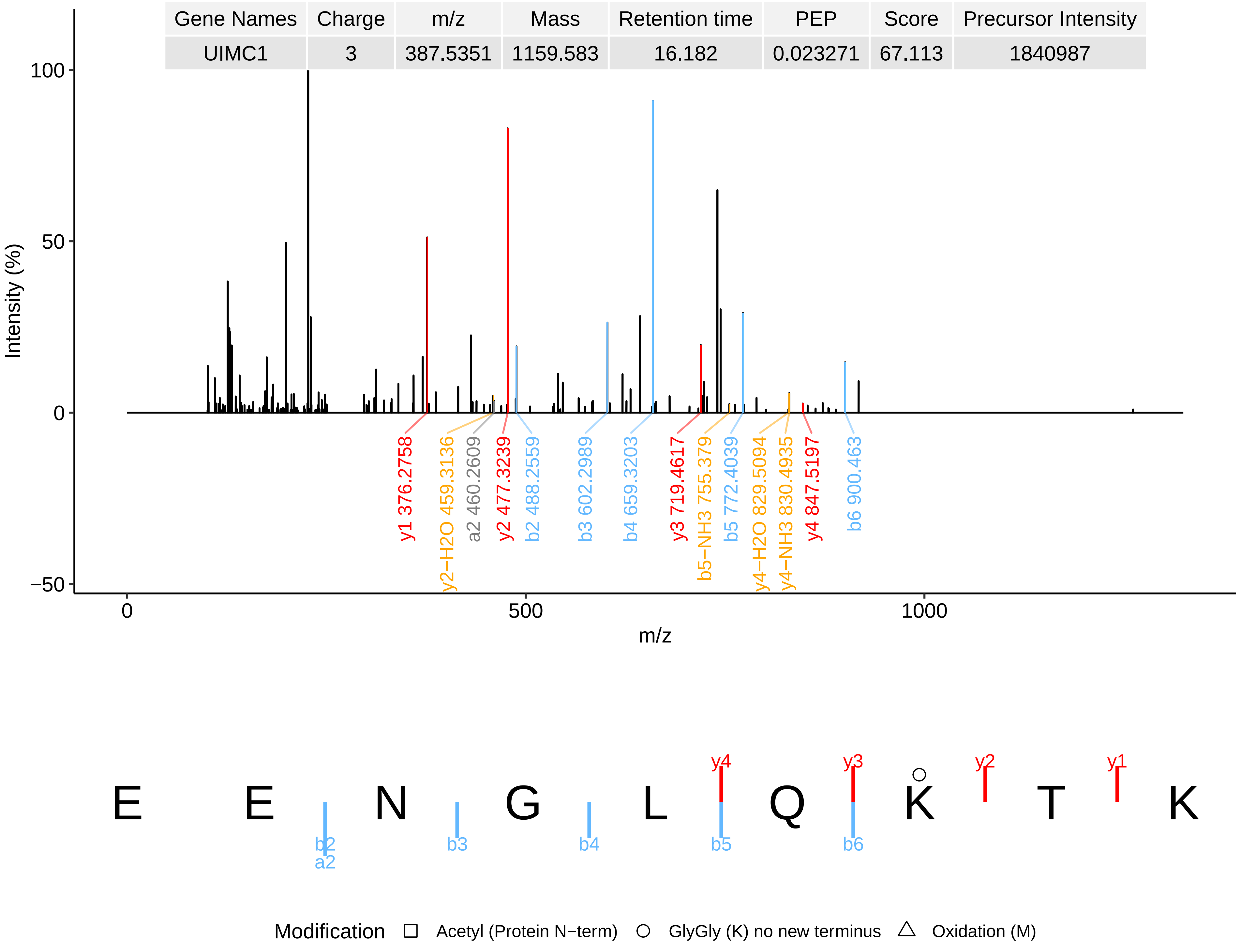

20200921_KIZ_MUb4_GlyGly_10plex_2 − Scan number: 16352

Gene Names	Charge	m/z	Mass	Retention time	PEP	Score	Precursor Intensity
BTN2A1	3	396.2274	1185.66	63.836	0.0065309	80.688	2706947

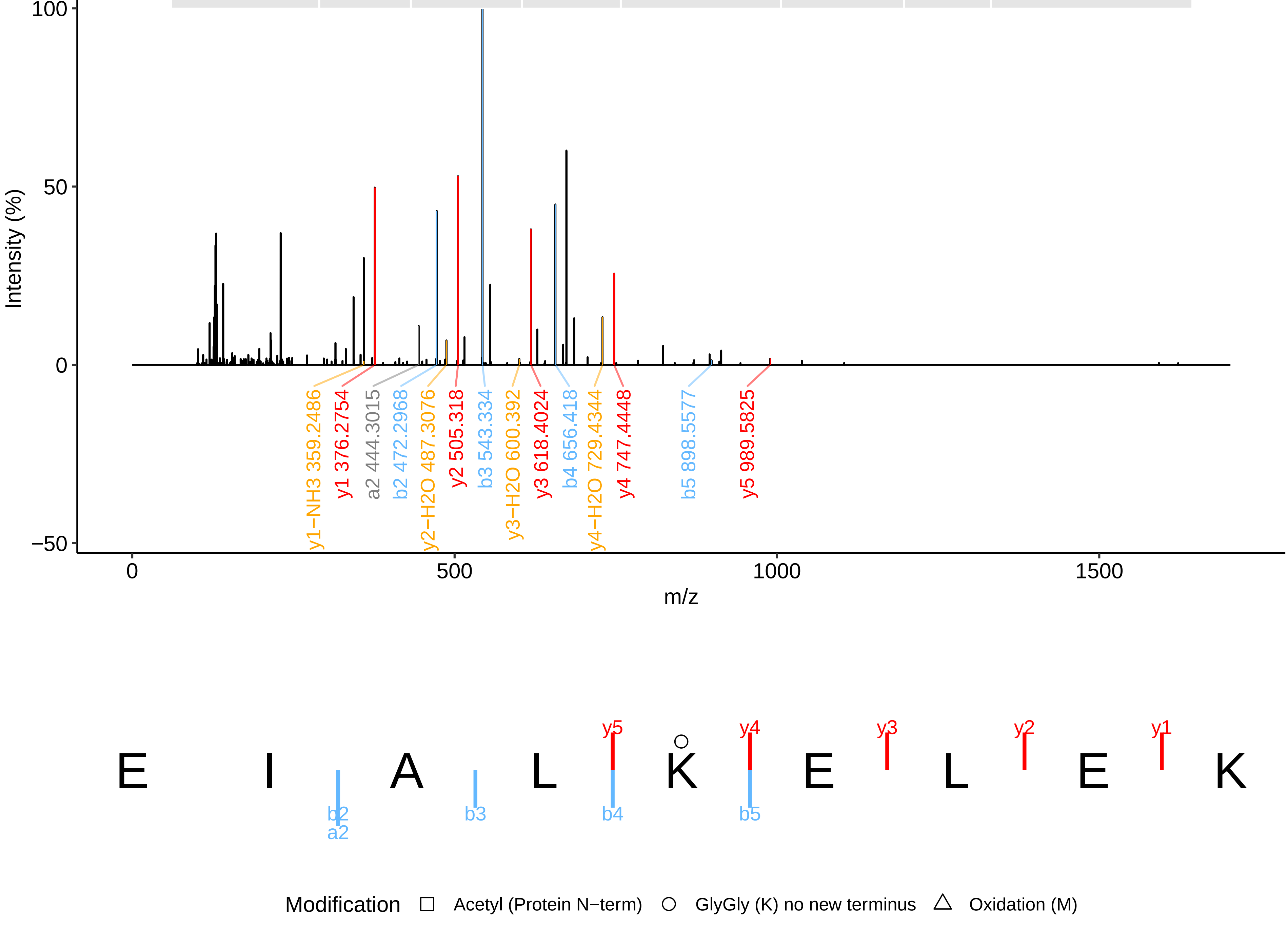

20201118_KIZ_MUb6_GlyGly_2 − Scan number: 19406

Gene Names	Charge	m/z	Mass	Retention time	PEP	Score	Precursor Intensity
BTN2A1	3	396.2274	1185.66	65.492	0.00051357	99.815	9919012

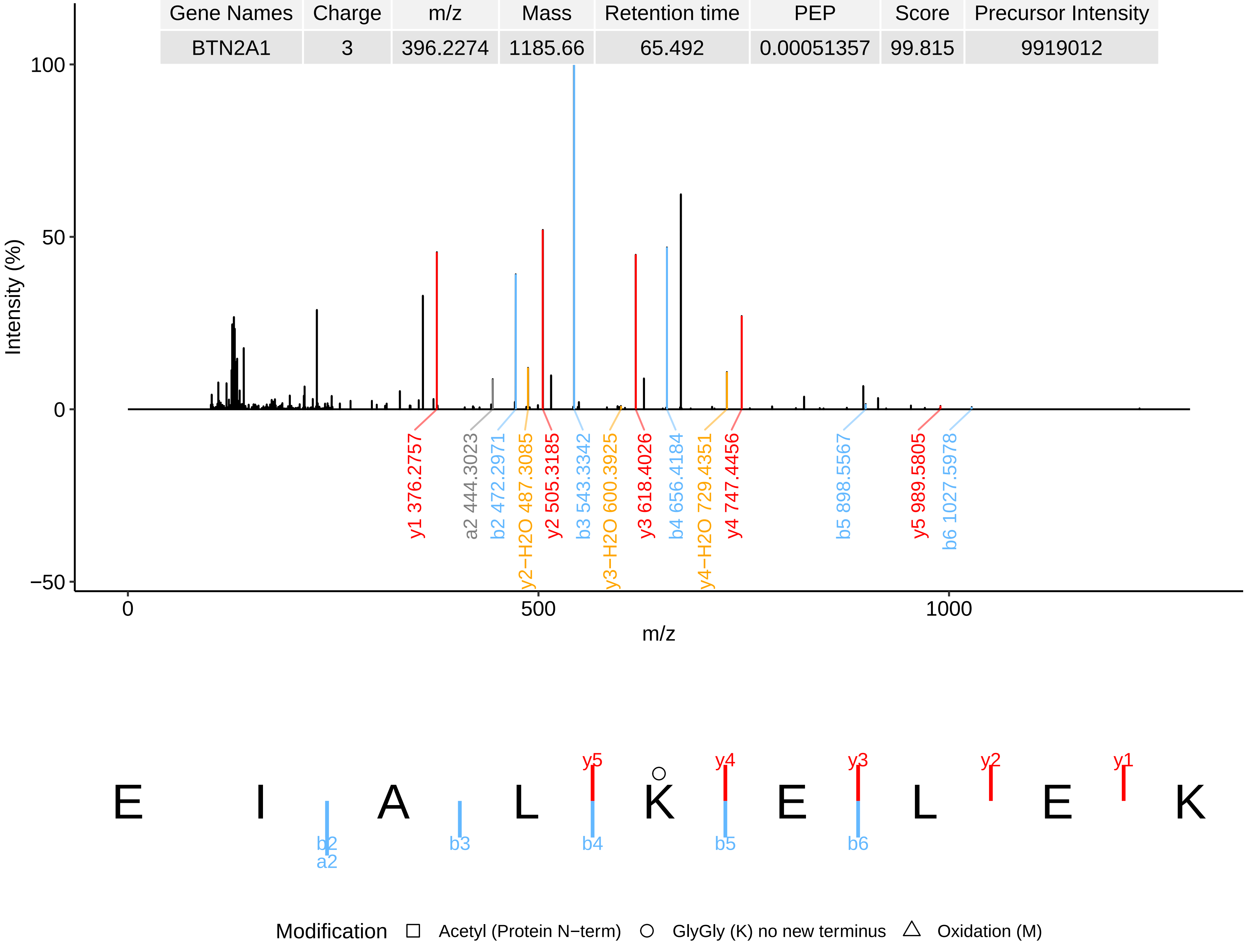

20200921_KIZ_MUb4_GlyGly_10plex − Scan number: 19994

Gene Names	Charge	m/z	Mass	Retention time	PEP	Score	Precursor Intensity
CYP51A1	3	390.5449	1168.613	72.188	0.13739	48.794	NA

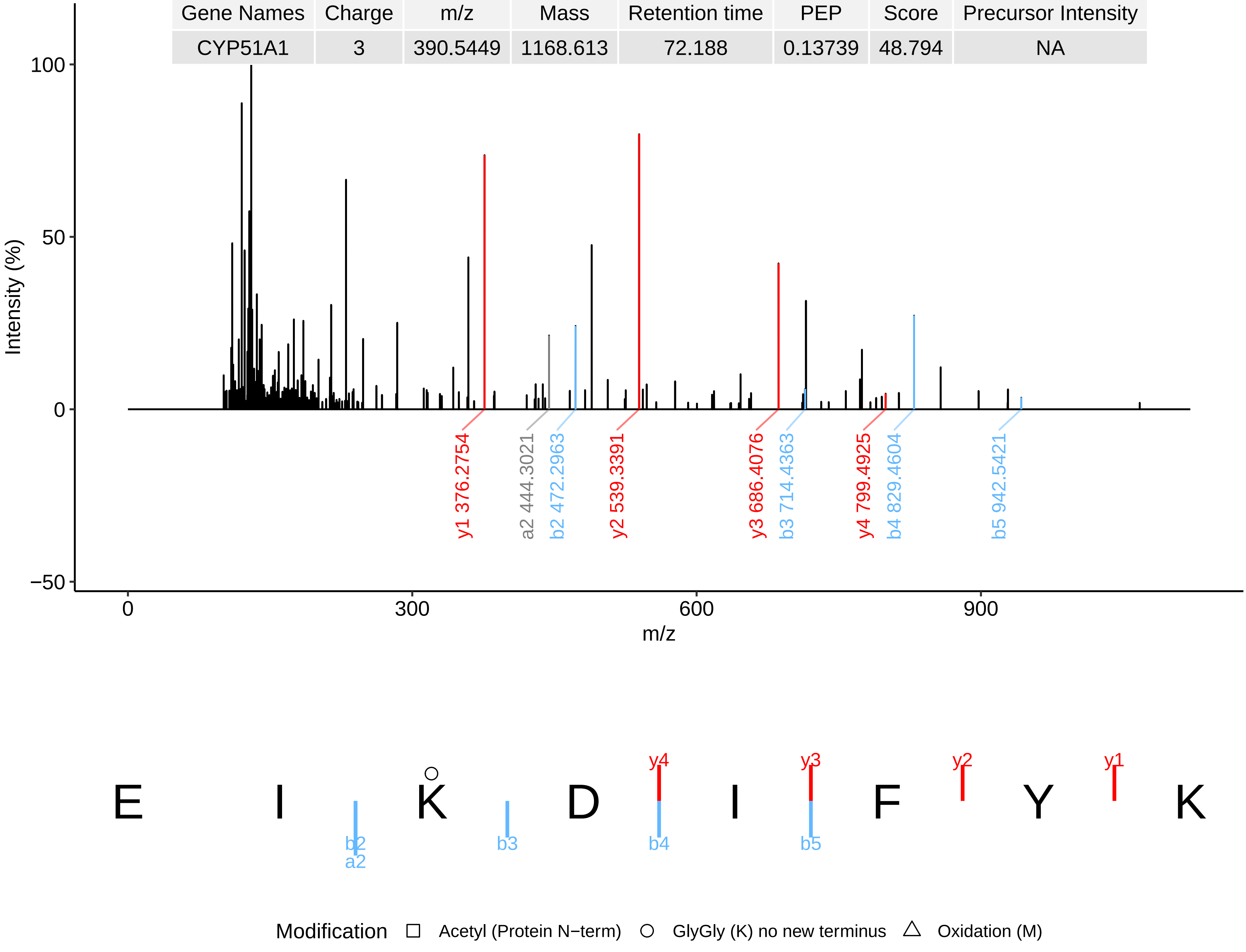

20200921_KIZ_MUb4_GlyGly_10plex_2 − Scan number: 3909

Gene Names	Charge	m/z	Mass	Retention time	PEP	Score	Precursor Intensity
SH3BGRL3	3	473.5793	1417.716	13.437	0.0014855	83.081	1131150

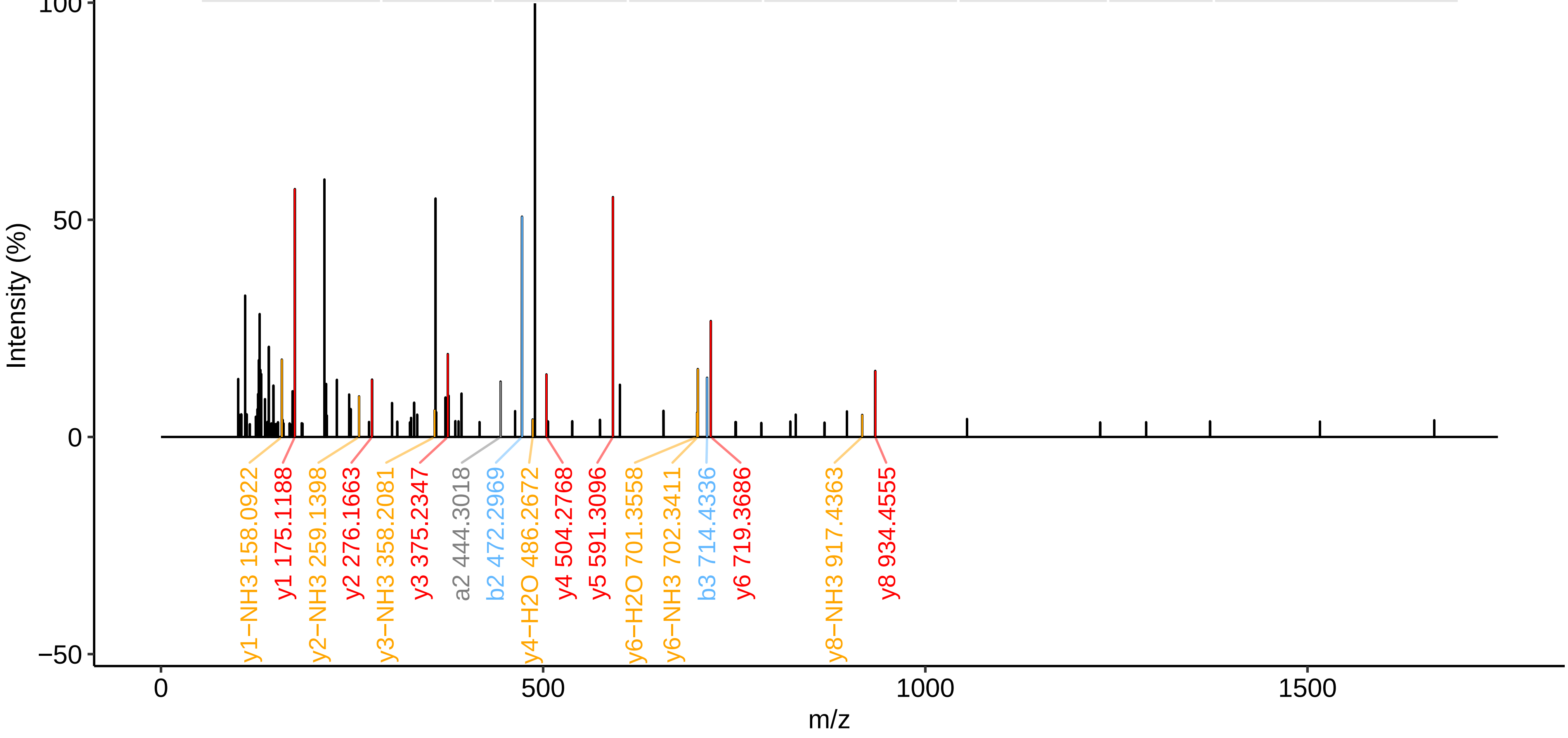

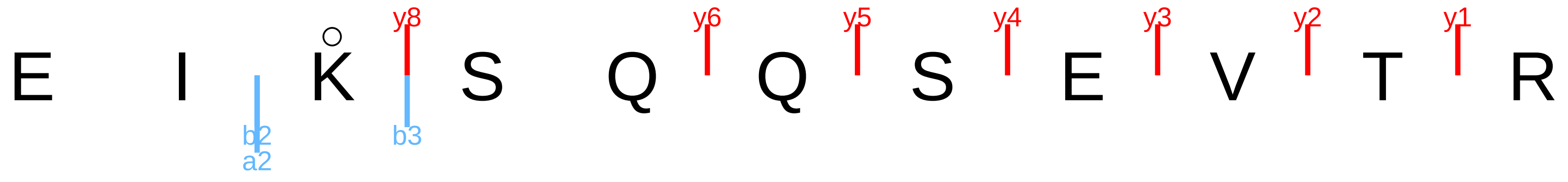

Modification □ Acetyl (Protein N−term) ○ GlyGly (K) no new terminus △ Oxidation (M)

20201118_KIZ_MUb6_GlyGly_2 − Scan number: 4197

Gene Names	Charge	m/z	Mass	Retention time	PEP	Score	Precursor Intensity
SH3BGRL3	3	473.5793	1417.716	14.226	0.016069	68.44	2021929

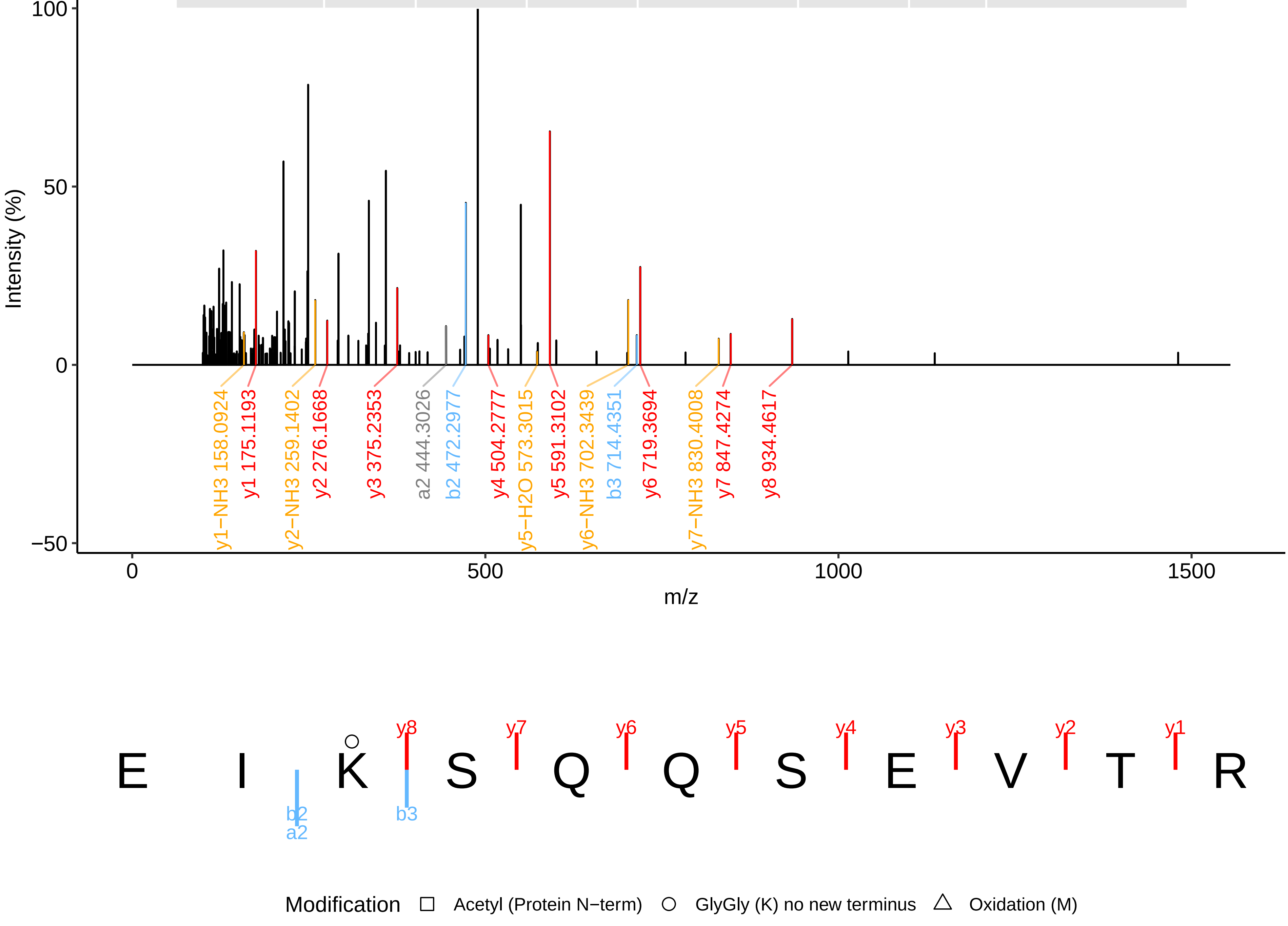

20200921_KIZ_MUb4_GlyGly_10plex − Scan number: 13896

Gene Names	Charge	m/z	Mass	Retention time	PEP	Score	Precursor Intensity
SYS1	2	691.8673	1381.72	49.182	0.052174	74.92	4348240

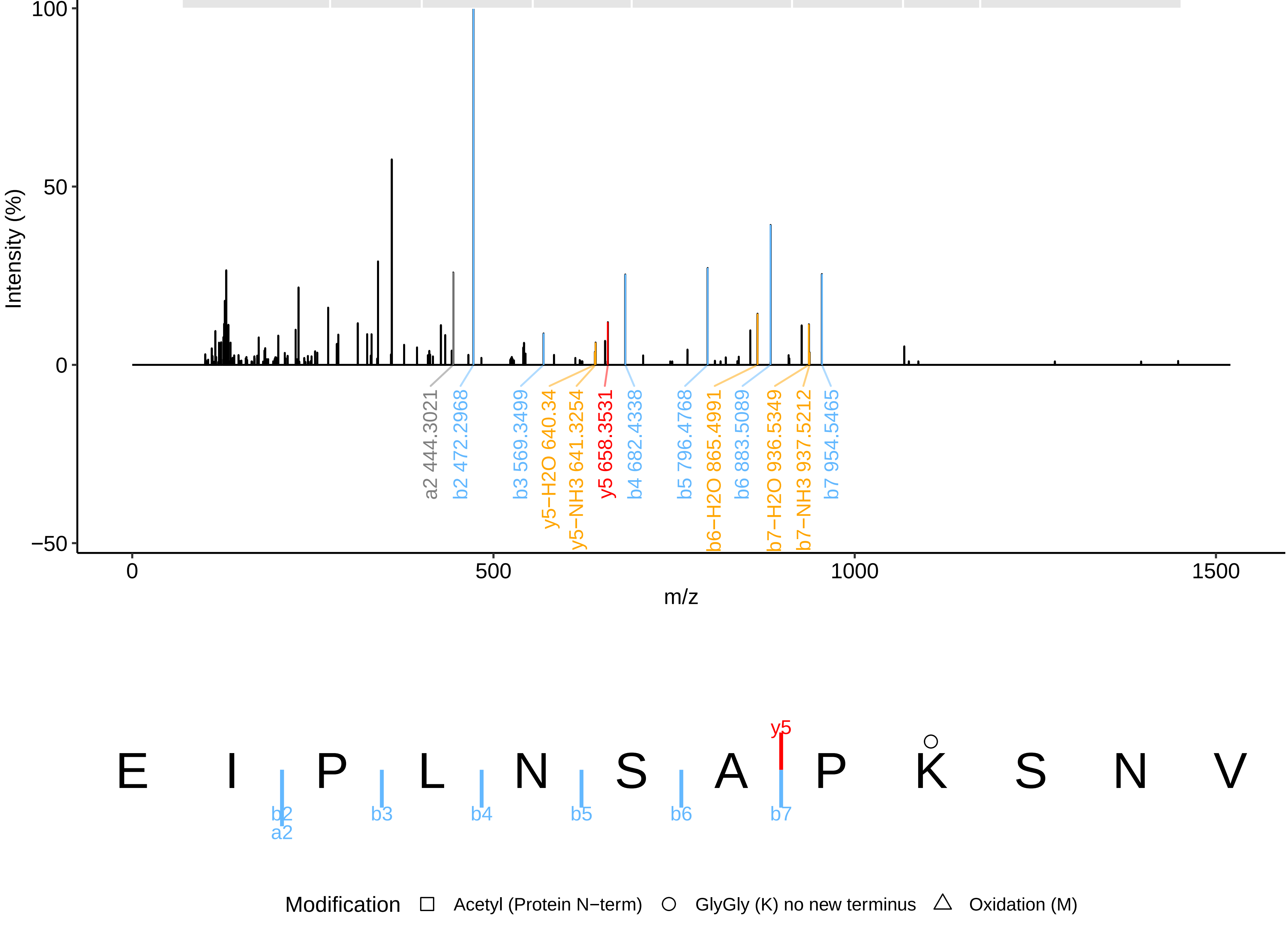

20200921_KIZ_MUb4_GlyGly_10plex_2 − Scan number: 12703

Gene Names	Charge	m/z	Mass	Retention time	PEP	Score	Precursor Intensity
SYS1	2	691.8673	1381.72	49.205	0.048772	75.378	655334.3

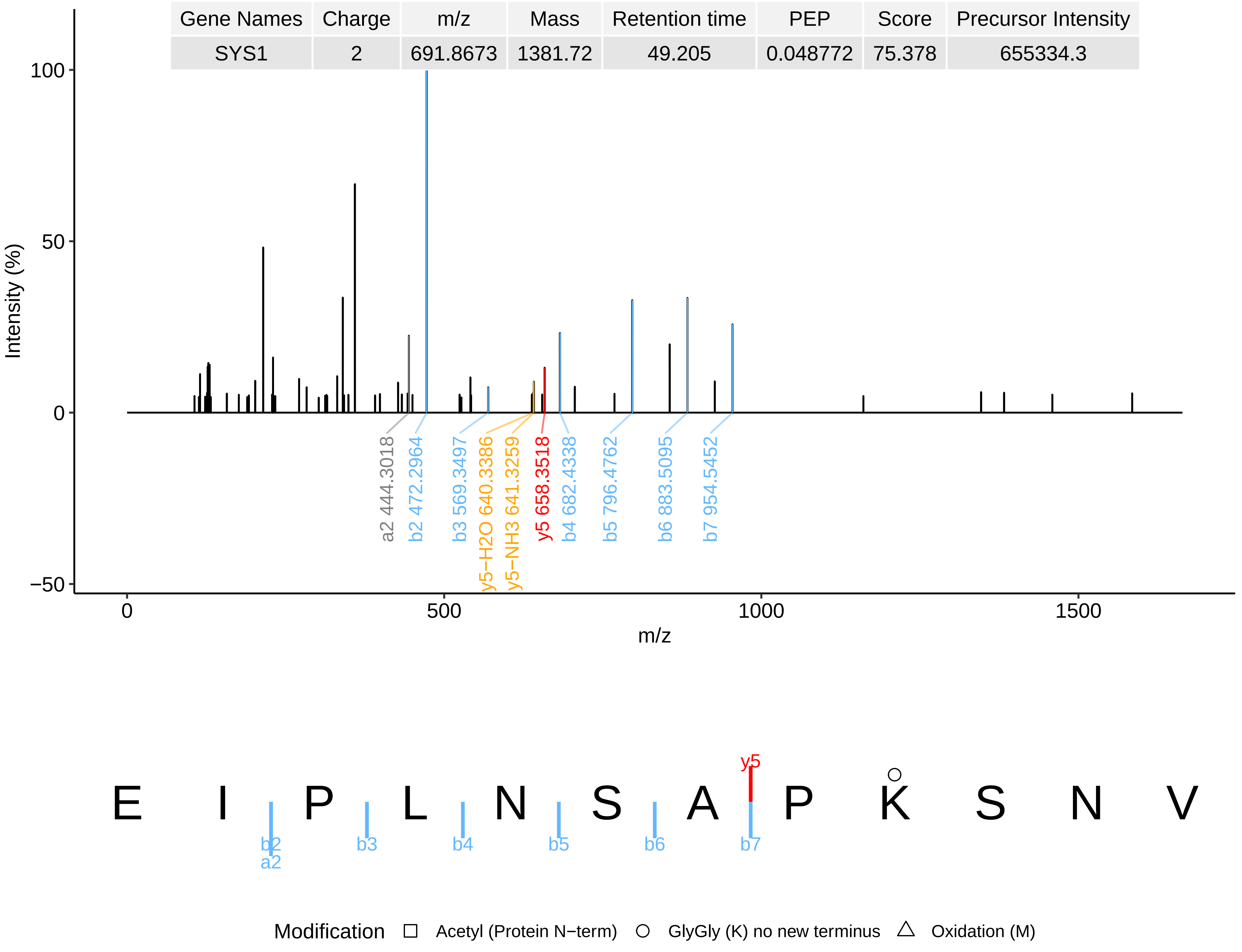

20200921_KIZ_MUb4_GlyGly_10plex − Scan number: 4048

Gene Names	Charge	m/z	Mass	Retention time	PEP	Score	Precursor Intensity
EIF3A	4	294.161	1172.615	13.158	0.031972	91.069	22105184

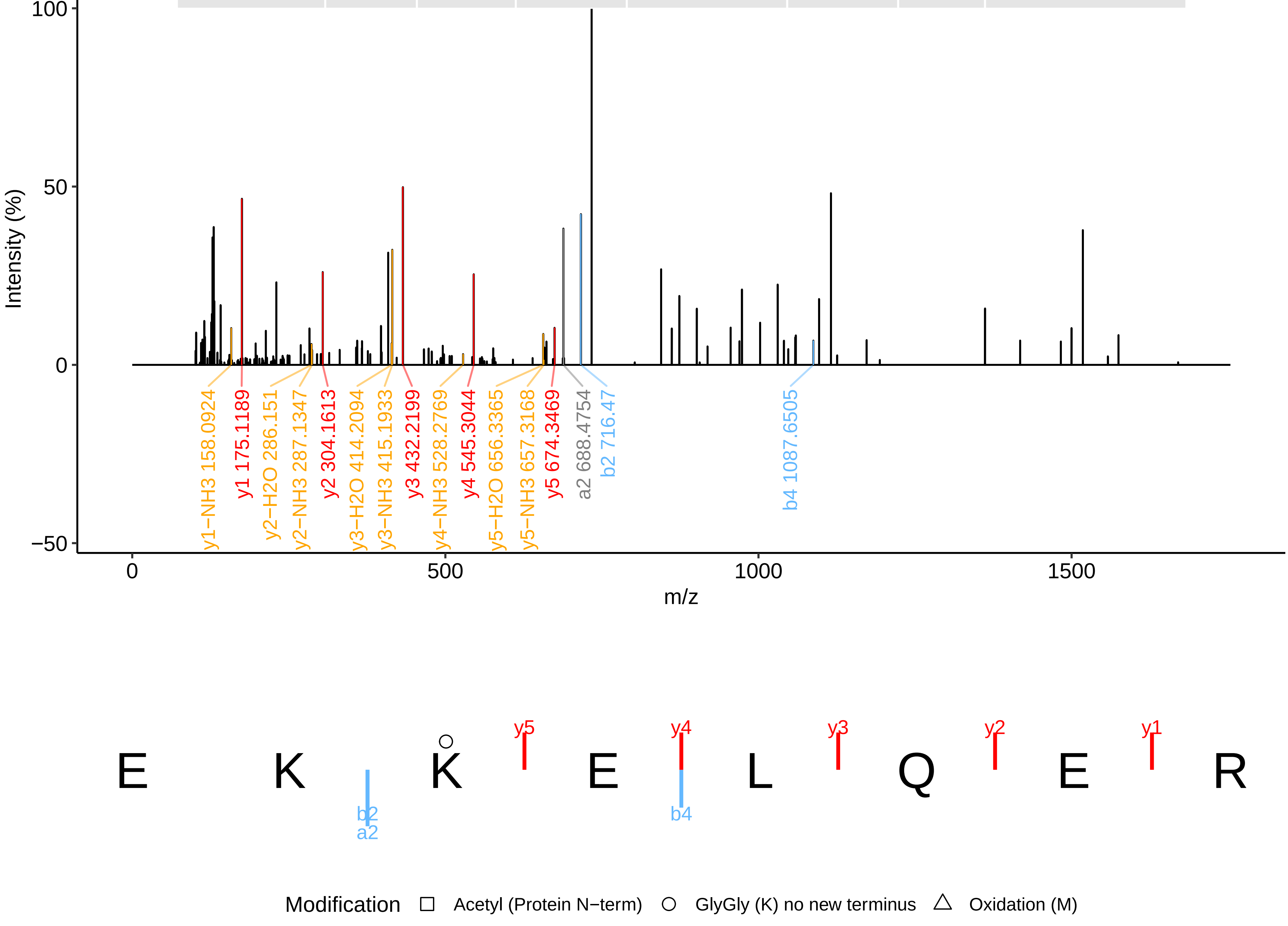

20200921_KIZ_MUb4_GlyGly_10plex − Scan number: 4043

Gene Names	Charge	m/z	Mass	Retention time	PEP	Score	Precursor Intensity
EIF3A	3	391.8789	1172.615	13.139	0.0302	75.387	2428951

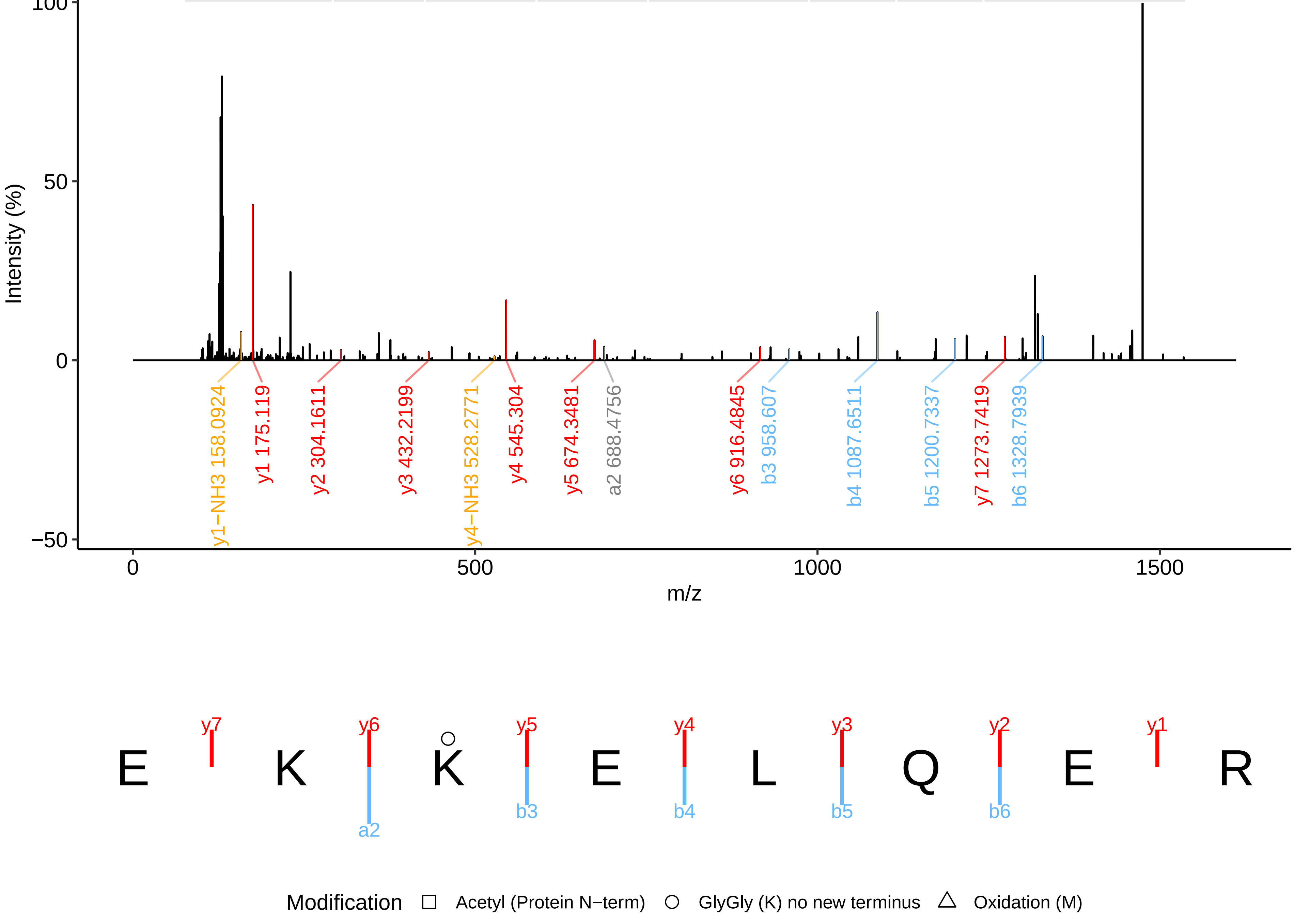

20200921_KIZ_MUb4_GlyGly_10plex_2 − Scan number: 3809

Gene Names	Charge	m/z	Mass	Retention time	PEP	Score	Precursor Intensity
EIF3A	4	294.161	1172.615	13.038	0.046978	85.161	669197.6

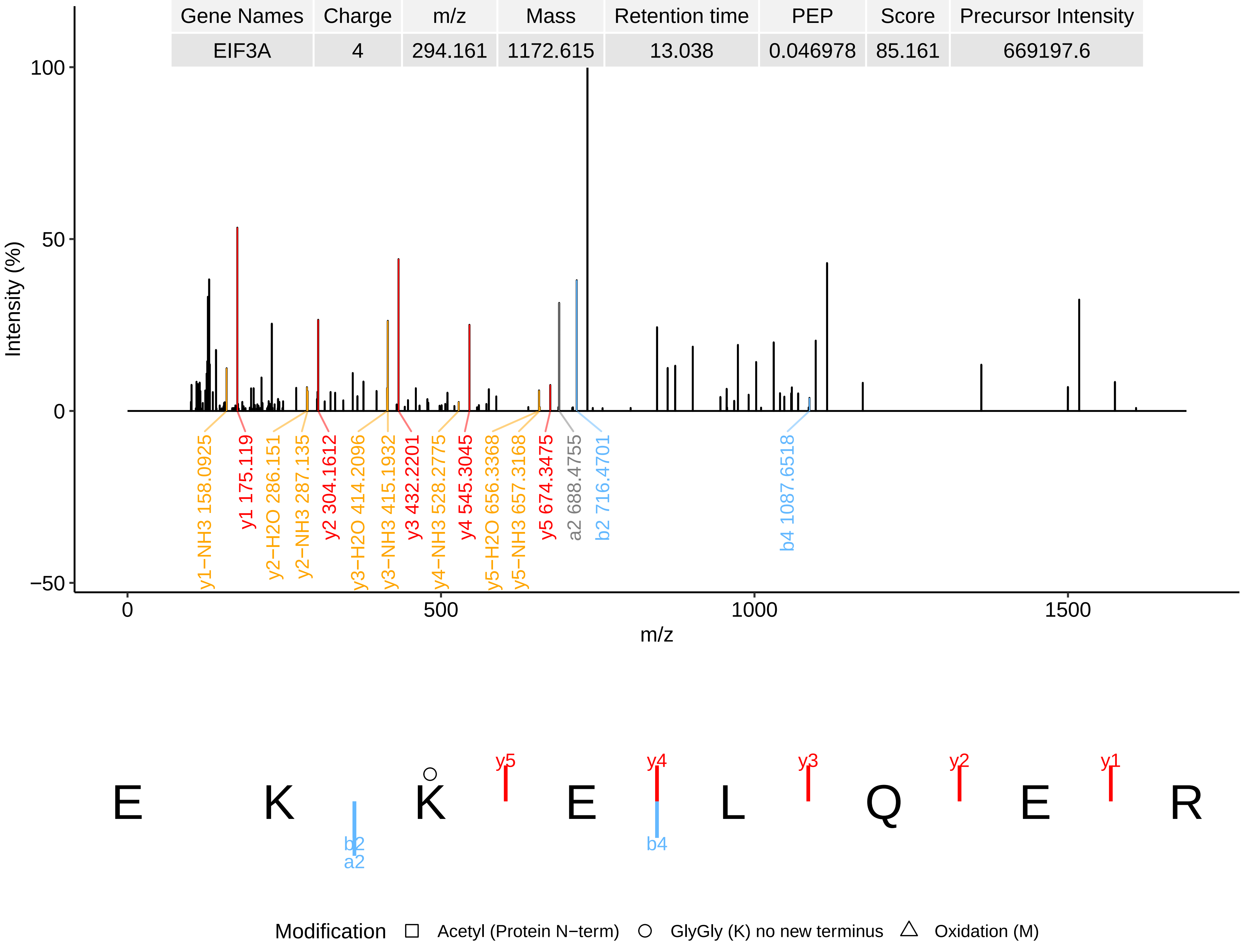

20200921_KIZ_MUb4_GlyGly_10plex − Scan number: 11003

Gene Names	Charge	m/z	Mass	Retention time	PEP	Score	Precursor Intensity
PABPC1;PABPC3	3	464.2528	1389.736	38.263	0.003542	79.07	2508828

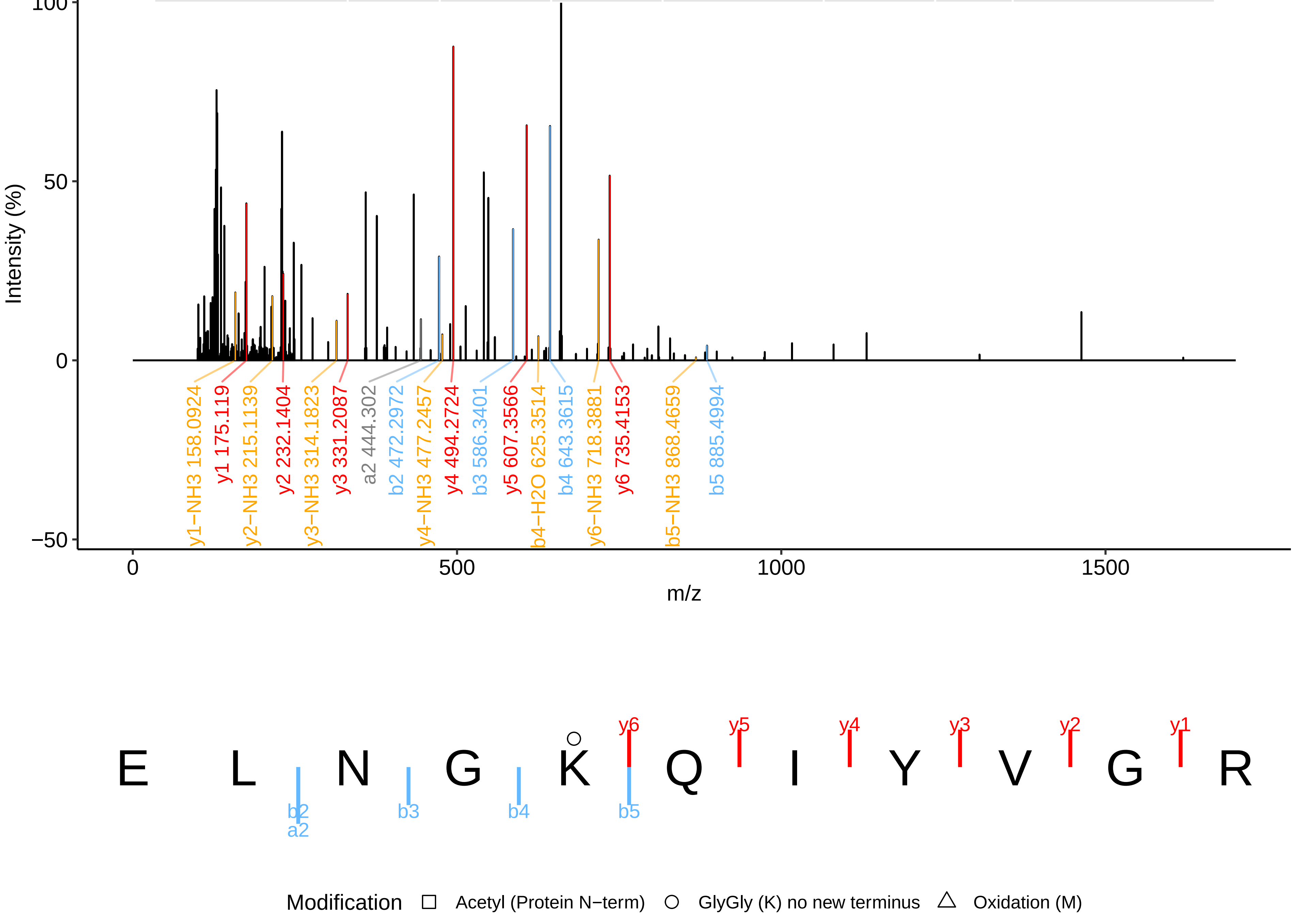

20200921_KIZ_MUb4_GlyGly_10plex − Scan number: 10236

Gene Names	Charge	m/z	Mass	Retention time	PEP	Score	Precursor Intensity
DNAJC13	3	475.272	1422.794	35.349	6.945e−09	130.63	4496641

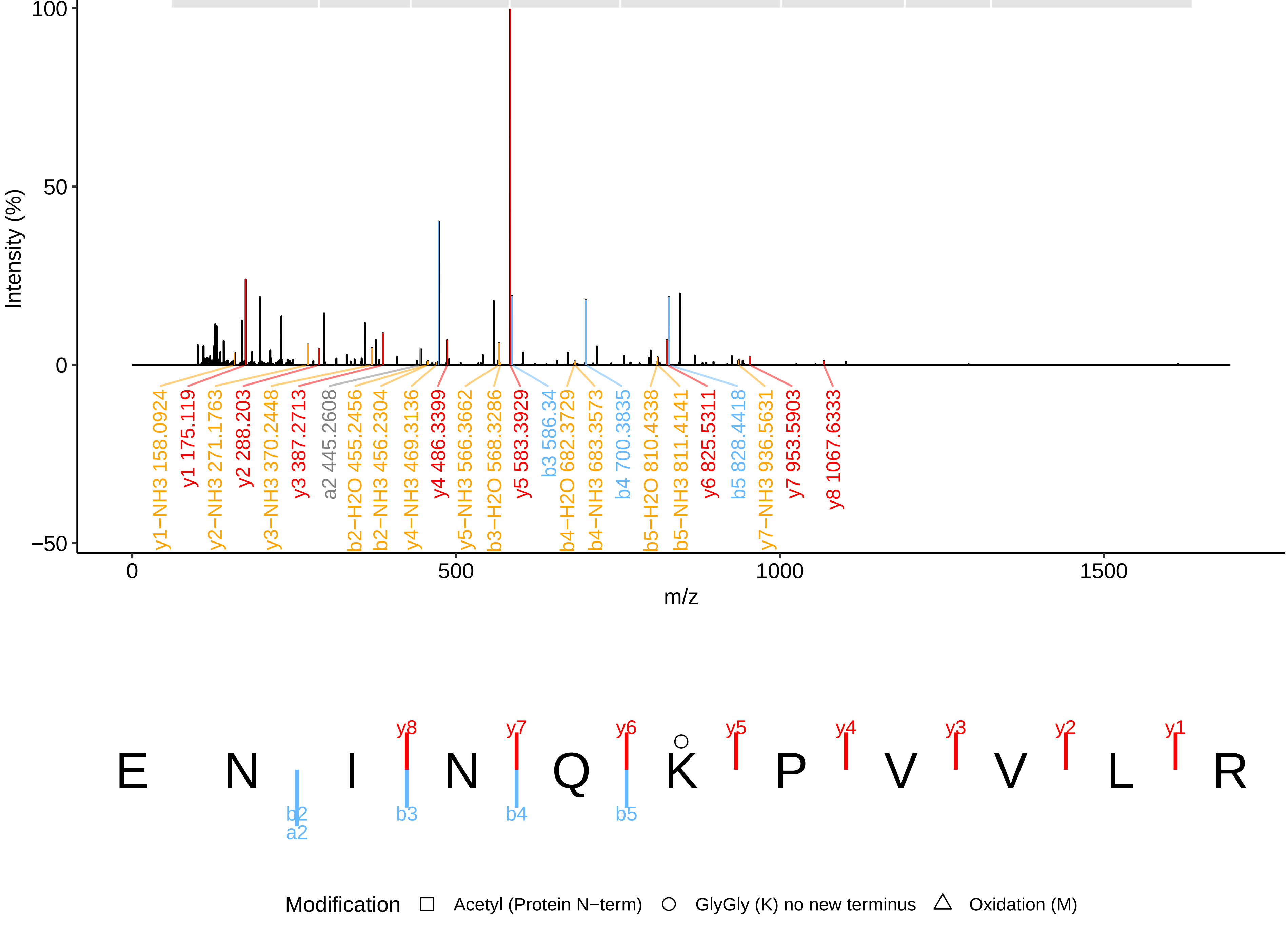

20200921_KIZ_MUb4_GlyGly_10plex_2 − Scan number: 9406

Gene Names	Charge	m/z	Mass	Retention time	PEP	Score	Precursor Intensity
DNAJC13	3	475.272	1422.794	35.741	0.011973	68.132	686521.2

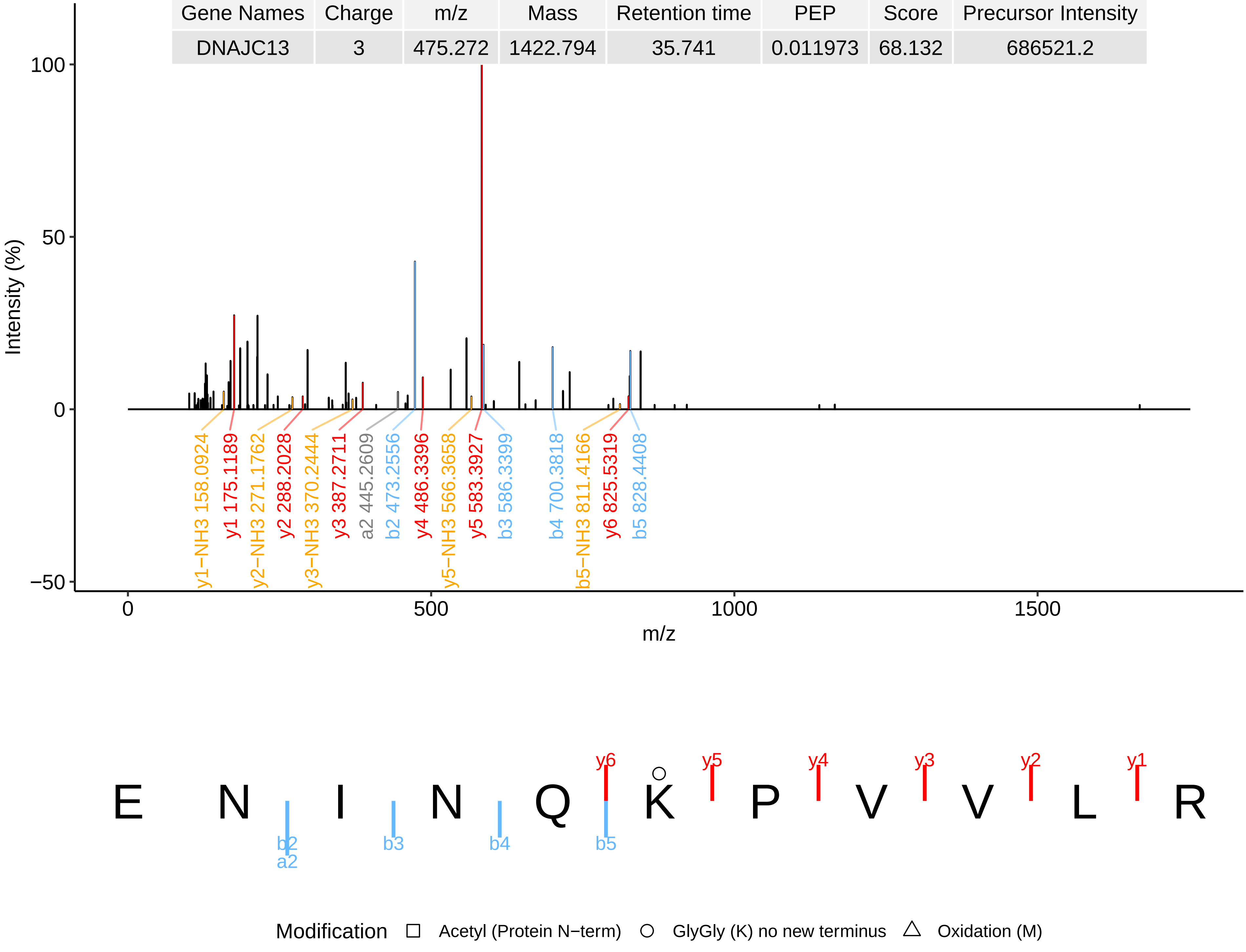

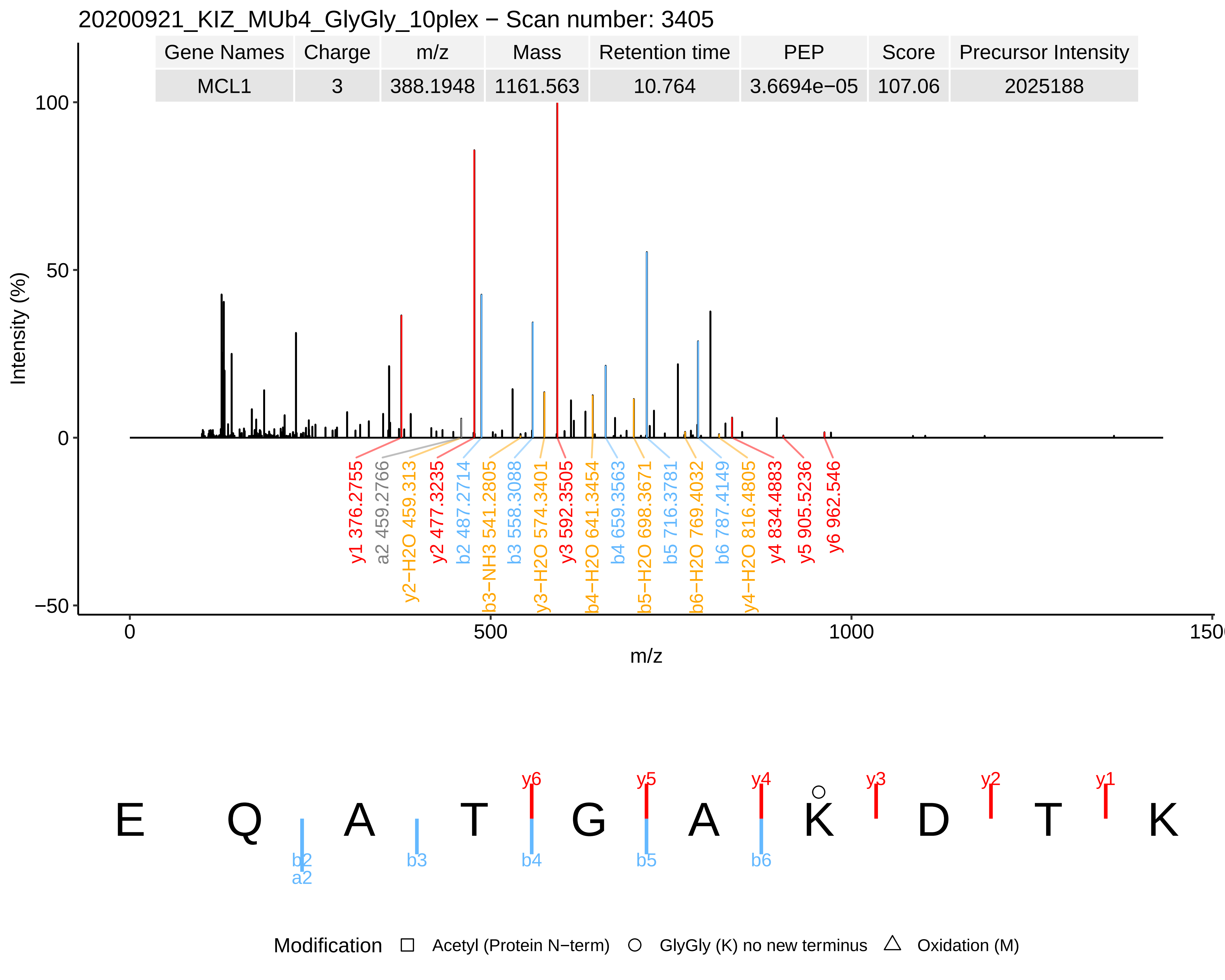
20200921_KIZ_MUb4_GlyGly_10plex − Scan number: 3405
Gene Names
Charge
m/z
Mass
Retention time
PEP
Score
Precursor Intensity
MCL1
3
388.1948
1161.563
10.764
3.6694e−05
107.06
2025188
Intensity (%)
m/z
y1 376.2755
a2 459.2766
y2−H2O 459.313
y2 477.3235
b2 487.2714
b3−NH3 541.2805
b3 558.3088
y3−H2O 574.3401
y3 592.3505
b4−H2O 641.3454
b4 659.3563
b5−H2O 698.3671
b5 716.3781
b6−H2O 769.4032
b6 787.4149
y4−H2O 816.4805
y4 834.4883
y5 905.5236
y6 962.546
E Q A T G A K D T K
y6 y5 y4 y3 y2 y1
b2 a2 b3 b4 b5 b6
Modification
Acetyl (Protein N−term)
GlyGly (K) no new terminus
Oxidation (M)

20200921_KIZ_MUb4_GlyGly_10plex_2 − Scan number: 3265

Gene Names	Charge	m/z	Mass	Retention time	PEP	Score	Precursor Intensity
MCL1	3	388.1948	1161.563	10.881	0.0018395	85.813	1915729

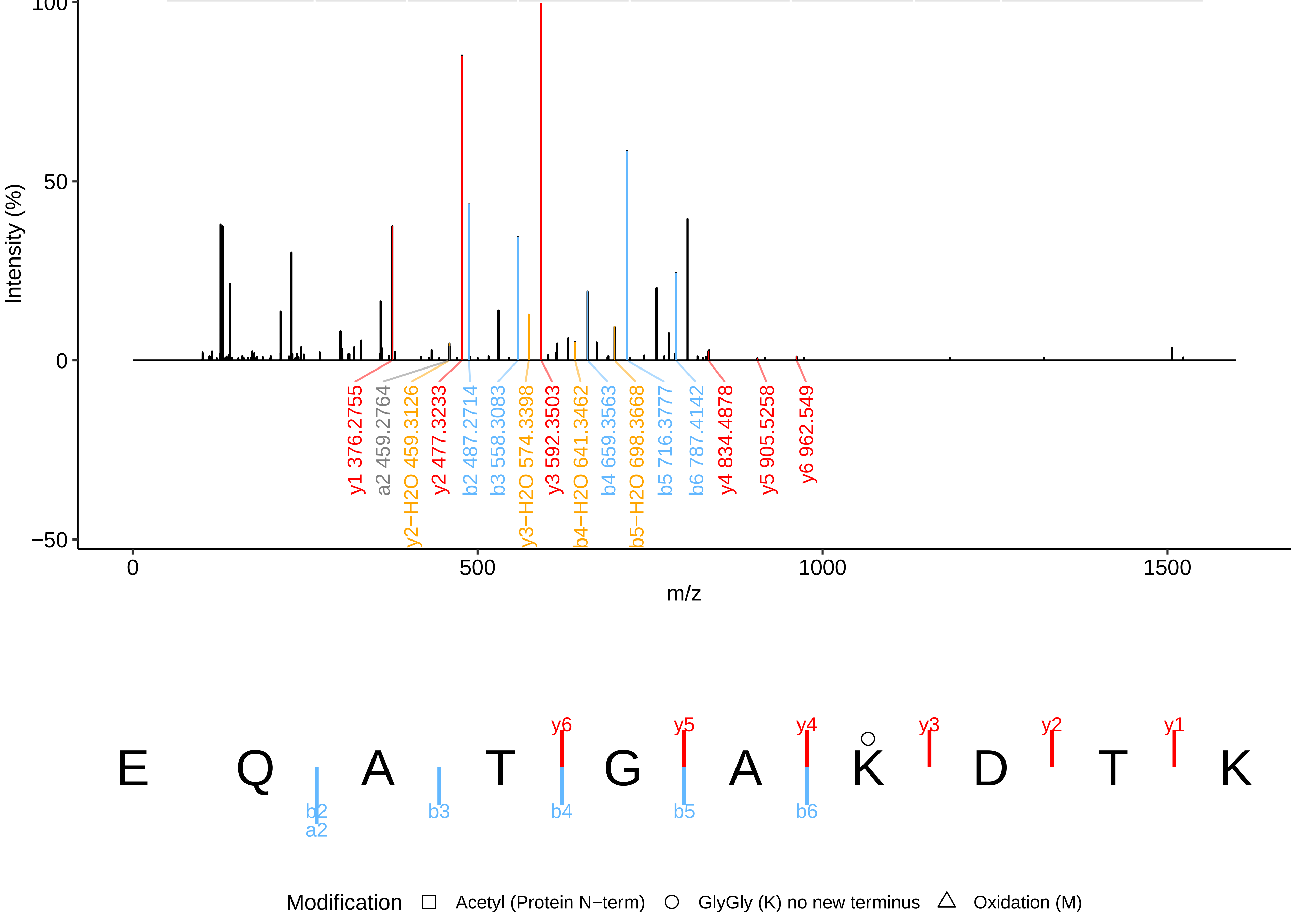

20200924_KIZ_MUb5_GlyGly_1 − Scan number: 3158

Gene Names	Charge	m/z	Mass	Retention time	PEP	Score	Precursor Intensity
MCL1	3	388.1948	1161.563	9.7437	0.00033194	96.229	19058458

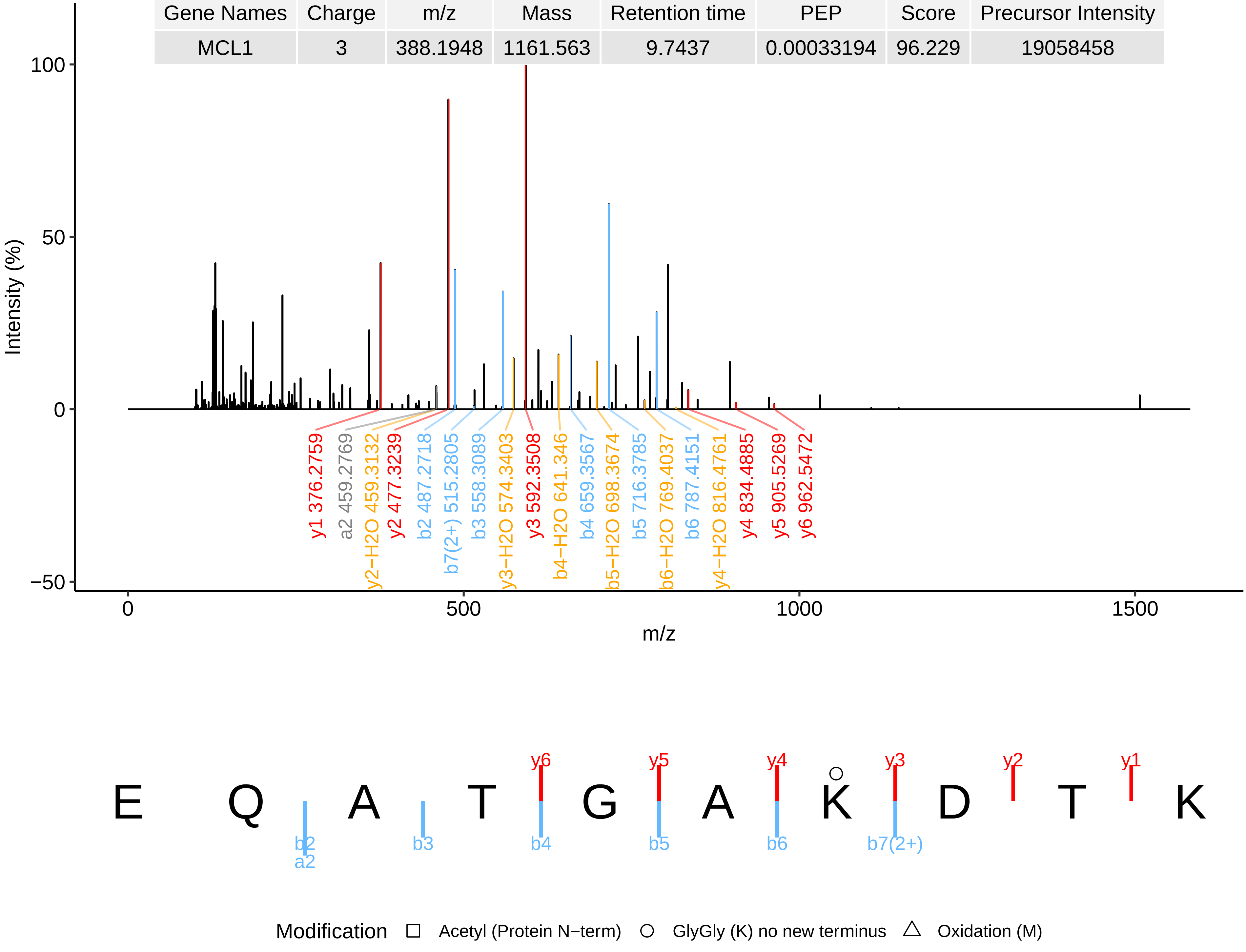

20200924_KIZ_MUb5_GlyGly_2 − Scan number: 2942

Gene Names	Charge	m/z	Mass	Retention time	PEP	Score	Precursor Intensity
MCL1	3	388.1948	1161.563	10.55	0.0011013	90.913	1954551

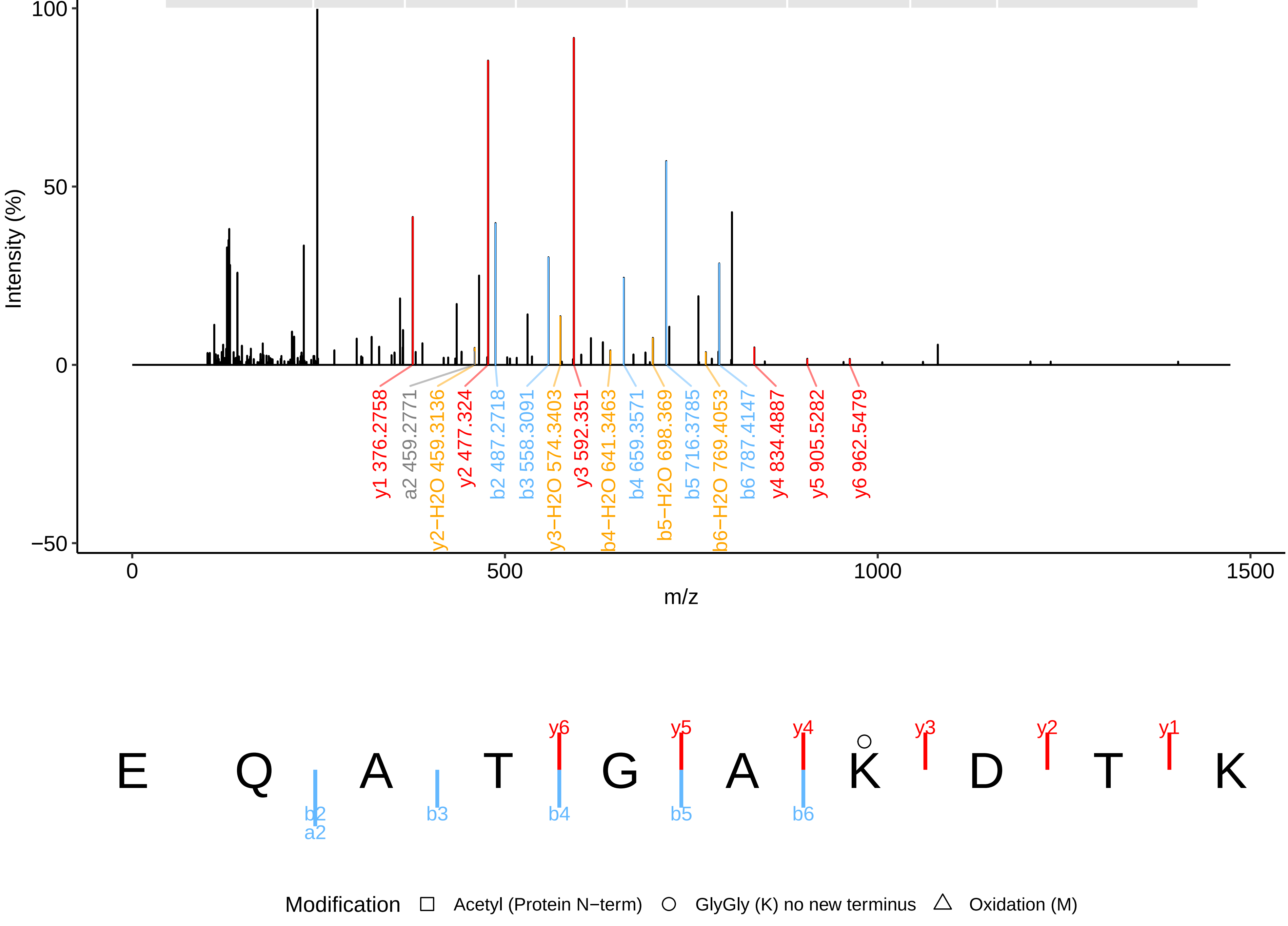

20200921_KIZ_MUb4_GlyGly_10plex_2 − Scan number: 10780

Gene Names	Charge	m/z	Mass	Retention time	PEP	Score	Precursor Intensity
TNIP1	3	467.9159	1400.726	41.317	0.0045248	77.185	1165211

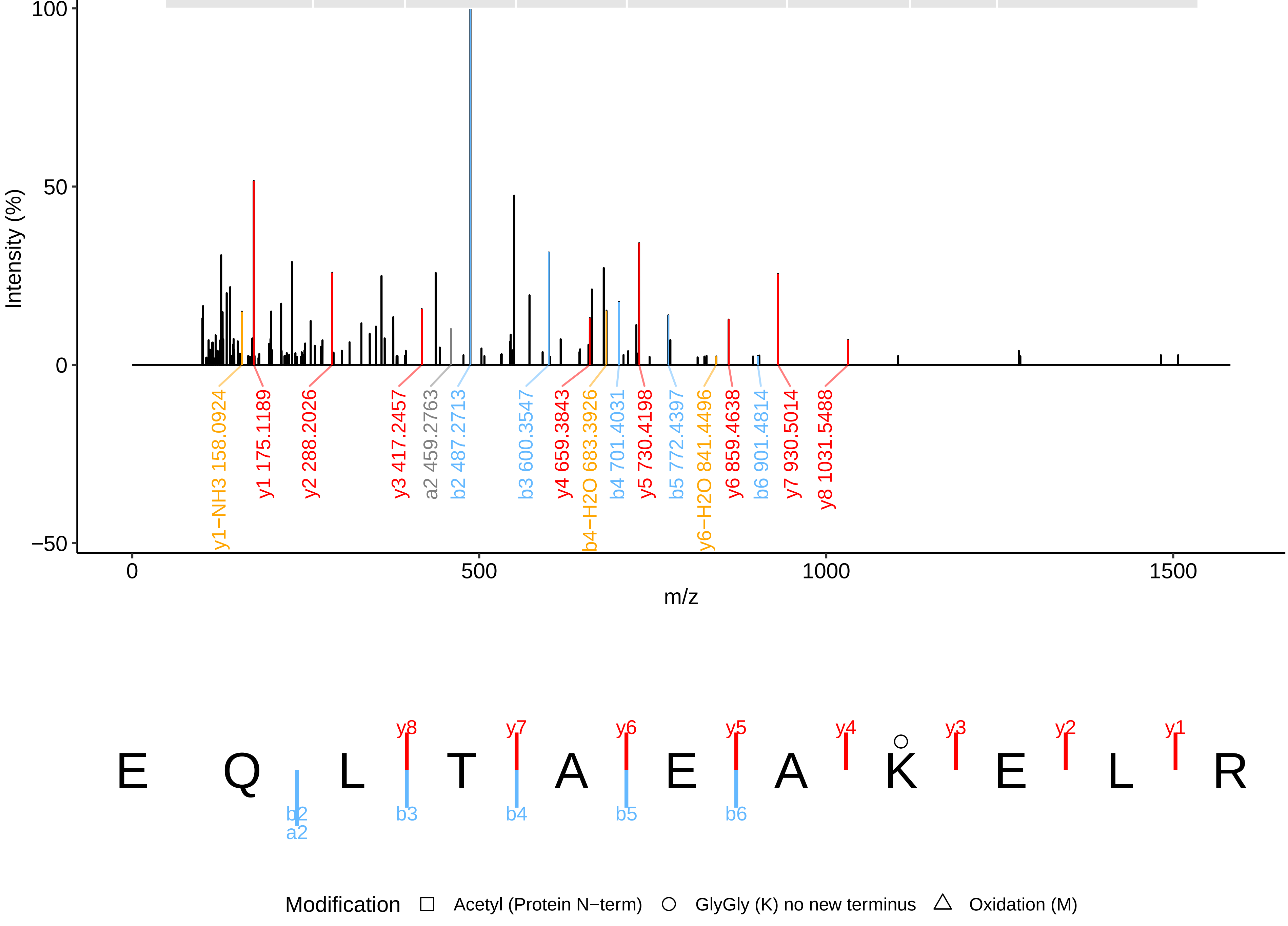

20200921_KIZ_MUb4_GlyGly_10plex − Scan number: 13424

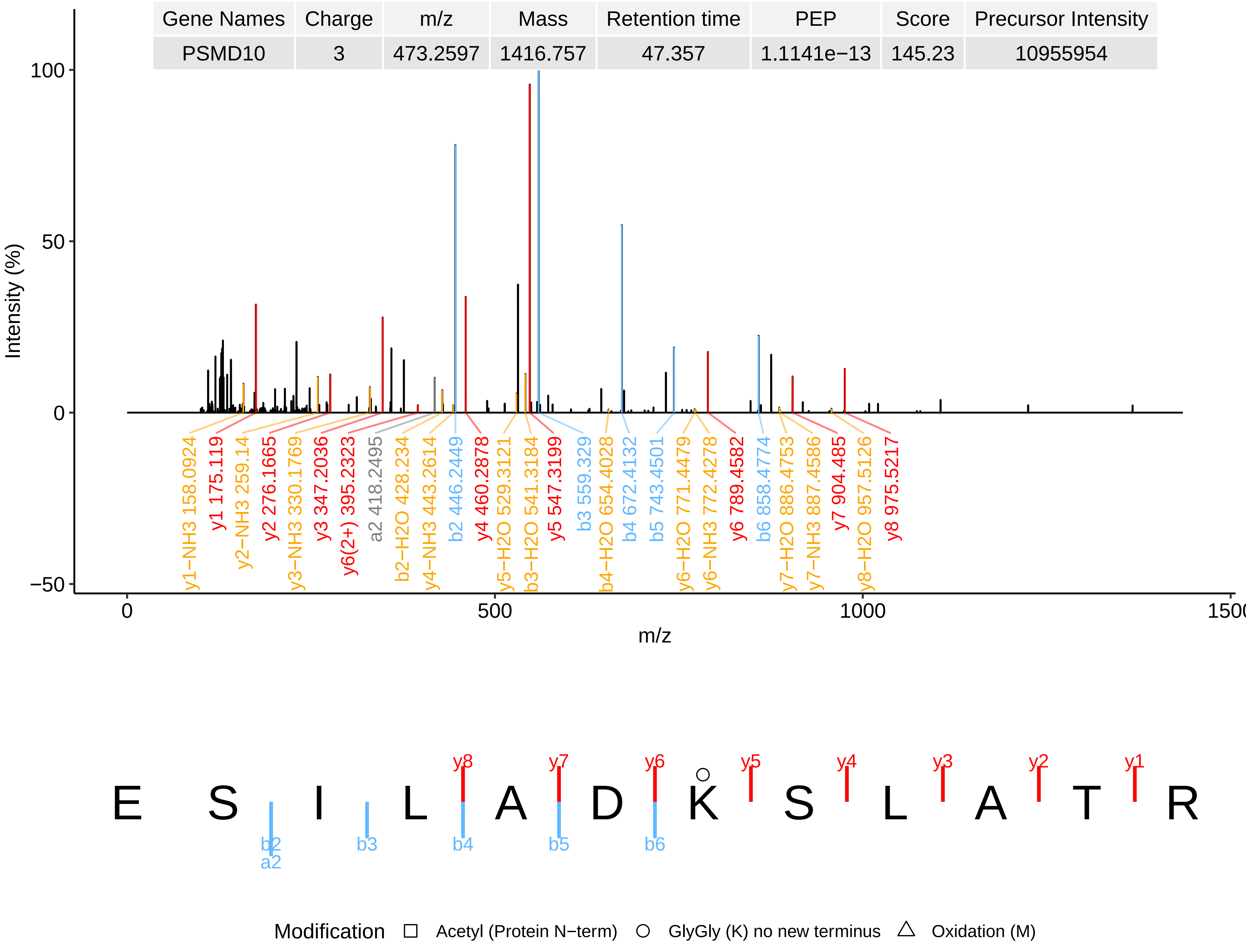

20200921_KIZ_MUb4_GlyGly_10plex_2 − Scan number: 12346

Gene Names	Charge	m/z	Mass	Retention time	PEP	Score	Precursor Intensity
PSMD10	3	473.2597	1416.757	47.746	1.2377e−08	117.79	1038388

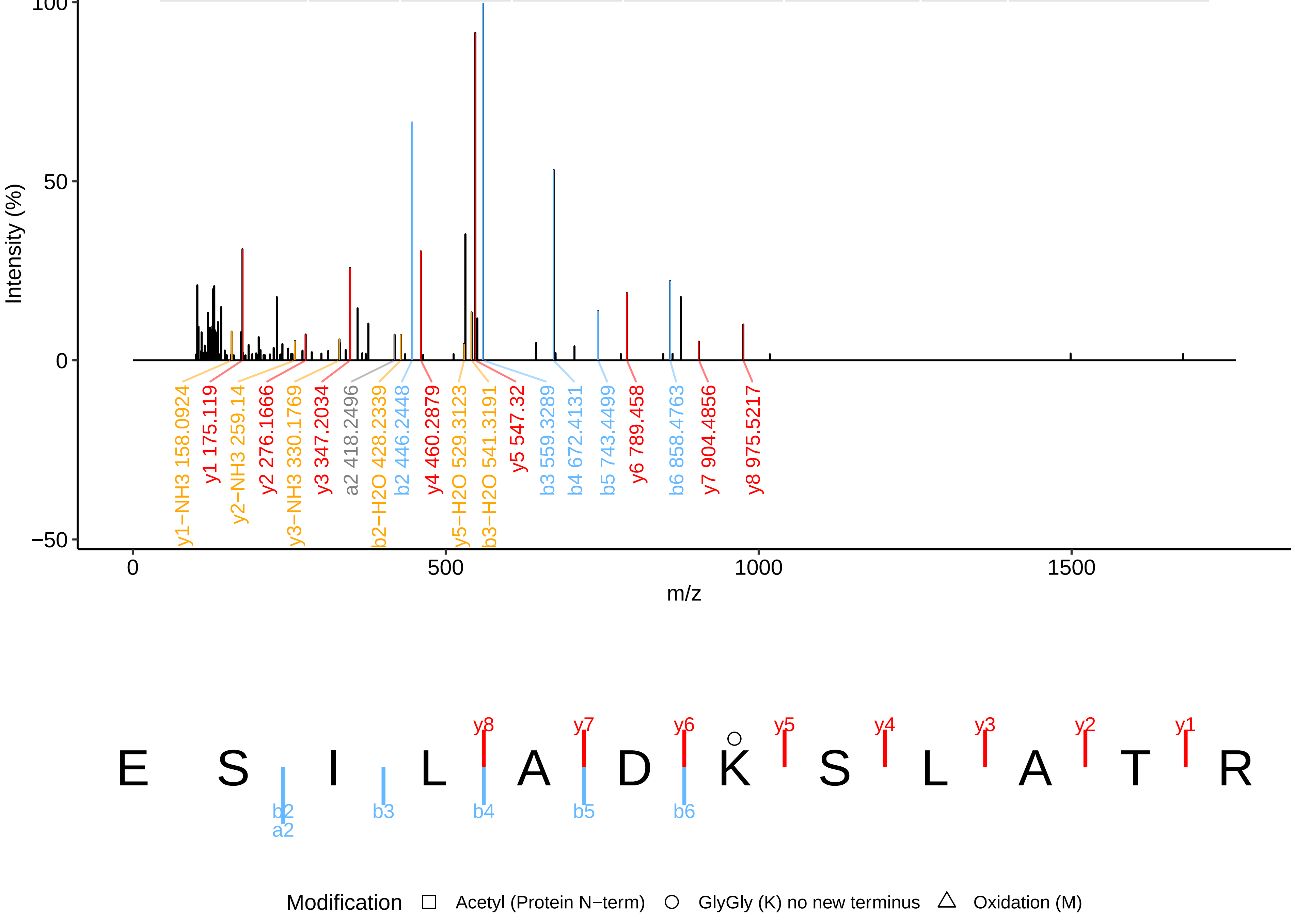

20200924_KIZ_MUb5_GlyGly_1 − Scan number: 12360

Gene Names	Charge	m/z	Mass	Retention time	PEP	Score	Precursor Intensity
PSMD10	3	473.2597	1416.757	40.21	0.0078824	70.622	9301506

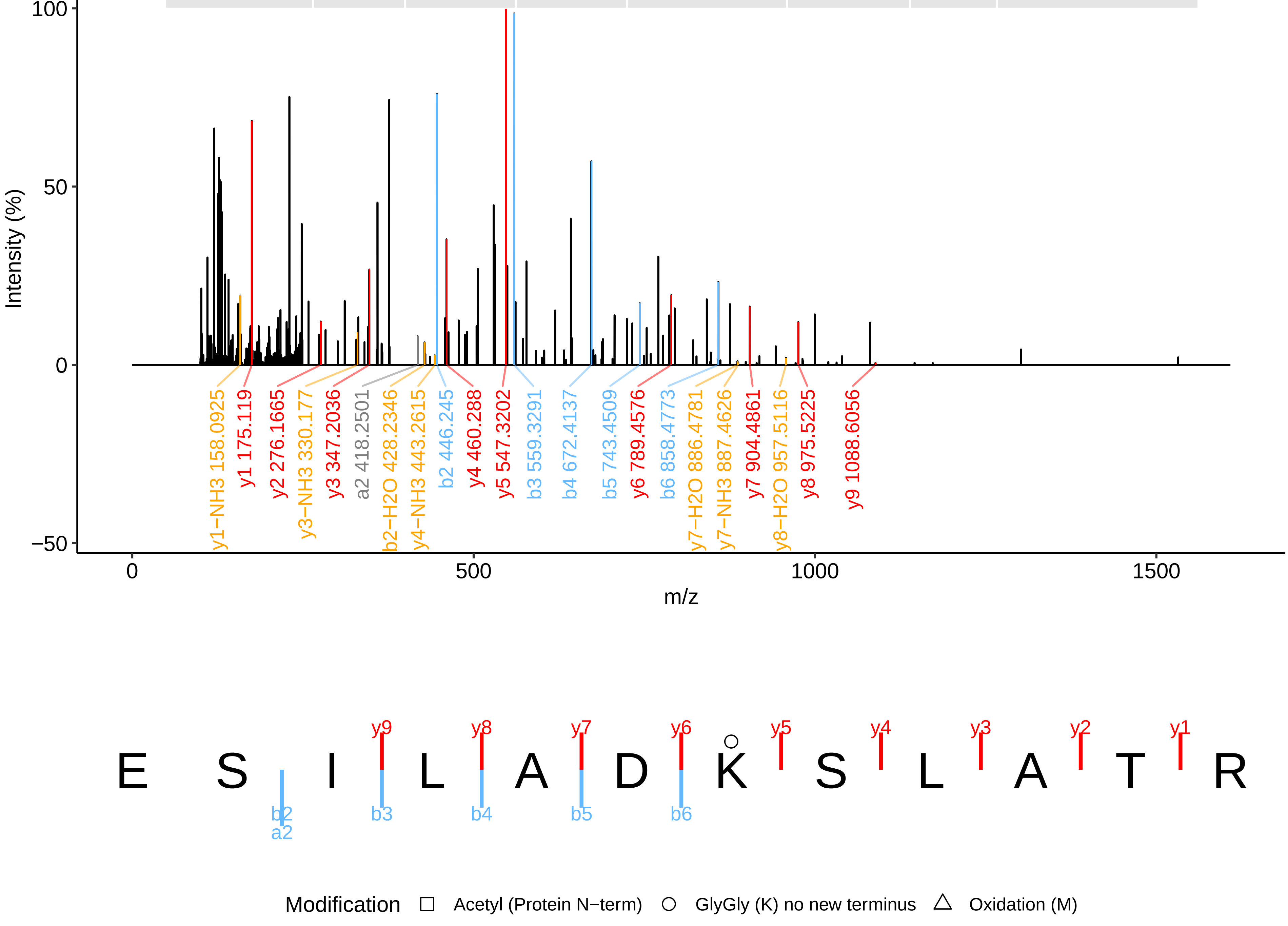

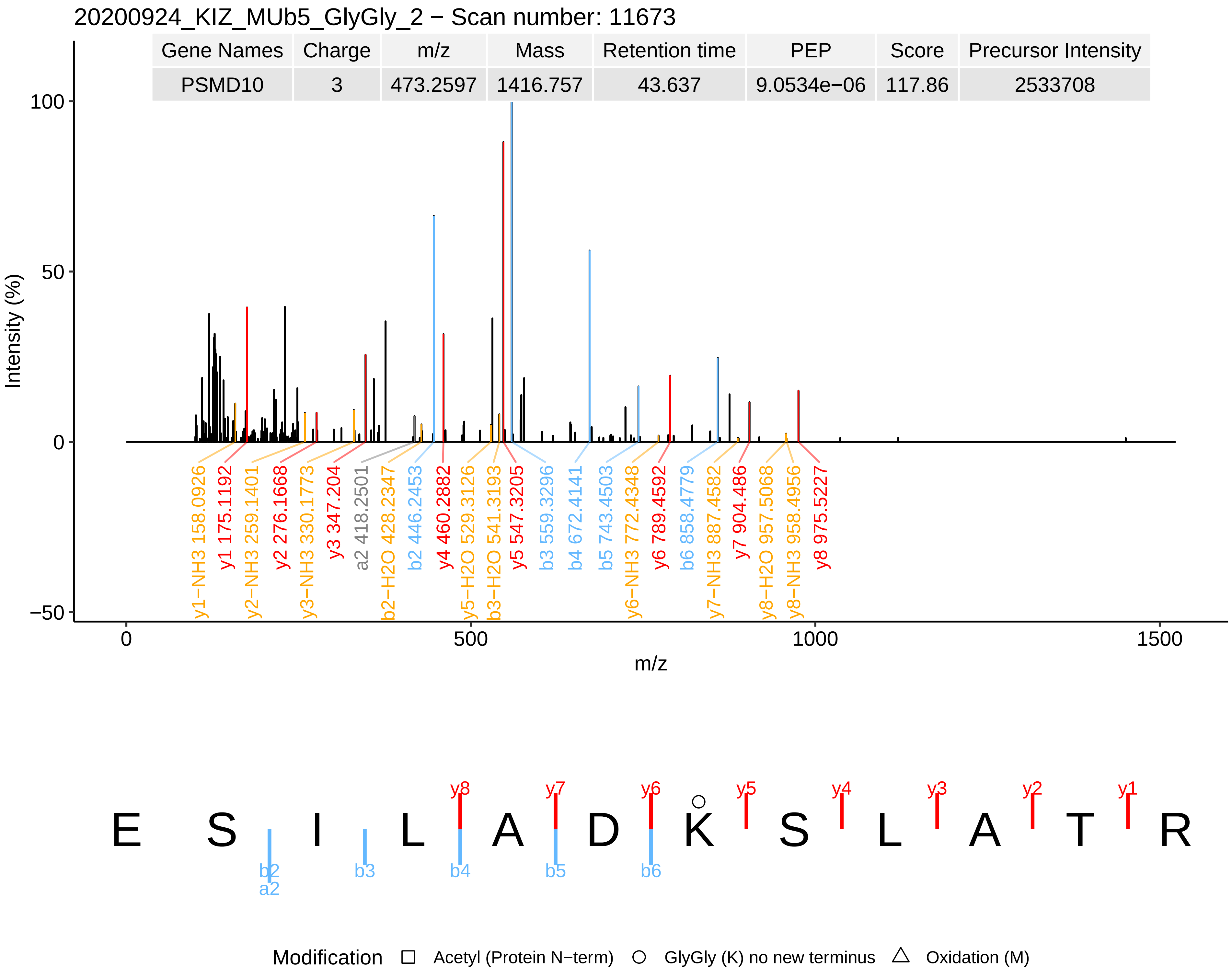

20200924_KIZ_MUb5_GlyGly_2 − Scan number: 11673
Gene Names | Charge | m/z | Mass | Retention time | PEP | Score | Precursor Intensity
PSMD10 | 3 | 473.2597 | 1416.757 | 43.637 | 9.0534e−06 | 117.86 | 2533708
Intensity (%)
m/z
y1−NH3 158.0926
y1 175.1192
y2−NH3 259.1401
y2 276.1668
y3−NH3 330.1773
y3 347.204
a2 418.2501
b2−H2O 428.2347
b2 446.2453
y4 460.2882
y5−H2O 529.3126
b3−H2O 541.3193
y5 547.3205
b3 559.3296
b4 672.4141
b5 743.4503
y6−NH3 772.4348
y6 789.4592
b6 858.4779
y7−NH3 887.4582
y7 904.486
y8−H2O 957.5068
y8−NH3 958.4956
y8 975.5227
E S I L A D K S L A T R
y8 y7 y6 y5 y4 y3 y2 y1
b2 a2 b3 b4 b5 b6
Modification
Acetyl (Protein N−term)
GlyGly (K) no new terminus
Oxidation (M)

20200921_KIZ_MUb4_GlyGly_10plex − Scan number: 6995

Gene Names	Charge	m/z	Mass	Retention time	PEP	Score	Precursor Intensity
AP2S1	3	383.2154	1146.624	23.351	0.0029587	83.948	10210415

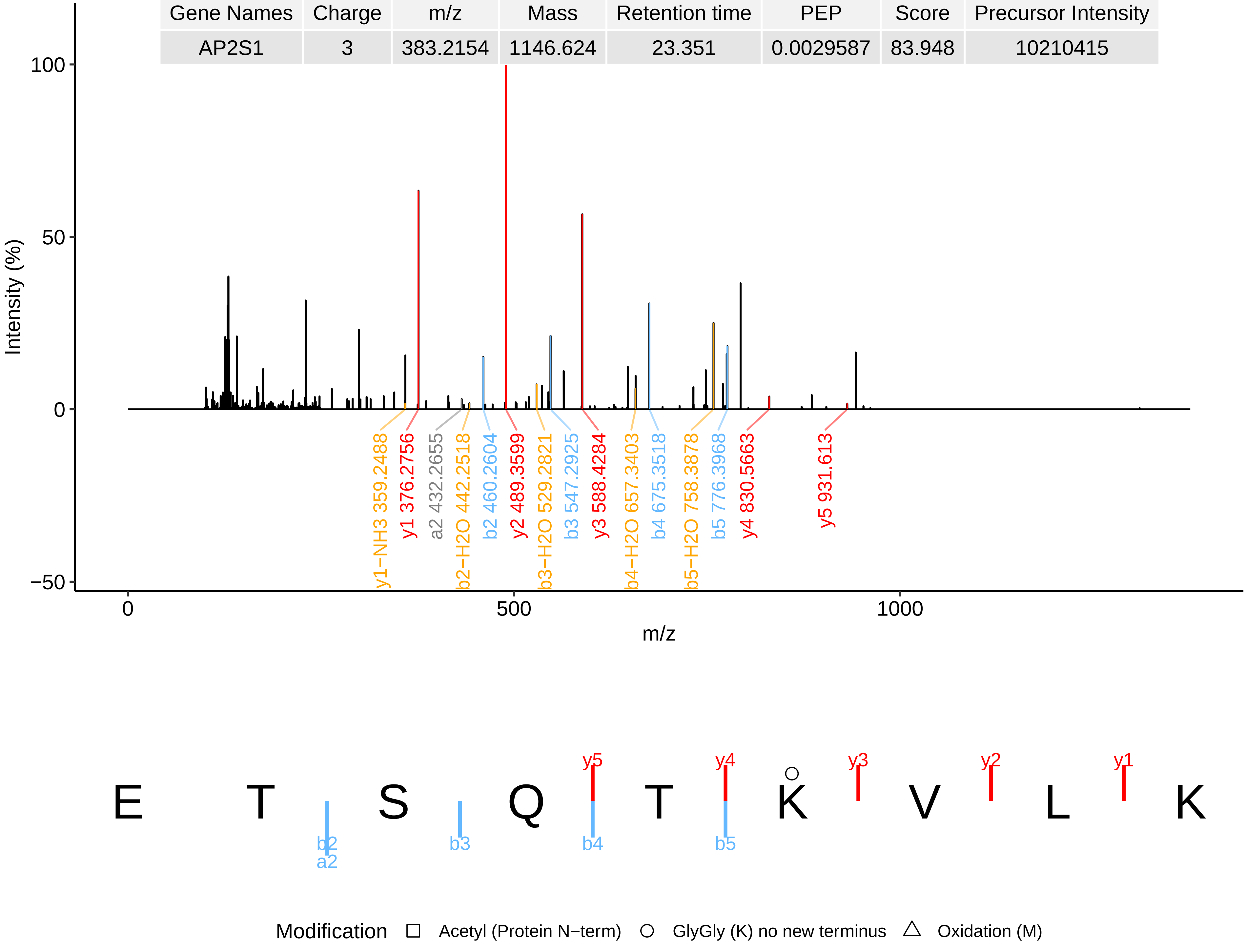

20200921_KIZ_MUb4_GlyGly_10plex_2 − Scan number: 6484

Gene Names	Charge	m/z	Mass	Retention time	PEP	Score	Precursor Intensity
AP2S1	3	383.2154	1146.624	23.847	0.018776	66.27	1057390

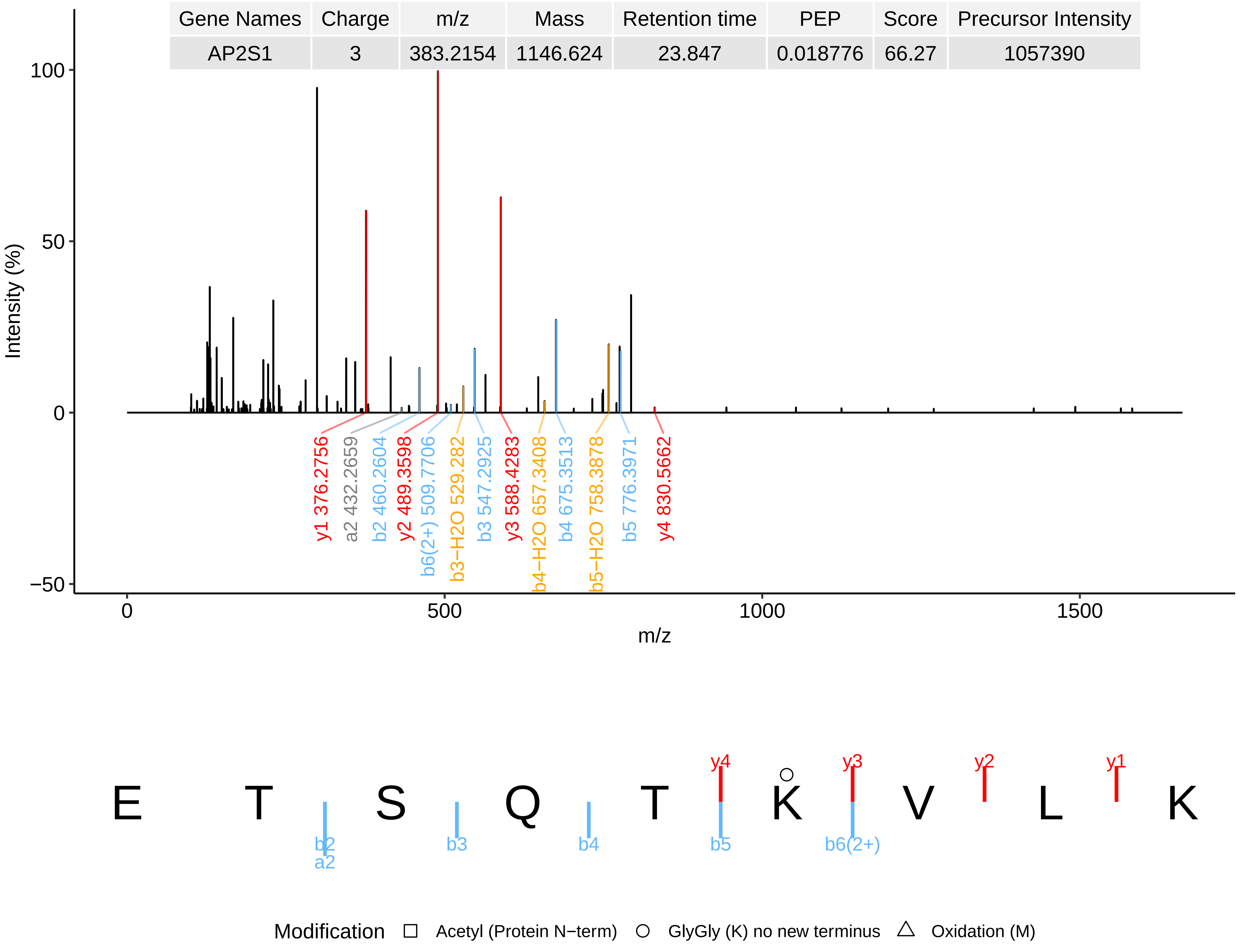

20200924_KIZ_MUb5_GlyGly_1 − Scan number: 6066

Gene Names	Charge	m/z	Mass	Retention time	PEP	Score	Precursor Intensity
AP2S1	3	383.2154	1146.624	19.003	0.059708	58.433	6561953

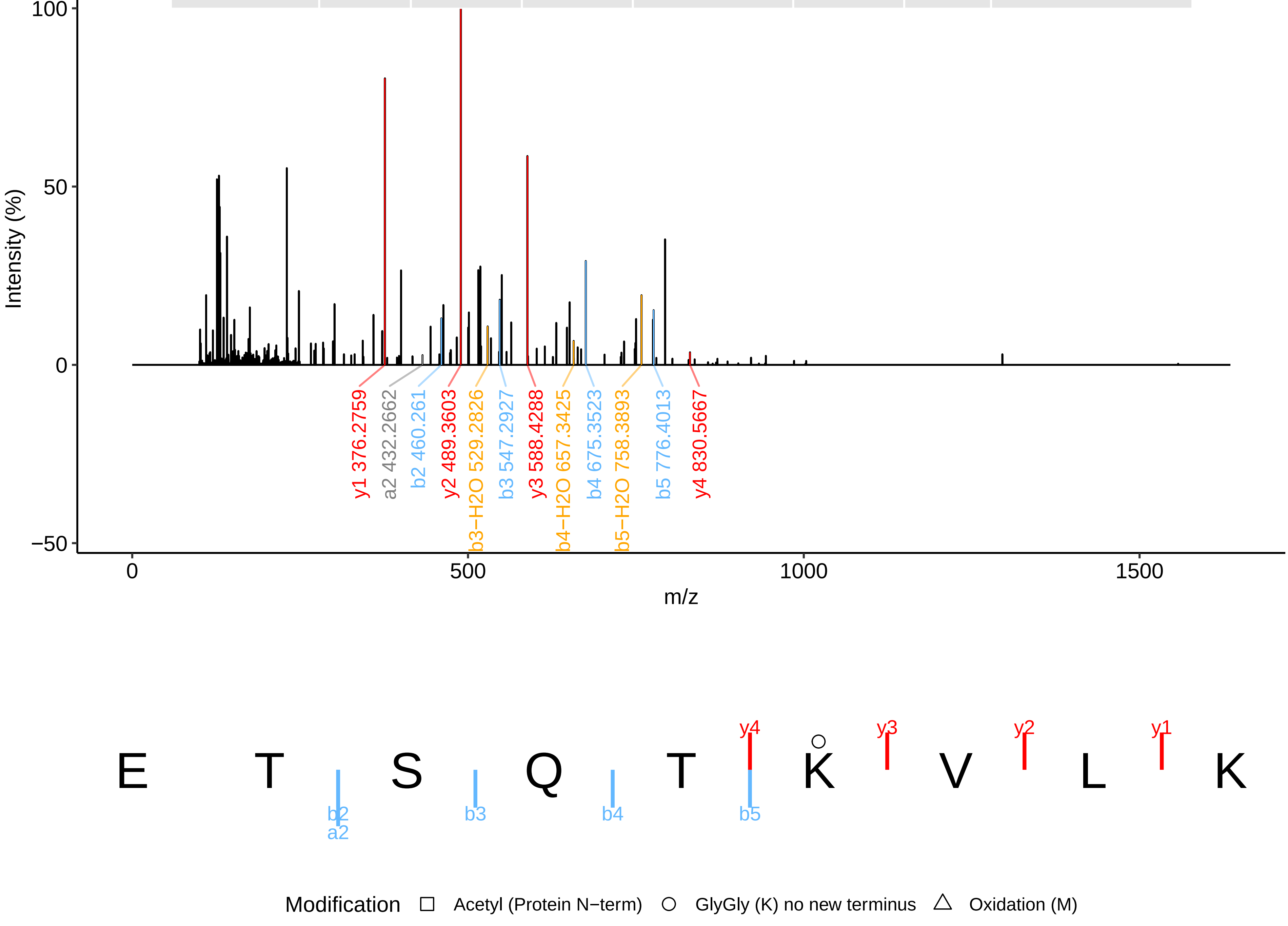

20200924_KIZ_MUb5_GlyGly_2 − Scan number: 7209

Gene Names	Charge	m/z	Mass	Retention time	PEP	Score	Precursor Intensity
CPS1	3	473.8893	1418.646	26.891	0.050242	58.098	NA

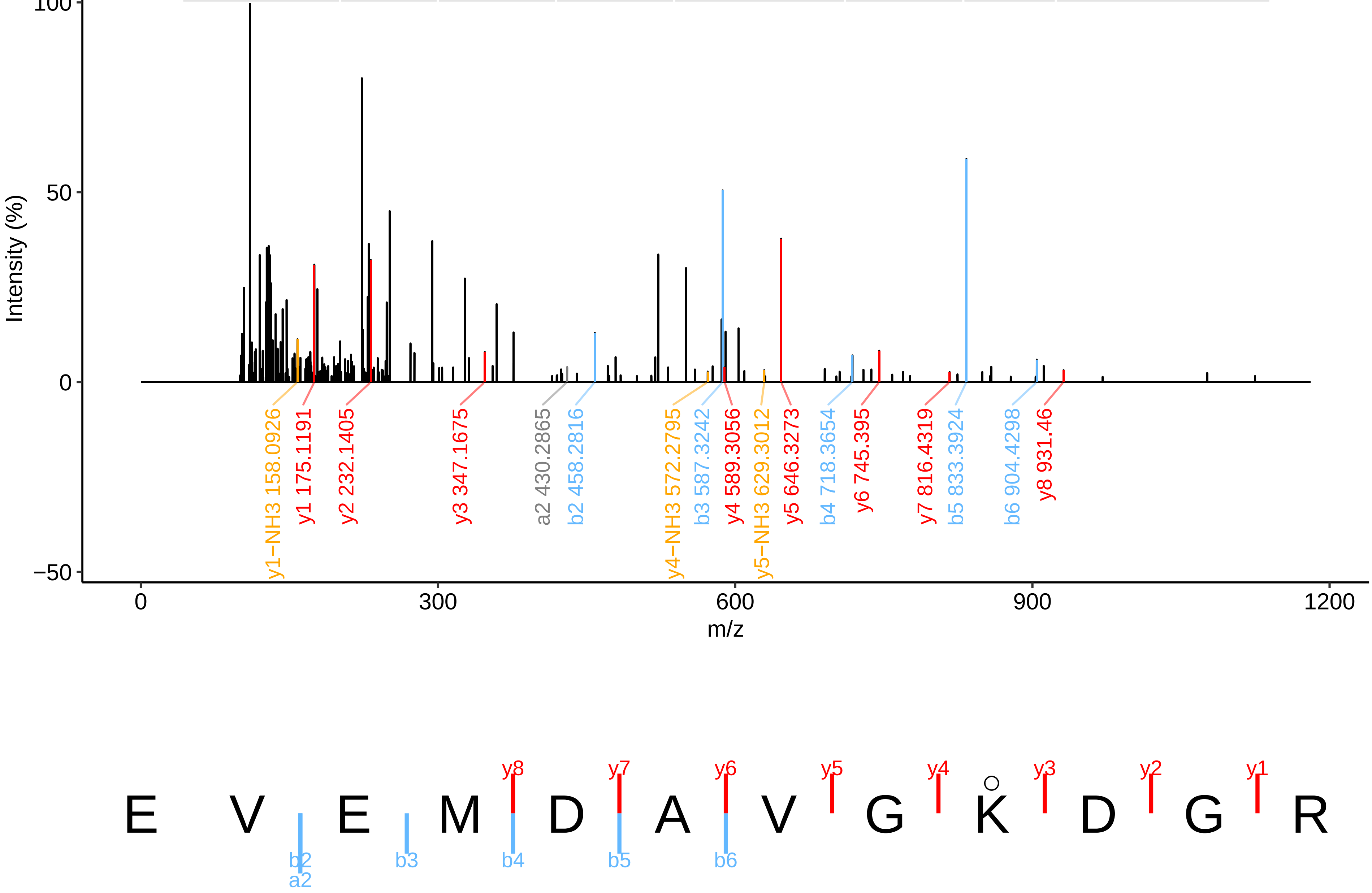

Modification □ Acetyl (Protein N−term) ○ GlyGly (K) no new terminus △ Oxidation (M)

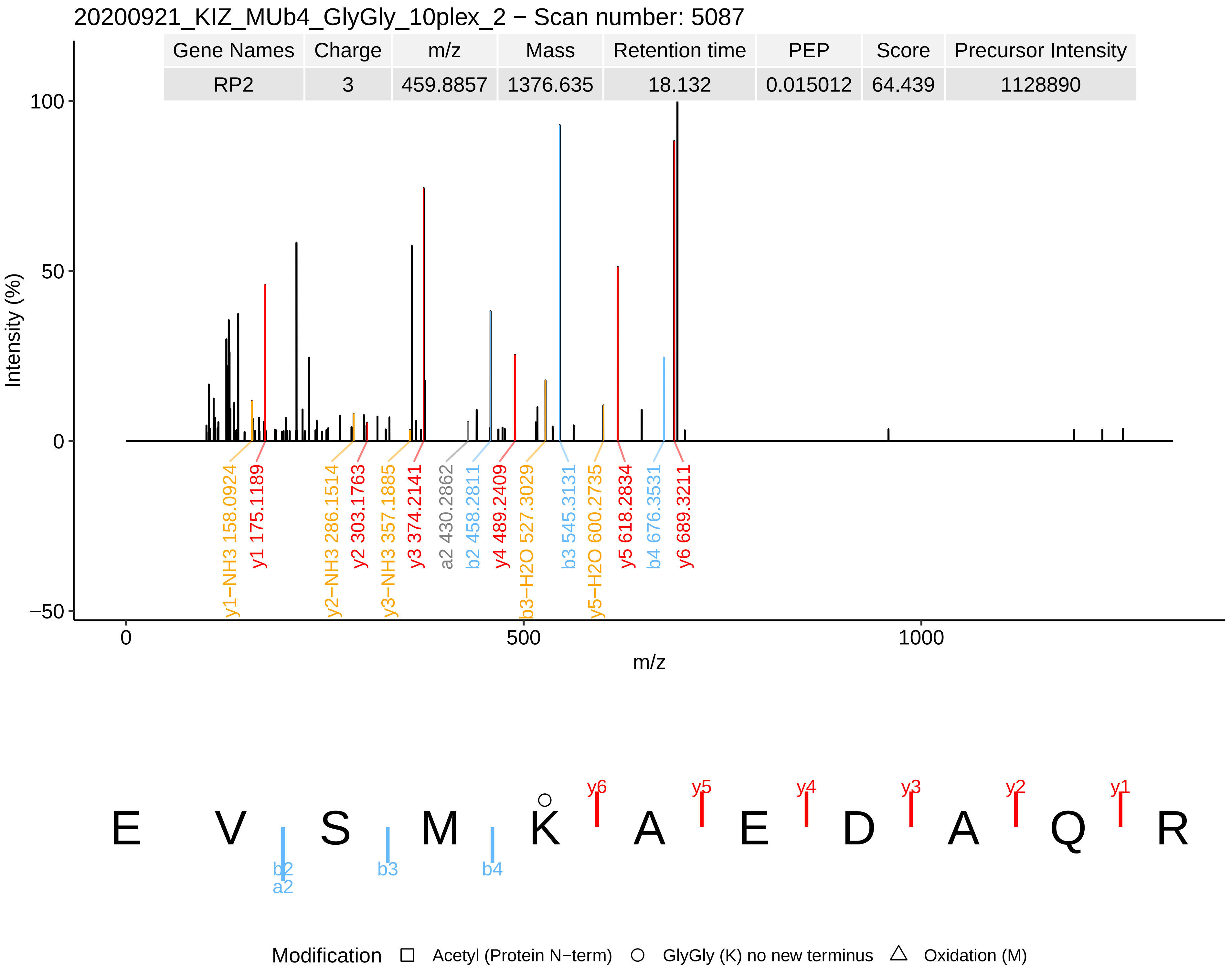
20200921_KIZ_MUb4_GlyGly_10plex_2 − Scan number: 5087
Gene Names | Charge | m/z | Mass | Retention time | PEP | Score | Precursor Intensity
RP2 | 3 | 459.8857 | 1376.635 | 18.132 | 0.015012 | 64.439 | 1128890
Intensity (%)
100
50
0
−50
0
500
1000
m/z
y1−NH3 158.0924
y1 175.1189
y2−NH3 286.1514
y2 303.1763
y3−NH3 357.1885
y3 374.2141
a2 430.2862
b2 458.2811
y4 489.2409
b3−H2O 527.3029
b3 545.3131
y5−H2O 600.2735
y5 618.2834
b4 676.3531
y6 689.3211
E V S M K A E D A Q R
y6 y5 y4 y3 y2 y1
b2 a2 b3 b4
Modification
Acetyl (Protein N−term)
GlyGly (K) no new terminus
Oxidation (M)

20200924_KIZ_MUb5_GlyGly_1 − Scan number: 4878

Gene Names	Charge	m/z	Mass	Retention time	PEP	Score	Precursor Intensity
RP2	3	459.8857	1376.635	15.103	0.0061661	76.843	5775762

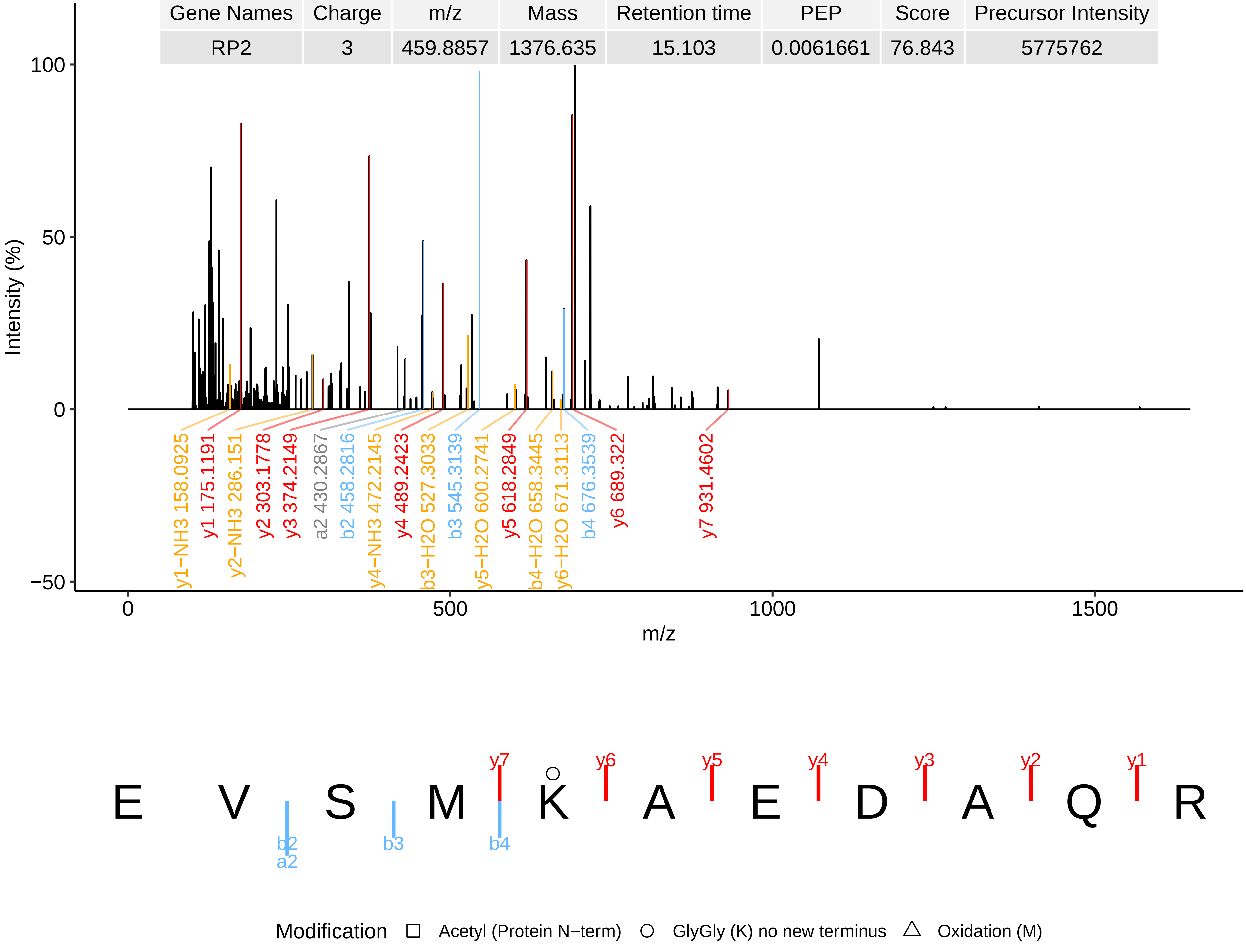

20200924_KIZ_MUb5_GlyGly_2 − Scan number: 4496

Gene Names	Charge	m/z	Mass	Retention time	PEP	Score	Precursor Intensity
RP2	3	459.8857	1376.635	16.507	0.036921	60.691	1852928

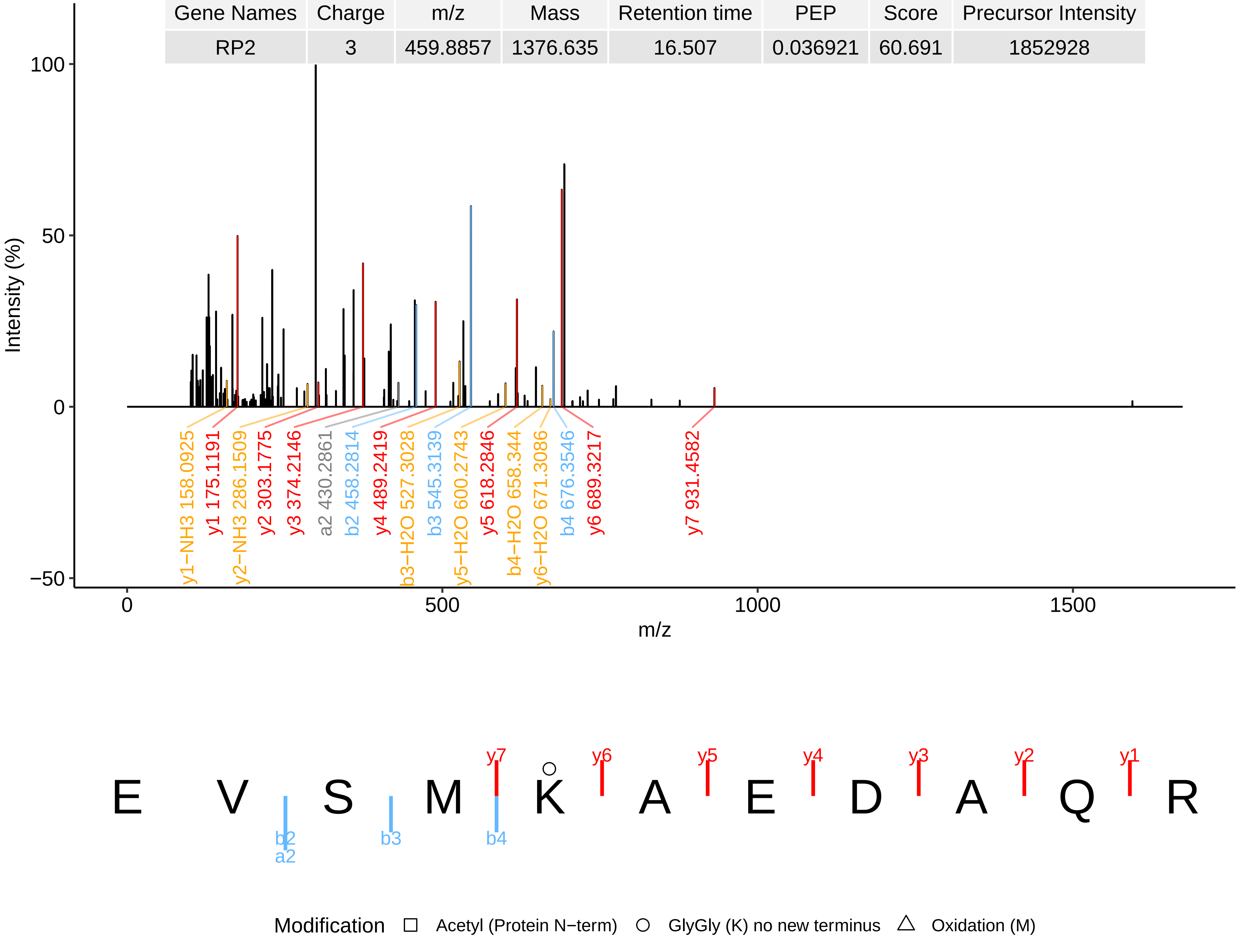

20200921_KIZ_MUb4_GlyGly_10plex − Scan number: 16231

Gene Names	Charge	m/z	Mass	Retention time	PEP	Score	Precursor Intensity
ATP1B1	3	389.2049	1164.593	58.238	0.010629	102.51	2964062

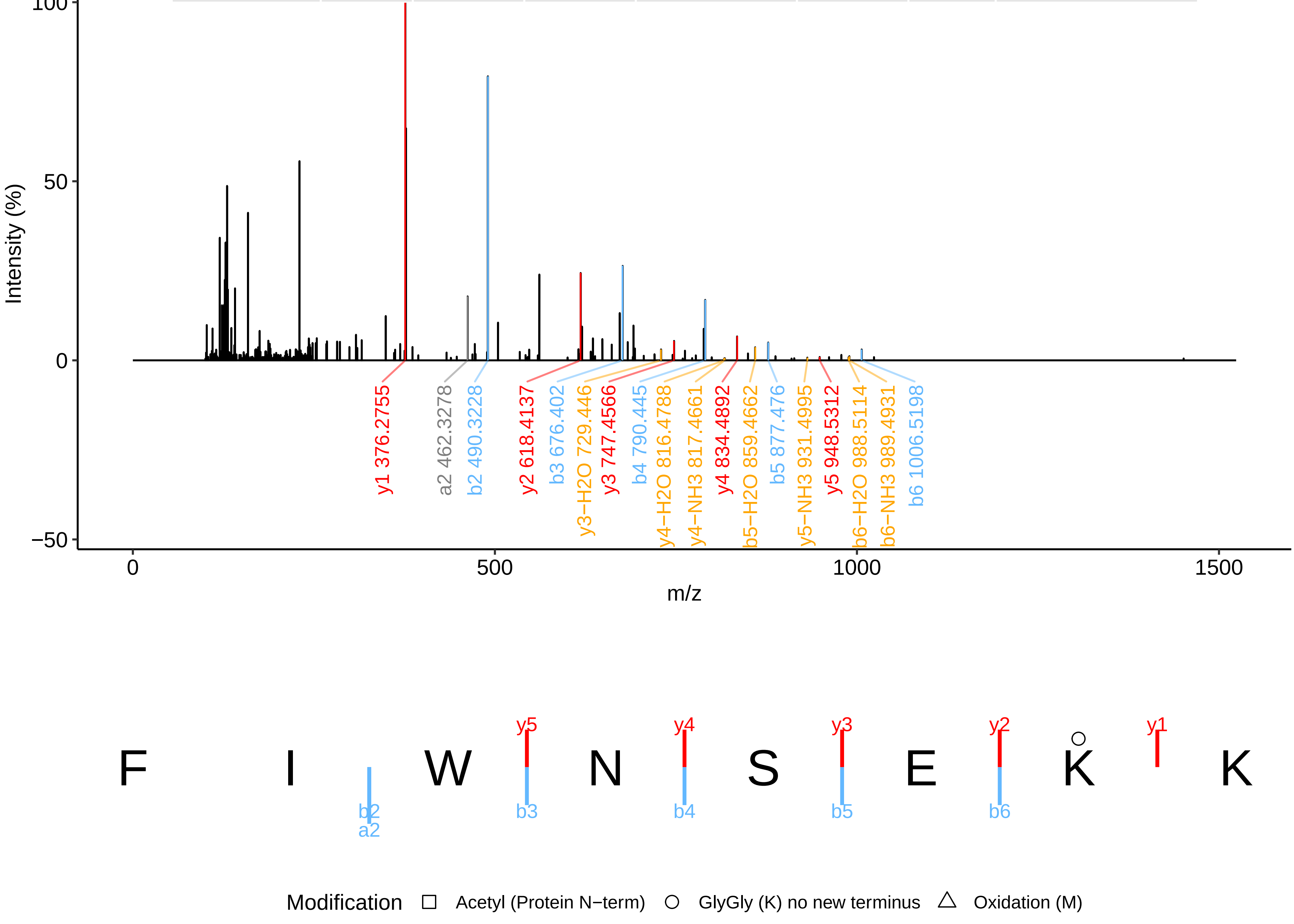

20200921_KIZ_MUb4_GlyGly_10plex − Scan number: 15639

Gene Names	Charge	m/z	Mass	Retention time	PEP	Score	Precursor Intensity
PPP2R1A;PPP2R1B	3	383.5523	1147.635	55.921	0.13701	47.603	3312010

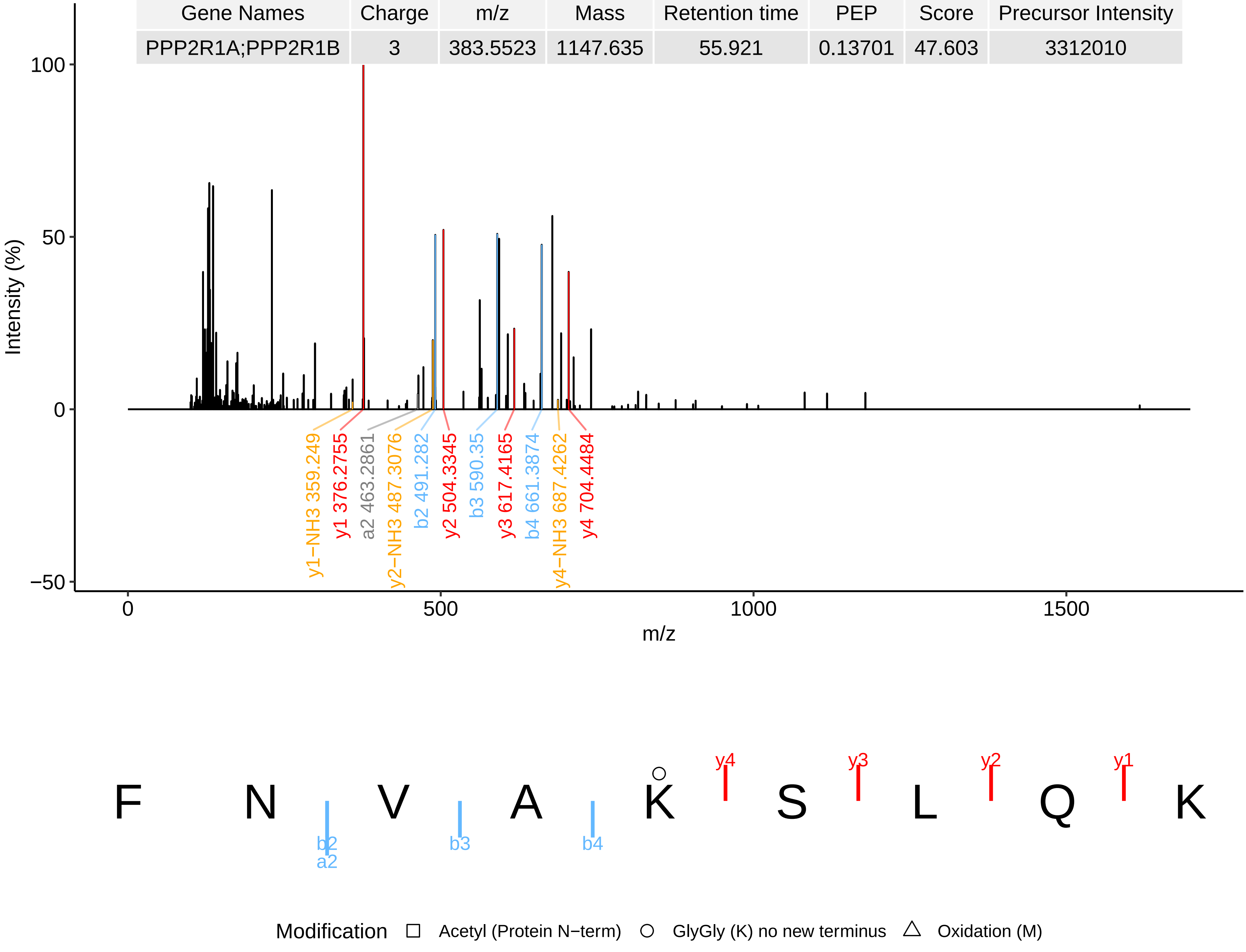

20200921_KIZ_MUb4_GlyGly_10plex − Scan number: 14150

Gene Names	Charge	m/z	Mass	Retention time	PEP	Score	Precursor Intensity
ADRM1	3	466.5772	1396.71	50.173	0.0012209	85.821	45031344

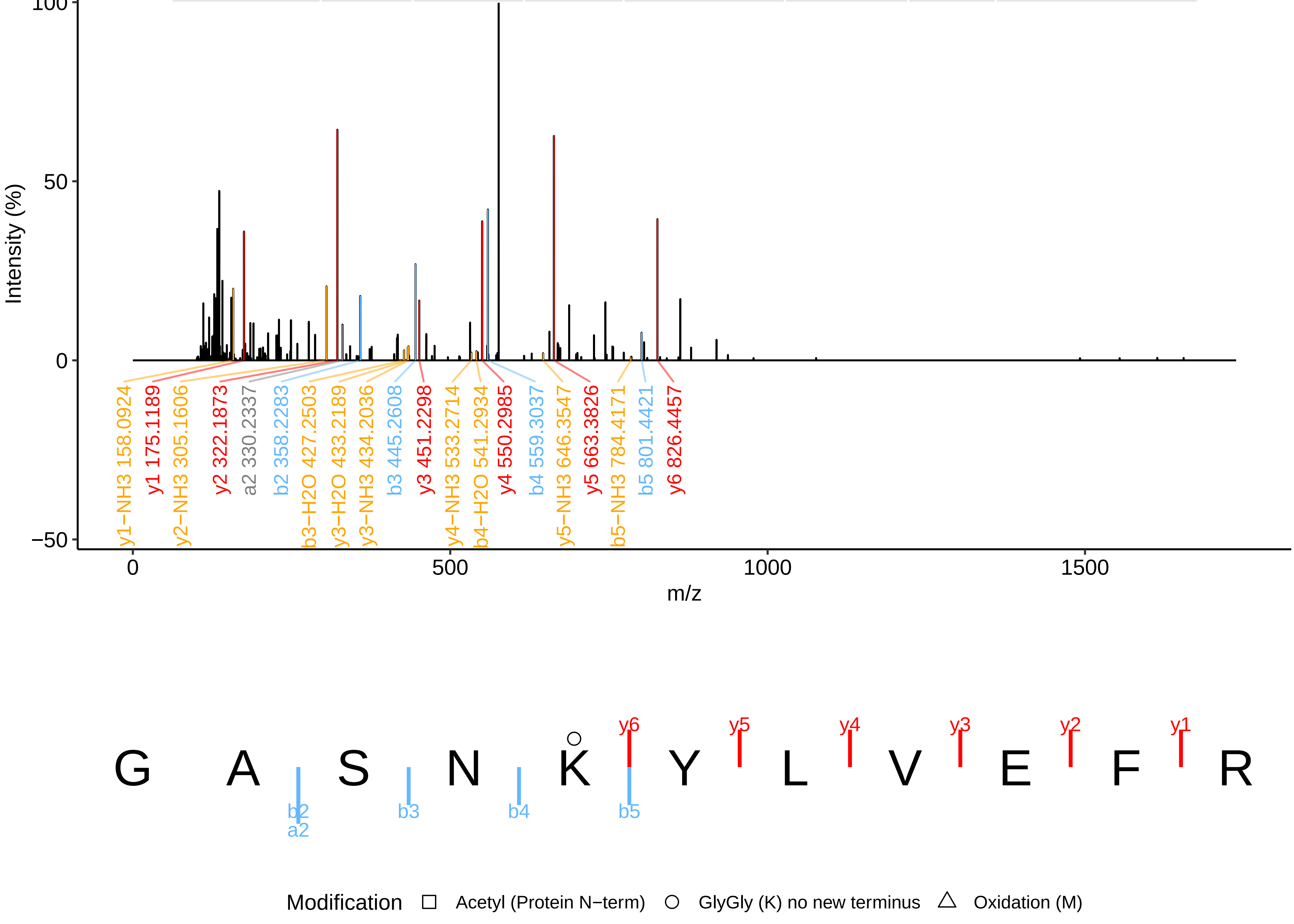

20200921_KIZ_MUb4_GlyGly_10plex_2 − Scan number: 13017

Gene Names	Charge	m/z	Mass	Retention time	PEP	Score	Precursor Intensity
ADRM1	3	466.5772	1396.71	50.508	0.012839	67.08	NA

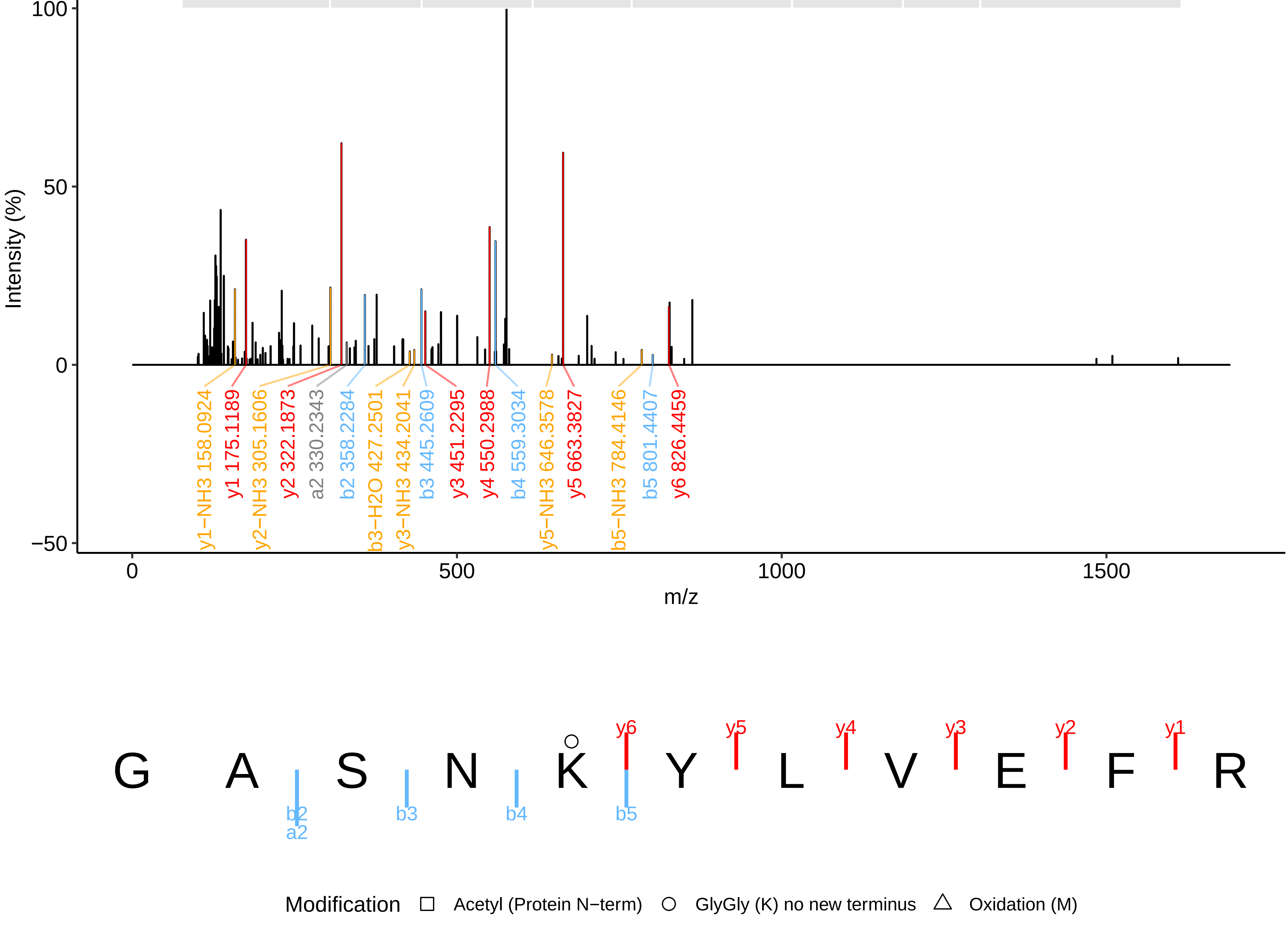

20200924_KIZ_MUb5_GlyGly_1 − Scan number: 12843

Gene Names	Charge	m/z	Mass	Retention time	PEP	Score	Precursor Intensity
ADRM1	3	466.5772	1396.71	41.848	0.025021	61.435	18262242

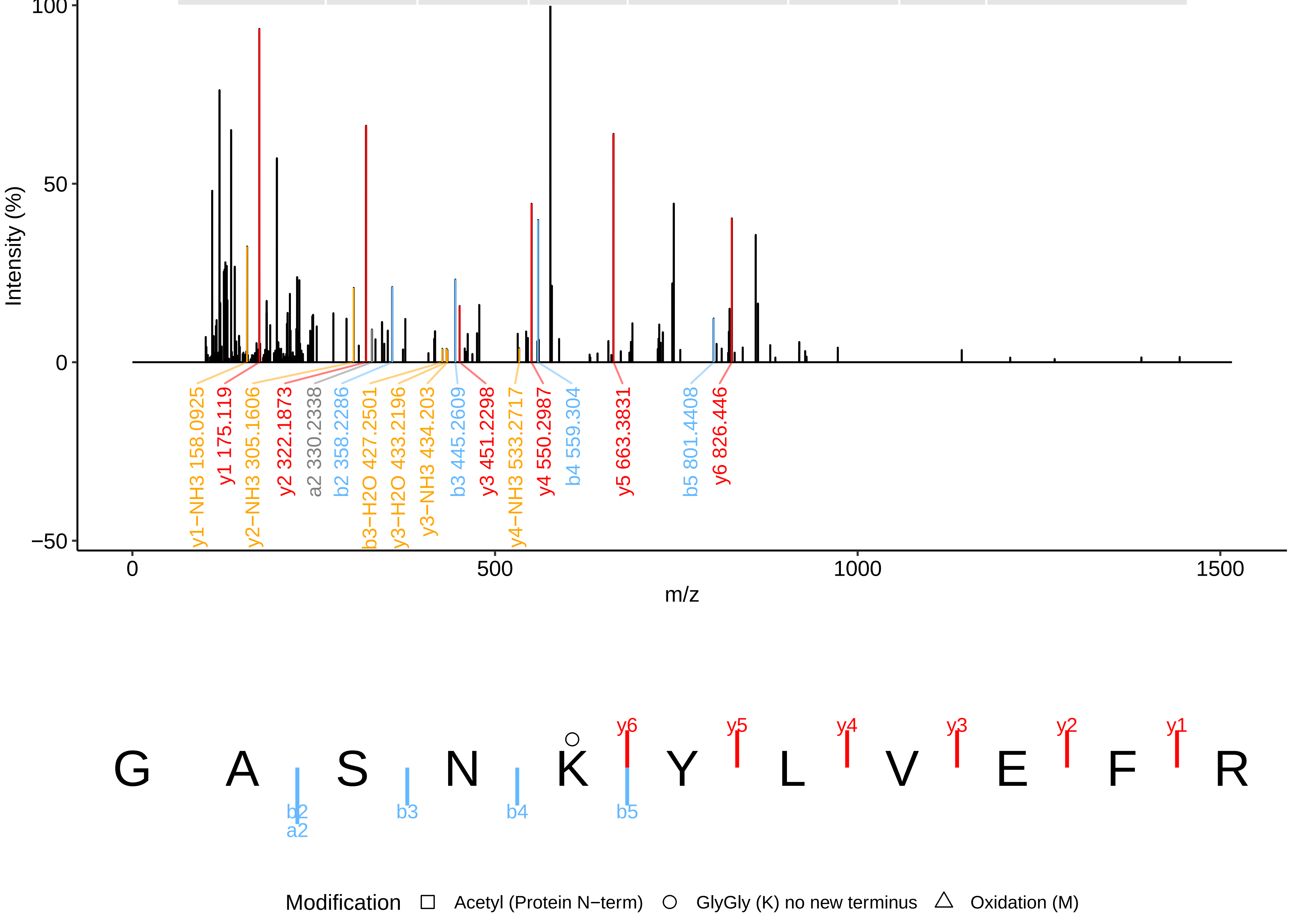

20200924_KIZ_MUb5_GlyGly_2 − Scan number: 12285

Gene Names	Charge	m/z	Mass	Retention time	PEP	Score	Precursor Intensity
ADRM1	3	466.5772	1396.71	45.921	0.01492	64.55	5791849

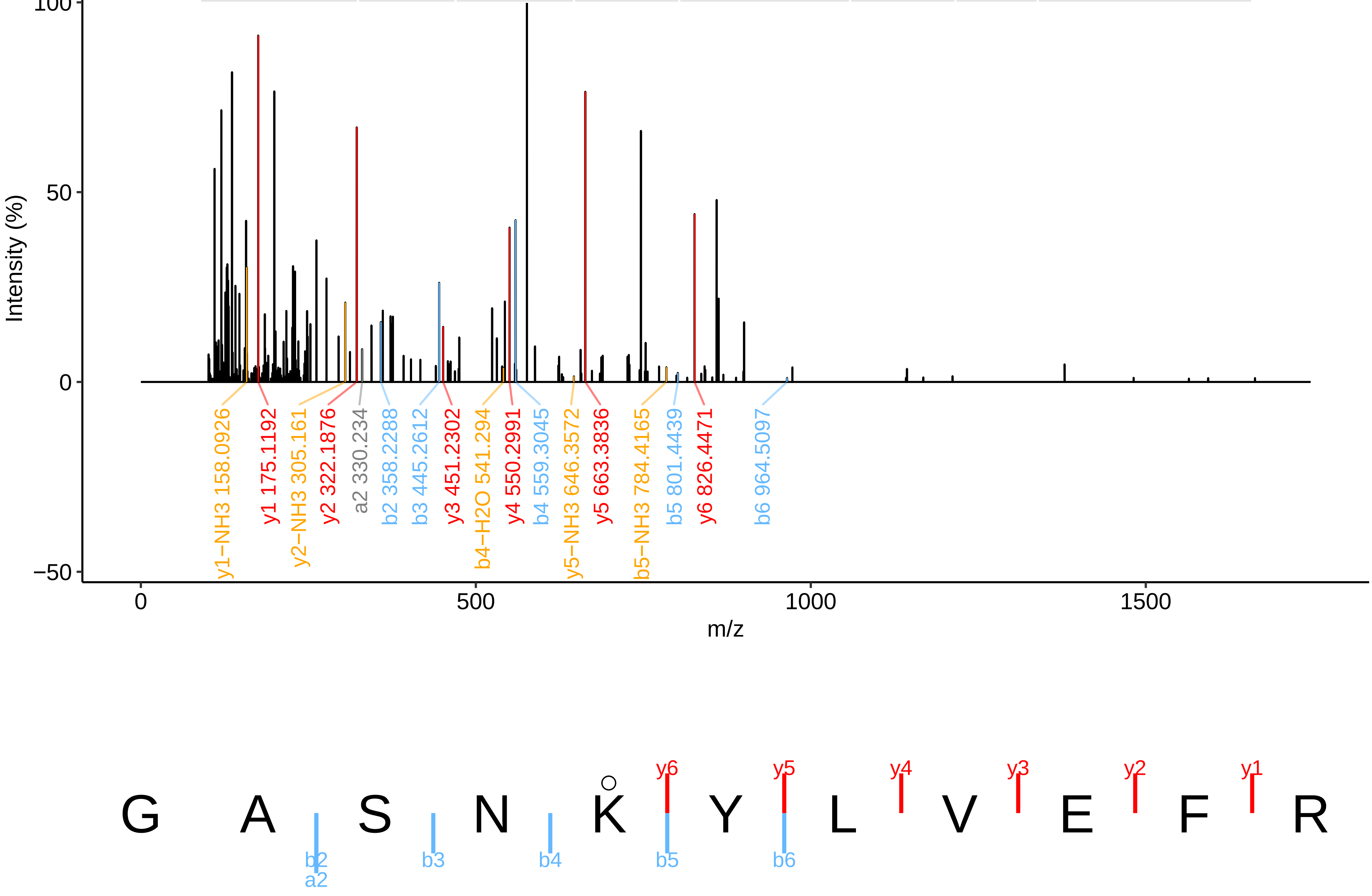

Modification □ Acetyl (Protein N−term) ○ GlyGly (K) no new terminus △ Oxidation (M)

20200921_KIZ_MUb4_GlyGly_10plex − Scan number: 9488

Gene Names	Charge	m/z	Mass	Retention time	PEP	Score	Precursor Intensity
ATF7IP	3	386.5473	1156.62	32.495	0.0018233	90.629	7520977

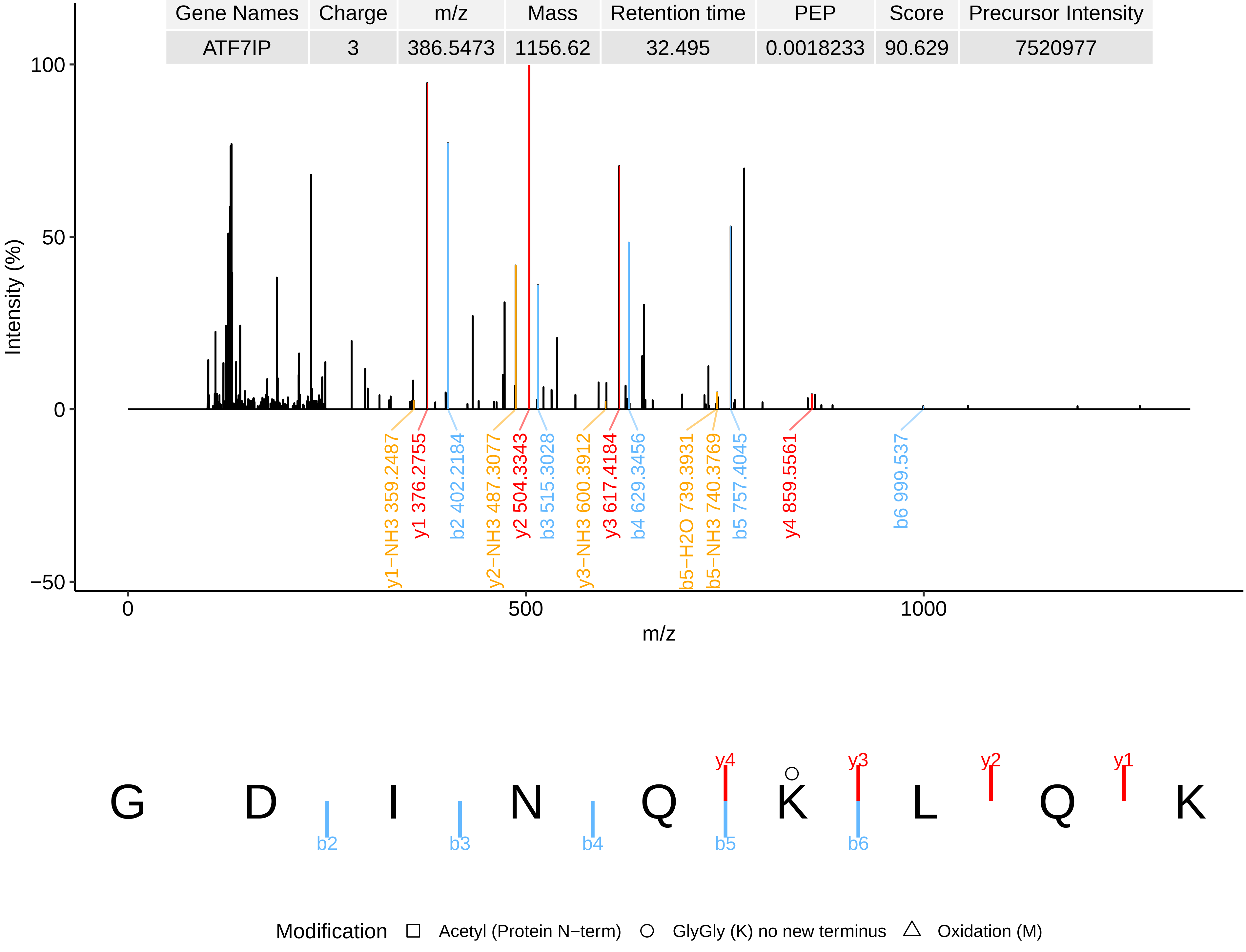

20200924_KIZ_MUb5_GlyGly_2 − Scan number: 8012

Gene Names	Charge	m/z	Mass	Retention time	PEP	Score	Precursor Intensity
ATF7IP	3	386.5473	1156.62	29.961	0.021211	68.735	NA

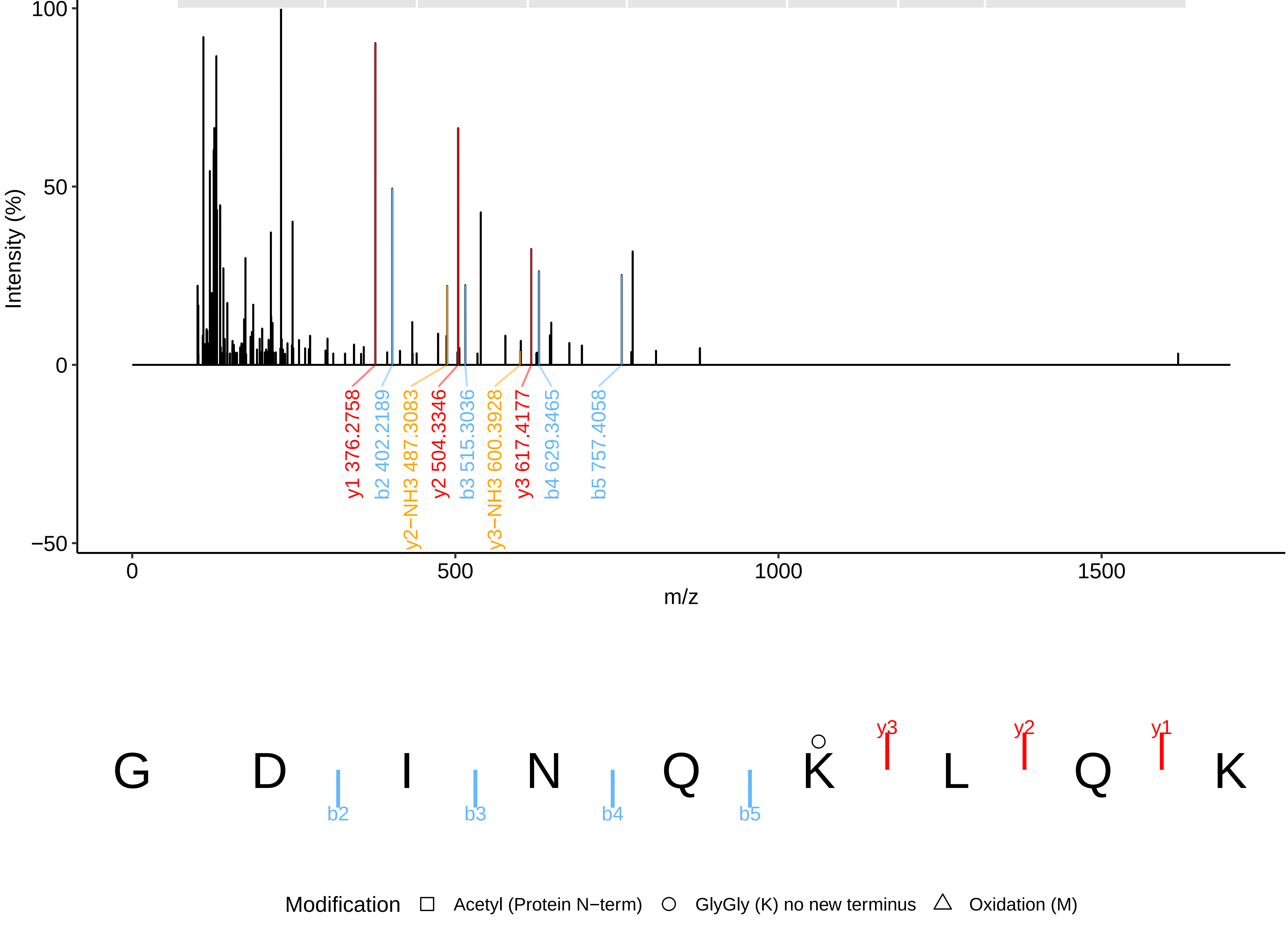

20200921_KIZ_MUb4_GlyGly_10plex_2 − Scan number: 8750

Gene Names	Charge	m/z	Mass	Retention time	PEP	Score	Precursor Intensity
UQCRC2	3	459.9126	1376.716	33.068	0.0013456	80.245	966327

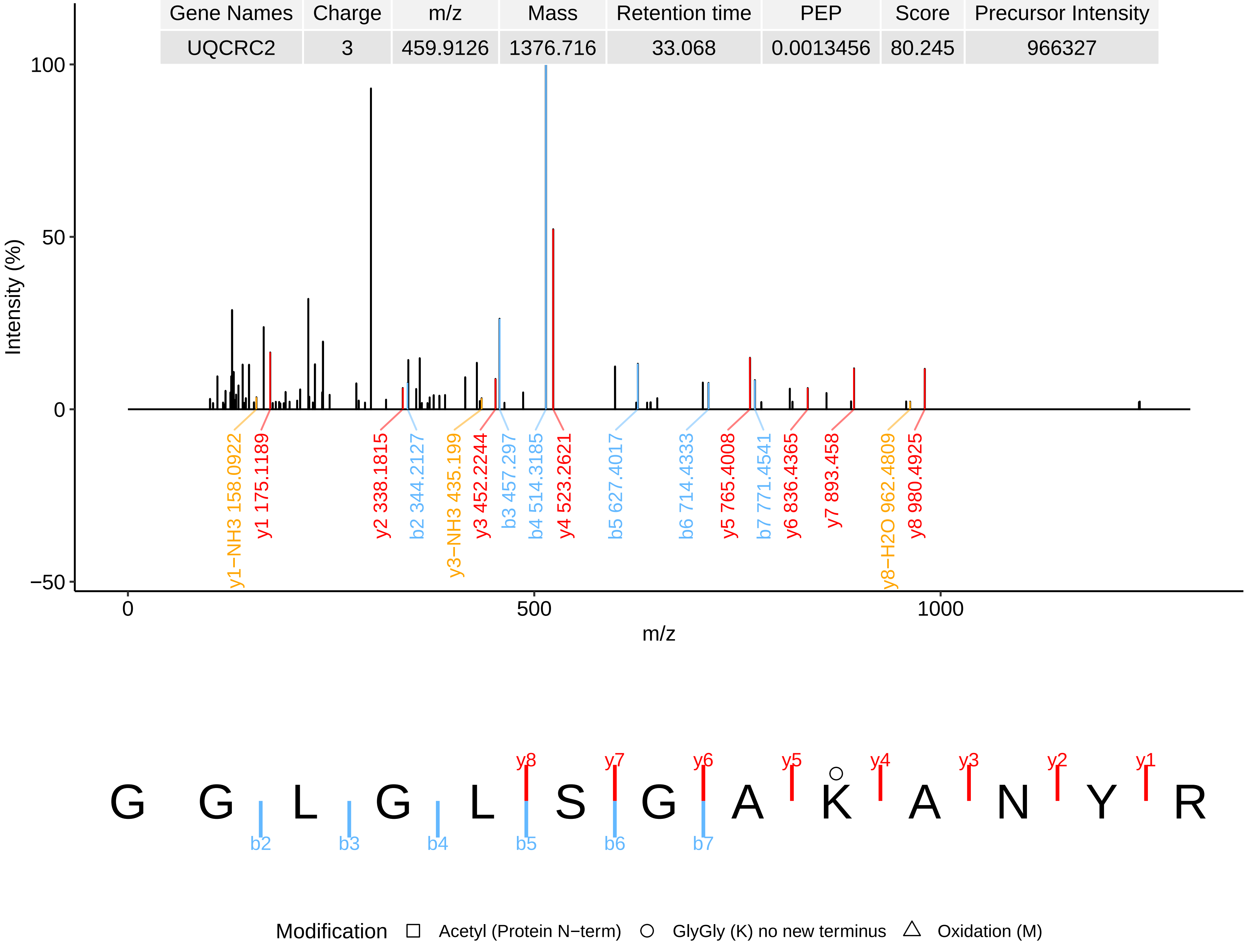

20200921_KIZ_MUb4_GlyGly_10plex_2 − Scan number: 3701

Gene Names	Charge	m/z	Mass	Retention time	PEP	Score	Precursor Intensity
GGCX	3	381.2194	1140.636	12.599	0.038591	62.303	1315272

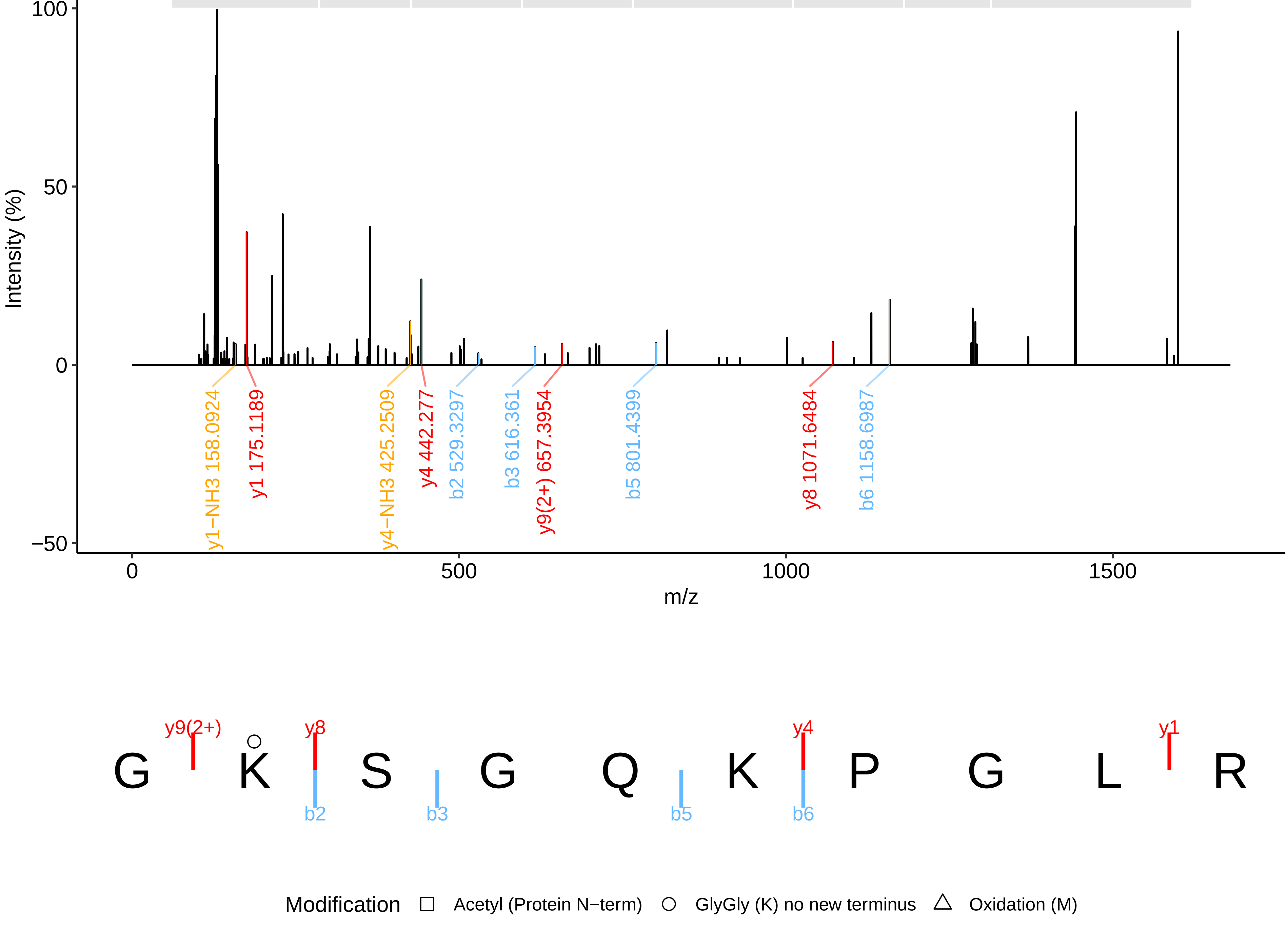

20200921_KIZ_MUb4_GlyGly_10plex − Scan number: 3270

Gene Names	Charge	m/z	Mass	Retention time	PEP	Score	Precursor Intensity
AP2M1	3	398.8581	1193.552	10.235	0.0010522	87.568	4195817

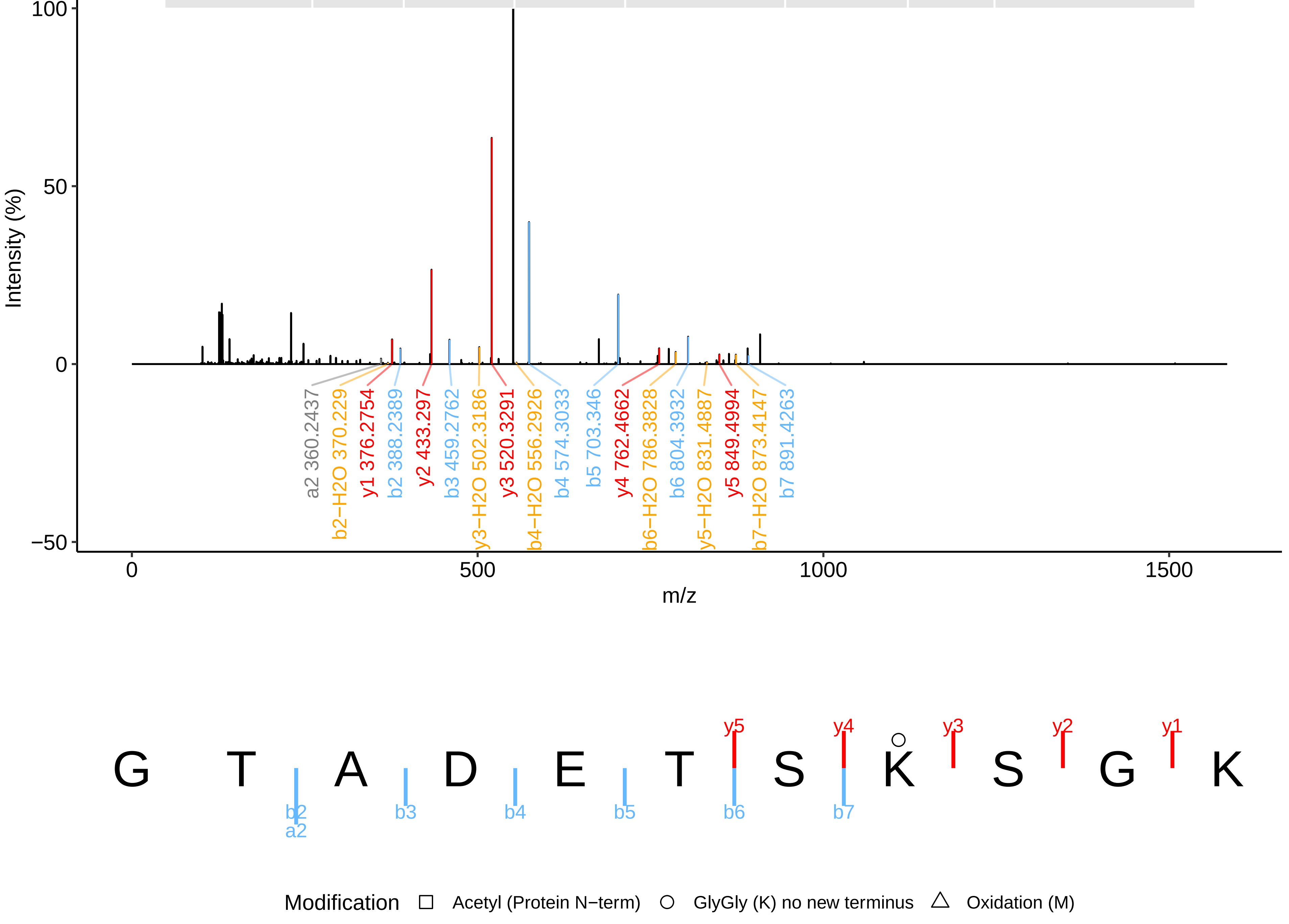

20200924_KIZ_MUb5_GlyGly_1 − Scan number: 3060

Gene Names	Charge	m/z	Mass	Retention time	PEP	Score	Precursor Intensity
AP2M1	3	398.8581	1193.552	9.4322	0.012839	67.08	17194818

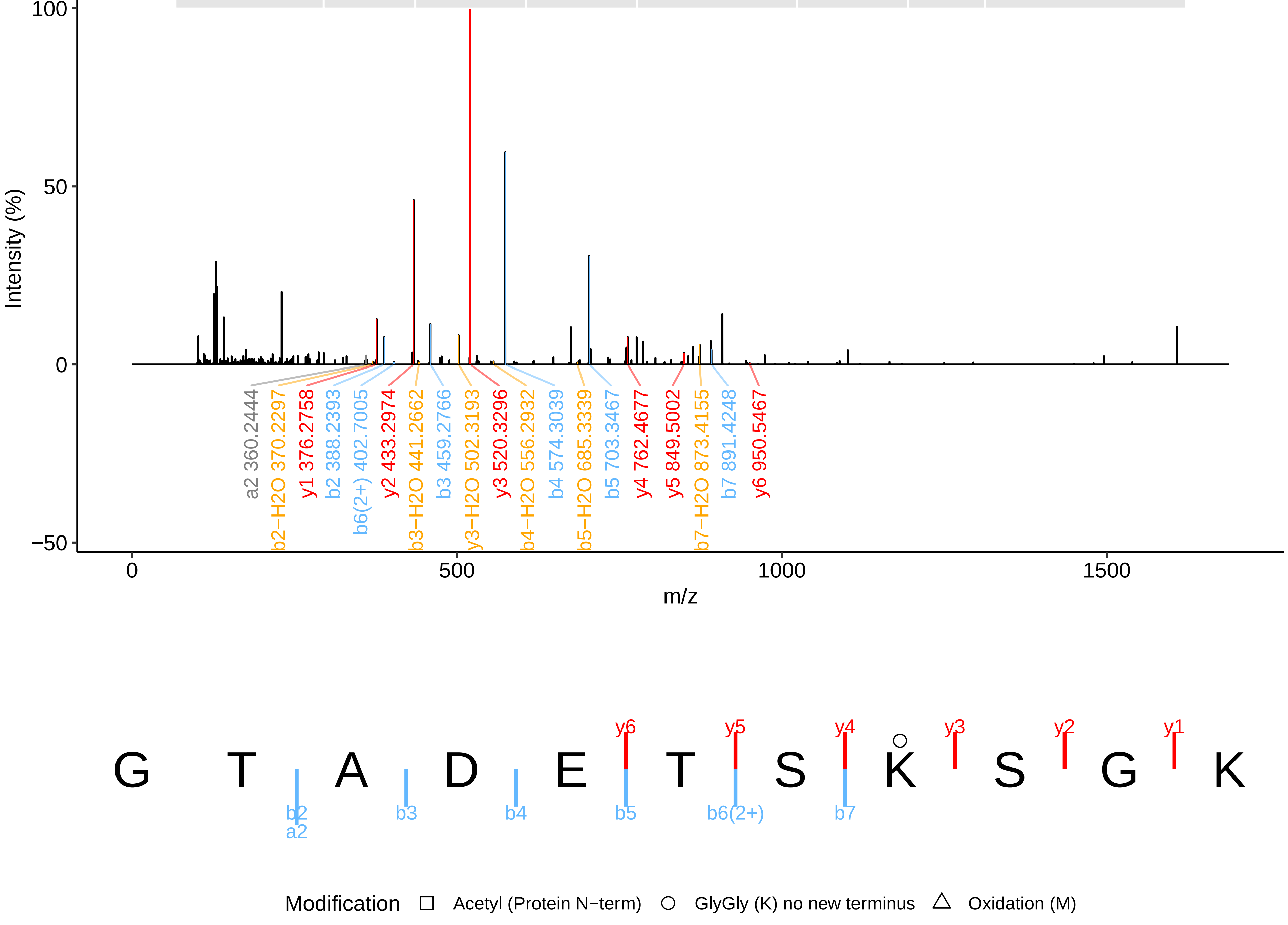

20201118_KIZ_MUb6_GlyGly_1 − Scan number: 3042

Gene Names	Charge	m/z	Mass	Retention time	PEP	Score	Precursor Intensity
AP2M1	3	398.8581	1193.552	9.3554	0.0024888	79.659	10165142

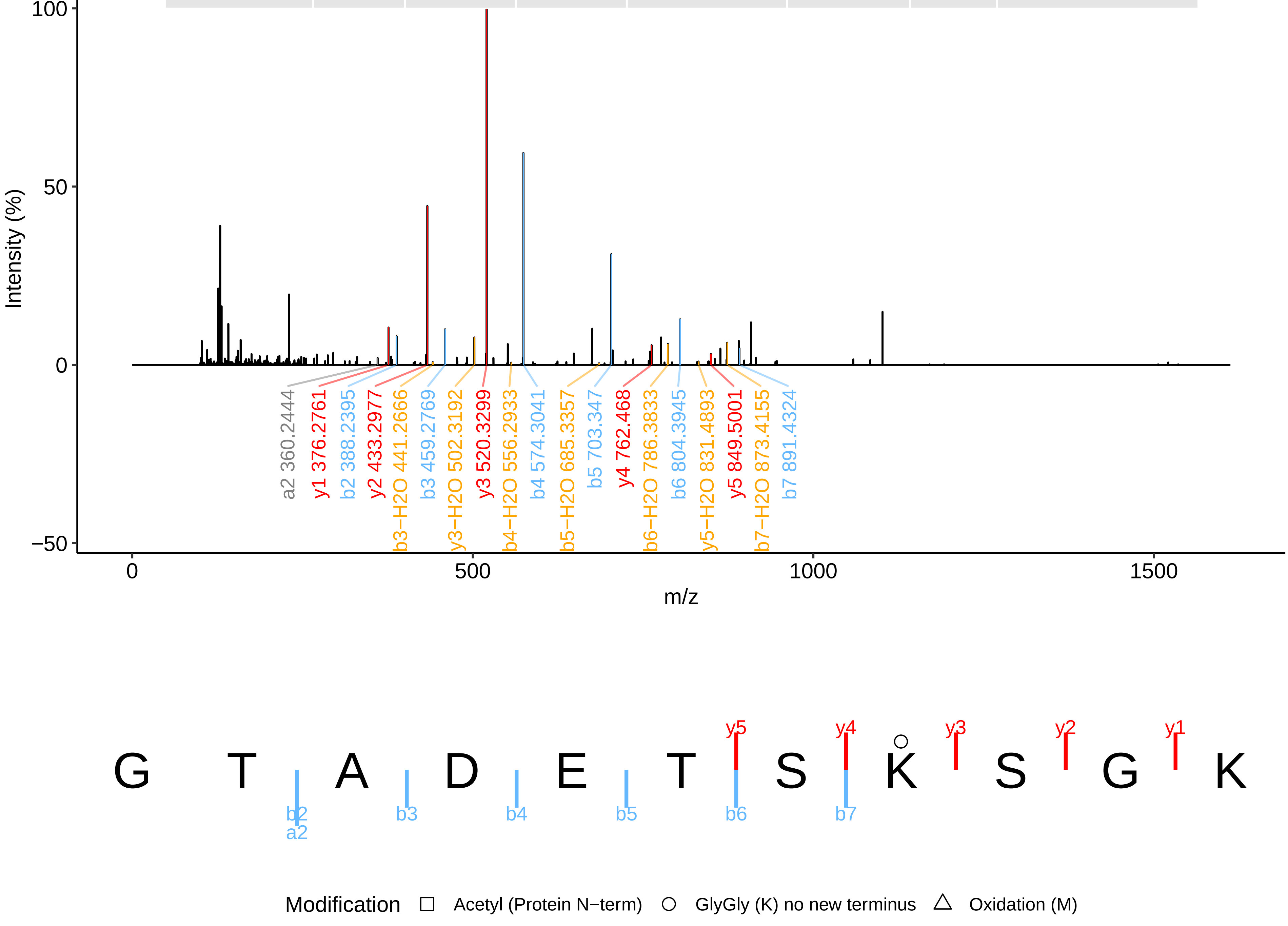

20201118_KIZ_MUb6_GlyGly_2 − Scan number: 3245

Gene Names	Charge	m/z	Mass	Retention time	PEP	Score	Precursor Intensity
AP2M1	3	398.8581	1193.552	10.569	0.00081248	90.05	7927119

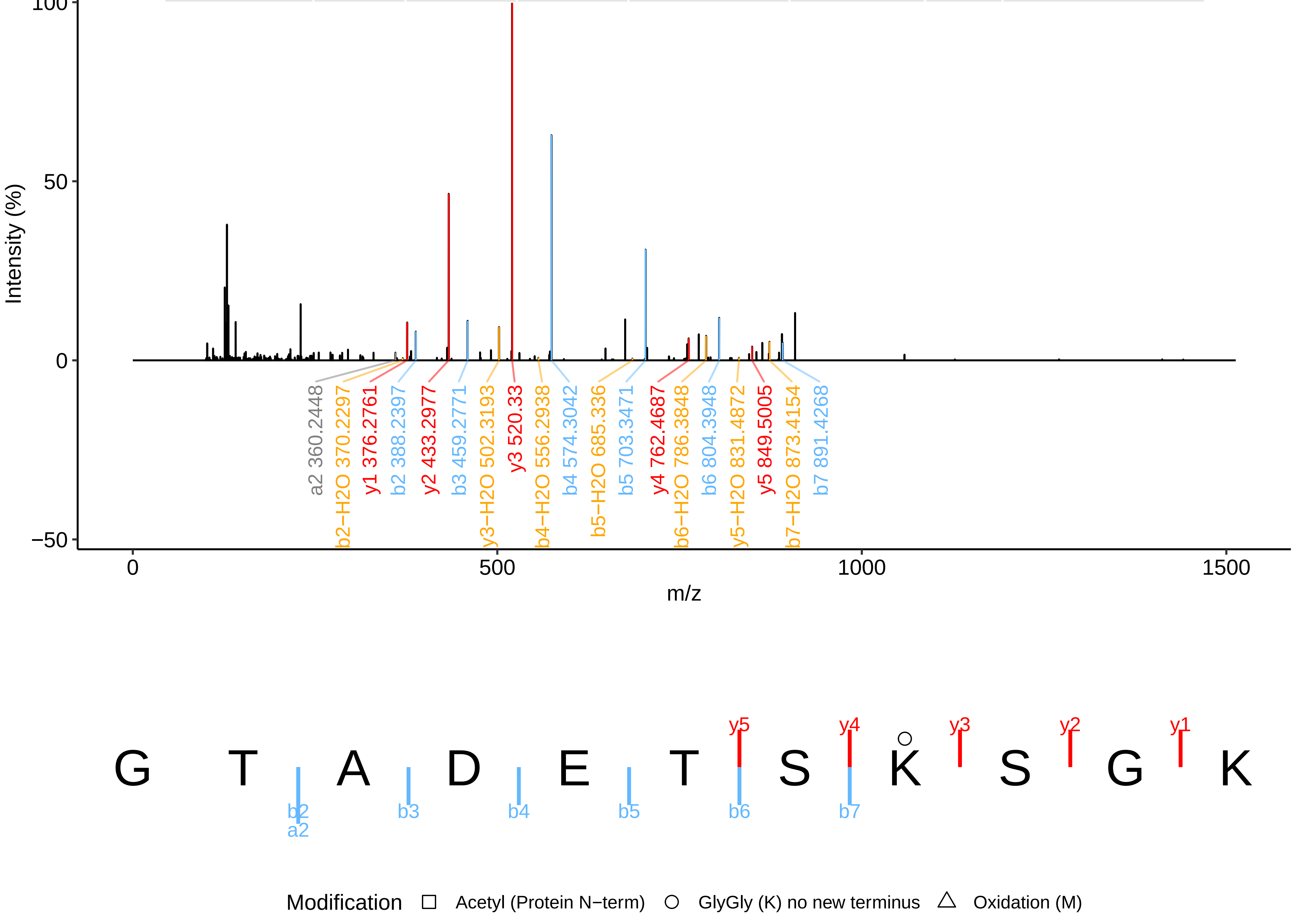

20200921_KIZ_MUb4_GlyGly_10plex − Scan number: 8613

Gene Names	Charge	m/z	Mass	Retention time	PEP	Score	Precursor Intensity
RUVBL2	3	458.5722	1372.695	29.284	4.1066e−06	115.18	2085868

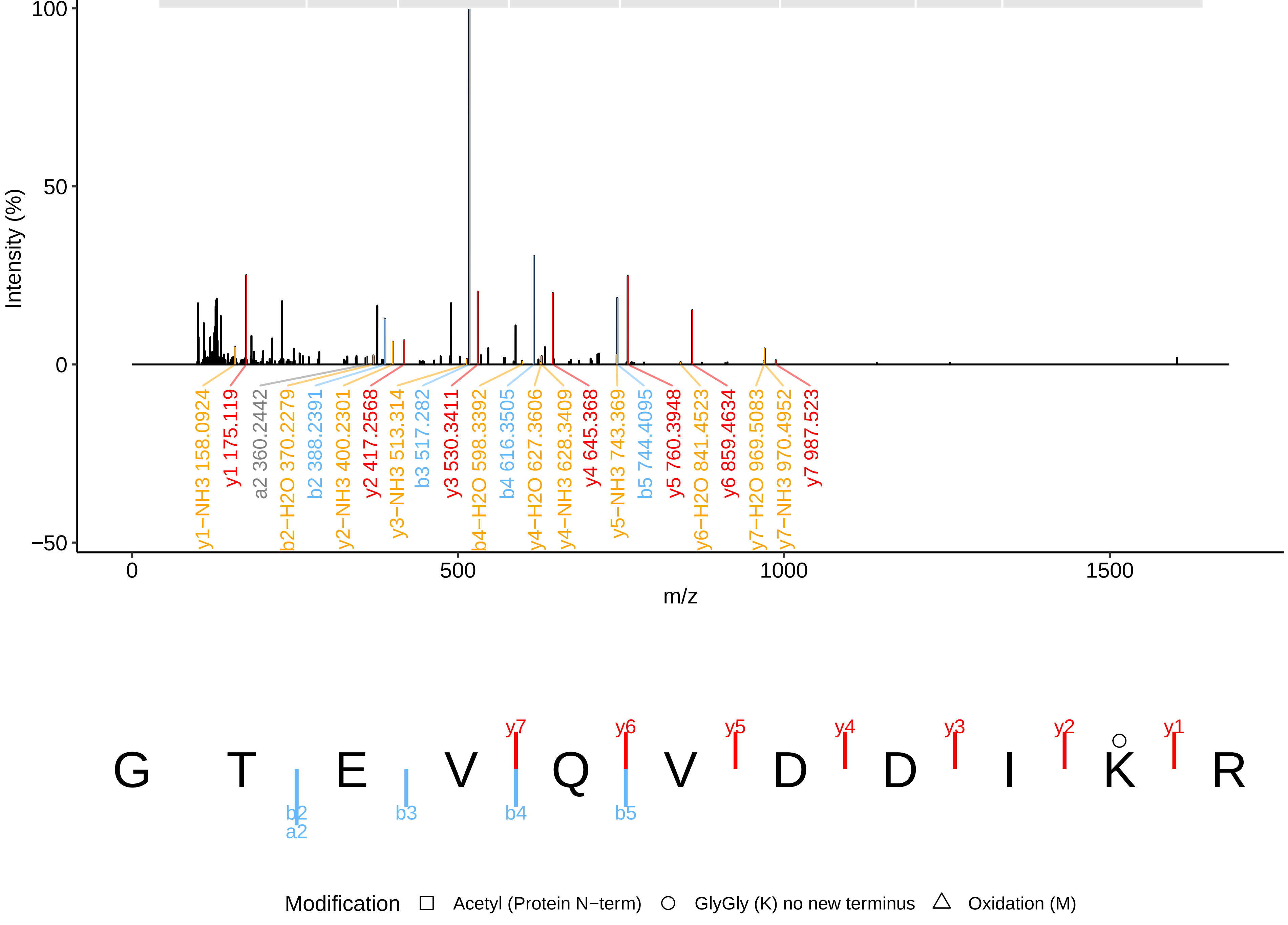

20200921_KIZ_MUb4_GlyGly_10plex − Scan number: 20362

Gene Names	Charge	m/z	Mass	Retention time	PEP	Score	Precursor Intensity
CDC2;CDK1	3	459.2613	1374.762	73.6	0.084325	49.423	4808702

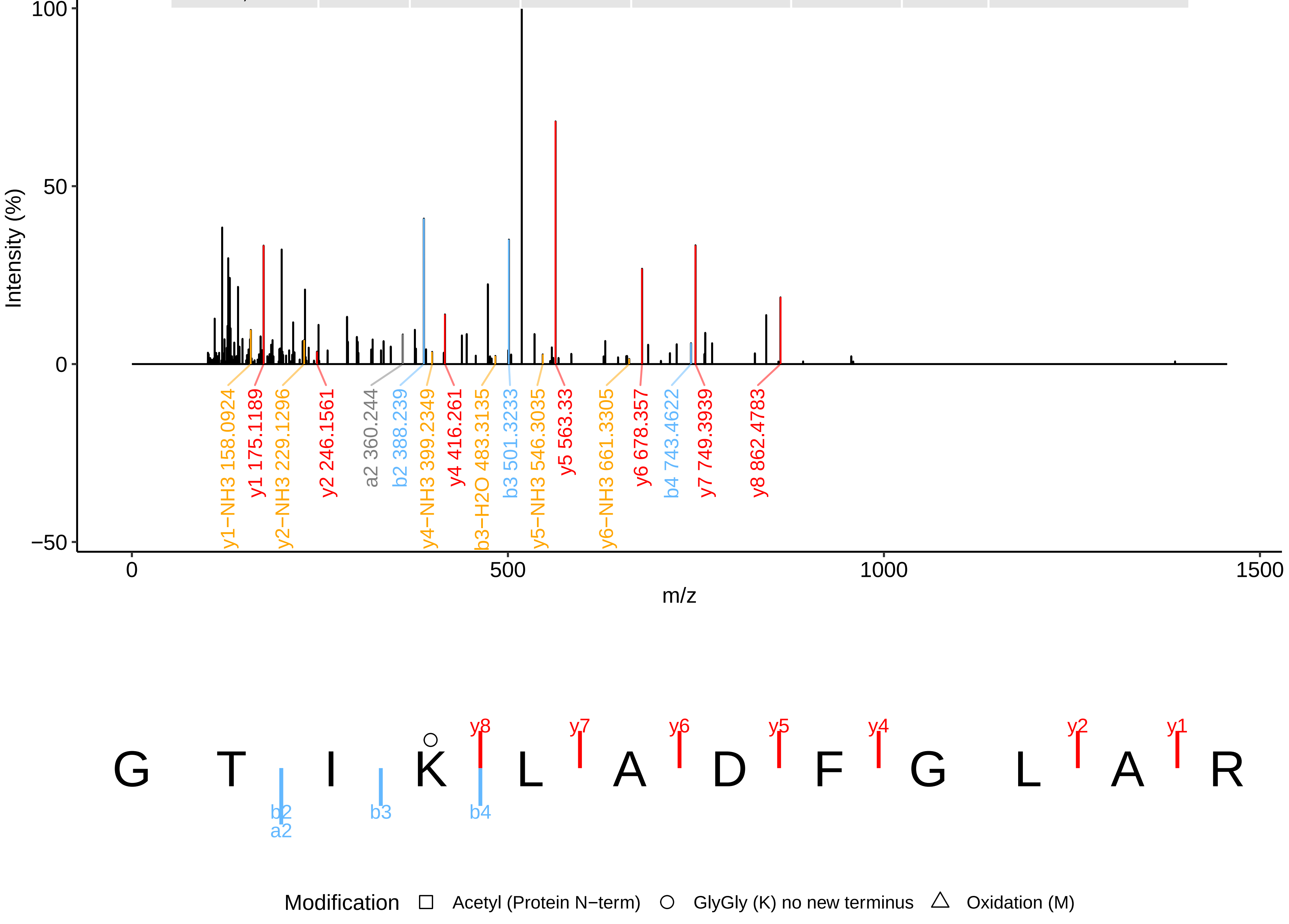

20200921_KIZ_MUb4_GlyGly_10plex_2 − Scan number: 18728

Gene Names	Charge	m/z	Mass	Retention time	PEP	Score	Precursor Intensity
CDC2;CDK1	3	459.2613	1374.762	73.403	0.0024846	78.692	447234.1

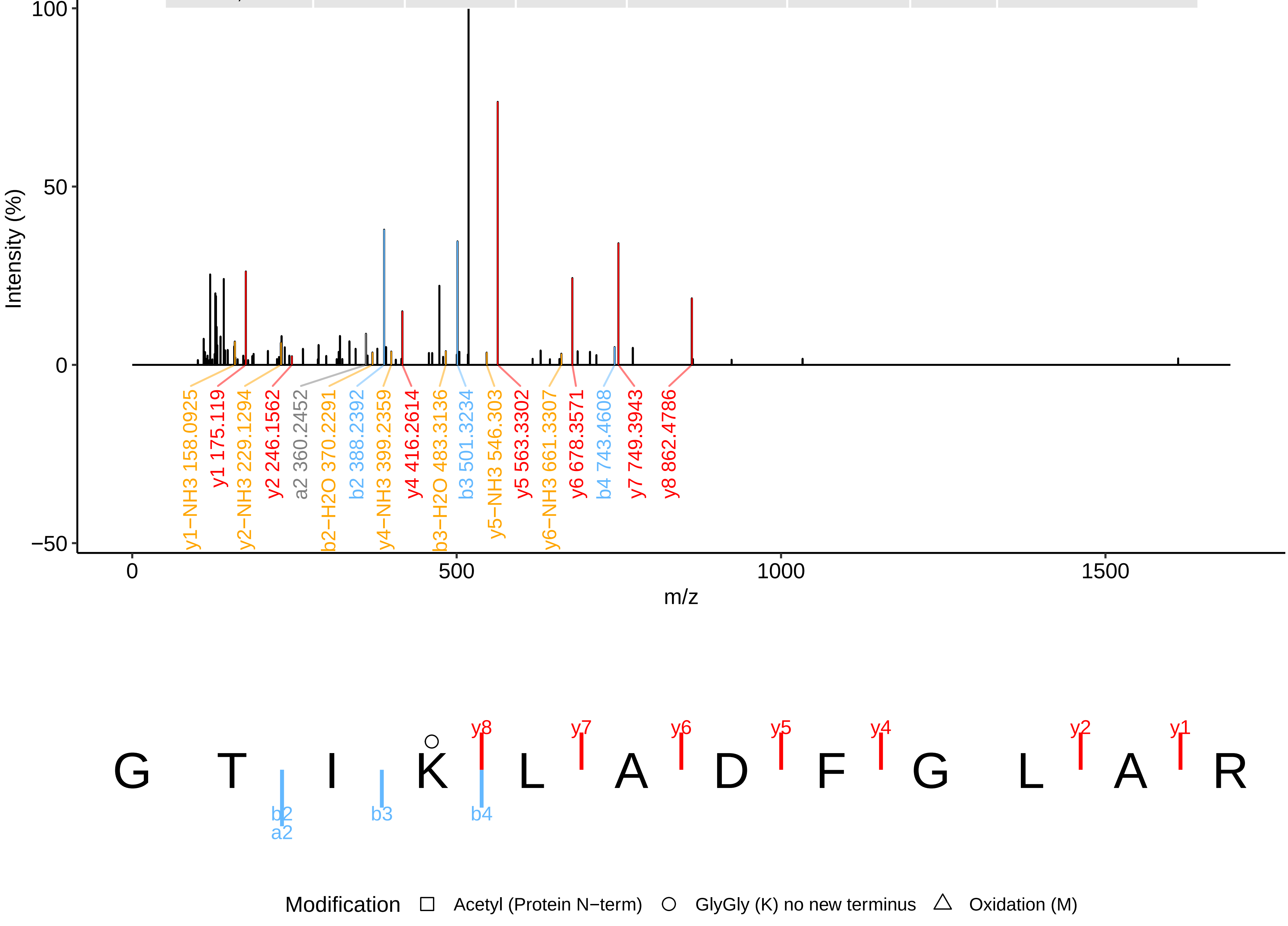

20200921_KIZ_MUb4_GlyGly_10plex − Scan number: 6348

Gene Names	Charge	m/z	Mass	Retention time	PEP	Score	Precursor Intensity
KCTD10	3	389.8717	1166.593	20.97	0.0011024	90.906	6602035

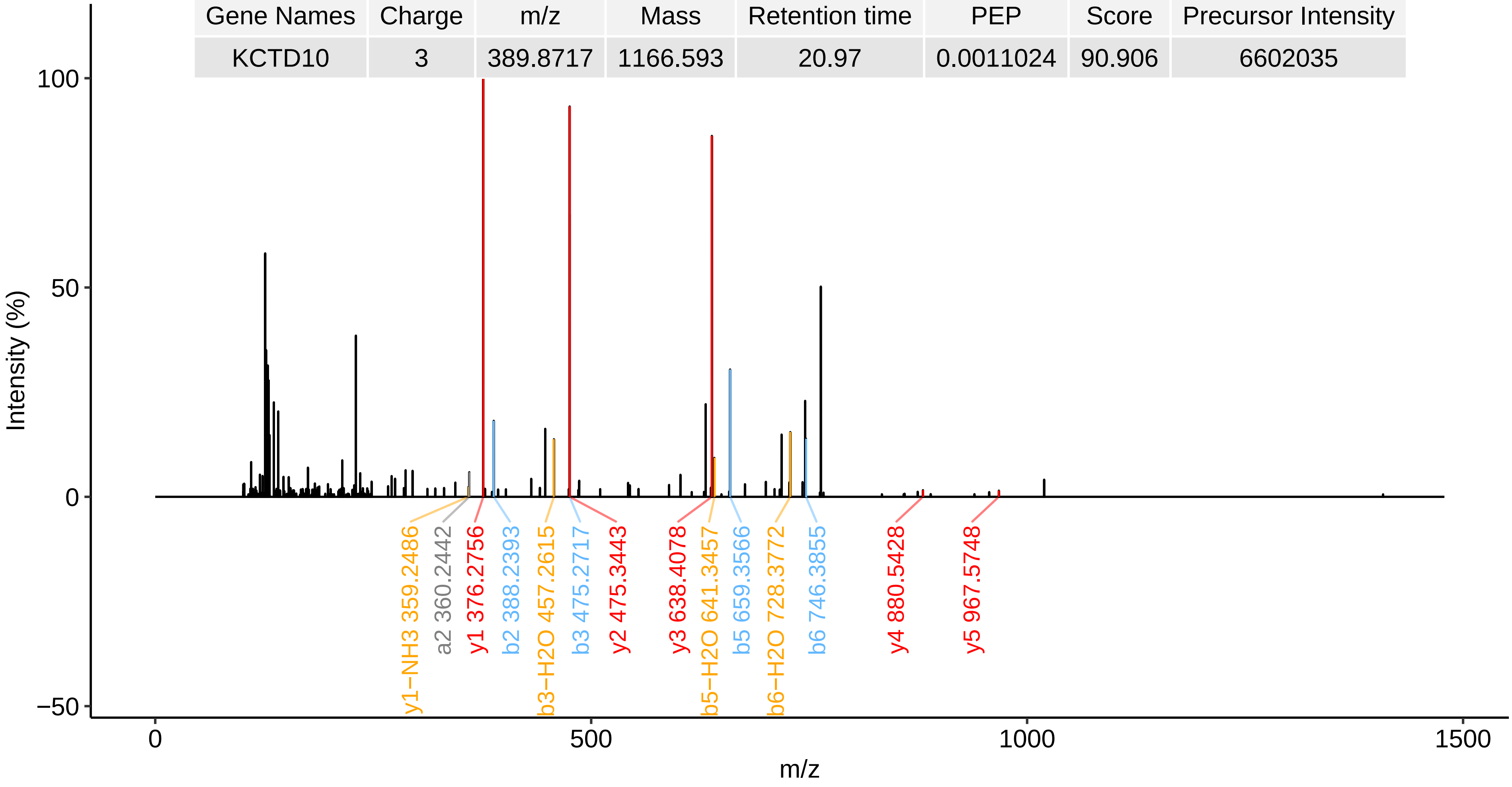

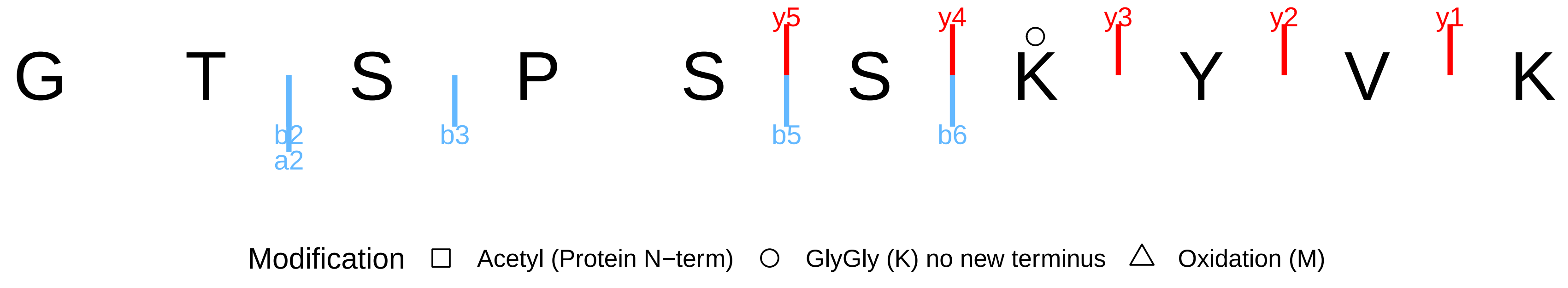

20200921_KIZ_MUb4_GlyGly_10plex_2 − Scan number: 5864

Gene Names	Charge	m/z	Mass	Retention time	PEP	Score	Precursor Intensity
KCTD10	3	389.8717	1166.593	21.295	0.11122	45.368	609637.8

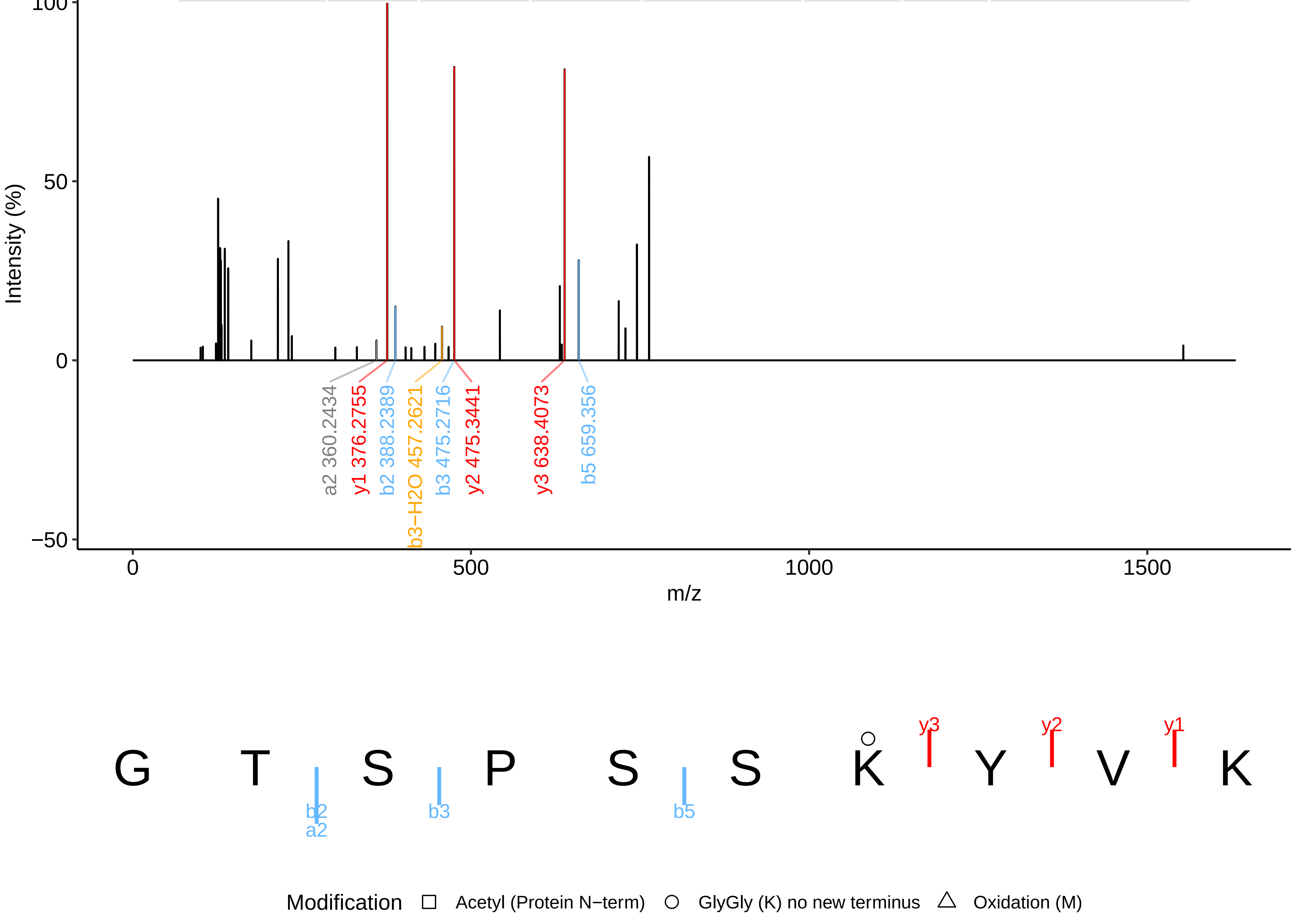

20200921_KIZ_MUb4_GlyGly_10plex − Scan number: 6597

Gene Names	Charge	m/z	Mass	Retention time	PEP	Score	Precursor Intensity
CSPG4	4	345.1954	1376.752	21.888	0.011411	65.842	8526726

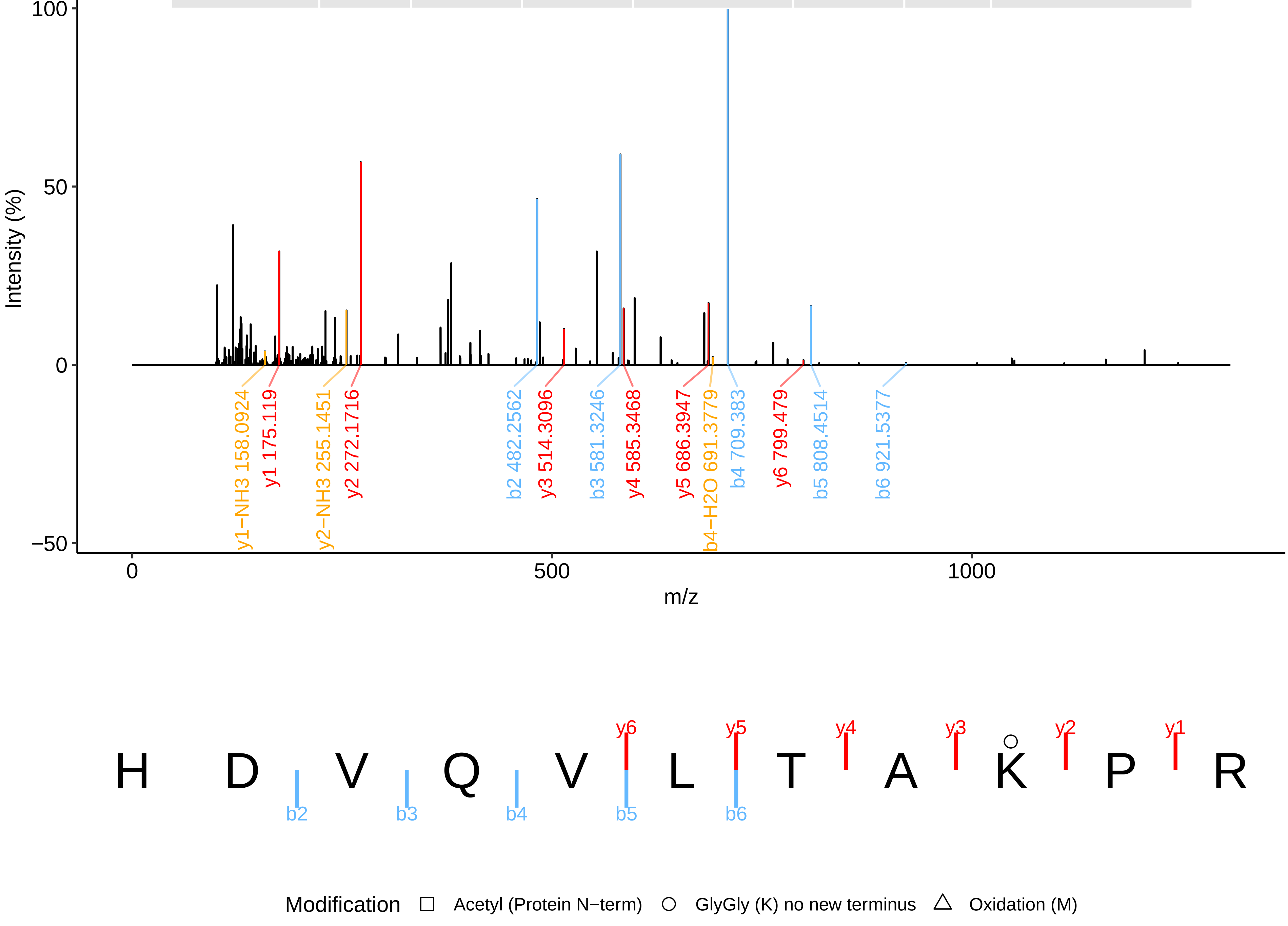

20200921_KIZ_MUb4_GlyGly_10plex − Scan number: 9572

Gene Names	Charge	m/z	Mass	Retention time	PEP	Score	Precursor Intensity
SUMO2;SUMO3	4	296.9169	1183.638	32.809	0.09705	44.377	1719789

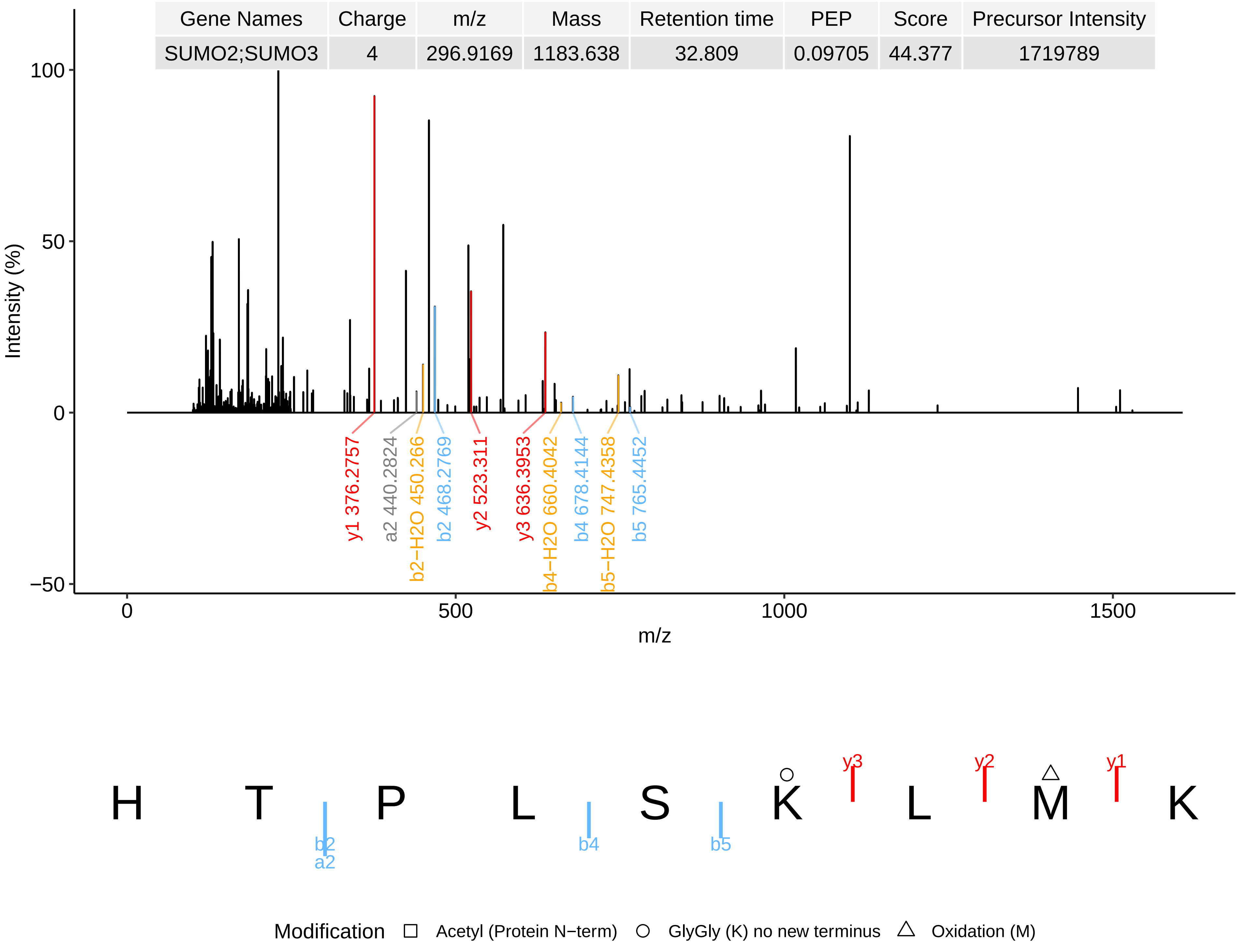

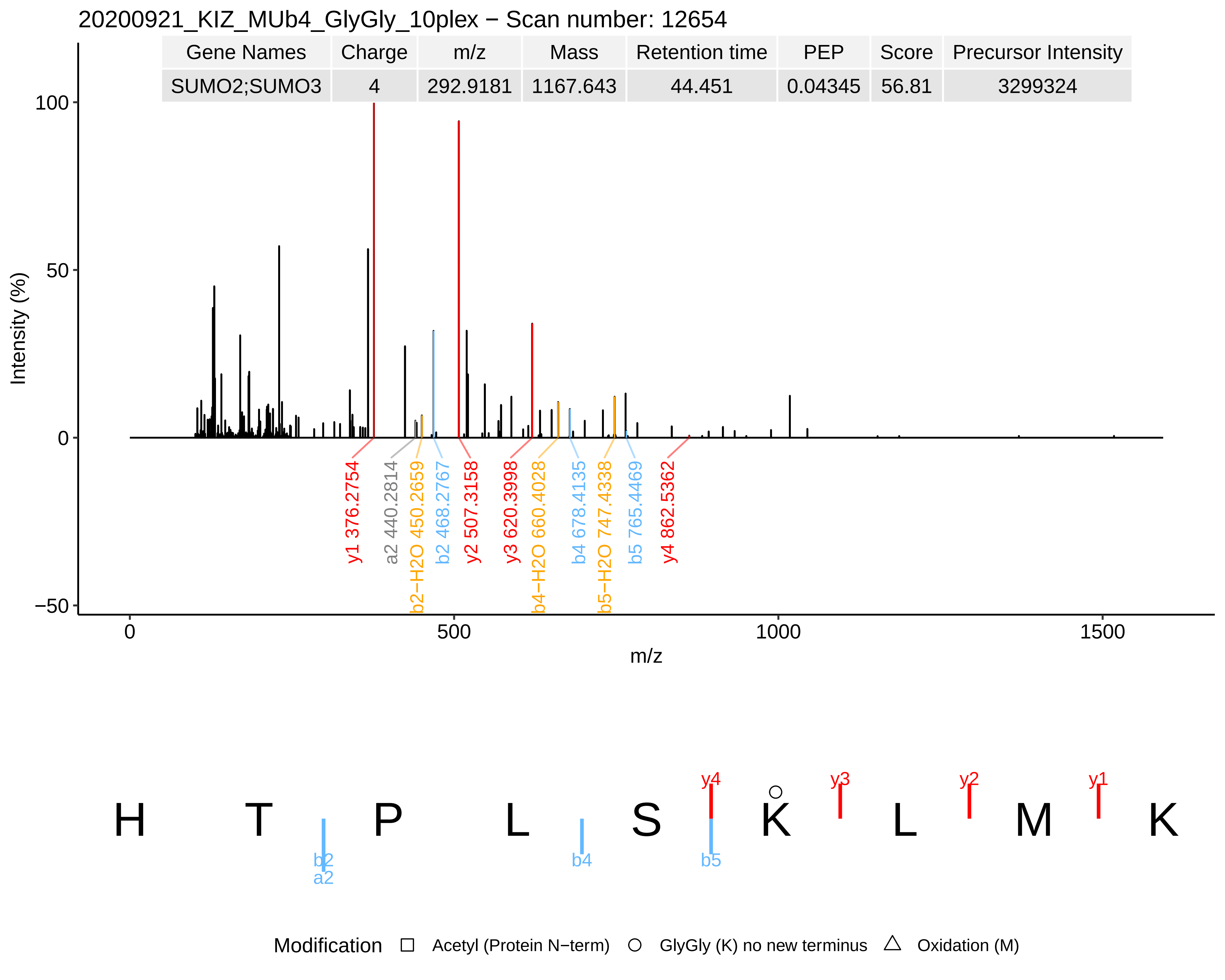

20200921_KIZ_MUb4_GlyGly_10plex − Scan number: 12654

Gene Names	Charge	m/z	Mass	Retention time	PEP	Score	Precursor Intensity
SUMO2;SUMO3	4	292.9181	1167.643	44.451	0.04345	56.81	3299324

Intensity (%)
m/z
y1 376.2754
a2 440.2814
b2−H2O 450.2659
b2 468.2767
y2 507.3158
y3 620.3998
b4−H2O 660.4028
b4 678.4135
b5−H2O 747.4338
b5 765.4469
y4 862.5362
H T P L S K L M K
y4 y3 y2 y1
b2 a2 b4 b5
Modification
Acetyl (Protein N−term)
GlyGly (K) no new terminus
Oxidation (M)

20200921_KIZ_MUb4_GlyGly_10plex − Scan number: 18609

Gene Names	Charge	m/z	Mass	Retention time	PEP	Score	Precursor Intensity
XRCC5	3	469.2648	1404.773	66.963	0.0042277	78.488	2178852

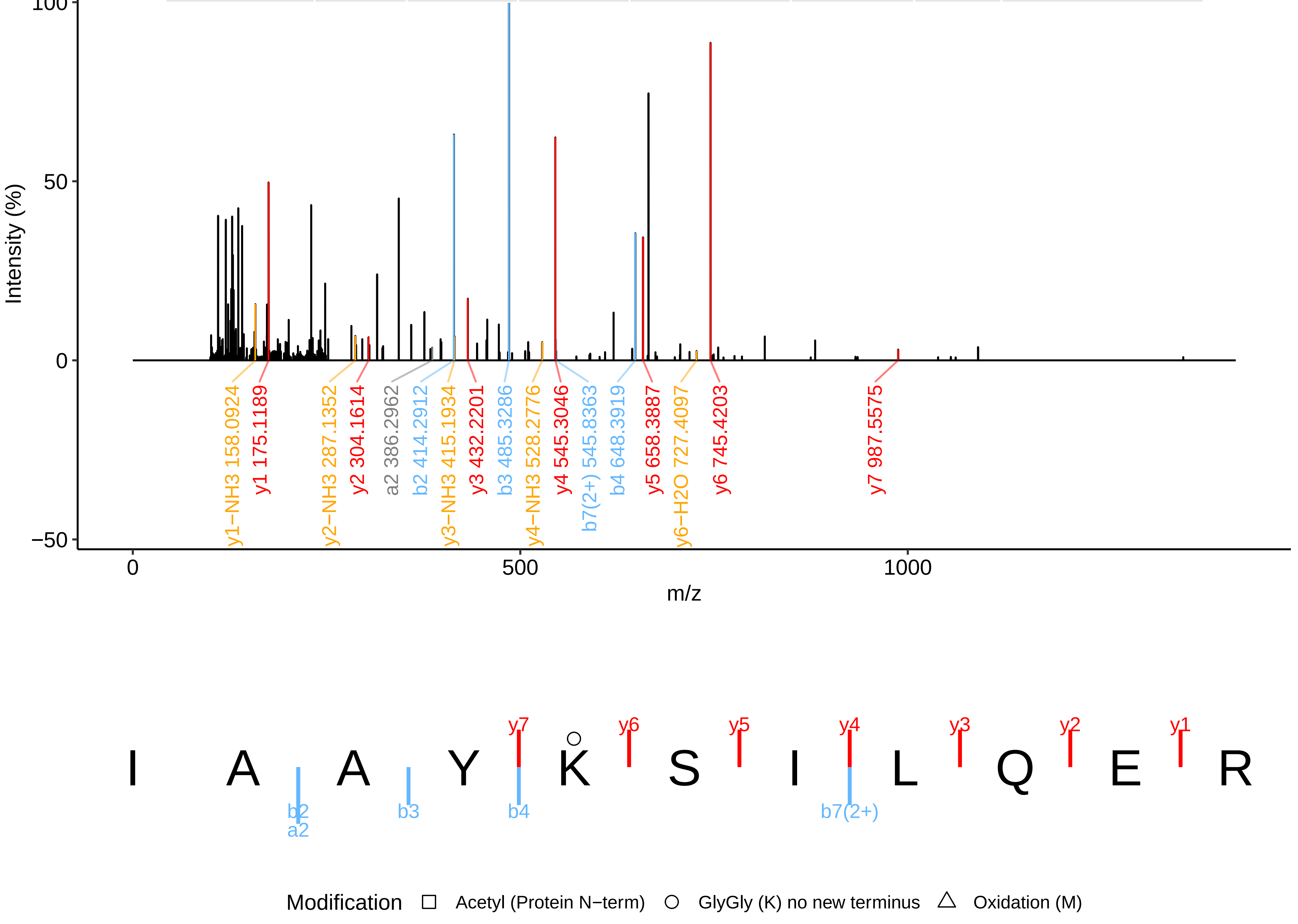

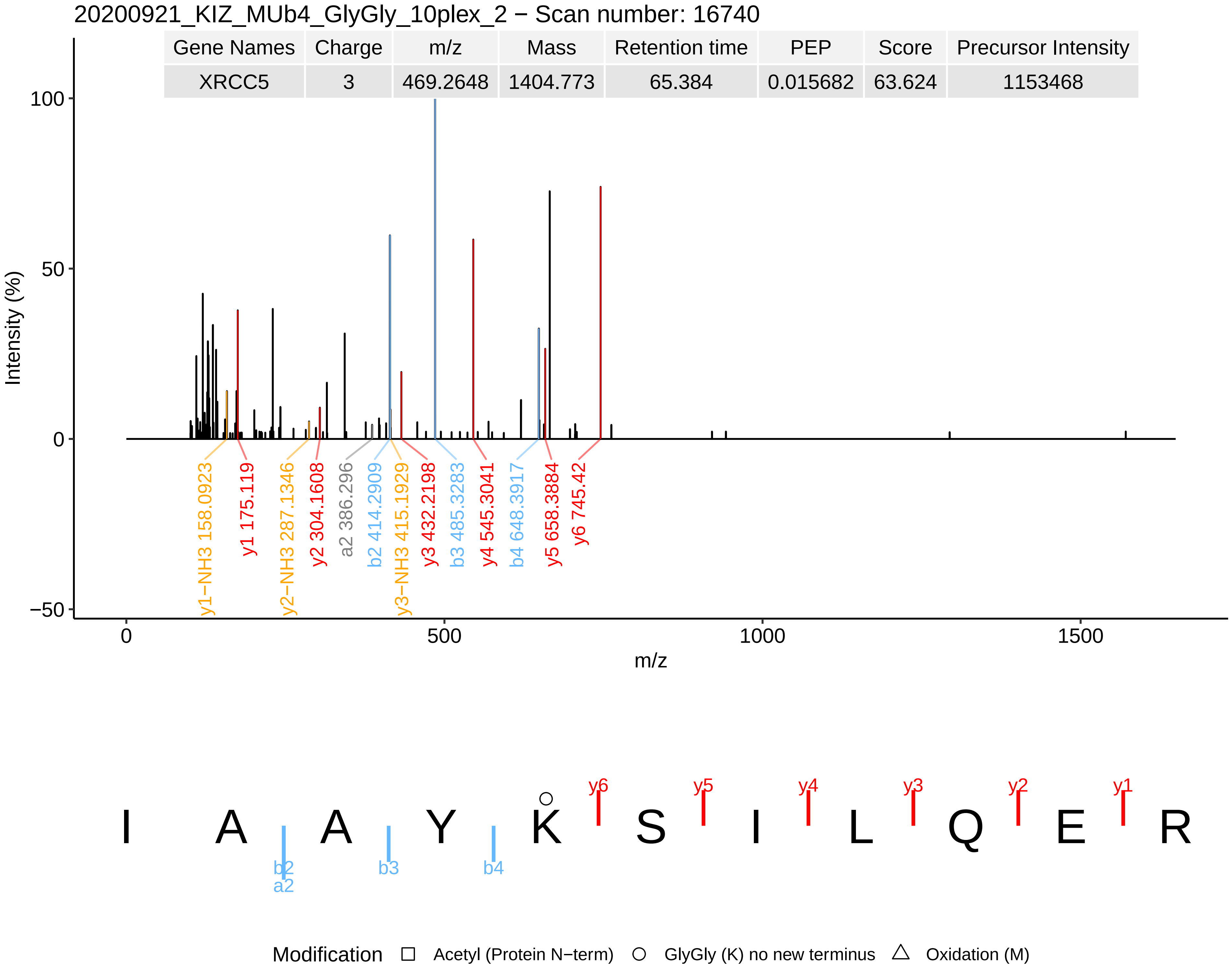
20200921_KIZ_MUb4_GlyGly_10plex_2 − Scan number: 16740
Gene Names | Charge | m/z | Mass | Retention time | PEP | Score | Precursor Intensity
XRCC5 | 3 | 469.2648 | 1404.773 | 65.384 | 0.015682 | 63.624 | 1153468
Intensity (%)
100
50
0
−50
0
500
1000
1500
m/z
y1−NH3 158.0923
y1 175.119
y2−NH3 287.1346
y2 304.1608
a2 386.296
b2 414.2909
y3−NH3 415.1929
y3 432.2198
b3 485.3283
y4 545.3041
b4 648.3917
y5 658.3884
y6 745.42
I A A Y K S I L Q E R
y6 y5 y4 y3 y2 y1
b2 a2 b3 b4
Modification
Acetyl (Protein N−term)
GlyGly (K) no new terminus
Oxidation (M)

20200921_KIZ_MUb4_GlyGly_10plex_2 − Scan number: 16755

Gene Names	Charge	m/z	Mass	Retention time	PEP	Score	Precursor Intensity
XRCC5	3	469.2648	1404.773	65.444	0.0010522	87.568	1800308

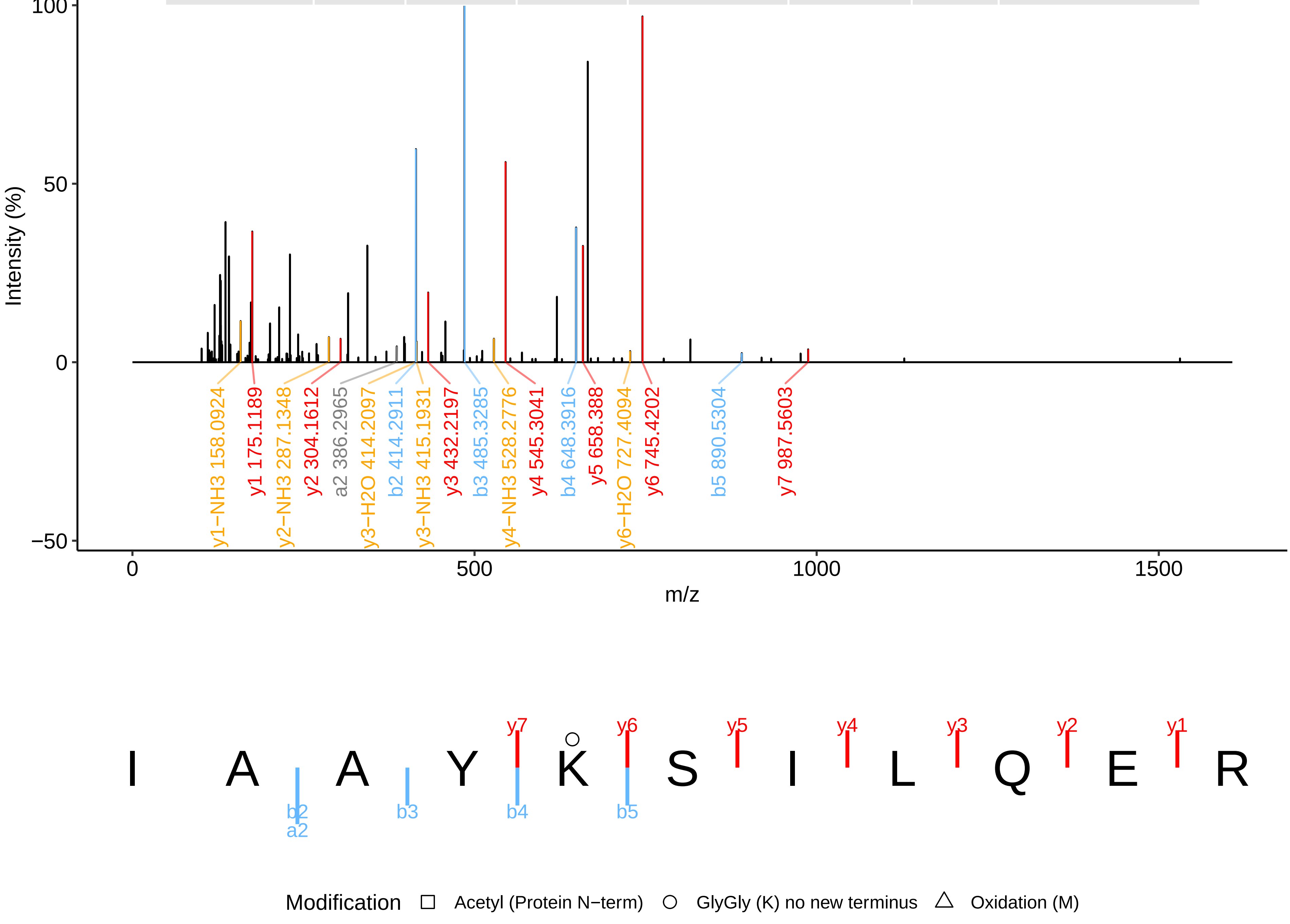

20200921_KIZ_MUb4_GlyGly_10plex − Scan number: 15120

Gene Names	Charge	m/z	Mass	Retention time	PEP	Score	Precursor Intensity
ZCCHC6	3	468.9209	1403.741	53.933	0.00175	97.456	NA

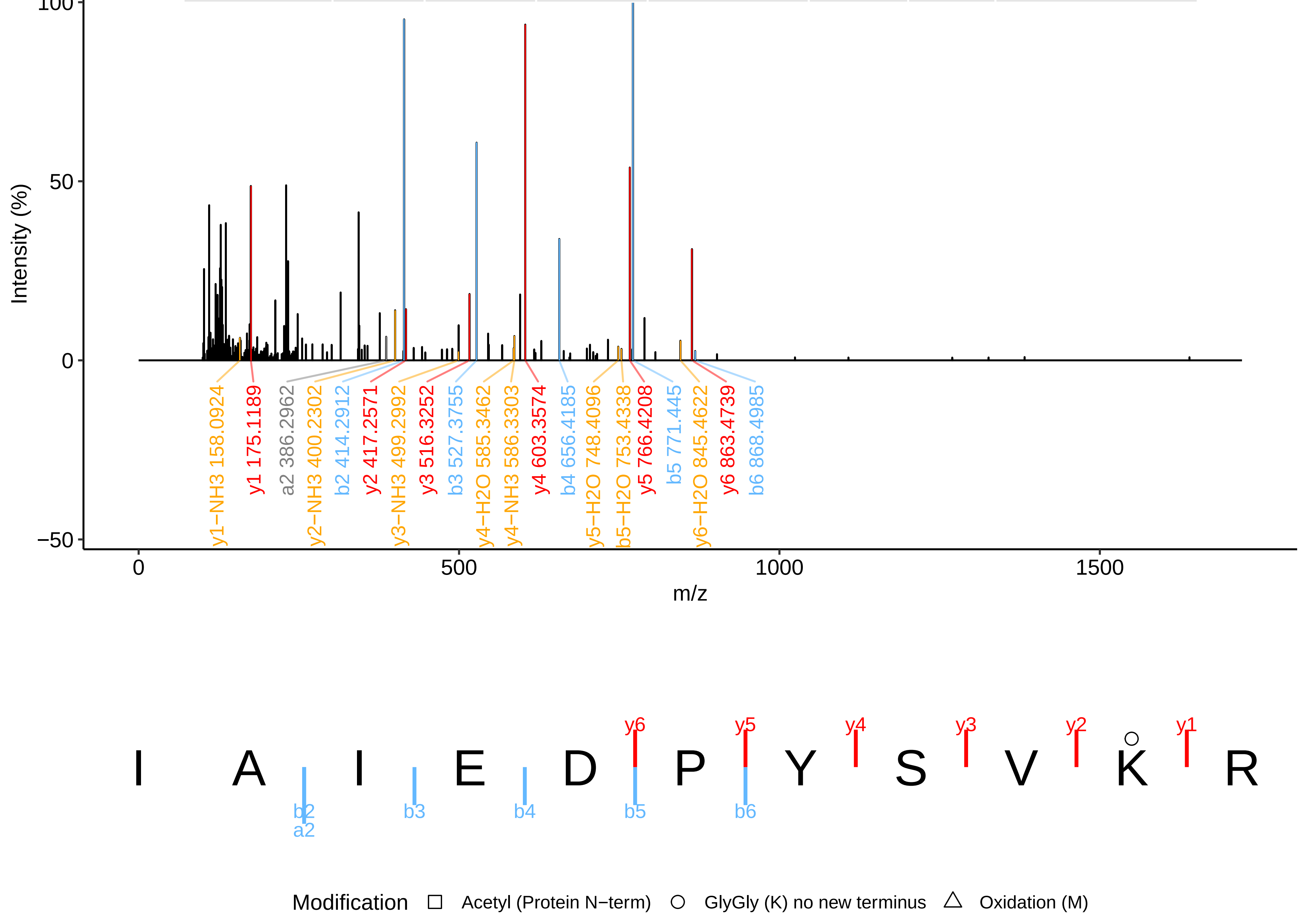

20200921_KIZ_MUb4_GlyGly_10plex − Scan number: 9560

Gene Names	Charge	m/z	Mass	Retention time	PEP	Score	Precursor Intensity
IGF2BP1	3	475.2562	1422.747	32.764	2.0328e−19	156.58	5786925

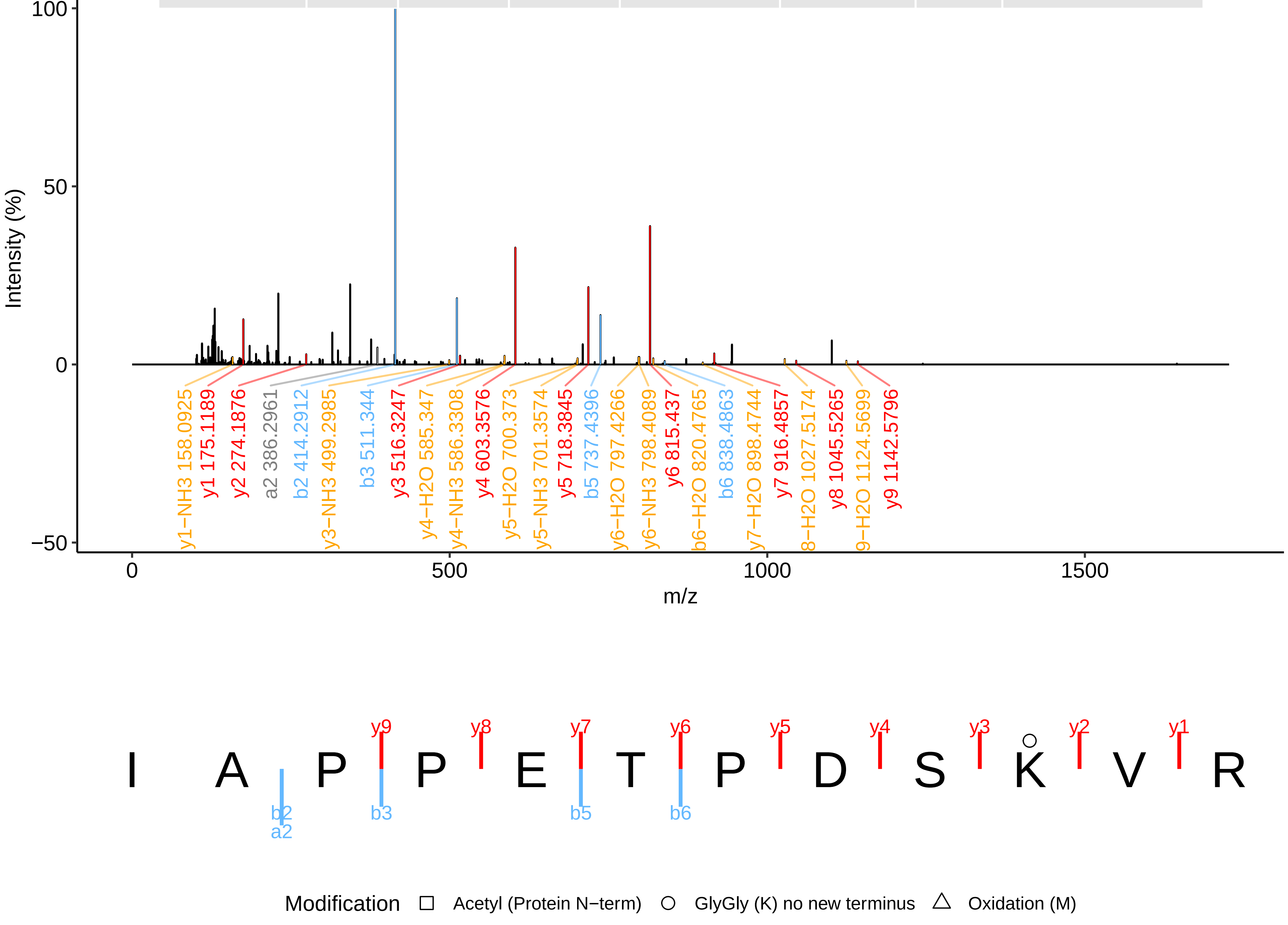

20200921_KIZ_MUb4_GlyGly_10plex_2 − Scan number: 8773

Gene Names	Charge	m/z	Mass	Retention time	PEP	Score	Precursor Intensity
IGF2BP1	3	475.2562	1422.747	33.163	0.11234	43.68	1028065

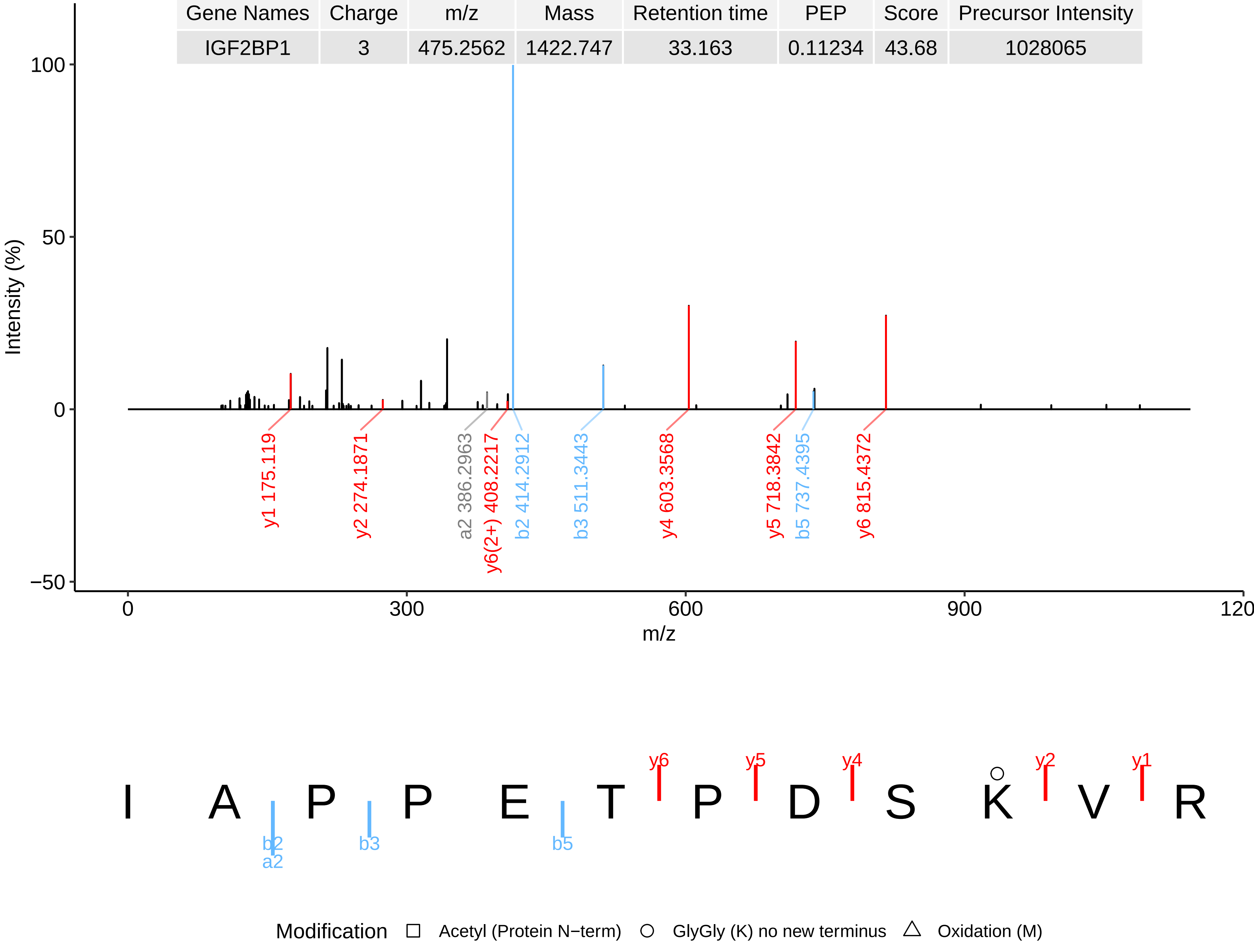

20200921_KIZ_MUb4_GlyGly_10plex_2 − Scan number: 8761

Gene Names	Charge	m/z	Mass	Retention time	PEP	Score	Precursor Intensity
IGF2BP1	3	475.2562	1422.747	33.114	0.01438	67.153	875922.1

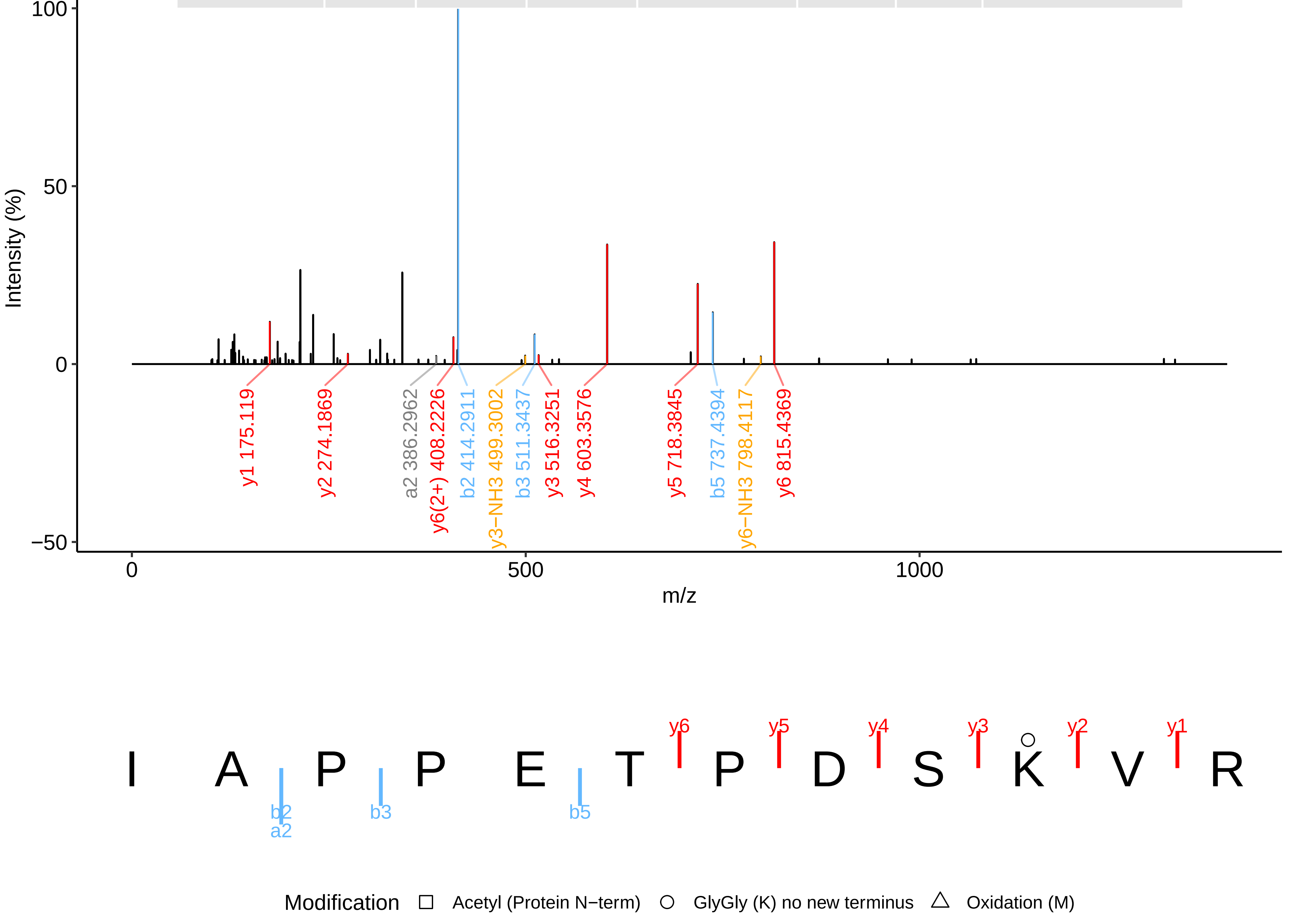

20200924_KIZ_MUb5_GlyGly_1 − Scan number: 8633

Gene Names	Charge	m/z	Mass	Retention time	PEP	Score	Precursor Intensity
IGF2BP1	3	475.2562	1422.747	27.633	4.5259e−05	100.09	5543723

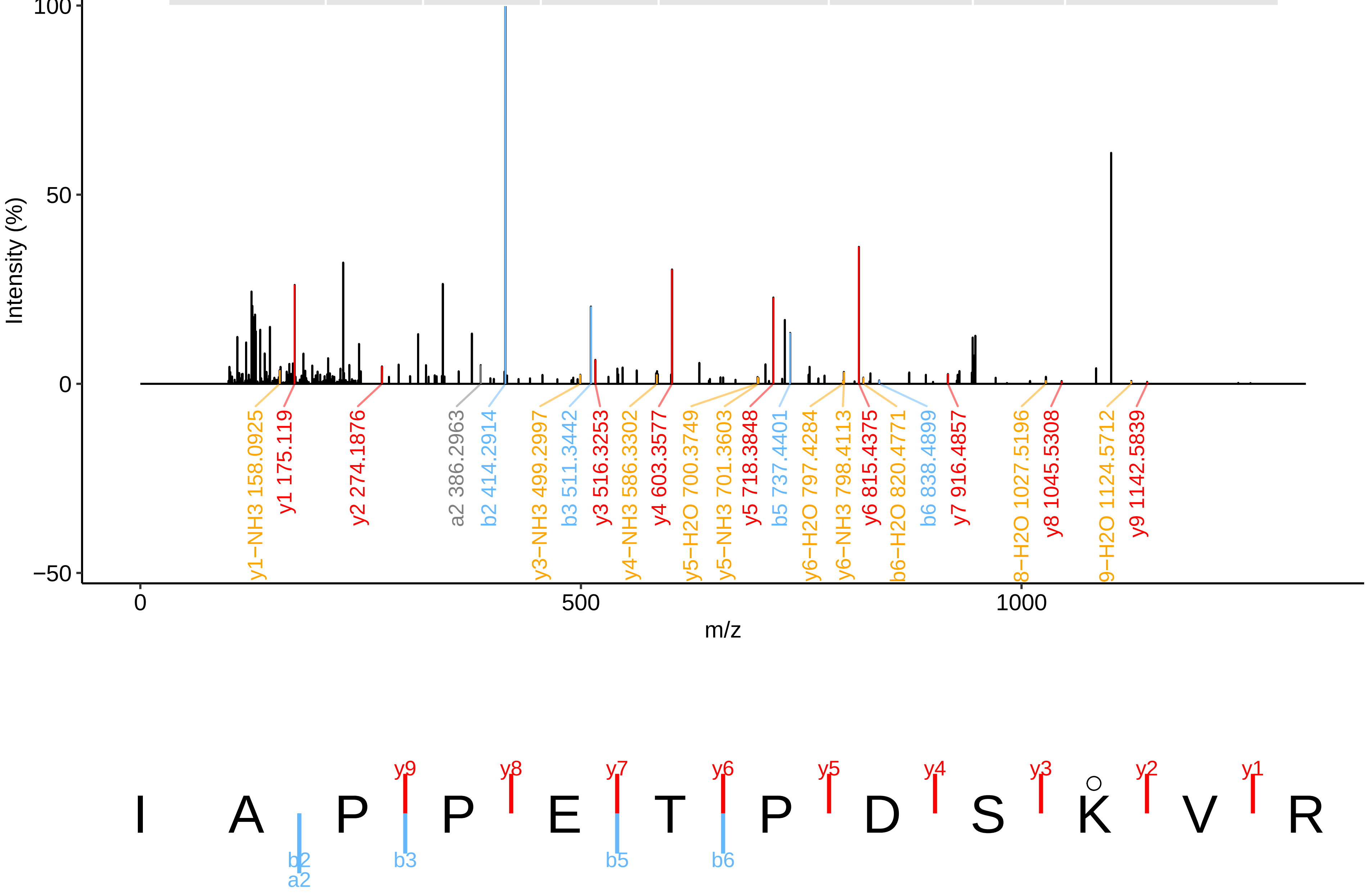

Modification □ Acetyl (Protein N−term) ○ GlyGly (K) no new terminus △ Oxidation (M)

20201118_KIZ_MUb6_GlyGly_2 − Scan number: 9949

Gene Names	Charge	m/z	Mass	Retention time	PEP	Score	Precursor Intensity
IGF2BP1	3	475.2562	1422.747	33.662	0.0042342	77.593	NA

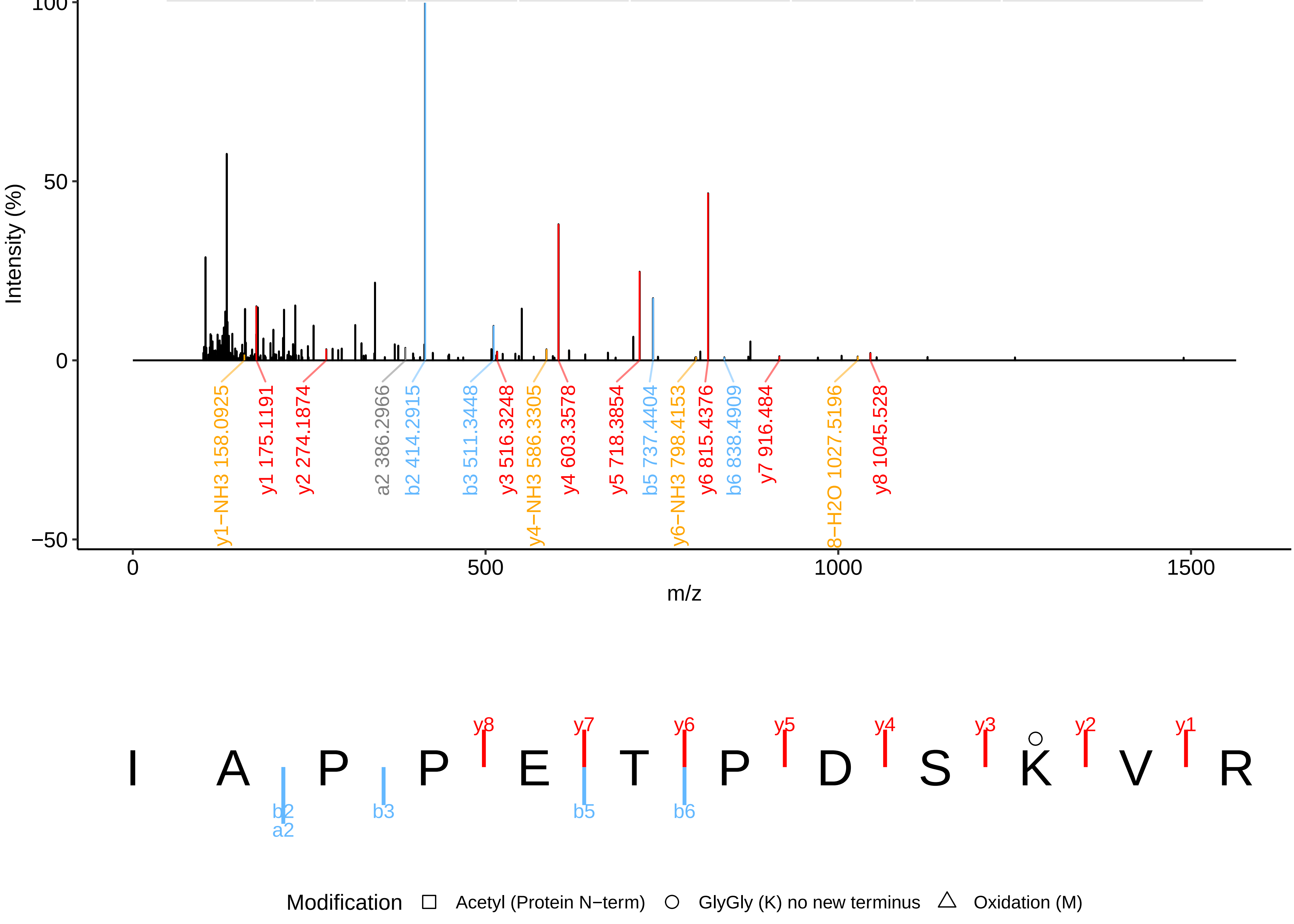

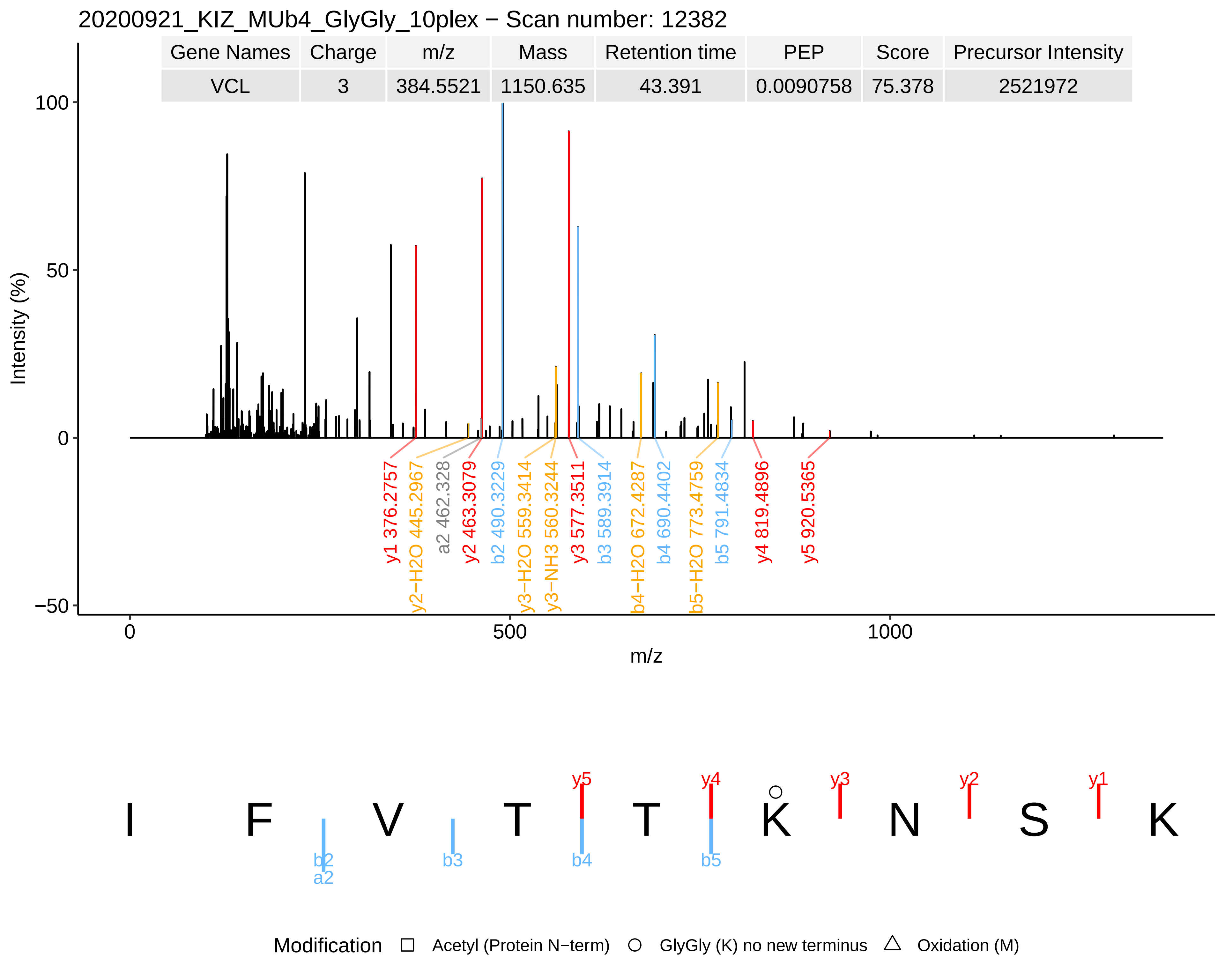
20200921_KIZ_MUb4_GlyGly_10plex − Scan number: 12382
Gene Names | Charge | m/z | Mass | Retention time | PEP | Score | Precursor Intensity
VCL | 3 | 384.5521 | 1150.635 | 43.391 | 0.0090758 | 75.378 | 2521972
Intensity (%)
m/z
y1 376.2757
y2−H2O 445.2967
a2 462.328
y2 463.3079
b2 490.3229
y3−H2O 559.3414
y3−NH3 560.3244
y3 577.3511
b3 589.3914
b4−H2O 672.4287
b4 690.4402
b5−H2O 773.4759
b5 791.4834
y4 819.4896
y5 920.5365
I F V T T K N S K
y5 y4 y3 y2 y1
b2 a2 b3 b4 b5
Modification □ Acetyl (Protein N−term) ○ GlyGly (K) no new terminus △ Oxidation (M)

20200921_KIZ_MUb4_GlyGly_10plex − Scan number: 15905

Gene Names	Charge	m/z	Mass	Retention time	PEP	Score	Precursor Intensity
VPS13A	3	473.2527	1416.736	56.968	0.00092528	93.258	6705673

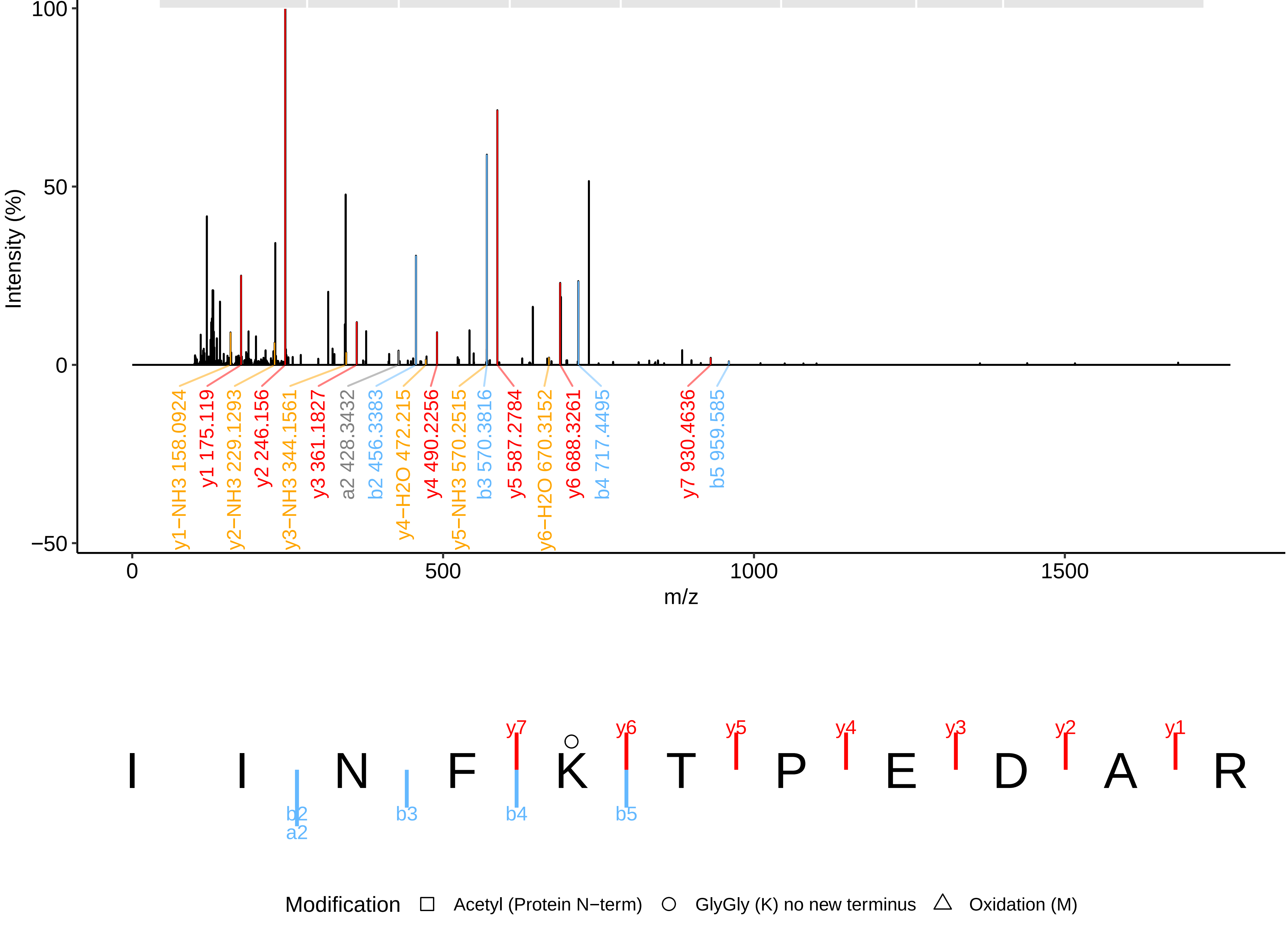

20200921_KIZ_MUb4_GlyGly_10plex − Scan number: 6214

Gene Names	Charge	m/z	Mass	Retention time	PEP	Score	Precursor Intensity
NEDD8;NEDD8−MDP1	3	382.2155	1143.625	20.484	0.015334	79.974	4115109

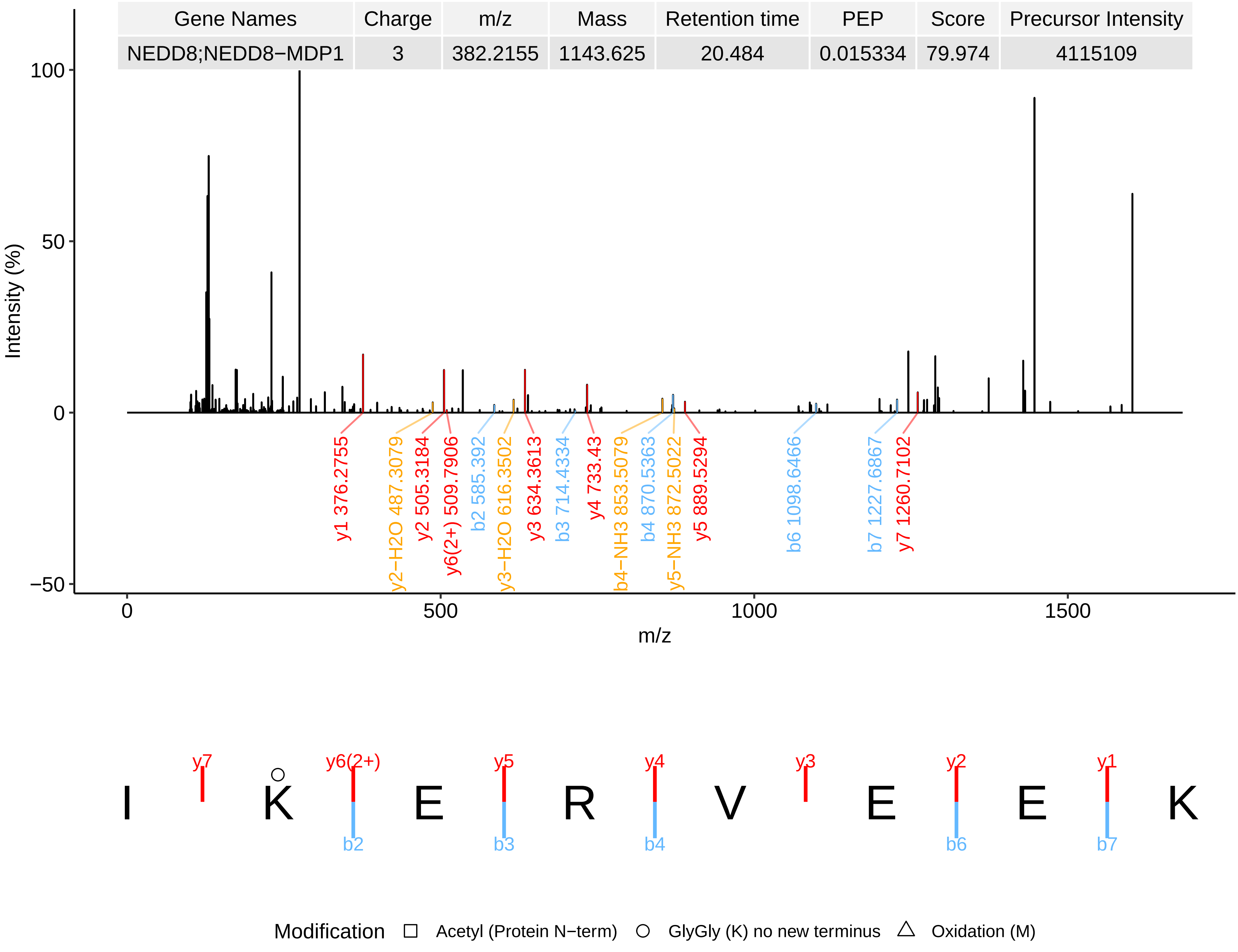

20200921_KIZ_MUb4_GlyGly_10plex − Scan number: 16756

Gene Names	Charge	m/z	Mass	Retention time	PEP	Score	Precursor Intensity
POR	3	462.5931	1384.757	60.299	0.00099013	92.611	4578013

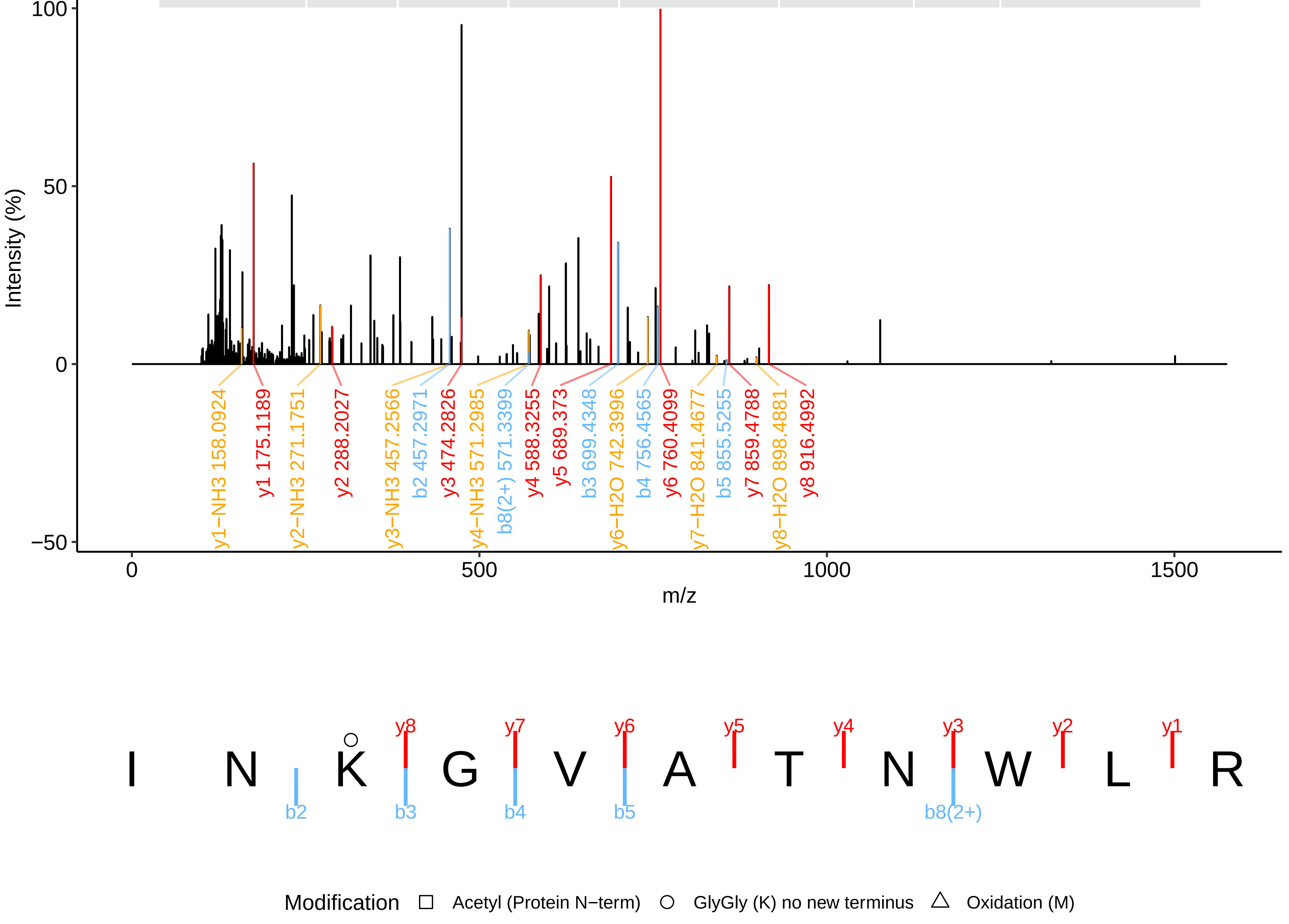

20200921_KIZ_MUb4_GlyGly_10plex_2 − Scan number: 15555

Gene Names	Charge	m/z	Mass	Retention time	PEP	Score	Precursor Intensity
POR	3	462.5931	1384.757	60.854	0.011641	68.536	498595.6

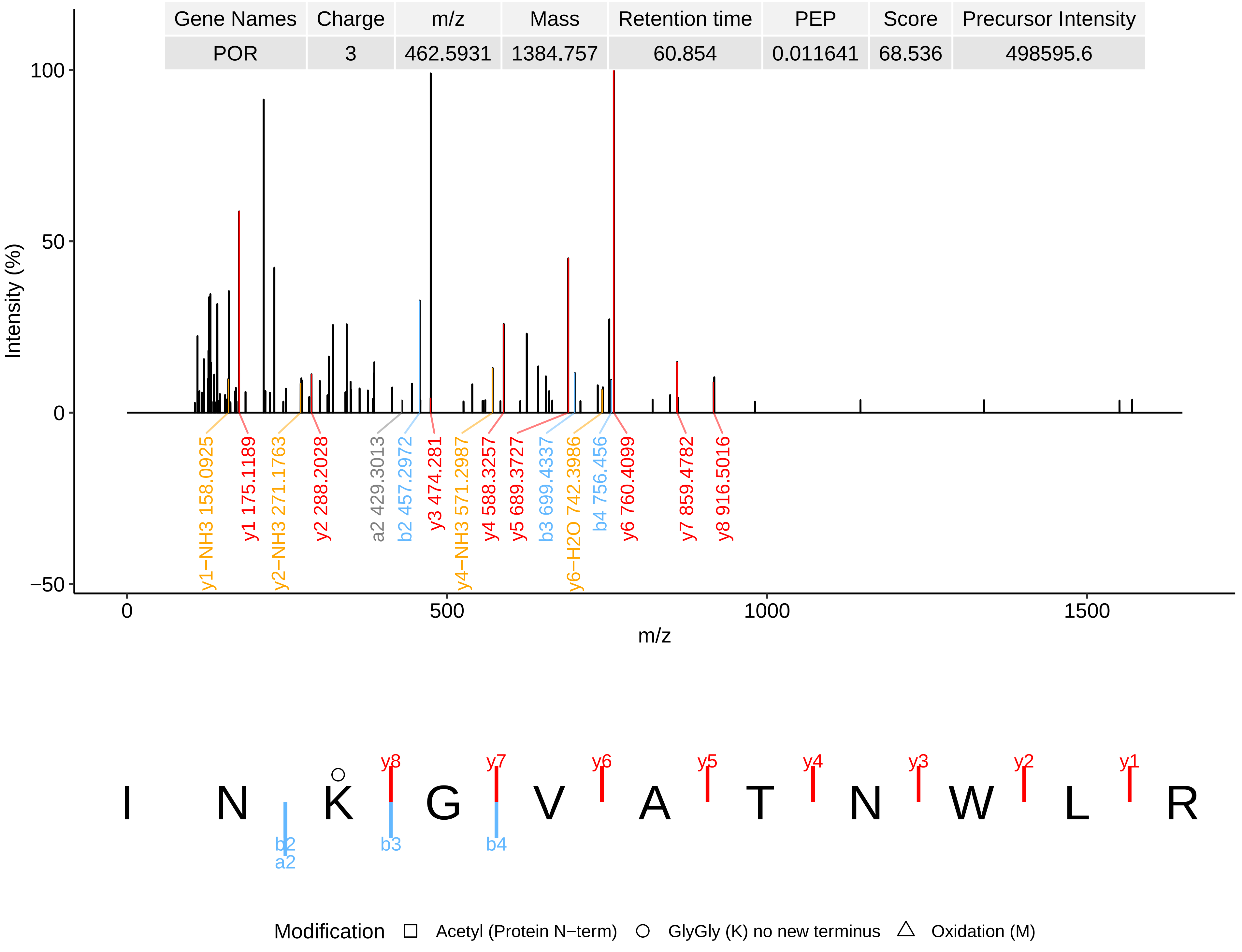

20200924_KIZ_MUb5_GlyGly_1 − Scan number: 15640

Gene Names	Charge	m/z	Mass	Retention time	PEP	Score	Precursor Intensity
POR	3	462.5931	1384.757	51.035	0.0014185	83.775	20109296

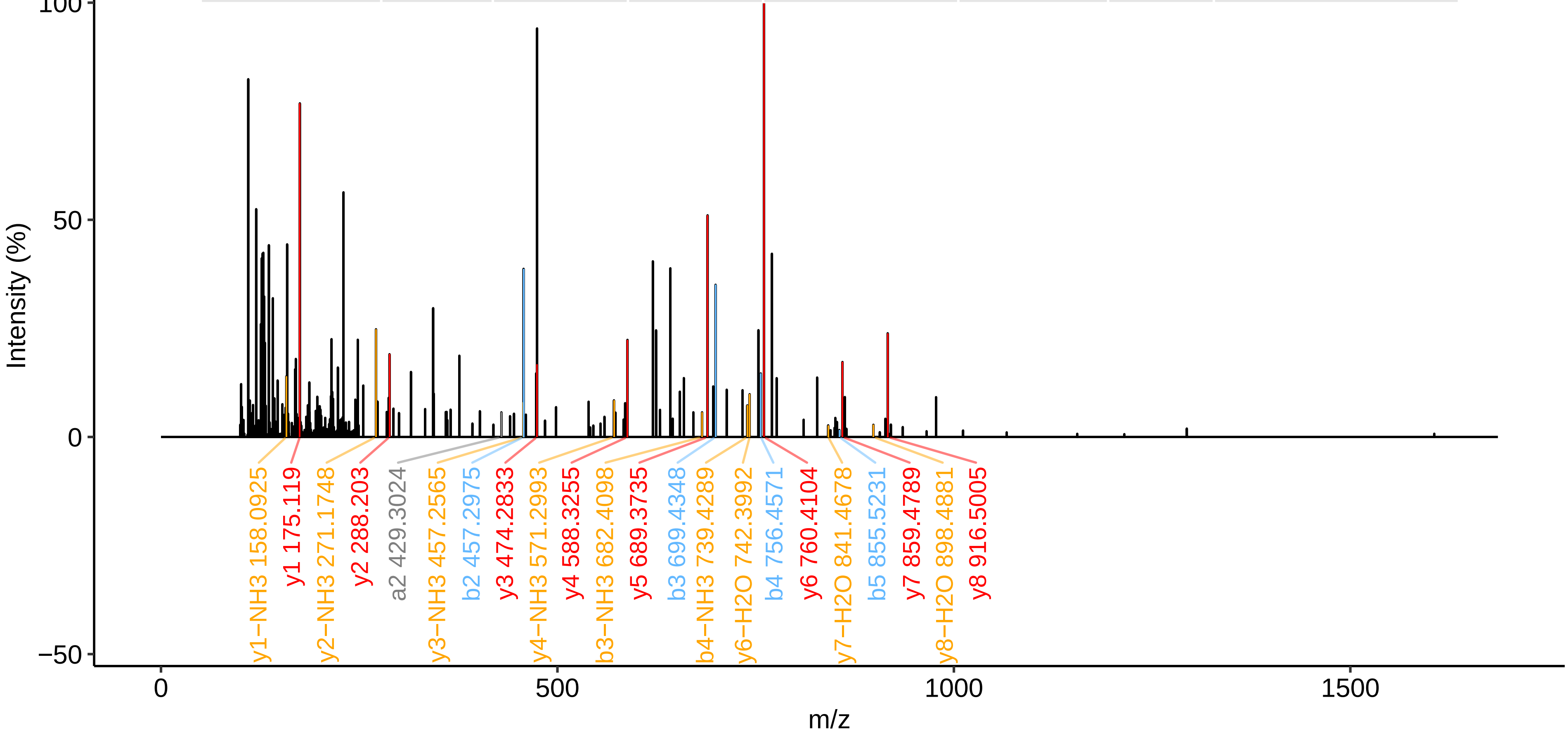

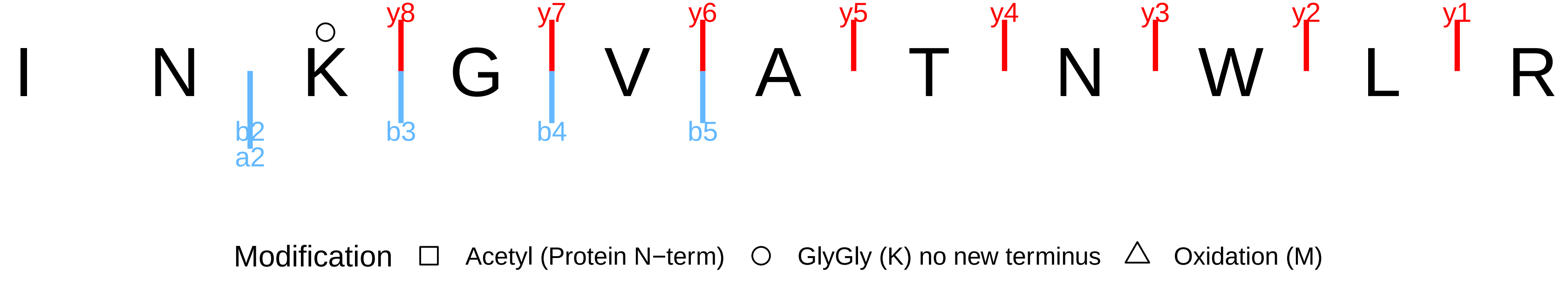

20200924_KIZ_MUb5_GlyGly_2 − Scan number: 14871

Gene Names	Charge	m/z	Mass	Retention time	PEP	Score	Precursor Intensity
POR	3	462.5931	1384.757	55.565	0.031836	61.435	2363432

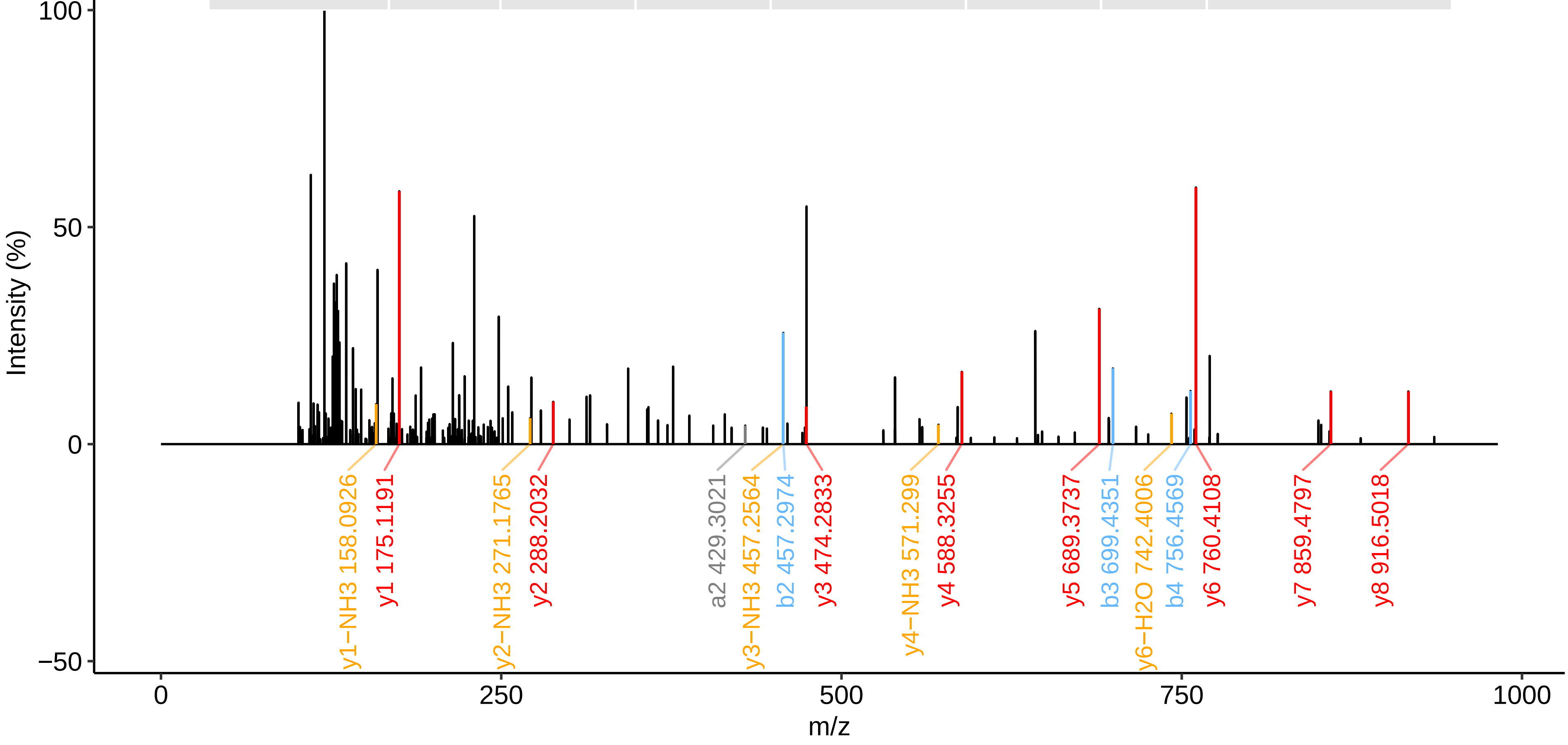

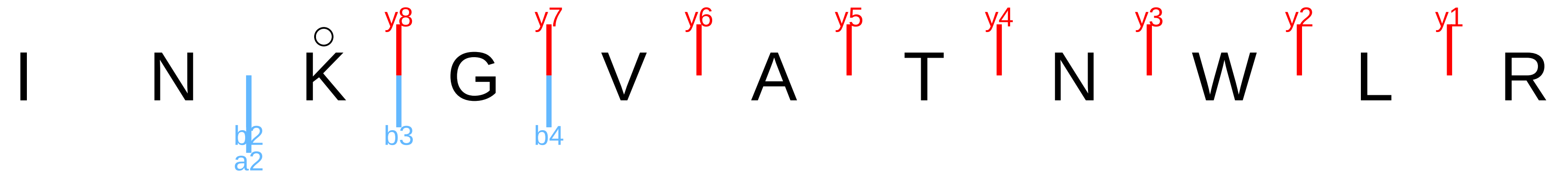

Modification □ Acetyl (Protein N−term) ○ GlyGly (K) no new terminus △ Oxidation (M)

20200921_KIZ_MUb4_GlyGly_10plex_2 − Scan number: 12910

Gene Names	Charge	m/z	Mass	Retention time	PEP	Score	Precursor Intensity
SLC9A8	3	397.5569	1189.649	50.061	0.018601	66.435	815128.9

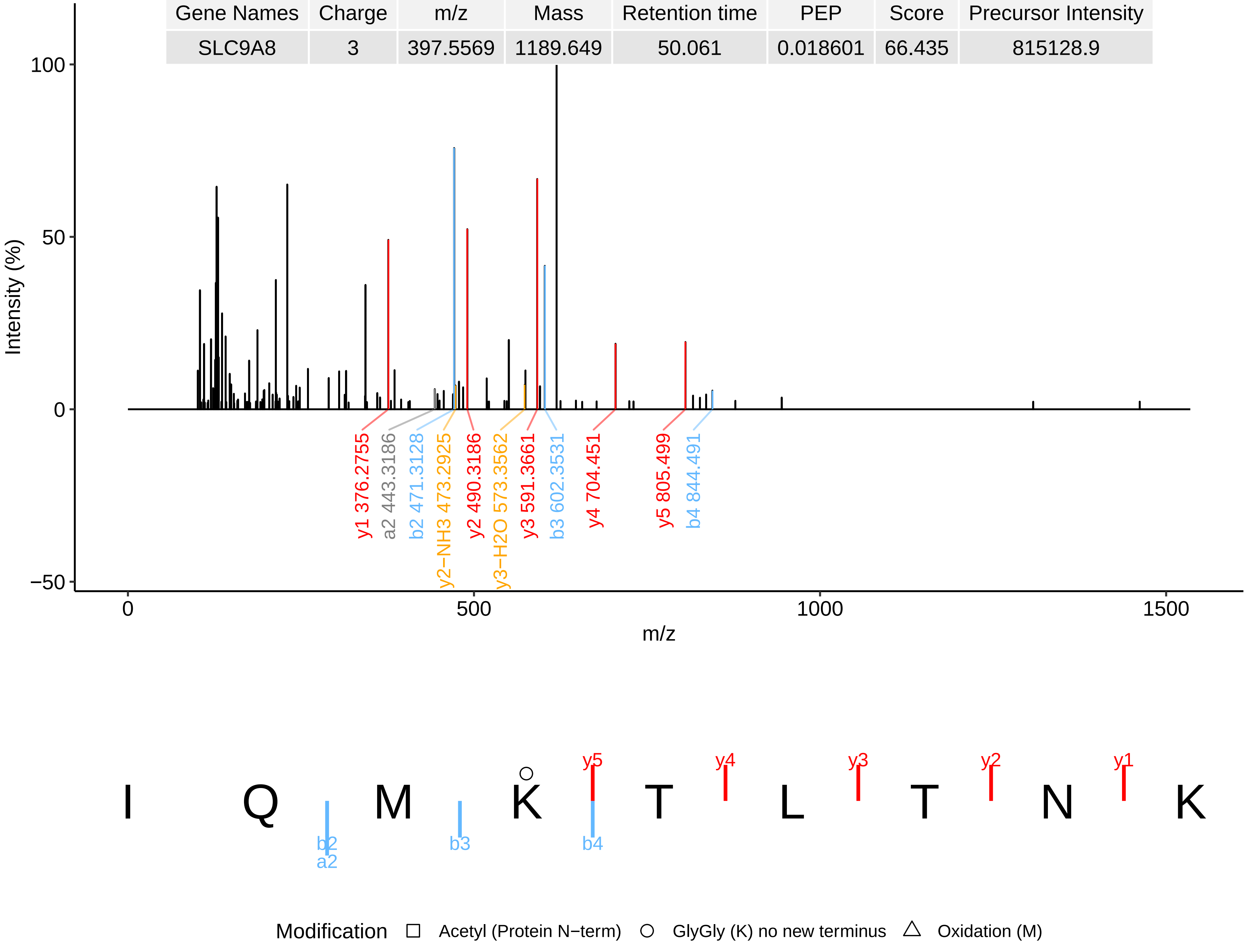

20200921_KIZ_MUb4_GlyGly_10plex − Scan number: 10244

Gene Names	Charge	m/z	Mass	Retention time	PEP	Score	Precursor Intensity
UBE2N	4	357.1954	1424.752	35.379	0.00060726	90.827	6477819

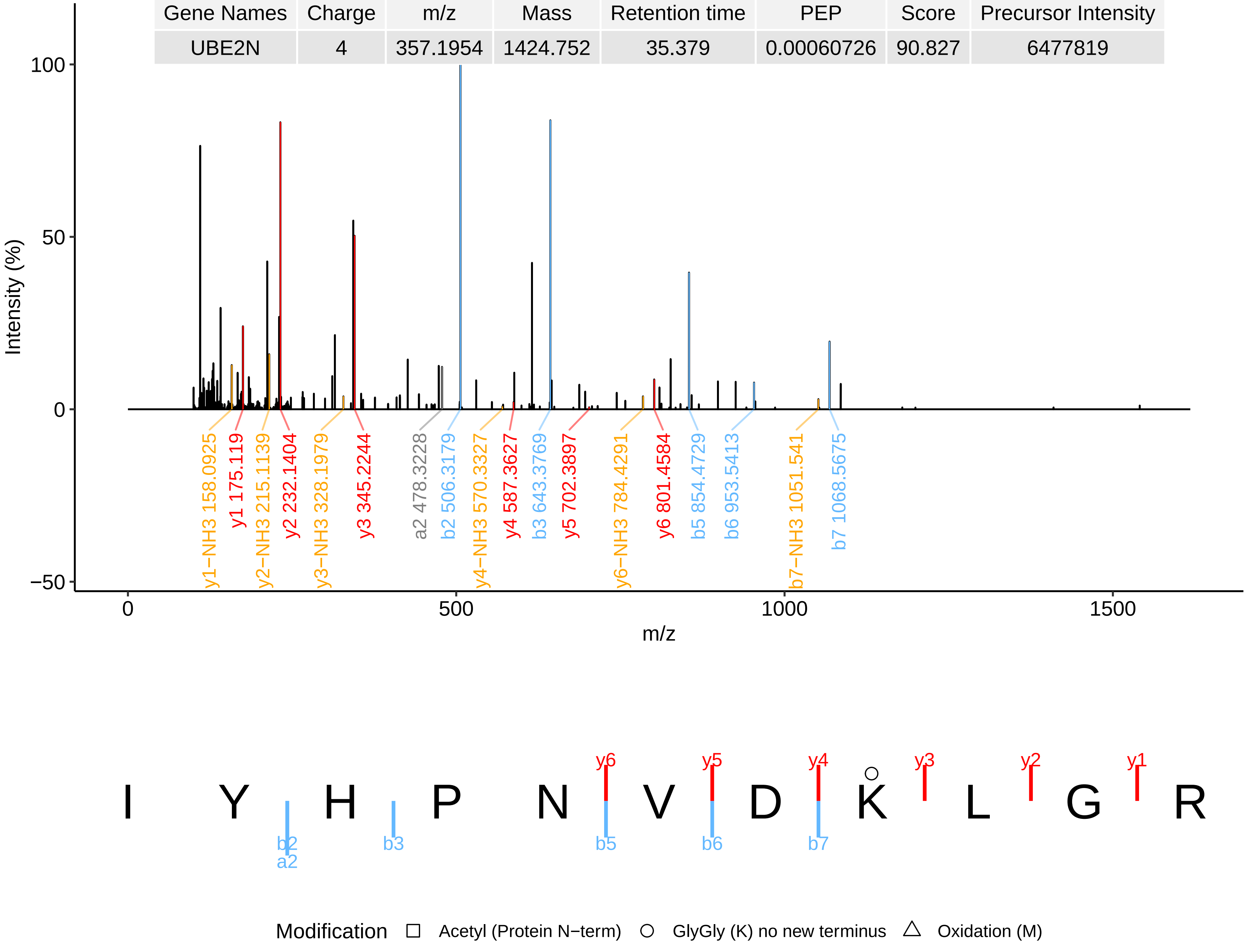

20200921_KIZ_MUb4_GlyGly_10plex_2 − Scan number: 9463

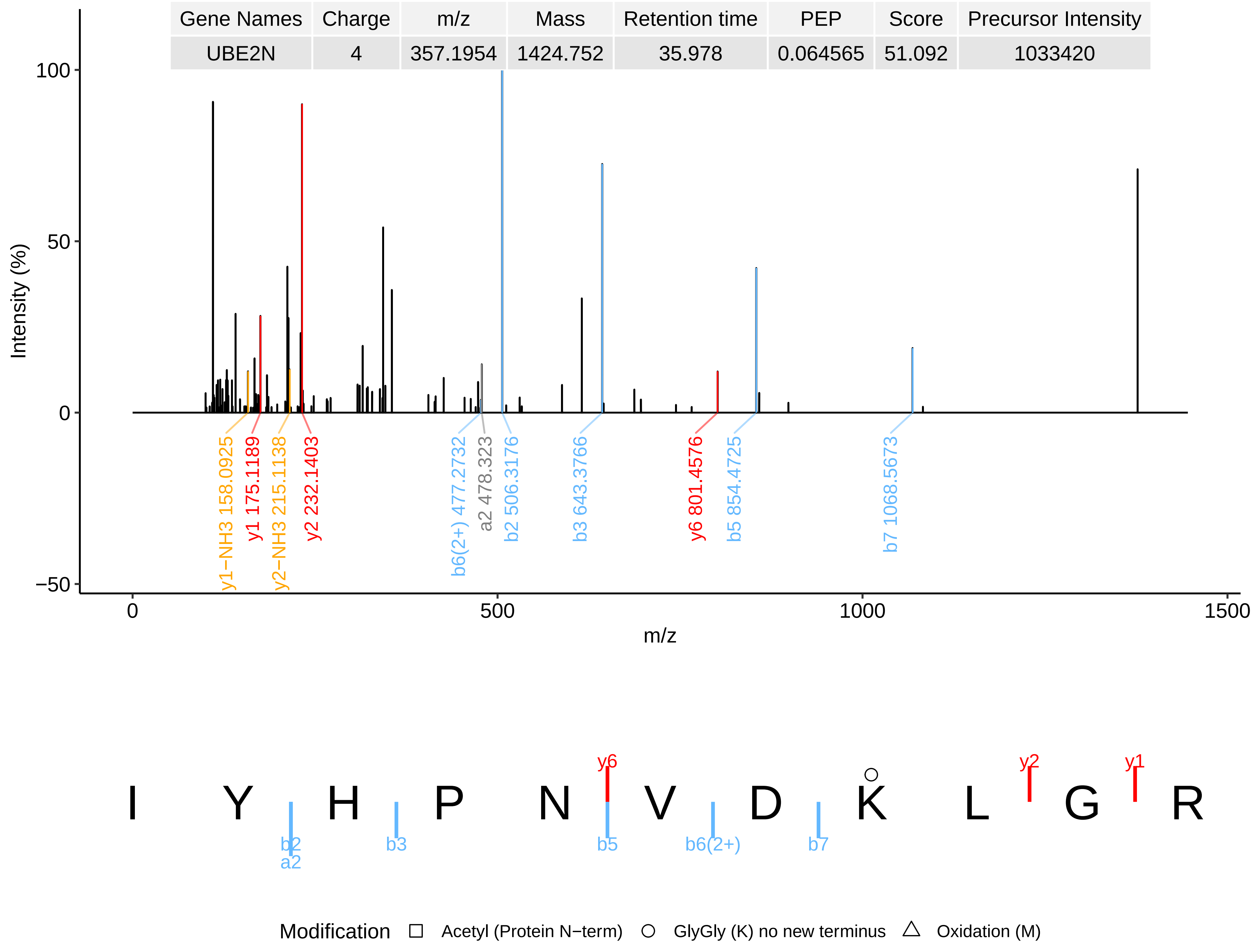

20200921_KIZ_MUb4_GlyGly_10plex − Scan number: 8740

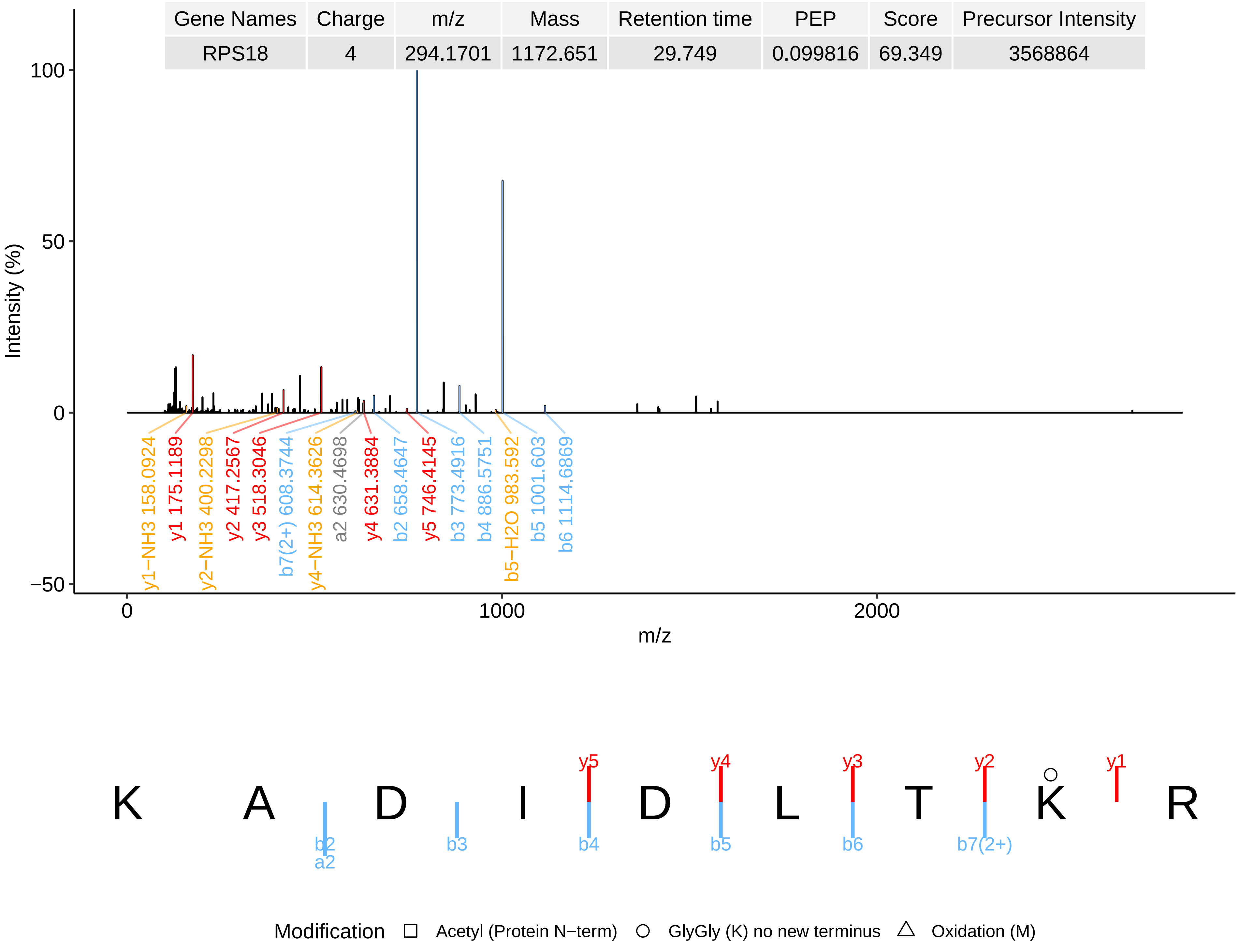

20200921_KIZ_MUb4_GlyGly_10plex − Scan number: 9137

Gene Names	Charge	m/z	Mass	Retention time	PEP	Score	Precursor Intensity
FANCI	3	465.5663	1393.677	31.253	0.00080504	85.924	NA

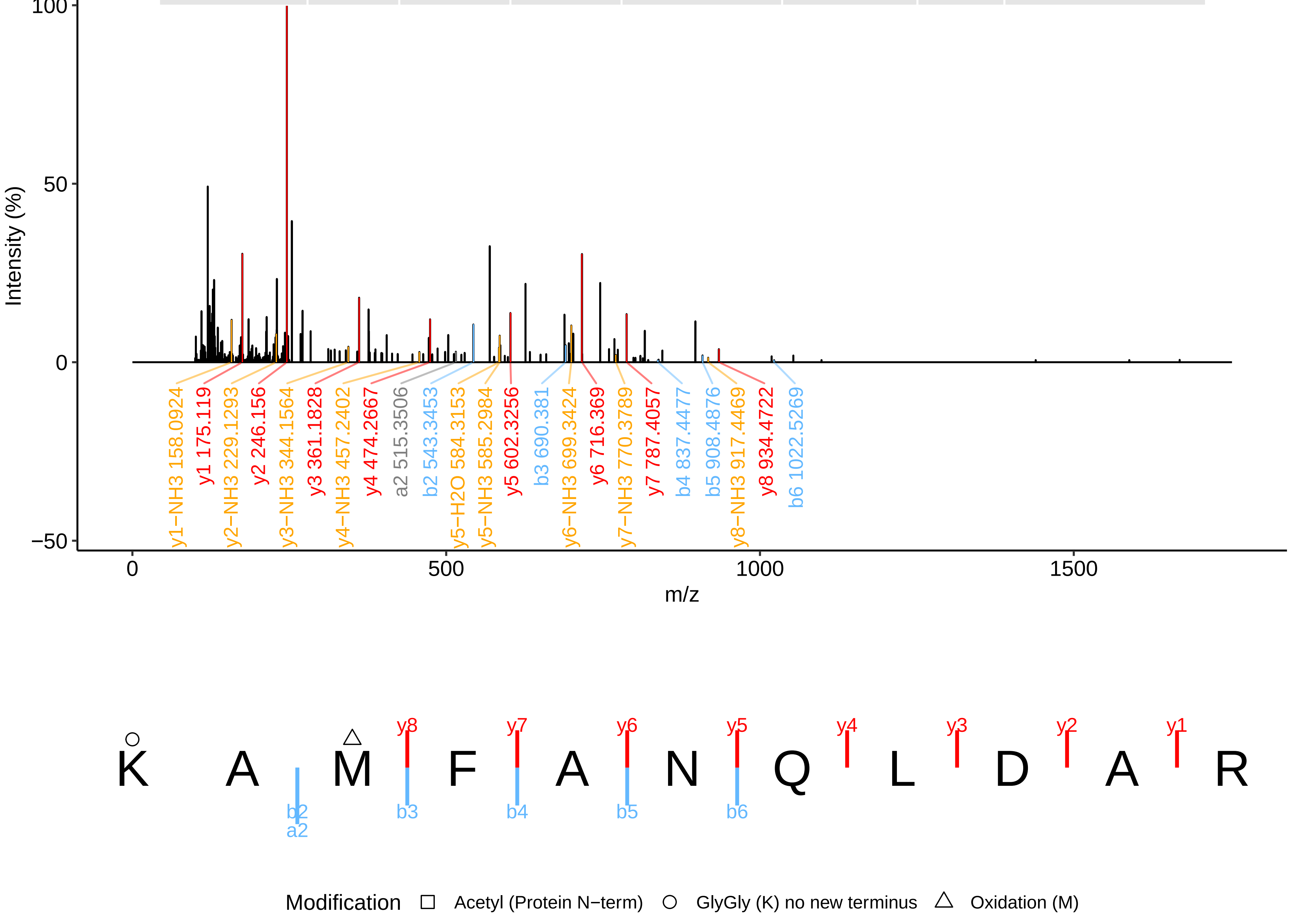

20200921_KIZ_MUb4_GlyGly_10plex_2 − Scan number: 8380

Gene Names	Charge	m/z	Mass	Retention time	PEP	Score	Precursor Intensity
FANCI	3	465.5663	1393.677	31.553	0.016947	61.344	258741.3

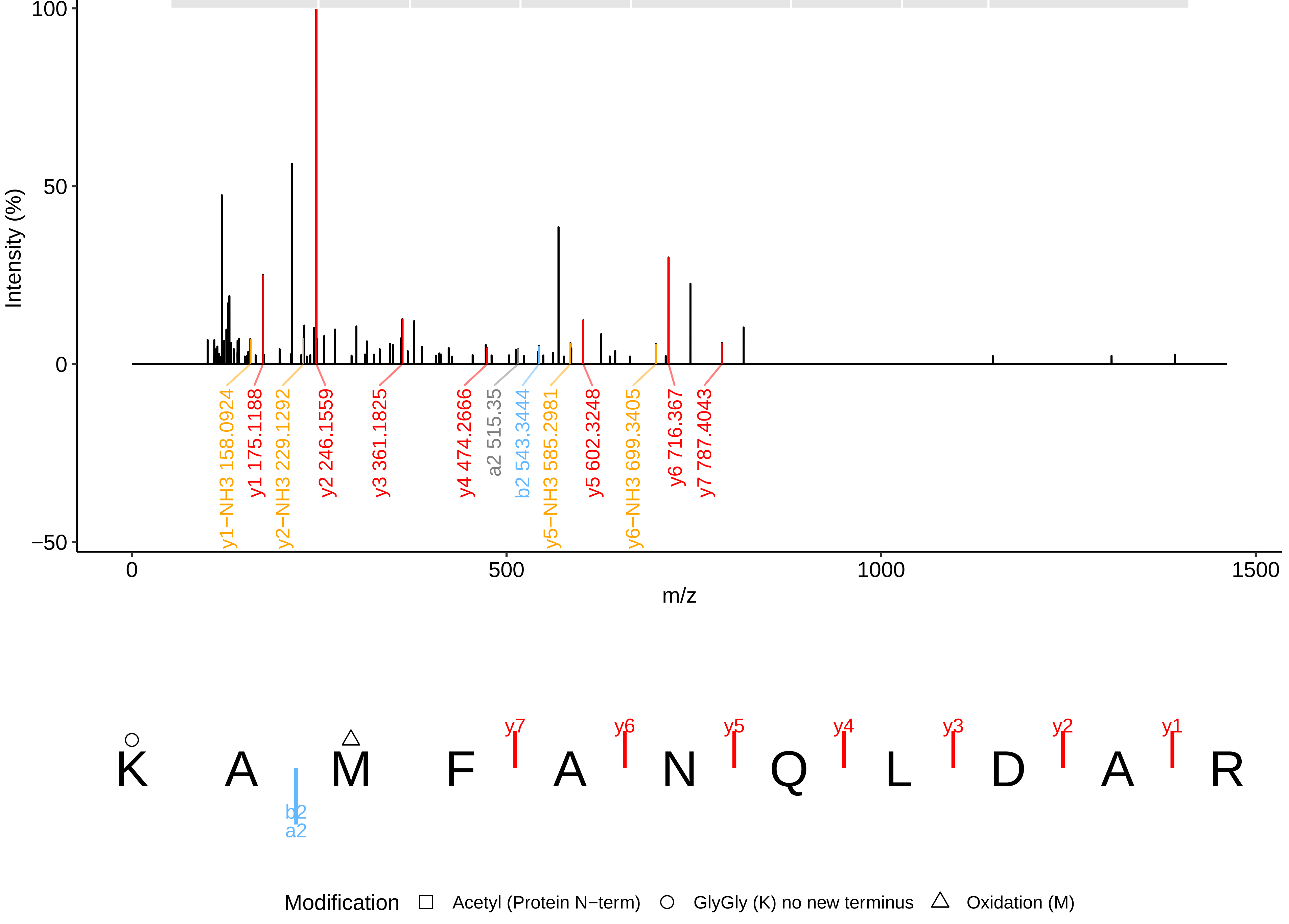

20200921_KIZ_MUb4_GlyGly_10plex − Scan number: 12262

Gene Names	Charge	m/z	Mass	Retention time	PEP	Score	Precursor Intensity
FANCI	3	460.2347	1377.682	42.938	0.005169	89.805	16512930

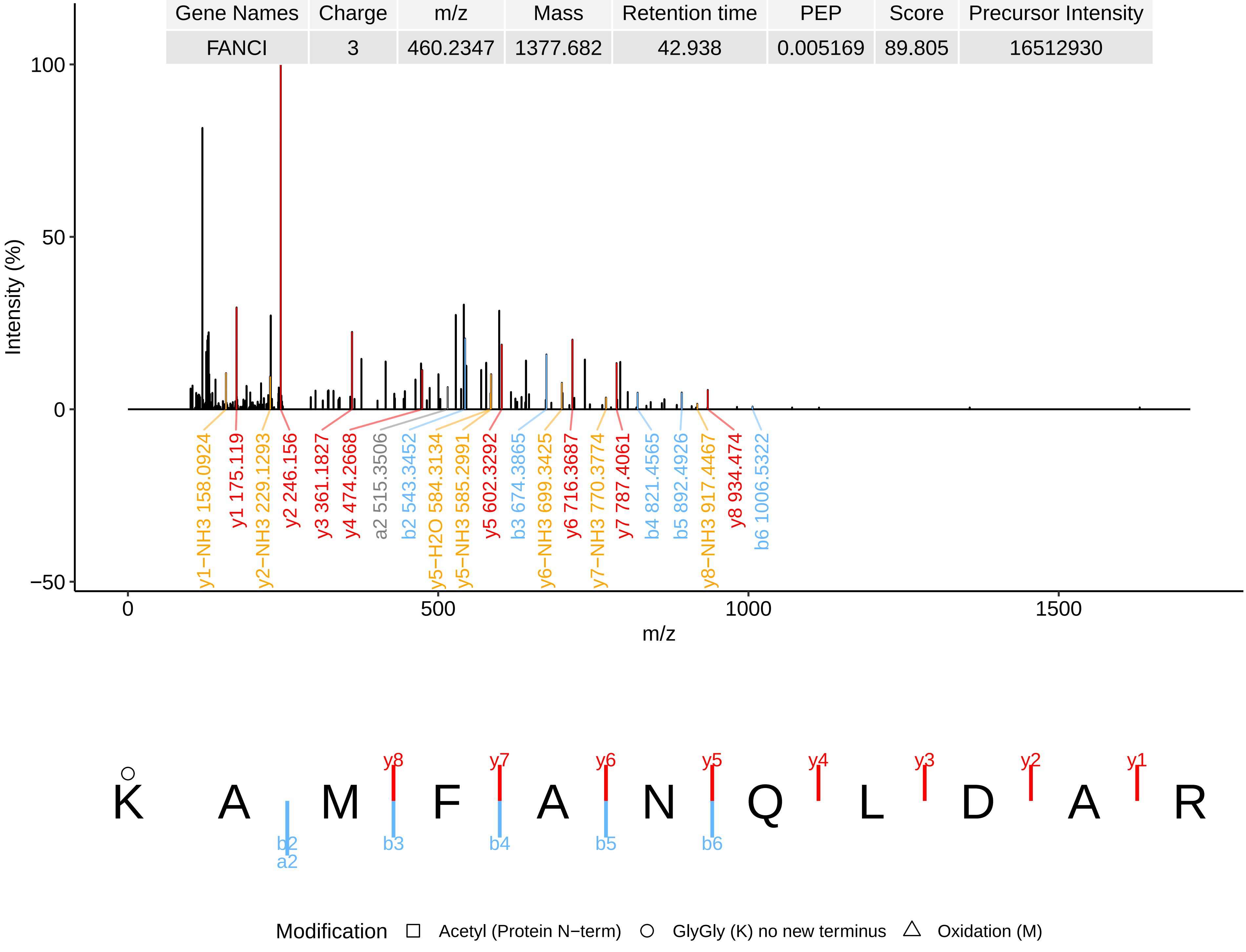

20200921_KIZ_MUb4_GlyGly_10plex_2 − Scan number: 11297

Gene Names	Charge	m/z	Mass	Retention time	PEP	Score	Precursor Intensity
FANCI	3	460.2347	1377.682	43.446	0.0054904	89.266	1319663

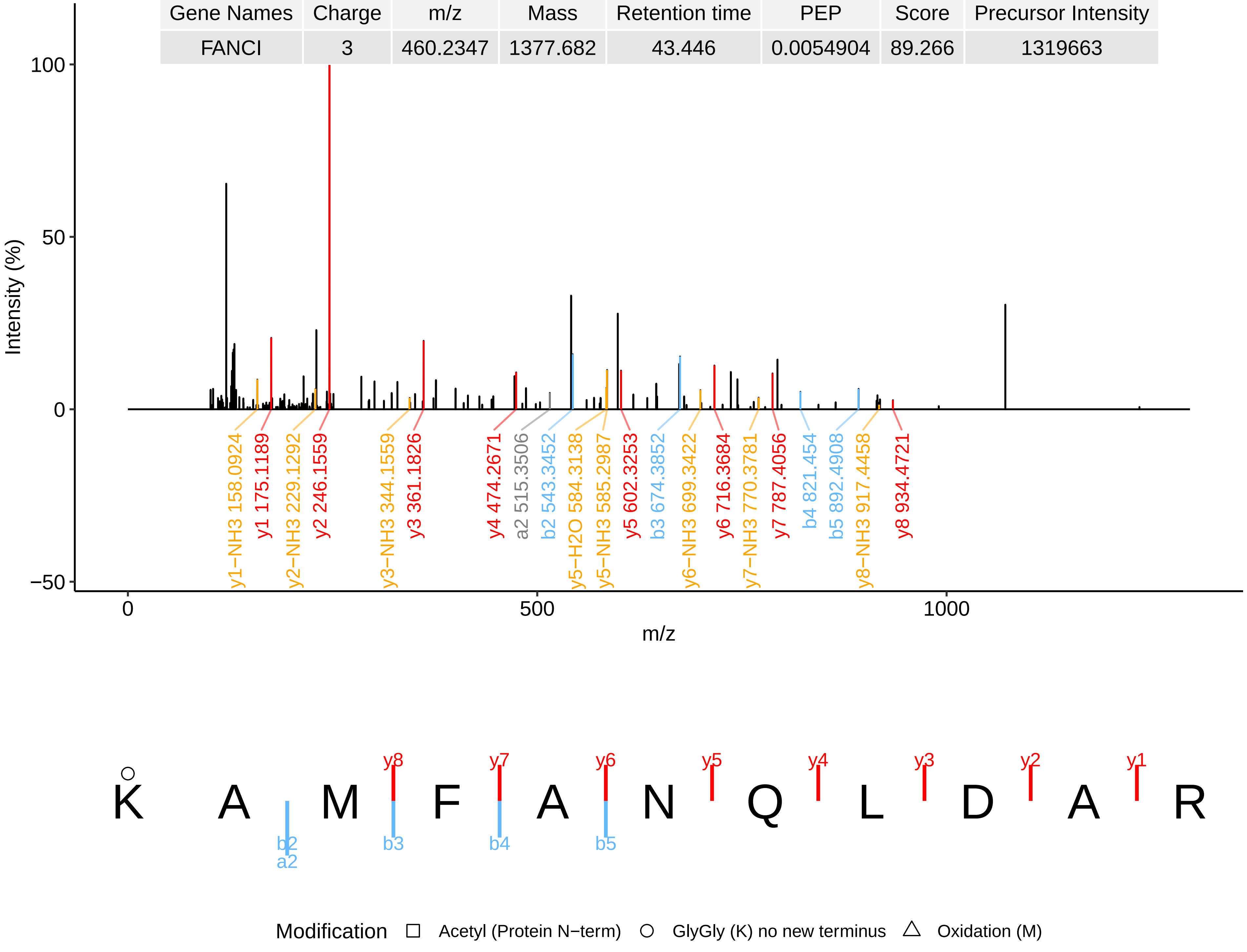

20200924_KIZ_MUb5_GlyGly_1 − Scan number: 10902

Gene Names	Charge	m/z	Mass	Retention time	PEP	Score	Precursor Intensity
FANCI	3	460.2347	1377.682	35.279	3.0444e−07	117.25	5137143

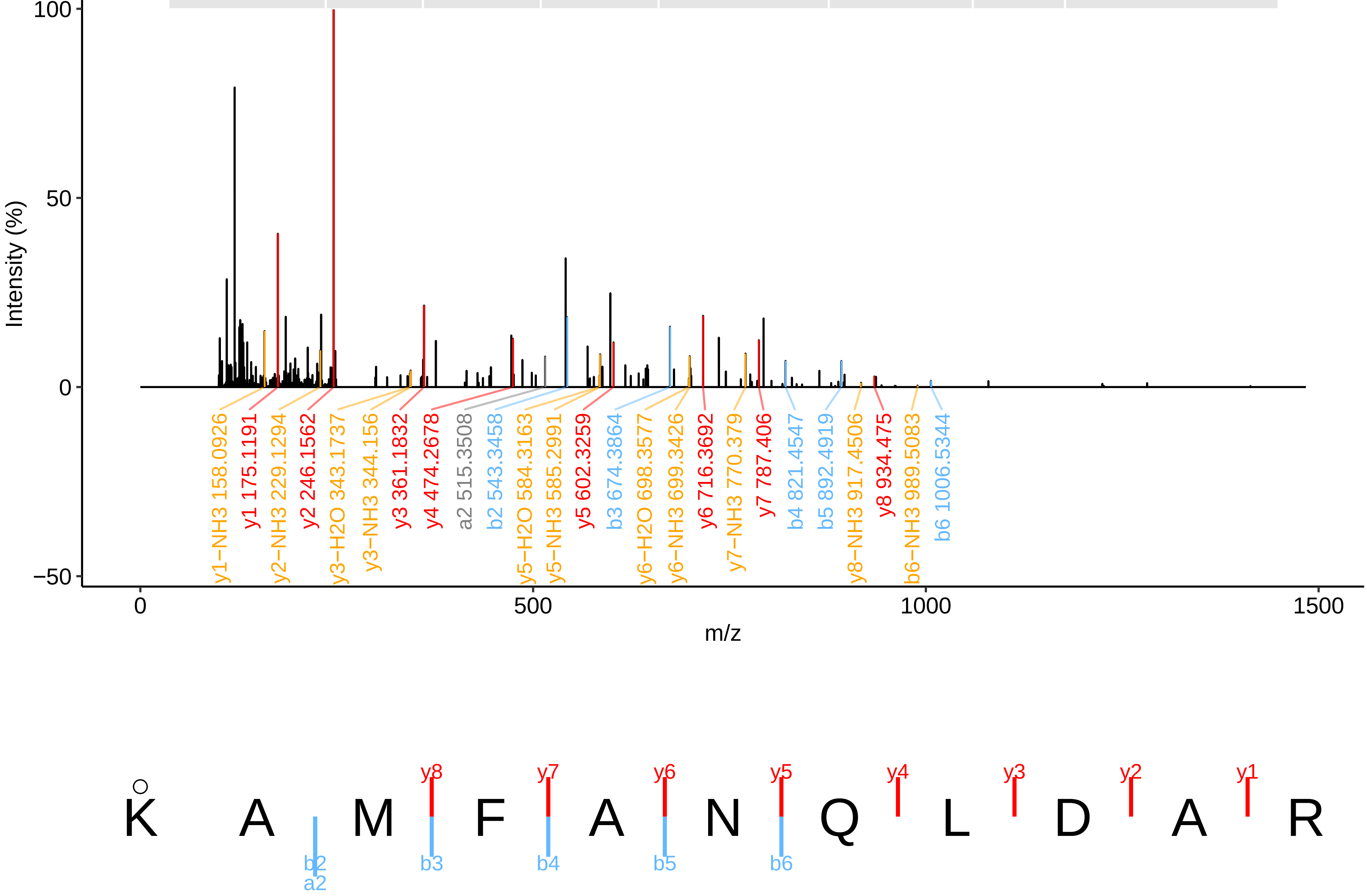

Modification □ Acetyl (Protein N−term) ○ GlyGly (K) no new terminus △ Oxidation (M)

20200924_KIZ_MUb5_GlyGly_2 − Scan number: 10535

Gene Names	Charge	m/z	Mass	Retention time	PEP	Score	Precursor Intensity
FANCI	3	460.2347	1377.682	39.322	0.021034	78.548	1280796

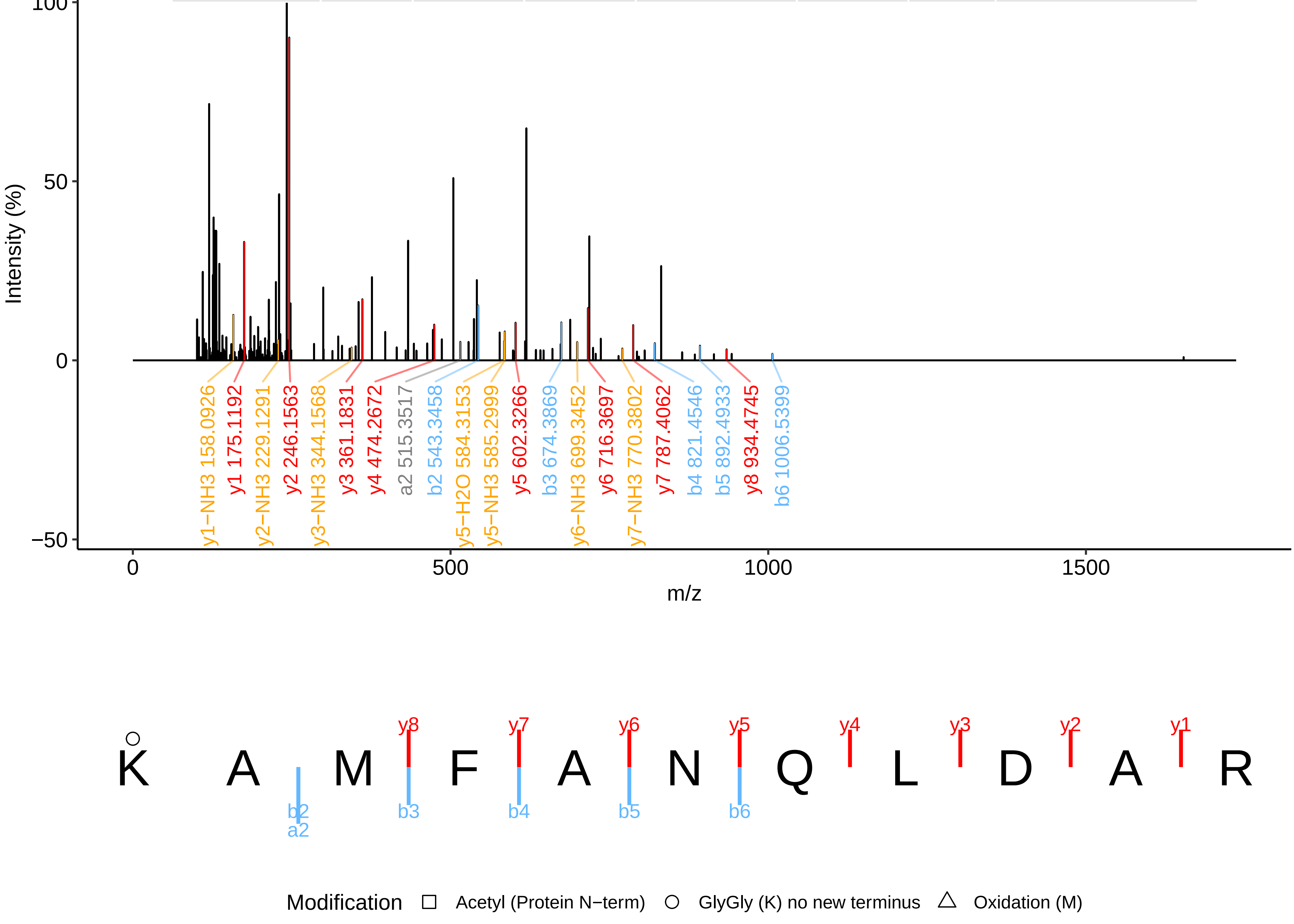

20201118_KIZ_MUb6_GlyGly_1 − Scan number: 10711

Gene Names	Charge	m/z	Mass	Retention time	PEP	Score	Precursor Intensity
FANCI	3	460.2347	1377.682	33.786	0.00030048	120.77	19572274

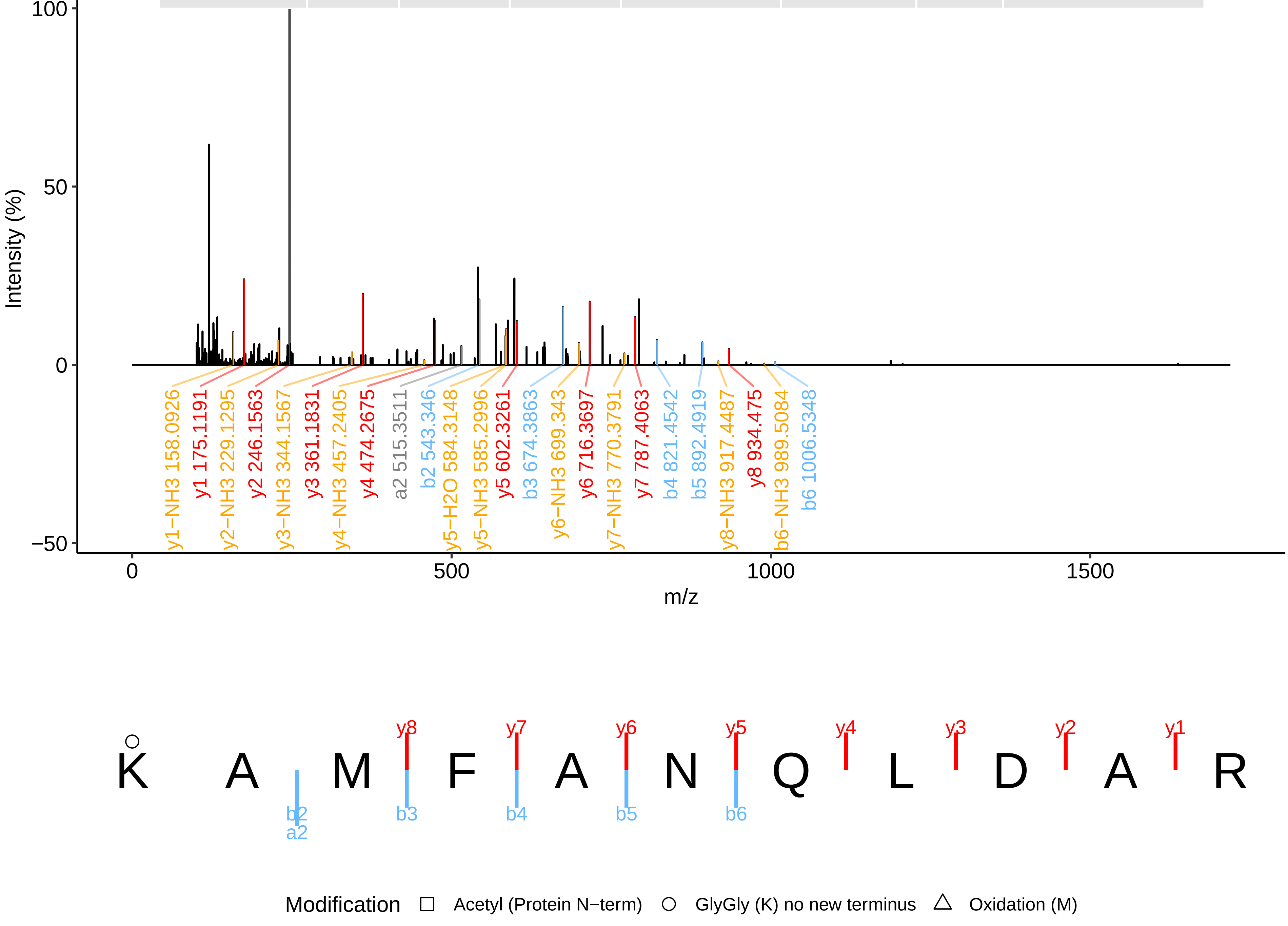

20200921_KIZ_MUb4_GlyGly_10plex − Scan number: 6013

Gene Names	Charge	m/z	Mass	Retention time	PEP	Score	Precursor Intensity
BASP1	3	394.1913	1179.552	19.751	0.018011	84.213	2071078

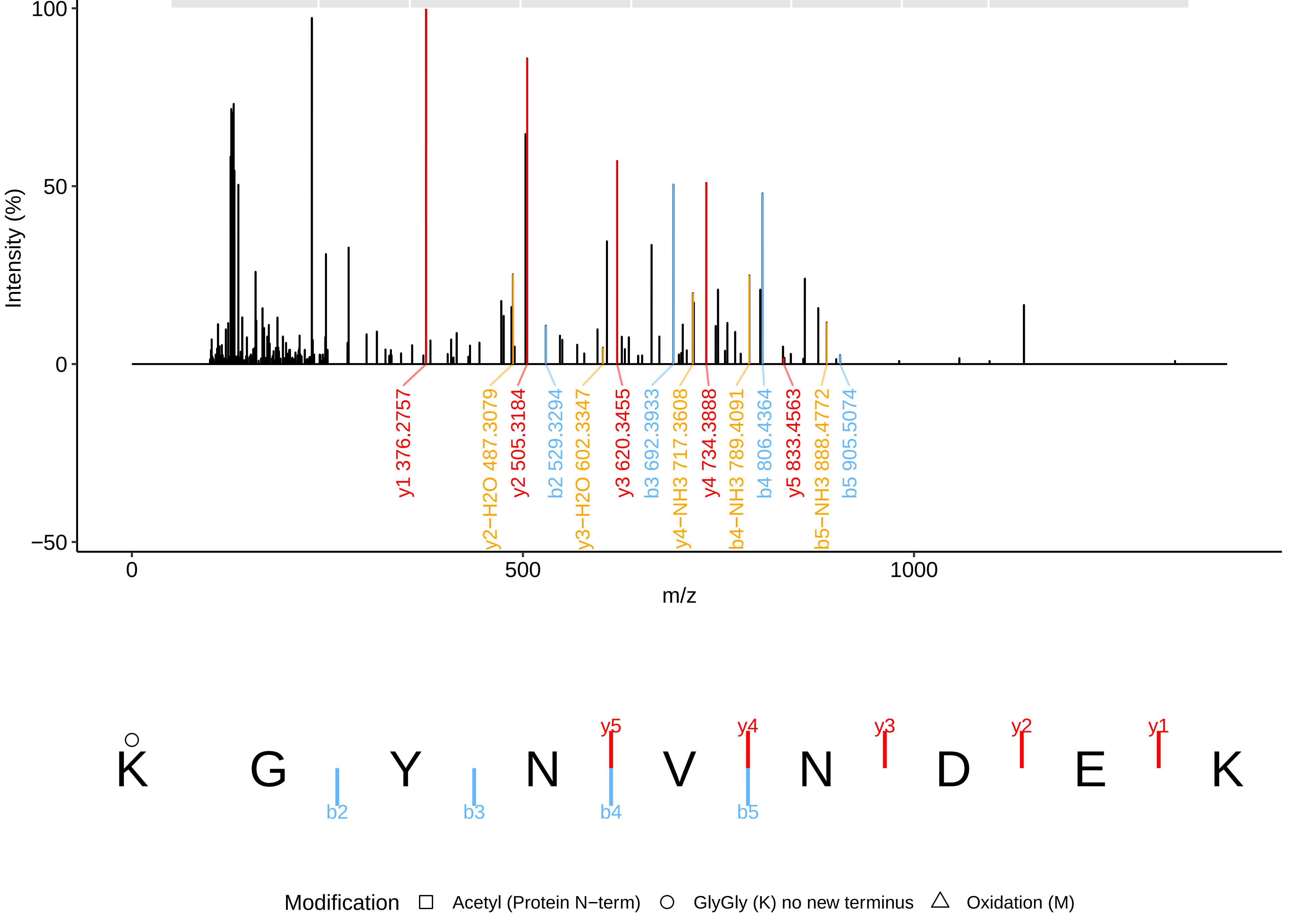

20200921_KIZ_MUb4_GlyGly_10plex − Scan number: 16241

Gene Names	Charge	m/z	Mass	Retention time	PEP	Score
B4A;TUBB;TUBB1;TUBB4B;TUBB2A;TUBB2B;TUBB8;TUBB6	3	467.9272	1400.76	58.278	0.0087112	66.758

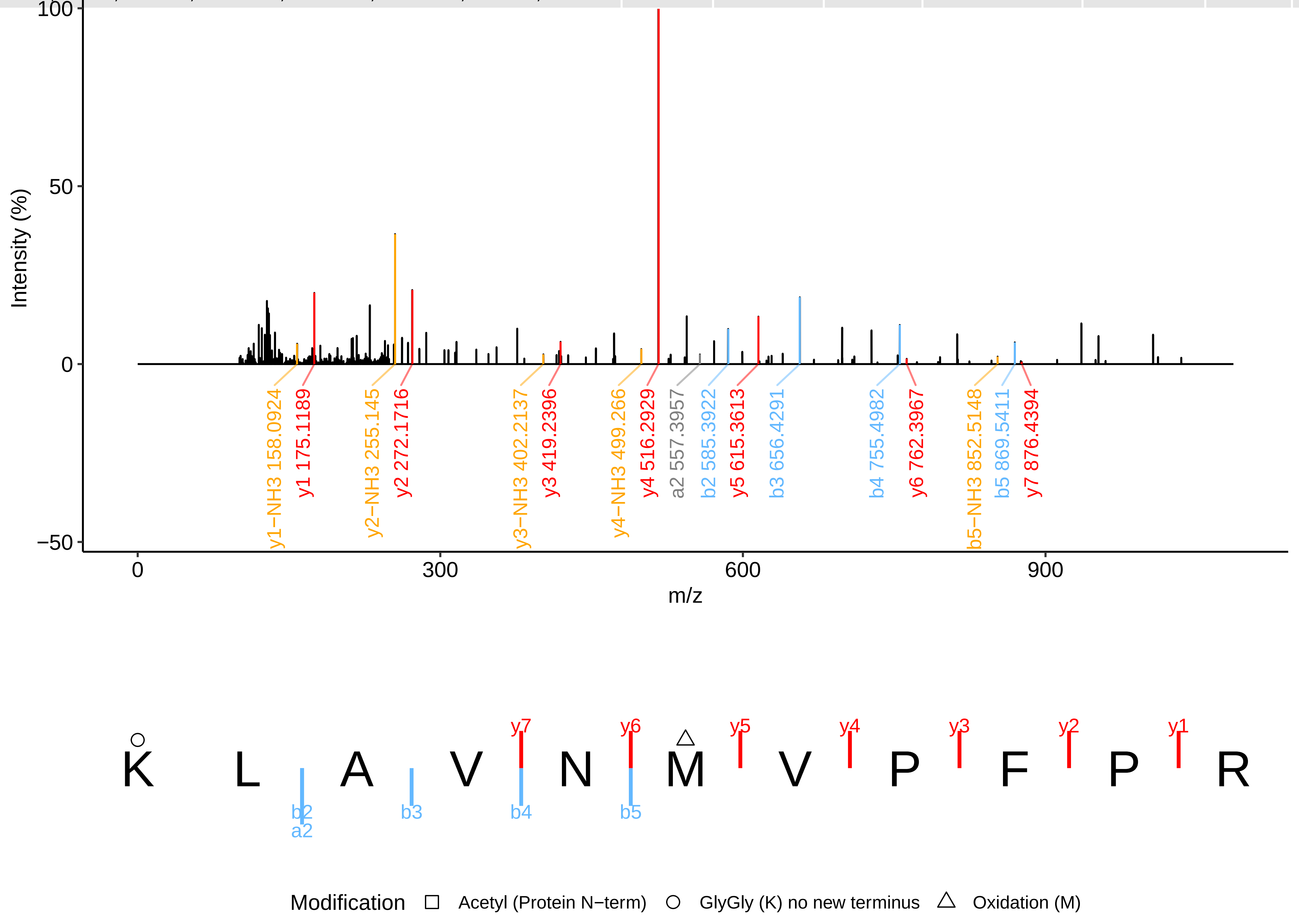

20200921_KIZ_MUb4_GlyGly_10plex_2 − Scan number: 15072

Gene Names	Charge	m/z	Mass	Retention time	PEP	Score
BB4A;TUBB;TUBB1;TUBB4B;TUBB2A;TUBB2B;TUBB8;TUBB6	3	467.9272	1400.76	58.868	0.0075432	68.893

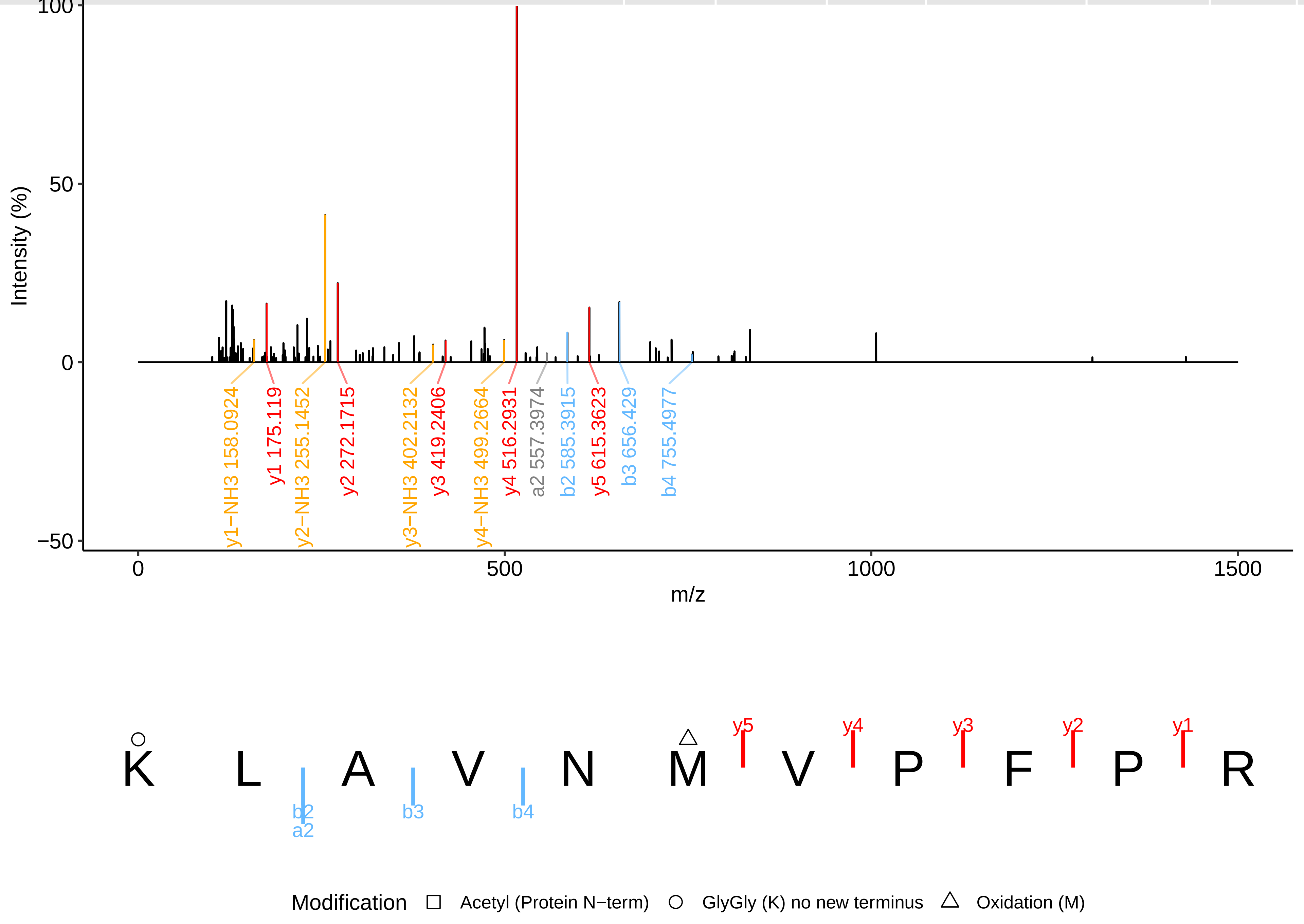

20200924_KIZ_MUb5_GlyGly_2 − Scan number: 14412

Gene Names	Charge	m/z	Mass	Retention time	PEP	Score
BB4A;TUBB;TUBB1;TUBB4B;TUBB2A;TUBB2B;TUBB8;TUBB6	3	467.9272	1400.76	53.866	0.033474	56.548

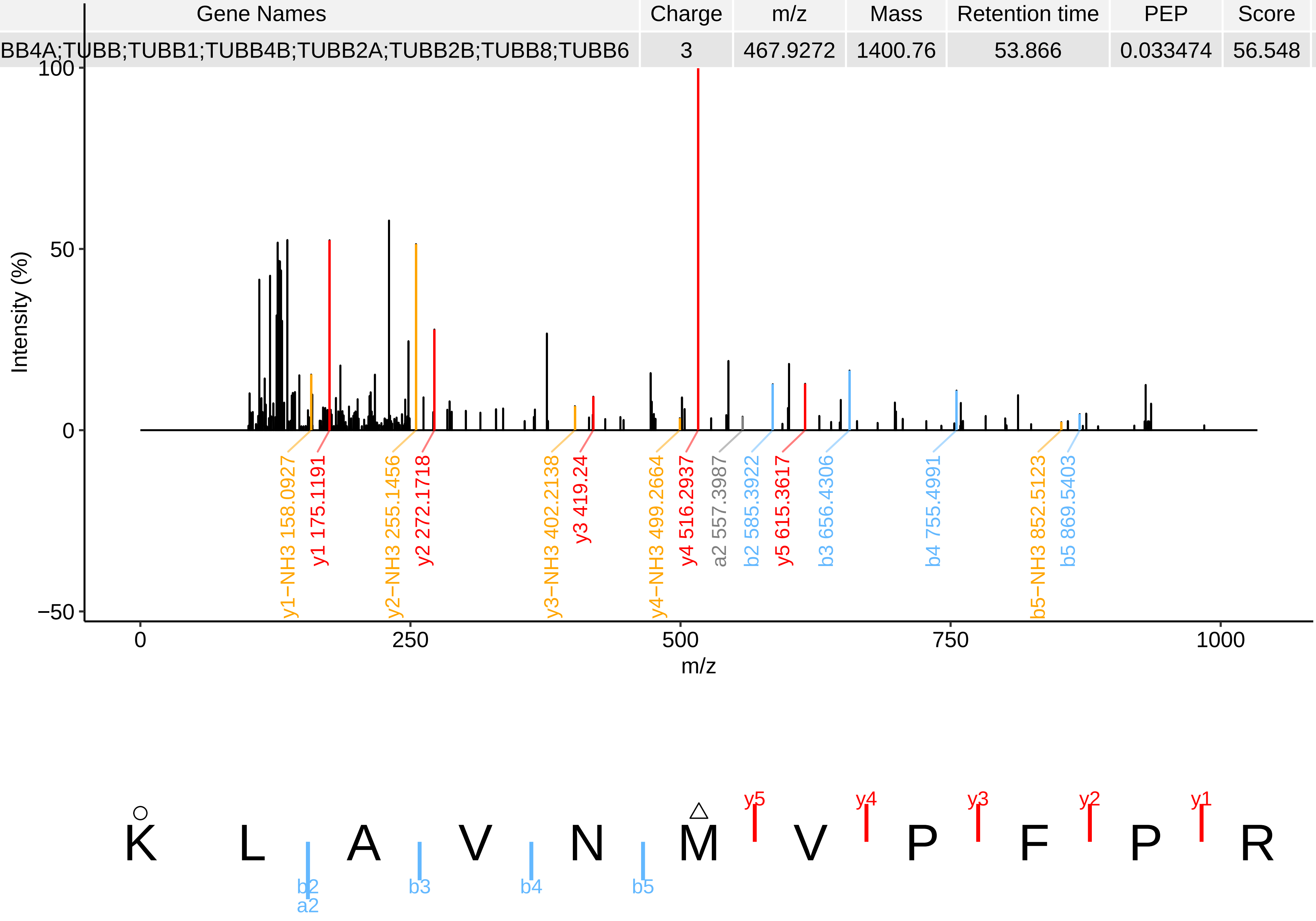

Modification □ Acetyl (Protein N−term) ○ GlyGly (K) no new terminus △ Oxidation (M)

20200921_KIZ_MUb4_GlyGly_10plex − Scan number: 19996

Gene Names	Charge	m/z	Mass	Retention time	PEP	Score
B4A;TUBB;TUBB1;TUBB4B;TUBB2A;TUBB2B;TUBB8;TUBB6	3	462.5956	1384.765	72.197	9.1165e−10	132.78

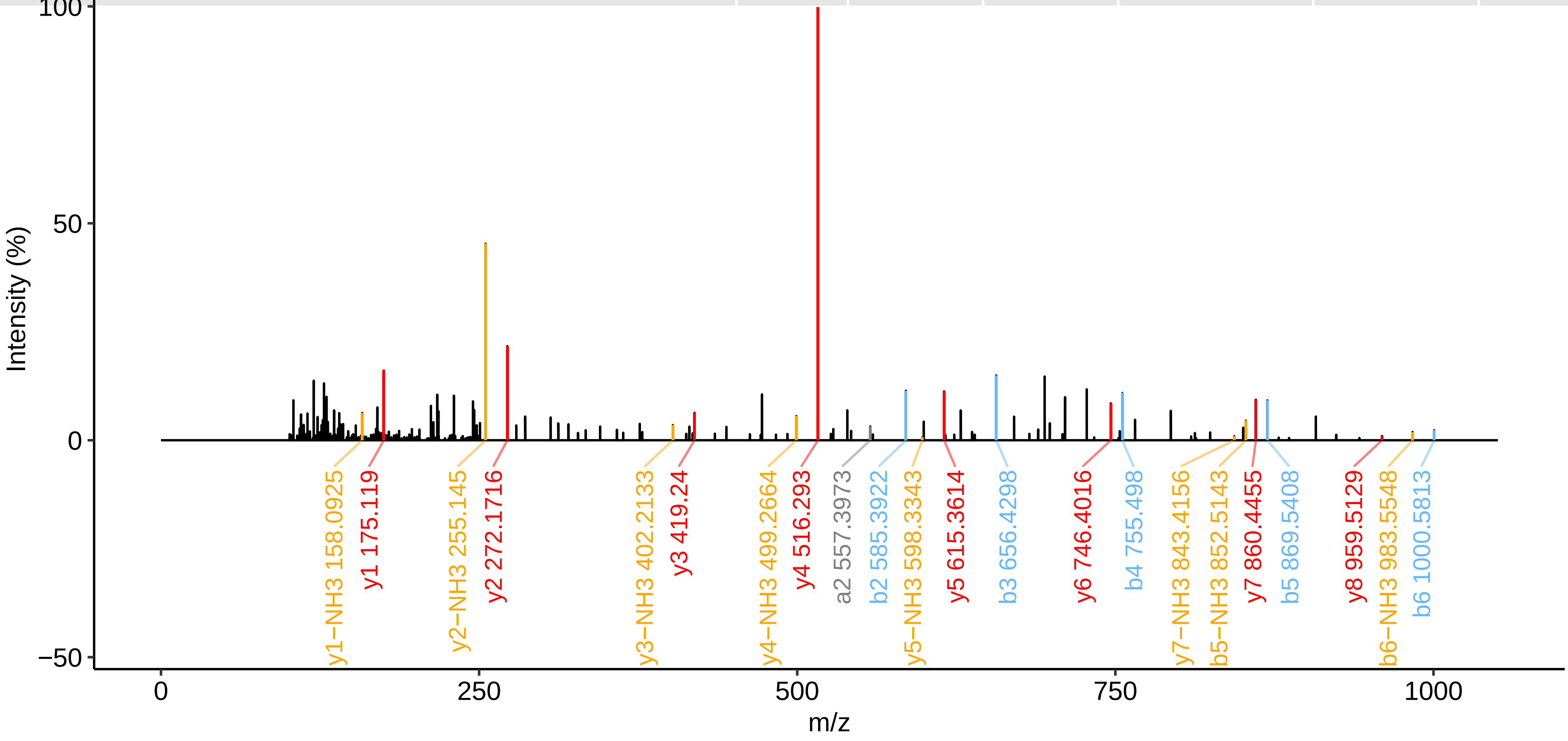

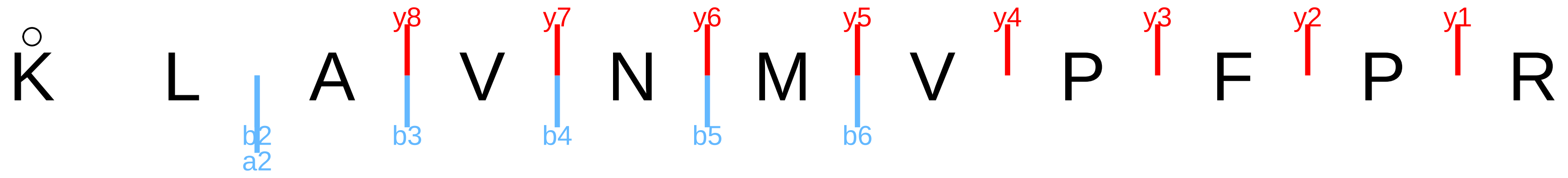

Modification □ Acetyl (Protein N−term) ○ GlyGly (K) no new terminus △ Oxidation (M)

20200921_KIZ_MUb4_GlyGly_10plex_2 − Scan number: 18423

Gene Names	Charge	m/z	Mass	Retention time	PEP	Score
B4A;TUBB;TUBB1;TUBB4B;TUBB2A;TUBB2B;TUBB8;TUBB6	3	462.5956	1384.765	72.14	0.0032045	93.096

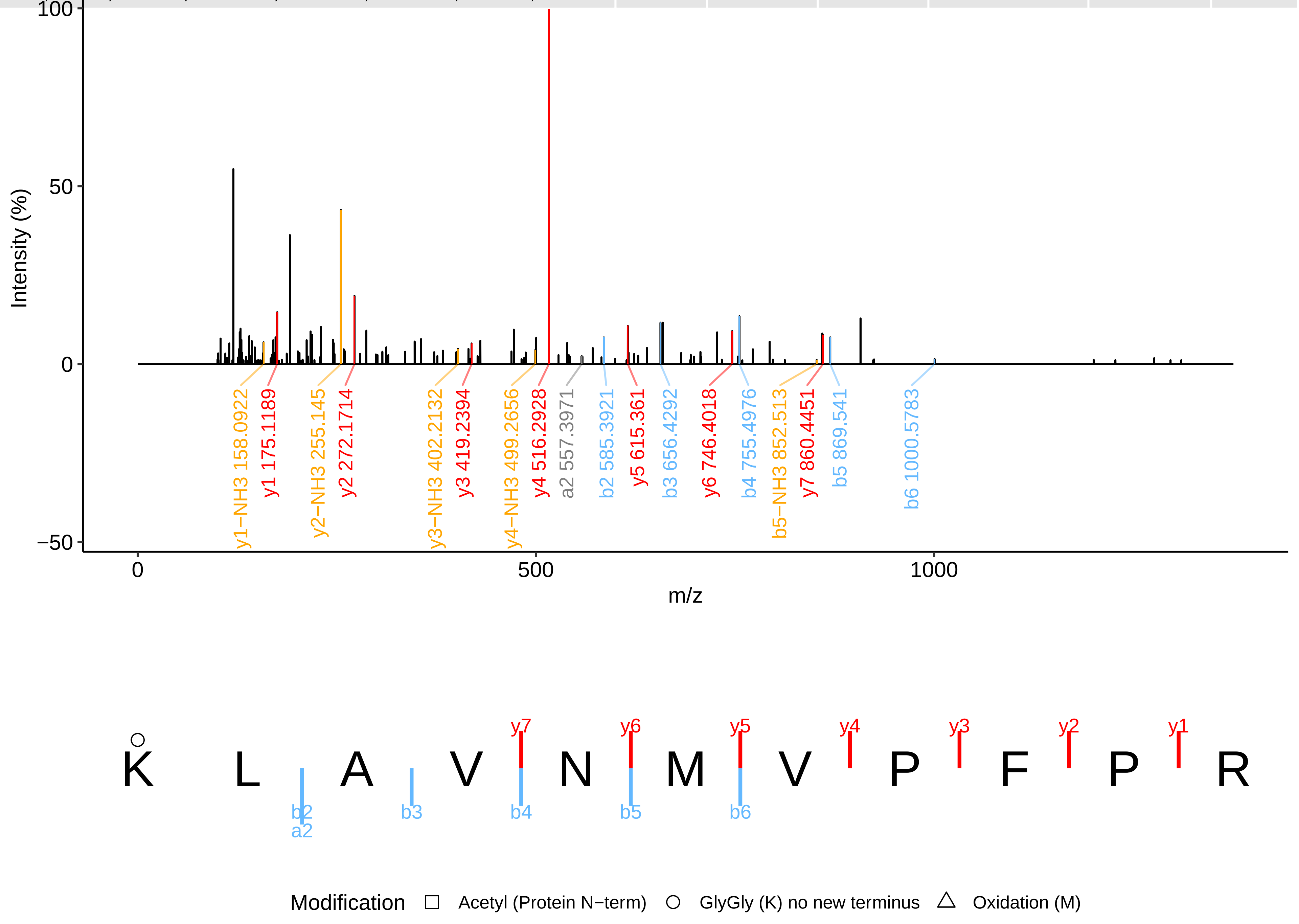

20200924_KIZ_MUb5_GlyGly_1 − Scan number: 19424

Gene Names	Charge	m/z	Mass	Retention time	PEP	Score
B4A;TUBB;TUBB1;TUBB4B;TUBB2A;TUBB2B;TUBB8;TUBB6	3	462.5956	1384.765	62.789	3.6029e−08	130.98

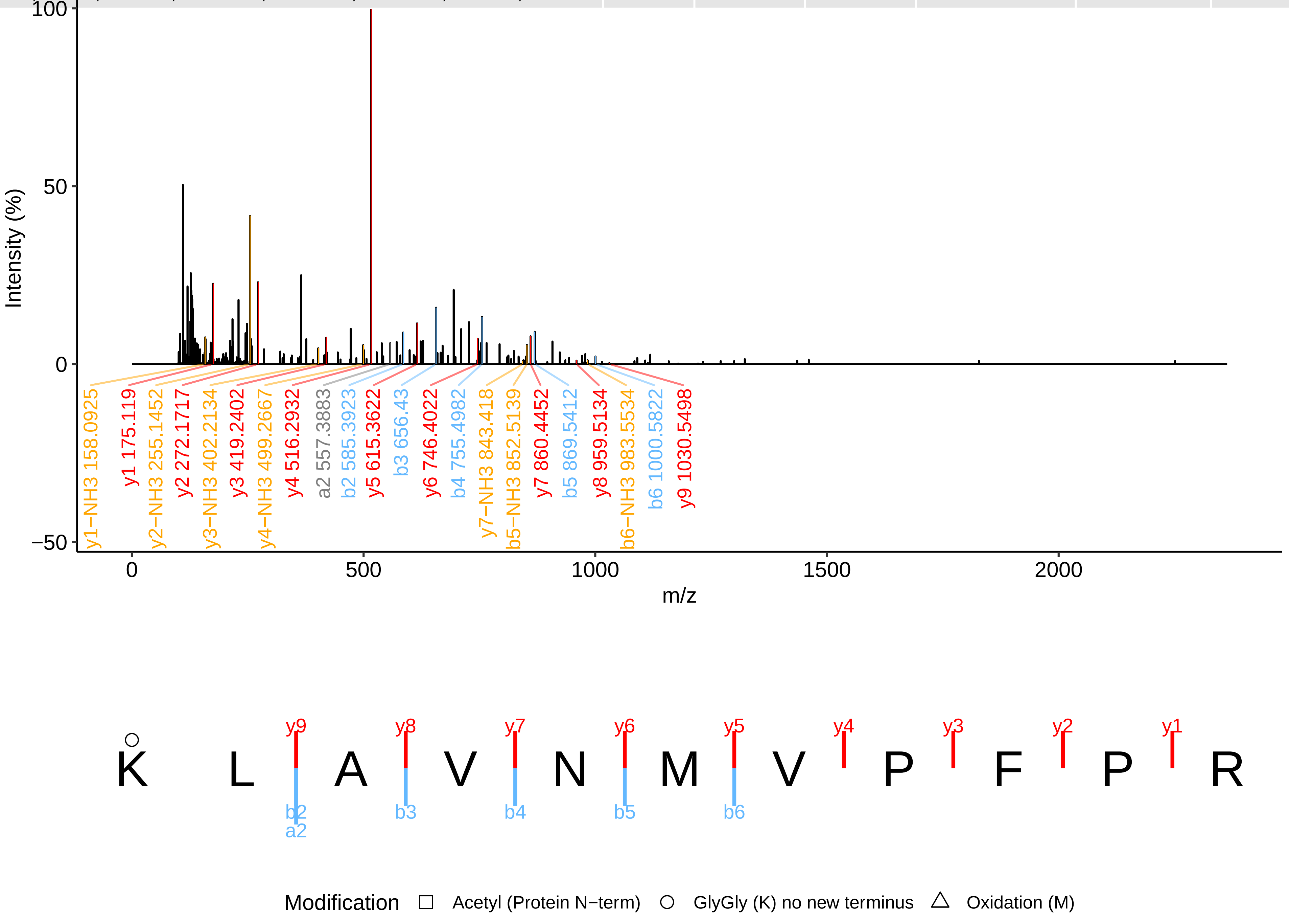

20200924_KIZ_MUb5_GlyGly_1 − Scan number: 19269

Gene Names	Charge	m/z	Mass	Retention time	PEP	Score
B4A;TUBB;TUBB1;TUBB4B;TUBB2A;TUBB2B;TUBB8;TUBB6	3	462.5956	1384.765	62.272	0.00017544	125.75

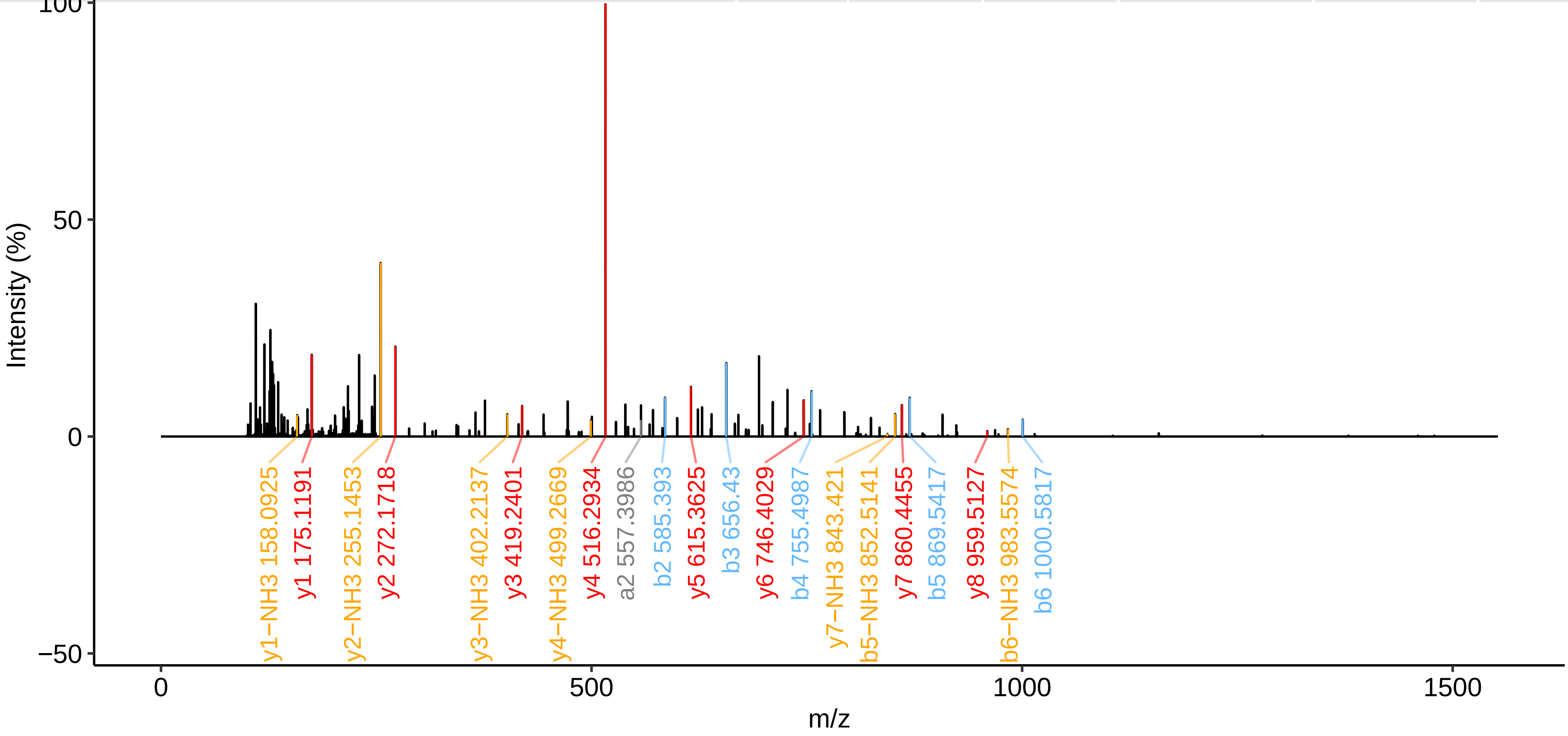

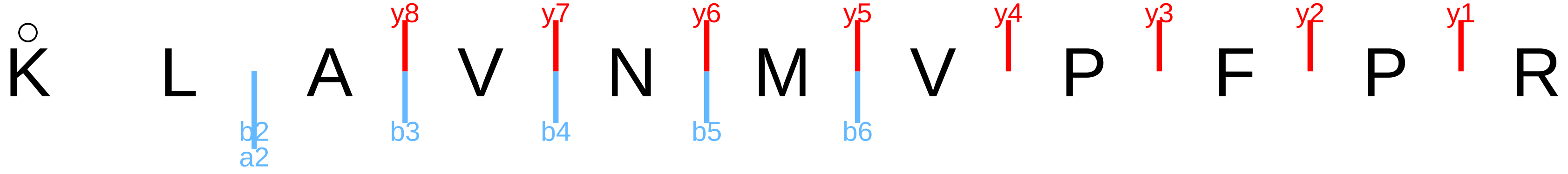

Modification □ Acetyl (Protein N−term) ○ GlyGly (K) no new terminus △ Oxidation (M)

20200924_KIZ_MUb5_GlyGly_2 − Scan number: 18079

Gene Names	Charge	m/z	Mass	Retention time	PEP	Score
B4A;TUBB;TUBB1;TUBB4B;TUBB2A;TUBB2B;TUBB8;TUBB6	3	462.5956	1384.765	67.034	1.3837e−07	125.75

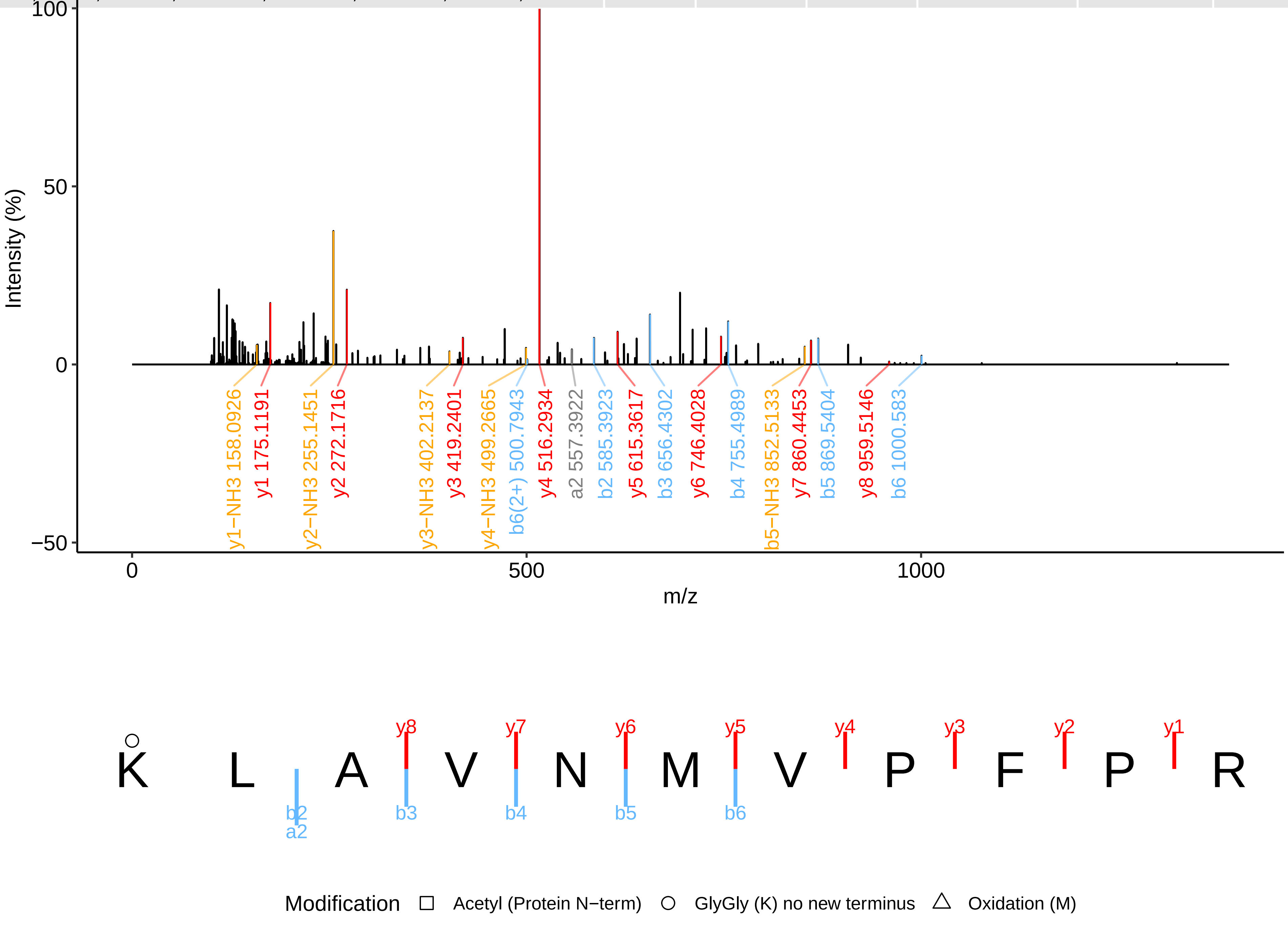

20200924_KIZ_MUb5_GlyGly_2 − Scan number: 17941

Gene Names	Charge	m/z	Mass	Retention time	PEP	Score
B4A;TUBB;TUBB1;TUBB4B;TUBB2A;TUBB2B;TUBB8;TUBB6	3	462.5956	1384.765	66.503	0.00052321	113.93

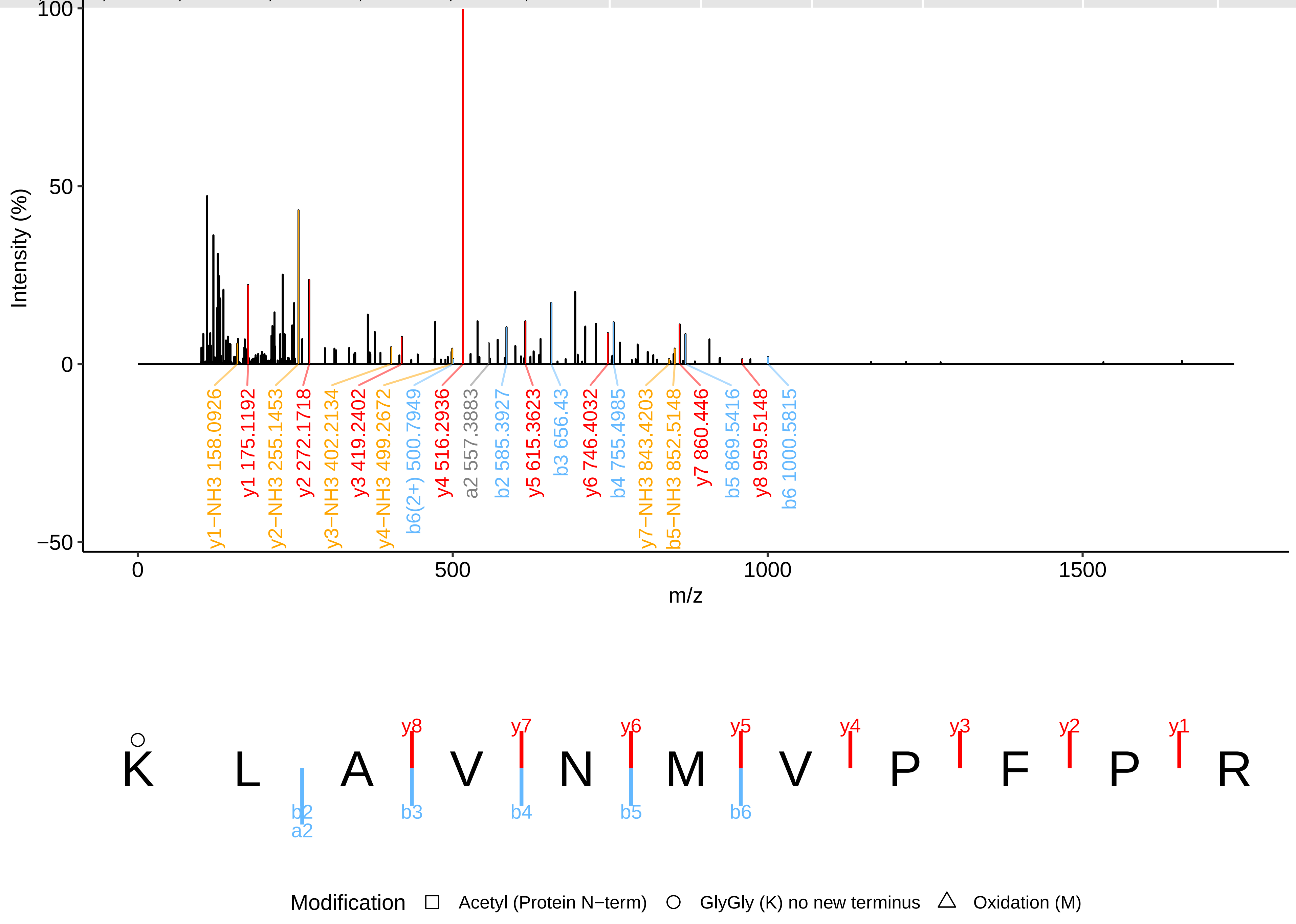

20201118_KIZ_MUb6_GlyGly_1 − Scan number: 19813

Gene Names	Charge	m/z	Mass	Retention time	PEP	Score
B4A;TUBB;TUBB1;TUBB4B;TUBB2A;TUBB2B;TUBB8;TUBB6	3	462.5956	1384.765	62.075	6.8024e−16	157.24

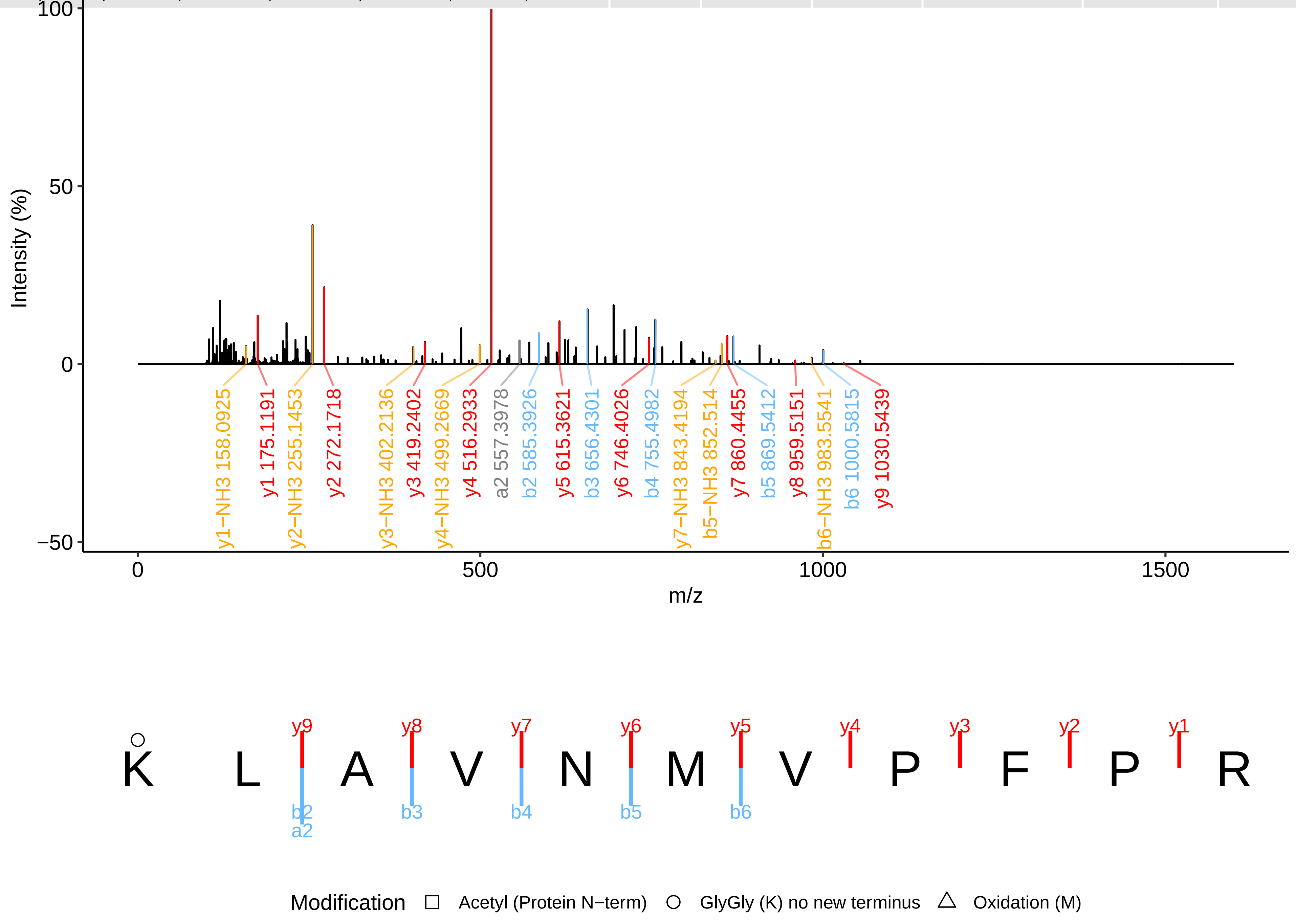

20201118_KIZ_MUb6_GlyGly_2 − Scan number: 22116

Gene Names	Charge	m/z	Mass	Retention time	PEP	Score
B4A;TUBB;TUBB1;TUBB4B;TUBB2A;TUBB2B;TUBB8;TUBB6	3	462.5956	1384.765	74.35	5.3935e−16	159.4

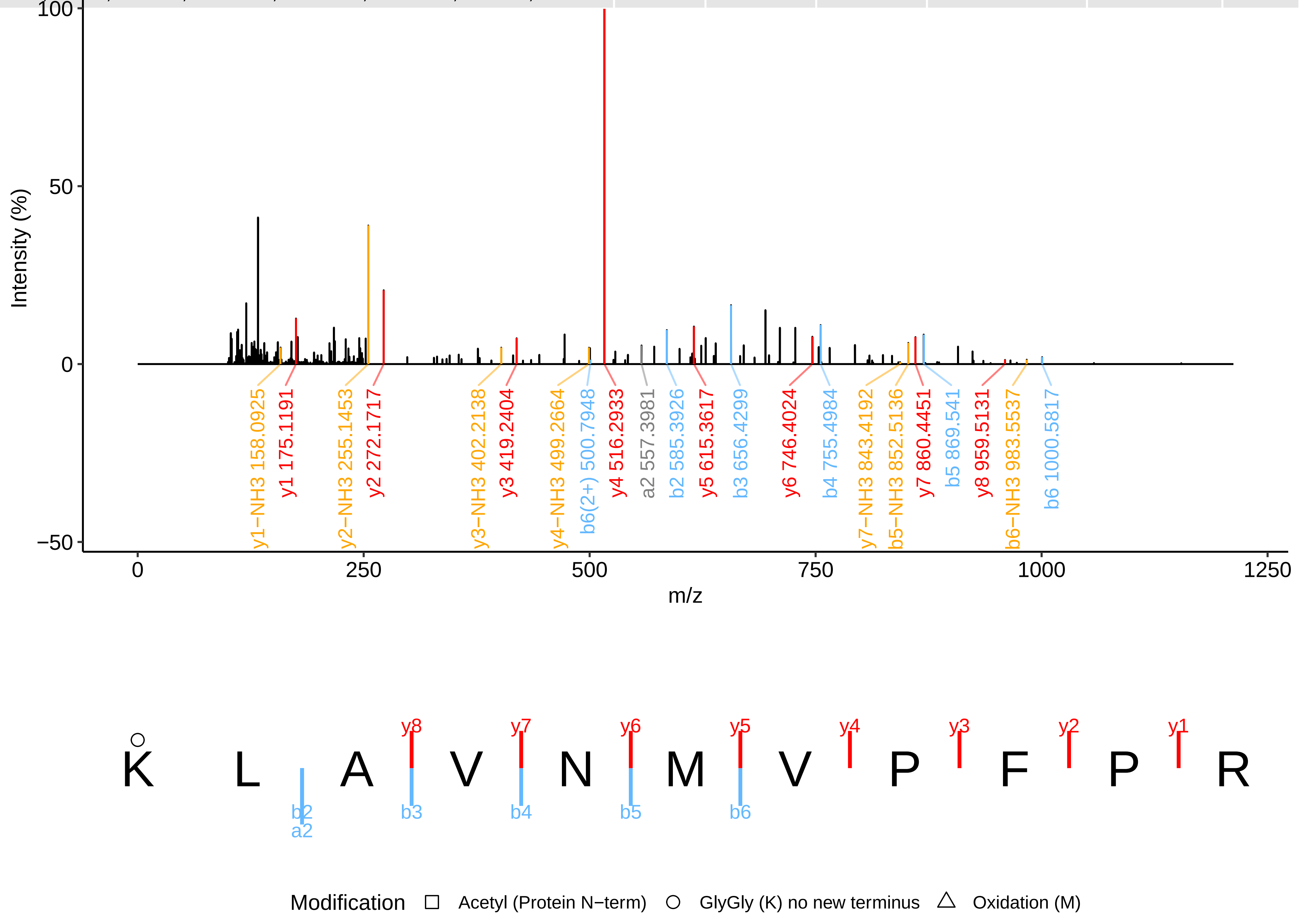

20200921_KIZ_MUb4_GlyGly_10plex − Scan number: 12910

Gene Names	Charge	m/z	Mass	Retention time	PEP	Score	Precursor Intensity
FANCD2	3	384.5521	1150.635	45.432	0.011818	90.108	14422329

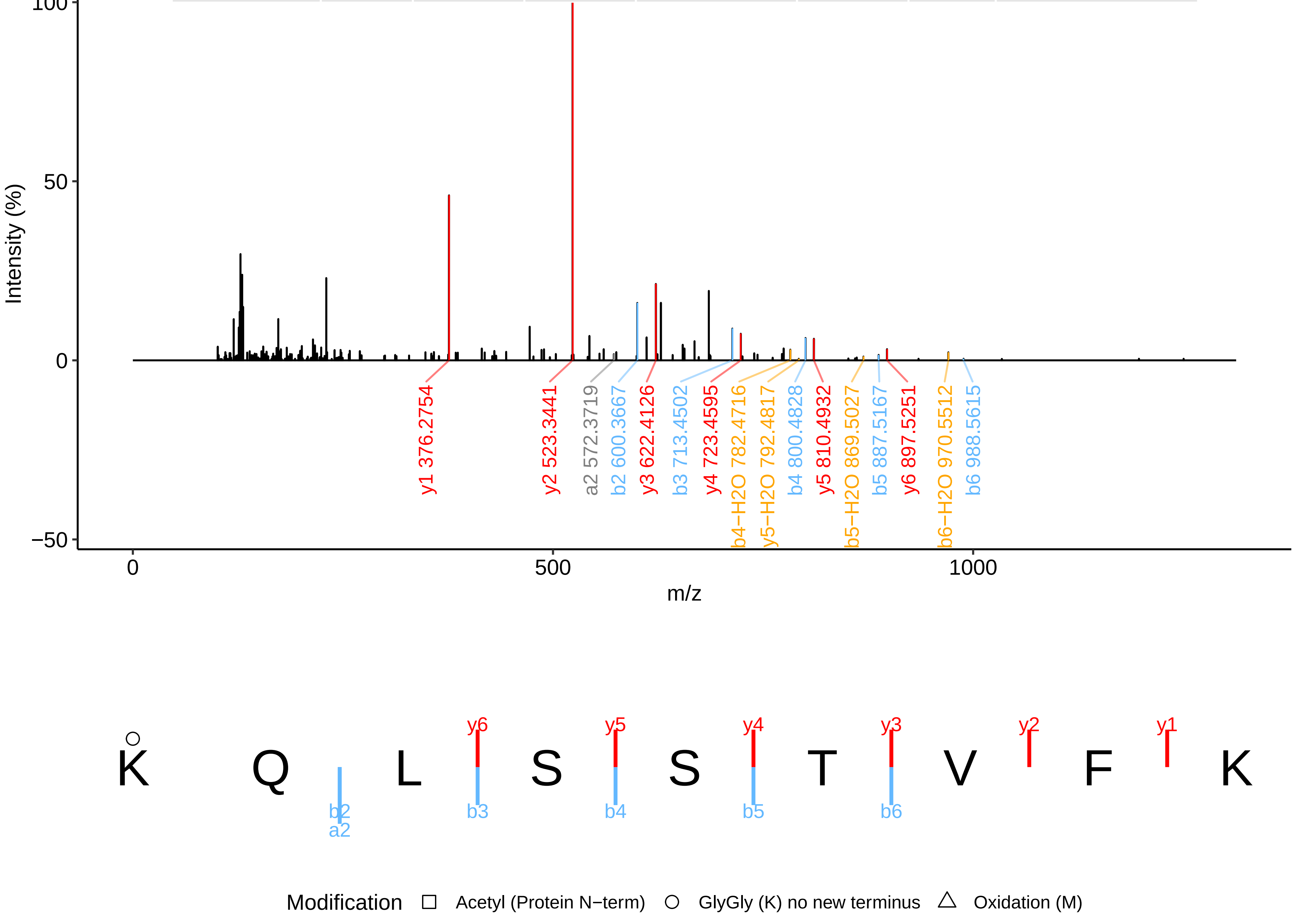

20201118_KIZ_MUb6_GlyGly_1 − Scan number: 11629

Gene Names	Charge	m/z	Mass	Retention time	PEP	Score	Precursor Intensity
FANCD2	3	384.5521	1150.635	36.695	0.011818	90.108	18816578

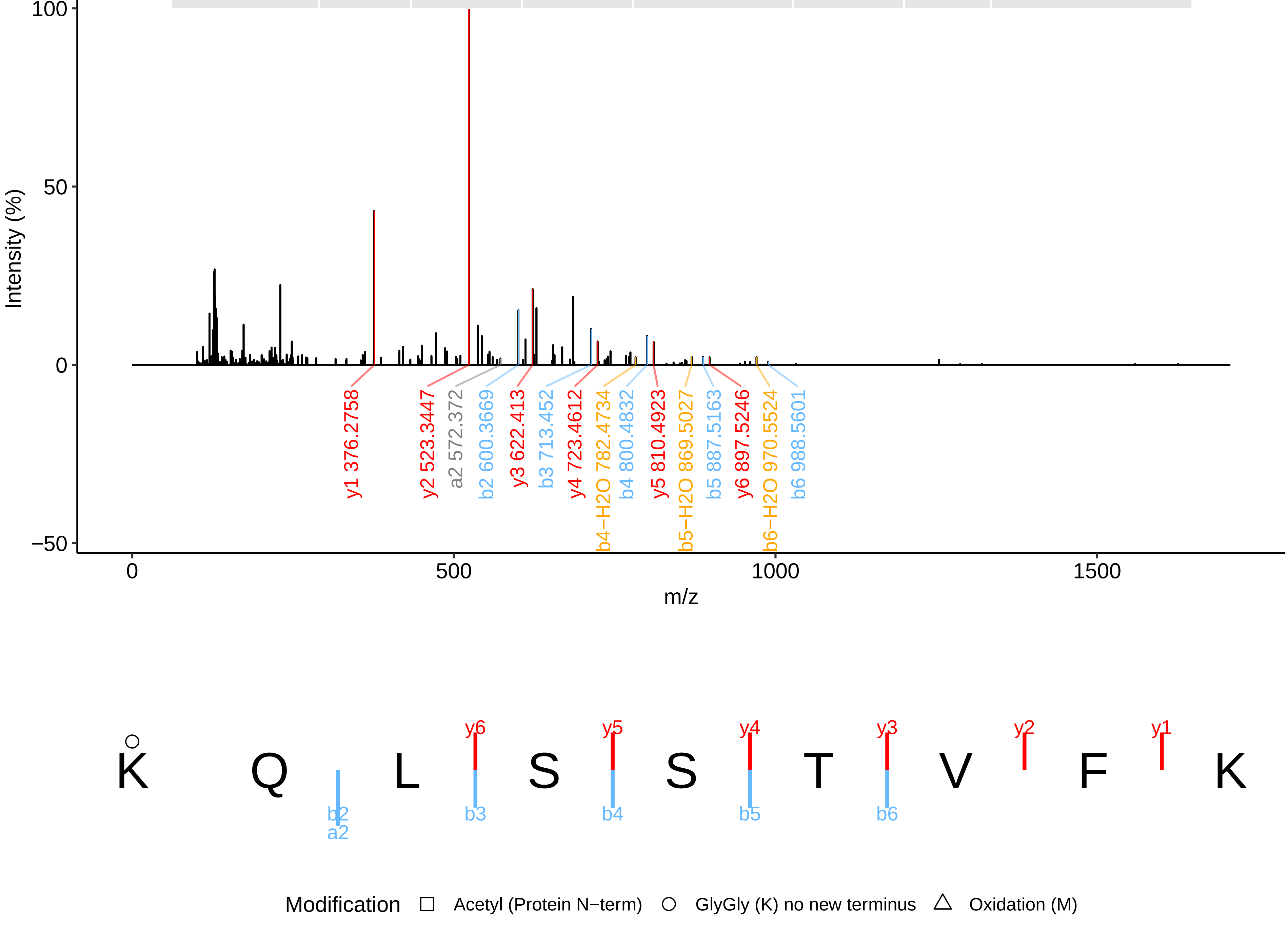

20200921_KIZ_MUb4_GlyGly_10plex_2 − Scan number: 14094

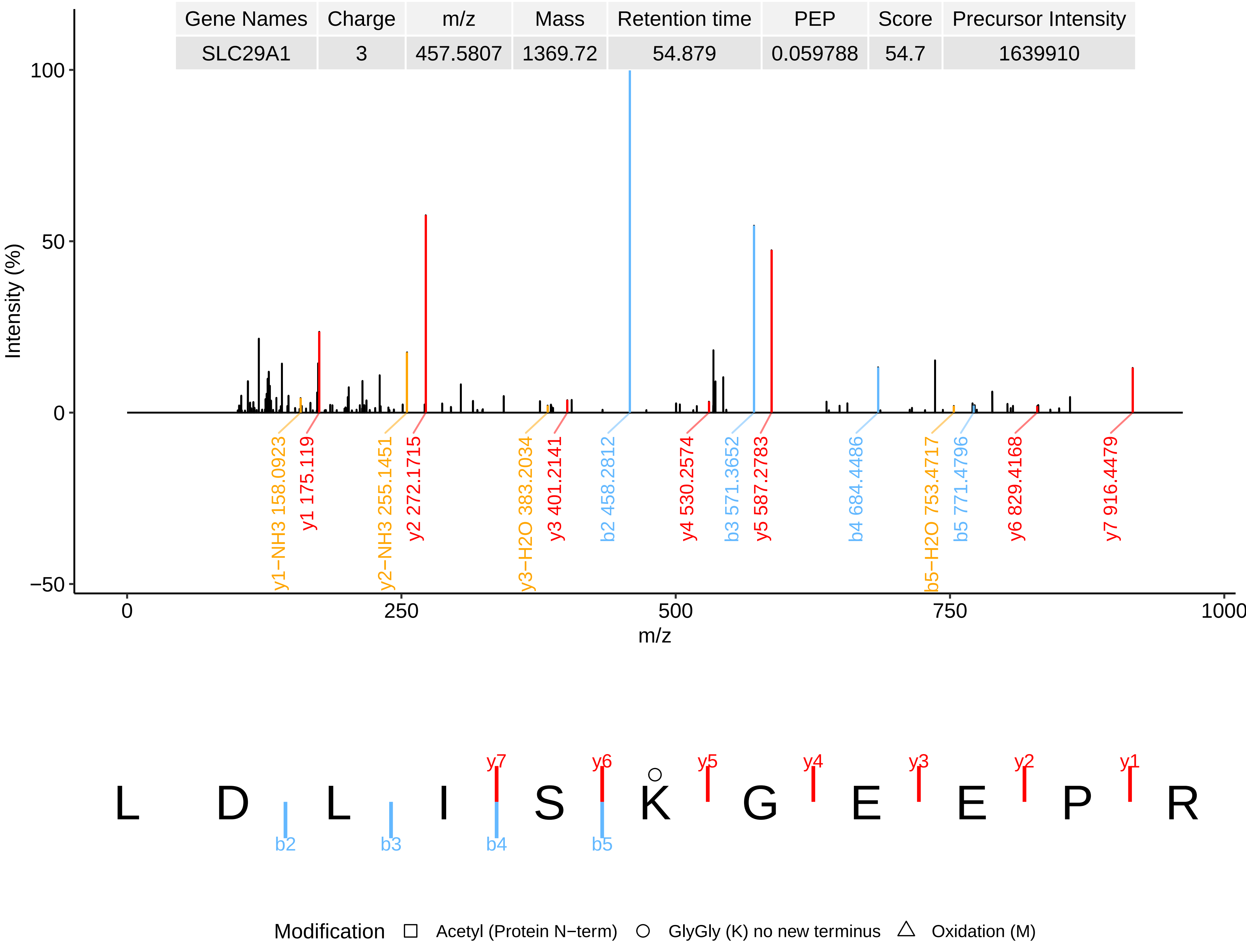

20200924_KIZ_MUb5_GlyGly_1 − Scan number: 14347

Gene Names	Charge	m/z	Mass	Retention time	PEP	Score	Precursor Intensity
SLC29A1	3	457.5807	1369.72	46.84	0.0094874	71.153	12571853

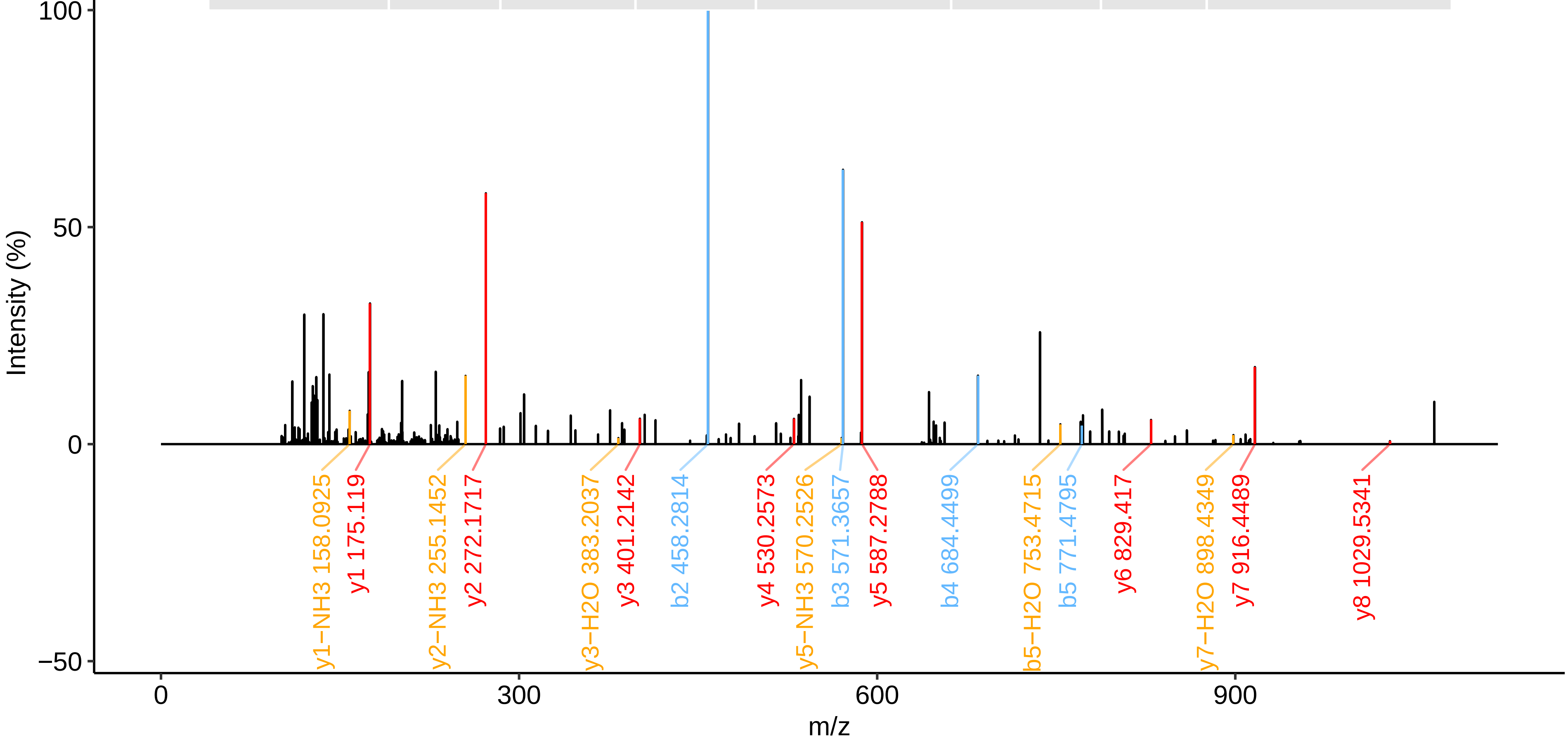

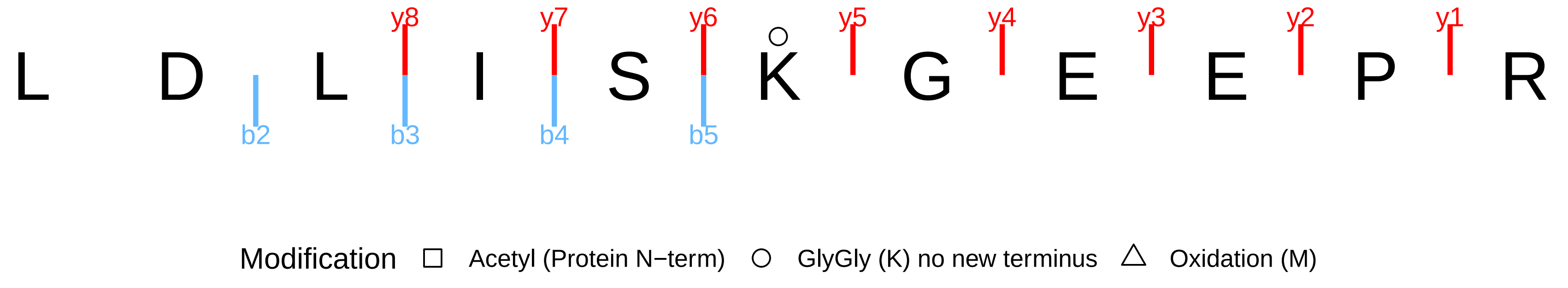

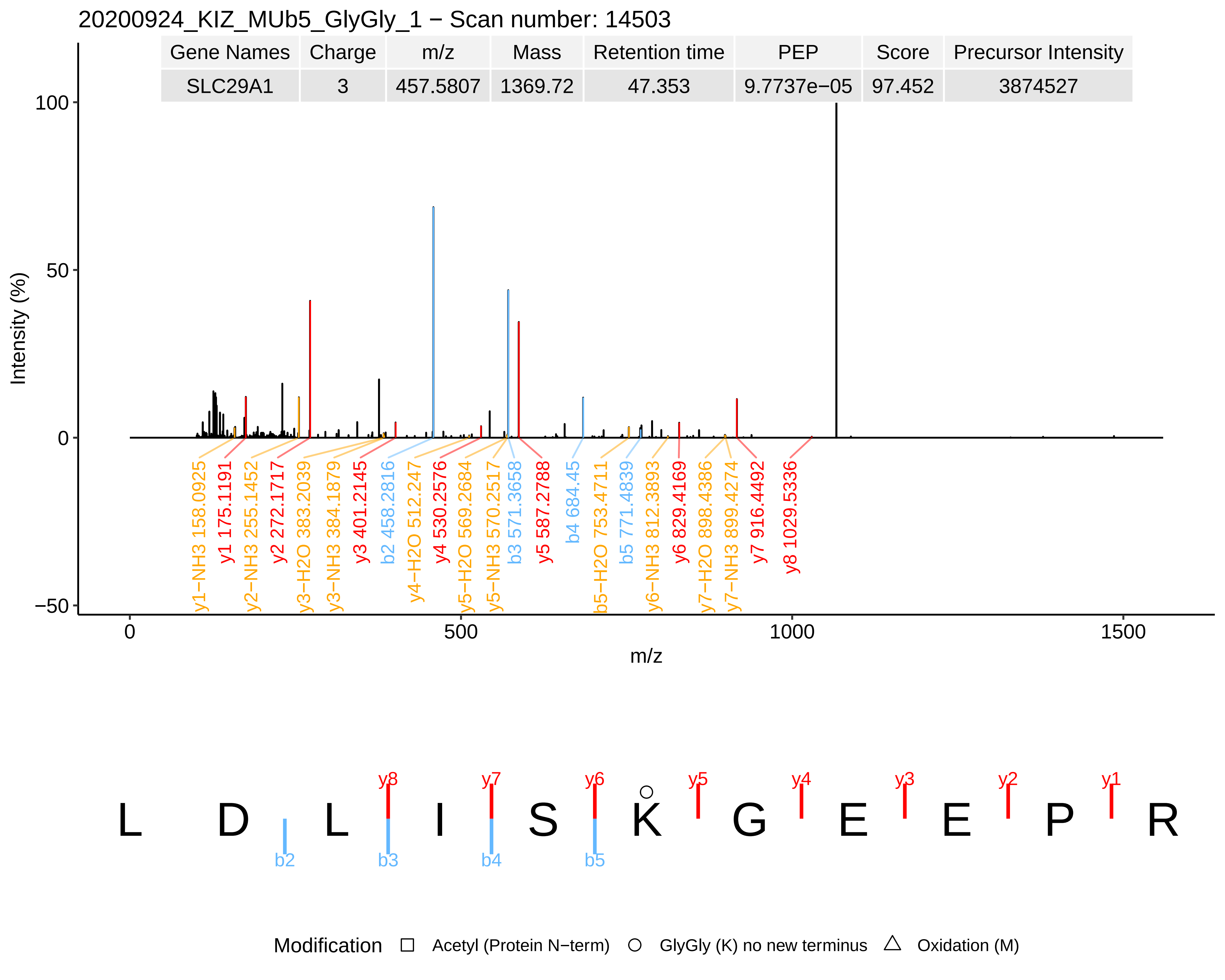
20200924_KIZ_MUb5_GlyGly_1 − Scan number: 14503
Gene Names | Charge | m/z | Mass | Retention time | PEP | Score | Precursor Intensity
SLC29A1 | 3 | 457.5807 | 1369.72 | 47.353 | 9.7737e−05 | 97.452 | 3874527
Intensity (%)
100
50
0
−50
0
500
1000
1500
m/z
y1−NH3 158.0925
y1 175.1191
y2−NH3 255.1452
y2 272.1717
y3−H2O 383.2039
y3−NH3 384.1879
y3 401.2145
b2 458.2816
y4−H2O 512.247
y4 530.2576
y5−H2O 569.2684
y5−NH3 570.2517
b3 571.3658
y5 587.2788
b4 684.45
b5−H2O 753.4711
b5 771.4839
y6−NH3 812.3893
y6 829.4169
y7−H2O 898.4386
y7−NH3 899.4274
y7 916.4492
y8 1029.5336
L D L I S K G E E P R
y8 y7 y6 y5 y4 y3 y2 y1
b2 b3 b4 b5
Modification
Acetyl (Protein N−term)
GlyGly (K) no new terminus
Oxidation (M)

20200924_KIZ_MUb5_GlyGly_2 − Scan number: 13467

Gene Names	Charge	m/z	Mass	Retention time	PEP	Score	Precursor Intensity
SLC29A1	3	457.5807	1369.72	50.364	0.0049605	76.655	5925766

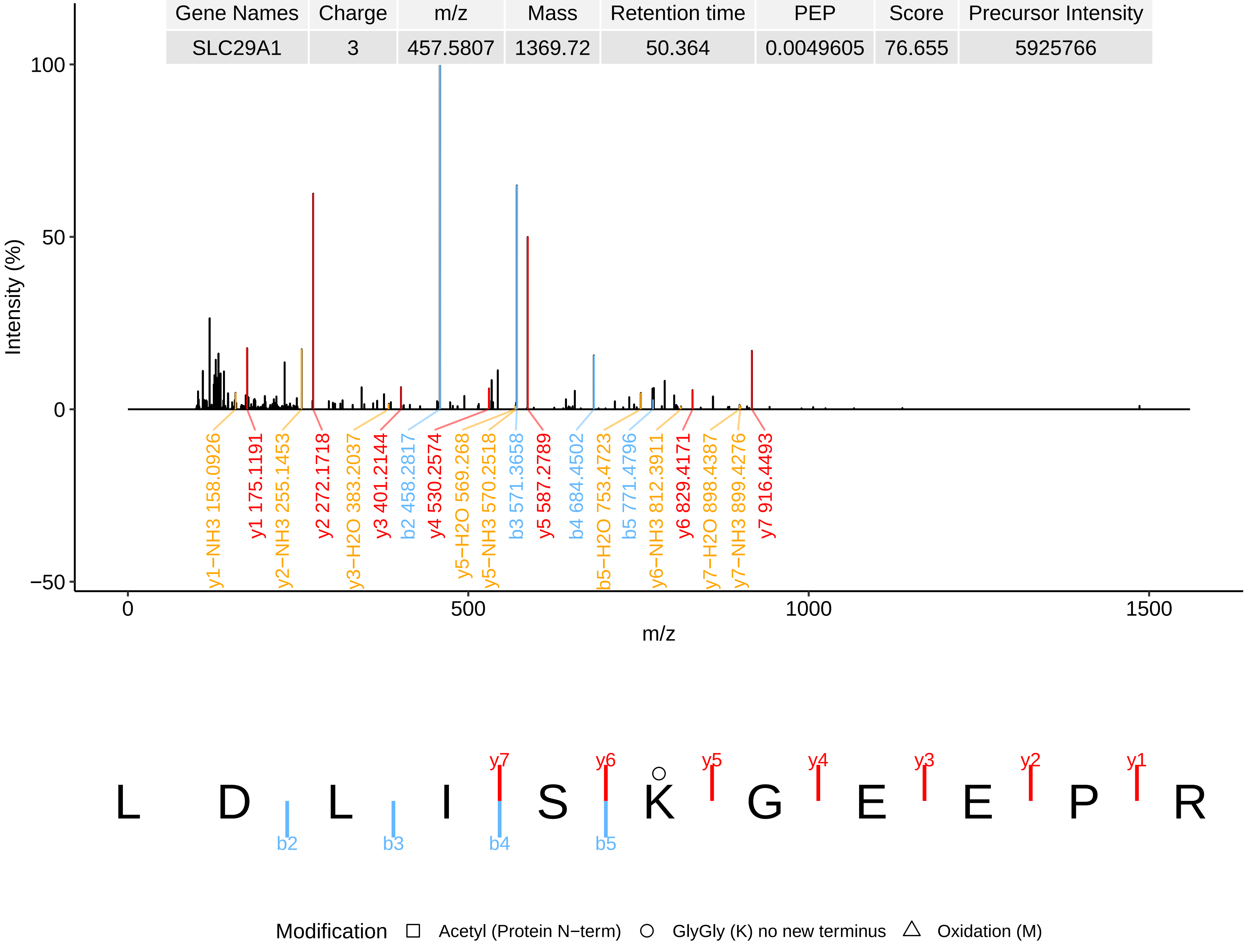

20200924_KIZ_MUb5_GlyGly_2 − Scan number: 13603

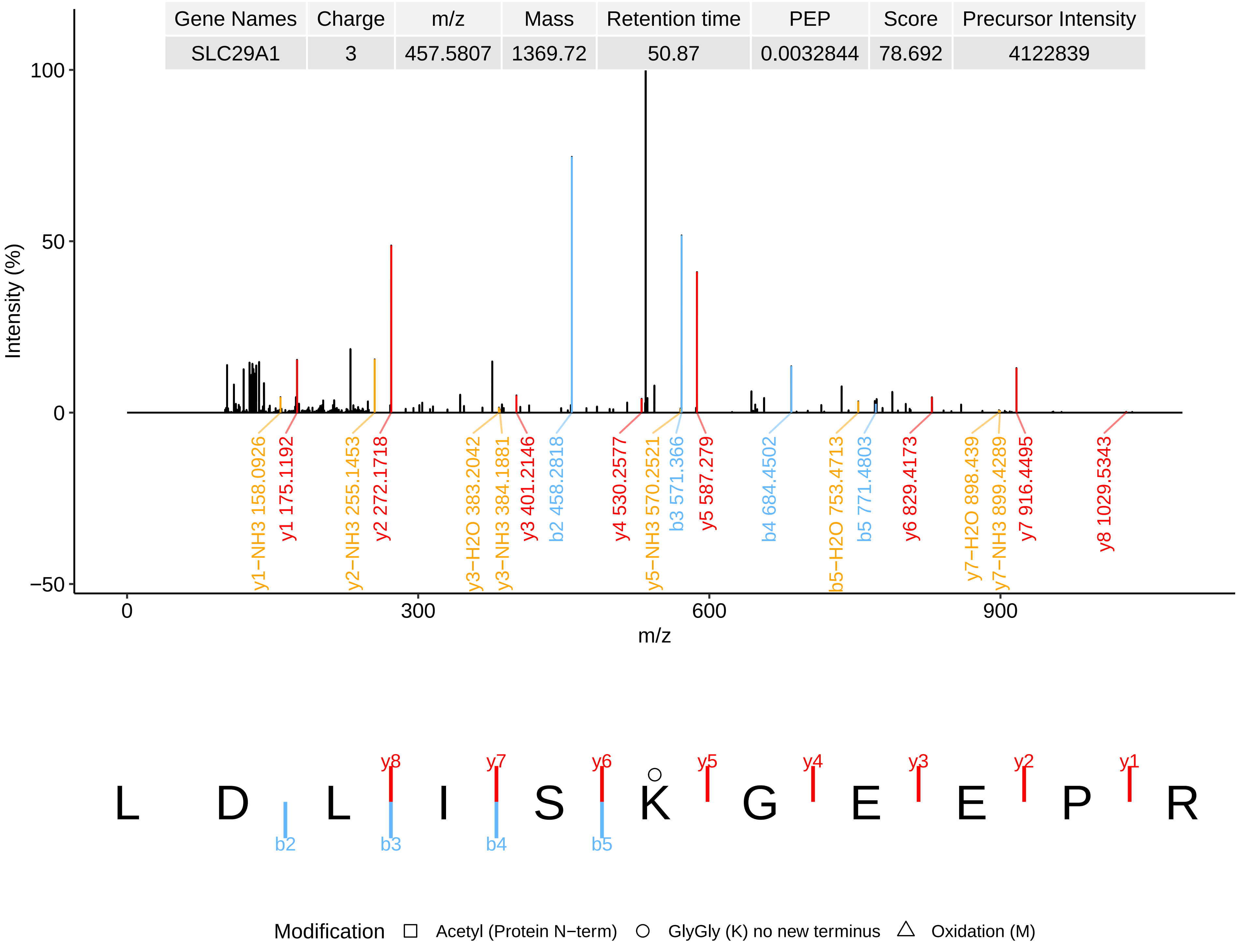

20201118_KIZ_MUb6_GlyGly_1 − Scan number: 14378

Gene Names	Charge	m/z	Mass	Retention time	PEP	Score	Precursor Intensity
SLC29A1	3	457.5807	1369.72	45.482	0.0049605	76.655	24446820

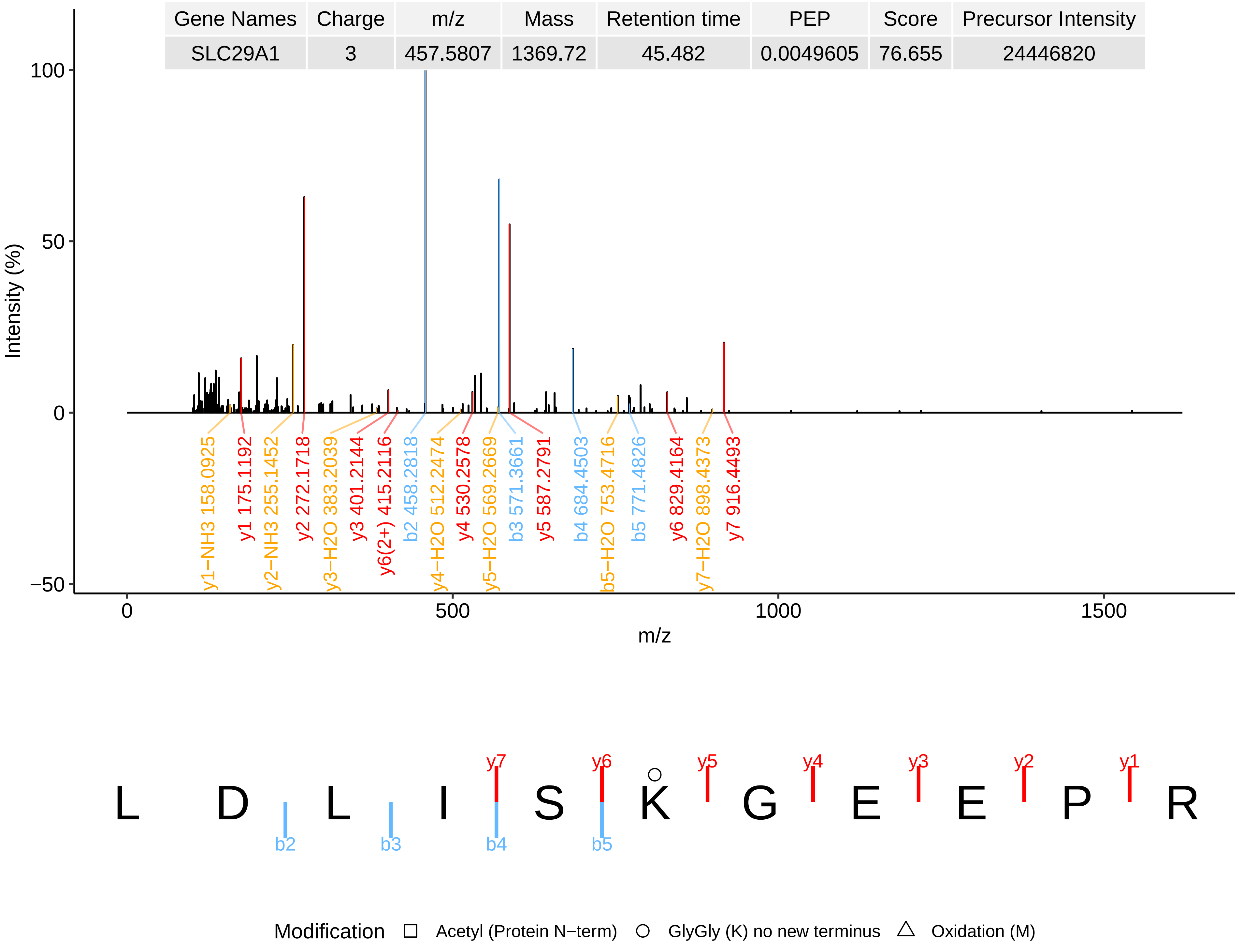

20201118_KIZ_MUb6_GlyGly_2 − Scan number: 16297

Gene Names	Charge	m/z	Mass	Retention time	PEP	Score	Precursor Intensity
SLC29A1	3	457.5807	1369.72	55.047	7.0501e−05	97.734	6689494

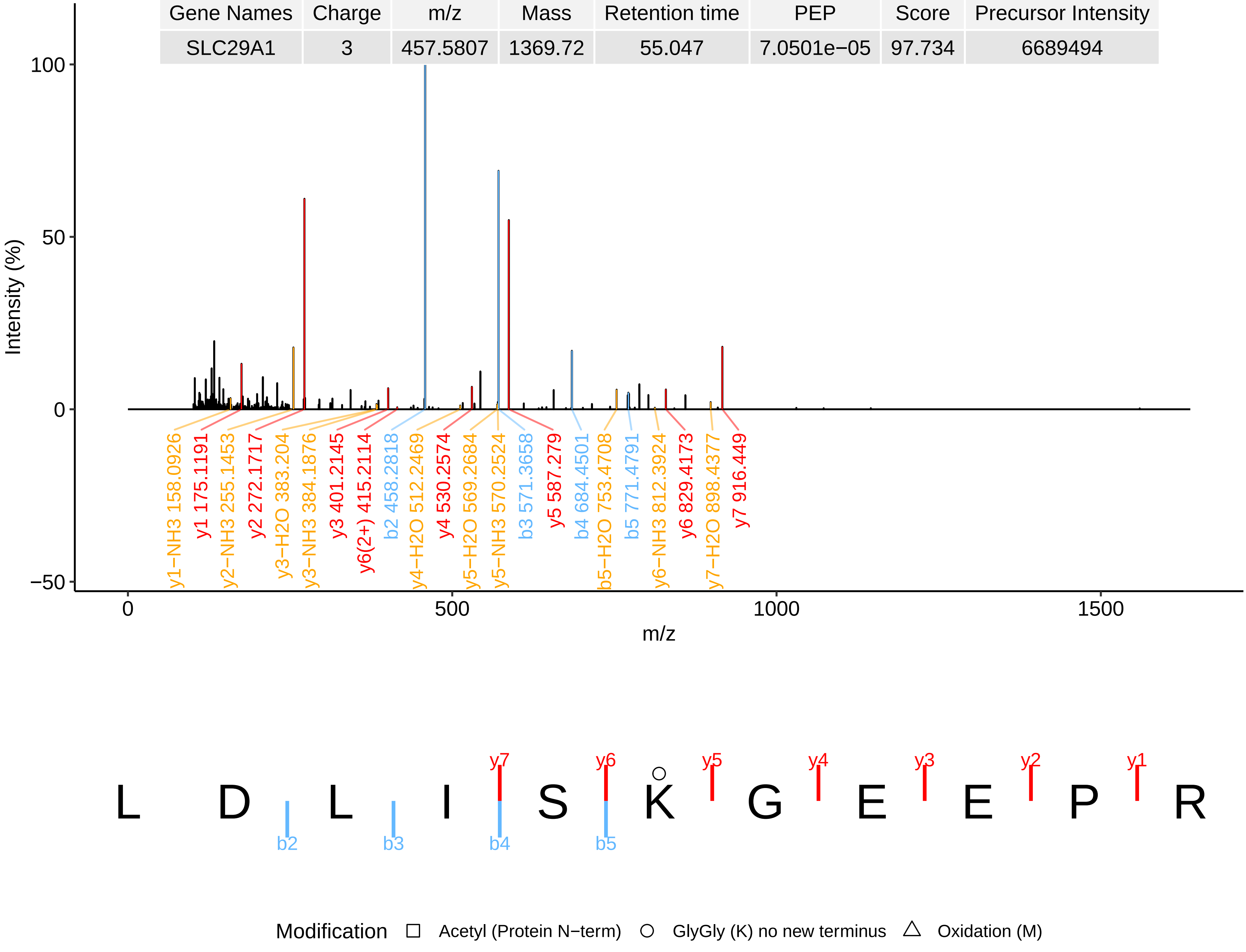

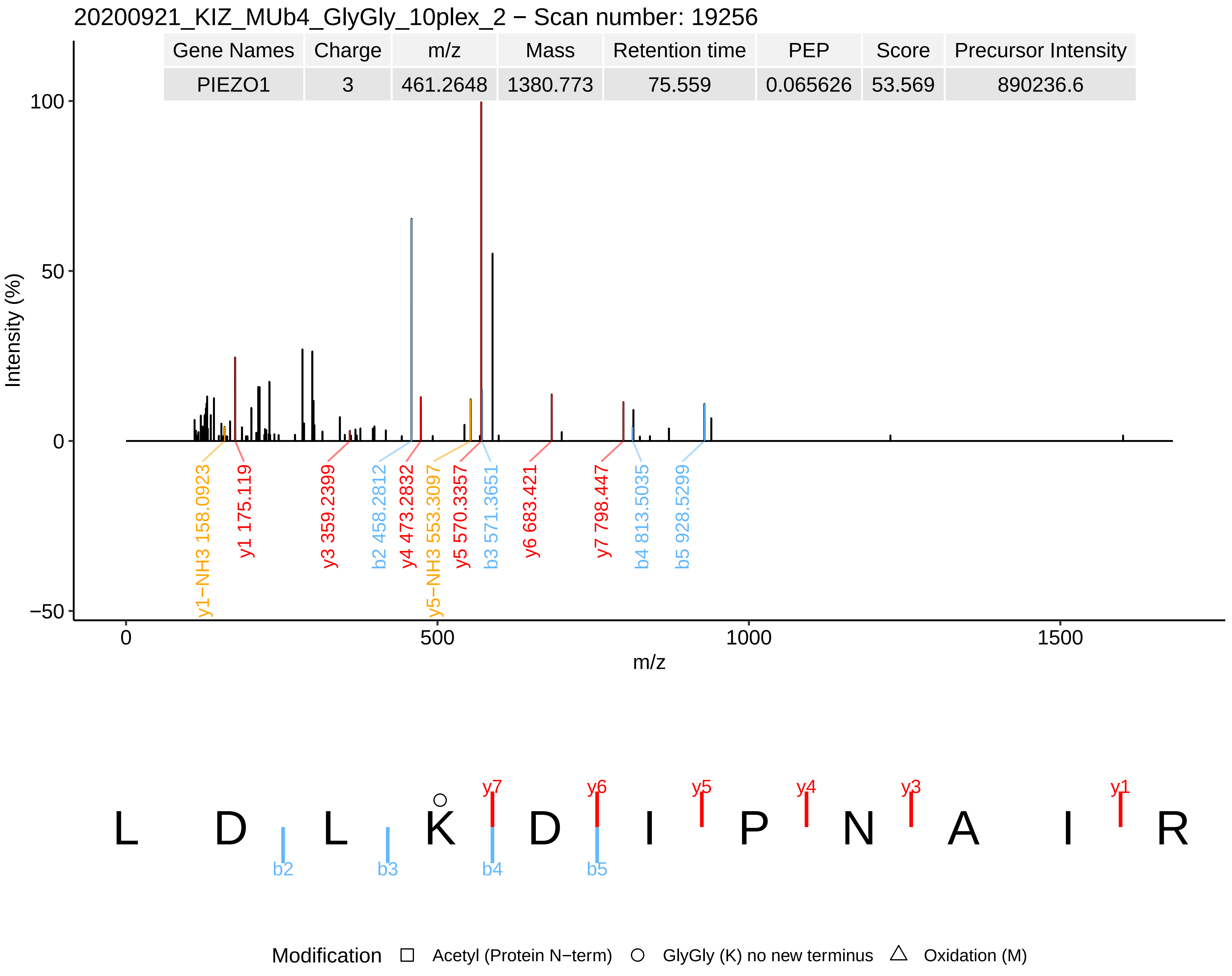
20200921_KIZ_MUb4_GlyGly_10plex_2 − Scan number: 19256
Gene Names
Charge
m/z
Mass
Retention time
PEP
Score
Precursor Intensity
PIEZO1
3
461.2648
1380.773
75.559
0.065626
53.569
890236.6
Intensity (%)
100
50
0
−50
m/z
0
500
1000
1500
y1−NH3 158.0923
y1 175.119
y3 359.2399
b2 458.2812
y4 473.2832
y5−NH3 553.3097
y5 570.3357
b3 571.3651
y6 683.421
y7 798.447
b4 813.5035
b5 928.5299
L D L K D I P N A I R
y7 y6 y5 y4 y3 y1
b2 b3 b4 b5
Modification
Acetyl (Protein N−term)
GlyGly (K) no new terminus
Oxidation (M)

20200921_KIZ_MUb4_GlyGly_10plex − Scan number: 4544

Gene Names	Charge	m/z	Mass	Retention time	PEP	Score	Precursor Intensity
TCP1	3	394.8632	1181.568	14.812	0.00029033	106.88	4361217

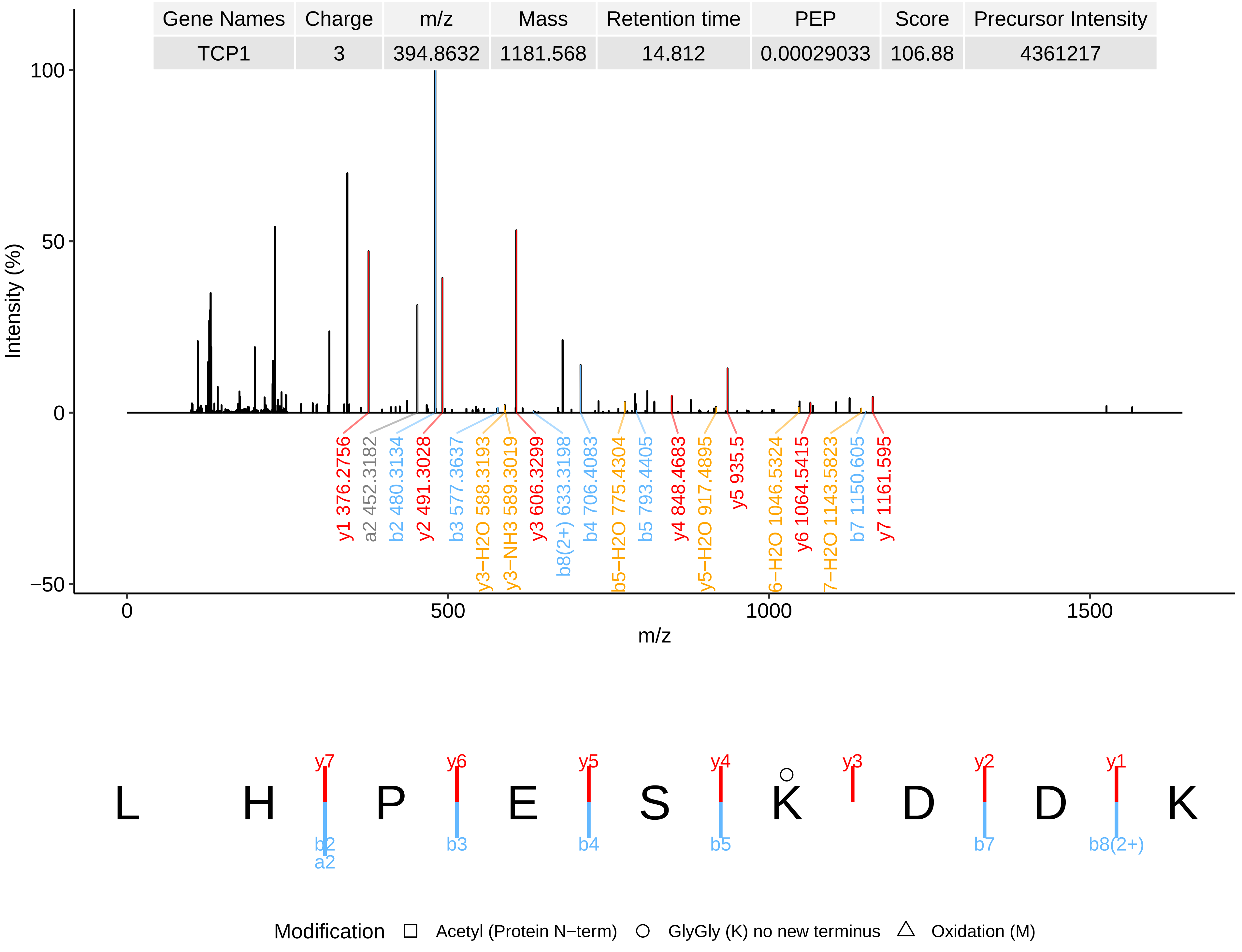

20200921_KIZ_MUb4_GlyGly_10plex − Scan number: 4545

Gene Names	Charge	m/z	Mass	Retention time	PEP	Score	Precursor Intensity
TCP1	4	296.3992	1181.568	14.815	0.038883	57.96	38879648

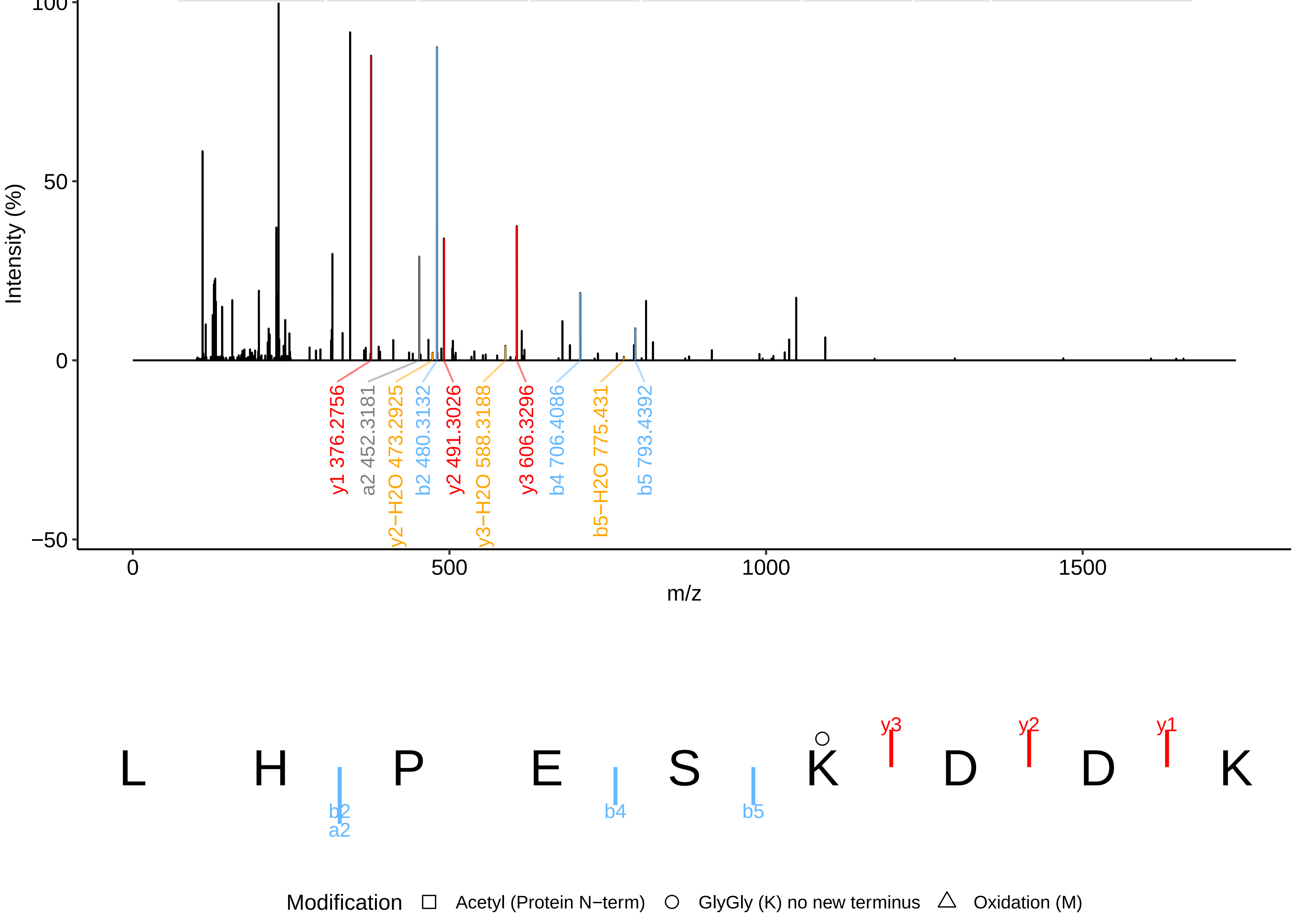

20200921_KIZ_MUb4_GlyGly_10plex_2 − Scan number: 4233

Gene Names	Charge	m/z	Mass	Retention time	PEP	Score	Precursor Intensity
TCP1	4	296.3992	1181.568	14.735	0.066266	51.066	884211.4

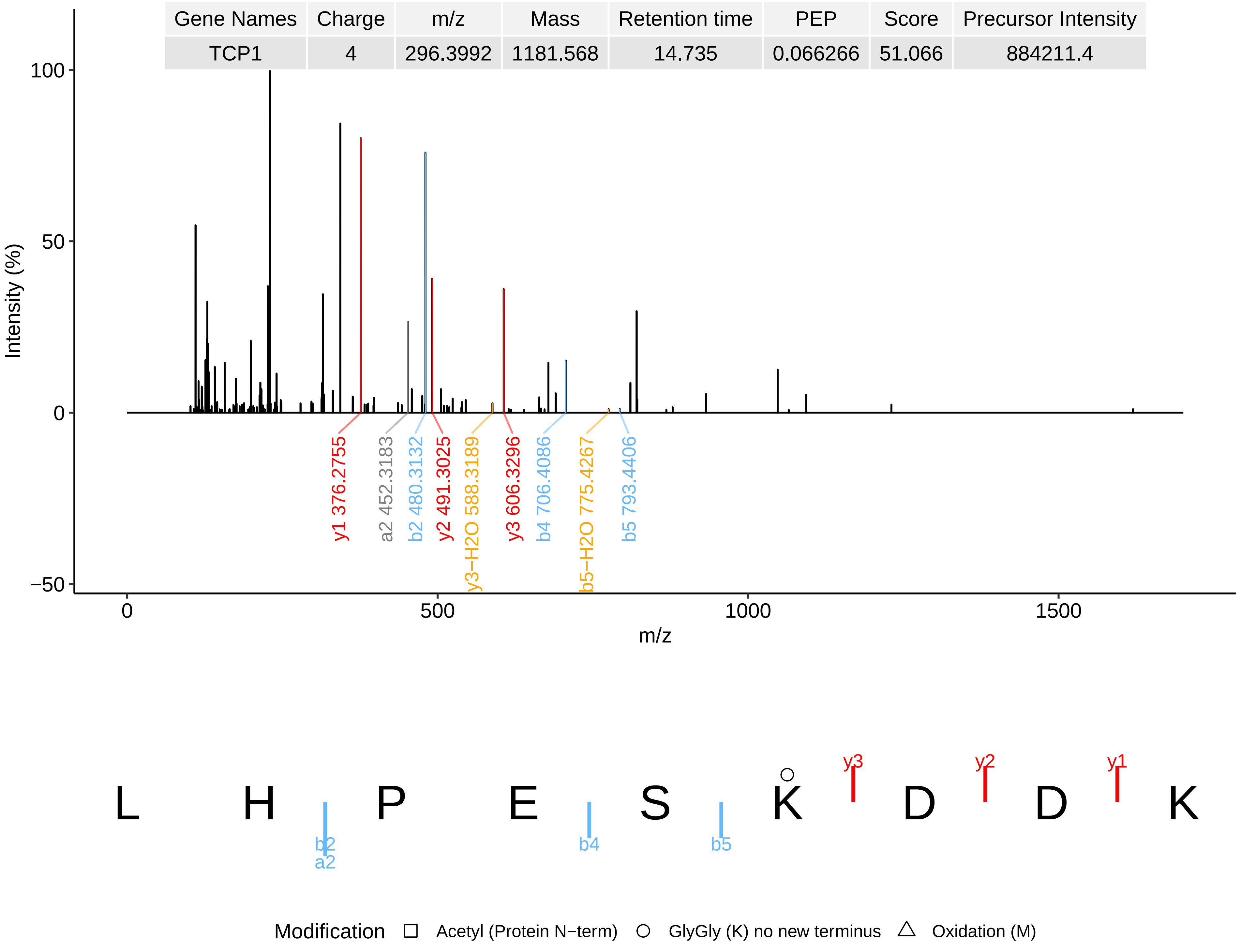

20200921_KIZ_MUb4_GlyGly_10plex_2 − Scan number: 4247

Gene Names	Charge	m/z	Mass	Retention time	PEP	Score	Precursor Intensity
TCP1	3	394.8632	1181.568	14.792	0.064314	54.416	842613

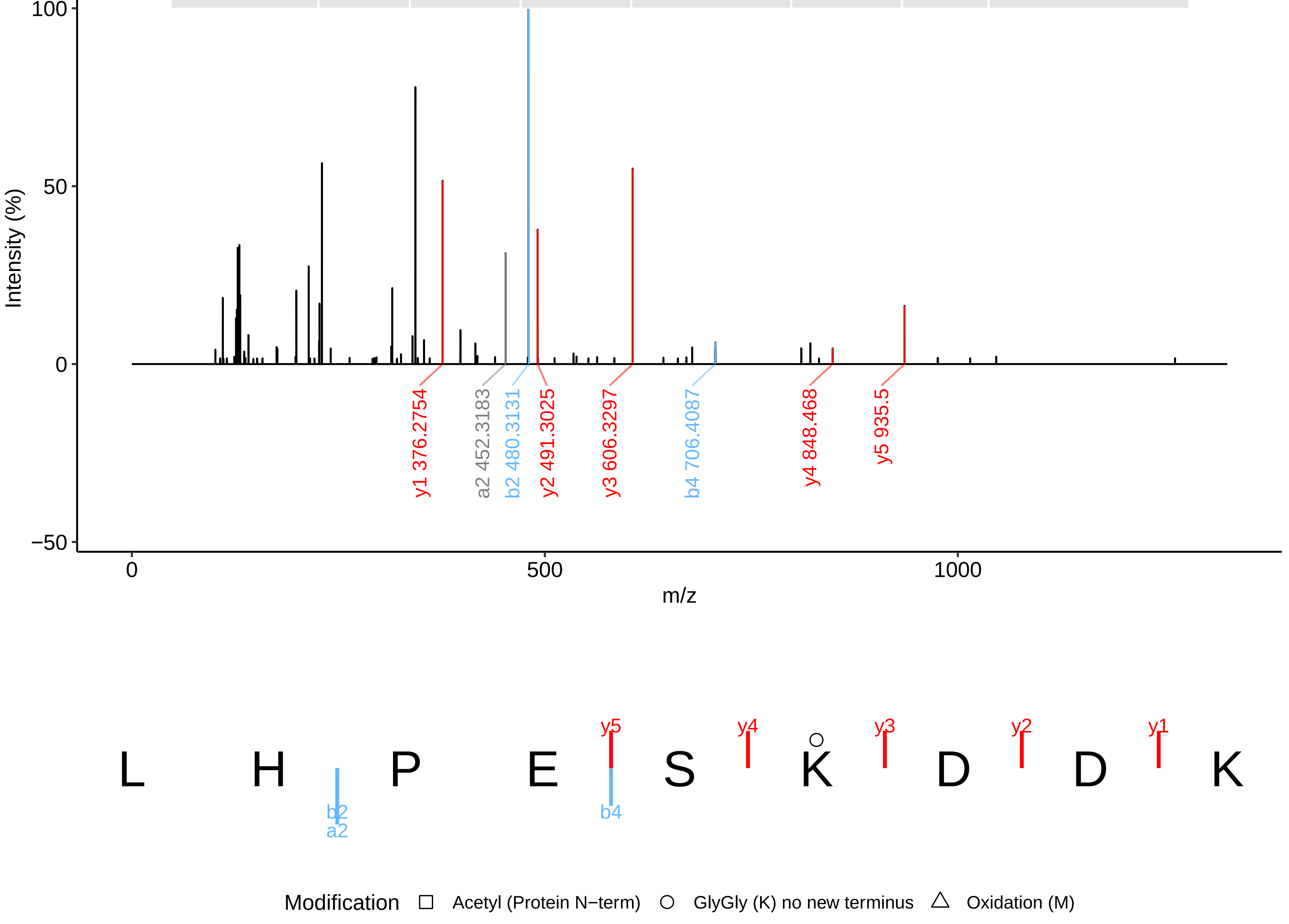

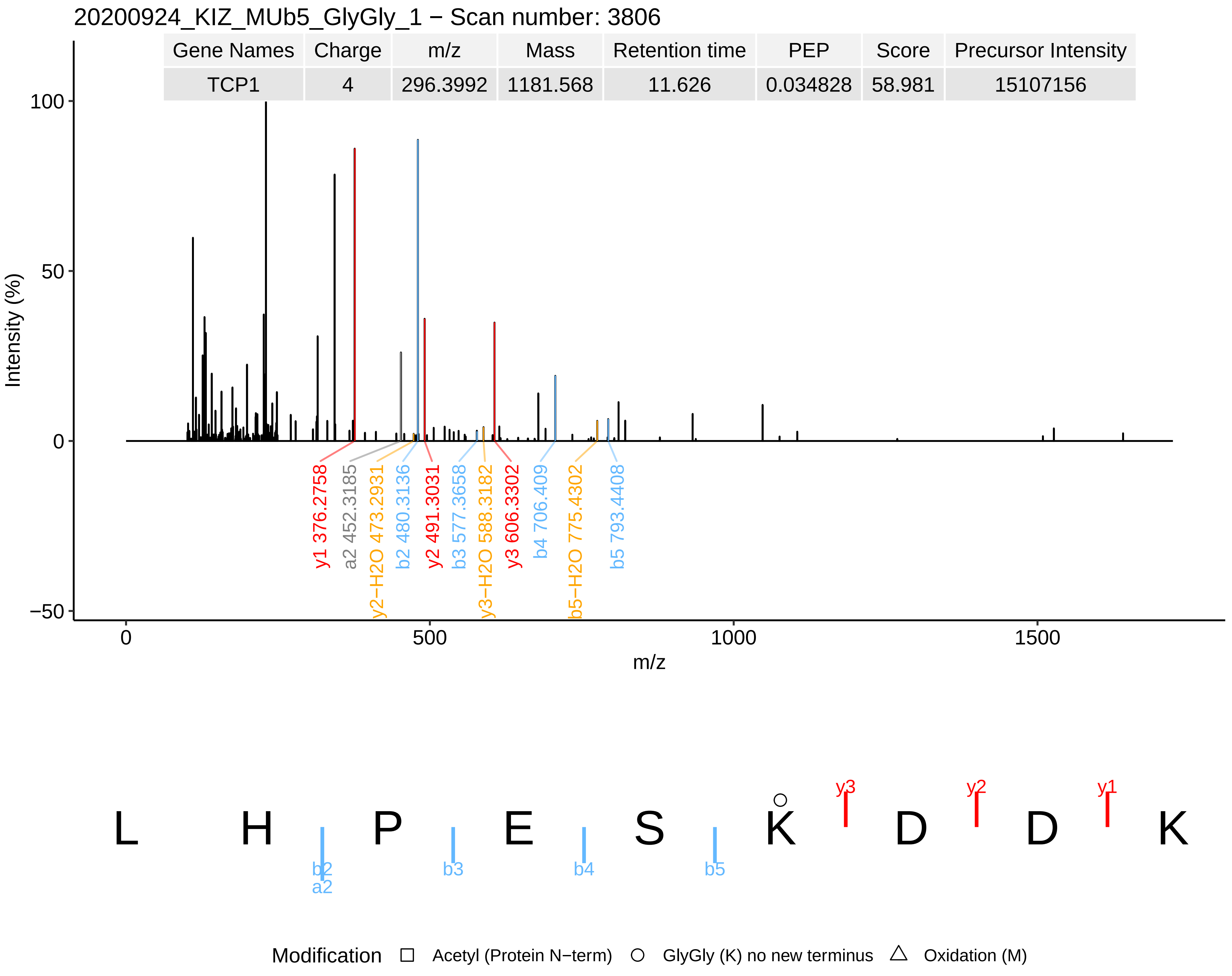
20200924_KIZ_MUb5_GlyGly_1 − Scan number: 3806
Gene Names
Charge
m/z
Mass
Retention time
PEP
Score
Precursor Intensity
TCP1
4
296.3992
1181.568
11.626
0.034828
58.981
15107156
Intensity (%)
100
50
0
−50
0
500
1000
1500
m/z
y1 376.2758
a2 452.3185
y2−H2O 473.2931
b2 480.3136
y2 491.3031
b3 577.3658
y3−H2O 588.3182
y3 606.3302
b4 706.409
b5−H2O 775.4302
b5 793.4408
L H P E S K D D K
y3
y2
y1
b2
a2
b3
b4
b5
Modification
Acetyl (Protein N−term)
GlyGly (K) no new terminus
Oxidation (M)

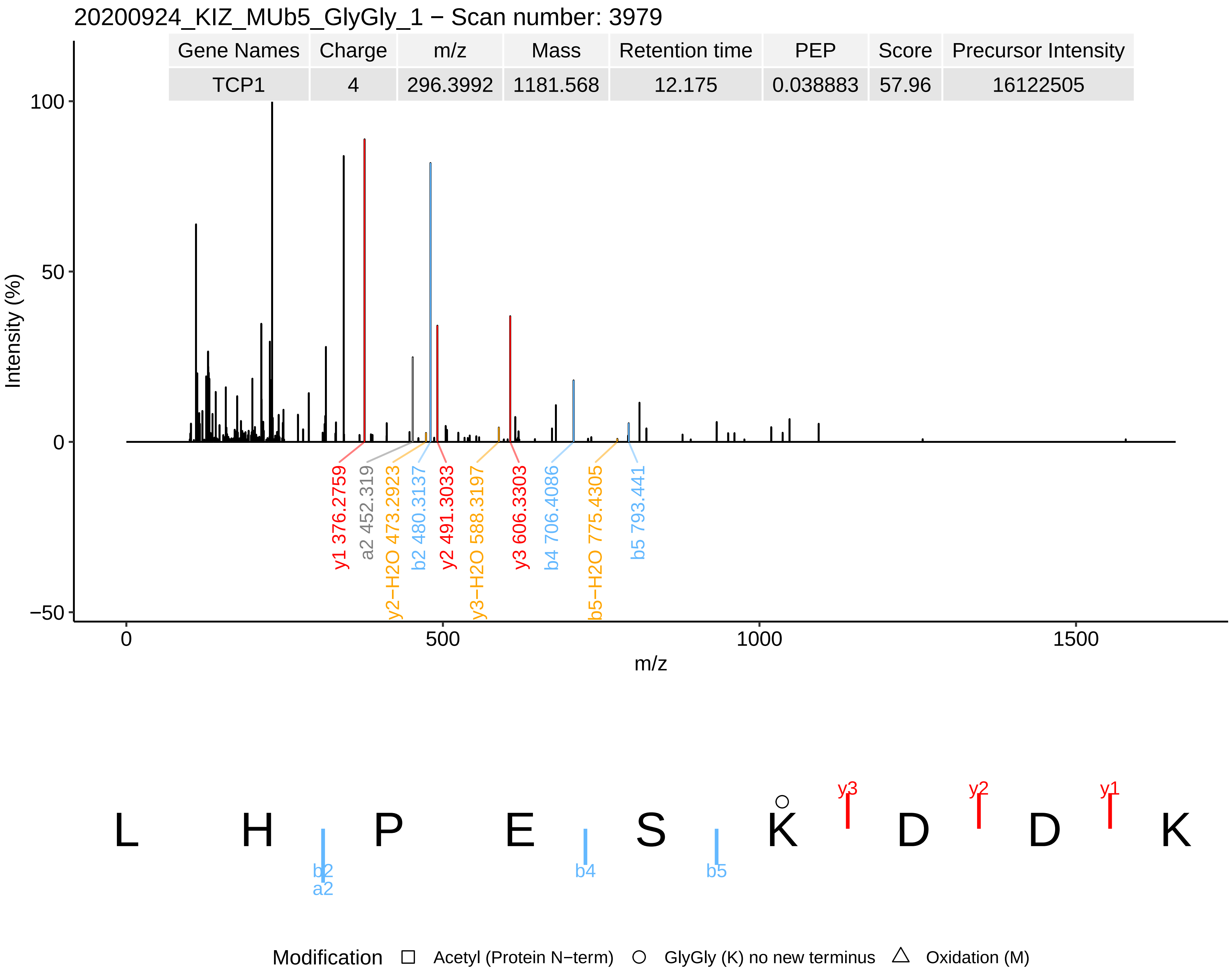
20200924_KIZ_MUb5_GlyGly_1 − Scan number: 3979
Gene Names | Charge | m/z | Mass | Retention time | PEP | Score | Precursor Intensity
TCP1 | 4 | 296.3992 | 1181.568 | 12.175 | 0.038883 | 57.96 | 16122505
Intensity (%)
m/z
y1 376.2759
a2 452.319
y2−H2O 473.2923
b2 480.3137
y2 491.3033
y3−H2O 588.3197
y3 606.3303
b4 706.4086
b5−H2O 775.4305
b5 793.441
L H P E S K D D K
y3 y2 y1
b2 a2 b4 b5
Modification Acetyl (Protein N−term) GlyGly (K) no new terminus Oxidation (M)

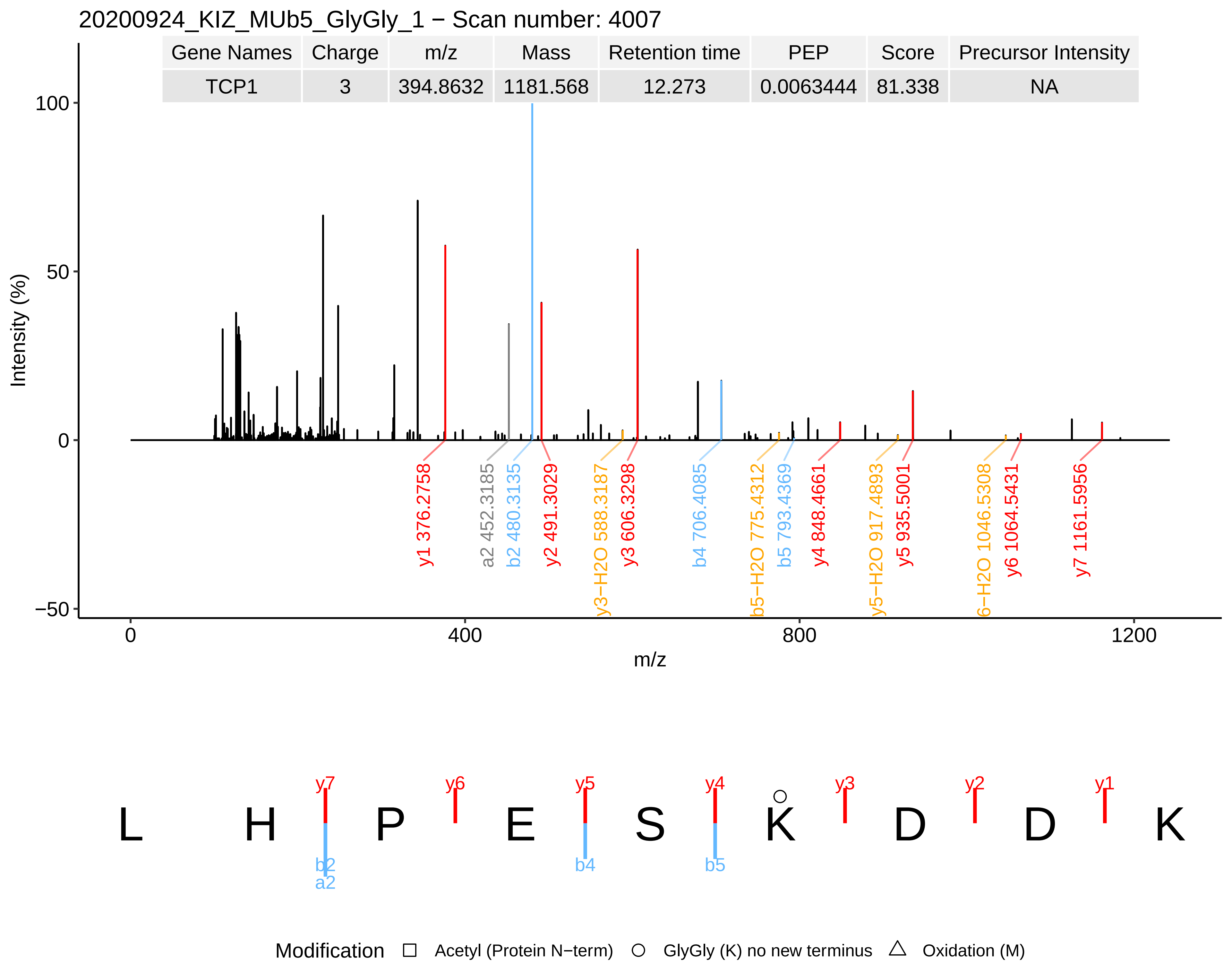

20200924_KIZ_MUb5_GlyGly_1 − Scan number: 4007
Gene Names | Charge | m/z | Mass | Retention time | PEP | Score | Precursor Intensity
TCP1 | 3 | 394.8632 | 1181.568 | 12.273 | 0.0063444 | 81.338 | NA
Intensity (%)
100
50
0
−50
0
400
800
1200
m/z
y1 376.2758
a2 452.3185
b2 480.3135
y2 491.3029
y3−H2O 588.3187
y3 606.3298
b4 706.4085
b5−H2O 775.4312
b5 793.4369
y4 848.4661
y5−H2O 917.4893
y5 935.5001
6−H2O 1046.5308
y6 1064.5431
y7 1161.5956
L H P E S K D D K
y7 y6 y5 y4 y3 y2 y1
b2 a2 b4 b5
Modification
Acetyl (Protein N−term)
GlyGly (K) no new terminus
Oxidation (M)

20200924_KIZ_MUb5_GlyGly_2 − Scan number: 3770

Gene Names	Charge	m/z	Mass	Retention time	PEP	Score	Precursor Intensity
TCP1	4	296.3992	1181.568	13.737	0.038883	57.96	5856235

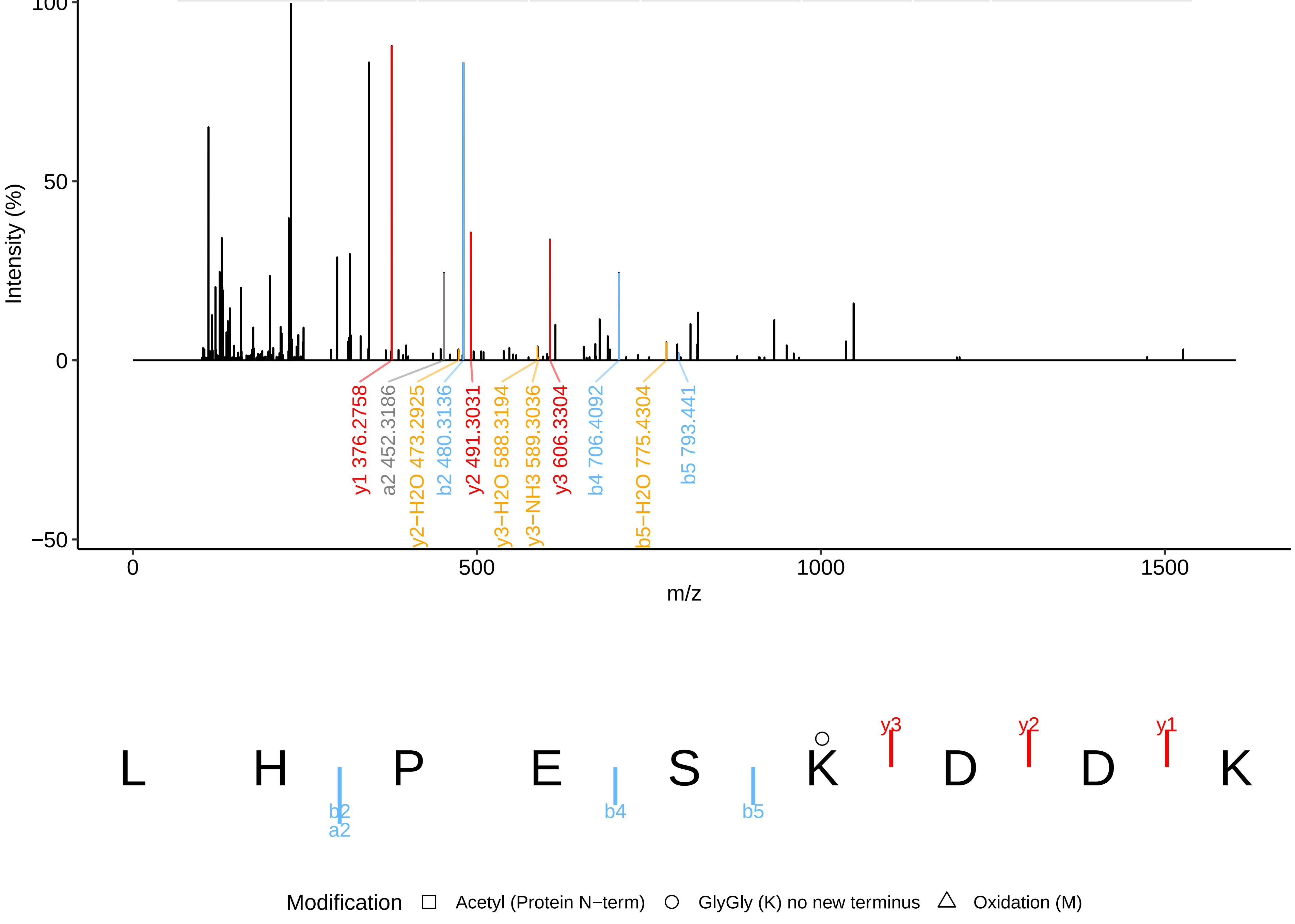

20200924_KIZ_MUb5_GlyGly_2 − Scan number: 3782

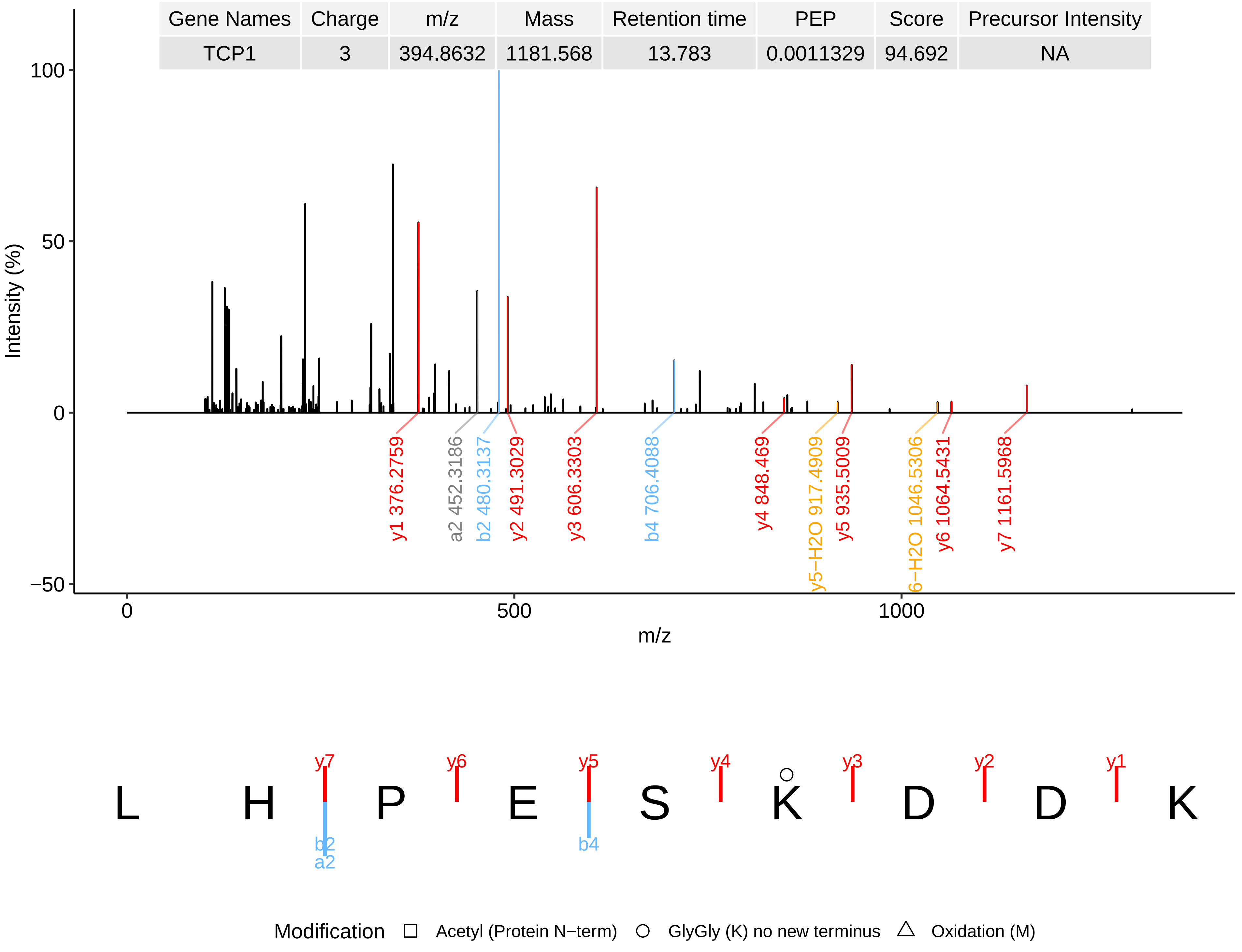

20200924_KIZ_MUb5_GlyGly_2 − Scan number: 3645

Gene Names	Charge	m/z	Mass	Retention time	PEP	Score	Precursor Intensity
TCP1	3	394.8632	1181.568	13.25	0.080166	55.567	NA

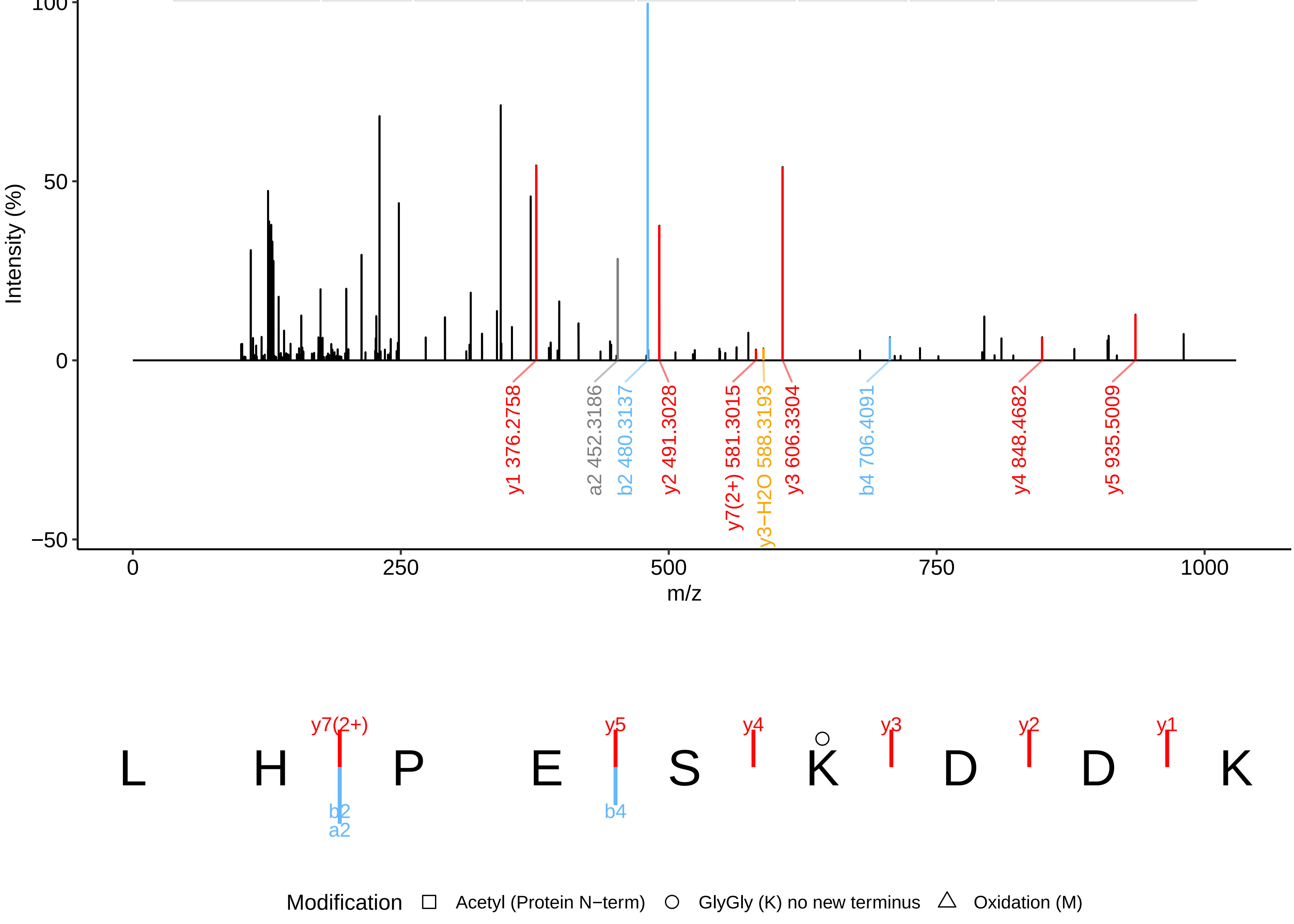

20201118_KIZ_MUb6_GlyGly_1 − Scan number: 3593

Gene Names	Charge	m/z	Mass	Retention time	PEP	Score	Precursor Intensity
TCP1	4	296.3992	1181.568	11.033	0.038883	57.96	12666677

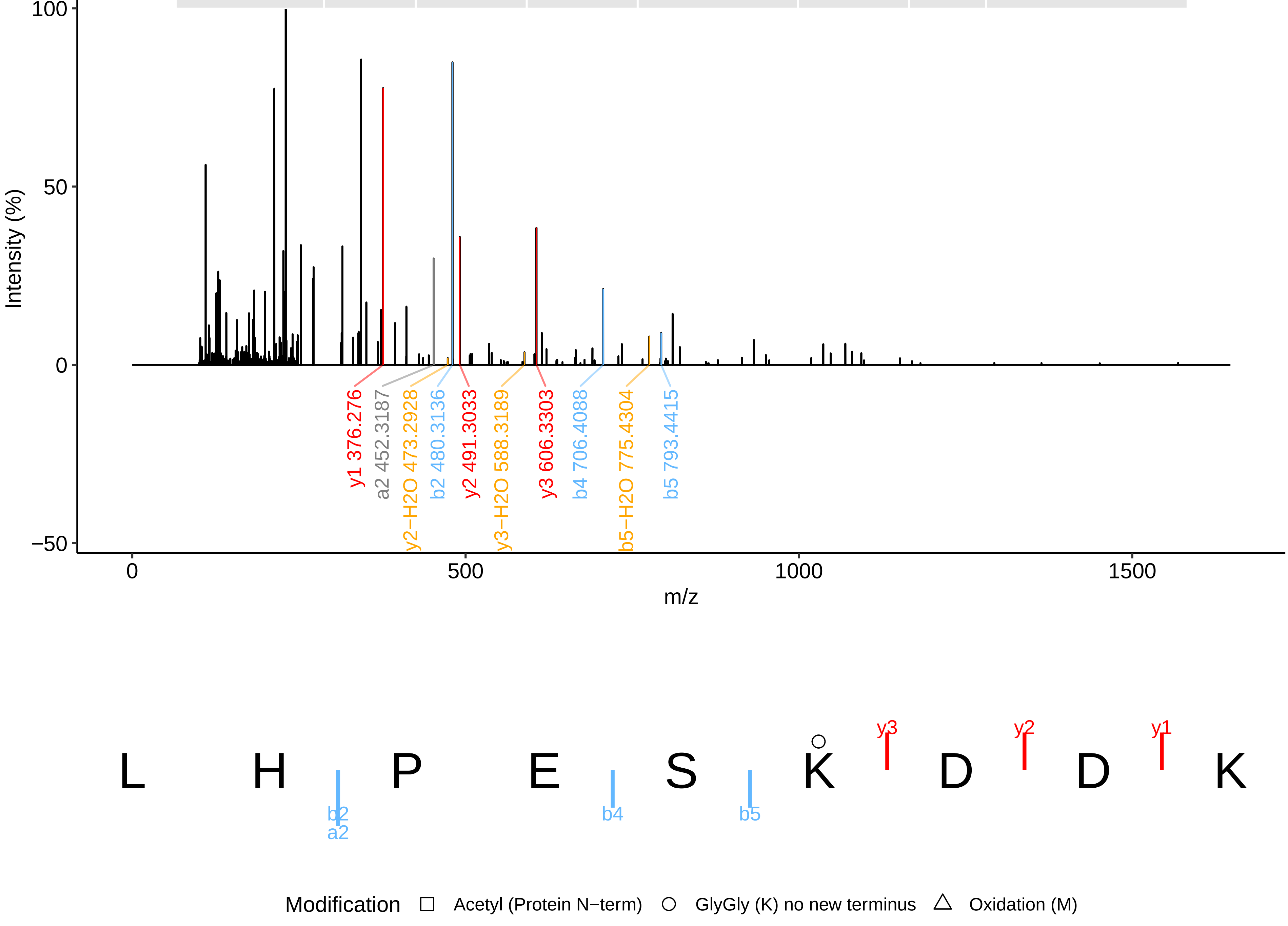

20201118_KIZ_MUb6_GlyGly_2 − Scan number: 4719

Gene Names	Charge	m/z	Mass	Retention time	PEP	Score	Precursor Intensity
TCP1	4	296.3992	1181.568	16.207	0.066266	51.066	7475307

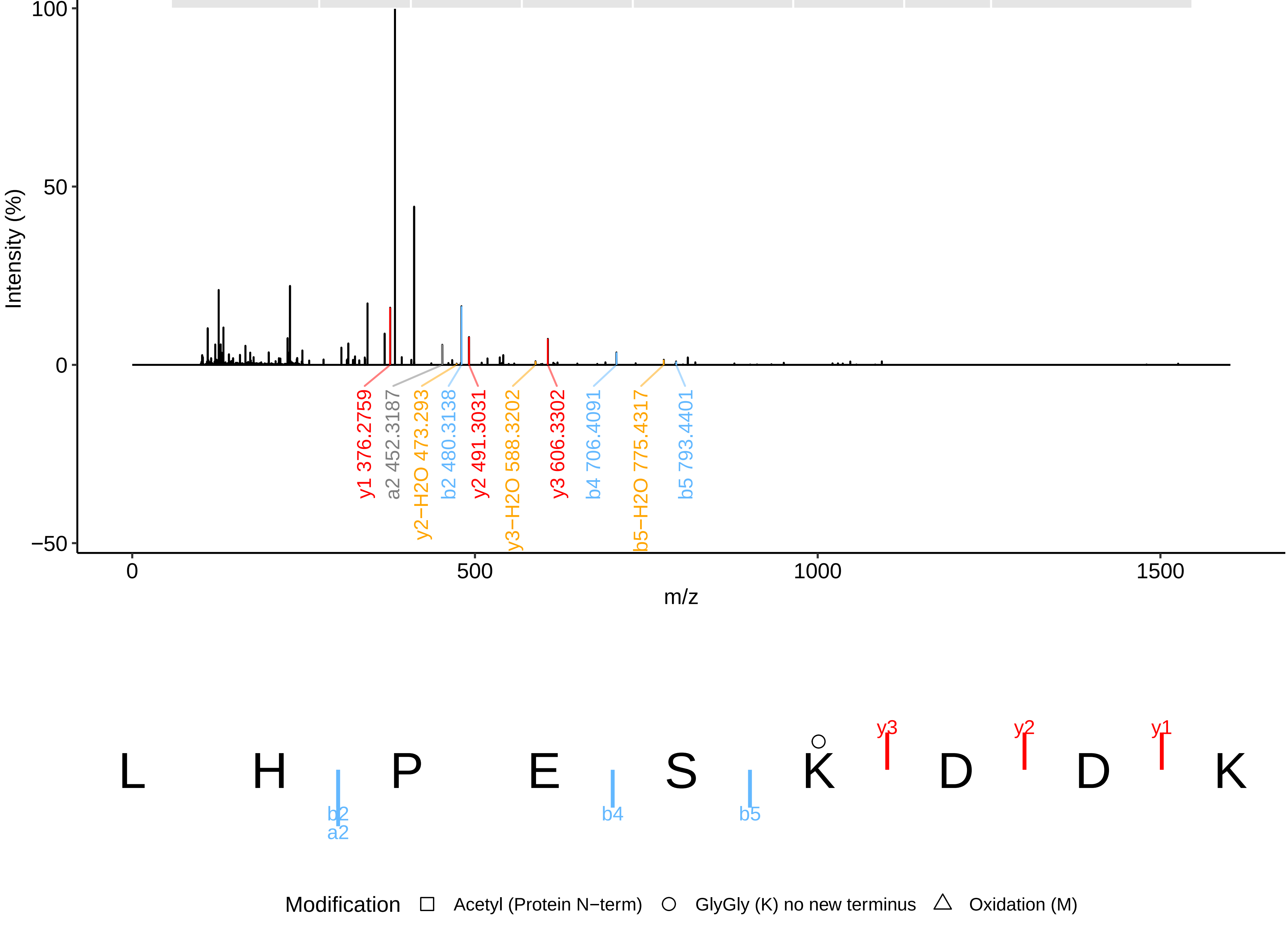

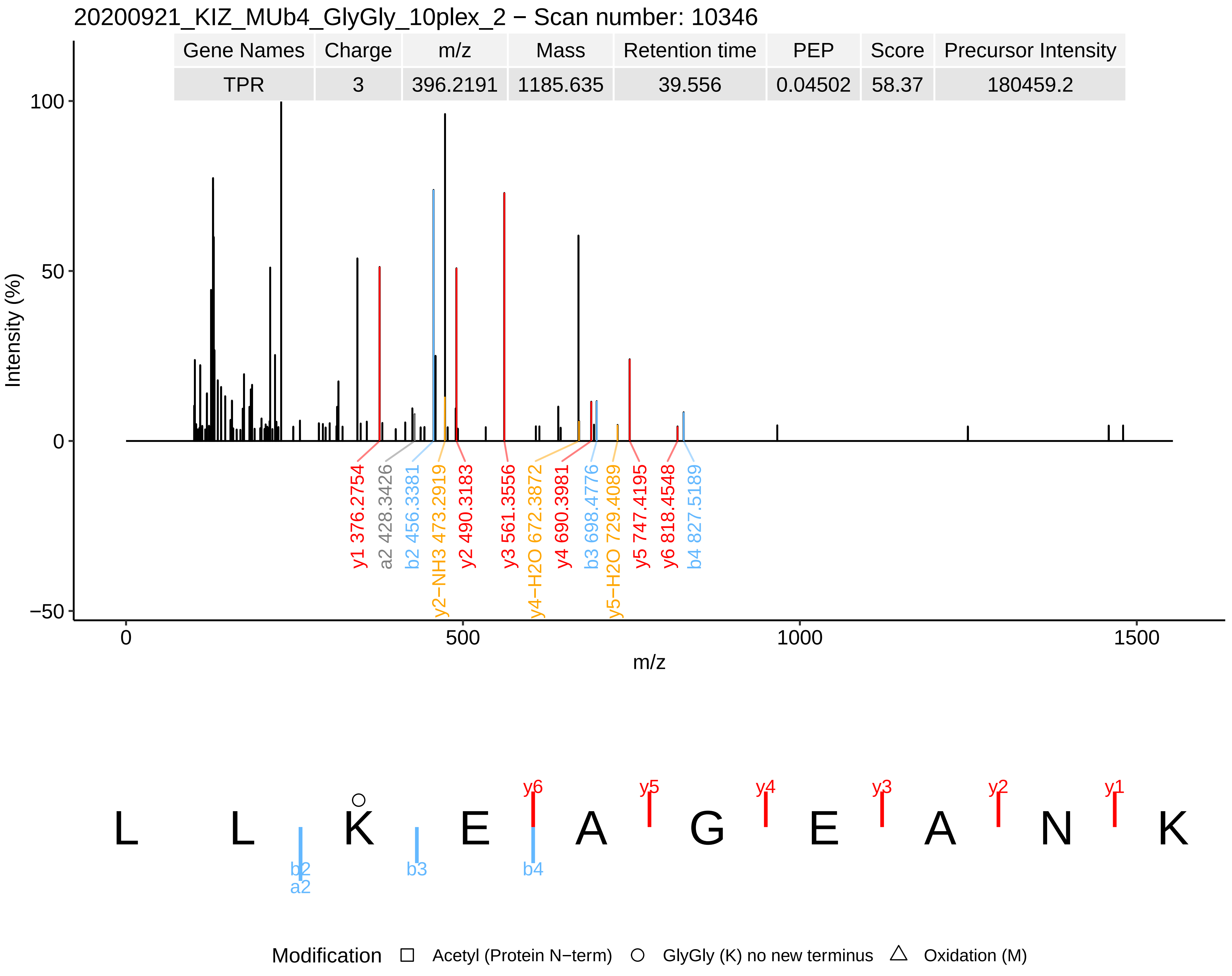
20200921_KIZ_MUb4_GlyGly_10plex_2 − Scan number: 10346
Gene Names
Charge
m/z
Mass
Retention time
PEP
Score
Precursor Intensity
TPR
3
396.2191
1185.635
39.556
0.04502
58.37
180459.2
Intensity (%)
100
50
0
−50
0
500
1000
1500
m/z
y1 376.2754
a2 428.3426
b2 456.3381
y2−NH3 473.2919
y2 490.3183
y3 561.3556
y4−H2O 672.3872
y4 690.3981
b3 698.4776
y5−H2O 729.4089
y5 747.4195
y6 818.4548
b4 827.5189
L
L
K
E
A
G
E
A
N
K
y6
y5
y4
y3
y2
y1
b2
a2
b3
b4
Modification
Acetyl (Protein N−term)
GlyGly (K) no new terminus
Oxidation (M)

20200921_KIZ_MUb4_GlyGly_10plex − Scan number: 14749

Gene Names	Charge	m/z	Mass	Retention time	PEP	Score	Precursor Intensity
CPT1A	3	474.9403	1421.799	52.483	7.841e−14	146.71	4261968

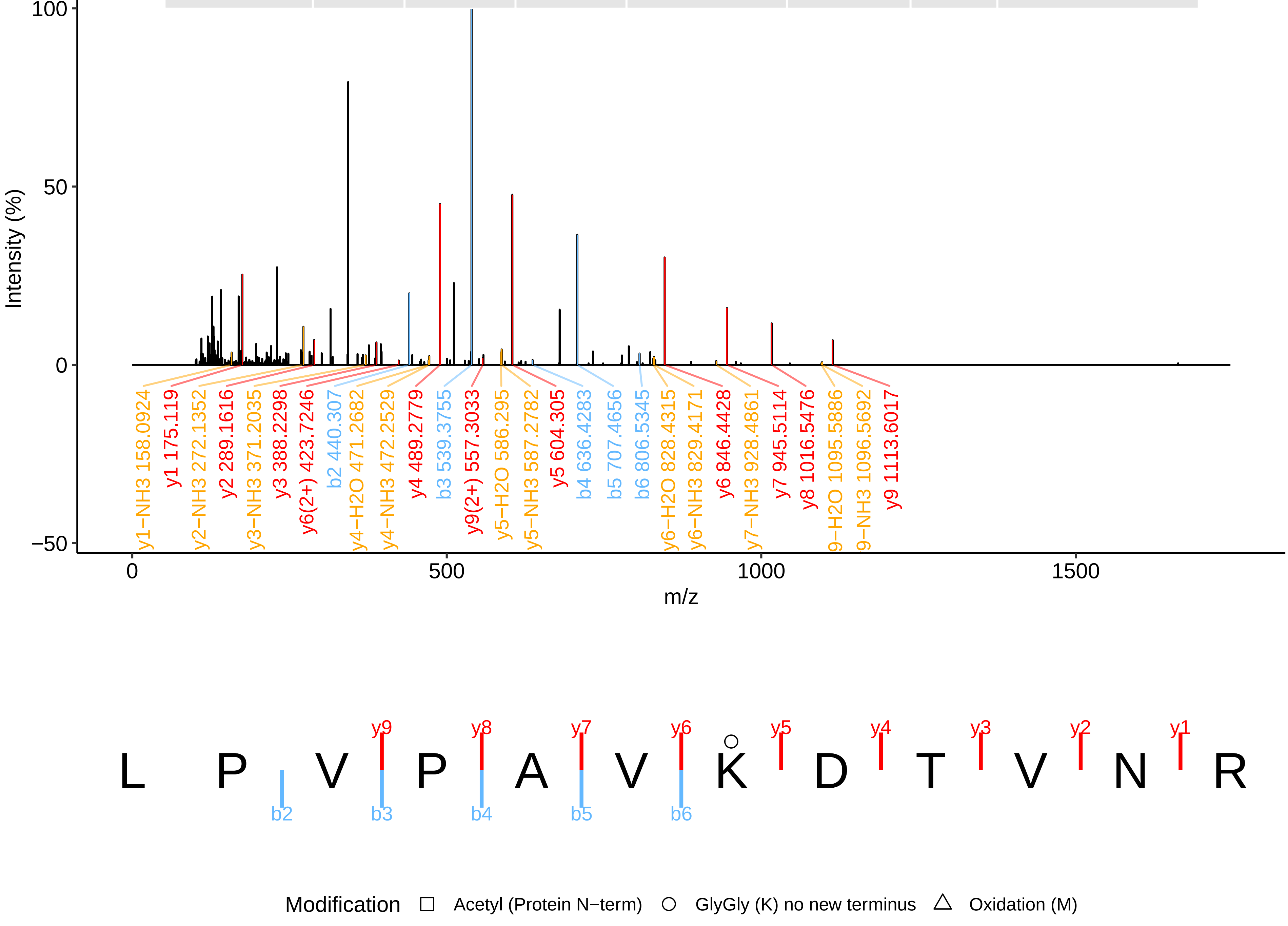

20200921_KIZ_MUb4_GlyGly_10plex − Scan number: 14881

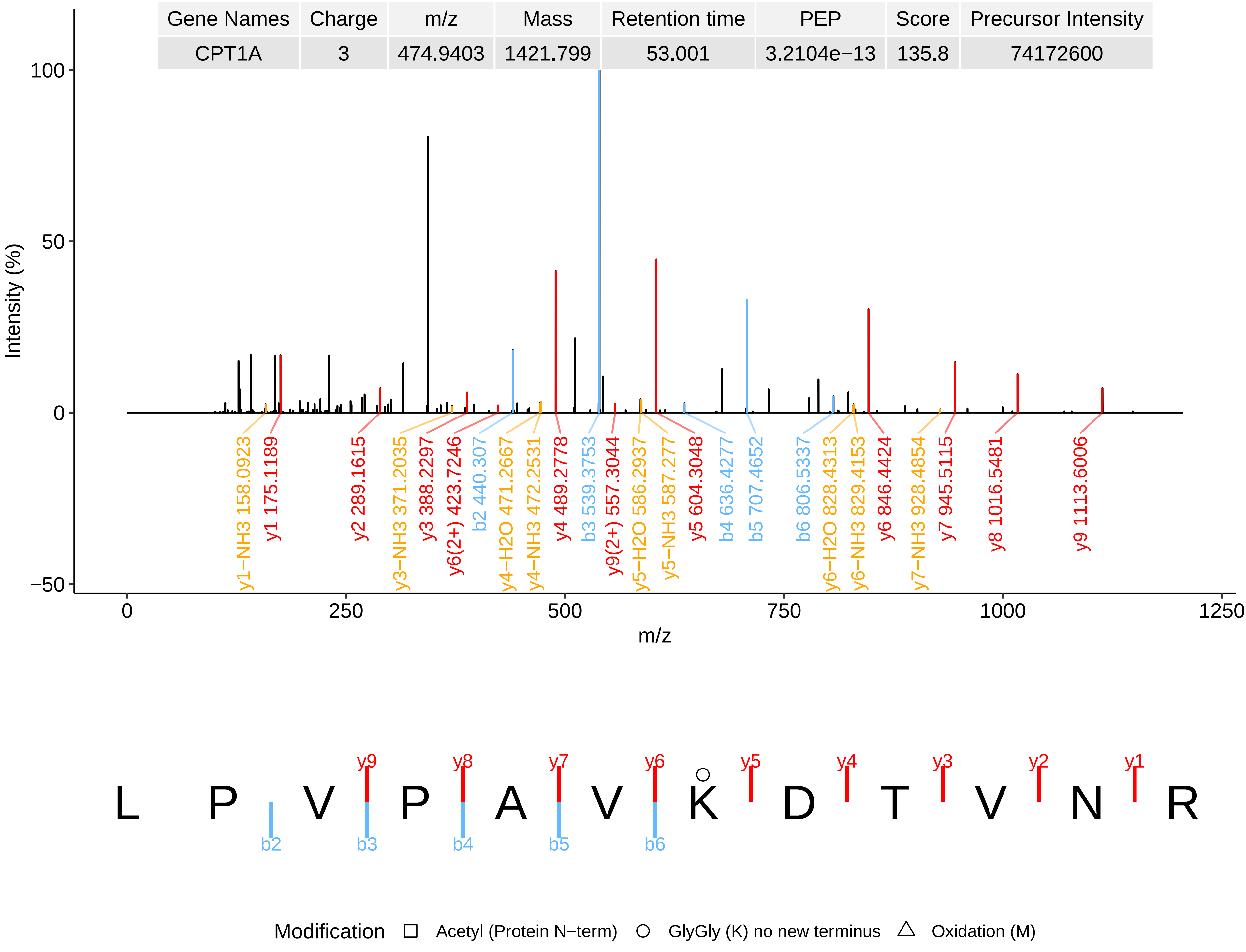

20200921_KIZ_MUb4_GlyGly_10plex − Scan number: 14833

Gene Names	Charge	m/z	Mass	Retention time	PEP	Score	Precursor Intensity
CPT1A	2	711.9068	1421.799	52.813	4.0513e−14	177.06	NA

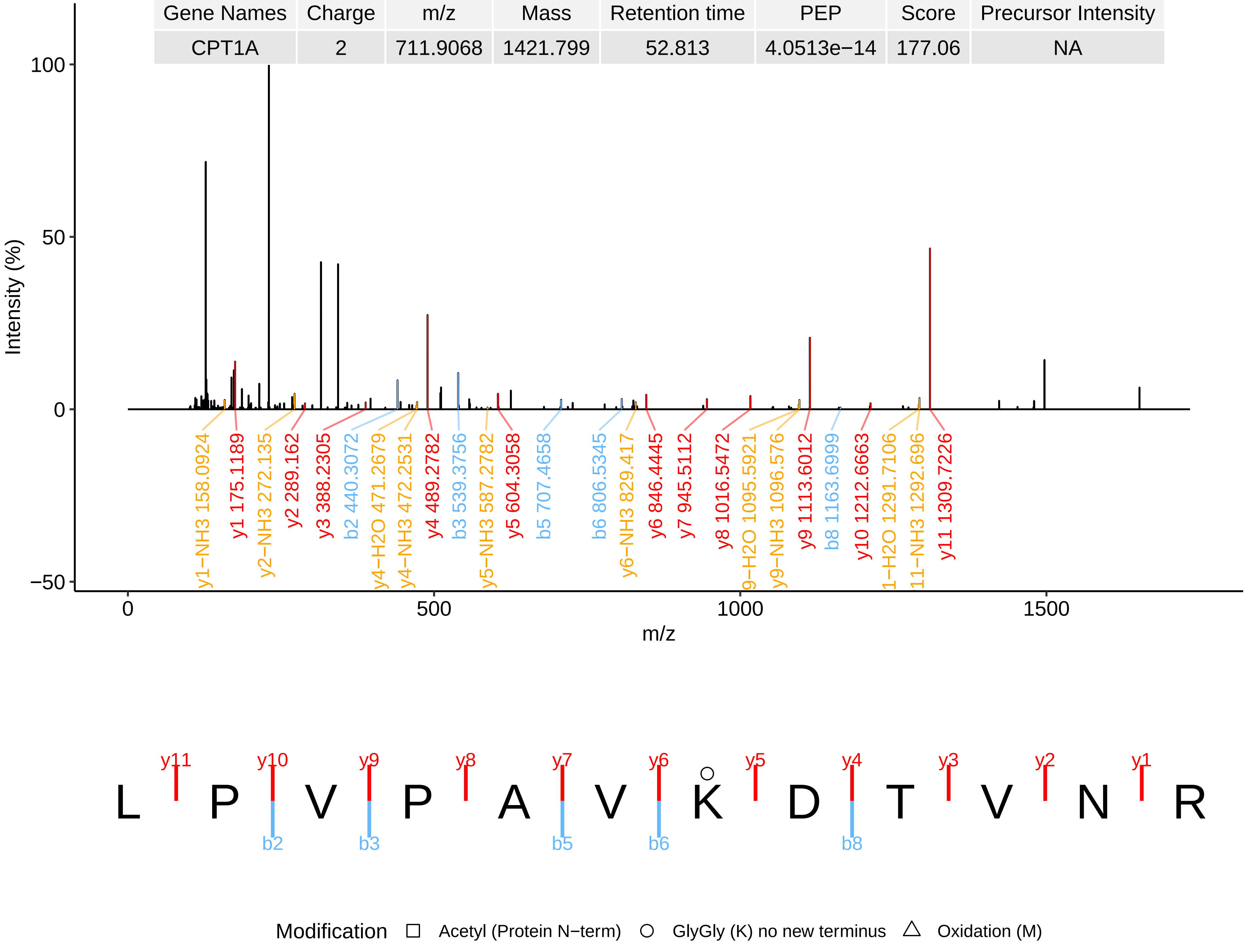

20200921_KIZ_MUb4_GlyGly_10plex_2 − Scan number: 13595

Gene Names	Charge	m/z	Mass	Retention time	PEP	Score	Precursor Intensity
CPT1A	3	474.9403	1421.799	52.823	2.5508e−13	138.76	1598629

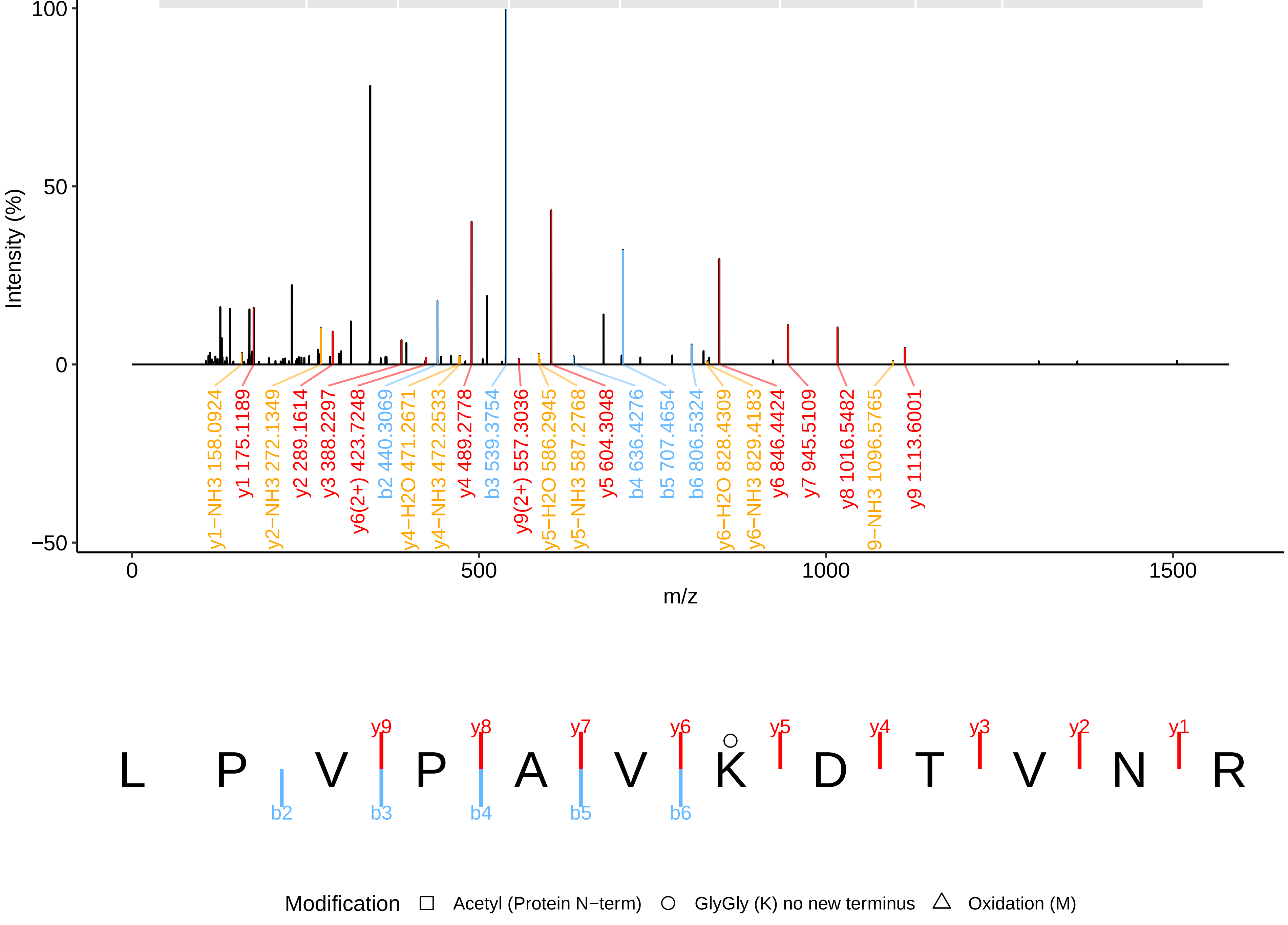

20200921_KIZ_MUb4_GlyGly_10plex_2 − Scan number: 13727

Gene Names	Charge	m/z	Mass	Retention time	PEP	Score	Precursor Intensity
CPT1A	3	474.9403	1421.799	53.358	2.0897e−06	113.67	4017840

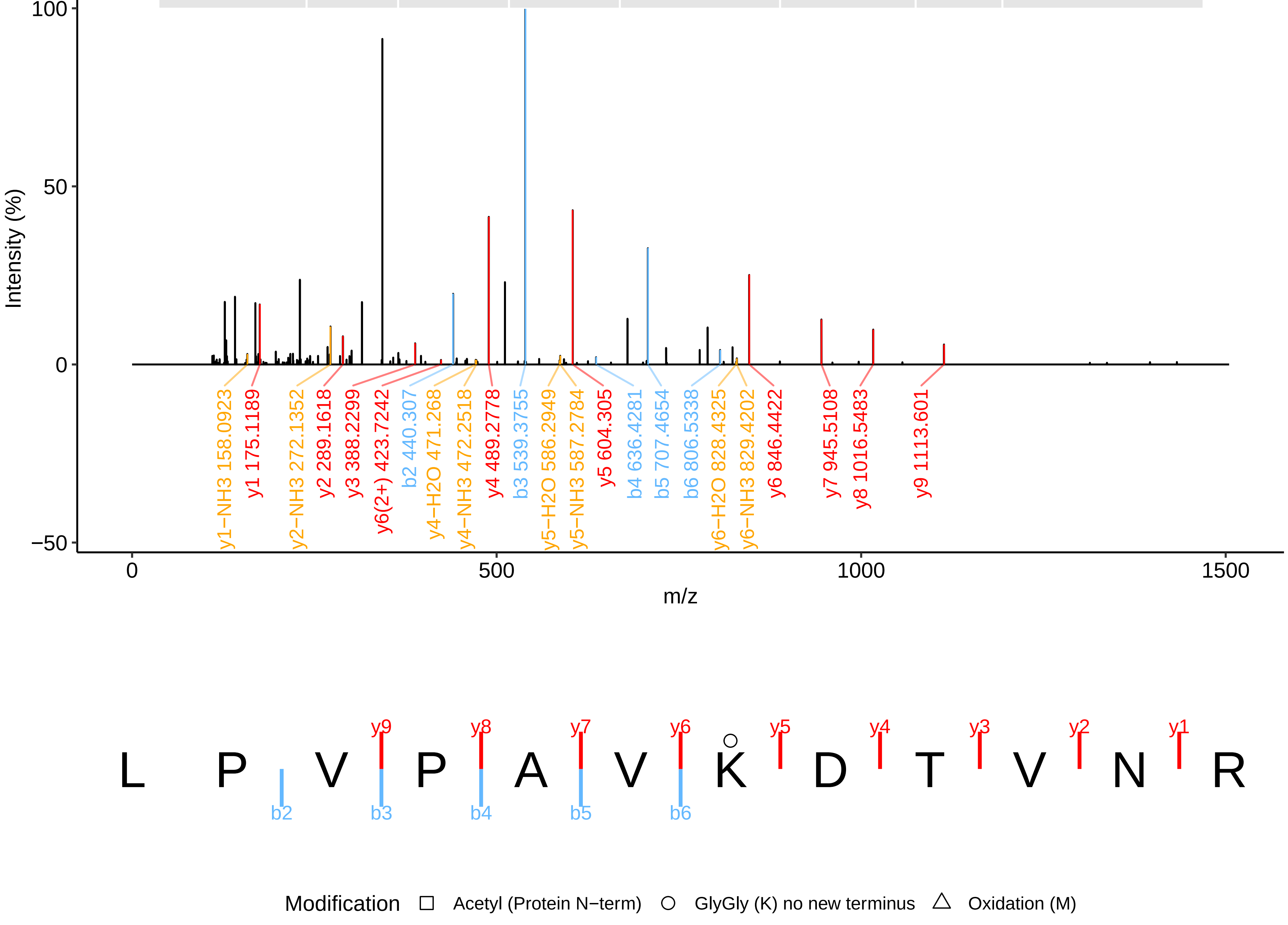

20200924_KIZ_MUb5_GlyGly_1 − Scan number: 13805

Gene Names	Charge	m/z	Mass	Retention time	PEP	Score	Precursor Intensity
CPT1A	3	474.9403	1421.799	45.027	2.497e−19	154.05	9093734

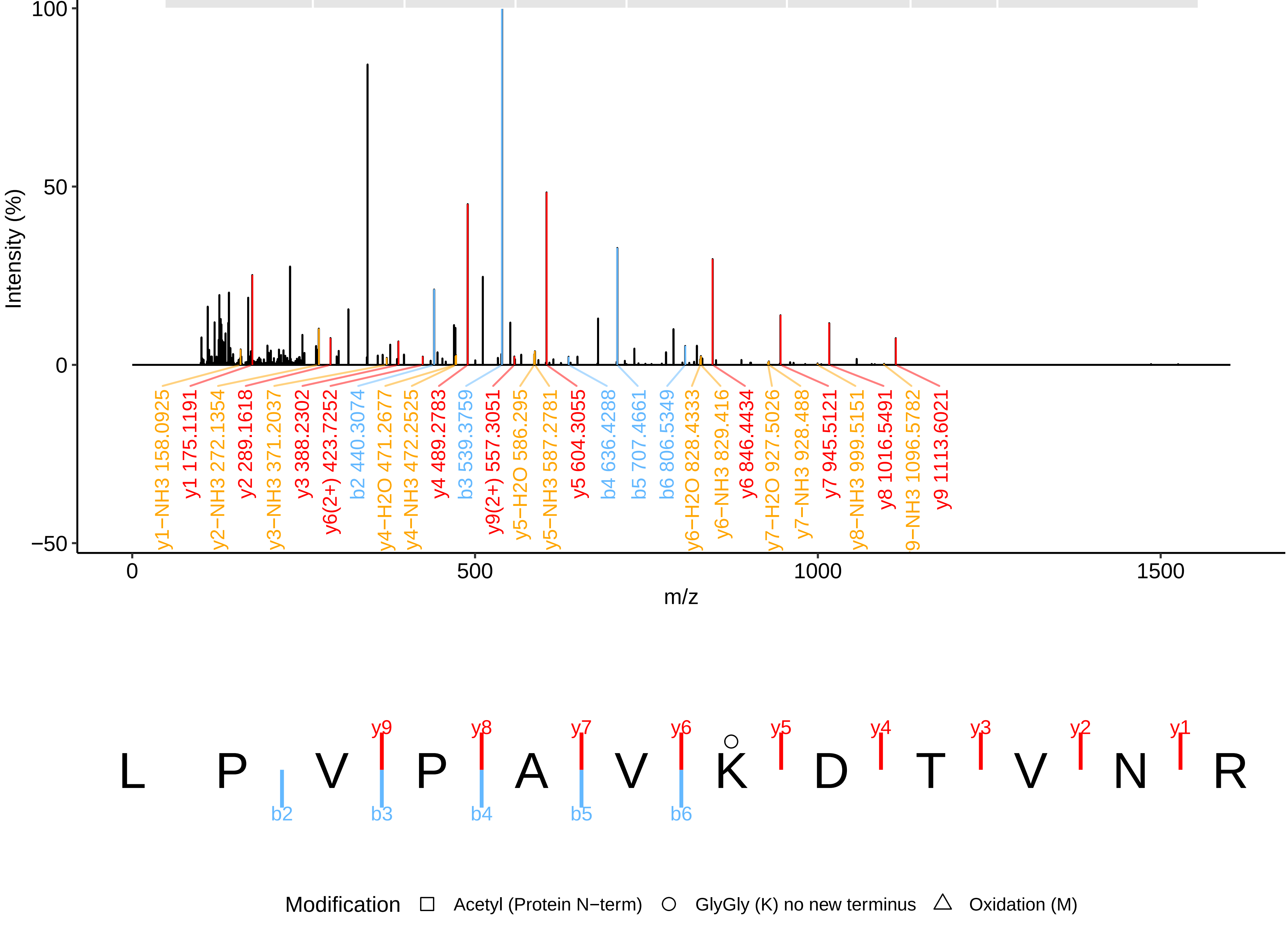

20200924_KIZ_MUb5_GlyGly_1 − Scan number: 13952

Gene Names	Charge	m/z	Mass	Retention time	PEP	Score	Precursor Intensity
CPT1A	3	474.9403	1421.799	45.532	6.2049e−15	149.96	102692424

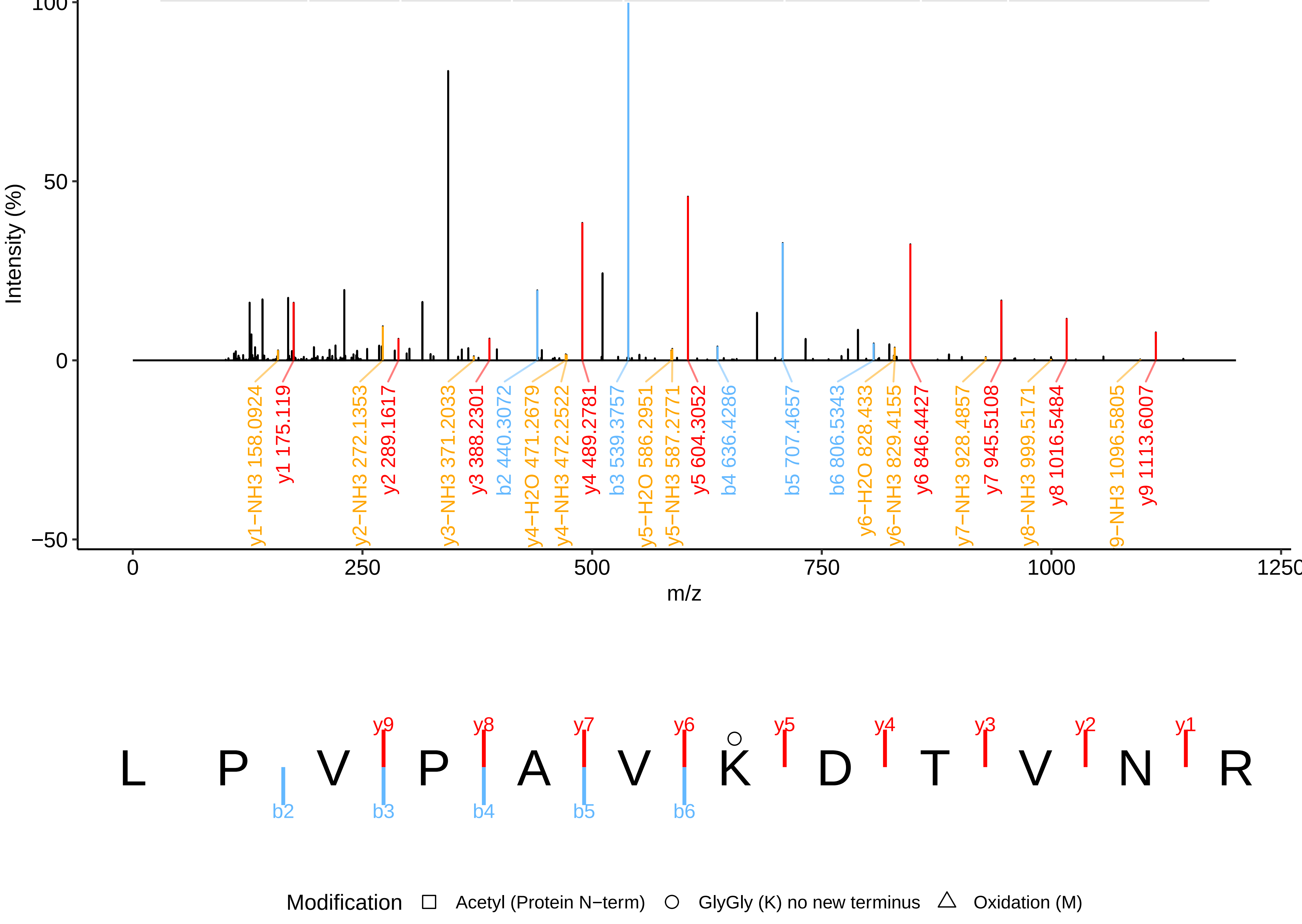

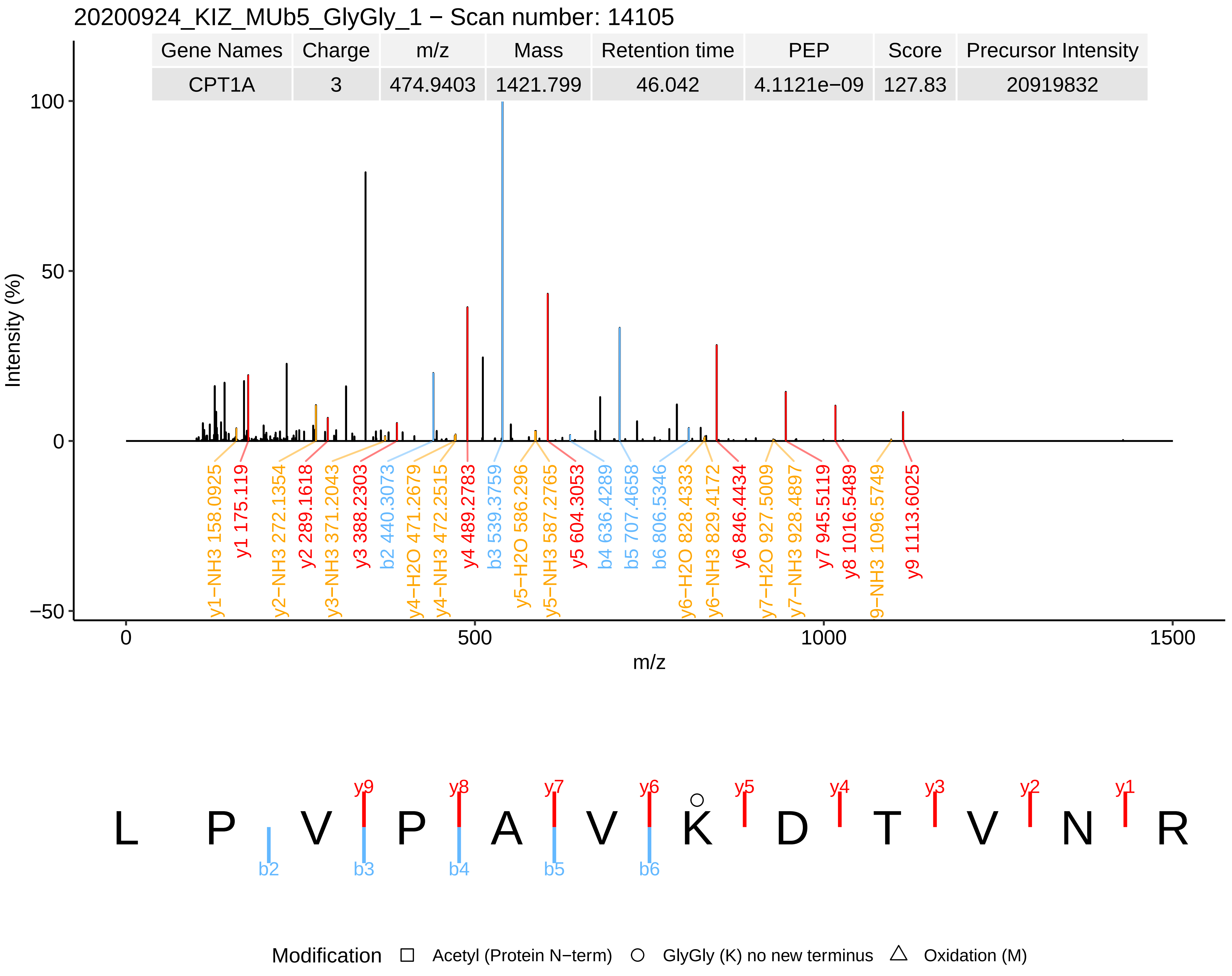
20200924_KIZ_MUb5_GlyGly_1 − Scan number: 14105
Gene Names | Charge | m/z | Mass | Retention time | PEP | Score | Precursor Intensity
CPT1A | 3 | 474.9403 | 1421.799 | 46.042 | 4.1121e−09 | 127.83 | 20919832
Intensity (%)
m/z
y1−NH3 158.0925
y1 175.119
y2−NH3 272.1354
y2 289.1618
y3−NH3 371.2043
y3 388.2303
b2 440.3073
y4−H2O 471.2679
y4−NH3 472.2515
y4 489.2783
b3 539.3759
y5−H2O 586.296
y5−NH3 587.2765
y5 604.3053
b4 636.4289
b5 707.4658
b6 806.5346
y6−H2O 828.4333
y6−NH3 829.4172
y6 846.4434
y7−H2O 927.5009
y7−NH3 928.4897
y7 945.5119
y8 1016.5489
y9−NH3 1096.5749
y9 1113.6025
L P V P A V K D T V N R
Modification
Acetyl (Protein N−term)
GlyGly (K) no new terminus
Oxidation (M)

20200924_KIZ_MUb5_GlyGly_2 − Scan number: 12926

Gene Names	Charge	m/z	Mass	Retention time	PEP	Score	Precursor Intensity
CPT1A	3	474.9403	1421.799	48.341	1.0984e−19	161.67	29676100

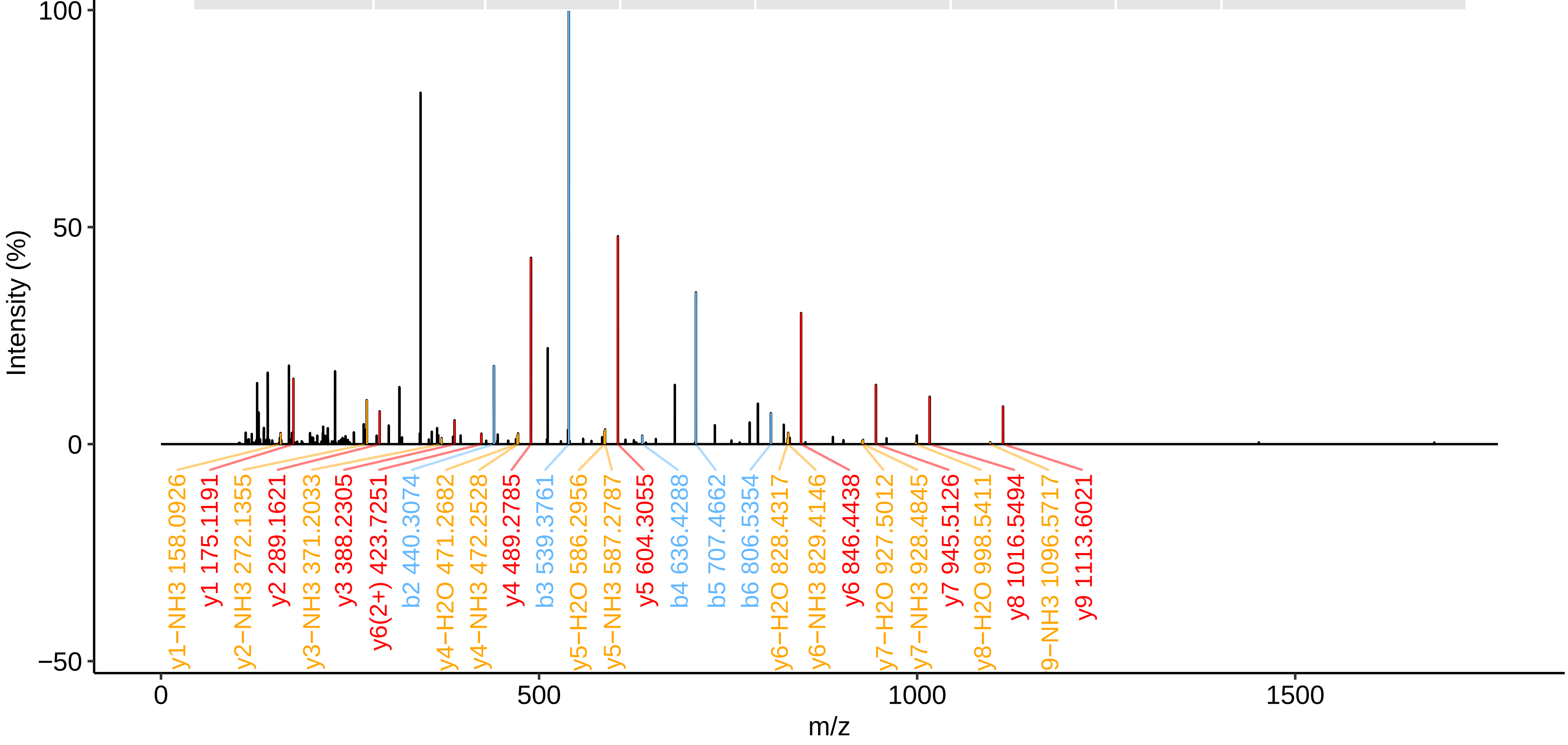

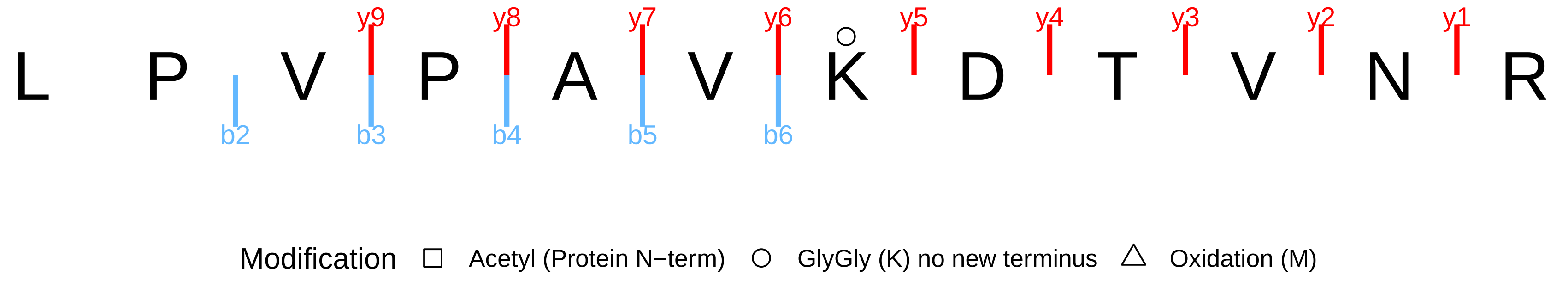

20200924_KIZ_MUb5_GlyGly_2 − Scan number: 13061

Gene Names	Charge	m/z	Mass	Retention time	PEP	Score	Precursor Intensity
CPT1A	3	474.9403	1421.799	48.851	7.841e−14	146.71	16533248

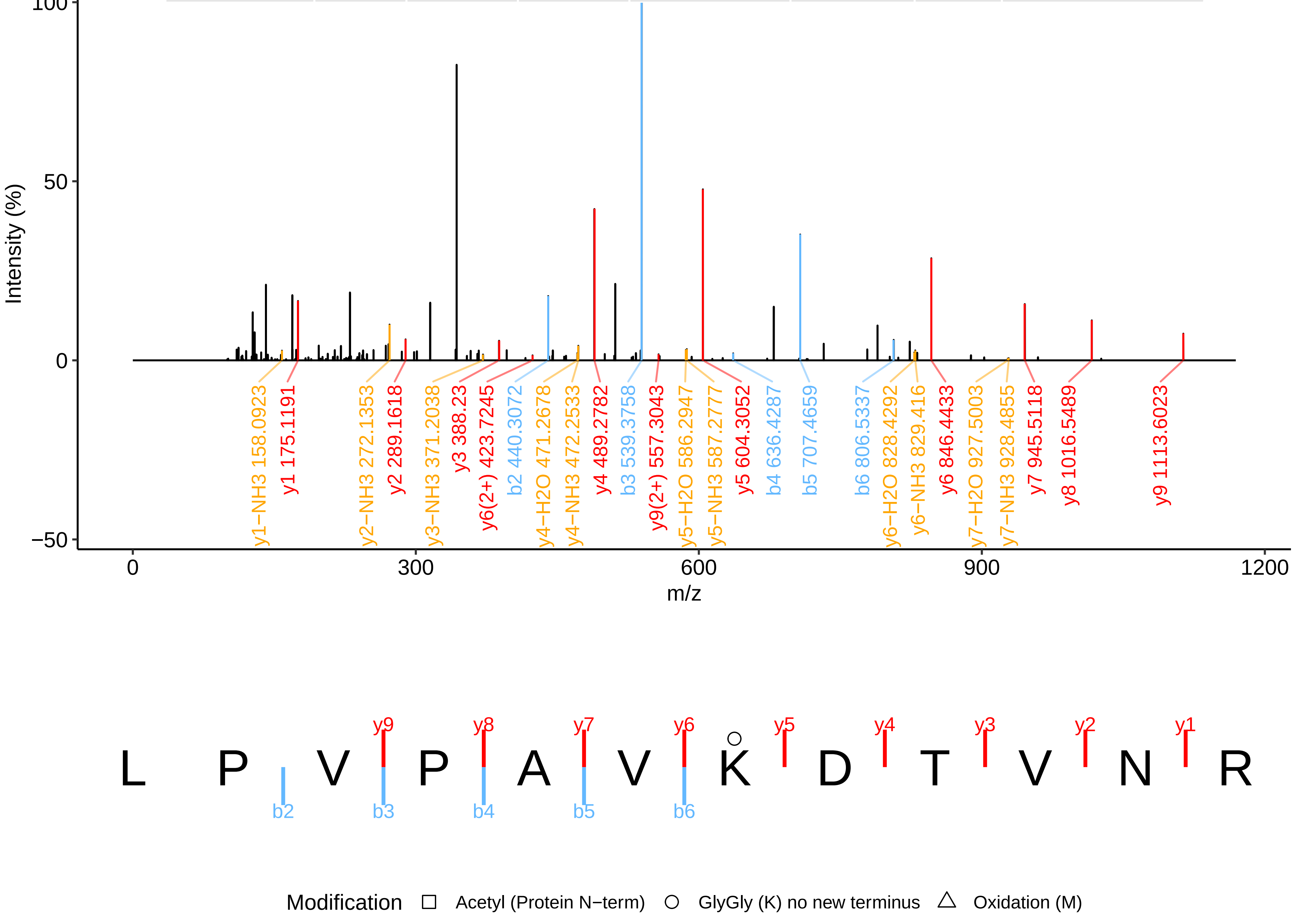

20201118_KIZ_MUb6_GlyGly_1 − Scan number: 13988

Gene Names	Charge	m/z	Mass	Retention time	PEP	Score	Precursor Intensity
CPT1A	3	474.9403	1421.799	44.224	1.0984e−19	161.67	NA

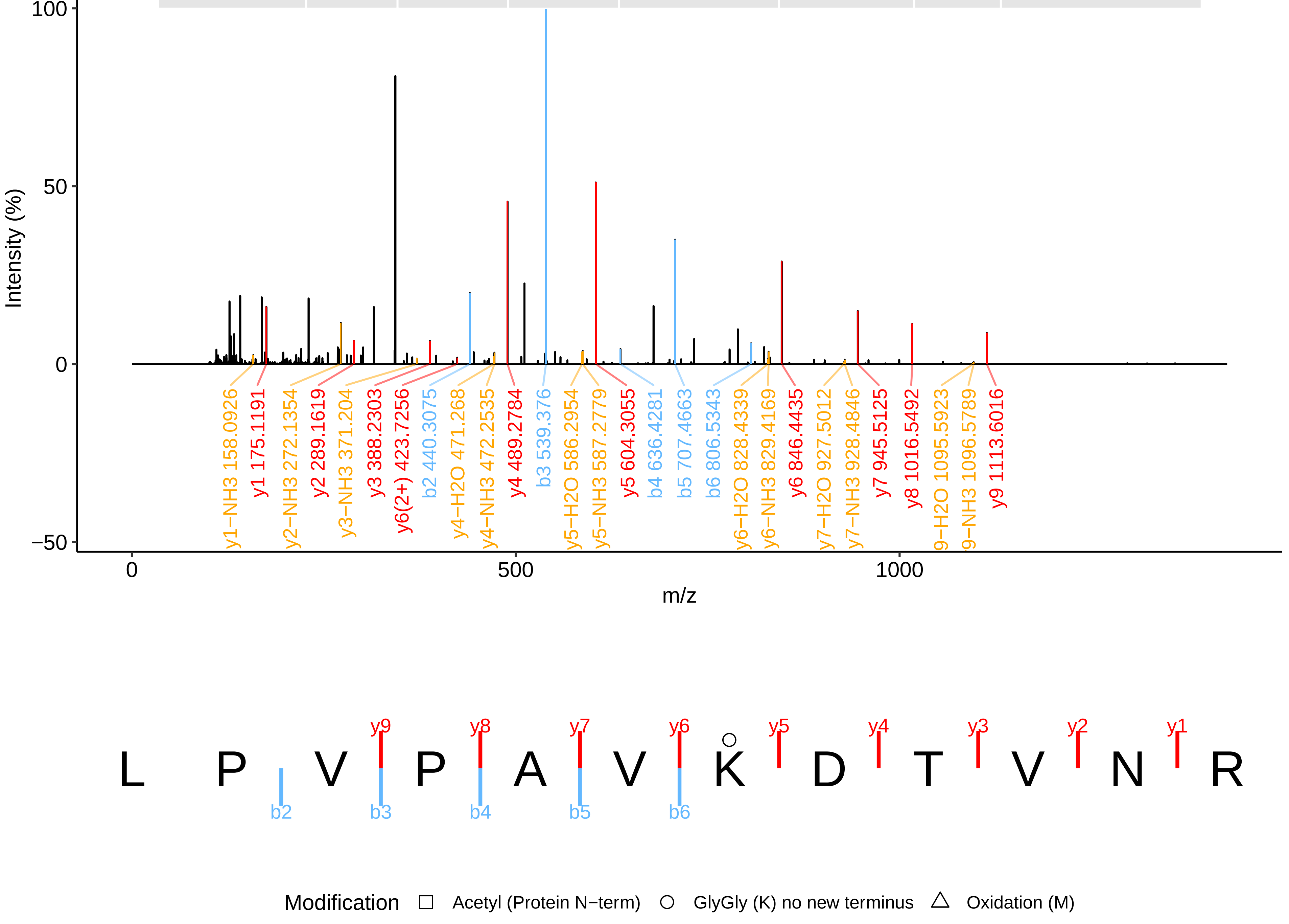

20201118_KIZ_MUb6_GlyGly_2 − Scan number: 15891

Gene Names	Charge	m/z	Mass	Retention time	PEP	Score	Precursor Intensity
CPT1A	3	474.9403	1421.799	53.597	6.2049e−15	149.96	3523491

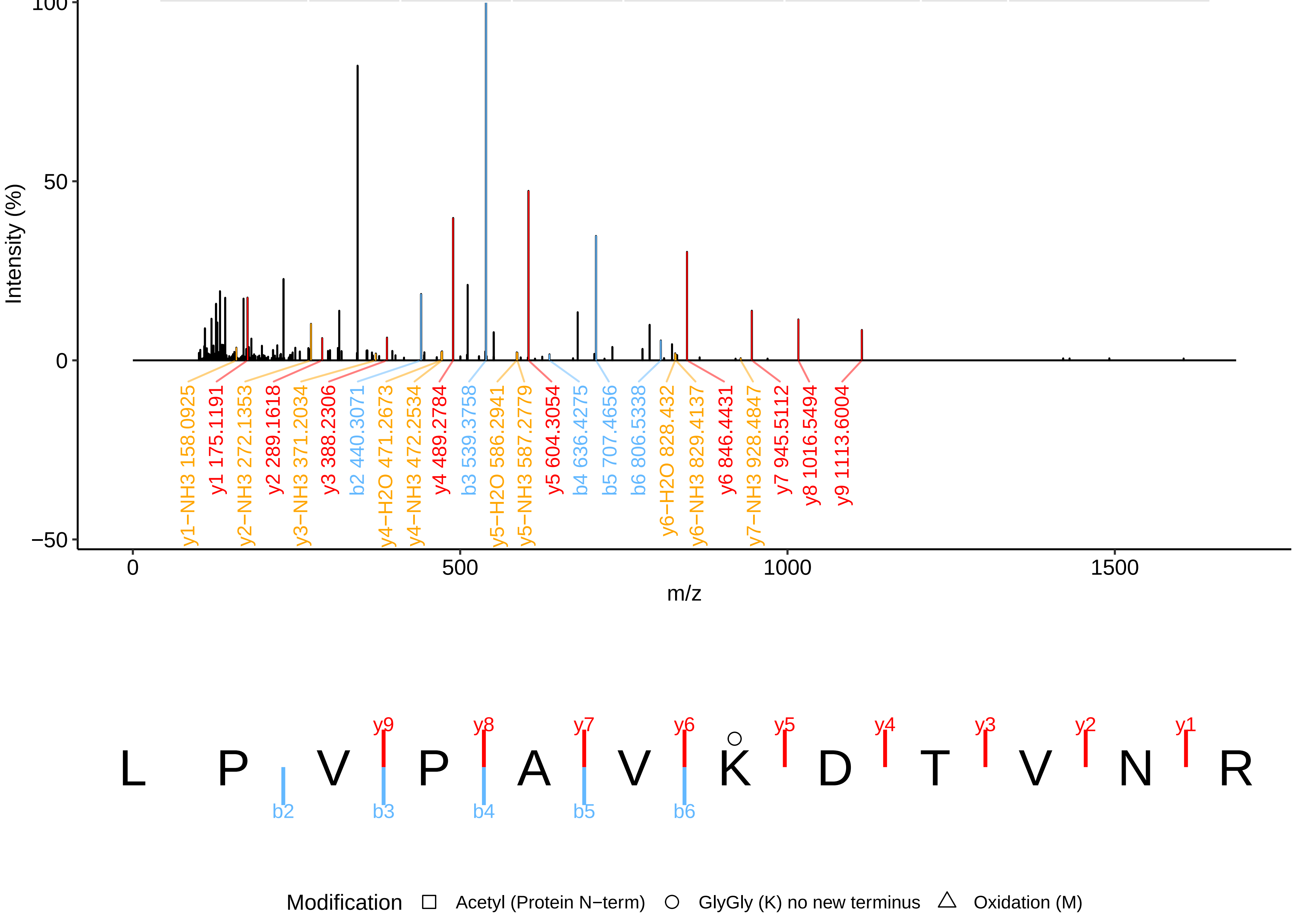

20201118_KIZ_MUb6_GlyGly_2 − Scan number: 15905

Gene Names	Charge	m/z	Mass	Retention time	PEP	Score	Precursor Intensity
CPT1A	3	474.9403	1421.799	53.652	0.00027078	94.363	2284096

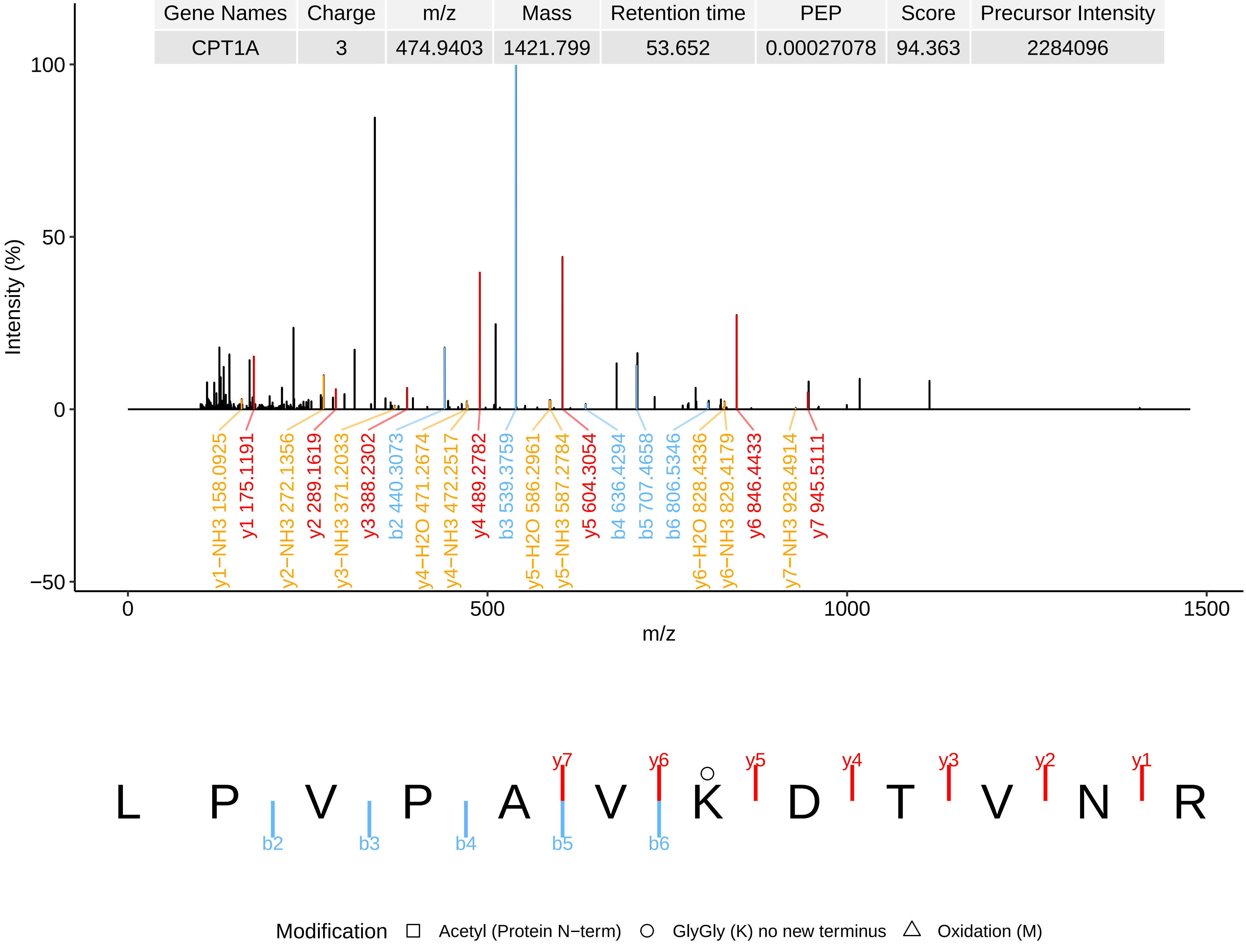

20200921_KIZ_MUb4_GlyGly_10plex_2 − Scan number: 17078

Gene Names	Charge	m/z	Mass	Retention time	PEP	Score	Precursor Intensity
MFAP3L;MFAP3	3	387.8924	1160.655	66.734	0.019804	65.305	1474204

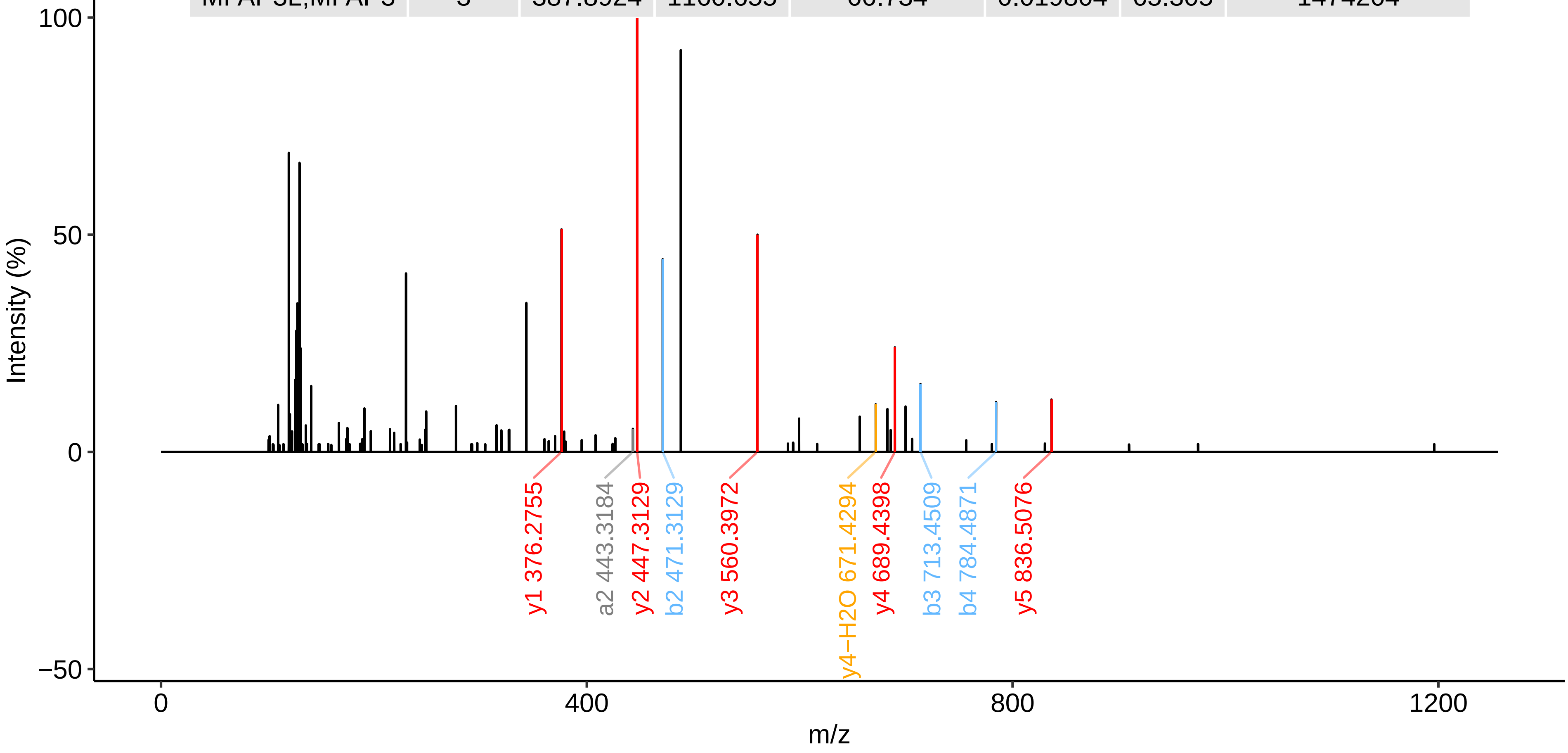

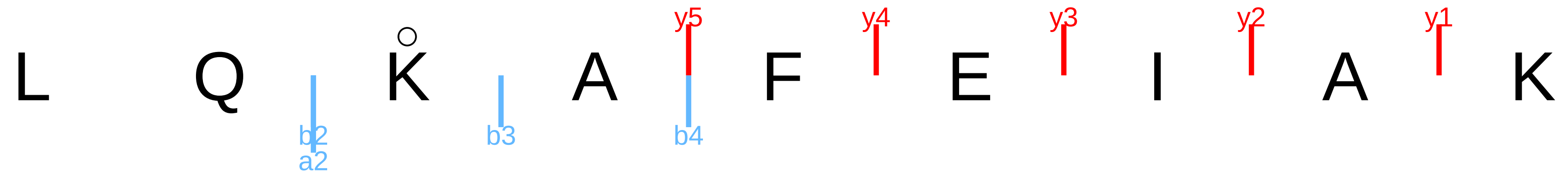

Modification □ Acetyl (Protein N−term) ○ GlyGly (K) no new terminus △ Oxidation (M)

20201118_KIZ_MUb6_GlyGly_1 − Scan number: 18079

Gene Names	Charge	m/z	Mass	Retention time	PEP	Score	Precursor Intensity
MFAP3L;MFAP3	3	387.8924	1160.655	56.859	0.0052837	78.939	14623260

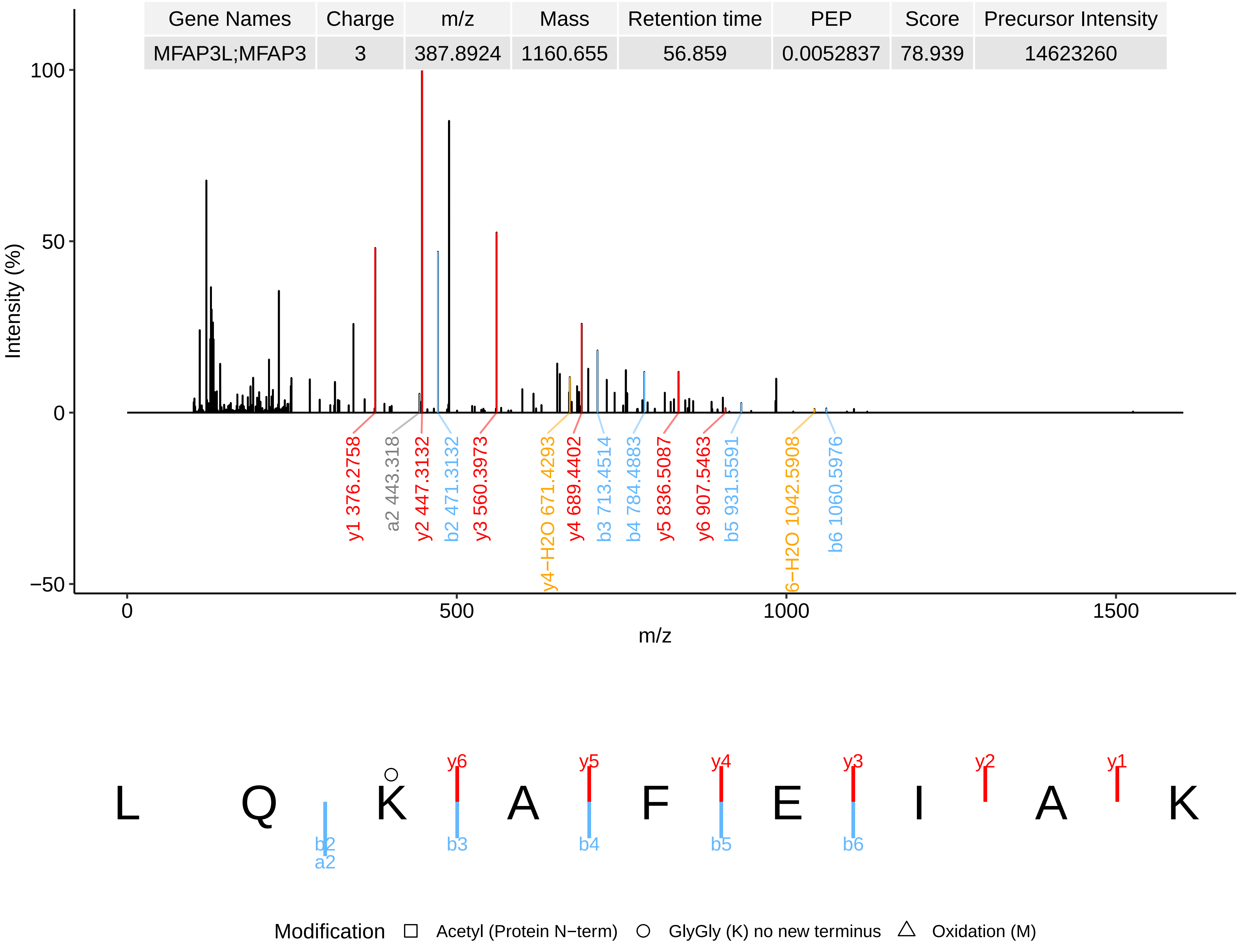

20200921_KIZ_MUb4_GlyGly_10plex_2 − Scan number: 15901

Gene Names	Charge	m/z	Mass	Retention time	PEP	Score	Precursor Intensity
ANKRD13A	3	396.8934	1187.658	62.272	0.076782	47.823	780495.6

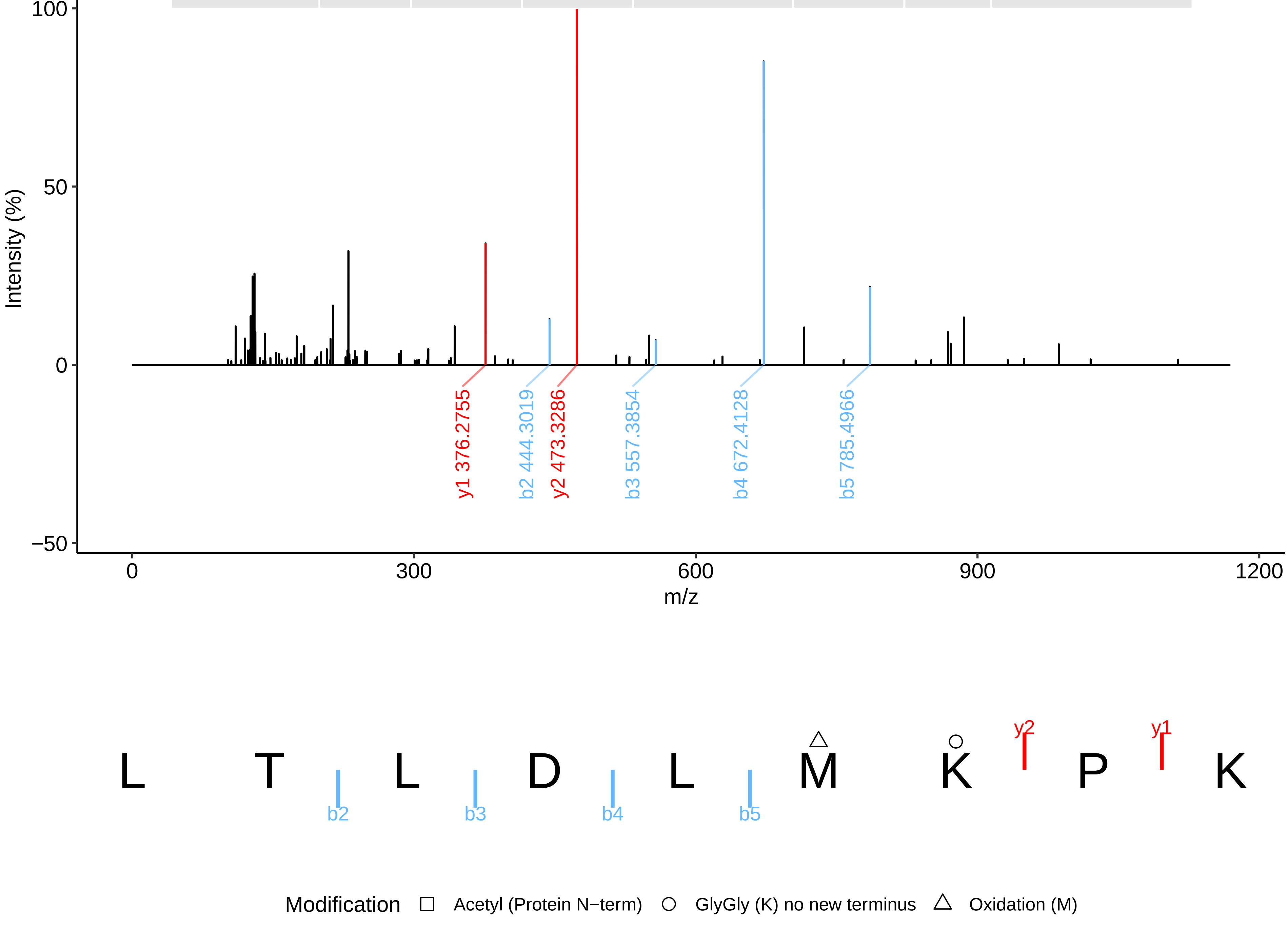

20200924_KIZ_MUb5_GlyGly_1 − Scan number: 27598

Gene Names	Charge	m/z	Mass	Retention time	PEP	Score	Precursor Intensity
	3	385.534	1153.58	88.17	0.014992	75.854	14247888

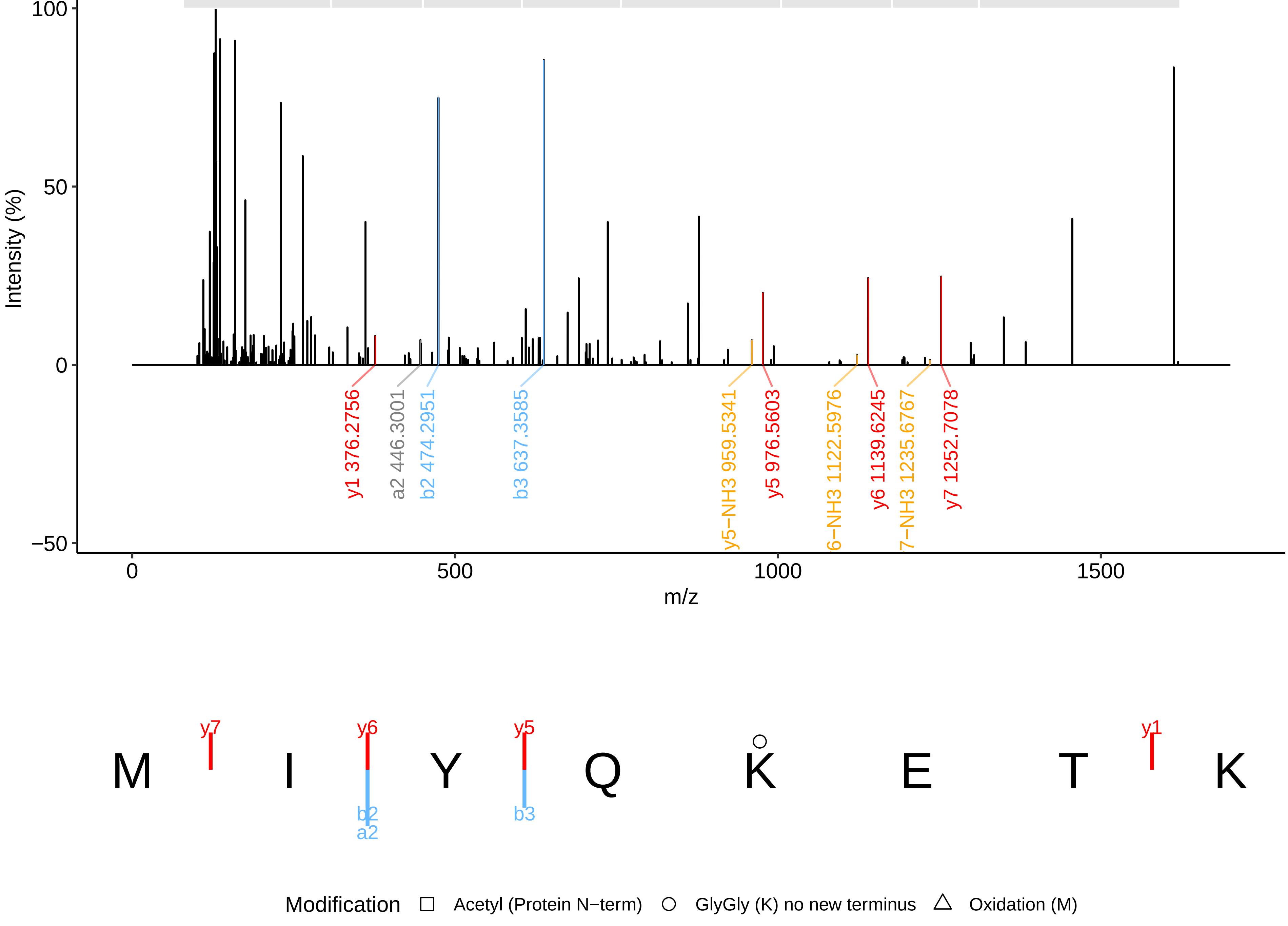

20200924_KIZ_MUb5_GlyGly_1 − Scan number: 27760

Gene Names	Charge	m/z	Mass	Retention time	PEP	Score	Precursor Intensity
	3	385.534	1153.58	88.683	0.028199	65.252	12873136

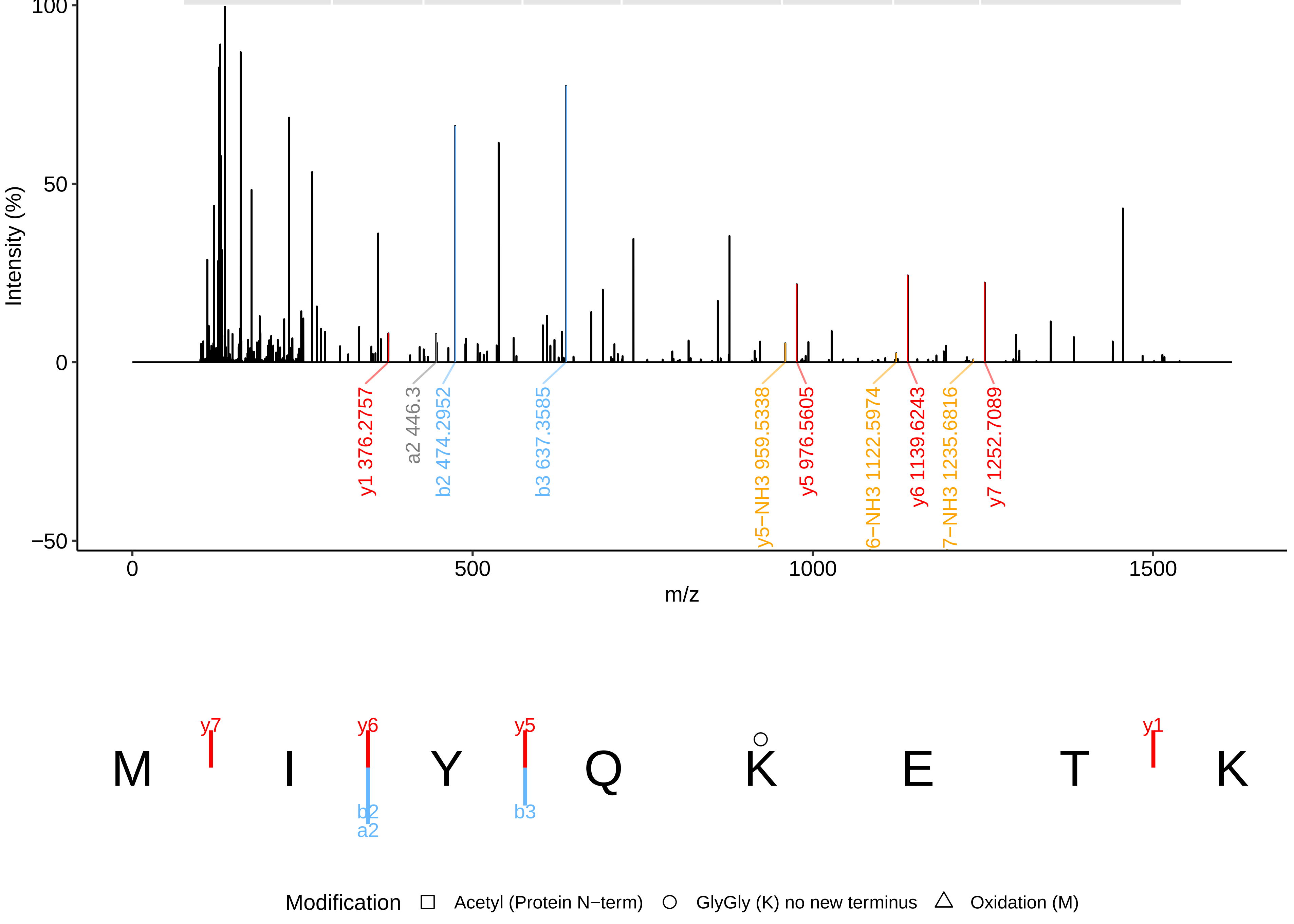

20200924_KIZ_MUb5_GlyGly_2 − Scan number: 25029

Gene Names	Charge	m/z	Mass	Retention time	PEP	Score	Precursor Intensity
	3	385.534	1153.58	92.694	0.028199	65.252	6650194

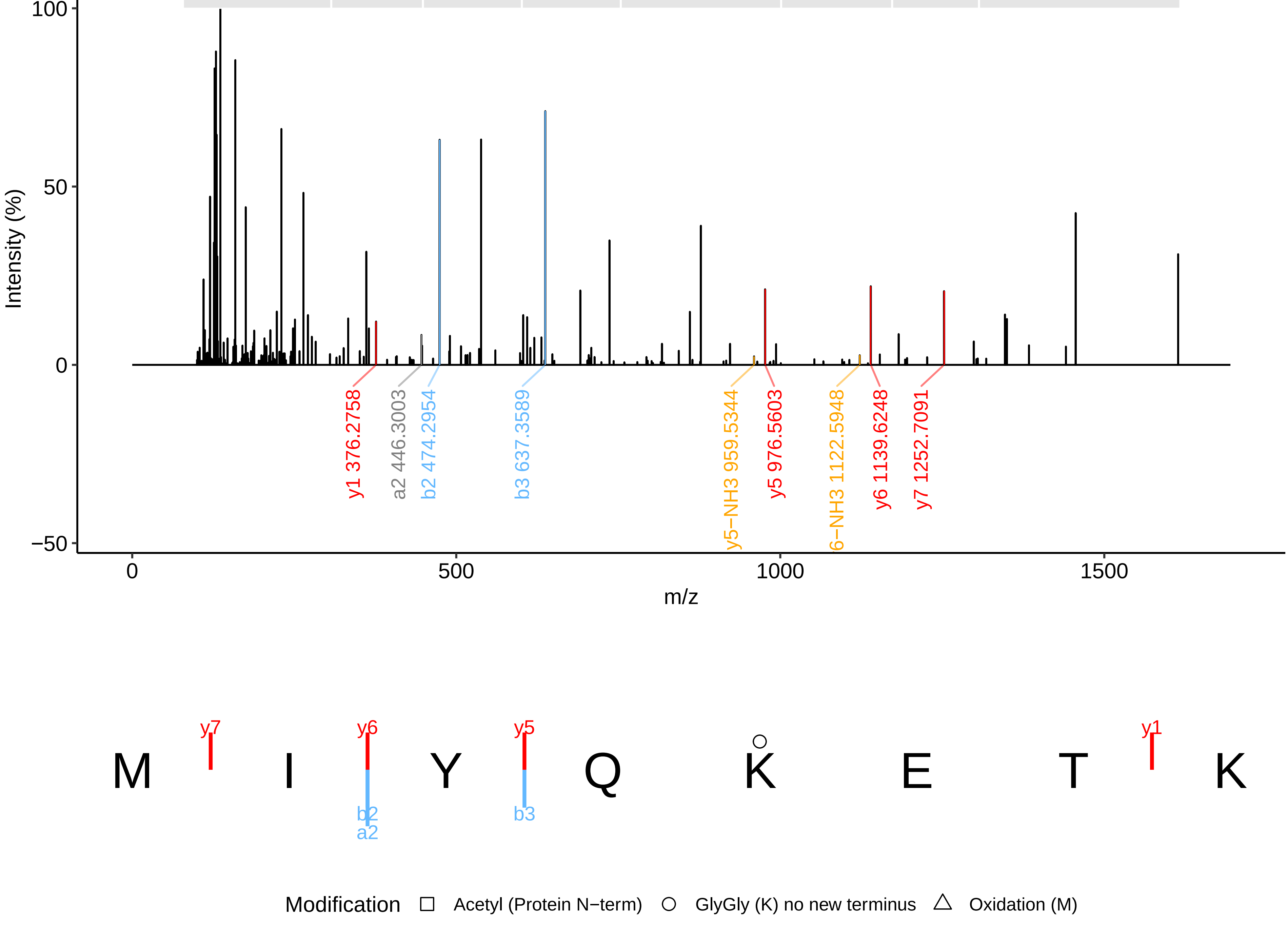

20200924_KIZ_MUb5_GlyGly_2 − Scan number: 24863

Gene Names	Charge	m/z	Mass	Retention time	PEP	Score	Precursor Intensity
	3	385.534	1153.58	92.073	0.12394	50.703	3822826

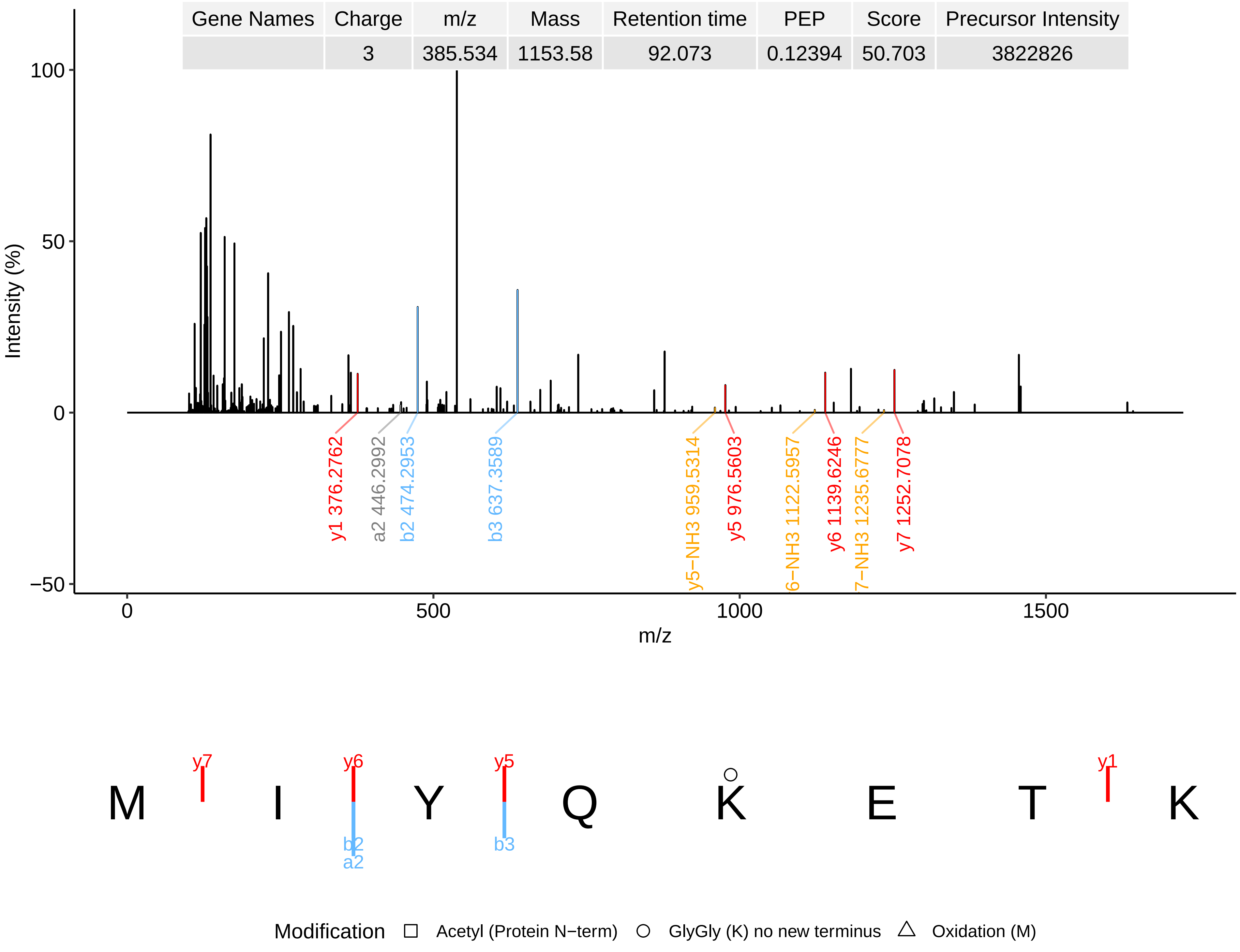

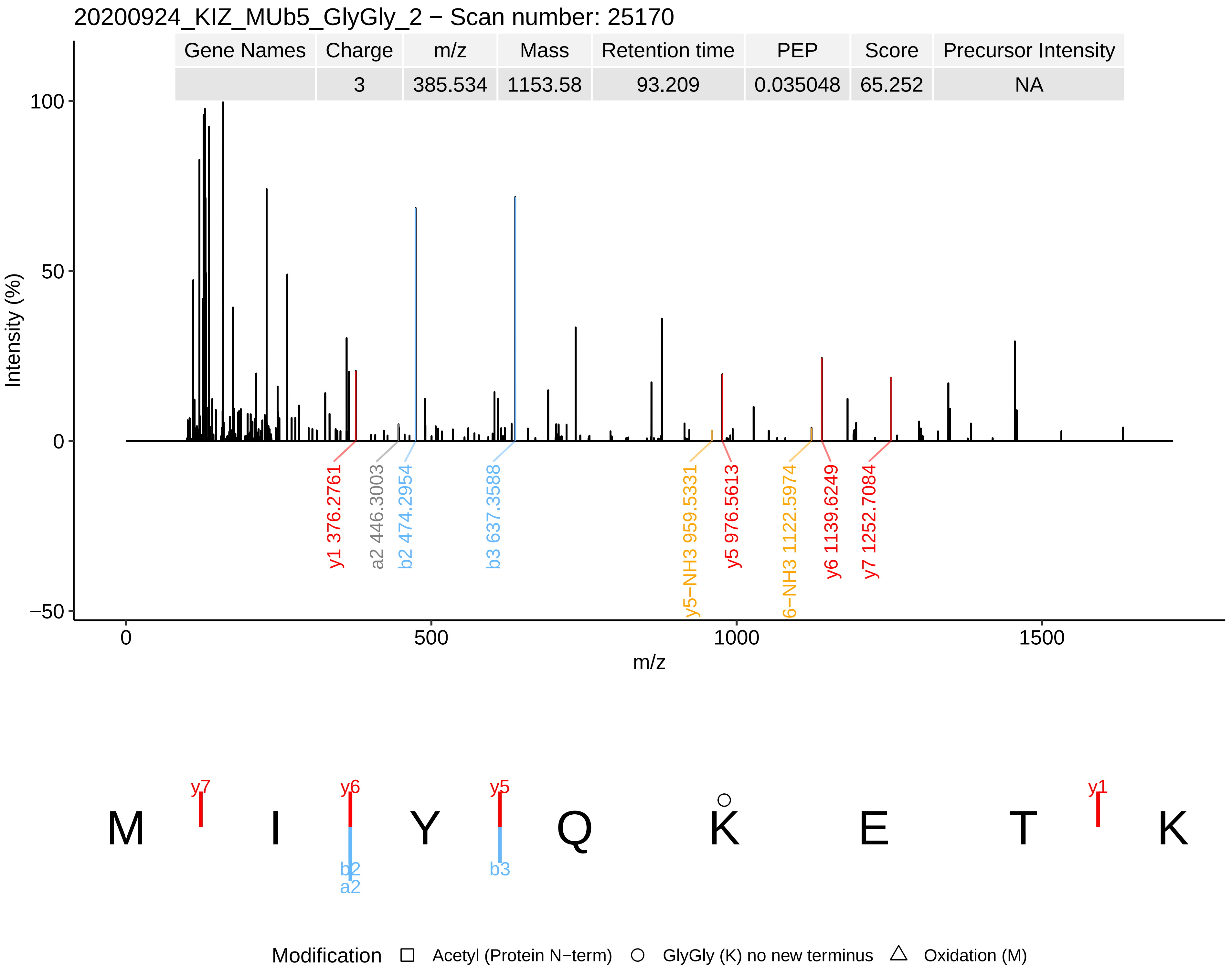
20200924_KIZ_MUb5_GlyGly_2 − Scan number: 25170
Gene Names
Charge
m/z
Mass
Retention time
PEP
Score
Precursor Intensity
3
385.534
1153.58
93.209
0.035048
65.252
NA
Intensity (%)
100
50
0
−50
0
500
1000
1500
m/z
y1 376.2761
a2 446.3003
b2 474.2954
b3 637.3588
y5−NH3 959.5331
y5 976.5613
y6−NH3 1122.5974
y6 1139.6249
y7 1252.7084
M
I
Y
Q
K
E
T
K
y7
y6
y5
y1
b2
a2
b3
Modification
Acetyl (Protein N−term)
GlyGly (K) no new terminus
Oxidation (M)

20201118_KIZ_MUb6_GlyGly_2 − Scan number: 31116

Gene Names	Charge	m/z	Mass	Retention time	PEP	Score	Precursor Intensity
	3	385.534	1153.58	102.43	0.025982	67.032	19930162

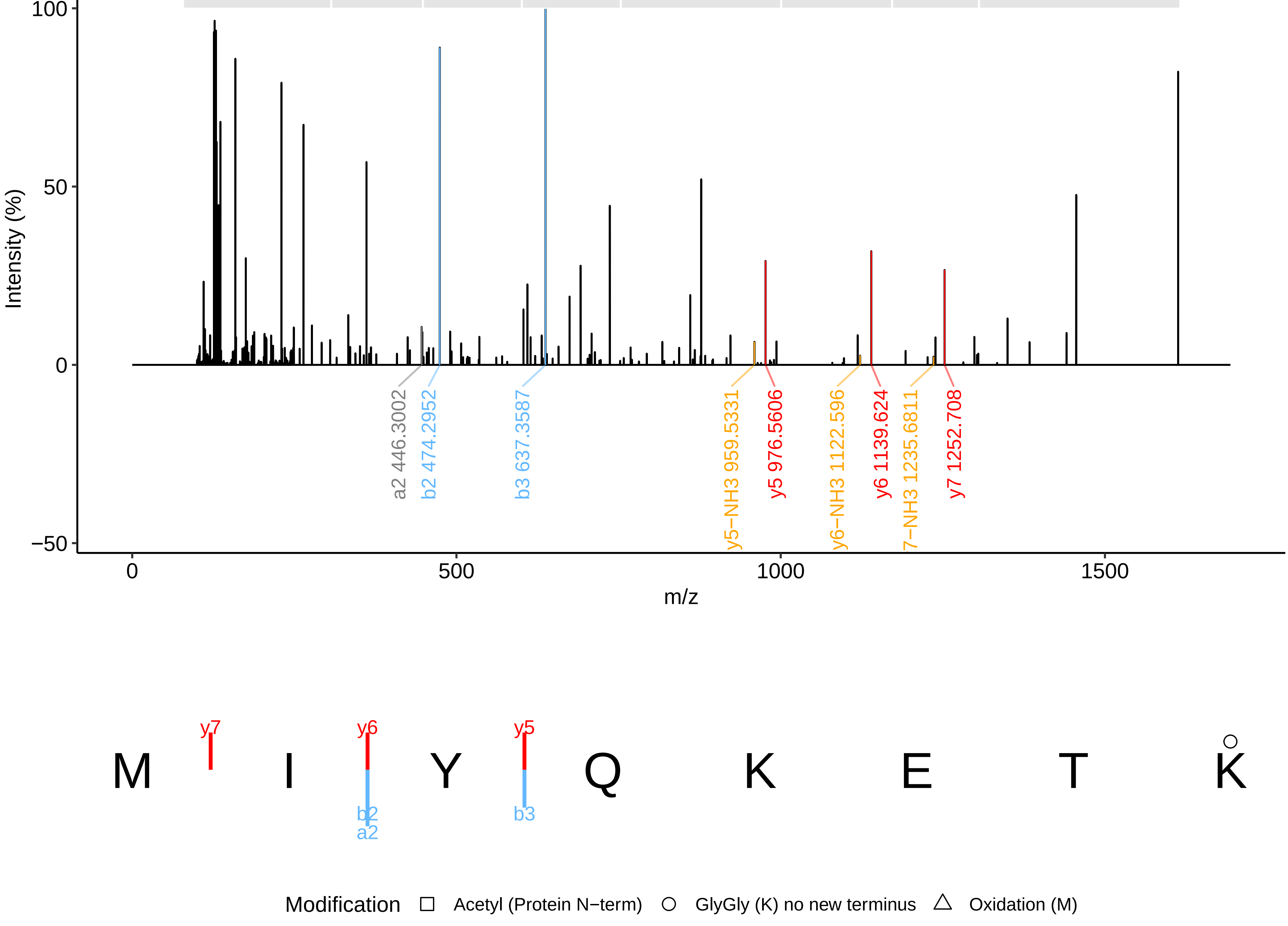

20201118_KIZ_MUb6_GlyGly_1 − Scan number: 23913

Gene Names	Charge	m/z	Mass	Retention time	PEP	Score	Precursor Intensity
	3	390.8656	1169.575	73.789	0.077354	47.082	32576510

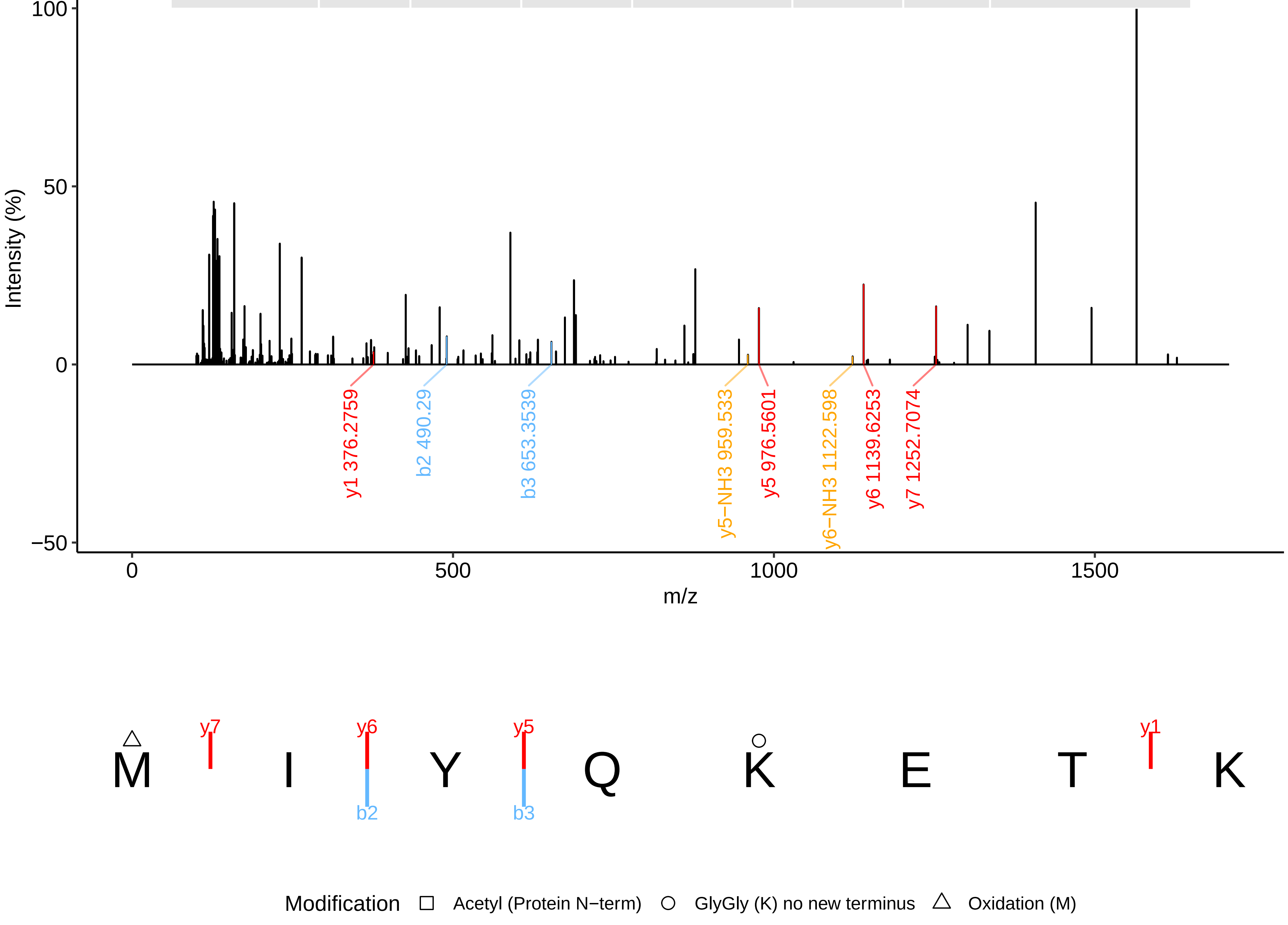

20200924_KIZ_MUb5_GlyGly_1 − Scan number: 3818

Gene Names	Charge	m/z	Mass	Retention time	PEP	Score	Precursor Intensity
CHCHD3	3	384.5169	1150.529	11.664	0.033732	61.363	6375977

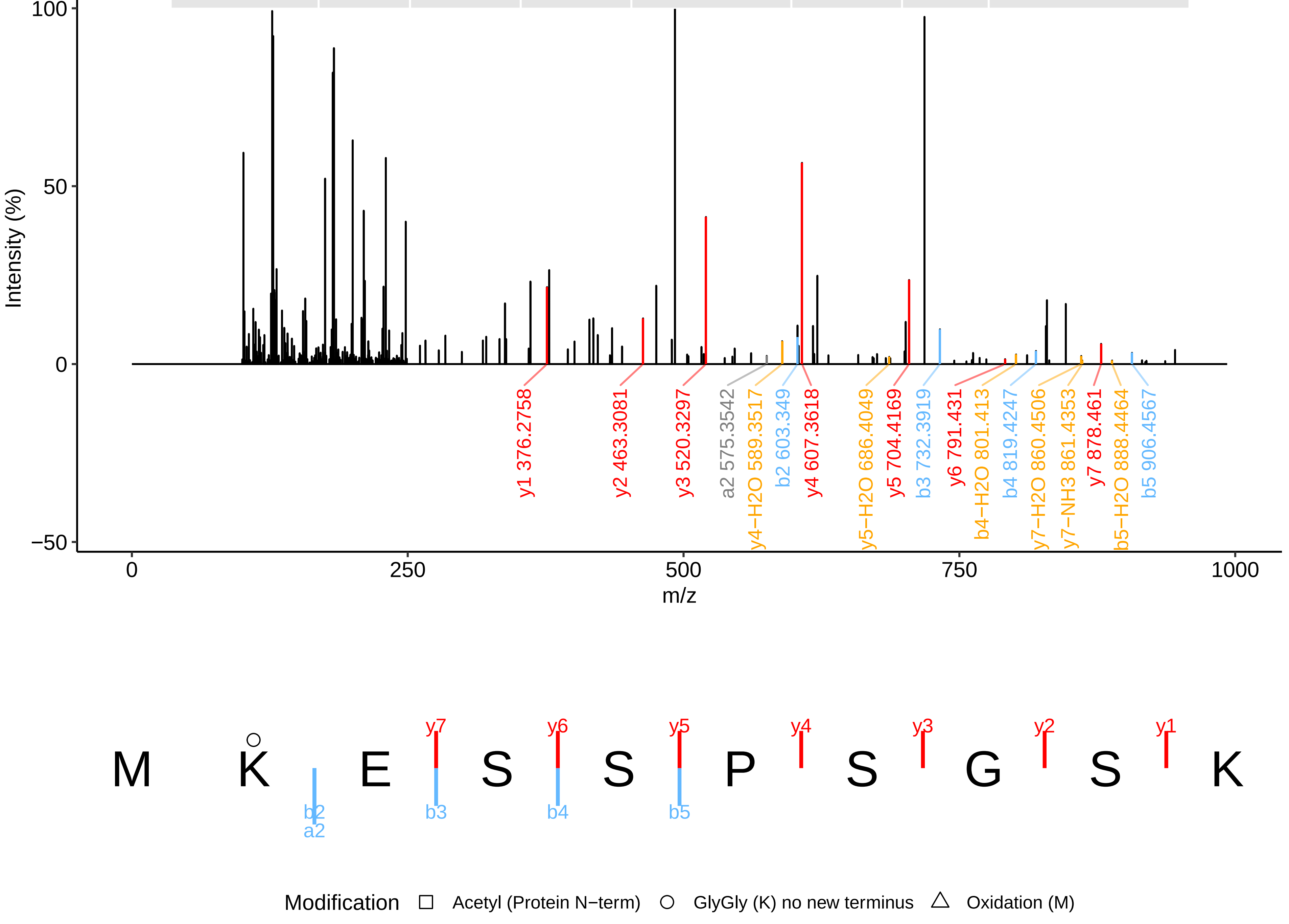

20200921_KIZ_MUb4_GlyGly_10plex_2 − Scan number: 6597

Gene Names	Charge	m/z	Mass	Retention time	PEP	Score	Precursor Intensity
IMMT	3	383.8693	1148.586	24.317	0.048881	55.064	794630

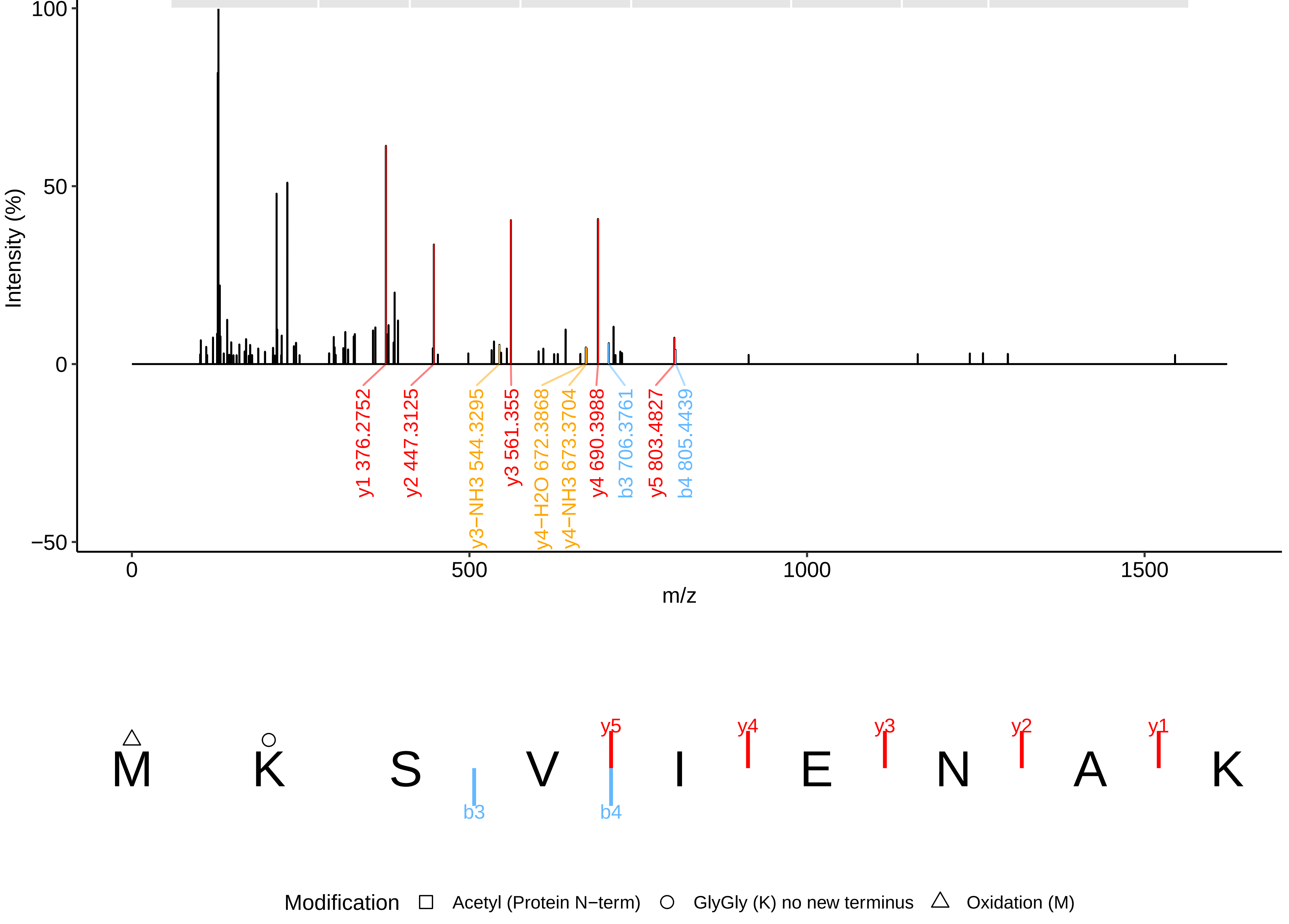

20200921_KIZ_MUb4_GlyGly_10plex − Scan number: 20665

Gene Names	Charge	m/z	Mass	Retention time	PEP	Score	Precursor Intensity
RPS27A;UBA52;UBC;UBB	2	711.3947	1420.775	74.734	1.2257e−08	86.275	3399837

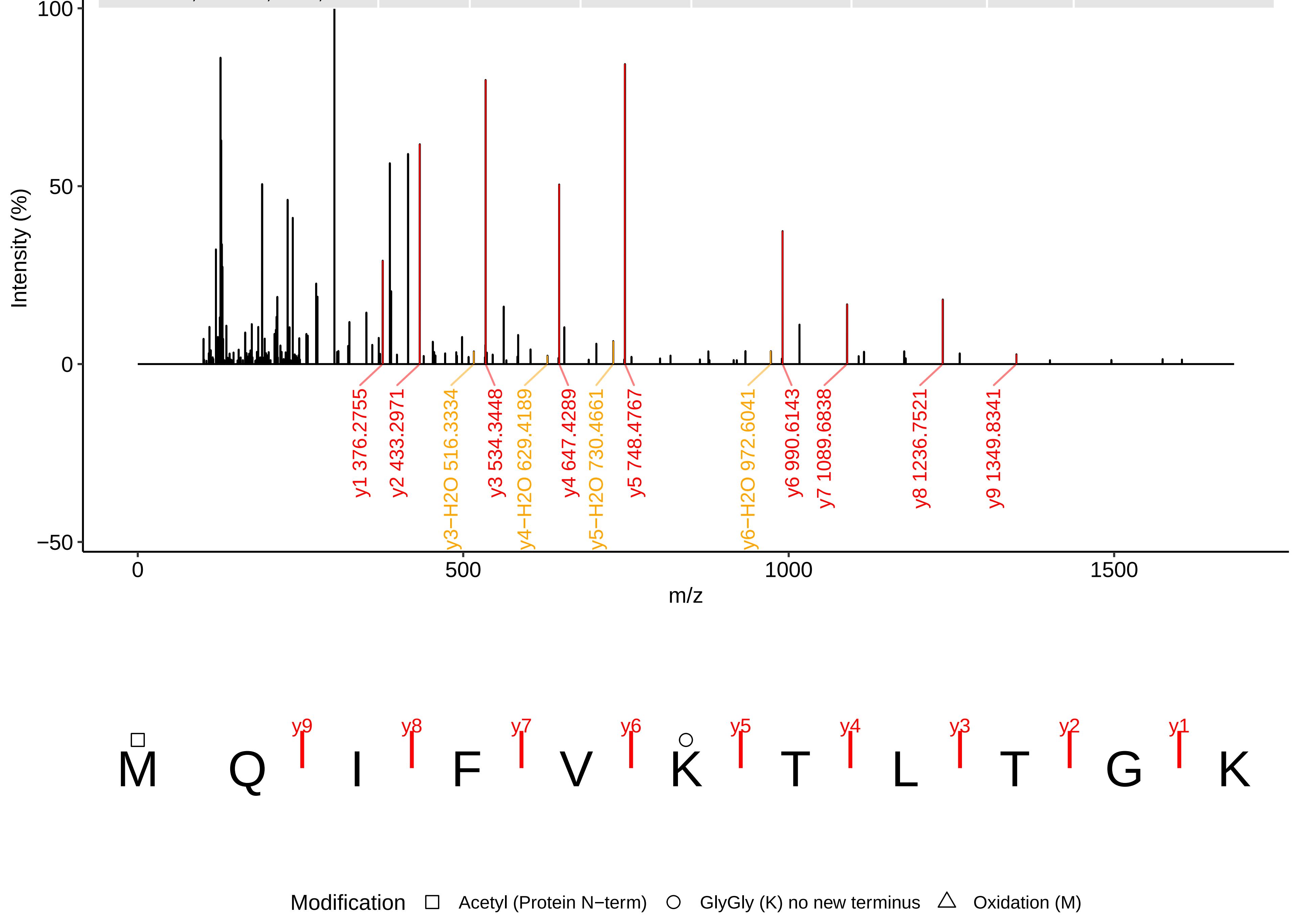

20200921_KIZ_MUb4_GlyGly_10plex − Scan number: 32552

Gene Names	Charge	m/z	Mass	Retention time	PEP	Score	Precursor Intensity
RPS27A;UBA52;UBC;UBB	2	711.3947	1420.775	120.12	1.3666e−09	133.57	NA

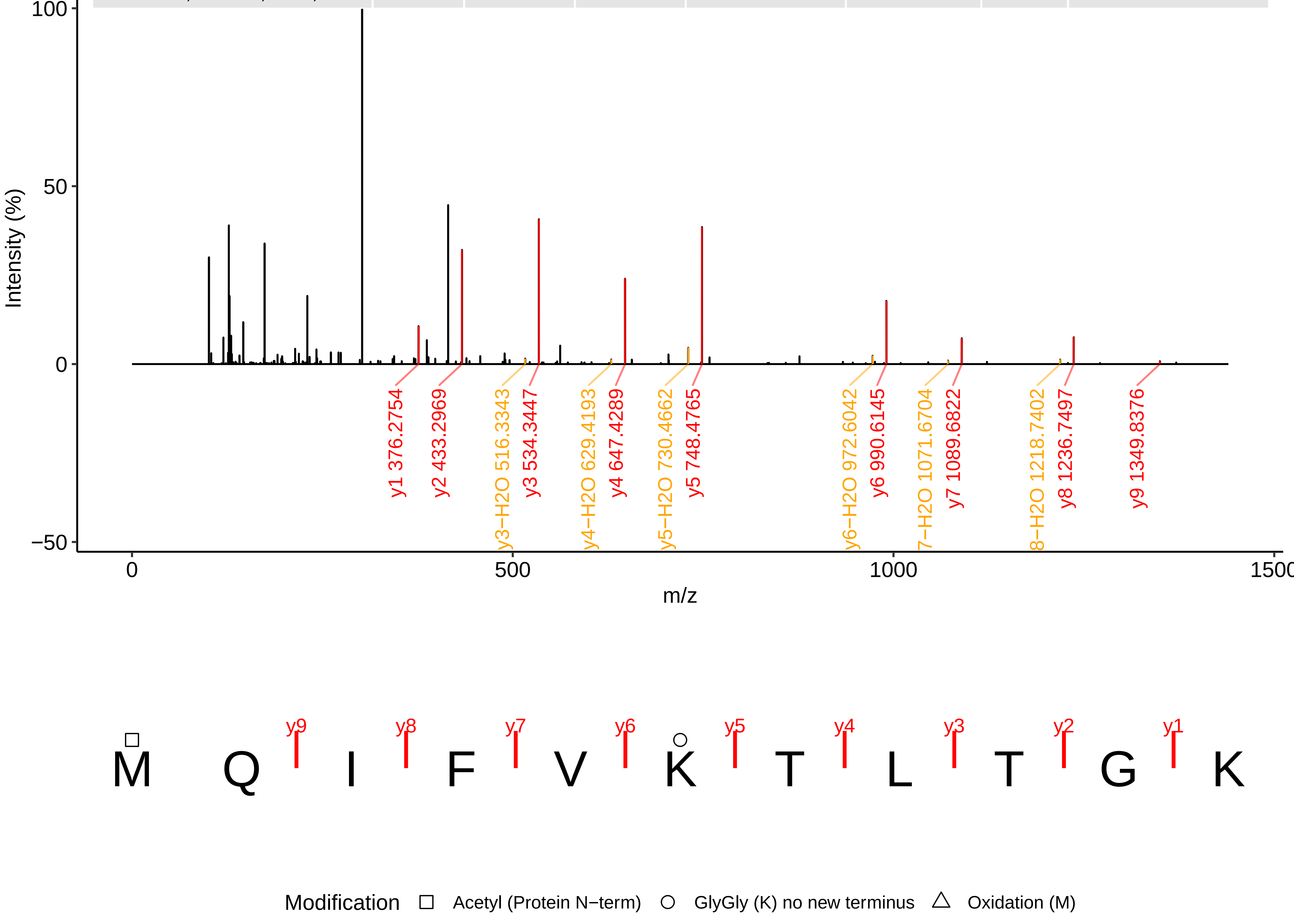

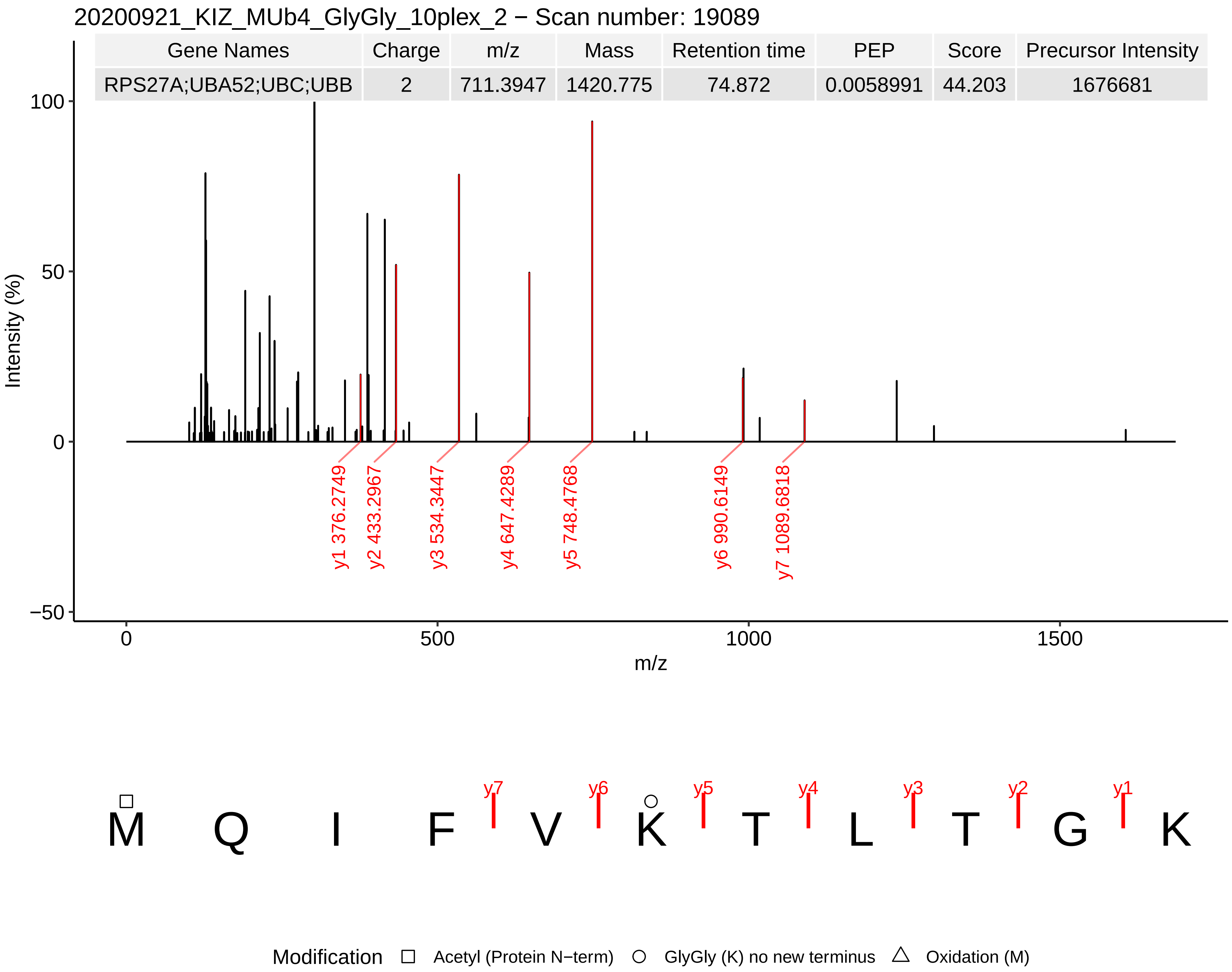
20200921_KIZ_MUb4_GlyGly_10plex_2 − Scan number: 19089
Gene Names | Charge | m/z | Mass | Retention time | PEP | Score | Precursor Intensity
RPS27A;UBA52;UBC;UBB | 2 | 711.3947 | 1420.775 | 74.872 | 0.0058991 | 44.203 | 1676681
Intensity (%)
100
50
0
−50
0
500
1000
1500
m/z
y1 376.2749
y2 433.2967
y3 534.3447
y4 647.4289
y5 748.4768
y6 990.6149
y7 1089.6818
M Q I F V K T L T G K
y7 y6 y5 y4 y3 y2 y1
Modification
Acetyl (Protein N−term)
GlyGly (K) no new terminus
Oxidation (M)

20200924_KIZ_MUb5_GlyGly_1 − Scan number: 36845

Gene Names	Charge	m/z	Mass	Retention time	PEP	Score	Precursor Intensity
RPS27A;UBA52;UBC;UBB	2	711.3947	1420.775	117.32	0.0027298	67.414	21116144

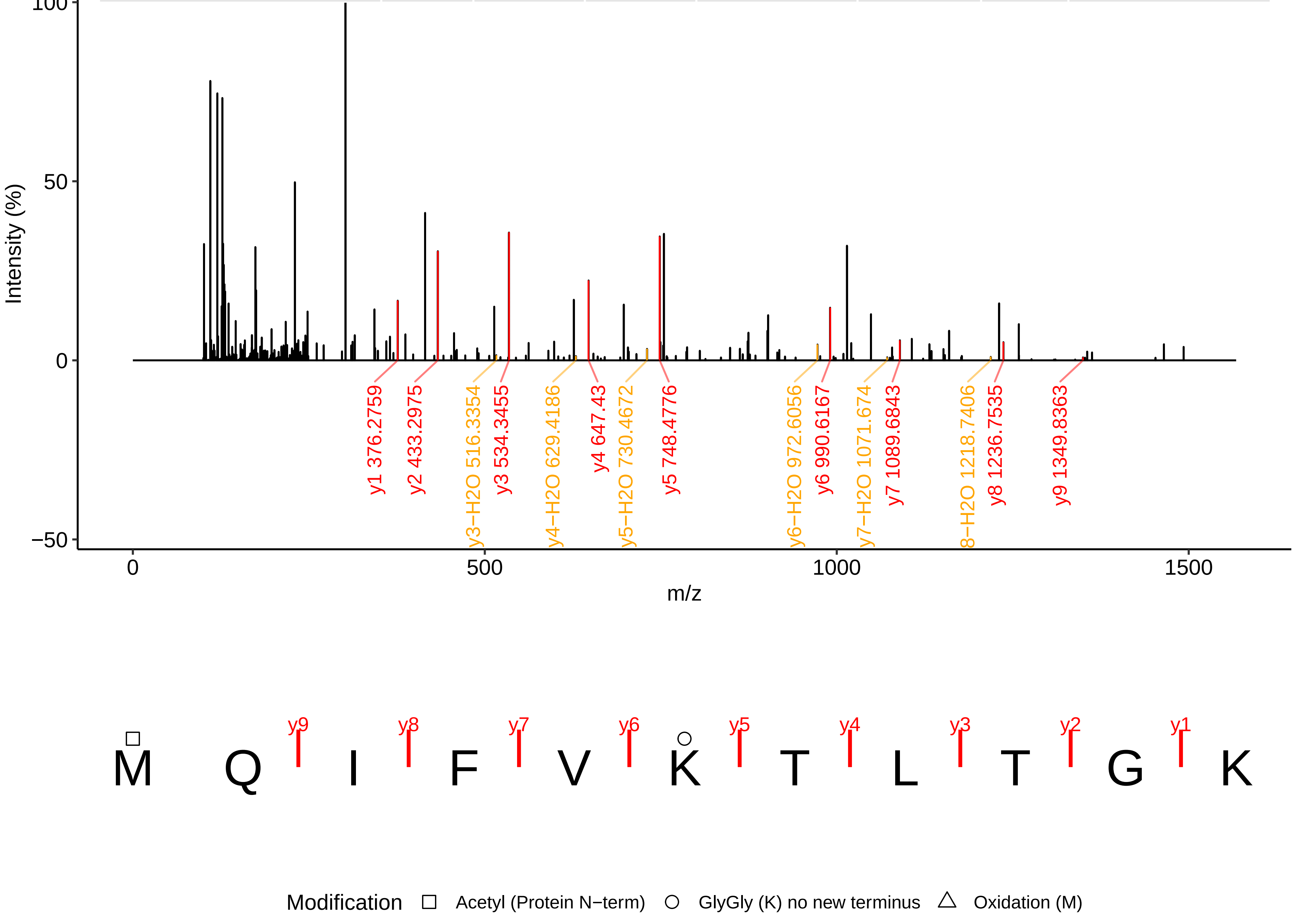

20200924_KIZ_MUb5_GlyGly_1 − Scan number: 36860

Gene Names	Charge	m/z	Mass	Retention time	PEP	Score	Precursor Intensity
RPS27A;UBA52;UBC;UBB	2	711.3947	1420.775	117.36	0.00026758	81.95	12642503

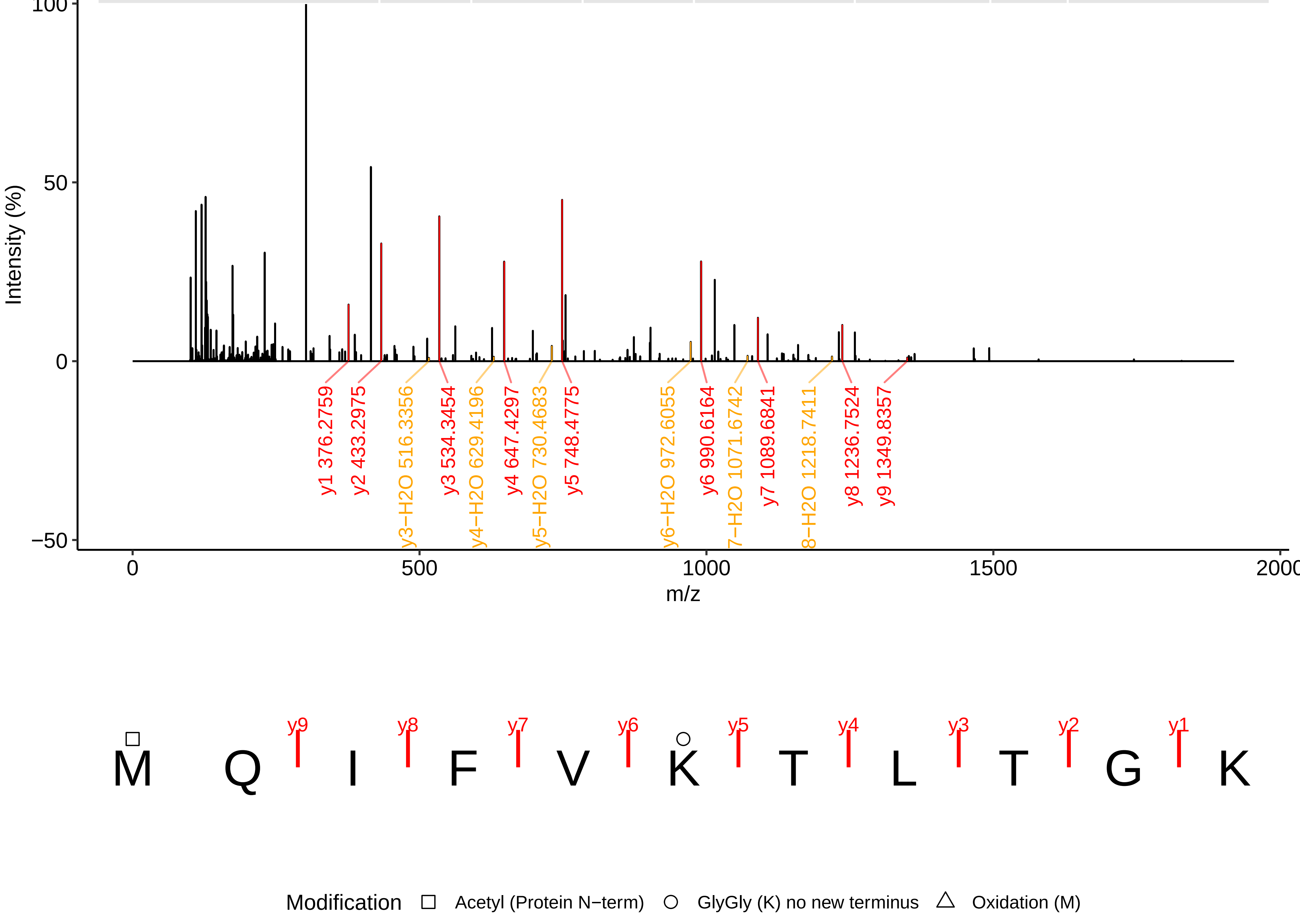

20200921_KIZ_MUb4_GlyGly_10plex − Scan number: 8823

Gene Names	Charge	m/z	Mass	Retention time	PEP	Score	Precursor Intensity
SNAP23	3	386.5244	1156.551	30.066	0.058764	55.567	2270599

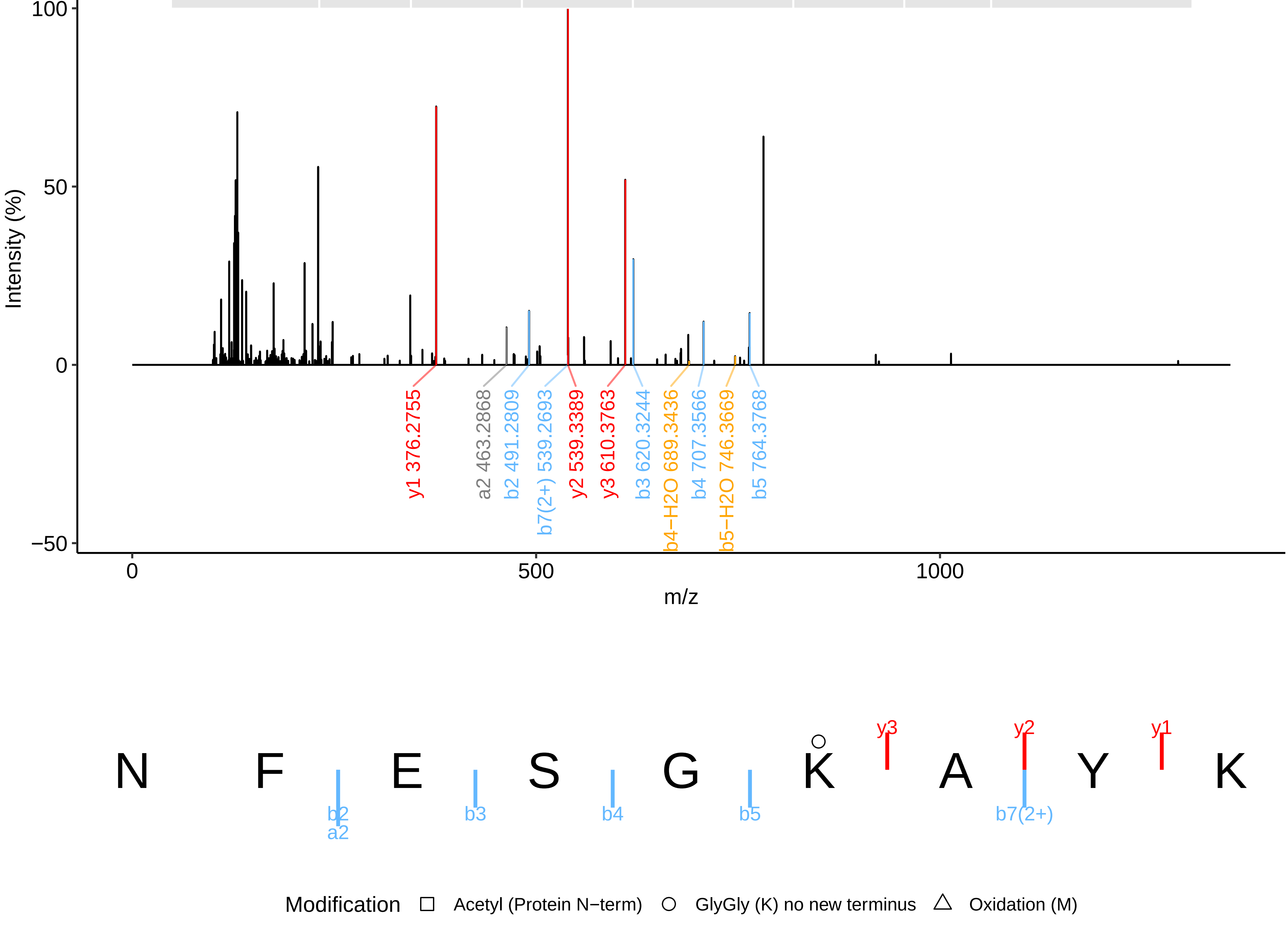

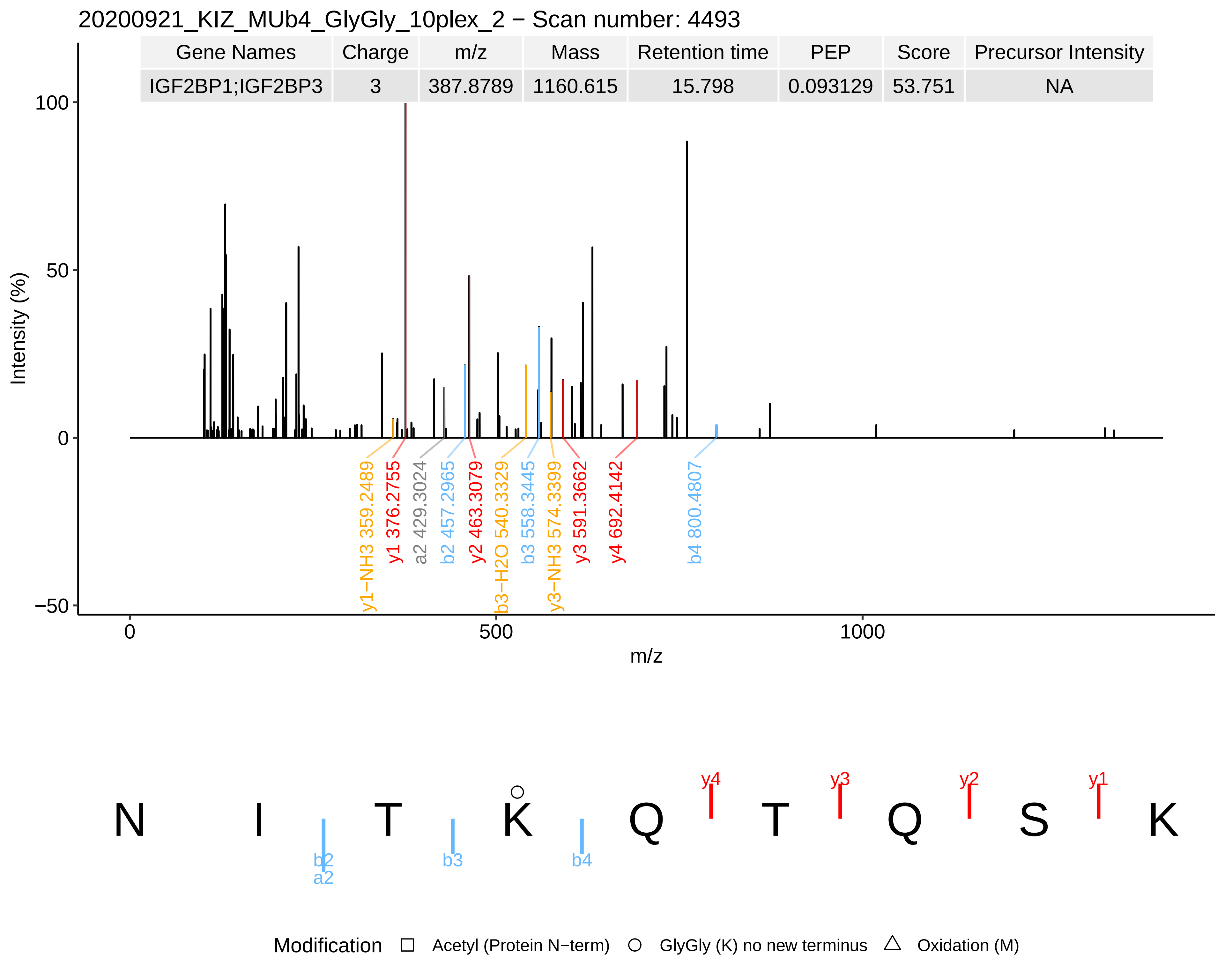
20200921_KIZ_MUb4_GlyGly_10plex_2 − Scan number: 4493
Gene Names | Charge | m/z | Mass | Retention time | PEP | Score | Precursor Intensity
IGF2BP1;IGF2BP3 | 3 | 387.8789 | 1160.615 | 15.798 | 0.093129 | 53.751 | NA
Intensity (%)
m/z
y1−NH3 359.2489
y1 376.2755
a2 429.3024
b2 457.2965
y2 463.3079
b3−H2O 540.3329
b3 558.3445
y3−NH3 574.3399
y3 591.3662
y4 692.4142
b4 800.4807
N I T K Q T Q S K
y4 y3 y2 y1
b2 a2 b3 b4
Modification
Acetyl (Protein N−term)
GlyGly (K) no new terminus
Oxidation (M)

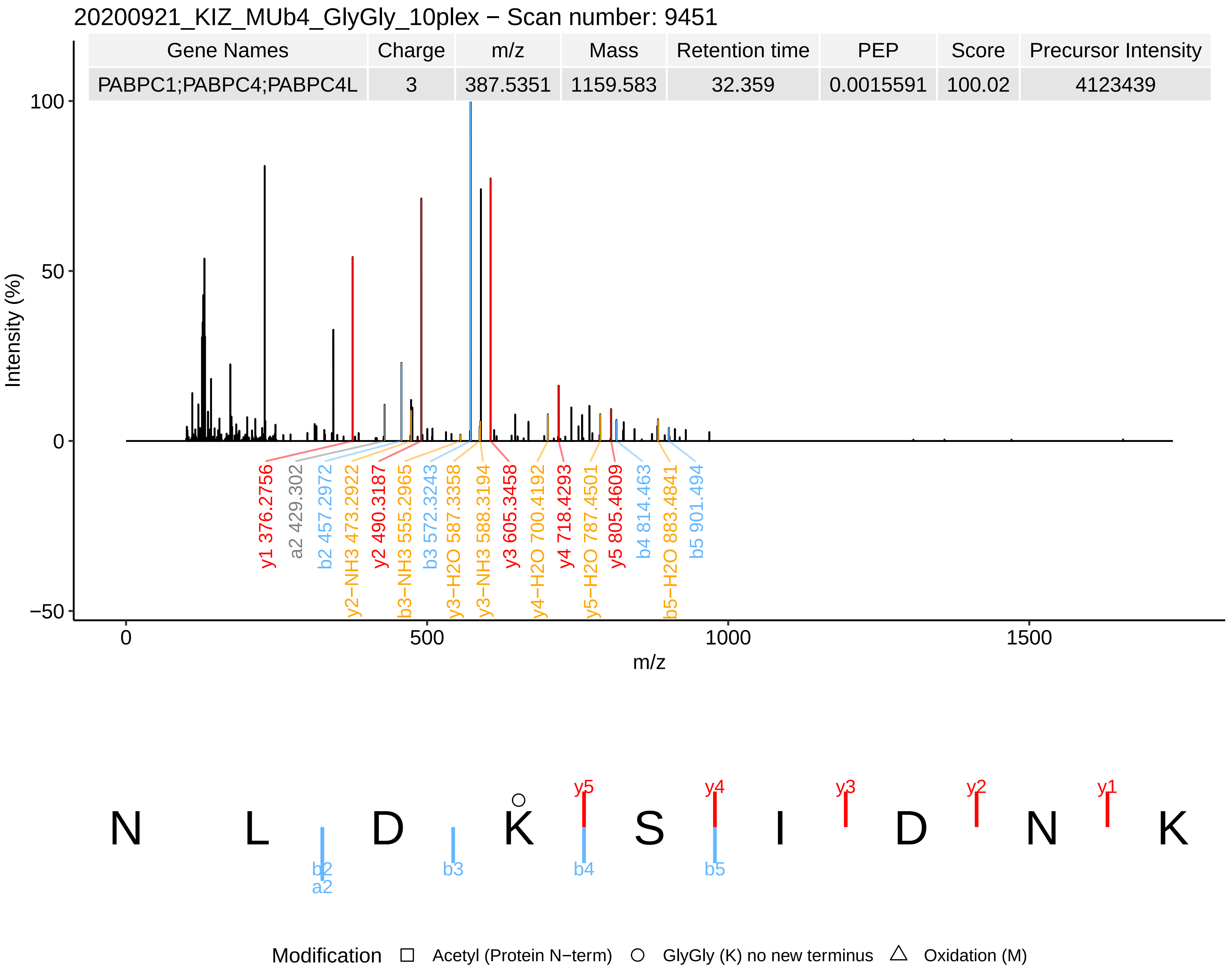
20200921_KIZ_MUb4_GlyGly_10plex − Scan number: 9451
Gene Names
Charge
m/z
Mass
Retention time
PEP
Score
Precursor Intensity
PABPC1;PABPC4;PABPC4L
3
387.5351
1159.583
32.359
0.0015591
100.02
4123439
Intensity (%)
100
50
0
−50
0
500
1000
1500
m/z
y1 376.2756
a2 429.302
b2 457.2972
y2−NH3 473.2922
y2 490.3187
b3−NH3 555.2965
b3 572.3243
y3−H2O 587.3358
y3−NH3 588.3194
y3 605.3458
y4−H2O 700.4192
y4 718.4293
y5−H2O 787.4501
y5 805.4609
b4 814.463
b5−H2O 883.4841
b5 901.494
N
L
D
K
S
I
D
N
K
y5
y4
y3
y2
y1
b2
a2
b3
b4
b5
Modification
Acetyl (Protein N−term)
GlyGly (K) no new terminus
Oxidation (M)

20200921_KIZ_MUb4_GlyGly_10plex_2 − Scan number: 8673

Gene Names	Charge	m/z	Mass	Retention time	PEP	Score	Precursor Intensity
PABPC1;PABPC4;PABPC4L	3	387.5351	1159.583	32.75	0.0029137	84.213	822917.3

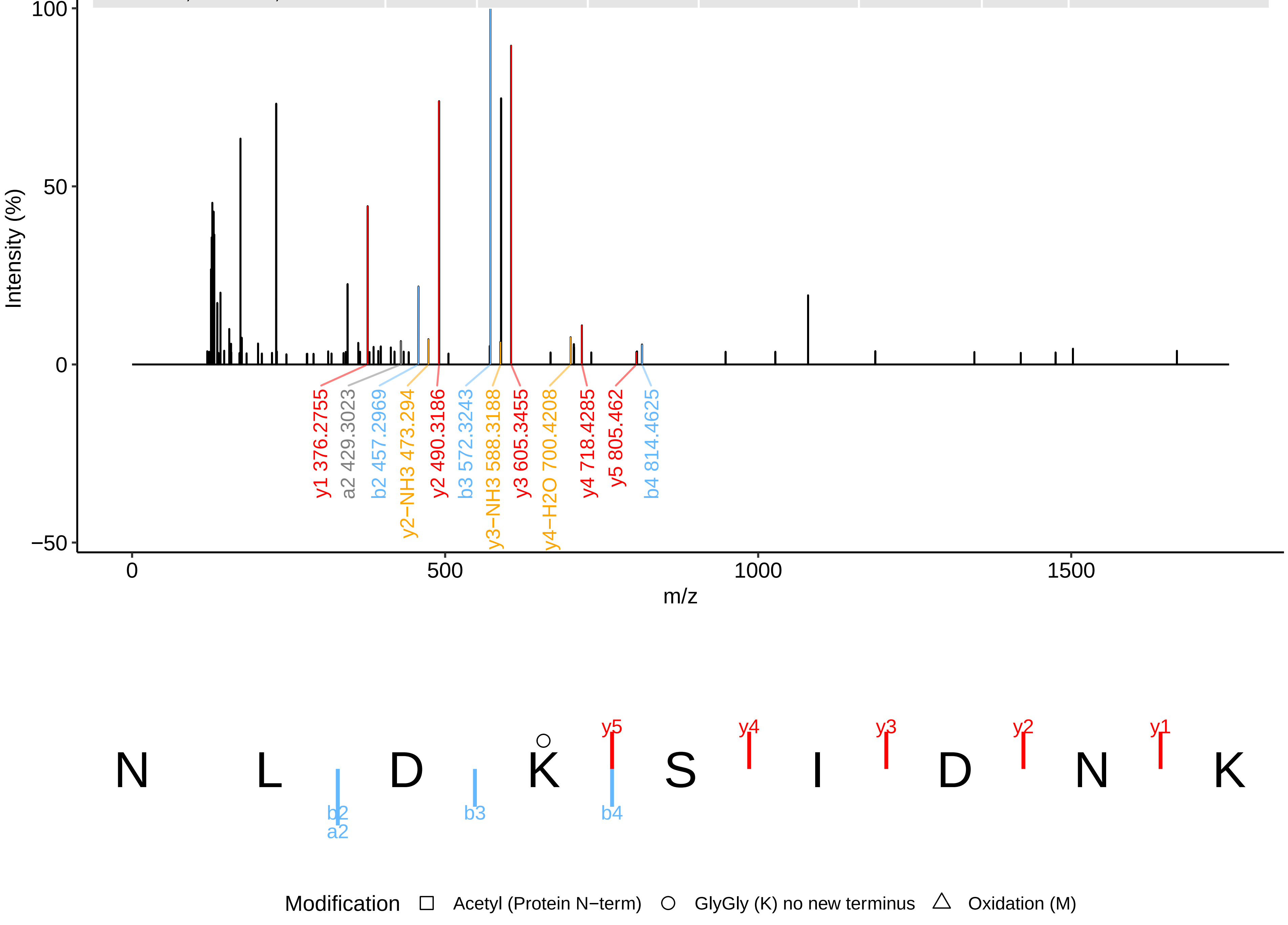

20200924_KIZ_MUb5_GlyGly_1 − Scan number: 8442

Gene Names	Charge	m/z	Mass	Retention time	PEP	Score	Precursor Intensity
PABPC1;PABPC4;PABPC4L	3	387.5351	1159.583	26.991	0.018904	70.552	4973365

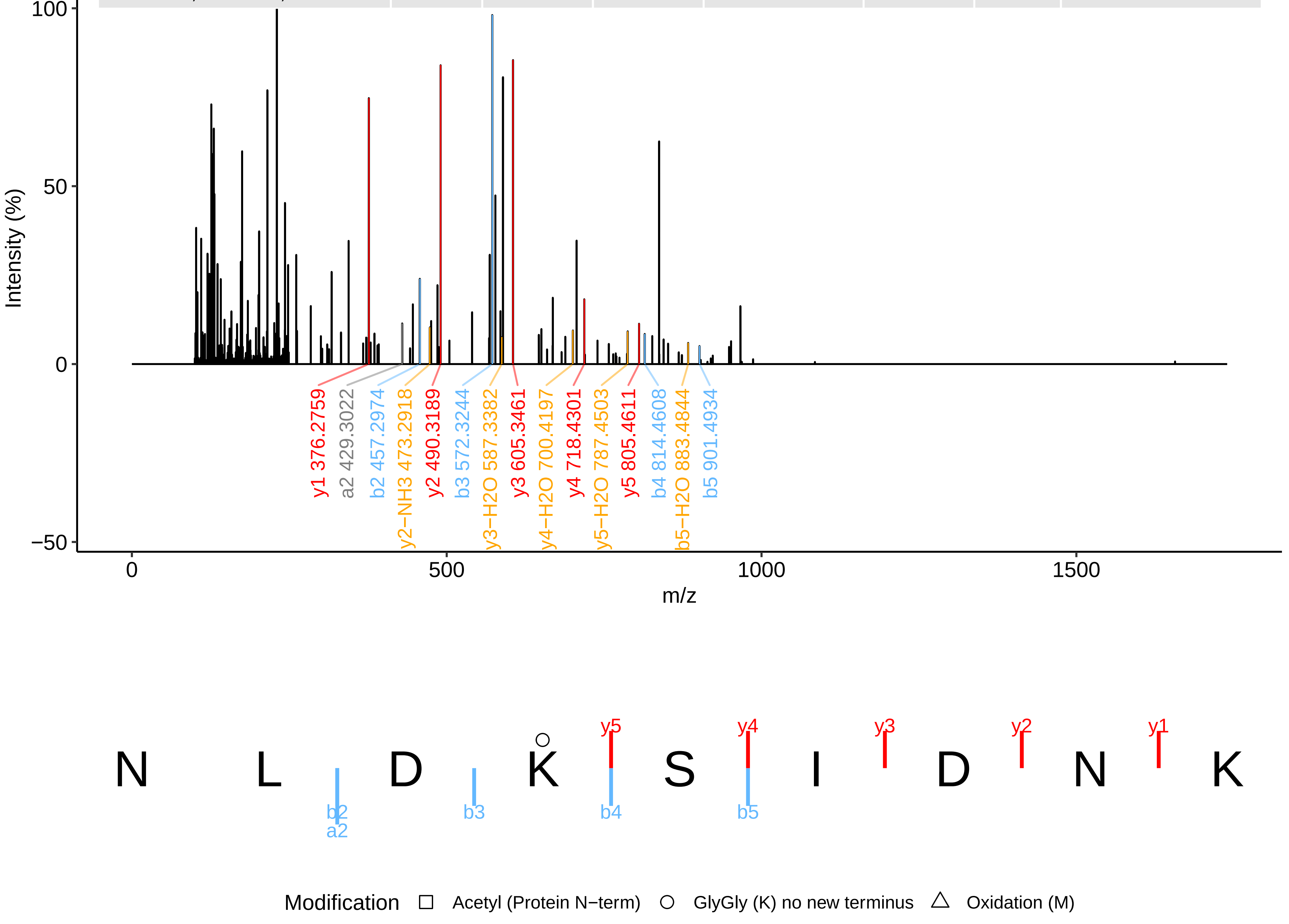

20201118_KIZ_MUb6_GlyGly_2 − Scan number: 2835

Gene Names	Charge	m/z	Mass	Retention time	PEP	Score	Precursor Intensity
SIRT1	3	464.5552	1390.644	9.0543	0.0013927	83.617	NA

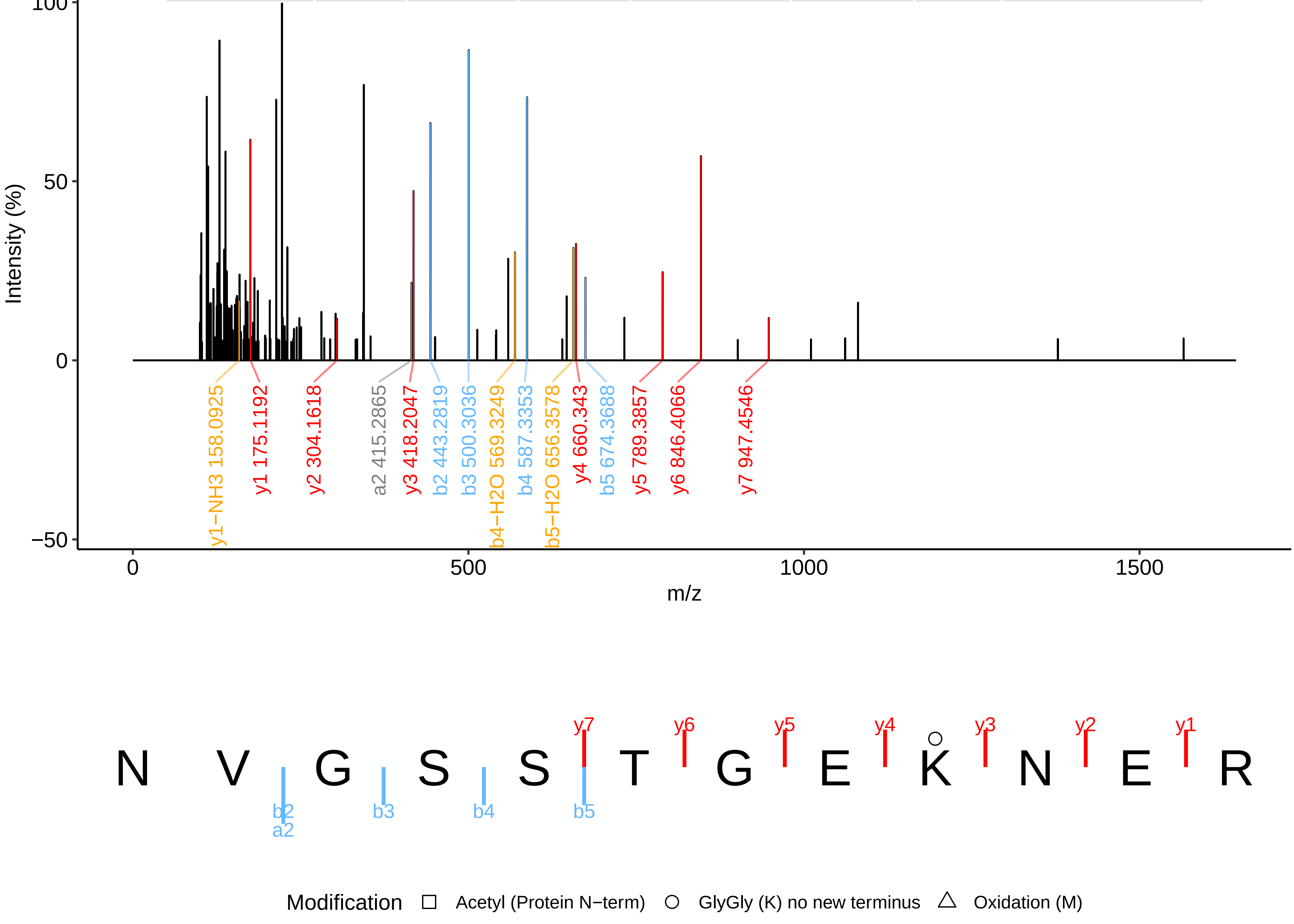

20200924_KIZ_MUb5_GlyGly_1 − Scan number: 26797

Gene Names	Charge	m/z	Mass	Retention time	PEP	Score	Precursor Intensity
MYH7B	2	696.3365	1390.659	85.72	0.06667	47.849	153278944

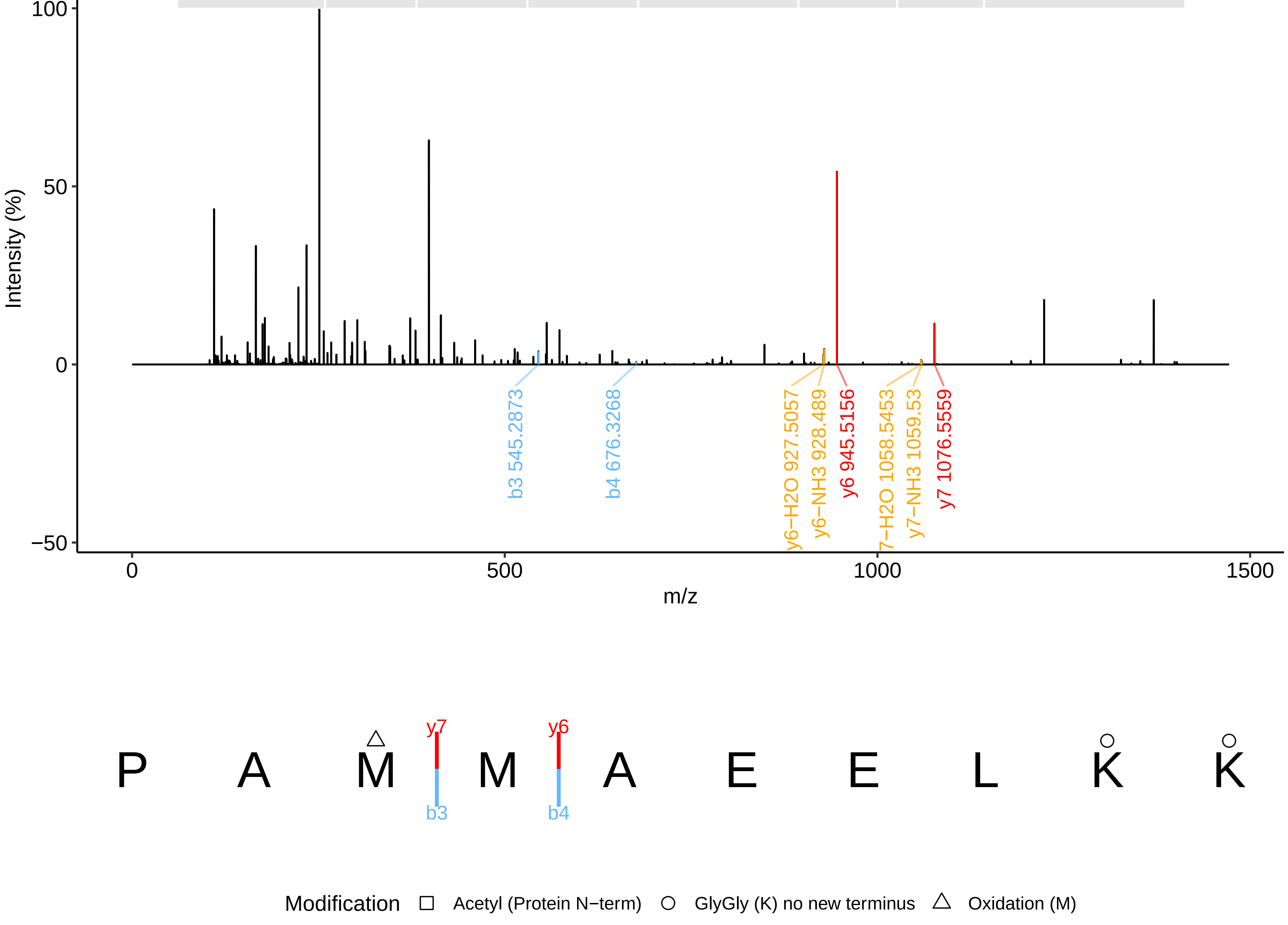

20200924_KIZ_MUb5_GlyGly_1 − Scan number: 26960

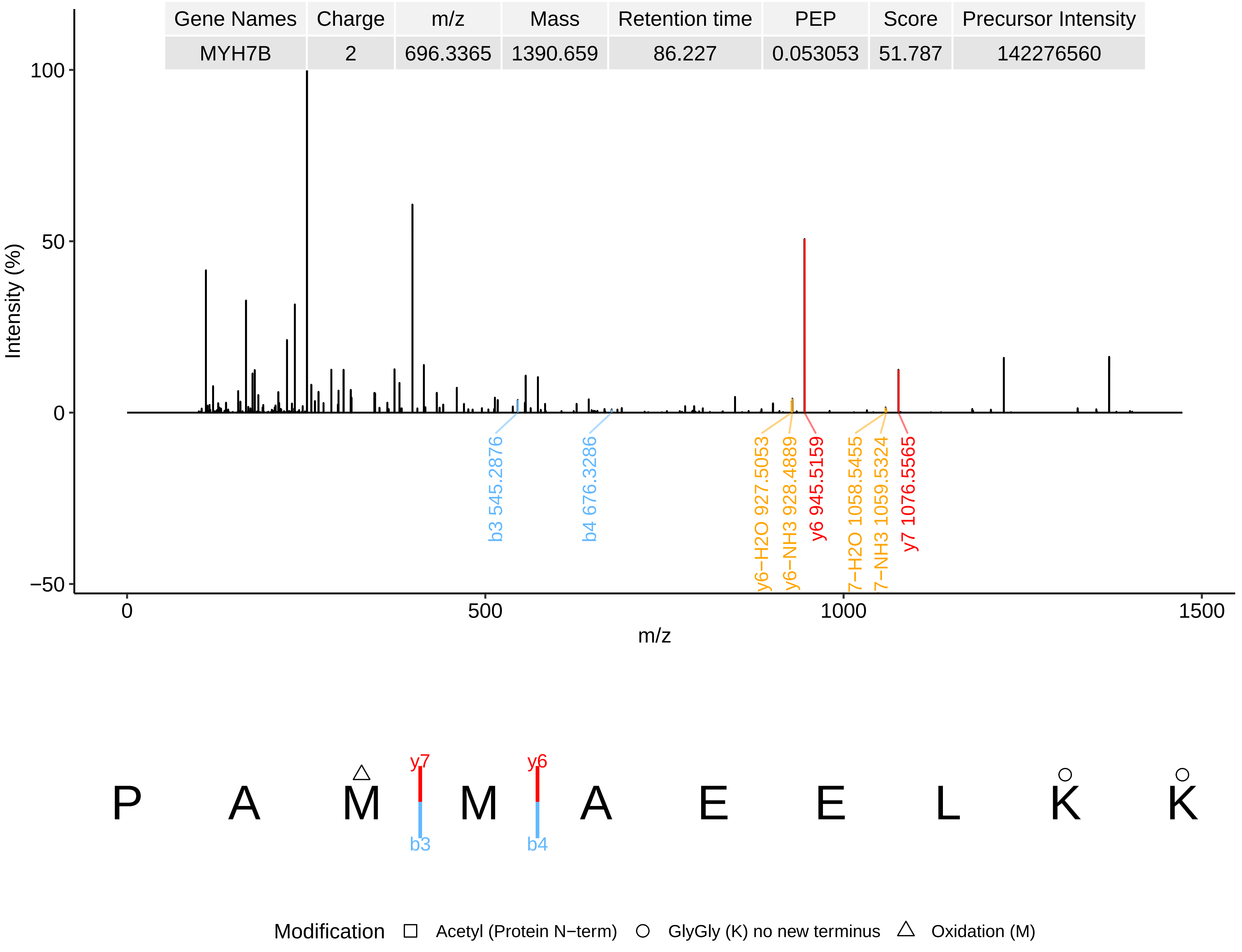

20200924_KIZ_MUb5_GlyGly_1 − Scan number: 27125

Gene Names	Charge	m/z	Mass	Retention time	PEP	Score	Precursor Intensity
MYH7B	2	696.3365	1390.659	86.736	0.06667	47.849	65649632

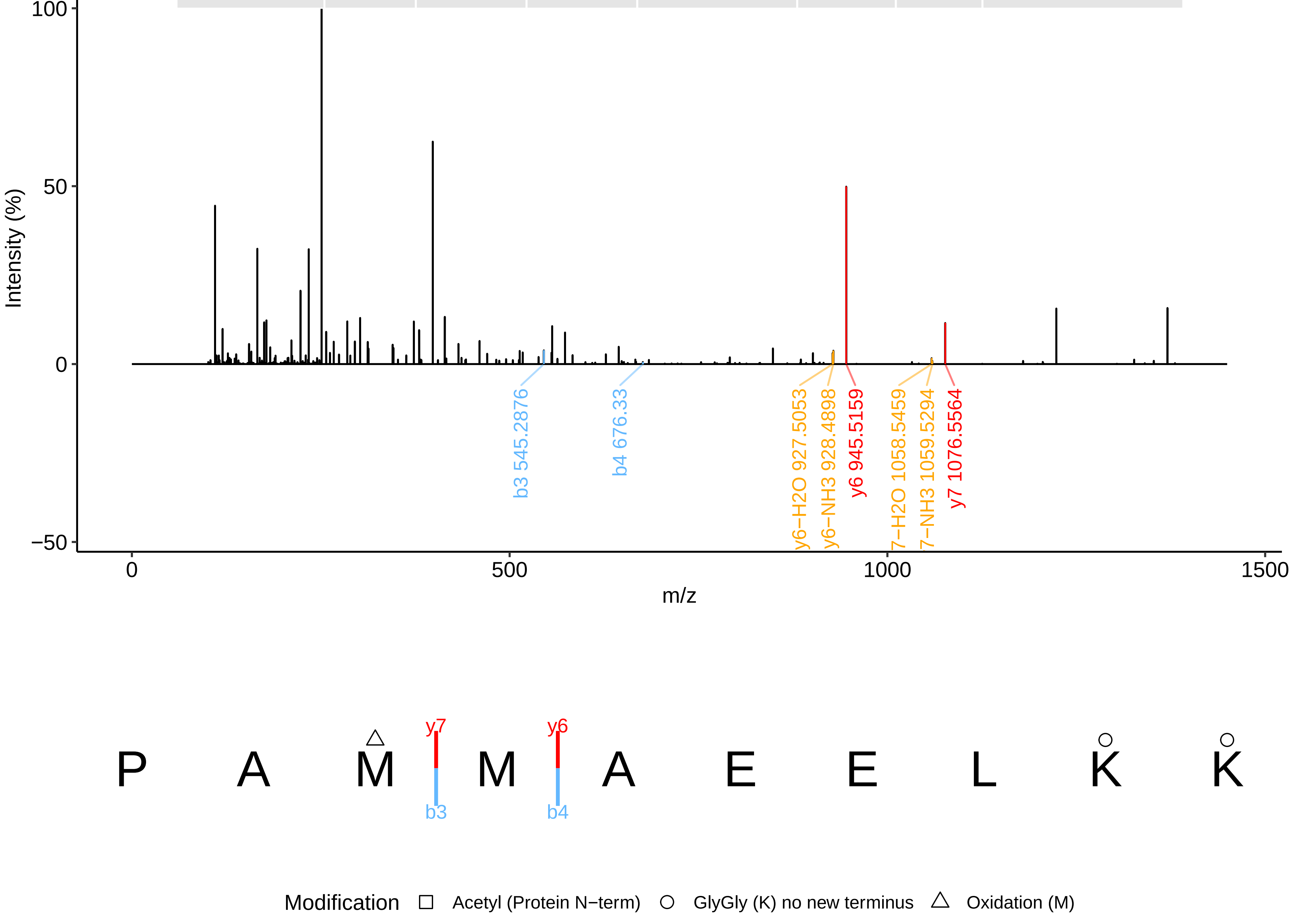

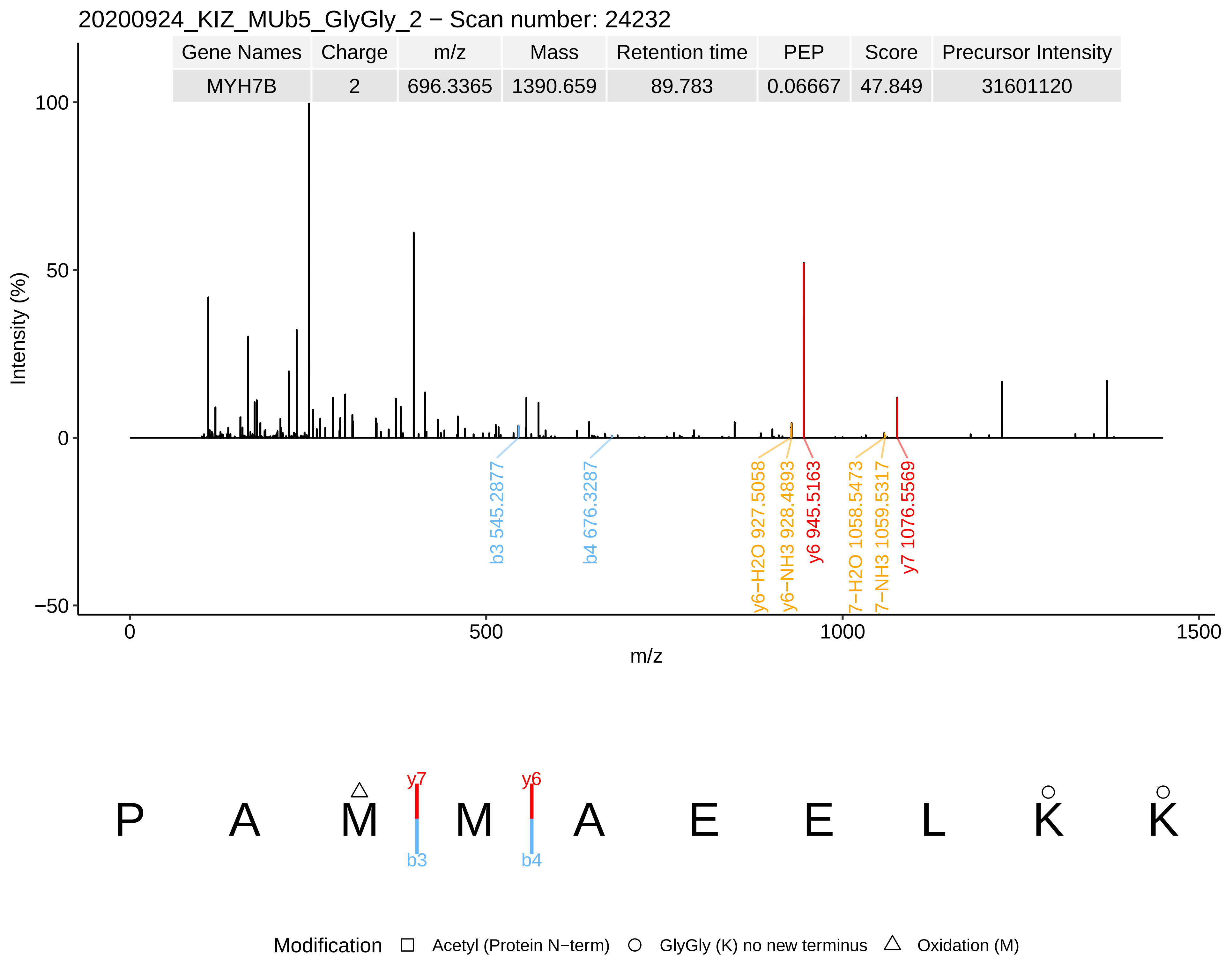
20200924_KIZ_MUb5_GlyGly_2 − Scan number: 24232
Gene Names | Charge | m/z | Mass | Retention time | PEP | Score | Precursor Intensity
MYH7B | 2 | 696.3365 | 1390.659 | 89.783 | 0.06667 | 47.849 | 31601120
Intensity (%)
100
50
0
−50
0
500
1000
1500
m/z
b3 545.2877
b4 676.3287
y6−H2O 927.5058
y6−NH3 928.4893
y6 945.5163
7−H2O 1058.5473
7−NH3 1059.5317
y7 1076.5569
P A M M A E E L K K
y7
y6
b3
b4
Modification
Acetyl (Protein N−term)
GlyGly (K) no new terminus
Oxidation (M)

20200924_KIZ_MUb5_GlyGly_2 − Scan number: 24371

Gene Names	Charge	m/z	Mass	Retention time	PEP	Score	Precursor Intensity
MYH7B	2	696.3365	1390.659	90.294	0.038451	56.011	2593123

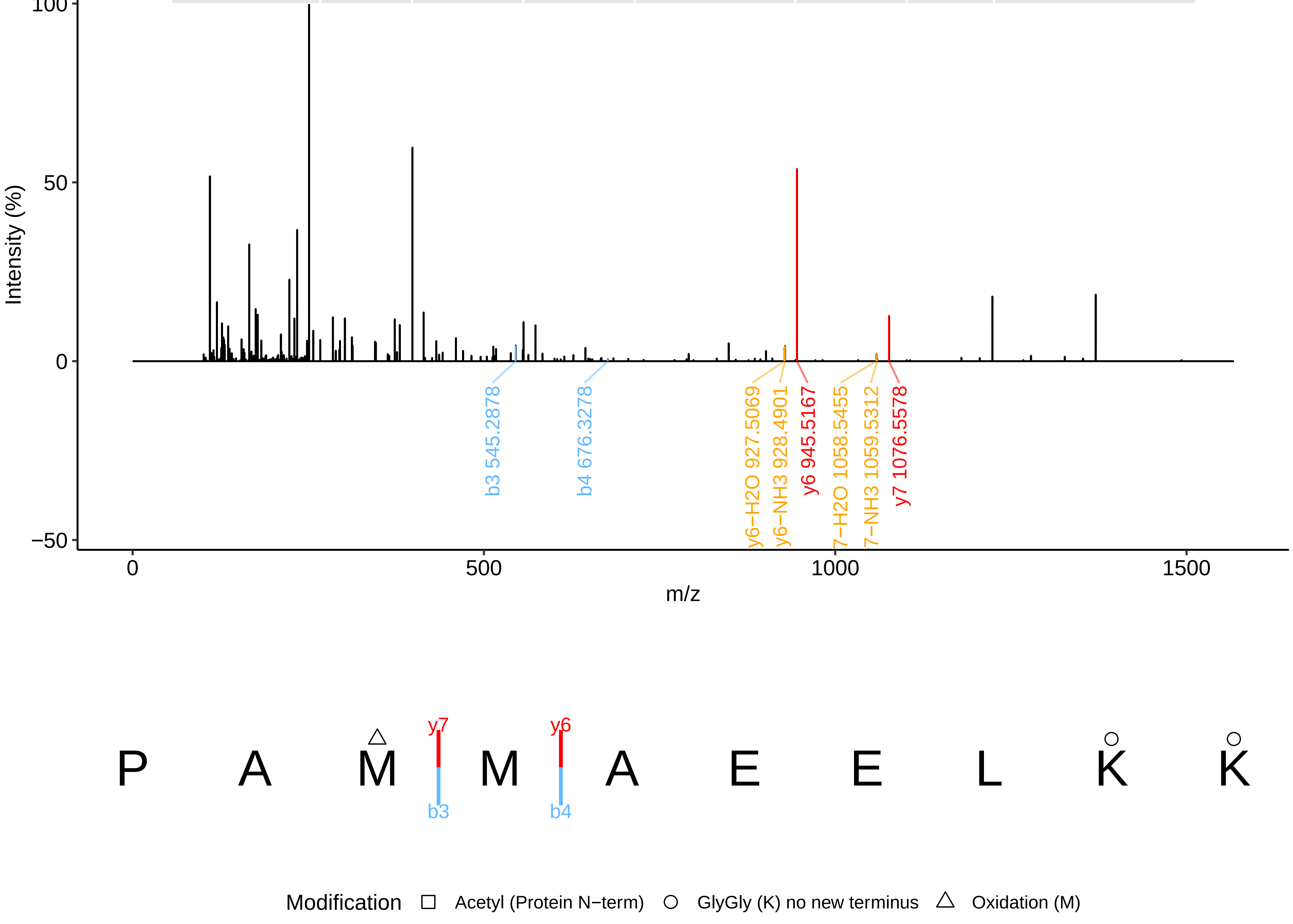

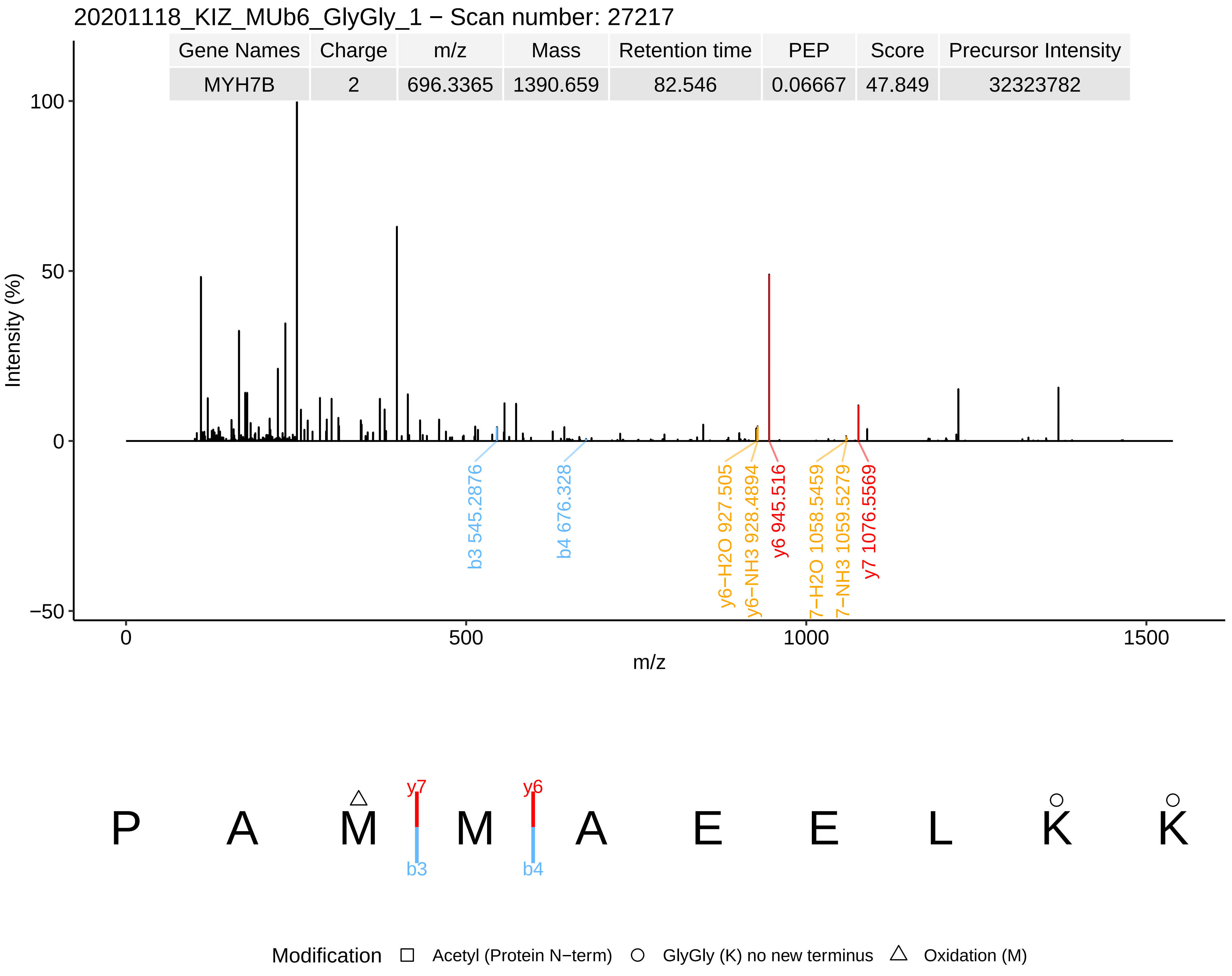

20201118_KIZ_MUb6_GlyGly_1 − Scan number: 27217
Gene Names | Charge | m/z | Mass | Retention time | PEP | Score | Precursor Intensity
MYH7B | 2 | 696.3365 | 1390.659 | 82.546 | 0.06667 | 47.849 | 32323782
Intensity (%)
100
50
0
−50
0
500
1000
1500
m/z
b3 545.2876
b4 676.328
y6−H2O 927.505
y6−NH3 928.4894
y6 945.516
7−H2O 1058.5459
7−NH3 1059.5279
y7 1076.5569
P A M M A E E L K K
y7
y6
b3
b4
Modification
Acetyl (Protein N−term)
GlyGly (K) no new terminus
Oxidation (M)

20201118_KIZ_MUb6_GlyGly_1 − Scan number: 27422

Gene Names	Charge	m/z	Mass	Retention time	PEP	Score	Precursor Intensity
MYH7B	2	696.3365	1390.659	83.05	0.06667	47.849	49978256

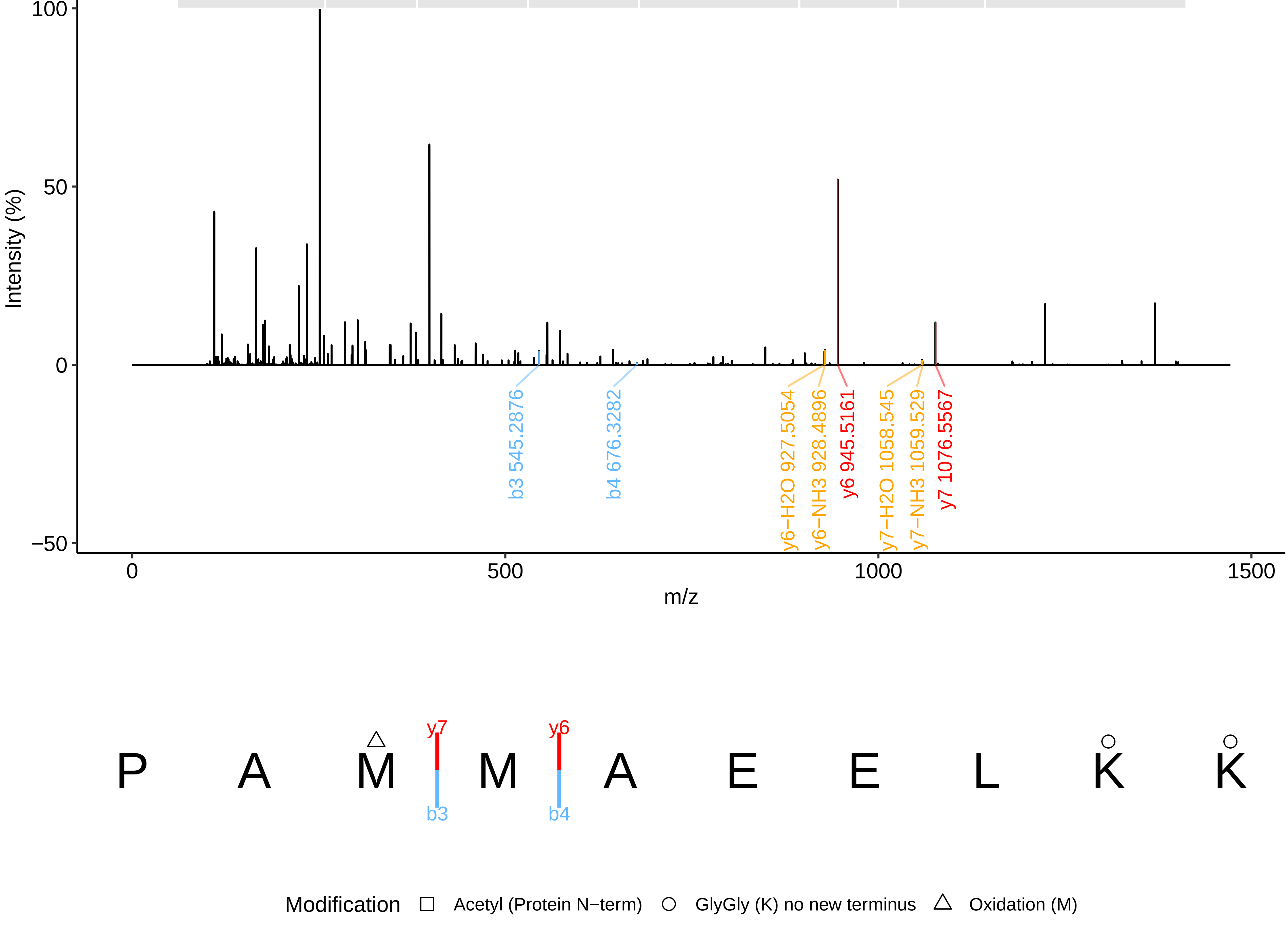

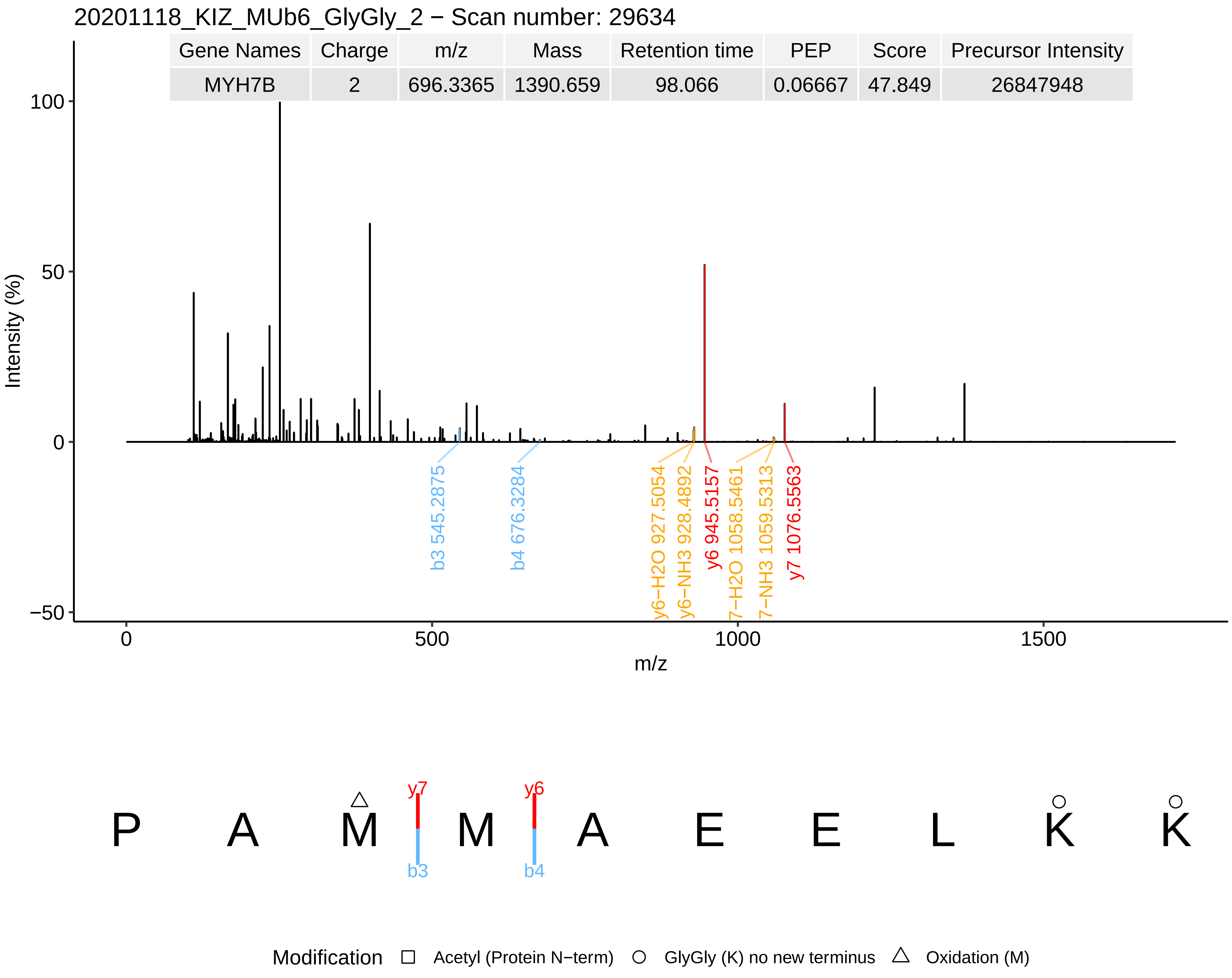
20201118_KIZ_MUb6_GlyGly_2 − Scan number: 29634
Gene Names | Charge | m/z | Mass | Retention time | PEP | Score | Precursor Intensity
MYH7B | 2 | 696.3365 | 1390.659 | 98.066 | 0.06667 | 47.849 | 26847948
Intensity (%)
100
50
0
−50
0
500
1000
1500
m/z
b3 545.2875
b4 676.3284
y6−H2O 927.5054
y6−NH3 928.4892
y6 945.5157
7−H2O 1058.5461
7−NH3 1059.5313
y7 1076.5563
P A M M A E E L K K
y7
y6
b3
b4
Modification
Acetyl (Protein N−term)
GlyGly (K) no new terminus
Oxidation (M)

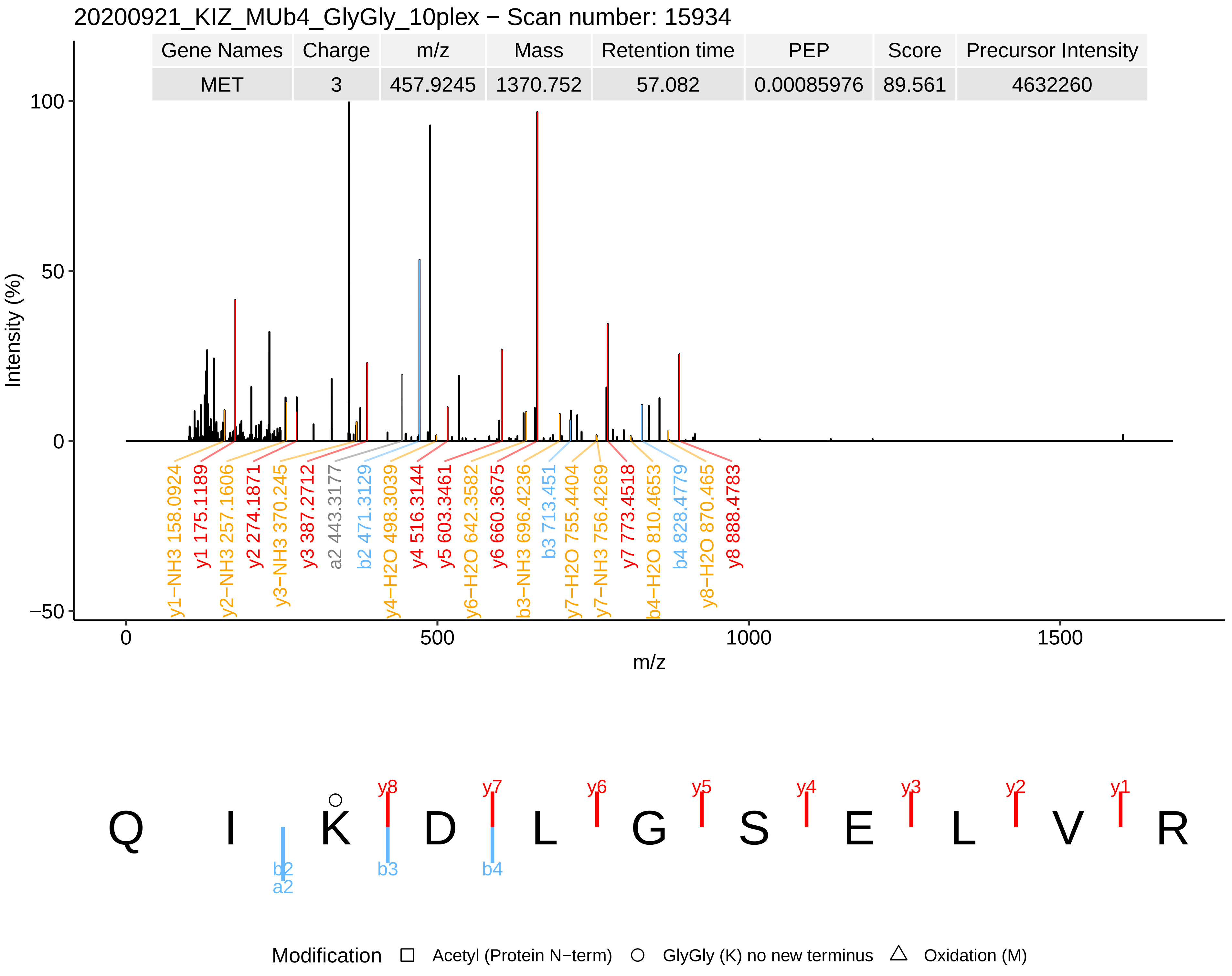
20200921_KIZ_MUb4_GlyGly_10plex − Scan number: 15934
Gene Names | Charge | m/z | Mass | Retention time | PEP | Score | Precursor Intensity
MET | 3 | 457.9245 | 1370.752 | 57.082 | 0.00085976 | 89.561 | 4632260
Intensity (%)
m/z
y1−NH3 158.0924
y1 175.1189
y2−NH3 257.1606
y2 274.1871
y3−NH3 370.245
y3 387.2712
a2 443.3177
b2 471.3129
y4−H2O 498.3039
y4 516.3144
y5 603.3461
y6−H2O 642.3582
y6 660.3675
b3−NH3 696.4236
b3 713.451
y7−H2O 755.4404
y7−NH3 756.4269
y7 773.4518
b4−H2O 810.4653
b4 828.4779
y8−H2O 870.465
y8 888.4783
Q I K D L G S E L V R
y8 y7 y6 y5 y4 y3 y2 y1
b2 a2 b3 b4
Modification
Acetyl (Protein N−term)
GlyGly (K) no new terminus
Oxidation (M)

20200921_KIZ_MUb4_GlyGly_10plex_2 − Scan number: 14755

Gene Names	Charge	m/z	Mass	Retention time	PEP	Score	Precursor Intensity
MET	3	457.9245	1370.752	57.581	0.0023561	79.82	829838.8

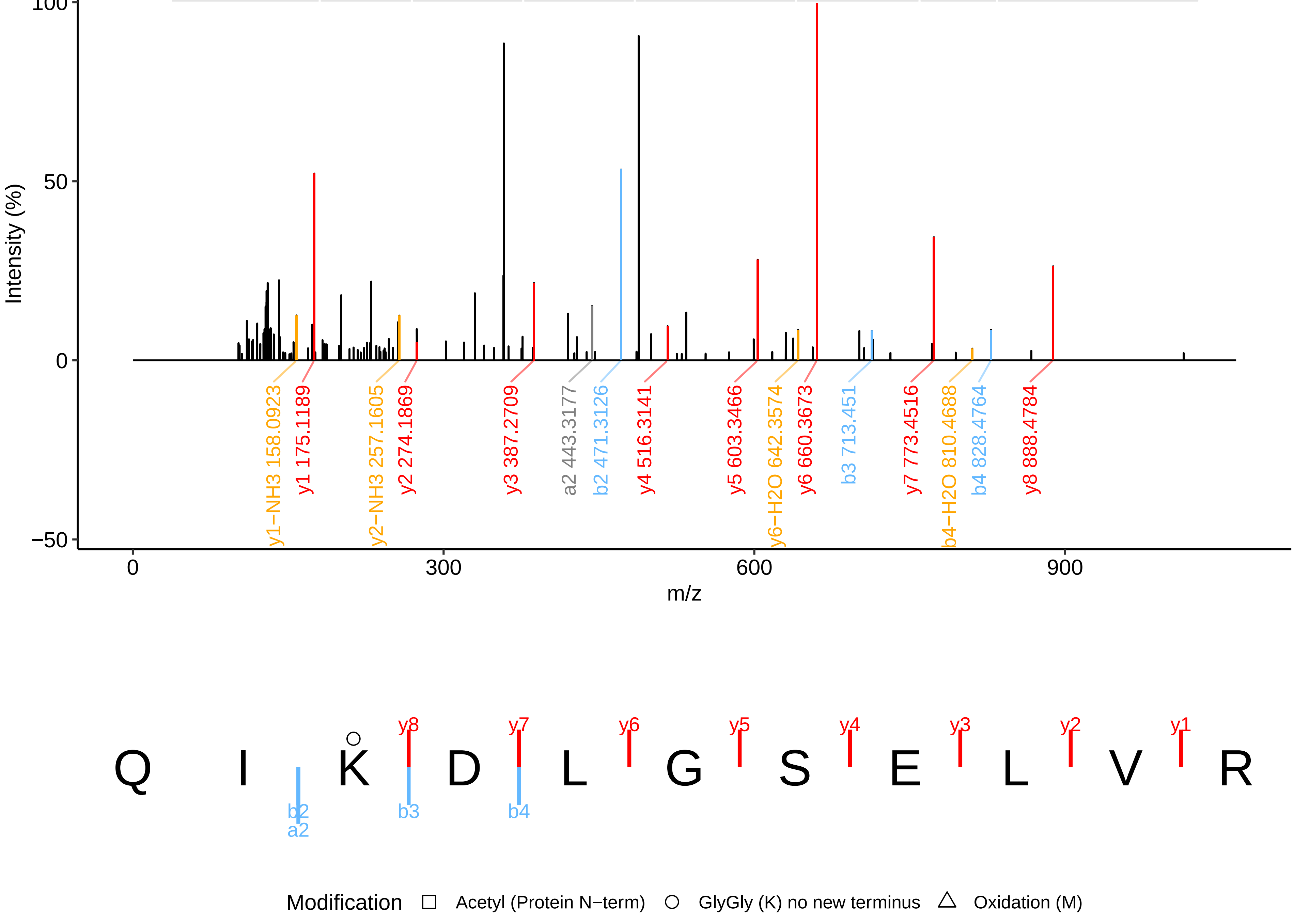

20200924_KIZ_MUb5_GlyGly_2 − Scan number: 14181

Gene Names	Charge	m/z	Mass	Retention time	PEP	Score	Precursor Intensity
MET	3	457.9245	1370.752	53.011	0.012952	71.085	4777139

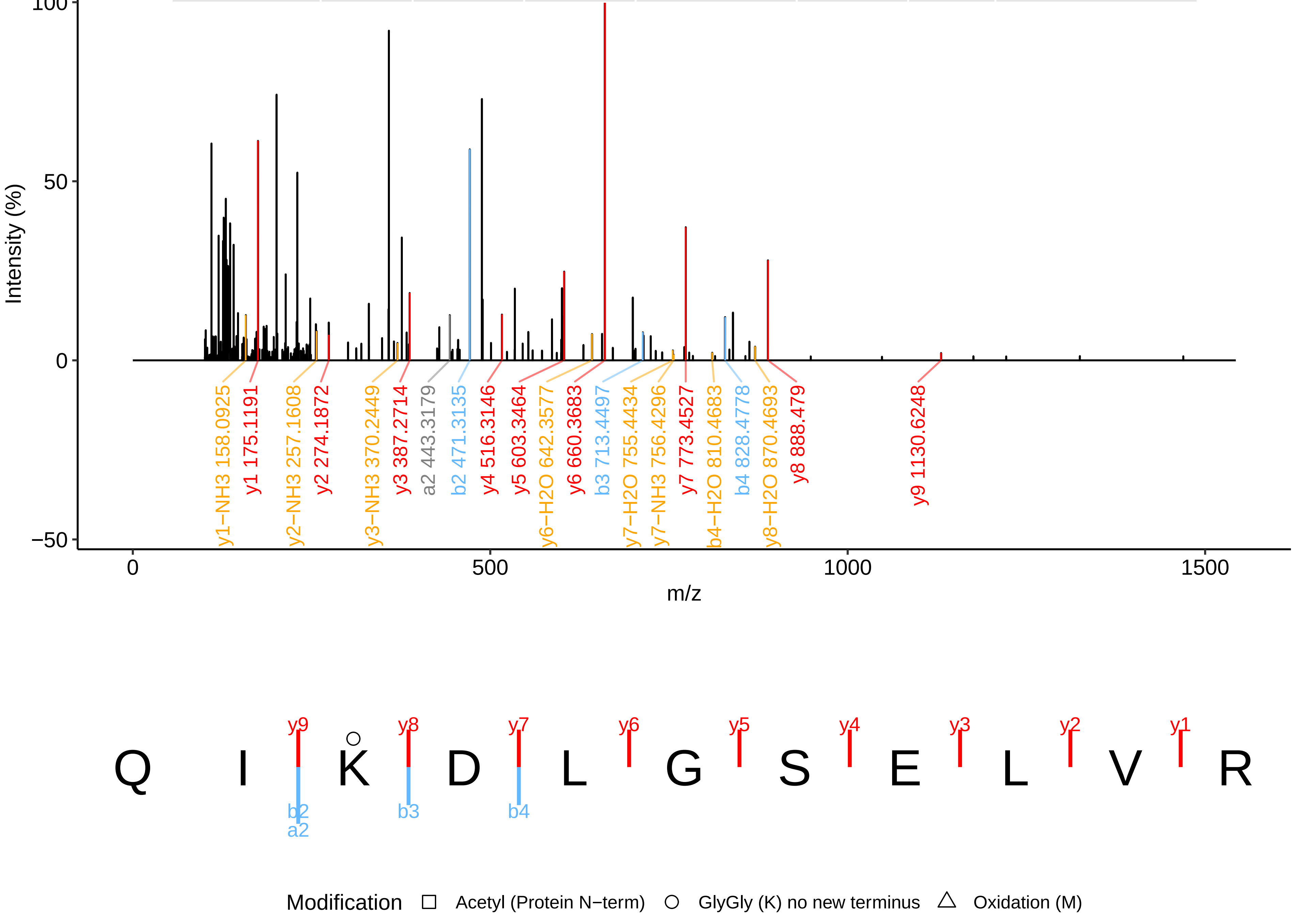

20201118_KIZ_MUb6_GlyGly_1 − Scan number: 15092

Gene Names	Charge	m/z	Mass	Retention time	PEP	Score	Precursor Intensity
MET	3	457.9245	1370.752	47.742	0.0021019	81.525	20997930

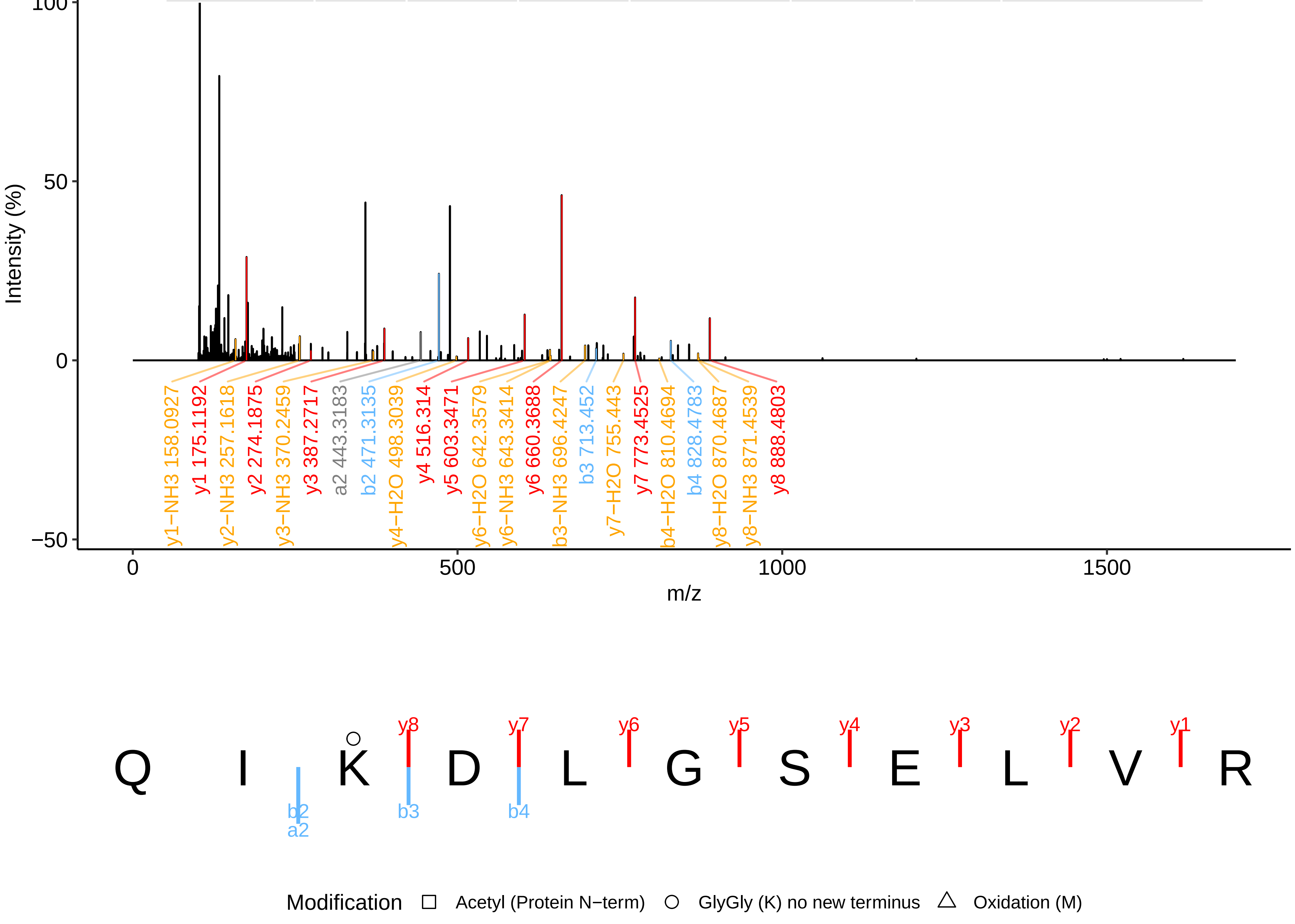

20200921_KIZ_MUb4_GlyGly_10plex − Scan number: 6375

Gene Names	Charge	m/z	Mass	Retention time	PEP	Score	Precursor Intensity
PRKAR1A	3	457.5881	1369.743	21.067	3.4125e−05	128.01	2292309

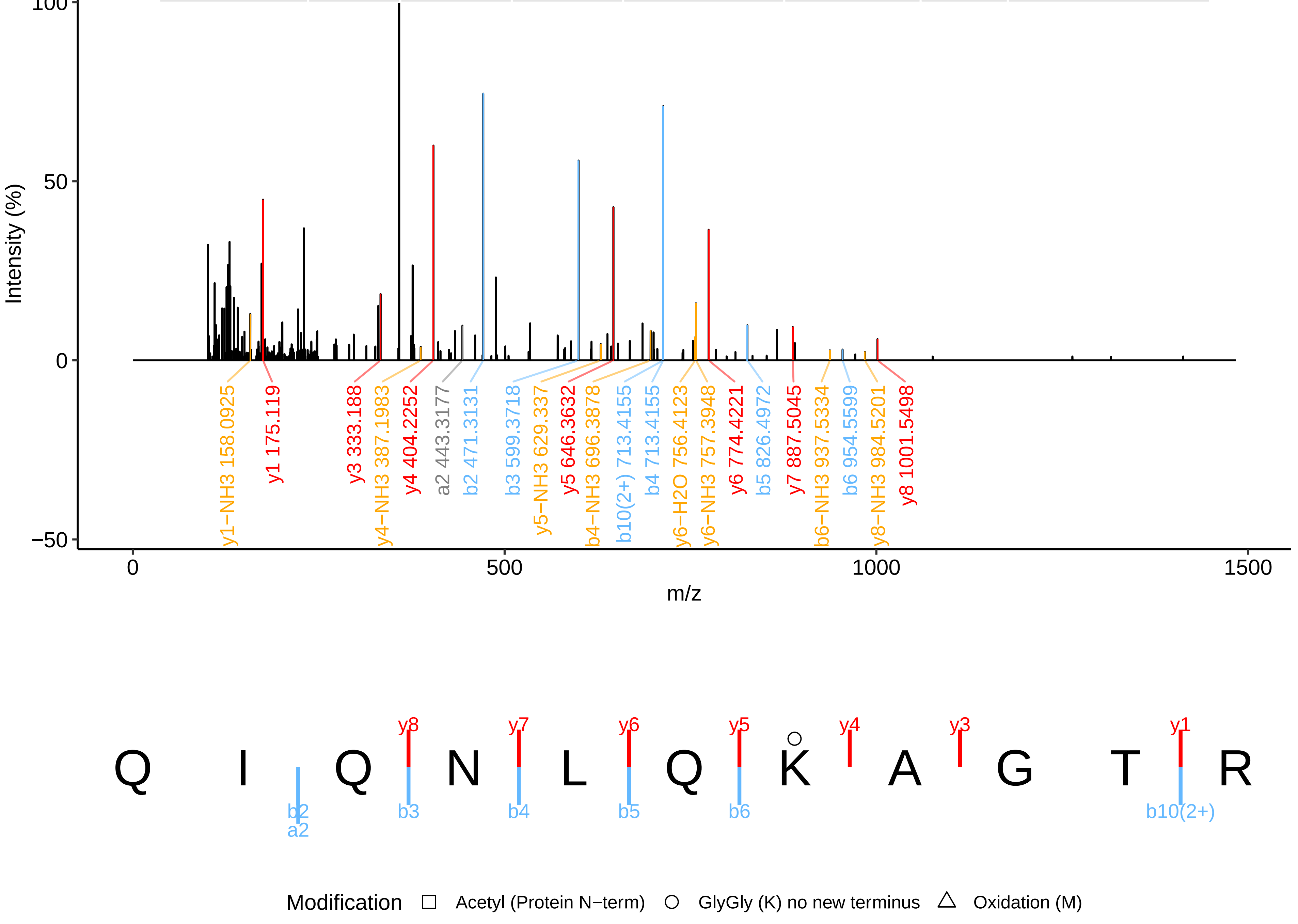

20200921_KIZ_MUb4_GlyGly_10plex_2 − Scan number: 5860

Gene Names	Charge	m/z	Mass	Retention time	PEP	Score	Precursor Intensity
PRKAR1A	3	457.5881	1369.743	21.277	0.0015164	87.363	895965.3

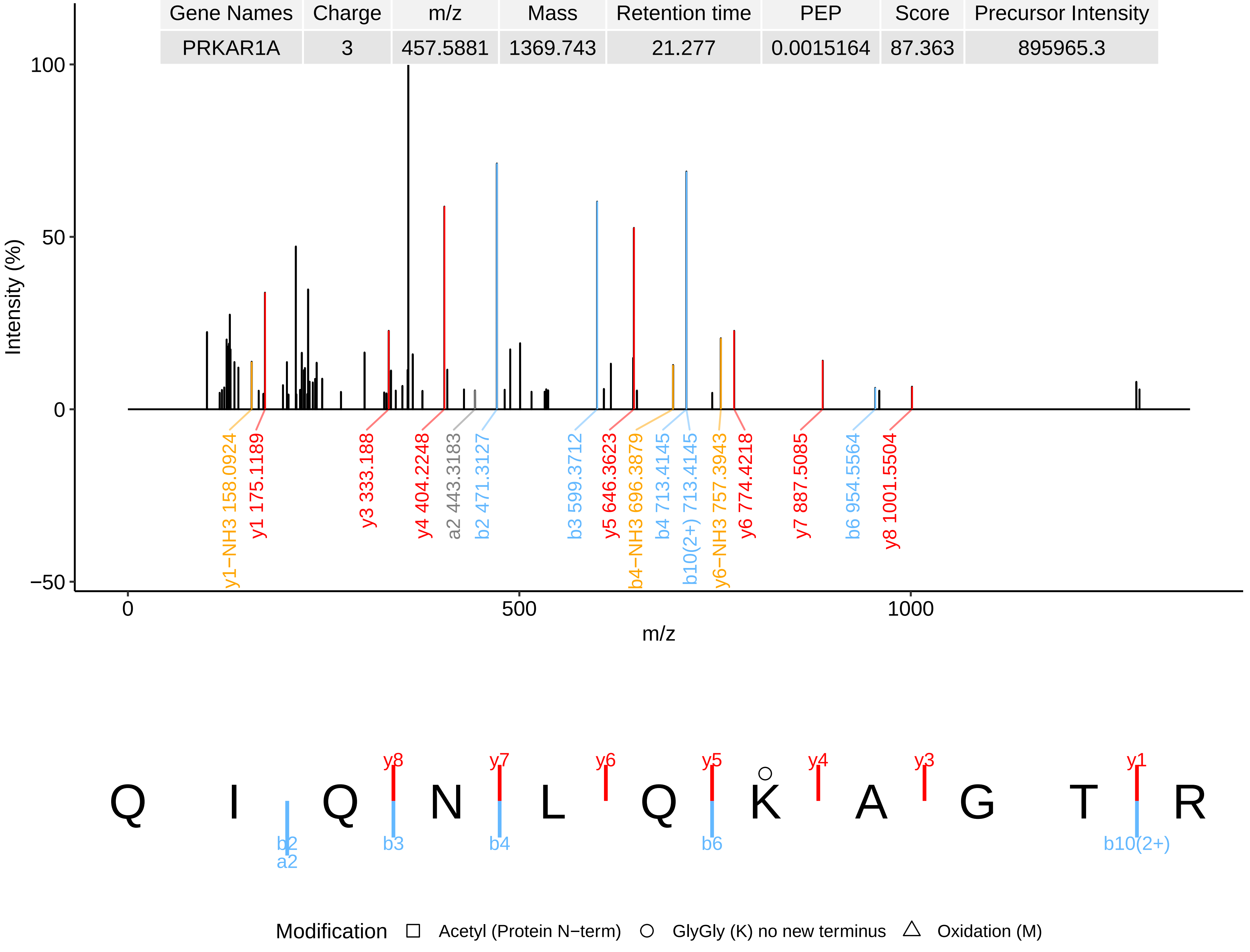

20200921_KIZ_MUb4_GlyGly_10plex_2 − Scan number: 18947

Gene Names	Charge	m/z	Mass	Retention time	PEP	Score	Precursor Intensity
PMAIP1	3	390.9117	1169.713	74.292	0.036748	61.65	998979.1

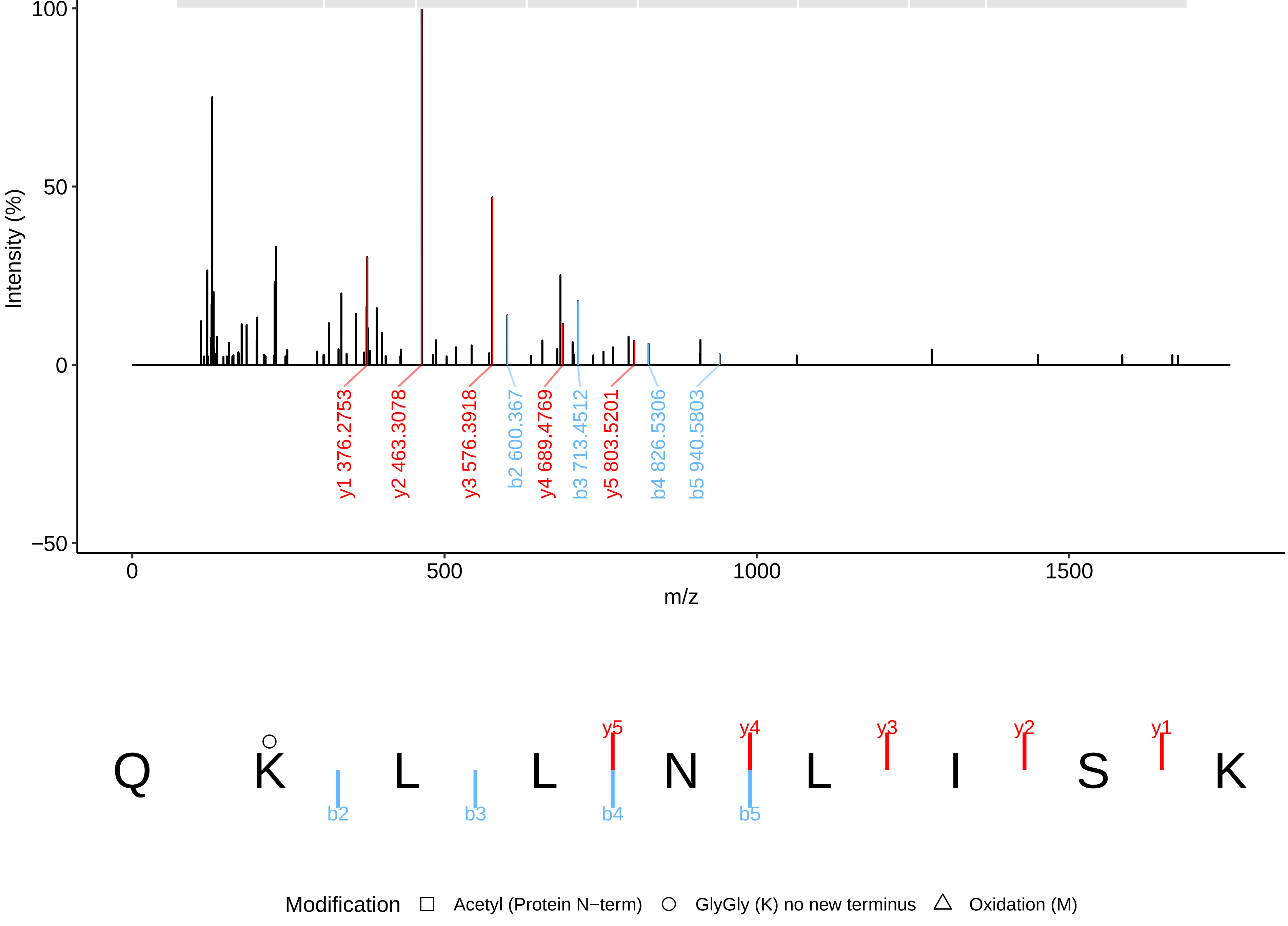

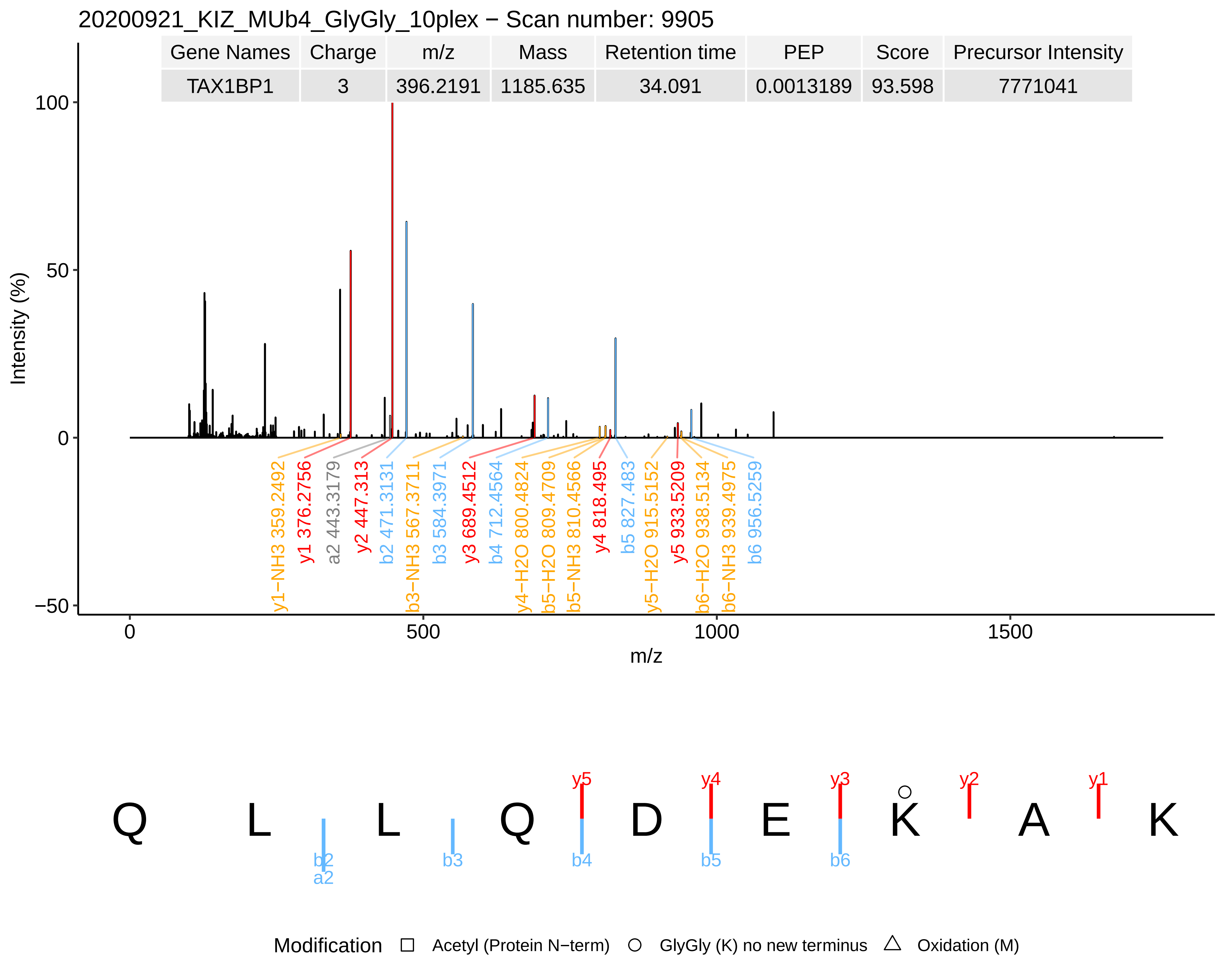

20200921_KIZ_MUb4_GlyGly_10plex − Scan number: 9905
Gene Names | Charge | m/z | Mass | Retention time | PEP | Score | Precursor Intensity
TAX1BP1 | 3 | 396.2191 | 1185.635 | 34.091 | 0.0013189 | 93.598 | 7771041
Intensity (%)
m/z
y1−NH3 359.2492
y1 376.2756
a2 443.3179
y2 447.313
b2 471.3131
b3−NH3 567.3711
b3 584.3971
y3 689.4512
b4 712.4564
y4−H2O 800.4824
b5−H2O 809.4709
b5−NH3 810.4566
y4 818.495
b5 827.483
y5−H2O 915.5152
y5 933.5209
b6−H2O 938.5134
b6−NH3 939.4975
b6 956.5259
Q L L Q D E K A K
y5 y4 y3 y2 y1
b2 a2 b3 b4 b5 b6
Modification
Acetyl (Protein N−term)
GlyGly (K) no new terminus
Oxidation (M)

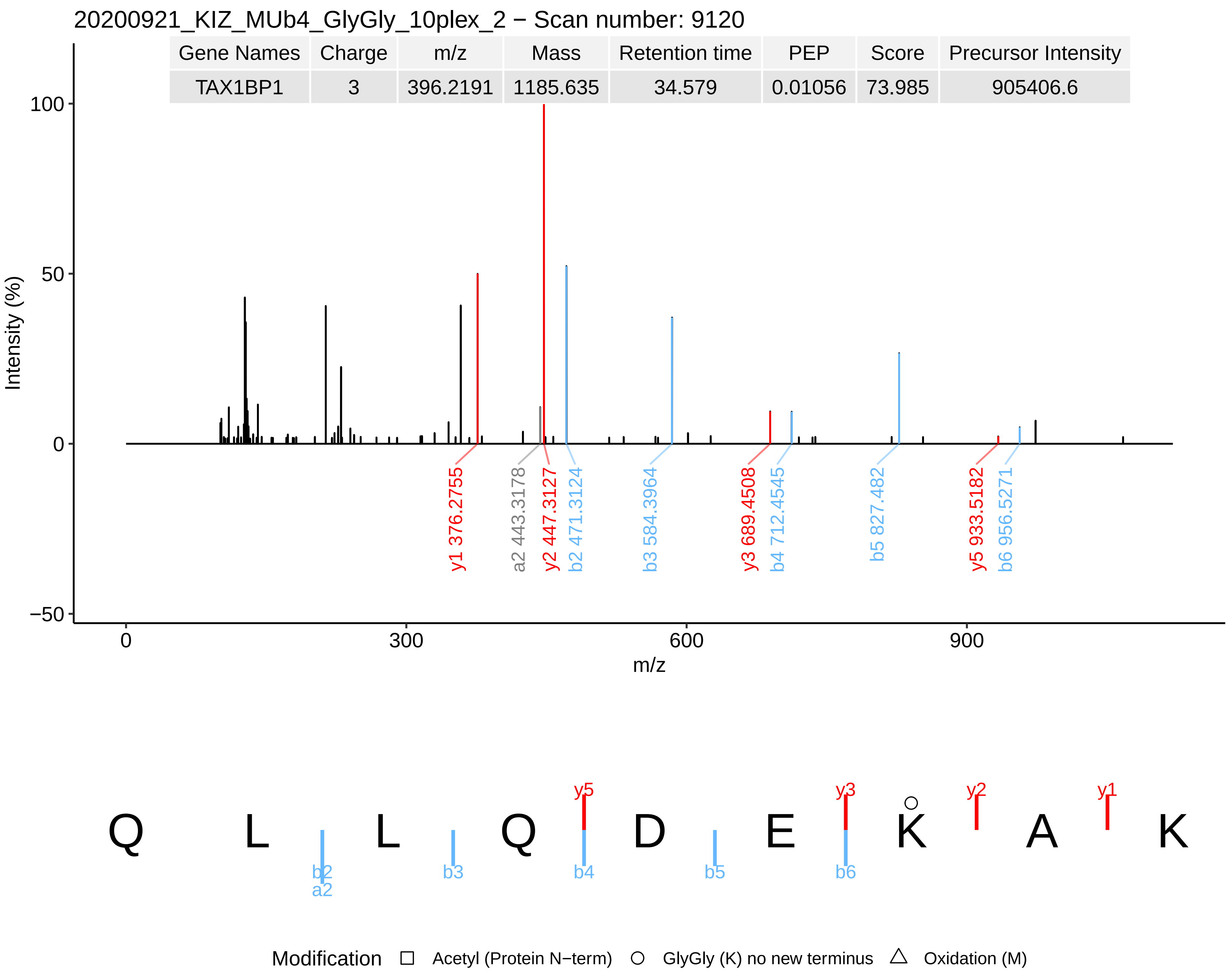

20200921_KIZ_MUb4_GlyGly_10plex_2 − Scan number: 9120
Gene Names | Charge | m/z | Mass | Retention time | PEP | Score | Precursor Intensity
TAX1BP1 | 3 | 396.2191 | 1185.635 | 34.579 | 0.01056 | 73.985 | 905406.6
Intensity (%)
m/z
y1 376.2755
a2 443.3178
y2 447.3127
b2 471.3124
b3 584.3964
y3 689.4508
b4 712.4545
b5 827.482
y5 933.5182
b6 956.5271
Q L L Q D E K A K
Modification
Acetyl (Protein N−term)
GlyGly (K) no new terminus
Oxidation (M)

20200921_KIZ_MUb4_GlyGly_10plex − Scan number: 16275

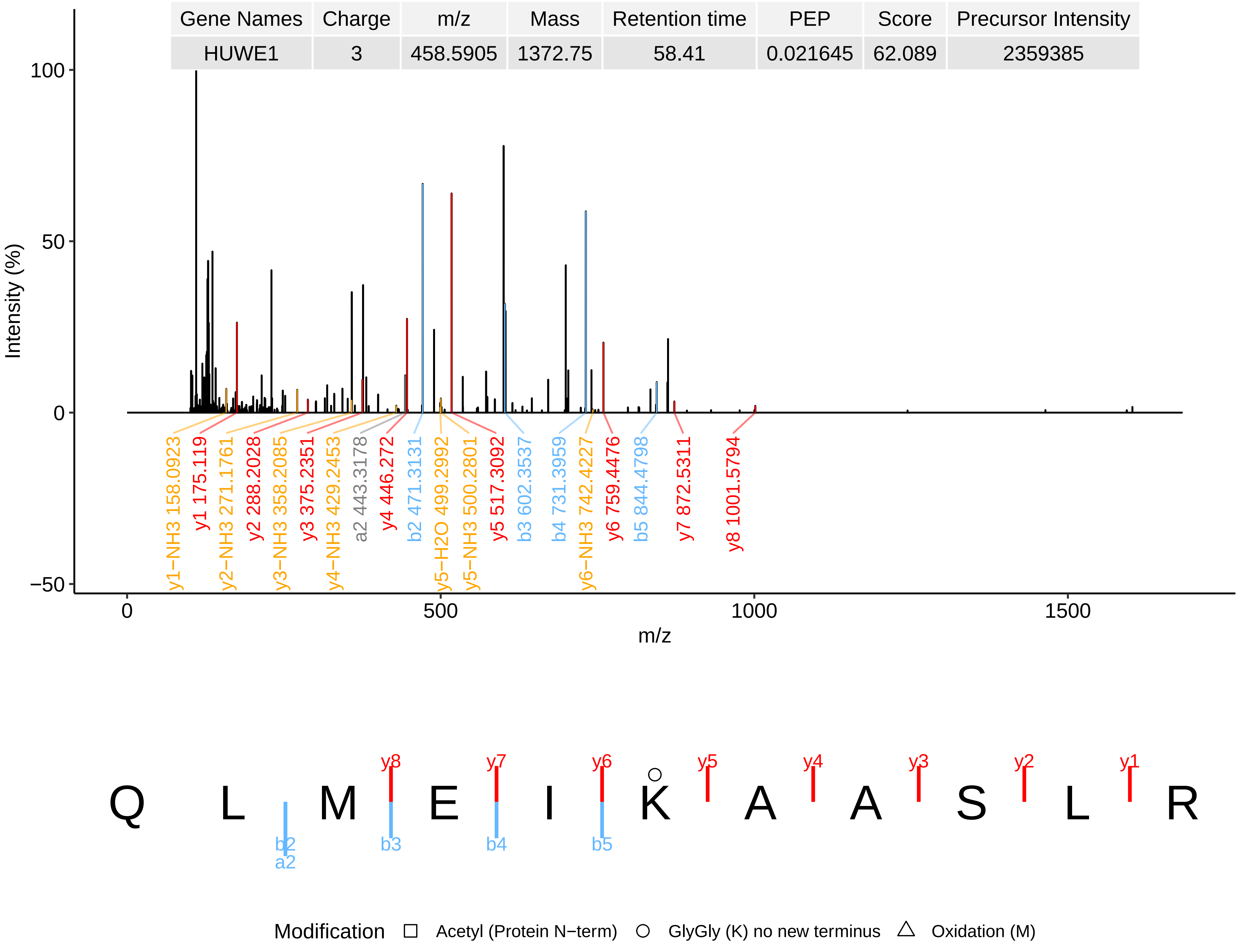

20200921_KIZ_MUb4_GlyGly_10plex − Scan number: 16291

Gene Names	Charge	m/z	Mass	Retention time	PEP	Score	Precursor Intensity
HUWE1	3	458.5905	1372.75	58.473	1.5971e−05	109.24	3547050

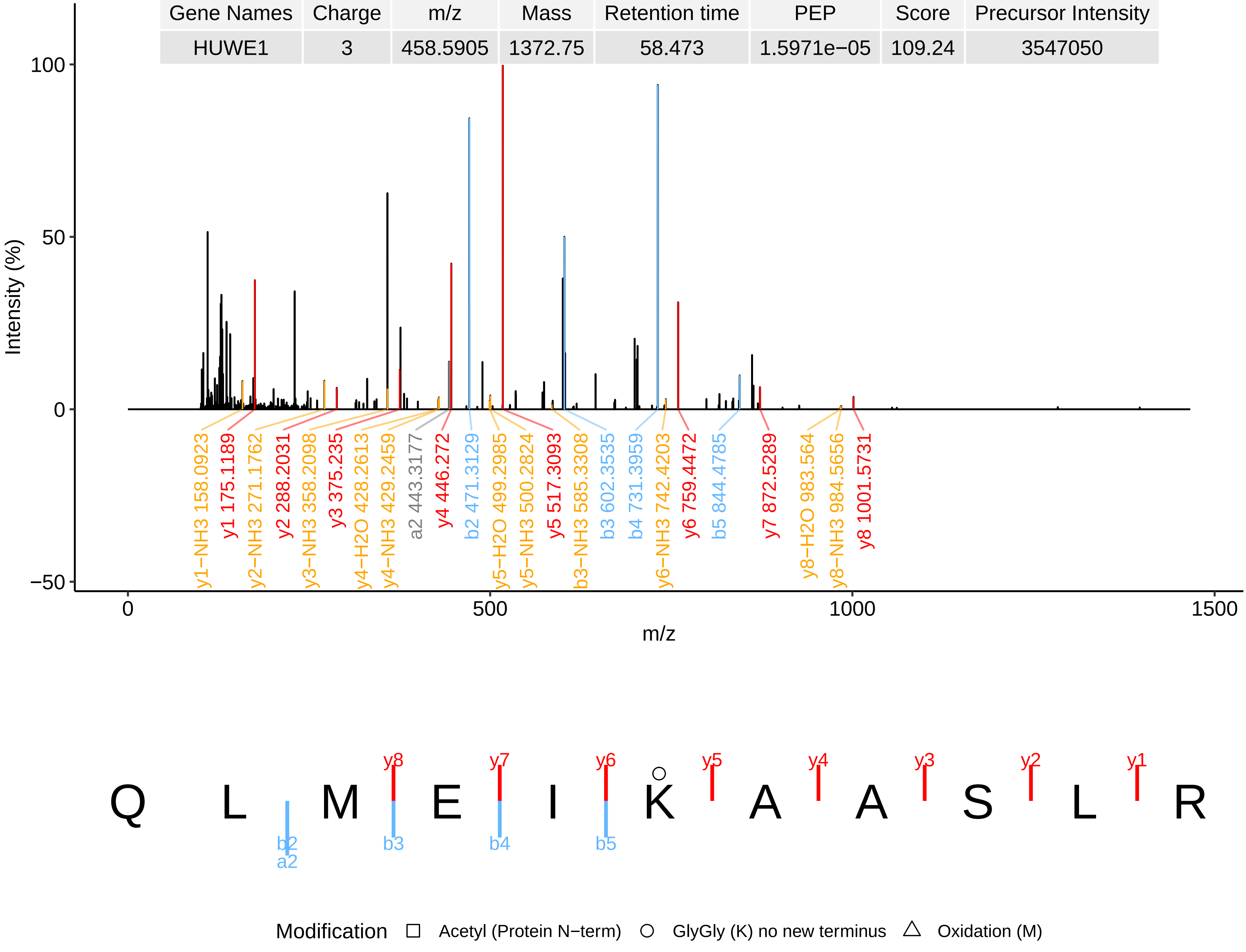

20200921_KIZ_MUb4_GlyGly_10plex_2 − Scan number: 15143

Gene Names	Charge	m/z	Mass	Retention time	PEP	Score	Precursor Intensity
HUWE1	3	458.5905	1372.75	59.157	0.13784	45.915	NA

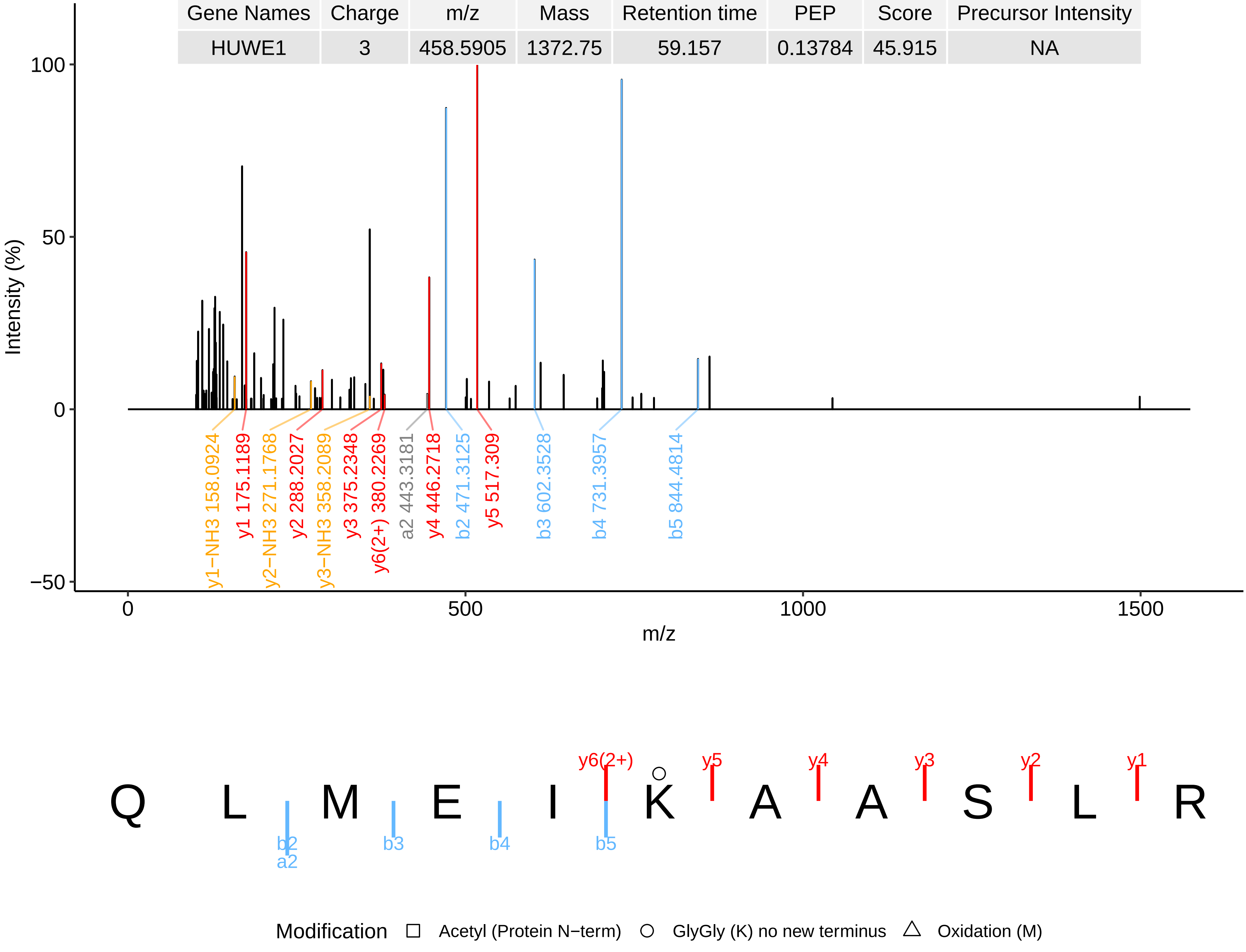

20200924_KIZ_MUb5_GlyGly_1 − Scan number: 14987

Gene Names	Charge	m/z	Mass	Retention time	PEP	Score	Precursor Intensity
GK5	3	395.5316	1183.573	48.961	0.058071	76.827	16025232

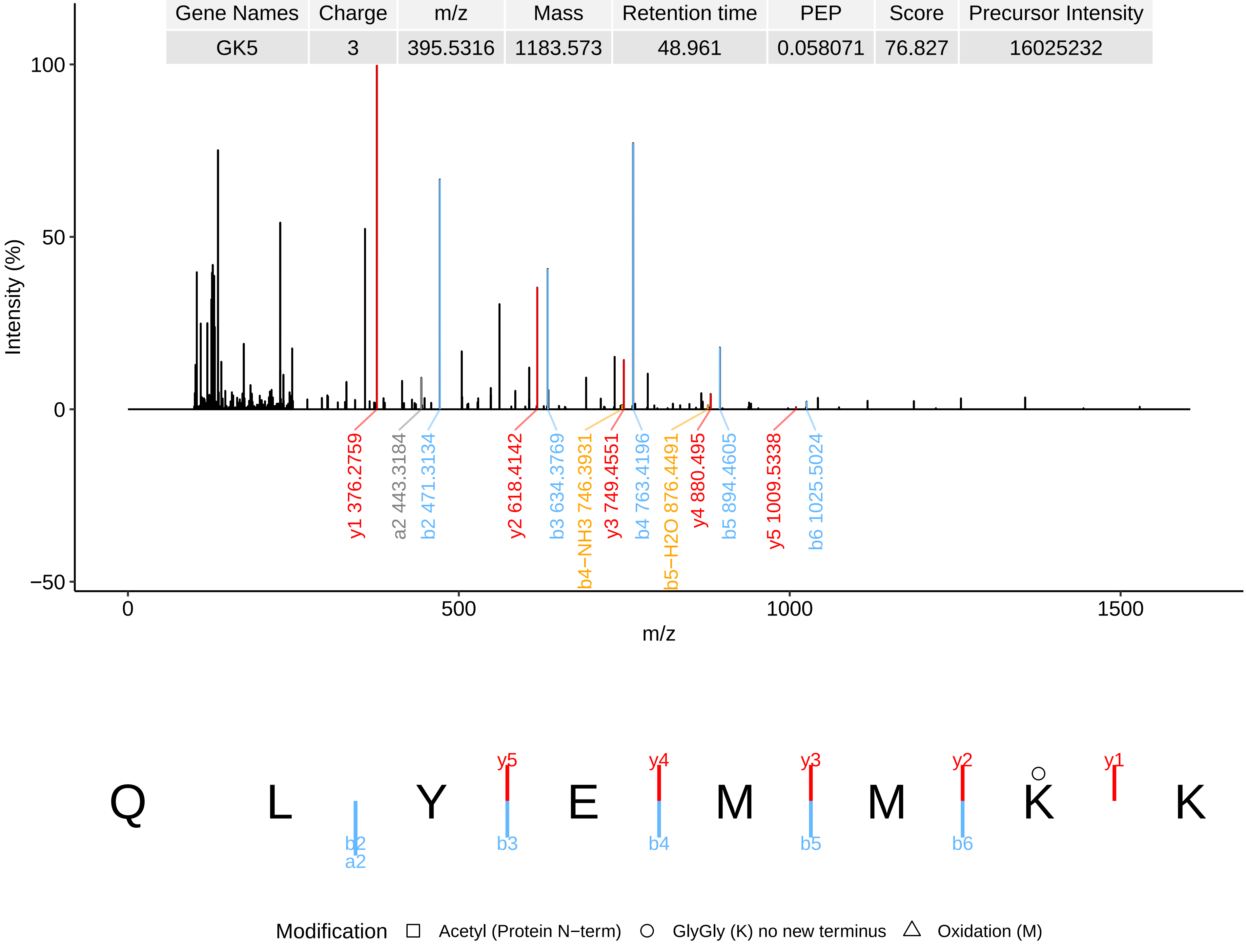

20200924_KIZ_MUb5_GlyGly_1 − Scan number: 4748

Gene Names	Charge	m/z	Mass	Retention time	PEP	Score	Precursor Intensity
HMGB2	3	392.8718	1175.593	14.689	0.080166	55.567	NA

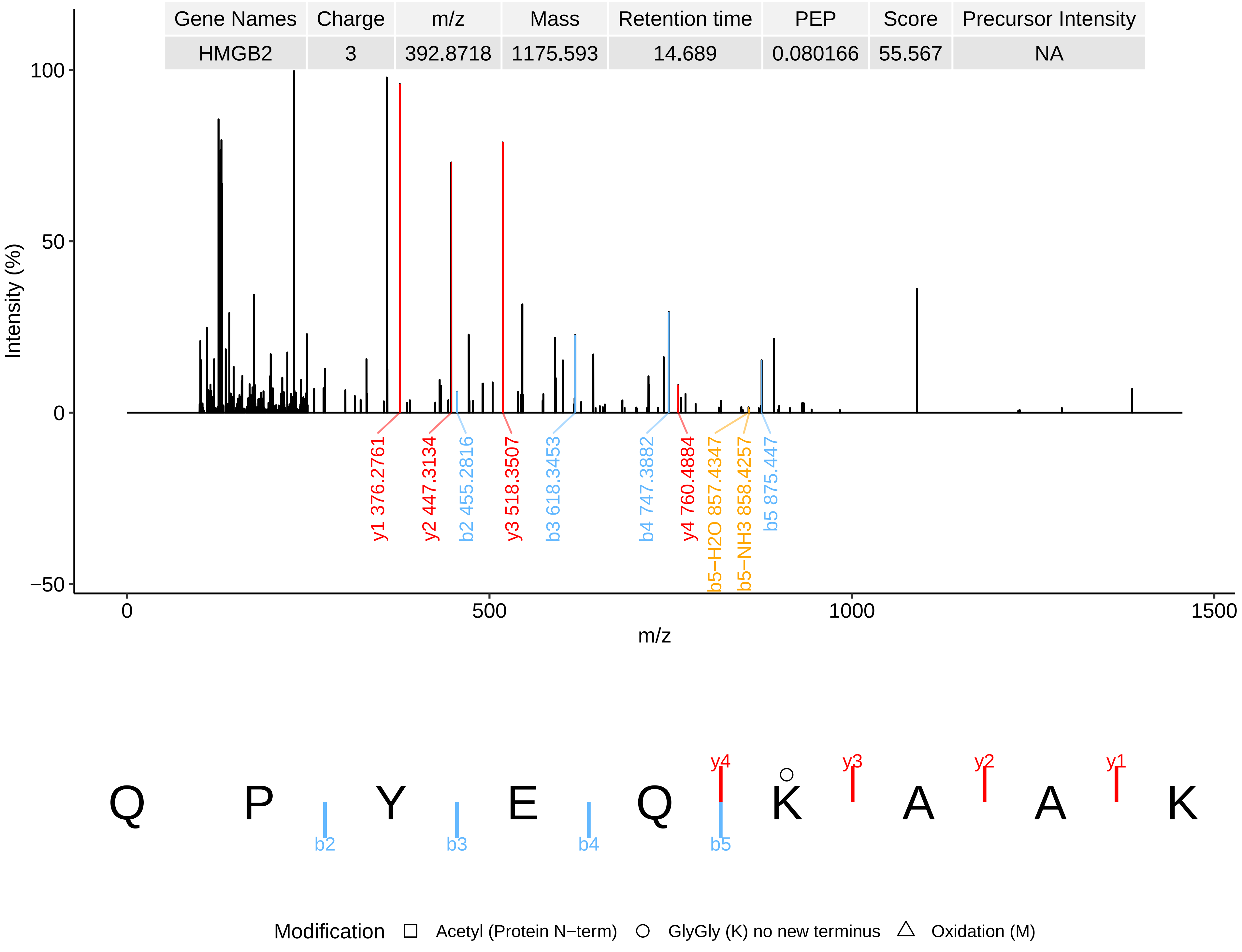

20200921_KIZ_MUb4_GlyGly_10plex − Scan number: 3678

Gene Names	Charge	m/z	Mass	Retention time	PEP	Score	Precursor Intensity
USP24	3	384.8596	1151.557	11.795	0.0096214	80.165	4486083

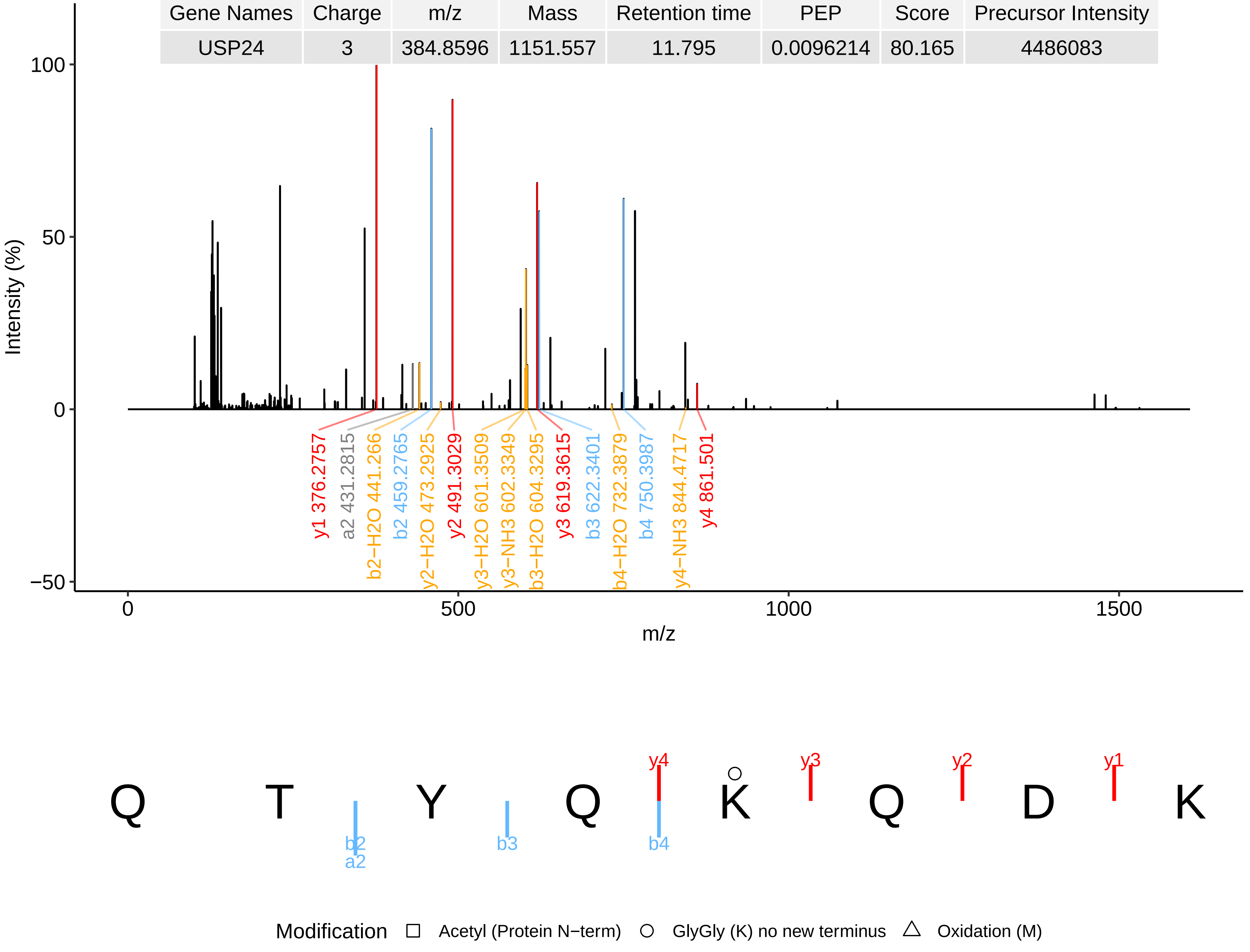

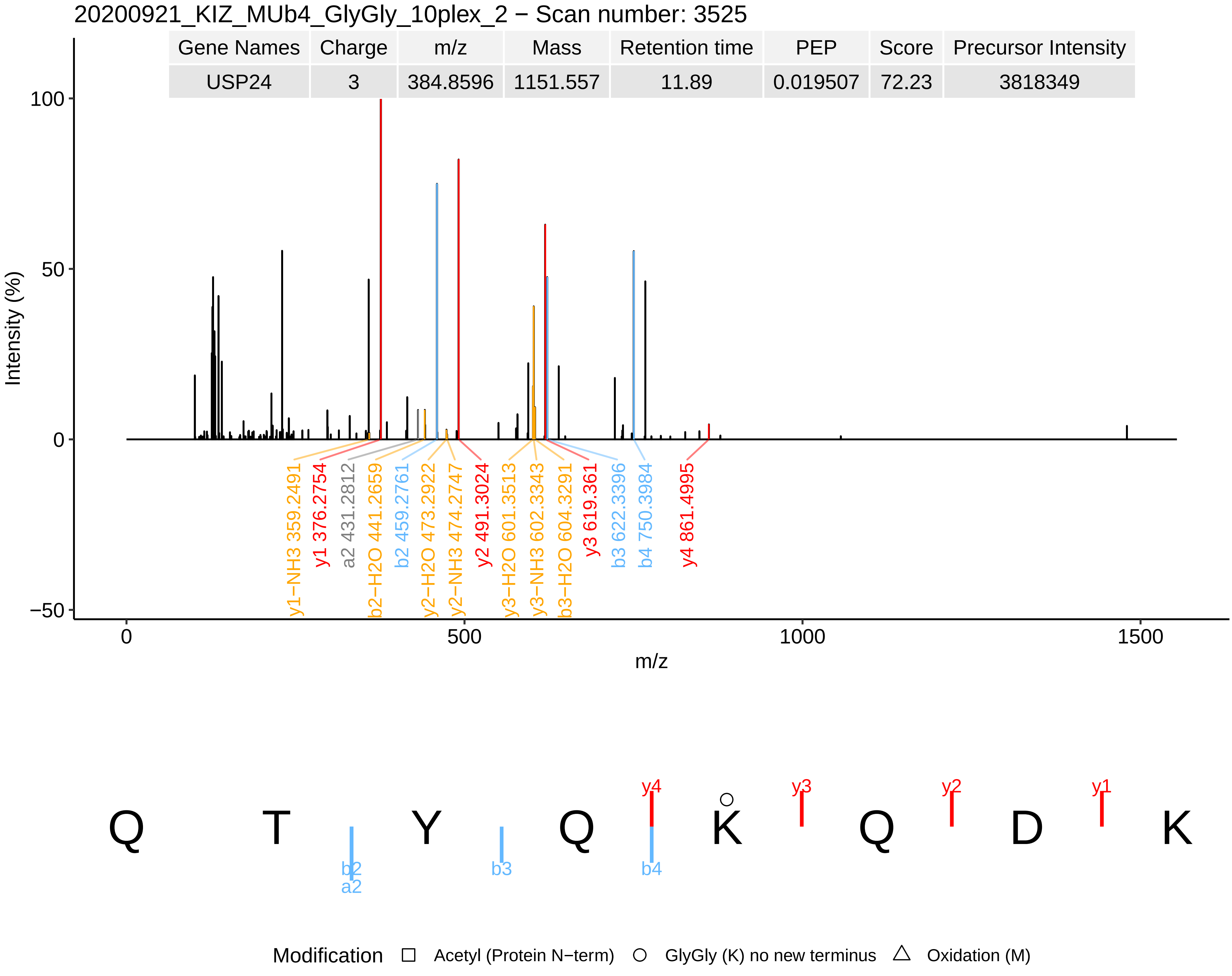
20200921_KIZ_MUb4_GlyGly_10plex_2 − Scan number: 3525
Gene Names
USP24
Charge
3
m/z
384.8596
Mass
1151.557
Retention time
11.89
PEP
0.019507
Score
72.23
Precursor Intensity
3818349
Intensity (%)
100
50
0
−50
0
500
1000
1500
m/z
y1−NH3 359.2491
y1 376.2754
a2 431.2812
b2−H2O 441.2659
b2 459.2761
y2−H2O 473.2922
y2−NH3 474.2747
y2 491.3024
y3−H2O 601.3513
y3−NH3 602.3343
b3−H2O 604.3291
y3 619.361
b3 622.3396
b4 750.3984
y4 861.4995
Q
T
Y
Q
K
Q
D
K
y4
y3
y2
y1
b2
a2
b3
b4
Modification
Acetyl (Protein N−term)
GlyGly (K) no new terminus
Oxidation (M)

20200924_KIZ_MUb5_GlyGly_1 − Scan number: 3289

Gene Names	Charge	m/z	Mass	Retention time	PEP	Score	Precursor Intensity
USP24	3	384.8596	1151.557	10.107	0.030113	68.371	30124702

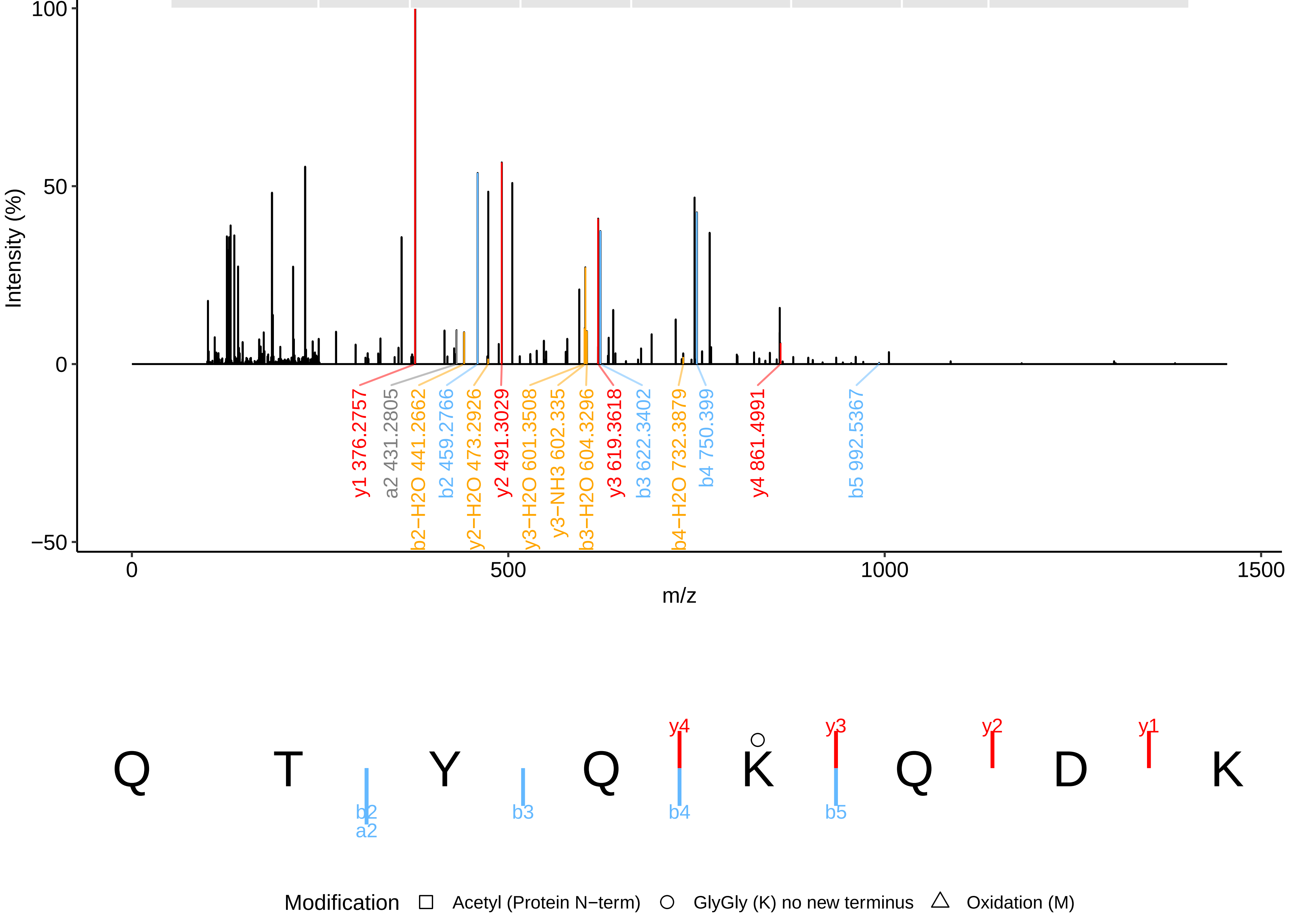

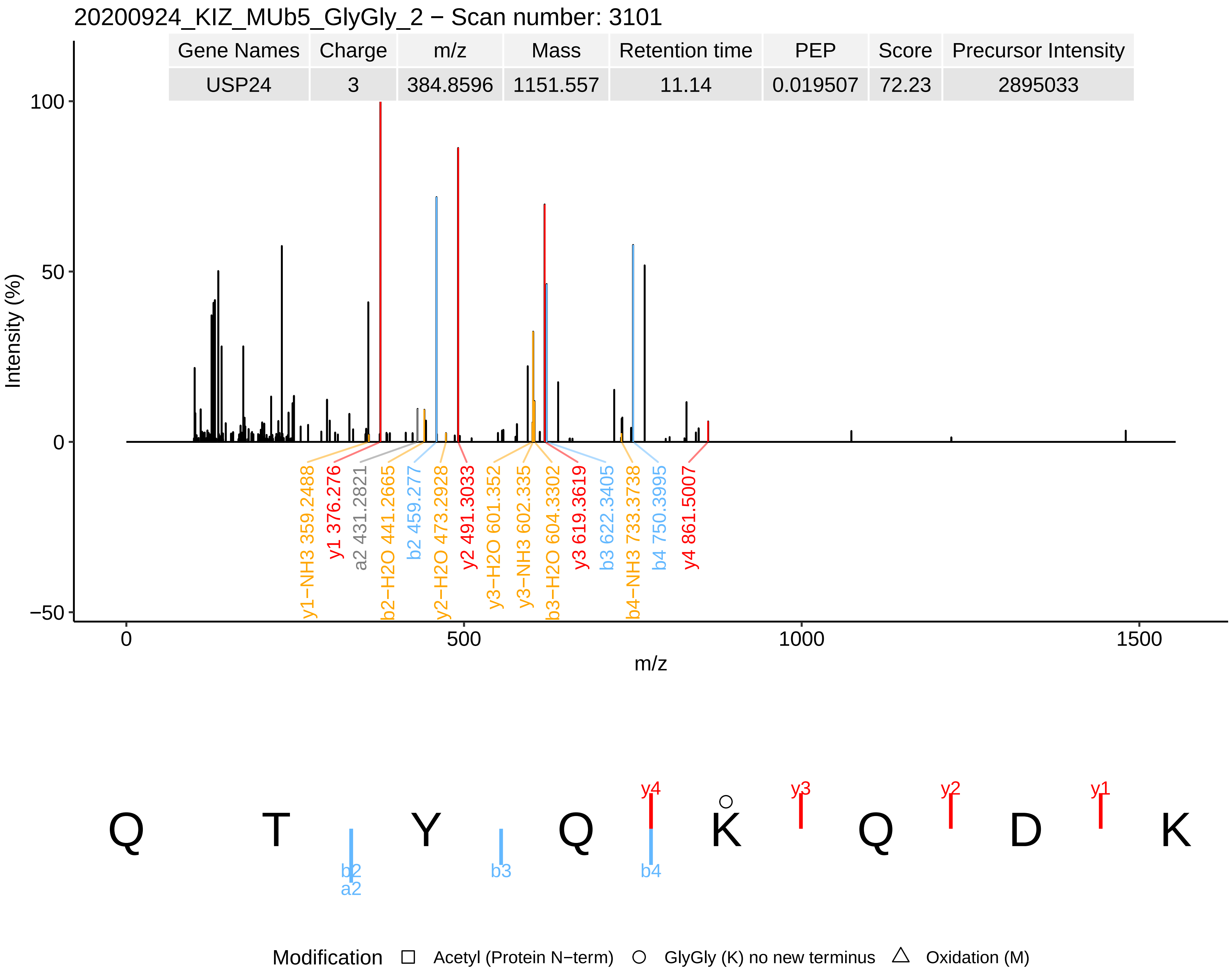

20200924_KIZ_MUb5_GlyGly_2 − Scan number: 3101
Gene Names | Charge | m/z | Mass | Retention time | PEP | Score | Precursor Intensity
USP24 | 3 | 384.8596 | 1151.557 | 11.14 | 0.019507 | 72.23 | 2895033
Intensity (%)
m/z
y1−NH3 359.2488
y1 376.276
a2 431.2821
b2−H2O 441.2665
b2 459.277
y2−H2O 473.2928
y2 491.3033
y3−H2O 601.352
y3−NH3 602.335
b3−H2O 604.3302
y3 619.3619
b3 622.3405
b4−NH3 733.3738
b4 750.3995
y4 861.5007
Q T Y Q K Q D K
y4 y3 y2 y1
b2 a2 b3 b4
Modification
Acetyl (Protein N−term)
GlyGly (K) no new terminus
Oxidation (M)

20201118_KIZ_MUb6_GlyGly_1 − Scan number: 3221

Gene Names	Charge	m/z	Mass	Retention time	PEP	Score	Precursor Intensity
USP24	3	384.8596	1151.557	9.9162	0.0072938	85.161	24000736

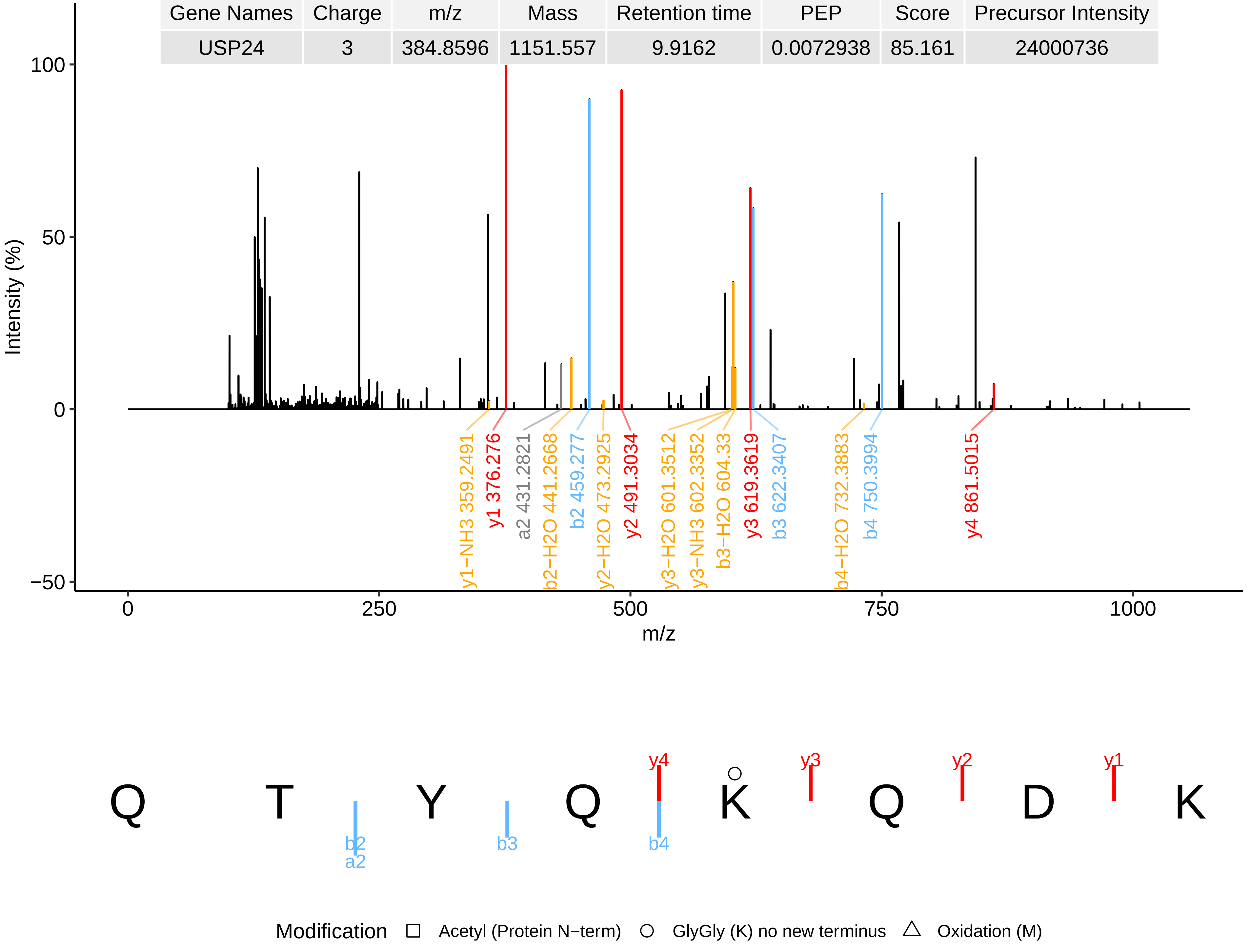

20201118_KIZ_MUb6_GlyGly_2 − Scan number: 3600

Gene Names	Charge	m/z	Mass	Retention time	PEP	Score	Precursor Intensity
USP24	3	384.8596	1151.557	11.991	0.022519	69.812	2968352

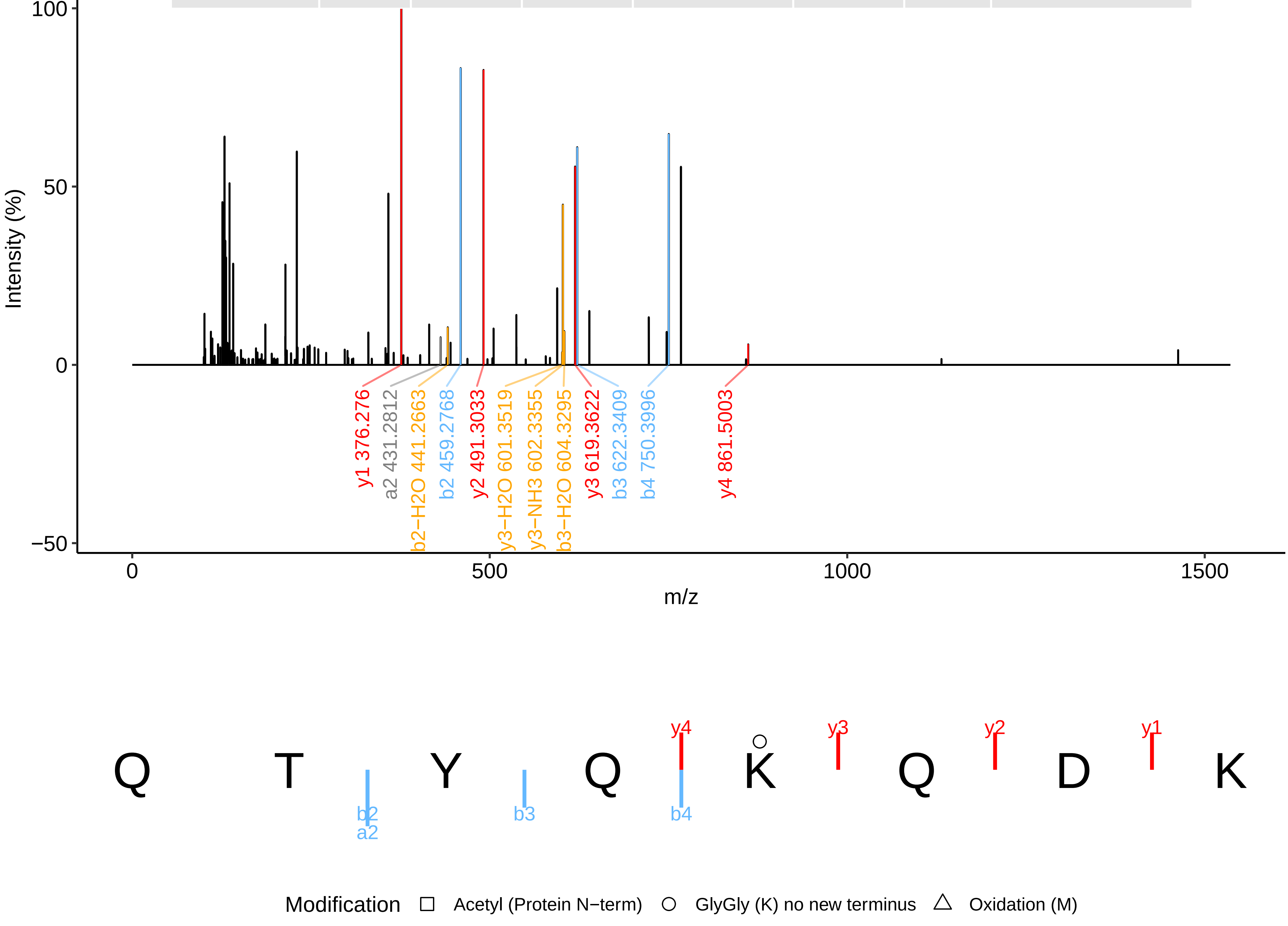

20200921_KIZ_MUb4_GlyGly_10plex − Scan number: 6194

Gene Names	Charge	m/z	Mass	Retention time	PEP	Score	Precursor Intensity
VIM	3	467.9075	1400.701	20.408	0.0014285	88.24	4458909

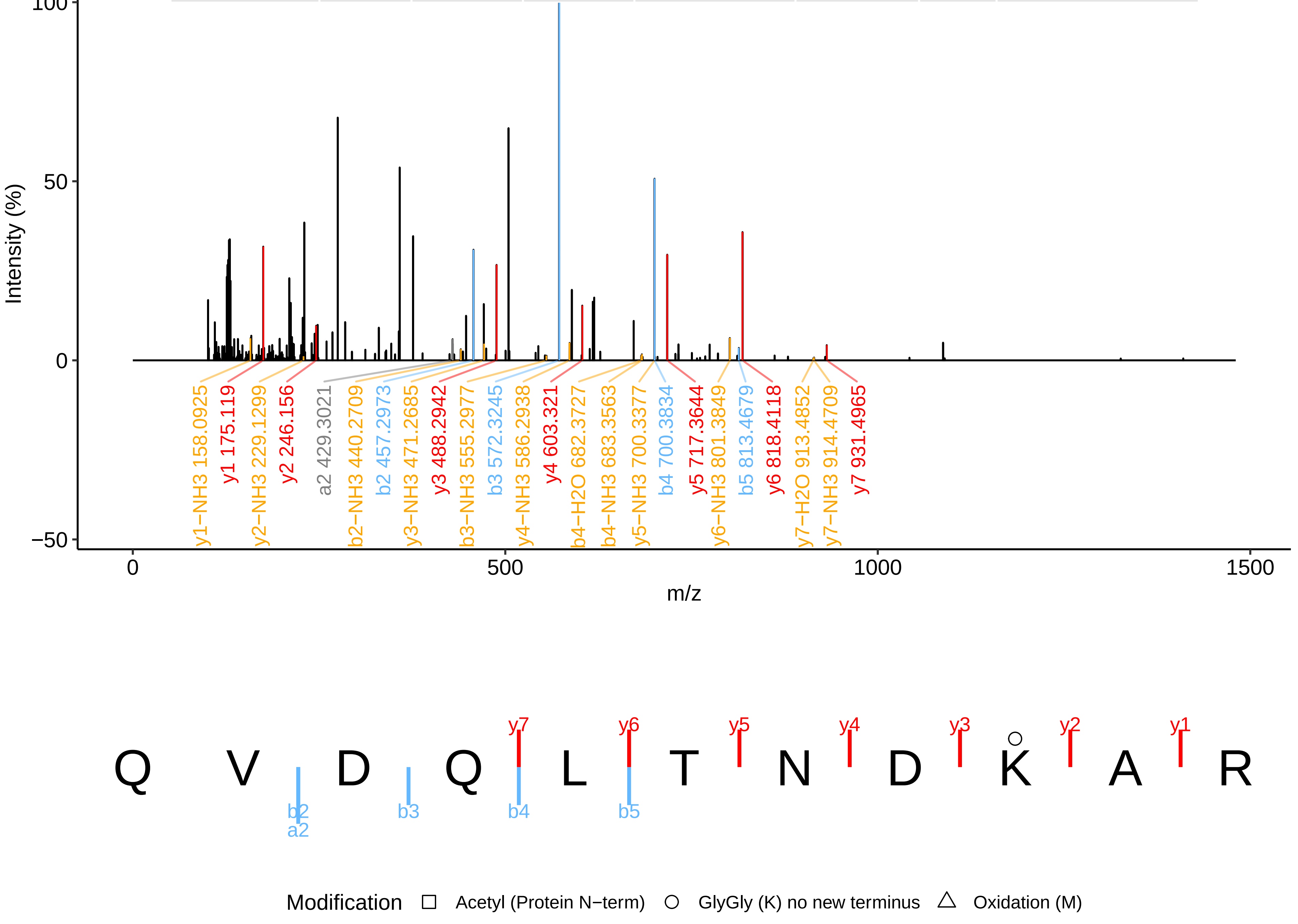

20200921_KIZ_MUb4_GlyGly_10plex_2 − Scan number: 5629

Gene Names	Charge	m/z	Mass	Retention time	PEP	Score	Precursor Intensity
VIM	3	467.9075	1400.701	20.332	0.0032844	78.692	526758.8

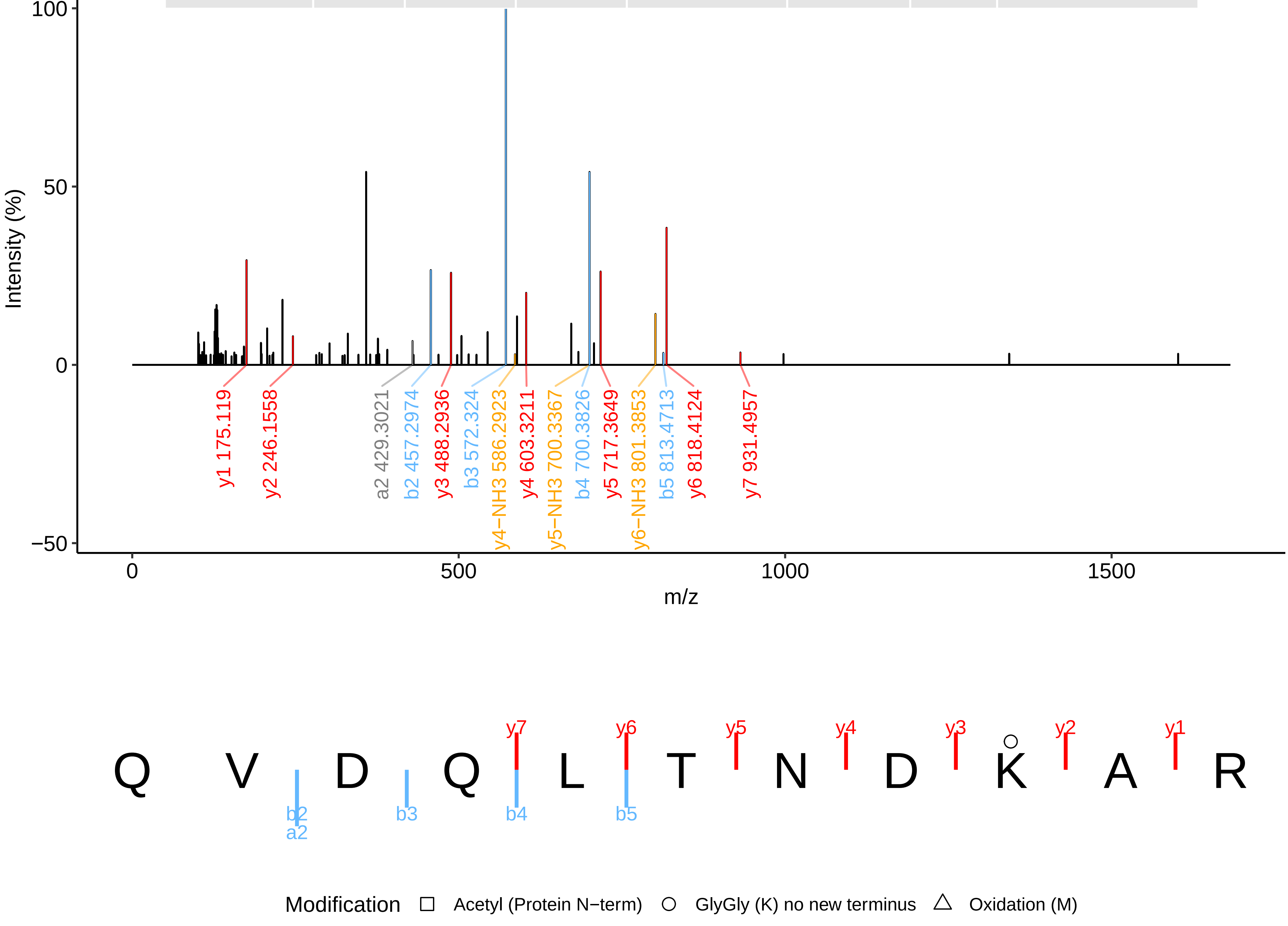

20200924_KIZ_MUb5_GlyGly_1 − Scan number: 5319

Gene Names	Charge	m/z	Mass	Retention time	PEP	Score	Precursor Intensity
VIM	3	467.9075	1400.701	16.517	0.13001	47.062	NA

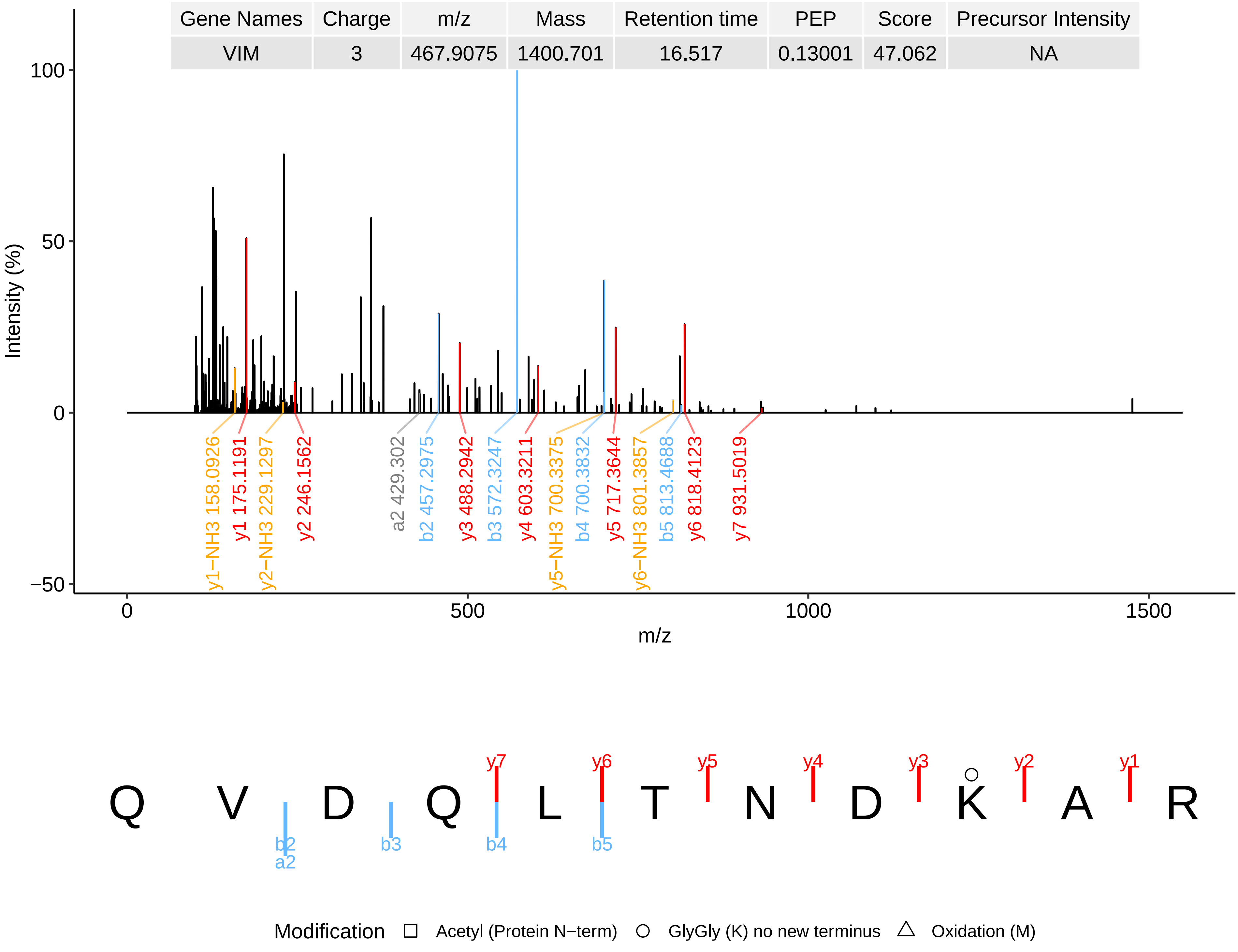

20200921_KIZ_MUb4_GlyGly_10plex − Scan number: 6467

Gene Names	Charge	m/z	Mass	Retention time	PEP	Score	Precursor Intensity
PACSIN3	4	294.1701	1172.651	21.411	0.012314	90.108	6217840

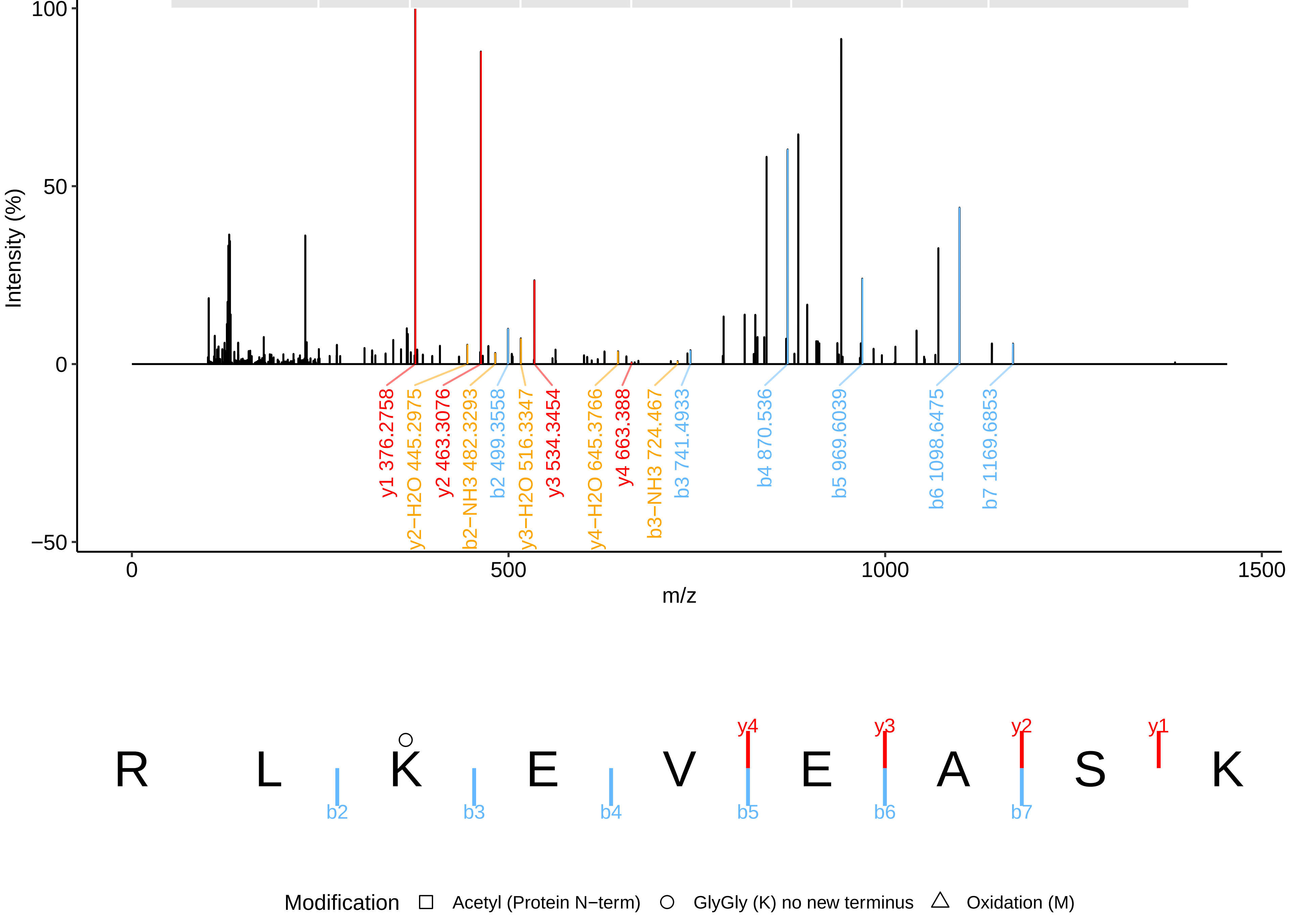

20200921_KIZ_MUb4_GlyGly_10plex − Scan number: 27536

Gene Names	Charge	m/z	Mass	Retention time	PEP	Score	Precursor Intensity
OGT	3	457.5965	1369.768	100.52	1.1808e−08	118.48	7376632

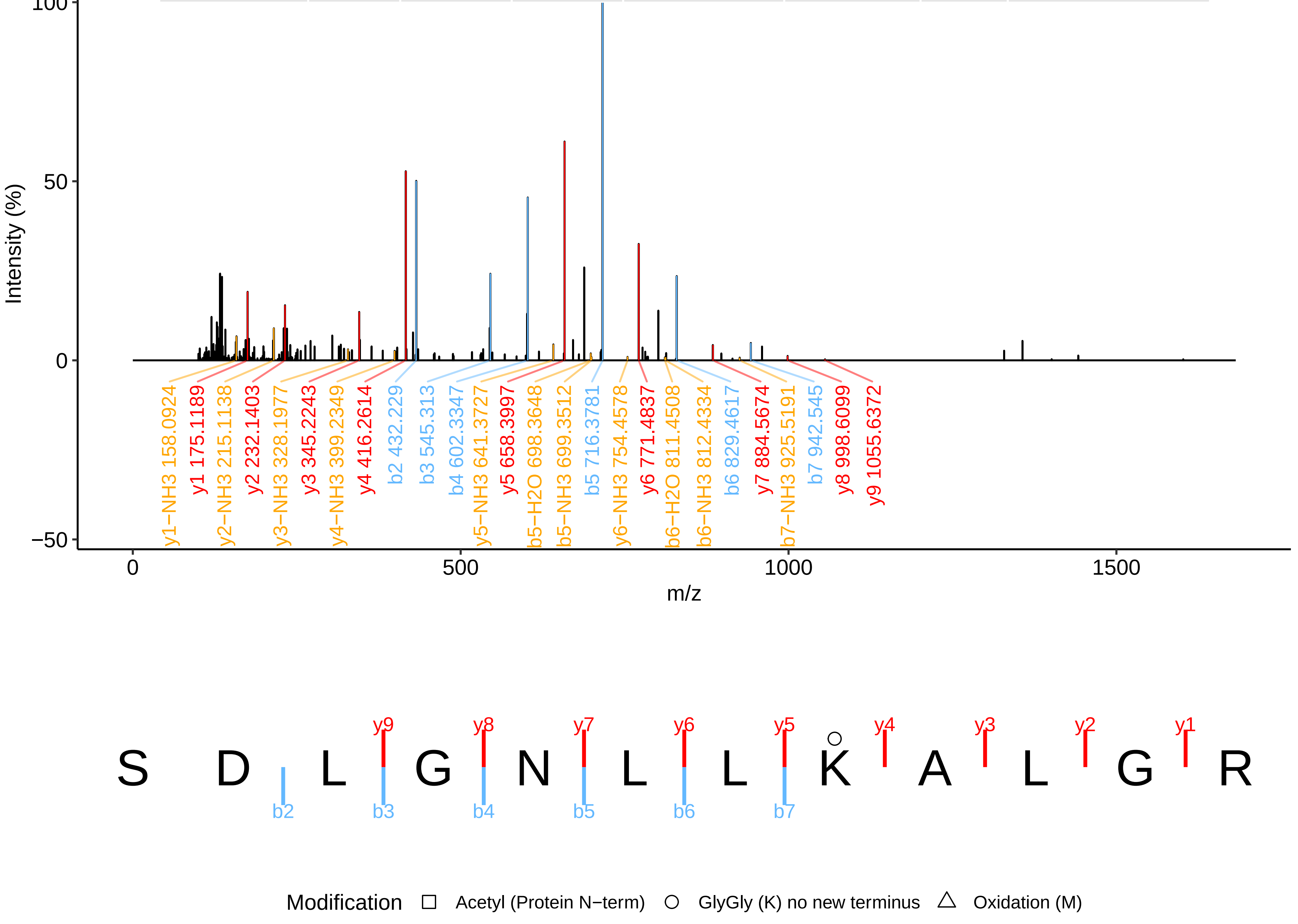

20200921_KIZ_MUb4_GlyGly_10plex_2 − Scan number: 23748

Gene Names	Charge	m/z	Mass	Retention time	PEP	Score	Precursor Intensity
OGT	3	457.5965	1369.768	93.76	7.4711e−05	97.214	1178406

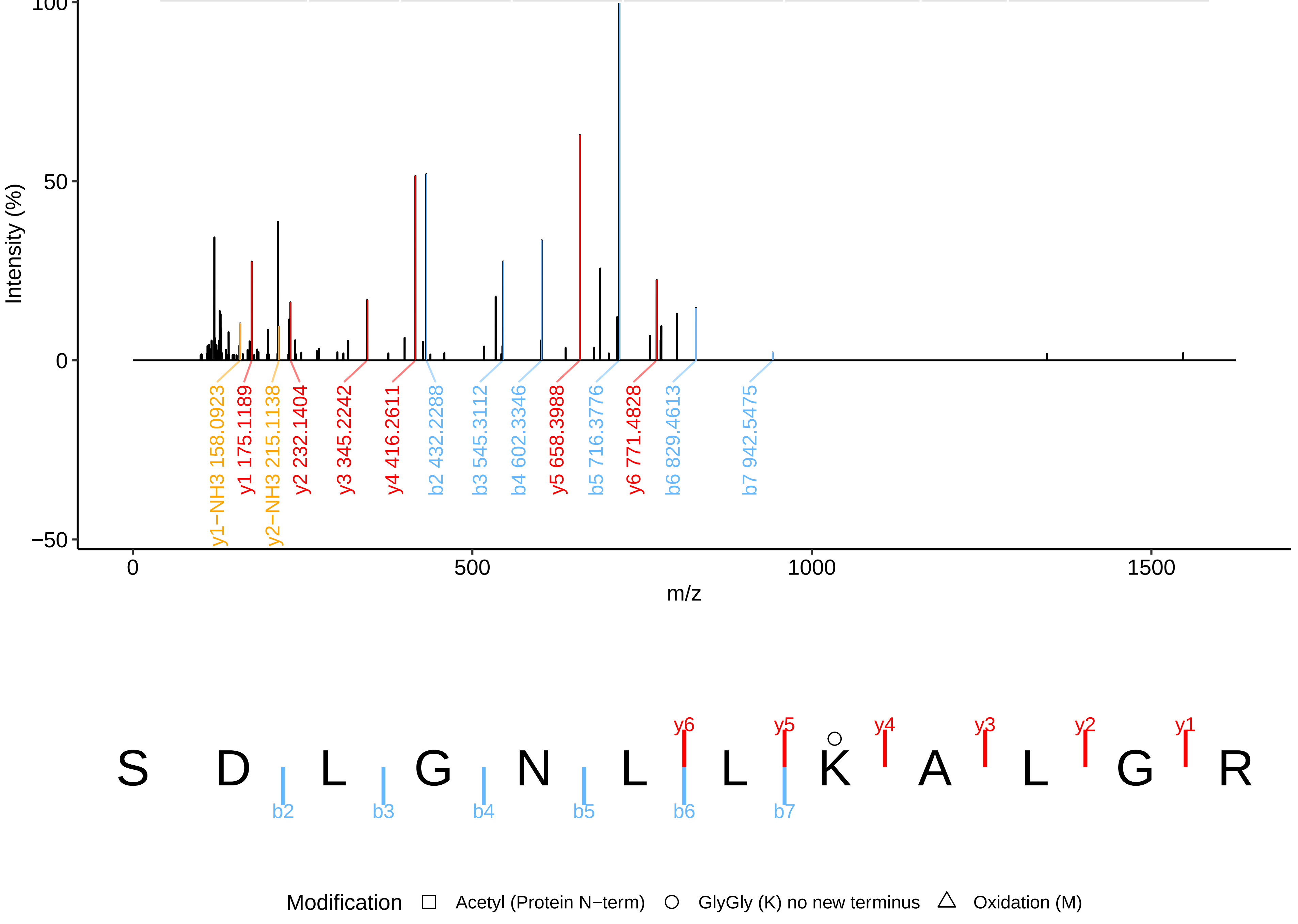

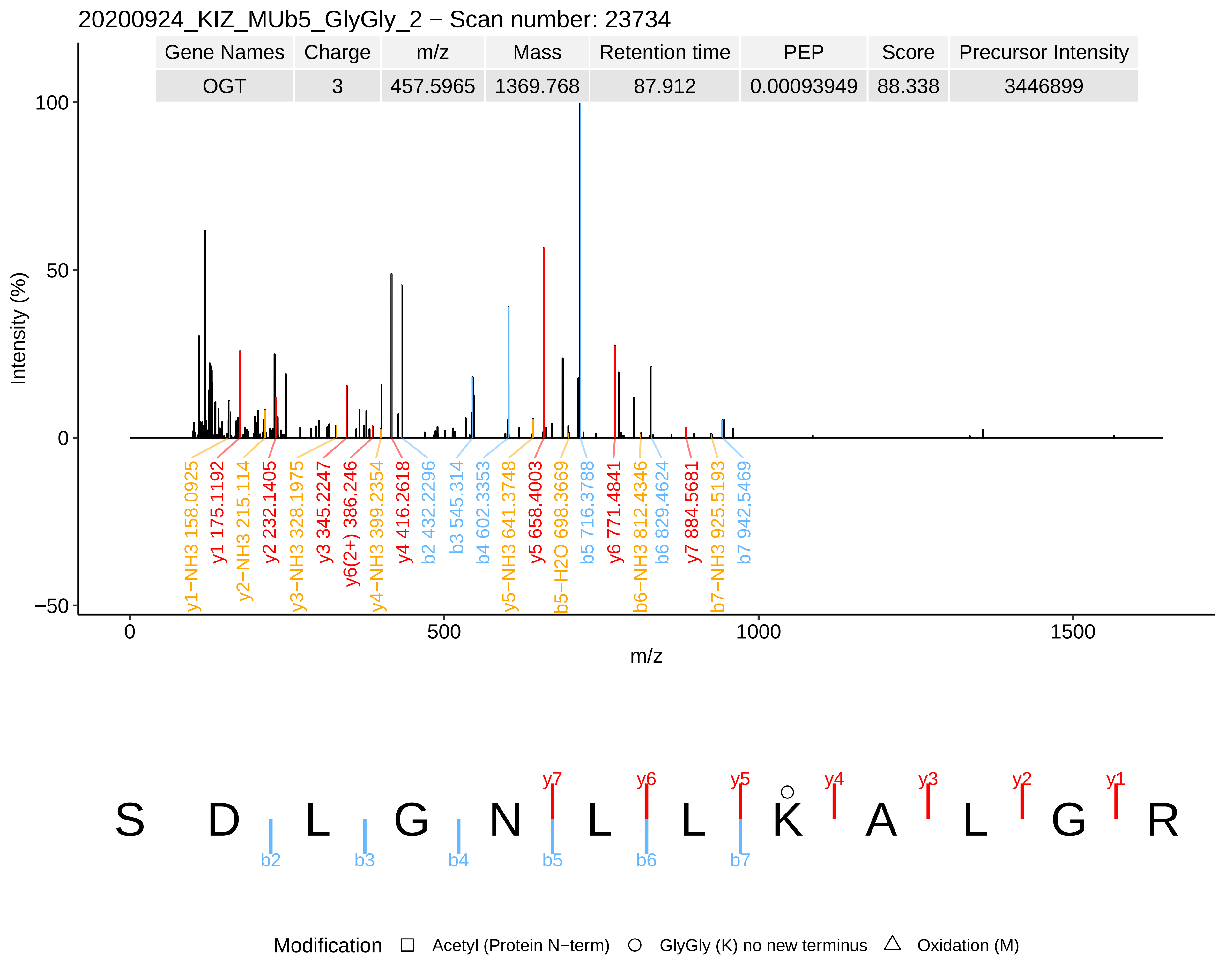
20200924_KIZ_MUb5_GlyGly_2 − Scan number: 23734
Gene Names | Charge | m/z | Mass | Retention time | PEP | Score | Precursor Intensity
OGT | 3 | 457.5965 | 1369.768 | 87.912 | 0.00093949 | 88.338 | 3446899
Intensity (%)
100
50
0
−50
0
500
1000
1500
m/z
y1−NH3 158.0925
y1 175.1192
y2−NH3 215.114
y2 232.1405
y3−NH3 328.1975
y3 345.2247
y6(2+) 386.246
y4−NH3 399.2354
y4 416.2618
b2 432.2296
b3 545.314
b4 602.3353
y5−NH3 641.3748
y5 658.4003
b5−H2O 698.3669
b5 716.3788
y6 771.4841
b6−NH3 812.4346
b6 829.4624
y7 884.5681
b7−NH3 925.5193
b7 942.5469
S D L G N L L K A L G R
y7 y6 y5 y4 y3 y2 y1
b2 b3 b4 b5 b6 b7
Modification
Acetyl (Protein N−term)
GlyGly (K) no new terminus
Oxidation (M)

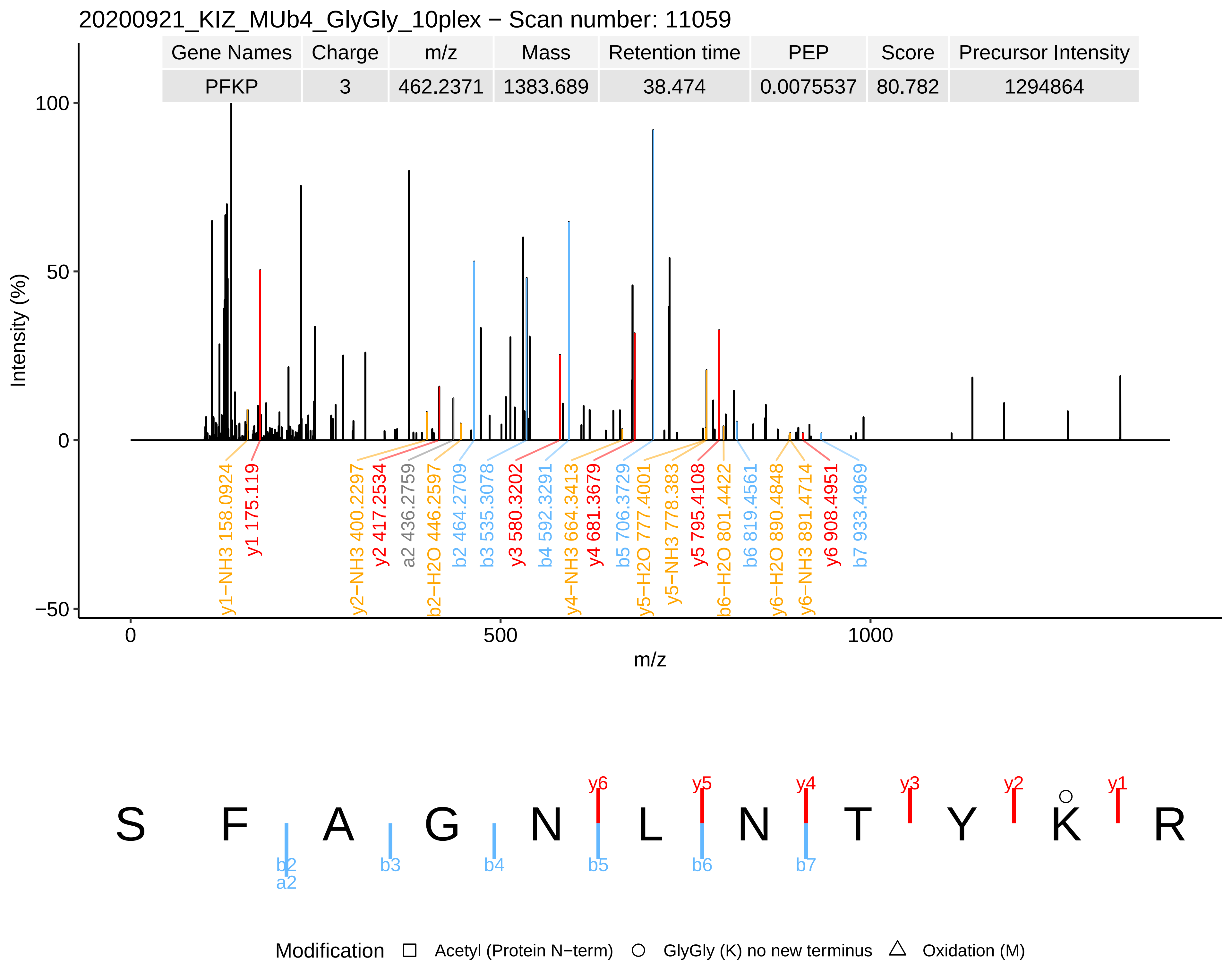

20200921_KIZ_MUb4_GlyGly_10plex − Scan number: 11059
Gene Names | Charge | m/z | Mass | Retention time | PEP | Score | Precursor Intensity
PFKP | 3 | 462.2371 | 1383.689 | 38.474 | 0.0075537 | 80.782 | 1294864
Intensity (%)
m/z
y1−NH3 158.0924
y1 175.119
y2−NH3 400.2297
y2 417.2534
a2 436.2759
b2−H2O 446.2597
b2 464.2709
b3 535.3078
y3 580.3202
b4 592.3291
y4−NH3 664.3413
y4 681.3679
b5 706.3729
y5−H2O 777.4001
y5−NH3 778.383
y5 795.4108
b6−H2O 801.4422
b6 819.4561
y6−H2O 890.4848
y6−NH3 891.4714
y6 908.4951
b7 933.4969
S F A G N L N T Y K R
y6 y5 y4 y3 y2 y1
b2 a2 b3 b4 b5 b6 b7
Modification
Acetyl (Protein N−term)
GlyGly (K) no new terminus
Oxidation (M)

20200921_KIZ_MUb4_GlyGly_10plex_2 − Scan number: 10246

Gene Names	Charge	m/z	Mass	Retention time	PEP	Score	Precursor Intensity
PFKP	3	462.2371	1383.689	39.145	0.0057522	85.958	1208695

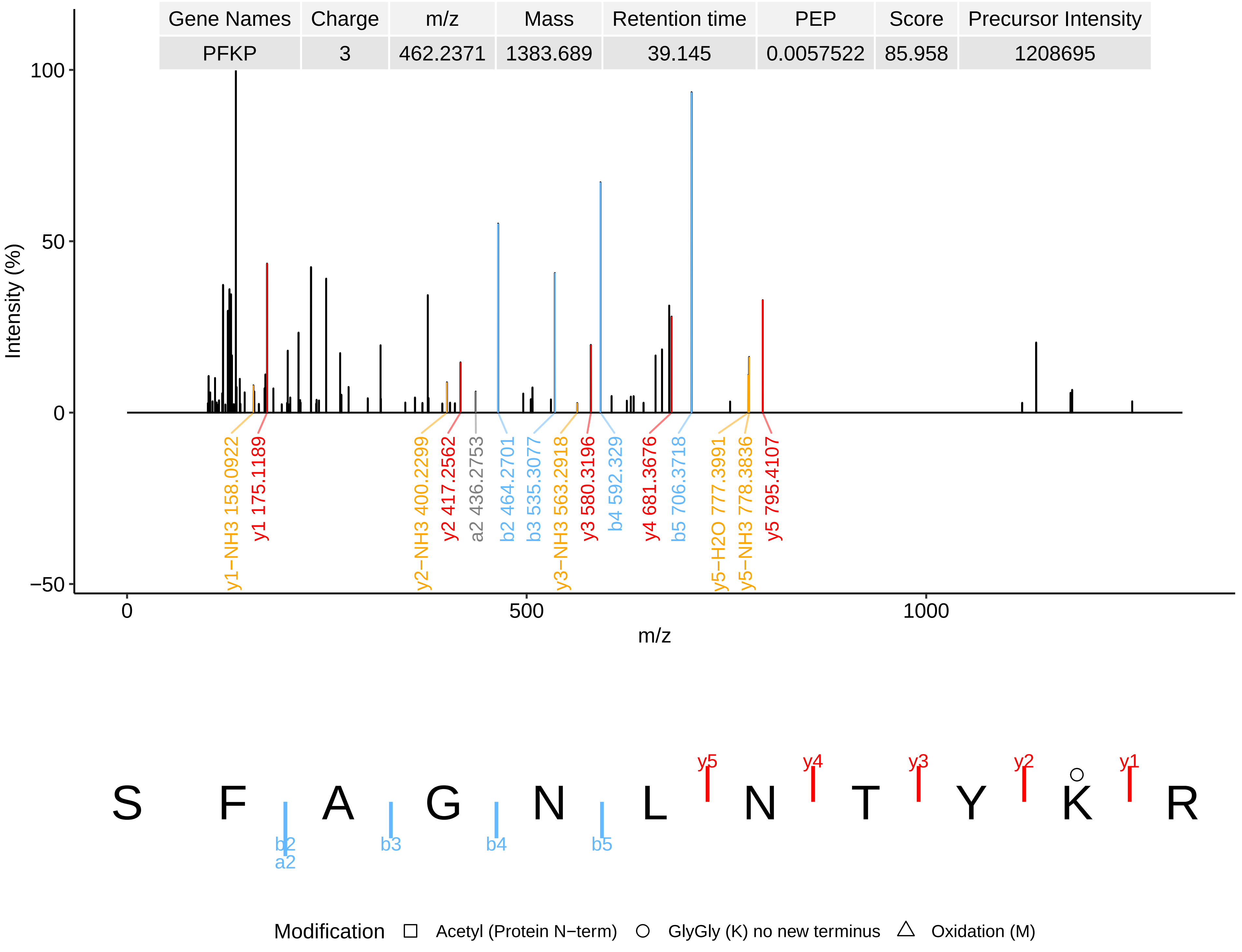

20200921_KIZ_MUb4_GlyGly_10plex − Scan number: 5291

Gene Names	Charge	m/z	Mass	Retention time	PEP	Score	Precursor Intensity
RAB13	3	458.9039	1373.69	17.304	0.010971	66.004	4348347

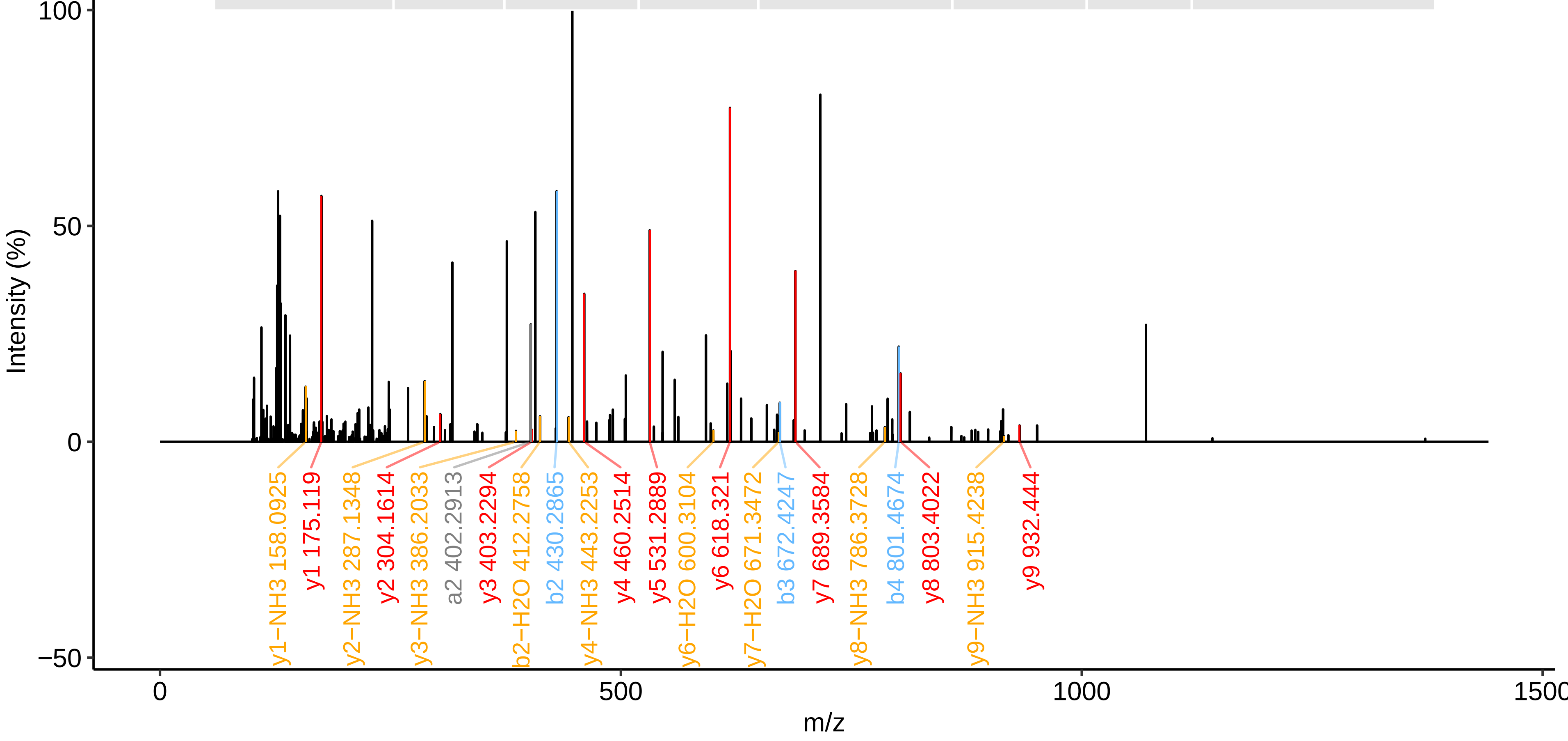

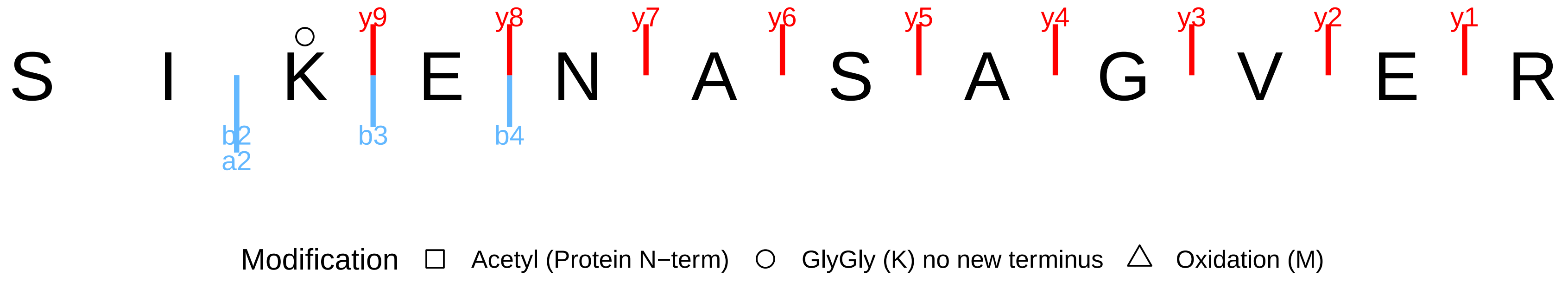

20200921_KIZ_MUb4_GlyGly_10plex − Scan number: 7399

Gene Names	Charge	m/z	Mass	Retention time	PEP	Score	Precursor Intensity
DNAJB6	3	465.5734	1393.698	24.825	0.00071715	90.653	NA

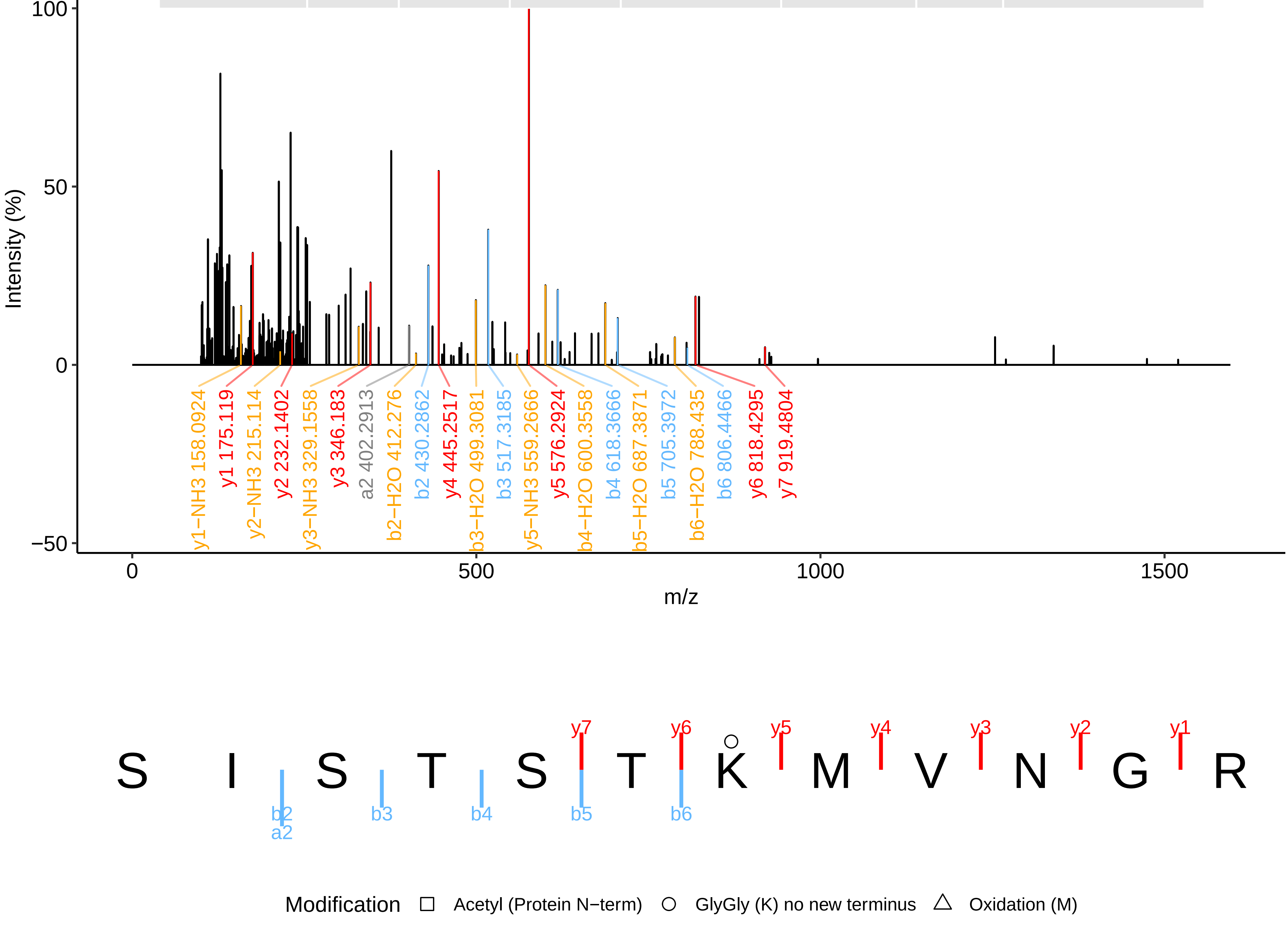

20200921_KIZ_MUb4_GlyGly_10plex_2 − Scan number: 6784

Gene Names	Charge	m/z	Mass	Retention time	PEP	Score	Precursor Intensity
DNAJB6	3	465.5734	1393.698	25.085	5.0508e−05	97.813	722528.2

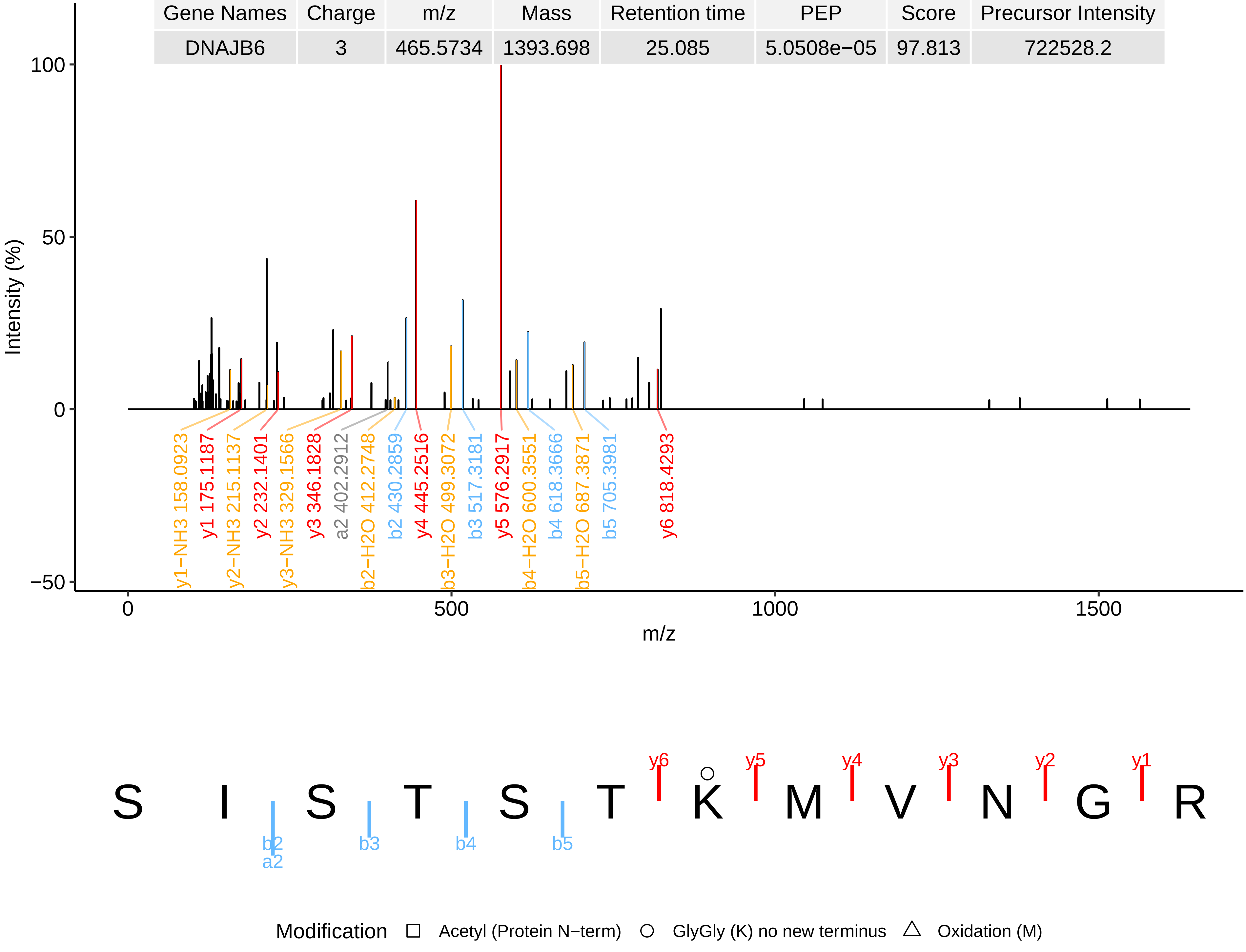

20200921_KIZ_MUb4_GlyGly_10plex − Scan number: 11418

Gene Names	Charge	m/z	Mass	Retention time	PEP	Score	Precursor Intensity
AHCY	3	458.5471	1372.619	39.773	0.023156	63.073	1274276

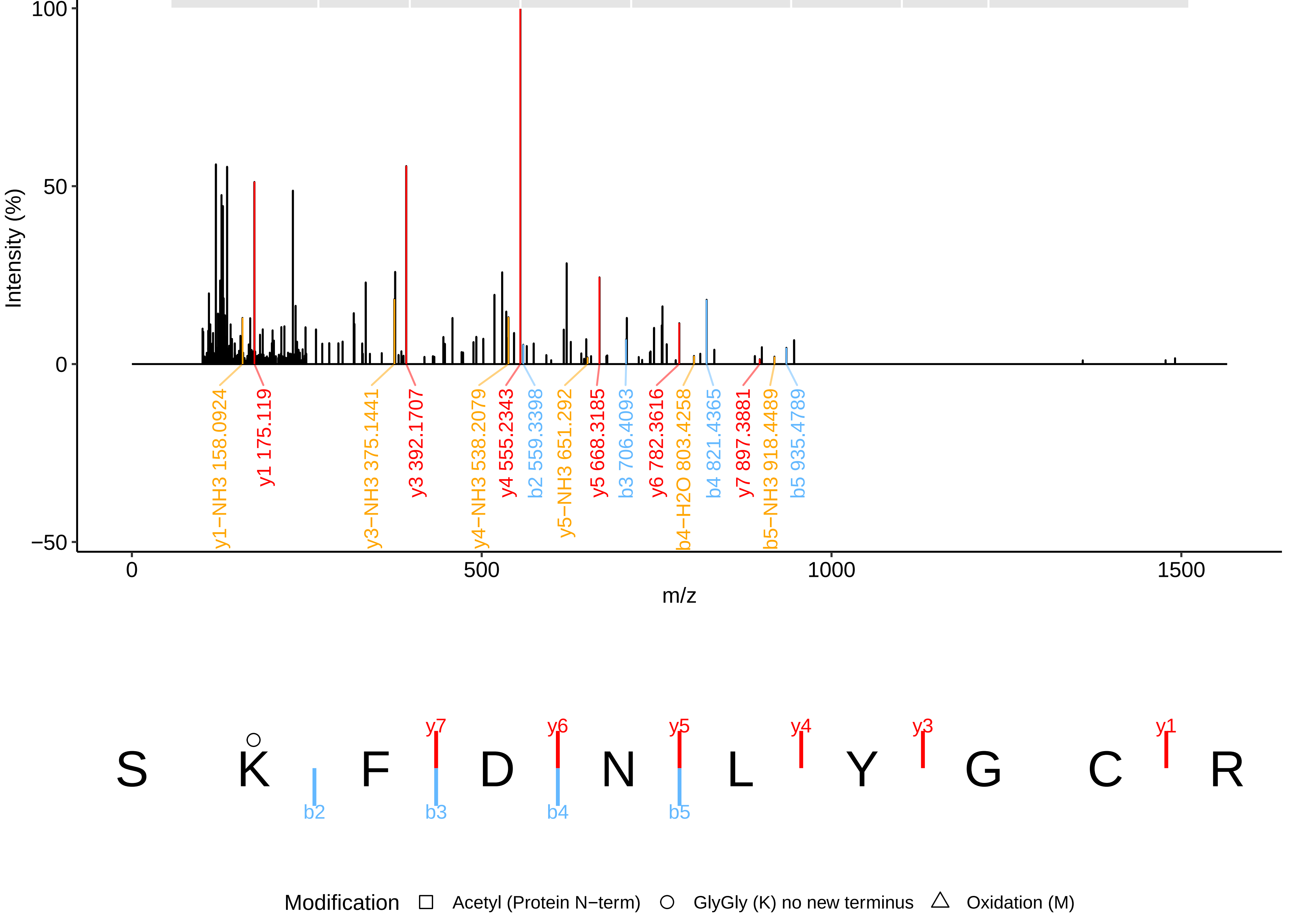

20200921_KIZ_MUb4_GlyGly_10plex − Scan number: 14788

Gene Names	Charge	m/z	Mass	Retention time	PEP	Score	Precursor Intensity
ATP1A1;ATP1A3;ATP1A4	3	390.8656	1169.575	52.639	0.10959	51.445	2696630

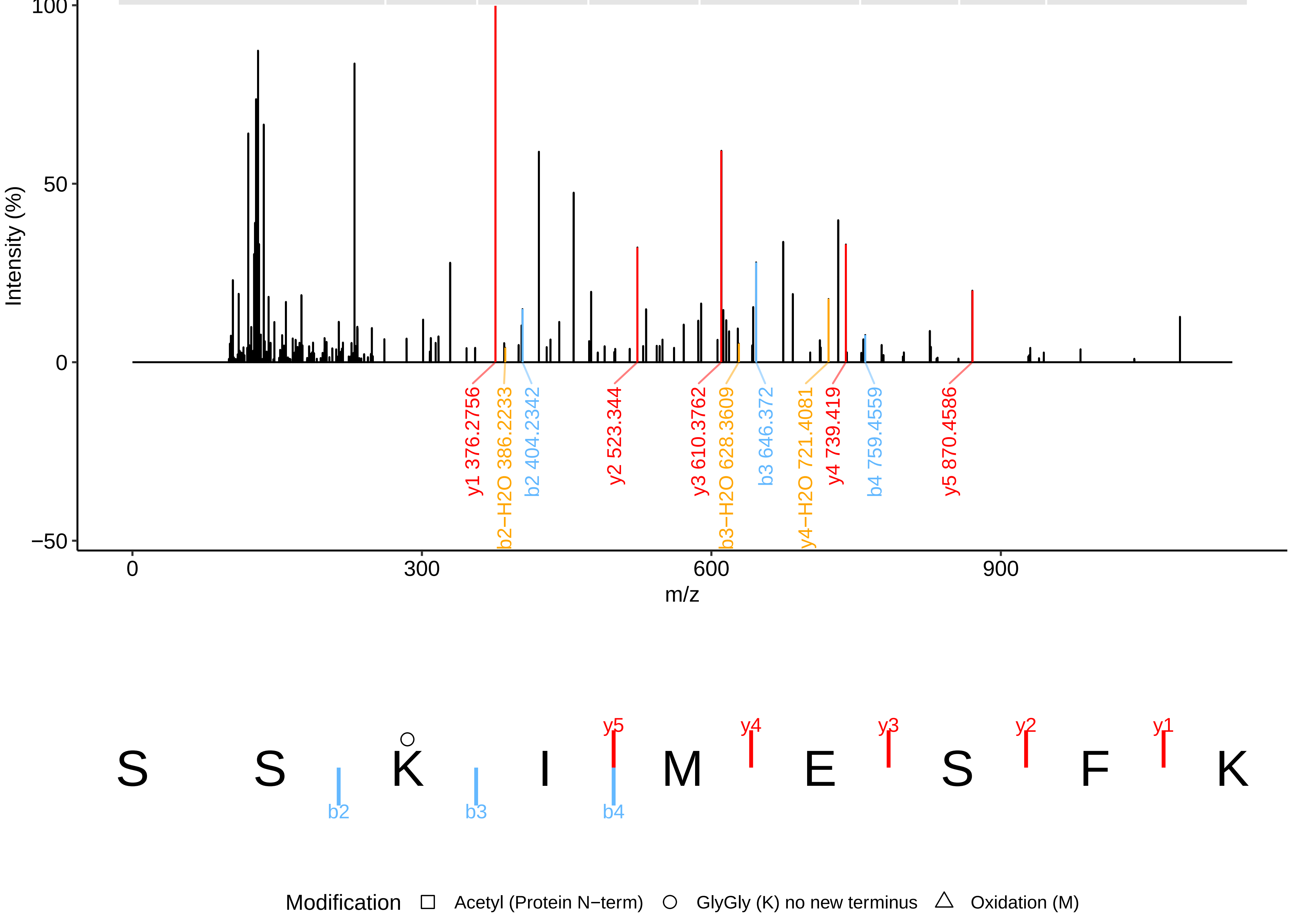

20200921_KIZ_MUb4_GlyGly_10plex − Scan number: 4270

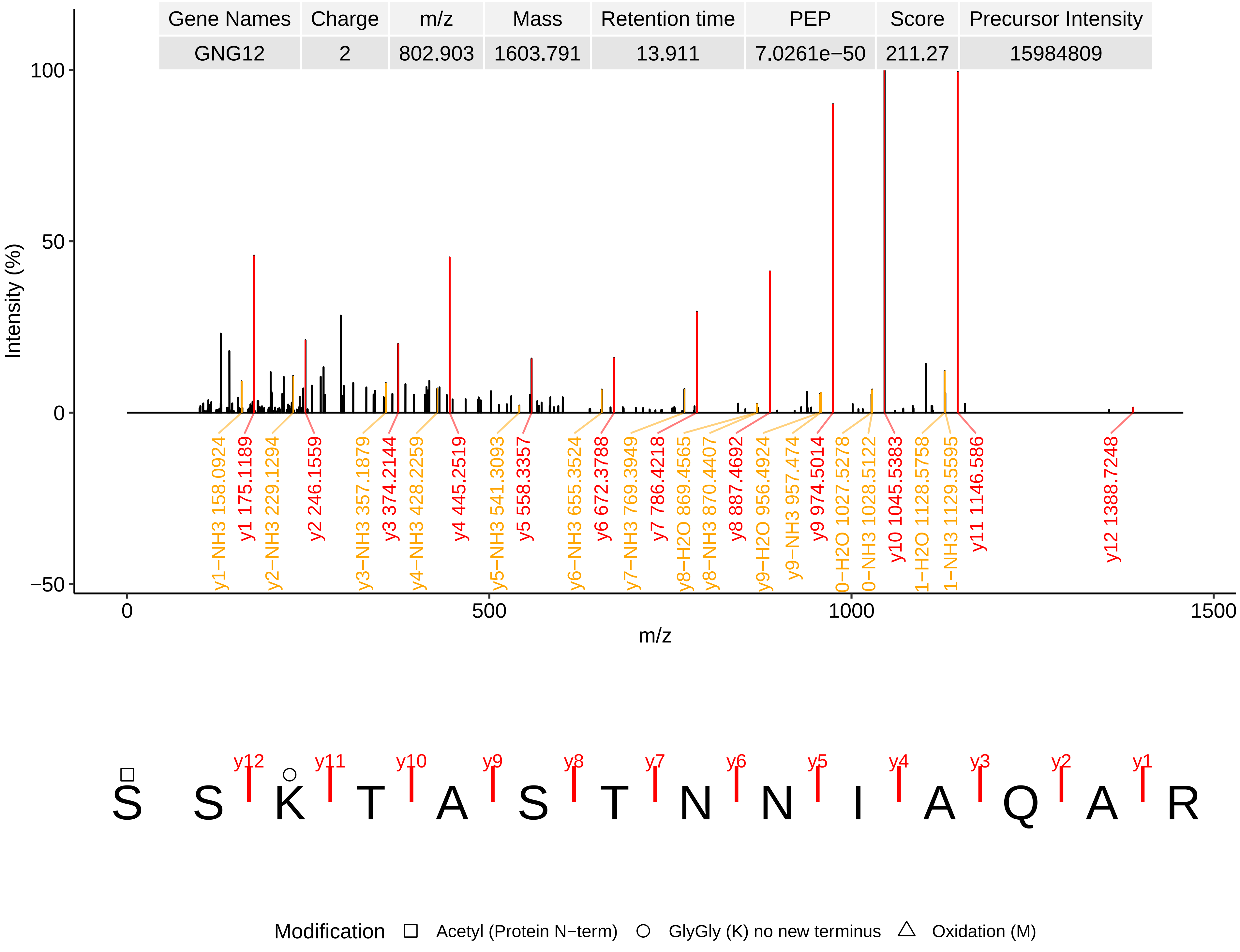

20200921_KIZ_MUb4_GlyGly_10plex_2 − Scan number: 3932

Gene Names	Charge	m/z	Mass	Retention time	PEP	Score	Precursor Intensity
GNG12	2	802.903	1603.791	13.531	2.1866e−13	136.63	1742861

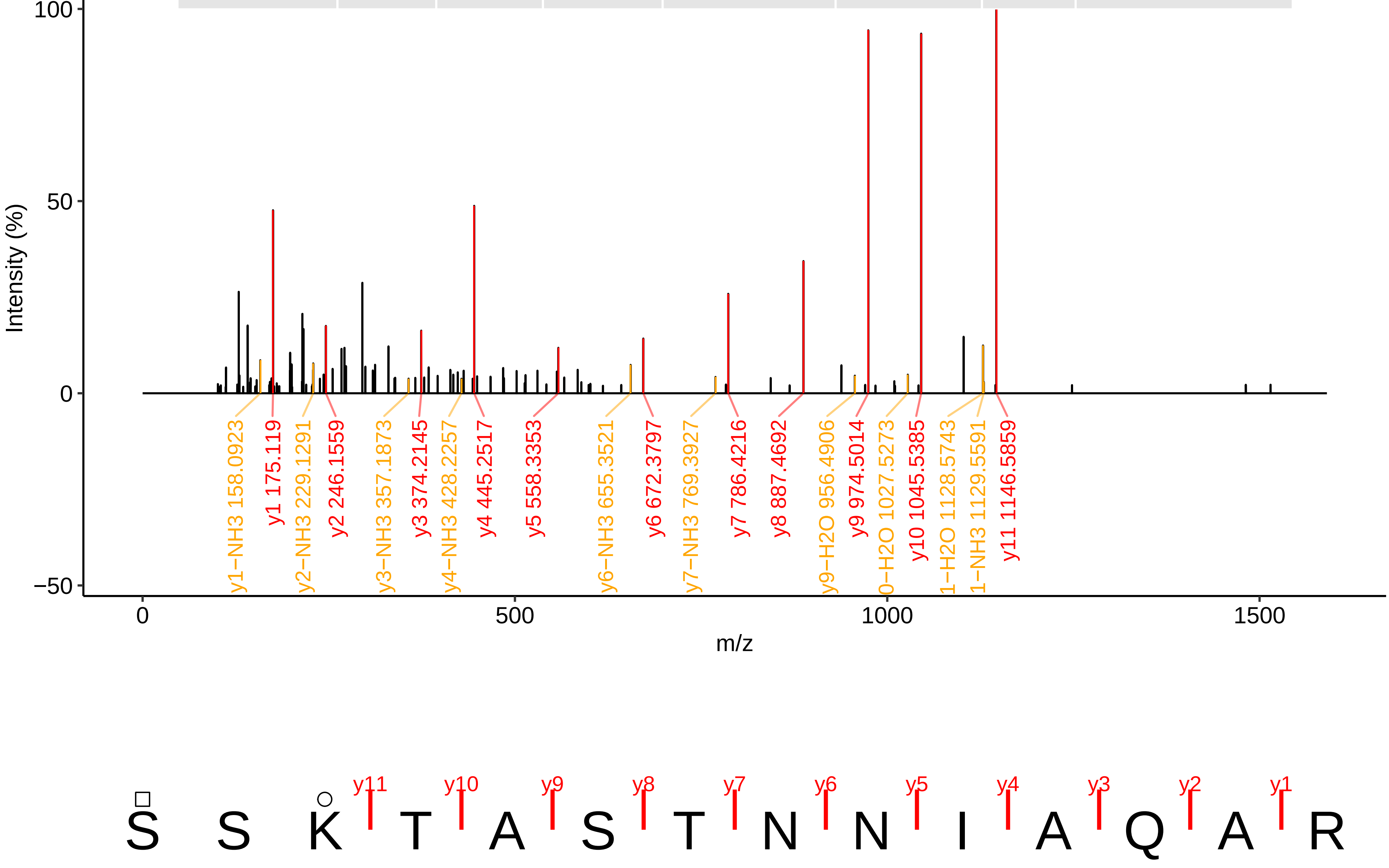

Modification □ Acetyl (Protein N−term) ○ GlyGly (K) no new terminus △ Oxidation (M)

20200924_KIZ_MUb5_GlyGly_1 − Scan number: 3673

Gene Names	Charge	m/z	Mass	Retention time	PEP	Score	Precursor Intensity
GNG12	2	802.903	1603.791	11.231	4.1072e−38	196.79	NA

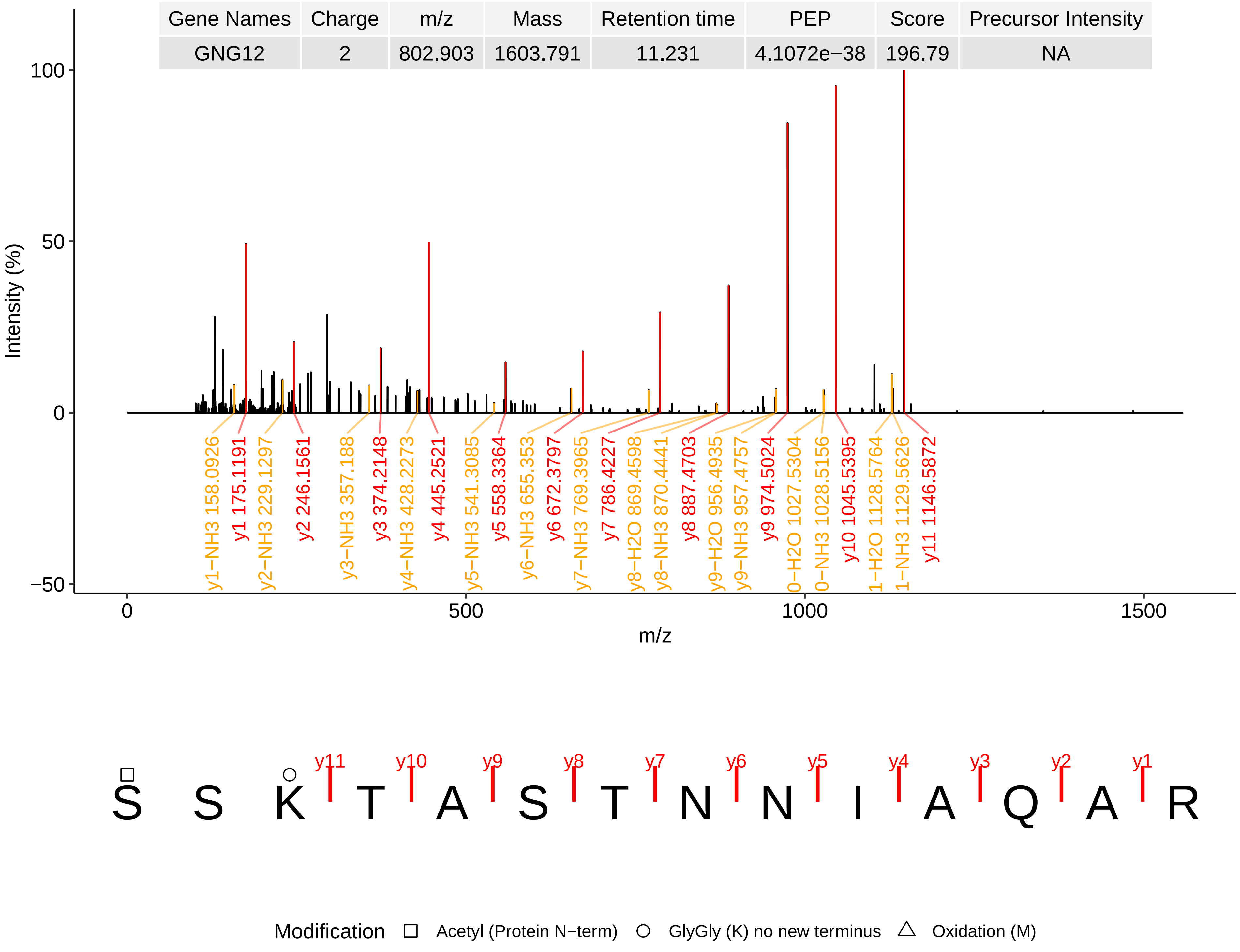

20200924_KIZ_MUb5_GlyGly_2 − Scan number: 3526

Gene Names	Charge	m/z	Mass	Retention time	PEP	Score	Precursor Intensity
GNG12	2	802.903	1603.791	12.798	5.403e−28	172.59	4000549

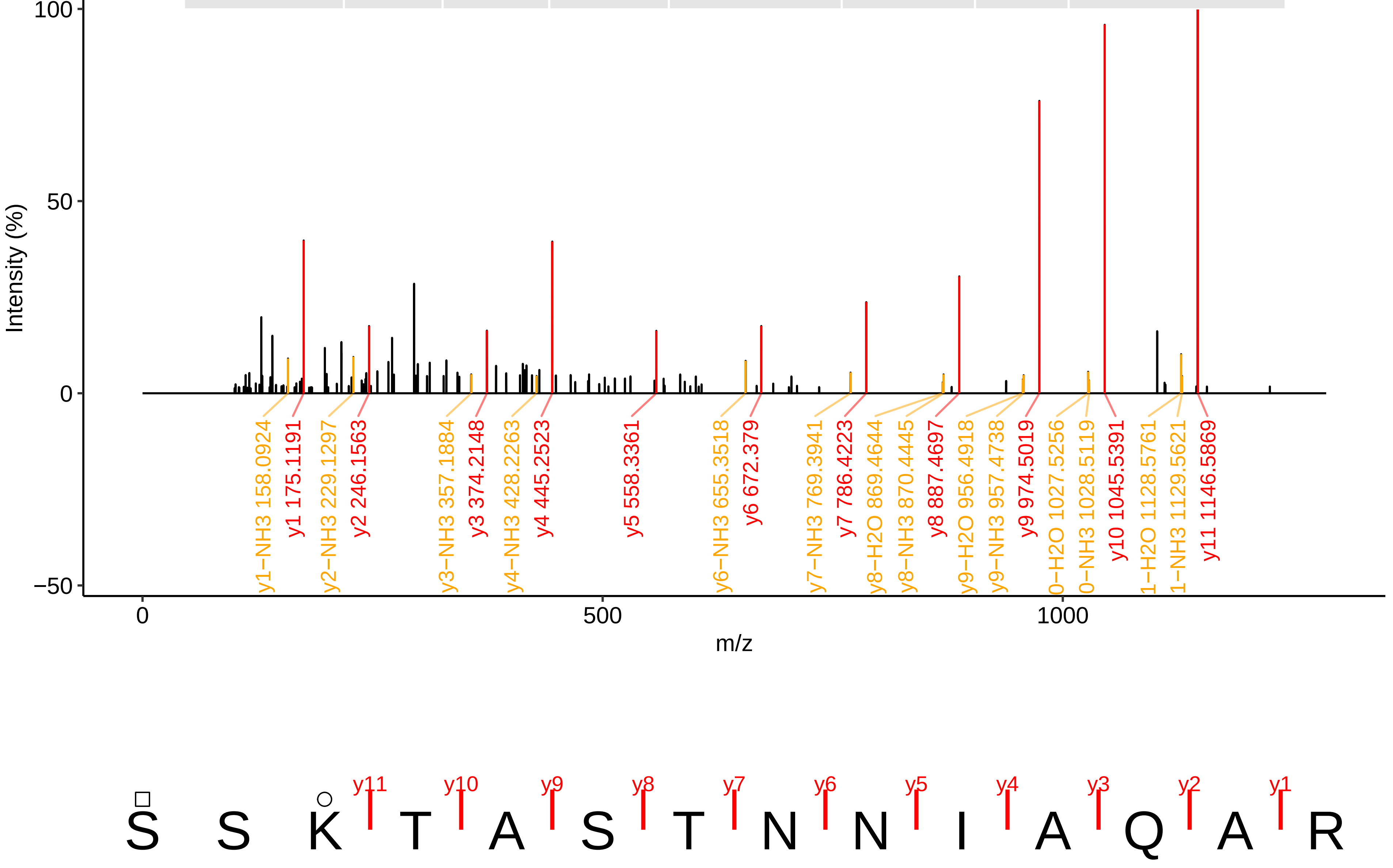

Modification □ Acetyl (Protein N−term) ○ GlyGly (K) no new terminus △ Oxidation (M)

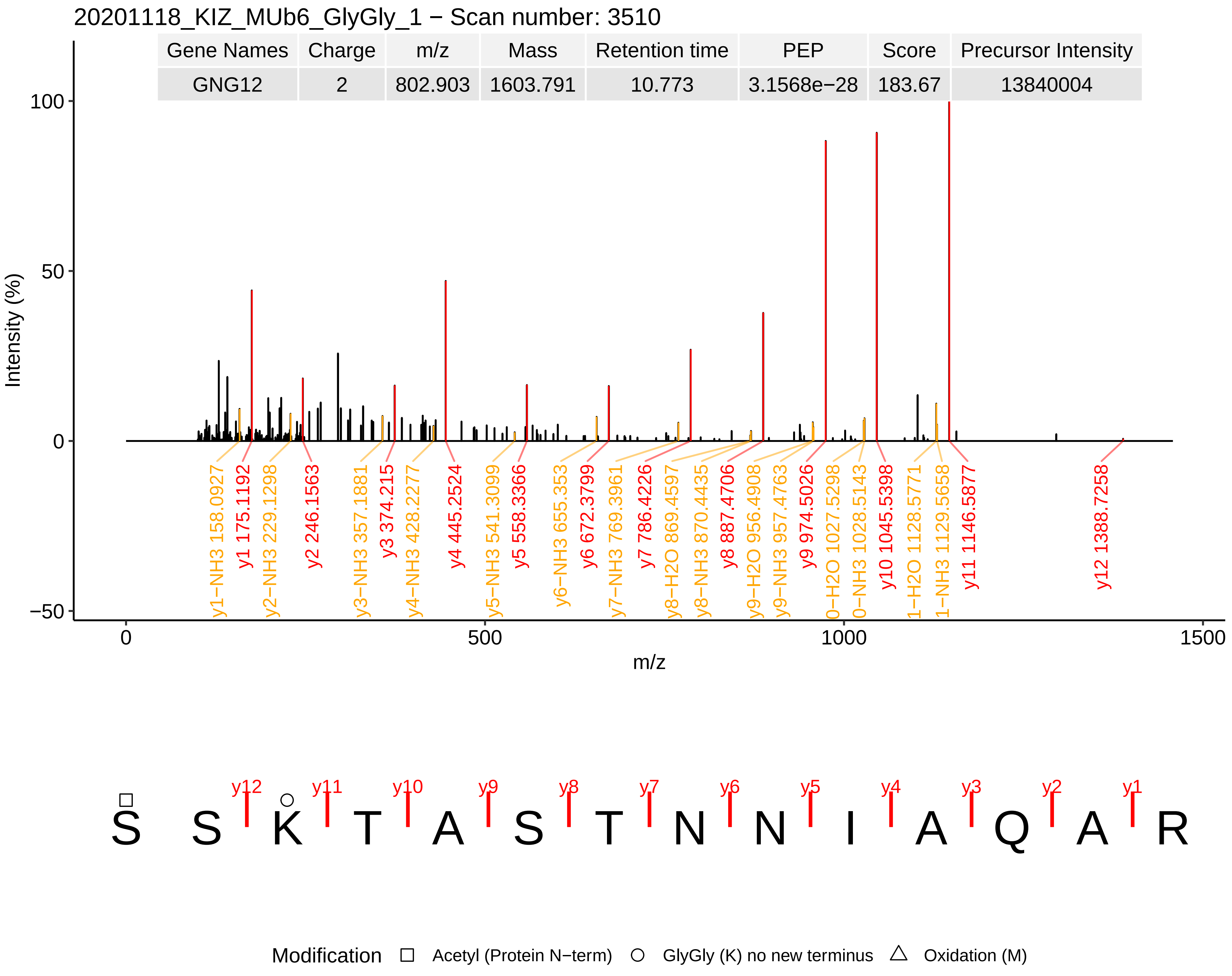
20201118_KIZ_MUb6_GlyGly_1 − Scan number: 3510
Gene Names
Charge
m/z
Mass
Retention time
PEP
Score
Precursor Intensity
GNG12
2
802.903
1603.791
10.773
3.1568e−28
183.67
13840004
Intensity (%)
m/z
y1−NH3 158.0927
y1 175.1192
y2−NH3 229.1298
y2 246.1563
y3−NH3 357.1881
y3 374.215
y4−NH3 428.2277
y4 445.2524
y5−NH3 541.3099
y5 558.3366
y6−NH3 655.353
y6 672.3799
y7−NH3 769.3961
y7 786.4226
y8−H2O 869.4597
y8−NH3 870.4435
y8 887.4706
y9−H2O 956.4908
y9−NH3 957.4763
y9 974.5026
0−H2O 1027.5298
0−NH3 1028.5143
y10 1045.5398
1−H2O 1128.5771
1−NH3 1129.5658
y11 1146.5877
y12 1388.7258
S S K T A S T N N I A Q A R
y12 y11 y10 y9 y8 y7 y6 y5 y4 y3 y2 y1
Modification
Acetyl (Protein N−term)
GlyGly (K) no new terminus
Oxidation (M)

20201118_KIZ_MUb6_GlyGly_2 − Scan number: 4051

Gene Names	Charge	m/z	Mass	Retention time	PEP	Score	Precursor Intensity
GNG12	2	802.903	1603.791	13.673	2.2796e−20	163.94	2084338

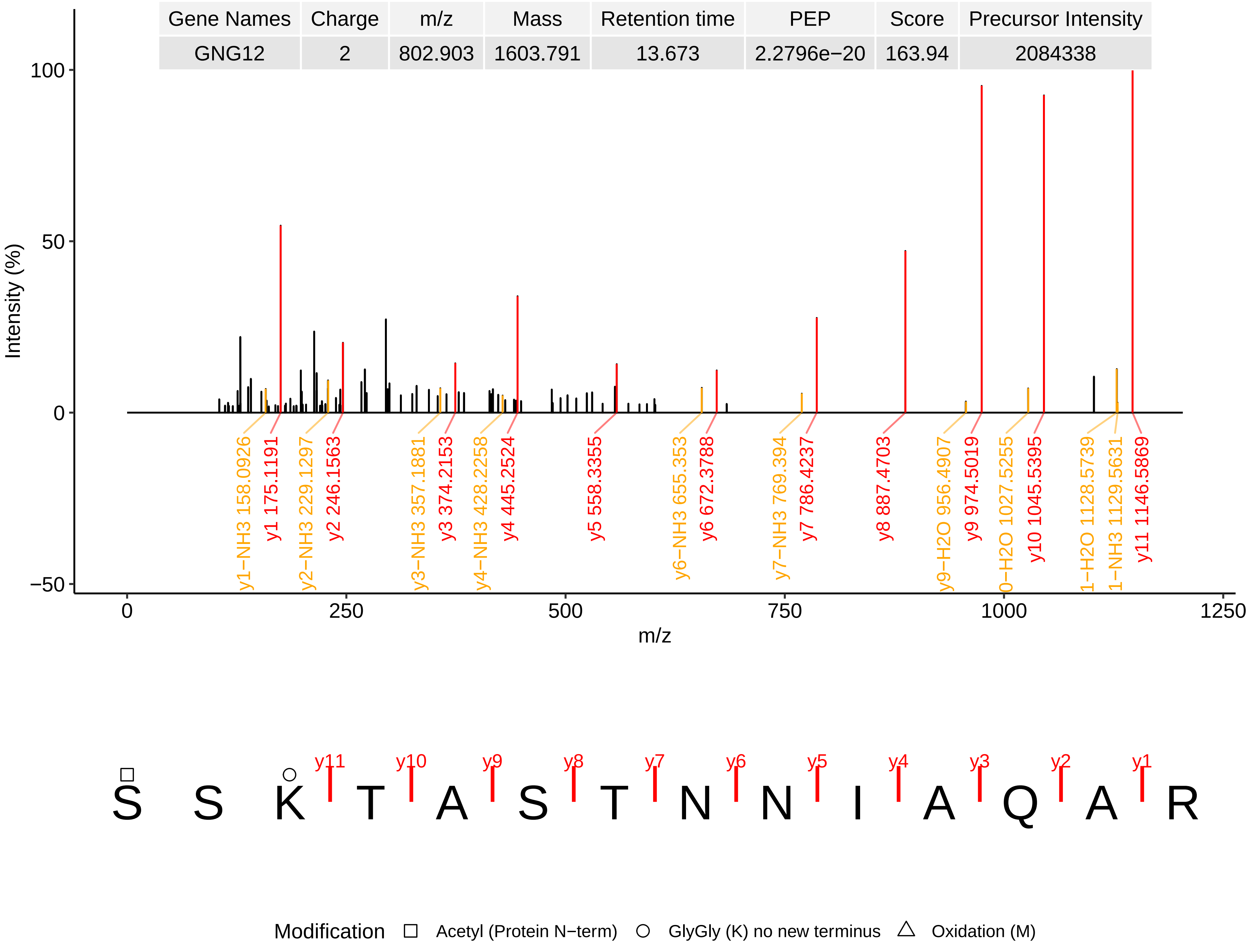

20200921_KIZ_MUb4_GlyGly_10plex_2 − Scan number: 6680

Gene Names	Charge	m/z	Mass	Retention time	PEP	Score	Precursor Intensity
YME1L1	3	387.5351	1159.583	24.66	0.038345	59.8	647058.7

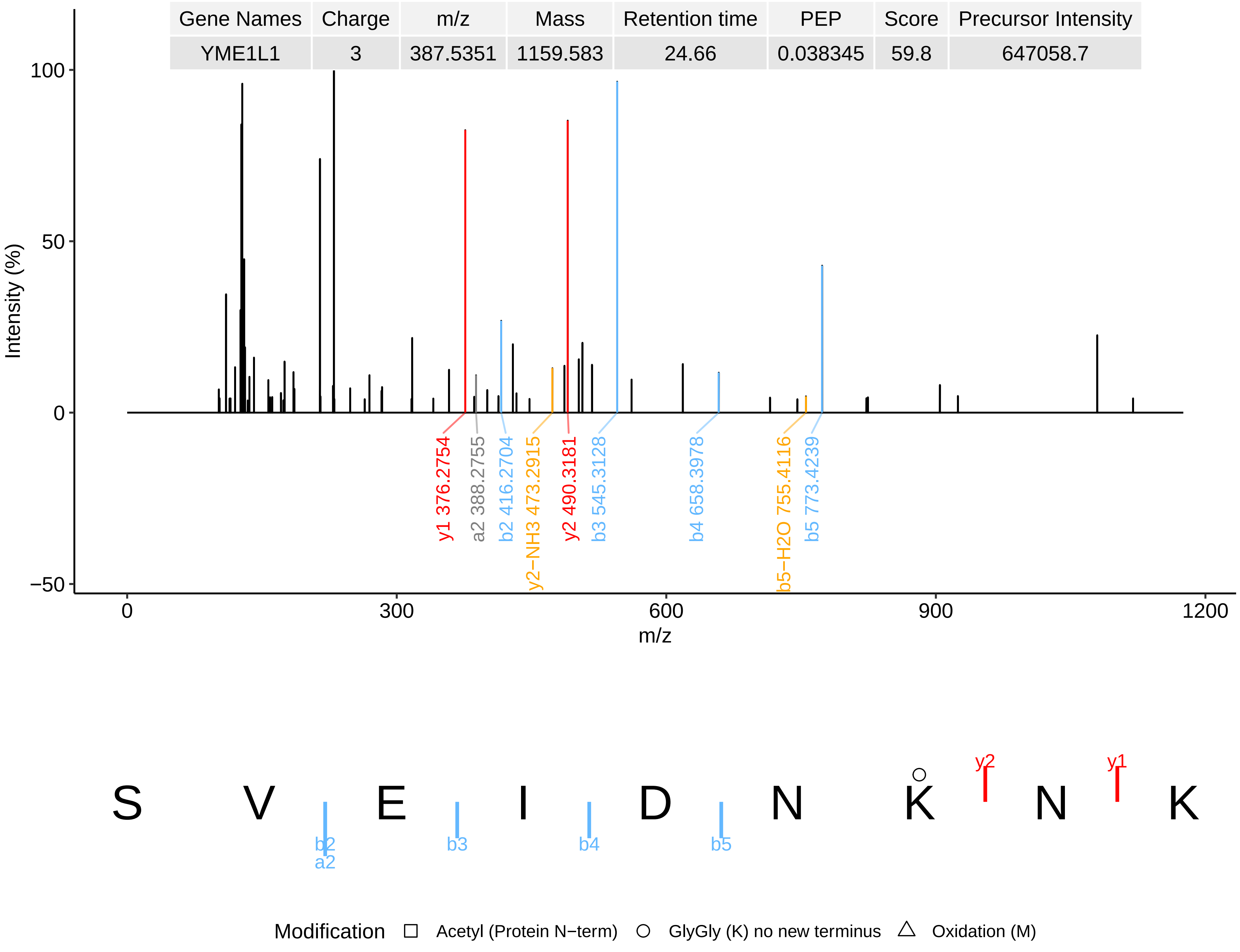

20200921_KIZ_MUb4_GlyGly_10plex_2 − Scan number: 15167

Gene Names	Charge	m/z	Mass	Retention time	PEP	Score	Precursor Intensity
RRM1	3	383.8924	1148.655	59.257	0.015232	69.598	1258641

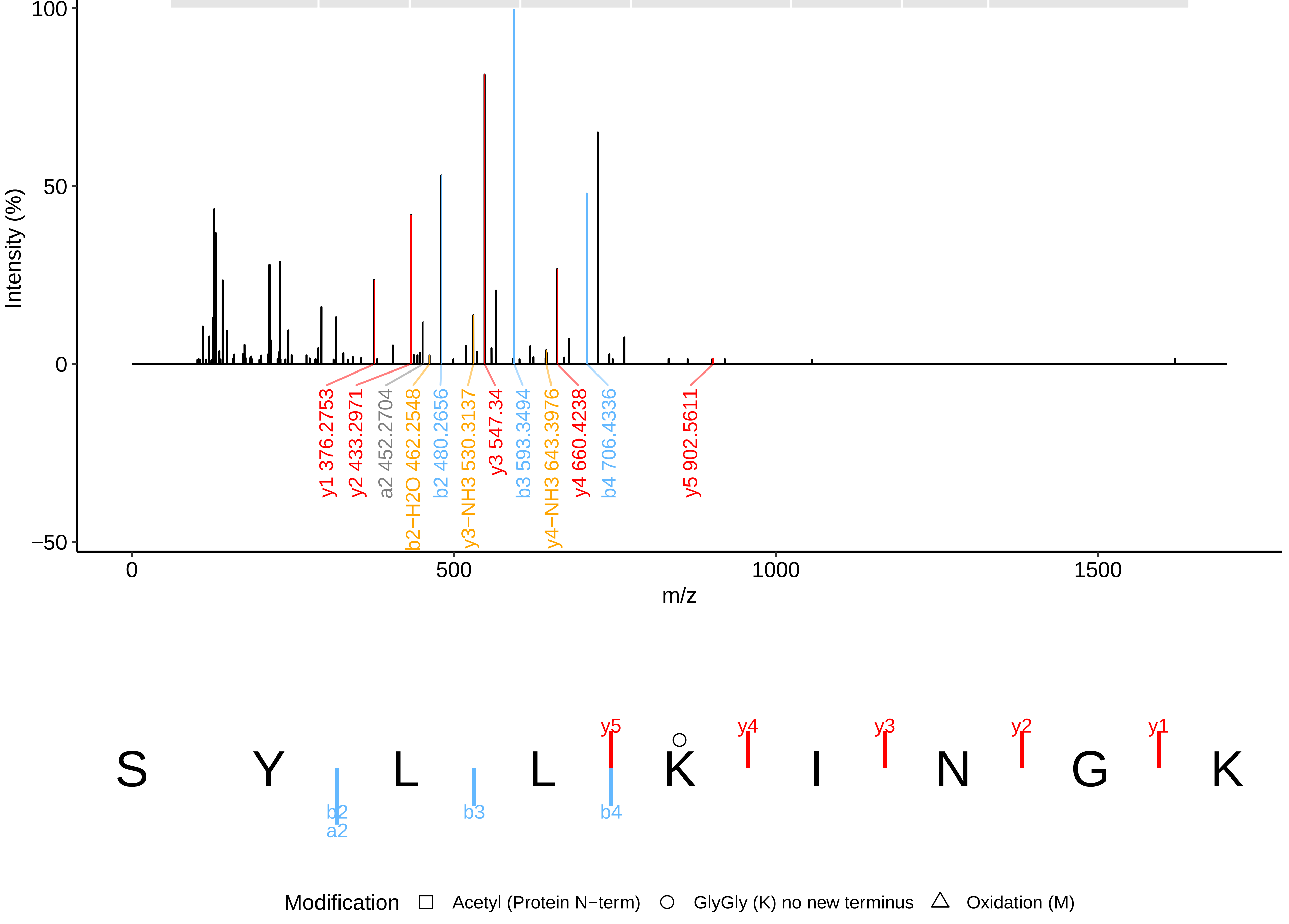

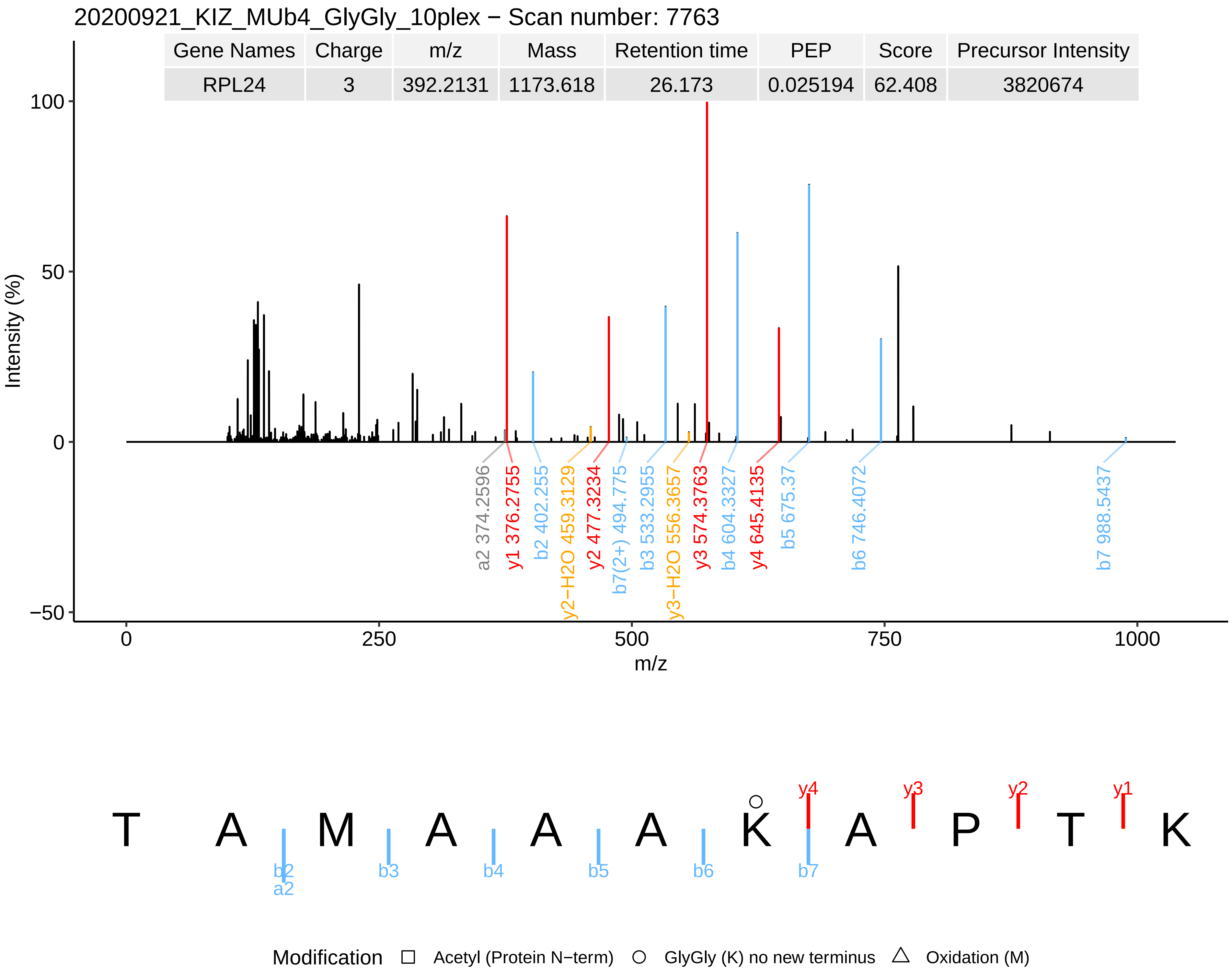

20200921_KIZ_MUb4_GlyGly_10plex − Scan number: 7763
Gene Names | Charge | m/z | Mass | Retention time | PEP | Score | Precursor Intensity
RPL24 | 3 | 392.2131 | 1173.618 | 26.173 | 0.025194 | 62.408 | 3820674
Intensity (%)
100
50
0
−50
0
250
500
750
1000
m/z
a2 374.2596
y1 376.2755
b2 402.255
y2−H2O 459.3129
y2 477.3234
b7(2+) 494.775
b3 533.2955
y3−H2O 556.3657
y3 574.3763
b4 604.3327
y4 645.4135
b5 675.37
b6 746.4072
b7 988.5437
T A M A A A K A P T K
y4 y3 y2 y1
b2 a2 b3 b4 b5 b6 b7
Modification
Acetyl (Protein N−term)
GlyGly (K) no new terminus
Oxidation (M)

20200921_KIZ_MUb4_GlyGly_10plex_2 − Scan number: 4415

Gene Names	Charge	m/z	Mass	Retention time	PEP	Score	Precursor Intensity
SRP9	3	382.8632	1145.568	15.485	0.062095	71.349	1049566

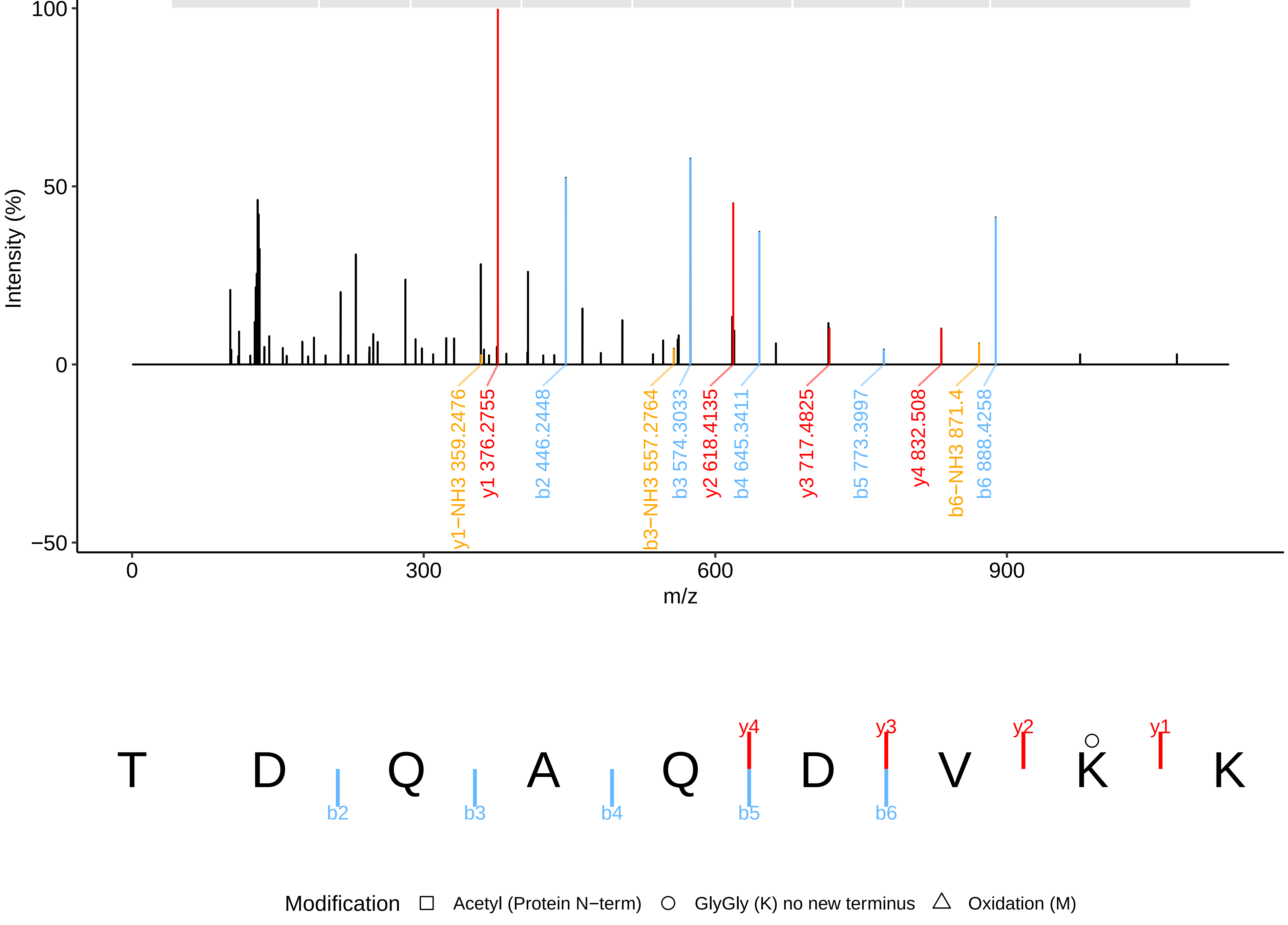

20200924_KIZ_MUb5_GlyGly_1 − Scan number: 28976

Gene Names	Charge	m/z	Mass	Retention time	PEP	Score	Precursor Intensity
IL1R2	2	704.8646	1407.715	92.518	0.030849	82.671	68396936

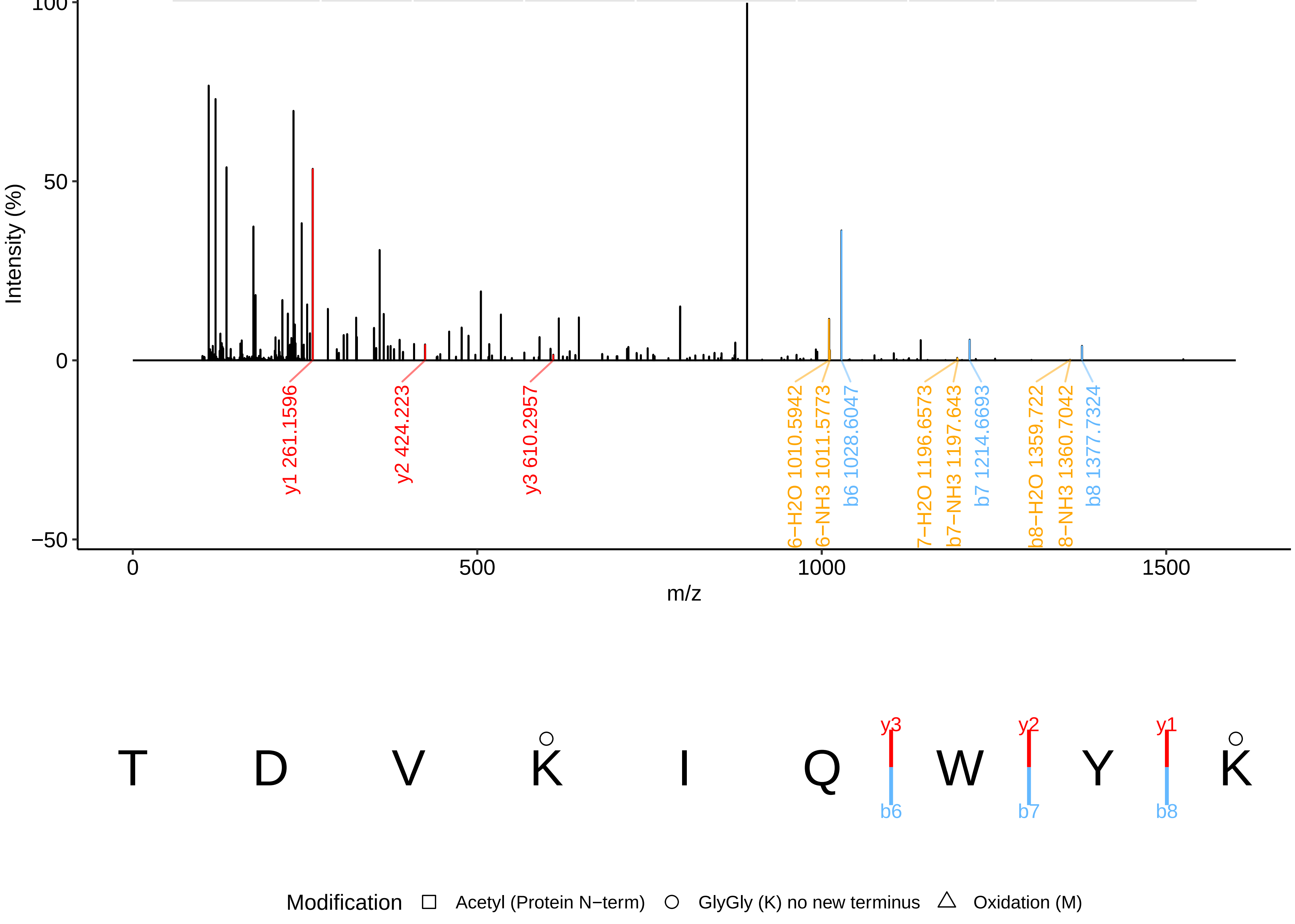

20200921_KIZ_MUb4_GlyGly_10plex_2 − Scan number: 14873

Gene Names	Charge	m/z	Mass	Retention time	PEP	Score	Precursor Intensity
TRAF2	3	396.5713	1186.692	58.056	0.015523	70.028	780505.6

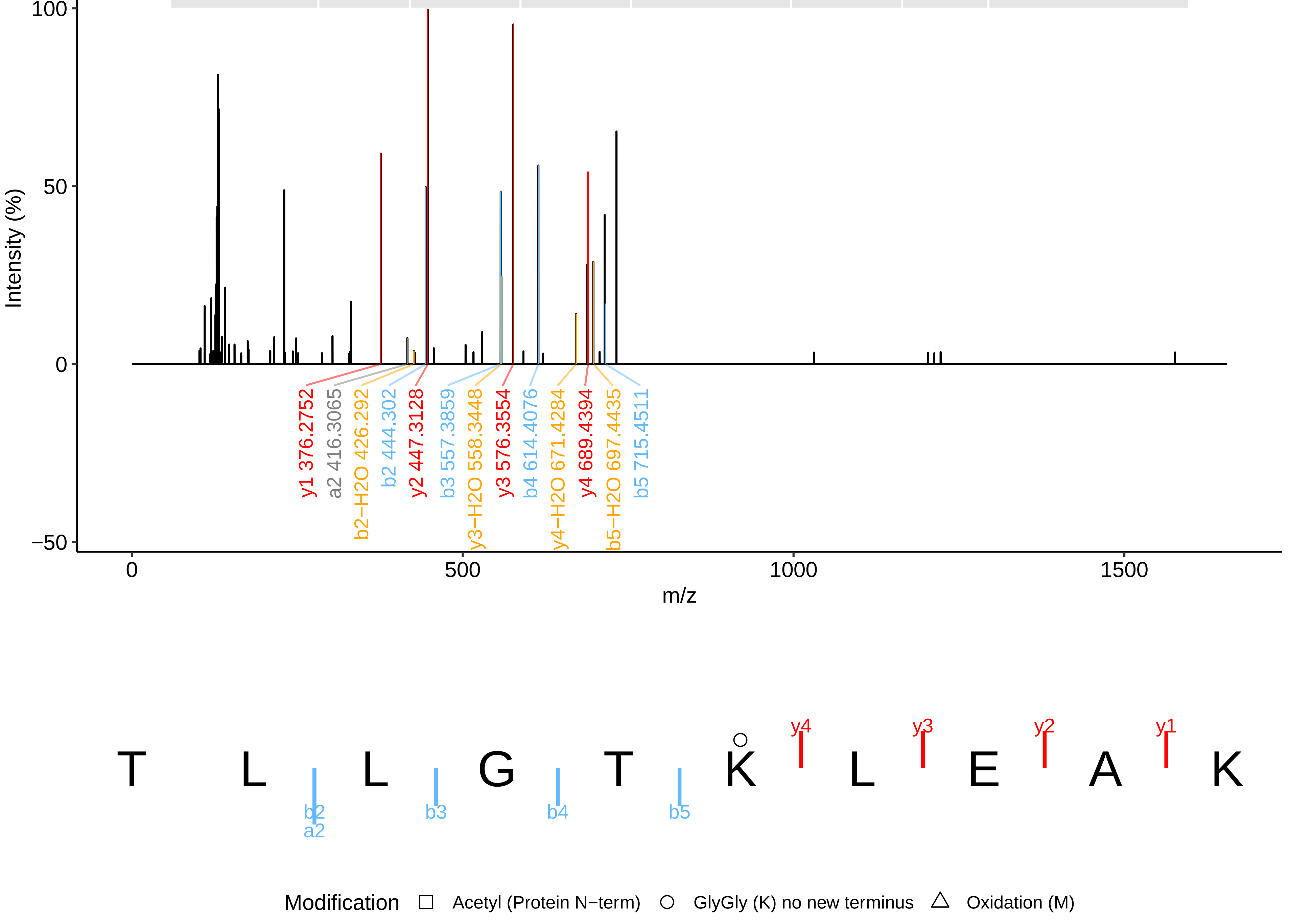

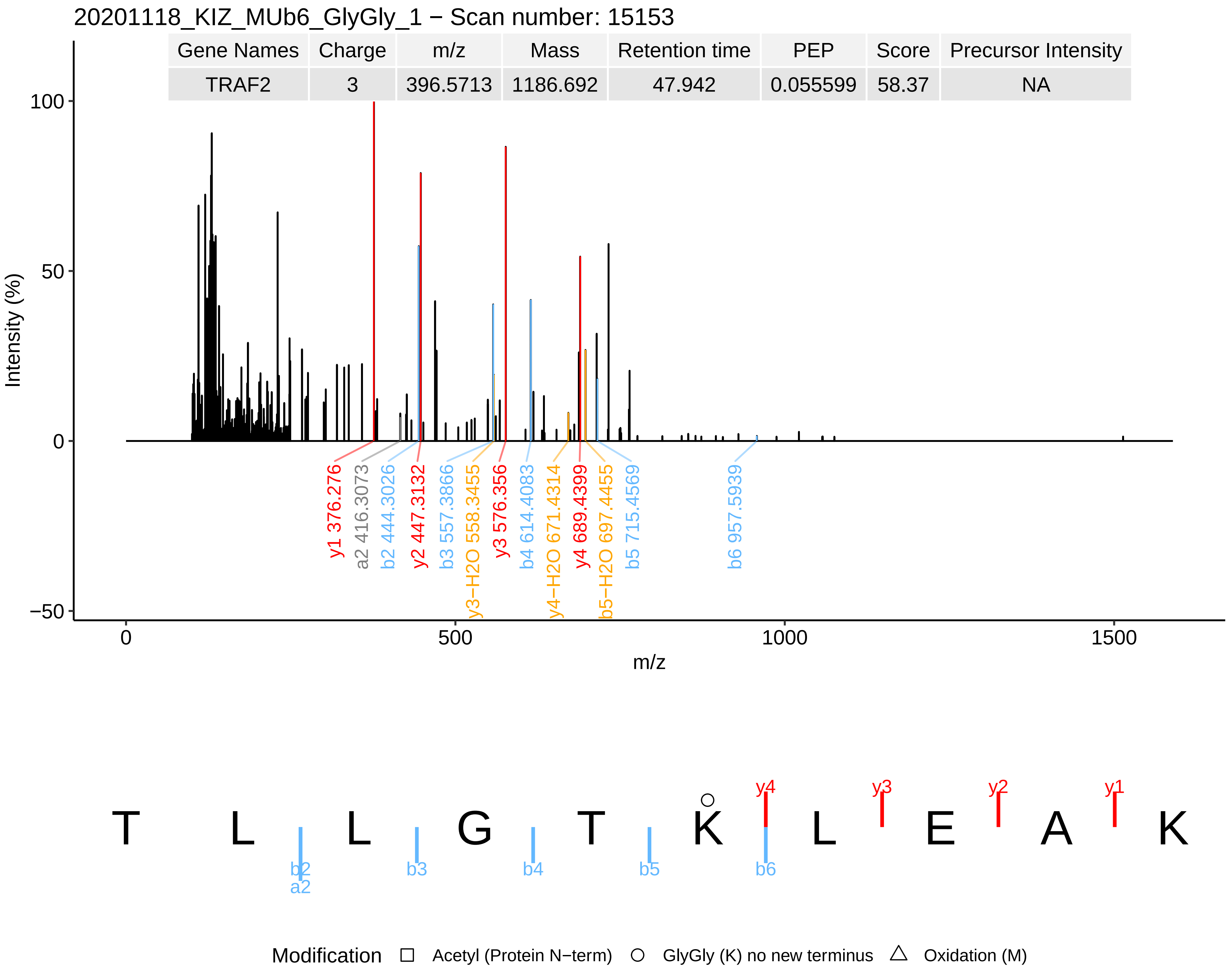
20201118_KIZ_MUb6_GlyGly_1 − Scan number: 15153
Gene Names | Charge | m/z | Mass | Retention time | PEP | Score | Precursor Intensity
TRAF2 | 3 | 396.5713 | 1186.692 | 47.942 | 0.055599 | 58.37 | NA
Intensity (%)
100
50
0
−50
0
500
1000
1500
m/z
y1 376.276
a2 416.3073
b2 444.3026
y2 447.3132
b3 557.3866
y3−H2O 558.3455
y3 576.356
b4 614.4083
y4−H2O 671.4314
y4 689.4399
b5−H2O 697.4455
b5 715.4569
b6 957.5939
T L L G T K L E A K
y4 y3 y2 y1
b2 a2 b3 b4 b5 b6
Modification
Acetyl (Protein N−term)
GlyGly (K) no new terminus
Oxidation (M)

20200921_KIZ_MUb4_GlyGly_10plex − Scan number: 6307

Gene Names	Charge	m/z	Mass	Retention time	PEP	Score	Precursor Intensity
RPL19	3	393.5348	1177.583	20.814	0.00050719	100.02	6810762

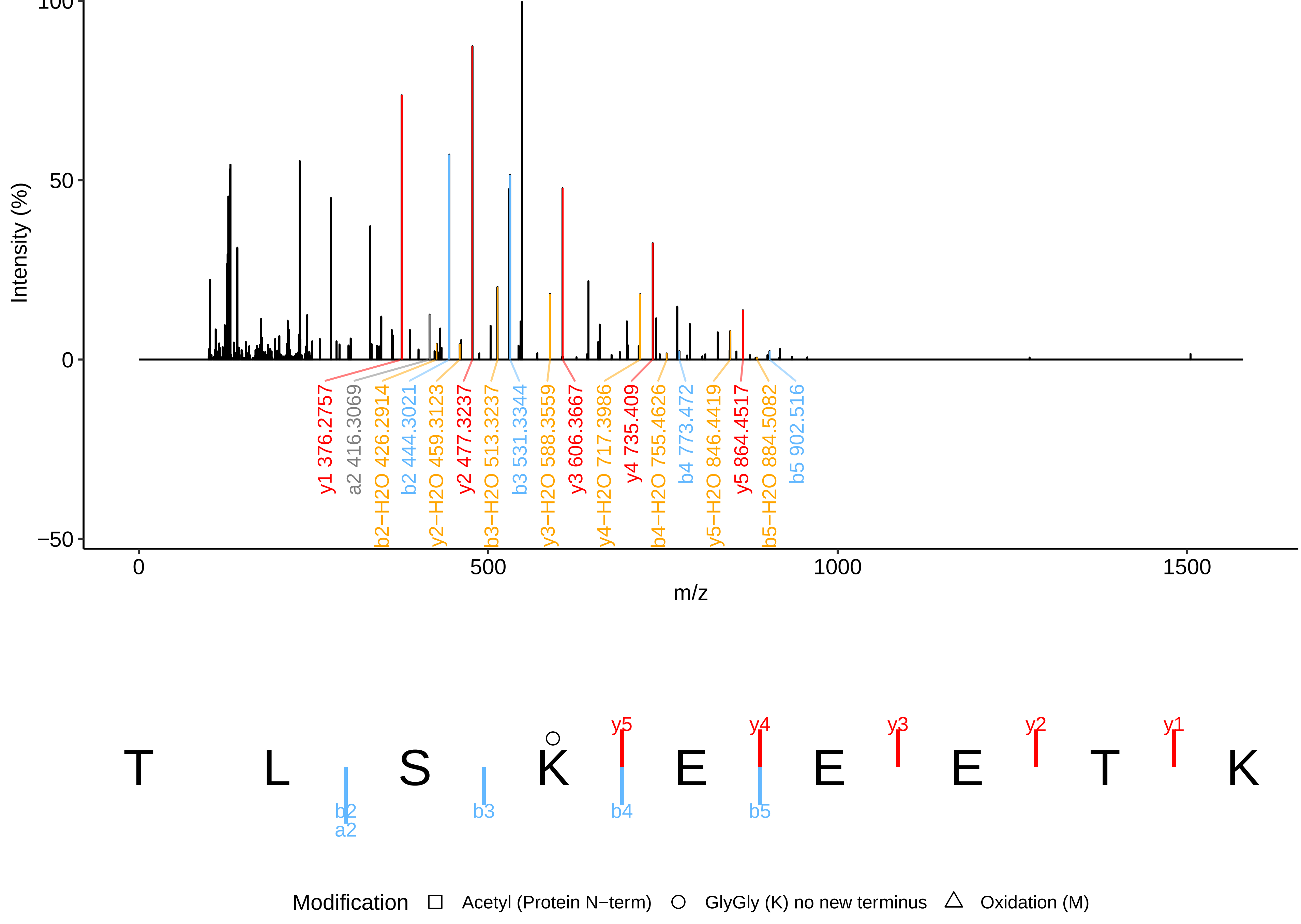

20200921_KIZ_MUb4_GlyGly_10plex_2 − Scan number: 5778

Gene Names	Charge	m/z	Mass	Retention time	PEP	Score	Precursor Intensity
RPL19	3	393.5348	1177.583	20.936	0.061192	55.064	419443.9

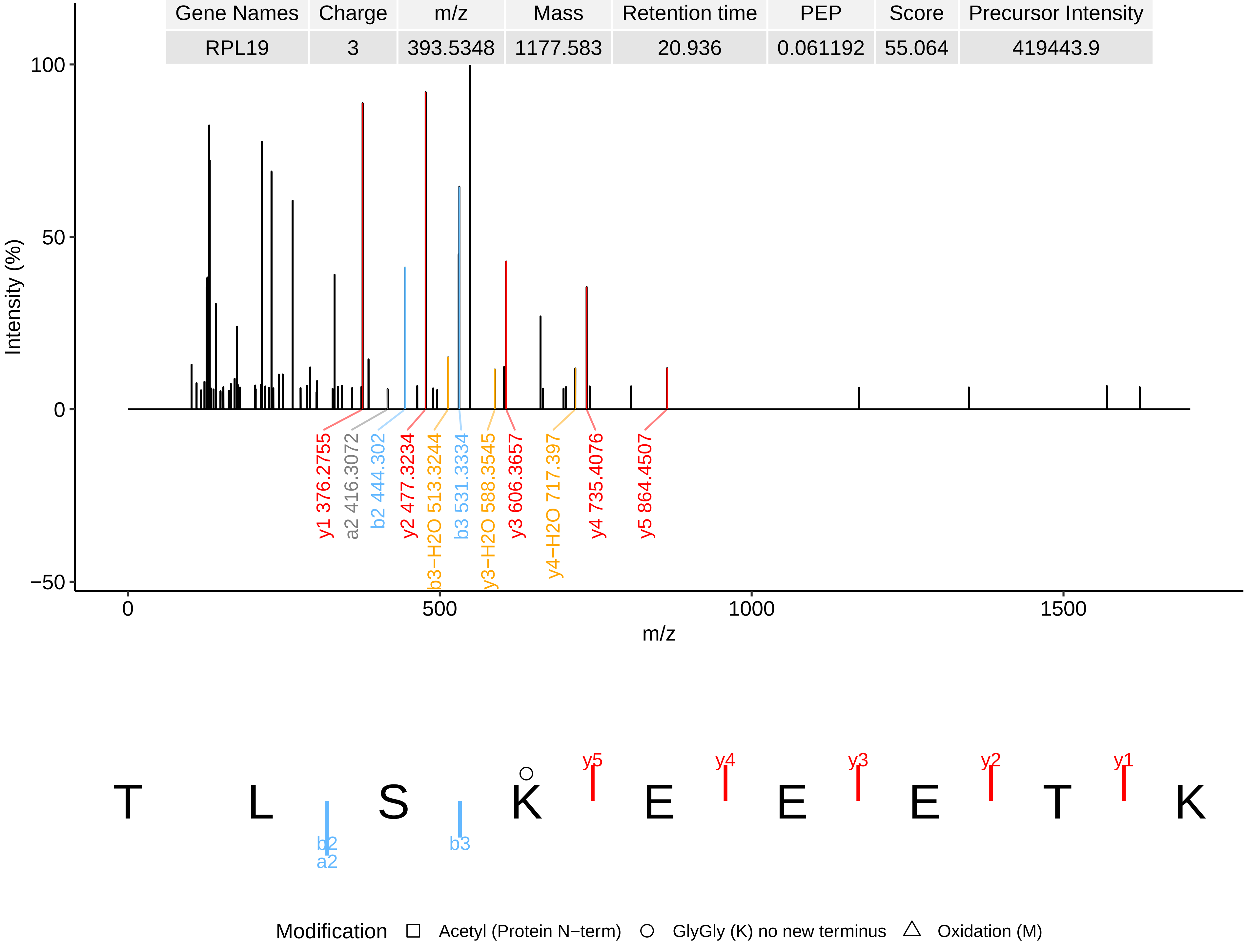

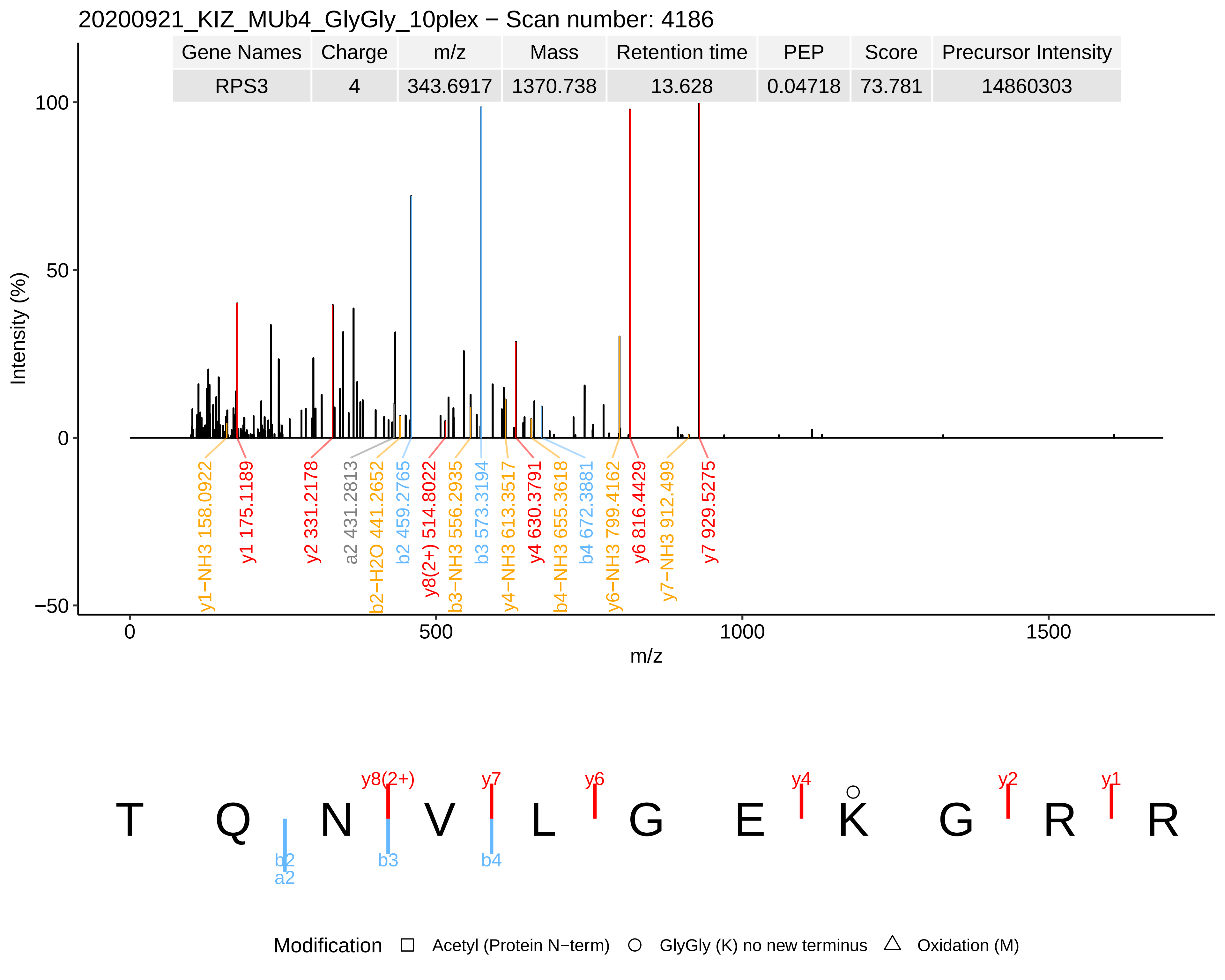

20200921_KIZ_MUb4_GlyGly_10plex − Scan number: 4186
Gene Names | Charge | m/z | Mass | Retention time | PEP | Score | Precursor Intensity
RPS3 | 4 | 343.6917 | 1370.738 | 13.628 | 0.04718 | 73.781 | 14860303
Intensity (%)
100
50
0
−50
y1−NH3 158.0922
y1 175.1189
y2 331.2178
a2 431.2813
b2−H2O 441.2652
b2 459.2765
y8(2+) 514.8022
b3−NH3 556.2935
b3 573.3194
y4−NH3 613.3517
y4 630.3791
b4−NH3 655.3618
b4 672.3881
y6−NH3 799.4162
y6 816.4429
y7−NH3 912.499
y7 929.5275
0
500
1000
1500
m/z
T Q N V L G E K G R R
y8(2+) y7 y6 y4 y2 y1
b2 a2 b3 b4
Modification
Acetyl (Protein N−term)
GlyGly (K) no new terminus
Oxidation (M)

20200921_KIZ_MUb4_GlyGly_10plex − Scan number: 4194

Gene Names	Charge	m/z	Mass	Retention time	PEP	Score	Precursor Intensity
RPS3	3	457.9199	1370.738	13.657	0.00972	75.088	8642696

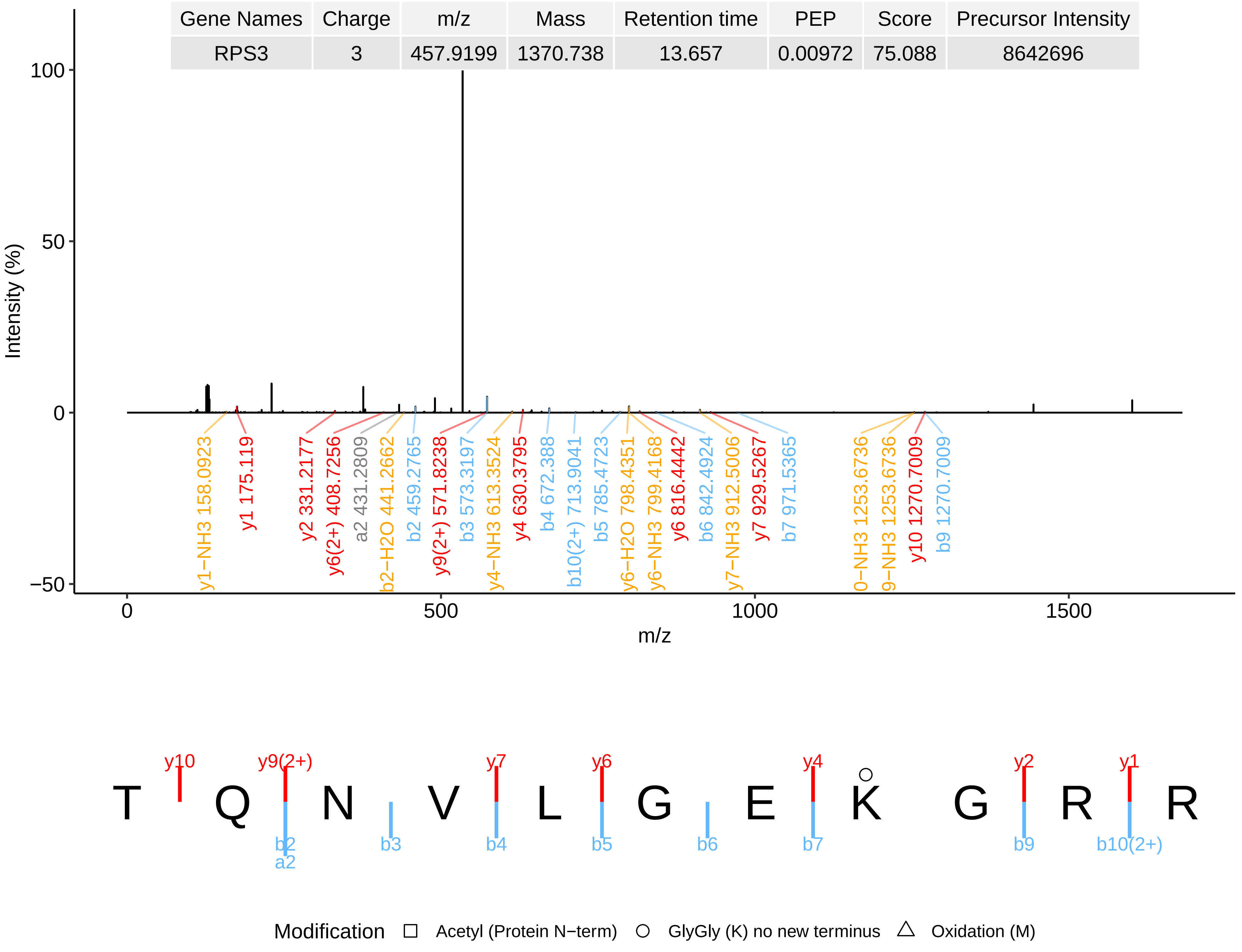

20200921_KIZ_MUb4_GlyGly_10plex_2 − Scan number: 3916

Gene Names	Charge	m/z	Mass	Retention time	PEP	Score	Precursor Intensity
RPS3	4	343.6917	1370.738	13.464	0.0078689	85.813	2625654

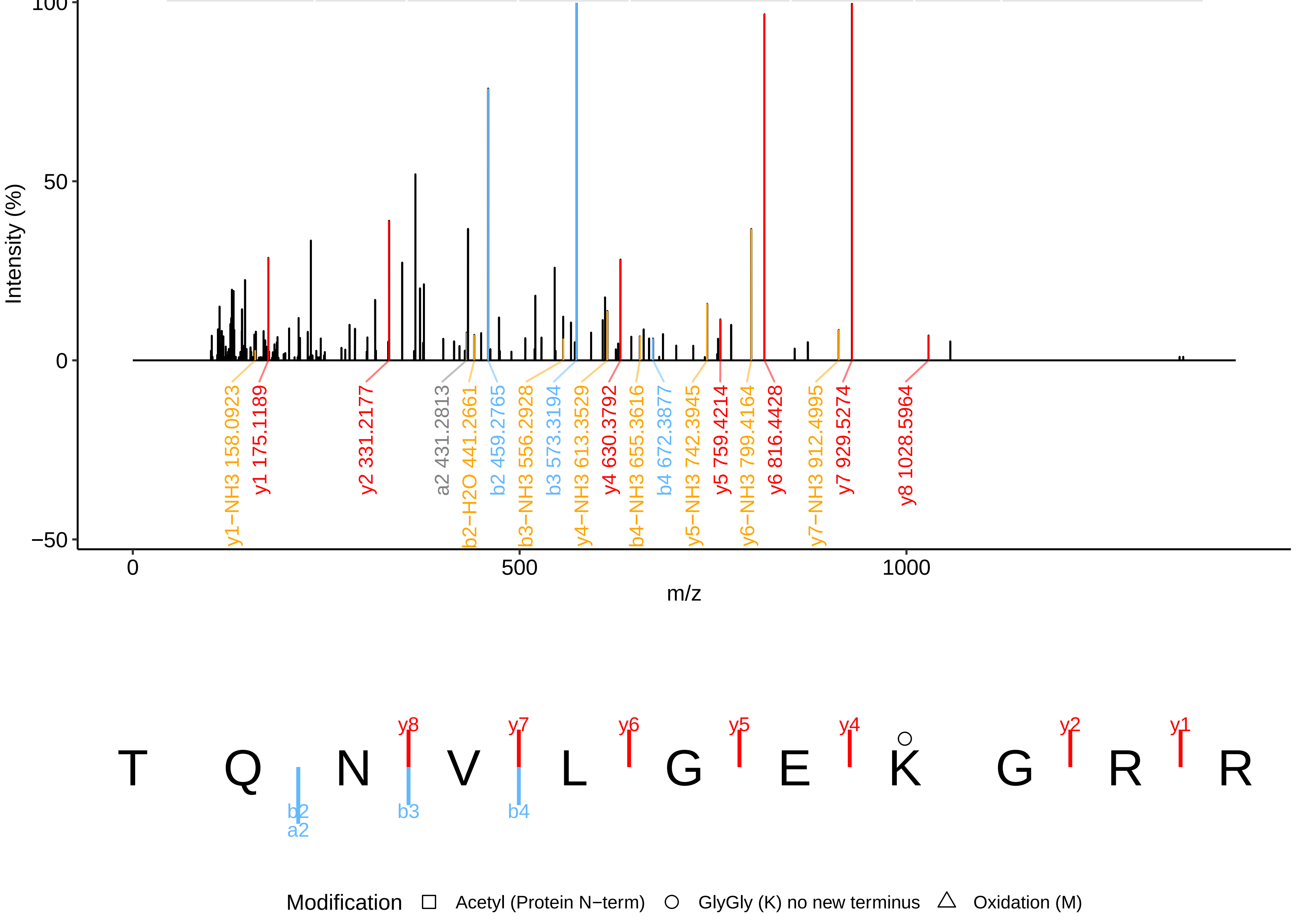

20200921_KIZ_MUb4_GlyGly_10plex_2 − Scan number: 4446

Gene Names	Charge	m/z	Mass	Retention time	PEP	Score	Precursor Intensity
GGCX	3	397.5386	1189.594	15.608	0.0079039	75.378	1115169

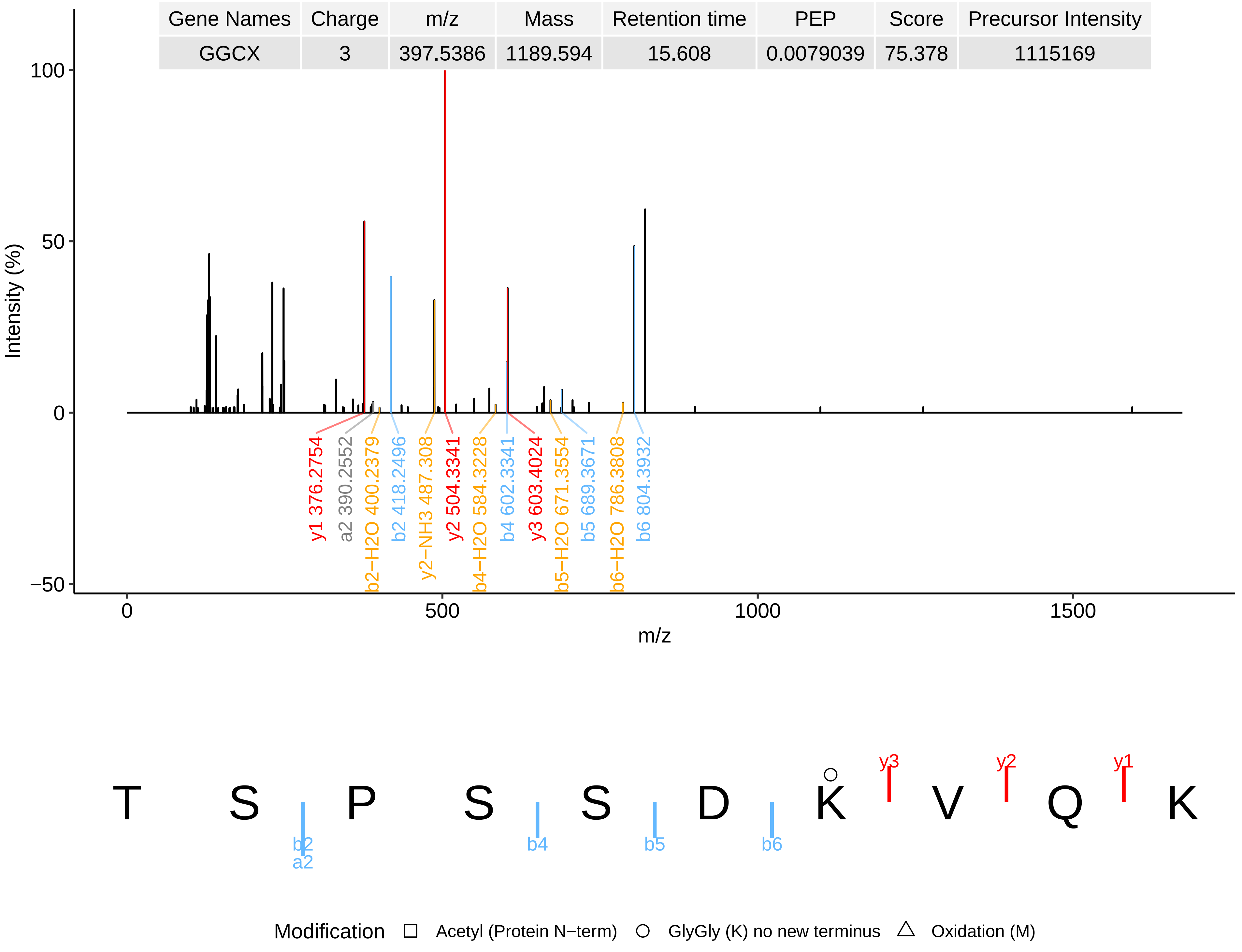

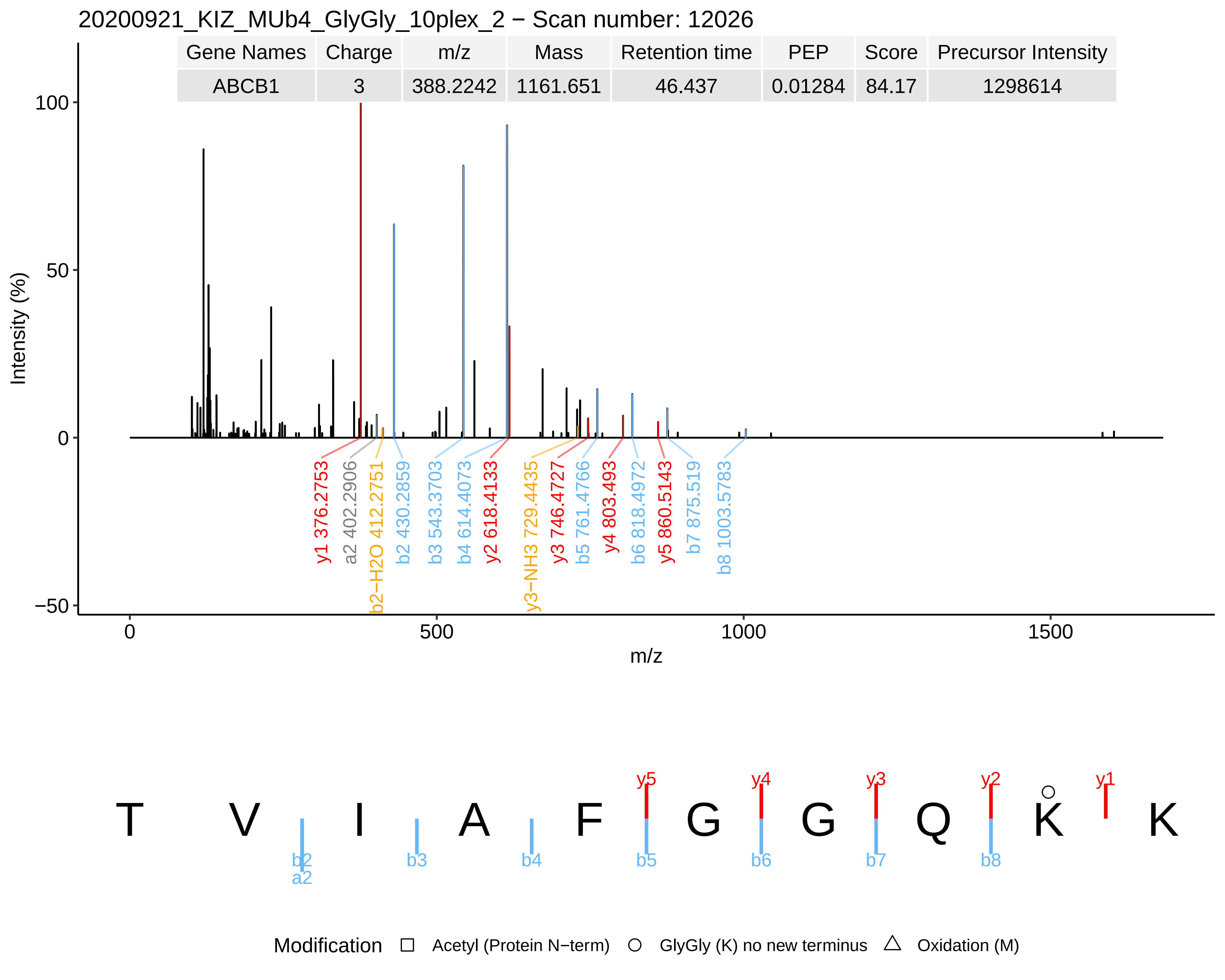

20200921_KIZ_MUb4_GlyGly_10plex_2 − Scan number: 12026
Gene Names | Charge | m/z | Mass | Retention time | PEP | Score | Precursor Intensity
ABCB1 | 3 | 388.2242 | 1161.651 | 46.437 | 0.01284 | 84.17 | 1298614
Intensity (%)
100
50
0
−50
0
500
1000
1500
m/z
y1 376.2753
a2 402.2906
b2−H2O 412.2751
b2 430.2859
b3 543.3703
b4 614.4073
y2 618.4133
y3−NH3 729.4435
y3 746.4727
b5 761.4766
y4 803.493
b6 818.4972
y5 860.5143
b7 875.519
b8 1003.5783
T V I A F G G Q K K
y5 y4 y3 y2 y1
b2 a2 b3 b4 b5 b6 b7 b8
Modification
Acetyl (Protein N−term)
GlyGly (K) no new terminus
Oxidation (M)

20200921_KIZ_MUb4_GlyGly_10plex − Scan number: 15285

Gene Names	Charge	m/z	Mass	Retention time	PEP	Score	Precursor Intensity
FLVCR1	2	704.361	1406.707	54.571	0.015169	79.906	7513344

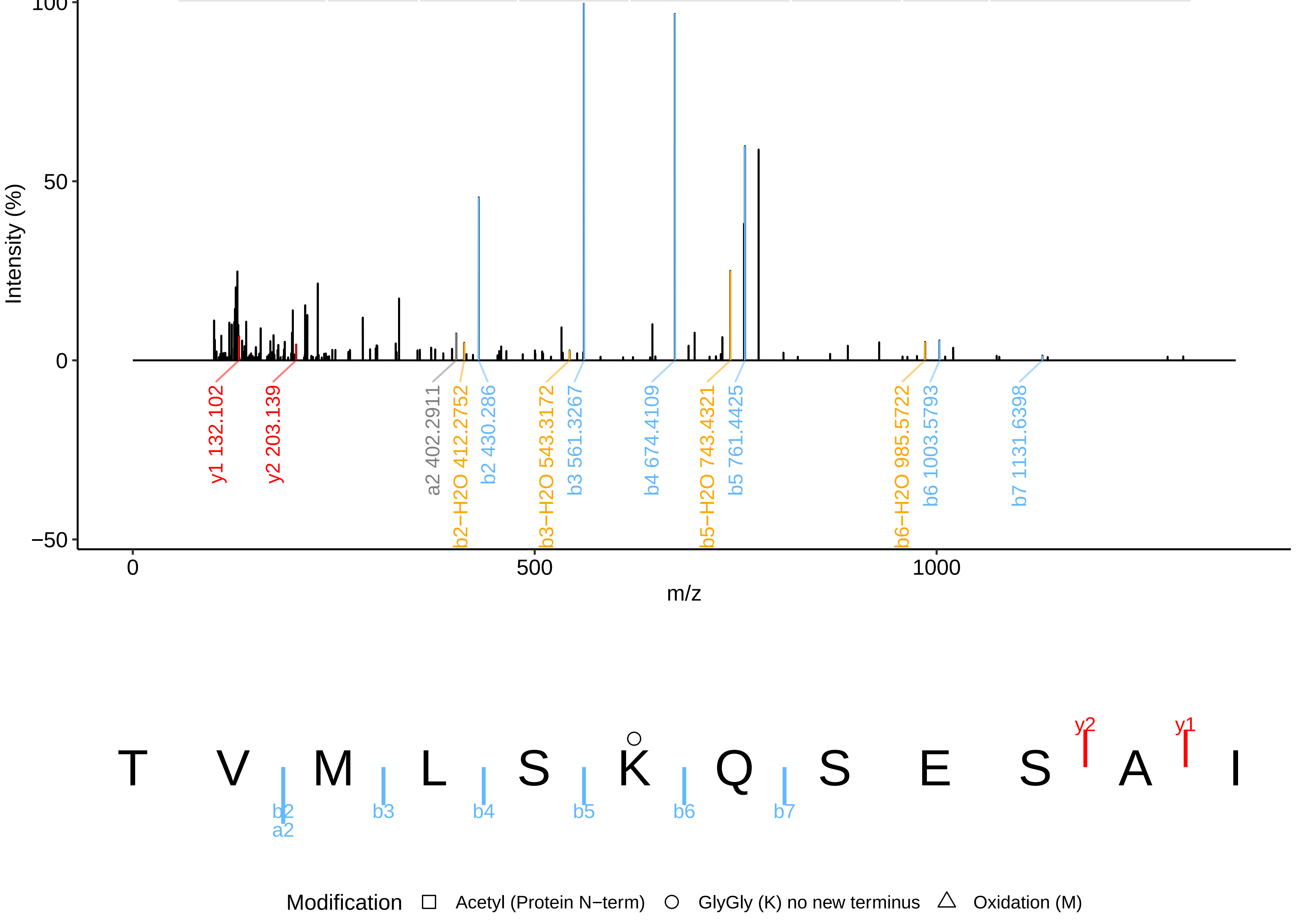

20200921_KIZ_MUb4_GlyGly_10plex_2 − Scan number: 4389

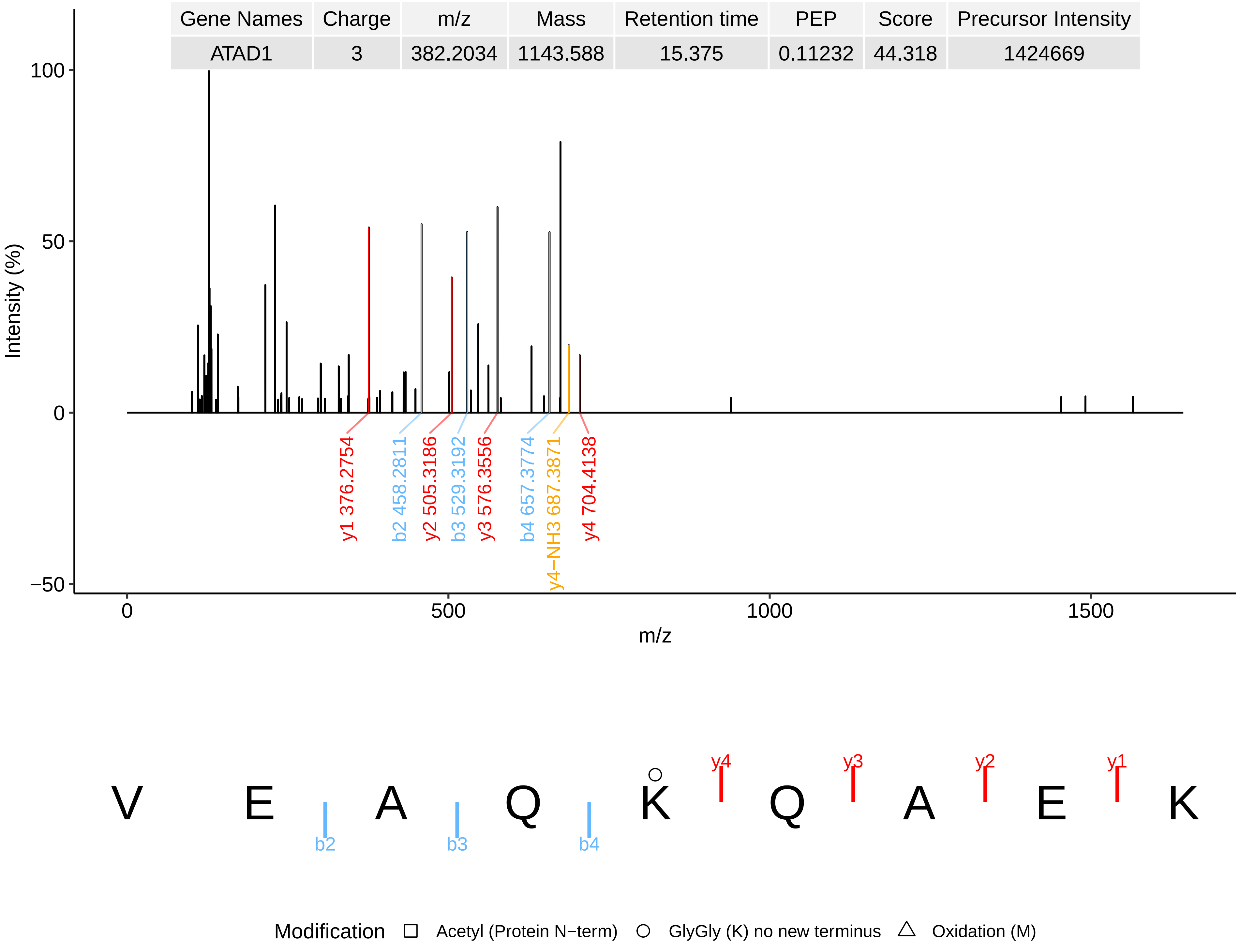

20200924_KIZ_MUb5_GlyGly_1 − Scan number: 11594

Gene Names	Charge	m/z	Mass	Retention time	PEP	Score	Precursor Intensity
POLR2A	3	457.5807	1369.72	37.591	0.016169	68.355	10238352

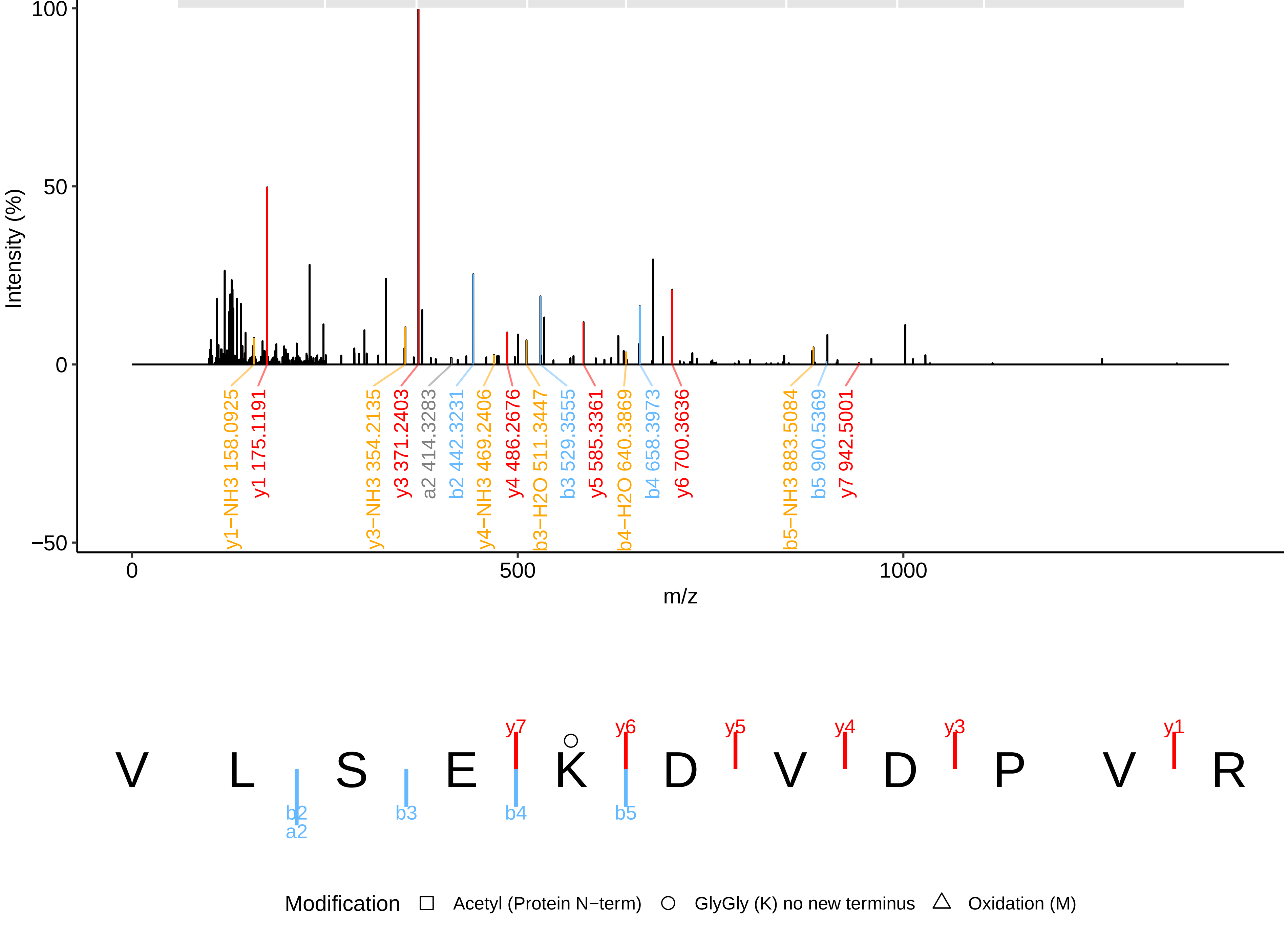

20200921_KIZ_MUb4_GlyGly_10plex − Scan number: 13465

Gene Names	Charge	m/z	Mass	Retention time	PEP	Score	Precursor Intensity
GNAS	3	395.8911	1184.651	47.522	0.0011329	94.692	7806346

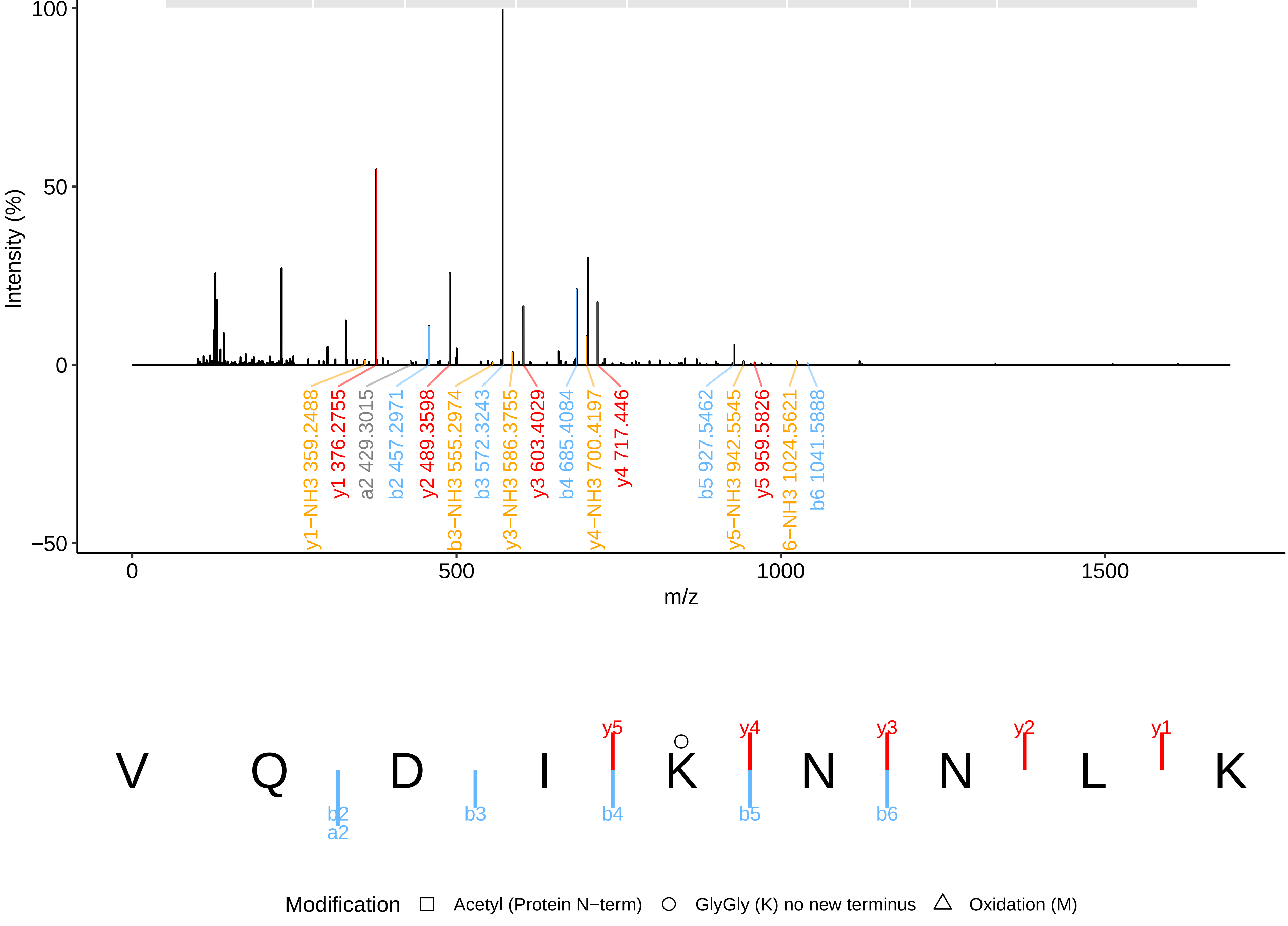

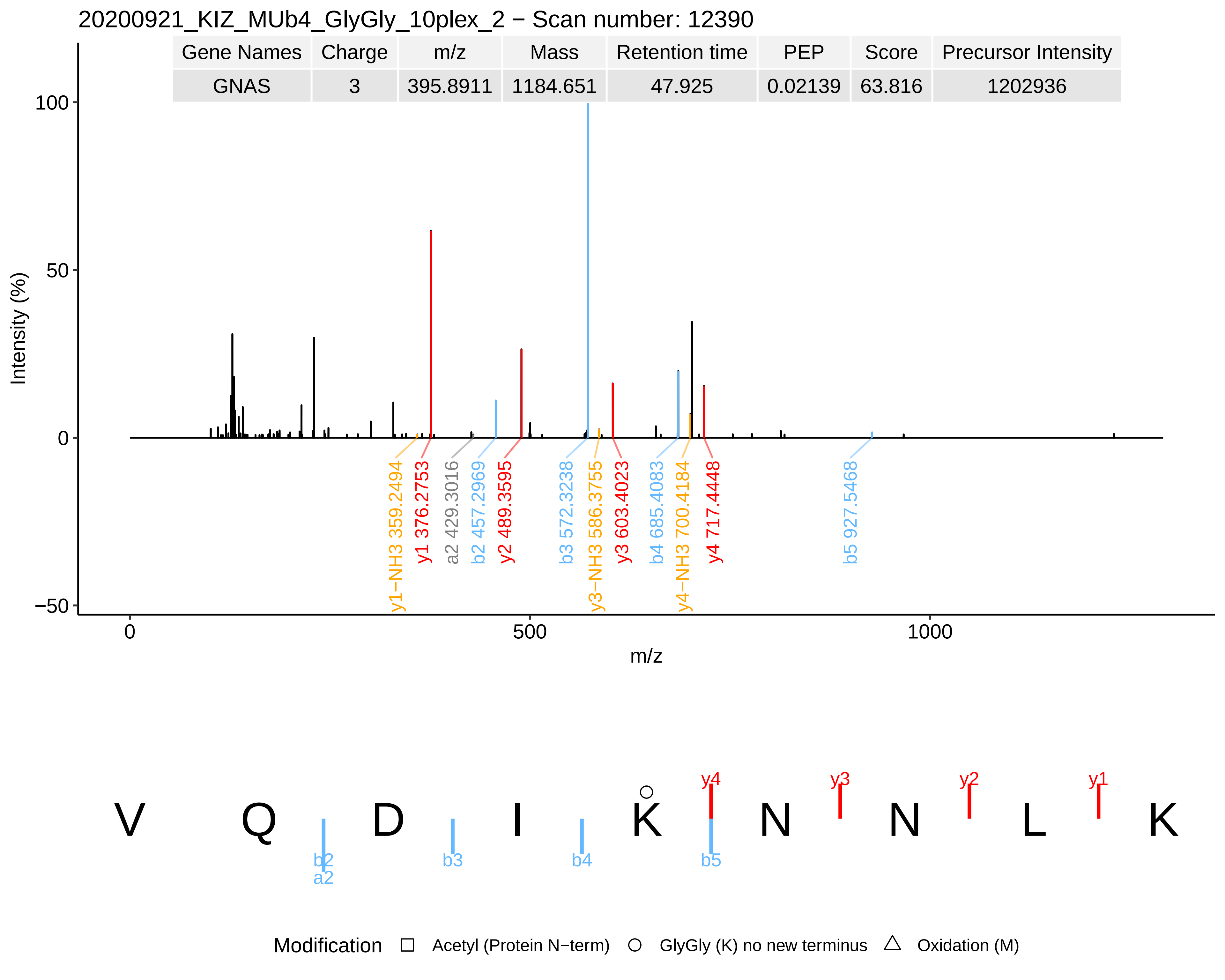
20200921_KIZ_MUb4_GlyGly_10plex_2 − Scan number: 12390
Gene Names | Charge | m/z | Mass | Retention time | PEP | Score | Precursor Intensity
GNAS | 3 | 395.8911 | 1184.651 | 47.925 | 0.02139 | 63.816 | 1202936
Intensity (%)
100
50
0
−50
0
500
1000
m/z
y1−NH3 359.2494
y1 376.2753
a2 429.3016
b2 457.2969
y2 489.3595
b3 572.3238
y3−NH3 586.3755
y3 603.4023
b4 685.4083
y4−NH3 700.4184
y4 717.4448
b5 927.5468
V Q D I K N N L K
y4 y3 y2 y1
b2 a2 b3 b4 b5
Modification
Acetyl (Protein N−term)
GlyGly (K) no new terminus
Oxidation (M)

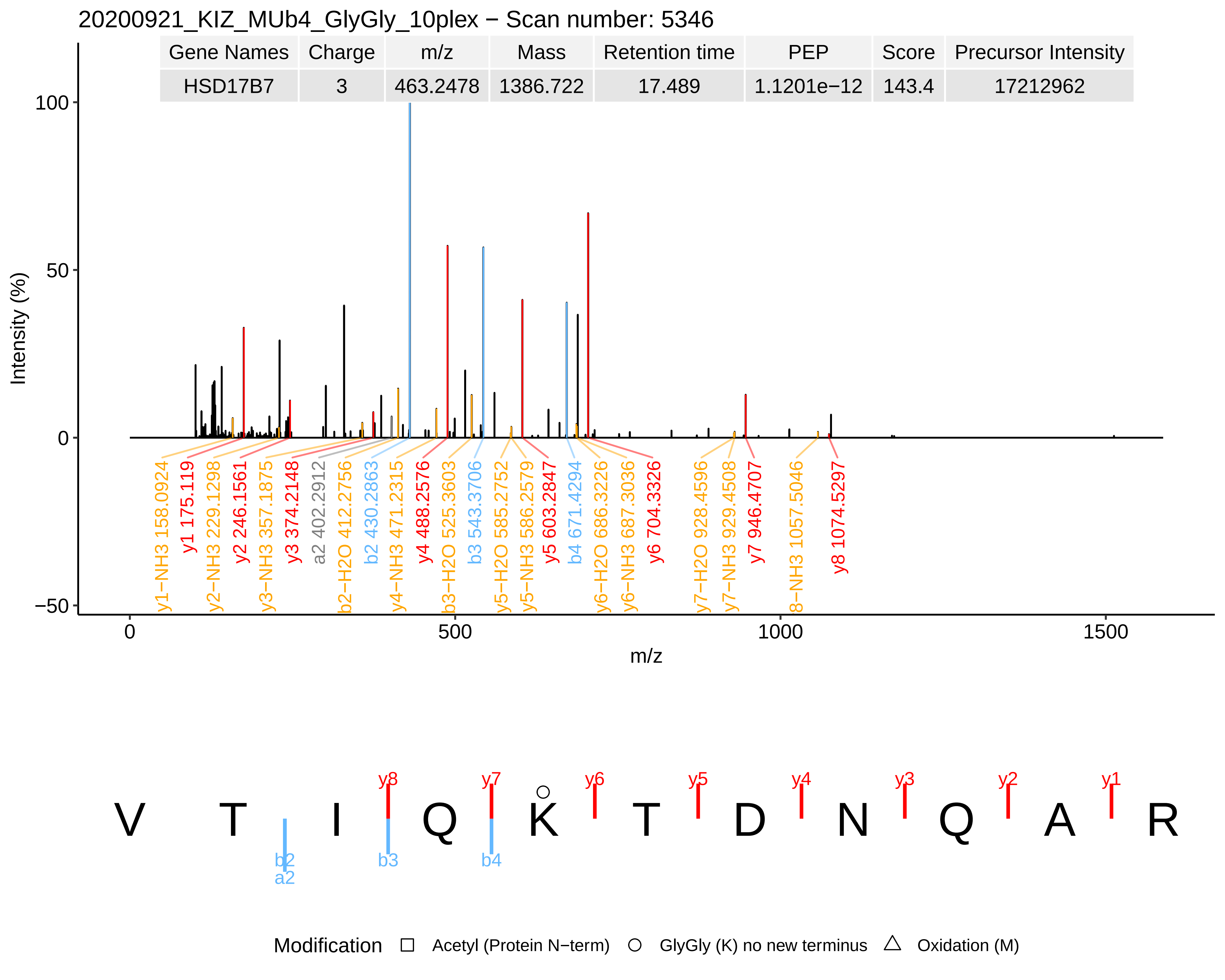
20200921_KIZ_MUb4_GlyGly_10plex − Scan number: 5346
Gene Names | Charge | m/z | Mass | Retention time | PEP | Score | Precursor Intensity
HSD17B7 | 3 | 463.2478 | 1386.722 | 17.489 | 1.1201e−12 | 143.4 | 17212962
Intensity (%)
m/z
y1−NH3 158.0924
y1 175.119
y2−NH3 229.1298
y2 246.1561
y3−NH3 357.1875
y3 374.2148
a2 402.2912
b2−H2O 412.2756
b2 430.2863
y4−NH3 471.2315
y4 488.2576
b3−H2O 525.3603
b3 543.3706
y5−H2O 585.2752
y5−NH3 586.2579
y5 603.2847
b4 671.4294
y6−H2O 686.3226
y6−NH3 687.3036
y6 704.3326
y7−H2O 928.4596
y7−NH3 929.4508
y7 946.4707
8−NH3 1057.5046
y8 1074.5297
V T I Q K T D N Q A R
Modification
Acetyl (Protein N−term)
GlyGly (K) no new terminus
Oxidation (M)

20200921_KIZ_MUb4_GlyGly_10plex_2 − Scan number: 4889

Gene Names	Charge	m/z	Mass	Retention time	PEP	Score	Precursor Intensity
HSD17B7	3	463.2478	1386.722	17.34	0.0010522	87.568	NA

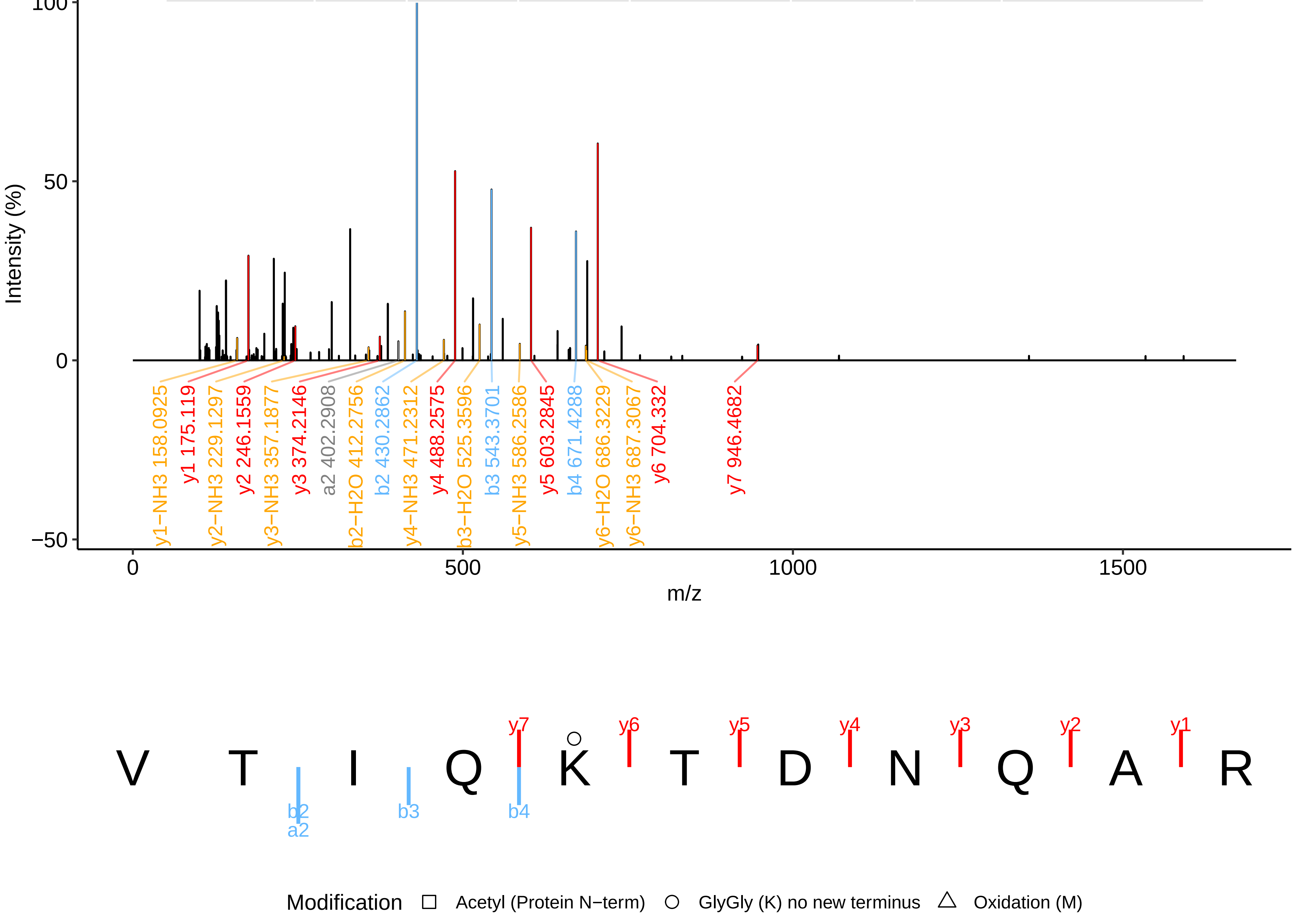

20200921_KIZ_MUb4_GlyGly_10plex − Scan number: 10670

Gene Names	Charge	m/z	Mass	Retention time	PEP	Score	Precursor Intensity
CYB5R1	4	289.4225	1153.661	36.993	0.011384	70.919	8944148

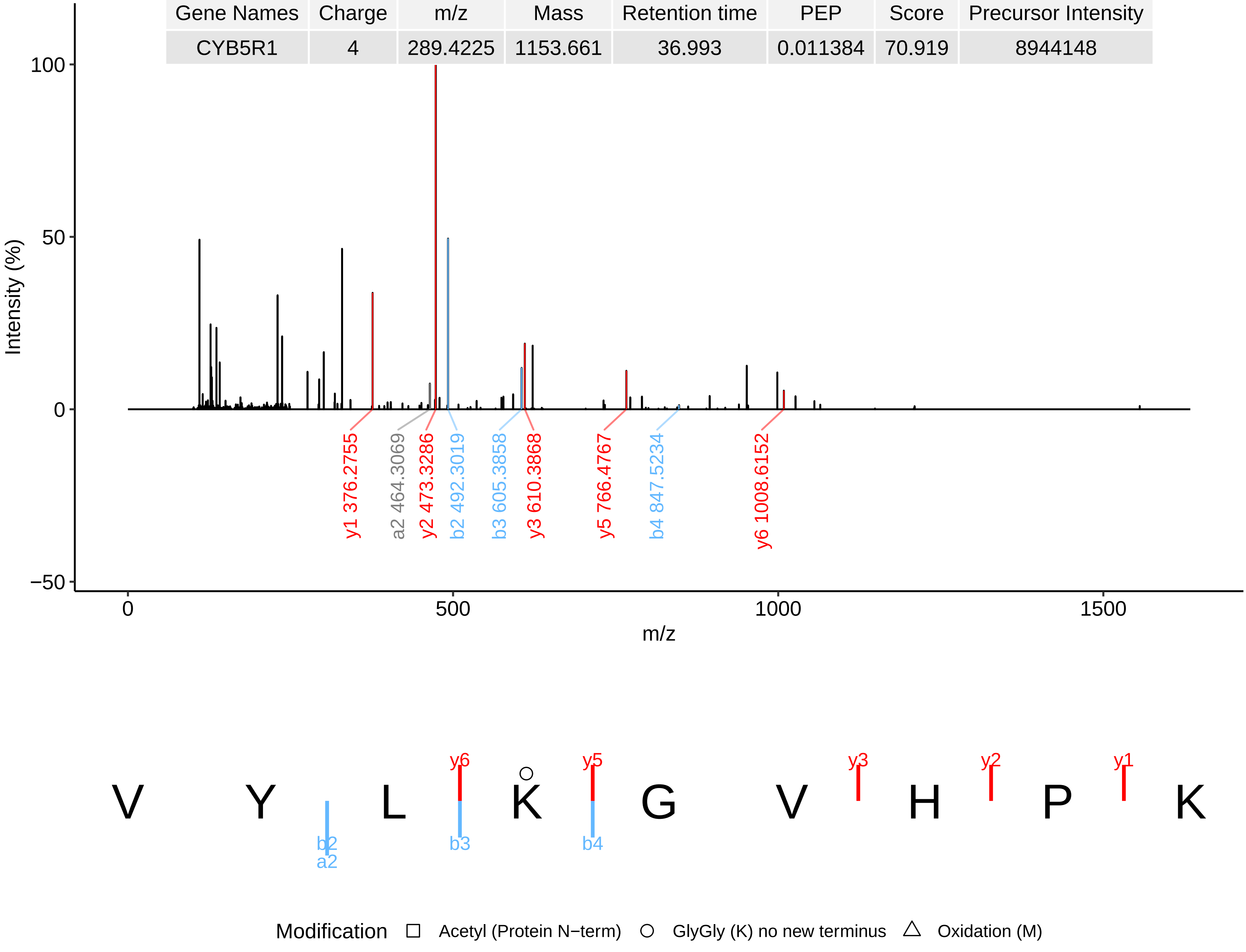

20200921_KIZ_MUb4_GlyGly_10plex_2 − Scan number: 9945

Gene Names	Charge	m/z	Mass	Retention time	PEP	Score	Precursor Intensity
CYB5R1	4	289.4225	1153.661	37.92	0.028436	60.59	2609808

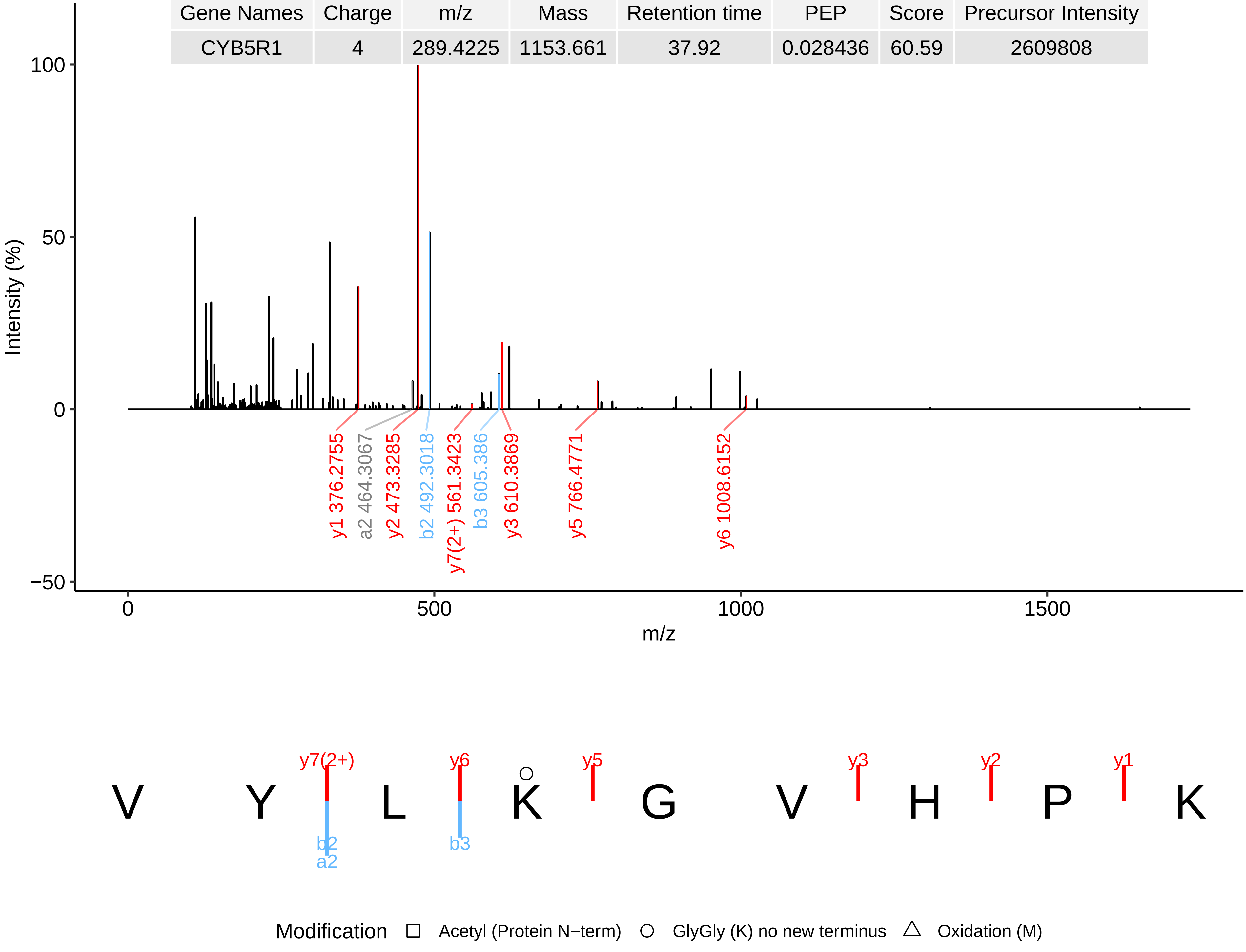

20200921_KIZ_MUb4_GlyGly_10plex − Scan number: 7515

Gene Names	Charge	m/z	Mass	Retention time	PEP	Score	Precursor Intensity
MORF4L1	3	460.2406	1377.7	25.246	1.0381e−05	144.77	2953134

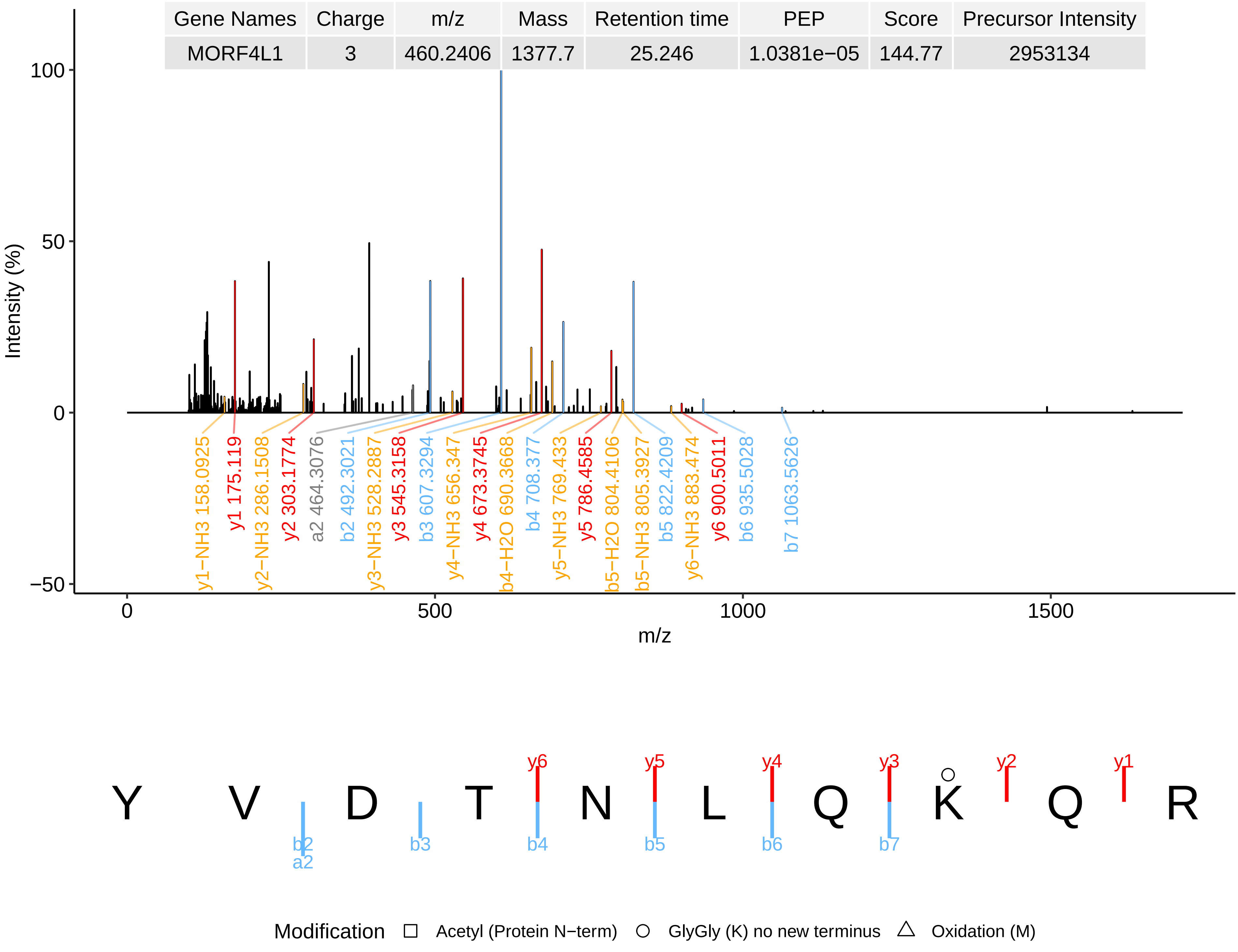

20200921_KIZ_MUb4_GlyGly_10plex − Scan number: 14266

Gene Names	Charge	m/z	Mass	Retention time	PEP	Score	Precursor Intensity
PPP2R1A;PPP2R1B	3	402.8874	1205.64	50.63	0.011436	68.786	4657614

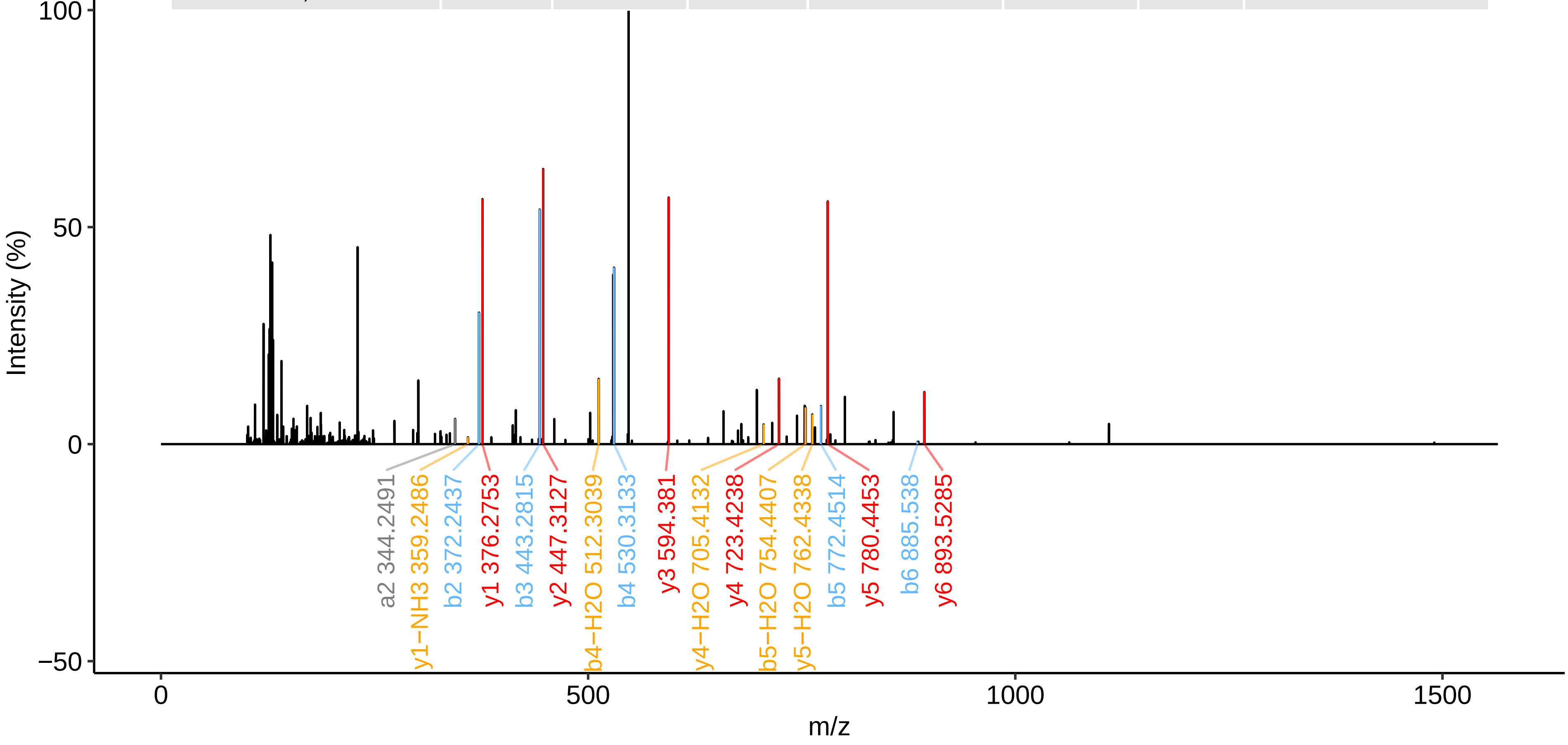

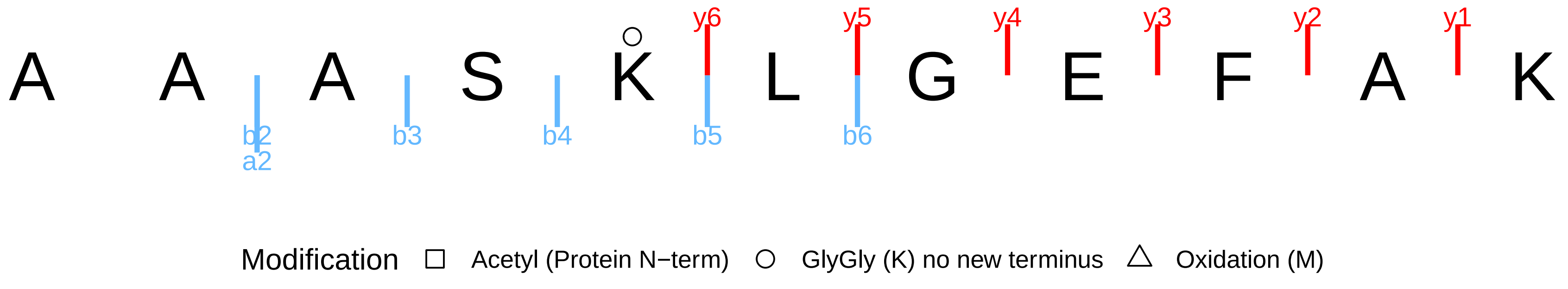

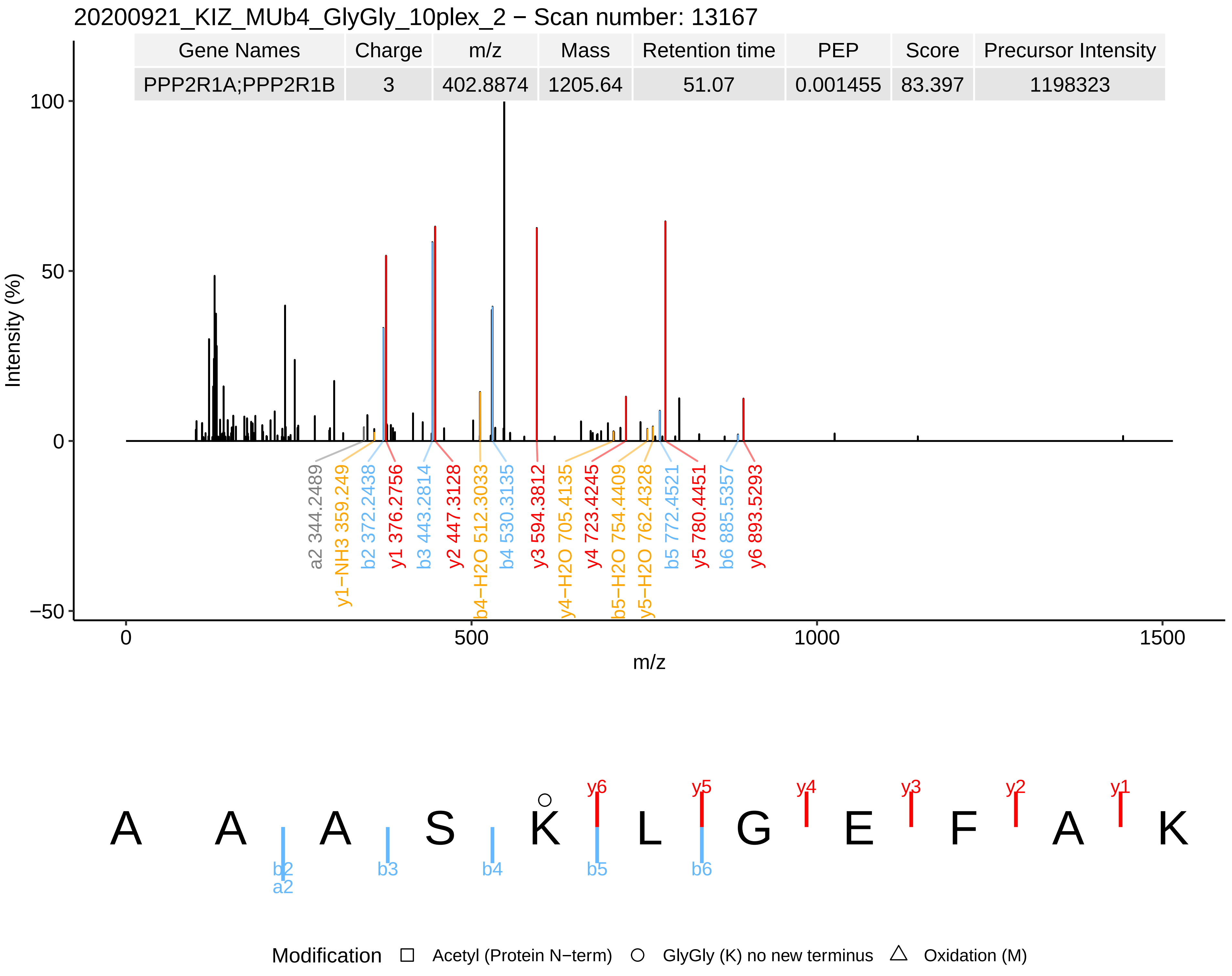

20200921_KIZ_MUb4_GlyGly_10plex_2 − Scan number: 13167
Gene Names | Charge | m/z | Mass | Retention time | PEP | Score | Precursor Intensity
PPP2R1A;PPP2R1B | 3 | 402.8874 | 1205.64 | 51.07 | 0.001455 | 83.397 | 1198323
Intensity (%)
100
50
0
−50
0
500
1000
1500
m/z
a2 344.2489
y1−NH3 359.249
b2 372.2438
y1 376.2756
b3 443.2814
y2 447.3128
b4−H2O 512.3033
b4 530.3135
y3 594.3812
y4−H2O 705.4135
y4 723.4245
b5−H2O 754.4409
y5−H2O 762.4328
b5 772.4521
y5 780.4451
b6 885.5357
y6 893.5293
A A A S K L G E F A K
y6 y5 y4 y3 y2 y1
b2 a2 b3 b4 b5 b6
Modification
Acetyl (Protein N−term)
GlyGly (K) no new terminus
Oxidation (M)

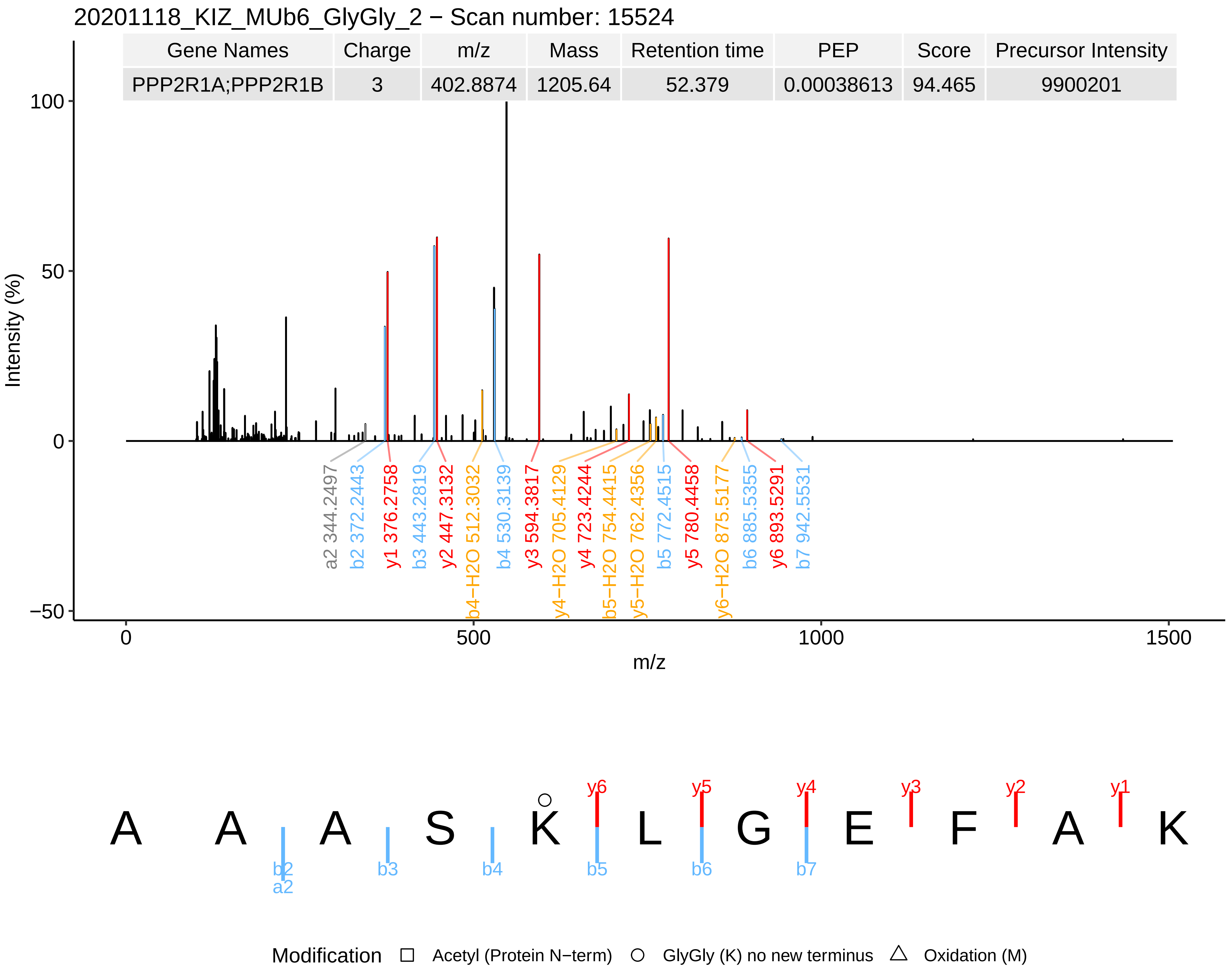
20201118_KIZ_MUb6_GlyGly_2 − Scan number: 15524
Gene Names | Charge | m/z | Mass | Retention time | PEP | Score | Precursor Intensity
PPP2R1A;PPP2R1B | 3 | 402.8874 | 1205.64 | 52.379 | 0.00038613 | 94.465 | 9900201
Intensity (%)
m/z
a2 344.2497
b2 372.2443
y1 376.2758
b3 443.2819
y2 447.3132
b4−H2O 512.3032
b4 530.3139
y3 594.3817
y4−H2O 705.4129
y4 723.4244
b5−H2O 754.4415
y5−H2O 762.4356
b5 772.4515
y5 780.4458
y6−H2O 875.5177
b6 885.5355
y6 893.5291
b7 942.5531
A A A S K L G E F A K
Modification
Acetyl (Protein N−term)
GlyGly (K) no new terminus
Oxidation (M)

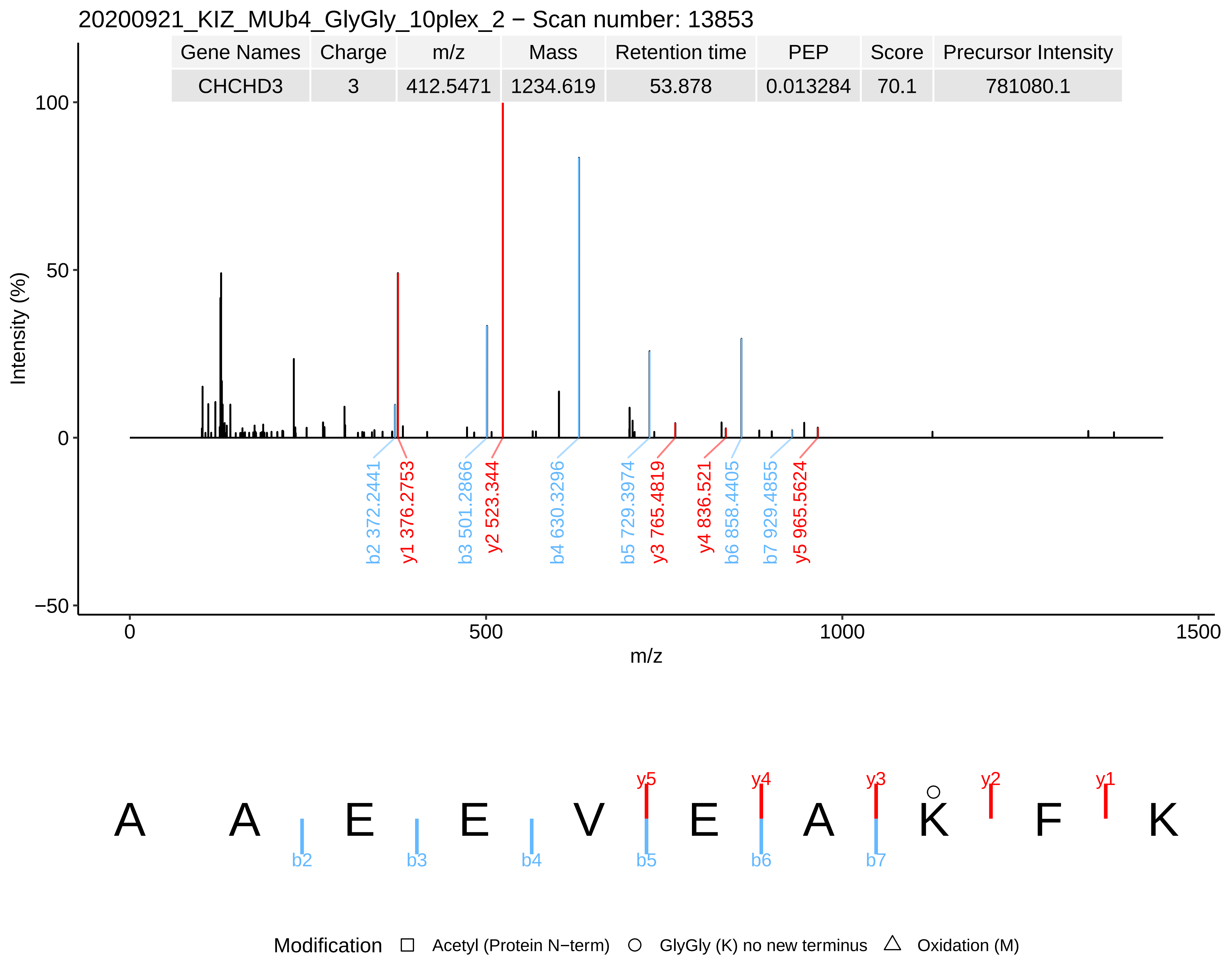
20200921_KIZ_MUb4_GlyGly_10plex_2 − Scan number: 13853
Gene Names
Charge
m/z
Mass
Retention time
PEP
Score
Precursor Intensity
CHCHD3
3
412.5471
1234.619
53.878
0.013284
70.1
781080.1
Intensity (%)
100
50
0
−50
0
500
1000
1500
m/z
b2 372.2441
y1 376.2753
b3 501.2866
y2 523.344
b4 630.3296
b5 729.3974
y3 765.4819
y4 836.521
b6 858.4405
b7 929.4855
y5 965.5624
A A E E V E A K F K
y5
y4
y3
y2
y1
b2
b3
b4
b5
b6
b7
Modification
Acetyl (Protein N−term)
GlyGly (K) no new terminus
Oxidation (M)

20200921_KIZ_MUb4_GlyGly_10plex − Scan number: 14246

Gene Names	Charge	m/z	Mass	Retention time	PEP	Score	Precursor Intensity
TRIM32	3	491.267	1470.779	50.554	3.5877e−06	112.64	26003578

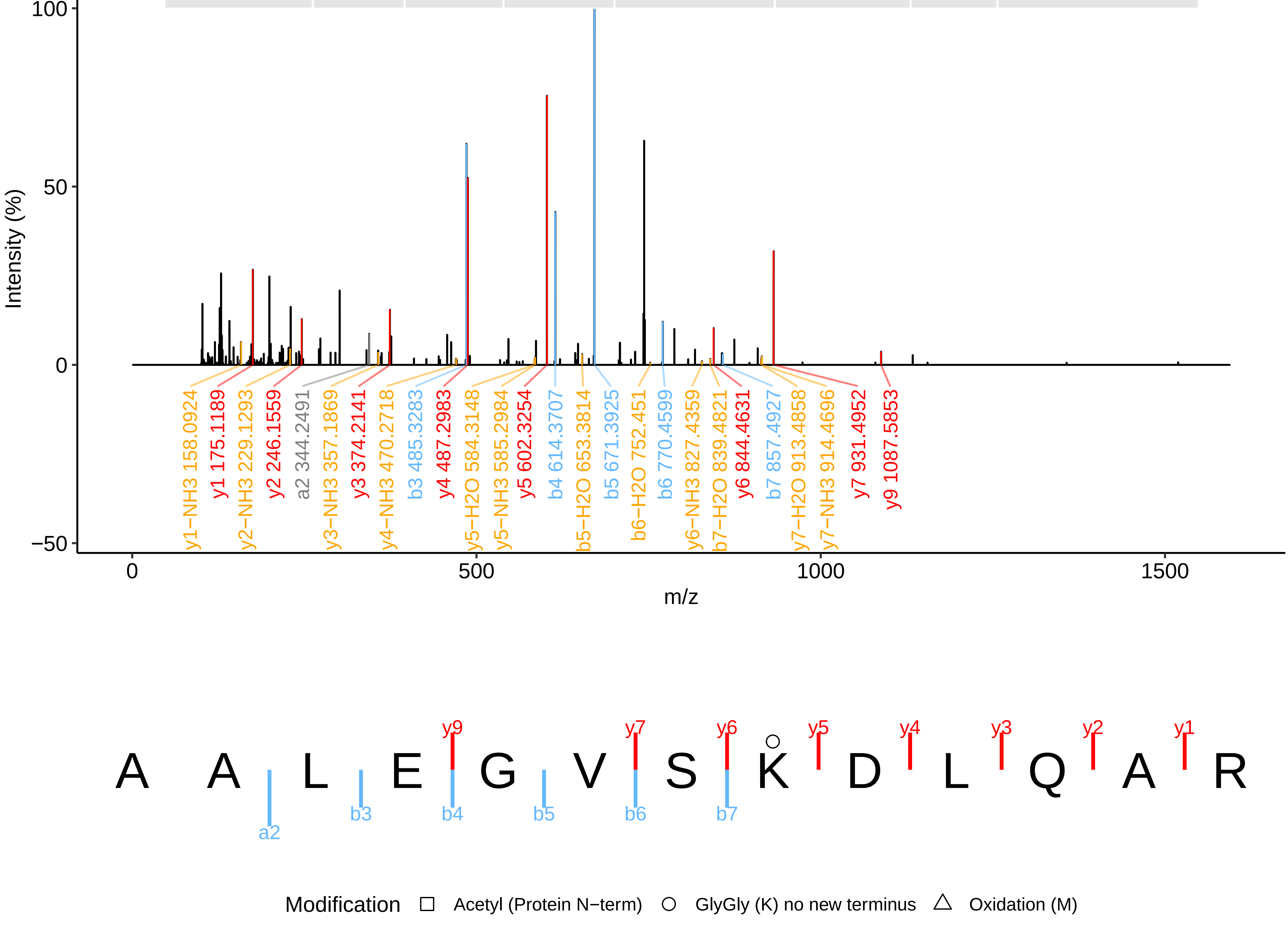

20200921_KIZ_MUb4_GlyGly_10plex_2 − Scan number: 13119

Gene Names	Charge	m/z	Mass	Retention time	PEP	Score	Precursor Intensity
TRIM32	3	491.267	1470.779	50.87	4.3882e−09	127.52	4101534

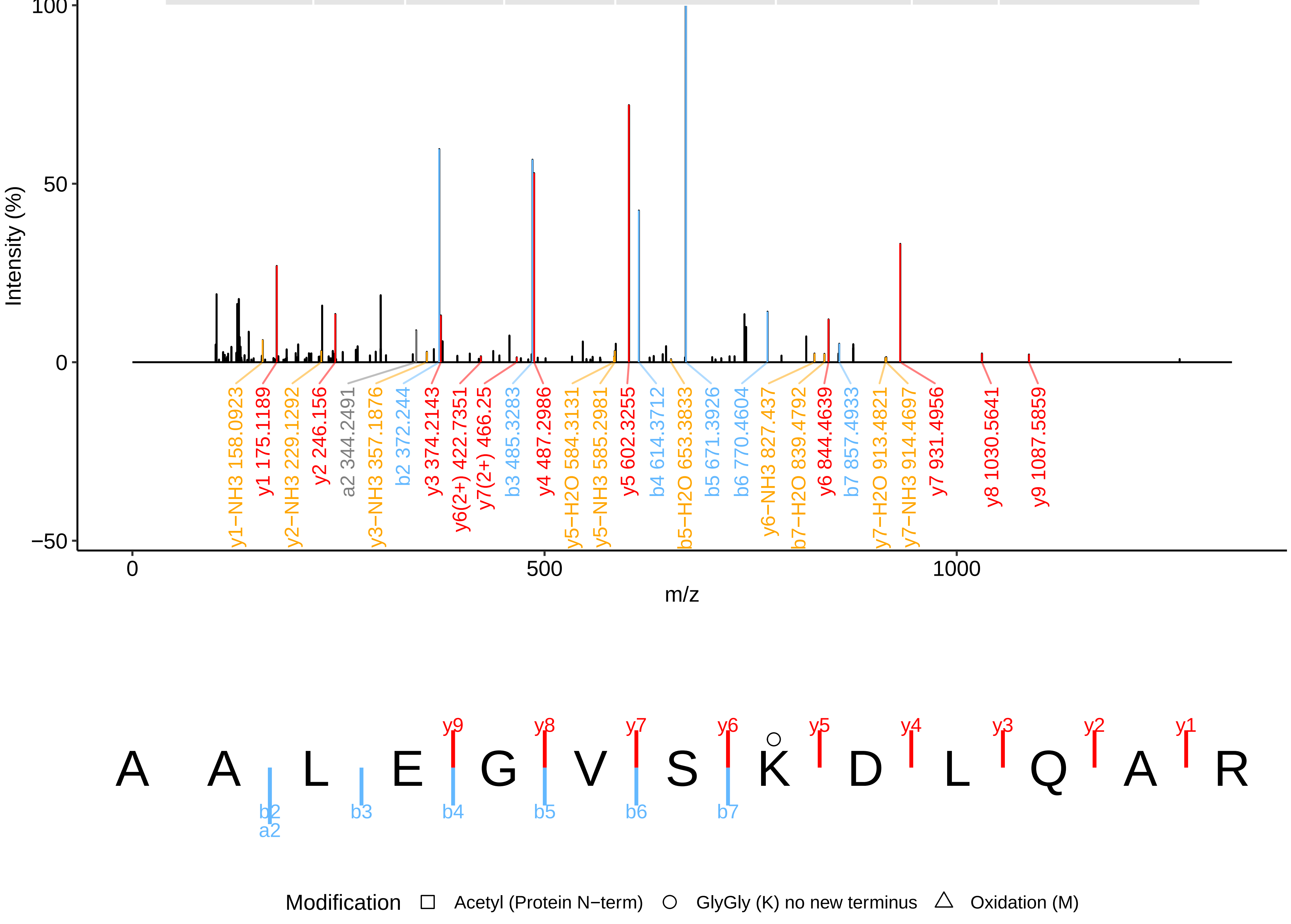

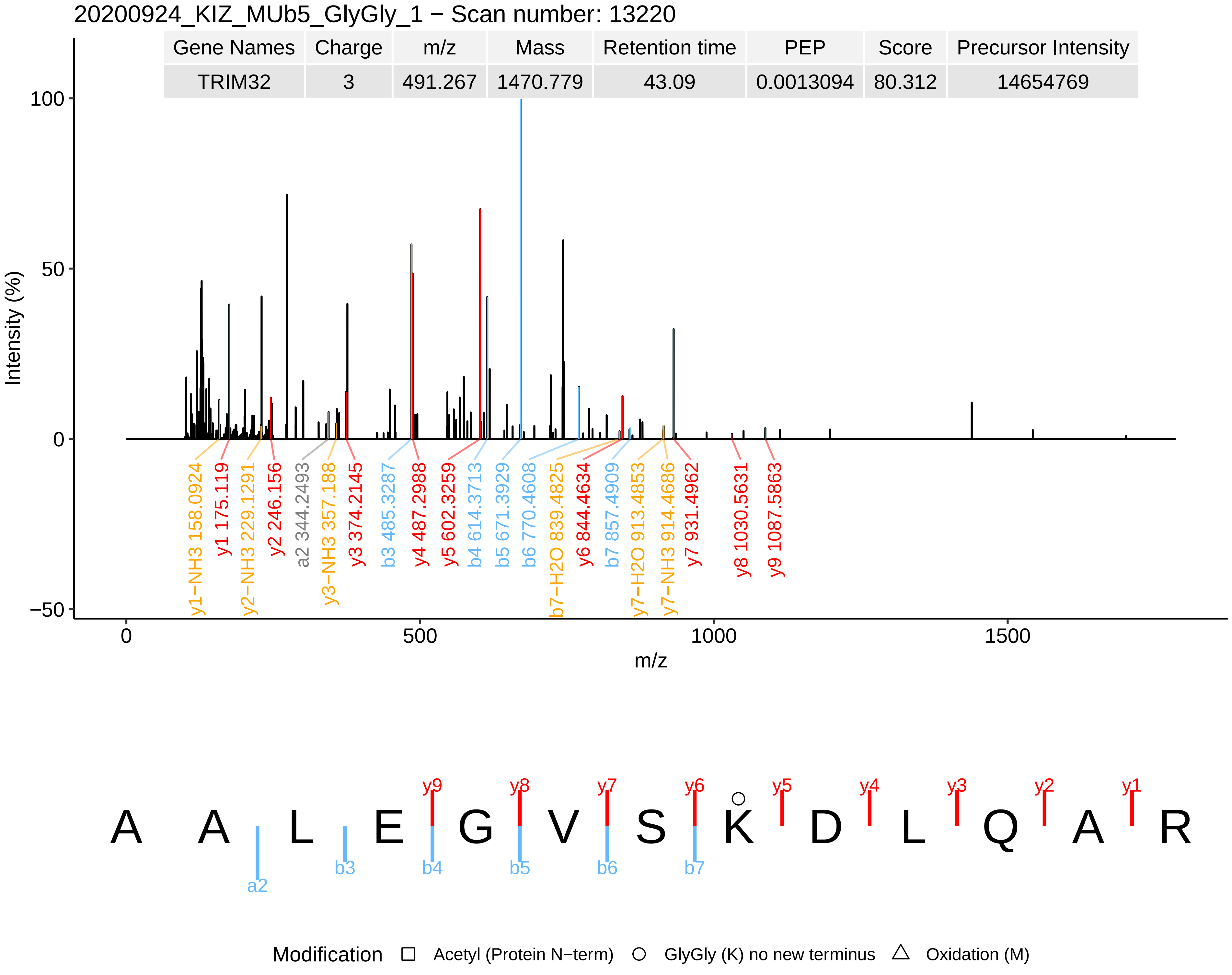
20200924_KIZ_MUb5_GlyGly_1 − Scan number: 13220
Gene Names | Charge | m/z | Mass | Retention time | PEP | Score | Precursor Intensity
TRIM32 | 3 | 491.267 | 1470.779 | 43.09 | 0.0013094 | 80.312 | 14654769
Intensity (%)
m/z
y1−NH3 158.0924
y1 175.119
y2−NH3 229.1291
y2 246.156
a2 344.2493
y3−NH3 357.188
y3 374.2145
b3 485.3287
y4 487.2988
y5 602.3259
b4 614.3713
b5 671.3929
b6 770.4608
b7−H2O 839.4825
y6 844.4634
b7 857.4909
y7−H2O 913.4853
y7−NH3 914.4686
y7 931.4962
y8 1030.5631
y9 1087.5863
A A L E G V S K D L Q A R
Modification
Acetyl (Protein N−term)
GlyGly (K) no new terminus
Oxidation (M)

20200924_KIZ_MUb5_GlyGly_1 − Scan number: 13055

Gene Names	Charge	m/z	Mass	Retention time	PEP	Score	Precursor Intensity
TRIM32	3	491.267	1470.779	42.567	0.0013788	83.53	NA

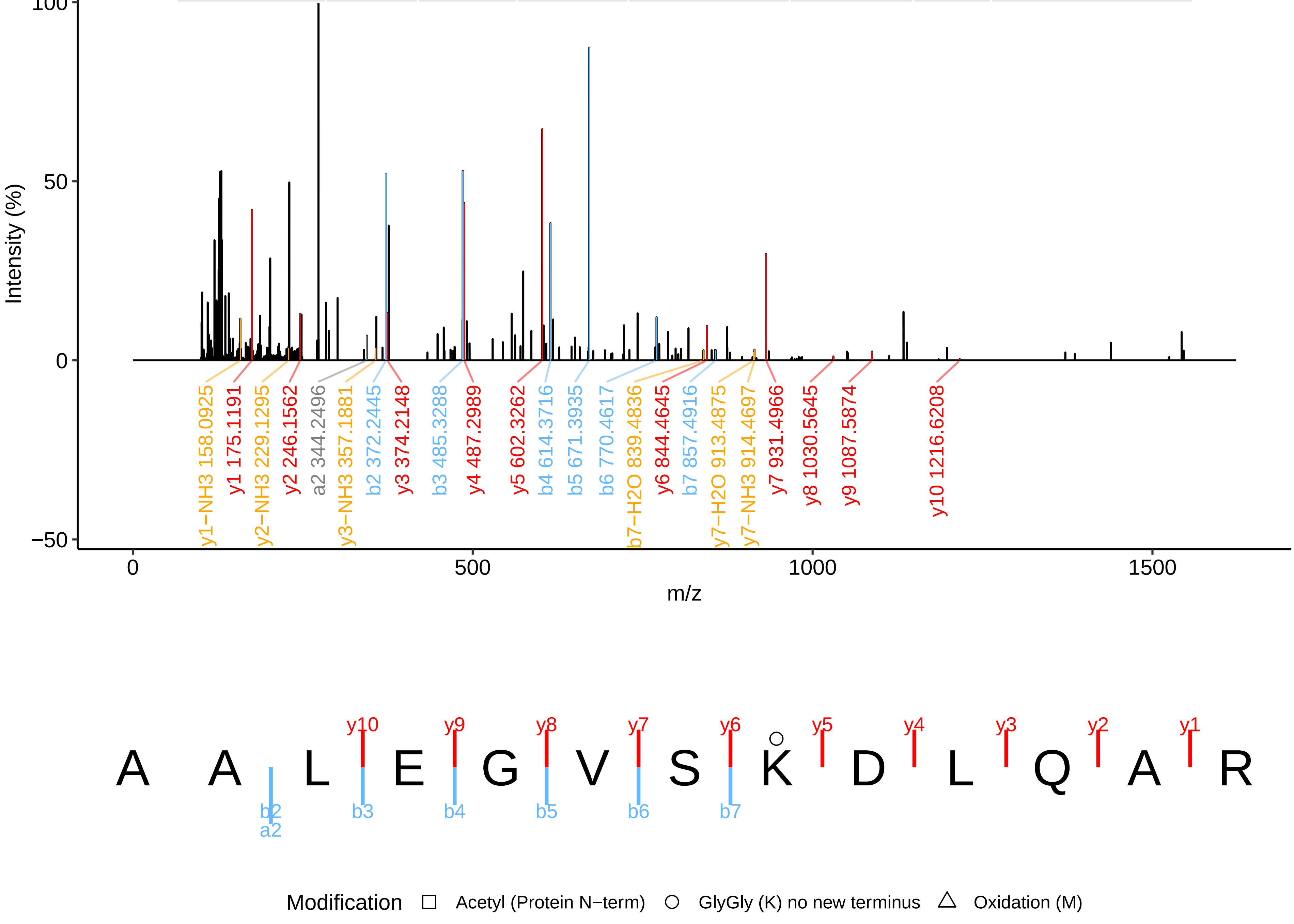

20200924_KIZ_MUb5_GlyGly_1 − Scan number: 13167

Gene Names	Charge	m/z	Mass	Retention time	PEP	Score	Precursor Intensity
TRIM32	3	491.267	1470.779	42.928	0.0047119	74.987	NA

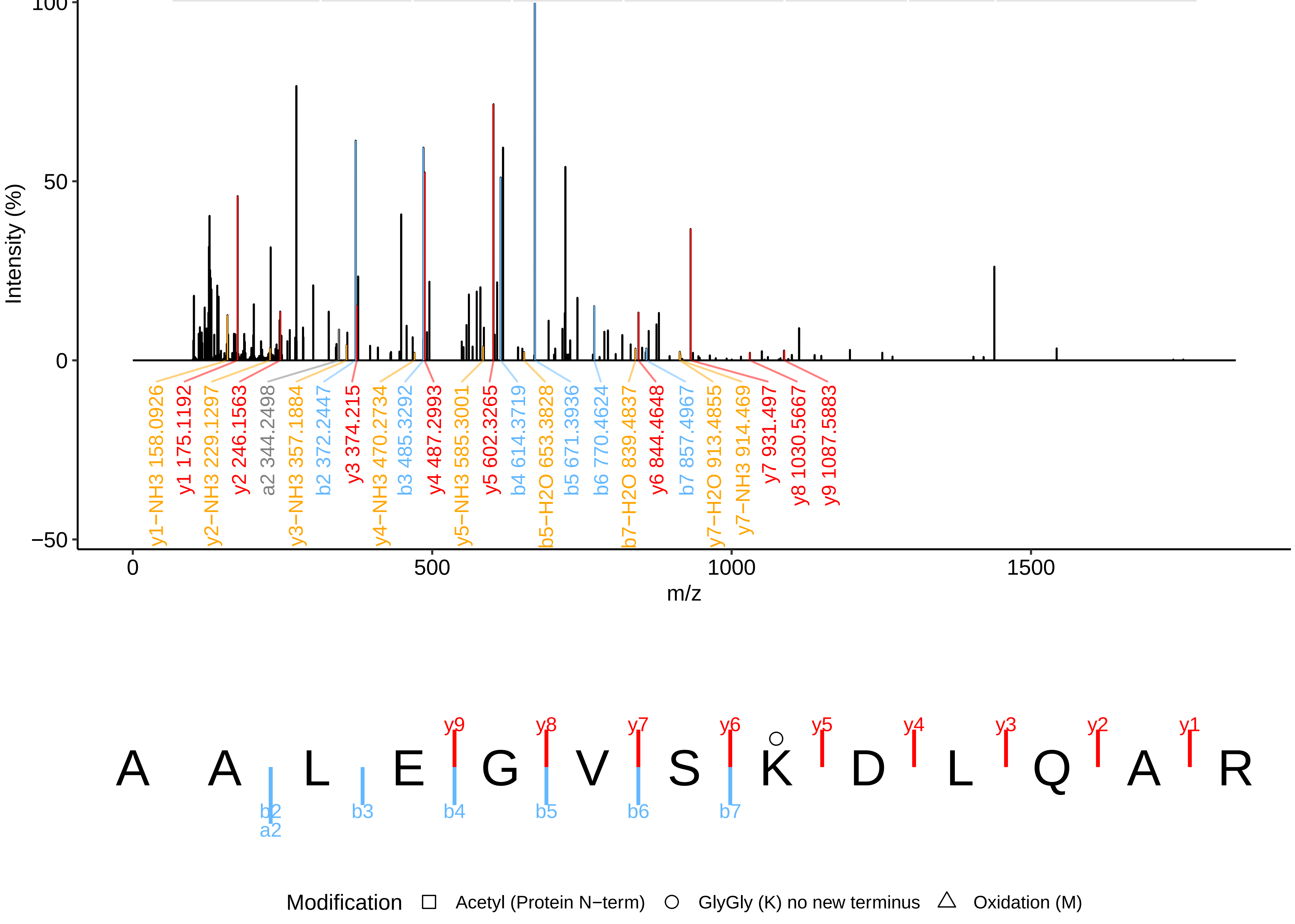

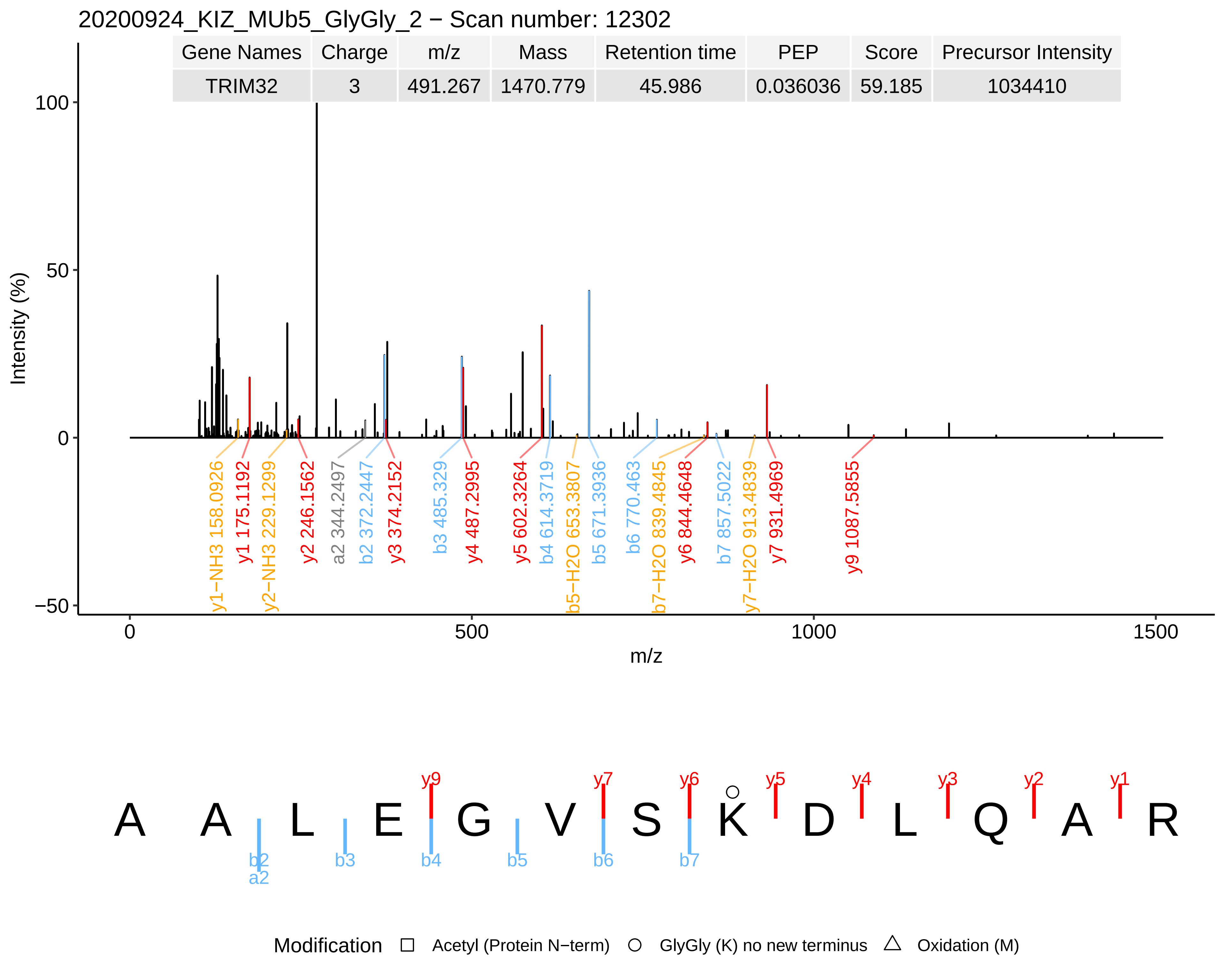
20200924_KIZ_MUb5_GlyGly_2 − Scan number: 12302
Gene Names | Charge | m/z | Mass | Retention time | PEP | Score | Precursor Intensity
TRIM32 | 3 | 491.267 | 1470.779 | 45.986 | 0.036036 | 59.185 | 1034410
Intensity (%)
100
50
0
−50
0
500
1000
1500
m/z
y1−NH3 158.0926
y1 175.1192
y2−NH3 229.1299
y2 246.1562
a2 344.2497
b2 372.2447
y3 374.2152
b3 485.329
y4 487.2995
y5 602.3264
b4 614.3719
b5−H2O 653.3807
b5 671.3936
b6 770.463
b7−H2O 839.4845
y6 844.4648
b7 857.5022
y7−H2O 913.4839
y7 931.4969
y9 1087.5855
A A L E G V S K D L Q A R
y9 y7 y6 y5 y4 y3 y2 y1
b2 a2 b3 b4 b5 b6 b7
Modification
Acetyl (Protein N−term)
GlyGly (K) no new terminus
Oxidation (M)

20201118_KIZ_MUb6_GlyGly_2 − Scan number: 15083

Gene Names	Charge	m/z	Mass	Retention time	PEP	Score	Precursor Intensity
TRIM32	3	491.267	1470.779	50.912	1.7696e−05	101.72	3004985

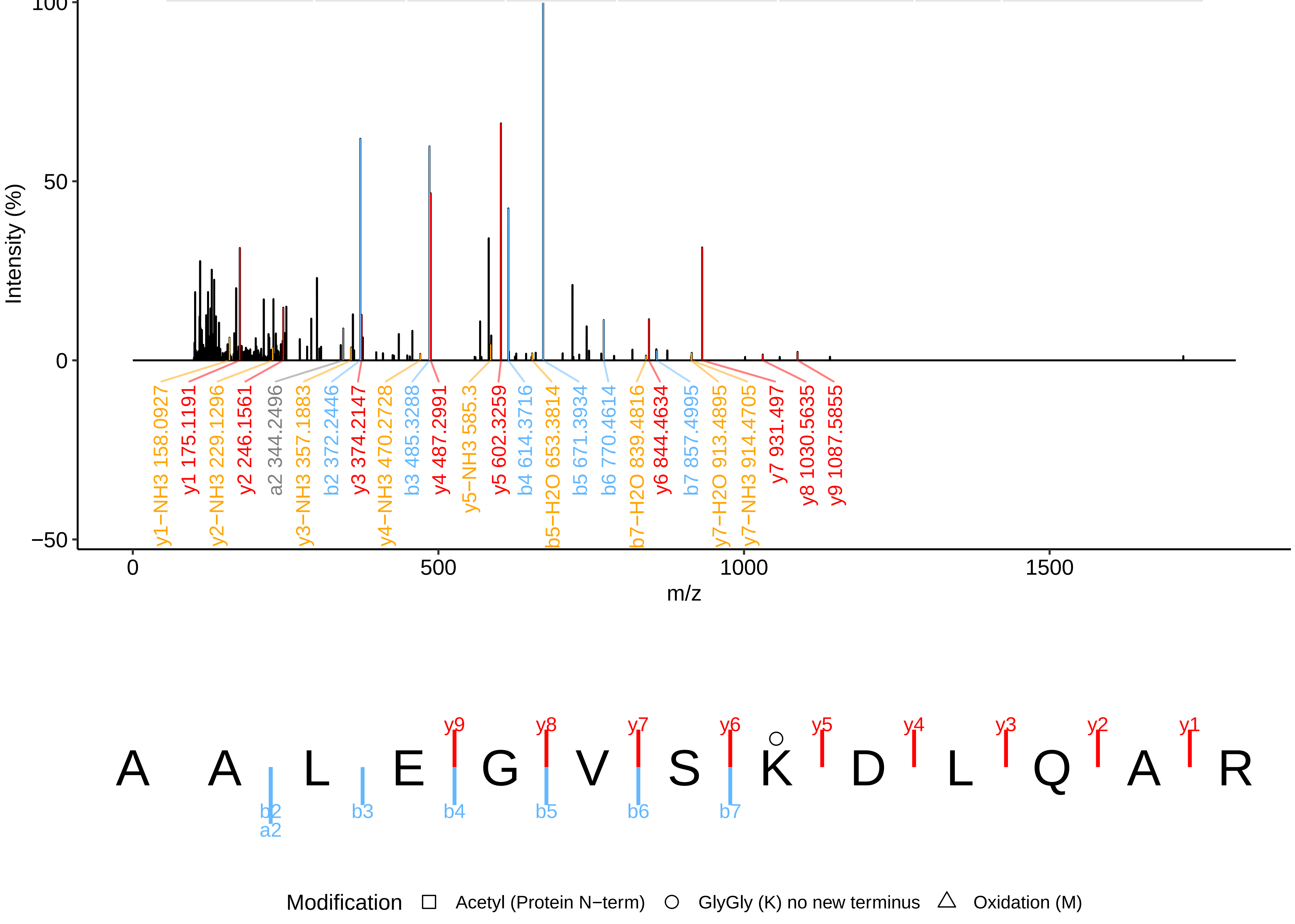

20200921_KIZ_MUb4_GlyGly_10plex − Scan number: 2675

Gene Names	Charge	m/z	Mass	Retention time	PEP	Score	Precursor Intensity
HSPA8	4	308.9021	1231.579	8.4273	0.0017045	107.69	12909322

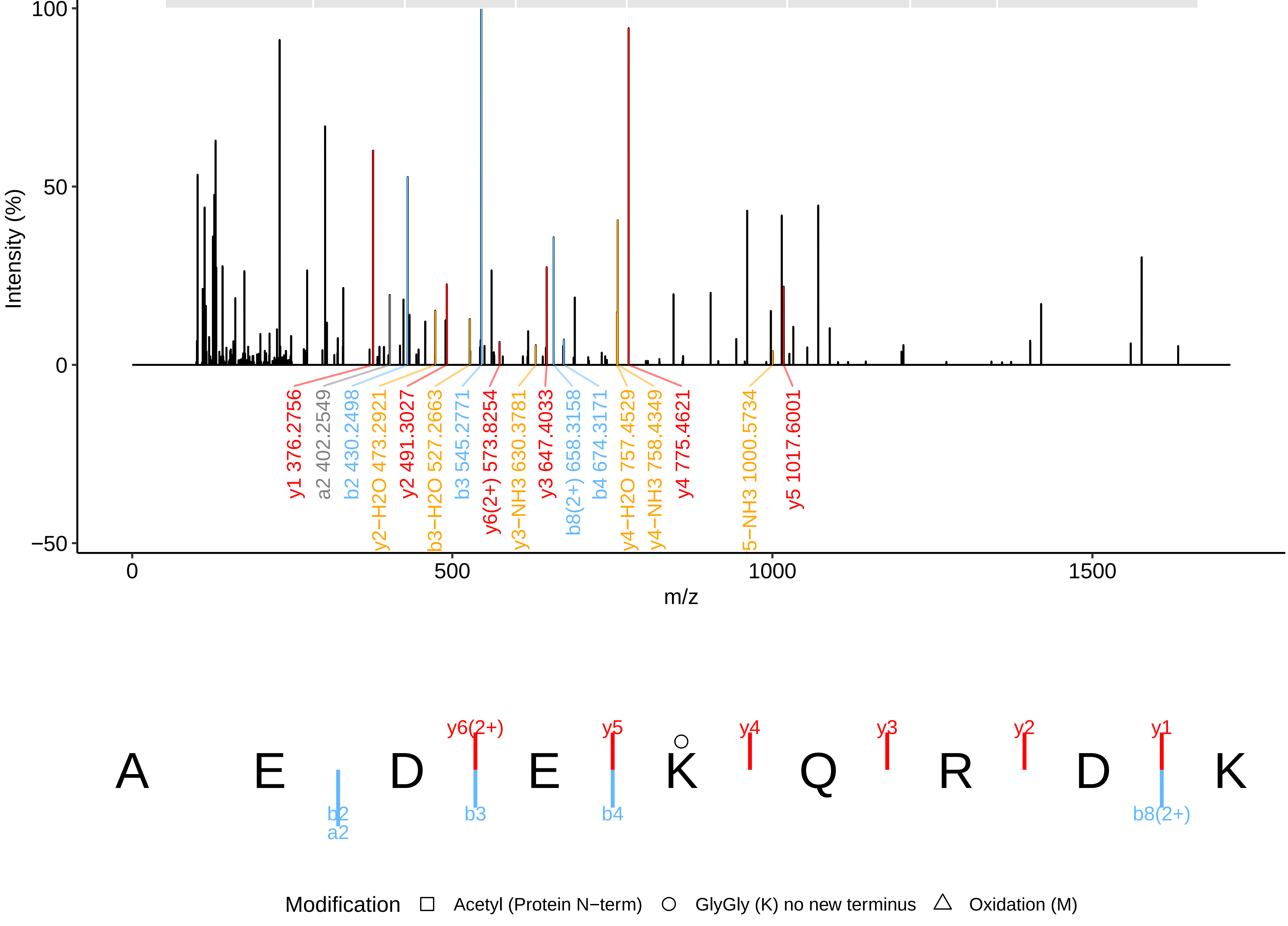

20201118_KIZ_MUb6_GlyGly_1 − Scan number: 2661

Gene Names	Charge	m/z	Mass	Retention time	PEP	Score	Precursor Intensity
HSPA8	4	308.9021	1231.579	8.143	0.022624	80.688	6634869

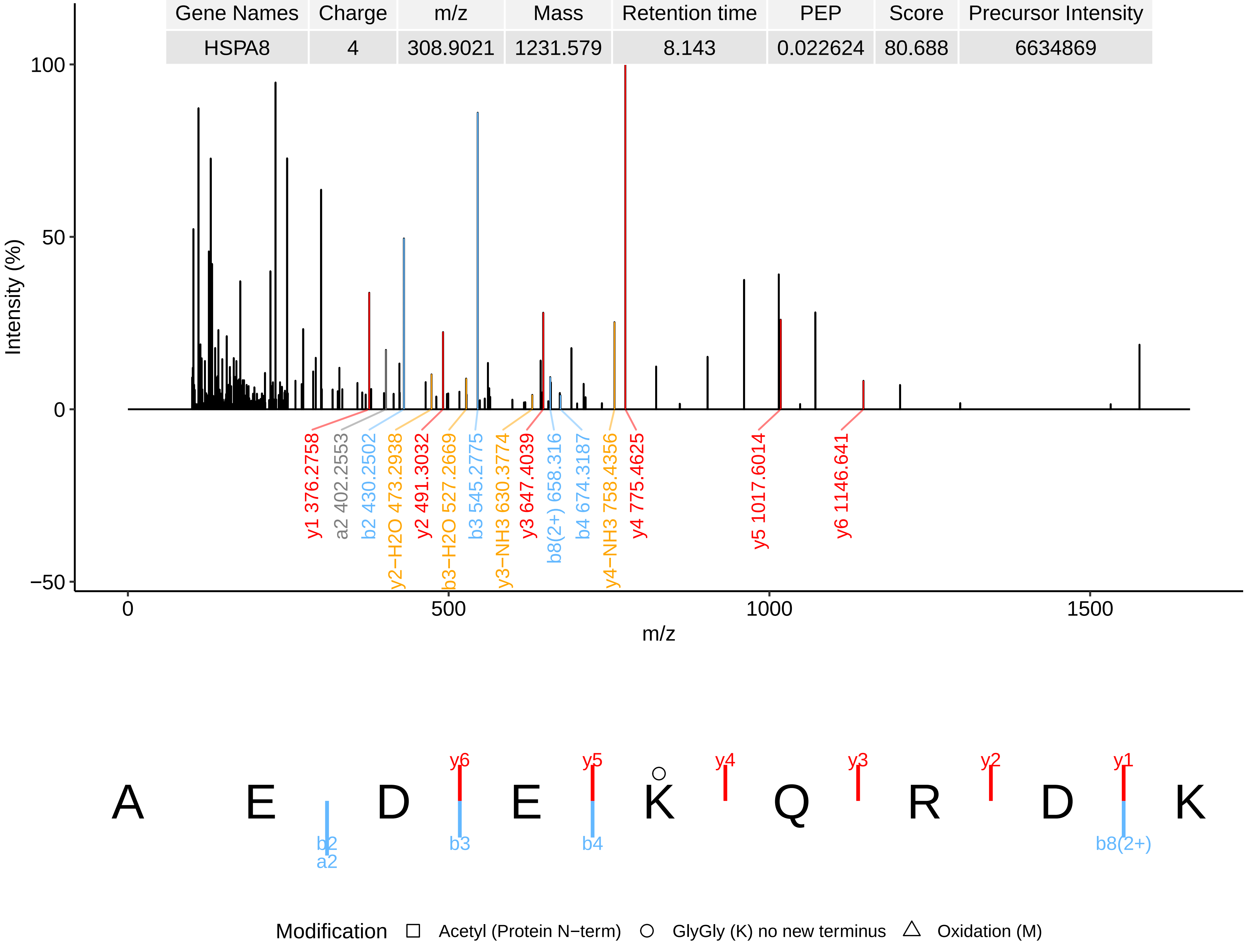

20201118_KIZ_MUb6_GlyGly_2 − Scan number: 2855

Gene Names	Charge	m/z	Mass	Retention time	PEP	Score	Precursor Intensity
HSPA8	4	308.9021	1231.579	9.1344	0.001963	106.42	5637164

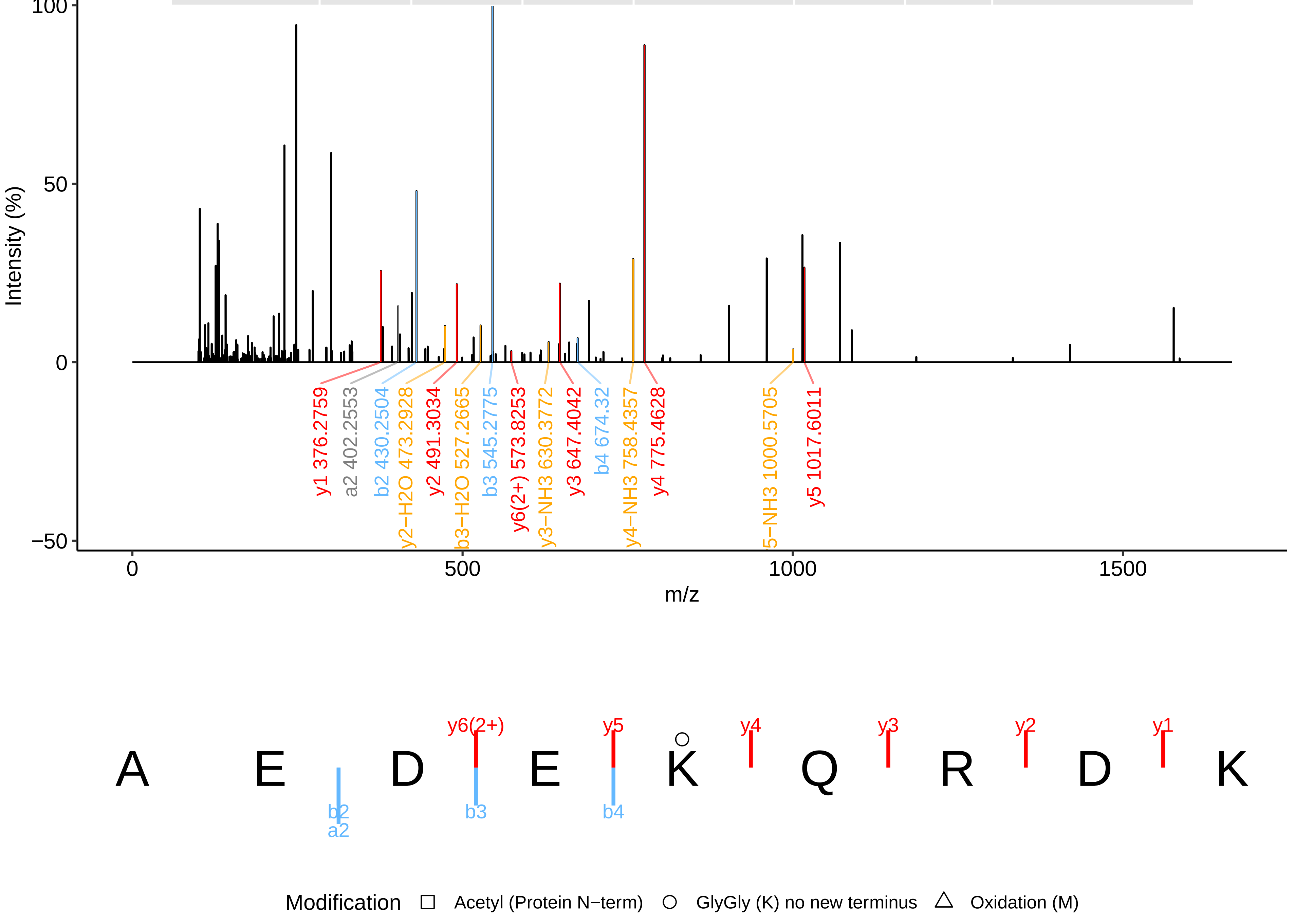

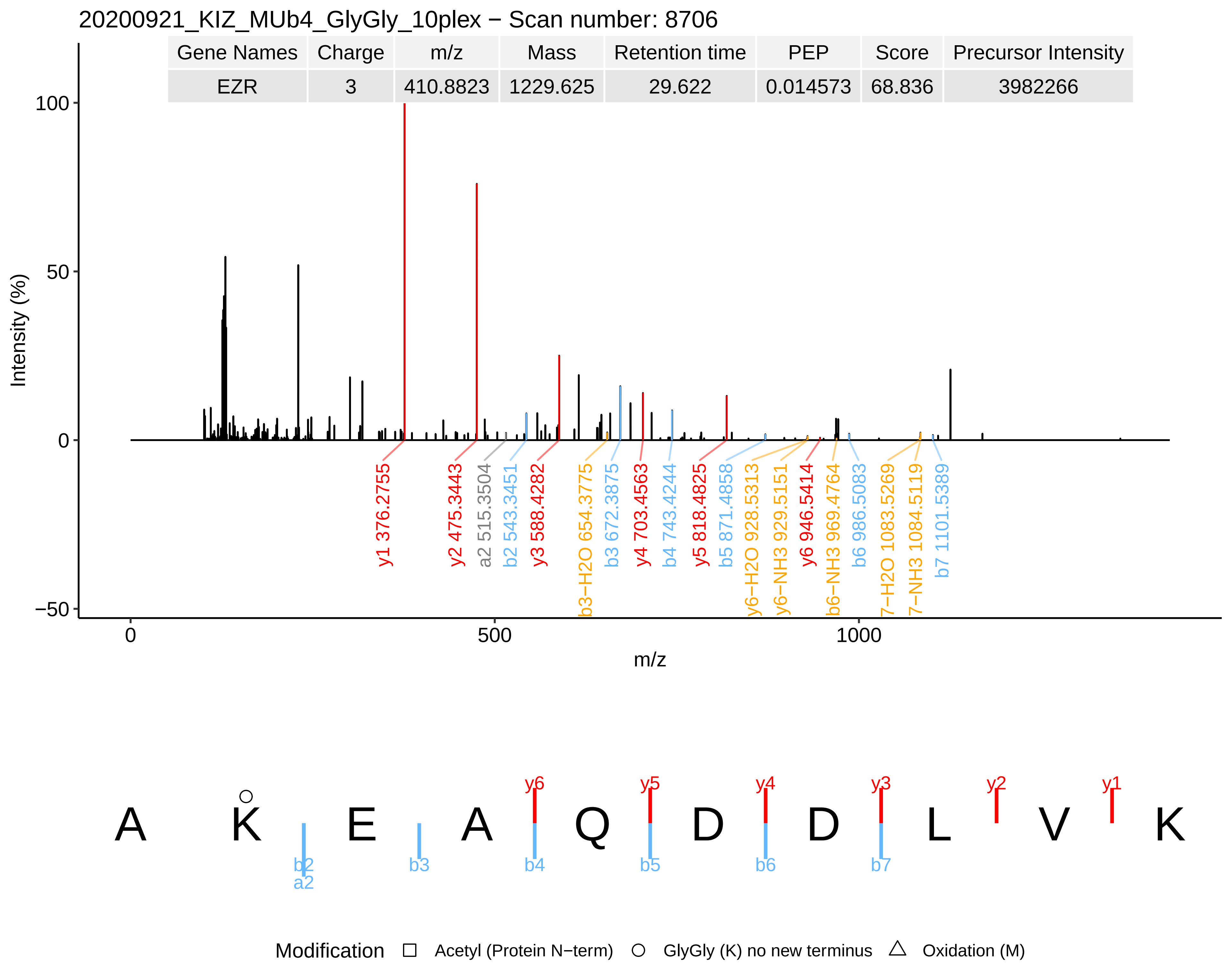
20200921_KIZ_MUb4_GlyGly_10plex − Scan number: 8706
Gene Names | Charge | m/z | Mass | Retention time | PEP | Score | Precursor Intensity
EZR | 3 | 410.8823 | 1229.625 | 29.622 | 0.014573 | 68.836 | 3982266
Intensity (%)
100
50
0
−50
0
500
1000
m/z
y1 376.2755
y2 475.3443
a2 515.3504
b2 543.3451
y3 588.4282
b3−H2O 654.3775
b3 672.3875
y4 703.4563
b4 743.4244
y5 818.4825
b5 871.4858
y6−H2O 928.5313
y6−NH3 929.5151
y6 946.5414
b6−NH3 969.4764
b6 986.5083
7−H2O 1083.5269
7−NH3 1084.5119
b7 1101.5389
A K E A Q D D L V K
y6 y5 y4 y3 y2 y1
b2 a2 b3 b4 b5 b6 b7
Modification
Acetyl (Protein N−term)
GlyGly (K) no new terminus
Oxidation (M)

20200921_KIZ_MUb4_GlyGly_10plex_2 − Scan number: 8014

Gene Names	Charge	m/z	Mass	Retention time	PEP	Score	Precursor Intensity
EZR	3	410.8823	1229.625	30.103	0.086566	50.284	410288.7

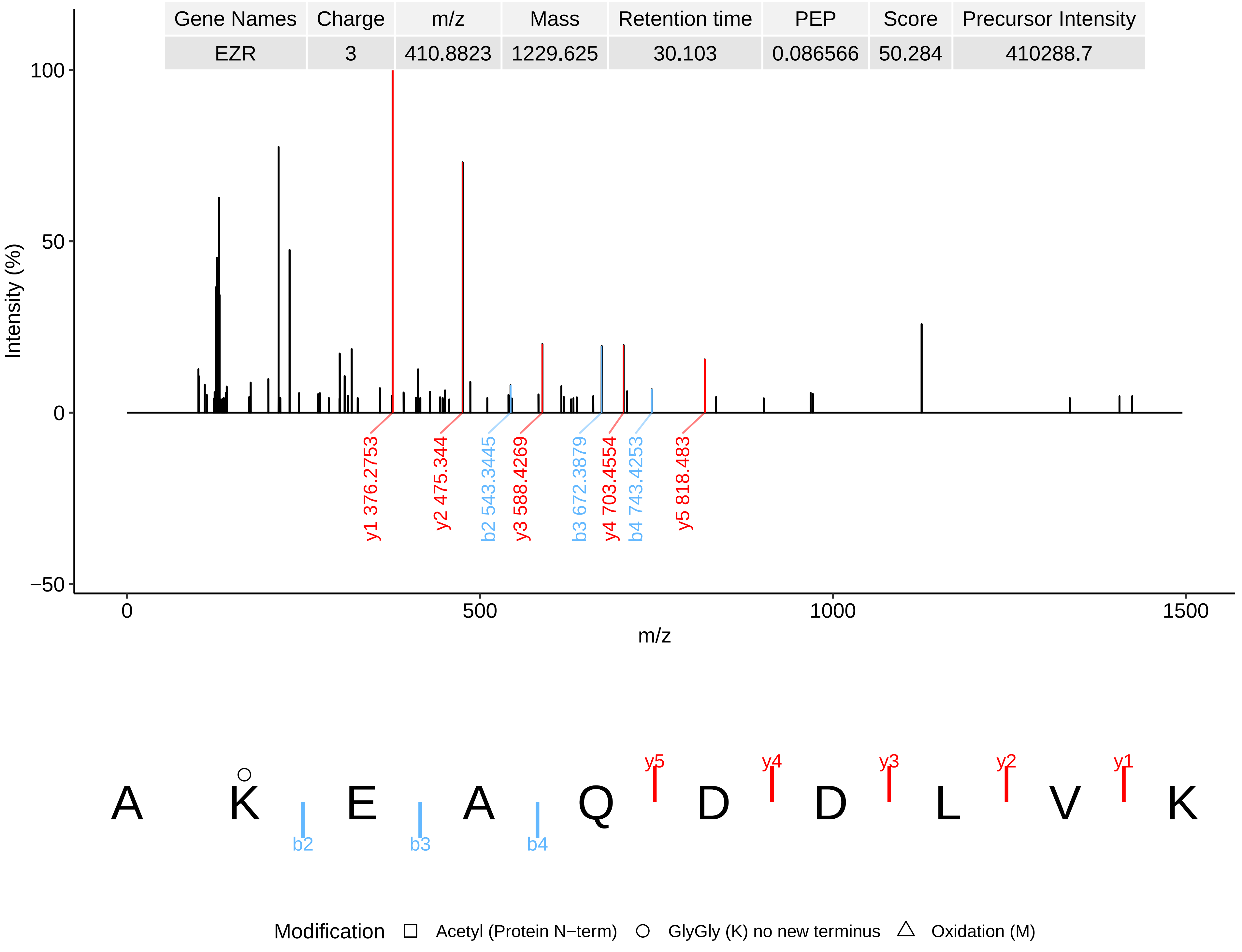

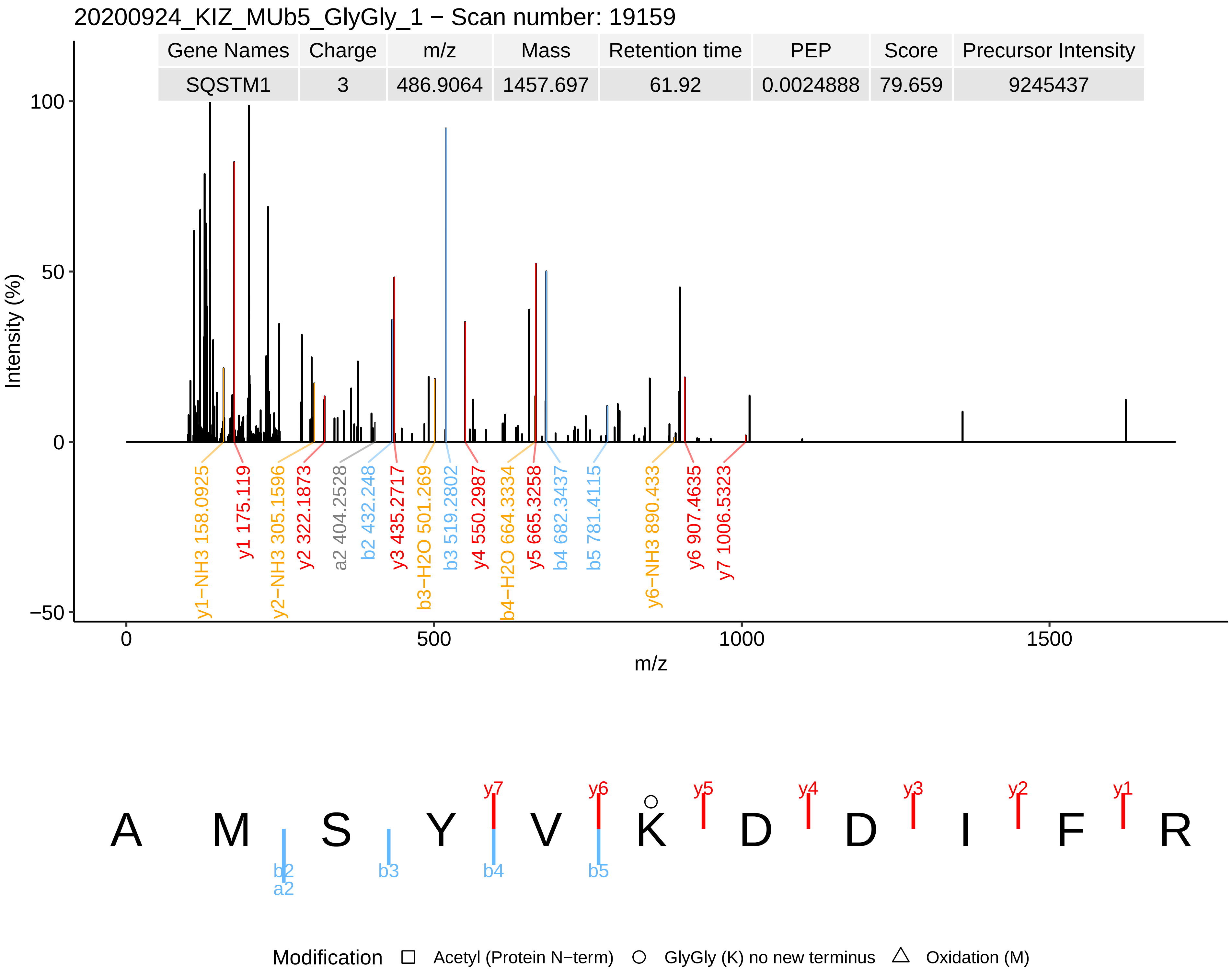
20200924_KIZ_MUb5_GlyGly_1 − Scan number: 19159
Gene Names | Charge | m/z | Mass | Retention time | PEP | Score | Precursor Intensity
SQSTM1 | 3 | 486.9064 | 1457.697 | 61.92 | 0.0024888 | 79.659 | 9245437
Intensity (%)
100
50
0
−50
0
500
1000
1500
m/z
y1−NH3 158.0925
y1 175.119
y2−NH3 305.1596
y2 322.1873
a2 404.2528
b2 432.248
y3 435.2717
b3−H2O 501.269
b3 519.2802
y4 550.2987
b4−H2O 664.3334
y5 665.3258
b4 682.3437
b5 781.4115
y6−NH3 890.433
y6 907.4635
y7 1006.5323
A M S Y V K D D I F R
y7 y6 y5 y4 y3 y2 y1
b2 a2 b3 b4 b5
Modification
Acetyl (Protein N−term)
GlyGly (K) no new terminus
Oxidation (M)

20201118_KIZ_MUb6_GlyGly_2 − Scan number: 21139

Gene Names	Charge	m/z	Mass	Retention time	PEP	Score	Precursor Intensity
SQSTM1	3	486.9064	1457.697	71.231	2.0359e−08	126.39	12086259

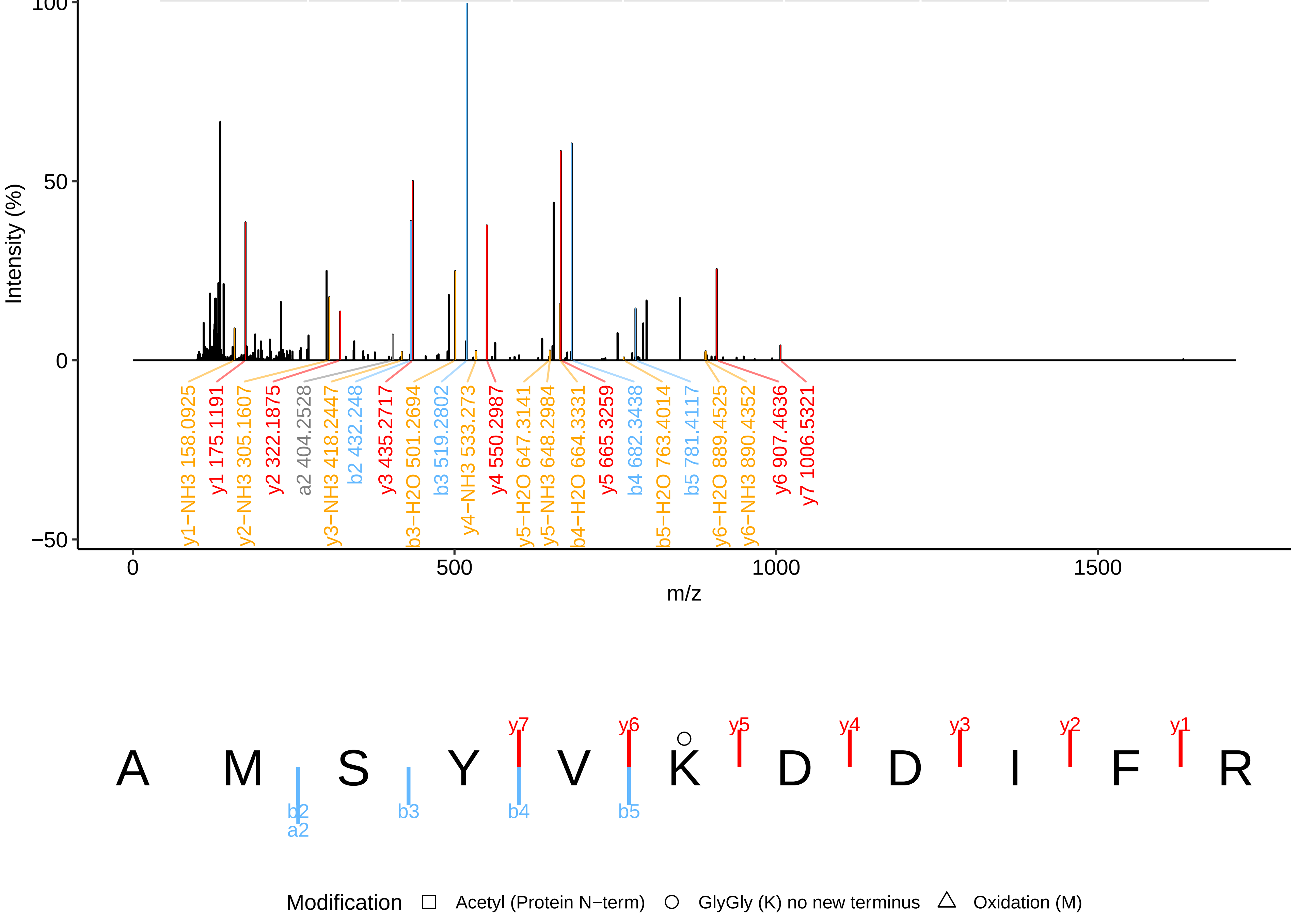

20200924_KIZ_MUb5_GlyGly_1 − Scan number: 13867

Gene Names	Charge	m/z	Mass	Retention time	PEP	Score	Precursor Intensity
SQSTM1	3	492.238	1473.692	45.24	0.02223	67.385	NA

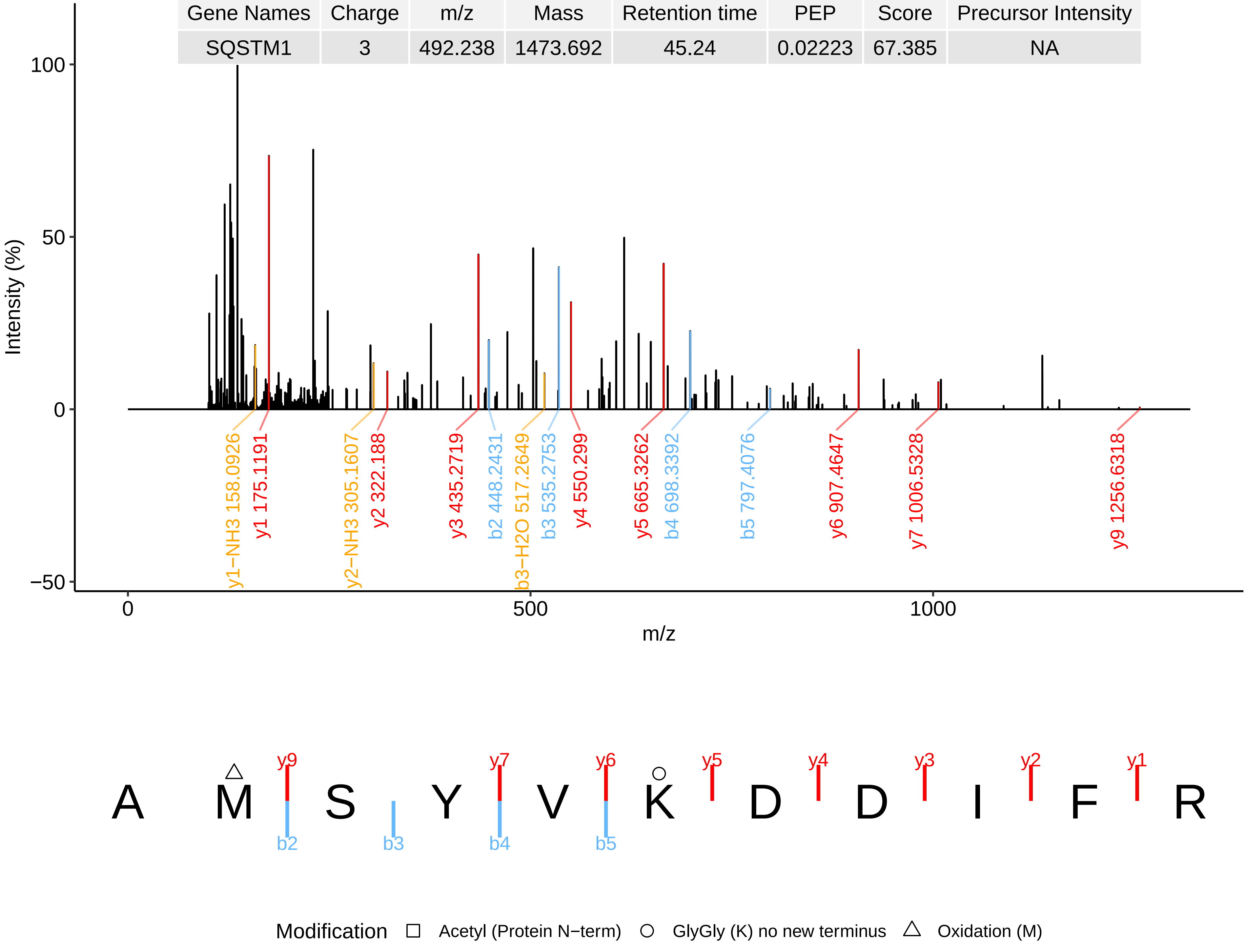

20200924_KIZ_MUb5_GlyGly_2 − Scan number: 13069

Gene Names	Charge	m/z	Mass	Retention time	PEP	Score	Precursor Intensity
SQSTM1	3	492.238	1473.692	48.882	0.051228	60.255	NA

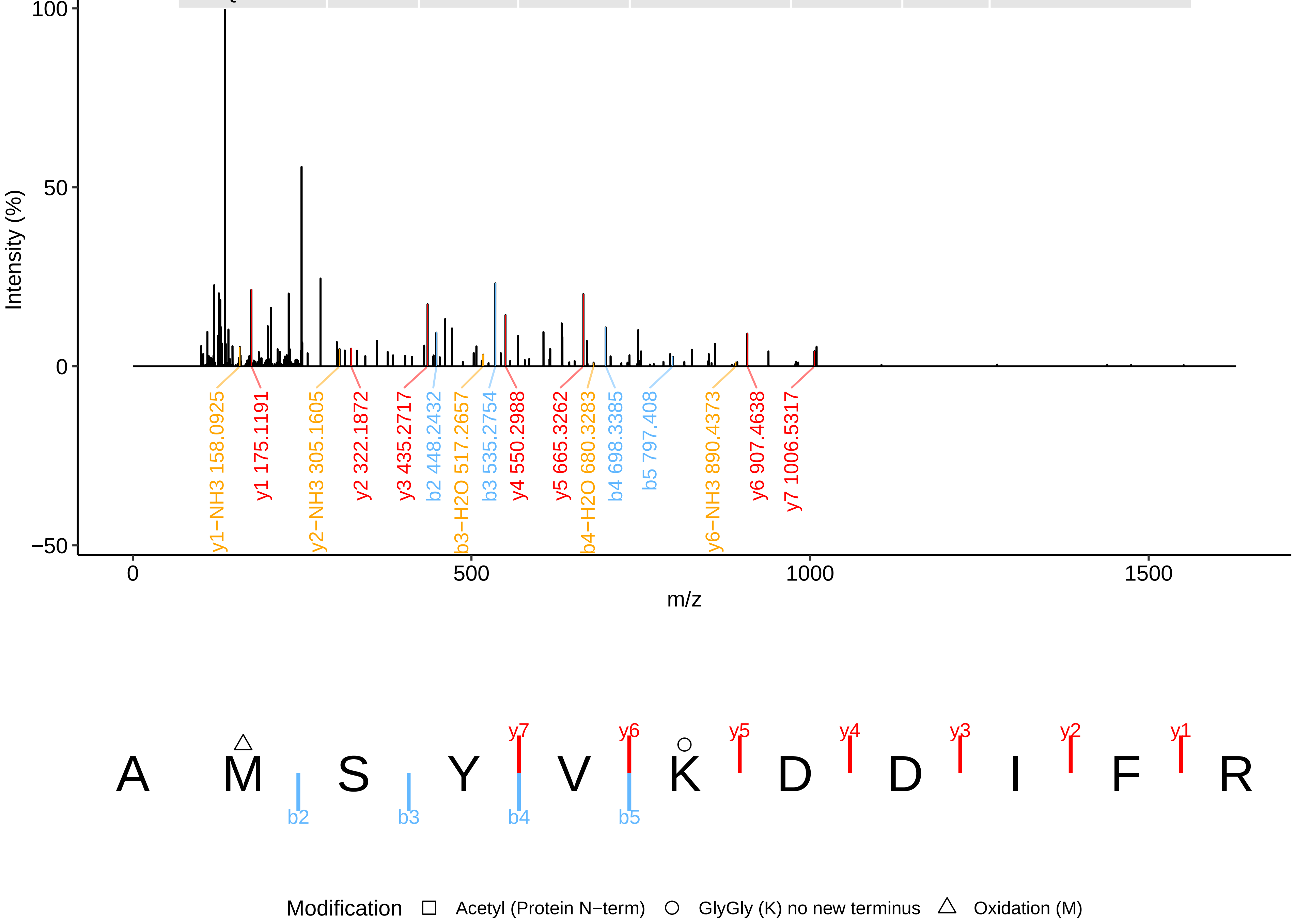

20200921_KIZ_MUb4_GlyGly_10plex − Scan number: 9878

Gene Names	Charge	m/z	Mass	Retention time	PEP	Score	Precursor Intensity
HSPA1B;HSPA1A;HSPA6	3	411.2262	1230.657	33.983	0.011143	72.2	6197807

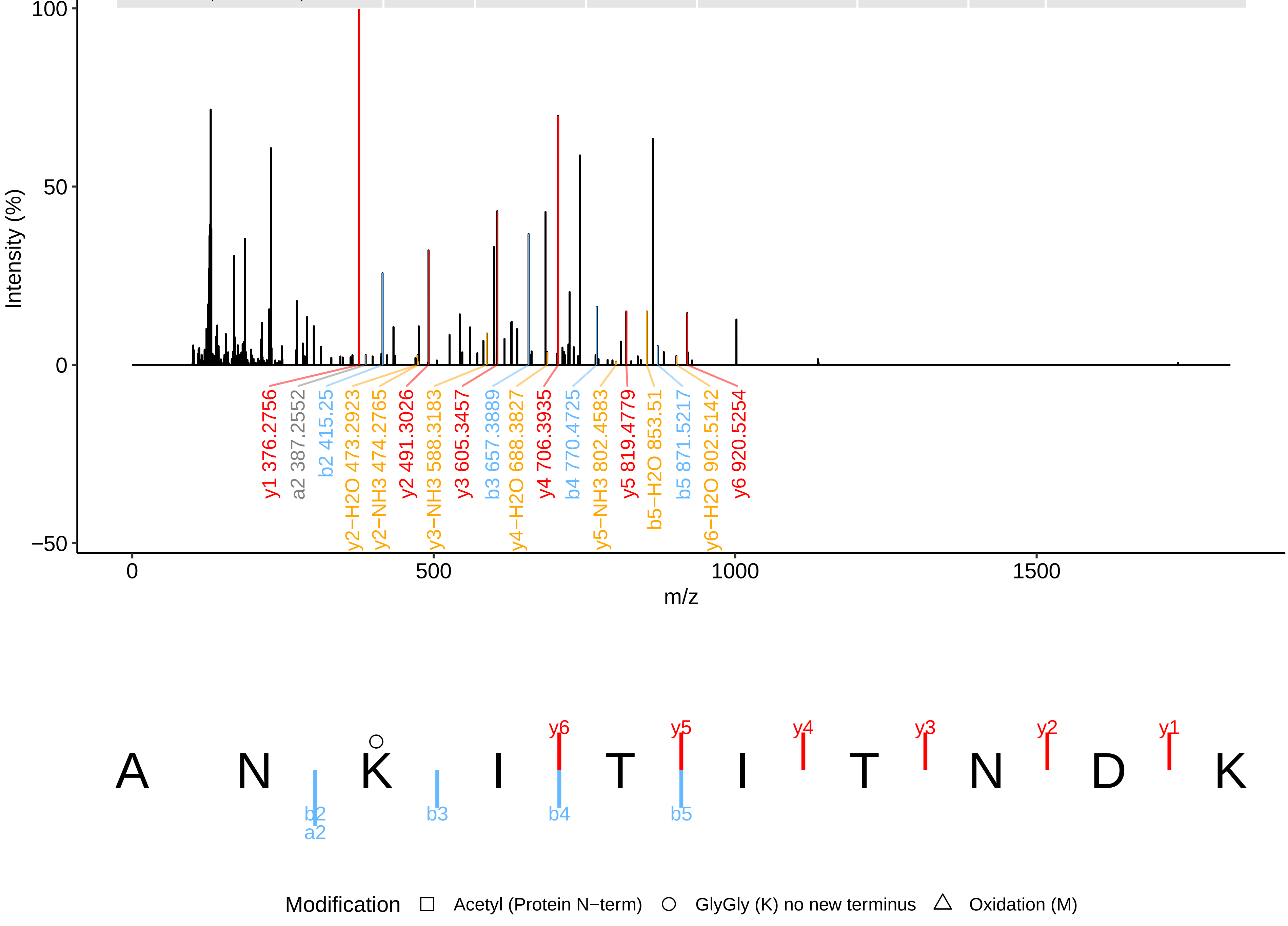

20200921_KIZ_MUb4_GlyGly_10plex_2 − Scan number: 9097

Gene Names	Charge	m/z	Mass	Retention time	PEP	Score	Precursor Intensity
HSPA1B;HSPA1A;HSPA6	3	411.2262	1230.657	34.485	0.0012586	89.827	1422155

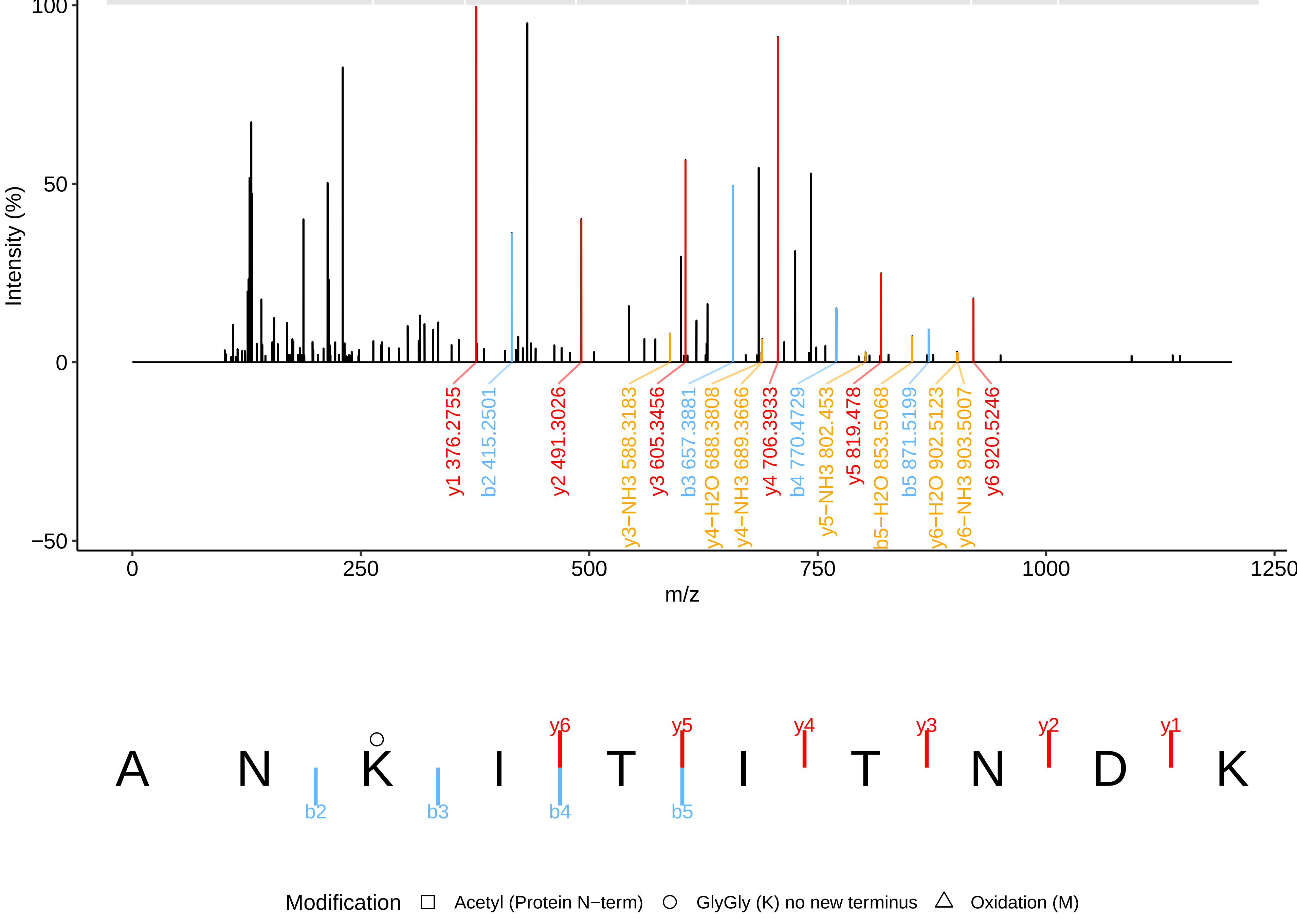

20200924_KIZ_MUb5_GlyGly_2 − Scan number: 8284

Gene Names	Charge	m/z	Mass	Retention time	PEP	Score	Precursor Intensity
HSPA1B;HSPA1A;HSPA6	3	411.2262	1230.657	30.943	0.021658	64.439	NA

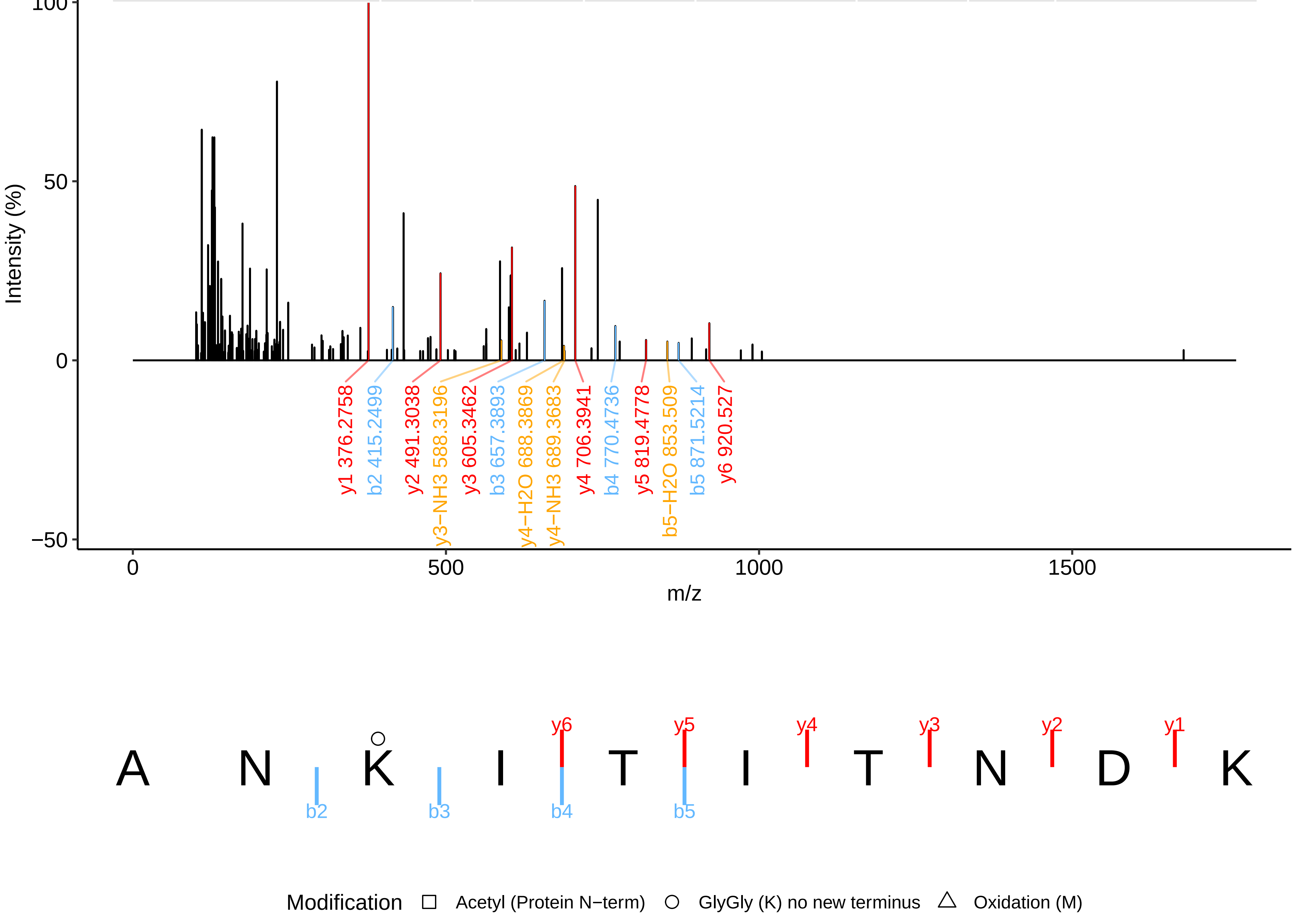

20200921_KIZ_MUb4_GlyGly_10plex − Scan number: 14082

Gene Names	Charge	m/z	Mass	Retention time	PEP	Score	Precursor Intensity
EPS15	3	486.2633	1455.768	49.922	1.2304e−16	156.01	6136344

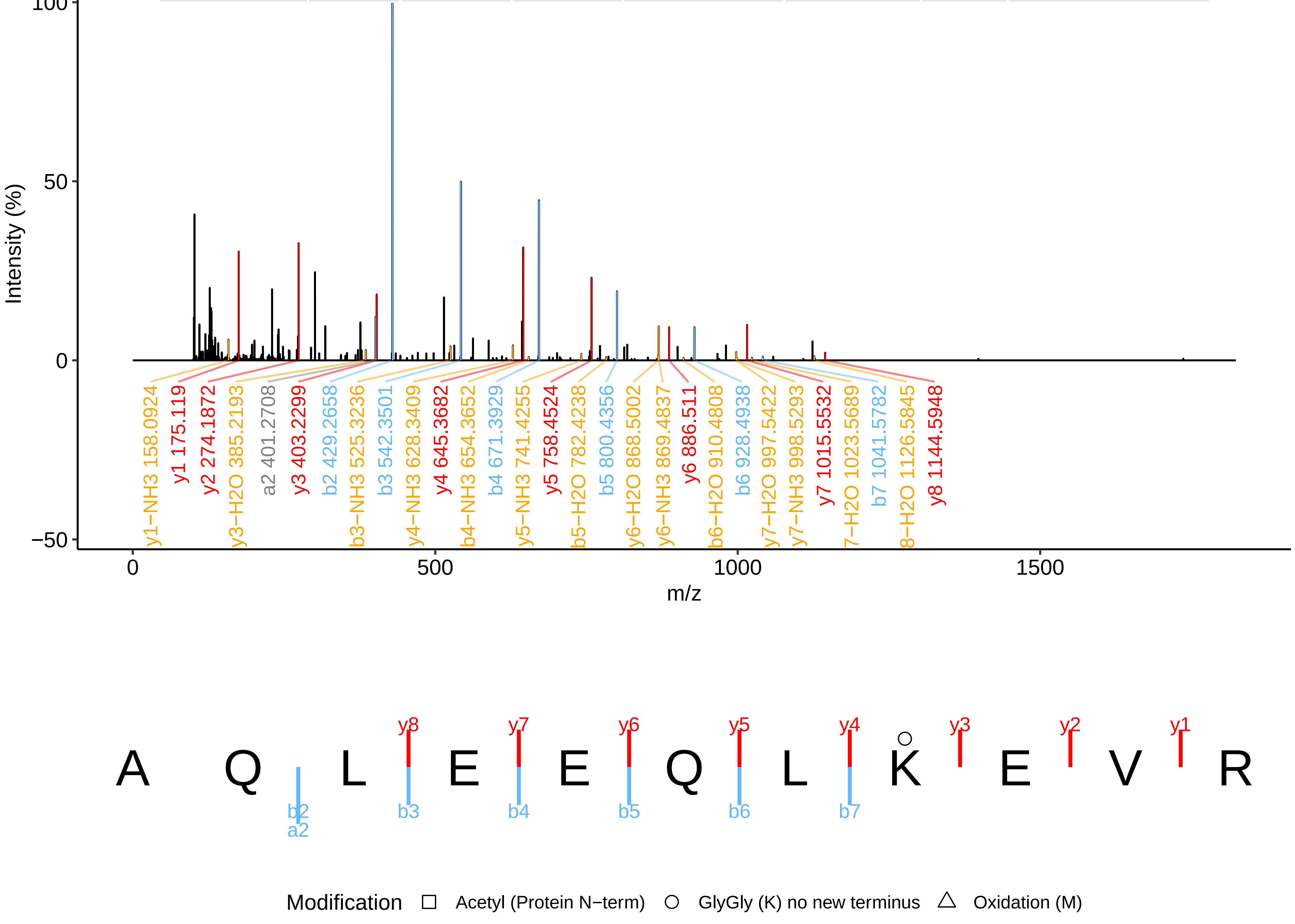

20200921_KIZ_MUb4_GlyGly_10plex_2 − Scan number: 12956

Gene Names	Charge	m/z	Mass	Retention time	PEP	Score	Precursor Intensity
EPS15	3	486.2633	1455.768	50.252	1.5971e−05	109.24	826431.9

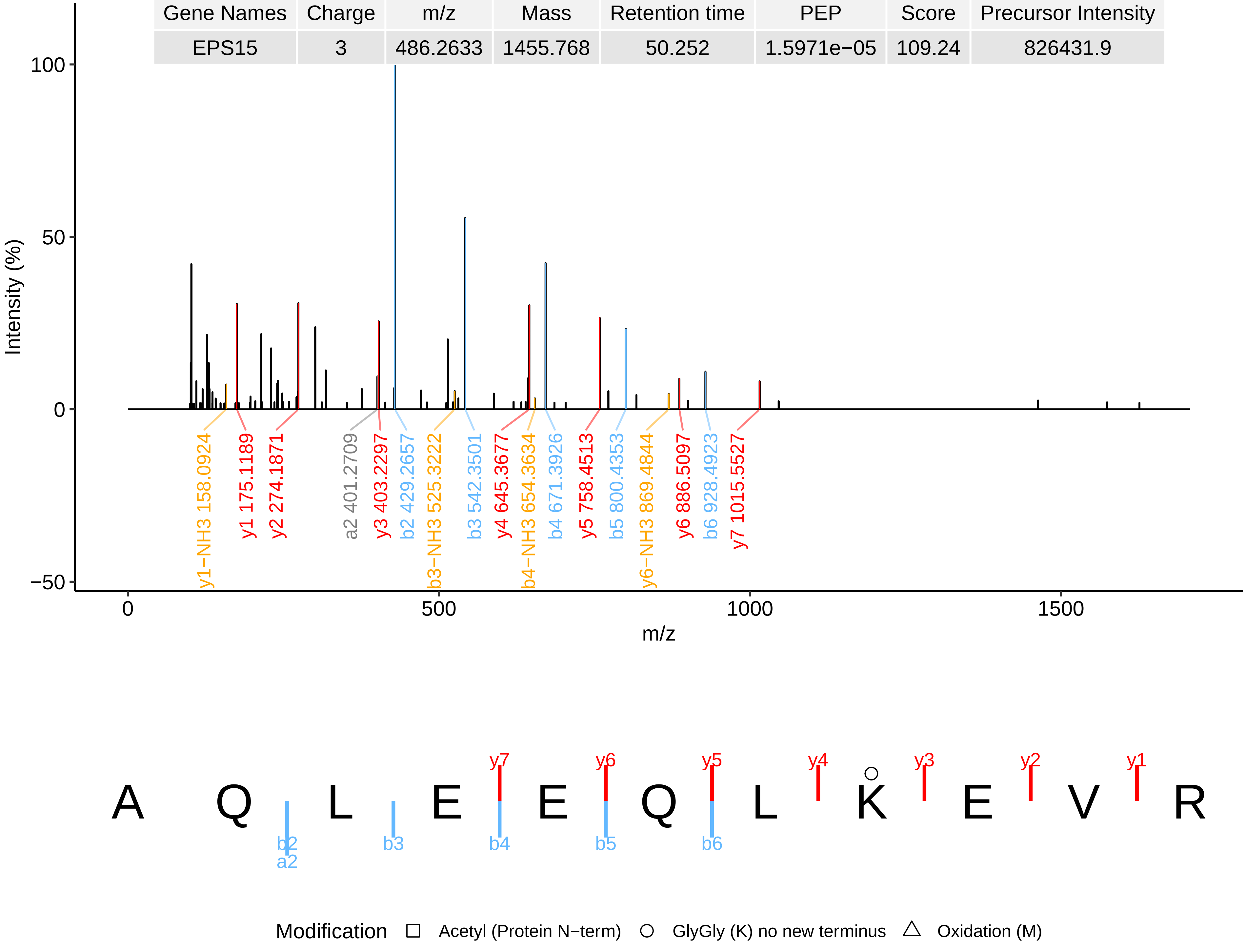

20200921_KIZ_MUb4_GlyGly_10plex_2 − Scan number: 7923

Gene Names	Charge	m/z	Mass	Retention time	PEP	Score	Precursor Intensity
VDAC3	3	408.8788	1223.615	29.72	0.011342	72.006	1453919

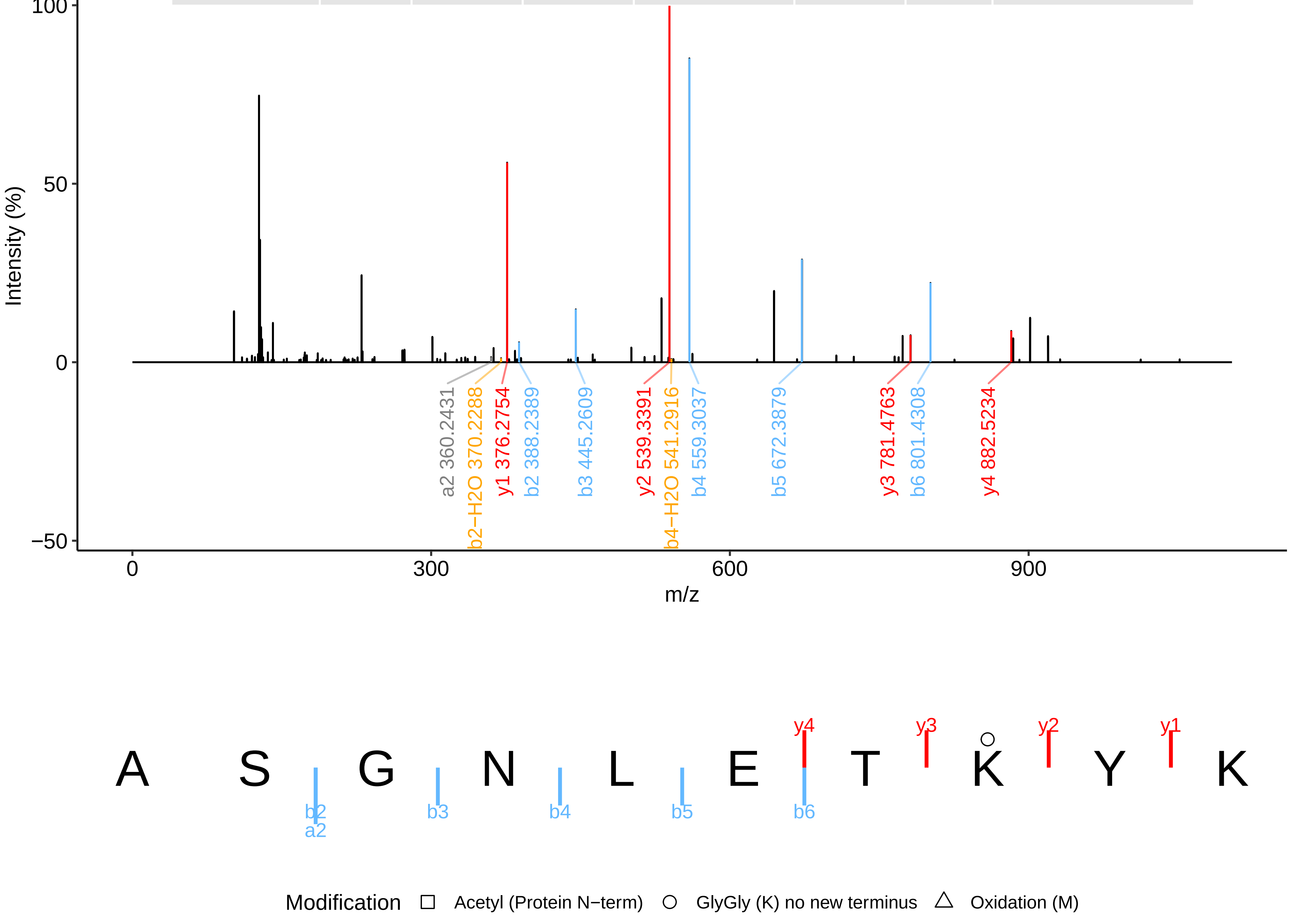

20200924_KIZ_MUb5_GlyGly_1 − Scan number: 7613

Gene Names	Charge	m/z	Mass	Retention time	PEP	Score	Precursor Intensity
VDAC3	3	408.8788	1223.615	24.205	0.011404	73.781	NA

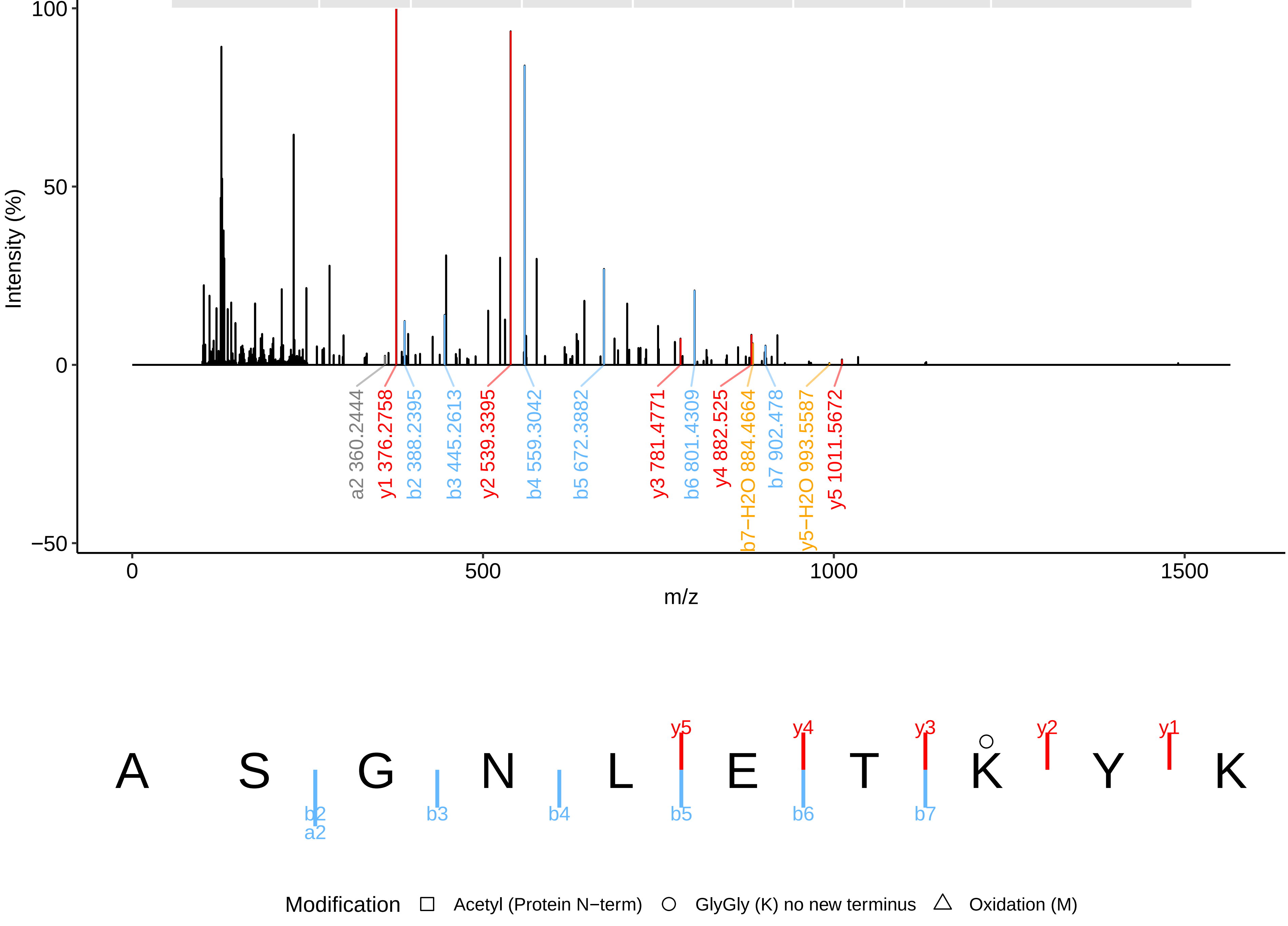

20200924_KIZ_MUb5_GlyGly_2 − Scan number: 7133

Gene Names	Charge	m/z	Mass	Retention time	PEP	Score	Precursor Intensity
VDAC3	3	408.8788	1223.615	26.594	0.085631	54.259	1405176

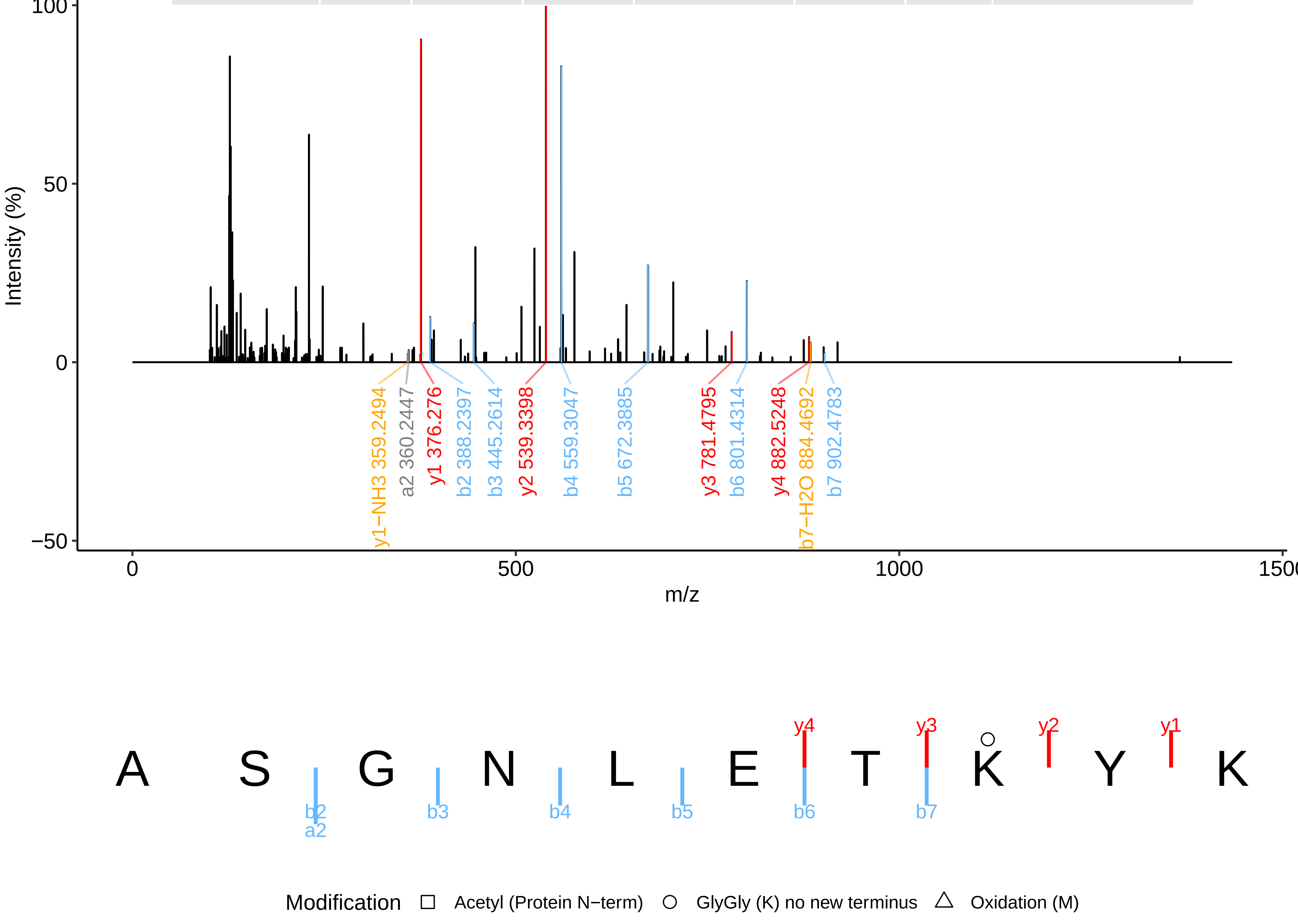

20201118_KIZ_MUb6_GlyGly_1 − Scan number: 7175

Gene Names	Charge	m/z	Mass	Retention time	PEP	Score	Precursor Intensity
VDAC3	3	408.8788	1223.615	22.737	0.077293	55.401	11175130

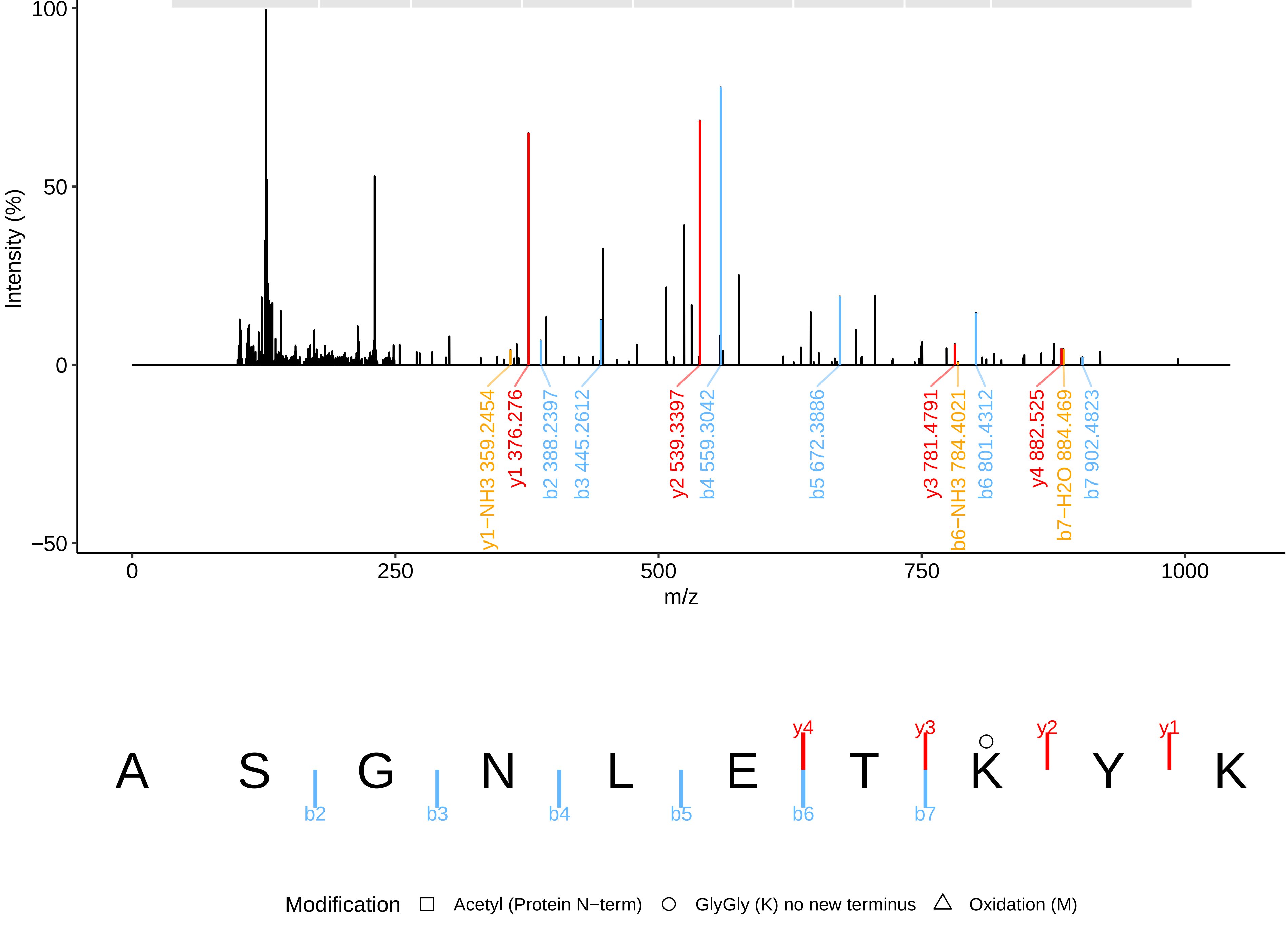

20200921_KIZ_MUb4_GlyGly_10plex − Scan number: 17020

Gene Names	Charge	m/z	Mass	Retention time	PEP	Score	Precursor Intensity
GK5	3	408.2242	1221.651	61.342	0.0034154	98.582	5130070

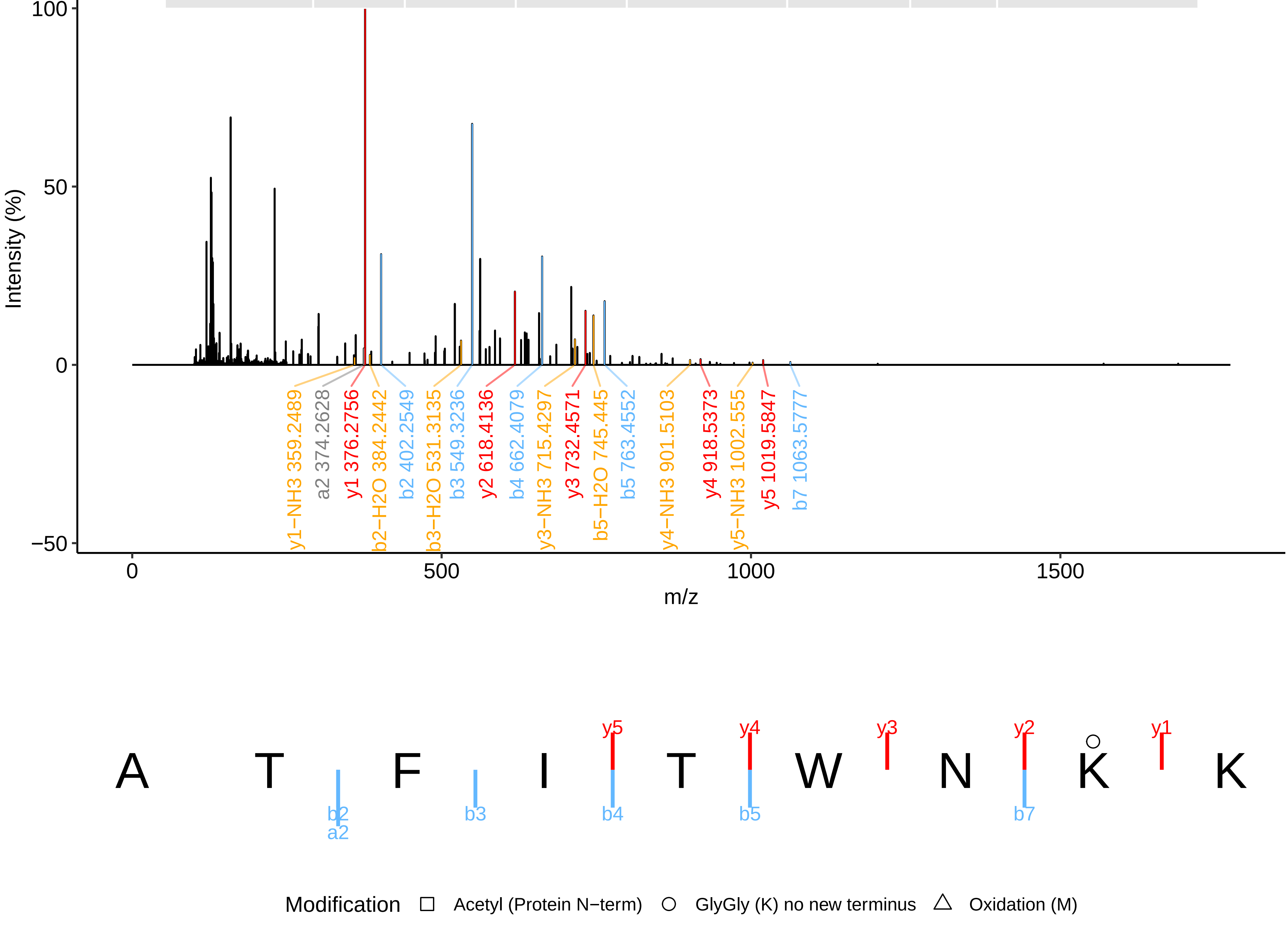

20200921_KIZ_MUb4_GlyGly_10plex − Scan number: 10507

Gene Names	Charge	m/z	Mass	Retention time	PEP	Score	Precursor Intensity
USP7	3	401.5507	1201.63	36.385	0.0051044	77.744	3066561

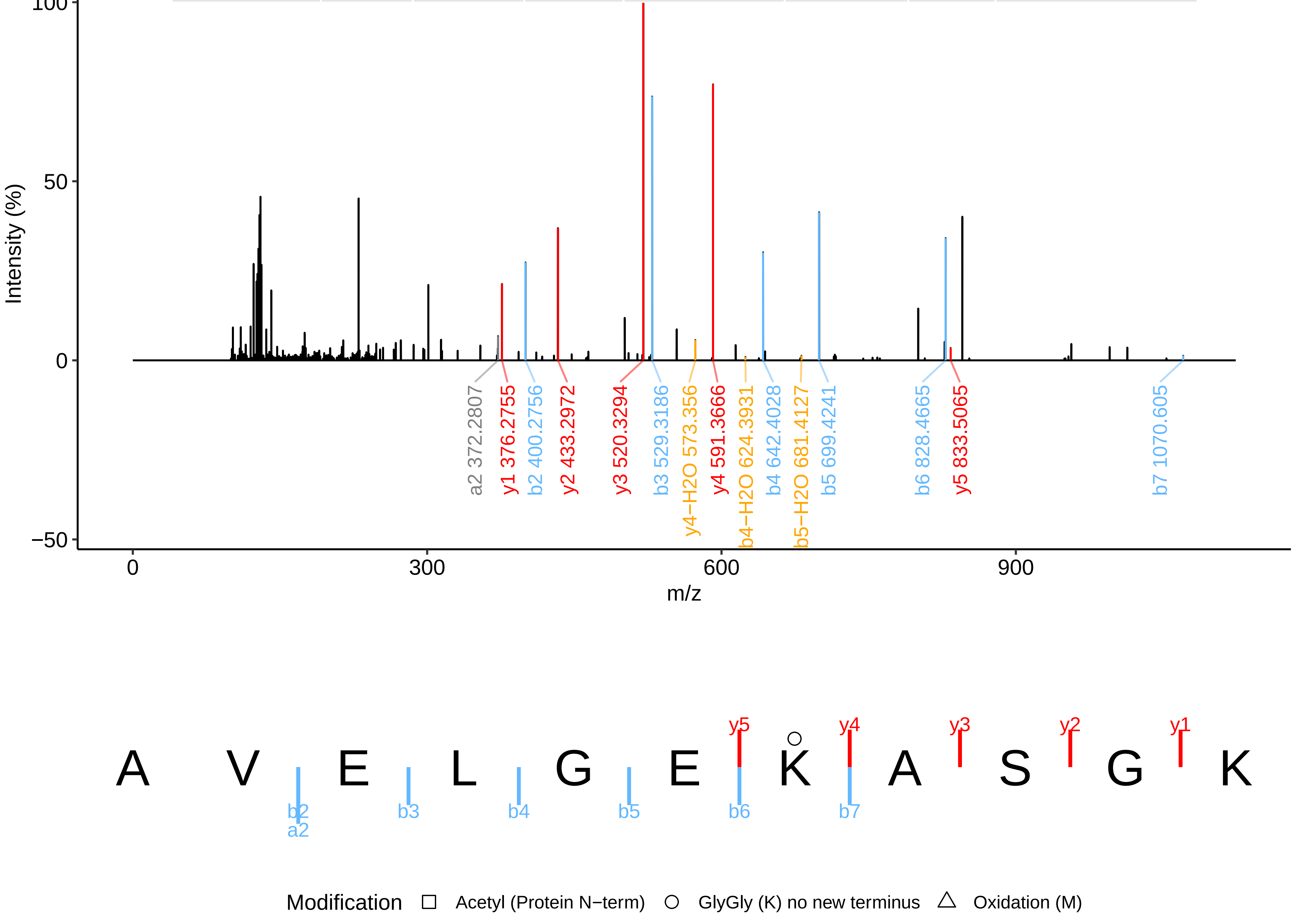

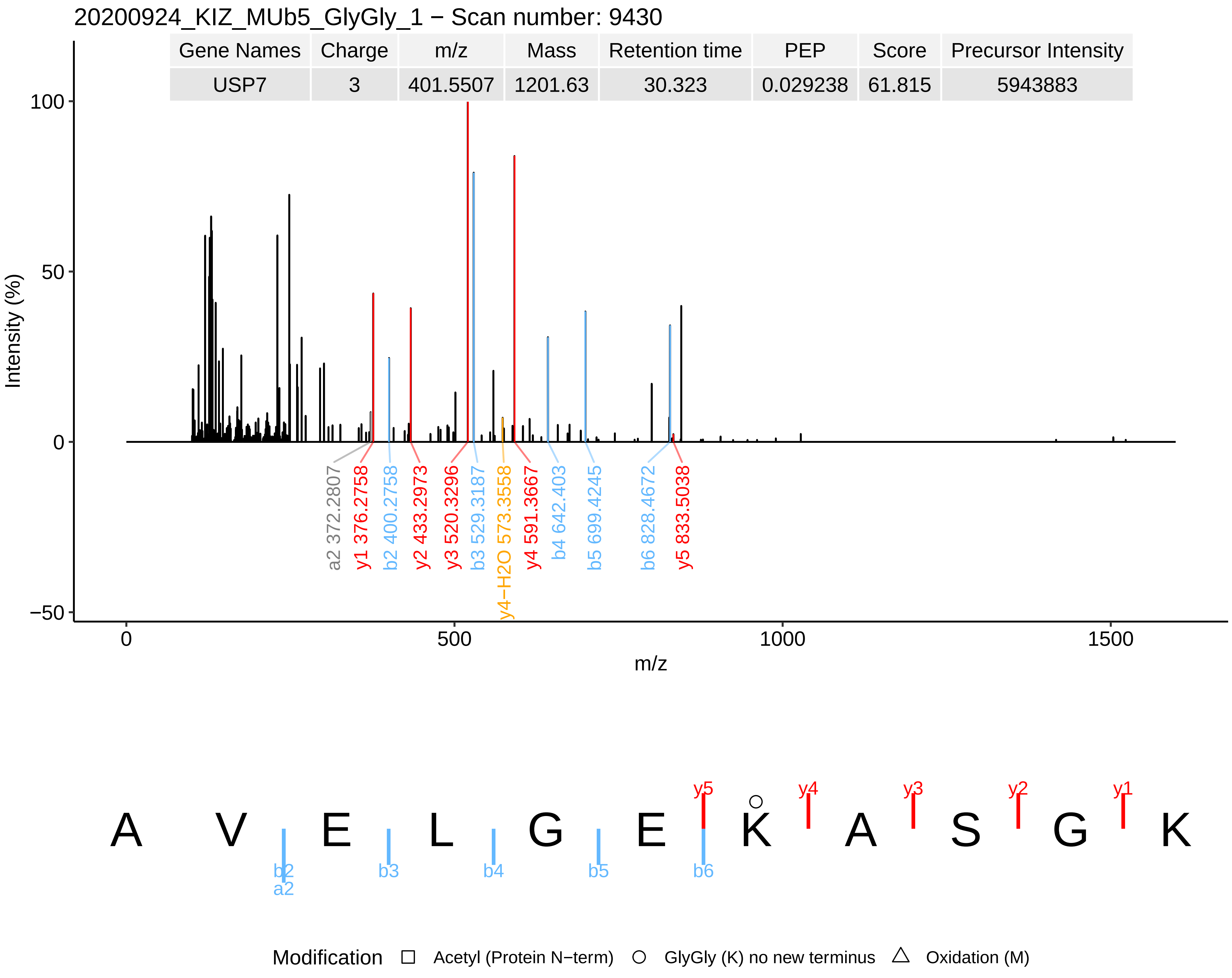
20200924_KIZ_MUb5_GlyGly_1 − Scan number: 9430
Gene Names | Charge | m/z | Mass | Retention time | PEP | Score | Precursor Intensity
USP7 | 3 | 401.5507 | 1201.63 | 30.323 | 0.029238 | 61.815 | 5943883
Intensity (%)
100
50
0
−50
a2 372.2807
y1 376.2758
b2 400.2758
y2 433.2973
y3 520.3296
b3 529.3187
y4−H2O 573.3558
y4 591.3667
b4 642.403
b5 699.4245
b6 828.4672
y5 833.5038
0
500
1000
1500
m/z
A V E L G E K A S G K
y5 y4 y3 y2 y1
b2 a2 b3 b4 b5 b6
Modification
Acetyl (Protein N−term)
GlyGly (K) no new terminus
Oxidation (M)

20200921_KIZ_MUb4_GlyGly_10plex − Scan number: 5826

Gene Names	Charge	m/z	Mass	Retention time	PEP	Score	Precursor Intensity
BASP1	3	405.5386	1213.594	19.112	0.01586	63.408	20705366

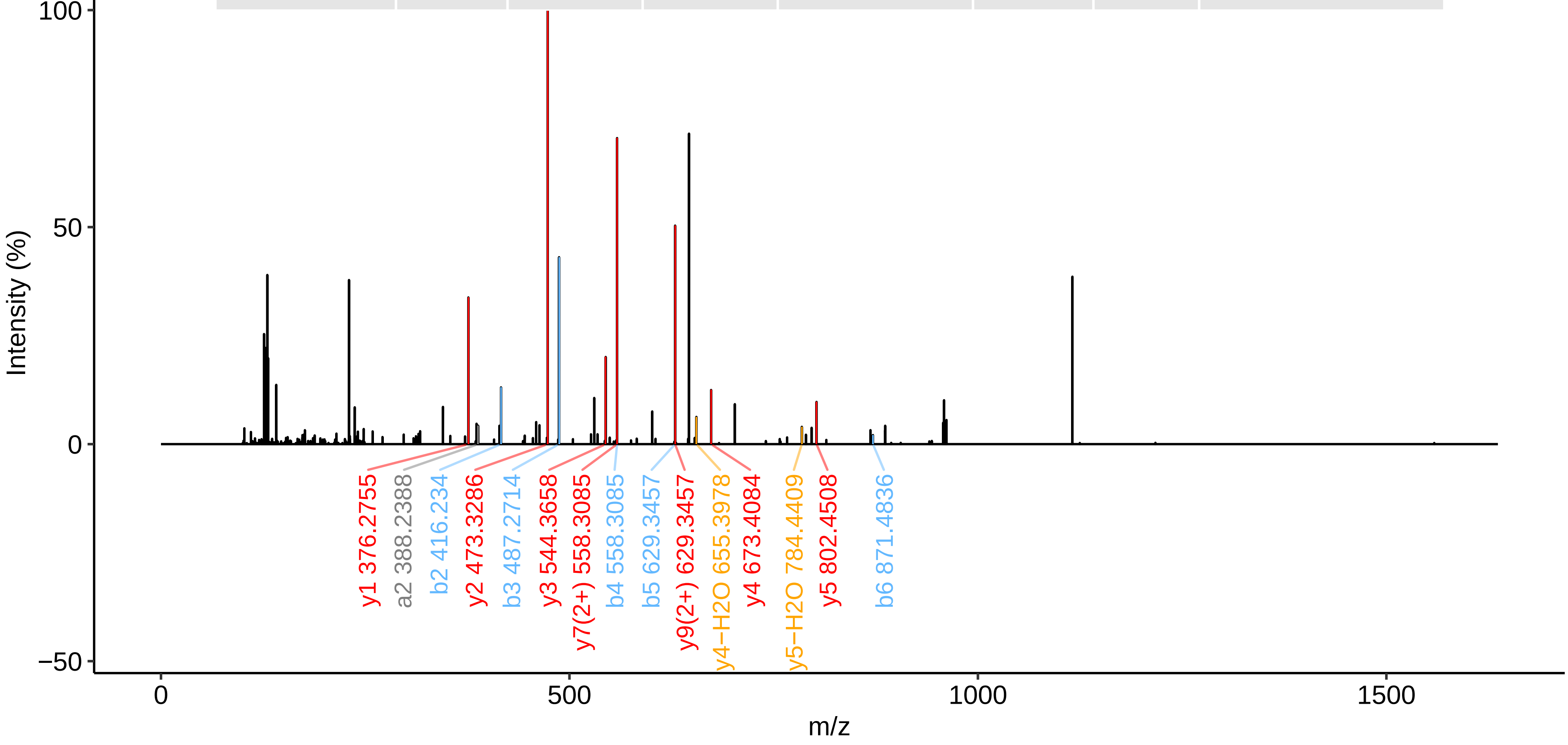

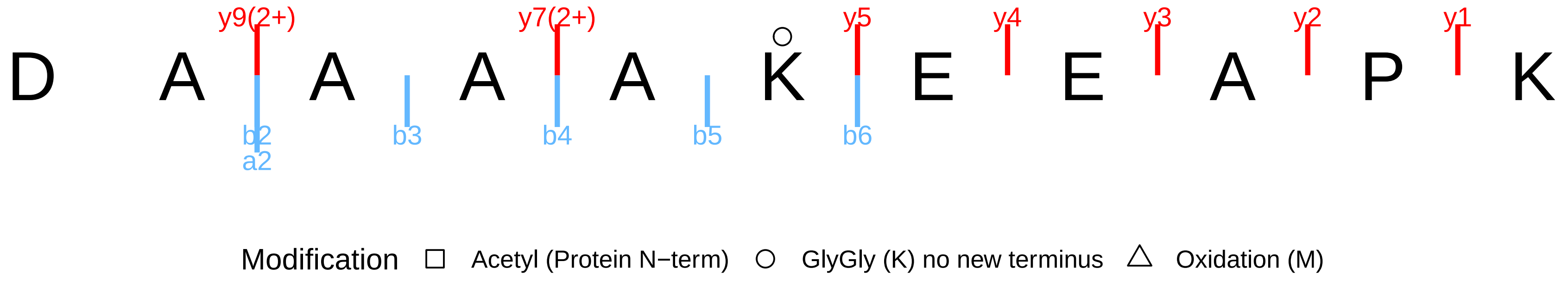

20200921_KIZ_MUb4_GlyGly_10plex_2 − Scan number: 5302

Gene Names	Charge	m/z	Mass	Retention time	PEP	Score	Precursor Intensity
BASP1	3	405.5386	1213.594	19.003	0.0016849	81.017	2002566

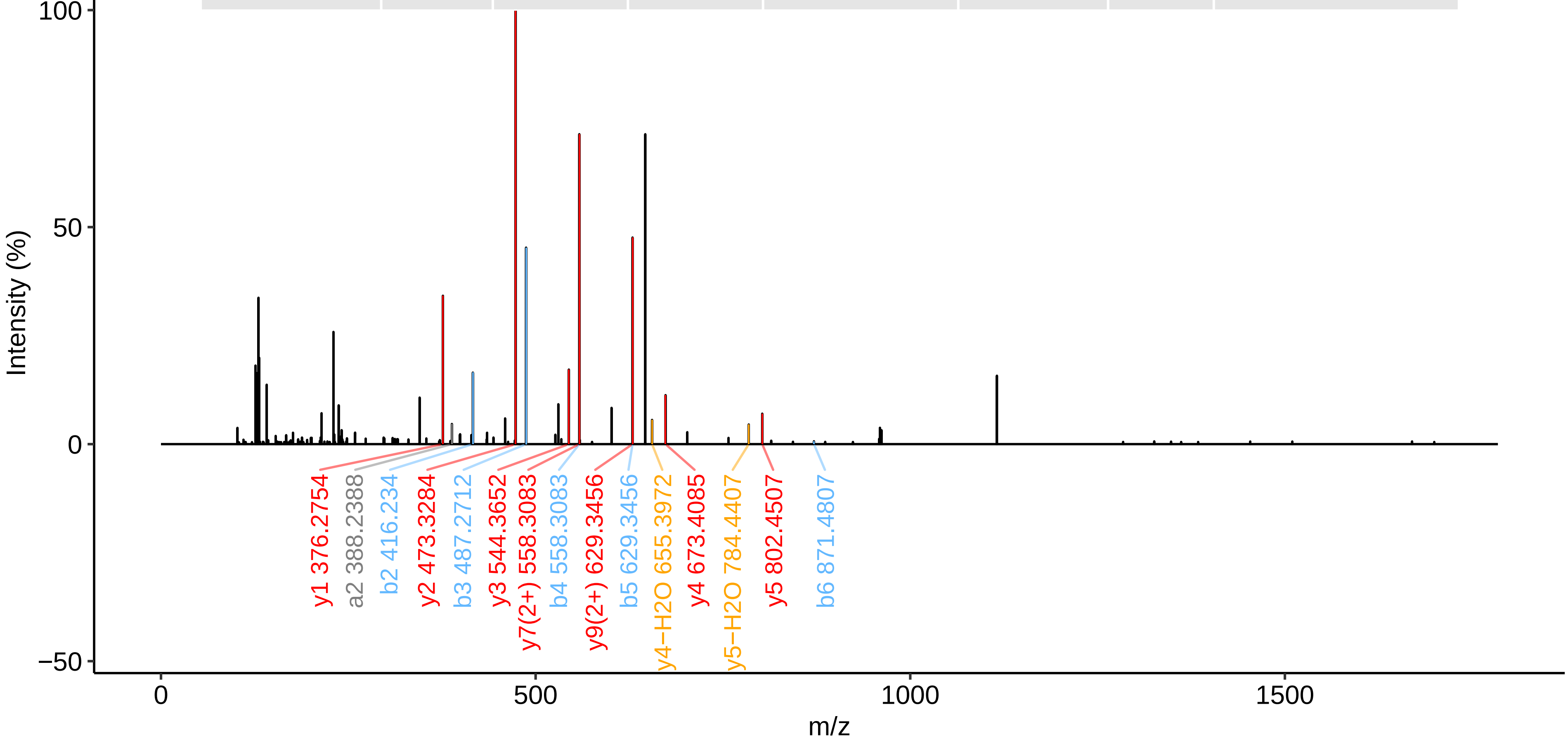

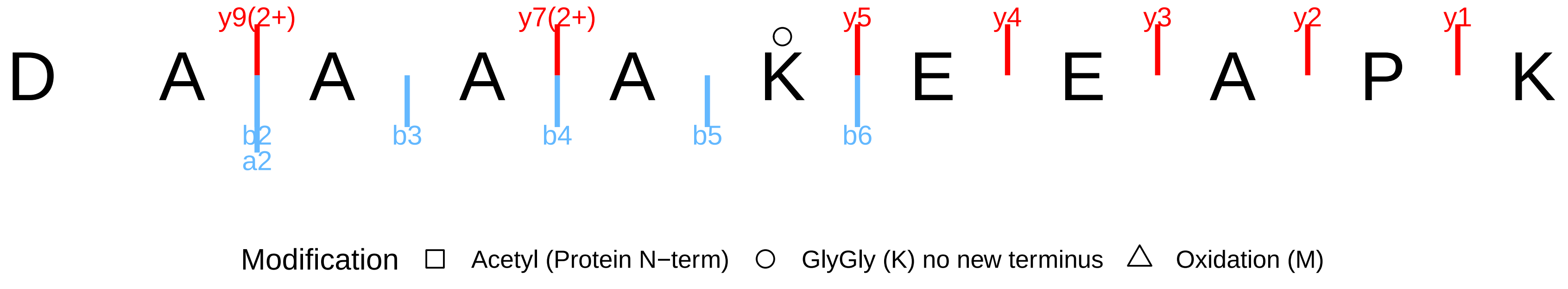

20200924_KIZ_MUb5_GlyGly_1 − Scan number: 5062

Gene Names	Charge	m/z	Mass	Retention time	PEP	Score	Precursor Intensity
BASP1	3	405.5386	1213.594	15.71	0.0013332	84.658	NA

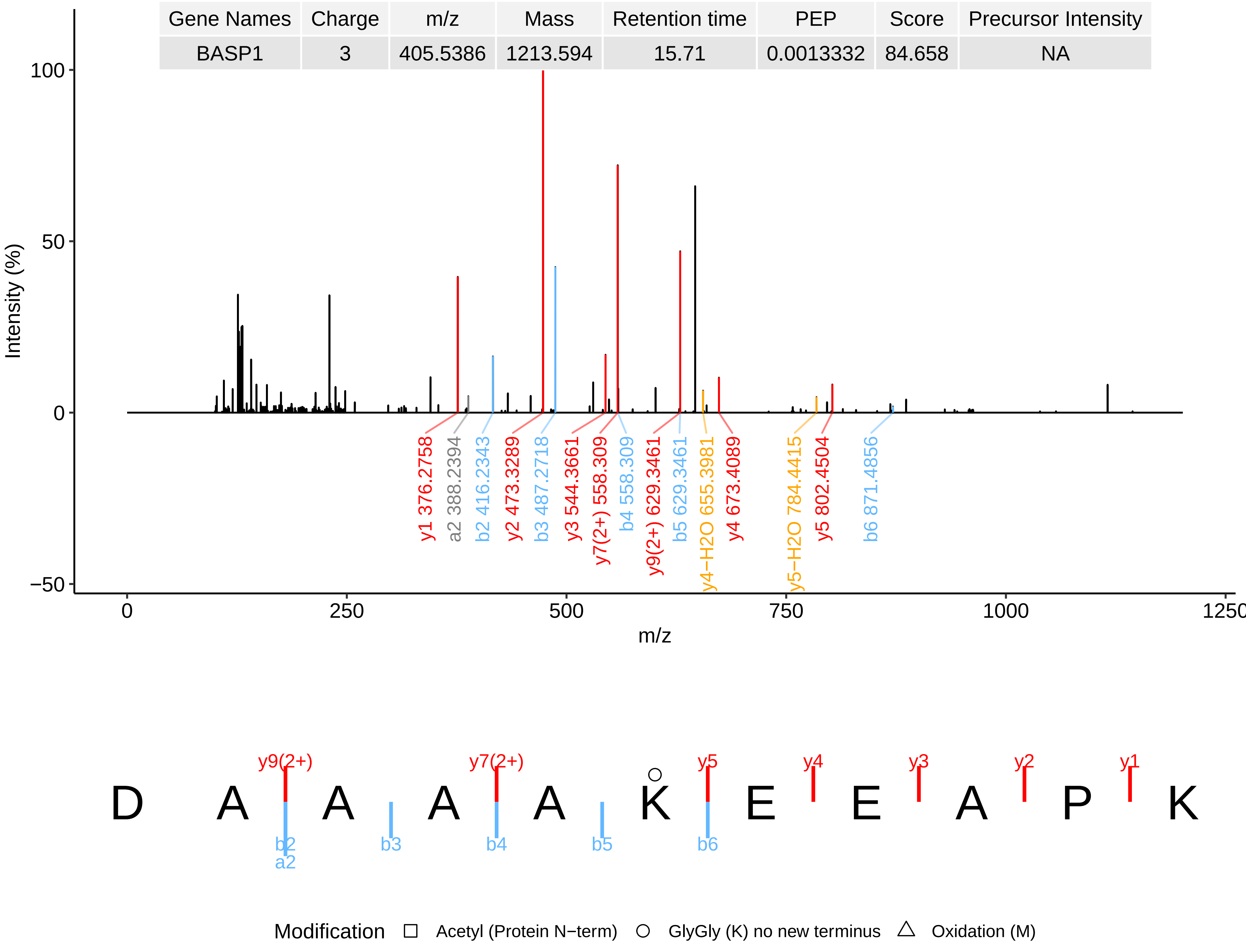

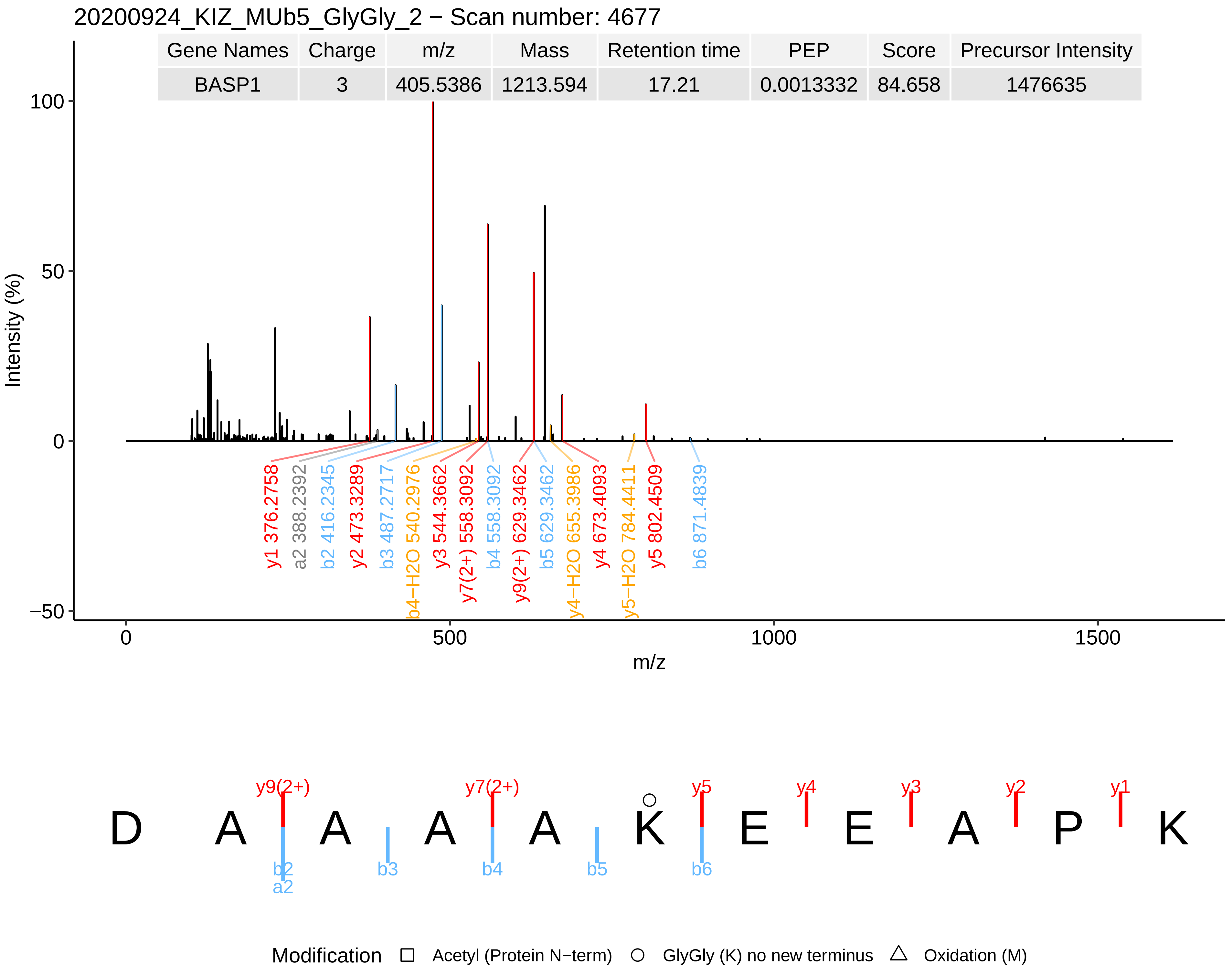
20200924_KIZ_MUb5_GlyGly_2 − Scan number: 4677
Gene Names | Charge | m/z | Mass | Retention time | PEP | Score | Precursor Intensity
BASP1 | 3 | 405.5386 | 1213.594 | 17.21 | 0.0013332 | 84.658 | 1476635
Intensity (%)
100
50
0
−50
0
500
1000
1500
m/z
y1 376.2758
a2 388.2392
b2 416.2345
y2 473.3289
b3 487.2717
b4−H2O 540.2976
y3 544.3662
y7(2+) 558.3092
b4 558.3092
y9(2+) 629.3462
b5 629.3462
y4−H2O 655.3986
y4 673.4093
y5−H2O 784.4411
y5 802.4509
b6 871.4839
D A A A A K E E A P K
y9(2+)
y7(2+)
y5
y4
y3
y2
y1
b2
a2
b3
b4
b5
b6
Modification
Acetyl (Protein N−term)
GlyGly (K) no new terminus
Oxidation (M)

20200924_KIZ_MUb5_GlyGly_2 − Scan number: 4806

Gene Names	Charge	m/z	Mass	Retention time	PEP	Score	Precursor Intensity
BASP1	3	405.5386	1213.594	17.72	0.0015734	82.171	4829308

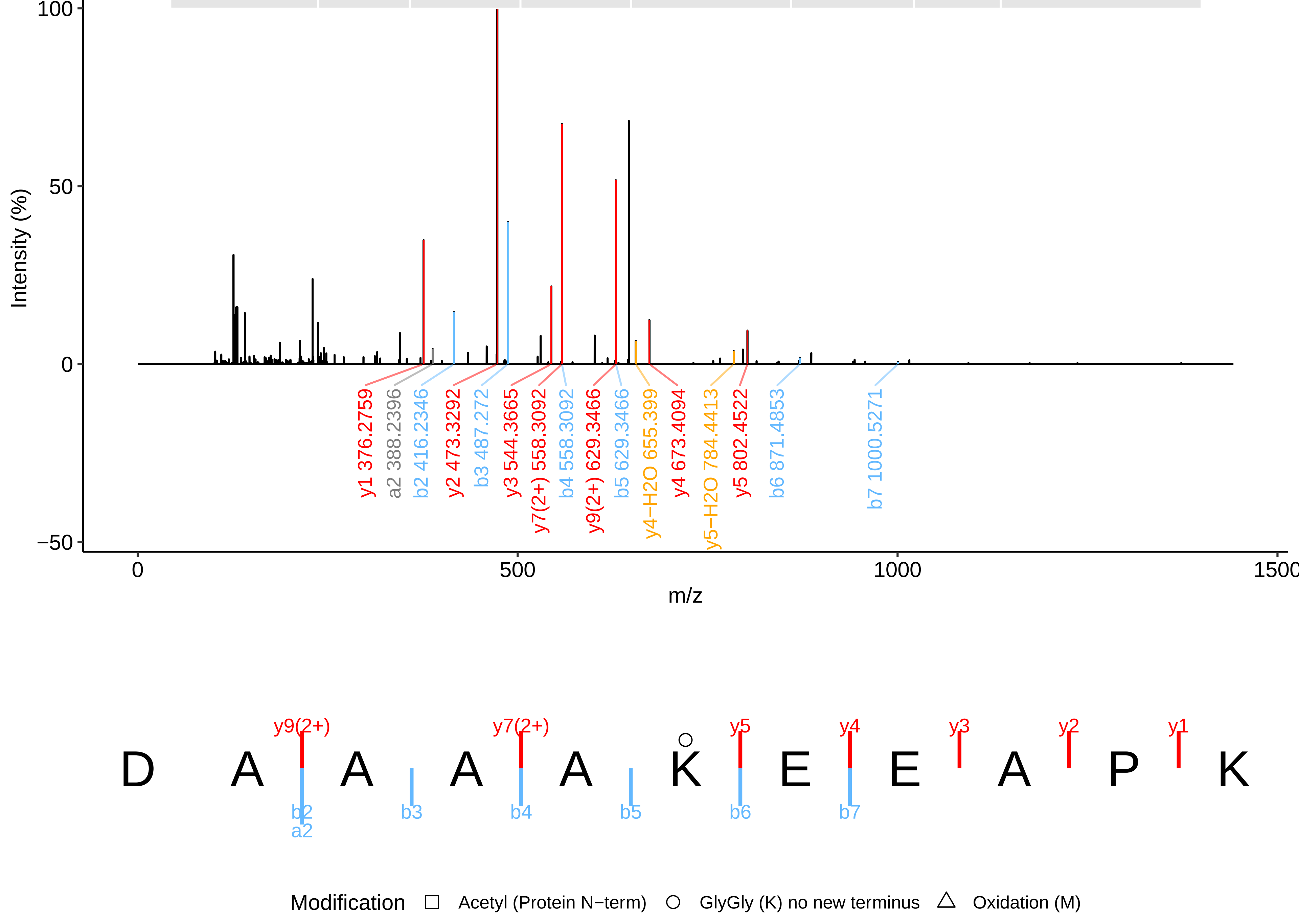

20201118_KIZ_MUb6_GlyGly_1 − Scan number: 4988

Gene Names	Charge	m/z	Mass	Retention time	PEP	Score	Precursor Intensity
BASP1	3	405.5386	1213.594	15.759	0.0096616	70.942	10515665

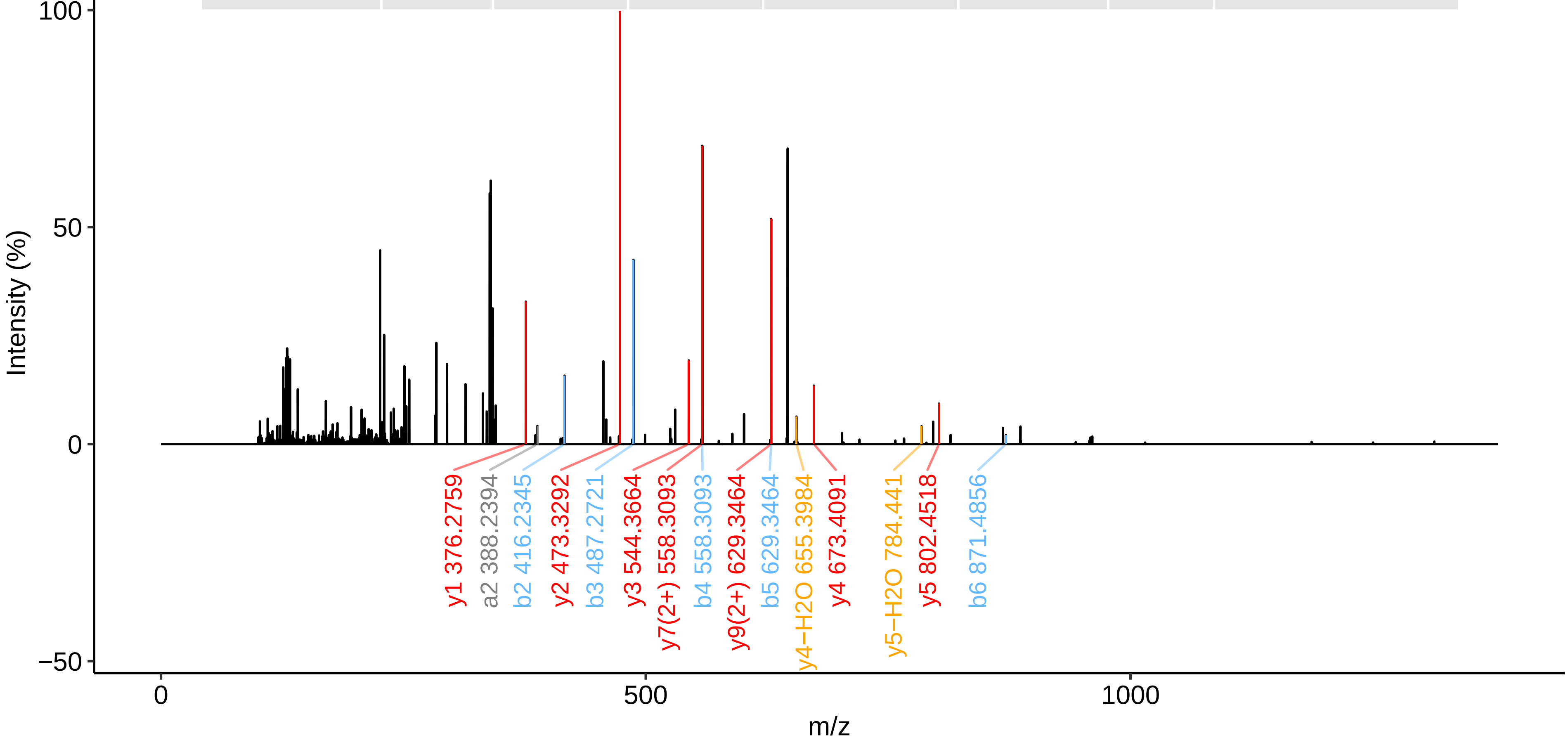

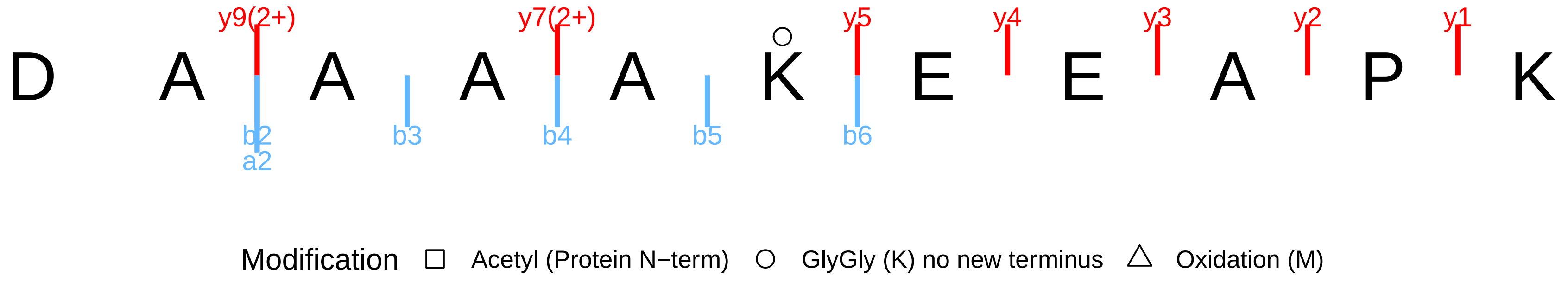

20200921_KIZ_MUb4_GlyGly_10plex − Scan number: 8317

Gene Names	Charge	m/z	Mass	Retention time	PEP	Score	Precursor Intensity
HLA−DQA1	3	412.5328	1234.576	28.188	0.05543	56.258	12812945

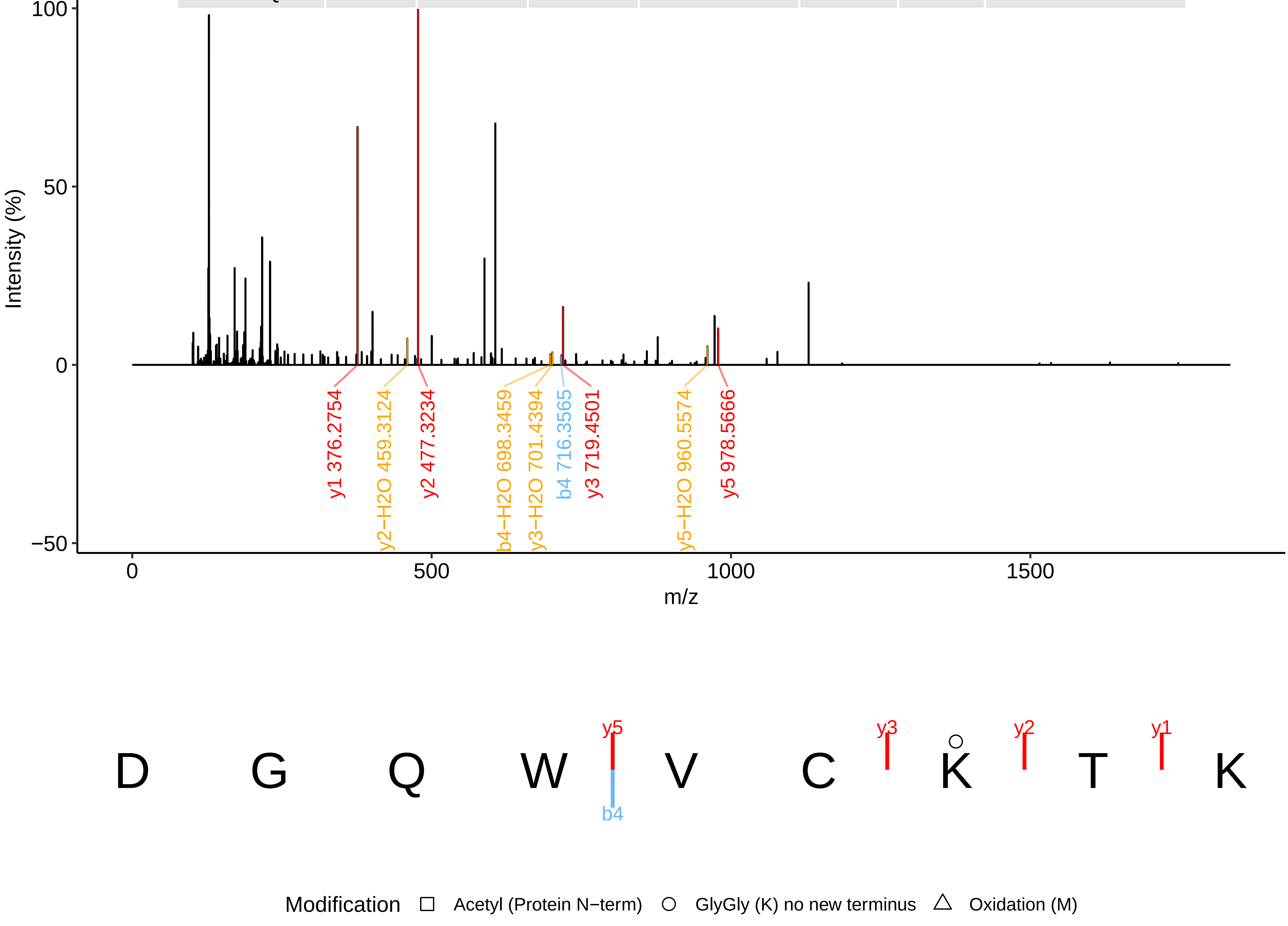

20200924_KIZ_MUb5_GlyGly_1 − Scan number: 7422

Gene Names	Charge	m/z	Mass	Retention time	PEP	Score	Precursor Intensity
HLA−DQA1	3	412.5328	1234.576	23.565	0.11653	50.473	21430160

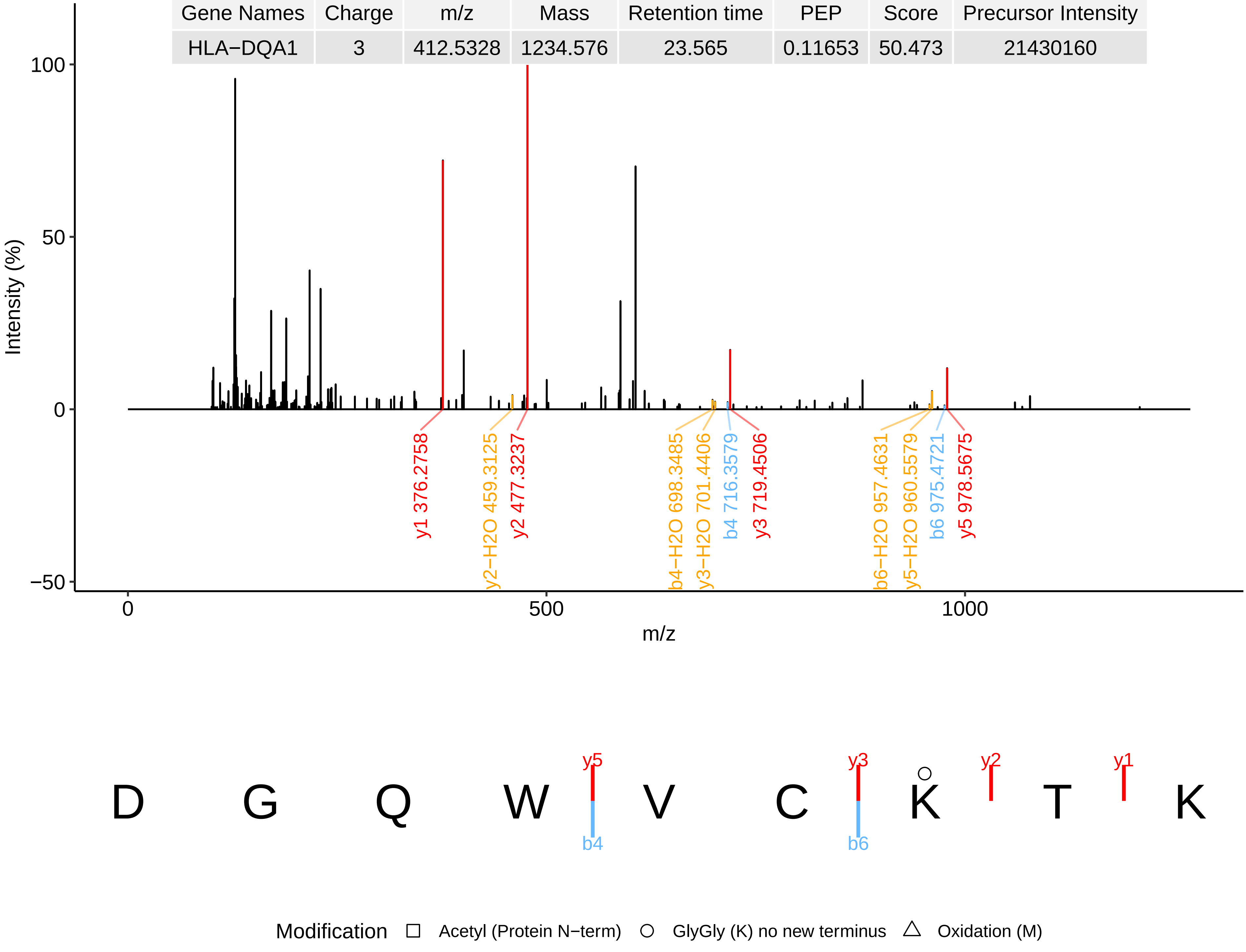

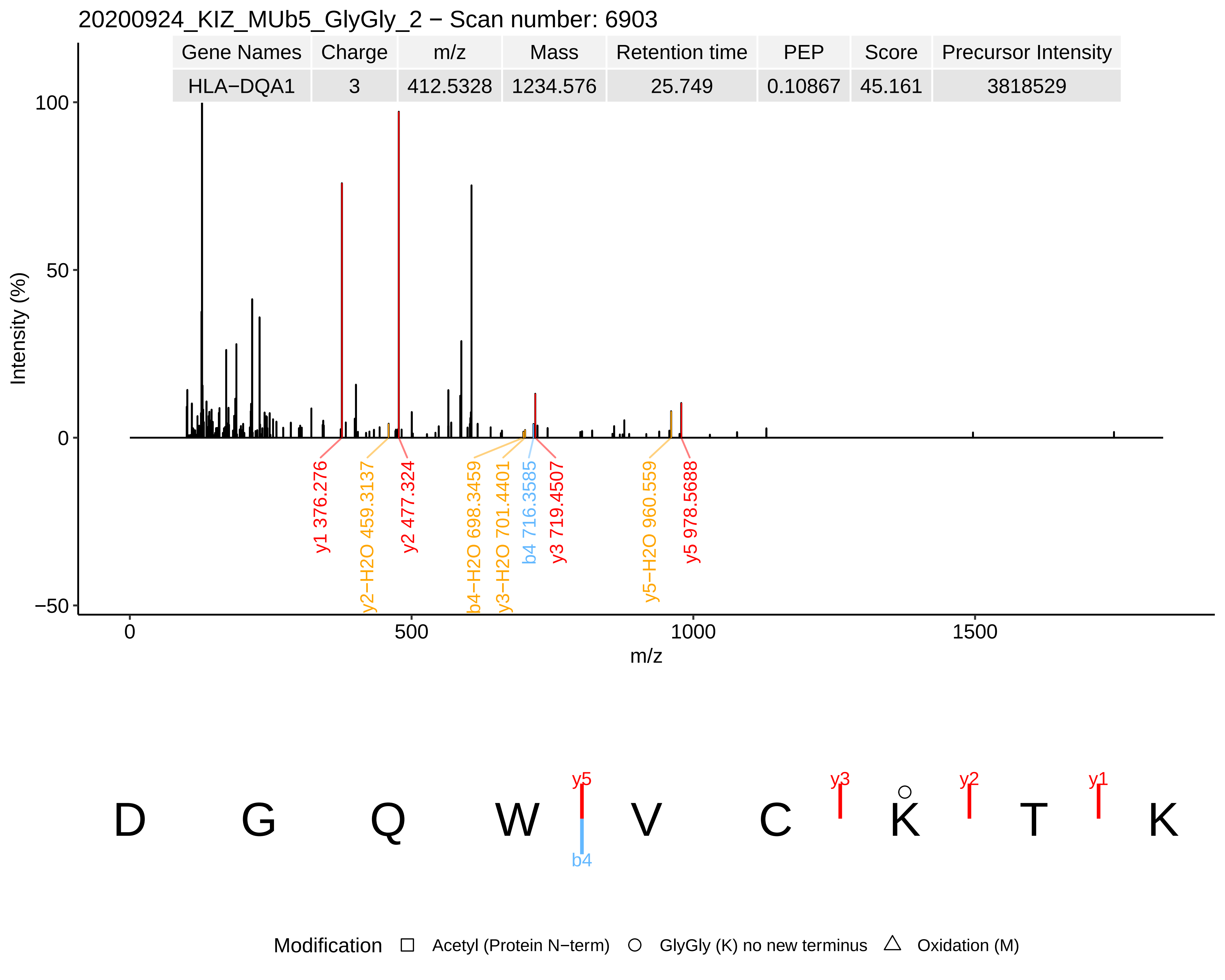
20200924_KIZ_MUb5_GlyGly_2 − Scan number: 6903
Gene Names | Charge | m/z | Mass | Retention time | PEP | Score | Precursor Intensity
HLA−DQA1 | 3 | 412.5328 | 1234.576 | 25.749 | 0.10867 | 45.161 | 3818529
Intensity (%)
100
50
0
−50
0
500
1000
1500
m/z
y1 376.276
y2−H2O 459.3137
y2 477.324
b4−H2O 698.3459
y3−H2O 701.4401
b4 716.3585
y3 719.4507
y5−H2O 960.559
y5 978.5688
D G Q W V C K T K
y5
b4
y3
y2
y1
Modification
Acetyl (Protein N−term)
GlyGly (K) no new terminus
Oxidation (M)

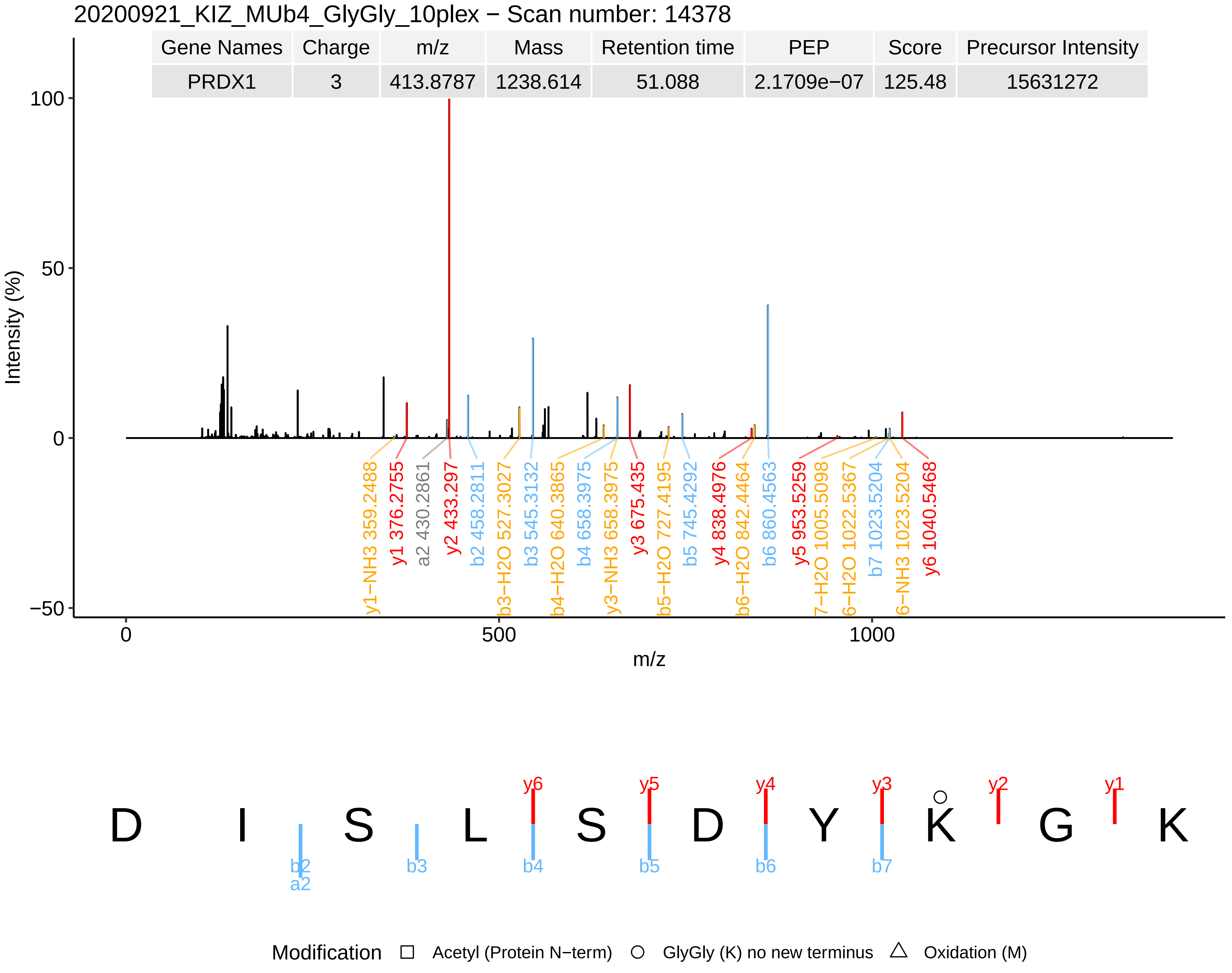

20200921_KIZ_MUb4_GlyGly_10plex − Scan number: 14378
Gene Names | Charge | m/z | Mass | Retention time | PEP | Score | Precursor Intensity
PRDX1 | 3 | 413.8787 | 1238.614 | 51.088 | 2.1709e−07 | 125.48 | 15631272
Intensity (%)
m/z
y1−NH3 359.2488
y1 376.2755
a2 430.2861
y2 433.297
b2 458.2811
b3−H2O 527.3027
b3 545.3132
b4−H2O 640.3865
b4 658.3975
y3−NH3 658.3975
y3 675.435
b5−H2O 727.4195
b5 745.4292
y4 838.4976
b6−H2O 842.4464
b6 860.4563
y5 953.5259
7−H2O 1005.5098
6−H2O 1022.5367
b7 1023.5204
6−NH3 1023.5204
y6 1040.5468
D I S L S D Y K G K
Modification
Acetyl (Protein N−term)
GlyGly (K) no new terminus
Oxidation (M)

20200921_KIZ_MUb4_GlyGly_10plex_2 − Scan number: 13223

Gene Names	Charge	m/z	Mass	Retention time	PEP	Score	Precursor Intensity
PRDX1	3	413.8787	1238.614	51.296	2.1709e−07	125.48	1123083

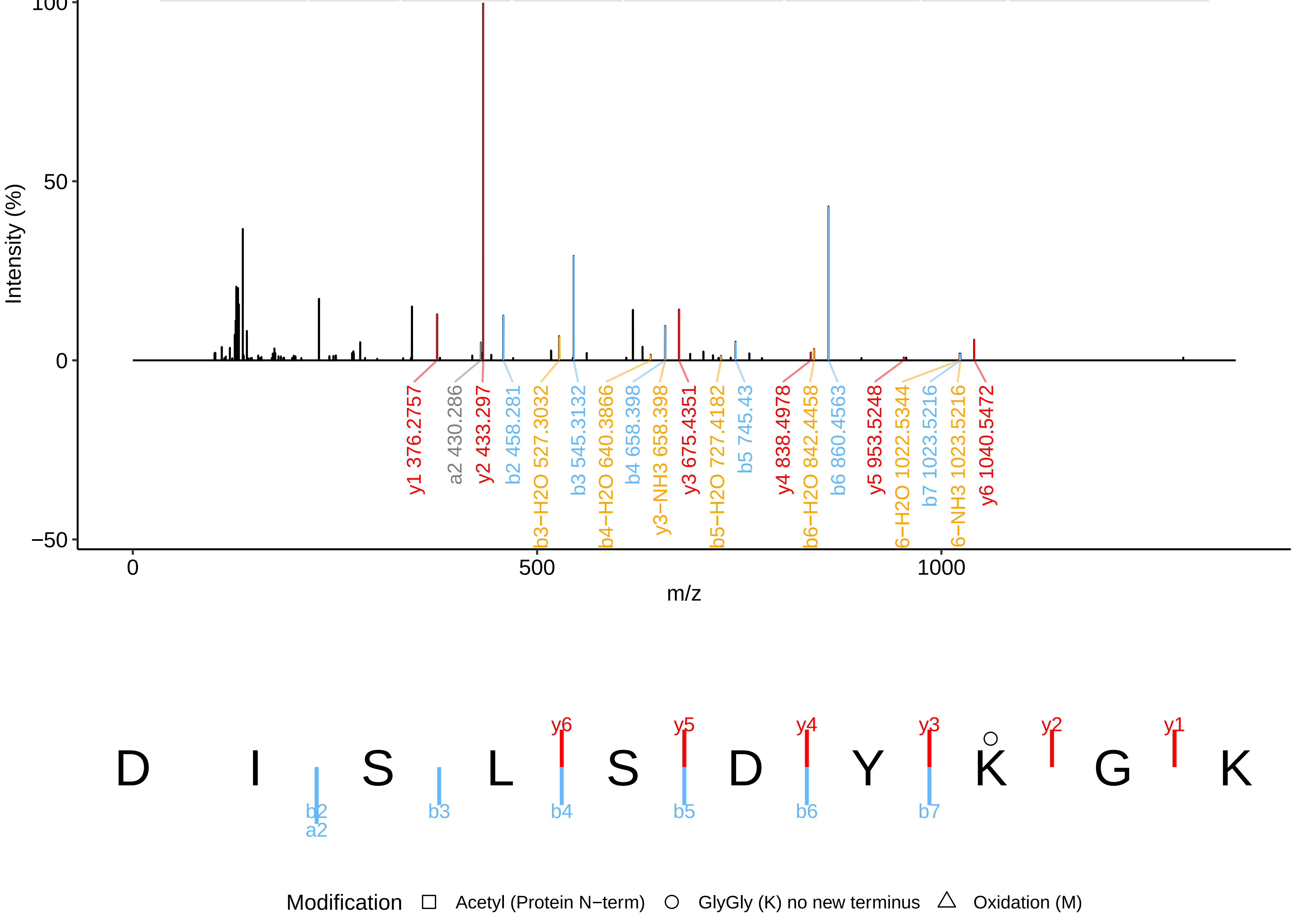

20200924_KIZ_MUb5_GlyGly_1 − Scan number: 13505

Gene Names	Charge	m/z	Mass	Retention time	PEP	Score	Precursor Intensity
PRDX1	3	413.8787	1238.614	44.044	3.8758e−07	119.68	18350886

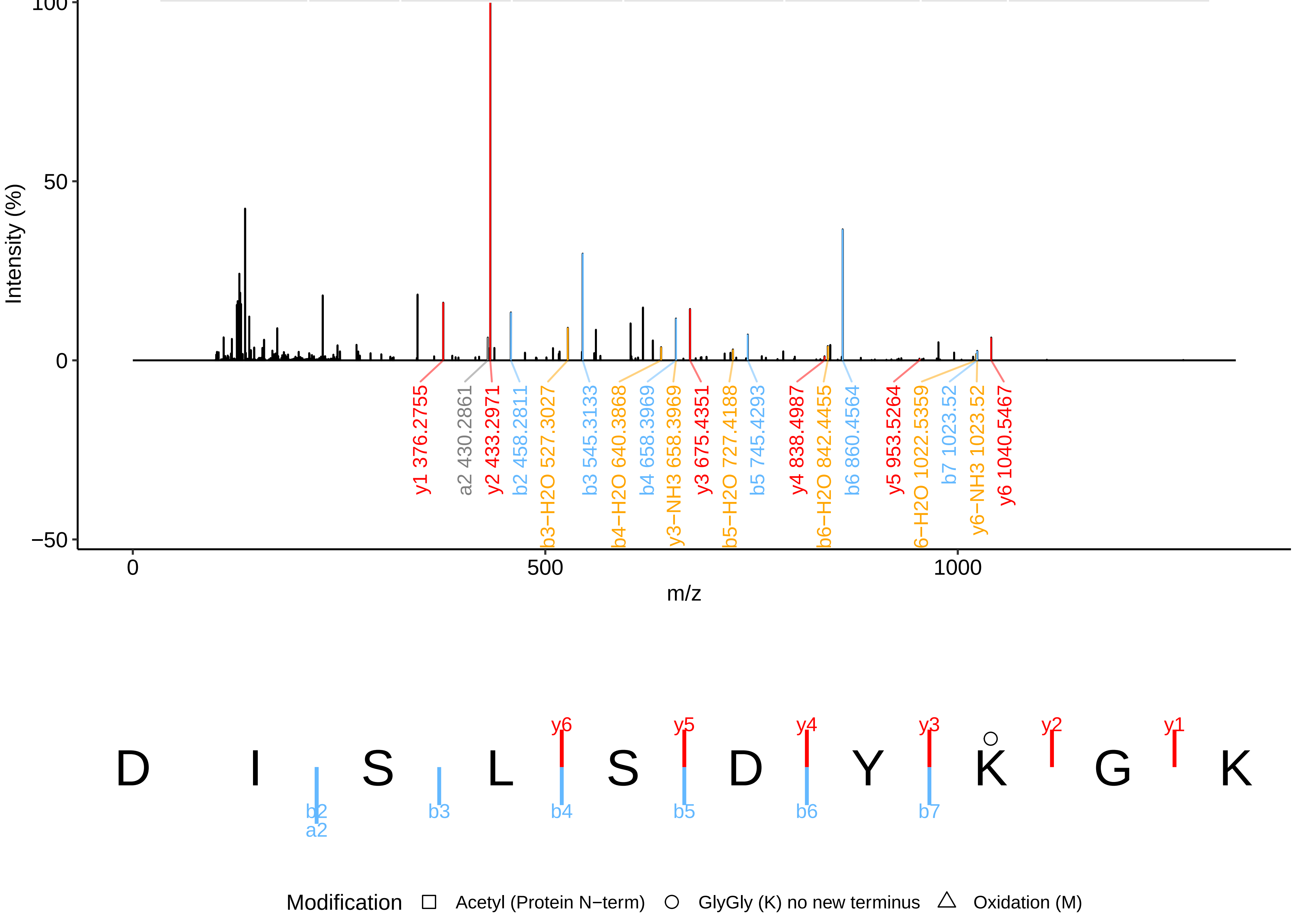

20200921_KIZ_MUb4_GlyGly_10plex − Scan number: 18584

Gene Names	Charge	m/z	Mass	Retention time	PEP	Score	Precursor Intensity
PRDX3	3	417.8787	1250.614	66.871	0.00096662	105.4	NA

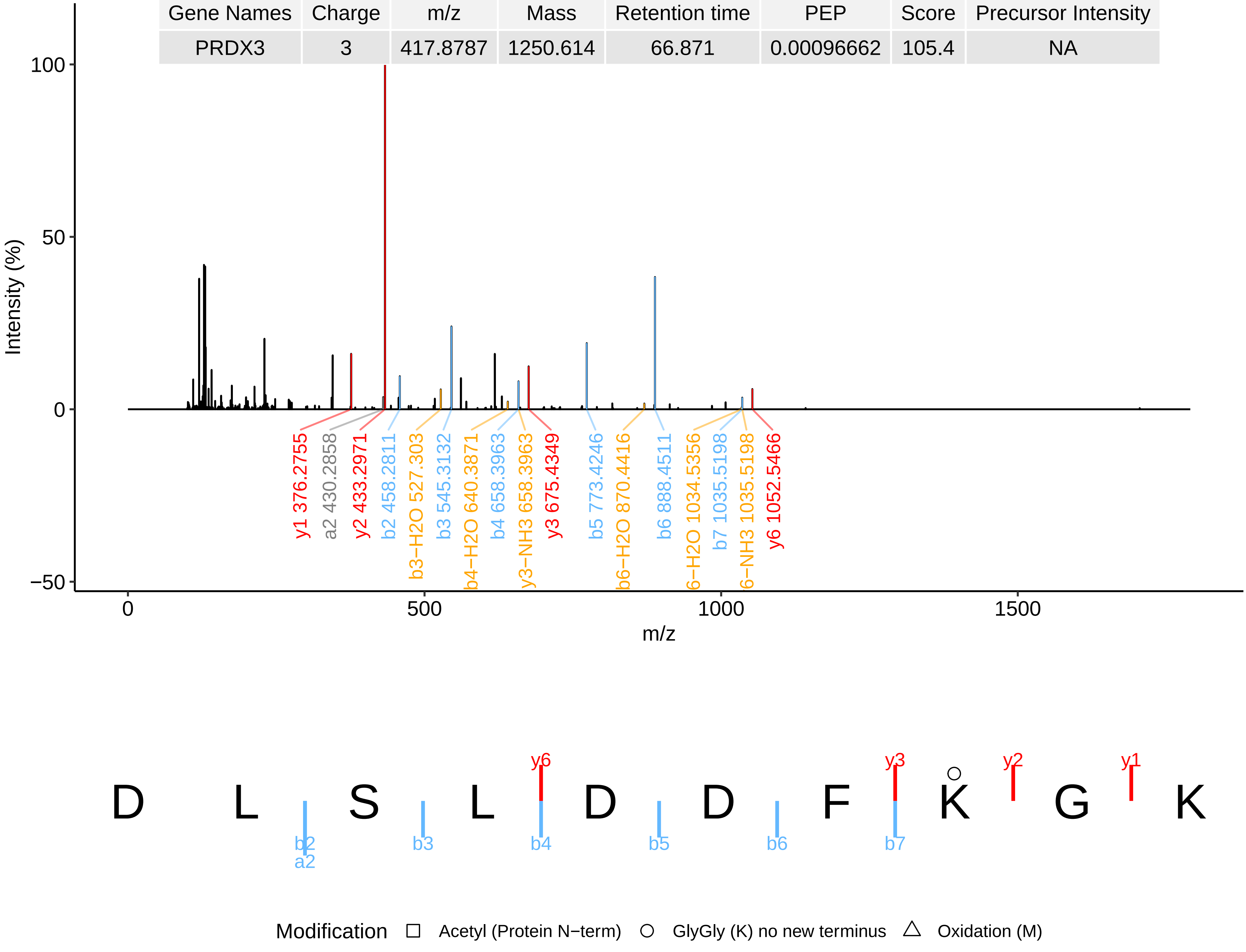

20200921_KIZ_MUb4_GlyGly_10plex_2 − Scan number: 14128

Gene Names	Charge	m/z	Mass	Retention time	PEP	Score	Precursor Intensity
RPLP0;RPLP0P6	3	495.2732	1482.798	55.015	0.015613	62.088	1007949

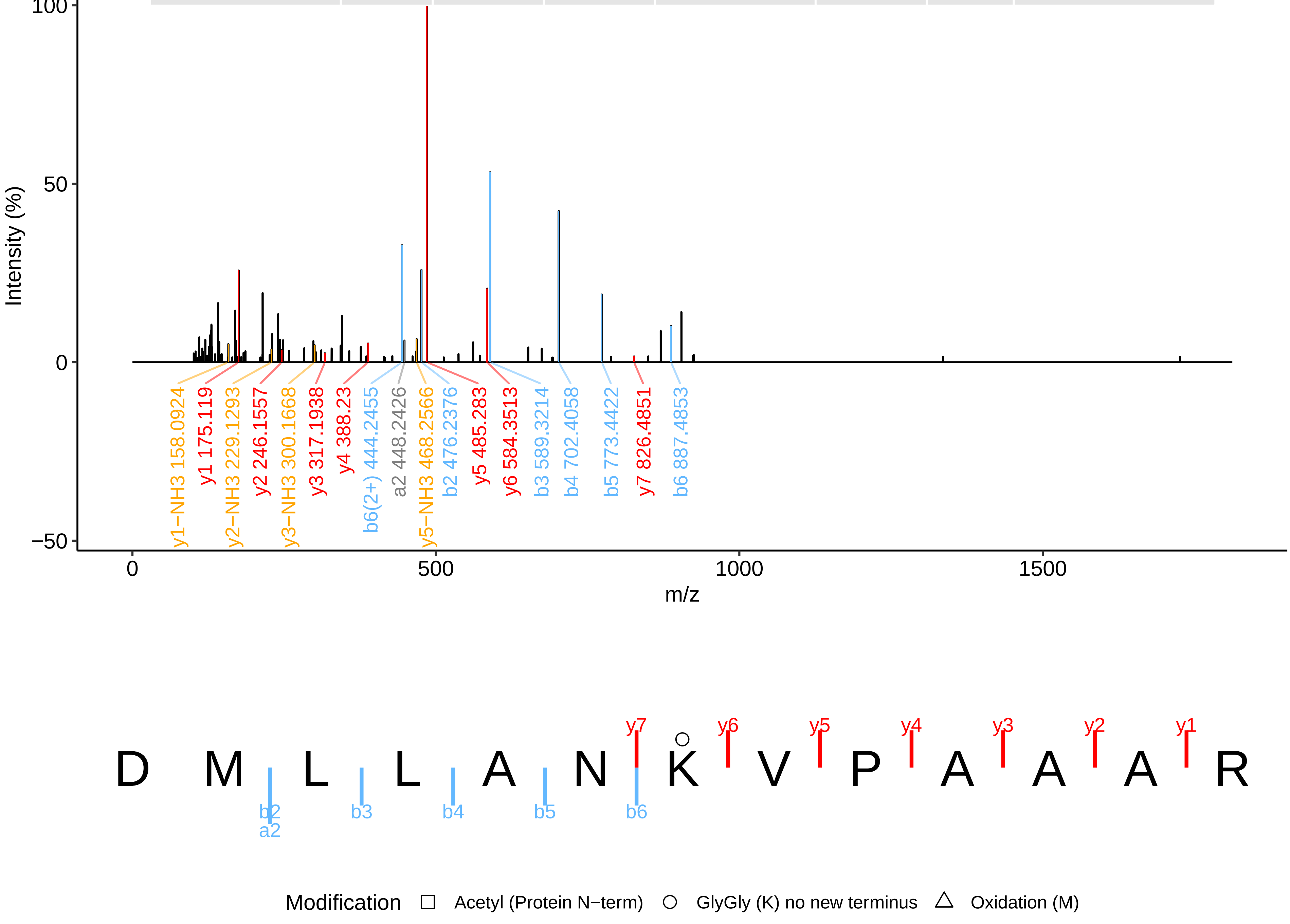

20200921_KIZ_MUb4_GlyGly_10plex − Scan number: 12159

Gene Names	Charge	m/z	Mass	Retention time	PEP	Score	Precursor Intensity
HIST1H4A	3	480.6037	1438.789	42.557	3.329e−08	145.31	4005886

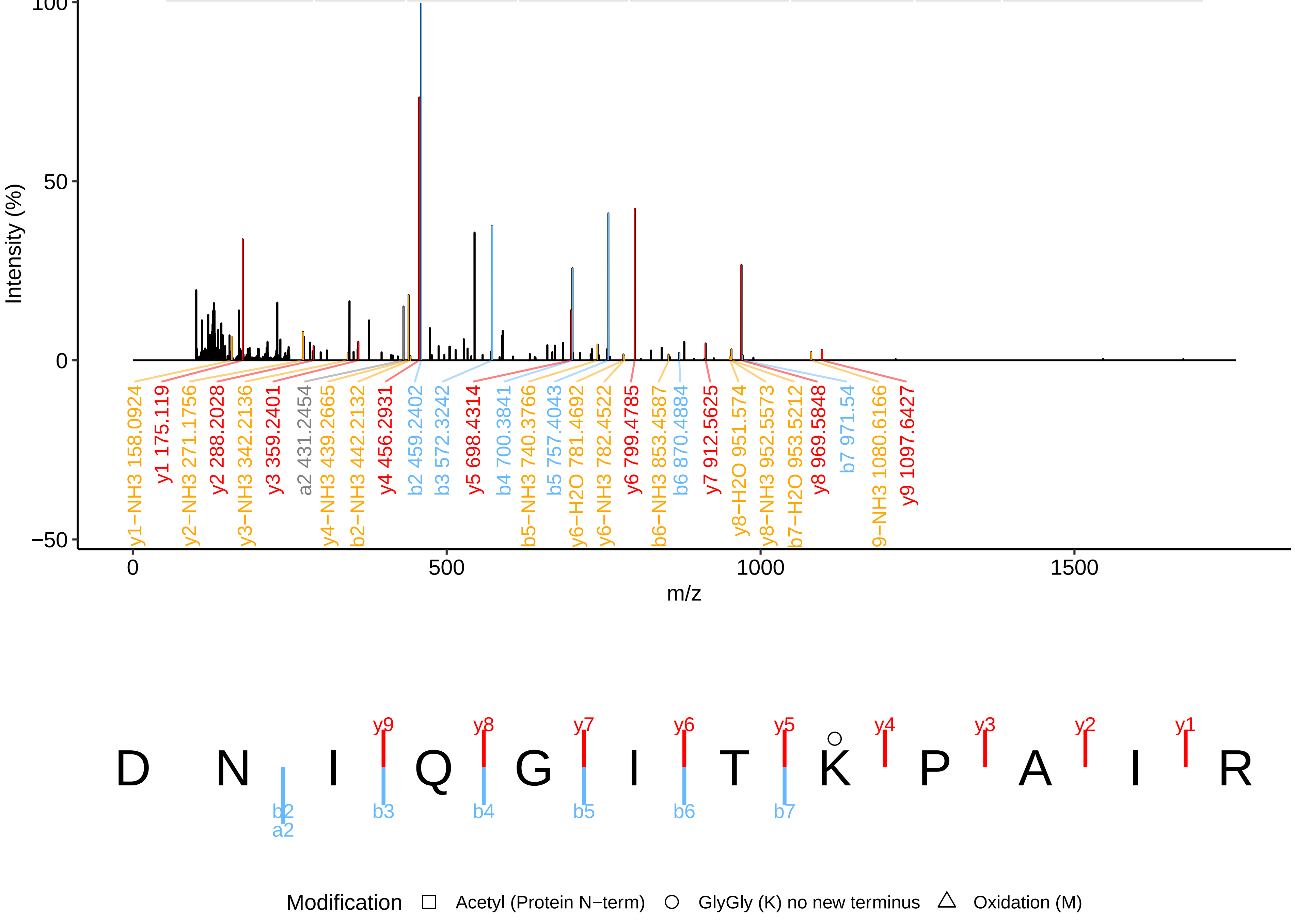

20200921_KIZ_MUb4_GlyGly_10plex_2 − Scan number: 11195

Gene Names	Charge	m/z	Mass	Retention time	PEP	Score	Precursor Intensity
HIST1H4A	3	480.6037	1438.789	43.028	1.8161e−05	100.19	1508631

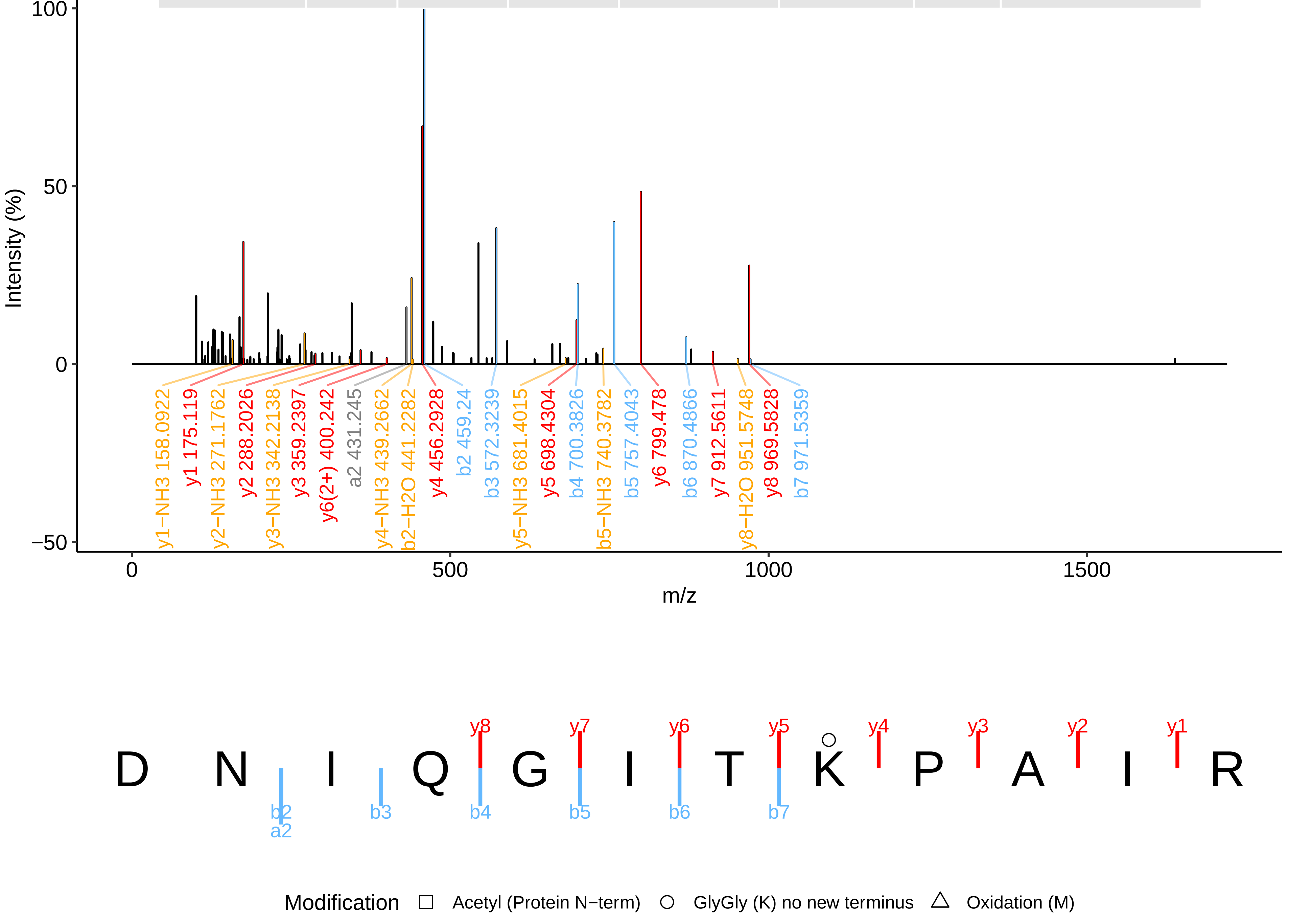

20200921_KIZ_MUb4_GlyGly_10plex − Scan number: 3167

Gene Names	Charge	m/z	Mass	Retention time	PEP	Score	Precursor Intensity
OLR1	3	411.1898	1230.548	9.9322	0.0069828	76.282	34215476

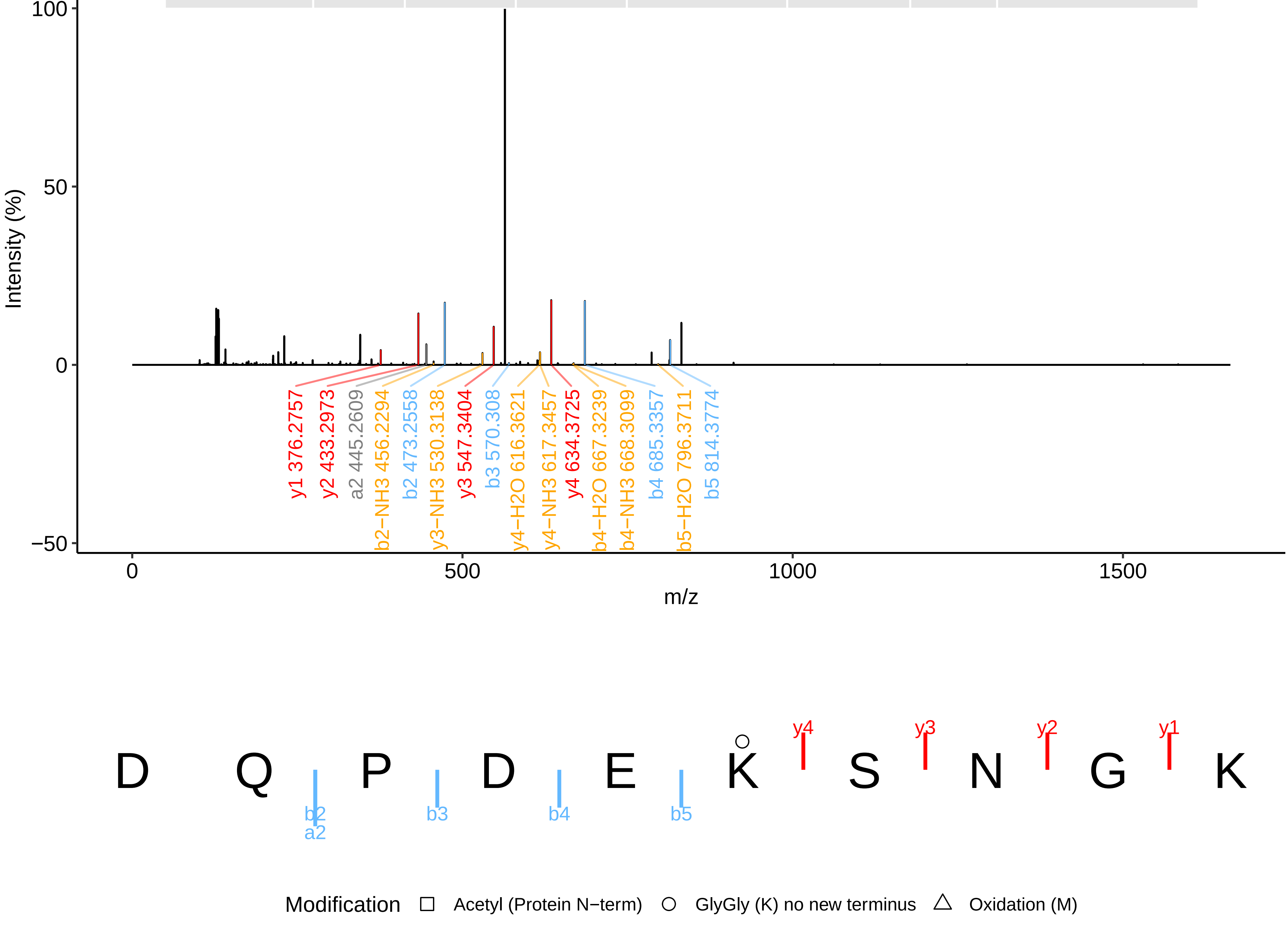

20200921_KIZ_MUb4_GlyGly_10plex_2 − Scan number: 3055

Gene Names	Charge	m/z	Mass	Retention time	PEP	Score	Precursor Intensity
OLR1	3	411.1898	1230.548	10.043	0.0022711	82.831	9284421

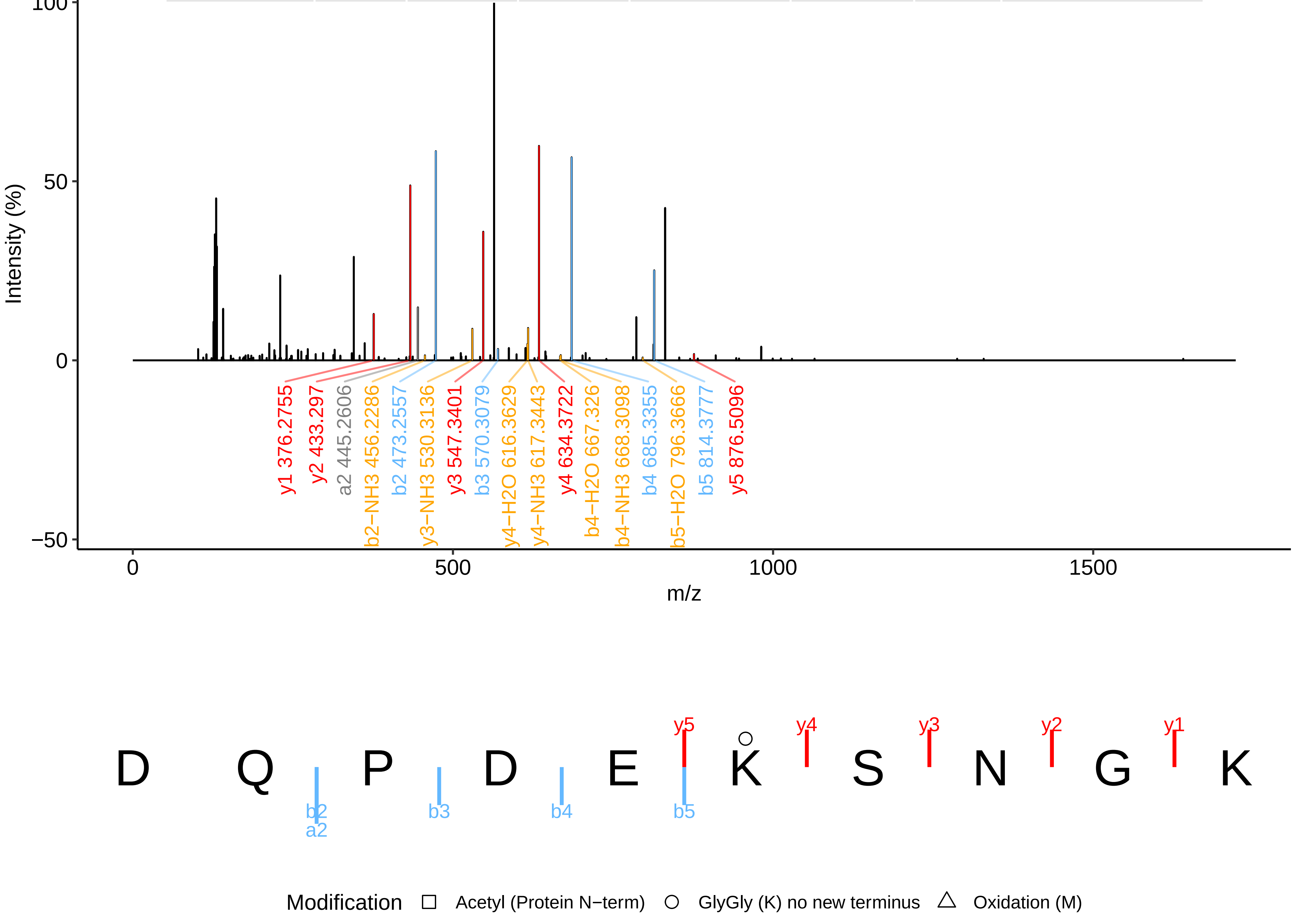

20200924_KIZ_MUb5_GlyGly_1 − Scan number: 3007

Gene Names	Charge	m/z	Mass	Retention time	PEP	Score	Precursor Intensity
OLR1	3	411.1898	1230.548	9.2724	0.0023535	82.261	29969142

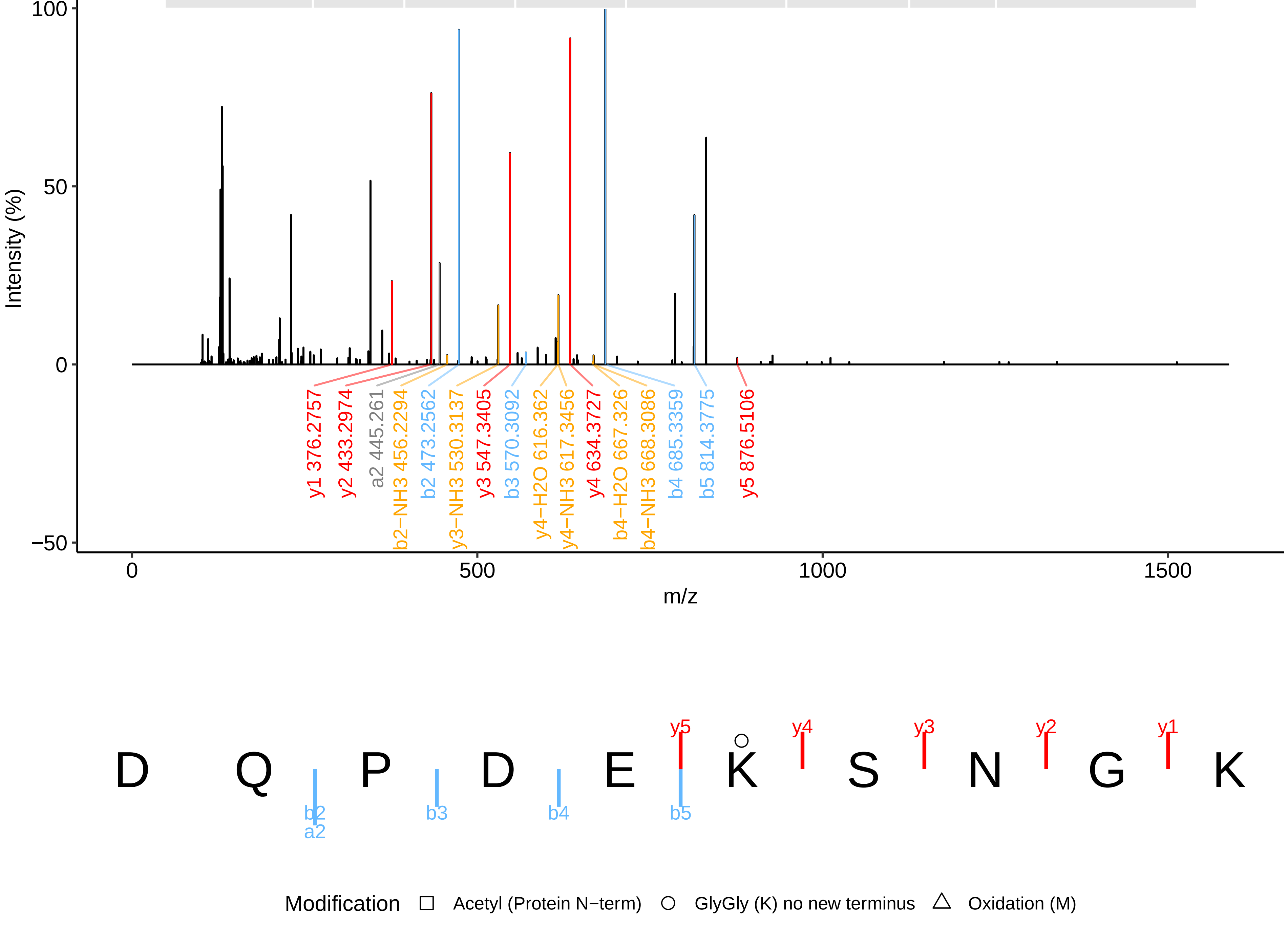

20200924_KIZ_MUb5_GlyGly_2 − Scan number: 2721

Gene Names	Charge	m/z	Mass	Retention time	PEP	Score	Precursor Intensity
OLR1	3	411.1898	1230.548	9.693	0.0016245	87.298	3421815

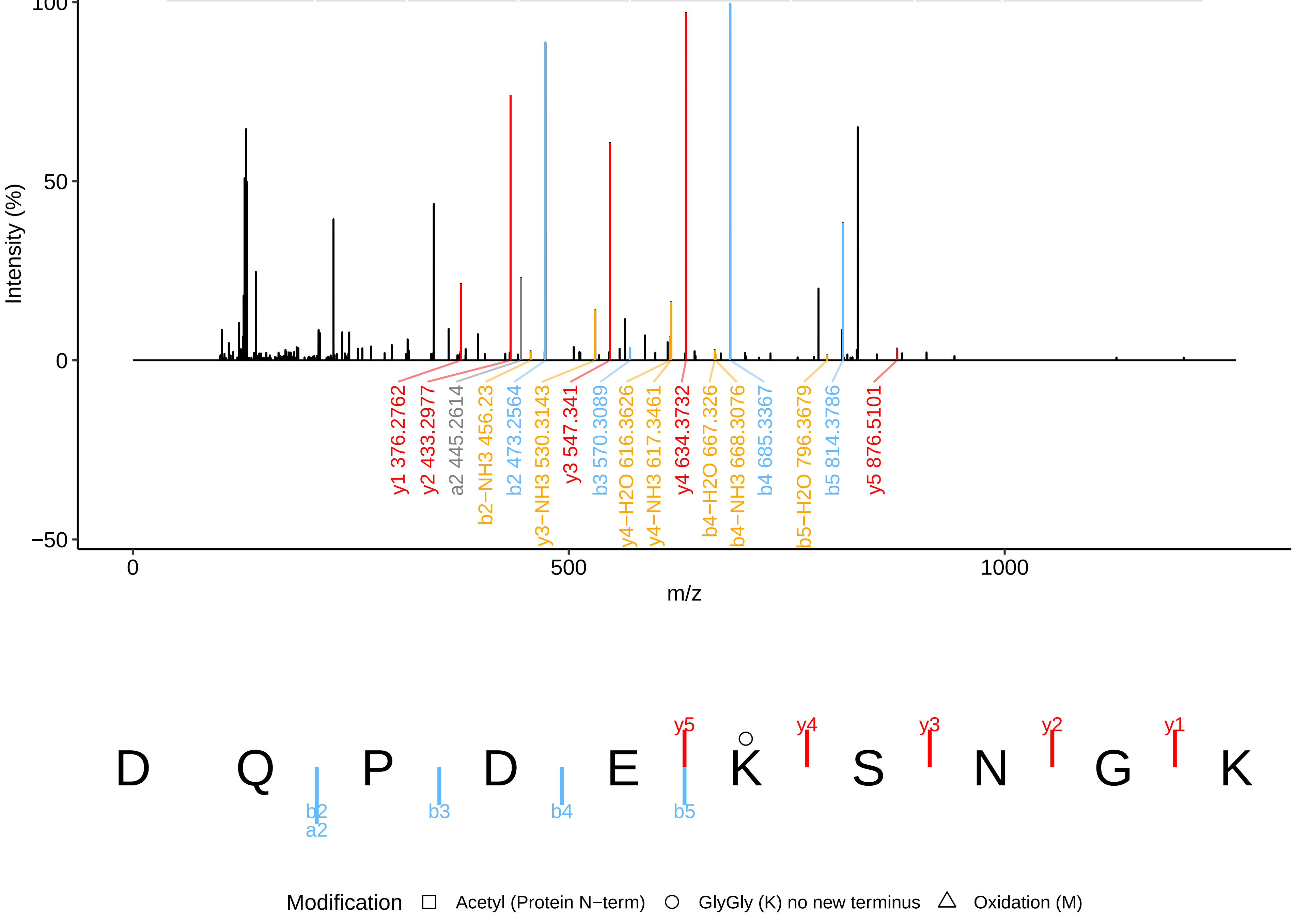

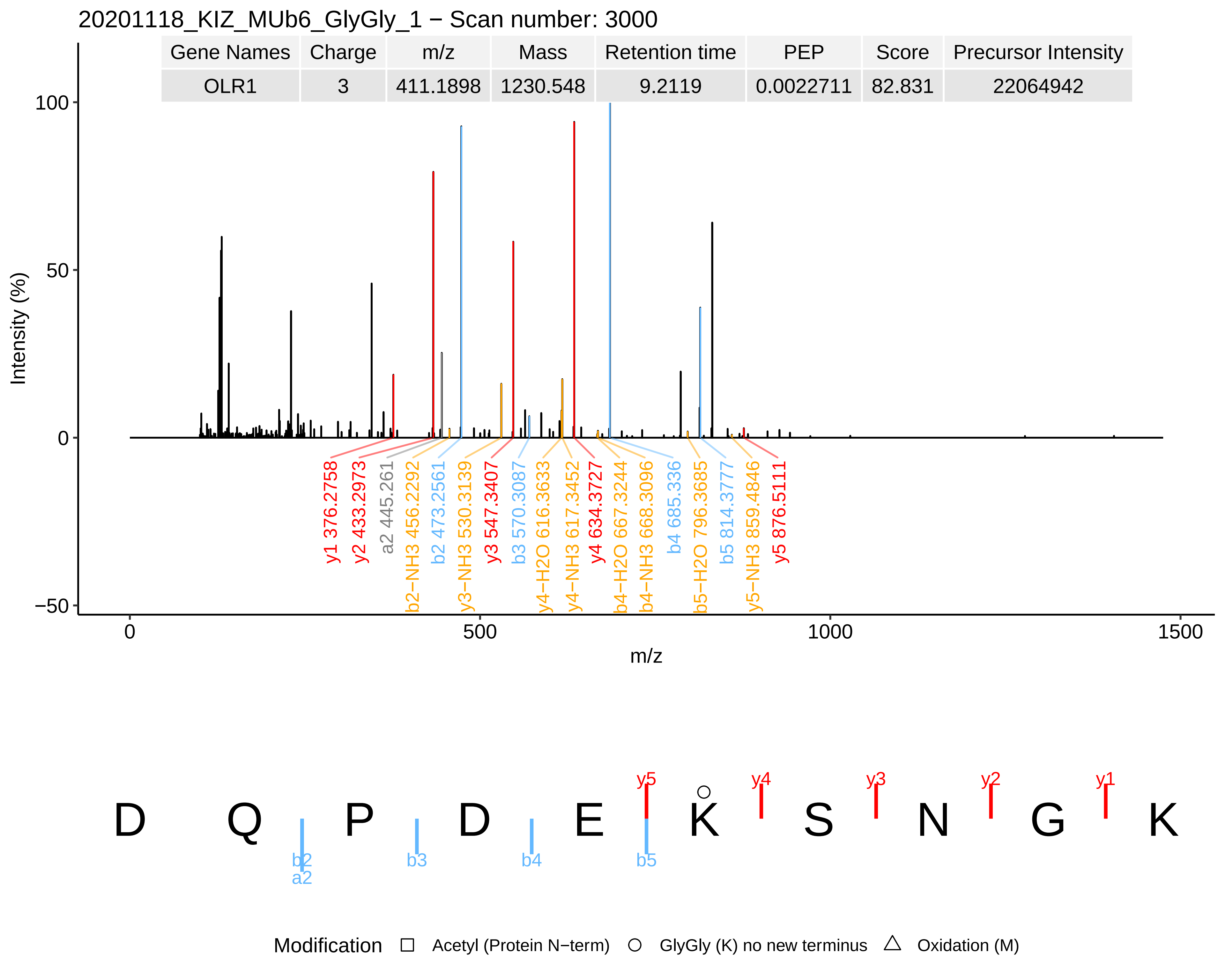
20201118_KIZ_MUb6_GlyGly_1 − Scan number: 3000
Gene Names
OLR1
Charge
3
m/z
411.1898
Mass
1230.548
Retention time
9.2119
PEP
0.0022711
Score
82.831
Precursor Intensity
22064942
Intensity (%)
100
50
0
−50
0
500
1000
1500
m/z
y1 376.2758
y2 433.2973
a2 445.261
b2−NH3 456.2292
b2 473.2561
y3−NH3 530.3139
y3 547.3407
b3 570.3087
y4−H2O 616.3633
y4−NH3 617.3452
y4 634.3727
b4−H2O 667.3244
b4−NH3 668.3096
b4 685.336
b5−H2O 796.3685
b5 814.3777
y5−NH3 859.4846
y5 876.5111
D Q P D E K S N G K
y5 y4 y3 y2 y1
b2 a2 b3 b4 b5
Modification
Acetyl (Protein N−term)
GlyGly (K) no new terminus
Oxidation (M)

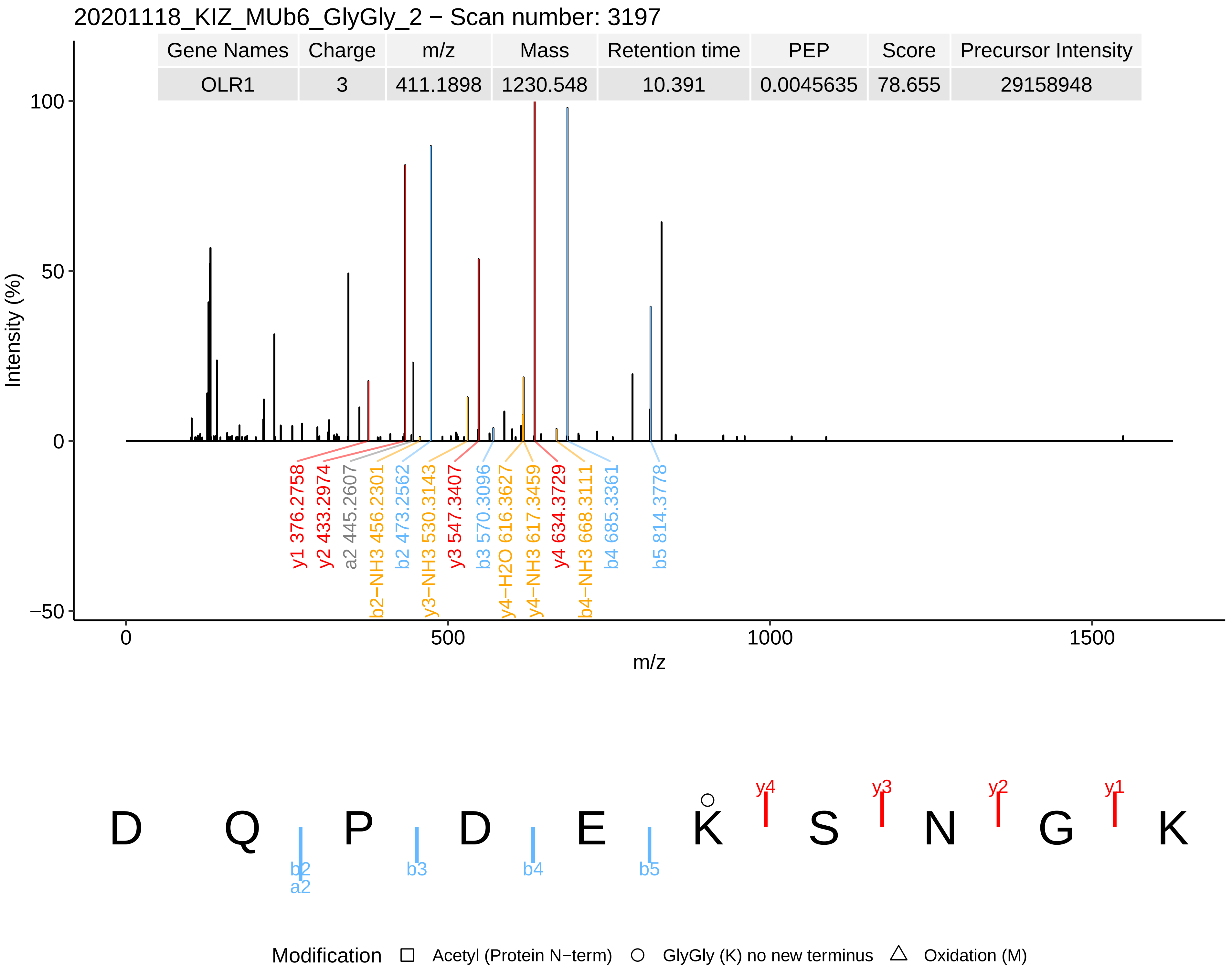
20201118_KIZ_MUb6_GlyGly_2 − Scan number: 3197
Gene Names | Charge | m/z | Mass | Retention time | PEP | Score | Precursor Intensity
OLR1 | 3 | 411.1898 | 1230.548 | 10.391 | 0.0045635 | 78.655 | 29158948
Intensity (%)
m/z
y1 376.2758
y2 433.2974
a2 445.2607
b2−NH3 456.2301
b2 473.2562
y3−NH3 530.3143
y3 547.3407
b3 570.3096
y4−H2O 616.3627
y4−NH3 617.3459
y4 634.3729
b4−NH3 668.3111
b4 685.3361
b5 814.3778
D Q P D E K S N G K
y4 y3 y2 y1
b2 a2 b3 b4 b5
Modification
Acetyl (Protein N−term)
GlyGly (K) no new terminus
Oxidation (M)

20200921_KIZ_MUb4_GlyGly_10plex − Scan number: 4157

Gene Names	Charge	m/z	Mass	Retention time	PEP	Score	Precursor Intensity
FARSB	3	485.2271	1452.659	13.532	9.0946e−06	117.79	NA

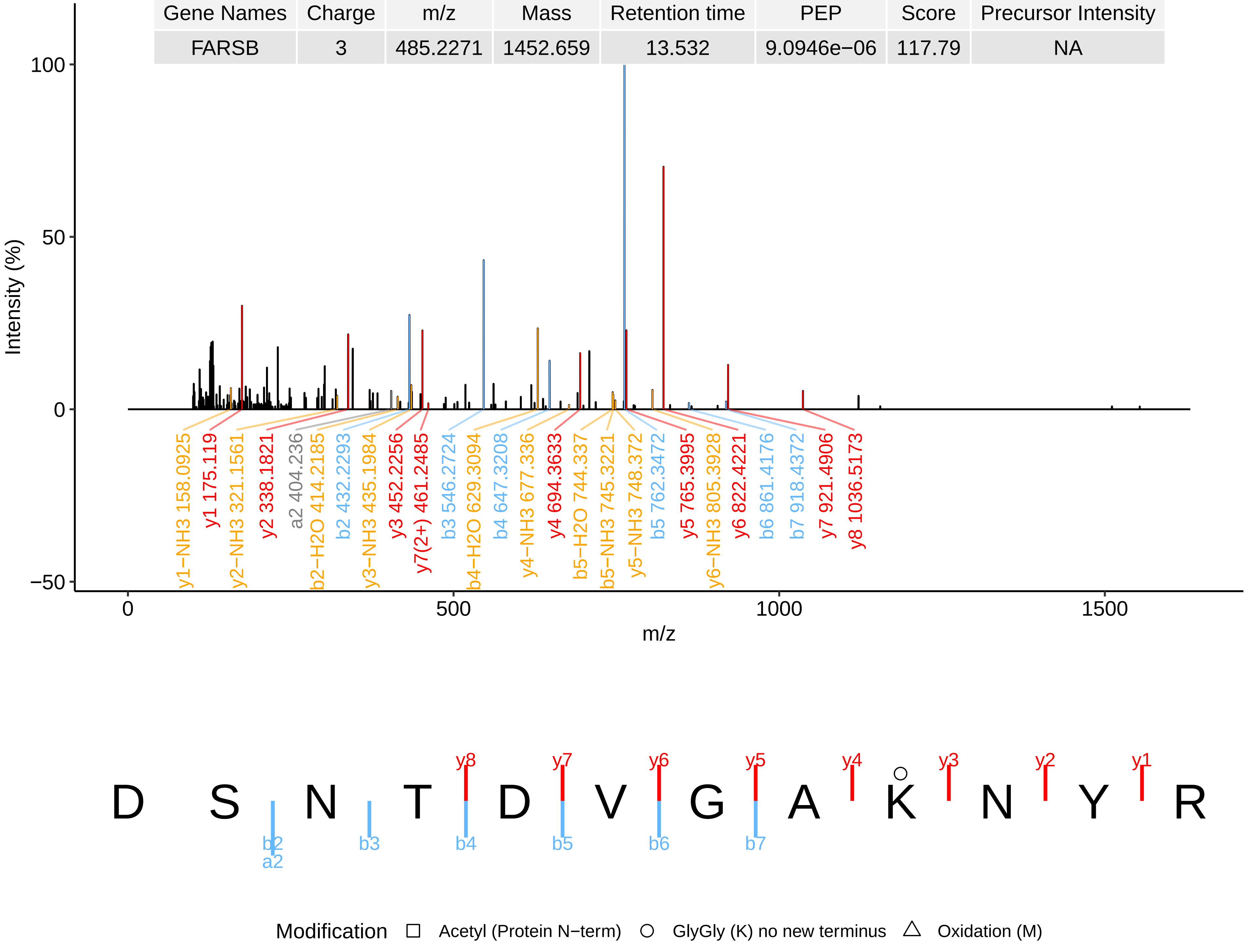

20200921_KIZ_MUb4_GlyGly_10plex − Scan number: 4903

Gene Names	Charge	m/z	Mass	Retention time	PEP	Score	Precursor Inte
ACTB;ACTG1;ACTA1;ACTC1;ACTG2;ACTA2	3	490.2269	1467.659	15.973	8.4298e−13	144.1	14088382

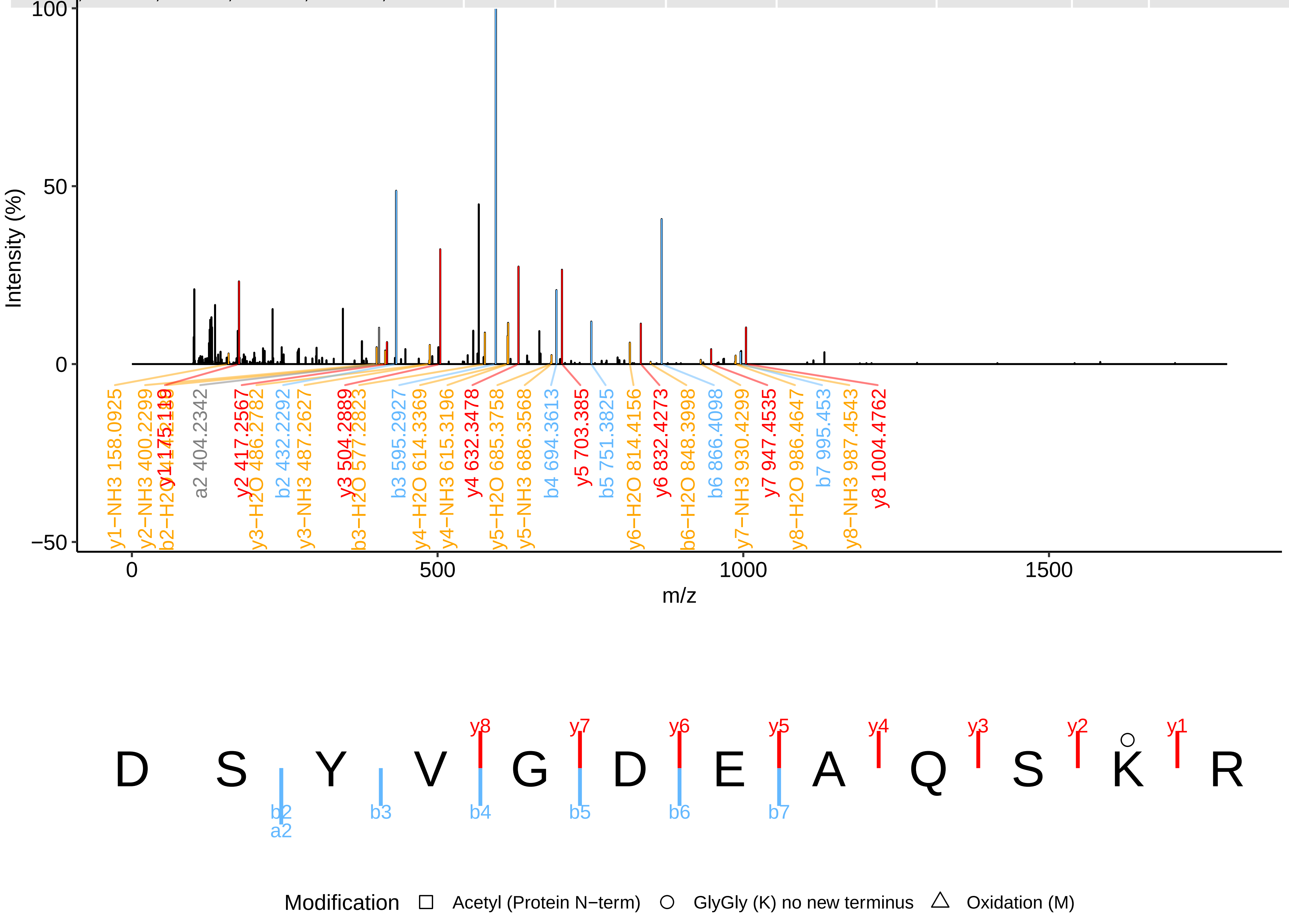

20200921_KIZ_MUb4_GlyGly_10plex_2 − Scan number: 4551

Gene Names	Charge	m/z	Mass	Retention time	PEP	Score	Precursor Inte
ACTB;ACTG1;ACTA1;ACTC1;ACTG2;ACTA2	3	490.2269	1467.659	15.992	2.7715e−25	169.65	8967743

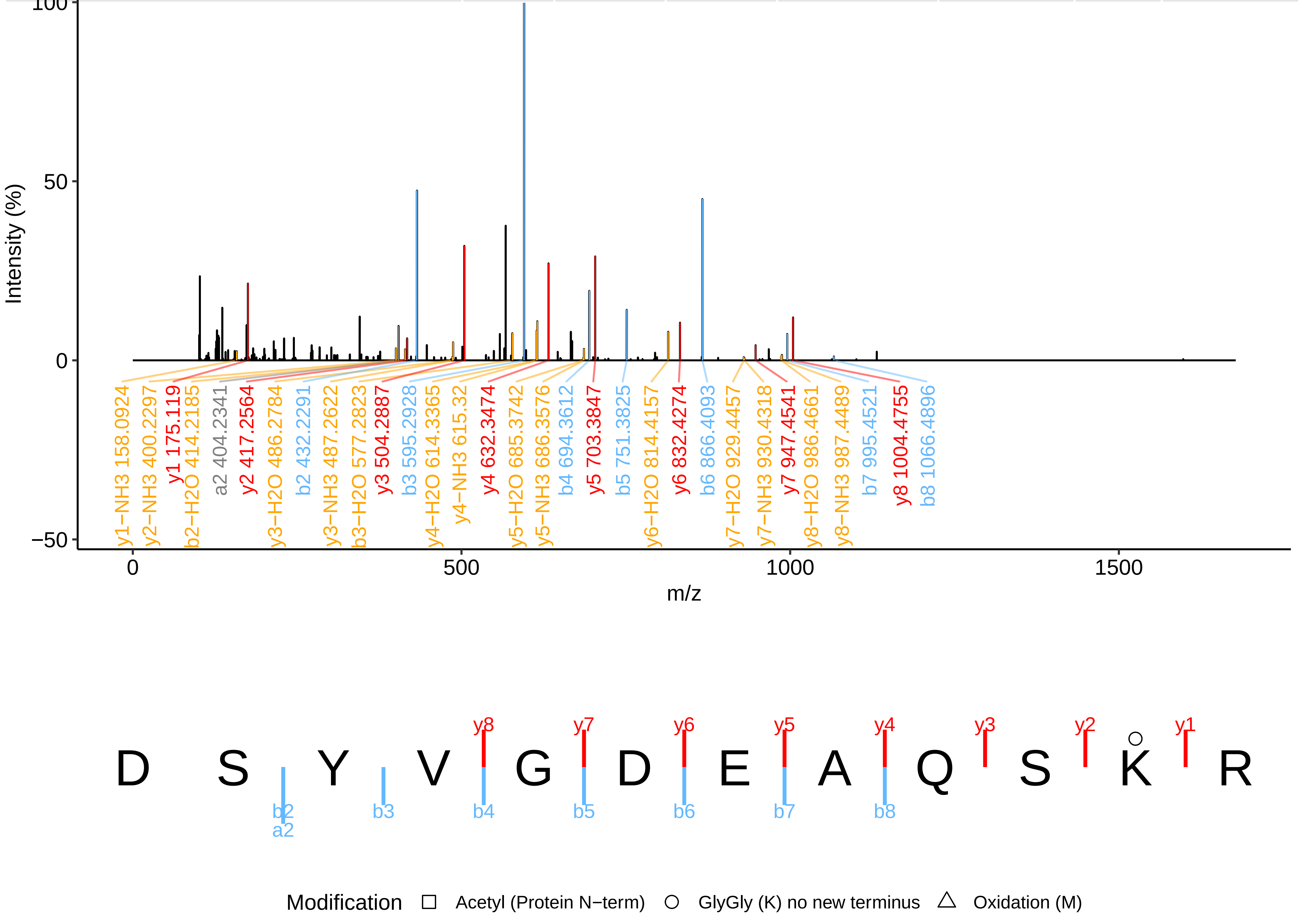

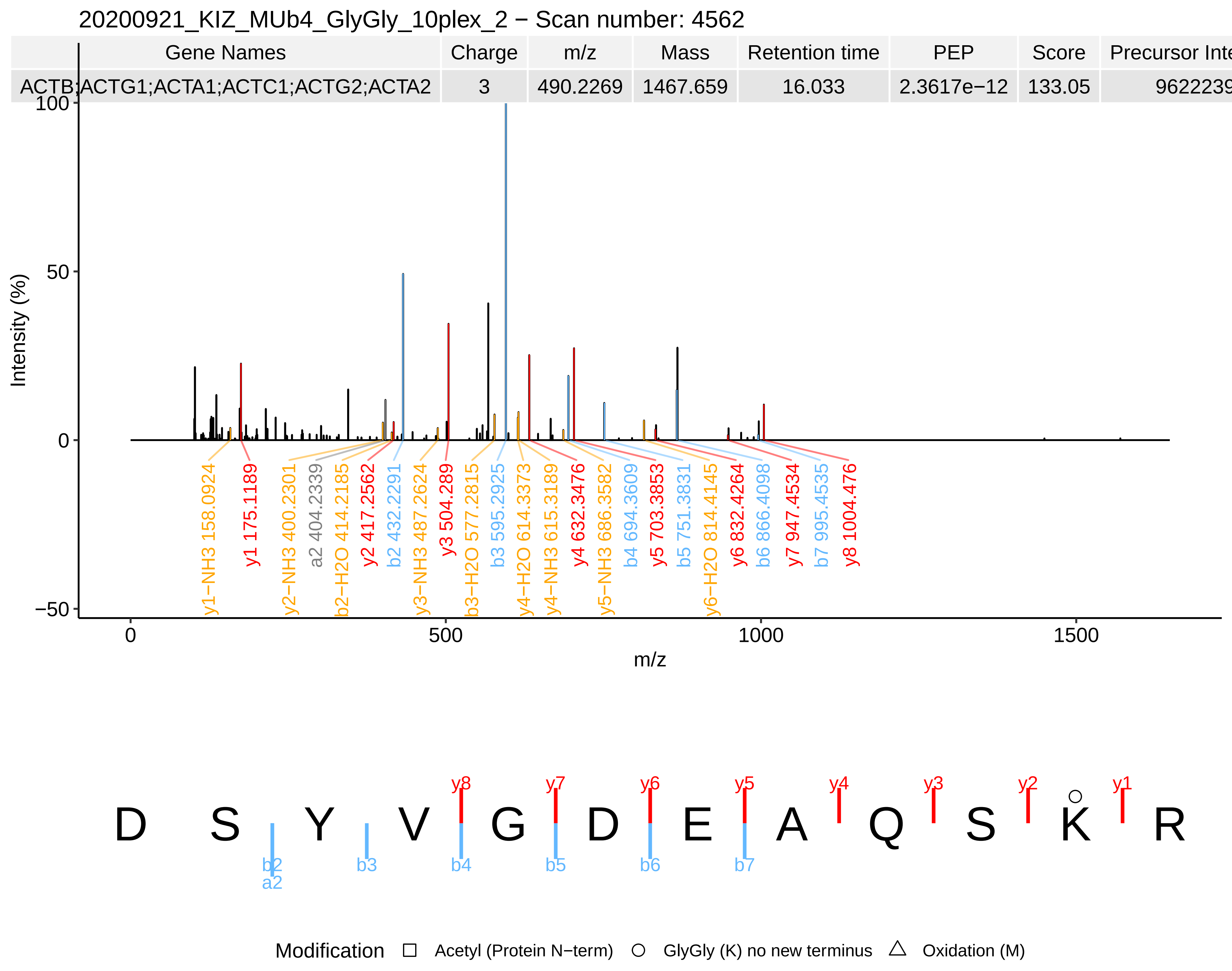
20200921_KIZ_MUb4_GlyGly_10plex_2 − Scan number: 4562
Gene Names
Charge
m/z
Mass
Retention time
PEP
Score
Precursor Inte
ACTB;ACTG1;ACTA1;ACTC1;ACTG2;ACTA2
3
490.2269
1467.659
16.033
2.3617e−12
133.05
9622239
Intensity (%)
100
50
0
−50
y1−NH3 158.0924
y1 175.1189
y2−NH3 400.2301
a2 404.2339
b2−H2O 414.2185
y2 417.2562
b2 432.2291
y3−NH3 487.2624
y3 504.289
b3−H2O 577.2815
b3 595.2925
y4−H2O 614.3373
y4−NH3 615.3189
y4 632.3476
y5−NH3 686.3582
b4 694.3609
y5 703.3853
b5 751.3831
y6−H2O 814.4145
y6 832.4264
b6 866.4098
y7 947.4534
b7 995.4535
y8 1004.476
0
500
1000
1500
m/z
D S Y V G D E A Q S K R
y8 y7 y6 y5 y4 y3 y2 y1
b2 a2 b3 b4 b5 b6 b7
Modification
Acetyl (Protein N−term)
GlyGly (K) no new terminus
Oxidation (M)

20200924_KIZ_MUb5_GlyGly_1 − Scan number: 4247

Gene Names	Charge	m/z	Mass	Retention time	PEP	Score	Precursor Inte
ACTB;ACTG1;ACTA1;ACTC1;ACTG2;ACTA2	3	490.2269	1467.659	13.081	1.6243e−18	153.34	5919700

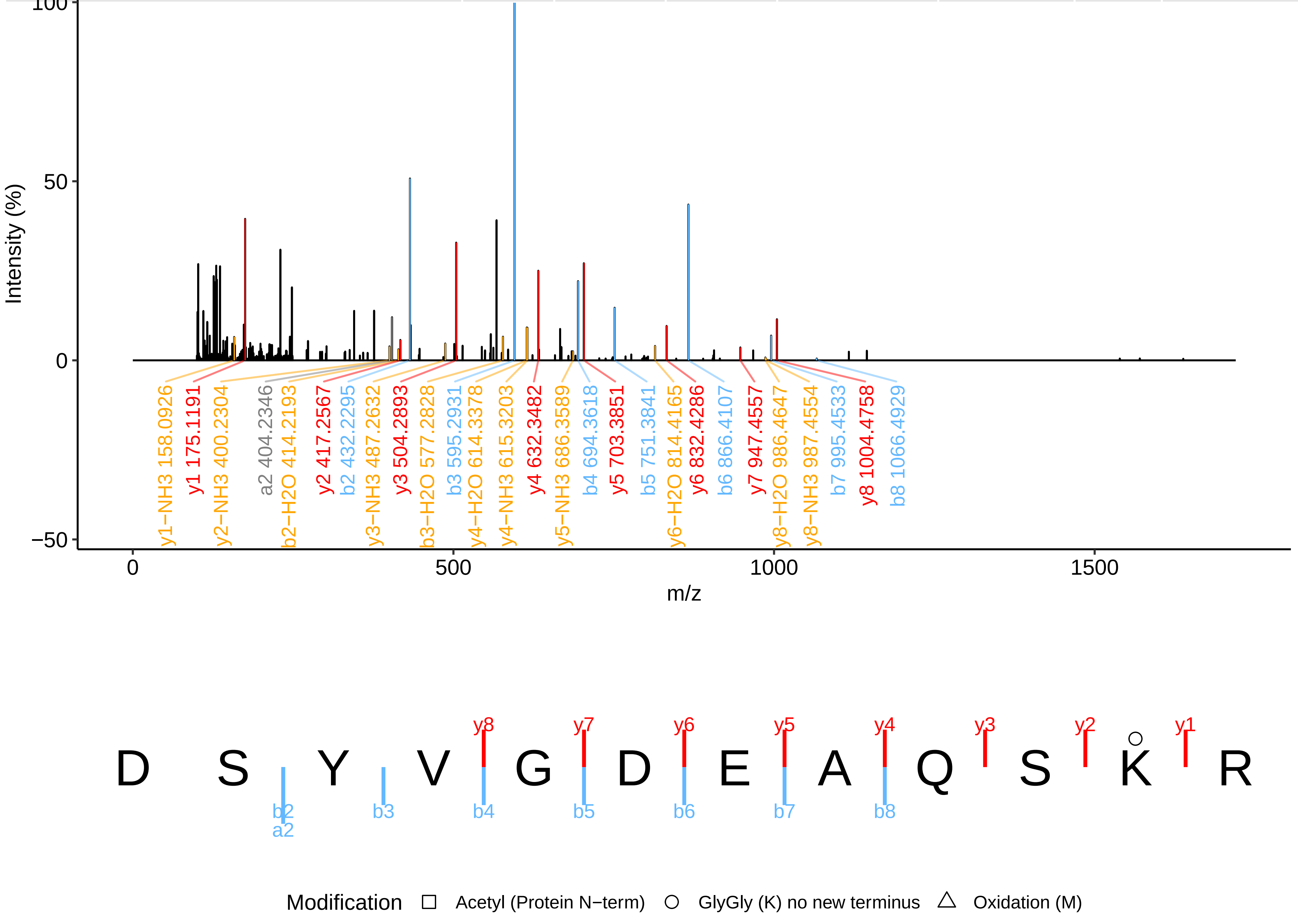

20200924_KIZ_MUb5_GlyGly_1 − Scan number: 4280

Gene Names	Charge	m/z	Mass	Retention time	PEP	Score	Precursor Inte
ACTB;ACTG1;ACTA1;ACTC1;ACTG2;ACTA2	3	490.2269	1467.659	13.194	7.6565e−05	105.03	1110718

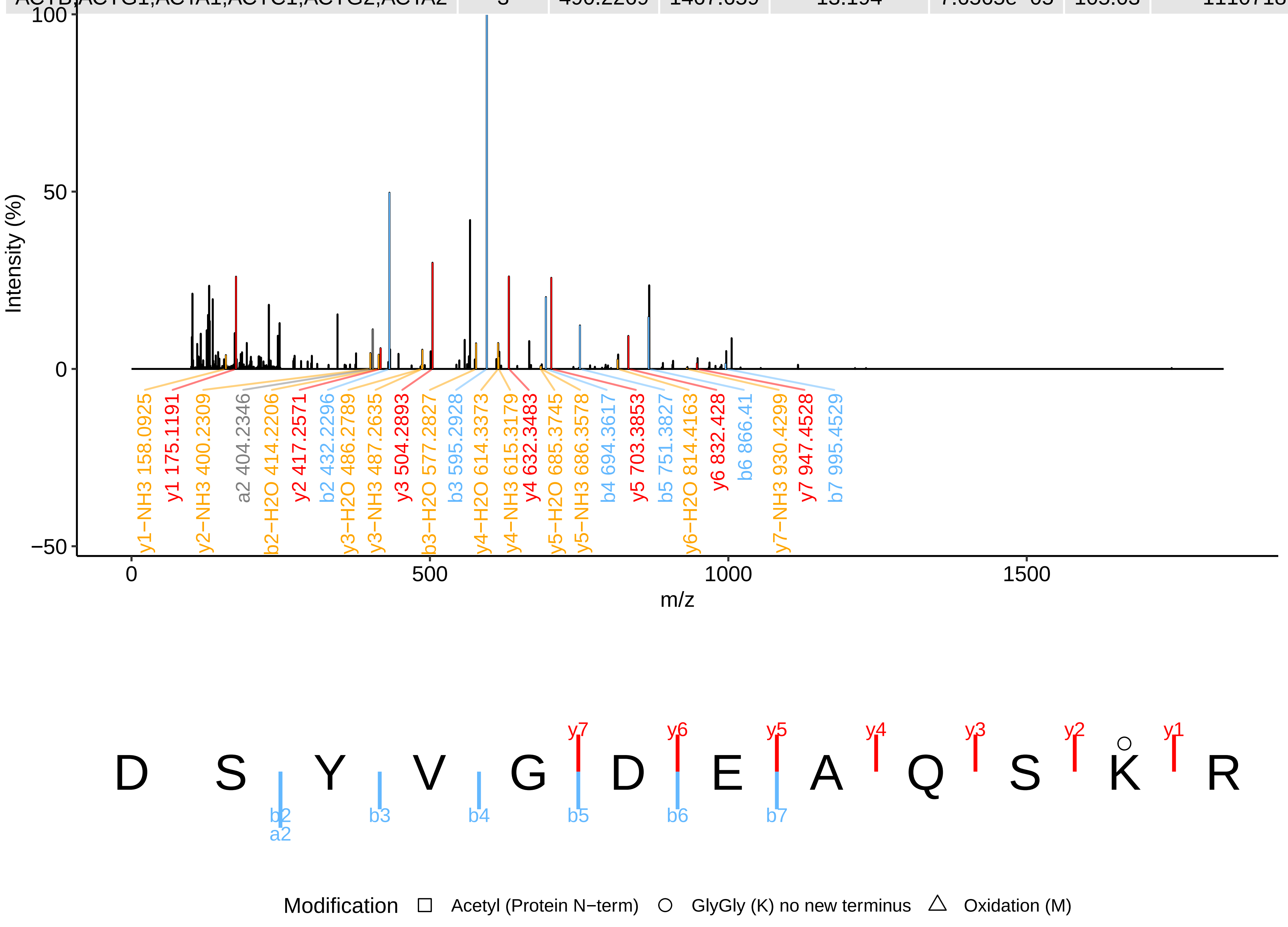

20200924_KIZ_MUb5_GlyGly_2 − Scan number: 4026

Gene Names	Charge	m/z	Mass	Retention time	PEP	Score	Precursor Inte
ACTB;ACTG1;ACTA1;ACTC1;ACTG2;ACTA2	3	490.2269	1467.659	14.714	6.7524e−26	181.31	5546100

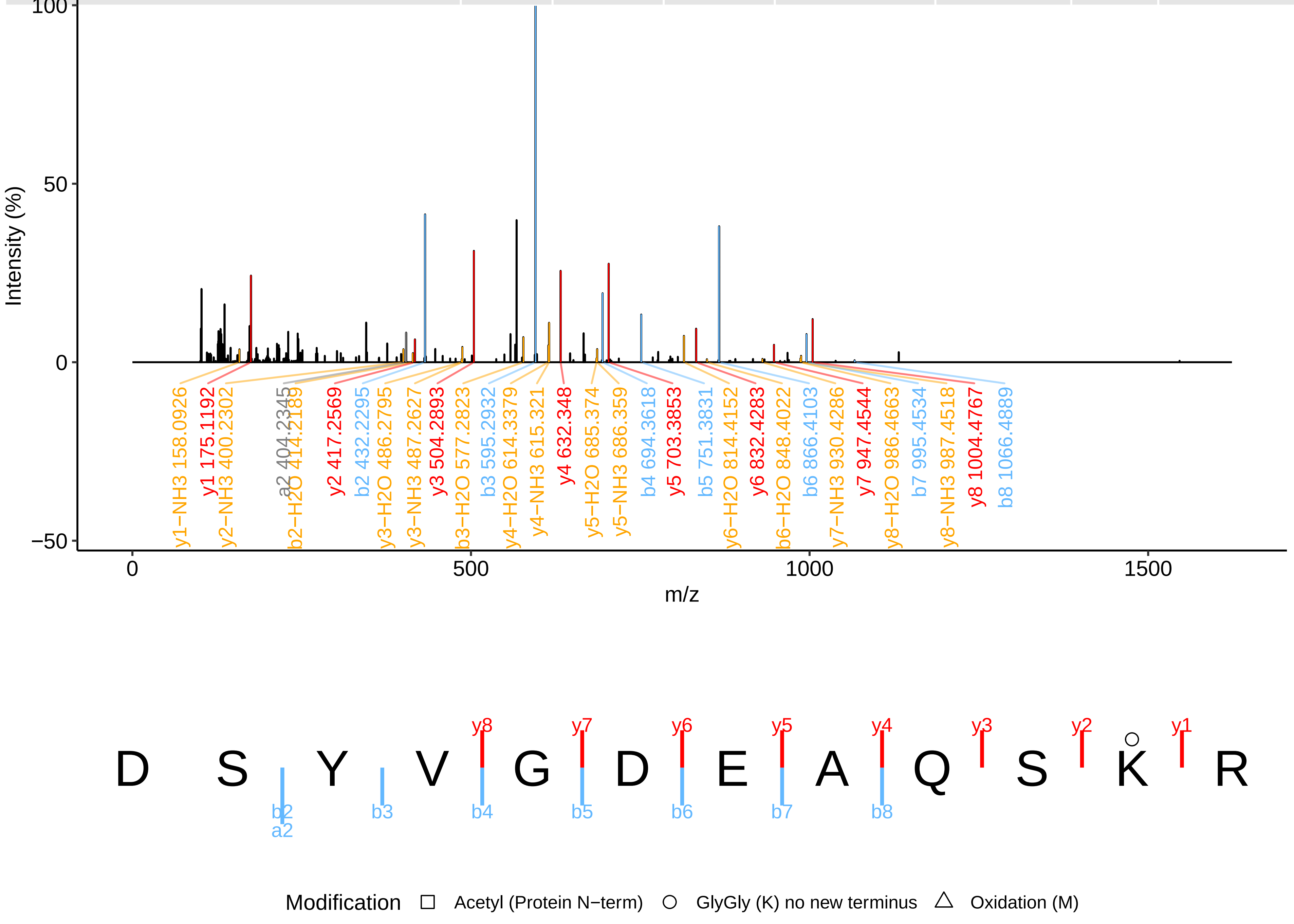

20200924_KIZ_MUb5_GlyGly_2 − Scan number: 4103

Gene Names	Charge	m/z	Mass	Retention time	PEP	Score	Precursor Inte
ACTB;ACTG1;ACTA1;ACTC1;ACTG2;ACTA2	3	490.2269	1467.659	15.01	0.00012538	98.407	3533875

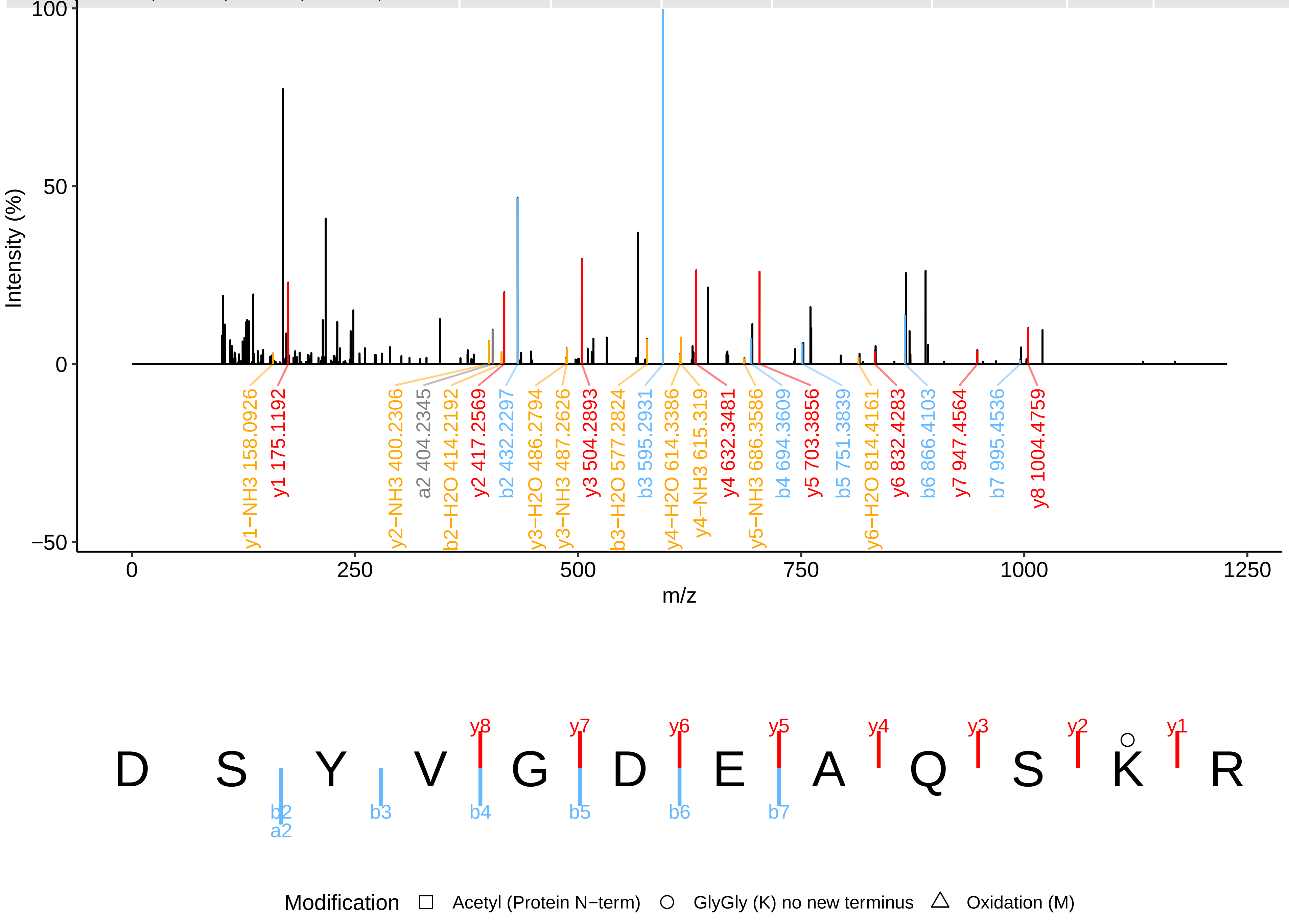

20201118_KIZ_MUb6_GlyGly_1 − Scan number: 3855

Gene Names	Charge	m/z	Mass	Retention time	PEP	Score	Precursor Inte
ACTB;ACTG1;ACTA1;ACTC1;ACTG2;ACTA2	3	490.2269	1467.659	11.936	1.6765e−25	175.74	6911705

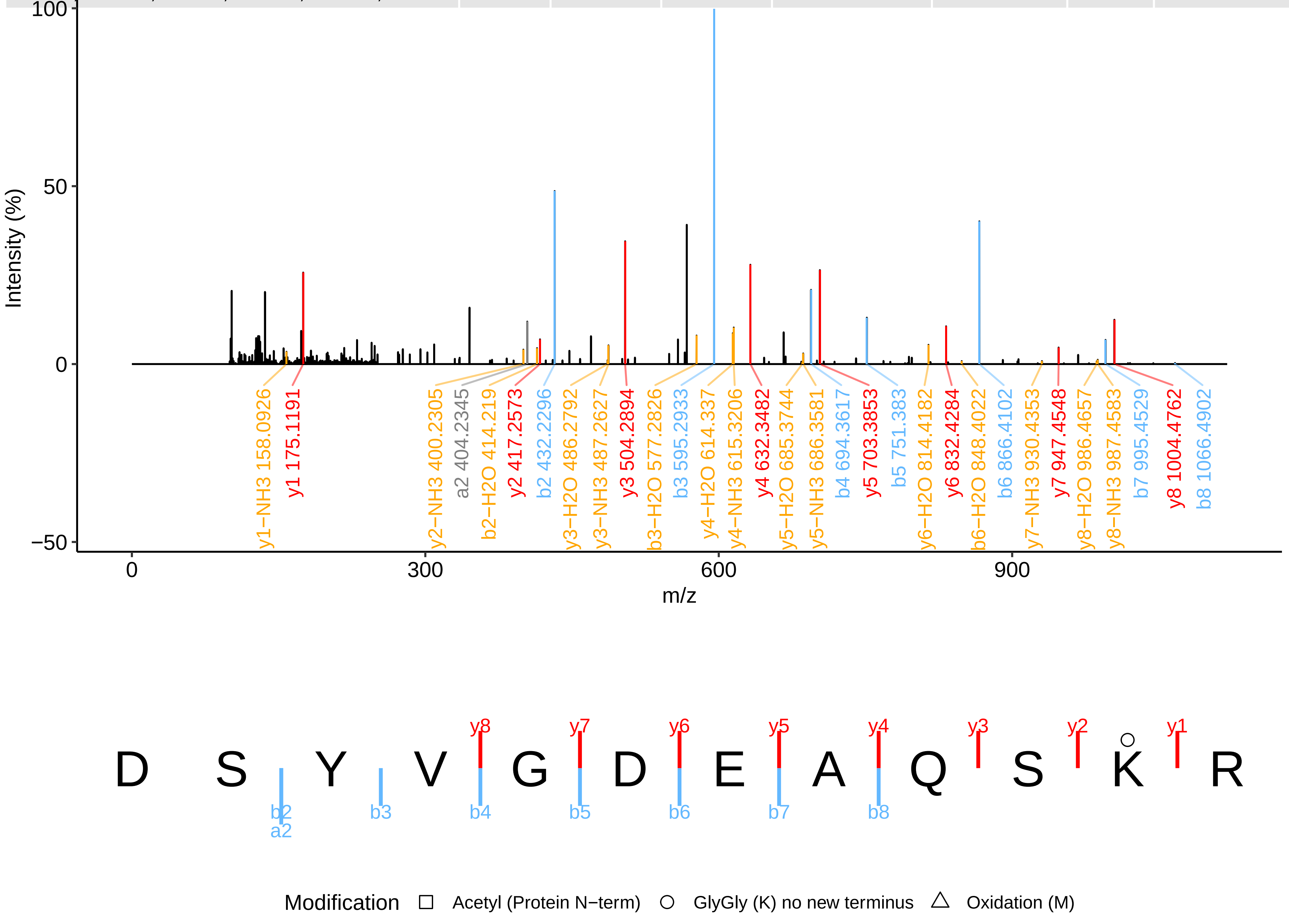

20201118_KIZ_MUb6_GlyGly_1 − Scan number: 3884

Gene Names	Charge	m/z	Mass	Retention time	PEP	Score	Precursor Inte
ACTB;ACTG1;ACTA1;ACTC1;ACTG2;ACTA2	3	490.2269	1467.659	12.036	4.1867e−08	124.6	6213027

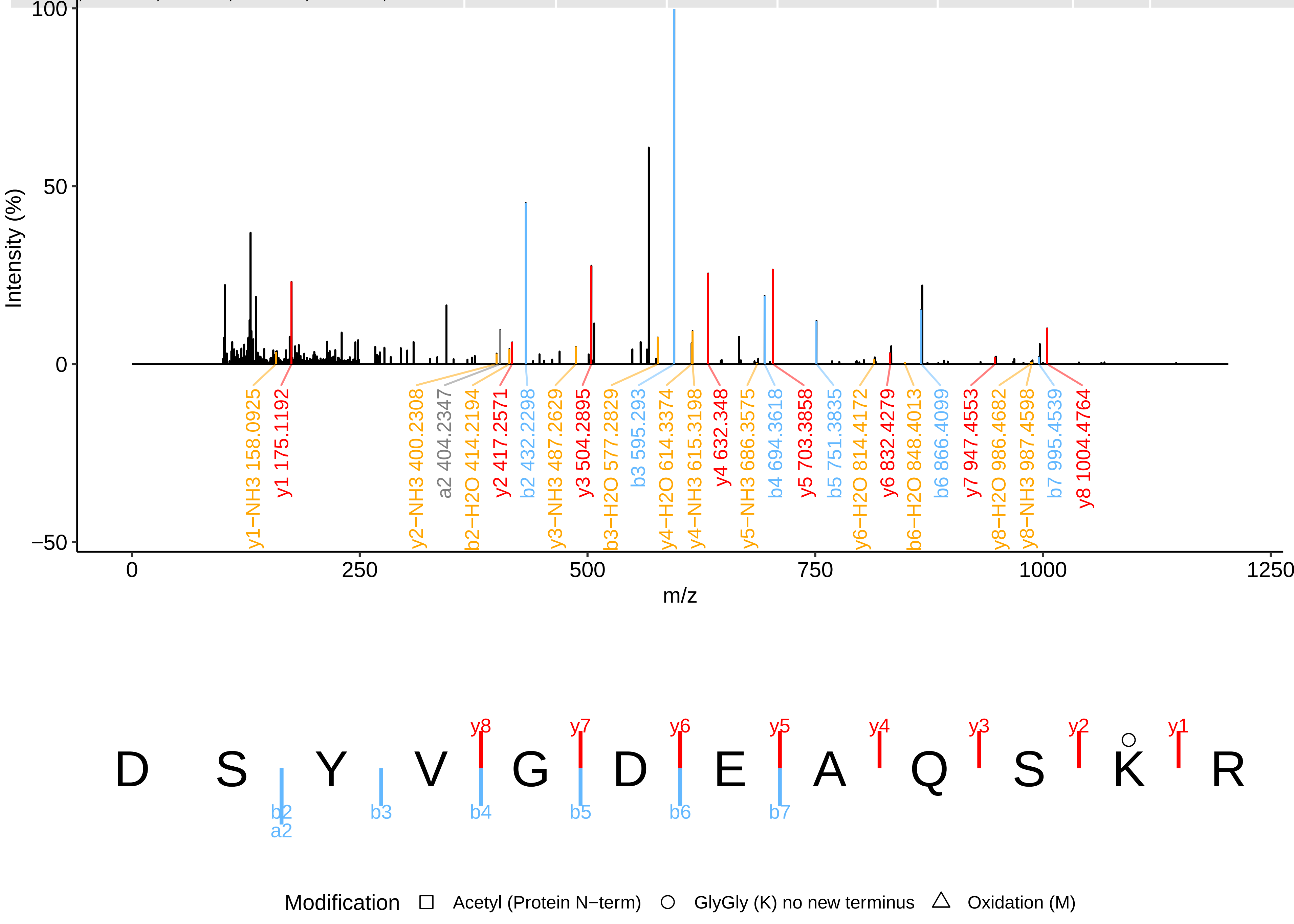

20201118_KIZ_MUb6_GlyGly_2 − Scan number: 4731

Gene Names	Charge	m/z	Mass	Retention time	PEP	Score	Precursor Inte
ACTB;ACTG1;ACTA1;ACTC1;ACTG2;ACTA2	3	490.2269	1467.659	16.246	1.3815e−18	155.48	3717801

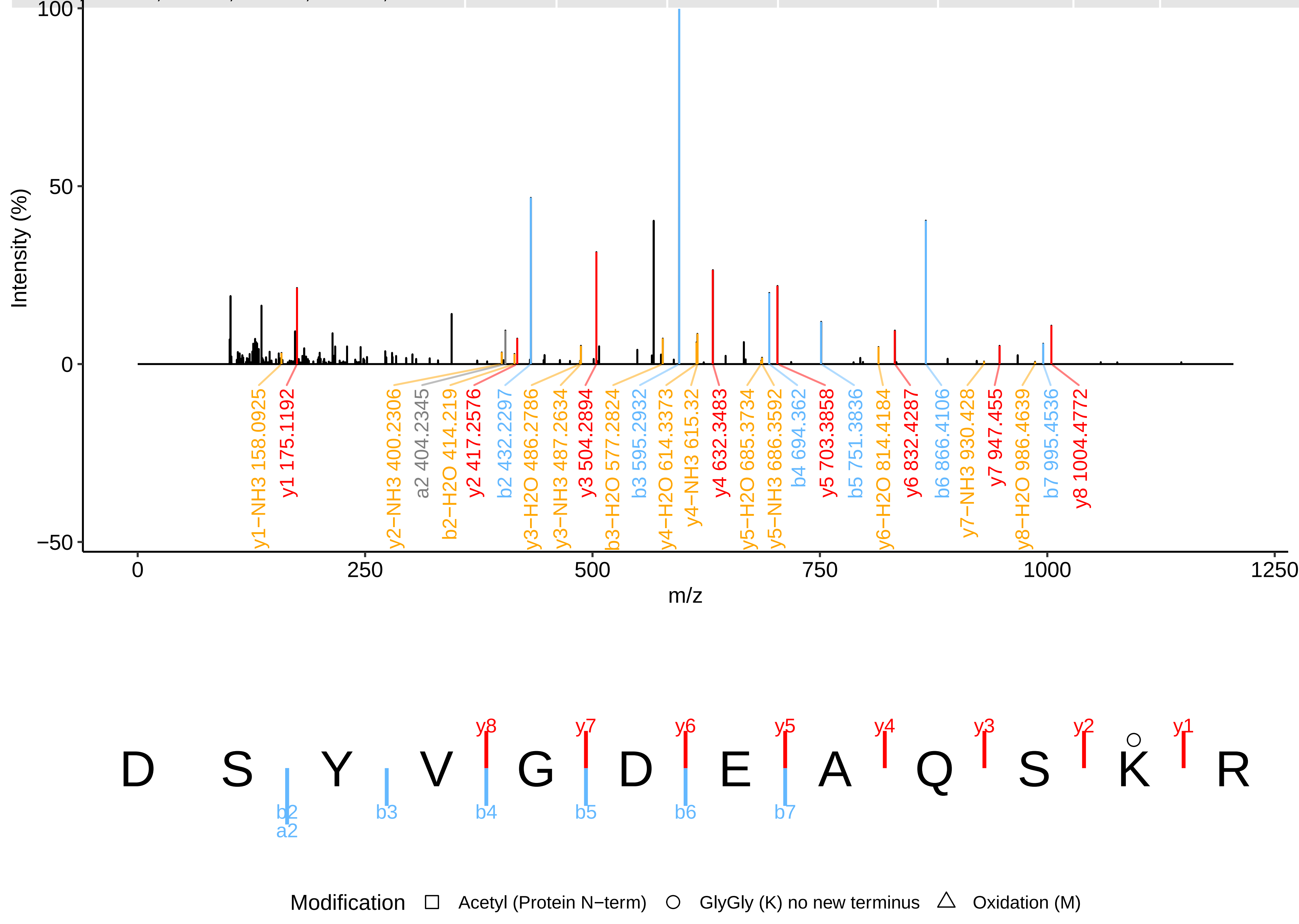

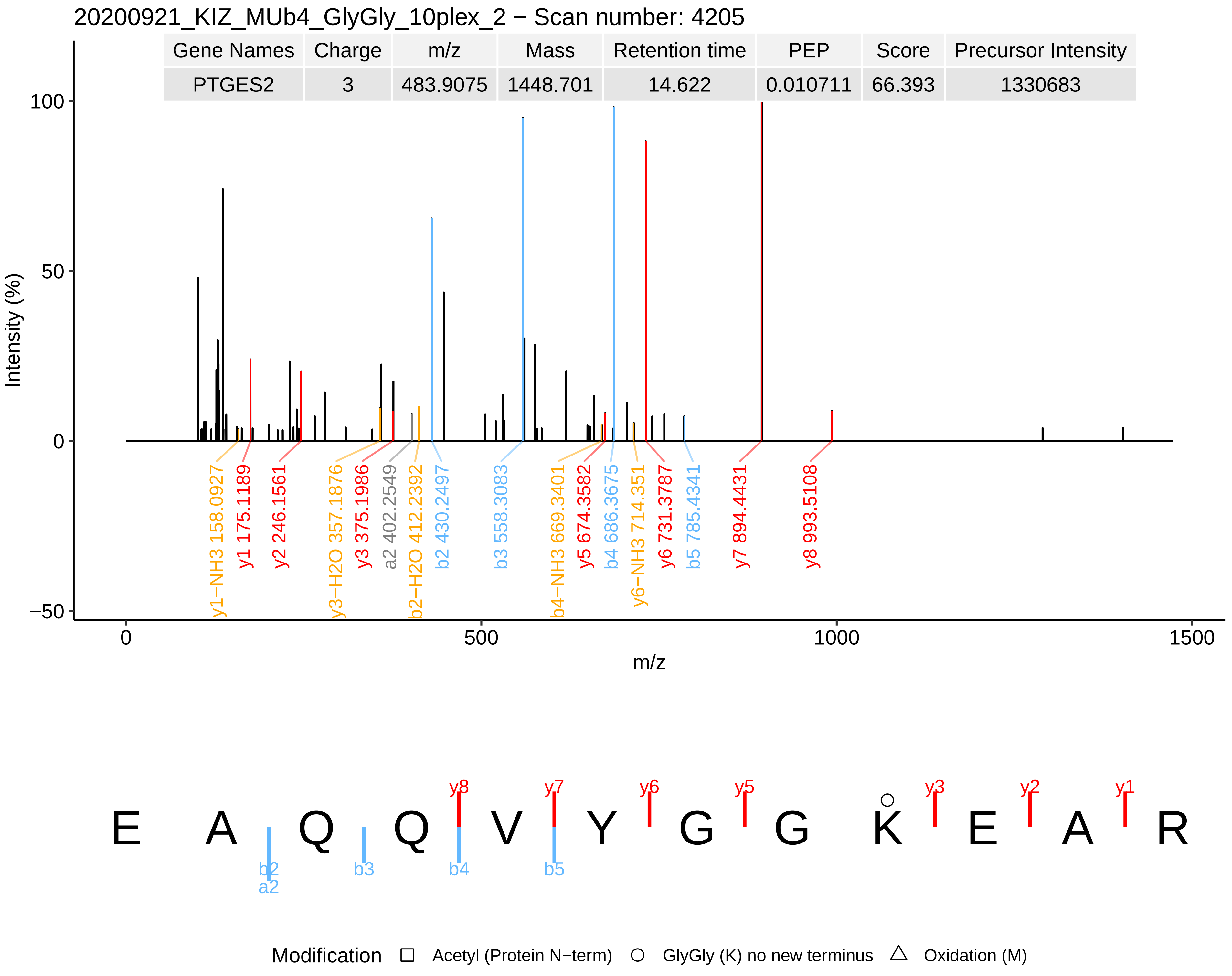
20200921_KIZ_MUb4_GlyGly_10plex_2 − Scan number: 4205
Gene Names | Charge | m/z | Mass | Retention time | PEP | Score | Precursor Intensity
PTGES2 | 3 | 483.9075 | 1448.701 | 14.622 | 0.010711 | 66.393 | 1330683
Intensity (%)
100
50
0
−50
y1−NH3 158.0927
y1 175.1189
y2 246.1561
y3−H2O 357.1876
y3 375.1986
a2 402.2549
b2−H2O 412.2392
b2 430.2497
b3 558.3083
b4−NH3 669.3401
y5 674.3582
b4 686.3675
y6−NH3 714.351
y6 731.3787
b5 785.4341
y7 894.4431
y8 993.5108
0
500
1000
1500
m/z
y8
y7
y6
y5
y3
y2
y1
E A Q Q V Y G G K E A R
b2
a2
b3
b4
b5
Modification
Acetyl (Protein N−term)
GlyGly (K) no new terminus
Oxidation (M)

20200921_KIZ_MUb4_GlyGly_10plex_2 − Scan number: 7849

Gene Names	Charge	m/z	Mass	Retention time	PEP	Score	Precursor Intensity
DNAJC11	3	489.9073	1466.7	29.419	0.0011091	82.171	712673.7

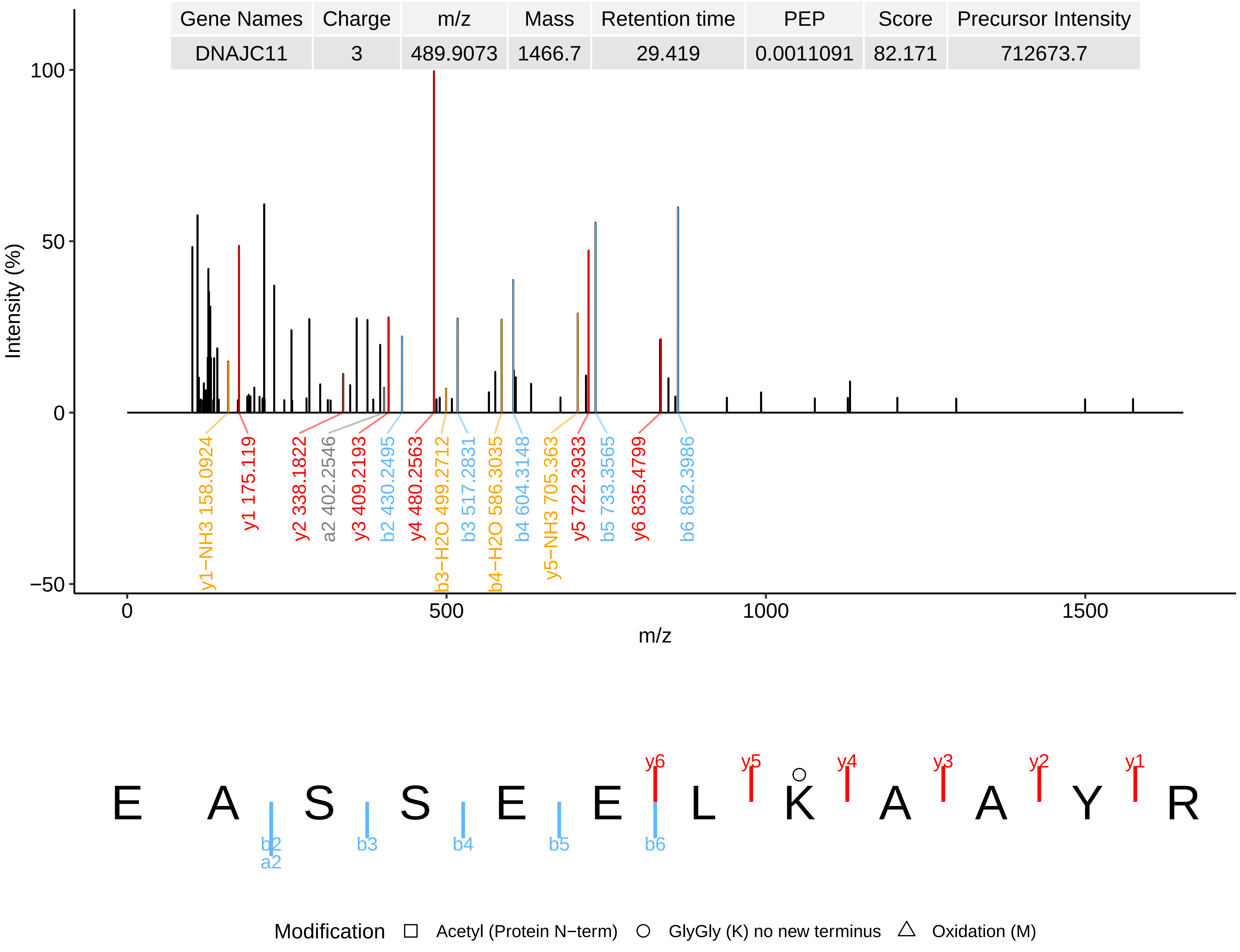

20200921_KIZ_MUb4_GlyGly_10plex − Scan number: 5497

Gene Names	Charge	m/z	Mass	Retention time	PEP	Score	Precursor Intensity
BASP1	3	414.5385	1240.593	18.011	0.00078547	93.096	4738852

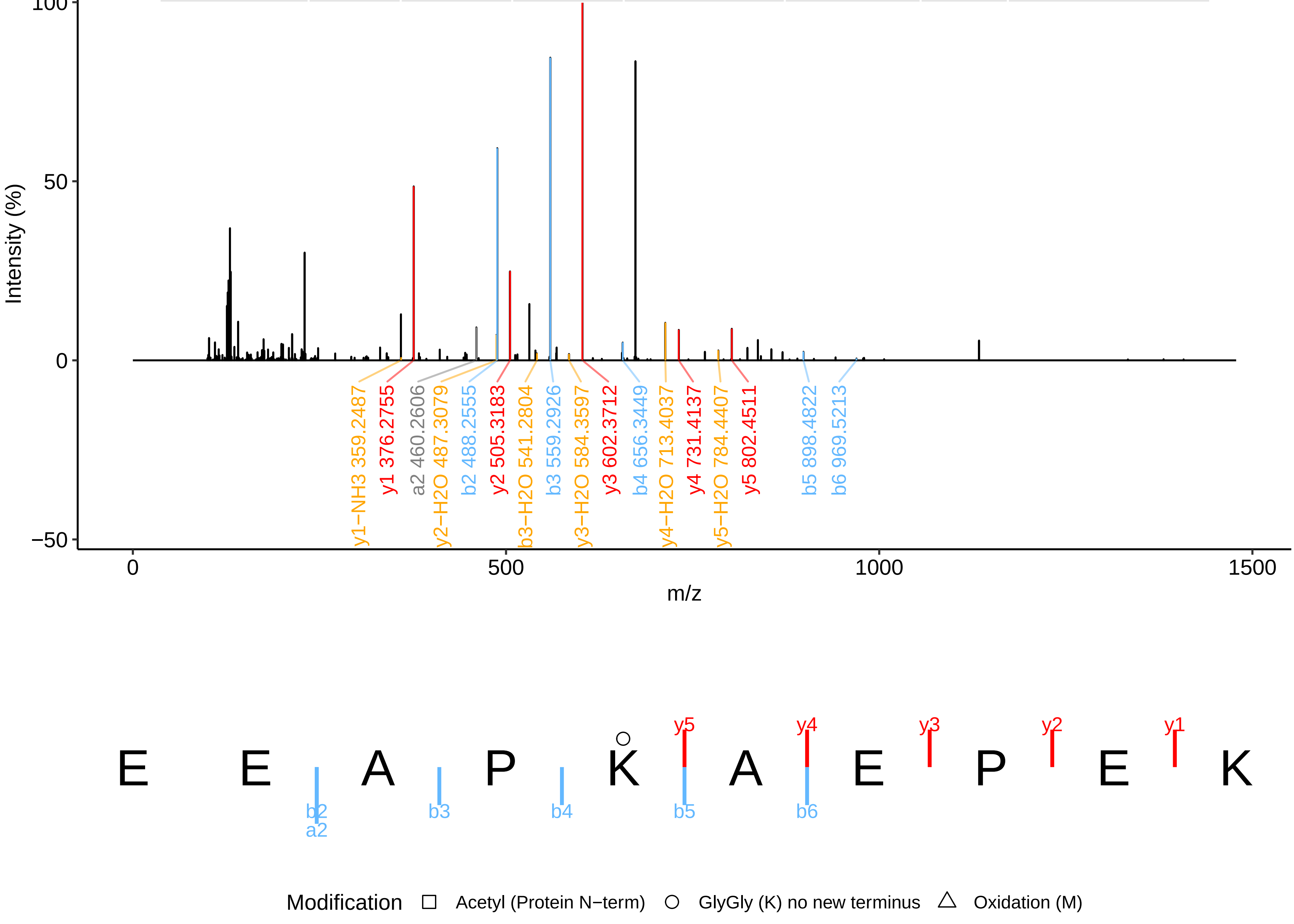

20200921_KIZ_MUb4_GlyGly_10plex − Scan number: 5525

Gene Names	Charge	m/z	Mass	Retention time	PEP	Score	Precursor Intensity
BASP1	3	414.5385	1240.593	18.106	0.018667	64.82	2918942

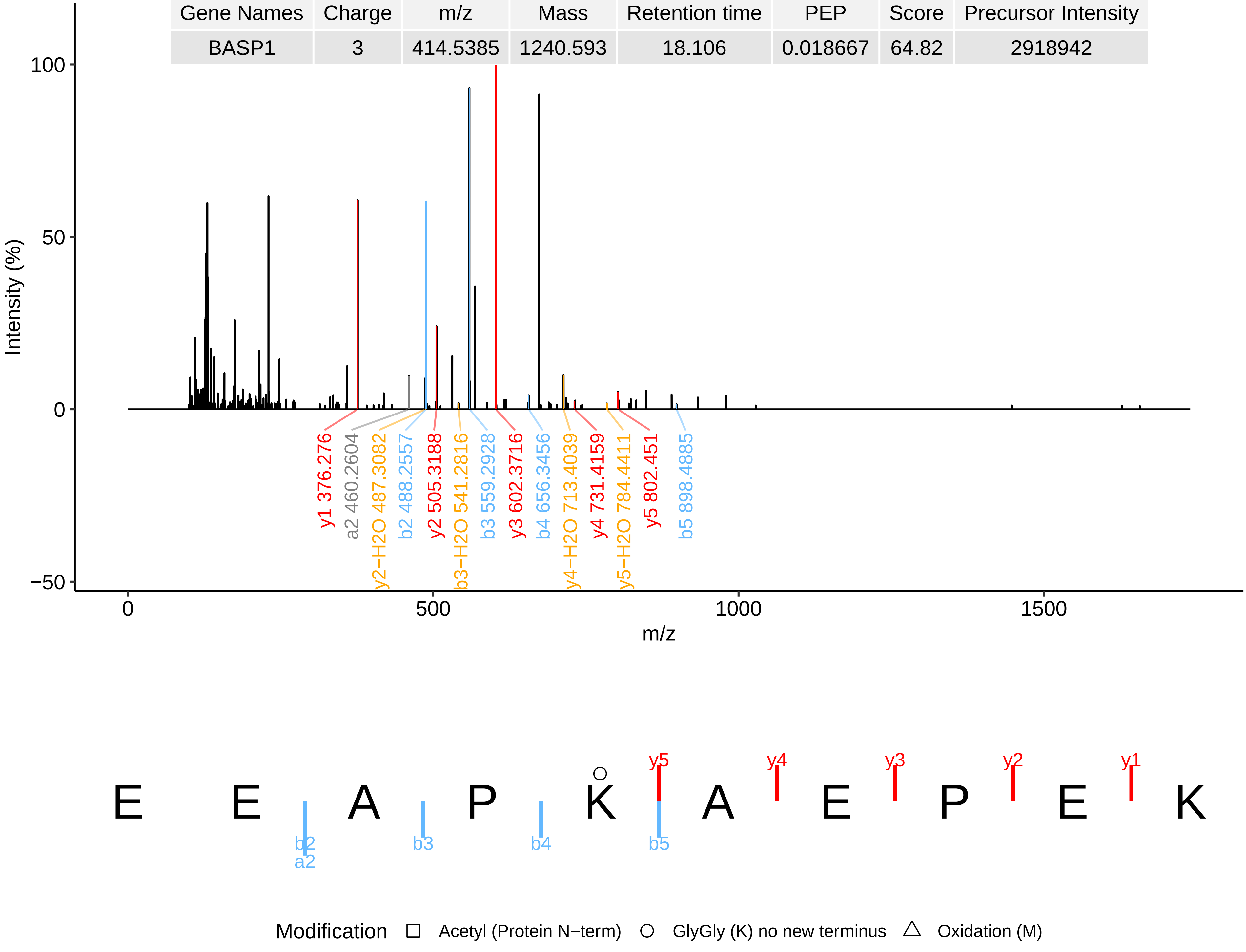

20200921_KIZ_MUb4_GlyGly_10plex_2 − Scan number: 5026

Gene Names	Charge	m/z	Mass	Retention time	PEP	Score	Precursor Intensity
BASP1	3	414.5385	1240.593	17.882	0.016329	67.113	628678.1

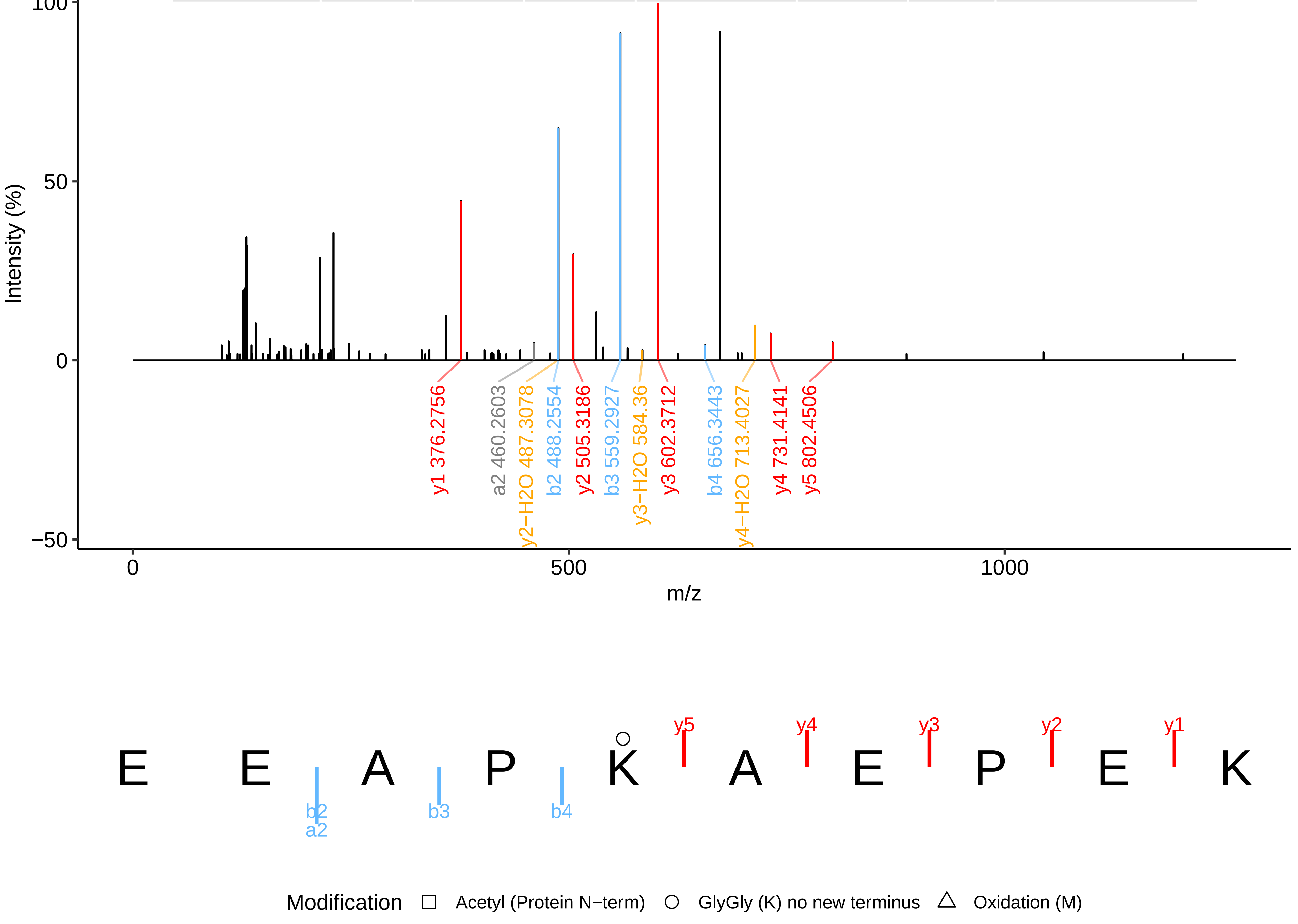

20200924_KIZ_MUb5_GlyGly_1 − Scan number: 4837

Gene Names	Charge	m/z	Mass	Retention time	PEP	Score	Precursor Intensity
BASP1	3	414.5385	1240.593	14.97	0.00081643	108.7	9466223

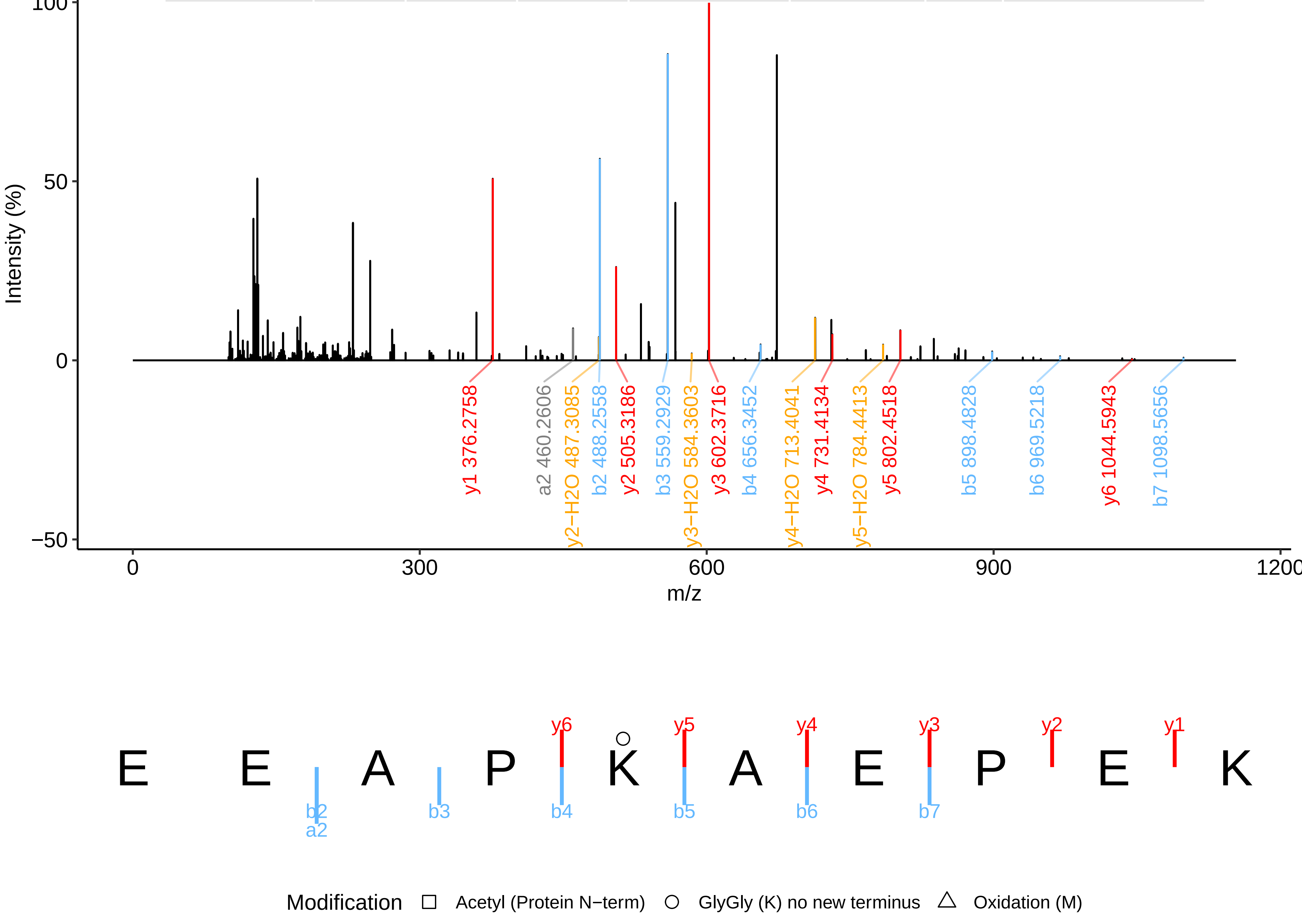

20200924_KIZ_MUb5_GlyGly_2 − Scan number: 4431

Gene Names	Charge	m/z	Mass	Retention time	PEP	Score	Precursor Intensity
BASP1	3	414.5385	1240.593	16.249	0.014515	68.893	1475819

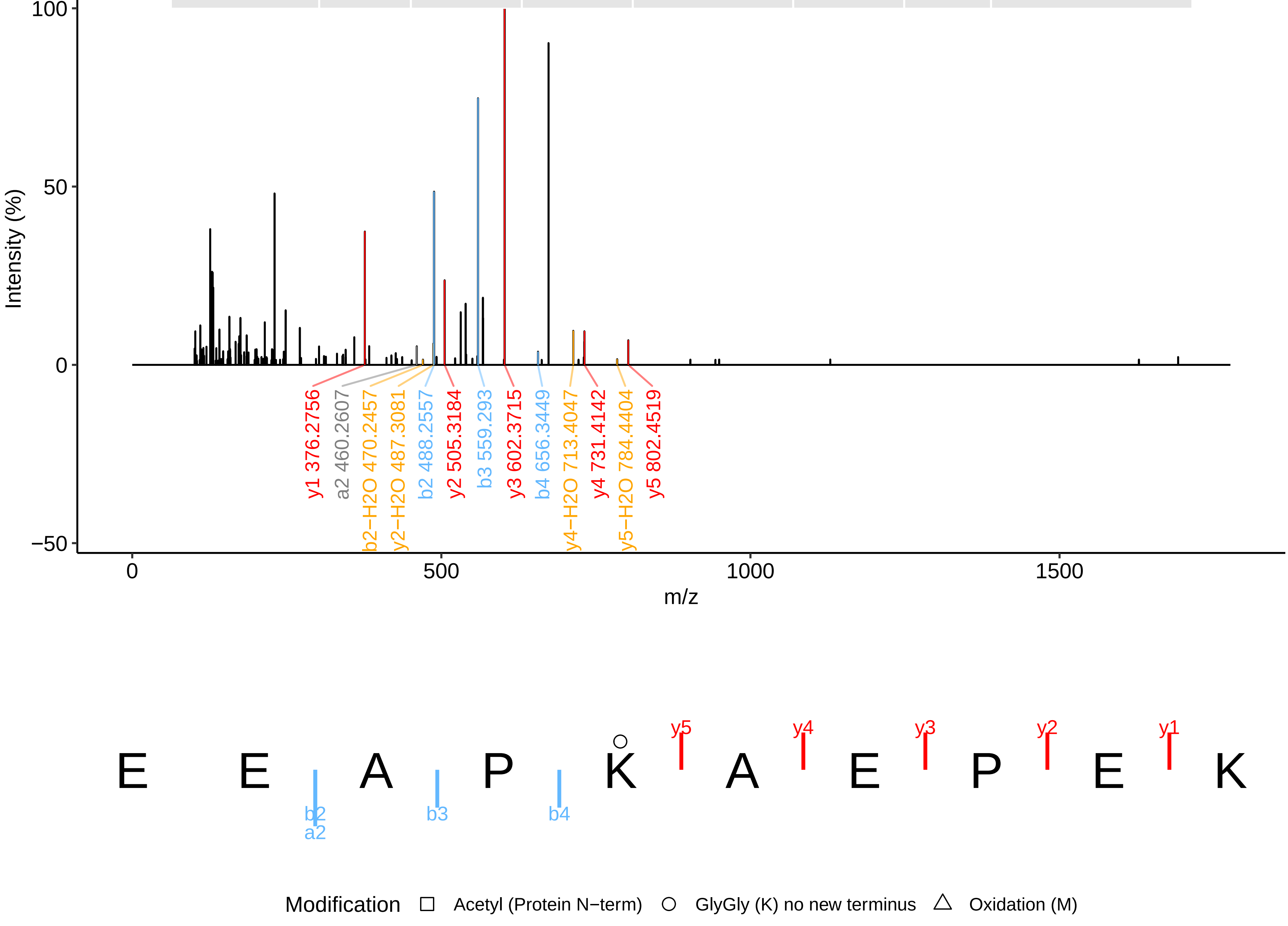

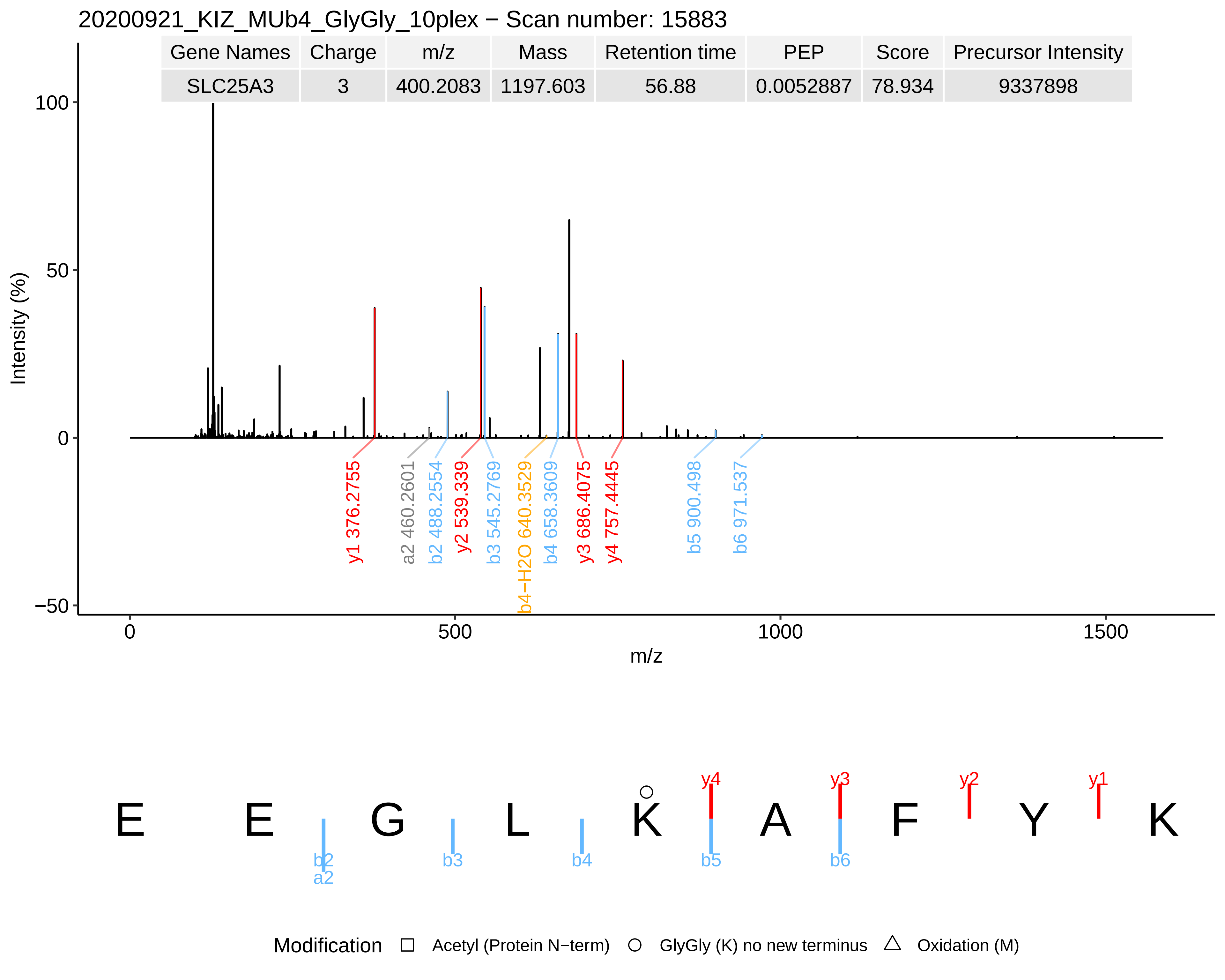

20200921_KIZ_MUb4_GlyGly_10plex − Scan number: 15883
Gene Names
Charge
m/z
Mass
Retention time
PEP
Score
Precursor Intensity
SLC25A3
3
400.2083
1197.603
56.88
0.0052887
78.934
9337898
Intensity (%)
100
50
0
−50
0
500
1000
1500
m/z
y1 376.2755
a2 460.2601
b2 488.2554
y2 539.339
b3 545.2769
b4−H2O 640.3529
b4 658.3609
y3 686.4075
y4 757.4445
b5 900.498
b6 971.537
E E G L K A F Y K
y4
y3
y2
y1
b2
a2
b3
b4
b5
b6
Modification
Acetyl (Protein N−term)
GlyGly (K) no new terminus
Oxidation (M)

20200921_KIZ_MUb4_GlyGly_10plex − Scan number: 28212

Gene Names	Charge	m/z	Mass	Retention time	PEP	Score	Precursor Intensity
MYH13	2	729.8922	1457.77	103.18	0.065084	50.607	3693589

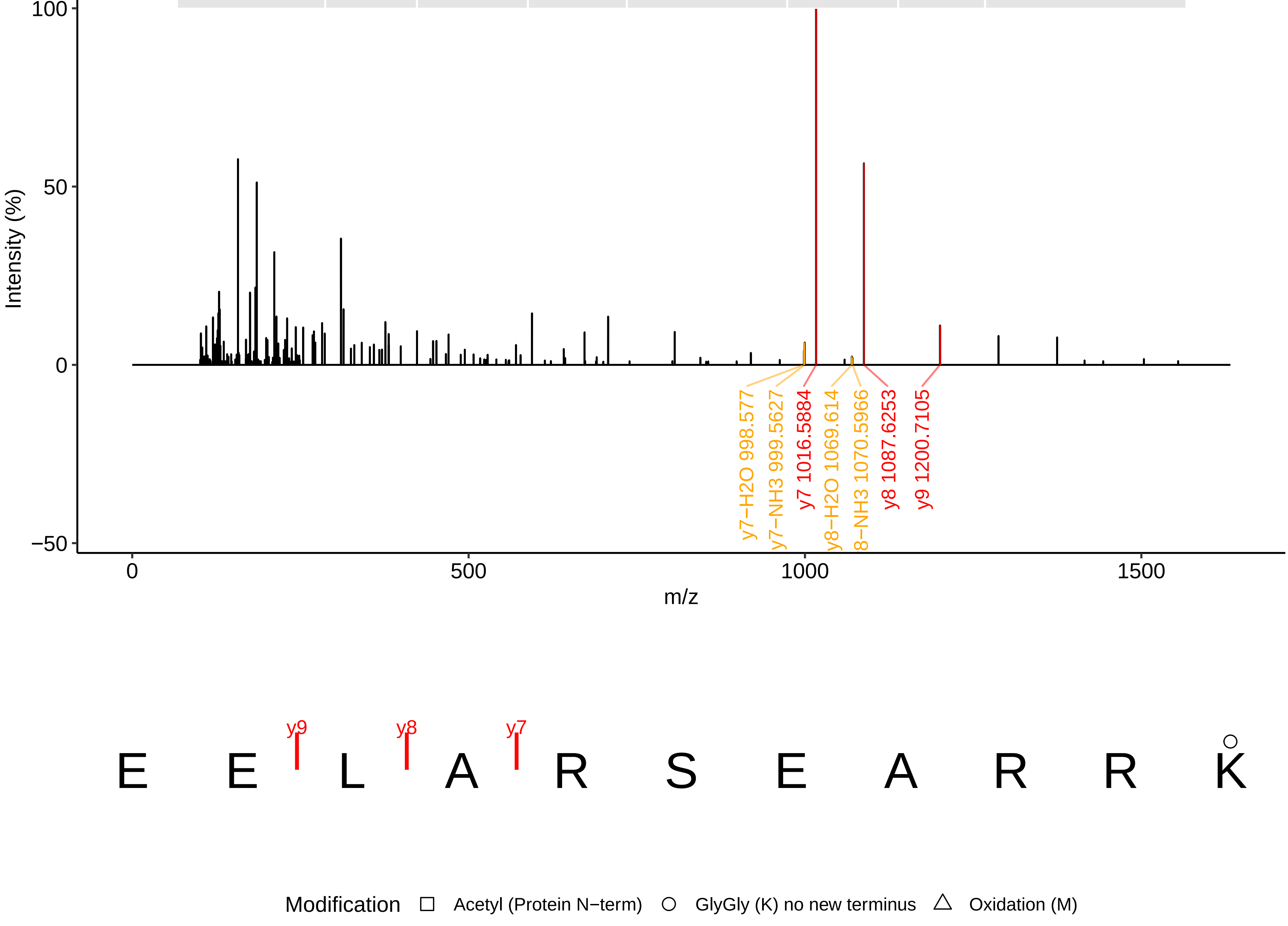

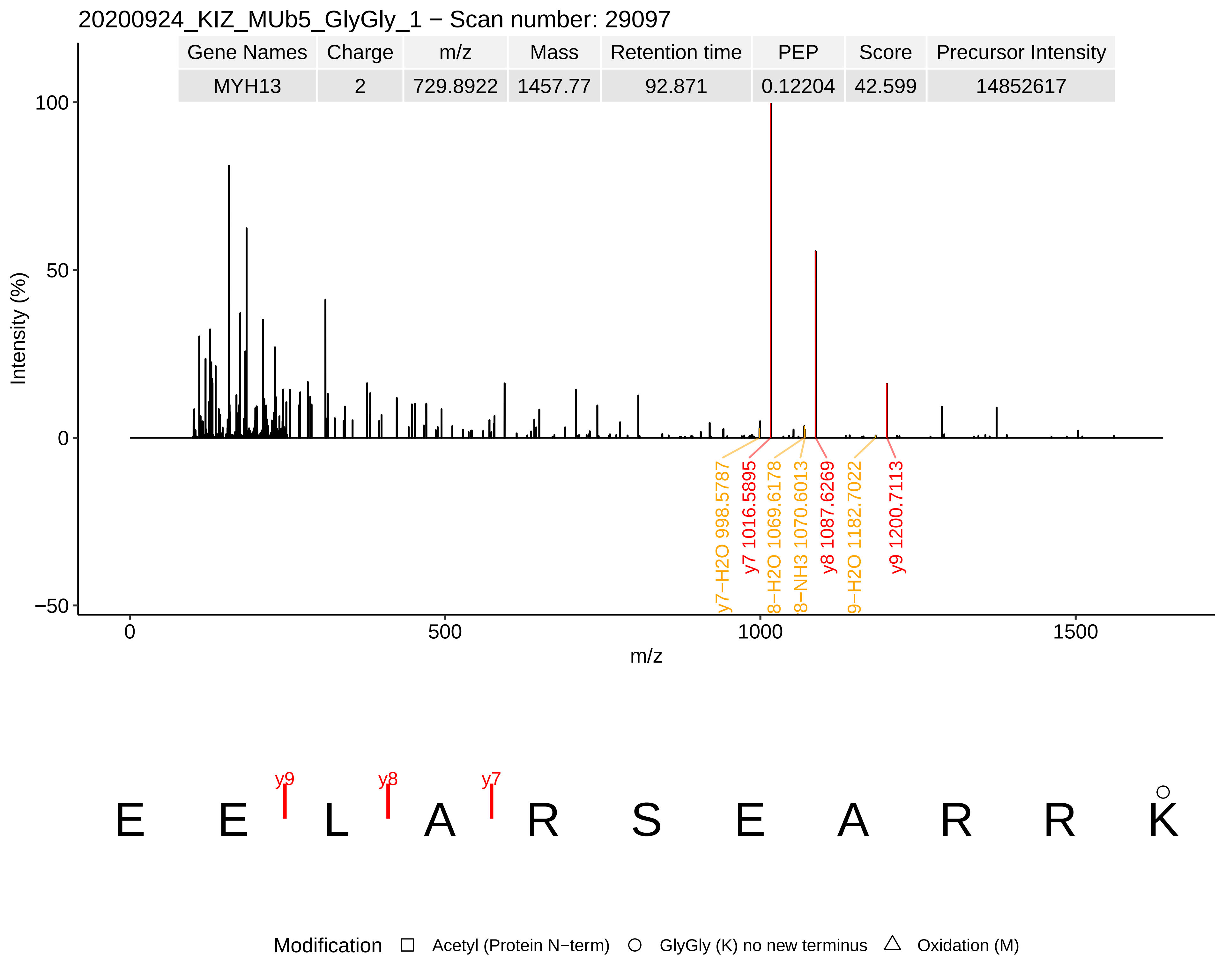
20200924_KIZ_MUb5_GlyGly_1 − Scan number: 29097
Gene Names | Charge | m/z | Mass | Retention time | PEP | Score | Precursor Intensity
MYH13 | 2 | 729.8922 | 1457.77 | 92.871 | 0.12204 | 42.599 | 14852617
Intensity (%)
100
50
0
−50
0
500
1000
1500
m/z
y7−H2O 998.5787
y7 1016.5895
y8−H2O 1069.6178
y8−NH3 1070.6013
y8 1087.6269
y9−H2O 1182.7022
y9 1200.7113
E E L A R S E A R R K
y9
y8
y7
Modification
Acetyl (Protein N−term)
GlyGly (K) no new terminus
Oxidation (M)

20200924_KIZ_MUb5_GlyGly_2 − Scan number: 25781

Gene Names	Charge	m/z	Mass	Retention time	PEP	Score	Precursor Intensity
MYH13	2	729.8922	1457.77	95.482	0.10437	40.002	4211435

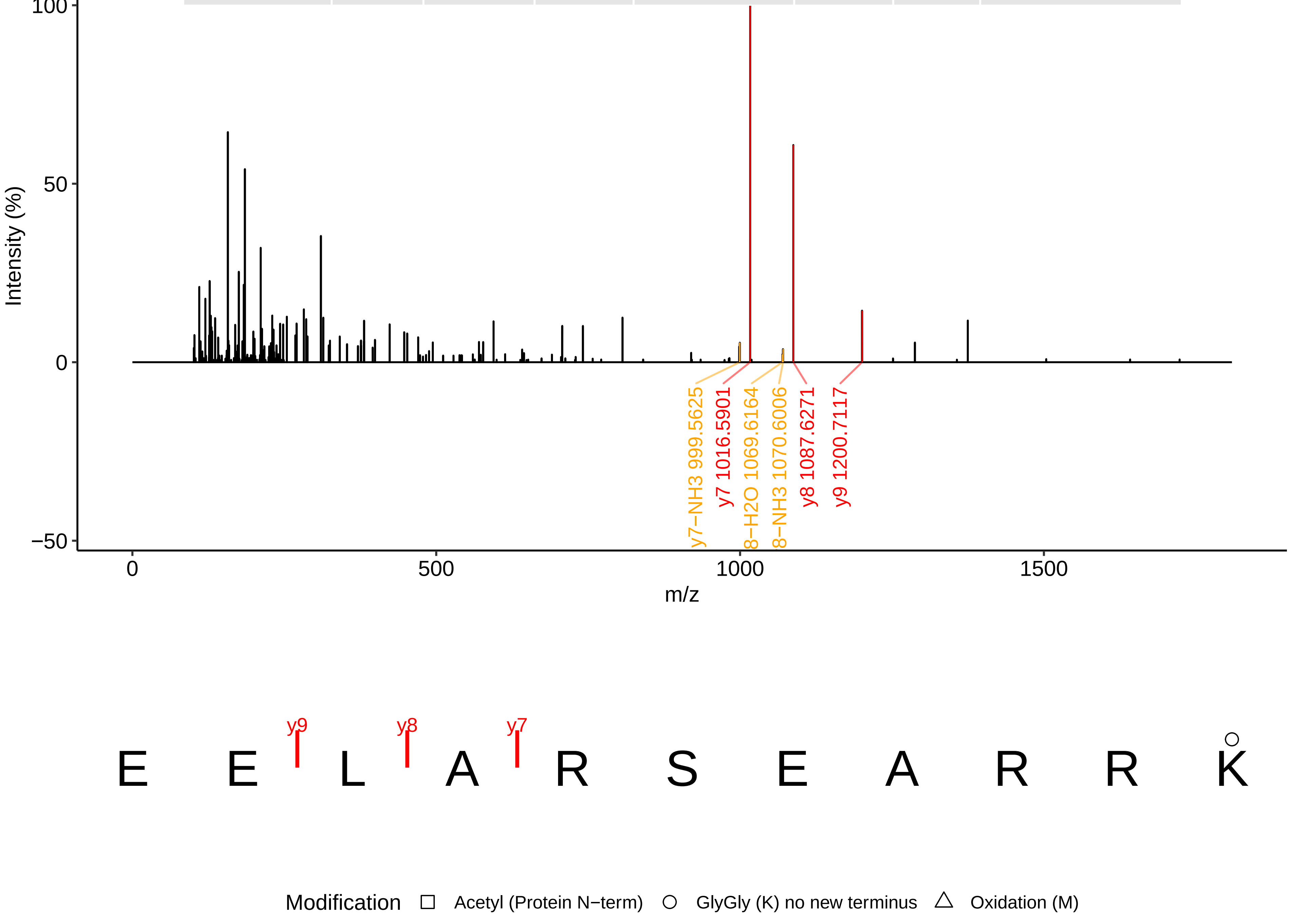

20201118_KIZ_MUb6_GlyGly_1 − Scan number: 3142

Gene Names	Charge	m/z	Mass	Retention time	PEP	Score	Precursor Intensity
SDR16C5	3	480.2065	1437.598	9.6855	8.6461e−09	130.09	7295352

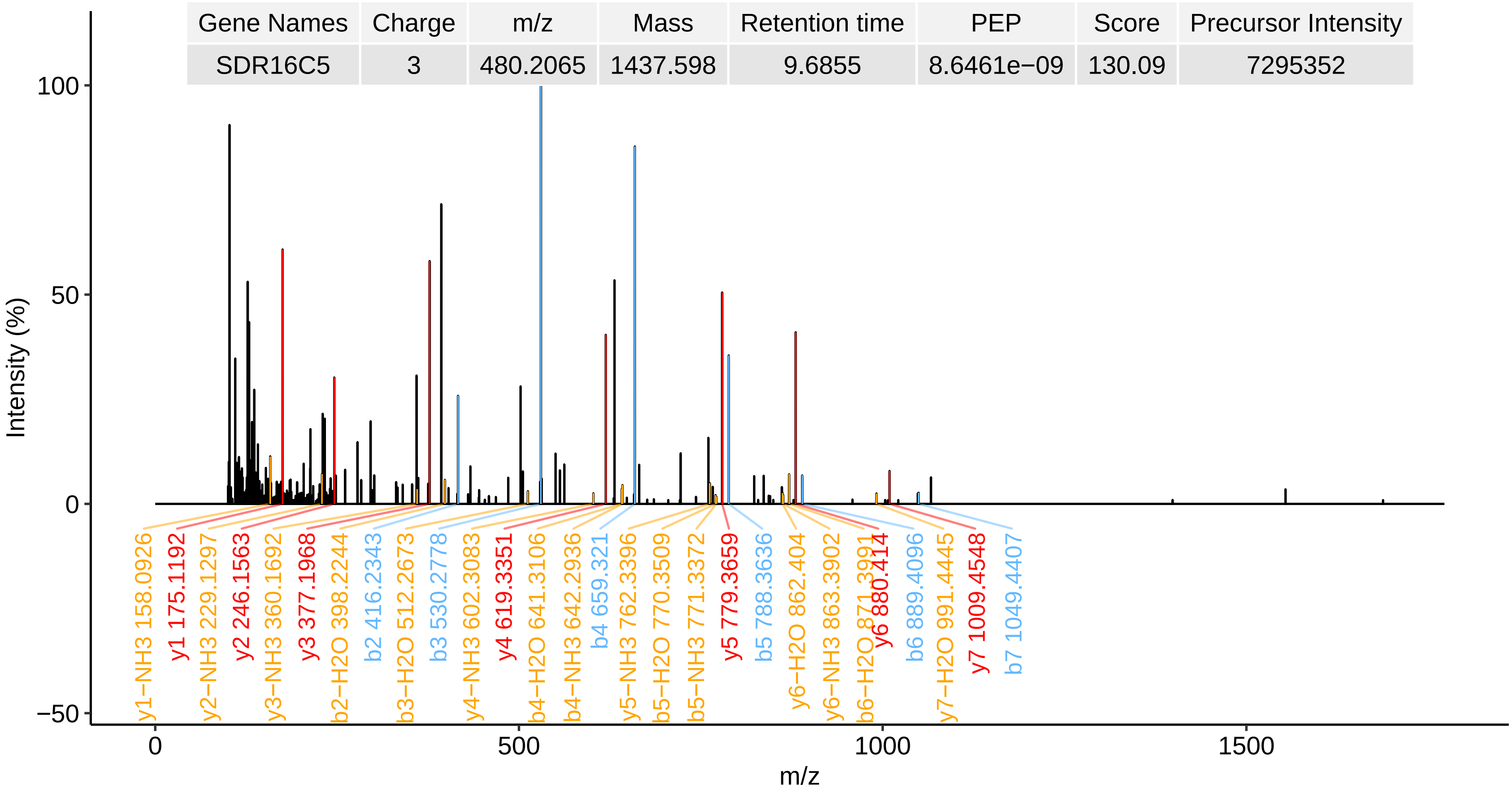

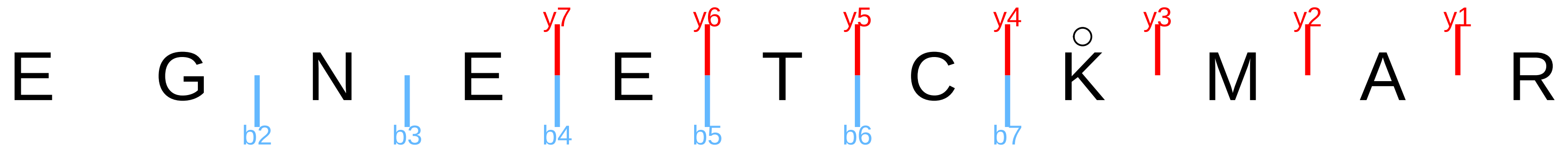

Modification □ Acetyl (Protein N−term) ○ GlyGly (K) no new terminus △ Oxidation (M)

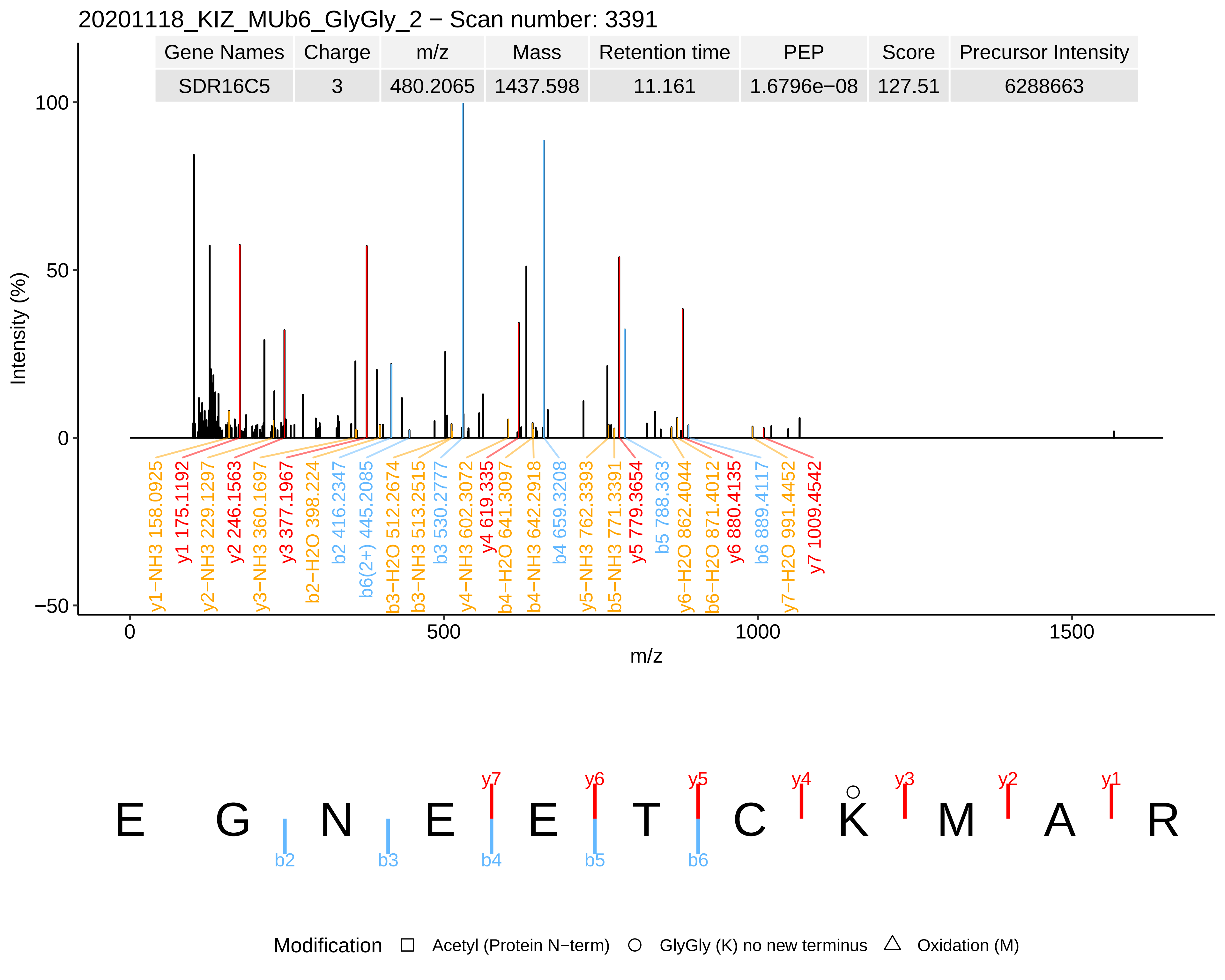

20201118_KIZ_MUb6_GlyGly_2 − Scan number: 3391
Gene Names | Charge | m/z | Mass | Retention time | PEP | Score | Precursor Intensity
SDR16C5 | 3 | 480.2065 | 1437.598 | 11.161 | 1.6796e−08 | 127.51 | 6288663
Intensity (%)
m/z
y1−NH3 158.0925
y1 175.1192
y2−NH3 229.1297
y2 246.1563
y3−NH3 360.1697
y3 377.1967
b2−H2O 398.224
b2 416.2347
b6(2+) 445.2085
b3−H2O 512.2674
b3−NH3 513.2515
b3 530.2777
y4−NH3 602.3072
y4 619.335
b4−H2O 641.3097
b4−NH3 642.2918
b4 659.3208
y5−NH3 762.3393
b5−NH3 771.3391
y5 779.3654
b5 788.363
y6−H2O 862.4044
b6−H2O 871.4012
y6 880.4135
b6 889.4117
y7−H2O 991.4452
y7 1009.4542
E G N E E T C K M A R
y7 y6 y5 y4 y3 y2 y1
b2 b3 b4 b5 b6
Modification
Acetyl (Protein N−term)
GlyGly (K) no new terminus
Oxidation (M)

20200921_KIZ_MUb4_GlyGly_10plex − Scan number: 15447

Gene Names	Charge	m/z	Mass	Retention time	PEP	Score	Precursor In
H3F3B;H3F3A;HIST1H3A;HIST2H3A;HIST3H3	3	483.9159	1448.726	55.174	1.2087e−05	110.74	735952

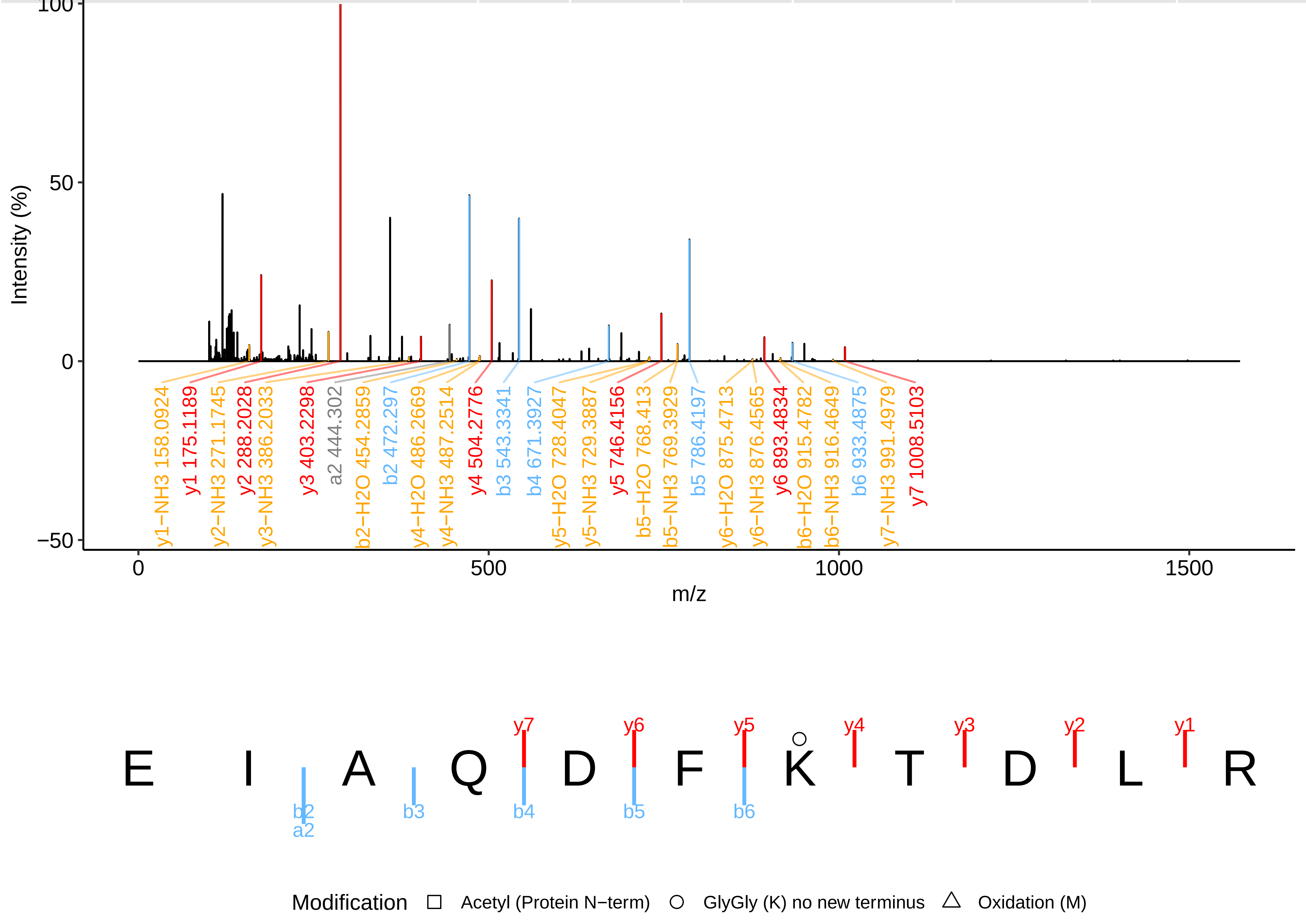

20200921_KIZ_MUb4_GlyGly_10plex_2 − Scan number: 14315

Gene Names	Charge	m/z	Mass	Retention time	PEP	Score	Precursor Inte
H3F3B;H3F3A;HIST1H3A;HIST2H3A;HIST3H3	3	483.9159	1448.726	55.791	0.001107	87.001	1896238

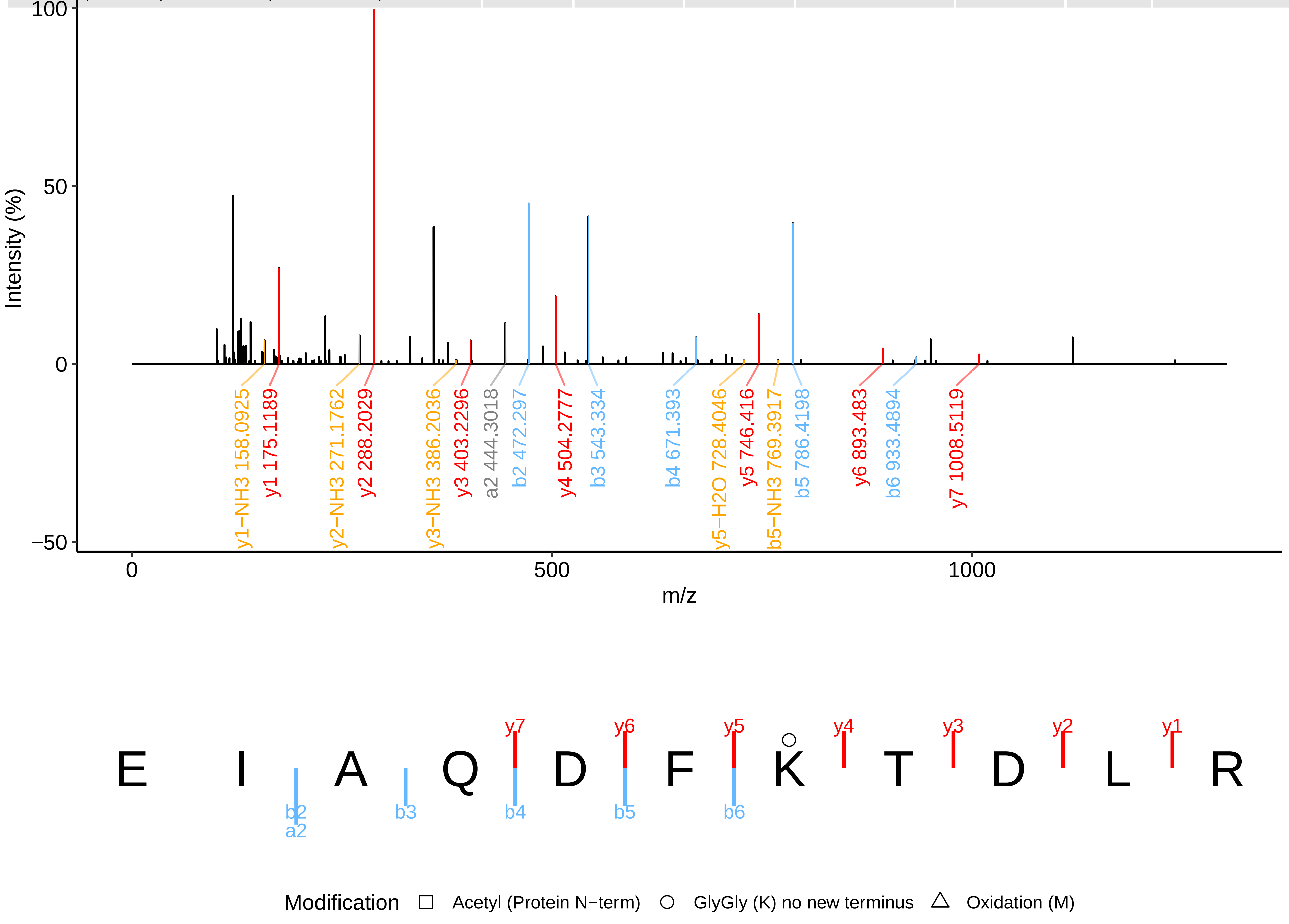

20200924_KIZ_MUb5_GlyGly_1 − Scan number: 14466

Gene Names	Charge	m/z	Mass	Retention time	PEP	Score	Precursor Int
H3F3B;H3F3A;HIST1H3A;HIST2H3A;HIST3H3	3	483.9159	1448.726	47.233	4.696e−08	117.98	2445804

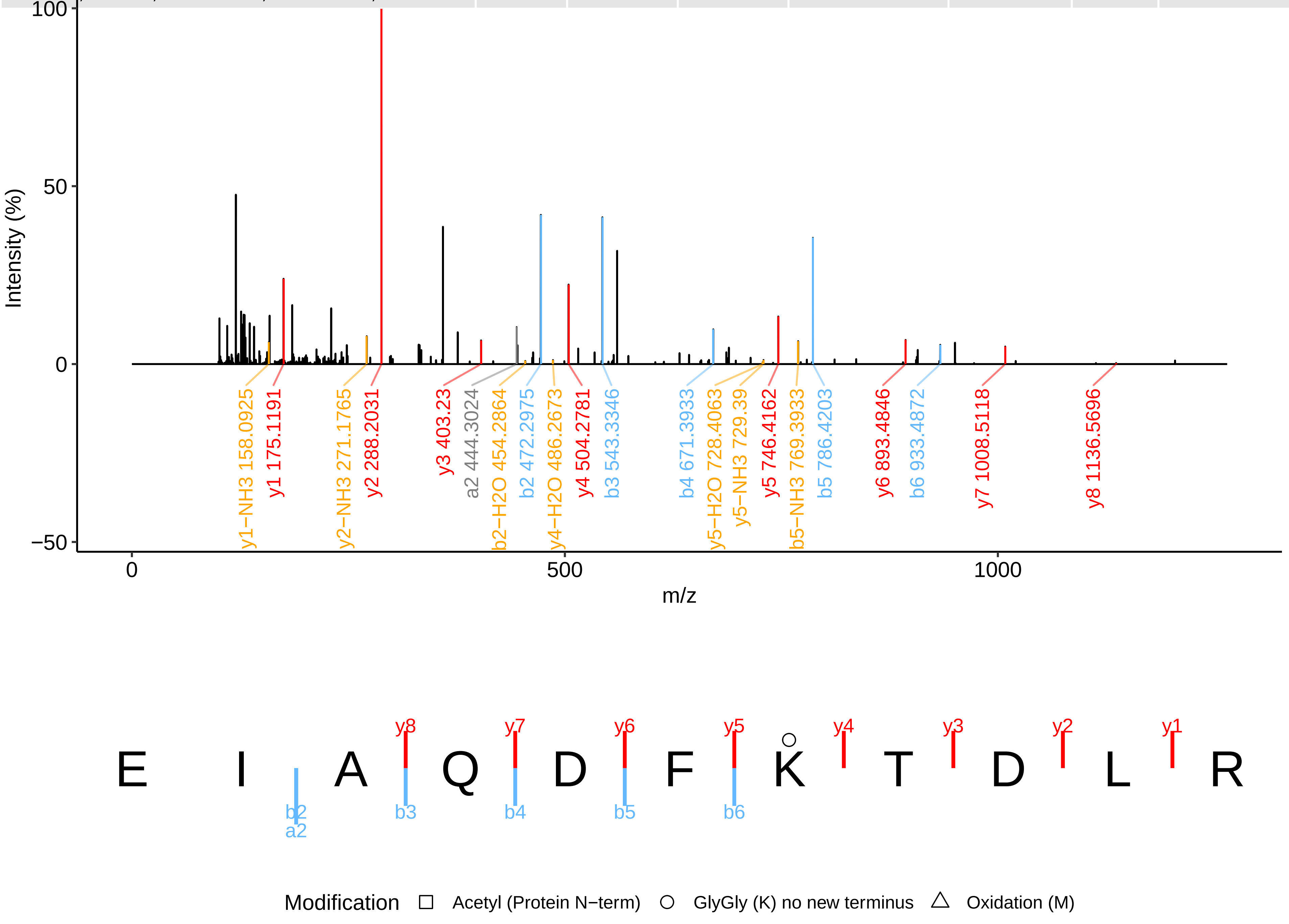

20200924_KIZ_MUb5_GlyGly_2 − Scan number: 13747

Gene Names	Charge	m/z	Mass	Retention time	PEP	Score	Precursor In
H3F3B;H3F3A;HIST1H3A;HIST2H3A;HIST3H3	3	483.9159	1448.726	51.411	1.5971e−05	109.24	653136

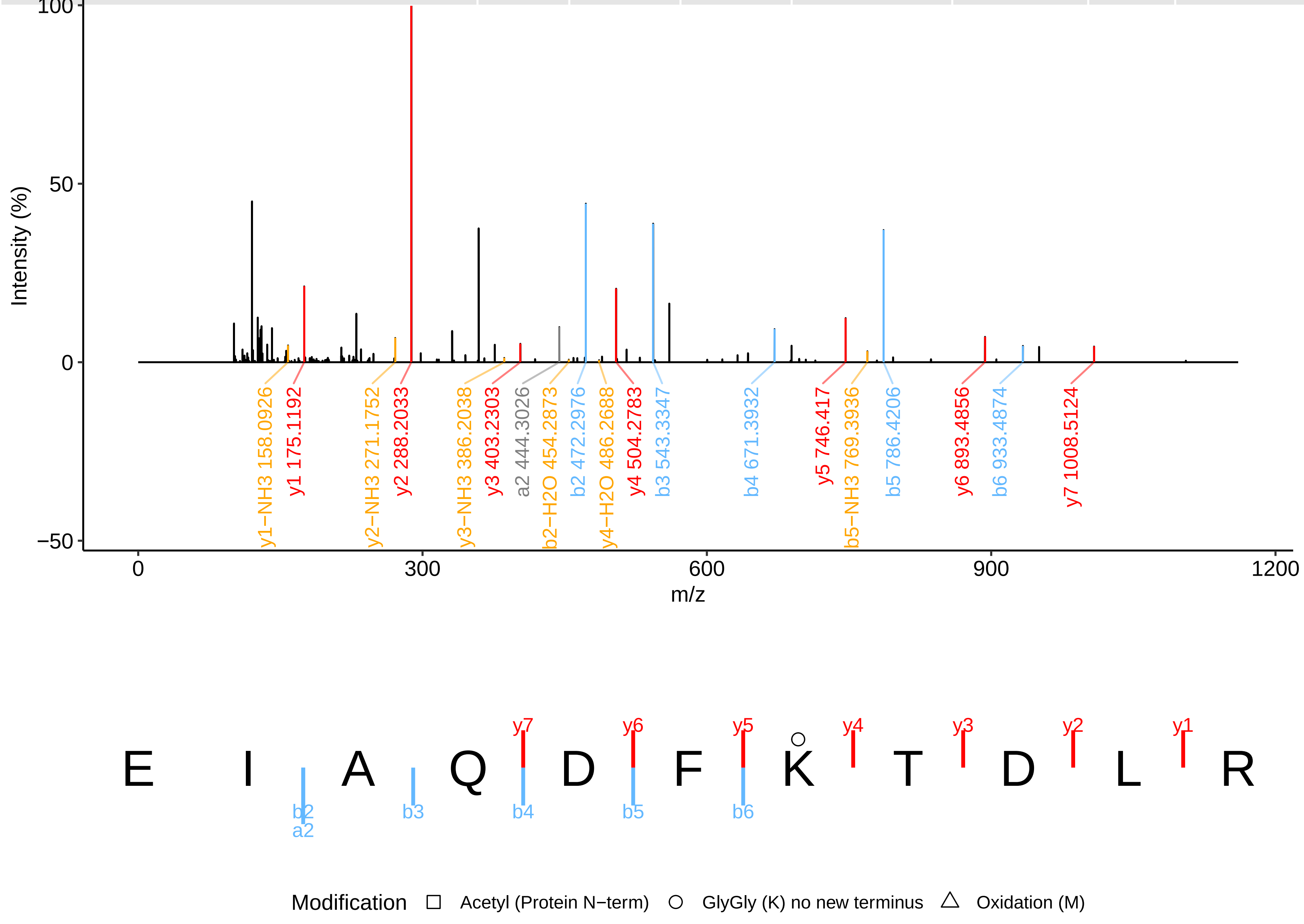

20201118_KIZ_MUb6_GlyGly_1 − Scan number: 14497

Gene Names	Charge	m/z	Mass	Retention time	PEP	Score	Precursor In
H3F3B;H3F3A;HIST1H3A;HIST2H3A;HIST3H3	3	483.9159	1448.726	45.86	5.3354e−05	97.911	2987888

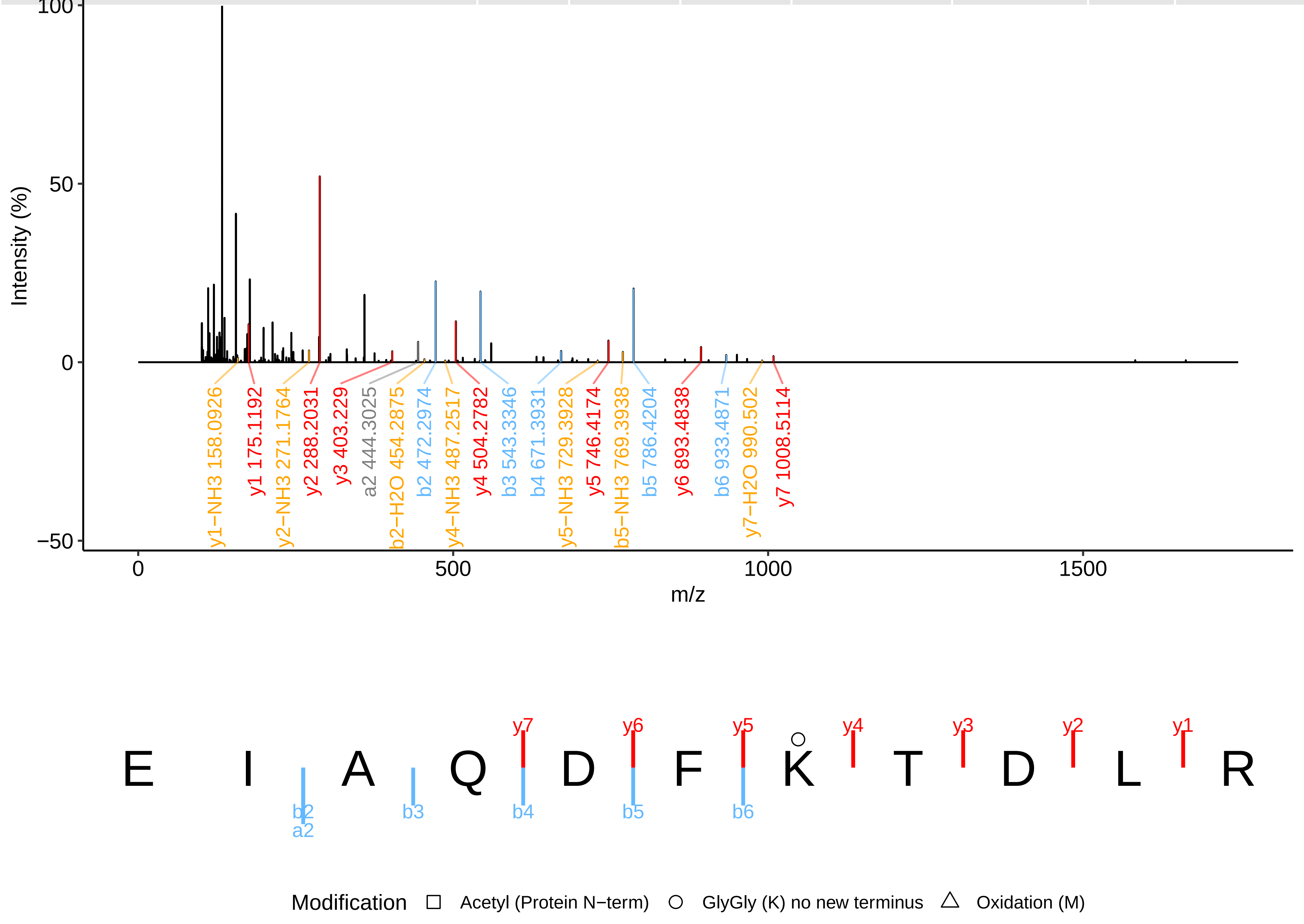

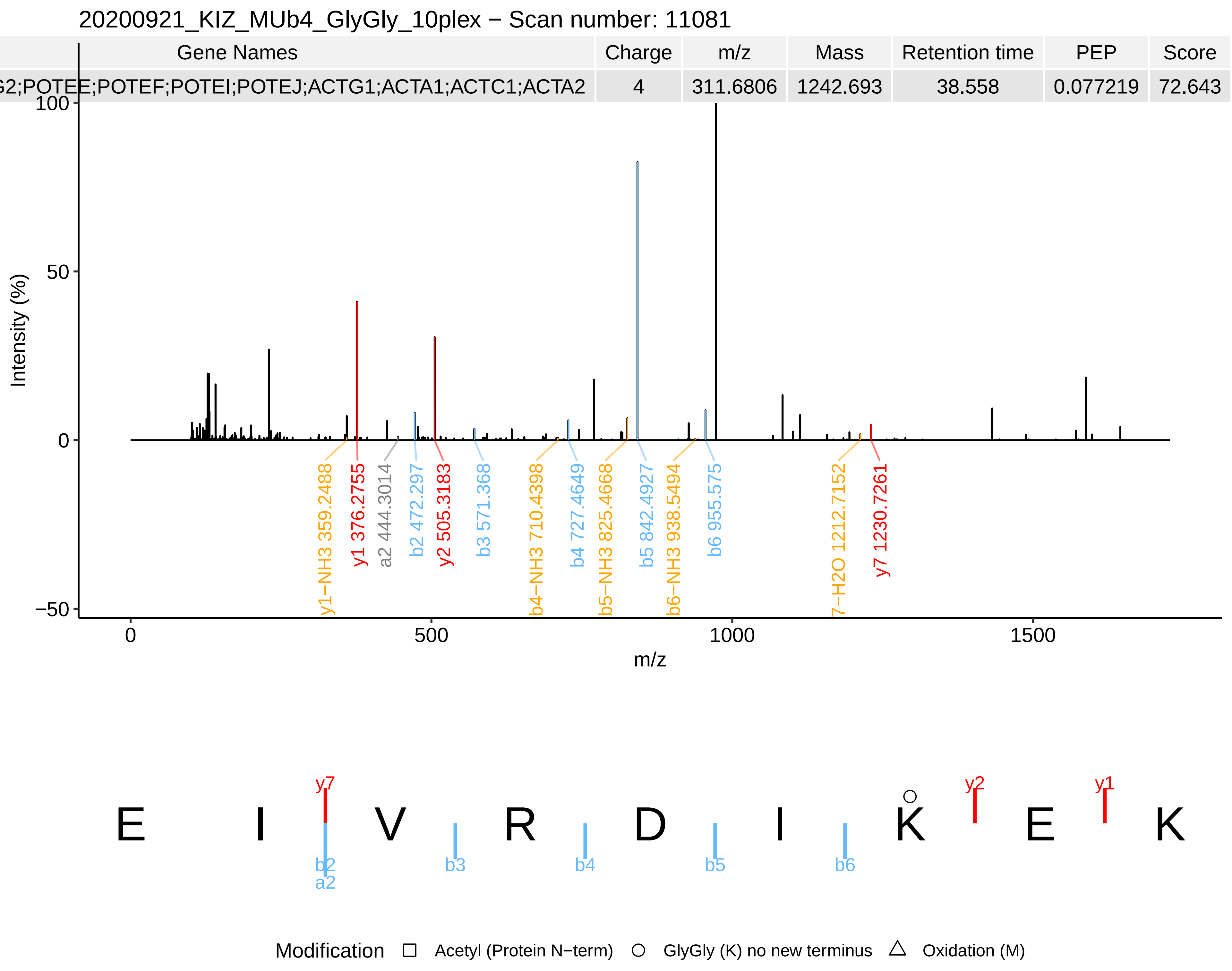
20200921_KIZ_MUb4_GlyGly_10plex − Scan number: 11081
Gene Names
Charge
m/z
Mass
Retention time
PEP
Score
G2;POTEE;POTEF;POTEI;POTEJ;ACTG1;ACTA1;ACTC1;ACTA2
4
311.6806
1242.693
38.558
0.077219
72.643
Intensity (%)
100
50
0
−50
0
500
1000
1500
m/z
y1−NH3 359.2488
y1 376.2755
a2 444.3014
b2 472.297
y2 505.3183
b3 571.368
b4−NH3 710.4398
b4 727.4649
b5−NH3 825.4668
b5 842.4927
b6−NH3 938.5494
b6 955.575
y7−H2O 1212.7152
y7 1230.7261
E I V R D I K E K
y7
y2
y1
b2
a2
b3
b4
b5
b6
Modification
Acetyl (Protein N−term)
GlyGly (K) no new terminus
Oxidation (M)

20200921_KIZ_MUb4_GlyGly_10plex_2 − Scan number: 10288

Gene Names	Charge	m/z	Mass	Retention time	PEP	Score
G2;POTEE;POTEF;POTEI;POTEJ;ACTG1;ACTA1;ACTC1;ACTA2	4	311.6806	1242.693	39.319	0.013256	89.247

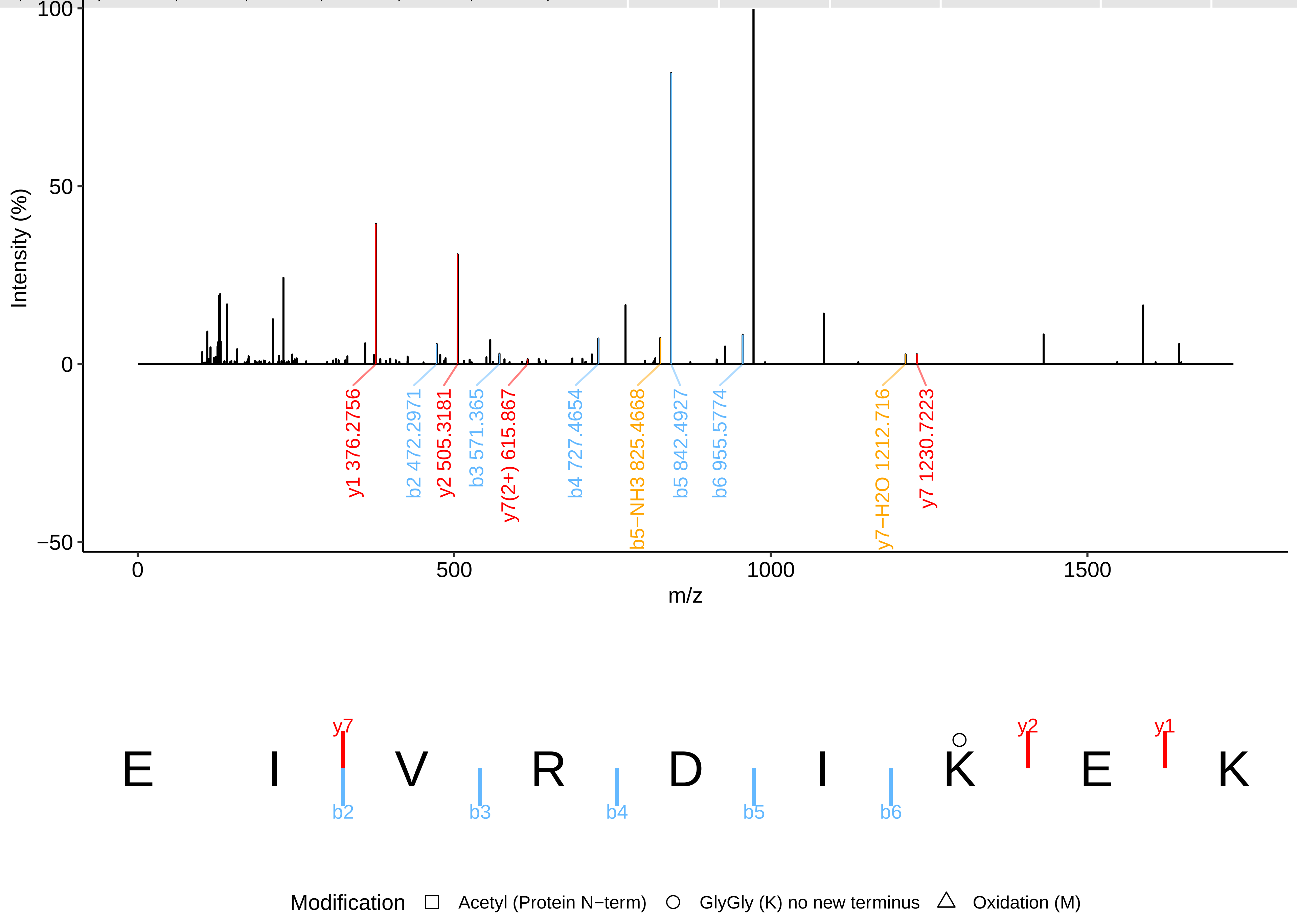

20200921_KIZ_MUb4_GlyGly_10plex − Scan number: 11646

Gene Names	Charge	m/z	Mass	Retention time	PEP	Score	Precursor Intensity
SLC25A3	3	411.5663	1231.677	40.639	0.0298	60.509	4842470

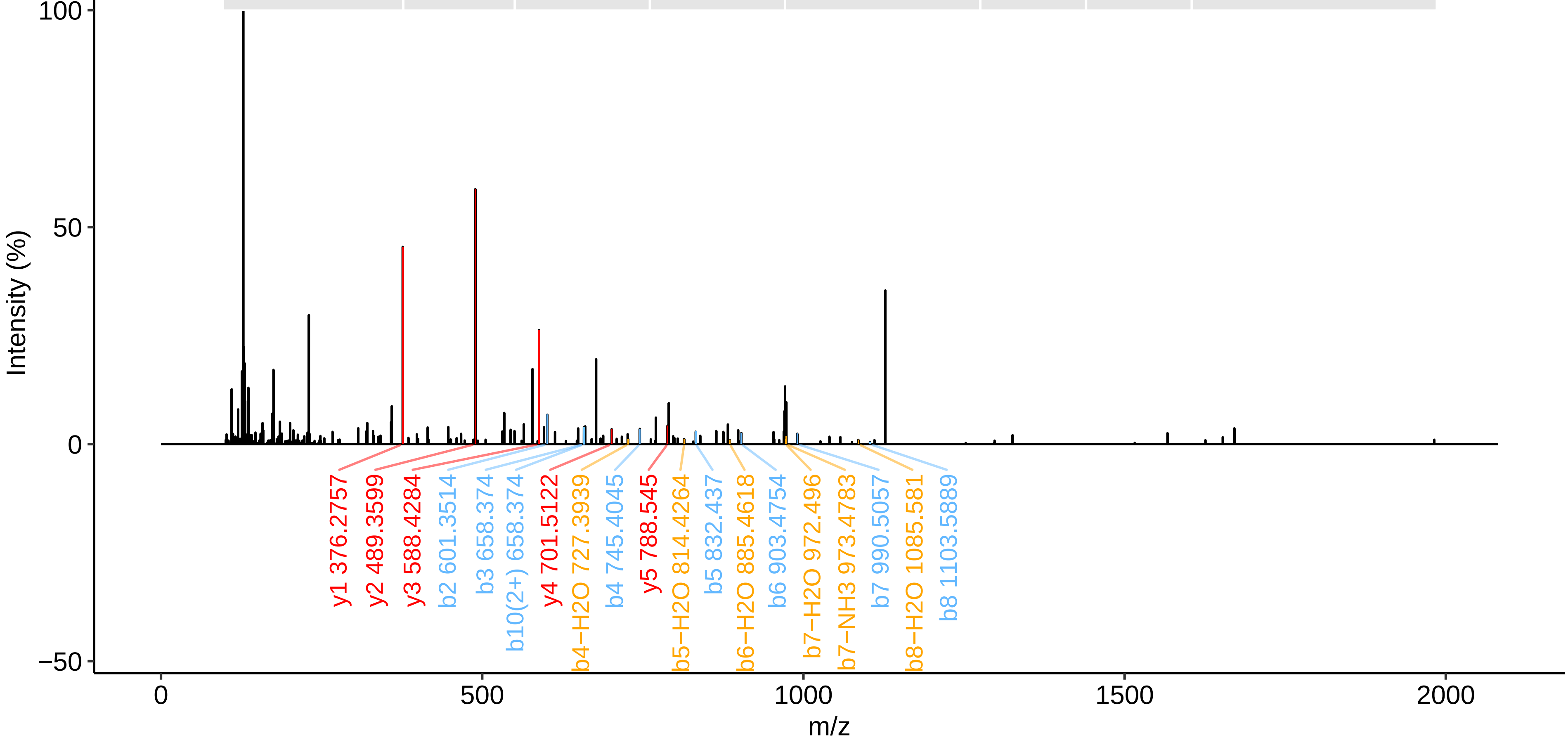

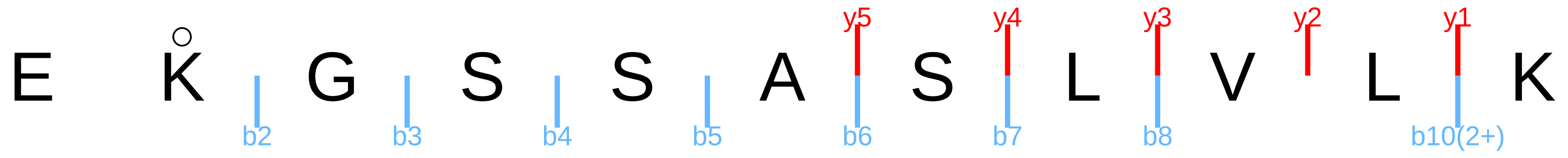

Modification □ Acetyl (Protein N−term) ○ GlyGly (K) no new terminus △ Oxidation (M)

20200921_KIZ_MUb4_GlyGly_10plex_2 − Scan number: 10764

Gene Names	Charge	m/z	Mass	Retention time	PEP	Score	Precursor Intensity
SLC25A3	3	411.5663	1231.677	41.252	0.001107	87.001	1144819

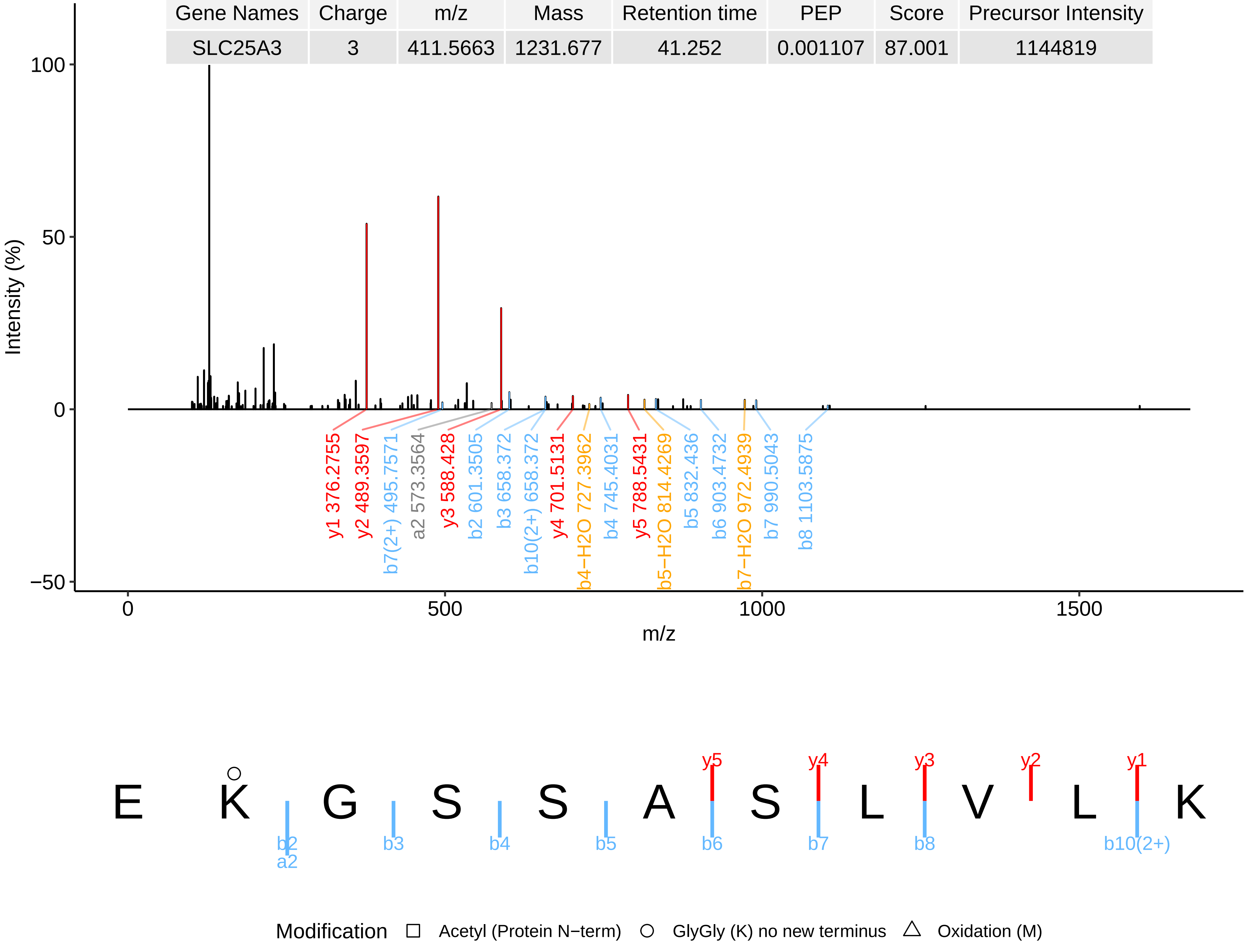

20200921_KIZ_MUb4_GlyGly_10plex_2 − Scan number: 3874

Gene Names	Charge	m/z	Mass	Retention time	PEP	Score	Precursor Intensity
BASP1	3	410.8581	1229.552	13.294	0.055351	56.359	2019822

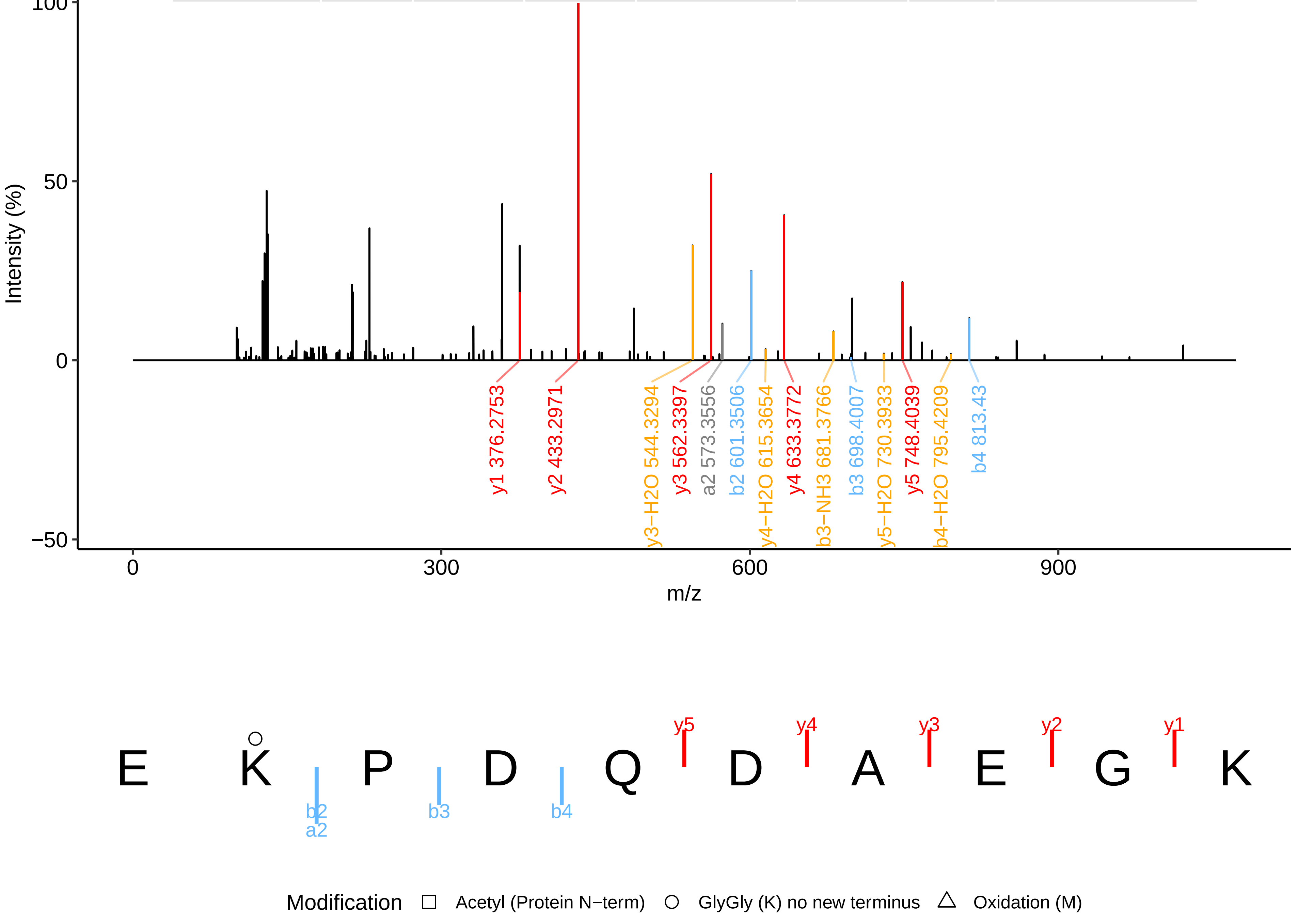

20200924_KIZ_MUb5_GlyGly_1 − Scan number: 3554

Gene Names	Charge	m/z	Mass	Retention time	PEP	Score	Precursor Intensity
BASP1	3	410.8581	1229.552	10.874	0.027317	61.815	9025699

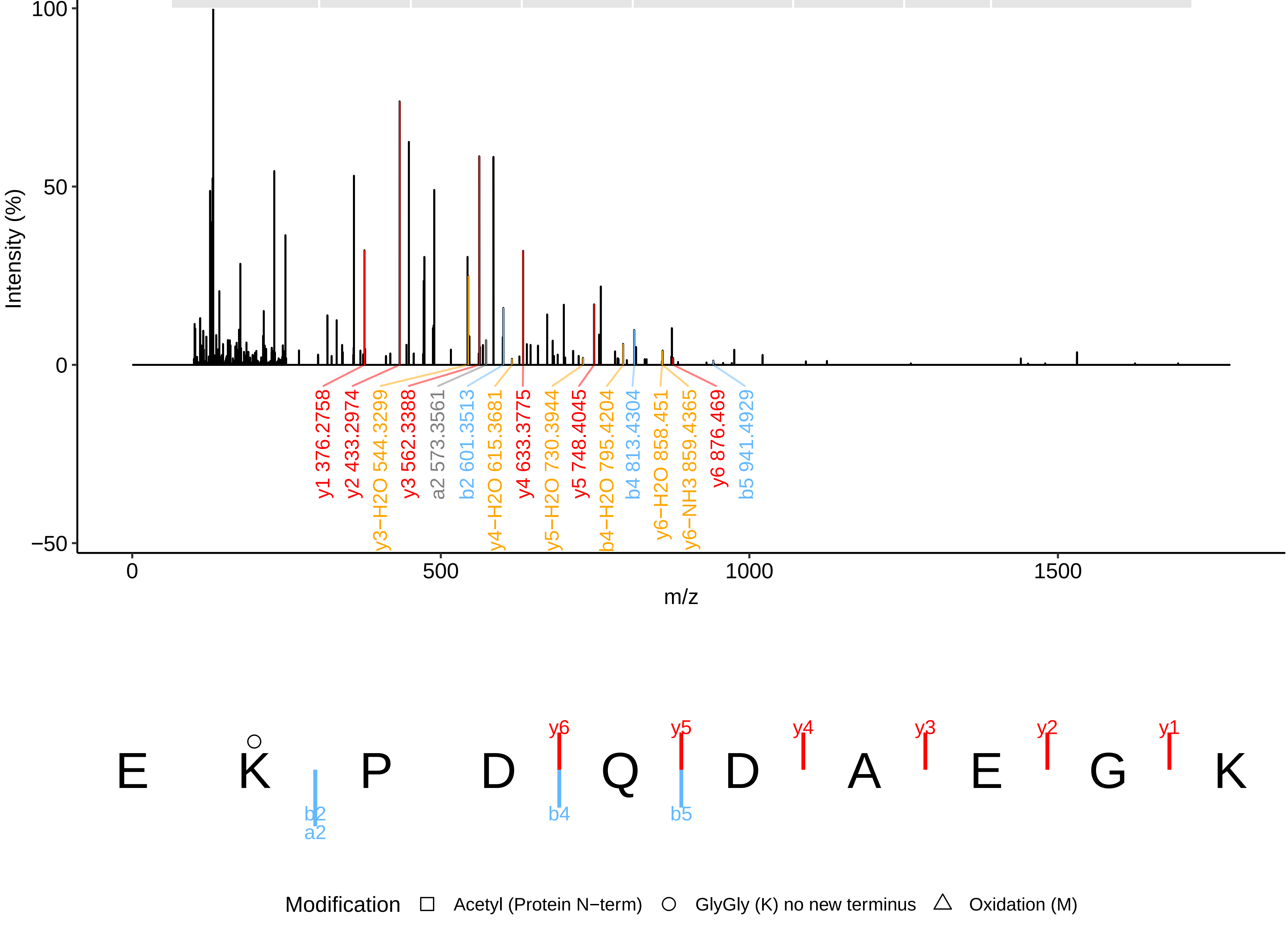

20200924_KIZ_MUb5_GlyGly_2 − Scan number: 3453

Gene Names	Charge	m/z	Mass	Retention time	PEP	Score	Precursor Intensity
BASP1	3	410.8581	1229.552	12.523	0.00099267	104.82	4644307

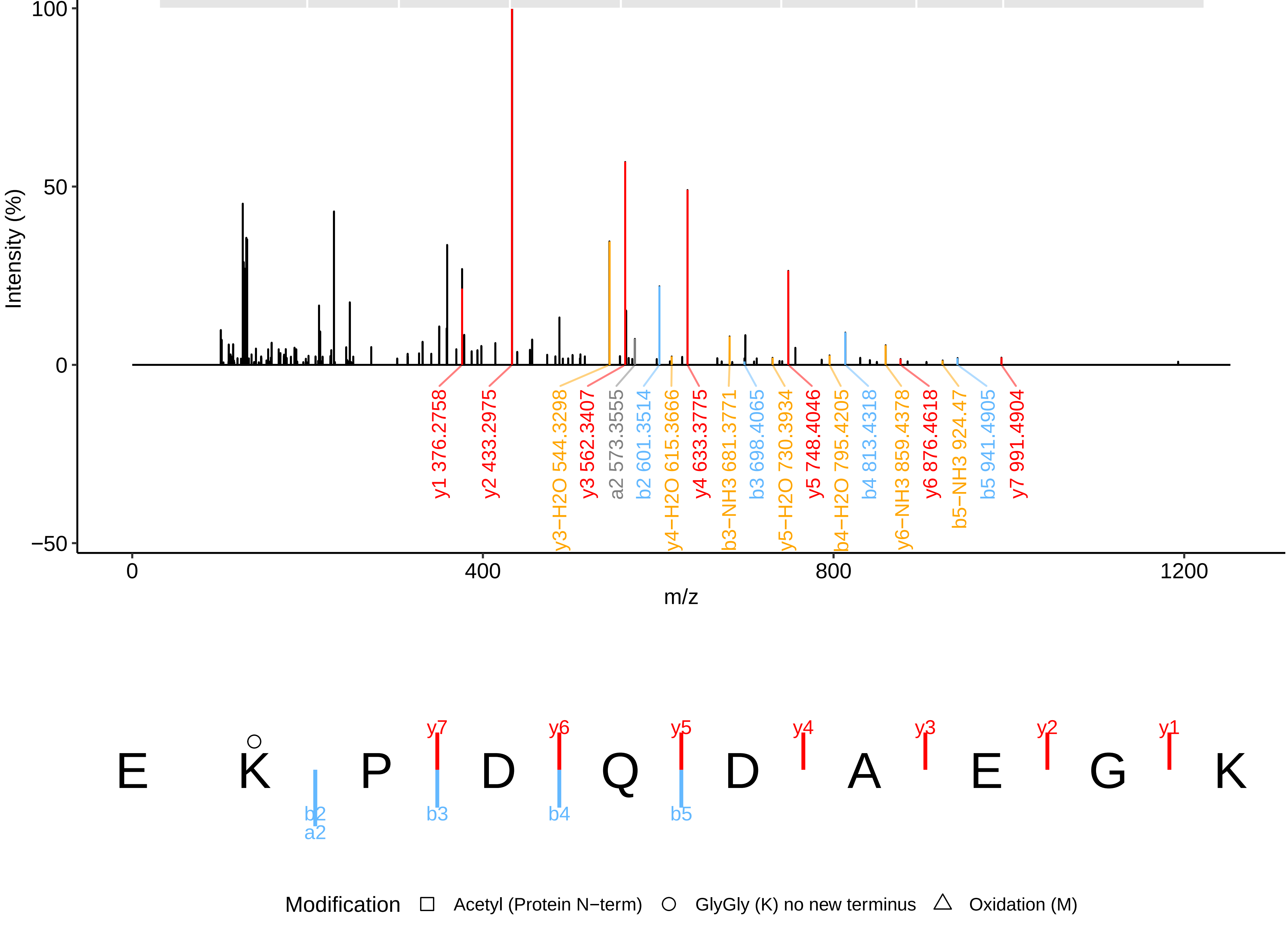

20201118_KIZ_MUb6_GlyGly_2 − Scan number: 4149

Gene Names	Charge	m/z	Mass	Retention time	PEP	Score	Precursor Intensity
BASP1	3	410.8581	1229.552	14.053	0.093259	48.981	1930761

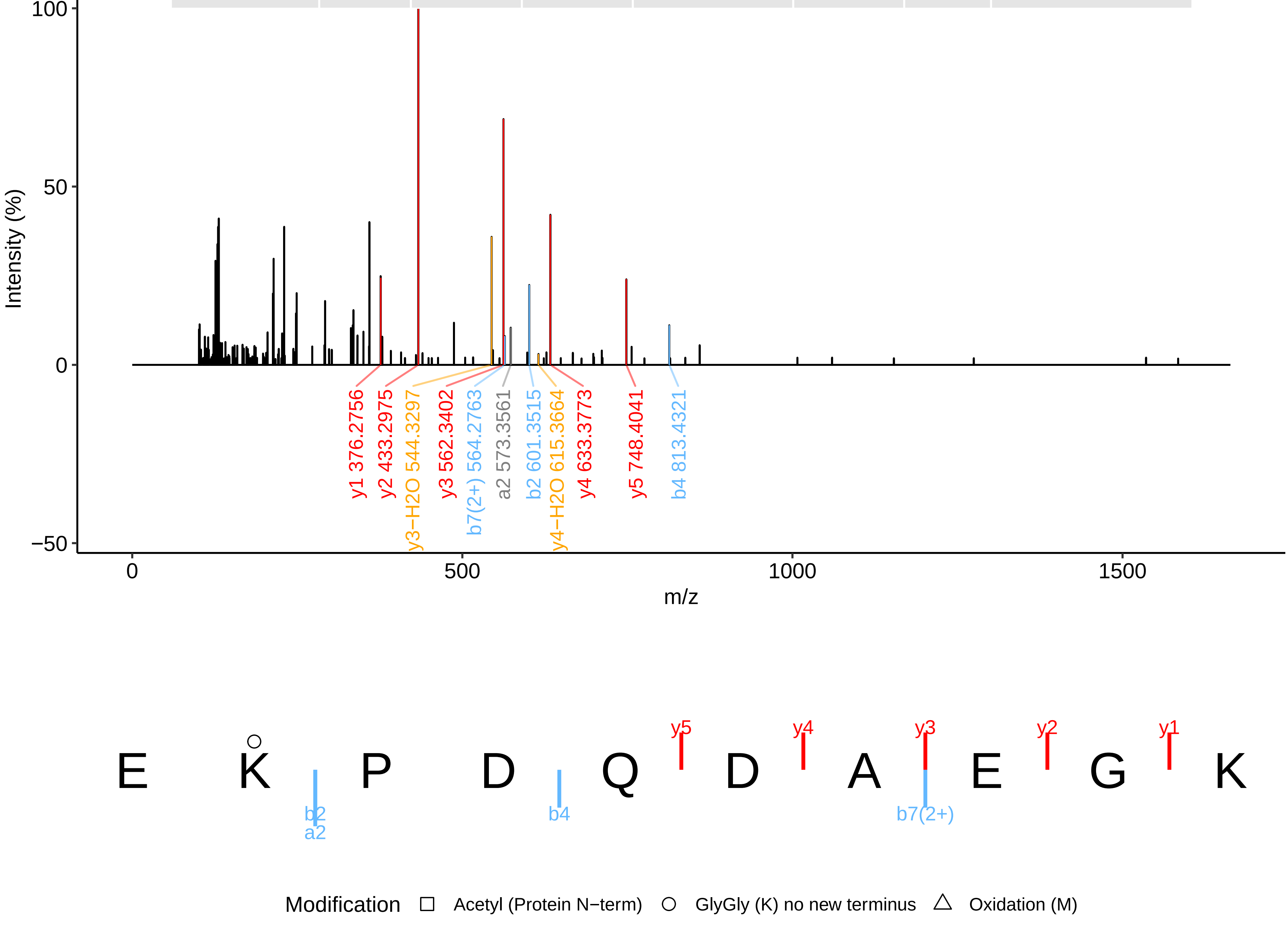

20200921_KIZ_MUb4_GlyGly_10plex − Scan number: 4100

Gene Names	Charge	m/z	Mass	Retention time	PEP	Score	Precursor Intensity
PTBP1	3	487.2427	1458.706	13.341	0.017409	62.298	6728638

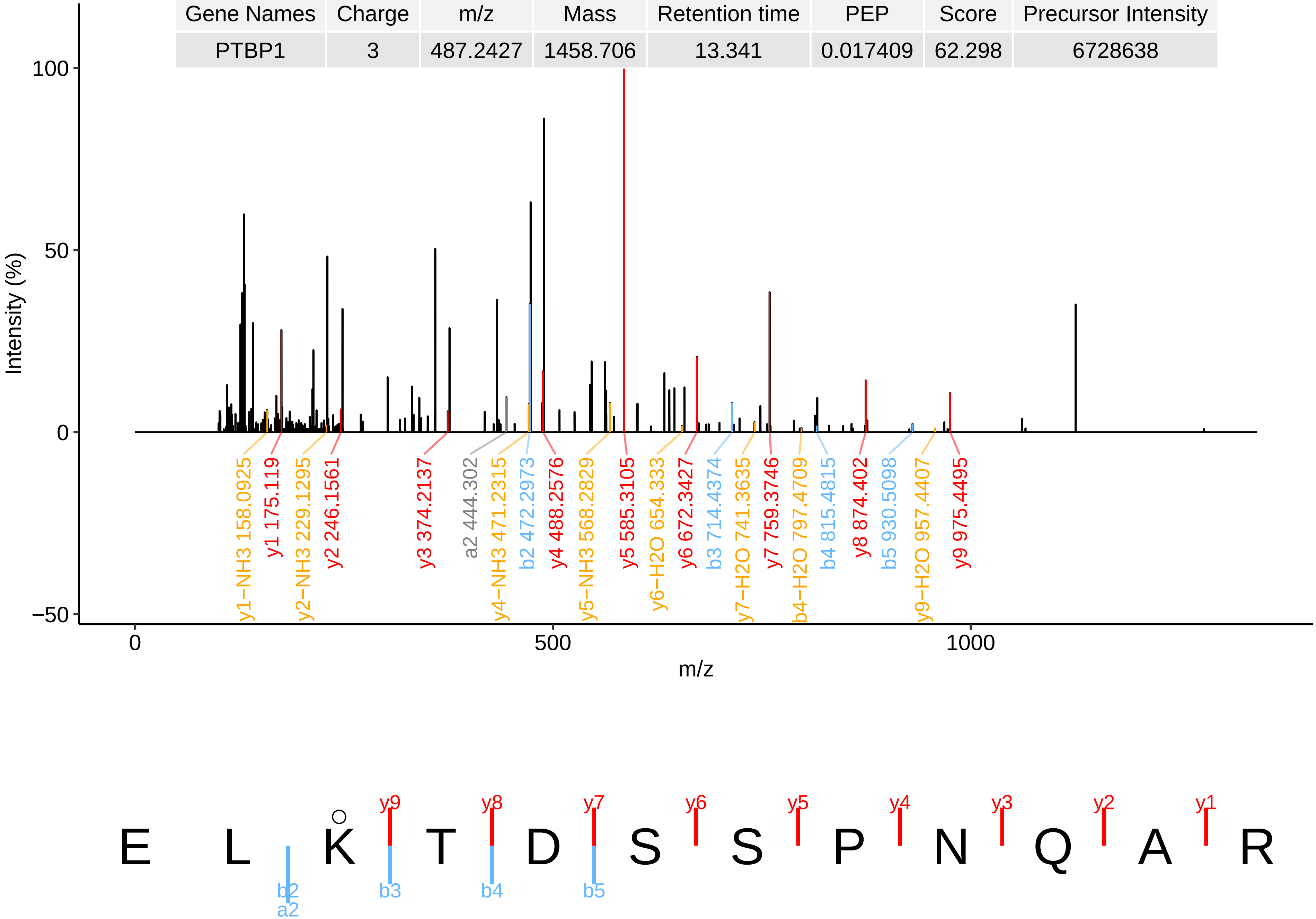

Modification □ Acetyl (Protein N−term) ○ GlyGly (K) no new terminus △ Oxidation (M)

20200921_KIZ_MUb4_GlyGly_10plex − Scan number: 6734

Gene Names	Charge	m/z	Mass	Retention time	PEP	Score	Precursor Intensity
MFSD1	3	401.5507	1201.63	22.393	0.0014959	88.187	4936619

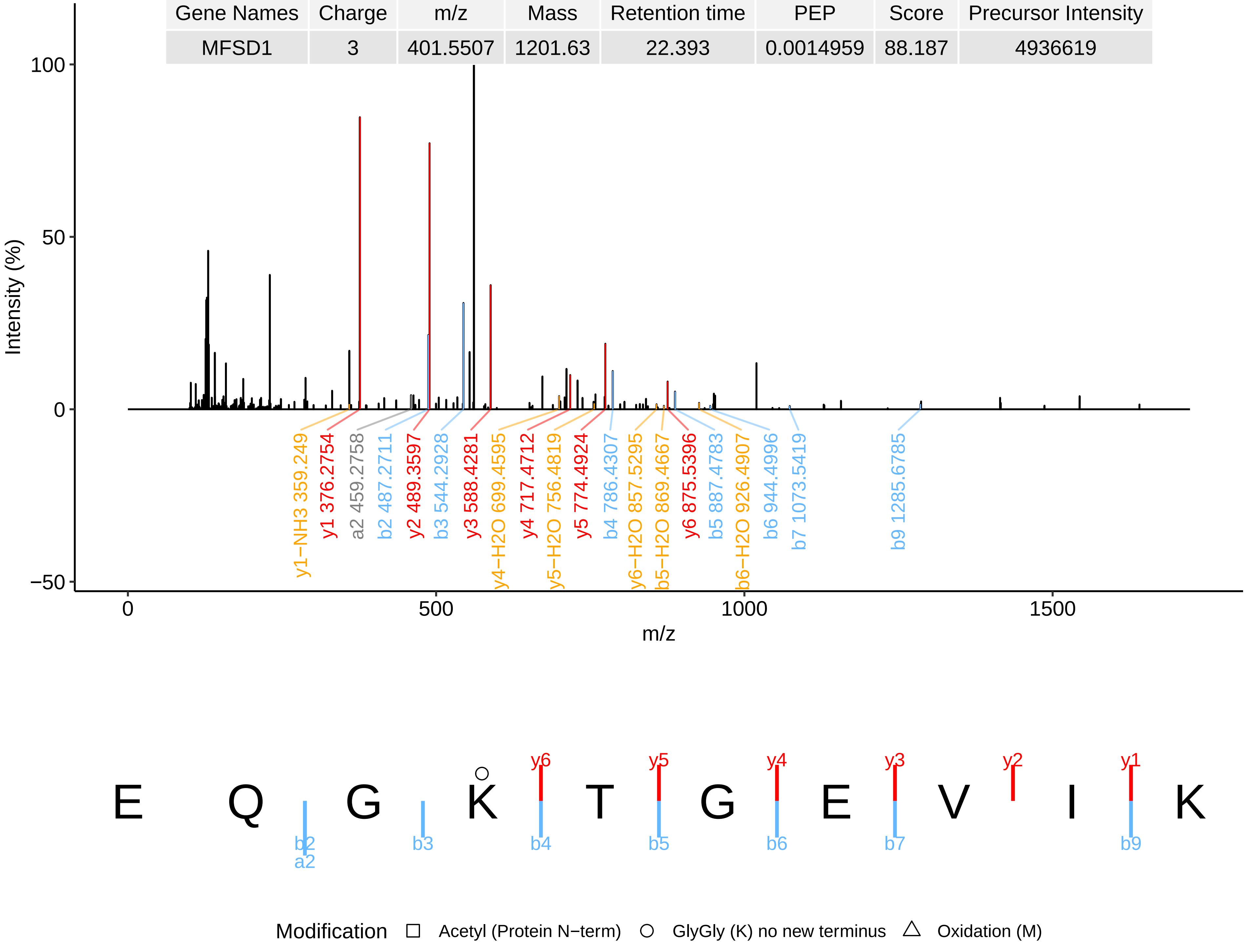

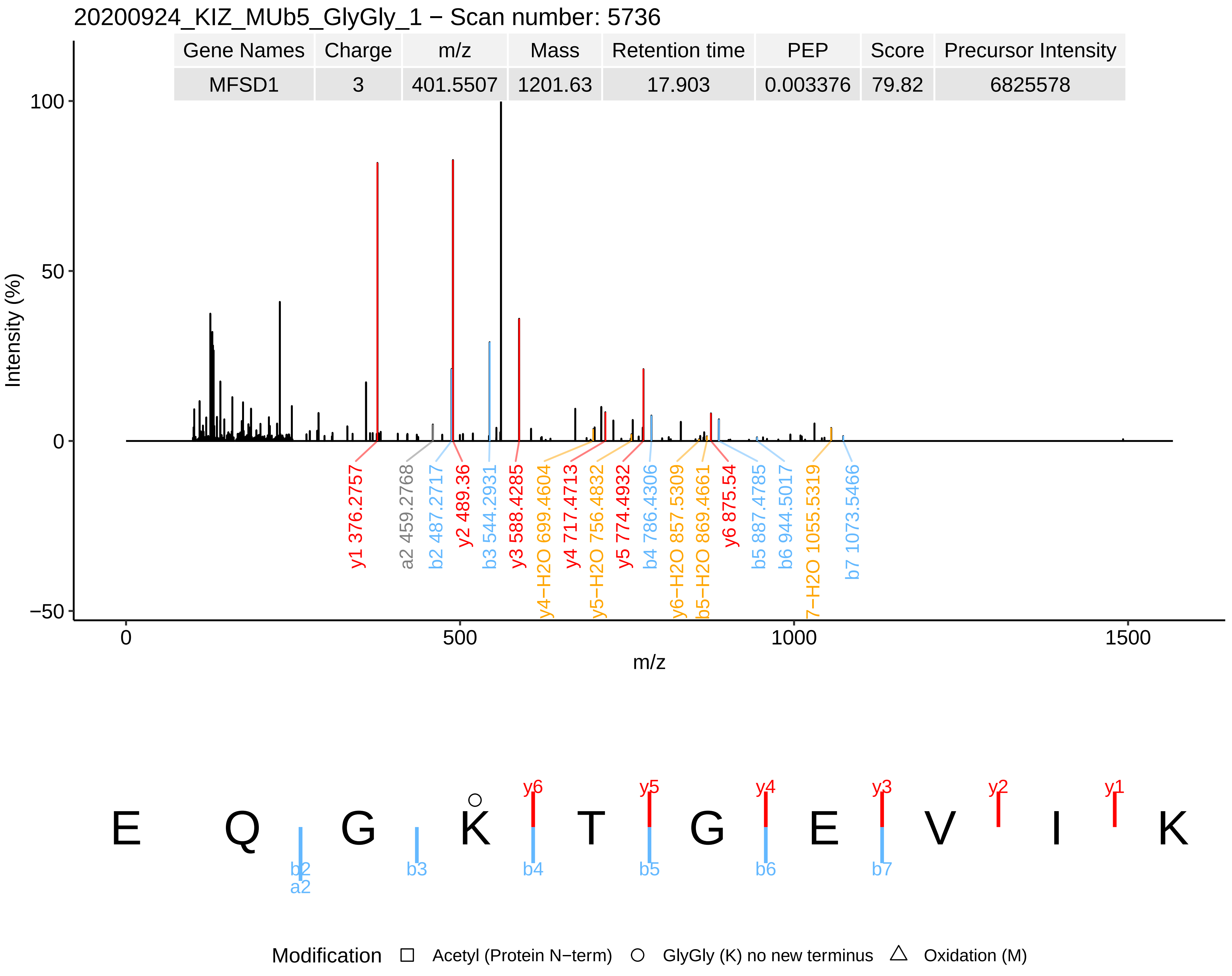
20200924_KIZ_MUb5_GlyGly_1 − Scan number: 5736
Gene Names | Charge | m/z | Mass | Retention time | PEP | Score | Precursor Intensity
MFSD1 | 3 | 401.5507 | 1201.63 | 17.903 | 0.003376 | 79.82 | 6825578
Intensity (%)
100
50
0
−50
0
500
1000
1500
m/z
y1 376.2757
a2 459.2768
b2 487.2717
y2 489.36
b3 544.2931
y3 588.4285
y4−H2O 699.4604
y4 717.4713
y5−H2O 756.4832
y5 774.4932
b4 786.4306
y6−H2O 857.5309
b5−H2O 869.4661
y6 875.54
b5 887.4785
b6 944.5017
7−H2O 1055.5319
b7 1073.5466
E Q G K T G E V I K
y6 y5 y4 y3 y2 y1
b2 a2 b3 b4 b5 b6 b7
Modification
Acetyl (Protein N−term)
GlyGly (K) no new terminus
Oxidation (M)

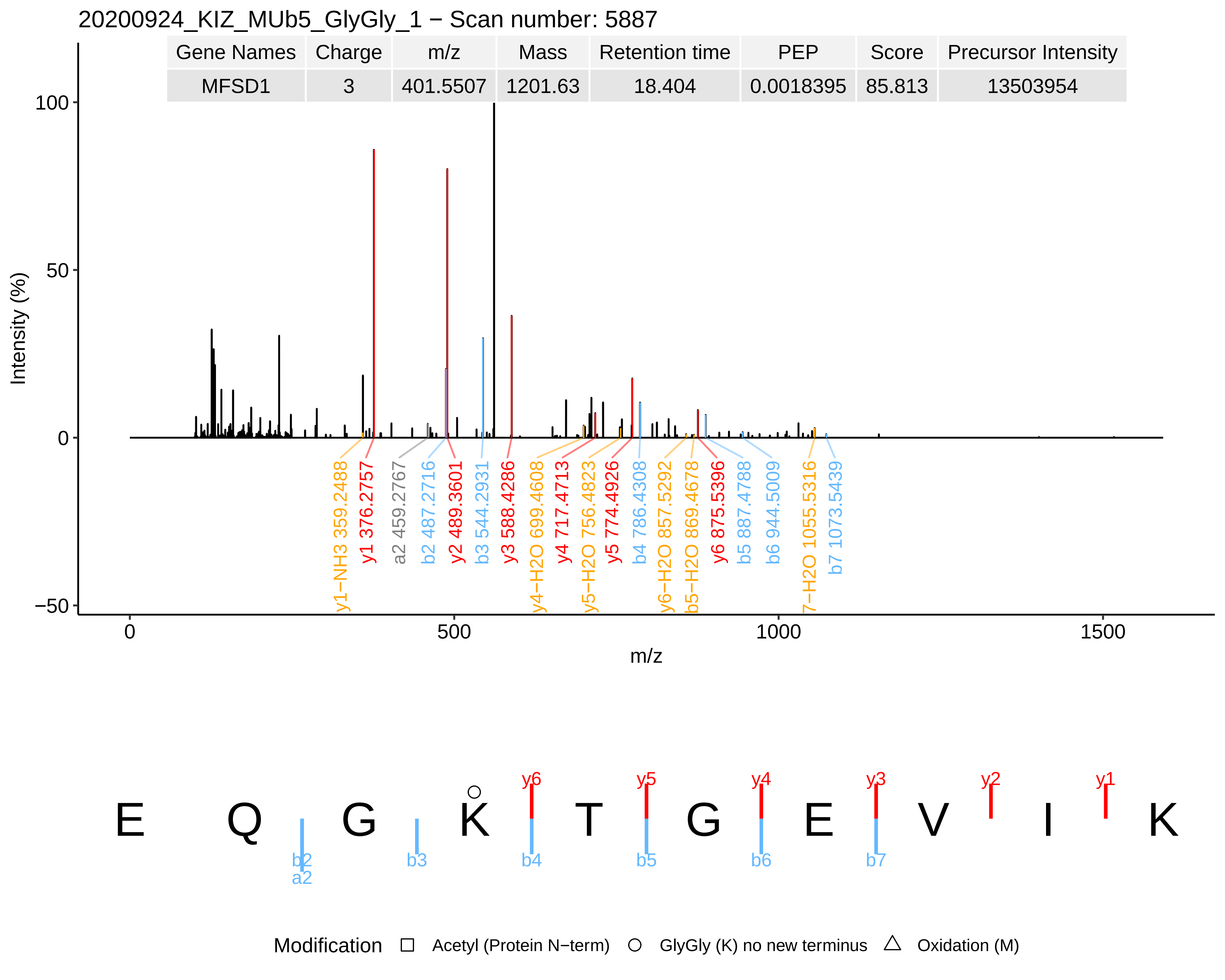
20200924_KIZ_MUb5_GlyGly_1 − Scan number: 5887
Gene Names | Charge | m/z | Mass | Retention time | PEP | Score | Precursor Intensity
MFSD1 | 3 | 401.5507 | 1201.63 | 18.404 | 0.0018395 | 85.813 | 13503954
Intensity (%)
100
50
0
−50
0
500
1000
1500
m/z
y1−NH3 359.2488
y1 376.2757
a2 459.2767
b2 487.2716
y2 489.3601
b3 544.2931
y3 588.4286
y4−H2O 699.4608
y4 717.4713
y5−H2O 756.4823
y5 774.4926
b4 786.4308
y6−H2O 857.5292
b5−H2O 869.4678
y6 875.5396
b5 887.4788
b6 944.5009
7−H2O 1055.5316
b7 1073.5439
E Q G K T G E V I K
y6 y5 y4 y3 y2 y1
b2 a2 b3 b4 b5 b6 b7
Modification
Acetyl (Protein N−term)
GlyGly (K) no new terminus
Oxidation (M)

20200924_KIZ_MUb5_GlyGly_2 − Scan number: 5385

Gene Names	Charge	m/z	Mass	Retention time	PEP	Score	Precursor Intensity
MFSD1	3	401.5507	1201.63	19.981	0.015355	68.069	2039286

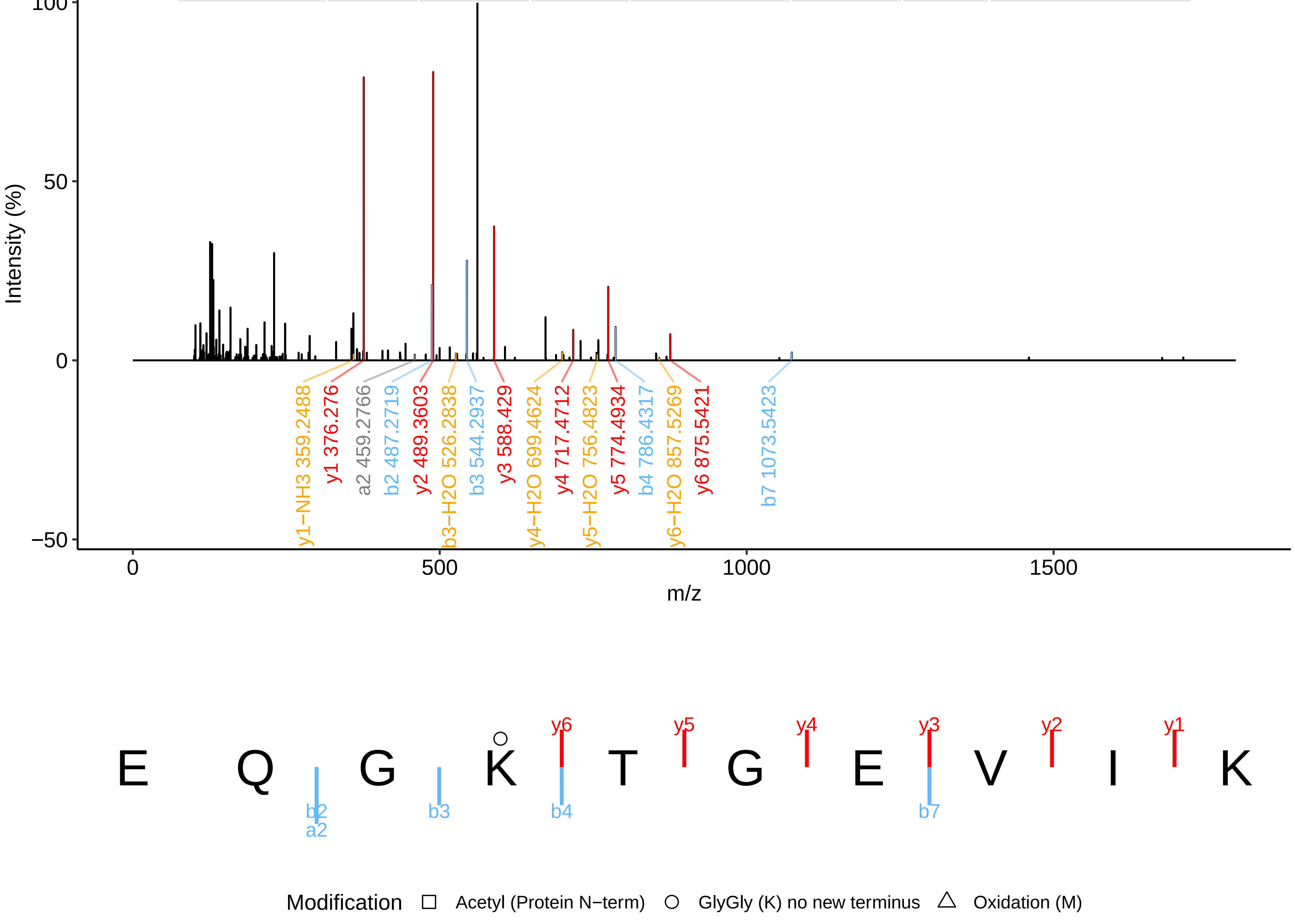

20200924_KIZ_MUb5_GlyGly_2 − Scan number: 5516

Gene Names	Charge	m/z	Mass	Retention time	PEP	Score	Precursor Intensity
MFSD1	3	401.5507	1201.63	20.485	0.0012479	89.9	2916120

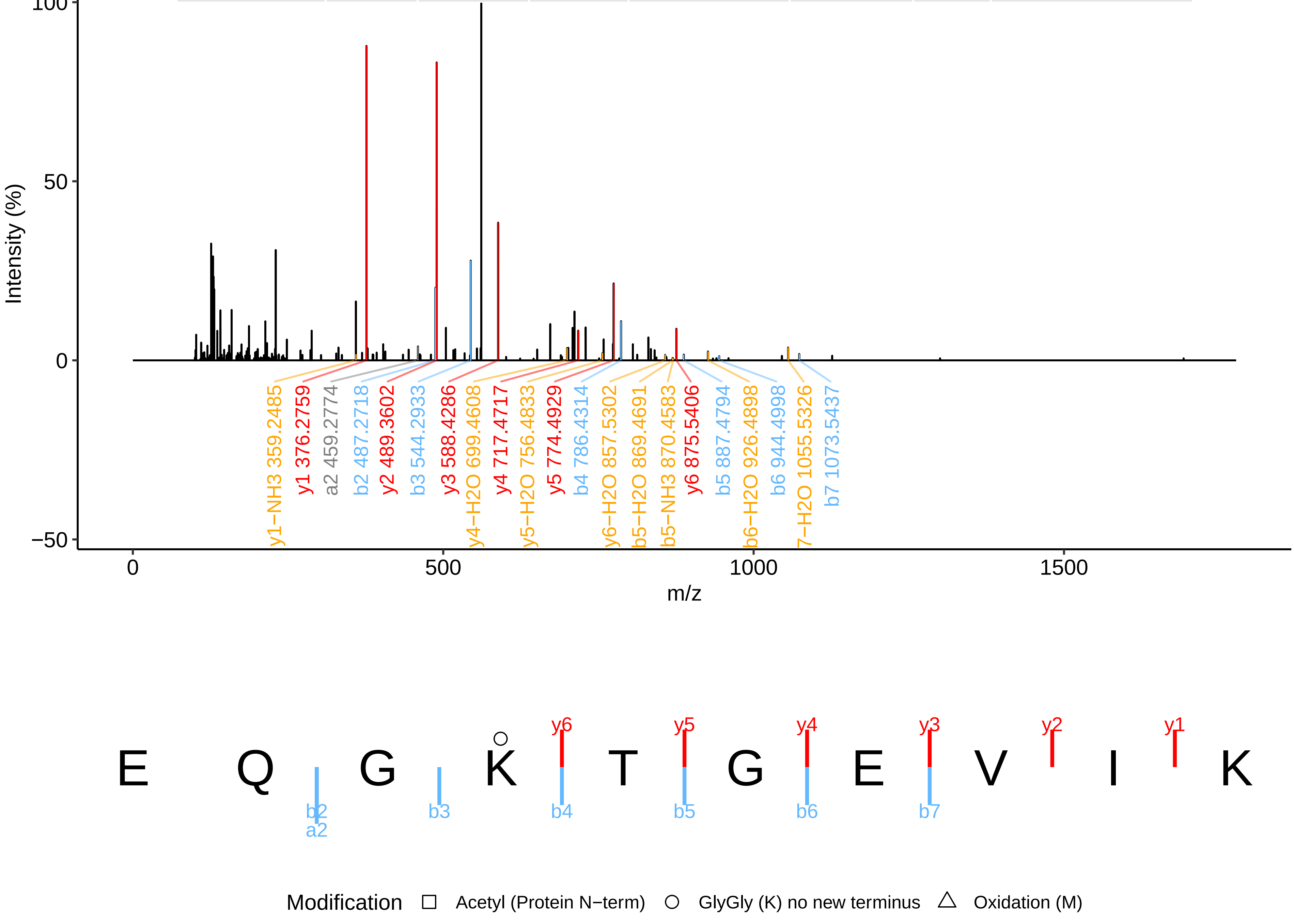

20201118_KIZ_MUb6_GlyGly_1 − Scan number: 5450

Gene Names	Charge	m/z	Mass	Retention time	PEP	Score	Precursor Intensity
MFSD1	3	401.5507	1201.63	17.264	0.0018395	85.813	8545708

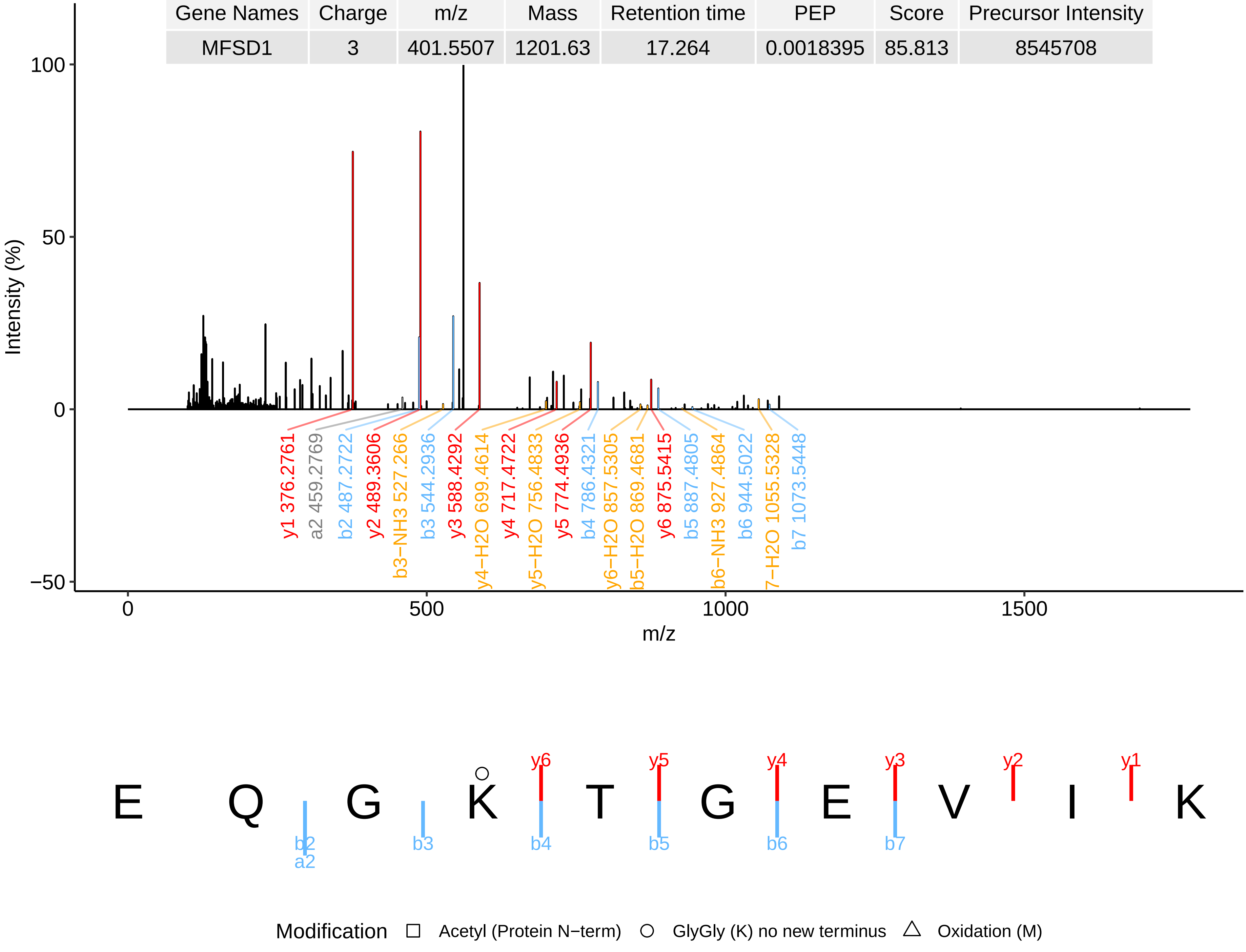

20201118_KIZ_MUb6_GlyGly_2 − Scan number: 7017

Gene Names	Charge	m/z	Mass	Retention time	PEP	Score	Precursor Intensity
MFSD1	3	401.5507	1201.63	23.944	0.0018395	85.813	4561749

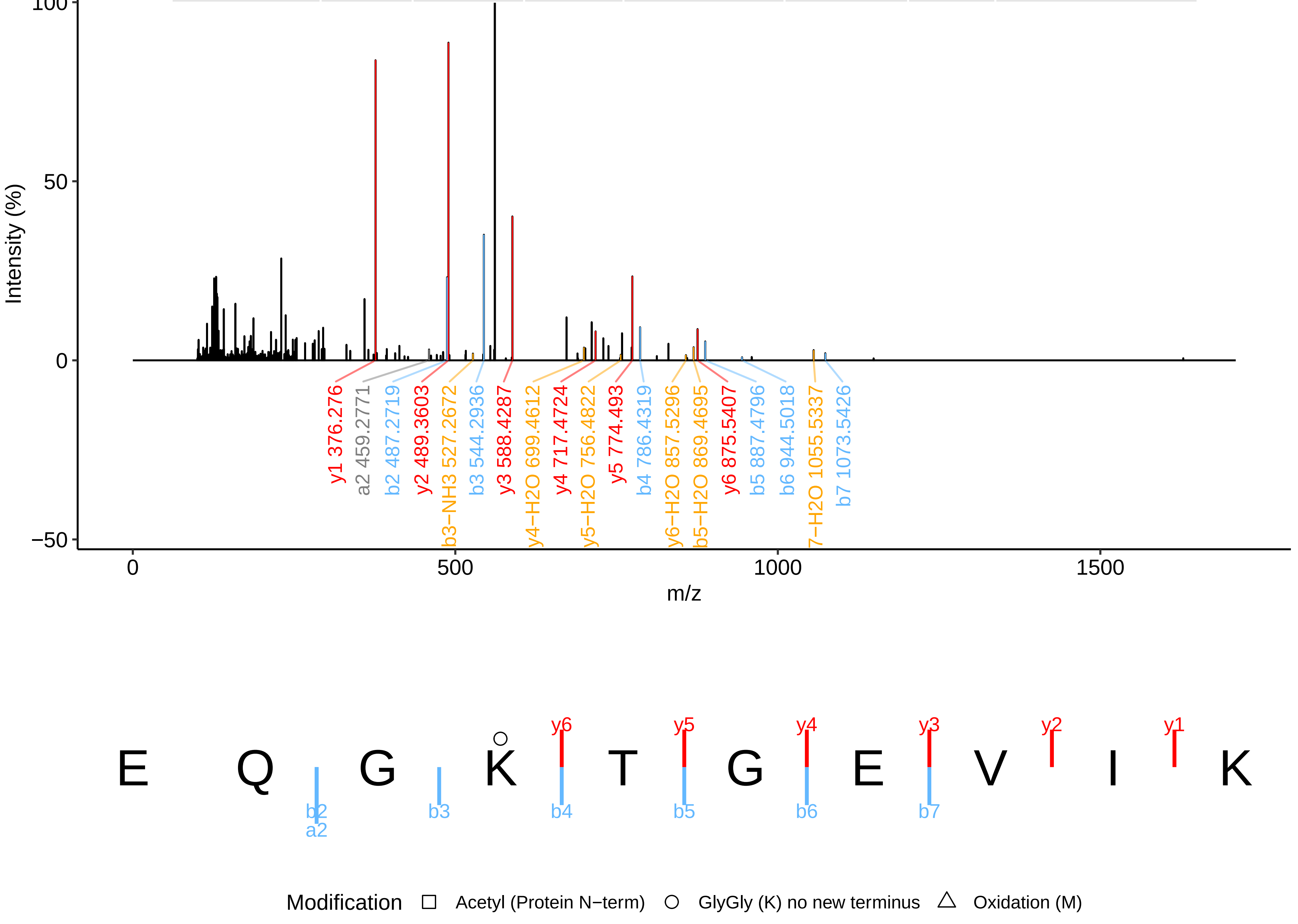

20200921_KIZ_MUb4_GlyGly_10plex − Scan number: 8687

Gene Names	Charge	m/z	Mass	Retention time	PEP	Score	Pr
T75;KRT76;KRT3;KRT84;KRT5;KRT6C;KRT6A;KRT2;KRT6B	3	401.2227	1200.646	29.559	0.067854	53.683	

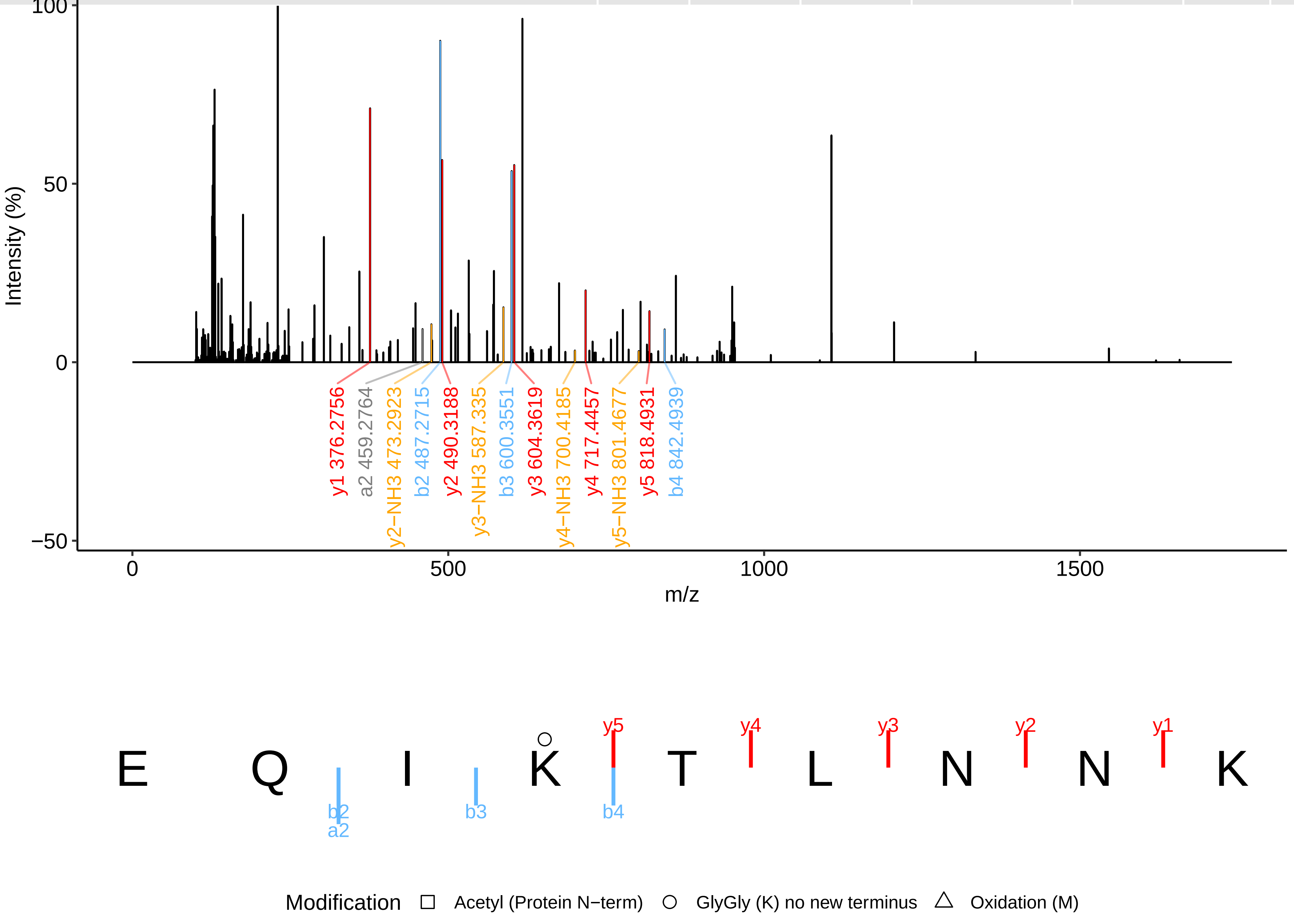

20200924_KIZ_MUb5_GlyGly_1 − Scan number: 7536

Gene Names	Charge	m/z	Mass	Retention time	PEP	Score	P
T75;KRT76;KRT3;KRT84;KRT5;KRT6C;KRT6A;KRT2;KRT6B	3	401.2227	1200.646	23.952	0.0031624	82.749	

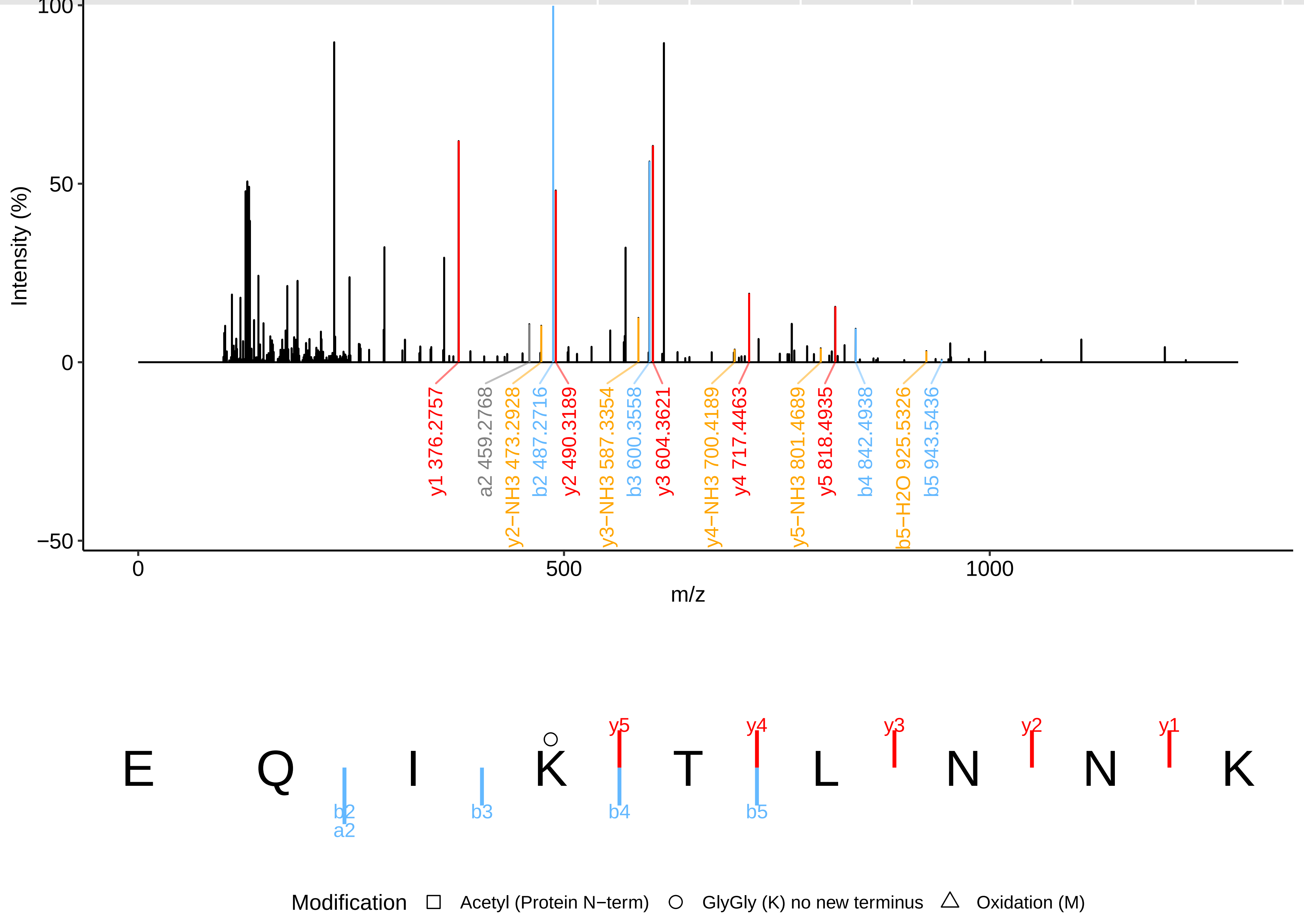

20200924_KIZ_MUb5_GlyGly_1 − Scan number: 7687

Gene Names	Charge	m/z	Mass	Retention time	PEP	Score	P
T75;KRT76;KRT3;KRT84;KRT5;KRT6C;KRT6A;KRT2;KRT6B	3	401.2227	1200.646	24.458	0.0032981	81.95	

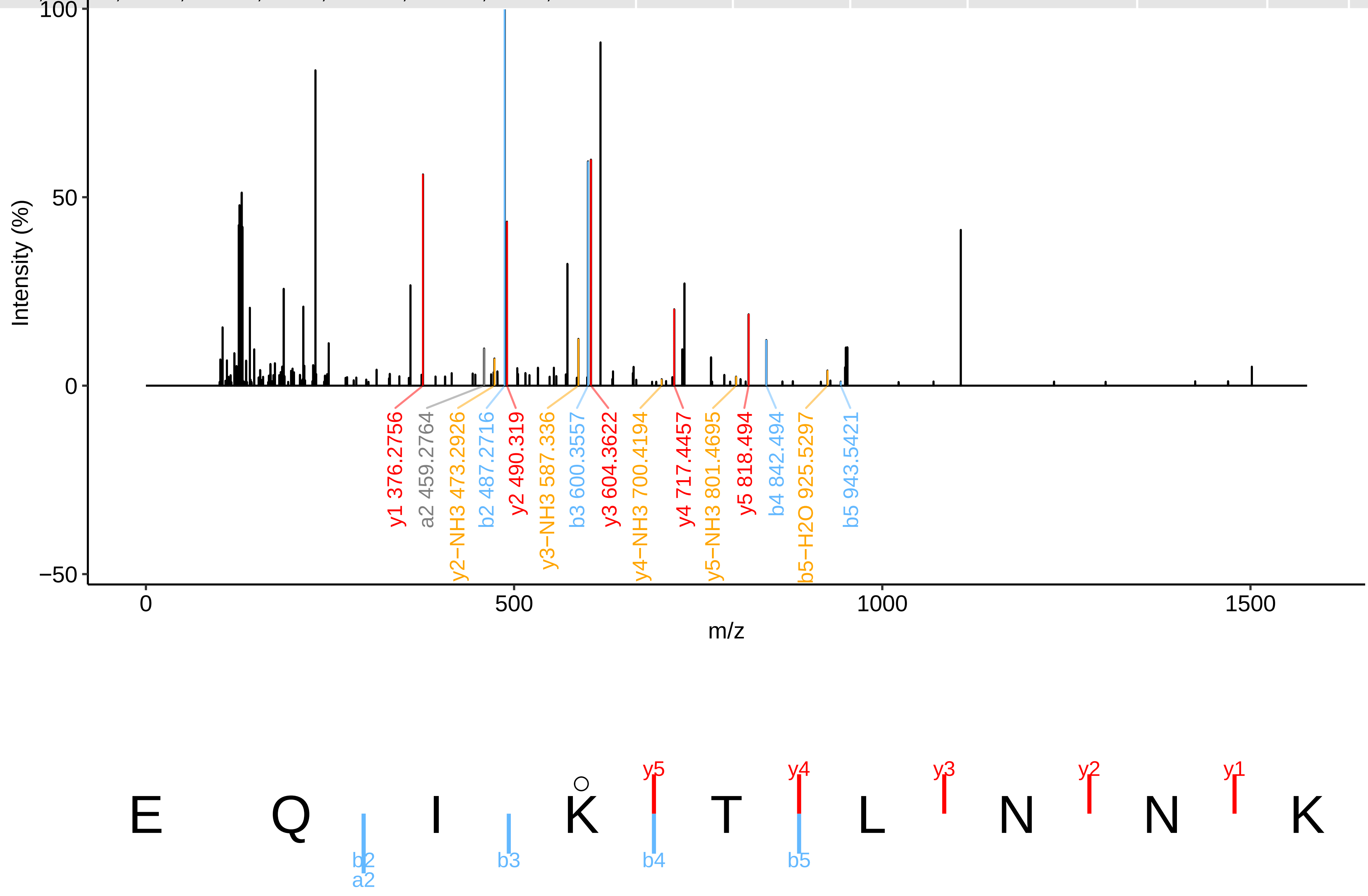

Modification □ Acetyl (Protein N−term) ○ GlyGly (K) no new terminus △ Oxidation (M)

20200924_KIZ_MUb5_GlyGly_1 − Scan number: 7837

Gene Names	Charge	m/z	Mass	Retention time	PEP	Score	P
T75;KRT76;KRT3;KRT84;KRT5;KRT6C;KRT6A;KRT2;KRT6B	3	401.2227	1200.646	24.967	0.0024147	87.149	

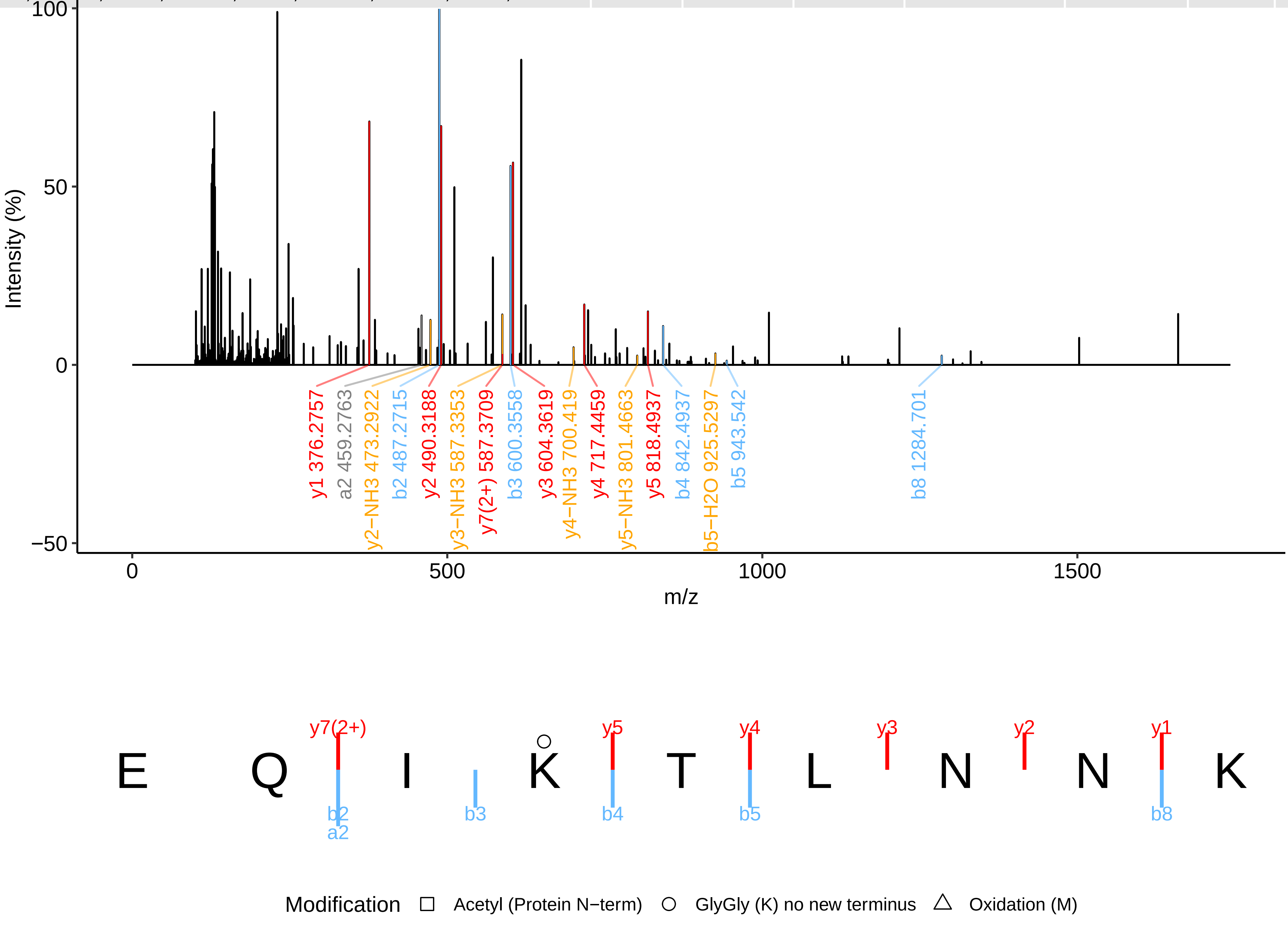

20200924_KIZ_MUb5_GlyGly_2 − Scan number: 7113

Gene Names	Charge	m/z	Mass	Retention time	PEP	Score	Pr
T75;KRT76;KRT3;KRT84;KRT5;KRT6C;KRT6A;KRT2;KRT6B	3	401.2227	1200.646	26.519	0.028135	63.283	

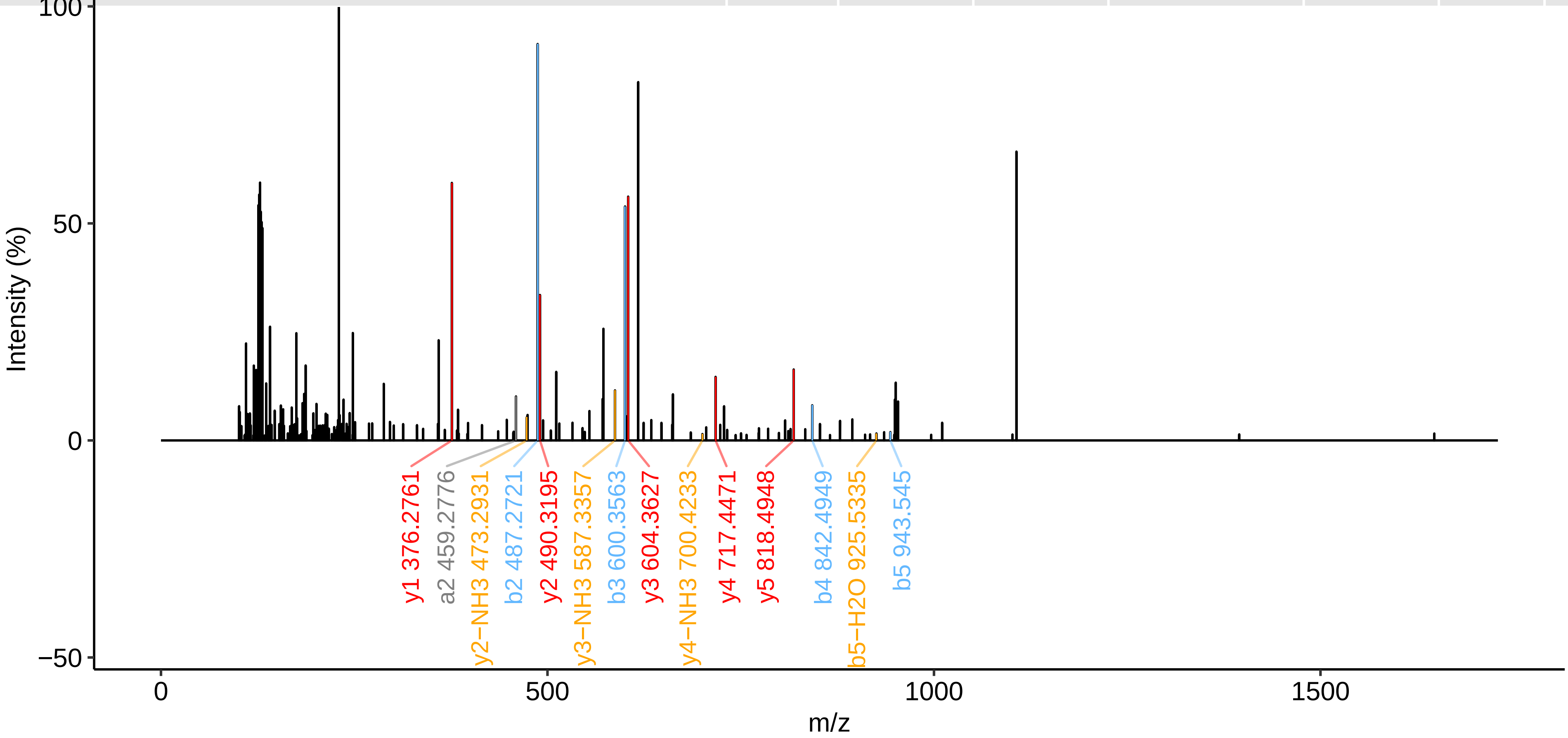

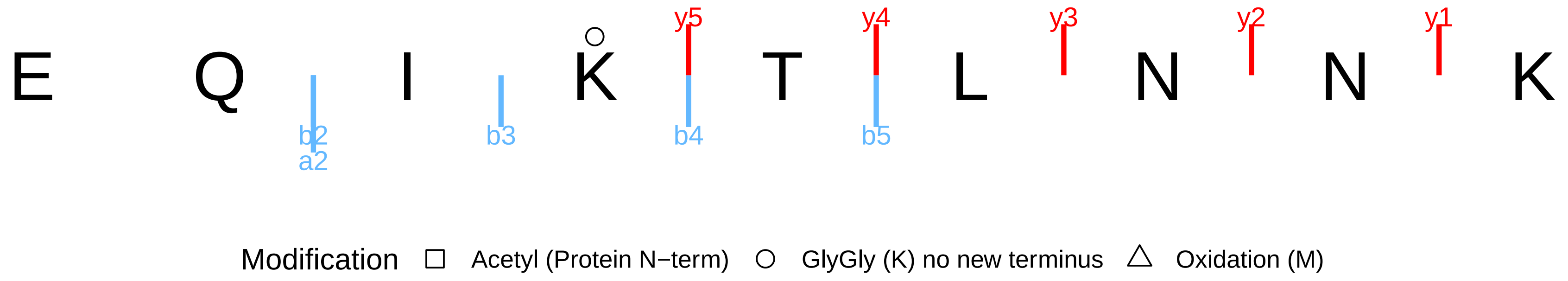

20200924_KIZ_MUb5_GlyGly_2 − Scan number: 7246

Gene Names	Charge	m/z	Mass	Retention time	PEP	Score	P
T75;KRT76;KRT3;KRT84;KRT5;KRT6C;KRT6A;KRT2;KRT6B	3	401.2227	1200.646	27.031	0.0032665	92.051	

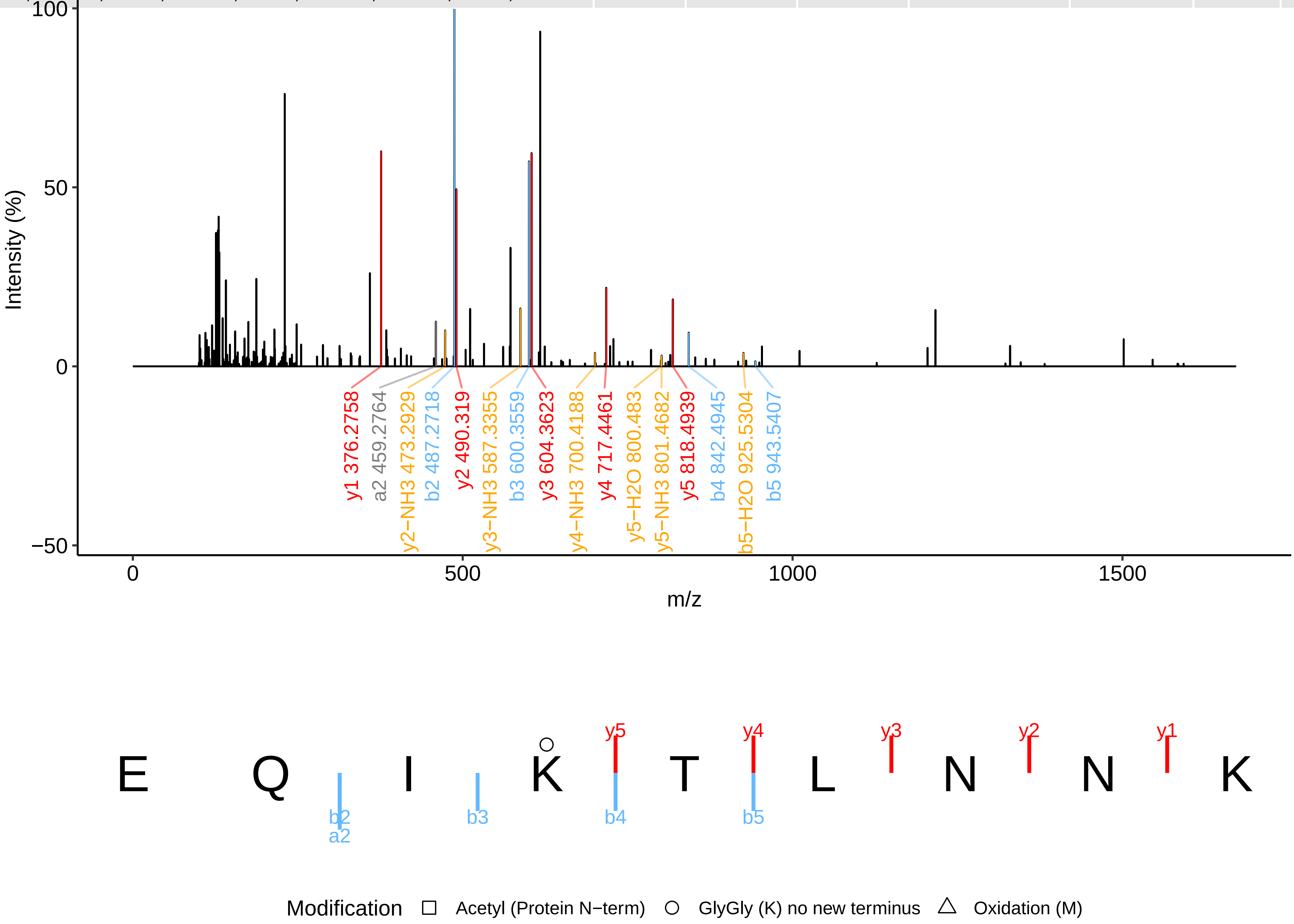

20200924_KIZ_MUb5_GlyGly_2 − Scan number: 7380

Gene Names	Charge	m/z	Mass	Retention time	PEP	Score	P
T75;KRT76;KRT3;KRT84;KRT5;KRT6C;KRT6A;KRT2;KRT6B	3	401.2227	1200.646	27.537	0.0014693	101.9	

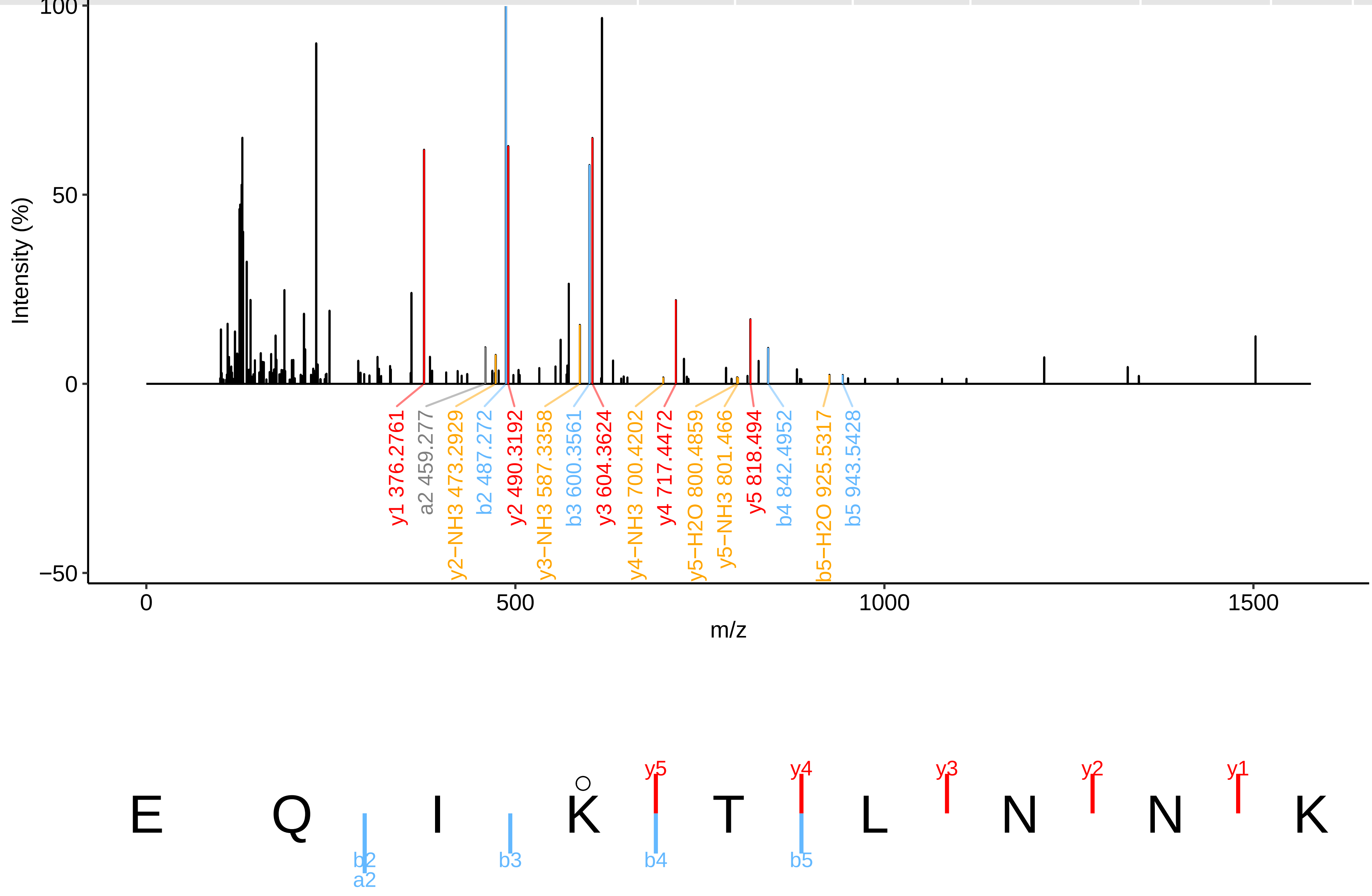

Modification □ Acetyl (Protein N−term) ○ GlyGly (K) no new terminus △ Oxidation (M)

20201118_KIZ_MUb6_GlyGly_1 − Scan number: 7338

Gene Names	Charge	m/z	Mass	Retention time	PEP	Score	P
T75;KRT76;KRT3;KRT84;KRT5;KRT6C;KRT6A;KRT2;KRT6B	3	401.2227	1200.646	23.25	0.0010053	111.61	

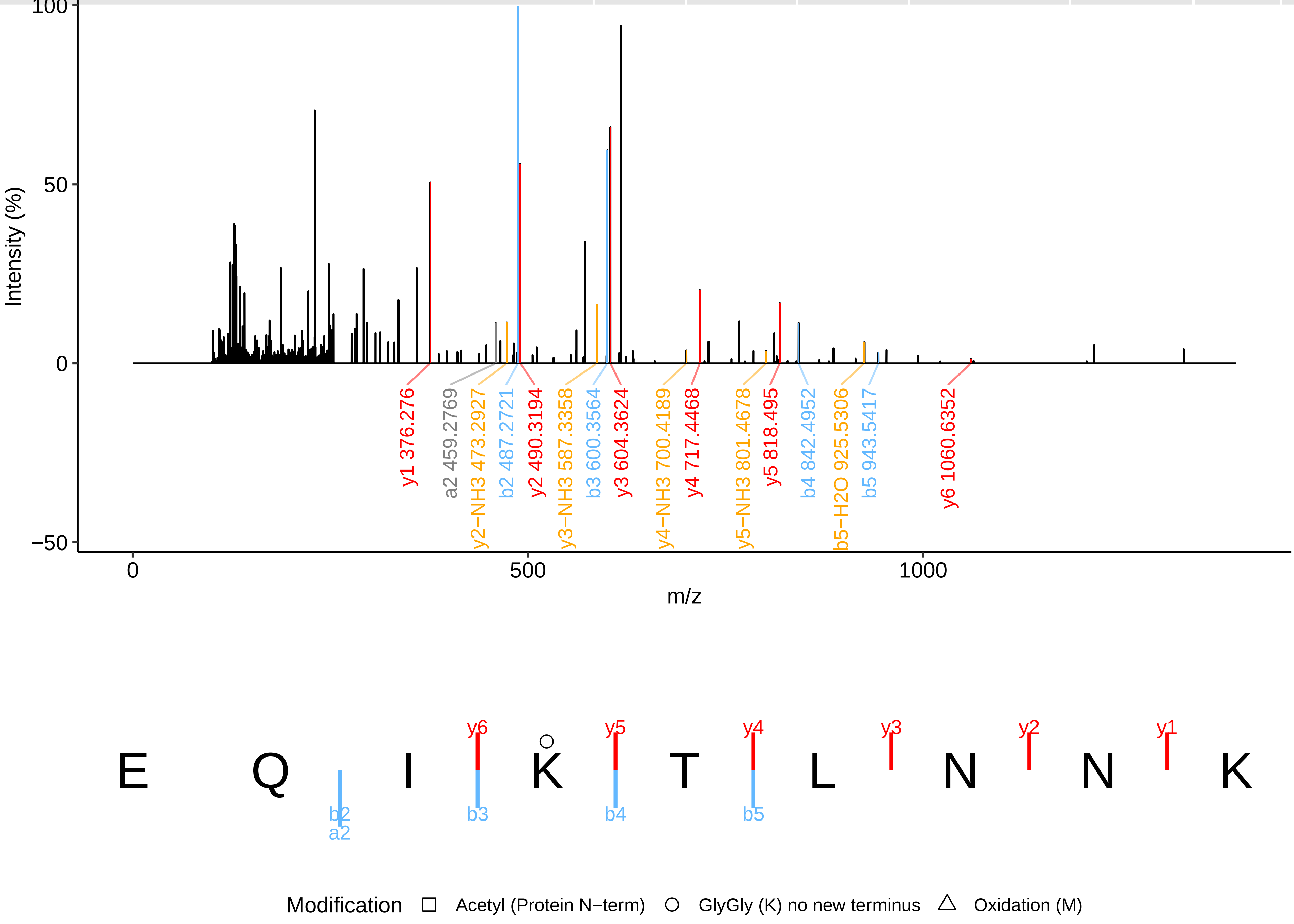

20200921_KIZ_MUb4_GlyGly_10plex_2 − Scan number: 10612

Gene Names	Charge	m/z	Mass	Retention time	PEP	Score	Precursor Intensity
PPP4C	3	416.8799	1247.618	40.635	0.020233	63.283	917242

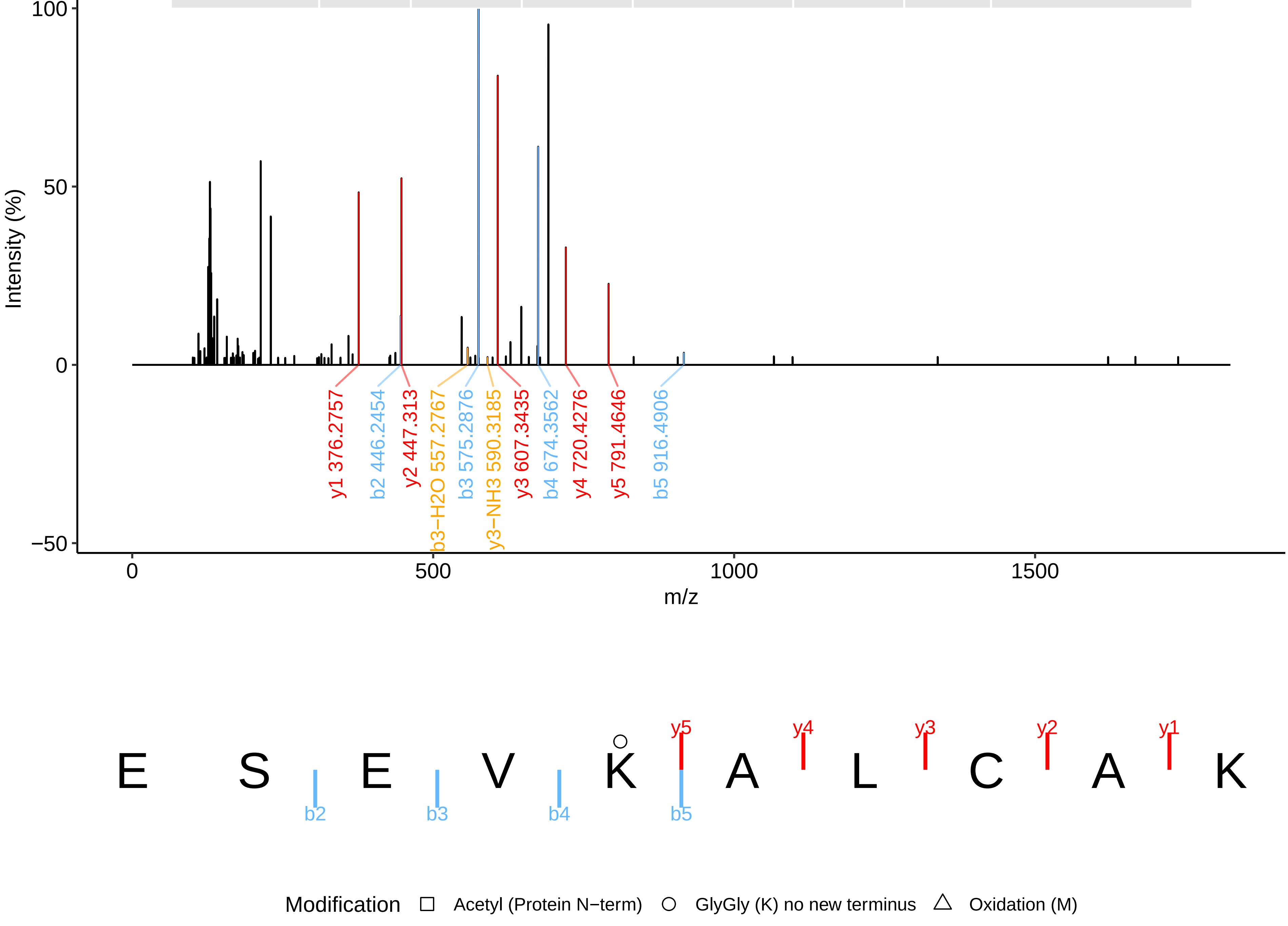

20200924_KIZ_MUb5_GlyGly_1 − Scan number: 5220

Gene Names	Charge	m/z	Mass	Retention time	PEP	Score	Precursor Intensity
DCUN1D1	4	308.9203	1231.652	16.205	0.00028822	105.2	9203468

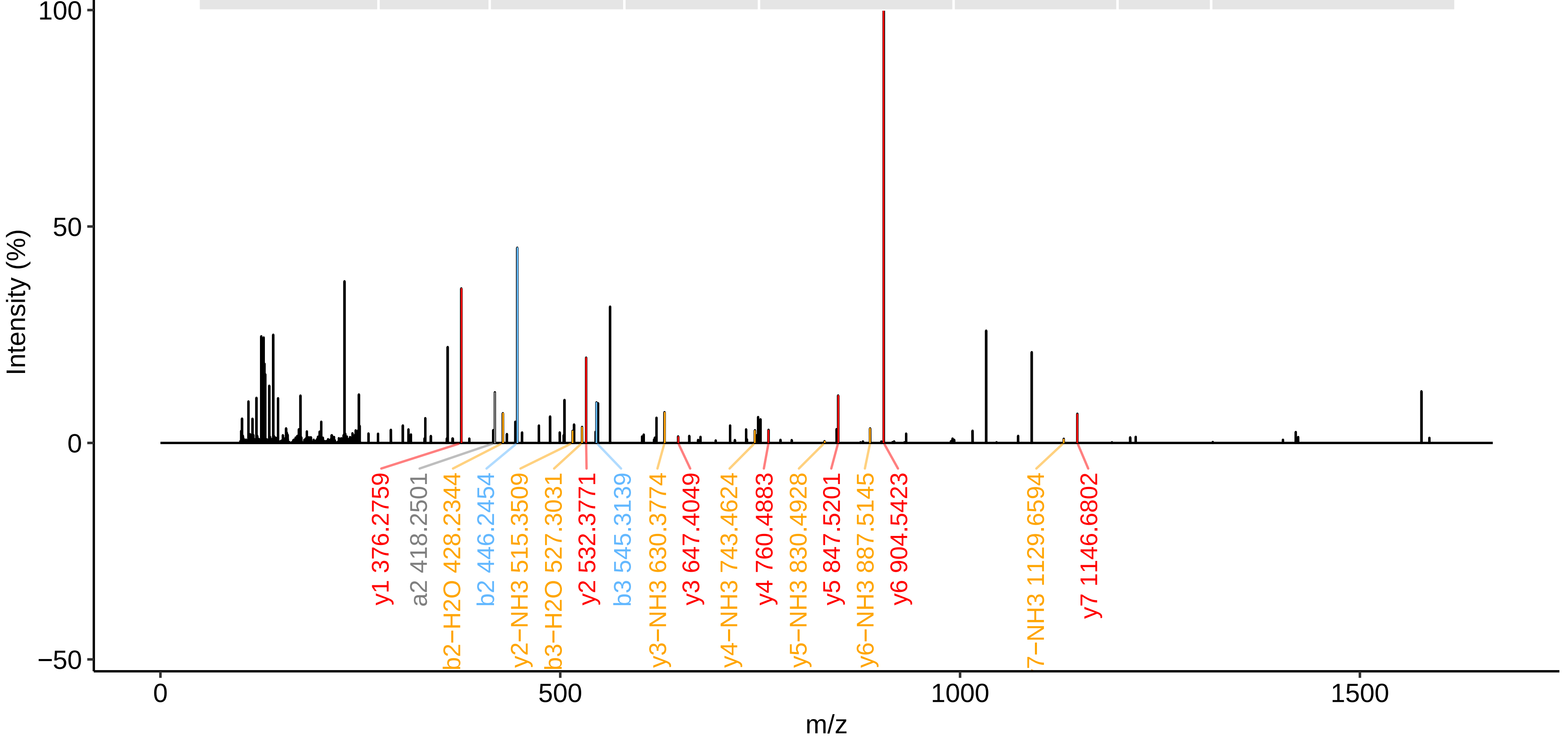

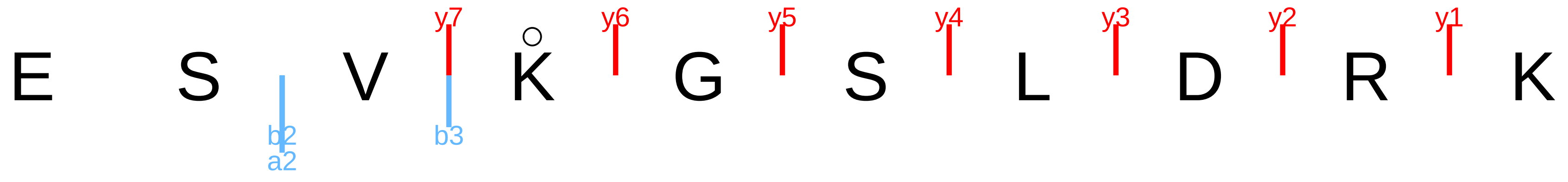

Modification □ Acetyl (Protein N−term) ○ GlyGly (K) no new terminus △ Oxidation (M)

20200924_KIZ_MUb5_GlyGly_1 − Scan number: 5228

Gene Names	Charge	m/z	Mass	Retention time	PEP	Score	Precursor Intensity
DCUN1D1	3	411.558	1231.652	16.227	0.0028886	94.767	NA

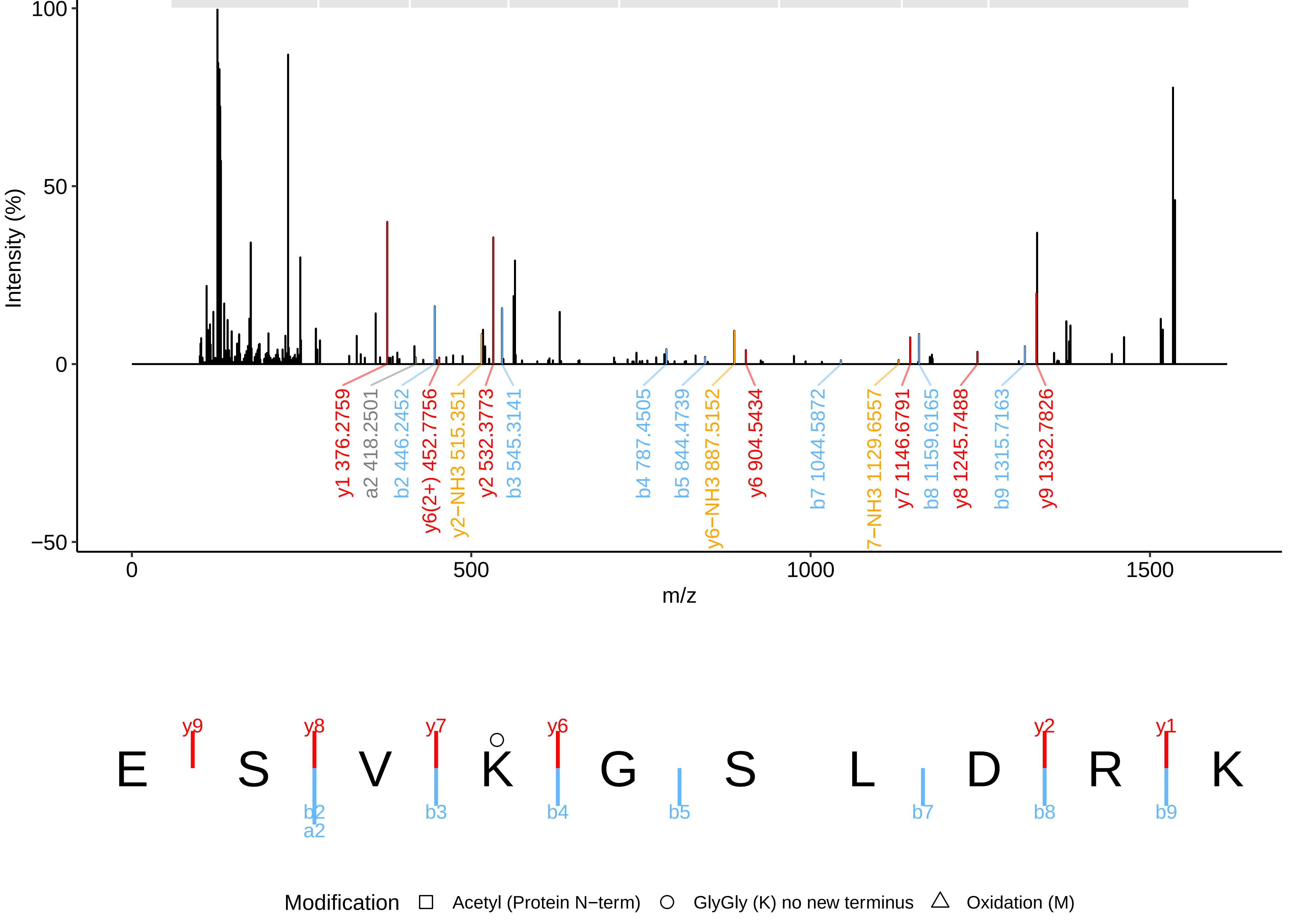

20200924_KIZ_MUb5_GlyGly_1 − Scan number: 21832

Gene Names	Charge	m/z	Mass	Retention time	PEP	Score	Precursor Intensity
	3	486.9231	1457.747	70.317	0.051914	56.225	55588048

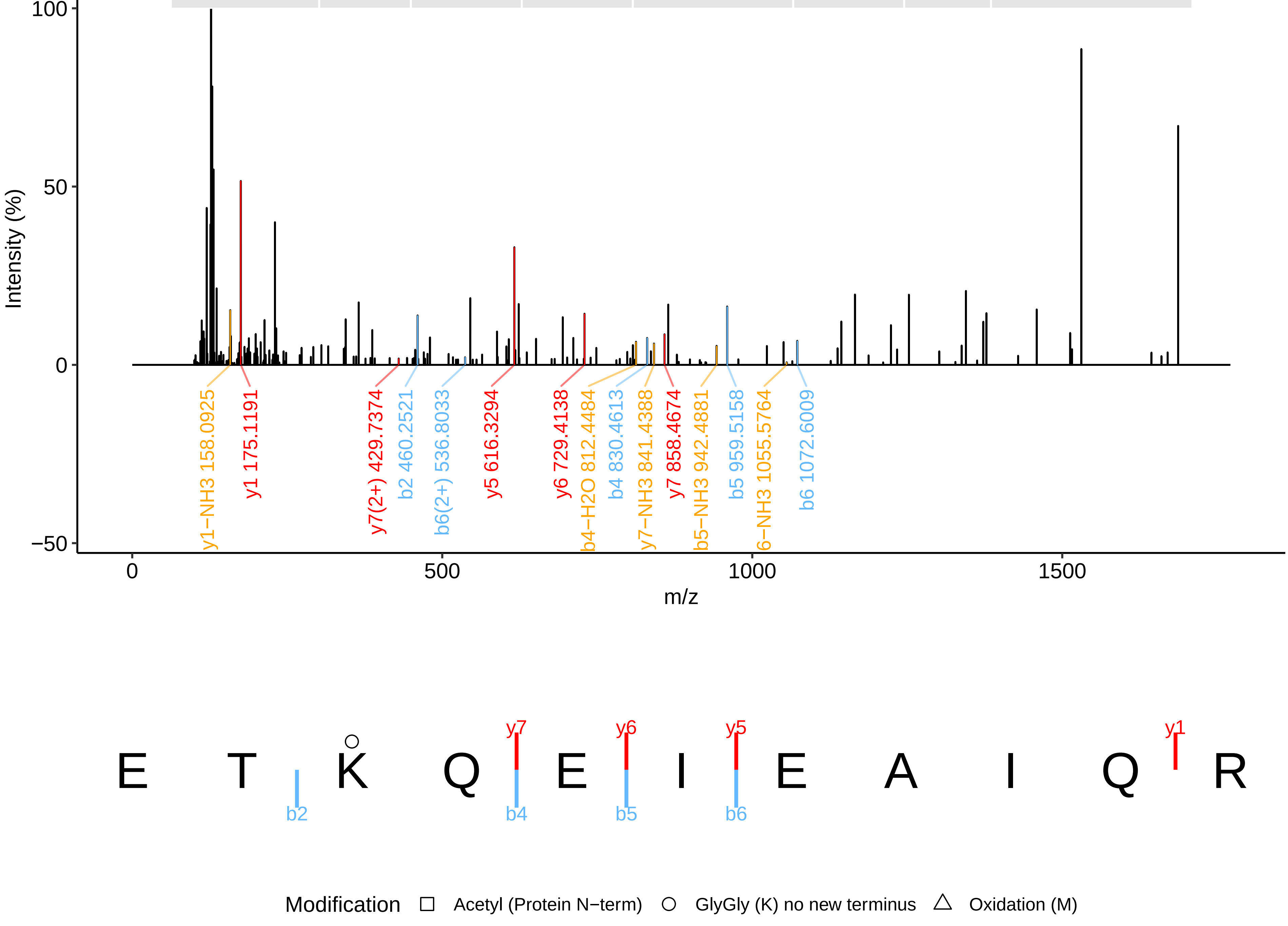

20200924_KIZ_MUb5_GlyGly_1 − Scan number: 22164

Gene Names	Charge	m/z	Mass	Retention time	PEP	Score	Precursor Intensity
	3	486.9231	1457.747	71.343	0.061998	57.019	19937070

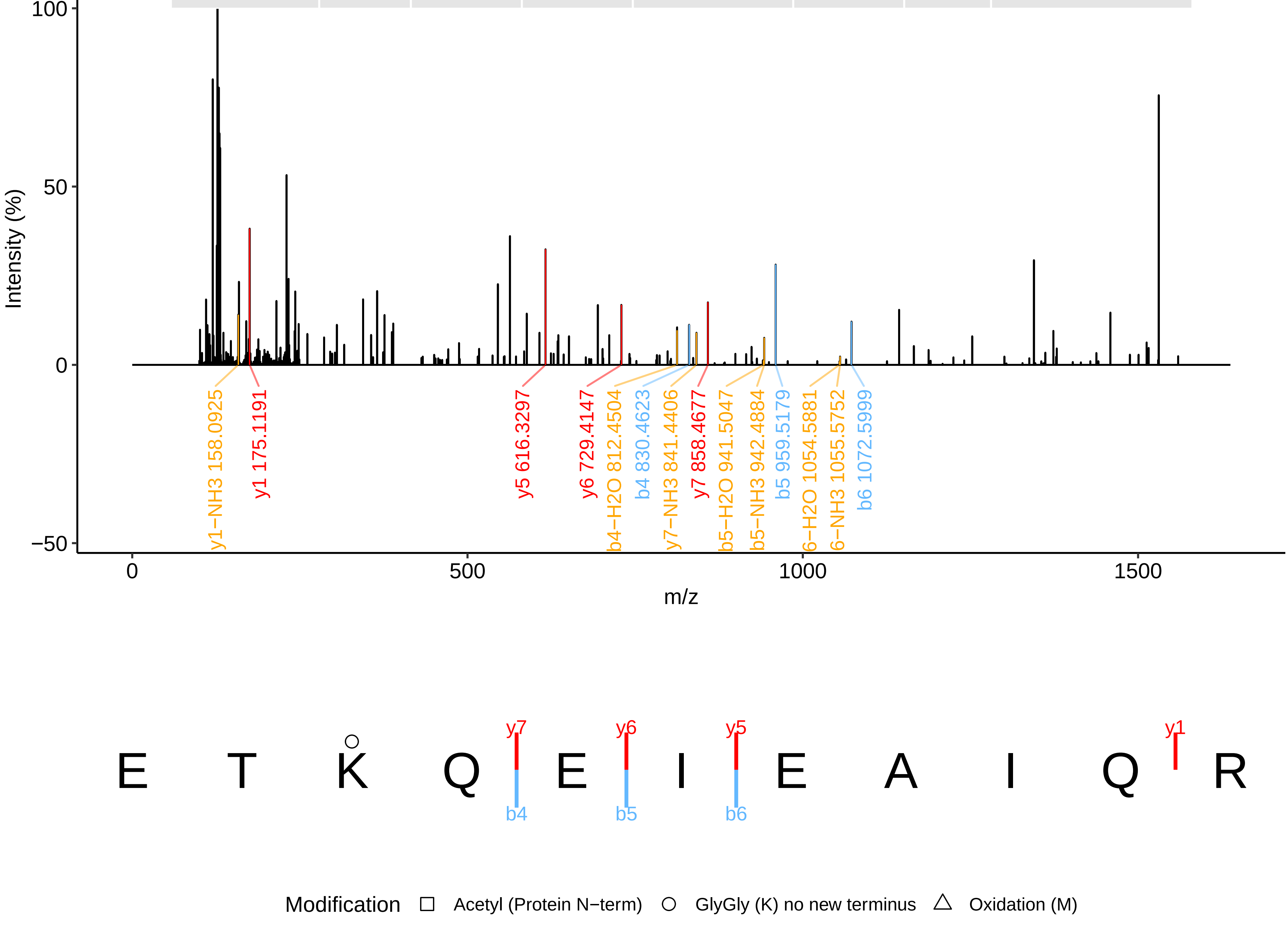

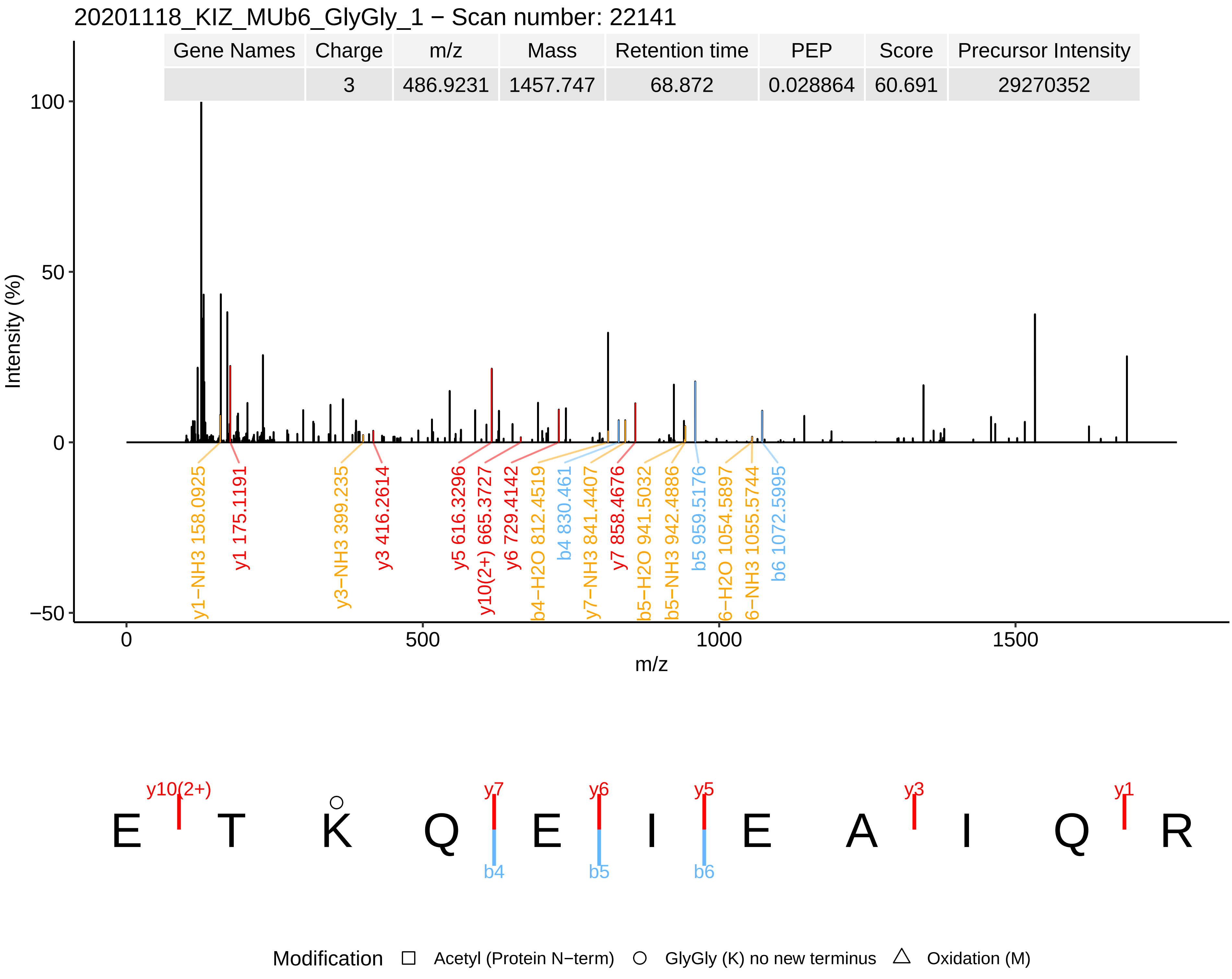

20201118_KIZ_MUb6_GlyGly_1 − Scan number: 22141
Gene Names | Charge | m/z | Mass | Retention time | PEP | Score | Precursor Intensity
3 | 486.9231 | 1457.747 | 68.872 | 0.028864 | 60.691 | 29270352
Intensity (%)
m/z
y1−NH3 158.0925
y1 175.1191
y3−NH3 399.235
y3 416.2614
y5 616.3296
y10(2+) 665.3727
y6 729.4142
b4−H2O 812.4519
b4 830.461
y7−NH3 841.4407
y7 858.4676
b5−H2O 941.5032
b5−NH3 942.4886
b5 959.5176
b6−H2O 1054.5897
b6−NH3 1055.5744
b6 1072.5995
E T K Q E I E A I Q R
y10(2+) y7 y6 y5 y3 y1
b4 b5 b6
Modification
Acetyl (Protein N−term)
GlyGly (K) no new terminus
Oxidation (M)

20200921_KIZ_MUb4_GlyGly_10plex − Scan number: 13551

Gene Names	Charge	m/z	Mass	Retention time	PEP	Score	Precursor Intensity
TPR	3	404.5713	1210.692	47.845	0.0046068	79.974	5185177

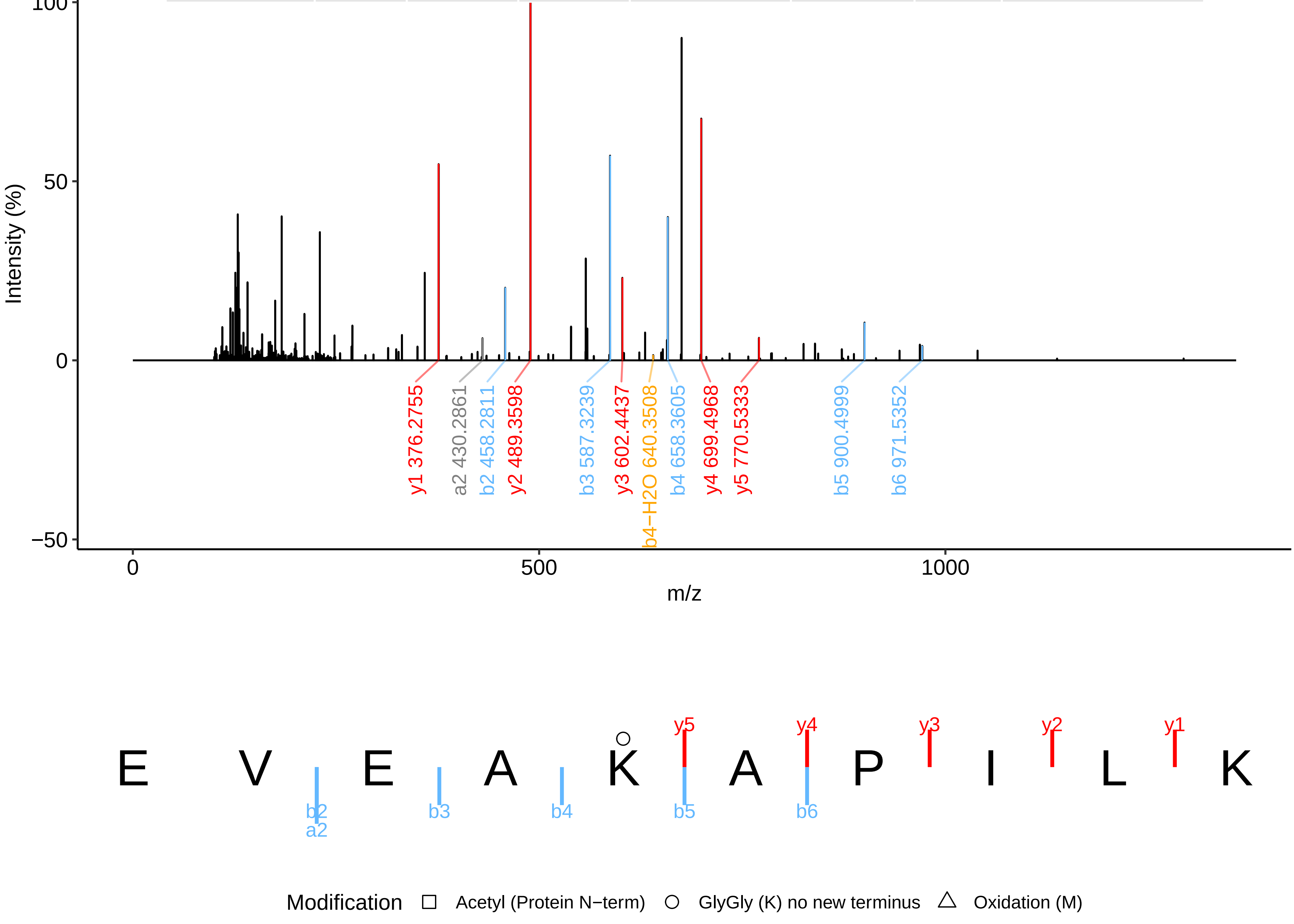

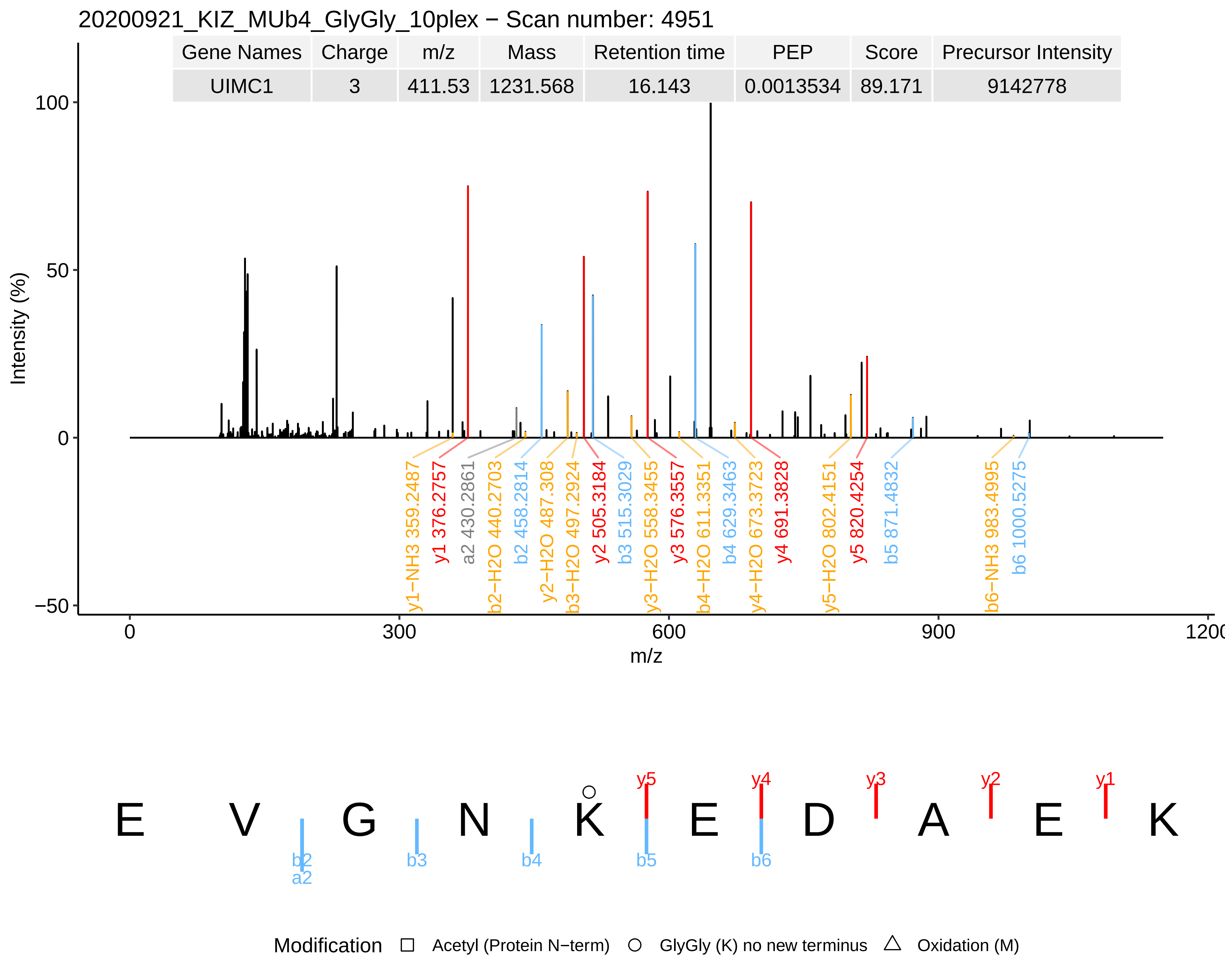
20200921_KIZ_MUb4_GlyGly_10plex − Scan number: 4951
Gene Names | Charge | m/z | Mass | Retention time | PEP | Score | Precursor Intensity
UIMC1 | 3 | 411.53 | 1231.568 | 16.143 | 0.0013534 | 89.171 | 9142778
Intensity (%)
m/z
y1−NH3 359.2487
y1 376.2757
a2 430.2861
b2−H2O 440.2703
b2 458.2814
y2−H2O 487.308
b3−H2O 497.2924
y2 505.3184
b3 515.3029
y3−H2O 558.3455
y3 576.3557
b4−H2O 611.3351
b4 629.3463
y4−H2O 673.3723
y4 691.3828
y5−H2O 802.4151
y5 820.4254
b5 871.4832
b6−NH3 983.4995
b6 1000.5275
E V G N K E D A E K
Modification □ Acetyl (Protein N−term) ○ GlyGly (K) no new terminus △ Oxidation (M)

20200924_KIZ_MUb5_GlyGly_2 − Scan number: 4074

Gene Names	Charge	m/z	Mass	Retention time	PEP	Score	Precursor Intensity
UIMC1	3	411.53	1231.568	14.901	0.0080887	75.197	4475474

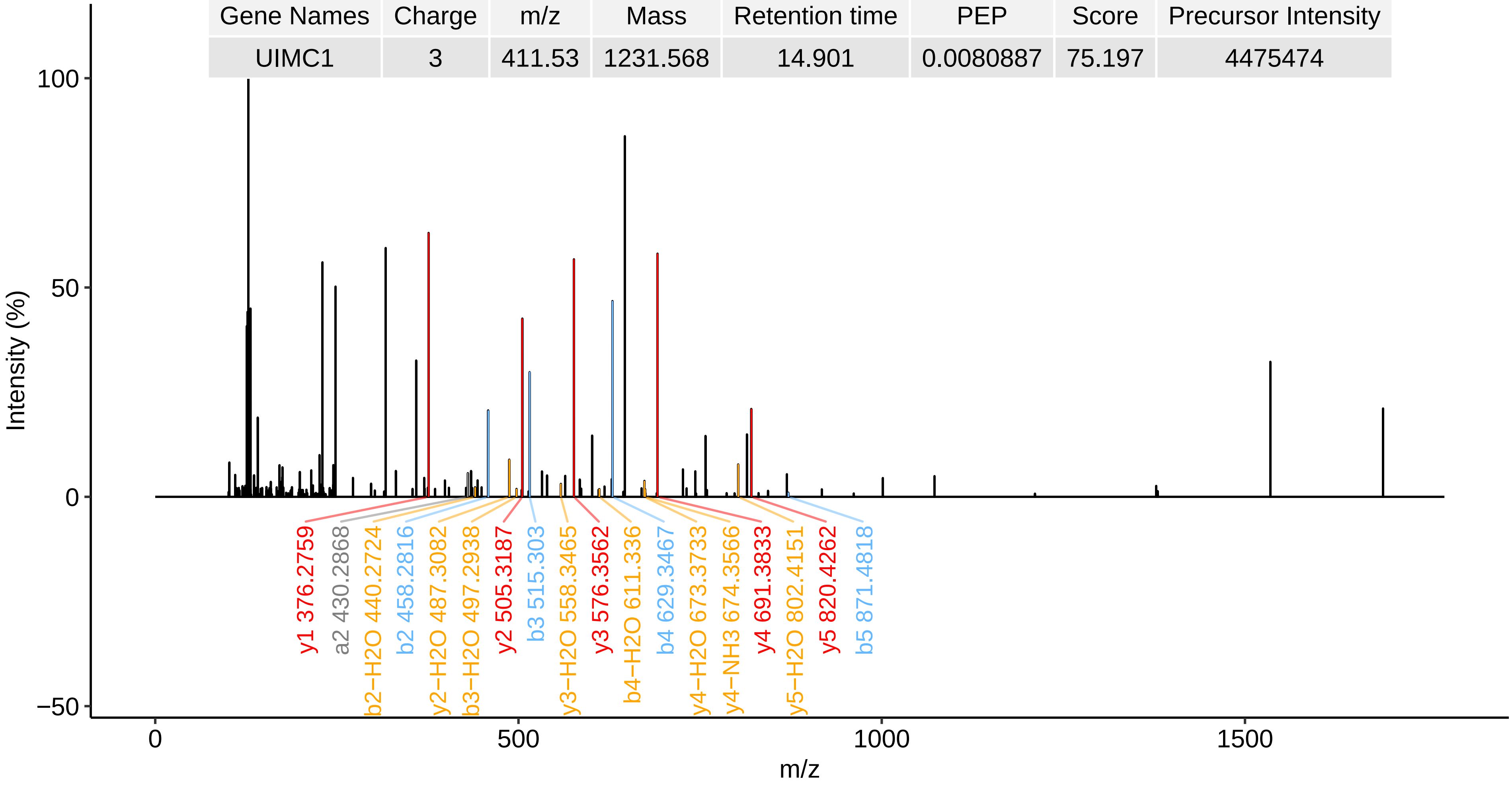

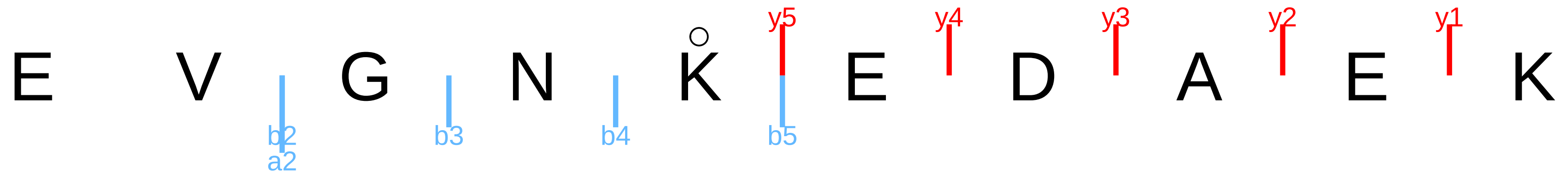

Modification □ Acetyl (Protein N−term) ○ GlyGly (K) no new terminus △ Oxidation (M)

20201118_KIZ_MUb6_GlyGly_1 − Scan number: 18403

Gene Names	Charge	m/z	Mass	Retention time	PEP	Score	Precursor Intensity
USP33	3	408.54	1222.598	57.879	0.00039514	103.56	12316713

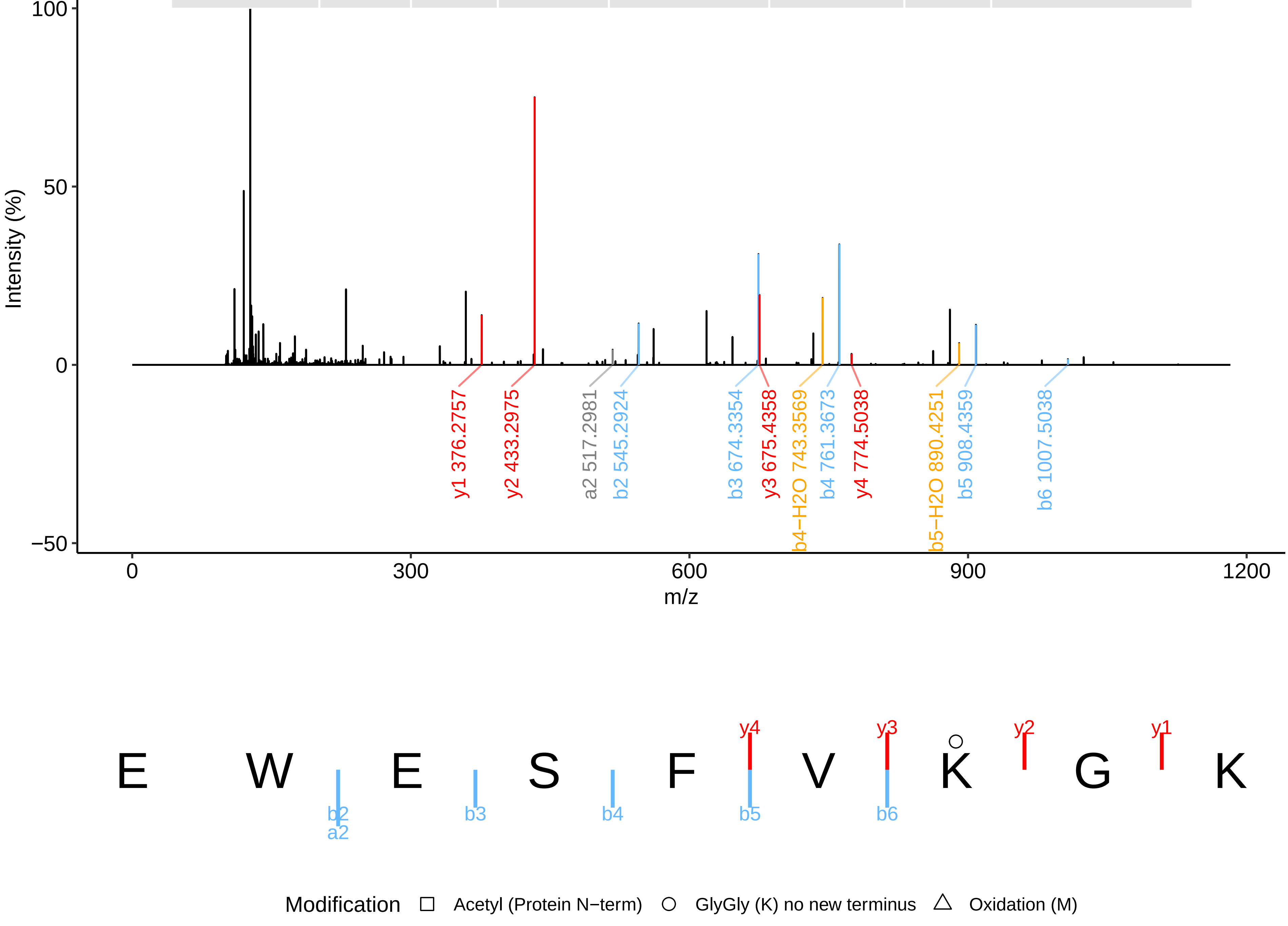

20201118_KIZ_MUb6_GlyGly_1 − Scan number: 18423

Gene Names	Charge	m/z	Mass	Retention time	PEP	Score	Precursor Intensity
USP33	3	408.54	1222.598	57.932	0.002109	88.948	14824772

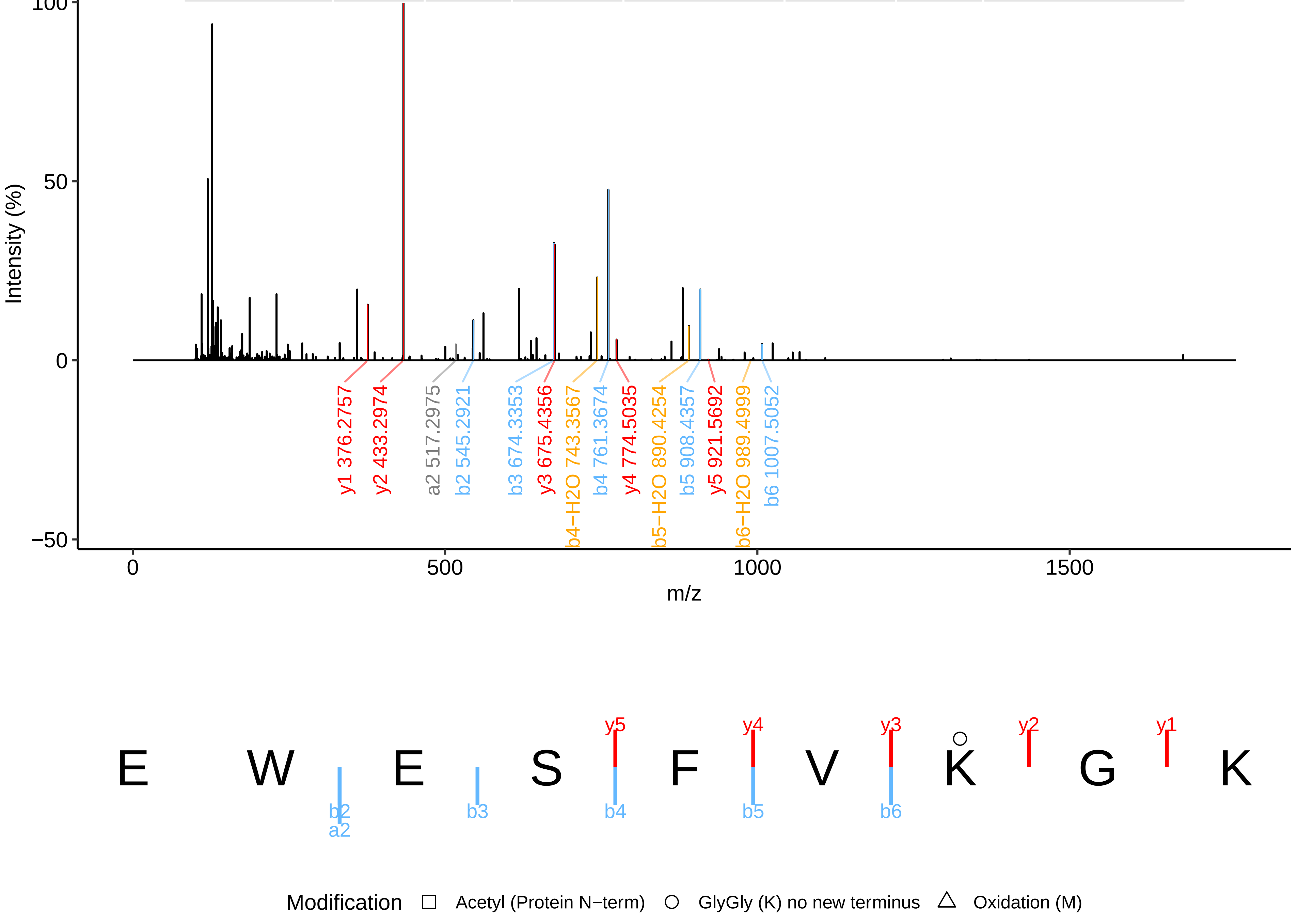

20200921_KIZ_MUb4_GlyGly_10plex − Scan number: 13512

Gene Names	Charge	m/z	Mass	Retention time	PEP	Score	Precursor Intensity
EEF1A1;EEF1A1P5;EEF1A2	3	418.5325	1252.576	47.7	0.0021891	83.397	12167218

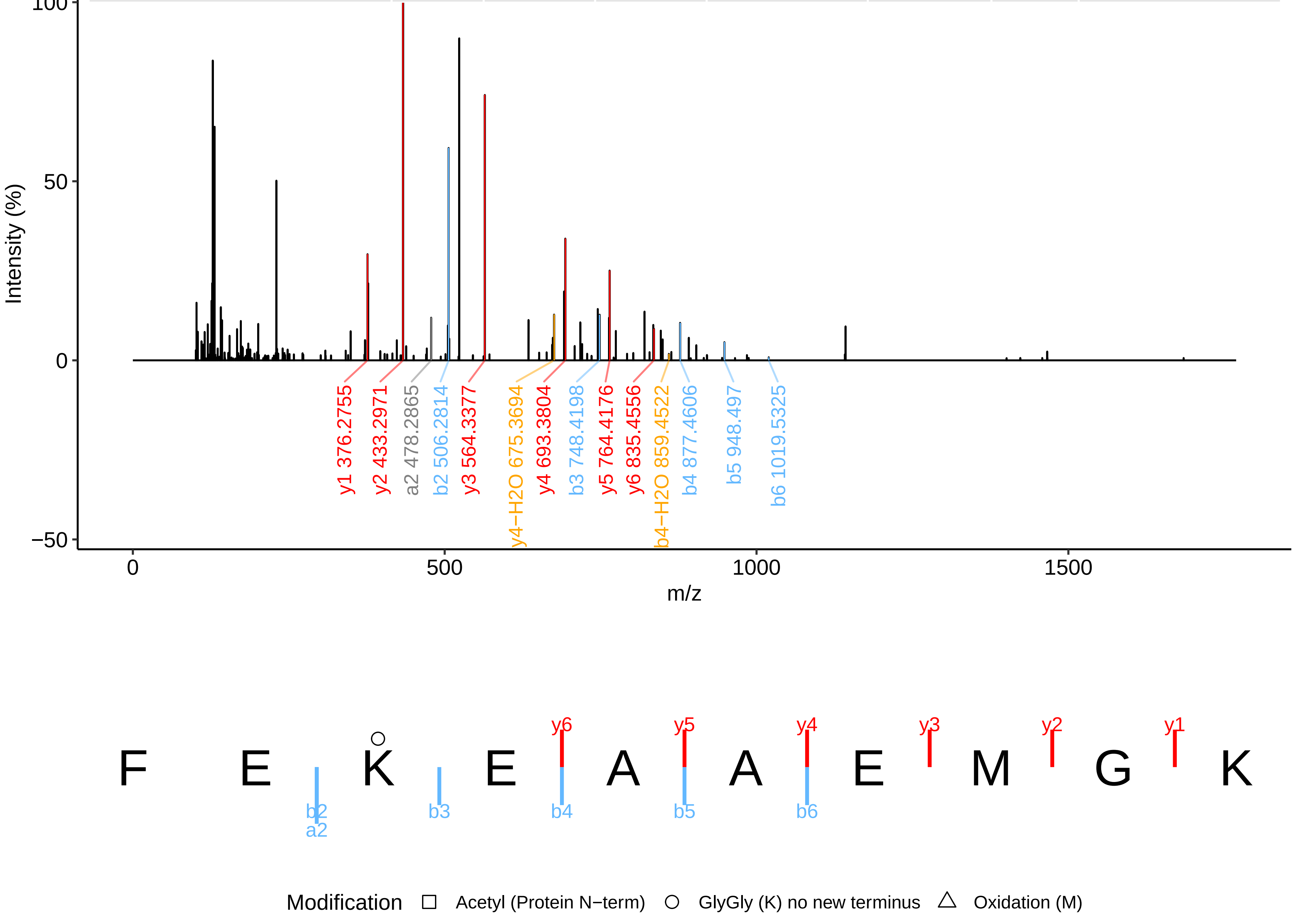

20200921_KIZ_MUb4_GlyGly_10plex_2 − Scan number: 12438

Gene Names	Charge	m/z	Mass	Retention time	PEP	Score	Precursor Intensity
EEF1A1;EEF1A1P5;EEF1A2	3	418.5325	1252.576	48.12	0.023974	62.466	1378221

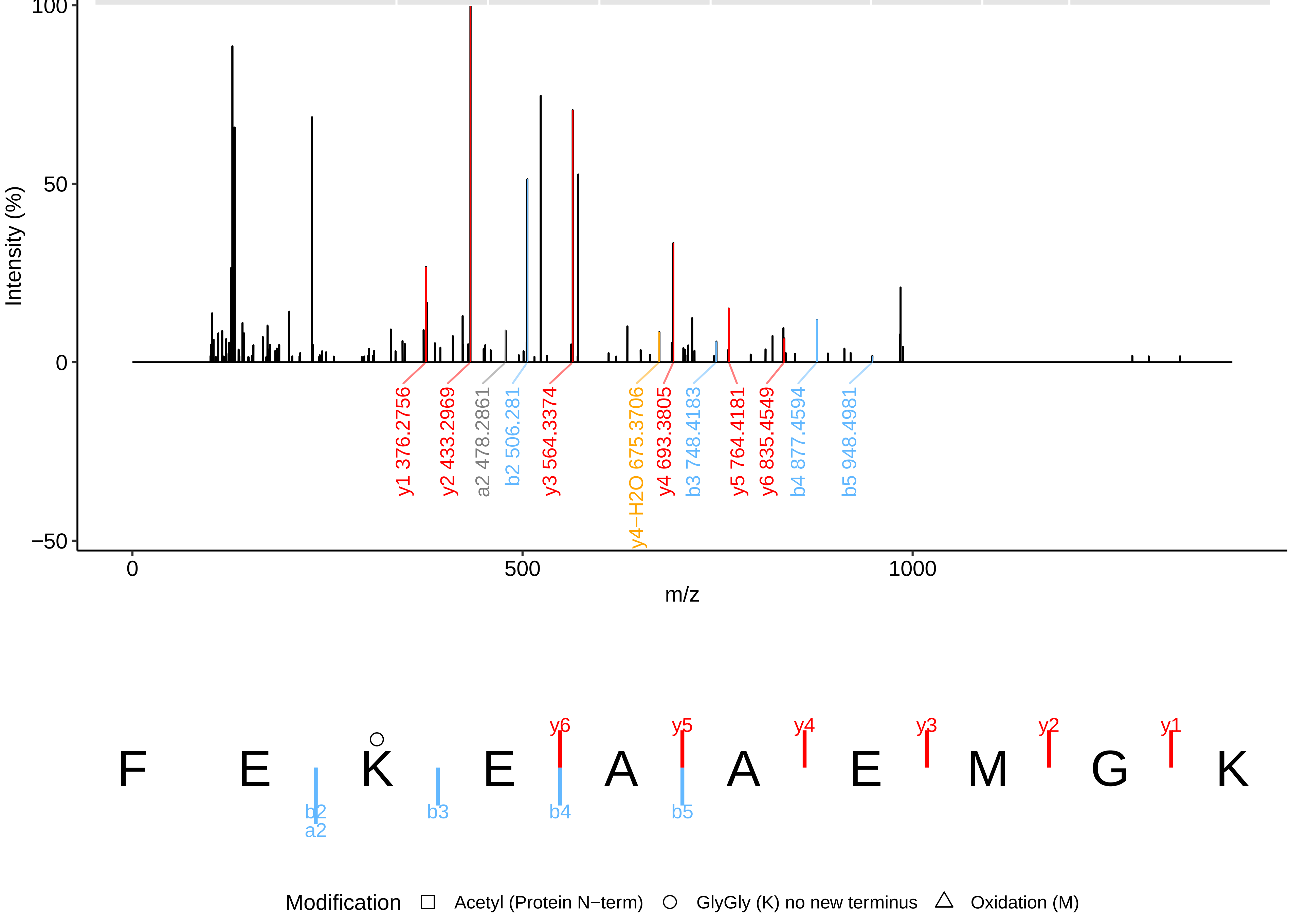

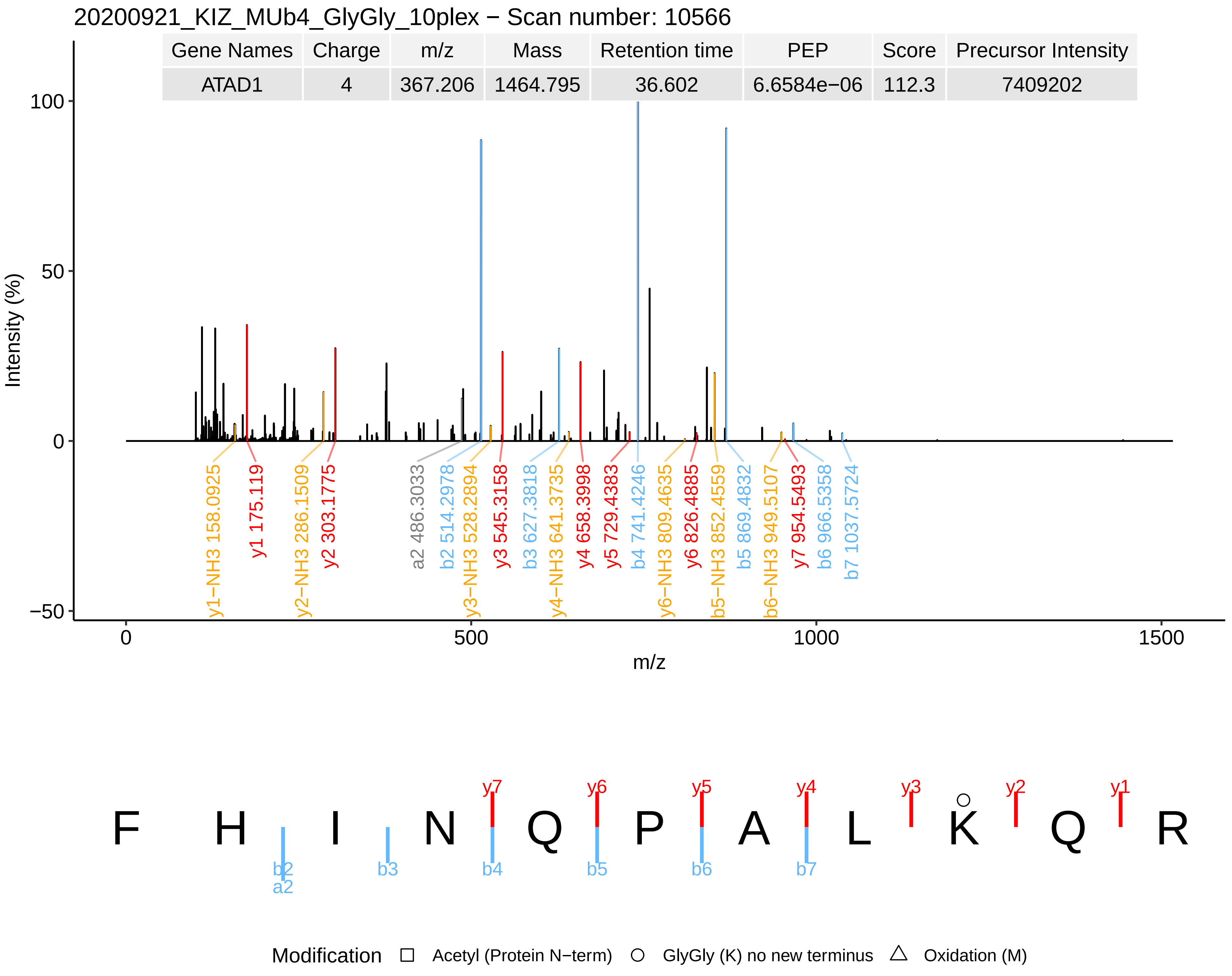
20200921_KIZ_MUb4_GlyGly_10plex − Scan number: 10566
Gene Names | Charge | m/z | Mass | Retention time | PEP | Score | Precursor Intensity
ATAD1 | 4 | 367.206 | 1464.795 | 36.602 | 6.6584e−06 | 112.3 | 7409202
Intensity (%)
100
50
0
−50
0
500
1000
1500
m/z
y1−NH3 158.0925
y1 175.119
y2−NH3 286.1509
y2 303.1775
a2 486.3033
b2 514.2978
y3−NH3 528.2894
y3 545.3158
b3 627.3818
y4−NH3 641.3735
y4 658.3998
y5 729.4383
b4 741.4246
y6−NH3 809.4635
y6 826.4885
b5−NH3 852.4559
b5 869.4832
b6−NH3 949.5107
y7 954.5493
b6 966.5358
b7 1037.5724
F H I N Q P A L K Q R
y7 y6 y5 y4 y3 y2 y1
b2 a2 b3 b4 b5 b6 b7
Modification
Acetyl (Protein N−term)
GlyGly (K) no new terminus
Oxidation (M)

20201118_KIZ_MUb6_GlyGly_1 − Scan number: 22087

Gene Names	Charge	m/z	Mass	Retention time	PEP	Score	Precursor Intensity
NPM1	3	492.2468	1473.719	68.711	7.8962e−06	113.71	23458484

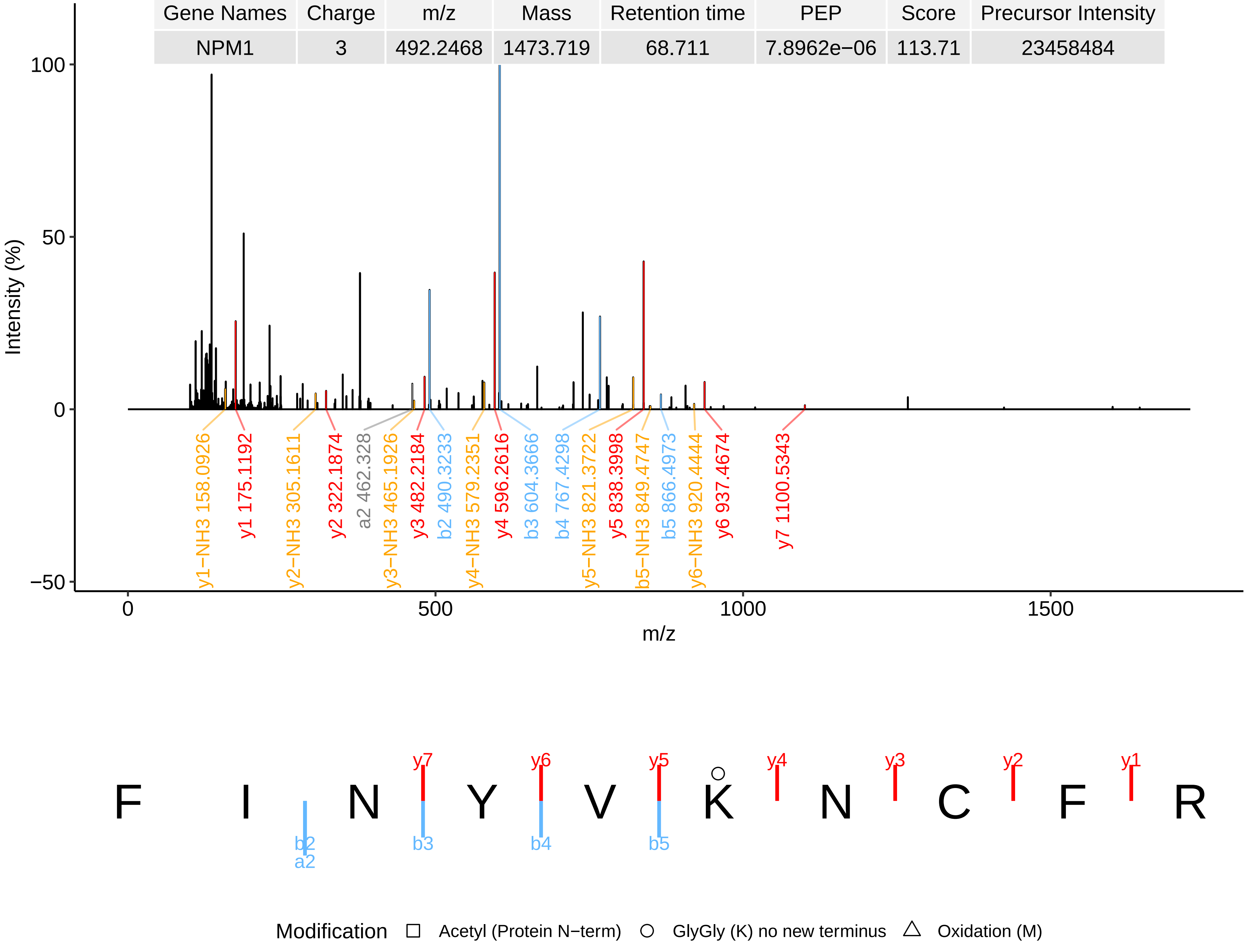

20200921_KIZ_MUb4_GlyGly_10plex − Scan number: 7868

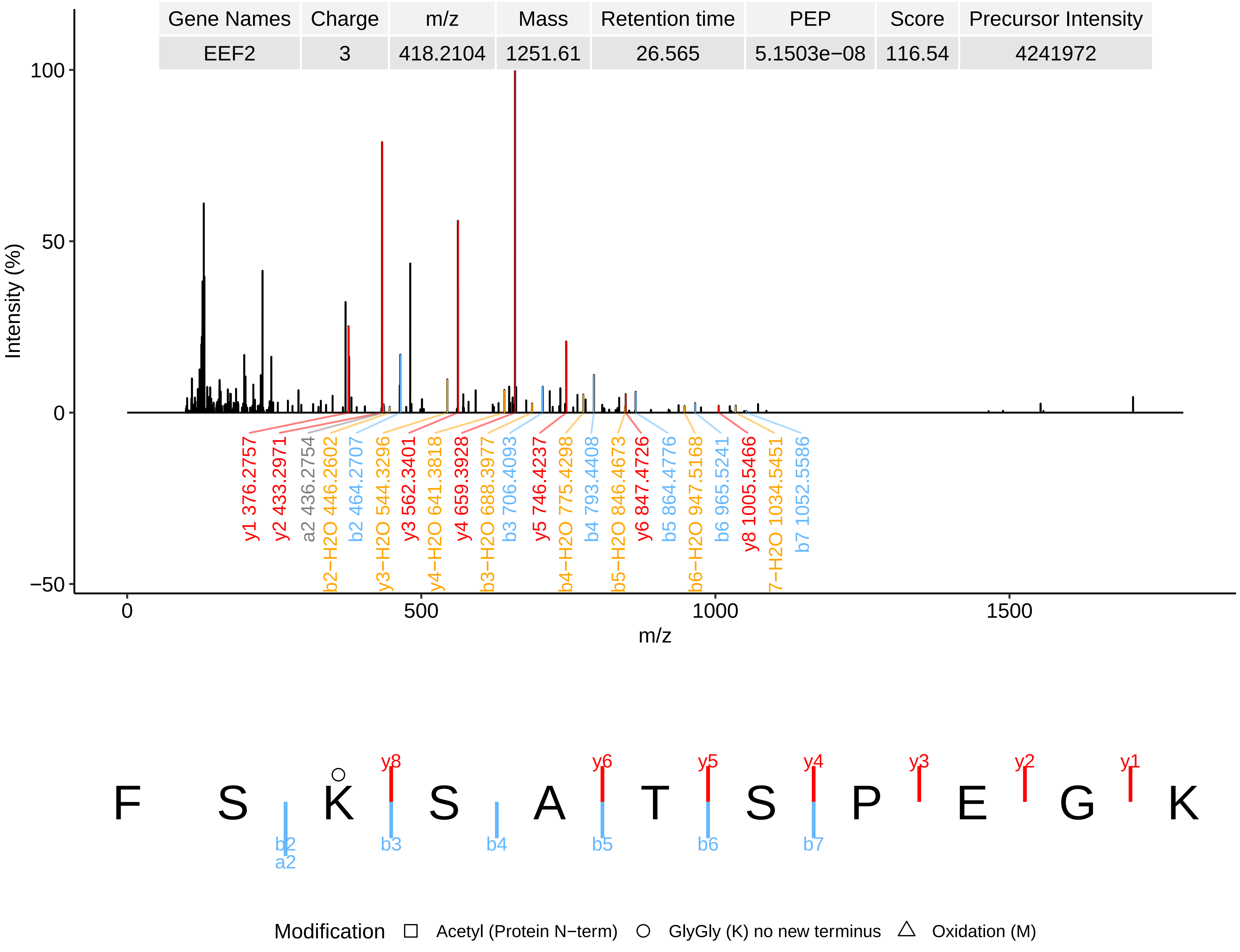

20200921_KIZ_MUb4_GlyGly_10plex_2 − Scan number: 7276

Gene Names	Charge	m/z	Mass	Retention time	PEP	Score	Precursor Intensity
EEF2	3	418.2104	1251.61	27.089	0.010888	69.451	1341150

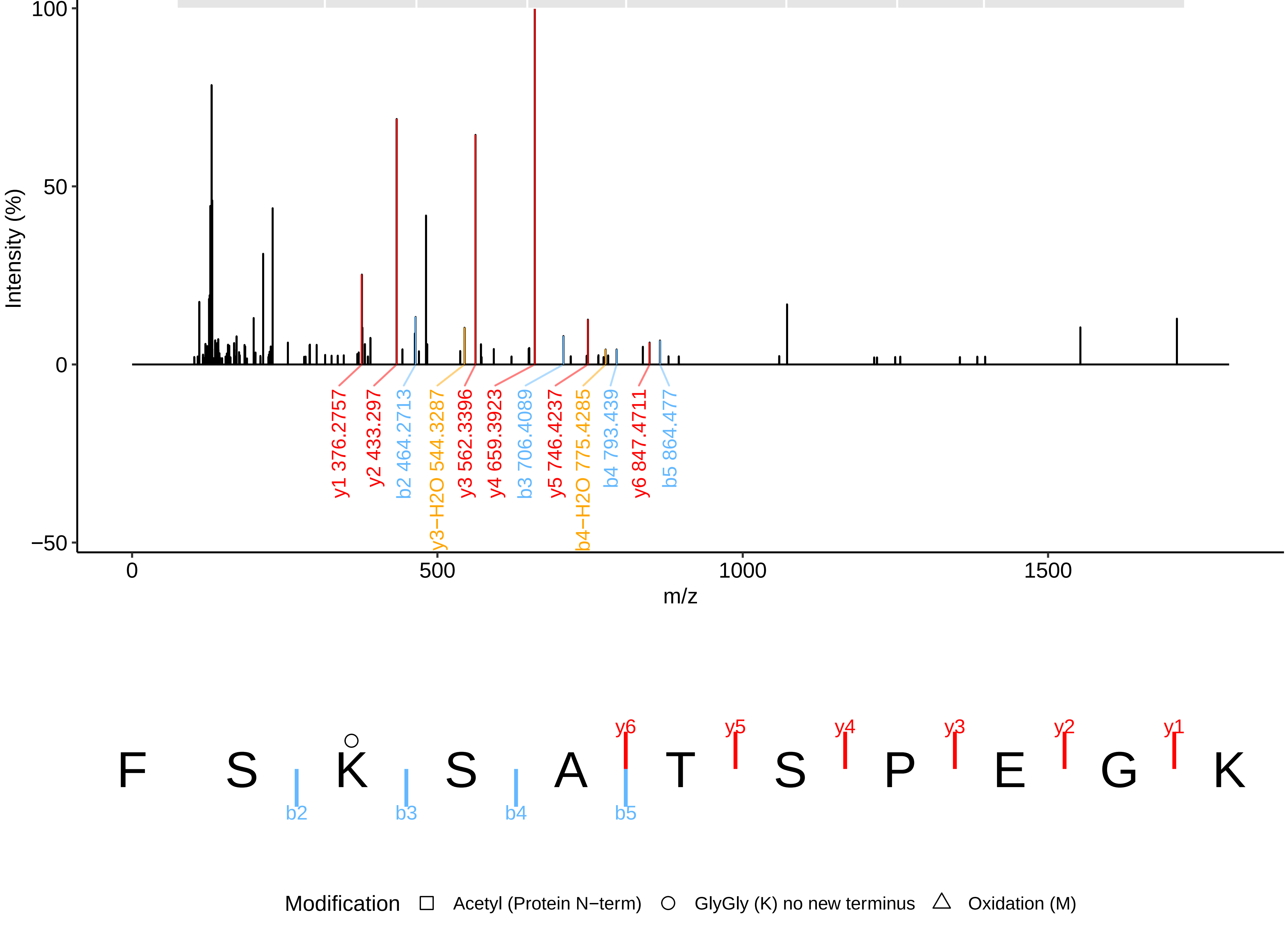

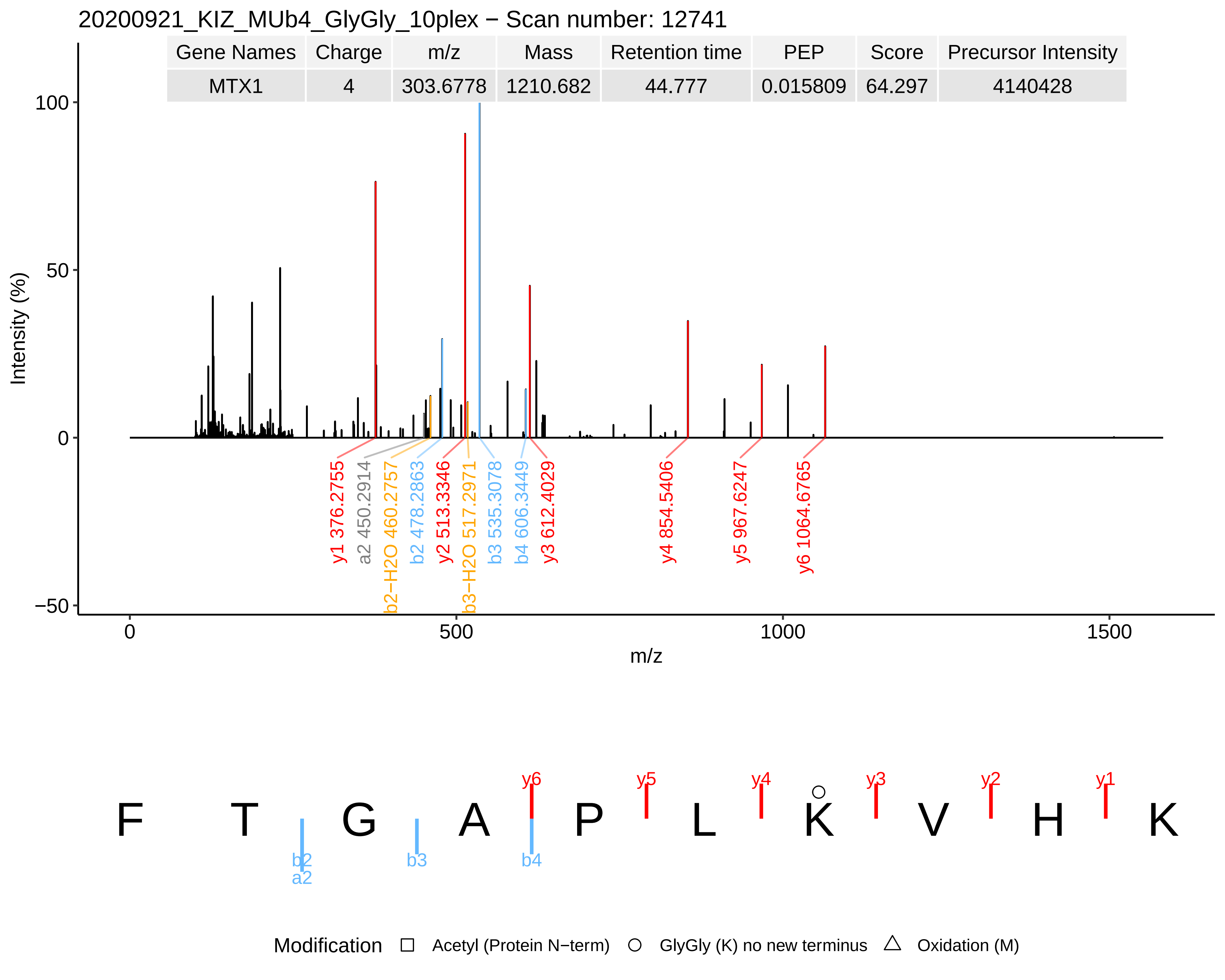
20200921_KIZ_MUb4_GlyGly_10plex − Scan number: 12741
Gene Names | Charge | m/z | Mass | Retention time | PEP | Score | Precursor Intensity
MTX1 | 4 | 303.6778 | 1210.682 | 44.777 | 0.015809 | 64.297 | 4140428
Intensity (%)
100
50
0
−50
0
500
1000
1500
m/z
y1 376.2755
a2 450.2914
b2−H2O 460.2757
b2 478.2863
y2 513.3346
b3−H2O 517.2971
b3 535.3078
b4 606.3449
y3 612.4029
y4 854.5406
y5 967.6247
y6 1064.6765
F T G A P L K V H K
y6
y5
y4
y3
y2
y1
b2
a2
b3
b4
Modification
Acetyl (Protein N−term)
GlyGly (K) no new terminus
Oxidation (M)

20200921_KIZ_MUb4_GlyGly_10plex − Scan number: 10357

Gene Names	Charge	m/z	Mass	Retention time	PEP	Score	Precursor Intensity
SPG20	3	410.5665	1228.678	35.827	0.00022138	96.171	6030862

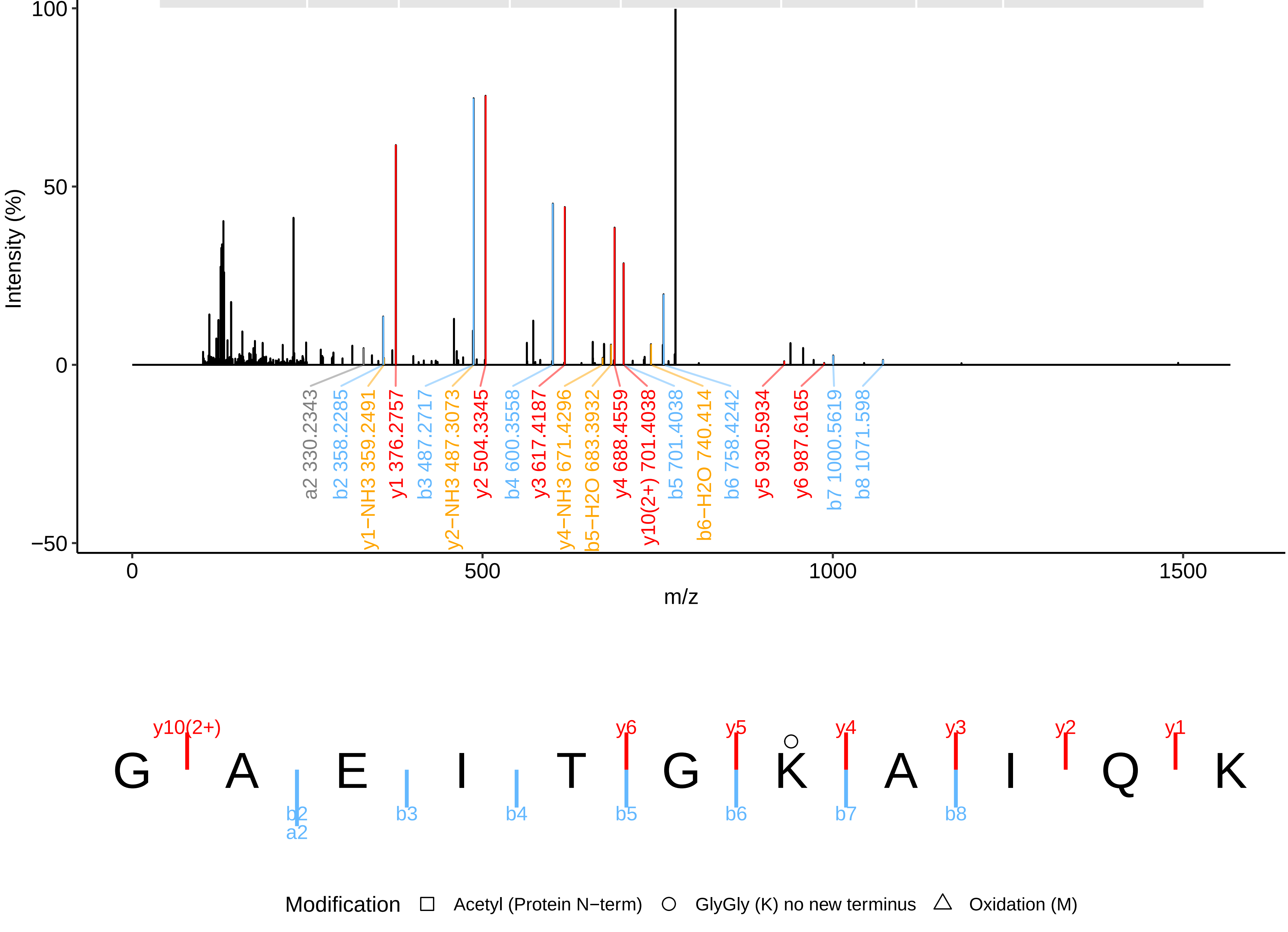

20200921_KIZ_MUb4_GlyGly_10plex − Scan number: 12617

Gene Names	Charge	m/z	Mass	Retention time	PEP	Score	Precursor Intensity
VPS13A	3	418.2193	1251.636	44.313	0.044092	57.164	7767182

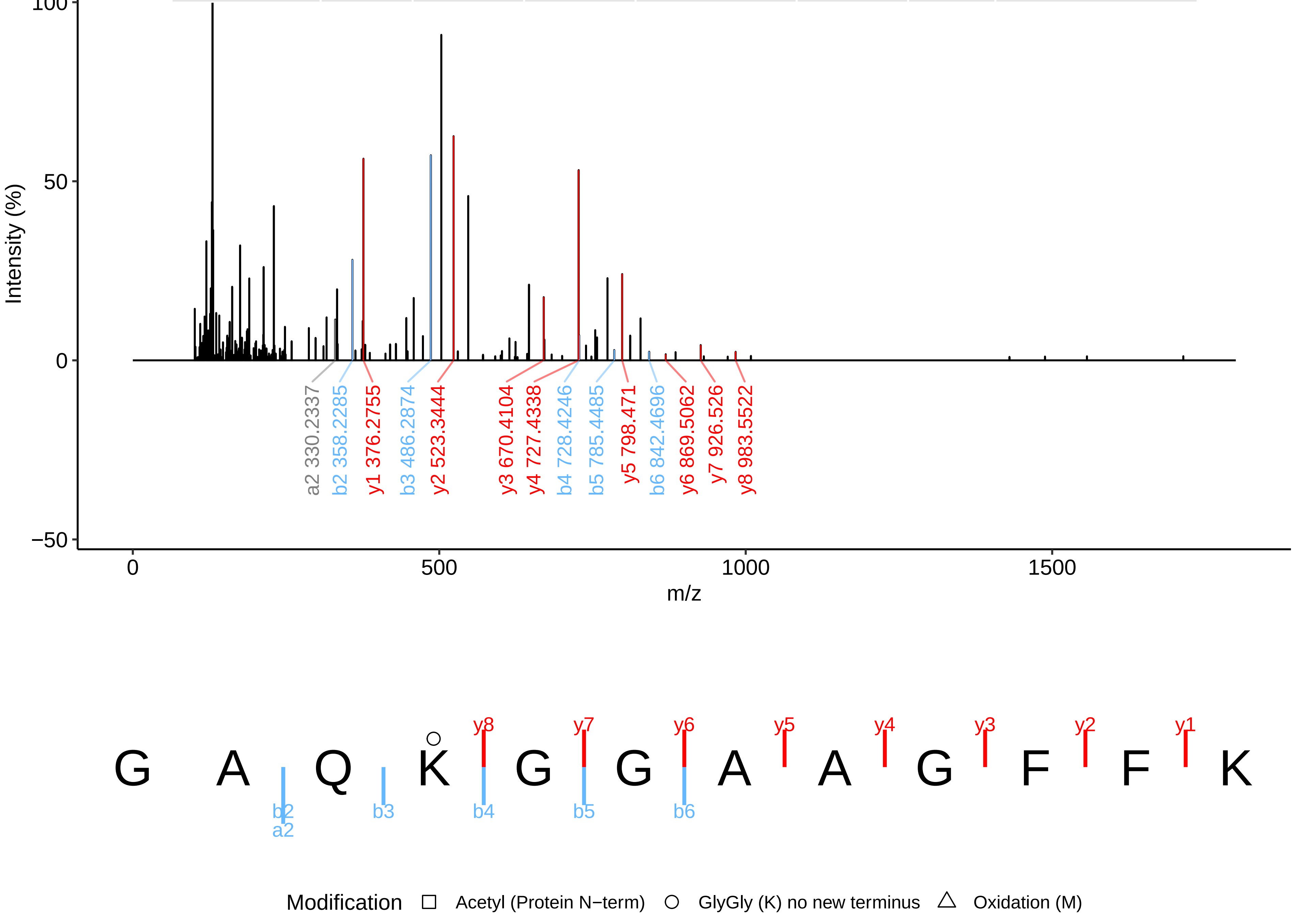

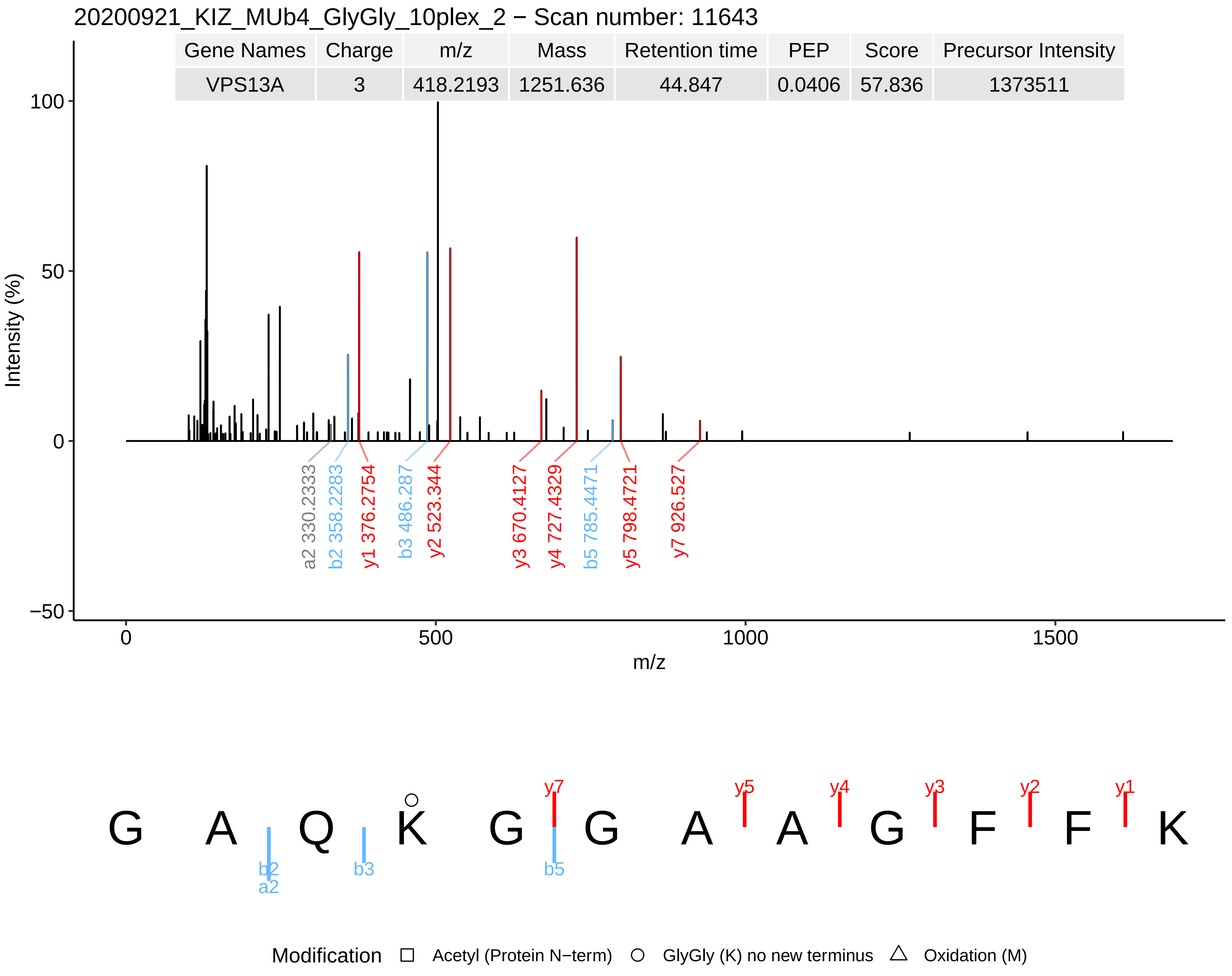
20200921_KIZ_MUb4_GlyGly_10plex_2 − Scan number: 11643
Gene Names | Charge | m/z | Mass | Retention time | PEP | Score | Precursor Intensity
VPS13A | 3 | 418.2193 | 1251.636 | 44.847 | 0.0406 | 57.836 | 1373511
Intensity (%)
m/z
a2 330.2333
b2 358.2283
y1 376.2754
b3 486.287
y2 523.344
y3 670.4127
y4 727.4329
b5 785.4471
y5 798.4721
y7 926.527
G A Q K G G A A G F F K
Modification
Acetyl (Protein N−term)
GlyGly (K) no new terminus
Oxidation (M)

20200921_KIZ_MUb4_GlyGly_10plex − Scan number: 3471

Gene Names	Charge	m/z	Mass	Retention time	PEP	Score	Precursor Intensity
GNAS	3	493.5599	1477.658	11.02	1.9319e−05	99.215	1306317

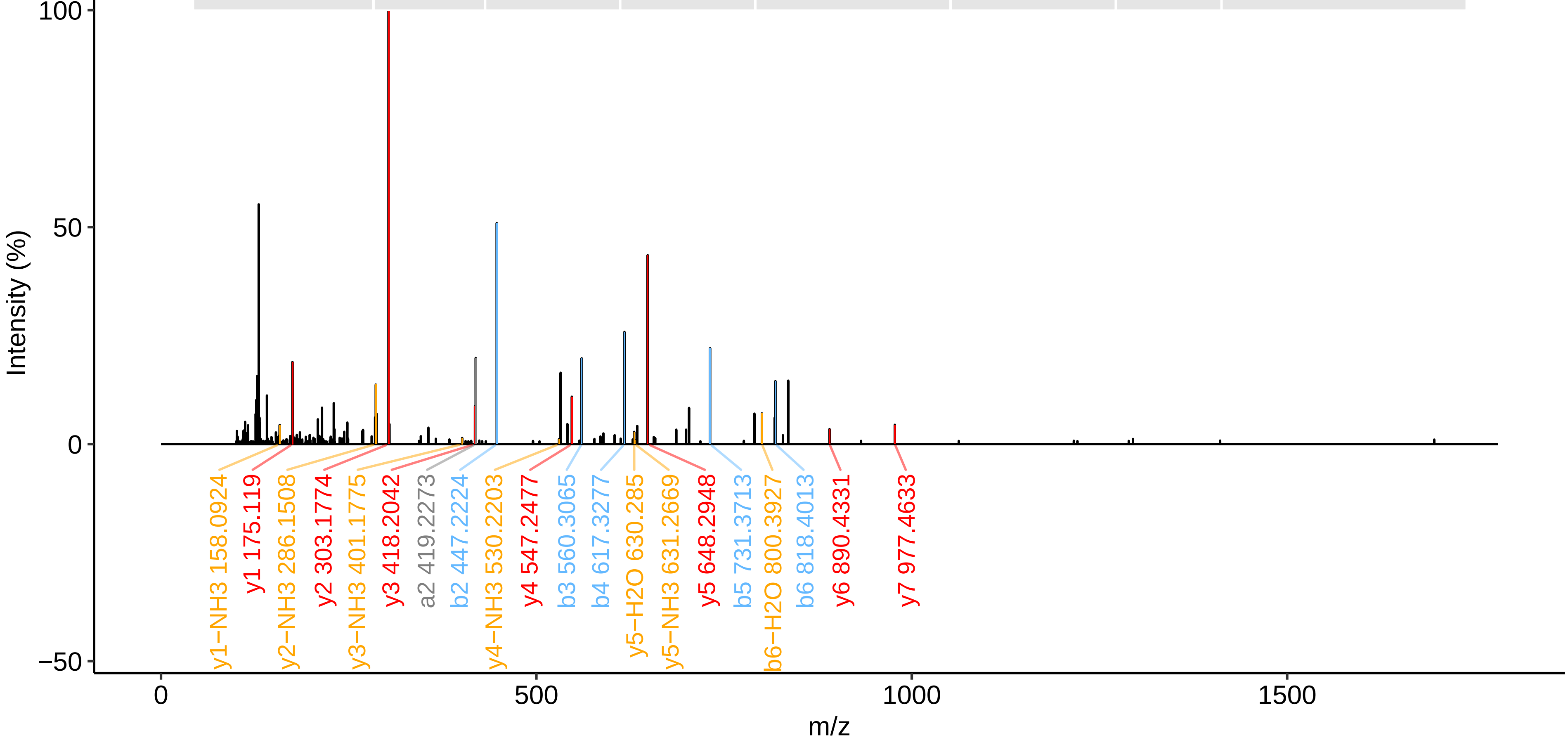

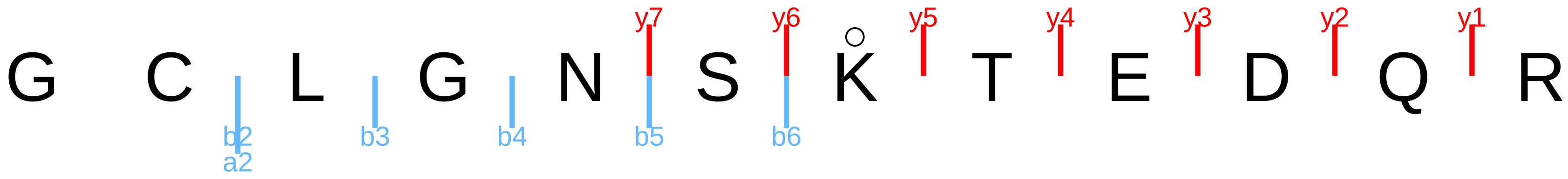

Modification □ Acetyl (Protein N−term) ○ GlyGly (K) no new terminus △ Oxidation (M)

20201118_KIZ_MUb6_GlyGly_1 − Scan number: 3129

Gene Names	Charge	m/z	Mass	Retention time	PEP	Score	Precursor Intensity
GNAS	3	493.5599	1477.658	9.6465	8.4926e−09	122.51	8137399

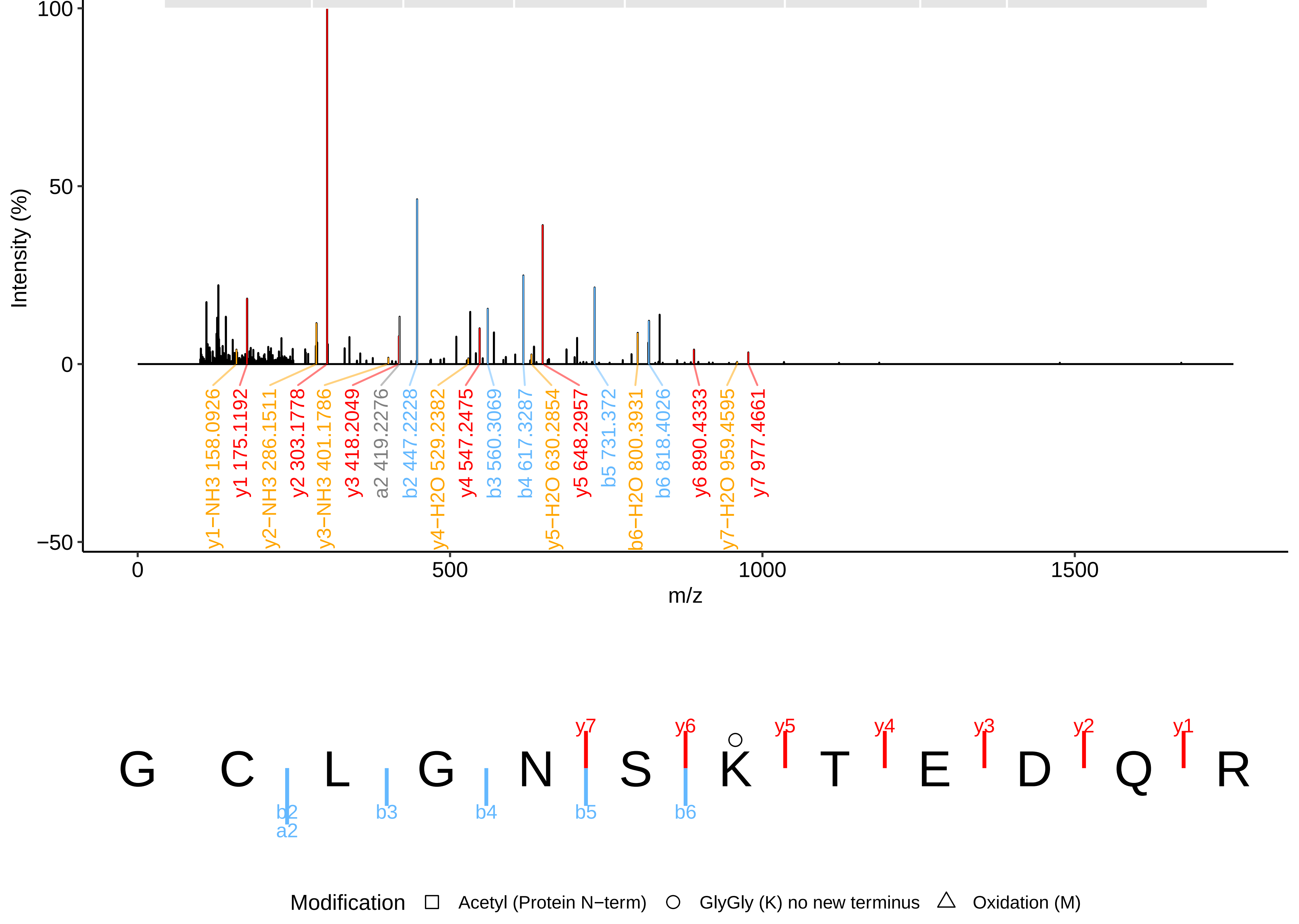

20201118_KIZ_MUb6_GlyGly_2 − Scan number: 3394

Gene Names	Charge	m/z	Mass	Retention time	PEP	Score	Precursor Intensity
GNAS	3	493.5599	1477.658	11.172	4.727e−05	99.215	NA

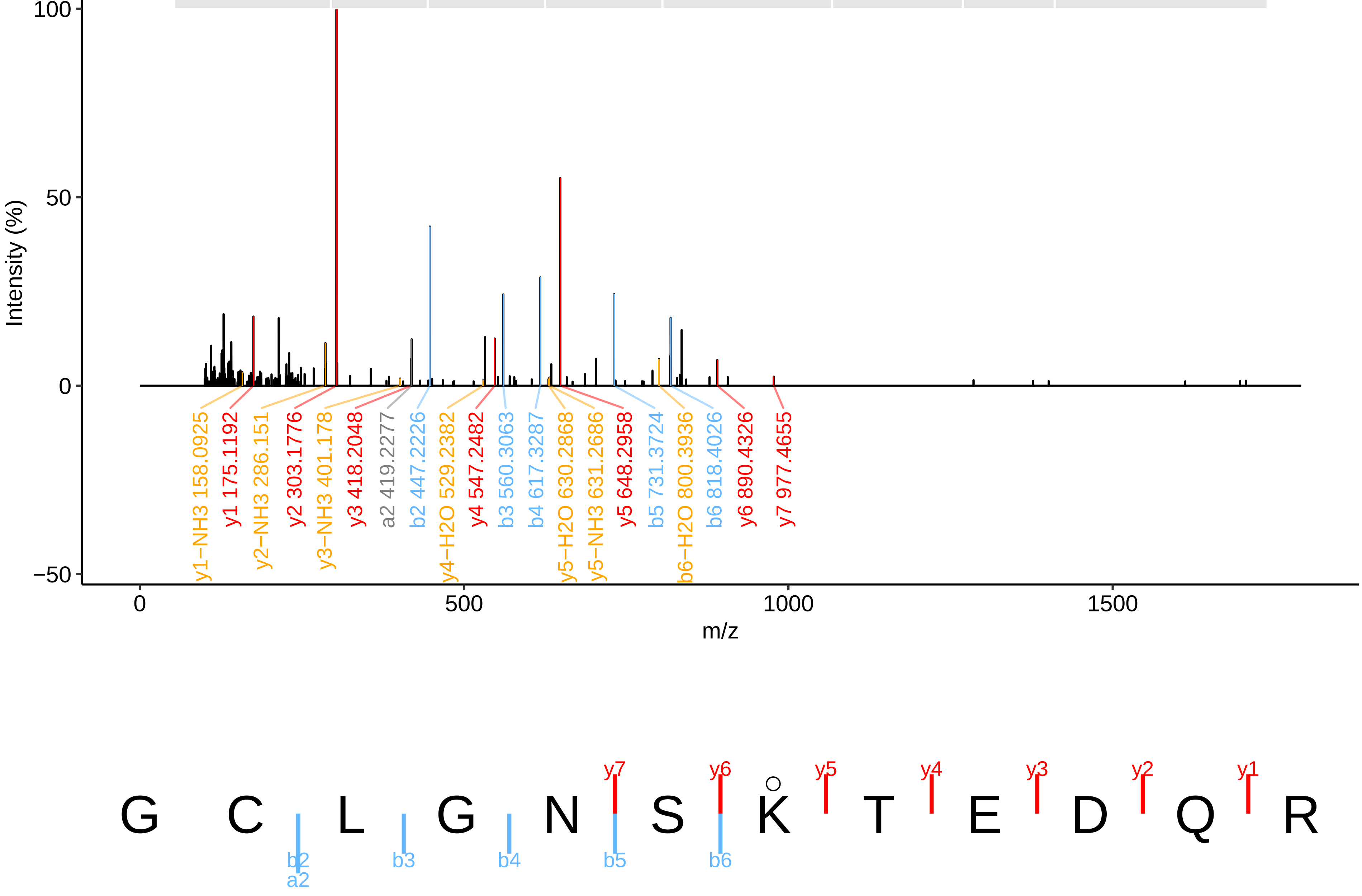

Modification □ Acetyl (Protein N−term) ○ GlyGly (K) no new terminus △ Oxidation (M)

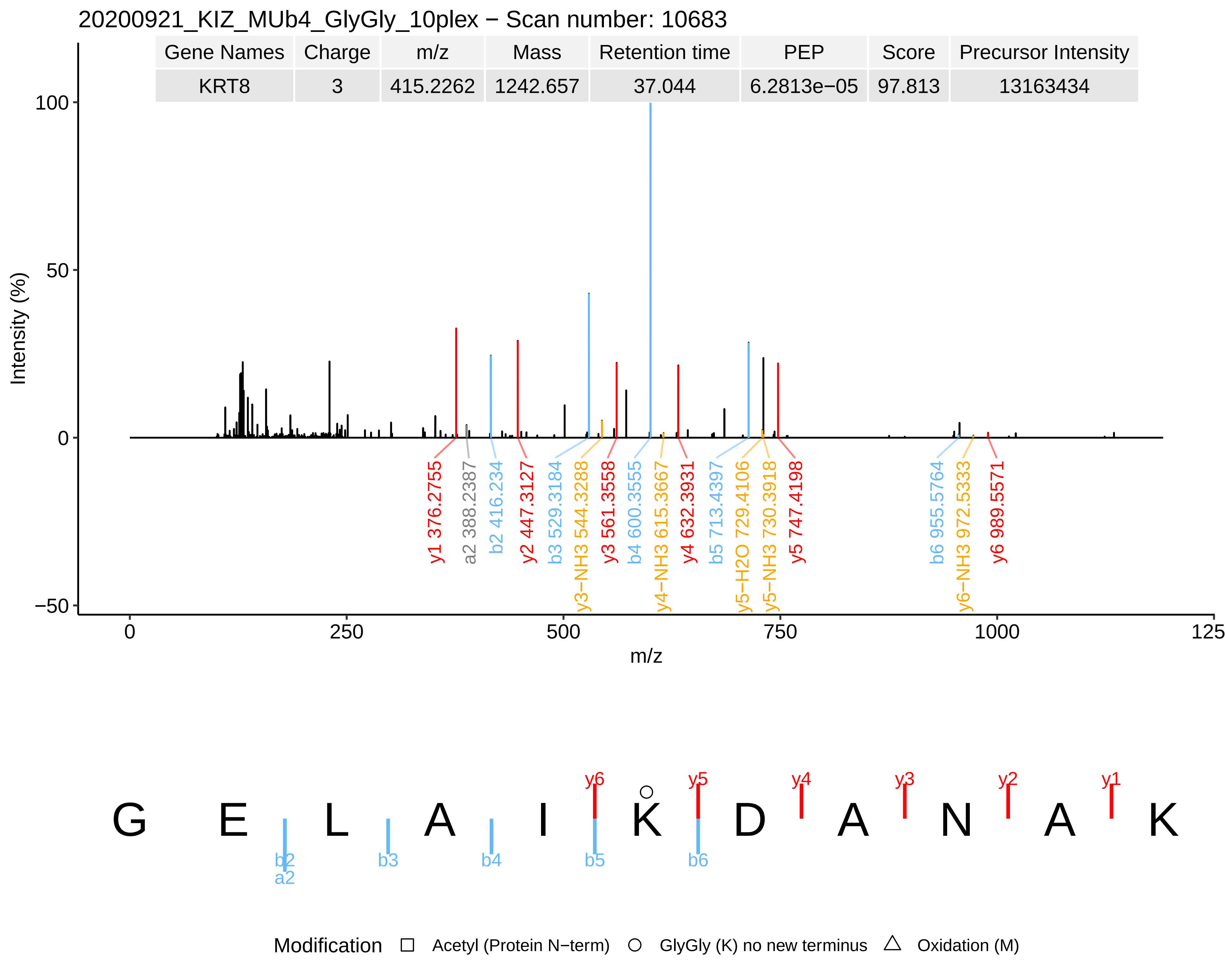
20200921_KIZ_MUb4_GlyGly_10plex − Scan number: 10683
Gene Names | Charge | m/z | Mass | Retention time | PEP | Score | Precursor Intensity
KRT8 | 3 | 415.2262 | 1242.657 | 37.044 | 6.2813e−05 | 97.813 | 13163434
Intensity (%)
m/z
y1 376.2755
a2 388.2387
b2 416.234
y2 447.3127
b3 529.3184
y3−NH3 544.3288
y3 561.3558
b4 600.3555
y4−NH3 615.3667
y4 632.3931
b5 713.4397
y5−H2O 729.4106
y5−NH3 730.3918
y5 747.4198
b6 955.5764
y6−NH3 972.5333
y6 989.5571
G E L A I K D A N A K
Modification Acetyl (Protein N−term) GlyGly (K) no new terminus Oxidation (M)

20200921_KIZ_MUb4_GlyGly_10plex − Scan number: 10568

Gene Names	Charge	m/z	Mass	Retention time	PEP	Score	Precursor Intensity
RAD23B	3	411.2192	1230.636	36.61	0.01586	63.408	3794529

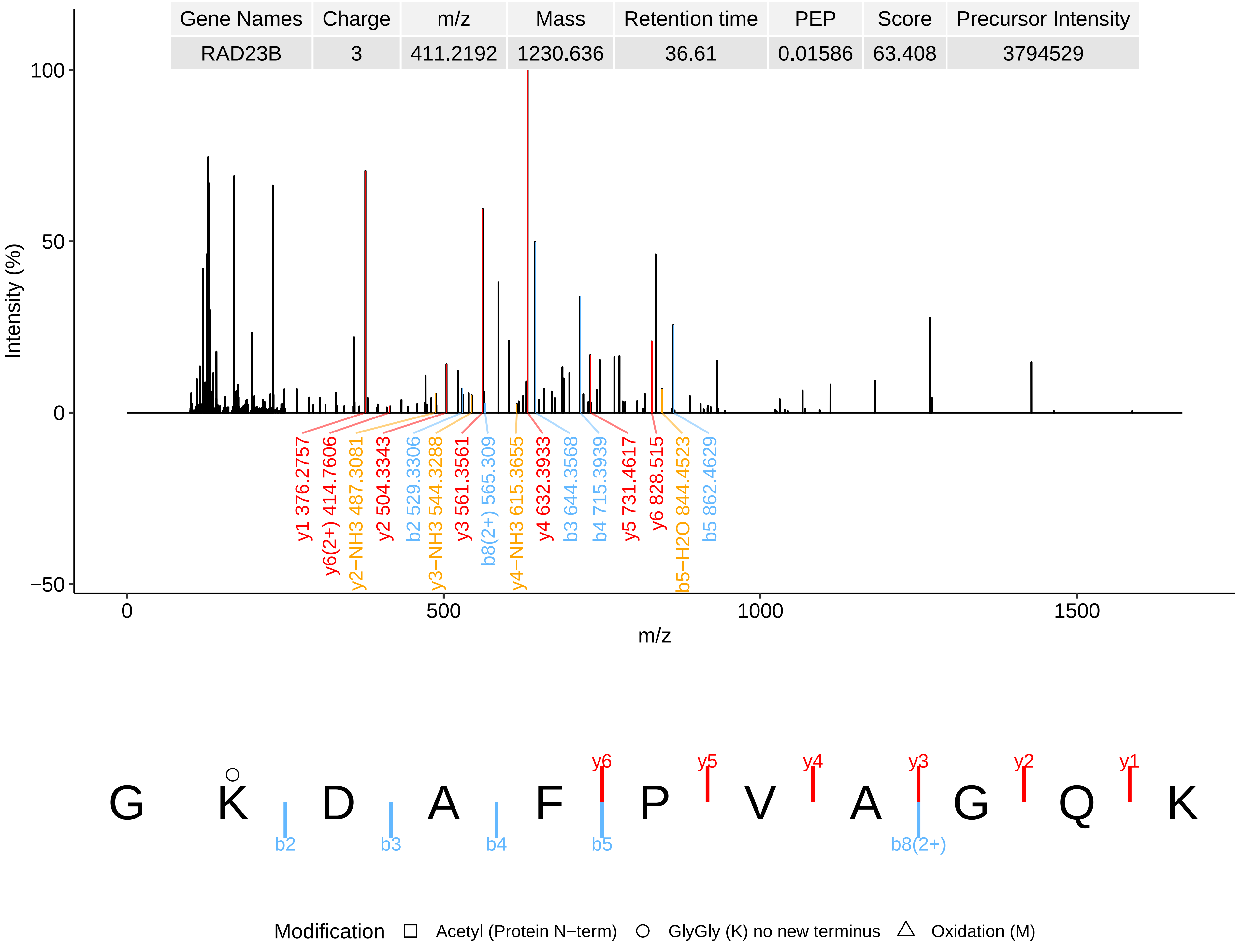

20200921_KIZ_MUb4_GlyGly_10plex − Scan number: 10570

Gene Names	Charge	m/z	Mass	Retention time	PEP	Score	Precursor Intensity
RAD23B	3	411.2192	1230.636	36.619	0.11364	43.897	6433136

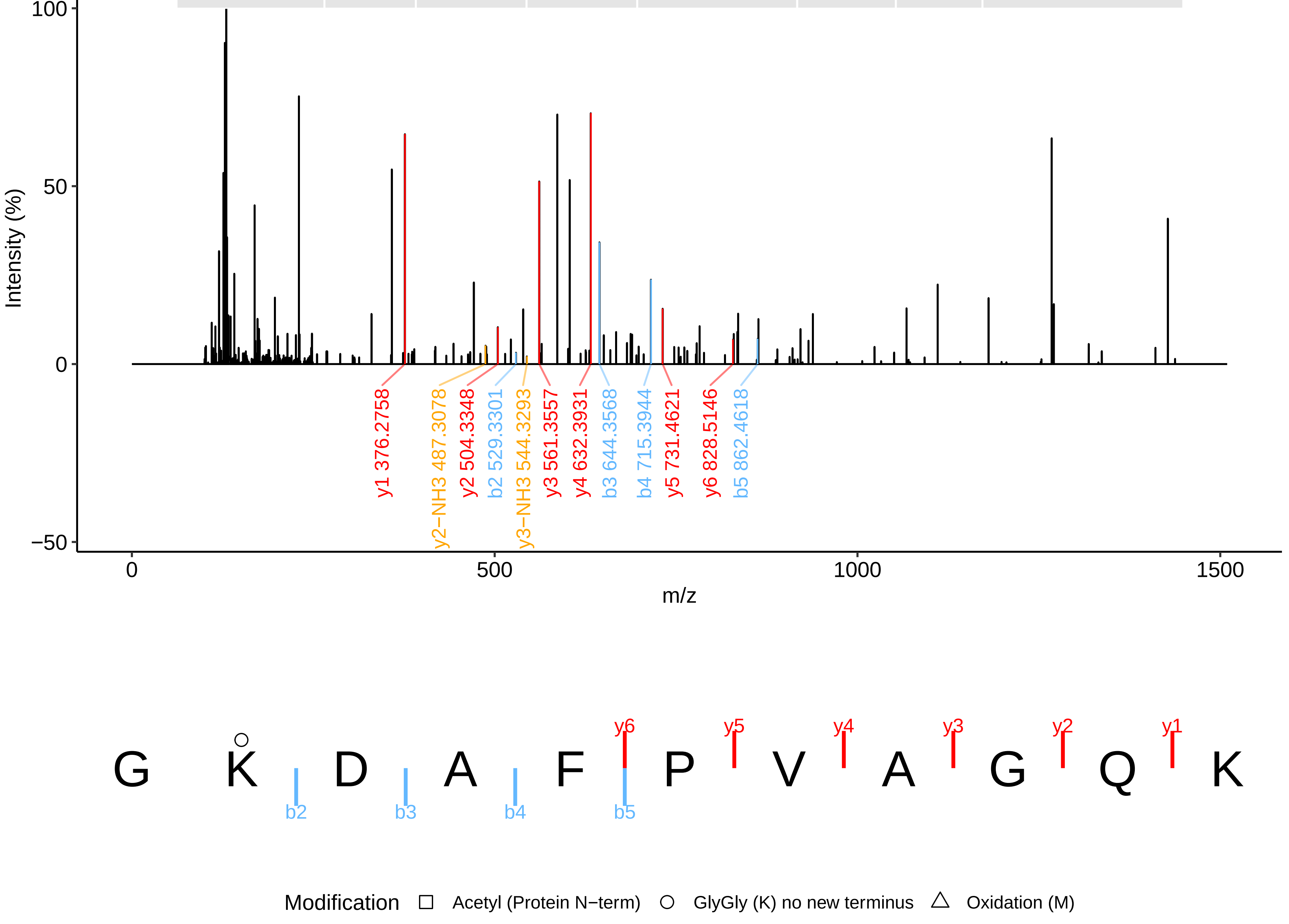

20200921_KIZ_MUb4_GlyGly_10plex_2 − Scan number: 9778

Gene Names	Charge	m/z	Mass	Retention time	PEP	Score	Precursor Intensity
RAD23B	3	411.2192	1230.636	37.278	0.01586	63.408	1384286

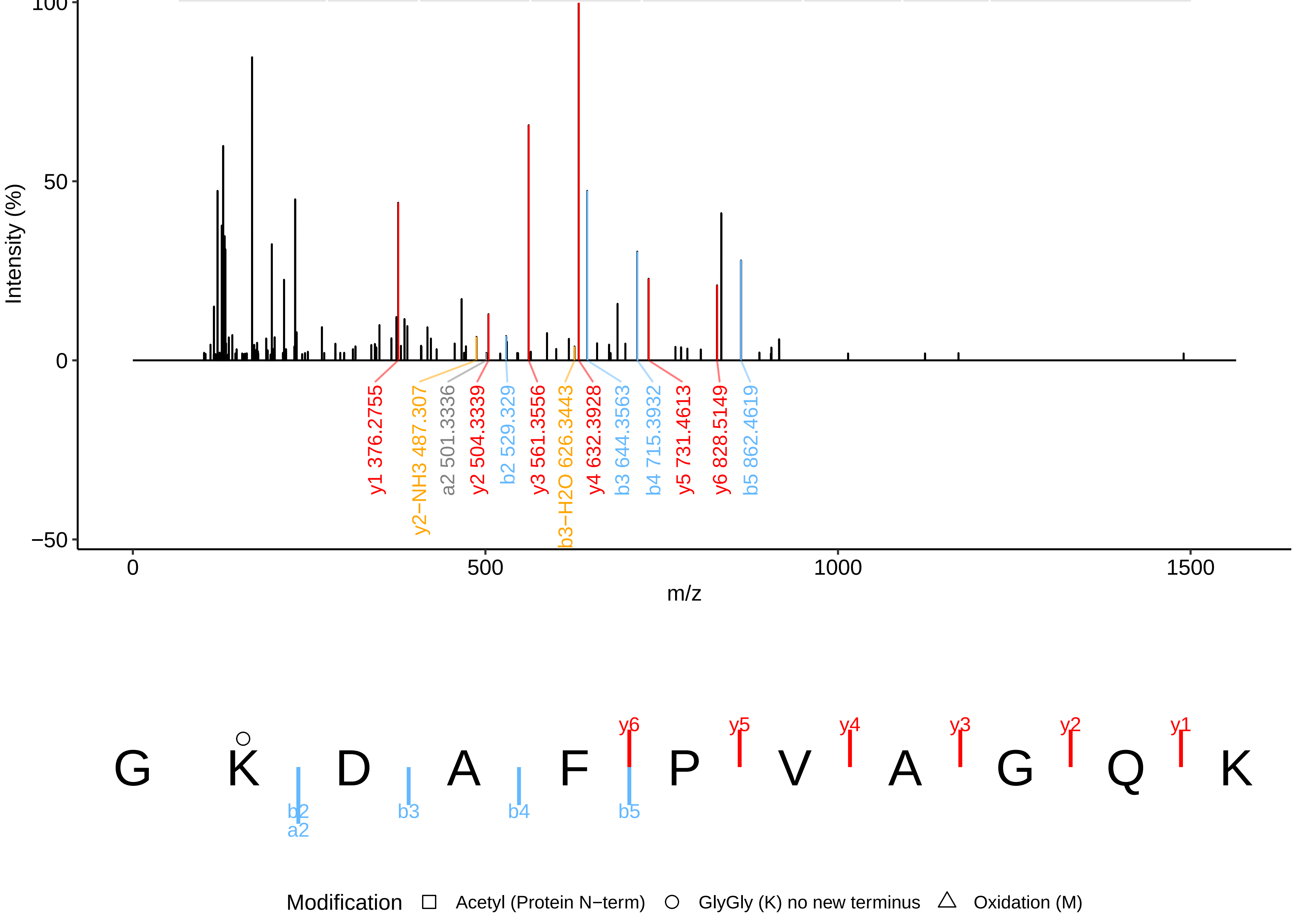

20200924_KIZ_MUb5_GlyGly_1 − Scan number: 9540

Gene Names	Charge	m/z	Mass	Retention time	PEP	Score	Precursor Intensity
RAD23B	3	411.2192	1230.636	30.687	0.016507	68.069	9101368

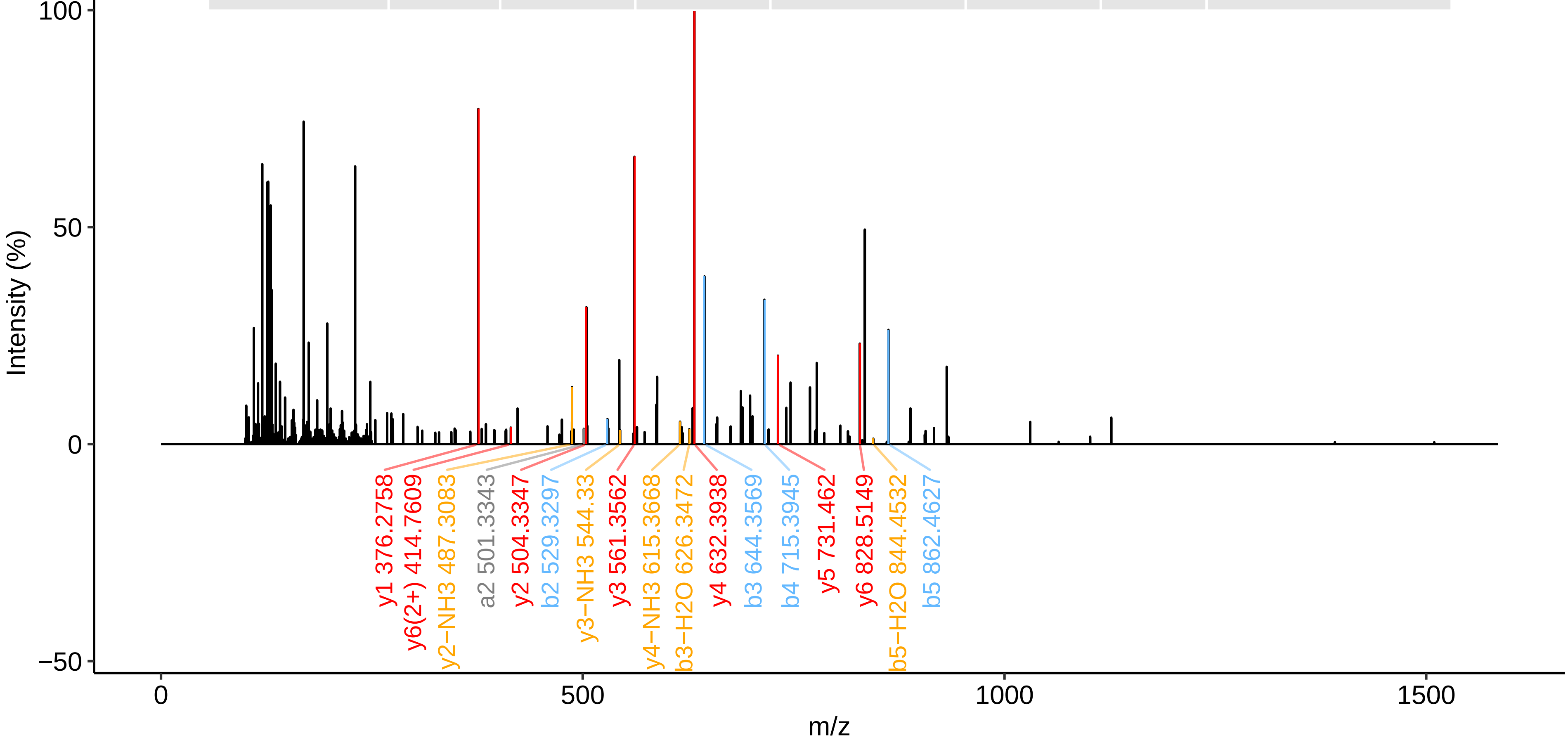

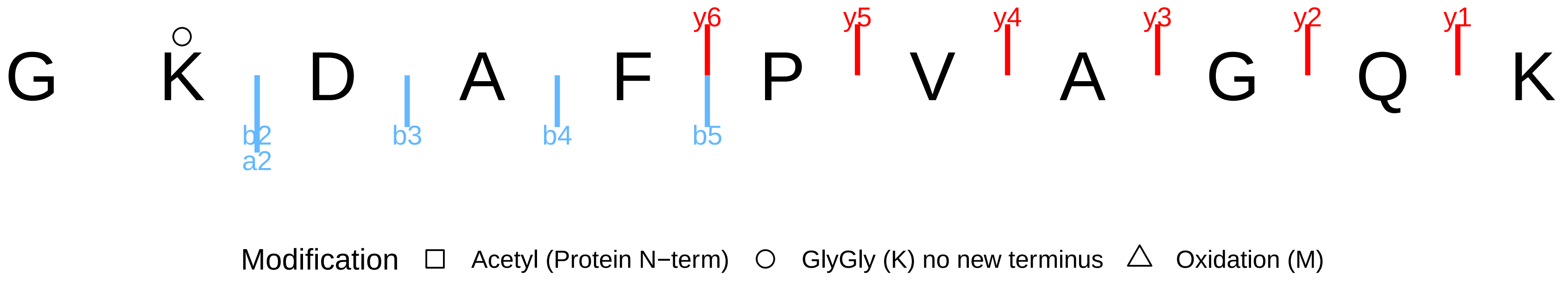

20200921_KIZ_MUb4_GlyGly_10plex − Scan number: 9233

Gene Names	Charge	m/z	Mass	Retention time	PEP	Score	Precursor Intensity
TM7SF3	3	491.9197	1472.737	31.597	2.0209e−05	110.93	3982912

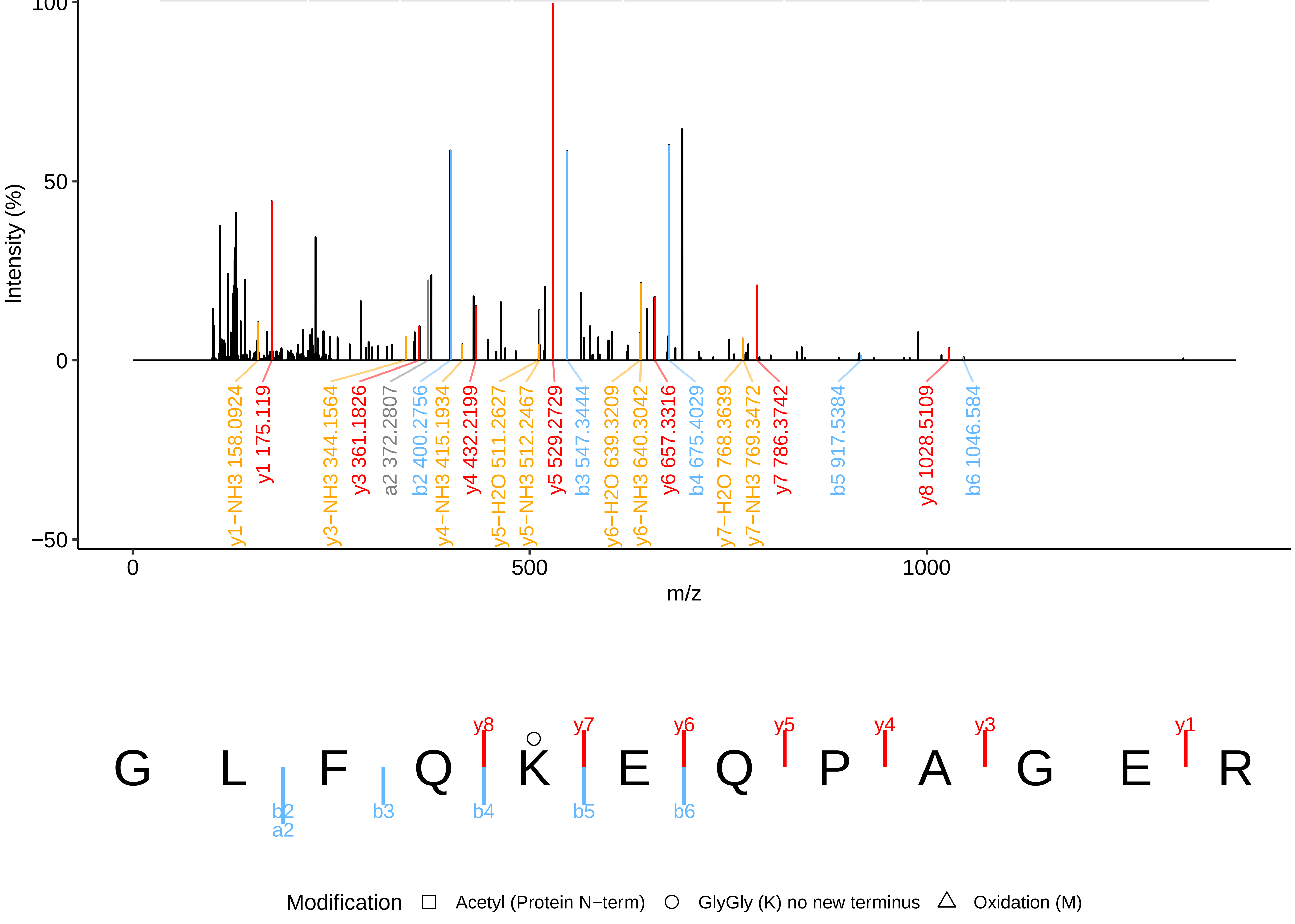

20200921_KIZ_MUb4_GlyGly_10plex − Scan number: 13849

Gene Names	Charge	m/z	Mass	Retention time	PEP	Score	Precursor Intensity
DPM1	3	479.2407	1434.7	48.991	5.0543e−05	108.56	2275913

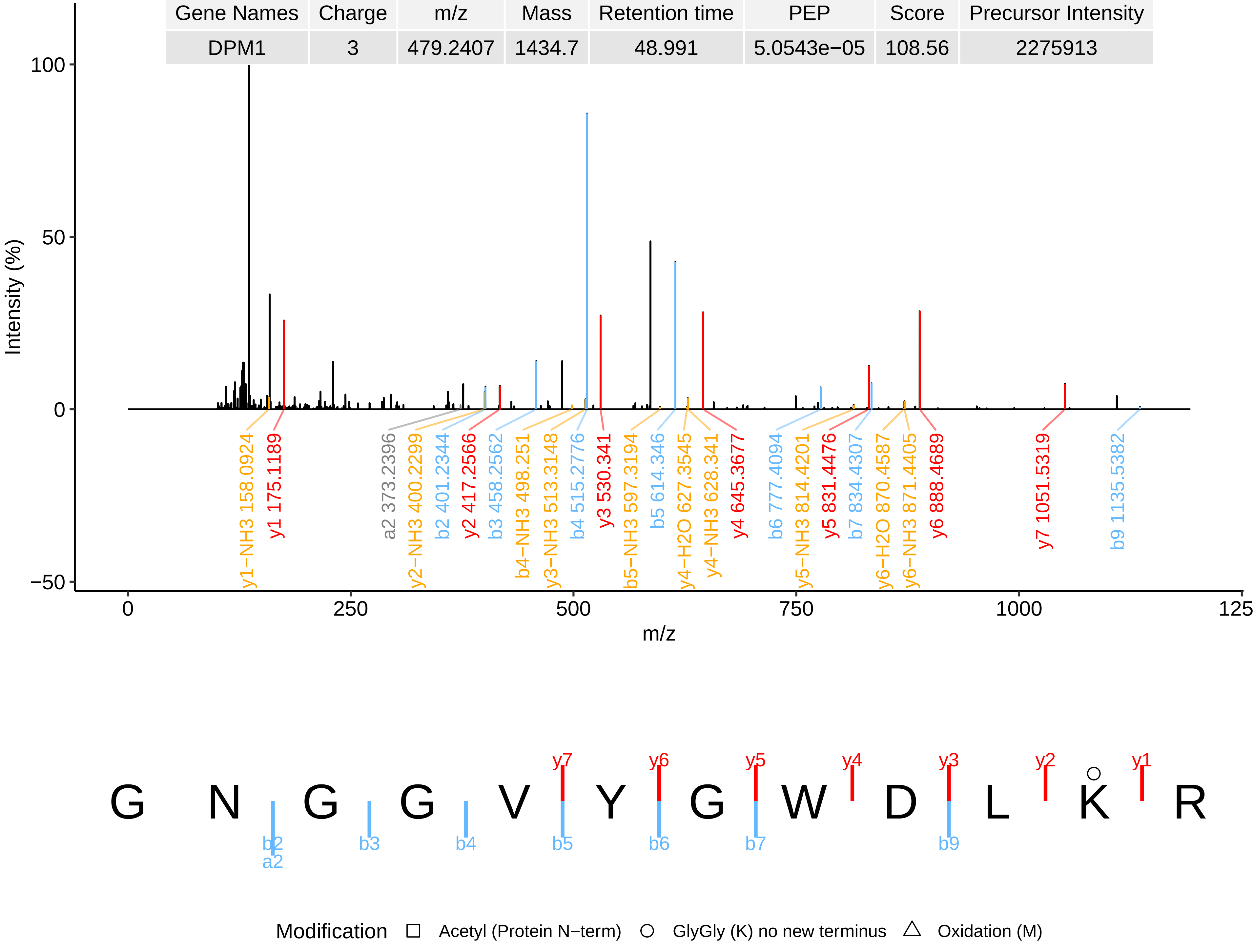

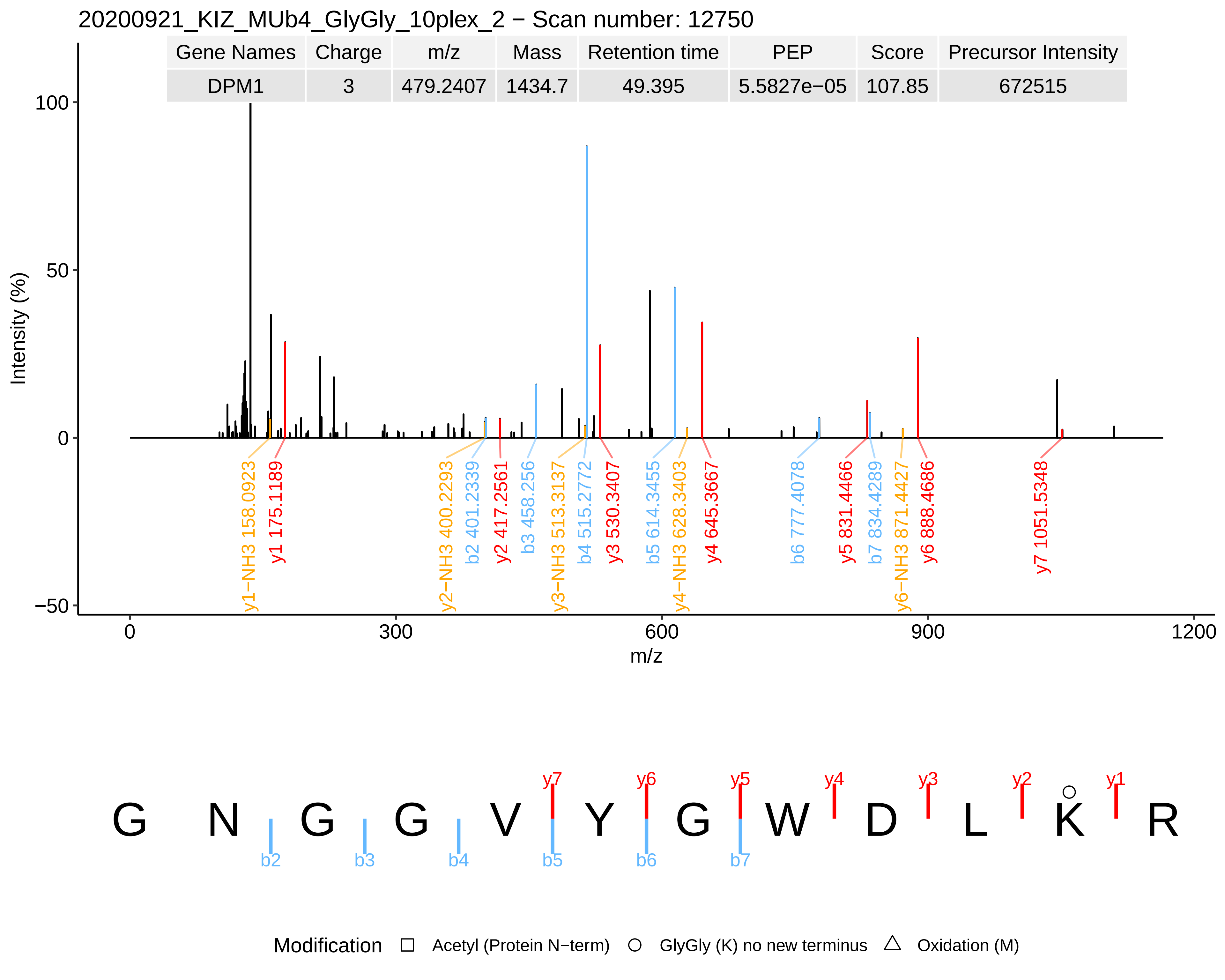
20200921_KIZ_MUb4_GlyGly_10plex_2 − Scan number: 12750
Gene Names | Charge | m/z | Mass | Retention time | PEP | Score | Precursor Intensity
DPM1 | 3 | 479.2407 | 1434.7 | 49.395 | 5.5827e−05 | 107.85 | 672515
Intensity (%)
100
50
0
−50
0
300
600
900
1200
m/z
y1−NH3 158.0923
y1 175.1189
y2−NH3 400.2293
b2 401.2339
y2 417.2561
b3 458.256
y3−NH3 513.3137
b4 515.2772
y3 530.3407
b5 614.3455
y4−NH3 628.3403
y4 645.3667
b6 777.4078
y5 831.4466
b7 834.4289
y6−NH3 871.4427
y6 888.4686
y7 1051.5348
G N G G V Y G W D L K R
y7 y6 y5 y4 y3 y2 y1
b2 b3 b4 b5 b6 b7
Modification
Acetyl (Protein N−term)
GlyGly (K) no new terminus
Oxidation (M)

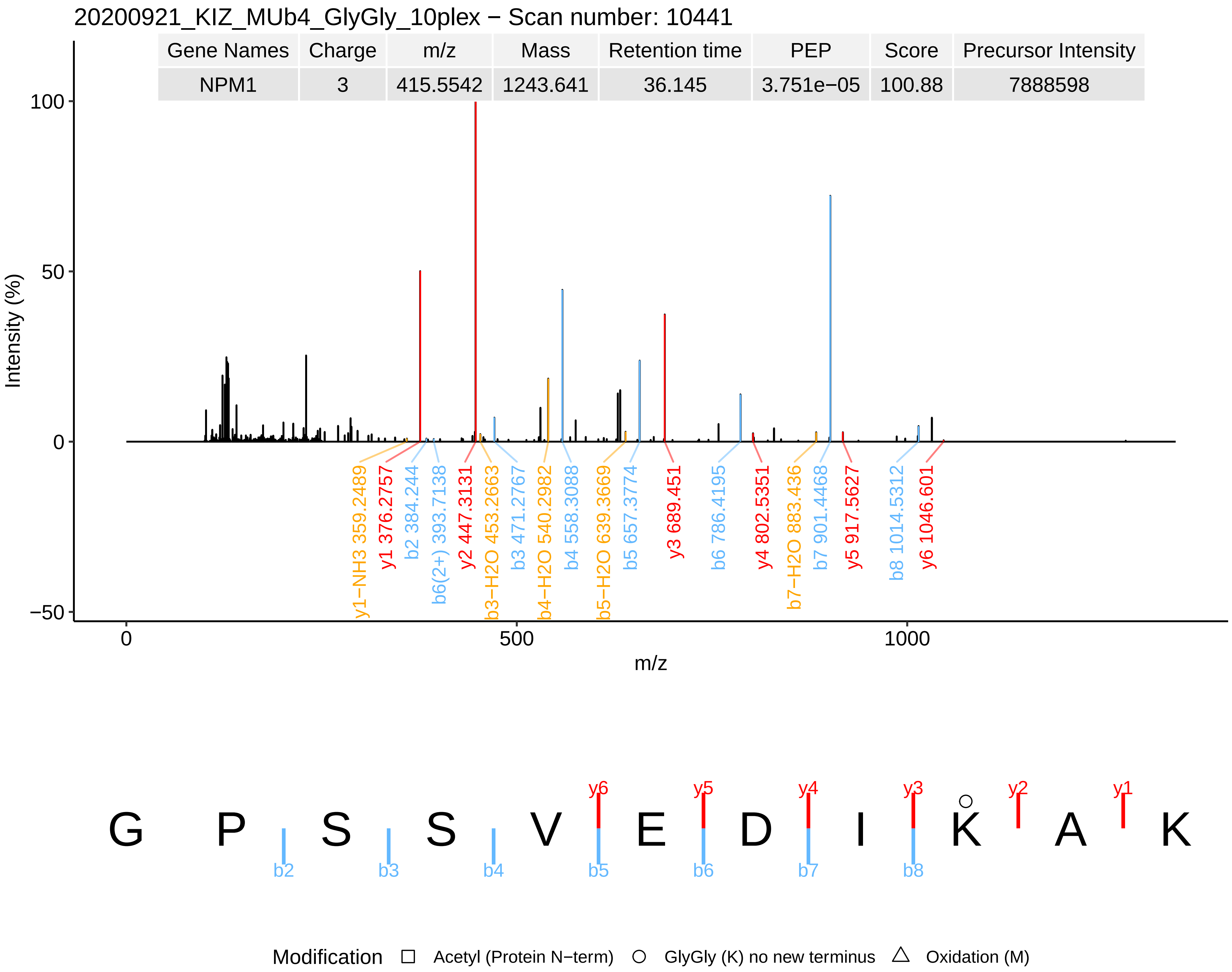
20200921_KIZ_MUb4_GlyGly_10plex − Scan number: 10441
Gene Names | Charge | m/z | Mass | Retention time | PEP | Score | Precursor Intensity
NPM1 | 3 | 415.5542 | 1243.641 | 36.145 | 3.751e−05 | 100.88 | 7888598
Intensity (%)
m/z
y1−NH3 359.2489
y1 376.2757
b2 384.244
b6(2+) 393.7138
y2 447.3131
b3−H2O 453.2663
b3 471.2767
b4−H2O 540.2982
b4 558.3088
b5−H2O 639.3669
b5 657.3774
y3 689.451
b6 786.4195
y4 802.5351
b7−H2O 883.436
b7 901.4468
y5 917.5627
b8 1014.5312
y6 1046.601
G P S S V E D I K A K
Modification □ Acetyl (Protein N−term) ○ GlyGly (K) no new terminus △ Oxidation (M)

20200921_KIZ_MUb4_GlyGly_10plex_2 − Scan number: 9651

Gene Names	Charge	m/z	Mass	Retention time	PEP	Score	Precursor Intensity
NPM1	3	415.5542	1243.641	36.759	2.8894e−05	104.22	2530329

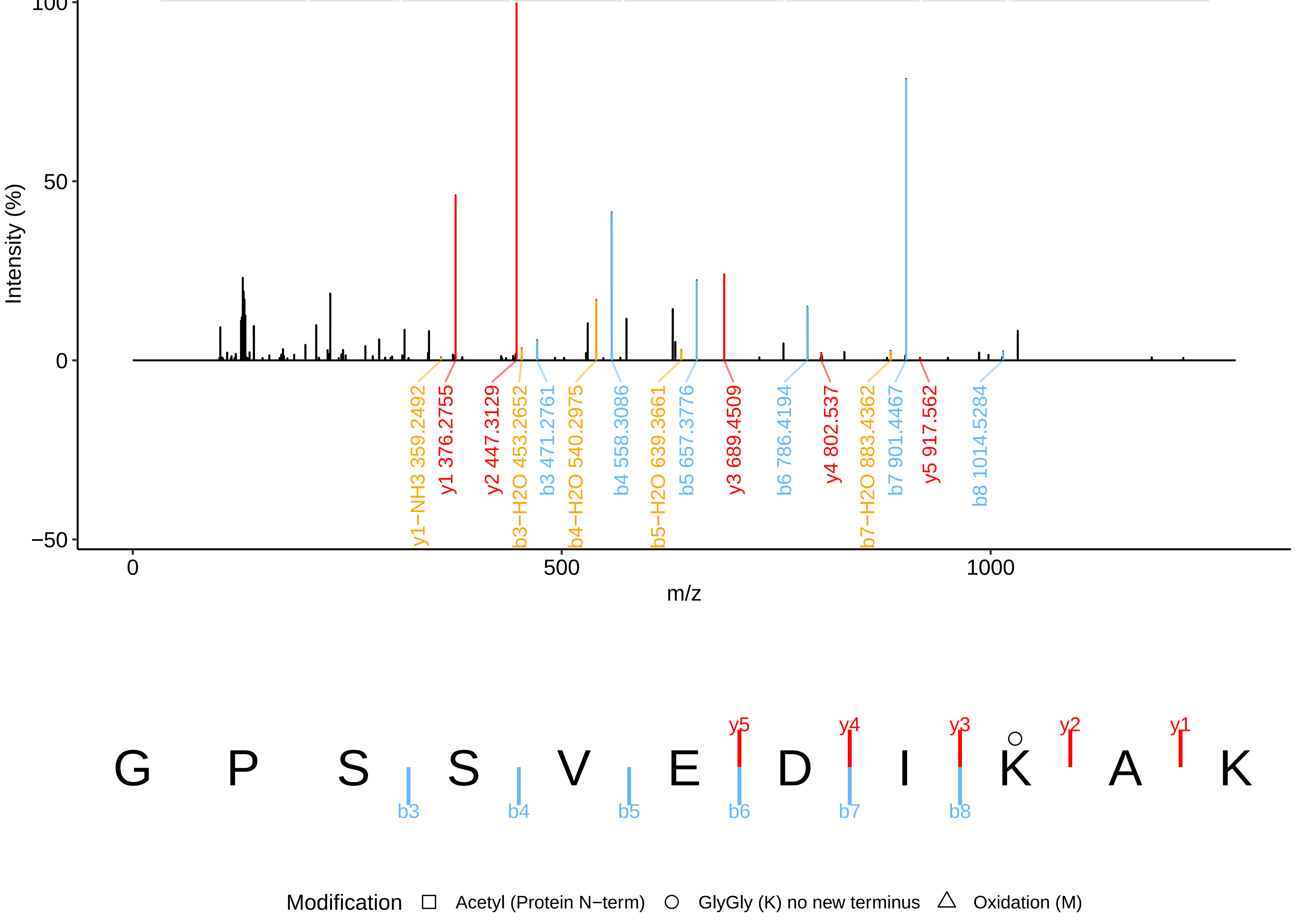

20200921_KIZ_MUb4_GlyGly_10plex − Scan number: 21654

Gene Names	Charge	m/z	Mass	Retention time	PEP	Score	Precursor Intensity
KCNJ9	2	739.3846	1476.755	78.46	0.097868	41.956	15934037

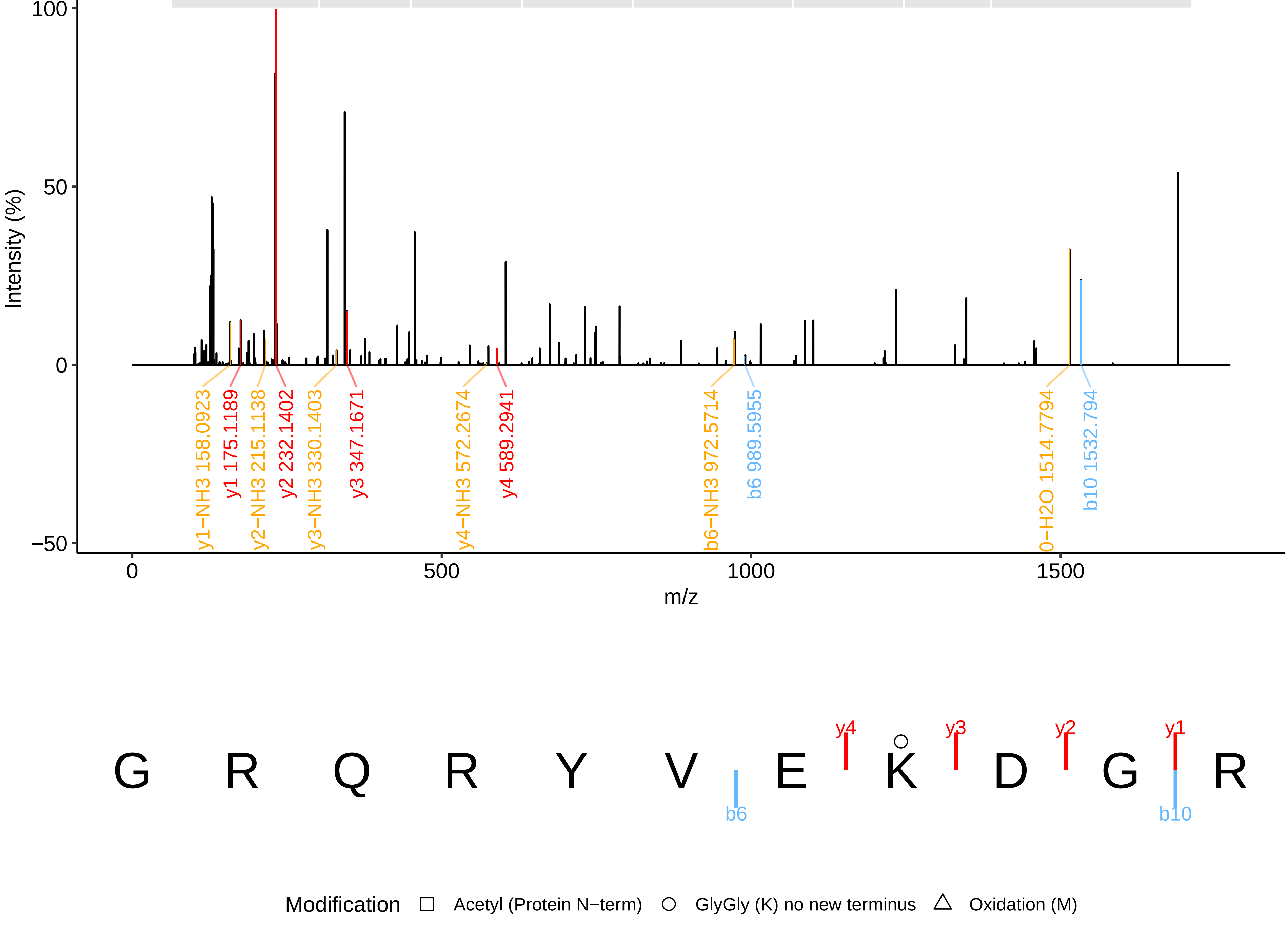

20200924_KIZ_MUb5_GlyGly_1 − Scan number: 21807

Gene Names	Charge	m/z	Mass	Retention time	PEP	Score	Precursor Intensity
KCNJ9	2	739.3846	1476.755	70.236	0.072609	48.794	16128538

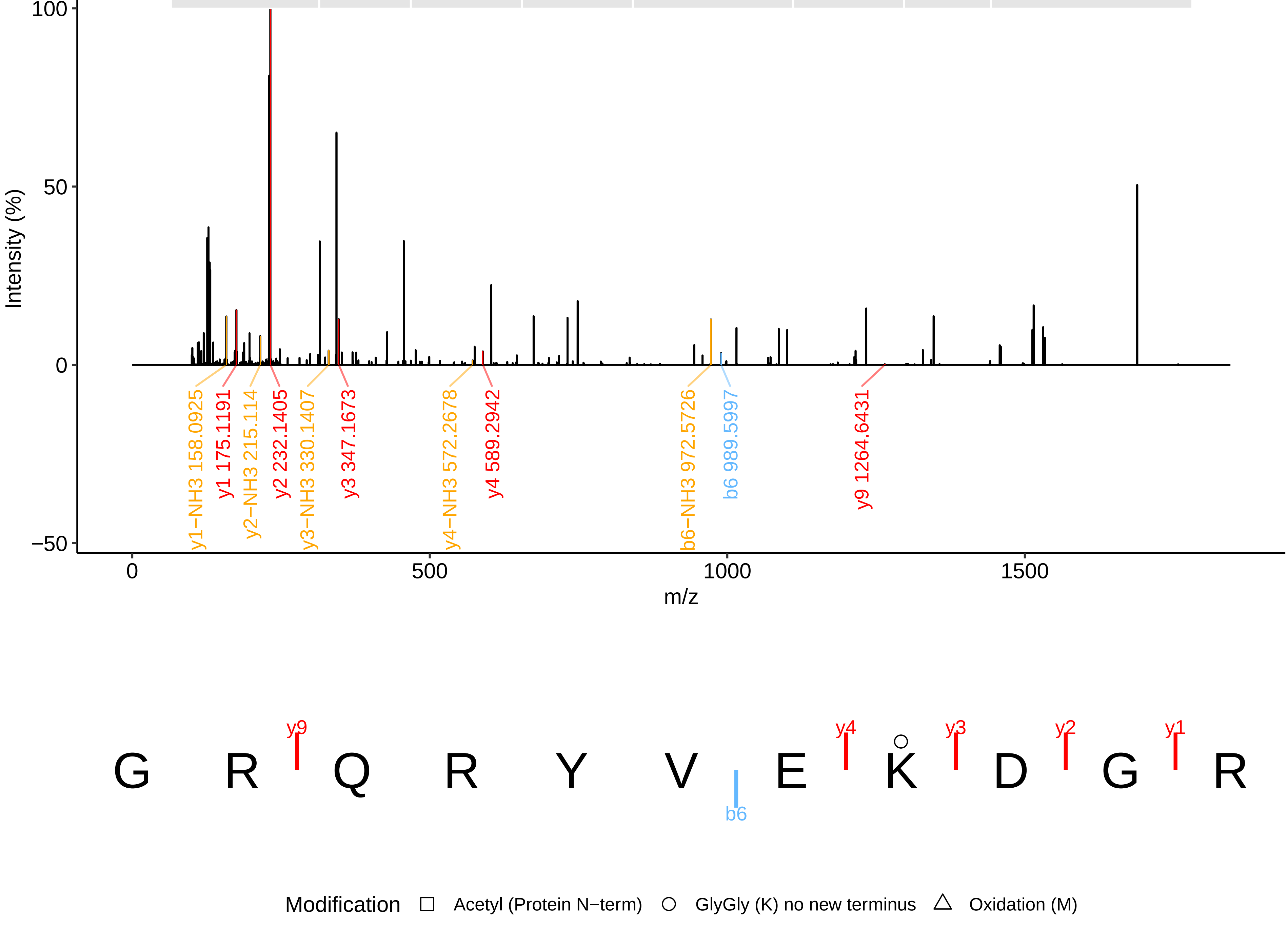

20200924_KIZ_MUb5_GlyGly_1 − Scan number: 21952

Gene Names	Charge	m/z	Mass	Retention time	PEP	Score	Precursor Intensity
KCNJ9	2	739.3846	1476.755	70.687	0.097868	41.956	17287382

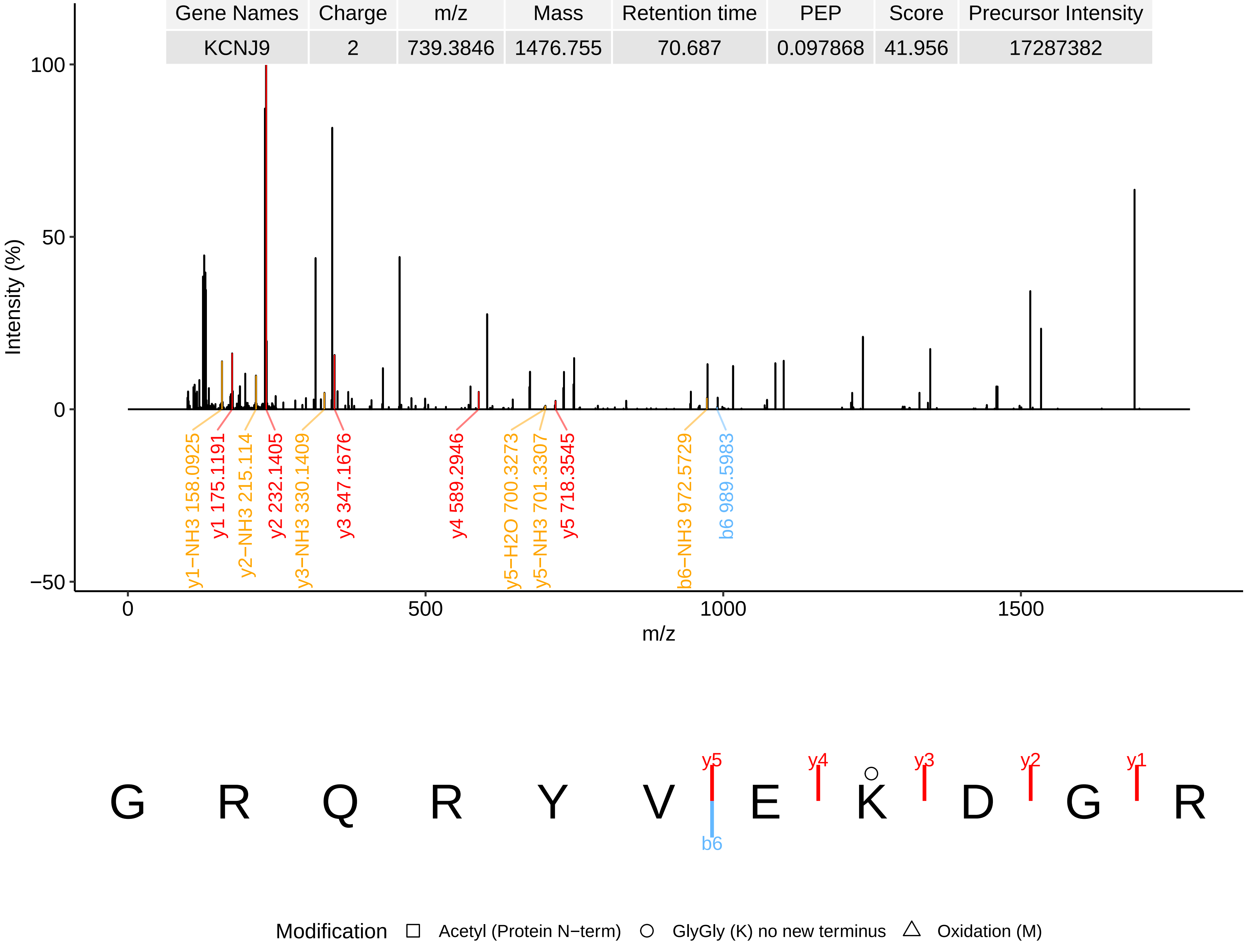

20200924_KIZ_MUb5_GlyGly_2 − Scan number: 19799

Gene Names	Charge	m/z	Mass	Retention time	PEP	Score	Precursor Intensity
KCNJ9	2	739.3846	1476.755	73.412	0.099556	41.448	6965992

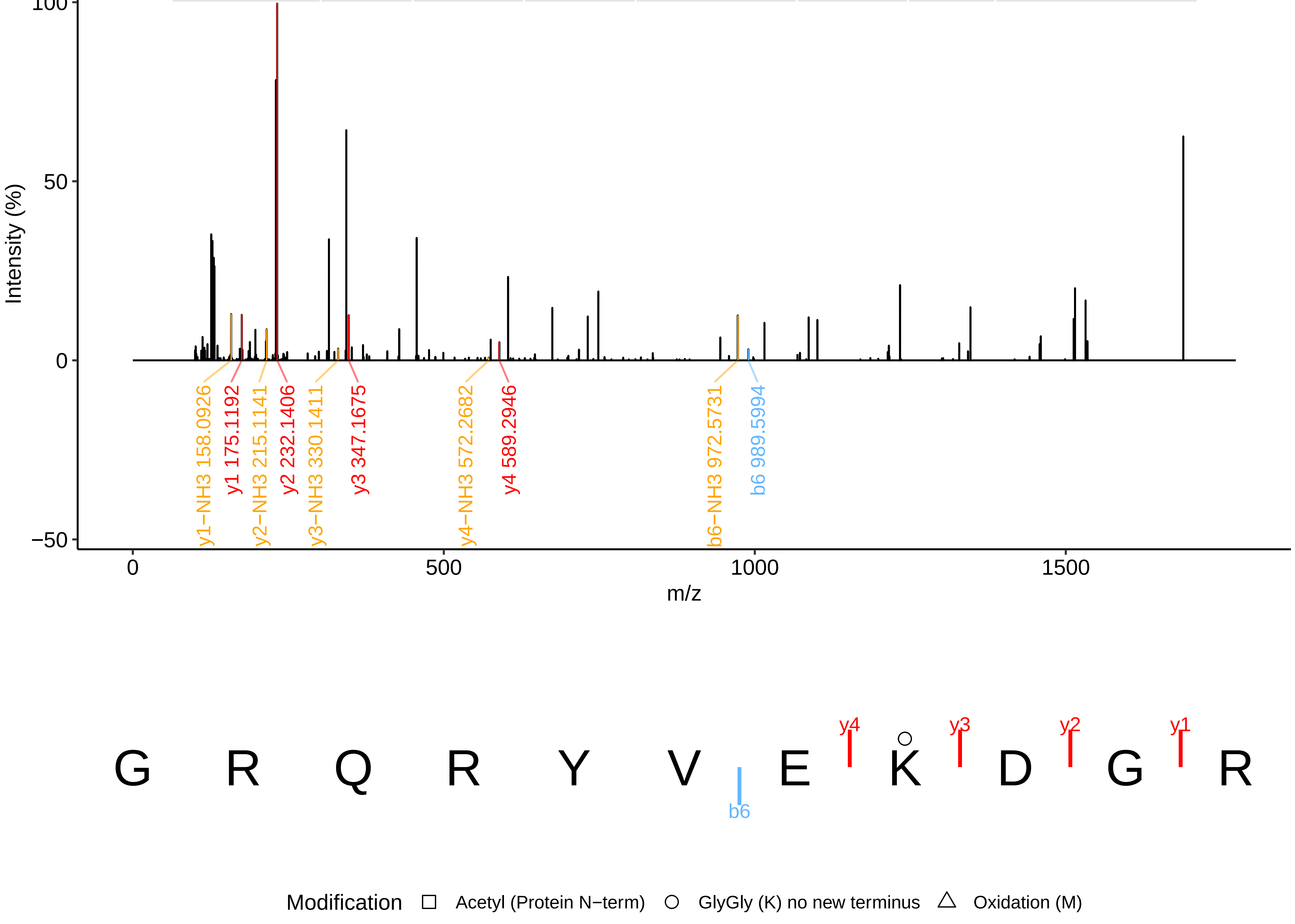

20201118_KIZ_MUb6_GlyGly_1 − Scan number: 21888

Gene Names	Charge	m/z	Mass	Retention time	PEP	Score	Precursor Intensity
KCNJ9	2	739.3846	1476.755	68.171	0.089902	44.349	26407260

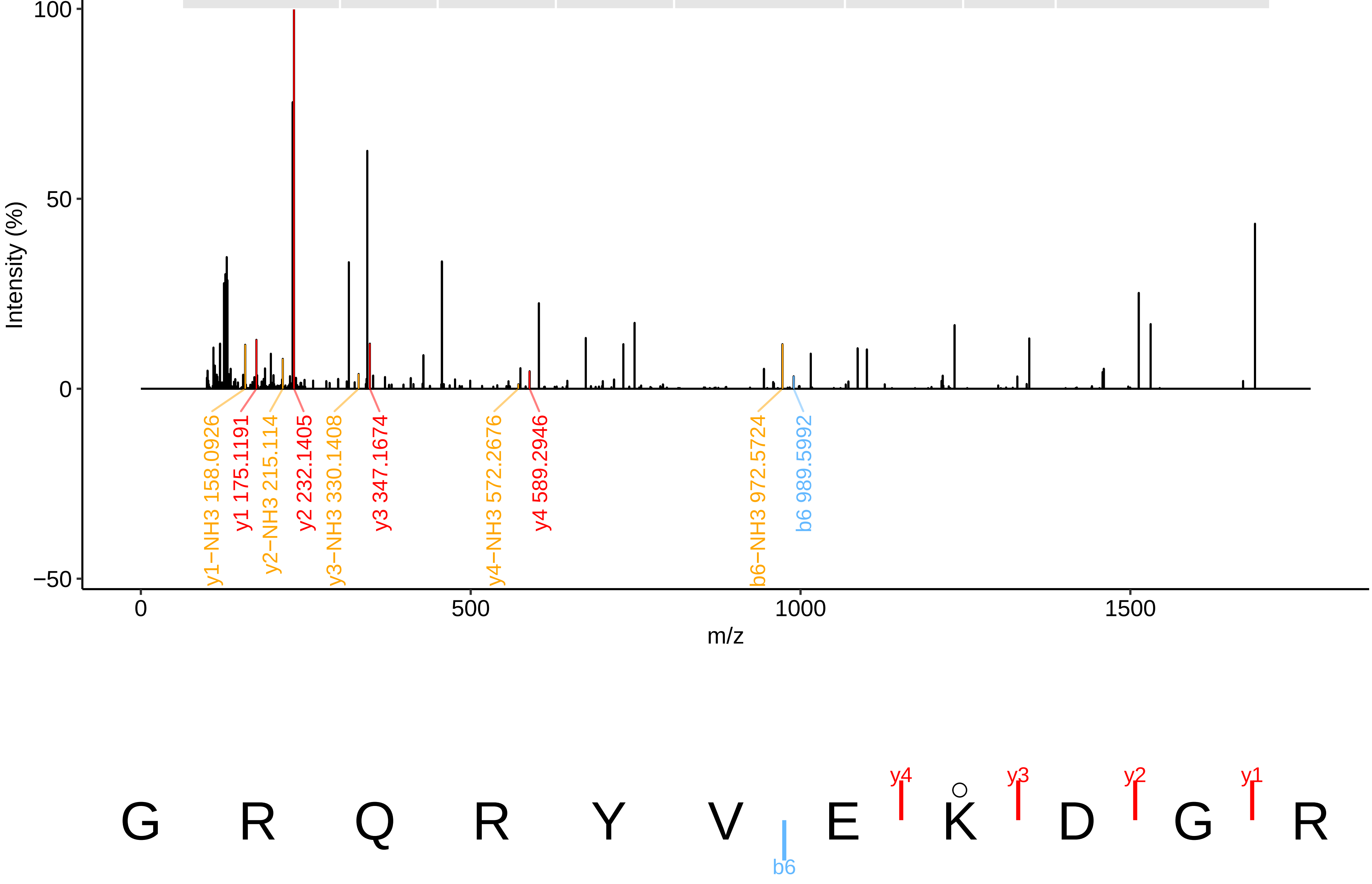

Modification □ Acetyl (Protein N−term) ○ GlyGly (K) no new terminus △ Oxidation (M)

20200921_KIZ_MUb4_GlyGly_10plex − Scan number: 5959

Gene Names	Charge	m/z	Mass	Retention time	PEP	Score	Precursor Intensity
TYMS	3	412.5457	1234.615	19.56	0.014846	64.64	5499953

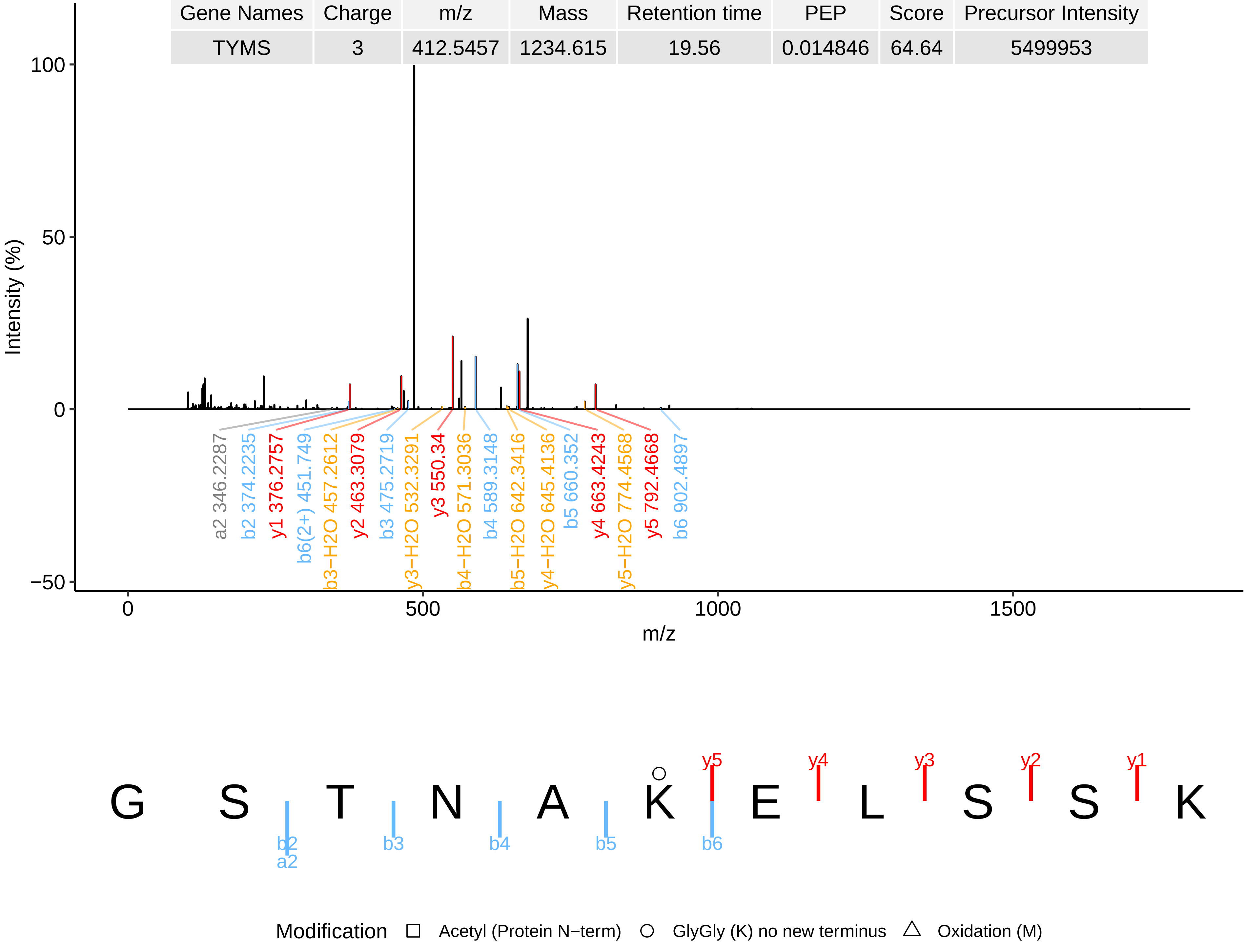

20200921_KIZ_MUb4_GlyGly_10plex_2 − Scan number: 5438

Gene Names	Charge	m/z	Mass	Retention time	PEP	Score	Precursor Intensity
TYMS	3	412.5457	1234.615	19.549	0.074544	51.841	488142.3

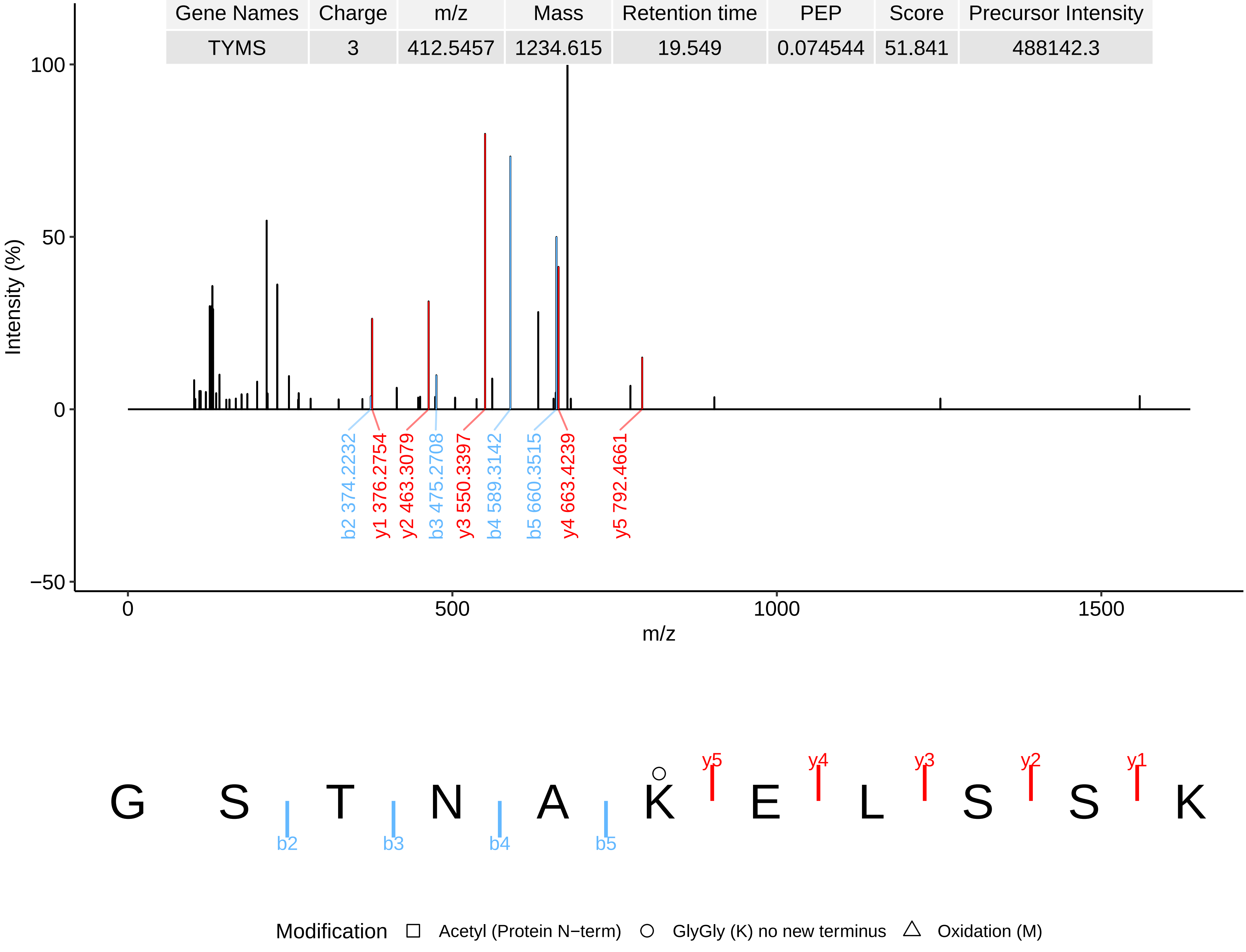

20200921_KIZ_MUb4_GlyGly_10plex − Scan number: 8467

Gene Names	Charge	m/z	Mass	Retention time	PEP	Score	Precursor Intensity
ICAM1	2	727.3588	1452.703	28.737	0.010067	84.17	NA

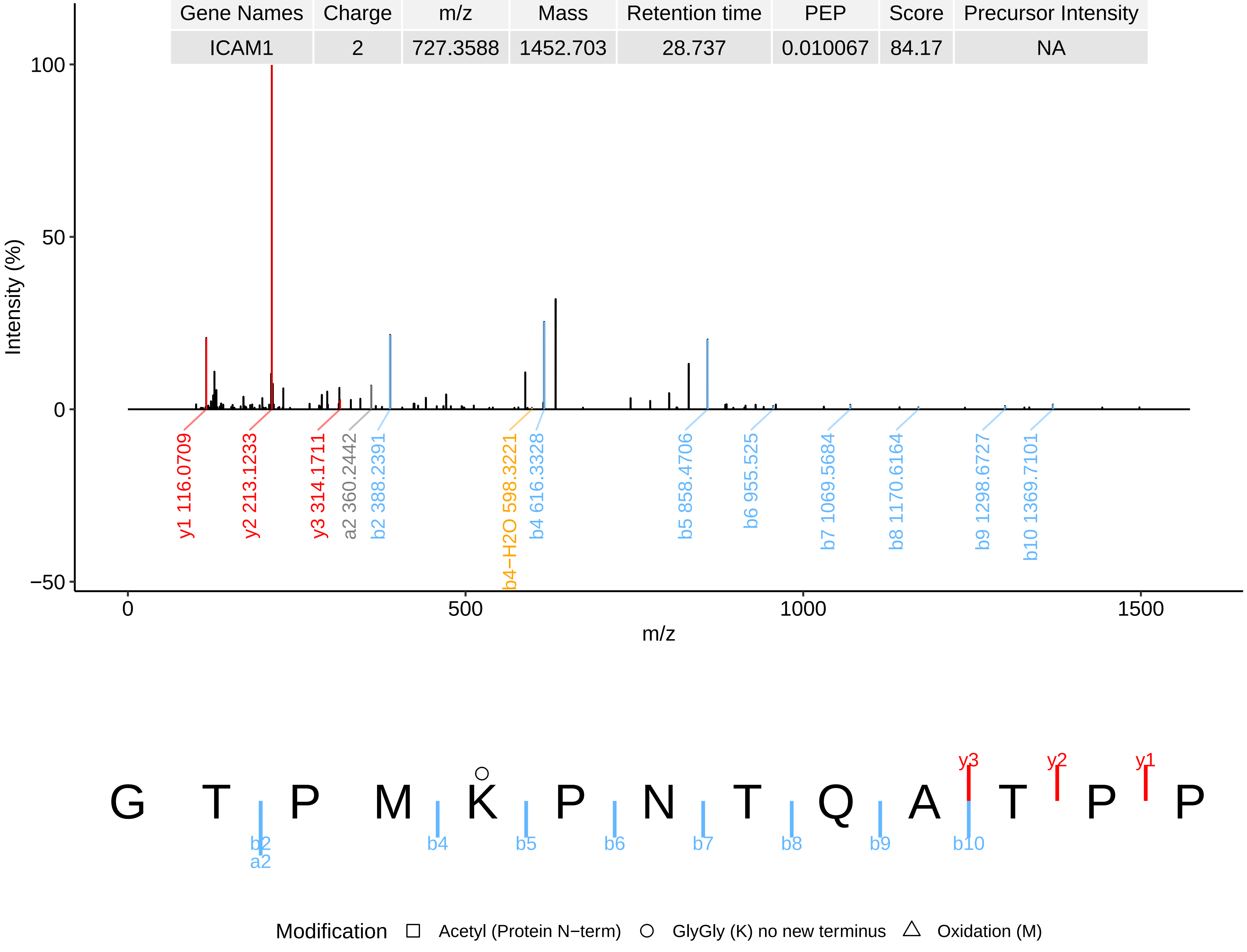

20200921_KIZ_MUb4_GlyGly_10plex − Scan number: 6585

Gene Names	Charge	m/z	Mass	Retention time	PEP	Score	Precursor Intensity
BASP1	3	417.8703	1250.589	21.845	2.646e−05	109.42	5475004

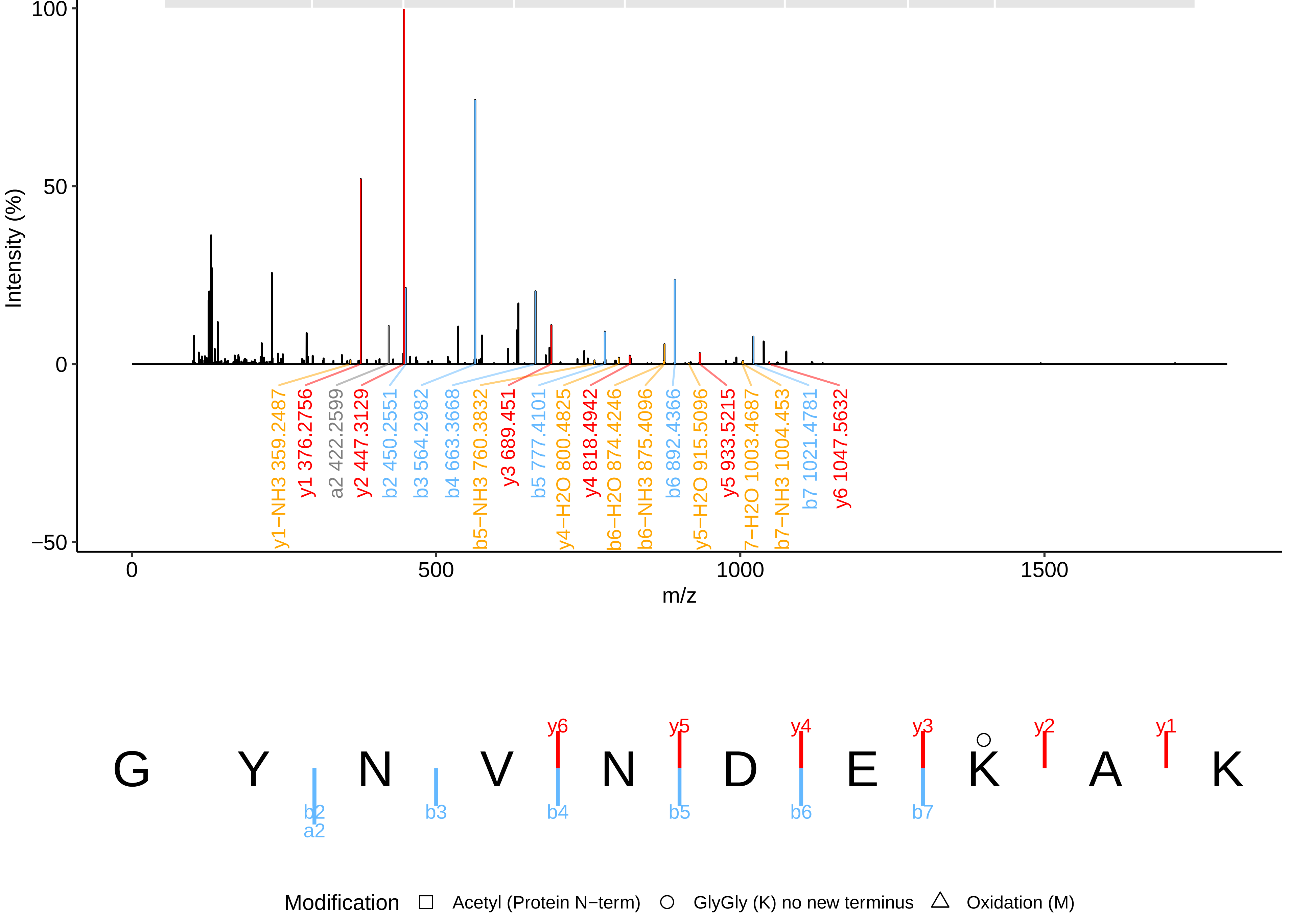

20200921_KIZ_MUb4_GlyGly_10plex_2 − Scan number: 6101

Gene Names	Charge	m/z	Mass	Retention time	PEP	Score	Precursor Intensity
BASP1	3	417.8703	1250.589	22.271	2.1709e−07	125.48	1726034

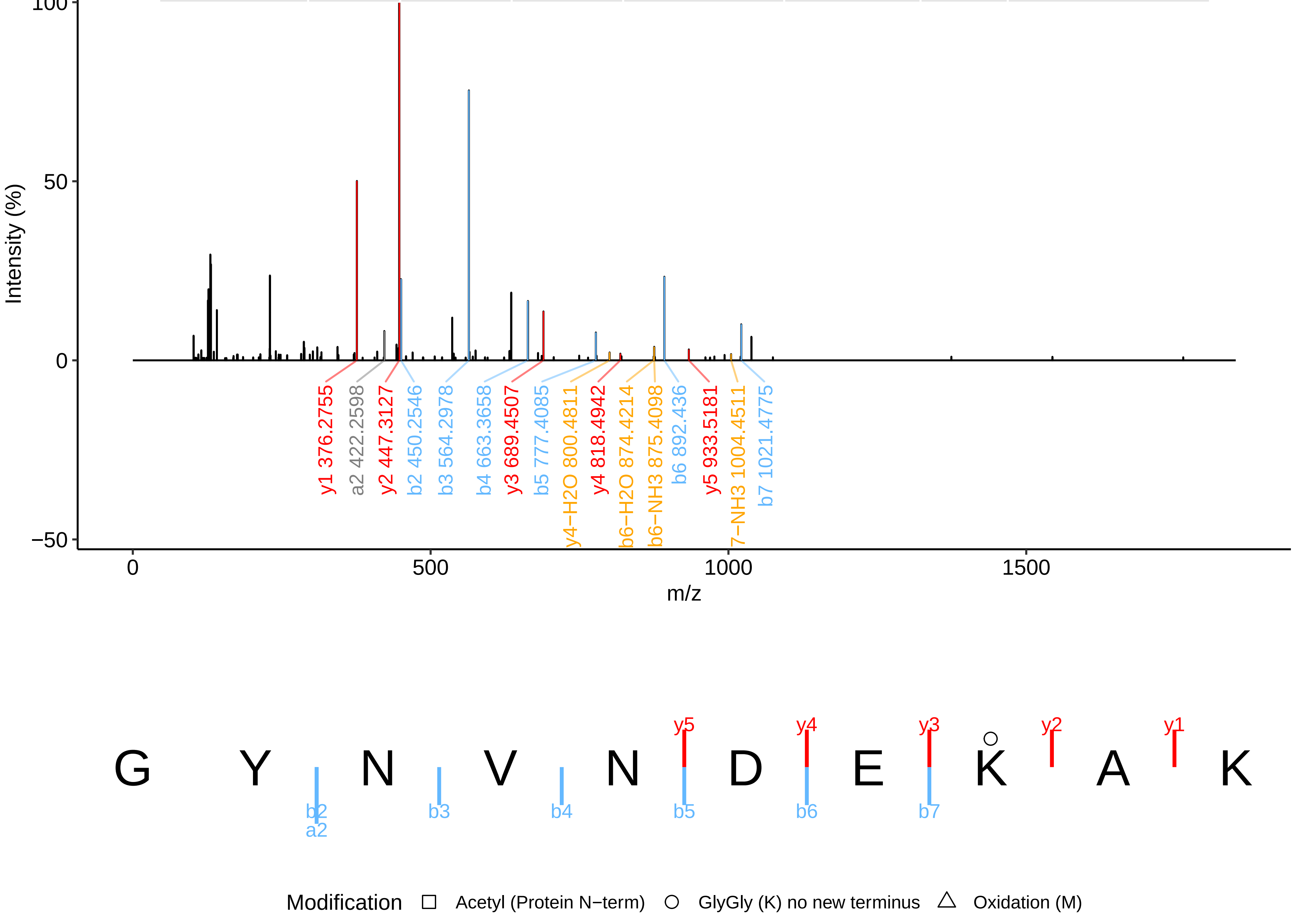

20200924_KIZ_MUb5_GlyGly_1 − Scan number: 5632

Gene Names	Charge	m/z	Mass	Retention time	PEP	Score	Precursor Intensity
BASP1	3	417.8703	1250.589	17.562	4.8964e−05	104.22	6996900

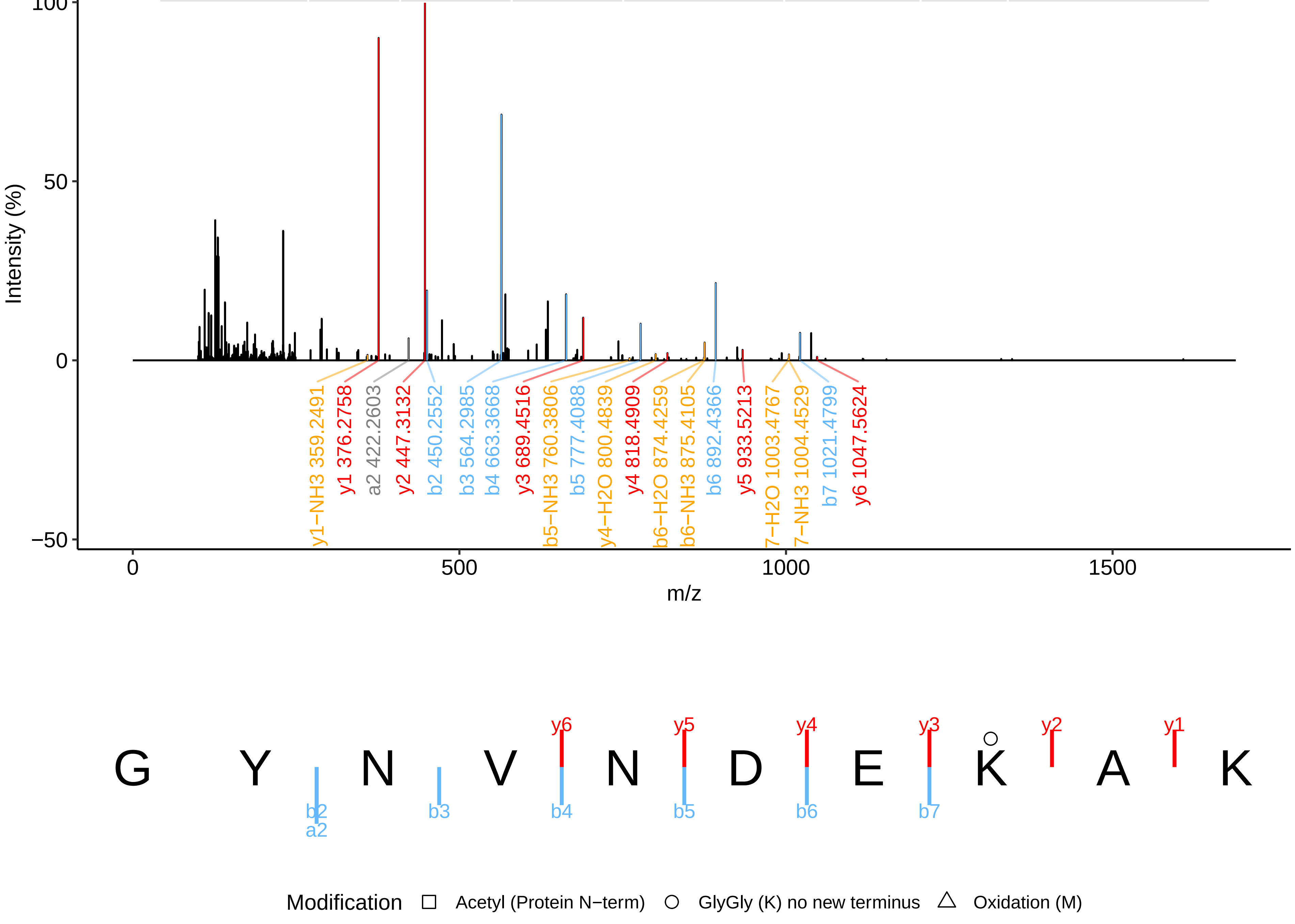

20200924_KIZ_MUb5_GlyGly_2 − Scan number: 5357

Gene Names	Charge	m/z	Mass	Retention time	PEP	Score	Precursor Intensity
BASP1	3	417.8703	1250.589	19.872	3.3523e−05	107.79	1528874

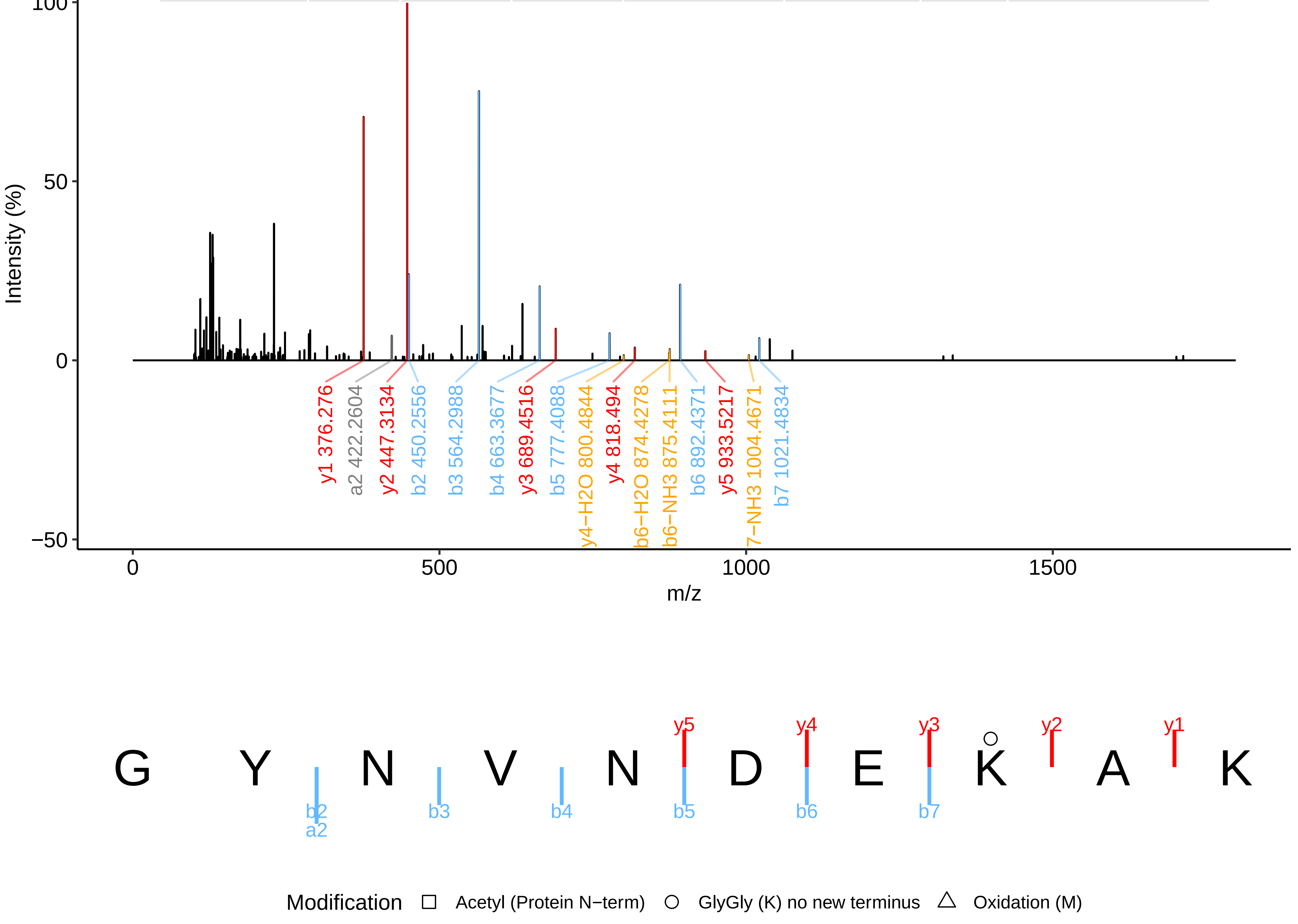

20200921_KIZ_MUb4_GlyGly_10plex_2 − Scan number: 20866

Gene Names	Charge	m/z	Mass	Retention time	PEP	Score	Precursor Intensity
UBE2N	3	411.2287	1230.664	82.083	0.058764	55.567	2664534

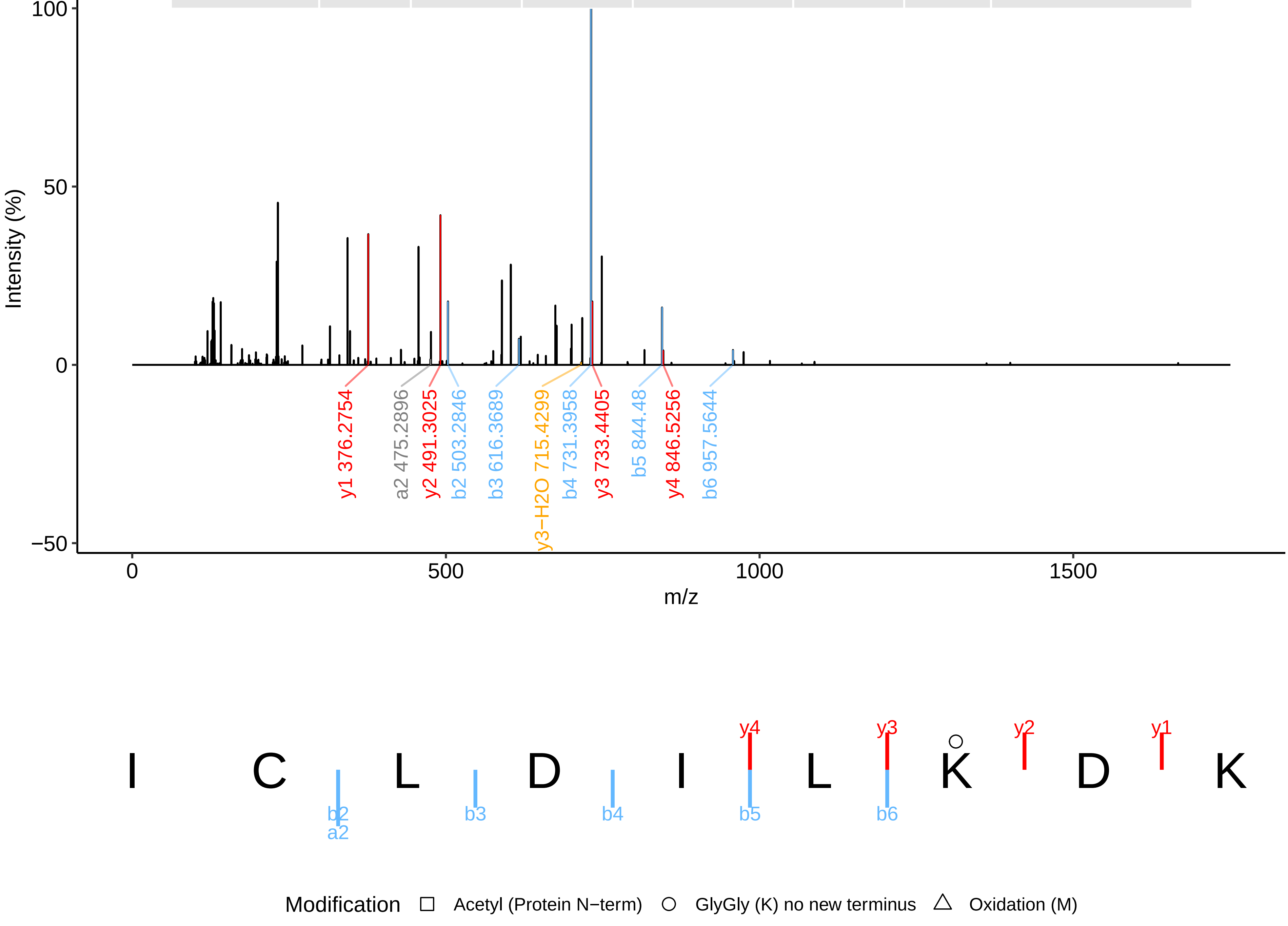

20200924_KIZ_MUb5_GlyGly_1 − Scan number: 22815

Gene Names	Charge	m/z	Mass	Retention time	PEP	Score	Precursor Intensity
UBE2N	3	411.2287	1230.664	73.377	0.0066273	77.677	15583137

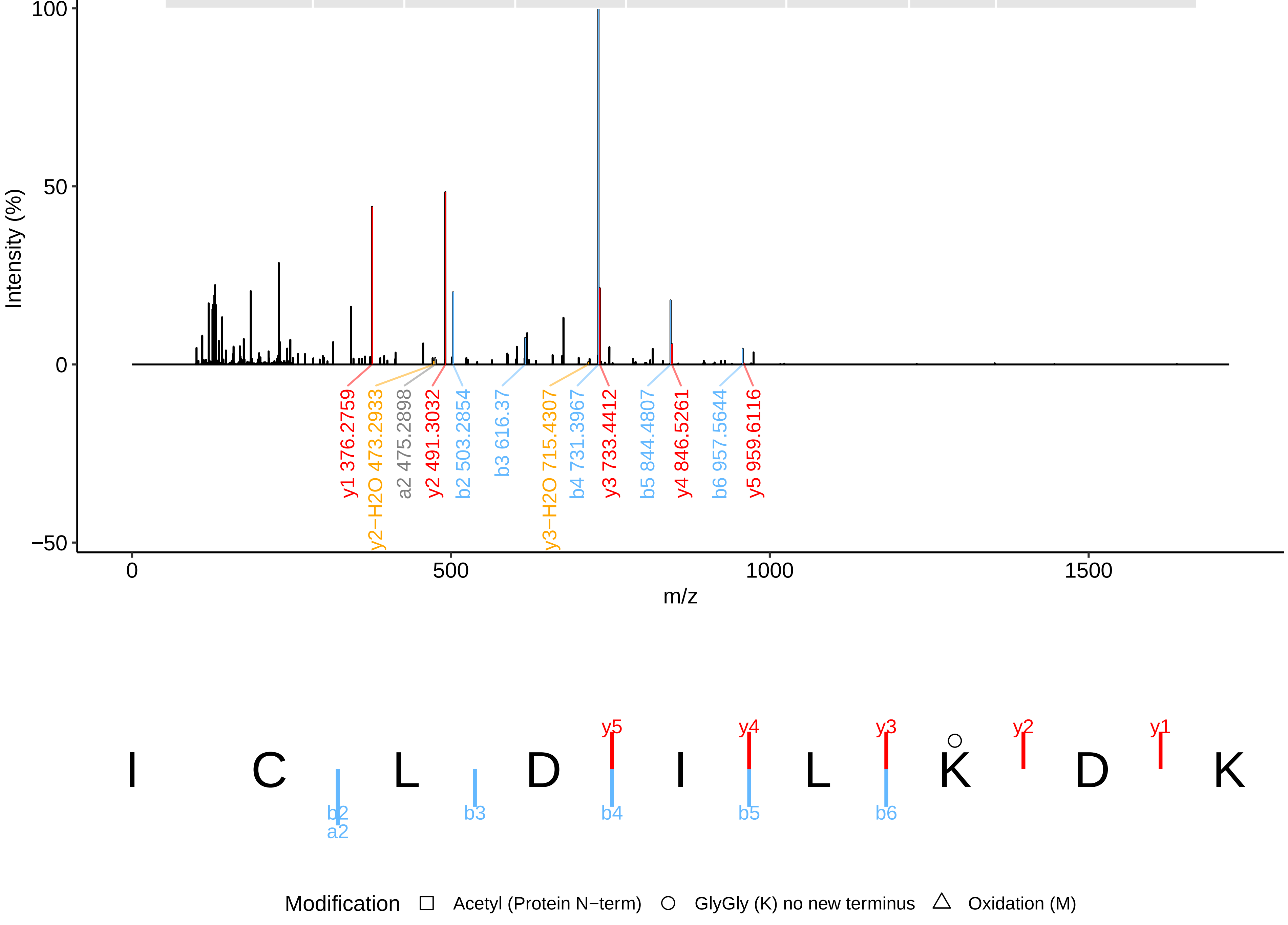

20200924_KIZ_MUb5_GlyGly_1 − Scan number: 23148

Gene Names	Charge	m/z	Mass	Retention time	PEP	Score	Precursor Intensity
UBE2N	3	411.2287	1230.664	74.432	0.025514	65.347	NA

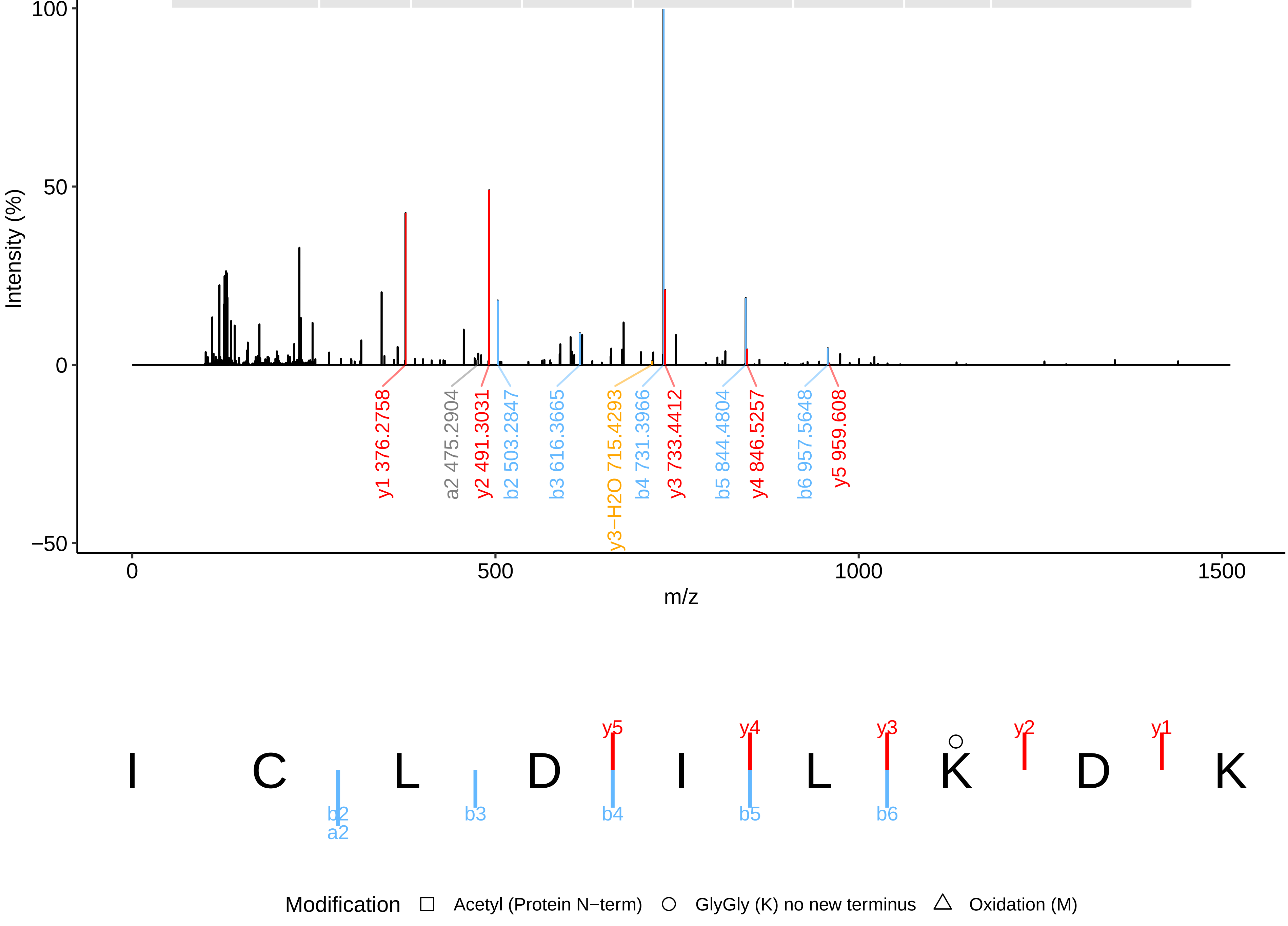

20200924_KIZ_MUb5_GlyGly_2 − Scan number: 20584

Gene Names	Charge	m/z	Mass	Retention time	PEP	Score	Precursor Intensity
UBE2N	3	411.2287	1230.664	76.22	0.0227	67.563	NA

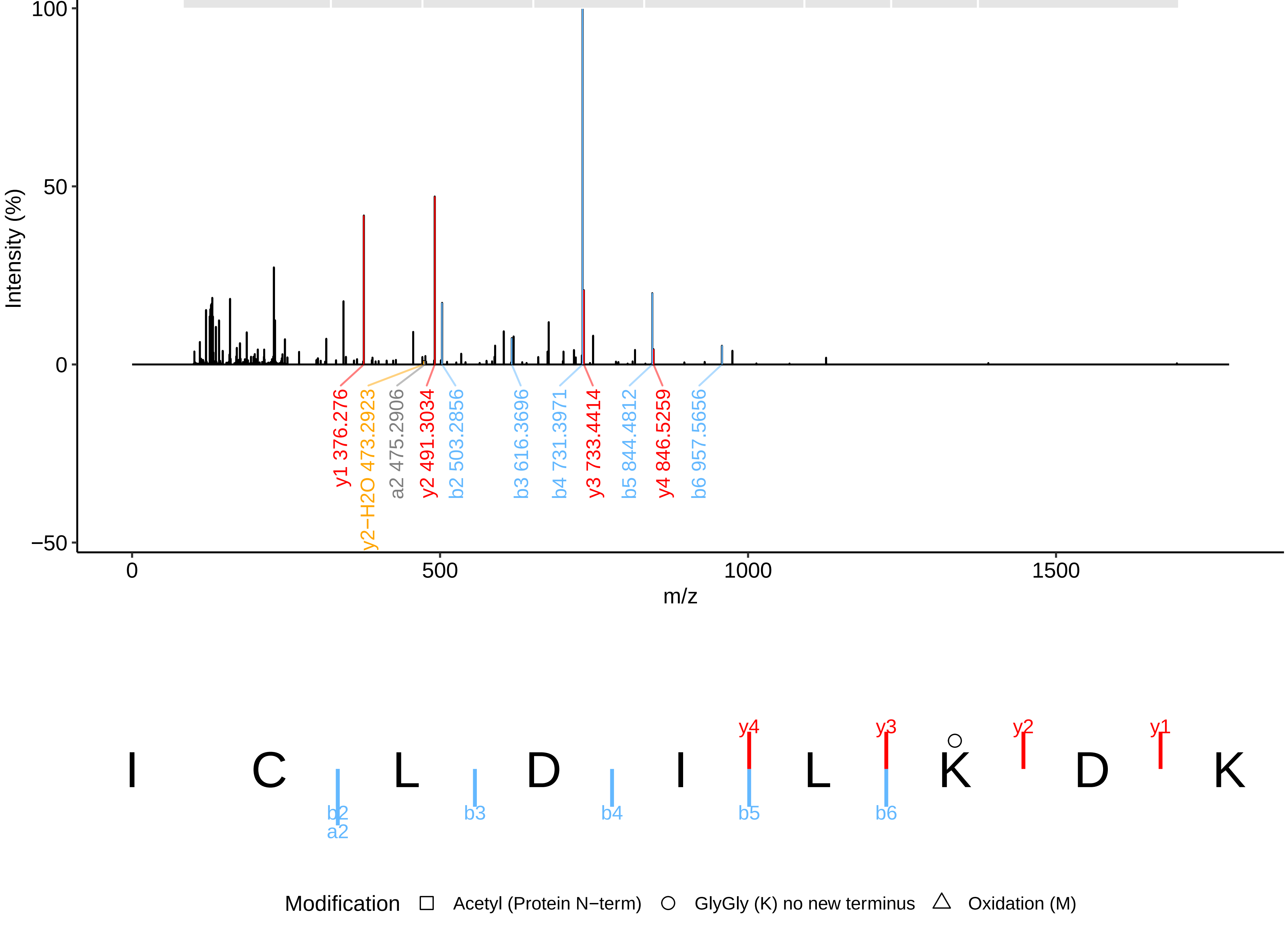

20200924_KIZ_MUb5_GlyGly_2 − Scan number: 20740

Gene Names	Charge	m/z	Mass	Retention time	PEP	Score	Precursor Intensity
UBE2N	3	411.2287	1230.664	76.779	0.009853	77.677	NA

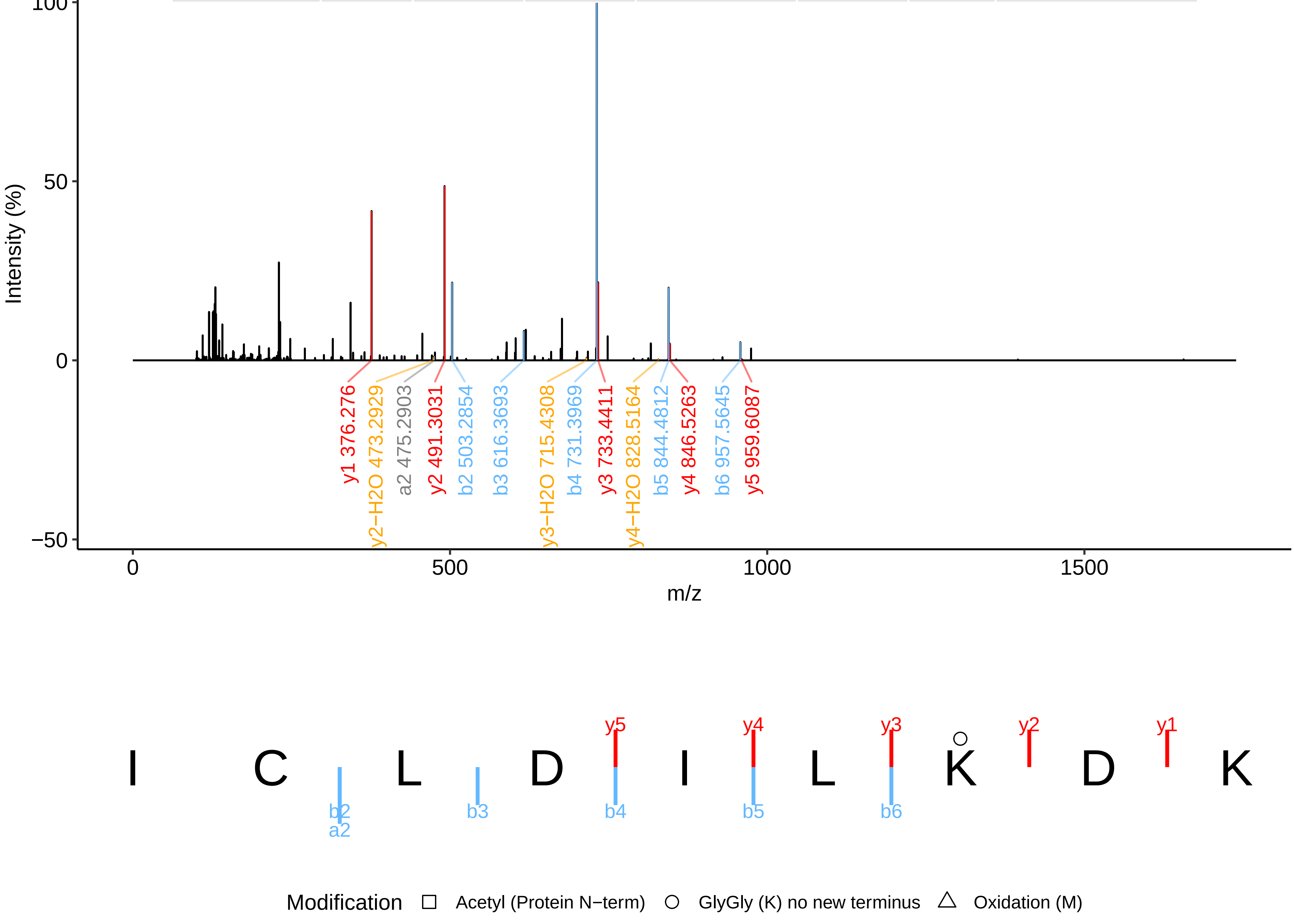

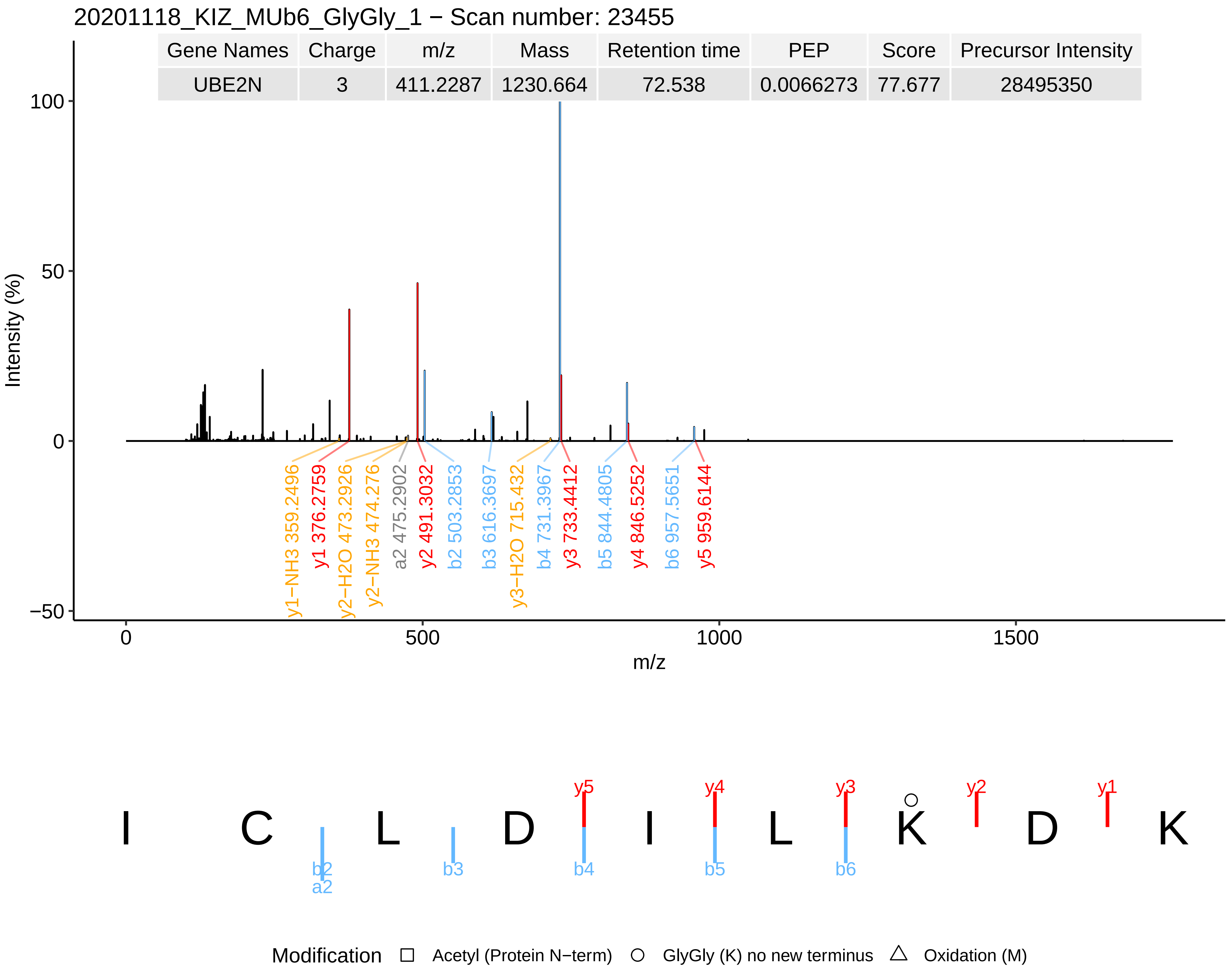
20201118_KIZ_MUb6_GlyGly_1 − Scan number: 23455
Gene Names | Charge | m/z | Mass | Retention time | PEP | Score | Precursor Intensity
UBE2N | 3 | 411.2287 | 1230.664 | 72.538 | 0.0066273 | 77.677 | 28495350
Intensity (%)
m/z
y1−NH3 359.2496
y1 376.2759
y2−H2O 473.2926
y2−NH3 474.276
a2 475.2902
y2 491.3032
b2 503.2853
b3 616.3697
y3−H2O 715.432
b4 731.3967
y3 733.4412
b5 844.4805
y4 846.5252
b6 957.5651
y5 959.6144
I C L D I L K D K
y5 y4 y3 y2 y1
b2 a2 b3 b4 b5 b6
Modification
Acetyl (Protein N−term)
GlyGly (K) no new terminus
Oxidation (M)

20201118_KIZ_MUb6_GlyGly_2 − Scan number: 25229

Gene Names	Charge	m/z	Mass	Retention time	PEP	Score	Precursor Intensity
UBE2N	3	411.2287	1230.664	84.33	0.0066273	77.677	32115990

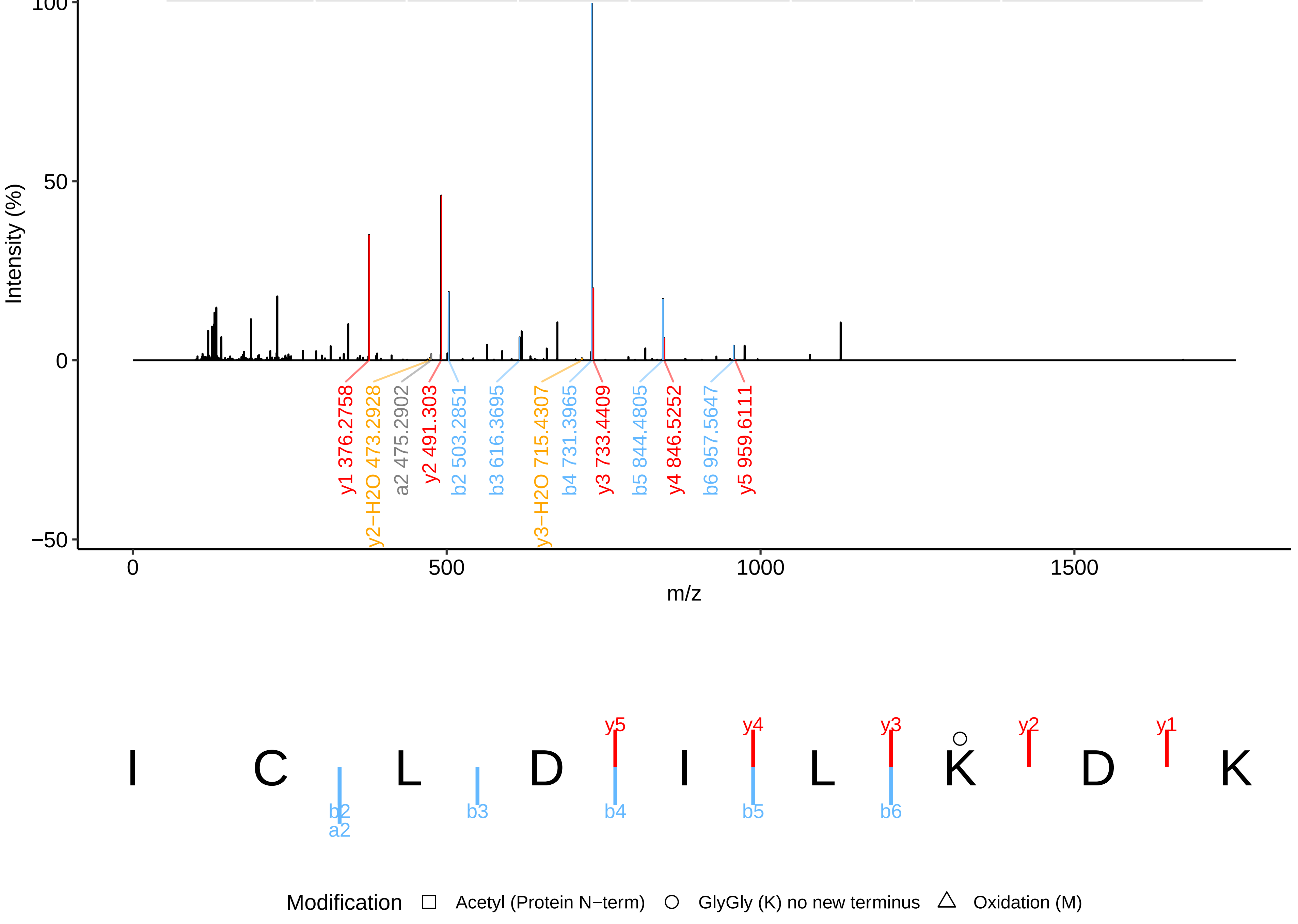

20200921_KIZ_MUb4_GlyGly_10plex − Scan number: 19686

Gene Names	Charge	m/z	Mass	Retention time	PEP	Score	Precursor Intensity
RPL6	3	414.2468	1239.719	71.042	0.0020772	84.17	4028643

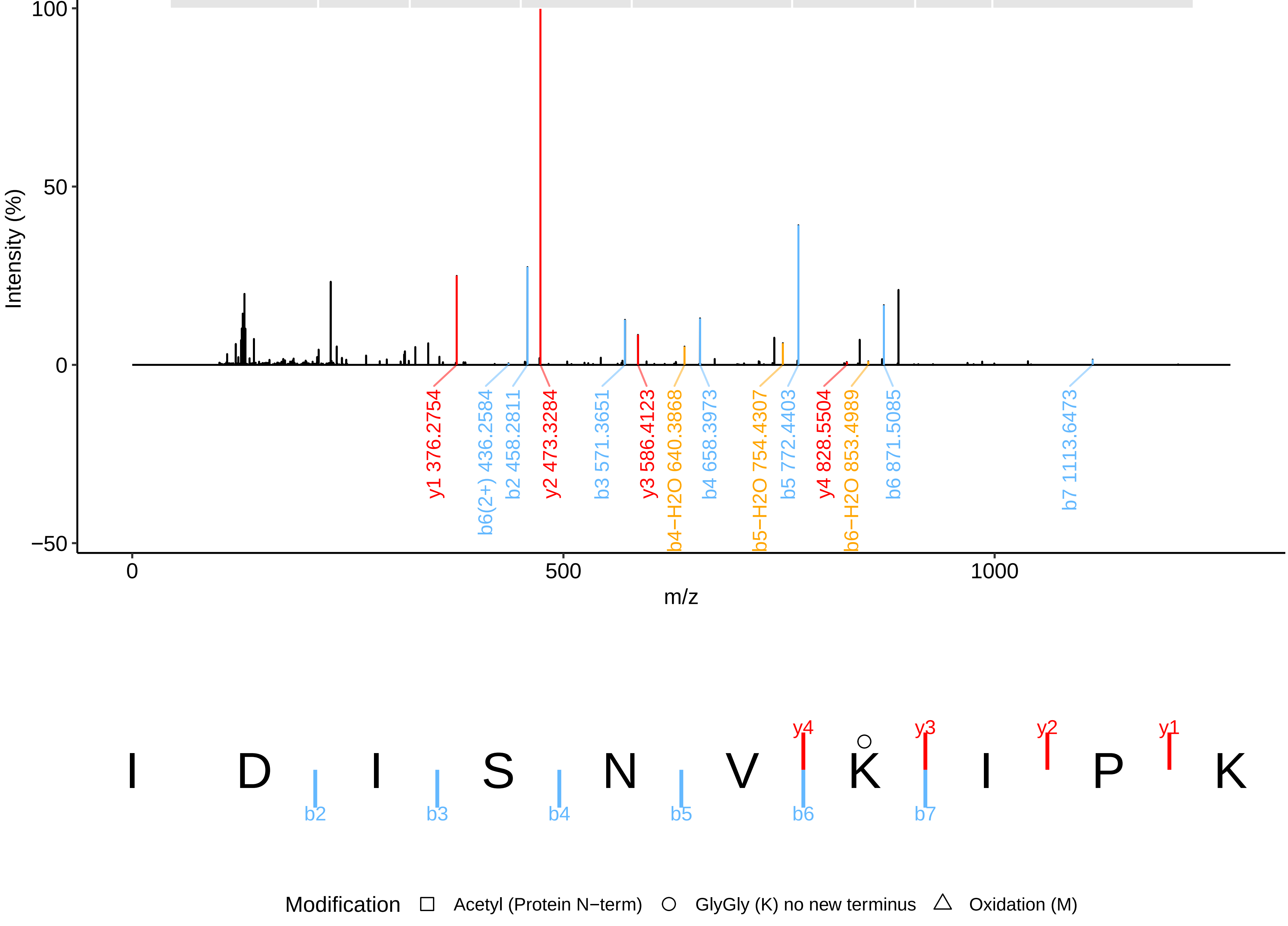

20200921_KIZ_MUb4_GlyGly_10plex_2 − Scan number: 18145

Gene Names	Charge	m/z	Mass	Retention time	PEP	Score	Precursor Intensity
RPL6	3	414.2468	1239.719	71.021	0.014755	68.657	447945

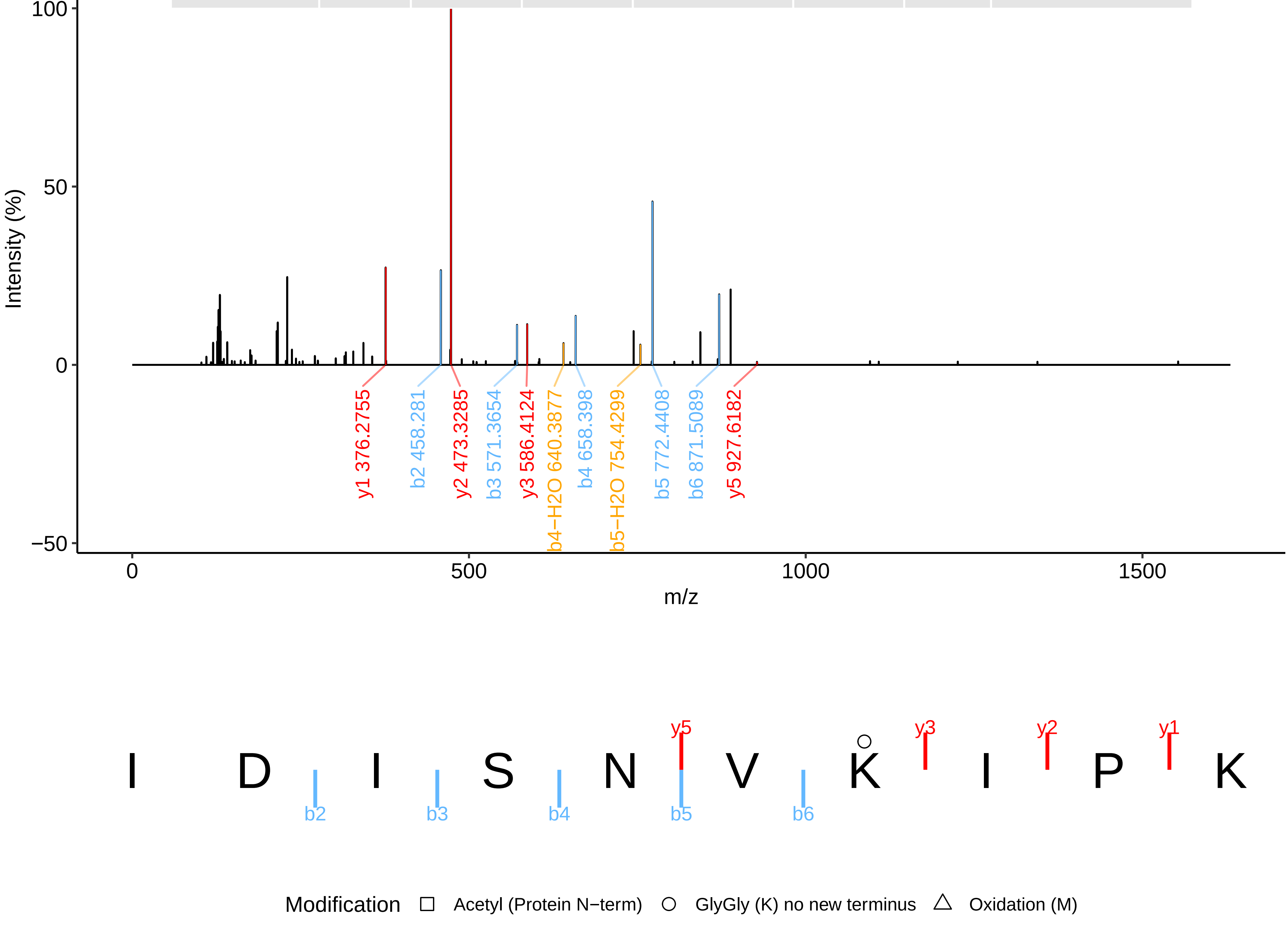

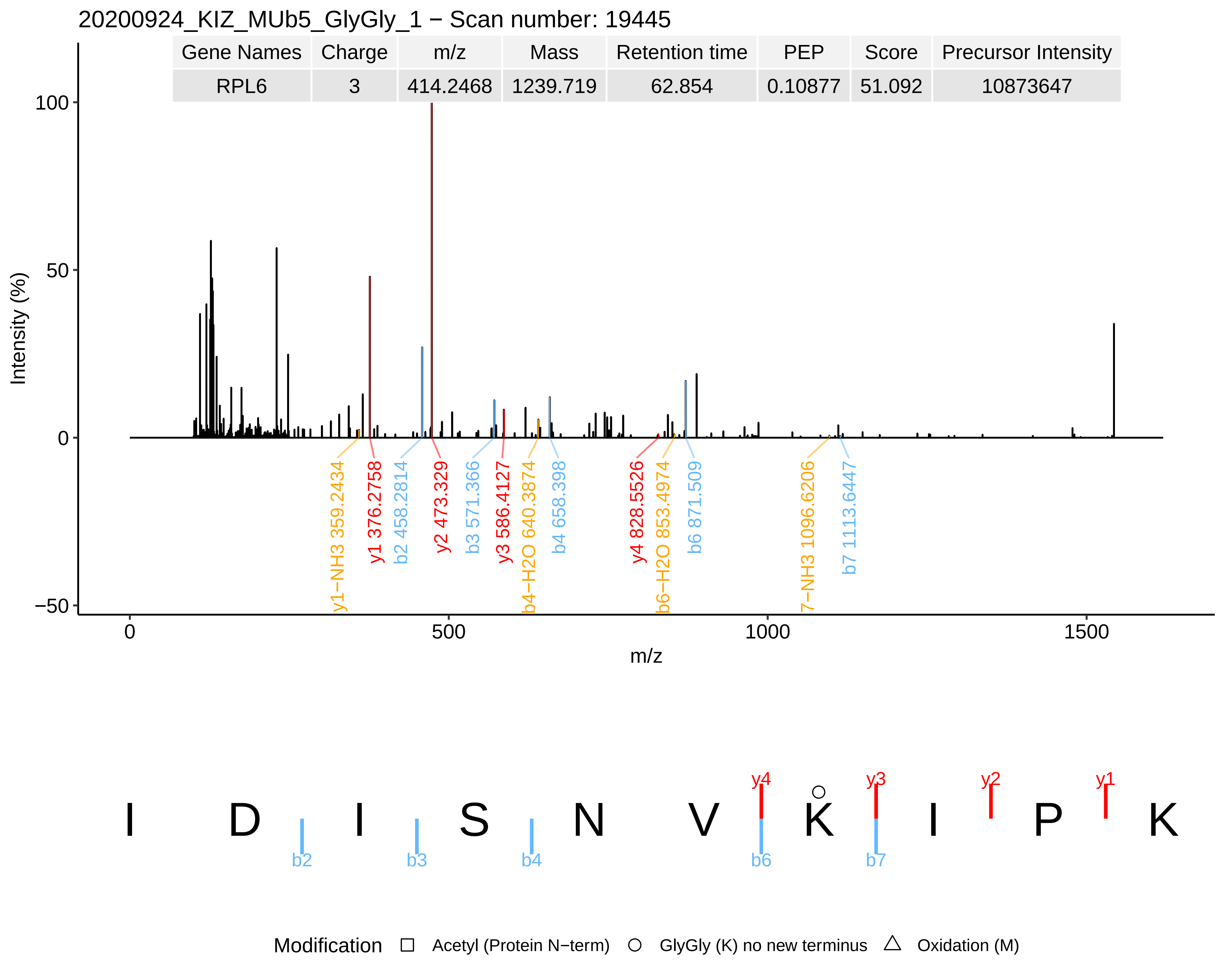
20200924_KIZ_MUb5_GlyGly_1 − Scan number: 19445
Gene Names | Charge | m/z | Mass | Retention time | PEP | Score | Precursor Intensity
RPL6 | 3 | 414.2468 | 1239.719 | 62.854 | 0.10877 | 51.092 | 10873647
Intensity (%)
100
50
0
−50
0
500
1000
1500
m/z
y1−NH3 359.2434
y1 376.2758
b2 458.2814
y2 473.329
b3 571.366
y3 586.4127
b4−H2O 640.3874
b4 658.398
y4 828.5526
b6−H2O 853.4974
b6 871.509
7−NH3 1096.6206
b7 1113.6447
I D I S N V K I P K
y4 y3 y2 y1
b2 b3 b4 b6 b7
Modification
Acetyl (Protein N−term)
GlyGly (K) no new terminus
Oxidation (M)

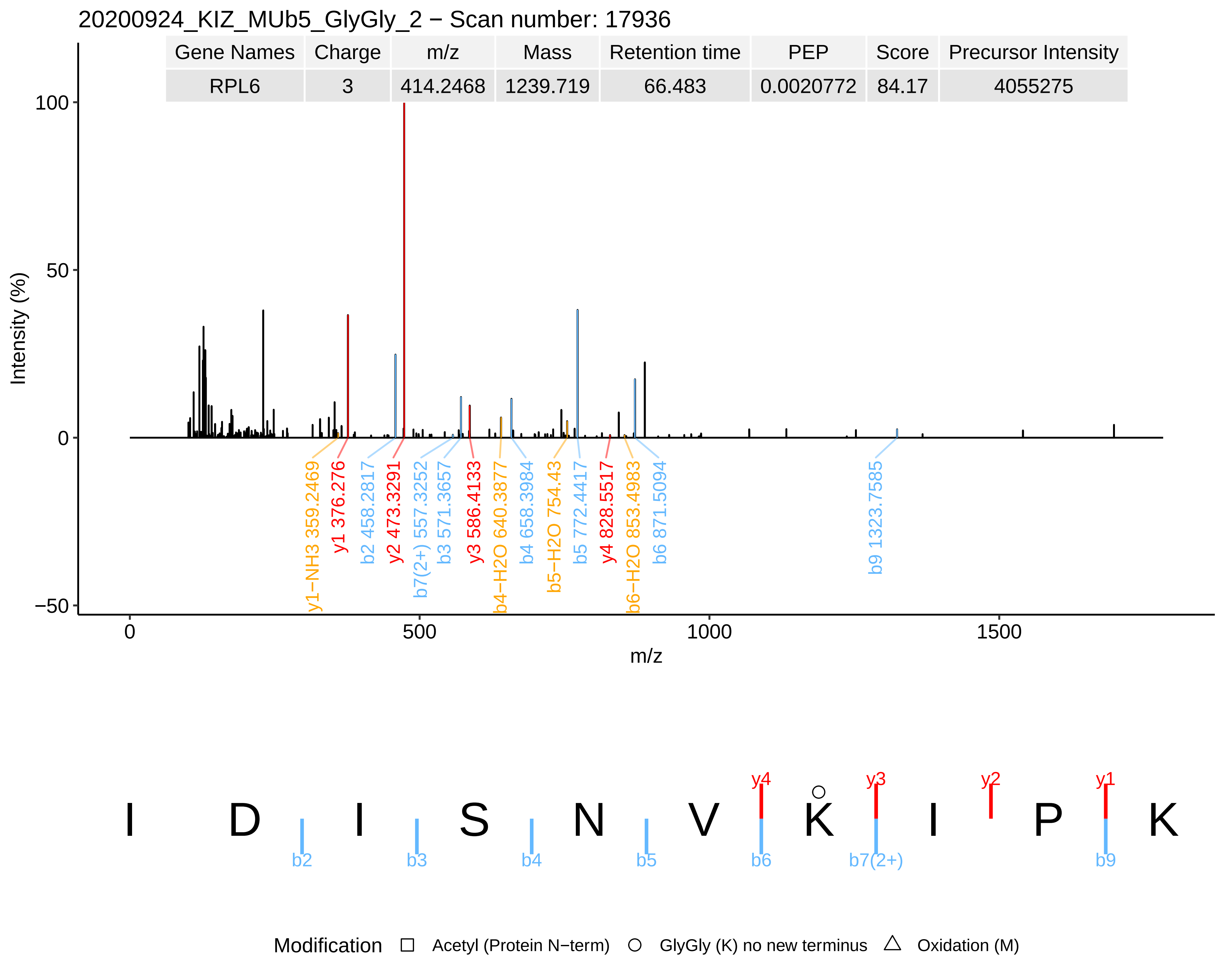
20200924_KIZ_MUb5_GlyGly_2 − Scan number: 17936
Gene Names | Charge | m/z | Mass | Retention time | PEP | Score | Precursor Intensity
RPL6 | 3 | 414.2468 | 1239.719 | 66.483 | 0.0020772 | 84.17 | 4055275
Intensity (%)
100
50
0
−50
y1−NH3 359.2469
y1 376.276
b2 458.2817
y2 473.3291
b7(2+) 557.3252
b3 571.3657
y3 586.4133
b4−H2O 640.3877
b4 658.3984
b5−H2O 754.43
b5 772.4417
y4 828.5517
b6−H2O 853.4983
b6 871.5094
b9 1323.7585
0
500
1000
1500
m/z
I D I S N V K I P K
y4 y3 y2 y1
b2 b3 b4 b5 b6 b7(2+) b9
Modification
Acetyl (Protein N−term)
GlyGly (K) no new terminus
Oxidation (M)

20201118_KIZ_MUb6_GlyGly_1 − Scan number: 20052

Gene Names	Charge	m/z	Mass	Retention time	PEP	Score	Precursor Intensity
RPL6	3	414.2468	1239.719	62.789	0.020252	65.719	19423506

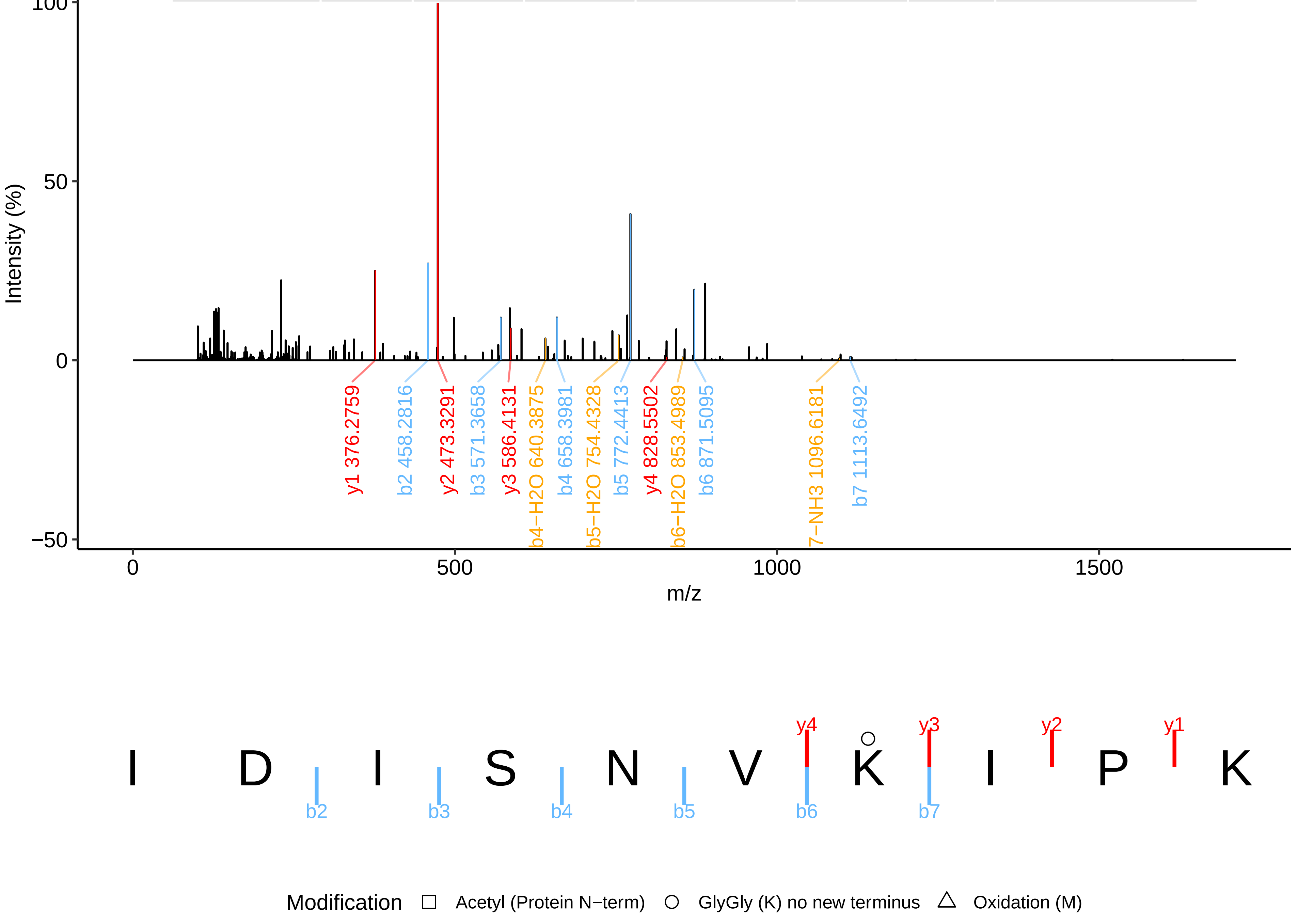

20200921_KIZ_MUb4_GlyGly_10plex − Scan number: 14466

Gene Names	Charge	m/z	Mass	Retention time	PEP	Score	Precursor Intensity
EIF2S3	3	415.5663	1243.677	51.421	0.0014244	88.681	7166916

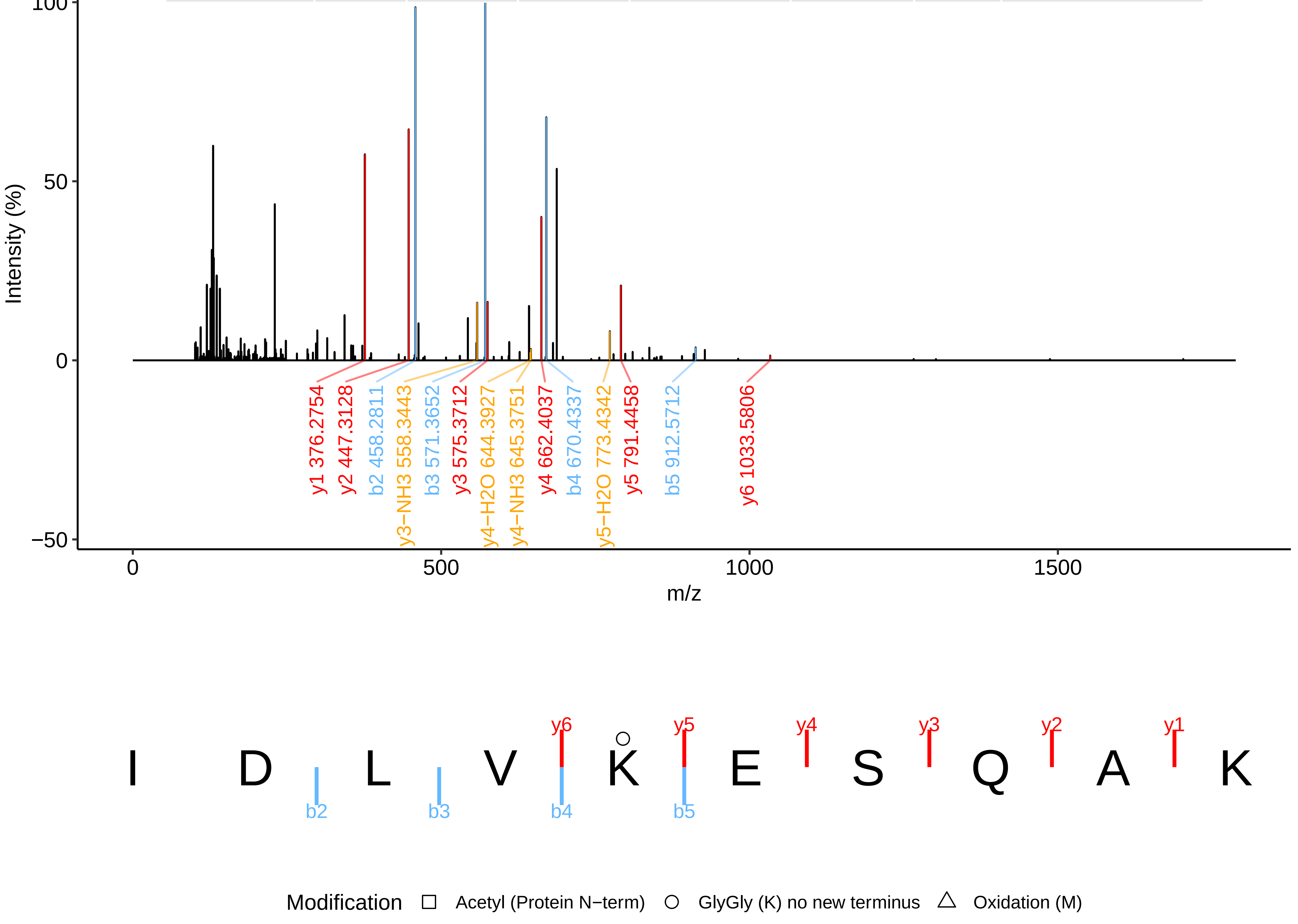

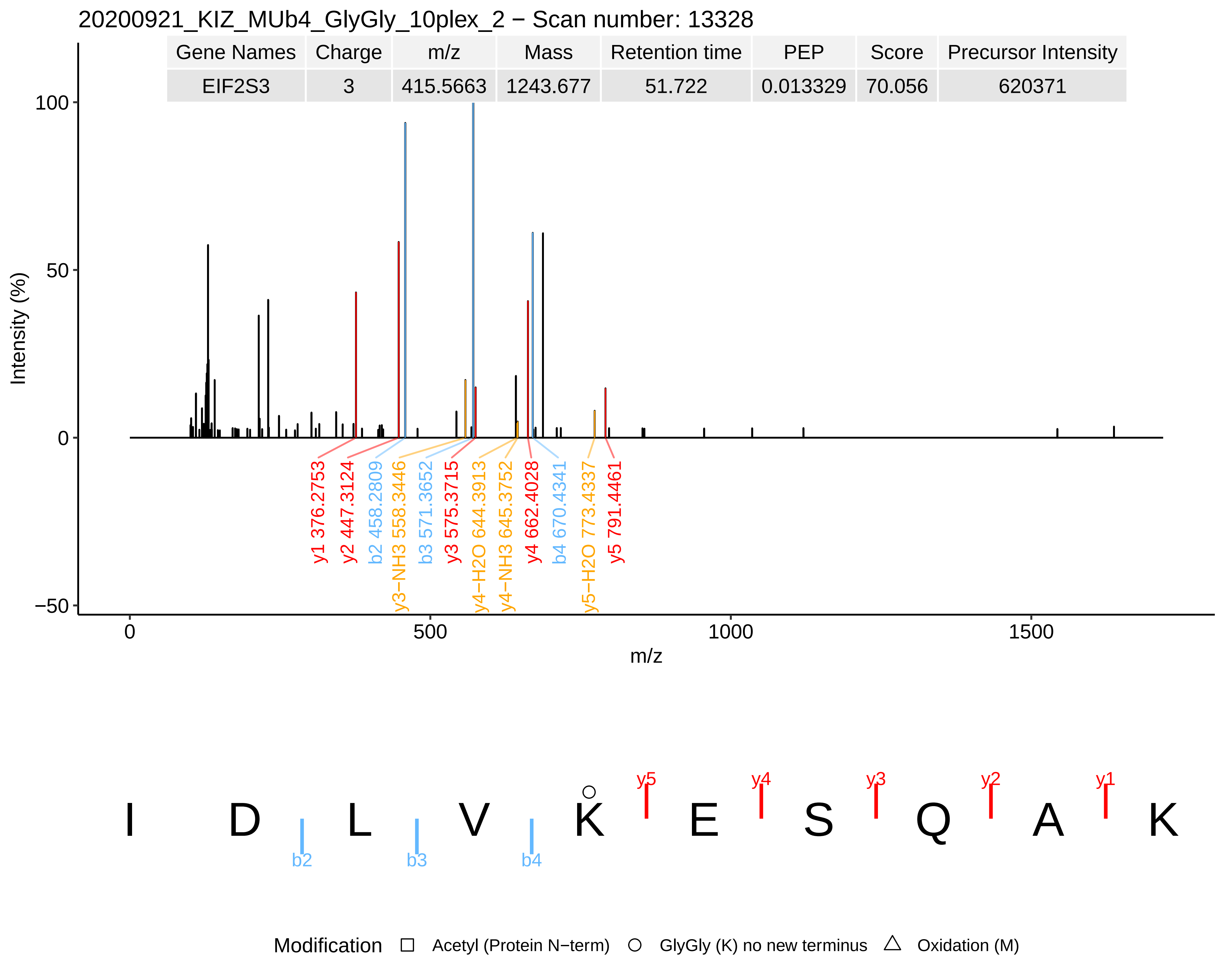

20200921_KIZ_MUb4_GlyGly_10plex_2 − Scan number: 13328
Gene Names | Charge | m/z | Mass | Retention time | PEP | Score | Precursor Intensity
EIF2S3 | 3 | 415.5663 | 1243.677 | 51.722 | 0.013329 | 70.056 | 620371
Intensity (%)
100
50
0
−50
0
500
1000
1500
m/z
y1 376.2753
y2 447.3124
b2 458.2809
y3−NH3 558.3446
b3 571.3652
y3 575.3715
y4−H2O 644.3913
y4−NH3 645.3752
y4 662.4028
b4 670.4341
y5−H2O 773.4337
y5 791.4461
I D L V K E S Q A K
y5 y4 y3 y2 y1
b2 b3 b4
Modification
Acetyl (Protein N−term)
GlyGly (K) no new terminus
Oxidation (M)

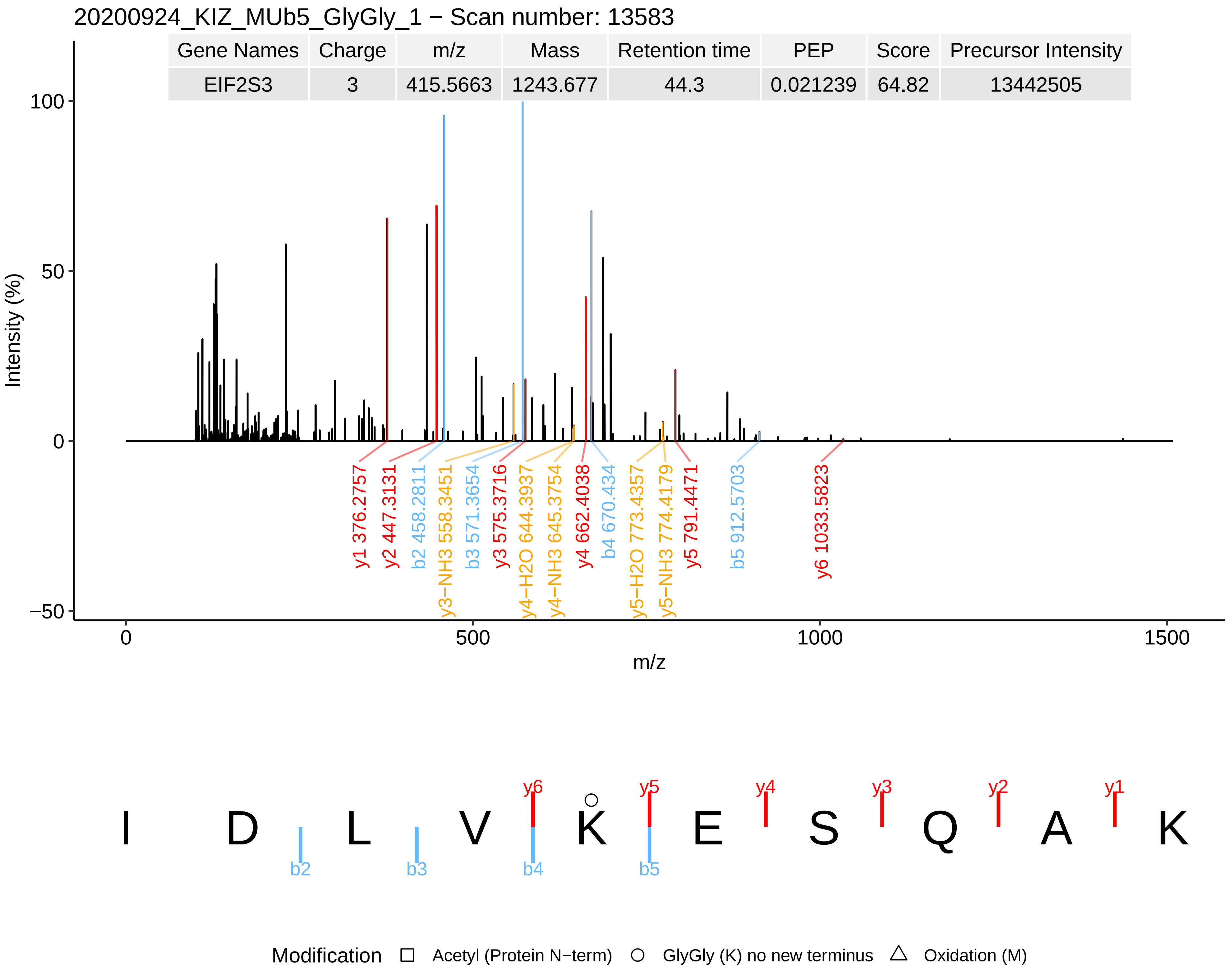

20200924_KIZ_MUb5_GlyGly_1 − Scan number: 13583
Gene Names | Charge | m/z | Mass | Retention time | PEP | Score | Precursor Intensity
EIF2S3 | 3 | 415.5663 | 1243.677 | 44.3 | 0.021239 | 64.82 | 13442505
Intensity (%)
100
50
0
−50
0
500
1000
1500
m/z
y1 376.2757
y2 447.3131
b2 458.2811
y3−NH3 558.3451
b3 571.3654
y3 575.3716
y4−H2O 644.3937
y4−NH3 645.3754
y4 662.4038
b4 670.434
y5−H2O 773.4357
y5−NH3 774.4179
y5 791.4471
b5 912.5703
y6 1033.5823
I D L V K E S Q A K
y6 y5 y4 y3 y2 y1
b2 b3 b4 b5
Modification
Acetyl (Protein N−term)
GlyGly (K) no new terminus
Oxidation (M)

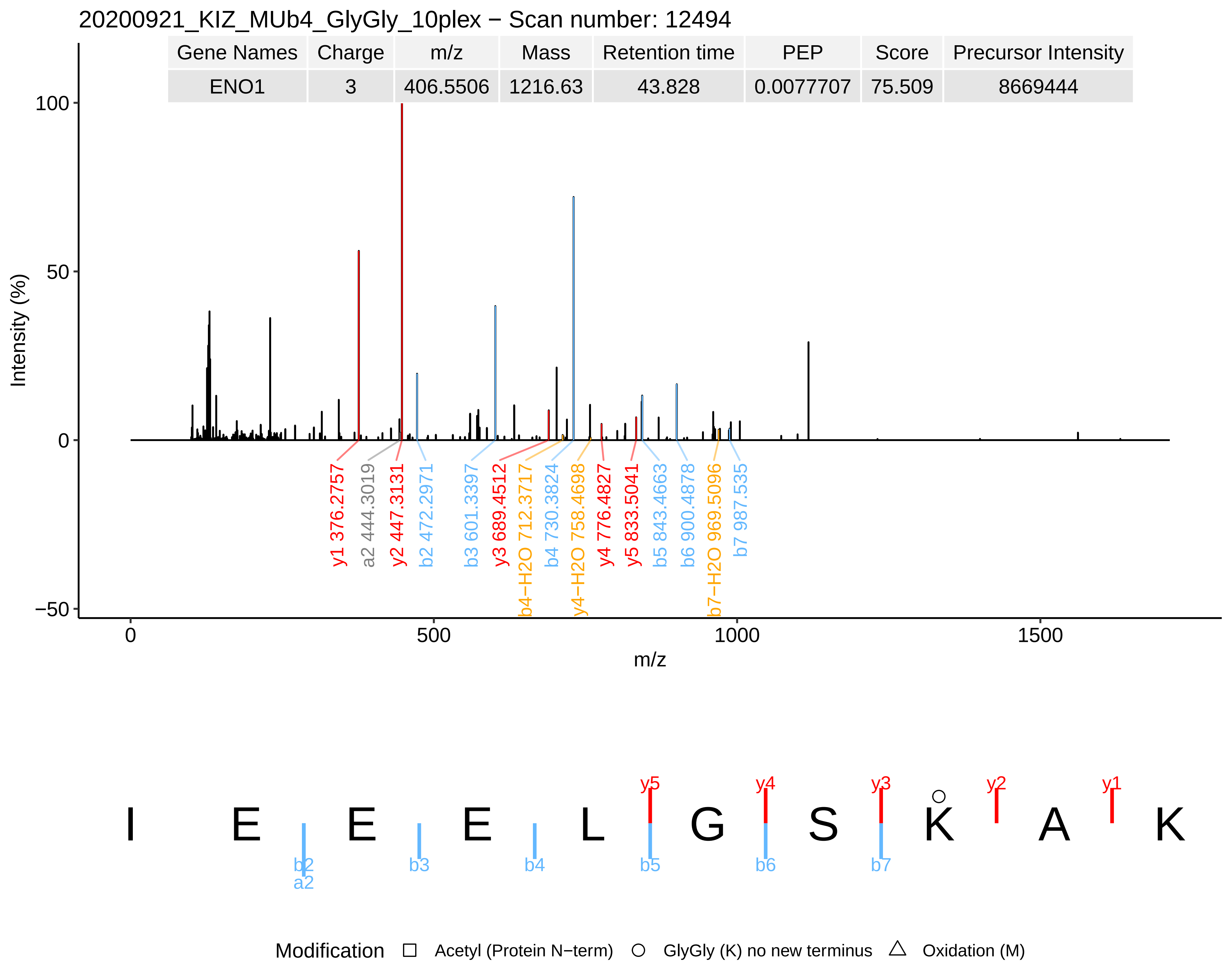

20200921_KIZ_MUb4_GlyGly_10plex − Scan number: 12494
Gene Names | Charge | m/z | Mass | Retention time | PEP | Score | Precursor Intensity
ENO1 | 3 | 406.5506 | 1216.63 | 43.828 | 0.0077707 | 75.509 | 8669444
Intensity (%)
100
50
0
−50
0
500
1000
1500
m/z
y1 376.2757
a2 444.3019
y2 447.3131
b2 472.2971
b3 601.3397
y3 689.4512
b4−H2O 712.3717
b4 730.3824
y4−H2O 758.4698
y4 776.4827
y5 833.5041
b5 843.4663
b6 900.4878
b7−H2O 969.5096
b7 987.535
I E E E L G S K A K
y5 y4 y3 y2 y1
b2 a2 b3 b4 b5 b6 b7
Modification
Acetyl (Protein N−term)
GlyGly (K) no new terminus
Oxidation (M)

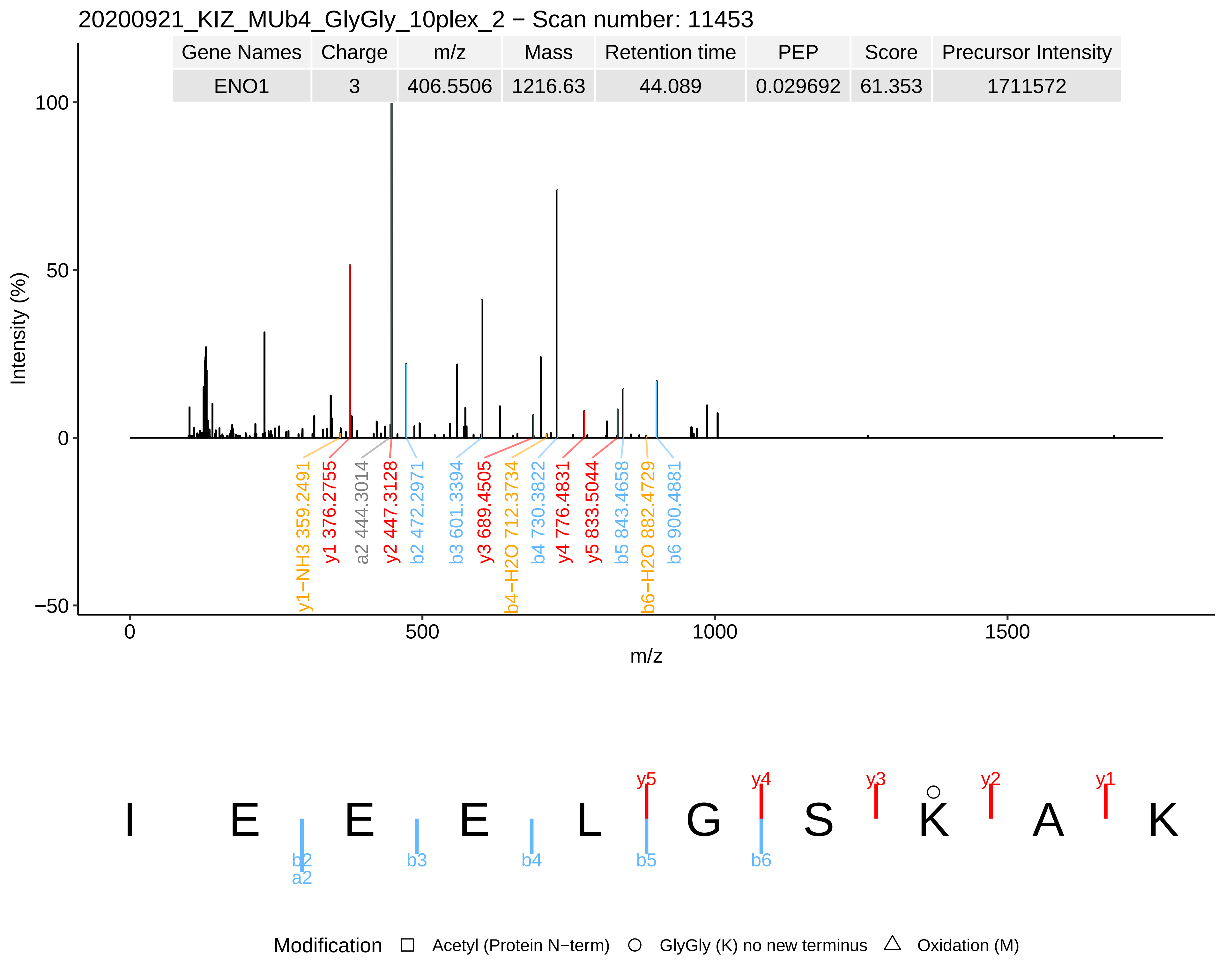
20200921_KIZ_MUb4_GlyGly_10plex_2 − Scan number: 11453
Gene Names | Charge | m/z | Mass | Retention time | PEP | Score | Precursor Intensity
ENO1 | 3 | 406.5506 | 1216.63 | 44.089 | 0.029692 | 61.353 | 1711572
Intensity (%)
m/z
y1−NH3 359.2491
y1 376.2755
a2 444.3014
y2 447.3128
b2 472.2971
b3 601.3394
y3 689.4505
b4−H2O 712.3734
b4 730.3822
y4 776.4831
y5 833.5044
b5 843.4658
b6−H2O 882.4729
b6 900.4881
I E E E L G S K A K
y5 y4 y3 y2 y1
b2 a2 b3 b4 b5 b6
Modification
Acetyl (Protein N−term)
GlyGly (K) no new terminus
Oxidation (M)

20200921_KIZ_MUb4_GlyGly_10plex − Scan number: 7730

Gene Names	Charge	m/z	Mass	Retention time	PEP	Score	Precursor Intensity
TOM1L1	3	410.2263	1227.657	26.051	0.01445	70.332	4195486

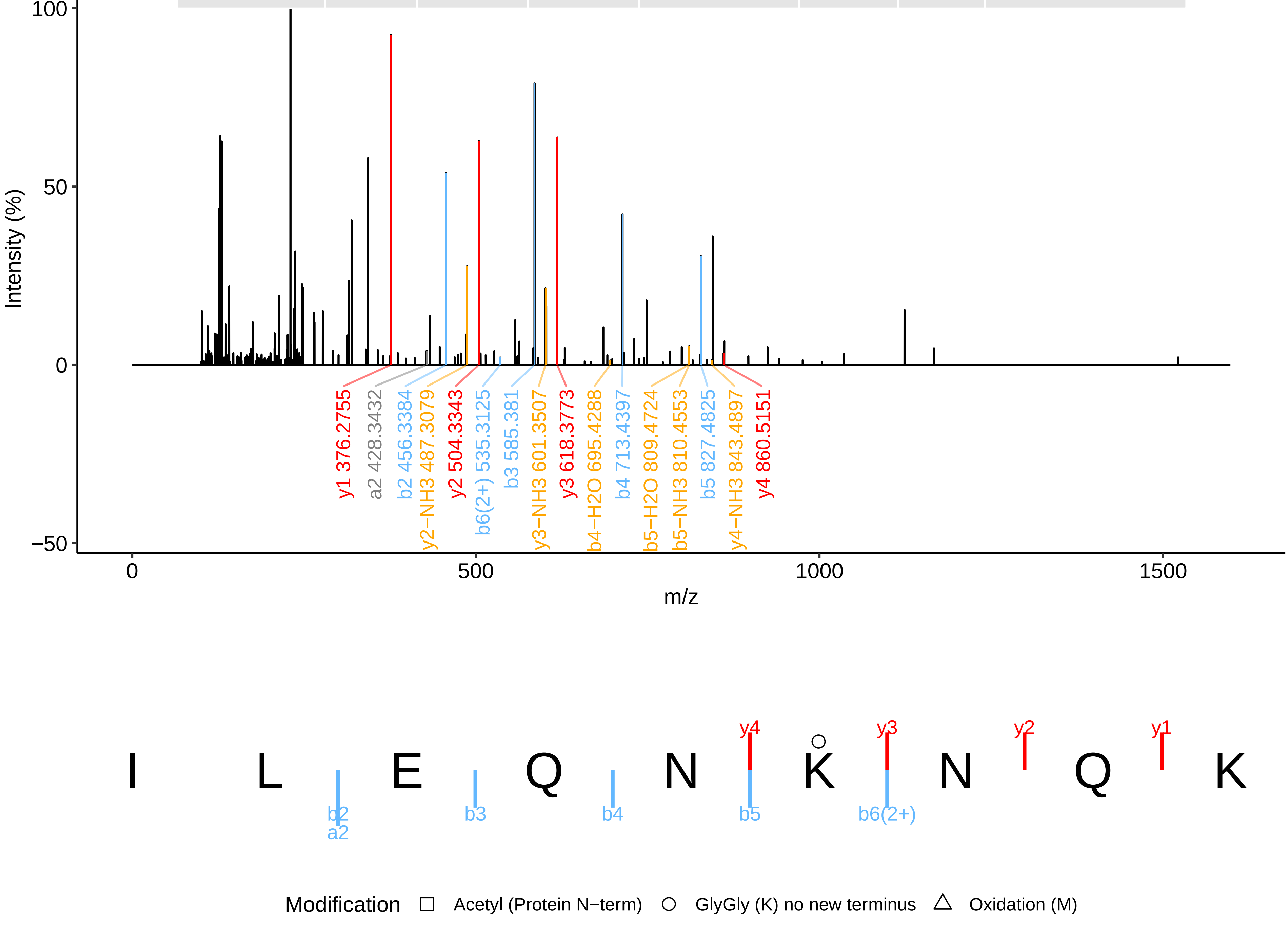

20200921_KIZ_MUb4_GlyGly_10plex_2 − Scan number: 7173

Gene Names	Charge	m/z	Mass	Retention time	PEP	Score	Precursor Intensity
TOM1L1	3	410.2263	1227.657	26.666	0.11611	43.442	969922.8

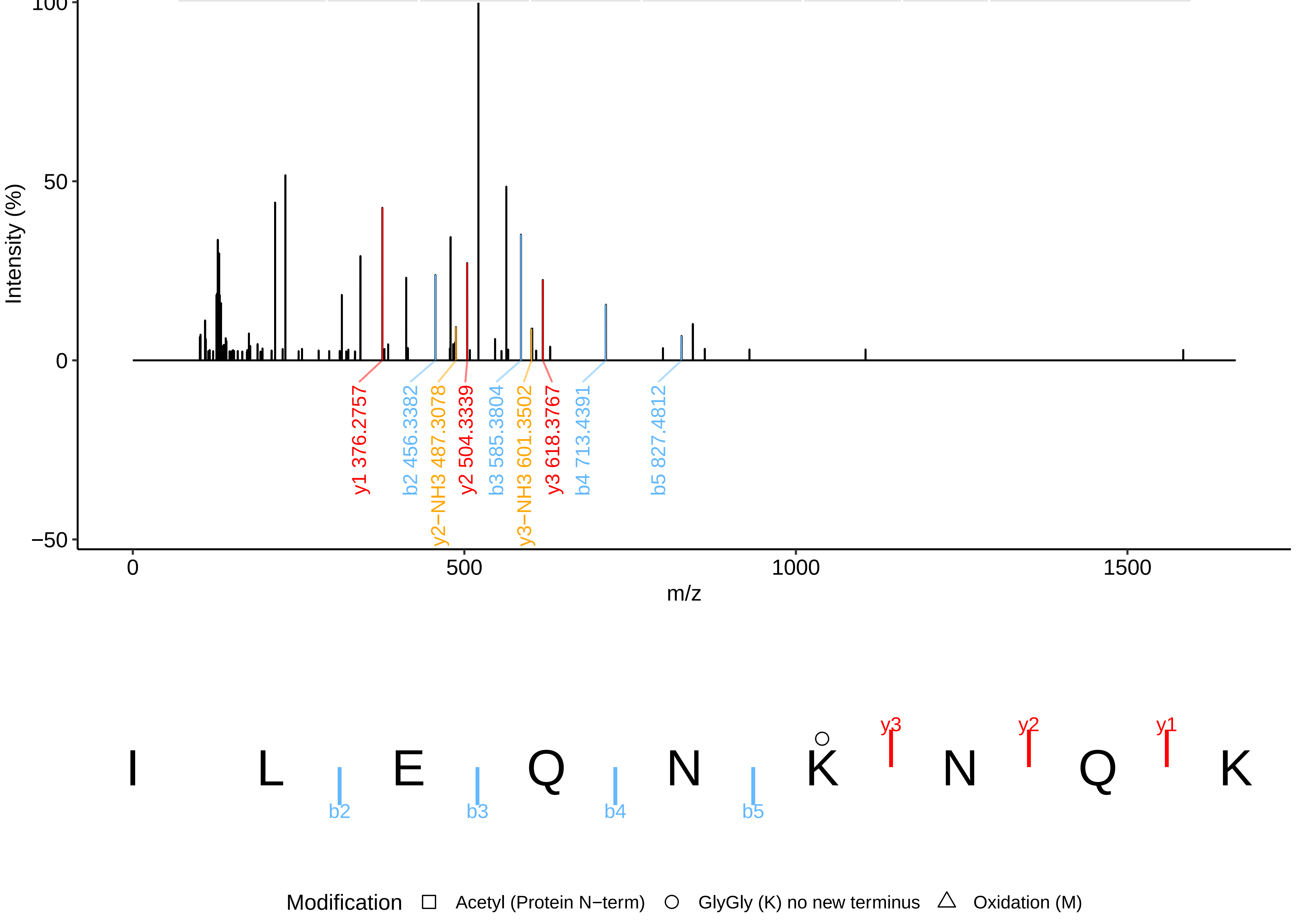

20201118_KIZ_MUb6_GlyGly_1 − Scan number: 24696

Gene Names	Charge	m/z	Mass	Retention time	PEP	Score	Precursor Intensity
NUDCD1	3	478.9401	1433.799	75.936	0.063708	45.764	17797872

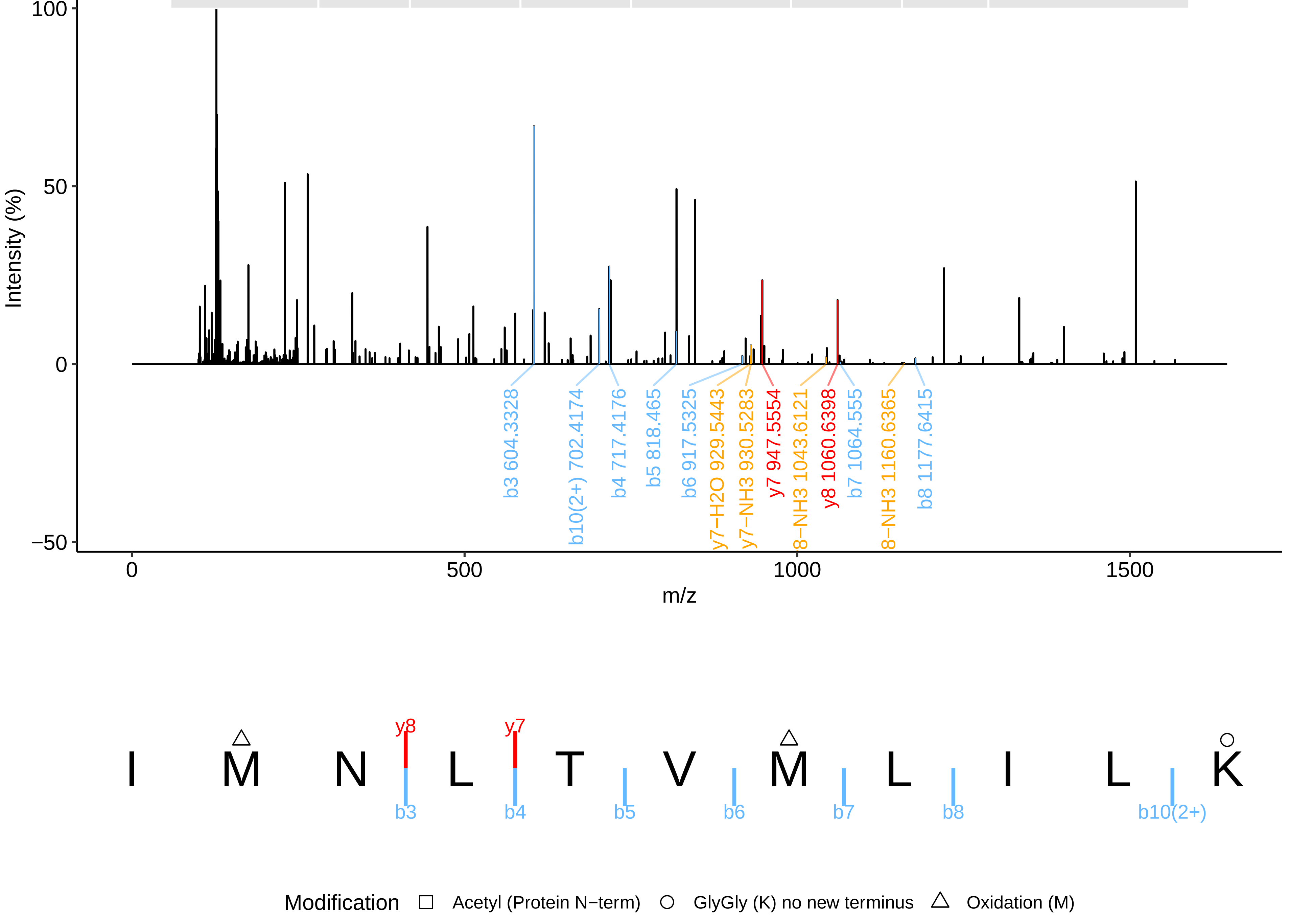

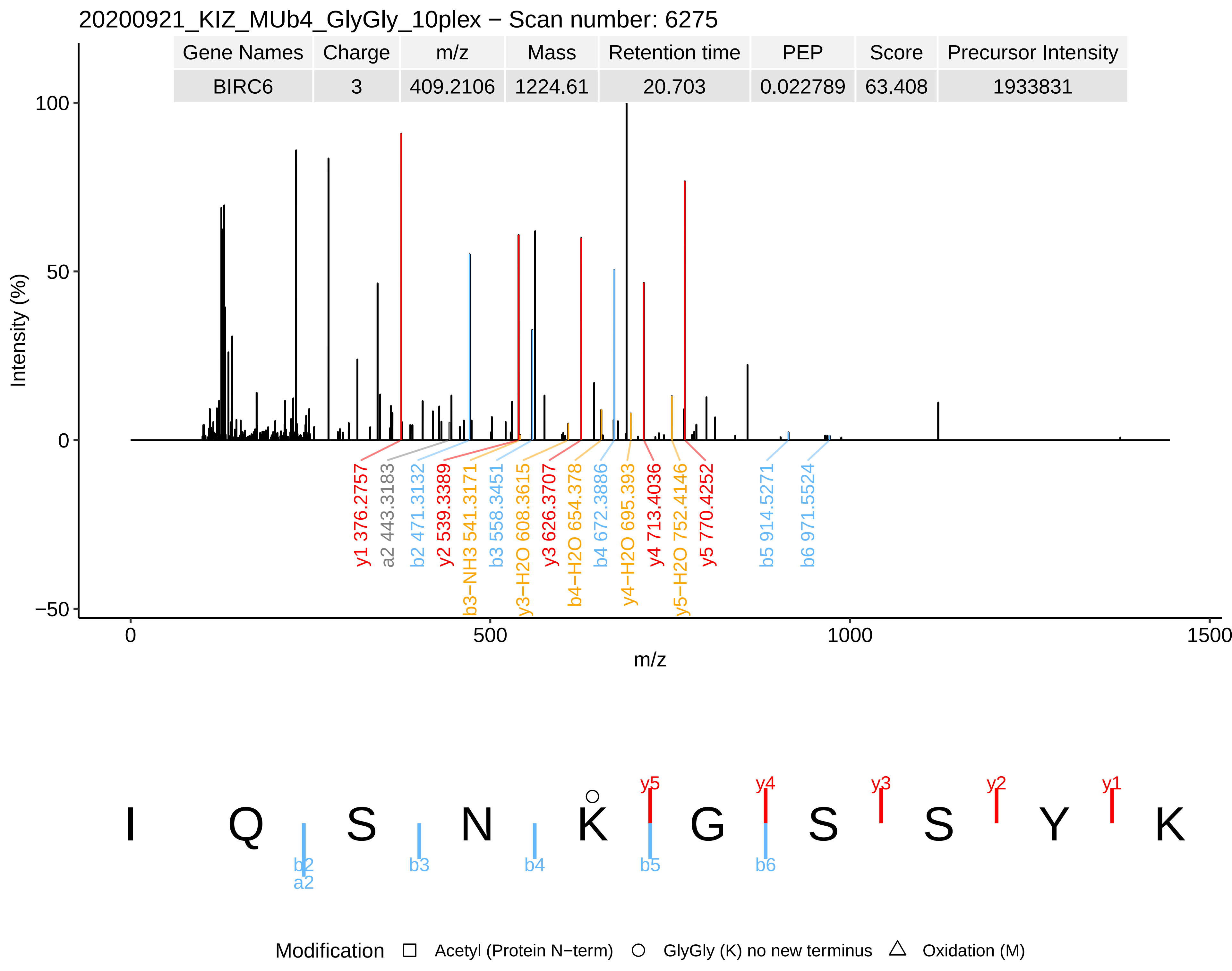
20200921_KIZ_MUb4_GlyGly_10plex − Scan number: 6275
Gene Names | Charge | m/z | Mass | Retention time | PEP | Score | Precursor Intensity
BIRC6 | 3 | 409.2106 | 1224.61 | 20.703 | 0.022789 | 63.408 | 1933831
Intensity (%)
100
50
0
−50
0
500
1000
1500
m/z
y1 376.2757
a2 443.3183
b2 471.3132
y2 539.3389
b3−NH3 541.3171
b3 558.3451
y3−H2O 608.3615
y3 626.3707
b4−H2O 654.378
b4 672.3886
y4−H2O 695.393
y4 713.4036
y5−H2O 752.4146
y5 770.4252
b5 914.5271
b6 971.5524
I Q S N K G S S Y K
y5 y4 y3 y2 y1
b2 a2 b3 b4 b5 b6
Modification
Acetyl (Protein N−term)
GlyGly (K) no new terminus
Oxidation (M)

20200921_KIZ_MUb4_GlyGly_10plex_2 − Scan number: 5733

Gene Names	Charge	m/z	Mass	Retention time	PEP	Score	Precursor Intensity
BIRC6	3	409.2106	1224.61	20.752	0.050269	57.348	457531.1

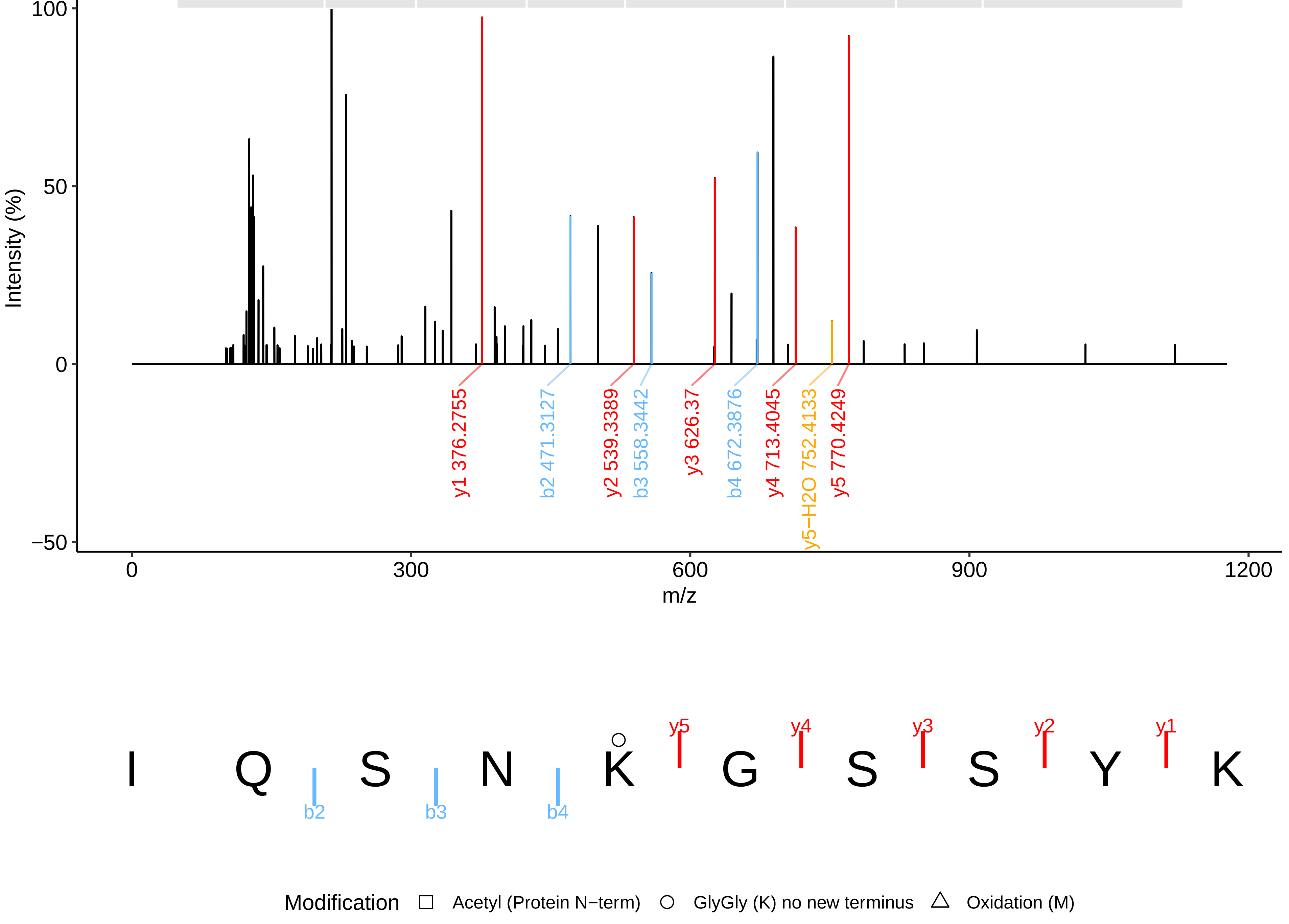

20200921_KIZ_MUb4_GlyGly_10plex − Scan number: 9421

Gene Names	Charge	m/z	Mass	Retention time	PEP	Score	Precursor Intensity
ATP5A1	3	411.5542	1231.641	32.245	0.0033265	86.189	11927588

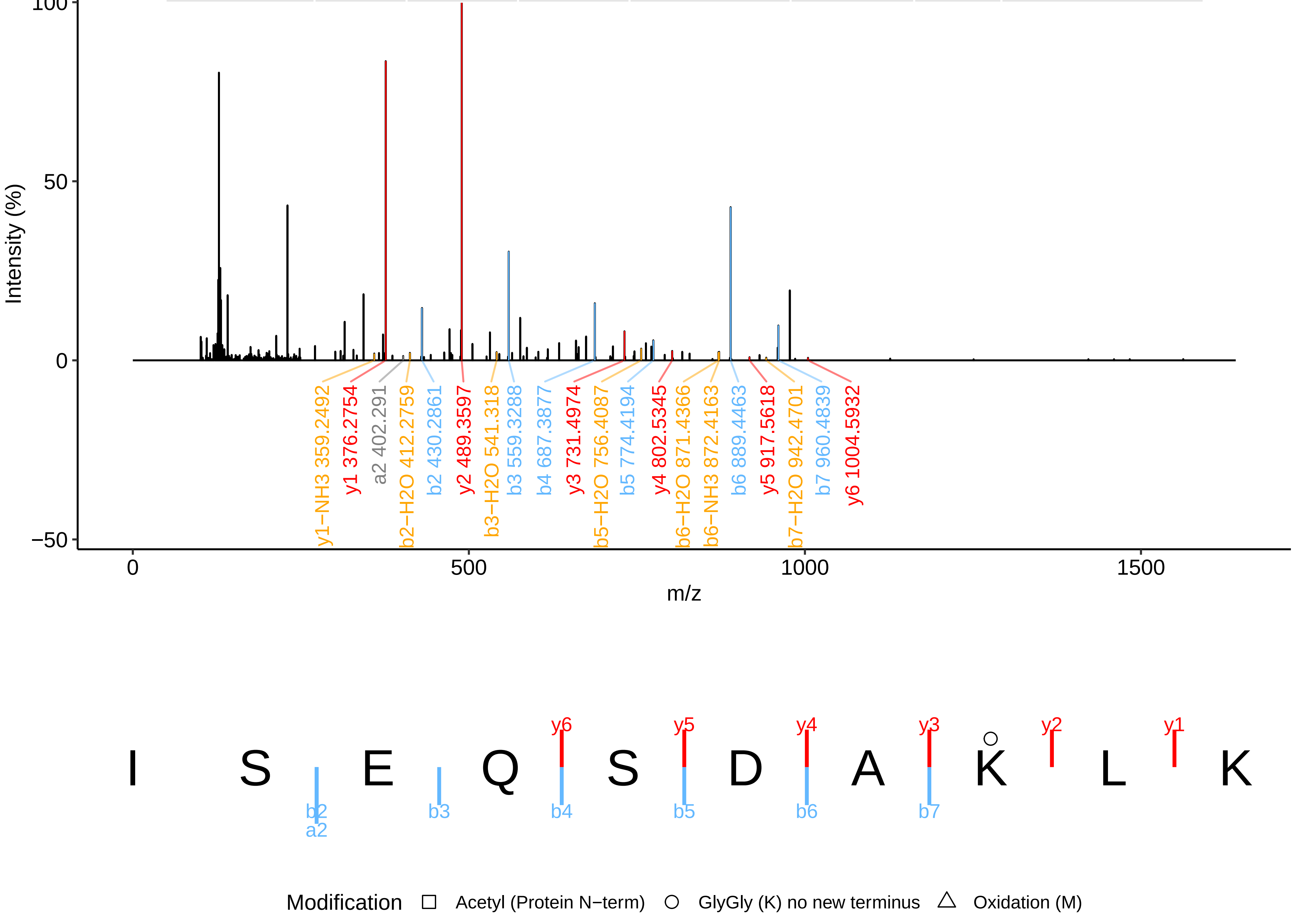

20200921_KIZ_MUb4_GlyGly_10plex_2 − Scan number: 8646

Gene Names	Charge	m/z	Mass	Retention time	PEP	Score	Precursor Intensity
ATP5A1	3	411.5542	1231.641	32.637	0.093259	48.981	586213.2

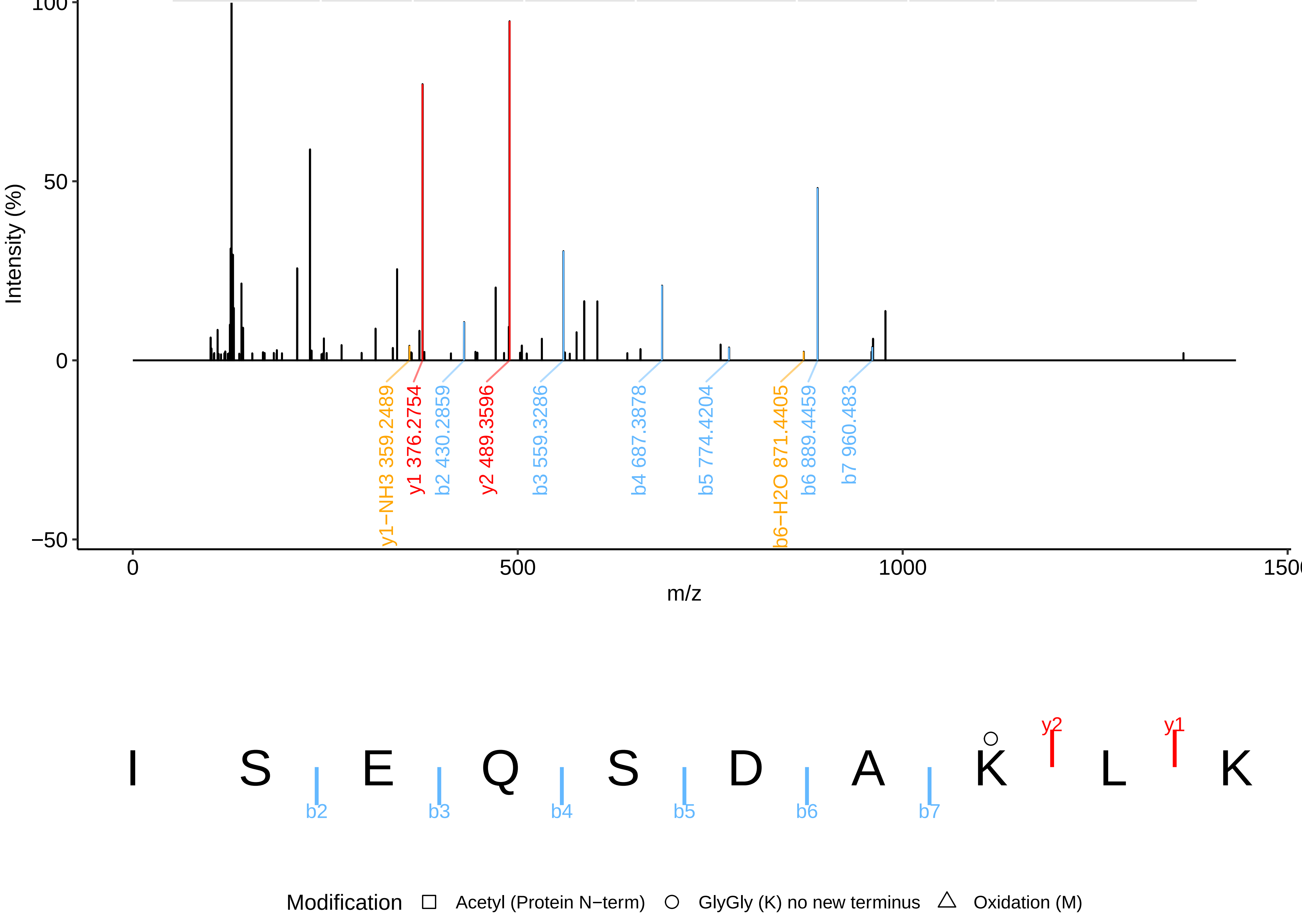

20200921_KIZ_MUb4_GlyGly_10plex − Scan number: 14123

Gene Names	Charge	m/z	Mass	Retention time	PEP	Score	Precursor Intensity
TCAF1	3	411.5714	1231.693	50.068	3.6267e−05	107.15	9744020

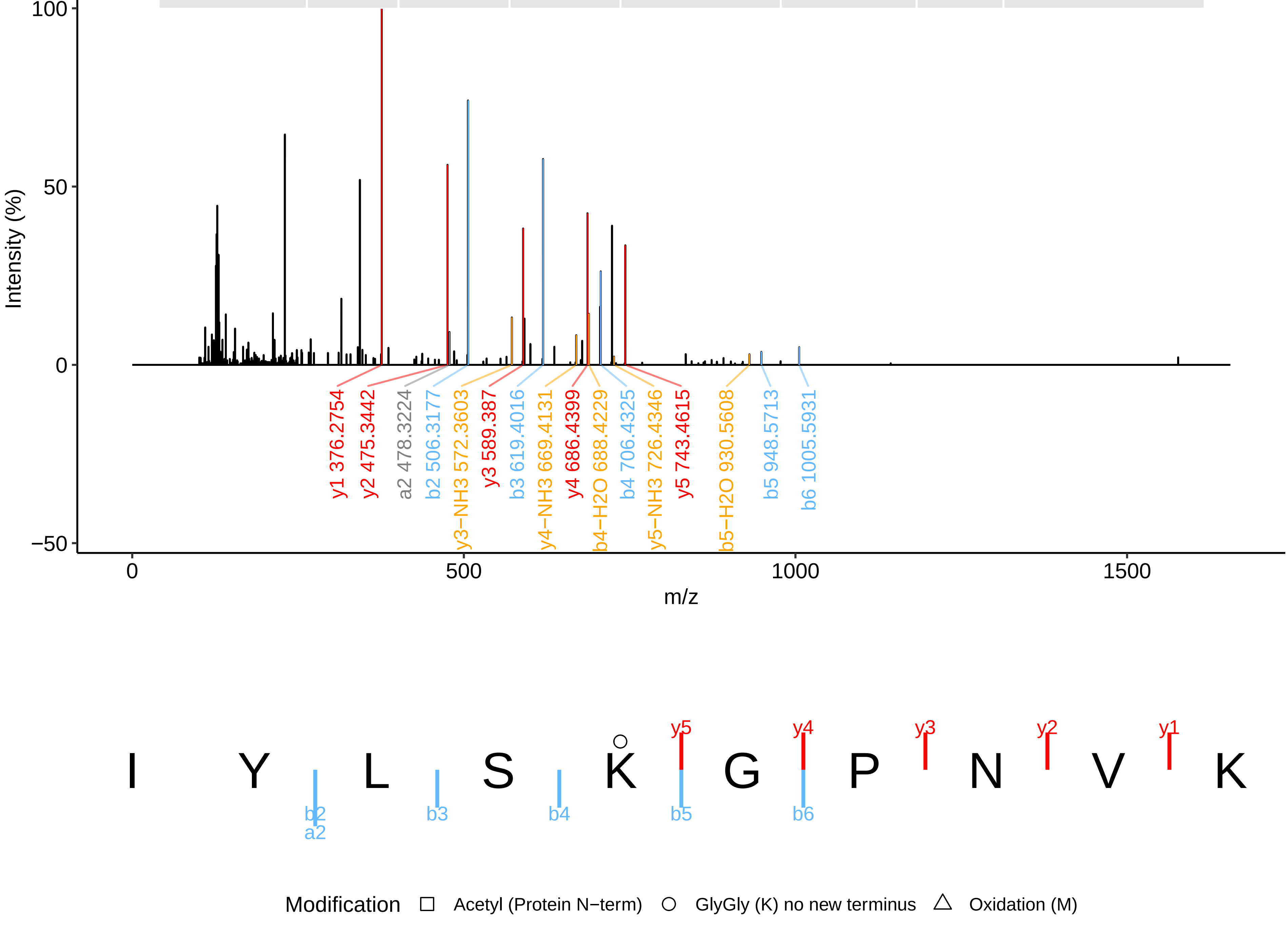

20200921_KIZ_MUb4_GlyGly_10plex_2 − Scan number: 13012

Gene Names	Charge	m/z	Mass	Retention time	PEP	Score	Precursor Intensity
TCAF1	3	411.5714	1231.693	50.486	0.044694	58.433	1253675

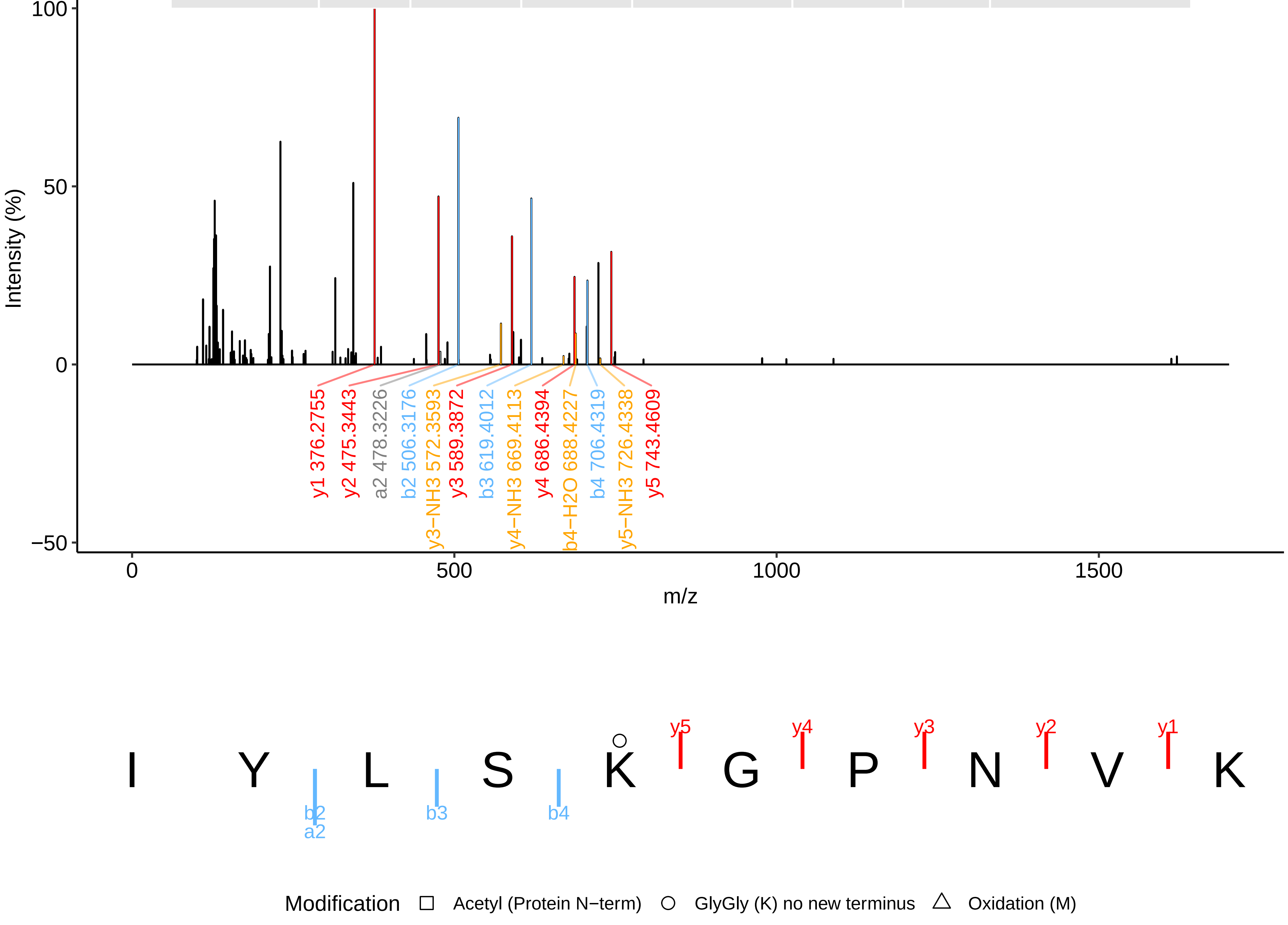

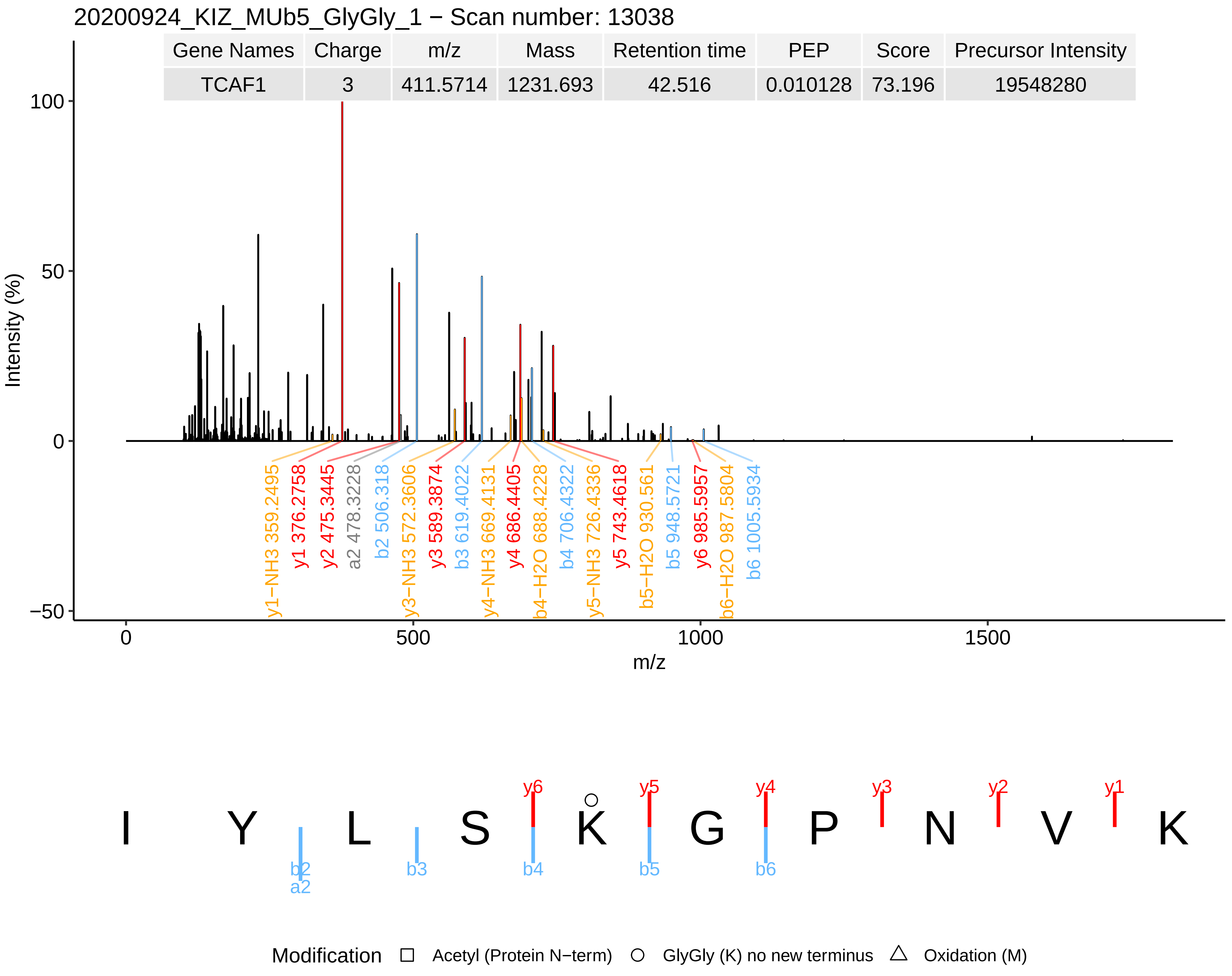
20200924_KIZ_MUb5_GlyGly_1 − Scan number: 13038
Gene Names | Charge | m/z | Mass | Retention time | PEP | Score | Precursor Intensity
TCAF1 | 3 | 411.5714 | 1231.693 | 42.516 | 0.010128 | 73.196 | 19548280
Intensity (%)
m/z
y1−NH3 359.2495
y1 376.2758
y2 475.3445
a2 478.3228
b2 506.318
y3−NH3 572.3606
y3 589.3874
b3 619.4022
y4−NH3 669.4131
y4 686.4405
b4−H2O 688.4228
b4 706.4322
y5−NH3 726.4336
y5 743.4618
b5−H2O 930.561
b5 948.5721
y6 985.5957
b6−H2O 987.5804
b6 1005.5934
I Y L S K G P N V K
y6 y5 y4 y3 y2 y1
b2 a2 b3 b4 b5 b6
Modification
Acetyl (Protein N−term)
GlyGly (K) no new terminus
Oxidation (M)

20200924_KIZ_MUb5_GlyGly_1 − Scan number: 13196

Gene Names	Charge	m/z	Mass	Retention time	PEP	Score	Precursor Intensity
TCAF1	3	411.5714	1231.693	43.019	2.067e−05	110.76	18918376

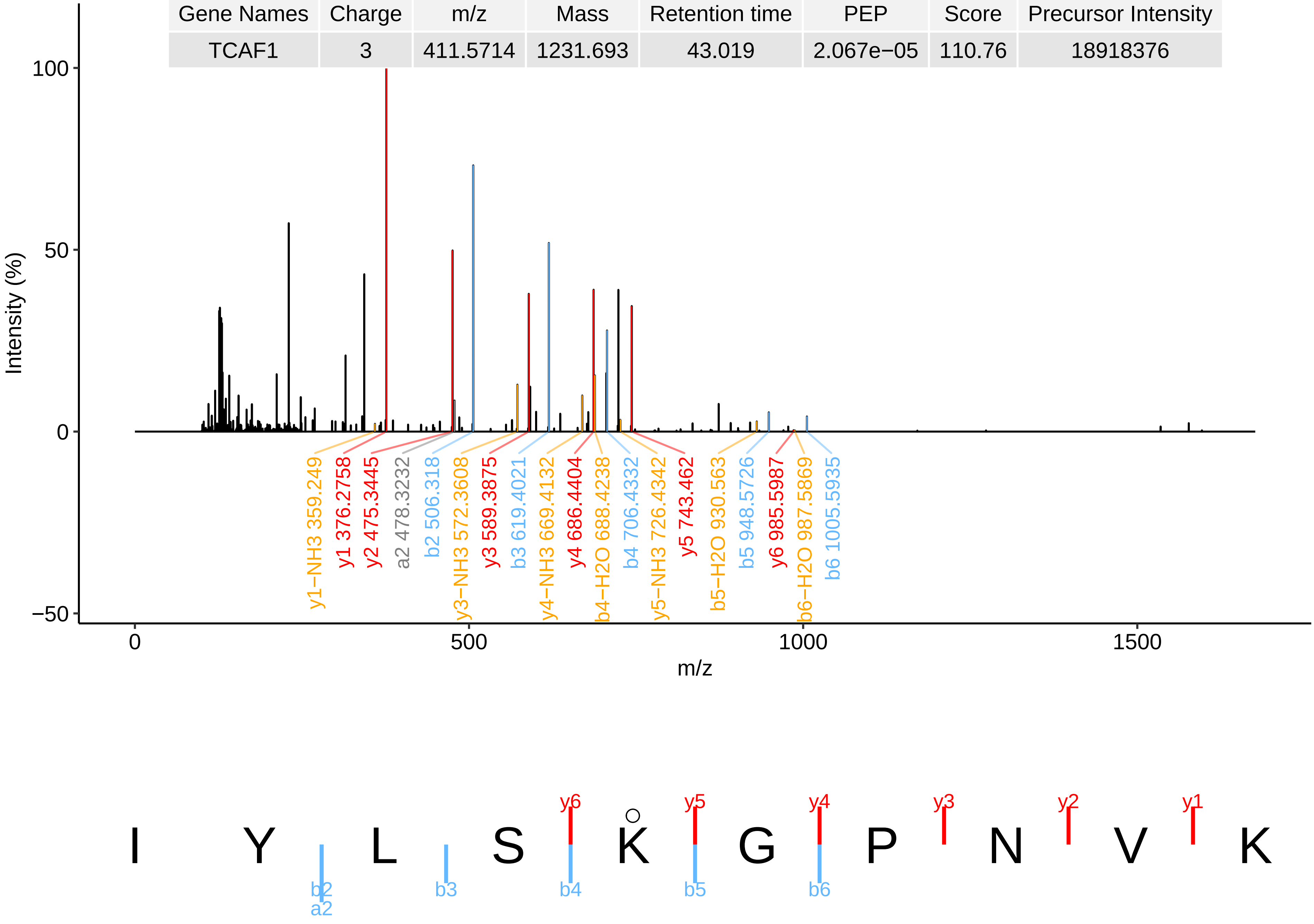

Modification □ Acetyl (Protein N−term) ○ GlyGly (K) no new terminus △ Oxidation (M)

20200924_KIZ_MUb5_GlyGly_1 − Scan number: 13020

Gene Names	Charge	m/z	Mass	Retention time	PEP	Score	Precursor Intensity
TCAF1	3	411.5714	1231.693	42.453	0.0039313	82.749	18732792

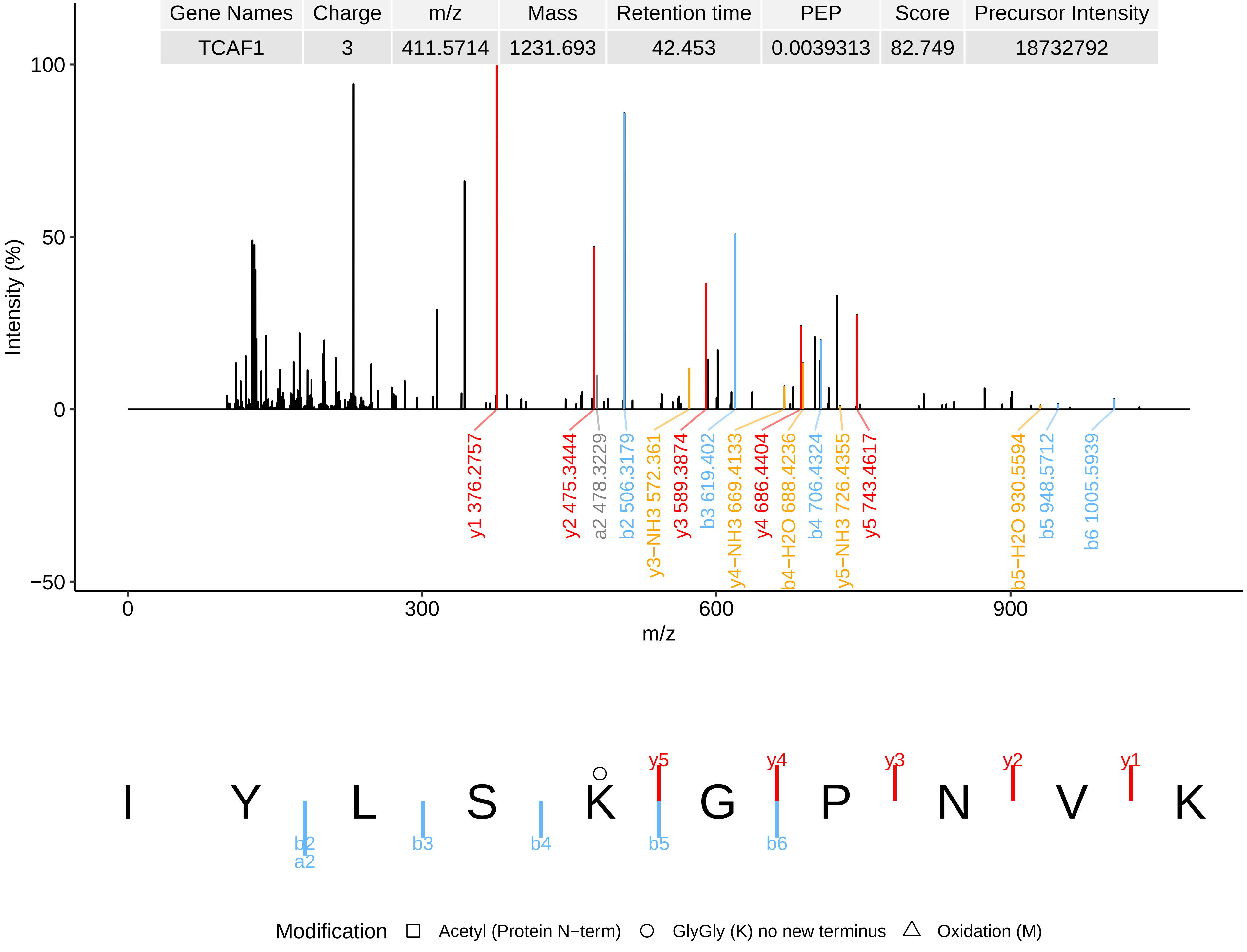

20200924_KIZ_MUb5_GlyGly_2 − Scan number: 12159

Gene Names	Charge	m/z	Mass	Retention time	PEP	Score	Precursor Intensity
TCAF1	3	411.5714	1231.693	45.457	0.00096332	91.867	2250002

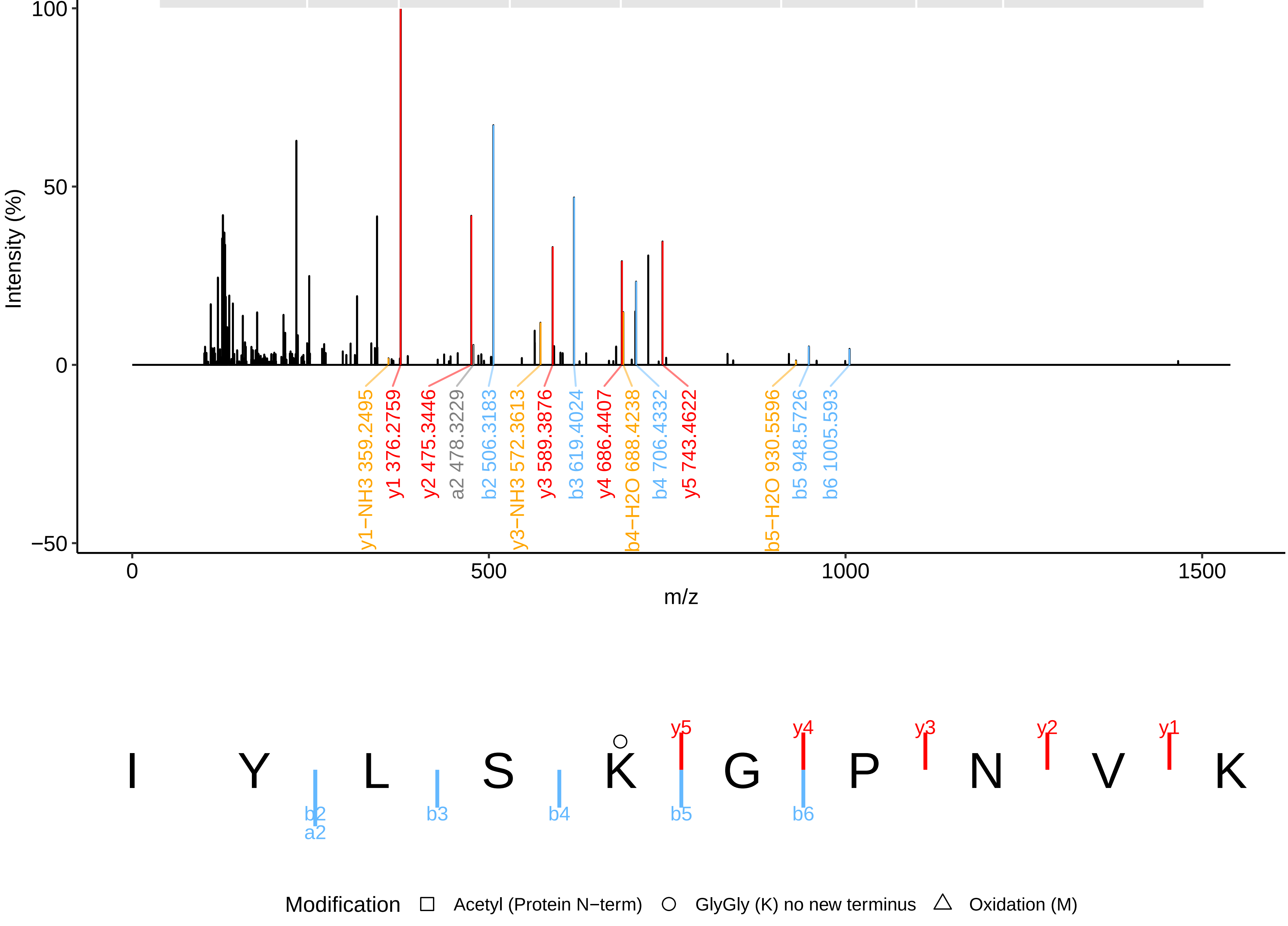

20200924_KIZ_MUb5_GlyGly_2 − Scan number: 12297

Gene Names	Charge	m/z	Mass	Retention time	PEP	Score	Precursor Intensity
TCAF1	3	411.5714	1231.693	45.967	5.1887e−05	103.55	5094329

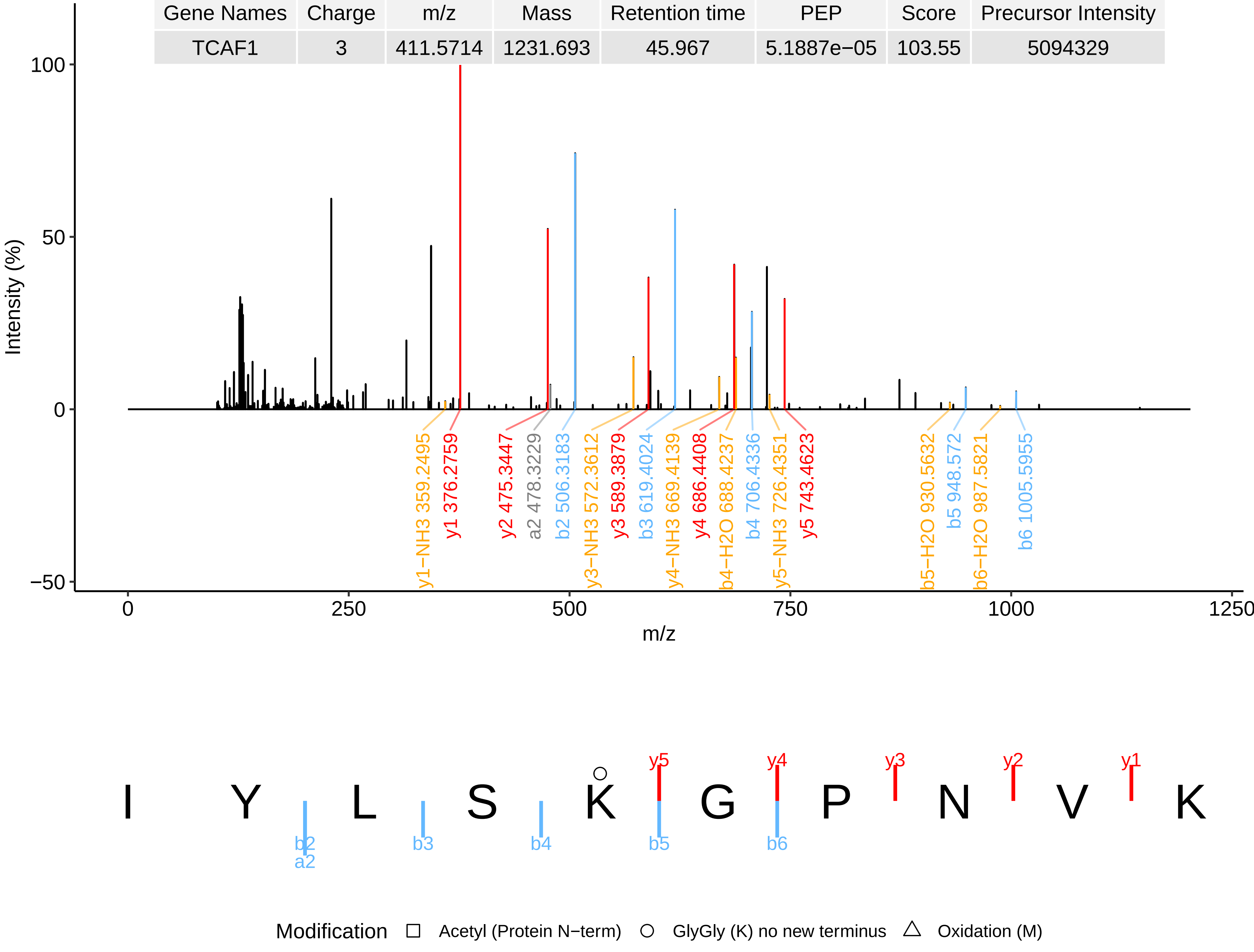

20200924_KIZ_MUb5_GlyGly_2 − Scan number: 12439

Gene Names	Charge	m/z	Mass	Retention time	PEP	Score	Precursor Intensity
TCAF1	3	411.5714	1231.693	46.48	0.00055448	94.692	4504174

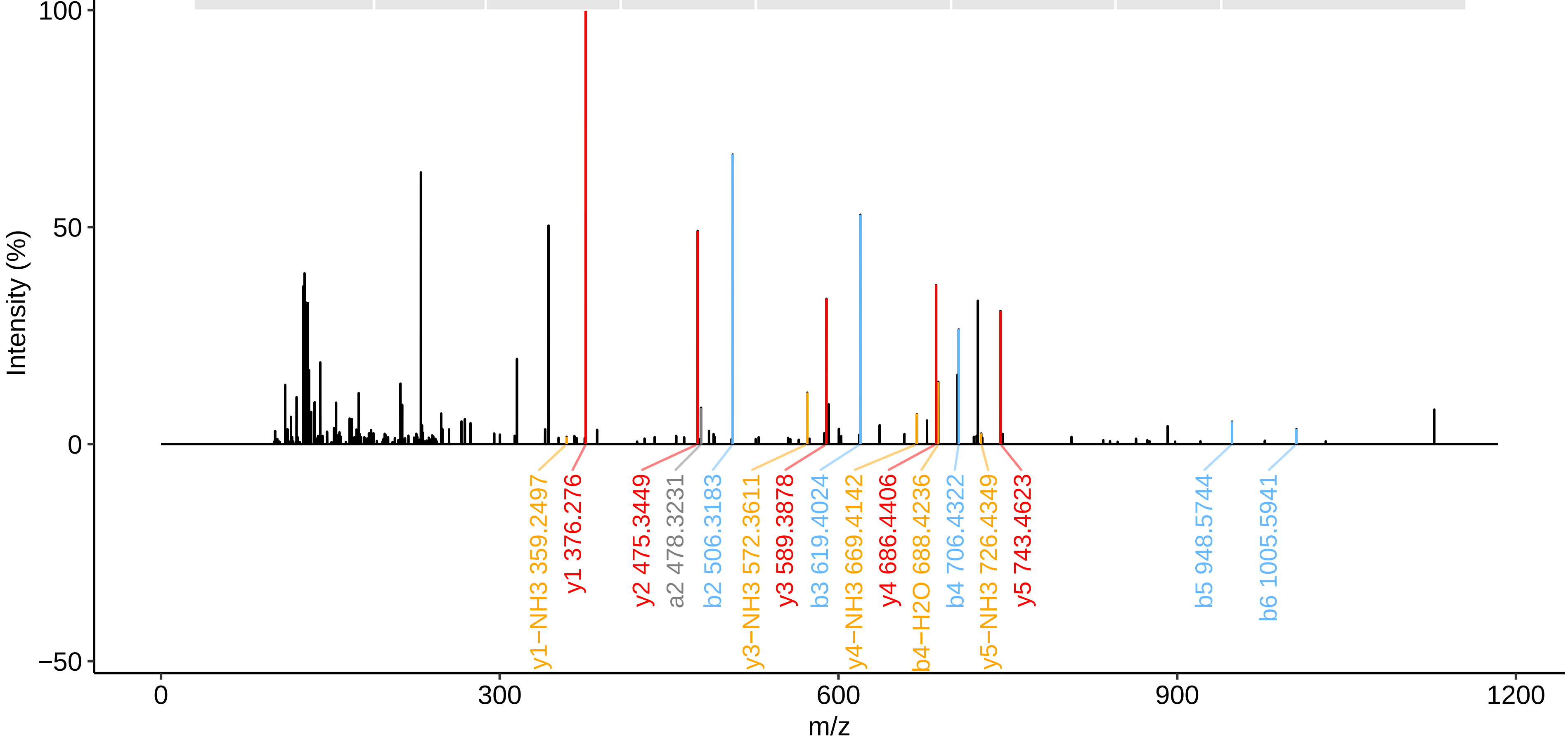

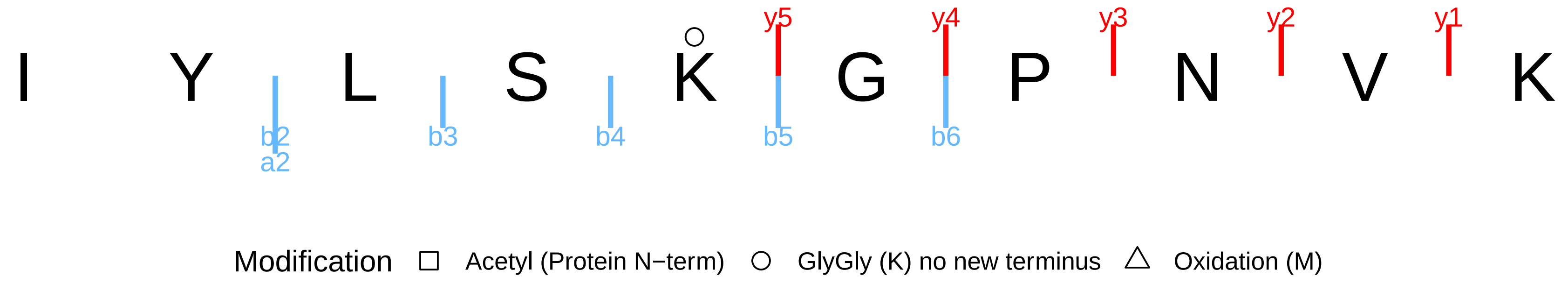

20200924_KIZ_MUb5_GlyGly_2 − Scan number: 12460

Gene Names	Charge	m/z	Mass	Retention time	PEP	Score	Precursor Intensity
TCAF1	3	411.5714	1231.693	46.561	0.11543	44.203	1962454

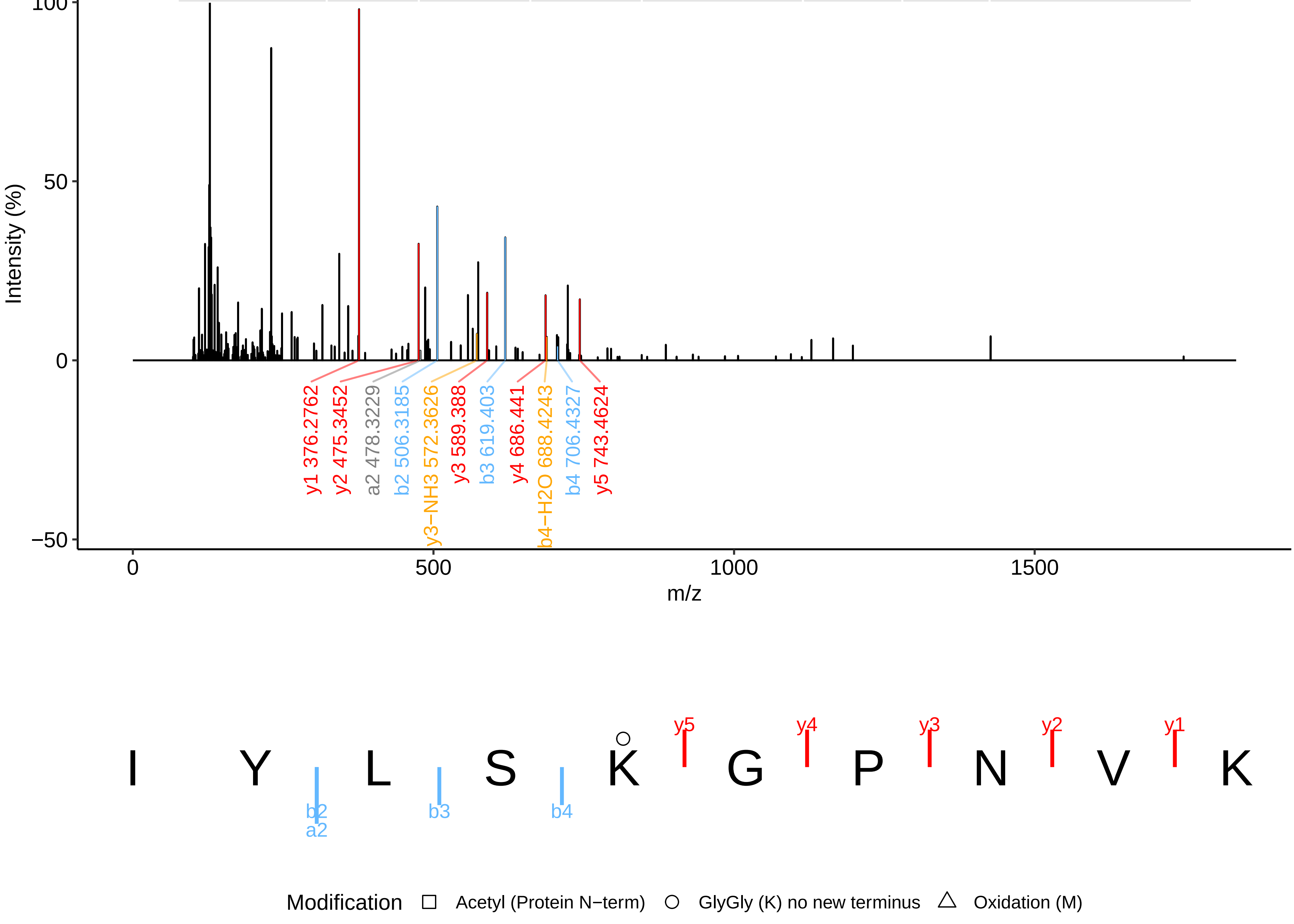

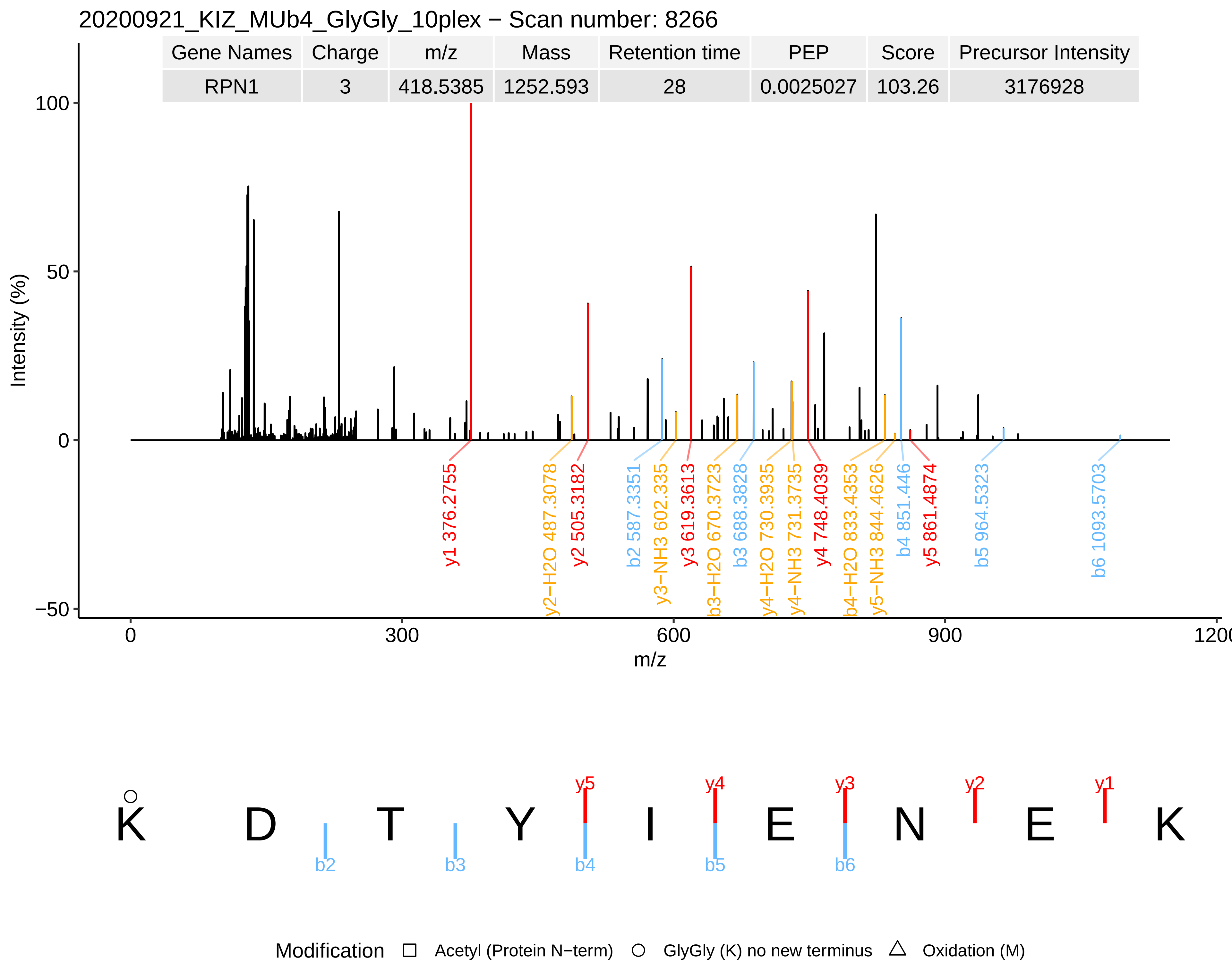
20200921_KIZ_MUb4_GlyGly_10plex − Scan number: 8266
Gene Names
RPN1
Charge
3
m/z
418.5385
Mass
1252.593
Retention time
28
PEP
0.0025027
Score
103.26
Precursor Intensity
3176928
Intensity (%)
100
50
0
−50
0
300
600
900
1200
m/z
y1 376.2755
y2−H2O 487.3078
y2 505.3182
b2 587.3351
y3−NH3 602.335
y3 619.3613
b3−H2O 670.3723
b3 688.3828
y4−H2O 730.3935
y4−NH3 731.3735
y4 748.4039
b4−H2O 833.4353
y5−NH3 844.4626
b4 851.446
y5 861.4874
b5 964.5323
b6 1093.5703
K D T Y I E N E K
y5 y4 y3 y2 y1
b2 b3 b4 b5 b6
Modification
Acetyl (Protein N−term)
GlyGly (K) no new terminus
Oxidation (M)

20201118_KIZ_MUb6_GlyGly_1 − Scan number: 3104

Gene Names	Charge	m/z	Mass	Retention time	PEP	Score	Precursor Intensity
DNAJA1	3	488.8762	1463.607	9.5722	0.0011829	104.79	NA

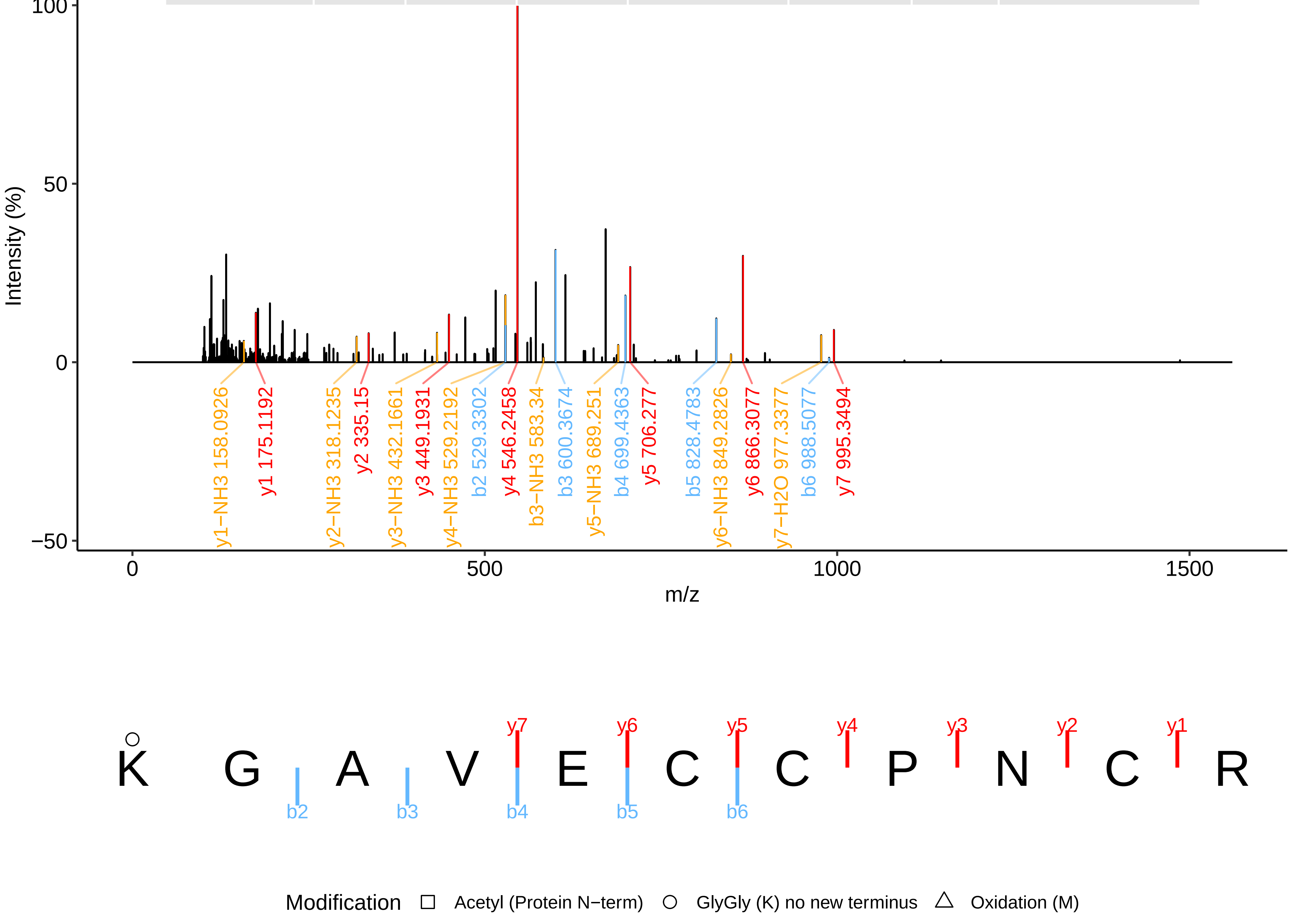

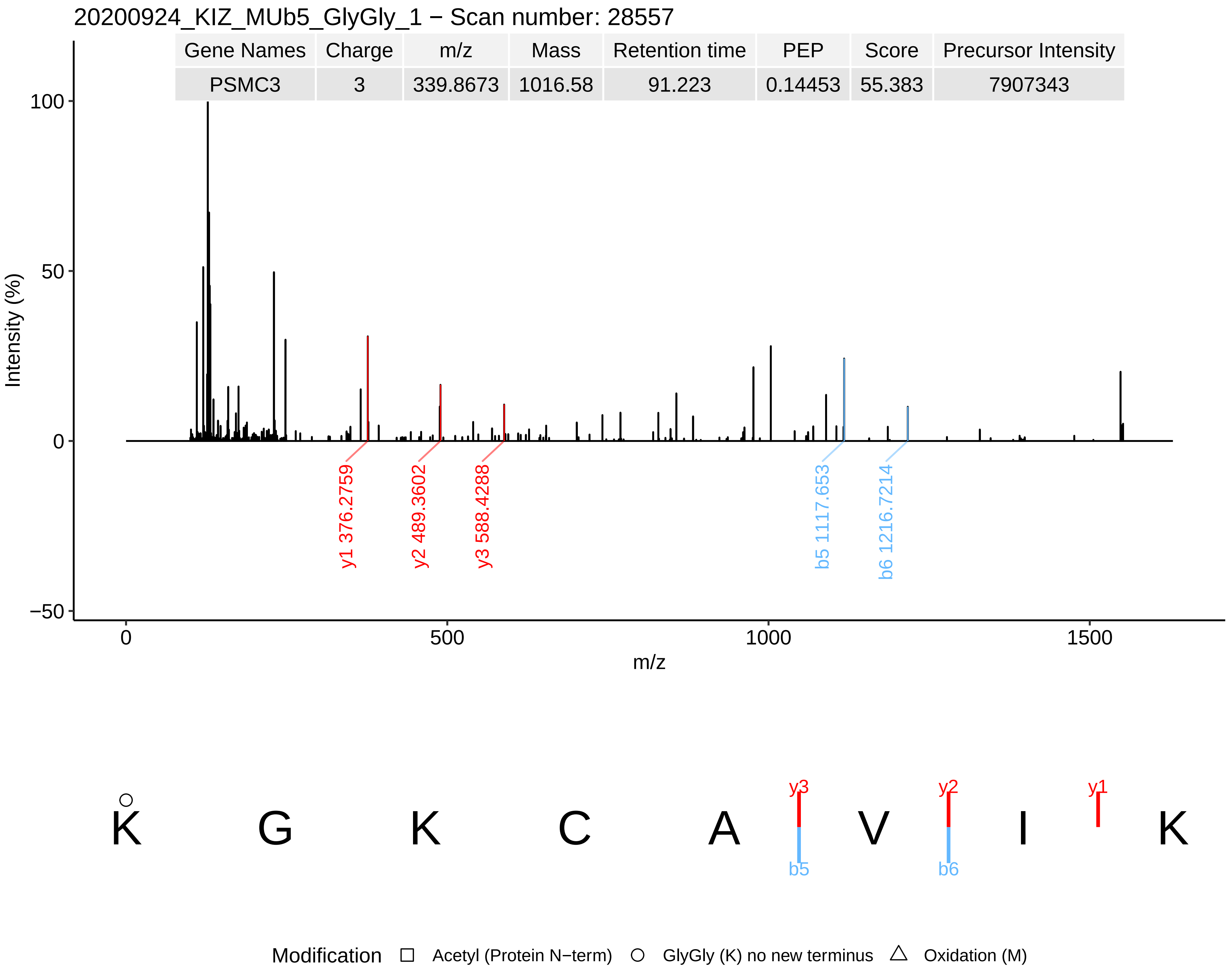

20200924_KIZ_MUb5_GlyGly_1 − Scan number: 28557

Gene Names	Charge	m/z	Mass	Retention time	PEP	Score	Precursor Intensity
PSMC3	3	339.8673	1016.58	91.223	0.14453	55.383	7907343

Intensity (%)
m/z
y1 376.2759
y2 489.3602
y3 588.4288
b5 1117.653
b6 1216.7214
K G K C A V I K
y3 y2 y1
b5 b6
Modification
Acetyl (Protein N−term)
GlyGly (K) no new terminus
Oxidation (M)

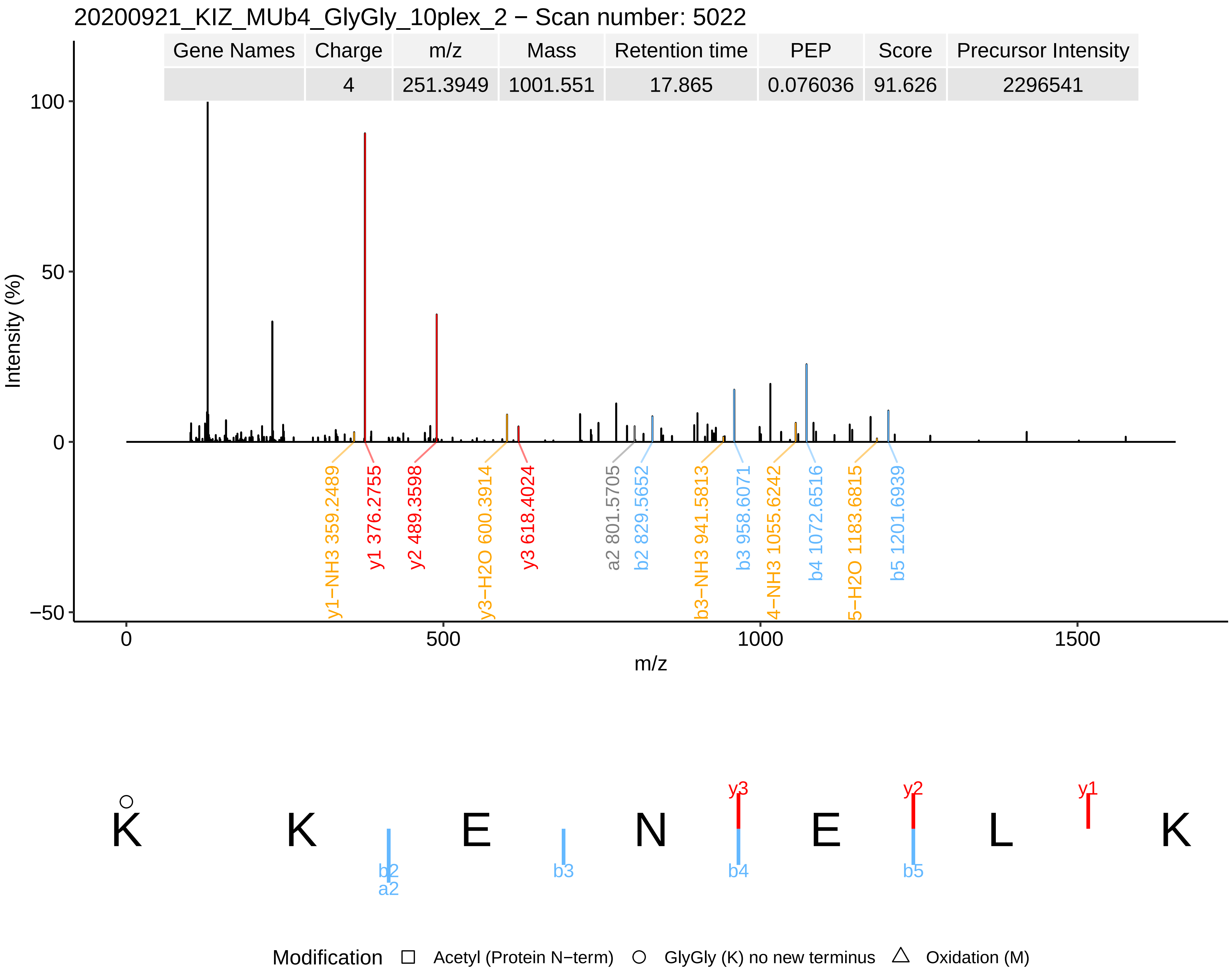

20200921_KIZ_MUb4_GlyGly_10plex_2 − Scan number: 5022
Gene Names | Charge | m/z | Mass | Retention time | PEP | Score | Precursor Intensity
4 | 251.3949 | 1001.551 | 17.865 | 0.076036 | 91.626 | 2296541
Intensity (%)
m/z
y1−NH3 359.2489
y1 376.2755
y2 489.3598
y3−H2O 600.3914
y3 618.4024
a2 801.5705
b2 829.5652
b3−NH3 941.5813
b3 958.6071
b4−NH3 1055.6242
b4 1072.6516
b5−H2O 1183.6815
b5 1201.6939
K K E N E L K
y3 y2 y1
b2 a2 b3 b4 b5
Modification
Acetyl (Protein N−term)
GlyGly (K) no new terminus
Oxidation (M)

20200921_KIZ_MUb4_GlyGly_10plex − Scan number: 5258

Gene Names	Charge	m/z	Mass	Retention time	PEP	Score	Precursor Intensity
ATP5I	3	334.8575	1001.551	17.193	0.0035583	114.89	7533899

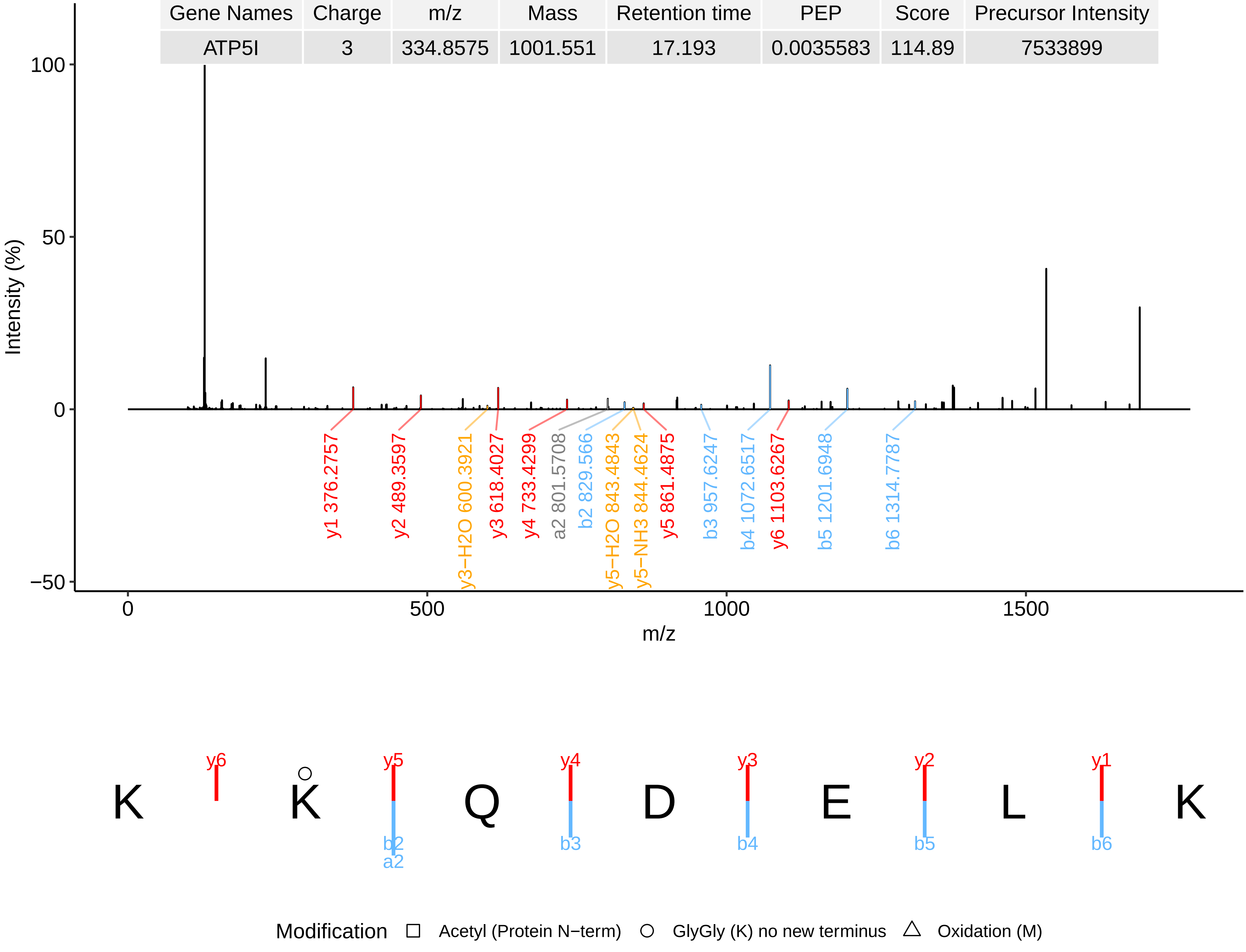

20200921_KIZ_MUb4_GlyGly_10plex − Scan number: 5429

Gene Names	Charge	m/z	Mass	Retention time	PEP	Score	Precursor Intensity
ATP5I	3	334.8575	1001.551	17.781	0.010253	100.02	2614563

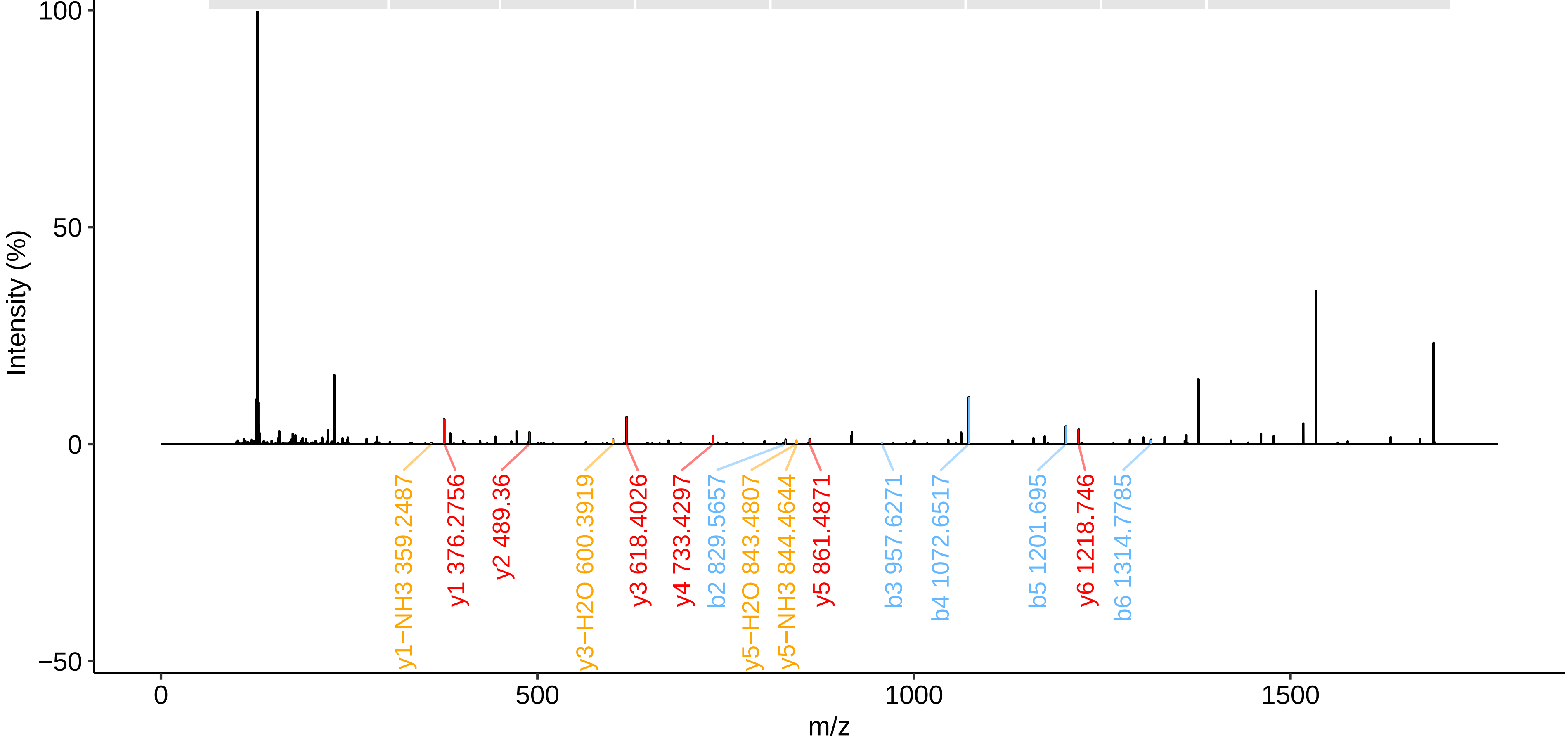

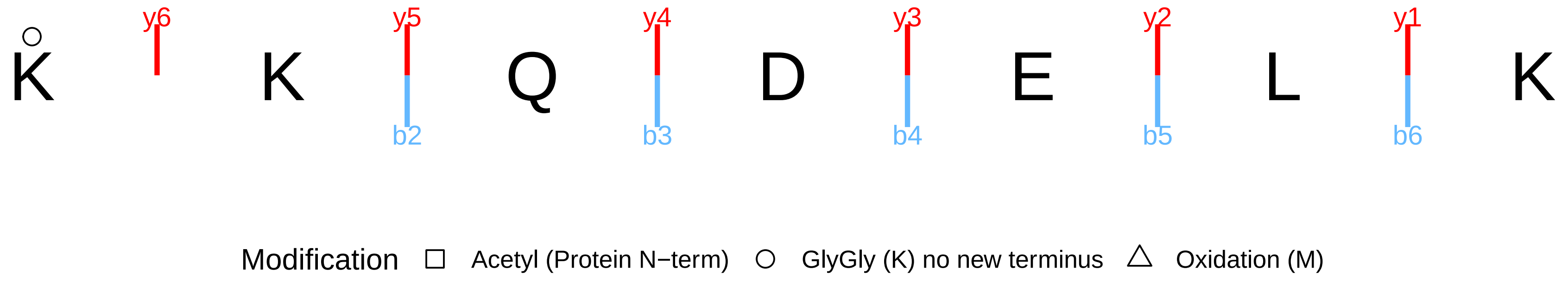

20200921_KIZ_MUb4_GlyGly_10plex_2 − Scan number: 4890

Gene Names	Charge	m/z	Mass	Retention time	PEP	Score	Precursor Intensity
ATP5I	3	334.8575	1001.551	17.344	0.0095672	101.54	1633824

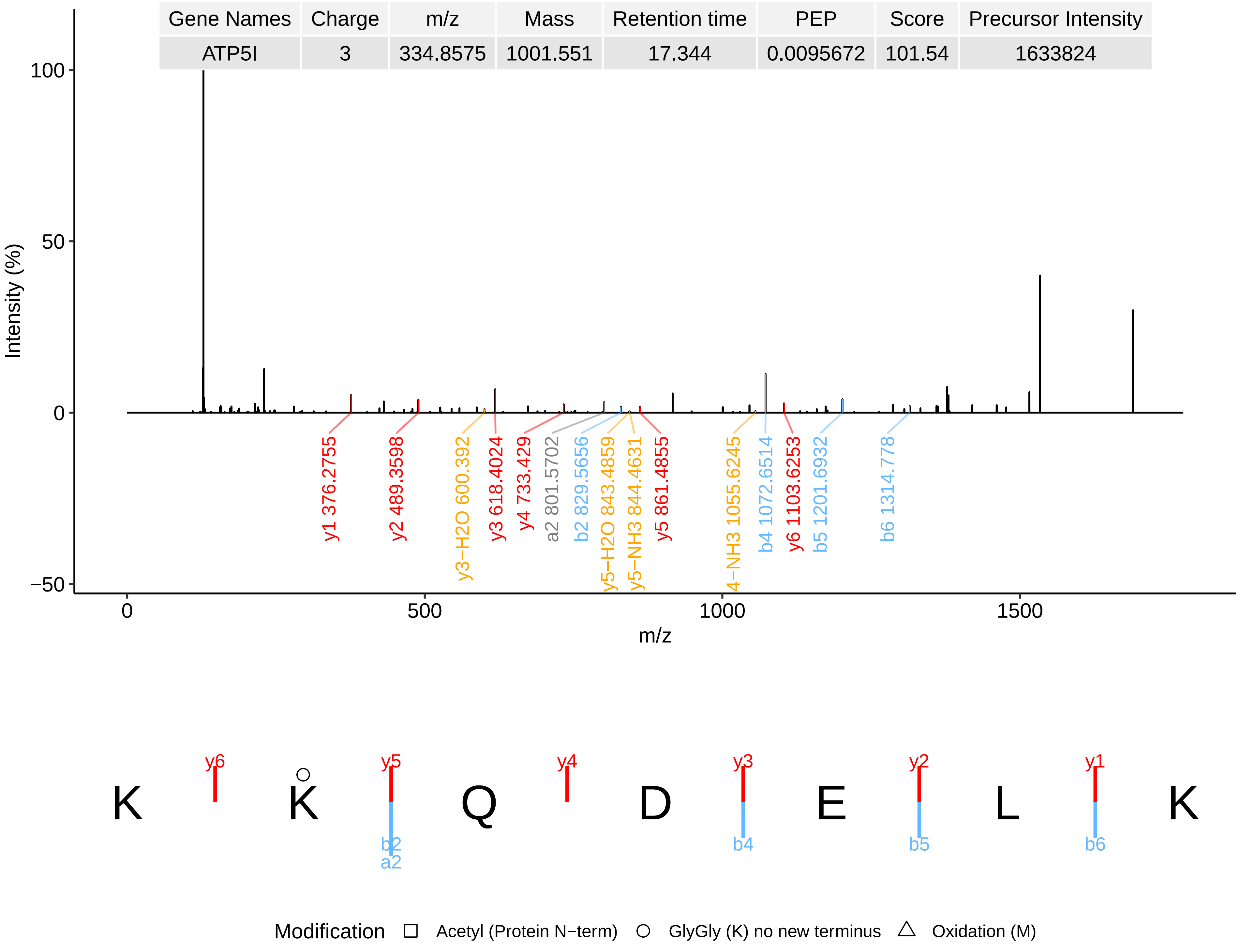

20200921_KIZ_MUb4_GlyGly_10plex_2 − Scan number: 5031

Gene Names	Charge	m/z	Mass	Retention time	PEP	Score	Precursor Intensity
ATP5I	3	334.8575	1001.551	17.902	0.062862	72.547	737644.6

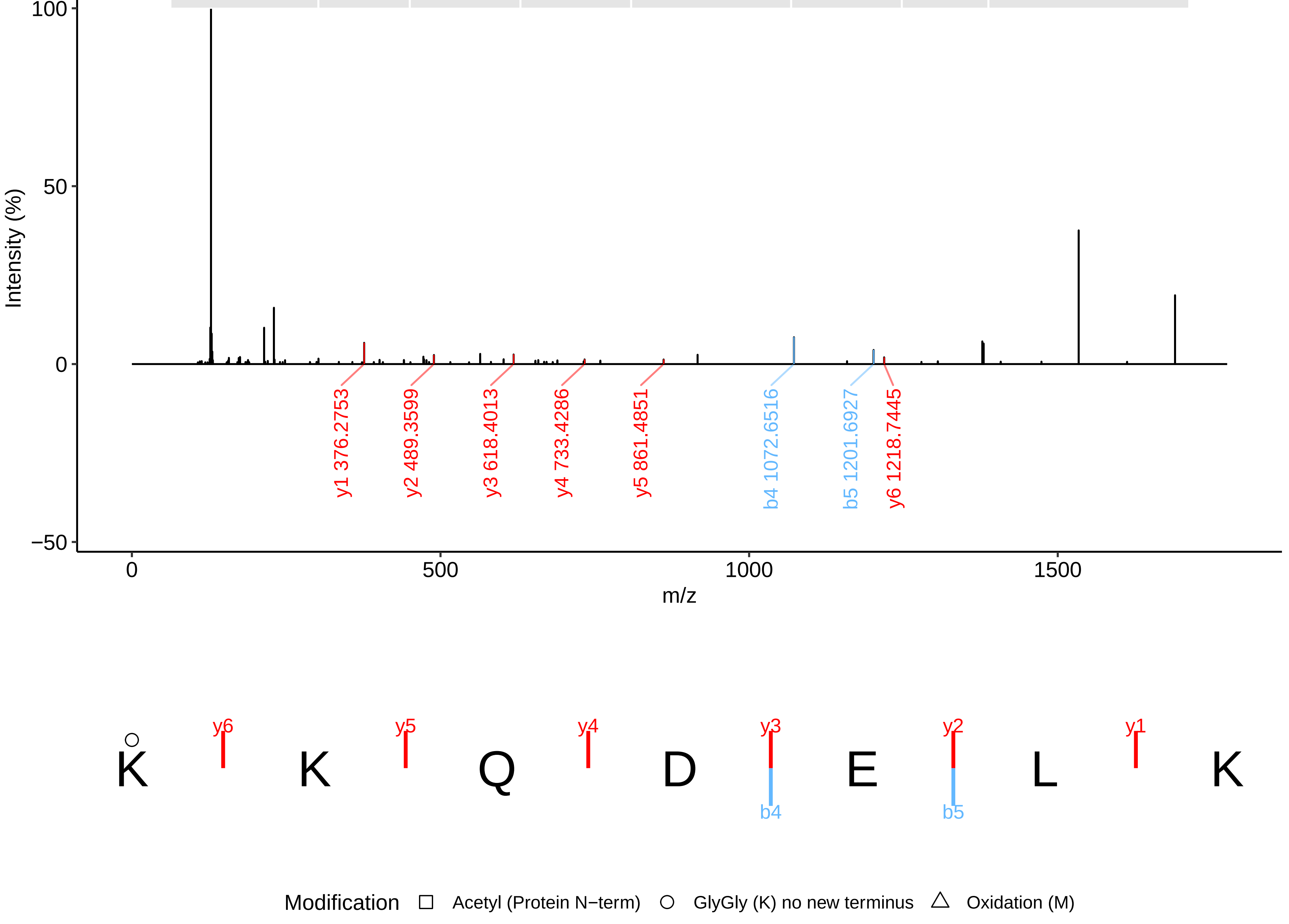

20200924_KIZ_MUb5_GlyGly_1 − Scan number: 4611

Gene Names	Charge	m/z	Mass	Retention time	PEP	Score	Precursor Intensity
ATP5I	3	334.8575	1001.551	14.251	0.0029882	121.96	14422835

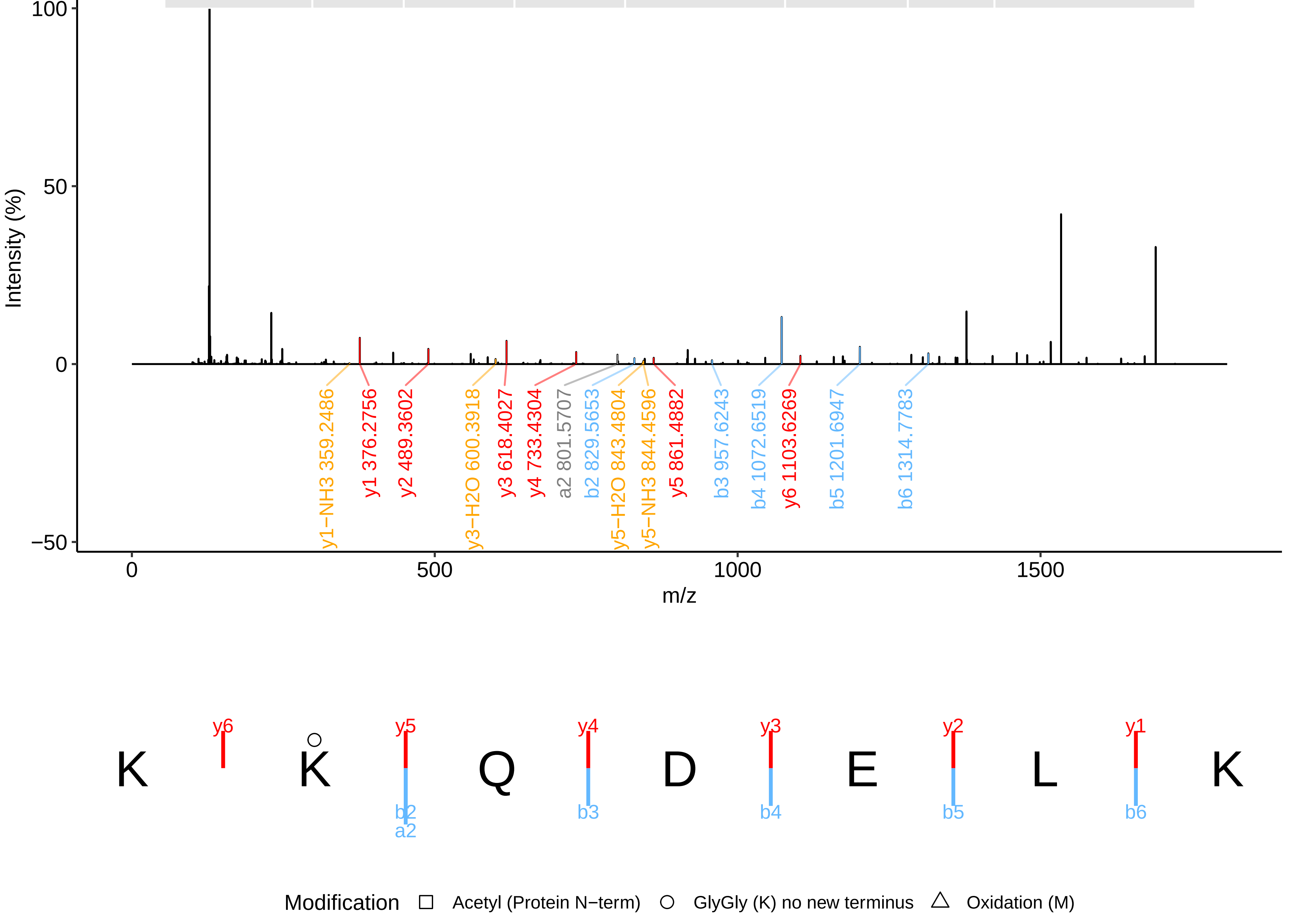

20200924_KIZ_MUb5_GlyGly_1 − Scan number: 4451

Gene Names	Charge	m/z	Mass	Retention time	PEP	Score	Precursor Intensity
ATP5I	3	334.8575	1001.551	13.737	0.023513	91.853	NA

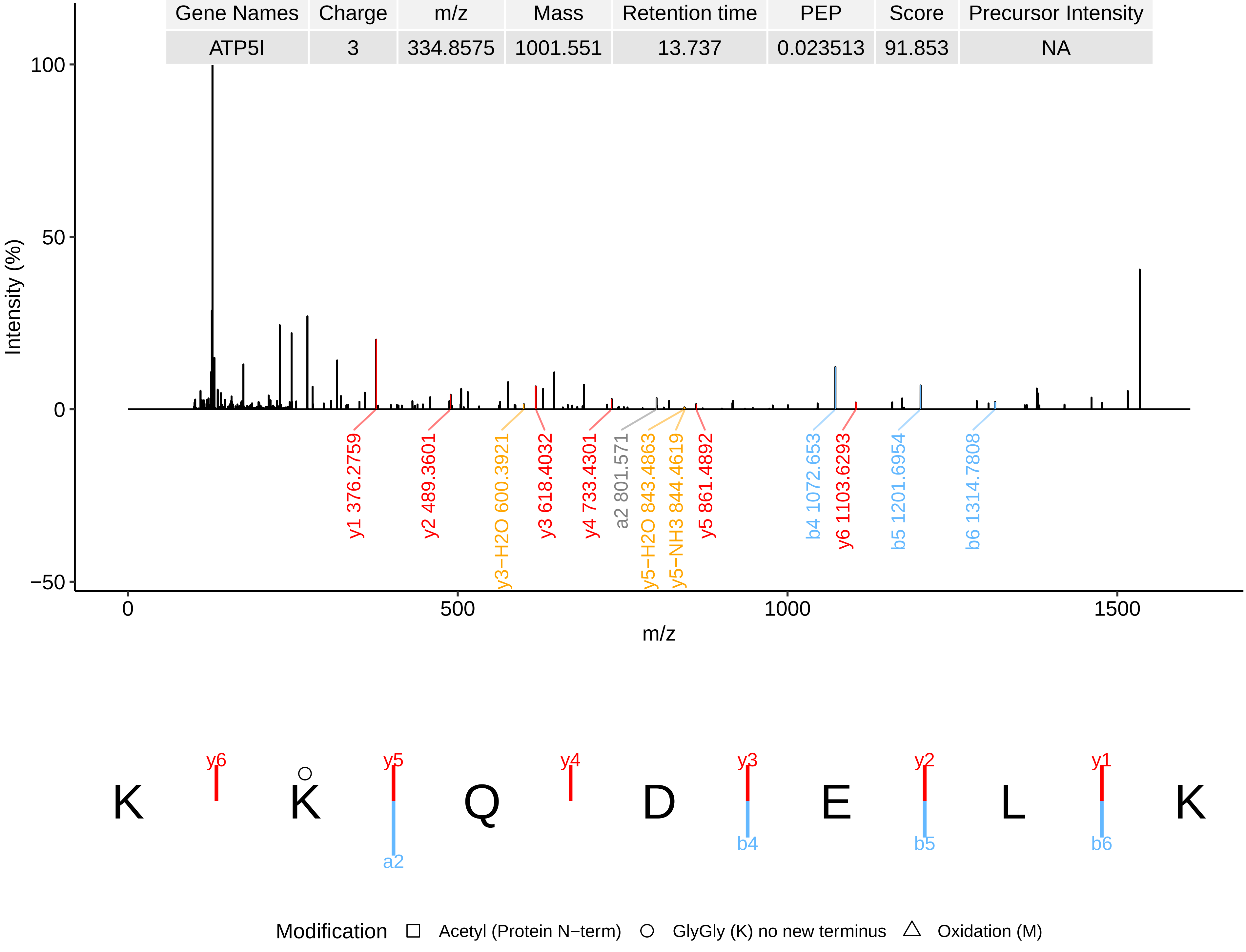

20200924_KIZ_MUb5_GlyGly_2 − Scan number: 4141

Gene Names	Charge	m/z	Mass	Retention time	PEP	Score	Precursor Intensity
ATP5I	3	334.8575	1001.551	15.154	0.019455	90.657	2832599

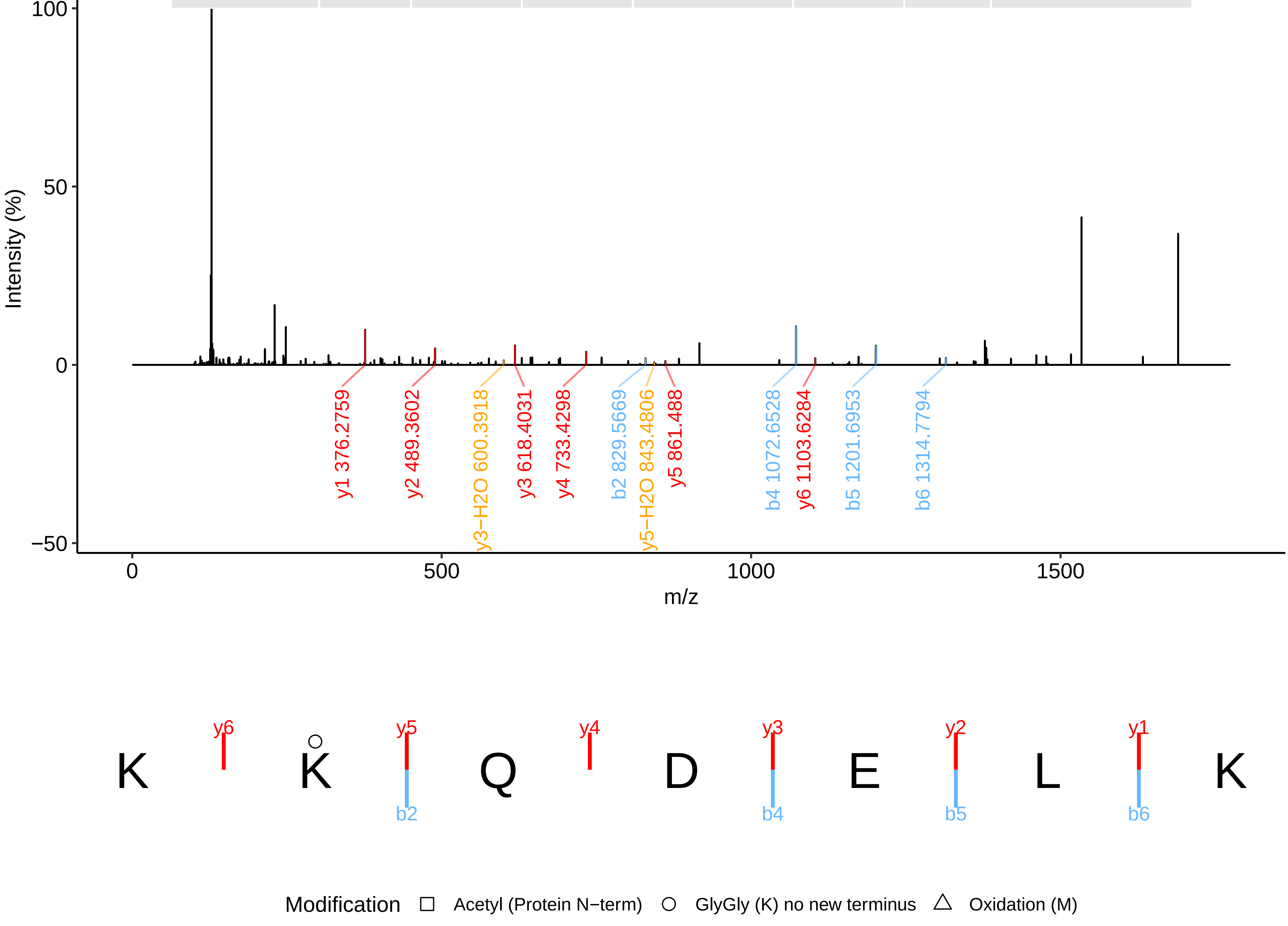

20200924_KIZ_MUb5_GlyGly_2 − Scan number: 4277

Gene Names	Charge	m/z	Mass	Retention time	PEP	Score	Precursor Intensity
ATP5I	3	334.8575	1001.551	15.672	0.0029882	121.96	4418753

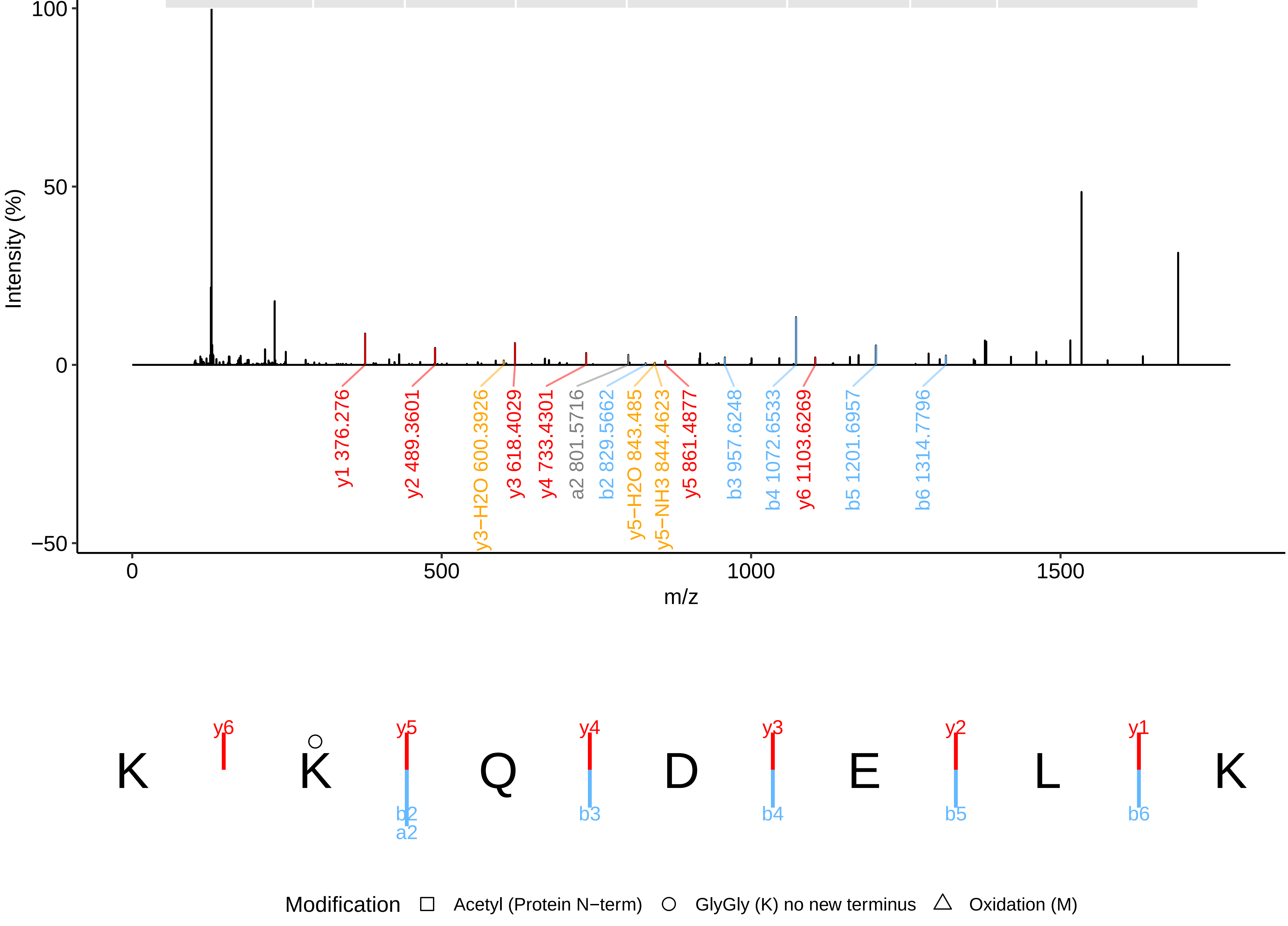

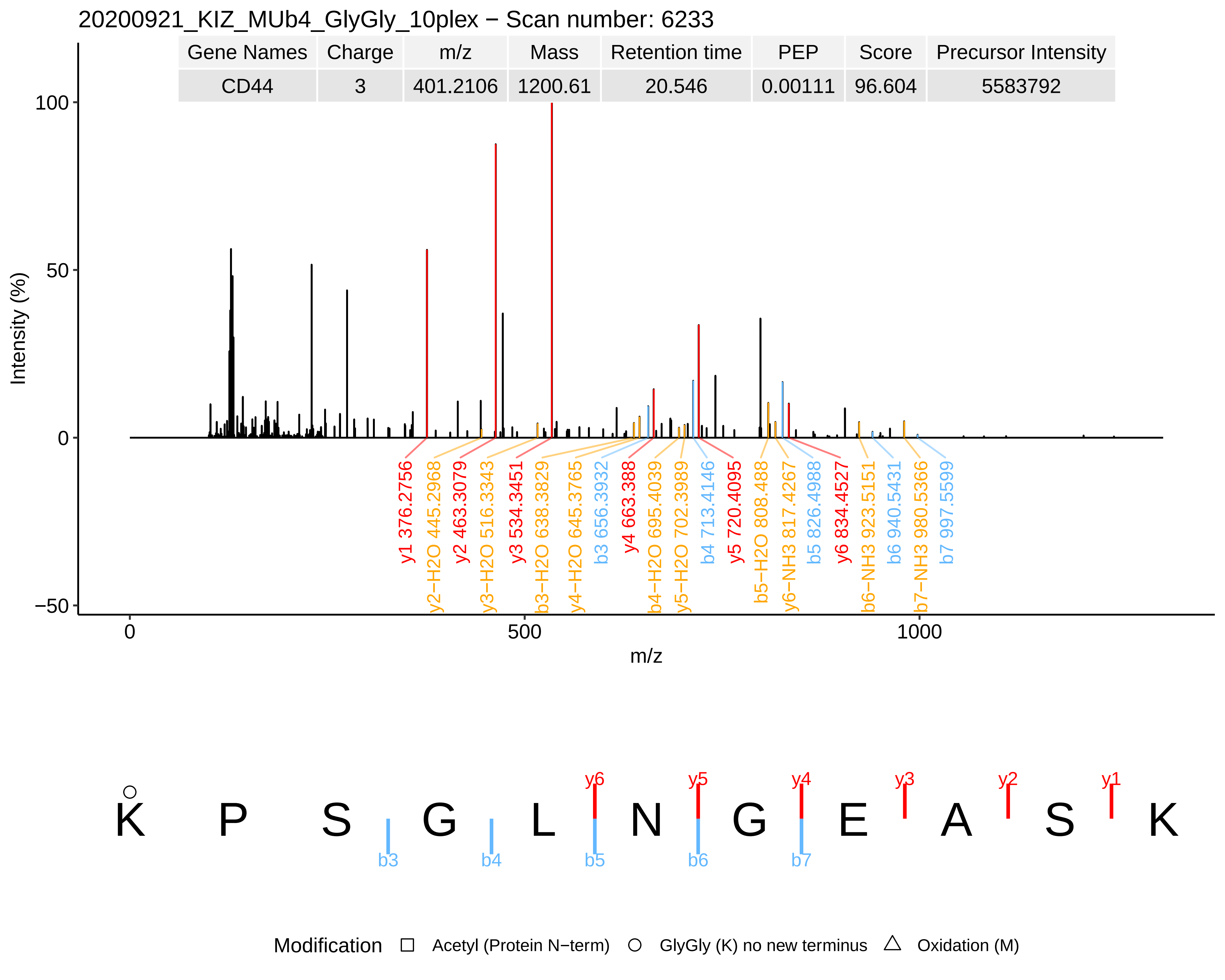

20200921_KIZ_MUb4_GlyGly_10plex − Scan number: 6233
Gene Names | Charge | m/z | Mass | Retention time | PEP | Score | Precursor Intensity
CD44 | 3 | 401.2106 | 1200.61 | 20.546 | 0.00111 | 96.604 | 5583792
Intensity (%)
100
50
0
−50
0
500
1000
m/z
y1 376.2756
y2−H2O 445.2968
y2 463.3079
y3−H2O 516.3343
y3 534.3451
b3−H2O 638.3829
y4−H2O 645.3765
b3 656.3932
y4 663.388
b4−H2O 695.4039
y5−H2O 702.3989
b4 713.4146
y5 720.4095
b5−H2O 808.488
y6−NH3 817.4267
b5 826.4988
y6 834.4527
b6−NH3 923.5151
b6 940.5431
b7−NH3 980.5366
b7 997.5599
K P S G L N G E A S K
y6 y5 y4 y3 y2 y1
b3 b4 b5 b6 b7
Modification
Acetyl (Protein N−term)
GlyGly (K) no new terminus
Oxidation (M)

20200921_KIZ_MUb4_GlyGly_10plex − Scan number: 3974

Gene Names	Charge	m/z	Mass	Retention time	PEP	Score	Precursor Intensity
BCAP31	3	411.8738	1232.6	12.887	0.065451	69.815	4379496

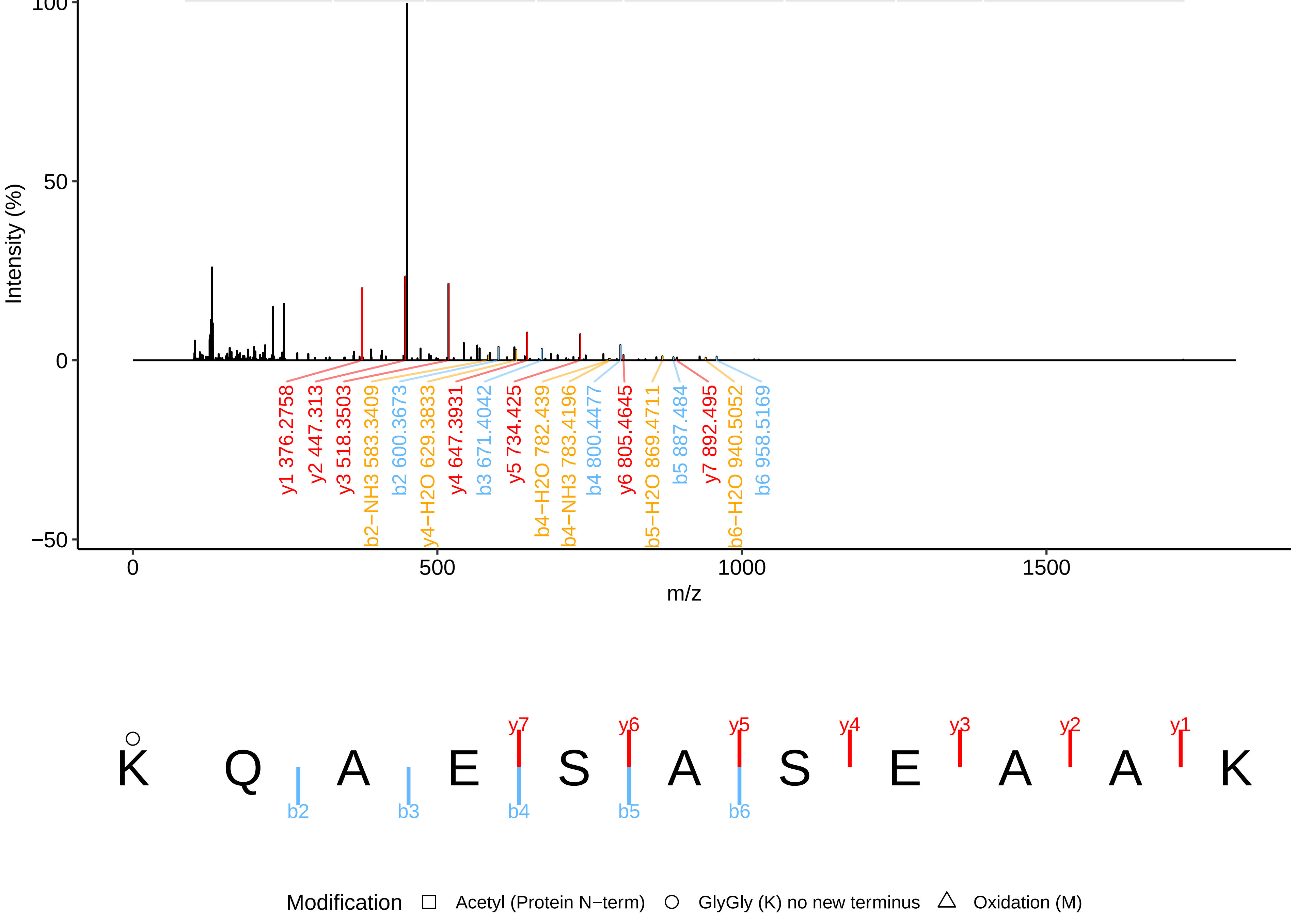

20200921_KIZ_MUb4_GlyGly_10plex_2 − Scan number: 3765

Gene Names	Charge	m/z	Mass	Retention time	PEP	Score	Precursor Intensity
BCAP31	3	411.8738	1232.6	12.859	0.035544	75.695	1465279

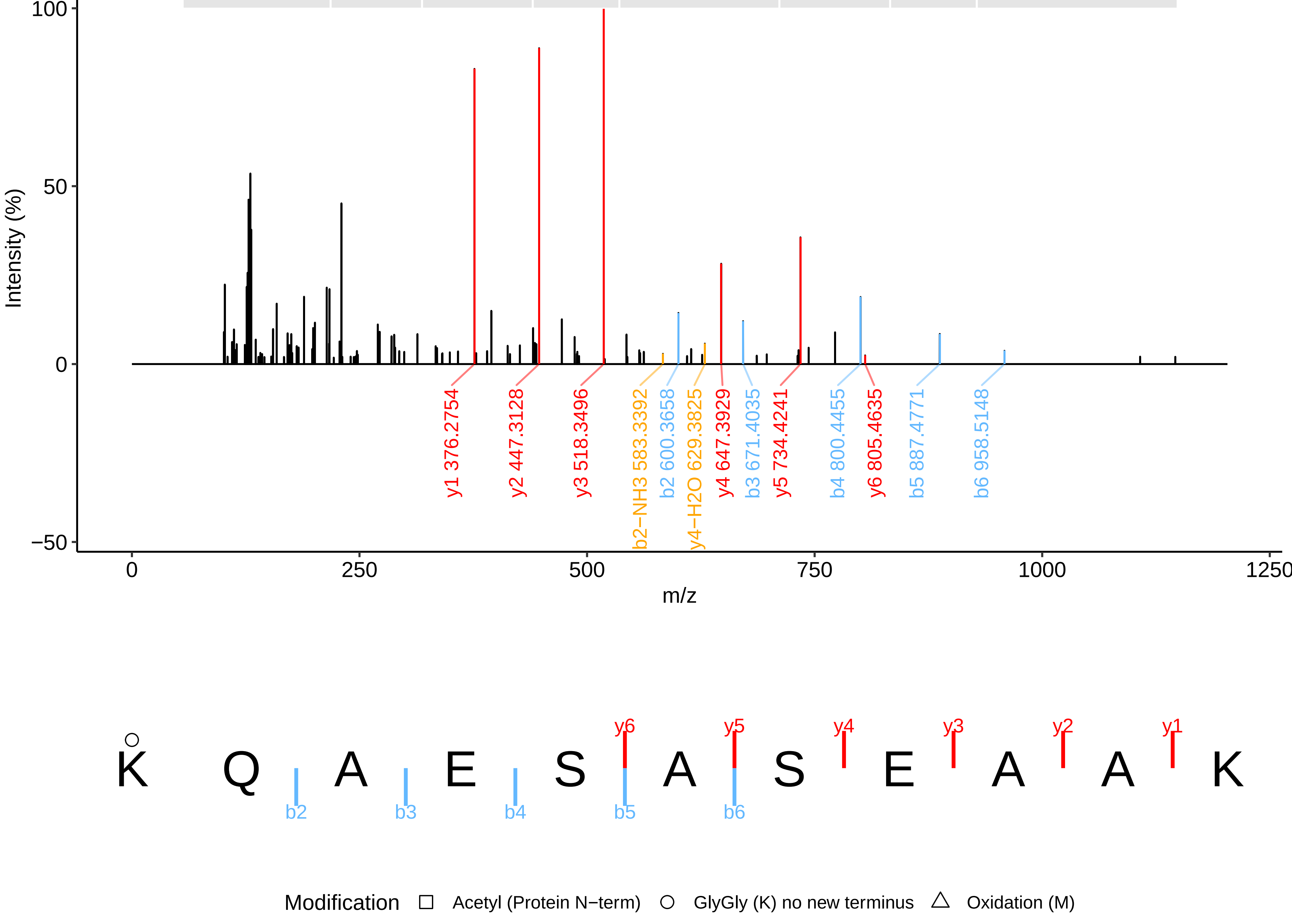

20200921_KIZ_MUb4_GlyGly_10plex − Scan number: 11613

Gene Names	Charge	m/z	Mass	Retention time	PEP	Score	Precursor Intensity
DNAJA1;DNAJA4;DNAJC5	4	300.4199	1197.651	40.526	0.01627	65.347	13440506

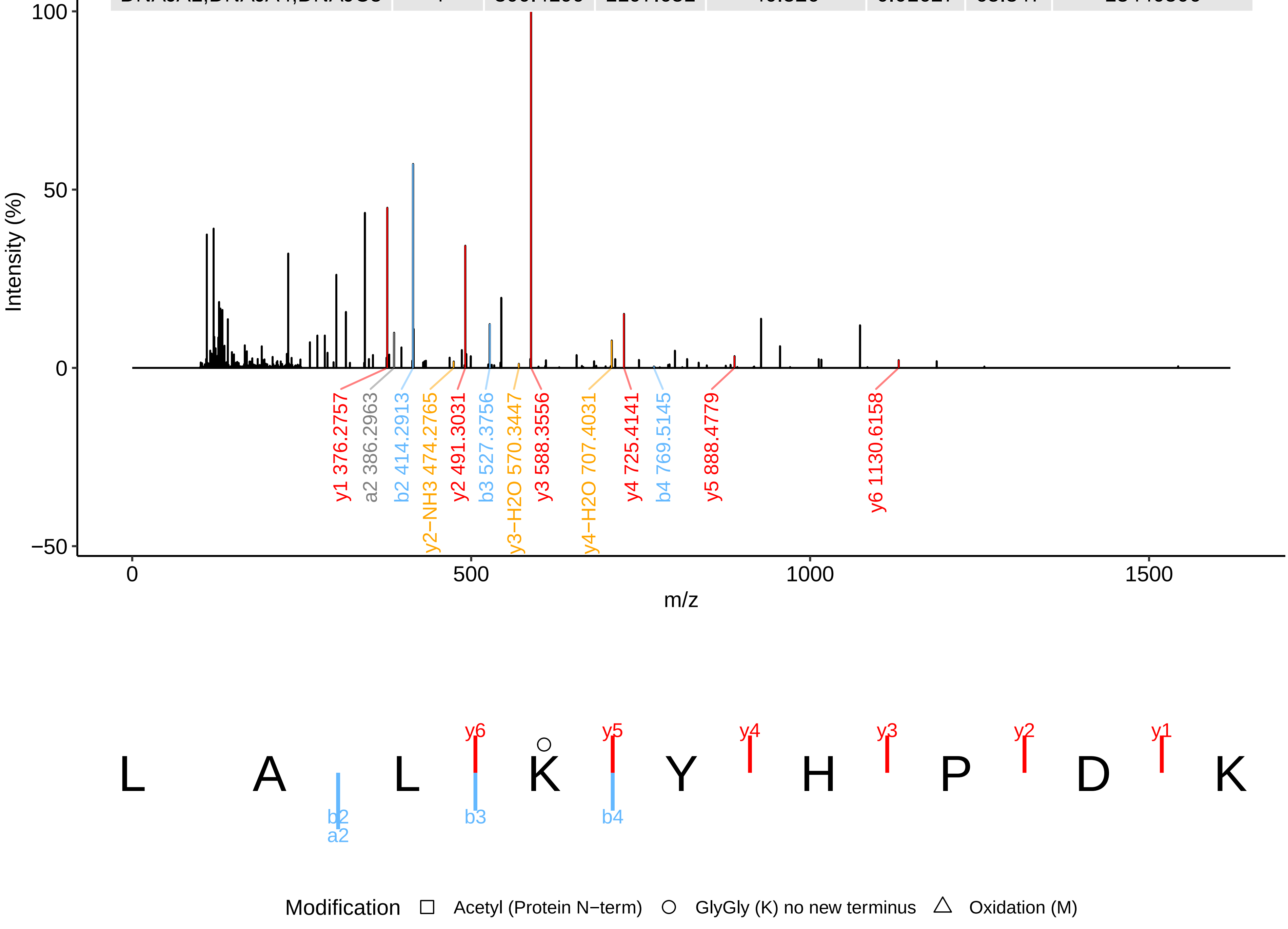

20200921_KIZ_MUb4_GlyGly_10plex − Scan number: 11620

Gene Names	Charge	m/z	Mass	Retention time	PEP	Score	Precursor Intensity
DNAJA1;DNAJA4;DNAJC5	3	400.2242	1197.651	40.55	0.02872	62.823	3446059

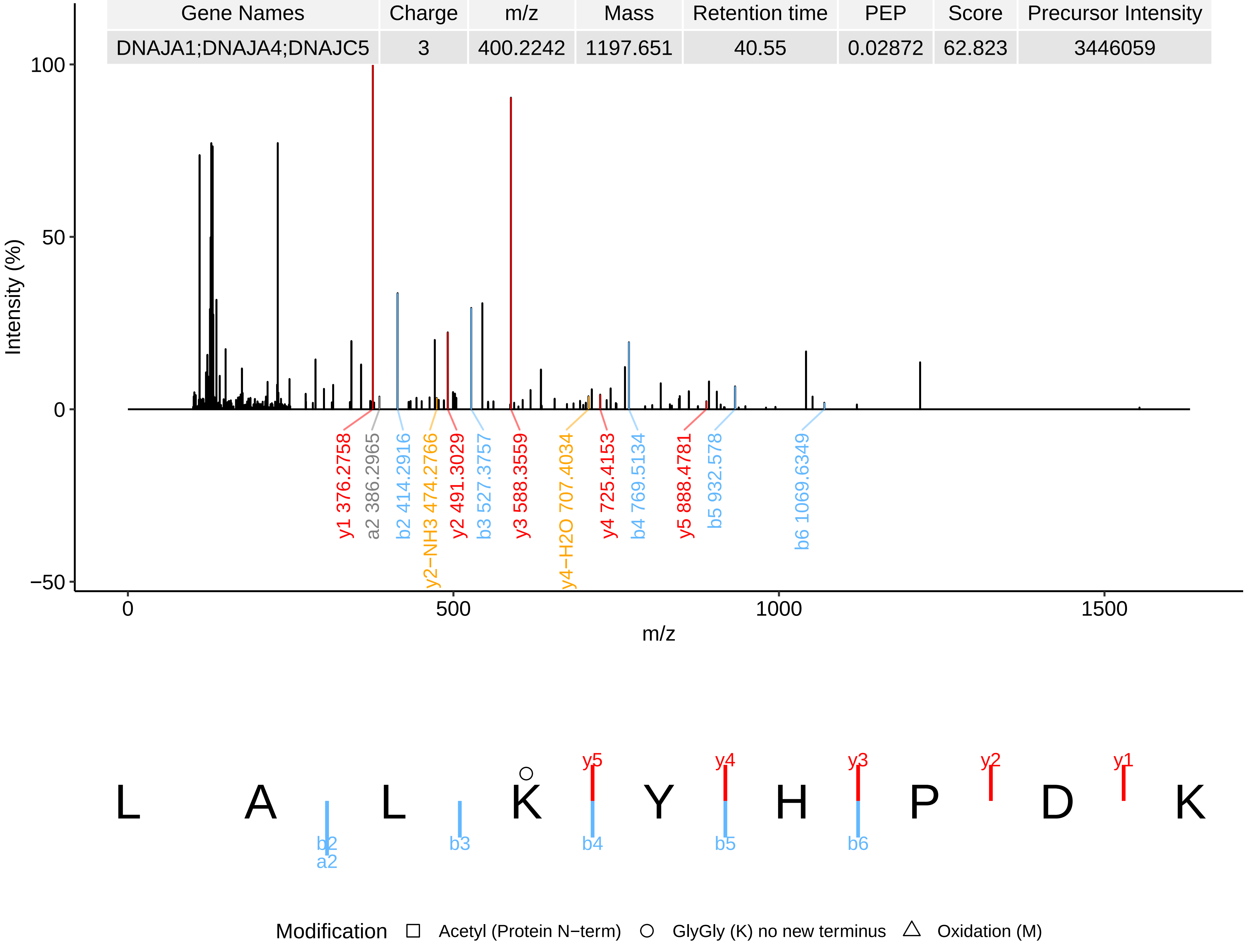

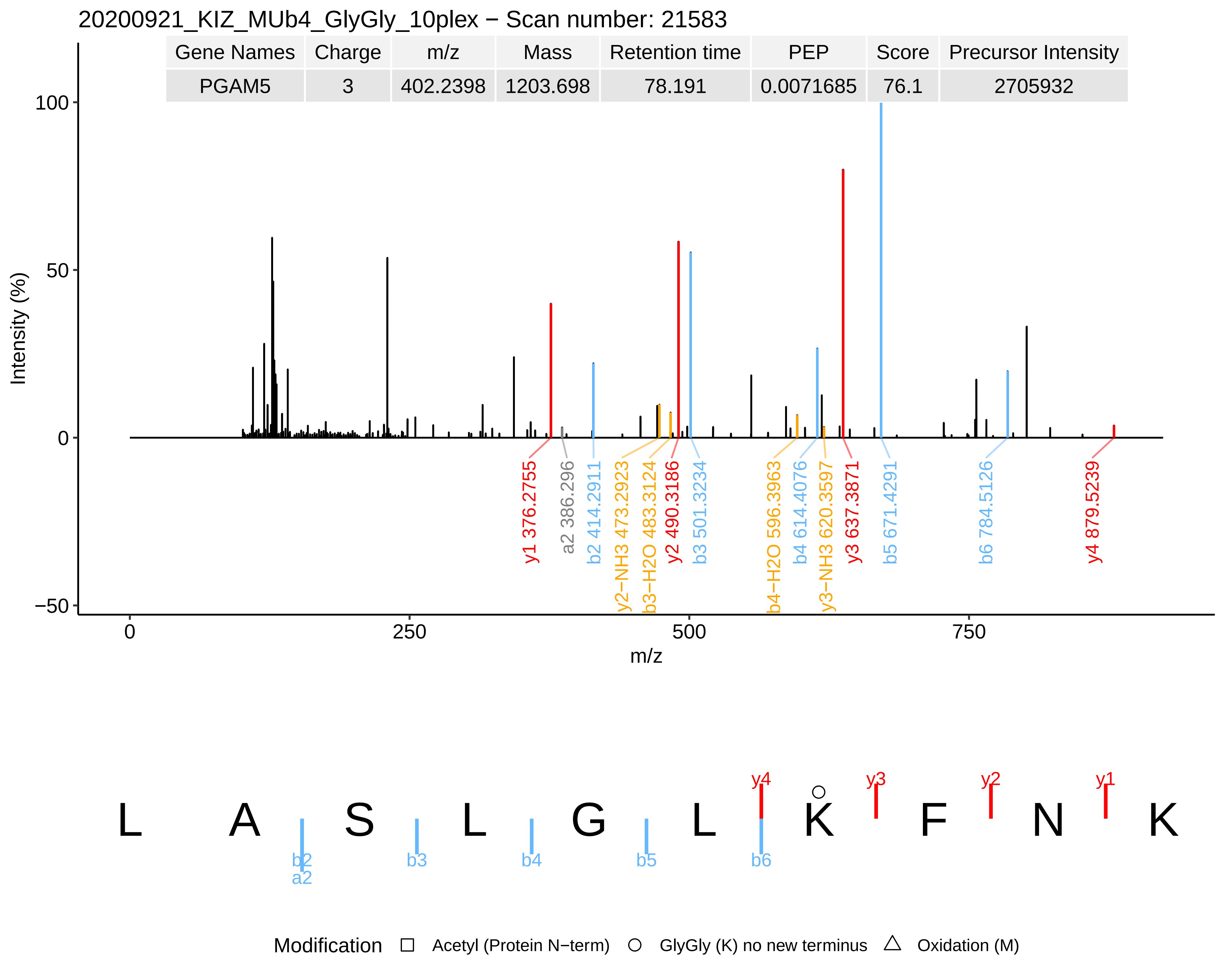
20200921_KIZ_MUb4_GlyGly_10plex − Scan number: 21583
Gene Names | Charge | m/z | Mass | Retention time | PEP | Score | Precursor Intensity
PGAM5 | 3 | 402.2398 | 1203.698 | 78.191 | 0.0071685 | 76.1 | 2705932
Intensity (%)
100
50
0
−50
0
250
500
750
m/z
y1 376.2755
a2 386.296
b2 414.2911
y2−NH3 473.2923
b3−H2O 483.3124
y2 490.3186
b3 501.3234
b4−H2O 596.3963
b4 614.4076
y3−NH3 620.3597
y3 637.3871
b5 671.4291
b6 784.5126
y4 879.5239
L A S L G L K F N K
y4 y3 y2 y1
b2 a2 b3 b4 b5 b6
Modification
Acetyl (Protein N−term)
GlyGly (K) no new terminus
Oxidation (M)

20200921_KIZ_MUb4_GlyGly_10plex − Scan number: 21718

Gene Names	Charge	m/z	Mass	Retention time	PEP	Score	Precursor Intensity
PGAM5	3	402.2398	1203.698	78.699	0.0020129	84.615	4202881

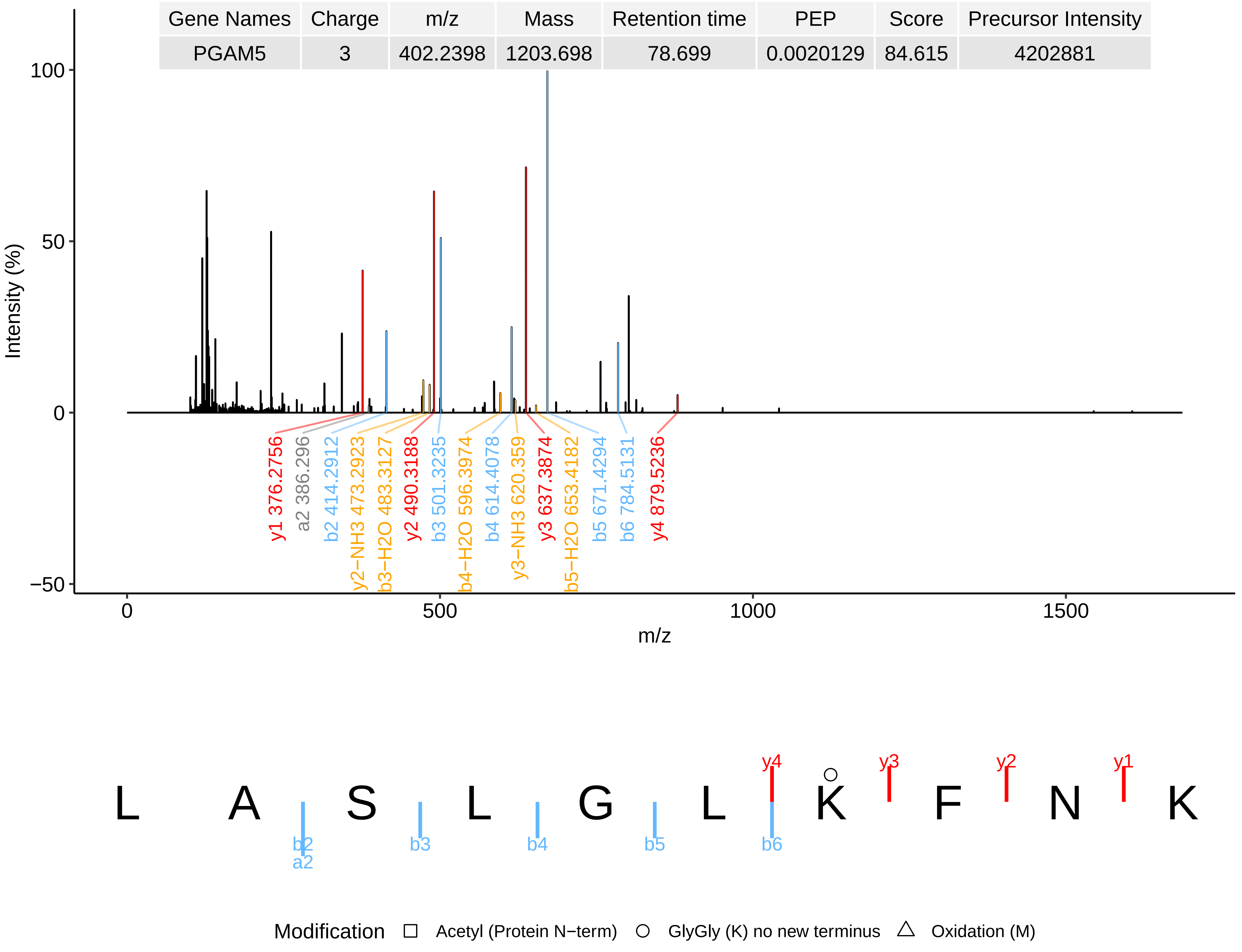

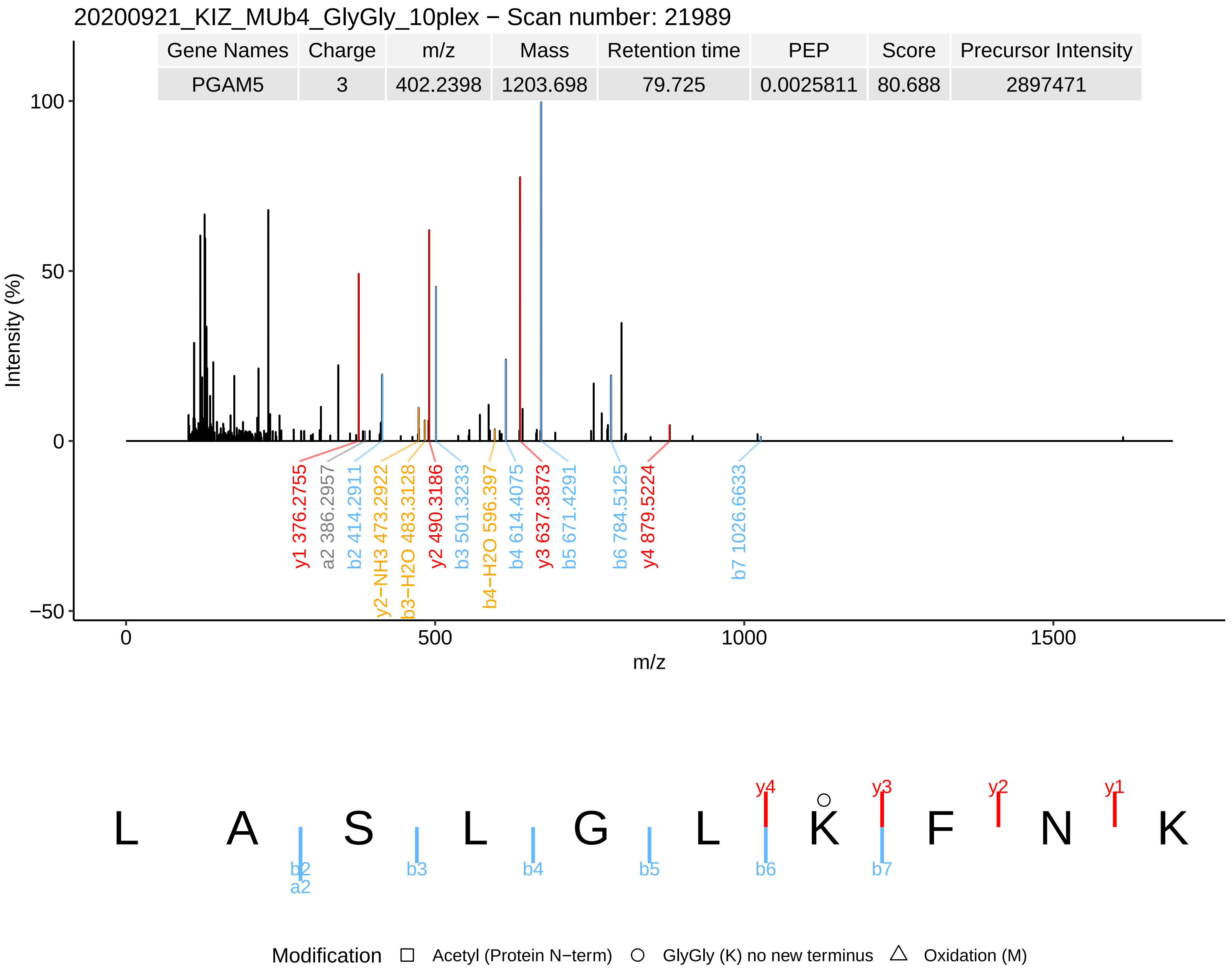
20200921_KIZ_MUb4_GlyGly_10plex − Scan number: 21989
Gene Names
Charge
m/z
Mass
Retention time
PEP
Score
Precursor Intensity
PGAM5
3
402.2398
1203.698
79.725
0.0025811
80.688
2897471
Intensity (%)
m/z
y1 376.2755
a2 386.2957
b2 414.2911
y2−NH3 473.2922
b3−H2O 483.3128
y2 490.3186
b3 501.3233
b4−H2O 596.397
b4 614.4075
y3 637.3873
b5 671.4291
b6 784.5125
y4 879.5224
b7 1026.6633
L A S L G L K F N K
Modification
Acetyl (Protein N−term)
GlyGly (K) no new terminus
Oxidation (M)

20200921_KIZ_MUb4_GlyGly_10plex − Scan number: 21887

Gene Names	Charge	m/z	Mass	Retention time	PEP	Score	Precursor Intensity
PGAM5	3	402.2398	1203.698	79.339	0.021422	64.654	NA

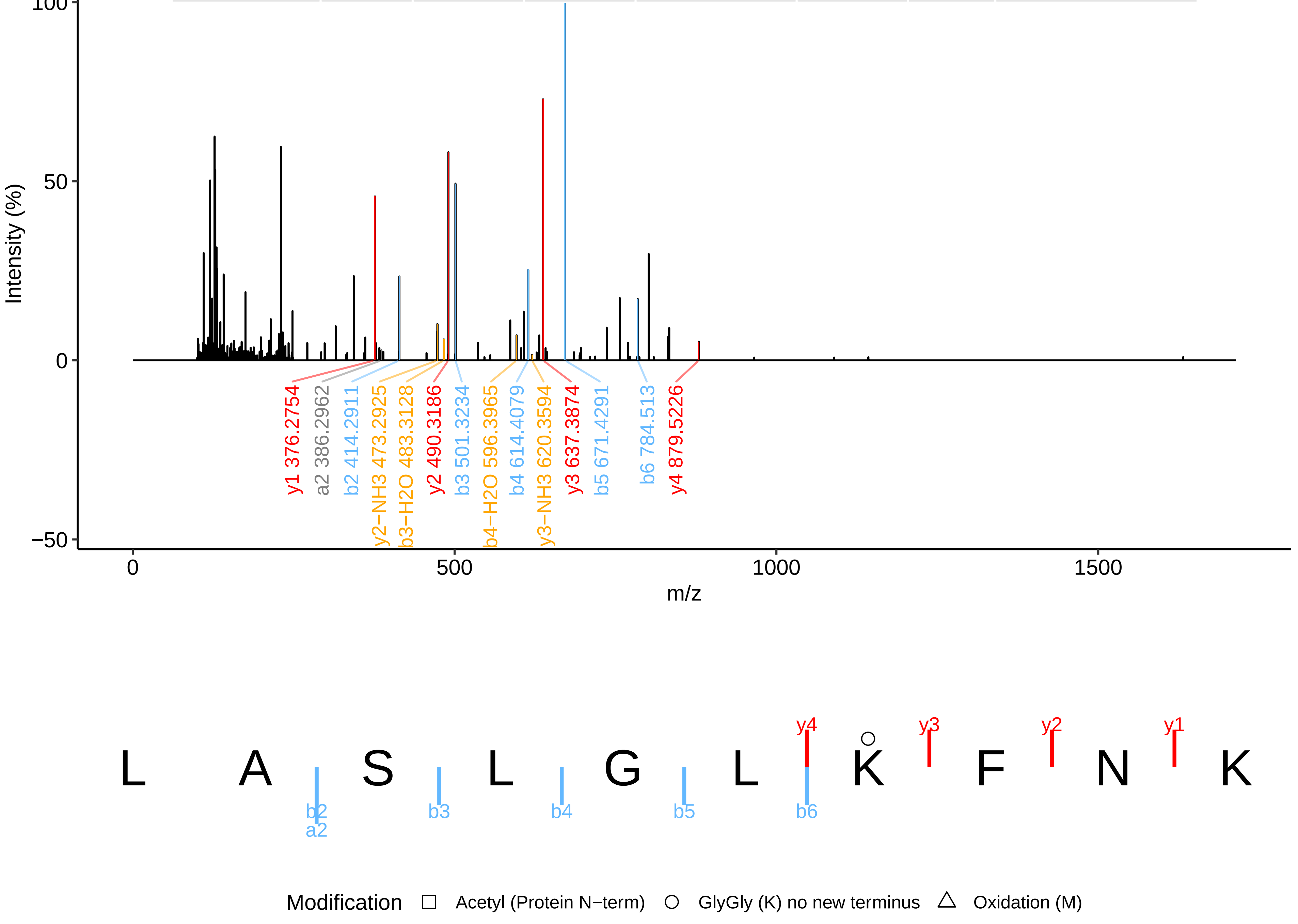

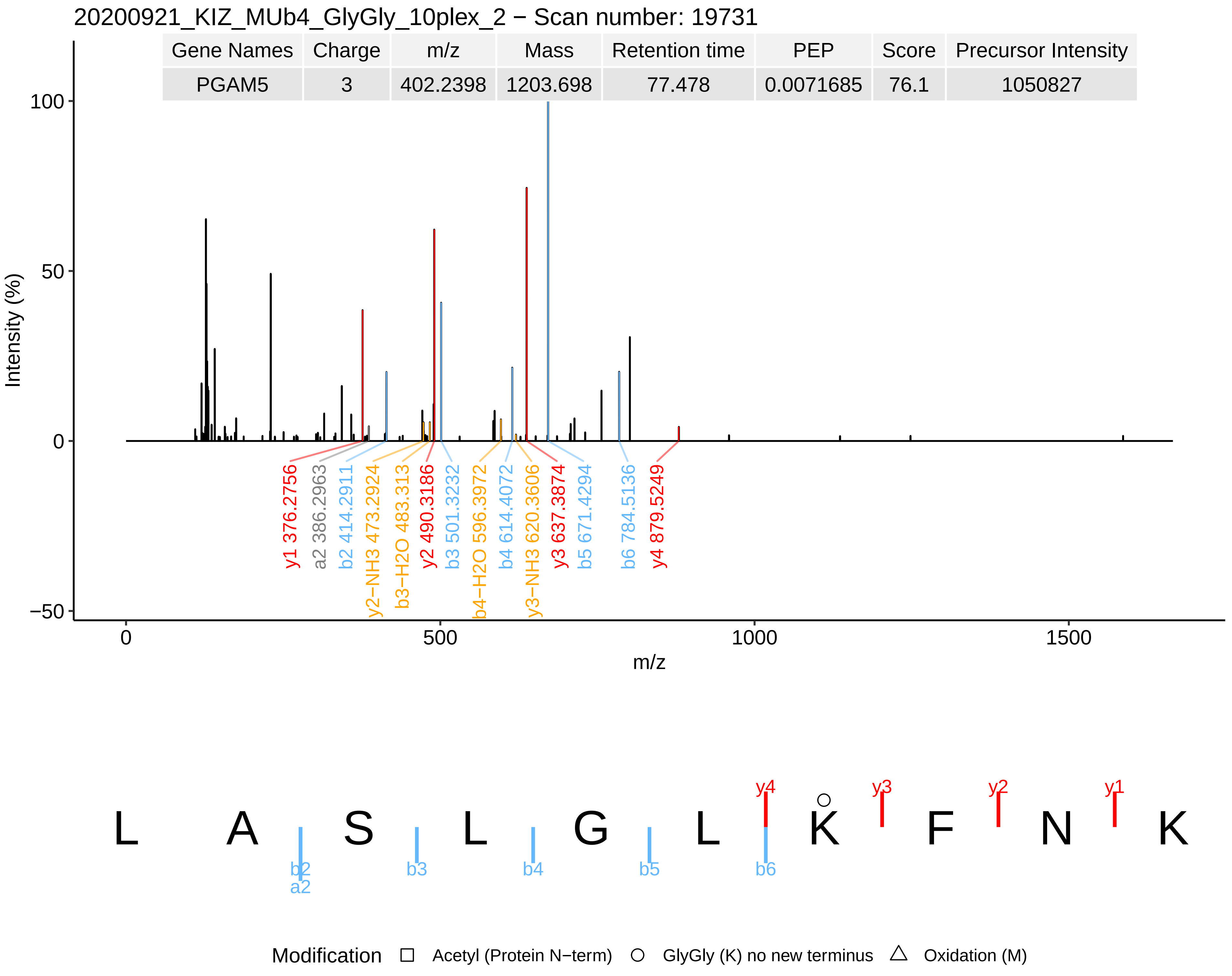
20200921_KIZ_MUb4_GlyGly_10plex_2 − Scan number: 19731
Gene Names | Charge | m/z | Mass | Retention time | PEP | Score | Precursor Intensity
PGAM5 | 3 | 402.2398 | 1203.698 | 77.478 | 0.0071685 | 76.1 | 1050827
Intensity (%)
m/z
y1 376.2756
a2 386.2963
b2 414.2911
y2−NH3 473.2924
b3−H2O 483.313
y2 490.3186
b3 501.3232
b4−H2O 596.3972
b4 614.4072
y3−NH3 620.3606
y3 637.3874
b5 671.4294
b6 784.5136
y4 879.5249
L A S L G L K F N K
y4 y3 y2 y1
b2 a2 b3 b4 b5 b6
Modification
Acetyl (Protein N−term)
GlyGly (K) no new terminus
Oxidation (M)

20200924_KIZ_MUb5_GlyGly_1 − Scan number: 21460

Gene Names	Charge	m/z	Mass	Retention time	PEP	Score	Precursor Intensity
PGAM5	3	402.2398	1203.698	69.127	0.0020129	84.615	11176441

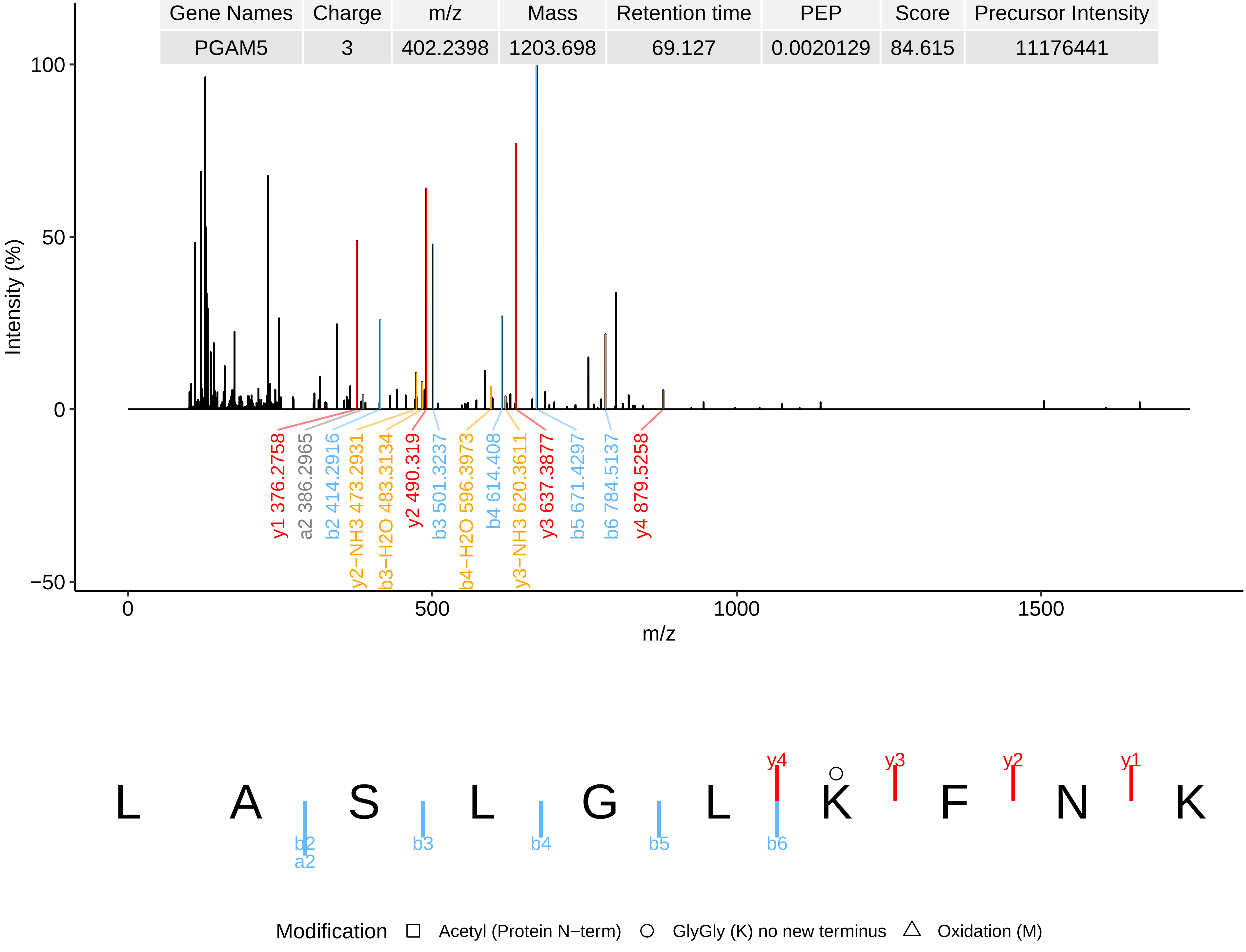

20200924_KIZ_MUb5_GlyGly_1 − Scan number: 21298

Gene Names	Charge	m/z	Mass	Retention time	PEP	Score	Precursor Intensity
PGAM5	3	402.2398	1203.698	68.613	0.010751	74.376	18523180

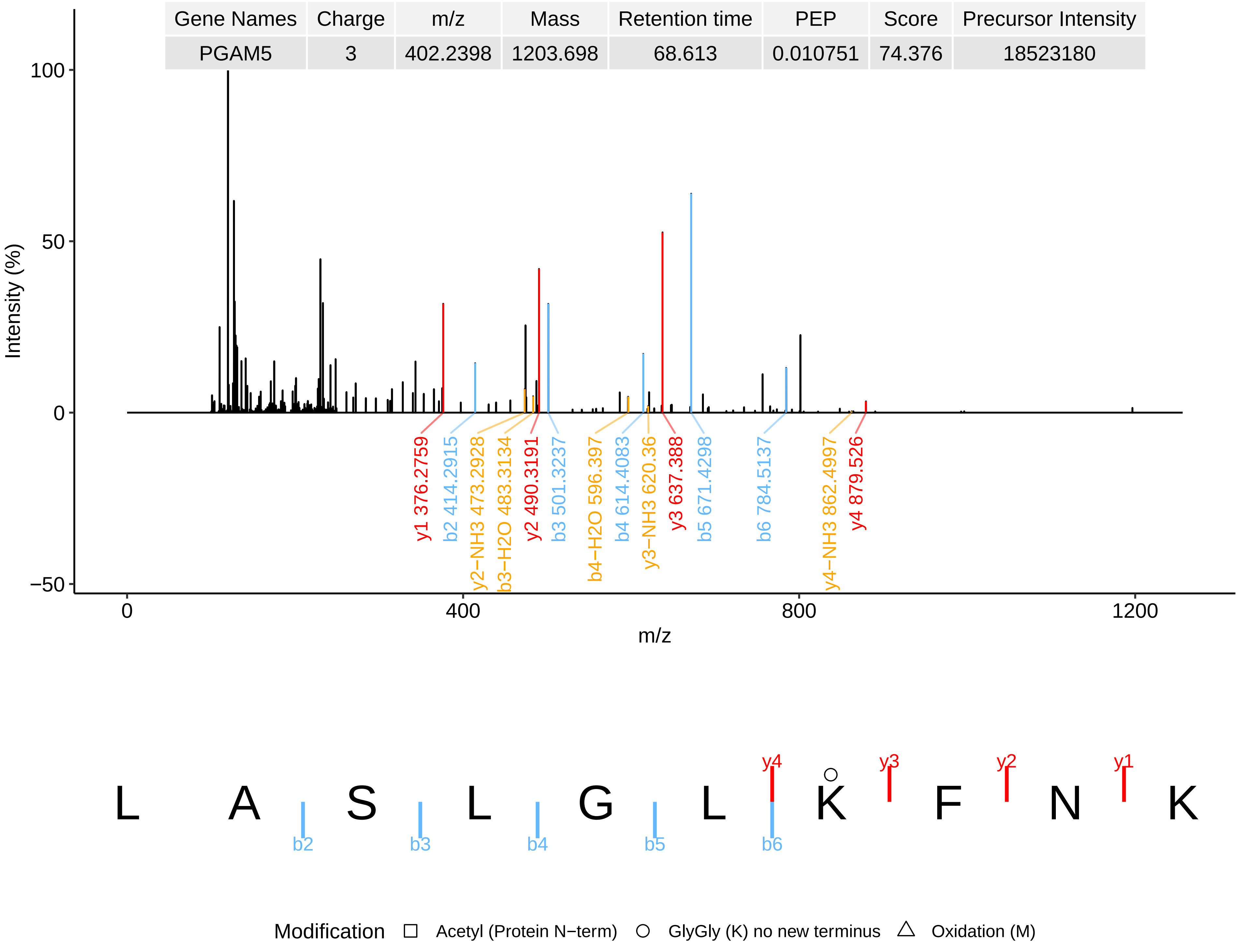

20200924_KIZ_MUb5_GlyGly_2 − Scan number: 19493

Gene Names	Charge	m/z	Mass	Retention time	PEP	Score	Precursor Intensity
PGAM5	3	402.2398	1203.698	72.255	0.0020129	84.615	4408971

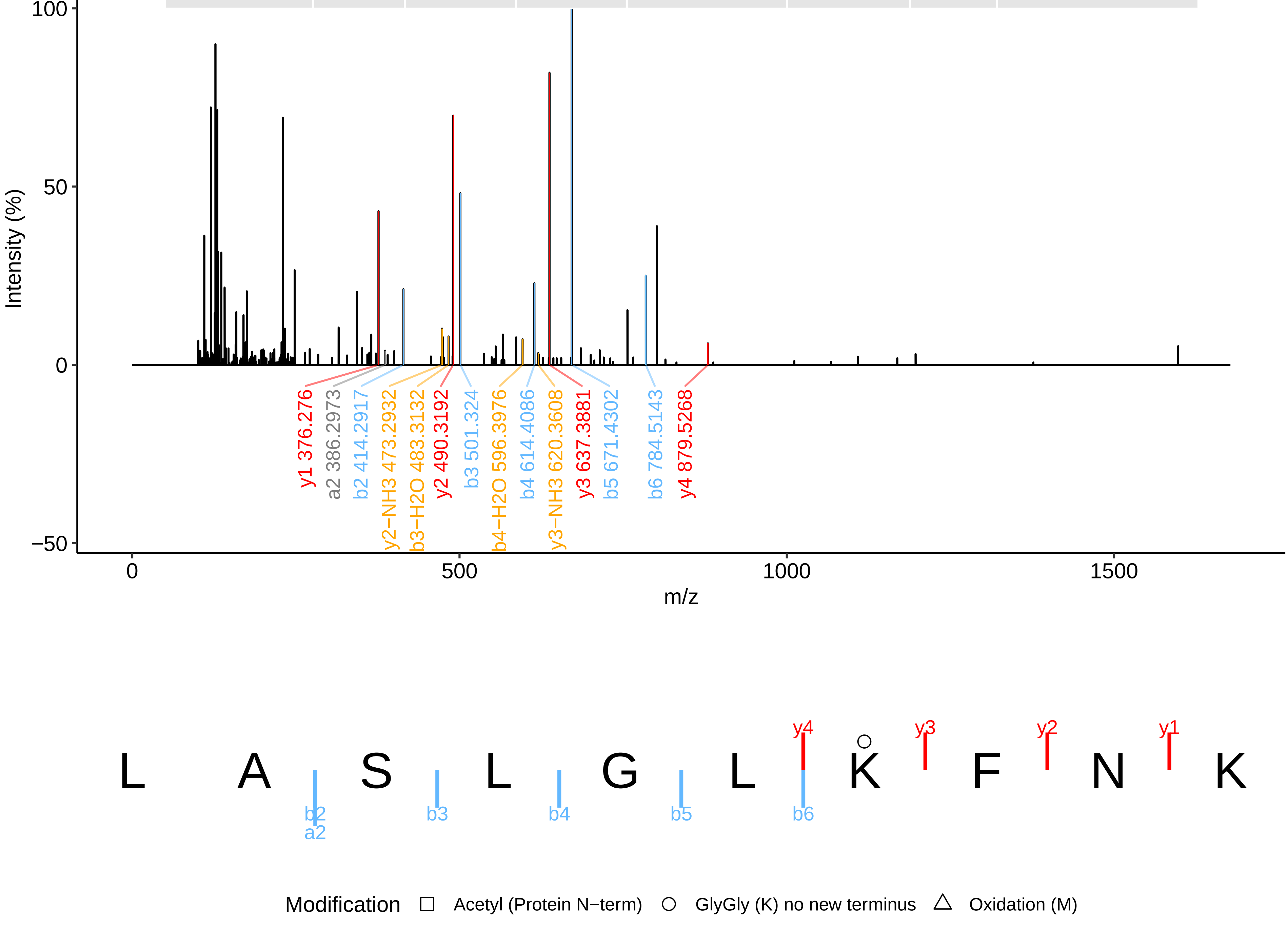

20201118_KIZ_MUb6_GlyGly_1 − Scan number: 21729

Gene Names	Charge	m/z	Mass	Retention time	PEP	Score	Precursor Intensity
PGAM5	3	402.2398	1203.698	67.744	5.8292e−05	102.07	27621036

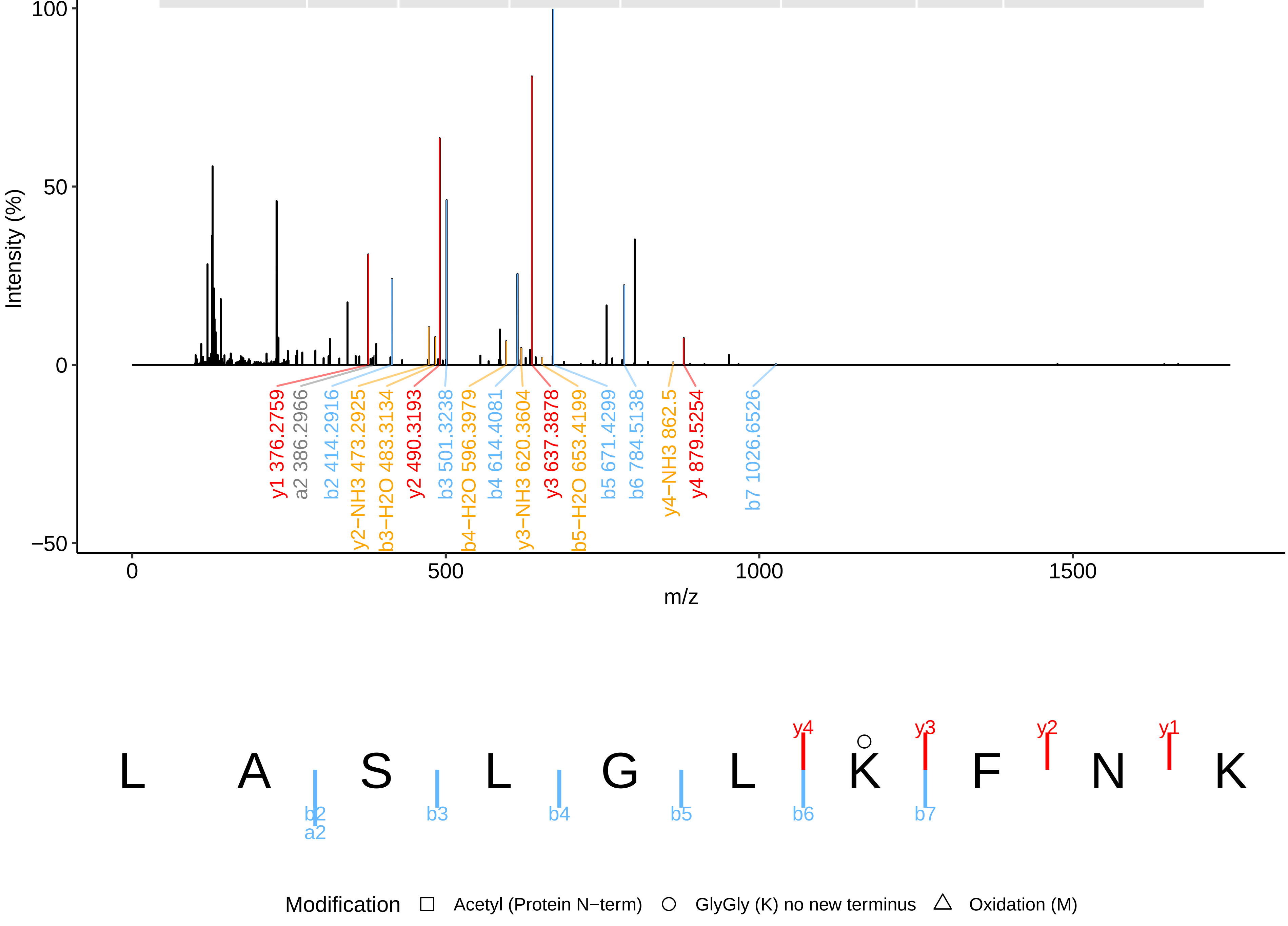

20201118_KIZ_MUb6_GlyGly_2 − Scan number: 23921

Gene Names	Charge	m/z	Mass	Retention time	PEP	Score	Precursor Intensity
PGAM5	3	402.2398	1203.698	80.177	0.0025811	80.688	15473399

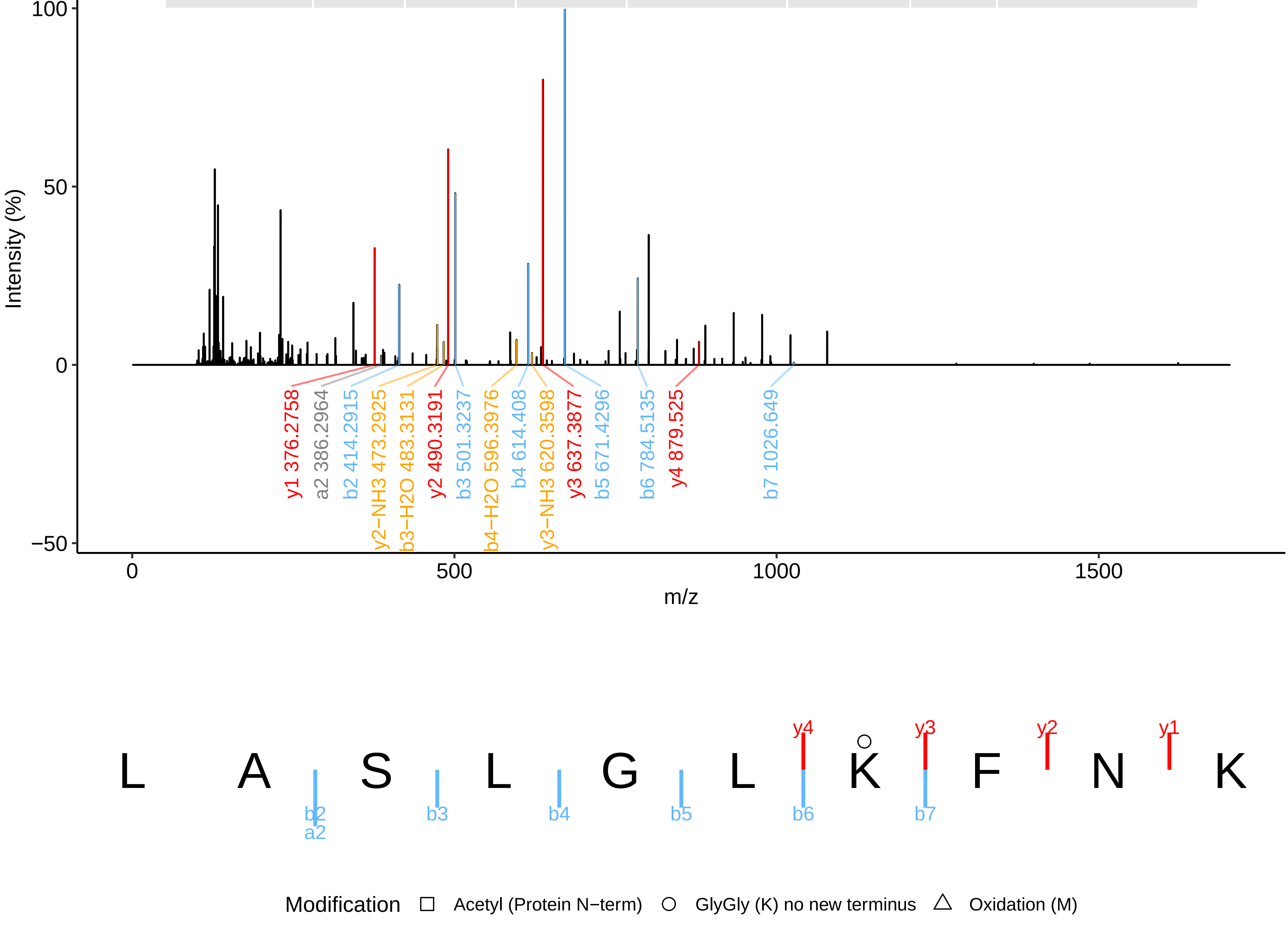

20200921_KIZ_MUb4_GlyGly_10plex_2 − Scan number: 14043

Gene Names	Charge	m/z	Mass	Retention time	PEP	Score	Precursor Intensity
RASA3	3	418.8727	1253.596	54.665	0.02928	61.679	1498815

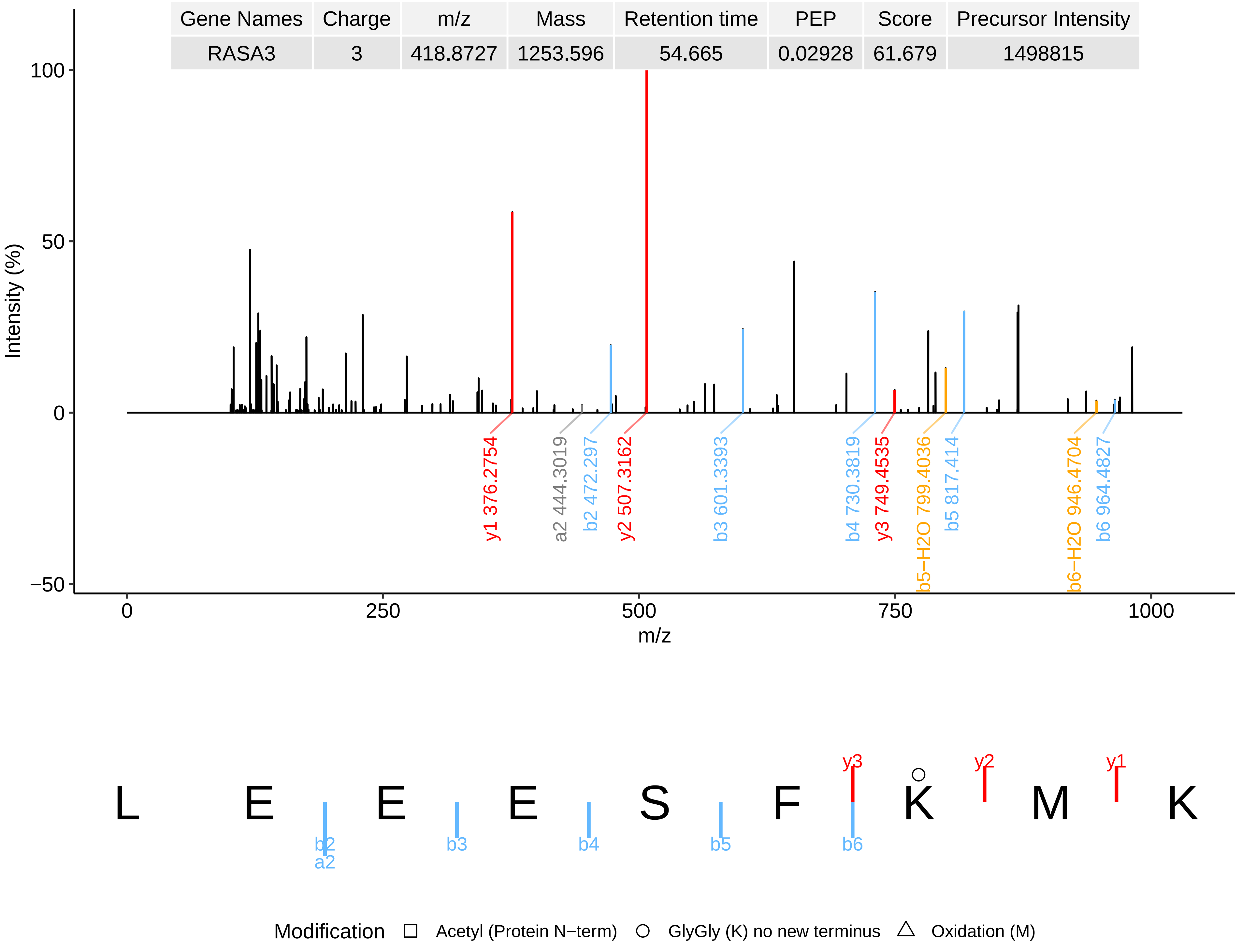

20200921_KIZ_MUb4_GlyGly_10plex − Scan number: 14402

Gene Names	Charge	m/z	Mass	Retention time	PEP	Score	Precursor Intensity
RPS27A;UBA52;UBC;UBB	3	487.6001	1459.778	51.177	0.079839	50.286	11143560

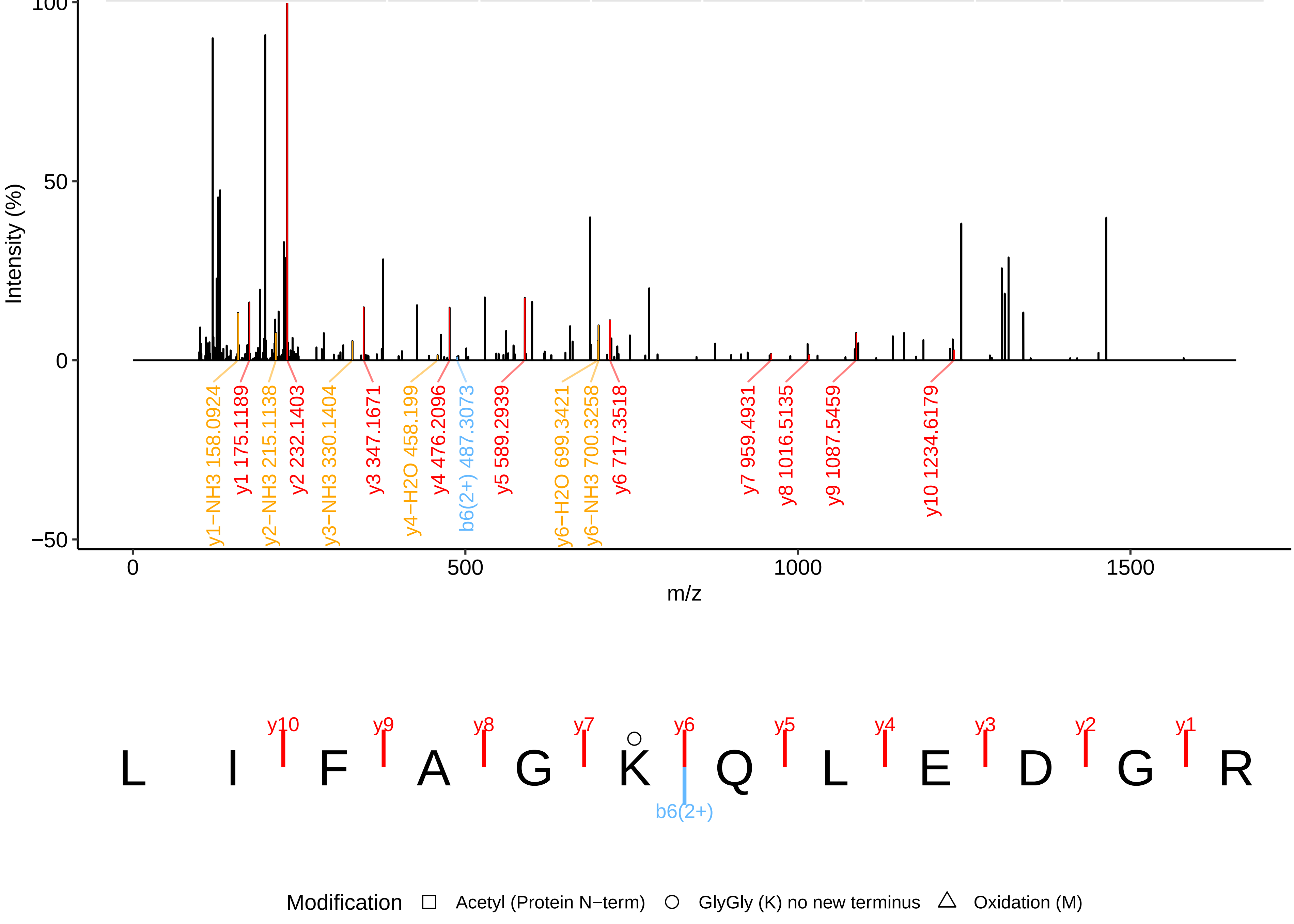

20200921_KIZ_MUb4_GlyGly_10plex − Scan number: 16927

Gene Names	Charge	m/z	Mass	Retention time	PEP	Score	Precursor Intensity
RPS27A;UBA52;UBC;UBB	3	487.6001	1459.778	60.98	0.00058422	89.805	NA

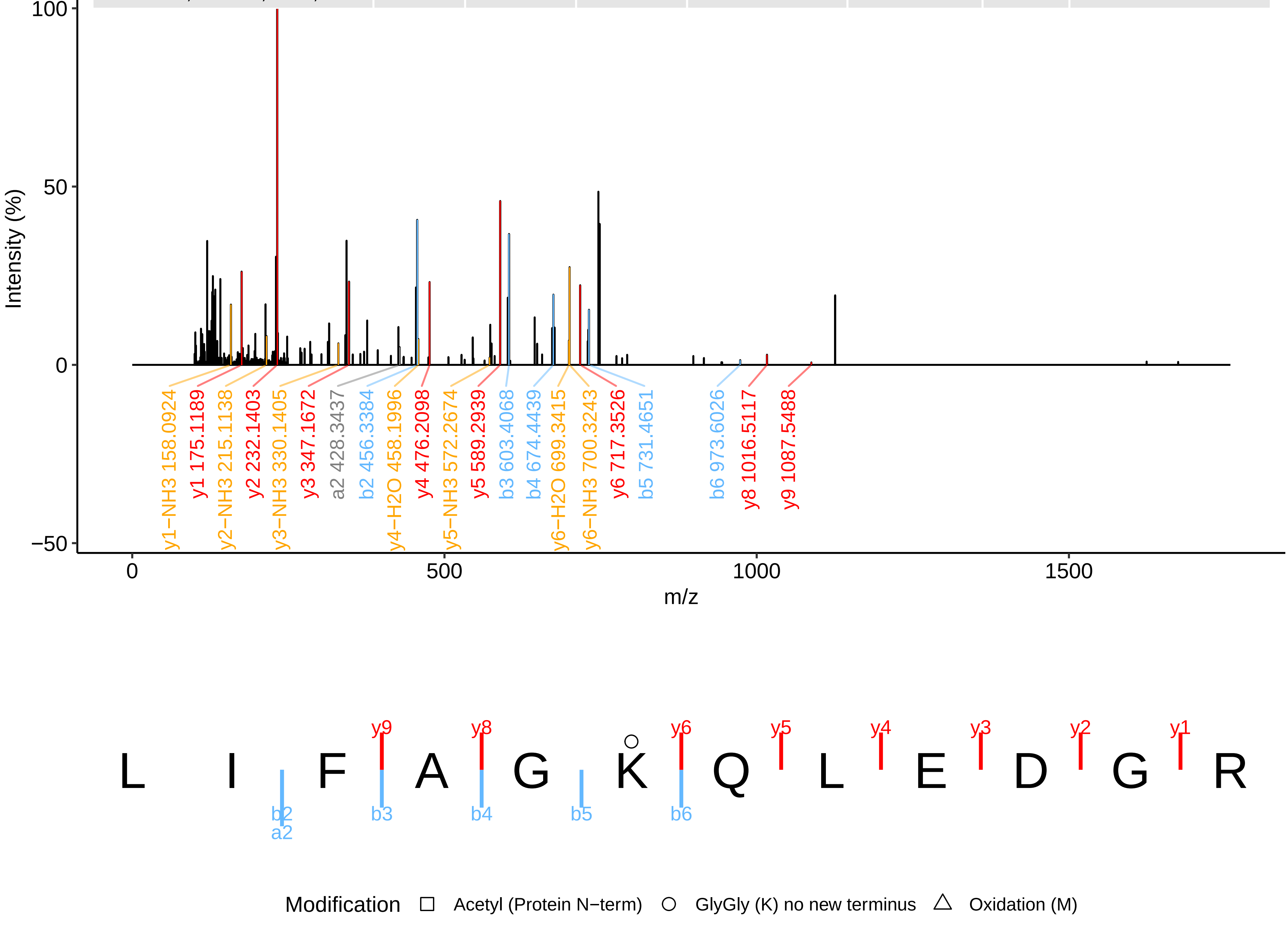

20200921_KIZ_MUb4_GlyGly_10plex − Scan number: 16978

Gene Names	Charge	m/z	Mass	Retention time	PEP	Score	Precursor Intensity
RPS27A;UBA52;UBC;UBB	3	487.6001	1459.778	61.183	0.00048047	91.313	4614896

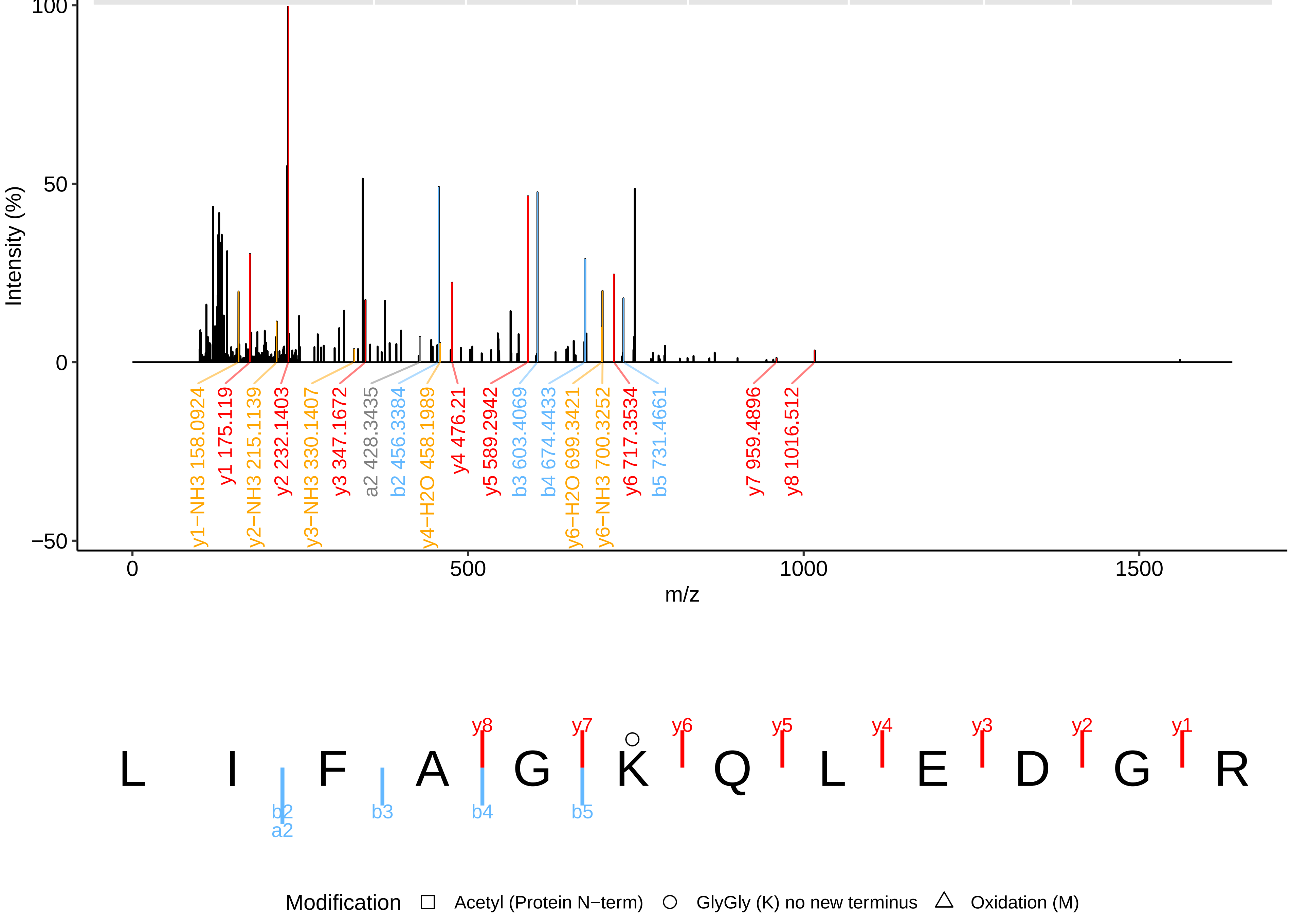

20200921_KIZ_MUb4_GlyGly_10plex − Scan number: 17032

Gene Names	Charge	m/z	Mass	Retention time	PEP	Score	Precursor Intensity
RPS27A;UBA52;UBC;UBB	2	730.8964	1459.778	61.392	0.00096055	94.309	NA

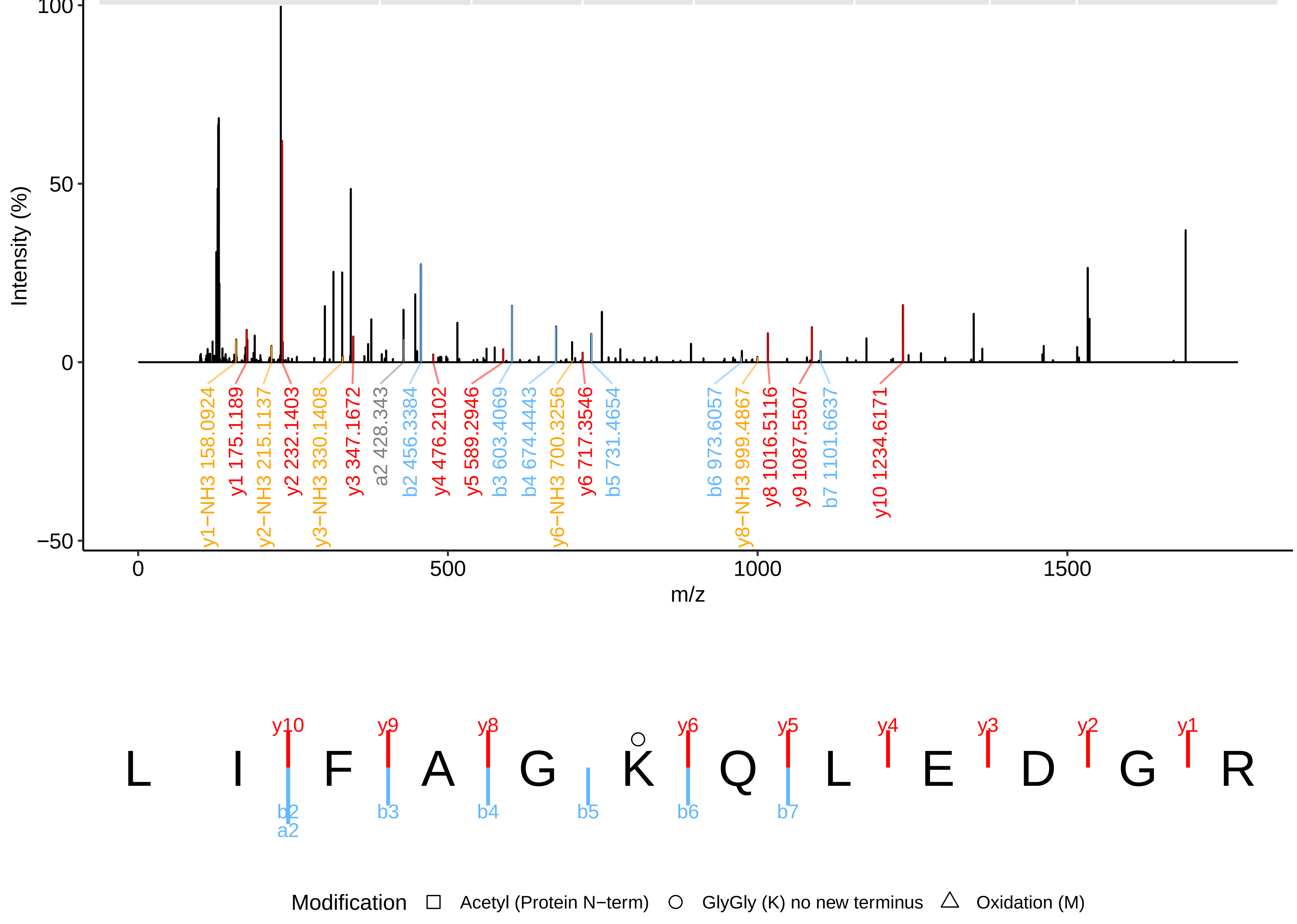

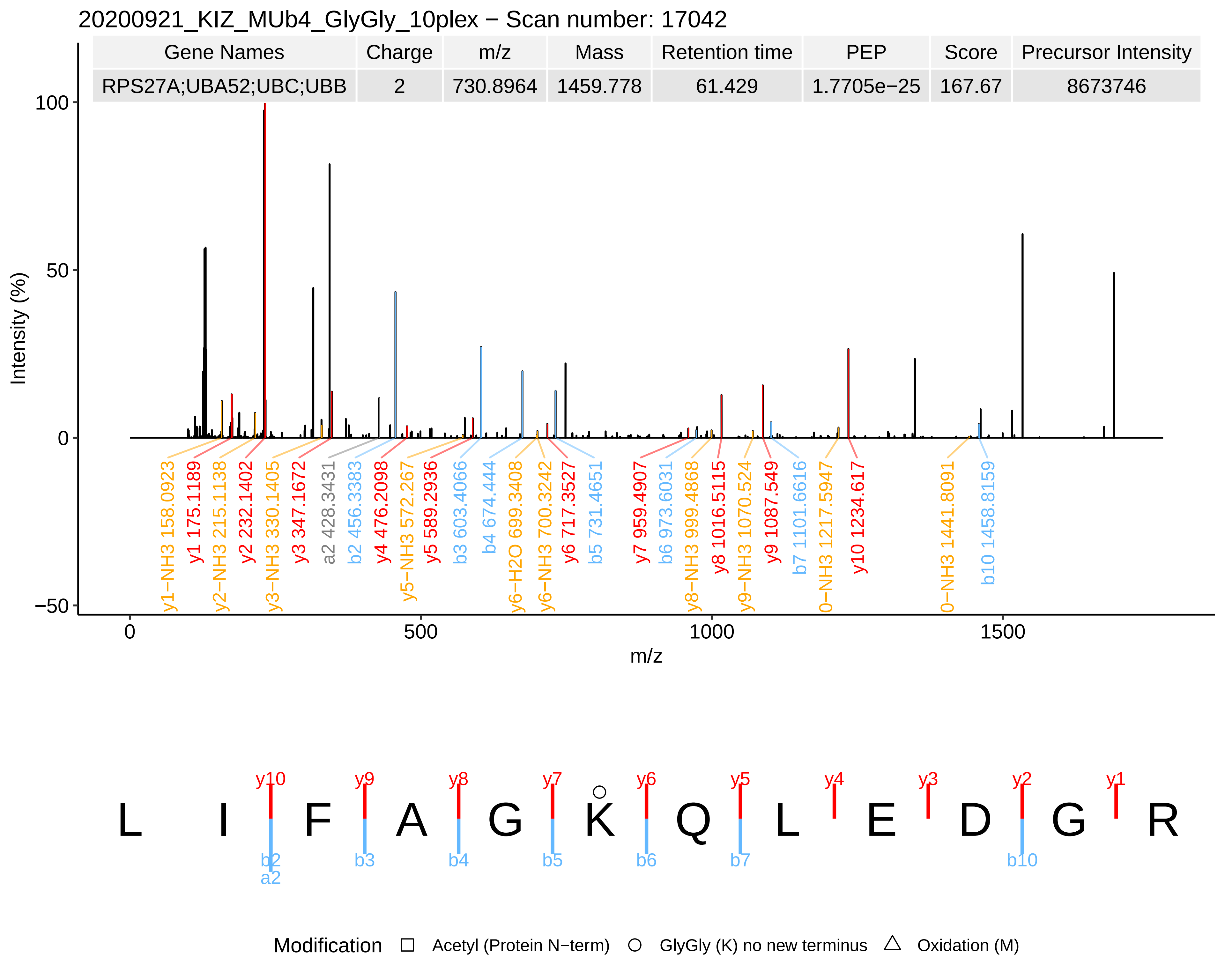

20200921_KIZ_MUb4_GlyGly_10plex − Scan number: 17042
Gene Names | Charge | m/z | Mass | Retention time | PEP | Score | Precursor Intensity
RPS27A;UBA52;UBC;UBB | 2 | 730.8964 | 1459.778 | 61.429 | 1.7705e−25 | 167.67 | 8673746
Intensity (%)
100
50
0
−50
y1−NH3 158.0923
y1 175.1189
y2−NH3 215.1138
y2 232.1402
y3−NH3 330.1405
y3 347.1672
a2 428.3431
b2 456.3383
y4 476.2098
y5−NH3 572.267
y5 589.2936
b3 603.4066
b4 674.444
y6−H2O 699.3408
y6−NH3 700.3242
y6 717.3527
b5 731.4651
y7 959.4907
b6 973.6031
y8−NH3 999.4868
y8 1016.5115
y9−NH3 1070.524
y9 1087.549
b7 1101.6616
0−NH3 1217.5947
y10 1234.617
0−NH3 1441.8091
b10 1458.8159
0
500
1000
1500
m/z
L I F A G K Q L E D G R
y10 y9 y8 y7 y6 y5 y4 y3 y2 y1
b2 a2 b3 b4 b5 b6 b7 b10
Modification
Acetyl (Protein N−term)
GlyGly (K) no new terminus
Oxidation (M)

20200921_KIZ_MUb4_GlyGly_10plex − Scan number: 17199

Gene Names	Charge	m/z	Mass	Retention time	PEP	Score	Precursor Intensity
RPS27A;UBA52;UBC;UBB	2	730.8964	1459.778	61.928	3.2634e−41	228.84	376506816

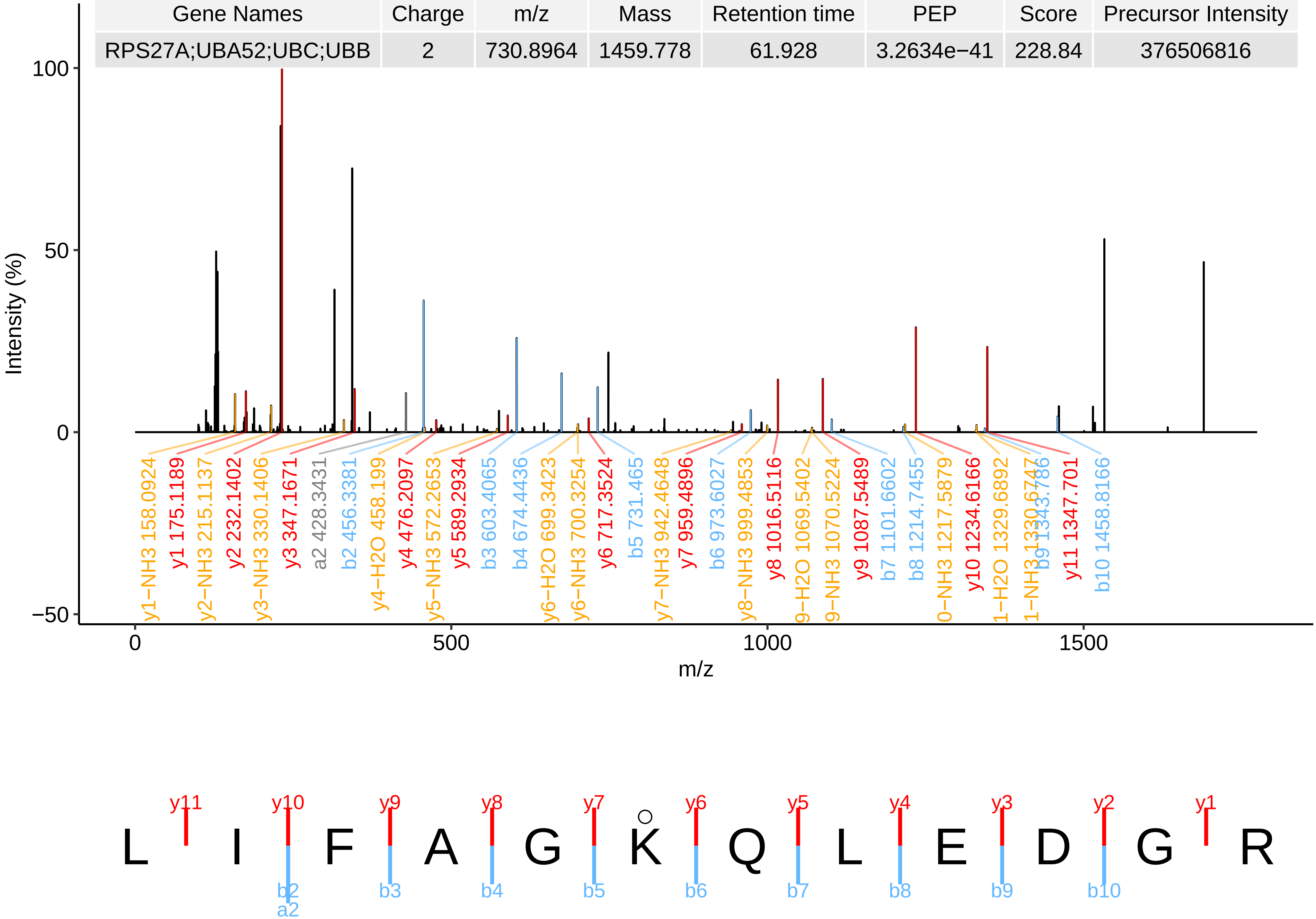

Modification □ Acetyl (Protein N−term) ○ GlyGly (K) no new terminus △ Oxidation (M)

20200921_KIZ_MUb4_GlyGly_10plex − Scan number: 17361

Gene Names	Charge	m/z	Mass	Retention time	PEP	Score	Precursor Intensity
RPS27A;UBA52;UBC;UBB	2	730.8964	1459.778	62.434	4.4229e−60	261.65	319739552

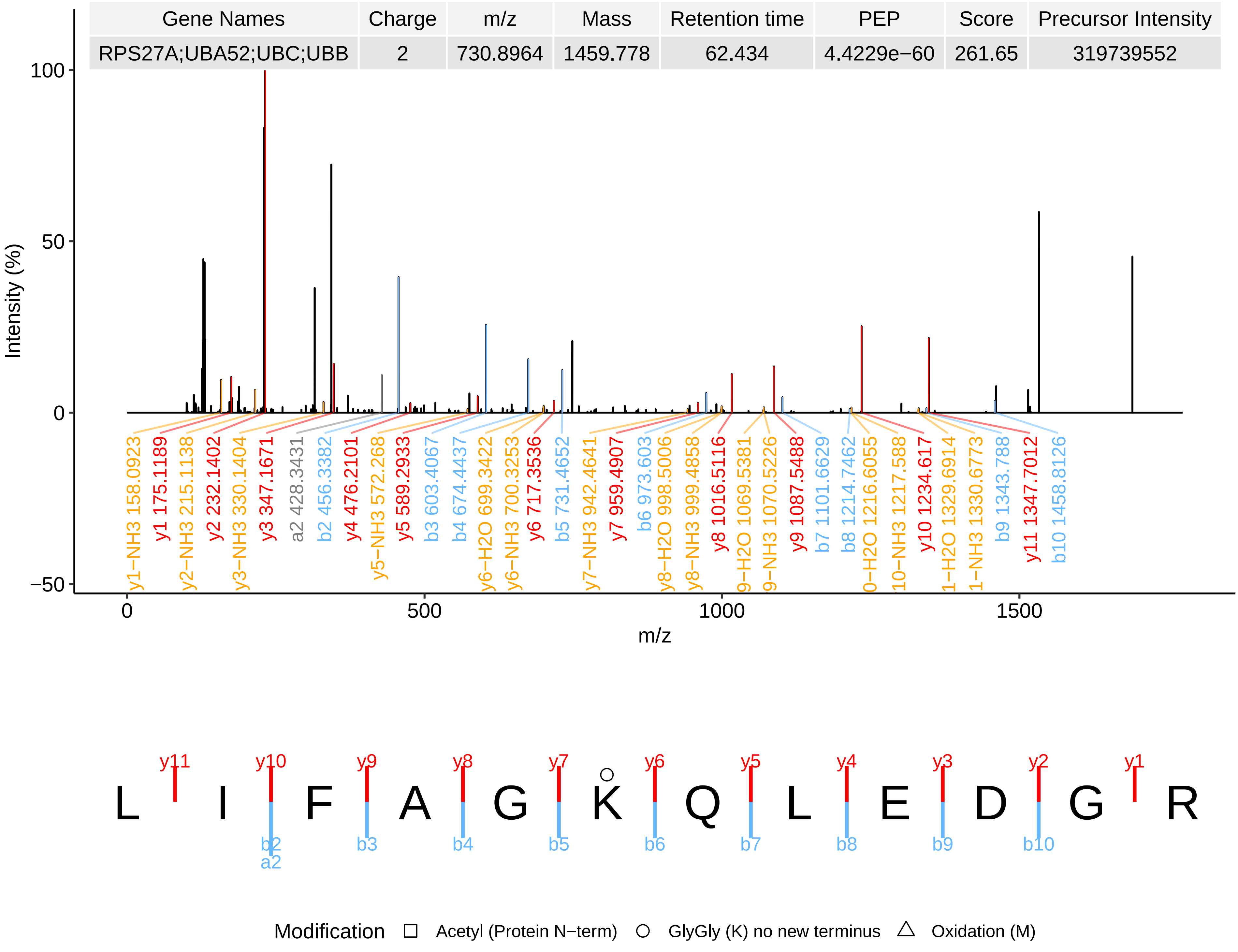

20200921_KIZ_MUb4_GlyGly_10plex − Scan number: 17515

Gene Names	Charge	m/z	Mass	Retention time	PEP	Score	Precursor Intensity
RPS27A;UBA52;UBC;UBB	2	730.8964	1459.778	62.942	1.2065e−49	239.54	242527616

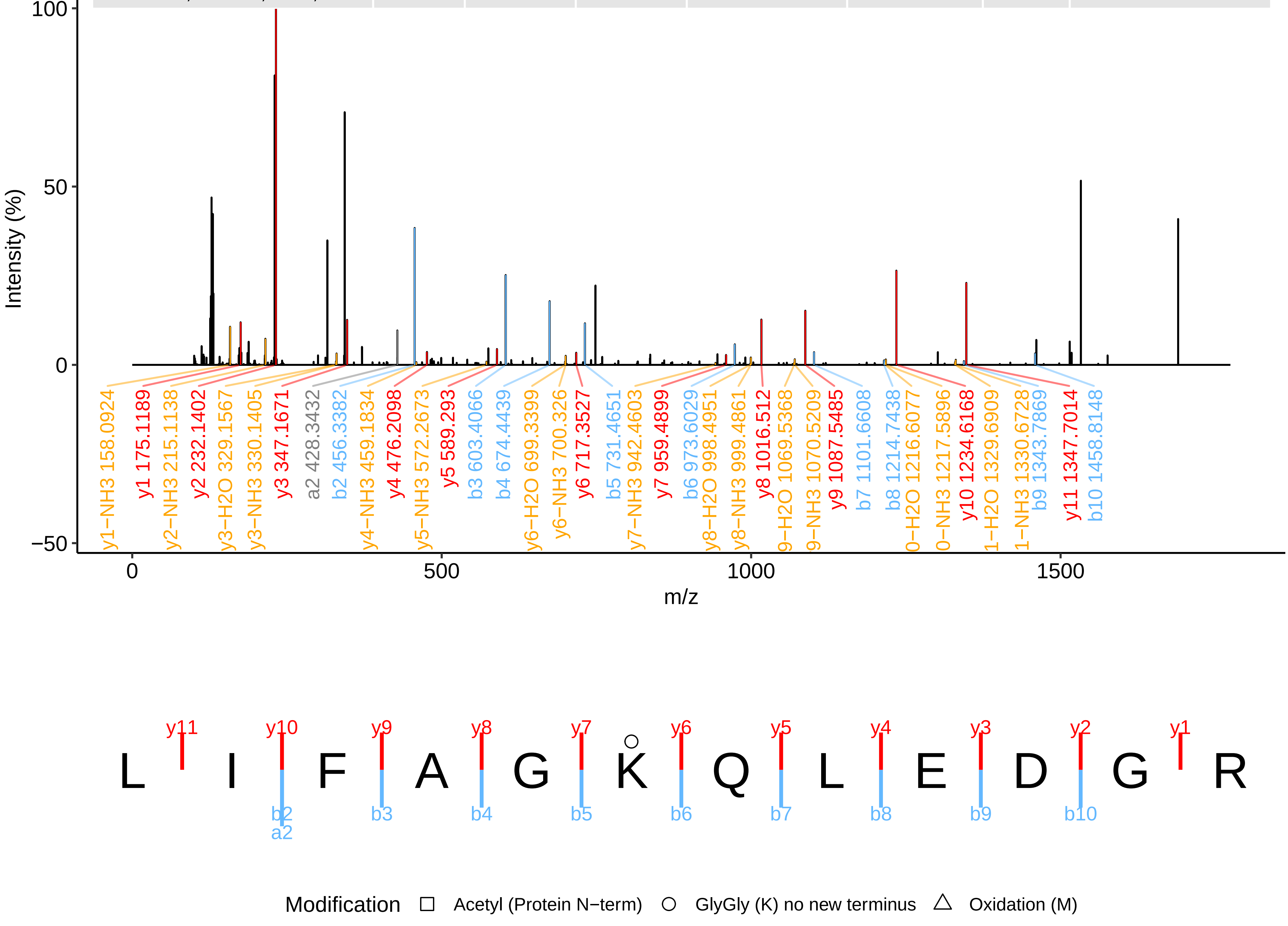

20200921_KIZ_MUb4_GlyGly_10plex − Scan number: 17665

Gene Names	Charge	m/z	Mass	Retention time	PEP	Score	Precursor Intensity
RPS27A;UBA52;UBC;UBB	2	730.8964	1459.778	63.443	4.7982e−60	260.76	148993632

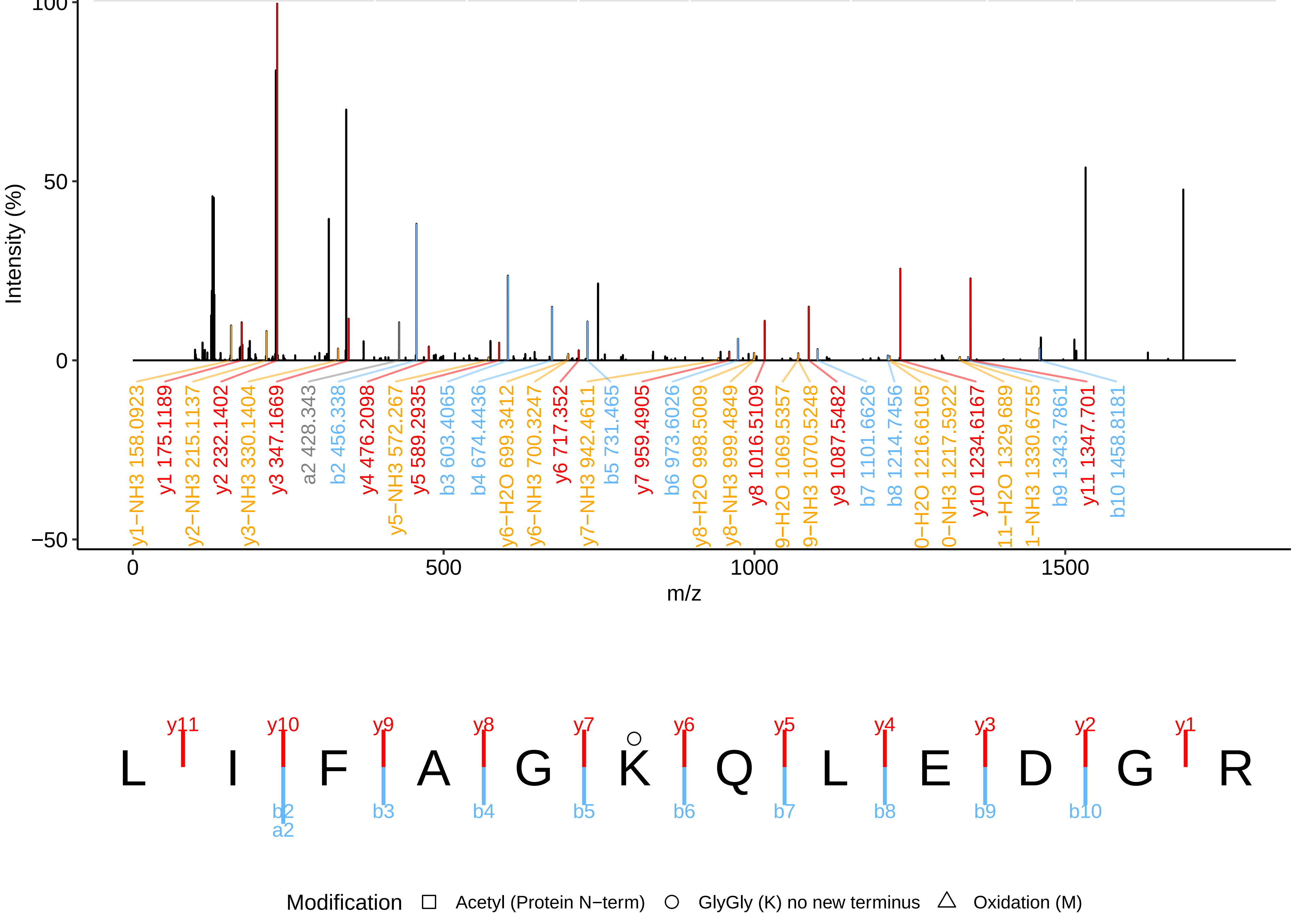

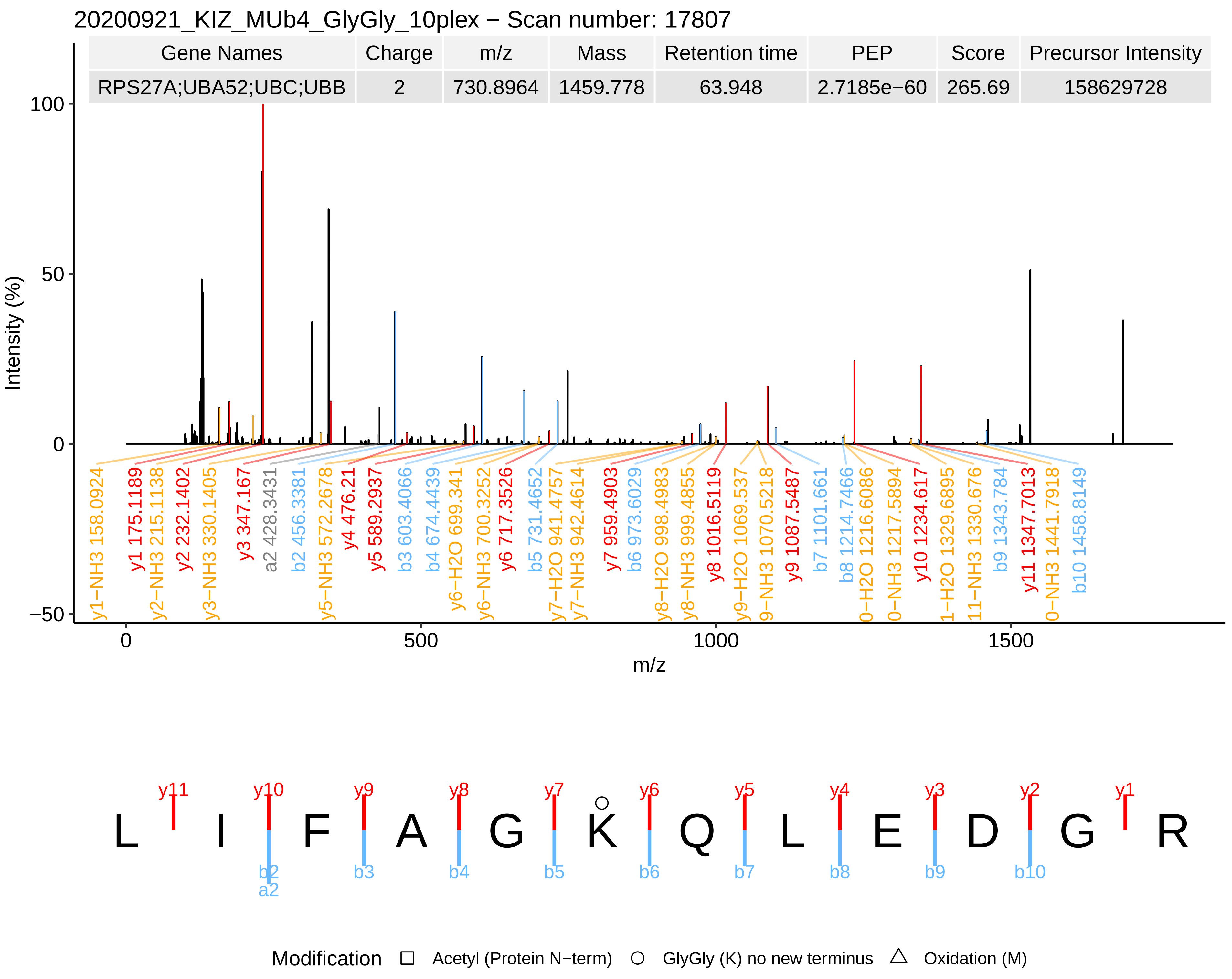

20200921_KIZ_MUb4_GlyGly_10plex − Scan number: 17807
Gene Names | Charge | m/z | Mass | Retention time | PEP | Score | Precursor Intensity
RPS27A;UBA52;UBC;UBB | 2 | 730.8964 | 1459.778 | 63.948 | 2.7185e−60 | 265.69 | 158629728
Intensity (%)
100
50
0
−50
0
500
1000
1500
m/z
y1−NH3 158.0924
y1 175.1189
y2−NH3 215.1138
y2 232.1402
y3−NH3 330.1405
y3 347.167
a2 428.3431
b2 456.3381
y5−NH3 572.2678
y4 476.21
y5 589.2937
b3 603.4066
b4 674.4439
y6−H2O 699.341
y6−NH3 700.3252
y6 717.3526
b5 731.4652
y7−H2O 941.4757
y7−NH3 942.4614
y7 959.4903
b6 973.6029
y8−H2O 998.4983
y8−NH3 999.4855
y8 1016.5119
y9−H2O 1069.537
9−NH3 1070.5218
y9 1087.5487
b7 1101.661
b8 1214.7466
0−H2O 1216.6086
0−NH3 1217.5894
y10 1234.617
1−H2O 1329.6895
11−NH3 1330.676
b9 1343.784
y11 1347.7013
0−NH3 1441.7918
b10 1458.8149
L I F A G K Q L E D G R
y11 y10 y9 y8 y7 y6 y5 y4 y3 y2 y1
b2 a2 b3 b4 b5 b6 b7 b8 b9 b10
Modification
Acetyl (Protein N−term)
GlyGly (K) no new terminus
Oxidation (M)

20200921_KIZ_MUb4_GlyGly_10plex − Scan number: 18085

Gene Names	Charge	m/z	Mass	Retention time	PEP	Score	Precursor Intensity
RPS27A;UBA52;UBC;UBB	2	730.8964	1459.778	64.961	4.4229e−60	261.65	57636548

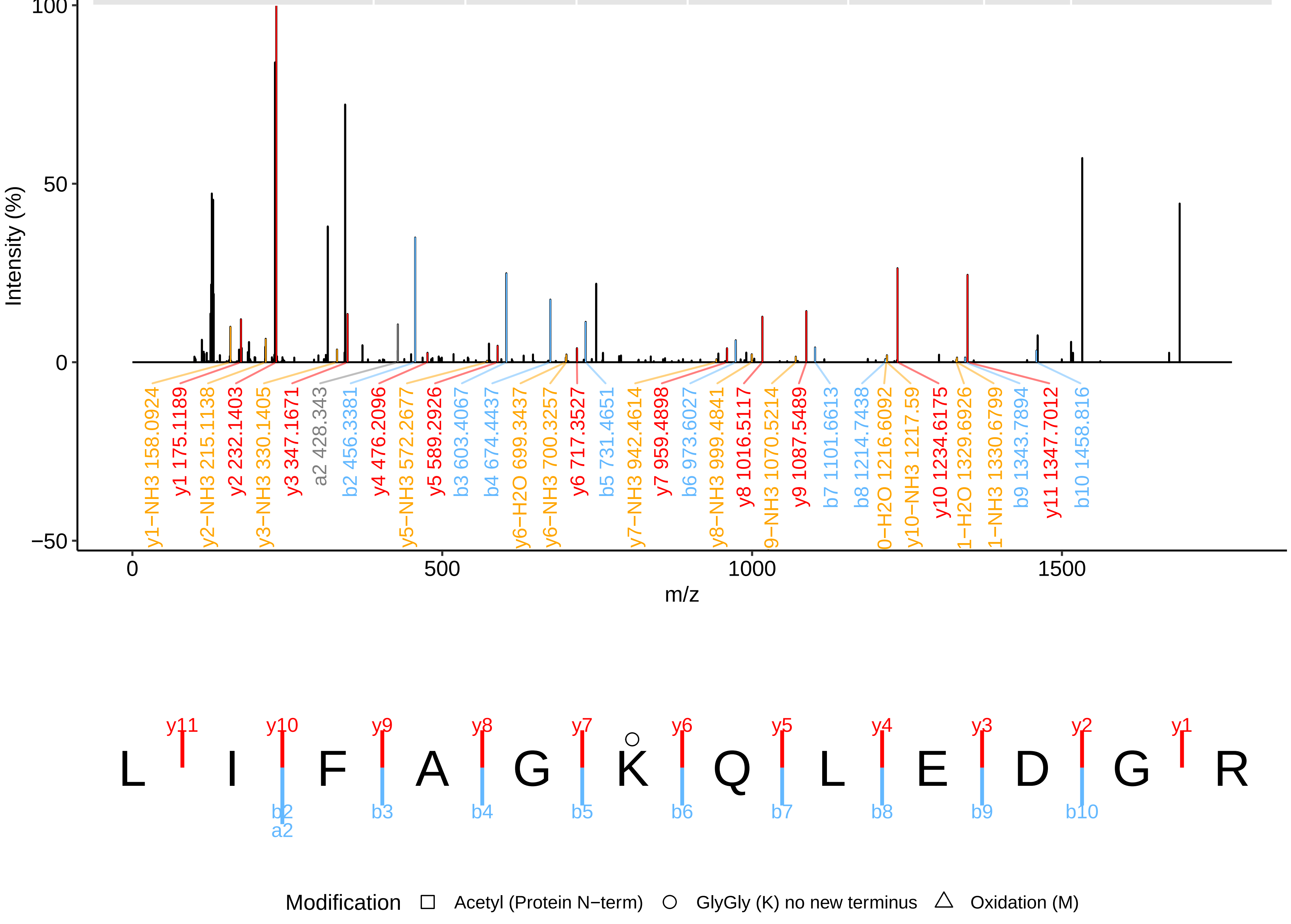

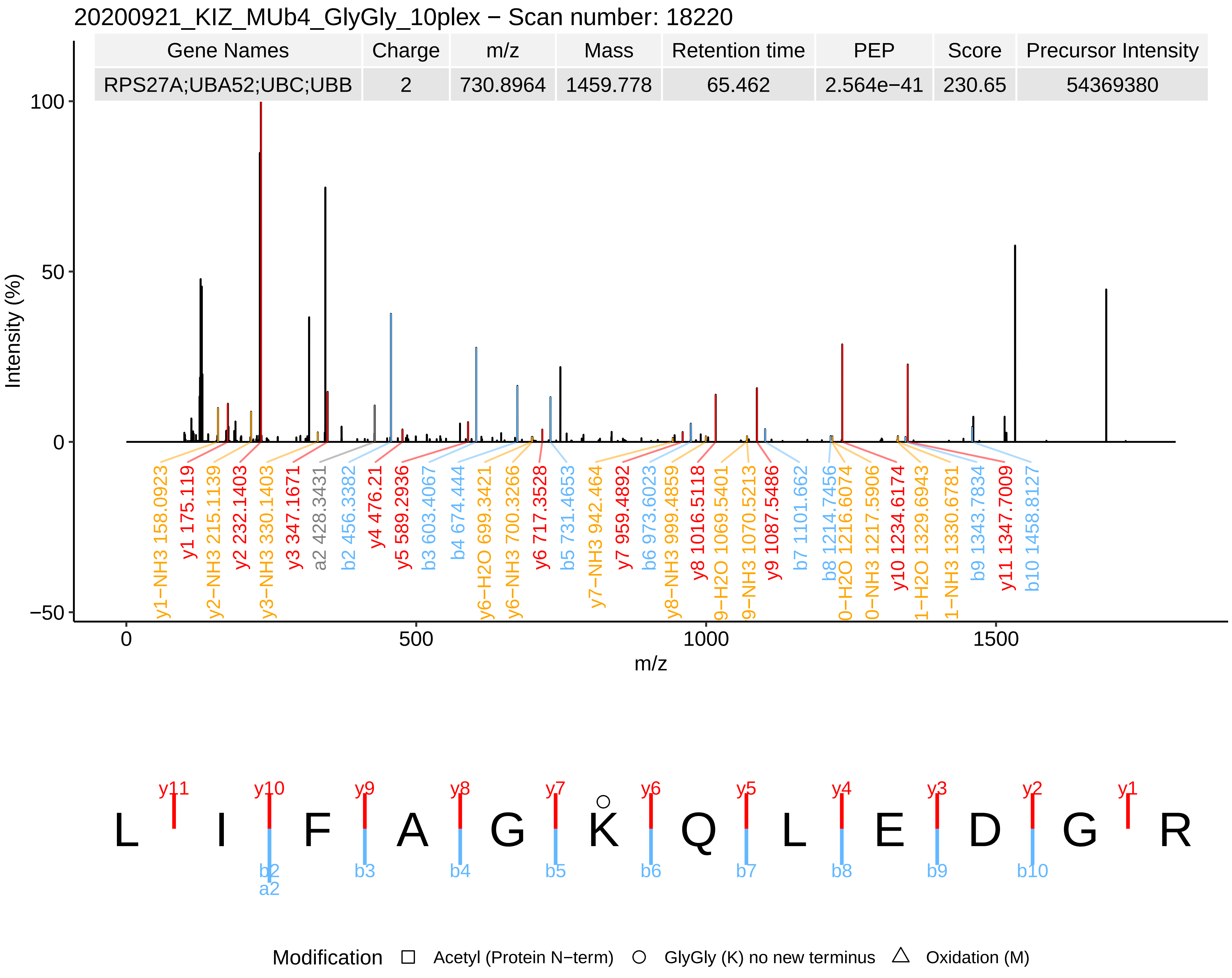

20200921_KIZ_MUb4_GlyGly_10plex − Scan number: 18220
Gene Names | Charge | m/z | Mass | Retention time | PEP | Score | Precursor Intensity
RPS27A;UBA52;UBC;UBB | 2 | 730.8964 | 1459.778 | 65.462 | 2.564e−41 | 230.65 | 54369380
Intensity (%)
100
50
0
−50
0
500
1000
1500
m/z
y1−NH3 158.0923
y1 175.119
y2−NH3 215.1139
y2 232.1403
y3−NH3 330.1403
y3 347.1671
a2 428.3431
b2 456.3382
y4 476.21
y5 589.2936
b3 603.4067
b4 674.444
y6−H2O 699.3421
y6−NH3 700.3266
y6 717.3528
b5 731.4653
y7−NH3 942.464
y7 959.4892
b6 973.6023
y8−NH3 999.4859
y8 1016.5118
9−H2O 1069.5401
9−NH3 1070.5213
y9 1087.5486
b7 1101.662
b8 1214.7456
0−H2O 1216.6074
0−NH3 1217.5906
y10 1234.6174
1−H2O 1329.6943
1−NH3 1330.6781
b9 1343.7834
y11 1347.7009
b10 1458.8127
L I F A G K Q L E D G R
y11 y10 y9 y8 y7 y6 y5 y4 y3 y2 y1
b2 a2 b3 b4 b5 b6 b7 b8 b9 b10
Modification
Acetyl (Protein N−term)
GlyGly (K) no new terminus
Oxidation (M)

20200921_KIZ_MUb4_GlyGly_10plex − Scan number: 18351

Gene Names	Charge	m/z	Mass	Retention time	PEP	Score	Precursor Intensity
RPS27A;UBA52;UBC;UBB	2	730.8964	1459.778	65.968	1.705e−41	232.88	56619168

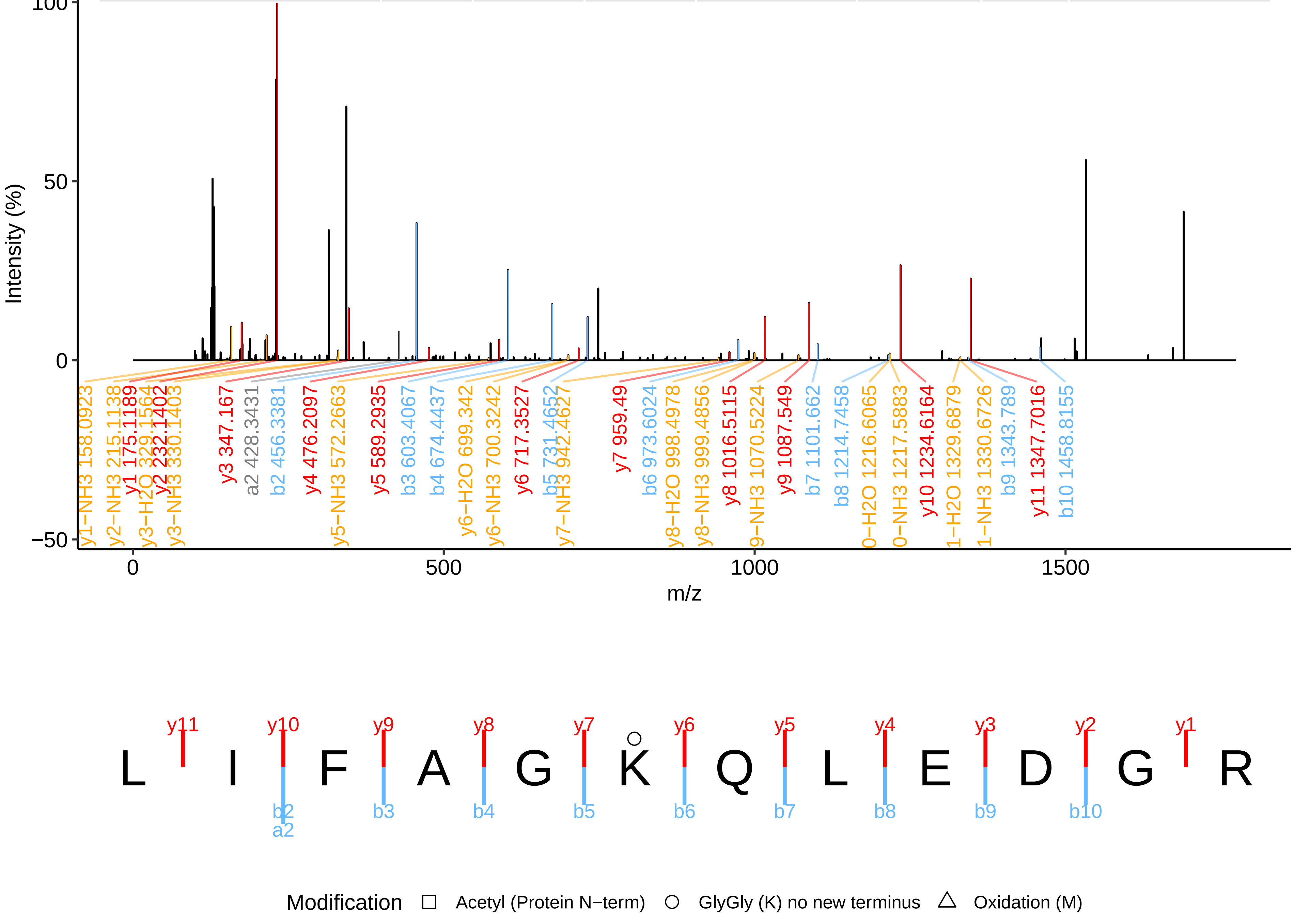

20200921_KIZ_MUb4_GlyGly_10plex − Scan number: 18482

Gene Names	Charge	m/z	Mass	Retention time	PEP	Score	Precursor Intensity
RPS27A;UBA52;UBC;UBB	2	730.8964	1459.778	66.484	6.0669e−50	247.08	38515636

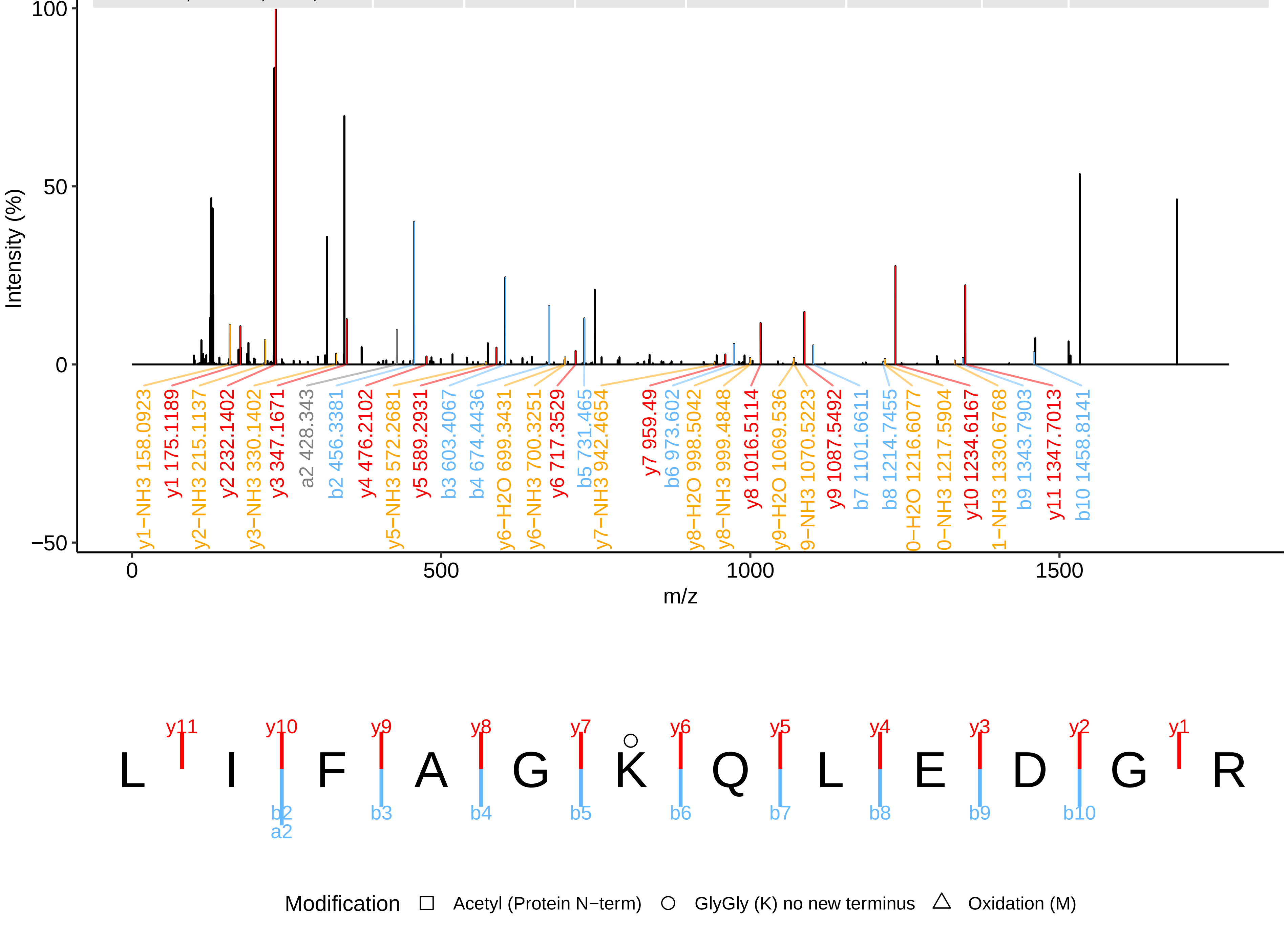

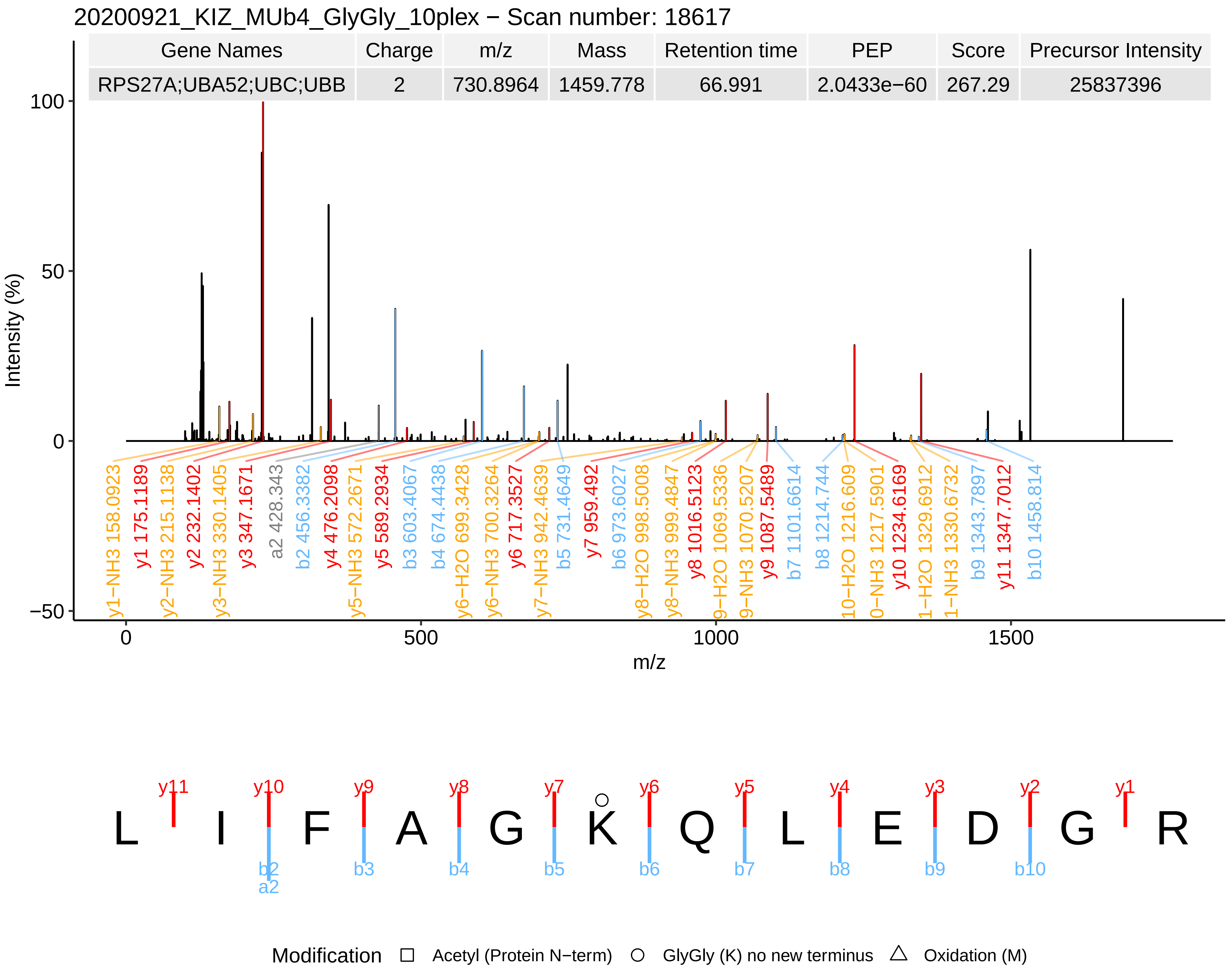
20200921_KIZ_MUb4_GlyGly_10plex − Scan number: 18617
Gene Names
RPS27A;UBA52;UBC;UBB
Charge
2
m/z
730.8964
Mass
1459.778
Retention time
66.991
PEP
2.0433e−60
Score
267.29
Precursor Intensity
25837396
Intensity (%)
100
50
0
−50
0
500
1000
1500
m/z
y1−NH3 158.0923
y1 175.1189
y2−NH3 215.1138
y2 232.1402
y3−NH3 330.1405
y3 347.1671
a2 428.343
b2 456.3382
y4 476.2098
y5−NH3 572.2671
y5 589.2934
b3 603.4067
b4 674.4438
y6−H2O 699.3428
y6−NH3 700.3264
y6 717.3527
y7−NH3 942.4639
b5 731.4649
y7 959.492
b6 973.6027
y8−H2O 998.5008
y8−NH3 999.4847
y8 1016.5123
b7 1101.6614
b8 1214.744
y9 1087.5489
y10 1234.6169
b9 1343.7897
y11 1347.7012
b10 1458.814
L I F A G K Q L E D G R
y11 y10 y9 y8 y7 y6 y5 y4 y3 y2 y1
b2 a2 b3 b4 b5 b6 b7 b8 b9 b10
Modification
Acetyl (Protein N−term)
GlyGly (K) no new terminus
Oxidation (M)

20200921_KIZ_MUb4_GlyGly_10plex − Scan number: 18751

Gene Names	Charge	m/z	Mass	Retention time	PEP	Score	Precursor Intensity
RPS27A;UBA52;UBC;UBB	2	730.8964	1459.778	67.508	6.4311e−50	246.63	16765273

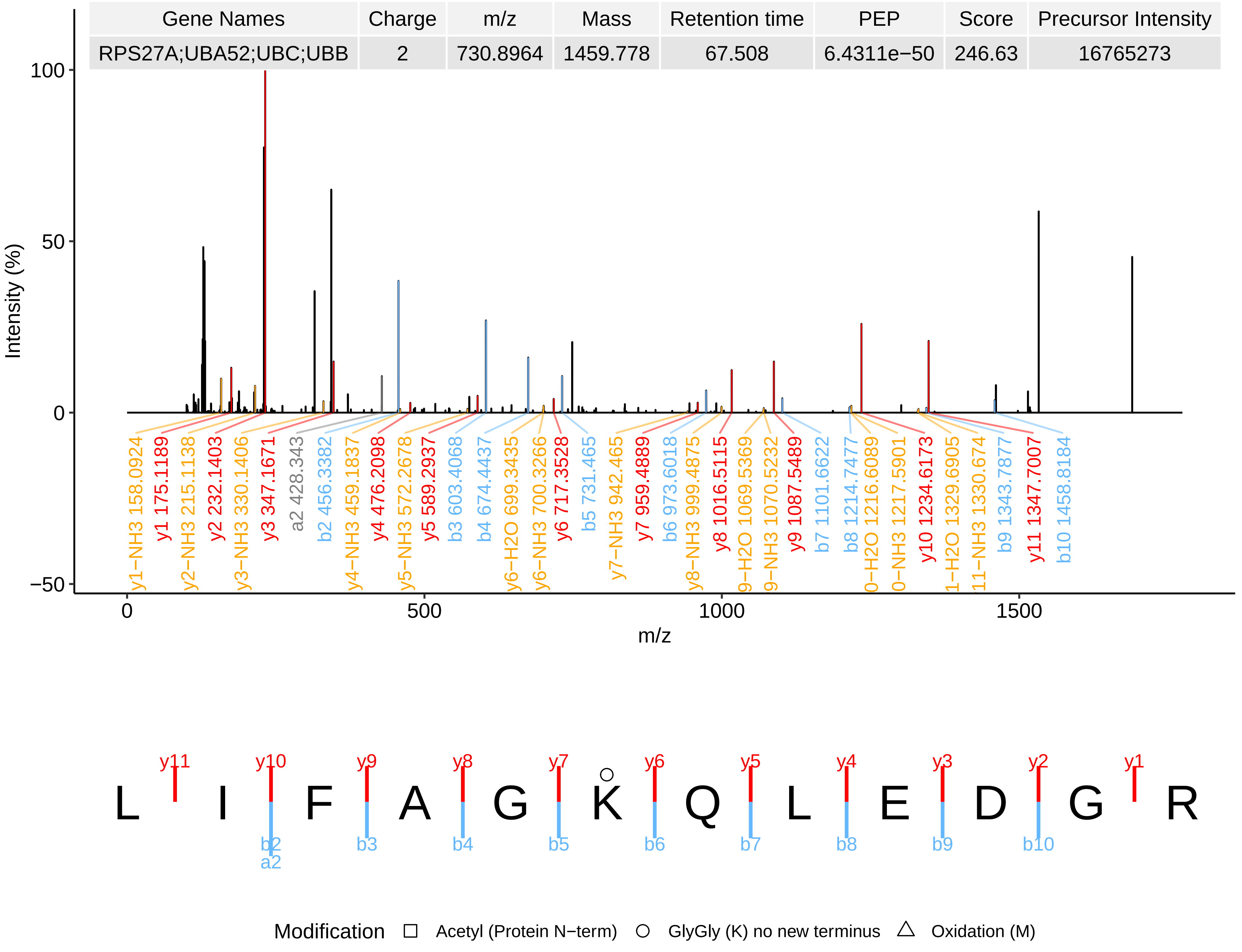

20200921_KIZ_MUb4_GlyGly_10plex − Scan number: 17125

Gene Names	Charge	m/z	Mass	Retention time	PEP	Score	Precursor Intensity
RPS27A;UBA52;UBC;UBB	3	487.6001	1459.778	61.691	2.5508e−13	138.76	9082460160

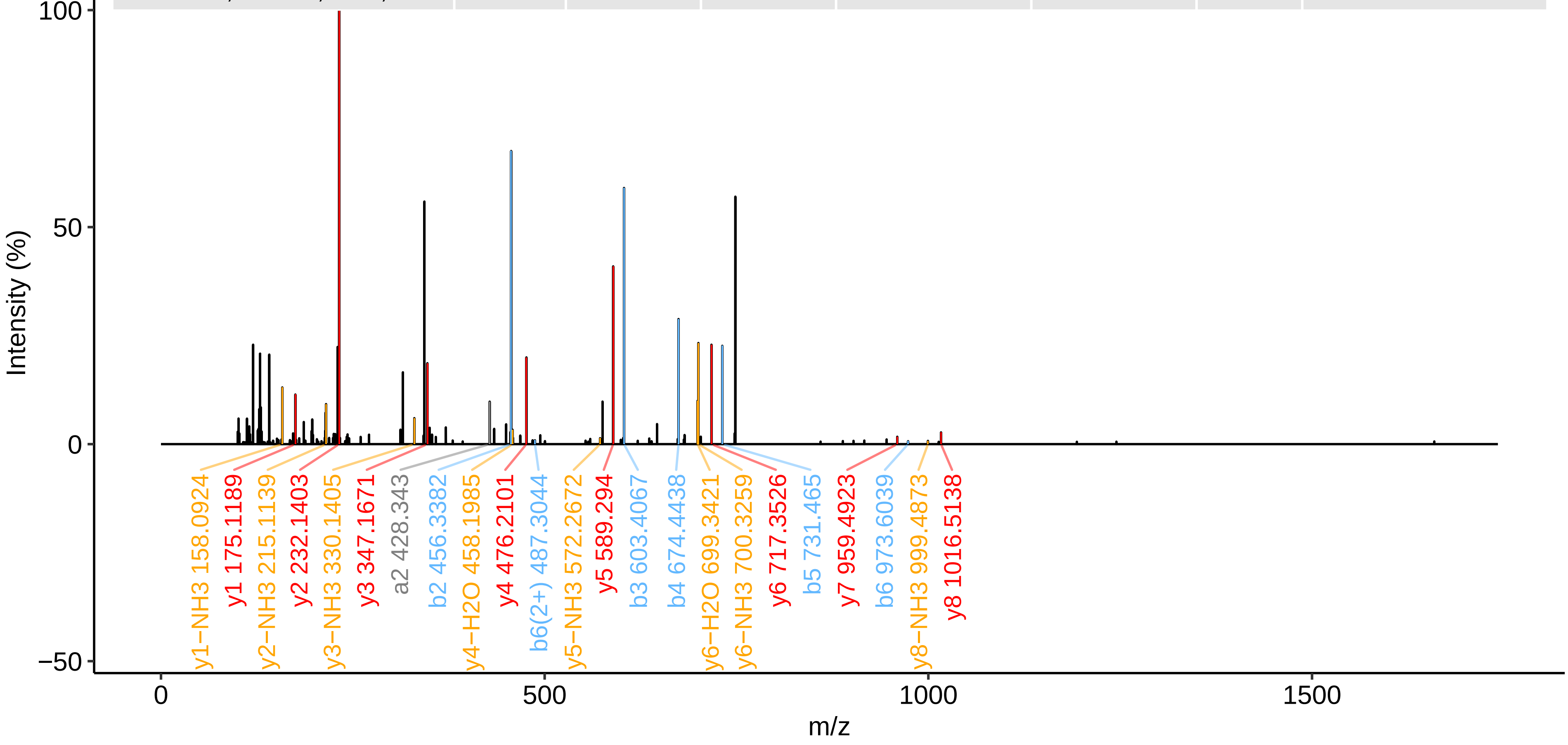

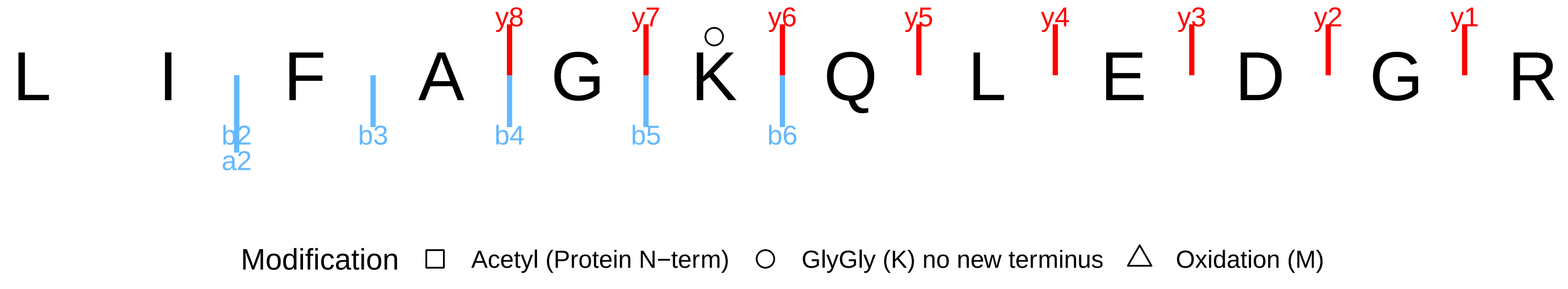

20200921_KIZ_MUb4_GlyGly_10plex − Scan number: 17287

Gene Names	Charge	m/z	Mass	Retention time	PEP	Score	Precursor Intensity
RPS27A;UBA52;UBC;UBB	3	487.6001	1459.778	62.197	2.0348e−13	141.08	7553702400

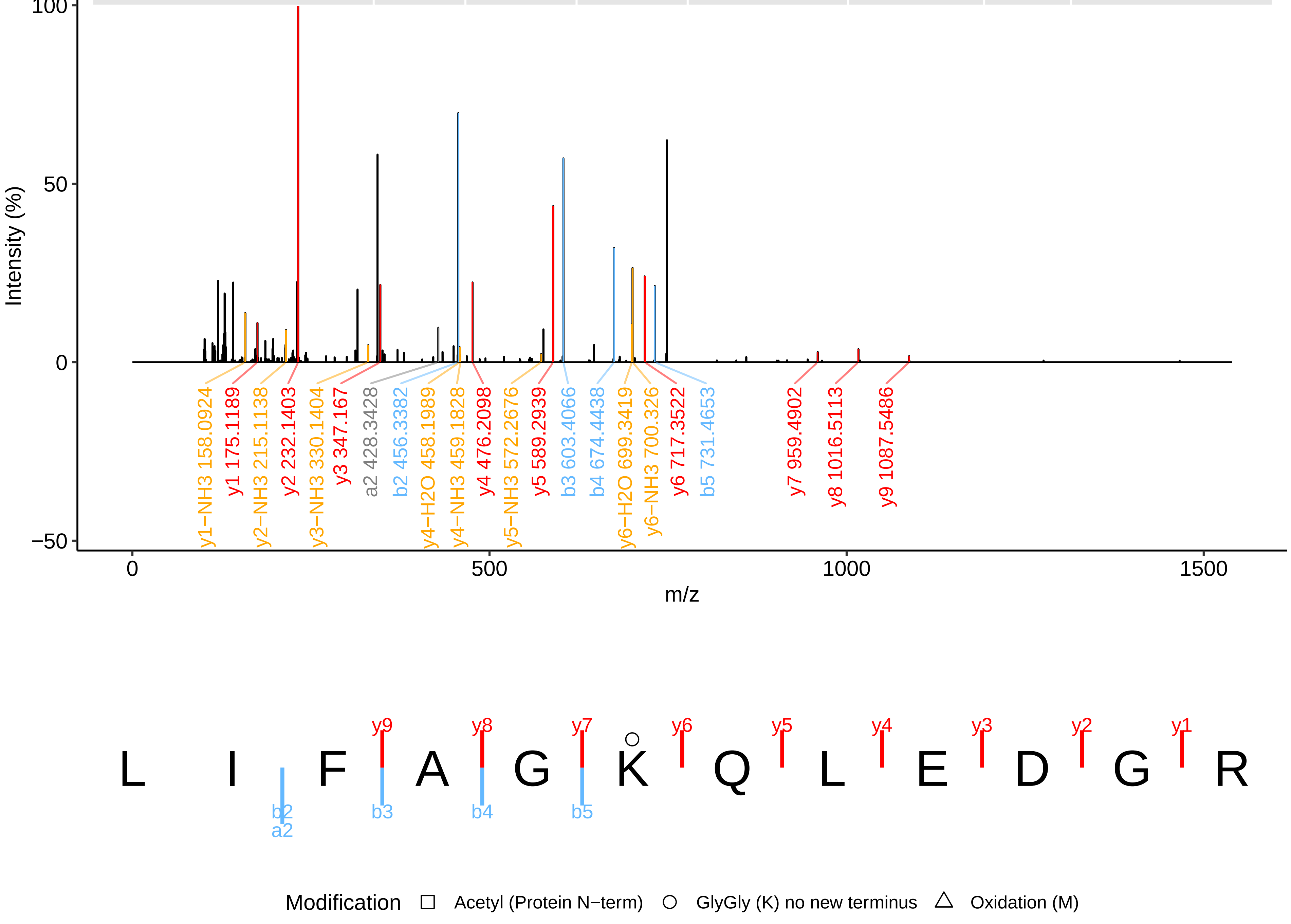

20200921_KIZ_MUb4_GlyGly_10plex − Scan number: 17442

Gene Names	Charge	m/z	Mass	Retention time	PEP	Score	Precursor Intensity
RPS27A;UBA52;UBC;UBB	3	487.6001	1459.778	62.698	9.899e−20	162.26	5322629632

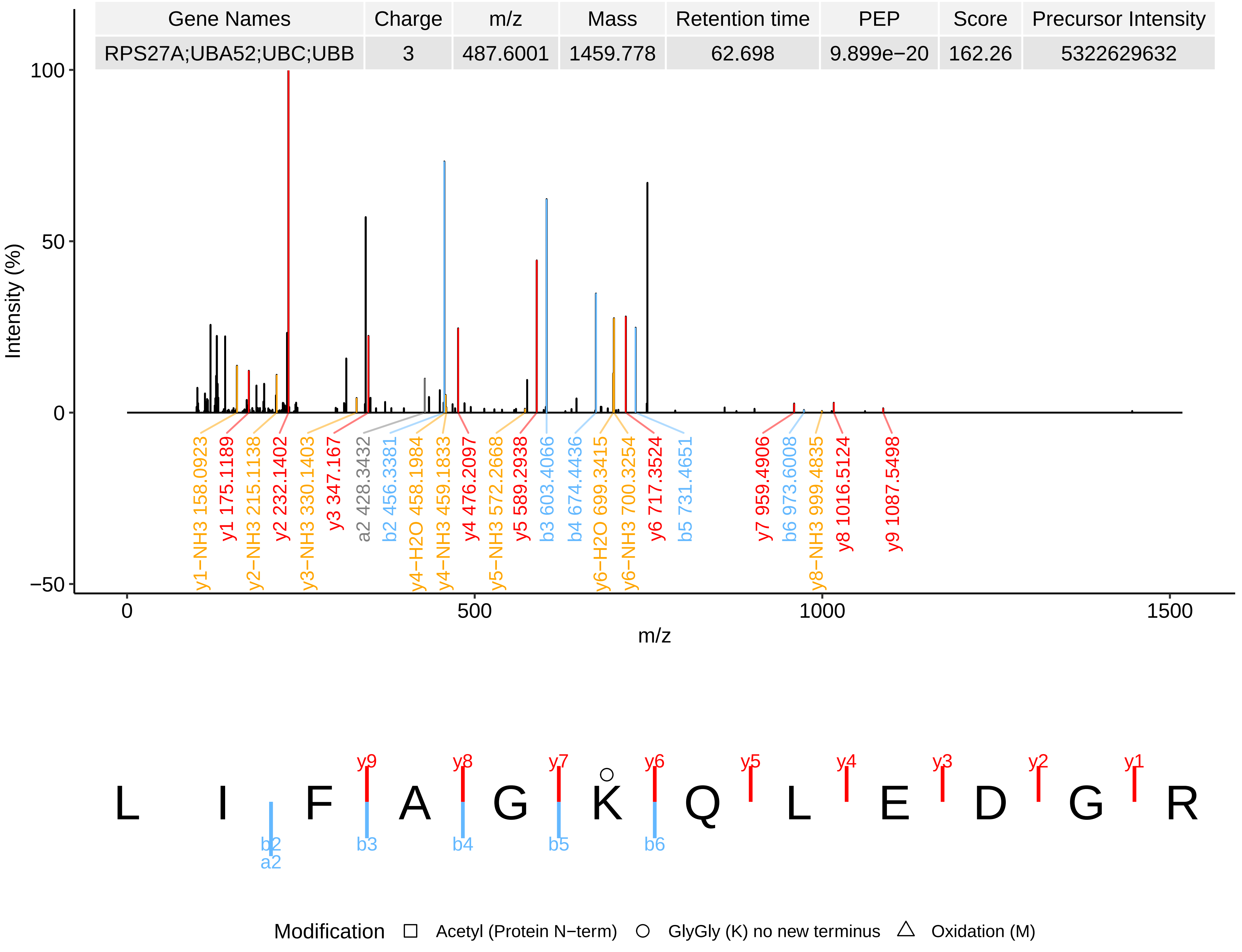

20200921_KIZ_MUb4_GlyGly_10plex − Scan number: 17590

Gene Names	Charge	m/z	Mass	Retention time	PEP	Score	Precursor Intensity
RPS27A;UBA52;UBC;UBB	3	487.6001	1459.778	63.201	3.1574e−19	150.46	4272533760

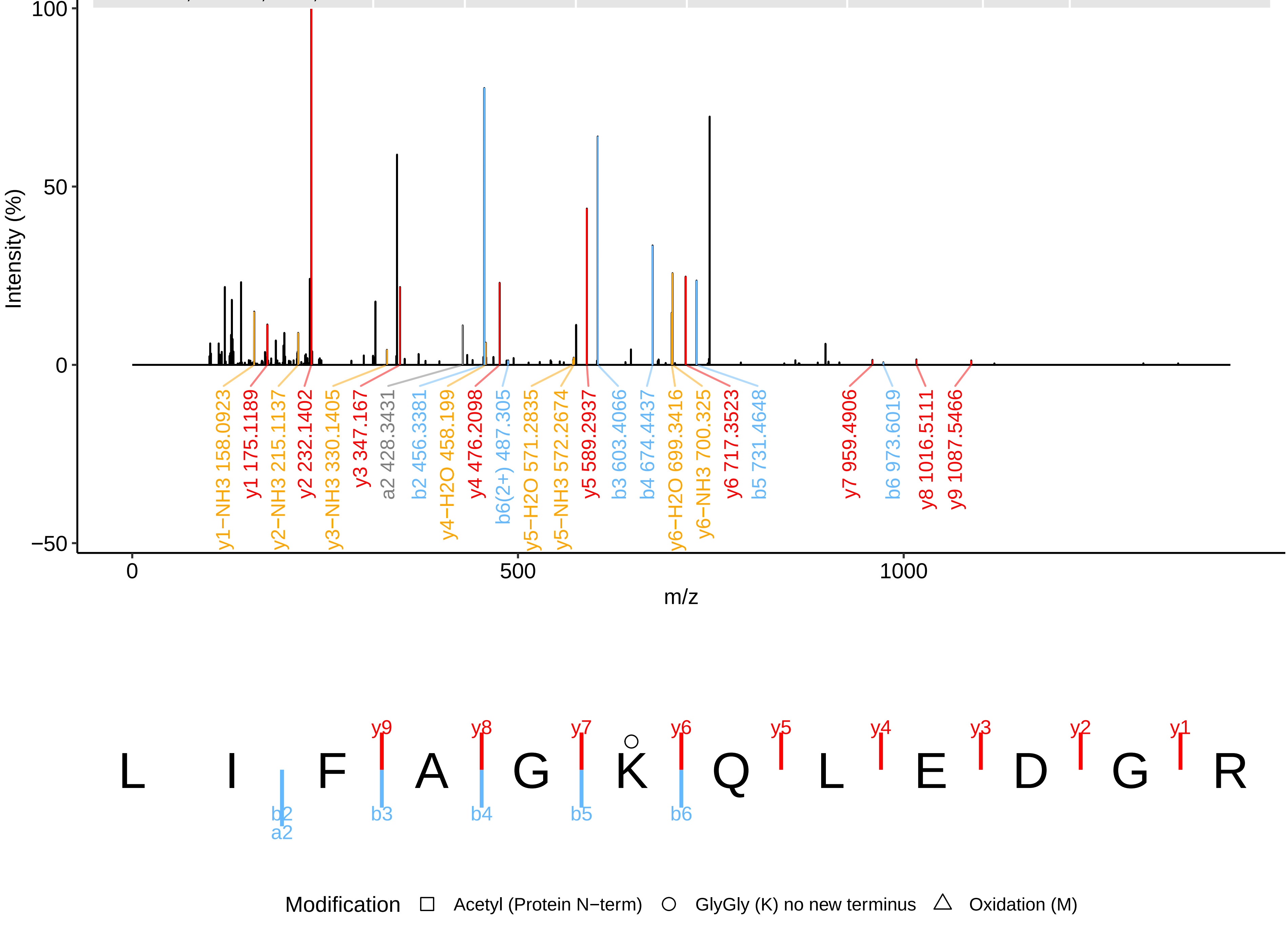

20200921_KIZ_MUb4_GlyGly_10plex − Scan number: 17739

Gene Names	Charge	m/z	Mass	Retention time	PEP	Score	Precursor Intensity
RPS27A;UBA52;UBC;UBB	3	487.6001	1459.778	63.707	3.1574e−19	150.46	3195756288

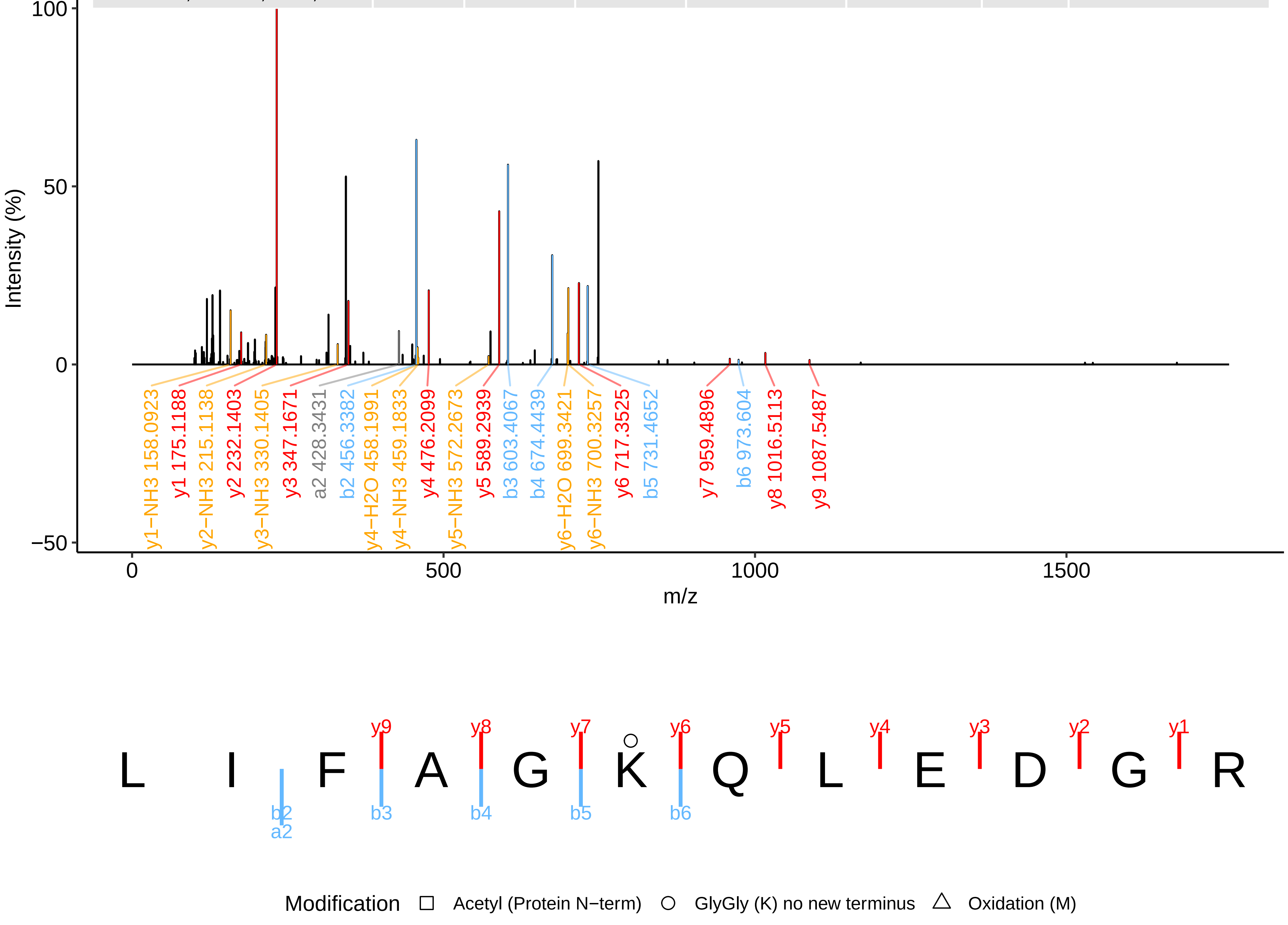

20200921_KIZ_MUb4_GlyGly_10plex − Scan number: 17880

Gene Names	Charge	m/z	Mass	Retention time	PEP	Score	Precursor Intensity
RPS27A;UBA52;UBC;UBB	3	487.6001	1459.778	64.213	3.1574e−19	150.46	2716582912

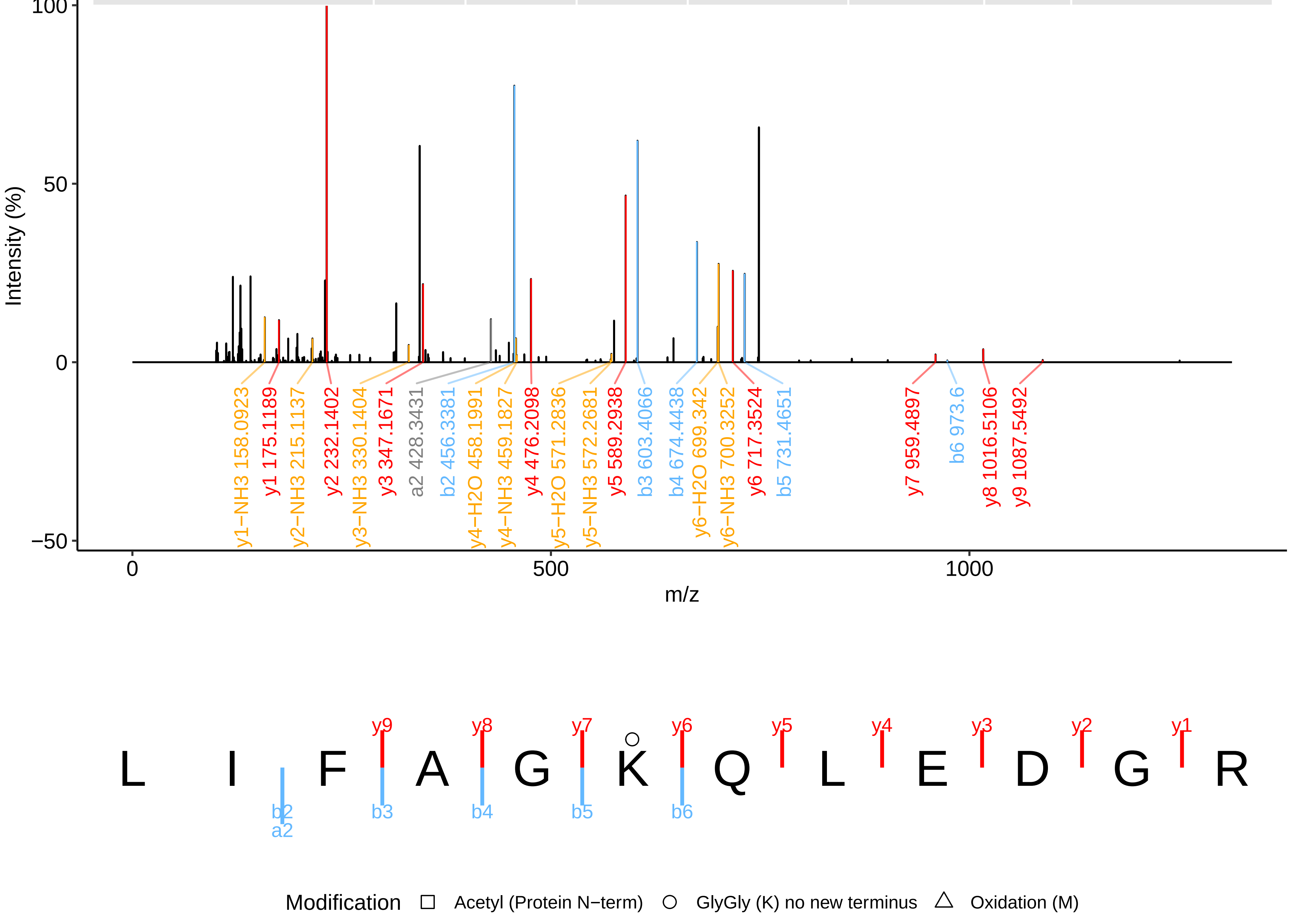

20200921_KIZ_MUb4_GlyGly_10plex − Scan number: 18022

Gene Names	Charge	m/z	Mass	Retention time	PEP	Score	Precursor Intensity
RPS27A;UBA52;UBC;UBB	3	487.6001	1459.778	64.725	3.1574e−19	150.46	2482566656

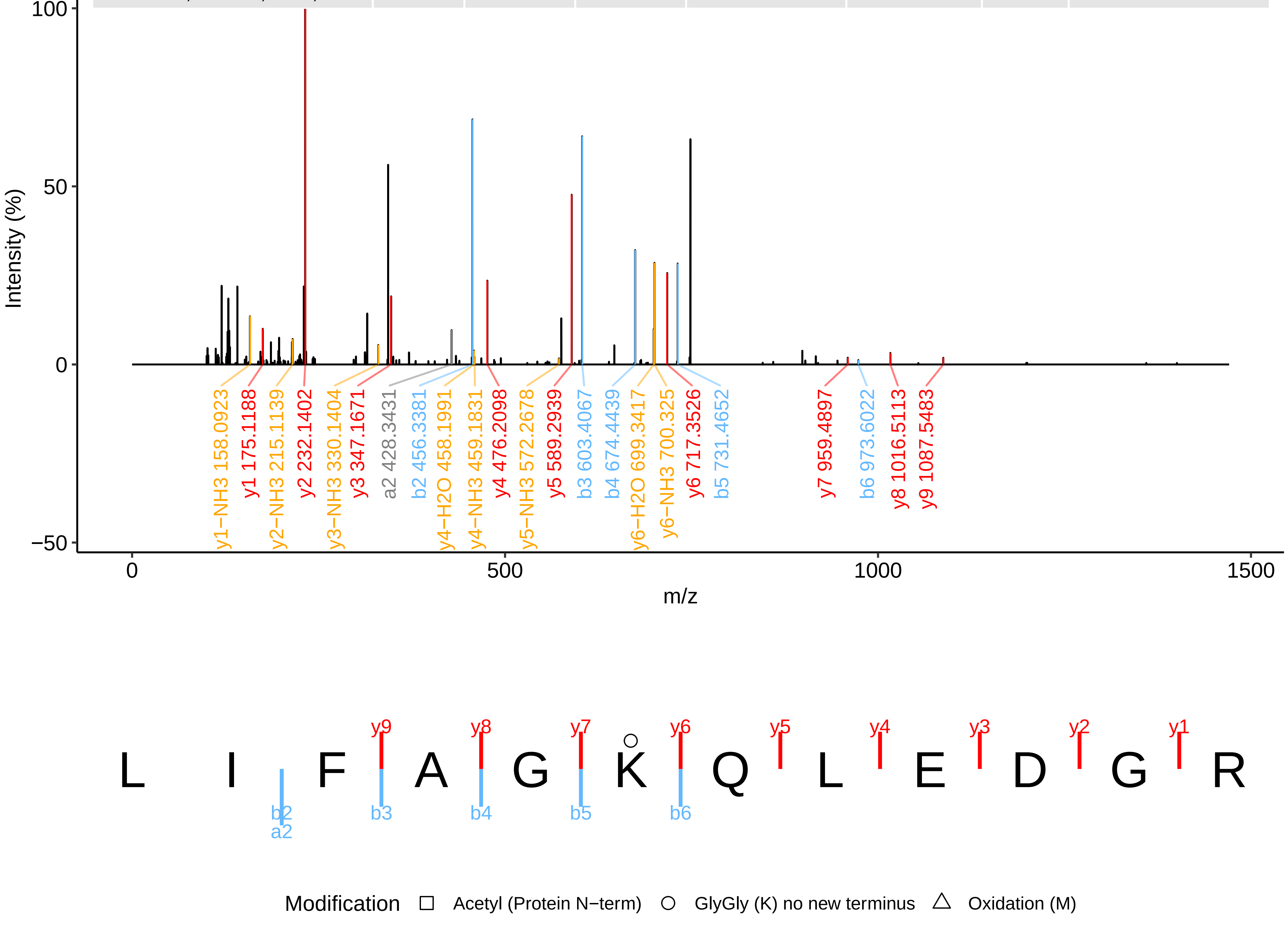

20200921_KIZ_MUb4_GlyGly_10plex − Scan number: 18159

Gene Names	Charge	m/z	Mass	Retention time	PEP	Score	Precursor Intensity
RPS27A;UBA52;UBC;UBB	3	487.6001	1459.778	65.235	1.0628e−13	145.46	1844011648

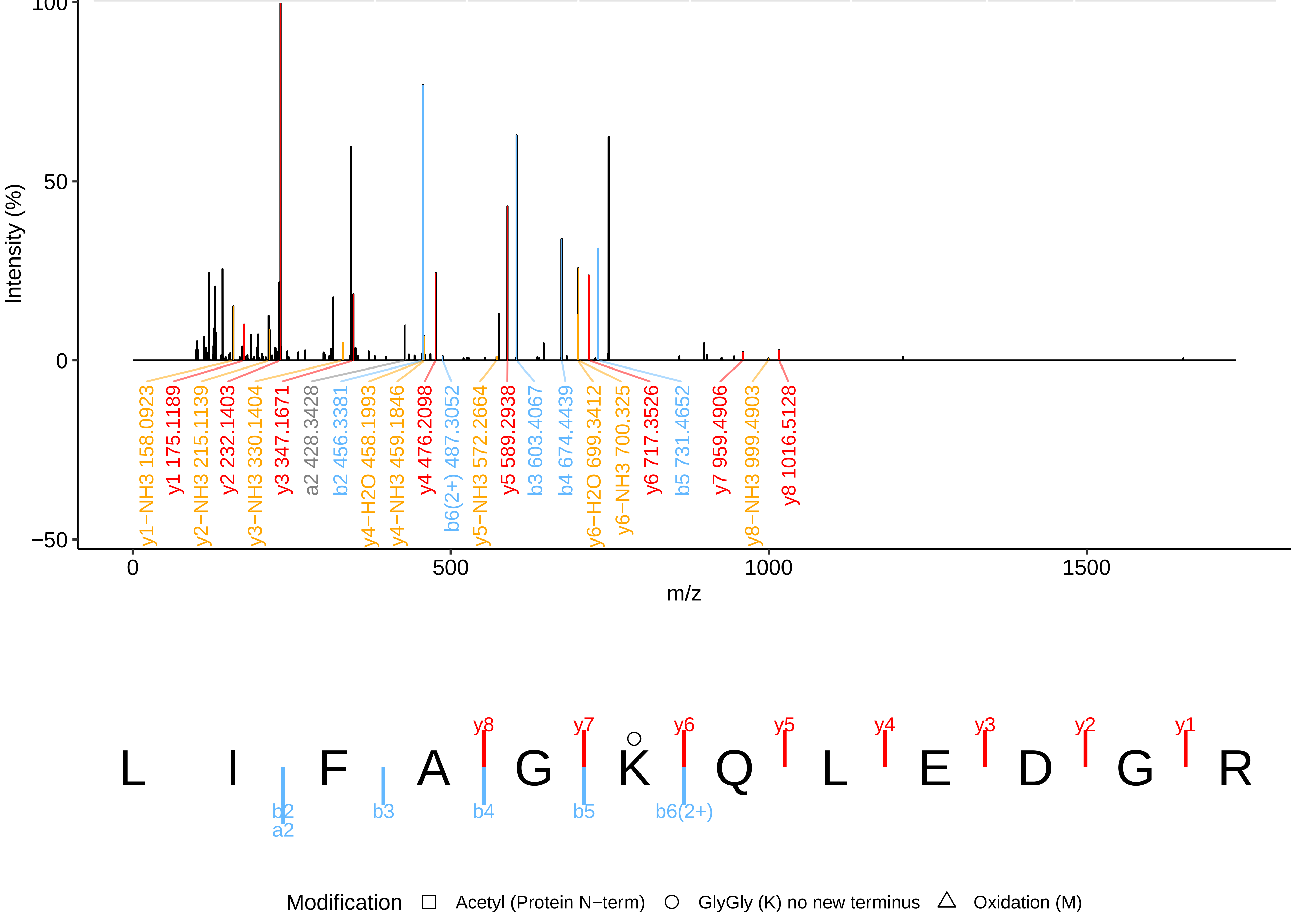

20200921_KIZ_MUb4_GlyGly_10plex − Scan number: 18294

Gene Names	Charge	m/z	Mass	Retention time	PEP	Score	Precursor Intensity
RPS27A;UBA52;UBC;UBB	3	487.6001	1459.778	65.748	2.7462e−19	152.7	1579387648

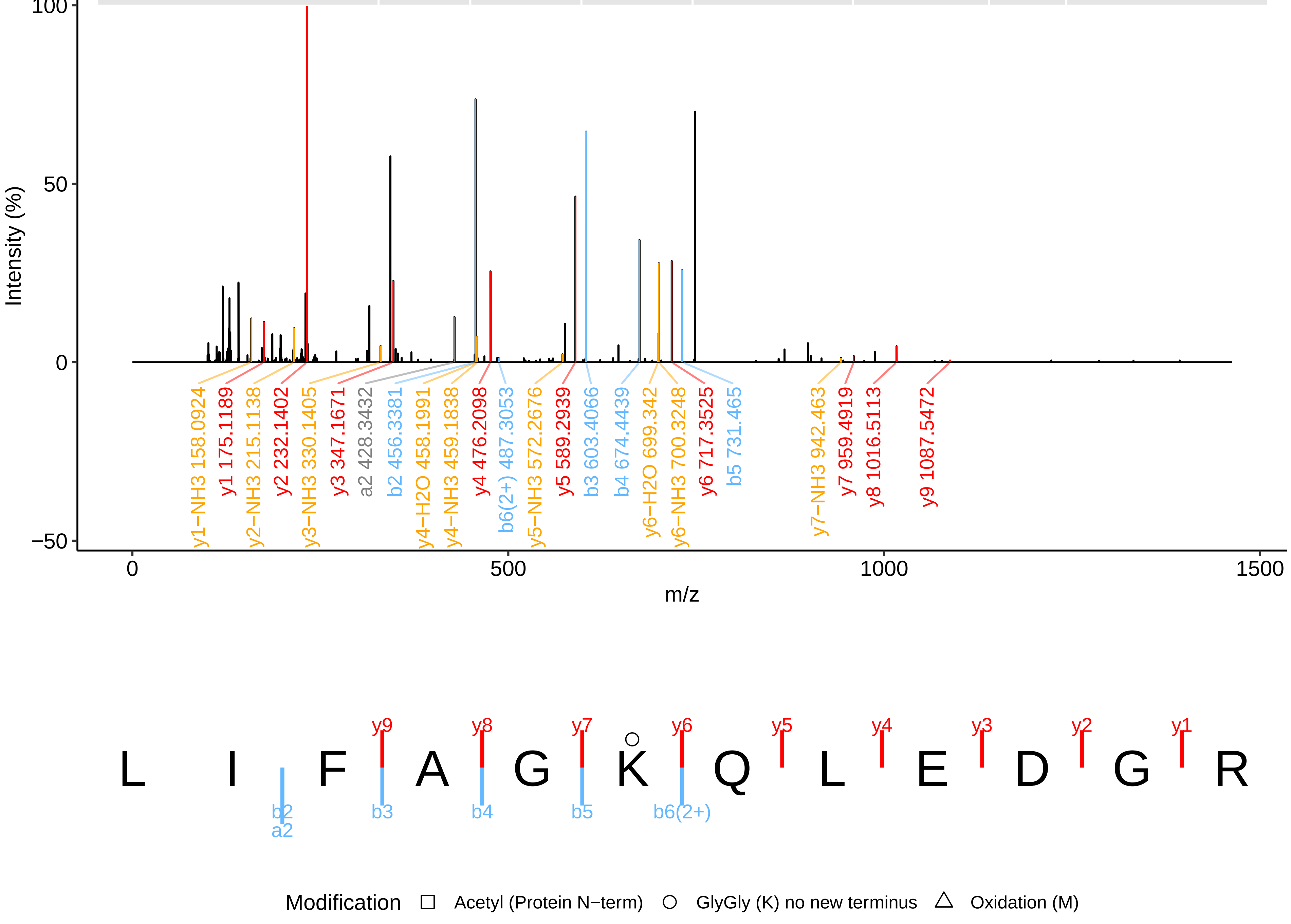

20200921_KIZ_MUb4_GlyGly_10plex − Scan number: 18424

Gene Names	Charge	m/z	Mass	Retention time	PEP	Score	Precursor Intensity
RPS27A;UBA52;UBC;UBB	3	487.6001	1459.778	66.254	3.1574e−19	150.46	989562240

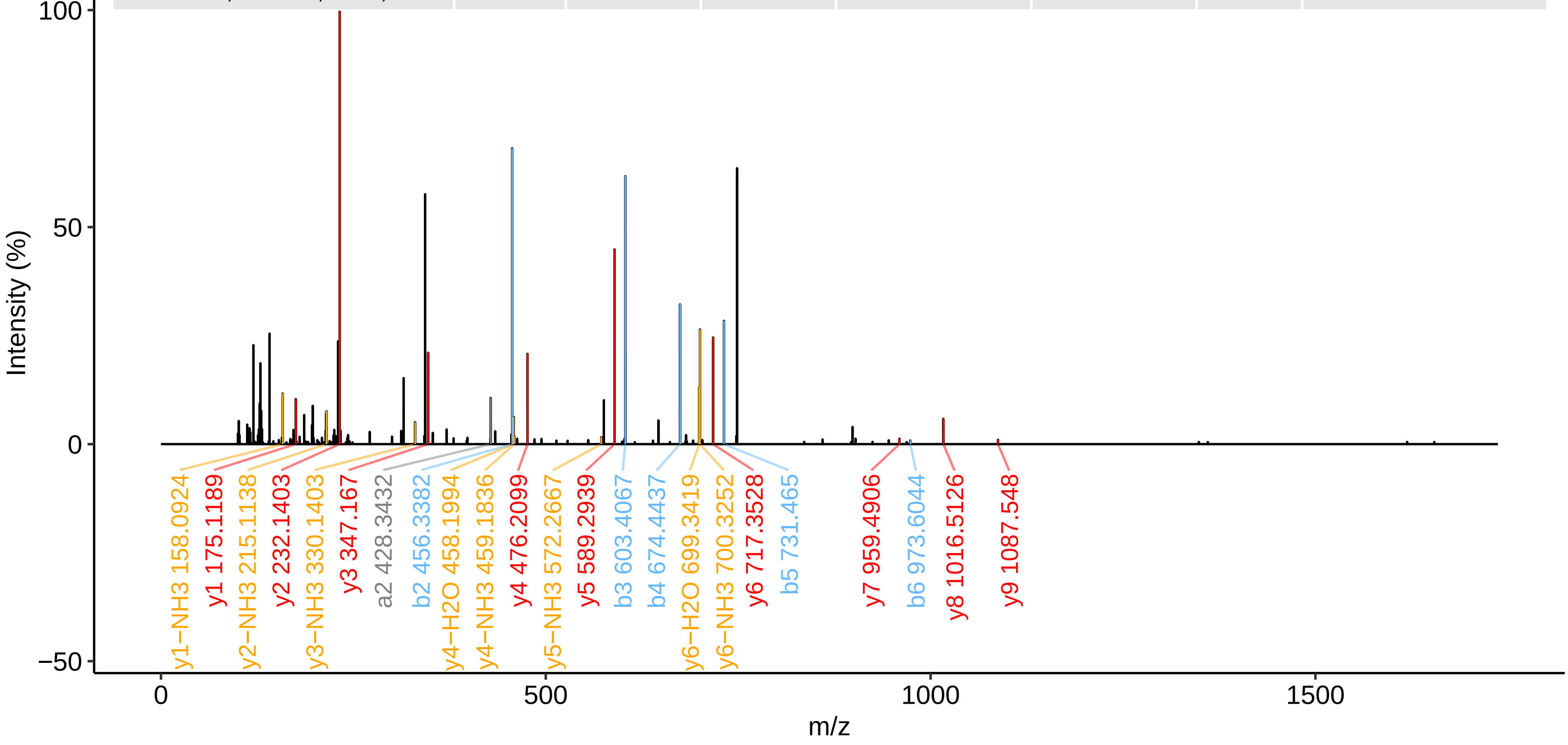

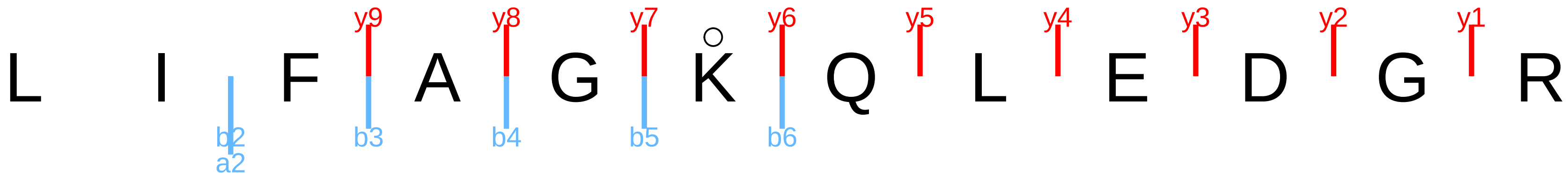

Modification □ Acetyl (Protein N−term) ○ GlyGly (K) no new terminus △ Oxidation (M)

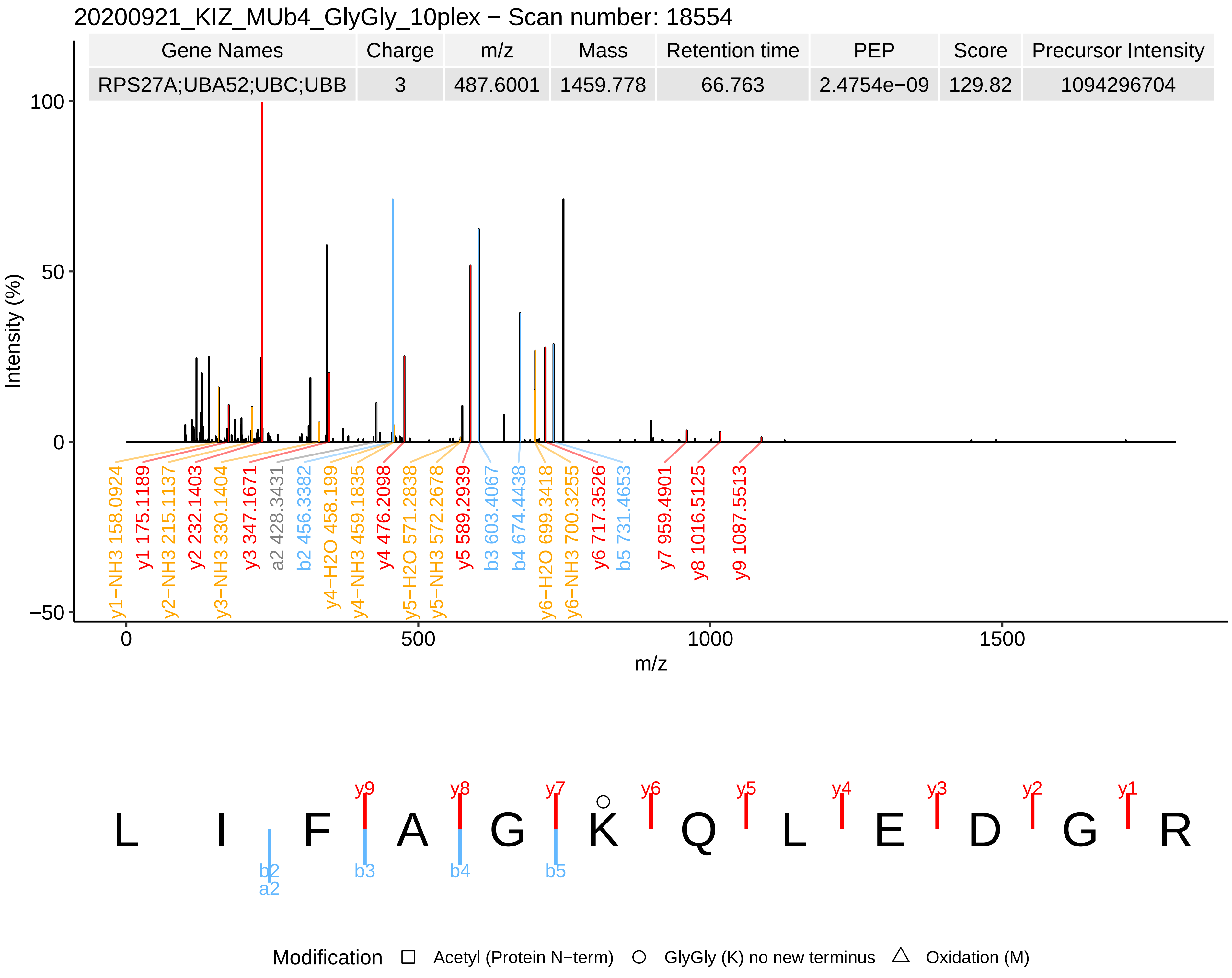

20200921_KIZ_MUb4_GlyGly_10plex − Scan number: 18554
Gene Names
RPS27A;UBA52;UBC;UBB
Charge
3
m/z
487.6001
Mass
1459.778
Retention time
66.763
PEP
2.4754e−09
Score
129.82
Precursor Intensity
1094296704
Intensity (%)
m/z
y1−NH3 158.0924
y1 175.1189
y2−NH3 215.1137
y2 232.1403
y3−NH3 330.1404
y3 347.1671
a2 428.3431
b2 456.3382
y4−H2O 458.199
y4−NH3 459.1835
y4 476.2098
y5−H2O 571.2838
y5−NH3 572.2678
y5 589.2939
b3 603.4067
b4 674.4438
y6−H2O 699.3418
y6−NH3 700.3255
y6 717.3526
b5 731.4653
y7 959.4901
y8 1016.5125
y9 1087.5513
L I F A G K Q L E D G R
Modification
Acetyl (Protein N−term)
GlyGly (K) no new terminus
Oxidation (M)

20200921_KIZ_MUb4_GlyGly_10plex − Scan number: 18687

Gene Names	Charge	m/z	Mass	Retention time	PEP	Score	Precursor Intensity
RPS27A;UBA52;UBC;UBB	3	487.6001	1459.778	67.268	3.5418e−13	134.3	723792832

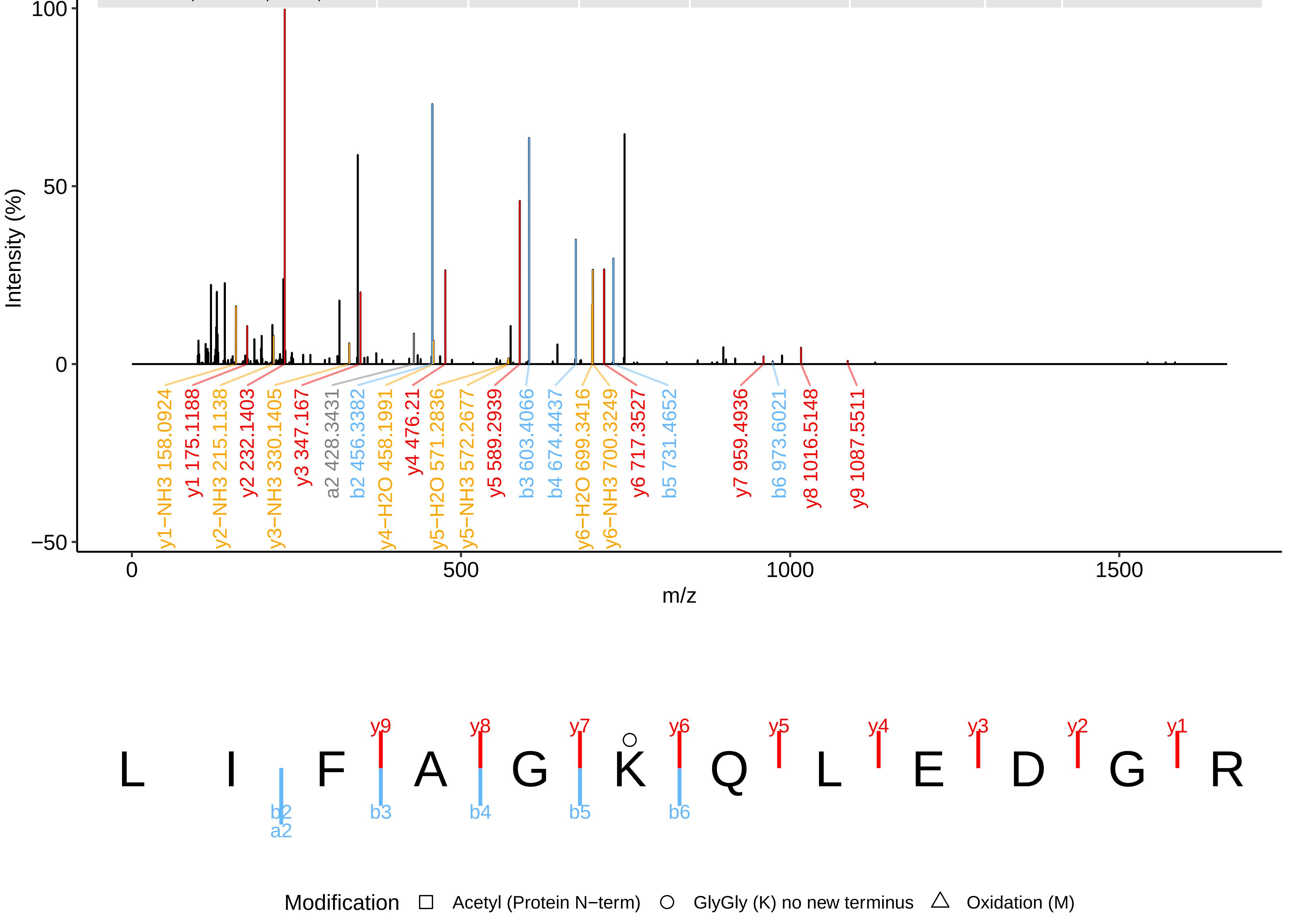

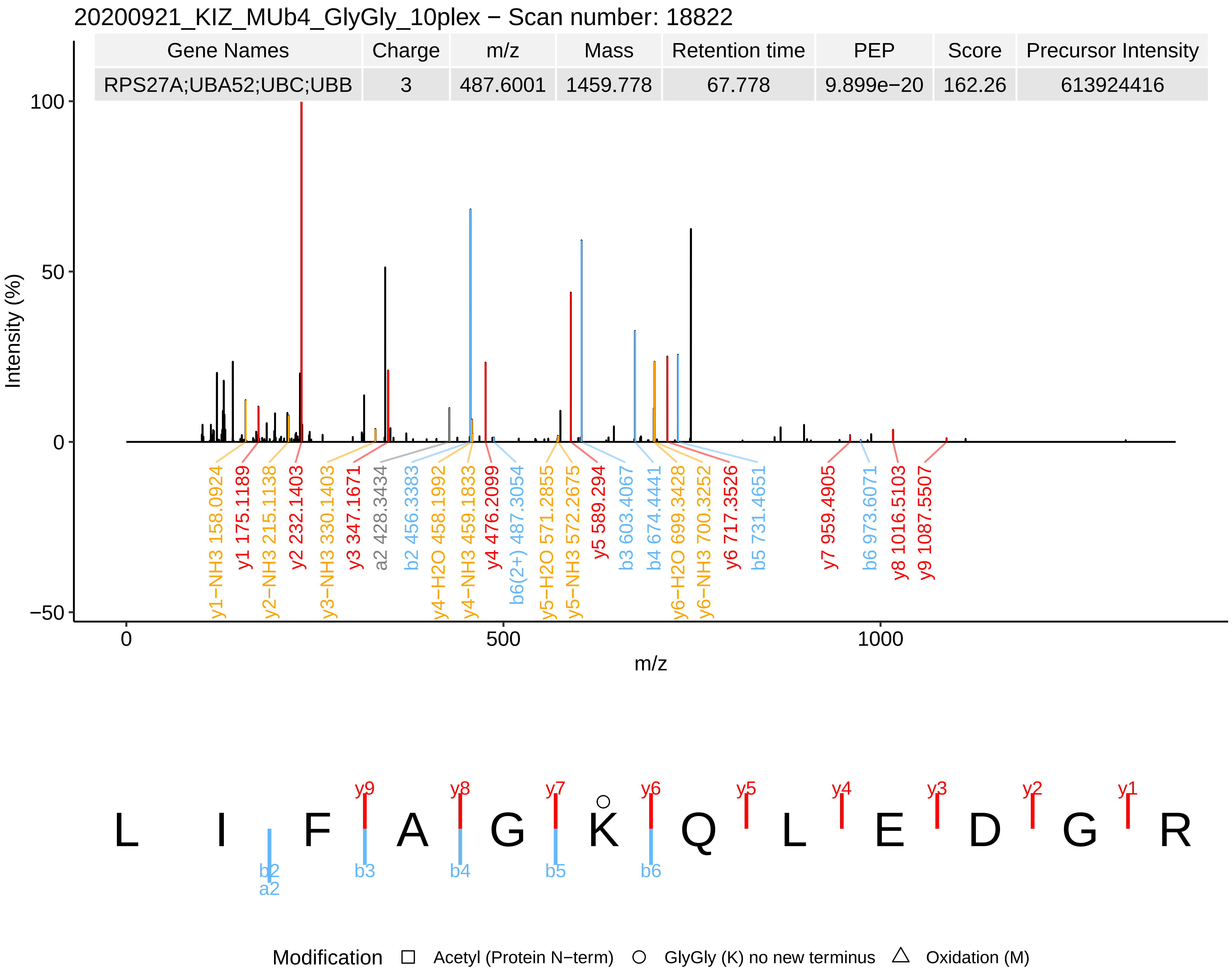
20200921_KIZ_MUb4_GlyGly_10plex − Scan number: 18822
Gene Names
Charge
m/z
Mass
Retention time
PEP
Score
Precursor Intensity
RPS27A;UBA52;UBC;UBB
3
487.6001
1459.778
67.778
9.899e−20
162.26
613924416
Intensity (%)
100
50
0
−50
0
500
1000
m/z
y1−NH3 158.0924
y1 175.1189
y2−NH3 215.1138
y2 232.1403
y3−NH3 330.1403
y3 347.1671
a2 428.3434
b2 456.3383
y4−H2O 458.1992
y4−NH3 459.1833
y4 476.2099
b6(2+) 487.3054
y5−H2O 571.2855
y5−NH3 572.2675
y5 589.294
b3 603.4067
b4 674.4441
y6−H2O 699.3428
y6−NH3 700.3252
y6 717.3526
b5 731.4651
y7 959.4905
b6 973.6071
y8 1016.5103
y9 1087.5507
L I F A G K Q L E D G R
y9 y8 y7 y6 y5 y4 y3 y2 y1
b2 a2 b3 b4 b5 b6
Modification
Acetyl (Protein N−term)
GlyGly (K) no new terminus
Oxidation (M)

20200921_KIZ_MUb4_GlyGly_10plex − Scan number: 18962

Gene Names	Charge	m/z	Mass	Retention time	PEP	Score	Precursor Intensity
RPS27A;UBA52;UBC;UBB	3	487.6001	1459.778	68.285	2.7462e−19	152.7	342623168

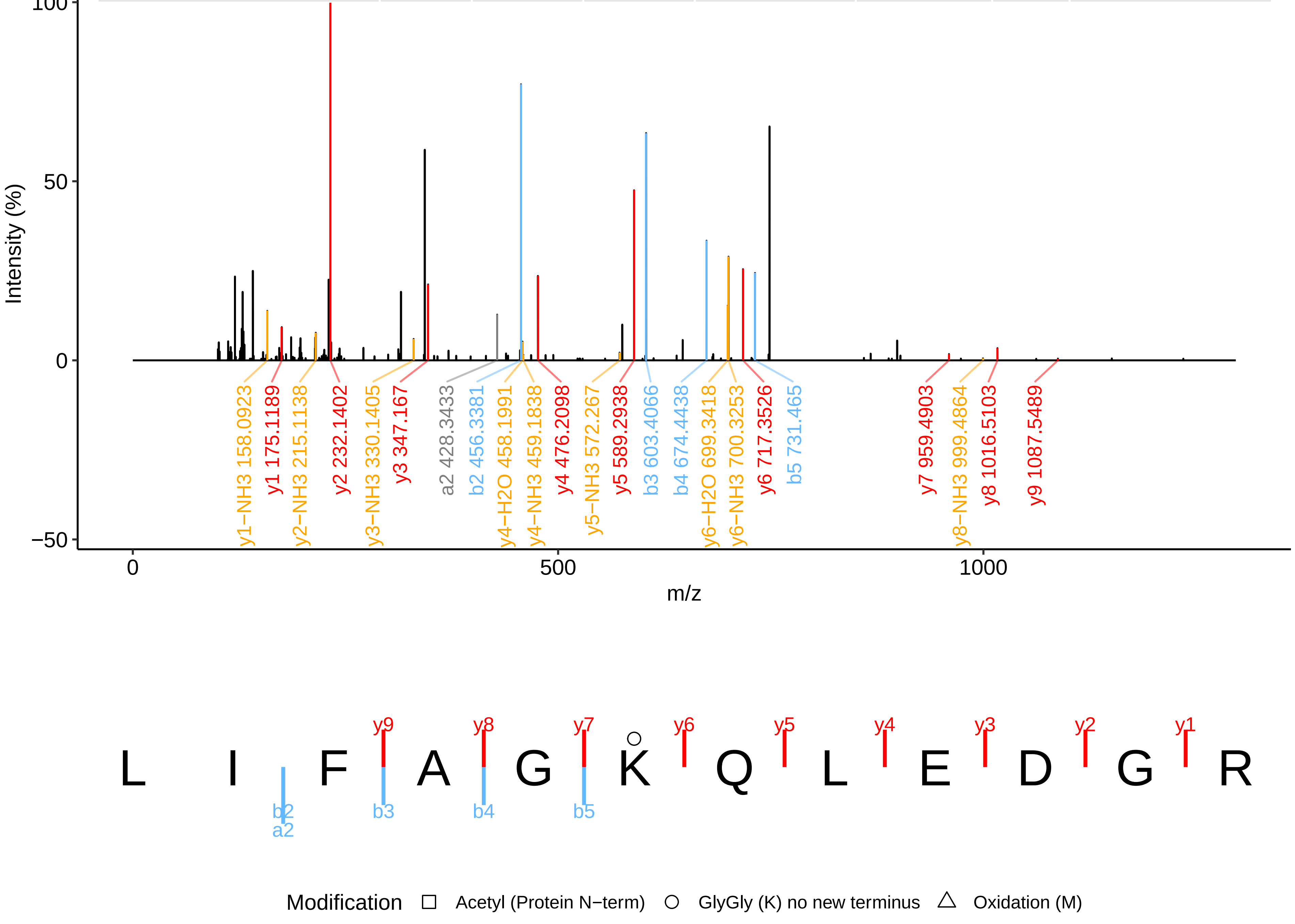

20200921_KIZ_MUb4_GlyGly_10plex − Scan number: 19105

Gene Names	Charge	m/z	Mass	Retention time	PEP	Score	Precursor Intensity
RPS27A;UBA52;UBC;UBB	3	487.6001	1459.778	68.79	3.1574e−19	150.46	251760896

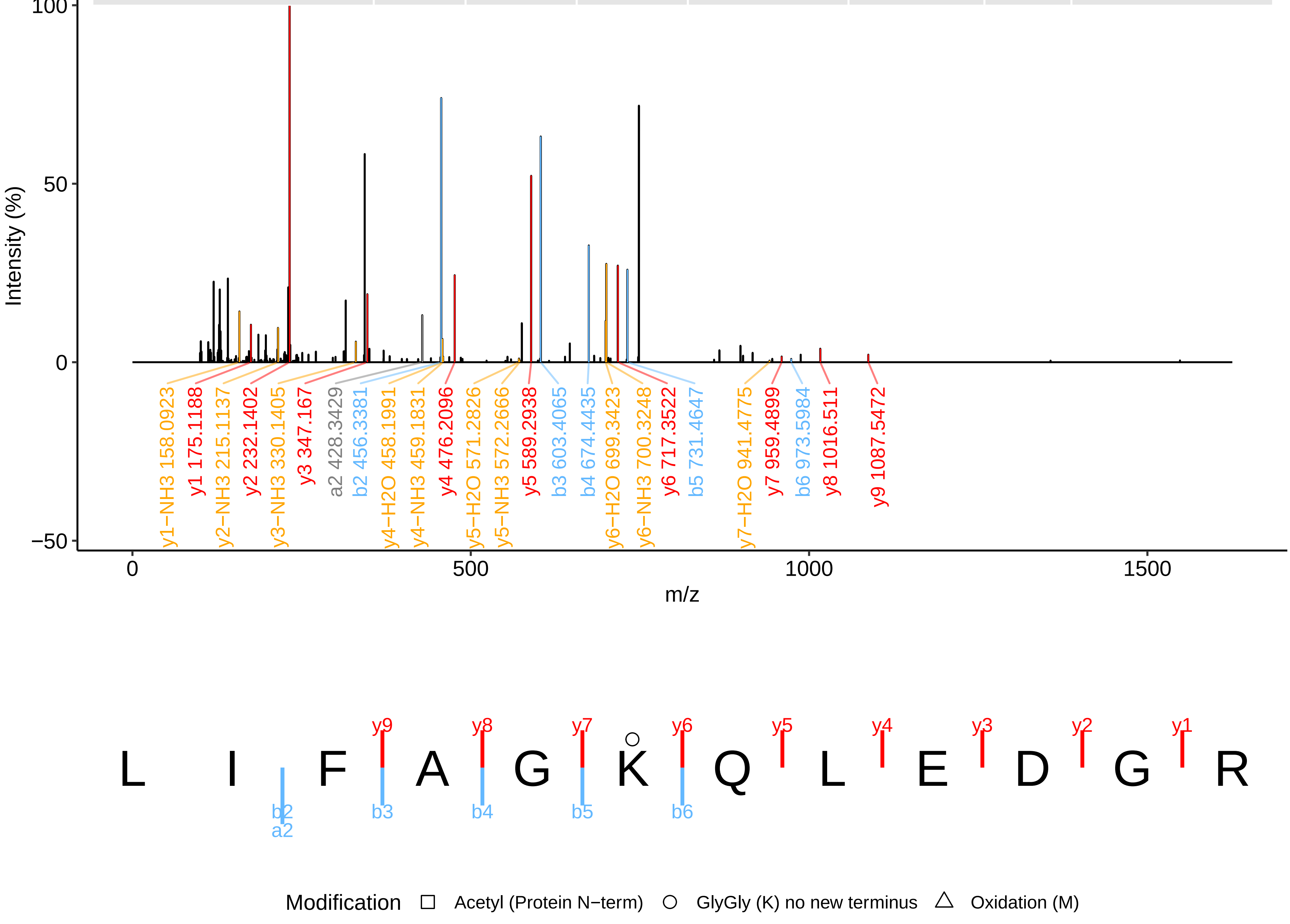

20200921_KIZ_MUb4_GlyGly_10plex − Scan number: 19239

Gene Names	Charge	m/z	Mass	Retention time	PEP	Score	Precursor Intensity
RPS27A;UBA52;UBC;UBB	3	487.6001	1459.778	69.311	9.899e−20	162.26	175486528

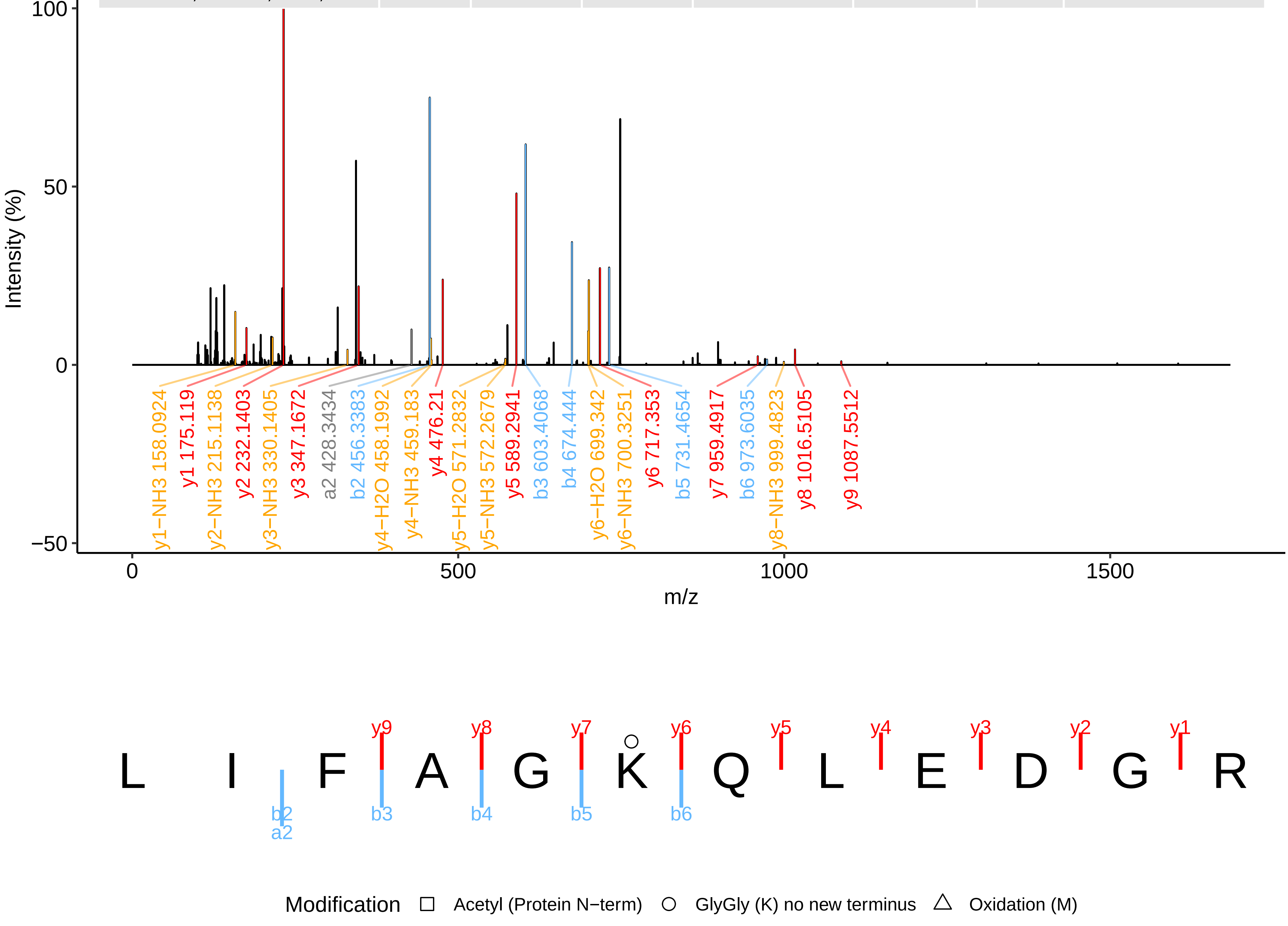

20200921_KIZ_MUb4_GlyGly_10plex − Scan number: 18883

Gene Names	Charge	m/z	Mass	Retention time	PEP	Score	Precursor Intensity
RPS27A;UBA52;UBC;UBB	2	730.8964	1459.778	68.009	4.7412e−41	225.01	19209738

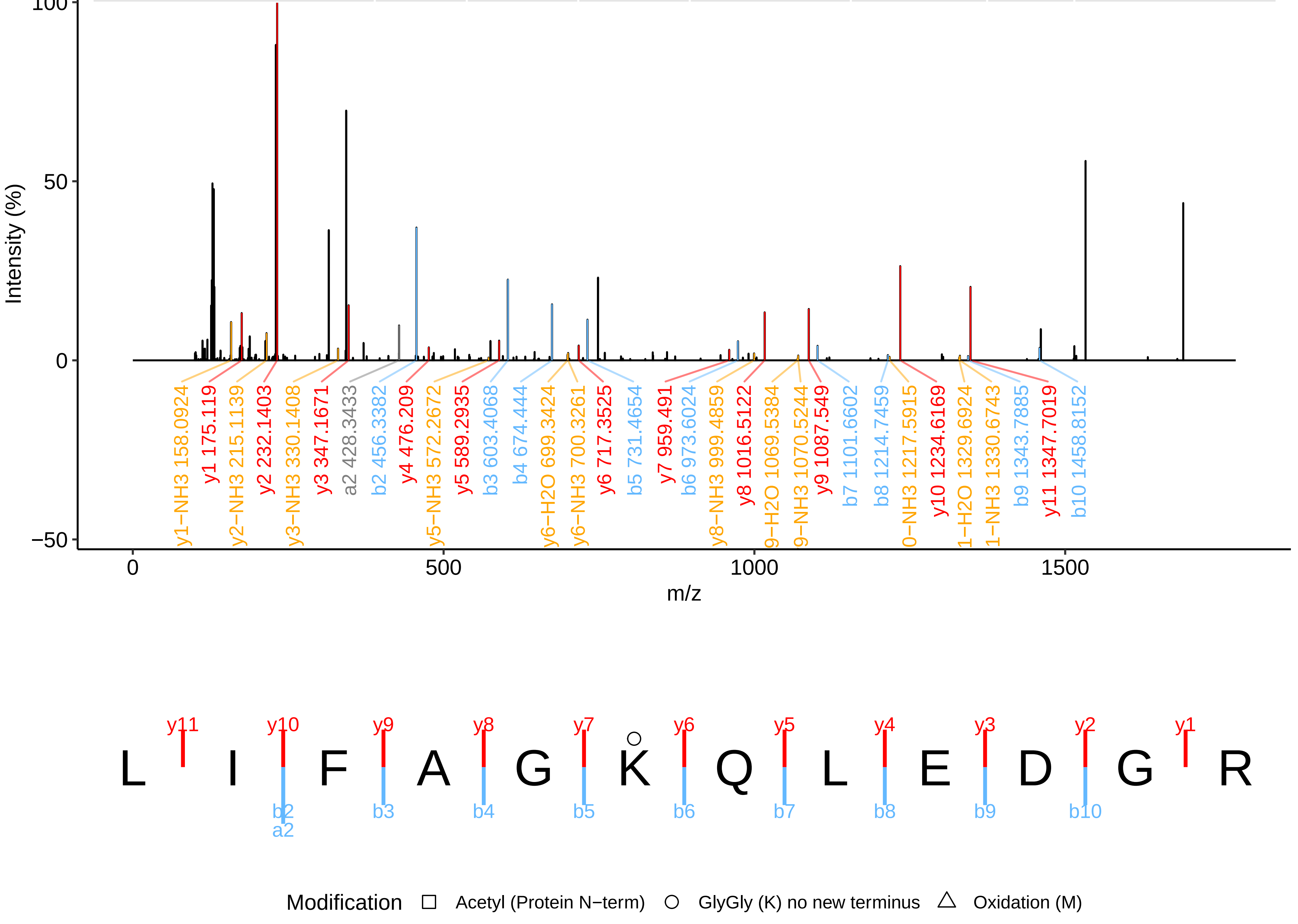

20200921_KIZ_MUb4_GlyGly_10plex − Scan number: 19165

Gene Names	Charge	m/z	Mass	Retention time	PEP	Score	Precursor Intensity
RPS27A;UBA52;UBC;UBB	2	730.8964	1459.778	69.027	3.2425e−34	205.53	4837570

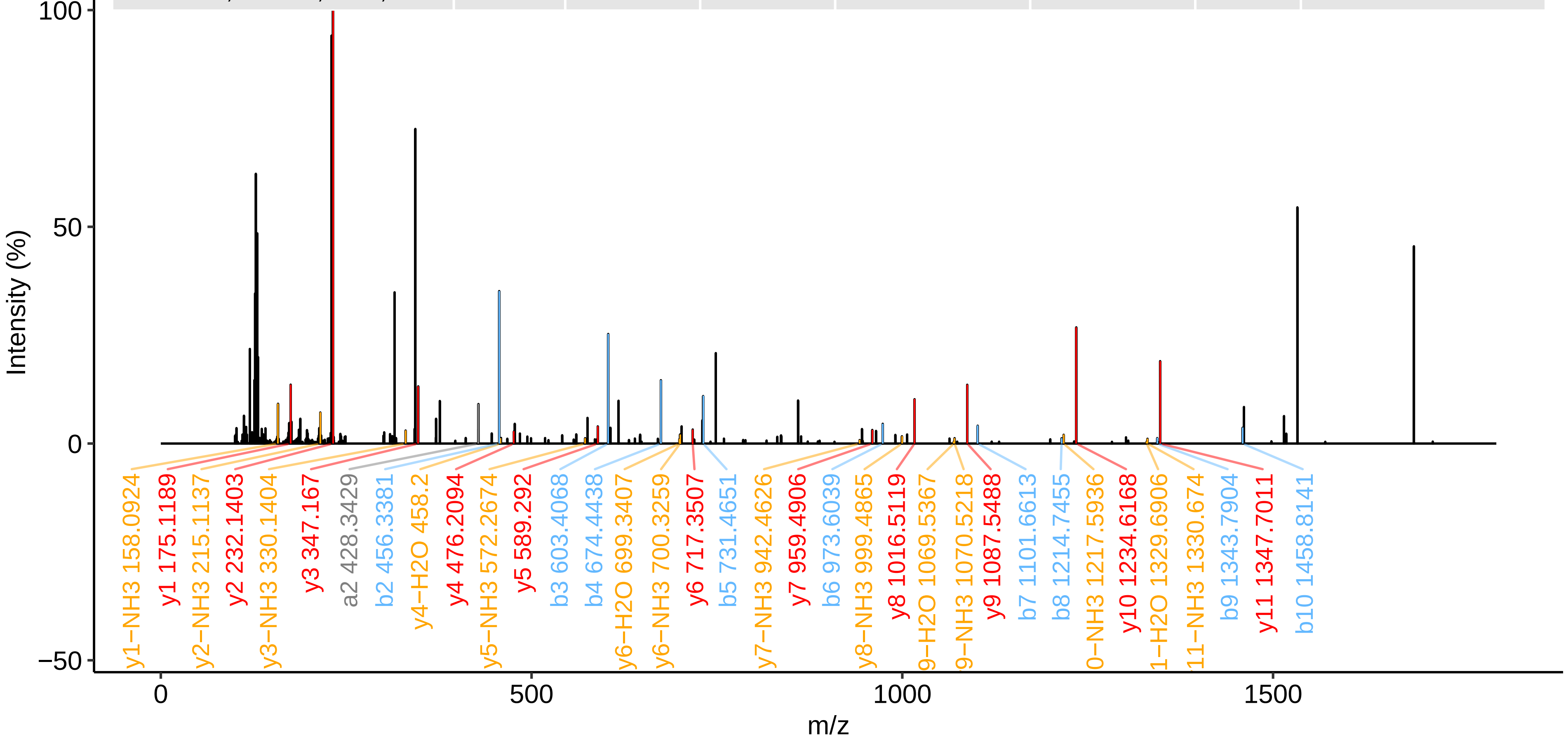

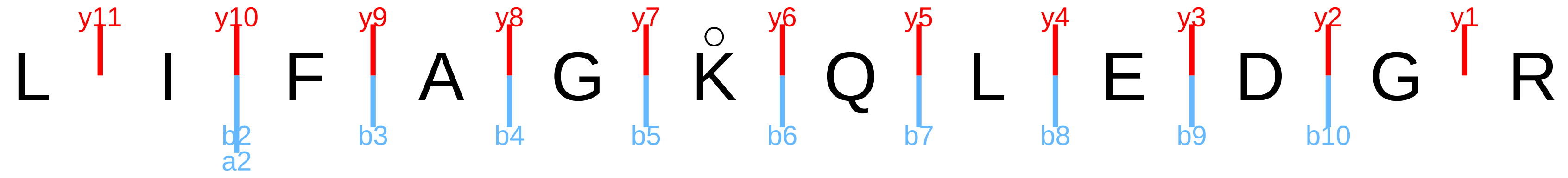

Modification □ Acetyl (Protein N−term) ○ GlyGly (K) no new terminus △ Oxidation (M)

20200921_KIZ_MUb4_GlyGly_10plex − Scan number: 19300

Gene Names	Charge	m/z	Mass	Retention time	PEP	Score	Precursor Intensity
RPS27A;UBA52;UBC;UBB	2	730.8964	1459.778	69.551	4.8161e−05	103.88	5407112

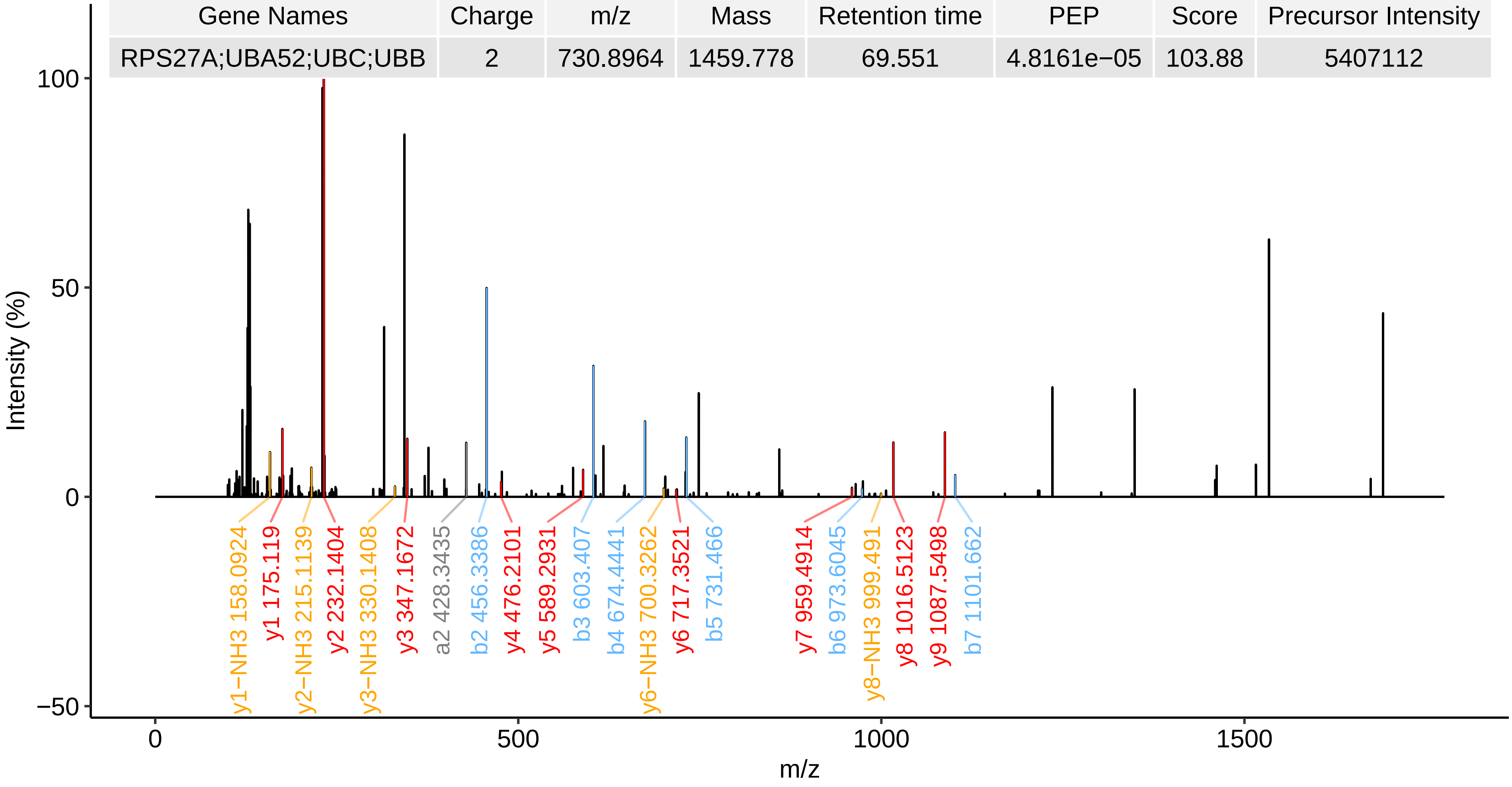

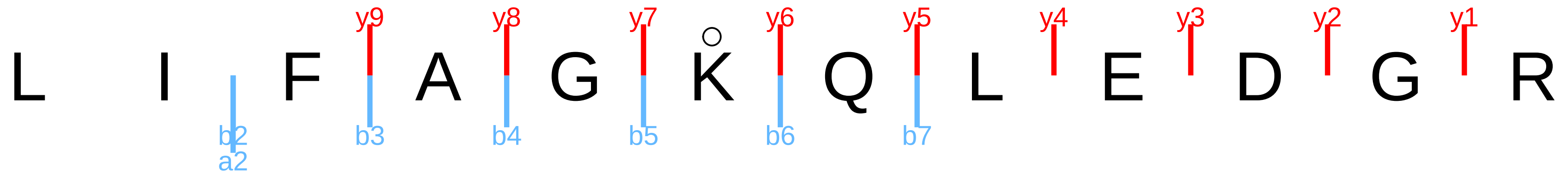

Modification □ Acetyl (Protein N−term) ○ GlyGly (K) no new terminus △ Oxidation (M)

20200921_KIZ_MUb4_GlyGly_10plex − Scan number: 19372

Gene Names	Charge	m/z	Mass	Retention time	PEP	Score	Precursor Intensity
RPS27A;UBA52;UBC;UBB	3	487.6001	1459.778	69.831	2.5e−13	138.99	110306128

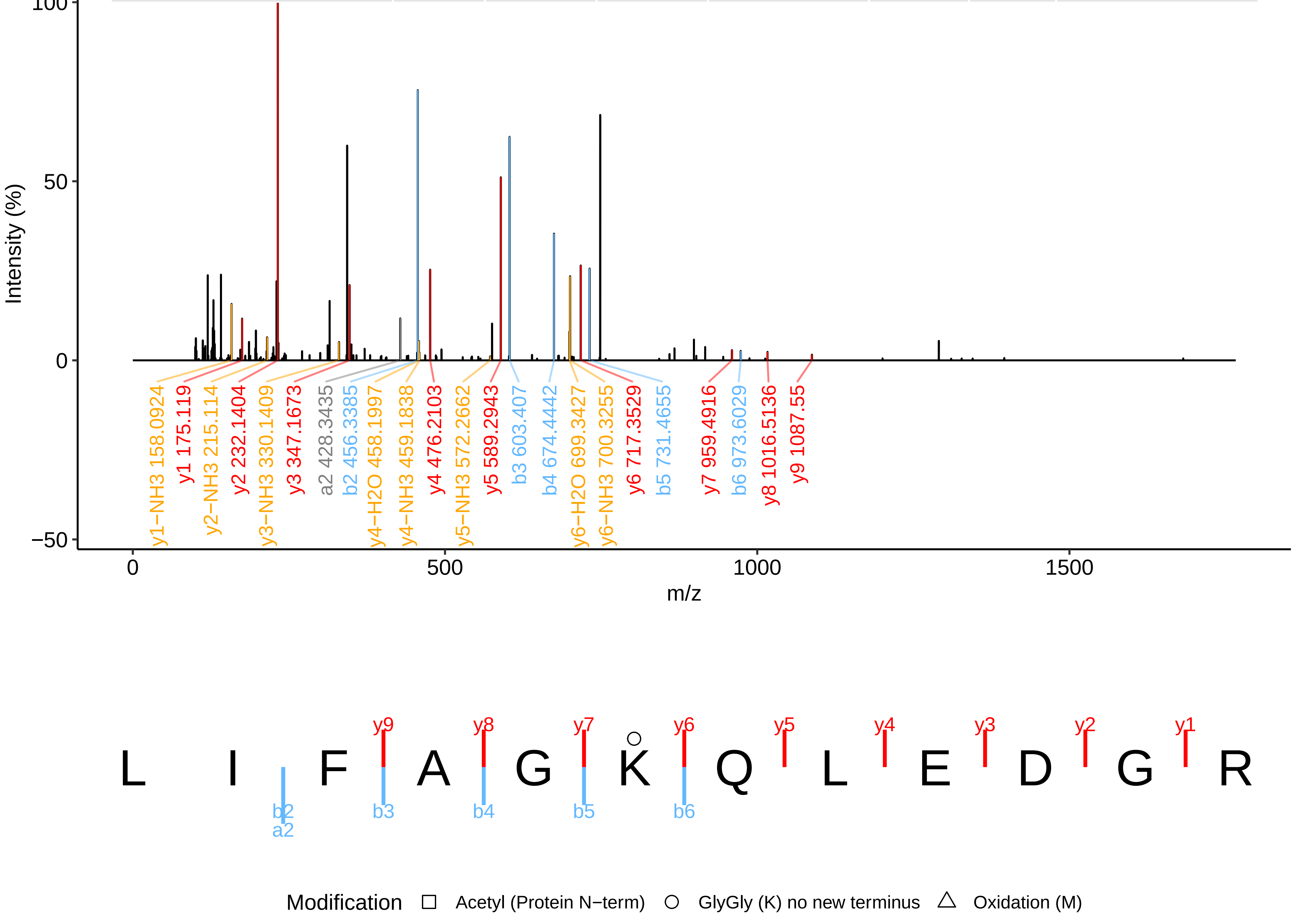

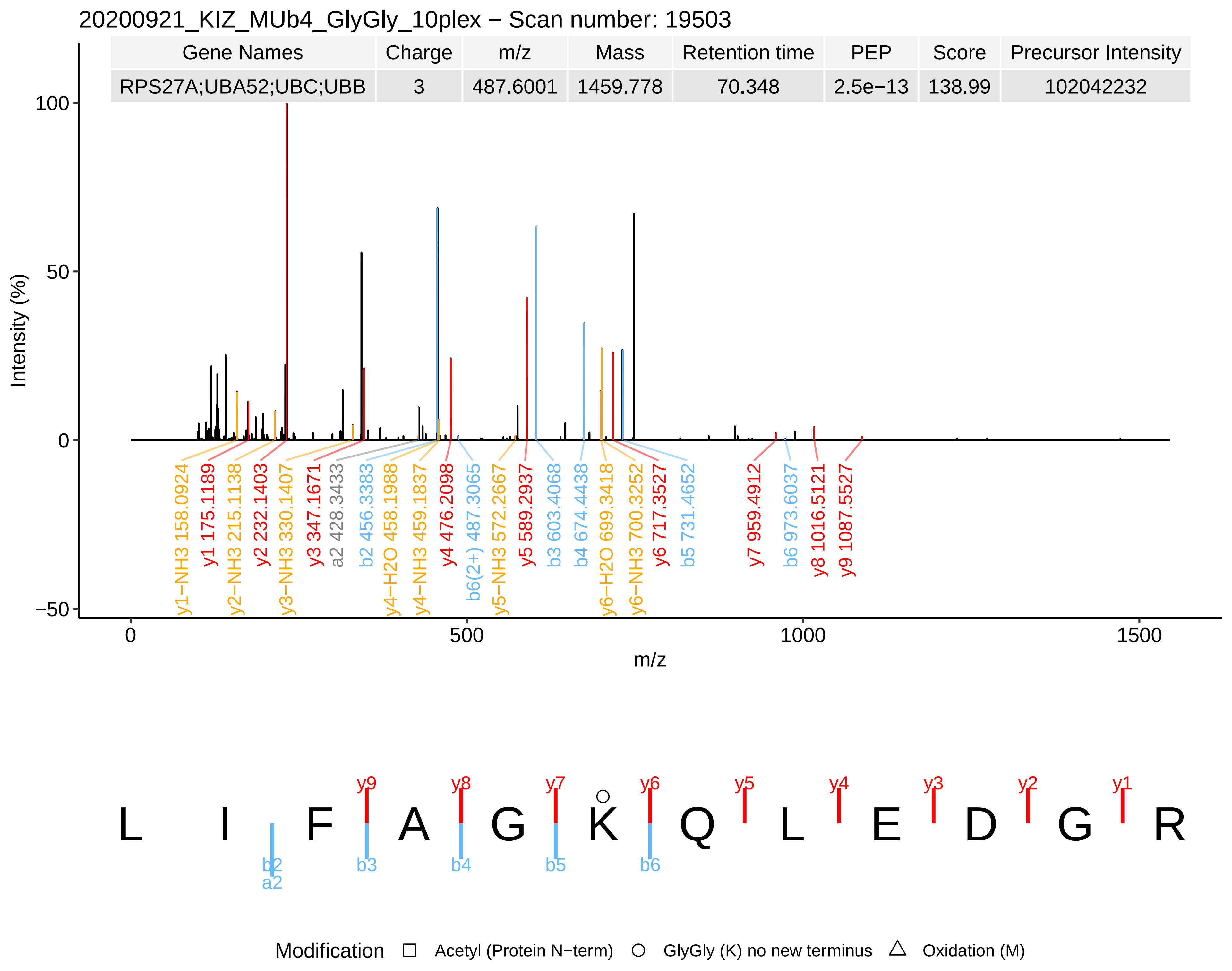
20200921_KIZ_MUb4_GlyGly_10plex − Scan number: 19503
Gene Names | Charge | m/z | Mass | Retention time | PEP | Score | Precursor Intensity
RPS27A;UBA52;UBC;UBB | 3 | 487.6001 | 1459.778 | 70.348 | 2.5e−13 | 138.99 | 102042232
Intensity (%)
m/z
y1−NH3 158.0924
y1 175.1189
y2−NH3 215.1138
y2 232.1403
y3−NH3 330.1407
y3 347.1671
a2 428.3433
b2 456.3383
y4−H2O 458.1988
y4−NH3 459.1837
y4 476.2098
b6(2+) 487.3065
y5−NH3 572.2667
y5 589.2937
b3 603.4068
b4 674.4438
y6−H2O 699.3418
y6−NH3 700.3252
y6 717.3527
b5 731.4652
y7 959.4912
b6 973.6037
y8 1016.5121
y9 1087.5527
L I F A G K Q L E D G R
Modification
Acetyl (Protein N−term)
GlyGly (K) no new terminus
Oxidation (M)

20200921_KIZ_MUb4_GlyGly_10plex − Scan number: 19634

Gene Names	Charge	m/z	Mass	Retention time	PEP	Score	Precursor Intensity
RPS27A;UBA52;UBC;UBB	3	487.6001	1459.778	70.851	3.1574e−19	150.46	94634520

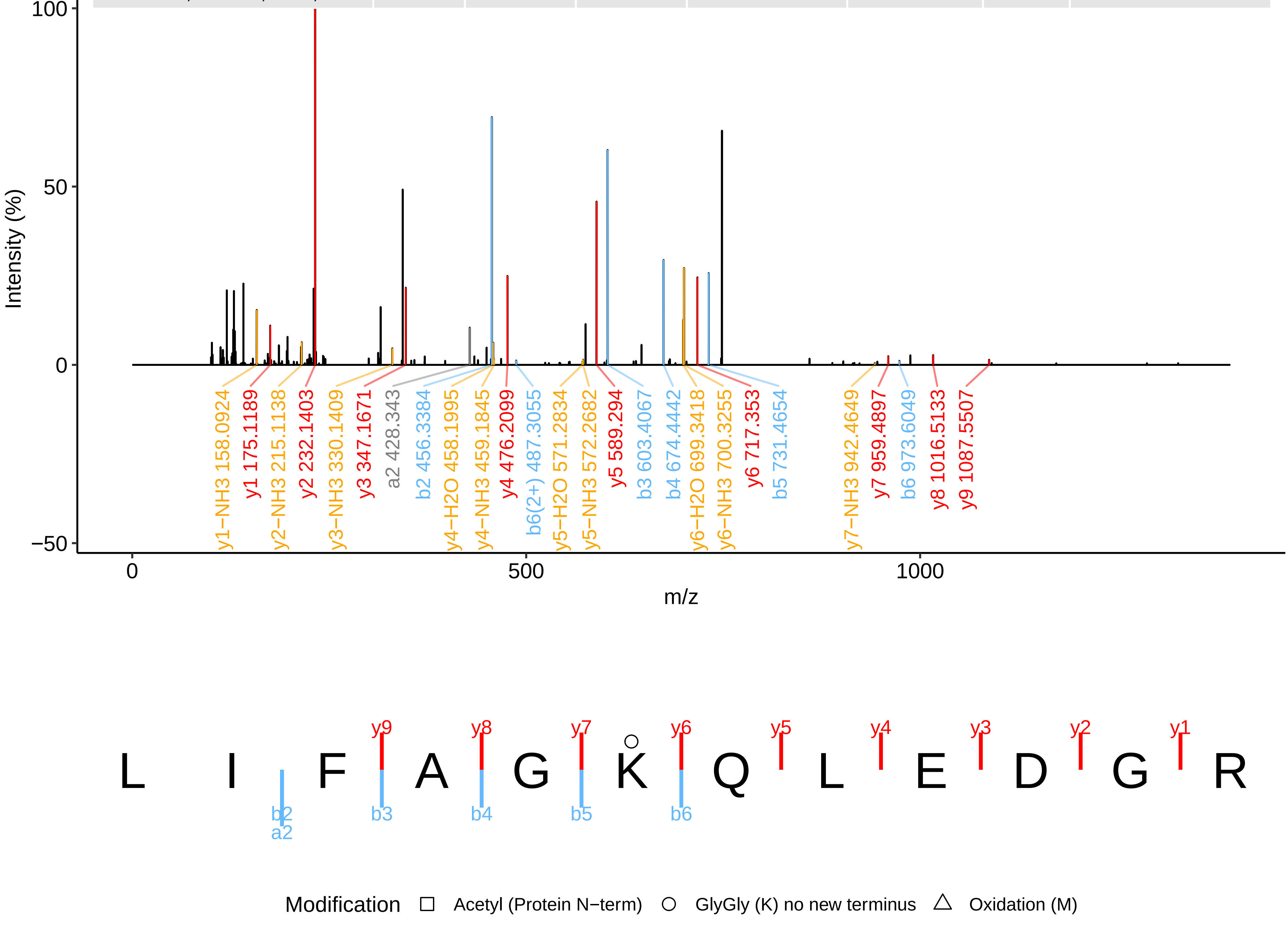

20200921_KIZ_MUb4_GlyGly_10plex − Scan number: 19770

Gene Names	Charge	m/z	Mass	Retention time	PEP	Score	Precursor Intensity
RPS27A;UBA52;UBC;UBB	3	487.6001	1459.778	71.355	9.899e−20	162.26	88899744

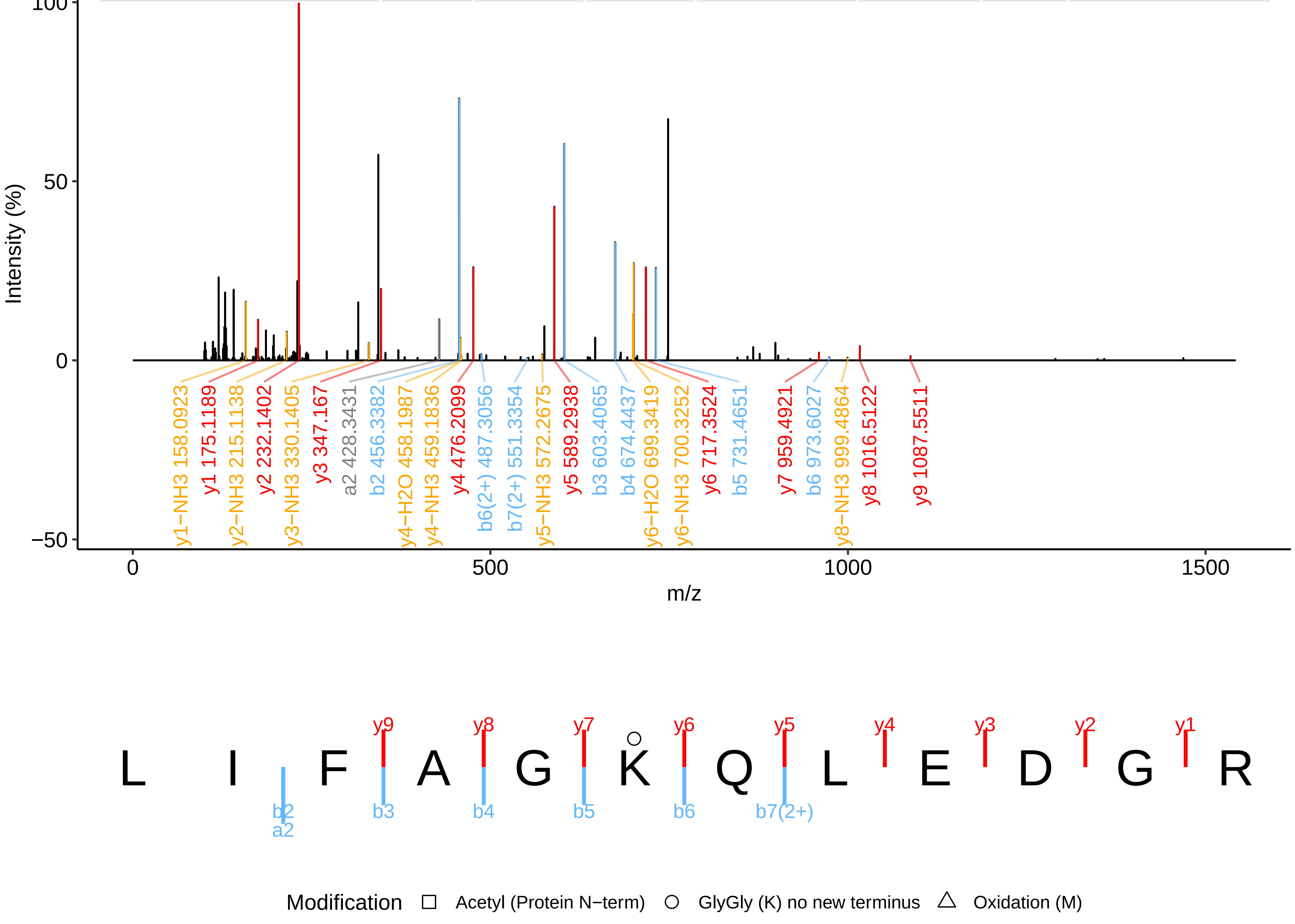

20200921_KIZ_MUb4_GlyGly_10plex − Scan number: 19906

Gene Names	Charge	m/z	Mass	Retention time	PEP	Score	Precursor Intensity
RPS27A;UBA52;UBC;UBB	3	487.6001	1459.778	71.862	2.3227e−19	155	89287128

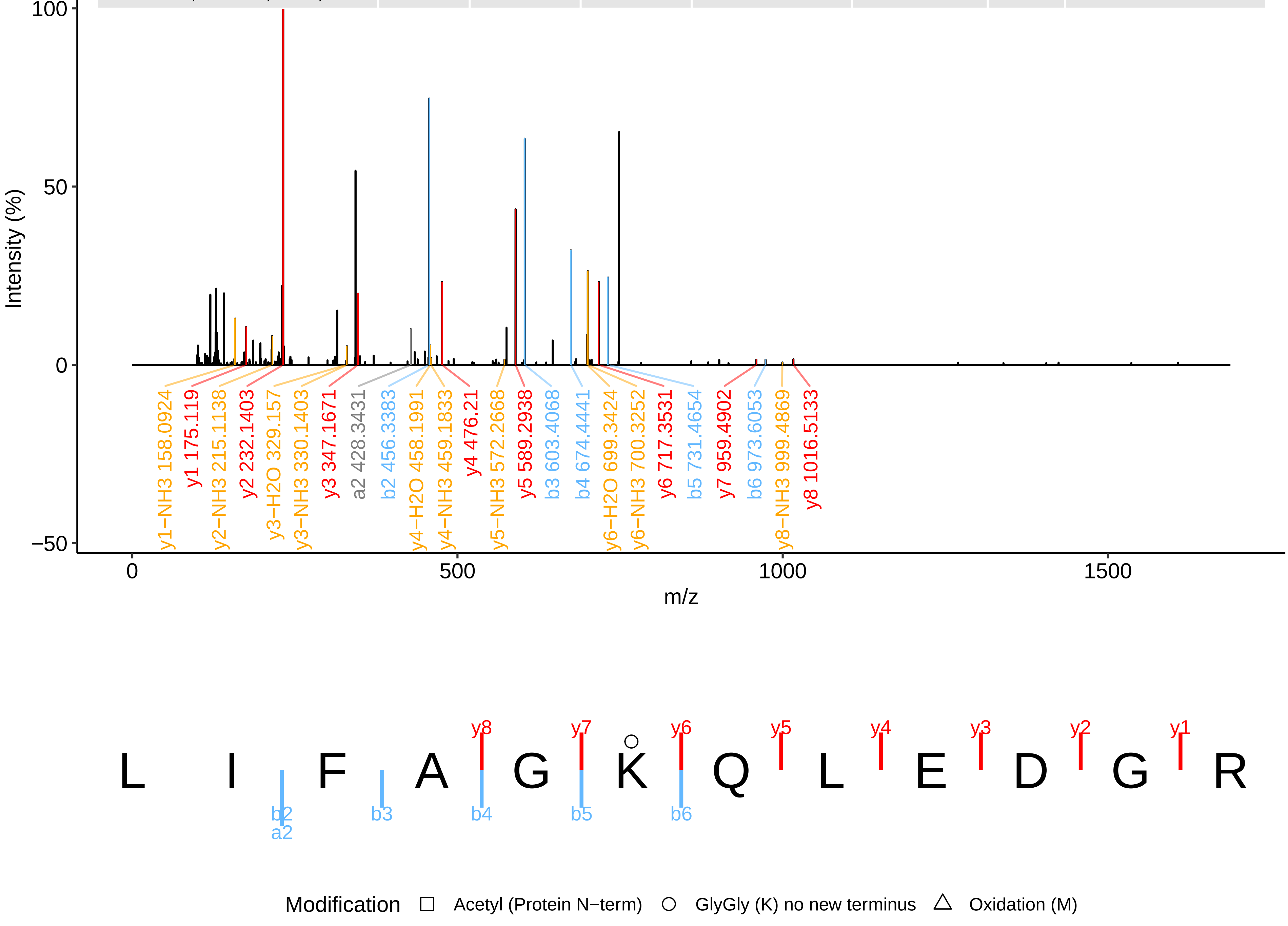

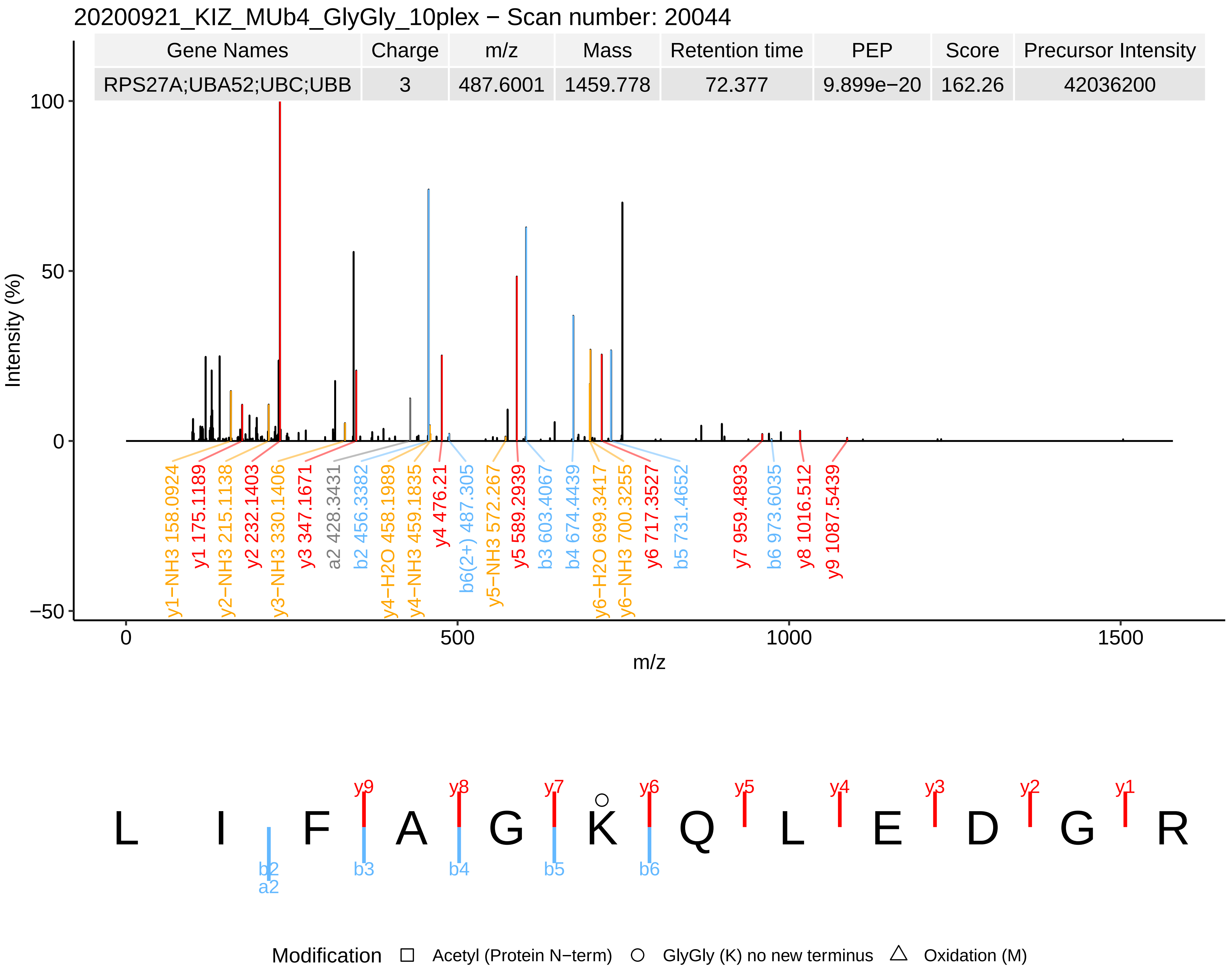
20200921_KIZ_MUb4_GlyGly_10plex − Scan number: 20044
Gene Names | Charge | m/z | Mass | Retention time | PEP | Score | Precursor Intensity
RPS27A;UBA52;UBC;UBB | 3 | 487.6001 | 1459.778 | 72.377 | 9.899e−20 | 162.26 | 42036200
Intensity (%)
m/z
y1−NH3 158.0924
y1 175.1189
y2−NH3 215.1138
y2 232.1403
y3−NH3 330.1406
y3 347.1671
a2 428.3431
b2 456.3382
y4−H2O 458.1989
y4−NH3 459.1835
y4 476.21
b6(2+) 487.305
y5−NH3 572.267
y5 589.2939
b3 603.4067
b4 674.4439
y6−H2O 699.3417
y6−NH3 700.3255
y6 717.3527
b5 731.4652
y7 959.4893
b6 973.6035
y8 1016.512
y9 1087.5439
L I F A G K Q L E D G R
Modification
Acetyl (Protein N−term)
GlyGly (K) no new terminus
Oxidation (M)

20200921_KIZ_MUb4_GlyGly_10plex − Scan number: 19618

Gene Names	Charge	m/z	Mass	Retention time	PEP	Score	Precursor Intensity
RPS27A;UBA52;UBC;UBB	2	730.8964	1459.778	70.795	2.0397e−08	125.75	2837780

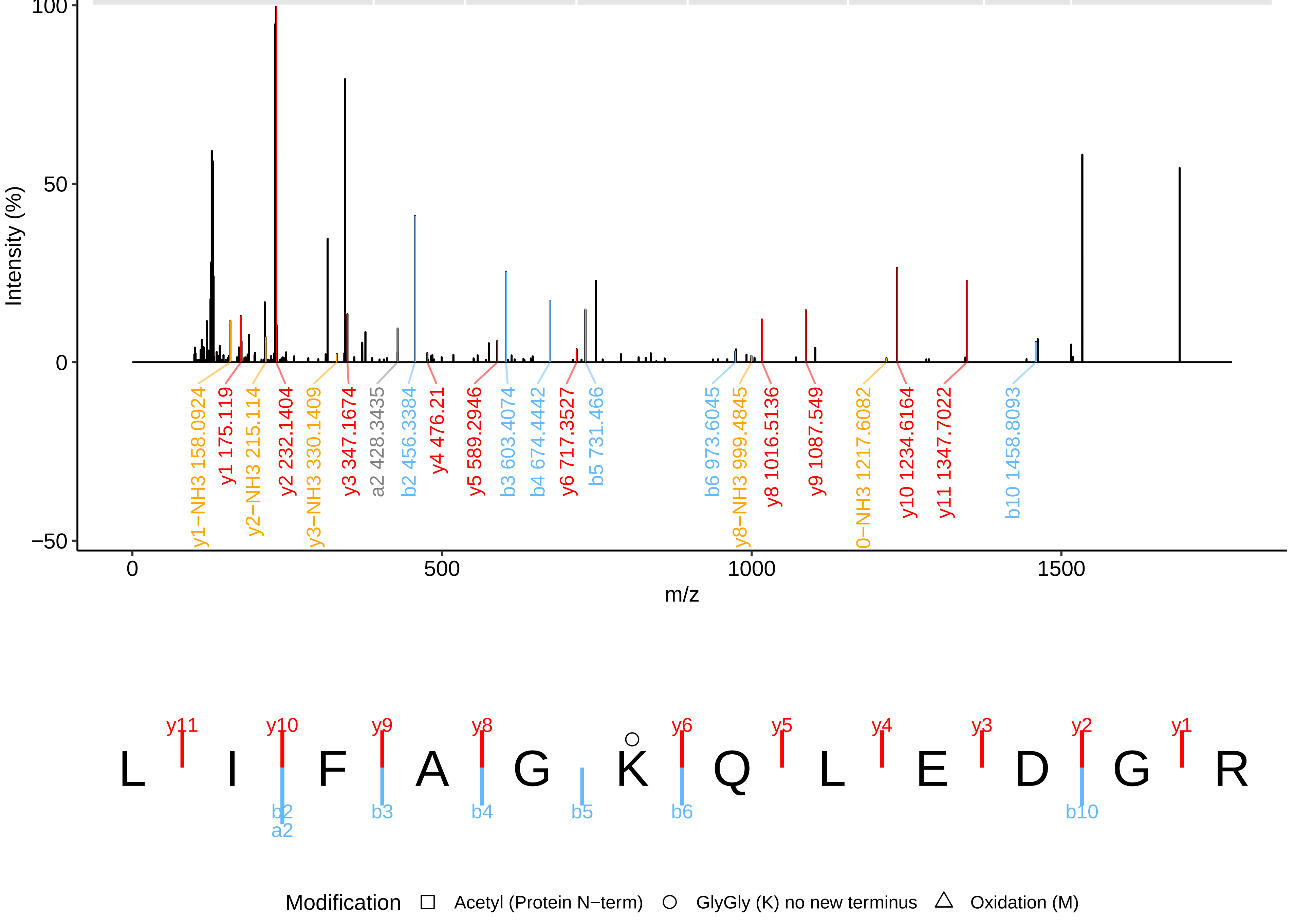

20200921_KIZ_MUb4_GlyGly_10plex − Scan number: 20178

Gene Names	Charge	m/z	Mass	Retention time	PEP	Score	Precursor Intensity
RPS27A;UBA52;UBC;UBB	3	487.6001	1459.778	72.888	4.2307e−26	170.52	NA

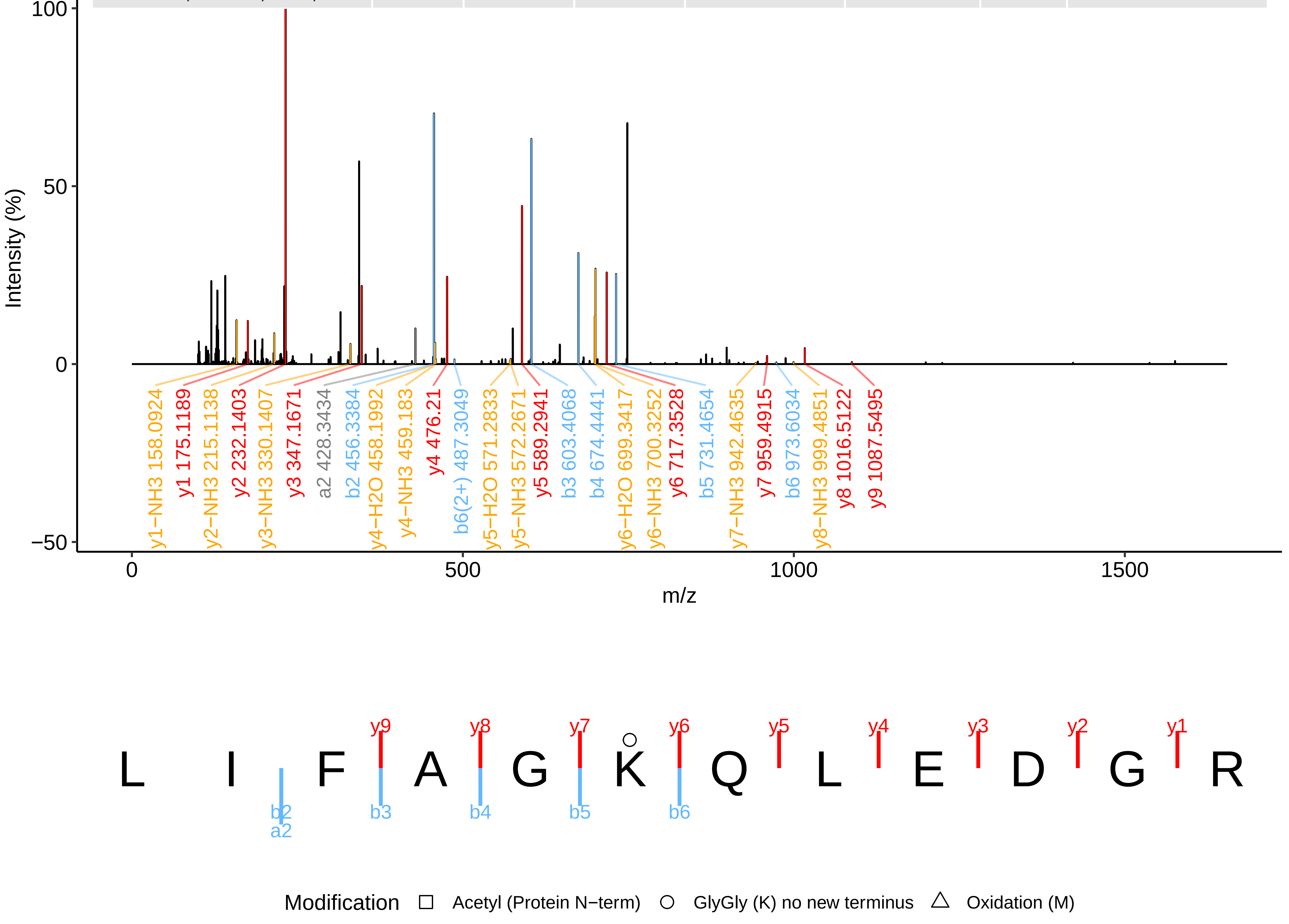

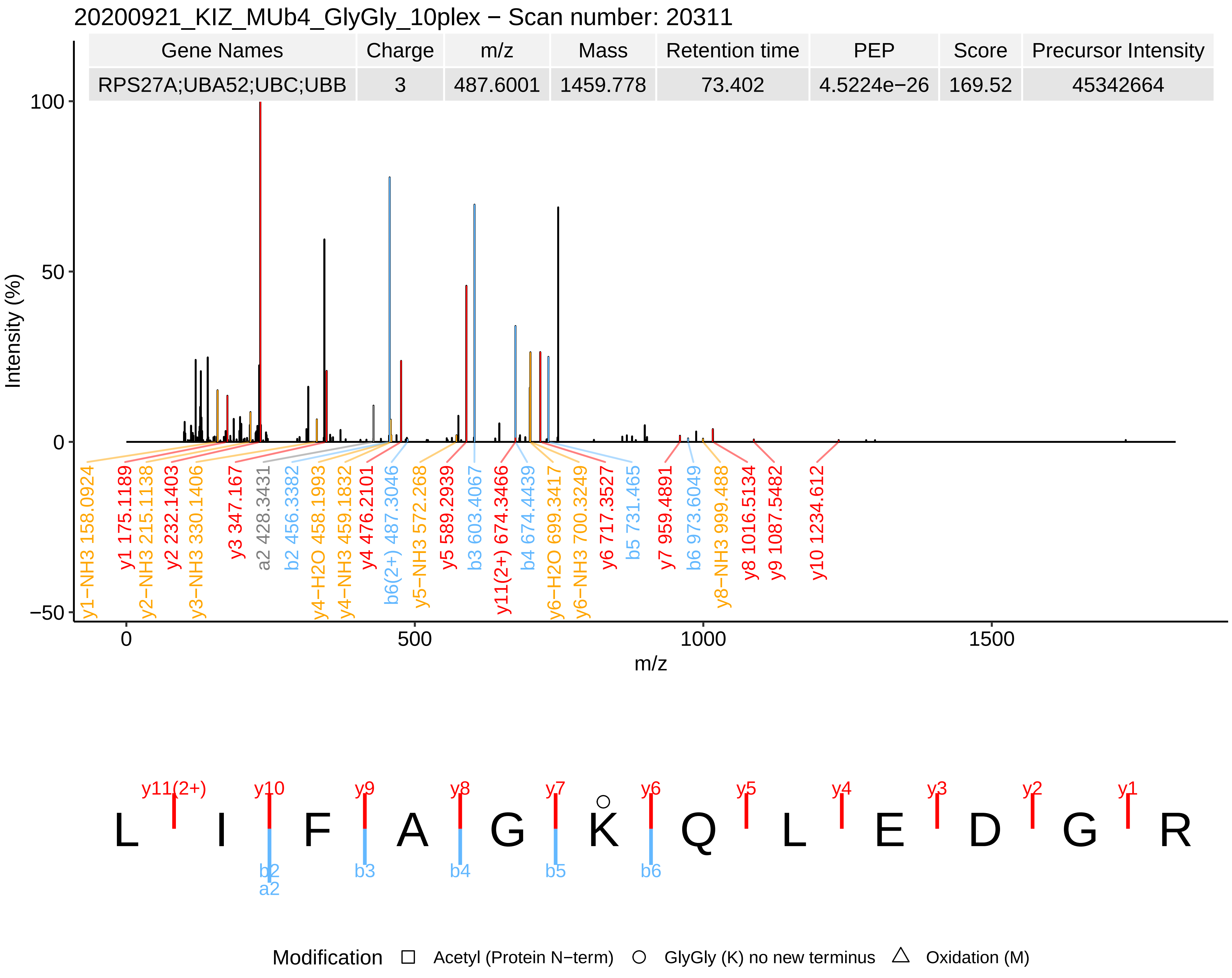
20200921_KIZ_MUb4_GlyGly_10plex − Scan number: 20311
Gene Names | Charge | m/z | Mass | Retention time | PEP | Score | Precursor Intensity
RPS27A;UBA52;UBC;UBB | 3 | 487.6001 | 1459.778 | 73.402 | 4.5224e−26 | 169.52 | 45342664
Intensity (%)
100
50
0
−50
0
500
1000
1500
m/z
y1−NH3 158.0924
y1 175.1189
y2−NH3 215.1138
y2 232.1403
y3−NH3 330.1406
y3 347.167
a2 428.3431
b2 456.3382
y4−H2O 458.1993
y4−NH3 459.1832
y4 476.2101
b6(2+) 487.3046
y5−NH3 572.268
y5 589.2939
b3 603.4067
y11(2+) 674.3466
b4 674.4439
y6−H2O 699.3417
y6−NH3 700.3249
y6 717.3527
b5 731.465
y7 959.4891
b6 973.6049
y8−NH3 999.488
y8 1016.5134
y9 1087.5482
y10 1234.612
y11(2+)
y10
y9
y8
y7
y6
y5
y4
y3
y2
y1
L I F A G K Q L E D G R
b2
a2
b3
b4
b5
b6
Modification
Acetyl (Protein N−term)
GlyGly (K) no new terminus
Oxidation (M)

20200921_KIZ_MUb4_GlyGly_10plex − Scan number: 20448

Gene Names	Charge	m/z	Mass	Retention time	PEP	Score	Precursor Intensity
RPS27A;UBA52;UBC;UBB	3	487.6001	1459.778	73.915	4.3409e−15	150.04	58598544

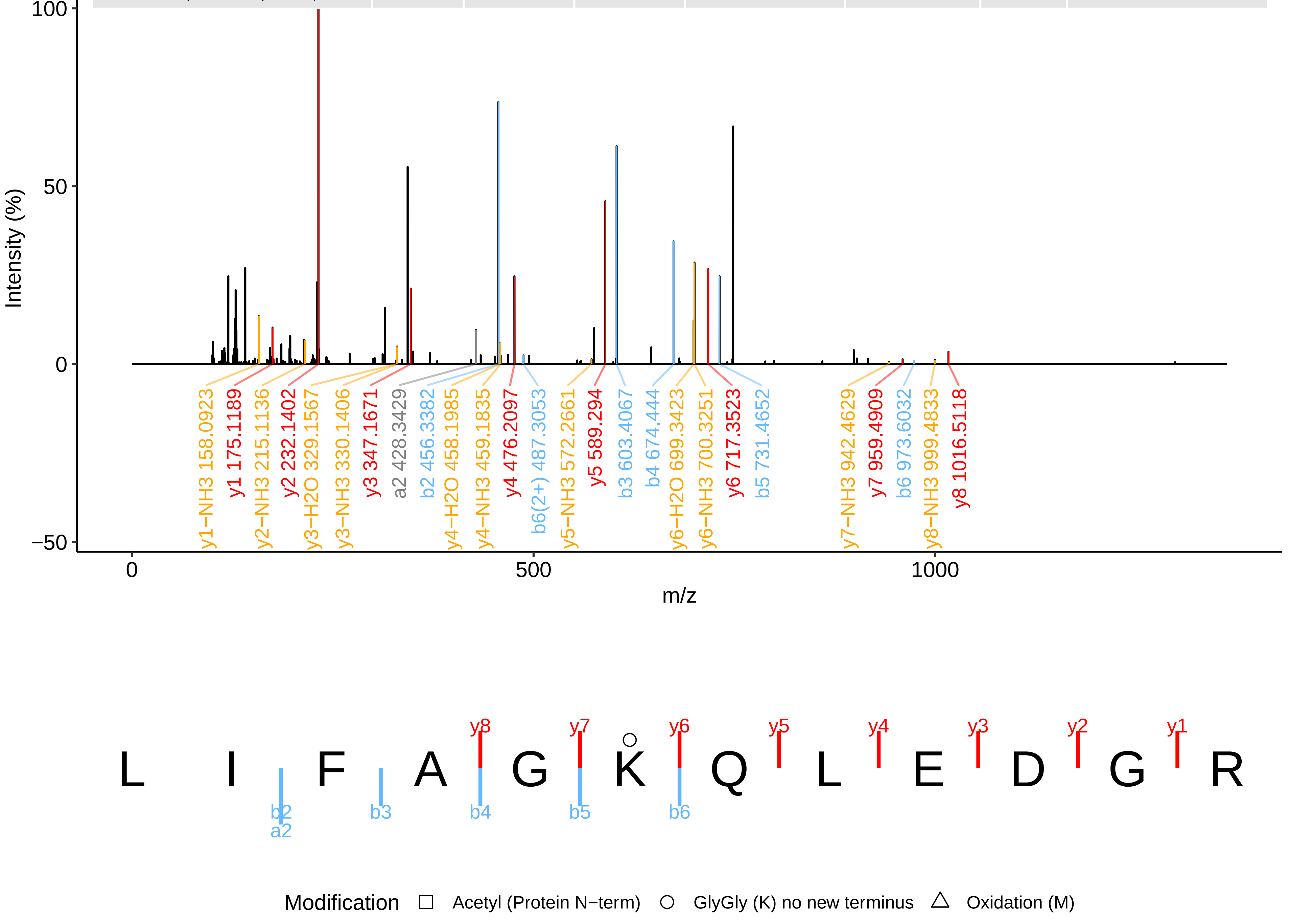

20200921_KIZ_MUb4_GlyGly_10plex − Scan number: 20582

Gene Names	Charge	m/z	Mass	Retention time	PEP	Score	Precursor Intensity
RPS27A;UBA52;UBC;UBB	3	487.6001	1459.778	74.423	2.7437e−13	137.89	49660356

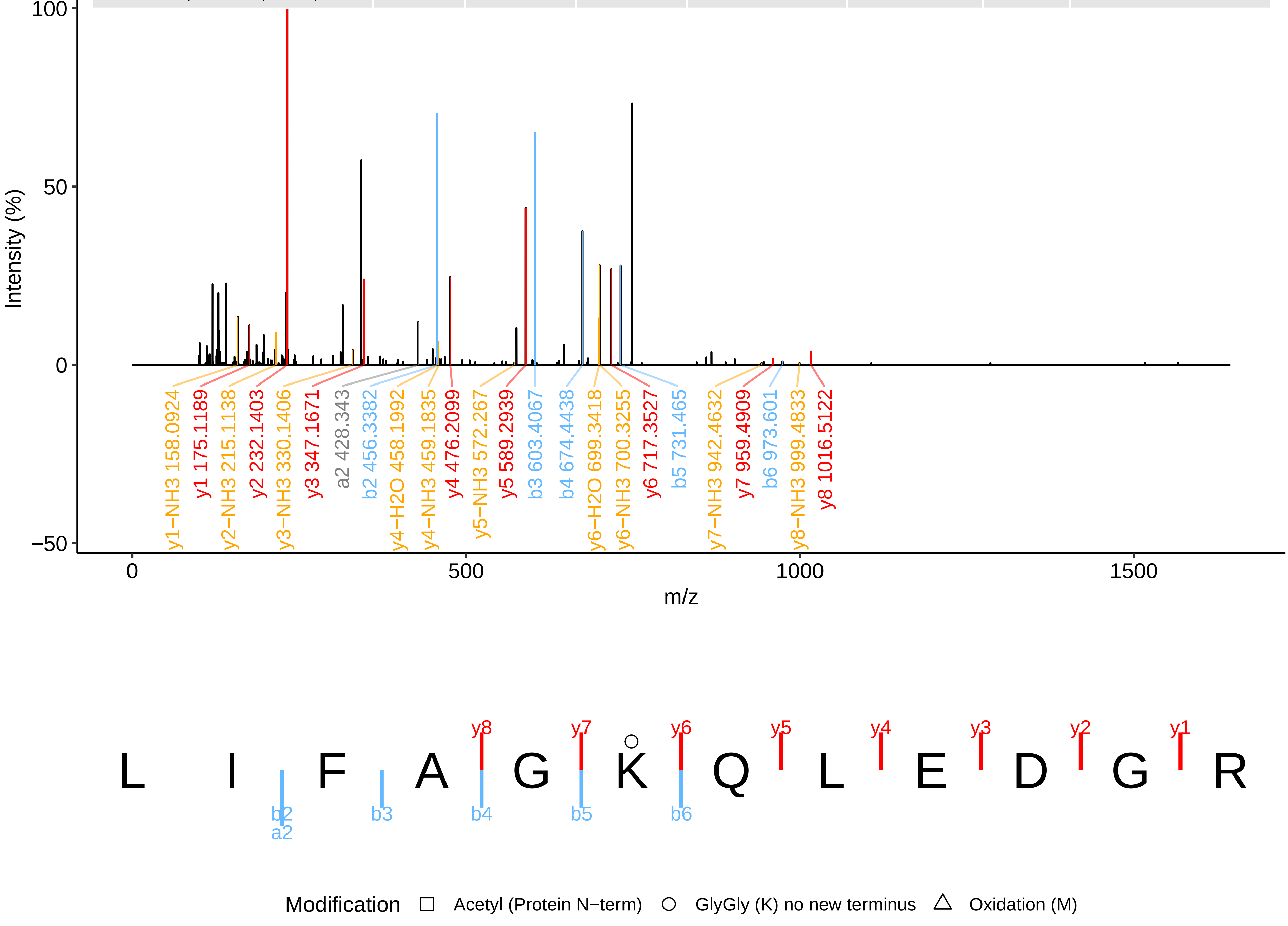

20200921_KIZ_MUb4_GlyGly_10plex − Scan number: 20718

Gene Names	Charge	m/z	Mass	Retention time	PEP	Score	Precursor Intensity
RPS27A;UBA52;UBC;UBB	3	487.6001	1459.778	74.929	9.899e−20	162.26	47723384

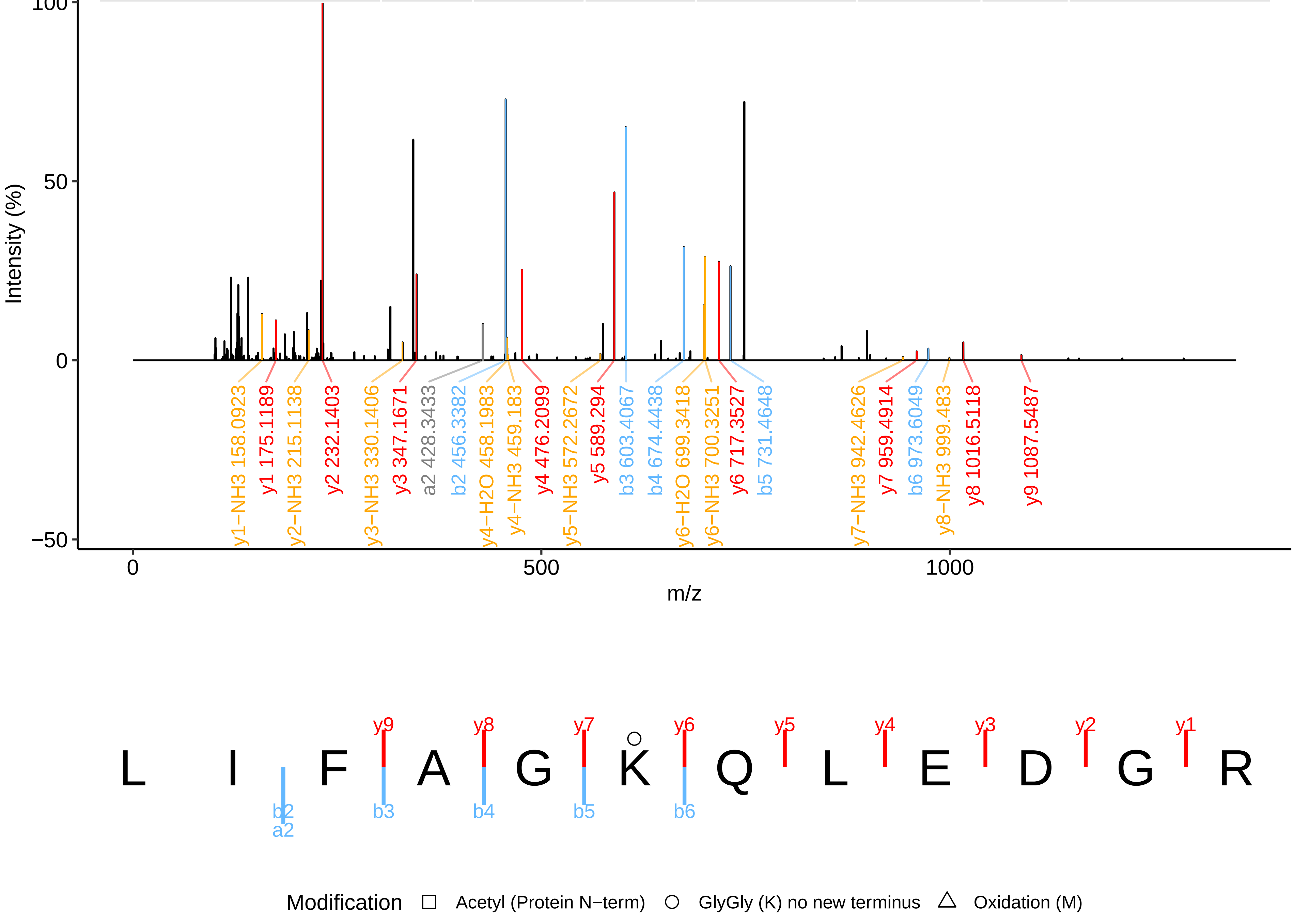

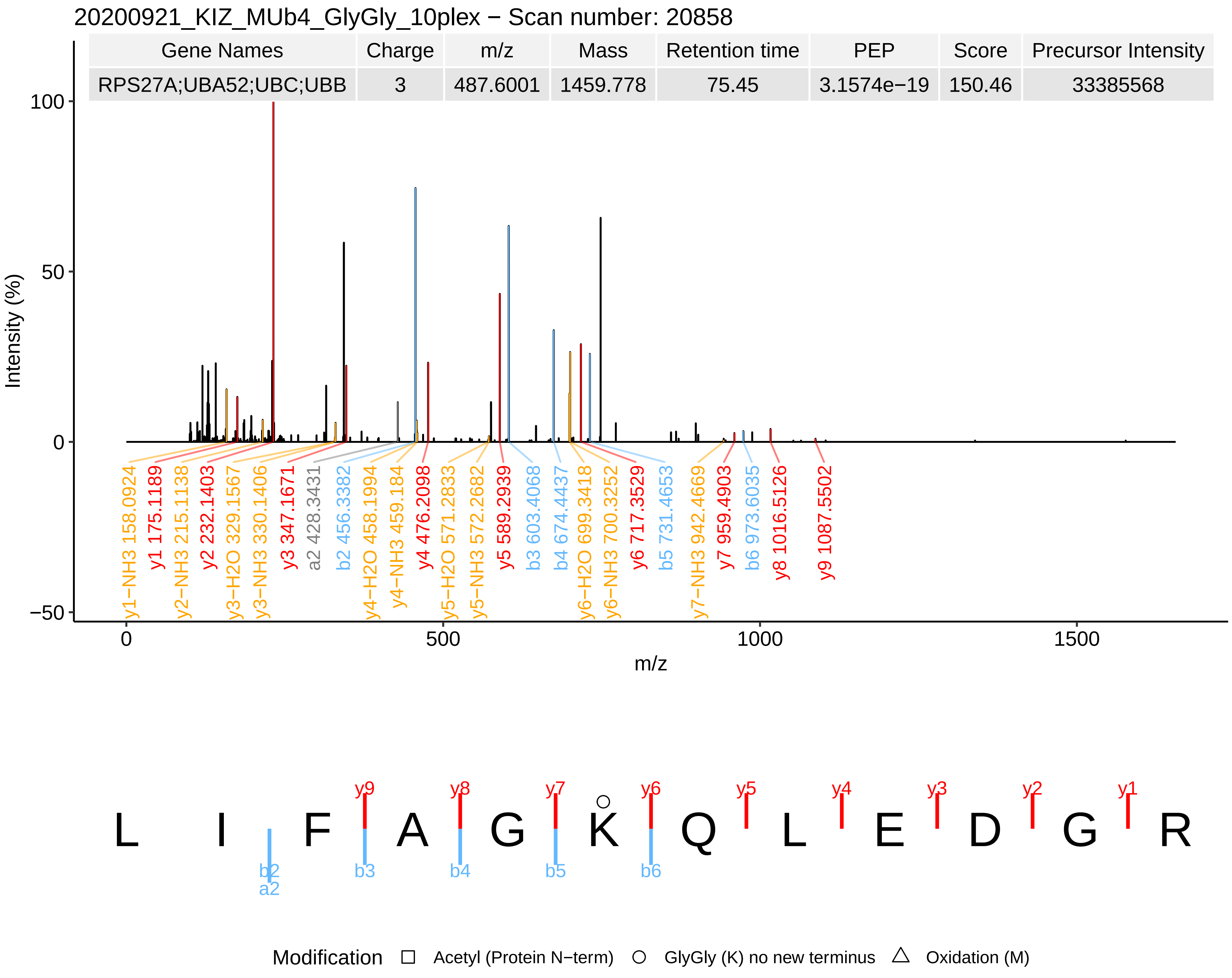
20200921_KIZ_MUb4_GlyGly_10plex − Scan number: 20858
Gene Names | Charge | m/z | Mass | Retention time | PEP | Score | Precursor Intensity
RPS27A;UBA52;UBC;UBB | 3 | 487.6001 | 1459.778 | 75.45 | 3.1574e−19 | 150.46 | 33385568
Intensity (%)
100
50
0
−50
0
500
1000
1500
m/z
y1−NH3 158.0924
y1 175.1189
y2−NH3 215.1138
y2 232.1403
y3−H2O 329.1567
y3−NH3 330.1406
y3 347.1671
a2 428.3431
b2 456.3382
y4−H2O 458.1994
y4−NH3 459.184
y4 476.2098
y5−H2O 571.2833
y5−NH3 572.2682
y5 589.2939
b3 603.4068
b4 674.4437
y6−H2O 699.3418
y6−NH3 700.3252
y6 717.3529
b5 731.4653
y7−NH3 942.4669
y7 959.4903
b6 973.6035
y8 1016.5126
y9 1087.5502
L I F A G K Q L E D G R
y9 y8 y7 y6 y5 y4 y3 y2 y1
b2 a2 b3 b4 b5 b6
Modification
Acetyl (Protein N−term)
GlyGly (K) no new terminus
Oxidation (M)

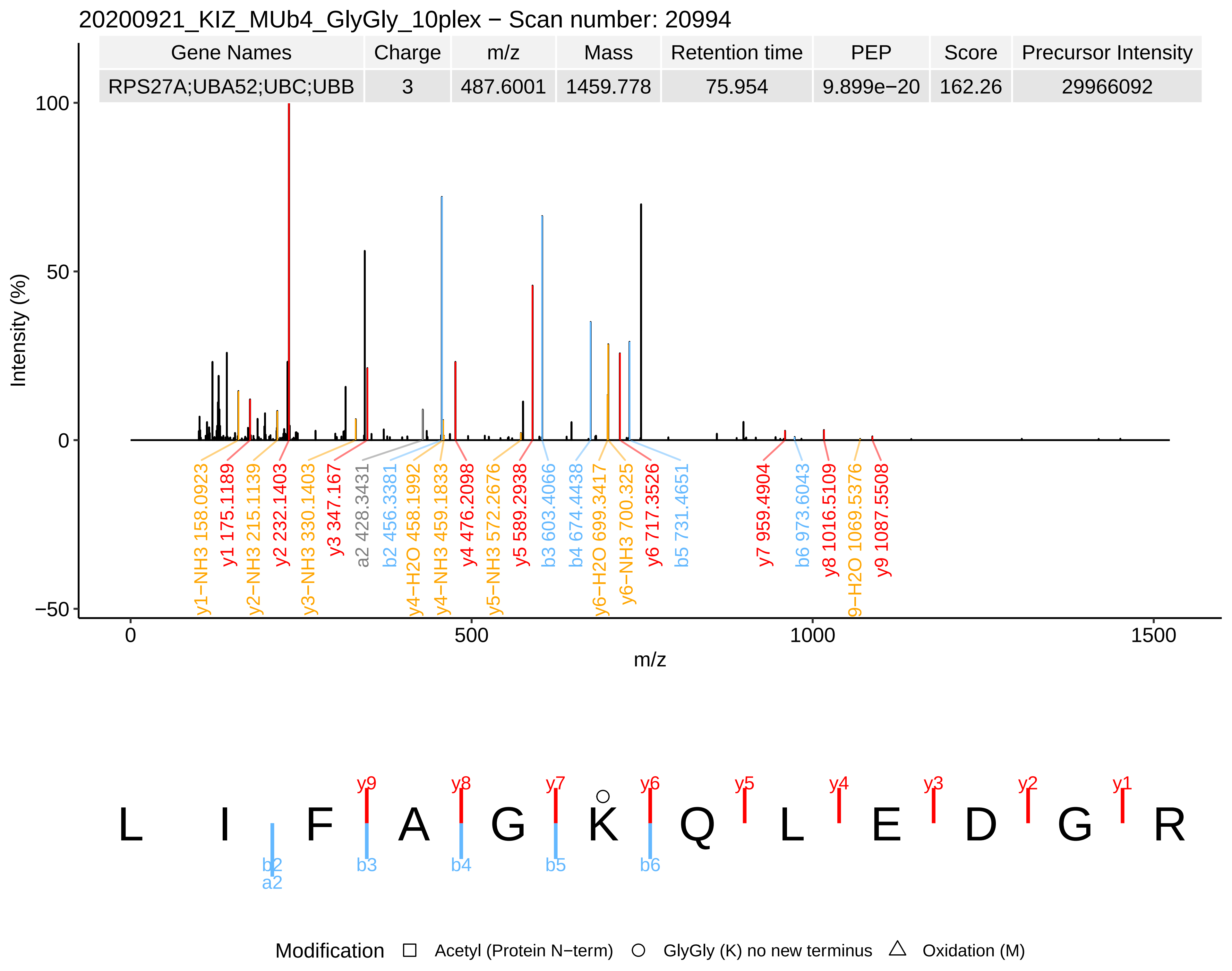
20200921_KIZ_MUb4_GlyGly_10plex − Scan number: 20994
Gene Names | Charge | m/z | Mass | Retention time | PEP | Score | Precursor Intensity
RPS27A;UBA52;UBC;UBB | 3 | 487.6001 | 1459.778 | 75.954 | 9.899e−20 | 162.26 | 29966092
Intensity (%)
100
50
0
−50
0
500
1000
1500
m/z
y1−NH3 158.0923
y1 175.1189
y2−NH3 215.1139
y2 232.1403
y3−NH3 330.1403
y3 347.167
a2 428.3431
b2 456.3381
y4−H2O 458.1992
y4−NH3 459.1833
y4 476.2098
y5−NH3 572.2676
y5 589.2938
b3 603.4066
b4 674.4438
y6−H2O 699.3417
y6−NH3 700.325
y6 717.3526
b5 731.4651
y7 959.4904
b6 973.6043
y8 1016.5109
y9−H2O 1069.5376
y9 1087.5508
L I F A G K Q L E D G R
y9 y8 y7 y6 y5 y4 y3 y2 y1
b2 a2 b3 b4 b5 b6
Modification
Acetyl (Protein N−term)
GlyGly (K) no new terminus
Oxidation (M)

20200921_KIZ_MUb4_GlyGly_10plex − Scan number: 21131

Gene Names	Charge	m/z	Mass	Retention time	PEP	Score	Precursor Intensity
RPS27A;UBA52;UBC;UBB	3	487.6001	1459.778	76.465	2.6215e−06	113.22	20252758

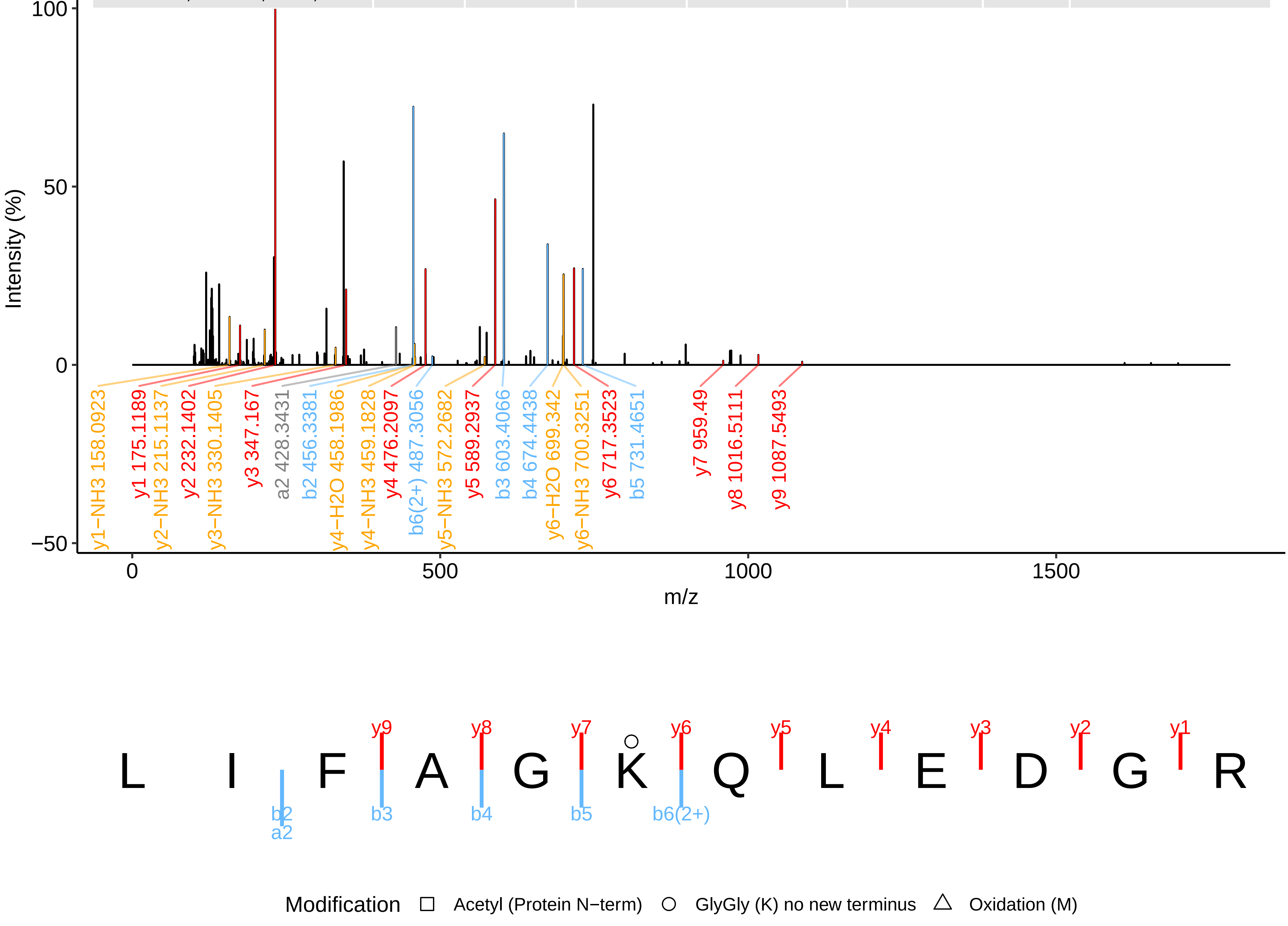

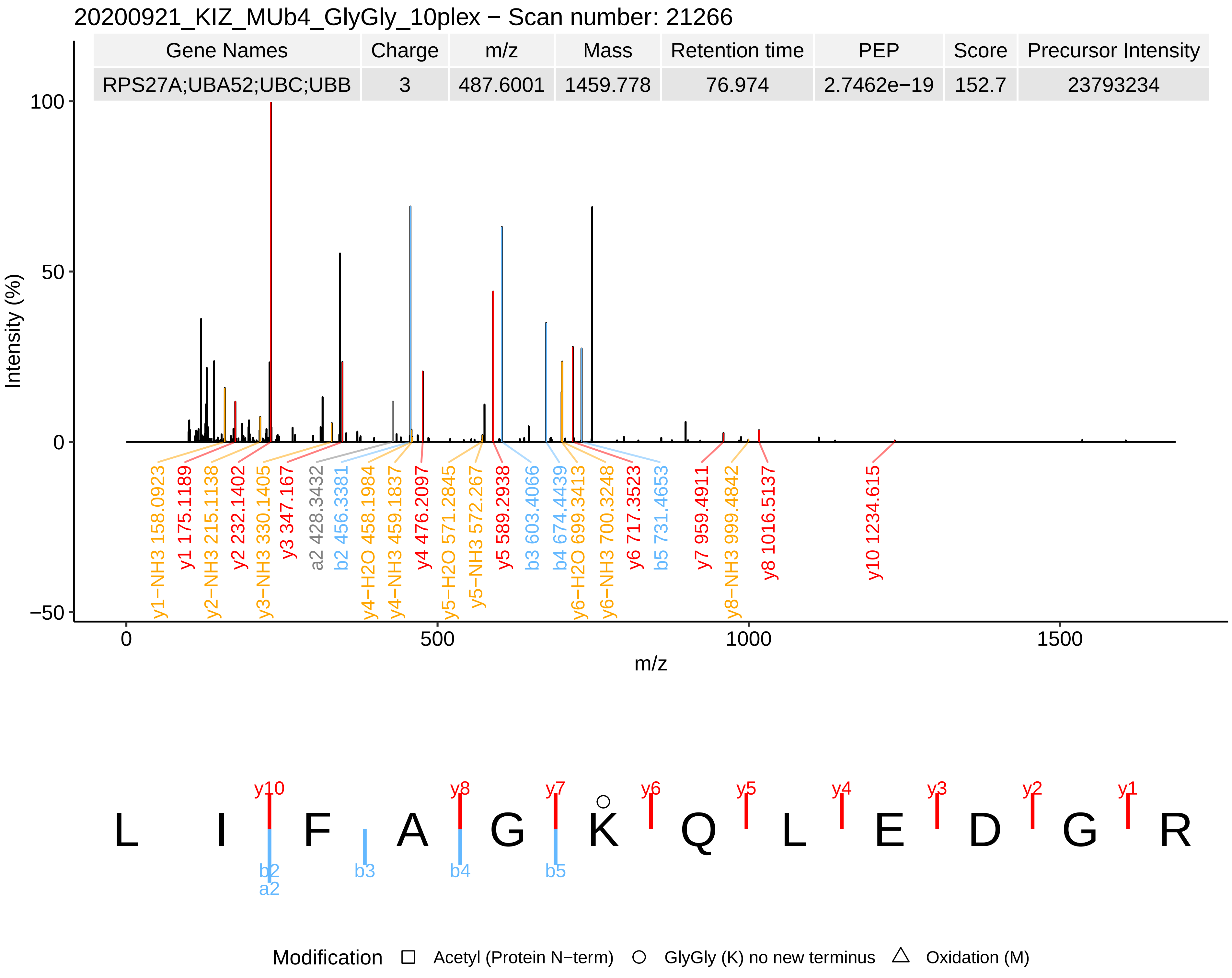
20200921_KIZ_MUb4_GlyGly_10plex − Scan number: 21266
Gene Names
RPS27A;UBA52;UBC;UBB
Charge
3
m/z
487.6001
Mass
1459.778
Retention time
76.974
PEP
2.7462e−19
Score
152.7
Precursor Intensity
23793234
Intensity (%)
100
50
0
−50
0
500
1000
1500
m/z
y1−NH3 158.0923
y1 175.1189
y2−NH3 215.1138
y2 232.1402
y3−NH3 330.1405
y3 347.167
a2 428.3432
b2 456.3381
y4−H2O 458.1984
y4−NH3 459.1837
y4 476.2097
y5−H2O 571.2845
y5−NH3 572.267
y5 589.2938
b3 603.4066
b4 674.4439
y6−H2O 699.3413
y6−NH3 700.3248
y6 717.3523
b5 731.4653
y7 959.4911
y8−NH3 999.4842
y8 1016.5137
y10 1234.615
L I F A G K Q L E D G R
y10 y8 y7 y6 y5 y4 y3 y2 y1
b2 a2 b3 b4 b5
Modification
Acetyl (Protein N−term)
GlyGly (K) no new terminus
Oxidation (M)

20200921_KIZ_MUb4_GlyGly_10plex − Scan number: 21403

Gene Names	Charge	m/z	Mass	Retention time	PEP	Score	Precursor Intensity
RPS27A;UBA52;UBC;UBB	3	487.6001	1459.778	77.51	1.8313e−19	157.68	20842224

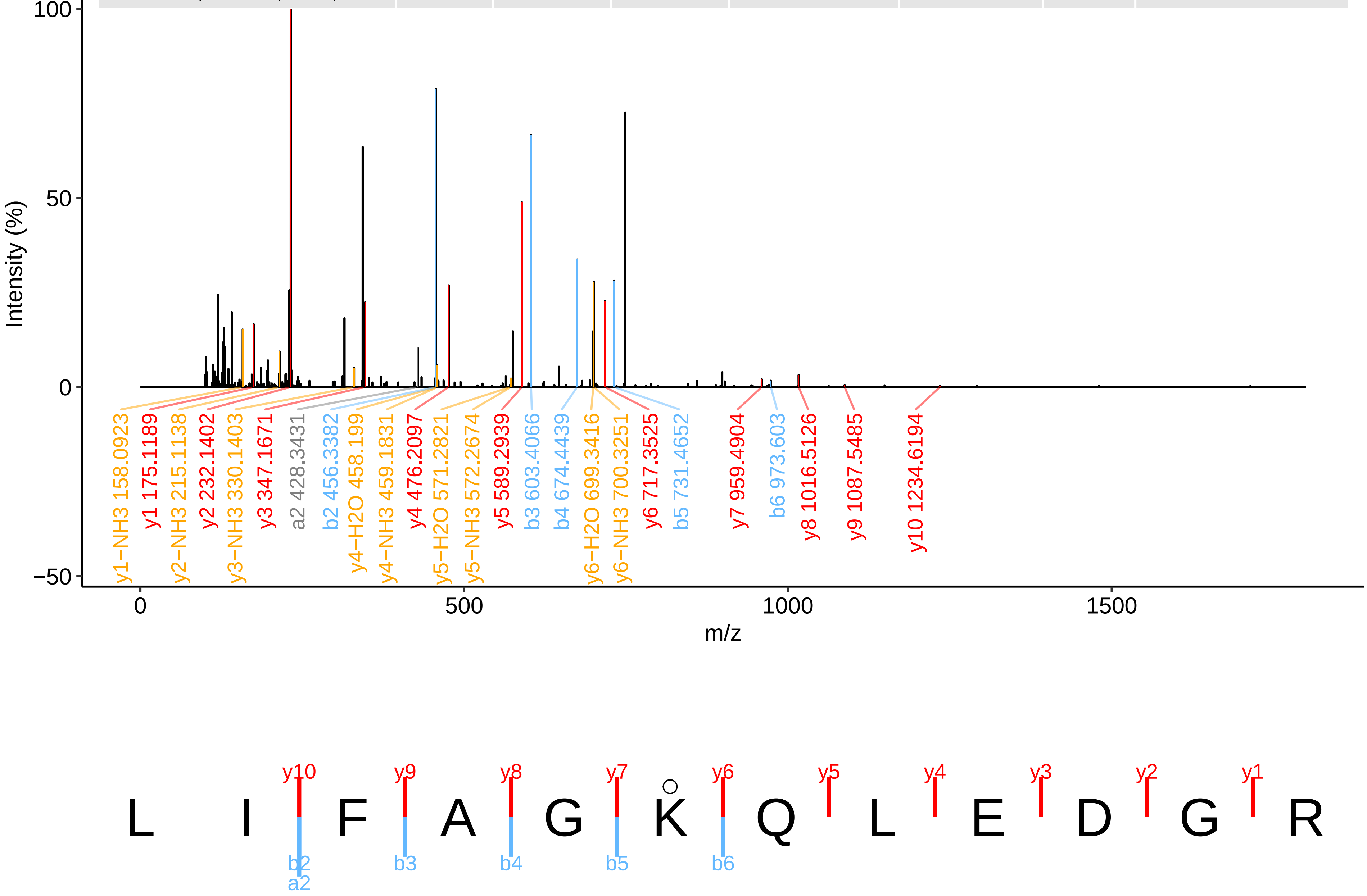

Modification □ Acetyl (Protein N−term) ○ GlyGly (K) no new terminus △ Oxidation (M)

20200921_KIZ_MUb4_GlyGly_10plex − Scan number: 21539

Gene Names	Charge	m/z	Mass	Retention time	PEP	Score	Precursor Intensity
RPS27A;UBA52;UBC;UBB	3	487.6001	1459.778	78.018	2.4754e−09	129.82	26200120

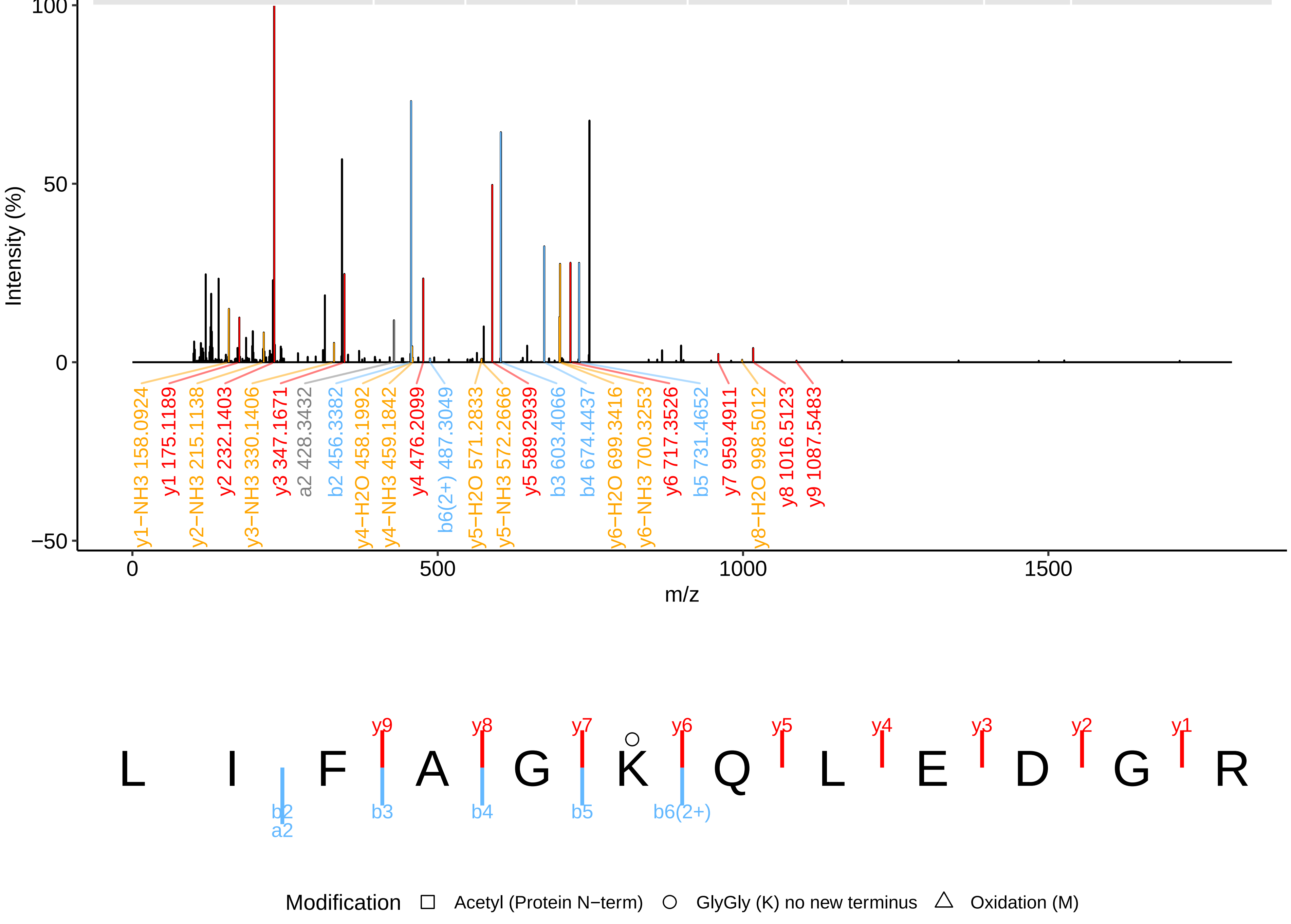

20200921_KIZ_MUb4_GlyGly_10plex − Scan number: 21671

Gene Names	Charge	m/z	Mass	Retention time	PEP	Score	Precursor Intensity
RPS27A;UBA52;UBC;UBB	3	487.6001	1459.778	78.522	2.5e−13	138.99	29290276

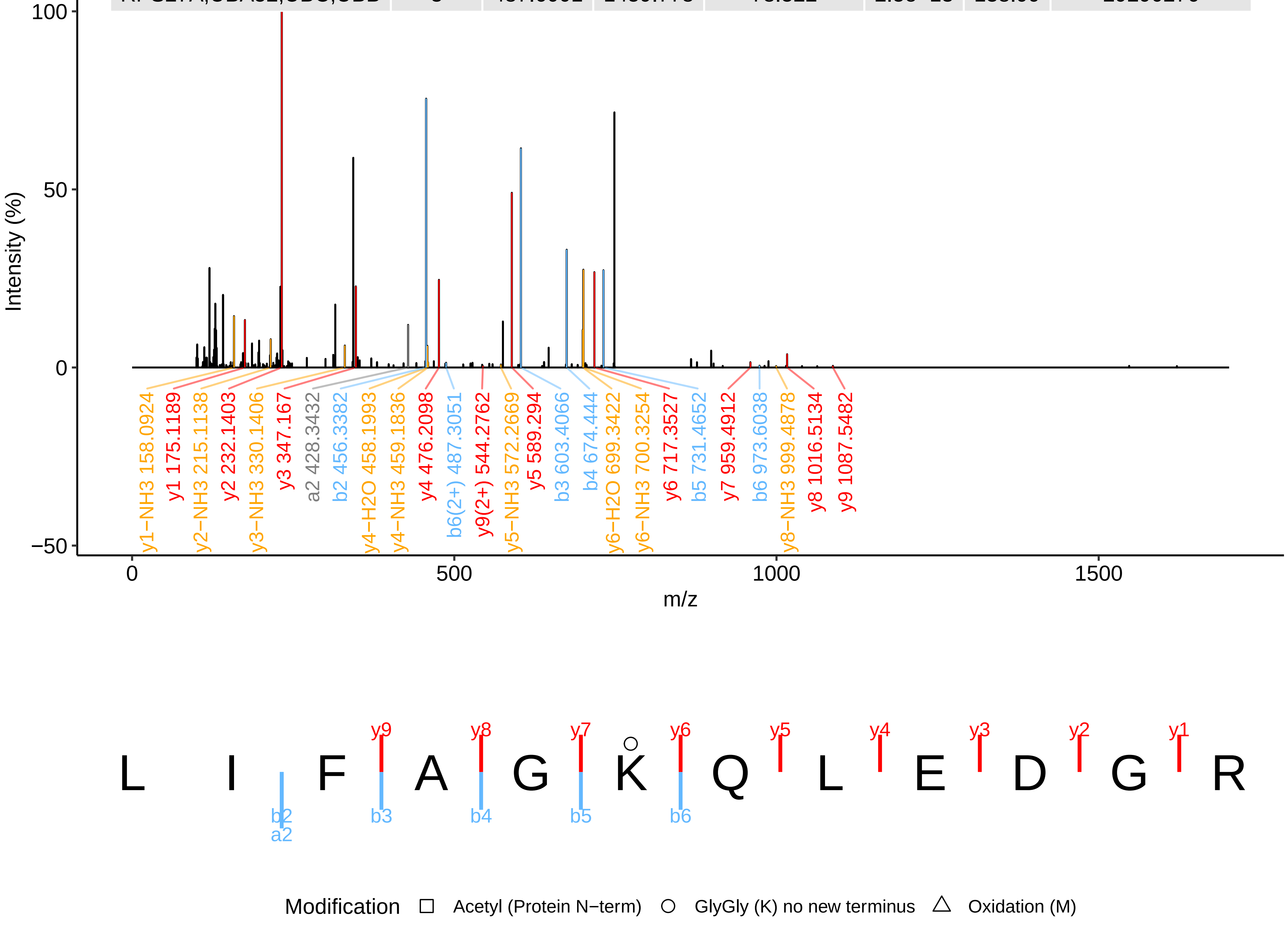

20200921_KIZ_MUb4_GlyGly_10plex − Scan number: 21804

Gene Names	Charge	m/z	Mass	Retention time	PEP	Score	Precursor Intensity
RPS27A;UBA52;UBC;UBB	3	487.6001	1459.778	79.029	4.1121e−09	127.83	14271172

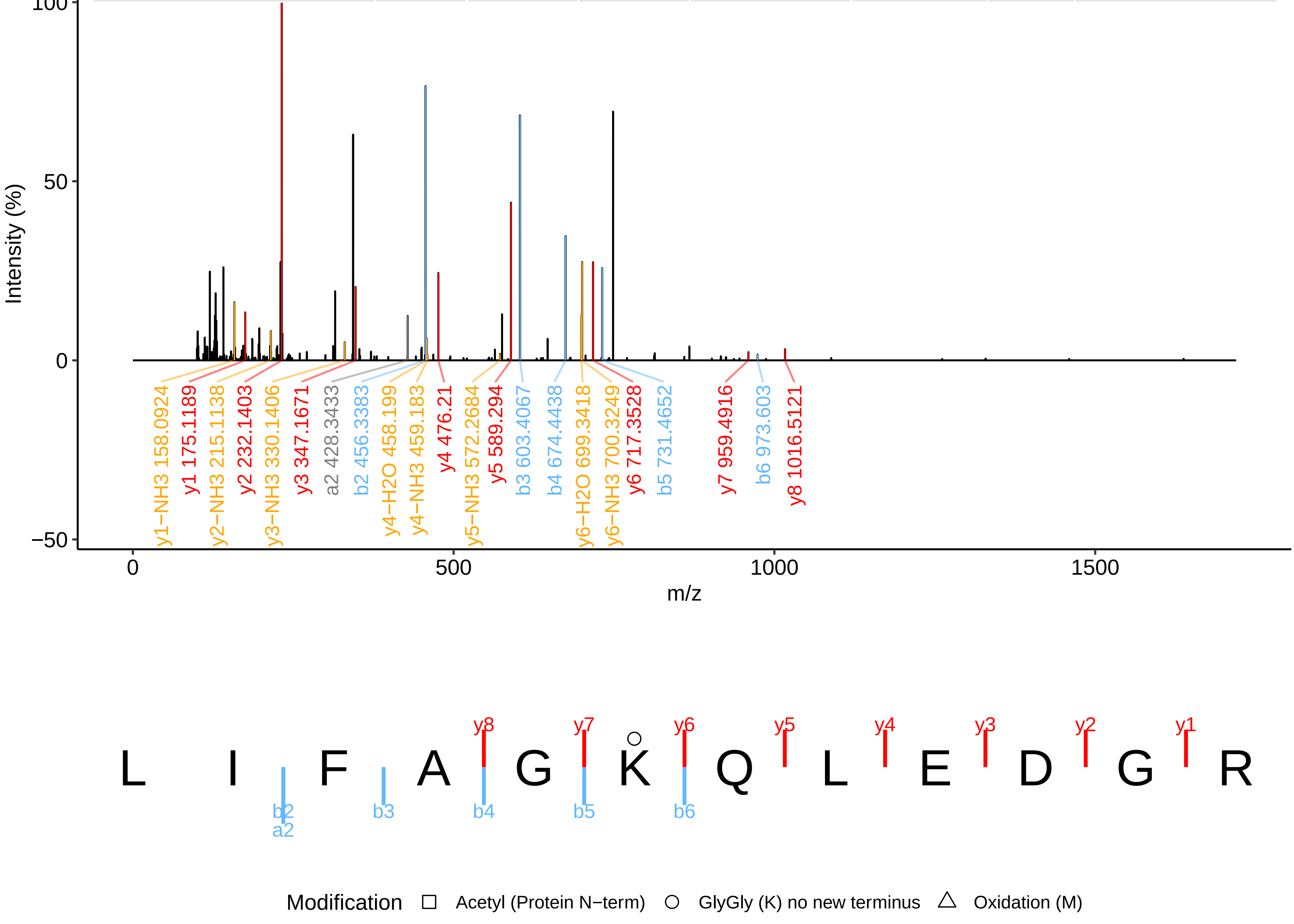

20200921_KIZ_MUb4_GlyGly_10plex − Scan number: 21942

Gene Names	Charge	m/z	Mass	Retention time	PEP	Score	Precursor Intensity
RPS27A;UBA52;UBC;UBB	3	487.6001	1459.778	79.538	9.6101e−14	145.91	17119312

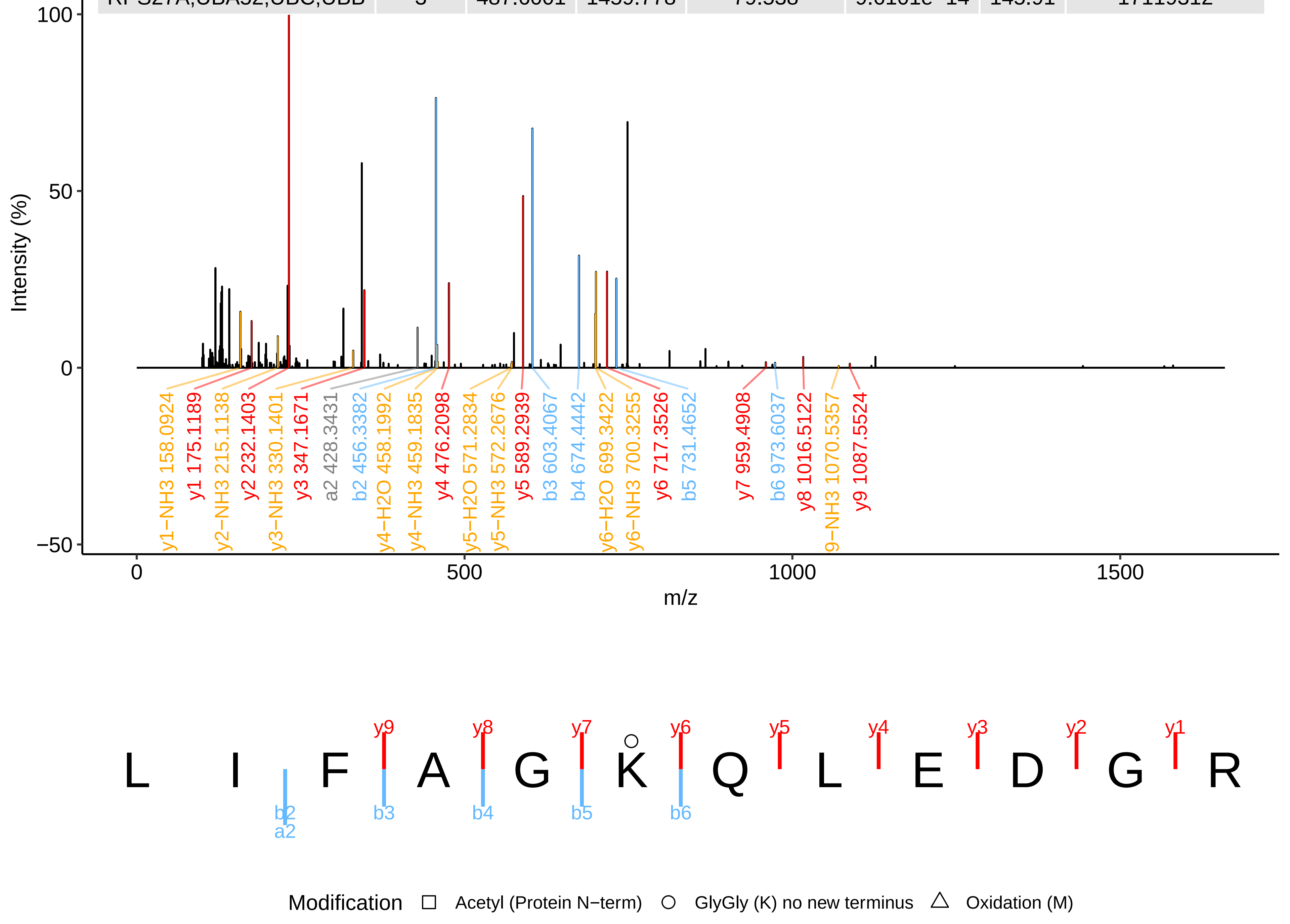

20200921_KIZ_MUb4_GlyGly_10plex − Scan number: 22074

Gene Names	Charge	m/z	Mass	Retention time	PEP	Score	Precursor Intensity
RPS27A;UBA52;UBC;UBB	3	487.6001	1459.778	80.05	2.5e−13	138.99	20784284

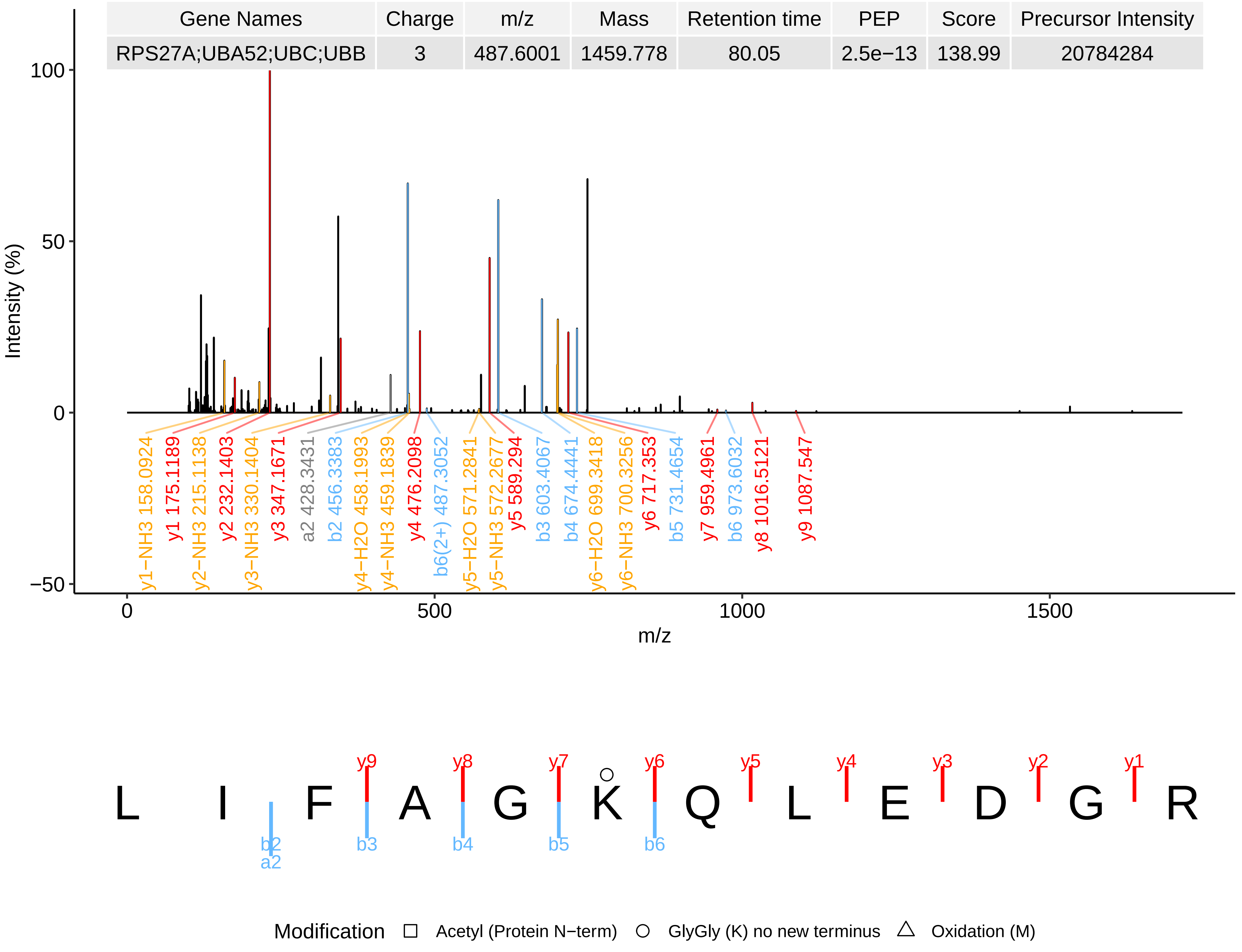

20200921_KIZ_MUb4_GlyGly_10plex − Scan number: 22216

Gene Names	Charge	m/z	Mass	Retention time	PEP	Score	Precursor Intensity
RPS27A;UBA52;UBC;UBB	3	487.6001	1459.778	80.562	3.5418e−13	134.3	23563696

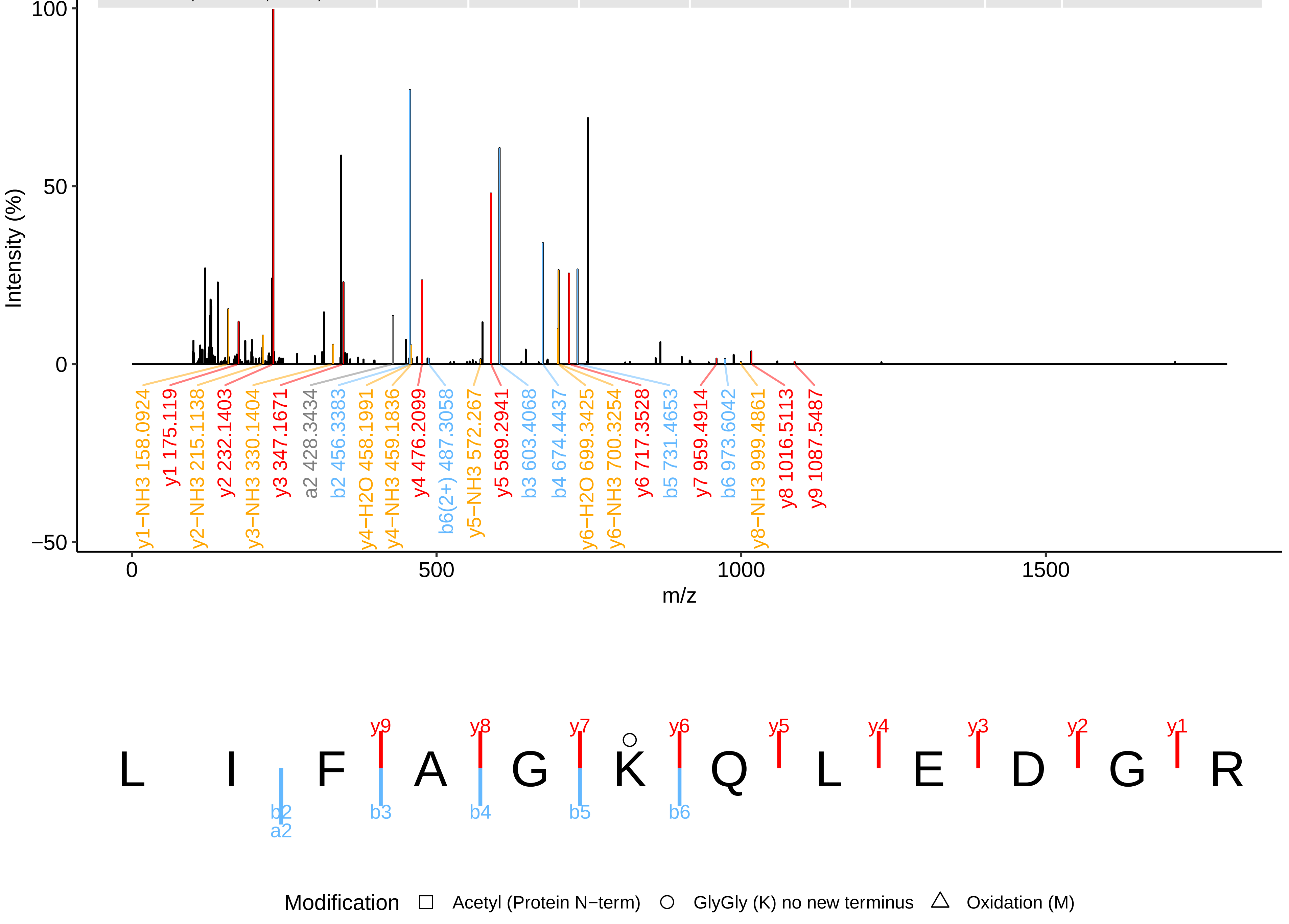

20200921_KIZ_MUb4_GlyGly_10plex − Scan number: 22358

Gene Names	Charge	m/z	Mass	Retention time	PEP	Score	Precursor Intensity
RPS27A;UBA52;UBC;UBB	3	487.6001	1459.778	81.075	9.6101e−14	145.91	17262590

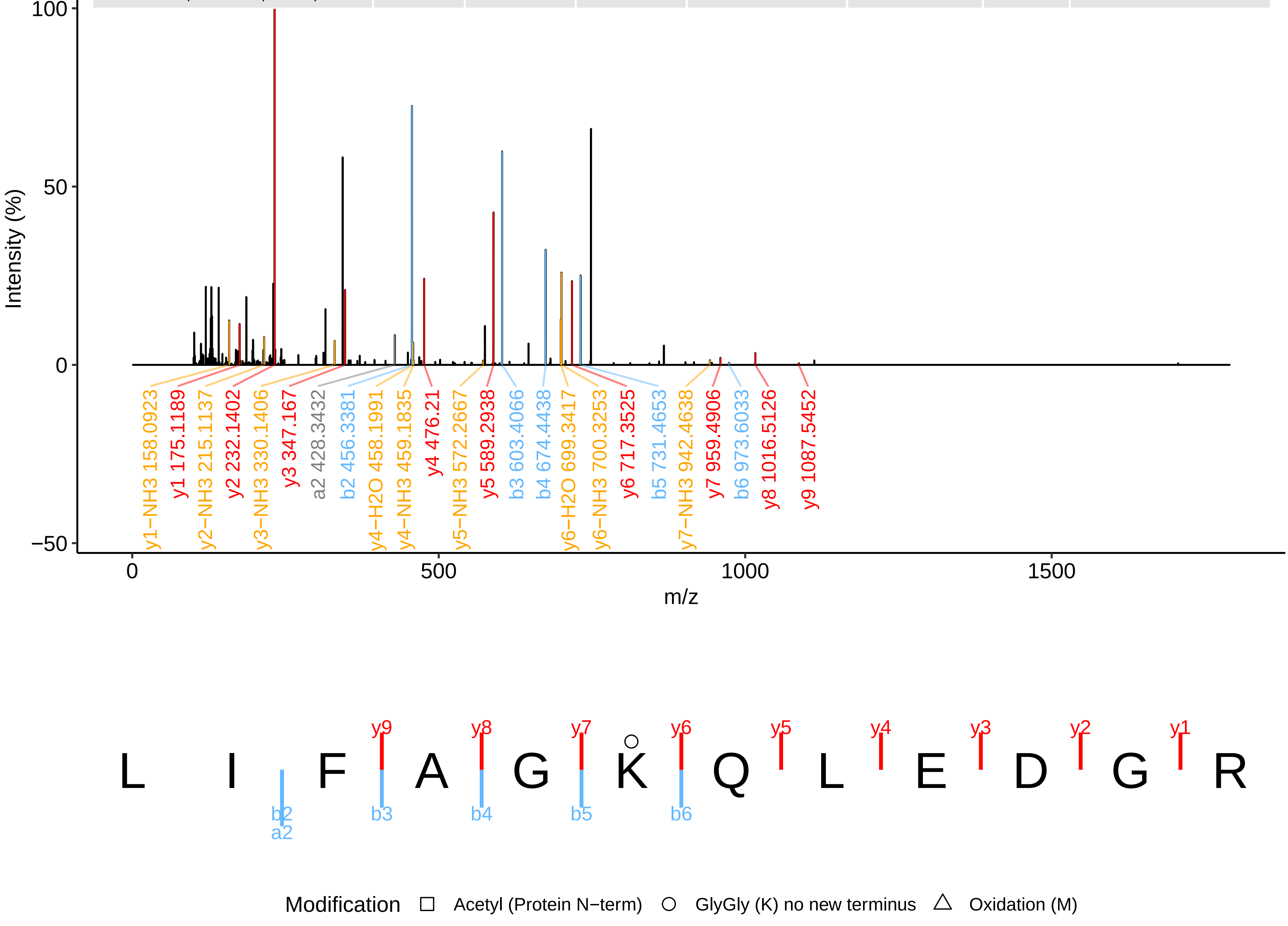

20200921_KIZ_MUb4_GlyGly_10plex − Scan number: 22495

Gene Names	Charge	m/z	Mass	Retention time	PEP	Score	Precursor Intensity
RPS27A;UBA52;UBC;UBB	3	487.6001	1459.778	81.592	2.7462e−19	152.7	19896808

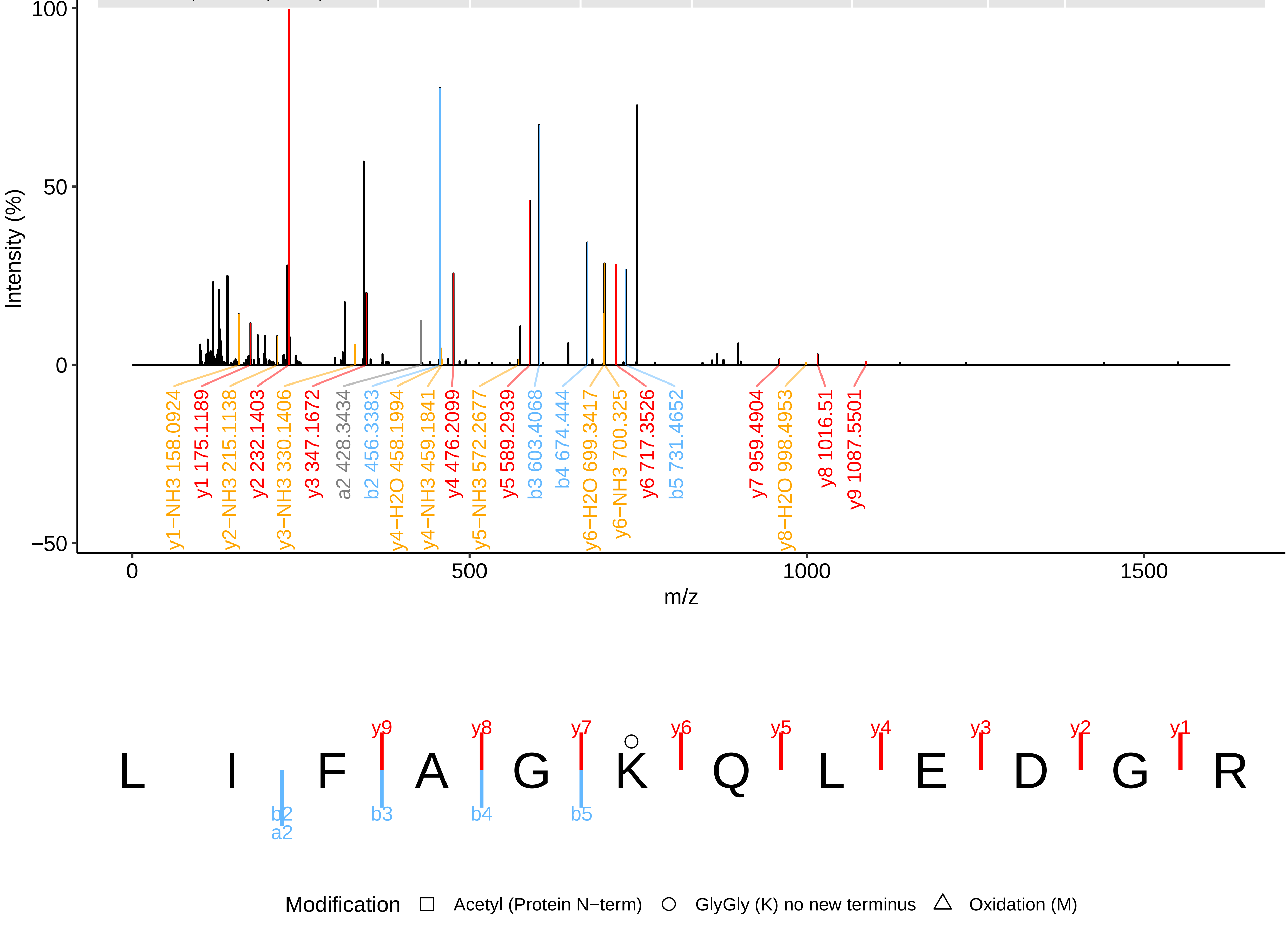

20200921_KIZ_MUb4_GlyGly_10plex − Scan number: 22629

Gene Names	Charge	m/z	Mass	Retention time	PEP	Score	Precursor Intensity
RPS27A;UBA52;UBC;UBB	3	487.6001	1459.778	82.104	1.1157e−08	119.27	24337606

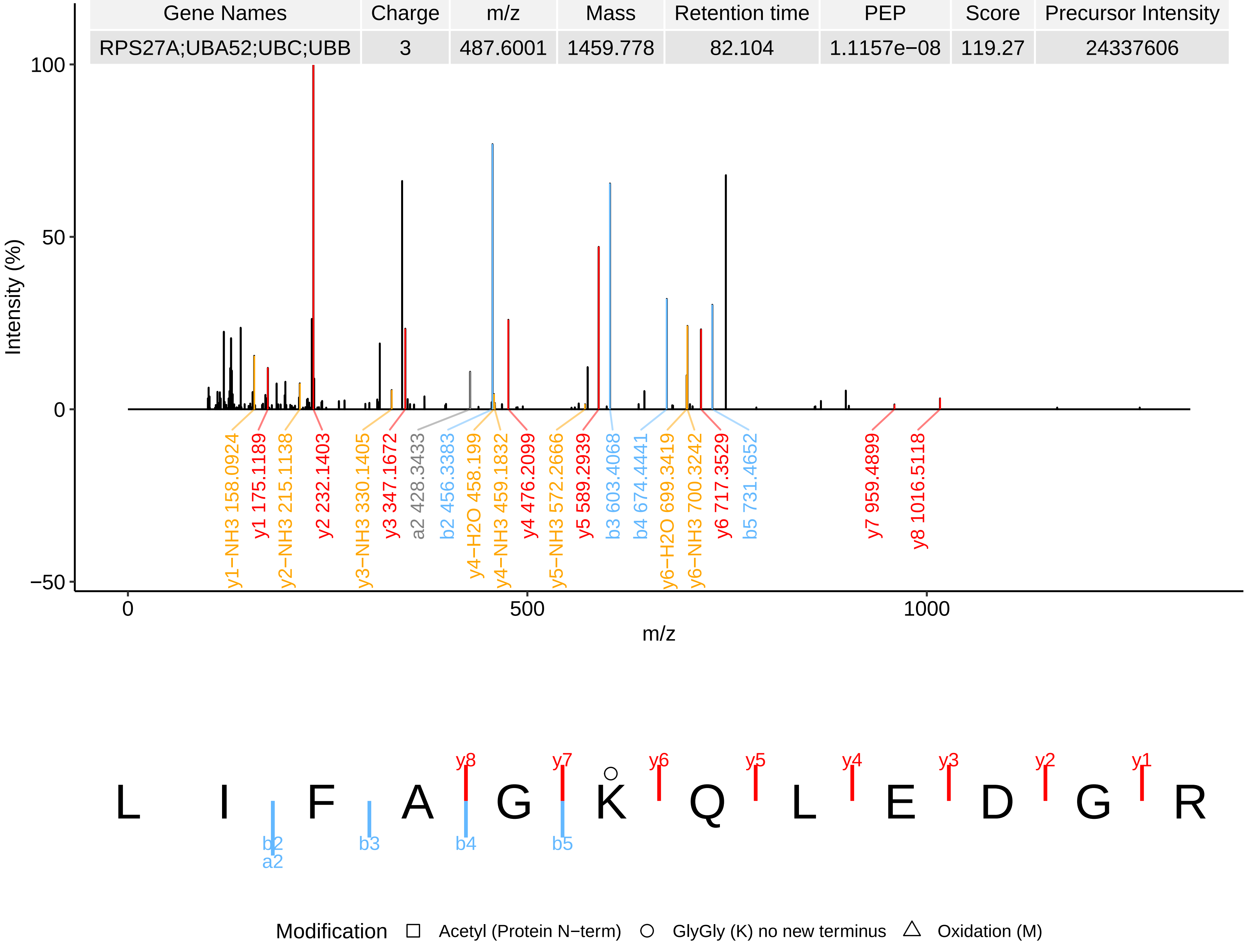

20200921_KIZ_MUb4_GlyGly_10plex − Scan number: 22763

Gene Names	Charge	m/z	Mass	Retention time	PEP	Score	Precursor Intensity
RPS27A;UBA52;UBC;UBB	3	487.6001	1459.778	82.612	1.4292e−05	103.43	17350644

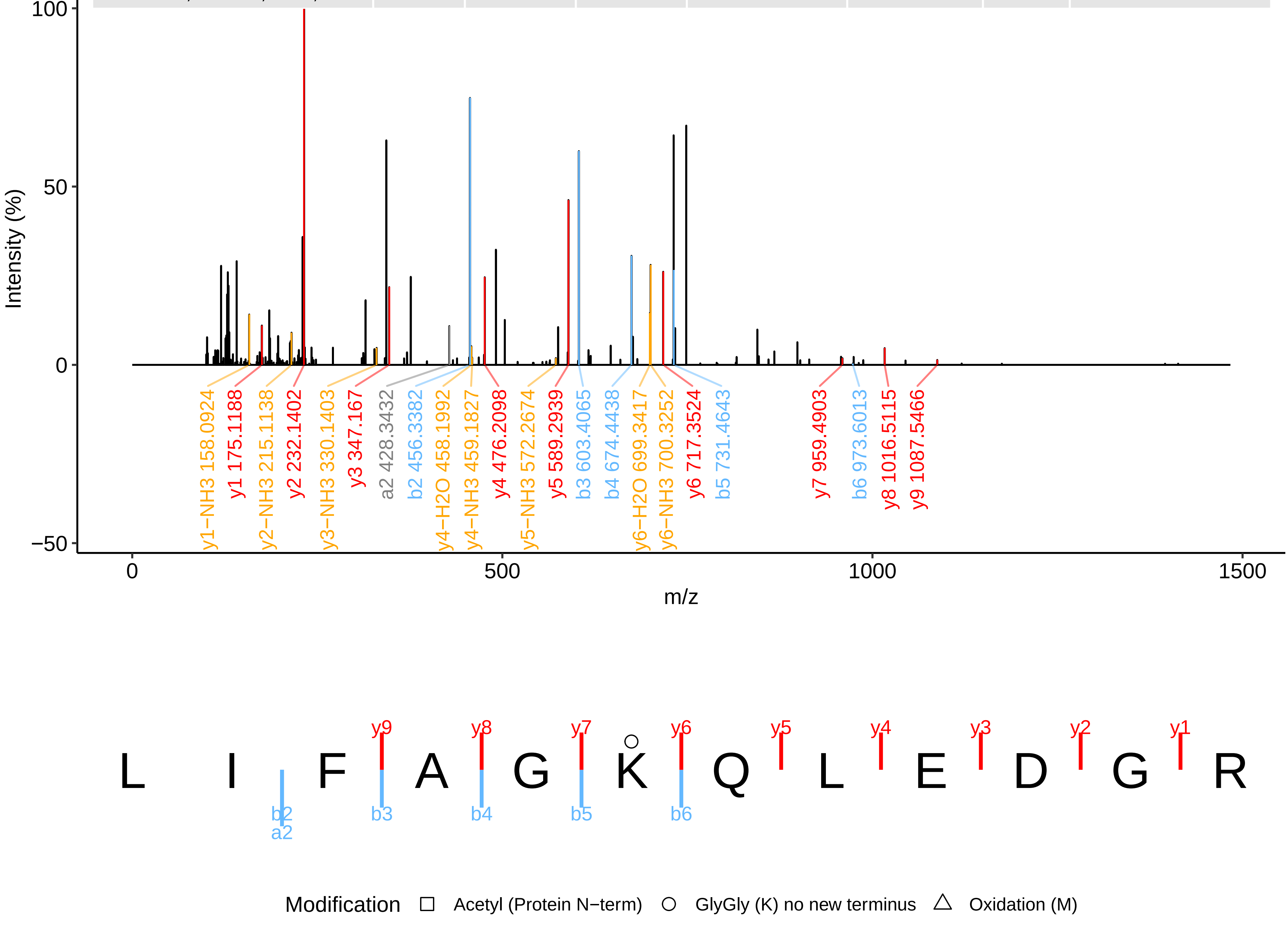

20200921_KIZ_MUb4_GlyGly_10plex − Scan number: 22904

Gene Names	Charge	m/z	Mass	Retention time	PEP	Score	Precursor Intensity
RPS27A;UBA52;UBC;UBB	3	487.6001	1459.778	83.122	4.9988e−07	115	8029070

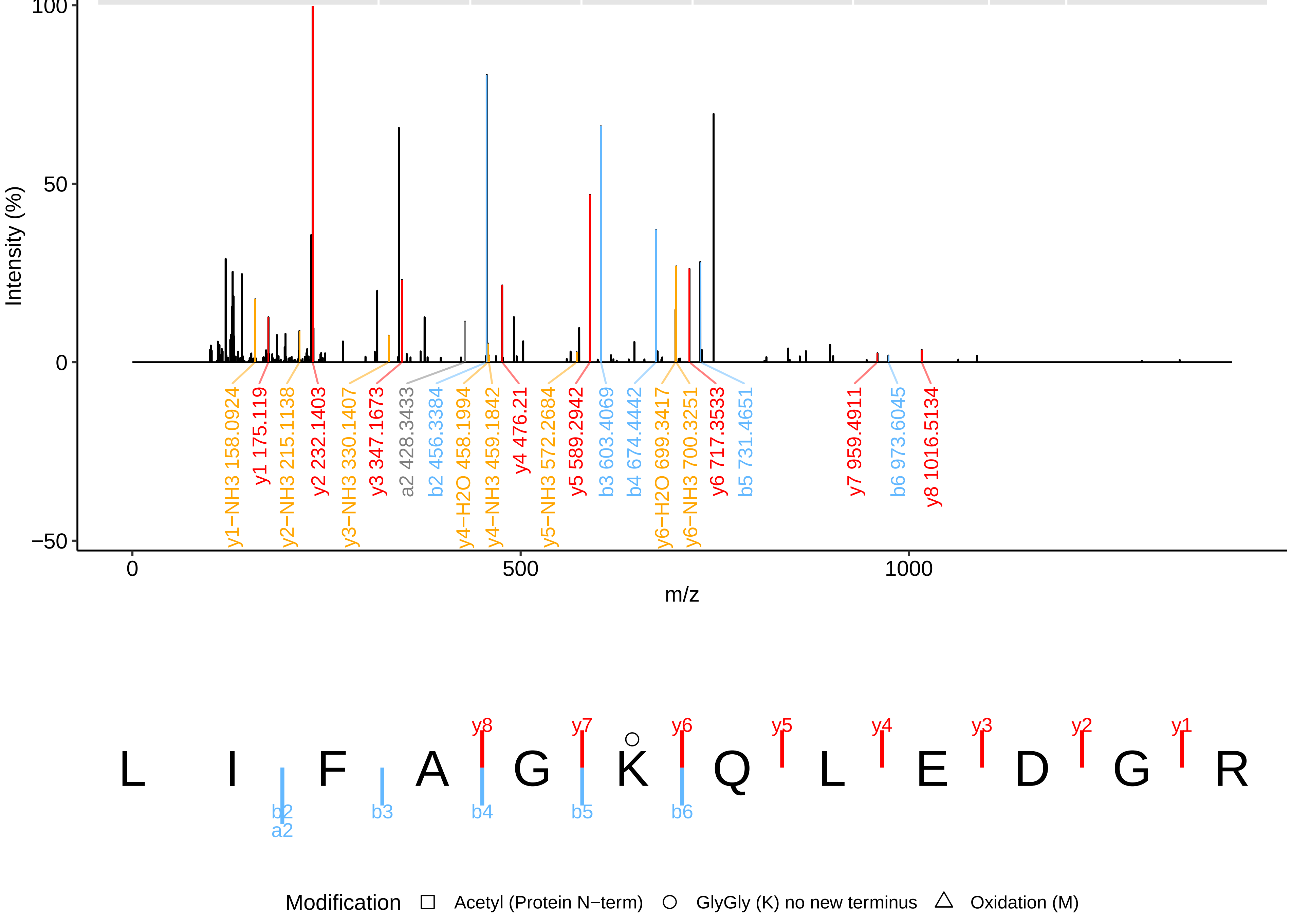

20200921_KIZ_MUb4_GlyGly_10plex − Scan number: 23046

Gene Names	Charge	m/z	Mass	Retention time	PEP	Score	Precursor Intensity
RPS27A;UBA52;UBC;UBB	3	487.6001	1459.778	83.627	1.0009e−05	107.03	10468769

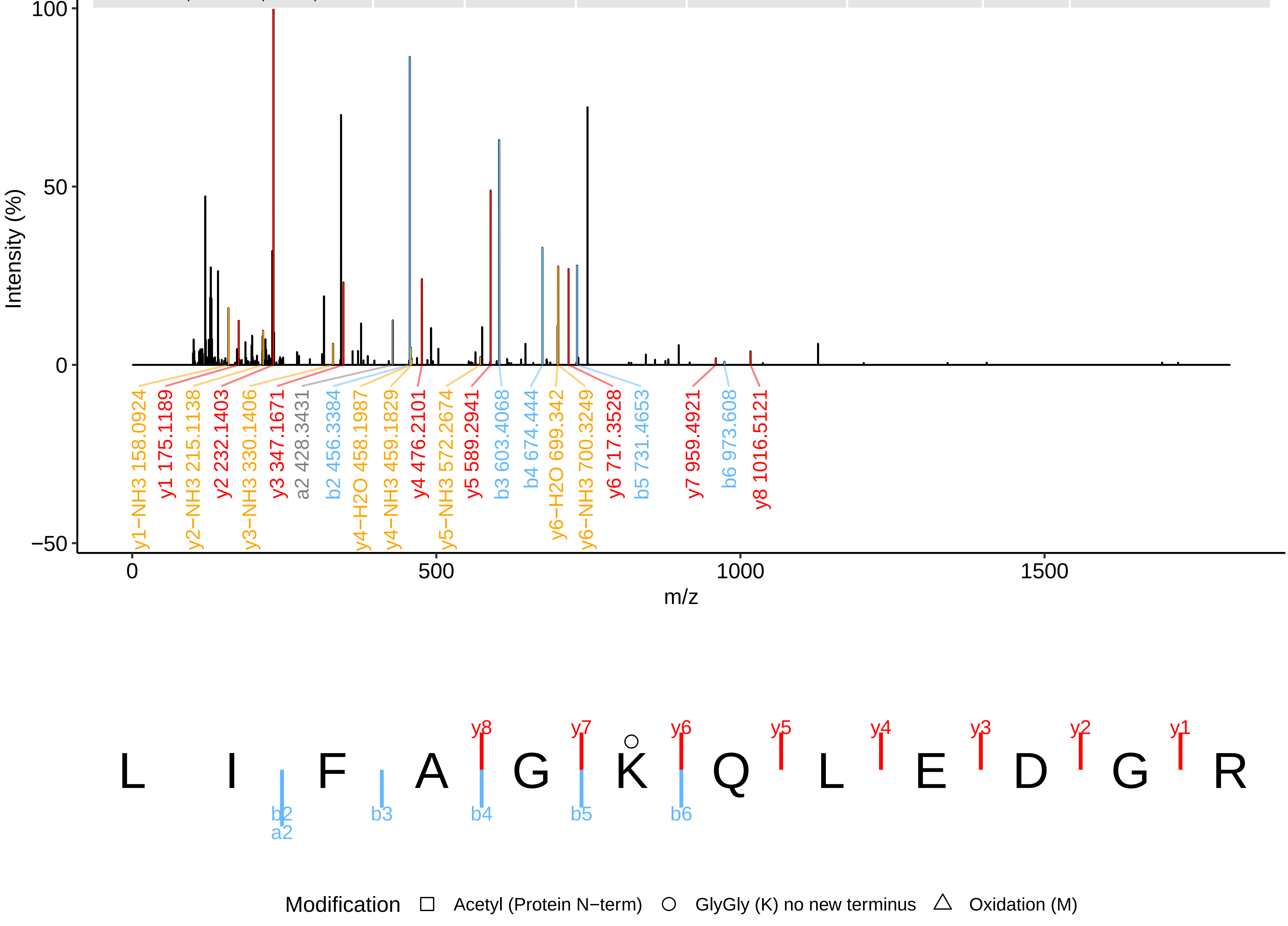

20200921_KIZ_MUb4_GlyGly_10plex − Scan number: 23181

Gene Names	Charge	m/z	Mass	Retention time	PEP	Score	Precursor Intensity
RPS27A;UBA52;UBC;UBB	3	487.6001	1459.778	84.128	1.136e−08	119.03	11614710

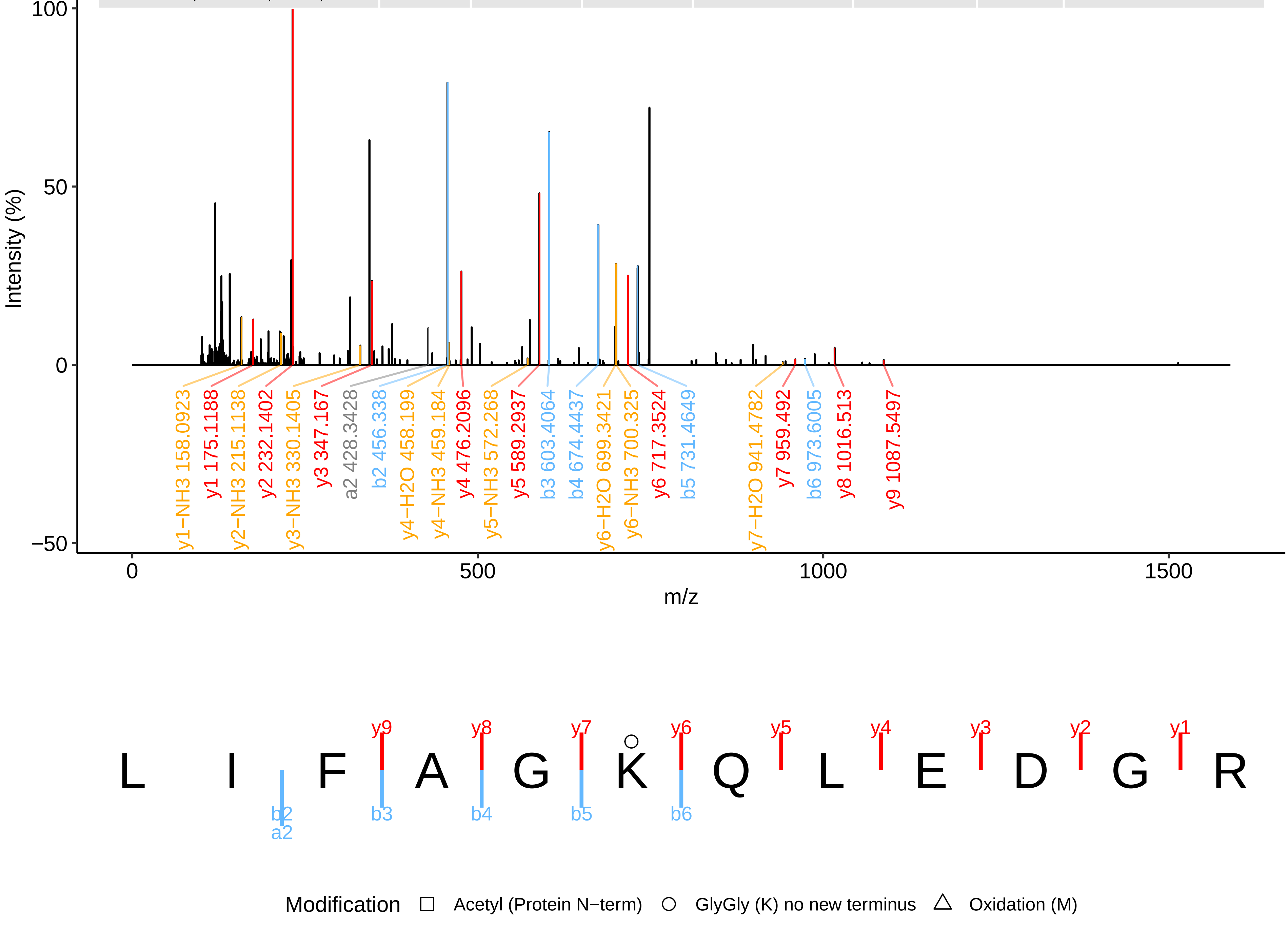

20200921_KIZ_MUb4_GlyGly_10plex − Scan number: 23317

Gene Names	Charge	m/z	Mass	Retention time	PEP	Score	Precursor Intensity
RPS27A;UBA52;UBC;UBB	3	487.6001	1459.778	84.636	1.2823e−08	117.25	9651425

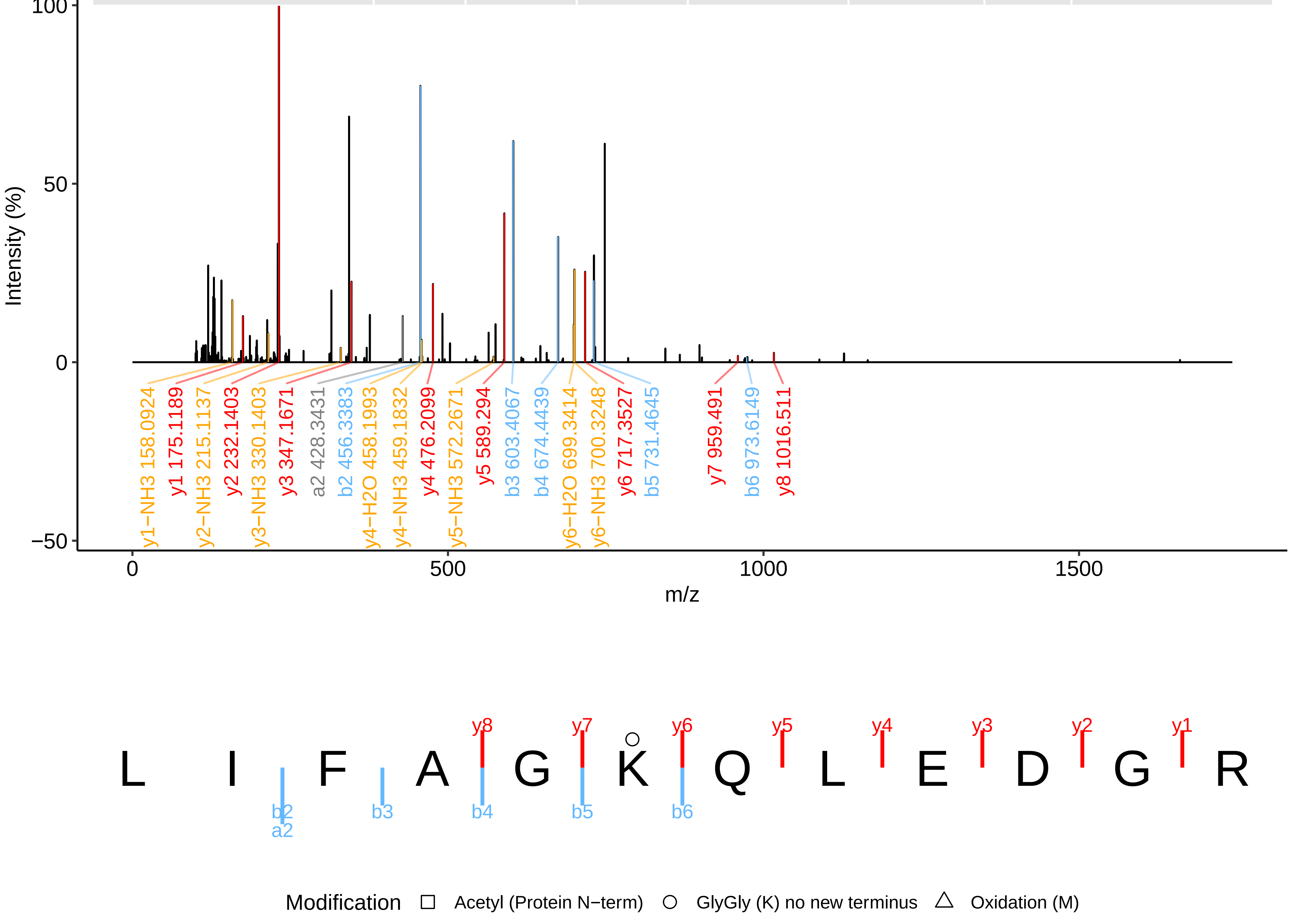

20200921_KIZ_MUb4_GlyGly_10plex − Scan number: 23455

Gene Names	Charge	m/z	Mass	Retention time	PEP	Score	Precursor Intensity
RPS27A;UBA52;UBC;UBB	3	487.6001	1459.778	85.142	6.5405e−15	149.94	8898411

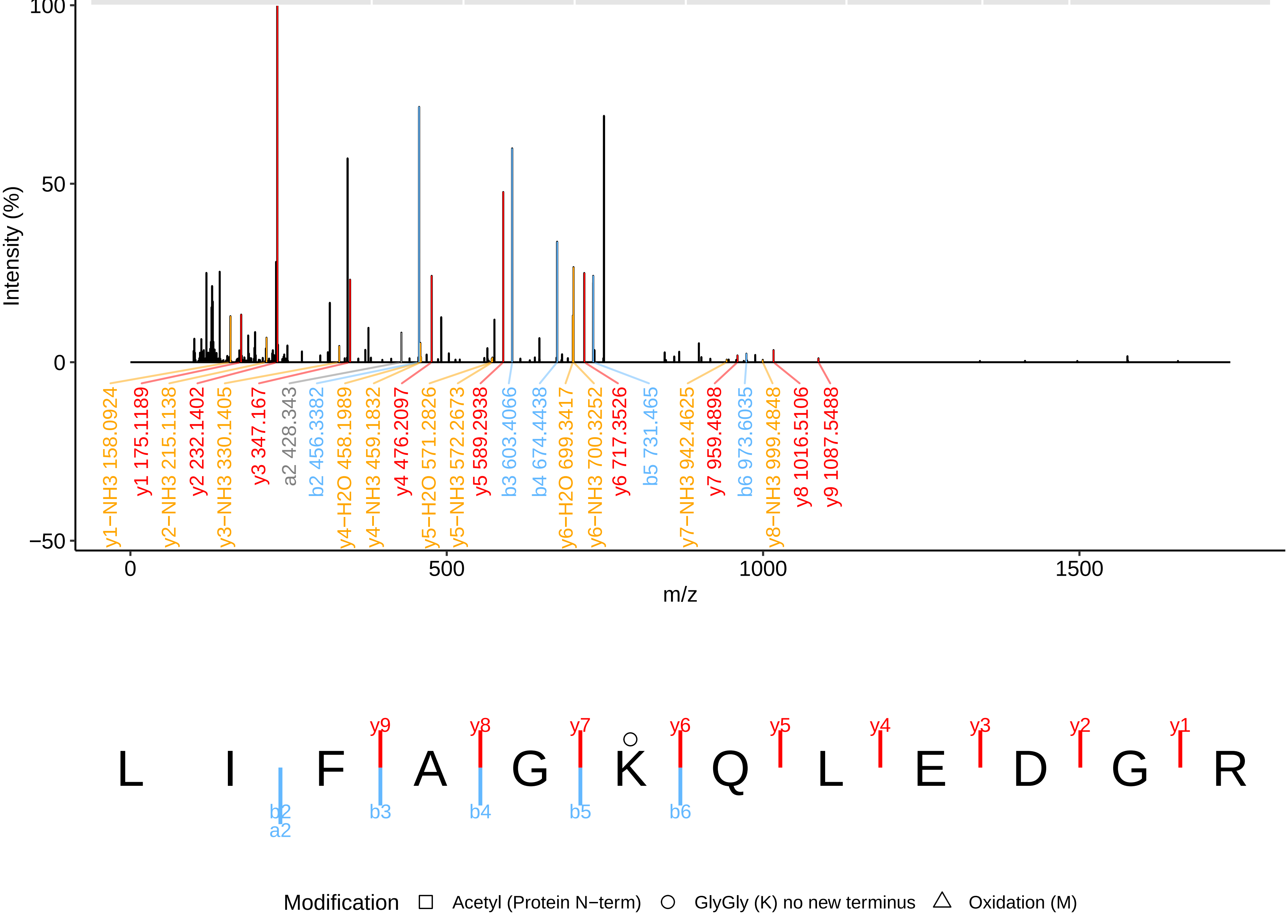

20200921_KIZ_MUb4_GlyGly_10plex − Scan number: 23597

Gene Names	Charge	m/z	Mass	Retention time	PEP	Score	Precursor Intensity
RPS27A;UBA52;UBC;UBB	3	487.6001	1459.778	85.661	4.9988e−07	115	14246455

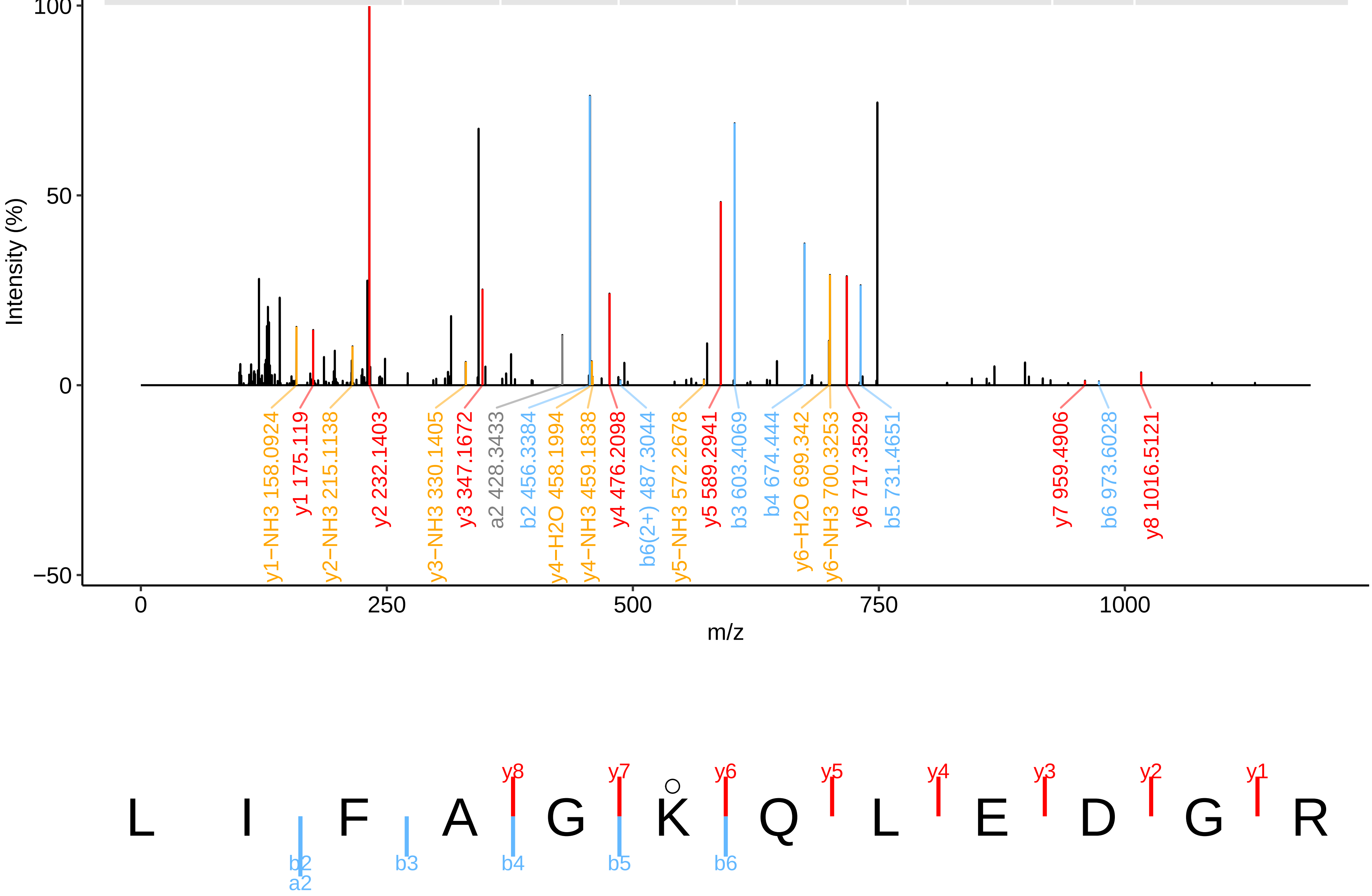

Modification □ Acetyl (Protein N−term) ○ GlyGly (K) no new terminus △ Oxidation (M)

20200921_KIZ_MUb4_GlyGly_10plex − Scan number: 23731

Gene Names	Charge	m/z	Mass	Retention time	PEP	Score	Precursor Intensity
RPS27A;UBA52;UBC;UBB	3	487.6001	1459.778	86.171	1.136e−08	119.03	9557504

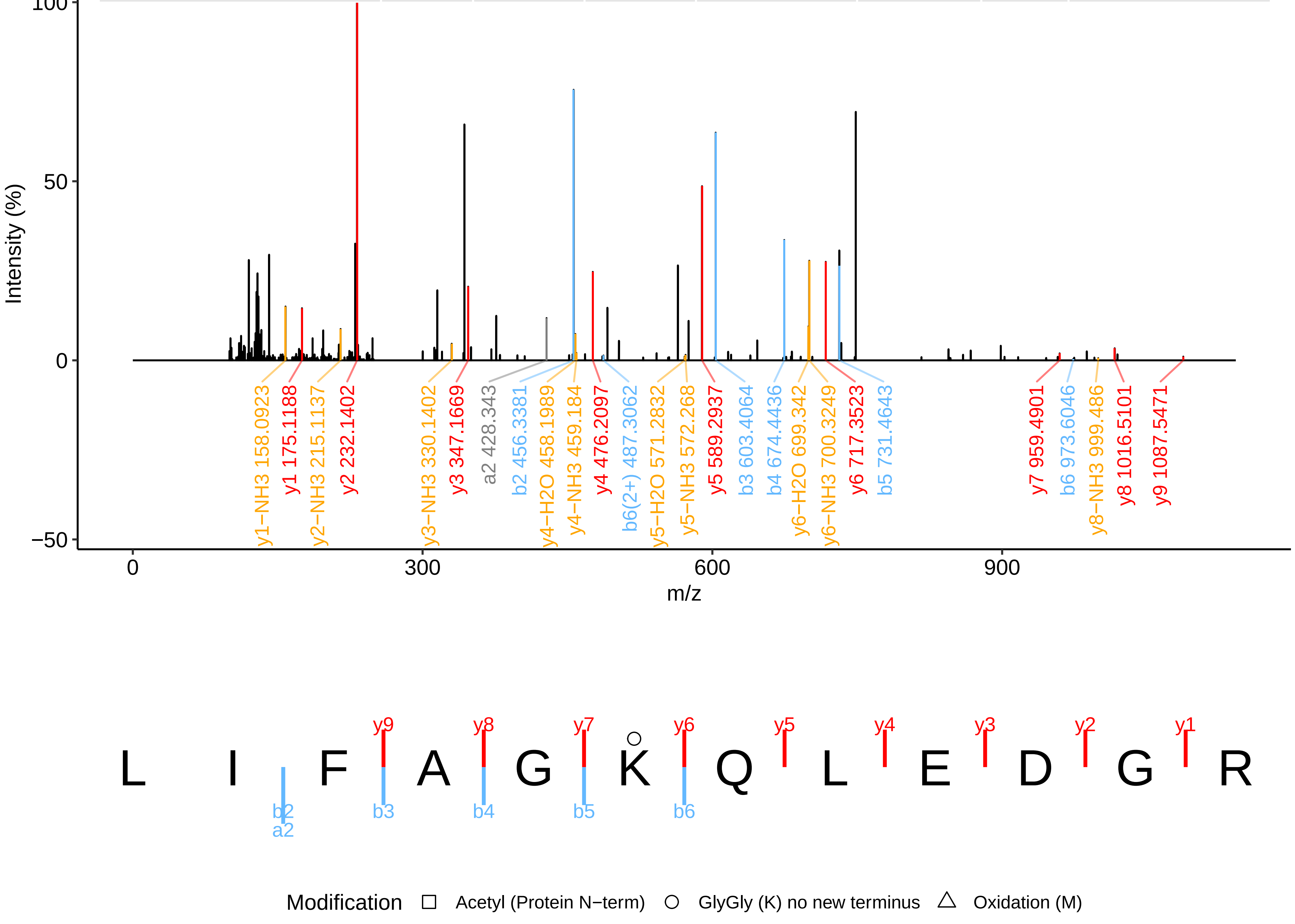

20200921_KIZ_MUb4_GlyGly_10plex − Scan number: 24004

Gene Names	Charge	m/z	Mass	Retention time	PEP	Score	Precursor Intensity
RPS27A;UBA52;UBC;UBB	3	487.6001	1459.778	87.179	2.4374e−06	113.38	12026343

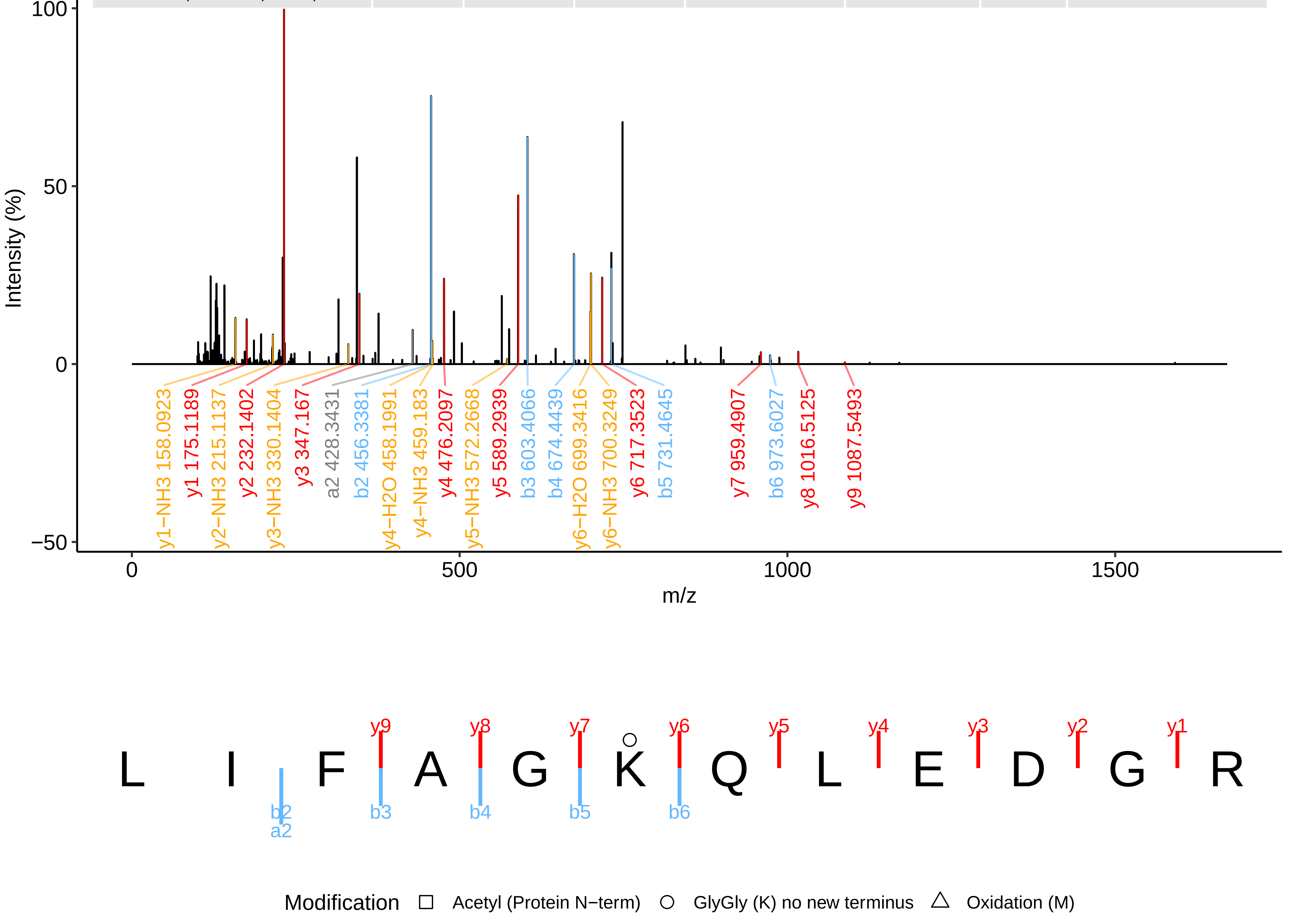

20200921_KIZ_MUb4_GlyGly_10plex − Scan number: 24139

Gene Names	Charge	m/z	Mass	Retention time	PEP	Score	Precursor Intensity
RPS27A;UBA52;UBC;UBB	3	487.6001	1459.778	87.689	4.3409e−15	150.04	10157250

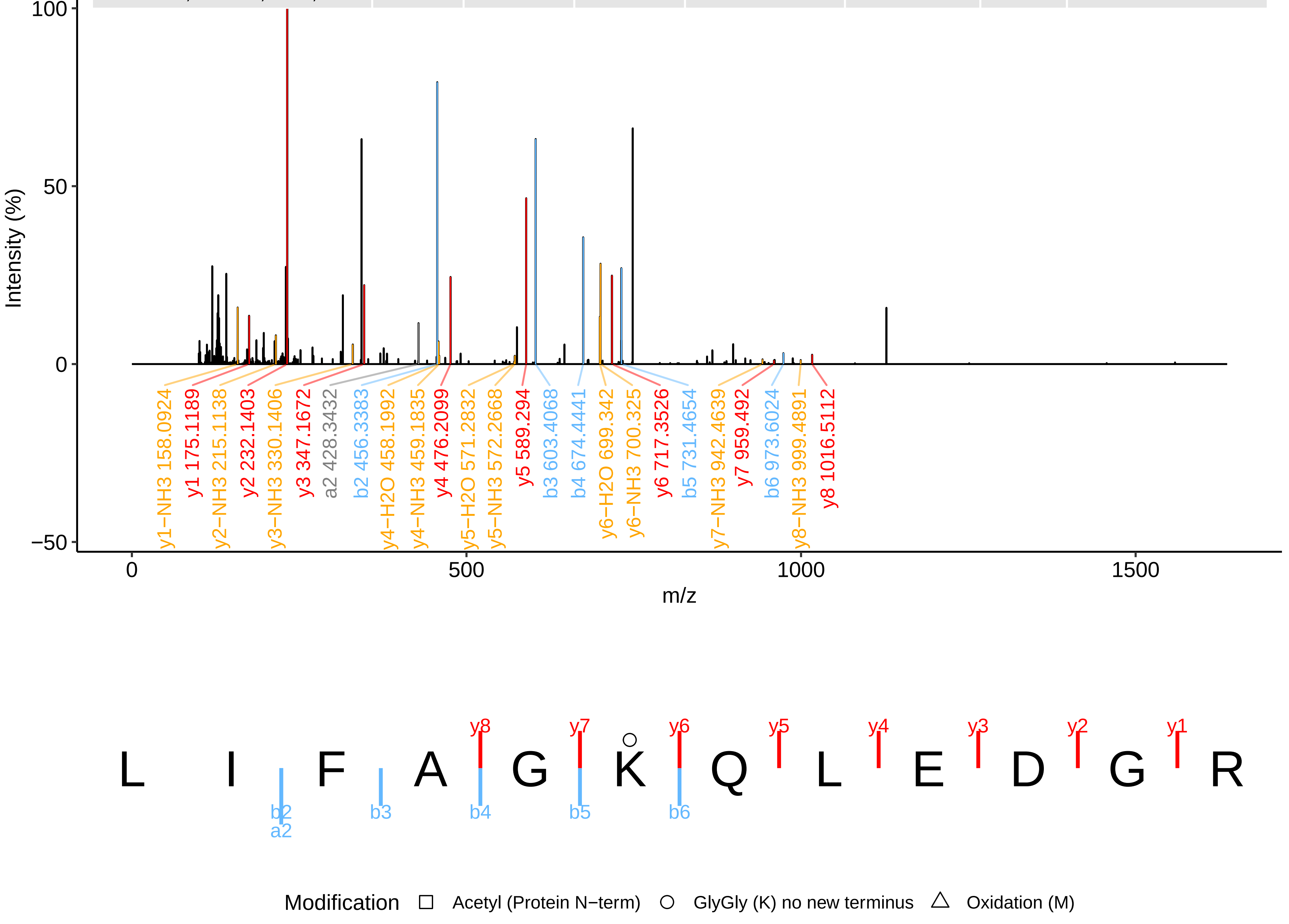

20200921_KIZ_MUb4_GlyGly_10plex − Scan number: 24269

Gene Names	Charge	m/z	Mass	Retention time	PEP	Score	Precursor Intensity
RPS27A;UBA52;UBC;UBB	3	487.6001	1459.778	88.197	3.0873e−06	112.83	13032787

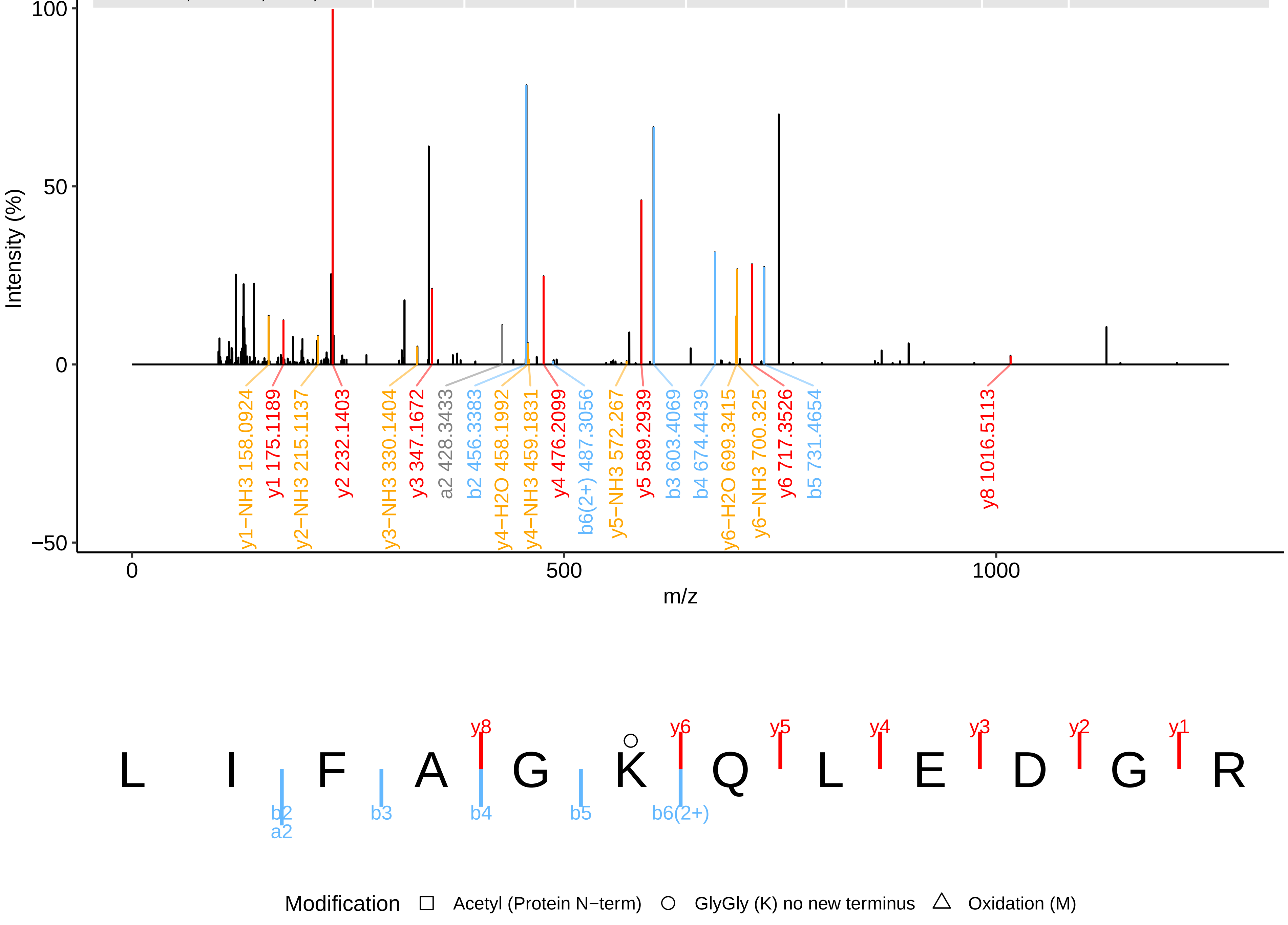

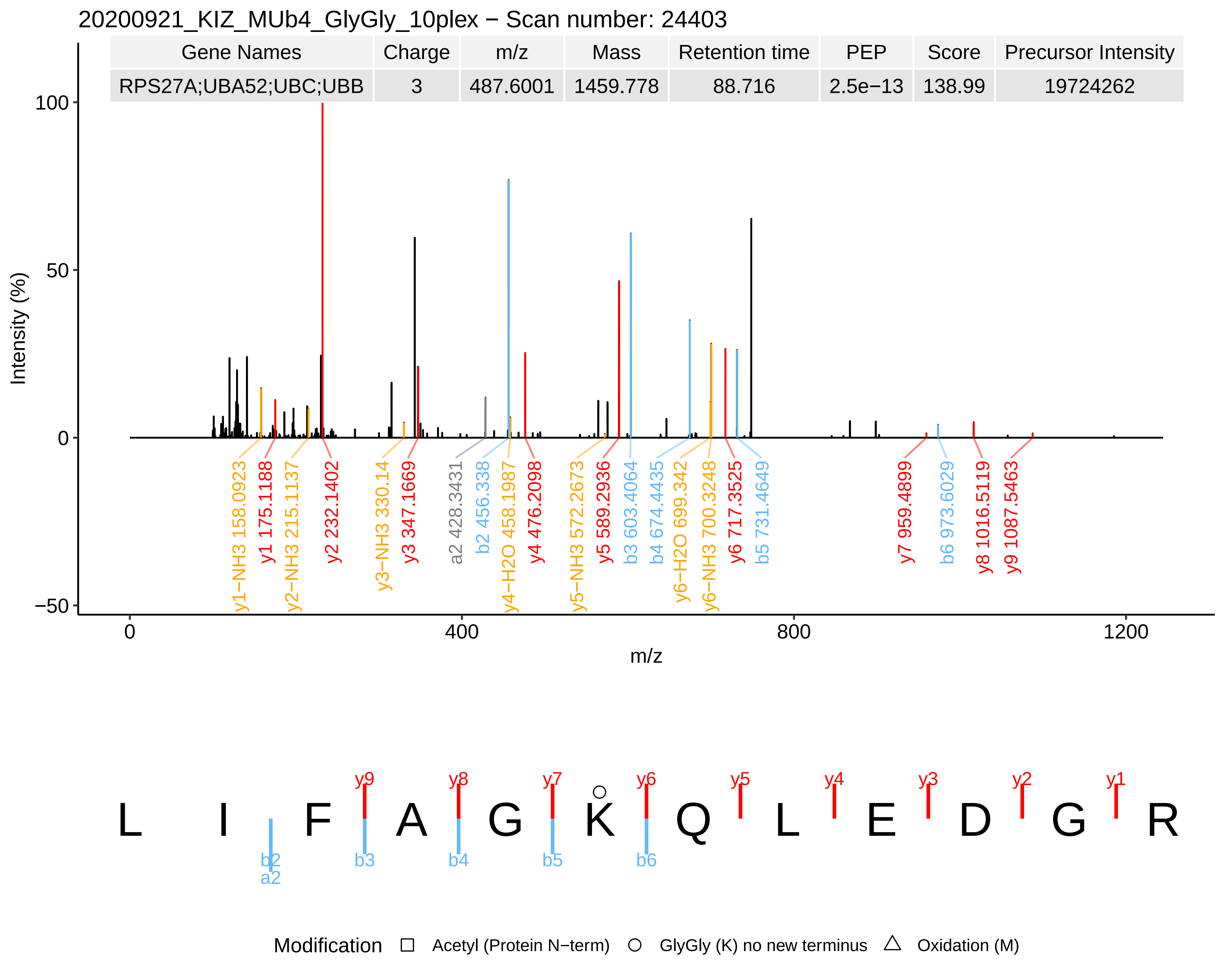

20200921_KIZ_MUb4_GlyGly_10plex − Scan number: 24403
Gene Names
Charge
m/z
Mass
Retention time
PEP
Score
Precursor Intensity
RPS27A;UBA52;UBC;UBB
3
487.6001
1459.778
88.716
2.5e−13
138.99
19724262
Intensity (%)
100
50
0
−50
0
400
800
1200
m/z
y1−NH3 158.0923
y1 175.1188
y2−NH3 215.1137
y2 232.1402
y3−NH3 330.14
y3 347.1669
a2 428.3431
b2 456.338
y4−H2O 458.1987
y4 476.2098
y5−NH3 572.2673
y5 589.2936
b3 603.4064
b4 674.4435
y6−H2O 699.342
y6−NH3 700.3248
y6 717.3525
b5 731.4649
y7 959.4899
b6 973.6029
y8 1016.5119
y9 1087.5463
y9 y8 y7 y6 y5 y4 y3 y2 y1
L I F A G K Q L E D G R
b2 b3 b4 b5 b6
a2
Modification
Acetyl (Protein N−term)
GlyGly (K) no new terminus
Oxidation (M)

20200921_KIZ_MUb4_GlyGly_10plex − Scan number: 24805

Gene Names	Charge	m/z	Mass	Retention time	PEP	Score	Precursor Intensity
RPS27A;UBA52;UBC;UBB	3	487.6001	1459.778	90.238	3.6333e−13	133.89	6859244

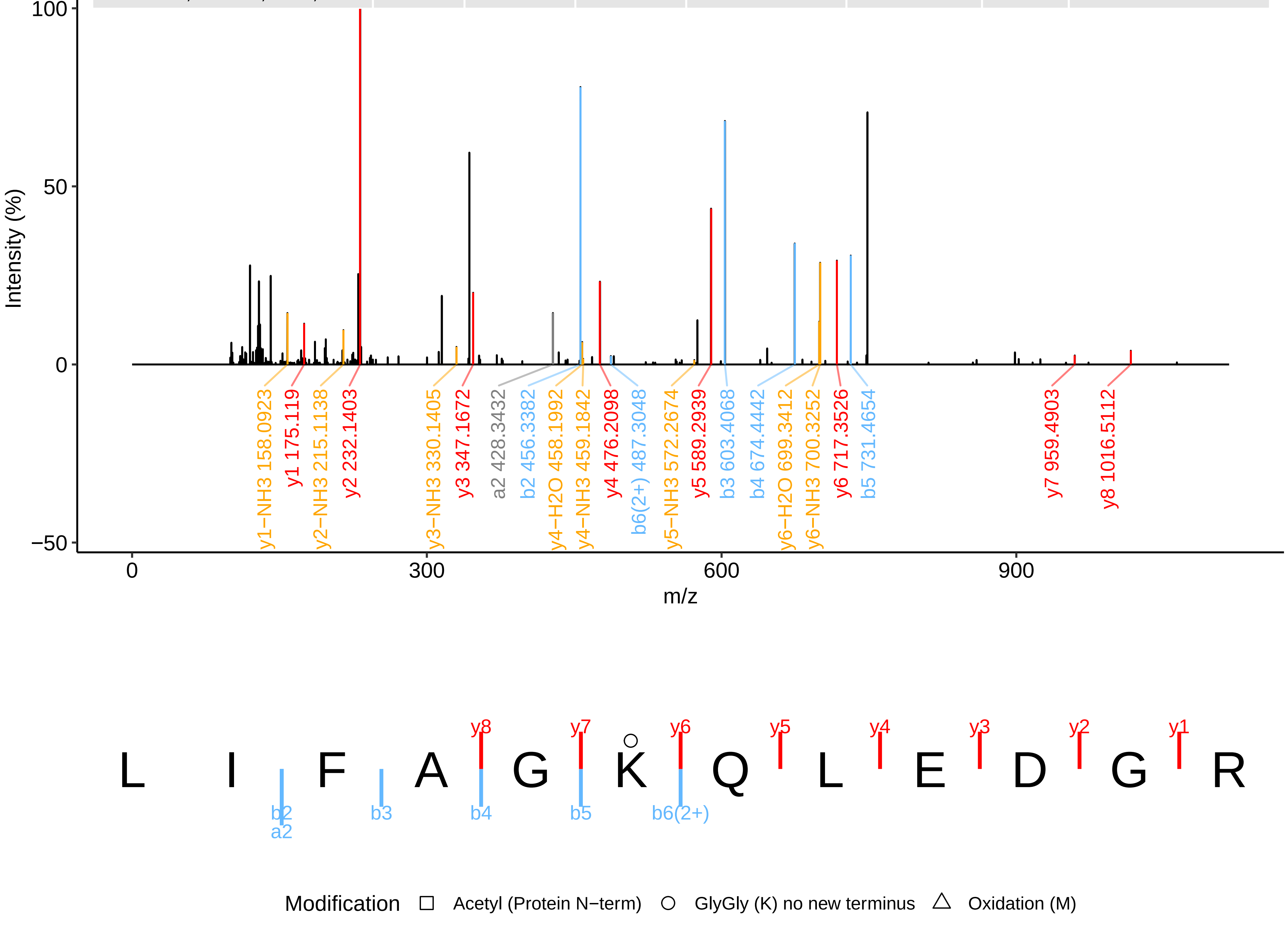

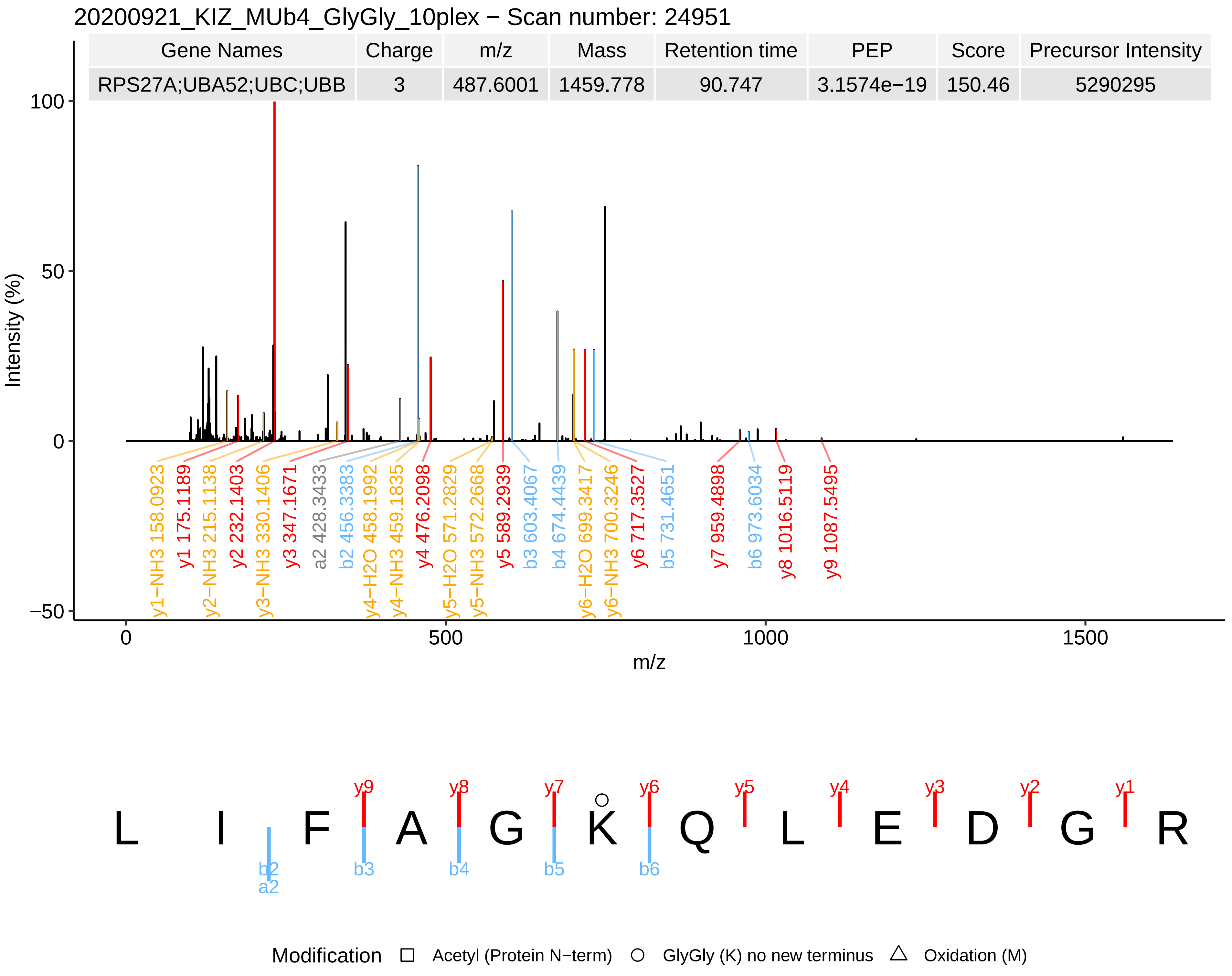
20200921_KIZ_MUb4_GlyGly_10plex − Scan number: 24951
Gene Names
Charge
m/z
Mass
Retention time
PEP
Score
Precursor Intensity
RPS27A;UBA52;UBC;UBB
3
487.6001
1459.778
90.747
3.1574e−19
150.46
5290295
Intensity (%)
100
50
0
−50
0
500
1000
1500
m/z
y1−NH3 158.0923
y1 175.1189
y2−NH3 215.1138
y2 232.1403
y3−NH3 330.1406
y3 347.1671
a2 428.3433
b2 456.3383
y4−H2O 458.1992
y4−NH3 459.1835
y4 476.2098
y5−H2O 571.2829
y5−NH3 572.2668
y5 589.2939
b3 603.4067
b4 674.4439
y6−H2O 699.3417
y6−NH3 700.3246
y6 717.3527
b5 731.4651
y7 959.4898
b6 973.6034
y8 1016.5119
y9 1087.5495
L I F A G K Q L E D G R
y9 y8 y7 y6 y5 y4 y3 y2 y1
b2 a2 b3 b4 b5 b6
Modification
Acetyl (Protein N−term)
GlyGly (K) no new terminus
Oxidation (M)

20200921_KIZ_MUb4_GlyGly_10plex − Scan number: 25089

Gene Names	Charge	m/z	Mass	Retention time	PEP	Score	Precursor Intensity
RPS27A;UBA52;UBC;UBB	3	487.6001	1459.778	91.255	3.1574e−19	150.46	15985397

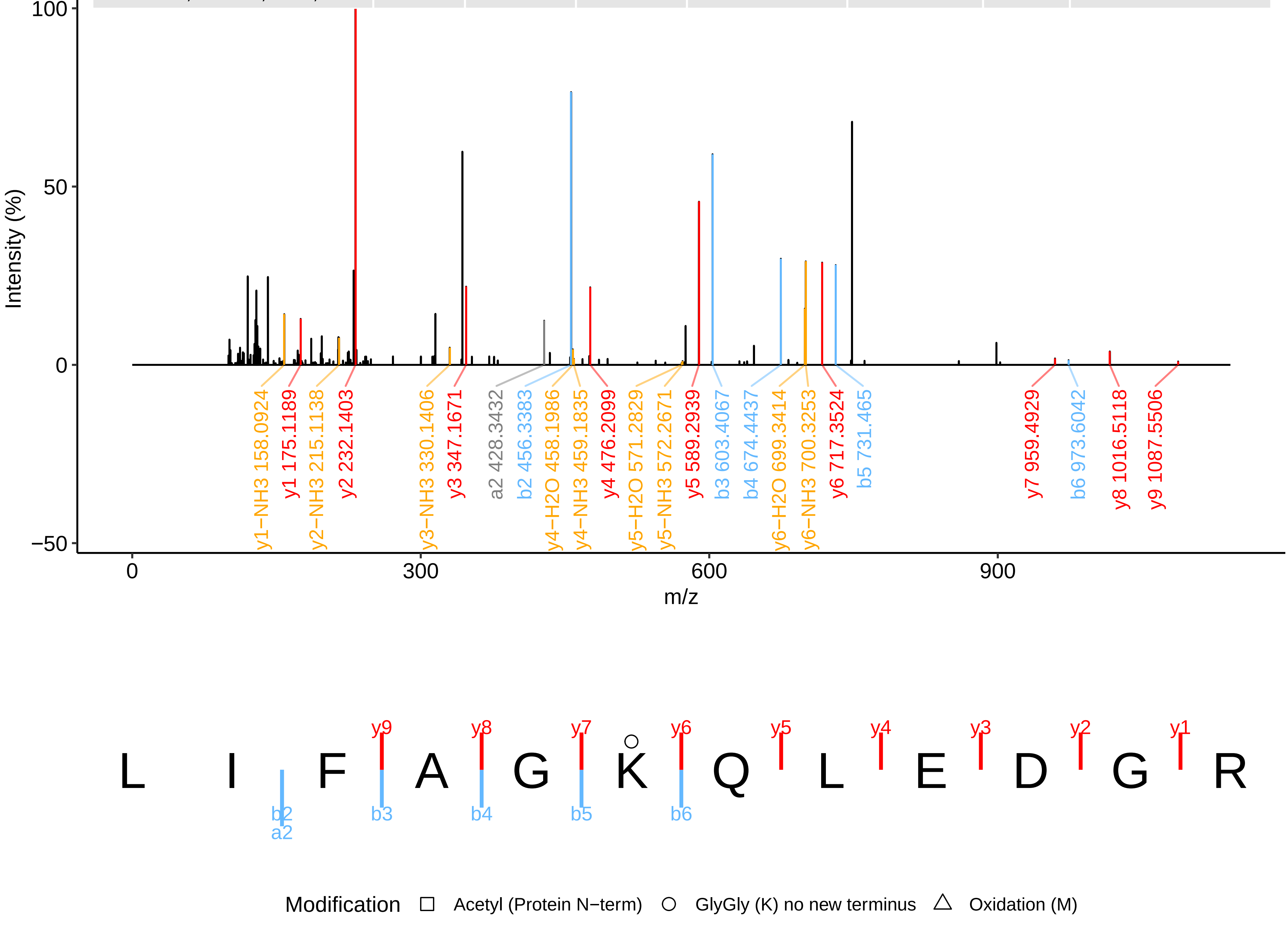

20200921_KIZ_MUb4_GlyGly_10plex − Scan number: 25227

Gene Names	Charge	m/z	Mass	Retention time	PEP	Score	Precursor Intensity
RPS27A;UBA52;UBC;UBB	3	487.6001	1459.778	91.764	1.2955e−08	117.09	5057215

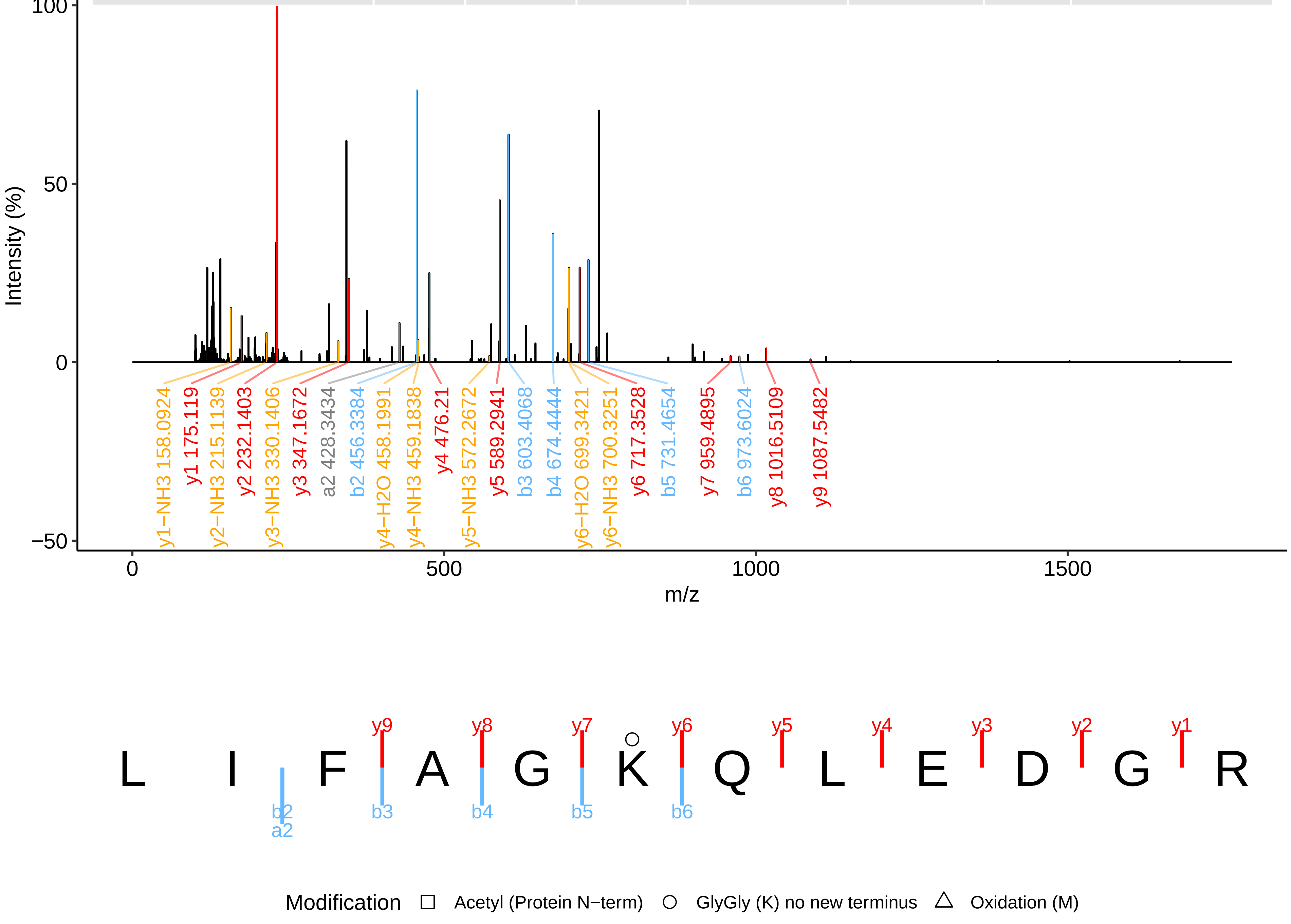

20200921_KIZ_MUb4_GlyGly_10plex − Scan number: 25500

Gene Names	Charge	m/z	Mass	Retention time	PEP	Score	Precursor Intensity
RPS27A;UBA52;UBC;UBB	3	487.6001	1459.778	92.776	2.5e−13	138.99	12473393

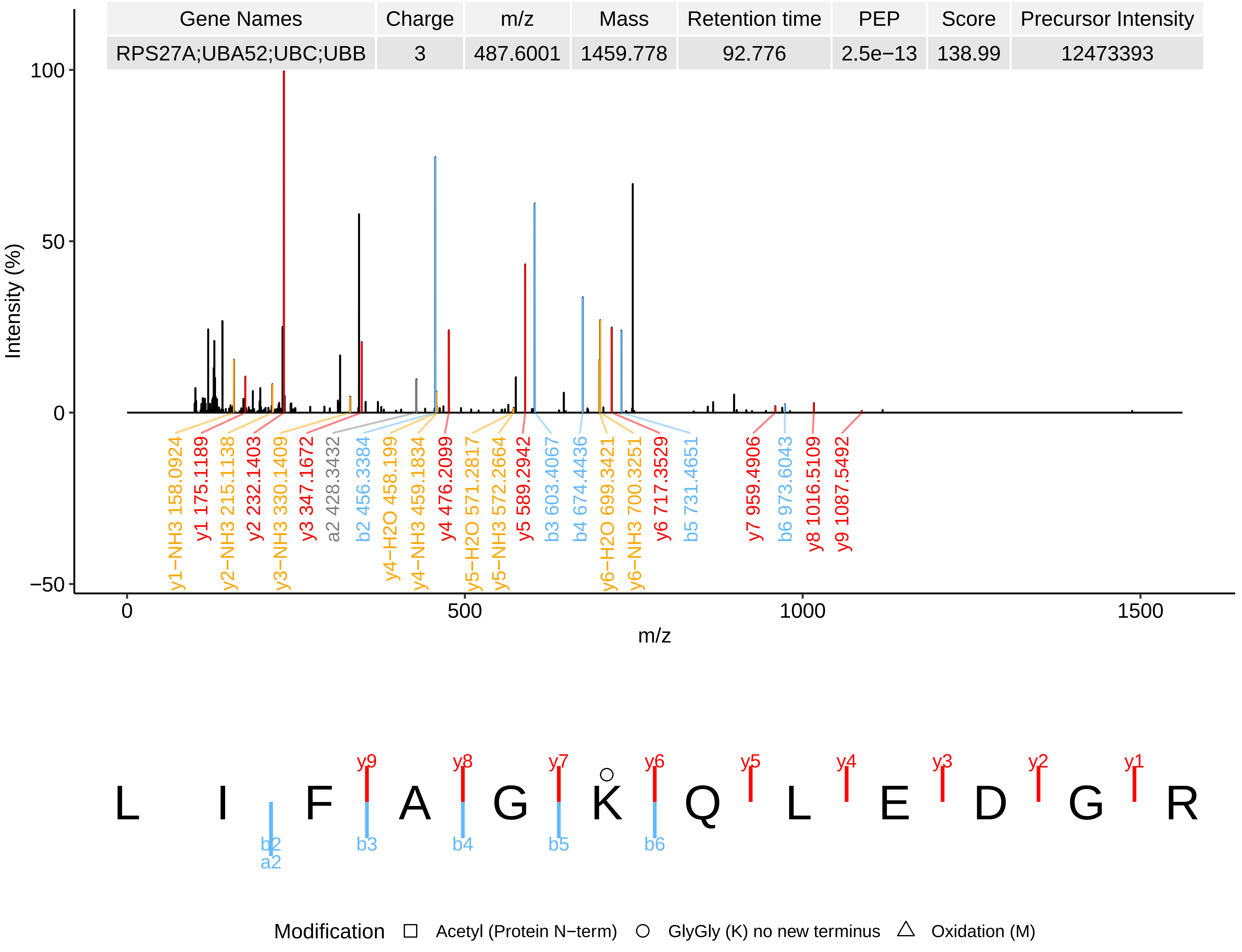

20200921_KIZ_MUb4_GlyGly_10plex − Scan number: 25636

Gene Names	Charge	m/z	Mass	Retention time	PEP	Score	Precursor Intensity
RPS27A;UBA52;UBC;UBB	3	487.6001	1459.778	93.285	9.6101e−14	145.91	8633438

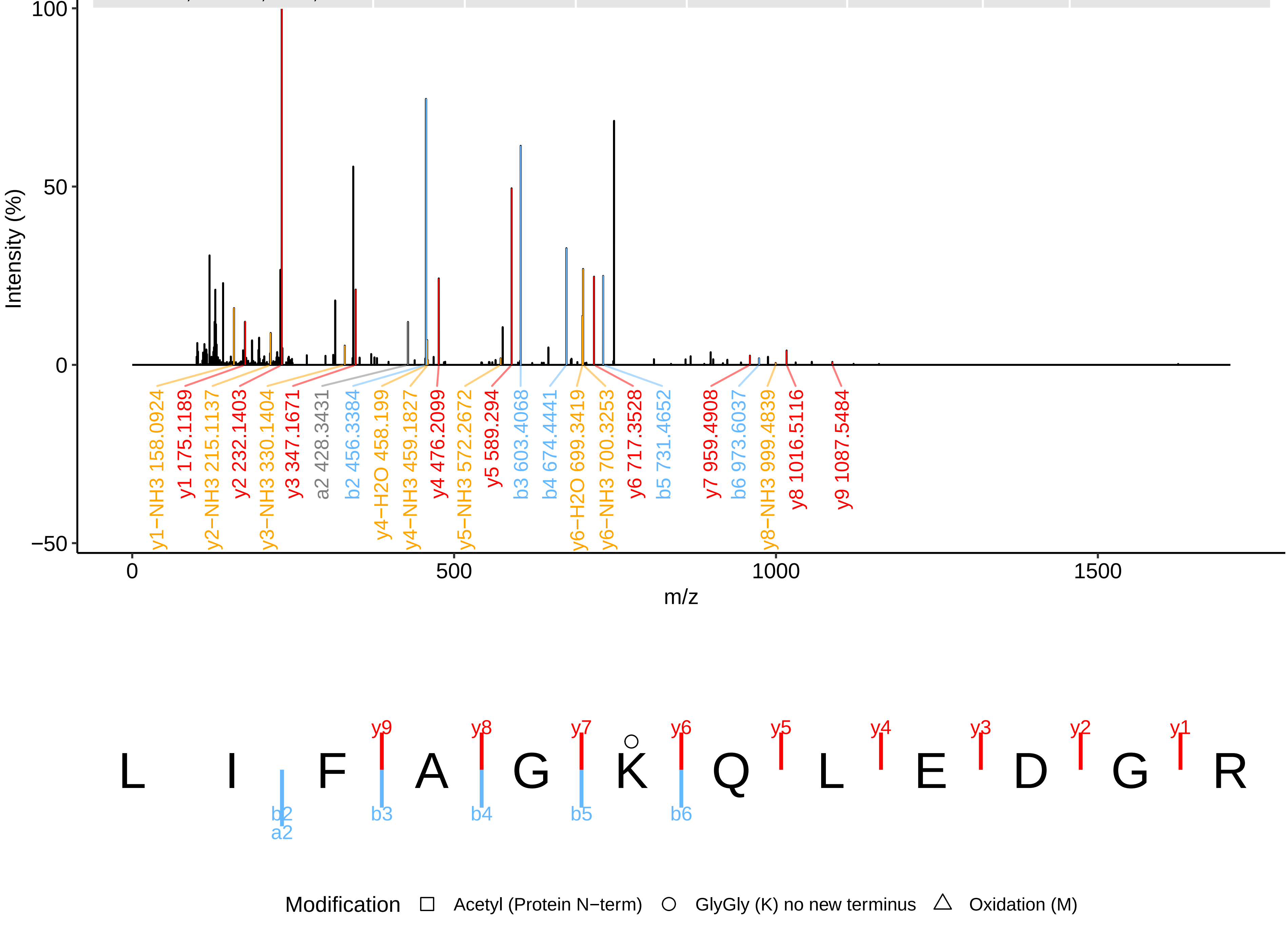

20200921_KIZ_MUb4_GlyGly_10plex − Scan number: 25769

Gene Names	Charge	m/z	Mass	Retention time	PEP	Score	Precursor Intensity
RPS27A;UBA52;UBC;UBB	3	487.6001	1459.778	93.799	2.0348e−13	141.08	11556150

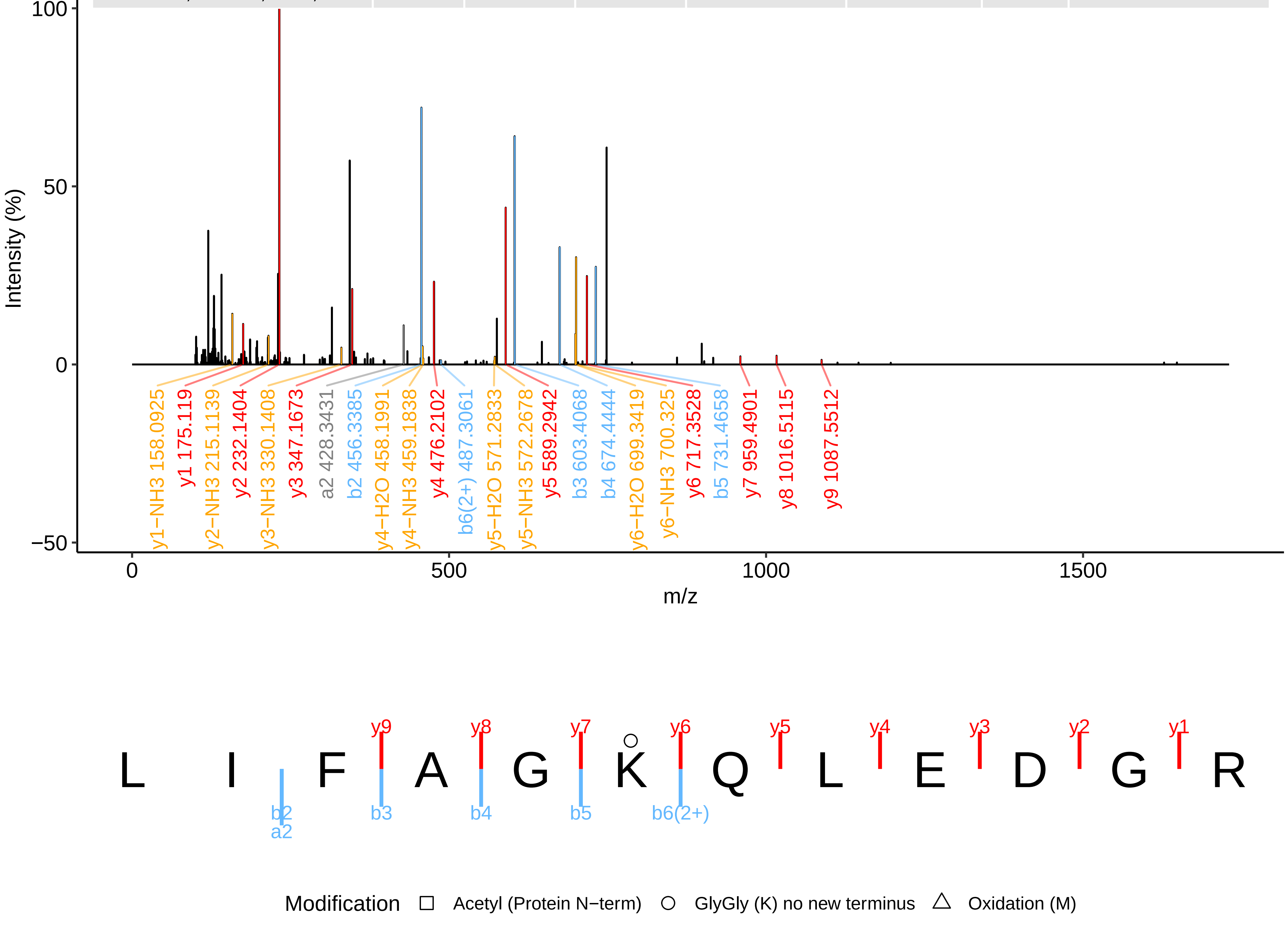

20200921_KIZ_MUb4_GlyGly_10plex − Scan number: 26042

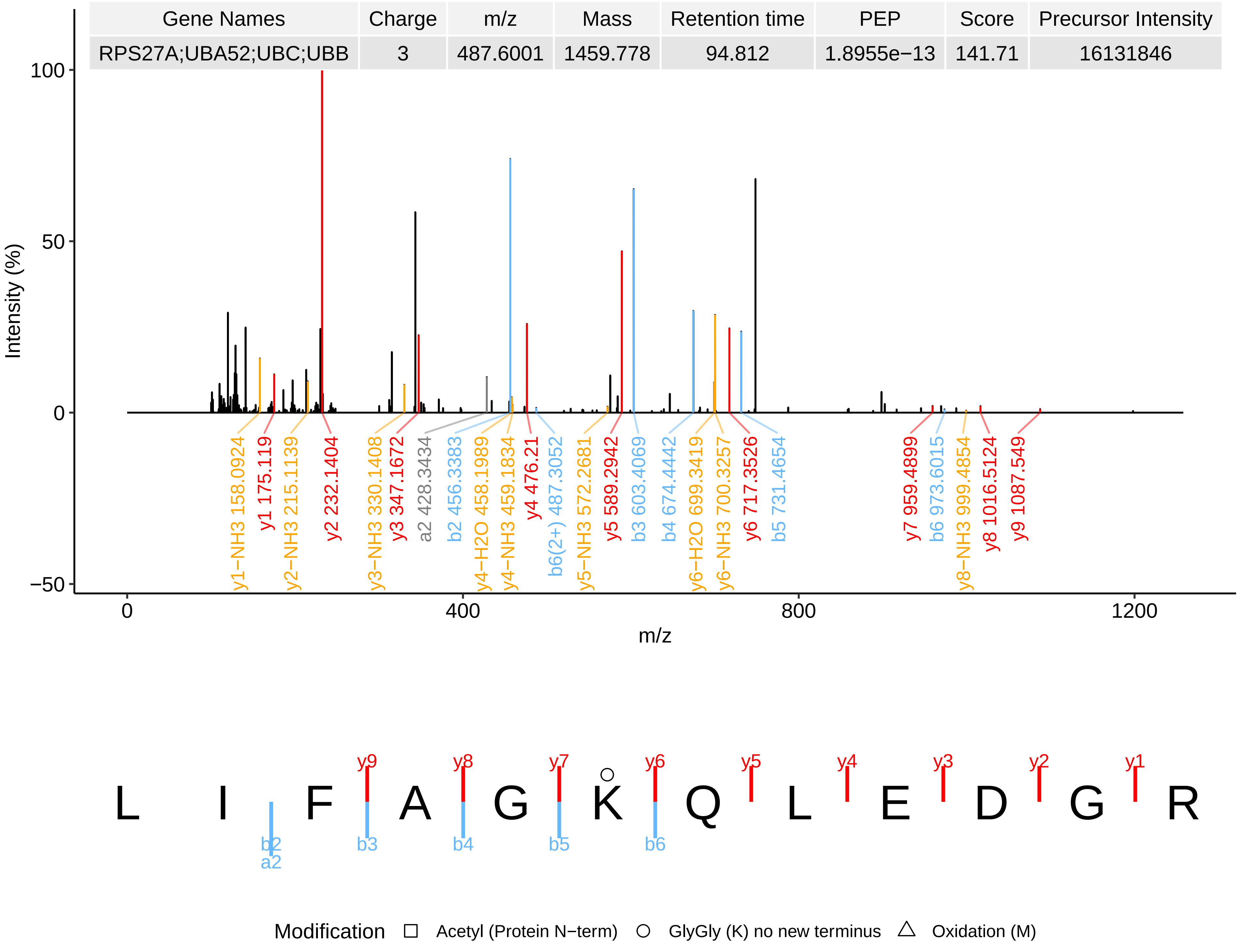

20200921_KIZ_MUb4_GlyGly_10plex − Scan number: 26179

Gene Names	Charge	m/z	Mass	Retention time	PEP	Score	Precursor Intensity
RPS27A;UBA52;UBC;UBB	3	487.6001	1459.778	95.328	3.5418e−13	134.3	8598458

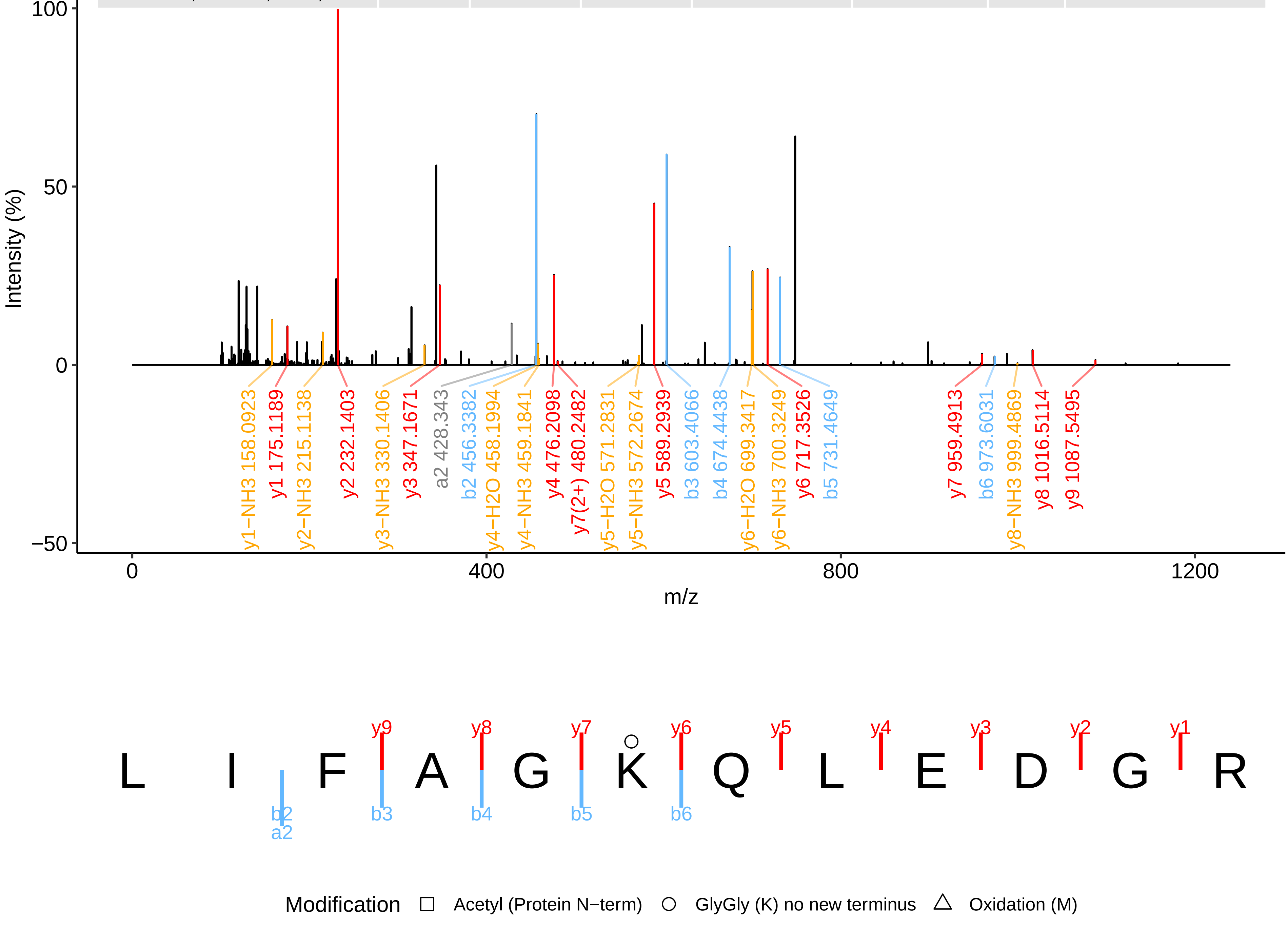

20200921_KIZ_MUb4_GlyGly_10plex − Scan number: 26312

Gene Names	Charge	m/z	Mass	Retention time	PEP	Score	Precursor Intensity
RPS27A;UBA52;UBC;UBB	3	487.6001	1459.778	95.841	3.0537e−13	136.5	6731764

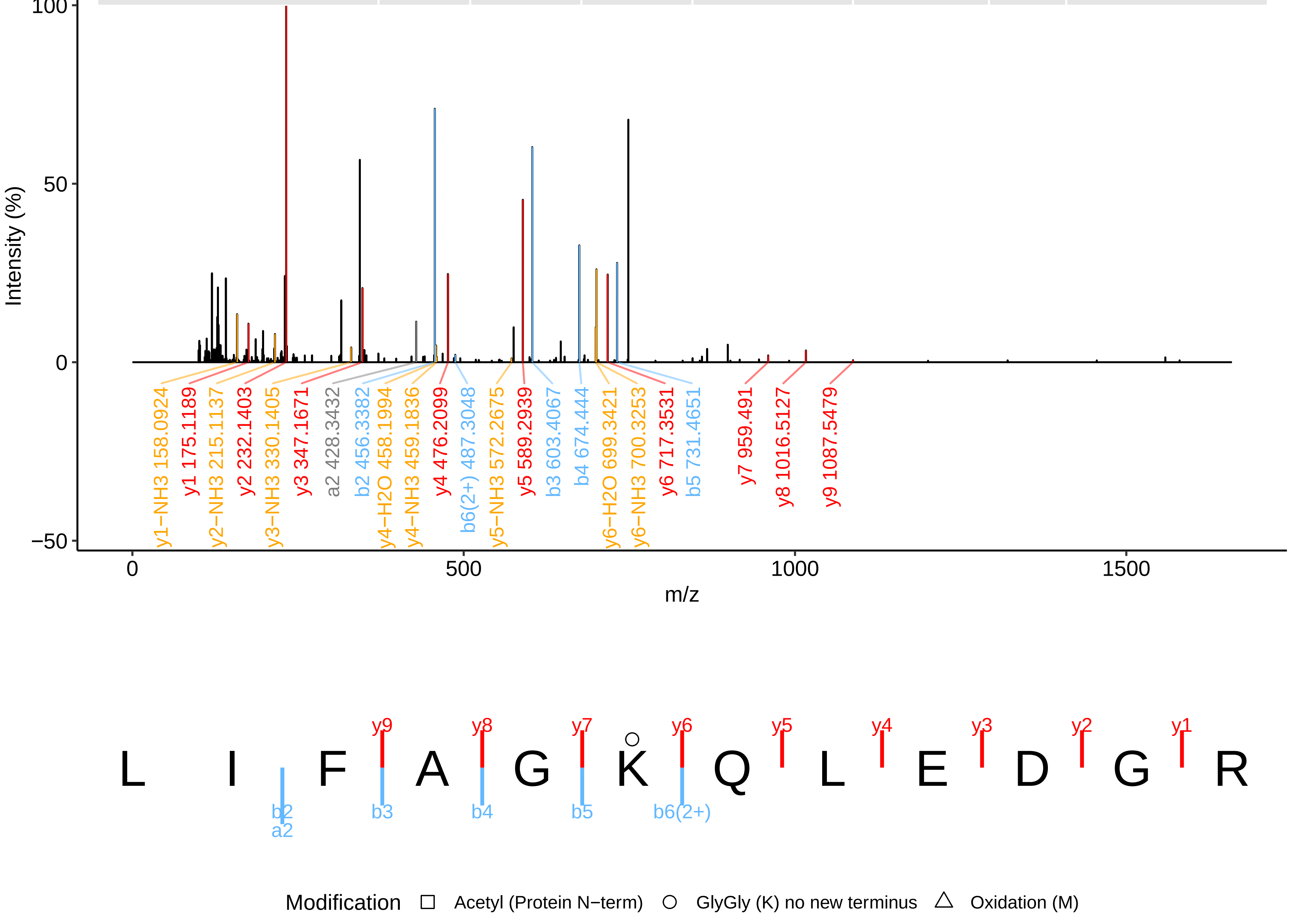

20200921_KIZ_MUb4_GlyGly_10plex − Scan number: 26443

Gene Names	Charge	m/z	Mass	Retention time	PEP	Score	Precursor Intensity
RPS27A;UBA52;UBC;UBB	3	487.6001	1459.778	96.352	5.8126e−14	147.62	8831504

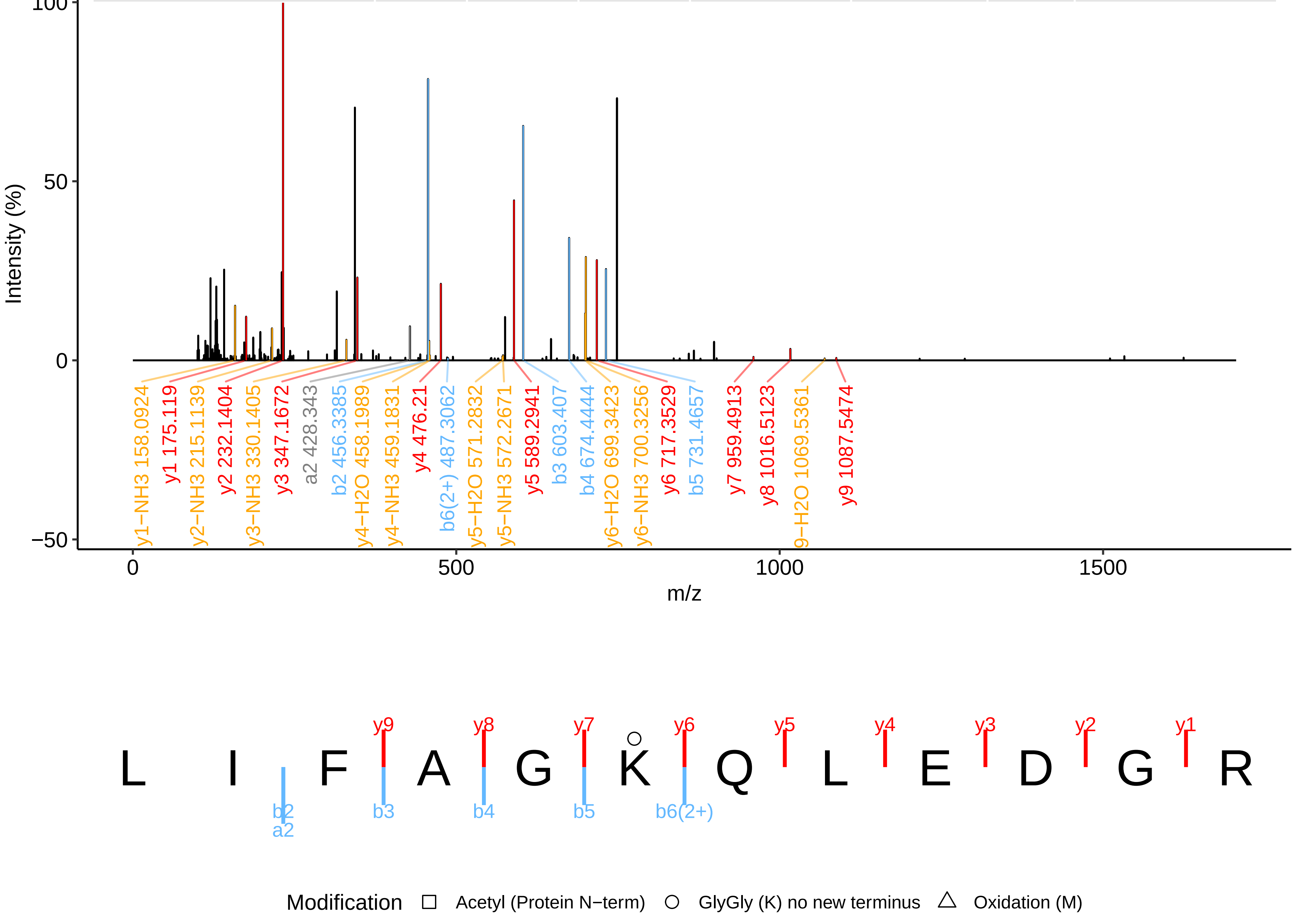

20200921_KIZ_MUb4_GlyGly_10plex − Scan number: 26580

Gene Names	Charge	m/z	Mass	Retention time	PEP	Score	Precursor Intensity
RPS27A;UBA52;UBC;UBB	3	487.6001	1459.778	96.862	9.899e−20	162.26	8787524

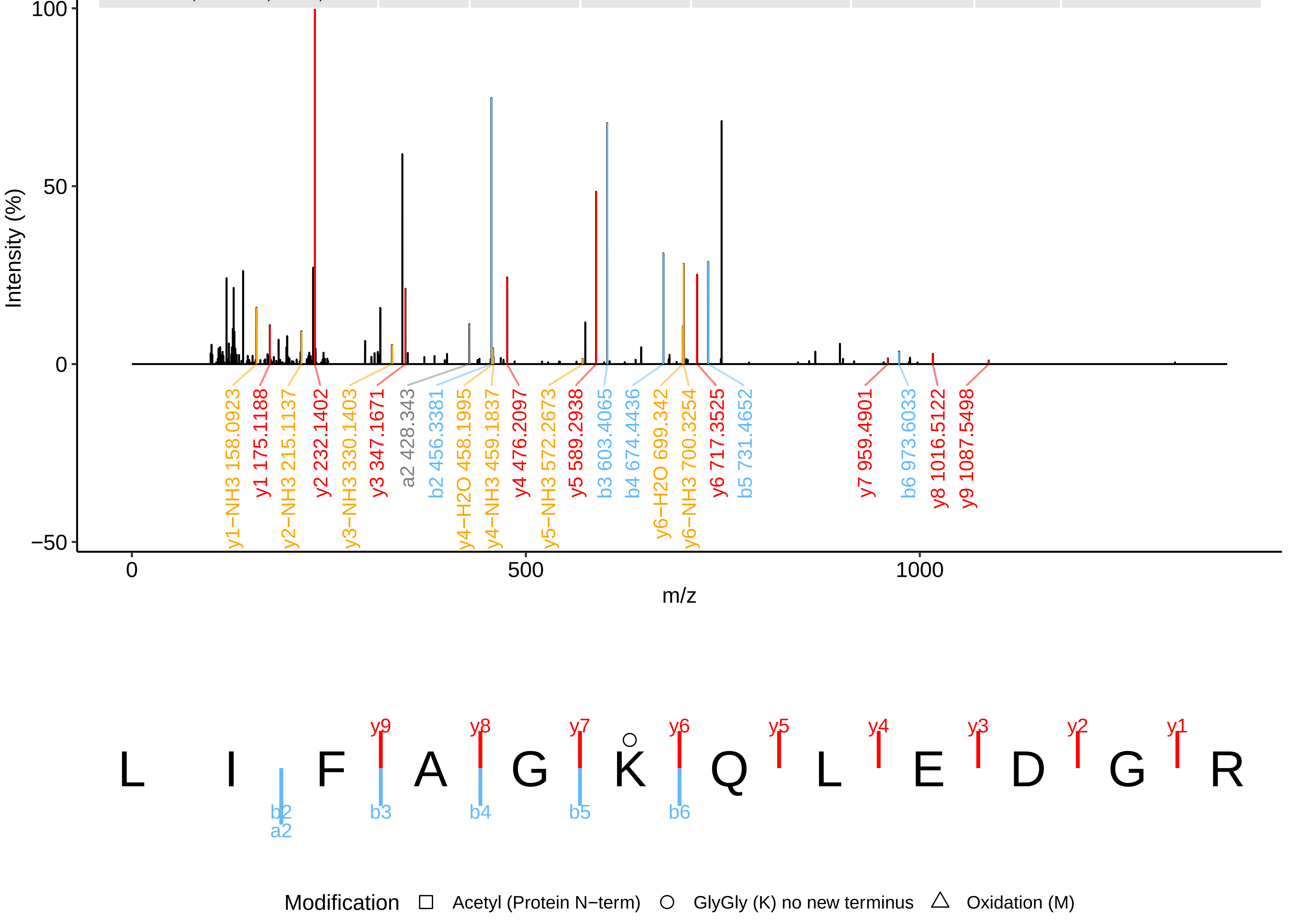

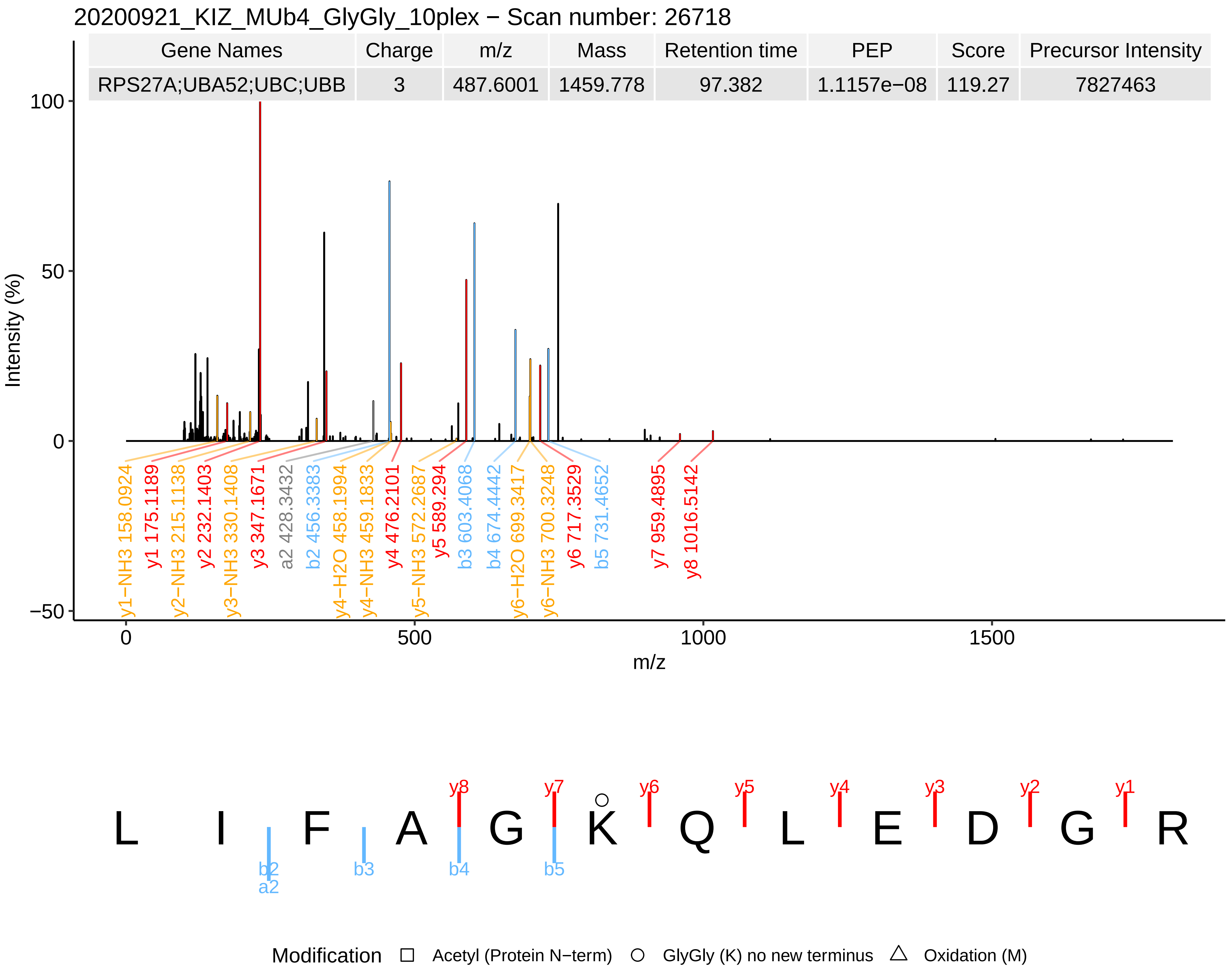

20200921_KIZ_MUb4_GlyGly_10plex − Scan number: 26718
Gene Names | Charge | m/z | Mass | Retention time | PEP | Score | Precursor Intensity
RPS27A;UBA52;UBC;UBB | 3 | 487.6001 | 1459.778 | 97.382 | 1.1157e−08 | 119.27 | 7827463
Intensity (%)
m/z
y1−NH3 158.0924
y1 175.1189
y2−NH3 215.1138
y2 232.1403
y3−NH3 330.1408
y3 347.1671
a2 428.3432
b2 456.3383
y4−H2O 458.1994
y4−NH3 459.1833
y4 476.2101
y5−NH3 572.2687
y5 589.294
b3 603.4068
b4 674.4442
y6−H2O 699.3417
y6−NH3 700.3248
y6 717.3529
b5 731.4652
y7 959.4895
y8 1016.5142
L I F A G K Q L E D G R
Modification
Acetyl (Protein N−term)
GlyGly (K) no new terminus
Oxidation (M)

20200921_KIZ_MUb4_GlyGly_10plex − Scan number: 26987

Gene Names	Charge	m/z	Mass	Retention time	PEP	Score	Precursor Intensity
RPS27A;UBA52;UBC;UBB	3	487.6001	1459.778	98.414	4.8999e−26	168.22	7595501

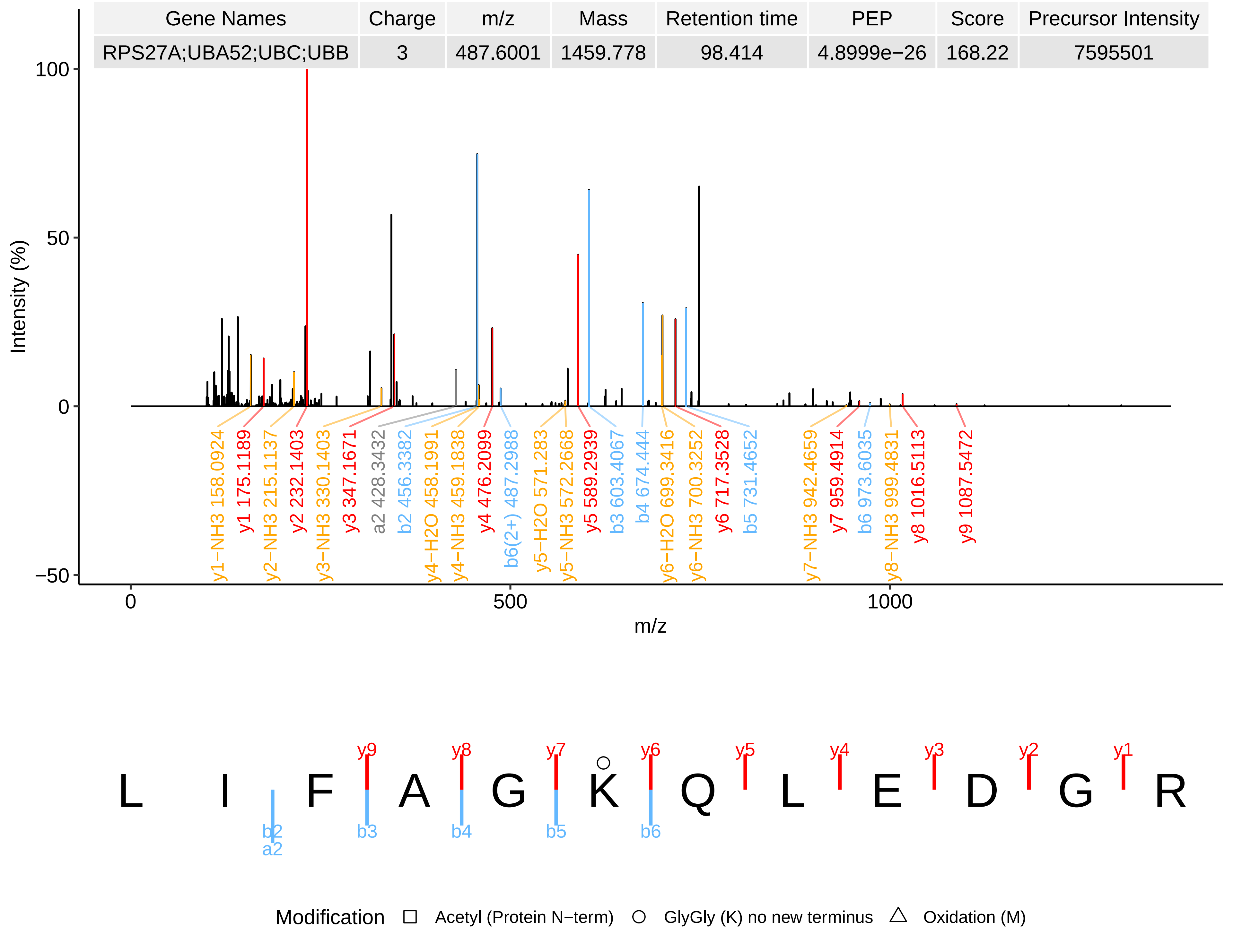

20200921_KIZ_MUb4_GlyGly_10plex − Scan number: 27387

Gene Names	Charge	m/z	Mass	Retention time	PEP	Score	Precursor Intensity
RPS27A;UBA52;UBC;UBB	3	487.6001	1459.778	99.951	3.1574e−19	150.46	8819615

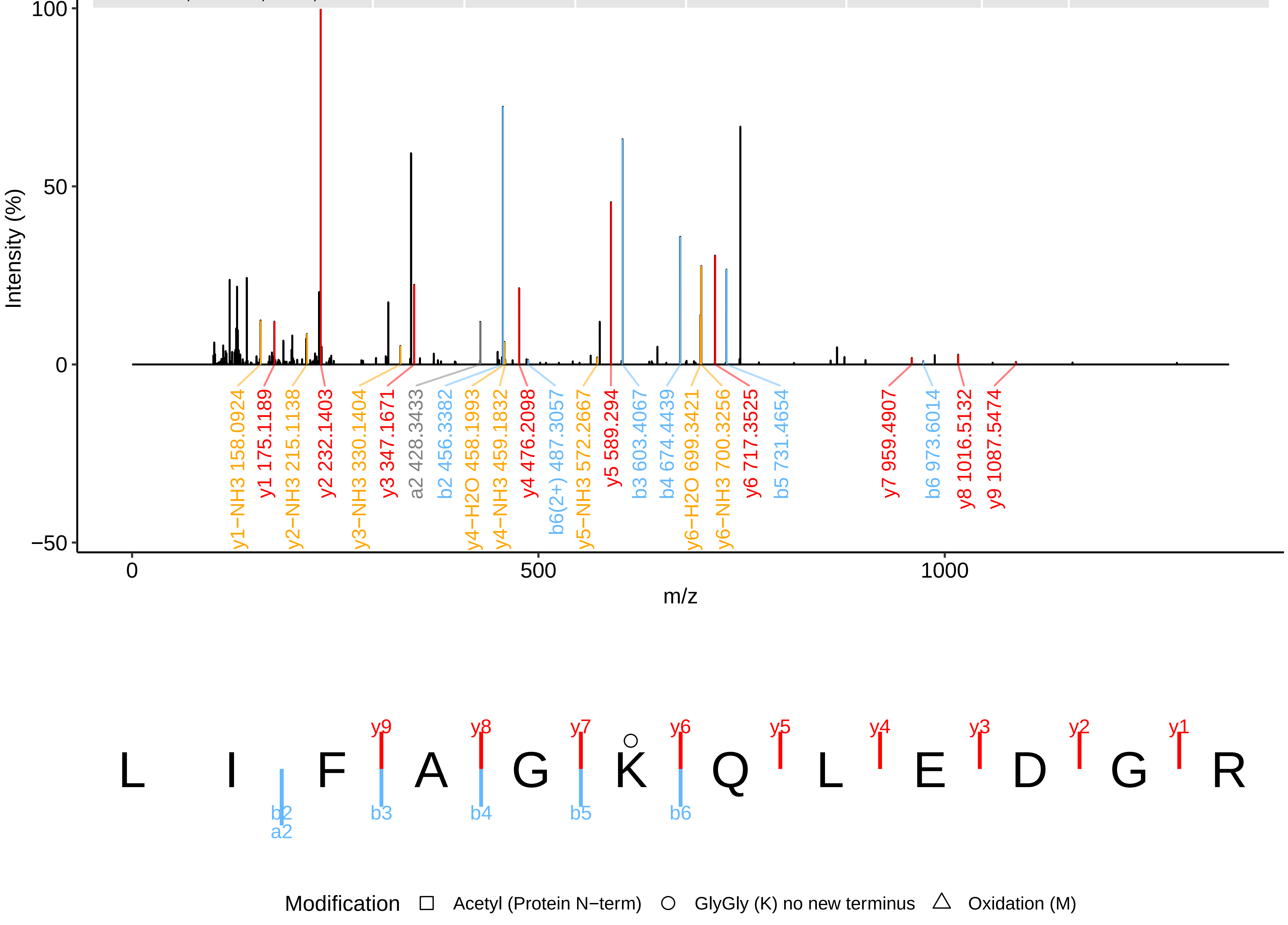

20200921_KIZ_MUb4_GlyGly_10plex − Scan number: 27522

Gene Names	Charge	m/z	Mass	Retention time	PEP	Score	Precursor Intensity
RPS27A;UBA52;UBC;UBB	3	487.6001	1459.778	100.47	4.3728e−09	127.51	8320944

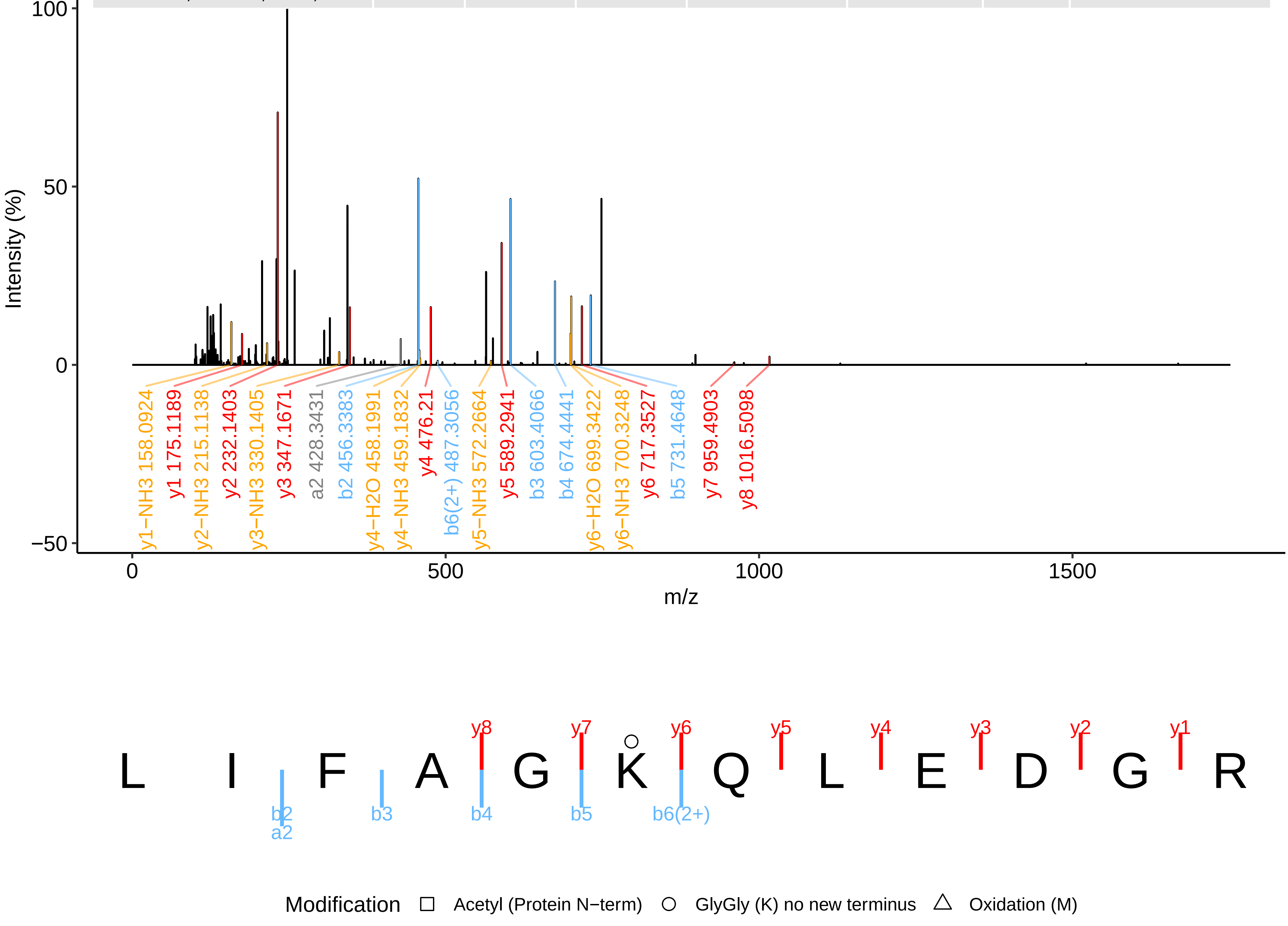

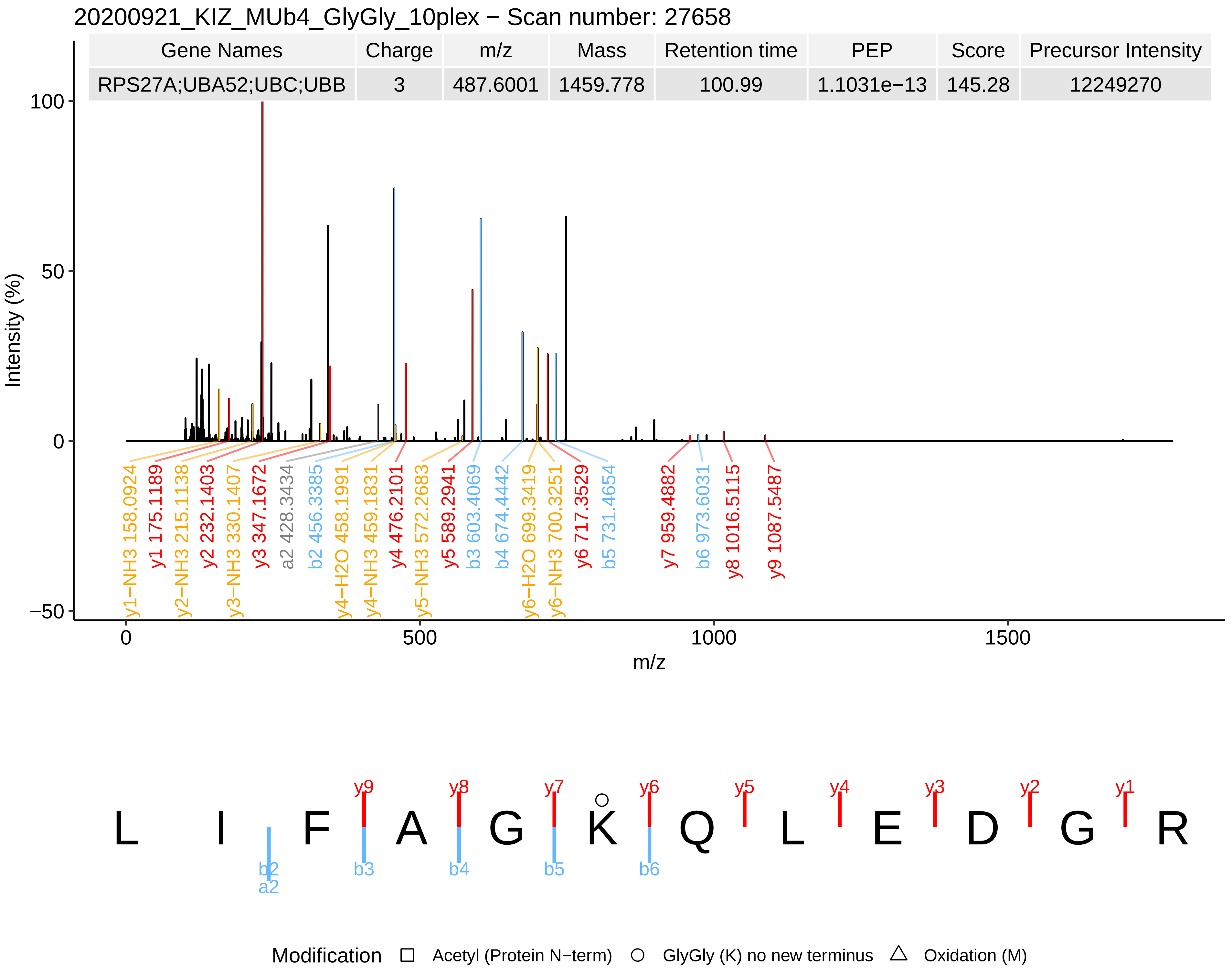
20200921_KIZ_MUb4_GlyGly_10plex − Scan number: 27658
Gene Names
Charge
m/z
Mass
Retention time
PEP
Score
Precursor Intensity
RPS27A;UBA52;UBC;UBB
3
487.6001
1459.778
100.99
1.1031e−13
145.28
12249270
Intensity (%)
100
50
0
−50
y1−NH3 158.0924
y1 175.1189
y2−NH3 215.1138
y2 232.1403
y3−NH3 330.1407
y3 347.1672
a2 428.3434
b2 456.3385
y4−H2O 458.1991
y4−NH3 459.1831
y4 476.2101
y5−NH3 572.2683
y5 589.2941
b3 603.4069
b4 674.4442
y6−H2O 699.3419
y6−NH3 700.3251
y6 717.3529
b5 731.4654
y7 959.4882
b6 973.6031
y8 1016.5115
y9 1087.5487
0
500
1000
1500
m/z
L I F A G K Q L E D G R
y9 y8 y7 y6 y5 y4 y3 y2 y1
b2 a2 b3 b4 b5 b6
Modification
Acetyl (Protein N−term)
GlyGly (K) no new terminus
Oxidation (M)

20200921_KIZ_MUb4_GlyGly_10plex − Scan number: 27790

Gene Names	Charge	m/z	Mass	Retention time	PEP	Score	Precursor Intensity
RPS27A;UBA52;UBC;UBB	3	487.6001	1459.778	101.5	2.4754e−09	129.82	9375183

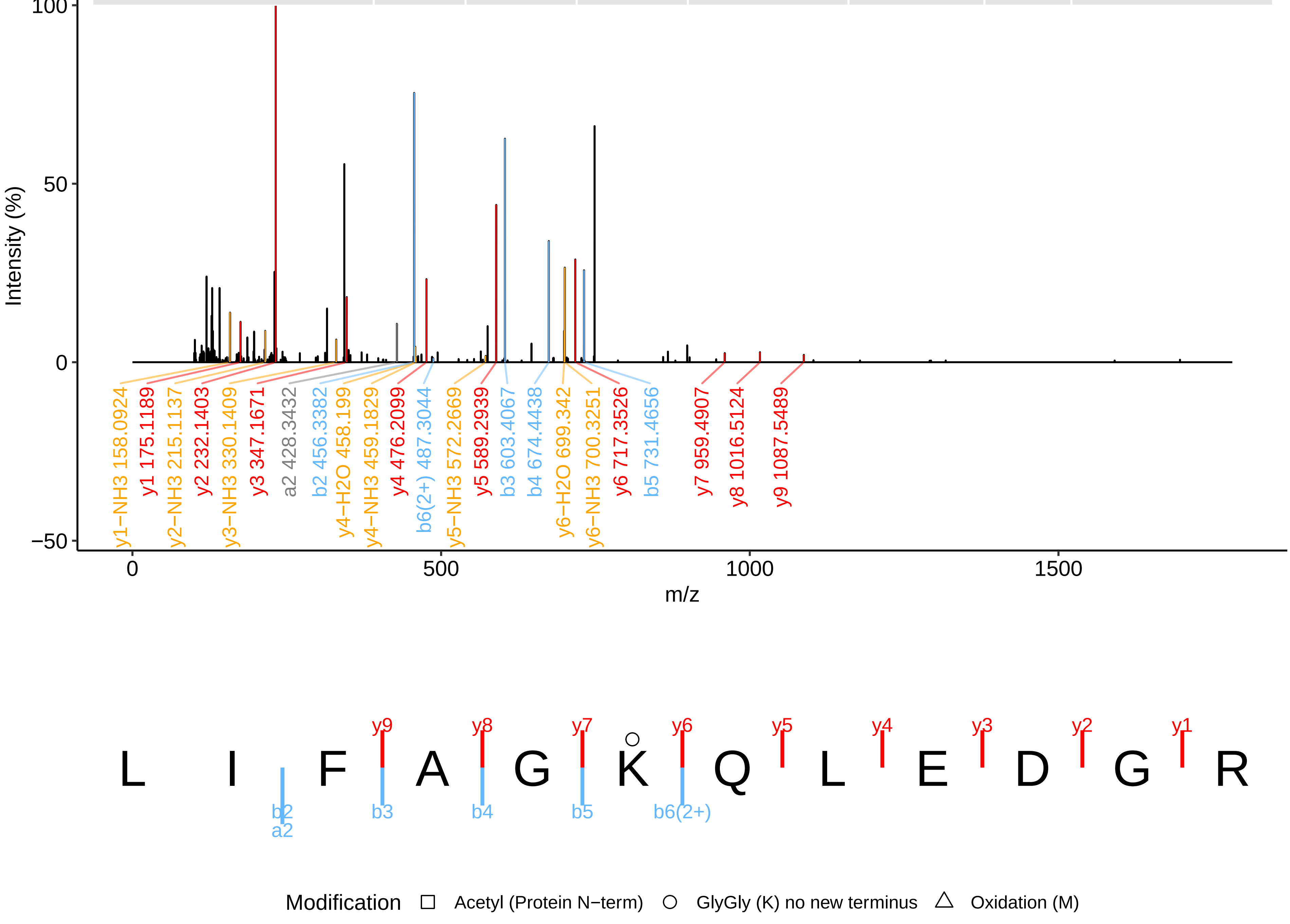

20200921_KIZ_MUb4_GlyGly_10plex − Scan number: 27915

Gene Names	Charge	m/z	Mass	Retention time	PEP	Score	Precursor Intensity
RPS27A;UBA52;UBC;UBB	3	487.6001	1459.778	102.01	2.7568e−19	152.64	8490841

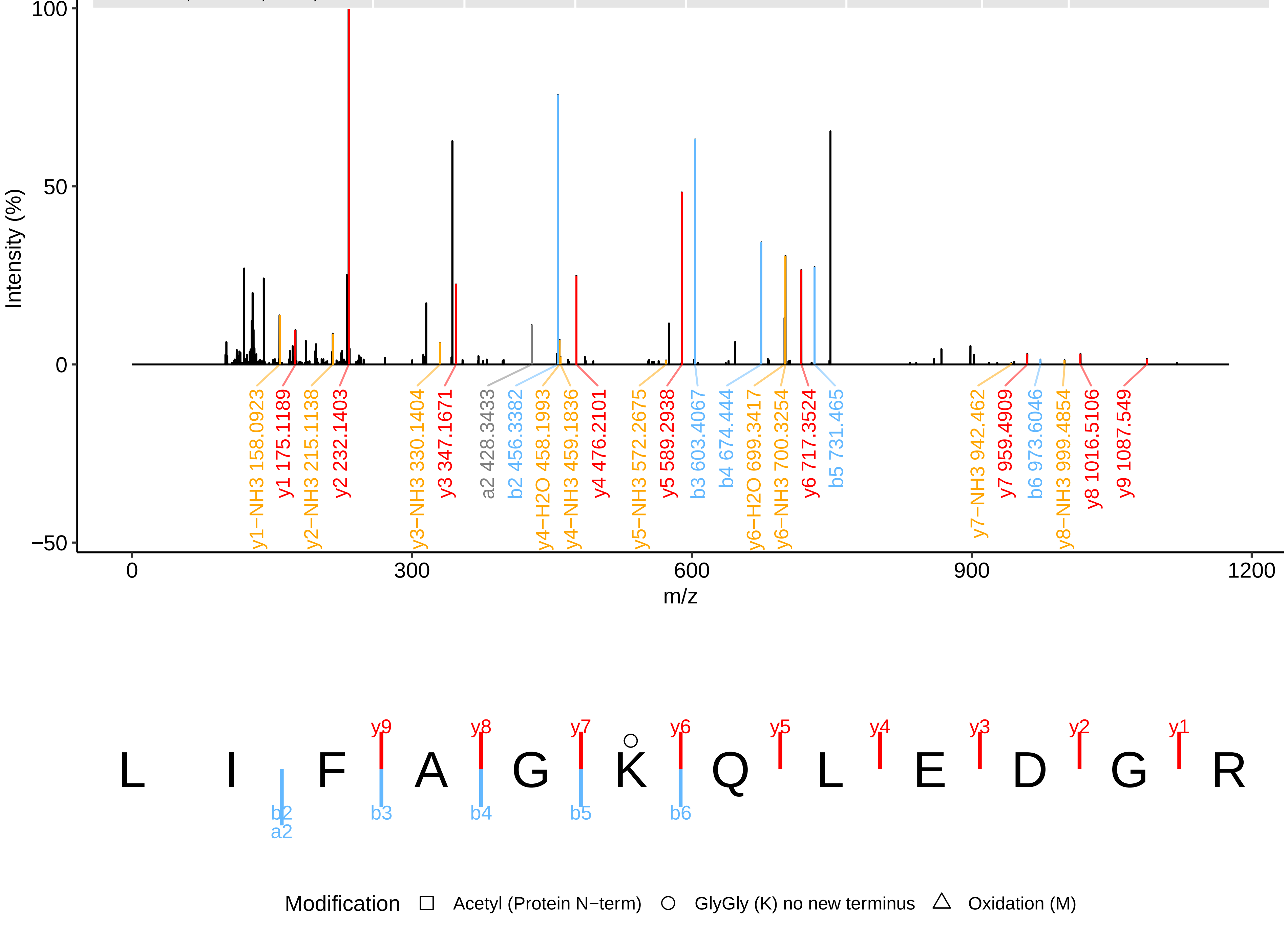

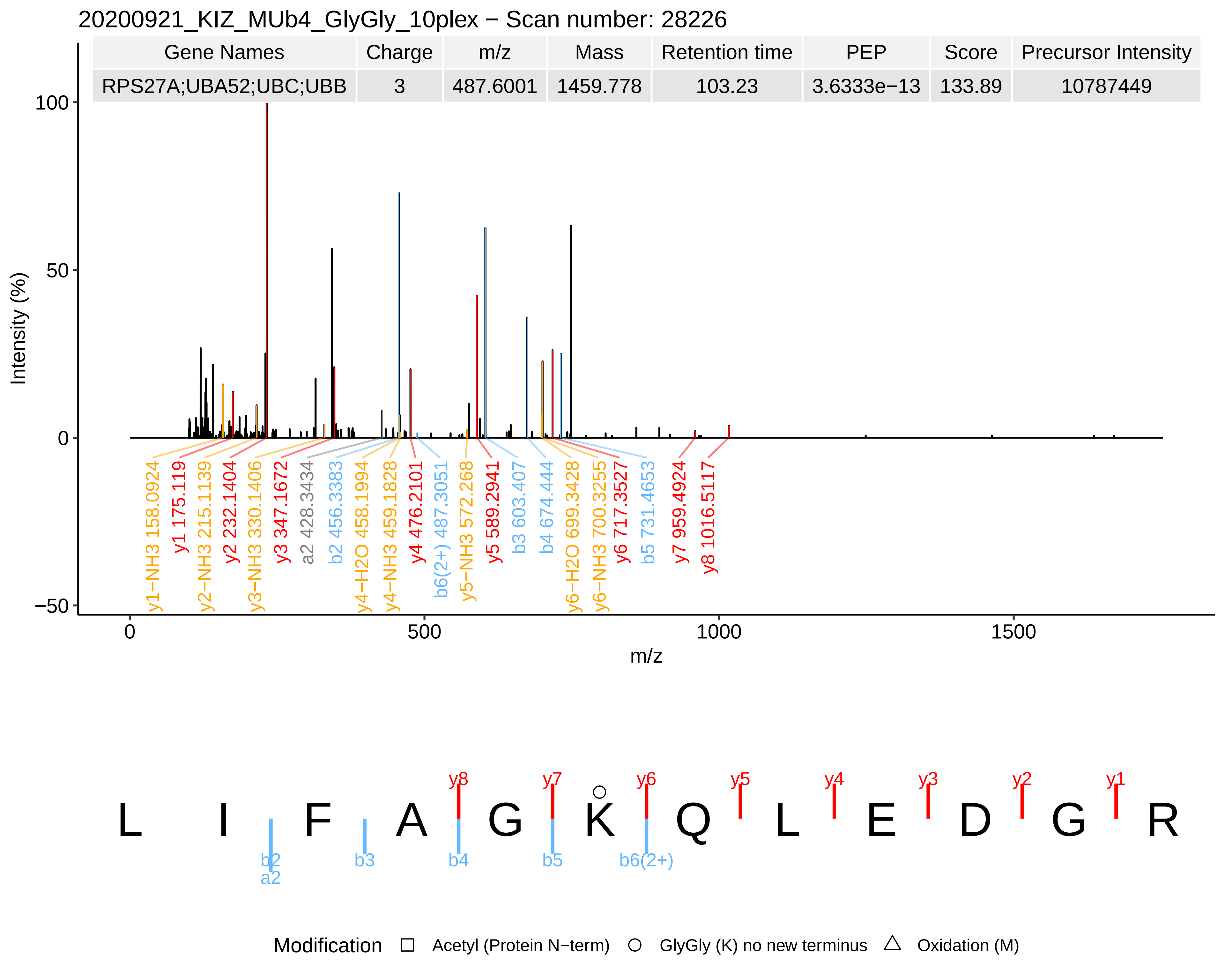

20200921_KIZ_MUb4_GlyGly_10plex − Scan number: 28226
Gene Names | Charge | m/z | Mass | Retention time | PEP | Score | Precursor Intensity
RPS27A;UBA52;UBC;UBB | 3 | 487.6001 | 1459.778 | 103.23 | 3.6333e−13 | 133.89 | 10787449
Intensity (%)
m/z
y1−NH3 158.0924
y1 175.119
y2−NH3 215.1139
y2 232.1404
y3−NH3 330.1406
y3 347.1672
a2 428.3434
b2 456.3383
y4−H2O 458.1994
y4−NH3 459.1828
y4 476.2101
b6(2+) 487.3051
y5−NH3 572.268
y5 589.2941
b3 603.407
b4 674.444
y6−H2O 699.3428
y6−NH3 700.3255
y6 717.3527
b5 731.4653
y7 959.4924
y8 1016.5117
L I F A G K Q L E D G R
y8 y7 y6 y5 y4 y3 y2 y1
b2 a2 b3 b4 b5 b6(2+)
Modification
Acetyl (Protein N−term)
GlyGly (K) no new terminus
Oxidation (M)

20200921_KIZ_MUb4_GlyGly_10plex − Scan number: 28358

Gene Names	Charge	m/z	Mass	Retention time	PEP	Score	Precursor Intensity
RPS27A;UBA52;UBC;UBB	3	487.6001	1459.778	103.74	8.2269e−10	131.83	6895078

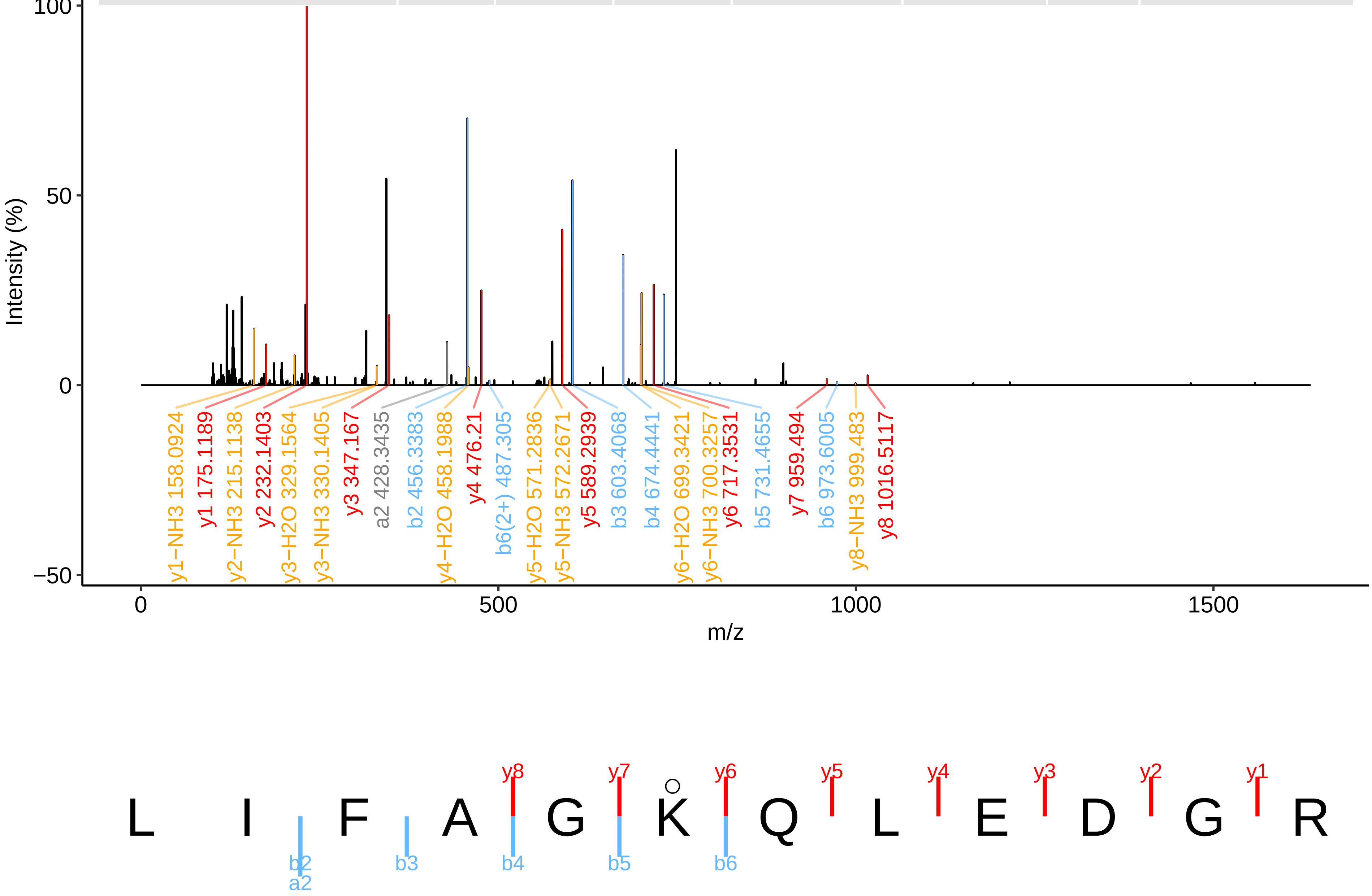

Modification □ Acetyl (Protein N−term) ○ GlyGly (K) no new terminus △ Oxidation (M)

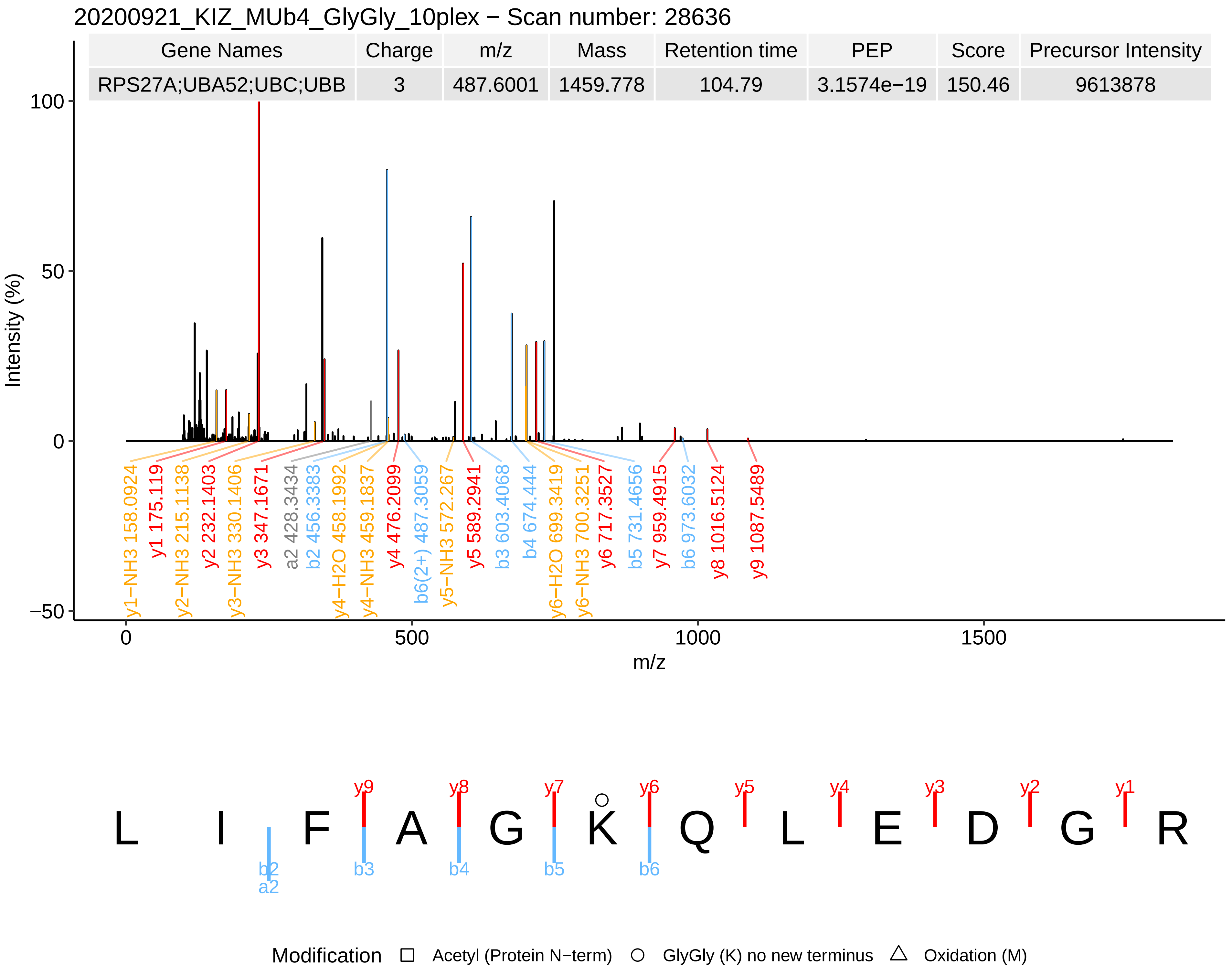
20200921_KIZ_MUb4_GlyGly_10plex − Scan number: 28636
Gene Names | Charge | m/z | Mass | Retention time | PEP | Score | Precursor Intensity
RPS27A;UBA52;UBC;UBB | 3 | 487.6001 | 1459.778 | 104.79 | 3.1574e−19 | 150.46 | 9613878
Intensity (%)
100
50
0
−50
0
500
1000
1500
m/z
y1−NH3 158.0924
y1 175.119
y2−NH3 215.1138
y2 232.1403
y3−NH3 330.1406
y3 347.1671
a2 428.3434
b2 456.3383
y4−H2O 458.1992
y4−NH3 459.1837
y4 476.2099
b6(2+) 487.3059
y5−NH3 572.267
y5 589.2941
b3 603.4068
b4 674.444
y6−H2O 699.3419
y6−NH3 700.3251
y6 717.3527
b5 731.4656
y7 959.4915
b6 973.6032
y8 1016.5124
y9 1087.5489
L I F A G K Q L E D G R
y9 y8 y7 y6 y5 y4 y3 y2 y1
b2 a2 b3 b4 b5 b6
Modification
Acetyl (Protein N−term)
GlyGly (K) no new terminus
Oxidation (M)

20200921_KIZ_MUb4_GlyGly_10plex − Scan number: 29180

Gene Names	Charge	m/z	Mass	Retention time	PEP	Score	Precursor Intensity
RPS27A;UBA52;UBC;UBB	3	487.6001	1459.778	106.91	2.0348e−13	141.08	6889255

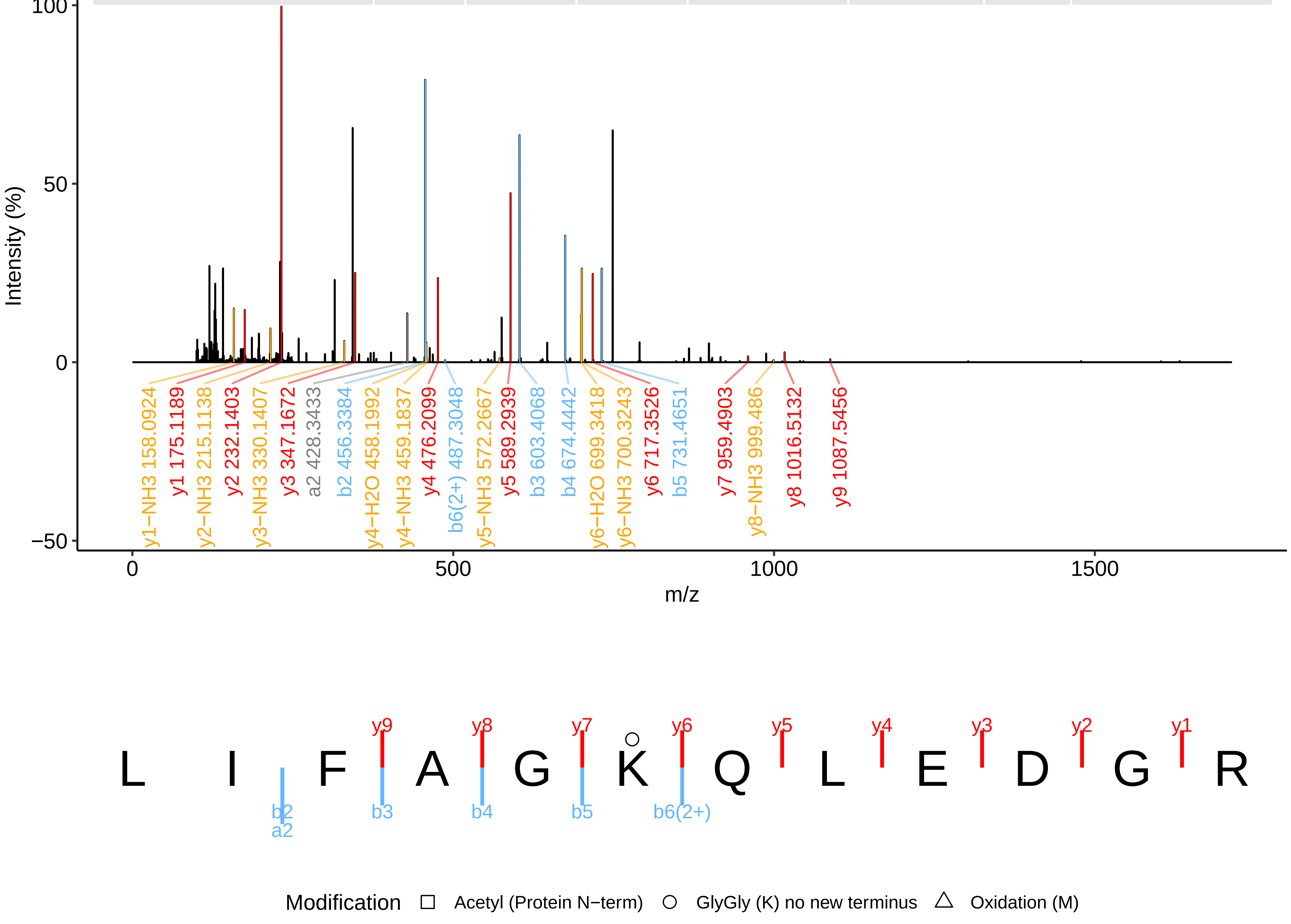

20200921_KIZ_MUb4_GlyGly_10plex − Scan number: 29317

Gene Names	Charge	m/z	Mass	Retention time	PEP	Score	Precursor Intensity
RPS27A;UBA52;UBC;UBB	3	487.6001	1459.778	107.45	2.7437e−13	137.89	5726512

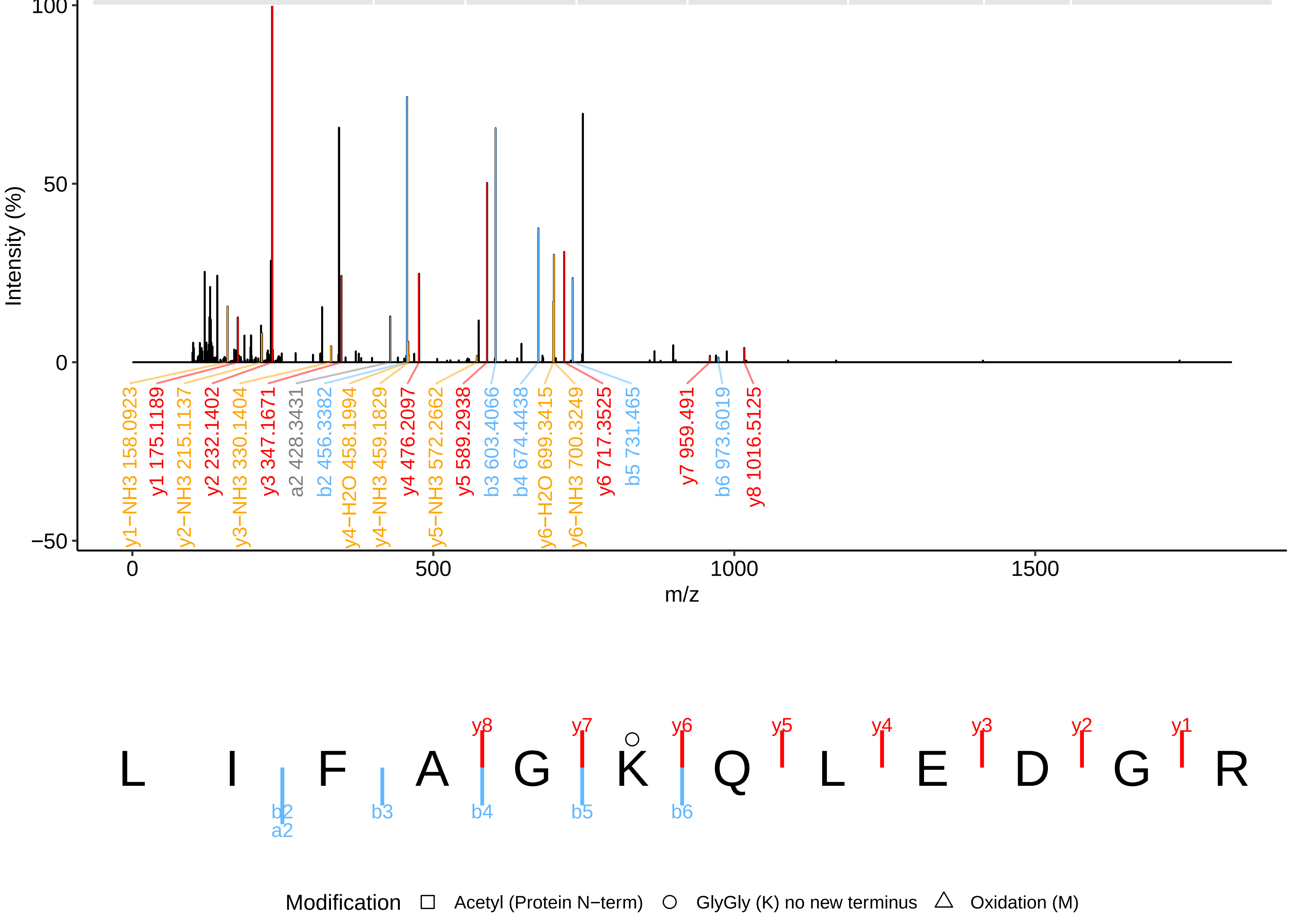

20200921_KIZ_MUb4_GlyGly_10plex − Scan number: 29446

Gene Names	Charge	m/z	Mass	Retention time	PEP	Score	Precursor Intensity
RPS27A;UBA52;UBC;UBB	3	487.6001	1459.778	107.97	2.0348e−13	141.08	7139298

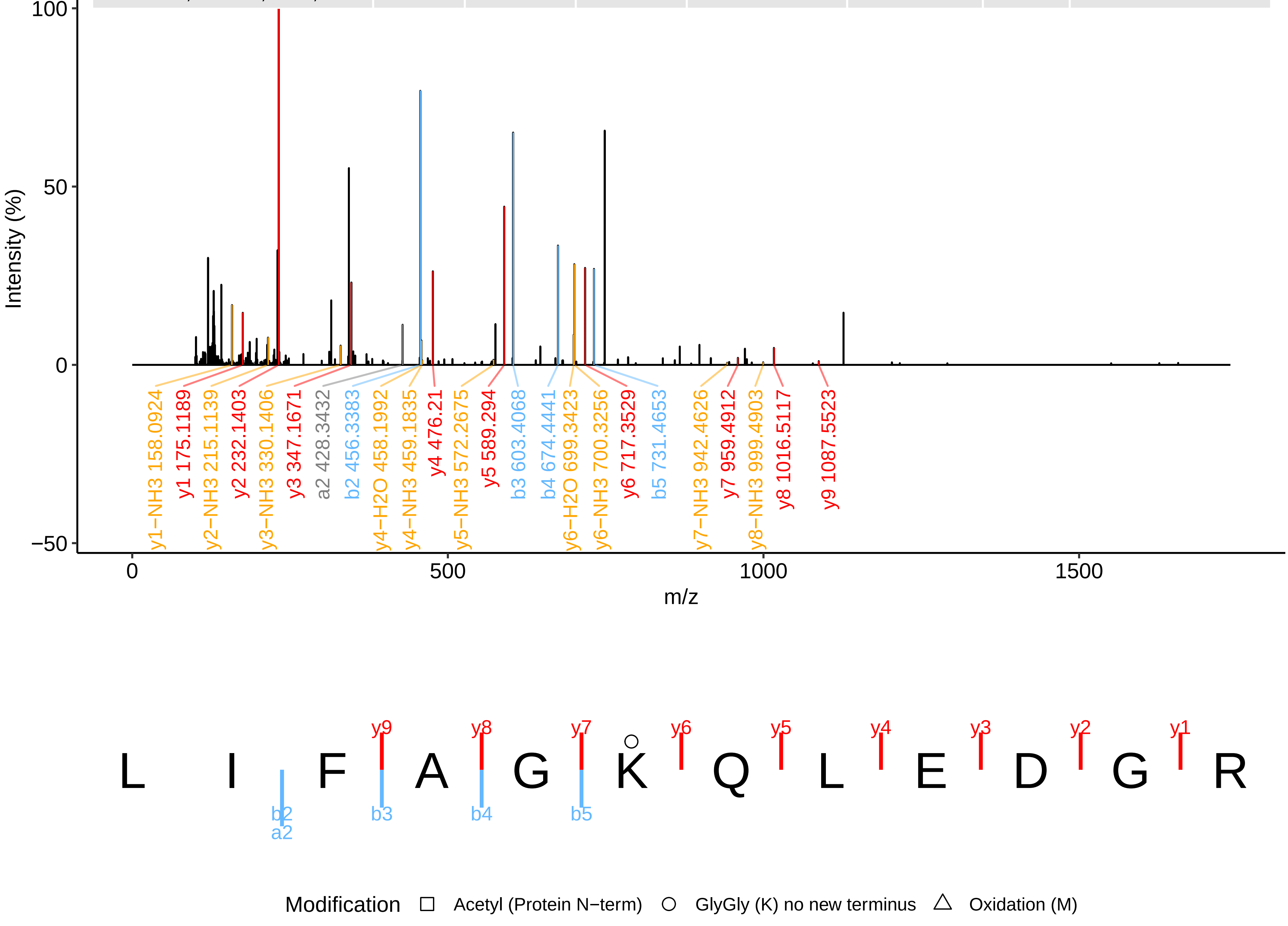

20200921_KIZ_MUb4_GlyGly_10plex − Scan number: 29958

Gene Names	Charge	m/z	Mass	Retention time	PEP	Score	Precursor Intensity
RPS27A;UBA52;UBC;UBB	3	487.6001	1459.778	110	2.0348e−13	141.08	7597353

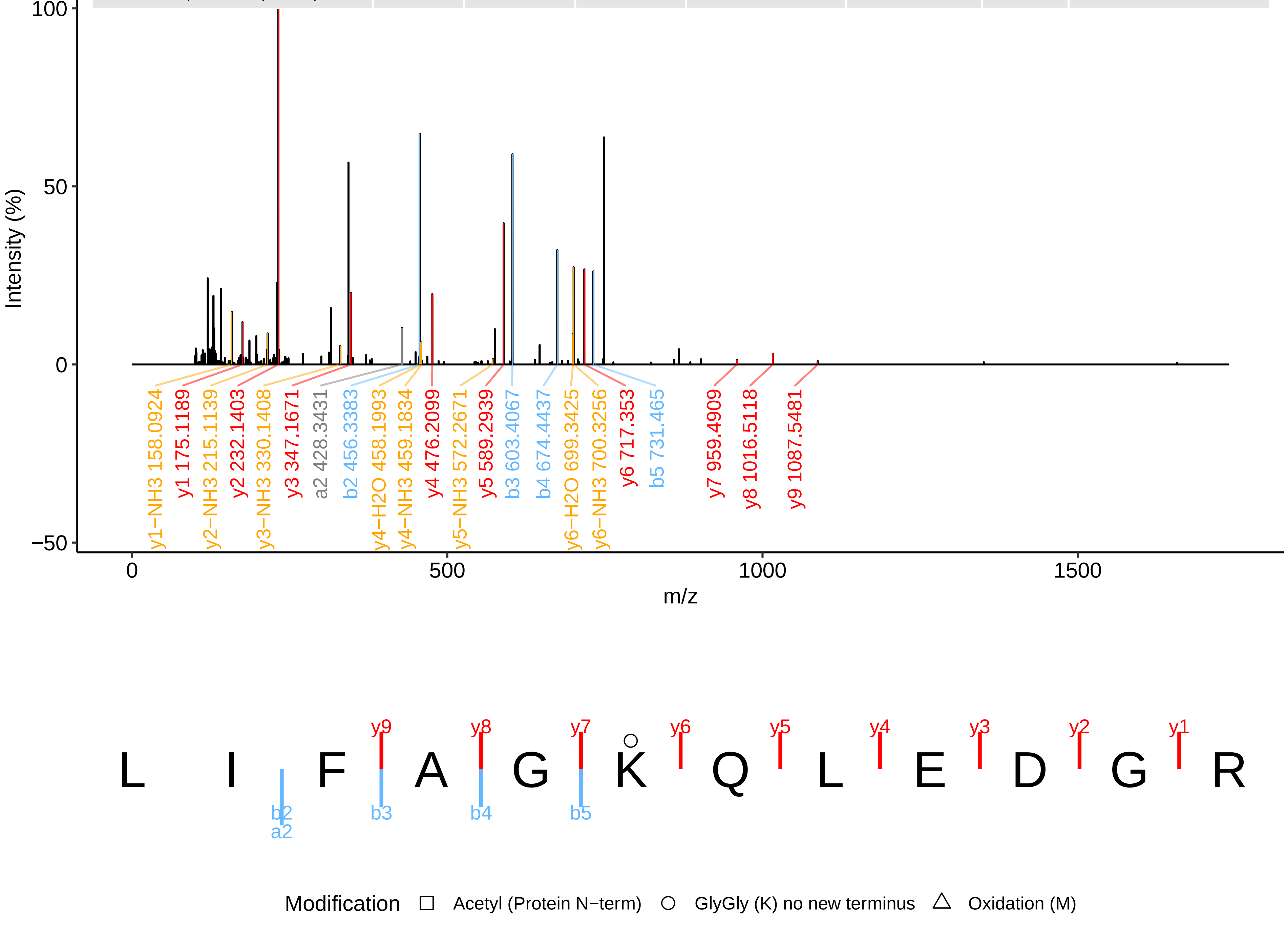

20200921_KIZ_MUb4_GlyGly_10plex − Scan number: 30089

Gene Names	Charge	m/z	Mass	Retention time	PEP	Score	Precursor Intensity
RPS27A;UBA52;UBC;UBB	3	487.6001	1459.778	110.52	9.899e−20	162.26	5315759

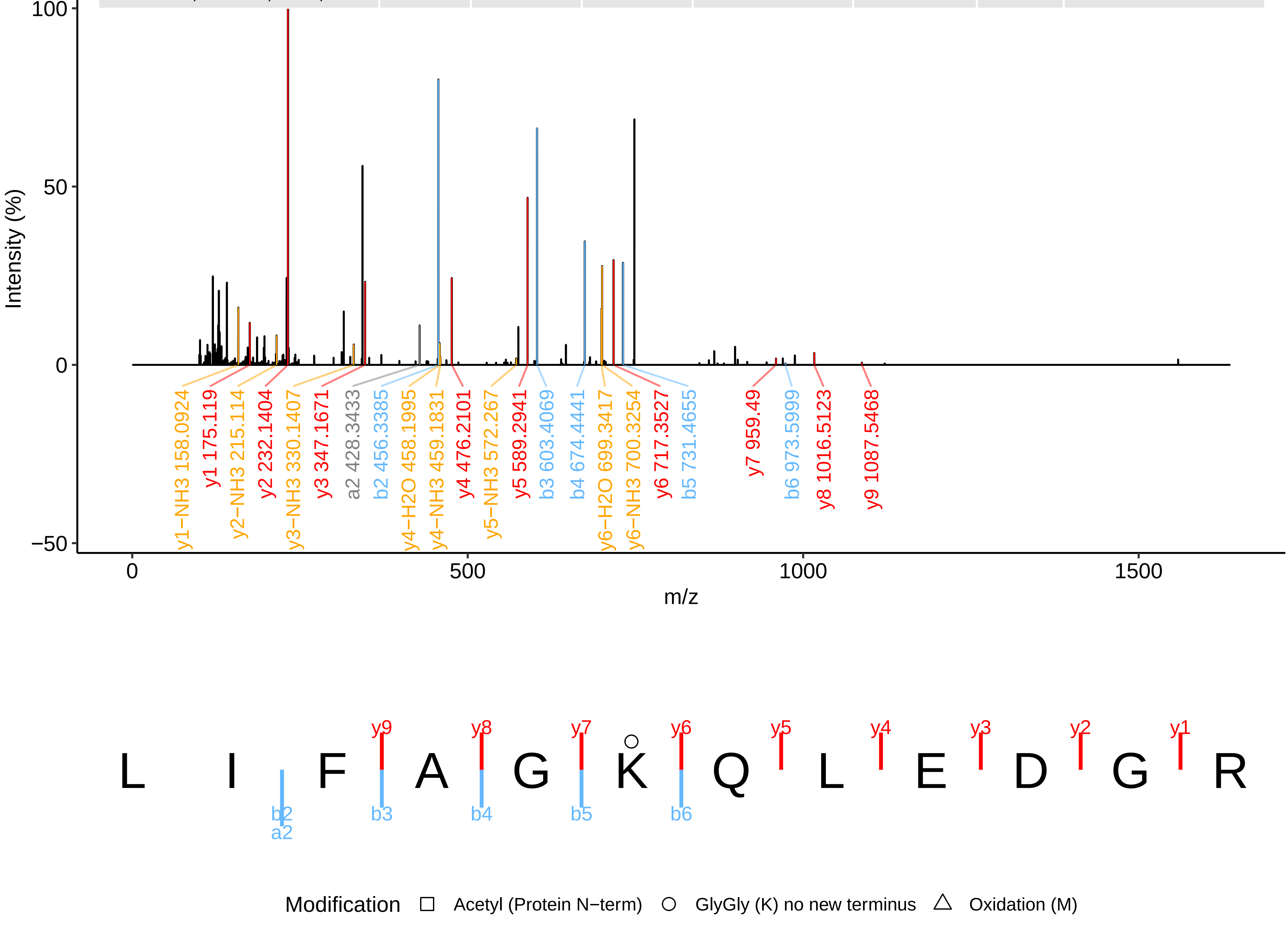

20200921_KIZ_MUb4_GlyGly_10plex − Scan number: 30217

Gene Names	Charge	m/z	Mass	Retention time	PEP	Score	Precursor Intensity
RPS27A;UBA52;UBC;UBB	3	487.6001	1459.778	111.03	8.2269e−10	131.83	7520955

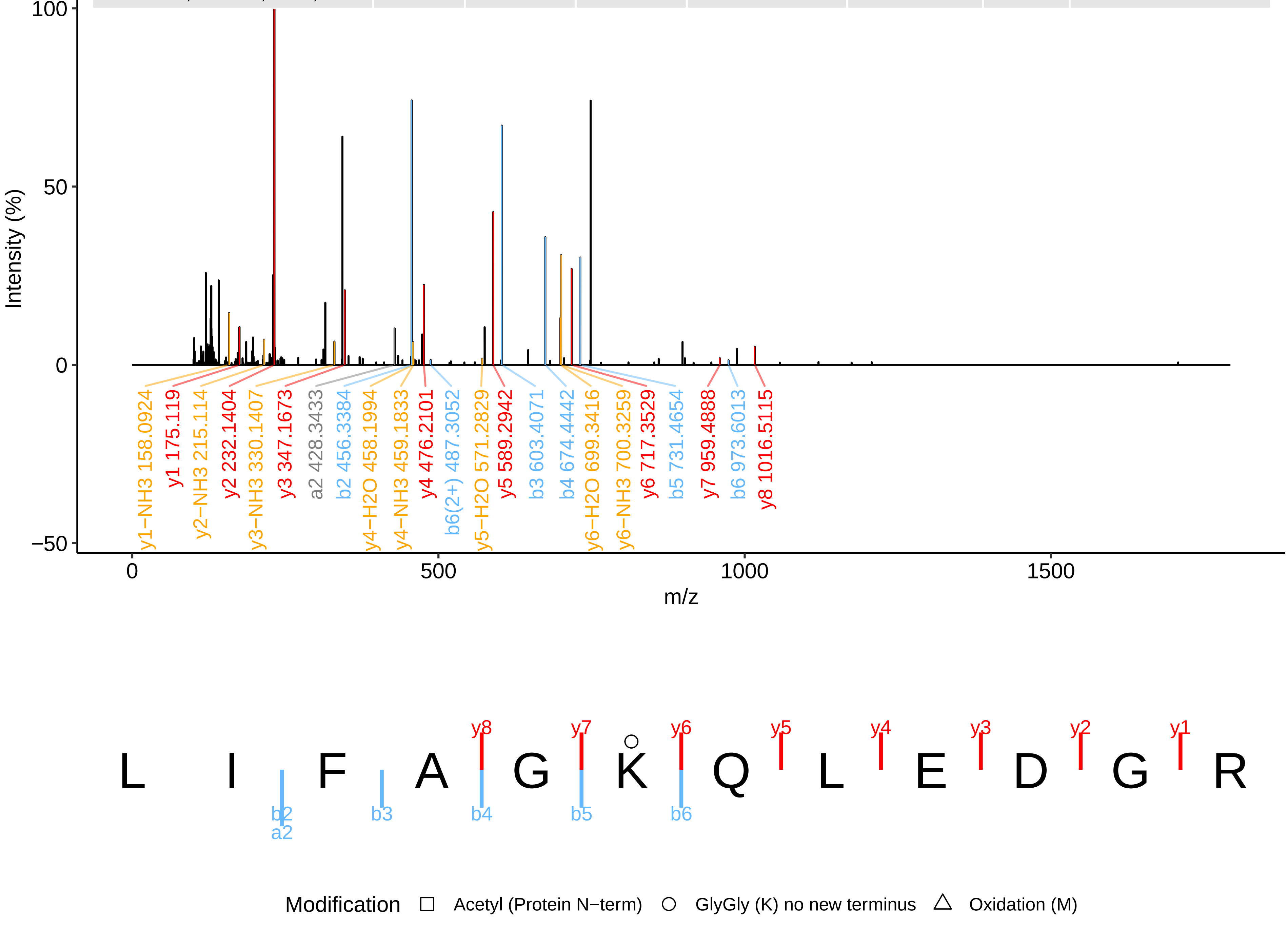

20200921_KIZ_MUb4_GlyGly_10plex − Scan number: 30345

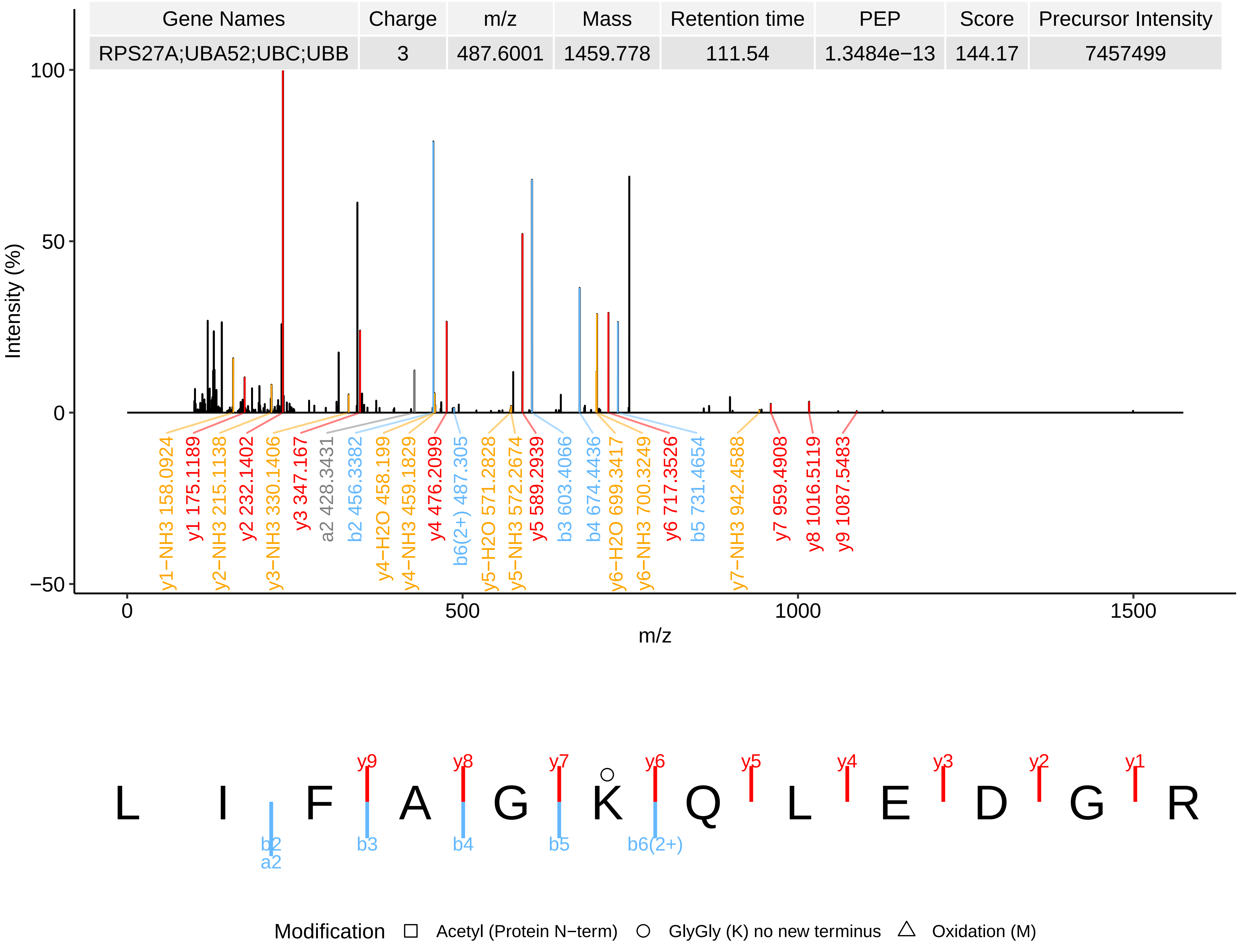

20200921_KIZ_MUb4_GlyGly_10plex − Scan number: 30867

Gene Names	Charge	m/z	Mass	Retention time	PEP	Score	Precursor Intensity
RPS27A;UBA52;UBC;UBB	3	487.6001	1459.778	113.57	2.0348e−13	141.08	5934997

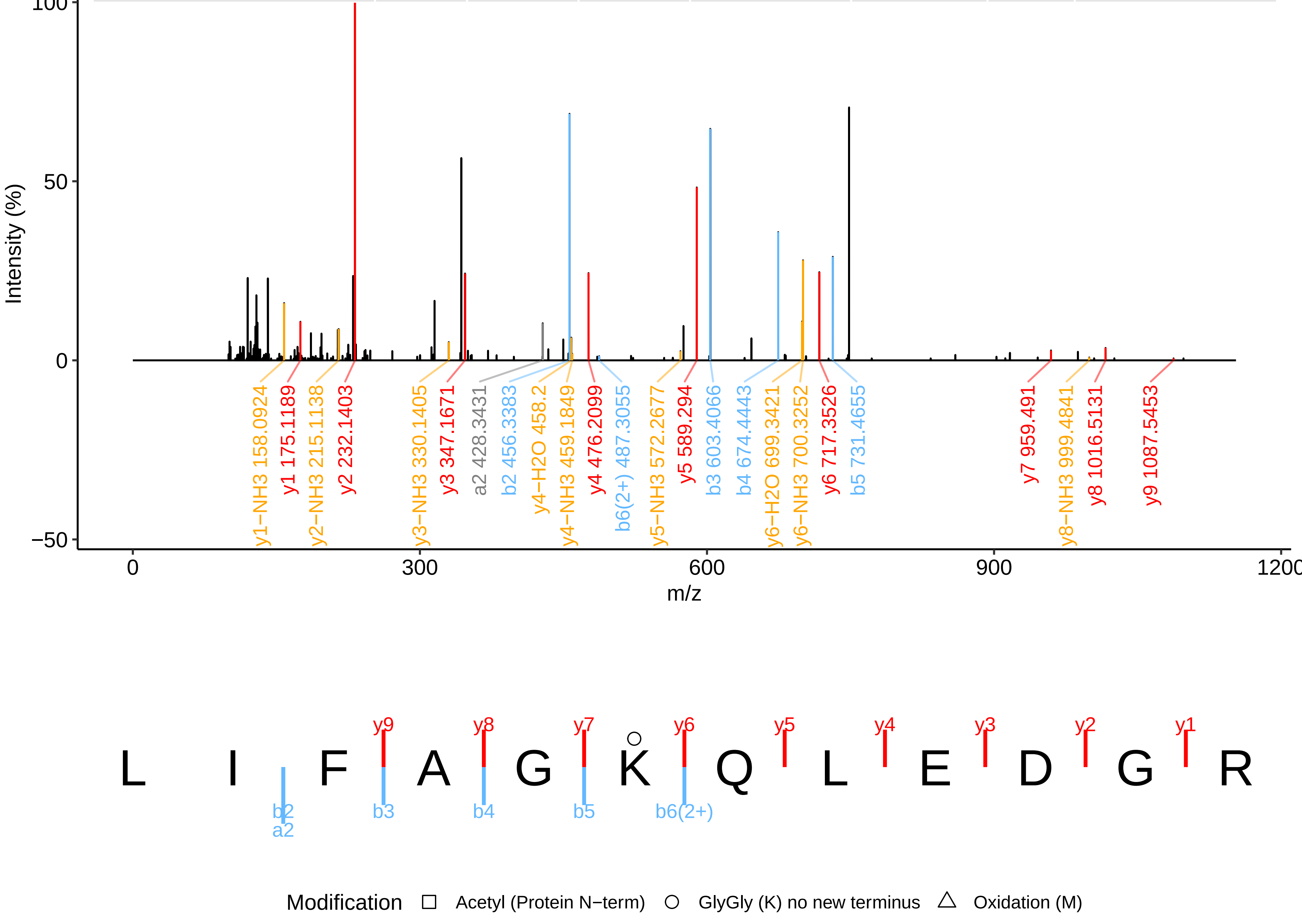

20200921_KIZ_MUb4_GlyGly_10plex − Scan number: 30991

Gene Names	Charge	m/z	Mass	Retention time	PEP	Score	Precursor Intensity
RPS27A;UBA52;UBC;UBB	3	487.6001	1459.778	114.08	3.1574e−19	150.46	5696641

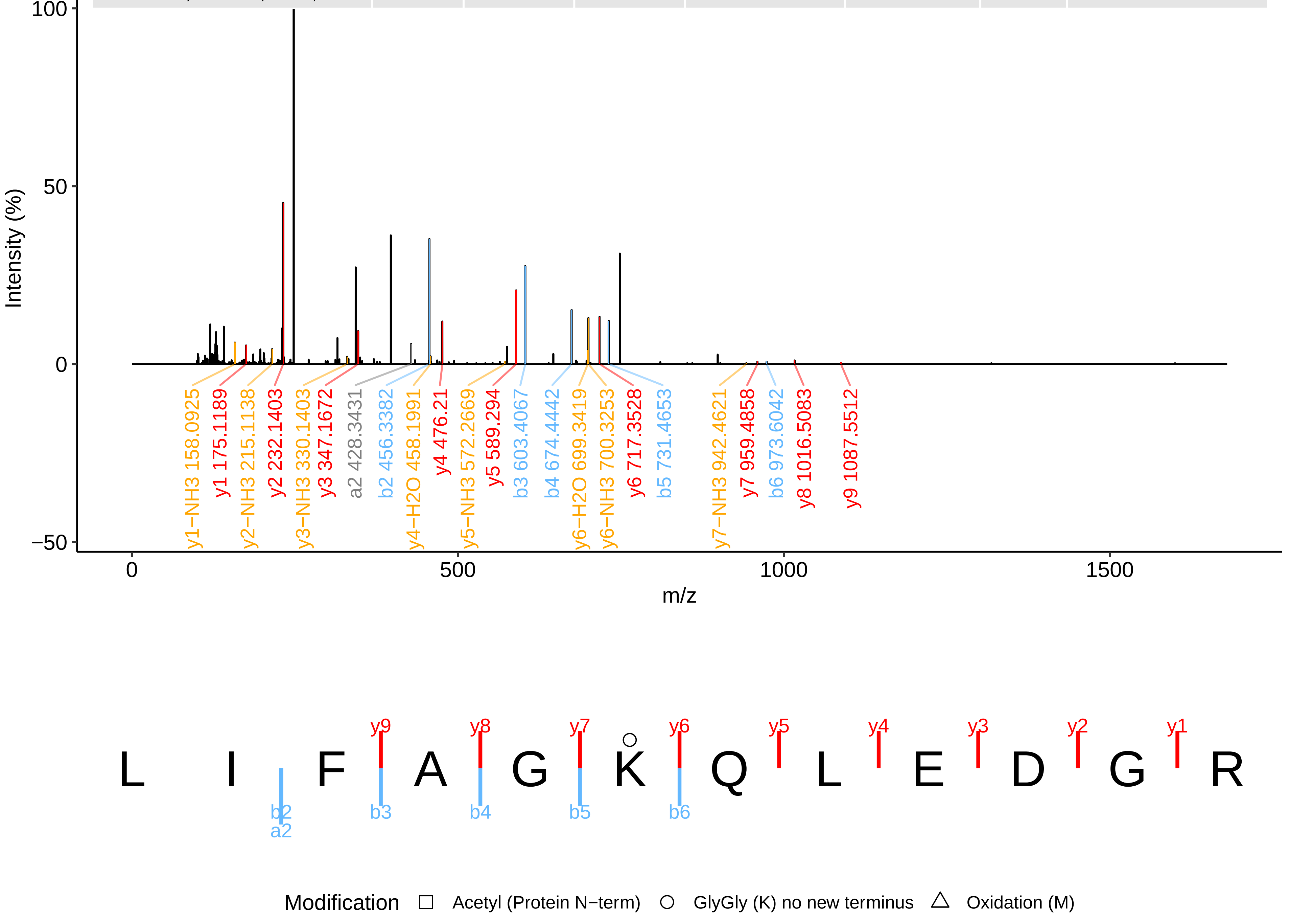

20200921_KIZ_MUb4_GlyGly_10plex − Scan number: 31113

Gene Names	Charge	m/z	Mass	Retention time	PEP	Score	Precursor Intensity
RPS27A;UBA52;UBC;UBB	3	487.6001	1459.778	114.59	2.0323e−13	141.1	3826041

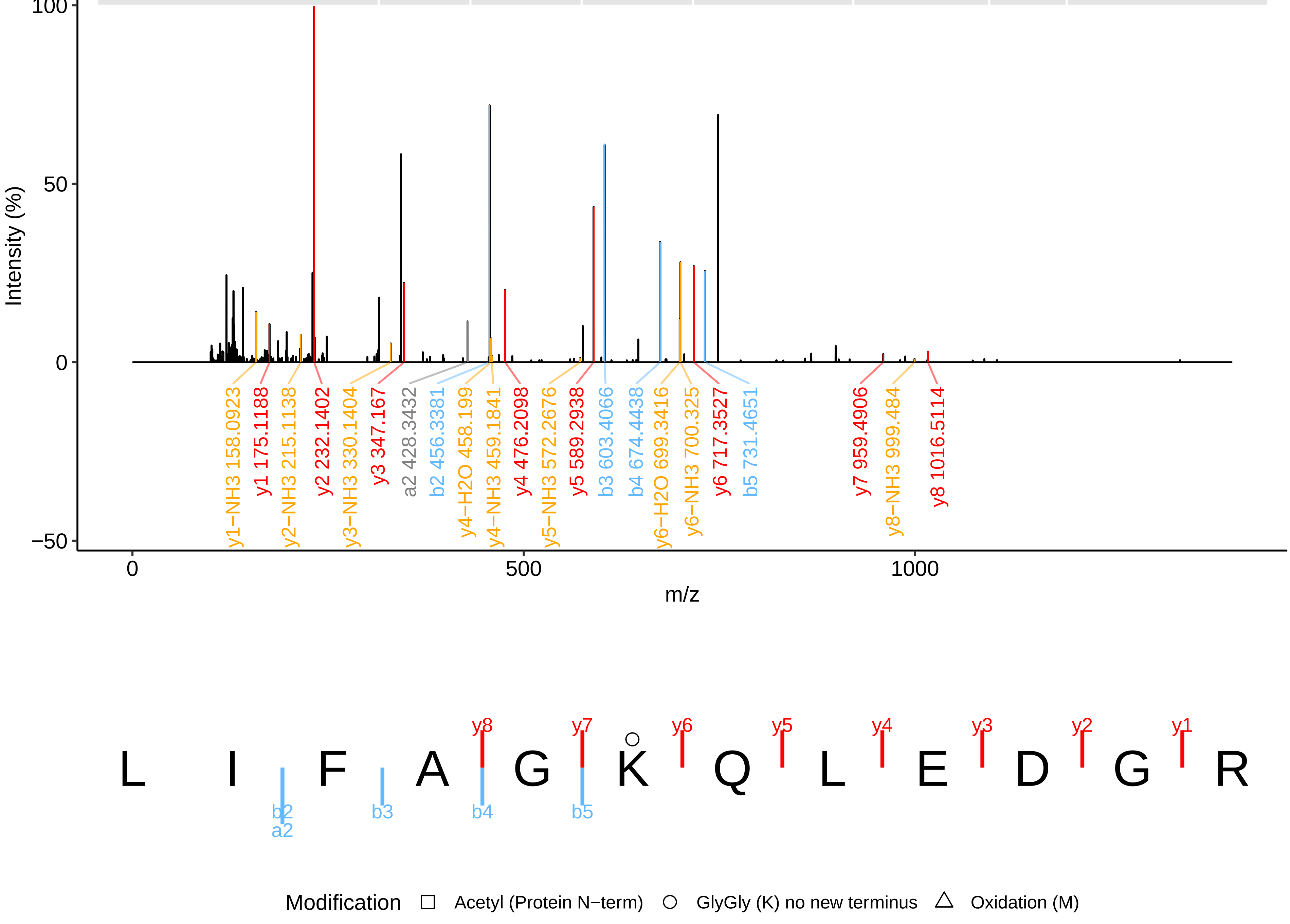

20200921_KIZ_MUb4_GlyGly_10plex − Scan number: 31630

Gene Names	Charge	m/z	Mass	Retention time	PEP	Score	Precursor Intensity
RPS27A;UBA52;UBC;UBB	3	487.6001	1459.778	116.65	2.4754e−09	129.82	5466824

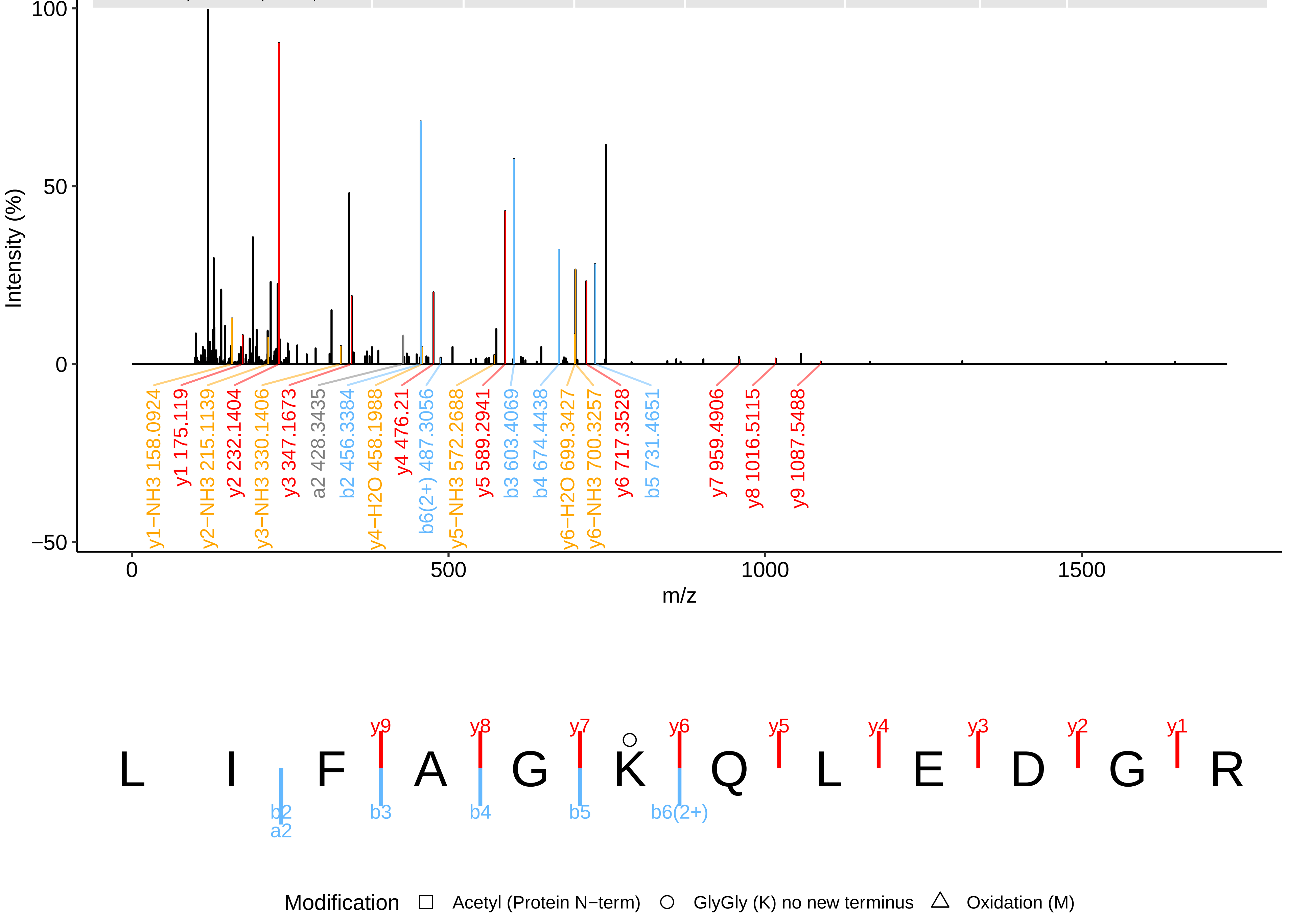

20200921_KIZ_MUb4_GlyGly_10plex − Scan number: 32060

Gene Names	Charge	m/z	Mass	Retention time	PEP	Score	Precursor Intensity
RPS27A;UBA52;UBC;UBB	3	487.6001	1459.778	118.39	9.899e−20	162.26	4418375

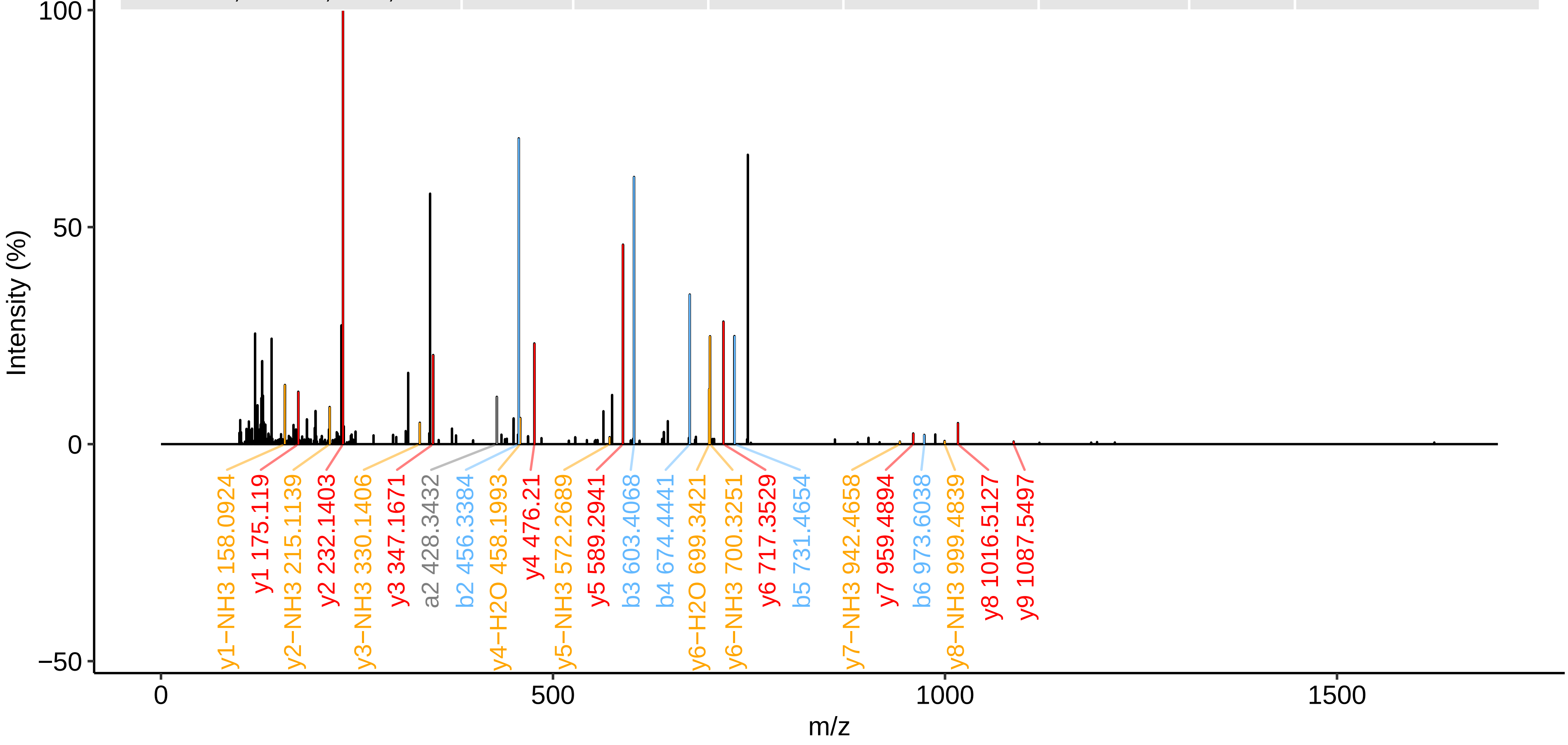

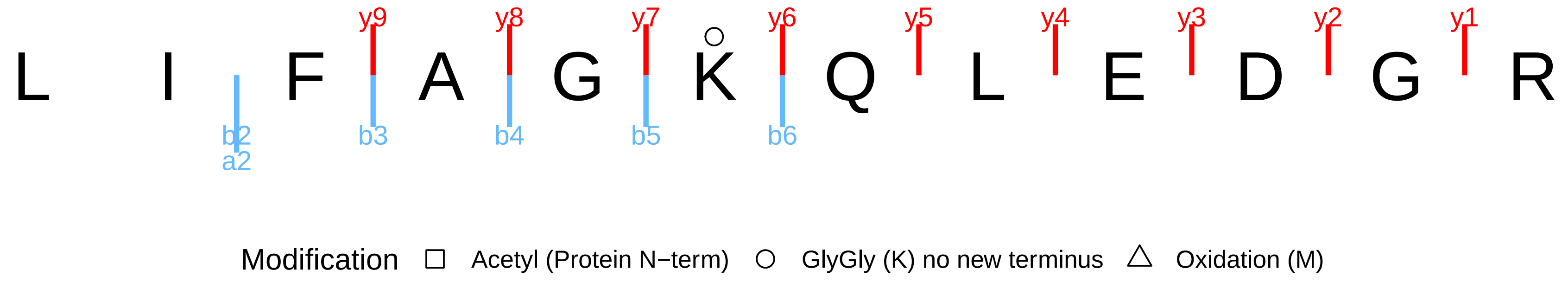

20200921_KIZ_MUb4_GlyGly_10plex − Scan number: 32183

Gene Names	Charge	m/z	Mass	Retention time	PEP	Score	Precursor Intensity
RPS27A;UBA52;UBC;UBB	3	487.6001	1459.778	118.89	3.5418e−13	134.3	6309799

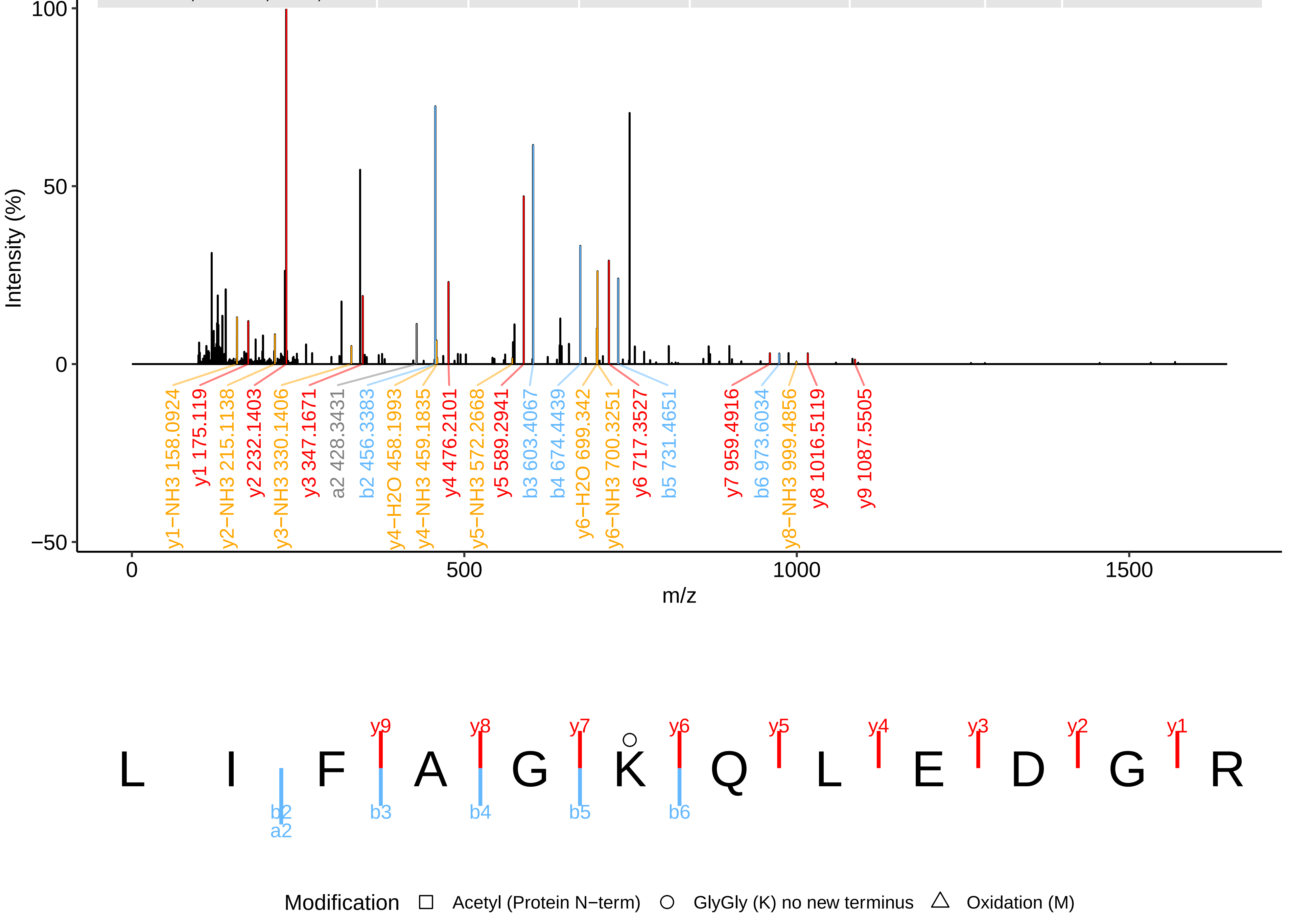

20200921_KIZ_MUb4_GlyGly_10plex − Scan number: 32312

Gene Names	Charge	m/z	Mass	Retention time	PEP	Score	Precursor Intensity
RPS27A;UBA52;UBC;UBB	3	487.6001	1459.778	119.4	1.8313e−19	157.68	4396463

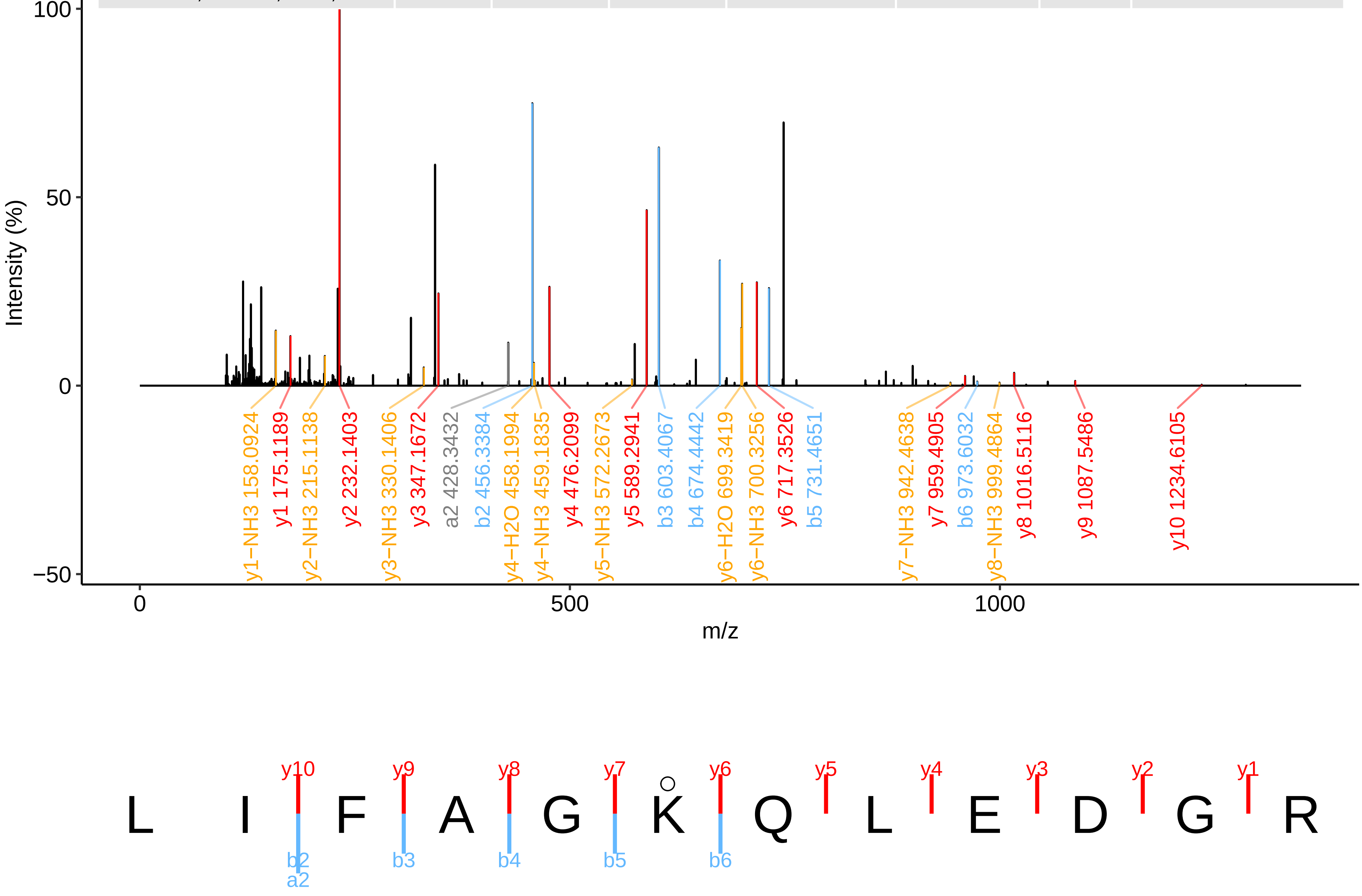

Modification □ Acetyl (Protein N−term) ○ GlyGly (K) no new terminus △ Oxidation (M)

20200921_KIZ_MUb4_GlyGly_10plex − Scan number: 34128

Gene Names	Charge	m/z	Mass	Retention time	PEP	Score	Precursor Intensity
RPS27A;UBA52;UBC;UBB	3	487.6001	1459.778	125.2	0.00014642	96.171	3679917

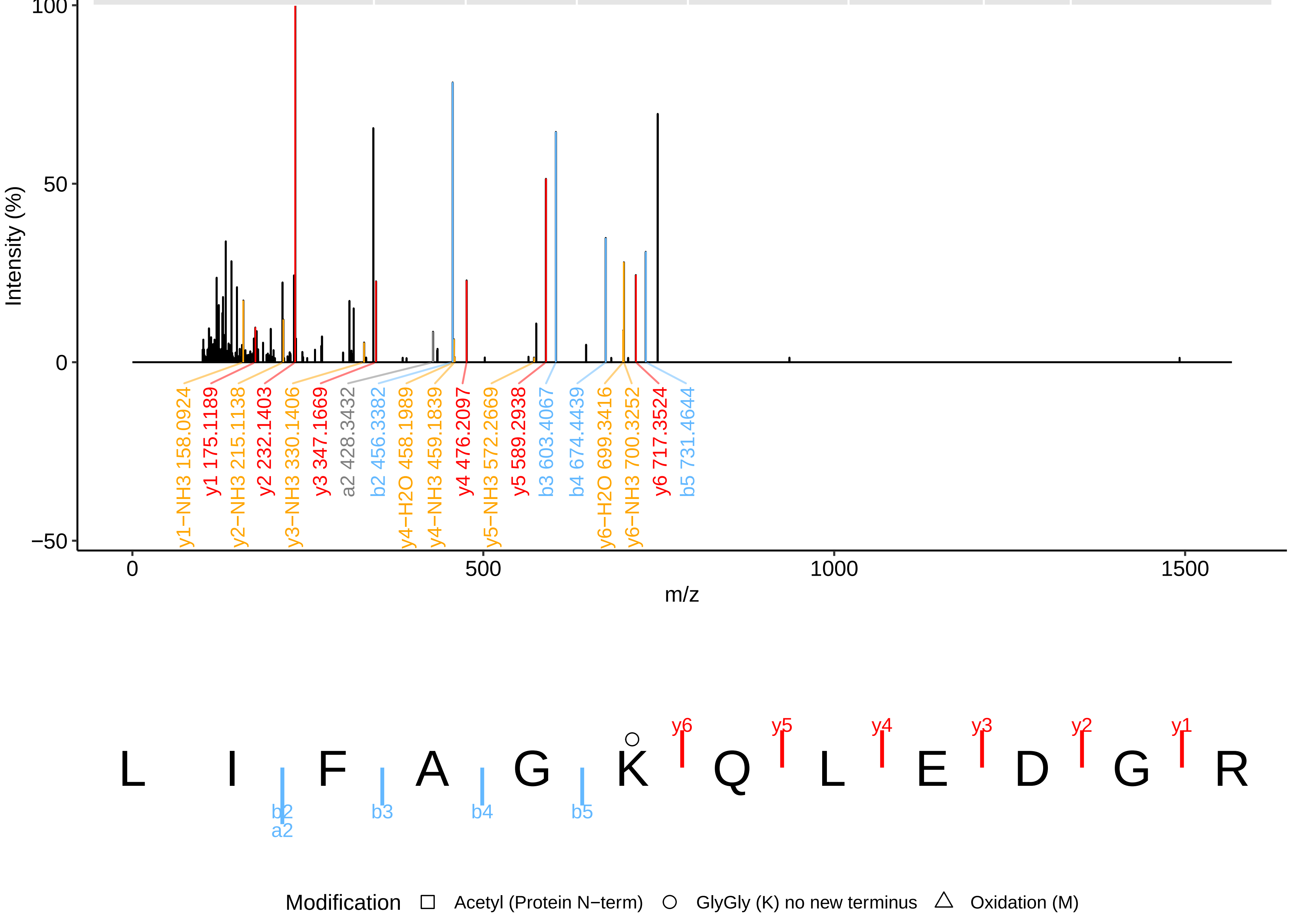

20200921_KIZ_MUb4_GlyGly_10plex − Scan number: 34368

Gene Names	Charge	m/z	Mass	Retention time	PEP	Score	Precursor Intensity
RPS27A;UBA52;UBC;UBB	3	487.6001	1459.778	126.12	0.019826	61.833	2480701

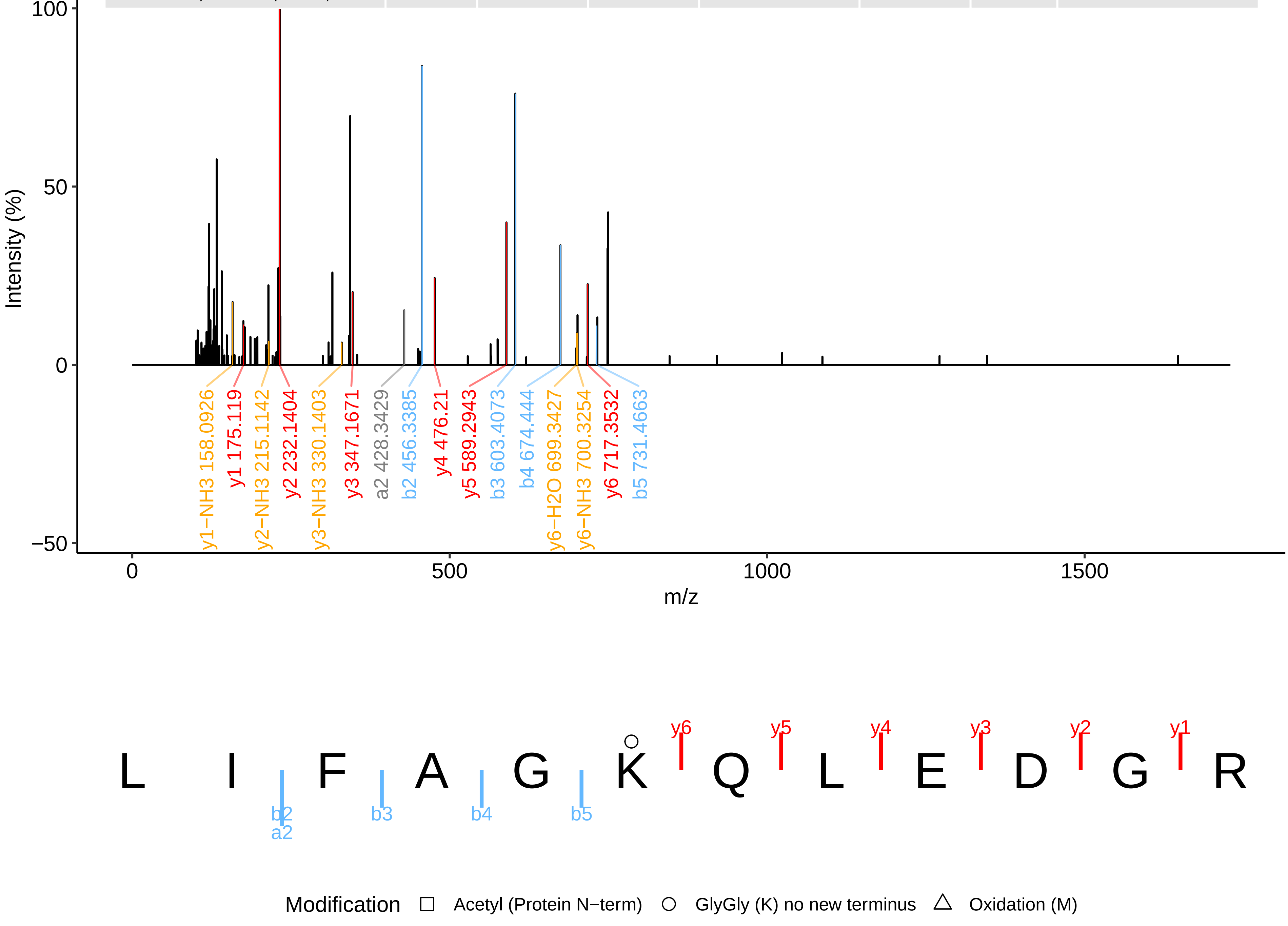

20200921_KIZ_MUb4_GlyGly_10plex − Scan number: 35061

Gene Names	Charge	m/z	Mass	Retention time	PEP	Score	Precursor Intensity
RPS27A;UBA52;UBC;UBB	3	487.6001	1459.778	128.48	2.4754e−09	129.82	818183.5

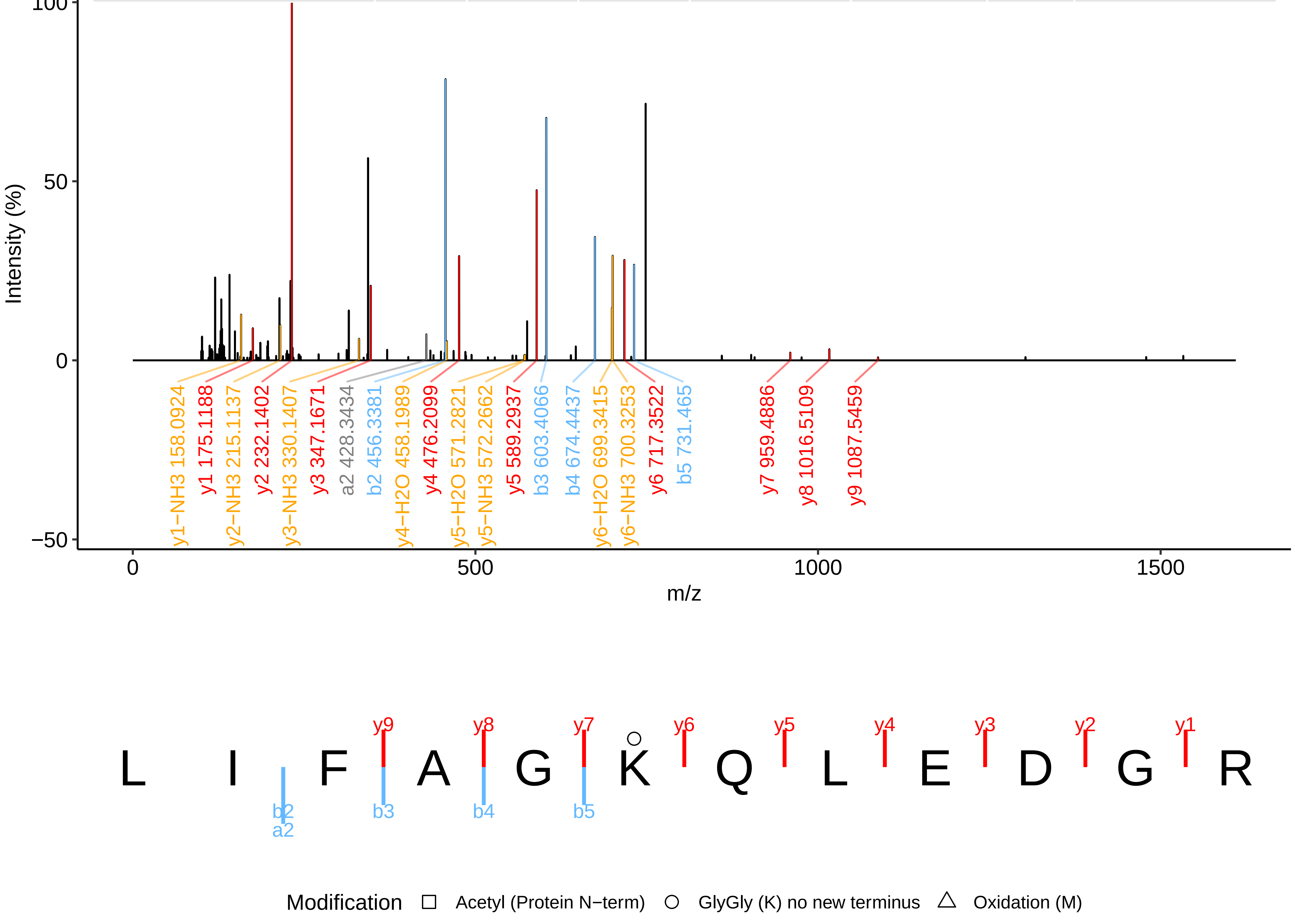

20200921_KIZ_MUb4_GlyGly_10plex − Scan number: 35191

Gene Names	Charge	m/z	Mass	Retention time	PEP	Score	Precursor Intensity
RPS27A;UBA52;UBC;UBB	3	487.6001	1459.778	128.98	9.6101e−14	145.91	2573672

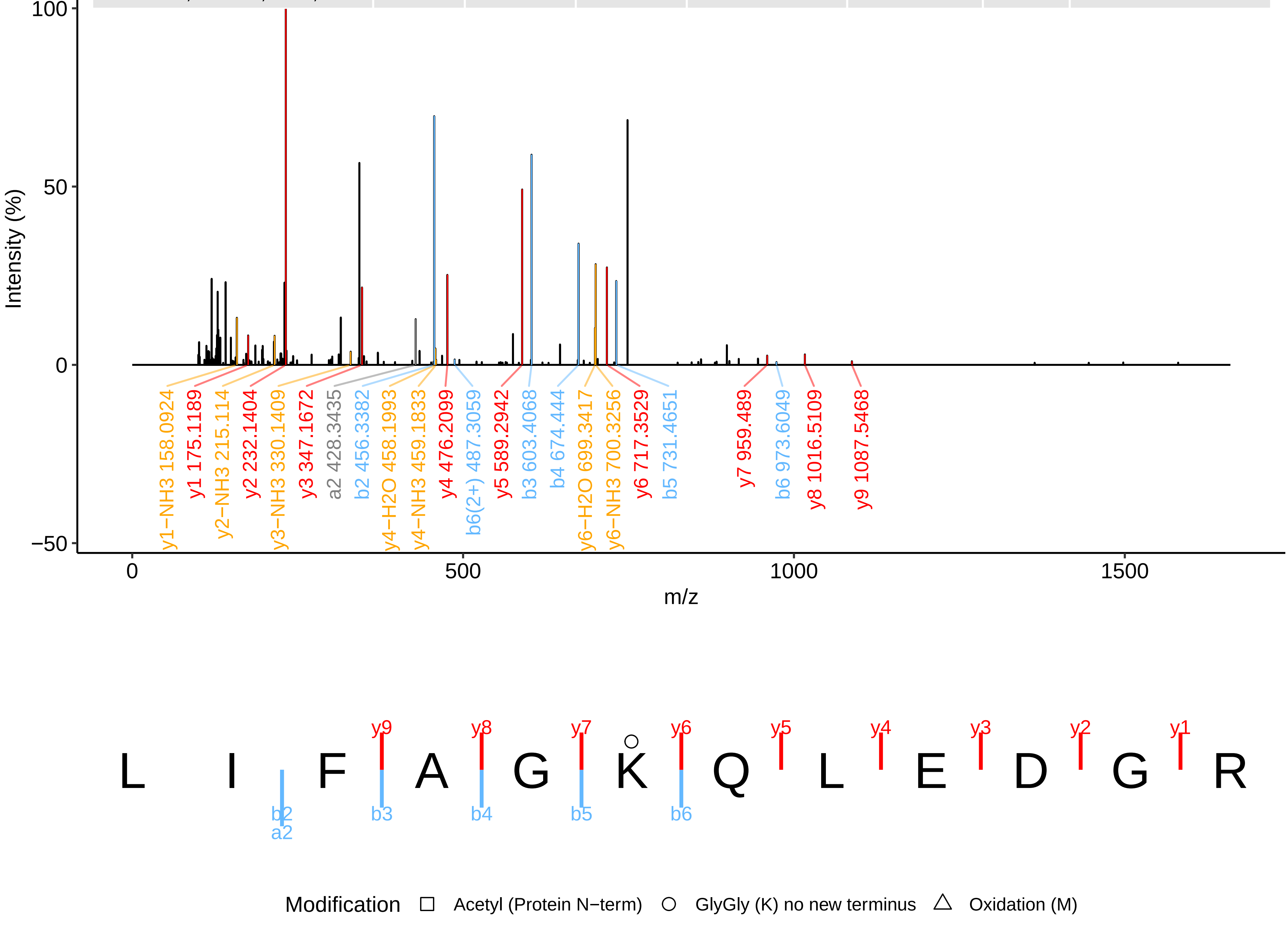

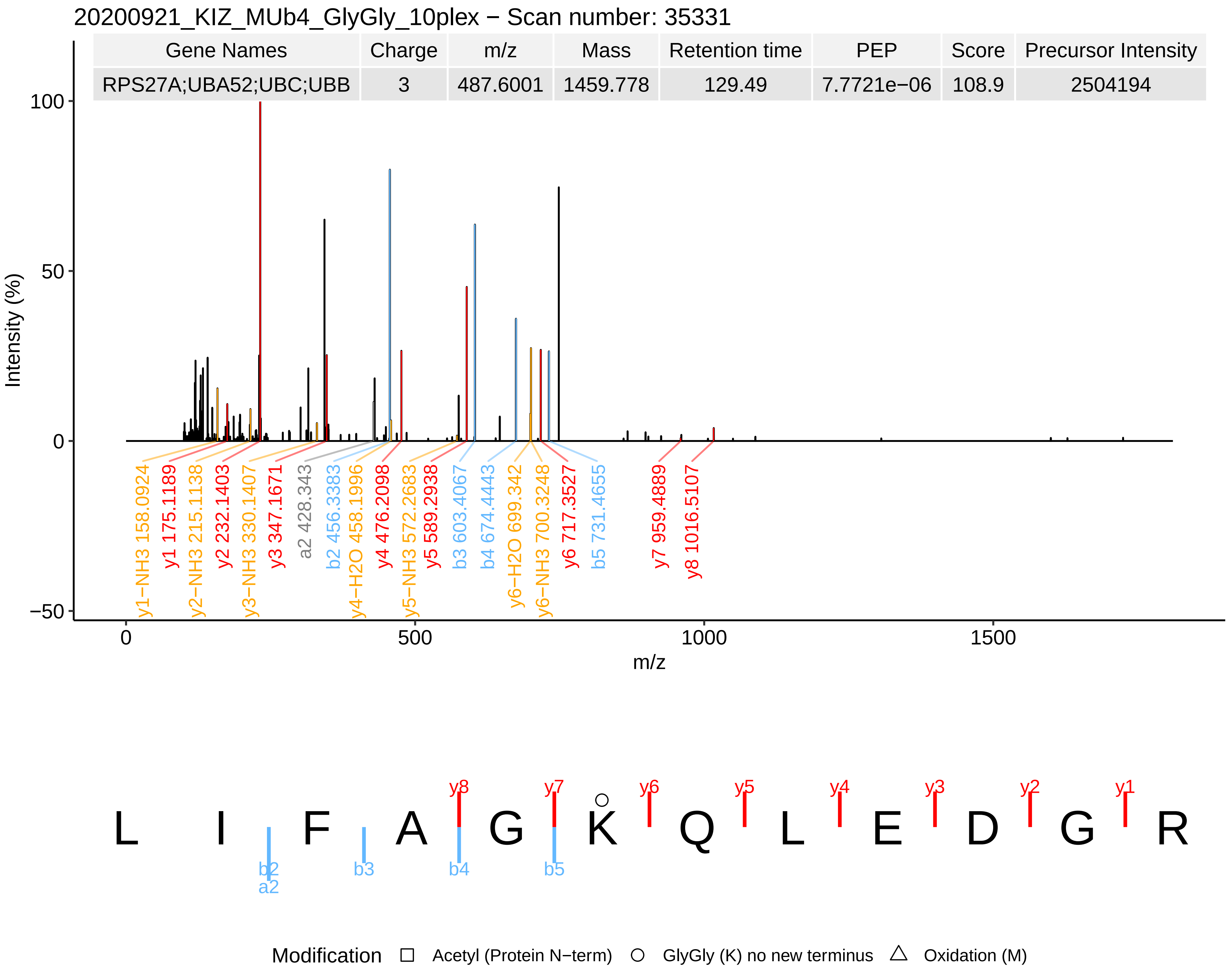

20200921_KIZ_MUb4_GlyGly_10plex − Scan number: 35331
Gene Names | Charge | m/z | Mass | Retention time | PEP | Score | Precursor Intensity
RPS27A;UBA52;UBC;UBB | 3 | 487.6001 | 1459.778 | 129.49 | 7.7721e−06 | 108.9 | 2504194
Intensity (%)
100
50
0
−50
y1−NH3 158.0924
y1 175.1189
y2−NH3 215.1138
y2 232.1403
y3−NH3 330.1407
y3 347.1671
a2 428.343
b2 456.3383
y4−H2O 458.1996
y4 476.2098
y5−NH3 572.2683
y5 589.2938
b3 603.4067
b4 674.4443
y6−H2O 699.342
y6−NH3 700.3248
y6 717.3527
b5 731.4655
y7 959.4889
y8 1016.5107
0
500
1000
1500
m/z
L I F A G K Q L E D G R
y8 y7 y6 y5 y4 y3 y2 y1
b2 a2 b3 b4 b5
Modification
Acetyl (Protein N−term)
GlyGly (K) no new terminus
Oxidation (M)

20200921_KIZ_MUb4_GlyGly_10plex − Scan number: 35469

Gene Names	Charge	m/z	Mass	Retention time	PEP	Score	Precursor Intensity
RPS27A;UBA52;UBC;UBB	3	487.6001	1459.778	129.99	2.4754e−09	129.82	NA

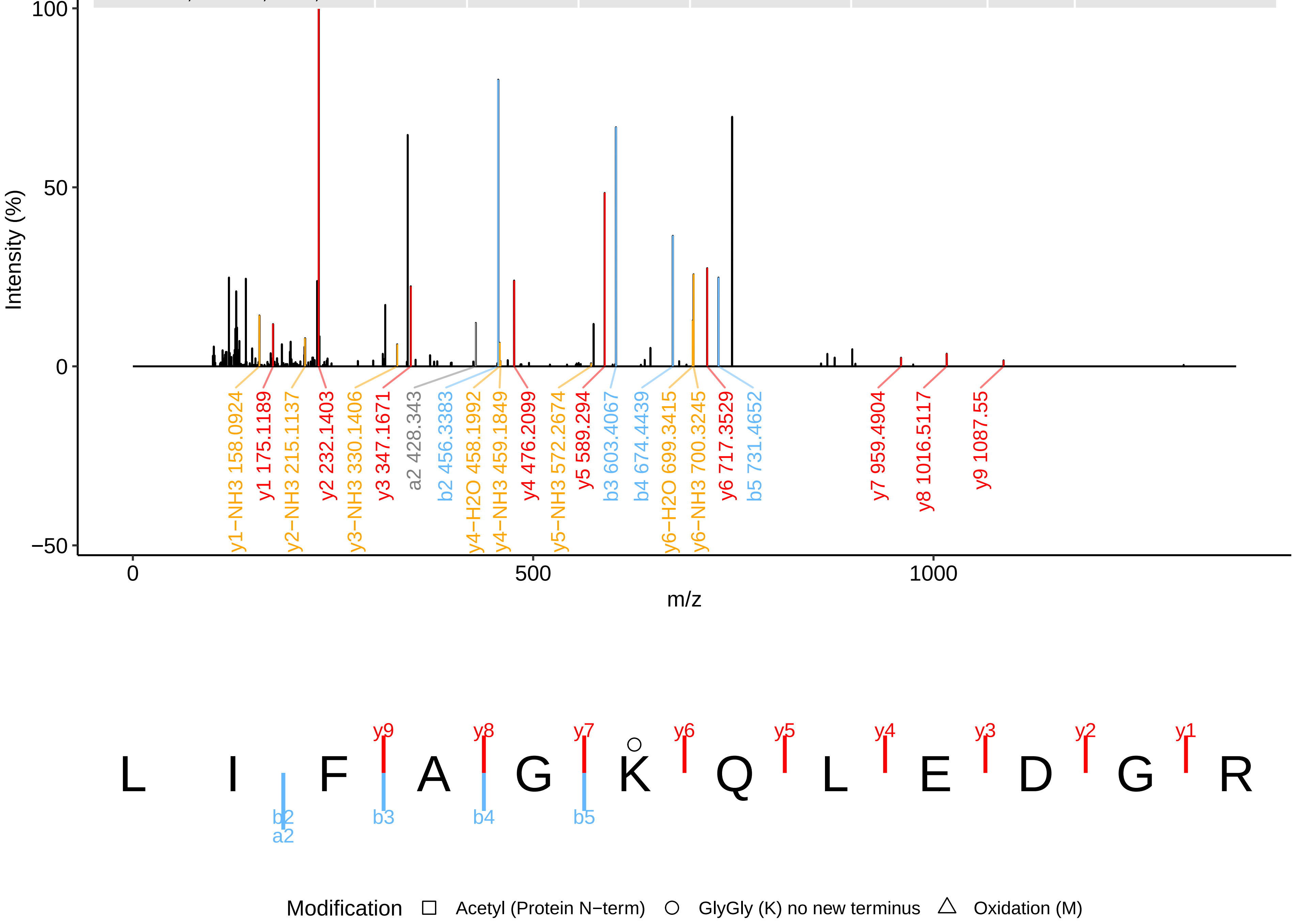

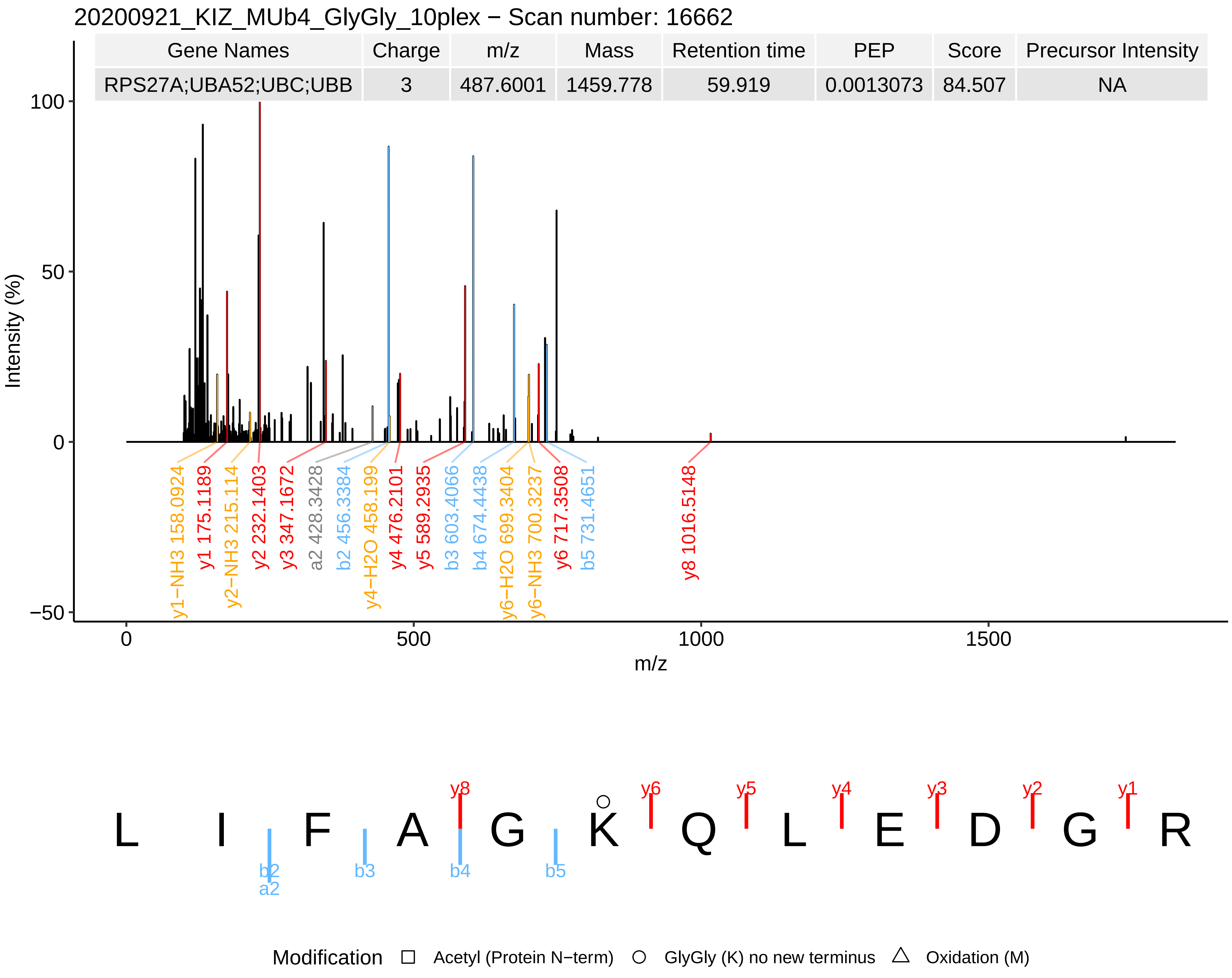
20200921_KIZ_MUb4_GlyGly_10plex − Scan number: 16662
Gene Names
Charge
m/z
Mass
Retention time
PEP
Score
Precursor Intensity
RPS27A;UBA52;UBC;UBB
3
487.6001
1459.778
59.919
0.0013073
84.507
NA
Intensity (%)
100
50
0
−50
y1−NH3 158.0924
y1 175.1189
y2−NH3 215.114
y2 232.1403
y3 347.1672
a2 428.3428
b2 456.3384
y4−H2O 458.199
y4 476.2101
y5 589.2935
b3 603.4066
b4 674.4438
y6−H2O 699.3404
y6−NH3 700.3237
y6 717.3508
b5 731.4651
y8 1016.5148
0
500
1000
1500
m/z
L I F A G K Q L E D G R
y8
y6
y5
y4
y3
y2
y1
b2
a2
b3
b4
b5
Modification
Acetyl (Protein N−term)
GlyGly (K) no new terminus
Oxidation (M)

20200921_KIZ_MUb4_GlyGly_10plex − Scan number: 19025

Gene Names	Charge	m/z	Mass	Retention time	PEP	Score	Precursor Intensity
RPS27A;UBA52;UBC;UBB	2	730.8964	1459.778	68.516	7.823e−25	228.84	NA

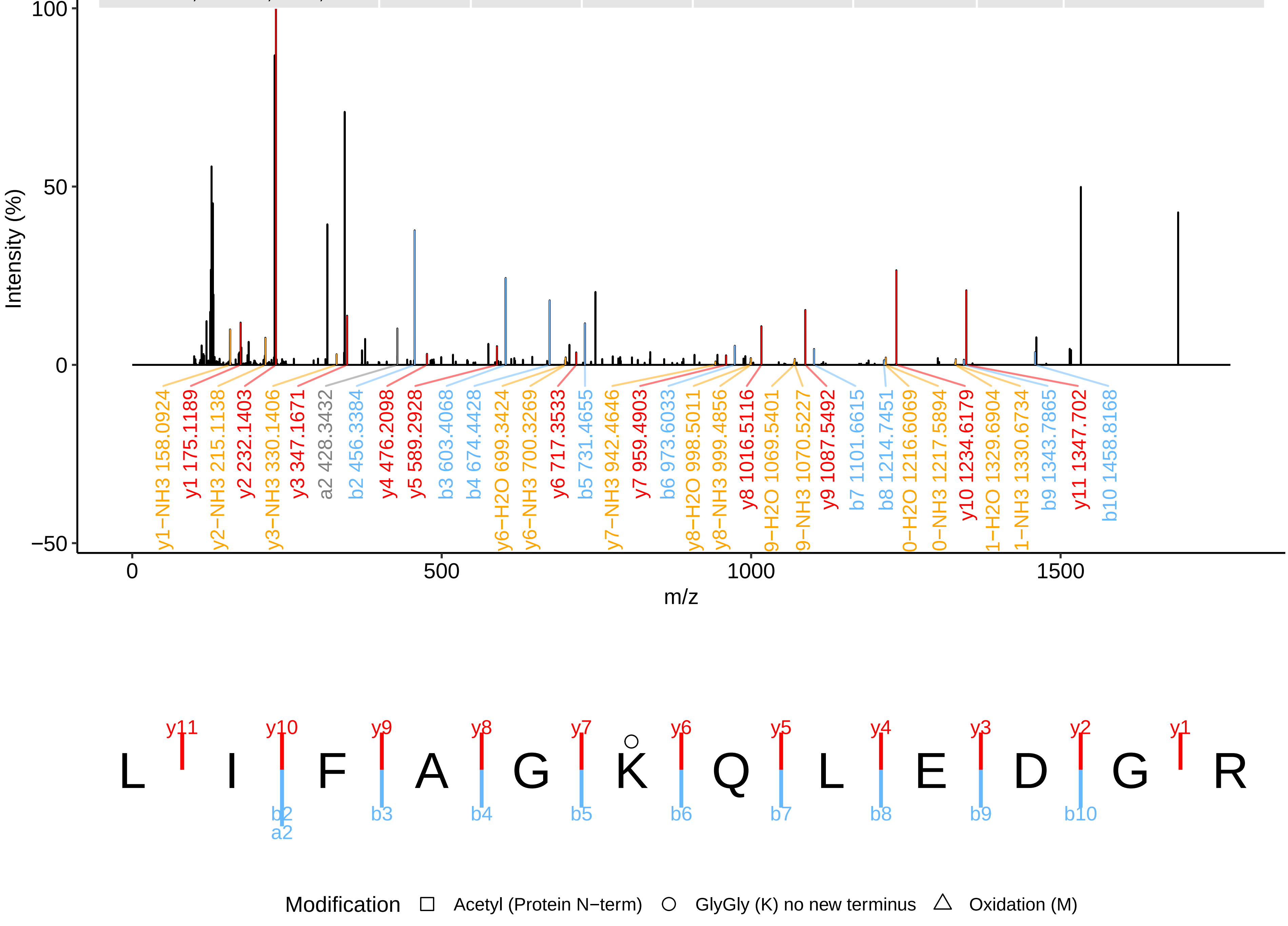

20200921_KIZ_MUb4_GlyGly_10plex − Scan number: 19759

Gene Names	Charge	m/z	Mass	Retention time	PEP	Score	Precursor Intensity
RPS27A;UBA52;UBC;UBB	2	730.8964	1459.778	71.318	2.5068e−14	180.07	NA

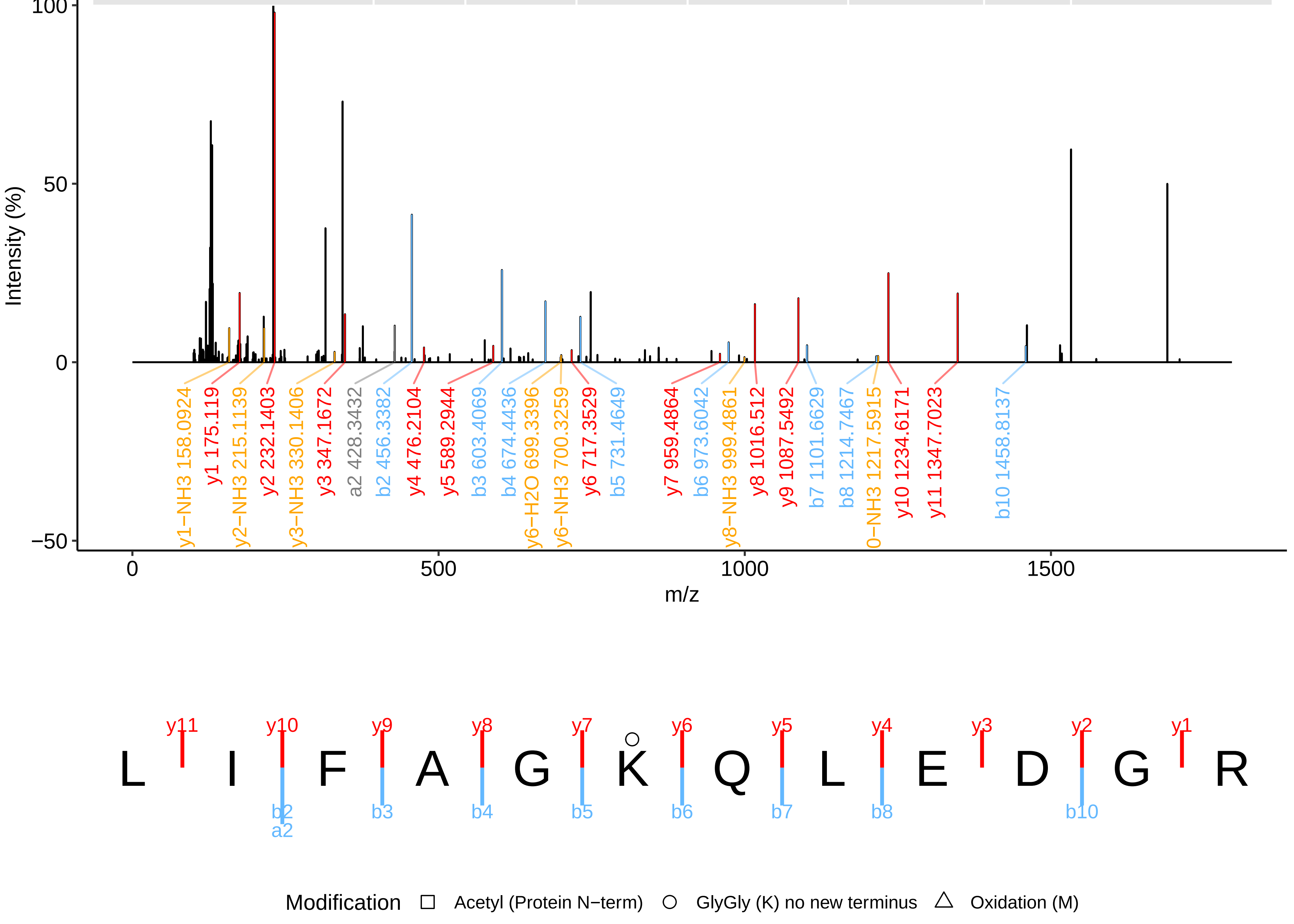

20200921_KIZ_MUb4_GlyGly_10plex − Scan number: 19916

Gene Names	Charge	m/z	Mass	Retention time	PEP	Score	Precursor Intensity
RPS27A;UBA52;UBC;UBB	2	730.8964	1459.778	71.894	1.0966e−15	188.99	4311145

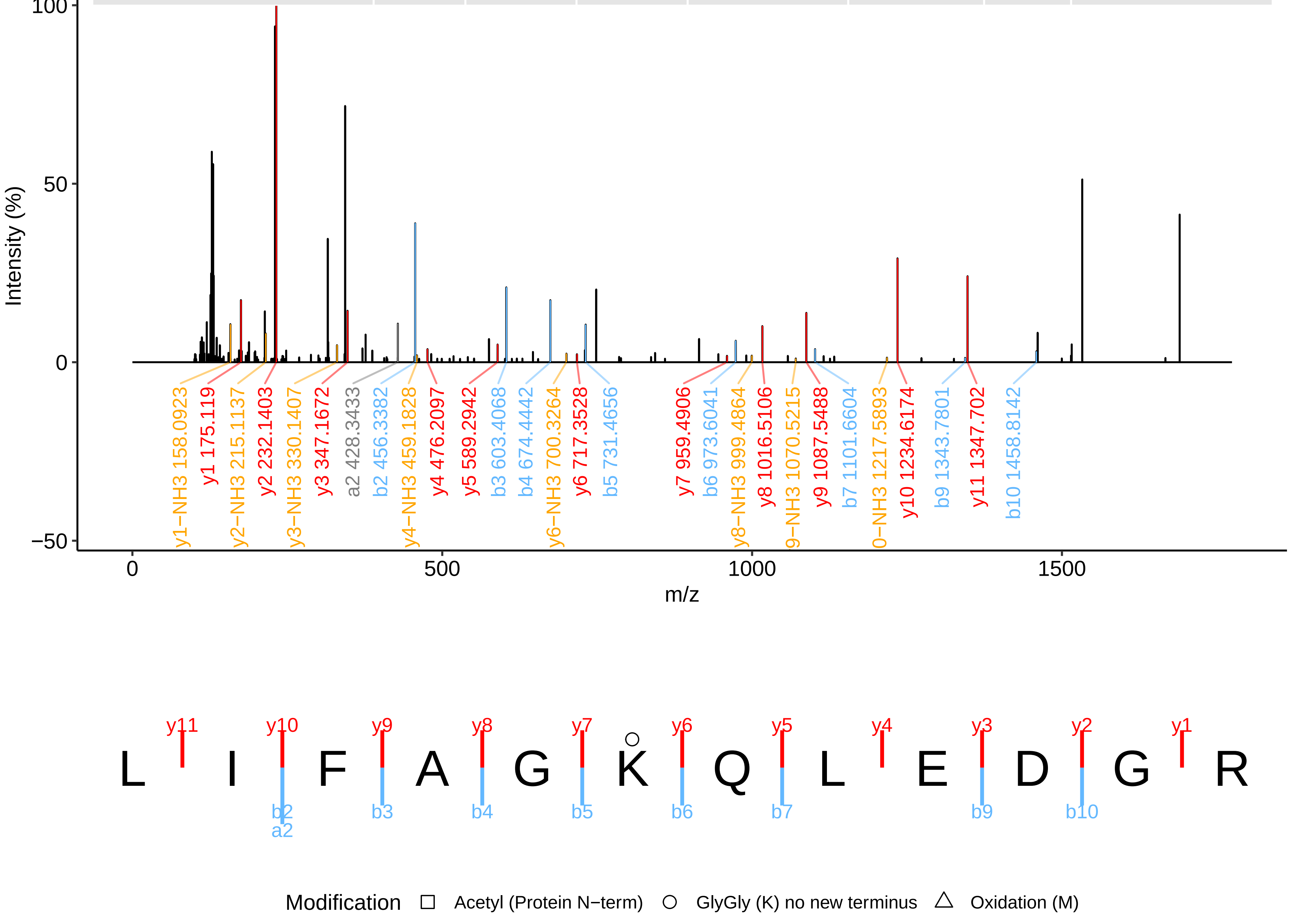

20200921_KIZ_MUb4_GlyGly_10plex − Scan number: 21555

Gene Names	Charge	m/z	Mass	Retention time	PEP	Score	Precursor Intensity
RPS27A;UBA52;UBC;UBB	2	730.8964	1459.778	78.078	0.0048195	91.549	NA

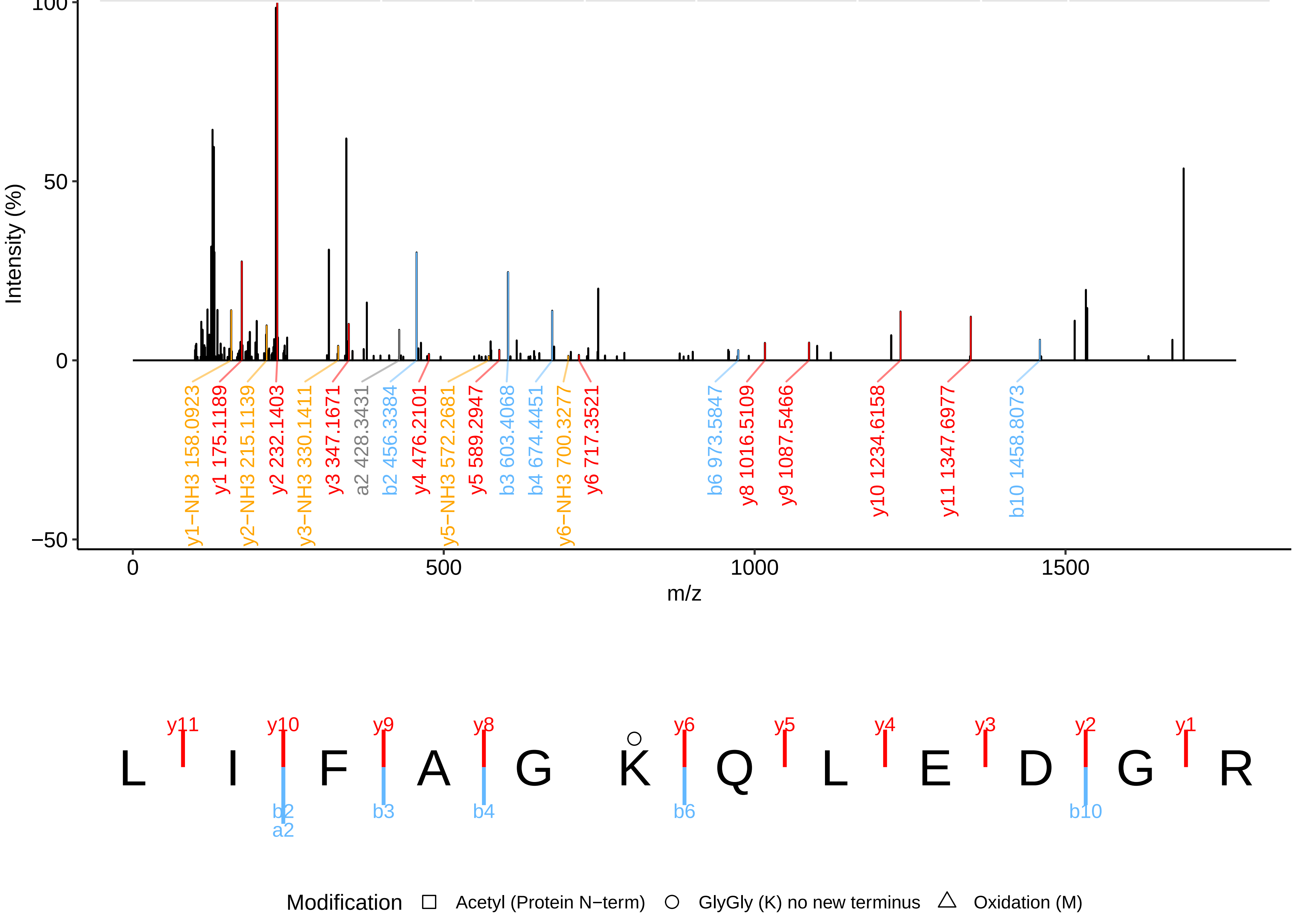

20200921_KIZ_MUb4_GlyGly_10plex − Scan number: 23866

Gene Names	Charge	m/z	Mass	Retention time	PEP	Score	Precursor Intensity
RPS27A;UBA52;UBC;UBB	3	487.6001	1459.778	86.674	5.4904e−06	123.67	18322132

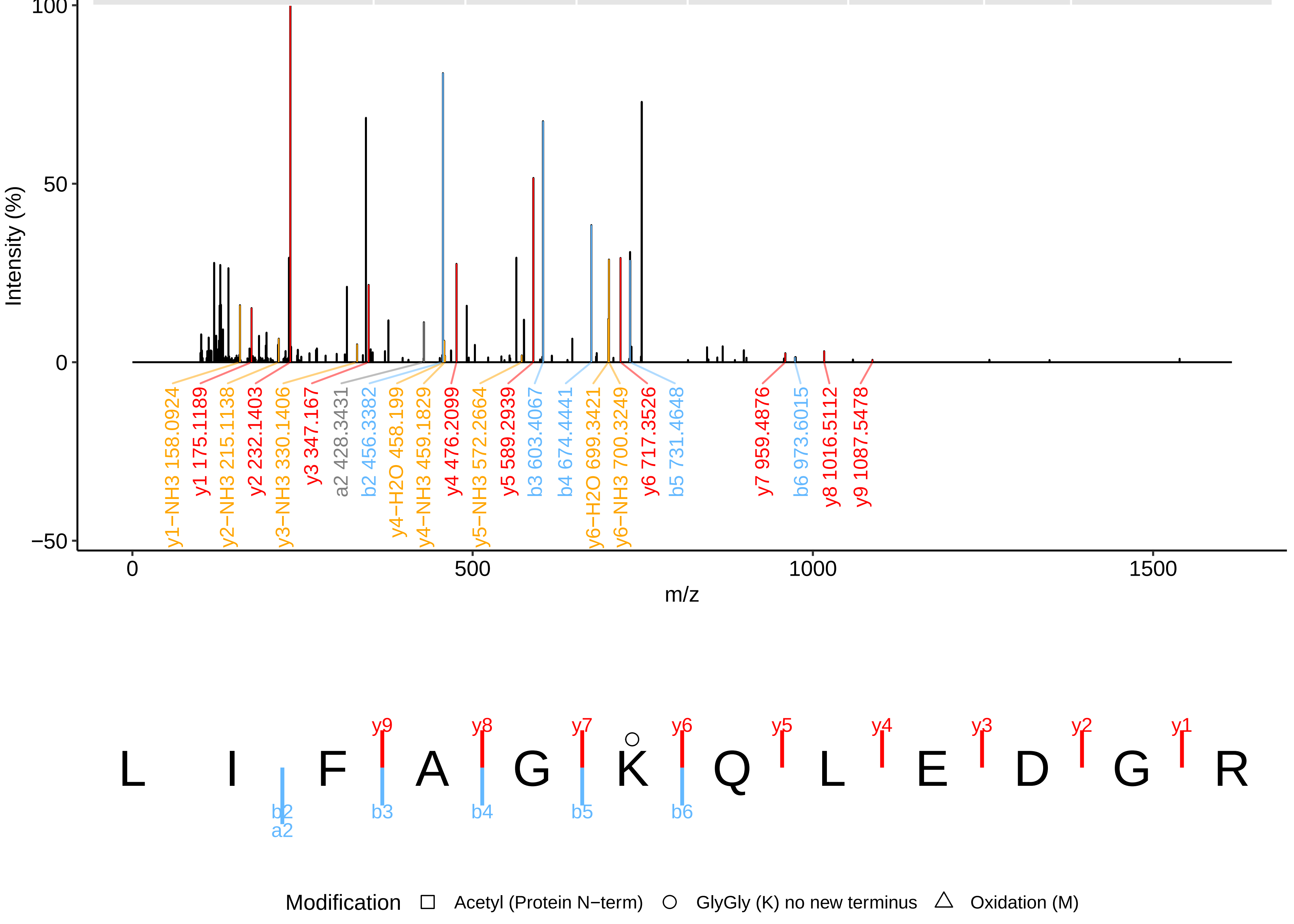

20200921_KIZ_MUb4_GlyGly_10plex − Scan number: 24536

Gene Names	Charge	m/z	Mass	Retention time	PEP	Score	Precursor Intensity
RPS27A;UBA52;UBC;UBB	3	487.6001	1459.778	89.227	5.9788e−08	141.71	14169513

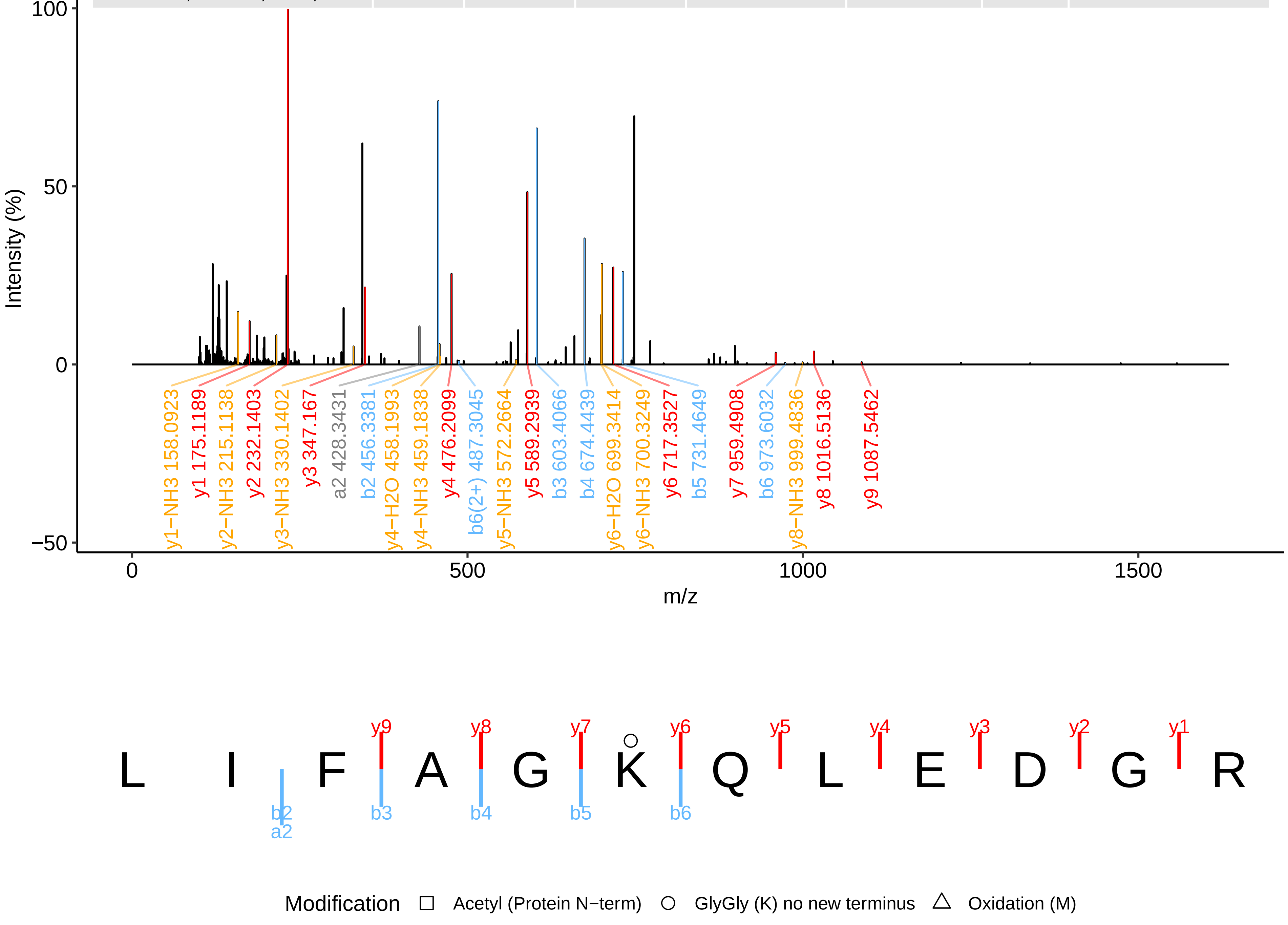

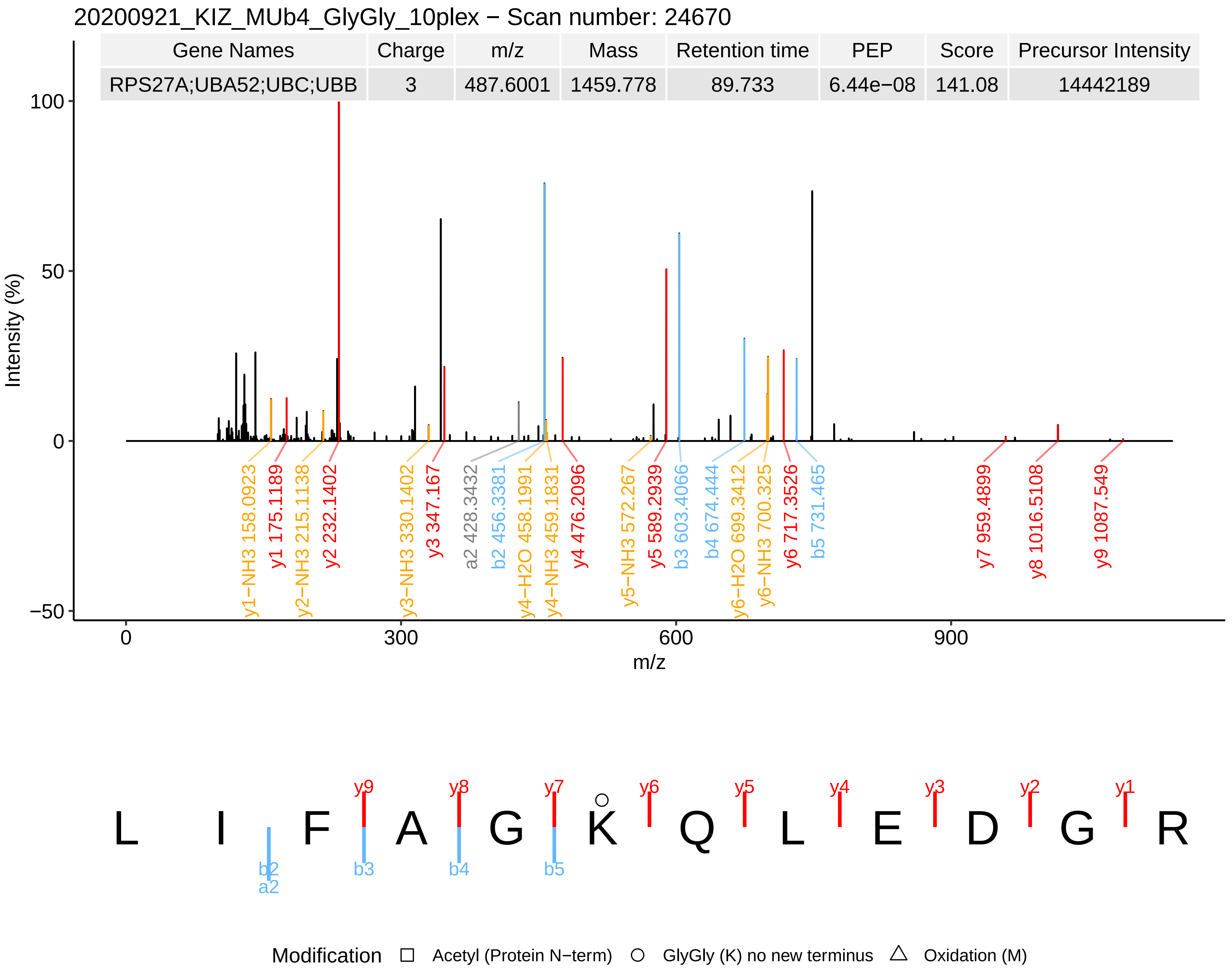
20200921_KIZ_MUb4_GlyGly_10plex − Scan number: 24670
Gene Names | Charge | m/z | Mass | Retention time | PEP | Score | Precursor Intensity
RPS27A;UBA52;UBC;UBB | 3 | 487.6001 | 1459.778 | 89.733 | 6.44e−08 | 141.08 | 14442189
Intensity (%)
100
50
0
−50
0
300
600
900
m/z
y1−NH3 158.0923
y1 175.1189
y2−NH3 215.1138
y2 232.1402
y3−NH3 330.1402
y3 347.167
a2 428.3432
b2 456.3381
y4−H2O 458.1991
y4−NH3 459.1831
y4 476.2096
y5−NH3 572.267
y5 589.2939
b3 603.4066
b4 674.444
y6−H2O 699.3412
y6−NH3 700.325
y6 717.3526
b5 731.465
y7 959.4899
y8 1016.5108
y9 1087.549
L I F A G K Q L E D G R
y9 y8 y7 y6 y5 y4 y3 y2 y1
b2 a2 b3 b4 b5
Modification
Acetyl (Protein N−term)
GlyGly (K) no new terminus
Oxidation (M)

20200921_KIZ_MUb4_GlyGly_10plex − Scan number: 27118

Gene Names	Charge	m/z	Mass	Retention time	PEP	Score	Precursor Intensity
RPS27A;UBA52;UBC;UBB	3	487.6001	1459.778	98.922	7.9801e−08	138.99	14833454

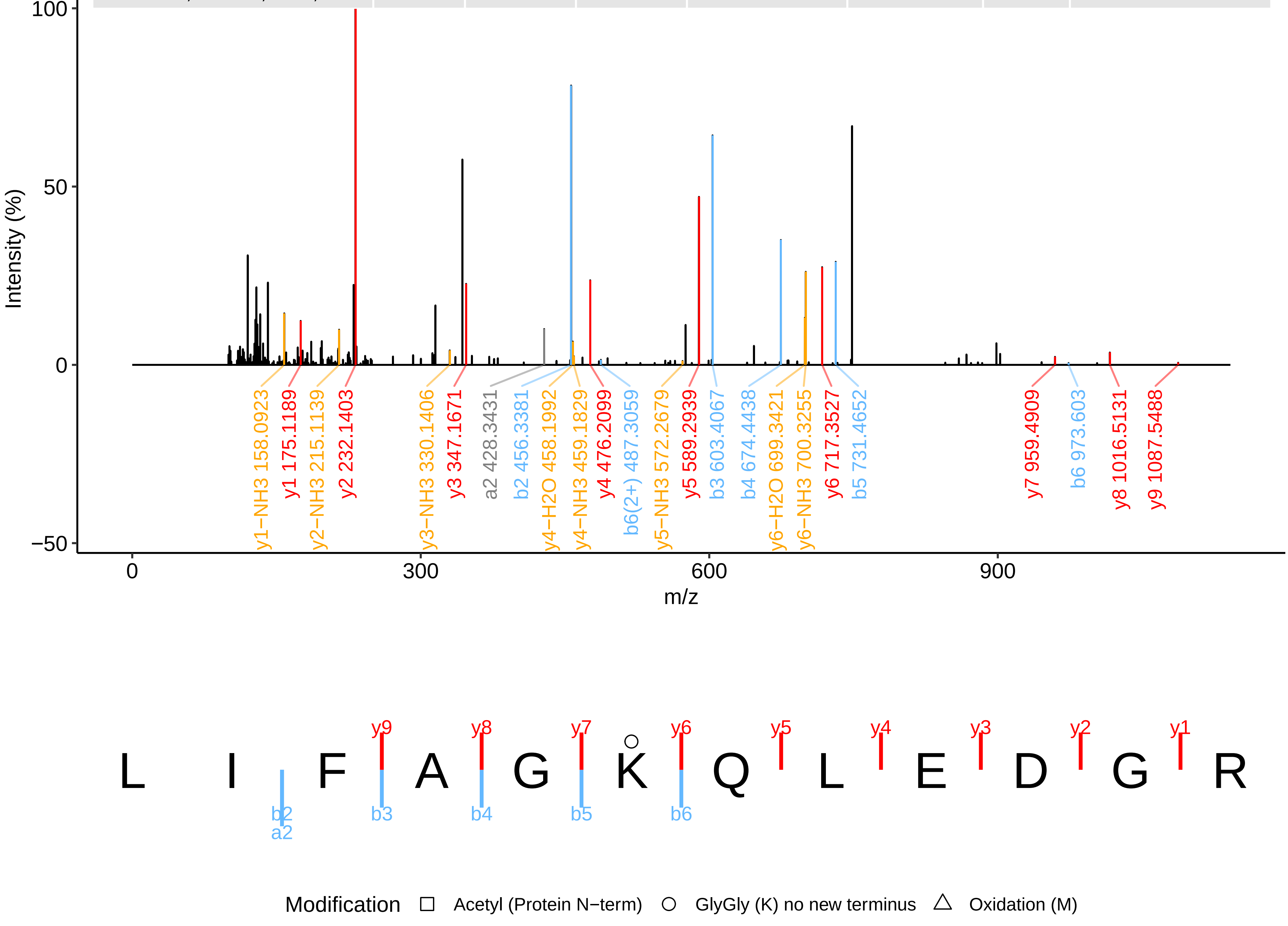

20200921_KIZ_MUb4_GlyGly_10plex − Scan number: 27254

Gene Names	Charge	m/z	Mass	Retention time	PEP	Score	Precursor Intensity
RPS27A;UBA52;UBC;UBB	3	487.6001	1459.778	99.437	7.9801e−08	138.99	14257809

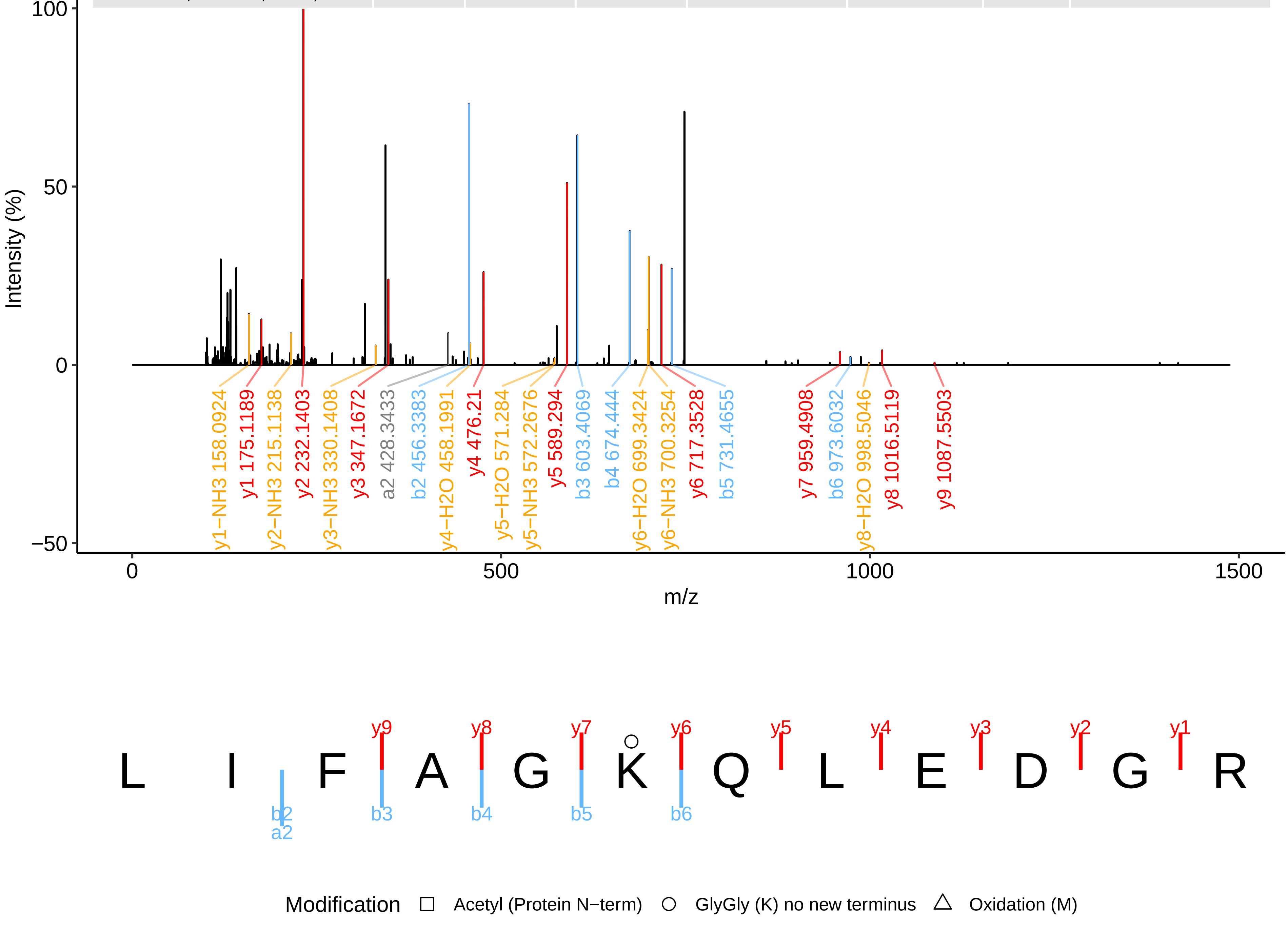

20200921_KIZ_MUb4_GlyGly_10plex − Scan number: 28052

Gene Names	Charge	m/z	Mass	Retention time	PEP	Score	Precursor Intensity
RPS27A;UBA52;UBC;UBB	3	487.6001	1459.778	102.54	3.6779e−11	150.46	7523231

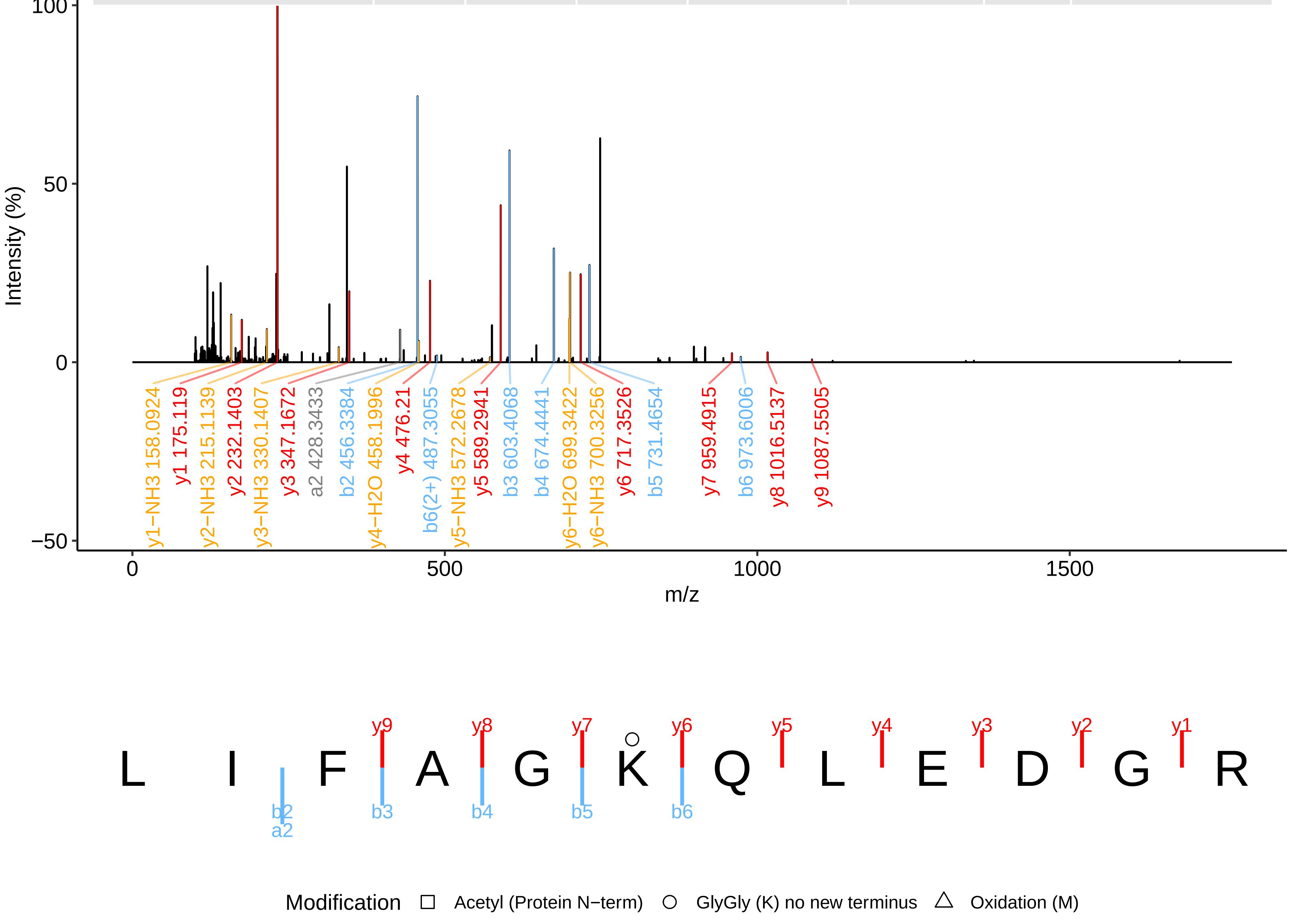

20200921_KIZ_MUb4_GlyGly_10plex − Scan number: 28792

Gene Names	Charge	m/z	Mass	Retention time	PEP	Score	Precursor Intensity
RPS27A;UBA52;UBC;UBB	3	487.6001	1459.778	105.38	7.9801e−08	138.99	10487963

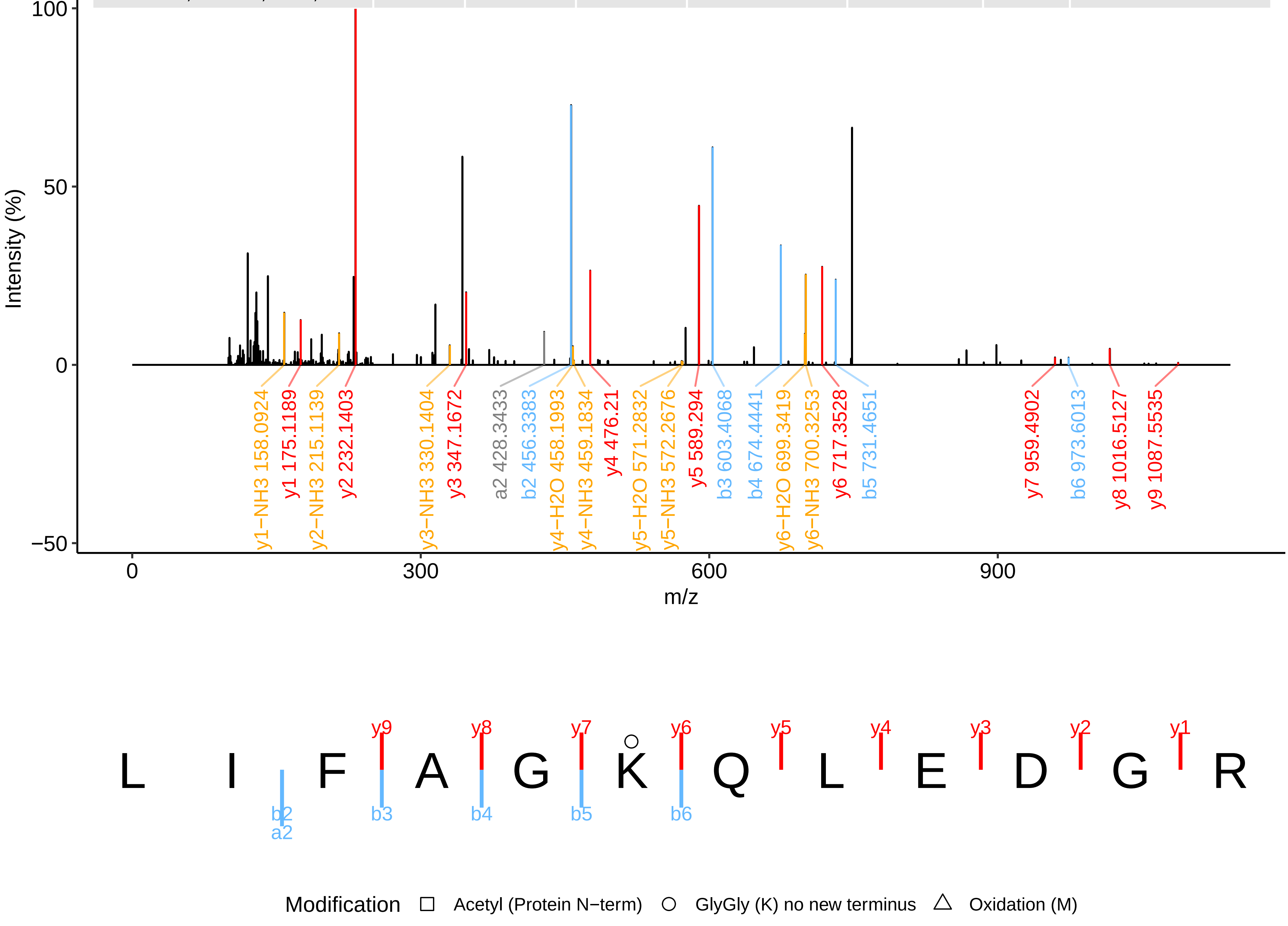

20200921_KIZ_MUb4_GlyGly_10plex − Scan number: 28923

Gene Names	Charge	m/z	Mass	Retention time	PEP	Score	Precursor Intensity
RPS27A;UBA52;UBC;UBB	3	487.6001	1459.778	105.89	3.6779e−11	150.46	6510435

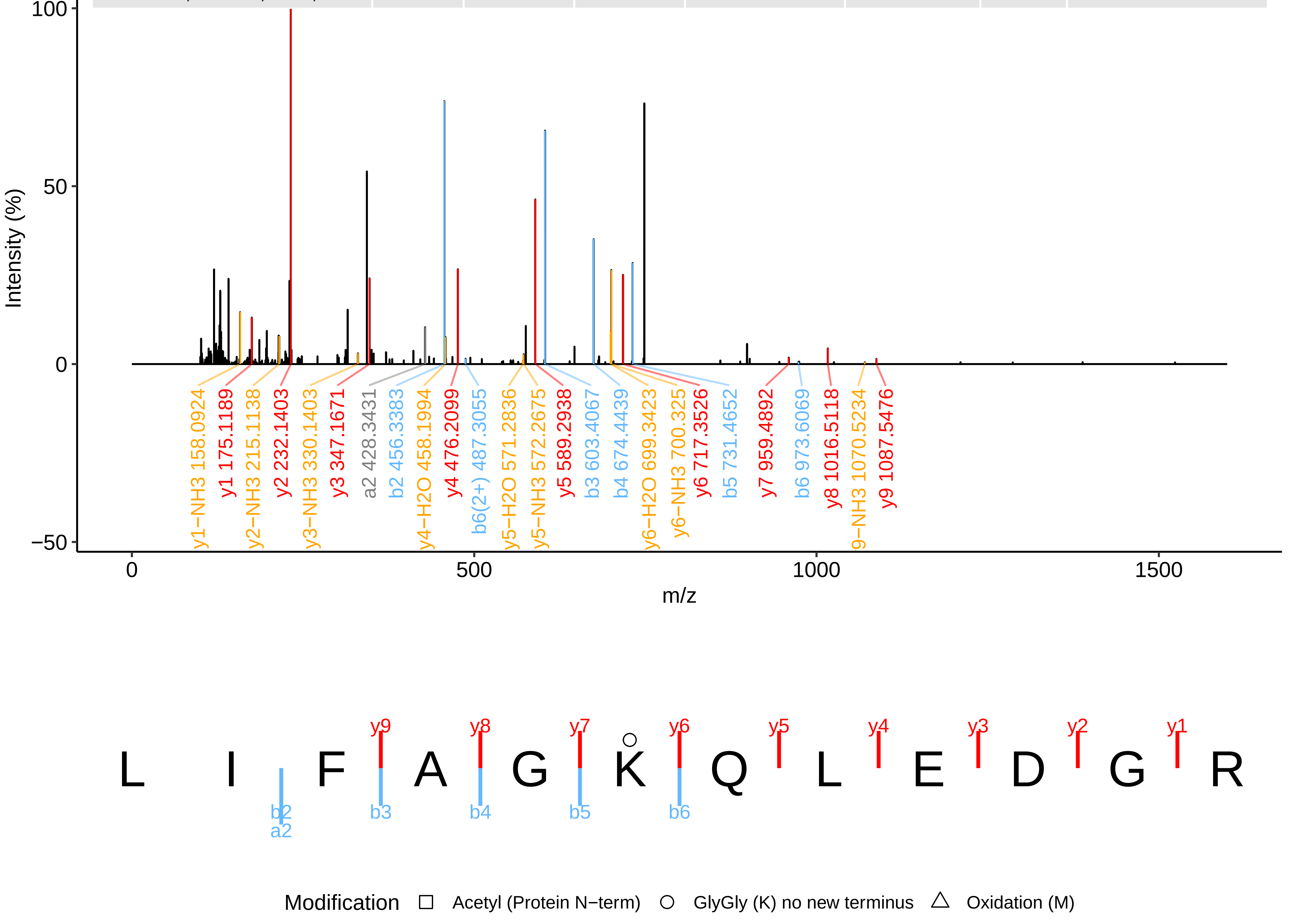

20200921_KIZ_MUb4_GlyGly_10plex − Scan number: 29705

Gene Names	Charge	m/z	Mass	Retention time	PEP	Score	Precursor Intensity
RPS27A;UBA52;UBC;UBB	3	487.6001	1459.778	108.99	6.0914e−06	122.69	7559102

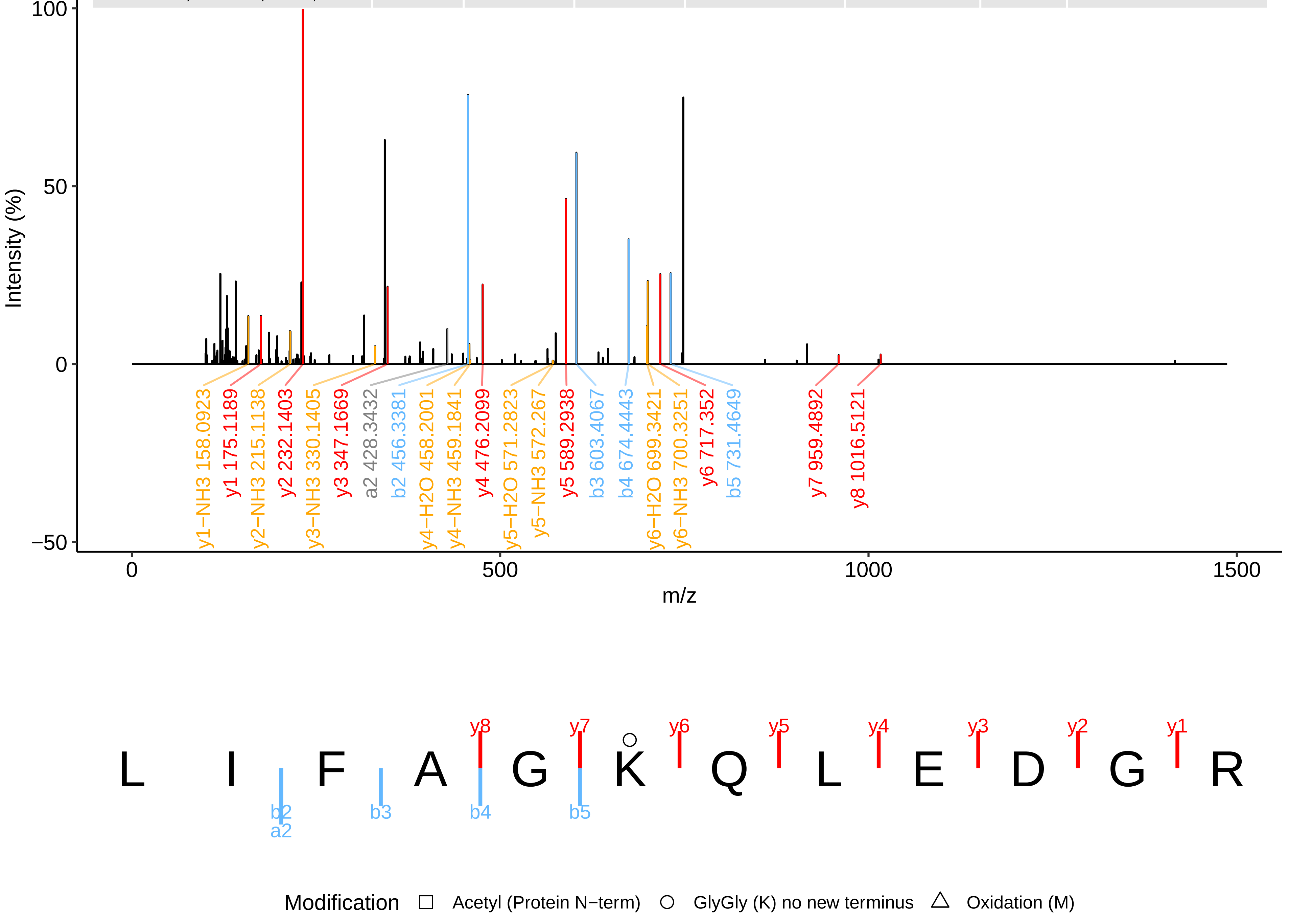

20200921_KIZ_MUb4_GlyGly_10plex − Scan number: 30468

Gene Names	Charge	m/z	Mass	Retention time	PEP	Score	Precursor Intensity
RPS27A;UBA52;UBC;UBB	3	487.6001	1459.778	112.04	2.8851e−08	145.91	7313169

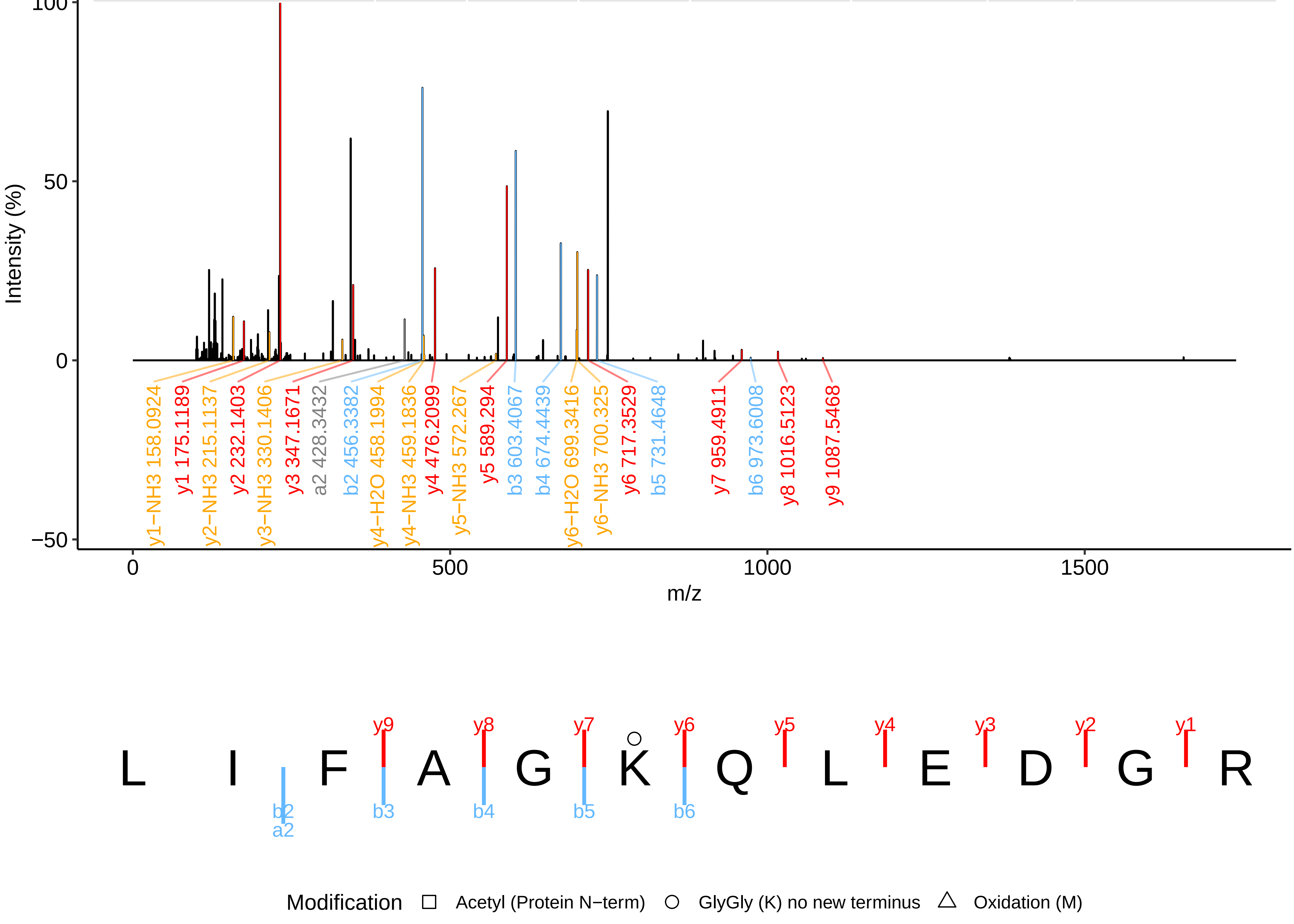

20200921_KIZ_MUb4_GlyGly_10plex − Scan number: 30603

Gene Names	Charge	m/z	Mass	Retention time	PEP	Score	Precursor Intensity
RPS27A;UBA52;UBC;UBB	3	487.6001	1459.778	112.55	7.0993e−15	174.4	5219931

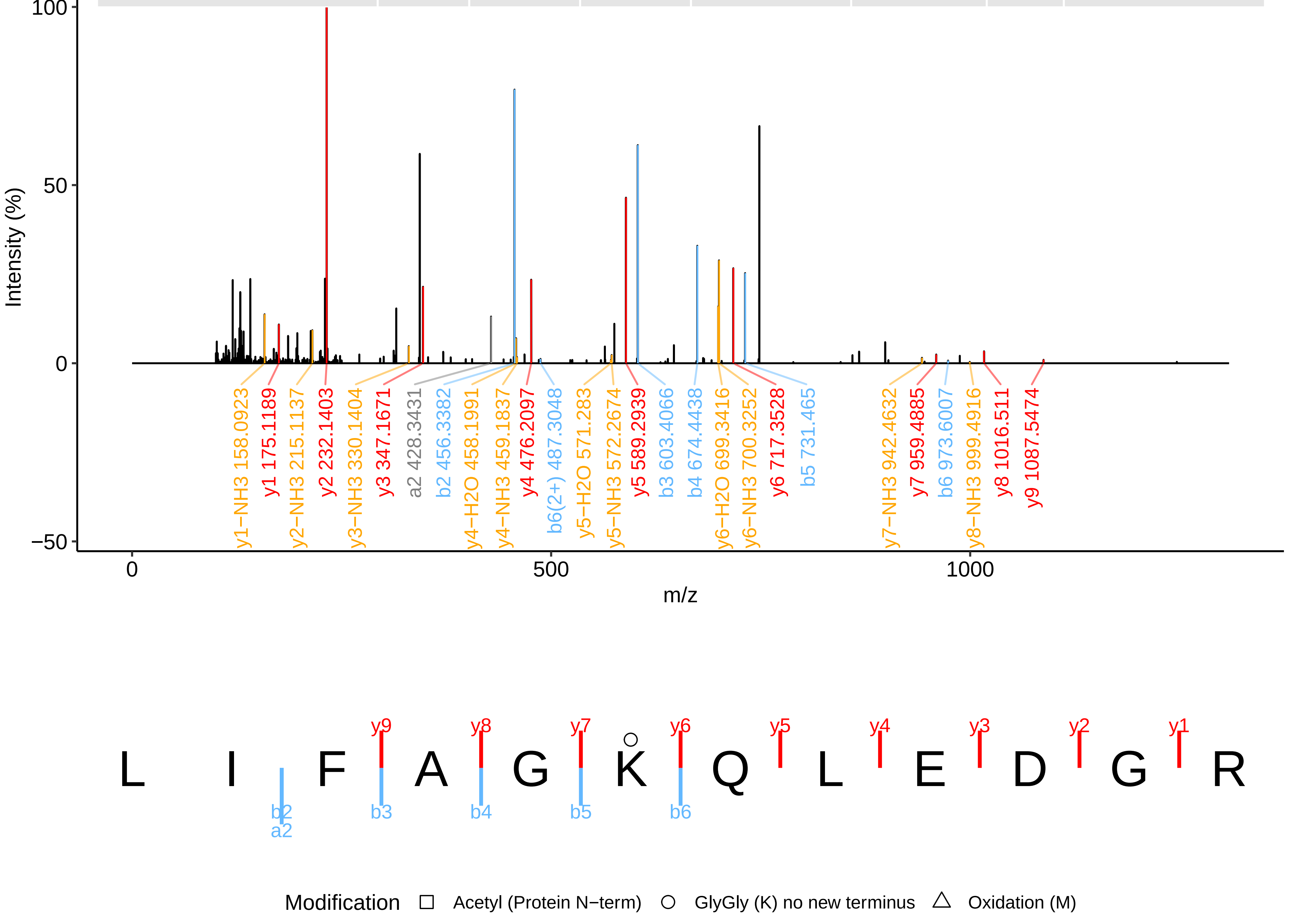

20200921_KIZ_MUb4_GlyGly_10plex − Scan number: 31756

Gene Names	Charge	m/z	Mass	Retention time	PEP	Score	Precursor Intensity
RPS27A;UBA52;UBC;UBB	3	487.6001	1459.778	117.15	4.2339e−06	125.72	2536870

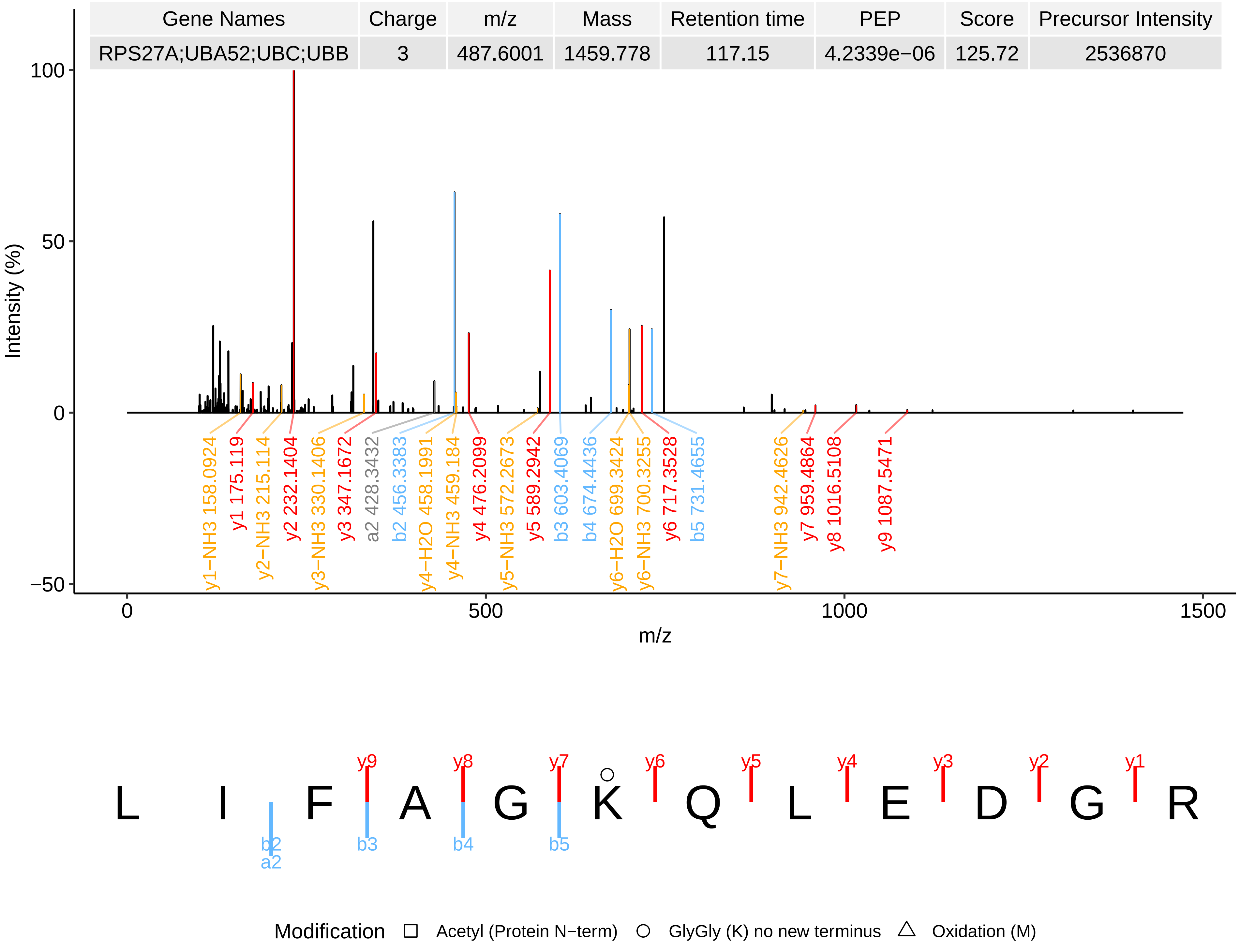

20200921_KIZ_MUb4_GlyGly_10plex − Scan number: 31934

Gene Names	Charge	m/z	Mass	Retention time	PEP	Score	Precursor Intensity
RPS27A;UBA52;UBC;UBB	3	487.6001	1459.778	117.87	2.6823e−11	155	6766476

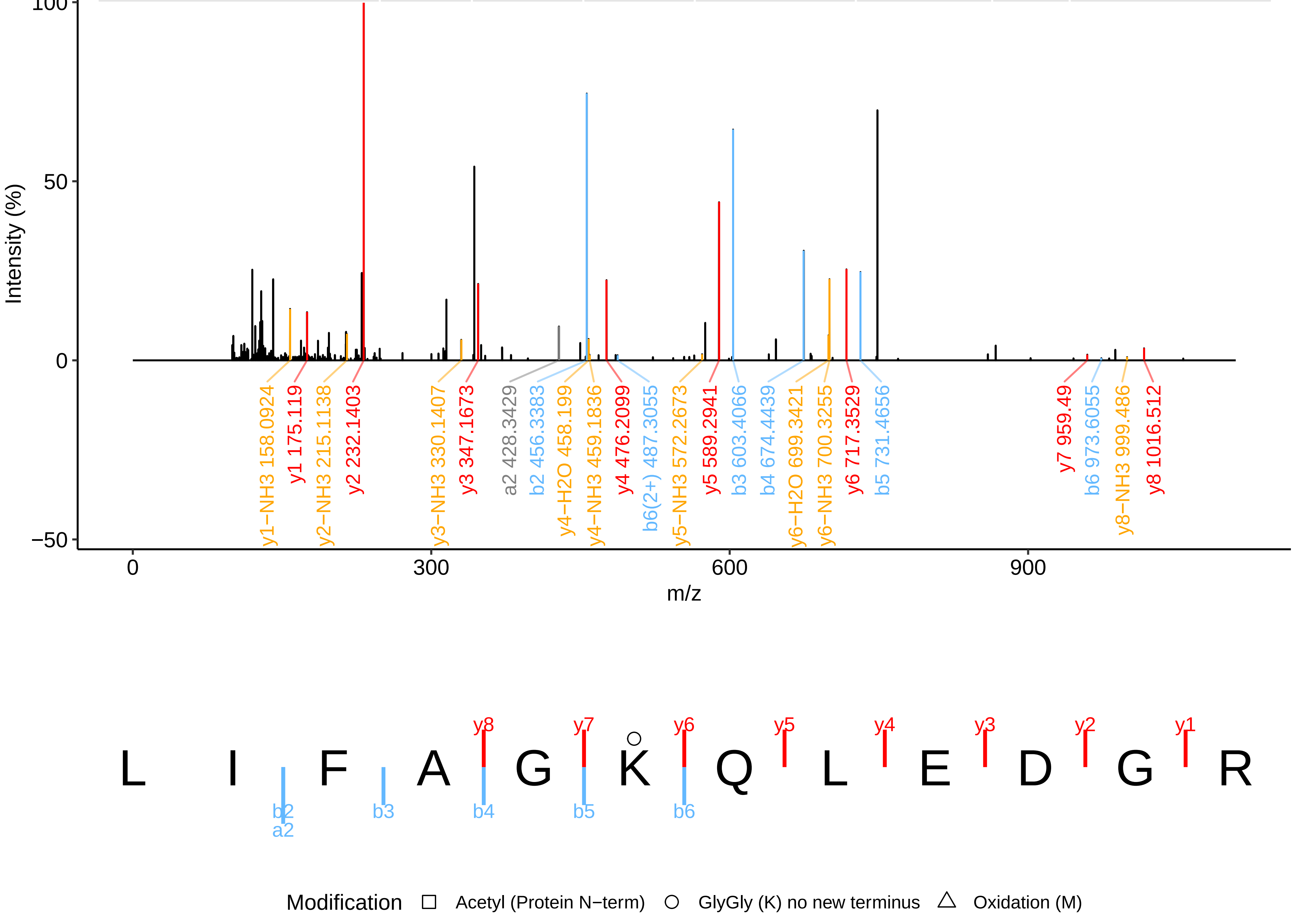

20200921_KIZ_MUb4_GlyGly_10plex − Scan number: 33309

Gene Names	Charge	m/z	Mass	Retention time	PEP	Score	Precursor Intensity
RPS27A;UBA52;UBC;UBB	3	487.6001	1459.778	122.36	0.013211	68.355	4631349

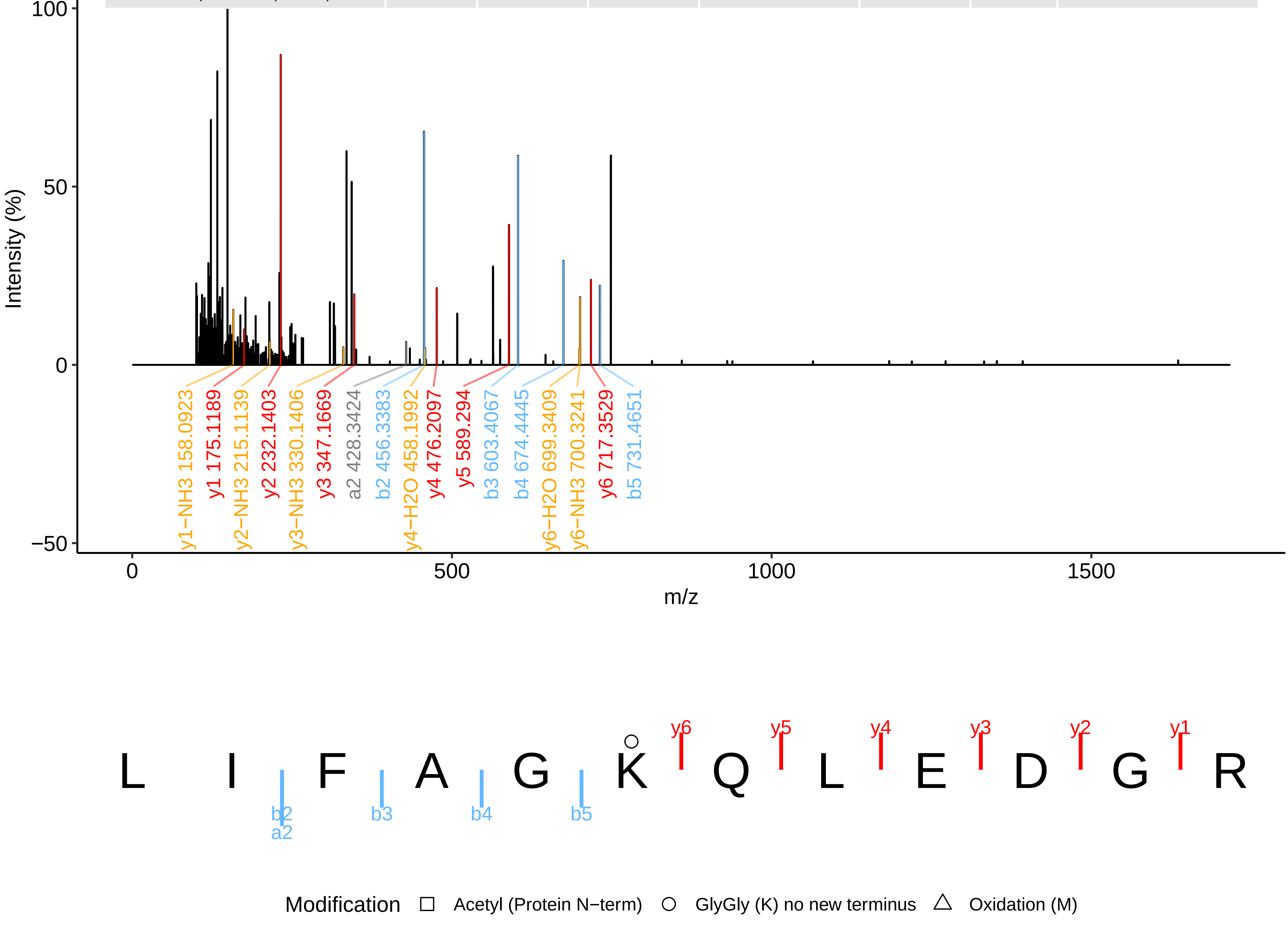

20200921_KIZ_MUb4_GlyGly_10plex − Scan number: 33501

Gene Names	Charge	m/z	Mass	Retention time	PEP	Score	Precursor Intensity
RPS27A;UBA52;UBC;UBB	3	487.6001	1459.778	122.99	4.8002e−05	98.898	2059836

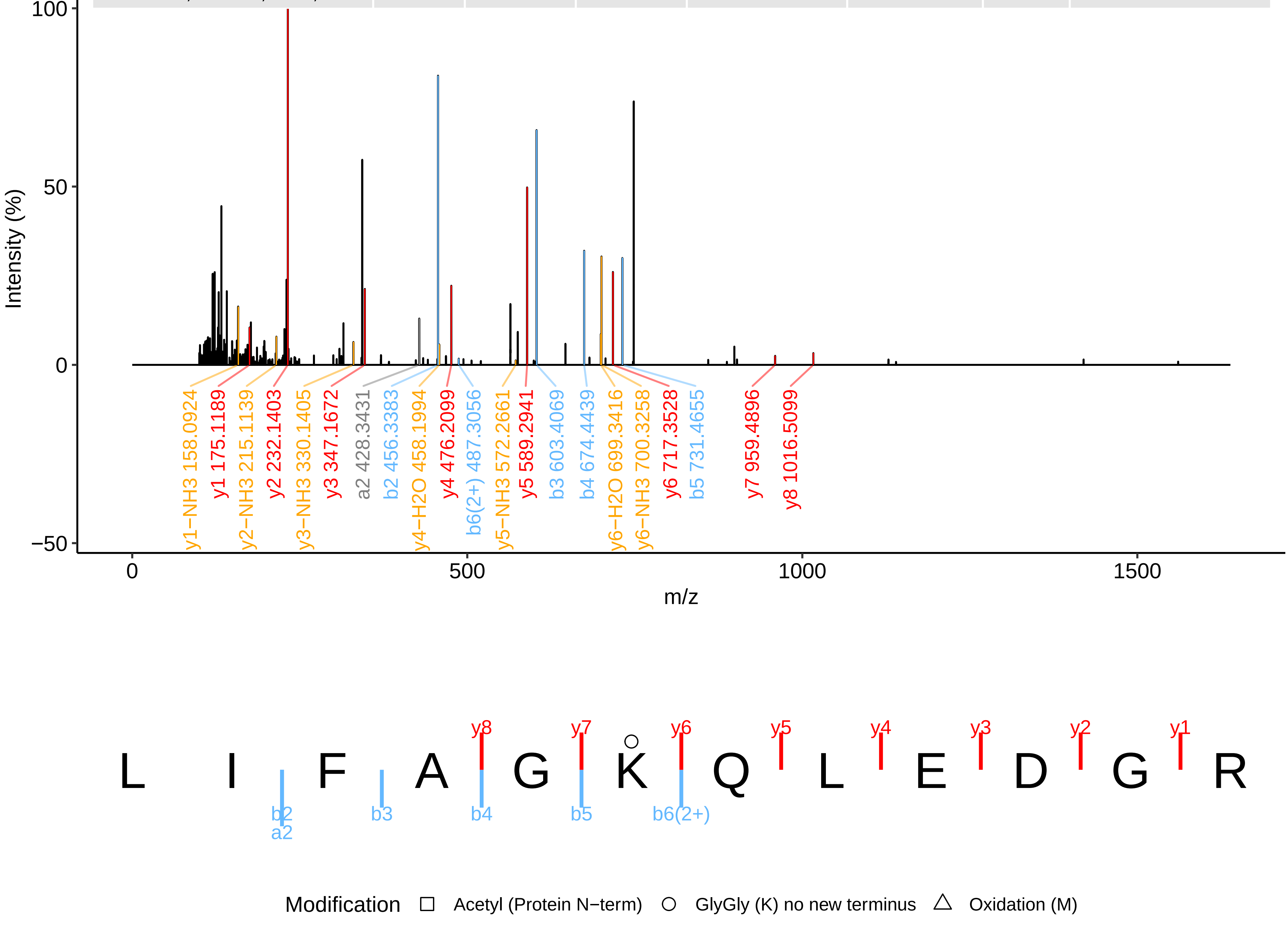

20200921_KIZ_MUb4_GlyGly_10plex − Scan number: 33642

Gene Names	Charge	m/z	Mass	Retention time	PEP	Score	Precursor Intensity
RPS27A;UBA52;UBC;UBB	3	487.6001	1459.778	123.52	6.4339e−05	97.452	3569184

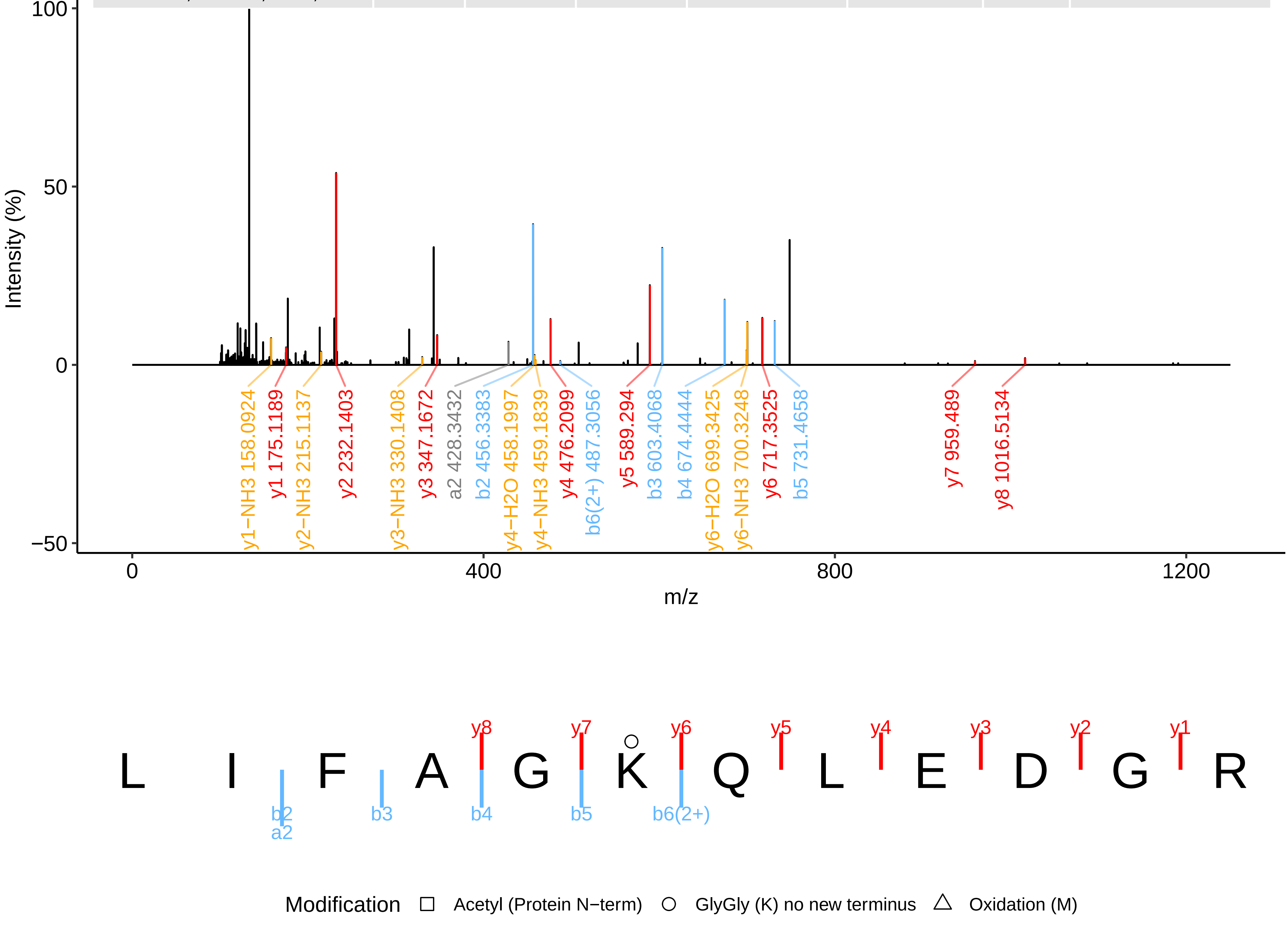

20200921_KIZ_MUb4_GlyGly_10plex − Scan number: 33837

Gene Names	Charge	m/z	Mass	Retention time	PEP	Score	Precursor Intensity
RPS27A;UBA52;UBC;UBB	3	487.6001	1459.778	124.28	8.1861e−06	119.27	NA

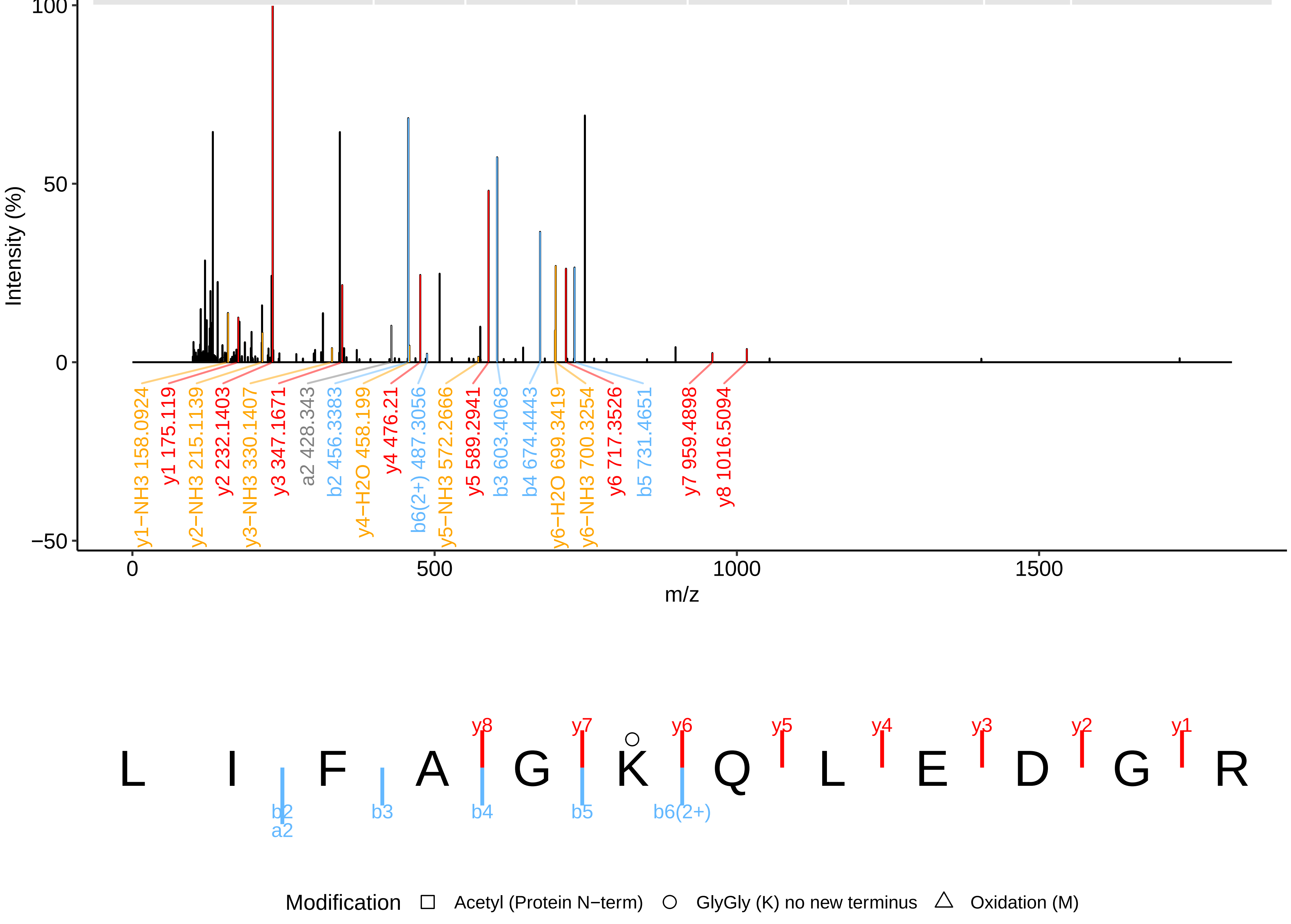

20200921_KIZ_MUb4_GlyGly_10plex − Scan number: 34257

Gene Names	Charge	m/z	Mass	Retention time	PEP	Score	Precursor Intensity
RPS27A;UBA52;UBC;UBB	3	487.6001	1459.778	125.72	2.923e−05	107.03	1659281

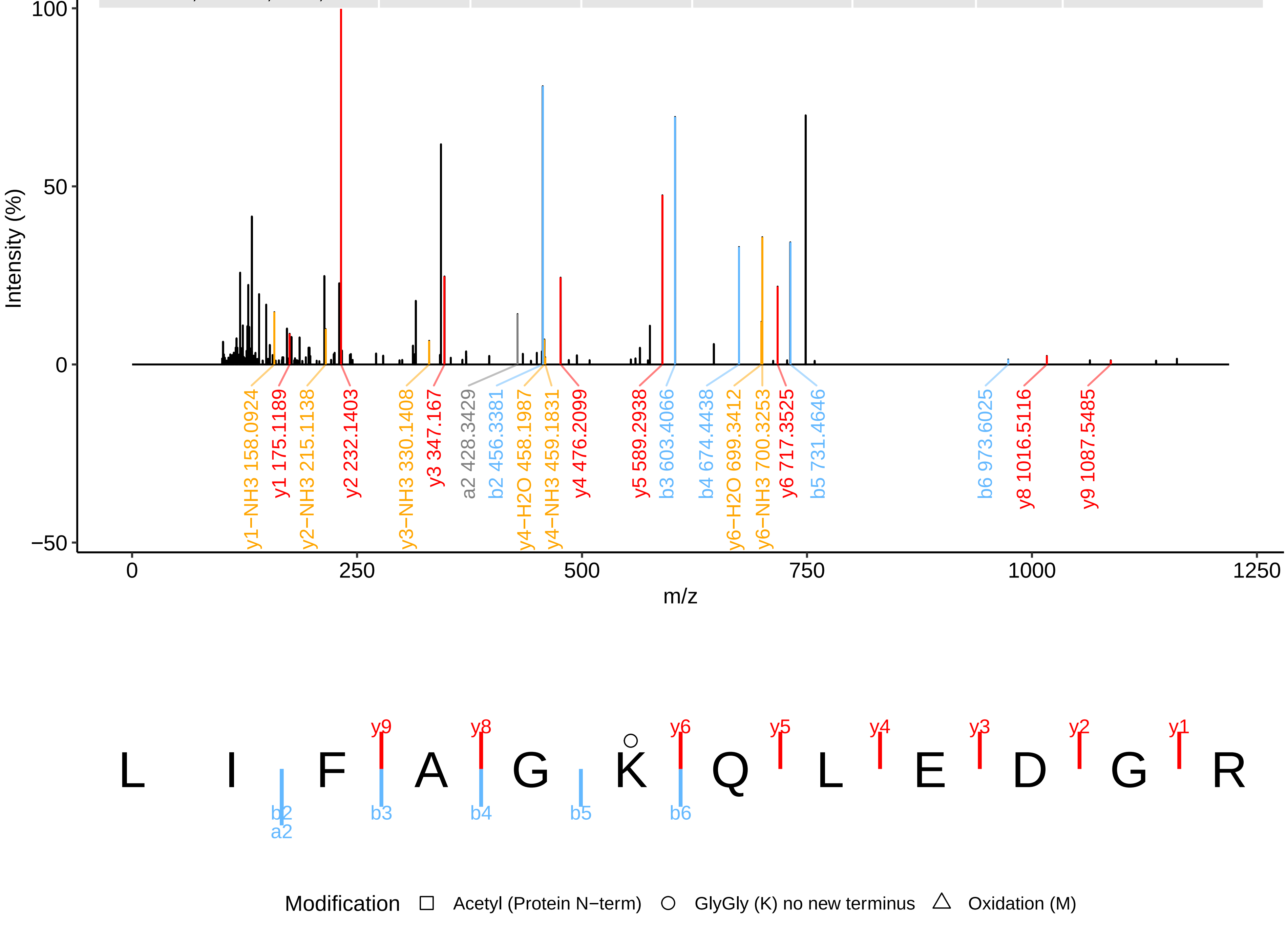

20200921_KIZ_MUb4_GlyGly_10plex − Scan number: 34414

Gene Names	Charge	m/z	Mass	Retention time	PEP	Score	Precursor Intensity
RPS27A;UBA52;UBC;UBB	3	487.6001	1459.778	126.29	0.0077734	73.951	2642471

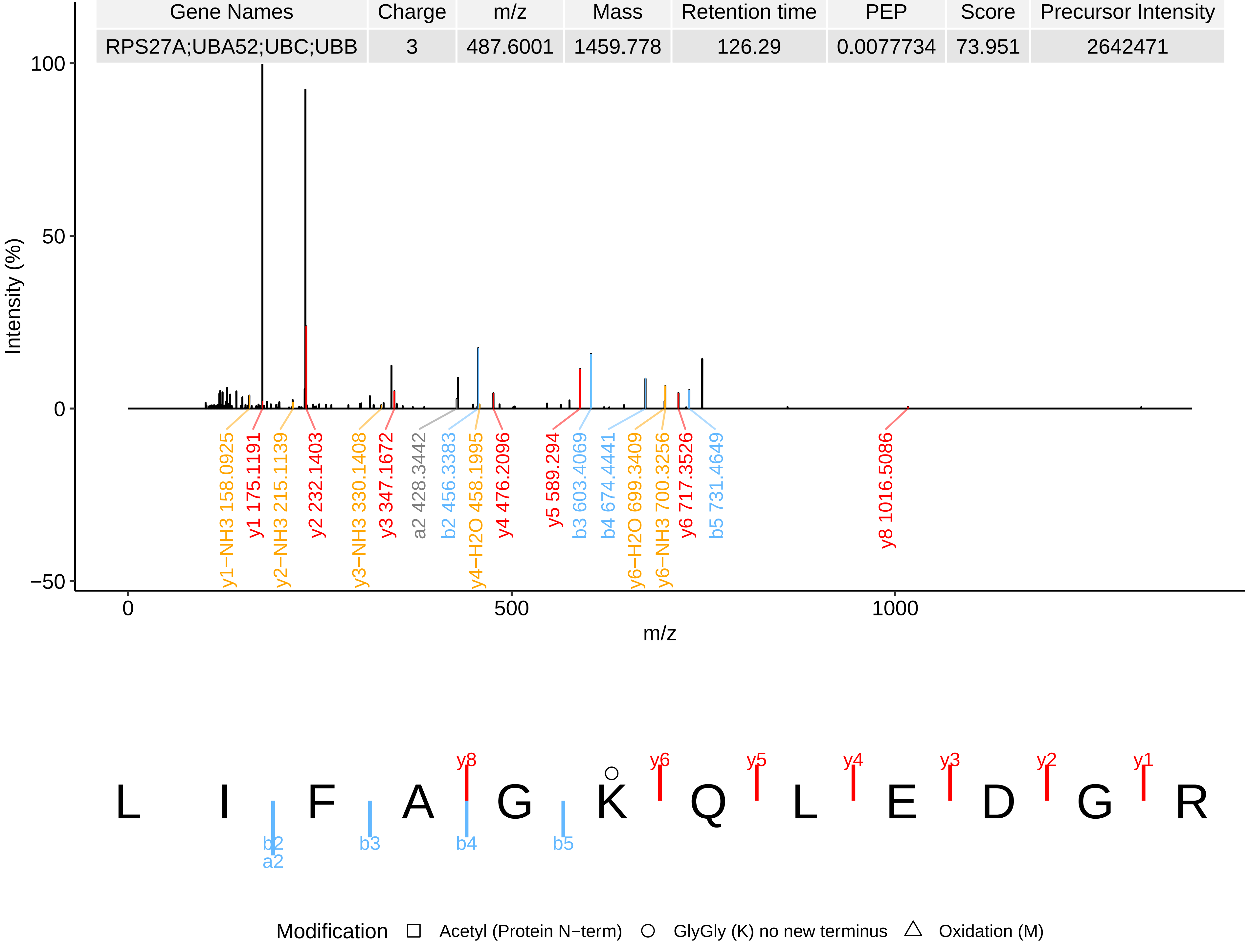

20200921_KIZ_MUb4_GlyGly_10plex − Scan number: 34567

Gene Names	Charge	m/z	Mass	Retention time	PEP	Score	Precursor Intensity
RPS27A;UBA52;UBC;UBB	3	487.6001	1459.778	126.81	0.00065376	91.313	NA

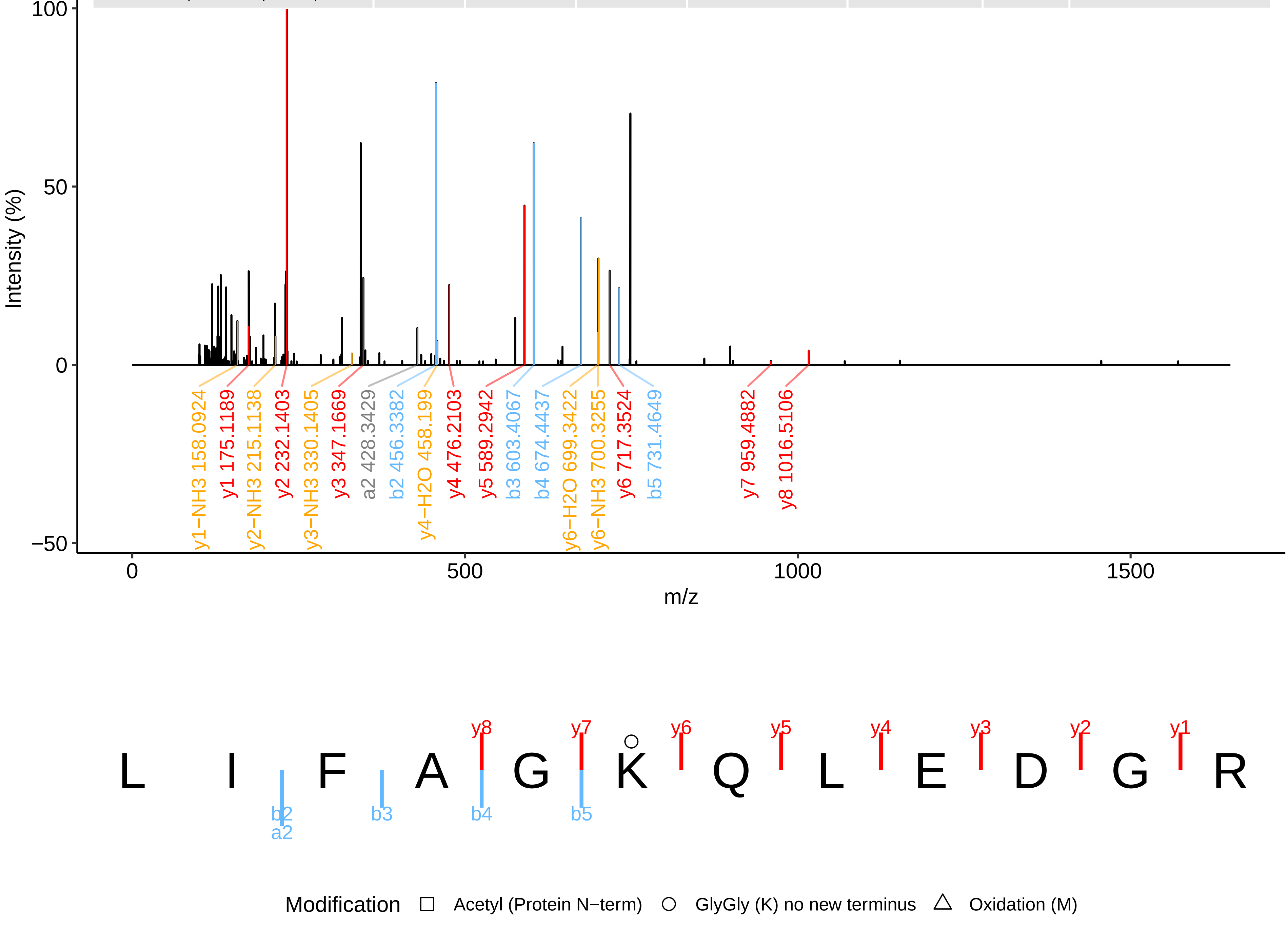

20200921_KIZ_MUb4_GlyGly_10plex_2 − Scan number: 12913

Gene Names	Charge	m/z	Mass	Retention time	PEP	Score	Precursor Intensity
RPS27A;UBA52;UBC;UBB	3	487.6001	1459.778	50.074	0.067333	52.693	NA

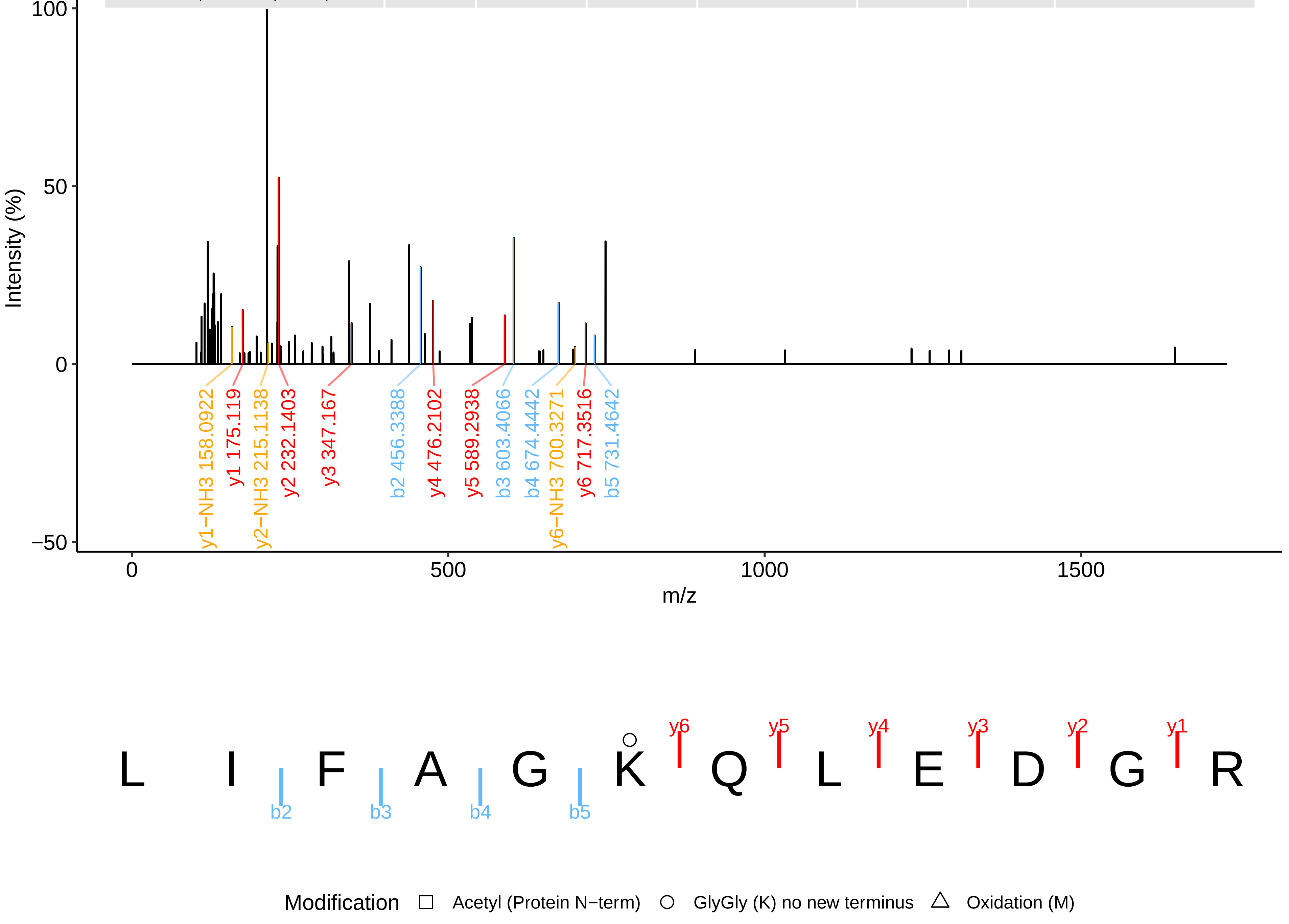

20200921_KIZ_MUb4_GlyGly_10plex_2 − Scan number: 13247

Gene Names	Charge	m/z	Mass	Retention time	PEP	Score	Precursor Intensity
RPS27A;UBA52;UBC;UBB	3	487.6001	1459.778	51.392	0.030186	59.84	427039.2

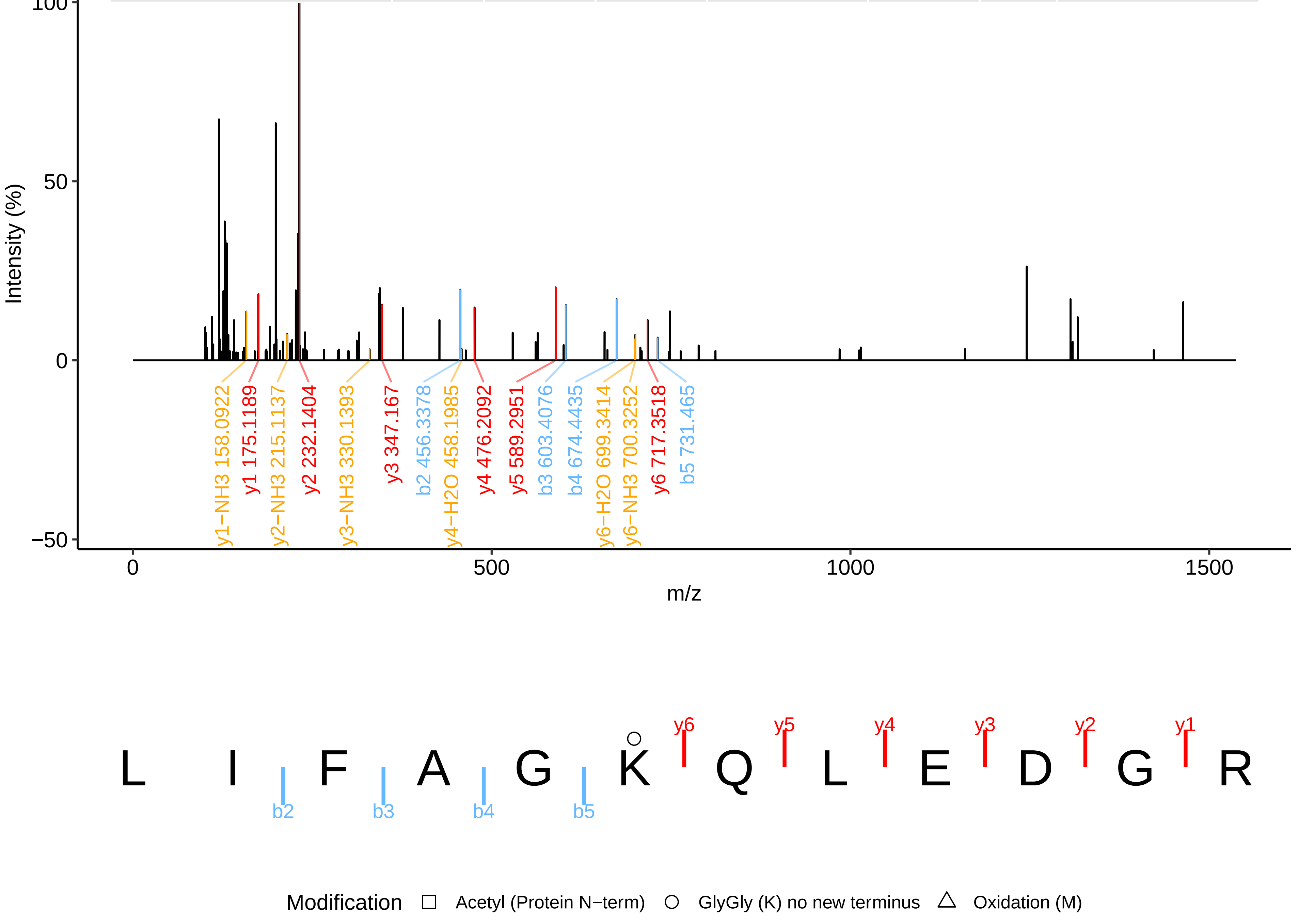

20200921_KIZ_MUb4_GlyGly_10plex_2 − Scan number: 14380

Gene Names	Charge	m/z	Mass	Retention time	PEP	Score	Precursor Intensity
RPS27A;UBA52;UBC;UBB	3	487.6001	1459.778	56.058	0.0058461	73.666	1060500

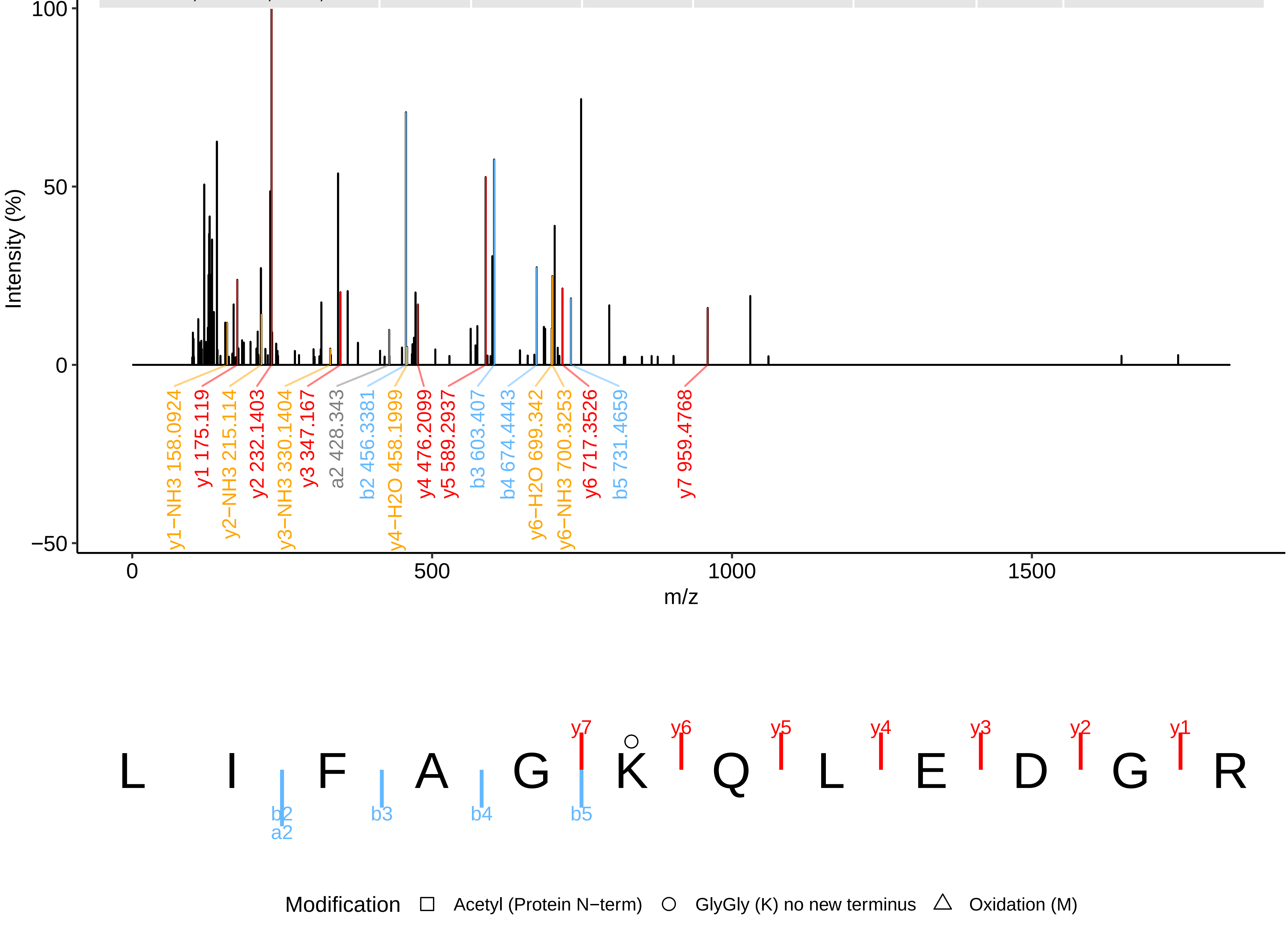

20200921_KIZ_MUb4_GlyGly_10plex_2 − Scan number: 15177

Gene Names	Charge	m/z	Mass	Retention time	PEP	Score	Precursor Intensity
RPS27A;UBA52;UBC;UBB	3	487.6001	1459.778	59.299	1.1157e−08	119.27	2782157

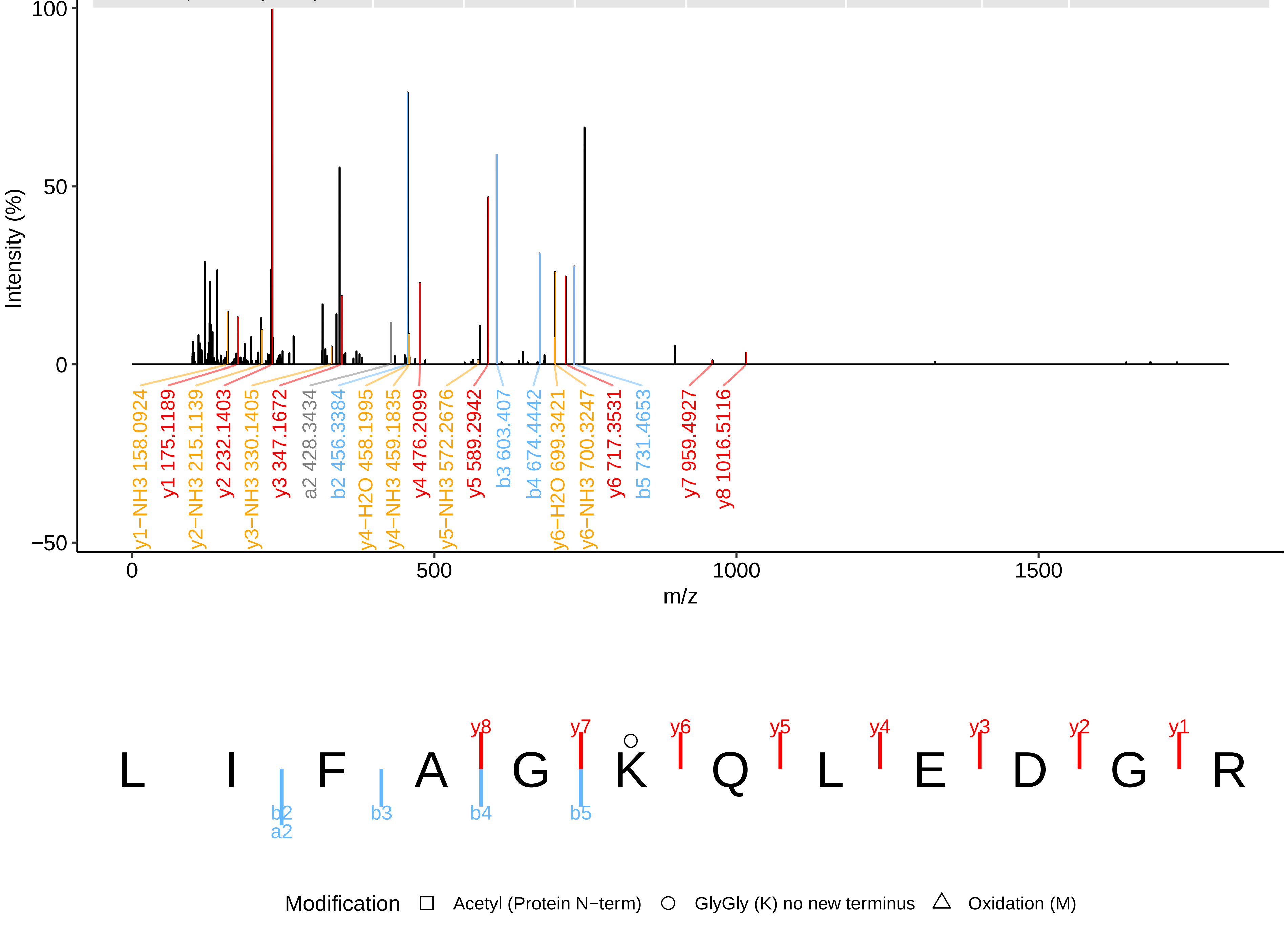

20200921_KIZ_MUb4_GlyGly_10plex_2 − Scan number: 15303

Gene Names	Charge	m/z	Mass	Retention time	PEP	Score	Precursor Intensity
RPS27A;UBA52;UBC;UBB	3	487.6001	1459.778	59.814	1.9697e−05	98.898	2257934

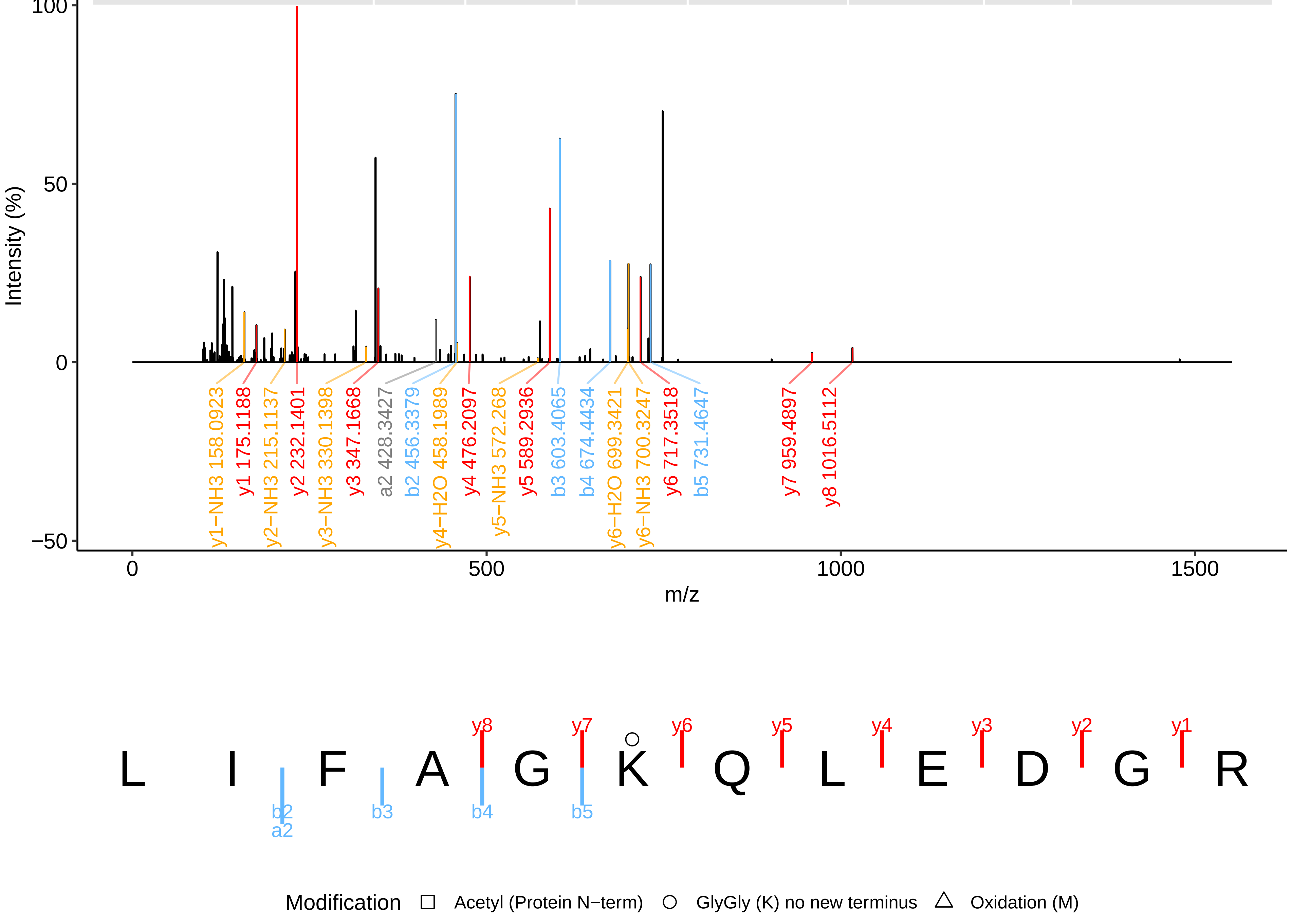

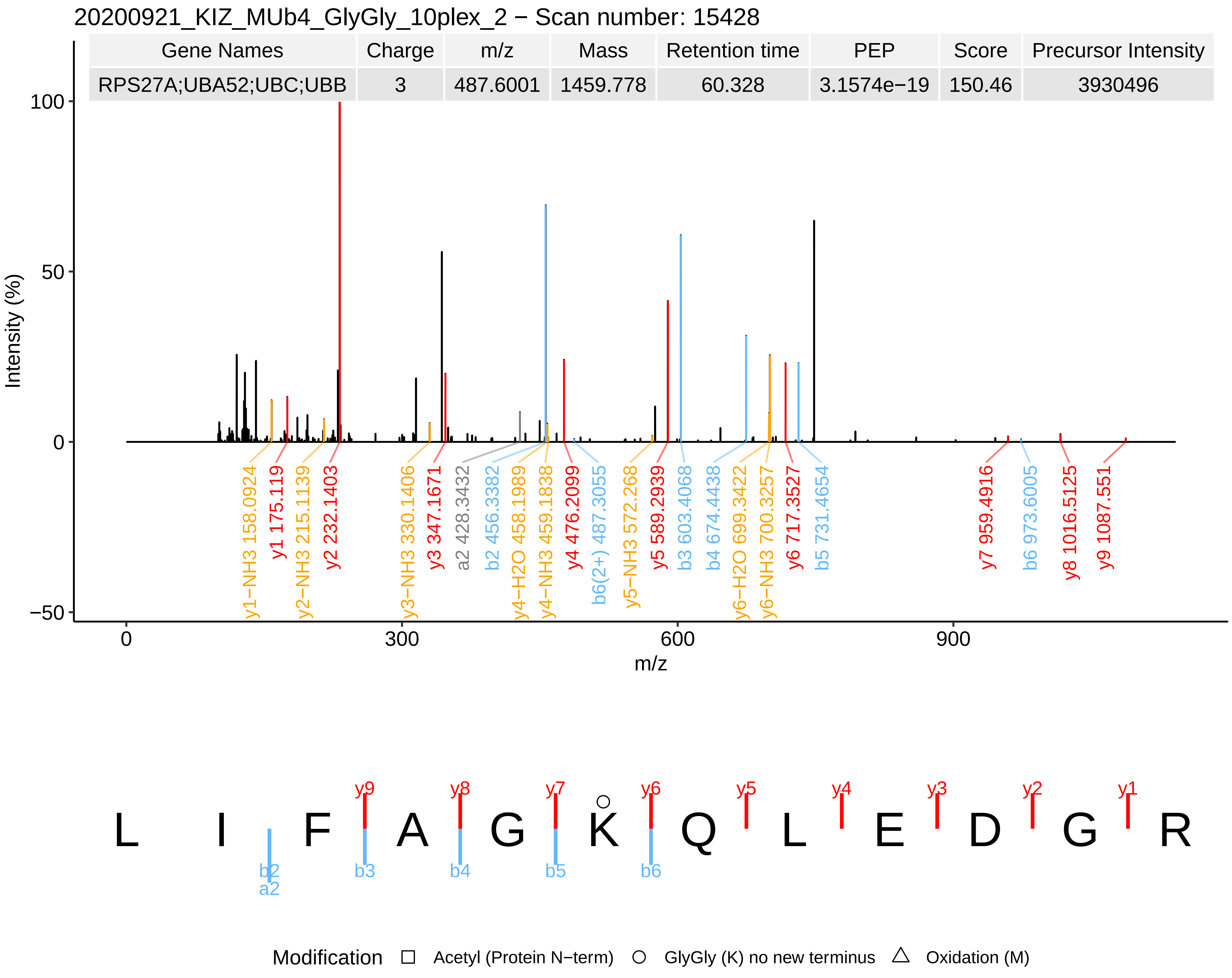
20200921_KIZ_MUb4_GlyGly_10plex_2 − Scan number: 15428
Gene Names | Charge | m/z | Mass | Retention time | PEP | Score | Precursor Intensity
RPS27A;UBA52;UBC;UBB | 3 | 487.6001 | 1459.778 | 60.328 | 3.1574e−19 | 150.46 | 3930496
Intensity (%)
m/z
y1−NH3 158.0924
y1 175.119
y2−NH3 215.1139
y2 232.1403
y3−NH3 330.1406
y3 347.1671
a2 428.3432
b2 456.3382
y4−H2O 458.1989
y4−NH3 459.1838
y4 476.2099
b6(2+) 487.3055
y5−NH3 572.268
y5 589.2939
b3 603.4068
b4 674.4438
y6−H2O 699.3422
y6−NH3 700.3257
y6 717.3527
b5 731.4654
y7 959.4916
b6 973.6005
y8 1016.5125
y9 1087.551
L I F A G K Q L E D G R
y9 y8 y7 y6 y5 y4 y3 y2 y1
b2 a2 b3 b4 b5 b6
Modification
Acetyl (Protein N−term)
GlyGly (K) no new terminus
Oxidation (M)

20200921_KIZ_MUb4_GlyGly_10plex_2 − Scan number: 15832

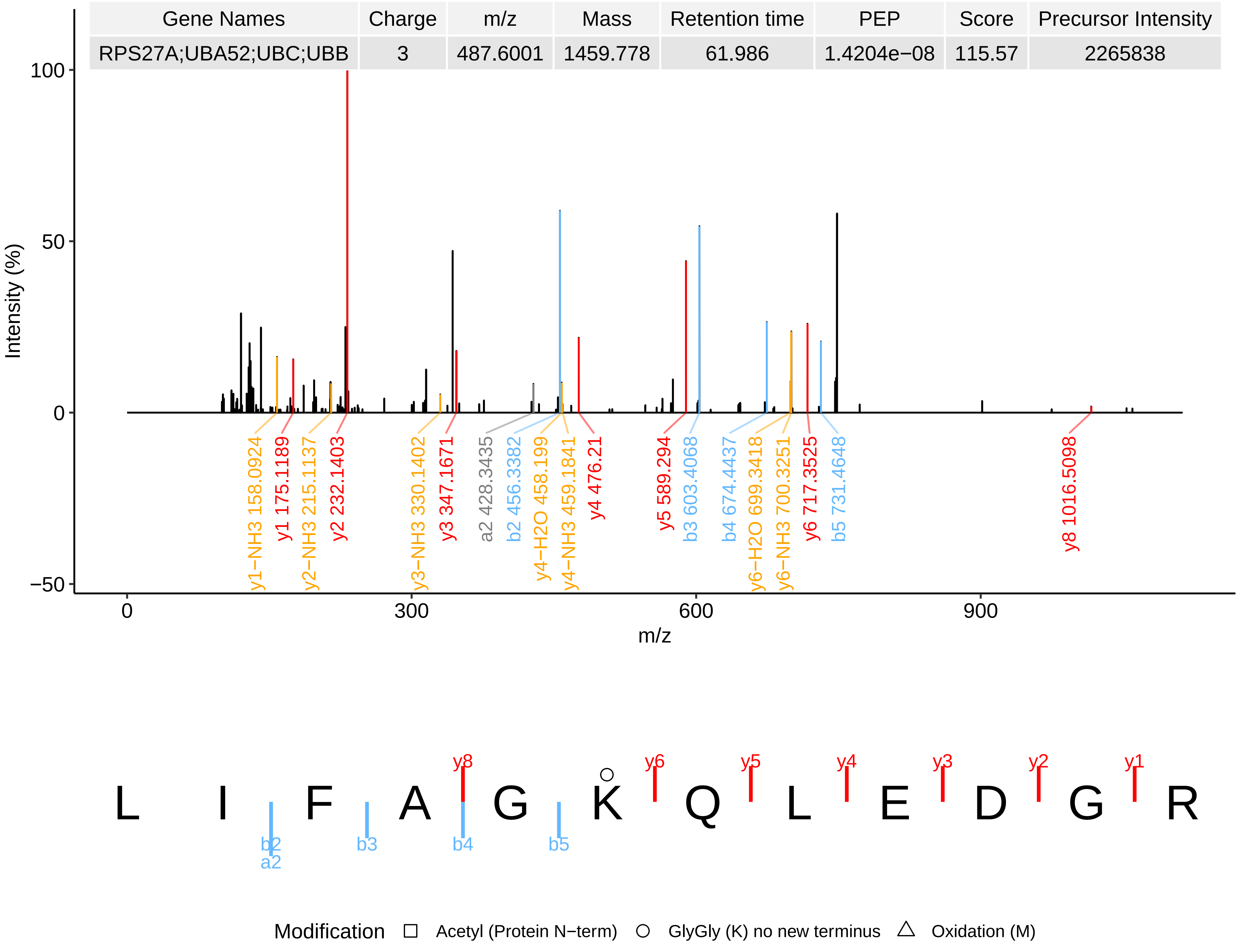

20200921_KIZ_MUb4_GlyGly_10plex_2 − Scan number: 15980

Gene Names	Charge	m/z	Mass	Retention time	PEP	Score	Precursor Intensity
RPS27A;UBA52;UBC;UBB	2	730.8964	1459.778	62.596	3.1853e−05	107.79	2717598

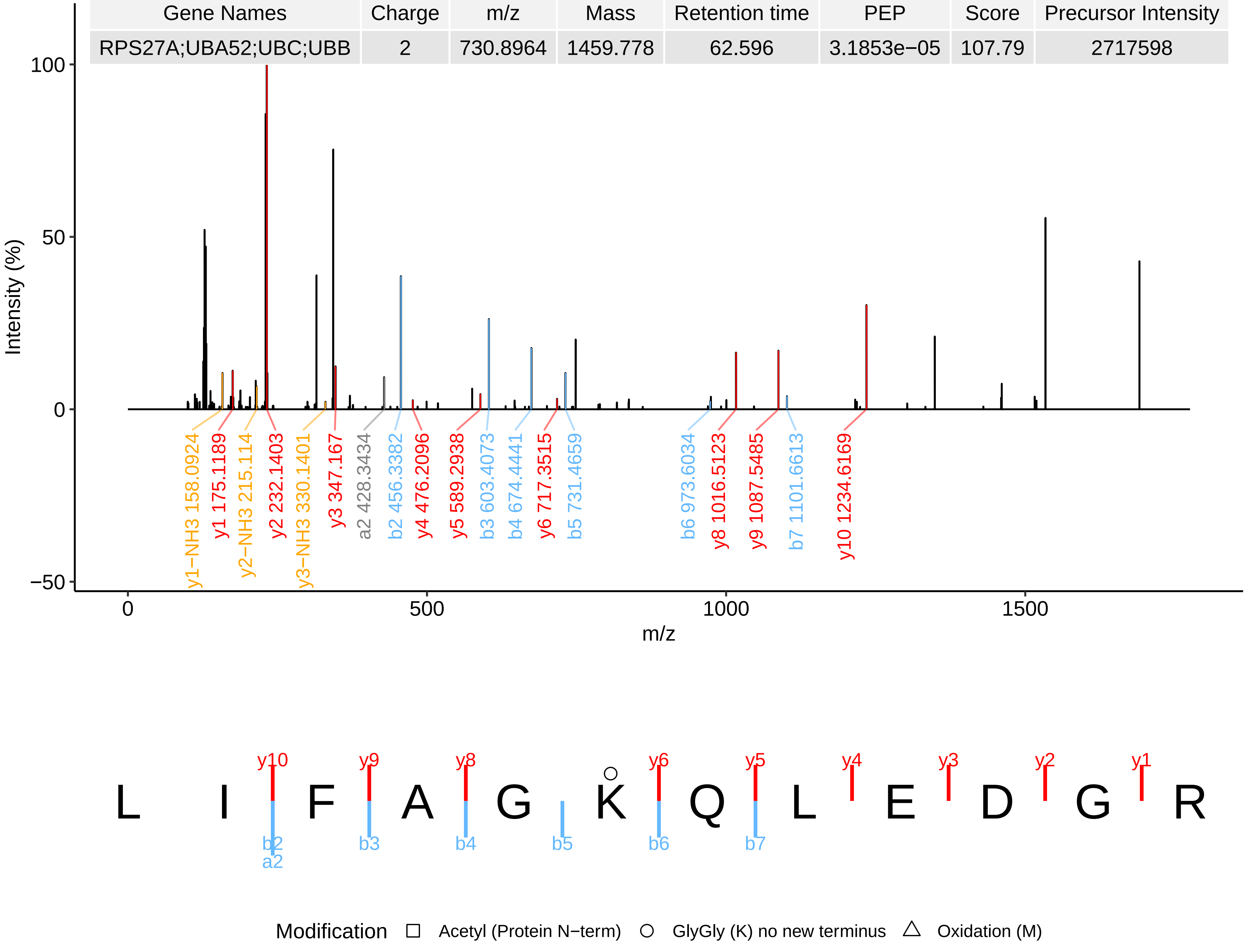

20200921_KIZ_MUb4_GlyGly_10plex_2 − Scan number: 16138

Gene Names	Charge	m/z	Mass	Retention time	PEP	Score	Precursor Intensity
RPS27A;UBA52;UBC;UBB	2	730.8964	1459.778	63.096	6.4124e−13	142	112340296

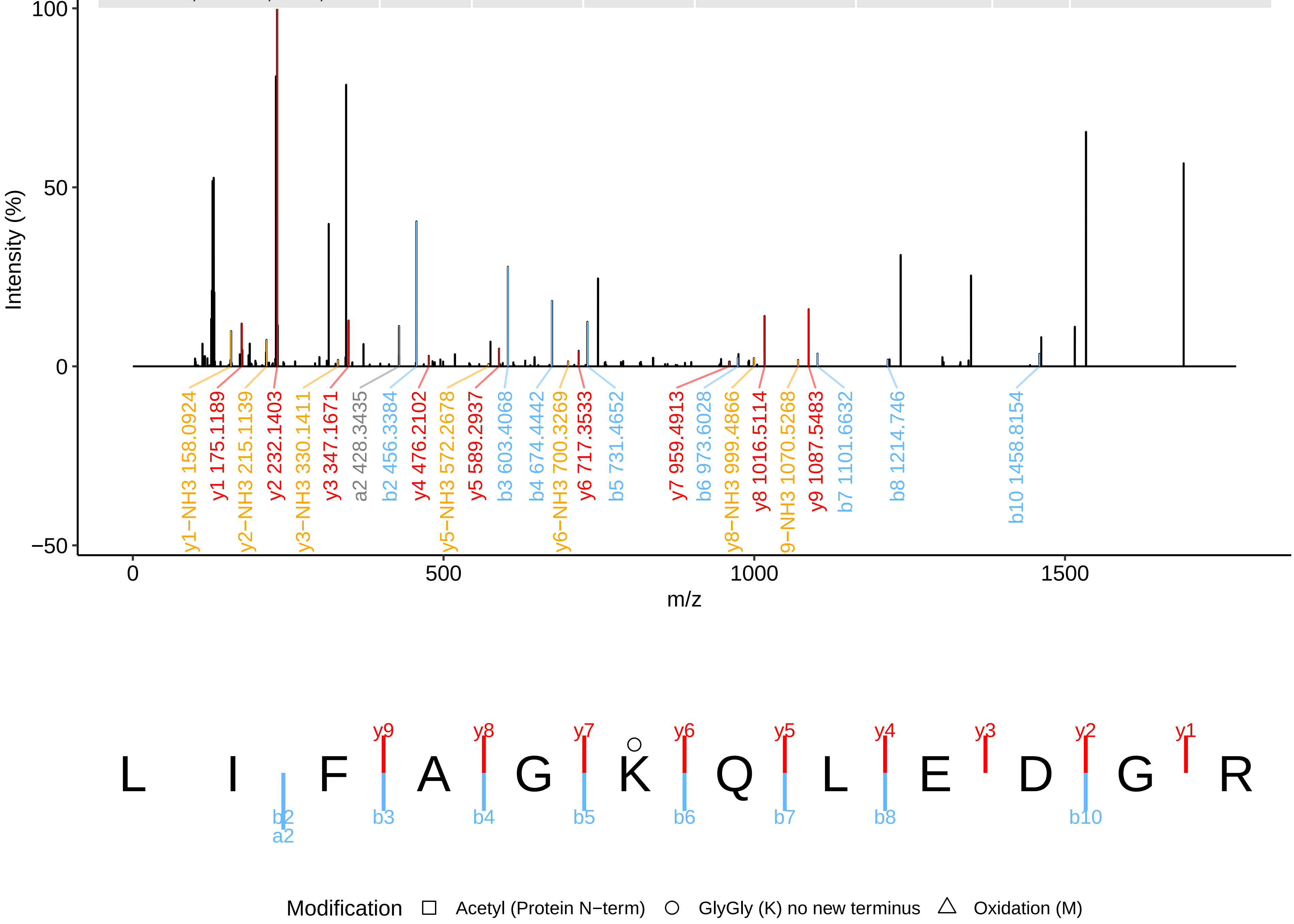

20200921_KIZ_MUb4_GlyGly_10plex_2 − Scan number: 16294

Gene Names	Charge	m/z	Mass	Retention time	PEP	Score	Precursor Intensity
RPS27A;UBA52;UBC;UBB	2	730.8964	1459.778	63.601	2.9221e−41	229.73	63347068

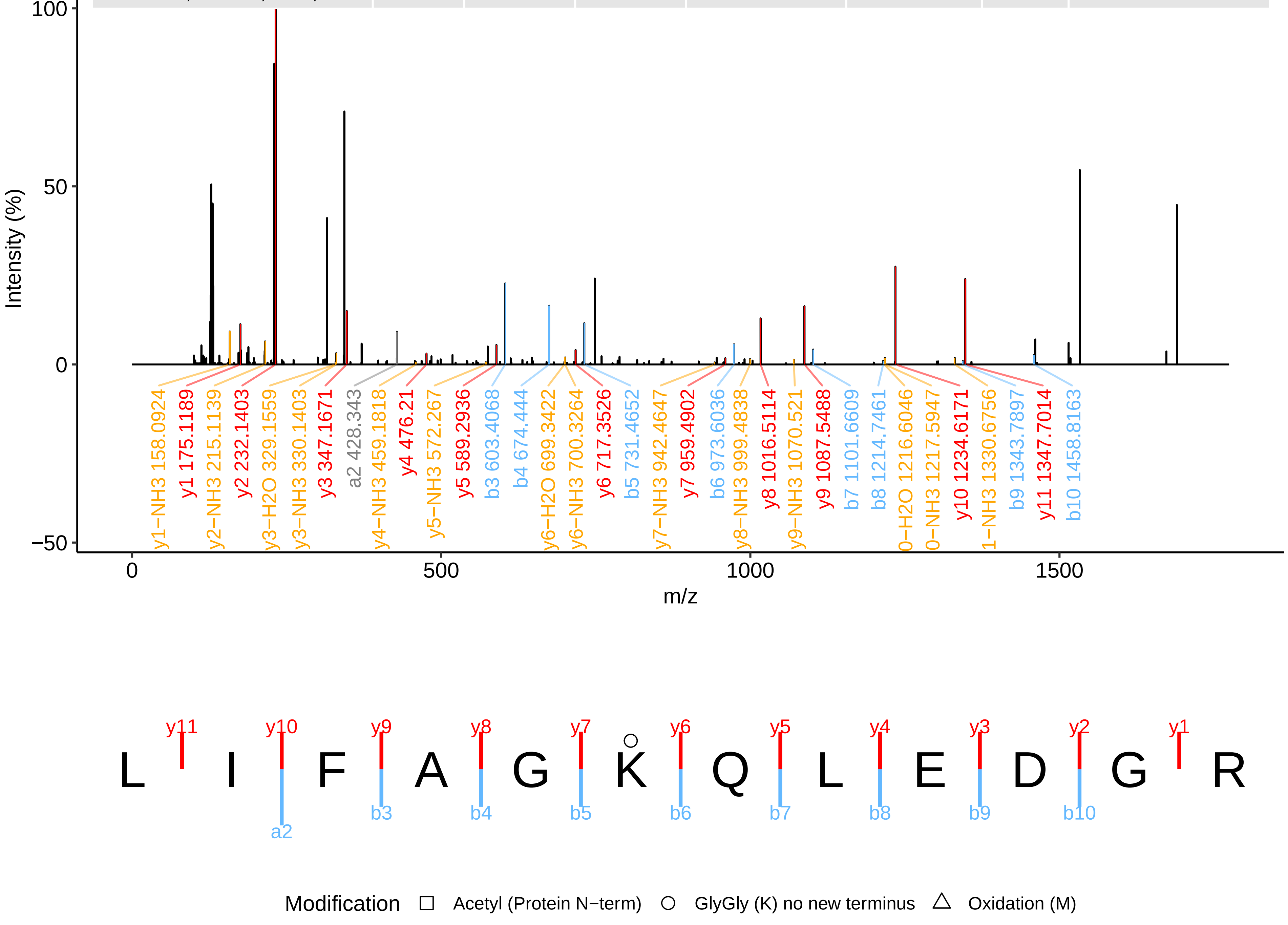

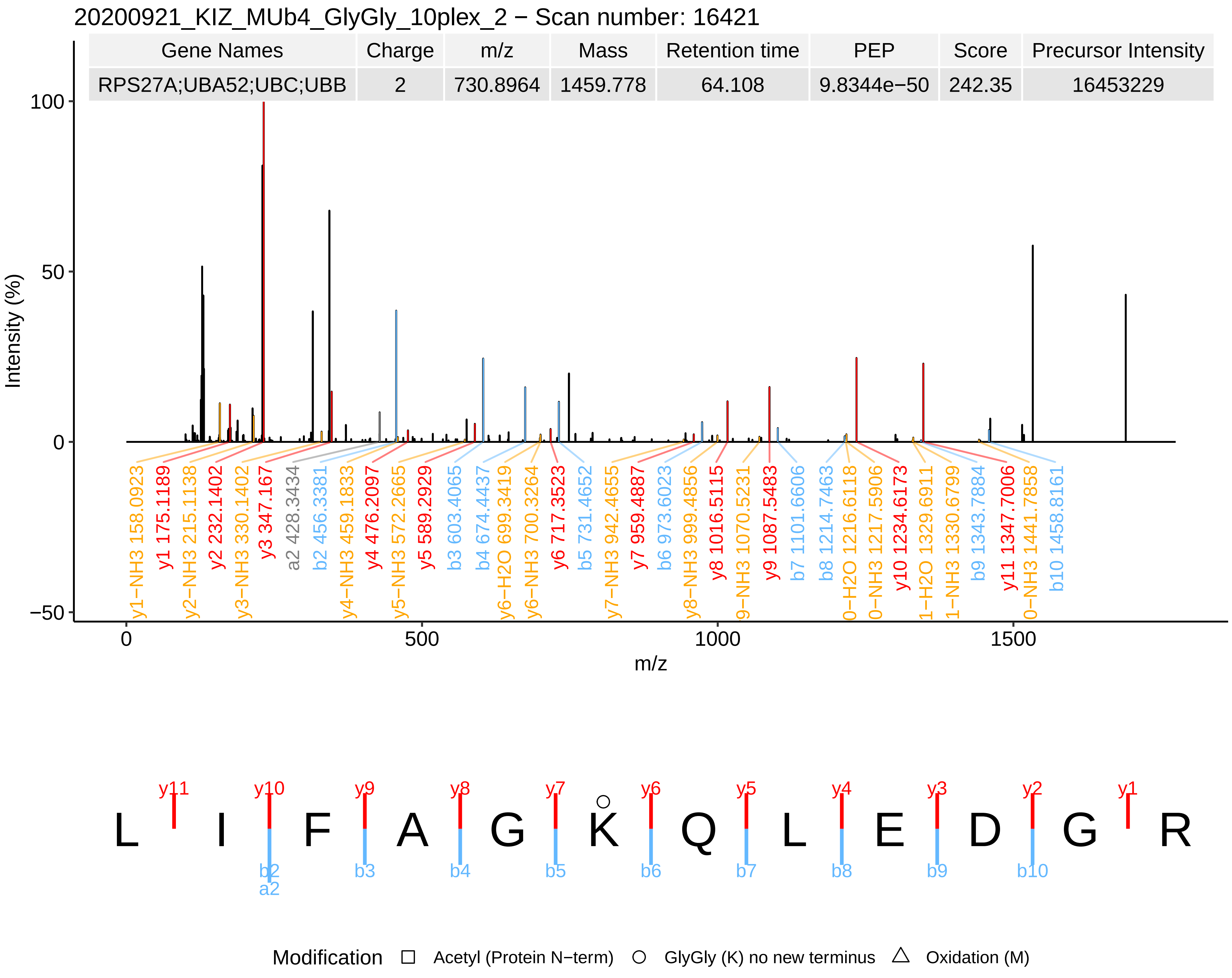

20200921_KIZ_MUb4_GlyGly_10plex_2 − Scan number: 16421
Gene Names | Charge | m/z | Mass | Retention time | PEP | Score | Precursor Intensity
RPS27A;UBA52;UBC;UBB | 2 | 730.8964 | 1459.778 | 64.108 | 9.8344e−50 | 242.35 | 16453229
Intensity (%)
m/z
y1−NH3 158.0923
y1 175.1189
y2−NH3 215.1138
y2 232.1402
y3−NH3 330.1402
y3 347.167
a2 428.3434
b2 456.3381
y4−NH3 459.1833
y4 476.2097
y5−NH3 572.2665
y5 589.2929
b3 603.4065
b4 674.4437
y6−H2O 699.3419
y6−NH3 700.3264
y6 717.3523
b5 731.4652
y7−NH3 942.4655
y7 959.4887
b6 973.6023
y8−NH3 999.4856
y8 1016.5115
9−NH3 1070.5231
y9 1087.5483
b7 1101.6606
b8 1214.7463
0−H2O 1216.6118
0−NH3 1217.5906
y10 1234.6173
1−H2O 1329.6911
1−NH3 1330.6799
b9 1343.7884
y11 1347.7006
0−NH3 1441.7858
b10 1458.8161
L I F A G K Q L E D G R
y11 y10 y9 y8 y7 y6 y5 y4 y3 y2 y1
b2 a2 b3 b4 b5 b6 b7 b8 b9 b10
Modification
Acetyl (Protein N−term)
GlyGly (K) no new terminus
Oxidation (M)

20200921_KIZ_MUb4_GlyGly_10plex_2 − Scan number: 16554

Gene Names	Charge	m/z	Mass	Retention time	PEP	Score	Precursor Intensity
RPS27A;UBA52;UBC;UBB	2	730.8964	1459.778	64.636	1.0903e−34	215.06	6292789

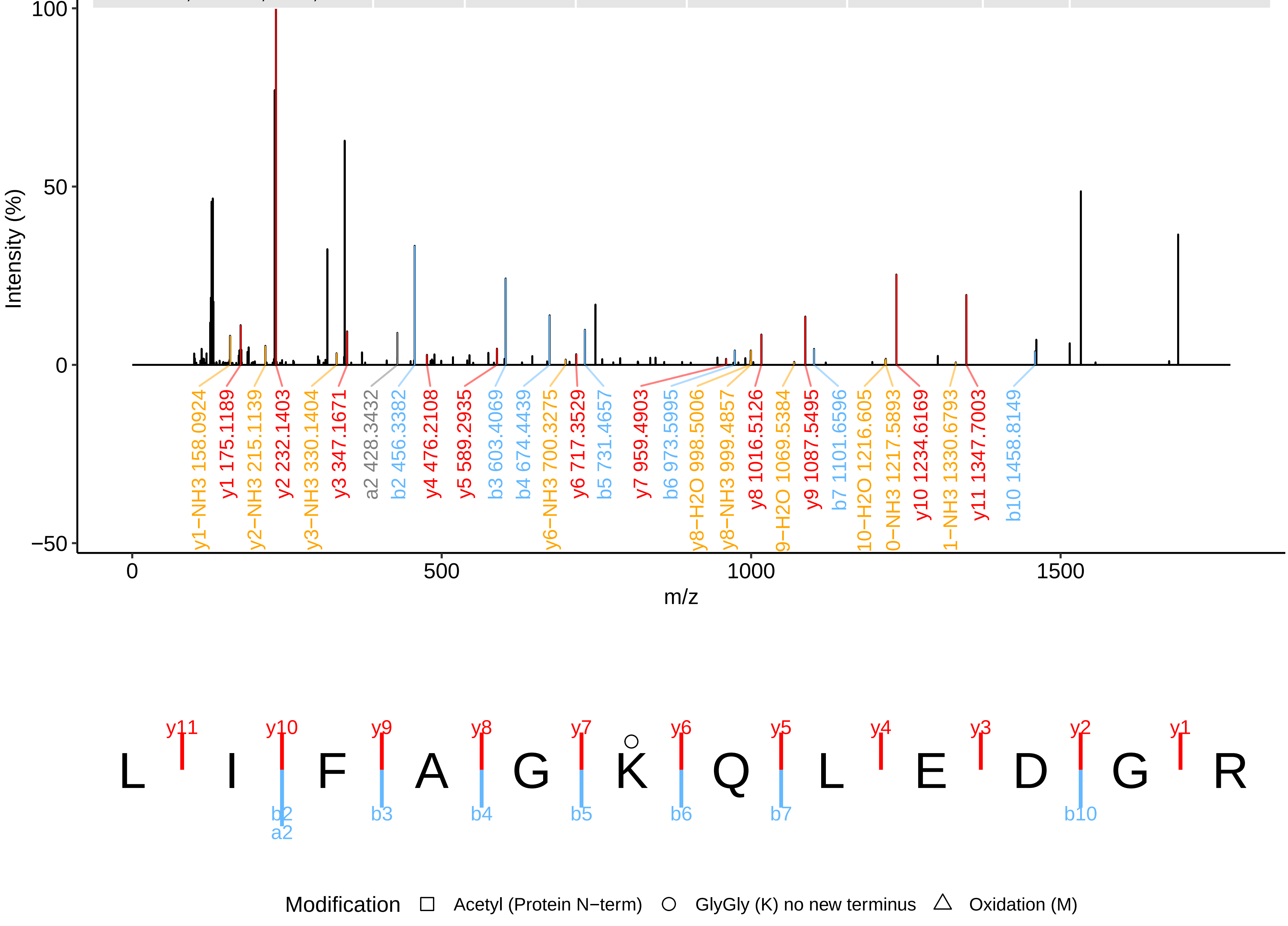

20200921_KIZ_MUb4_GlyGly_10plex_2 − Scan number: 16098

Gene Names	Charge	m/z	Mass	Retention time	PEP	Score	Precursor Intensity
RPS27A;UBA52;UBC;UBB	3	487.6001	1459.778	62.969	5.46e−09	126.19	5205687808

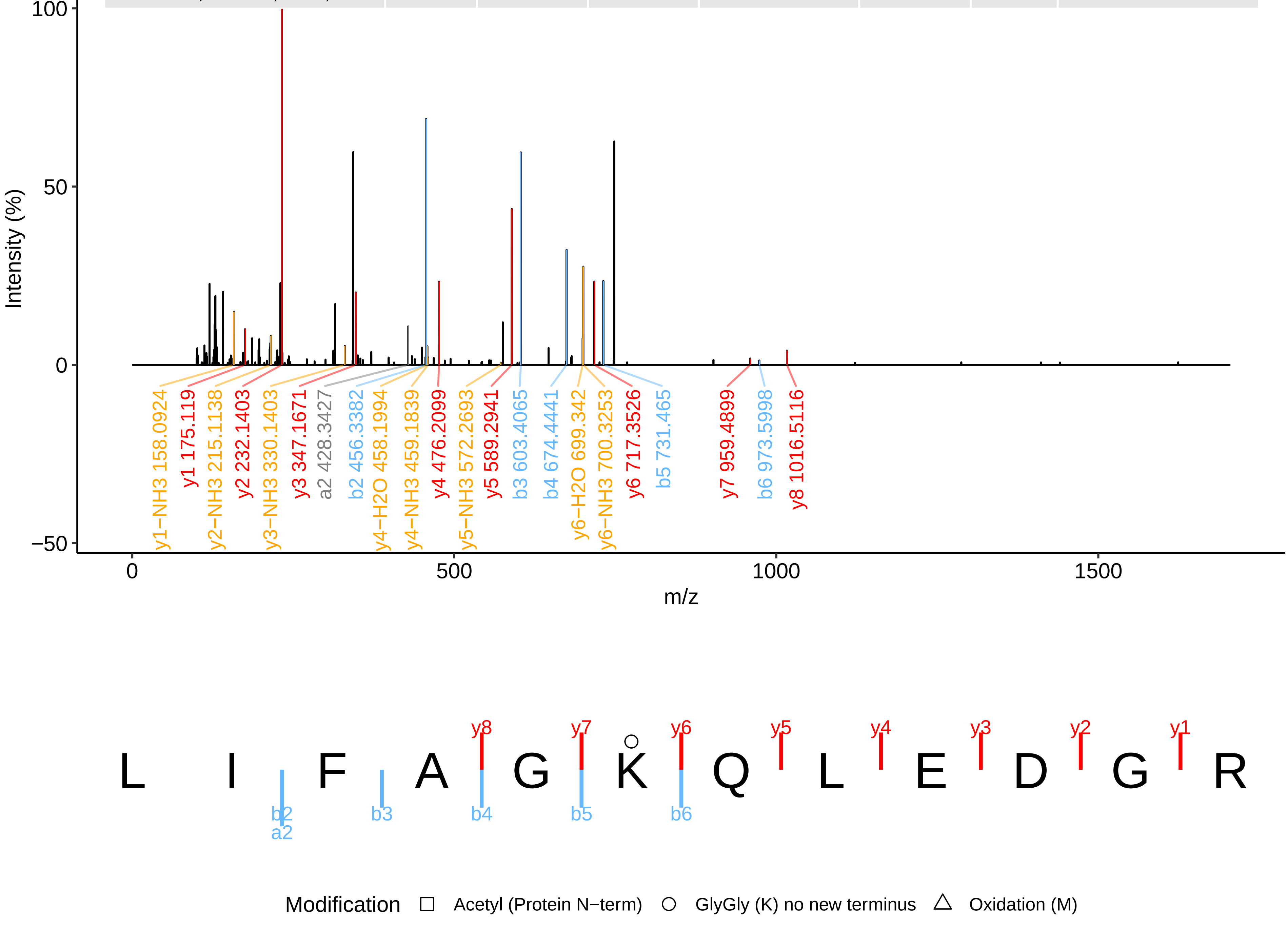

20200921_KIZ_MUb4_GlyGly_10plex_2 − Scan number: 16255

Gene Names	Charge	m/z	Mass	Retention time	PEP	Score	Precursor Intensity
RPS27A;UBA52;UBC;UBB	3	487.6001	1459.778	63.477	2.7462e−19	152.7	2680802816

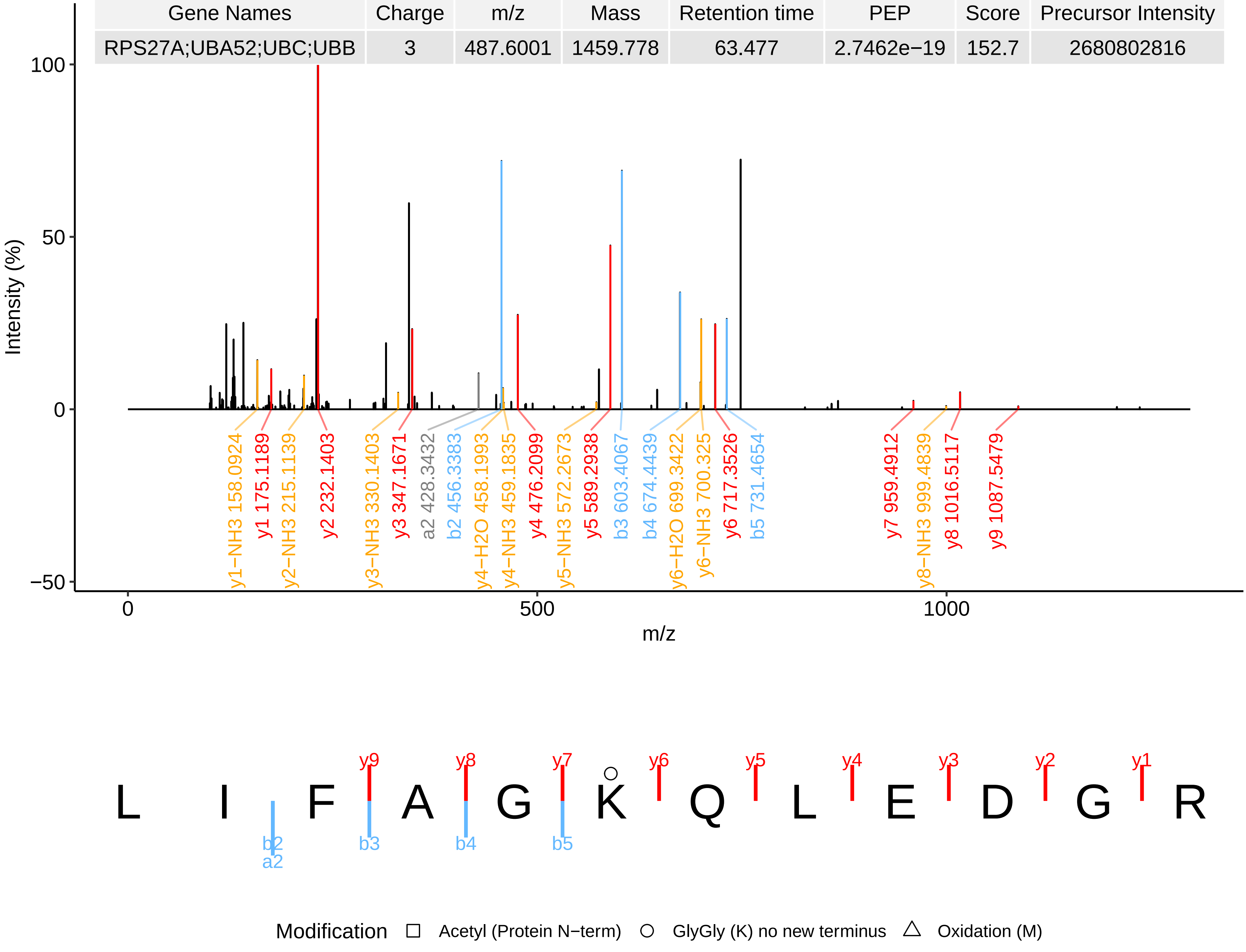

20200921_KIZ_MUb4_GlyGly_10plex_2 − Scan number: 16390

Gene Names	Charge	m/z	Mass	Retention time	PEP	Score	Precursor Intensity
RPS27A;UBA52;UBC;UBB	3	487.6001	1459.778	63.986	3.9606e−09	128.01	700446208

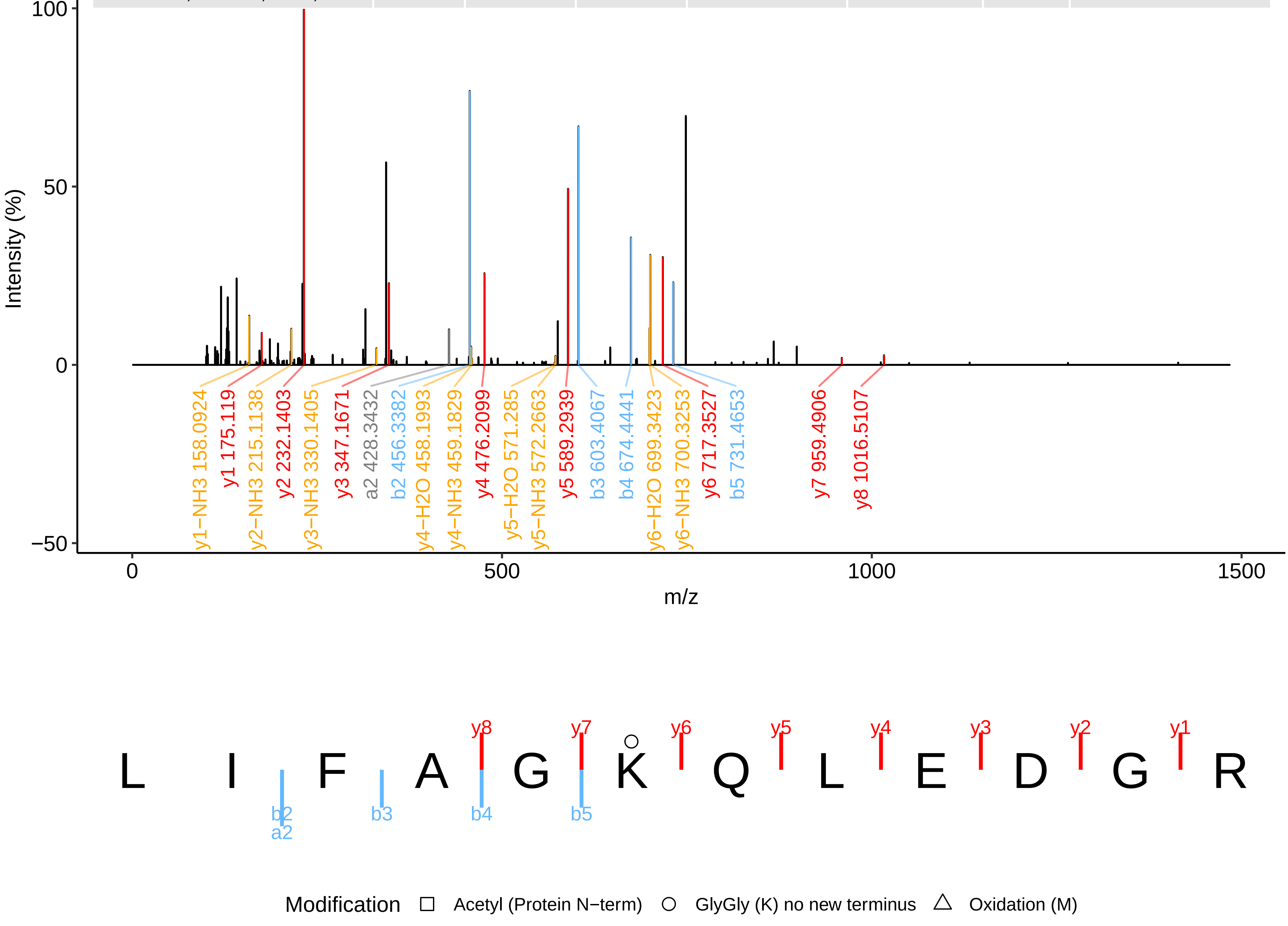

20200921_KIZ_MUb4_GlyGly_10plex_2 − Scan number: 16521

Gene Names	Charge	m/z	Mass	Retention time	PEP	Score	Precursor Intensity
RPS27A;UBA52;UBC;UBB	3	487.6001	1459.778	64.498	3.1574e−19	150.46	134542800

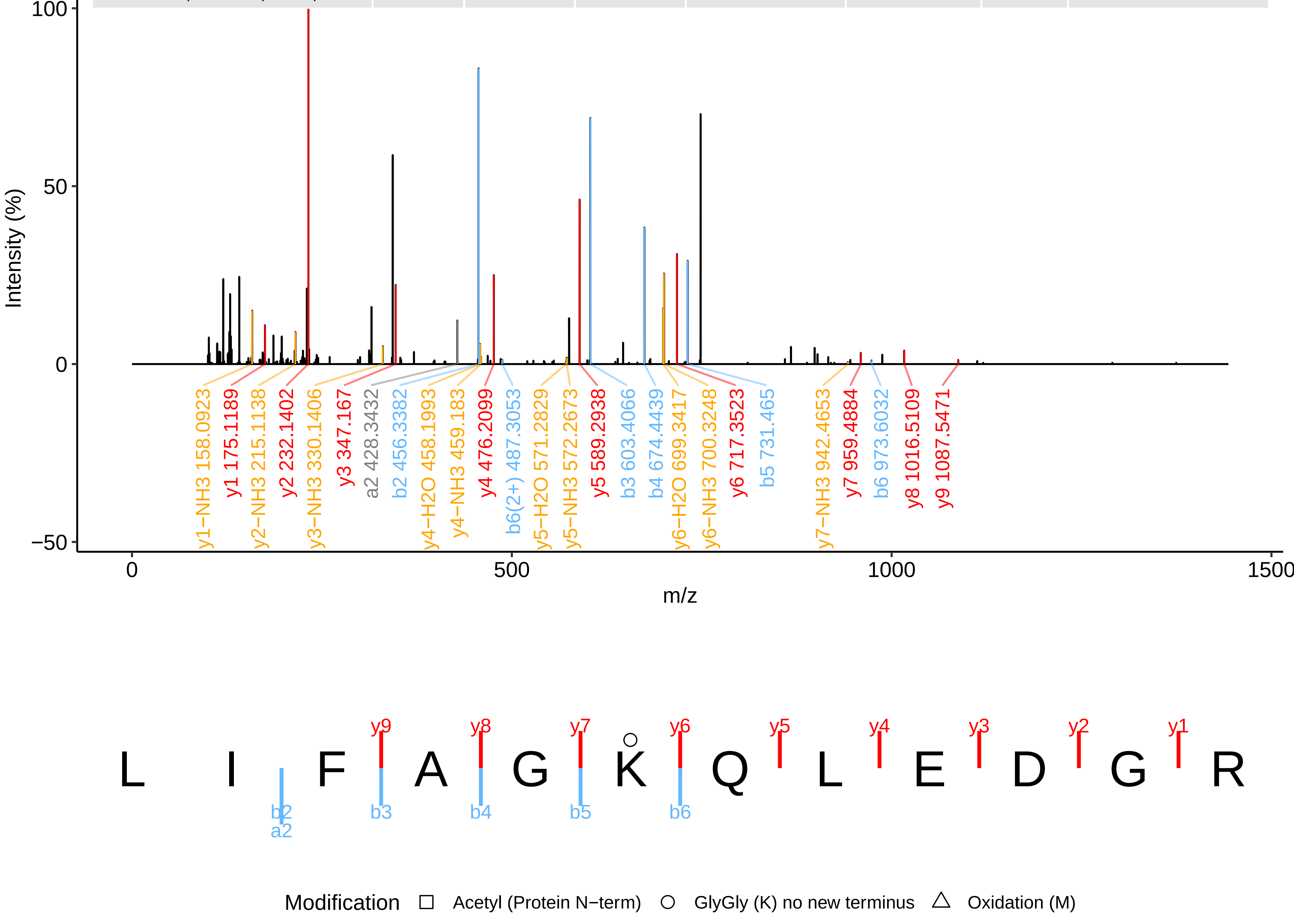

20200921_KIZ_MUb4_GlyGly_10plex_2 − Scan number: 16646

Gene Names	Charge	m/z	Mass	Retention time	PEP	Score	Precursor Intensity
RPS27A;UBA52;UBC;UBB	3	487.6001	1459.778	65.011	1.556e−13	143.24	142743664

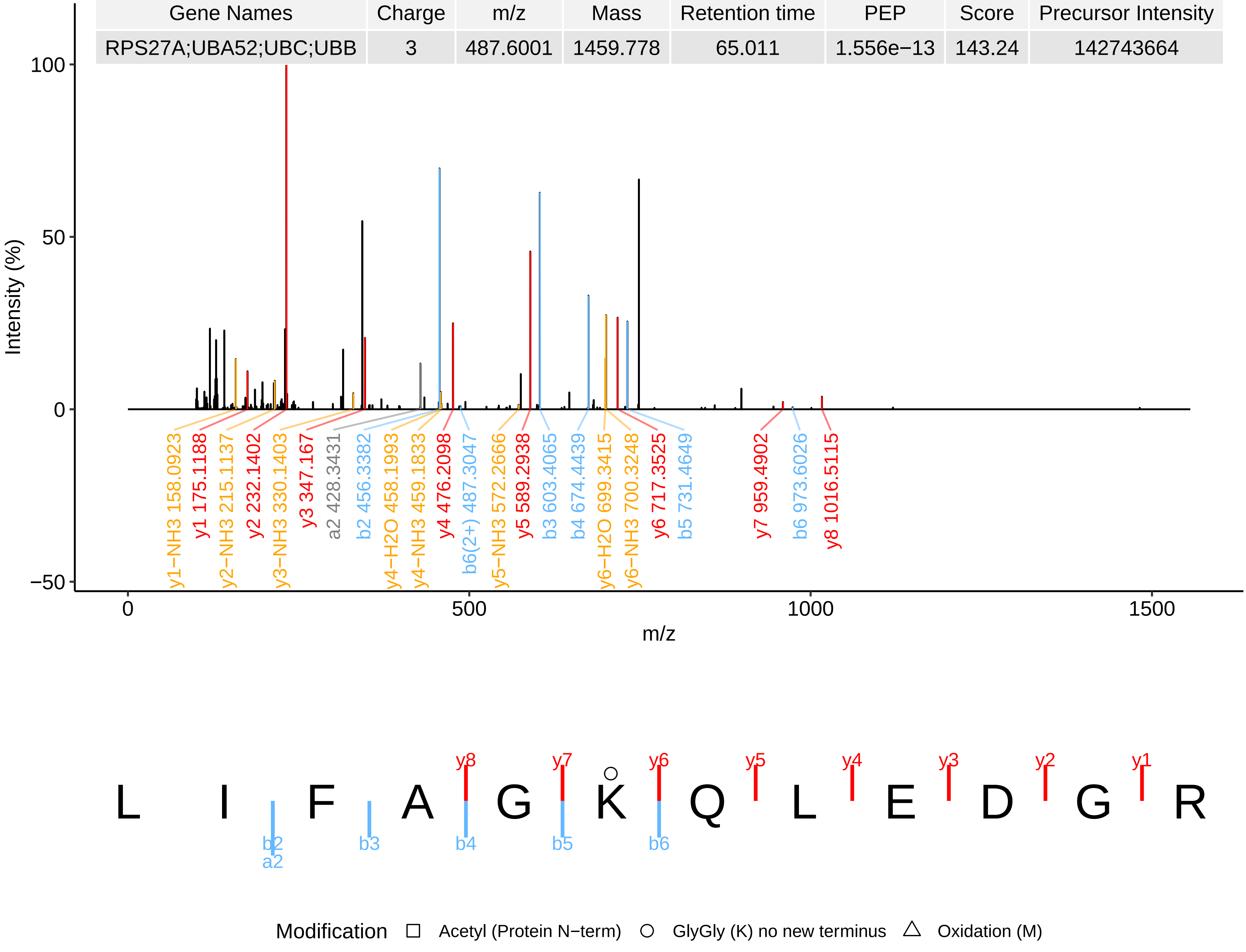

20200921_KIZ_MUb4_GlyGly_10plex_2 − Scan number: 16779

Gene Names	Charge	m/z	Mass	Retention time	PEP	Score	Precursor Intensity
RPS27A;UBA52;UBC;UBB	3	487.6001	1459.778	65.539	2.0348e−13	141.08	50614876

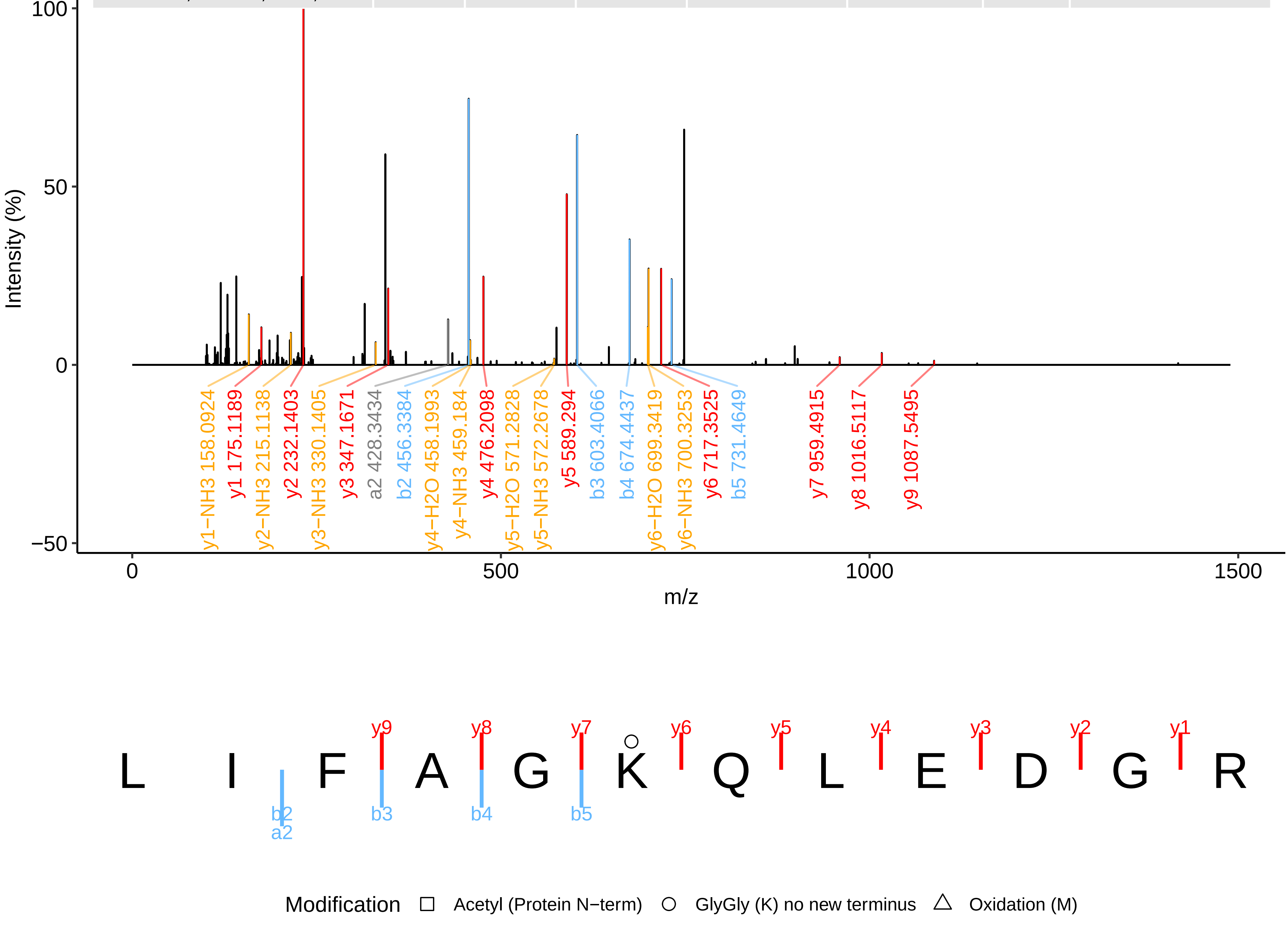

20200921_KIZ_MUb4_GlyGly_10plex_2 − Scan number: 16912

Gene Names	Charge	m/z	Mass	Retention time	PEP	Score	Precursor Intensity
RPS27A;UBA52;UBC;UBB	3	487.6001	1459.778	66.071	3.1574e−19	150.46	31326500

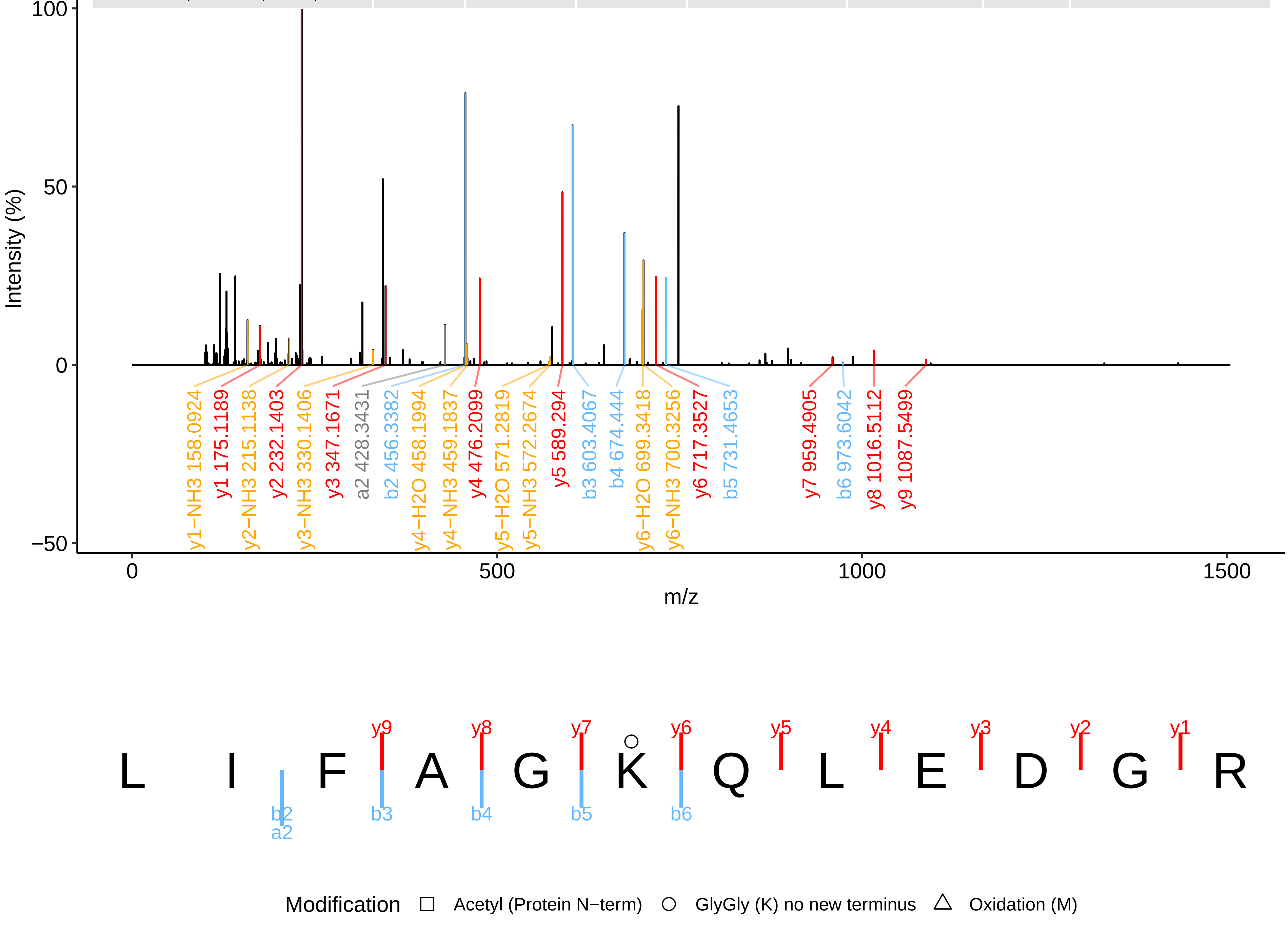

20200921_KIZ_MUb4_GlyGly_10plex_2 − Scan number: 17041

Gene Names	Charge	m/z	Mass	Retention time	PEP	Score	Precursor Intensity
RPS27A;UBA52;UBC;UBB	3	487.6001	1459.778	66.584	3.6333e−13	133.89	23635322

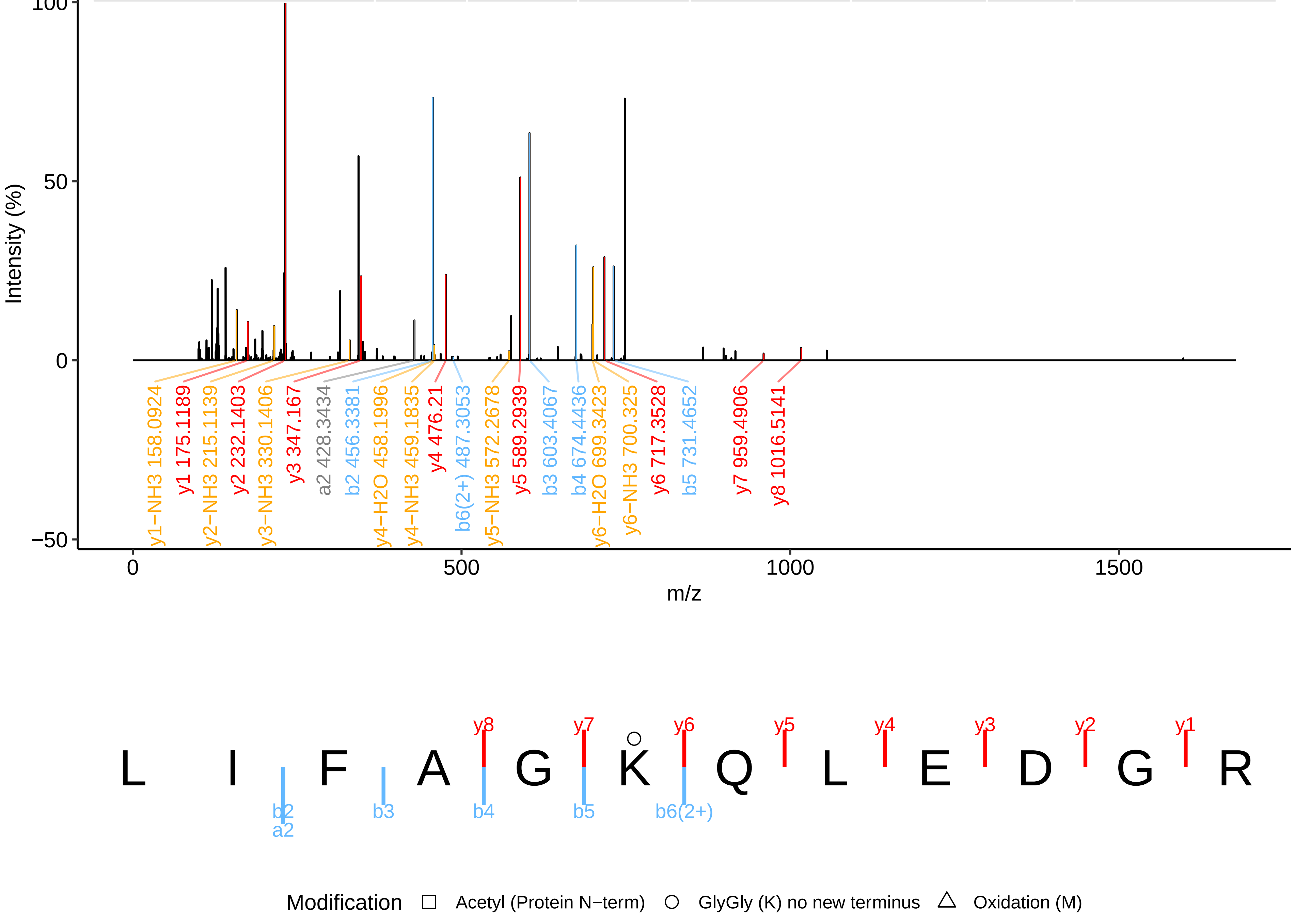

20200921_KIZ_MUb4_GlyGly_10plex_2 − Scan number: 17168

Gene Names	Charge	m/z	Mass	Retention time	PEP	Score	Precursor Intensity
RPS27A;UBA52;UBC;UBB	3	487.6001	1459.778	67.094	2.0328e−19	156.58	18020440

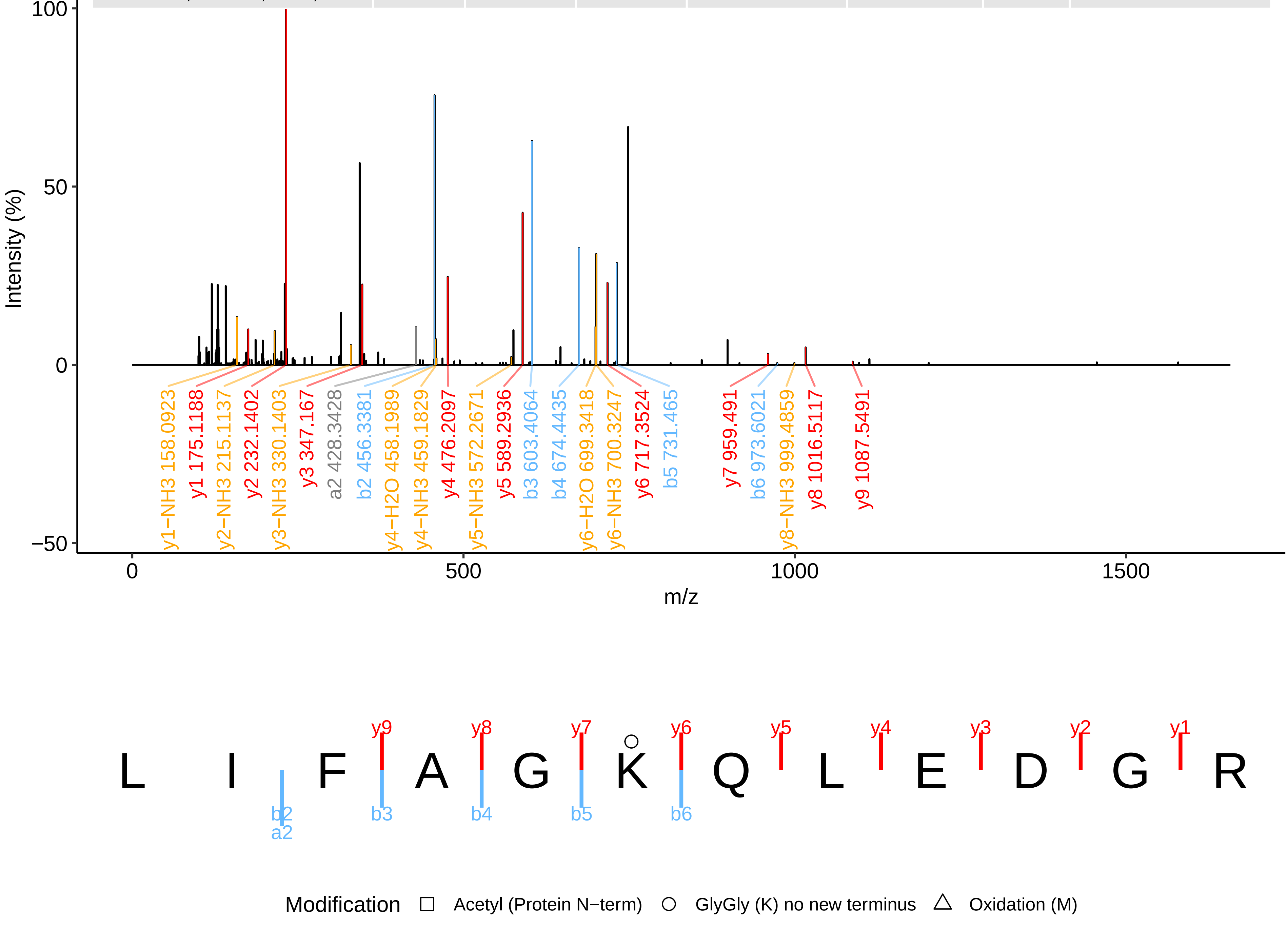

20200921_KIZ_MUb4_GlyGly_10plex_2 − Scan number: 17294

Gene Names	Charge	m/z	Mass	Retention time	PEP	Score	Precursor Intensity
RPS27A;UBA52;UBC;UBB	3	487.6001	1459.778	67.614	7.841e−14	146.71	13429578

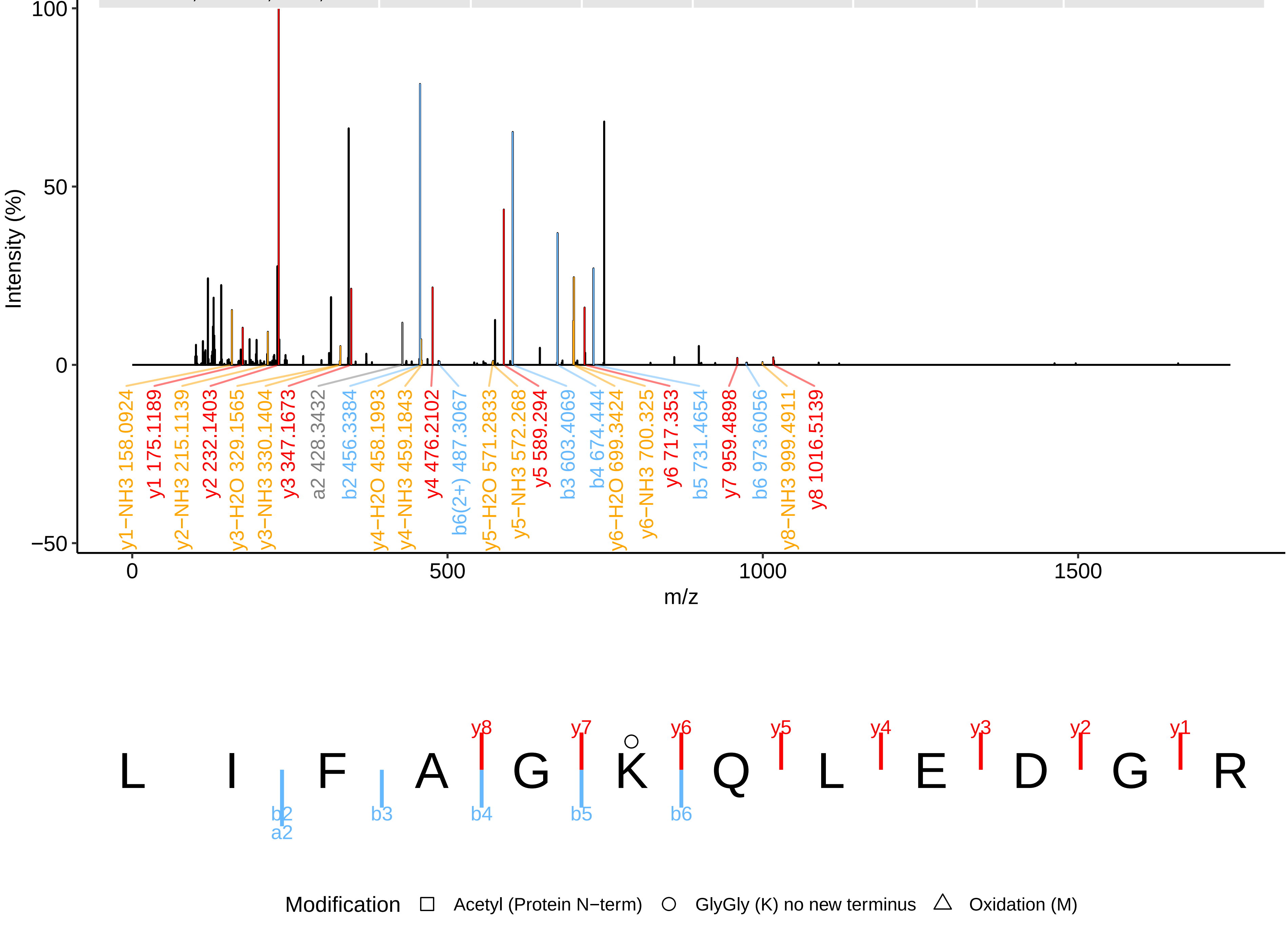

20200921_KIZ_MUb4_GlyGly_10plex_2 − Scan number: 16678

Gene Names	Charge	m/z	Mass	Retention time	PEP	Score	Precursor Intensity
RPS27A;UBA52;UBC;UBB	2	730.8964	1459.778	65.139	4.1623e−19	161.17	984662.1

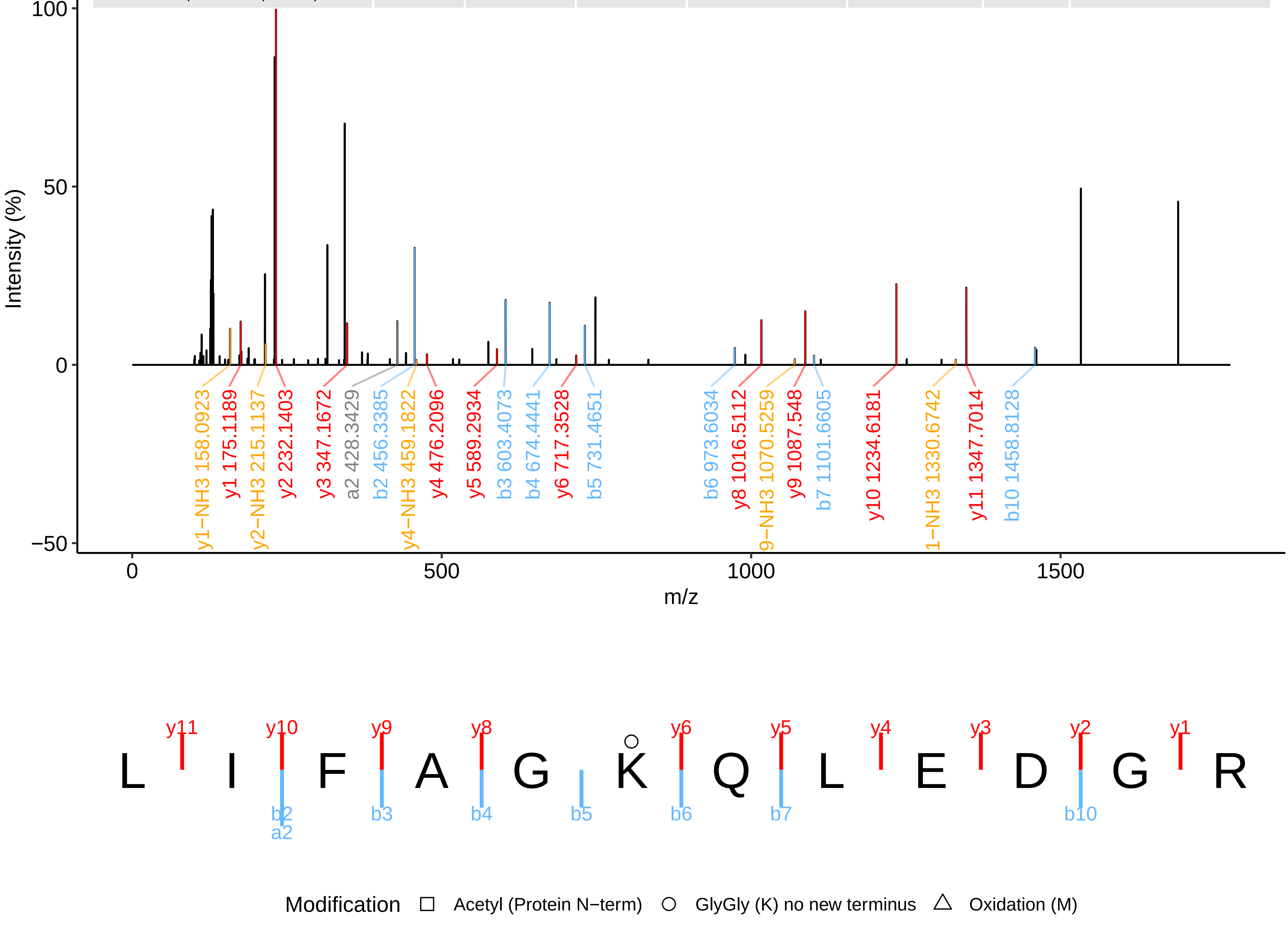

20200921_KIZ_MUb4_GlyGly_10plex_2 − Scan number: 16808

Gene Names	Charge	m/z	Mass	Retention time	PEP	Score	Precursor Intensity
RPS27A;UBA52;UBC;UBB	2	730.8964	1459.778	65.655	0.0016164	91.584	1746812

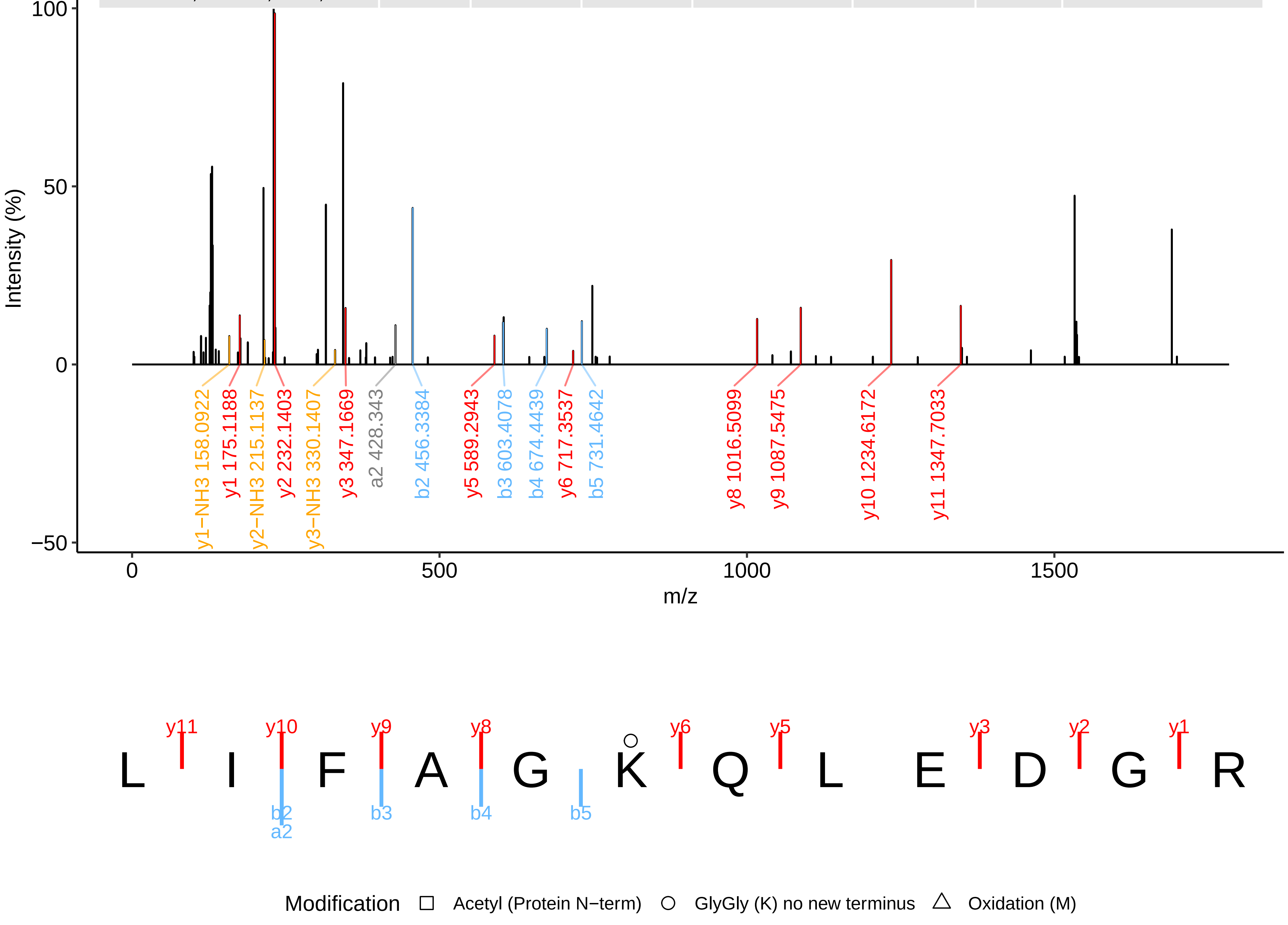

20200921_KIZ_MUb4_GlyGly_10plex_2 − Scan number: 16937

Gene Names	Charge	m/z	Mass	Retention time	PEP	Score	Precursor Intensity
RPS27A;UBA52;UBC;UBB	2	730.8964	1459.778	66.17	6.5787e−05	99.653	NA

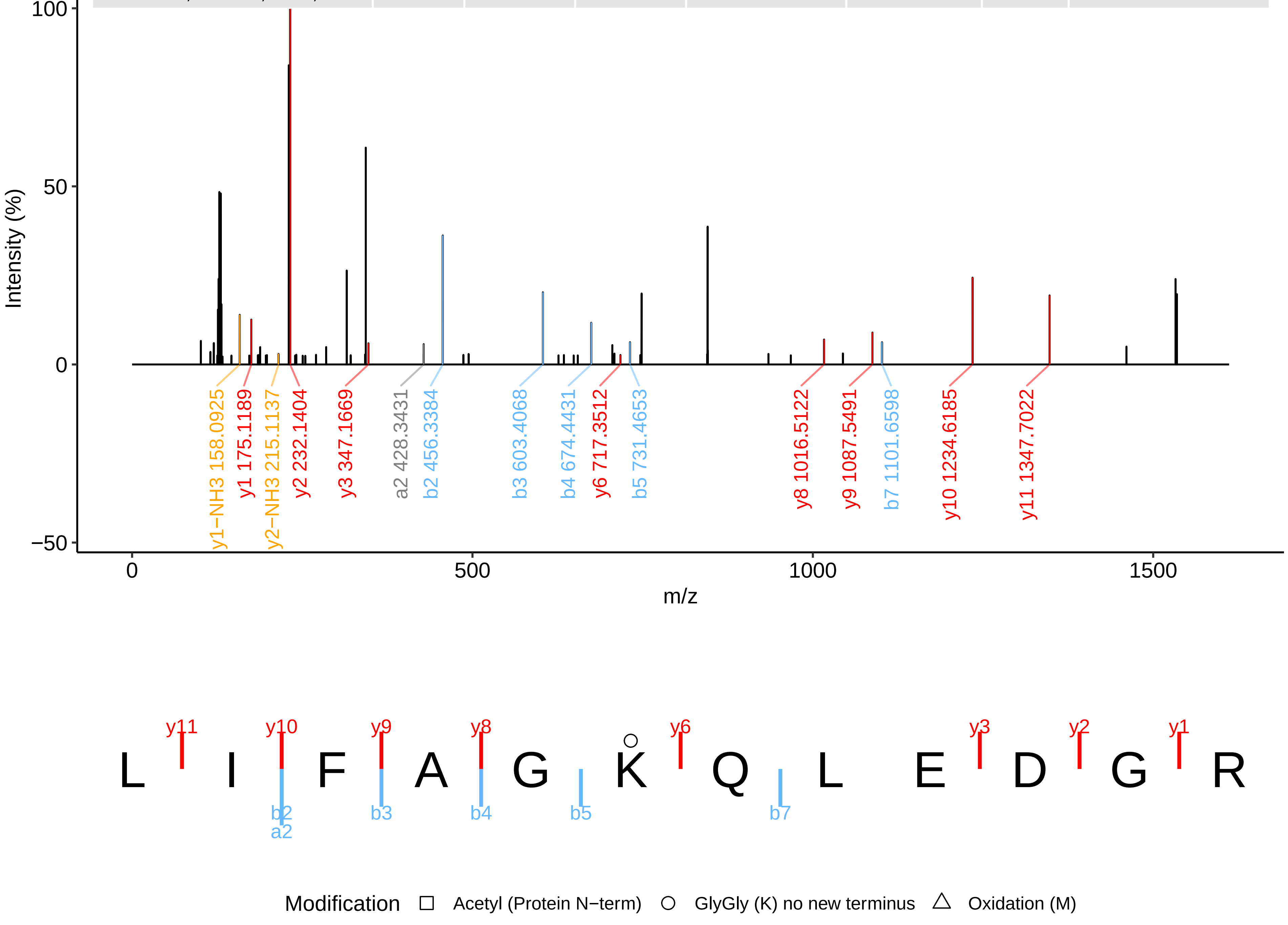

20200921_KIZ_MUb4_GlyGly_10plex_2 − Scan number: 17420

Gene Names	Charge	m/z	Mass	Retention time	PEP	Score	Precursor Intensity
RPS27A;UBA52;UBC;UBB	3	487.6001	1459.778	68.127	1.1157e−08	119.27	18296204

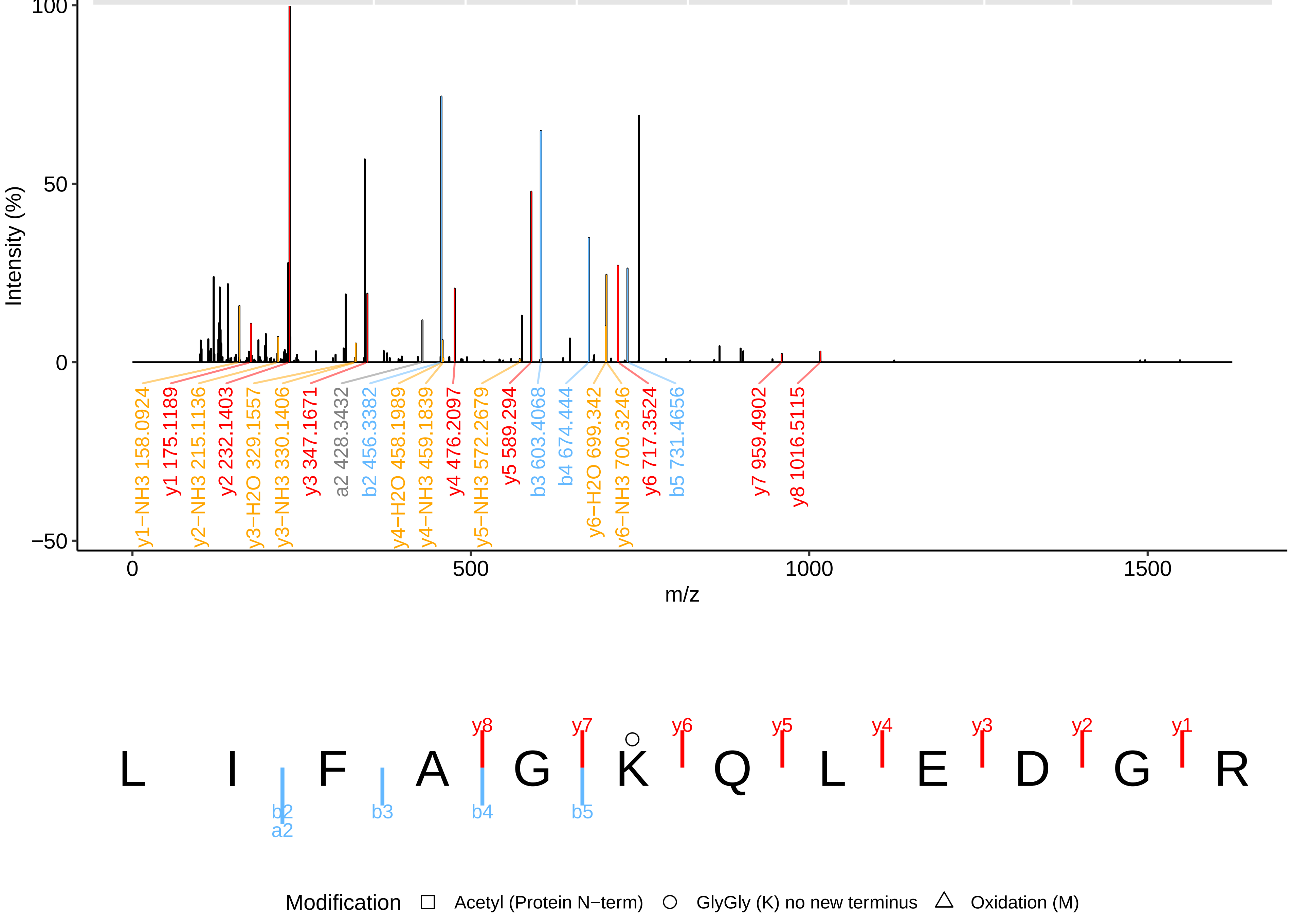

20200921_KIZ_MUb4_GlyGly_10plex_2 − Scan number: 17554

Gene Names	Charge	m/z	Mass	Retention time	PEP	Score	Precursor Intensity
RPS27A;UBA52;UBC;UBB	3	487.6001	1459.778	68.652	2.7462e−19	152.7	13375617

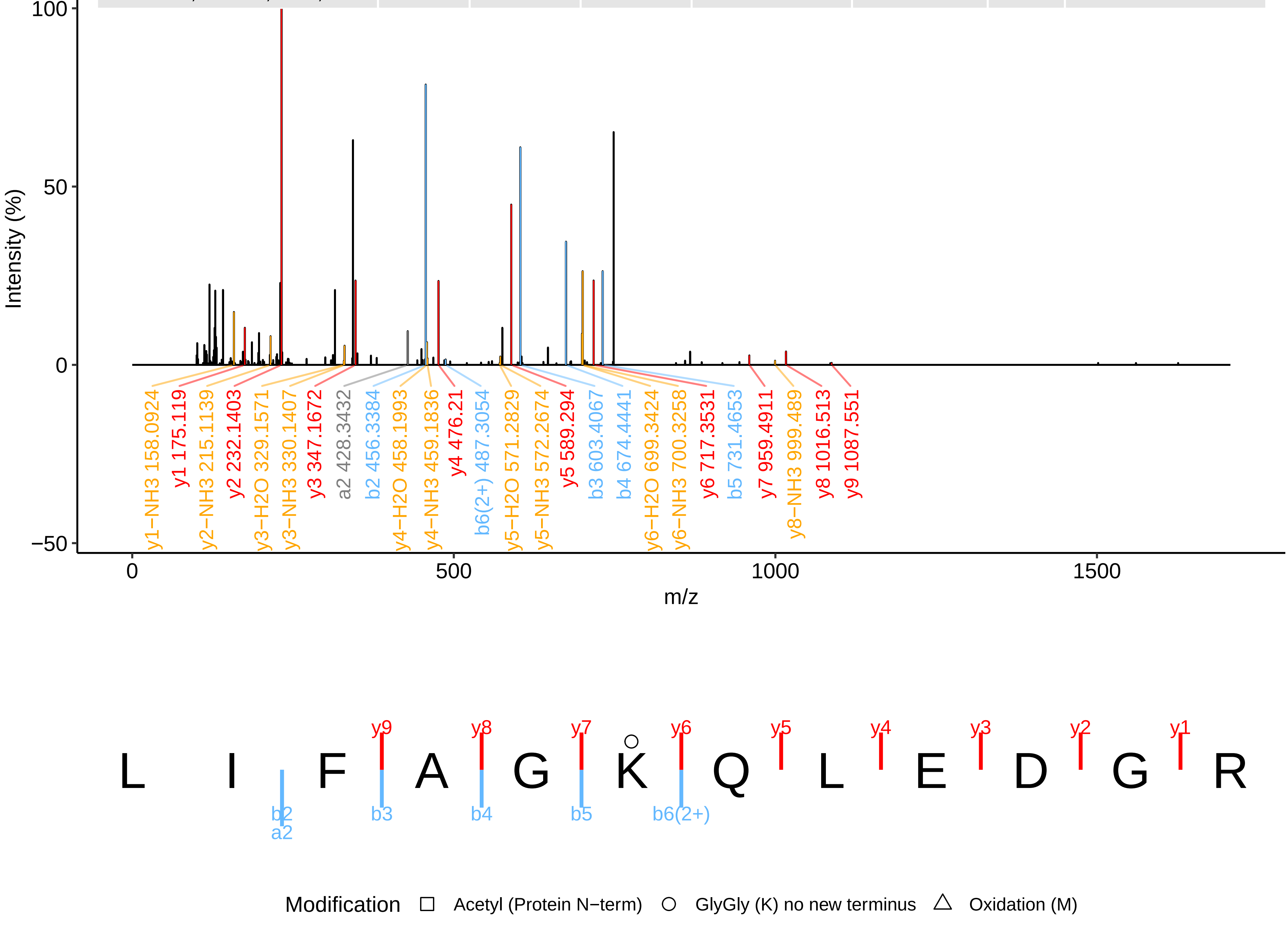

20200921_KIZ_MUb4_GlyGly_10plex_2 − Scan number: 17680

Gene Names	Charge	m/z	Mass	Retention time	PEP	Score	Precursor Intensity
RPS27A;UBA52;UBC;UBB	3	487.6001	1459.778	69.156	2.4793e−19	154.15	15718334

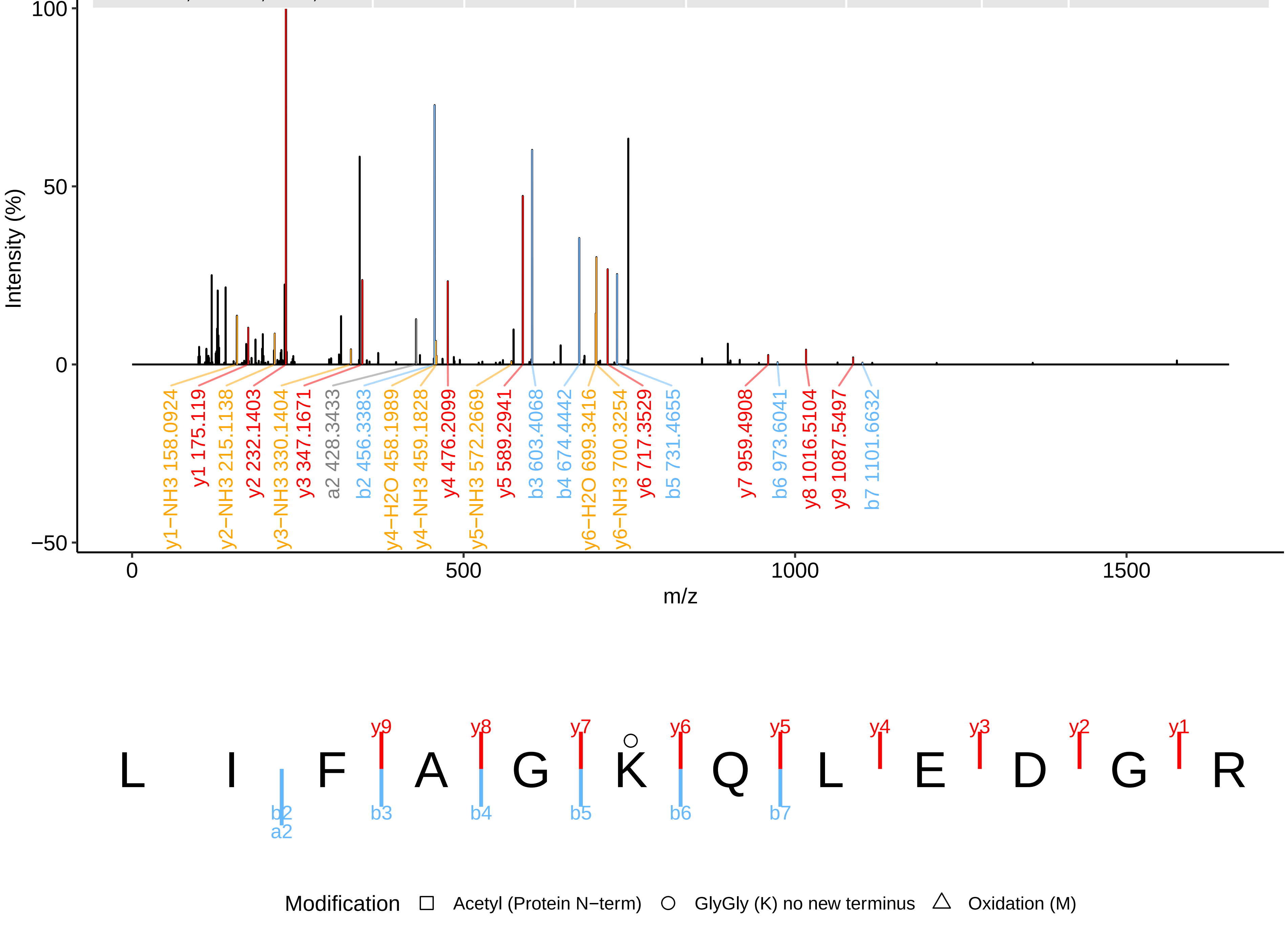

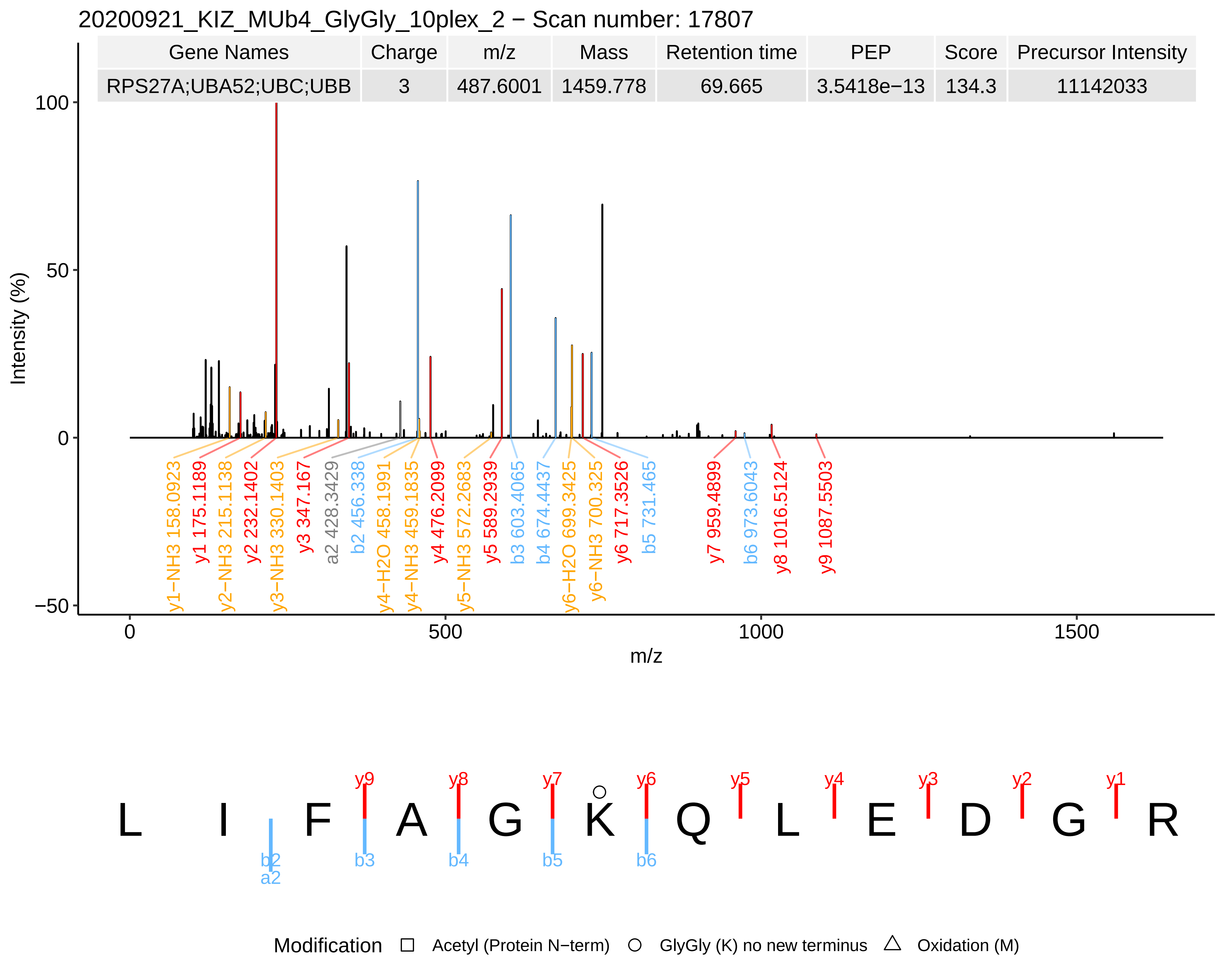
20200921_KIZ_MUb4_GlyGly_10plex_2 − Scan number: 17807
Gene Names
Charge
m/z
Mass
Retention time
PEP
Score
Precursor Intensity
RPS27A;UBA52;UBC;UBB
3
487.6001
1459.778
69.665
3.5418e−13
134.3
11142033
Intensity (%)
m/z
y1−NH3 158.0923
y1 175.1189
y2−NH3 215.1138
y2 232.1402
y3−NH3 330.1403
y3 347.167
a2 428.3429
b2 456.338
y4−H2O 458.1991
y4−NH3 459.1835
y4 476.2099
y5−NH3 572.2683
y5 589.2939
b3 603.4065
b4 674.4437
y6−H2O 699.3425
y6−NH3 700.325
y6 717.3526
b5 731.465
y7 959.4899
b6 973.6043
y8 1016.5124
y9 1087.5503
L I F A G K Q L E D G R
Modification
Acetyl (Protein N−term)
GlyGly (K) no new terminus
Oxidation (M)

20200921_KIZ_MUb4_GlyGly_10plex_2 − Scan number: 17934

Gene Names	Charge	m/z	Mass	Retention time	PEP	Score	Precursor Intensity
RPS27A;UBA52;UBC;UBB	3	487.6001	1459.778	70.177	3.1574e−19	150.46	9232999

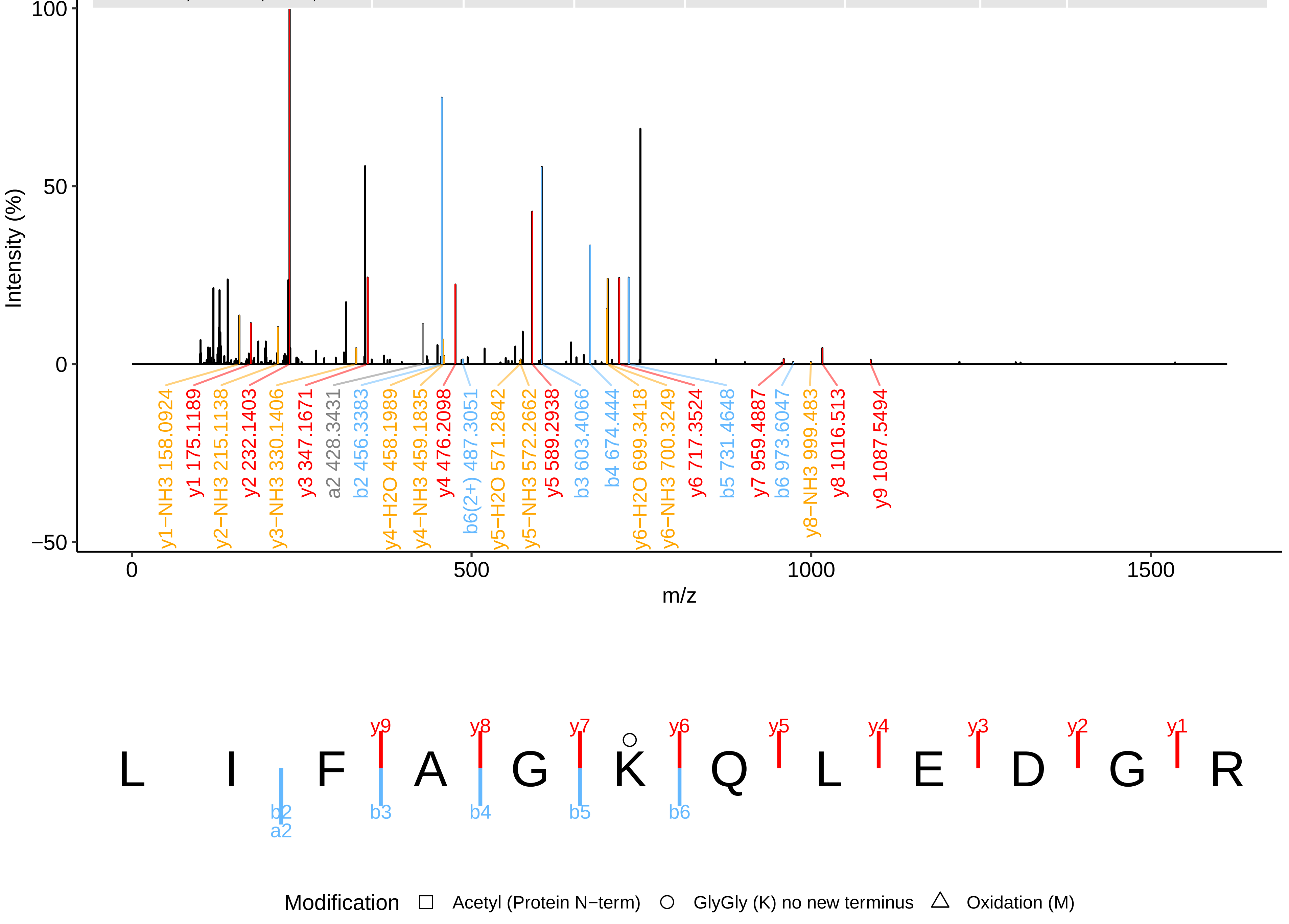

20200921_KIZ_MUb4_GlyGly_10plex_2 − Scan number: 18063

Gene Names	Charge	m/z	Mass	Retention time	PEP	Score	Precursor Intensity
RPS27A;UBA52;UBC;UBB	3	487.6001	1459.778	70.693	9.899e−20	162.26	9670218

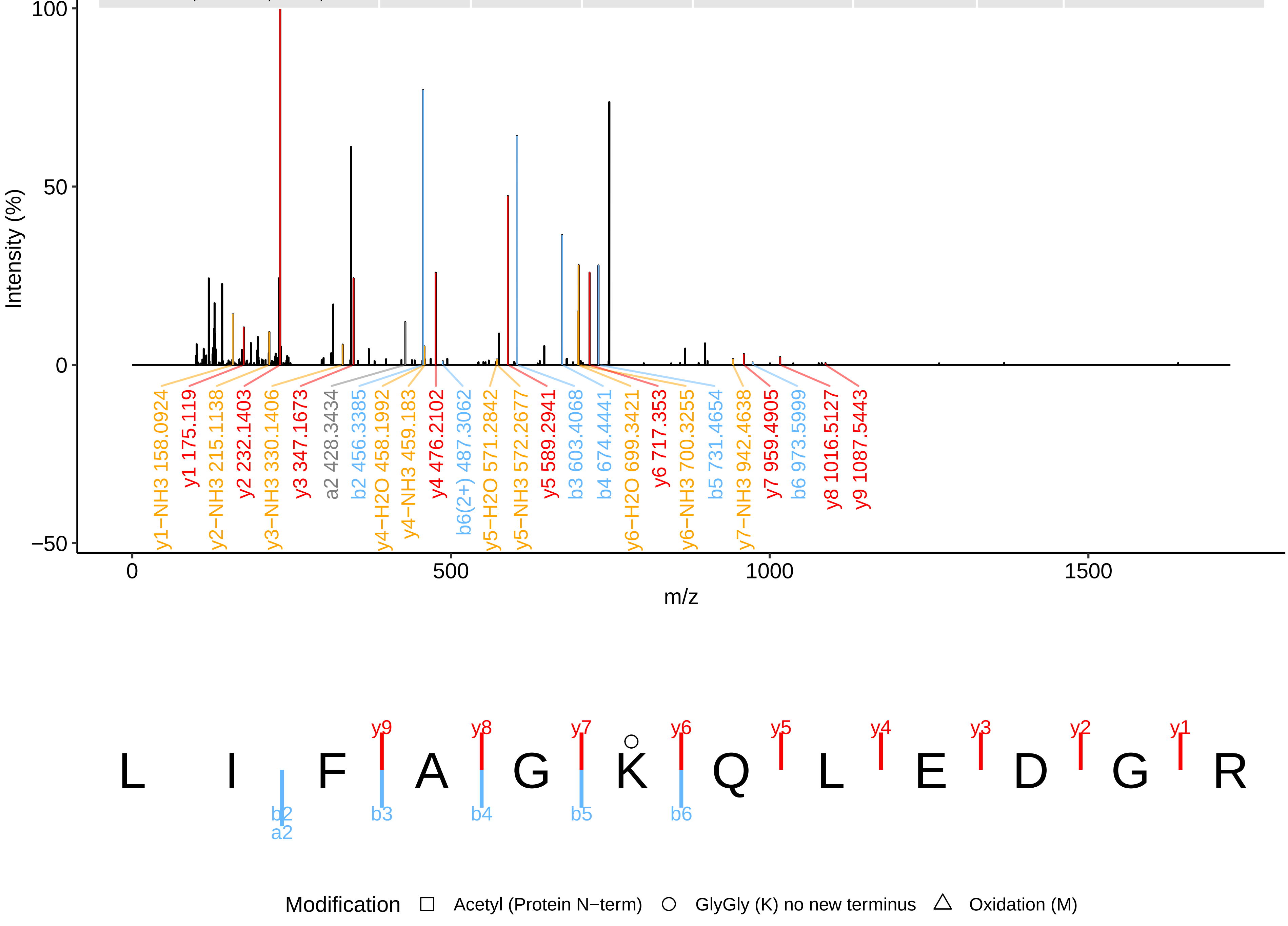

20200921_KIZ_MUb4_GlyGly_10plex_2 − Scan number: 18189

Gene Names	Charge	m/z	Mass	Retention time	PEP	Score	Precursor Intensity
RPS27A;UBA52;UBC;UBB	3	487.6001	1459.778	71.2	3.1574e−19	150.46	7227704

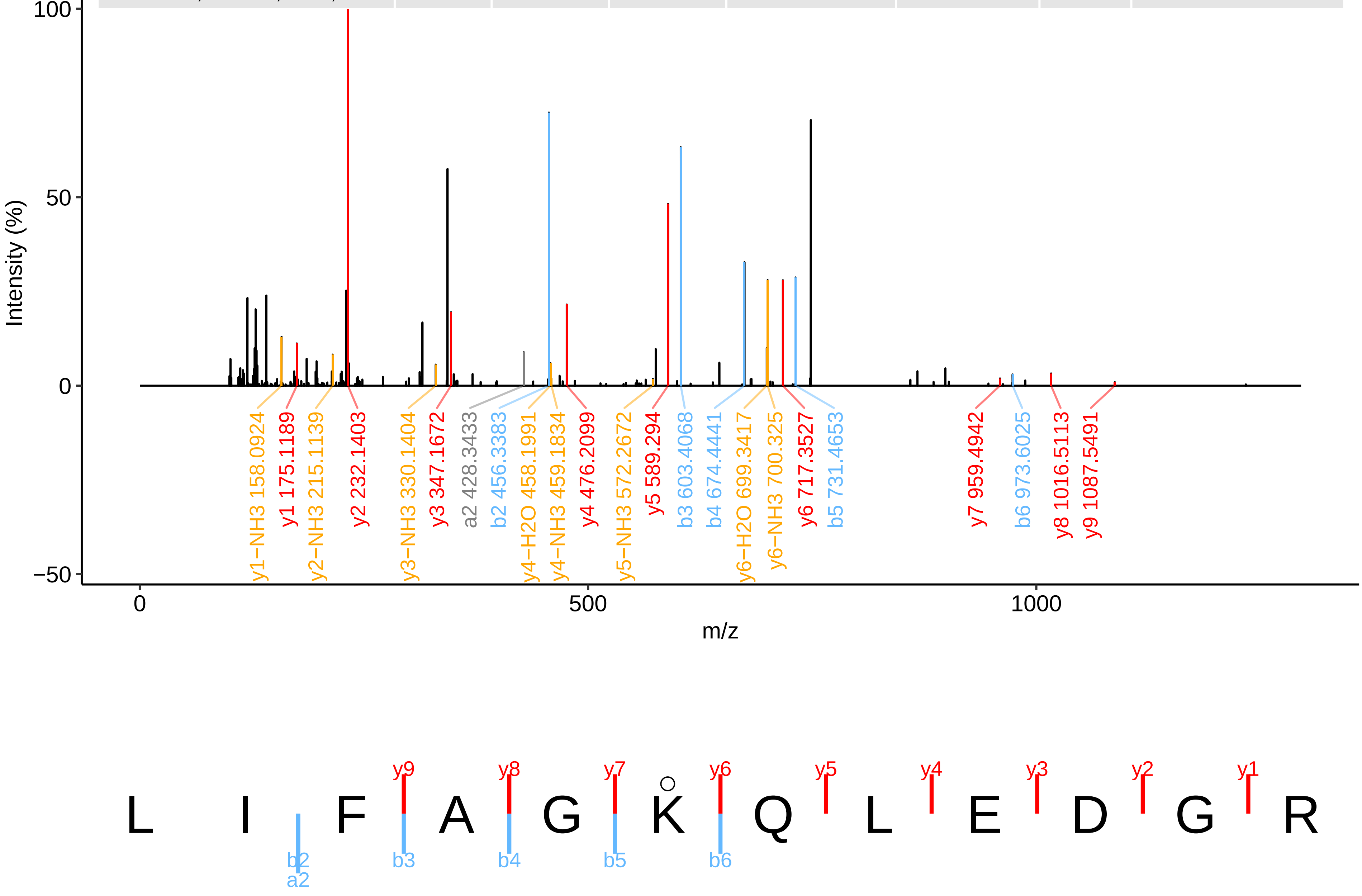

Modification □ Acetyl (Protein N−term) ○ GlyGly (K) no new terminus △ Oxidation (M)

20200921_KIZ_MUb4_GlyGly_10plex_2 − Scan number: 18316

Gene Names	Charge	m/z	Mass	Retention time	PEP	Score	Precursor Intensity
RPS27A;UBA52;UBC;UBB	3	487.6001	1459.778	71.711	3.1679e−13	135.99	6954110

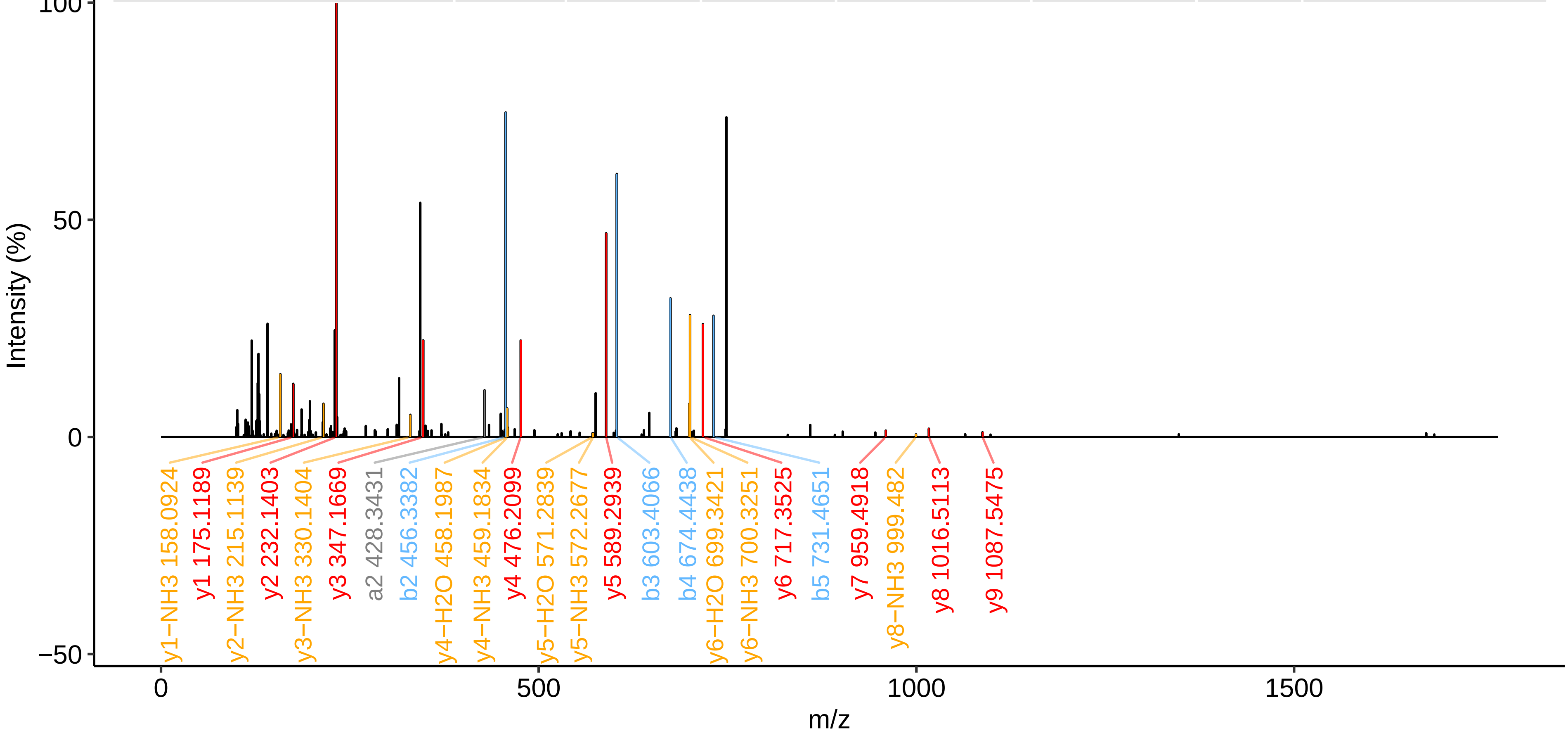

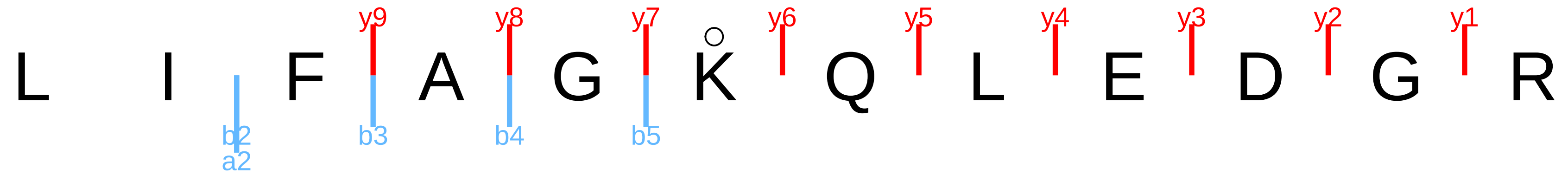

Modification □ Acetyl (Protein N−term) ○ GlyGly (K) no new terminus △ Oxidation (M)

20200921_KIZ_MUb4_GlyGly_10plex_2 − Scan number: 18445

Gene Names	Charge	m/z	Mass	Retention time	PEP	Score	Precursor Intensity
RPS27A;UBA52;UBC;UBB	3	487.6001	1459.778	72.228	1.1157e−08	119.27	7341280

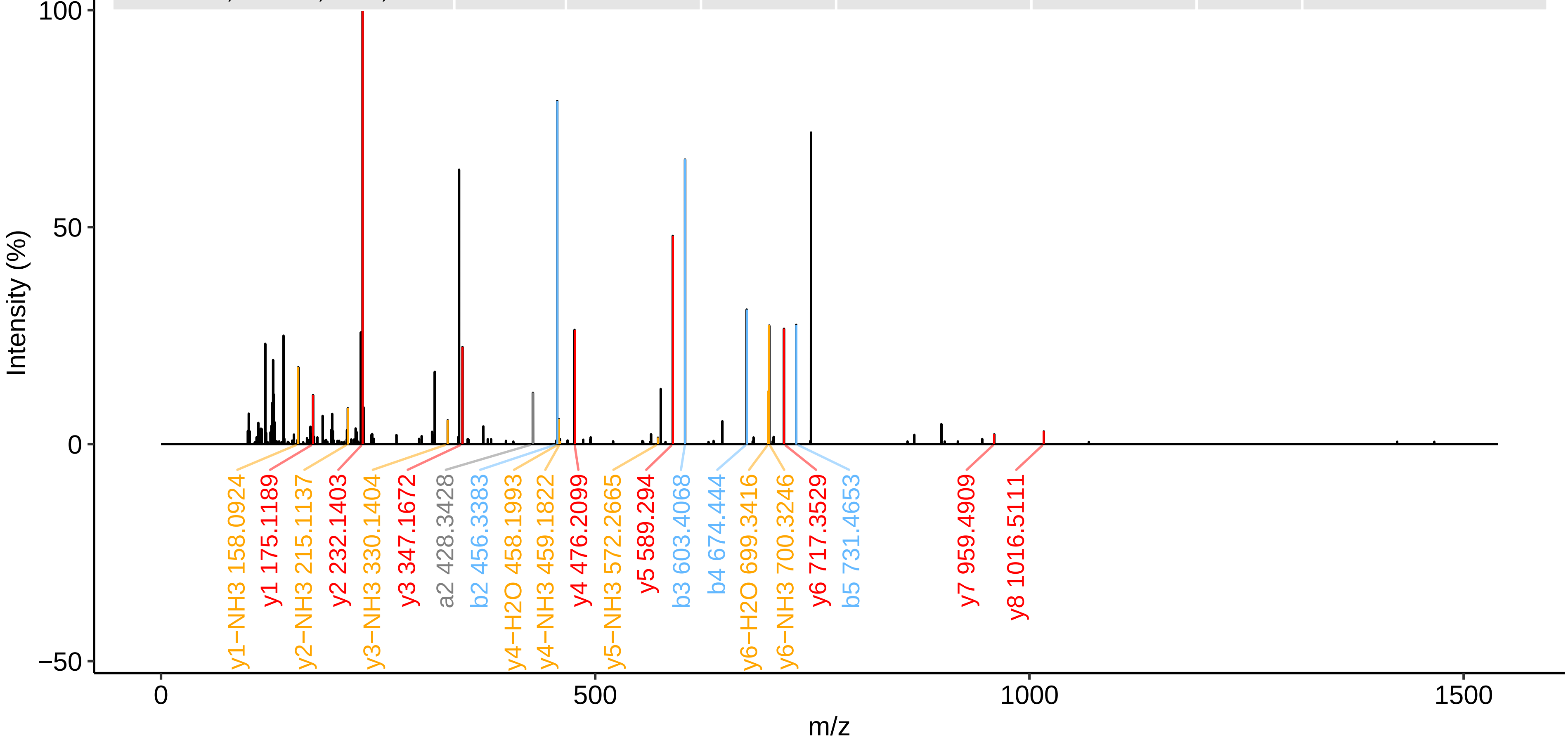

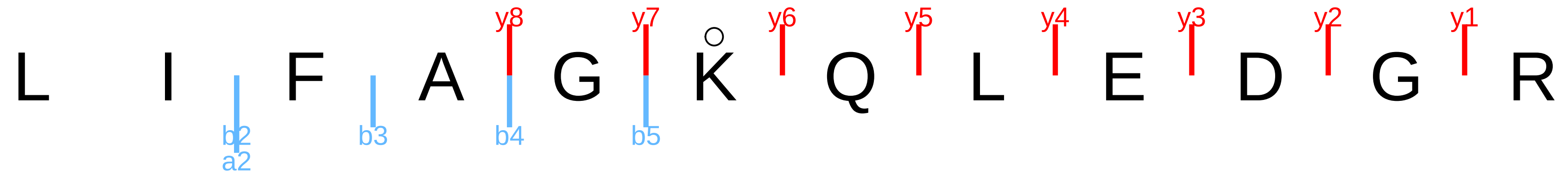

Modification □ Acetyl (Protein N−term) ○ GlyGly (K) no new terminus △ Oxidation (M)

20200921_KIZ_MUb4_GlyGly_10plex_2 − Scan number: 17535

Gene Names	Charge	m/z	Mass	Retention time	PEP	Score	Precursor Intensity
RPS27A;UBA52;UBC;UBB	2	730.8964	1459.778	68.576	0.034746	67.563	927551.8

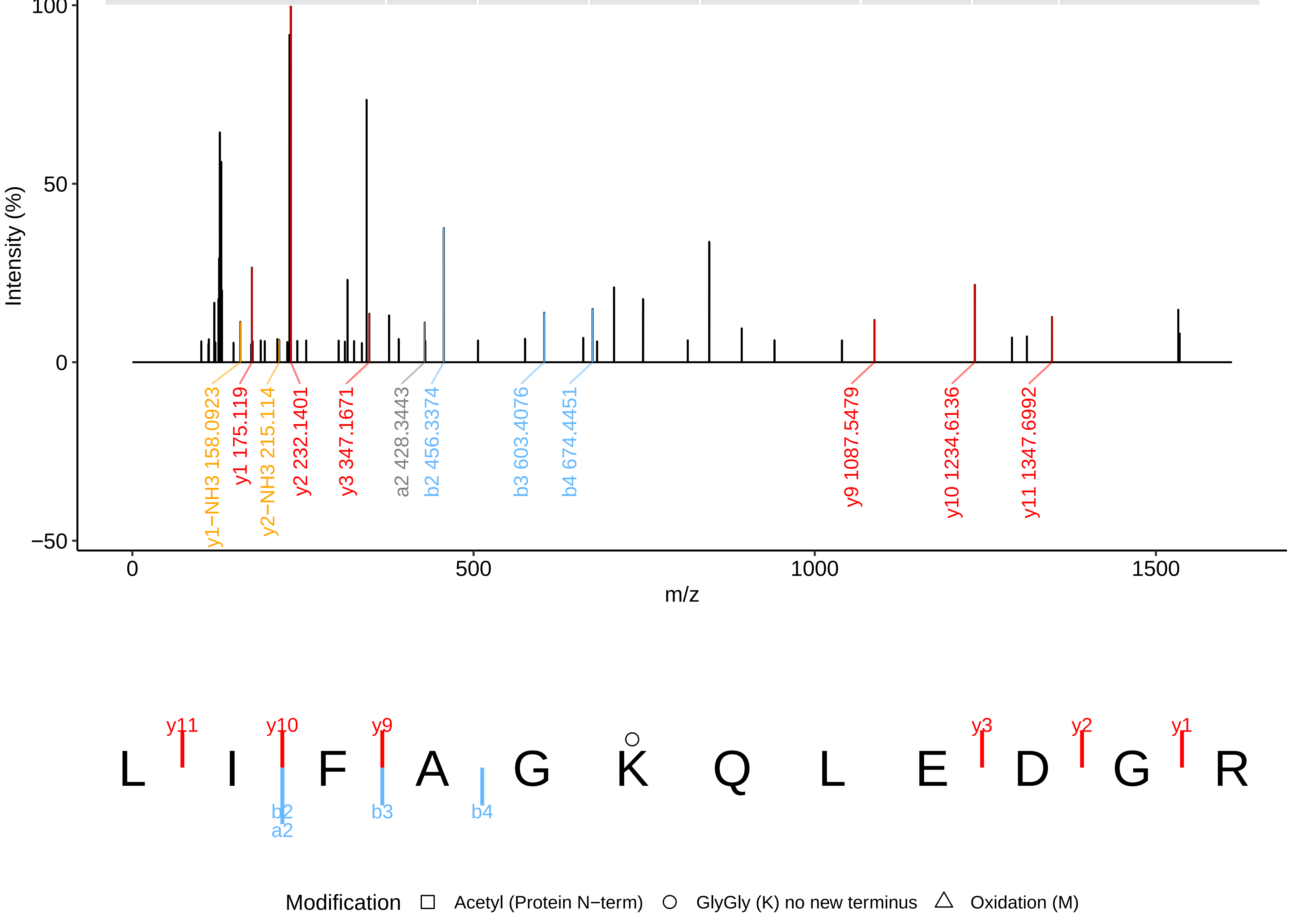

20200921_KIZ_MUb4_GlyGly_10plex_2 − Scan number: 18694

Gene Names	Charge	m/z	Mass	Retention time	PEP	Score	Precursor Intensity
RPS27A;UBA52;UBC;UBB	3	487.6001	1459.778	73.267	3.5418e−13	134.3	7151561

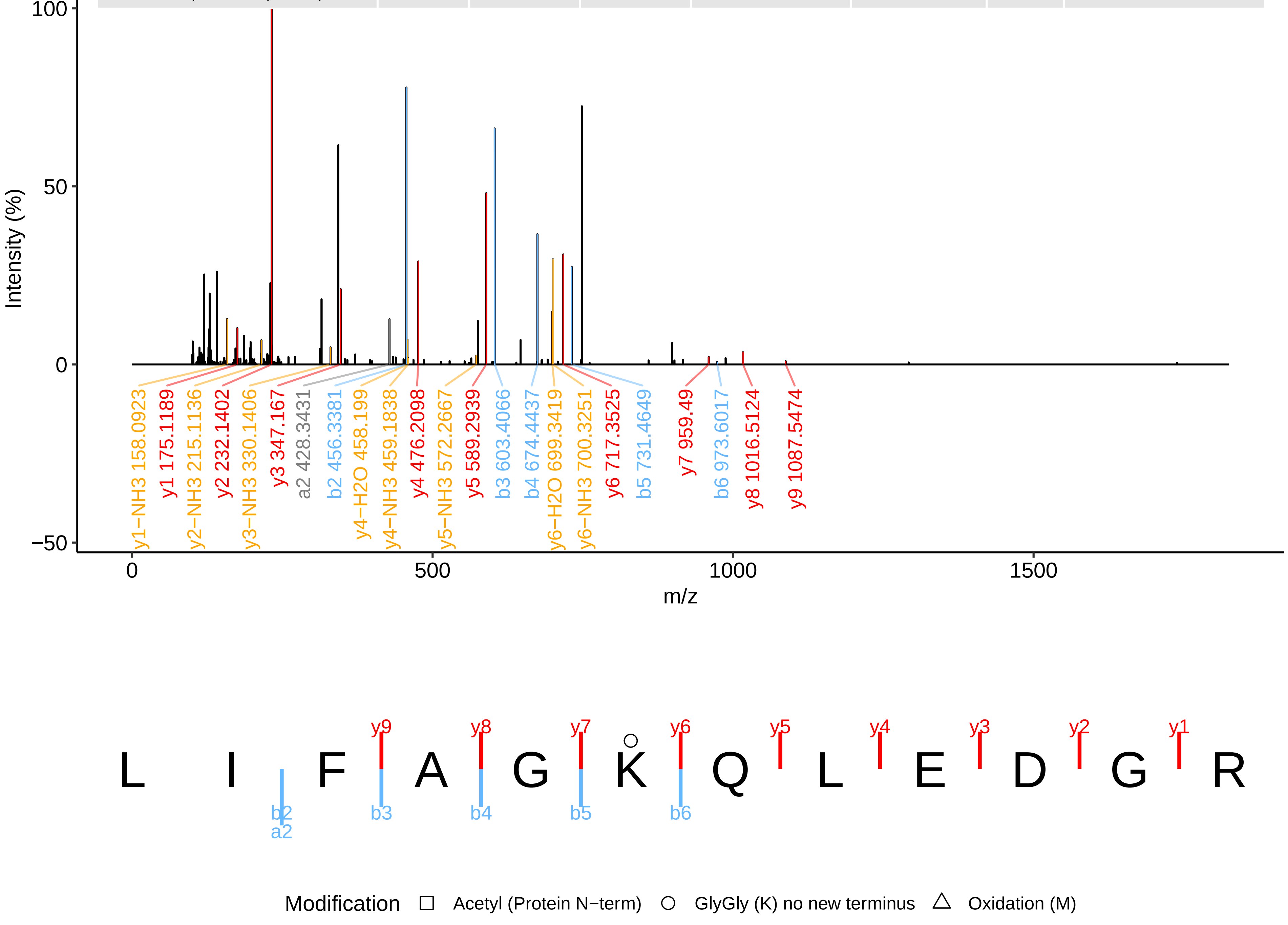

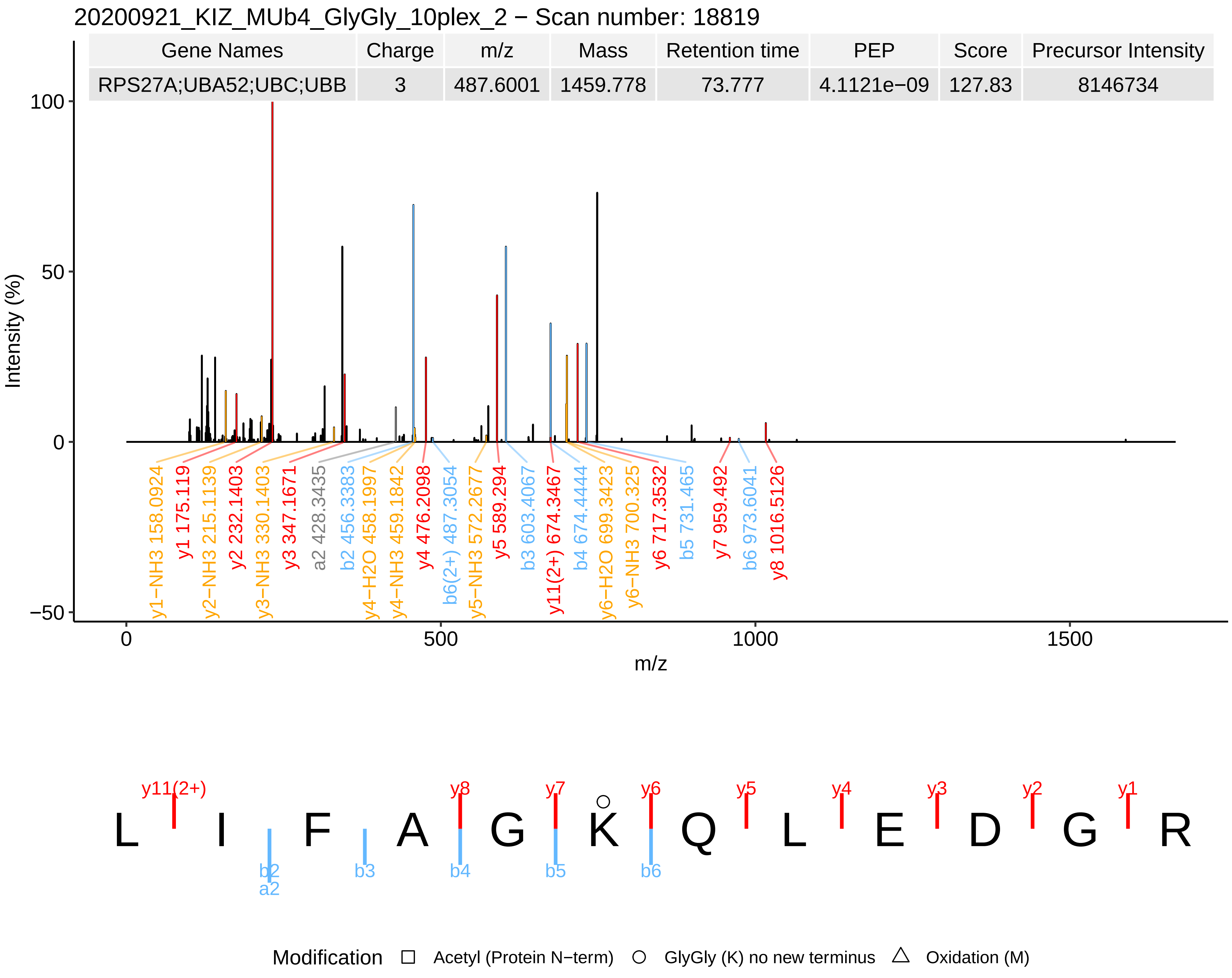
20200921_KIZ_MUb4_GlyGly_10plex_2 − Scan number: 18819
Gene Names
Charge
m/z
Mass
Retention time
PEP
Score
Precursor Intensity
RPS27A;UBA52;UBC;UBB
3
487.6001
1459.778
73.777
4.1121e−09
127.83
8146734
Intensity (%)
m/z
y1−NH3 158.0924
y1 175.119
y2−NH3 215.1139
y2 232.1403
y3−NH3 330.1403
y3 347.1671
a2 428.3435
b2 456.3383
y4−H2O 458.1997
y4−NH3 459.1842
y4 476.2098
b6(2+) 487.3054
y5−NH3 572.2677
y5 589.294
b3 603.4067
y11(2+) 674.3467
b4 674.4444
y6−H2O 699.3423
y6−NH3 700.325
y6 717.3532
b5 731.465
y7 959.492
b6 973.6041
y8 1016.5126
L I F A G K Q L E D G R
y11(2+) y8 y7 y6 y5 y4 y3 y2 y1
b2 a2 b3 b4 b5 b6
Modification
Acetyl (Protein N−term)
GlyGly (K) no new terminus
Oxidation (M)

20200921_KIZ_MUb4_GlyGly_10plex_2 − Scan number: 19073

Gene Names	Charge	m/z	Mass	Retention time	PEP	Score	Precursor Intensity
RPS27A;UBA52;UBC;UBB	3	487.6001	1459.778	74.805	2.3227e−19	155	8367433

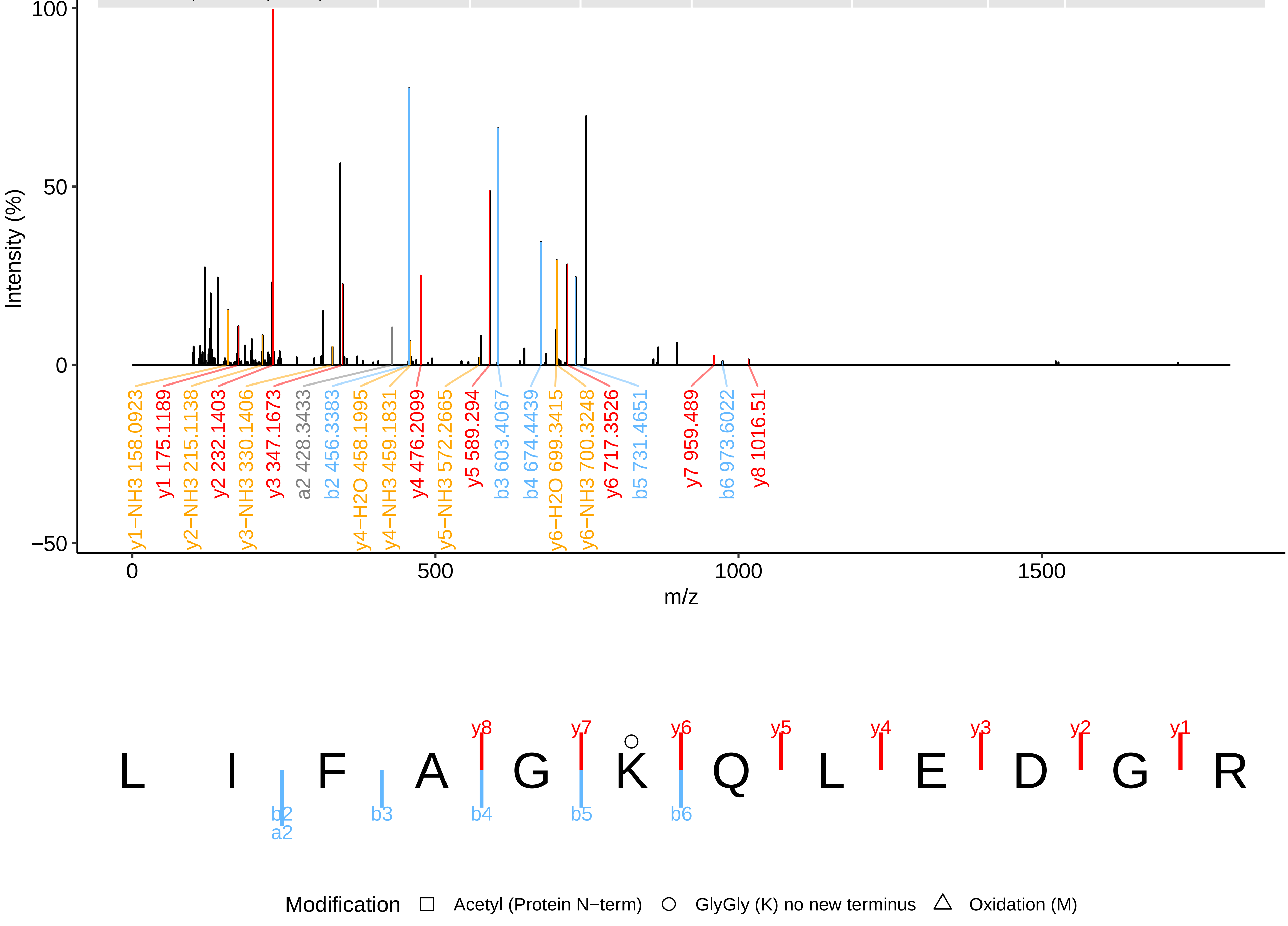

20200921_KIZ_MUb4_GlyGly_10plex_2 − Scan number: 19200

Gene Names	Charge	m/z	Mass	Retention time	PEP	Score	Precursor Intensity
RPS27A;UBA52;UBC;UBB	3	487.6001	1459.778	75.332	9.6101e−14	145.91	6022158

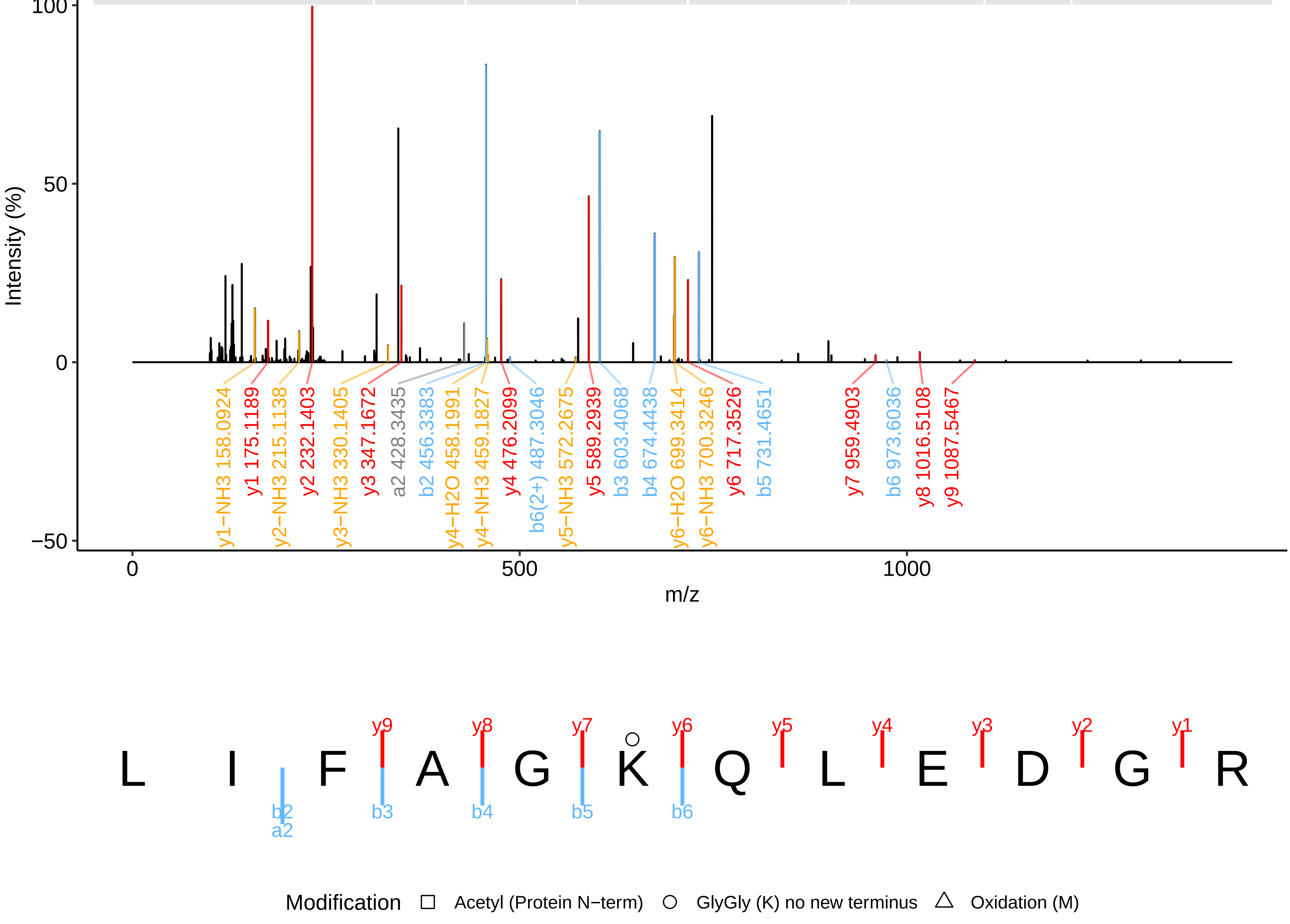

20200921_KIZ_MUb4_GlyGly_10plex_2 − Scan number: 19325

Gene Names	Charge	m/z	Mass	Retention time	PEP	Score	Precursor Intensity
RPS27A;UBA52;UBC;UBB	3	487.6001	1459.778	75.84	2.3212e−09	130.01	6001798

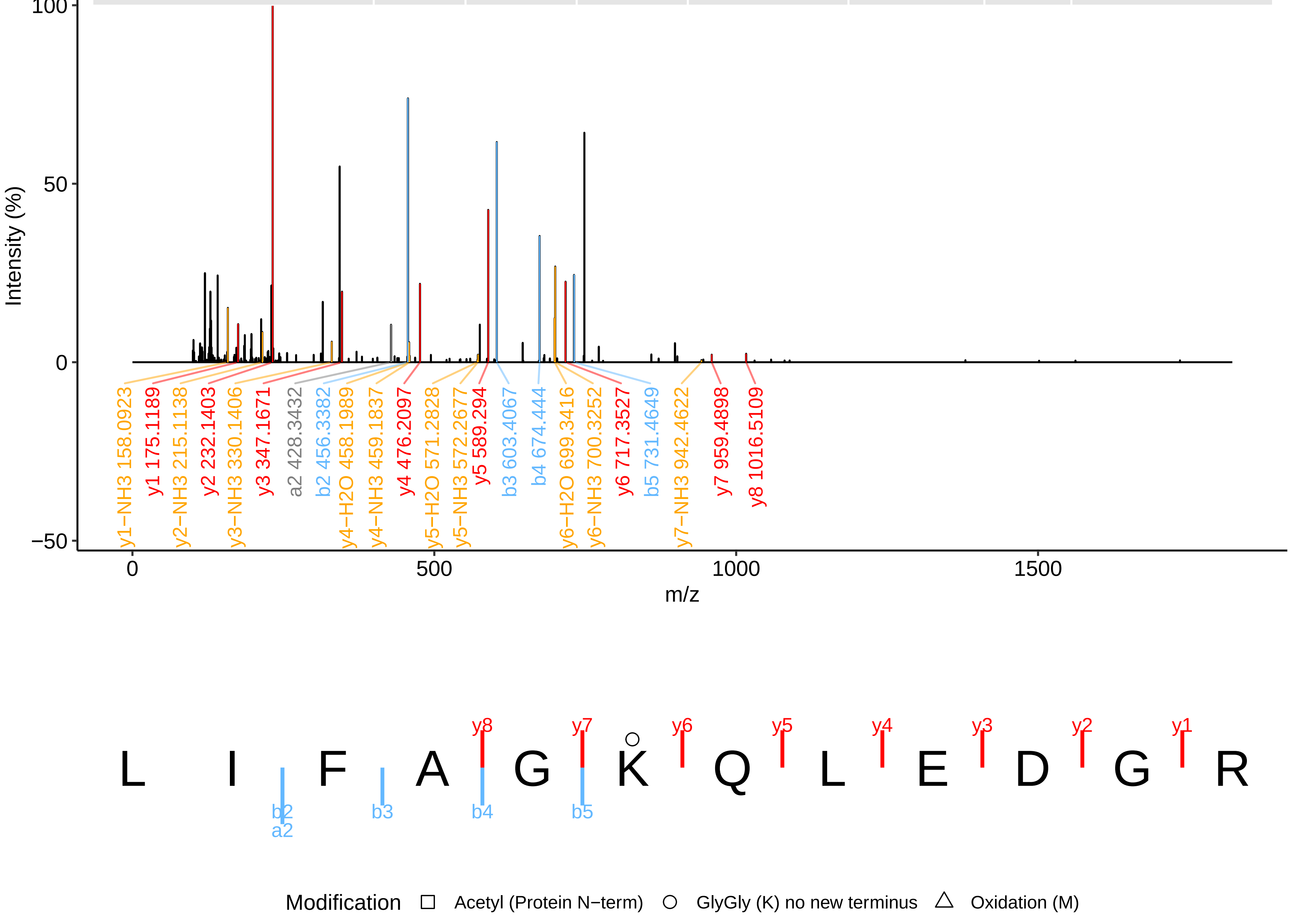

20200921_KIZ_MUb4_GlyGly_10plex_2 − Scan number: 19585

Gene Names	Charge	m/z	Mass	Retention time	PEP	Score	Precursor Intensity
RPS27A;UBA52;UBC;UBB	3	487.6001	1459.778	76.889	6.257e−09	125.23	4384161

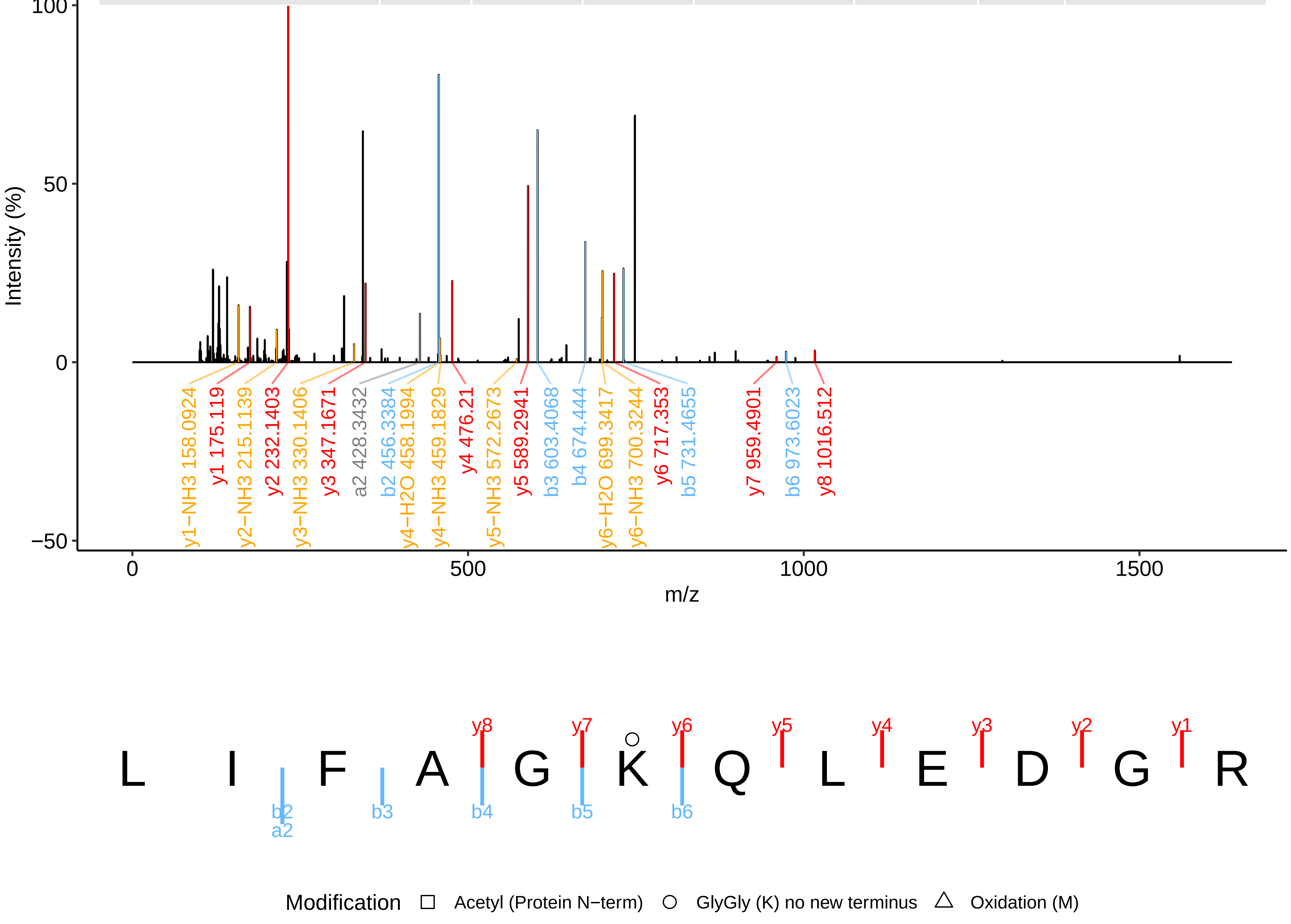

20200921_KIZ_MUb4_GlyGly_10plex_2 − Scan number: 19968

Gene Names	Charge	m/z	Mass	Retention time	PEP	Score	Precursor Intensity
RPS27A;UBA52;UBC;UBB	3	487.6001	1459.778	78.435	1.1031e−13	145.28	5975704

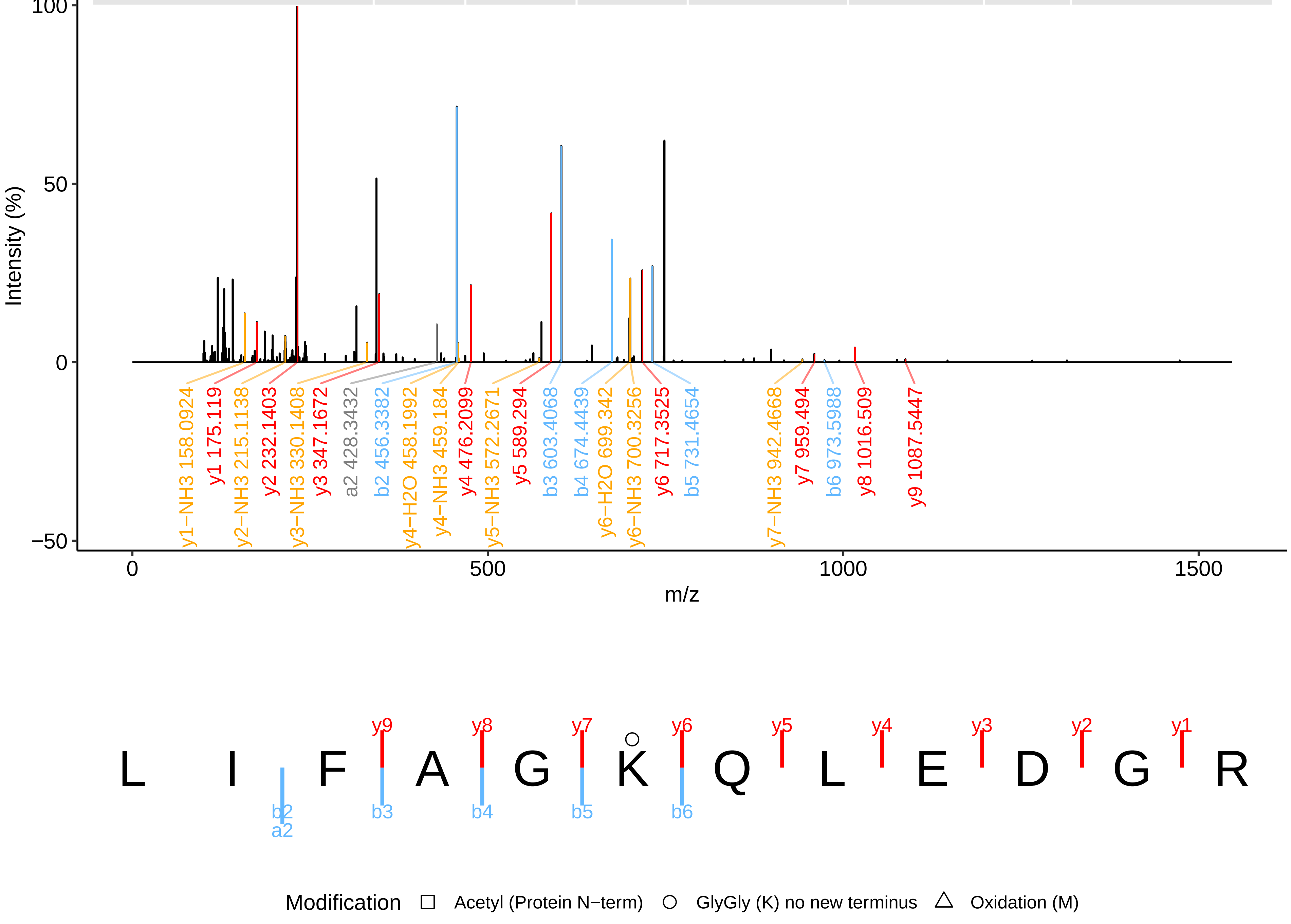

20200921_KIZ_MUb4_GlyGly_10plex_2 − Scan number: 20312

Gene Names	Charge	m/z	Mass	Retention time	PEP	Score	Precursor Intensity
RPS27A;UBA52;UBC;UBB	3	487.6001	1459.778	79.827	0.00099886	83.775	2959988

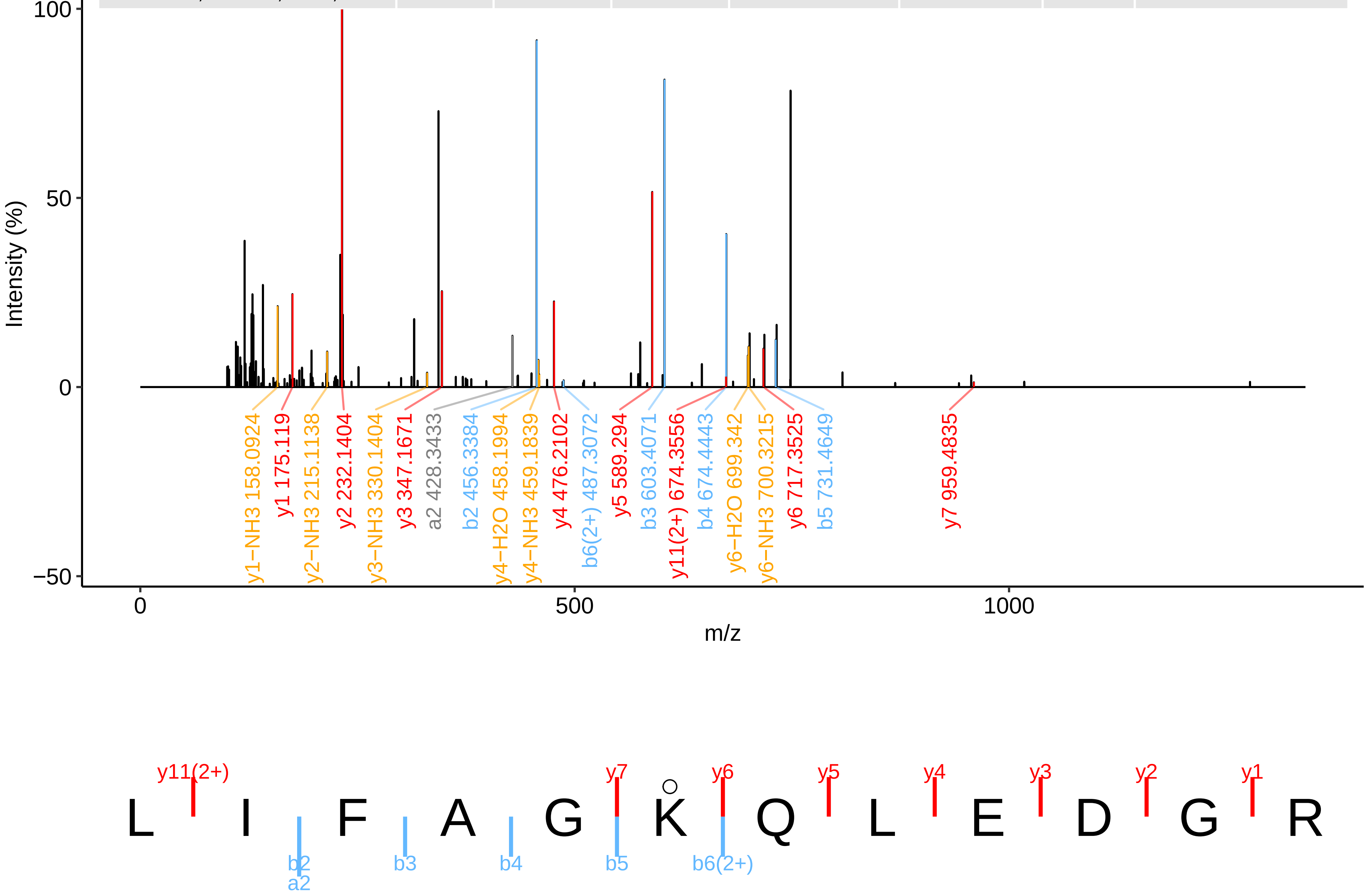

Modification □ Acetyl (Protein N−term) ○ GlyGly (K) no new terminus △ Oxidation (M)

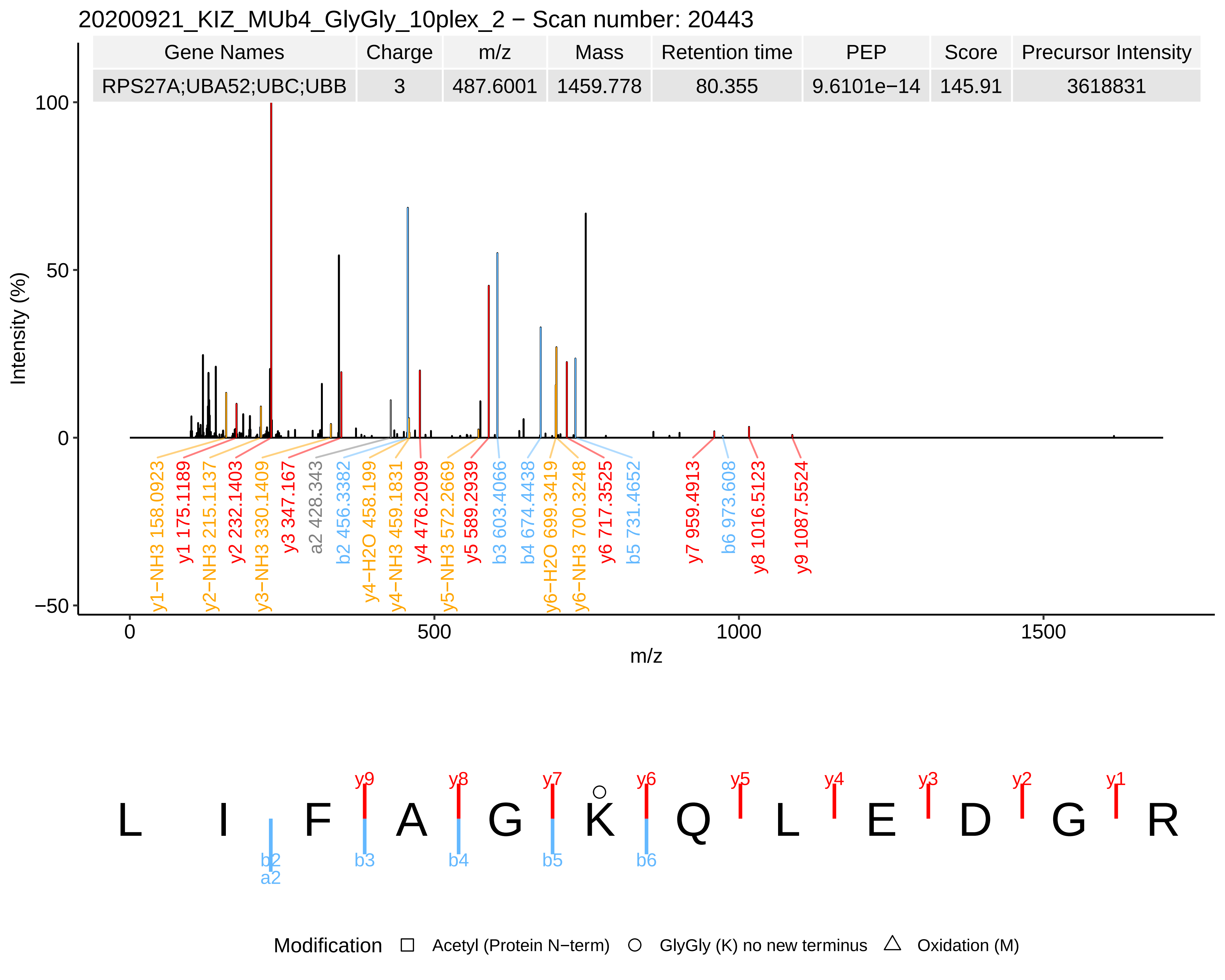

20200921_KIZ_MUb4_GlyGly_10plex_2 − Scan number: 20443
Gene Names | Charge | m/z | Mass | Retention time | PEP | Score | Precursor Intensity
RPS27A;UBA52;UBC;UBB | 3 | 487.6001 | 1459.778 | 80.355 | 9.6101e−14 | 145.91 | 3618831
Intensity (%)
100
50
0
−50
m/z
0
500
1000
1500
y1−NH3 158.0923
y1 175.1189
y2−NH3 215.1137
y2 232.1403
y3−NH3 330.1409
y3 347.167
a2 428.343
b2 456.3382
y4−H2O 458.199
y4−NH3 459.1831
y4 476.2099
y5−NH3 572.2669
y5 589.2939
b3 603.4066
b4 674.4438
y6−H2O 699.3419
y6−NH3 700.3248
y6 717.3525
b5 731.4652
y7 959.4913
b6 973.608
y8 1016.5123
y9 1087.5524
L I F A G K Q L E D G R
y9 y8 y7 y6 y5 y4 y3 y2 y1
b2 a2 b3 b4 b5 b6
Modification
Acetyl (Protein N−term)
GlyGly (K) no new terminus
Oxidation (M)

20200921_KIZ_MUb4_GlyGly_10plex_2 − Scan number: 20570

Gene Names	Charge	m/z	Mass	Retention time	PEP	Score	Precursor Intensity
RPS27A;UBA52;UBC;UBB	3	487.6001	1459.778	80.868	8.2269e−10	131.83	3361433

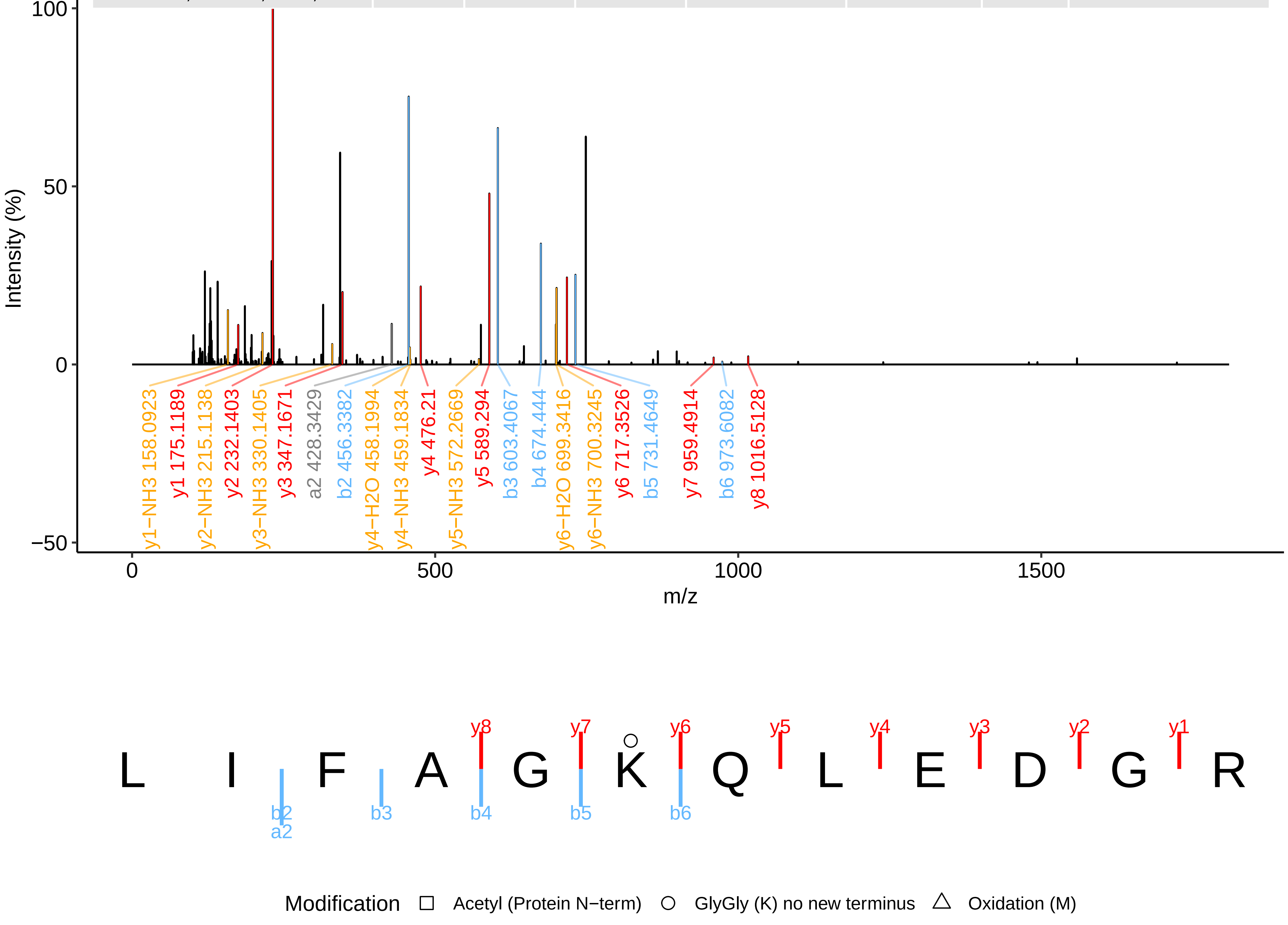

20200921_KIZ_MUb4_GlyGly_10plex_2 − Scan number: 20694

Gene Names	Charge	m/z	Mass	Retention time	PEP	Score	Precursor Intensity
RPS27A;UBA52;UBC;UBB	3	487.6001	1459.778	81.381	1.1157e−08	119.27	3583301

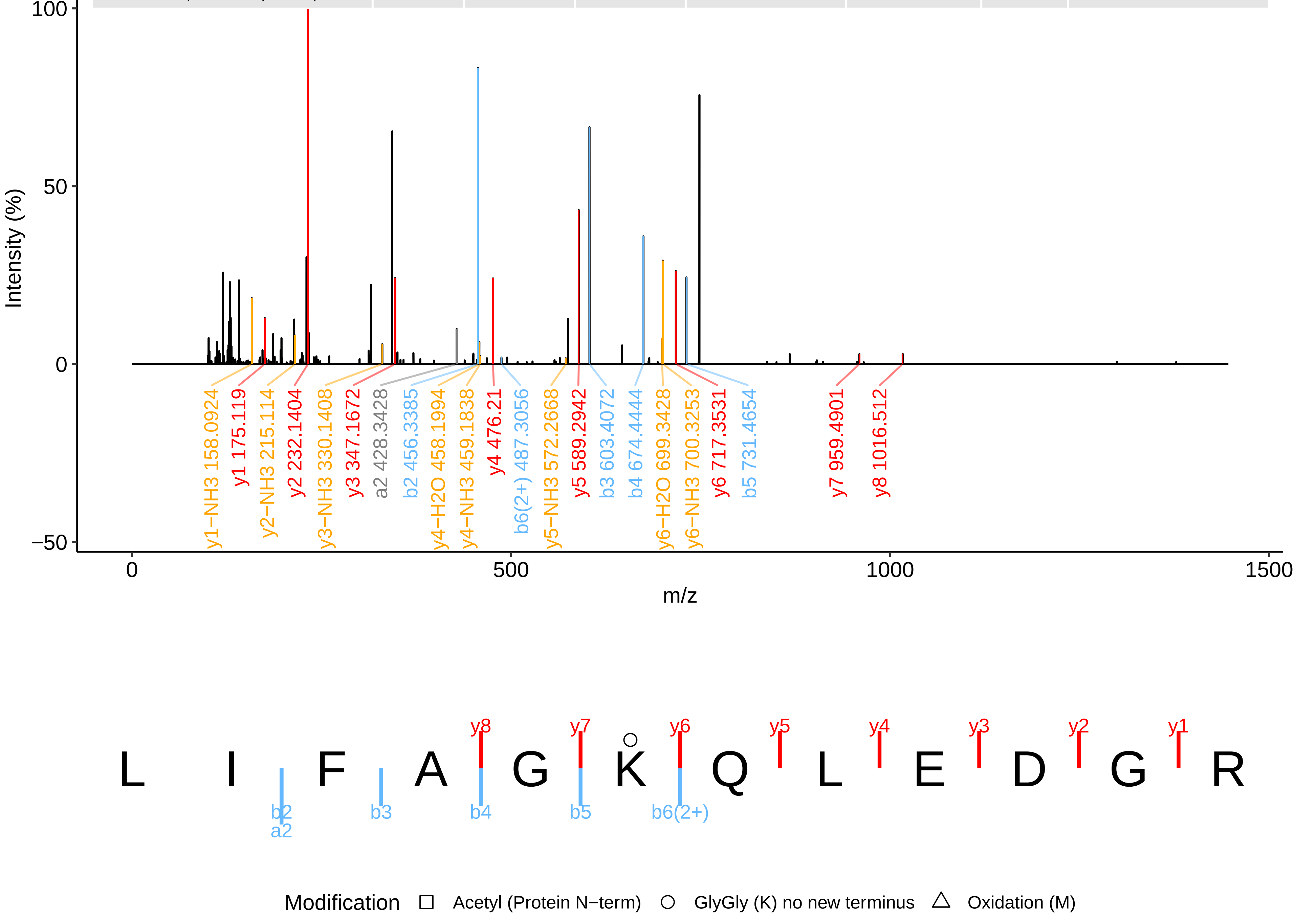

20200921_KIZ_MUb4_GlyGly_10plex_2 − Scan number: 20955

Gene Names	Charge	m/z	Mass	Retention time	PEP	Score	Precursor Intensity
RPS27A;UBA52;UBC;UBB	3	487.6001	1459.778	82.444	3.5805e−13	134.13	3280819

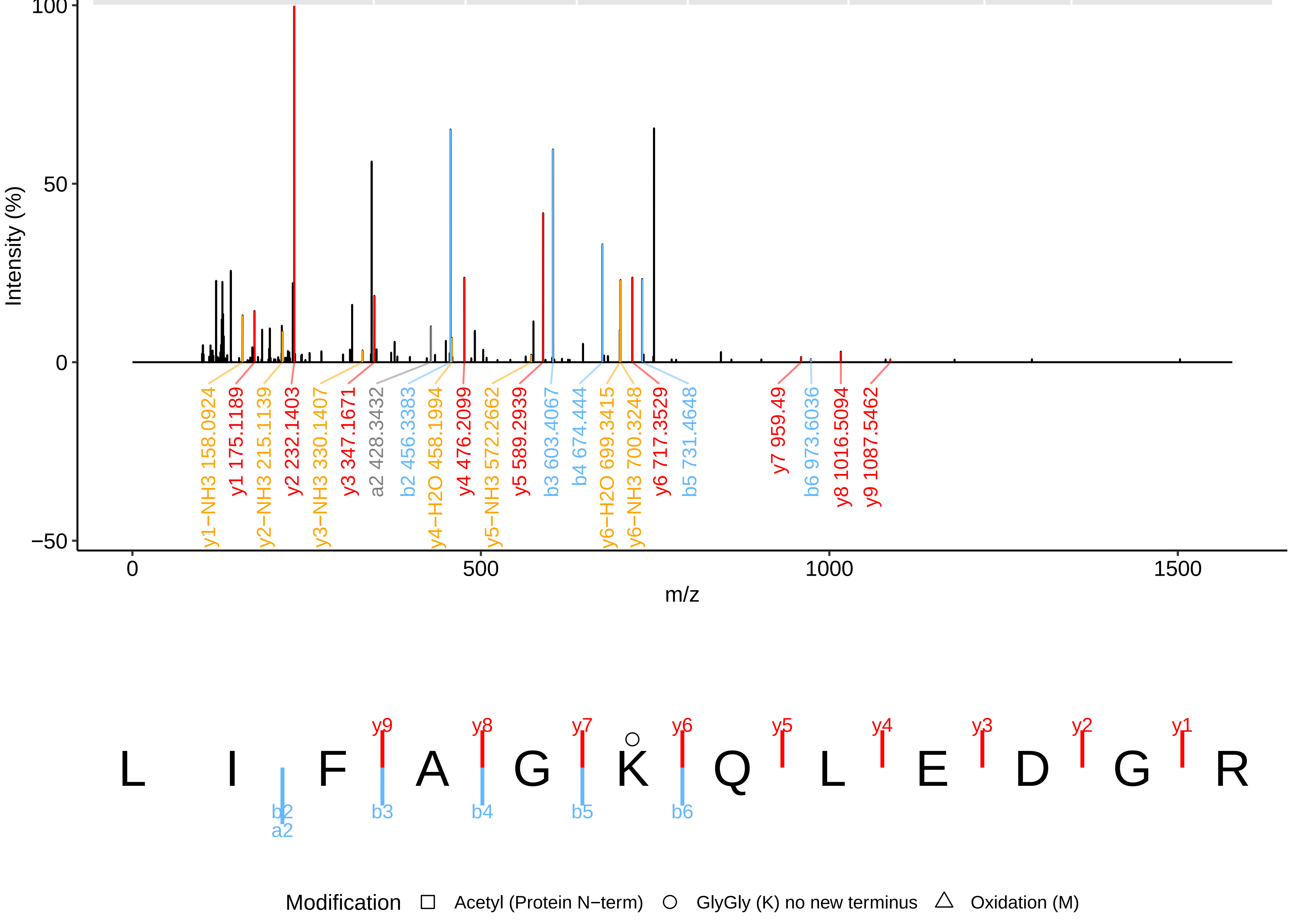

20200921_KIZ_MUb4_GlyGly_10plex_2 − Scan number: 21081

Gene Names	Charge	m/z	Mass	Retention time	PEP	Score	Precursor Intensity
RPS27A;UBA52;UBC;UBB	3	487.6001	1459.778	82.952	1.1031e−13	145.28	4339184

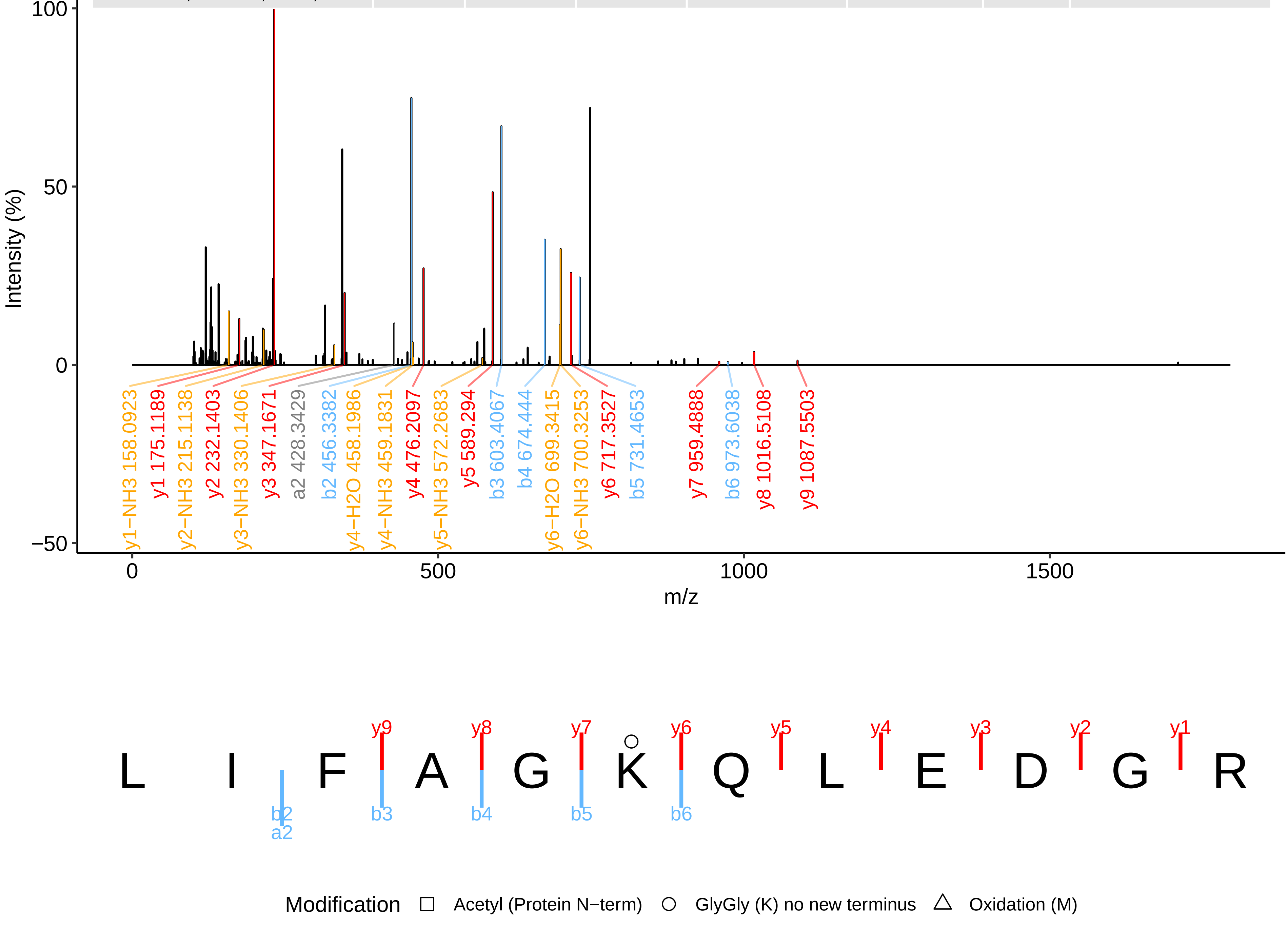

20200921_KIZ_MUb4_GlyGly_10plex_2 − Scan number: 21209

Gene Names	Charge	m/z	Mass	Retention time	PEP	Score	Precursor Intensity
RPS27A;UBA52;UBC;UBB	3	487.6001	1459.778	83.473	1.3403e−08	116.55	2846360

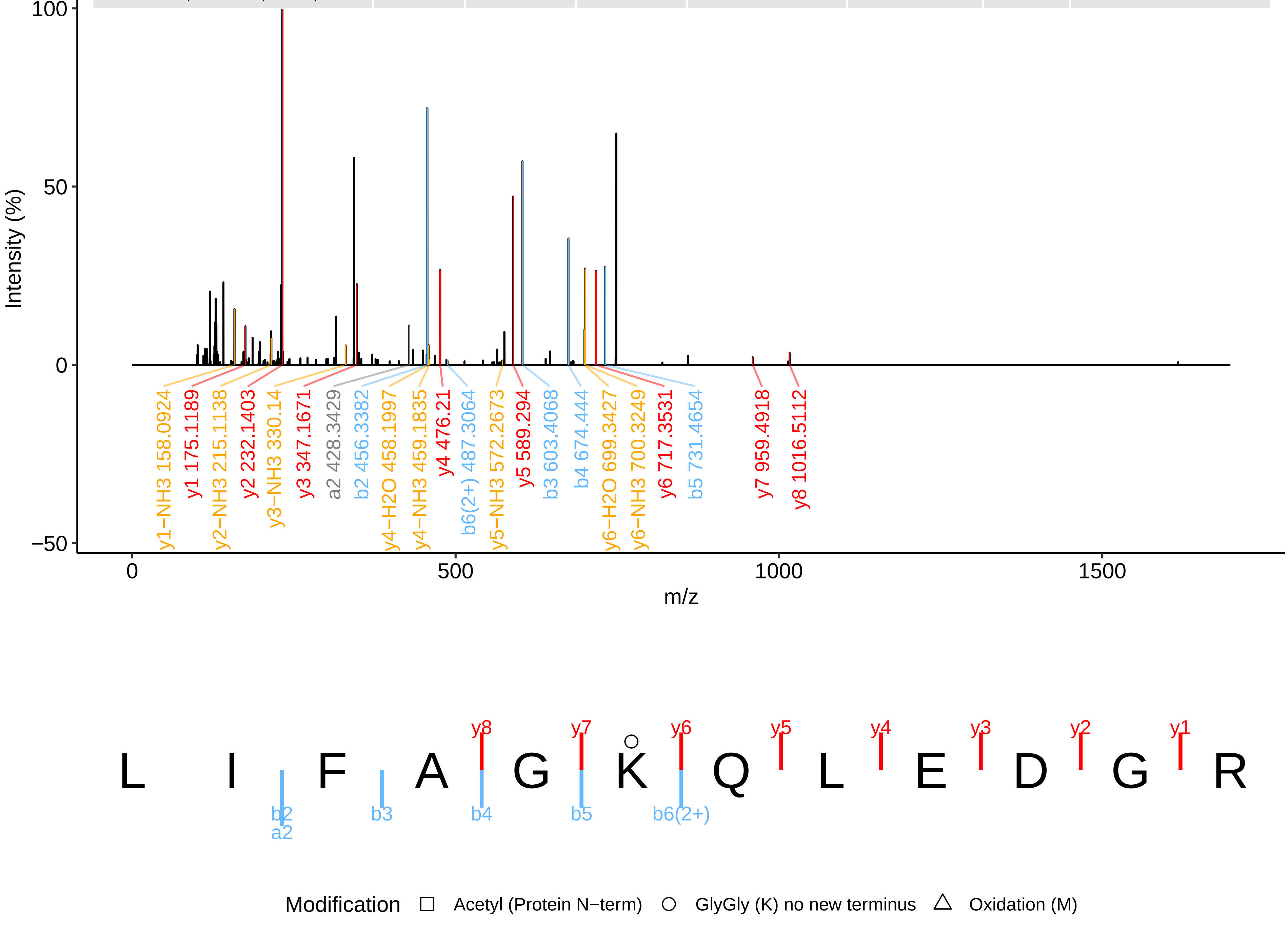

20200921_KIZ_MUb4_GlyGly_10plex_2 − Scan number: 21361

Gene Names	Charge	m/z	Mass	Retention time	PEP	Score	Precursor Intensity
RPS27A;UBA52;UBC;UBB	3	487.6001	1459.778	84.097	3.6333e−13	133.89	3425911

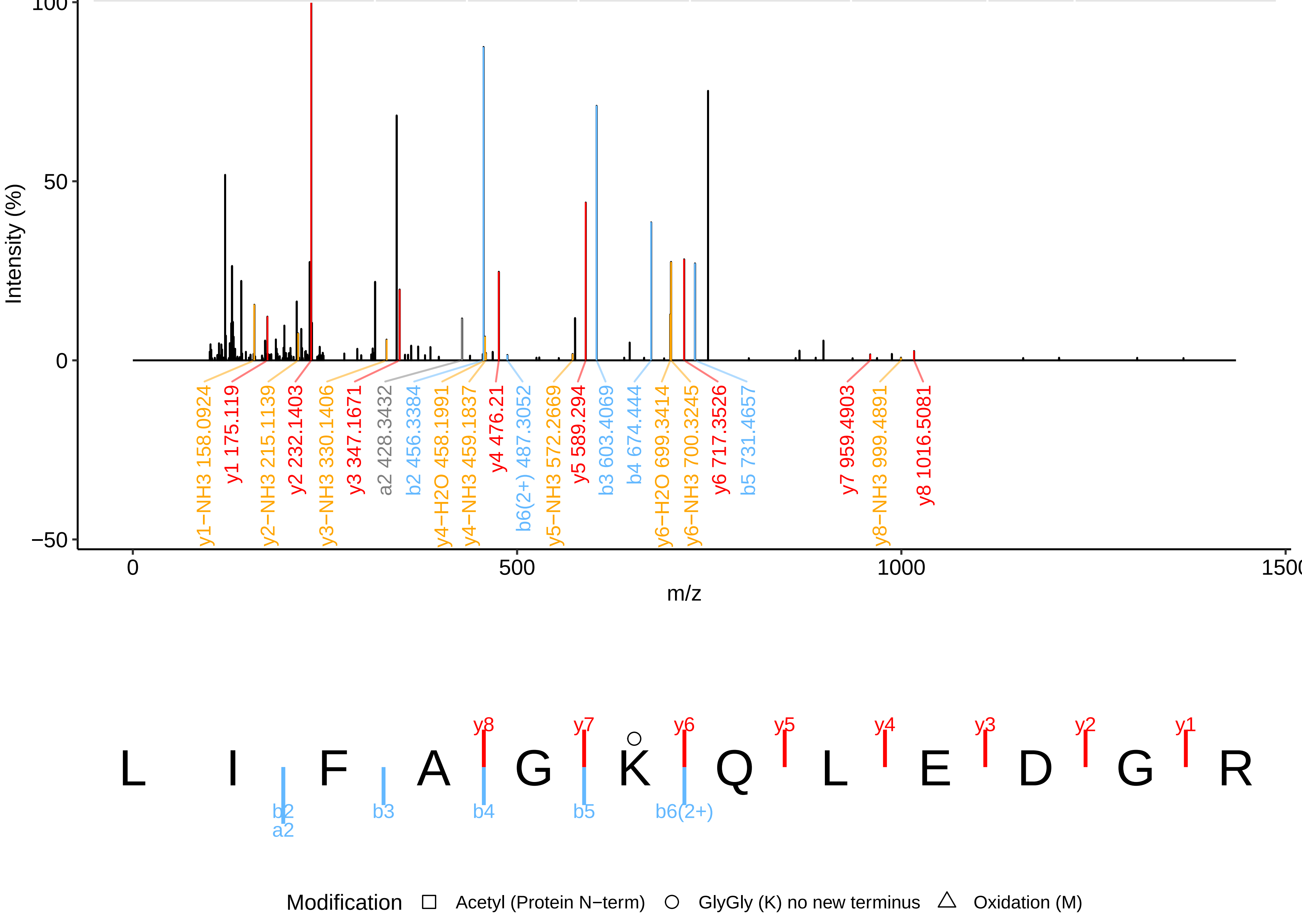

20200921_KIZ_MUb4_GlyGly_10plex_2 − Scan number: 21489

Gene Names	Charge	m/z	Mass	Retention time	PEP	Score	Precursor Intensity
RPS27A;UBA52;UBC;UBB	3	487.6001	1459.778	84.607	1.4204e−08	115.57	4238031

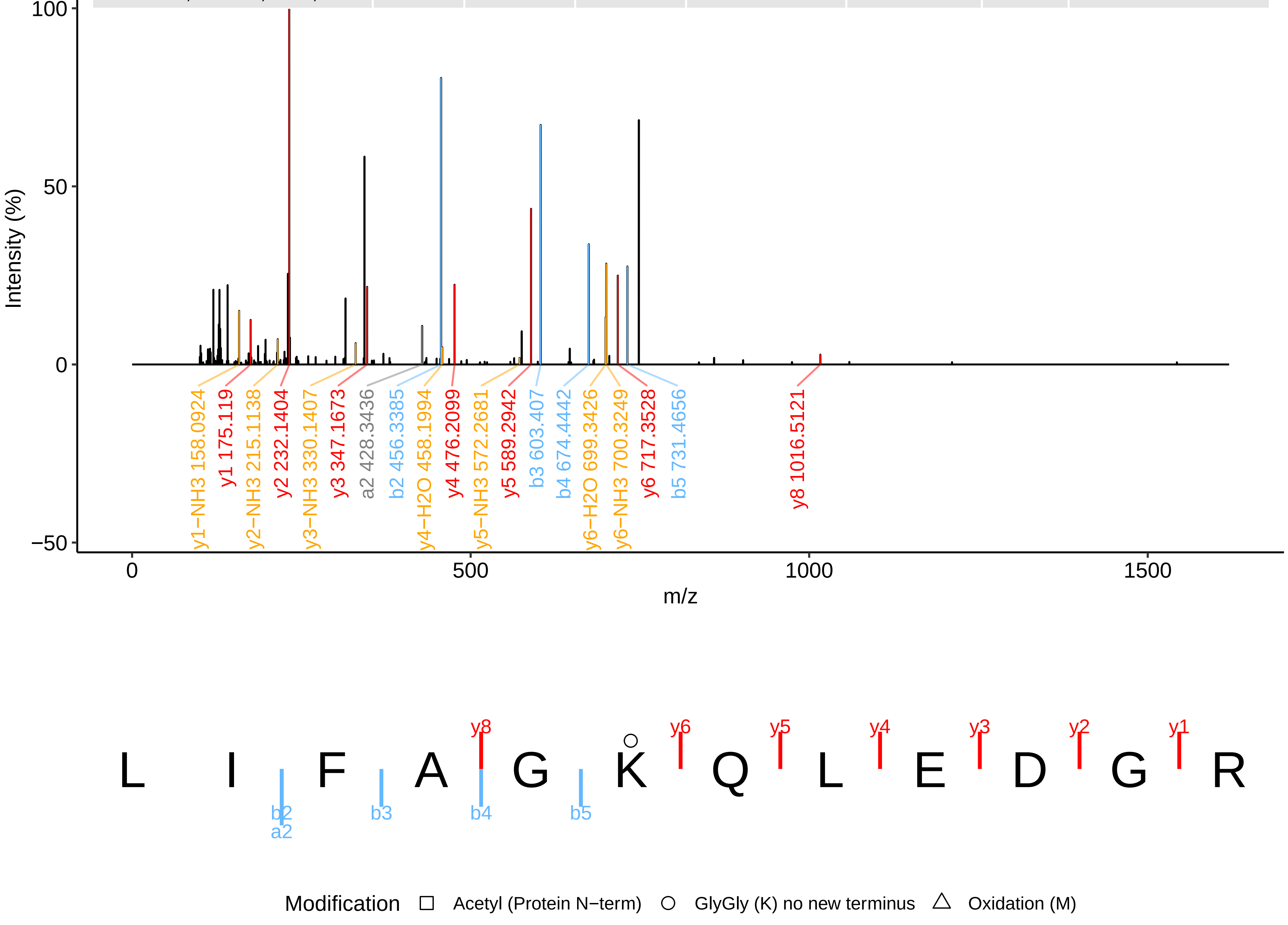

20200921_KIZ_MUb4_GlyGly_10plex_2 − Scan number: 21617

Gene Names	Charge	m/z	Mass	Retention time	PEP	Score	Precursor Intensity
RPS27A;UBA52;UBC;UBB	3	487.6001	1459.778	85.127	3.6333e−13	133.89	NA

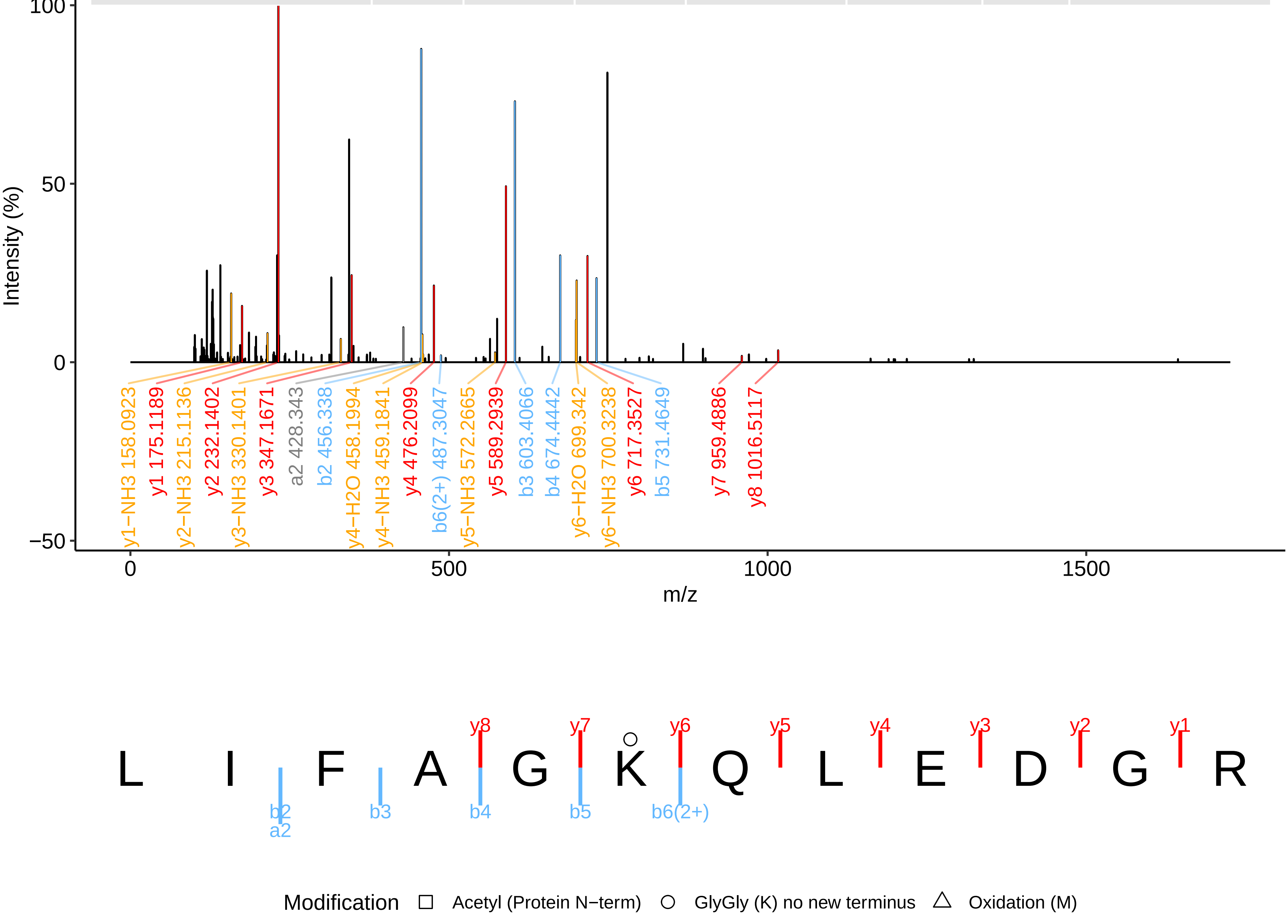

20200921_KIZ_MUb4_GlyGly_10plex_2 − Scan number: 21743

Gene Names	Charge	m/z	Mass	Retention time	PEP	Score	Precursor Intensity
RPS27A;UBA52;UBC;UBB	3	487.6001	1459.778	85.633	2.0348e−13	141.08	2581310

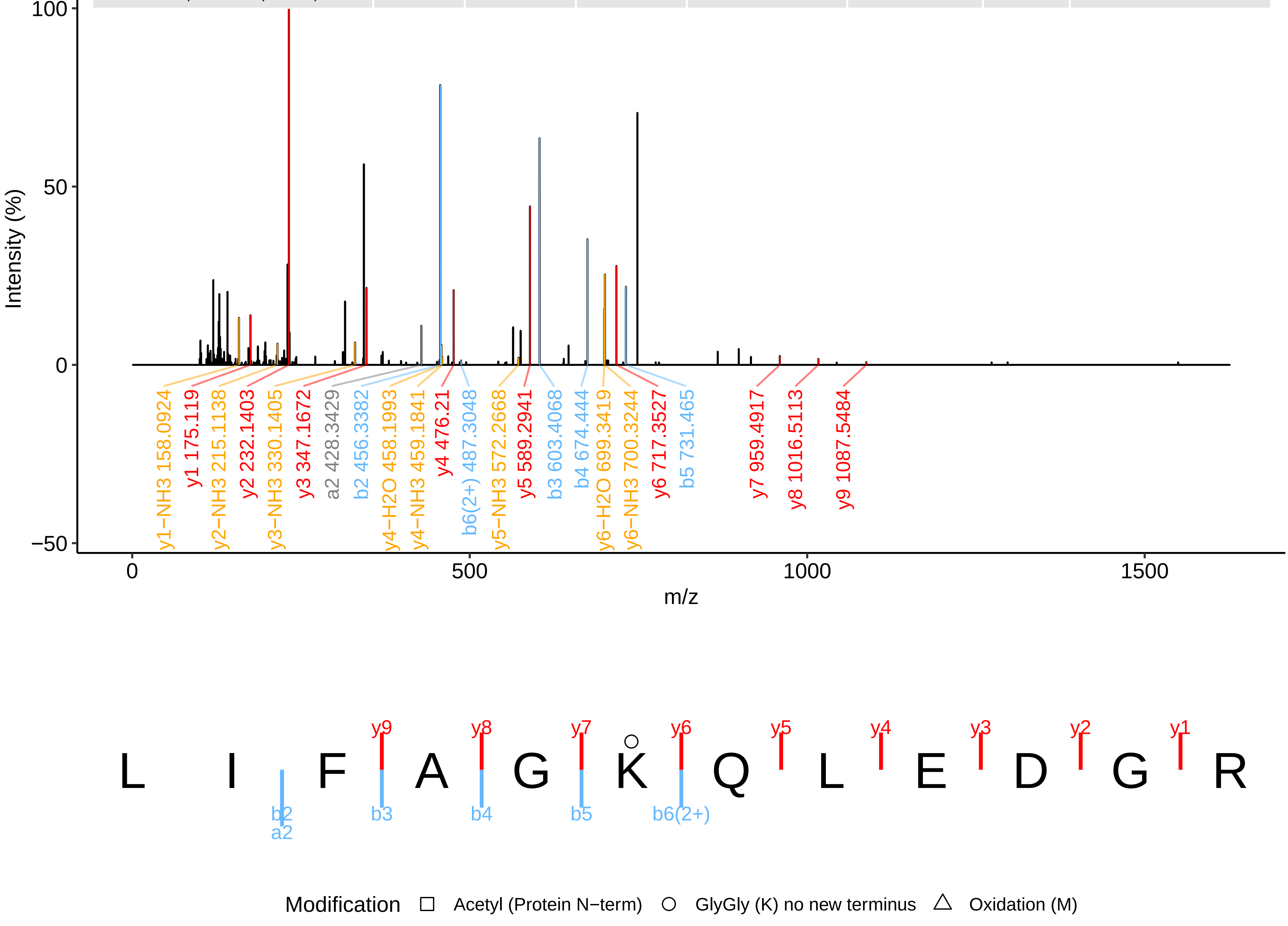

20200921_KIZ_MUb4_GlyGly_10plex_2 − Scan number: 21868

Gene Names	Charge	m/z	Mass	Retention time	PEP	Score	Precursor Intensity
RPS27A;UBA52;UBC;UBB	3	487.6001	1459.778	86.137	1.4204e−08	115.57	1713081

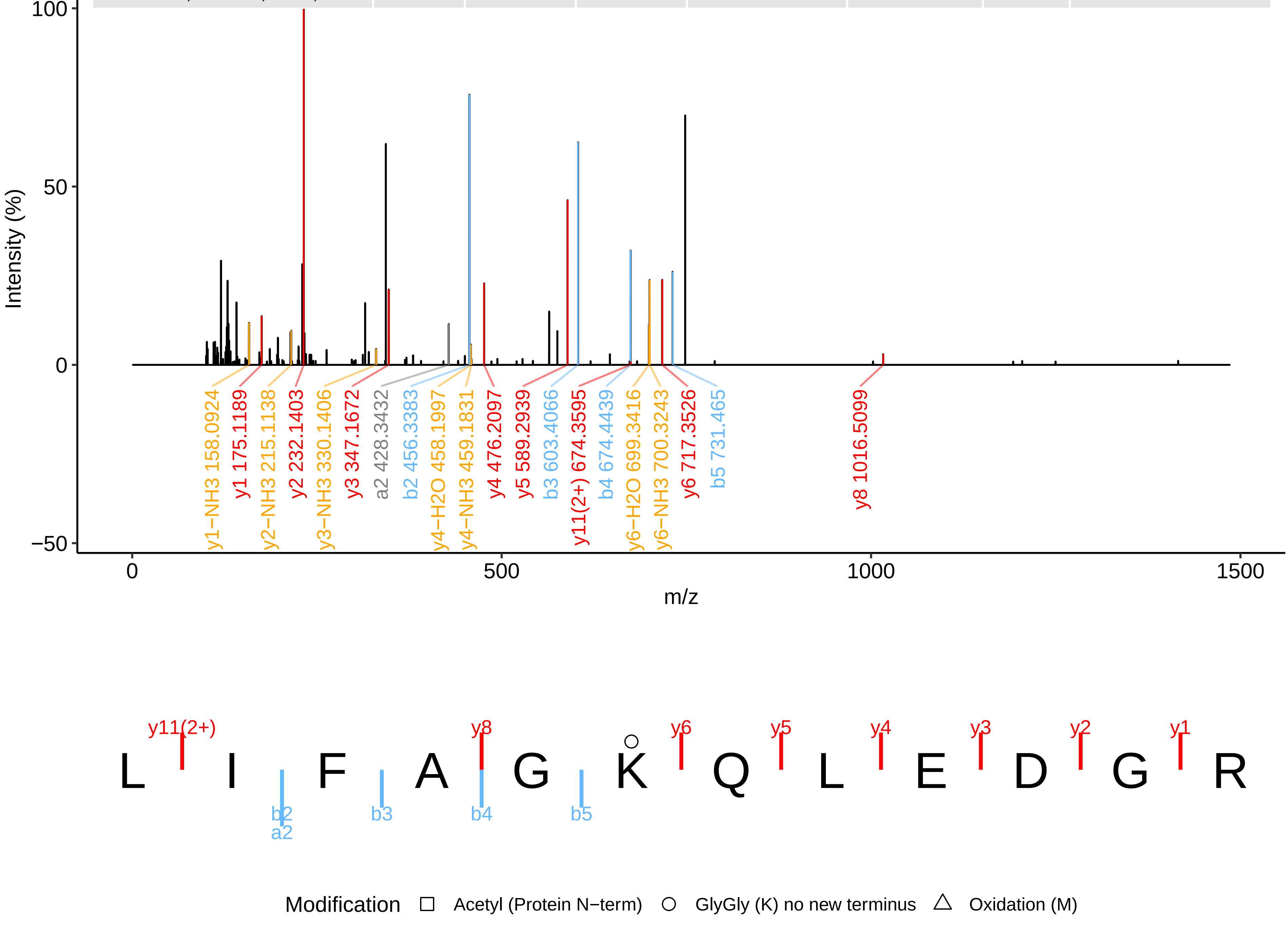

20200921_KIZ_MUb4_GlyGly_10plex_2 − Scan number: 21994

Gene Names	Charge	m/z	Mass	Retention time	PEP	Score	Precursor Intensity
RPS27A;UBA52;UBC;UBB	3	487.6001	1459.778	86.653	2.4754e−09	129.82	3025186

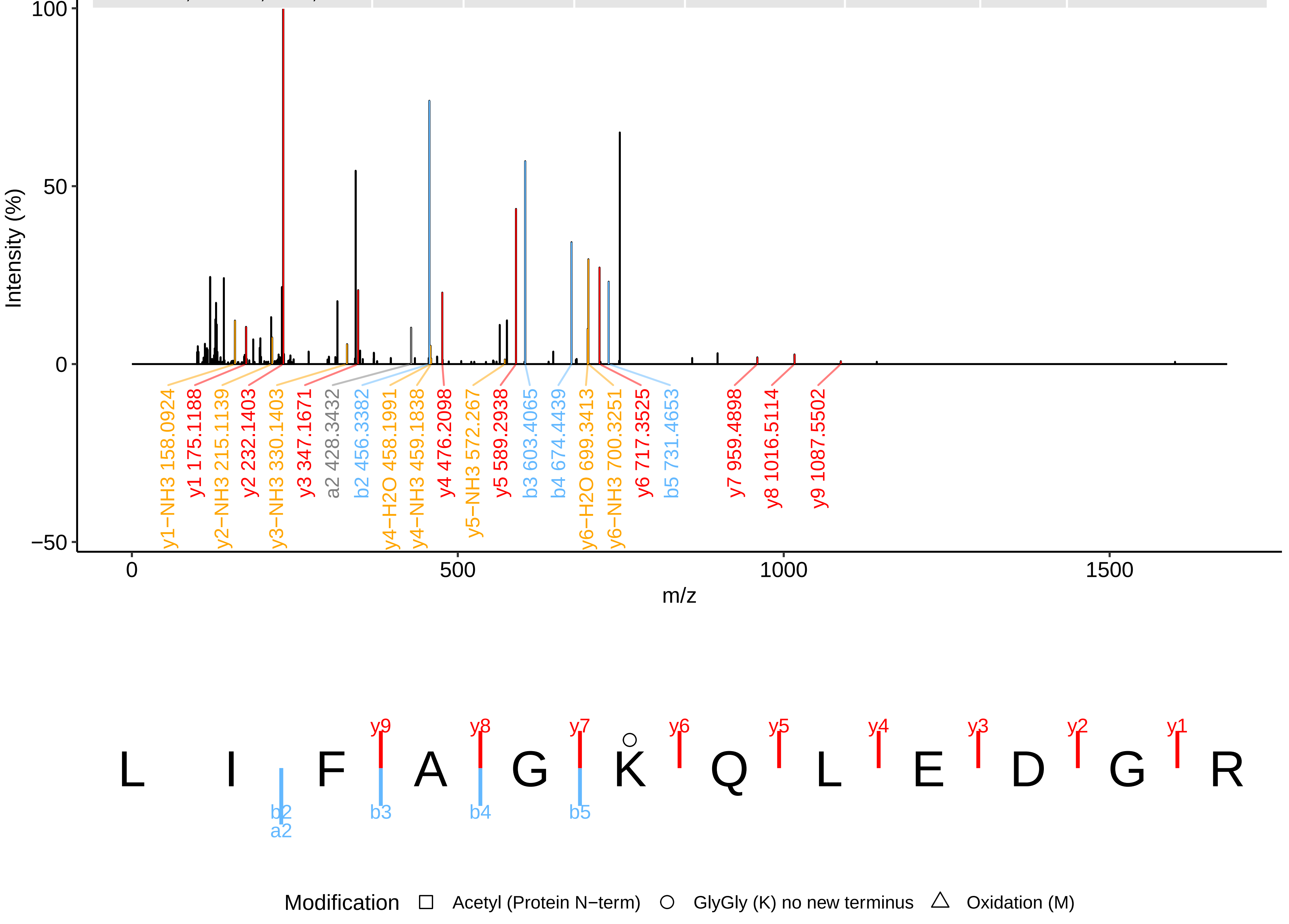

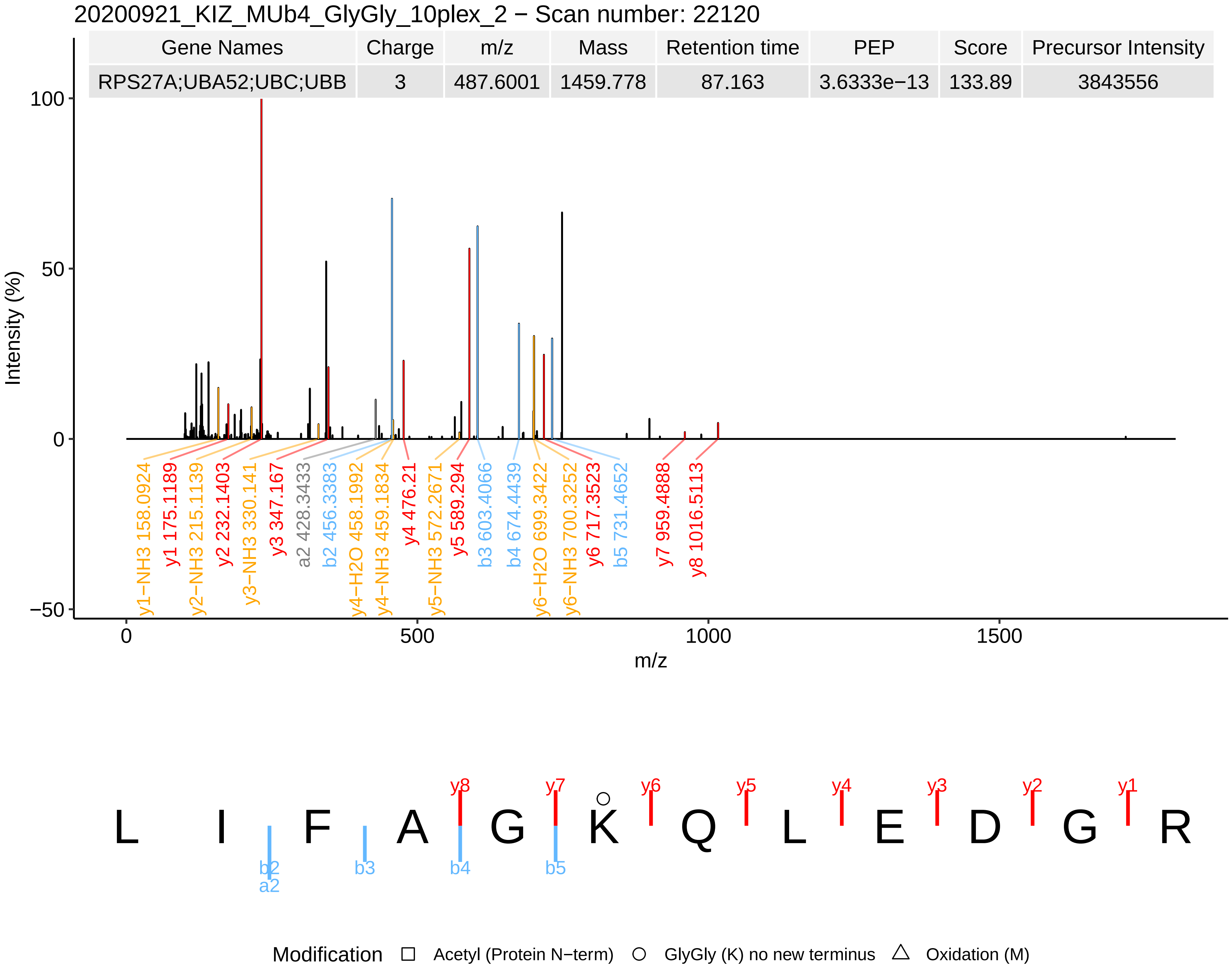

20200921_KIZ_MUb4_GlyGly_10plex_2 − Scan number: 22120
Gene Names
RPS27A;UBA52;UBC;UBB
Charge
3
m/z
487.6001
Mass
1459.778
Retention time
87.163
PEP
3.6333e−13
Score
133.89
Precursor Intensity
3843556
Intensity (%)
100
50
0
−50
0
500
1000
1500
m/z
y1−NH3 158.0924
y1 175.1189
y2−NH3 215.1139
y2 232.1403
y3−NH3 330.141
y3 347.167
a2 428.3433
b2 456.3383
y4−H2O 458.1992
y4−NH3 459.1834
y4 476.21
y5−NH3 572.2671
y5 589.294
b3 603.4066
b4 674.4439
y6−H2O 699.3422
y6−NH3 700.3252
y6 717.3523
b5 731.4652
y7 959.4888
y8 1016.5113
L I F A G K Q L E D G R
y8 y7 y6 y5 y4 y3 y2 y1
b2 a2 b3 b4 b5
Modification
Acetyl (Protein N−term)
GlyGly (K) no new terminus
Oxidation (M)

20200921_KIZ_MUb4_GlyGly_10plex_2 − Scan number: 22250

Gene Names	Charge	m/z	Mass	Retention time	PEP	Score	Precursor Intensity
RPS27A;UBA52;UBC;UBB	3	487.6001	1459.778	87.682	1.1157e−08	119.27	1686474

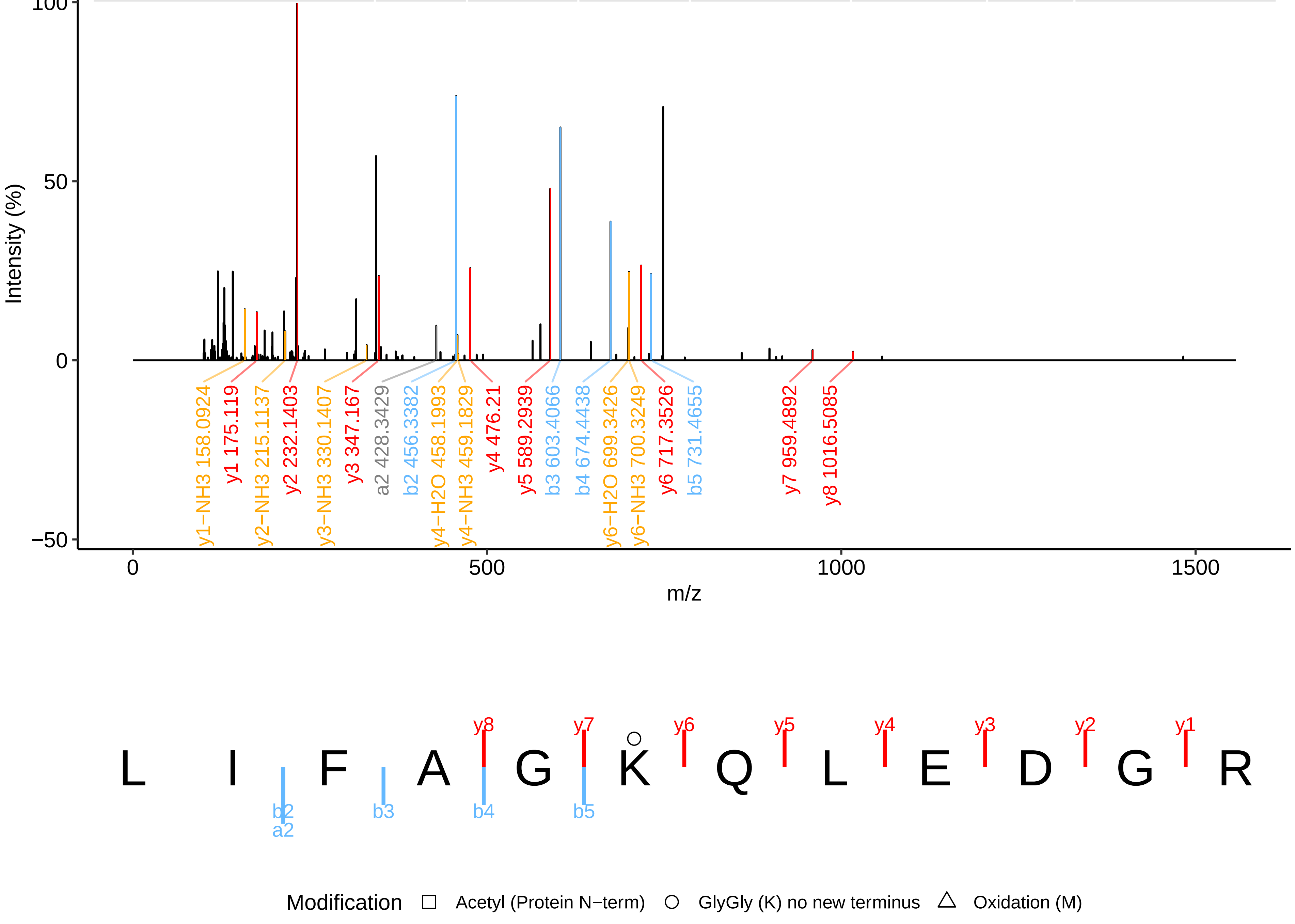

20200921_KIZ_MUb4_GlyGly_10plex_2 − Scan number: 22374

Gene Names	Charge	m/z	Mass	Retention time	PEP	Score	Precursor Intensity
RPS27A;UBA52;UBC;UBB	3	487.6001	1459.778	88.19	5.8499e−09	125.72	2847708

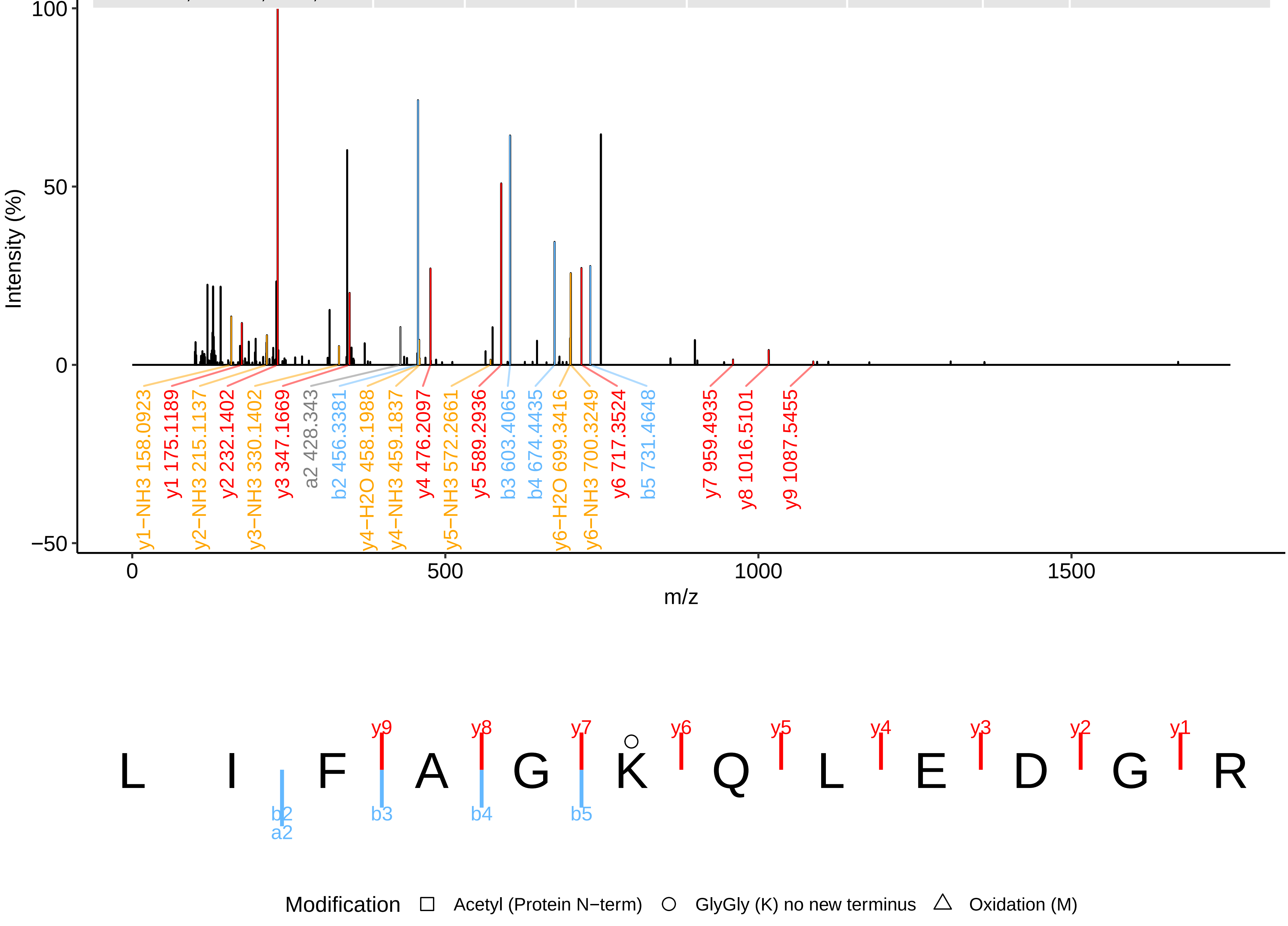

20200921_KIZ_MUb4_GlyGly_10plex_2 − Scan number: 22499

Gene Names	Charge	m/z	Mass	Retention time	PEP	Score	Precursor Intensity
RPS27A;UBA52;UBC;UBB	3	487.6001	1459.778	88.707	1.1157e−08	119.27	2396251

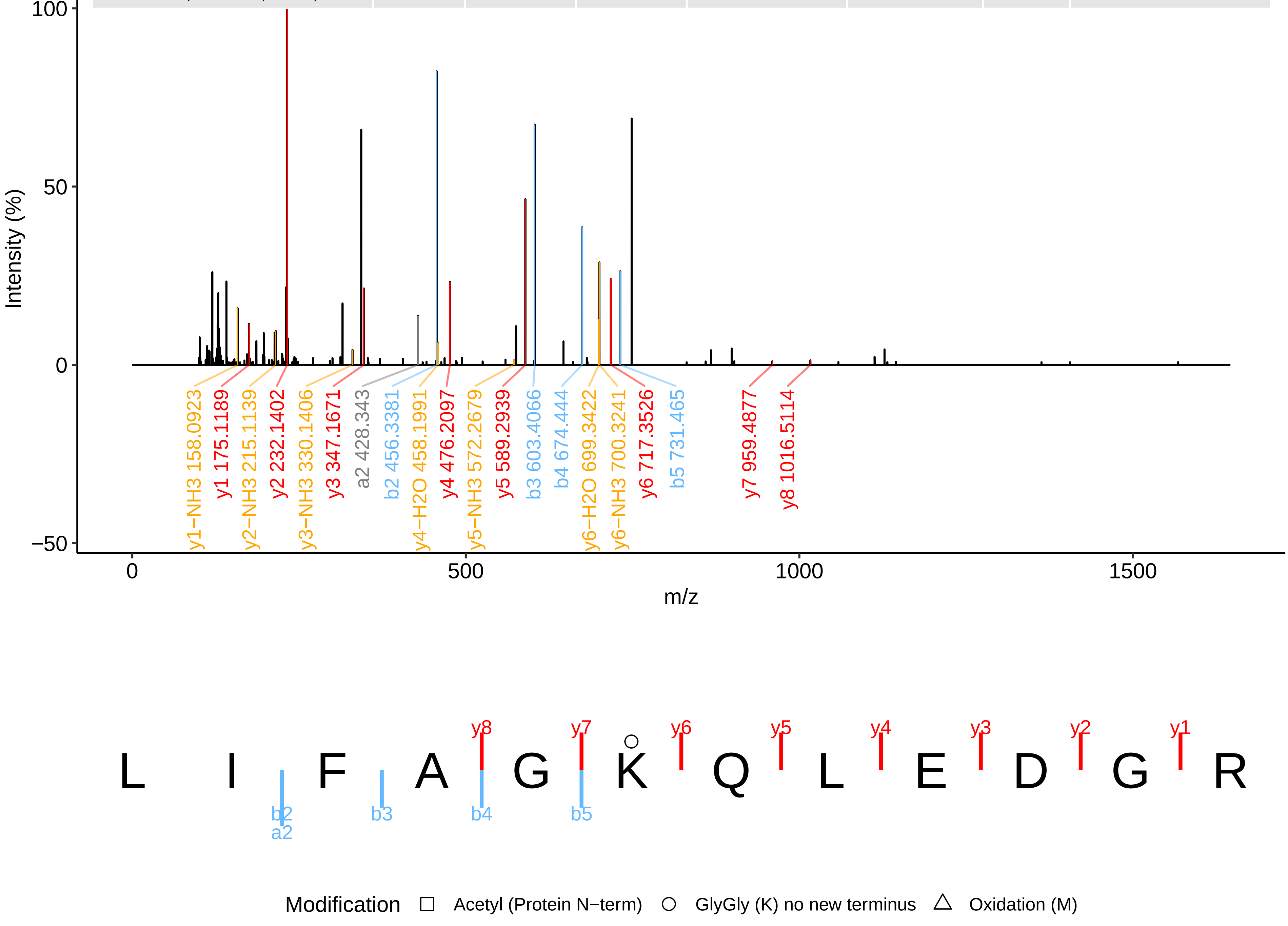

20200921_KIZ_MUb4_GlyGly_10plex_2 − Scan number: 22623

Gene Names	Charge	m/z	Mass	Retention time	PEP	Score	Precursor Intensity
RPS27A;UBA52;UBC;UBB	3	487.6001	1459.778	89.215	3.6333e−13	133.89	2360029

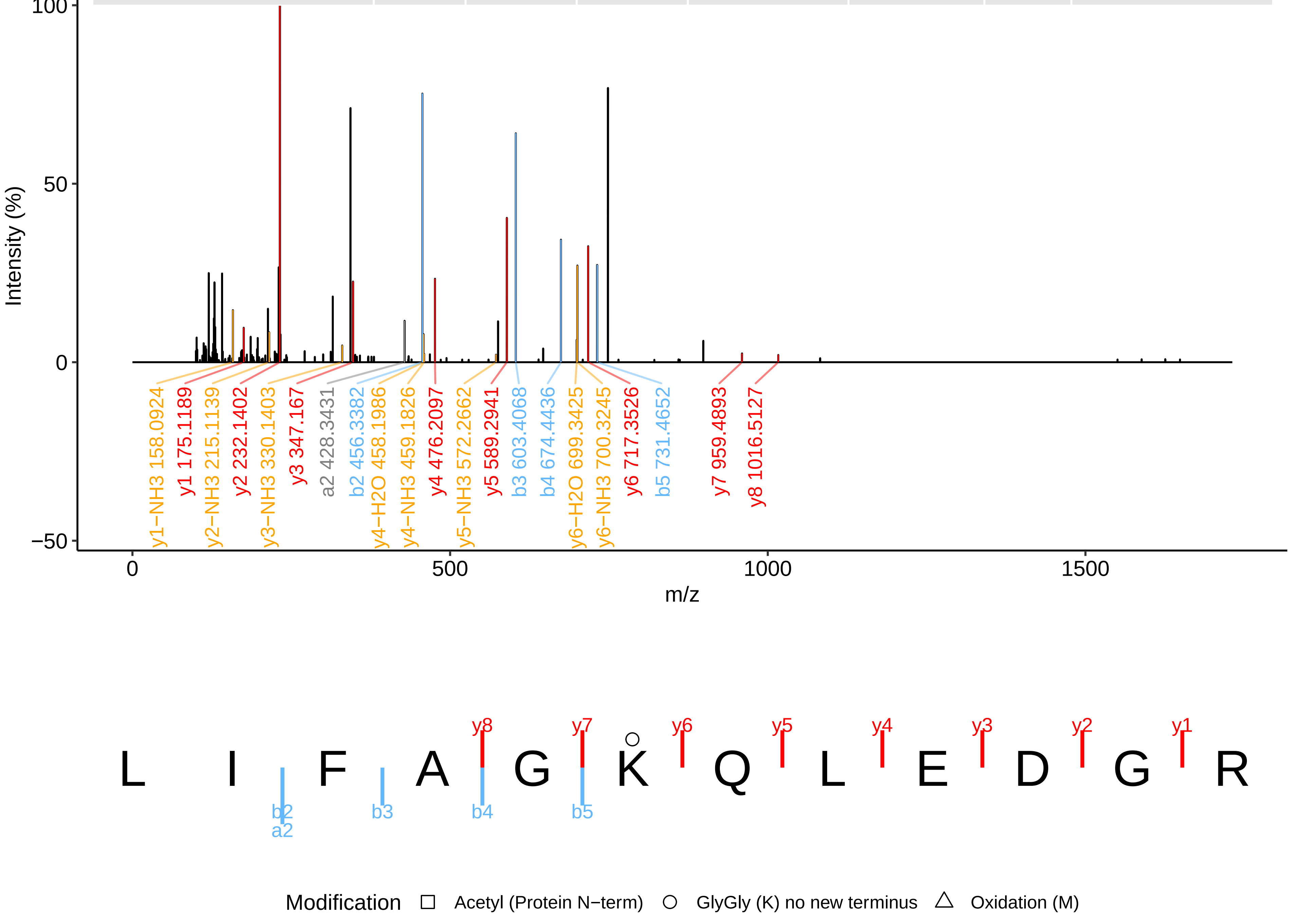

20200921_KIZ_MUb4_GlyGly_10plex_2 − Scan number: 22750

Gene Names	Charge	m/z	Mass	Retention time	PEP	Score	Precursor Intensity
RPS27A;UBA52;UBC;UBB	3	487.6001	1459.778	89.732	1.0628e−13	145.46	2916913

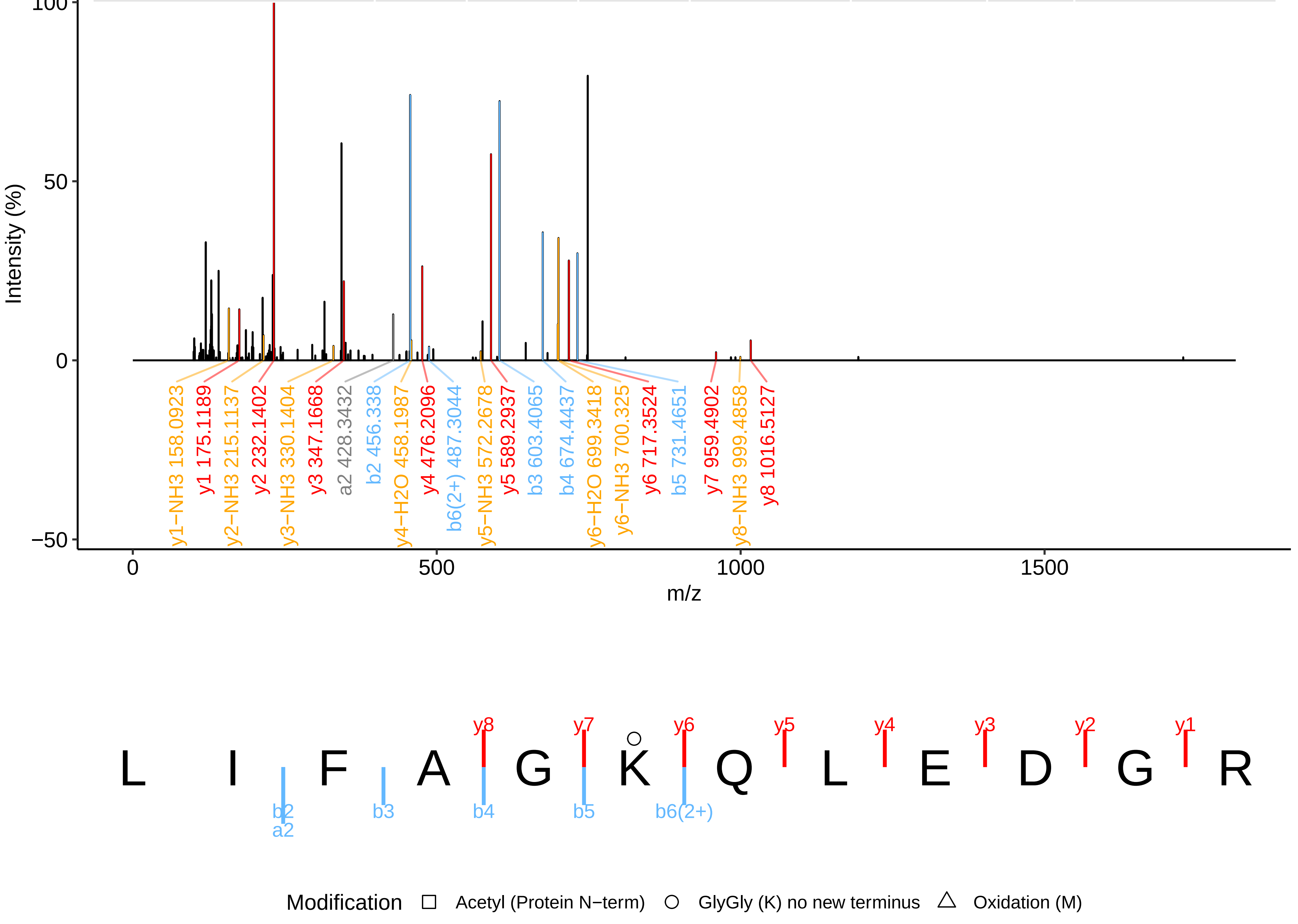

20200921_KIZ_MUb4_GlyGly_10plex_2 − Scan number: 22876

Gene Names	Charge	m/z	Mass	Retention time	PEP	Score	Precursor Intensity
RPS27A;UBA52;UBC;UBB	3	487.6001	1459.778	90.236	8.2269e−10	131.83	1632972

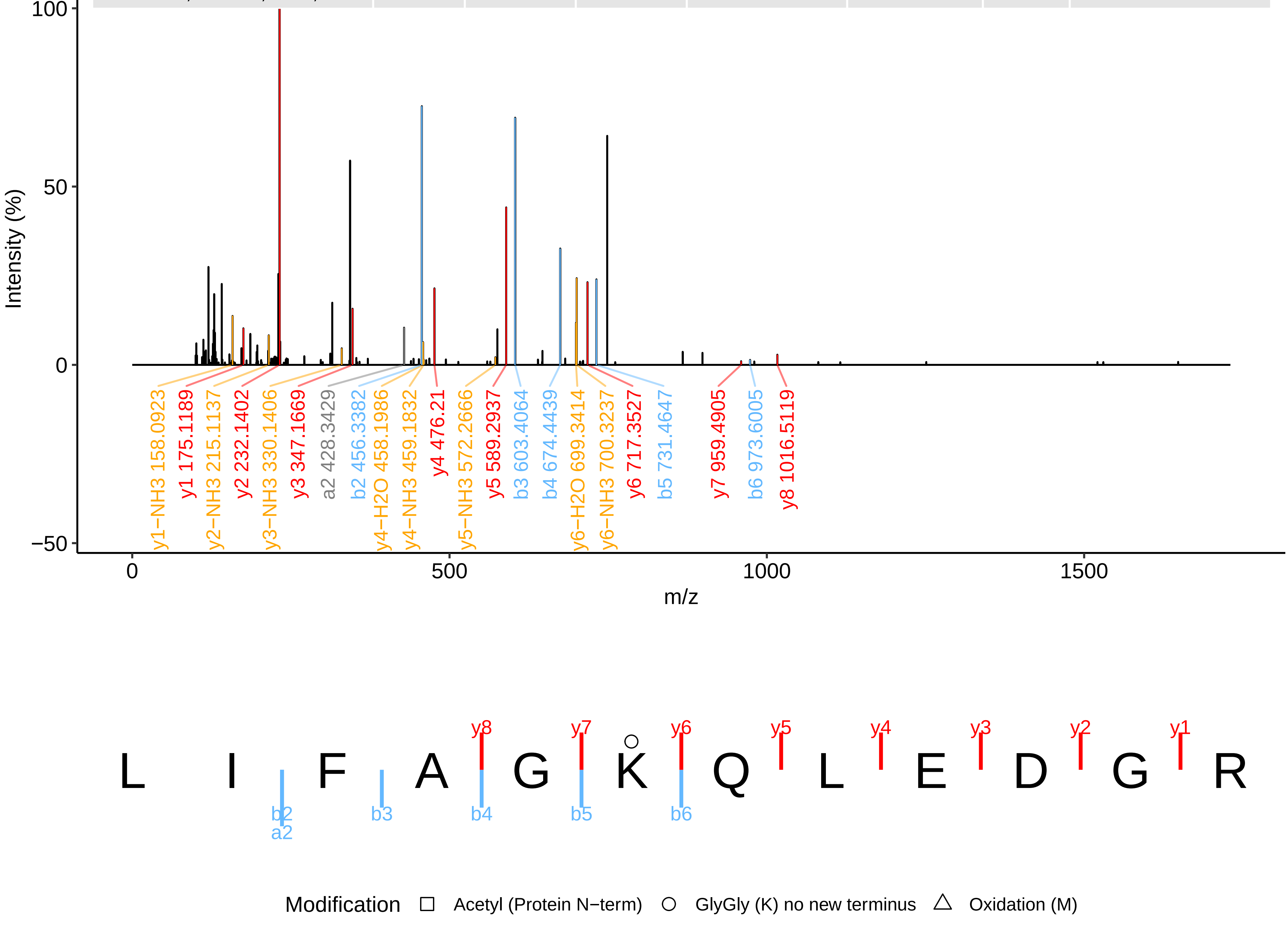

20200921_KIZ_MUb4_GlyGly_10plex_2 − Scan number: 23006

Gene Names	Charge	m/z	Mass	Retention time	PEP	Score	Precursor Intensity
RPS27A;UBA52;UBC;UBB	3	487.6001	1459.778	90.744	4.1121e−09	127.83	3309466

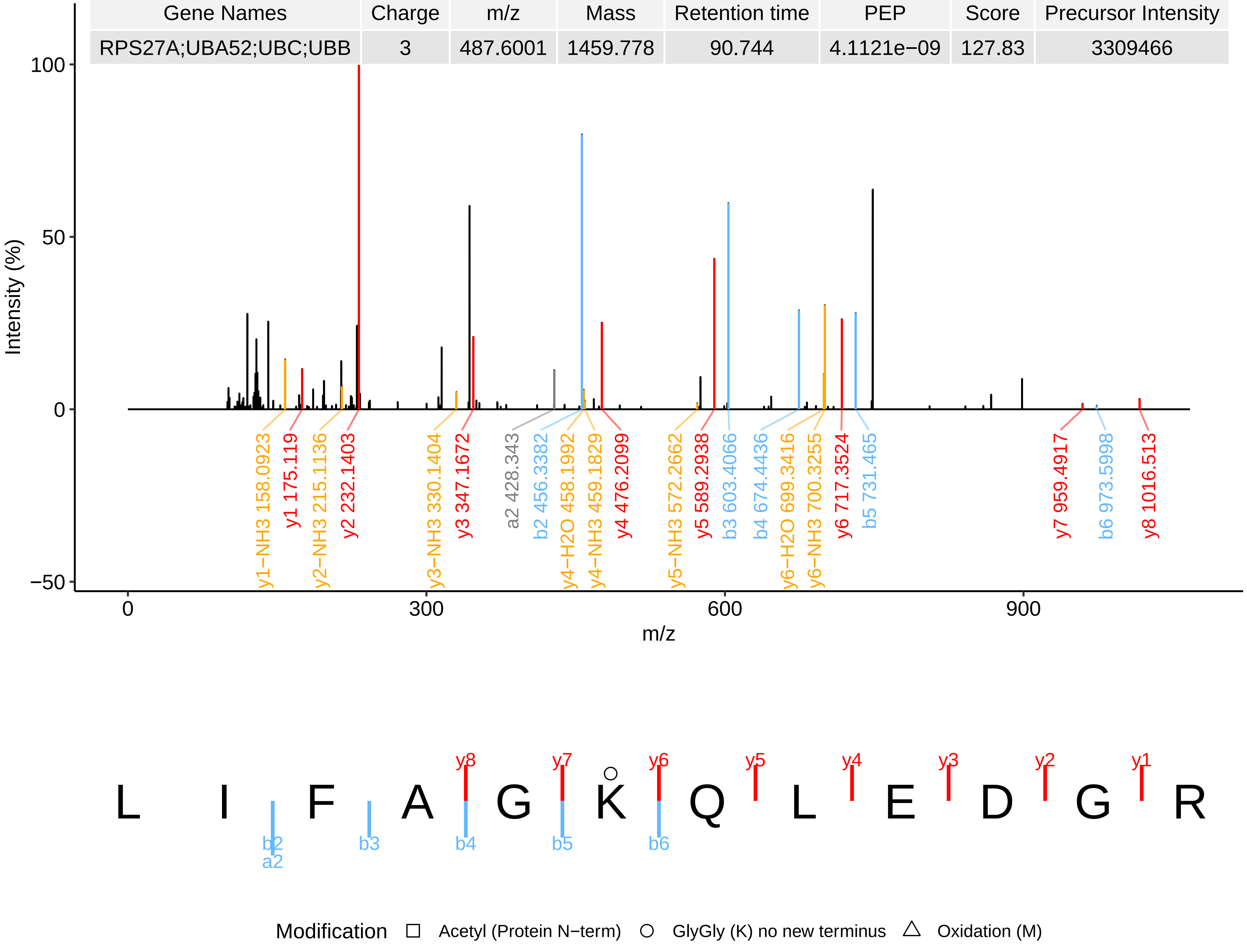

20200921_KIZ_MUb4_GlyGly_10plex_2 − Scan number: 23130

Gene Names	Charge	m/z	Mass	Retention time	PEP	Score	Precursor Intensity
RPS27A;UBA52;UBC;UBB	3	487.6001	1459.778	91.255	3.2481e−09	128.88	5929820

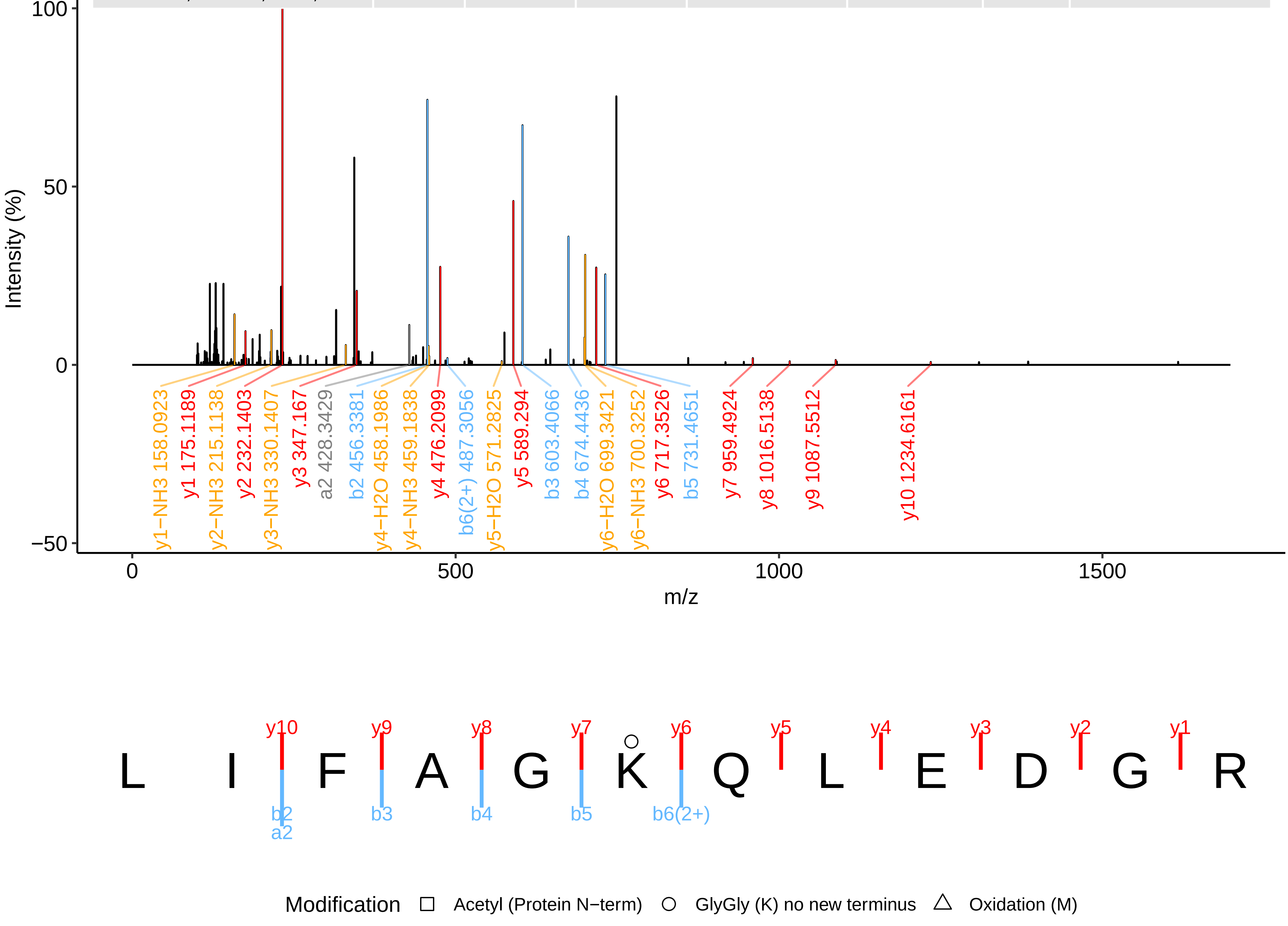

20200921_KIZ_MUb4_GlyGly_10plex_2 − Scan number: 23512

Gene Names	Charge	m/z	Mass	Retention time	PEP	Score	Precursor Intensity
RPS27A;UBA52;UBC;UBB	3	487.6001	1459.778	92.803	4.2533e−06	111.86	3534993

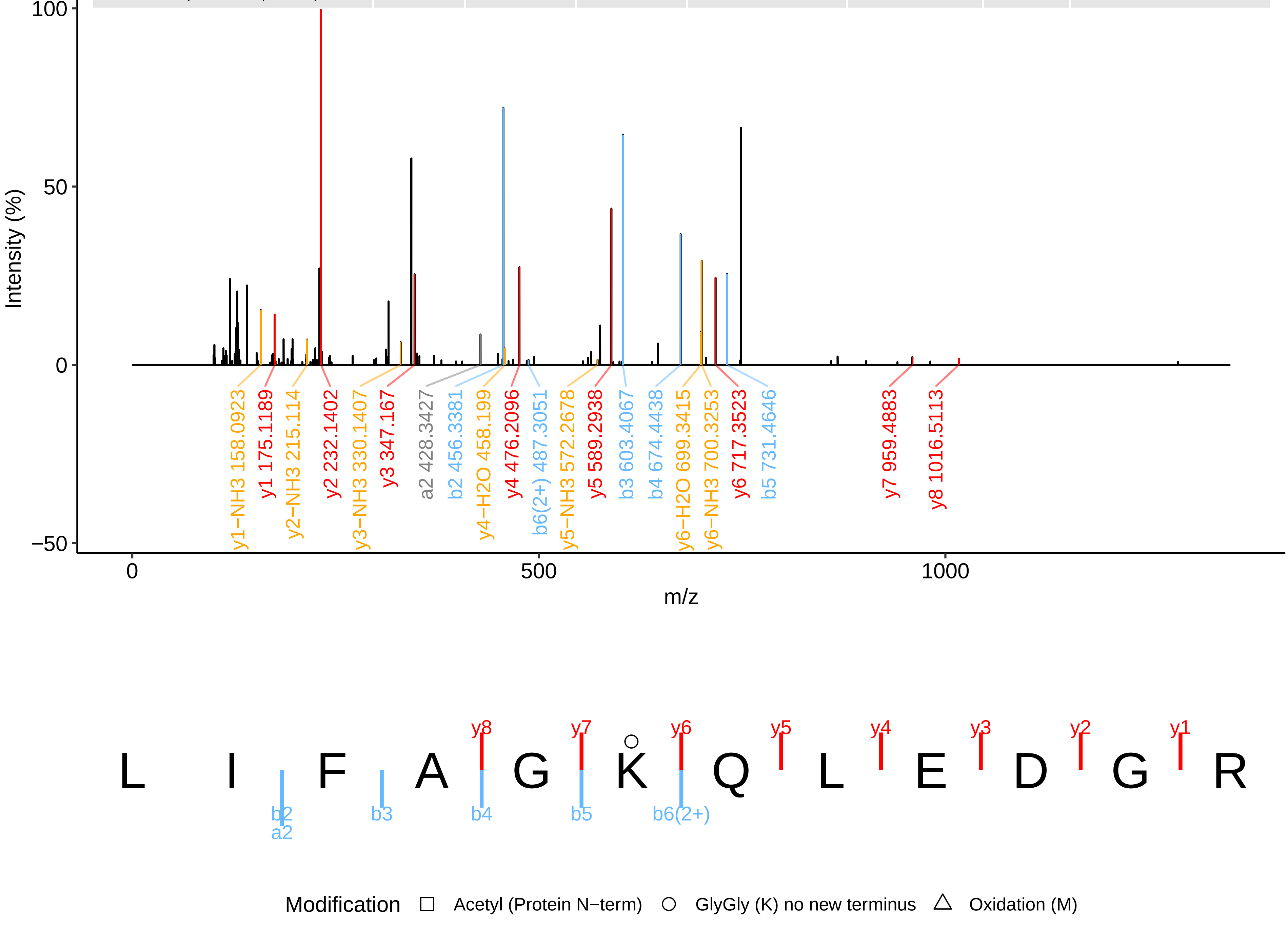

20200921_KIZ_MUb4_GlyGly_10plex_2 − Scan number: 24018

Gene Names	Charge	m/z	Mass	Retention time	PEP	Score	Precursor Intensity
RPS27A;UBA52;UBC;UBB	3	487.6001	1459.778	94.856	8.3443e−09	122.69	2783051

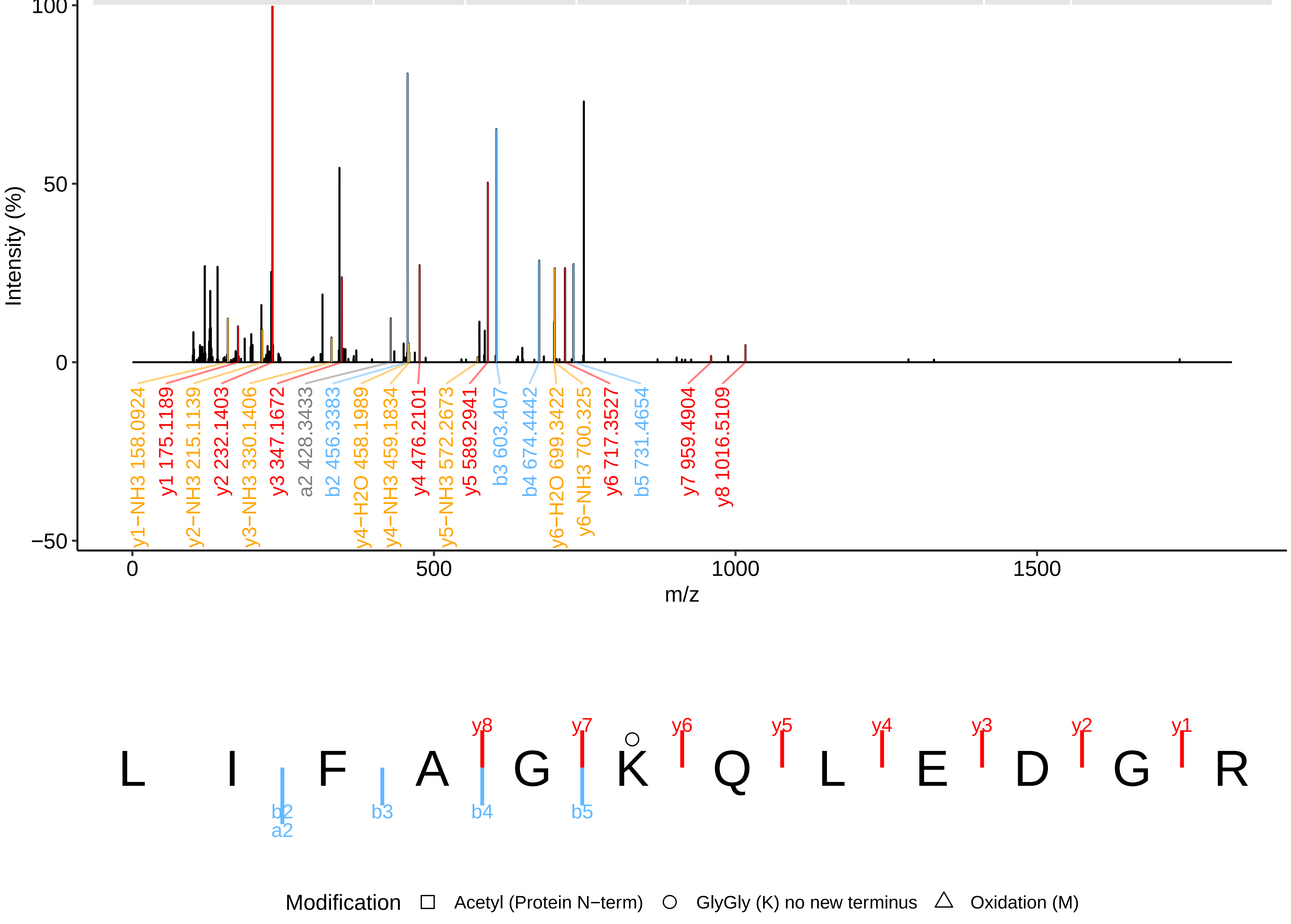

20200921_KIZ_MUb4_GlyGly_10plex_2 − Scan number: 24142

Gene Names	Charge	m/z	Mass	Retention time	PEP	Score	Precursor Intensity
RPS27A;UBA52;UBC;UBB	3	487.6001	1459.778	95.367	9.4395e−09	121.36	2189693

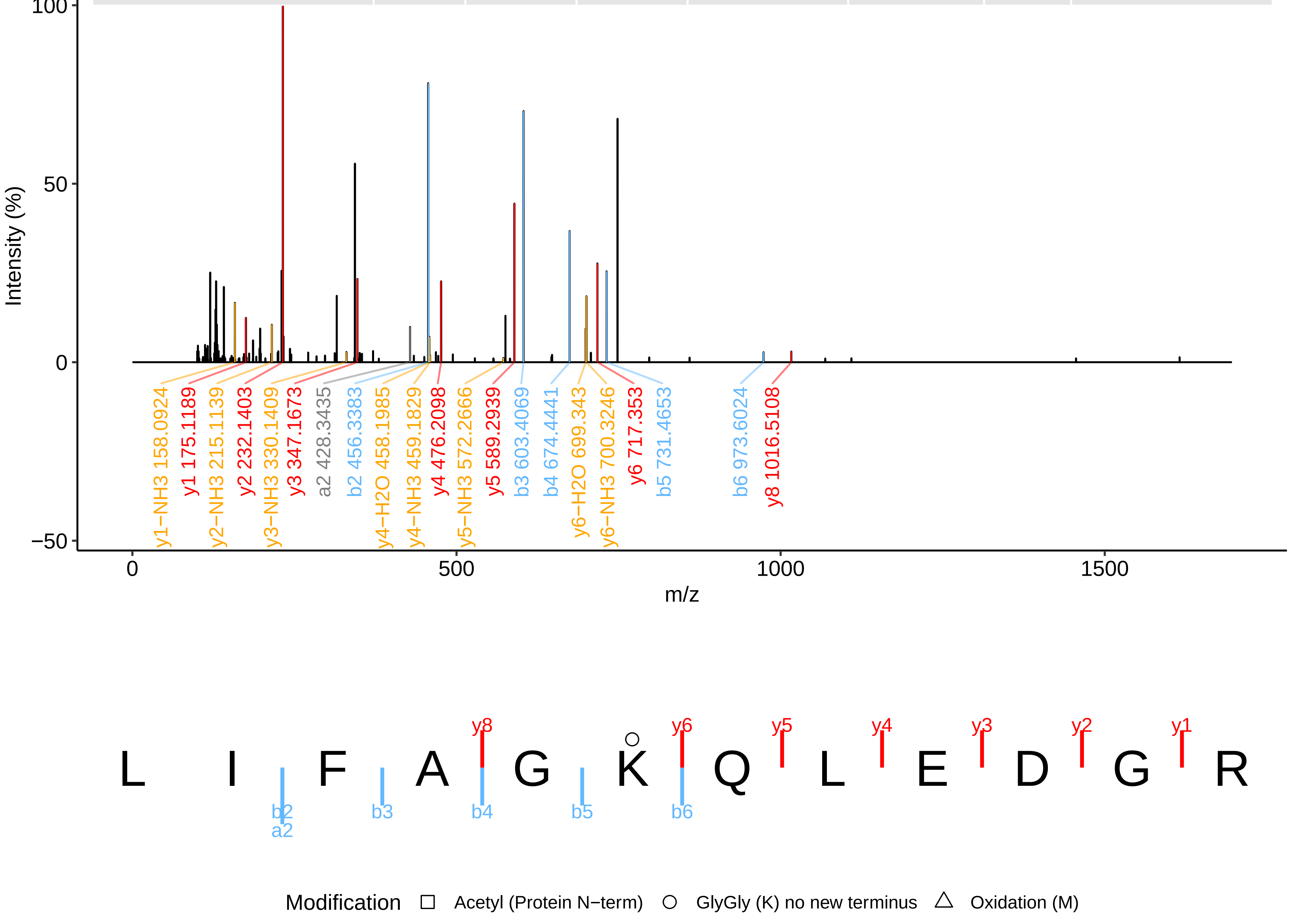

20200921_KIZ_MUb4_GlyGly_10plex_2 − Scan number: 24271

Gene Names	Charge	m/z	Mass	Retention time	PEP	Score	Precursor Intensity
RPS27A;UBA52;UBC;UBB	3	487.6001	1459.778	95.885	8.3443e−09	122.69	2700753

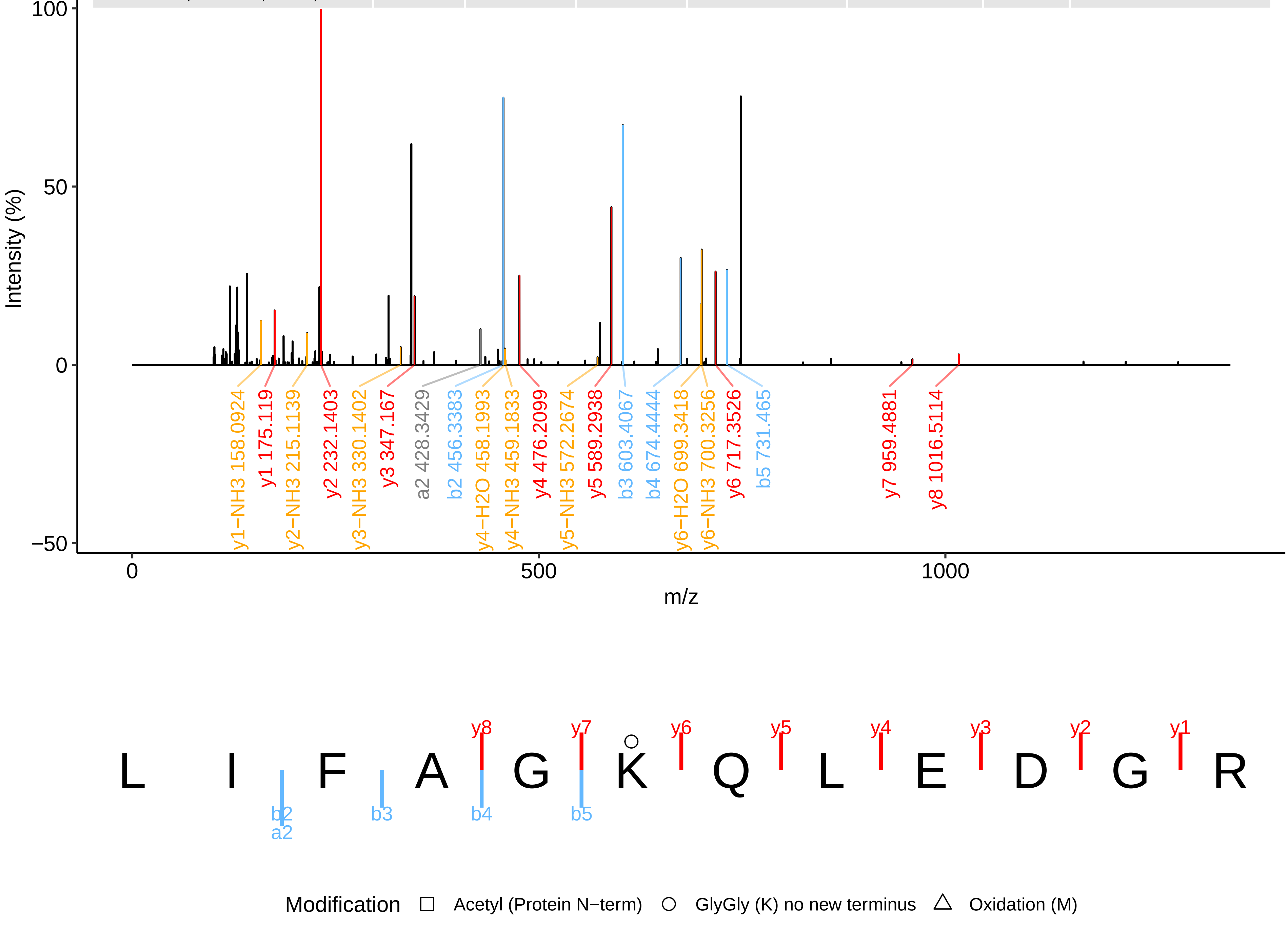

20200921_KIZ_MUb4_GlyGly_10plex_2 − Scan number: 24394

Gene Names	Charge	m/z	Mass	Retention time	PEP	Score	Precursor Intensity
RPS27A;UBA52;UBC;UBB	3	487.6001	1459.778	96.392	8.3443e−09	122.69	2757415

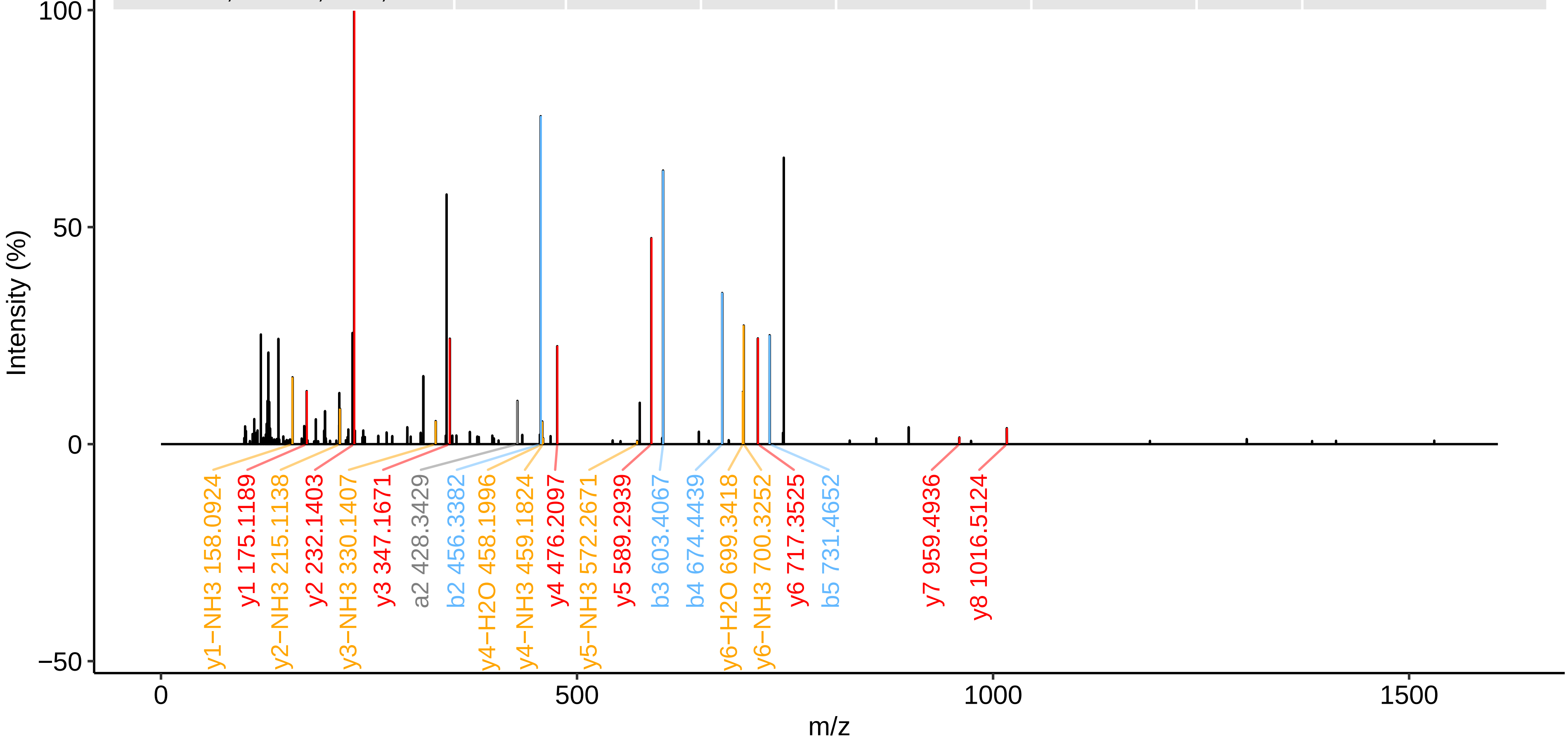

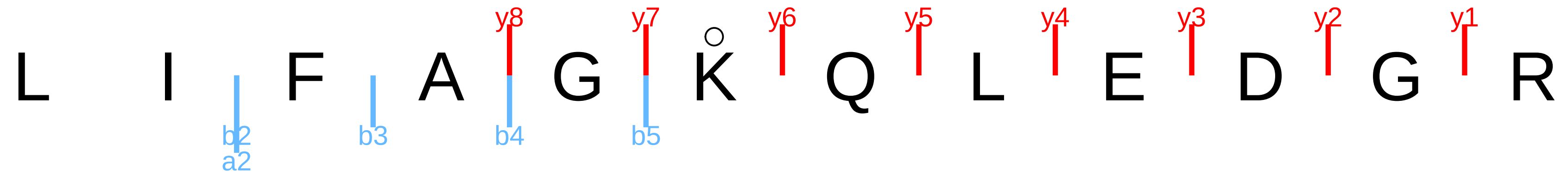

Modification □ Acetyl (Protein N−term) ○ GlyGly (K) no new terminus △ Oxidation (M)

20200921_KIZ_MUb4_GlyGly_10plex_2 − Scan number: 24519

Gene Names	Charge	m/z	Mass	Retention time	PEP	Score	Precursor Intensity
RPS27A;UBA52;UBC;UBB	3	487.6001	1459.778	96.897	8.0835e−09	123.01	2368025

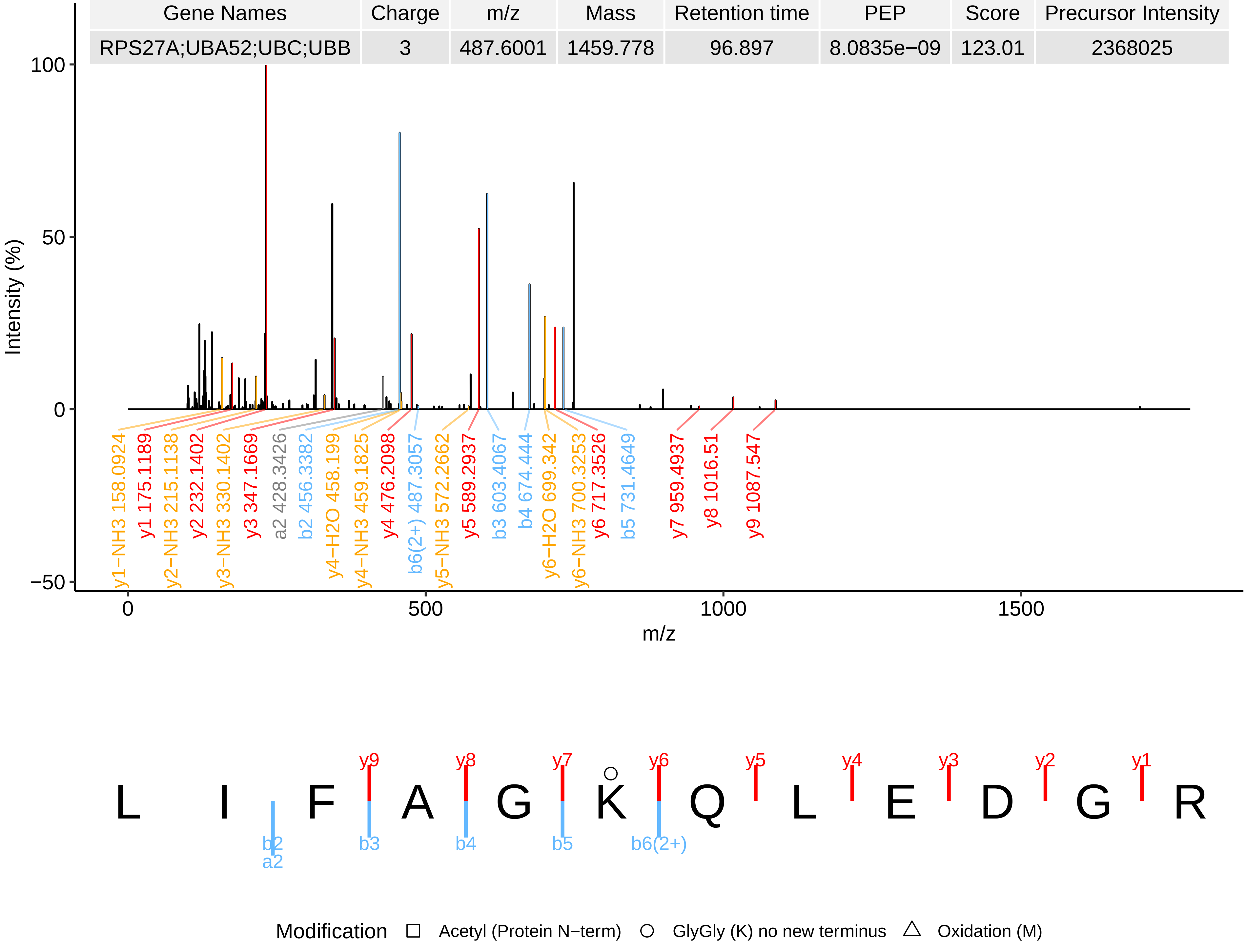

20200921_KIZ_MUb4_GlyGly_10plex_2 − Scan number: 24780

Gene Names	Charge	m/z	Mass	Retention time	PEP	Score	Precursor Intensity
RPS27A;UBA52;UBC;UBB	3	487.6001	1459.778	97.932	4.2533e−06	111.86	2044795

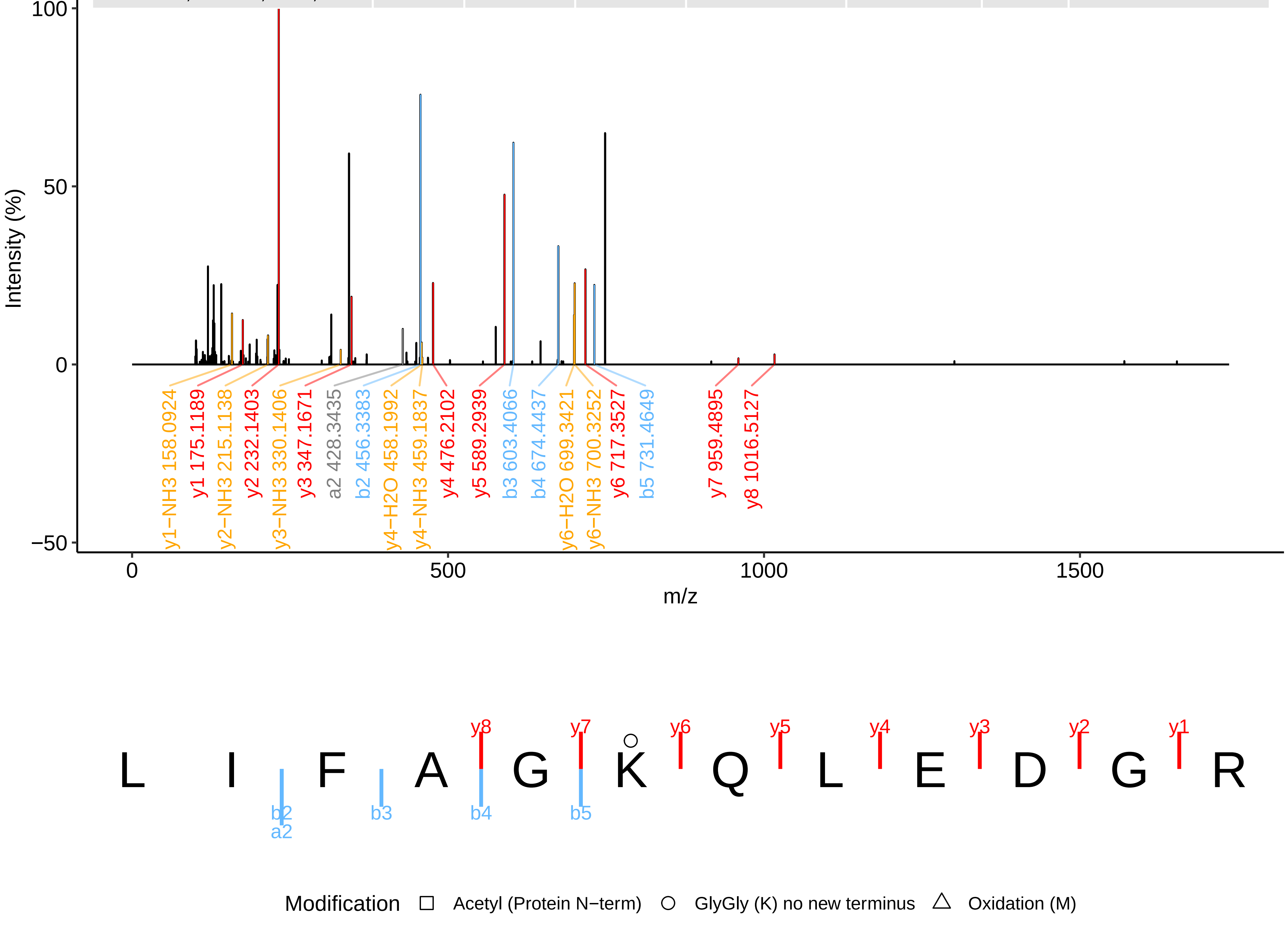

20200921_KIZ_MUb4_GlyGly_10plex_2 − Scan number: 24903

Gene Names	Charge	m/z	Mass	Retention time	PEP	Score	Precursor Intensity
RPS27A;UBA52;UBC;UBB	3	487.6001	1459.778	98.436	3.6333e−13	133.89	2216930

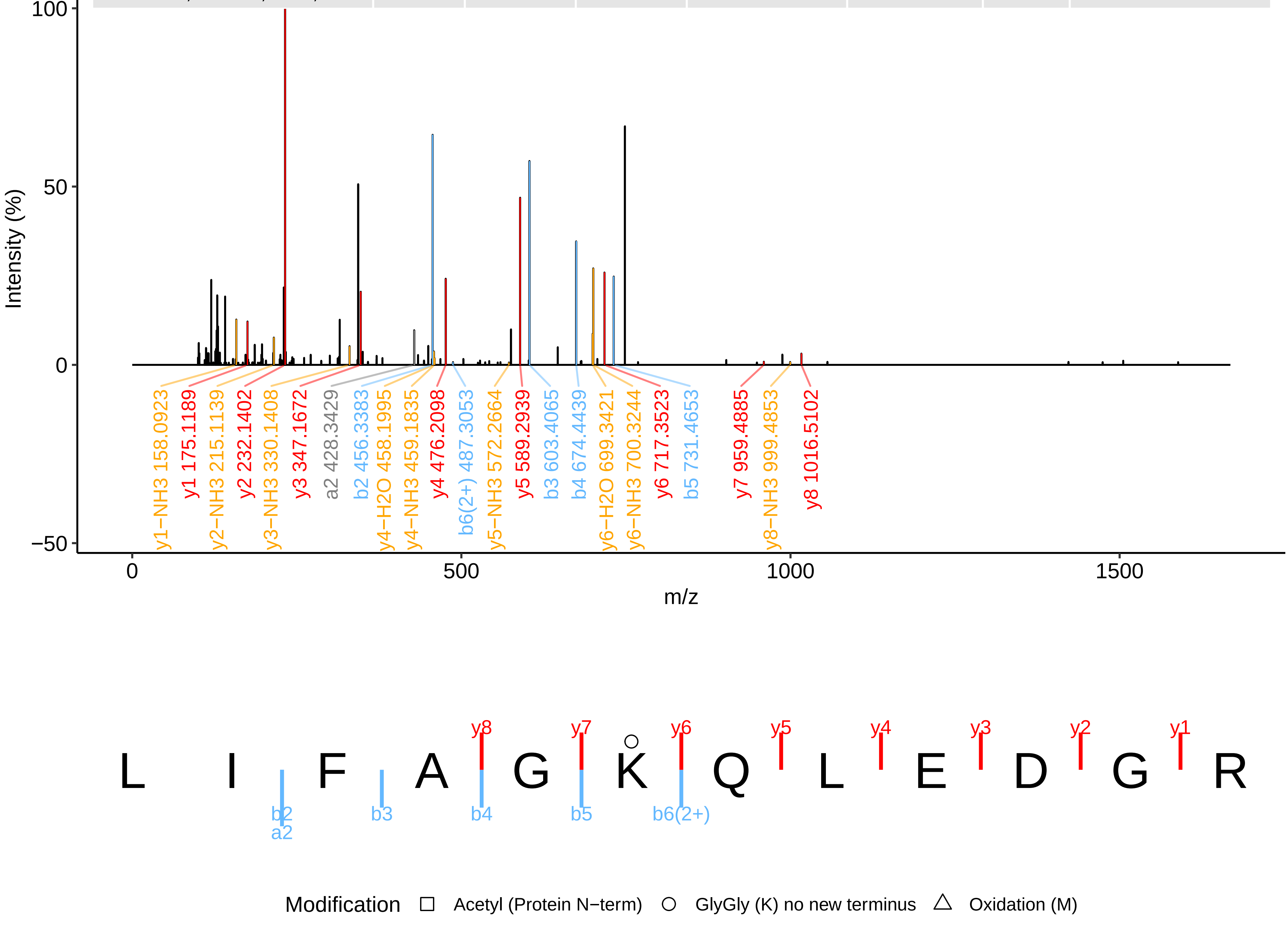

20200921_KIZ_MUb4_GlyGly_10plex_2 − Scan number: 25026

Gene Names	Charge	m/z	Mass	Retention time	PEP	Score	Precursor Intensity
RPS27A;UBA52;UBC;UBB	3	487.6001	1459.778	98.944	4.2533e−06	111.86	2061230

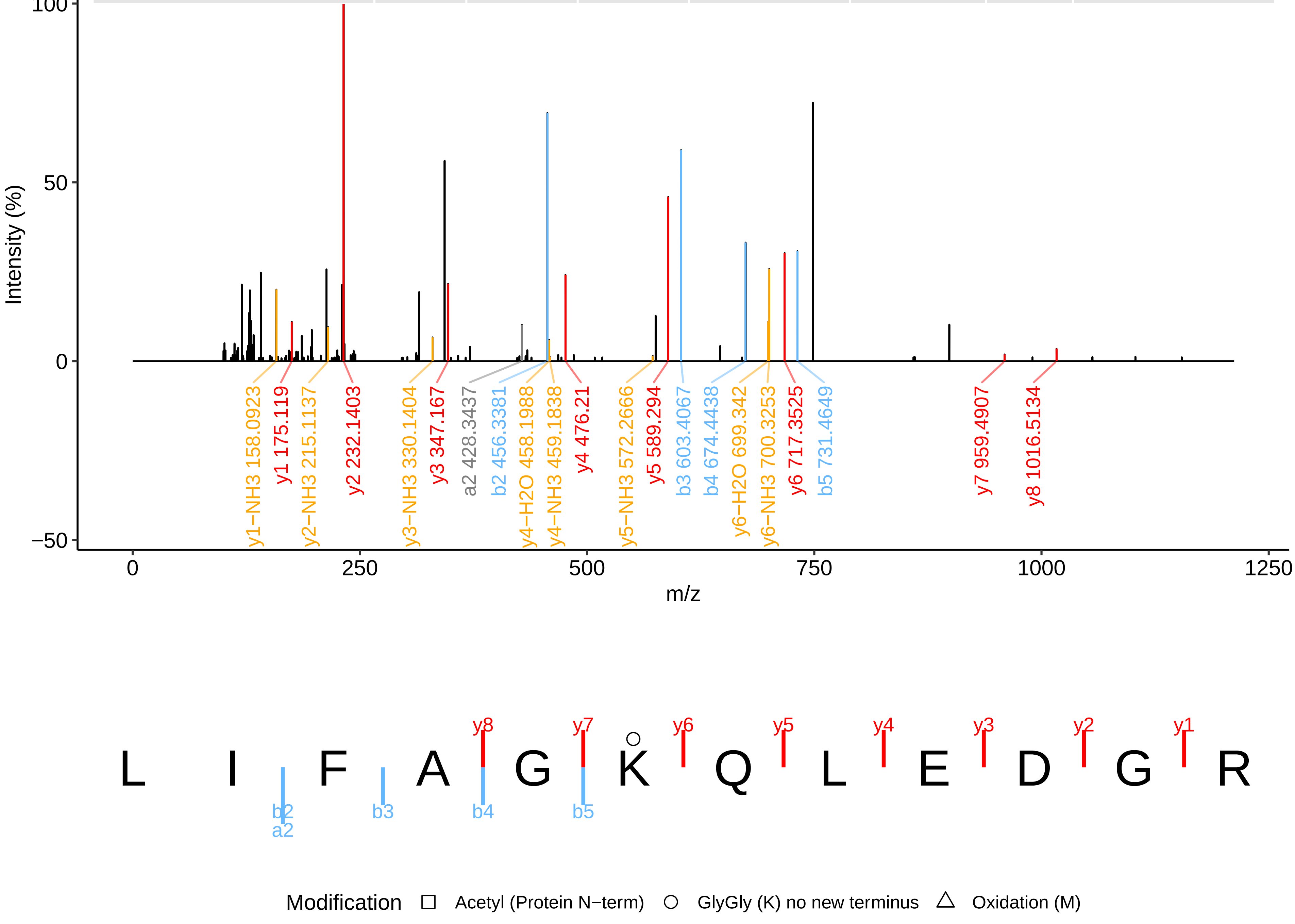

20200921_KIZ_MUb4_GlyGly_10plex_2 − Scan number: 25149

Gene Names	Charge	m/z	Mass	Retention time	PEP	Score	Precursor Intensity
RPS27A;UBA52;UBC;UBB	3	487.6001	1459.778	99.45	0.00062125	89.266	2523349

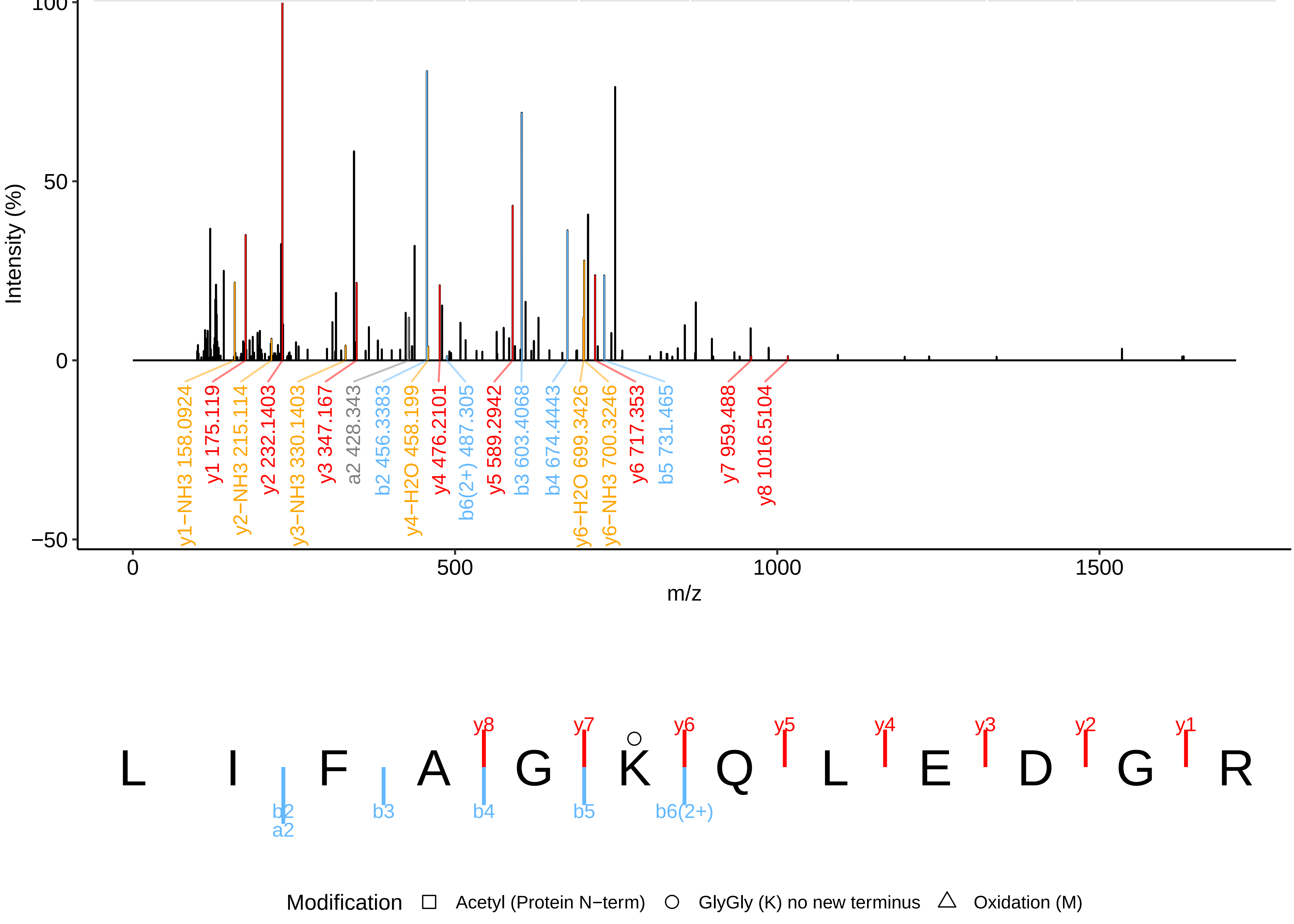

20200921_KIZ_MUb4_GlyGly_10plex_2 − Scan number: 25533

Gene Names	Charge	m/z	Mass	Retention time	PEP	Score	Precursor Intensity
RPS27A;UBA52;UBC;UBB	3	487.6001	1459.778	101.01	4.2533e−06	111.86	2538132

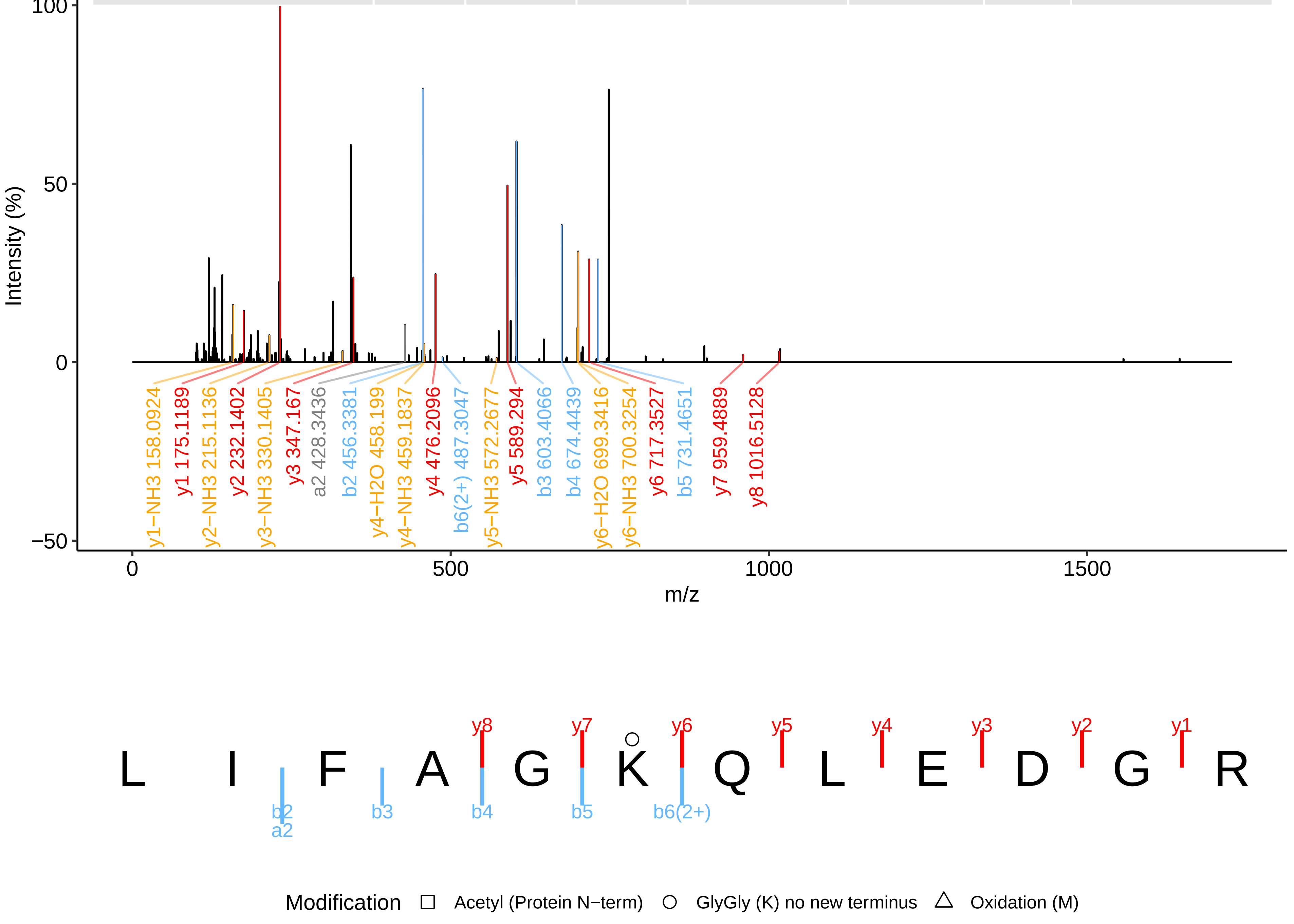

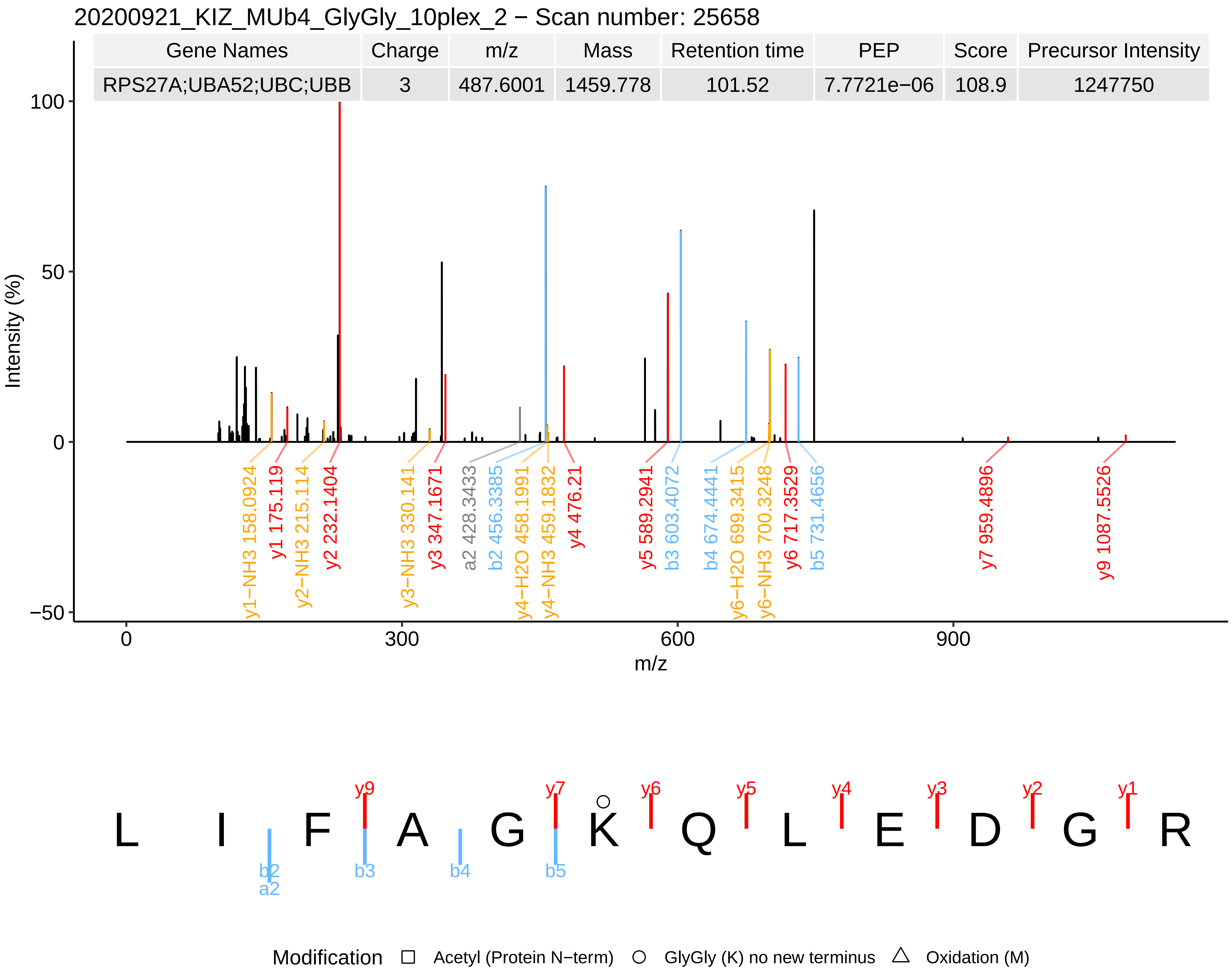
20200921_KIZ_MUb4_GlyGly_10plex_2 − Scan number: 25658
Gene Names | Charge | m/z | Mass | Retention time | PEP | Score | Precursor Intensity
RPS27A;UBA52;UBC;UBB | 3 | 487.6001 | 1459.778 | 101.52 | 7.7721e−06 | 108.9 | 1247750
Intensity (%)
100
50
0
−50
0
300
600
900
m/z
y1−NH3 158.0924
y1 175.119
y2−NH3 215.114
y2 232.1404
y3−NH3 330.141
y3 347.1671
a2 428.3433
b2 456.3385
y4−H2O 458.1991
y4−NH3 459.1832
y4 476.21
y5 589.2941
b3 603.4072
b4 674.4441
y6−H2O 699.3415
y6−NH3 700.3248
y6 717.3529
b5 731.4656
y7 959.4896
y9 1087.5526
L I F A G K Q L E D G R
y9 y7 y6 y5 y4 y3 y2 y1
b2 a2 b3 b4 b5
Modification
Acetyl (Protein N−term)
GlyGly (K) no new terminus
Oxidation (M)

20200921_KIZ_MUb4_GlyGly_10plex_2 − Scan number: 25780

Gene Names	Charge	m/z	Mass	Retention time	PEP	Score	Precursor Intensity
RPS27A;UBA52;UBC;UBB	3	487.6001	1459.778	102.02	4.2533e−06	111.86	2366110

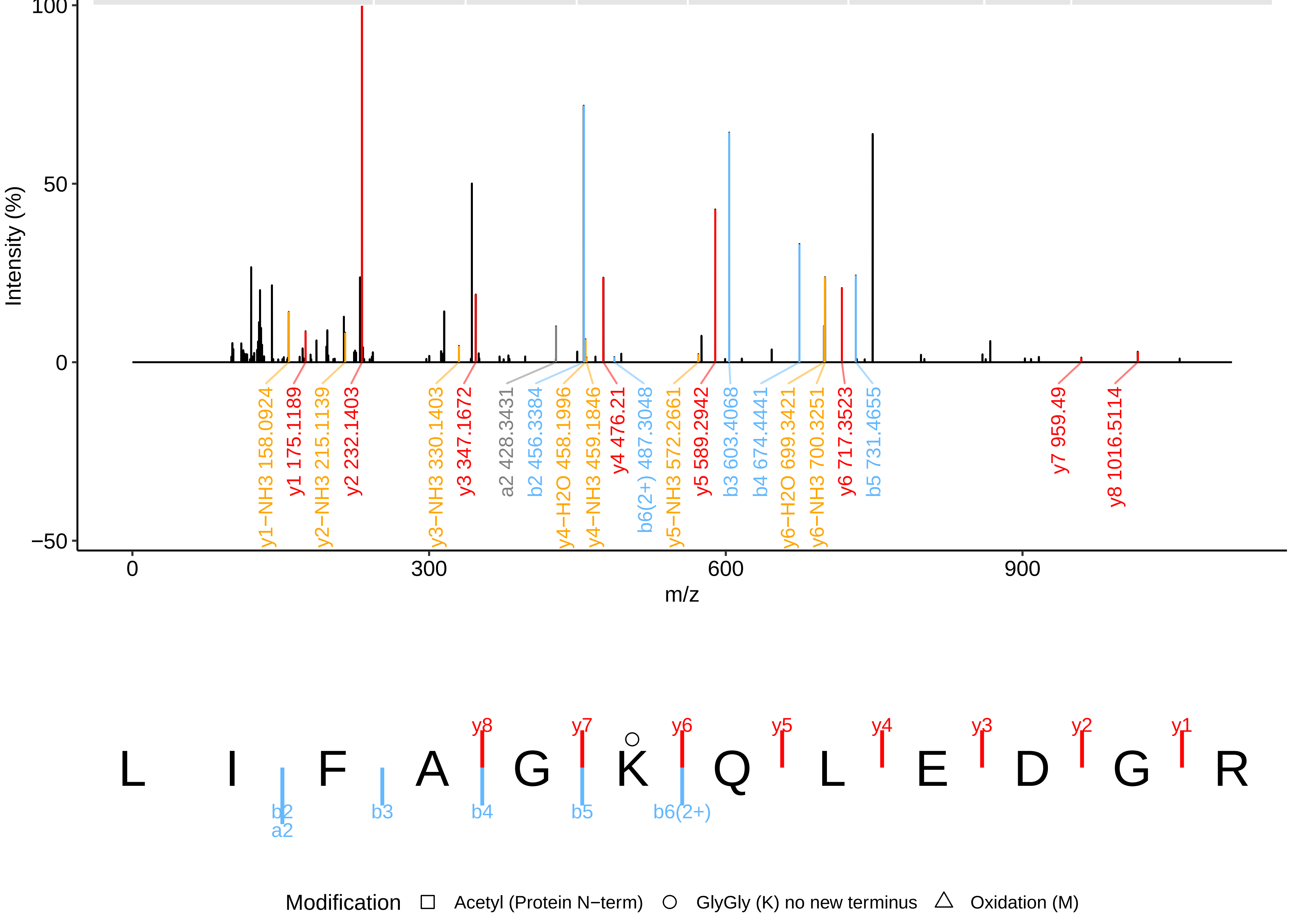

20200921_KIZ_MUb4_GlyGly_10plex_2 − Scan number: 26030

Gene Names	Charge	m/z	Mass	Retention time	PEP	Score	Precursor Intensity
RPS27A;UBA52;UBC;UBB	3	487.6001	1459.778	103.05	8.3443e−09	122.69	2160037

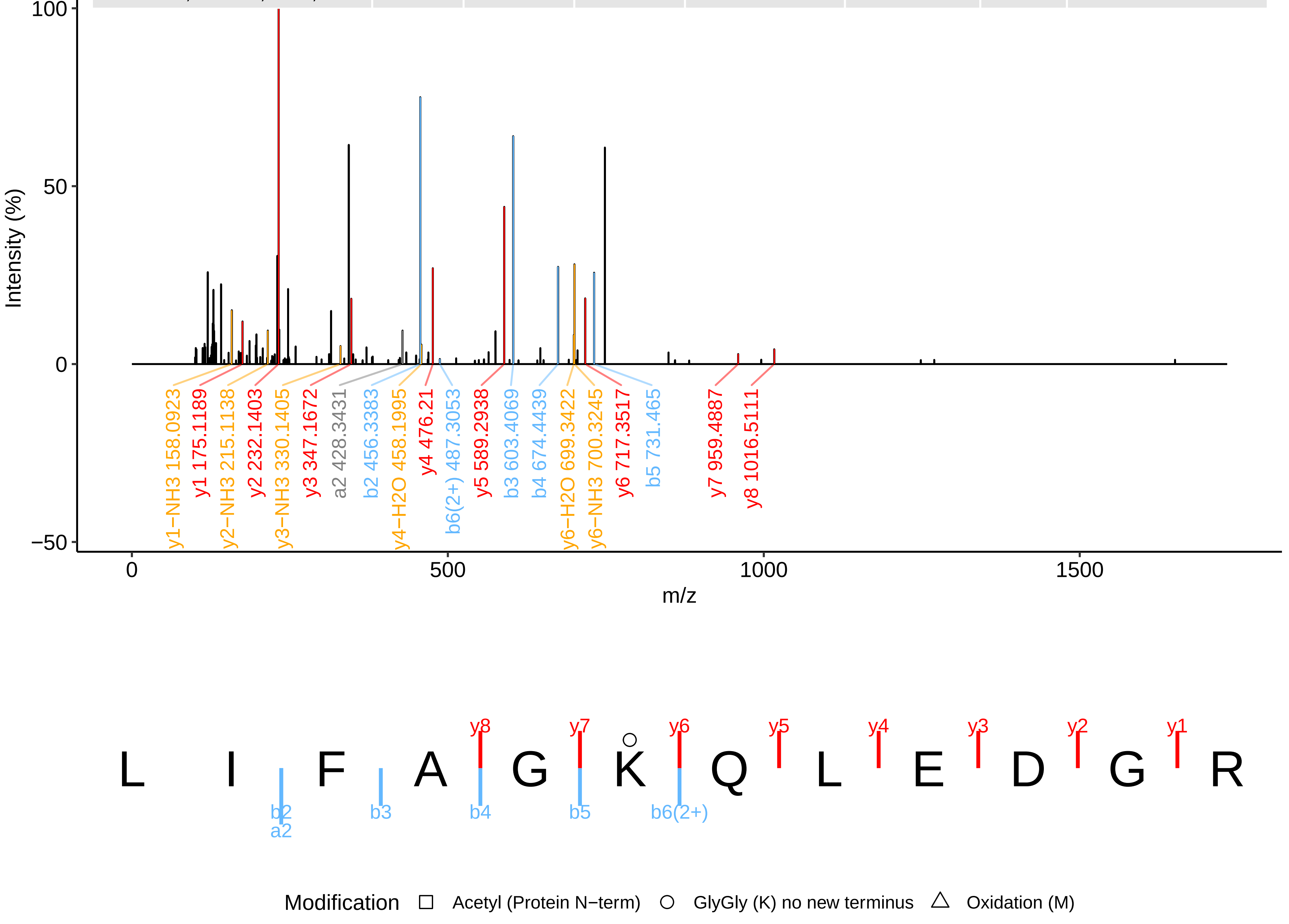

20200921_KIZ_MUb4_GlyGly_10plex_2 − Scan number: 26159

Gene Names	Charge	m/z	Mass	Retention time	PEP	Score	Precursor Intensity
RPS27A;UBA52;UBC;UBB	3	487.6001	1459.778	103.58	1.6723e−05	101.39	2471379

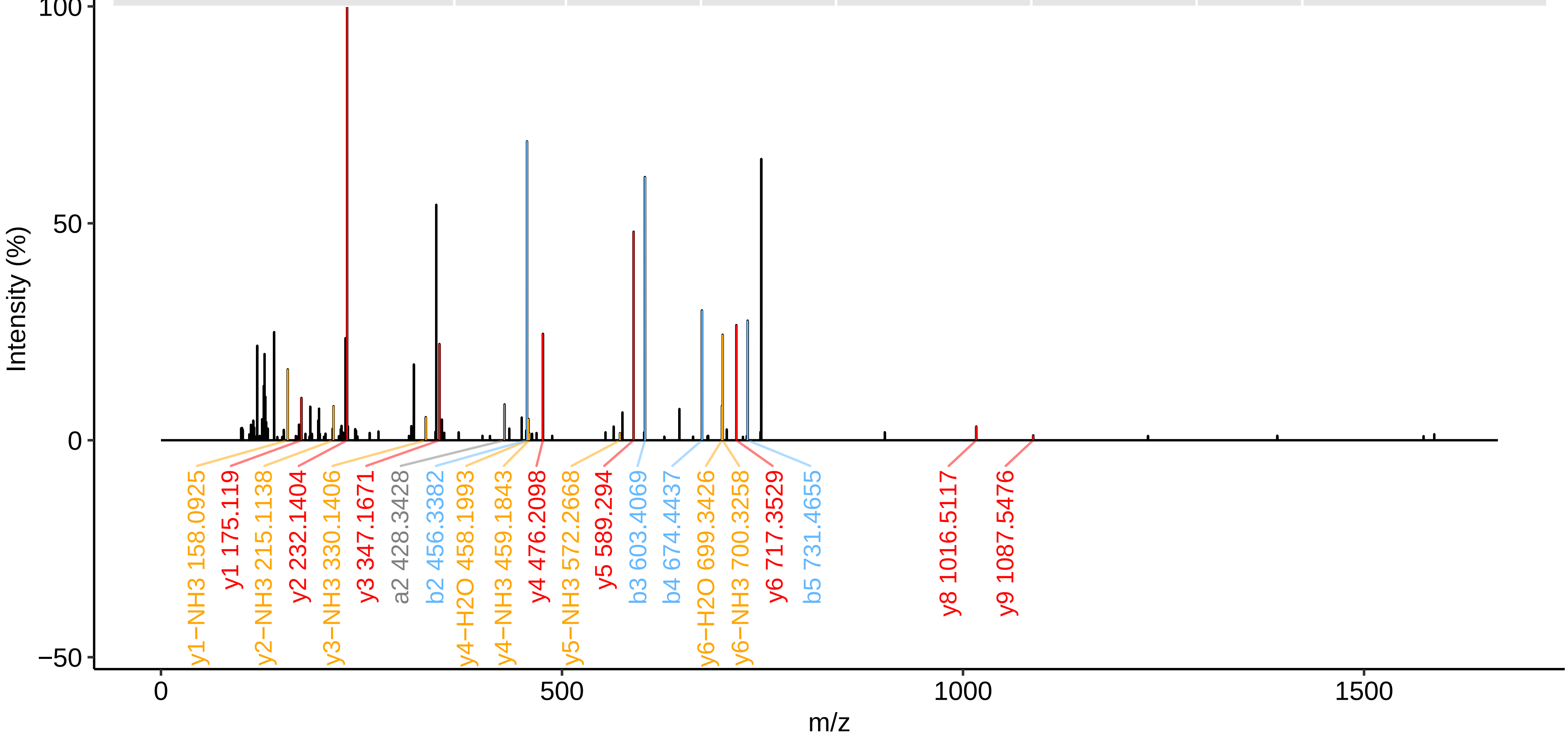

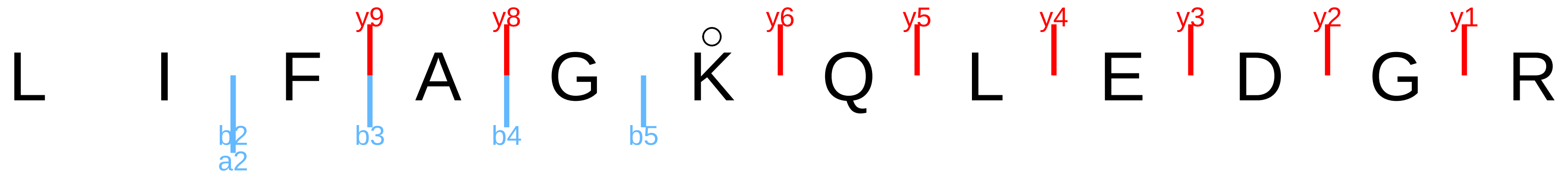

Modification □ Acetyl (Protein N−term) ○ GlyGly (K) no new terminus △ Oxidation (M)

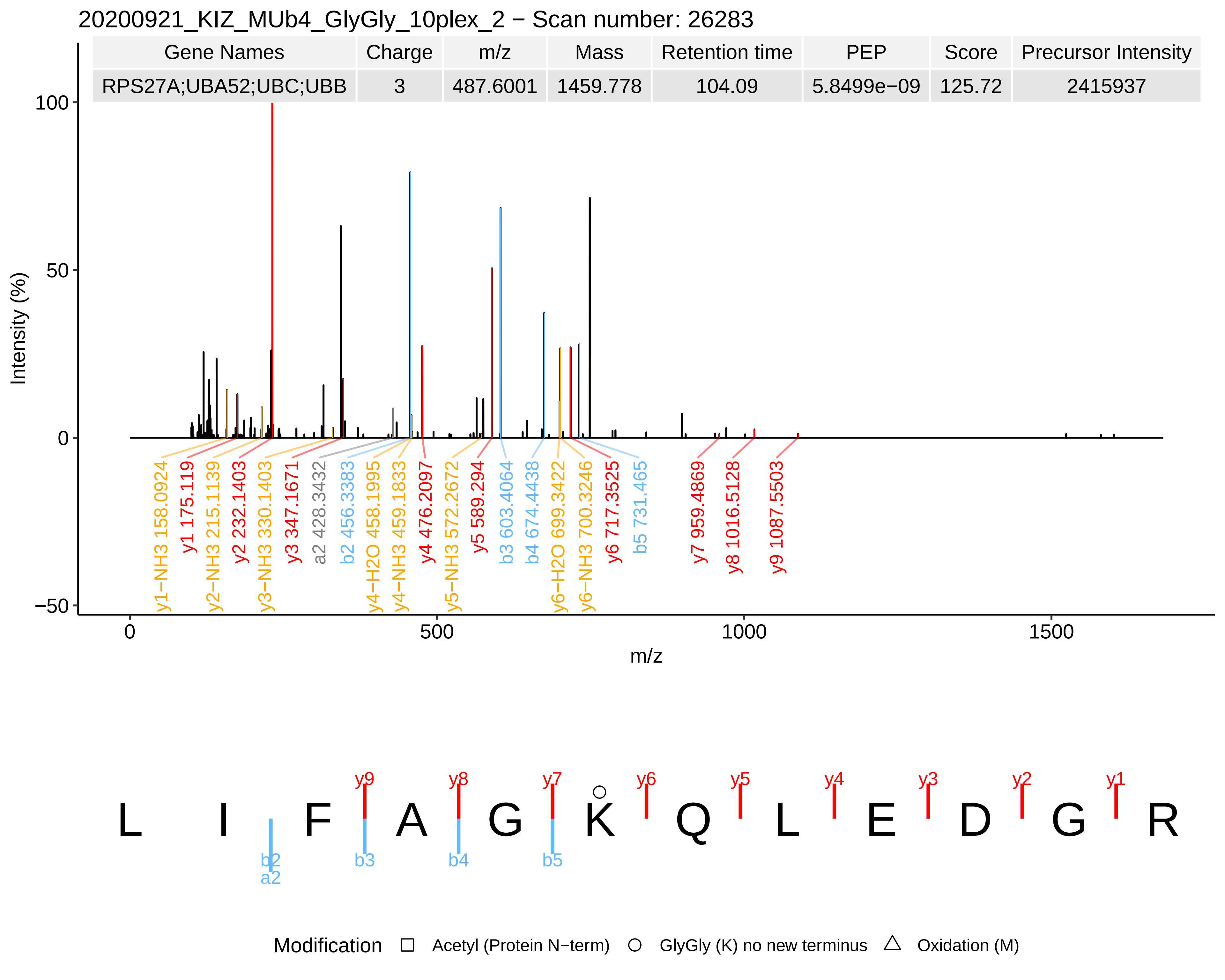
20200921_KIZ_MUb4_GlyGly_10plex_2 − Scan number: 26283
Gene Names | Charge | m/z | Mass | Retention time | PEP | Score | Precursor Intensity
RPS27A;UBA52;UBC;UBB | 3 | 487.6001 | 1459.778 | 104.09 | 5.8499e−09 | 125.72 | 2415937
Intensity (%)
m/z
y1−NH3 158.0924
y1 175.119
y2−NH3 215.1139
y2 232.1403
y3−NH3 330.1403
y3 347.1671
a2 428.3432
b2 456.3383
y4−H2O 458.1995
y4−NH3 459.1833
y4 476.2097
y5−NH3 572.2672
y5 589.294
b3 603.4064
b4 674.4438
y6−H2O 699.3422
y6−NH3 700.3246
y6 717.3525
b5 731.465
y7 959.4869
y8 1016.5128
y9 1087.5503
L I F A G K Q L E D G R
Modification
Acetyl (Protein N−term)
GlyGly (K) no new terminus
Oxidation (M)

20200921_KIZ_MUb4_GlyGly_10plex_2 − Scan number: 26407

Gene Names	Charge	m/z	Mass	Retention time	PEP	Score	Precursor Intensity
RPS27A;UBA52;UBC;UBB	3	487.6001	1459.778	104.6	4.2533e−06	111.86	1692613

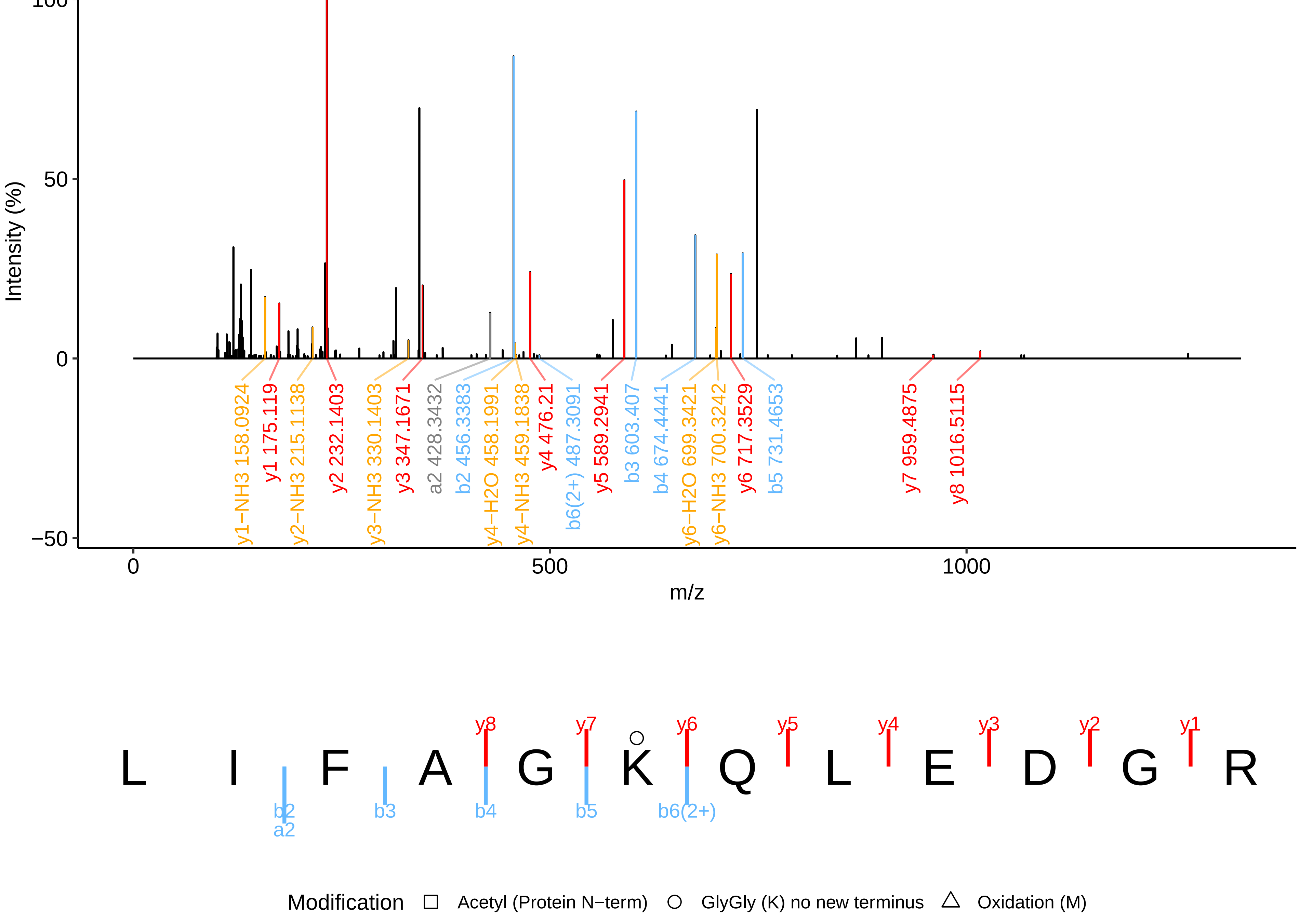

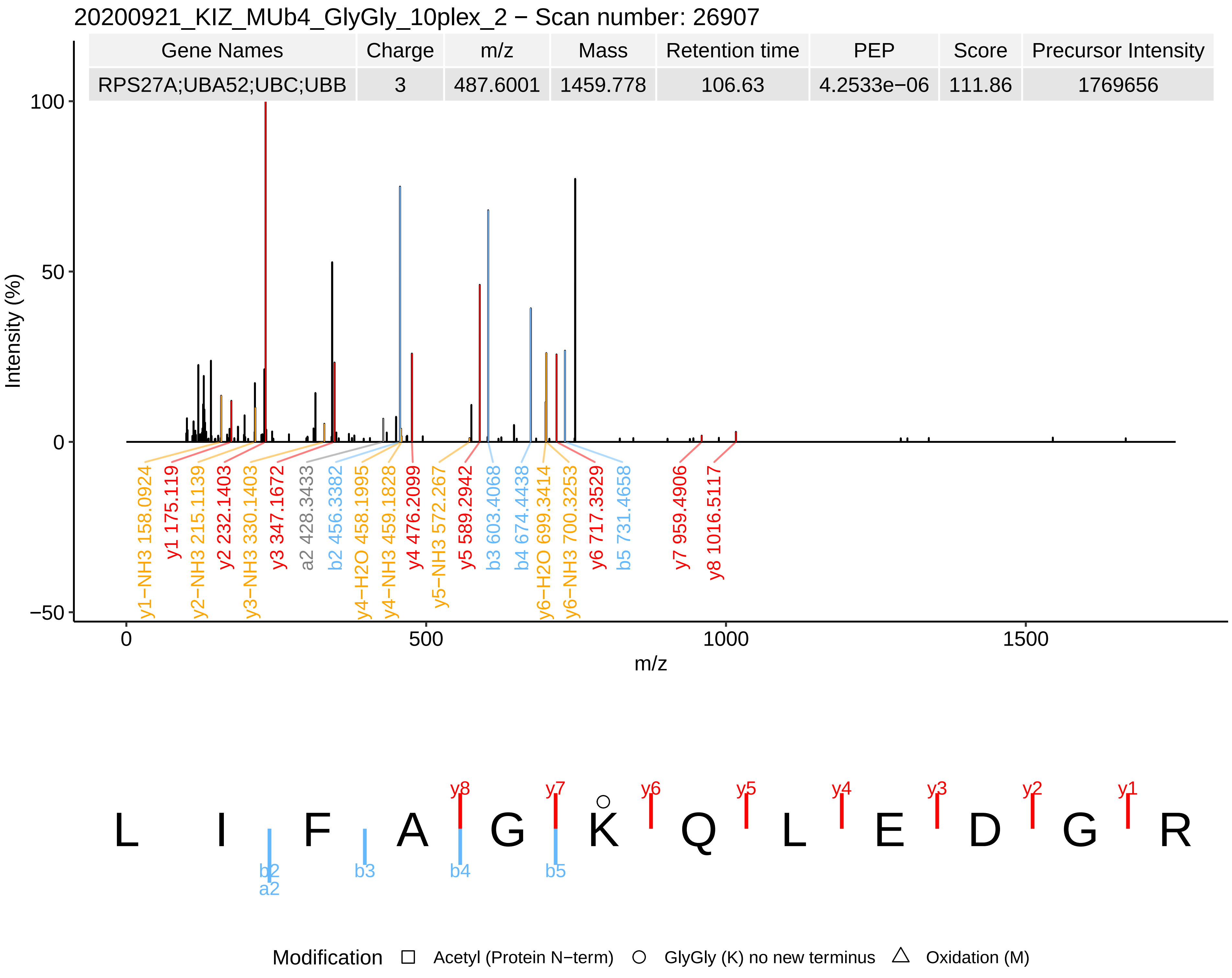
20200921_KIZ_MUb4_GlyGly_10plex_2 − Scan number: 26907
Gene Names | Charge | m/z | Mass | Retention time | PEP | Score | Precursor Intensity
RPS27A;UBA52;UBC;UBB | 3 | 487.6001 | 1459.778 | 106.63 | 4.2533e−06 | 111.86 | 1769656
Intensity (%)
100
50
0
−50
y1−NH3 158.0924
y1 175.119
y2−NH3 215.1139
y2 232.1403
y3−NH3 330.1403
y3 347.1672
a2 428.3433
b2 456.3382
y4−H2O 458.1995
y4−NH3 459.1828
y4 476.2099
y5−NH3 572.267
y5 589.2942
b3 603.4068
b4 674.4438
y6−H2O 699.3414
y6−NH3 700.3253
y6 717.3529
b5 731.4658
y7 959.4906
y8 1016.5117
0
500
1000
1500
m/z
L I F A G K Q L E D G R
y8 y7 y6 y5 y4 y3 y2 y1
b2 a2 b3 b4 b5
Modification
Acetyl (Protein N−term)
GlyGly (K) no new terminus
Oxidation (M)

20200921_KIZ_MUb4_GlyGly_10plex_2 − Scan number: 27031

Gene Names	Charge	m/z	Mass	Retention time	PEP	Score	Precursor Intensity
RPS27A;UBA52;UBC;UBB	3	487.6001	1459.778	107.14	1.6351e−07	115.29	2287271

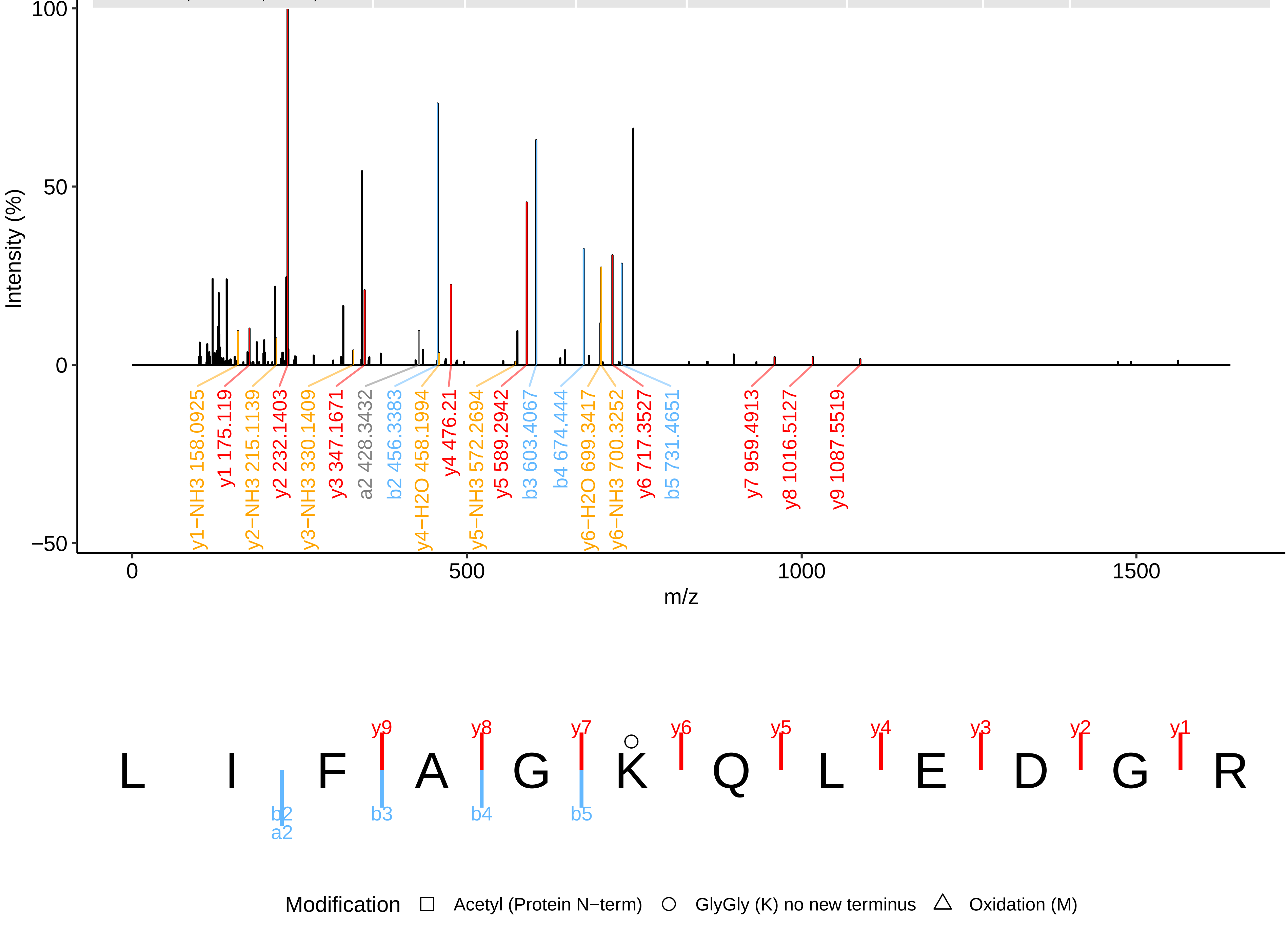

20200921_KIZ_MUb4_GlyGly_10plex_2 − Scan number: 27153

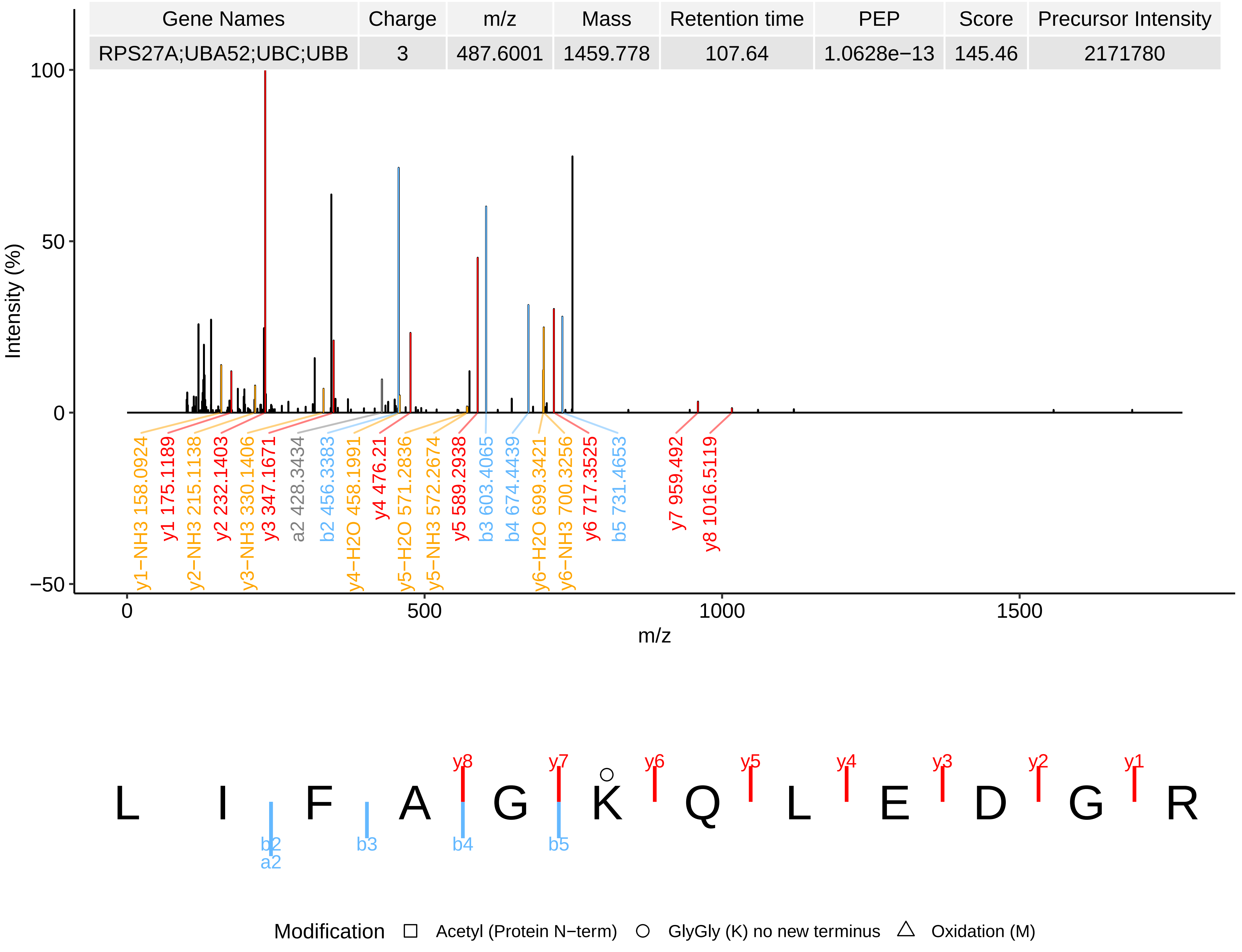

20200921_KIZ_MUb4_GlyGly_10plex_2 − Scan number: 27277

Gene Names	Charge	m/z	Mass	Retention time	PEP	Score	Precursor Intensity
RPS27A;UBA52;UBC;UBB	3	487.6001	1459.778	108.14	1.556e−13	143.24	1752469

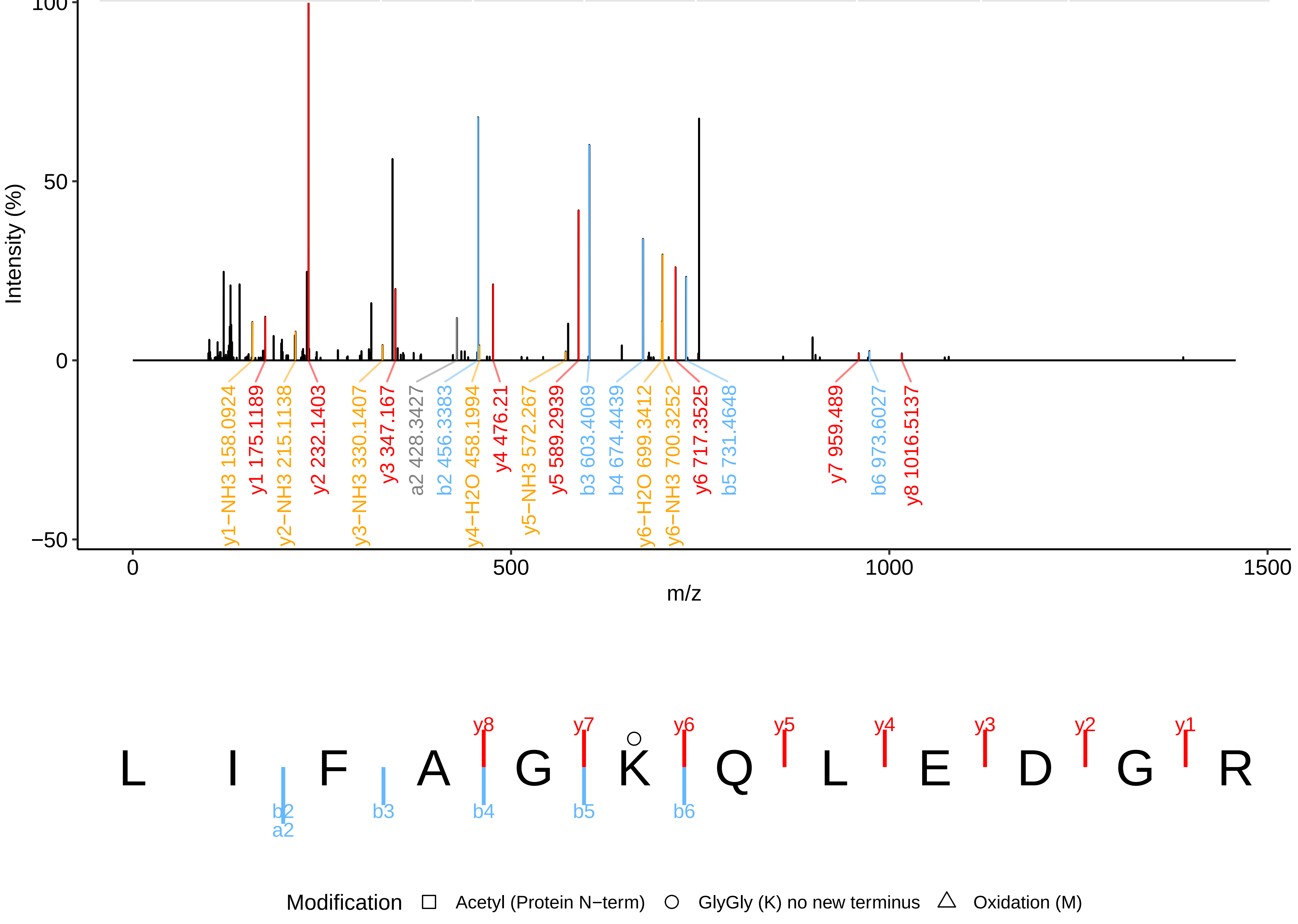

20200921_KIZ_MUb4_GlyGly_10plex_2 − Scan number: 27402

Gene Names	Charge	m/z	Mass	Retention time	PEP	Score	Precursor Intensity
RPS27A;UBA52;UBC;UBB	3	487.6001	1459.778	108.65	1.6351e−07	115.29	1900017

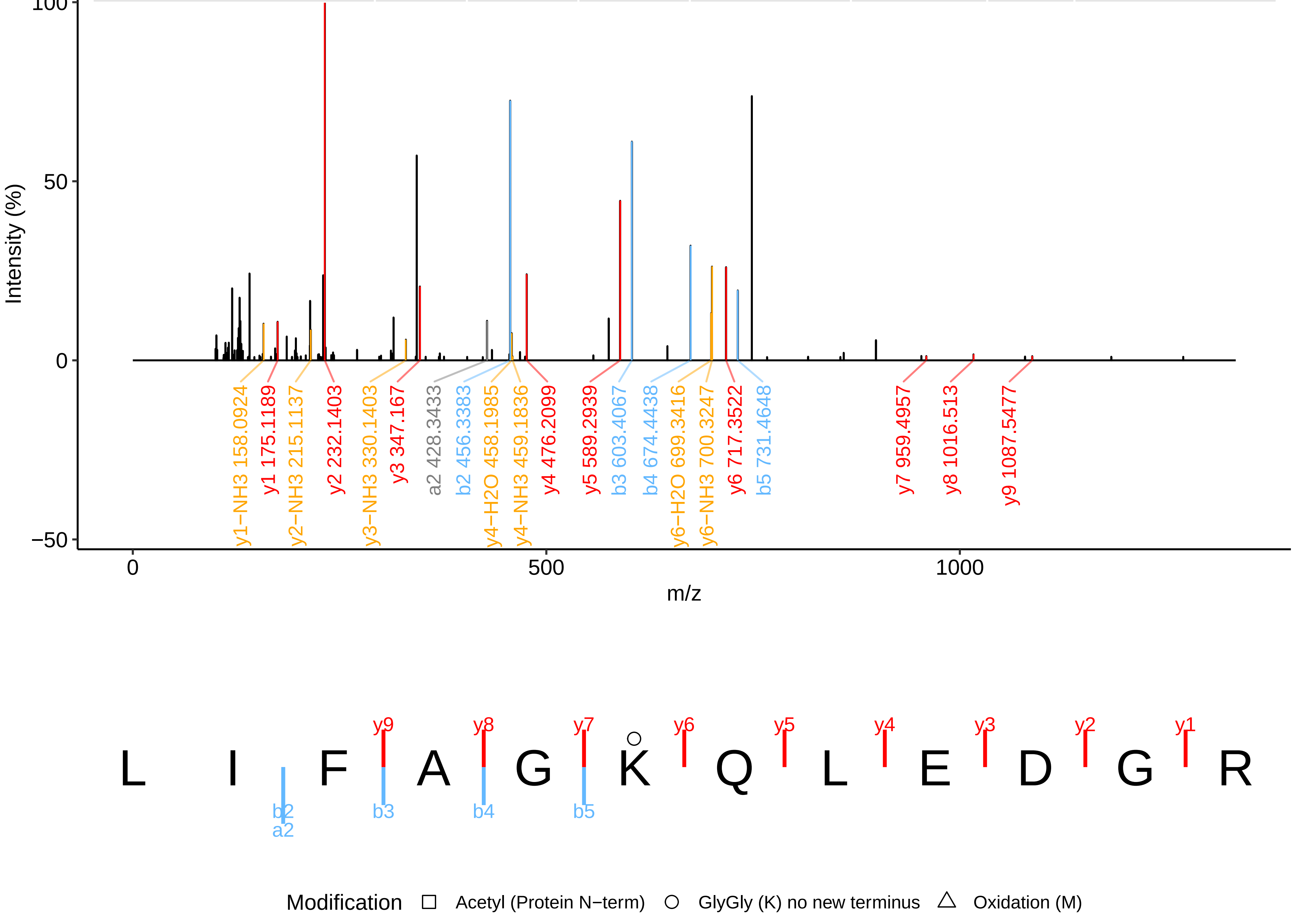

20200921_KIZ_MUb4_GlyGly_10plex_2 − Scan number: 27528

Gene Names	Charge	m/z	Mass	Retention time	PEP	Score	Precursor Intensity
RPS27A;UBA52;UBC;UBB	3	487.6001	1459.778	109.15	1.6723e−05	101.39	2445543

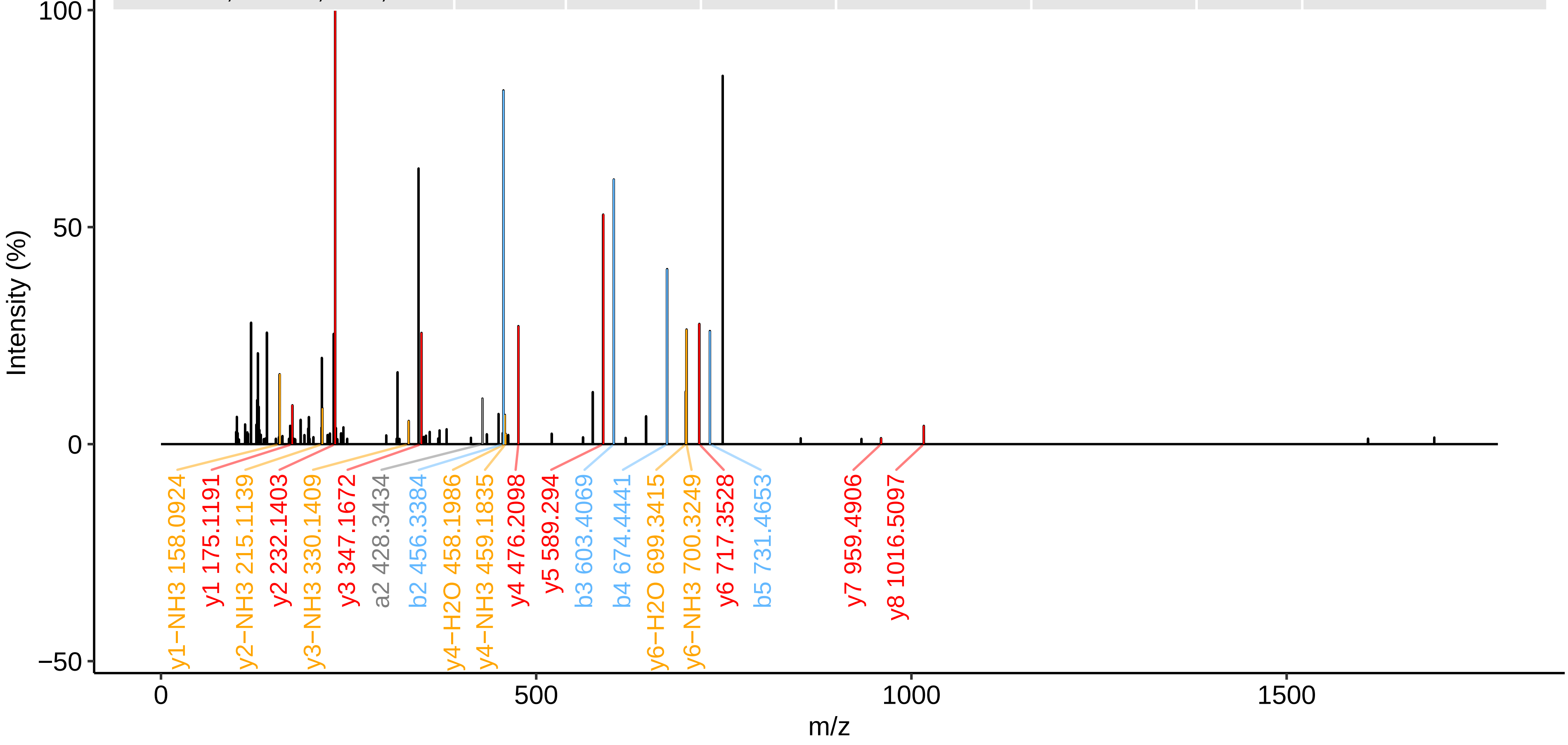

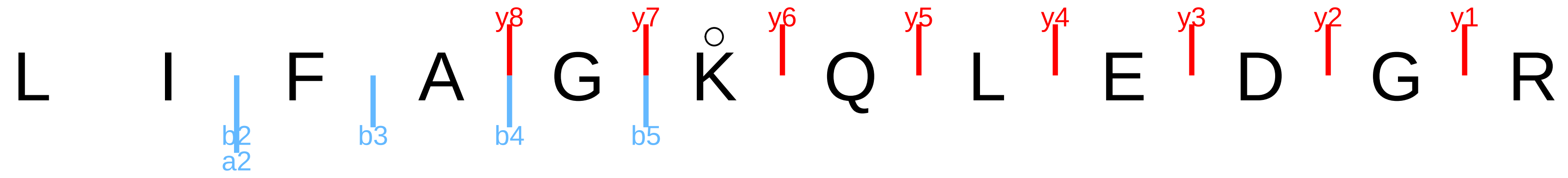

Modification □ Acetyl (Protein N−term) ○ GlyGly (K) no new terminus △ Oxidation (M)

20200921_KIZ_MUb4_GlyGly_10plex_2 − Scan number: 27652

Gene Names	Charge	m/z	Mass	Retention time	PEP	Score	Precursor Intensity
RPS27A;UBA52;UBC;UBB	3	487.6001	1459.778	109.65	3.1599e−13	136.02	1751357

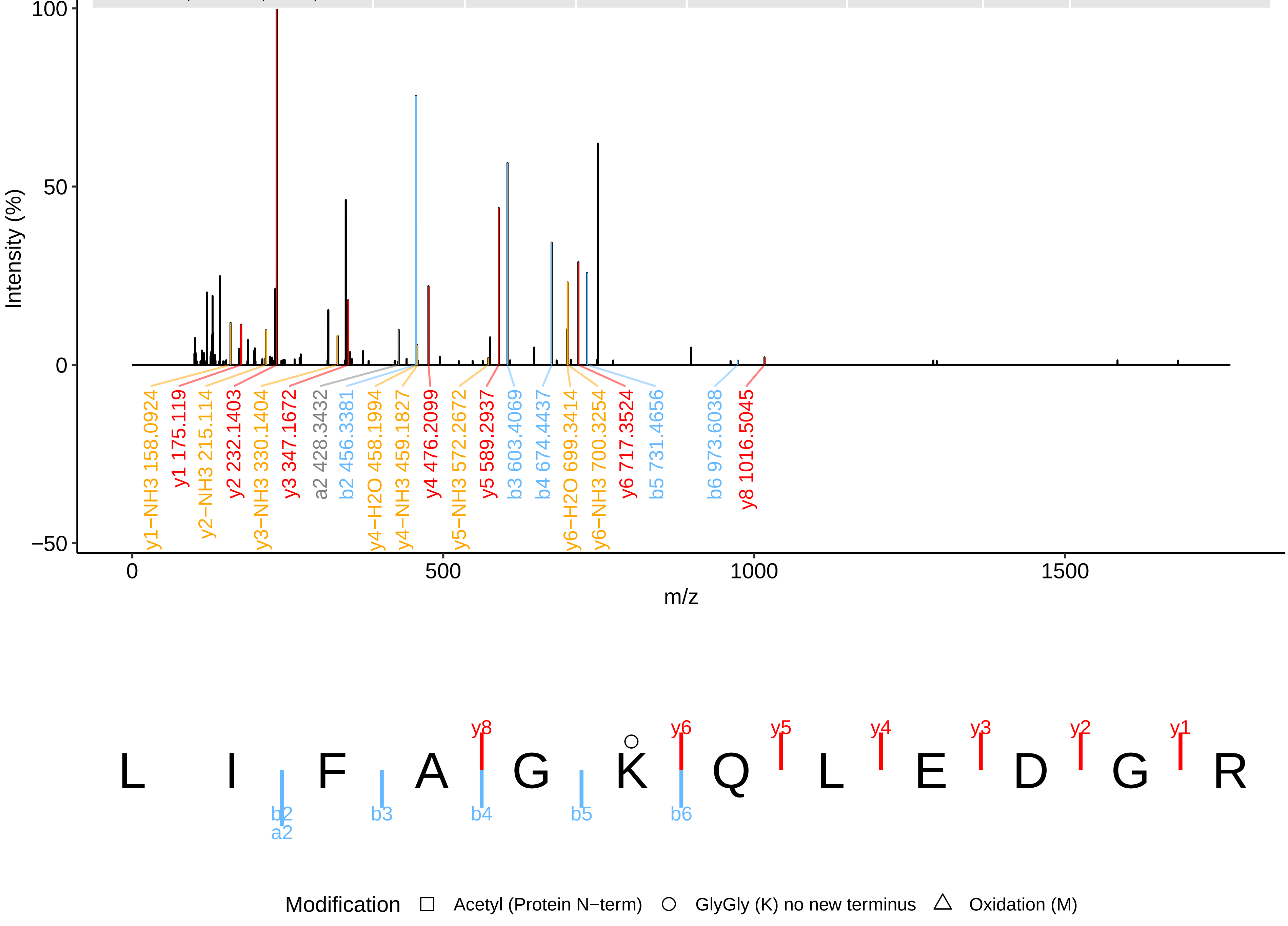

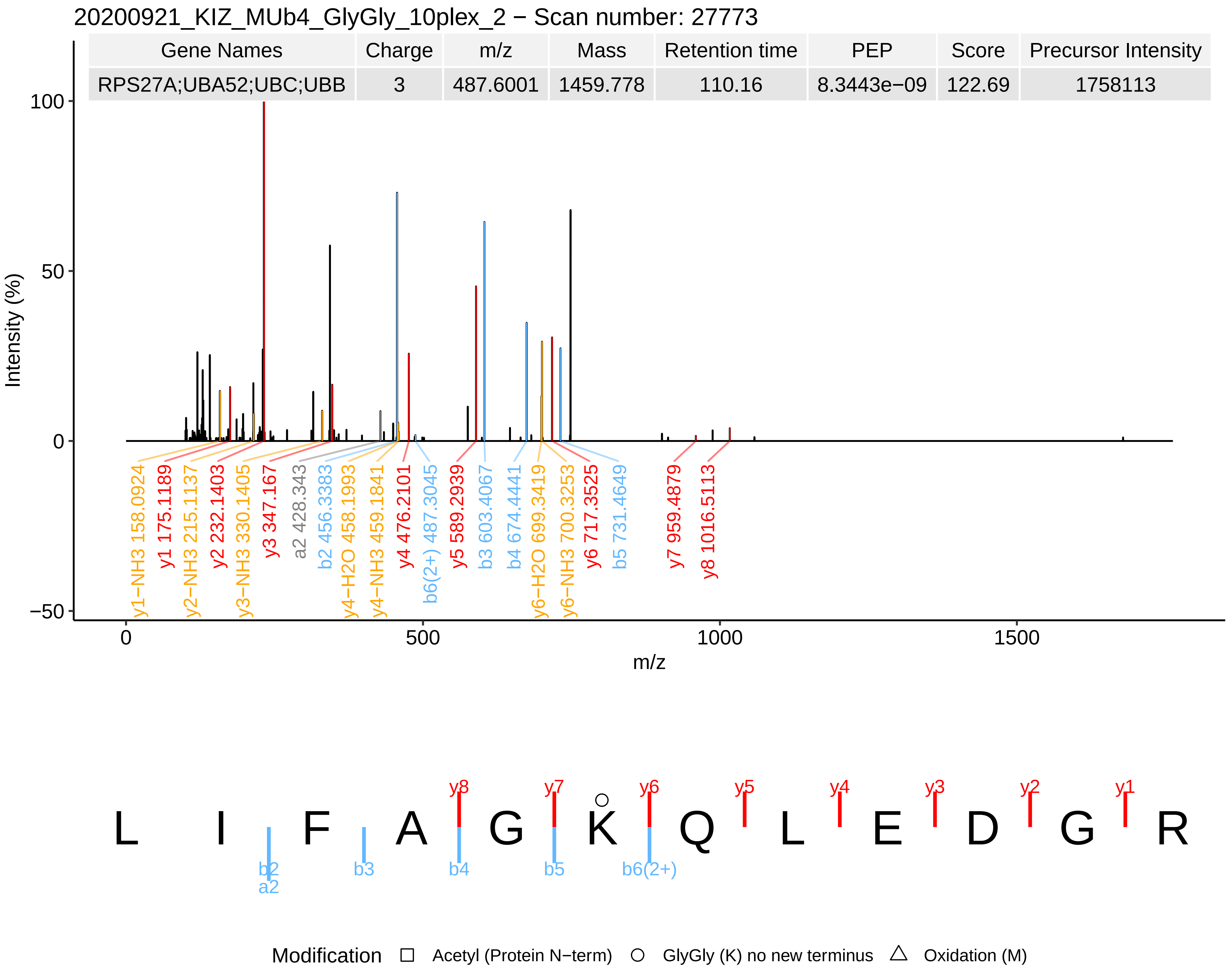

20200921_KIZ_MUb4_GlyGly_10plex_2 − Scan number: 27773
Gene Names | Charge | m/z | Mass | Retention time | PEP | Score | Precursor Intensity
RPS27A;UBA52;UBC;UBB | 3 | 487.6001 | 1459.778 | 110.16 | 8.3443e−09 | 122.69 | 1758113
Intensity (%)
m/z
y1−NH3 158.0924
y1 175.1189
y2−NH3 215.1137
y2 232.1403
y3−NH3 330.1405
y3 347.167
a2 428.343
b2 456.3383
y4−H2O 458.1993
y4−NH3 459.1841
y4 476.2101
b6(2+) 487.3045
y5 589.2939
b3 603.4067
b4 674.4441
y6−H2O 699.3419
y6−NH3 700.3253
y6 717.3525
b5 731.4649
y7 959.4879
y8 1016.5113
L I F A G K Q L E D G R
y8 y7 y6 y5 y4 y3 y2 y1
b2 a2 b3 b4 b5 b6(2+)
Modification
Acetyl (Protein N−term)
GlyGly (K) no new terminus
Oxidation (M)

20200921_KIZ_MUb4_GlyGly_10plex_2 − Scan number: 27899

Gene Names	Charge	m/z	Mass	Retention time	PEP	Score	Precursor Intensity
RPS27A;UBA52;UBC;UBB	3	487.6001	1459.778	110.66	7.7721e−06	108.9	2746036

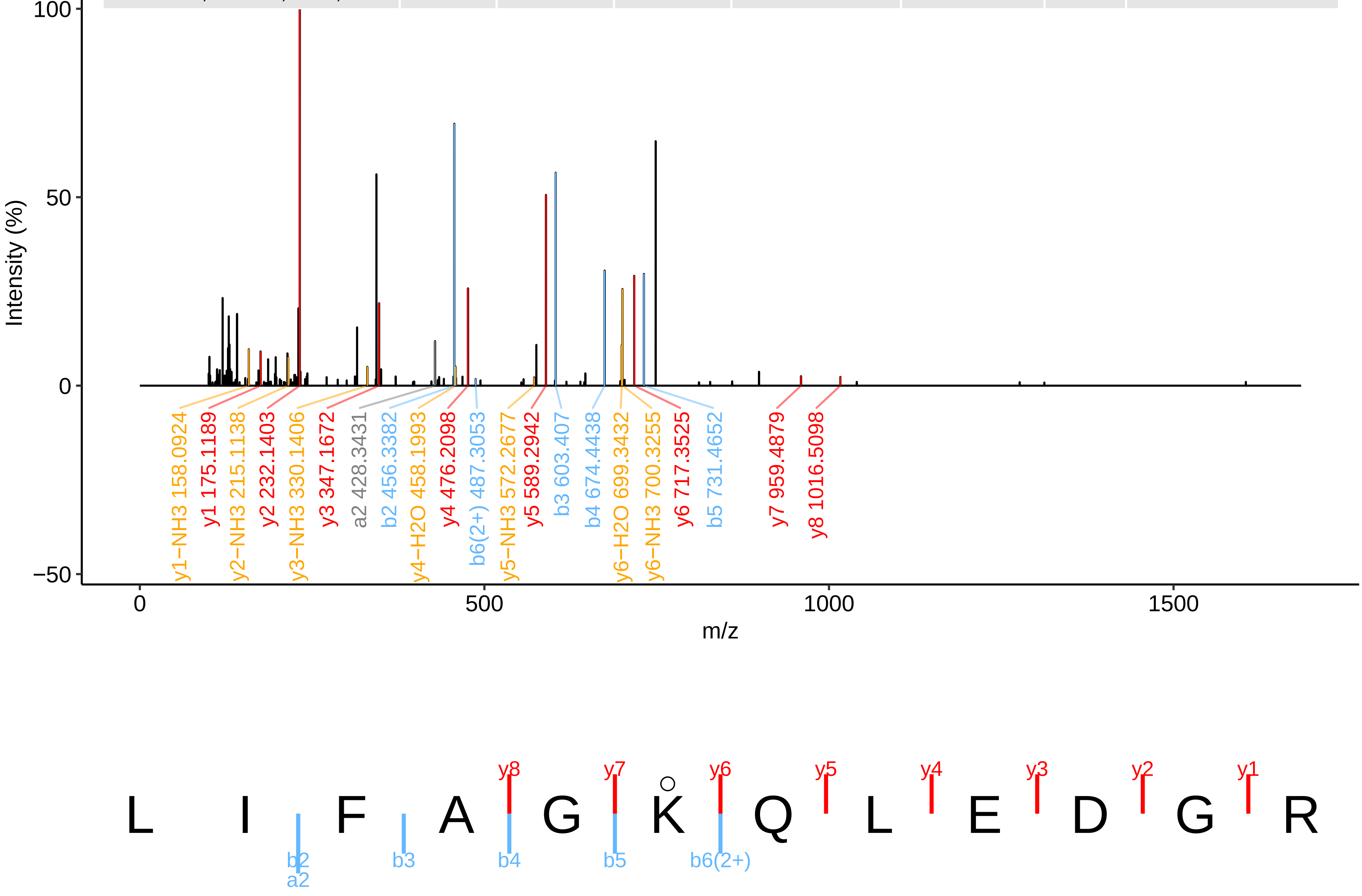

Modification □ Acetyl (Protein N−term) ○ GlyGly (K) no new terminus △ Oxidation (M)

20200921_KIZ_MUb4_GlyGly_10plex_2 − Scan number: 28024

Gene Names	Charge	m/z	Mass	Retention time	PEP	Score	Precursor Intensity
RPS27A;UBA52;UBC;UBB	3	487.6001	1459.778	111.17	1.2602e−08	117.52	2270599

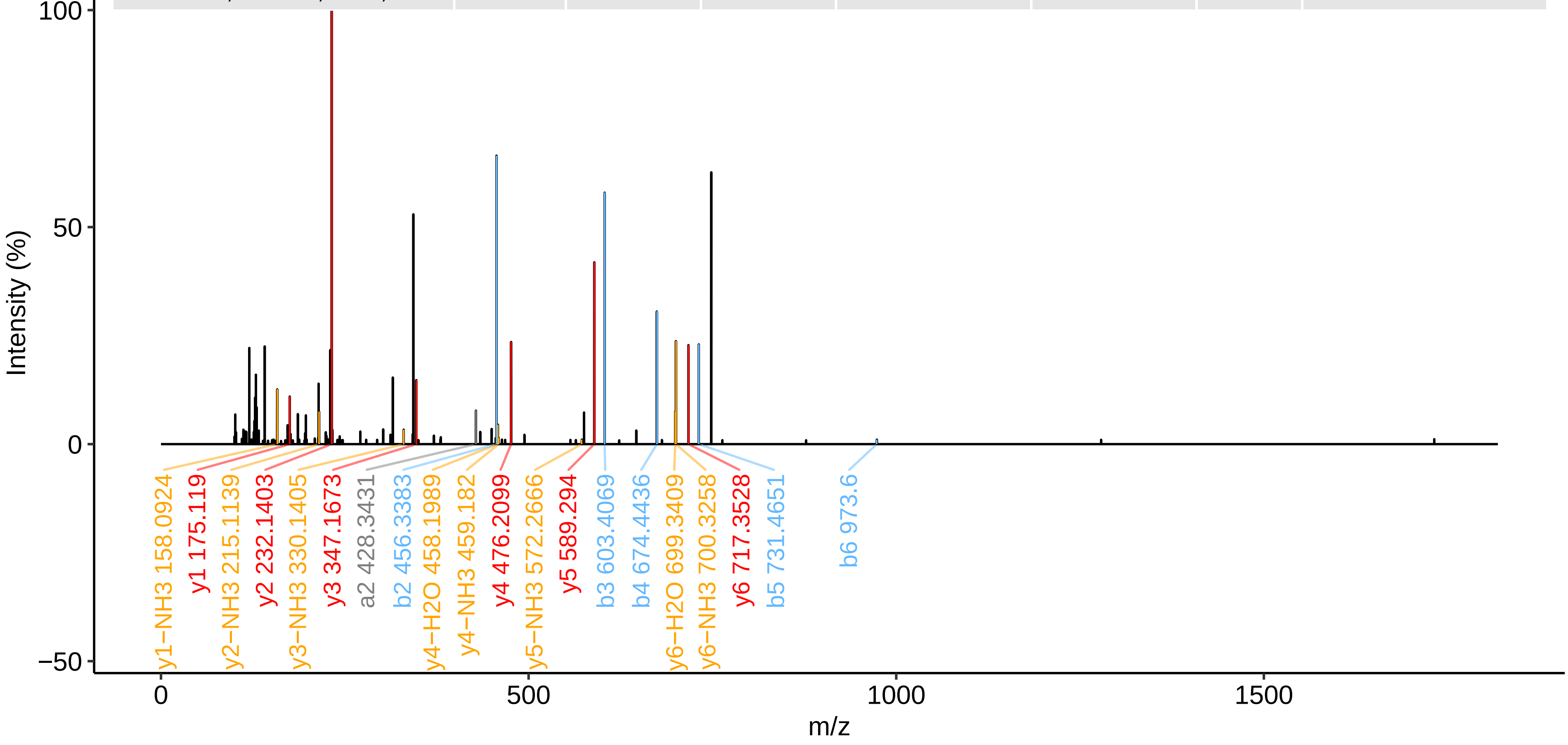

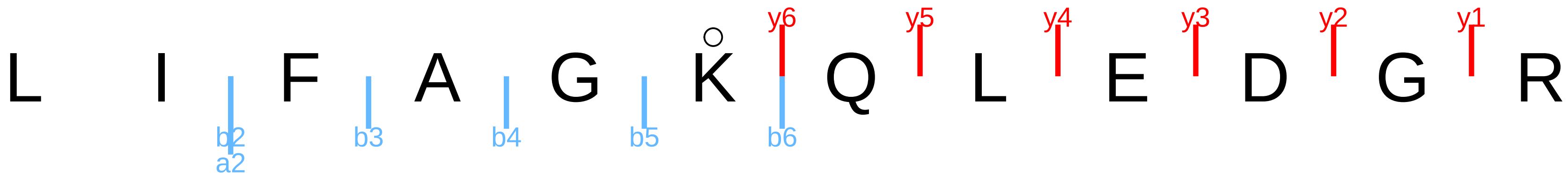

Modification □ Acetyl (Protein N−term) ○ GlyGly (K) no new terminus △ Oxidation (M)

20200921_KIZ_MUb4_GlyGly_10plex_2 − Scan number: 28280

Gene Names	Charge	m/z	Mass	Retention time	PEP	Score	Precursor Intensity
RPS27A;UBA52;UBC;UBB	3	487.6001	1459.778	112.19	8.3443e−09	122.69	2043165

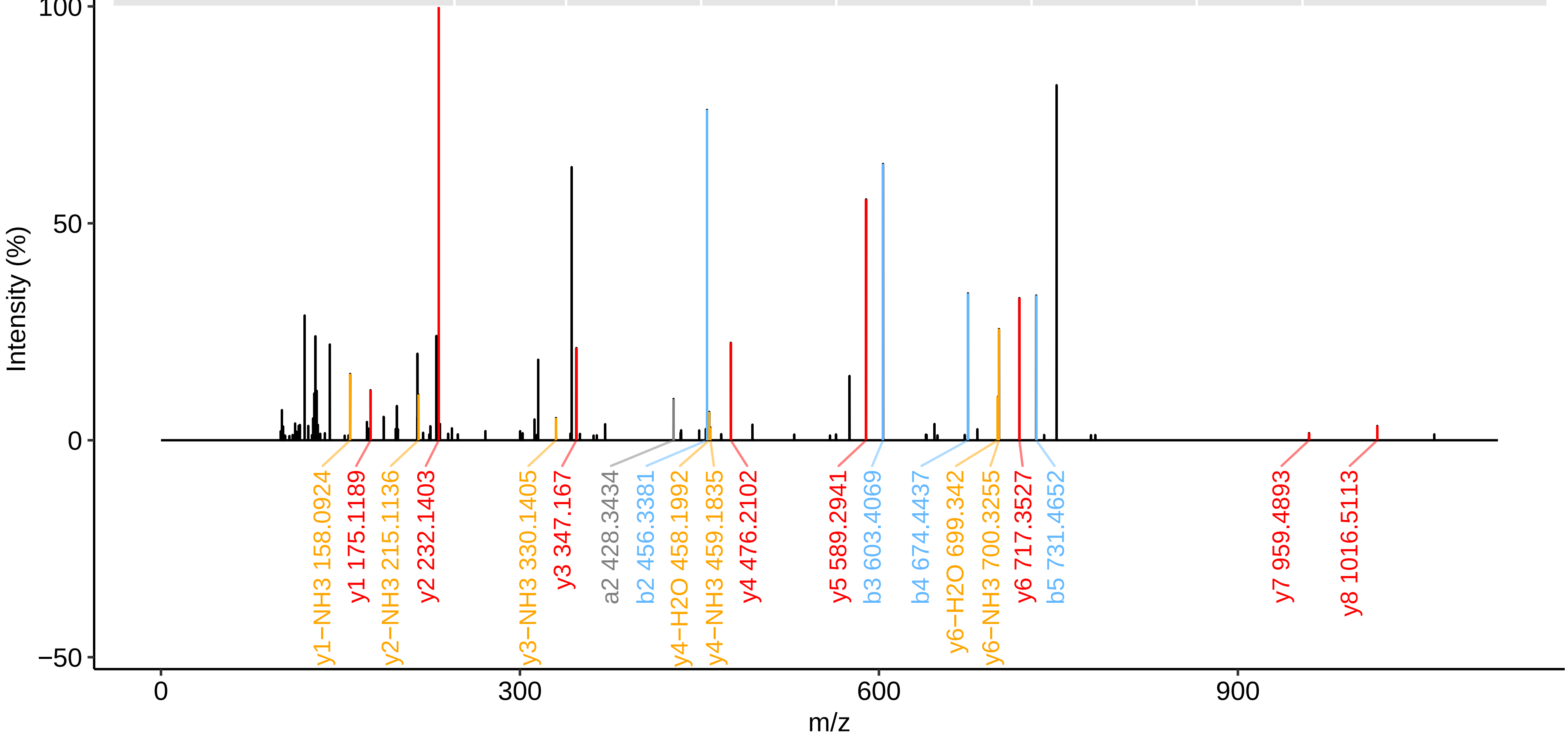

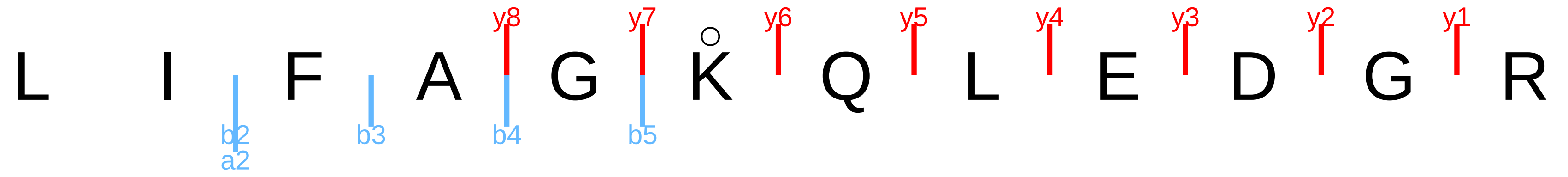

Modification □ Acetyl (Protein N−term) ○ GlyGly (K) no new terminus △ Oxidation (M)

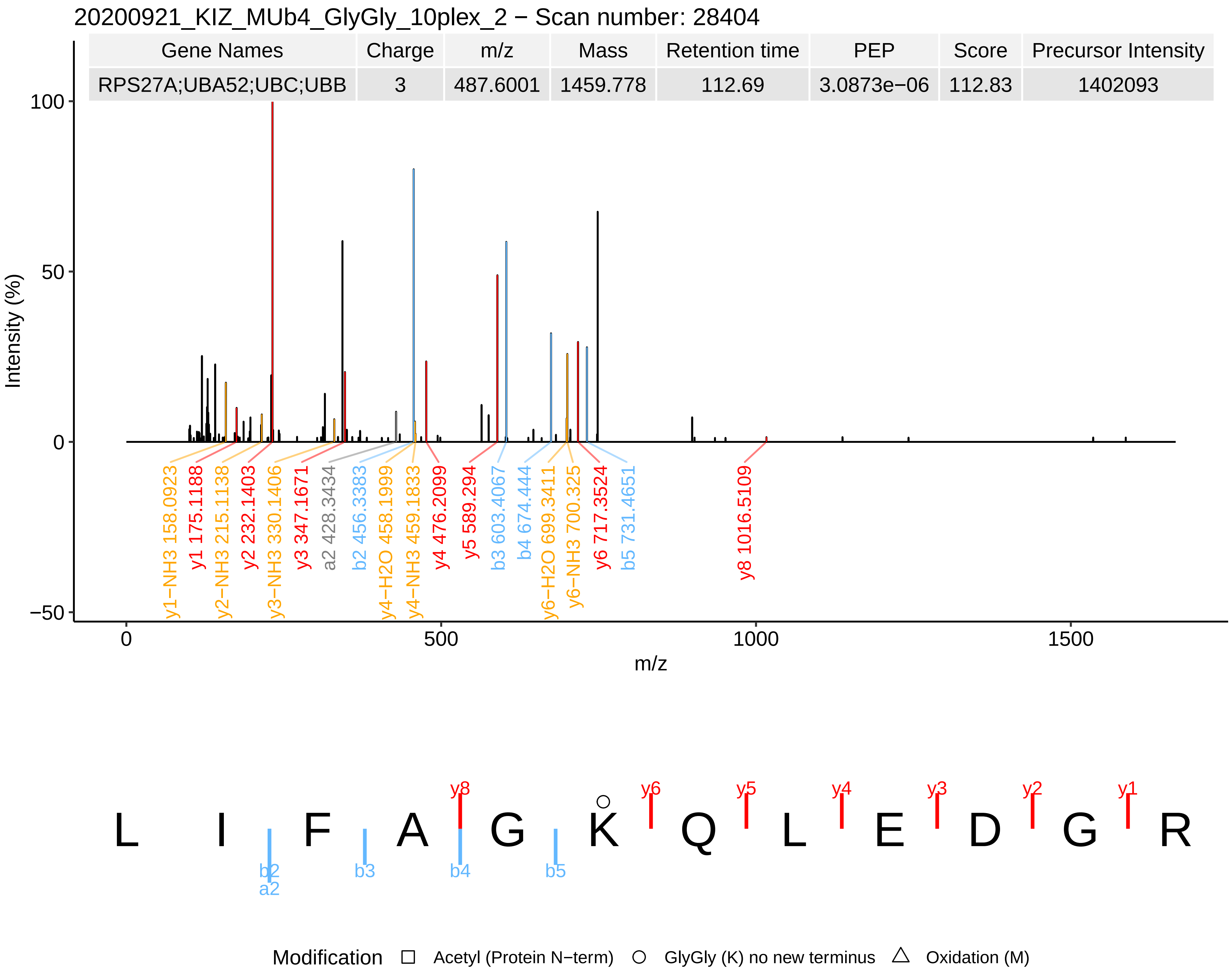

20200921_KIZ_MUb4_GlyGly_10plex_2 − Scan number: 28404
Gene Names
Charge
m/z
Mass
Retention time
PEP
Score
Precursor Intensity
RPS27A;UBA52;UBC;UBB
3
487.6001
1459.778
112.69
3.0873e−06
112.83
1402093
Intensity (%)
m/z
y1−NH3 158.0923
y1 175.1188
y2−NH3 215.1138
y2 232.1403
y3−NH3 330.1406
y3 347.1671
a2 428.3434
b2 456.3383
y4−H2O 458.1999
y4−NH3 459.1833
y4 476.2099
y5 589.294
b3 603.4067
b4 674.444
y6−H2O 699.3411
y6−NH3 700.325
y6 717.3524
b5 731.4651
y8 1016.5109
L I F A G K Q L E D G R
y8 y6 y5 y4 y3 y2 y1
b2 a2 b3 b4 b5
Modification
Acetyl (Protein N−term)
GlyGly (K) no new terminus
Oxidation (M)

20200921_KIZ_MUb4_GlyGly_10plex_2 − Scan number: 28906

Gene Names	Charge	m/z	Mass	Retention time	PEP	Score	Precursor Intensity
RPS27A;UBA52;UBC;UBB	3	487.6001	1459.778	114.73	9.4395e−09	121.36	1727399

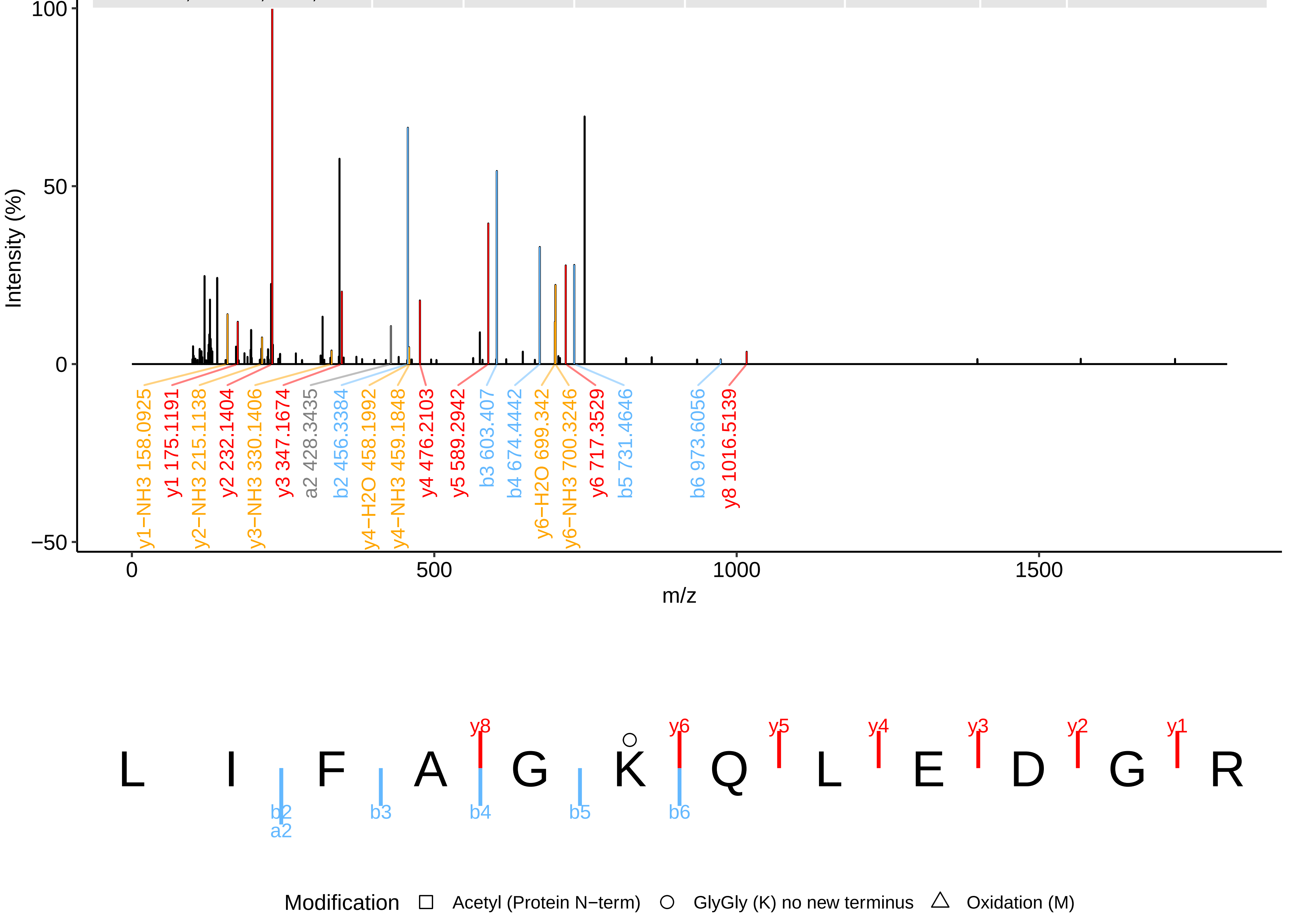

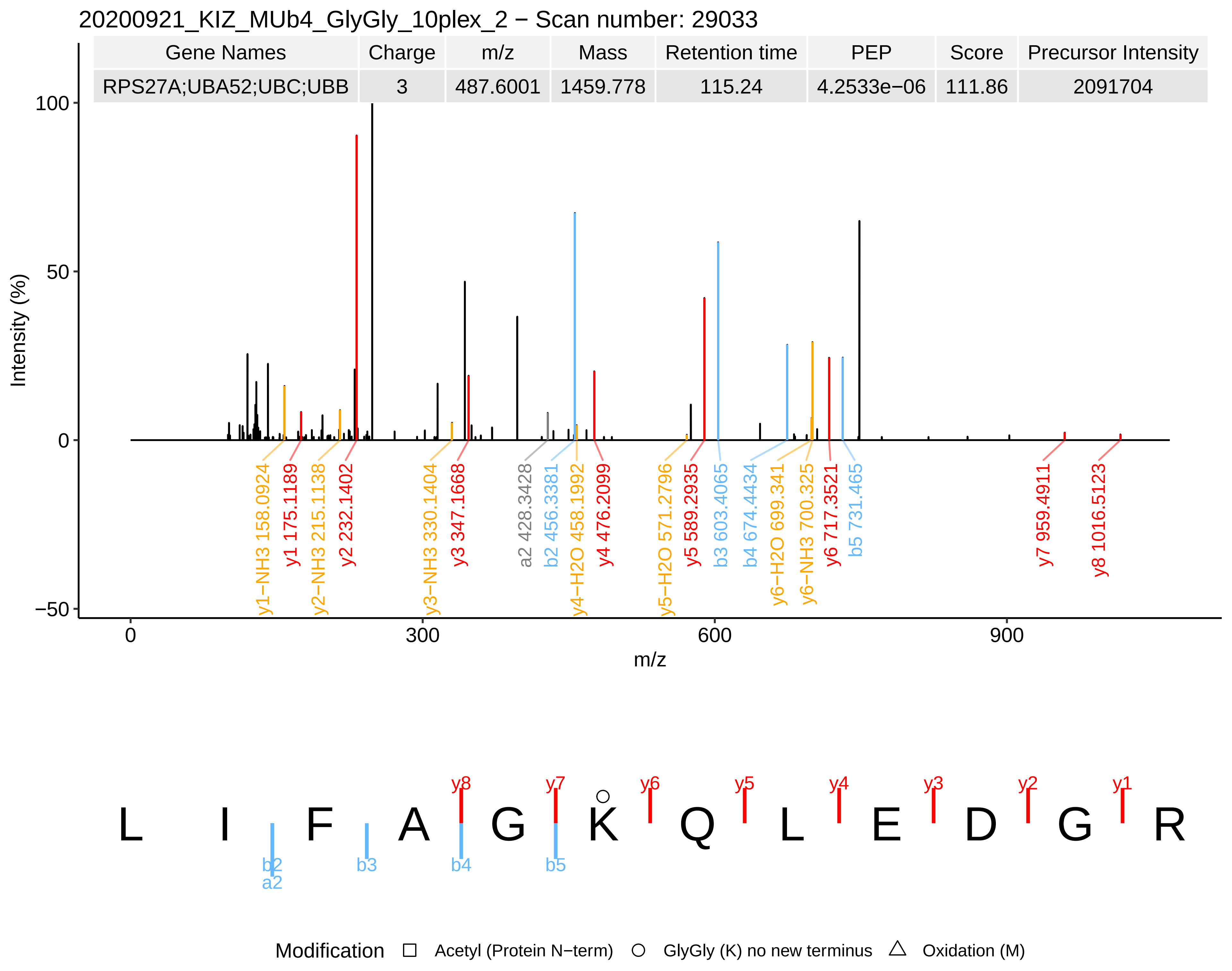

20200921_KIZ_MUb4_GlyGly_10plex_2 − Scan number: 29033
Gene Names | Charge | m/z | Mass | Retention time | PEP | Score | Precursor Intensity
RPS27A;UBA52;UBC;UBB | 3 | 487.6001 | 1459.778 | 115.24 | 4.2533e−06 | 111.86 | 2091704
Intensity (%)
m/z
y1−NH3 158.0924
y1 175.1189
y2−NH3 215.1138
y2 232.1402
y3−NH3 330.1404
y3 347.1668
a2 428.3428
b2 456.3381
y4−H2O 458.1992
y4 476.2099
y5−H2O 571.2796
y5 589.2935
b3 603.4065
b4 674.4434
y6−H2O 699.341
y6−NH3 700.325
y6 717.3521
b5 731.465
y7 959.4911
y8 1016.5123
L I F A G K Q L E D G R
y8 y7 y6 y5 y4 y3 y2 y1
b2 a2 b3 b4 b5
Modification
Acetyl (Protein N−term)
GlyGly (K) no new terminus
Oxidation (M)

20200921_KIZ_MUb4_GlyGly_10plex_2 − Scan number: 29159

Gene Names	Charge	m/z	Mass	Retention time	PEP	Score	Precursor Intensity
RPS27A;UBA52;UBC;UBB	3	487.6001	1459.778	115.74	1.6351e−07	115.29	1571765

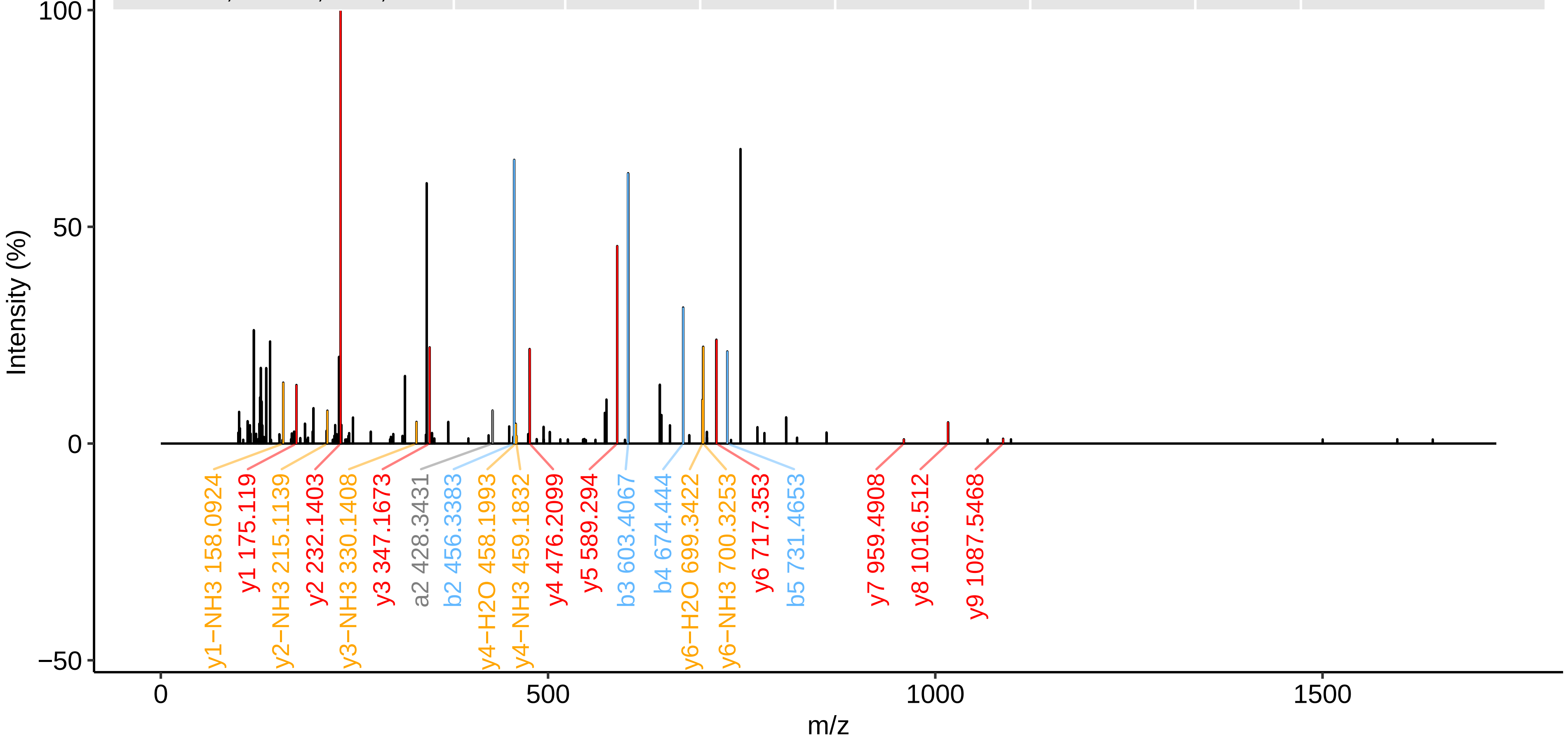

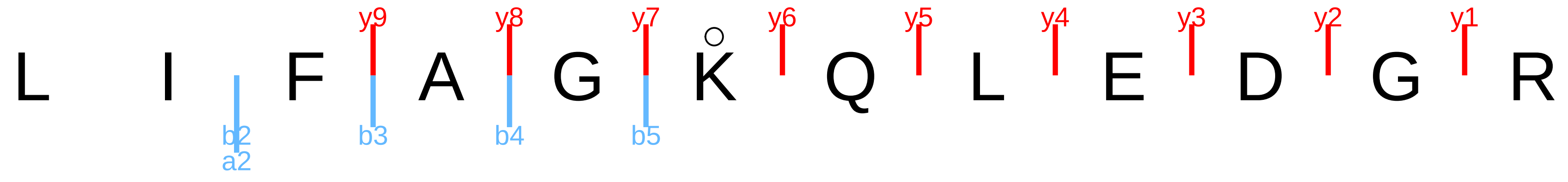

Modification □ Acetyl (Protein N−term) ○ GlyGly (K) no new terminus △ Oxidation (M)

20200921_KIZ_MUb4_GlyGly_10plex_2 − Scan number: 29284

Gene Names	Charge	m/z	Mass	Retention time	PEP	Score	Precursor Intensity
RPS27A;UBA52;UBC;UBB	3	487.6001	1459.778	116.25	1.2634e−05	104.82	1707437

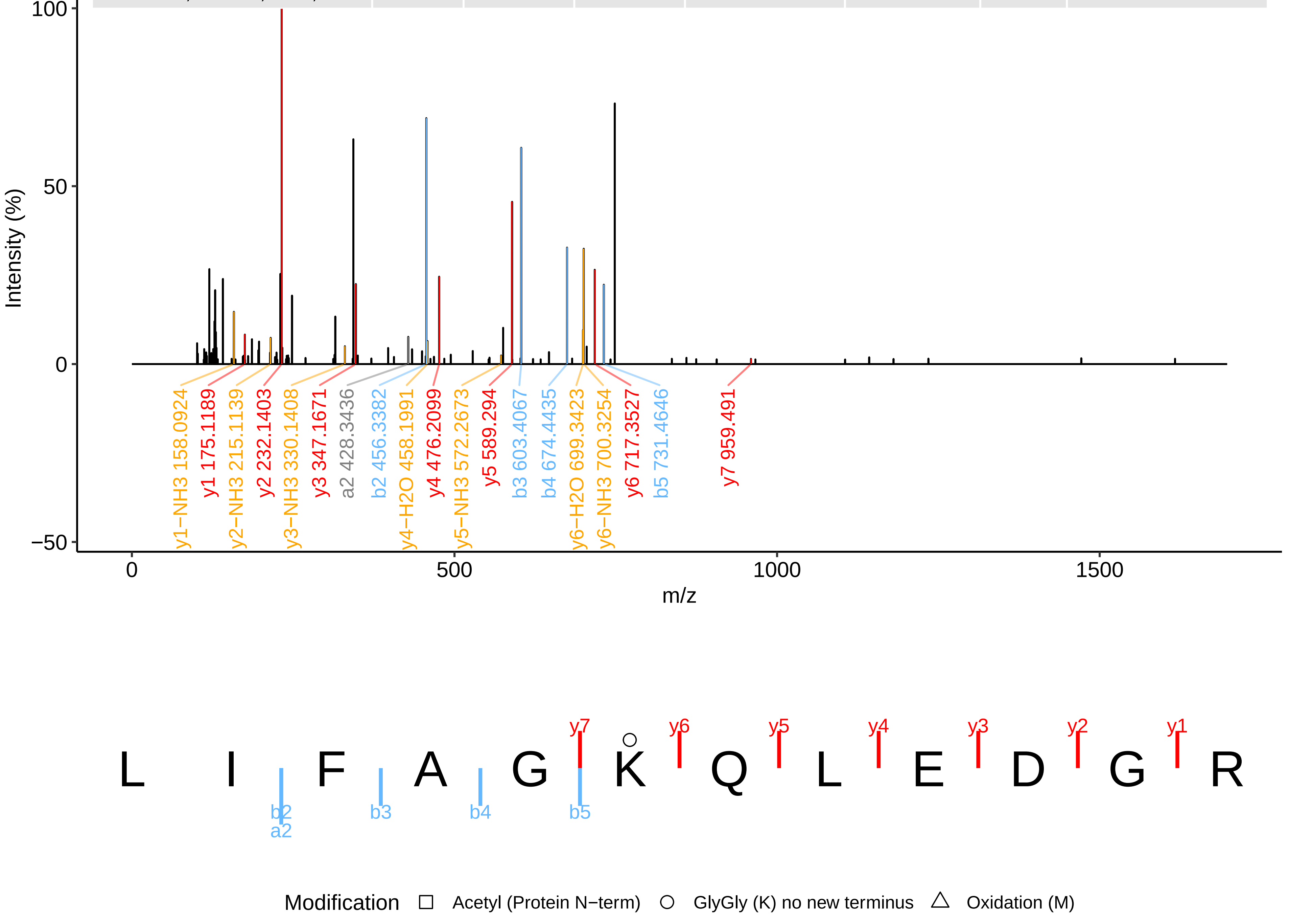

20200921_KIZ_MUb4_GlyGly_10plex_2 − Scan number: 29409

Gene Names	Charge	m/z	Mass	Retention time	PEP	Score	Precursor Intensity
RPS27A;UBA52;UBC;UBB	3	487.6001	1459.778	116.76	7.5372e−09	123.67	1254416

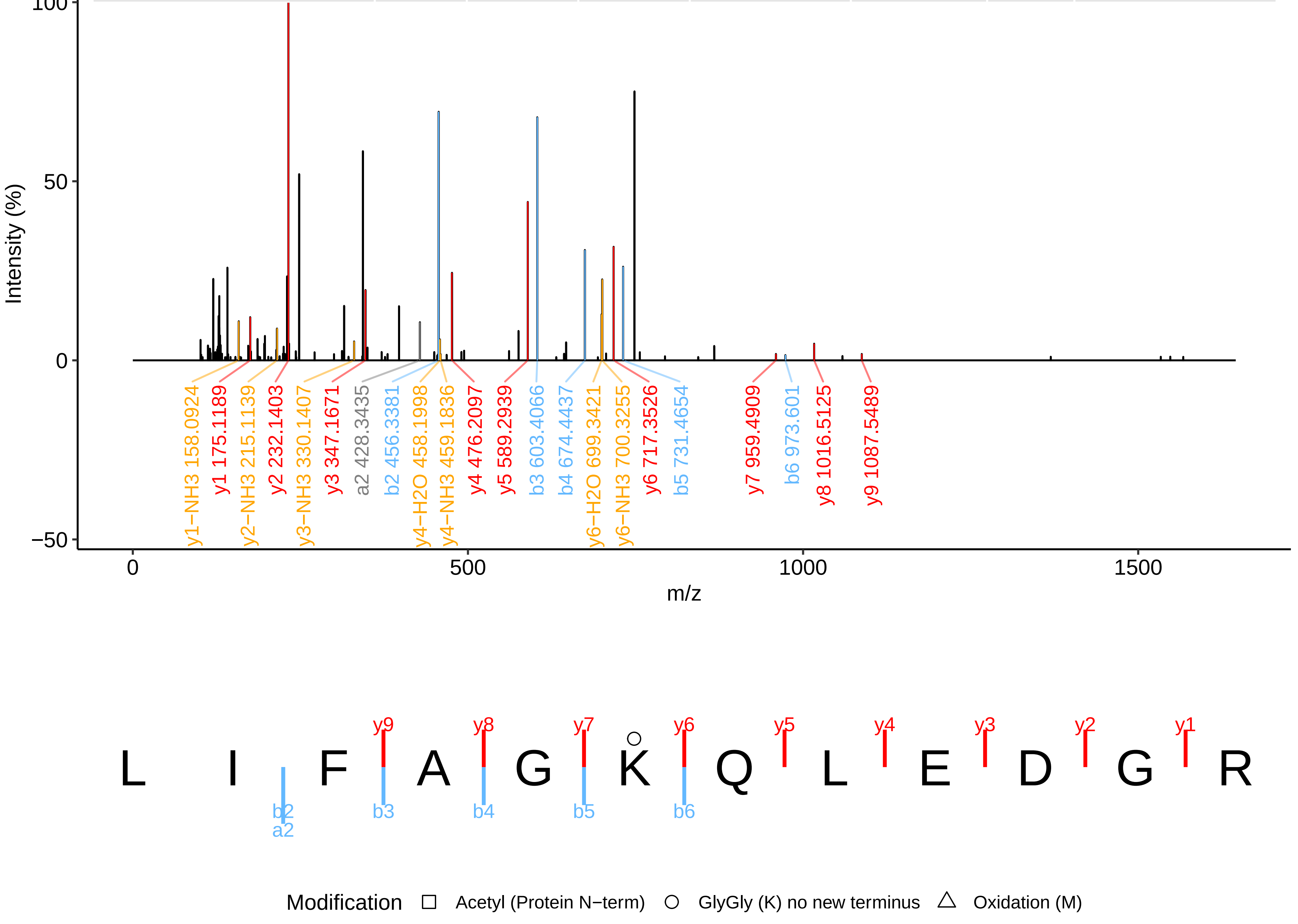

20200921_KIZ_MUb4_GlyGly_10plex_2 − Scan number: 29535

Gene Names	Charge	m/z	Mass	Retention time	PEP	Score	Precursor Intensity
RPS27A;UBA52;UBC;UBB	3	487.6001	1459.778	117.26	3.5418e−13	134.3	1959960

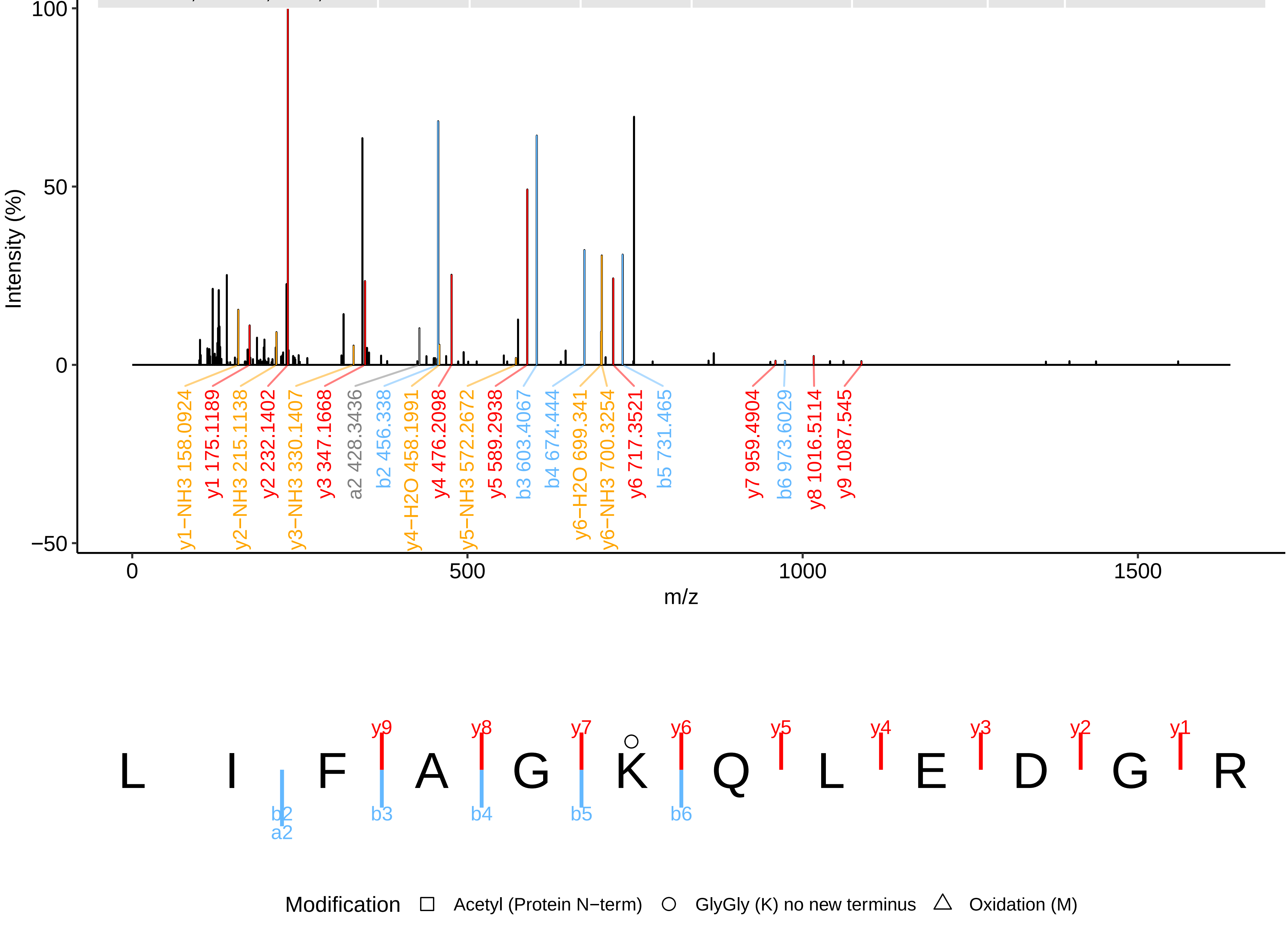

20200921_KIZ_MUb4_GlyGly_10plex_2 − Scan number: 29663

Gene Names	Charge	m/z	Mass	Retention time	PEP	Score	Precursor Intensity
RPS27A;UBA52;UBC;UBB	3	487.6001	1459.778	117.77	8.3443e−09	122.69	2599934

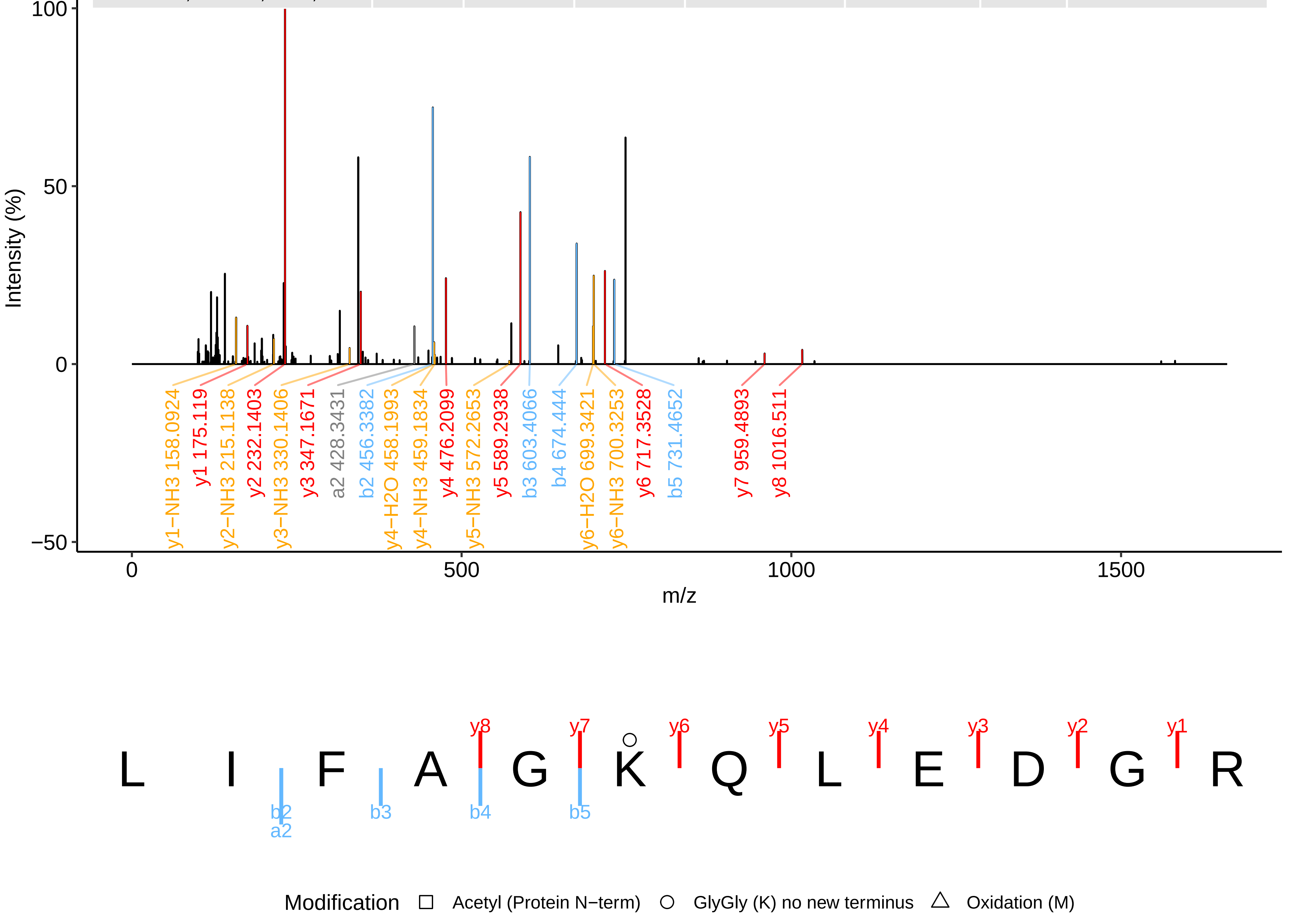

20200921_KIZ_MUb4_GlyGly_10plex_2 − Scan number: 29796

Gene Names	Charge	m/z	Mass	Retention time	PEP	Score	Precursor Intensity
RPS27A;UBA52;UBC;UBB	3	487.6001	1459.778	118.28	1.0628e−13	145.46	3177212

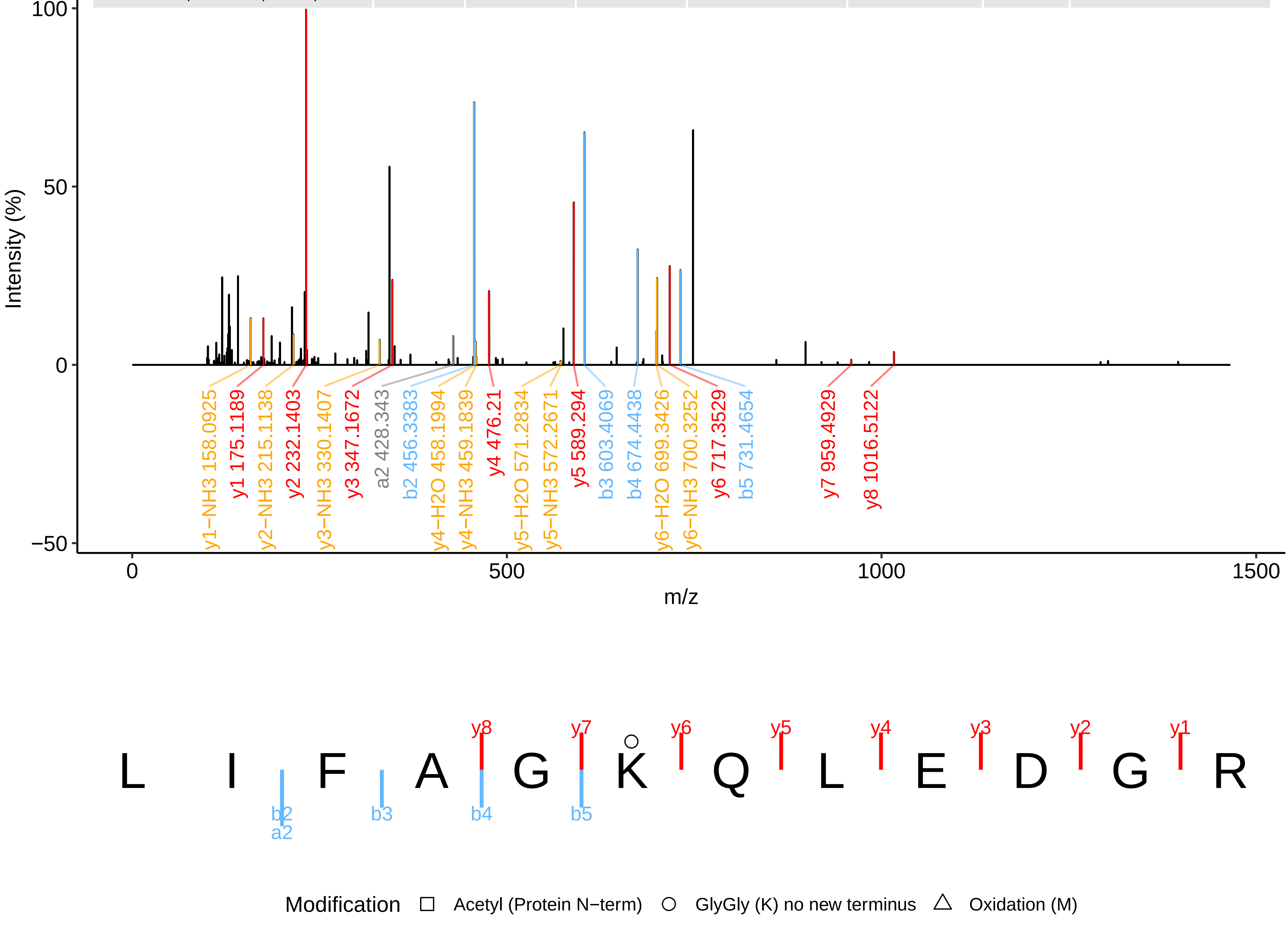

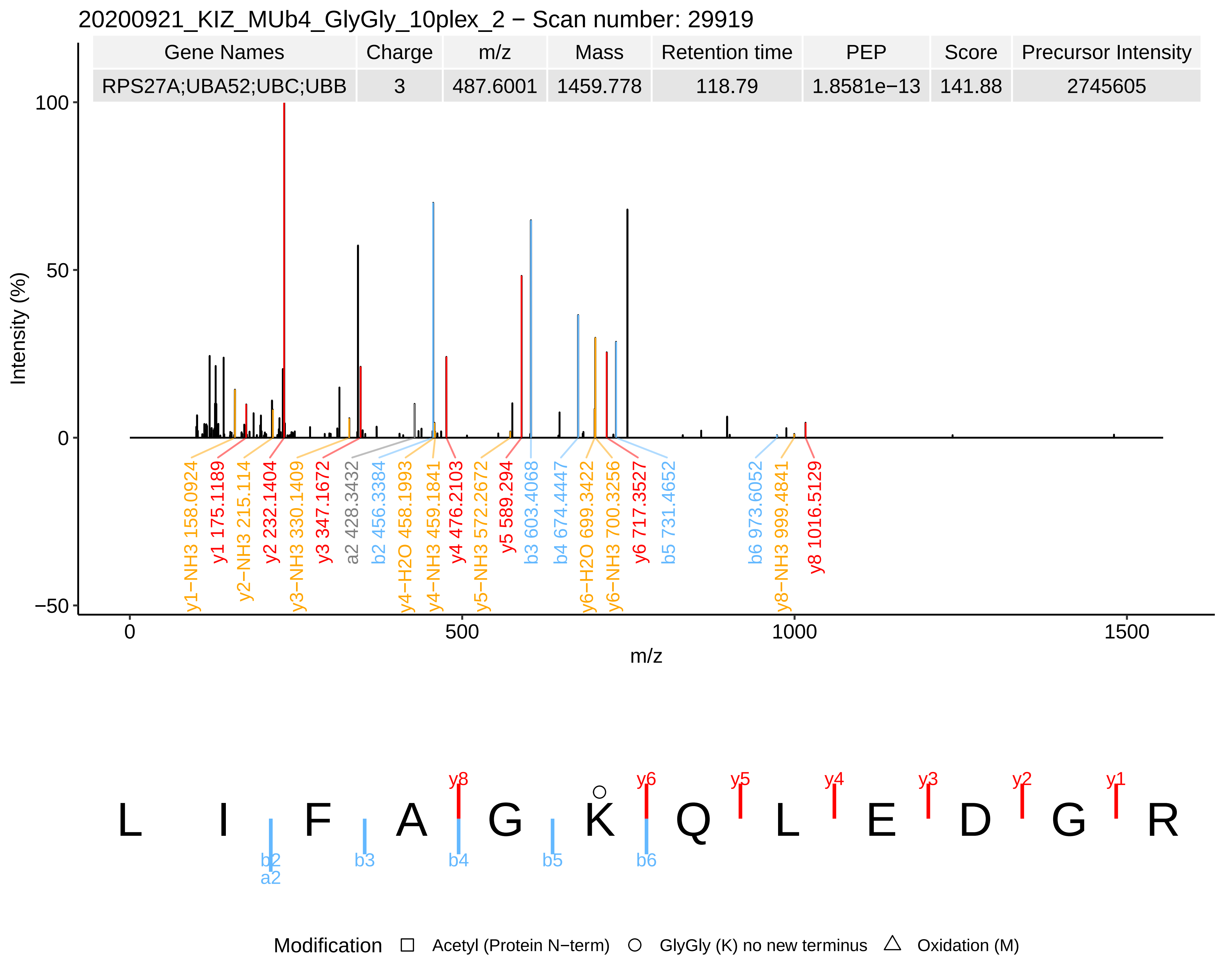
20200921_KIZ_MUb4_GlyGly_10plex_2 − Scan number: 29919
Gene Names | Charge | m/z | Mass | Retention time | PEP | Score | Precursor Intensity
RPS27A;UBA52;UBC;UBB | 3 | 487.6001 | 1459.778 | 118.79 | 1.8581e−13 | 141.88 | 2745605
Intensity (%)
100
50
0
−50
m/z
0
500
1000
1500
y1−NH3 158.0924
y1 175.1189
y2−NH3 215.114
y2 232.1404
y3−NH3 330.1409
y3 347.1672
a2 428.3432
b2 456.3384
y4−H2O 458.1993
y4−NH3 459.1841
y4 476.2103
y5−NH3 572.2672
y5 589.294
b3 603.4068
b4 674.4447
y6−H2O 699.3422
y6−NH3 700.3256
y6 717.3527
b5 731.4652
b6 973.6052
y8−NH3 999.4841
y8 1016.5129
L I F A G K Q L E D G R
y8 y6 y5 y4 y3 y2 y1
b2 a2 b3 b4 b5 b6
Modification
Acetyl (Protein N−term)
GlyGly (K) no new terminus
Oxidation (M)

20200921_KIZ_MUb4_GlyGly_10plex_2 − Scan number: 30040

Gene Names	Charge	m/z	Mass	Retention time	PEP	Score	Precursor Intensity
RPS27A;UBA52;UBC;UBB	3	487.6001	1459.778	119.3	5.0371e−09	126.71	2305711

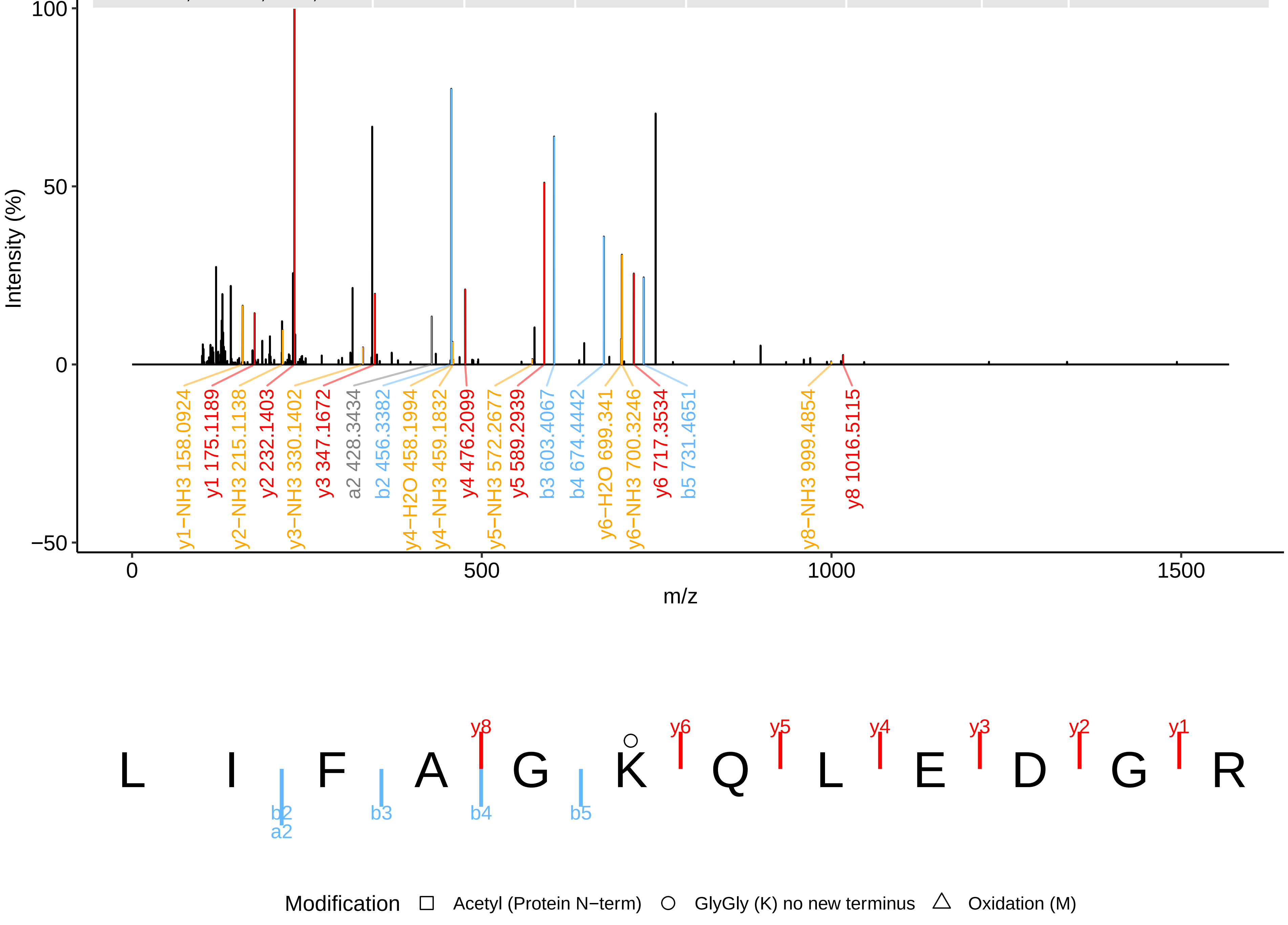

20200921_KIZ_MUb4_GlyGly_10plex_2 − Scan number: 30931

Gene Names	Charge	m/z	Mass	Retention time	PEP	Score	Precursor Intensity
RPS27A;UBA52;UBC;UBB	3	487.6001	1459.778	122.34	0.0096824	67.93	1751233

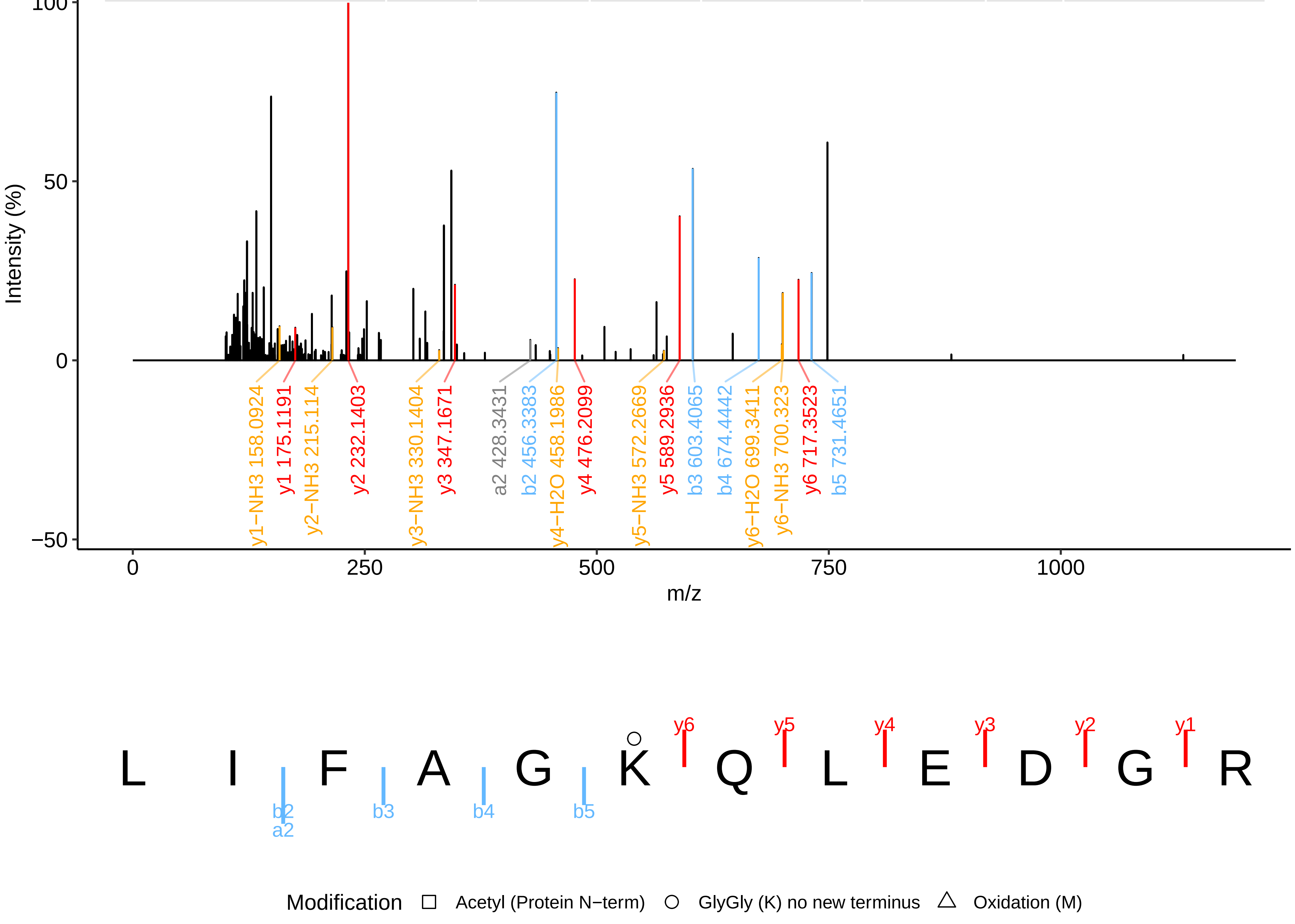

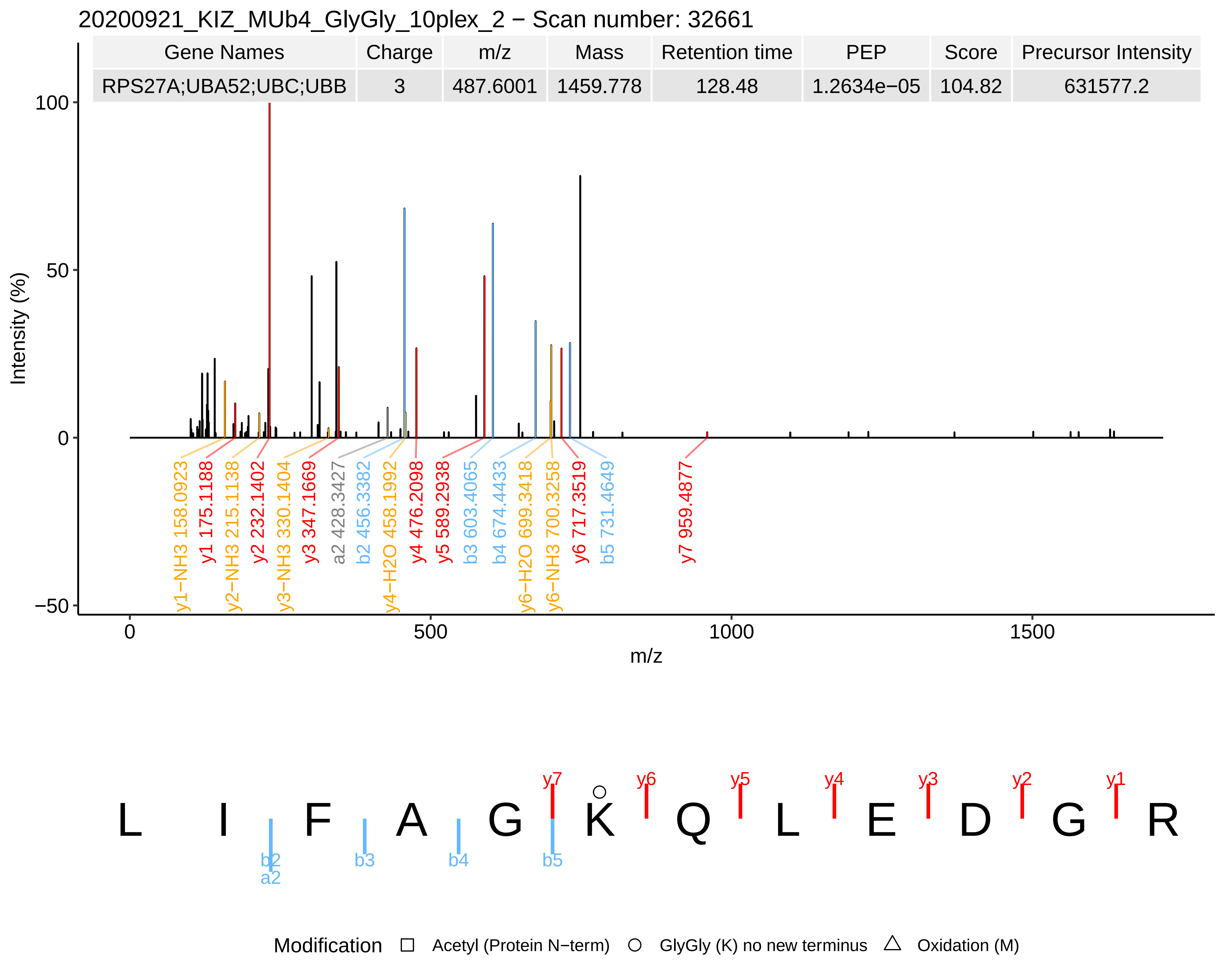
20200921_KIZ_MUb4_GlyGly_10plex_2 − Scan number: 32661
Gene Names
Charge
m/z
Mass
Retention time
PEP
Score
Precursor Intensity
RPS27A;UBA52;UBC;UBB
3
487.6001
1459.778
128.48
1.2634e−05
104.82
631577.2
Intensity (%)
100
50
0
−50
0
500
1000
1500
m/z
y1−NH3 158.0923
y1 175.1188
y2−NH3 215.1138
y2 232.1402
y3−NH3 330.1404
y3 347.1669
a2 428.3427
b2 456.3382
y4−H2O 458.1992
y4 476.2098
y5 589.2938
b3 603.4065
b4 674.4433
y6−H2O 699.3418
y6−NH3 700.3258
y6 717.3519
b5 731.4649
y7 959.4877
L I F A G K Q L E D G R
y7 y6 y5 y4 y3 y2 y1
b2 a2 b3 b4 b5
Modification
Acetyl (Protein N−term)
GlyGly (K) no new terminus
Oxidation (M)

20200921_KIZ_MUb4_GlyGly_10plex_2 − Scan number: 32804

Gene Names	Charge	m/z	Mass	Retention time	PEP	Score	Precursor Intensity
RPS27A;UBA52;UBC;UBB	3	487.6001	1459.778	128.99	0.00094847	84.507	1372980

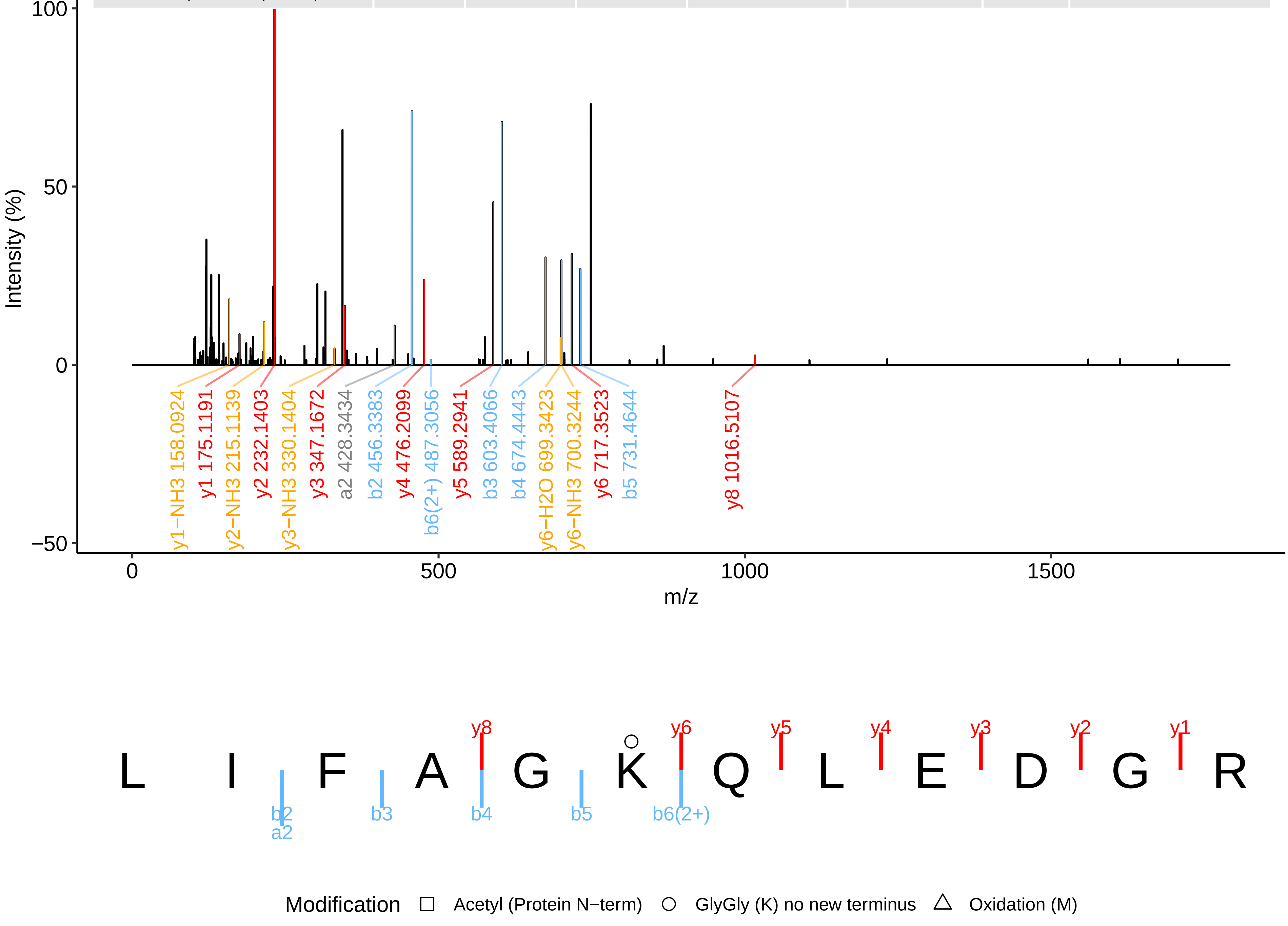

20200921_KIZ_MUb4_GlyGly_10plex_2 − Scan number: 32946

Gene Names	Charge	m/z	Mass	Retention time	PEP	Score	Precursor Intensity
RPS27A;UBA52;UBC;UBB	3	487.6001	1459.778	129.5	0.0004538	91.701	1096462

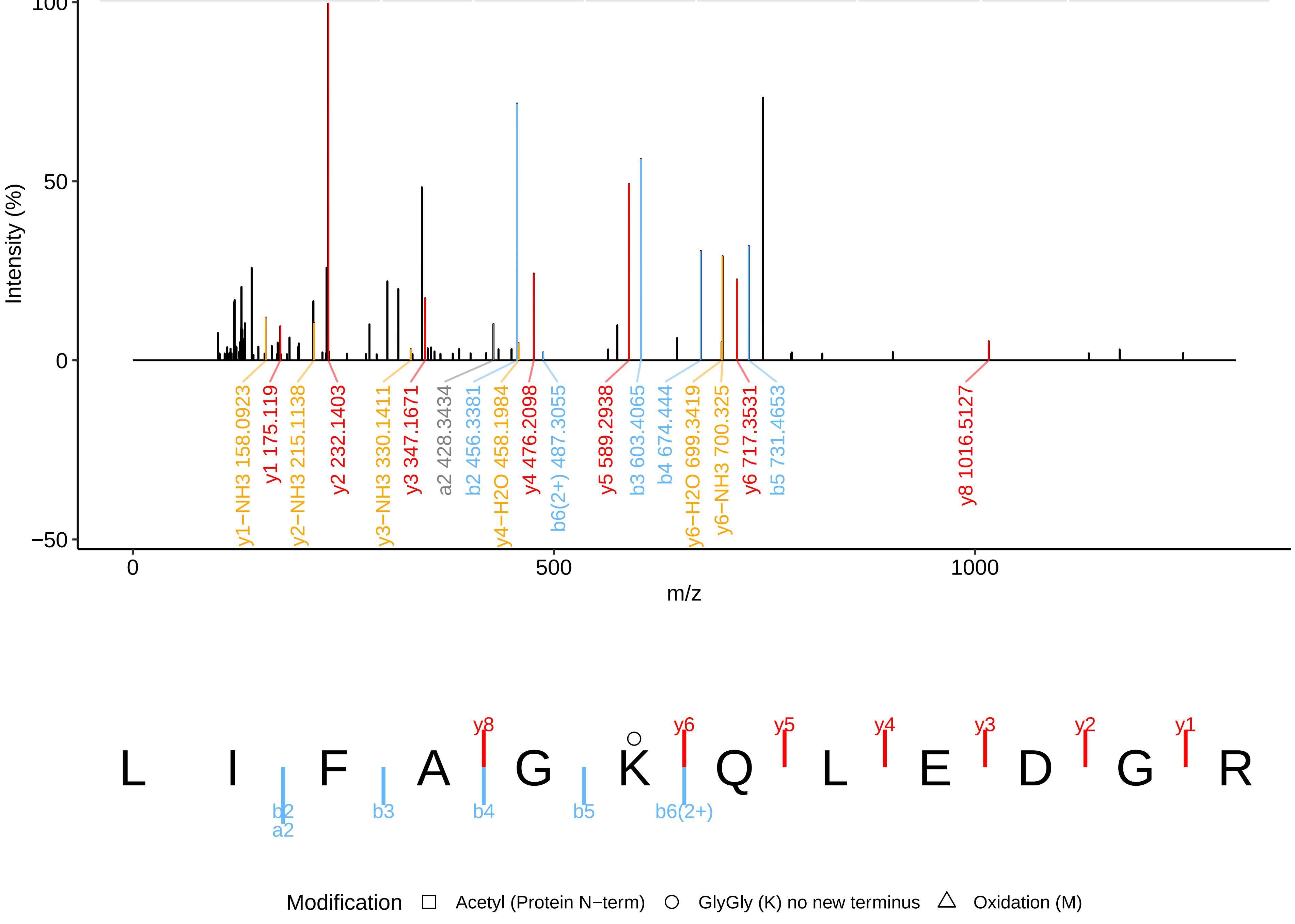

20200921_KIZ_MUb4_GlyGly_10plex_2 − Scan number: 13875

Gene Names	Charge	m/z	Mass	Retention time	PEP	Score	Precursor Intensity
RPS27A;UBA52;UBC;UBB	3	487.6001	1459.778	53.969	0.0013073	84.507	510401

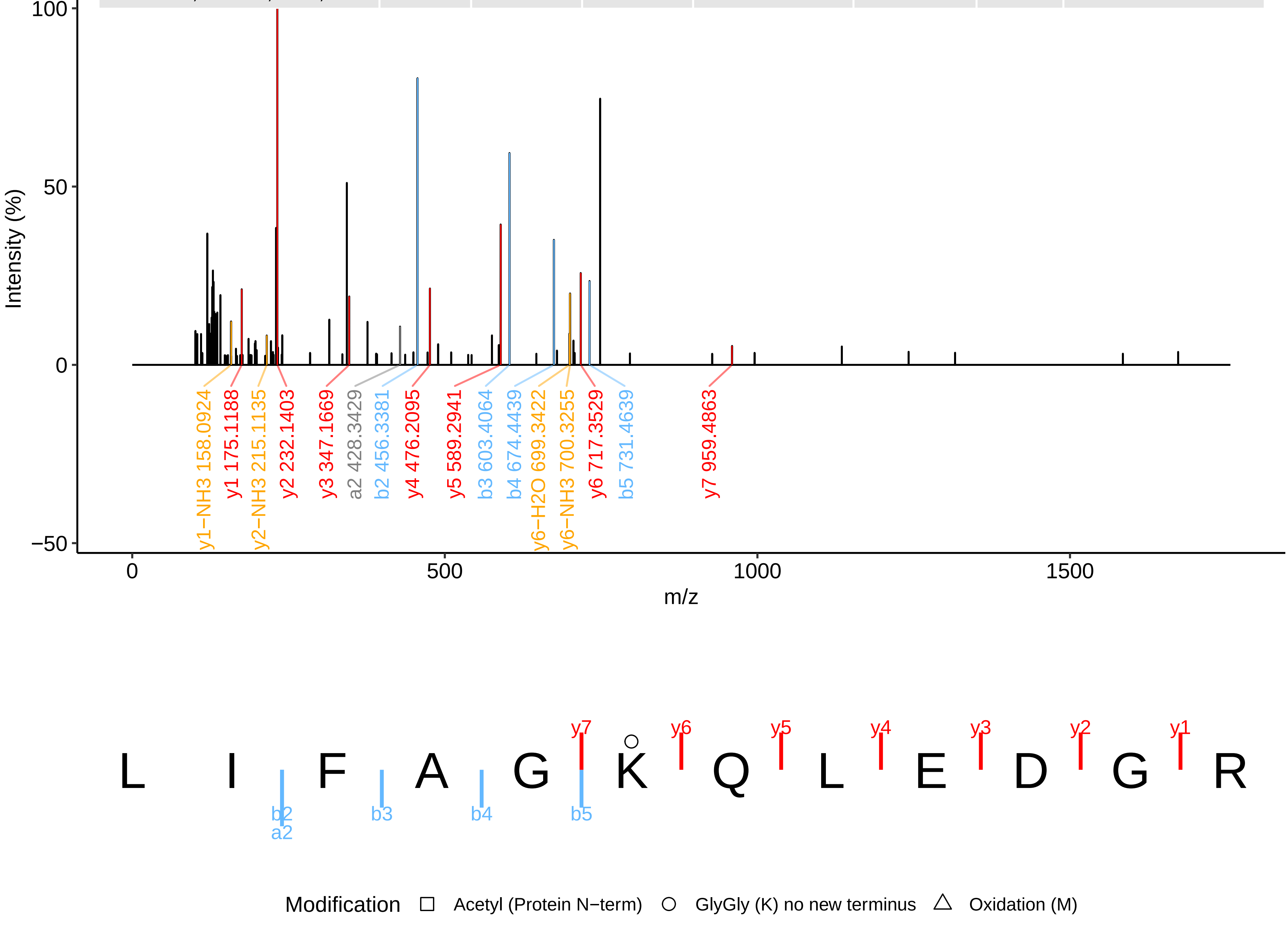

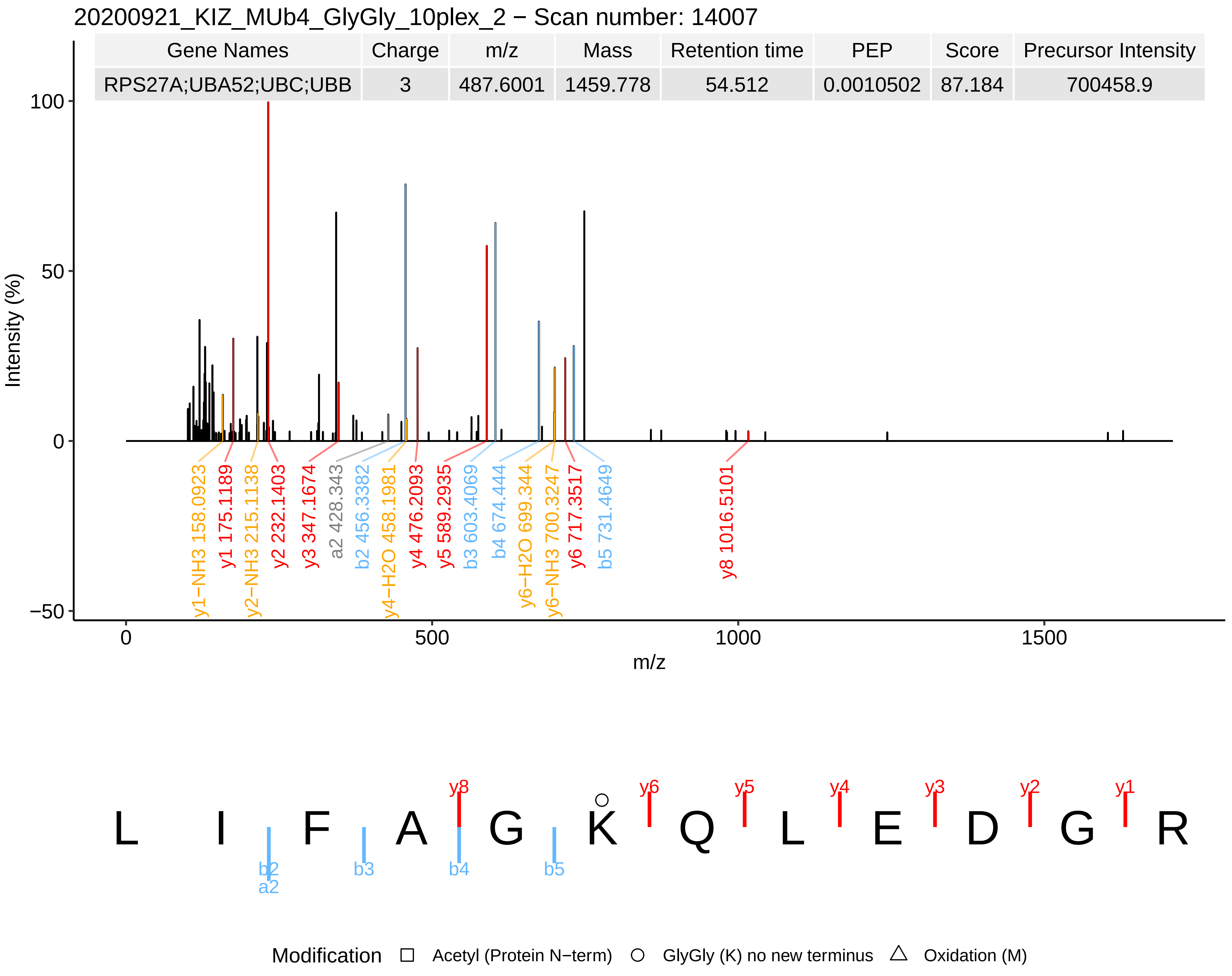
20200921_KIZ_MUb4_GlyGly_10plex_2 − Scan number: 14007
Gene Names | Charge | m/z | Mass | Retention time | PEP | Score | Precursor Intensity
RPS27A;UBA52;UBC;UBB | 3 | 487.6001 | 1459.778 | 54.512 | 0.0010502 | 87.184 | 700458.9
Intensity (%)
100
50
0
−50
y1−NH3 158.0923
y1 175.1189
y2−NH3 215.1138
y2 232.1403
y3 347.1674
a2 428.343
b2 456.3382
y4−H2O 458.1981
y4 476.2093
y5 589.2935
b3 603.4069
b4 674.444
y6−H2O 699.344
y6−NH3 700.3247
y6 717.3517
b5 731.4649
y8 1016.5101
0
500
1000
1500
m/z
L I F A G K Q L E D G R
y8 y6 y5 y4 y3 y2 y1
b2 a2 b3 b4 b5
Modification
Acetyl (Protein N−term)
GlyGly (K) no new terminus
Oxidation (M)

20200921_KIZ_MUb4_GlyGly_10plex_2 − Scan number: 14236

Gene Names	Charge	m/z	Mass	Retention time	PEP	Score	Precursor Intensity
RPS27A;UBA52;UBC;UBB	3	487.6001	1459.778	55.466	0.0010134	87.568	673598.9

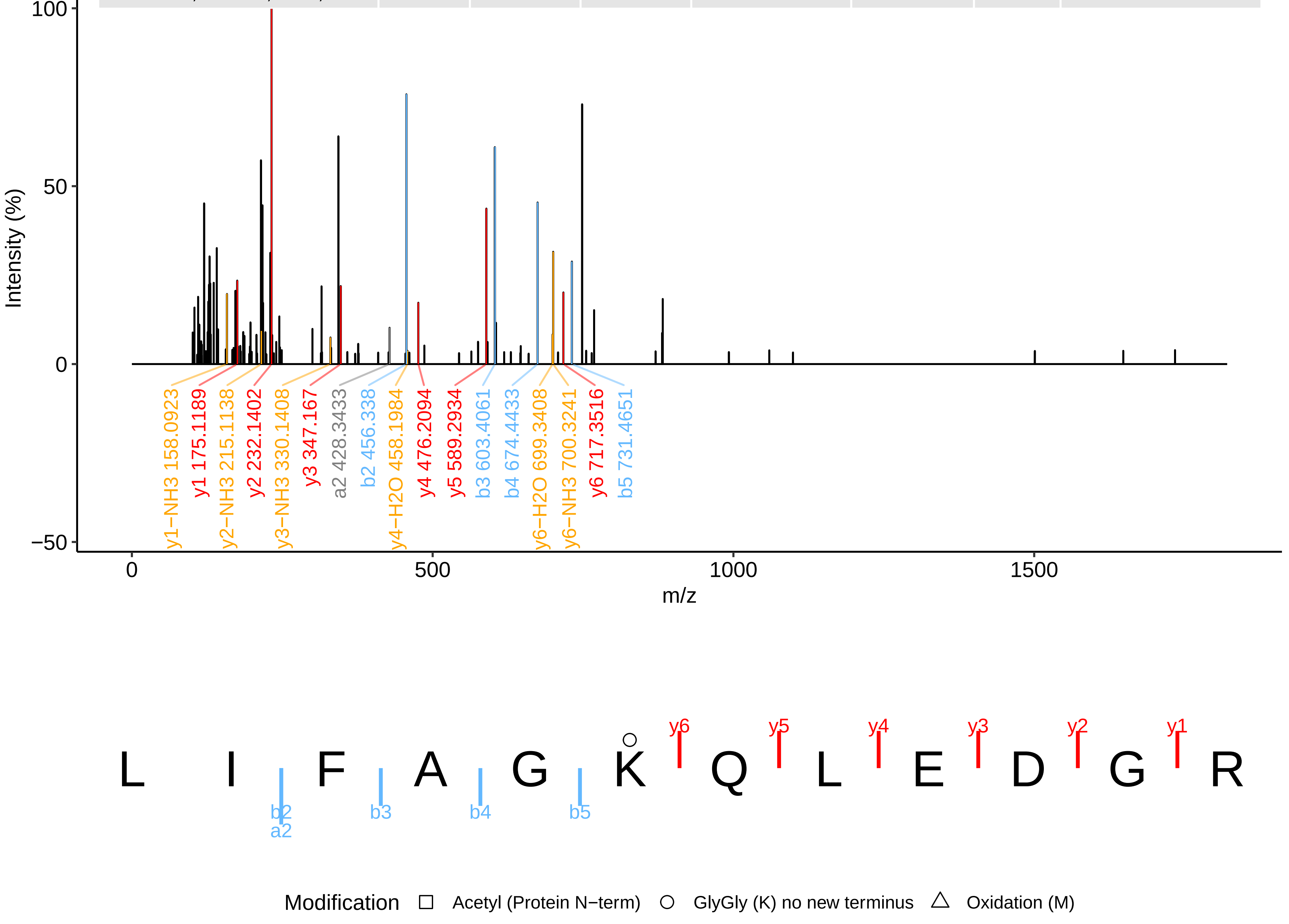

20200921_KIZ_MUb4_GlyGly_10plex_2 − Scan number: 14650

Gene Names	Charge	m/z	Mass	Retention time	PEP	Score	Precursor Intensity
RPS27A;UBA52;UBC;UBB	3	487.6001	1459.778	57.148	0.00052916	92.611	1851769

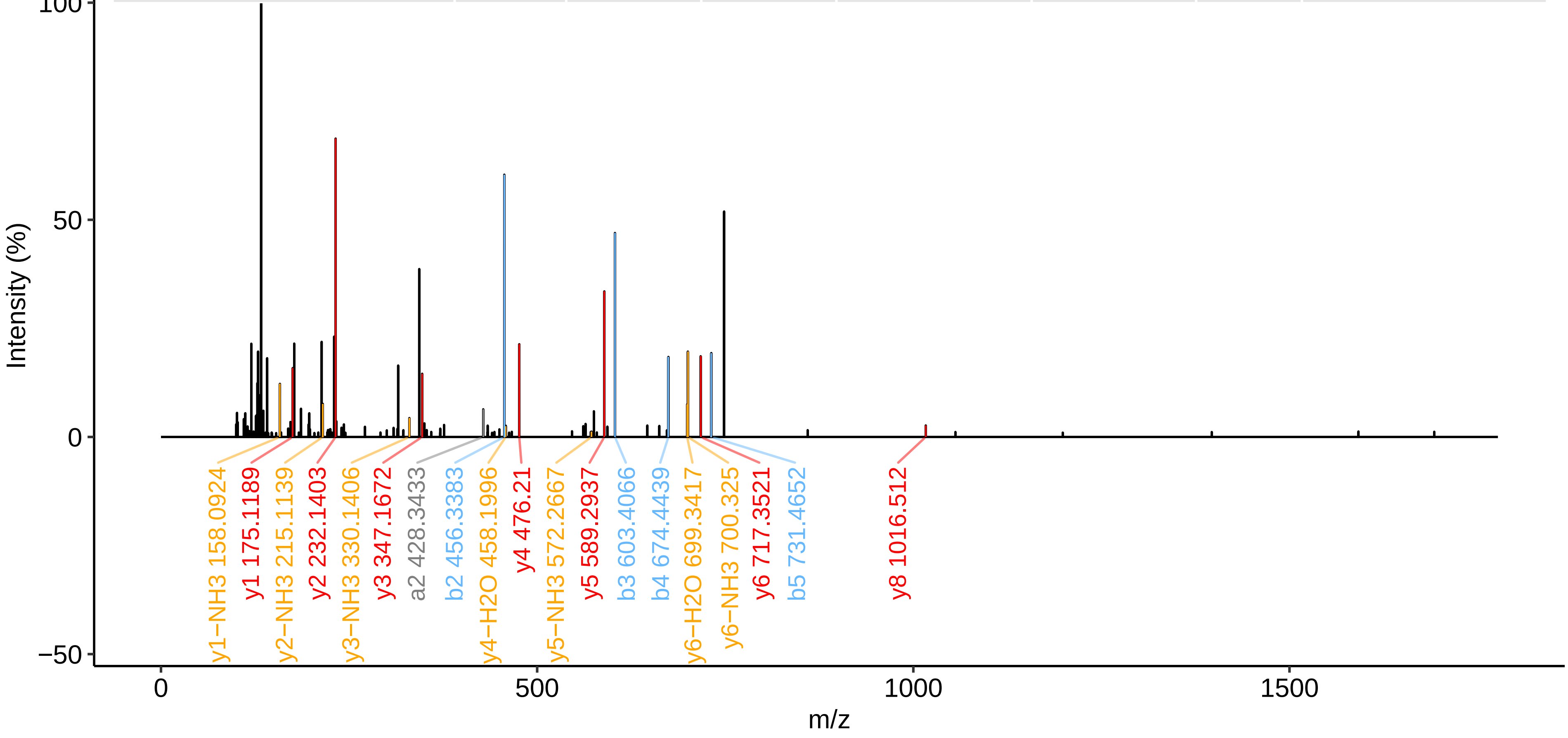

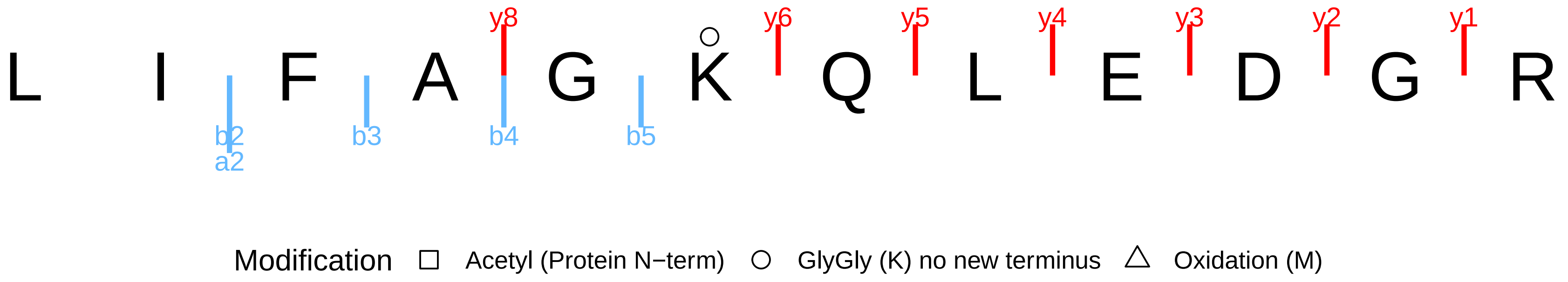

20200921_KIZ_MUb4_GlyGly_10plex_2 − Scan number: 14776

Gene Names	Charge	m/z	Mass	Retention time	PEP	Score	Precursor Intensity
RPS27A;UBA52;UBC;UBB	3	487.6001	1459.778	57.667	5.0692e−05	97.734	1695379

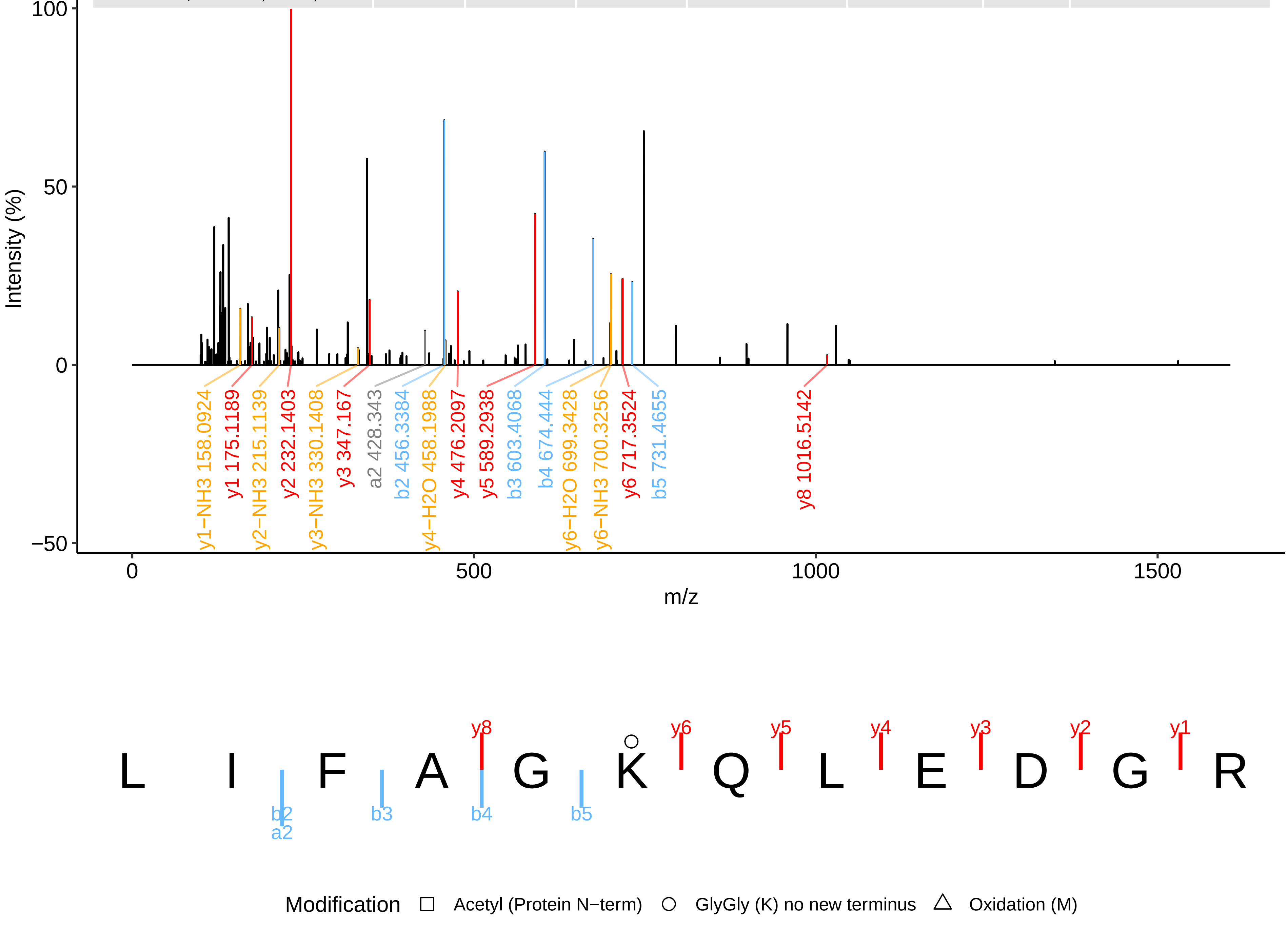

20200921_KIZ_MUb4_GlyGly_10plex_2 − Scan number: 14907

Gene Names	Charge	m/z	Mass	Retention time	PEP	Score	Precursor Intensity
RPS27A;UBA52;UBC;UBB	3	487.6001	1459.778	58.193	3.3439e−05	105.2	2190878

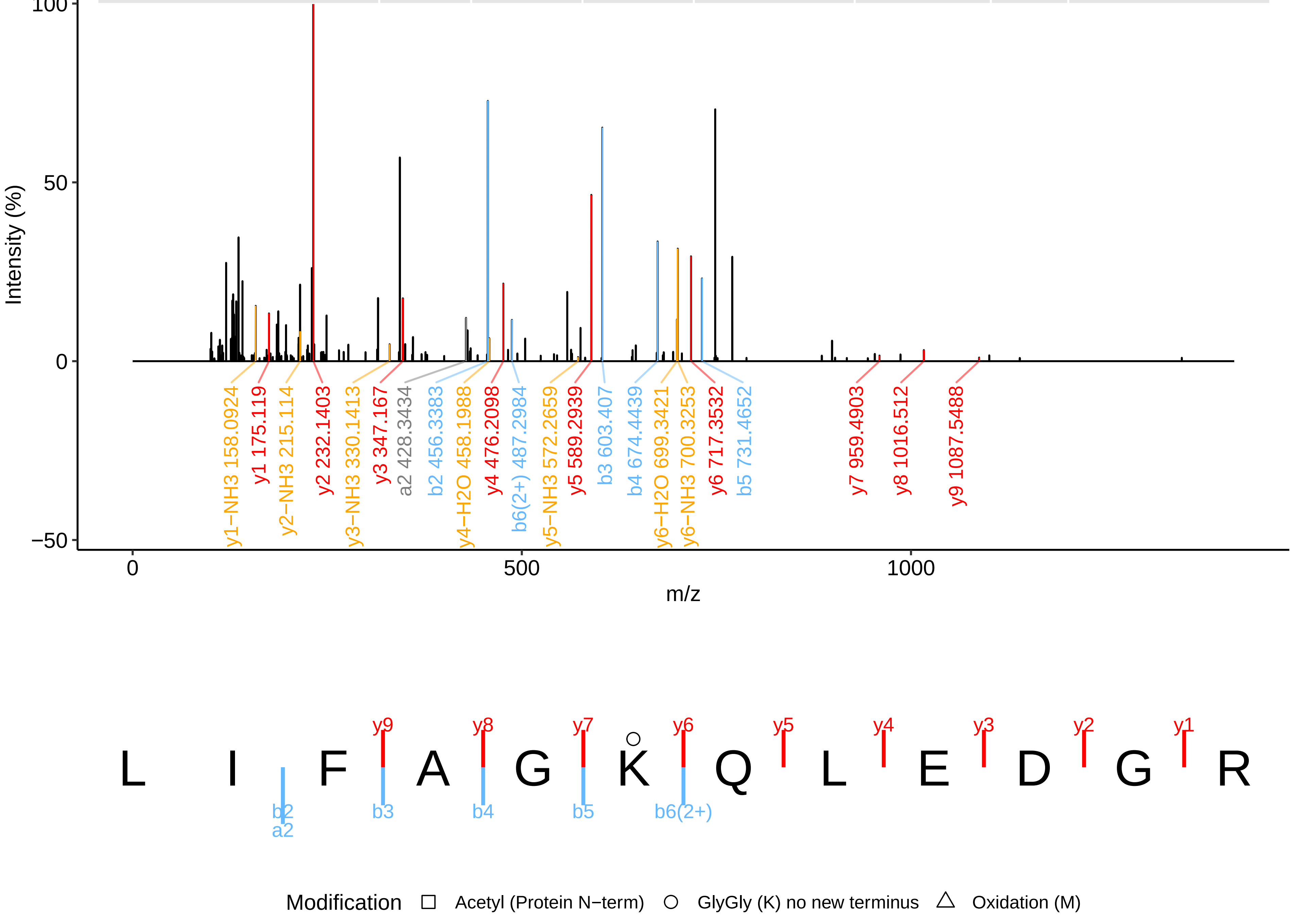

20200921_KIZ_MUb4_GlyGly_10plex_2 − Scan number: 15053

Gene Names	Charge	m/z	Mass	Retention time	PEP	Score	Precursor Intensity
RPS27A;UBA52;UBC;UBB	3	487.6001	1459.778	58.789	1.3883e−05	113.67	1888706

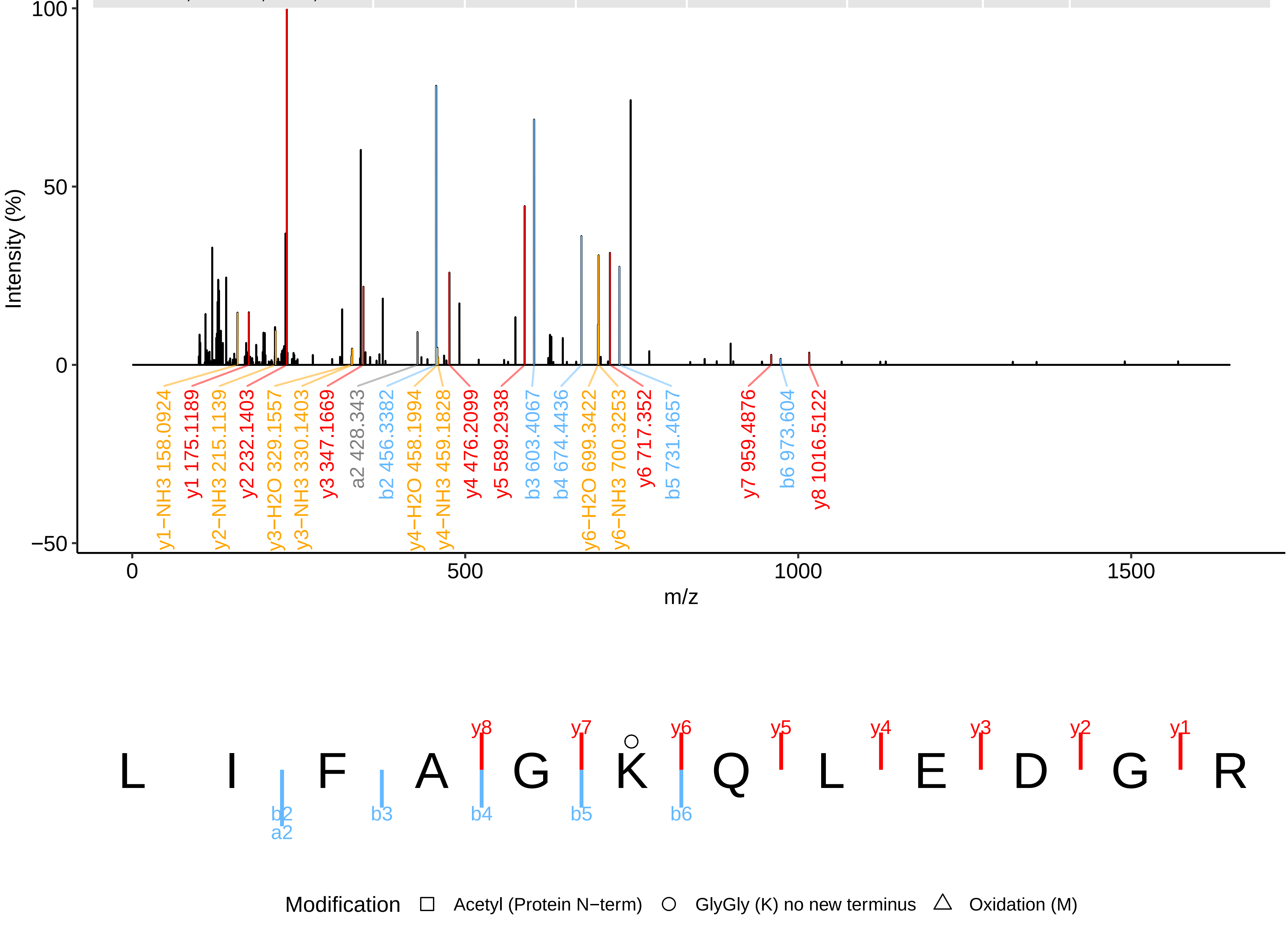

20200921_KIZ_MUb4_GlyGly_10plex_2 − Scan number: 17249

Gene Names	Charge	m/z	Mass	Retention time	PEP	Score	Precursor Intensity
RPS27A;UBA52;UBC;UBB	2	730.8964	1459.778	67.43	0.0054943	90.629	931938.6

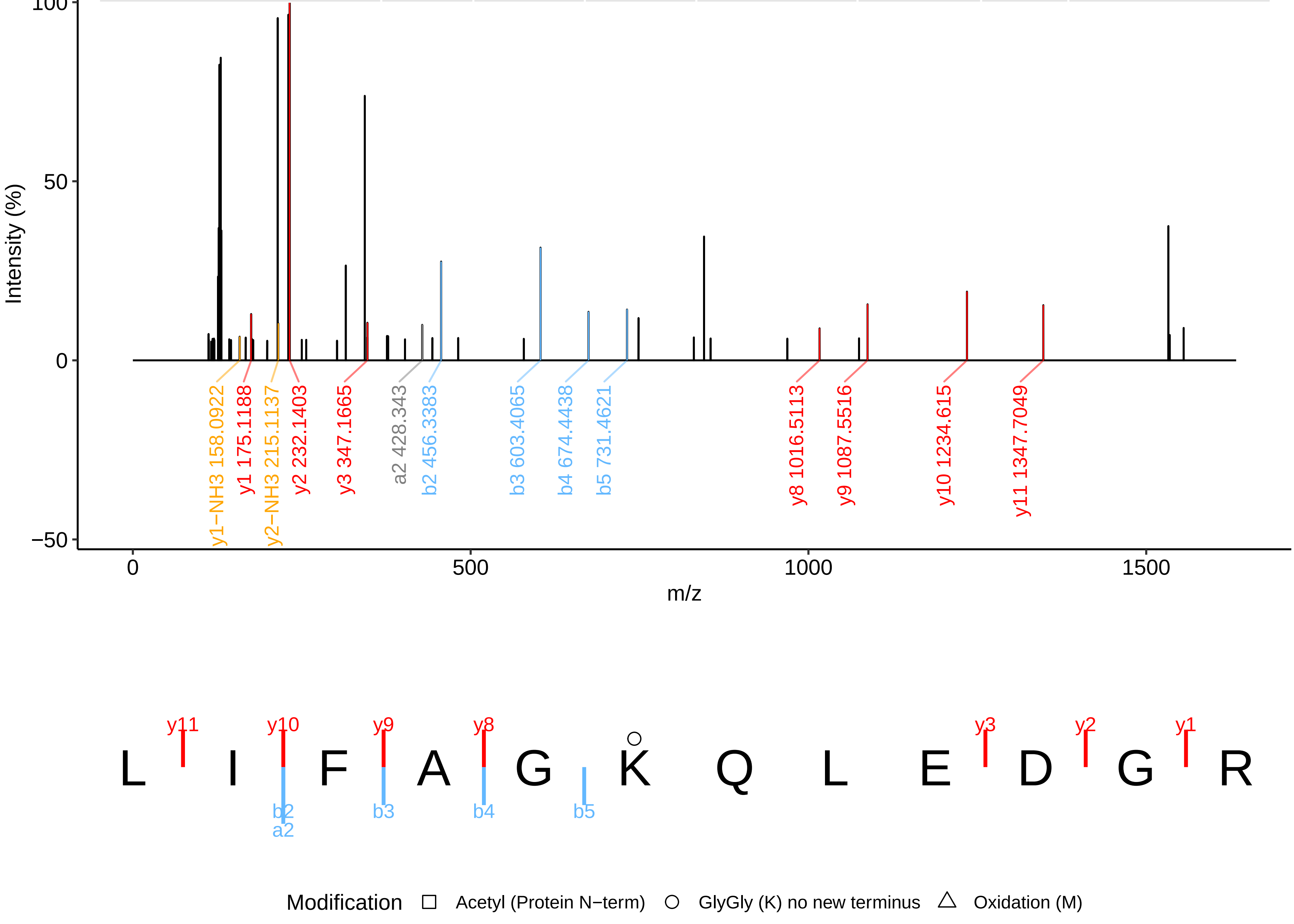

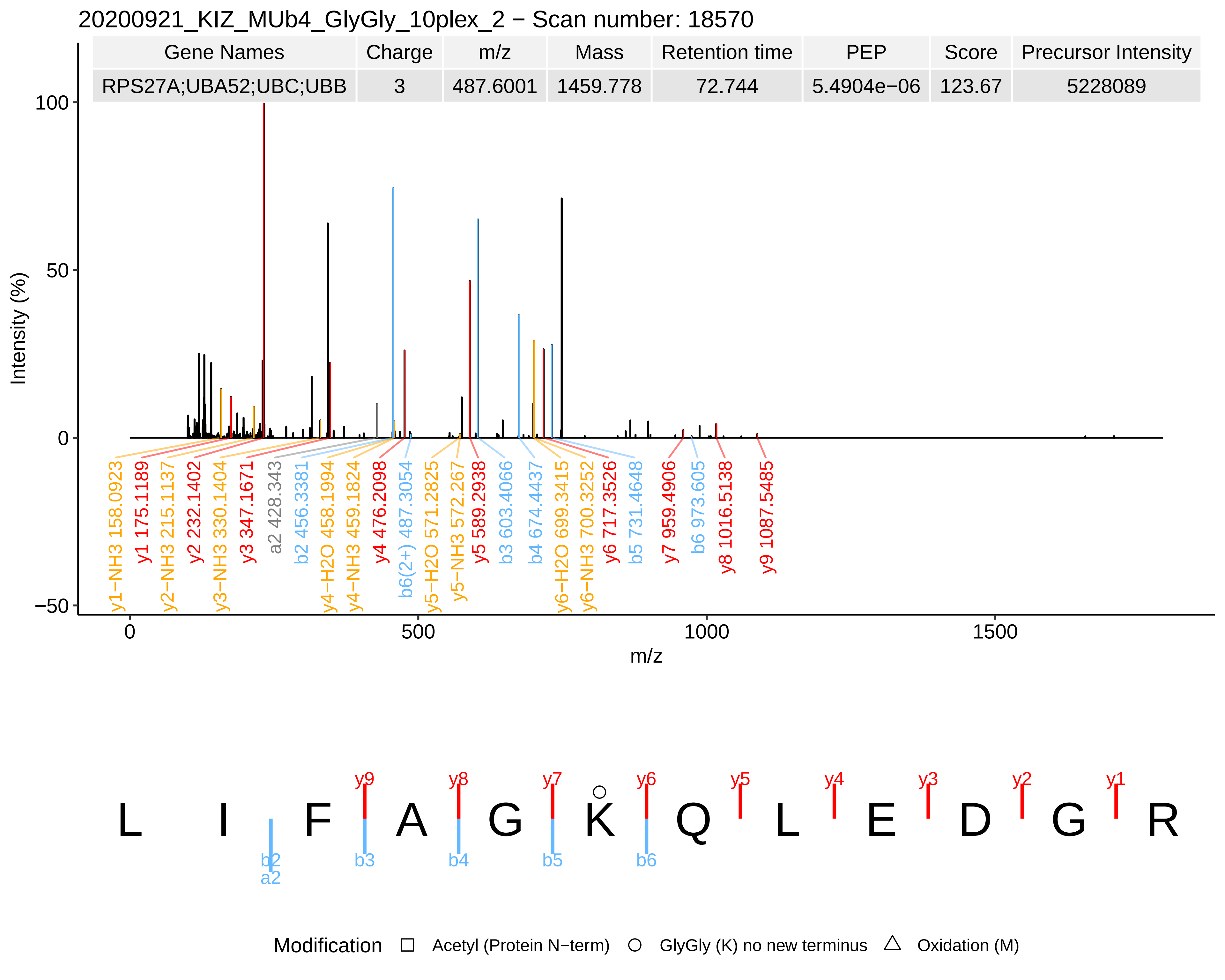
20200921_KIZ_MUb4_GlyGly_10plex_2 − Scan number: 18570
Gene Names
RPS27A;UBA52;UBC;UBB
Charge
3
m/z
487.6001
Mass
1459.778
Retention time
72.744
PEP
5.4904e−06
Score
123.67
Precursor Intensity
5228089
Intensity (%)
100
50
0
−50
0
500
1000
1500
m/z
y1−NH3 158.0923
y1 175.1189
y2−NH3 215.1137
y2 232.1402
y3−NH3 330.1404
y3 347.1671
a2 428.343
b2 456.3381
y4−H2O 458.1994
y4−NH3 459.1824
y4 476.2098
b6(2+) 487.3054
y5−H2O 571.2825
y5−NH3 572.267
y5 589.2938
b3 603.4066
b4 674.4437
y6−H2O 699.3415
y6−NH3 700.3252
y6 717.3526
b5 731.4648
y7 959.4906
b6 973.605
y8 1016.5138
y9 1087.5485
L I F A G K Q L E D G R
y9 y8 y7 y6 y5 y4 y3 y2 y1
b2 a2 b3 b4 b5 b6
Modification
Acetyl (Protein N−term)
GlyGly (K) no new terminus
Oxidation (M)

20200921_KIZ_MUb4_GlyGly_10plex_2 − Scan number: 19453

Gene Names	Charge	m/z	Mass	Retention time	PEP	Score	Precursor Intensity
RPS27A;UBA52;UBC;UBB	3	487.6001	1459.778	76.356	2.9172e−06	127.87	5343912

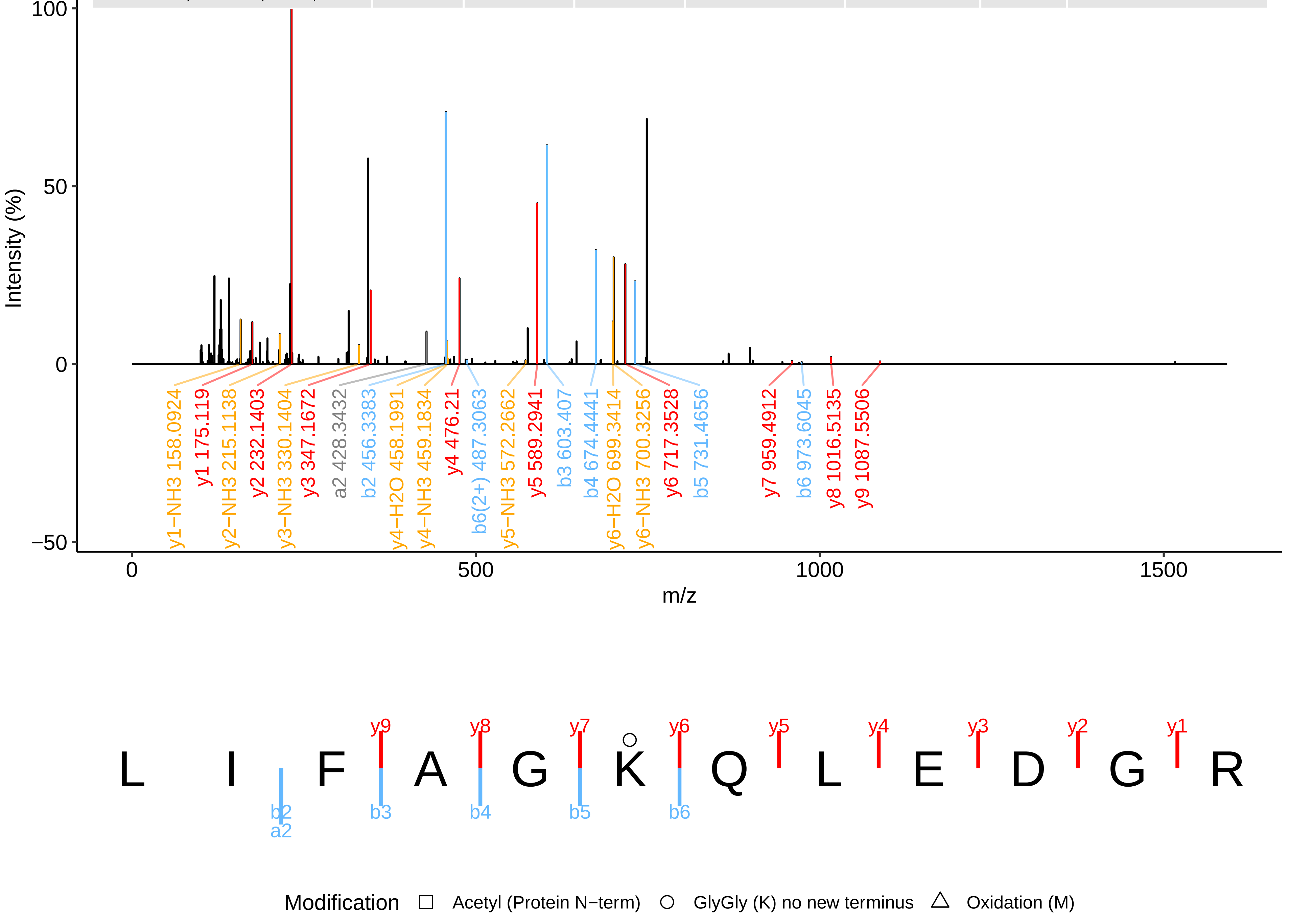

20200921_KIZ_MUb4_GlyGly_10plex_2 − Scan number: 19673

Gene Names	Charge	m/z	Mass	Retention time	PEP	Score	Precursor Intensity
RPS27A;UBA52;UBC;UBB	3	487.6001	1459.778	77.242	0.00052916	92.611	NA

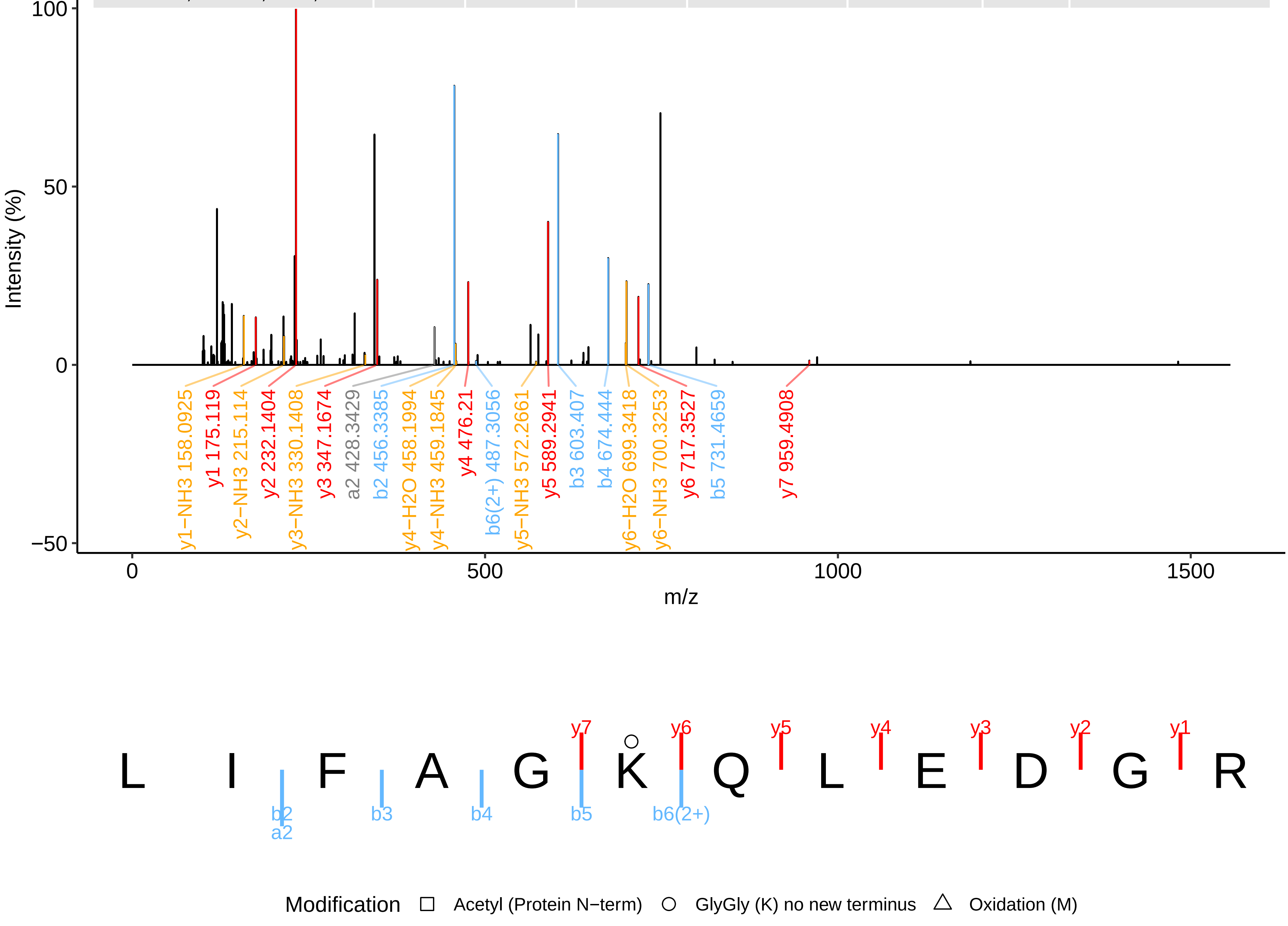

20200921_KIZ_MUb4_GlyGly_10plex_2 − Scan number: 19714

Gene Names	Charge	m/z	Mass	Retention time	PEP	Score	Precursor Intensity
RPS27A;UBA52;UBC;UBB	3	487.6001	1459.778	77.409	9.4972e−06	117.14	6684238

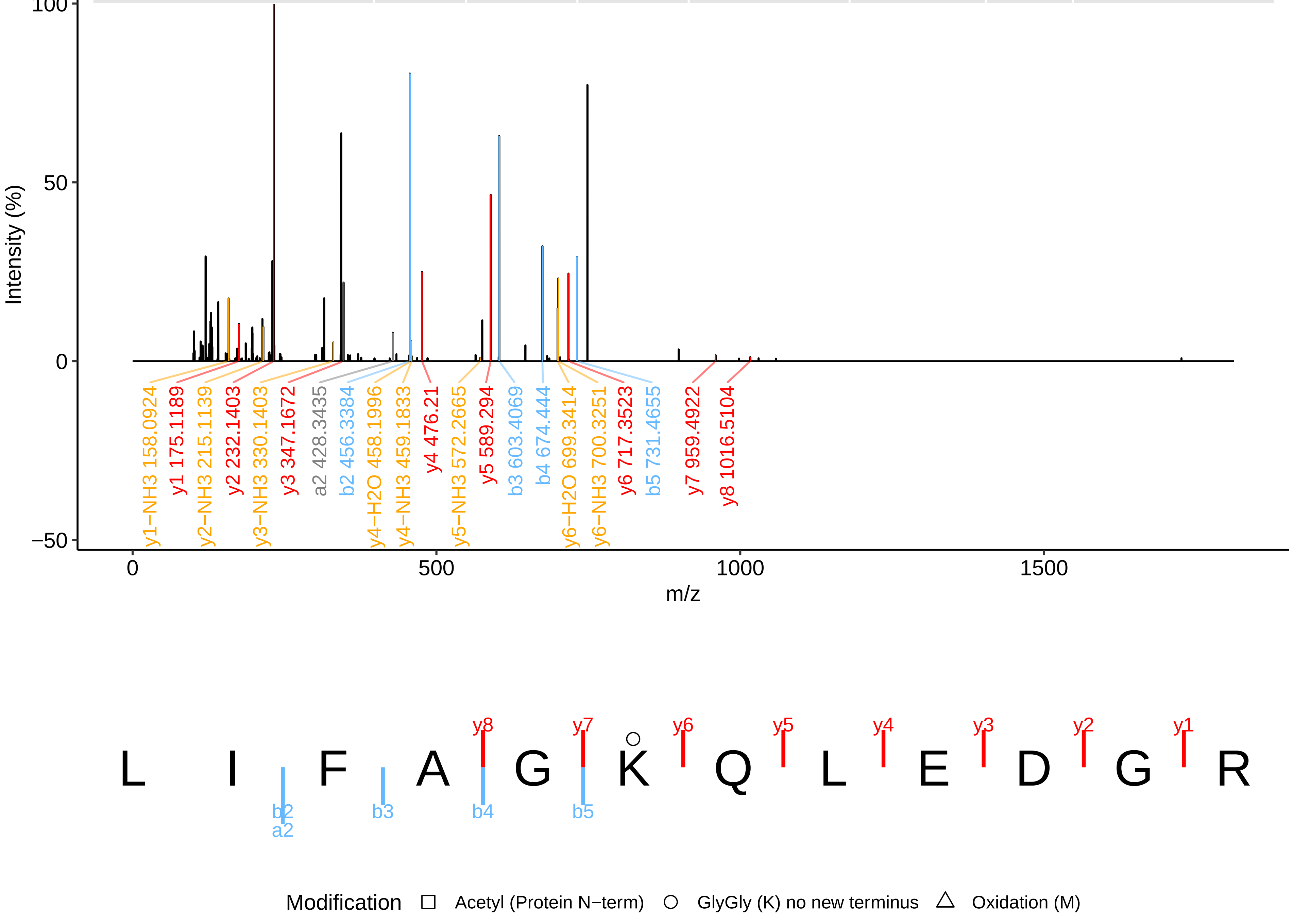

20200921_KIZ_MUb4_GlyGly_10plex_2 − Scan number: 19840

Gene Names	Charge	m/z	Mass	Retention time	PEP	Score	Precursor Intensity
RPS27A;UBA52;UBC;UBB	3	487.6001	1459.778	77.929	3.7869e−11	149.96	5737094

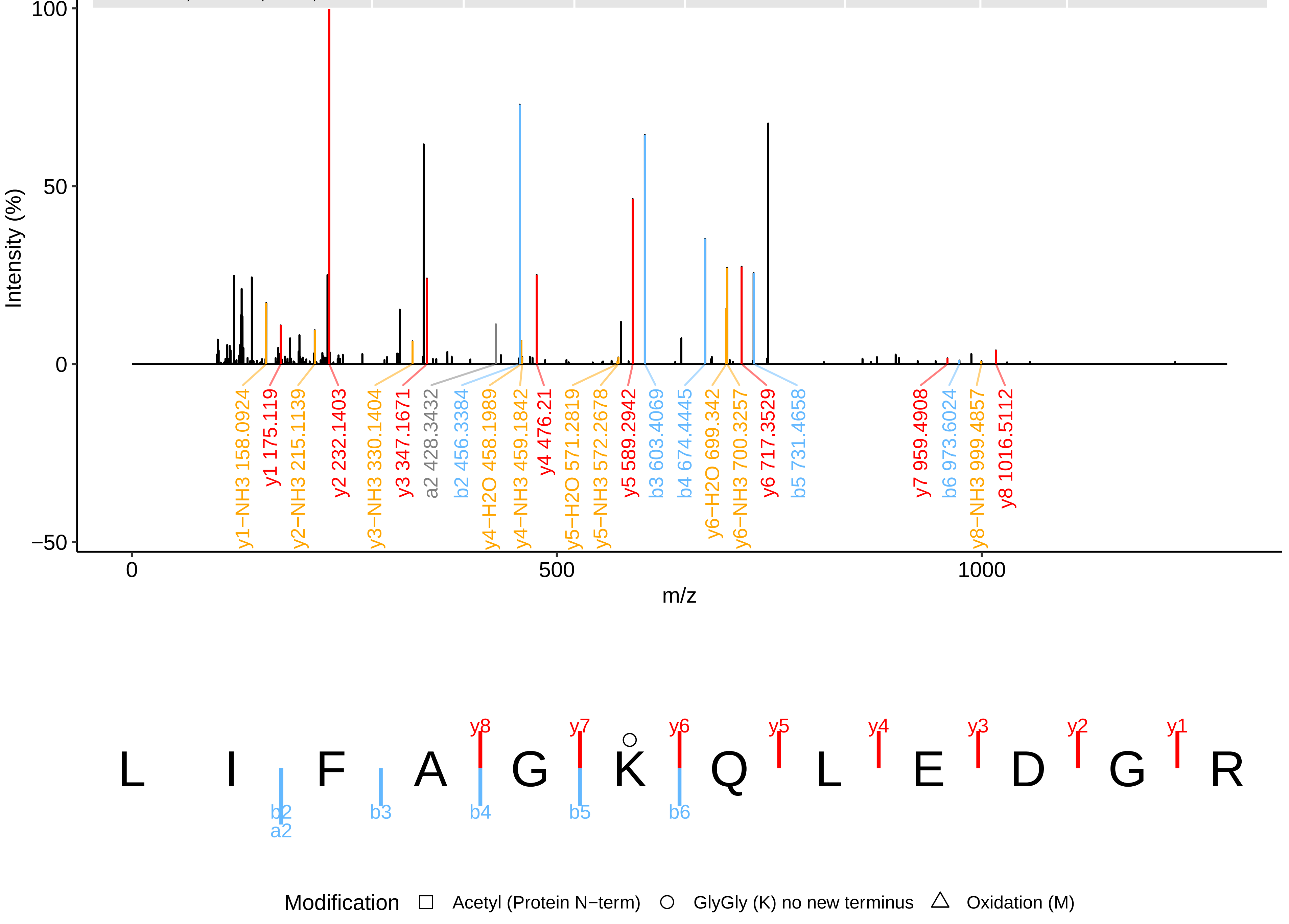

20200921_KIZ_MUb4_GlyGly_10plex_2 − Scan number: 20824

Gene Names	Charge	m/z	Mass	Retention time	PEP	Score	Precursor Intensity
RPS27A;UBA52;UBC;UBB	3	487.6001	1459.778	81.912	5.8972e−06	123.01	3633734

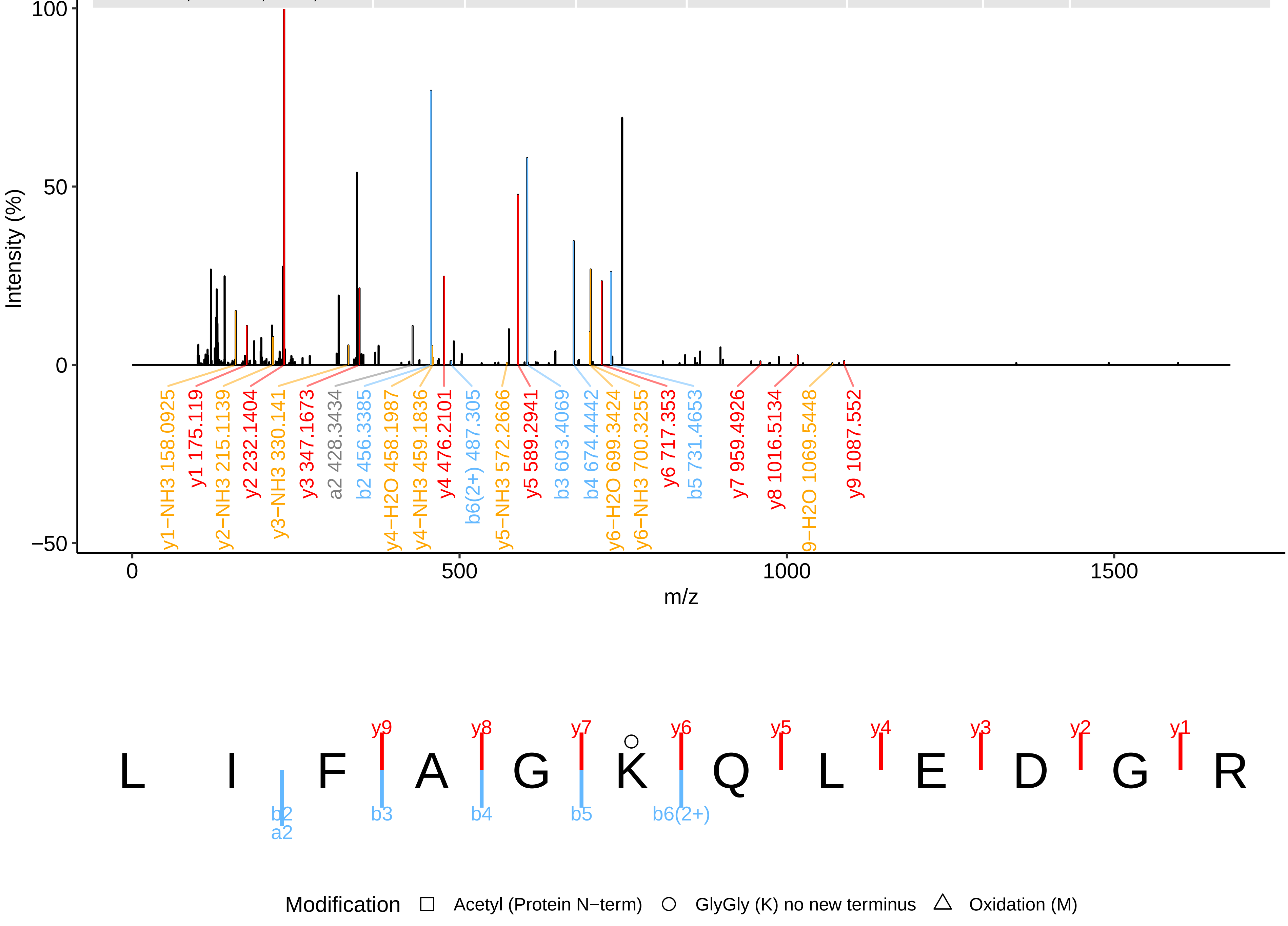

20200921_KIZ_MUb4_GlyGly_10plex_2 − Scan number: 23257

Gene Names	Charge	m/z	Mass	Retention time	PEP	Score	Precursor Intensity
RPS27A;UBA52;UBC;UBB	3	487.6001	1459.778	91.777	1.8076e−05	111.86	2054215

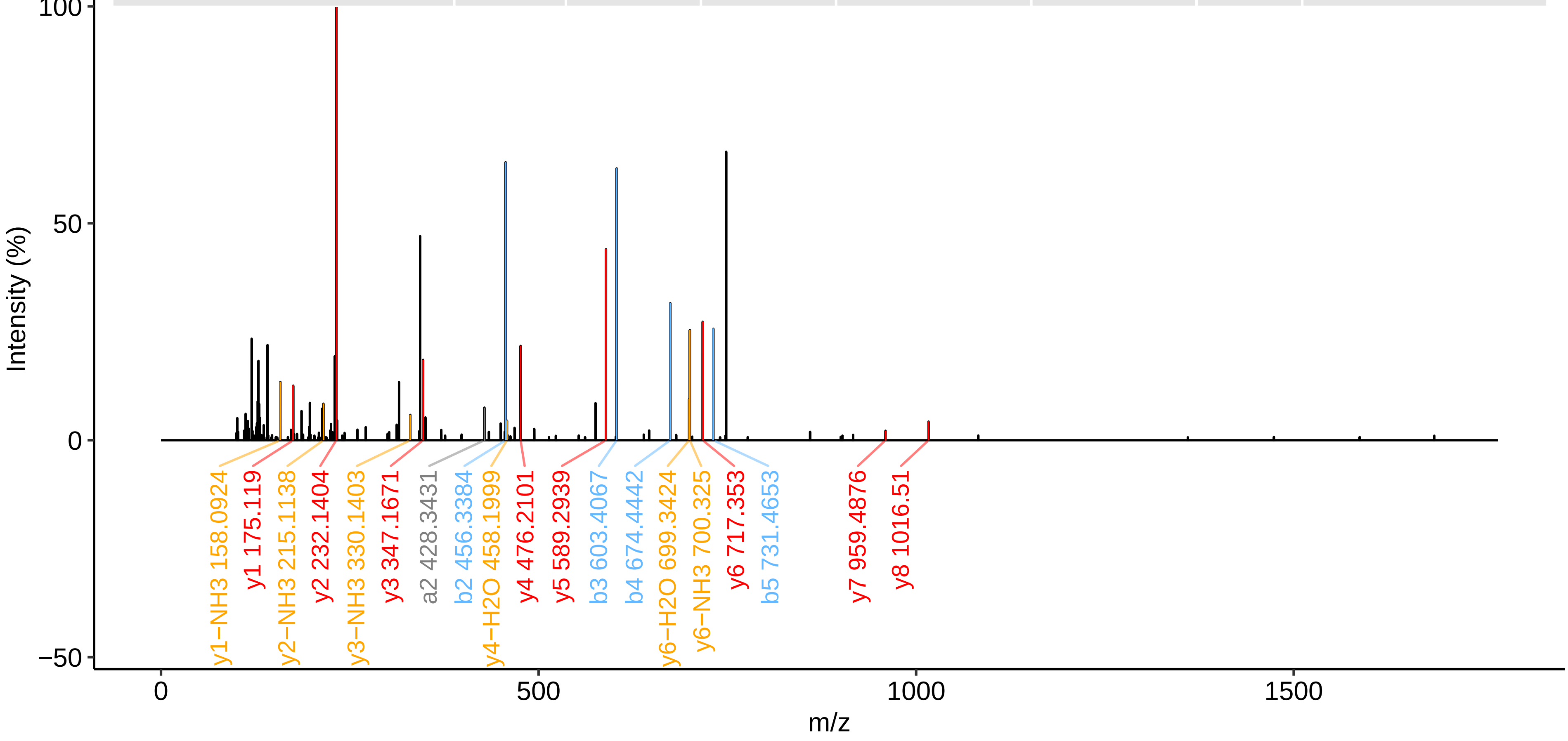

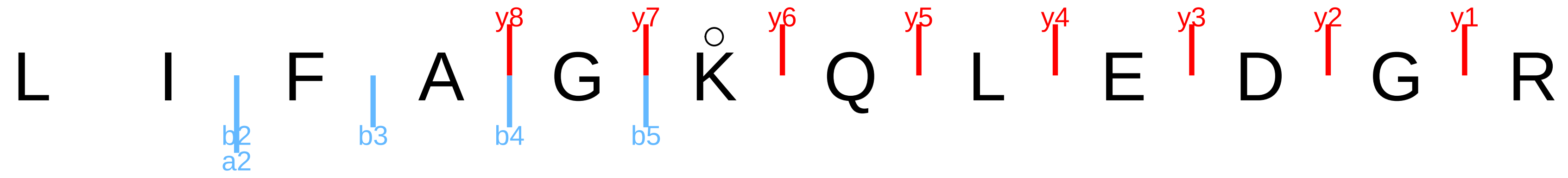

Modification □ Acetyl (Protein N−term) ○ GlyGly (K) no new terminus △ Oxidation (M)

20200921_KIZ_MUb4_GlyGly_10plex_2 − Scan number: 23385

Gene Names	Charge	m/z	Mass	Retention time	PEP	Score	Precursor Intensity
RPS27A;UBA52;UBC;UBB	3	487.6001	1459.778	92.289	4.8548e−08	143.24	4400253

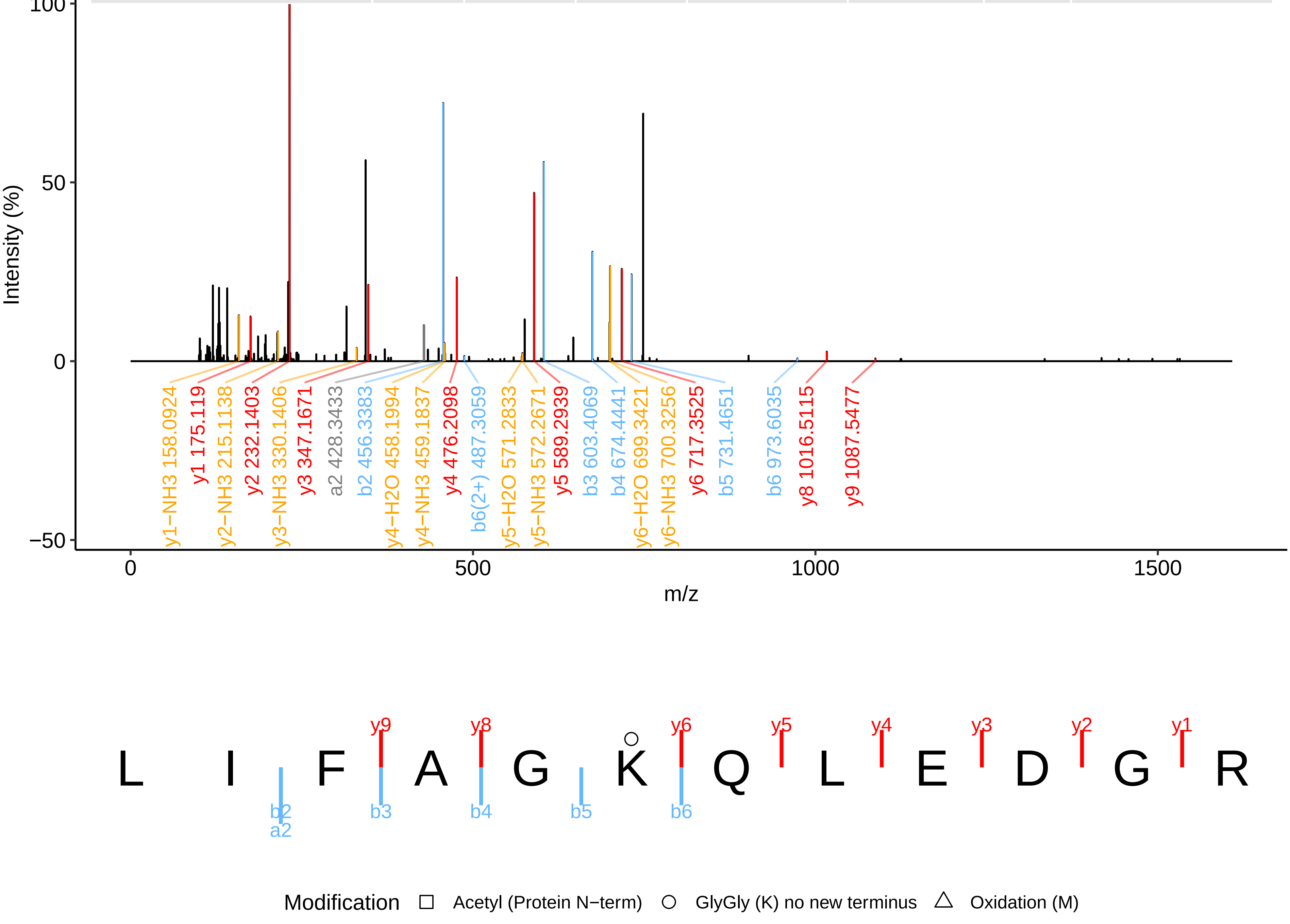

20200921_KIZ_MUb4_GlyGly_10plex_2 − Scan number: 23638

Gene Names	Charge	m/z	Mass	Retention time	PEP	Score	Precursor Intensity
RPS27A;UBA52;UBC;UBB	3	487.6001	1459.778	93.312	3.6779e−11	150.46	3588673

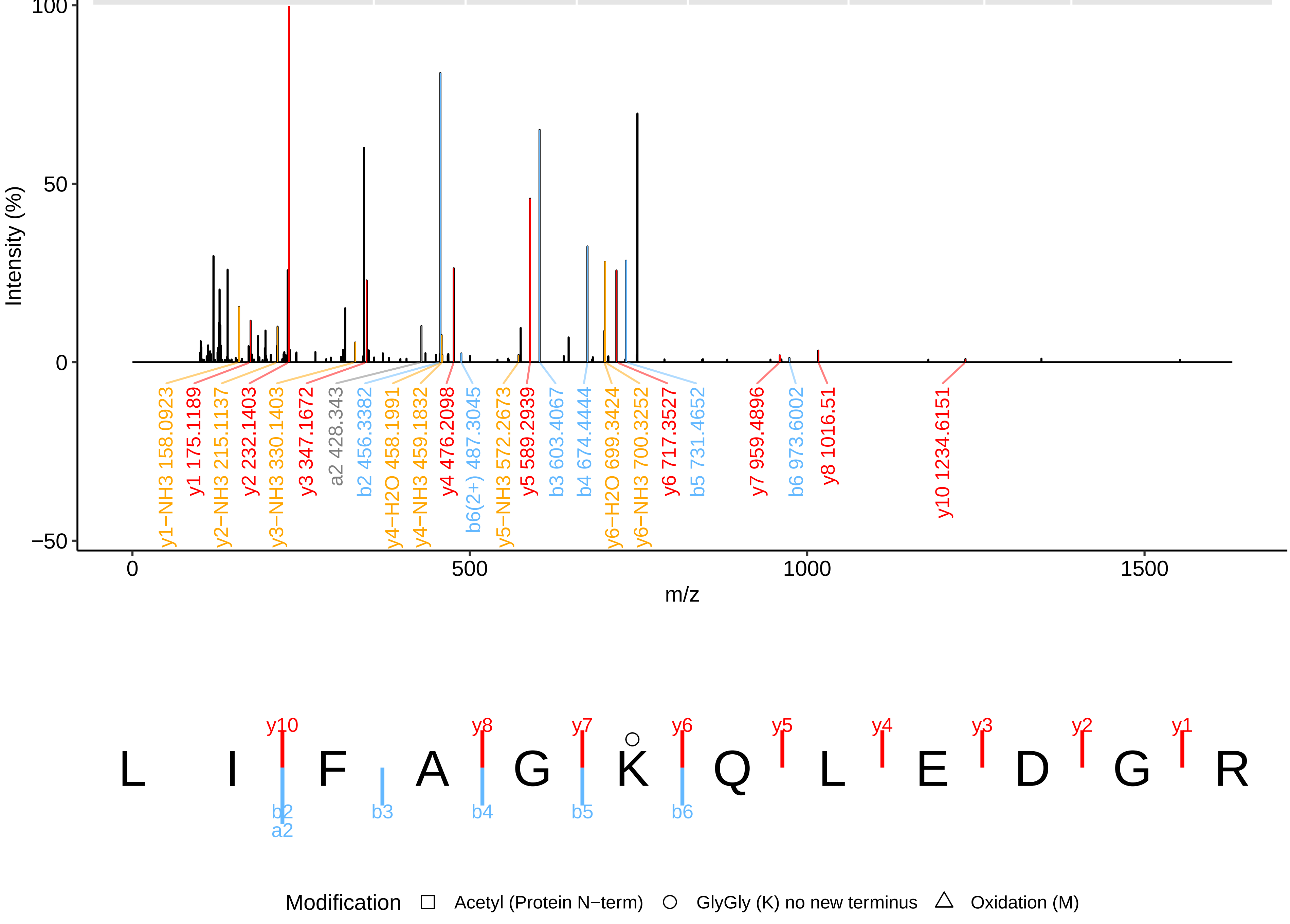

20200921_KIZ_MUb4_GlyGly_10plex_2 − Scan number: 23889

Gene Names	Charge	m/z	Mass	Retention time	PEP	Score	Precursor Intensity
RPS27A;UBA52;UBC;UBB	3	487.6001	1459.778	94.331	1.1732e−07	133.89	2952961

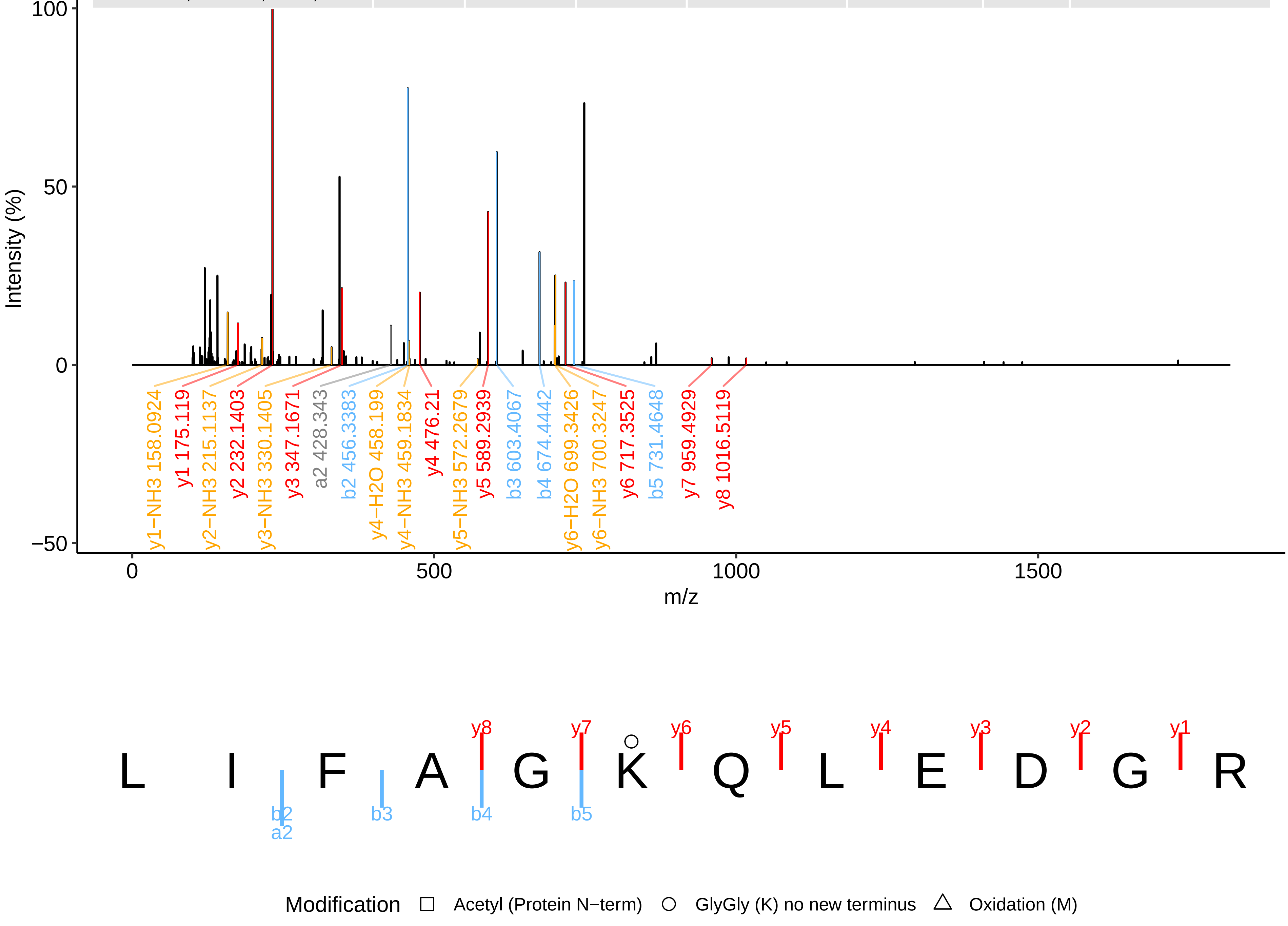

20200921_KIZ_MUb4_GlyGly_10plex_2 − Scan number: 24649

Gene Names	Charge	m/z	Mass	Retention time	PEP	Score	Precursor Intensity
RPS27A;UBA52;UBC;UBB	3	487.6001	1459.778	97.404	9.4268e−06	117.25	NA

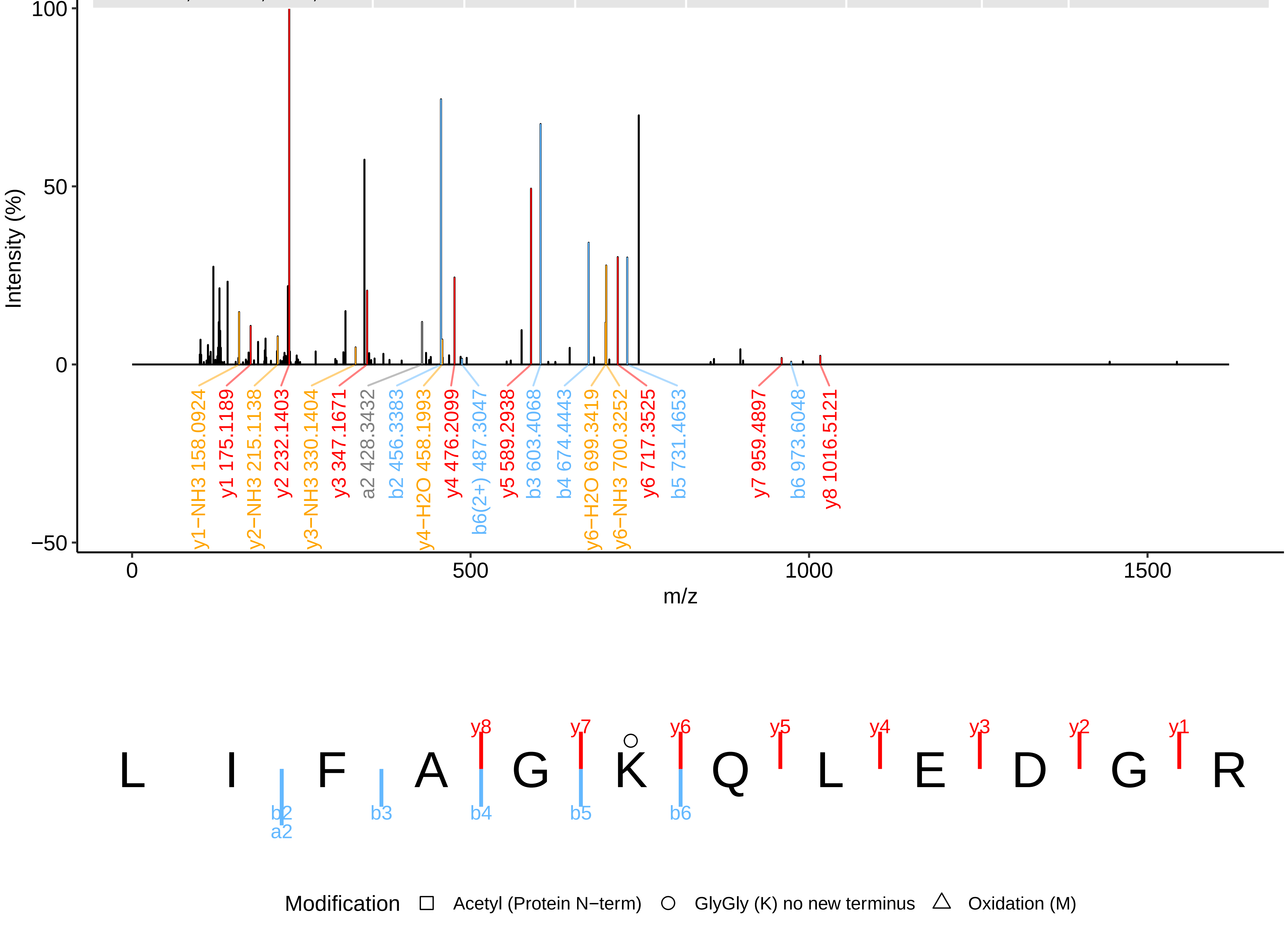

20200921_KIZ_MUb4_GlyGly_10plex_2 − Scan number: 25274

Gene Names	Charge	m/z	Mass	Retention time	PEP	Score	Precursor Intensity
RPS27A;UBA52;UBC;UBB	3	487.6001	1459.778	99.961	7.9801e−08	138.99	2146461

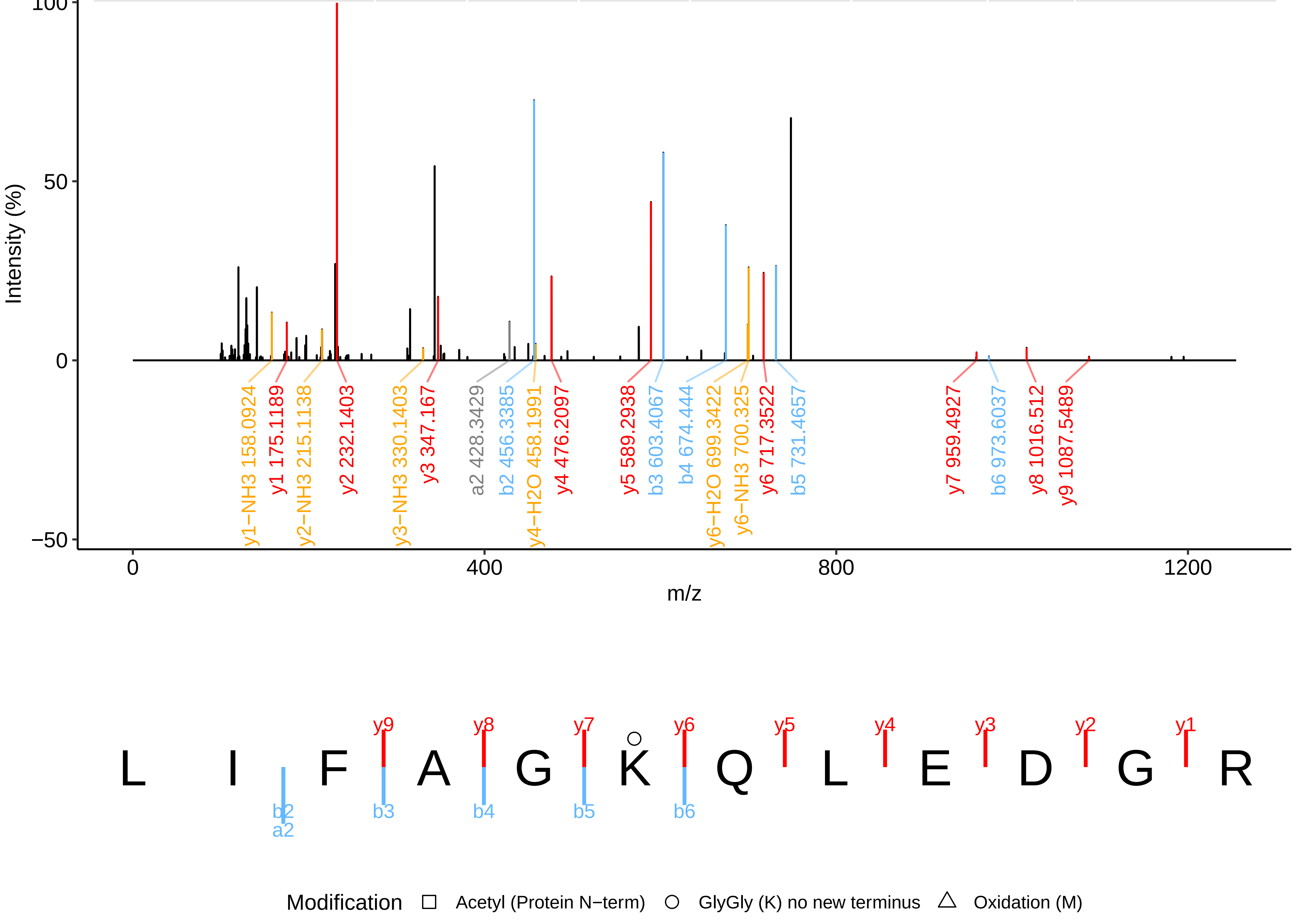

20200921_KIZ_MUb4_GlyGly_10plex_2 − Scan number: 25403

Gene Names	Charge	m/z	Mass	Retention time	PEP	Score	Precursor Intensity
RPS27A;UBA52;UBC;UBB	3	487.6001	1459.778	100.47	8.1861e−06	119.27	2344469

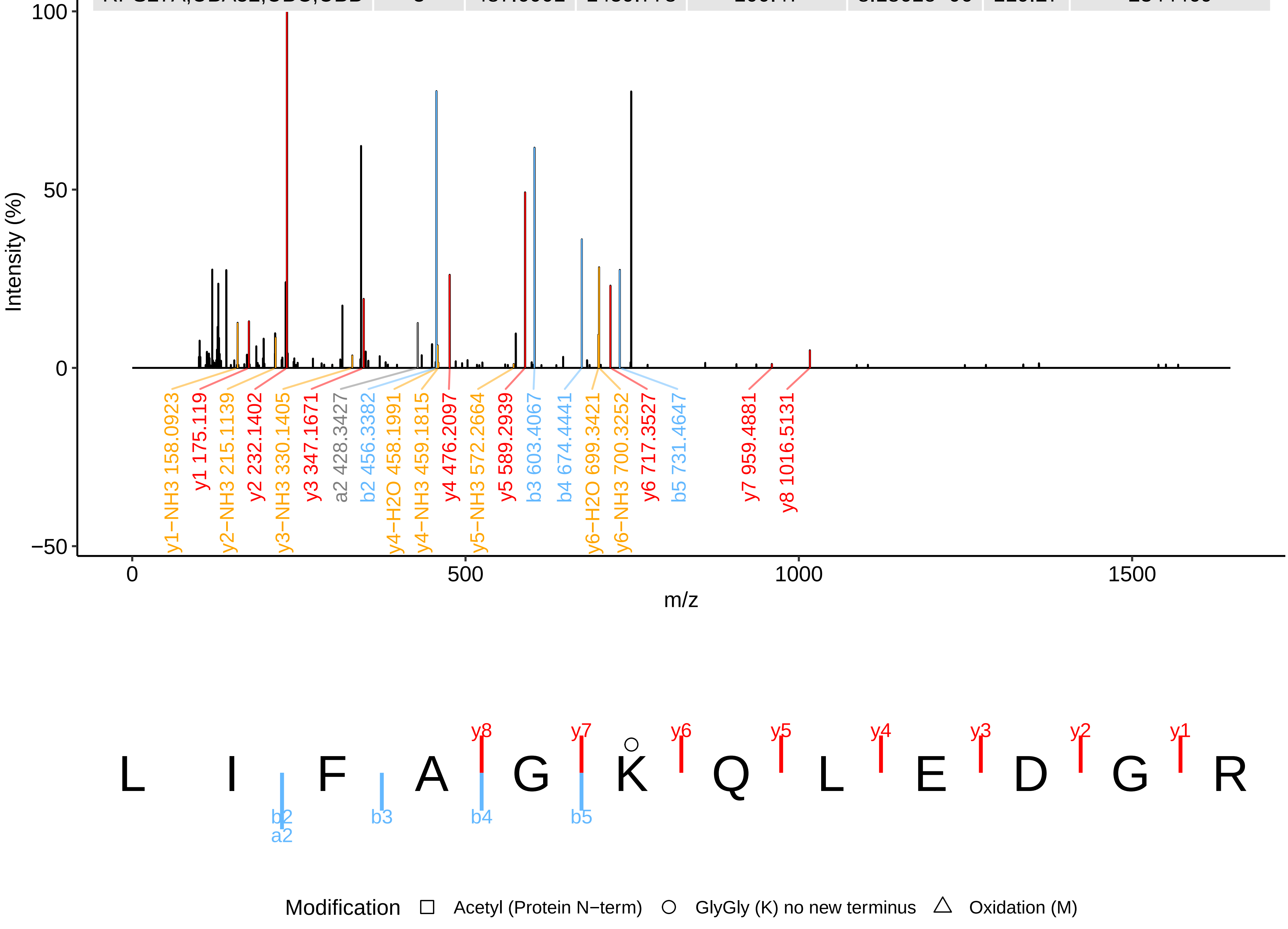

20200921_KIZ_MUb4_GlyGly_10plex_2 − Scan number: 26653

Gene Names	Charge	m/z	Mass	Retention time	PEP	Score	Precursor Intensity
RPS27A;UBA52;UBC;UBB	3	487.6001	1459.778	105.61	5.0692e−05	97.734	1975262

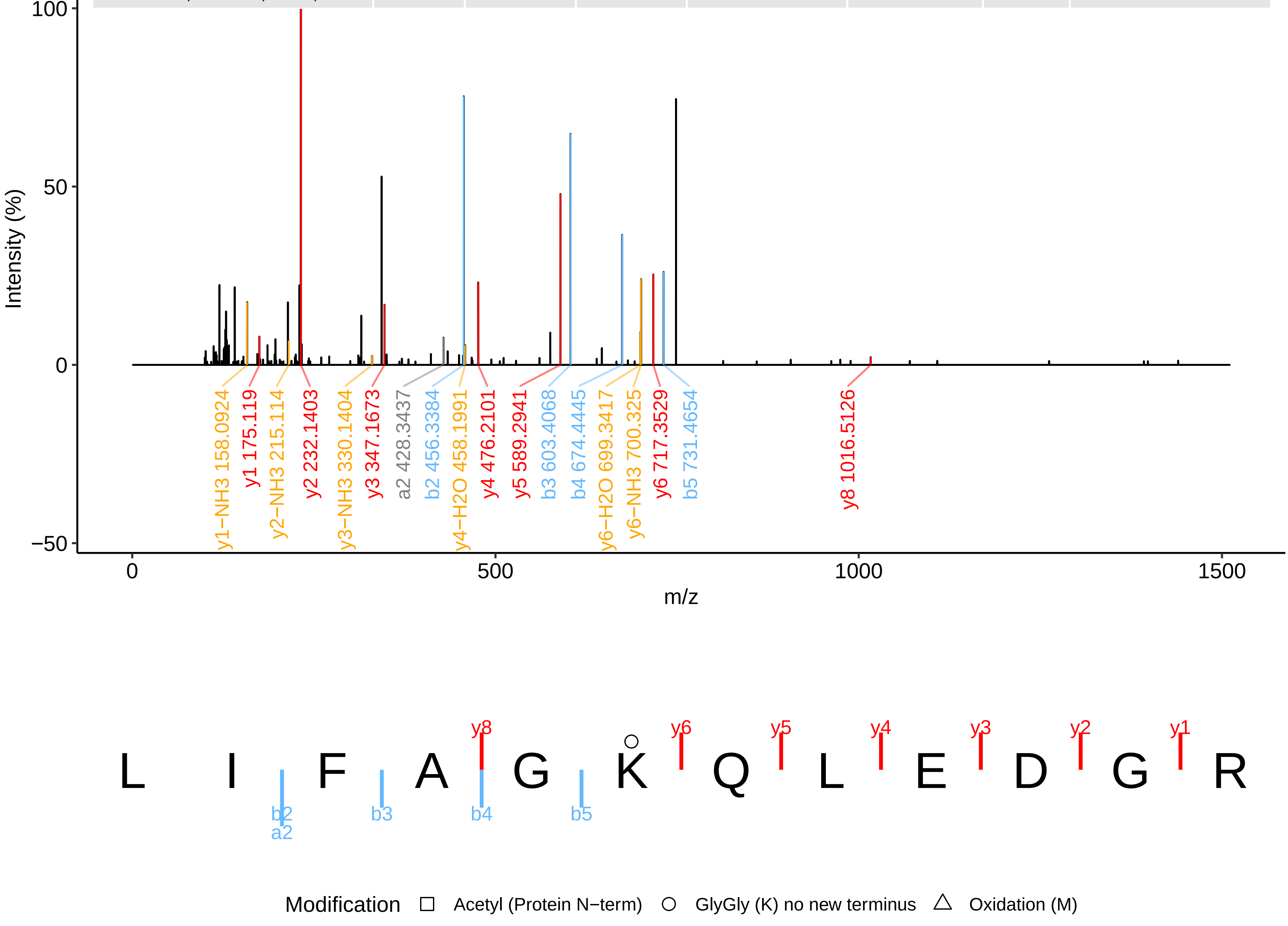

20200921_KIZ_MUb4_GlyGly_10plex_2 − Scan number: 28152

Gene Names	Charge	m/z	Mass	Retention time	PEP	Score	Precursor Intensity
RPS27A;UBA52;UBC;UBB	3	487.6001	1459.778	111.68	1.8076e−05	111.86	1242046

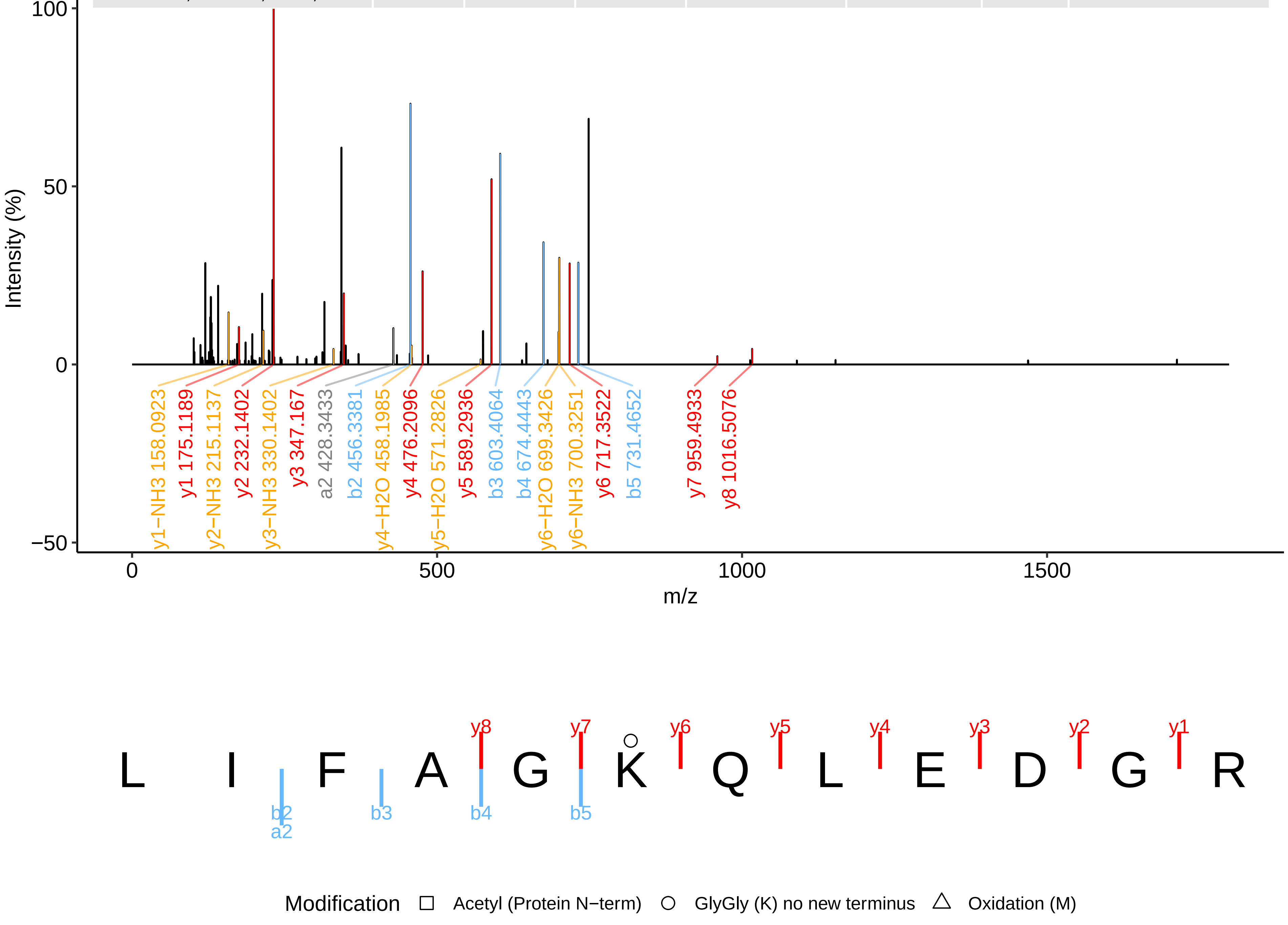

20200921_KIZ_MUb4_GlyGly_10plex_2 − Scan number: 28531

Gene Names	Charge	m/z	Mass	Retention time	PEP	Score	Precursor Intensity
RPS27A;UBA52;UBC;UBB	3	487.6001	1459.778	113.2	6.0914e−06	122.69	988028.2

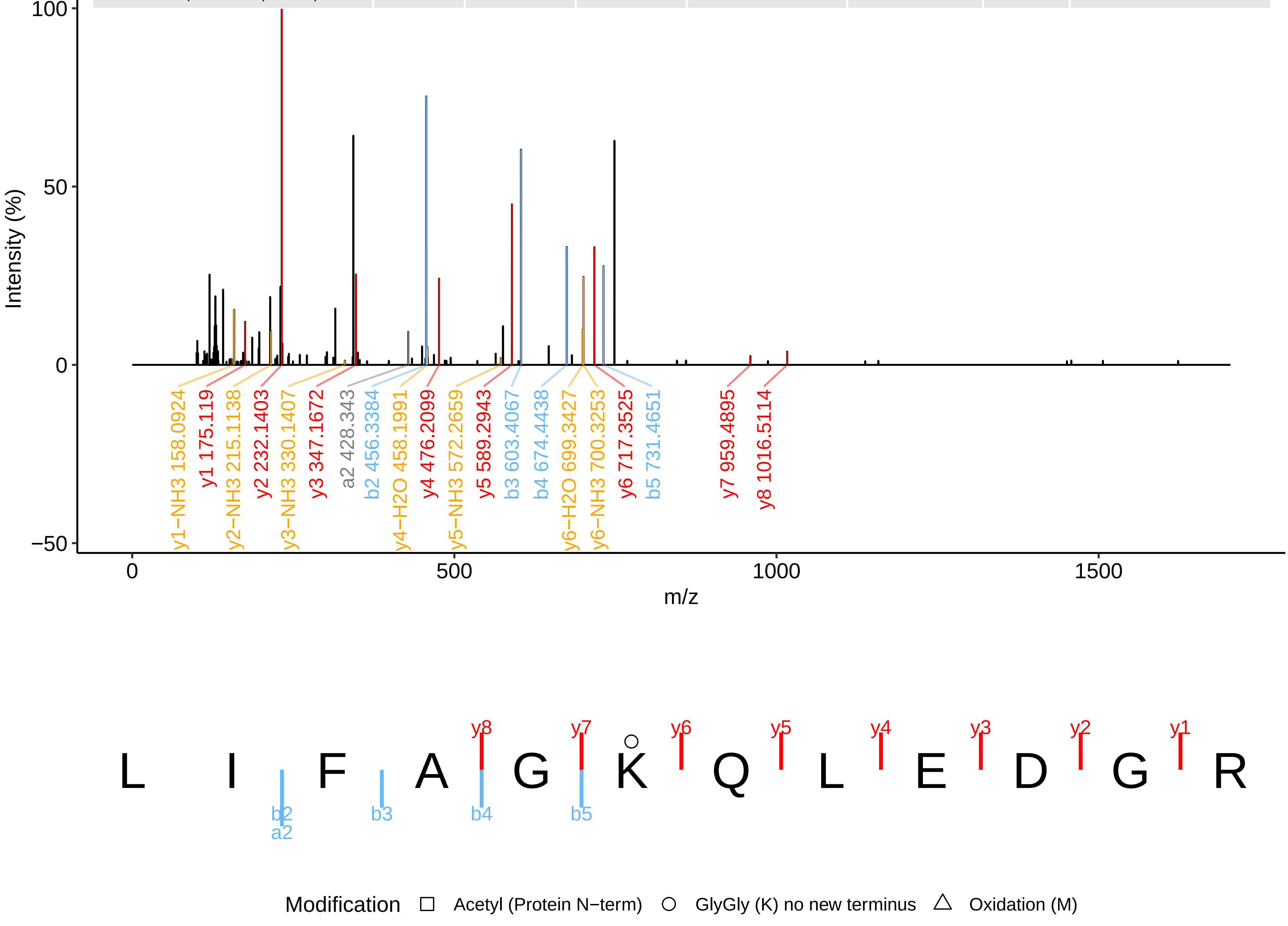

20200921_KIZ_MUb4_GlyGly_10plex_2 − Scan number: 28660

Gene Names	Charge	m/z	Mass	Retention time	PEP	Score	Precursor Intensity
RPS27A;UBA52;UBC;UBB	3	487.6001	1459.778	113.71	6.0914e−06	122.69	NA

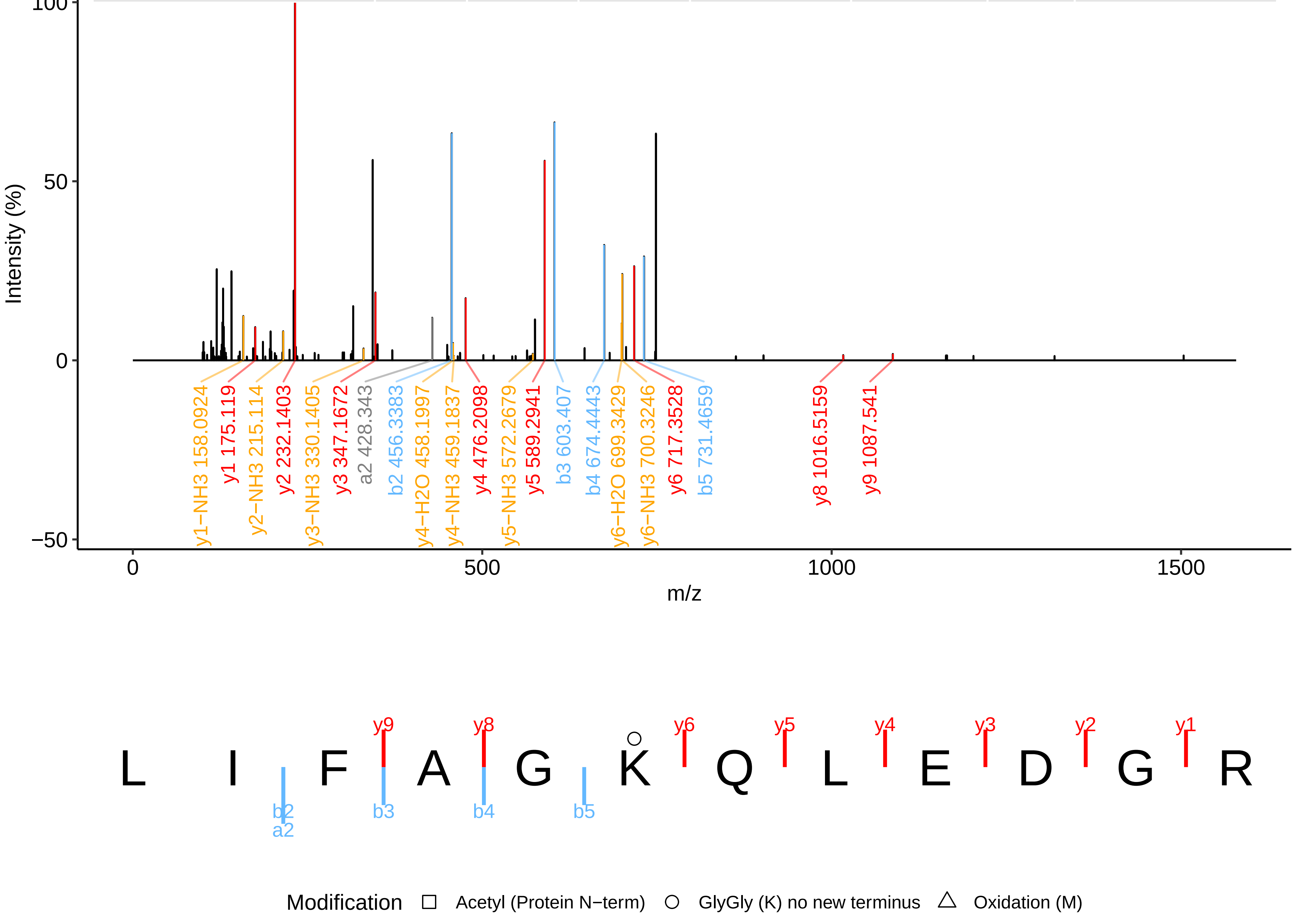

20200921_KIZ_MUb4_GlyGly_10plex_2 − Scan number: 28783

Gene Names	Charge	m/z	Mass	Retention time	PEP	Score	Precursor Intensity
RPS27A;UBA52;UBC;UBB	3	487.6001	1459.778	114.22	2.4894e−05	108.9	1541102

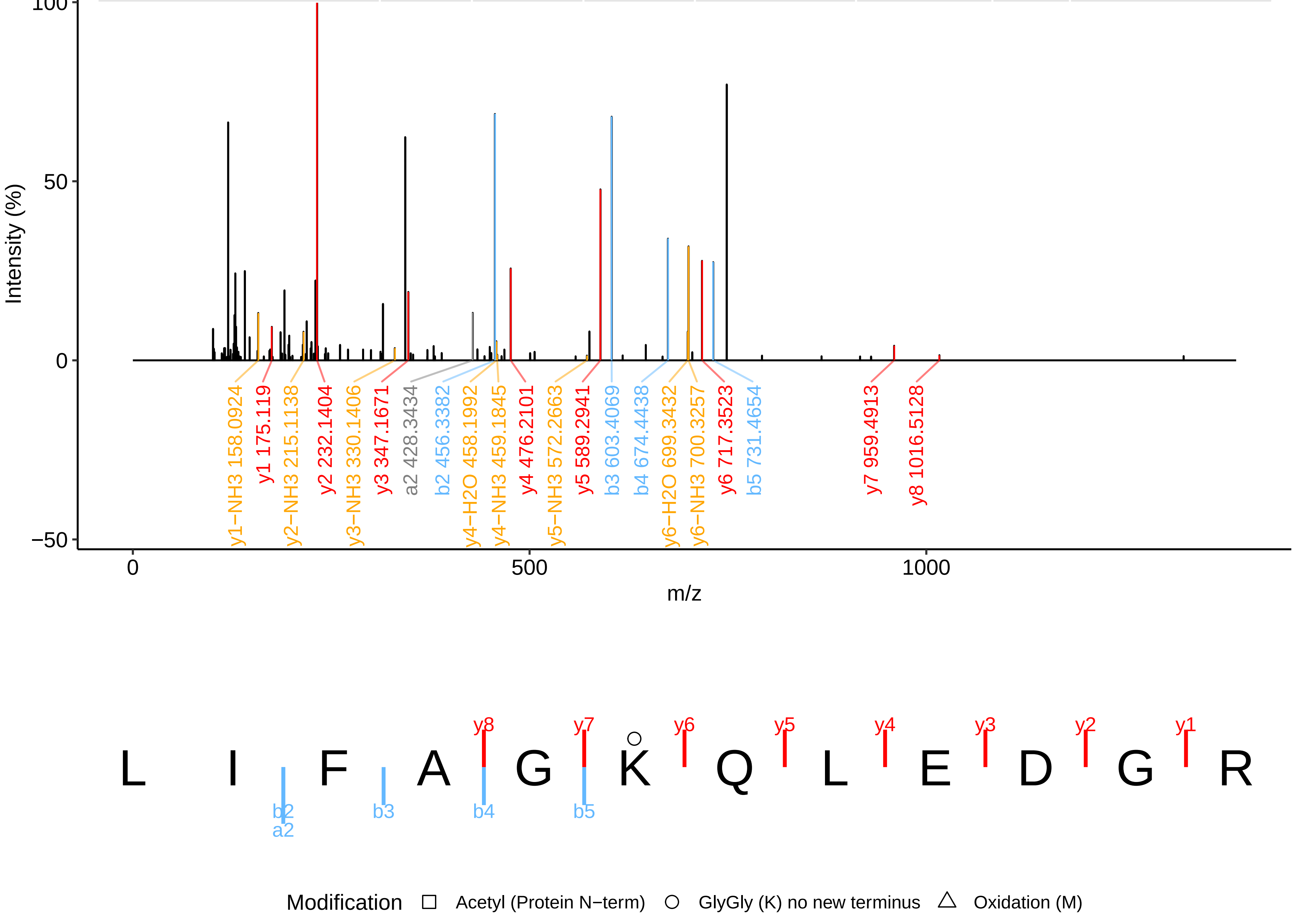

20200921_KIZ_MUb4_GlyGly_10plex_2 − Scan number: 30328

Gene Names	Charge	m/z	Mass	Retention time	PEP	Score	Precursor Intensity
RPS27A;UBA52;UBC;UBB	3	487.6001	1459.778	120.34	4.224e−05	101.39	NA

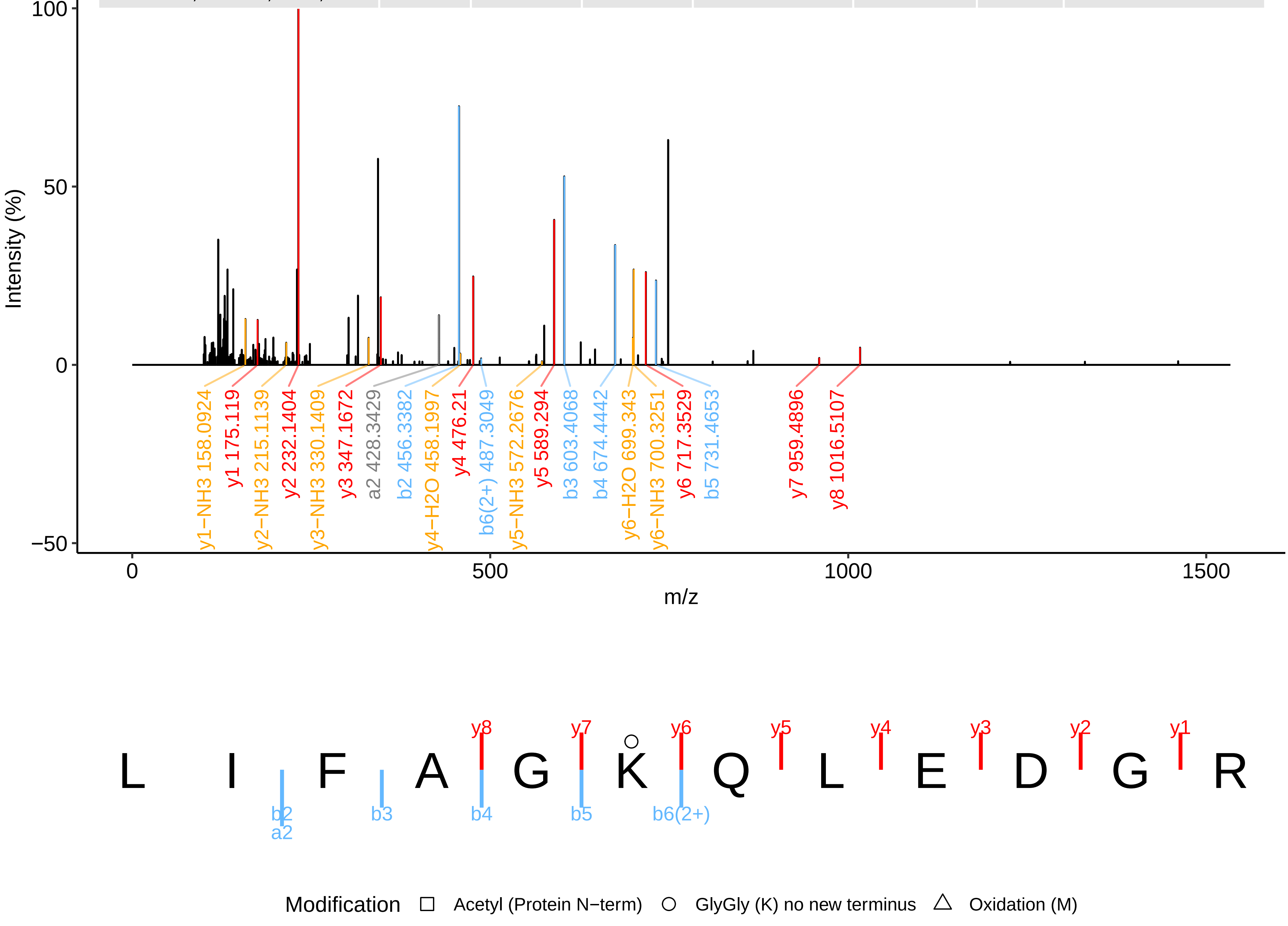

20200921_KIZ_MUb4_GlyGly_10plex_2 − Scan number: 31236

Gene Names	Charge	m/z	Mass	Retention time	PEP	Score	Precursor Intensity
RPS27A;UBA52;UBC;UBB	3	487.6001	1459.778	123.47	0.0014442	83.081	1316934

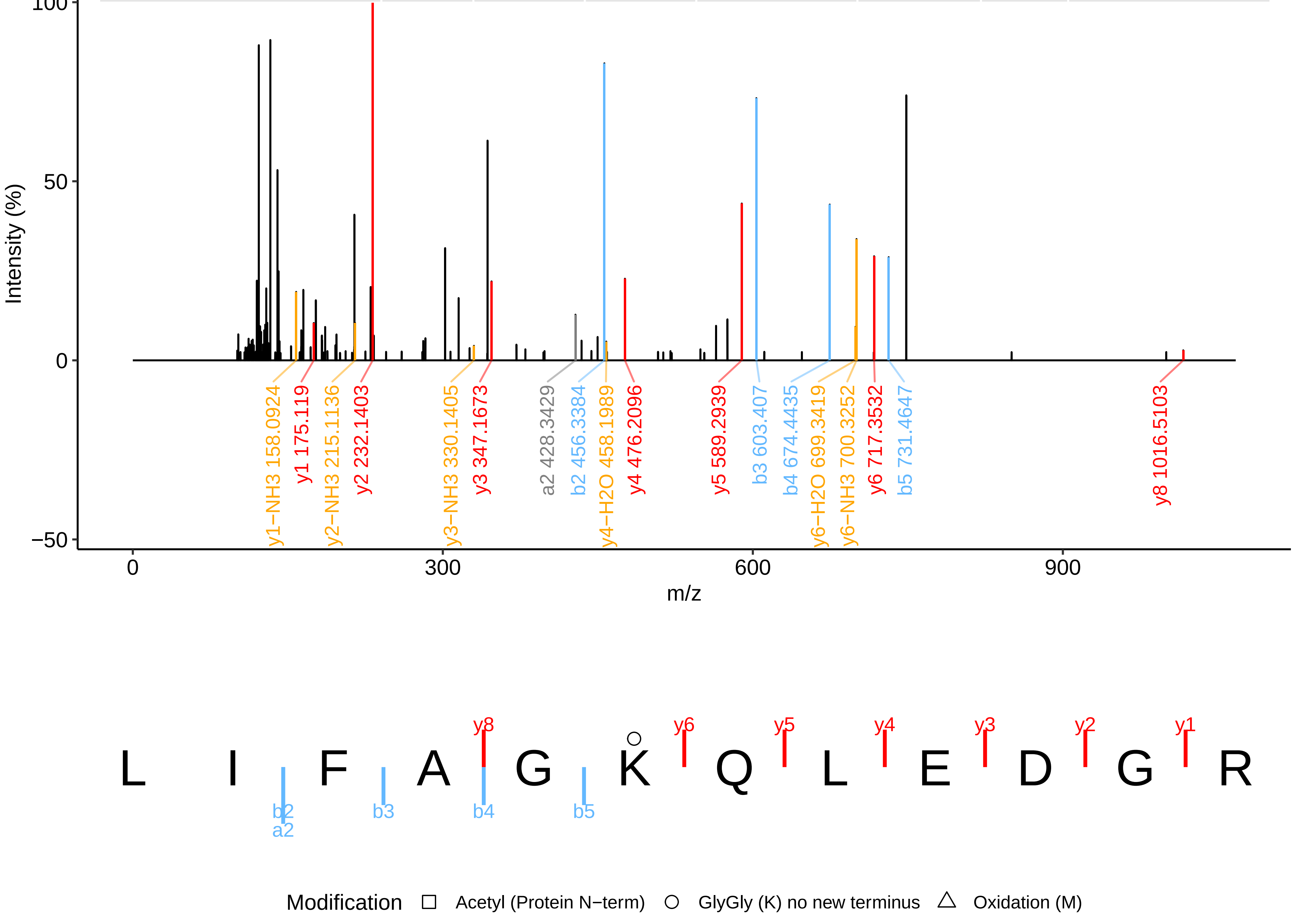

20200921_KIZ_MUb4_GlyGly_10plex_2 − Scan number: 31365

Gene Names	Charge	m/z	Mass	Retention time	PEP	Score	Precursor Intensity
RPS27A;UBA52;UBC;UBB	3	487.6001	1459.778	123.97	0.0040875	77.744	930021.6

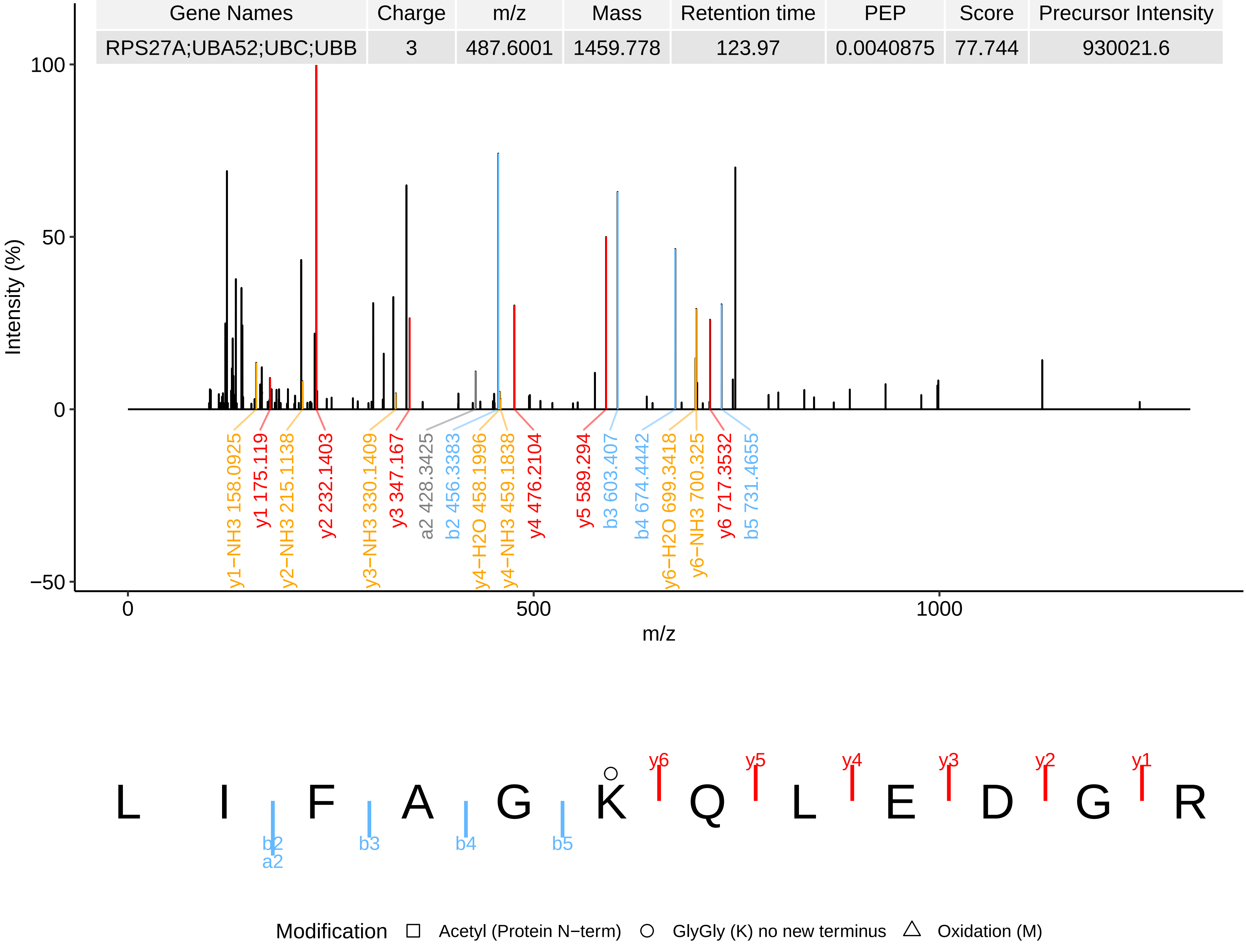

20200924_KIZ_MUb5_GlyGly_1 − Scan number: 15744

Gene Names	Charge	m/z	Mass	Retention time	PEP	Score	Precursor Intensity
RPS27A;UBA52;UBC;UBB	3	487.6001	1459.778	51.313	1.8973e−05	99.506	41298304

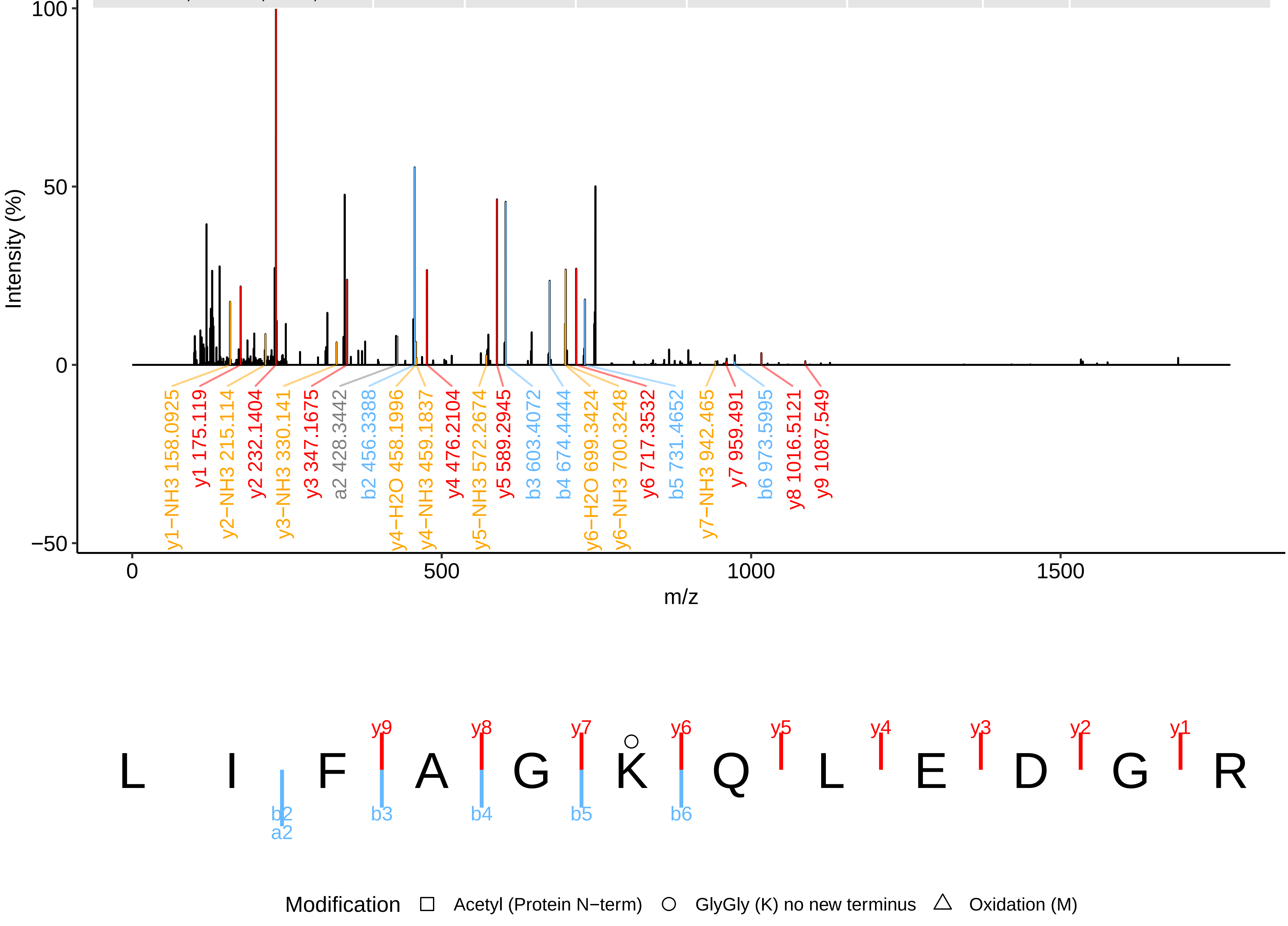

20200924_KIZ_MUb5_GlyGly_1 − Scan number: 15872

Gene Names	Charge	m/z	Mass	Retention time	PEP	Score	Precursor Intensity
RPS27A;UBA52;UBC;UBB	3	487.6001	1459.778	51.615	3.6333e−13	133.89	6779444224

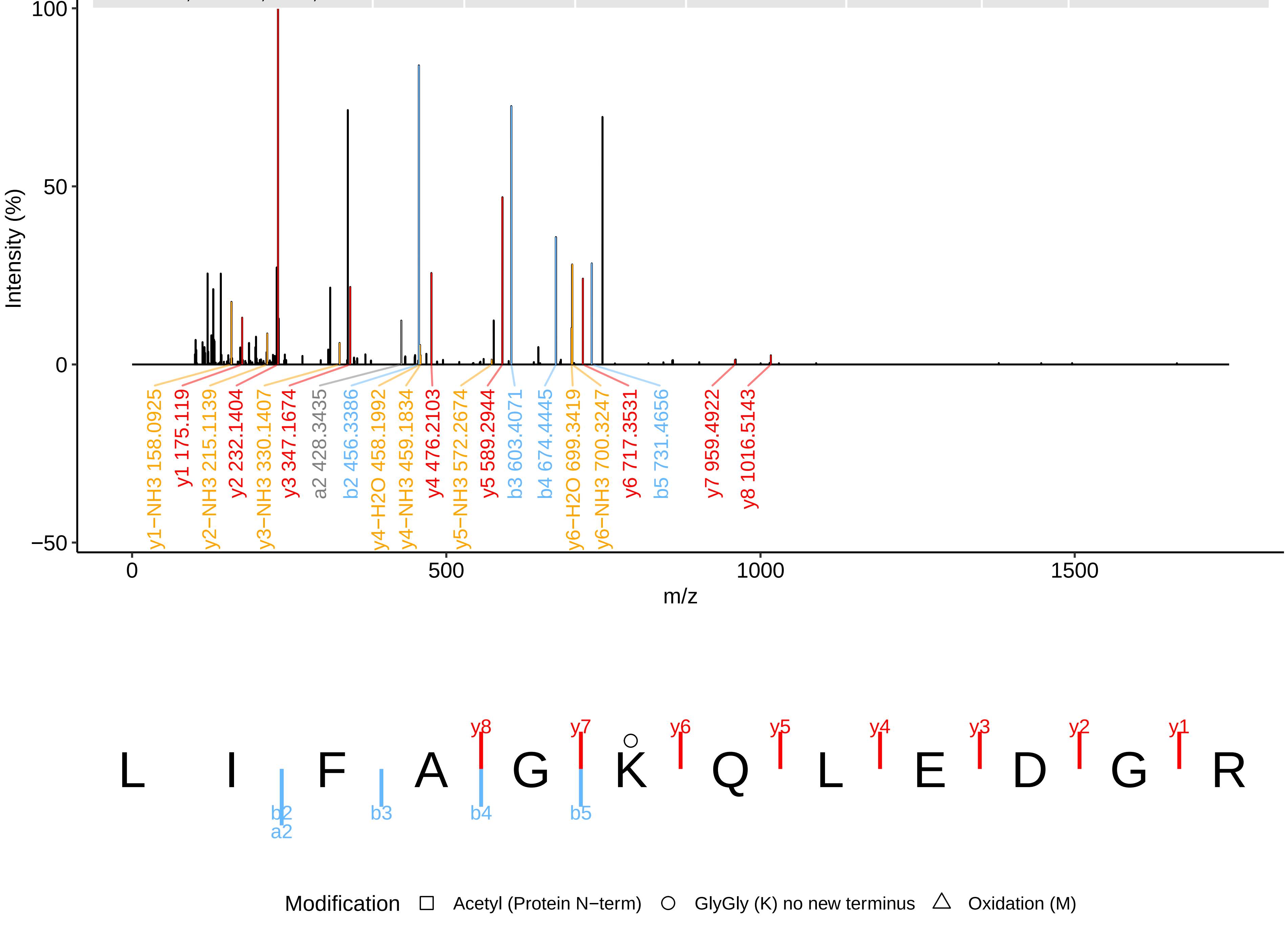

20200924_KIZ_MUb5_GlyGly_1 − Scan number: 15943

Gene Names	Charge	m/z	Mass	Retention time	PEP	Score	Precursor Intensity
RPS27A;UBA52;UBC;UBB	3	487.6001	1459.778	51.824	3.1574e−19	150.46	19886145536

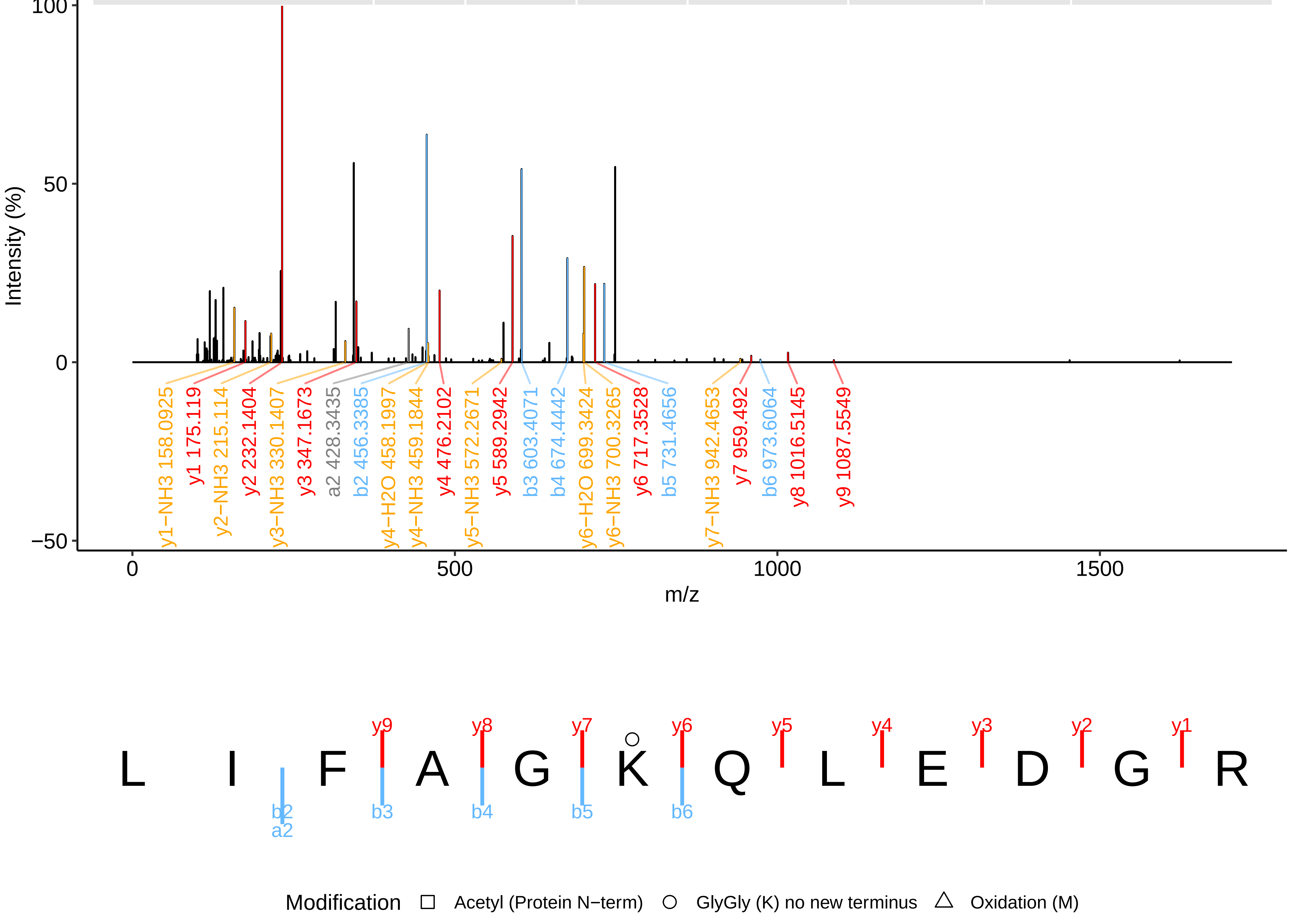

20200924_KIZ_MUb5_GlyGly_1 − Scan number: 16117

Gene Names	Charge	m/z	Mass	Retention time	PEP	Score	Precursor Intensity
RPS27A;UBA52;UBC;UBB	3	487.6001	1459.778	52.335	3.1574e−19	150.46	15374178304

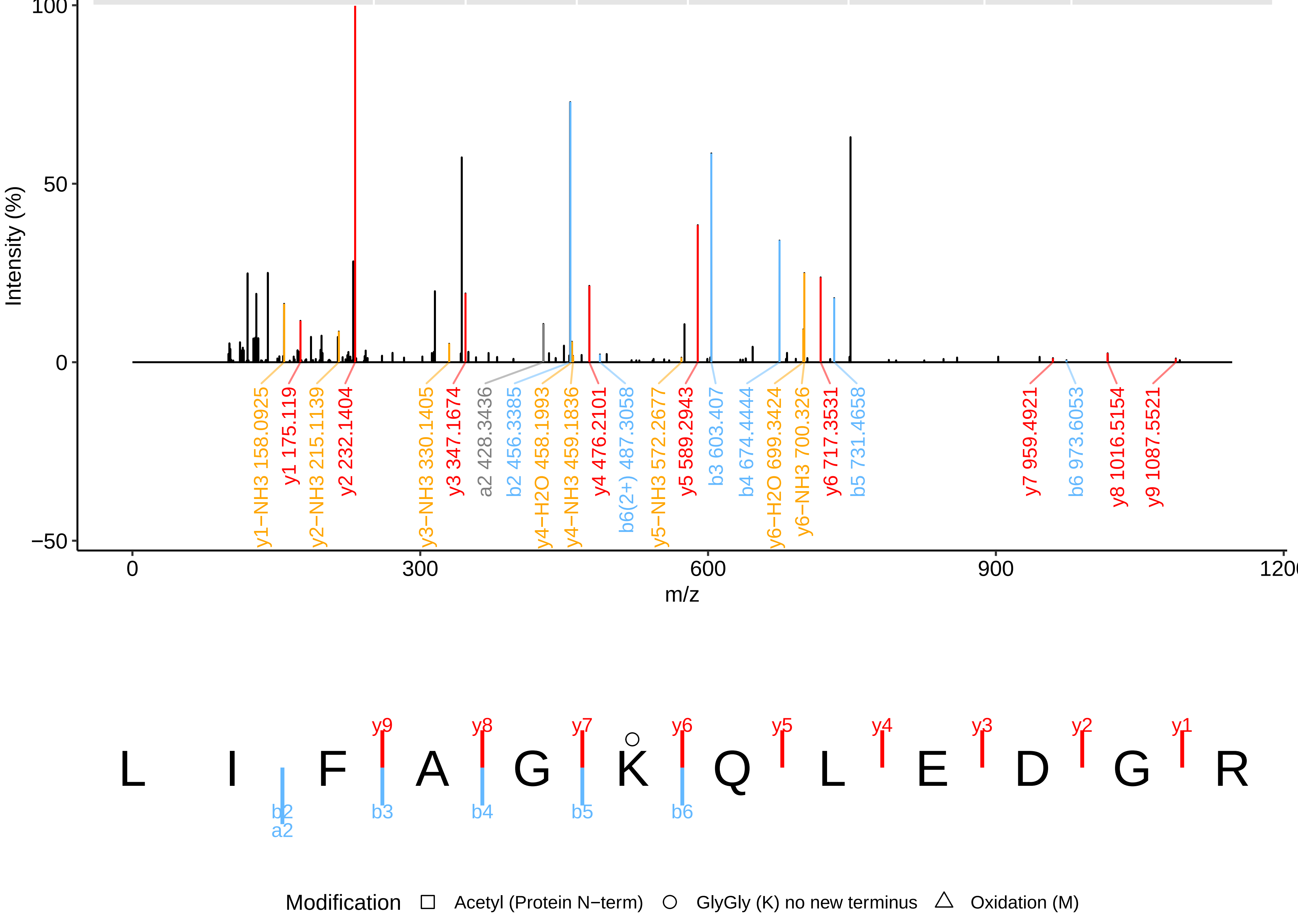

20200924_KIZ_MUb5_GlyGly_1 − Scan number: 16294

Gene Names	Charge	m/z	Mass	Retention time	PEP	Score	Precursor Intensity
RPS27A;UBA52;UBC;UBB	3	487.6001	1459.778	52.841	3.1574e−19	150.46	16147339264

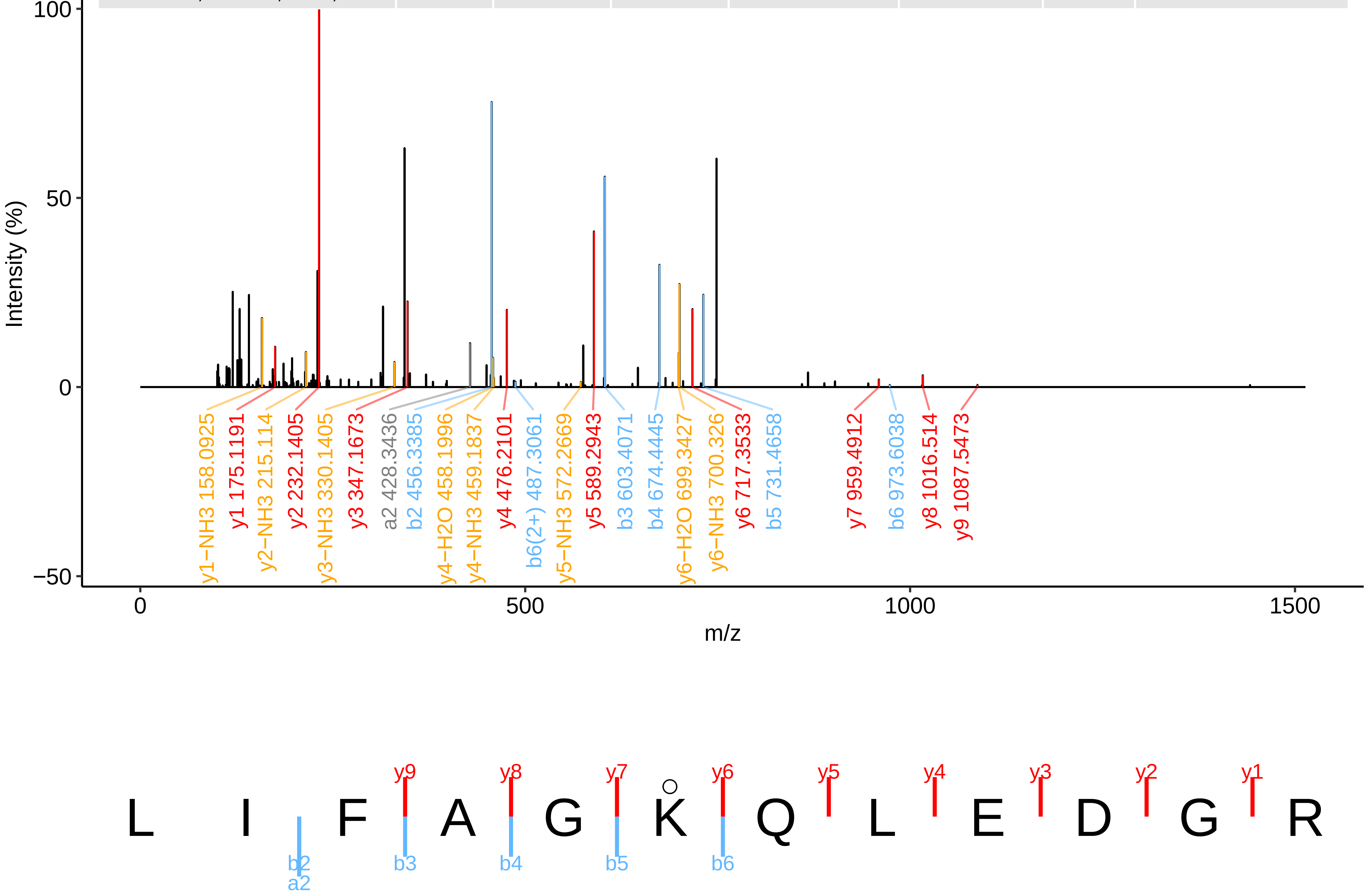

Modification □ Acetyl (Protein N−term) ○ GlyGly (K) no new terminus △ Oxidation (M)

20200924_KIZ_MUb5_GlyGly_1 − Scan number: 16469

Gene Names	Charge	m/z	Mass	Retention time	PEP	Score	Precursor Intensity
RPS27A;UBA52;UBC;UBB	3	487.6001	1459.778	53.345	2.3212e−09	130.01	14699272192

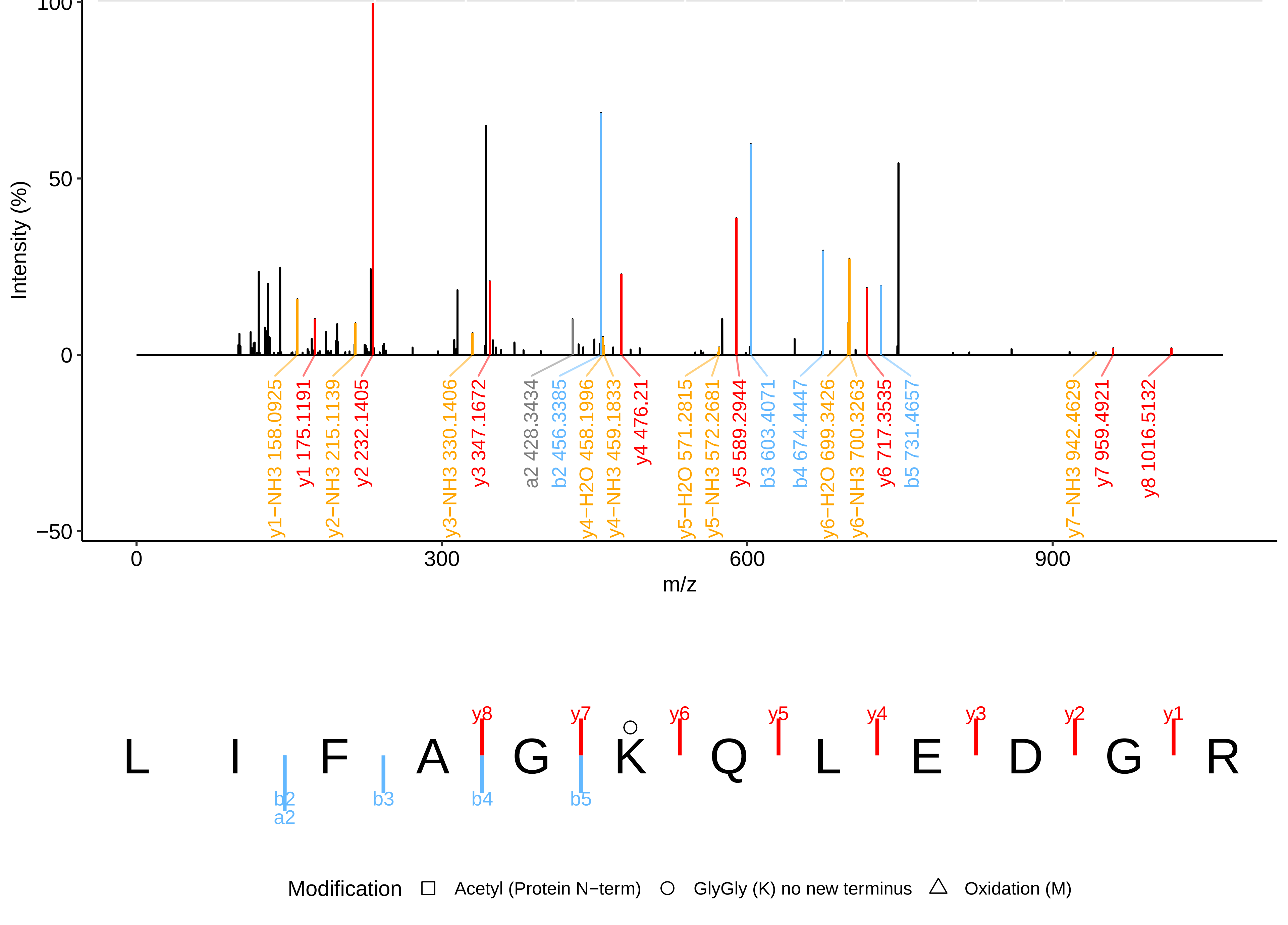

20200924_KIZ_MUb5_GlyGly_1 − Scan number: 16645

Gene Names	Charge	m/z	Mass	Retention time	PEP	Score	Precursor Intensity
RPS27A;UBA52;UBC;UBB	3	487.6001	1459.778	53.855	3.0537e−13	136.5	13198663680

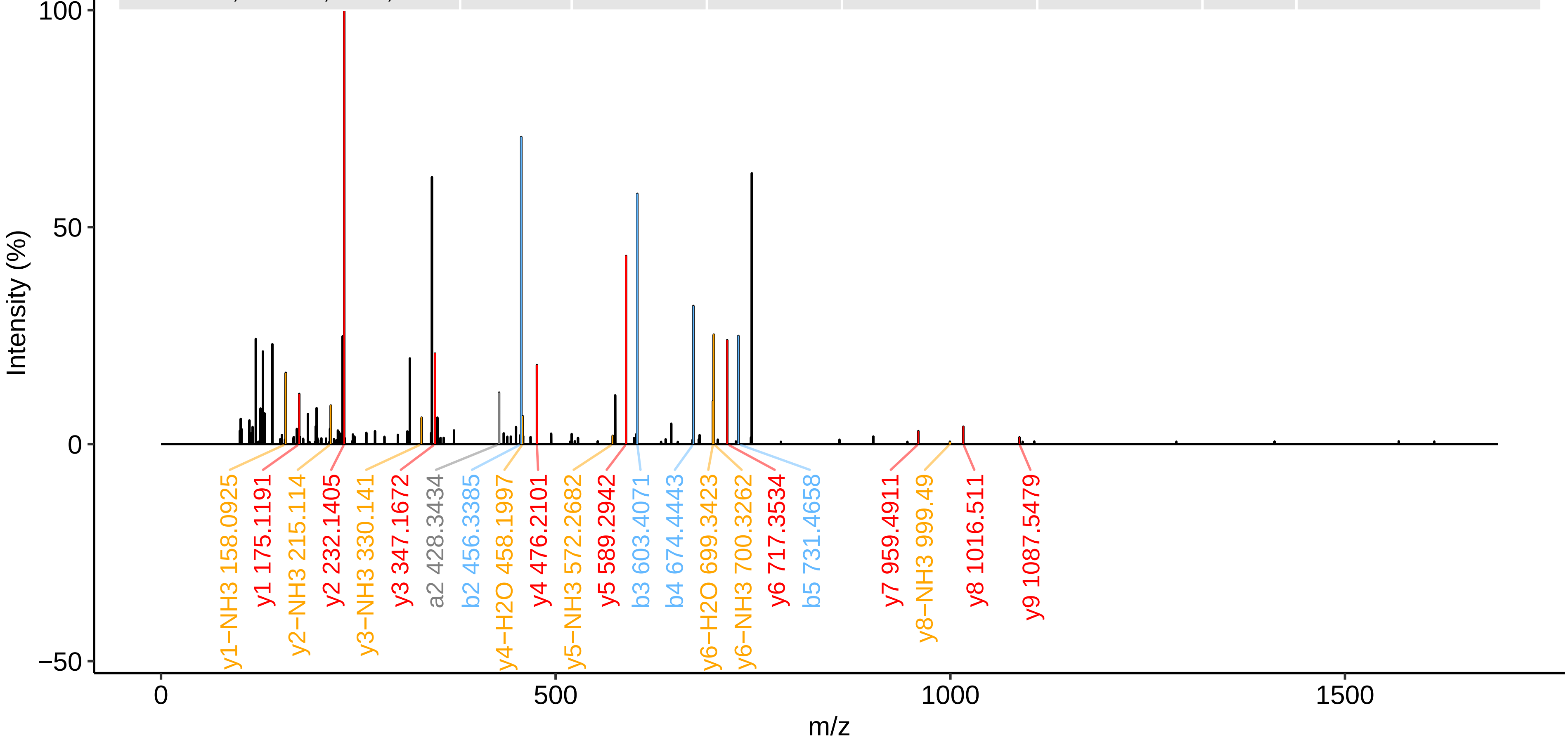

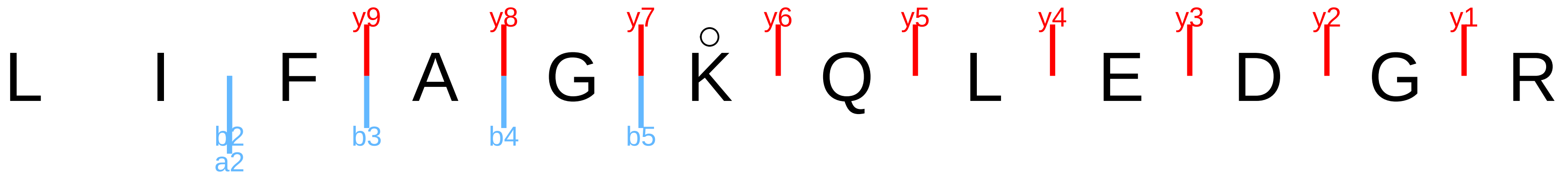

Modification □ Acetyl (Protein N−term) ○ GlyGly (K) no new terminus △ Oxidation (M)

20200924_KIZ_MUb5_GlyGly_1 − Scan number: 16816

Gene Names	Charge	m/z	Mass	Retention time	PEP	Score	Precursor Intensity
RPS27A;UBA52;UBC;UBB	3	487.6001	1459.778	54.361	9.899e−20	162.26	10251172864

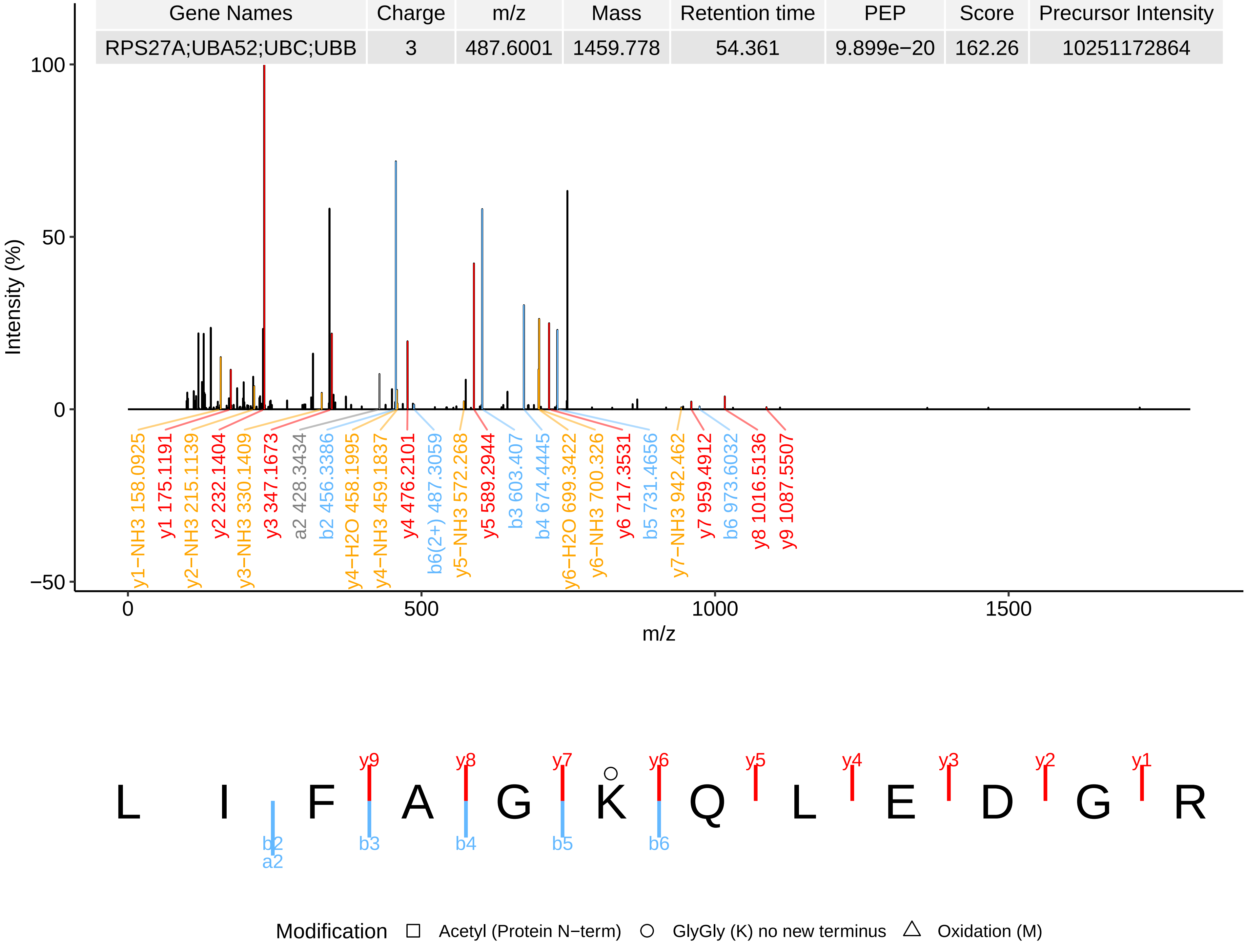

20200924_KIZ_MUb5_GlyGly_1 − Scan number: 16985

Gene Names	Charge	m/z	Mass	Retention time	PEP	Score	Precursor Intensity
RPS27A;UBA52;UBC;UBB	3	487.6001	1459.778	54.867	9.8422e−21	167.12	5339959296

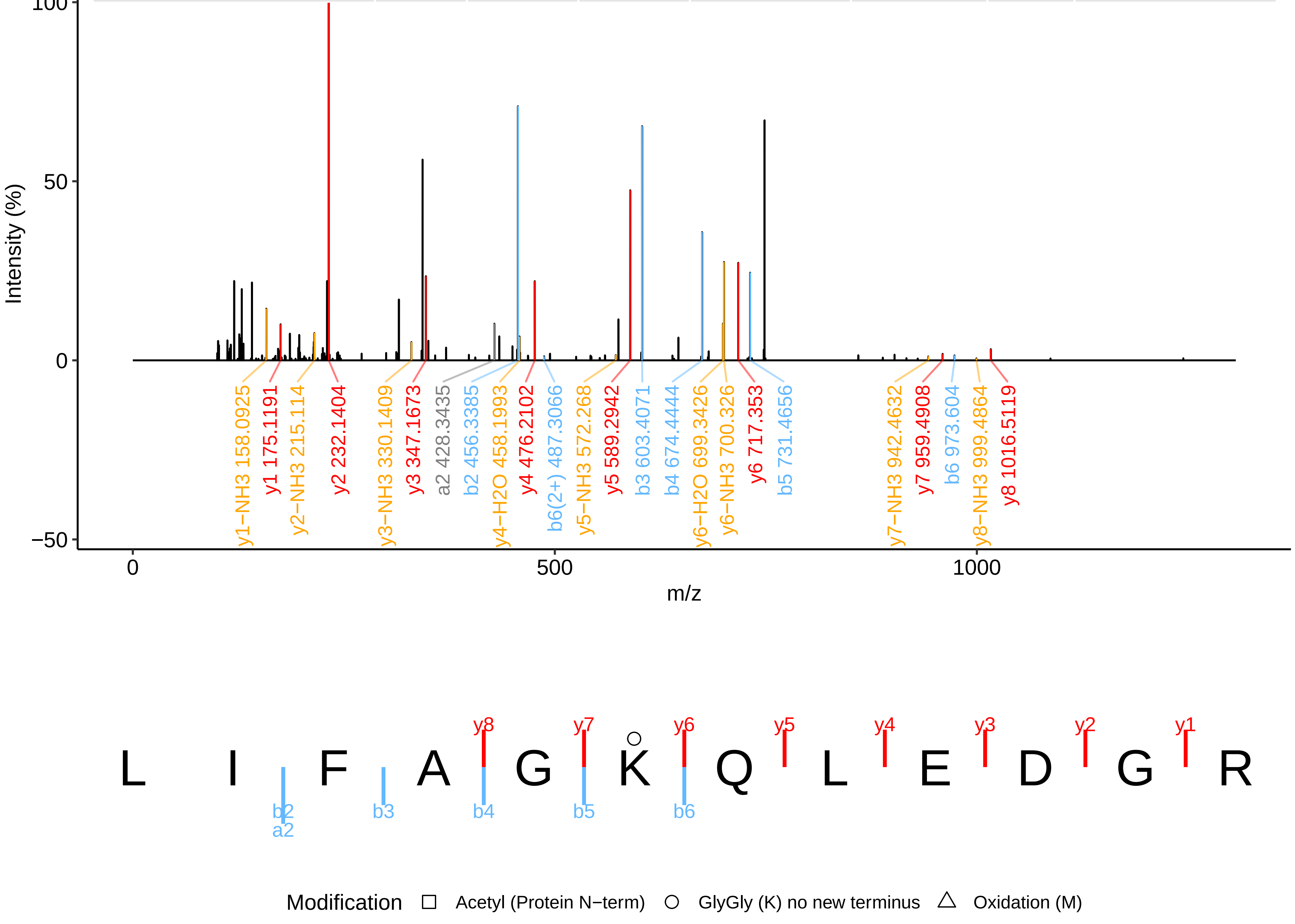

20200924_KIZ_MUb5_GlyGly_1 − Scan number: 17146

Gene Names	Charge	m/z	Mass	Retention time	PEP	Score	Precursor Intensity
RPS27A;UBA52;UBC;UBB	3	487.6001	1459.778	55.378	9.9253e−09	120.77	2326561792

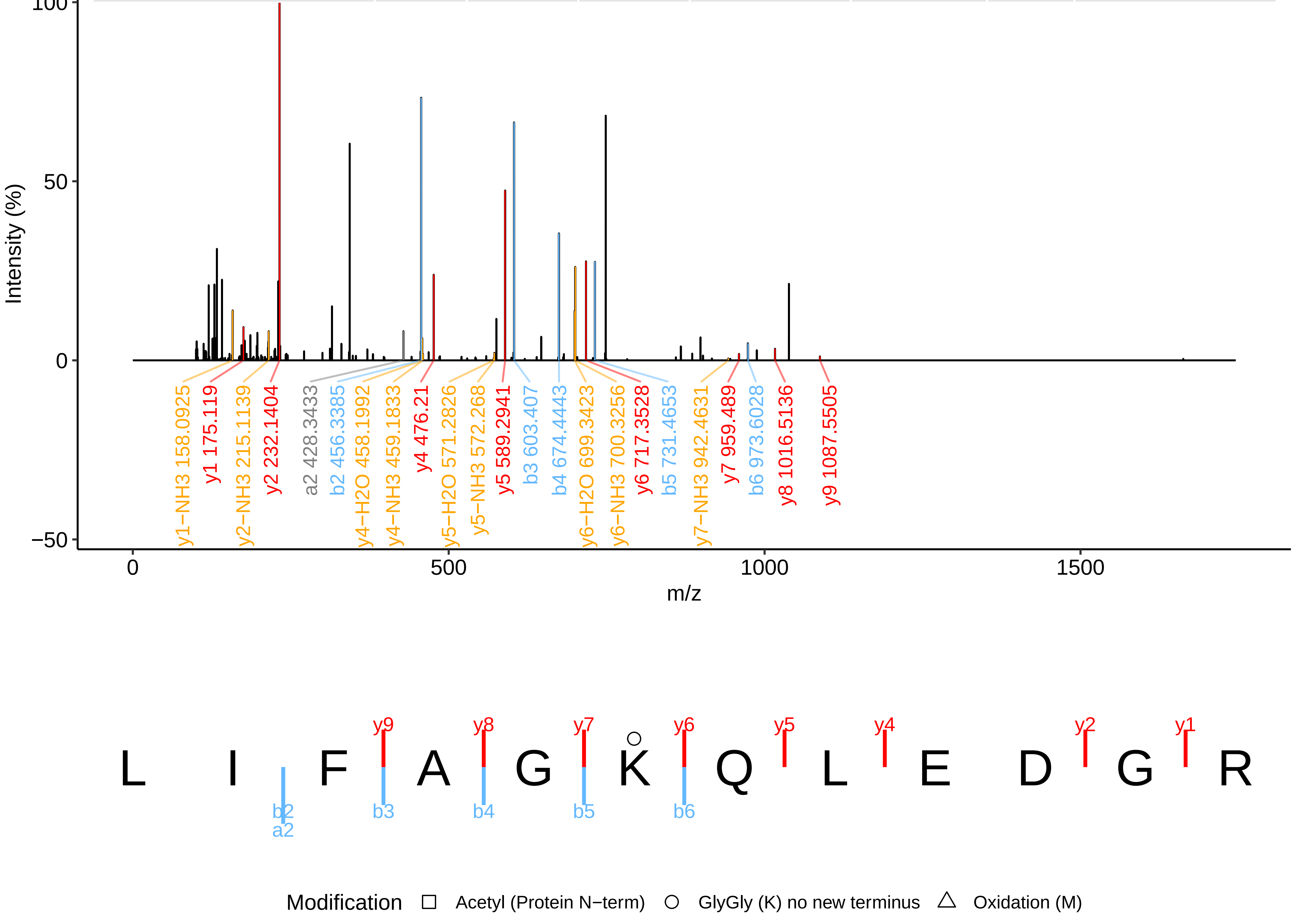

20200924_KIZ_MUb5_GlyGly_1 − Scan number: 17318

Gene Names	Charge	m/z	Mass	Retention time	PEP	Score	Precursor Intensity
RPS27A;UBA52;UBC;UBB	3	487.6001	1459.778	55.91	1.1031e−13	145.28	182915280

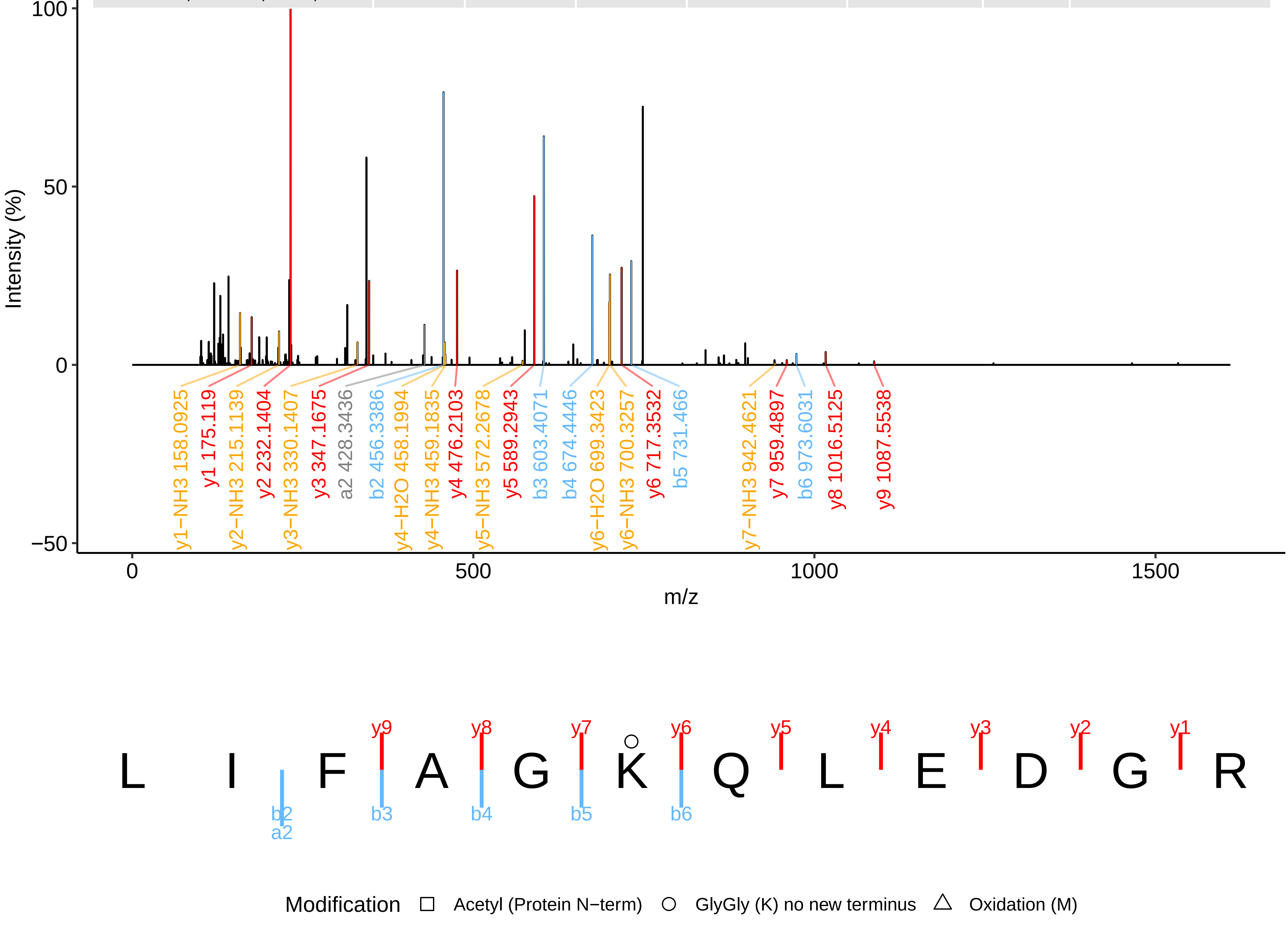

20200924_KIZ_MUb5_GlyGly_1 − Scan number: 17571

Gene Names	Charge	m/z	Mass	Retention time	PEP	Score	Precursor Intensity
RPS27A;UBA52;UBC;UBB	3	487.6001	1459.778	56.734	3.5805e−13	134.13	71289992

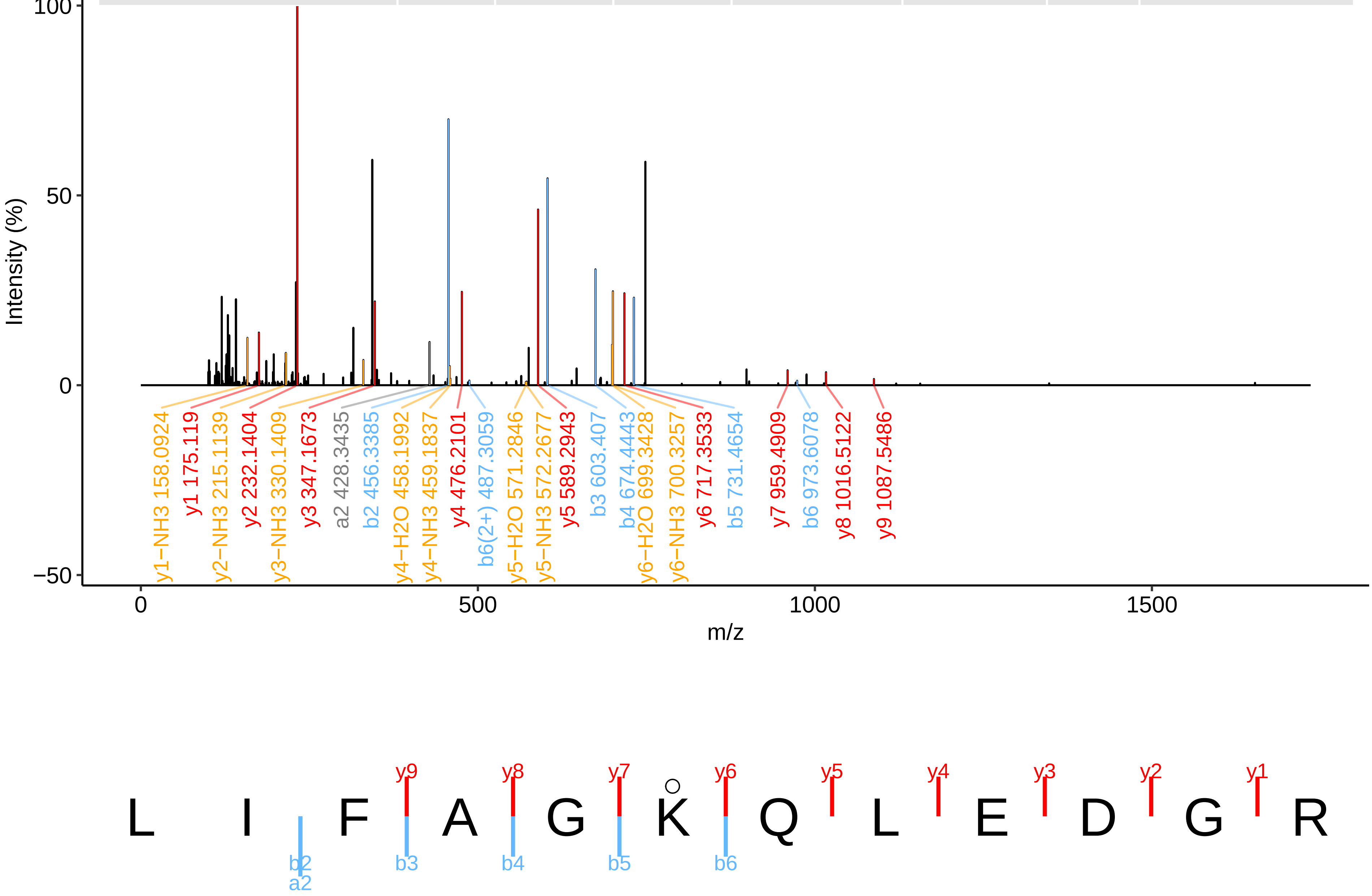

Modification □ Acetyl (Protein N−term) ○ GlyGly (K) no new terminus △ Oxidation (M)

20200924_KIZ_MUb5_GlyGly_1 − Scan number: 17718

Gene Names	Charge	m/z	Mass	Retention time	PEP	Score	Precursor Intensity
RPS27A;UBA52;UBC;UBB	3	487.6001	1459.778	57.241	3.5418e−13	134.3	47717872

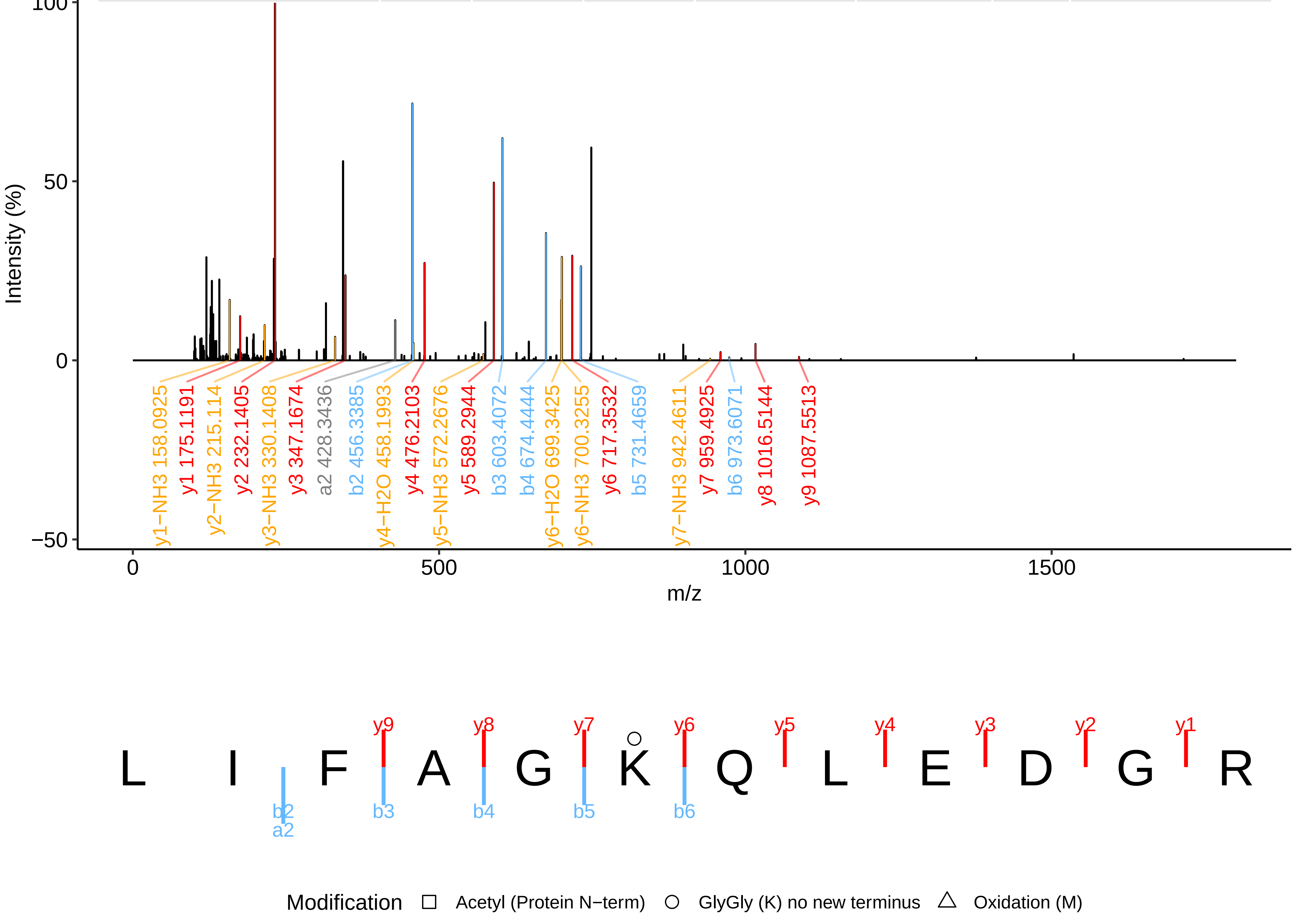

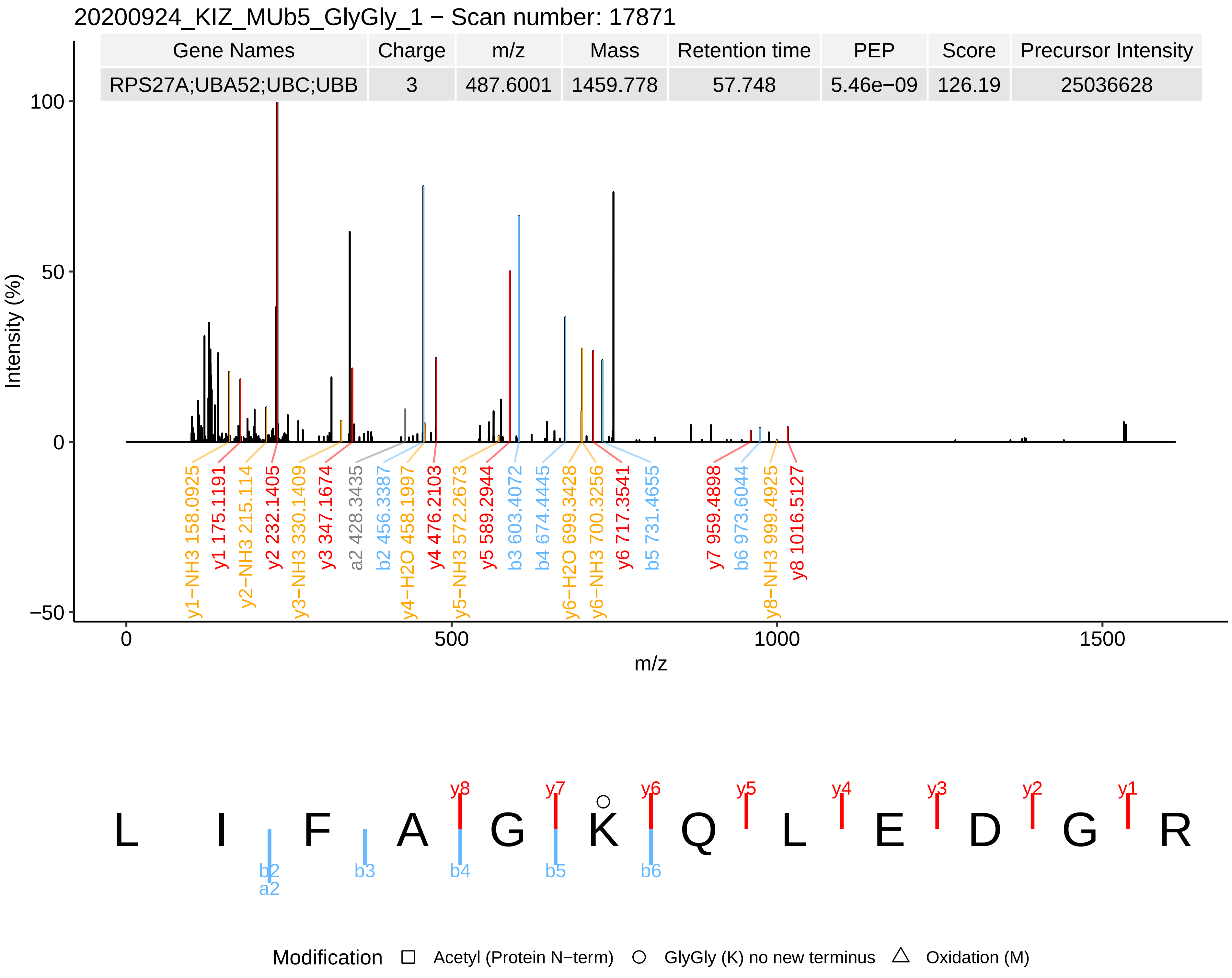
20200924_KIZ_MUb5_GlyGly_1 − Scan number: 17871
Gene Names | Charge | m/z | Mass | Retention time | PEP | Score | Precursor Intensity
RPS27A;UBA52;UBC;UBB | 3 | 487.6001 | 1459.778 | 57.748 | 5.46e−09 | 126.19 | 25036628
Intensity (%)
100
50
0
−50
0
500
1000
1500
m/z
y1−NH3 158.0925
y1 175.1191
y2−NH3 215.114
y2 232.1405
y3−NH3 330.1409
y3 347.1674
a2 428.3435
b2 456.3387
y4−H2O 458.1997
y4 476.2103
y5−NH3 572.2673
y5 589.2944
b3 603.4072
b4 674.4445
y6−H2O 699.3428
y6−NH3 700.3256
y6 717.3541
b5 731.4655
y7 959.4898
b6 973.6044
y8−NH3 999.4925
y8 1016.5127
L I F A G K Q L E D G R
y8 y7 y6 y5 y4 y3 y2 y1
b2 a2 b3 b4 b5 b6
Modification
Acetyl (Protein N−term)
GlyGly (K) no new terminus
Oxidation (M)

20200924_KIZ_MUb5_GlyGly_1 − Scan number: 15784

Gene Names	Charge	m/z	Mass	Retention time	PEP	Score	Precursor Intensity
RPS27A;UBA52;UBC;UBB	2	730.8964	1459.778	51.409	1.3667e−49	237.52	44580448

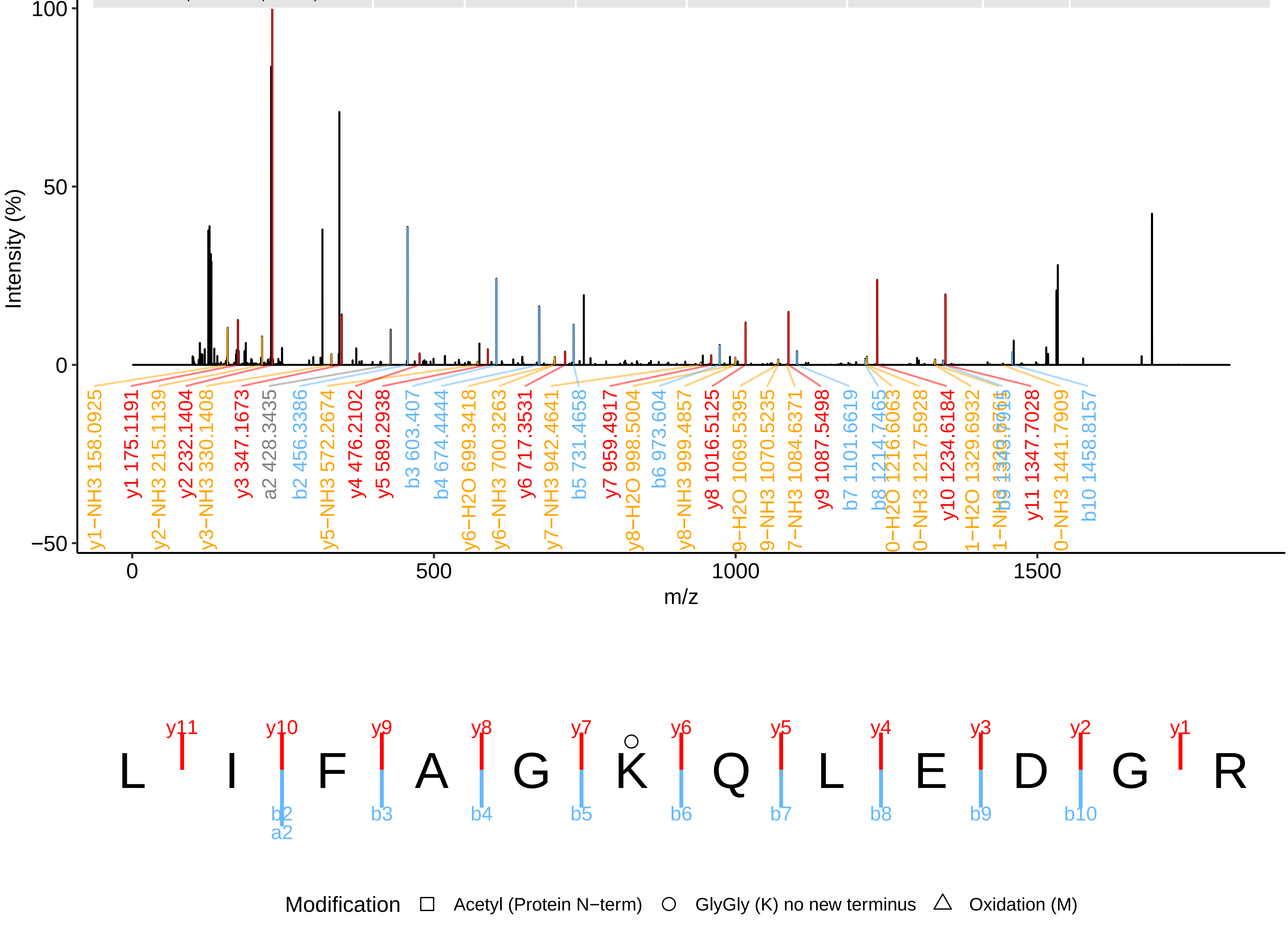

20200924_KIZ_MUb5_GlyGly_1 − Scan number: 15974

Gene Names	Charge	m/z	Mass	Retention time	PEP	Score	Precursor Intensity
RPS27A;UBA52;UBC;UBB	2	730.8964	1459.778	51.917	1.3667e−49	237.52	1336354944

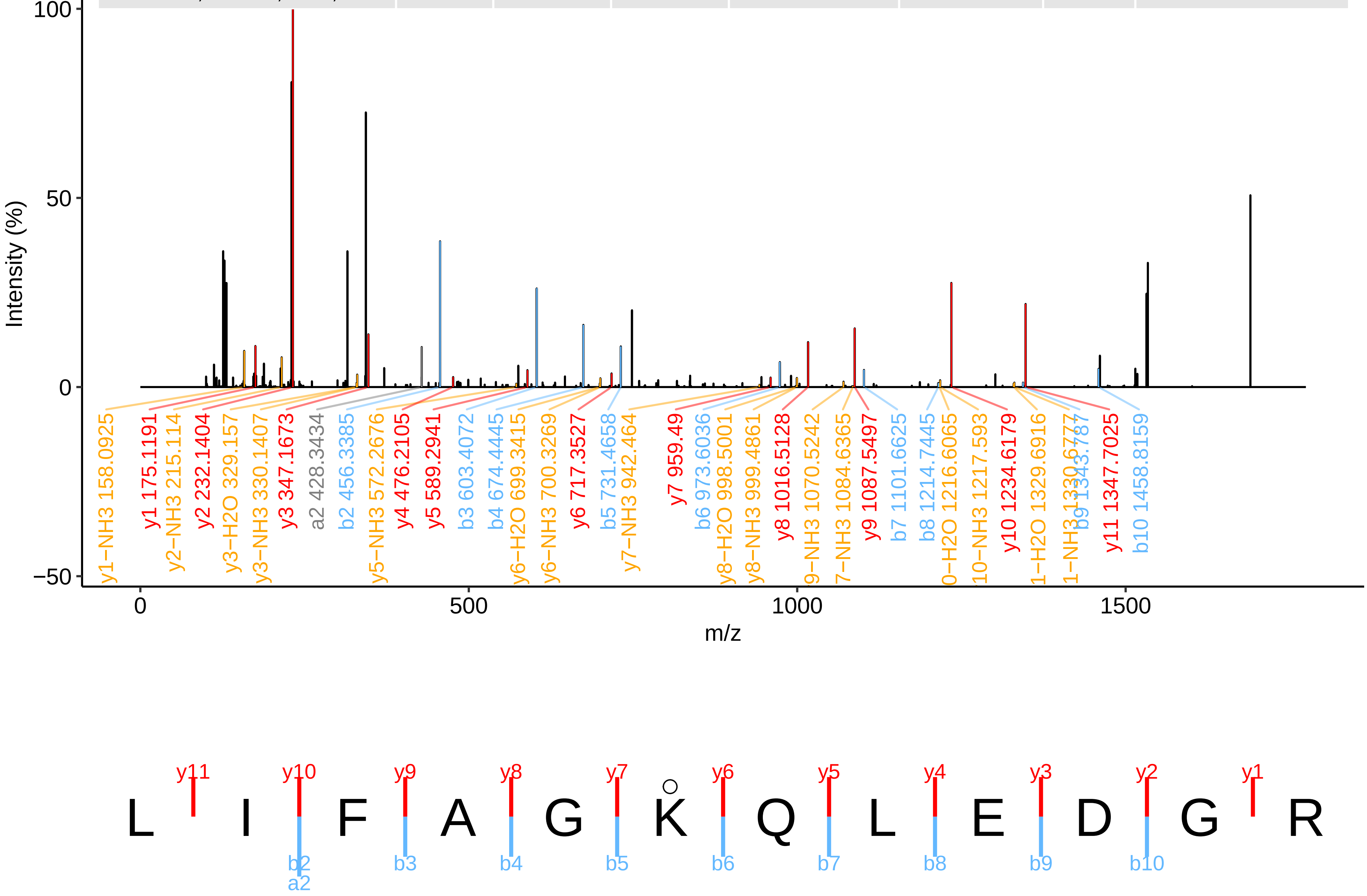

Modification □ Acetyl (Protein N−term) ○ GlyGly (K) no new terminus △ Oxidation (M)

20200924_KIZ_MUb5_GlyGly_1 − Scan number: 16148

Gene Names	Charge	m/z	Mass	Retention time	PEP	Score	Precursor Intensity
RPS27A;UBA52;UBC;UBB	2	730.8964	1459.778	52.423	6.4311e−50	246.63	1300054016

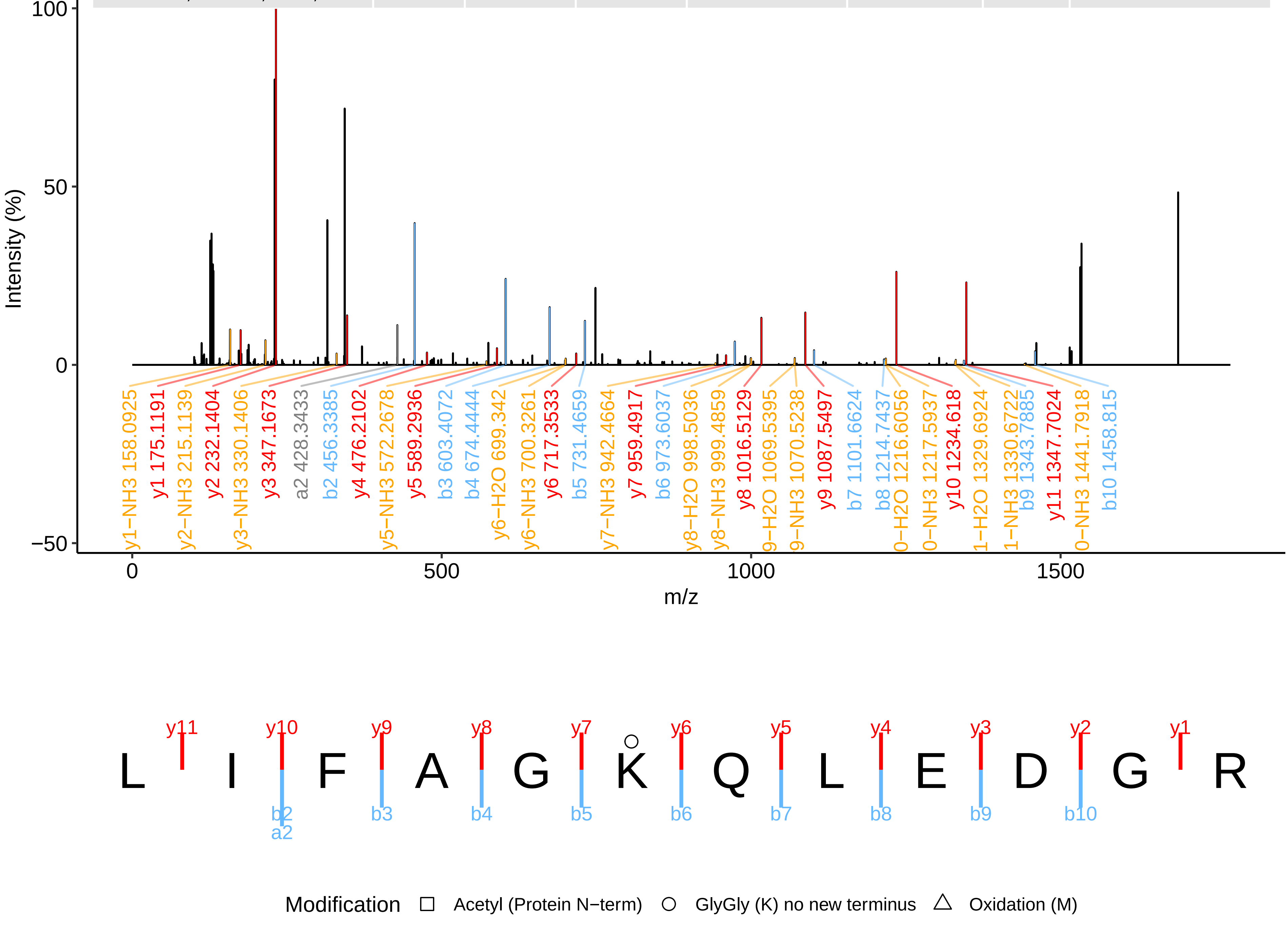

20200924_KIZ_MUb5_GlyGly_1 − Scan number: 18327

Gene Names	Charge	m/z	Mass	Retention time	PEP	Score	Precursor Intensity
RPS27A;UBA52;UBC;UBB	3	487.6001	1459.778	59.259	4.2533e−06	111.86	10646595

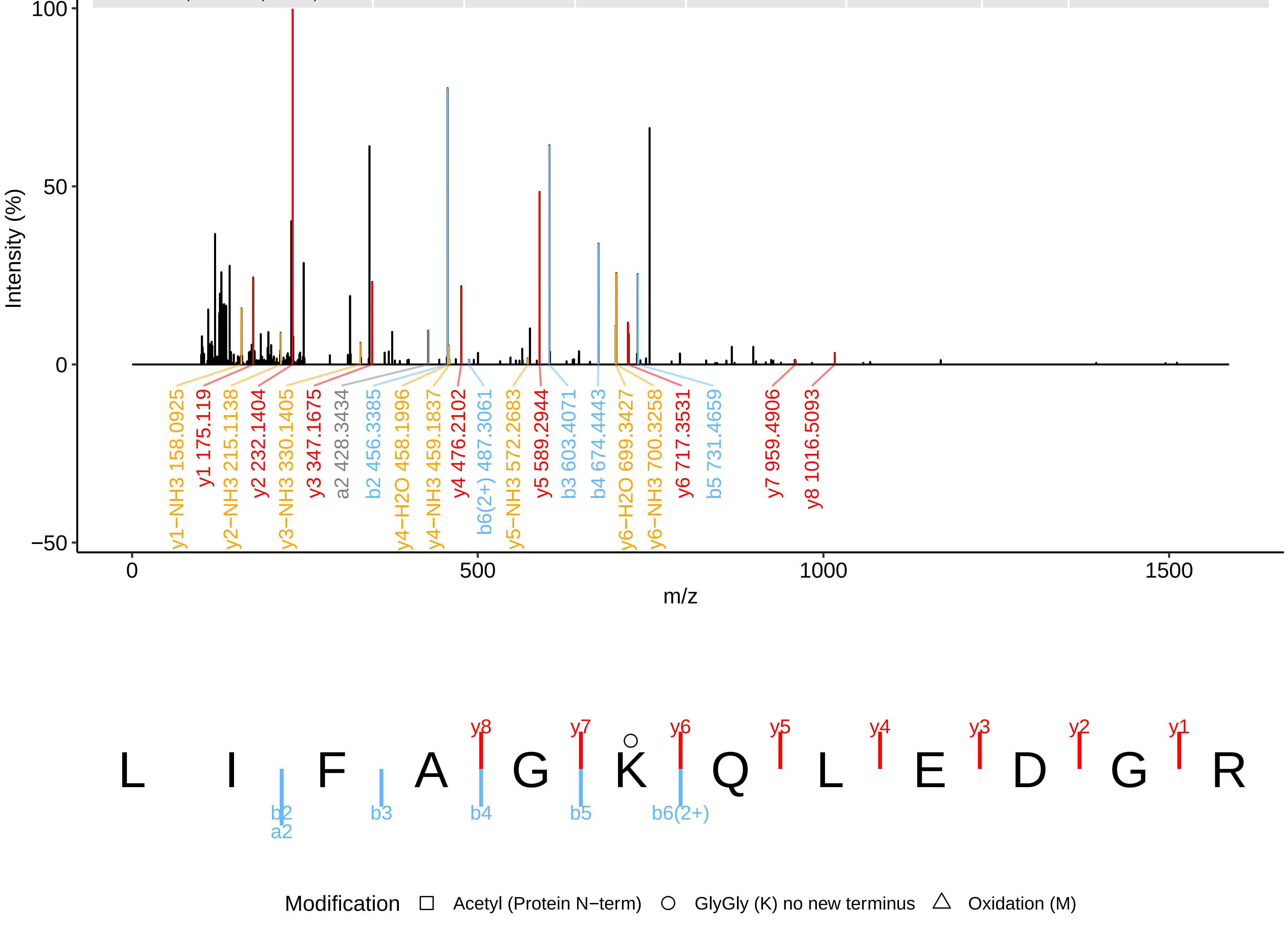

20200924_KIZ_MUb5_GlyGly_1 − Scan number: 18349

Gene Names	Charge	m/z	Mass	Retention time	PEP	Score	Precursor Intensity
RPS27A;UBA52;UBC;UBB	3	487.6001	1459.778	59.326	1.6723e−05	101.39	18821106

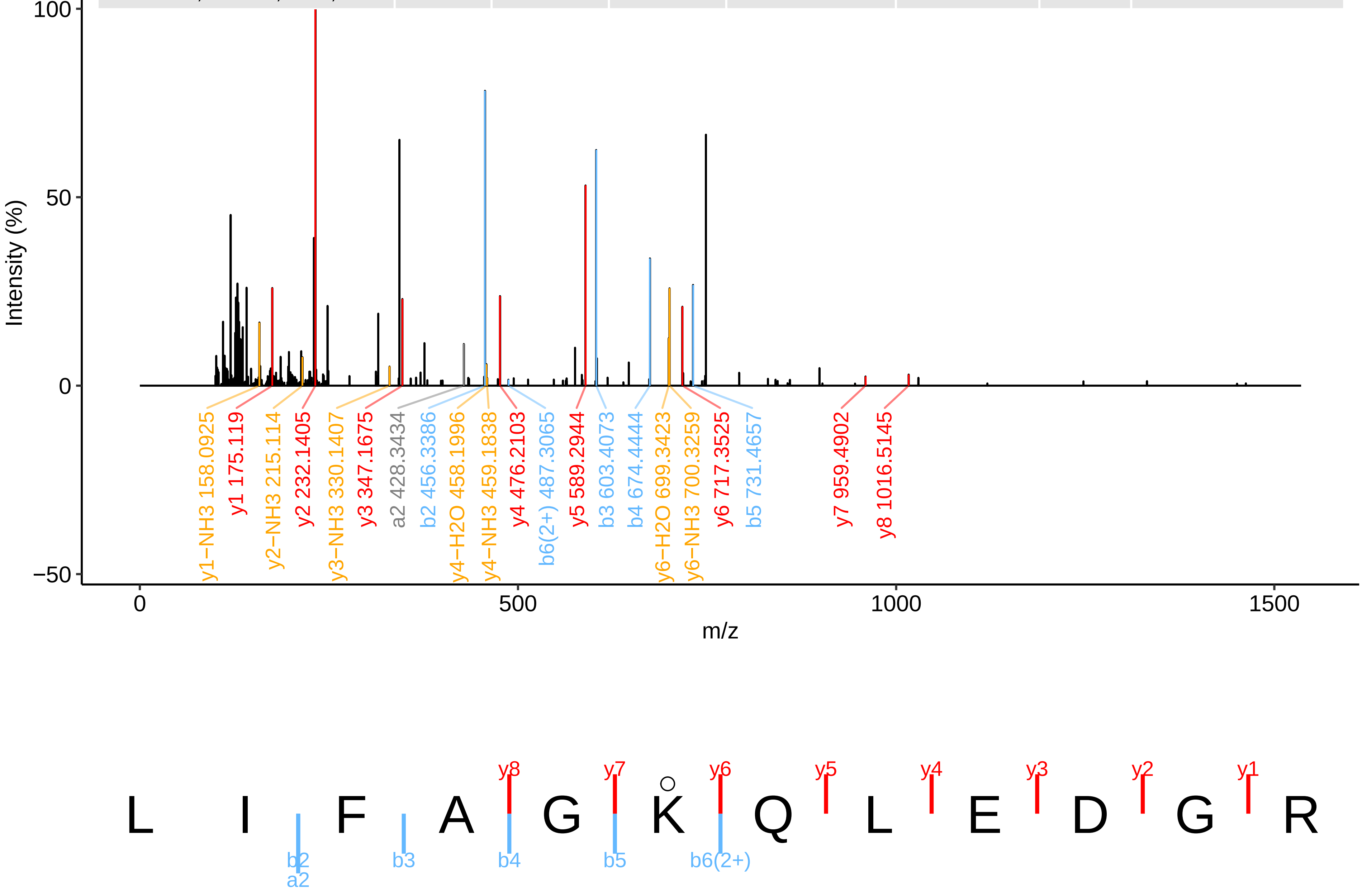

Modification □ Acetyl (Protein N−term) ○ GlyGly (K) no new terminus △ Oxidation (M)

20200924_KIZ_MUb5_GlyGly_1 − Scan number: 19131

Gene Names	Charge	m/z	Mass	Retention time	PEP	Score	Precursor Intensity
RPS27A;UBA52;UBC;UBB	3	487.6001	1459.778	61.839	0.0098035	67.749	16581664

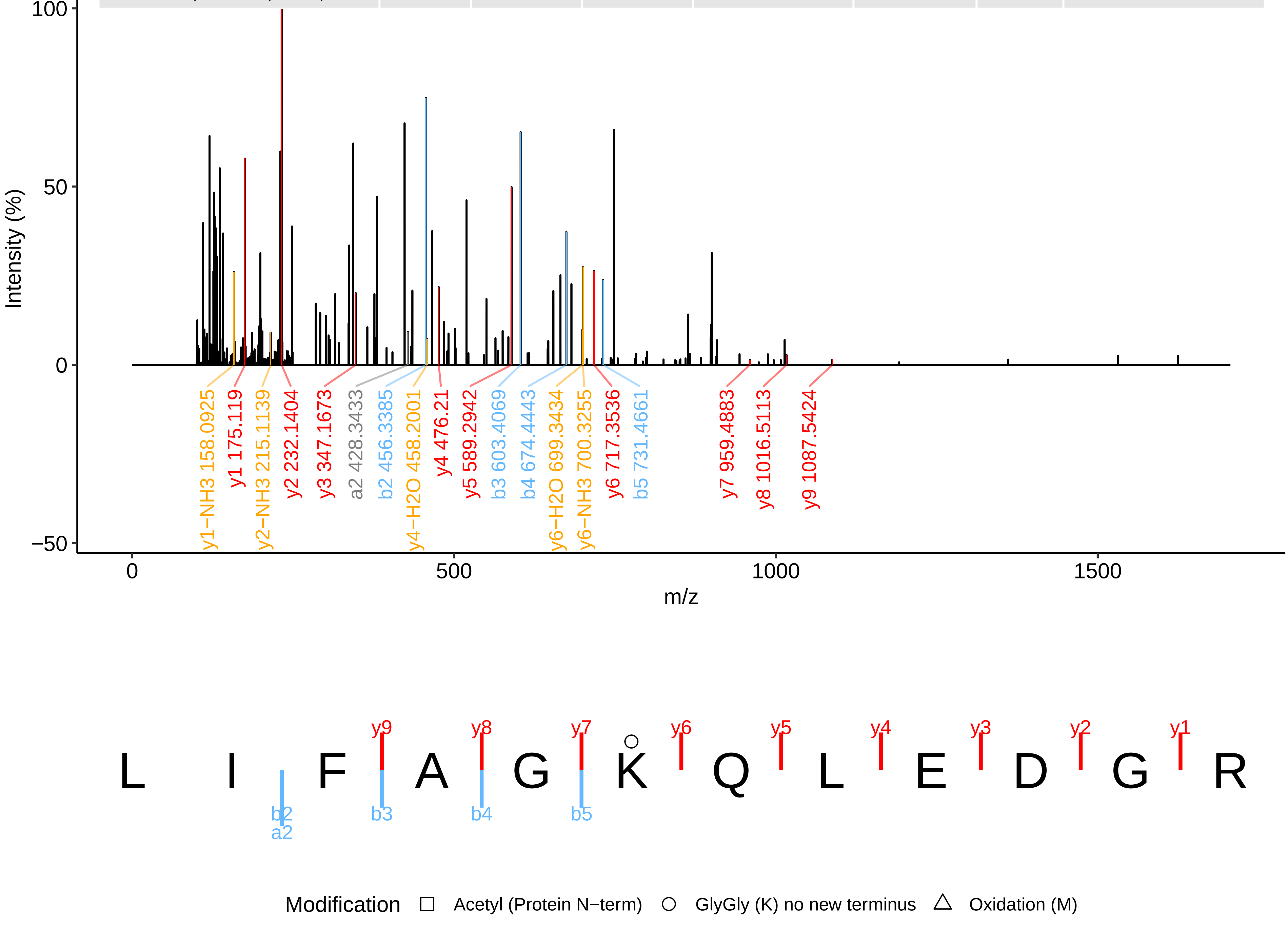

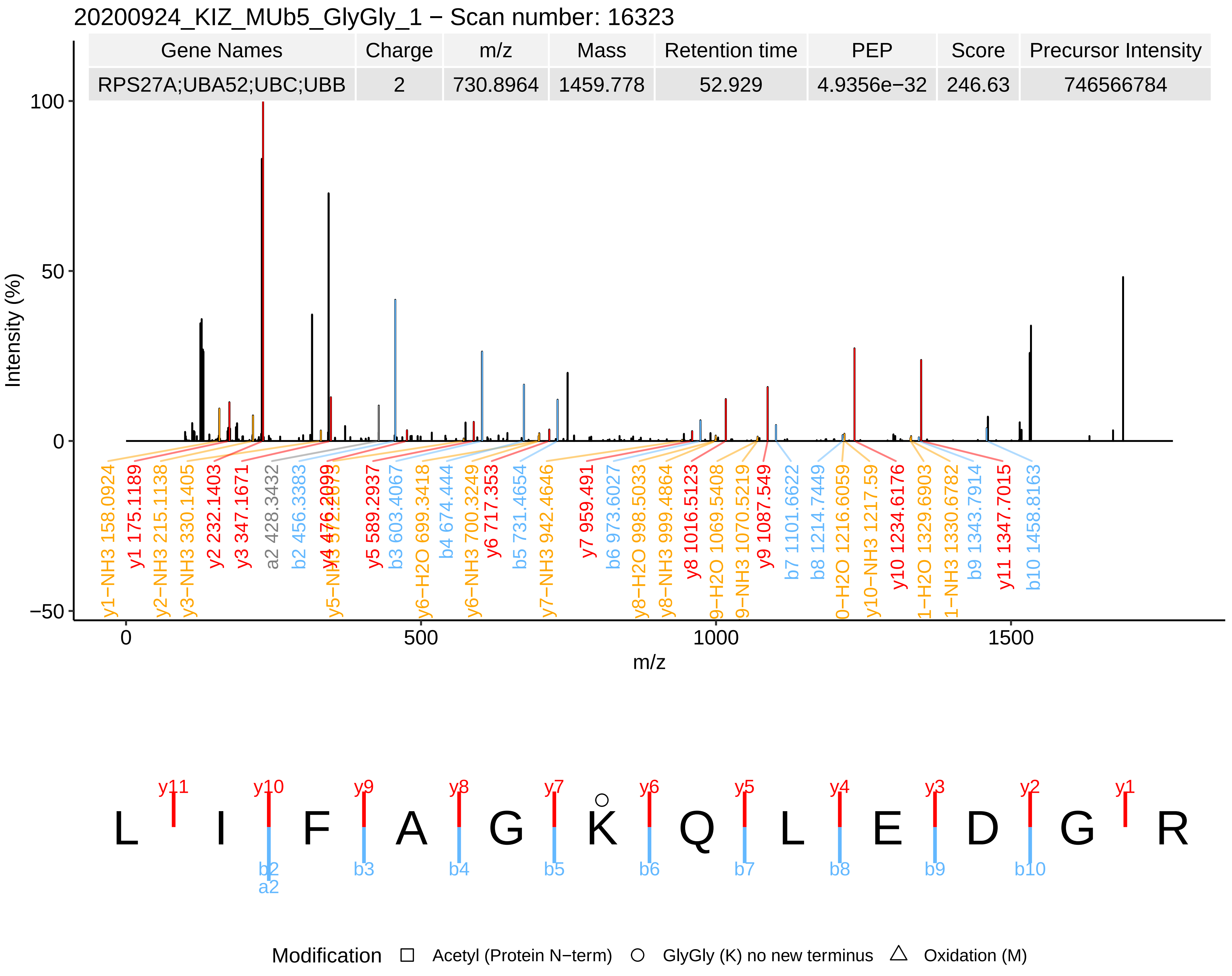

20200924_KIZ_MUb5_GlyGly_1 − Scan number: 16323
Gene Names
Charge
m/z
Mass
Retention time
PEP
Score
Precursor Intensity
RPS27A;UBA52;UBC;UBB
2
730.8964
1459.778
52.929
4.9356e−32
246.63
746566784
Intensity (%)
m/z
y1−NH3 158.0924
y1 175.1189
y2−NH3 215.1138
y3−NH3 330.1405
y2 232.1403
y3 347.1671
a2 428.3432
b2 456.3383
y4 476.2099
y5 589.2937
b3 603.4067
y6−H2O 699.3418
b4 674.444
y6−NH3 700.3249
y6 717.353
b5 731.4654
y7−NH3 942.4646
y7 959.491
b6 973.6027
y8−H2O 998.5033
y8−NH3 999.4864
y8 1016.5123
y9 1087.549
b7 1101.6622
b8 1214.7449
y10−NH3 1217.59
y10 1234.6176
b9 1343.7914
y11 1347.7015
b10 1458.8163
L I F A G K Q L E D G R
Modification
Acetyl (Protein N−term)
GlyGly (K) no new terminus
Oxidation (M)

20200924_KIZ_MUb5_GlyGly_1 − Scan number: 16500

Gene Names	Charge	m/z	Mass	Retention time	PEP	Score	Precursor Intensity
RPS27A;UBA52;UBC;UBB	2	730.8964	1459.778	53.435	9.036e−42	270.54	641235968

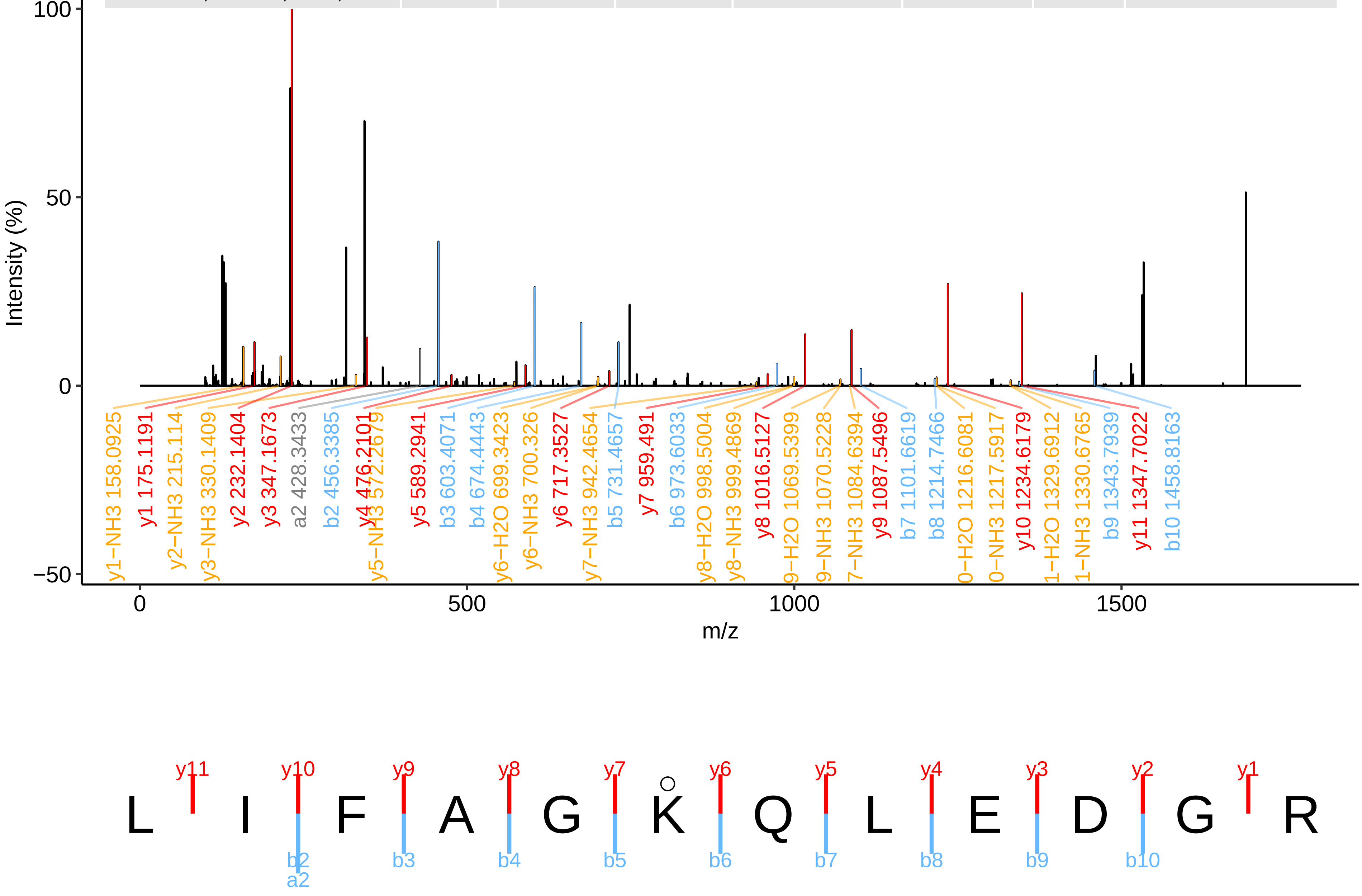

Modification □ Acetyl (Protein N−term) ○ GlyGly (K) no new terminus △ Oxidation (M)

20200924_KIZ_MUb5_GlyGly_1 − Scan number: 16677

Gene Names	Charge	m/z	Mass	Retention time	PEP	Score	Precursor Intensity
RPS27A;UBA52;UBC;UBB	2	730.8964	1459.778	53.946	1.0007e−31	238.73	503304672

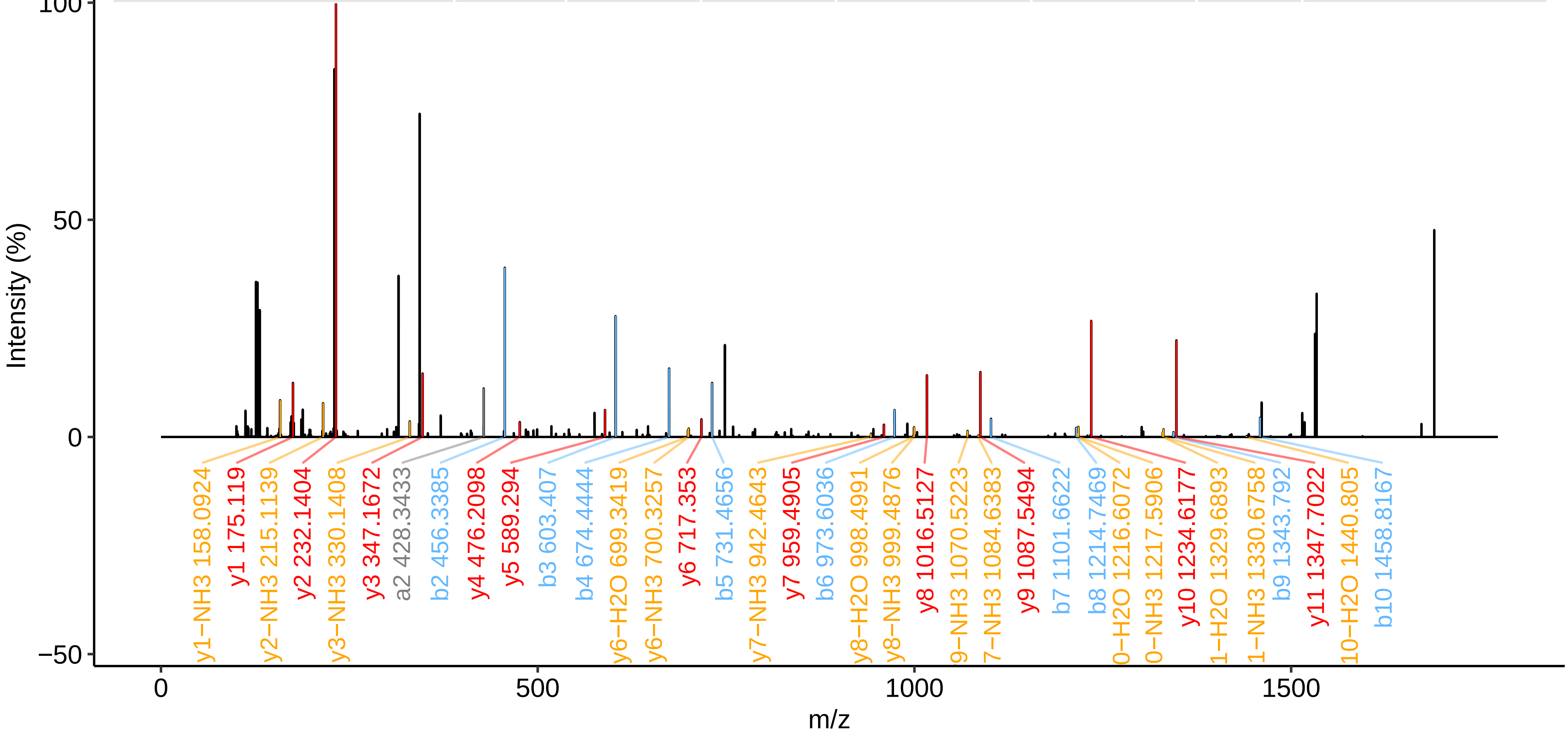

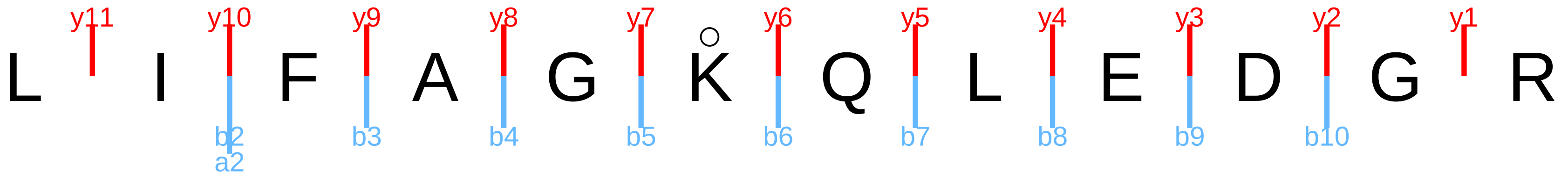

Modification □ Acetyl (Protein N−term) ○ GlyGly (K) no new terminus △ Oxidation (M)

20200924_KIZ_MUb5_GlyGly_1 − Scan number: 16849

Gene Names	Charge	m/z	Mass	Retention time	PEP	Score	Precursor Intensity
RPS27A;UBA52;UBC;UBB	2	730.8964	1459.778	54.456	1.0779e−63	295.32	266719664

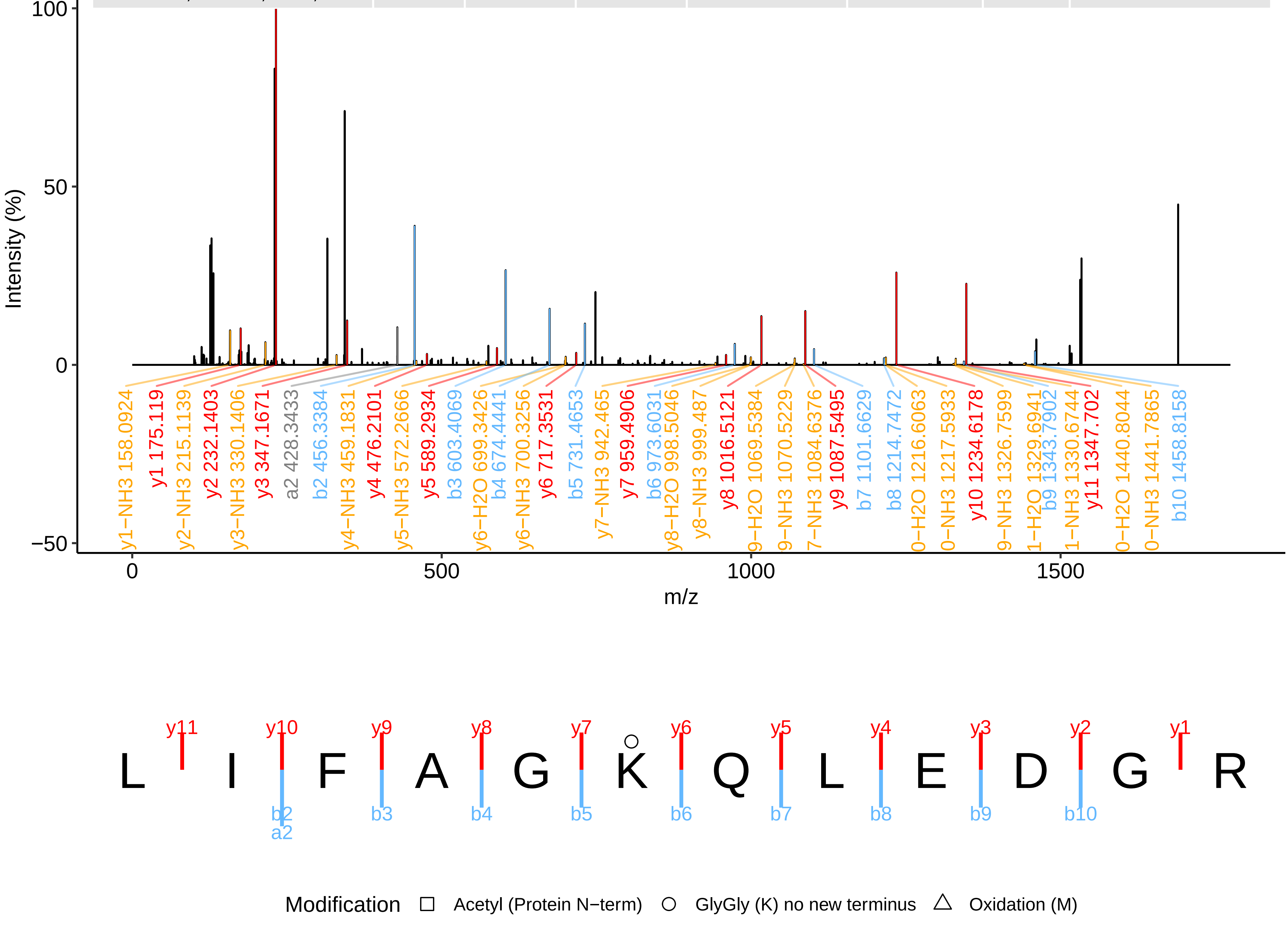

20200924_KIZ_MUb5_GlyGly_1 − Scan number: 17016

Gene Names	Charge	m/z	Mass	Retention time	PEP	Score	Precursor Intensity
RPS27A;UBA52;UBC;UBB	2	730.8964	1459.778	54.959	1.9946e−32	251.2	115827920

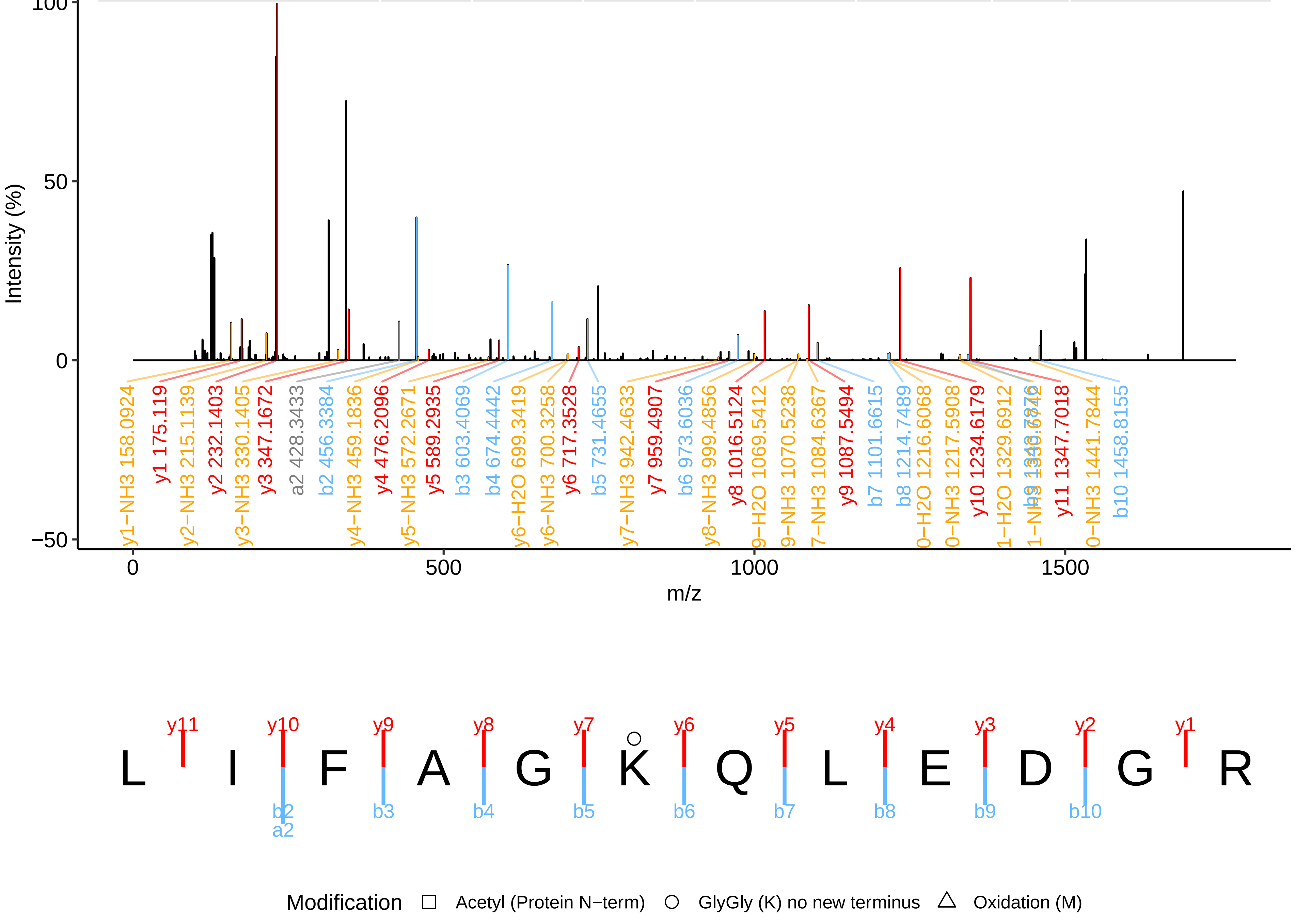

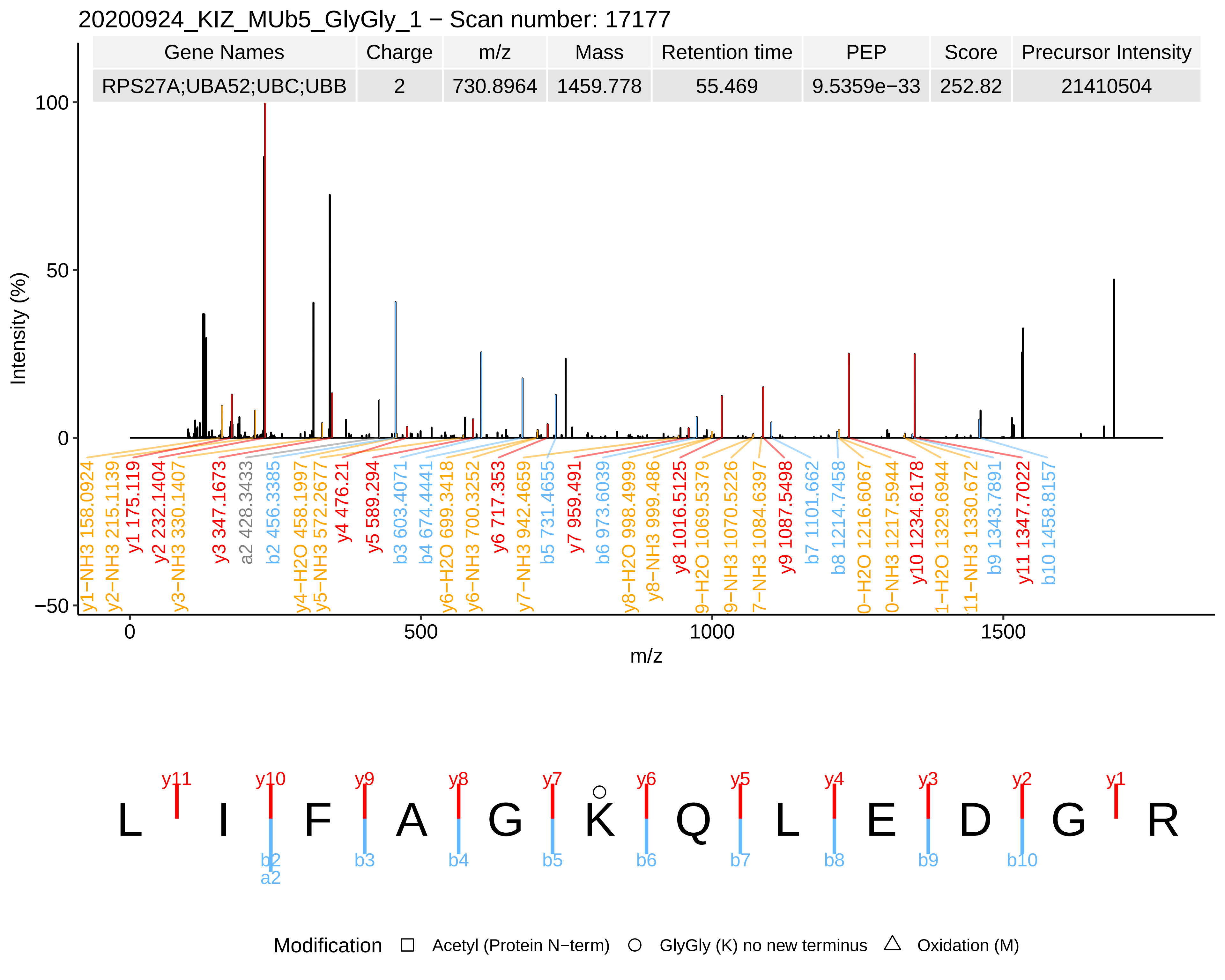

20200924_KIZ_MUb5_GlyGly_1 − Scan number: 17177
Gene Names | Charge | m/z | Mass | Retention time | PEP | Score | Precursor Intensity
RPS27A;UBA52;UBC;UBB | 2 | 730.8964 | 1459.778 | 55.469 | 9.5359e−33 | 252.82 | 21410504
Intensity (%)
100
50
0
−50
y1−NH3 158.0924
y2−NH3 215.1139
y1 175.119
y2 232.1404
y3−NH3 330.1407
y3 347.1673
a2 428.3433
b2 456.3385
y4−H2O 458.1997
y5−NH3 572.2677
y4 476.21
y5 589.294
b3 603.4071
b4 674.4441
y6−H2O 699.3418
y6−NH3 700.3252
y6 717.353
y7−NH3 942.4659
b5 731.4655
y7 959.491
b6 973.6039
y8−H2O 998.4999
y8−NH3 999.486
y8 1016.5125
9−H2O 1069.5379
9−NH3 1070.5226
7−NH3 1084.6397
y9 1087.5498
b7 1101.662
b8 1214.7458
0−H2O 1216.6067
0−NH3 1217.5944
y10 1234.6178
1−H2O 1329.6944
11−NH3 1330.672
b9 1343.7891
y11 1347.7022
b10 1458.8157
0
500
1000
1500
m/z
y11 y10 y9 y8 y7 y6 y5 y4 y3 y2 y1
L I F A G K Q L E D G R
b2 b3 b4 b5 b6 b7 b8 b9 b10
a2
Modification
Acetyl (Protein N−term)
GlyGly (K) no new terminus
Oxidation (M)

20200924_KIZ_MUb5_GlyGly_1 − Scan number: 17339

Gene Names	Charge	m/z	Mass	Retention time	PEP	Score	Precursor Intensity
RPS27A;UBA52;UBC;UBB	2	730.8964	1459.778	55.975	3.4778e−19	202.96	NA

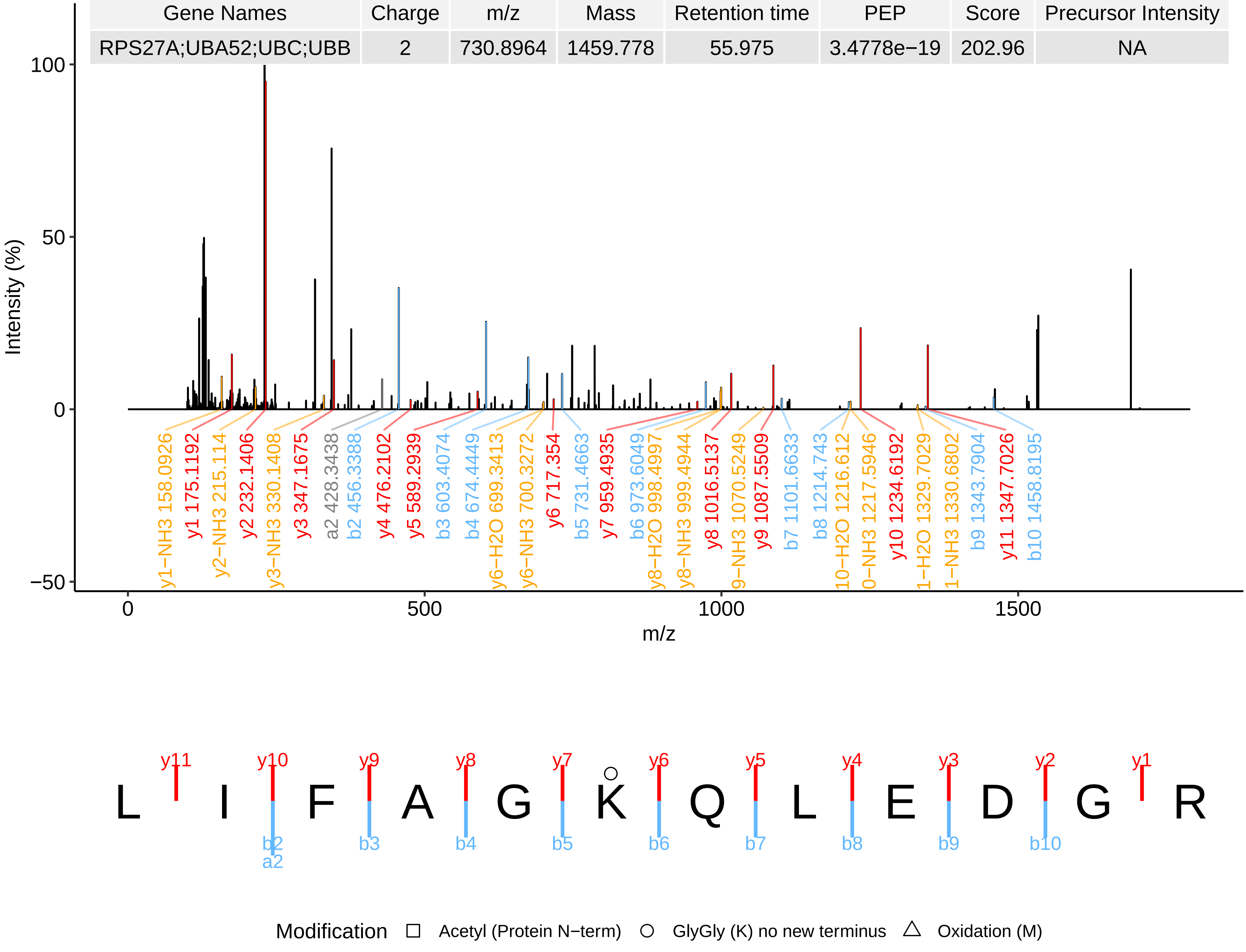

20200924_KIZ_MUb5_GlyGly_1 − Scan number: 18607

Gene Names	Charge	m/z	Mass	Retention time	PEP	Score	Precursor Intensity
RPS27A;UBA52;UBC;UBB	3	487.6001	1459.778	60.138	0.00041122	93.839	12275844

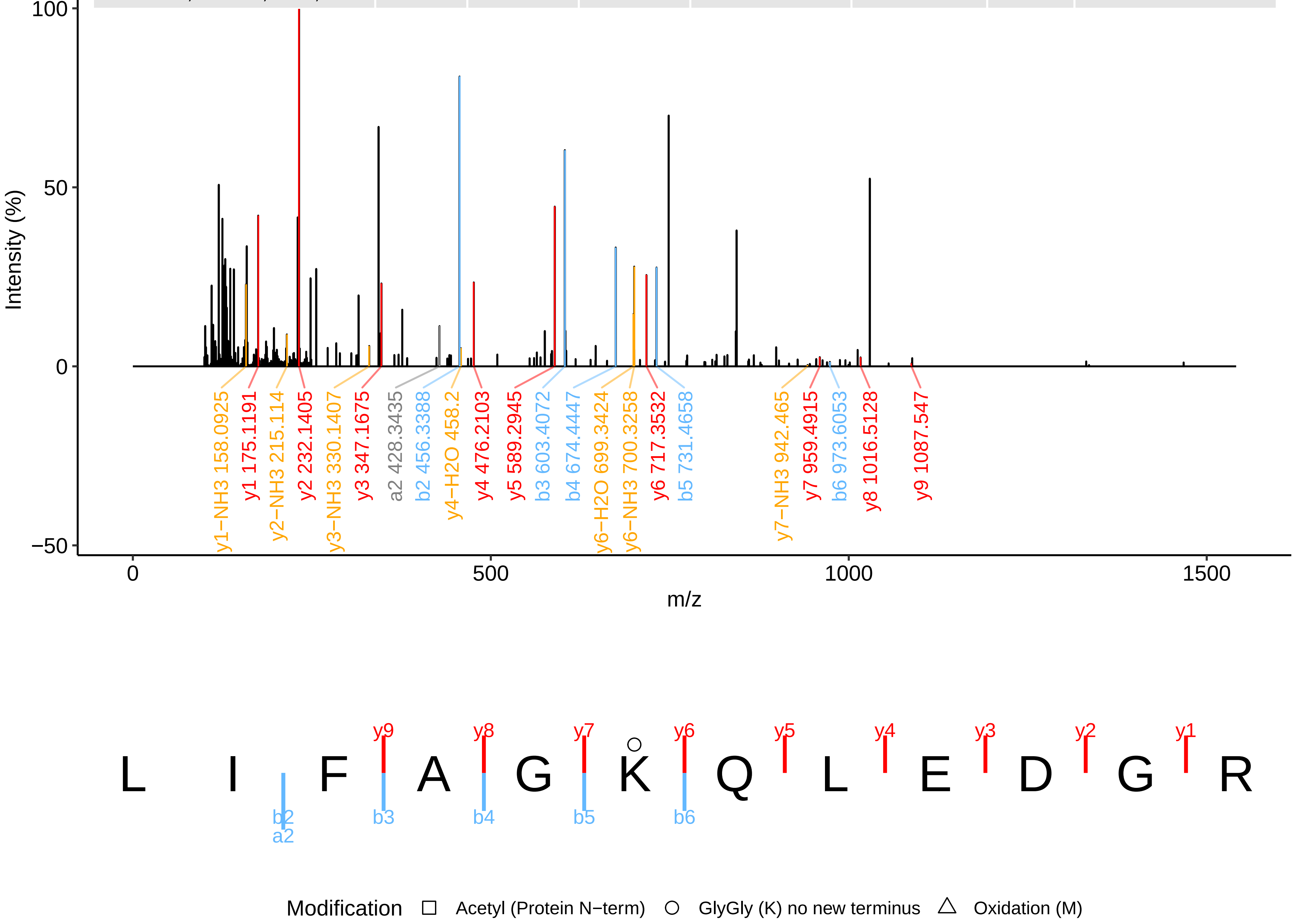

20200924_KIZ_MUb5_GlyGly_2 − Scan number: 15272

Gene Names	Charge	m/z	Mass	Retention time	PEP	Score	Precursor Intensity
RPS27A;UBA52;UBC;UBB	2	730.8964	1459.778	57.086	8.738e−19	154.05	30650178

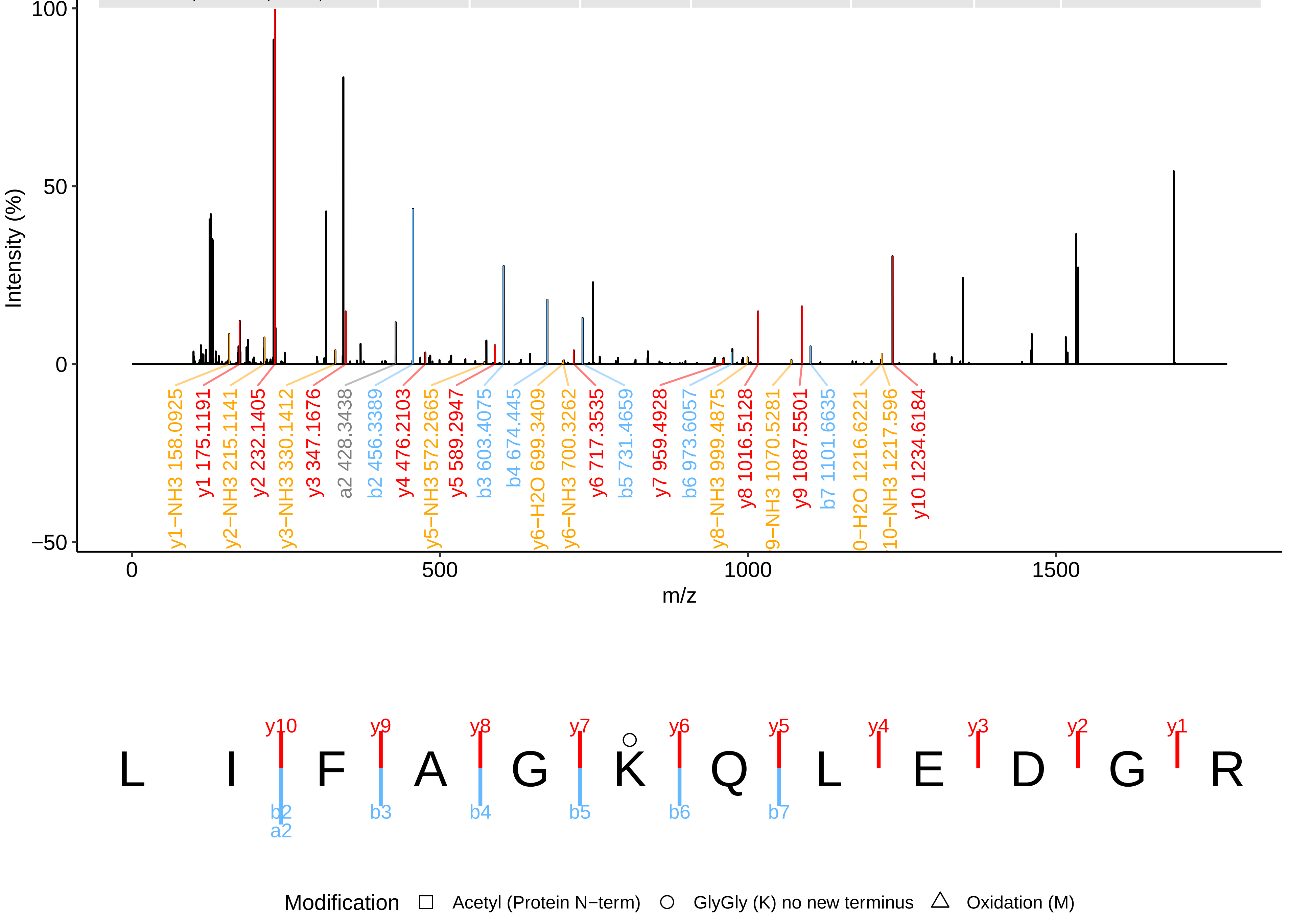

20200924_KIZ_MUb5_GlyGly_2 − Scan number: 15439

Gene Names	Charge	m/z	Mass	Retention time	PEP	Score	Precursor Intensity
RPS27A;UBA52;UBC;UBB	2	730.8964	1459.778	57.587	6.4311e−50	246.63	283318112

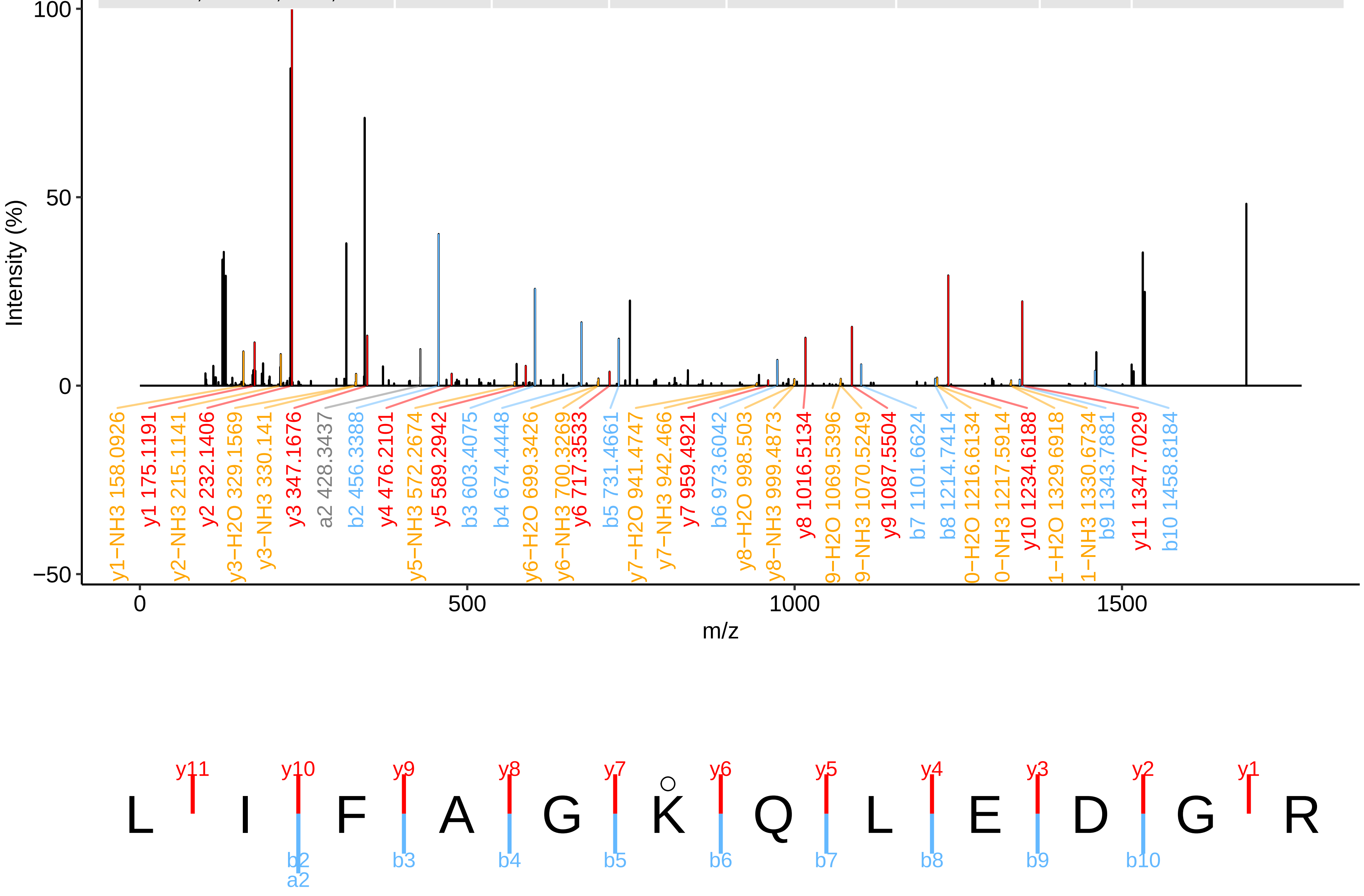

Modification □ Acetyl (Protein N−term) ○ GlyGly (K) no new terminus △ Oxidation (M)

20200924_KIZ_MUb5_GlyGly_2 − Scan number: 15297

Gene Names	Charge	m/z	Mass	Retention time	PEP	Score	Precursor Intensity
RPS27A;UBA52;UBC;UBB	3	487.6001	1459.778	57.154	3.1574e−19	150.46	10028980224

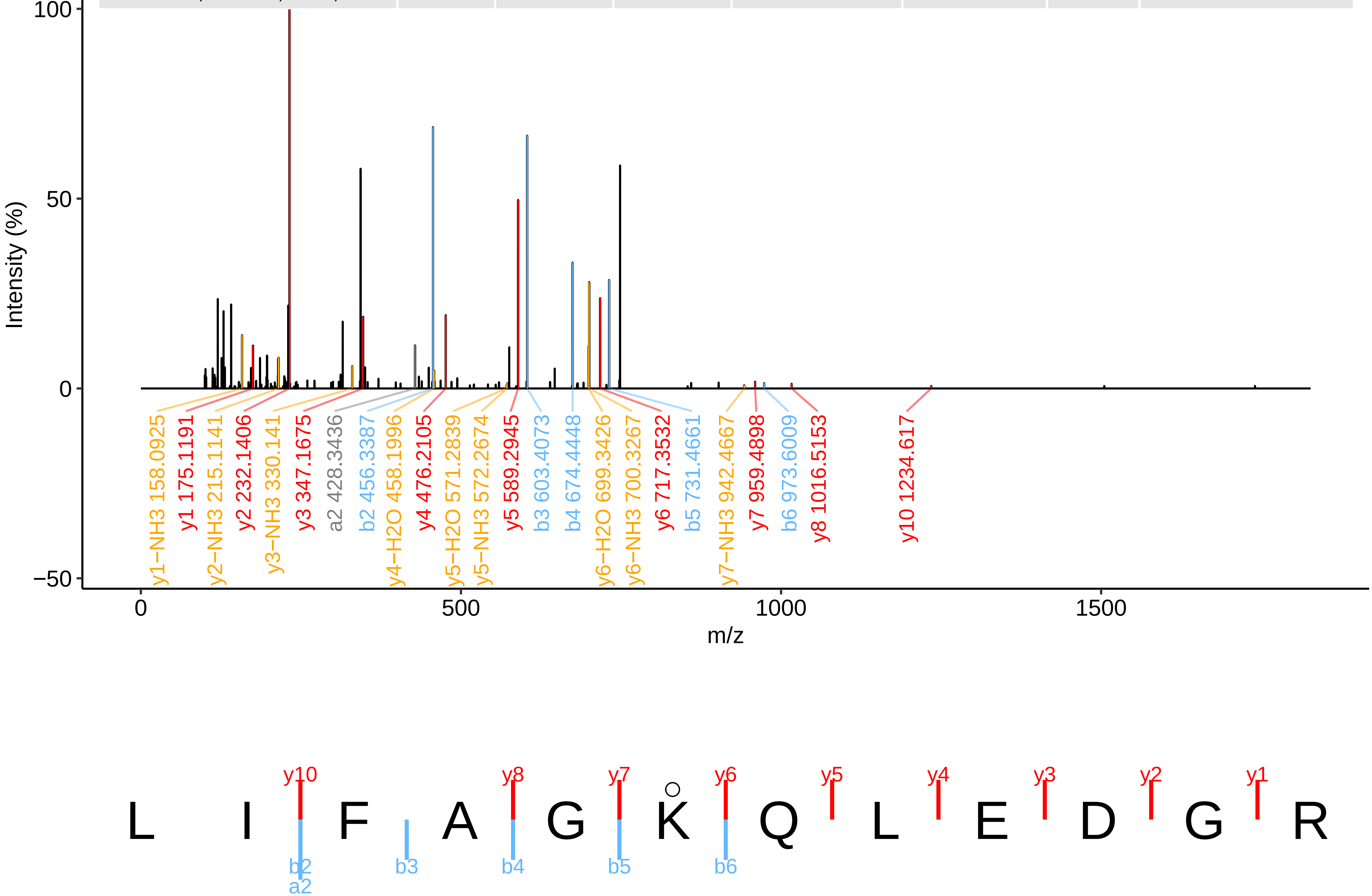

Modification □ Acetyl (Protein N−term) ○ GlyGly (K) no new terminus △ Oxidation (M)

20200924_KIZ_MUb5_GlyGly_2 − Scan number: 15465

Gene Names	Charge	m/z	Mass	Retention time	PEP	Score	Precursor Intensity
RPS27A;UBA52;UBC;UBB	3	487.6001	1459.778	57.661	3.6333e−13	133.89	11488747520

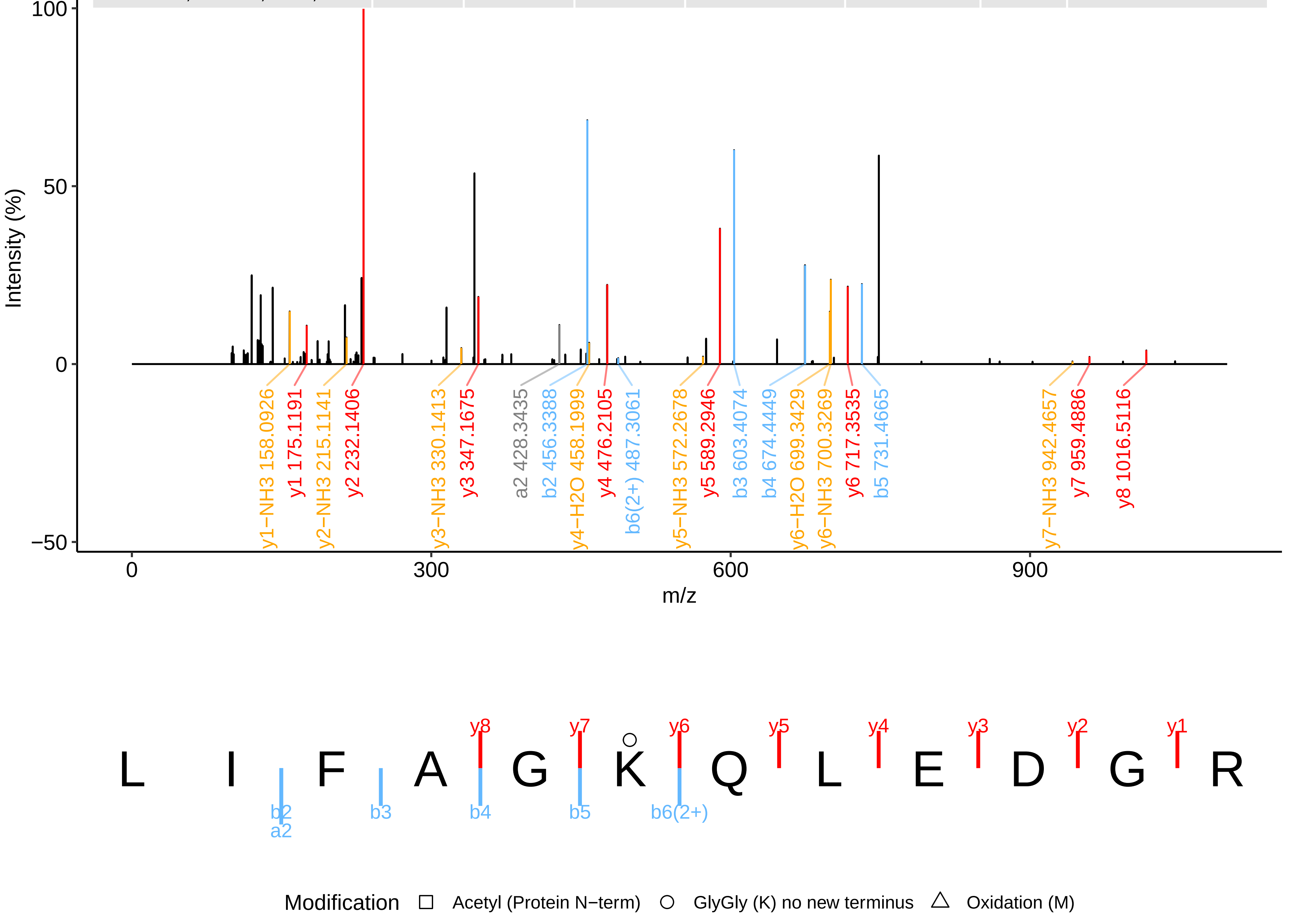

20200924_KIZ_MUb5_GlyGly_2 − Scan number: 15631

Gene Names	Charge	m/z	Mass	Retention time	PEP	Score	Precursor Intensity
RPS27A;UBA52;UBC;UBB	3	487.6001	1459.778	58.164	3.1574e−19	150.46	7613999104

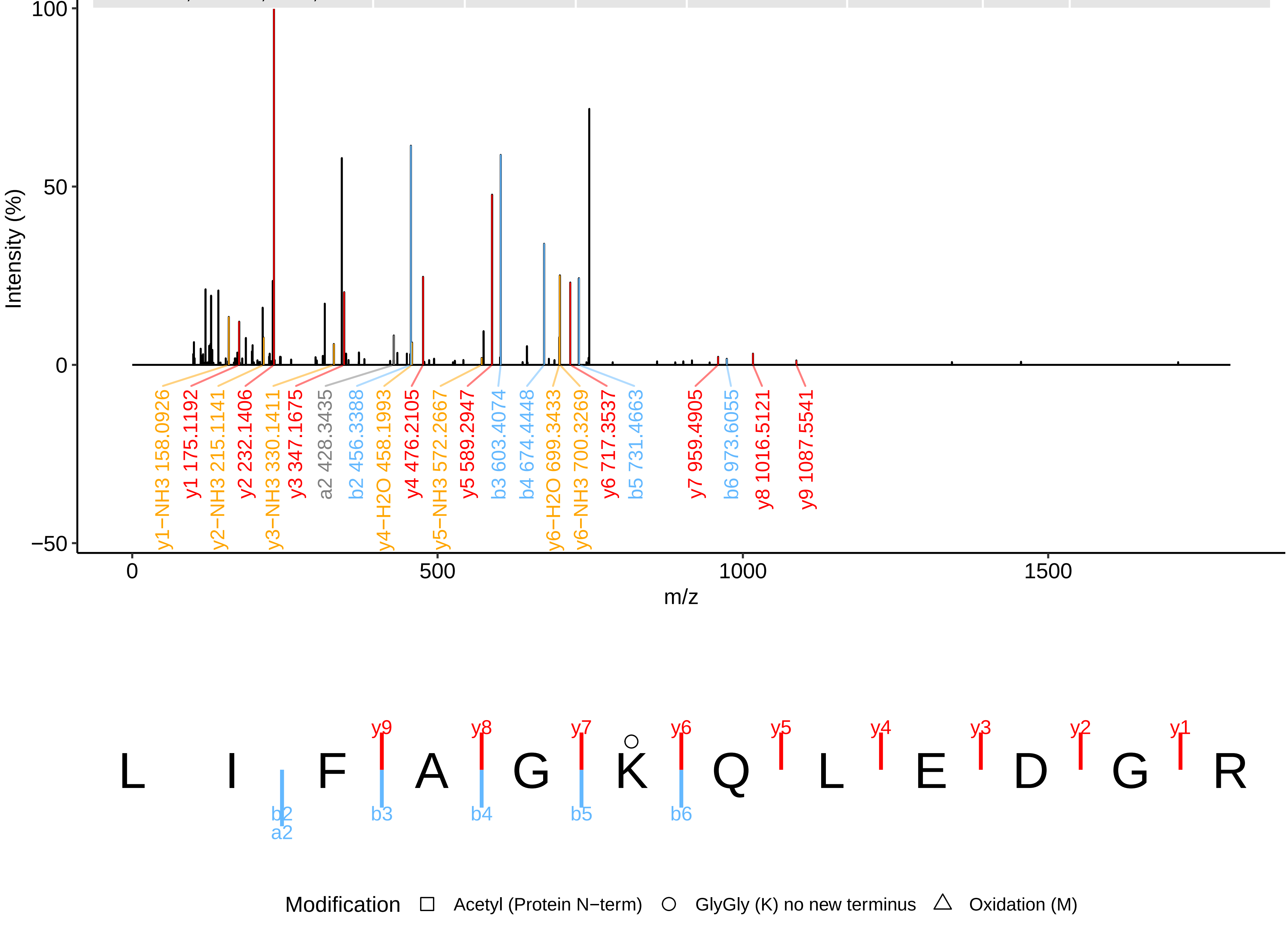

20200924_KIZ_MUb5_GlyGly_2 − Scan number: 15789

Gene Names	Charge	m/z	Mass	Retention time	PEP	Score	Precursor Intensity
RPS27A;UBA52;UBC;UBB	3	487.6001	1459.778	58.667	1.2223e−29	186.87	5944741376

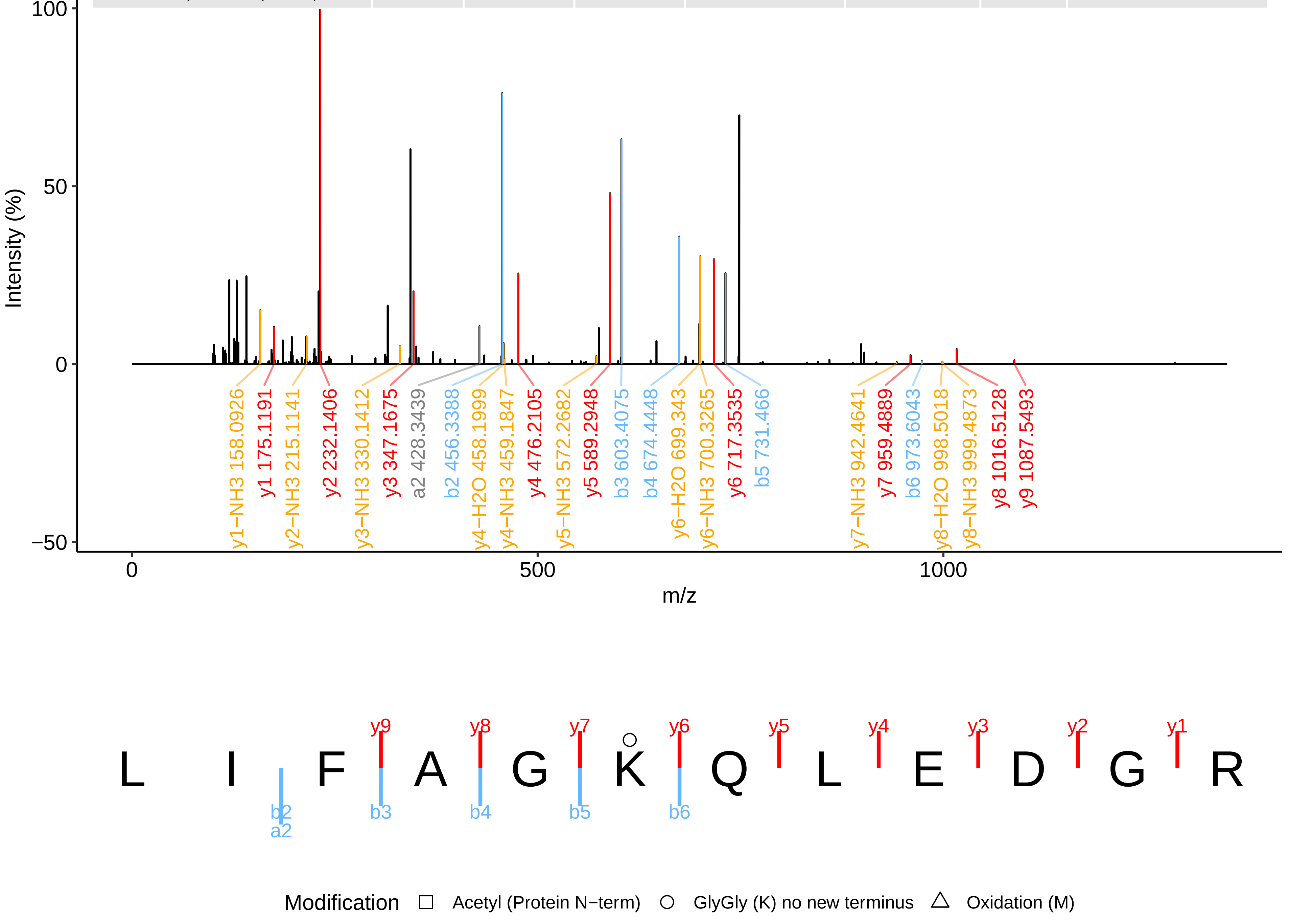

20200924_KIZ_MUb5_GlyGly_2 − Scan number: 15855

Gene Names	Charge	m/z	Mass	Retention time	PEP	Score	Precursor Intensity
RPS27A;UBA52;UBC;UBB	3	487.6001	1459.778	58.901	0.011782	64.792	158753472

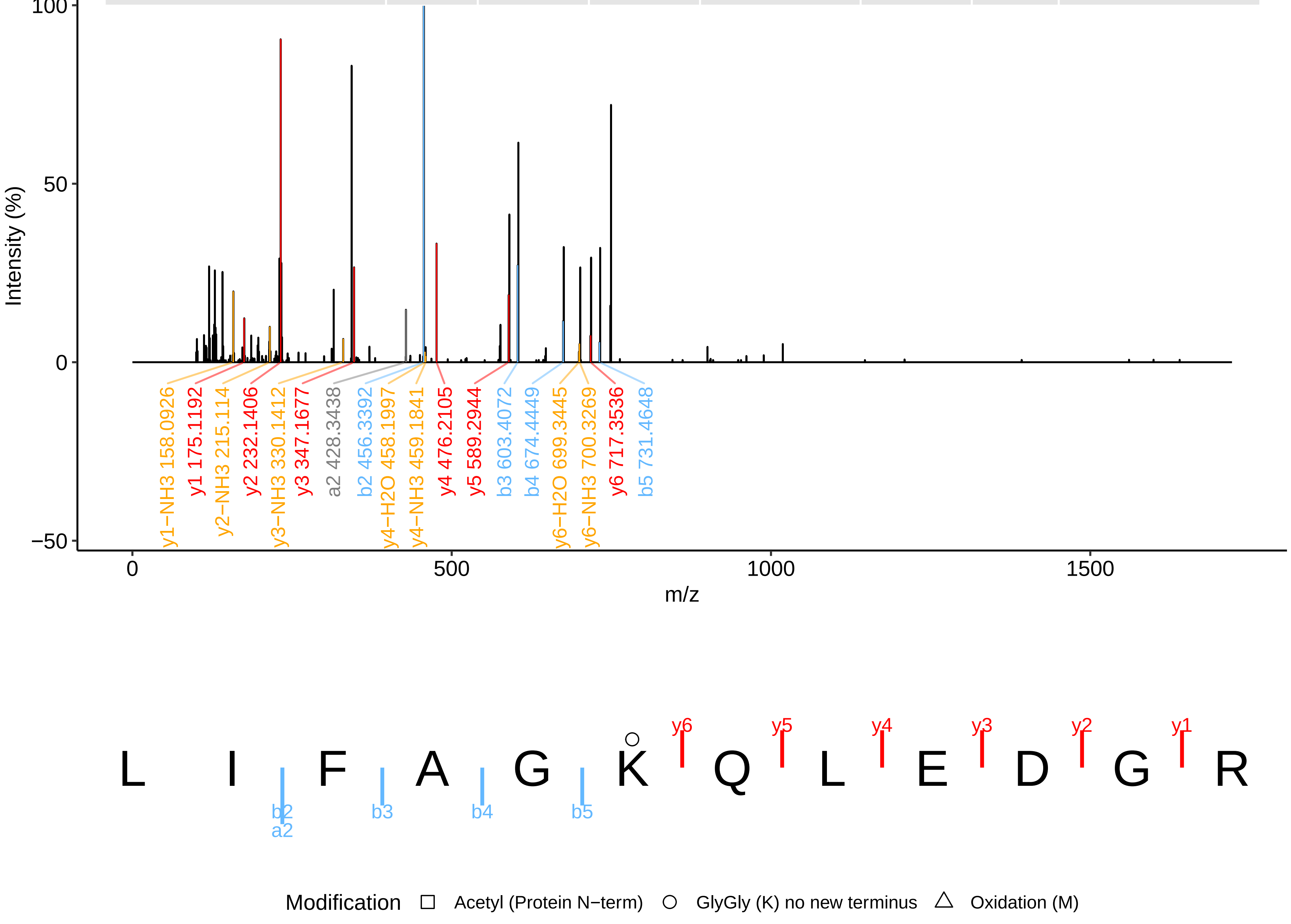

20200924_KIZ_MUb5_GlyGly_2 − Scan number: 15993

Gene Names	Charge	m/z	Mass	Retention time	PEP	Score	Precursor Intensity
RPS27A;UBA52;UBC;UBB	3	487.6001	1459.778	59.41	3.1574e−19	150.46	1487622912

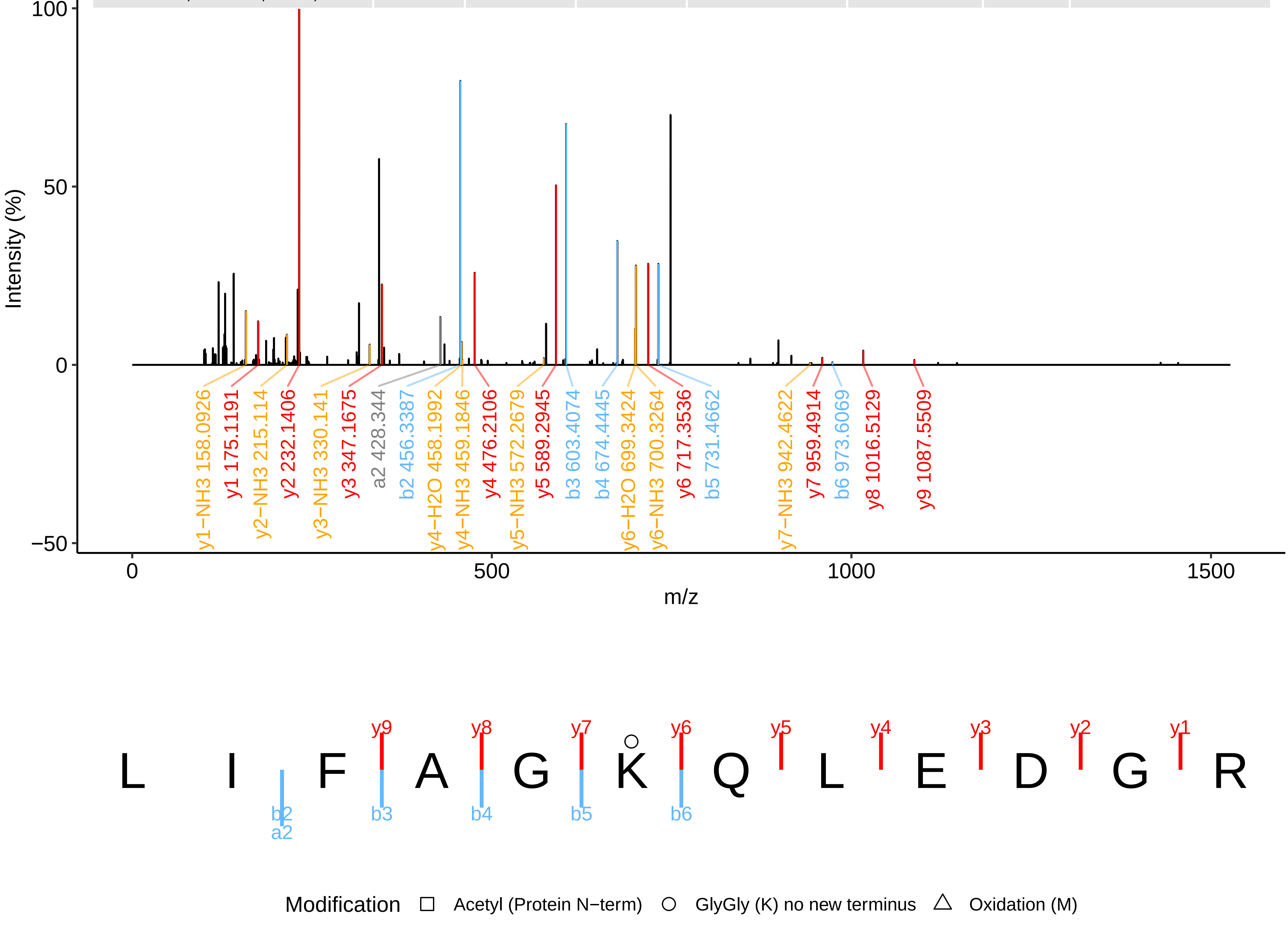

20200924_KIZ_MUb5_GlyGly_2 − Scan number: 16005

Gene Names	Charge	m/z	Mass	Retention time	PEP	Score	Precursor Intensity
RPS27A;UBA52;UBC;UBB	3	487.6001	1459.778	59.45	9.899e−20	162.26	40330928

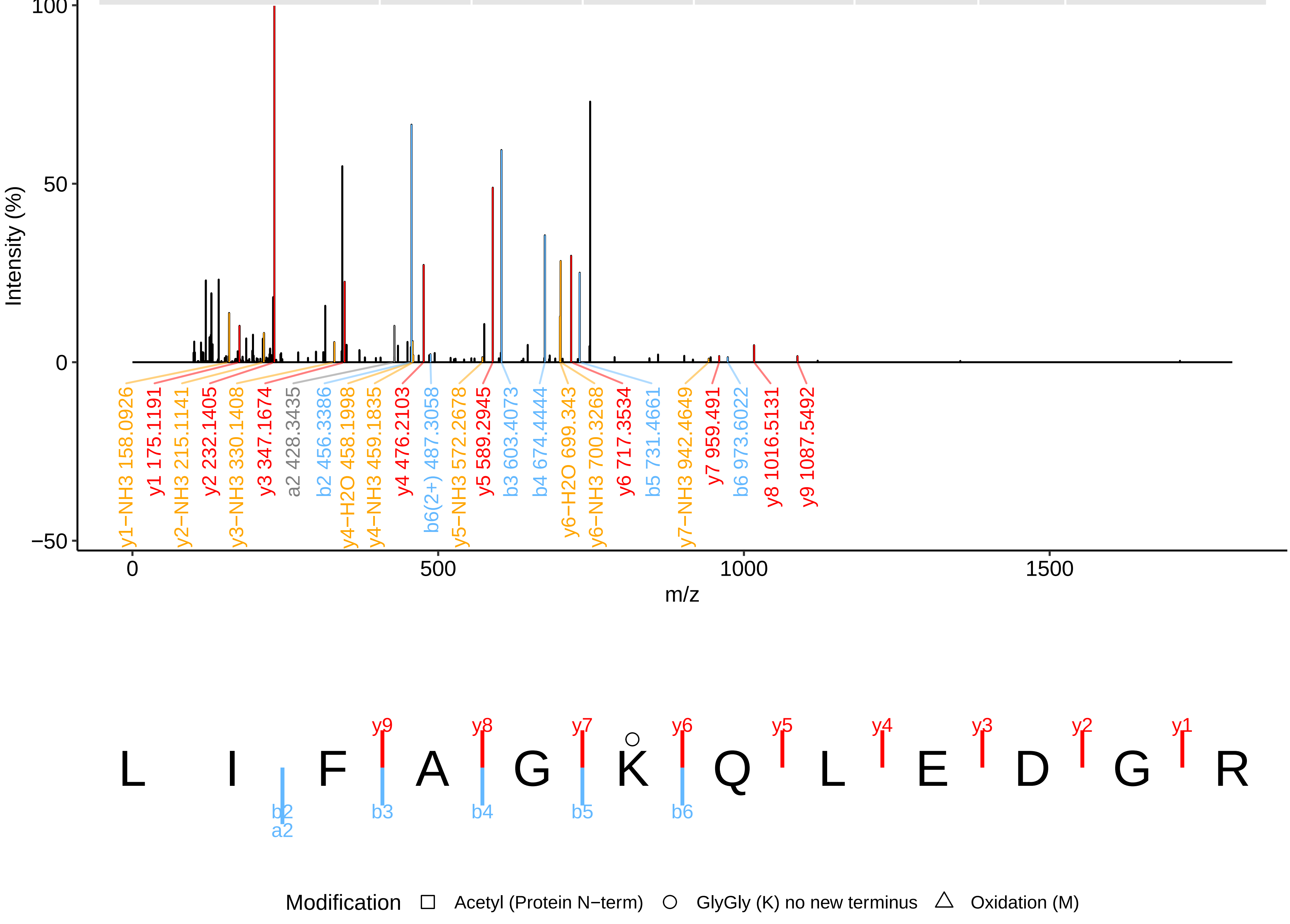

20200924_KIZ_MUb5_GlyGly_2 − Scan number: 16151

Gene Names	Charge	m/z	Mass	Retention time	PEP	Score	Precursor Intensity
RPS27A;UBA52;UBC;UBB	3	487.6001	1459.778	59.96	3.1574e−19	150.46	137349120

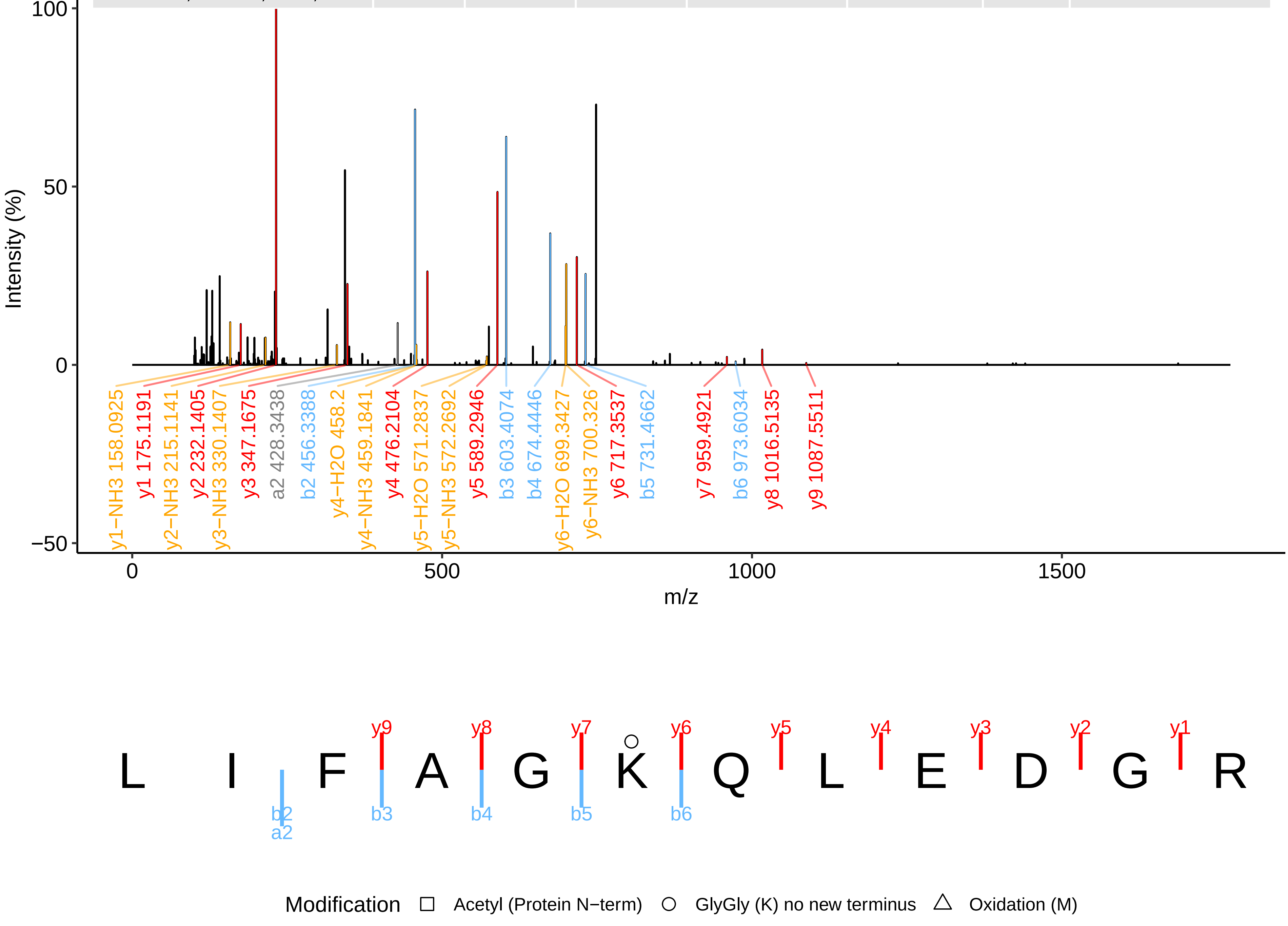

20200924_KIZ_MUb5_GlyGly_2 − Scan number: 16296

Gene Names	Charge	m/z	Mass	Retention time	PEP	Score	Precursor Intensity
RPS27A;UBA52;UBC;UBB	3	487.6001	1459.778	60.466	2.4754e−09	129.82	22133514

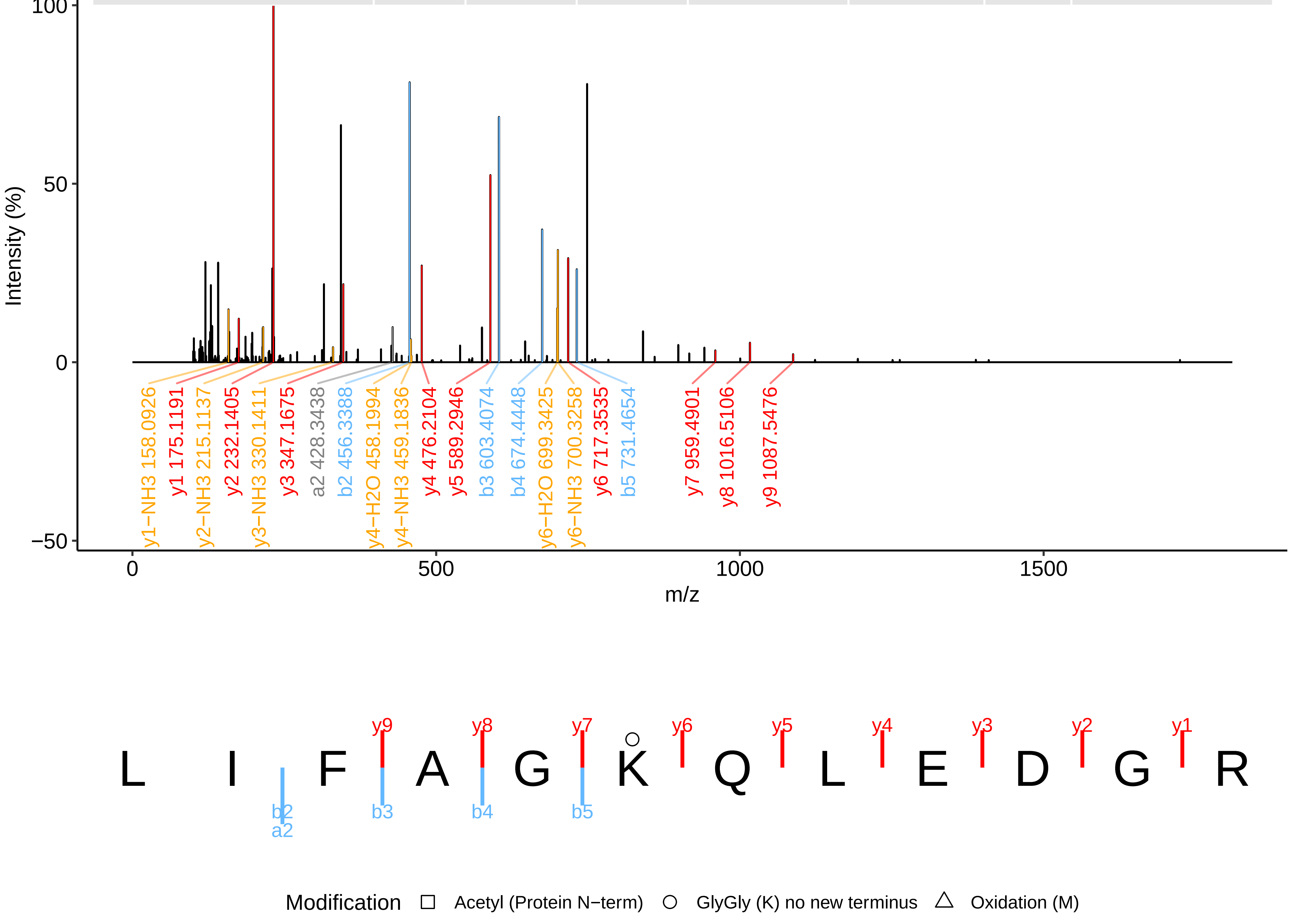

20200924_KIZ_MUb5_GlyGly_2 − Scan number: 16449

Gene Names	Charge	m/z	Mass	Retention time	PEP	Score	Precursor Intensity
RPS27A;UBA52;UBC;UBB	3	487.6001	1459.778	61.033	1.0628e−13	145.46	28214502

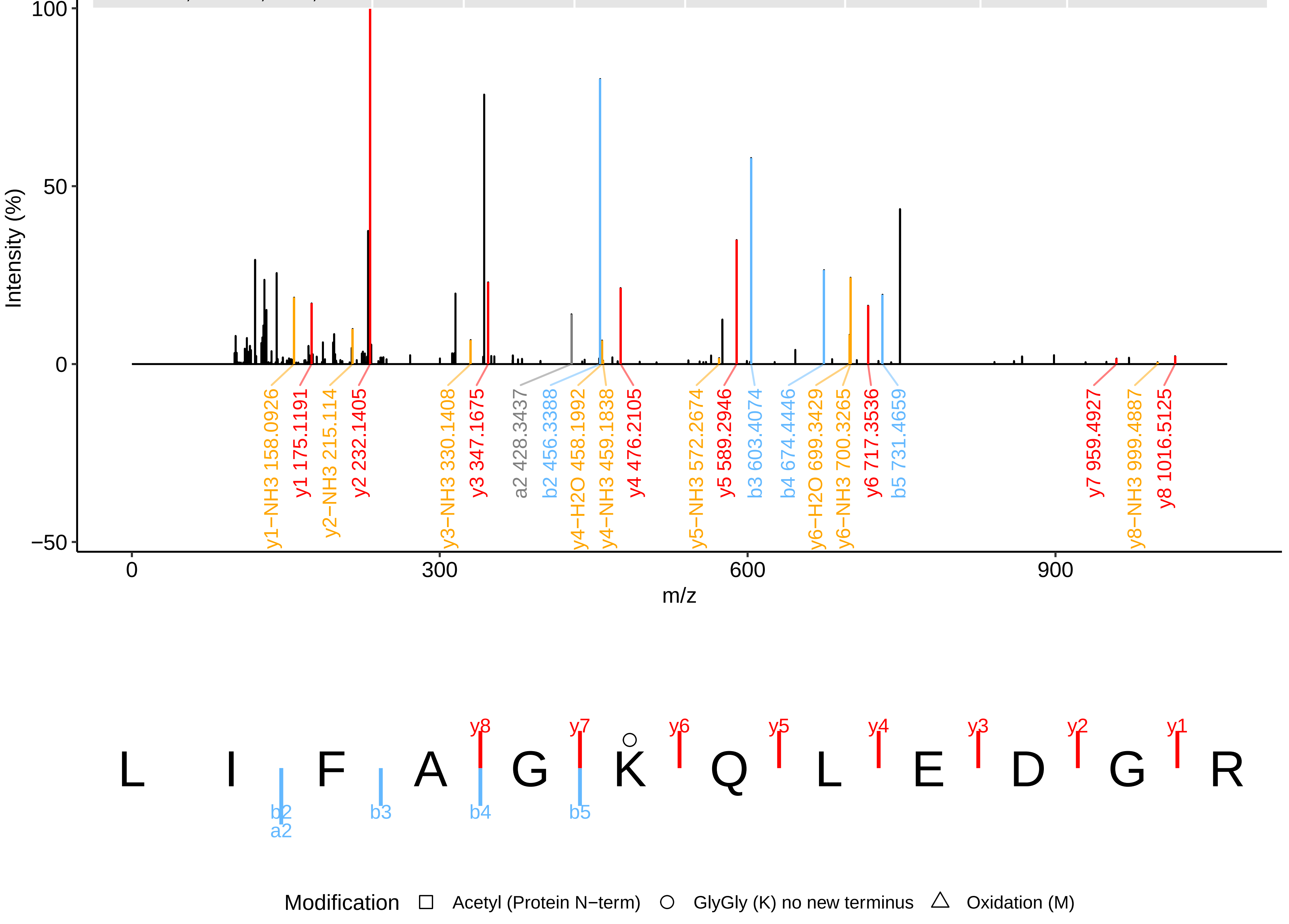

20200924_KIZ_MUb5_GlyGly_2 − Scan number: 15606

Gene Names	Charge	m/z	Mass	Retention time	PEP	Score	Precursor Intensity
RPS27A;UBA52;UBC;UBB	2	730.8964	1459.778	58.088	1.705e−41	232.88	156711424

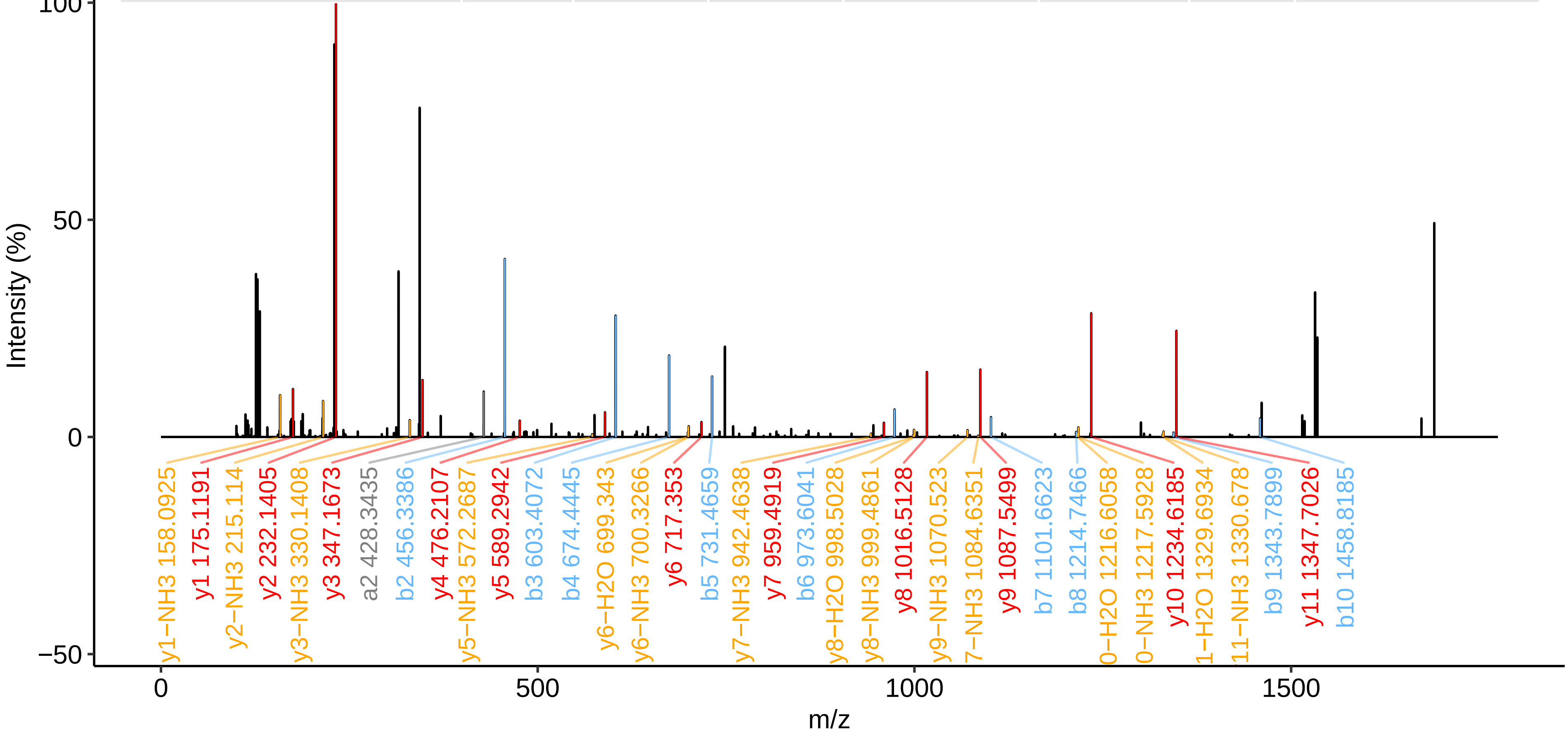

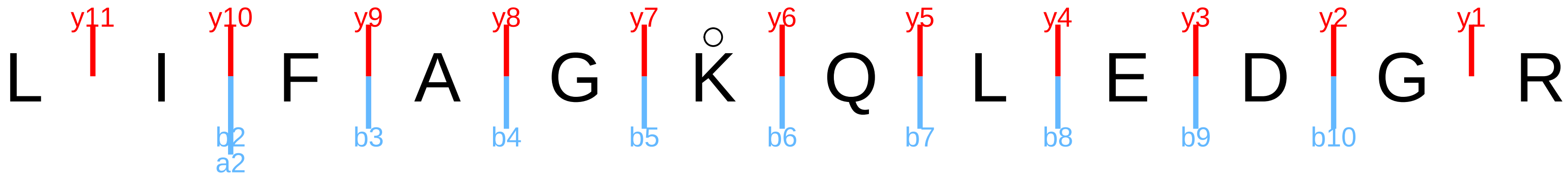

Modification □ Acetyl (Protein N−term) ○ GlyGly (K) no new terminus △ Oxidation (M)

20200924_KIZ_MUb5_GlyGly_2 − Scan number: 15765

Gene Names	Charge	m/z	Mass	Retention time	PEP	Score	Precursor Intensity
RPS27A;UBA52;UBC;UBB	2	730.8964	1459.778	58.594	8.0931e−19	155.06	110683712

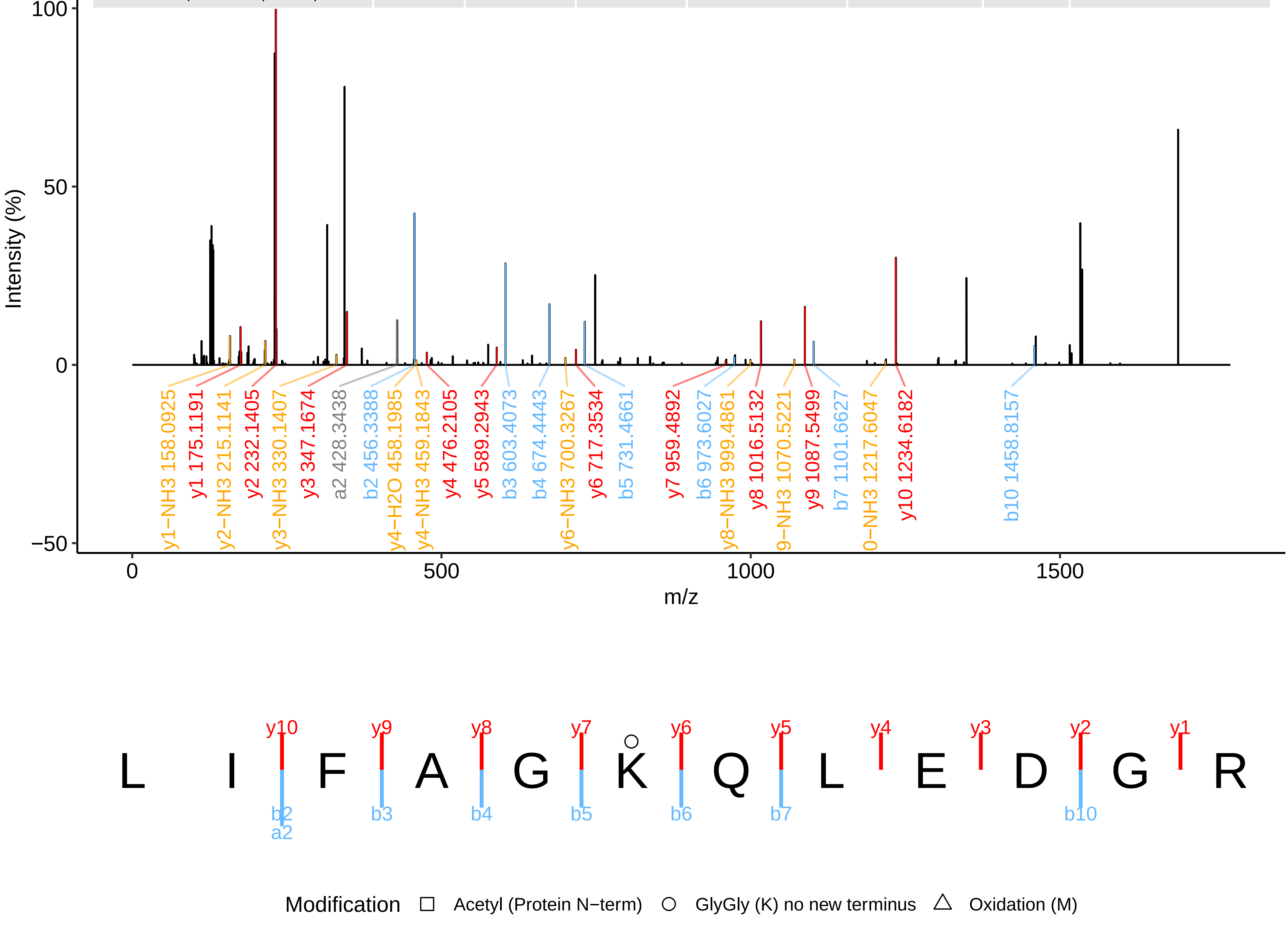

20200924_KIZ_MUb5_GlyGly_2 − Scan number: 15908

Gene Names	Charge	m/z	Mass	Retention time	PEP	Score	Precursor Intensity
RPS27A;UBA52;UBC;UBB	2	730.8964	1459.778	59.101	5.1416e−72	276.57	46600544

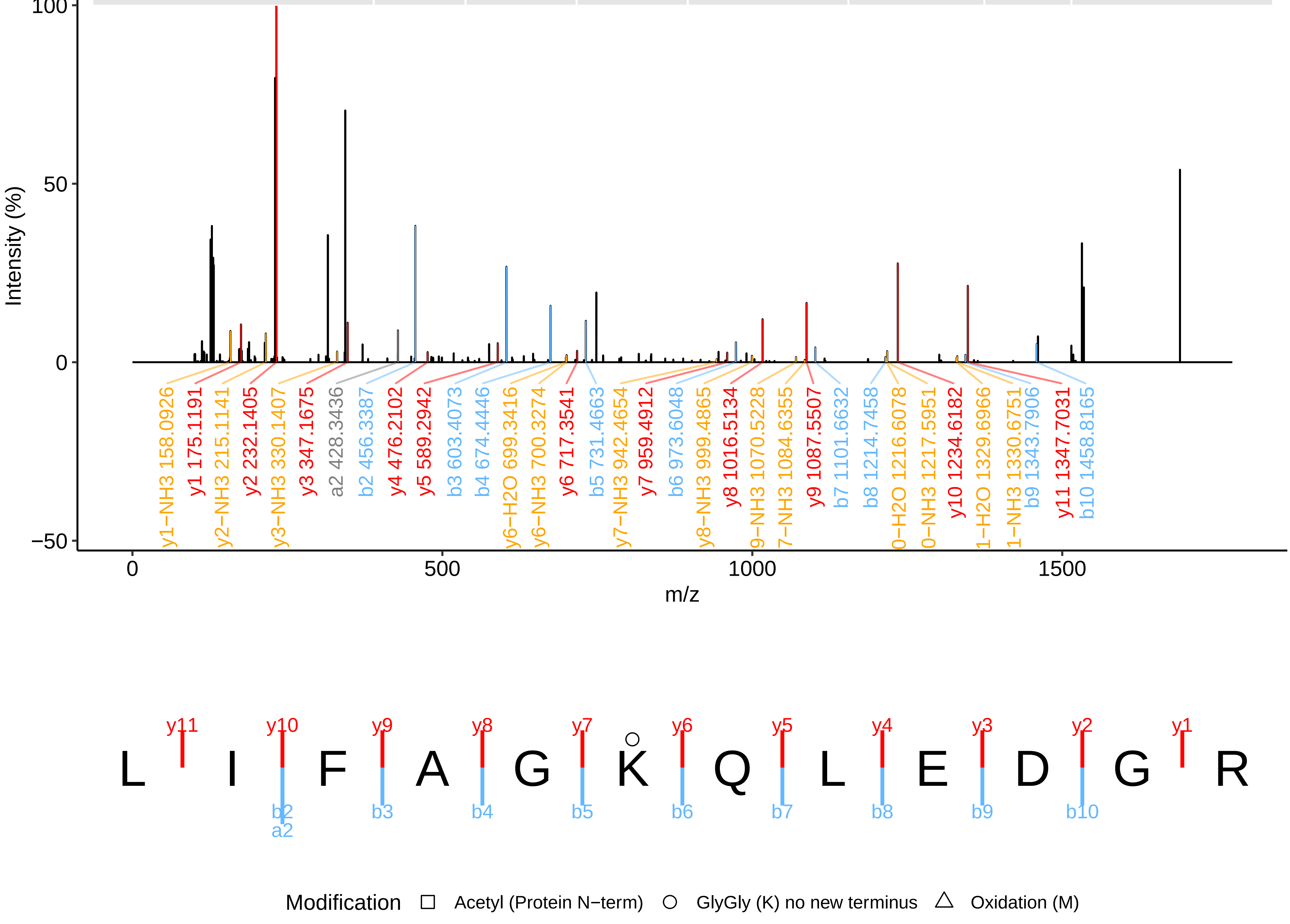

20200924_KIZ_MUb5_GlyGly_2 − Scan number: 16050

Gene Names	Charge	m/z	Mass	Retention time	PEP	Score	Precursor Intensity
RPS27A;UBA52;UBC;UBB	2	730.8964	1459.778	59.611	6.0669e−50	247.08	11443751

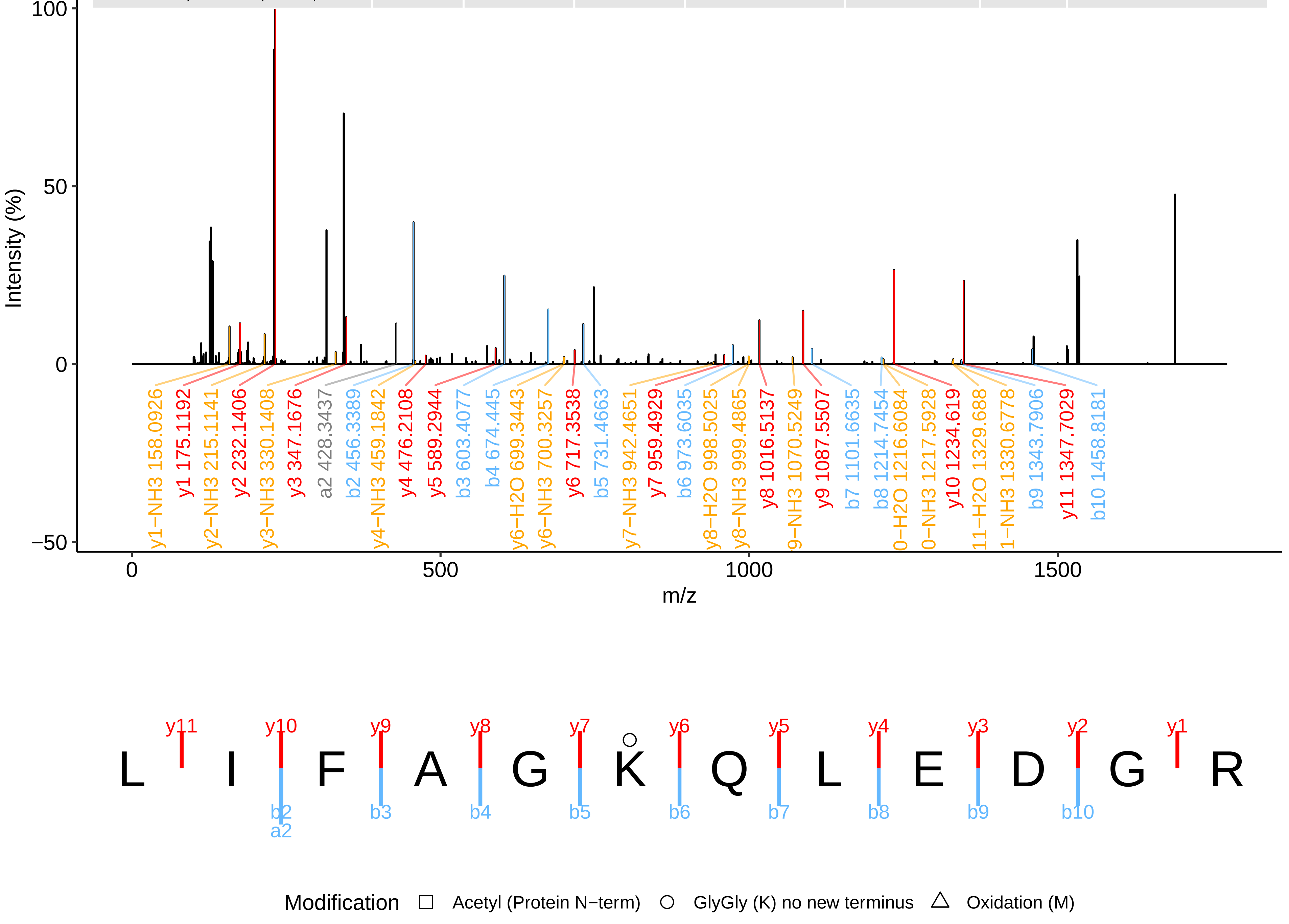

20200924_KIZ_MUb5_GlyGly_2 − Scan number: 14743

Gene Names	Charge	m/z	Mass	Retention time	PEP	Score	Precursor Intensity
RPS27A;UBA52;UBC;UBB	3	487.6001	1459.778	55.083	0.041652	59.367	NA

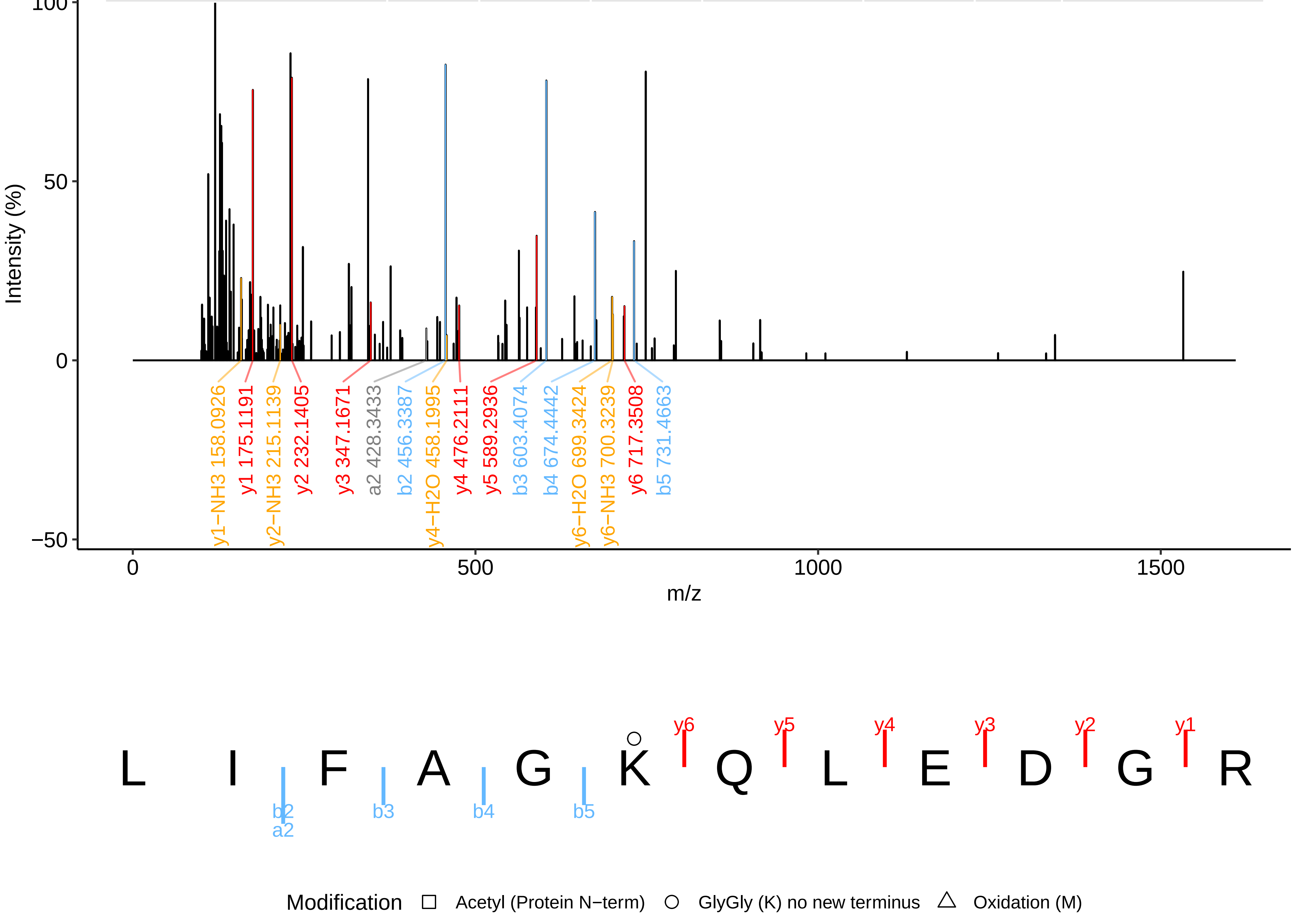

20200924_KIZ_MUb5_GlyGly_2 − Scan number: 16610

Gene Names	Charge	m/z	Mass	Retention time	PEP	Score	Precursor Intensity
RPS27A;UBA52;UBC;UBB	3	487.6001	1459.778	61.605	1.721e−06	129.82	15498530

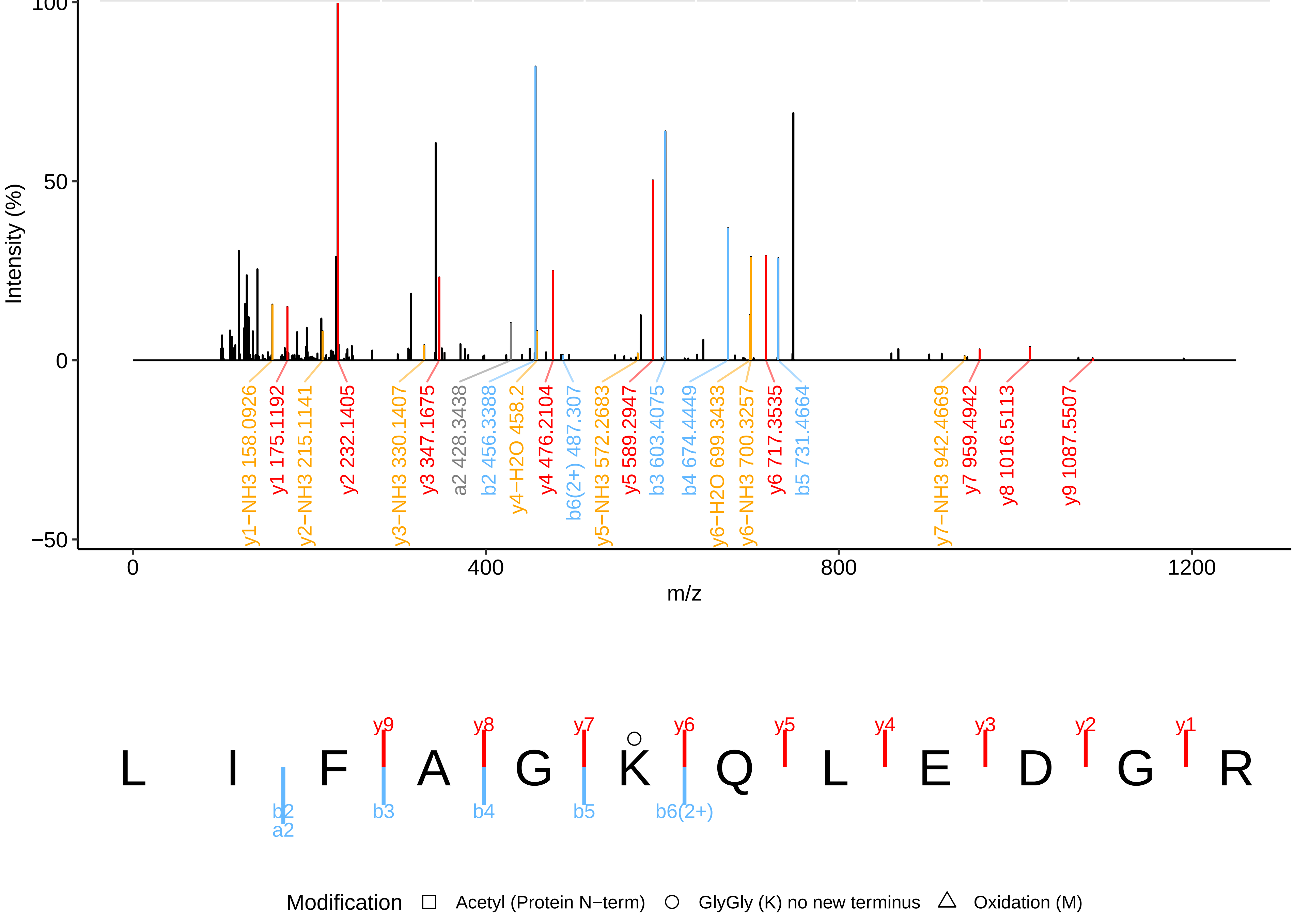

20200924_KIZ_MUb5_GlyGly_2 − Scan number: 16745

Gene Names	Charge	m/z	Mass	Retention time	PEP	Score	Precursor Intensity
RPS27A;UBA52;UBC;UBB	3	487.6001	1459.778	62.124	7.9801e−08	138.99	5886922

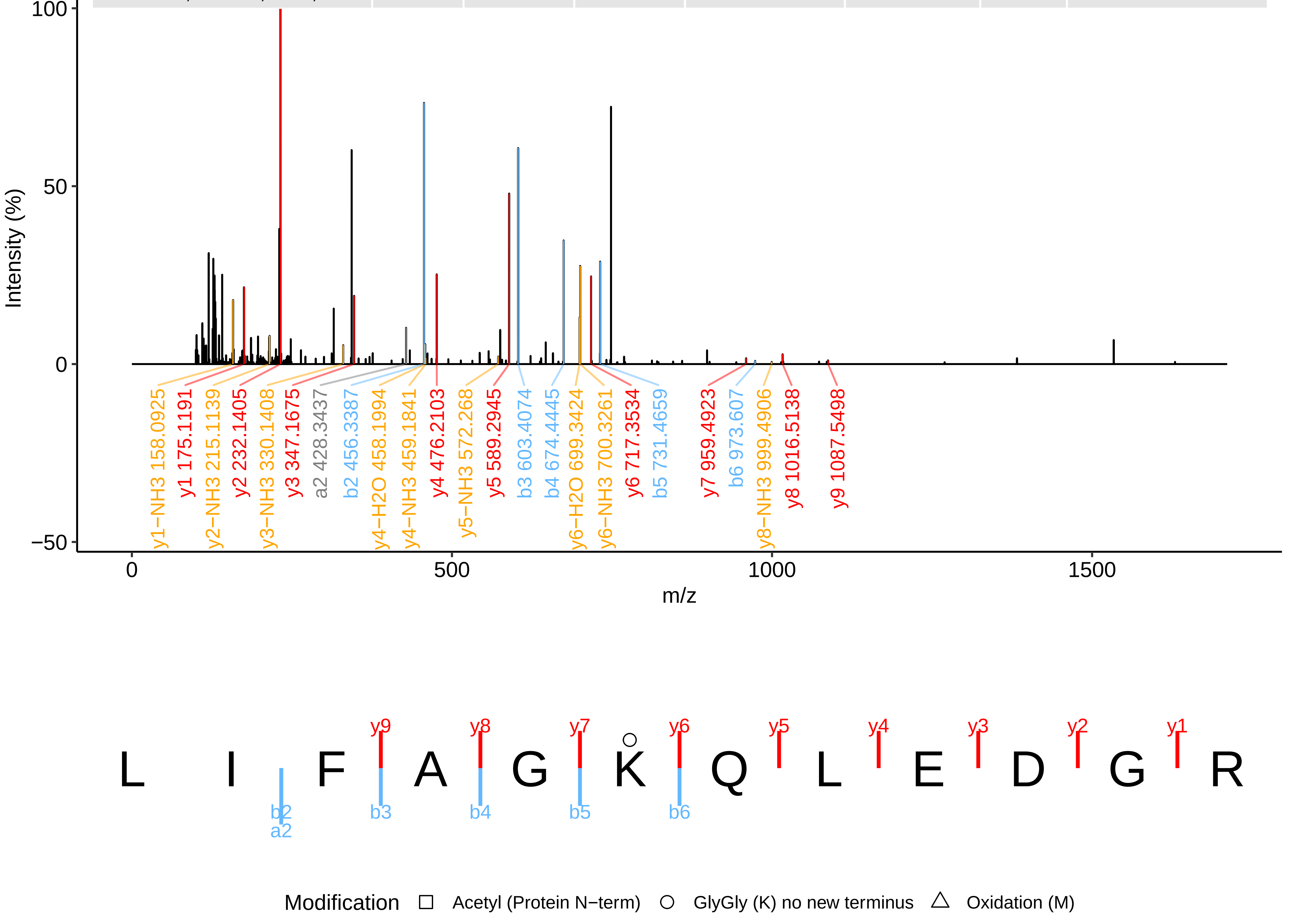

20200924_KIZ_MUb5_GlyGly_2 − Scan number: 17834

Gene Names	Charge	m/z	Mass	Retention time	PEP	Score	Precursor Intensity
RPS27A;UBA52;UBC;UBB	3	487.6001	1459.778	66.094	3.0463e−05	106.49	4895580

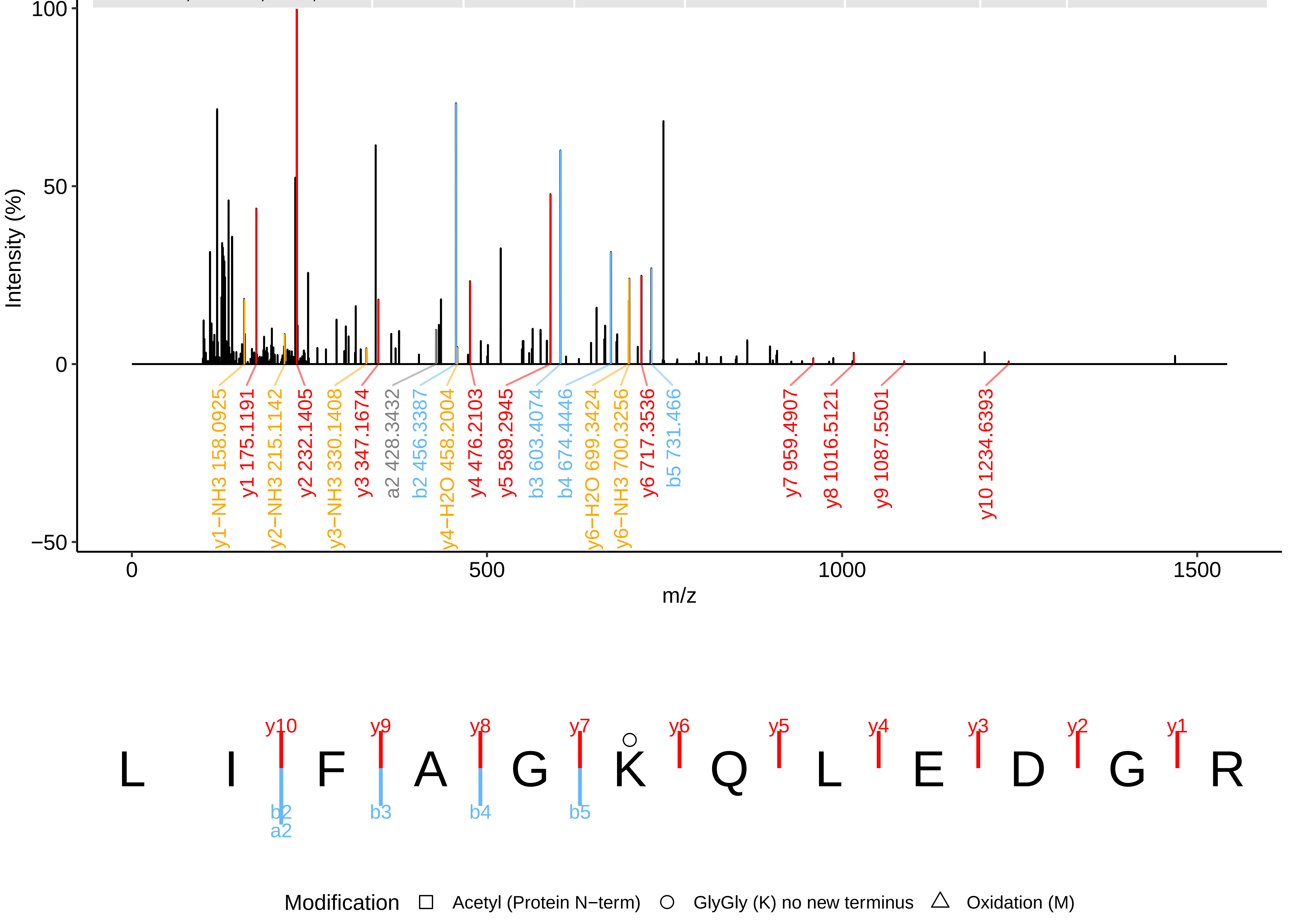

20200924_KIZ_MUb5_GlyGly_2 − Scan number: 17998

Gene Names	Charge	m/z	Mass	Retention time	PEP	Score	Precursor Intensity
RPS27A;UBA52;UBC;UBB	3	487.6001	1459.778	66.717	2.4894e−05	108.9	2109935

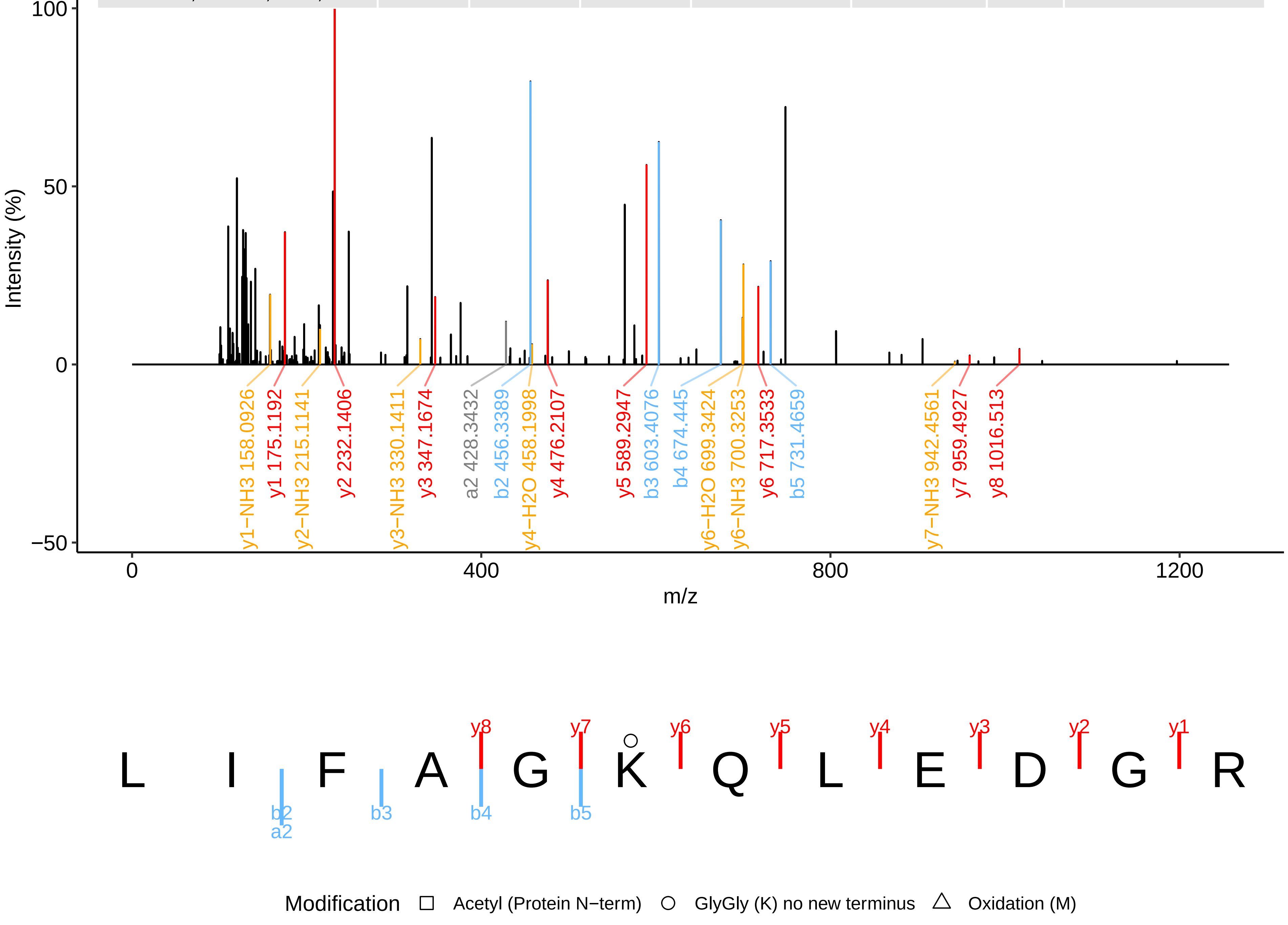

20200924_KIZ_MUb5_GlyGly_2 − Scan number: 18155

Gene Names	Charge	m/z	Mass	Retention time	PEP	Score	Precursor Intensity
RPS27A;UBA52;UBC;UBB	3	487.6001	1459.778	67.33	0.0067915	74.962	3212790

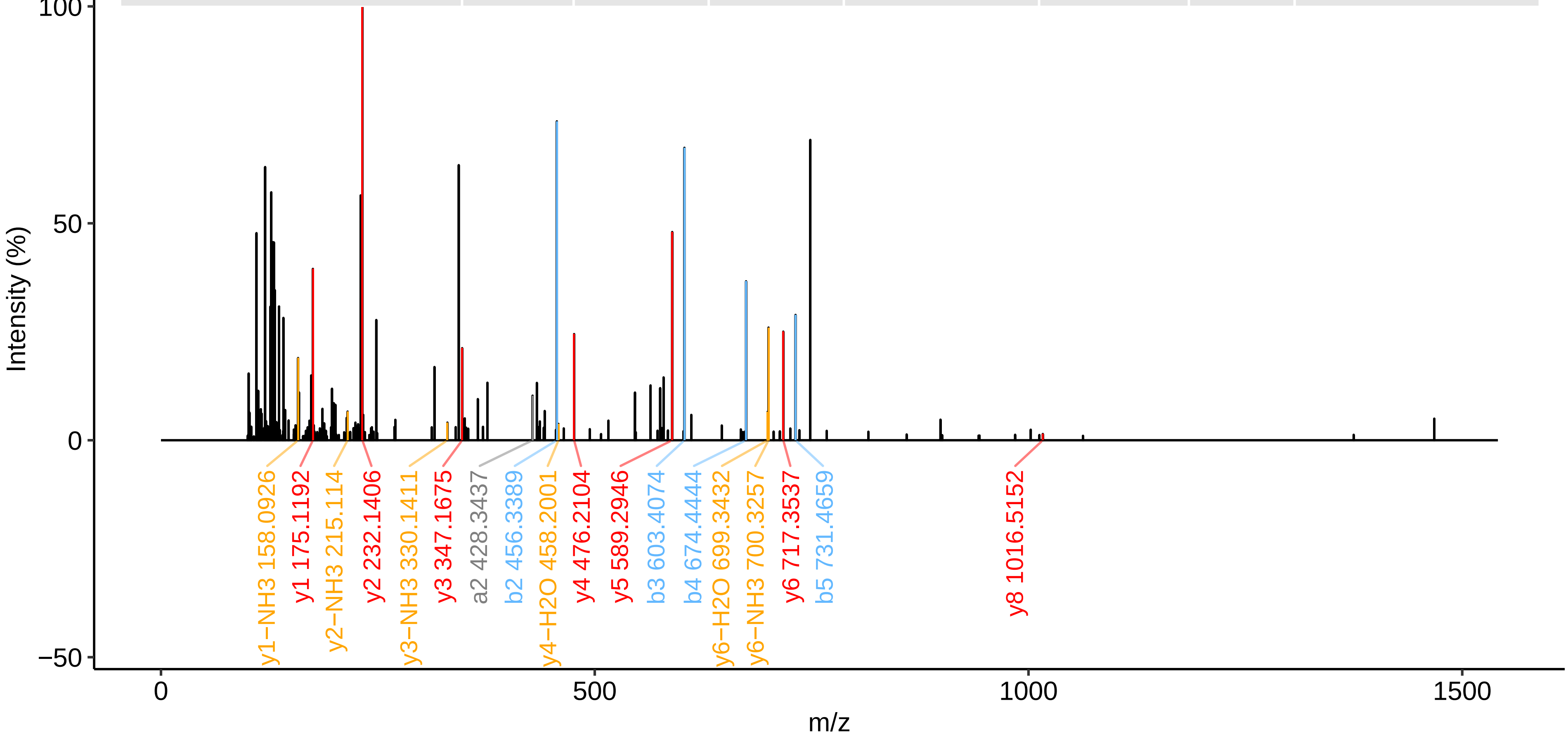

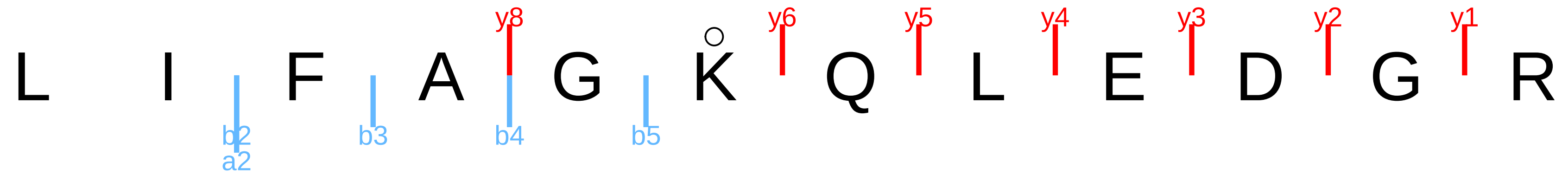

Modification □ Acetyl (Protein N−term) ○ GlyGly (K) no new terminus △ Oxidation (M)

20200924_KIZ_MUb5_GlyGly_2 − Scan number: 18663

Gene Names	Charge	m/z	Mass	Retention time	PEP	Score	Precursor Intensity
RPS27A;UBA52;UBC;UBB	3	487.6001	1459.778	69.219	0.00032211	94.767	3351371

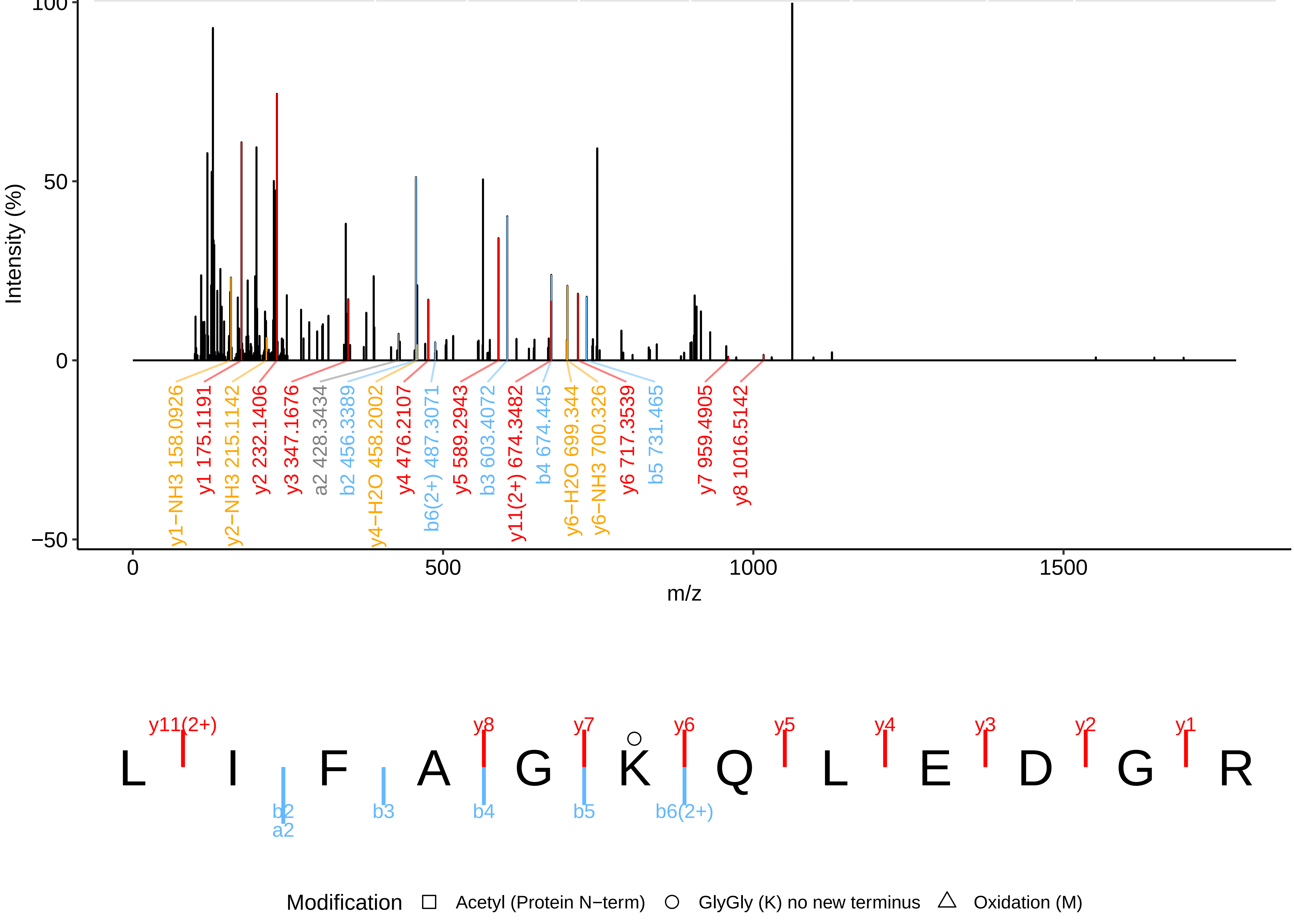

20200924_KIZ_MUb5_GlyGly_2 − Scan number: 18679

Gene Names	Charge	m/z	Mass	Retention time	PEP	Score	Precursor Intensity
RPS27A;UBA52;UBC;UBB	3	487.6001	1459.778	69.28	0.013996	67.548	NA

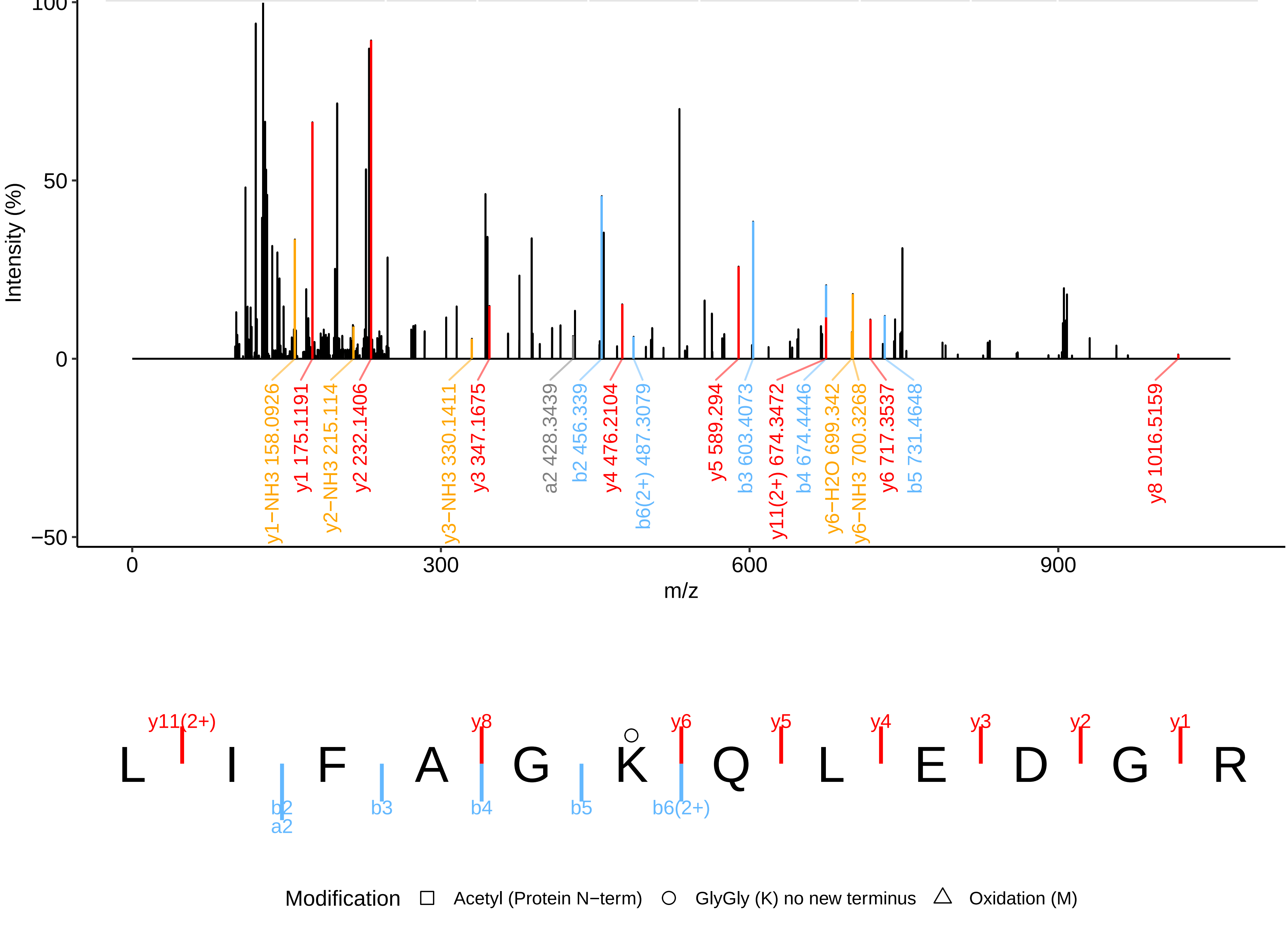

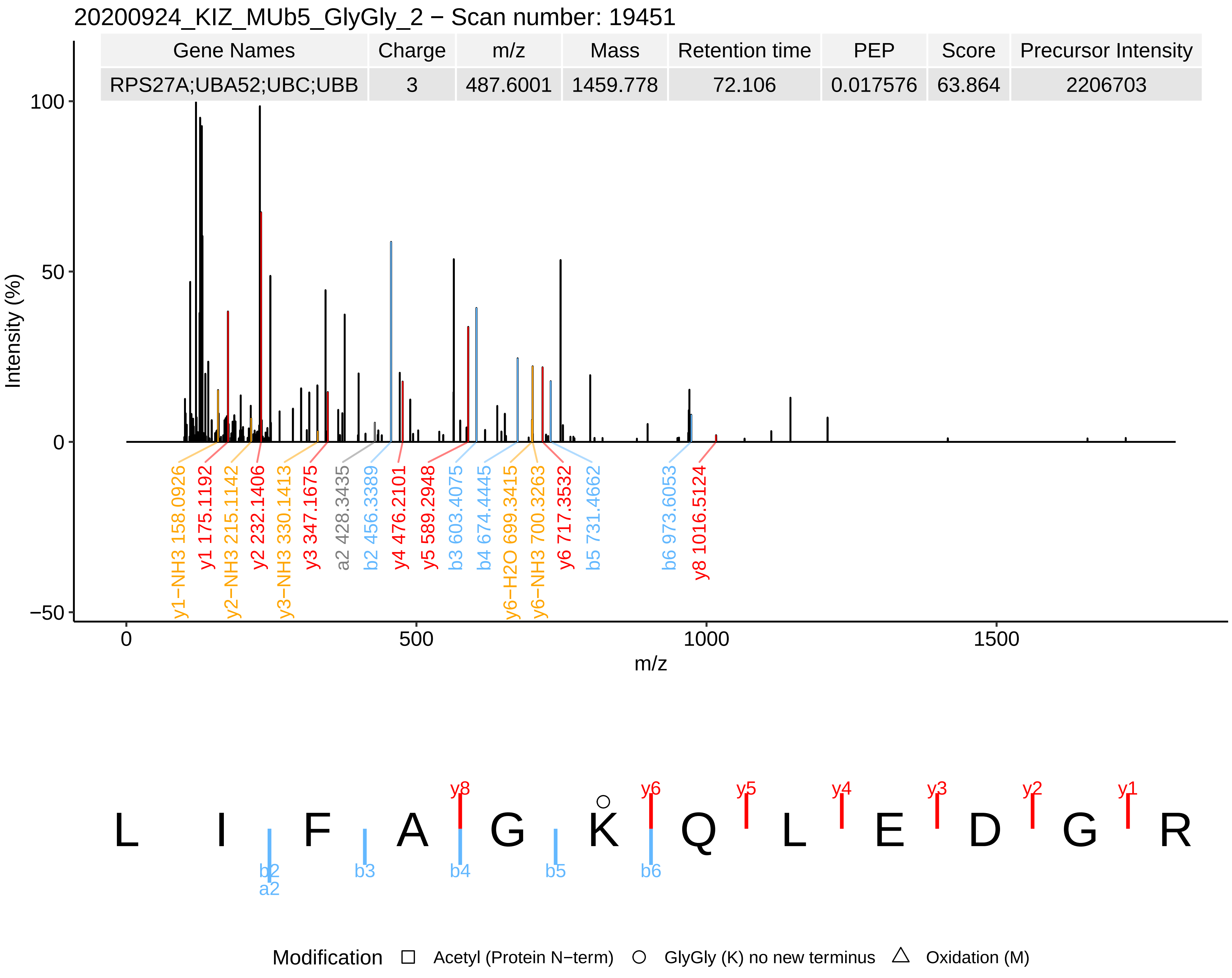
20200924_KIZ_MUb5_GlyGly_2 − Scan number: 19451
Gene Names | Charge | m/z | Mass | Retention time | PEP | Score | Precursor Intensity
RPS27A;UBA52;UBC;UBB | 3 | 487.6001 | 1459.778 | 72.106 | 0.017576 | 63.864 | 2206703
Intensity (%)
100
50
0
−50
0
500
1000
1500
m/z
y1−NH3 158.0926
y1 175.1192
y2−NH3 215.1142
y2 232.1406
y3−NH3 330.1413
y3 347.1675
a2 428.3435
b2 456.3389
y4 476.2101
y5 589.2948
b3 603.4075
b4 674.4445
y6−H2O 699.3415
y6−NH3 700.3263
y6 717.3532
b5 731.4662
b6 973.6053
y8 1016.5124
L I F A G K Q L E D G R
y8 y6 y5 y4 y3 y2 y1
b2 a2 b3 b4 b5 b6
Modification
Acetyl (Protein N−term)
GlyGly (K) no new terminus
Oxidation (M)

20201118_KIZ_MUb6_GlyGly_1 − Scan number: 16057

Gene Names	Charge	m/z	Mass	Retention time	PEP	Score	Precursor Intensity
RPS27A;UBA52;UBC;UBB	2	730.8964	1459.778	50.732	1.2065e−49	239.54	37323776

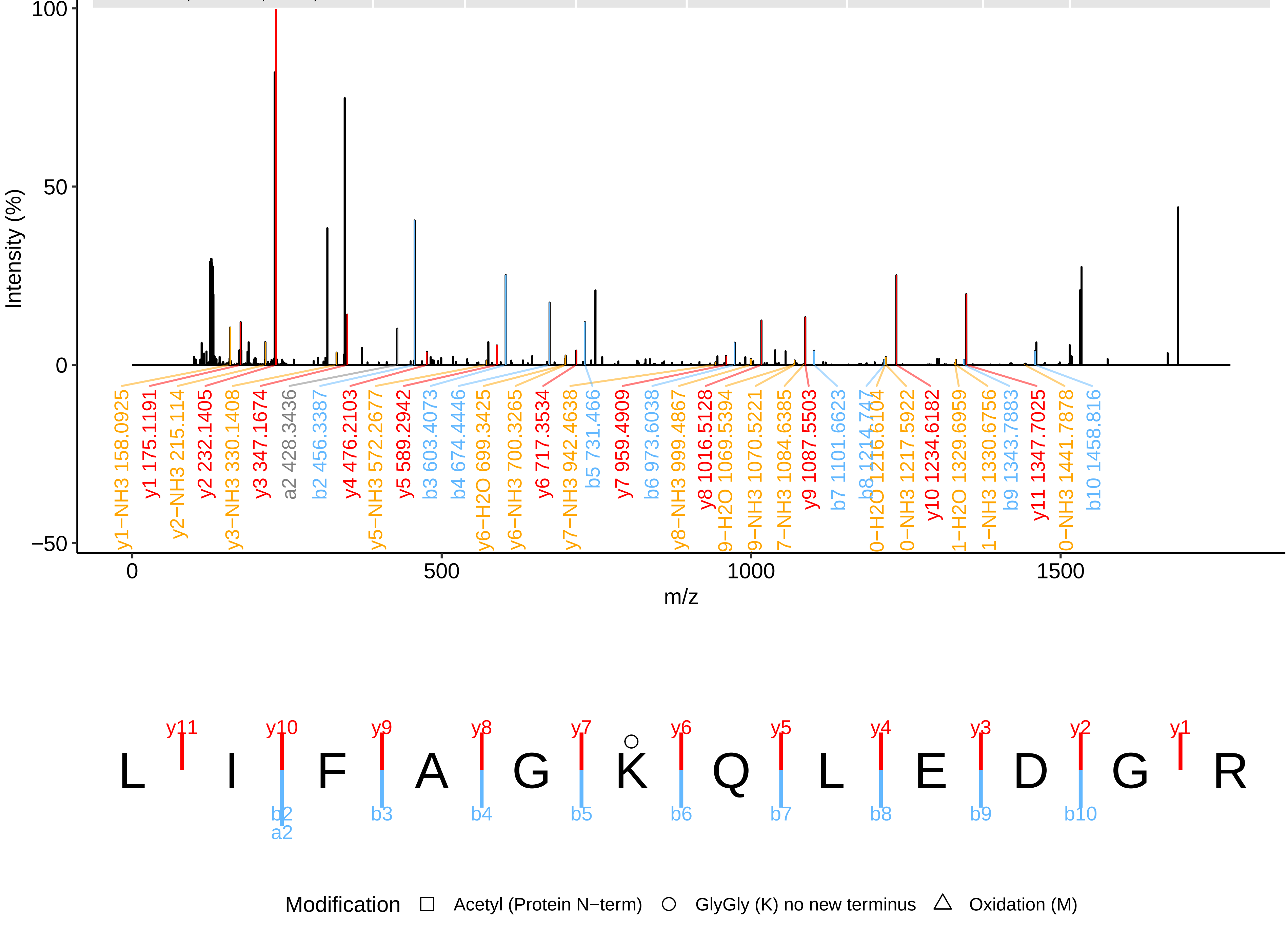

20201118_KIZ_MUb6_GlyGly_1 − Scan number: 16065

Gene Names	Charge	m/z	Mass	Retention time	PEP	Score	Precursor Intensity
RPS27A;UBA52;UBC;UBB	3	487.6001	1459.778	50.751	1.1157e−08	119.27	2797183488

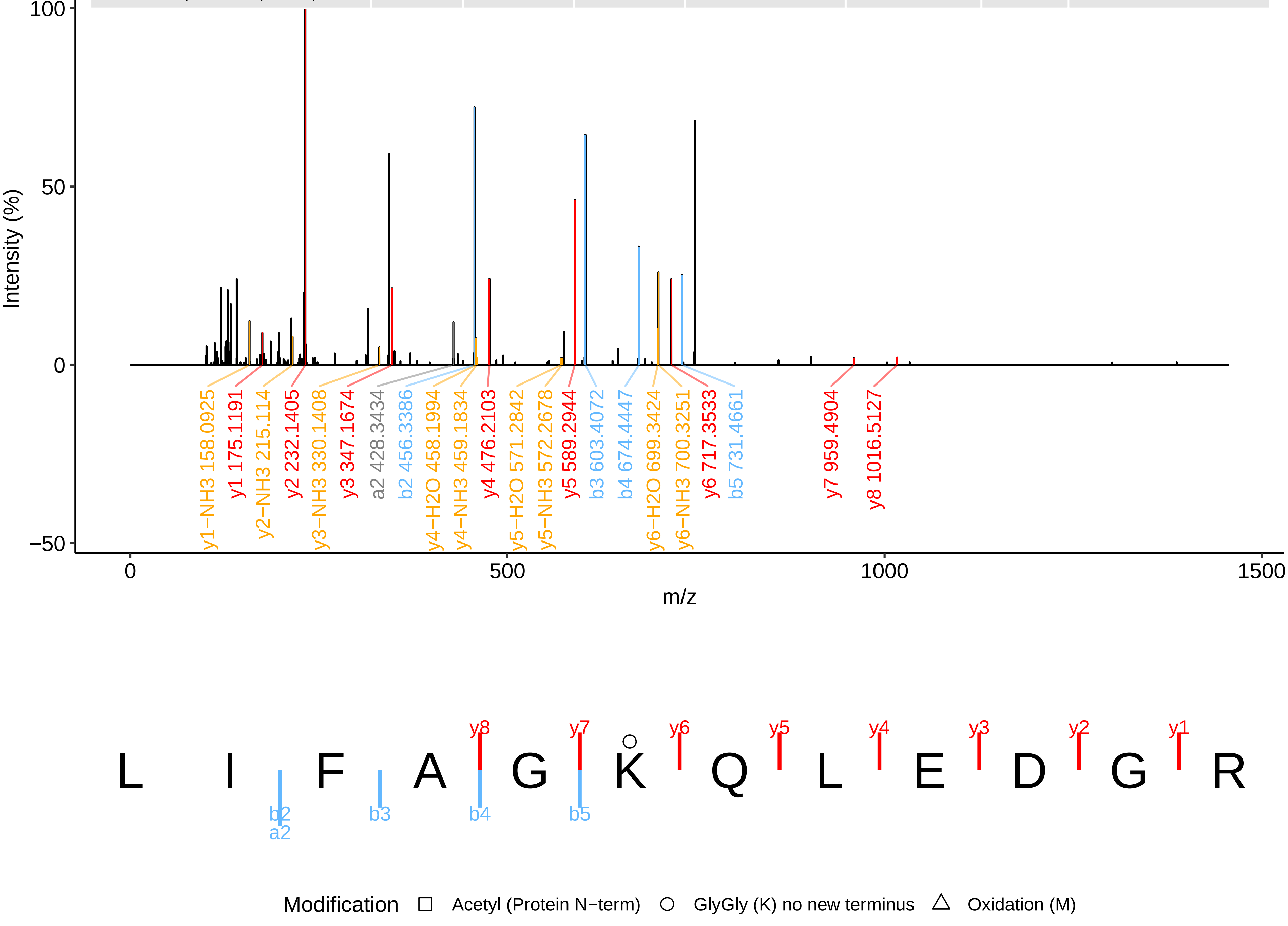

20201118_KIZ_MUb6_GlyGly_1 − Scan number: 16244

Gene Names	Charge	m/z	Mass	Retention time	PEP	Score	Precursor Intensity
RPS27A;UBA52;UBC;UBB	3	487.6001	1459.778	51.259	2.7462e−19	152.7	6717381120

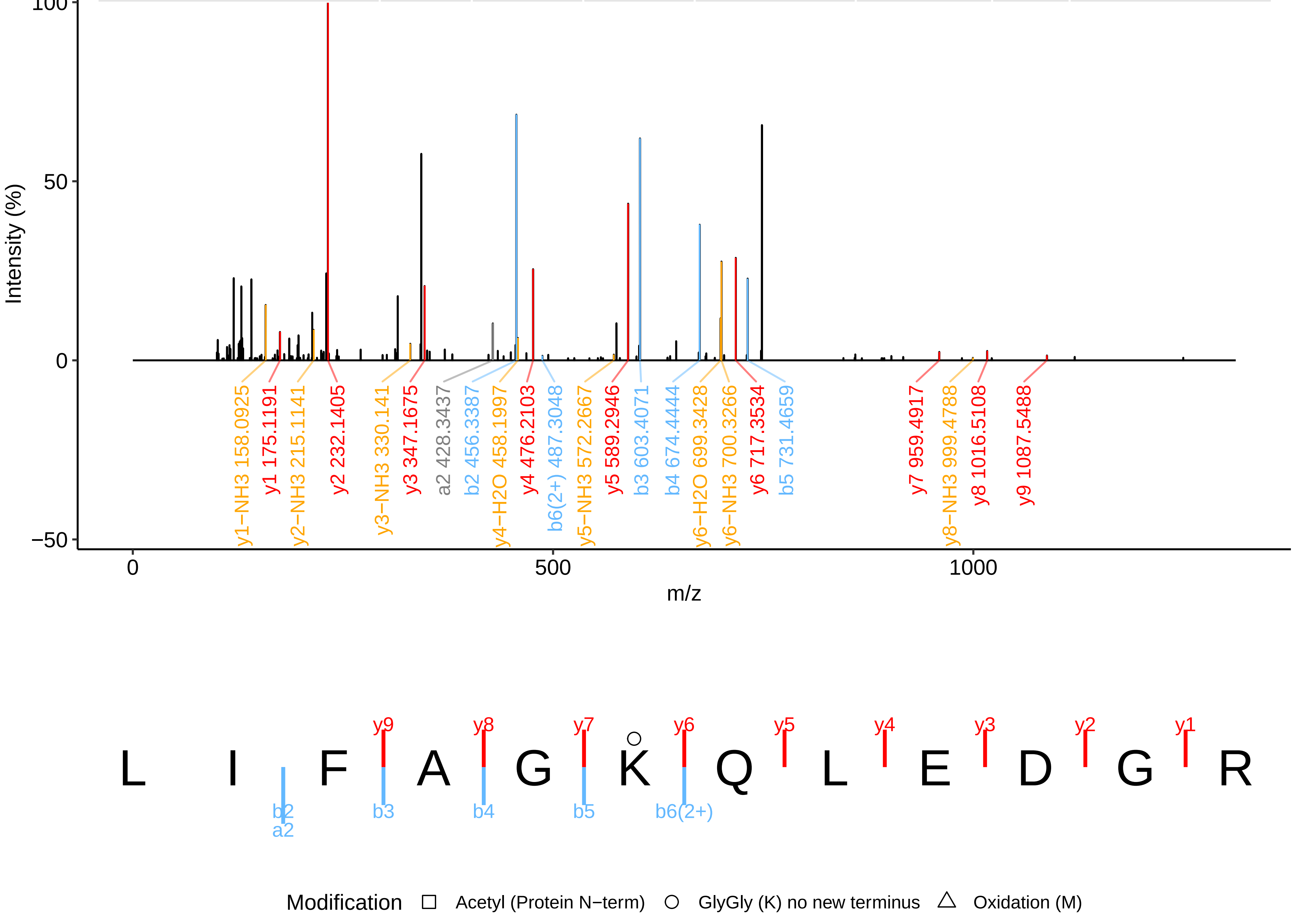

20201118_KIZ_MUb6_GlyGly_1 − Scan number: 16235

Gene Names	Charge	m/z	Mass	Retention time	PEP	Score	Precursor Intensity
RPS27A;UBA52;UBC;UBB	2	730.8964	1459.778	51.236	1.4685e−42	236.92	161344864

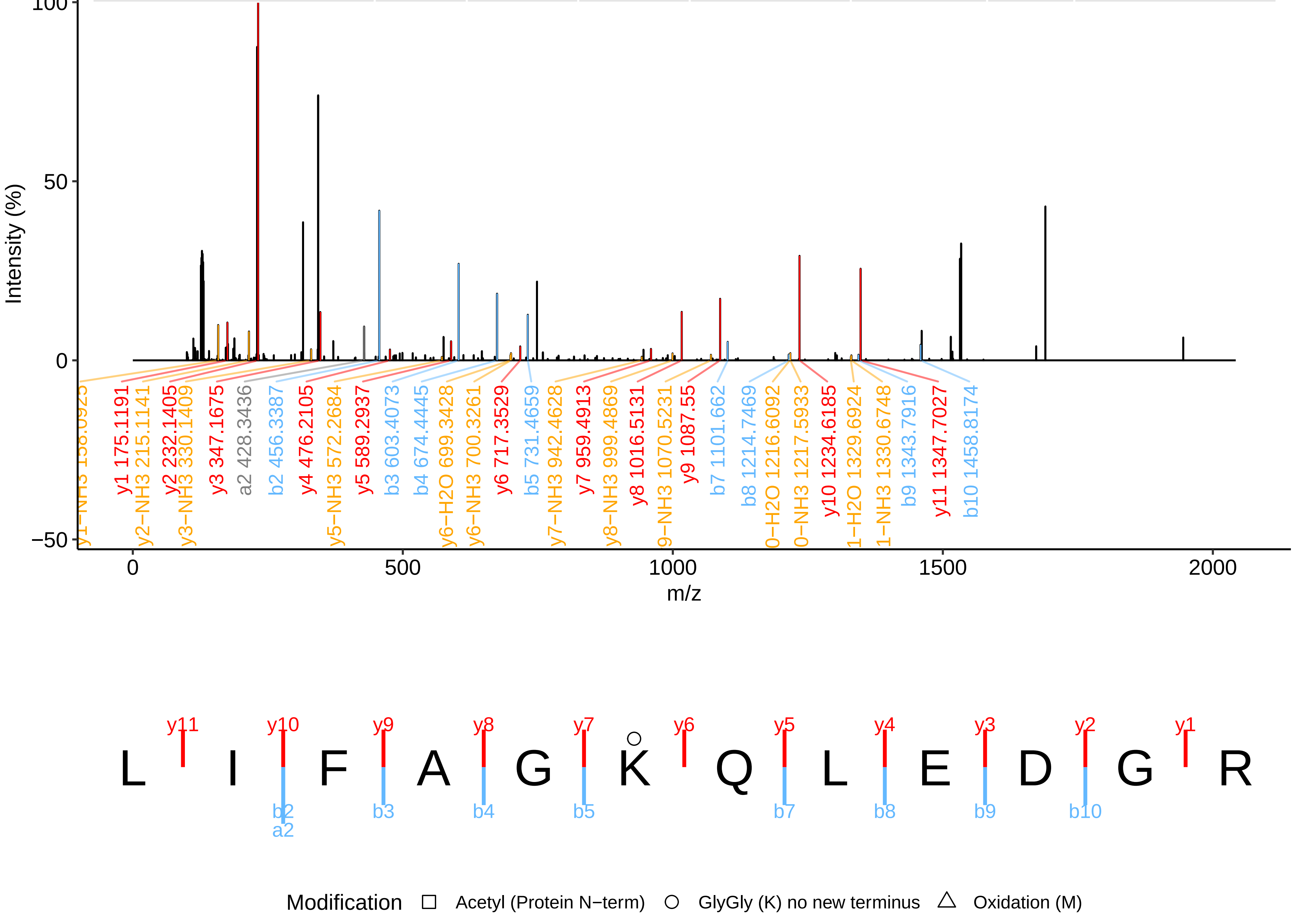

20201118_KIZ_MUb6_GlyGly_1 − Scan number: 16407

Gene Names	Charge	m/z	Mass	Retention time	PEP	Score	Precursor Intensity
RPS27A;UBA52;UBC;UBB	2	730.8964	1459.778	51.742	1.5043e−50	252.82	98554568

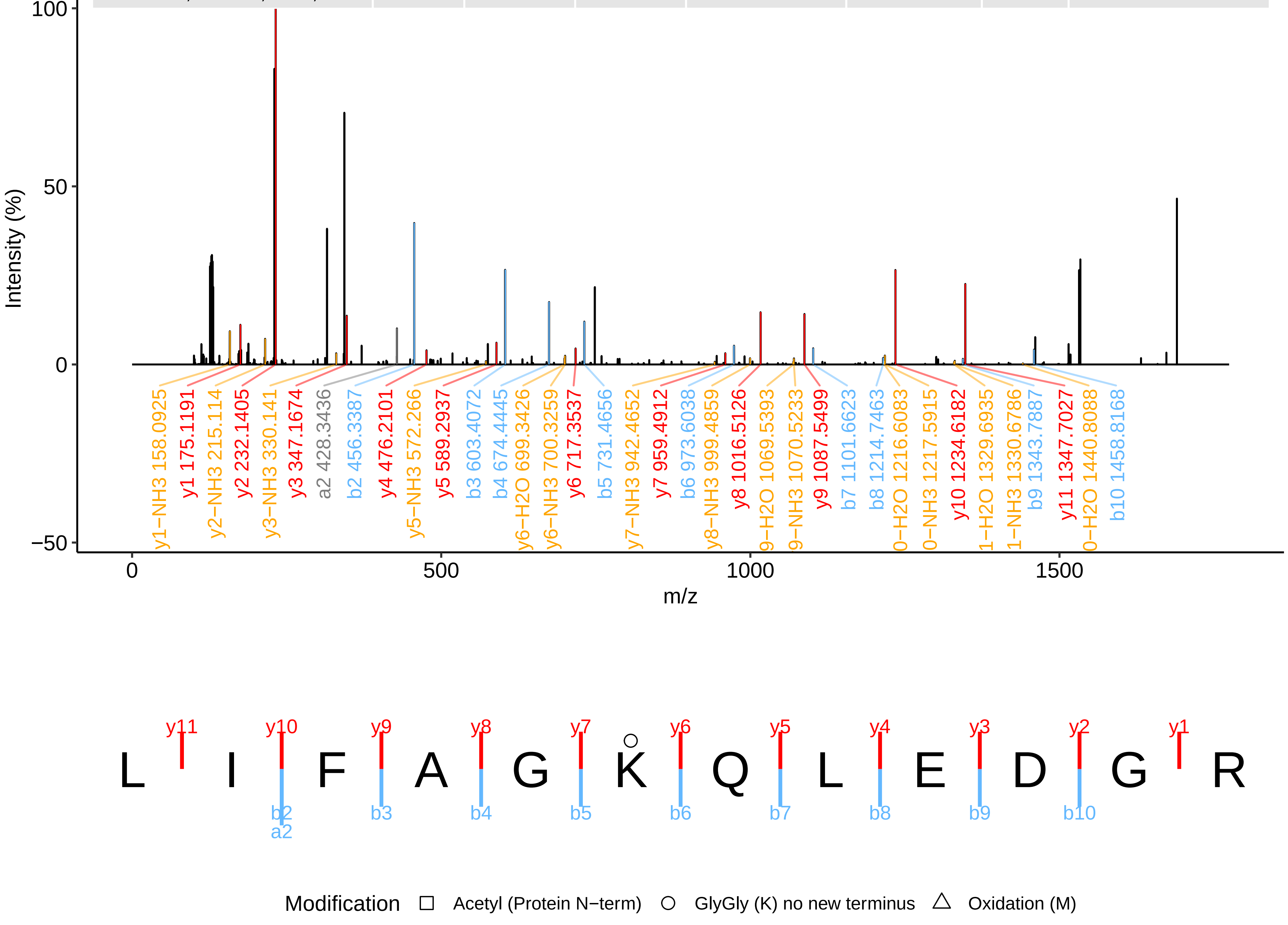

20201118_KIZ_MUb6_GlyGly_1 − Scan number: 16415

Gene Names	Charge	m/z	Mass	Retention time	PEP	Score	Precursor Intensity
RPS27A;UBA52;UBC;UBB	3	487.6001	1459.778	51.765	3.6333e−13	133.89	6838753280

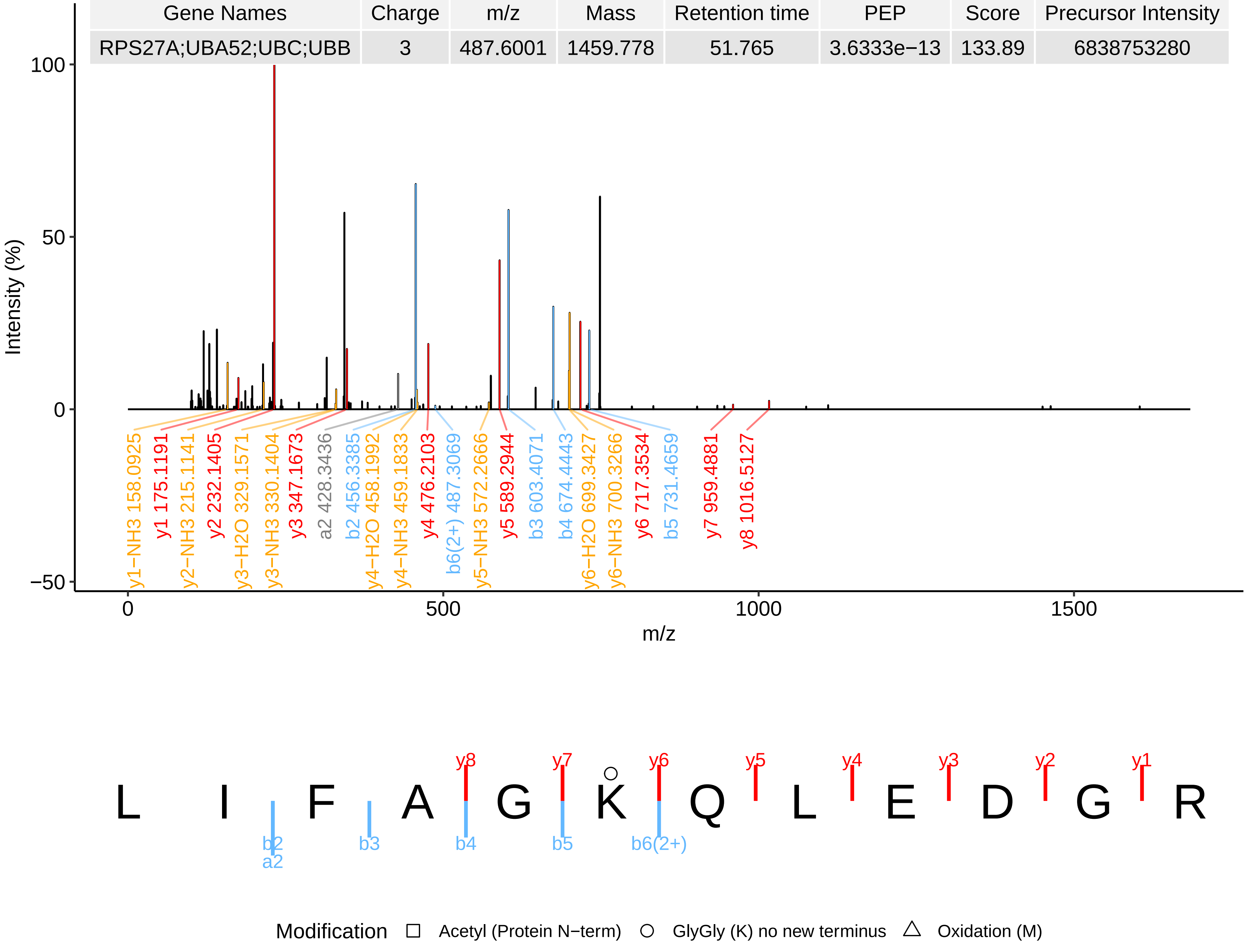

20201118_KIZ_MUb6_GlyGly_1 − Scan number: 16573

Gene Names	Charge	m/z	Mass	Retention time	PEP	Score	Precursor Intensity
RPS27A;UBA52;UBC;UBB	2	730.8964	1459.778	52.25	1.2705e−49	238.73	126269416

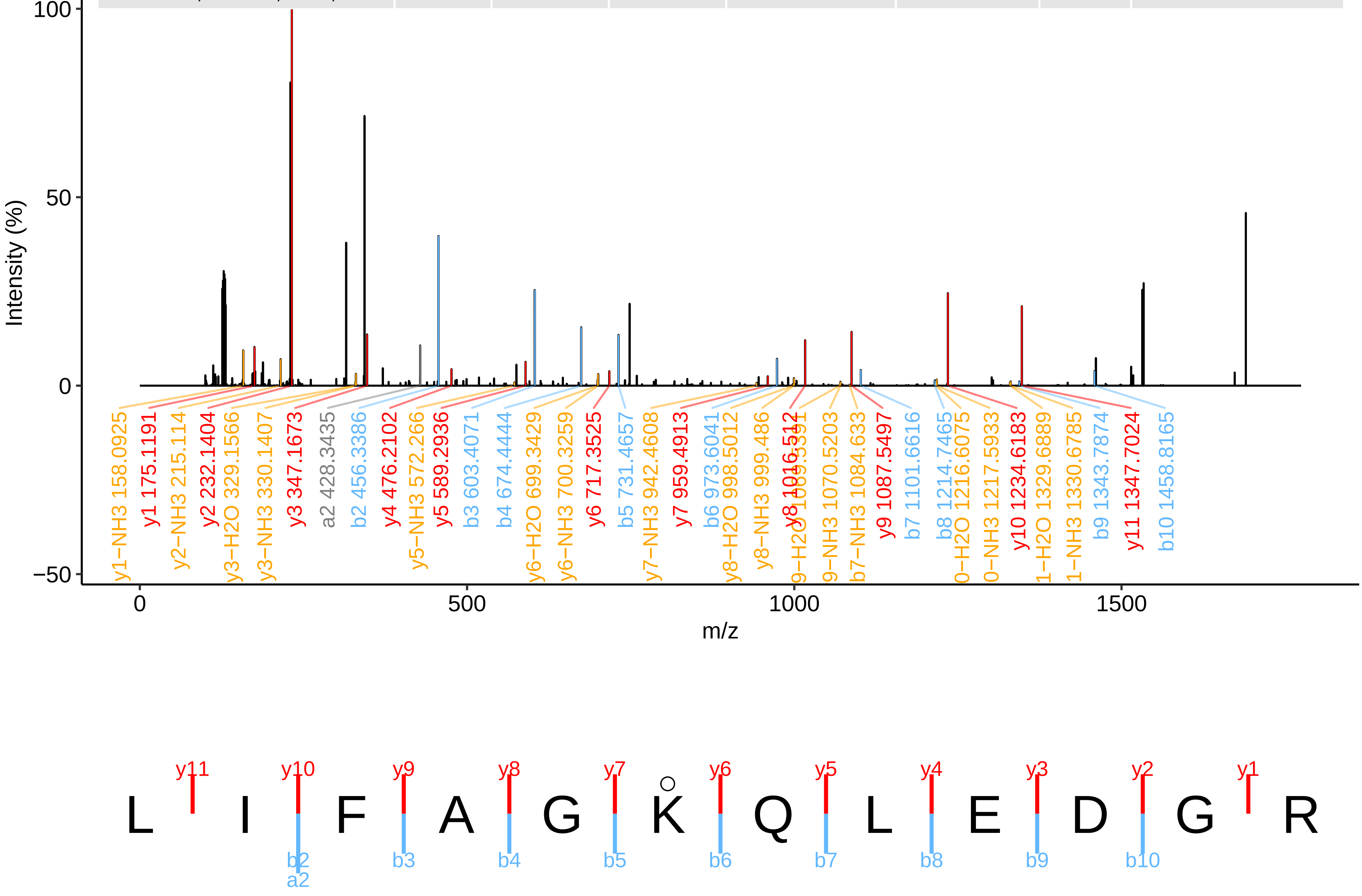

Modification □ Acetyl (Protein N−term) ○ GlyGly (K) no new terminus △ Oxidation (M)

20201118_KIZ_MUb6_GlyGly_1 − Scan number: 16580

Gene Names	Charge	m/z	Mass	Retention time	PEP	Score	Precursor Intensity
RPS27A;UBA52;UBC;UBB	3	487.6001	1459.778	52.271	4.9564e−06	111.27	6321429504

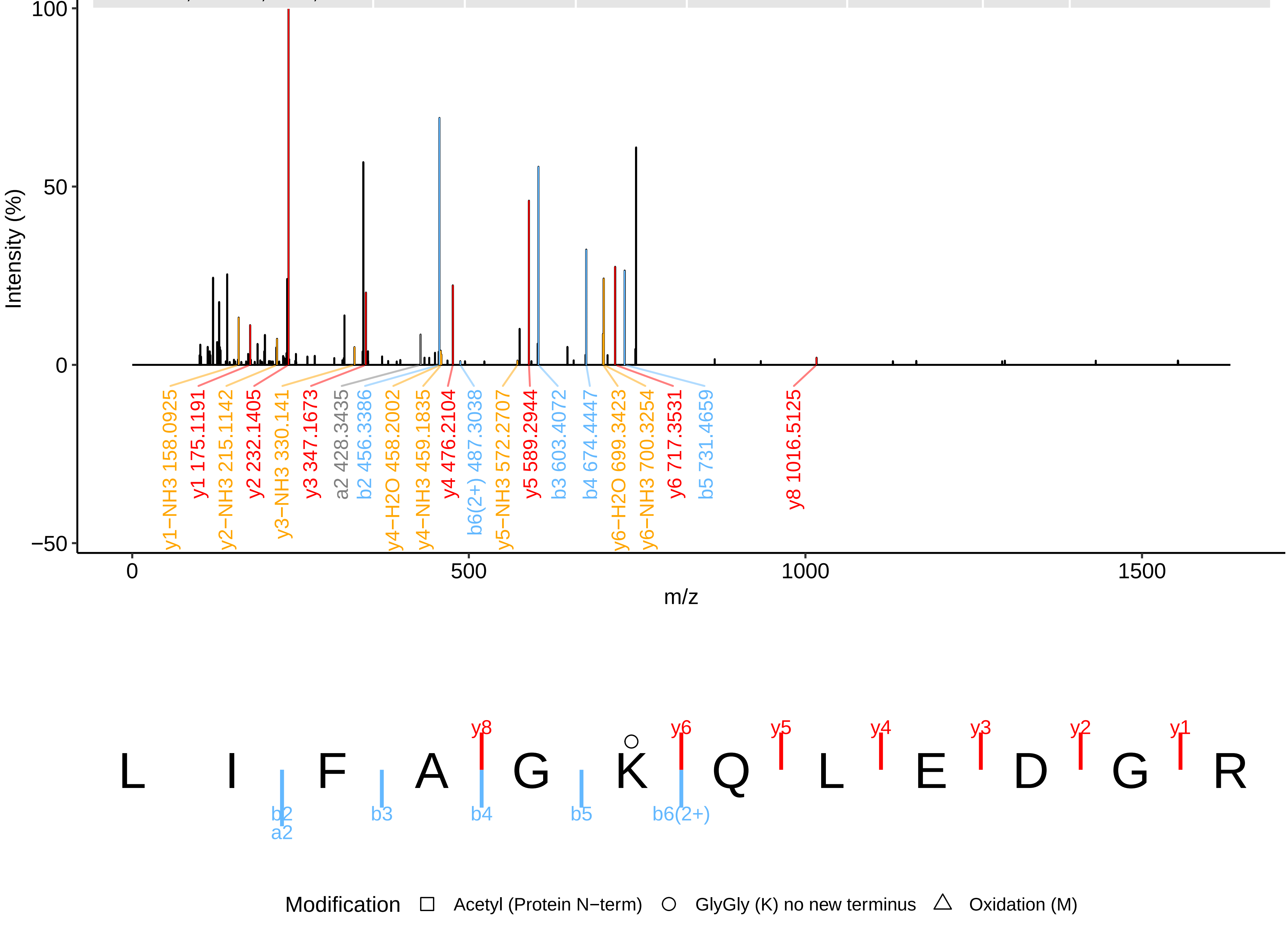

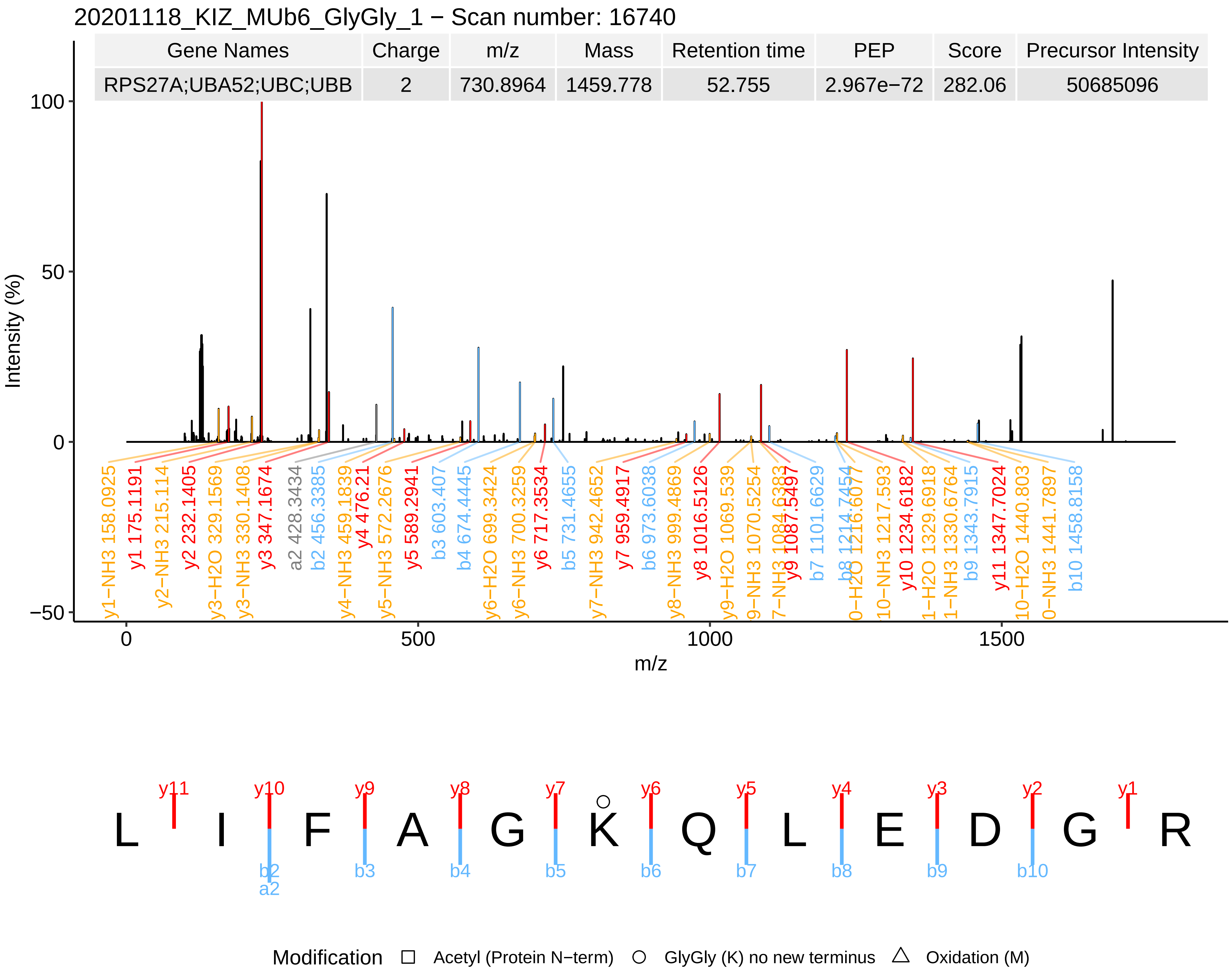
20201118_KIZ_MUb6_GlyGly_1 − Scan number: 16740
Gene Names
Charge
m/z
Mass
Retention time
PEP
Score
Precursor Intensity
RPS27A;UBA52;UBC;UBB
2
730.8964
1459.778
52.755
2.967e−72
282.06
50685096
Intensity (%)
m/z
y1−NH3 158.0925
y1 175.1191
y2−NH3 215.114
y2 232.1405
y3−H2O 329.1569
y3−NH3 330.1408
y3 347.1674
a2 428.3434
b2 456.3385
y4−NH3 459.1839
y4 476.21
y5−NH3 572.2676
y5 589.2941
b3 603.407
b4 674.4445
y6−H2O 699.3424
y6−NH3 700.3259
y6 717.3534
b5 731.4655
y7−NH3 942.4652
y7 959.4917
b6 973.6038
y8−NH3 999.4869
y8 1016.5126
y9−H2O 1069.539
b7 1101.6629
y10 1234.6182
b9 1343.7915
y11 1347.7024
b10 1458.8158
L I F A G K Q L E D G R
Modification
Acetyl (Protein N−term)
GlyGly (K) no new terminus
Oxidation (M)

20201118_KIZ_MUb6_GlyGly_1 − Scan number: 16747

Gene Names	Charge	m/z	Mass	Retention time	PEP	Score	Precursor Intensity
RPS27A;UBA52;UBC;UBB	3	487.6001	1459.778	52.772	4.0818e−09	127.87	NA

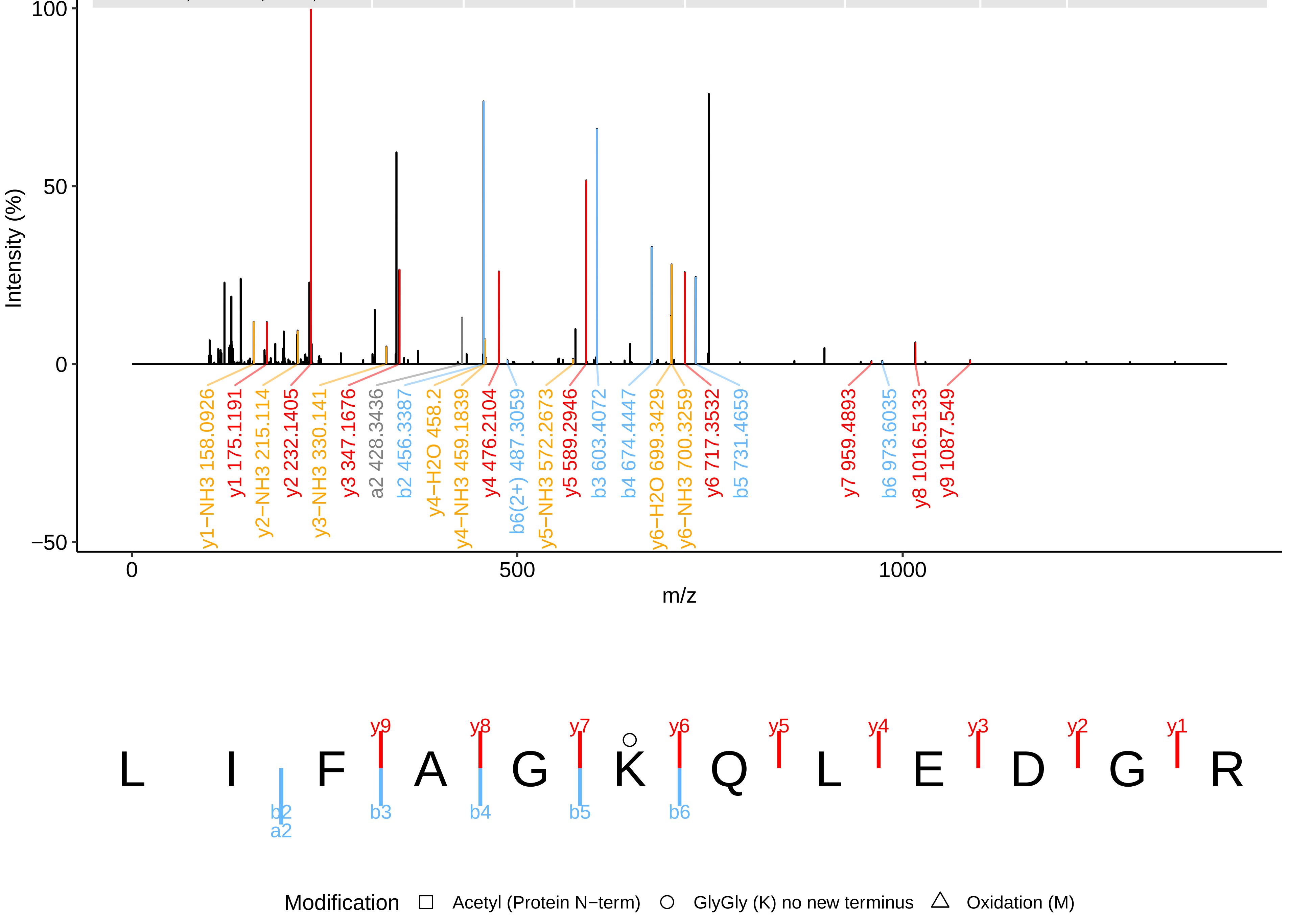

20201118_KIZ_MUb6_GlyGly_1 − Scan number: 16917

Gene Names	Charge	m/z	Mass	Retention time	PEP	Score	Precursor Intensity
RPS27A;UBA52;UBC;UBB	3	487.6001	1459.778	53.28	4.0818e−09	127.87	1208844800

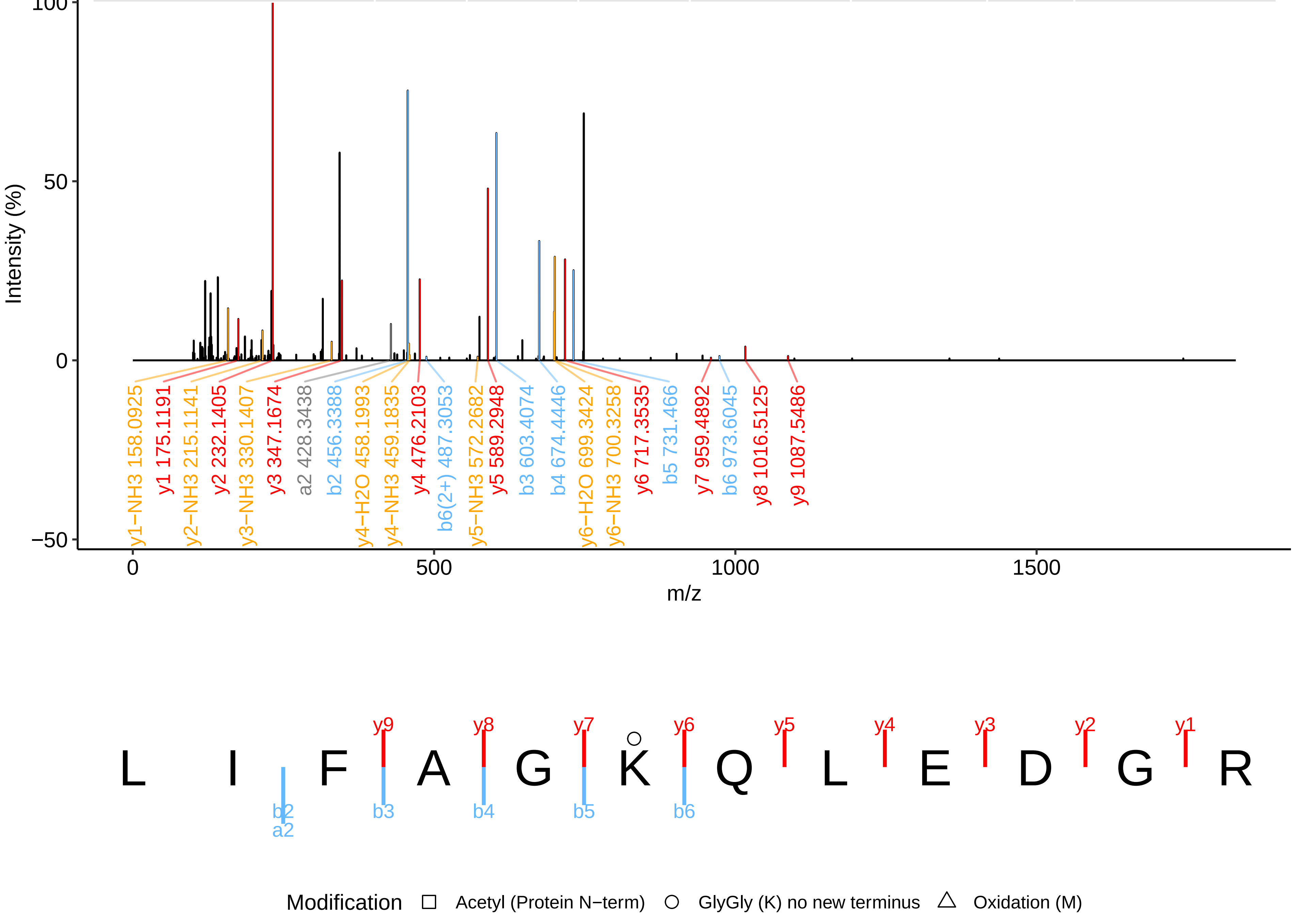

20201118_KIZ_MUb6_GlyGly_1 − Scan number: 16912

Gene Names	Charge	m/z	Mass	Retention time	PEP	Score	Precursor Intensity
RPS27A;UBA52;UBC;UBB	2	730.8964	1459.778	53.269	1.7599e−29	196.06	28316922

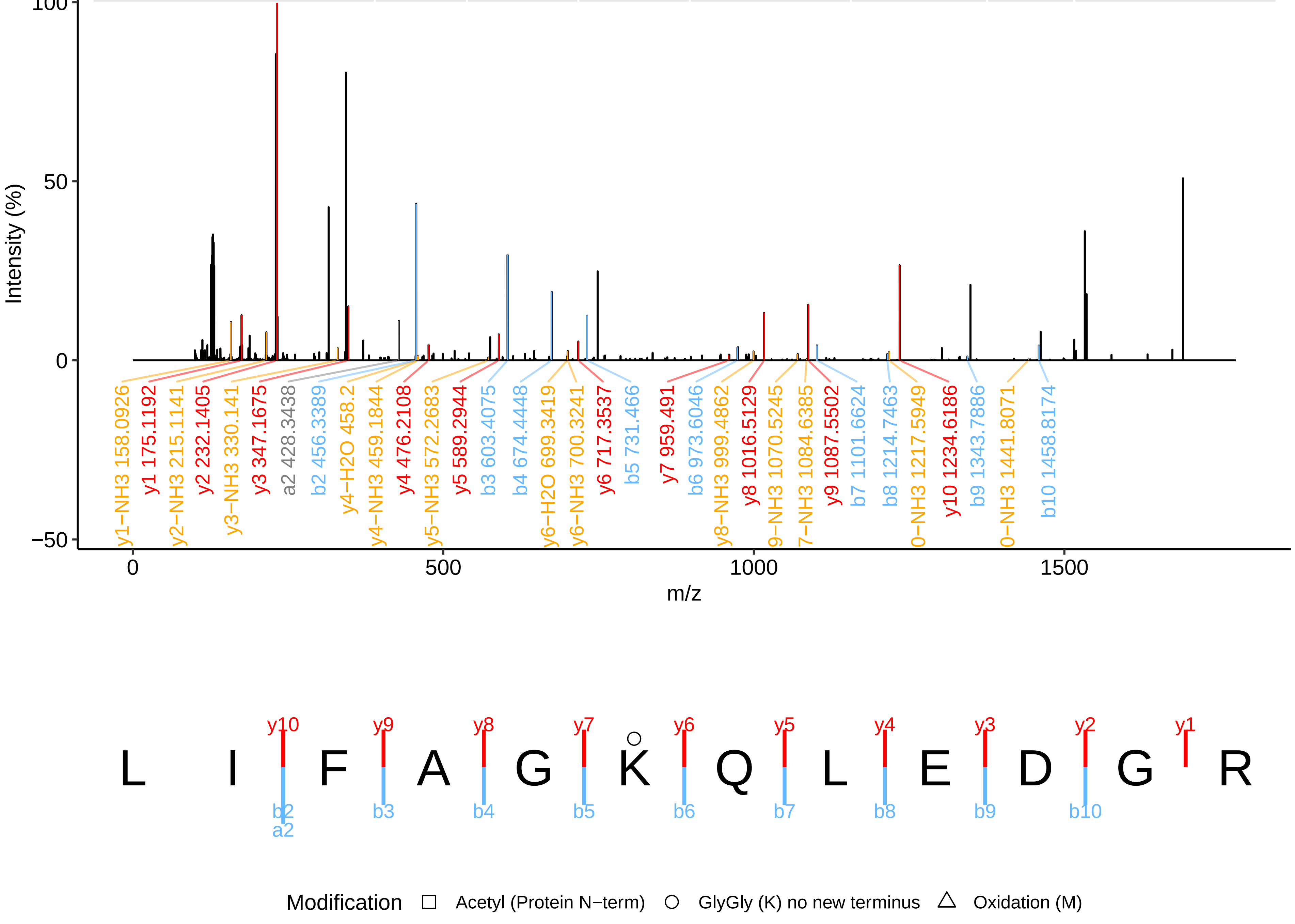

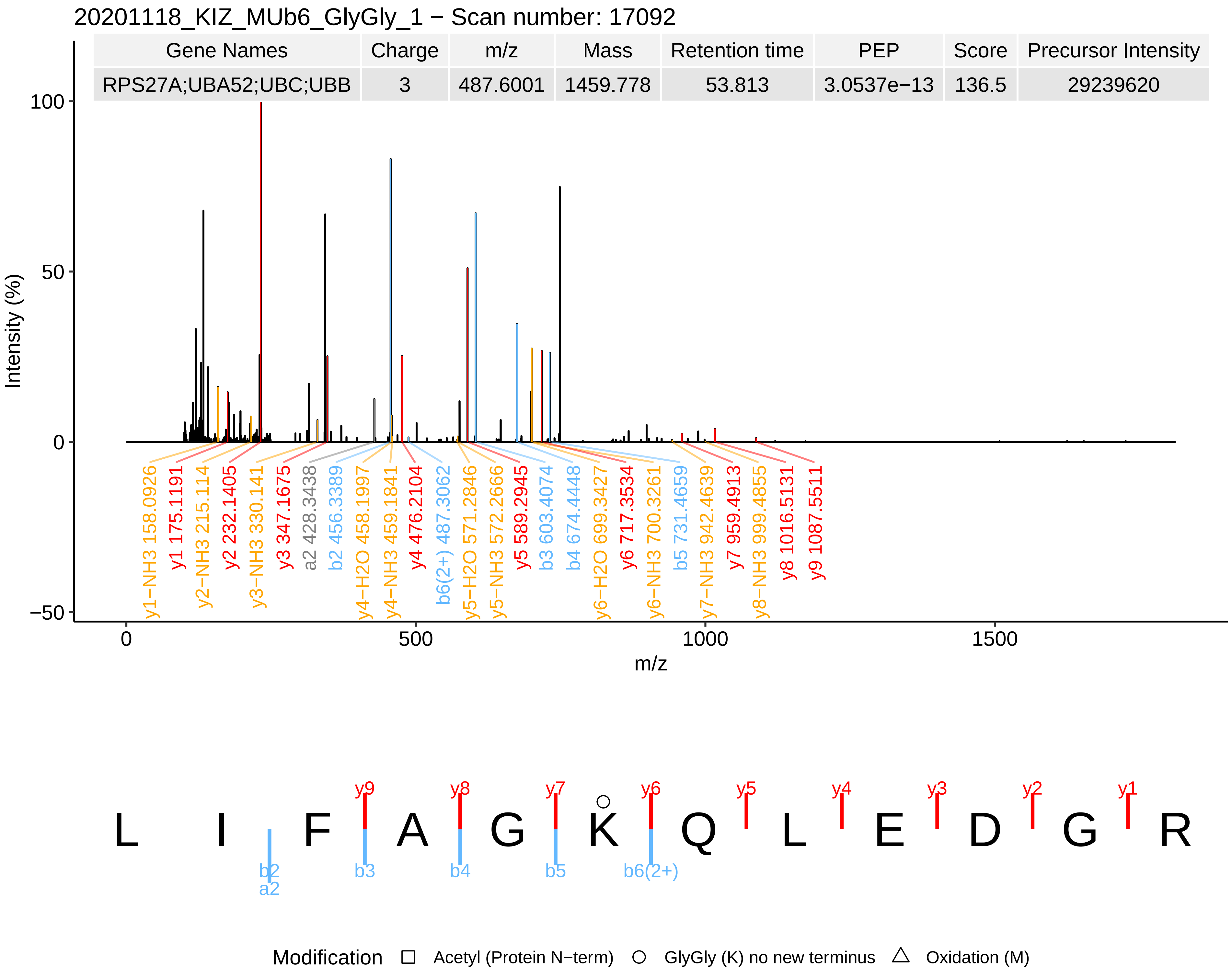

20201118_KIZ_MUb6_GlyGly_1 − Scan number: 17092
Gene Names | Charge | m/z | Mass | Retention time | PEP | Score | Precursor Intensity
RPS27A;UBA52;UBC;UBB | 3 | 487.6001 | 1459.778 | 53.813 | 3.0537e−13 | 136.5 | 29239620
Intensity (%)
100
50
0
−50
0
500
1000
1500
m/z
y1−NH3 158.0926
y1 175.1191
y2−NH3 215.114
y2 232.1405
y3−NH3 330.141
y3 347.1675
a2 428.3438
b2 456.3389
y4−H2O 458.1997
y4−NH3 459.1841
y4 476.2104
b6(2+) 487.3062
y5−H2O 571.2846
y5−NH3 572.2666
y5 589.2945
b3 603.4074
b4 674.4448
y6−H2O 699.3427
y6 717.3534
y6−NH3 700.3261
b5 731.4659
y7−NH3 942.4639
y7 959.4913
y8−NH3 999.4855
y8 1016.5131
y9 1087.5511
L I F A G K Q L E D G R
y9 y8 y7 y6 y5 y4 y3 y2 y1
b2 a2 b3 b4 b5 b6(2+)
Modification
Acetyl (Protein N−term)
GlyGly (K) no new terminus
Oxidation (M)

20201118_KIZ_MUb6_GlyGly_1 − Scan number: 17439

Gene Names	Charge	m/z	Mass	Retention time	PEP	Score	Precursor Intensity
RPS27A;UBA52;UBC;UBB	3	487.6001	1459.778	54.883	2.913e−19	151.79	10003289

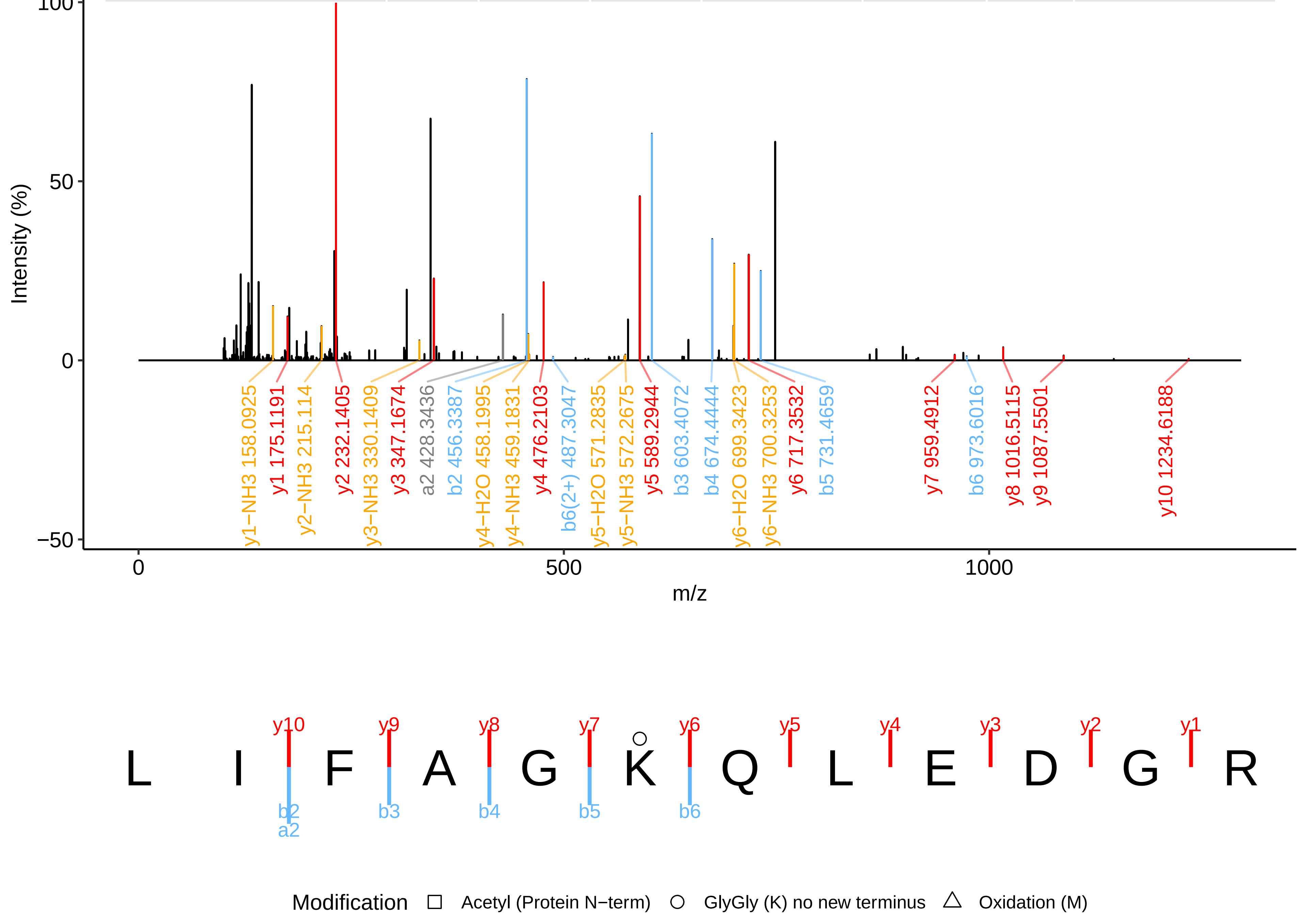

20201118_KIZ_MUb6_GlyGly_2 − Scan number: 18622

Gene Names	Charge	m/z	Mass	Retention time	PEP	Score	Precursor Intensity
RPS27A;UBA52;UBC;UBB	2	730.8964	1459.778	62.931	4.4089e−26	180.74	24064782

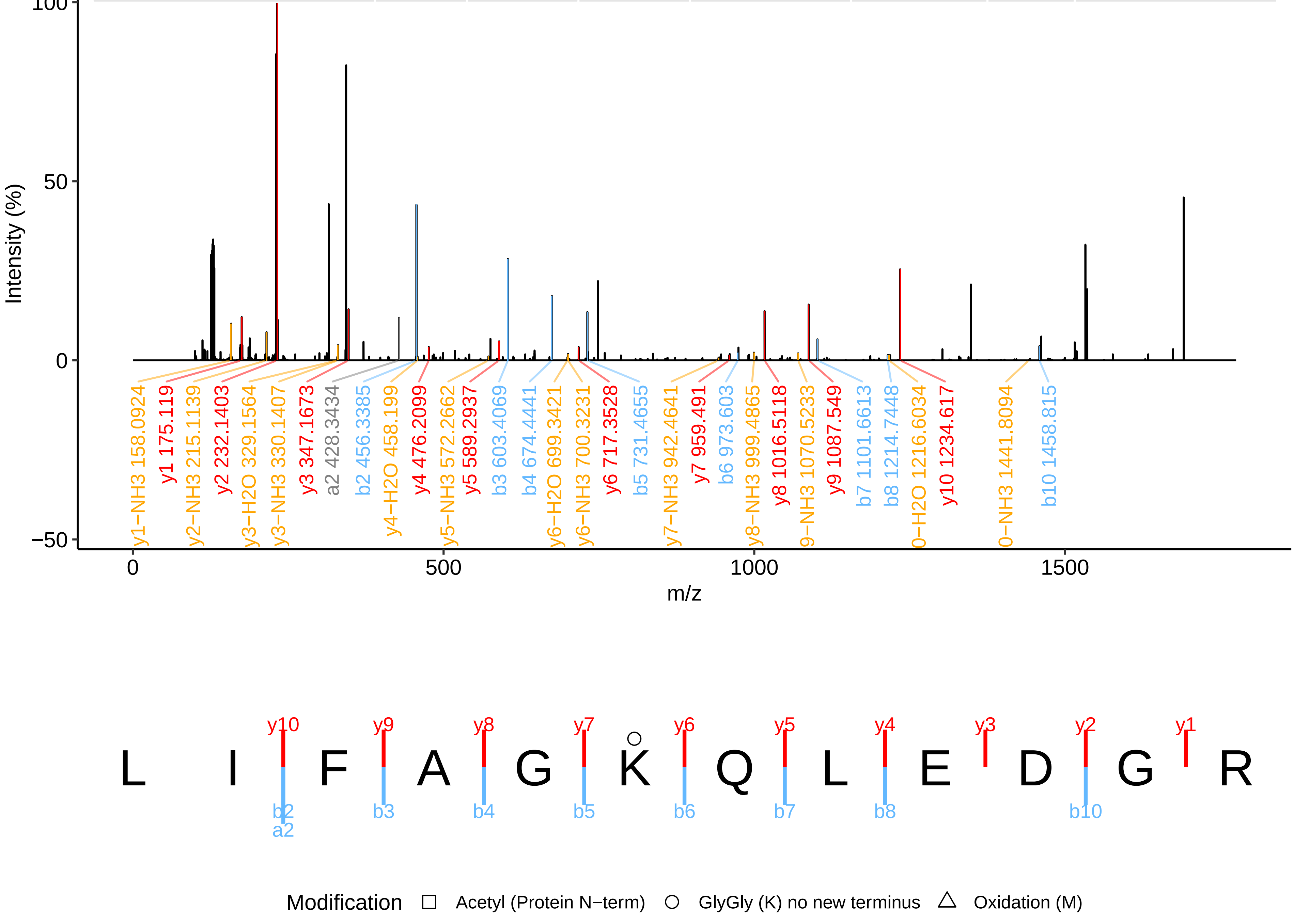

20201118_KIZ_MUb6_GlyGly_2 − Scan number: 18637

Gene Names	Charge	m/z	Mass	Retention time	PEP	Score	Precursor Intensity
RPS27A;UBA52;UBC;UBB	3	487.6001	1459.778	62.978	2.4754e−09	129.82	3703764736

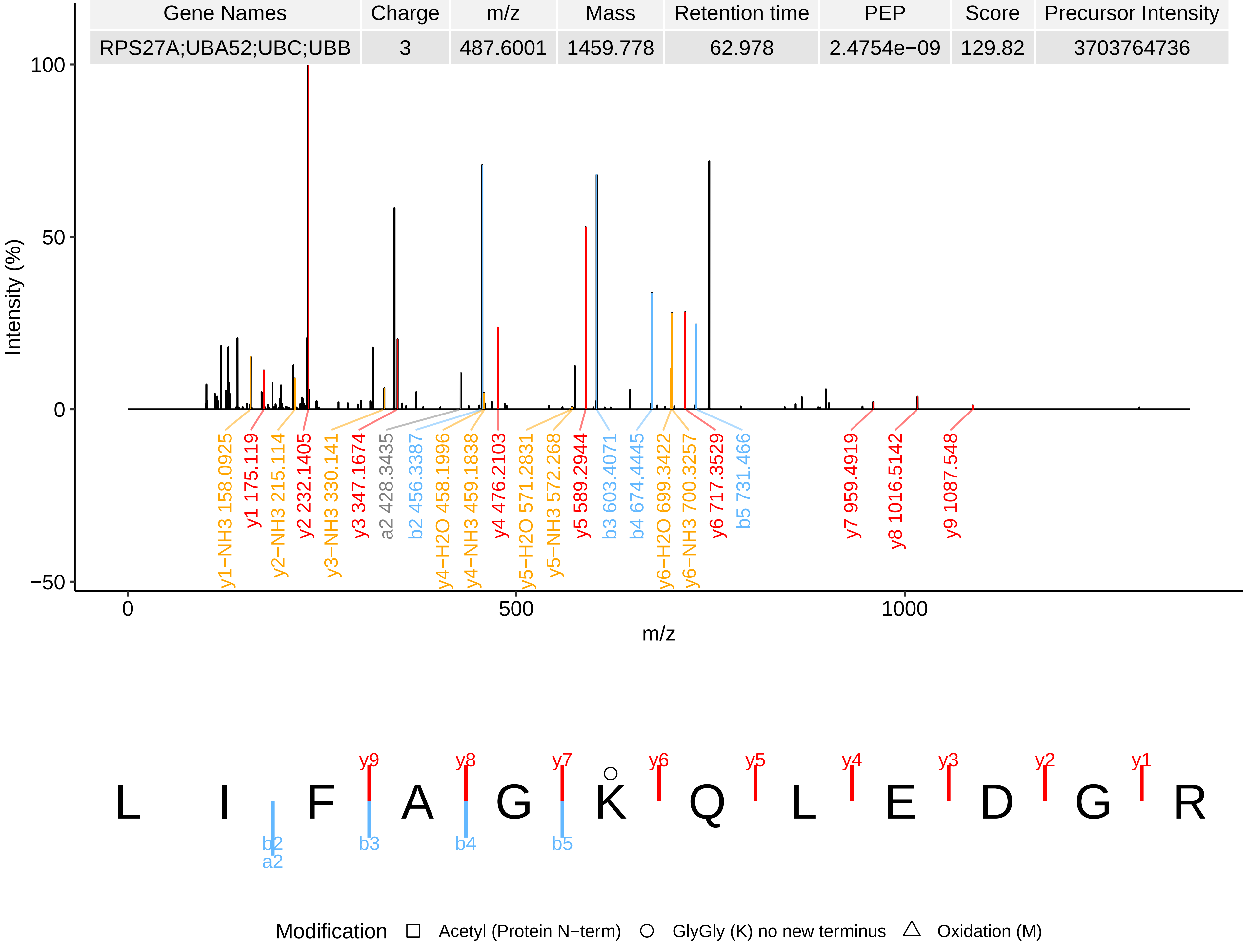

20201118_KIZ_MUb6_GlyGly_2 − Scan number: 18801

Gene Names	Charge	m/z	Mass	Retention time	PEP	Score	Precursor Intensity
RPS27A;UBA52;UBC;UBB	3	487.6001	1459.778	63.486	8.3443e−09	122.69	4453406720

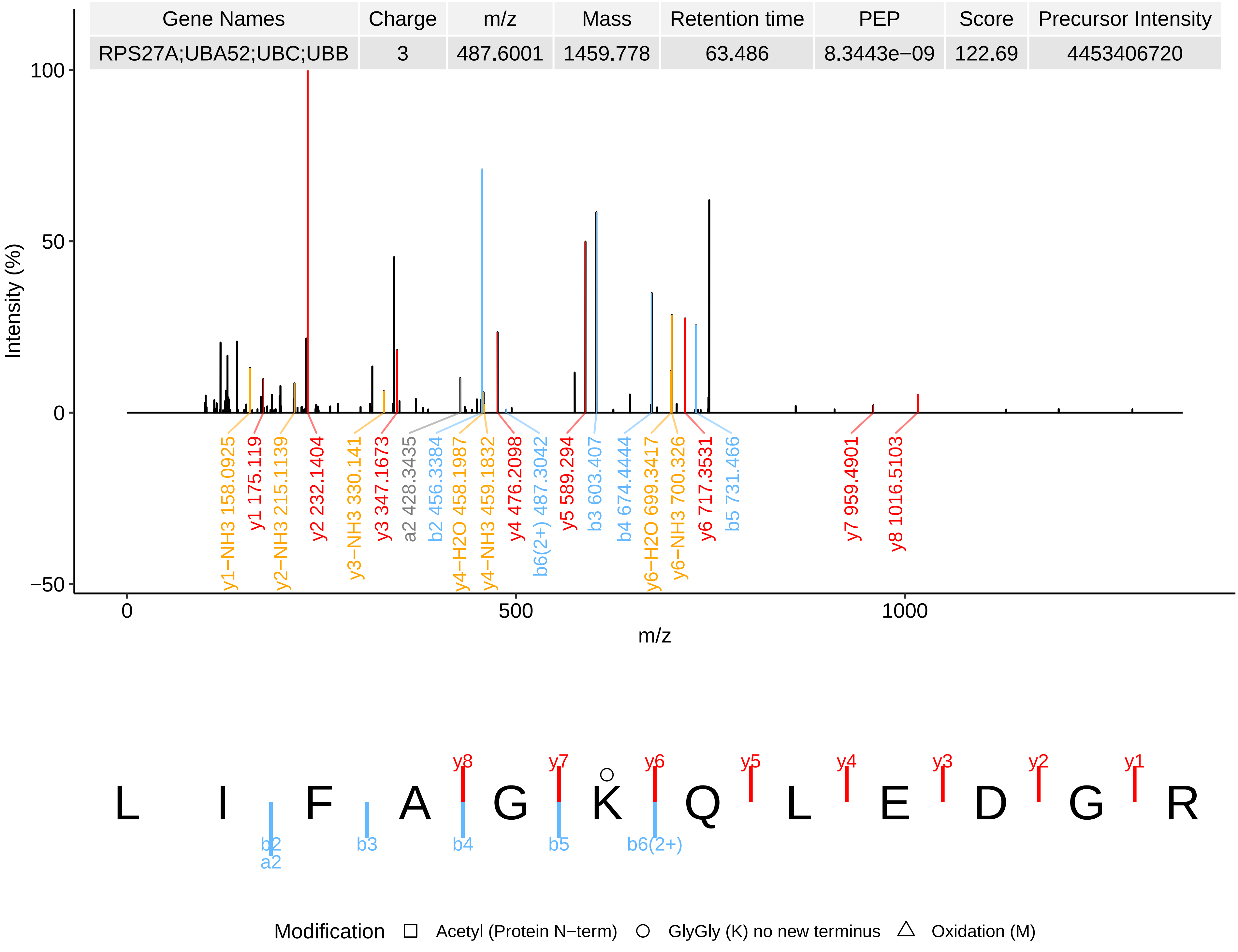

20201118_KIZ_MUb6_GlyGly_2 − Scan number: 18794

Gene Names	Charge	m/z	Mass	Retention time	PEP	Score	Precursor Intensity
RPS27A;UBA52;UBC;UBB	2	730.8964	1459.778	63.466	2.0433e−60	267.29	120765120

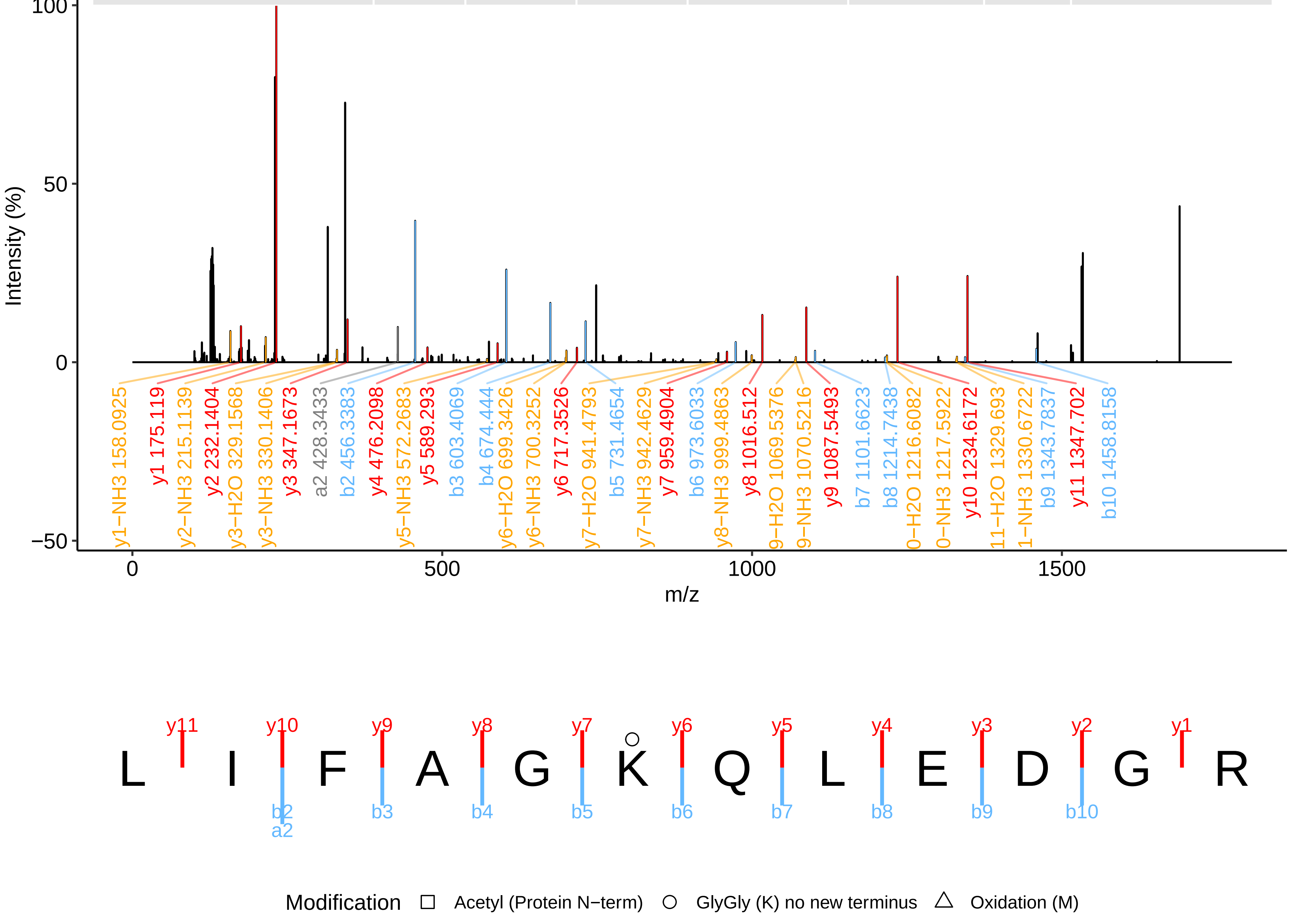

20201118_KIZ_MUb6_GlyGly_2 − Scan number: 18962

Gene Names	Charge	m/z	Mass	Retention time	PEP	Score	Precursor Intensity
RPS27A;UBA52;UBC;UBB	3	487.6001	1459.778	63.994	2.3227e−19	155	2602600704

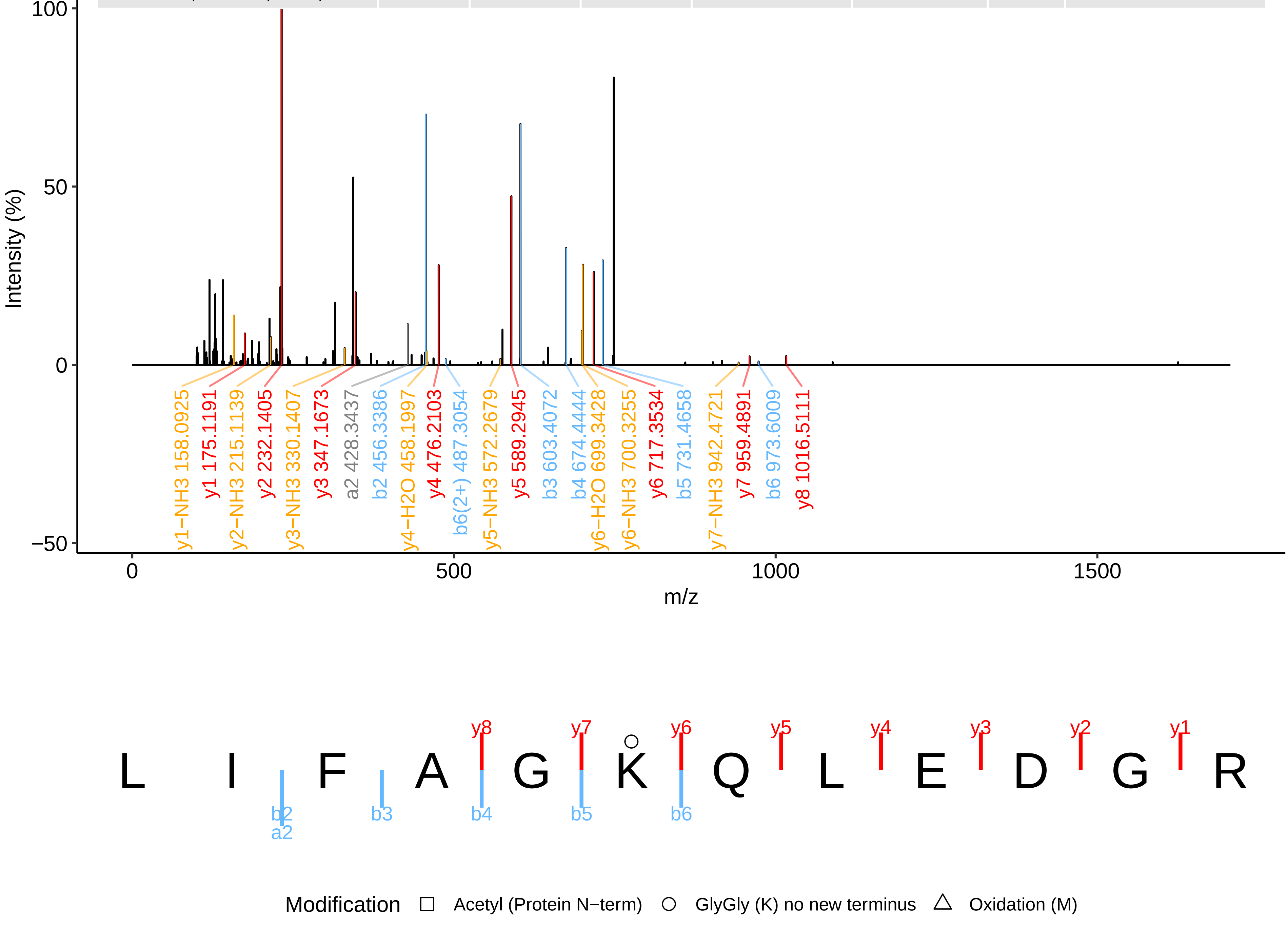

20201118_KIZ_MUb6_GlyGly_2 − Scan number: 19115

Gene Names	Charge	m/z	Mass	Retention time	PEP	Score	Precursor Intensity
RPS27A;UBA52;UBC;UBB	2	730.8964	1459.778	64.491	3.1566e−29	190.96	25432042

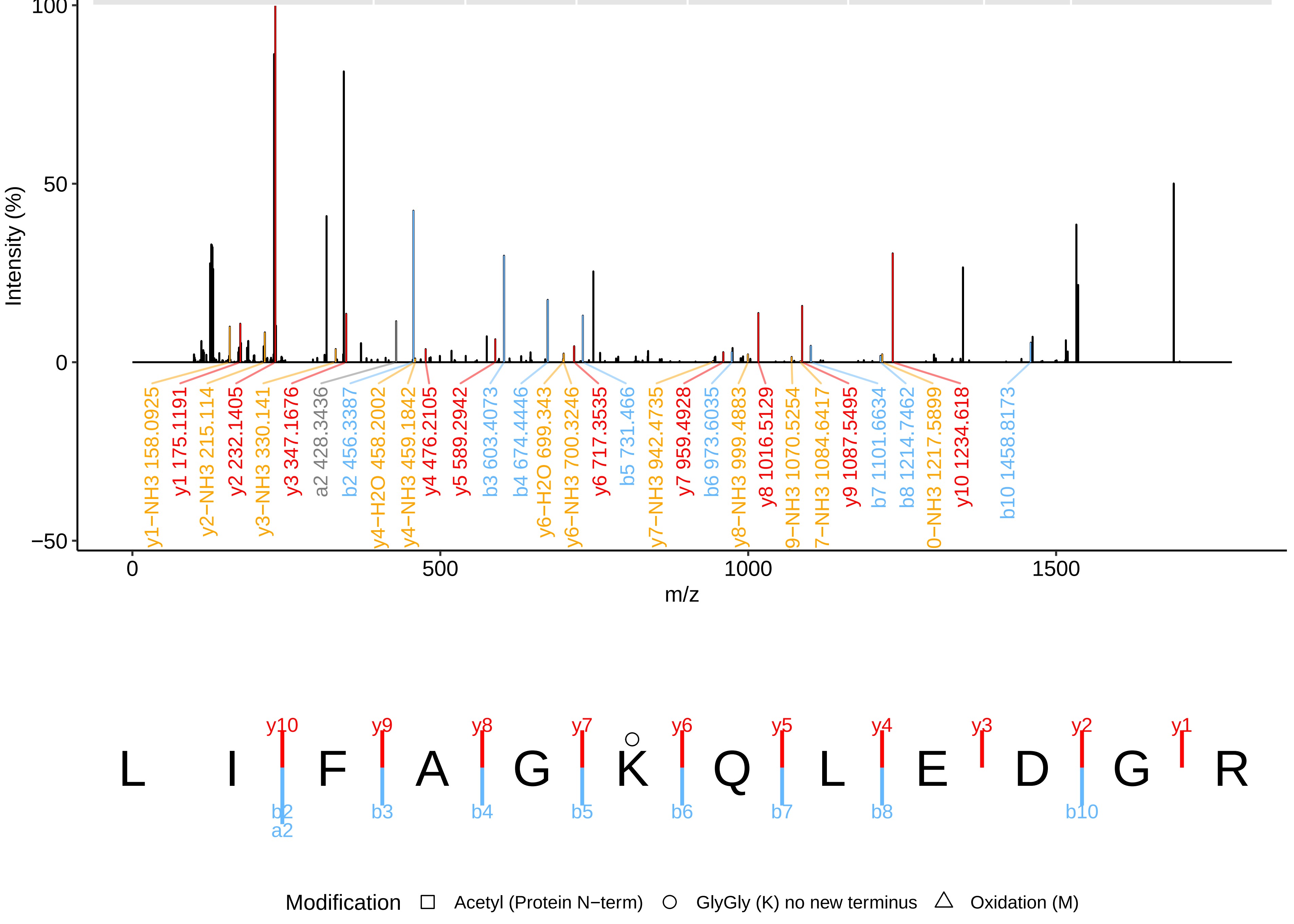

20201118_KIZ_MUb6_GlyGly_2 − Scan number: 19119

Gene Names	Charge	m/z	Mass	Retention time	PEP	Score	Precursor Intensity
RPS27A;UBA52;UBC;UBB	3	487.6001	1459.778	64.502	3.6333e−13	133.89	2163529472

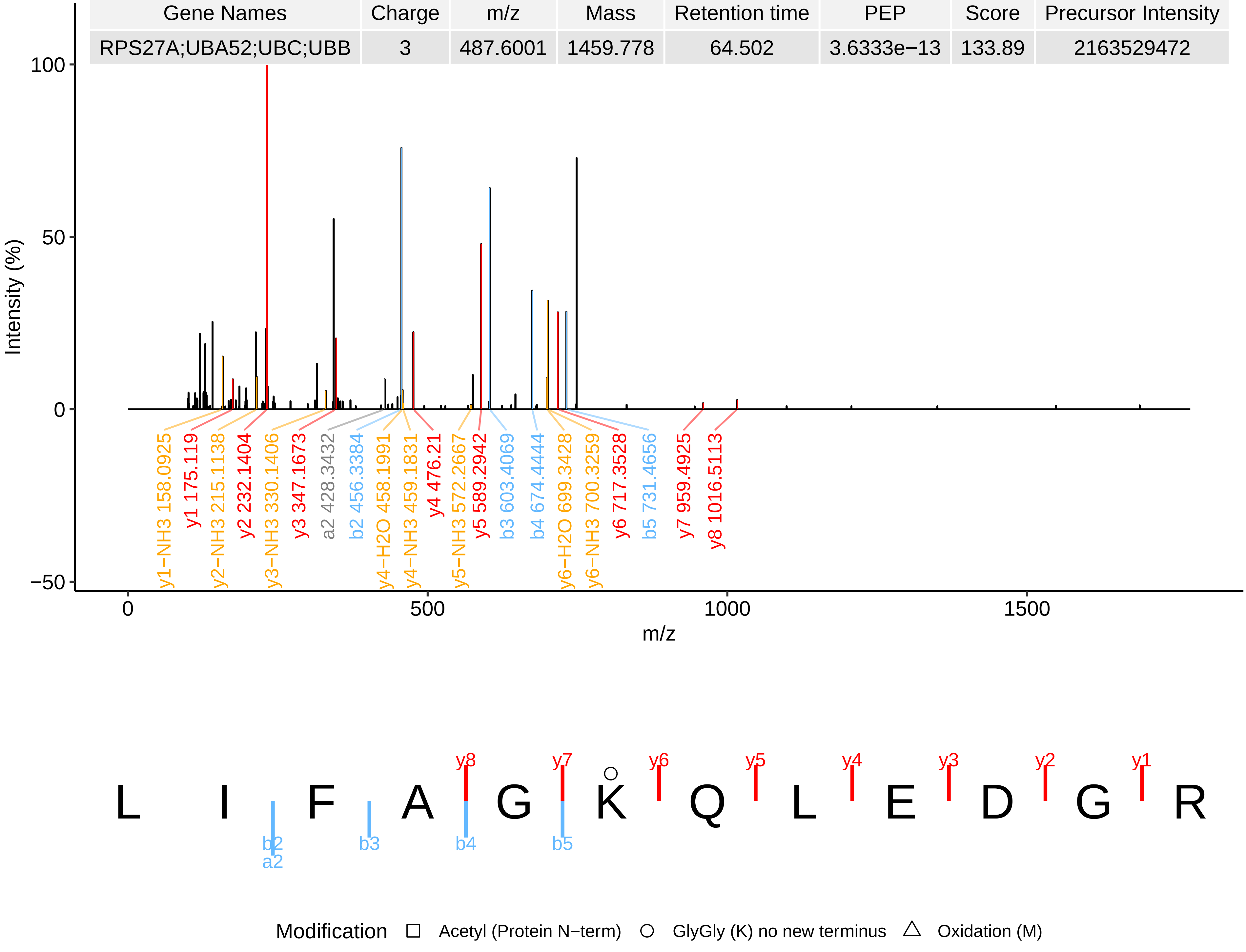

20201118_KIZ_MUb6_GlyGly_2 − Scan number: 19275

Gene Names	Charge	m/z	Mass	Retention time	PEP	Score	Precursor Intensity
RPS27A;UBA52;UBC;UBB	3	487.6001	1459.778	65.036	3.1574e−19	150.46	781895808

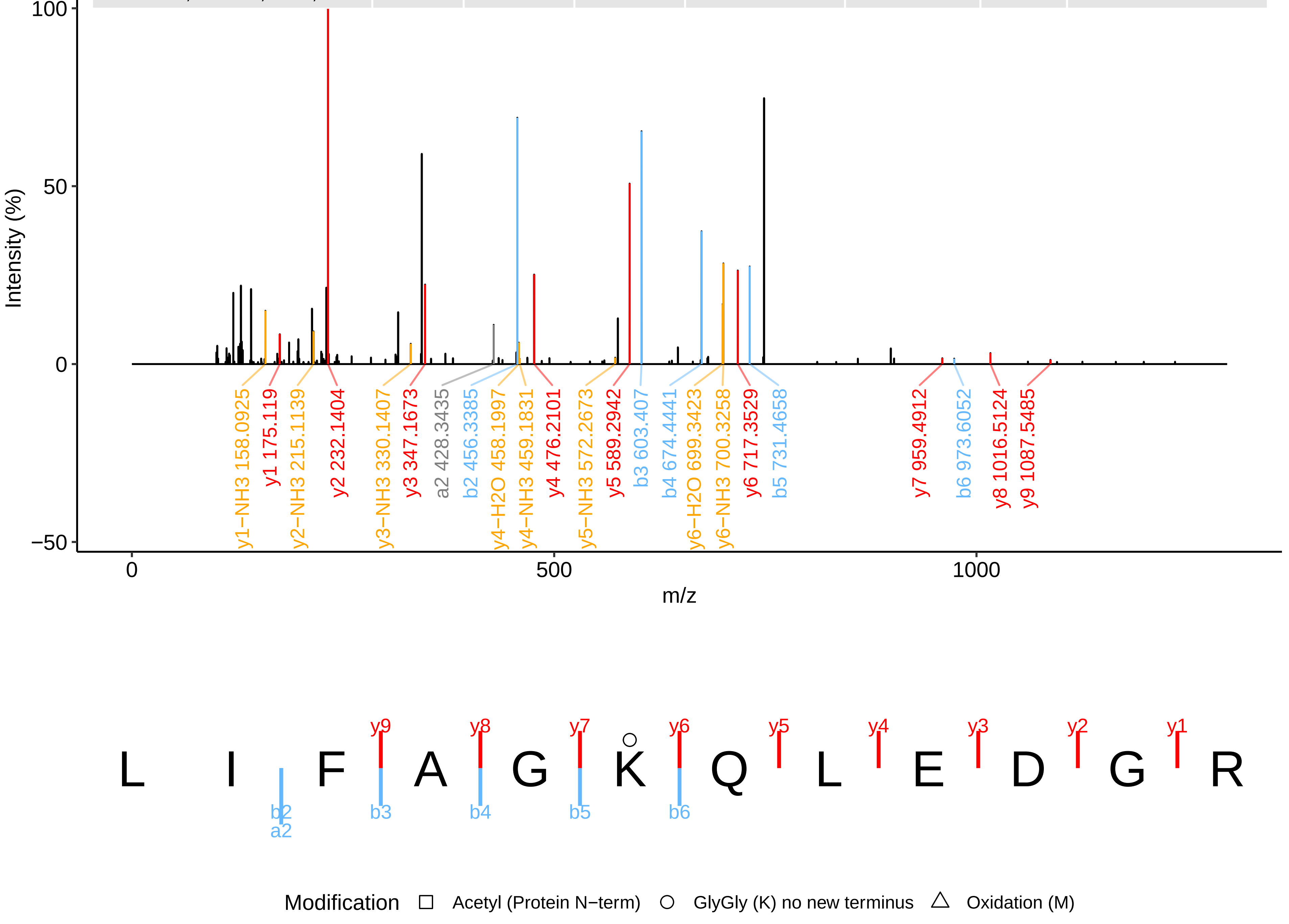

20201118_KIZ_MUb6_GlyGly_2 − Scan number: 19423

Gene Names	Charge	m/z	Mass	Retention time	PEP	Score	Precursor Intensity
RPS27A;UBA52;UBC;UBB	3	487.6001	1459.778	65.547	1.2918e−08	117.14	26522516

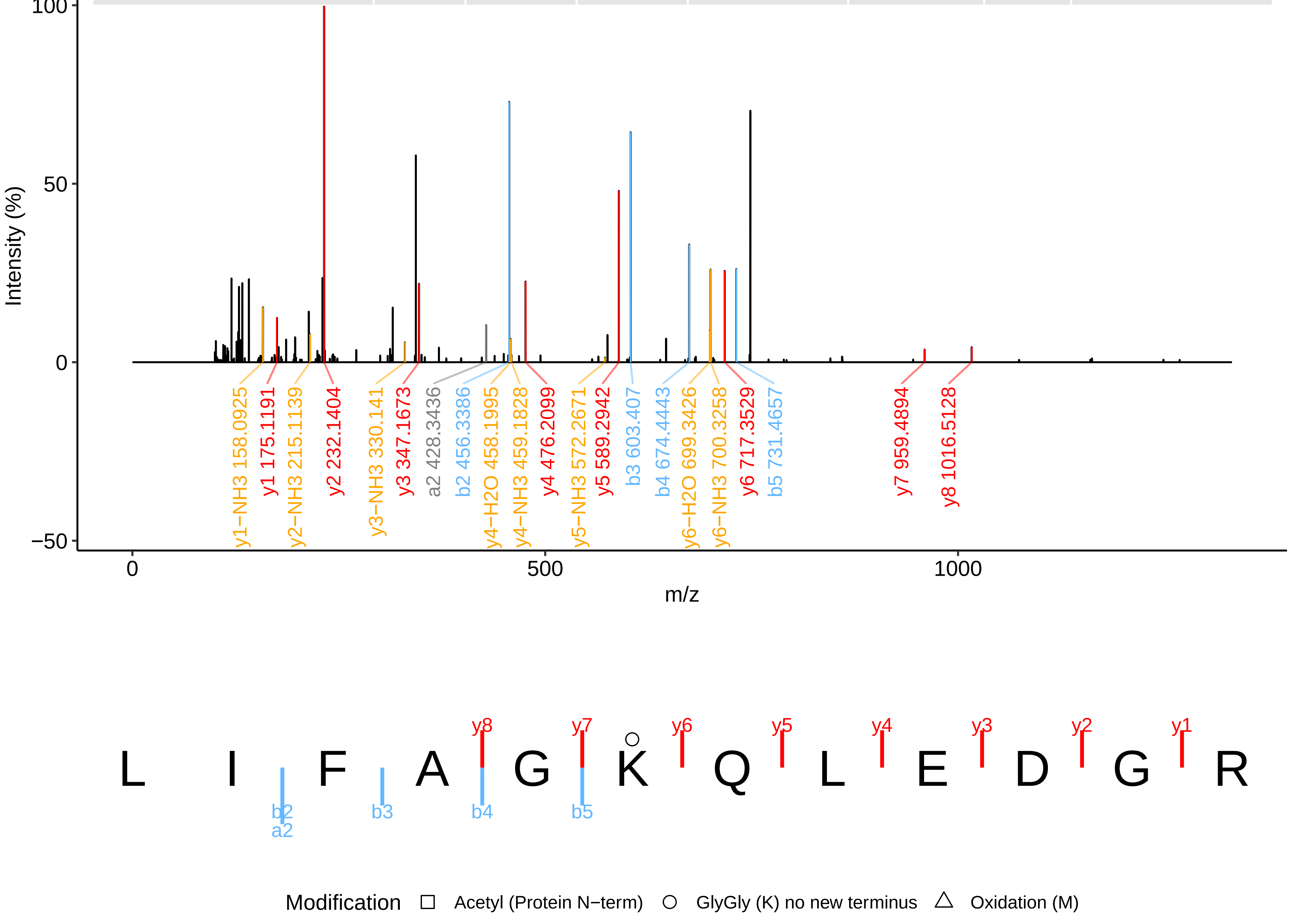

20201118_KIZ_MUb6_GlyGly_2 − Scan number: 18633

Gene Names	Charge	m/z	Mass	Retention time	PEP	Score	Precursor Intensity
RPS27A;UBA52;UBC;UBB	2	730.8964	1459.778	62.966	1.9946e−32	251.2	37259604

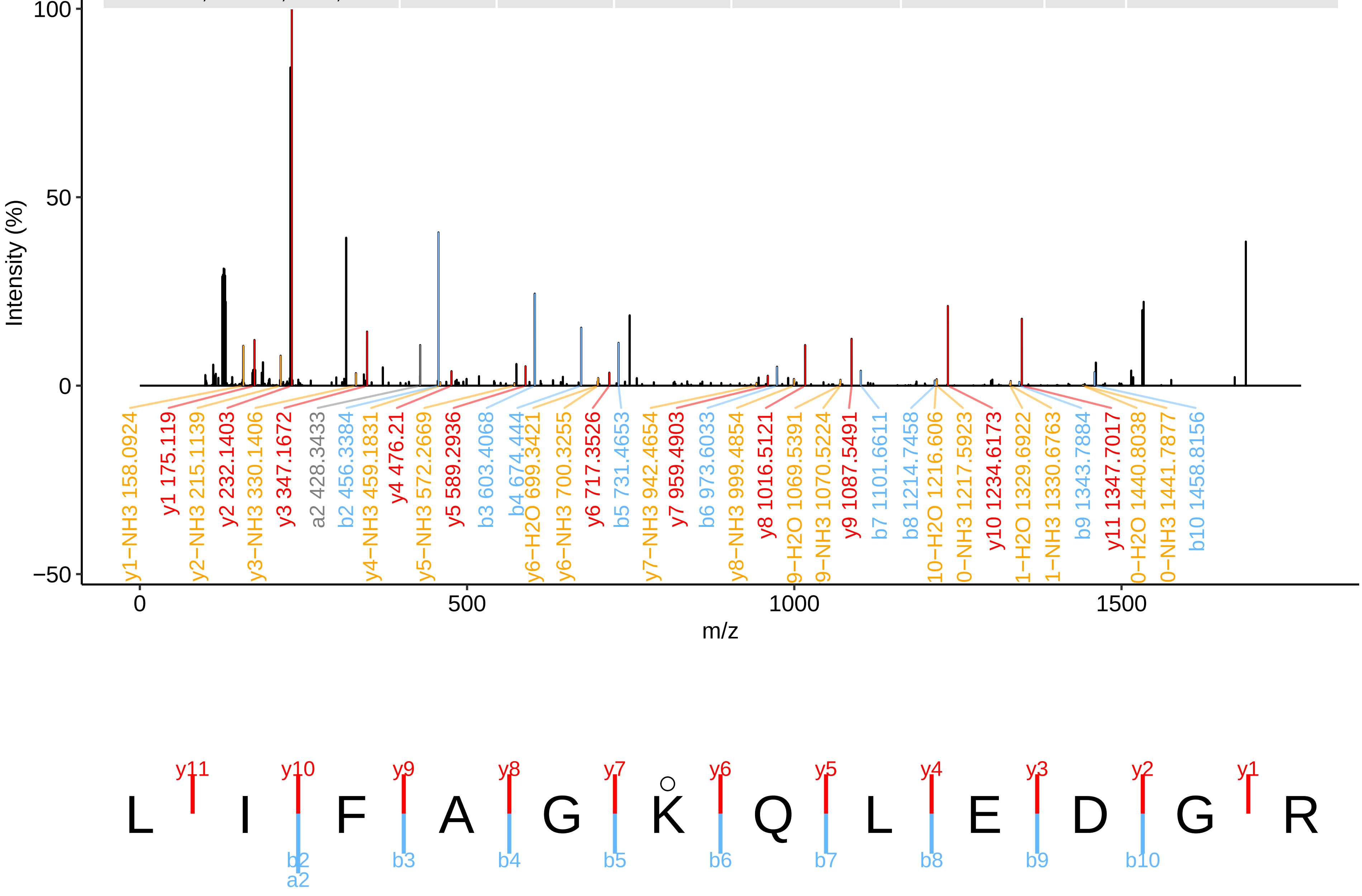

Modification □ Acetyl (Protein N−term) ○ GlyGly (K) no new terminus △ Oxidation (M)

20201118_KIZ_MUb6_GlyGly_2 − Scan number: 19267

Gene Names	Charge	m/z	Mass	Retention time	PEP	Score	Precursor Intensity
RPS27A;UBA52;UBC;UBB	2	730.8964	1459.778	65.012	3.9016e−25	232.88	13188582

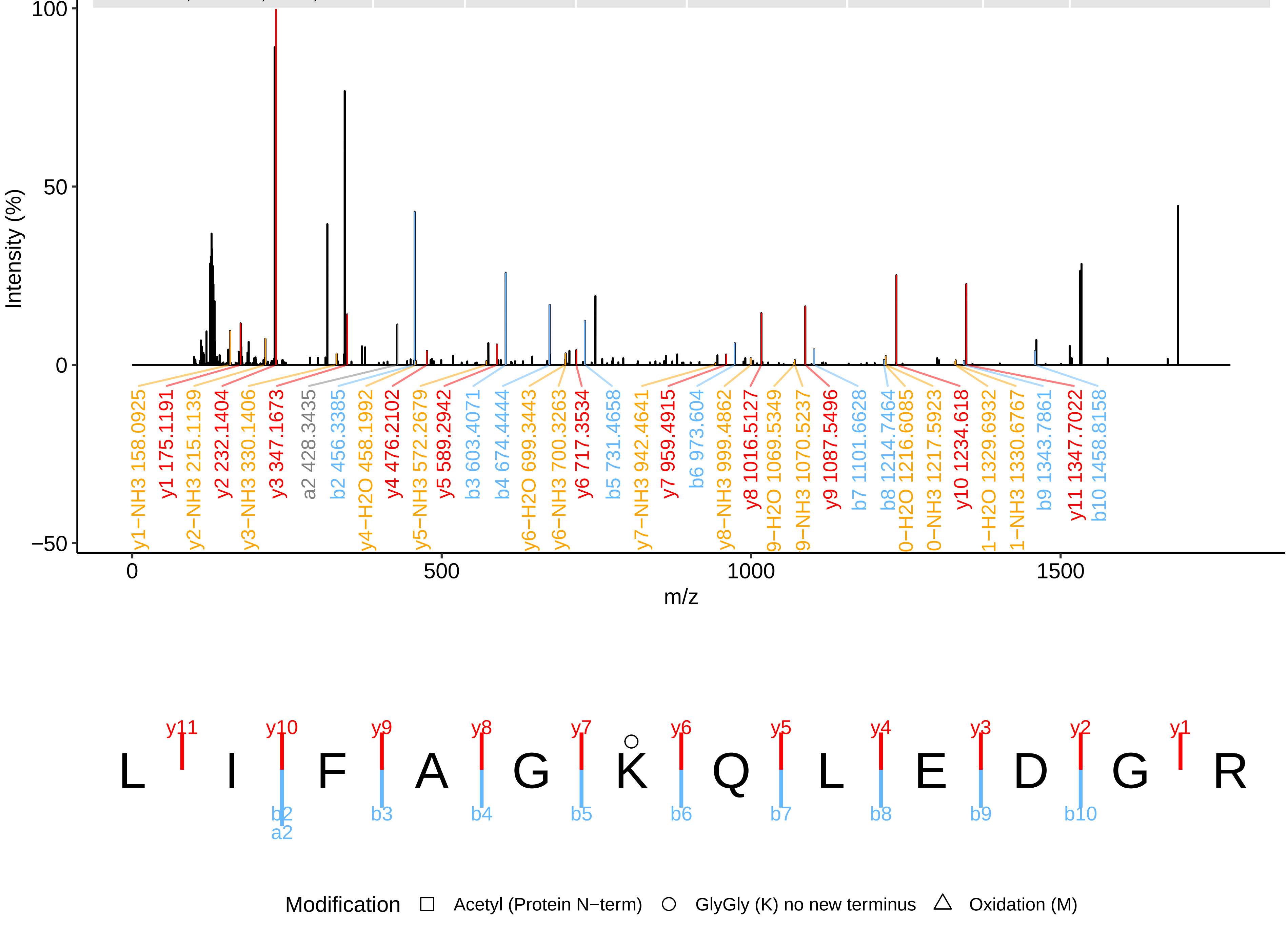

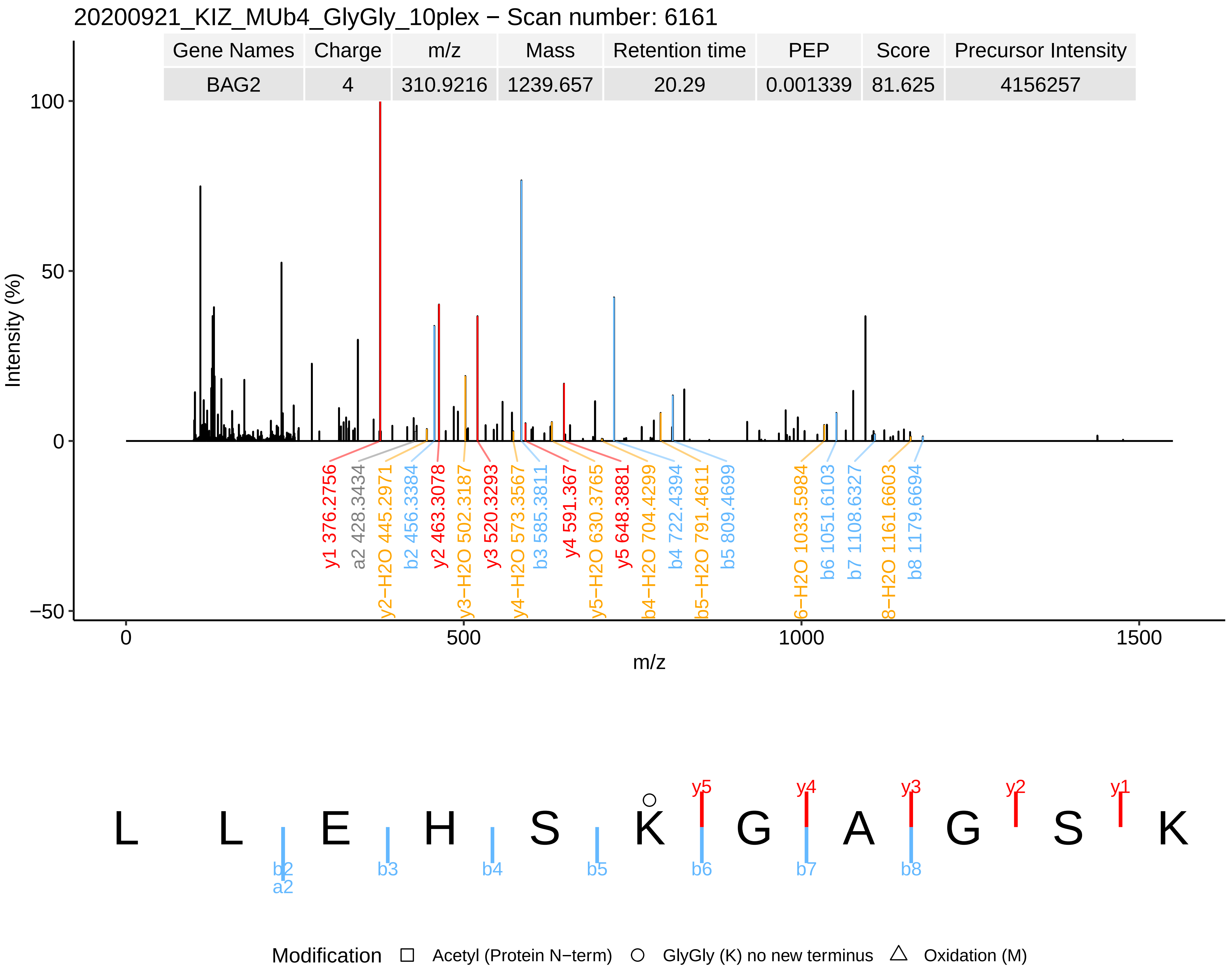
20200921_KIZ_MUb4_GlyGly_10plex − Scan number: 6161
Gene Names | Charge | m/z | Mass | Retention time | PEP | Score | Precursor Intensity
BAG2 | 4 | 310.9216 | 1239.657 | 20.29 | 0.001339 | 81.625 | 4156257
Intensity (%)
100
50
0
−50
0
500
1000
1500
m/z
y1 376.2756
a2 428.3434
y2−H2O 445.2971
b2 456.3384
y2 463.3078
y3−H2O 502.3187
y3 520.3293
y4−H2O 573.3567
b3 585.3811
y4 591.367
y5−H2O 630.3765
y5 648.3881
b4−H2O 704.4299
b4 722.4394
b5−H2O 791.4611
b5 809.4699
6−H2O 1033.5984
b6 1051.6103
b7 1108.6327
8−H2O 1161.6603
b8 1179.6694
L L E H S K G A G S K
y5 y4 y3 y2 y1
b2 a2 b3 b4 b5 b6 b7 b8
Modification
Acetyl (Protein N−term)
GlyGly (K) no new terminus
Oxidation (M)

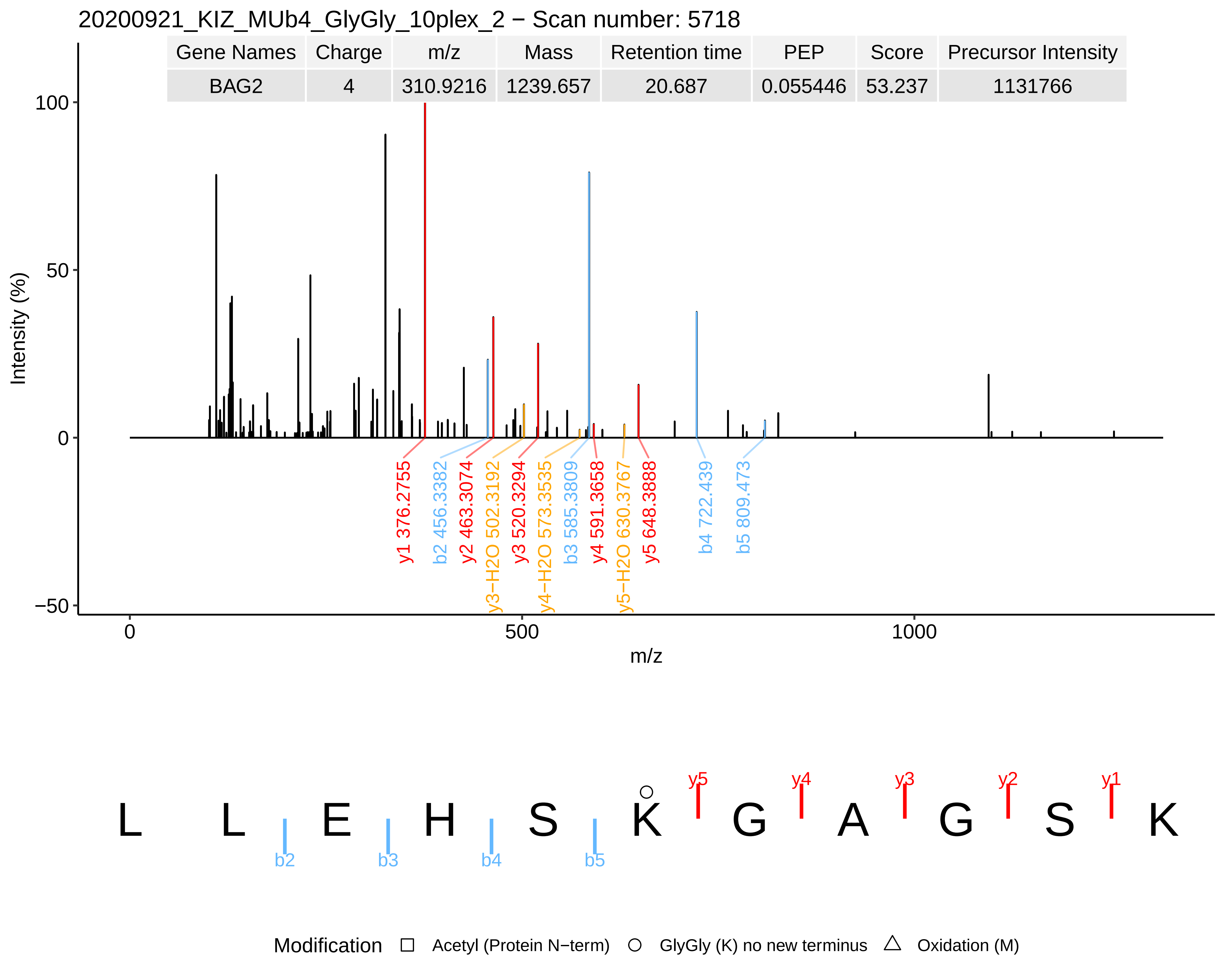

20200921_KIZ_MUb4_GlyGly_10plex_2 − Scan number: 5718
Gene Names | Charge | m/z | Mass | Retention time | PEP | Score | Precursor Intensity
BAG2 | 4 | 310.9216 | 1239.657 | 20.687 | 0.055446 | 53.237 | 1131766
Intensity (%)
100
50
0
−50
0
500
1000
m/z
y1 376.2755
b2 456.3382
y2 463.3074
y3−H2O 502.3192
y3 520.3294
y4−H2O 573.3535
b3 585.3809
y4 591.3658
y5−H2O 630.3767
y5 648.3888
b4 722.439
b5 809.473
L L E H S K G A G S K
y5 y4 y3 y2 y1
b2 b3 b4 b5
Modification
Acetyl (Protein N−term)
GlyGly (K) no new terminus
Oxidation (M)

20200924_KIZ_MUb5_GlyGly_1 − Scan number: 5194

Gene Names	Charge	m/z	Mass	Retention time	PEP	Score	Precursor Intensity
BAG2	3	414.2263	1239.657	16.123	0.0013333	89.189	NA

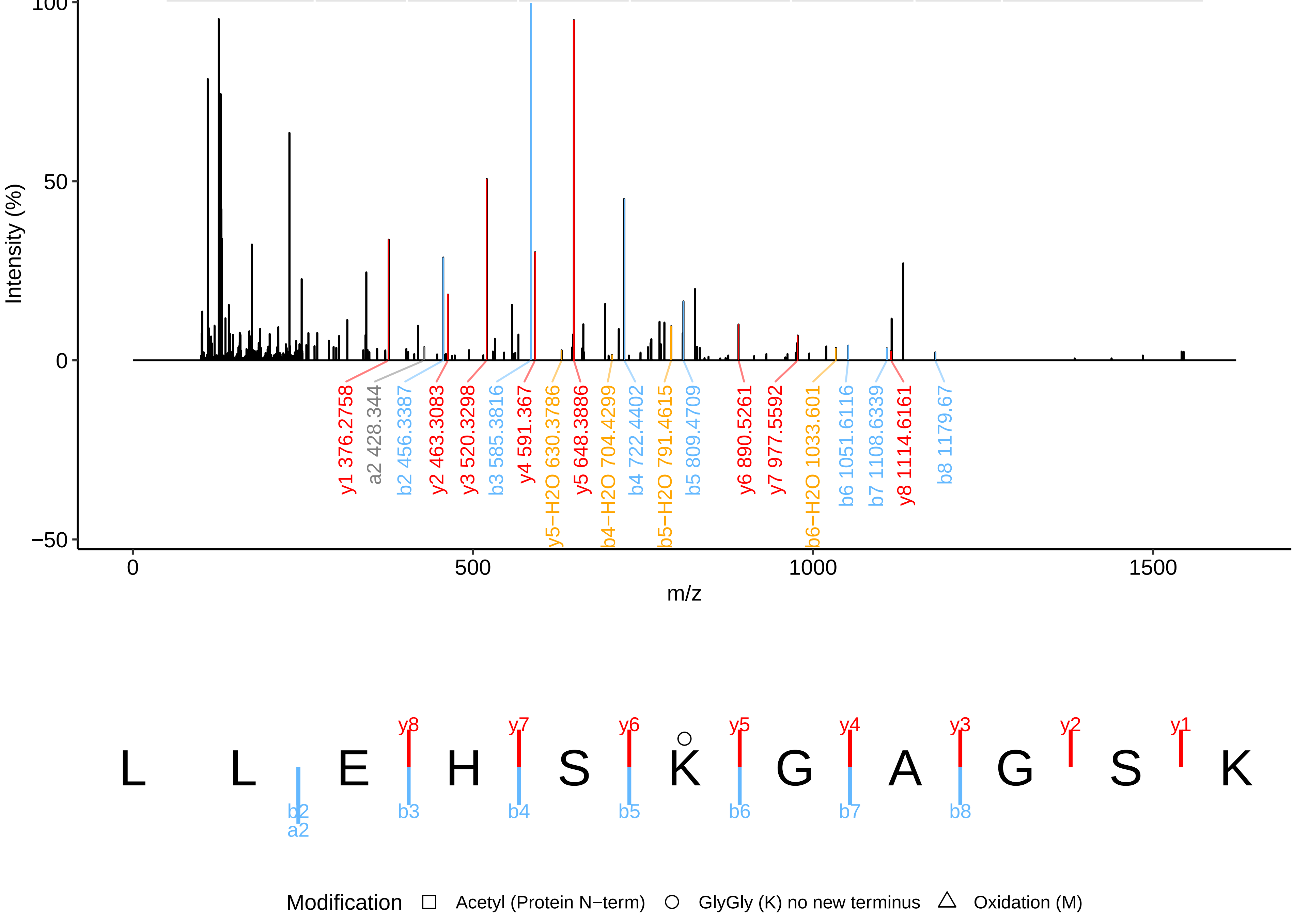

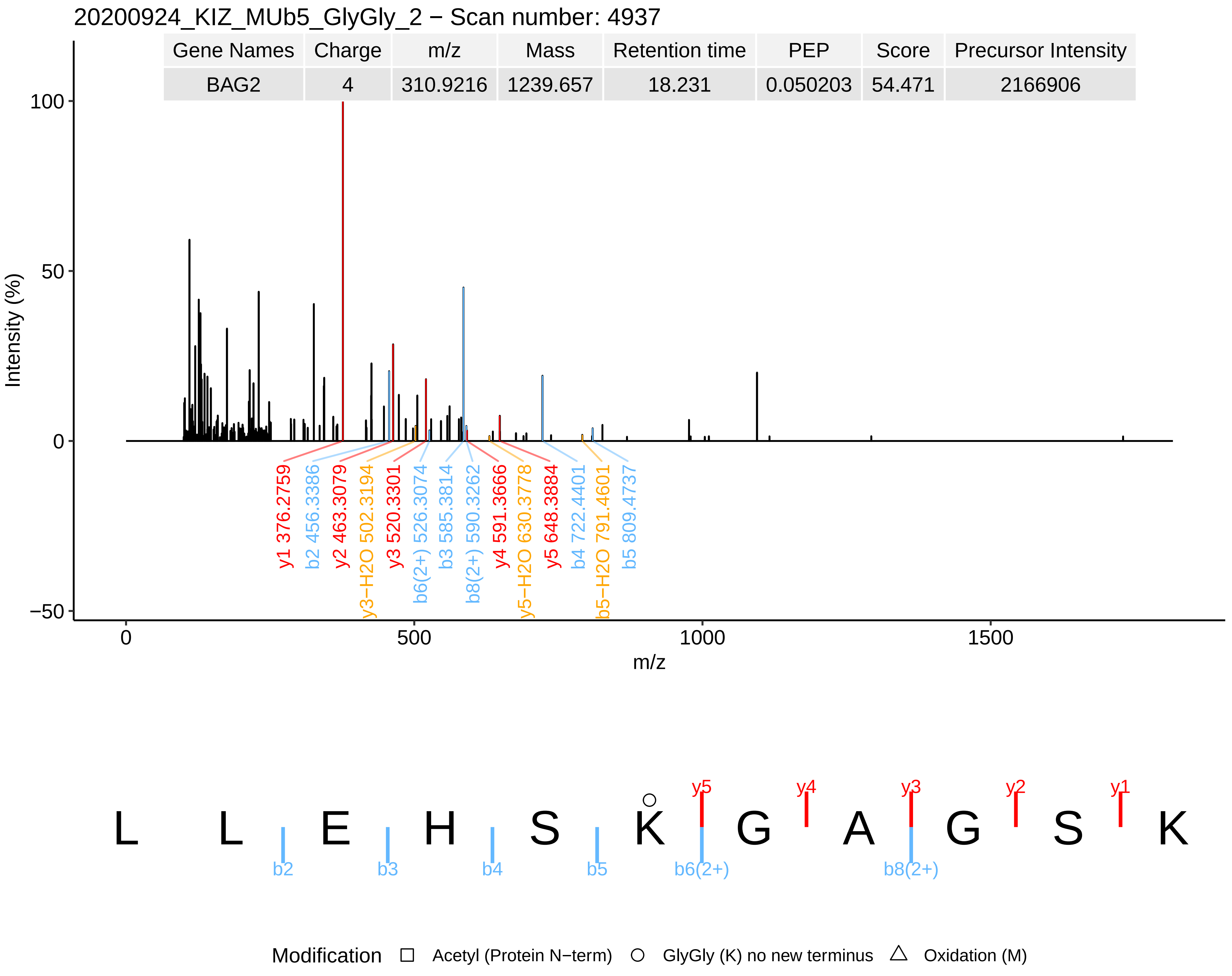
20200924_KIZ_MUb5_GlyGly_2 − Scan number: 4937
Gene Names
Charge
m/z
Mass
Retention time
PEP
Score
Precursor Intensity
BAG2
4
310.9216
1239.657
18.231
0.050203
54.471
2166906
Intensity (%)
m/z
y1 376.2759
b2 456.3386
y2 463.3079
y3−H2O 502.3194
y3 520.3301
b6(2+) 526.3074
b3 585.3814
b8(2+) 590.3262
y4 591.3666
y5−H2O 630.3778
y5 648.3884
b4 722.4401
b5−H2O 791.4601
b5 809.4737
L L E H S K G A G S K
y5 y4 y3 y2 y1
b2 b3 b4 b5 b6(2+) b8(2+)
Modification
Acetyl (Protein N−term)
GlyGly (K) no new terminus
Oxidation (M)

20200921_KIZ_MUb4_GlyGly_10plex_2 − Scan number: 17561

Gene Names	Charge	m/z	Mass	Retention time	PEP	Score	Precursor Intensity
TELO2	3	404.5872	1210.74	68.68	0.019467	66.435	316213.9

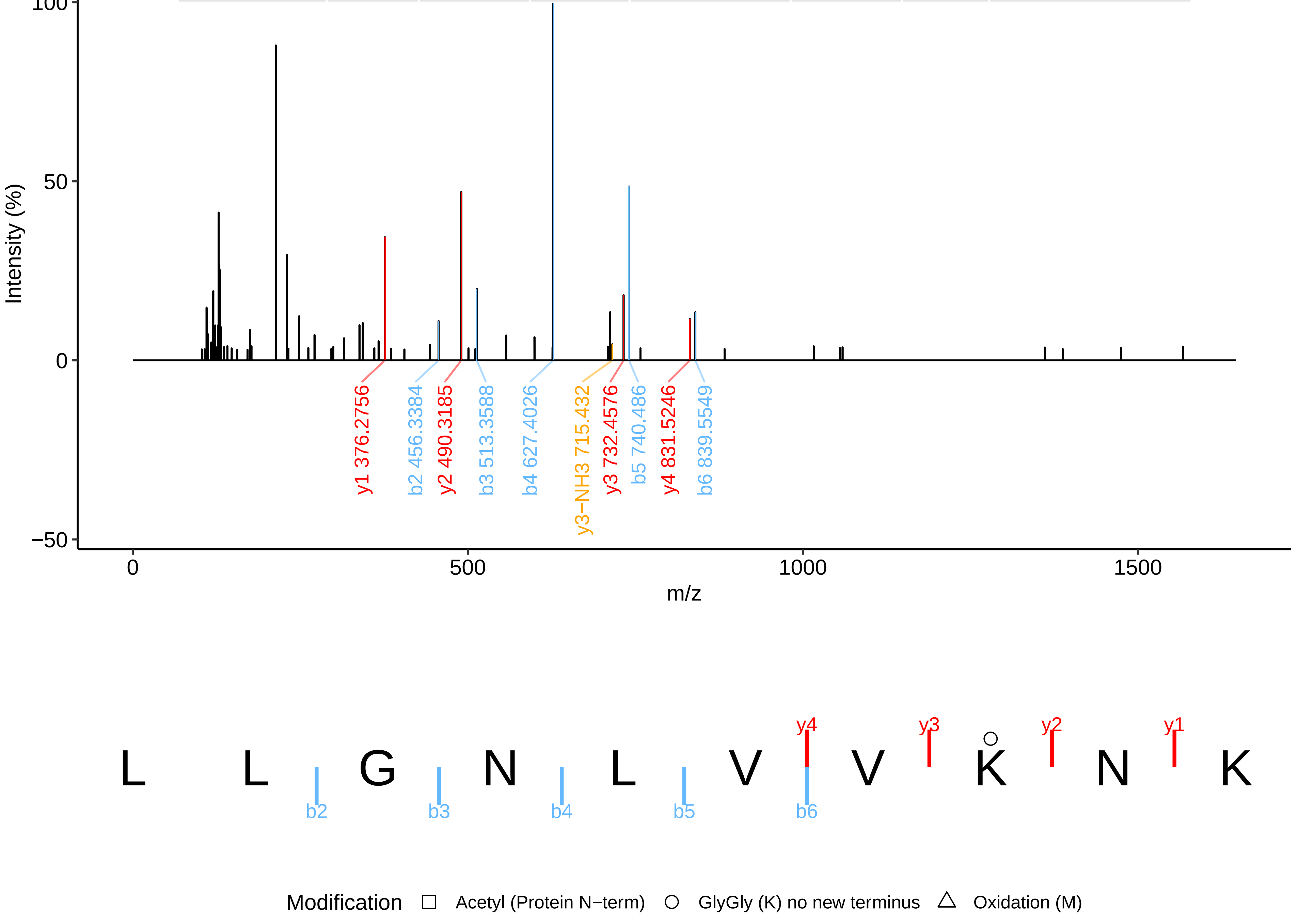

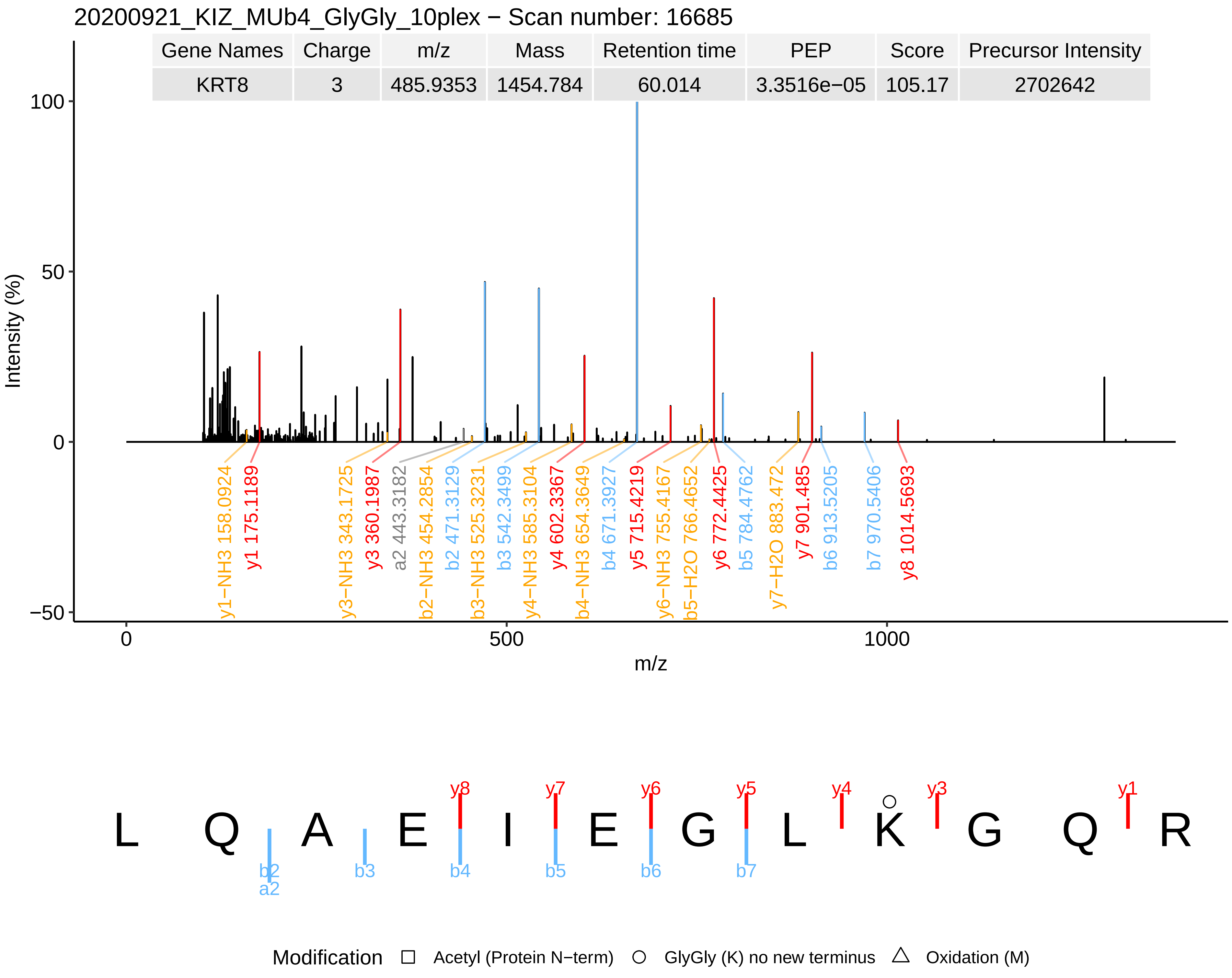
20200921_KIZ_MUb4_GlyGly_10plex − Scan number: 16685
Gene Names | Charge | m/z | Mass | Retention time | PEP | Score | Precursor Intensity
KRT8 | 3 | 485.9353 | 1454.784 | 60.014 | 3.3516e−05 | 105.17 | 2702642
Intensity (%)
m/z
y1−NH3 158.0924
y1 175.1189
y3−NH3 343.1725
y3 360.1987
a2 443.3182
b2−NH3 454.2854
b2 471.3129
b3−NH3 525.3231
b3 542.3499
y4−NH3 585.3104
y4 602.3367
b4−NH3 654.3649
b4 671.3927
y5 715.4219
y6−NH3 755.4167
b5−H2O 766.4652
y6 772.4425
b5 784.4762
y7−H2O 883.472
y7 901.485
b6 913.5205
b7 970.5406
y8 1014.5693
L Q A E I E G L K G Q R
y8 y7 y6 y5 y4 y3 y1
b2 a2 b3 b4 b5 b6 b7
Modification
Acetyl (Protein N−term)
GlyGly (K) no new terminus
Oxidation (M)

20200921_KIZ_MUb4_GlyGly_10plex_2 − Scan number: 15413

Gene Names	Charge	m/z	Mass	Retention time	PEP	Score	Precursor Intensity
KRT8	3	485.9353	1454.784	60.27	3.9062e−09	128.08	1791989

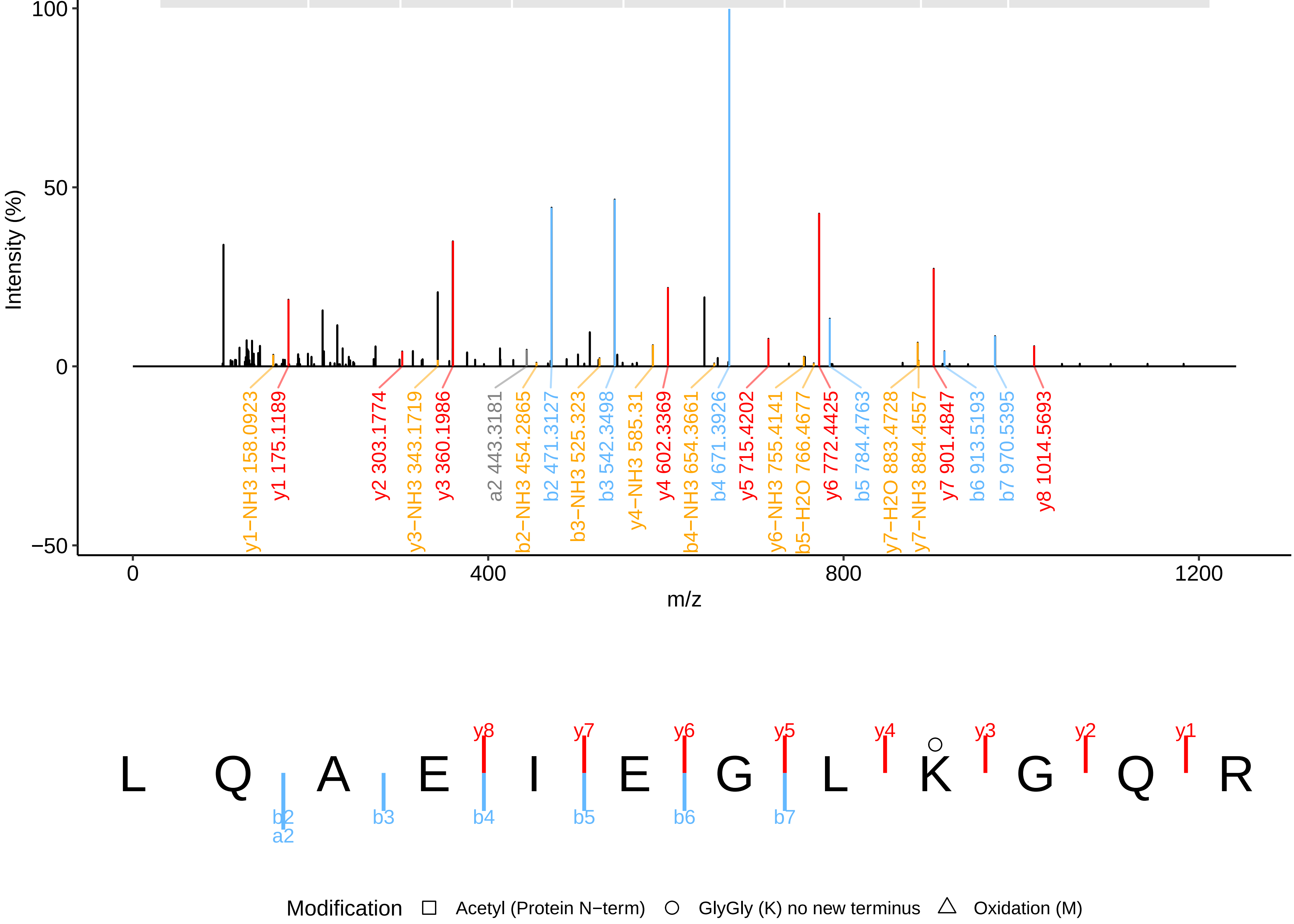

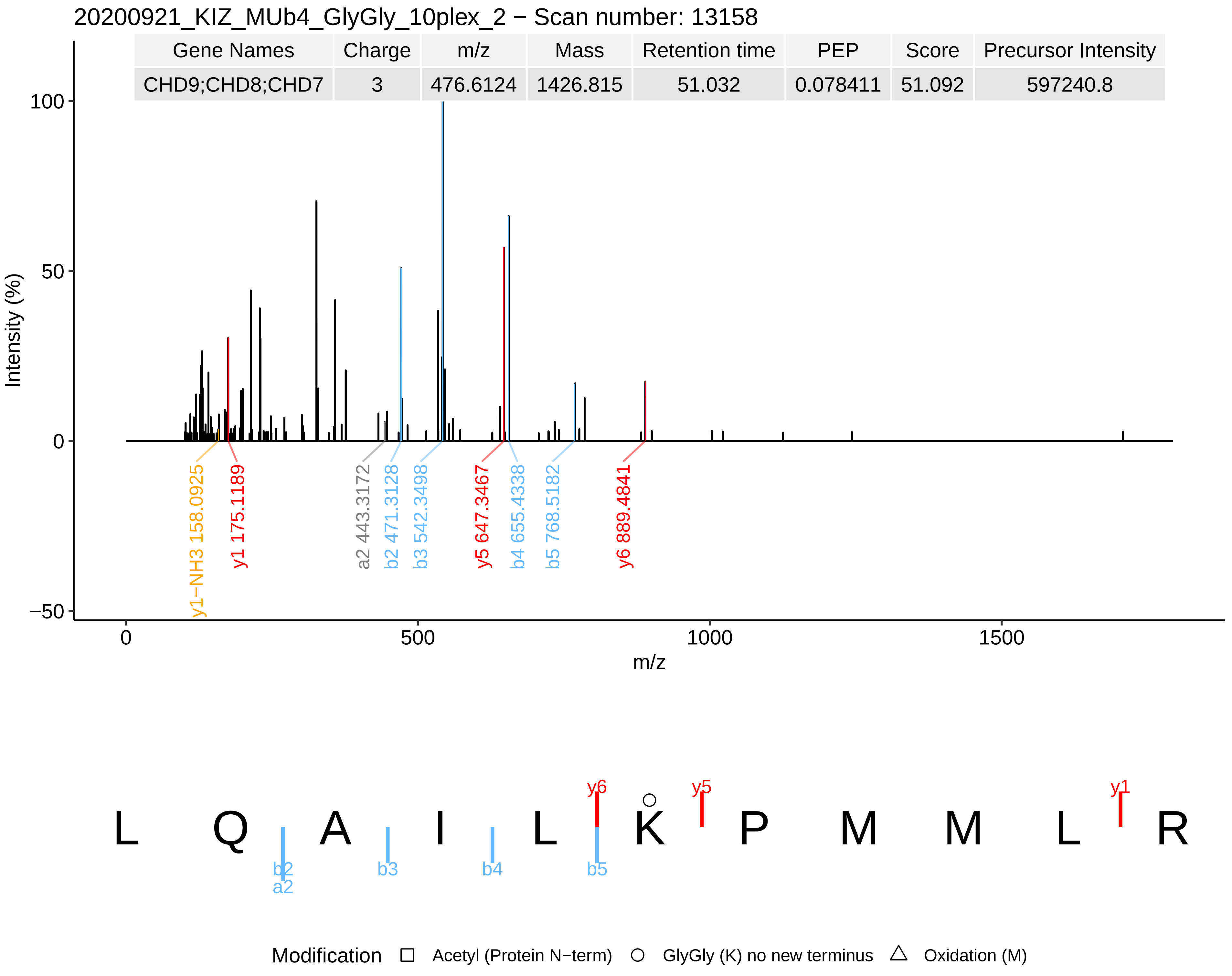
20200921_KIZ_MUb4_GlyGly_10plex_2 − Scan number: 13158
Gene Names | Charge | m/z | Mass | Retention time | PEP | Score | Precursor Intensity
CHD9;CHD8;CHD7 | 3 | 476.6124 | 1426.815 | 51.032 | 0.078411 | 51.092 | 597240.8
Intensity (%)
100
50
0
−50
y1−NH3 158.0925
y1 175.1189
a2 443.3172
b2 471.3128
b3 542.3498
y5 647.3467
b4 655.4338
b5 768.5182
y6 889.4841
0
500
1000
1500
m/z
L Q A I L K P M M L R
y6 y5 y1
b2 a2 b3 b4 b5
Modification
Acetyl (Protein N−term)
GlyGly (K) no new terminus
Oxidation (M)

20200921_KIZ_MUb4_GlyGly_10plex − Scan number: 9937

Gene Names	Charge	m/z	Mass	Retention time	PEP	Score	Precursor Intensity
VPS13A	4	362.1884	1444.725	34.212	3.32e−05	99.788	4414464

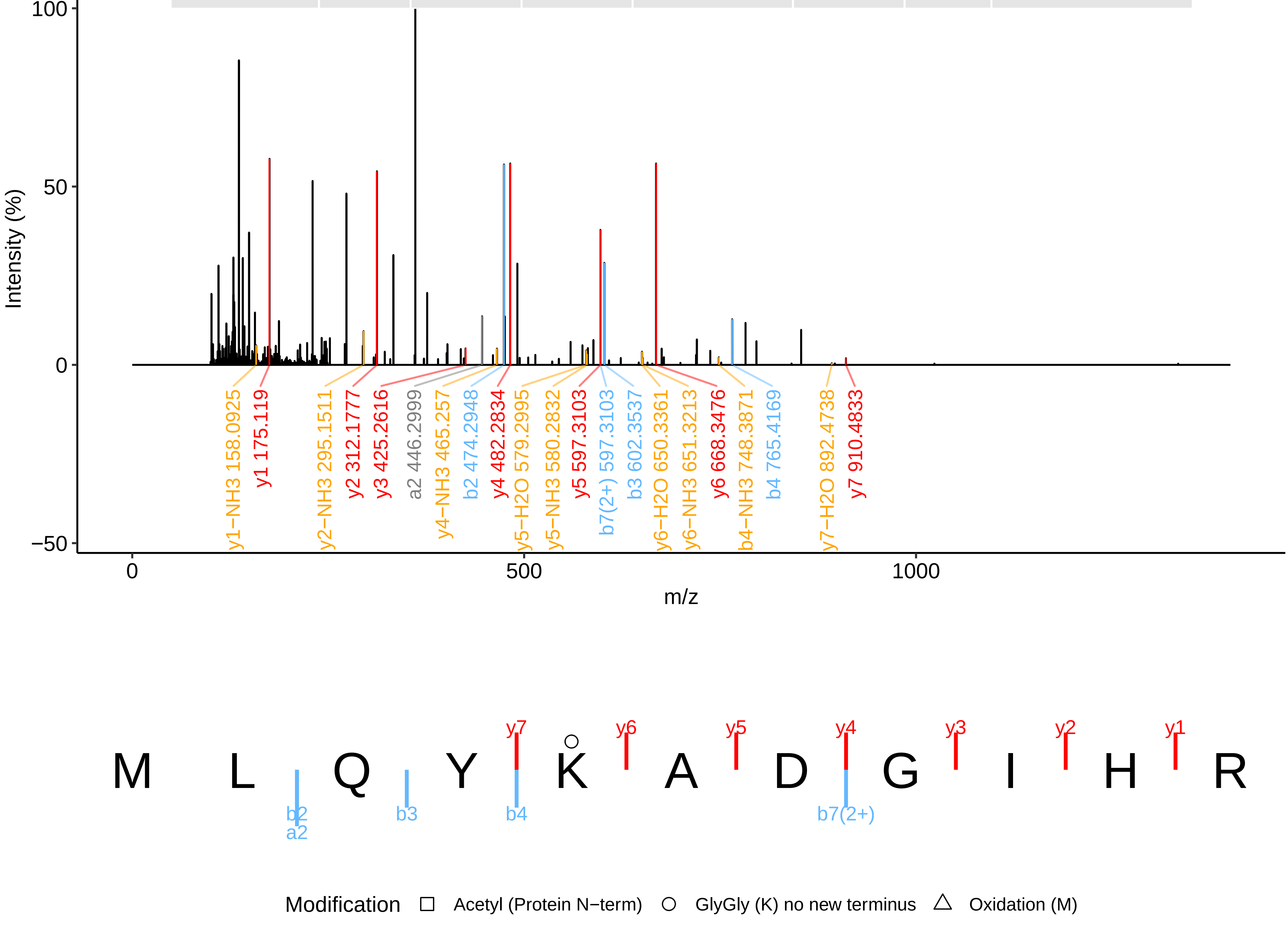

20200921_KIZ_MUb4_GlyGly_10plex_2 − Scan number: 22445

Gene Names	Charge	m/z	Mass	Retention time	PEP	Score	Precursor Intensity
RPS27A;UBA52;UBC;UBB	2	719.3921	1436.77	88.482	0.0096932	47.938	488652

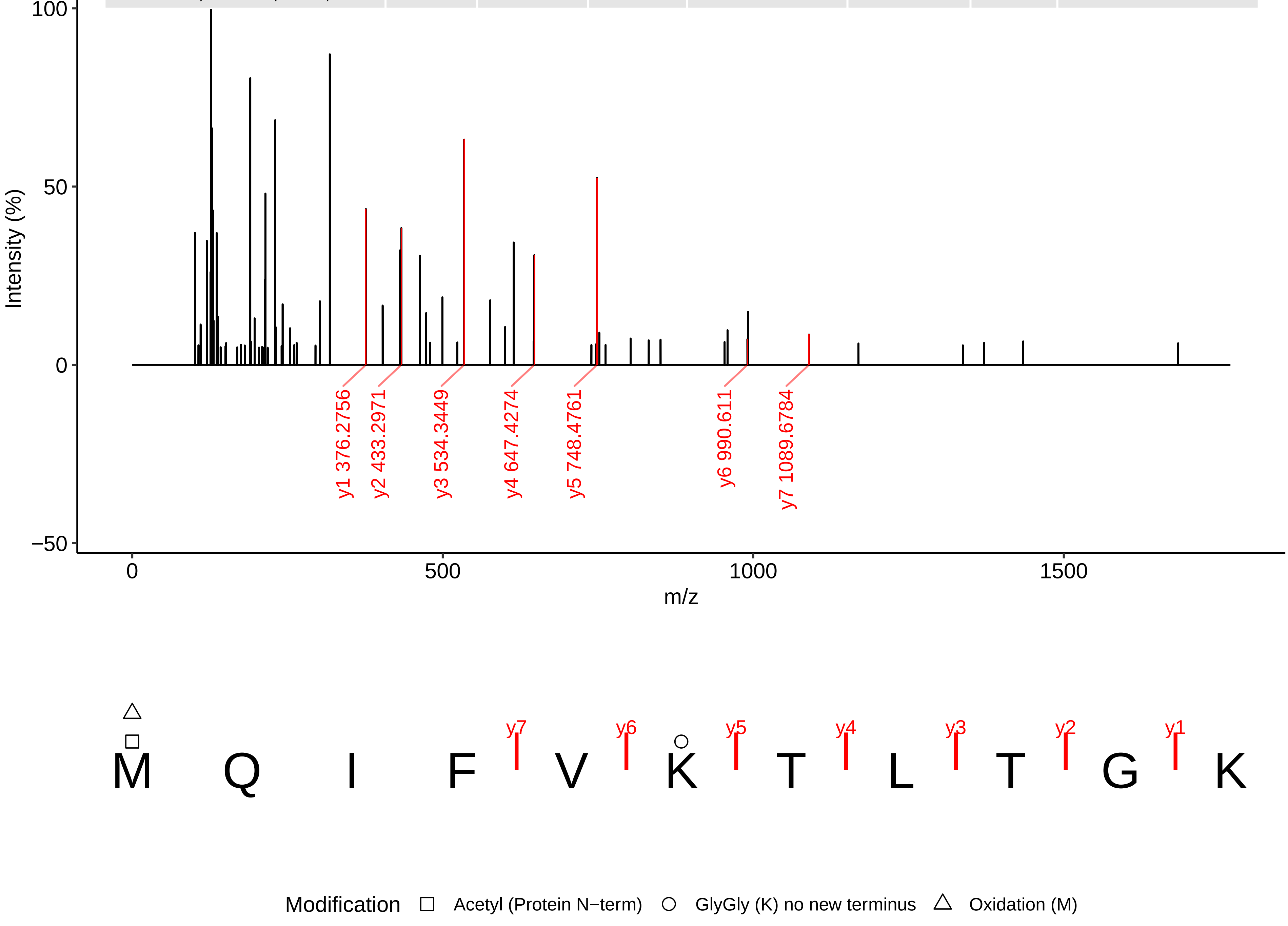

20200924_KIZ_MUb5_GlyGly_1 − Scan number: 22623

Gene Names	Charge	m/z	Mass	Retention time	PEP	Score	Precursor Intensity
DHX9	2	731.3796	1460.745	72.777	0.0006453	100.19	18679514

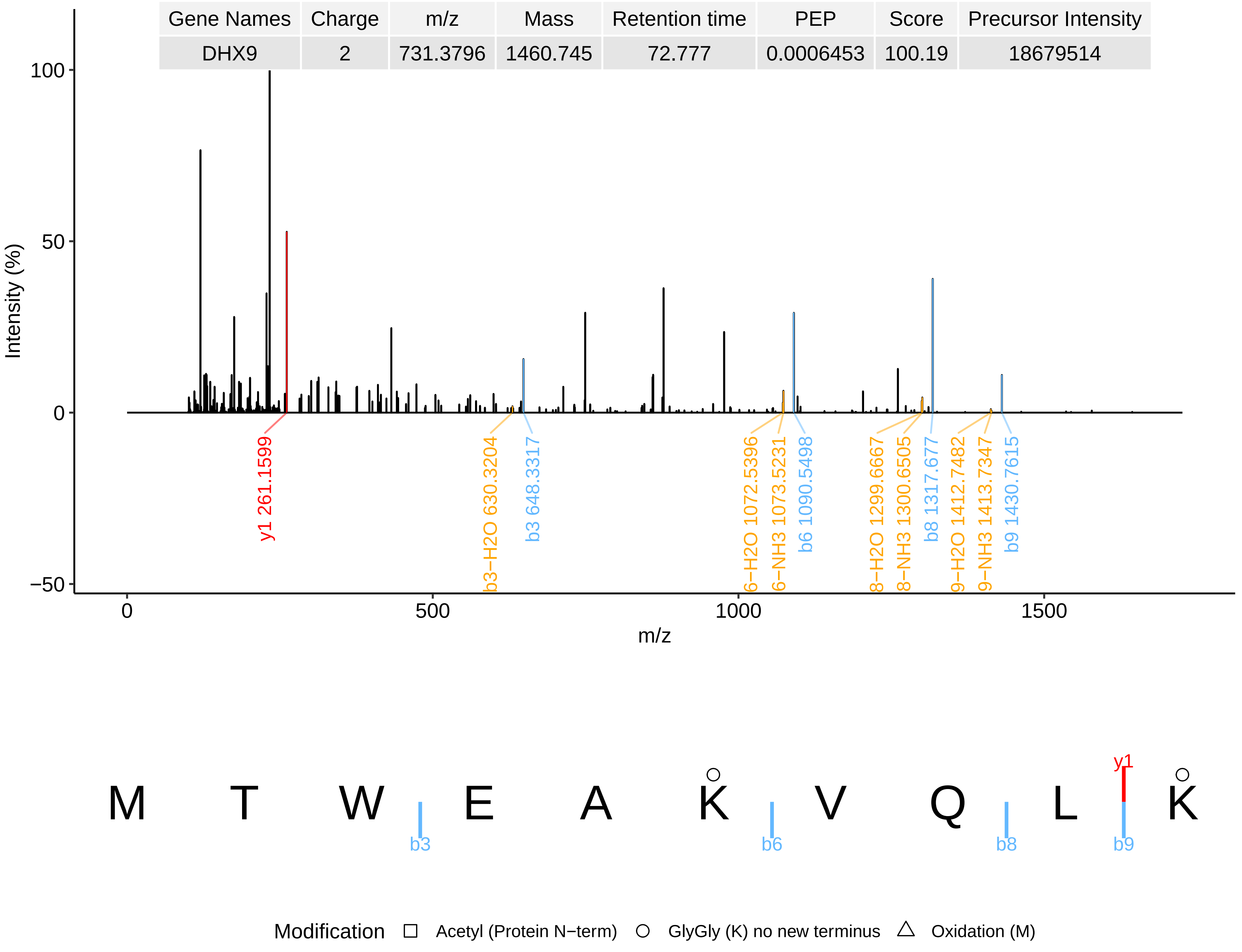

20200921_KIZ_MUb4_GlyGly_10plex − Scan number: 12842

Gene Names	Charge	m/z	Mass	Retention time	PEP	Score	Precursor Intensity
HSPA8;HSPA1B;HSPA1A	3	413.8728	1238.597	45.17	0.0091655	75.294	3954184

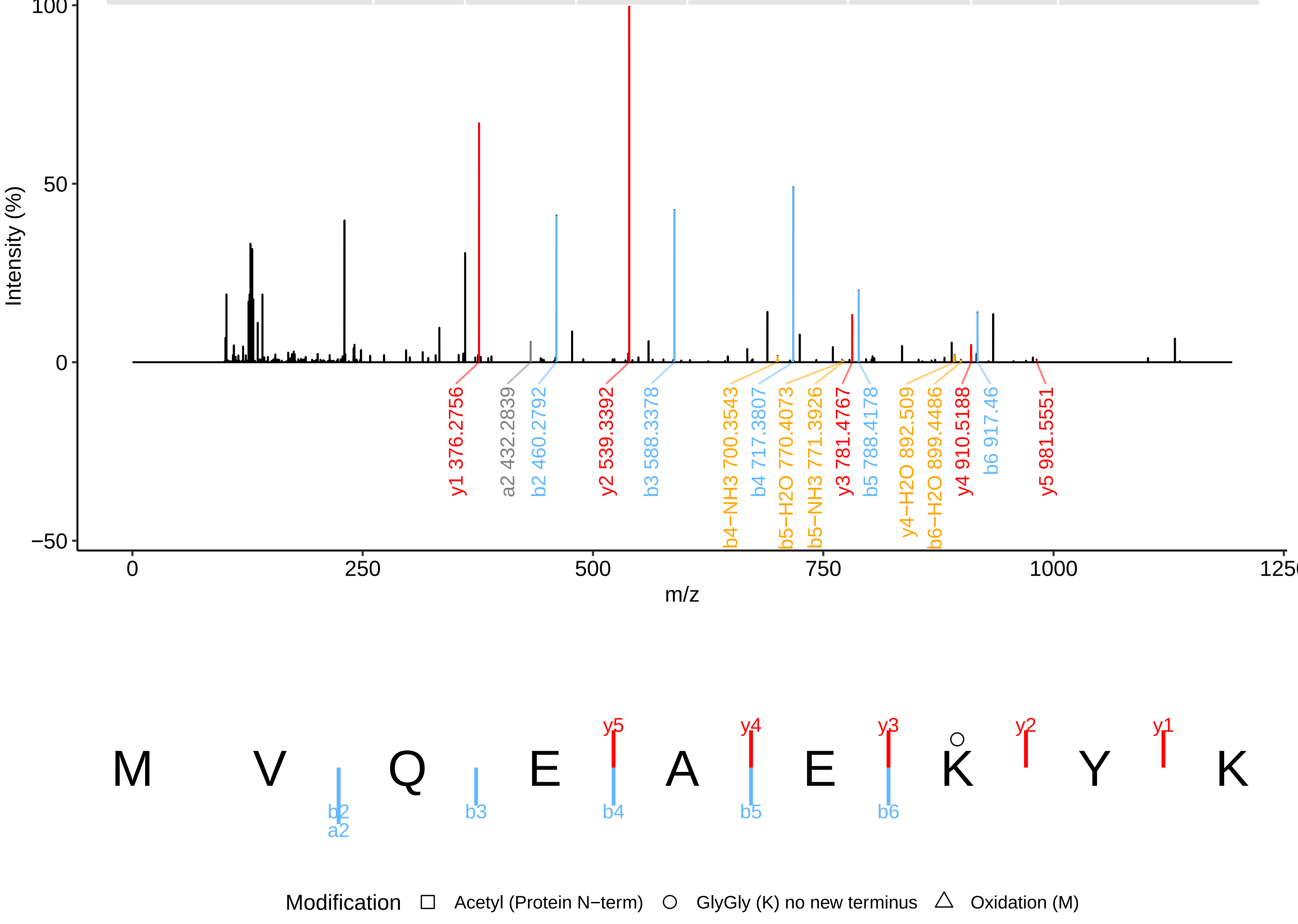

20200921_KIZ_MUb4_GlyGly_10plex_2 − Scan number: 11821

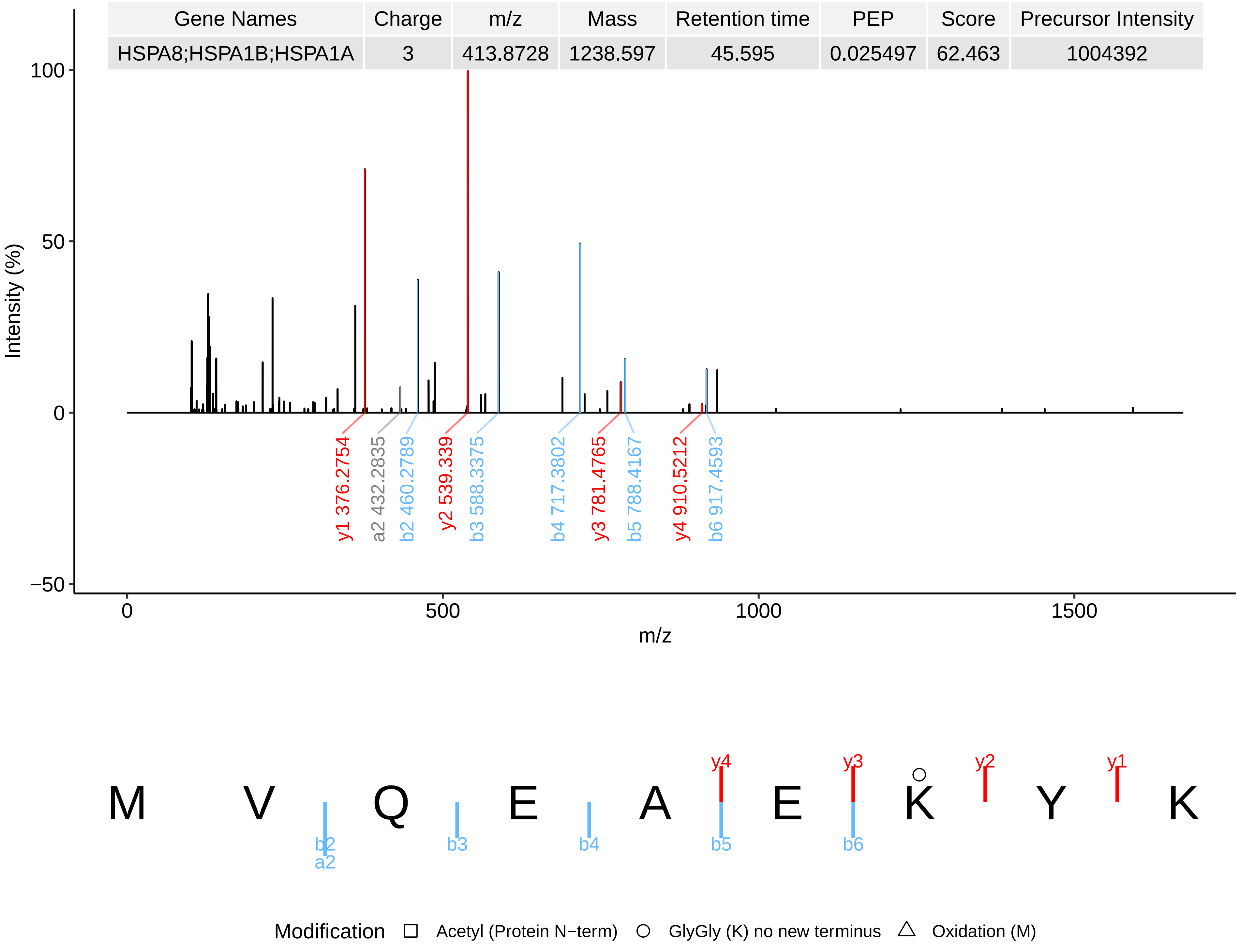

20200924_KIZ_MUb5_GlyGly_1 − Scan number: 11513

Gene Names	Charge	m/z	Mass	Retention time	PEP	Score	Precursor Intensity
HSPA8;HSPA1B;HSPA1A	3	413.8728	1238.597	37.315	0.0091655	75.294	11592449

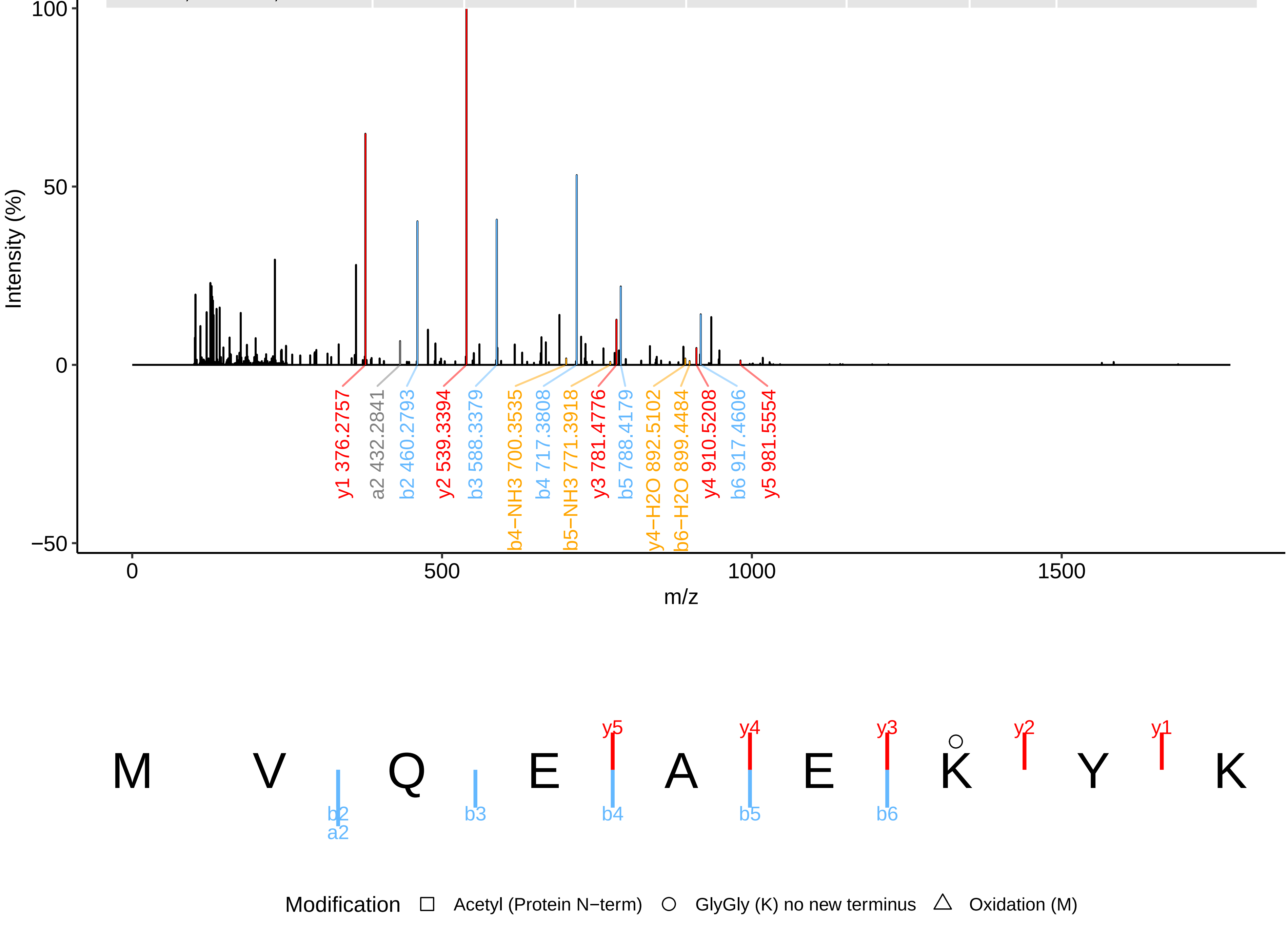

20200924_KIZ_MUb5_GlyGly_1 − Scan number: 11660

Gene Names	Charge	m/z	Mass	Retention time	PEP	Score	Precursor Intensity
HSPA8;HSPA1B;HSPA1A	3	413.8728	1238.597	37.821	0.00056953	98.044	80951632

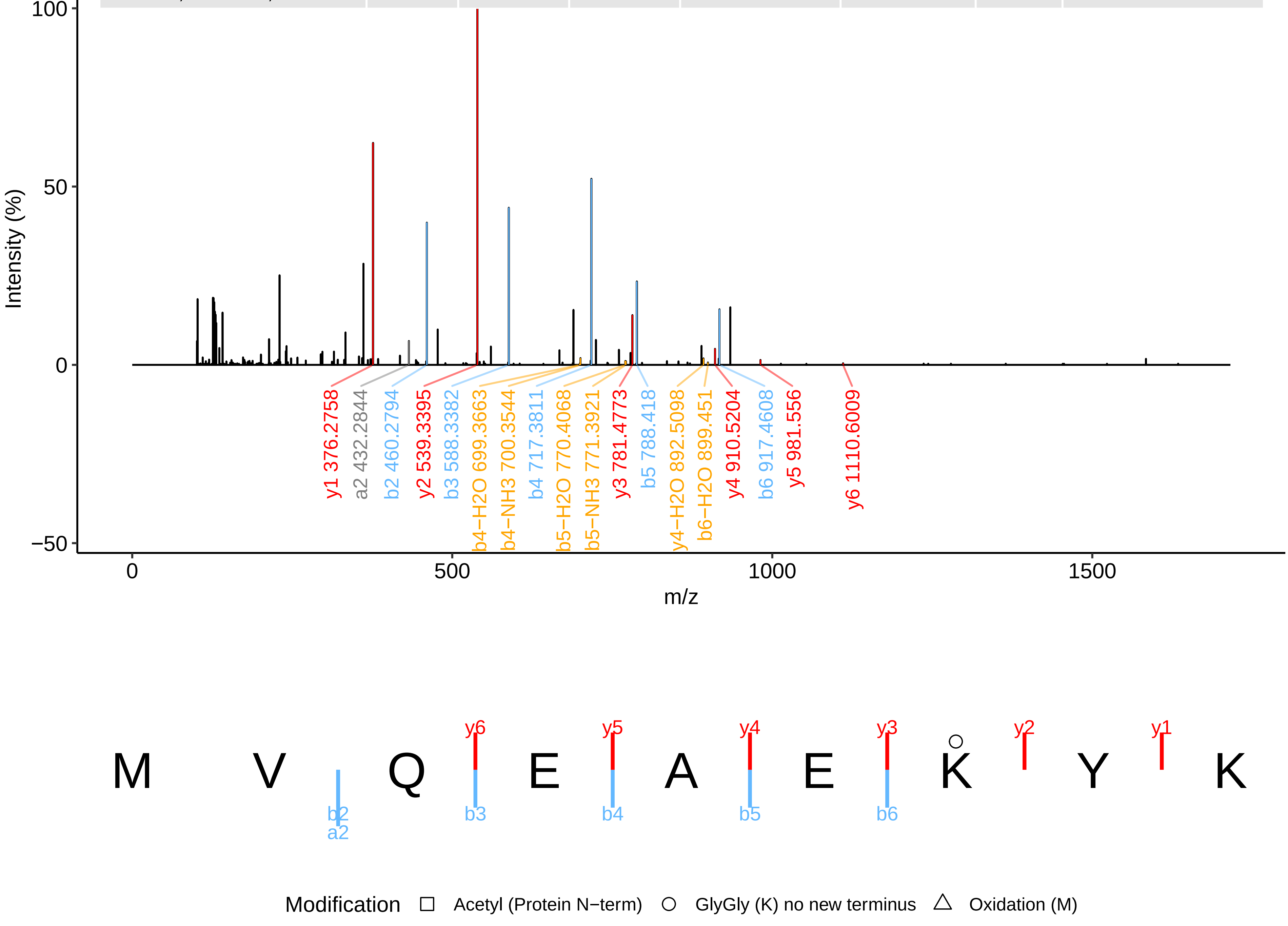

20200924_KIZ_MUb5_GlyGly_1 − Scan number: 11817

Gene Names	Charge	m/z	Mass	Retention time	PEP	Score	Precursor Intensity
HSPA8;HSPA1B;HSPA1A	3	413.8728	1238.597	38.352	0.0091655	75.294	29529054

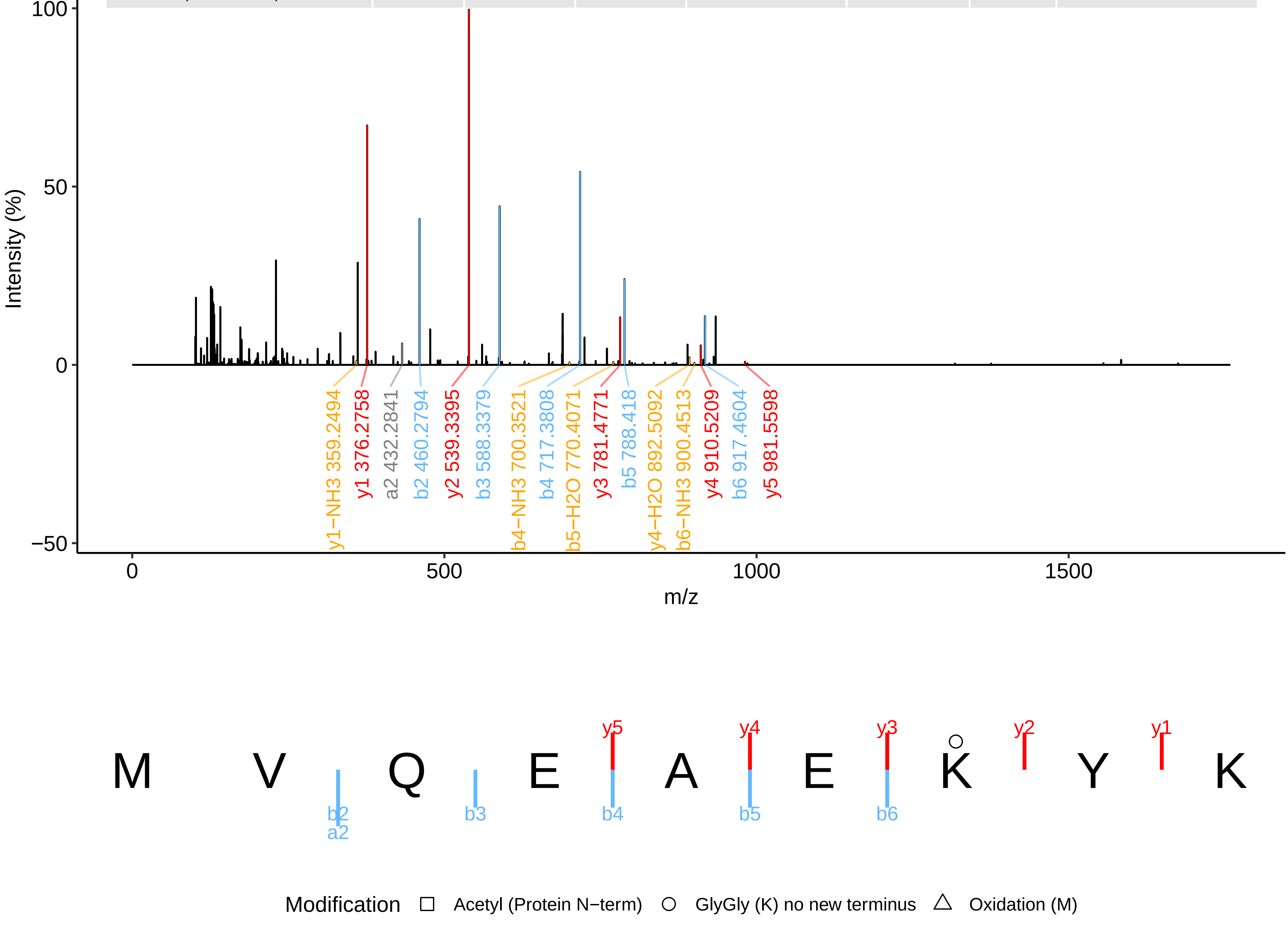

20200924_KIZ_MUb5_GlyGly_2 − Scan number: 10917

Gene Names	Charge	m/z	Mass	Retention time	PEP	Score	Precursor Intensity
HSPA8;HSPA1B;HSPA1A	3	413.8728	1238.597	40.763	0.0091655	75.294	4634388

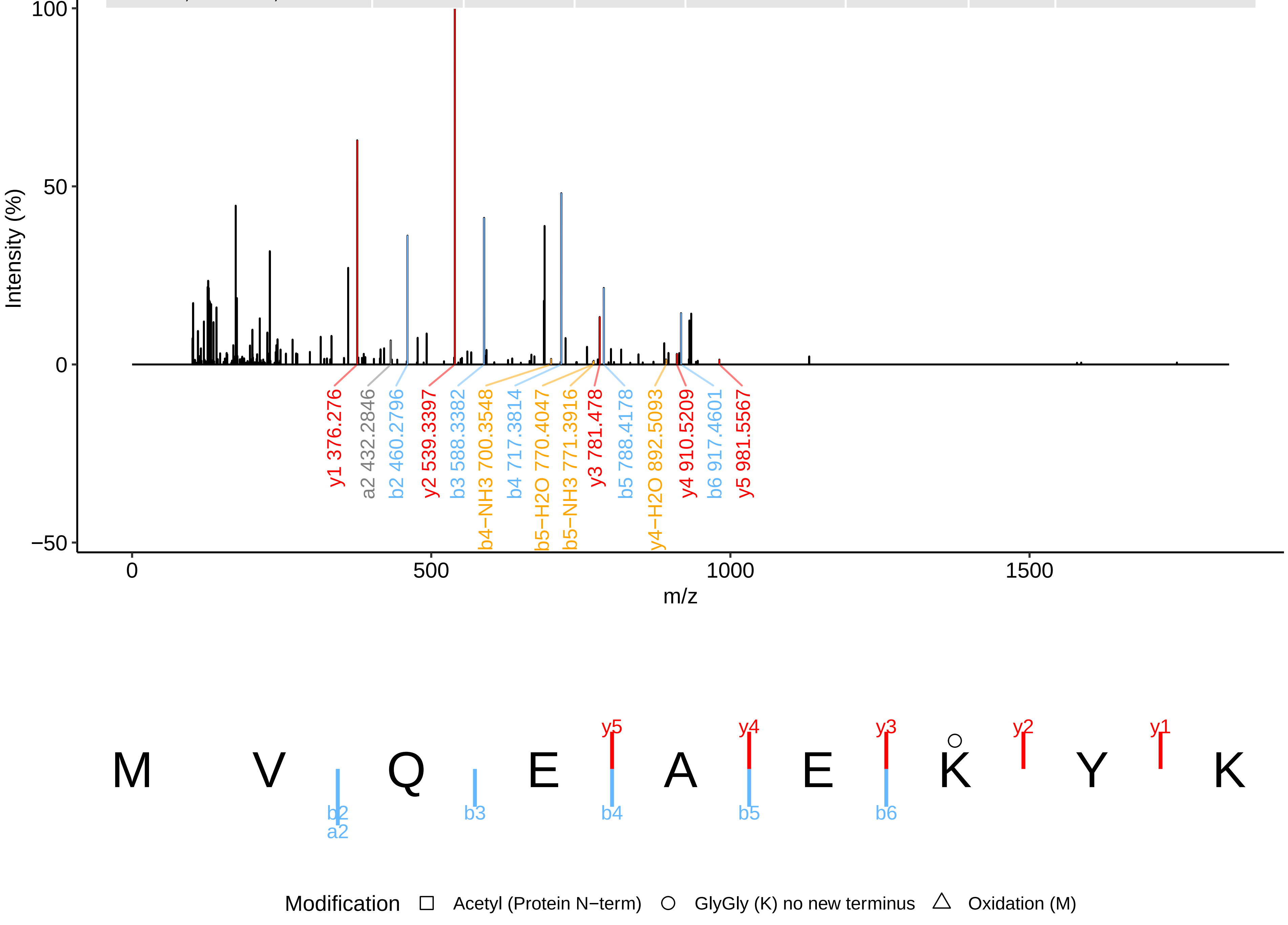

20200924_KIZ_MUb5_GlyGly_2 − Scan number: 10928

Gene Names	Charge	m/z	Mass	Retention time	PEP	Score	Precursor Intensity
HSPA8;HSPA1B;HSPA1A	3	413.8728	1238.597	40.805	0.0031624	82.749	4731107

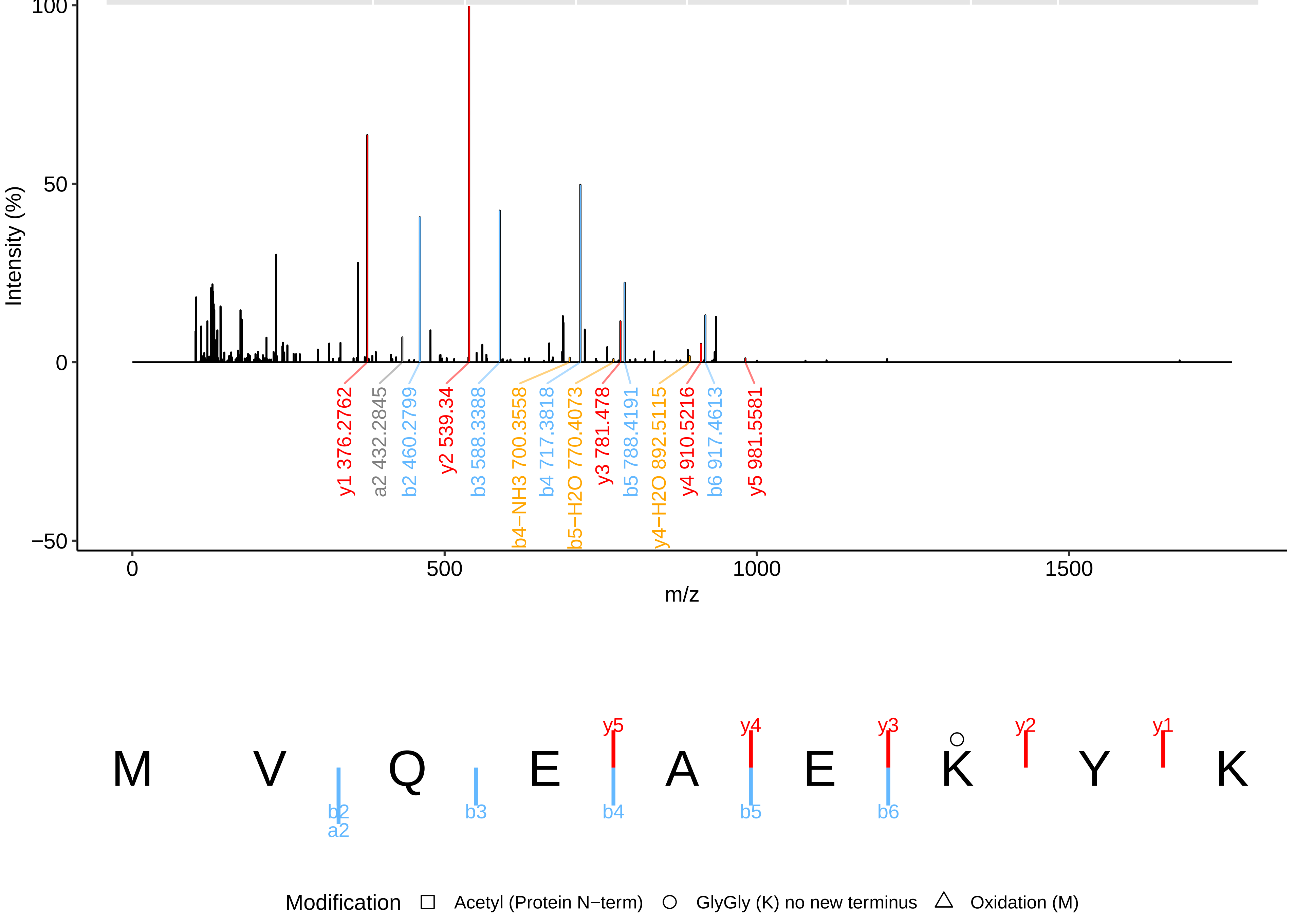

20200924_KIZ_MUb5_GlyGly_2 − Scan number: 11062

Gene Names	Charge	m/z	Mass	Retention time	PEP	Score	Precursor Intensity
HSPA8;HSPA1B;HSPA1A	3	413.8728	1238.597	41.314	0.0039074	80.231	11322015

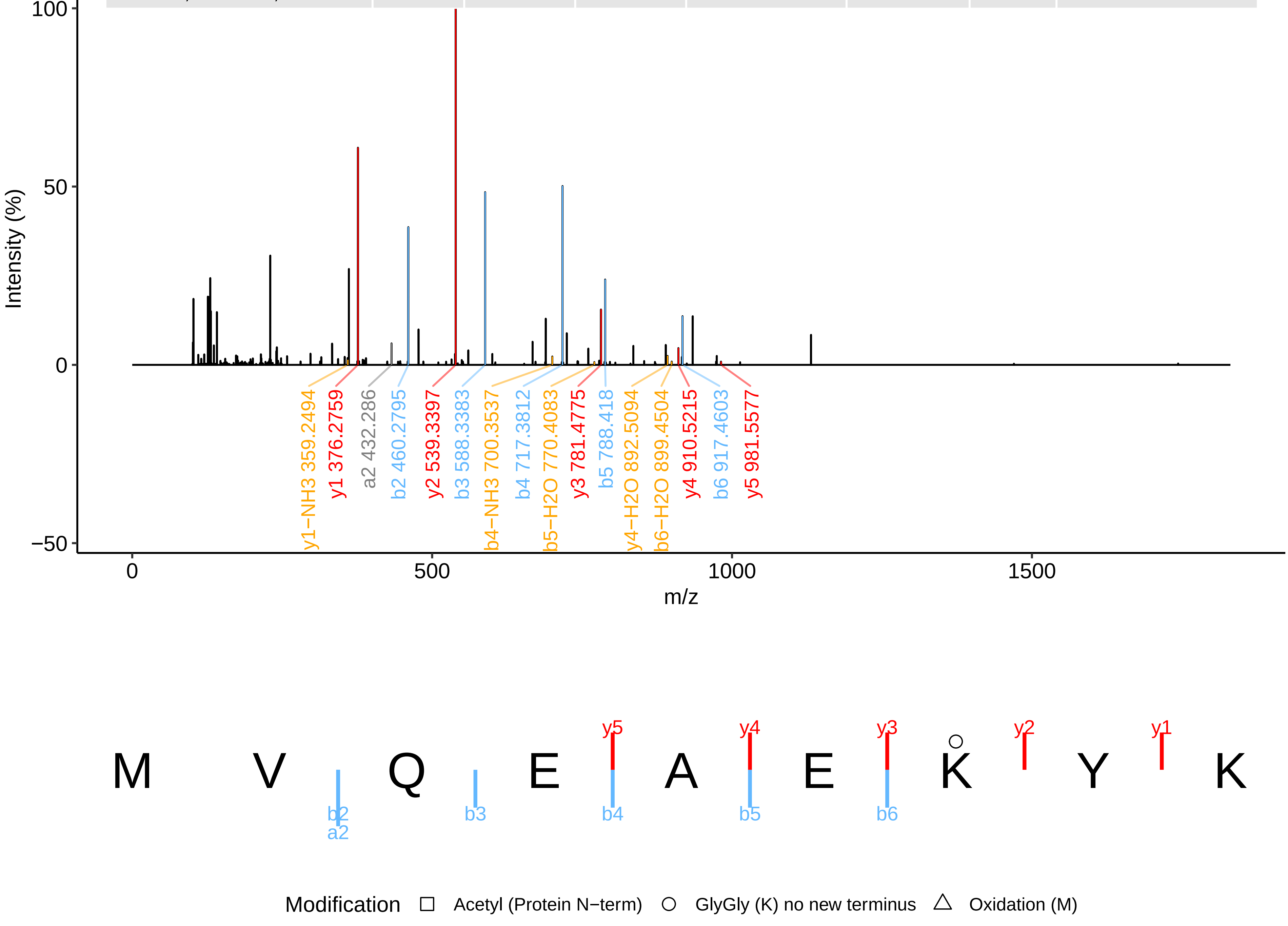

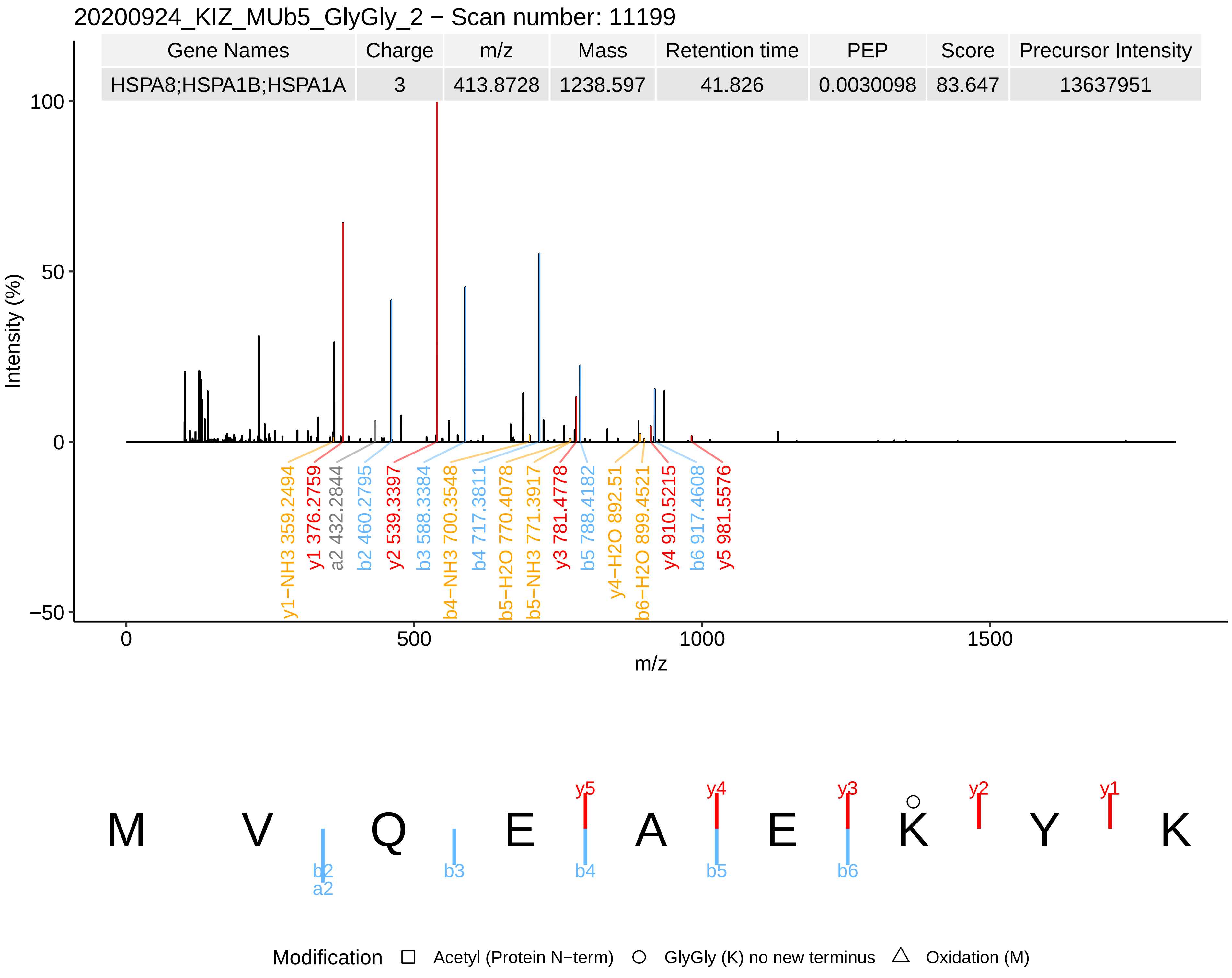
20200924_KIZ_MUb5_GlyGly_2 − Scan number: 11199
Gene Names | Charge | m/z | Mass | Retention time | PEP | Score | Precursor Intensity
HSPA8;HSPA1B;HSPA1A | 3 | 413.8728 | 1238.597 | 41.826 | 0.0030098 | 83.647 | 13637951
Intensity (%)
100
50
0
−50
0
500
1000
1500
m/z
y1−NH3 359.2494
y1 376.2759
a2 432.2844
b2 460.2795
y2 539.3397
b3 588.3384
b4−NH3 700.3548
b4 717.3811
b5−H2O 770.4078
b5−NH3 771.3917
y3 781.4778
b5 788.4182
y4−H2O 892.51
b6−H2O 899.4521
y4 910.5215
b6 917.4608
y5 981.5576
M V Q E A E K Y K
y5 y4 y3 y2 y1
b2 a2 b3 b4 b5 b6
Modification
Acetyl (Protein N−term)
GlyGly (K) no new terminus
Oxidation (M)

20200924_KIZ_MUb5_GlyGly_2 − Scan number: 11335

Gene Names	Charge	m/z	Mass	Retention time	PEP	Score	Precursor Intensity
HSPA8;HSPA1B;HSPA1A	3	413.8728	1238.597	42.353	0.0091655	75.294	3330020

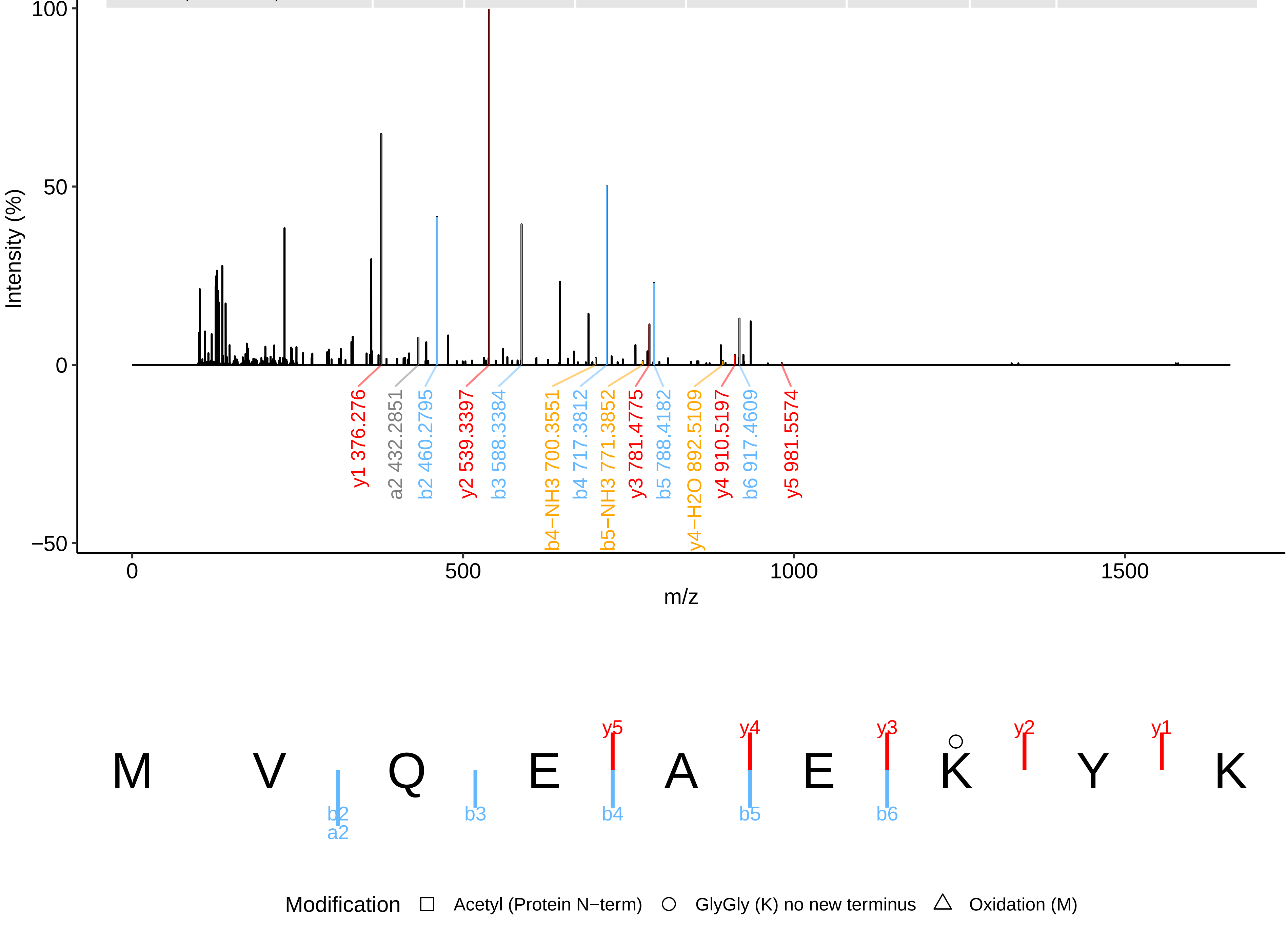

20200921_KIZ_MUb4_GlyGly_10plex − Scan number: 21730

Gene Names	Charge	m/z	Mass	Retention time	PEP	Score	Precursor Intensity
TOMM40	2	728.3561	1454.698	78.748	0.033199	68.224	4094478

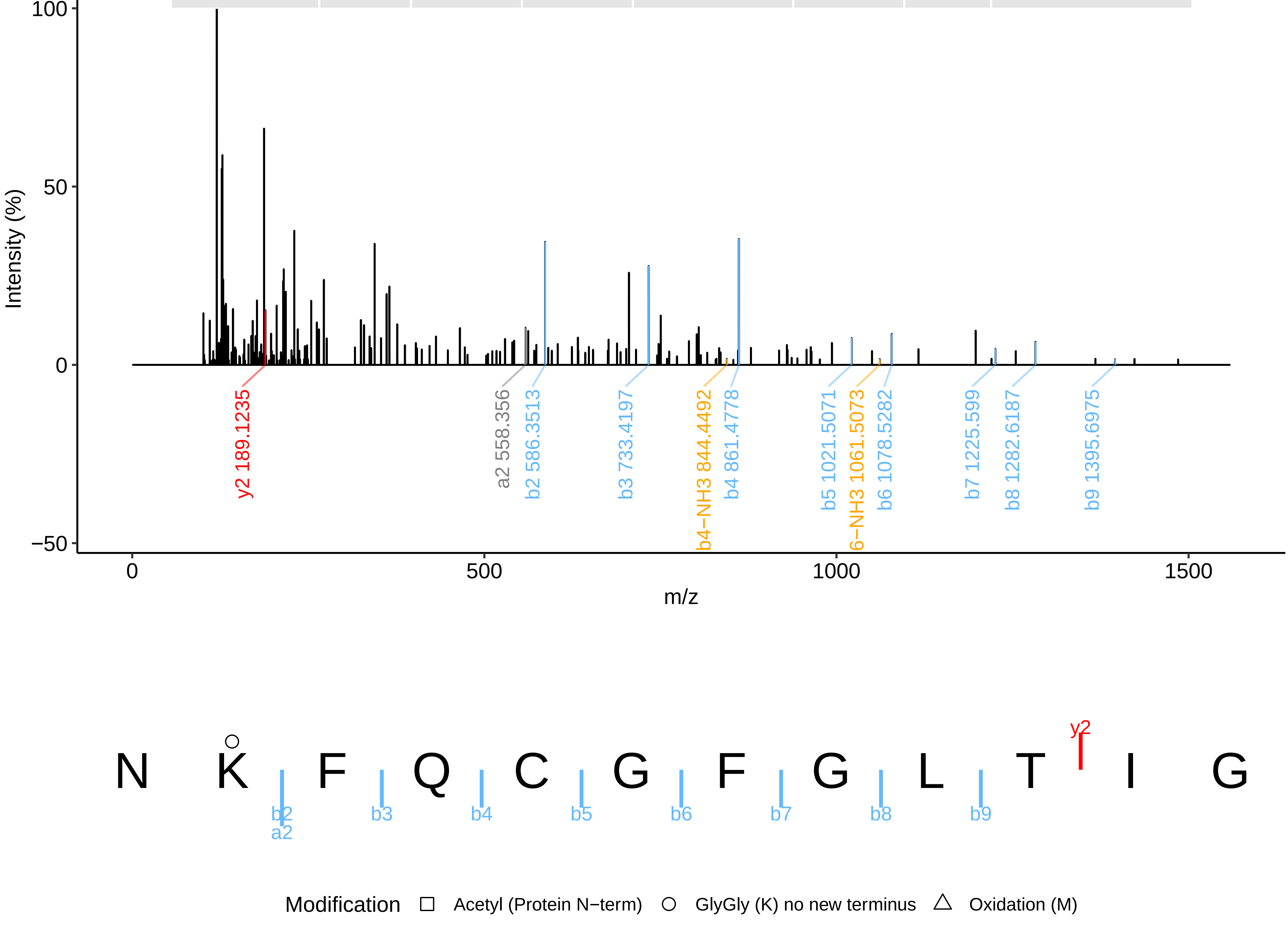

20201118_KIZ_MUb6_GlyGly_1 − Scan number: 22165

Gene Names	Charge	m/z	Mass	Retention time	PEP	Score	Precursor Intensity
TOMM40	2	728.3561	1454.698	68.946	0.042873	64.091	28255236

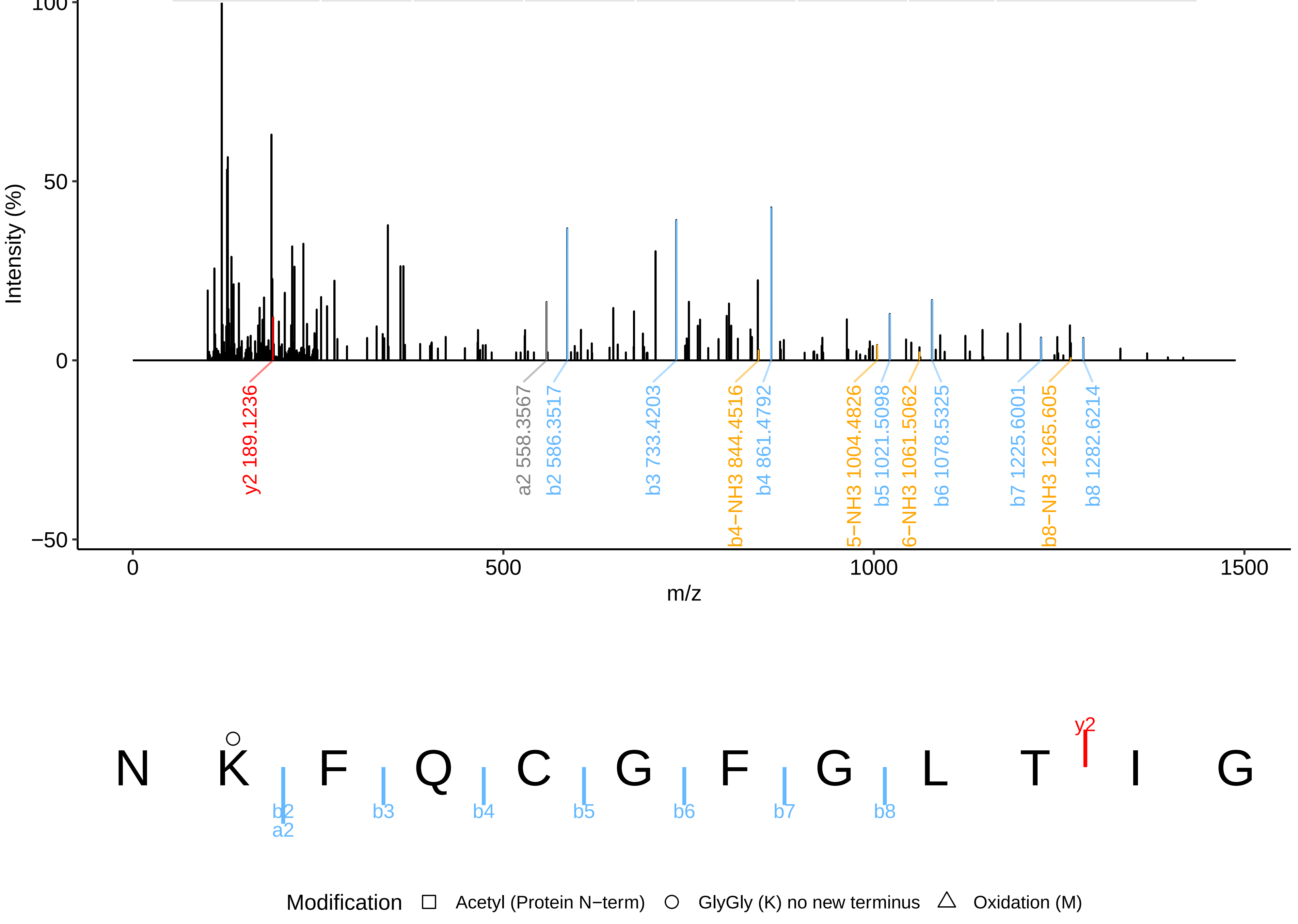

20201118_KIZ_MUb6_GlyGly_2 − Scan number: 23605

Gene Names	Charge	m/z	Mass	Retention time	PEP	Score	Precursor Intensity
TOMM40	2	728.3561	1454.698	79.156	0.078448	71.379	12010997

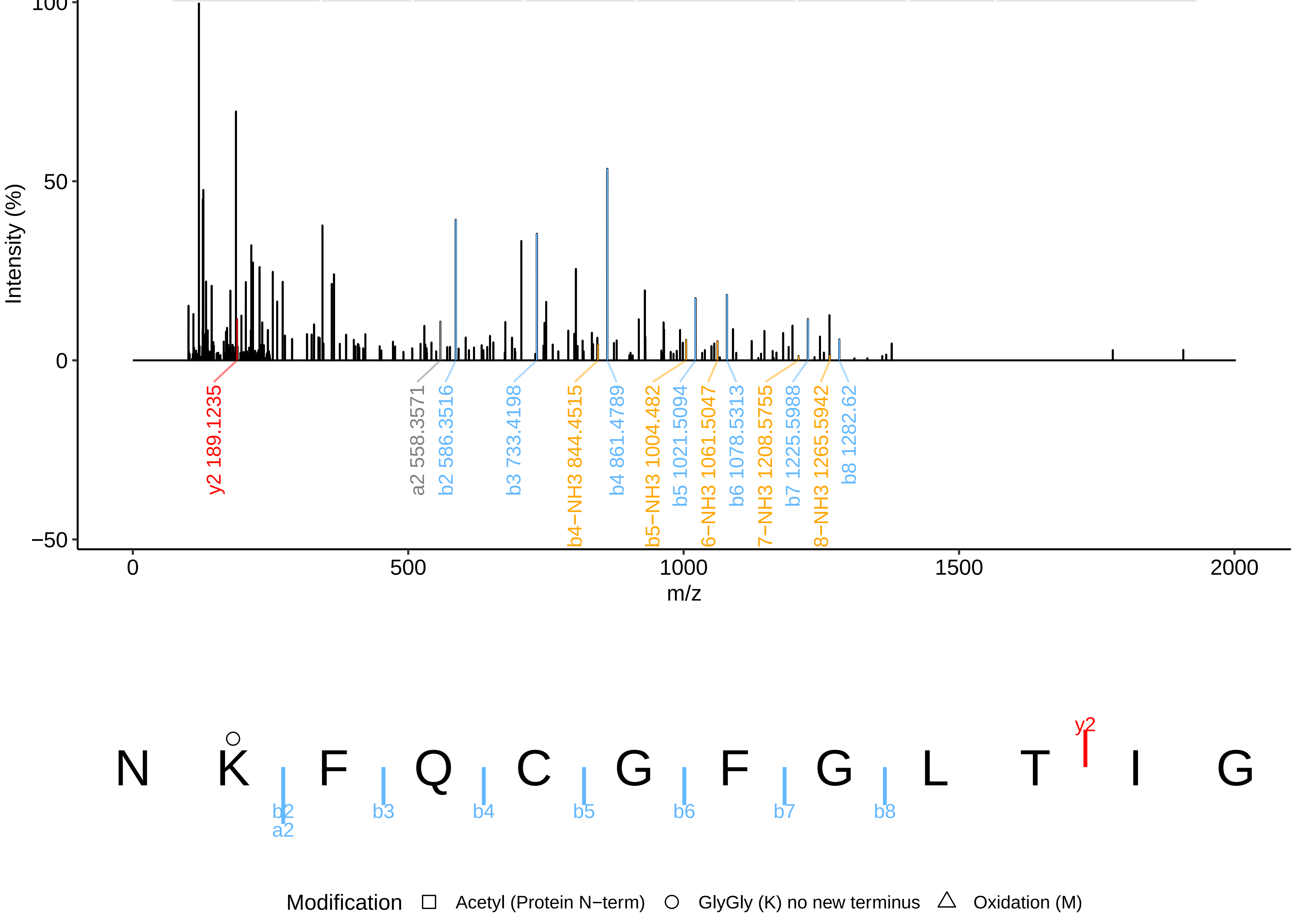

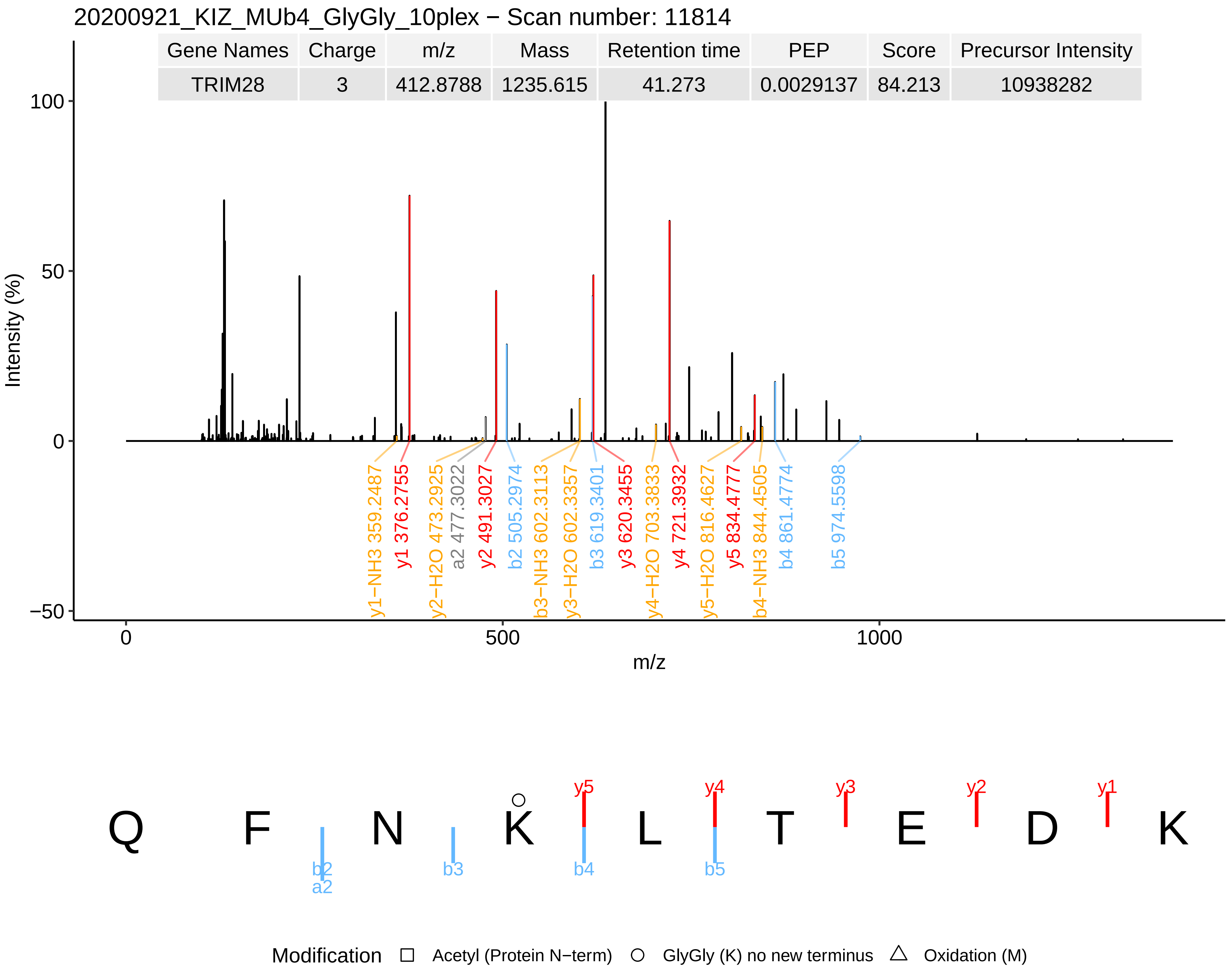
20200921_KIZ_MUb4_GlyGly_10plex − Scan number: 11814
Gene Names | Charge | m/z | Mass | Retention time | PEP | Score | Precursor Intensity
TRIM28 | 3 | 412.8788 | 1235.615 | 41.273 | 0.0029137 | 84.213 | 10938282
Intensity (%)
100
50
0
−50
0
500
1000
m/z
y1−NH3 359.2487
y1 376.2755
y2−H2O 473.2925
a2 477.3022
y2 491.3027
b2 505.2974
b3−NH3 602.3113
y3−H2O 602.3357
b3 619.3401
y3 620.3455
y4−H2O 703.3833
y4 721.3932
y5−H2O 816.4627
y5 834.4777
b4−NH3 844.4505
b4 861.4774
b5 974.5598
Q F N K L T E D K
y5 y4 y3 y2 y1
b2 a2 b3 b4 b5
Modification
Acetyl (Protein N−term)
GlyGly (K) no new terminus
Oxidation (M)

20200924_KIZ_MUb5_GlyGly_1 − Scan number: 10740

Gene Names	Charge	m/z	Mass	Retention time	PEP	Score	Precursor Intensity
TRIM28	3	412.8788	1235.615	34.706	0.10599	51.949	6969395

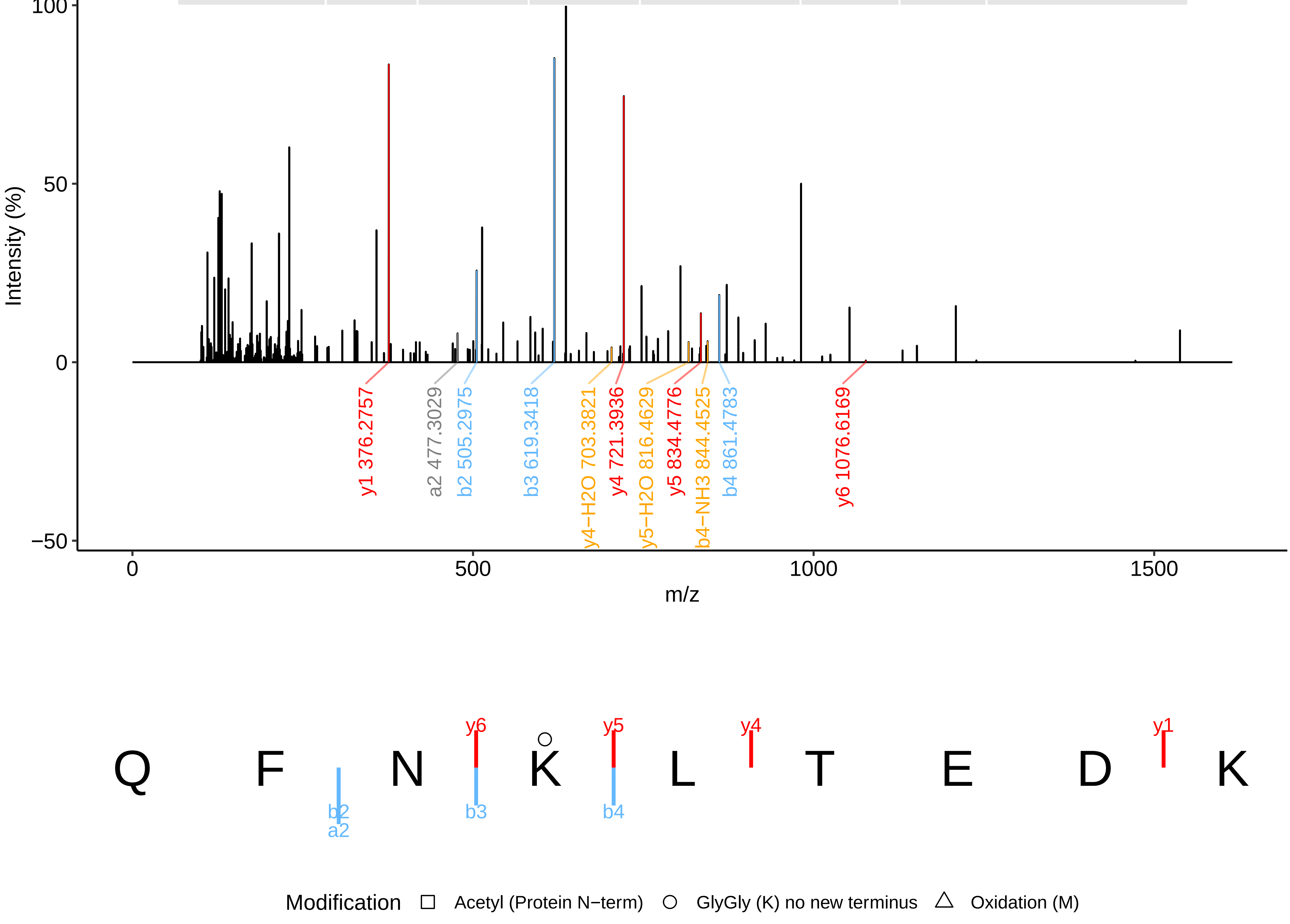

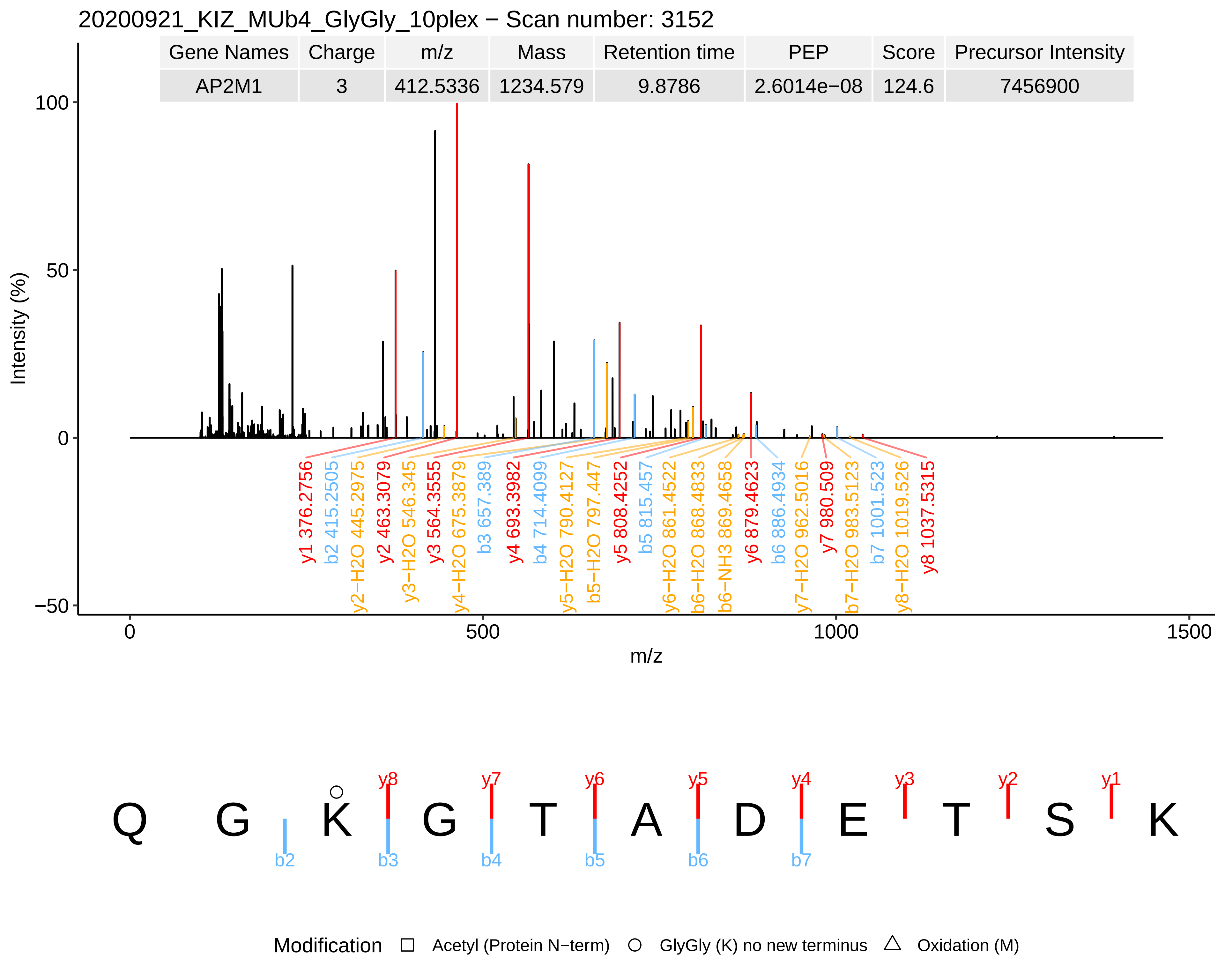

20200921_KIZ_MUb4_GlyGly_10plex − Scan number: 3152
Gene Names | Charge | m/z | Mass | Retention time | PEP | Score | Precursor Intensity
AP2M1 | 3 | 412.5336 | 1234.579 | 9.8786 | 2.6014e−08 | 124.6 | 7456900
Intensity (%)
100
50
0
−50
0
500
1000
1500
m/z
y1 376.2756
b2 415.2505
y2−H2O 445.2975
y2 463.3079
y3−H2O 546.345
y3 564.3555
y4−H2O 675.3879
b3 657.389
y4 693.3982
b4 714.4099
y5−H2O 790.4127
b5−H2O 797.447
y5 808.4252
b5 815.457
y6−H2O 861.4522
b6−H2O 868.4833
b6−NH3 869.4658
y6 879.4623
b6 886.4934
y7−H2O 962.5016
y7 980.509
b7−H2O 983.5123
b7 1001.523
y8−H2O 1019.526
y8 1037.5315
Q G K G T A D E T S K
y8 y7 y6 y5 y4 y3 y2 y1
b2 b3 b4 b5 b6 b7
Modification
Acetyl (Protein N−term)
GlyGly (K) no new terminus
Oxidation (M)

20200921_KIZ_MUb4_GlyGly_10plex_2 − Scan number: 3027

Gene Names	Charge	m/z	Mass	Retention time	PEP	Score	Precursor Intensity
AP2M1	3	412.5336	1234.579	9.9287	0.01172	68.44	960059.5

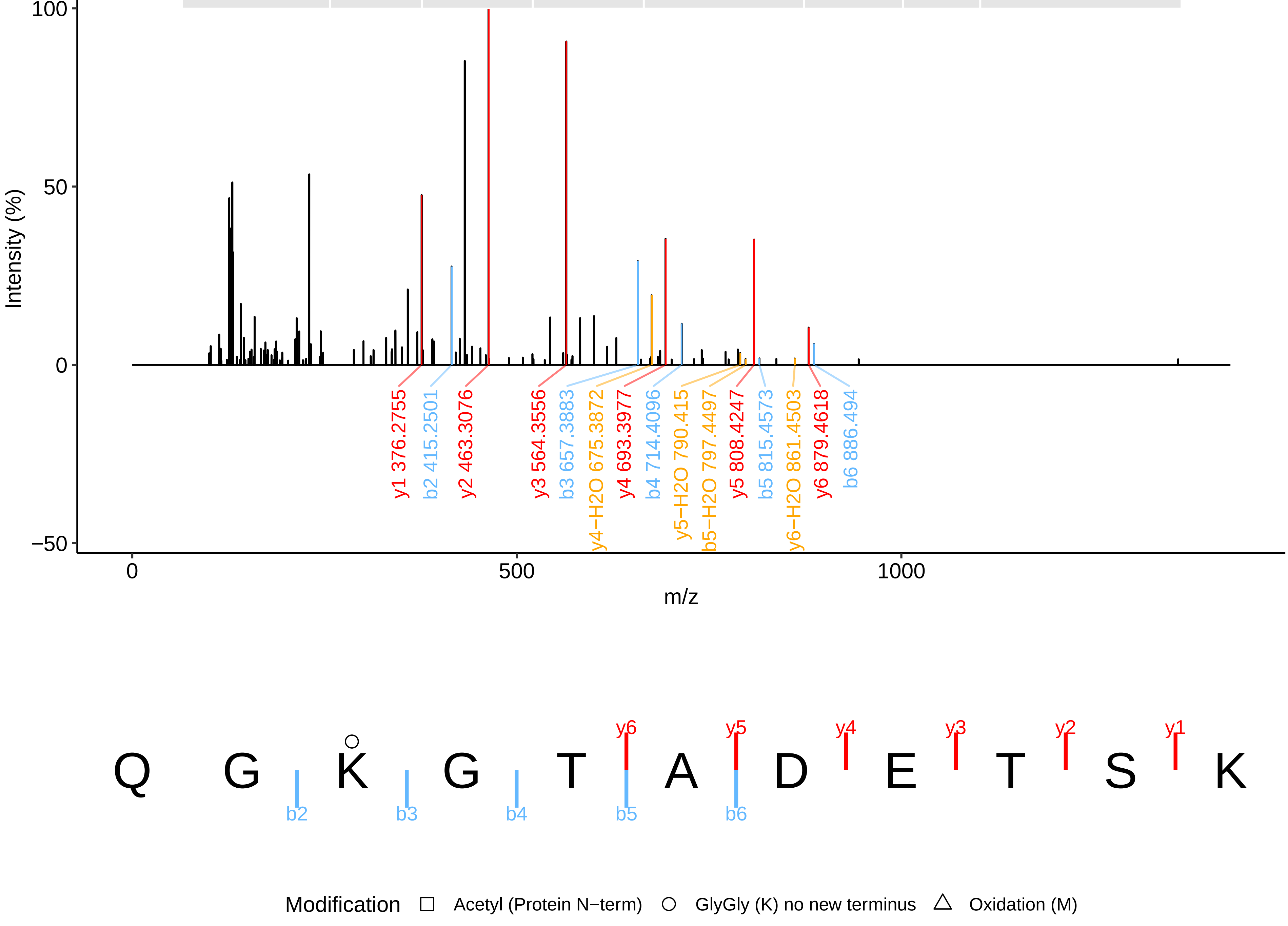

20200924_KIZ_MUb5_GlyGly_1 − Scan number: 2981

Gene Names	Charge	m/z	Mass	Retention time	PEP	Score	Precursor Intensity
AP2M1	3	412.5336	1234.579	9.2043	2.6805e−05	105.03	21406216

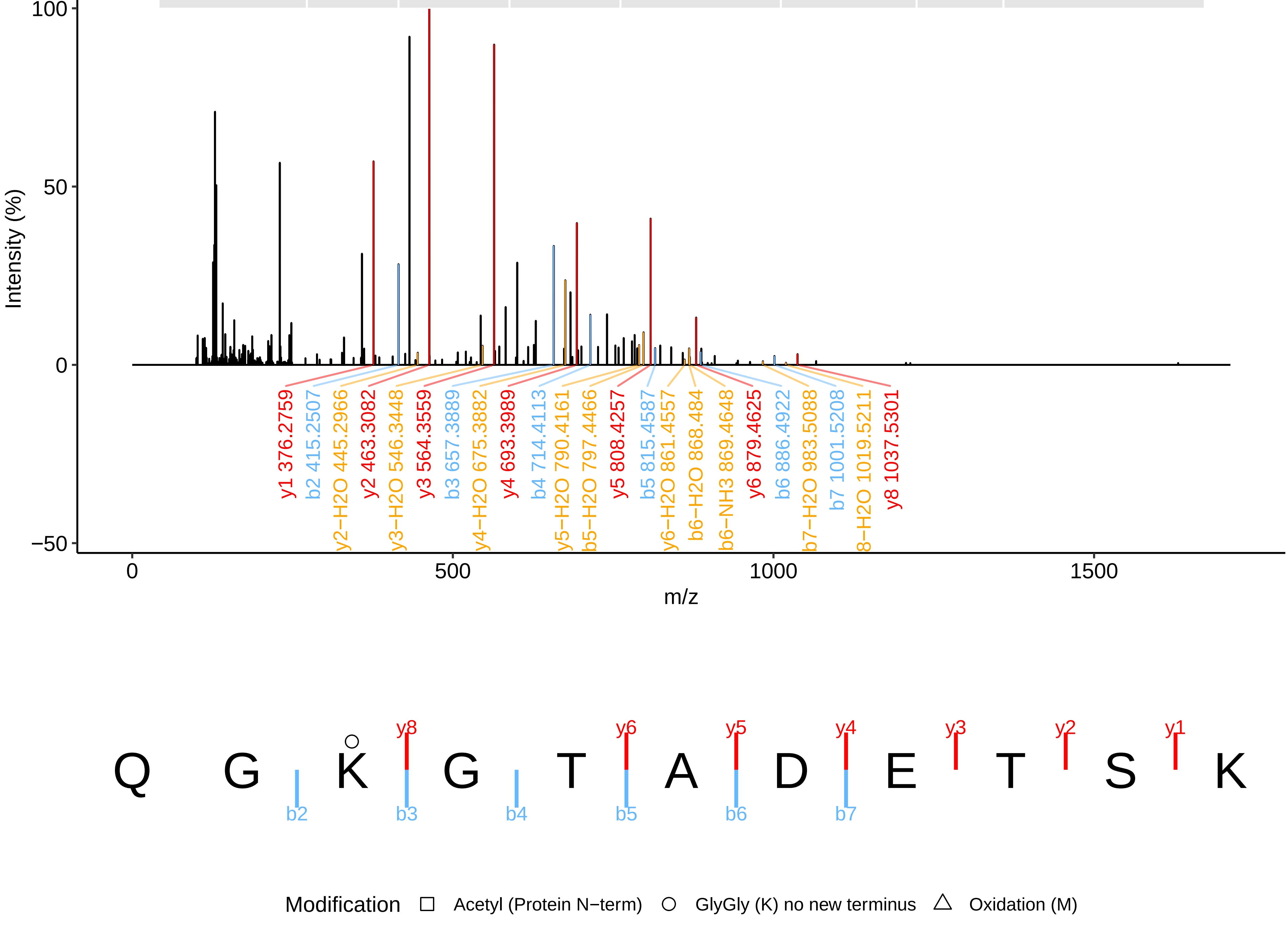

20201118_KIZ_MUb6_GlyGly_1 − Scan number: 2973

Gene Names	Charge	m/z	Mass	Retention time	PEP	Score	Precursor Intensity
AP2M1	3	412.5336	1234.579	9.1199	0.00052659	93.011	10269566

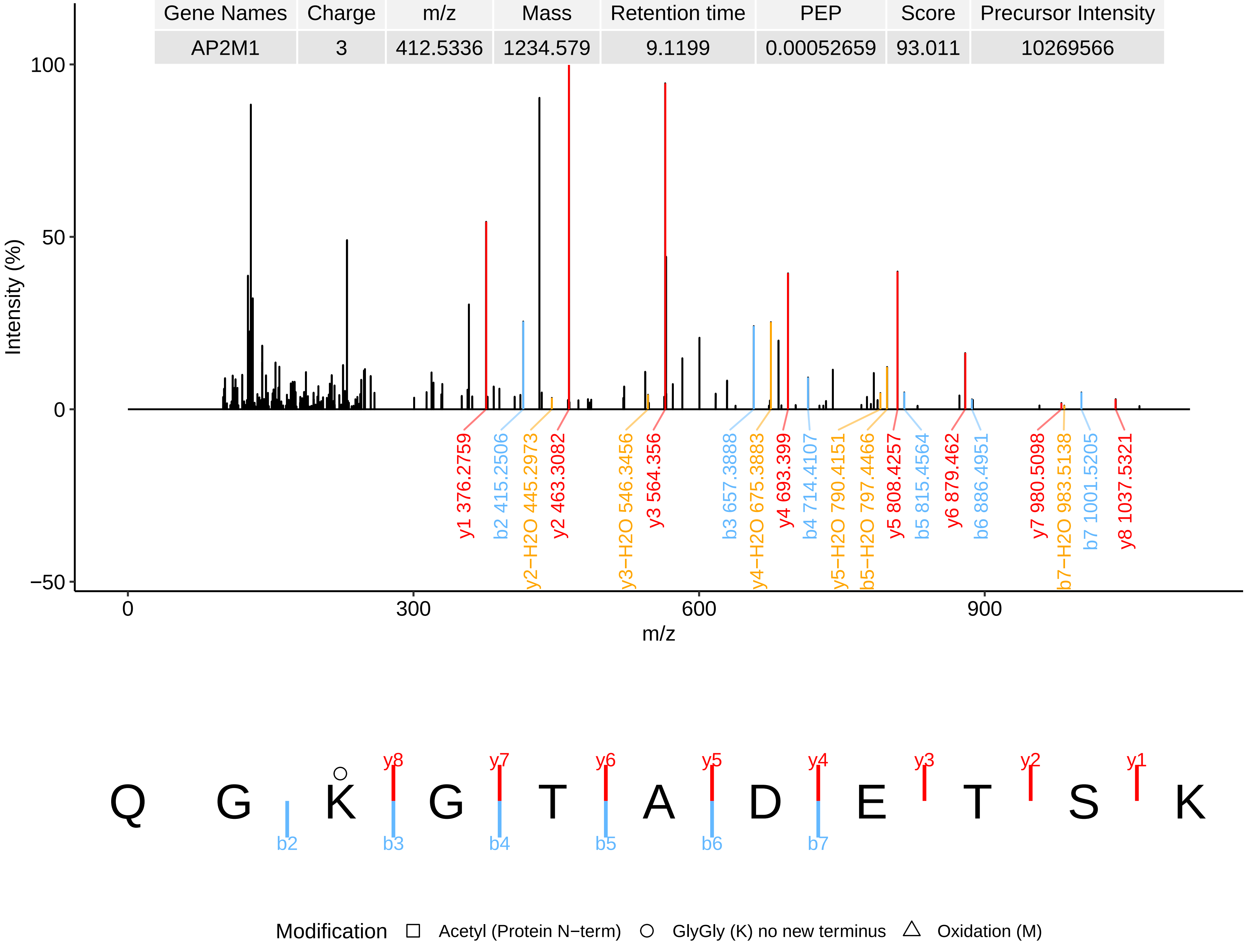

20201118_KIZ_MUb6_GlyGly_2 − Scan number: 3188

Gene Names	Charge	m/z	Mass	Retention time	PEP	Score	Precursor Intensity
AP2M1	3	412.5336	1234.579	10.36	4.3878e−05	98.407	12111651

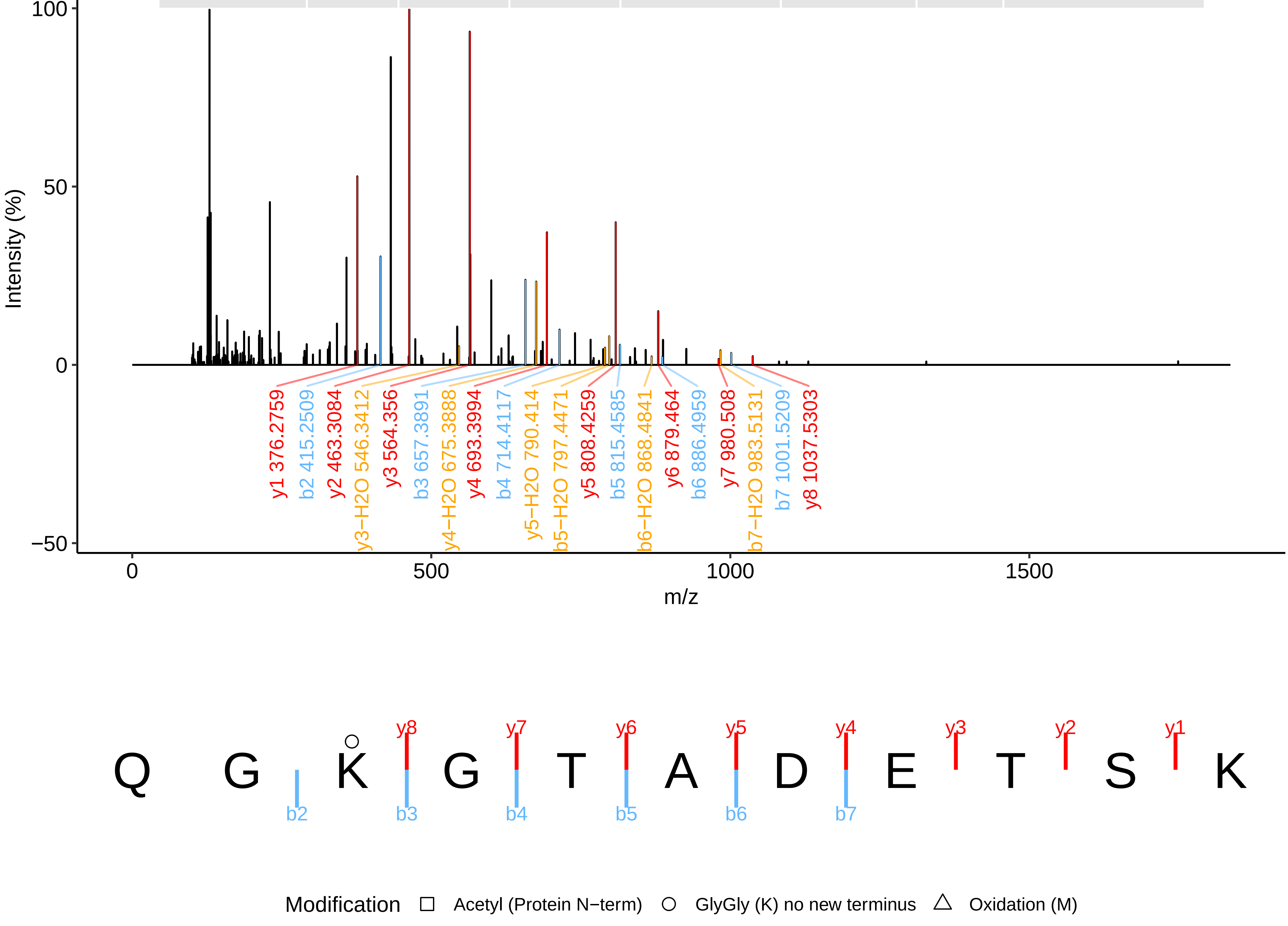

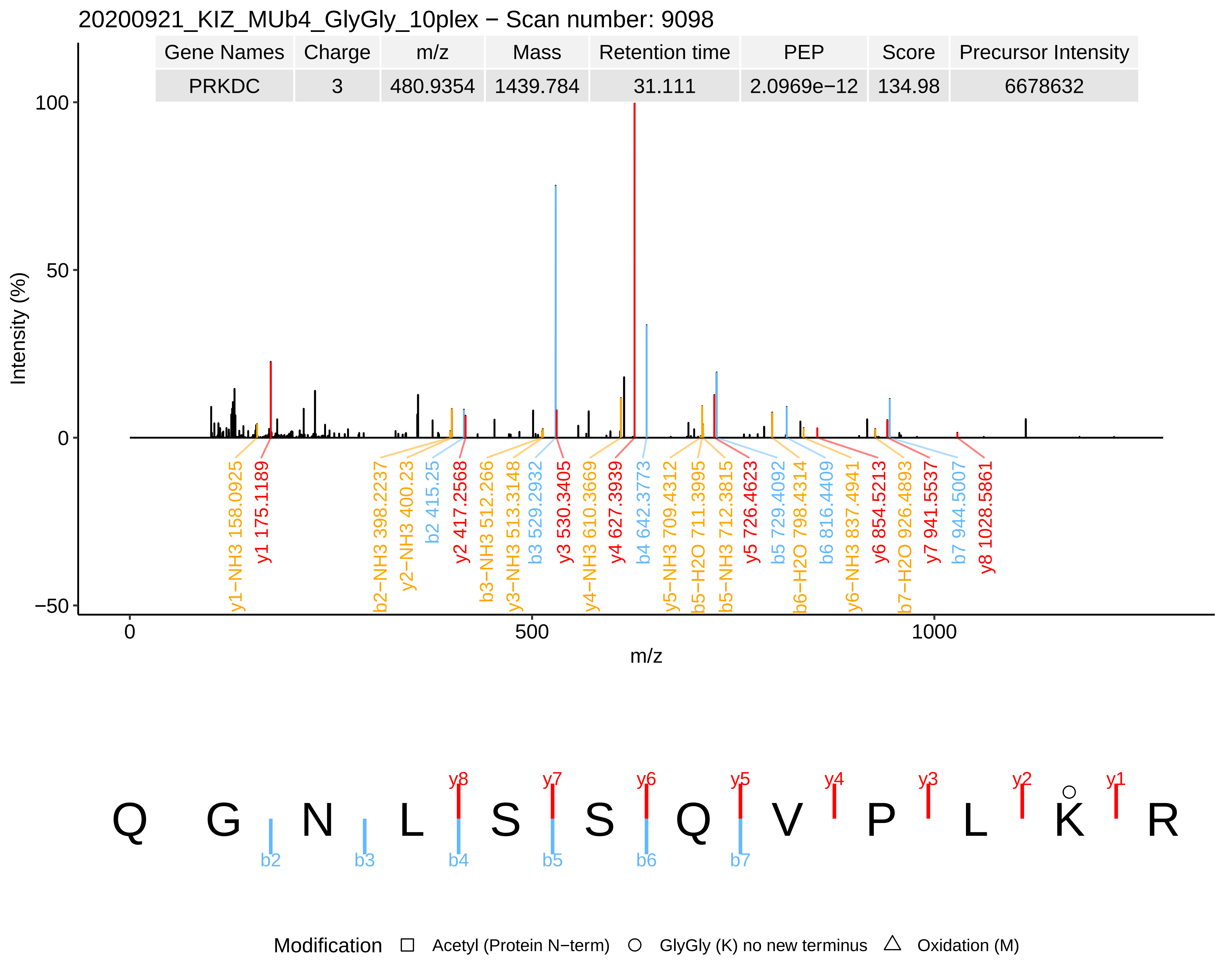

20200921_KIZ_MUb4_GlyGly_10plex − Scan number: 9098
Gene Names | Charge | m/z | Mass | Retention time | PEP | Score | Precursor Intensity
PRKDC | 3 | 480.9354 | 1439.784 | 31.111 | 2.0969e−12 | 134.98 | 6678632
Intensity (%)
m/z
y1−NH3 158.0925
y1 175.1189
b2−NH3 398.2237
y2−NH3 400.23
b2 415.25
y2 417.2568
b3−NH3 512.266
y3−NH3 513.3148
b3 529.2932
y3 530.3405
y4−NH3 610.3669
y4 627.3939
b4 642.3773
y5−NH3 709.4312
b5−H2O 711.3995
b5−NH3 712.3815
y5 726.4623
b5 729.4092
b6−H2O 798.4314
b6 816.4409
y6−NH3 837.4941
y6 854.5213
b7−H2O 926.4893
y7 941.5537
b7 944.5007
y8 1028.5861
Q G N L S S Q V P L K R
Modification
Acetyl (Protein N−term)
GlyGly (K) no new terminus
Oxidation (M)

20200921_KIZ_MUb4_GlyGly_10plex_2 − Scan number: 8377

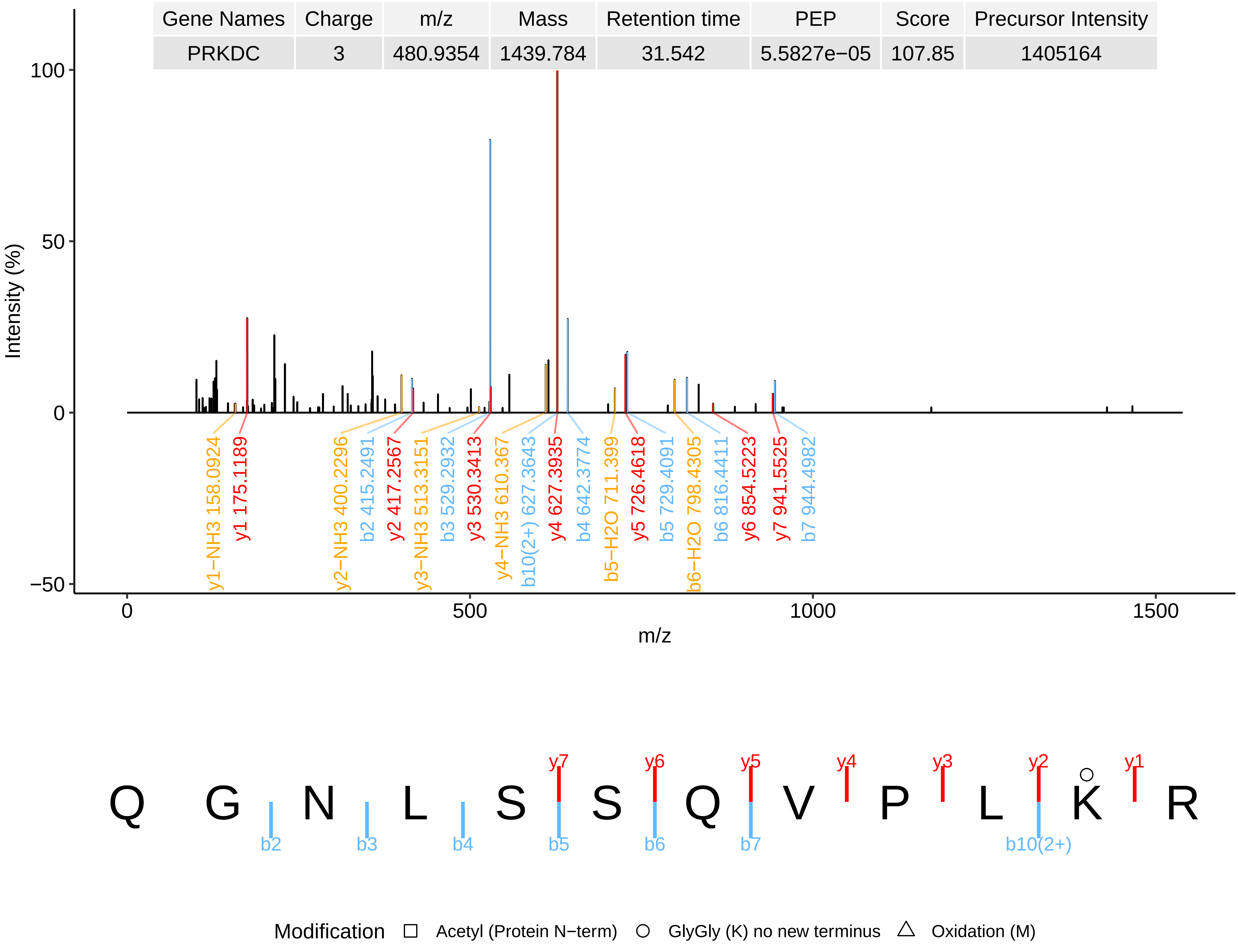

20200921_KIZ_MUb4_GlyGly_10plex − Scan number: 14491

Gene Names	Charge	m/z	Mass	Retention time	PEP	Score	Precursor Intensity
PSMA4	3	410.2347	1227.682	51.52	0.0020921	89.047	2759965

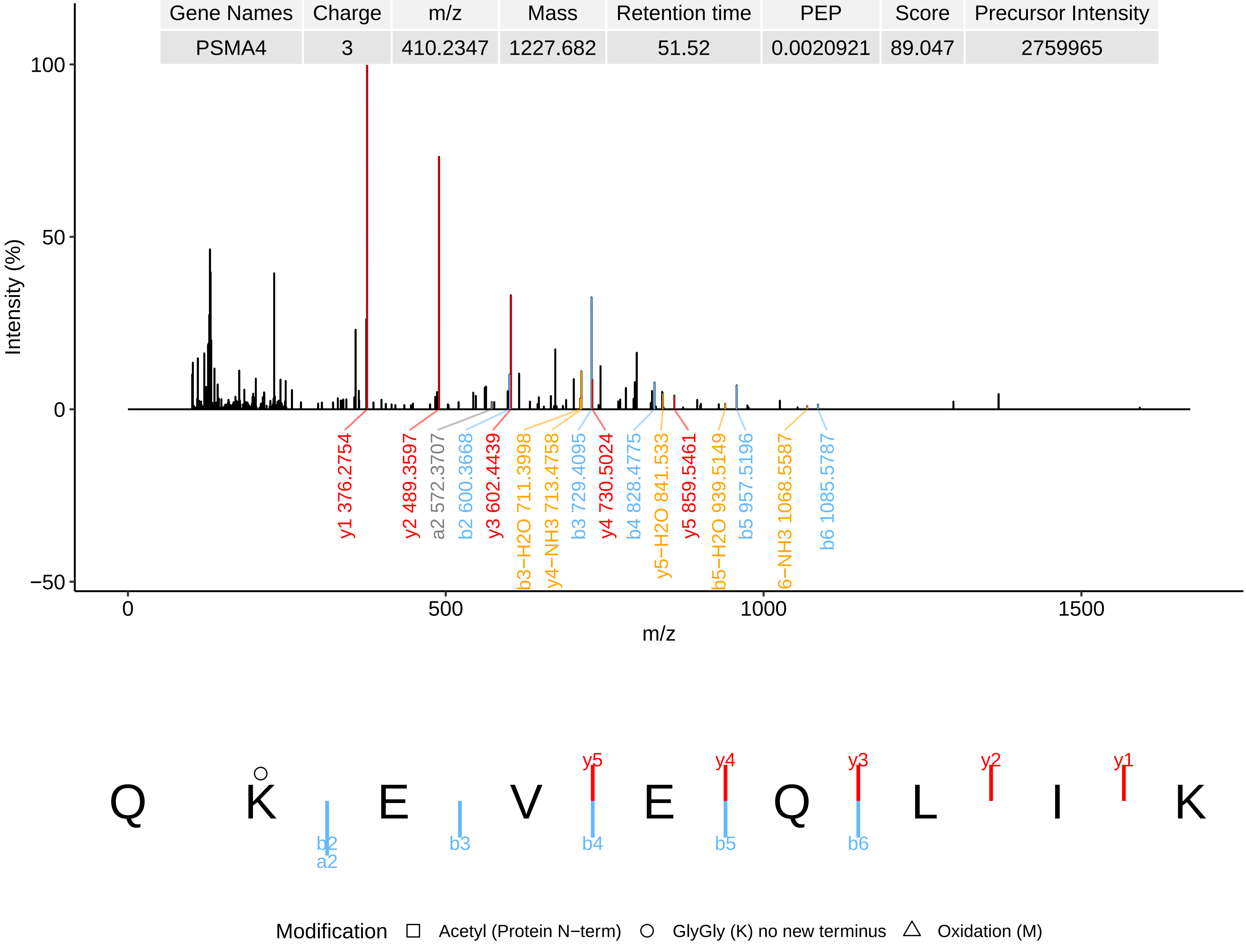

20200921_KIZ_MUb4_GlyGly_10plex − Scan number: 19295

Gene Names	Charge	m/z	Mass	Retention time	PEP	Score	Precursor Intensity
MORF4L1	3	407.9045	1220.692	69.532	0.020761	81.594	19853762

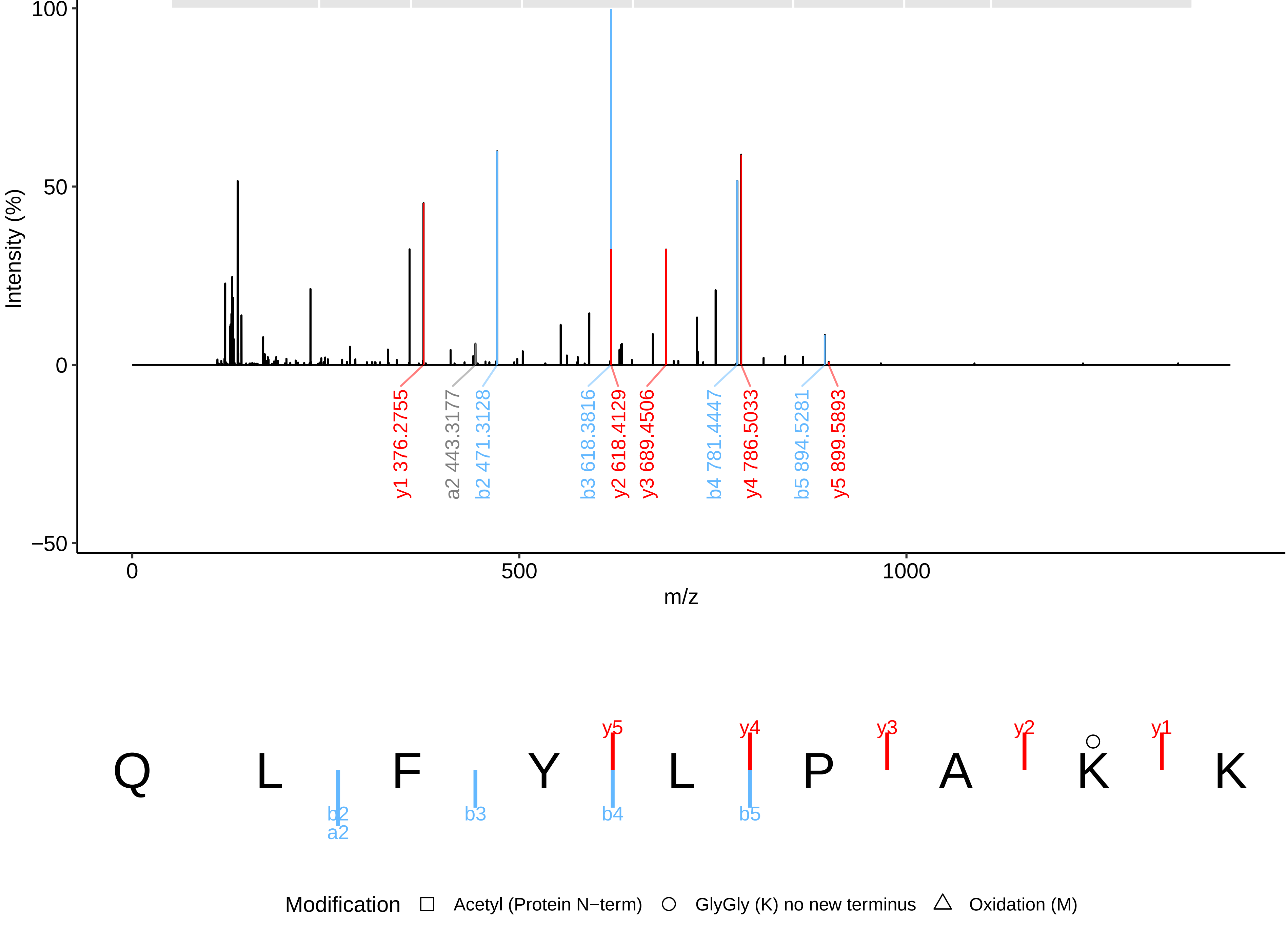

20200921_KIZ_MUb4_GlyGly_10plex_2 − Scan number: 17836

Gene Names	Charge	m/z	Mass	Retention time	PEP	Score	Precursor Intensity
MORF4L1	3	407.9045	1220.692	69.784	0.019279	83.005	1479742

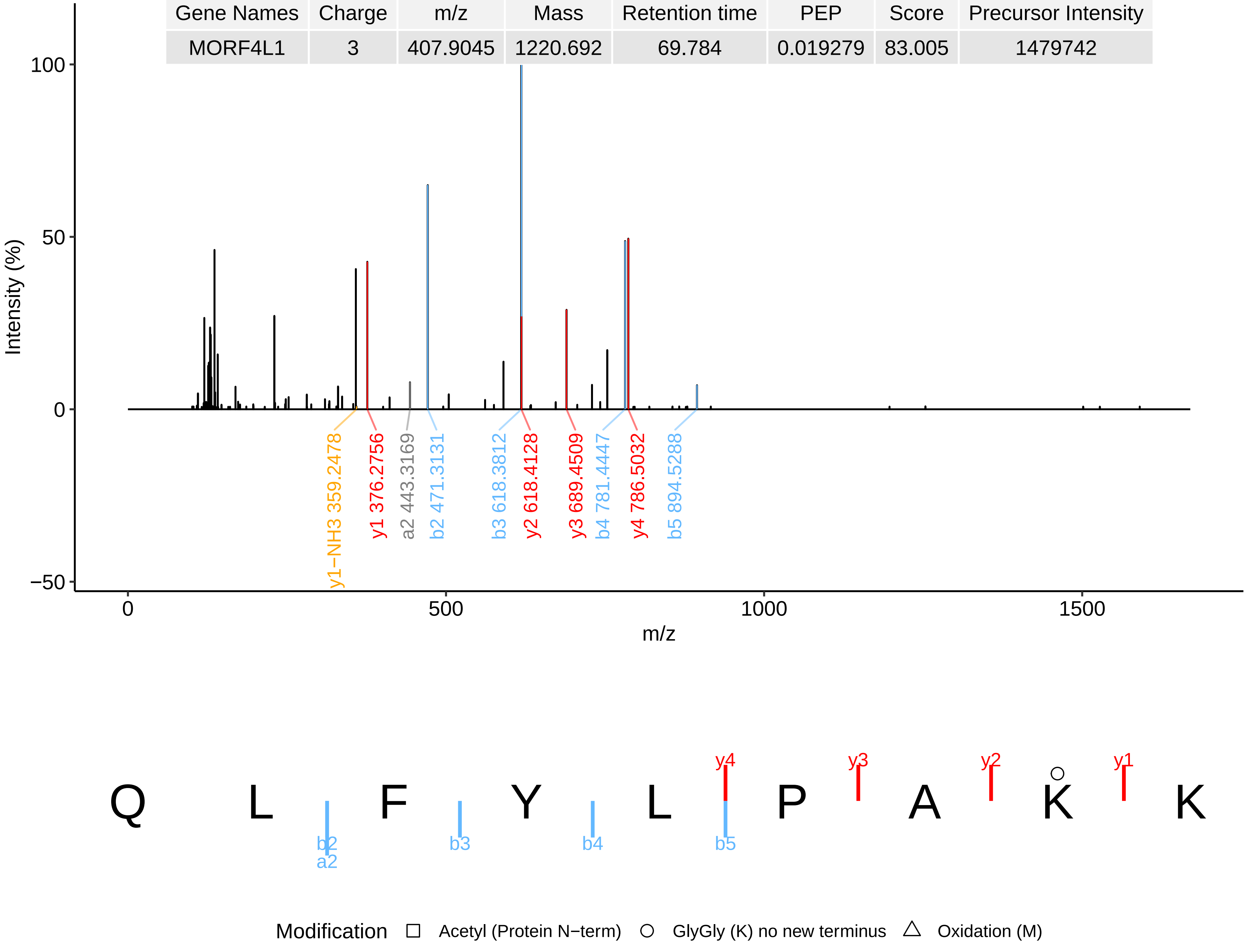

20200924_KIZ_MUb5_GlyGly_1 − Scan number: 18574

Gene Names	Charge	m/z	Mass	Retention time	PEP	Score	Precursor Intensity
MORF4L1	3	407.9045	1220.692	60.03	0.07362	68.735	NA

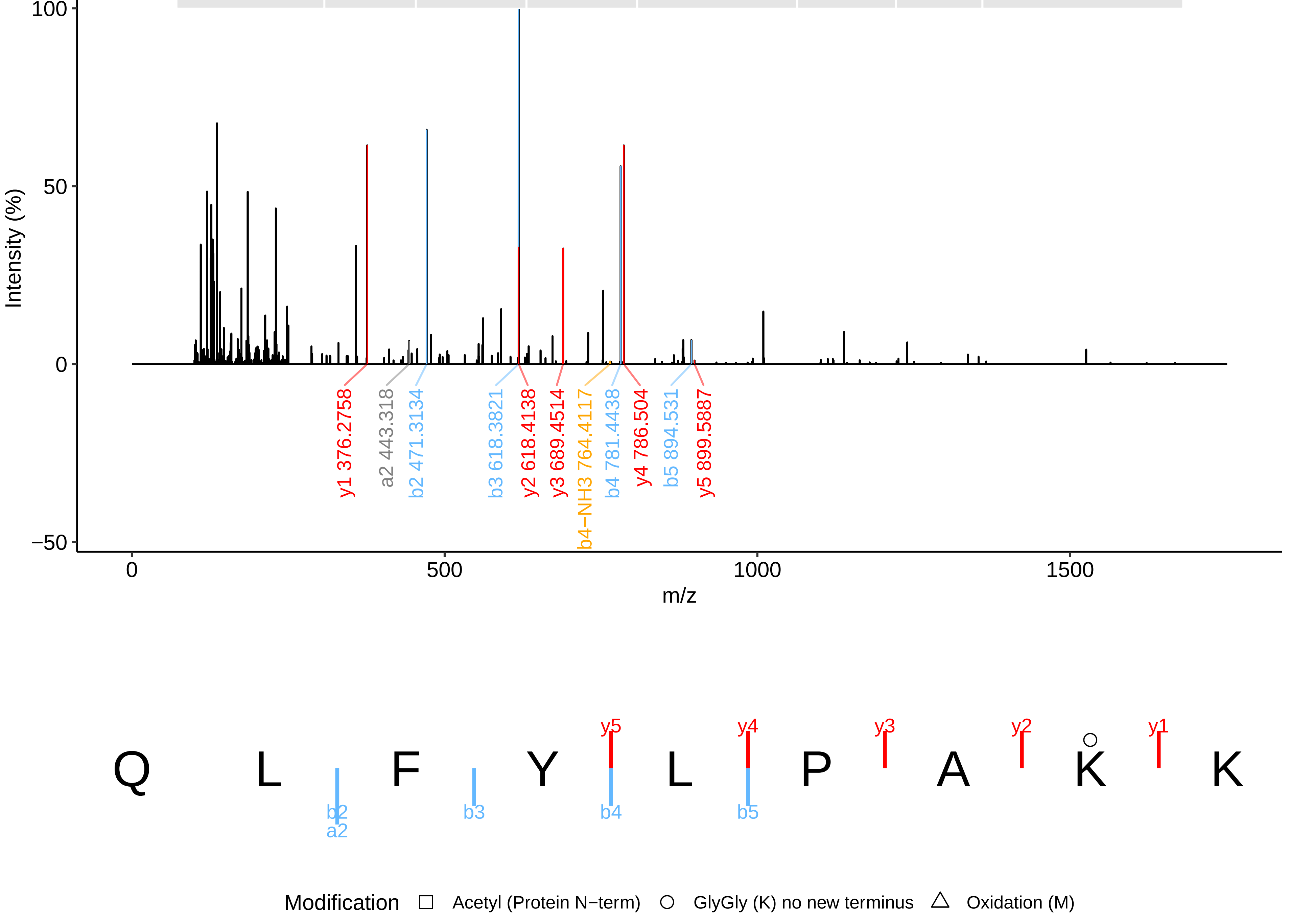

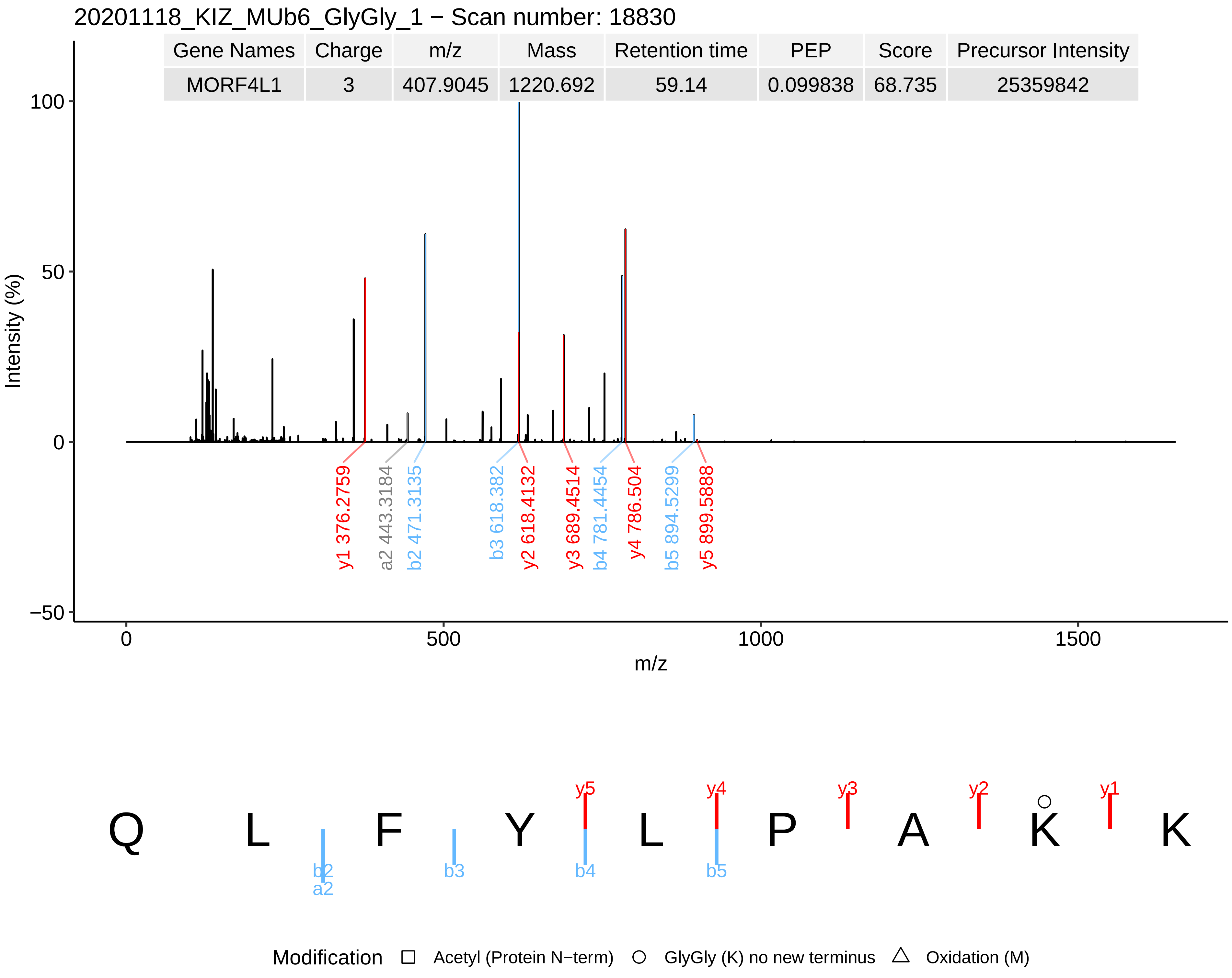
20201118_KIZ_MUb6_GlyGly_1 − Scan number: 18830
Gene Names | Charge | m/z | Mass | Retention time | PEP | Score | Precursor Intensity
MORF4L1 | 3 | 407.9045 | 1220.692 | 59.14 | 0.099838 | 68.735 | 25359842
Intensity (%)
100
50
0
−50
0
500
1000
1500
m/z
y1 376.2759
a2 443.3184
b2 471.3135
b3 618.382
y2 618.4132
y3 689.4514
b4 781.4454
y4 786.504
b5 894.5299
y5 899.5888
Q L F Y L P A K K
y5 y4 y3 y2 y1
b2 a2 b3 b4 b5
Modification
Acetyl (Protein N−term)
GlyGly (K) no new terminus
Oxidation (M)

20201118_KIZ_MUb6_GlyGly_2 − Scan number: 21258

Gene Names	Charge	m/z	Mass	Retention time	PEP	Score	Precursor Intensity
MORF4L1	3	407.9045	1220.692	71.631	0.021764	81.594	8023567

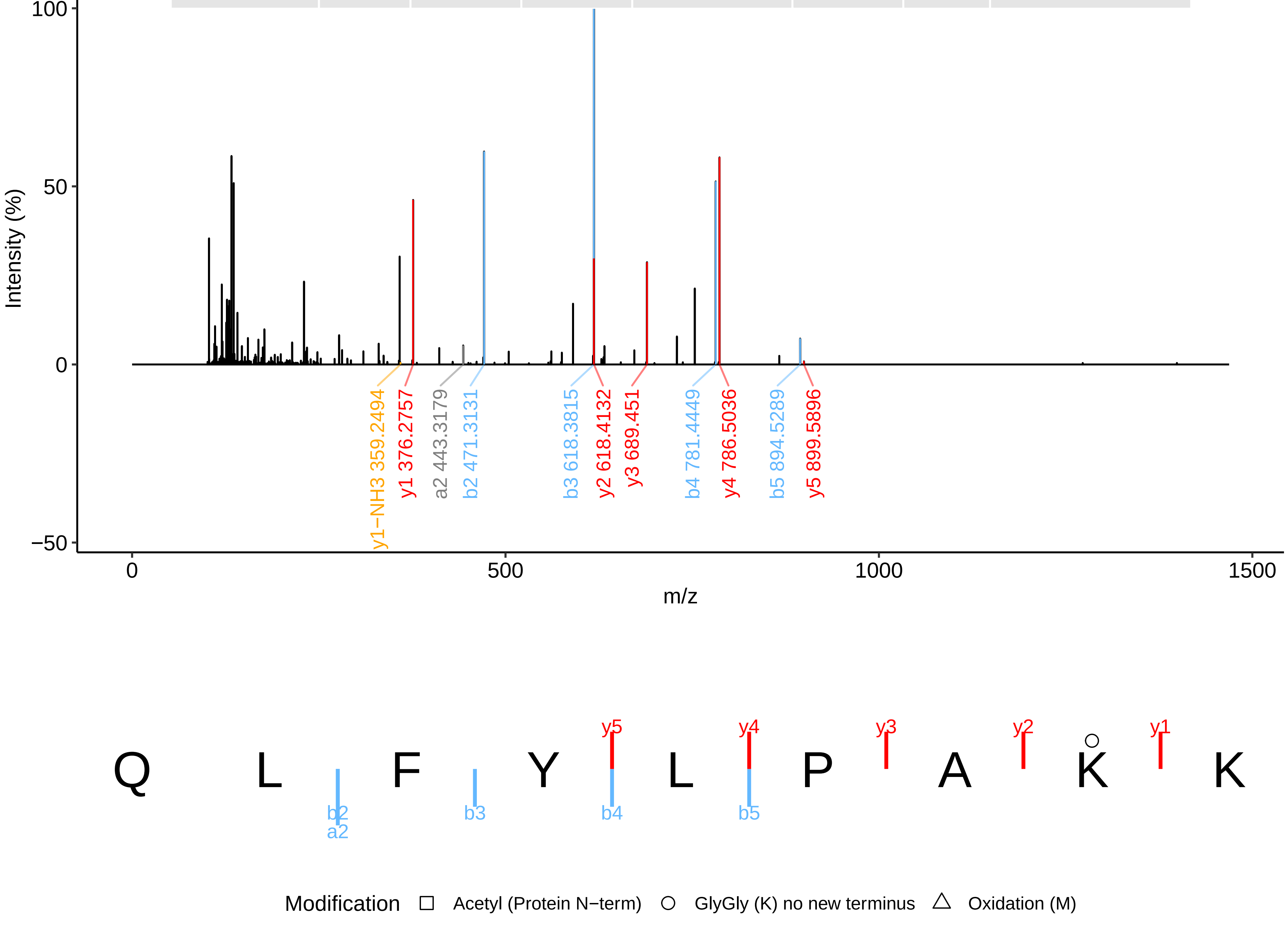

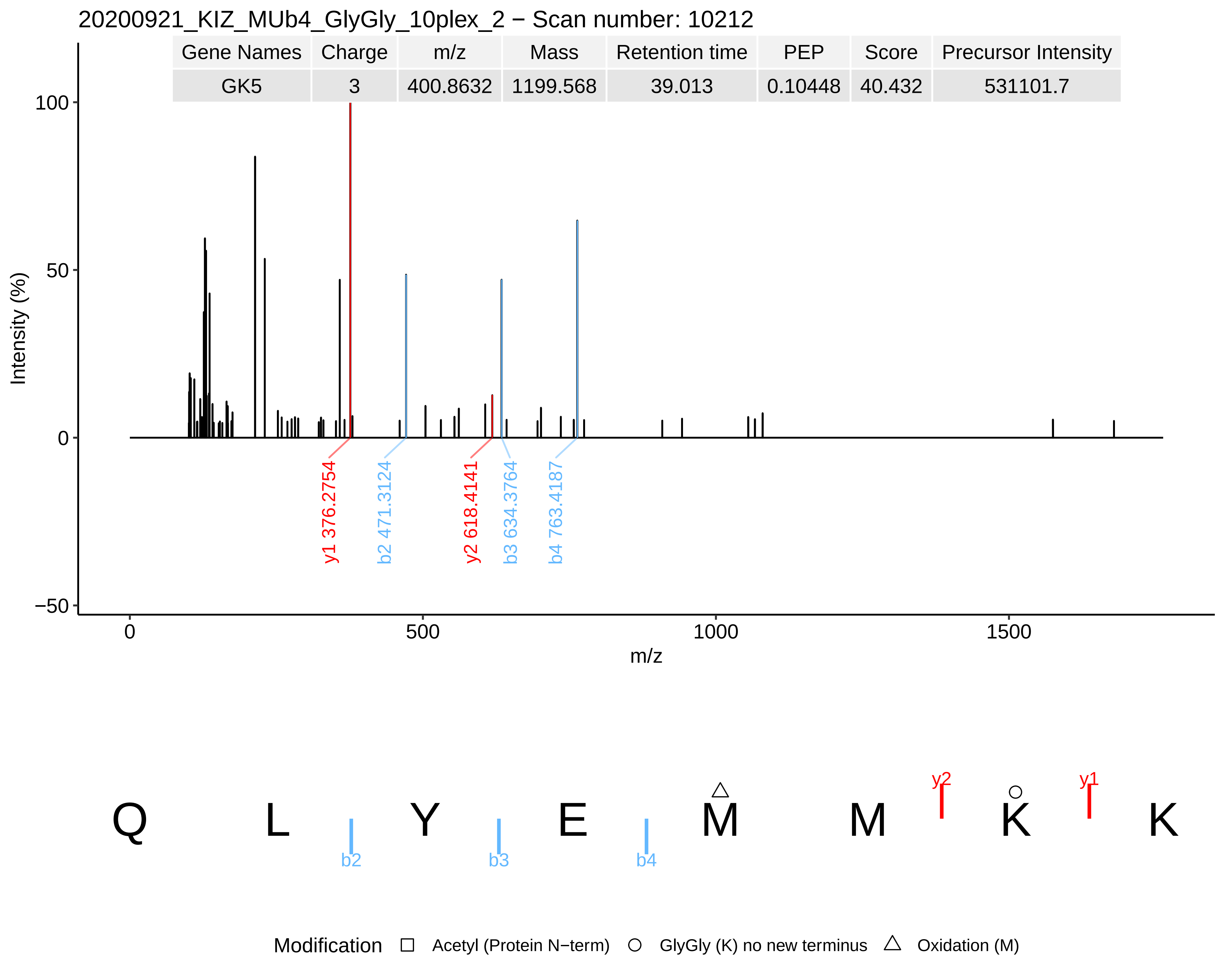
20200921_KIZ_MUb4_GlyGly_10plex_2 − Scan number: 10212
Gene Names | Charge | m/z | Mass | Retention time | PEP | Score | Precursor Intensity
GK5 | 3 | 400.8632 | 1199.568 | 39.013 | 0.10448 | 40.432 | 531101.7
Intensity (%)
100
50
0
−50
0
500
1000
1500
m/z
y1 376.2754
b2 471.3124
y2 618.4141
b3 634.3764
b4 763.4187
Q L Y E M M K K
b2
b3
b4
y2
y1
Modification
Acetyl (Protein N−term)
GlyGly (K) no new terminus
Oxidation (M)

20200921_KIZ_MUb4_GlyGly_10plex_2 − Scan number: 9793

Gene Names	Charge	m/z	Mass	Retention time	PEP	Score	Precursor Intensity
NCAPD2	3	492.5916	1474.753	37.338	0.0057246	73.848	323380.8

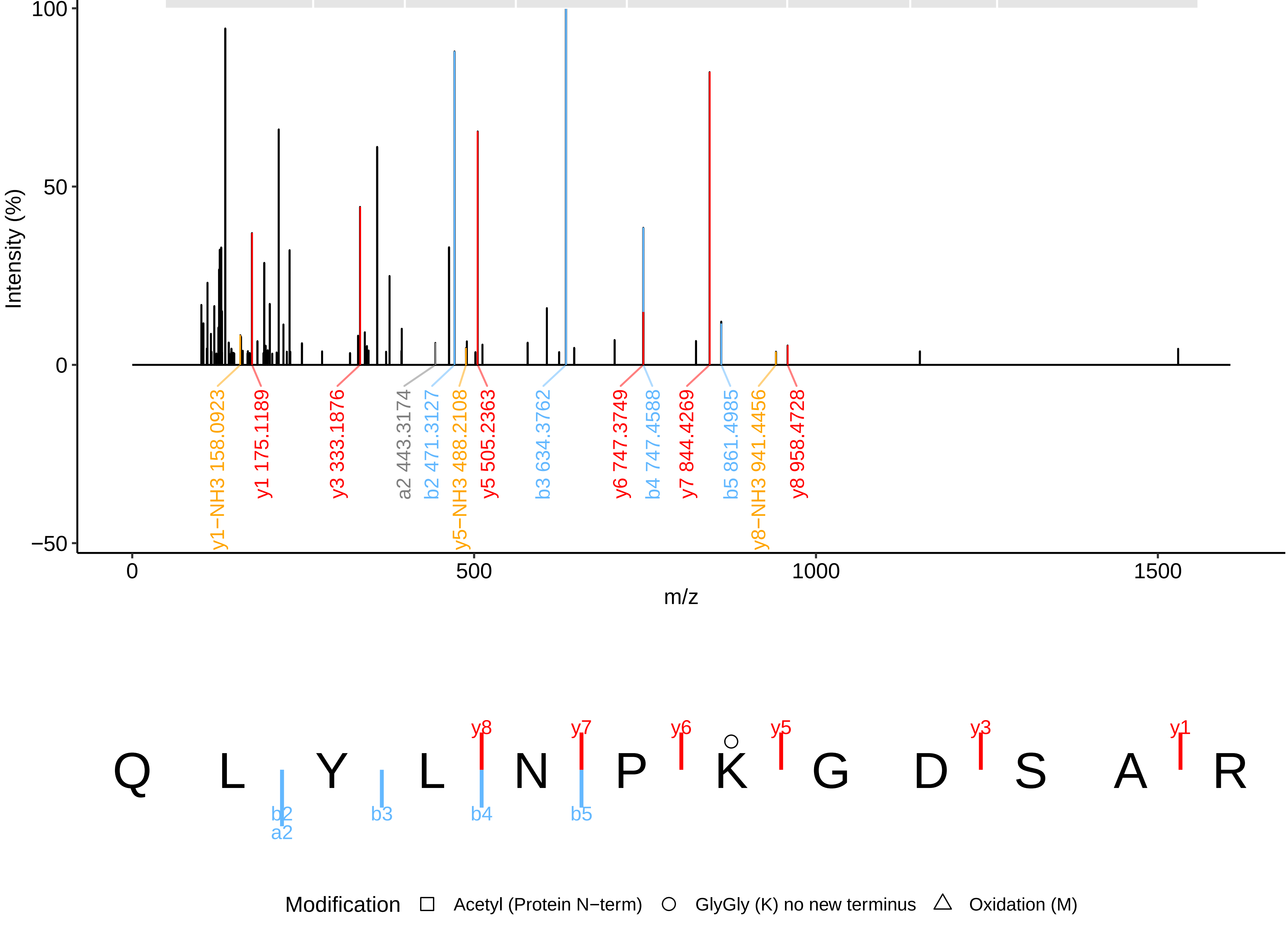

20200921_KIZ_MUb4_GlyGly_10plex_2 − Scan number: 5435

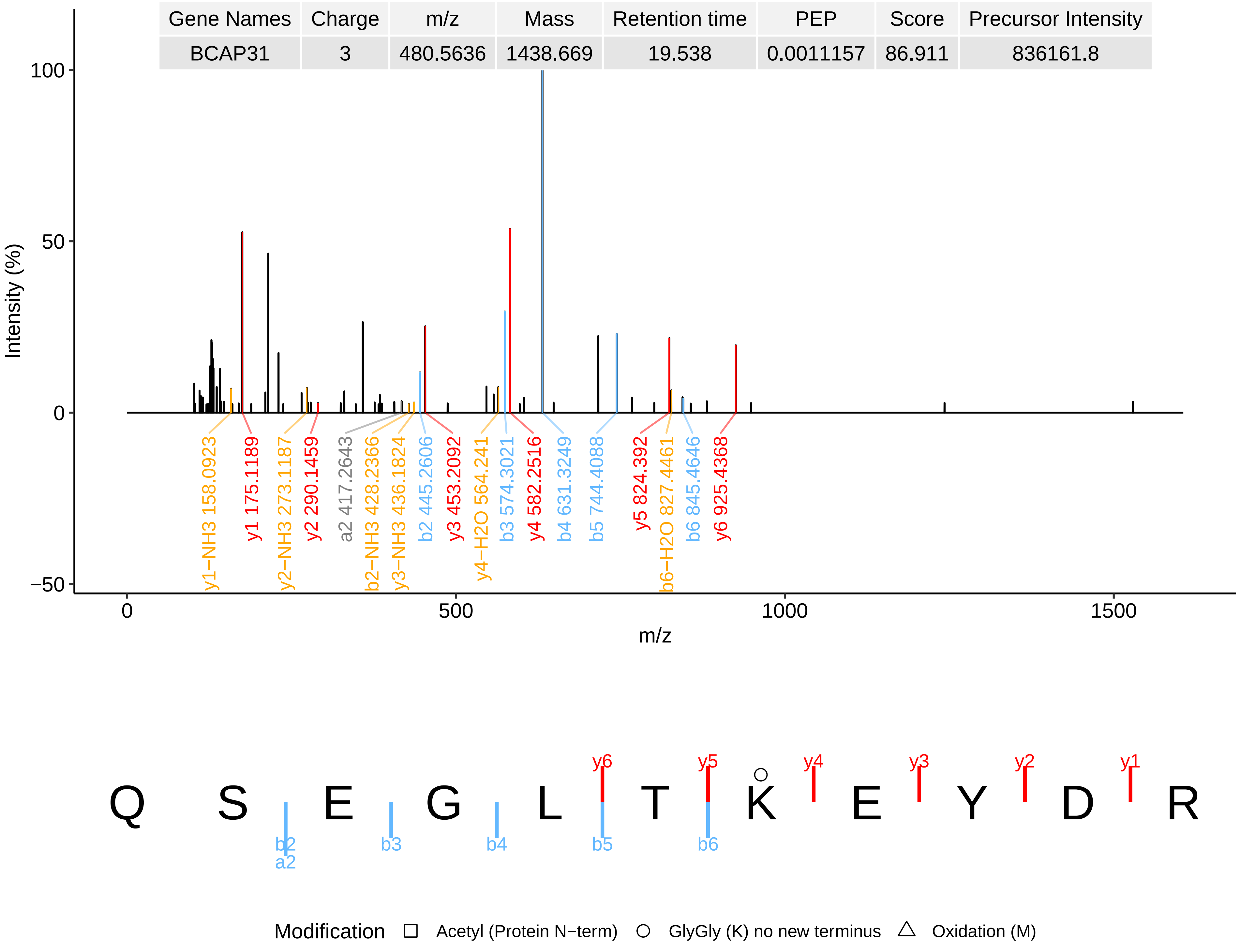

20200921_KIZ_MUb4_GlyGly_10plex − Scan number: 10846

Gene Names	Charge	m/z	Mass	Retention time	PEP	Score	Precursor Intensity
EIF3A	3	413.219	1236.635	37.678	0.0012001	94.297	13043759

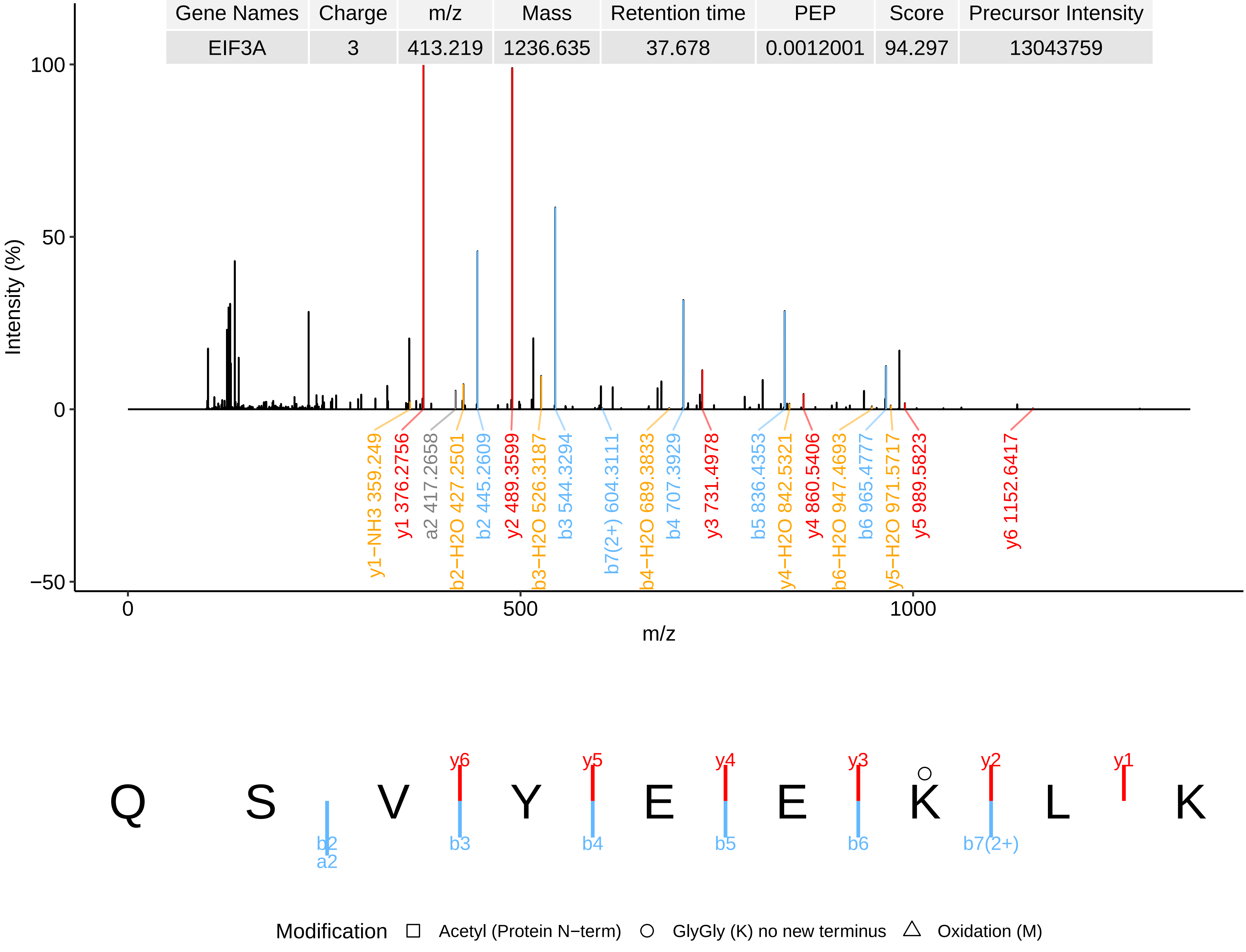

20200921_KIZ_MUb4_GlyGly_10plex_2 − Scan number: 10036

Gene Names	Charge	m/z	Mass	Retention time	PEP	Score	Precursor Intensity
EIF3A	3	413.219	1236.635	38.288	0.0019118	90.108	3380622

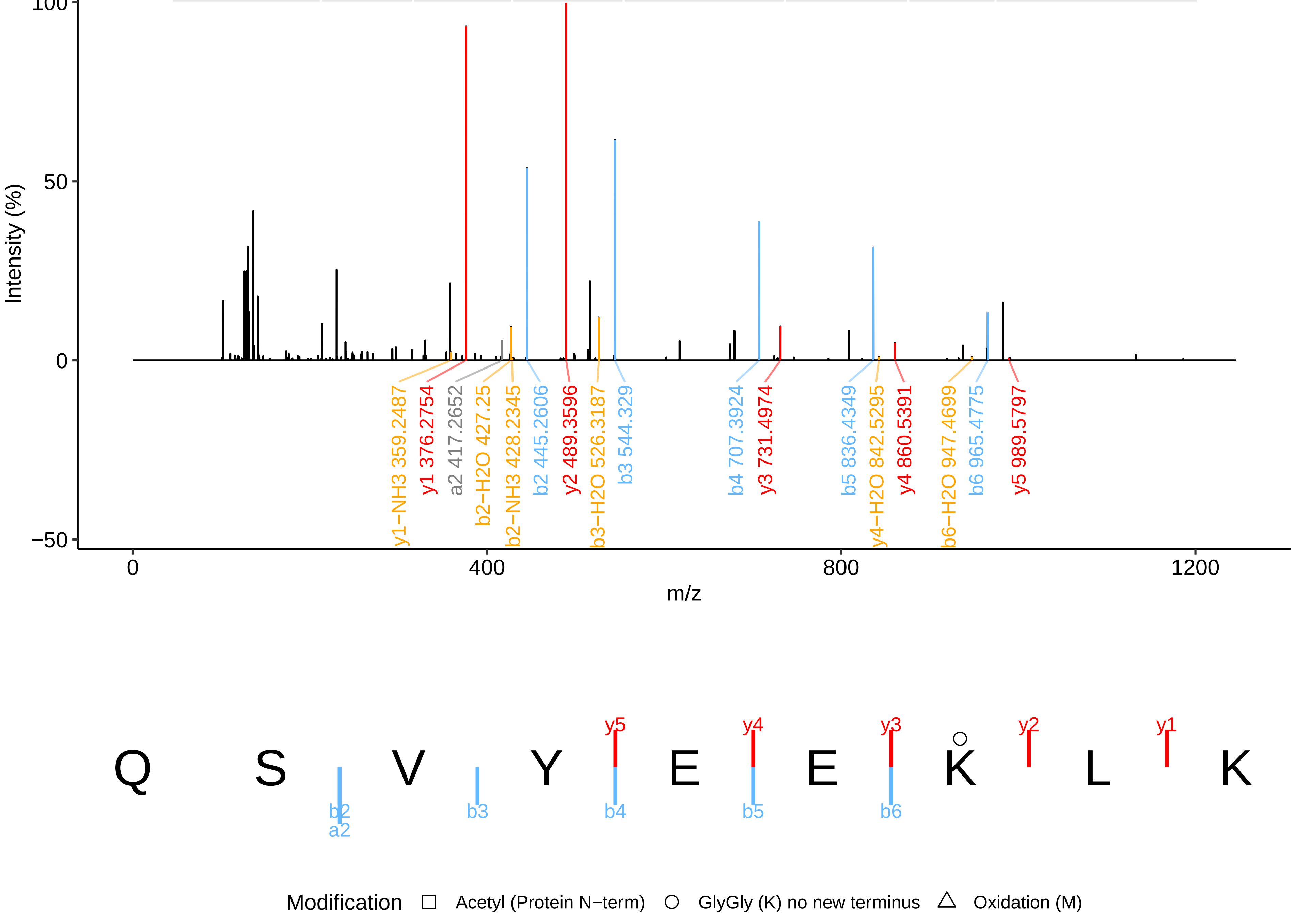

20200924_KIZ_MUb5_GlyGly_1 − Scan number: 9757

Gene Names	Charge	m/z	Mass	Retention time	PEP	Score	Precursor Intensity
EIF3A	3	413.219	1236.635	31.423	0.00045514	101.66	38985488

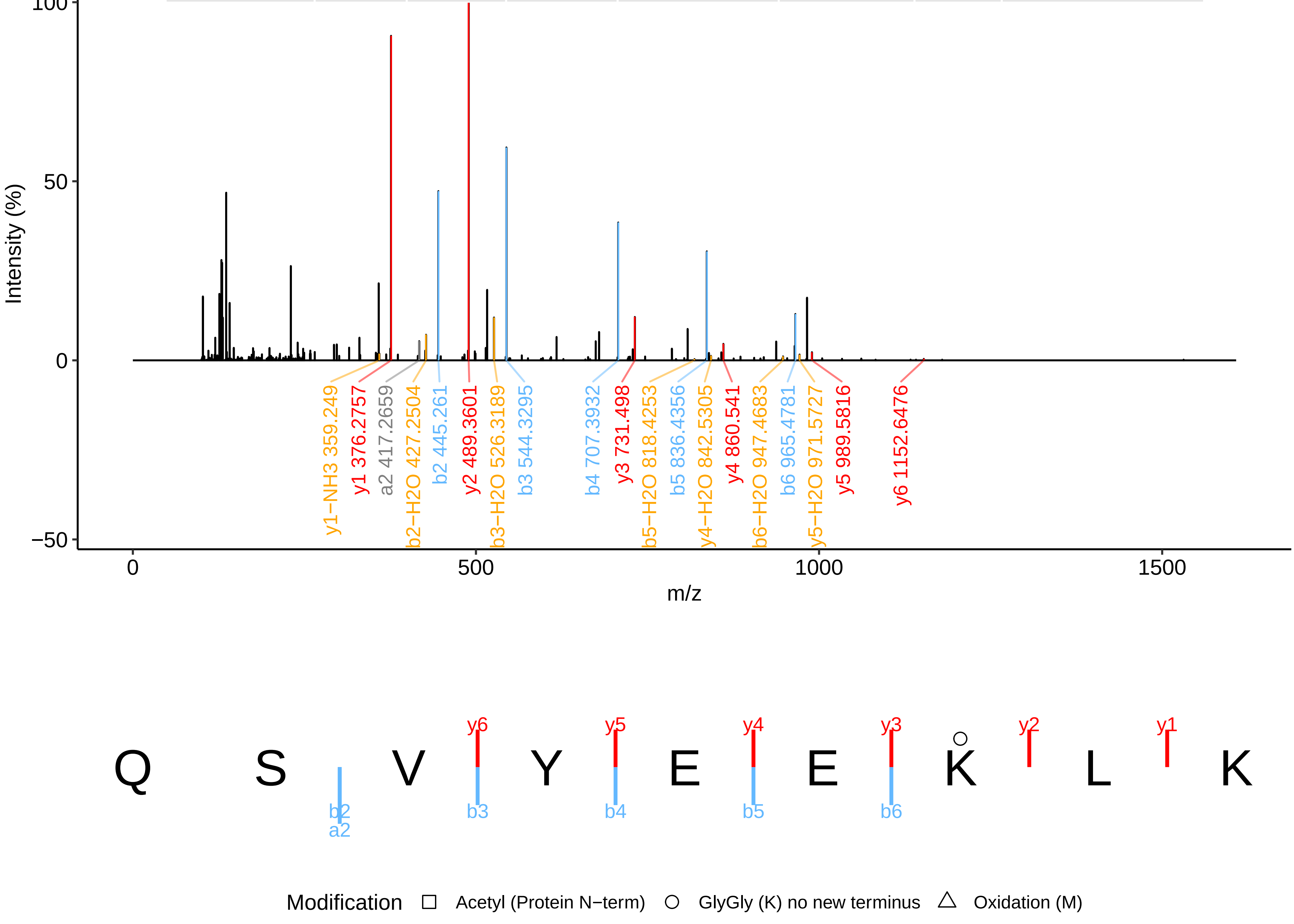

20200924_KIZ_MUb5_GlyGly_1 − Scan number: 9914

Gene Names	Charge	m/z	Mass	Retention time	PEP	Score	Precursor Intensity
EIF3A	3	413.219	1236.635	31.937	0.0009489	95.775	22535016

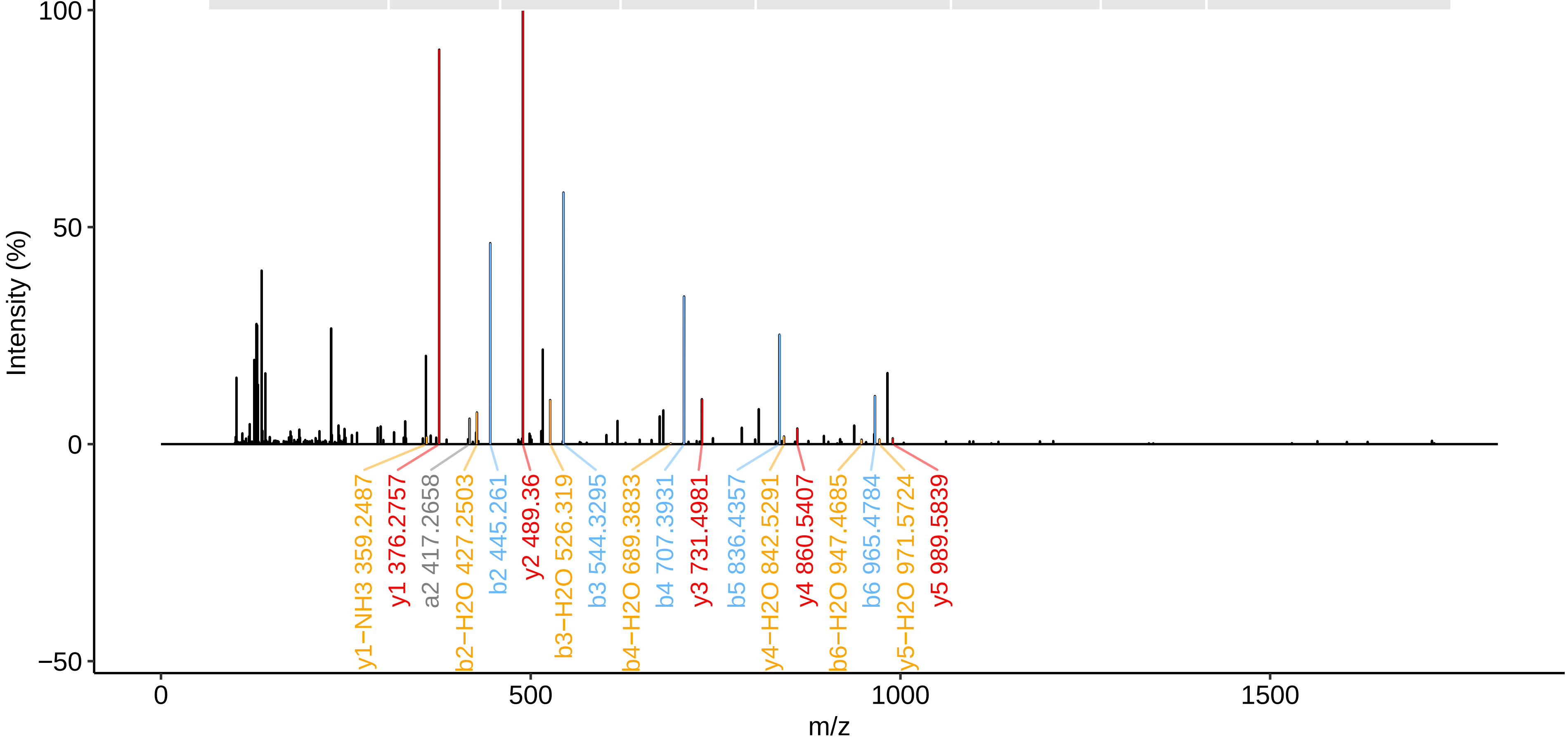

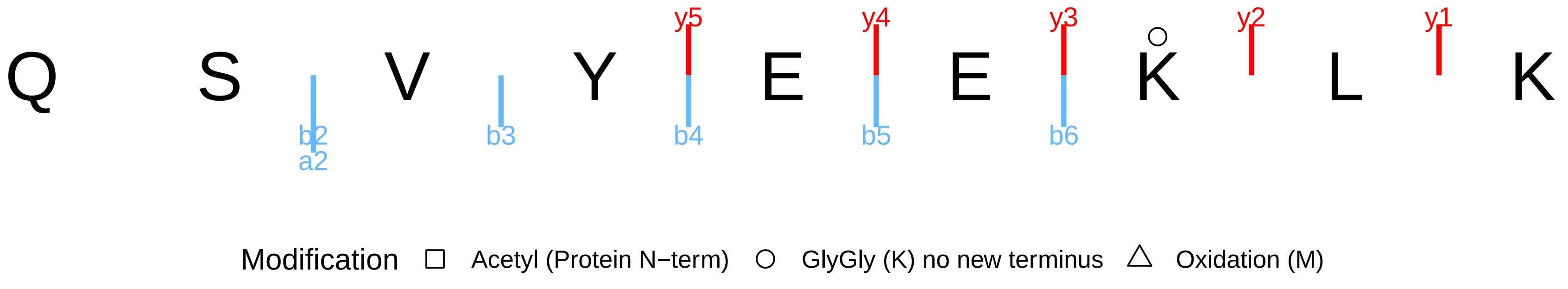

20200924_KIZ_MUb5_GlyGly_2 − Scan number: 9062

Gene Names	Charge	m/z	Mass	Retention time	PEP	Score	Precursor Intensity
EIF3A	3	413.219	1236.635	33.865	0.0026893	85.533	2116476

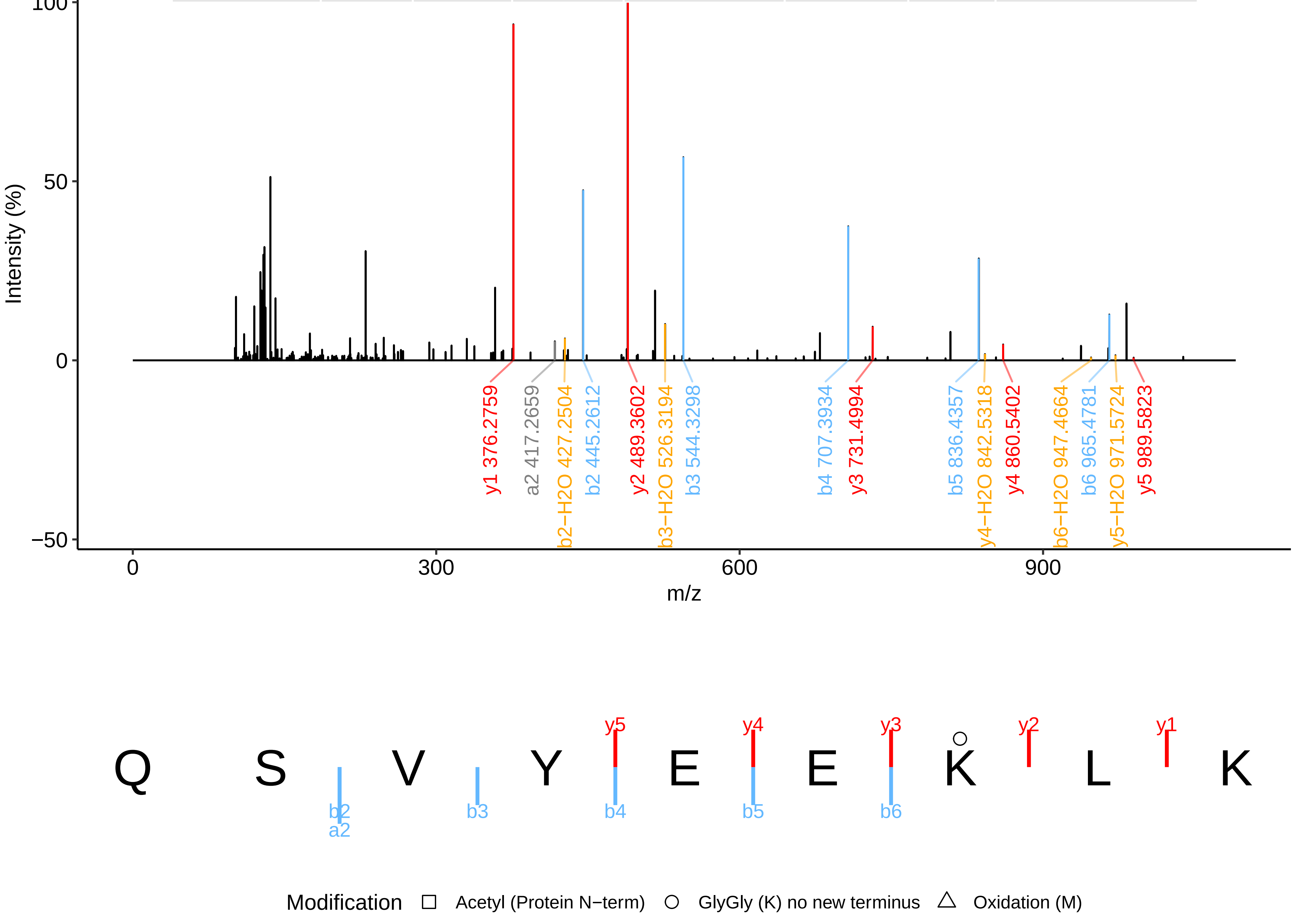

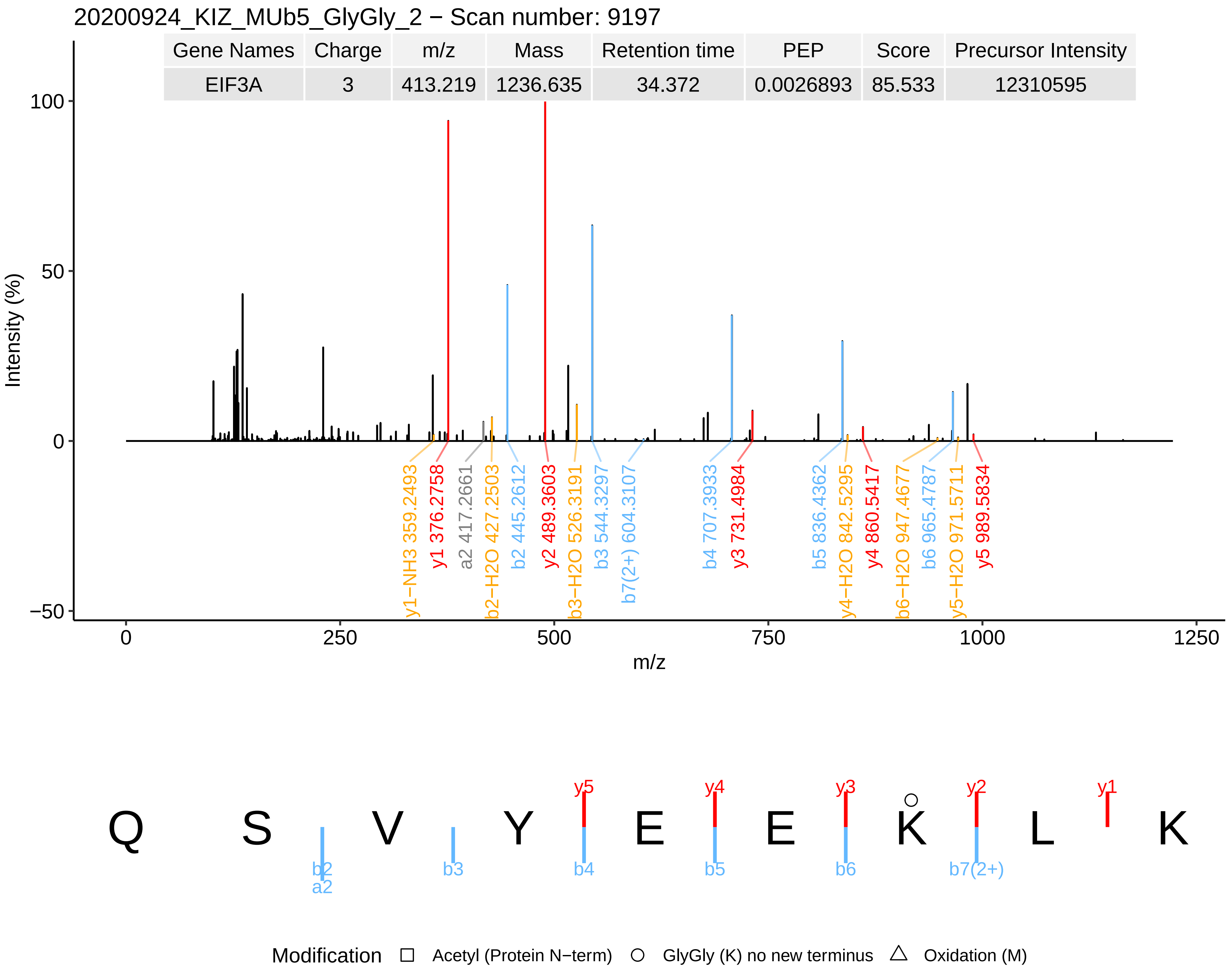
20200924_KIZ_MUb5_GlyGly_2 − Scan number: 9197
Gene Names
Charge
m/z
Mass
Retention time
PEP
Score
Precursor Intensity
EIF3A
3
413.219
1236.635
34.372
0.0026893
85.533
12310595
Intensity (%)
m/z
y1−NH3 359.2493
y1 376.2758
a2 417.2661
b2−H2O 427.2503
b2 445.2612
y2 489.3603
b3−H2O 526.3191
b3 544.3297
b7(2+) 604.3107
b4 707.3933
y3 731.4984
b5 836.4362
y4−H2O 842.5295
y4 860.5417
b6−H2O 947.4677
b6 965.4787
y5−H2O 971.5711
y5 989.5834
Q S V Y E E K L K
y5 y4 y3 y2 y1
b2 a2 b3 b4 b5 b6 b7(2+)
Modification
Acetyl (Protein N−term)
GlyGly (K) no new terminus
Oxidation (M)

20200924_KIZ_MUb5_GlyGly_2 − Scan number: 9333

Gene Names	Charge	m/z	Mass	Retention time	PEP	Score	Precursor Intensity
EIF3A	3	413.219	1236.635	34.883	0.0026427	85.807	1766180

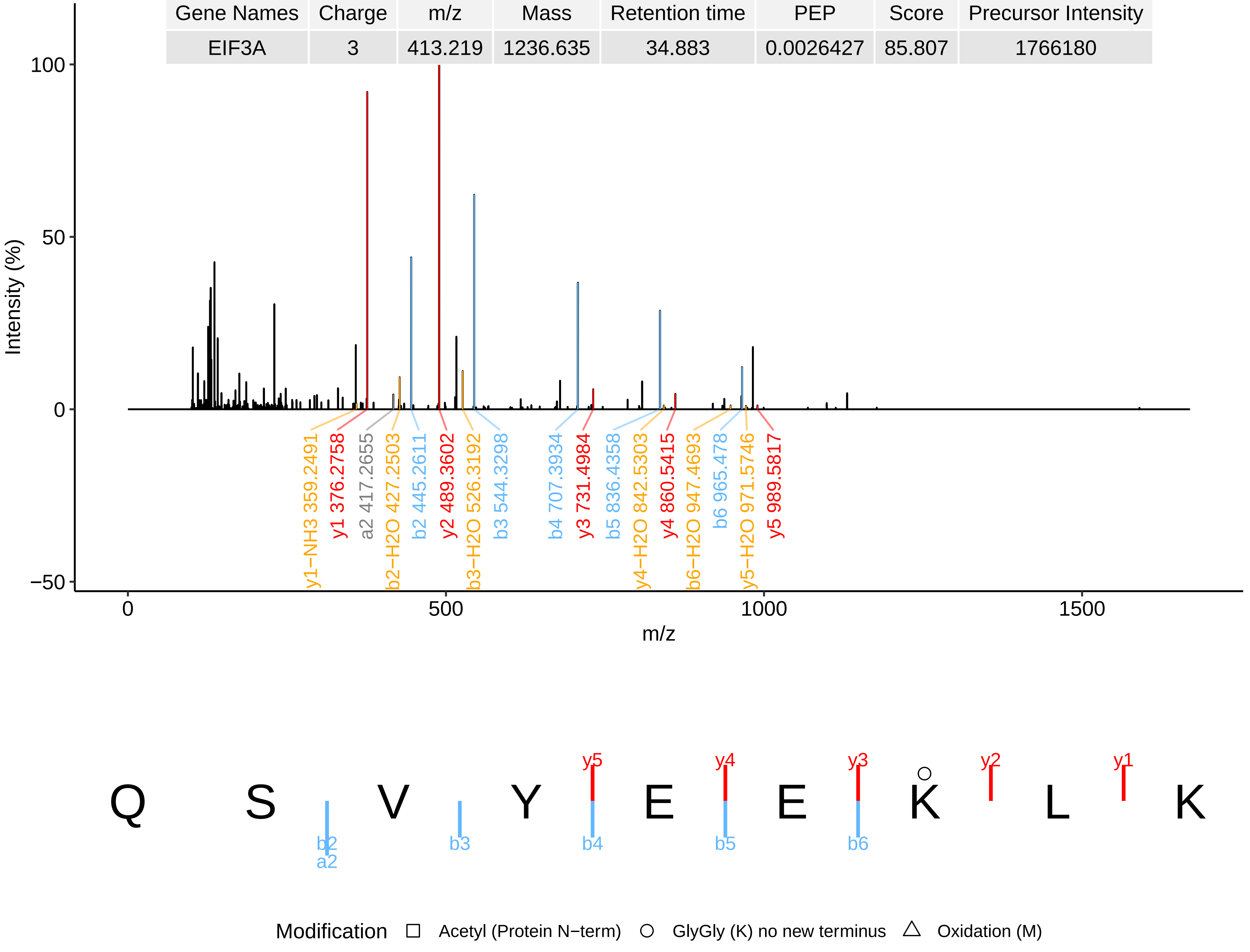

20201118_KIZ_MUb6_GlyGly_2 − Scan number: 11483

Gene Names	Charge	m/z	Mass	Retention time	PEP	Score	Precursor Intensity
EIF3A	3	413.219	1236.635	38.781	0.014697	70.1	2209720

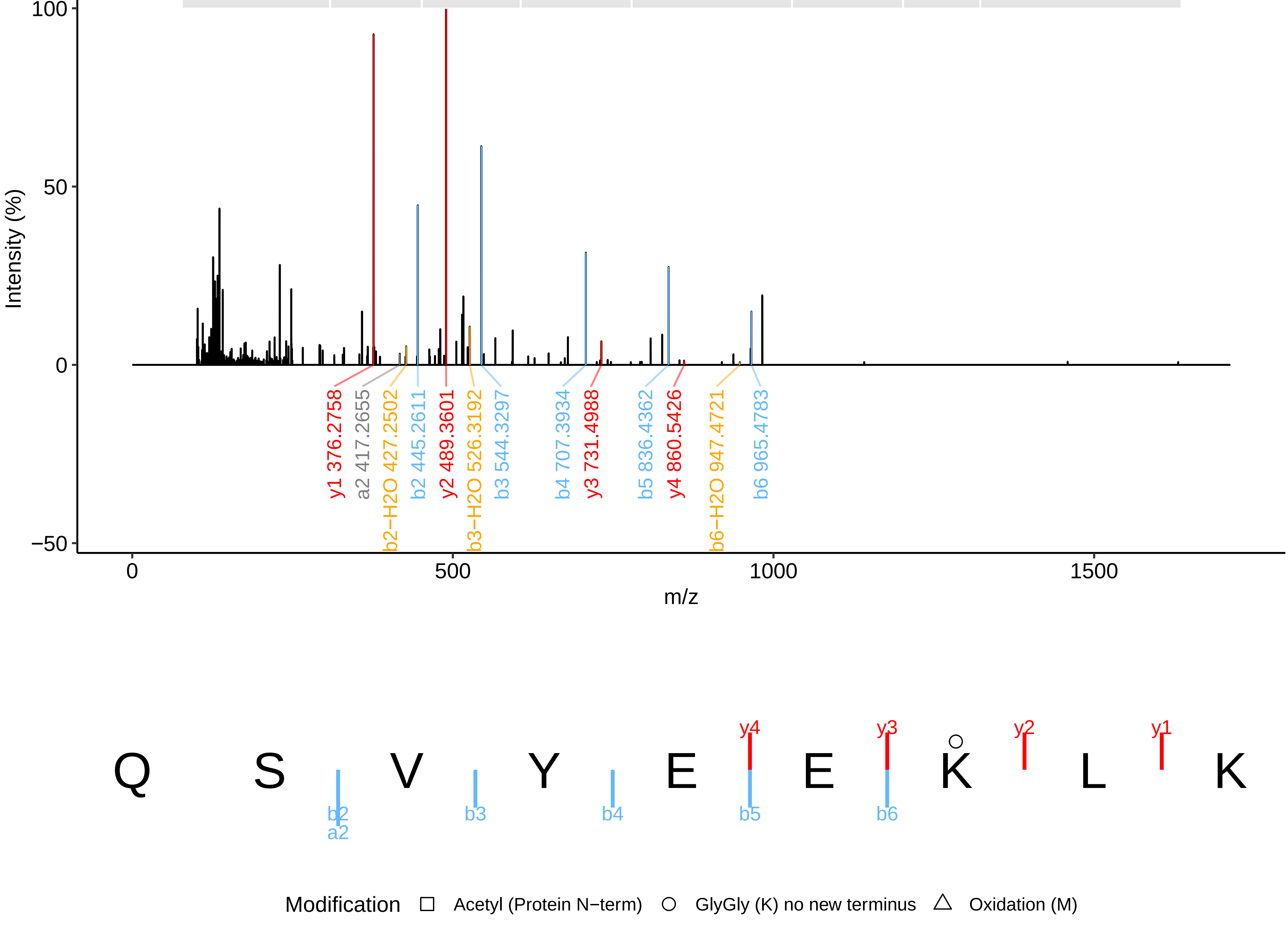

20200921_KIZ_MUb4_GlyGly_10plex − Scan number: 5654

Gene Names	Charge	m/z	Mass	Retention time	PEP	Score	Precursor Intensity
BUB3	3	410.8823	1229.625	18.546	4.8964e−05	104.22	6345960

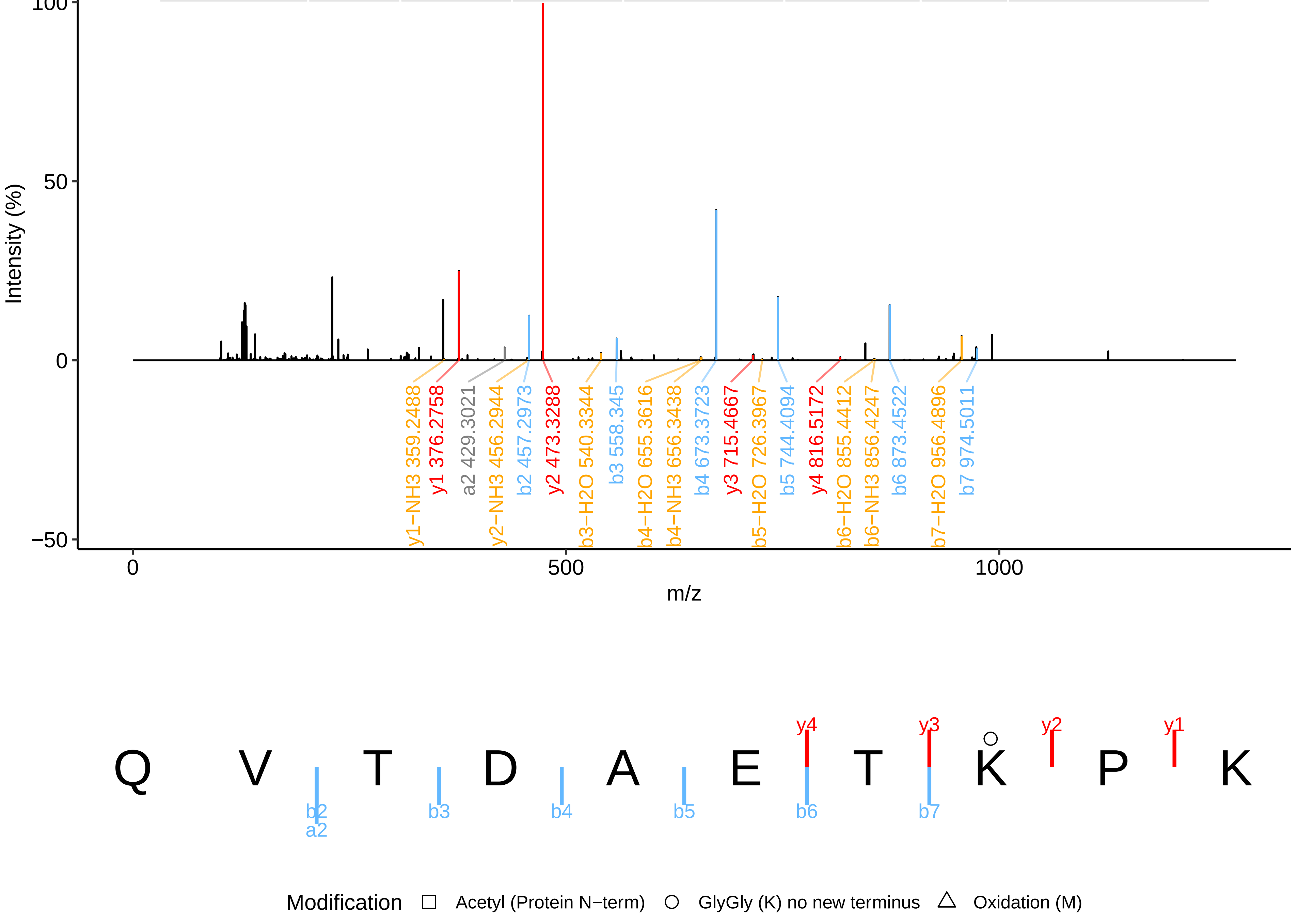

20200921_KIZ_MUb4_GlyGly_10plex_2 − Scan number: 5131

Gene Names	Charge	m/z	Mass	Retention time	PEP	Score	Precursor Intensity
BUB3	3	410.8823	1229.625	18.309	0.016857	66.595	839120.4

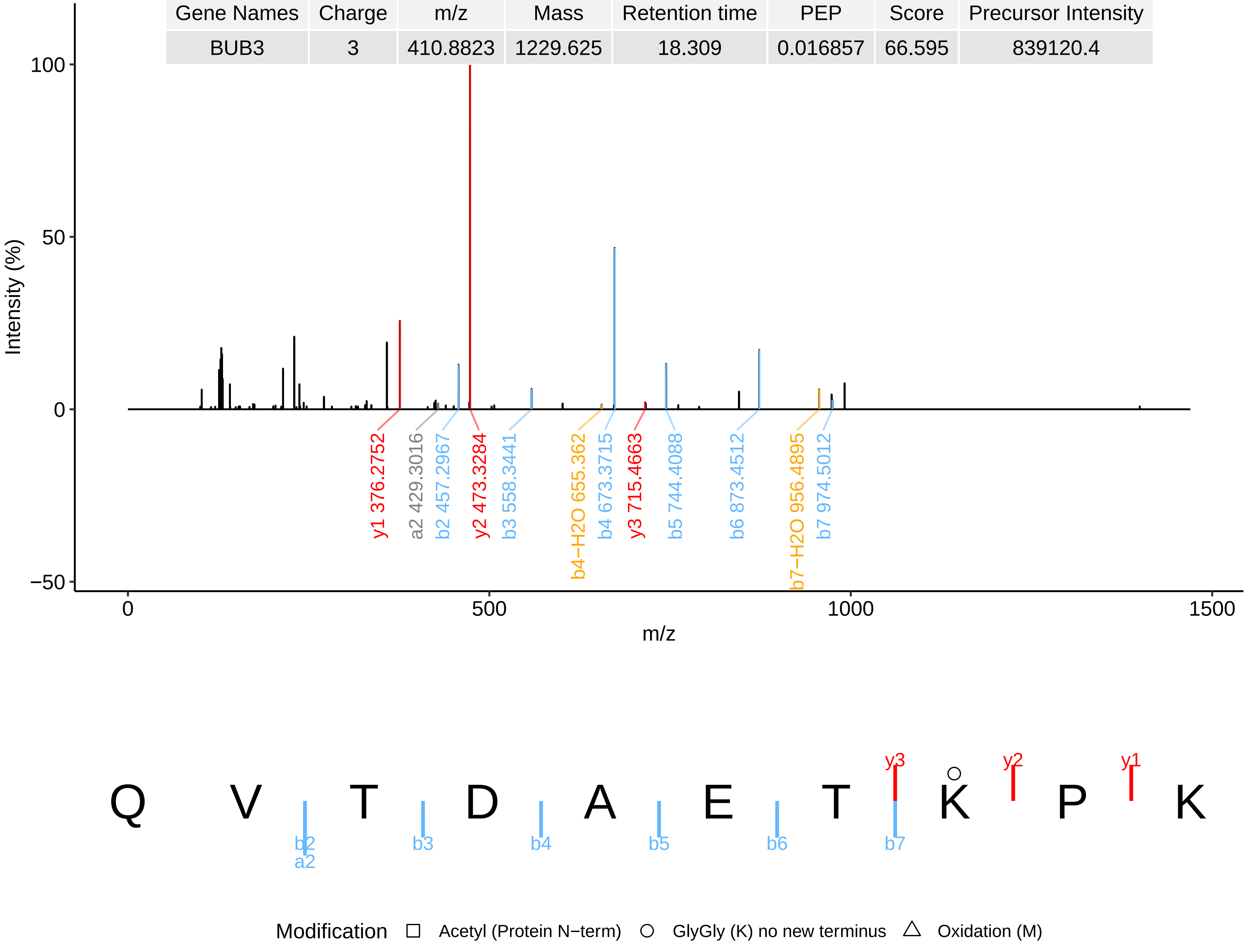

20200924_KIZ_MUb5_GlyGly_1 − Scan number: 4965

Gene Names	Charge	m/z	Mass	Retention time	PEP	Score	Precursor Intensity
BUB3	3	410.8823	1229.625	15.387	0.00078547	93.096	9278138

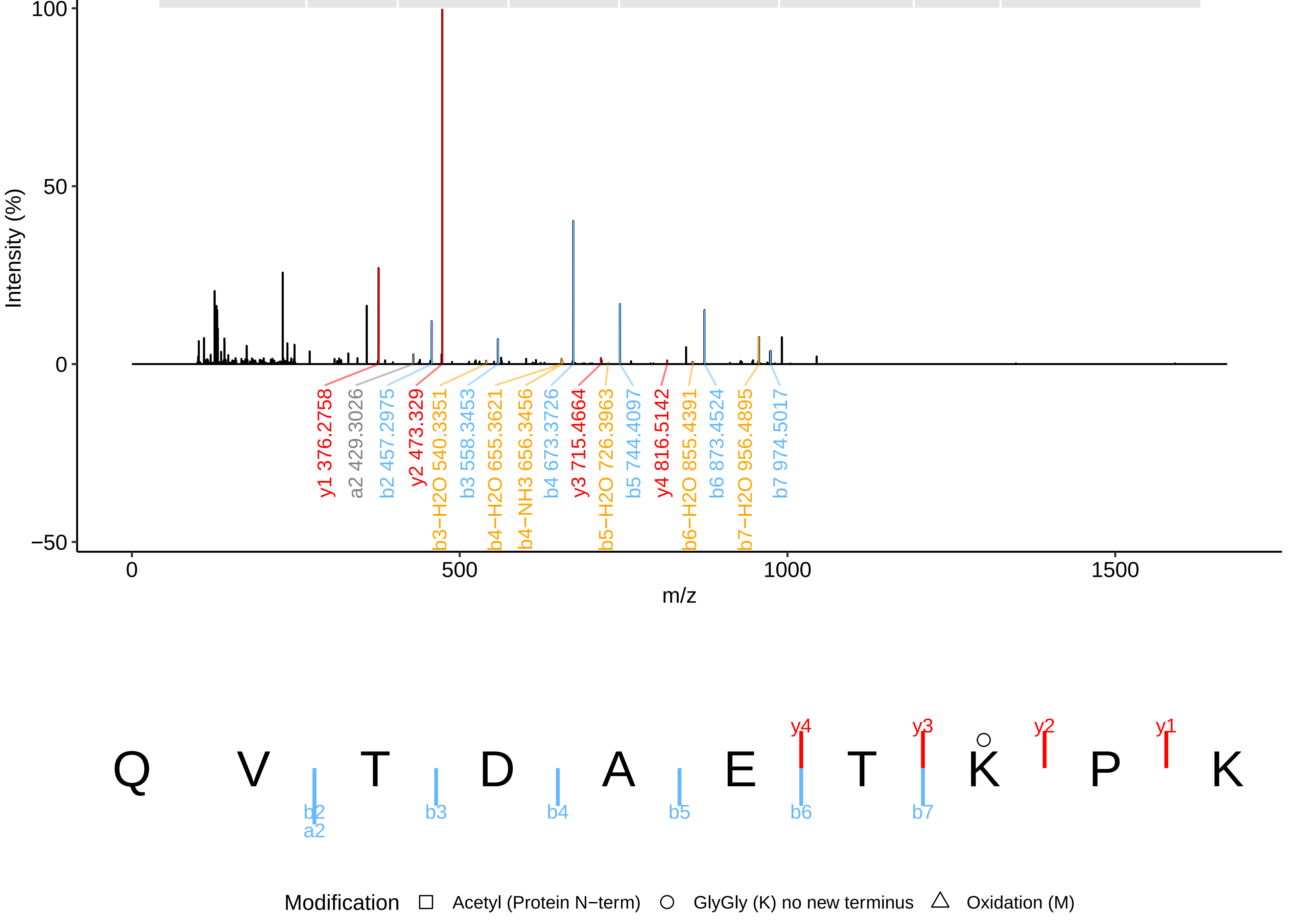

20200924_KIZ_MUb5_GlyGly_2 − Scan number: 4558

Gene Names	Charge	m/z	Mass	Retention time	PEP	Score	Precursor Intensity
BUB3	3	410.8823	1229.625	16.745	0.021239	64.82	3654055

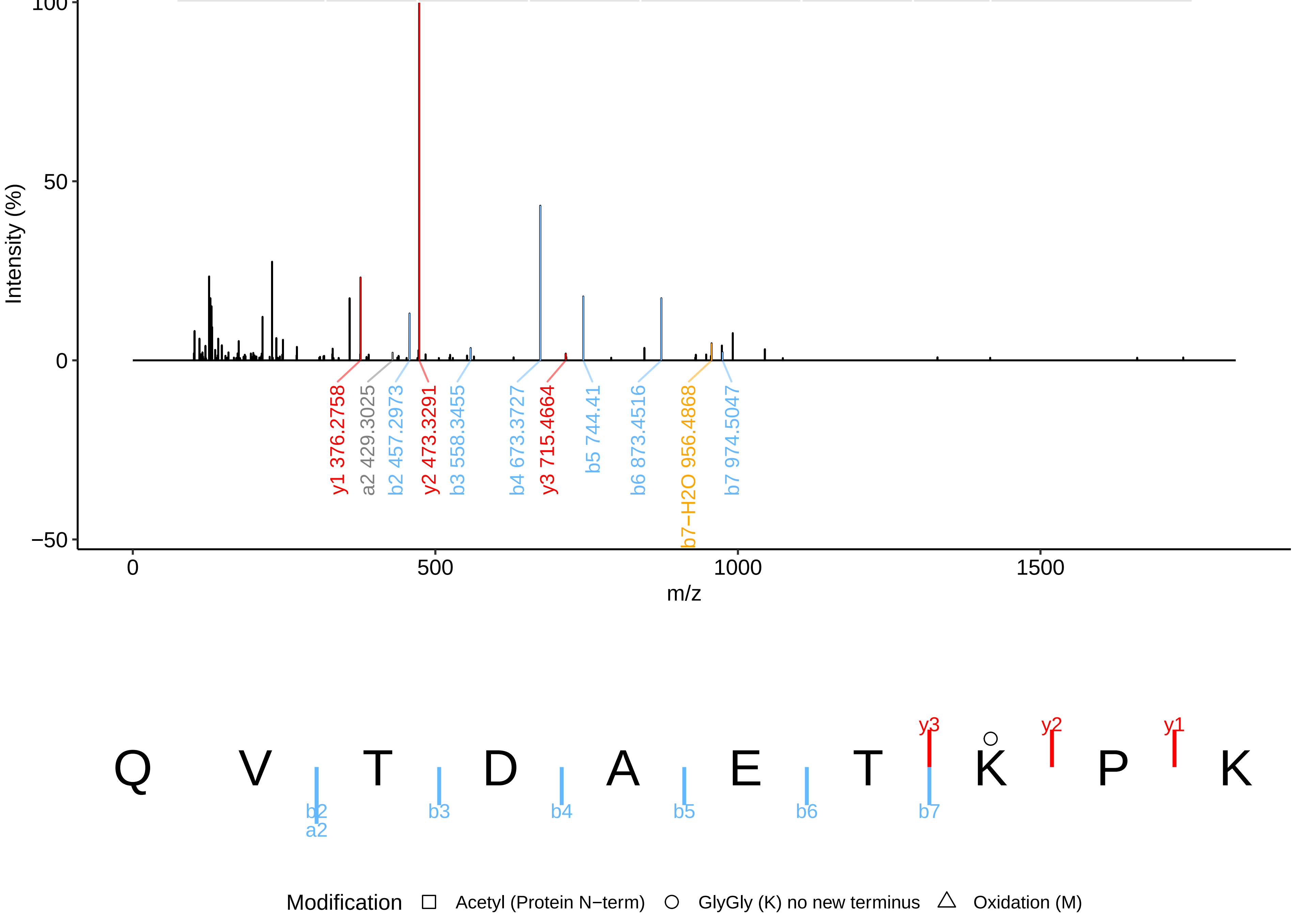

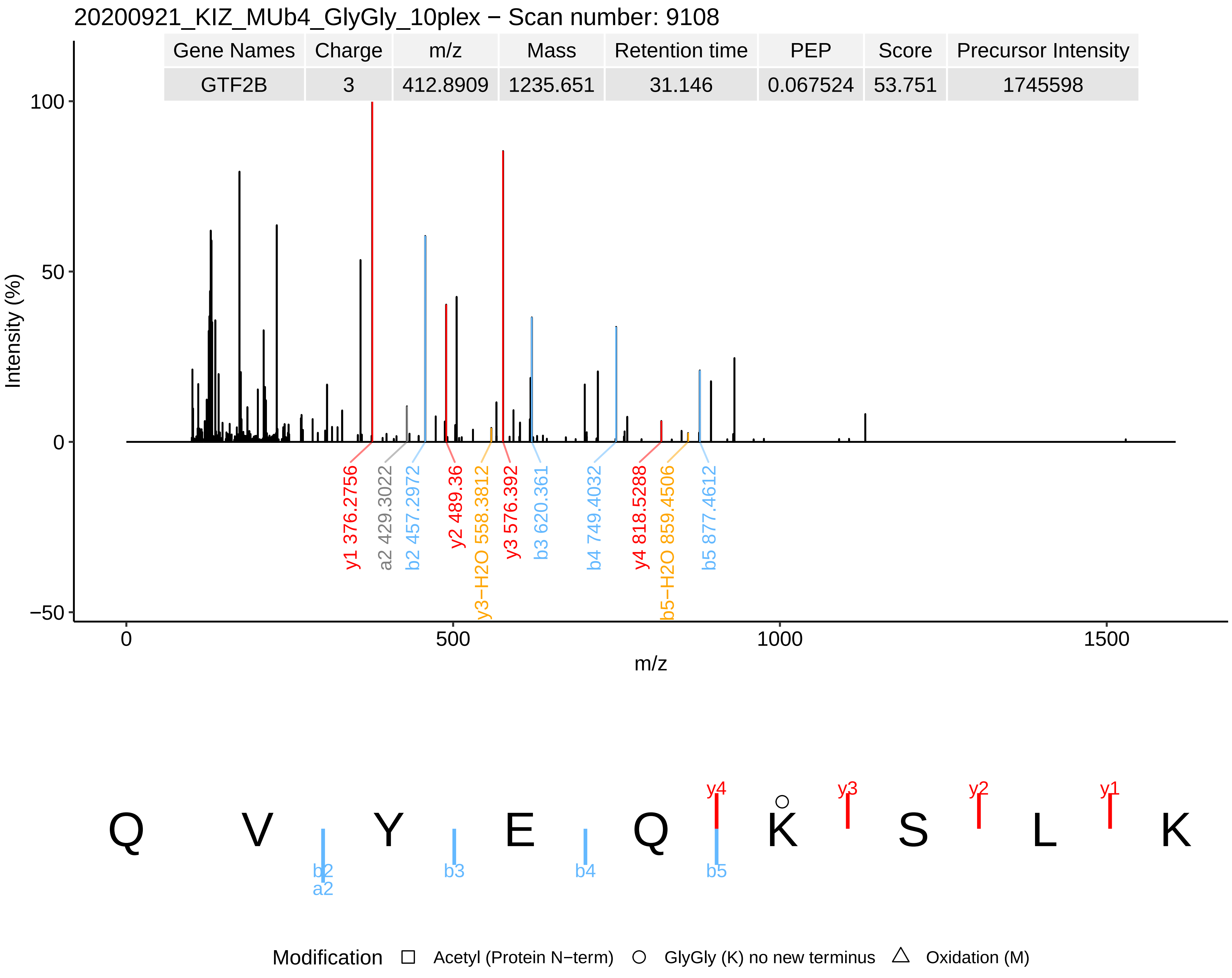
20200921_KIZ_MUb4_GlyGly_10plex − Scan number: 9108
Gene Names | Charge | m/z | Mass | Retention time | PEP | Score | Precursor Intensity
GTF2B | 3 | 412.8909 | 1235.651 | 31.146 | 0.067524 | 53.751 | 1745598
Intensity (%)
100
50
0
−50
0
500
1000
1500
m/z
y1 376.2756
a2 429.3022
b2 457.2972
y2 489.36
y3−H2O 558.3812
y3 576.392
b3 620.361
b4 749.4032
y4 818.5288
b5−H2O 859.4506
b5 877.4612
Q V Y E Q K S L K
y4
y3
y2
y1
b2
a2
b3
b4
b5
Modification
Acetyl (Protein N−term)
GlyGly (K) no new terminus
Oxidation (M)

20200921_KIZ_MUb4_GlyGly_10plex − Scan number: 2739

Gene Names	Charge	m/z	Mass	Retention time	PEP	Score	Precursor Intensity
RHOT2	4	368.9446	1471.749	8.6094	0.00021416	102.53	23330494

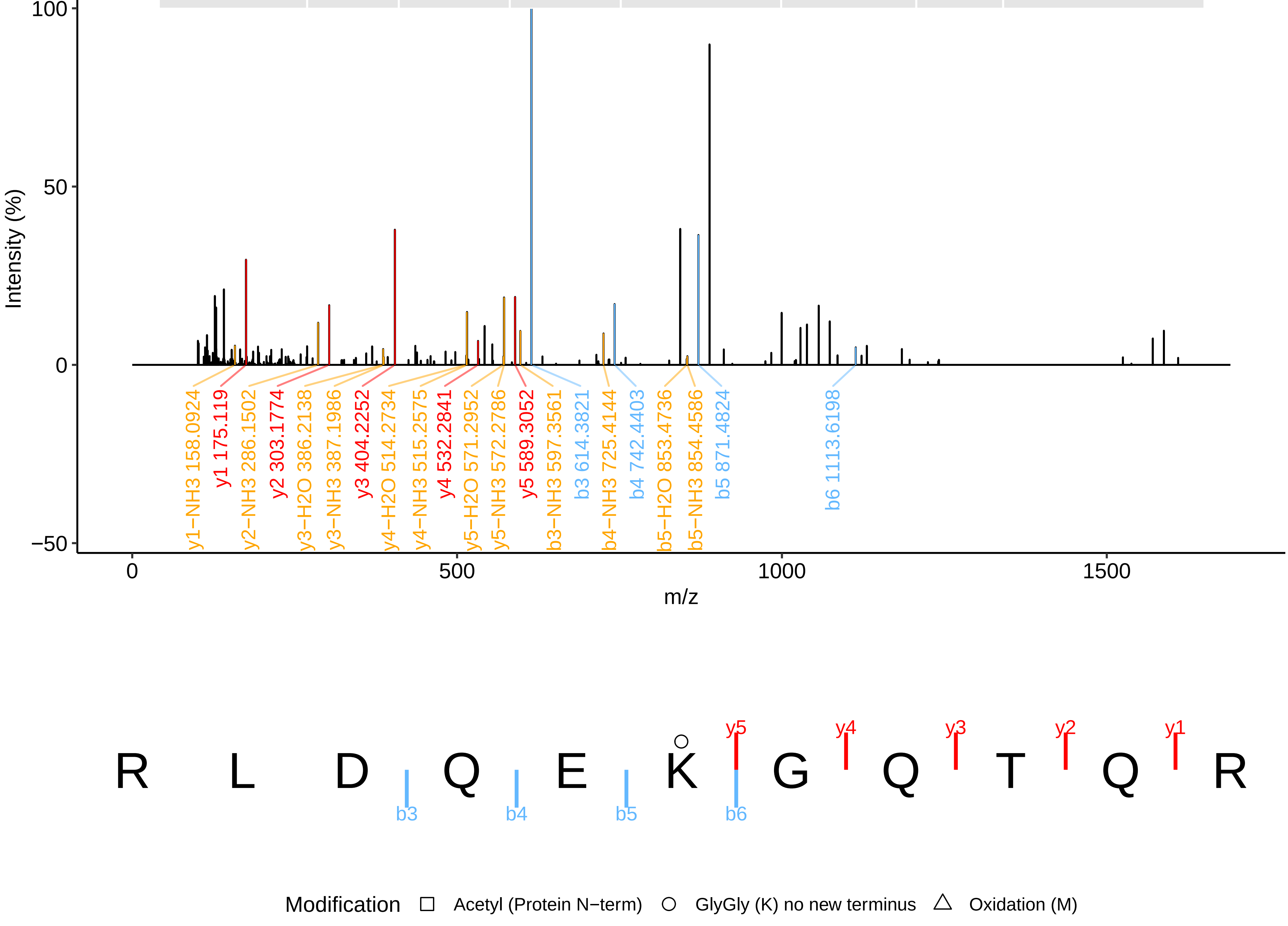

20200921_KIZ_MUb4_GlyGly_10plex − Scan number: 2750

Gene Names	Charge	m/z	Mass	Retention time	PEP	Score	Precursor Intensity
RHOT2	3	491.5903	1471.749	8.6368	2.0106e−17	166.45	4818662

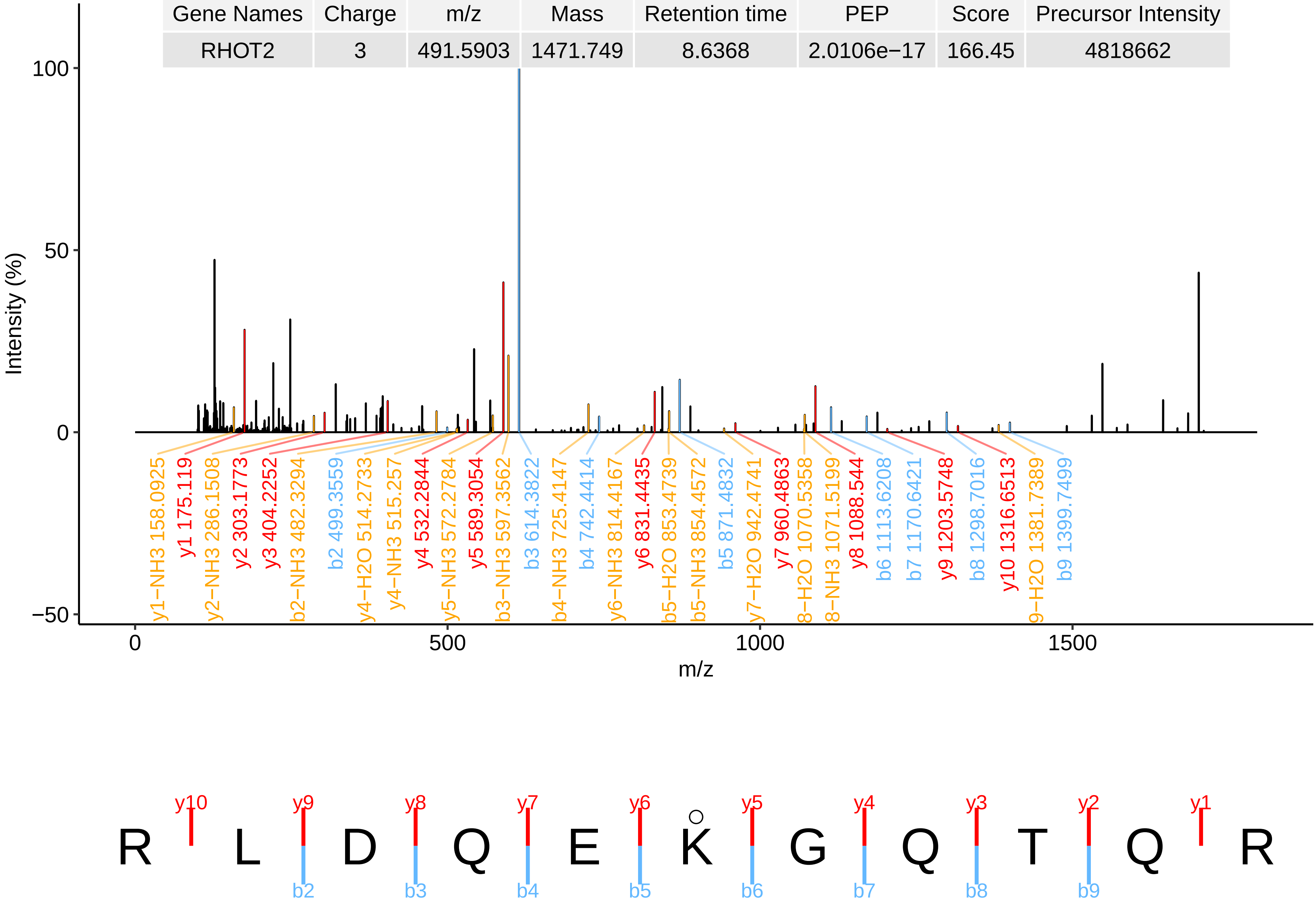

Modification □ Acetyl (Protein N−term) ○ GlyGly (K) no new terminus △ Oxidation (M)

20200921_KIZ_MUb4_GlyGly_10plex_2 − Scan number: 2698

Gene Names	Charge	m/z	Mass	Retention time	PEP	Score	Precursor Intensity
RHOT2	3	491.5903	1471.749	8.6723	1.5806e−16	158.04	2681442

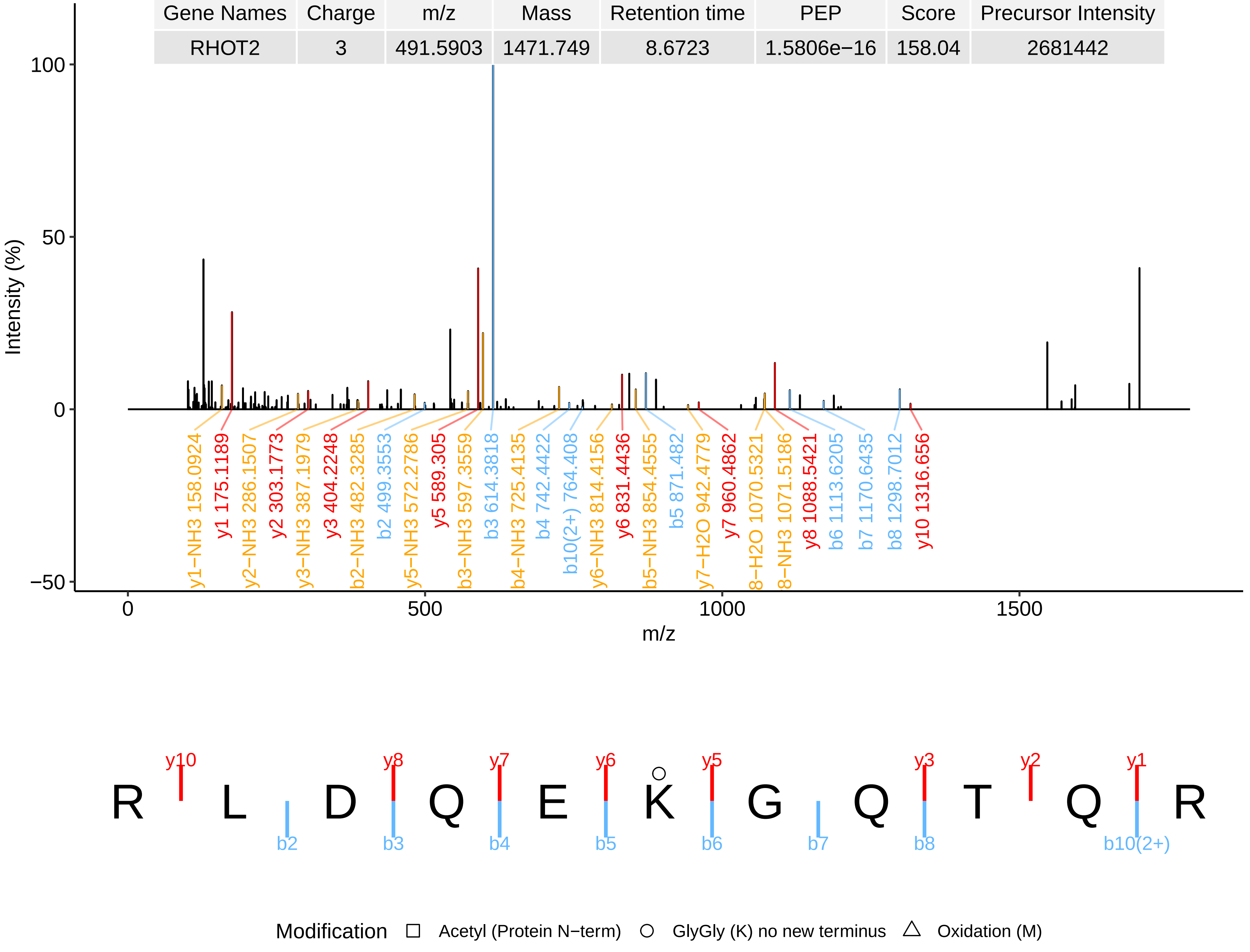

20200921_KIZ_MUb4_GlyGly_10plex_2 − Scan number: 2699

Gene Names	Charge	m/z	Mass	Retention time	PEP	Score	Precursor Intensity
RHOT2	4	368.9446	1471.749	8.6752	0.021364	78.653	4831404

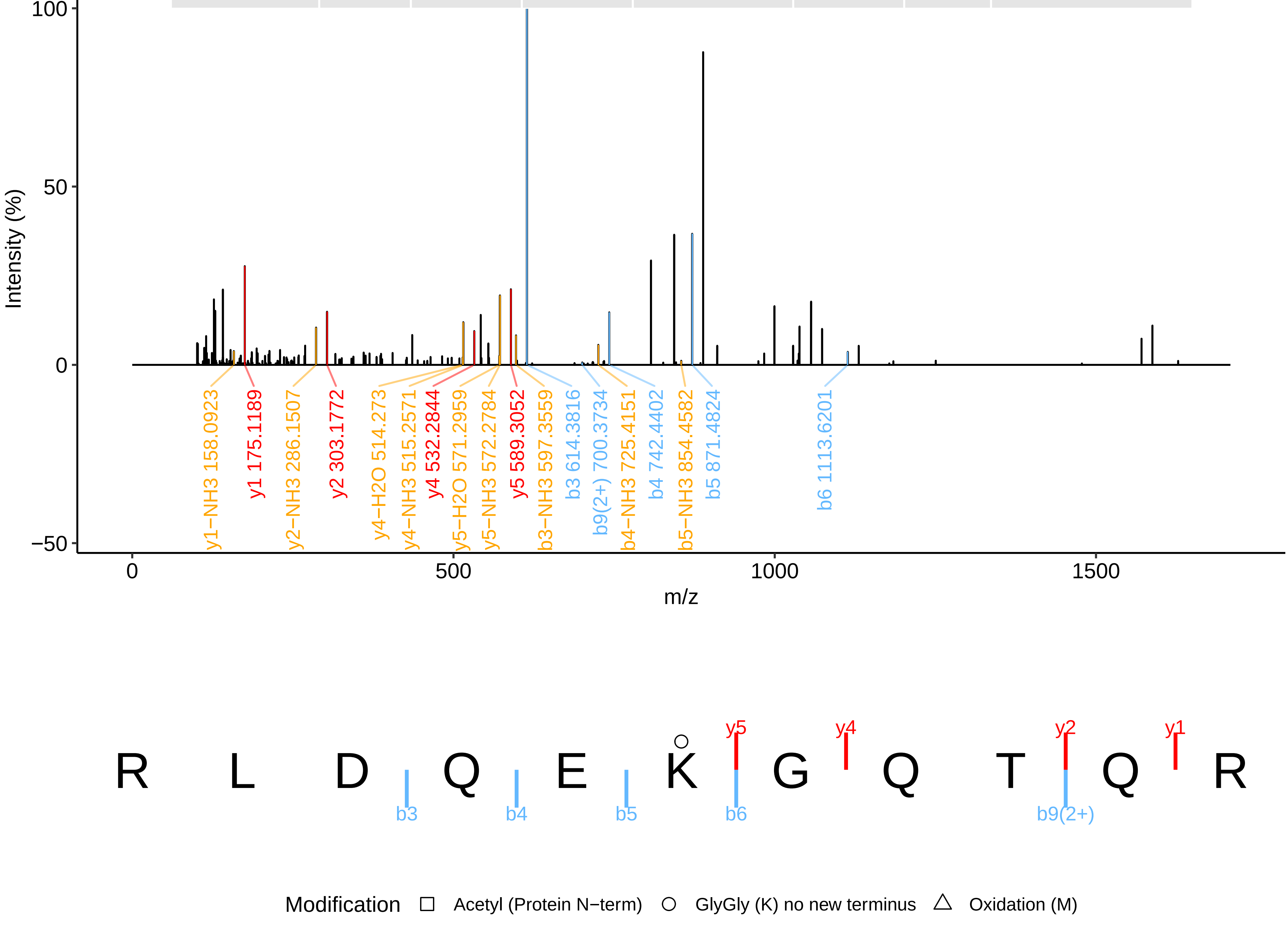

20200924_KIZ_MUb5_GlyGly_1 − Scan number: 2615

Gene Names	Charge	m/z	Mass	Retention time	PEP	Score	Precursor Intensity
RHOT2	3	491.5903	1471.749	8.1021	2.6464e−16	151.55	8878770

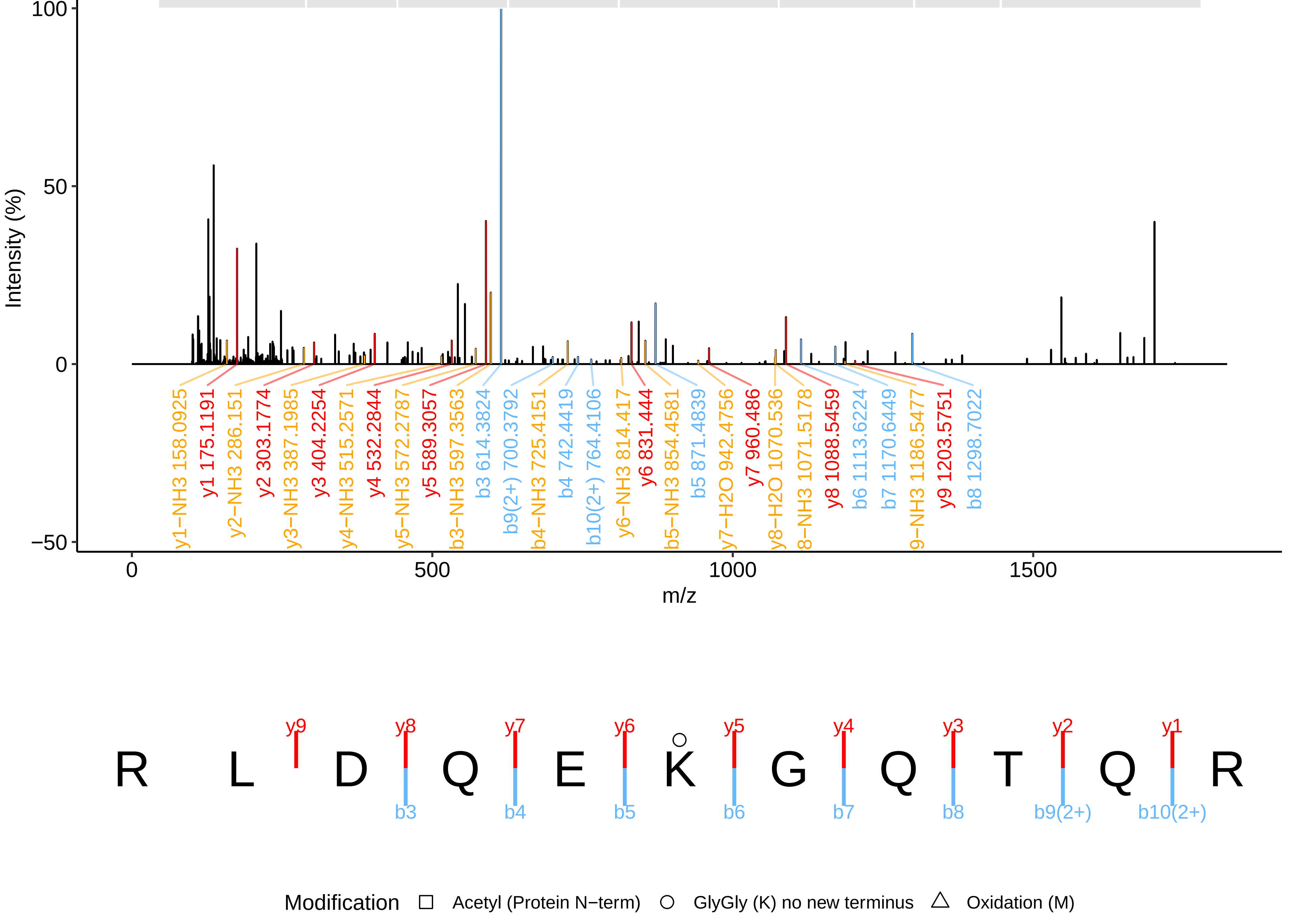

20200924_KIZ_MUb5_GlyGly_1 − Scan number: 2617

Gene Names	Charge	m/z	Mass	Retention time	PEP	Score	Precursor Intensity
RHOT2	4	368.9446	1471.749	8.108	0.0095903	83.045	29747490

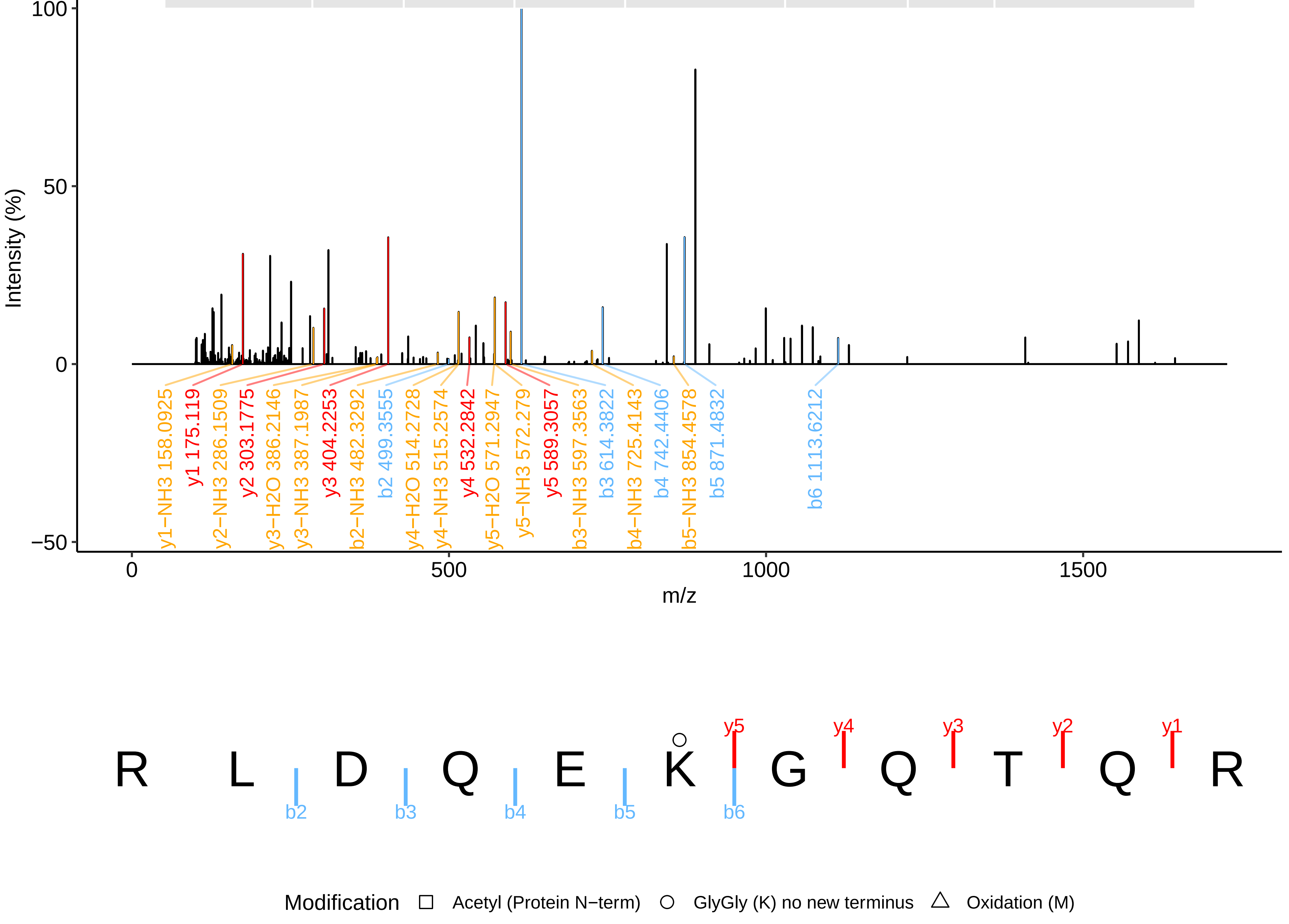

20201118_KIZ_MUb6_GlyGly_2 − Scan number: 3219

Gene Names	Charge	m/z	Mass	Retention time	PEP	Score	Precursor Intensity
RNF19A	3	492.9039	1475.69	10.475	0.023197	60.196	1611928

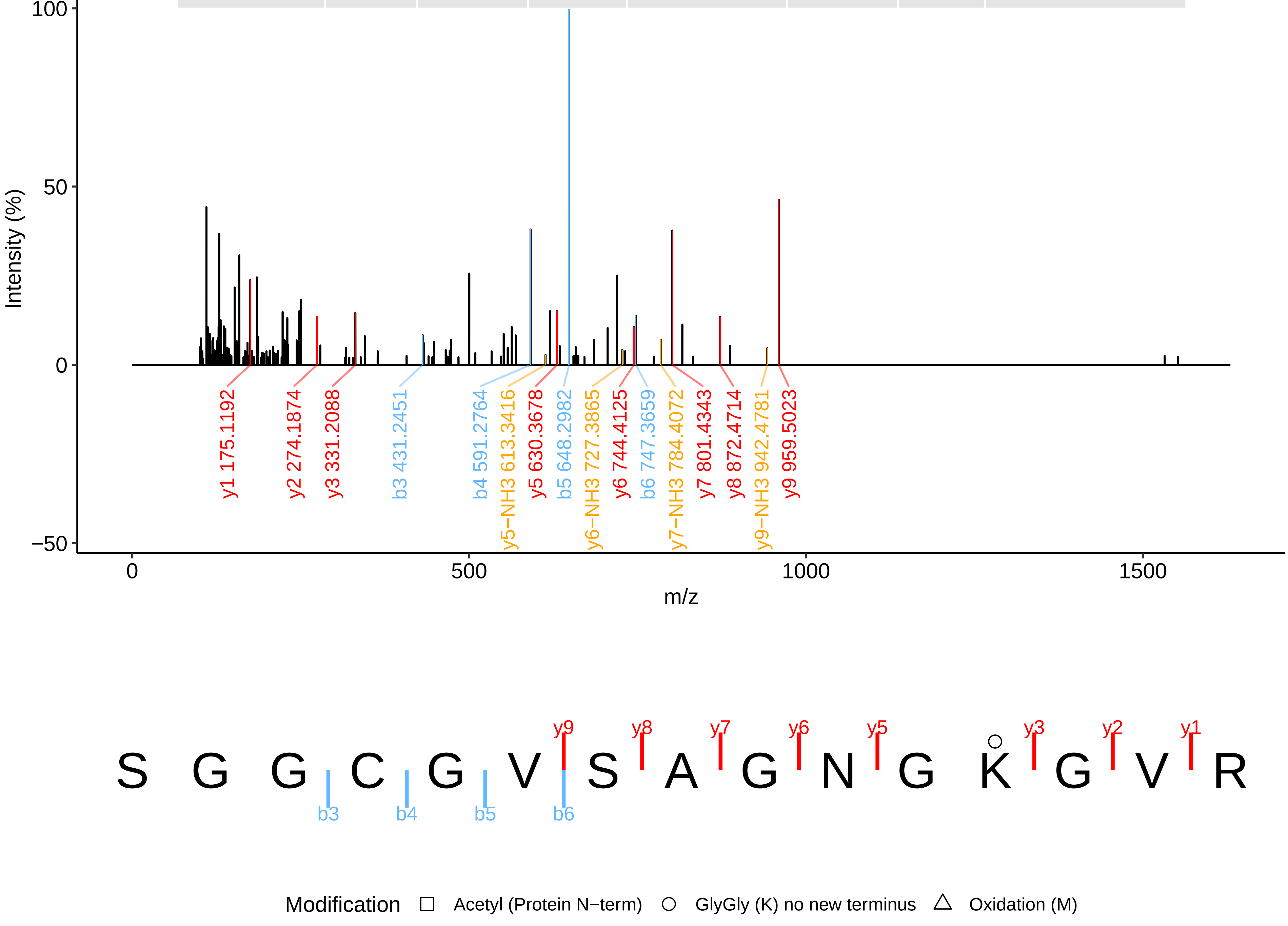

20201118_KIZ_MUb6_GlyGly_2 − Scan number: 34723

Gene Names	Charge	m/z	Mass	Retention time	PEP	Score	Precursor Intensity
TTF1	2	610.3377	1218.661	112.81	0.076088	63.486	16309456

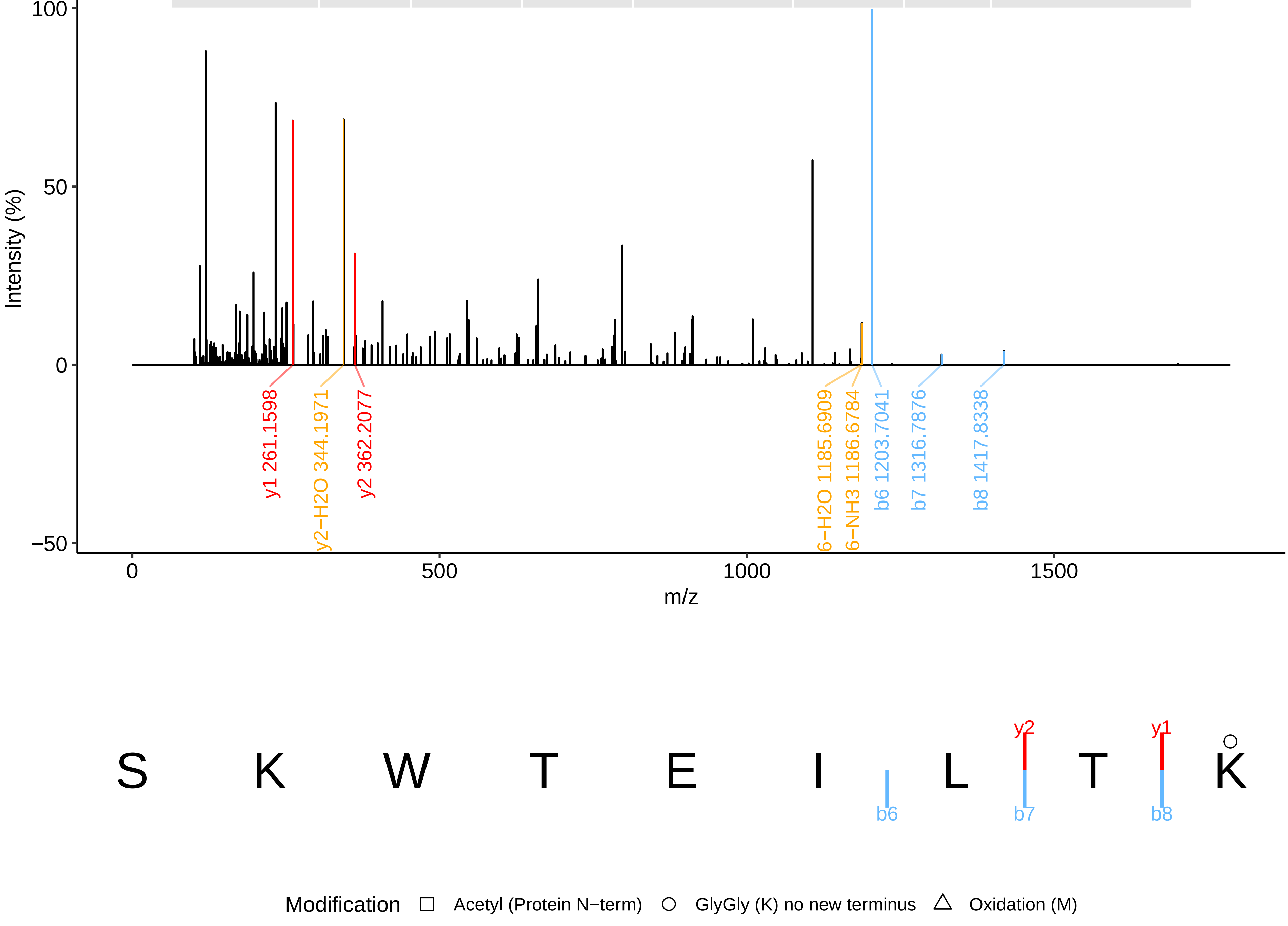

20200921_KIZ_MUb4_GlyGly_10plex − Scan number: 8939

Gene Names	Charge	m/z	Mass	Retention time	PEP	Score	Precursor Intensity
PKP4;CTNND2	3	486.2512	1455.732	30.496	2.163e−06	113.61	5099616

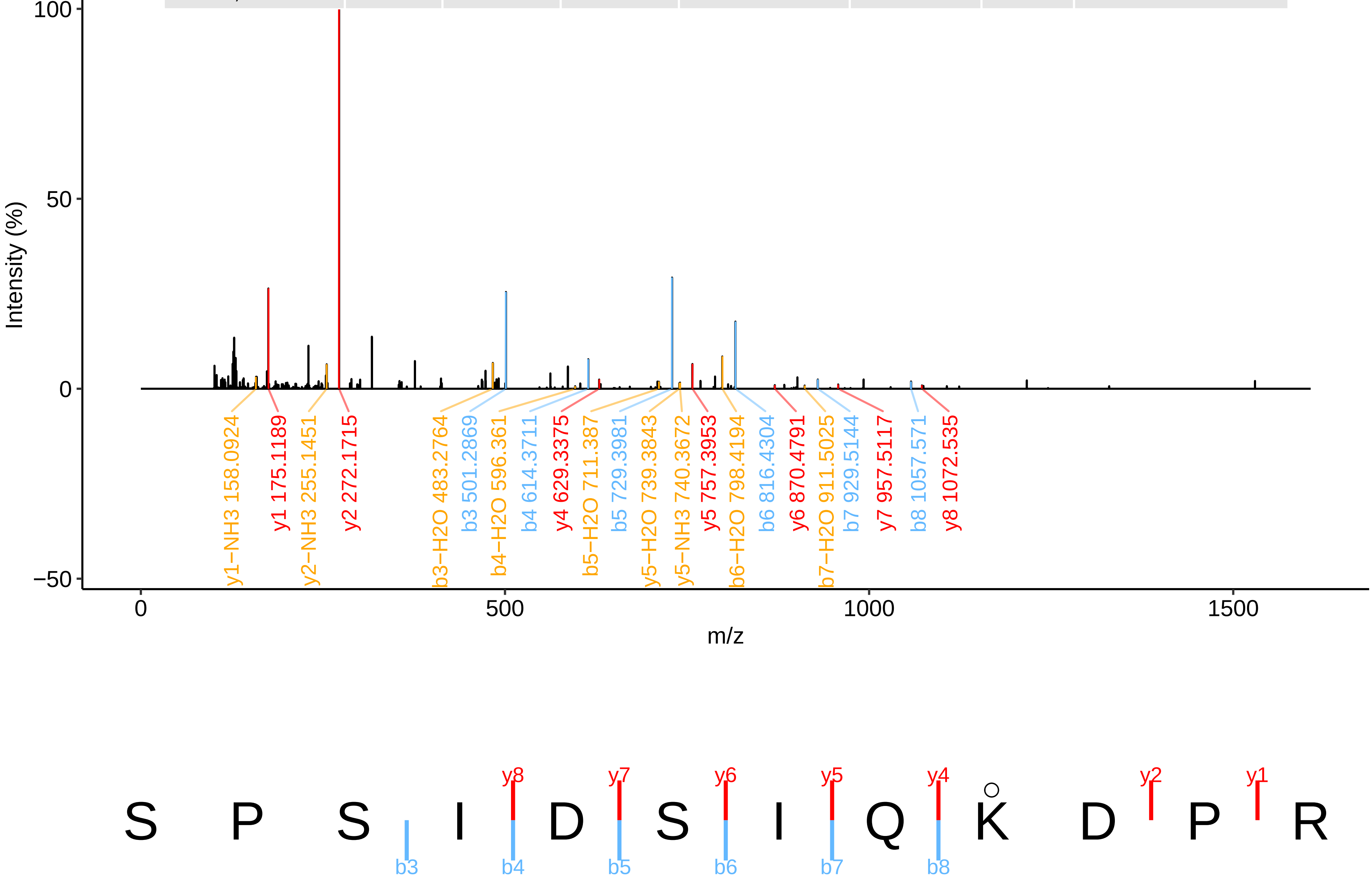

Modification □ Acetyl (Protein N−term) ○ GlyGly (K) no new terminus △ Oxidation (M)

20200924_KIZ_MUb5_GlyGly_2 − Scan number: 7532

Gene Names	Charge	m/z	Mass	Retention time	PEP	Score	Precursor Intensity
PKP4;CTNND2	3	486.2512	1455.732	28.121	0.001576	81.709	NA

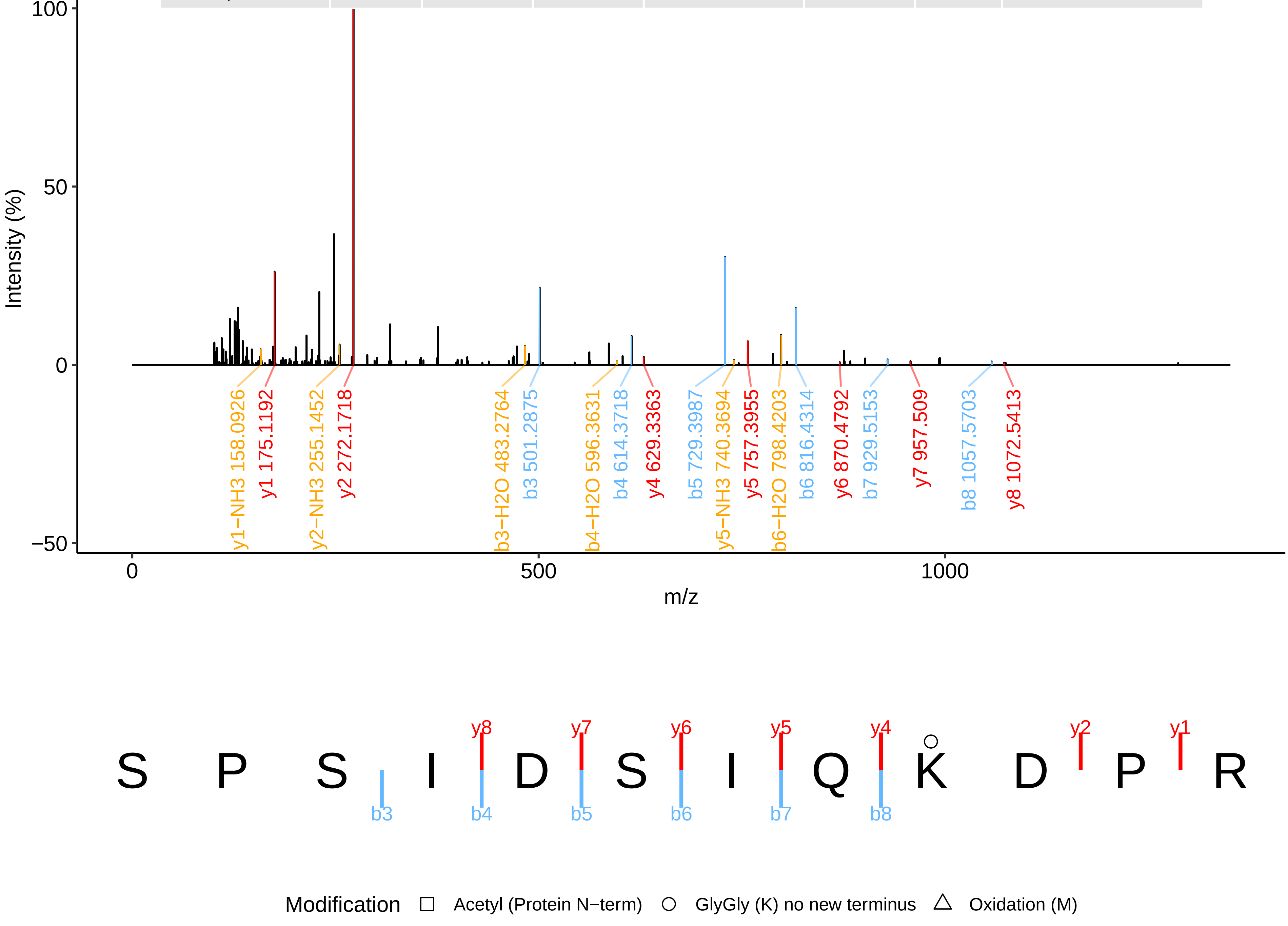

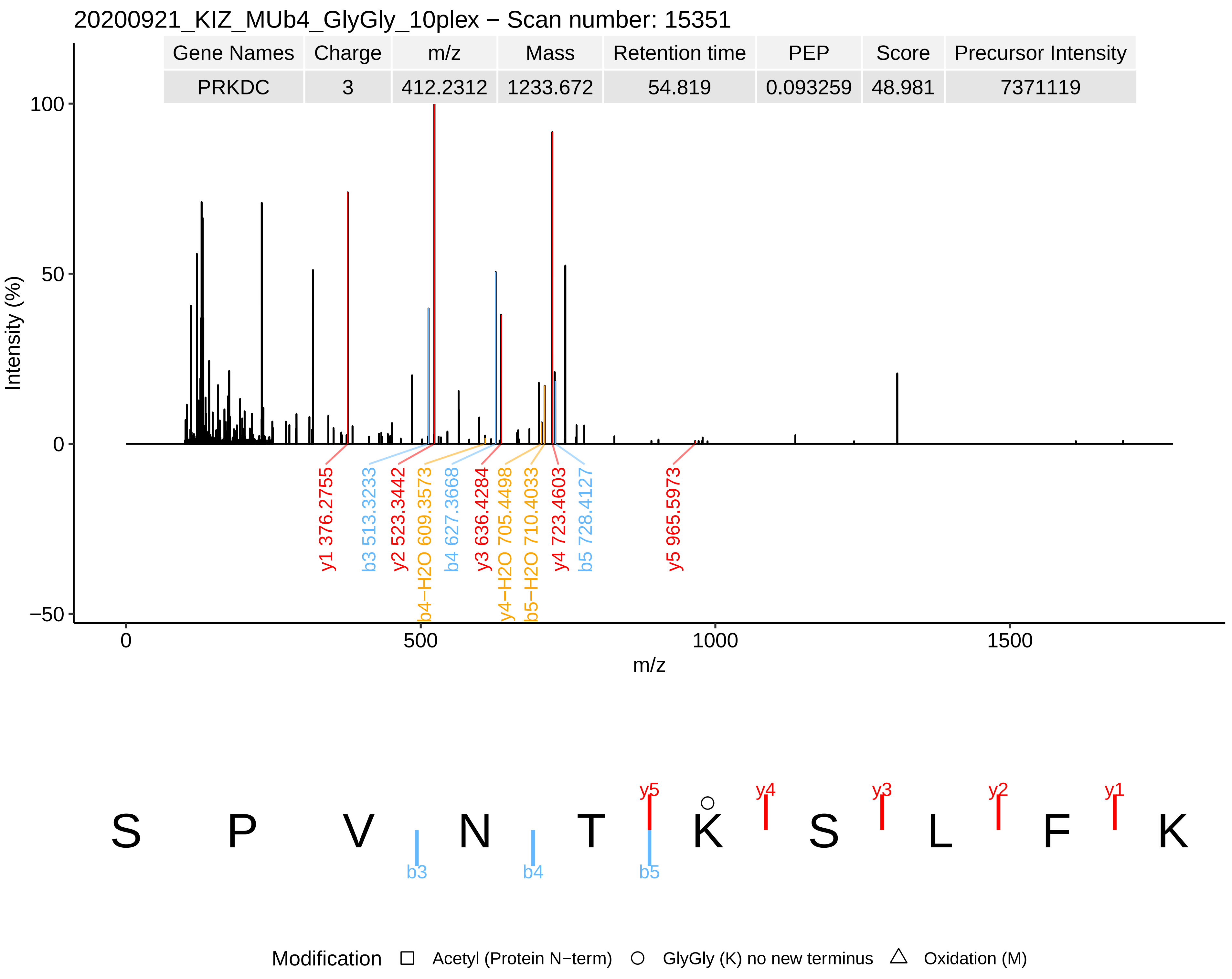

20200921_KIZ_MUb4_GlyGly_10plex − Scan number: 15351
Gene Names | Charge | m/z | Mass | Retention time | PEP | Score | Precursor Intensity
PRKDC | 3 | 412.2312 | 1233.672 | 54.819 | 0.093259 | 48.981 | 7371119
Intensity (%)
100
50
0
−50
0
500
1000
1500
m/z
y1 376.2755
b3 513.3233
y2 523.3442
b4−H2O 609.3573
b4 627.3668
y3 636.4284
y4−H2O 705.4498
b5−H2O 710.4033
y4 723.4603
b5 728.4127
y5 965.5973
S P V N T K S L F K
y5 y4 y3 y2 y1
b3 b4 b5
Modification
Acetyl (Protein N−term)
GlyGly (K) no new terminus
Oxidation (M)

20200921_KIZ_MUb4_GlyGly_10plex_2 − Scan number: 20802

Gene Names	Charge	m/z	Mass	Retention time	PEP	Score	Precursor Intensity
NEXN	3	477.5906	1429.75	81.823	0.076551	46.891	936937.6

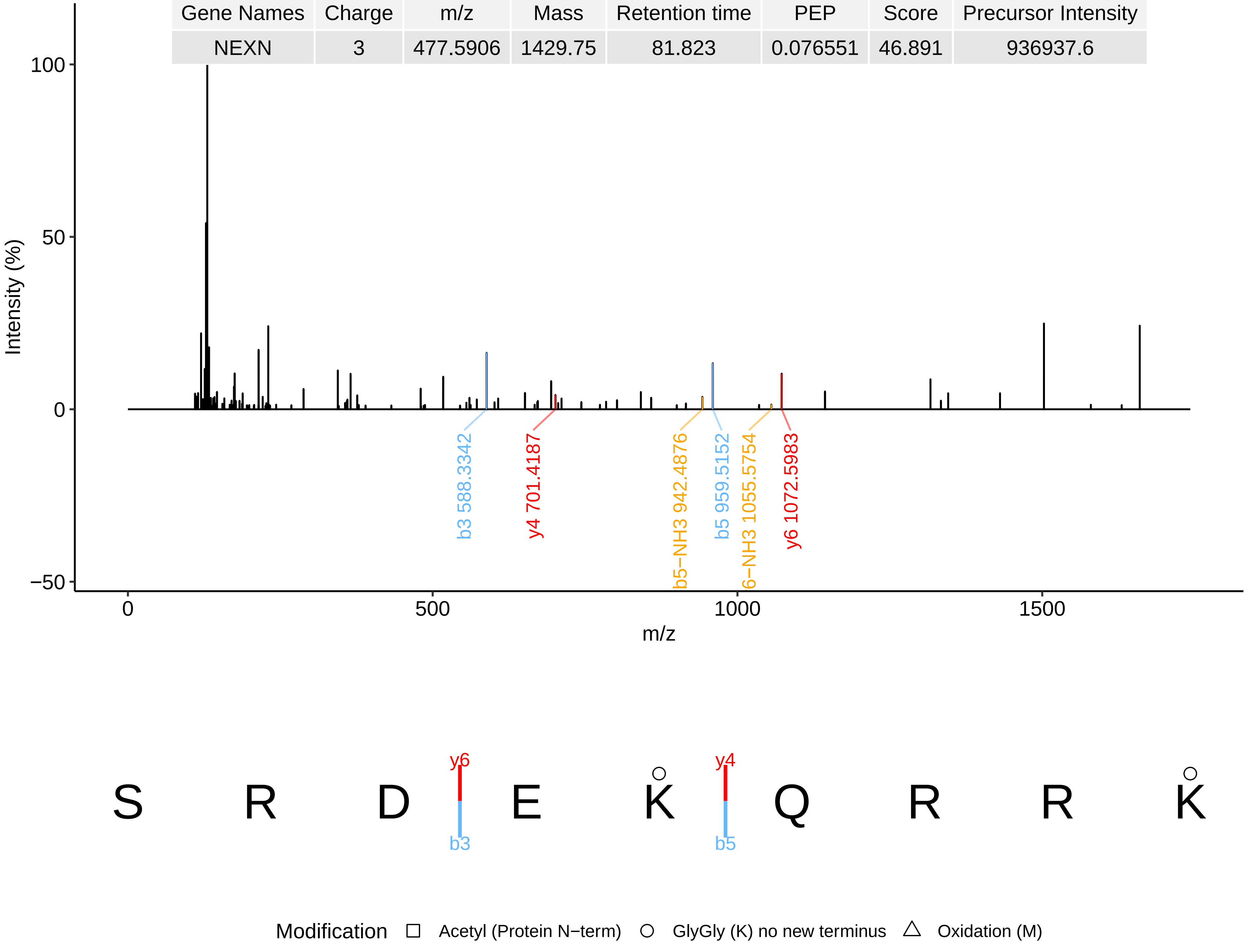

20200924_KIZ_MUb5_GlyGly_1 − Scan number: 22448

Gene Names	Charge	m/z	Mass	Retention time	PEP	Score	Precursor Intensity
NEXN	3	477.5906	1429.75	72.241	0.074846	46.891	NA

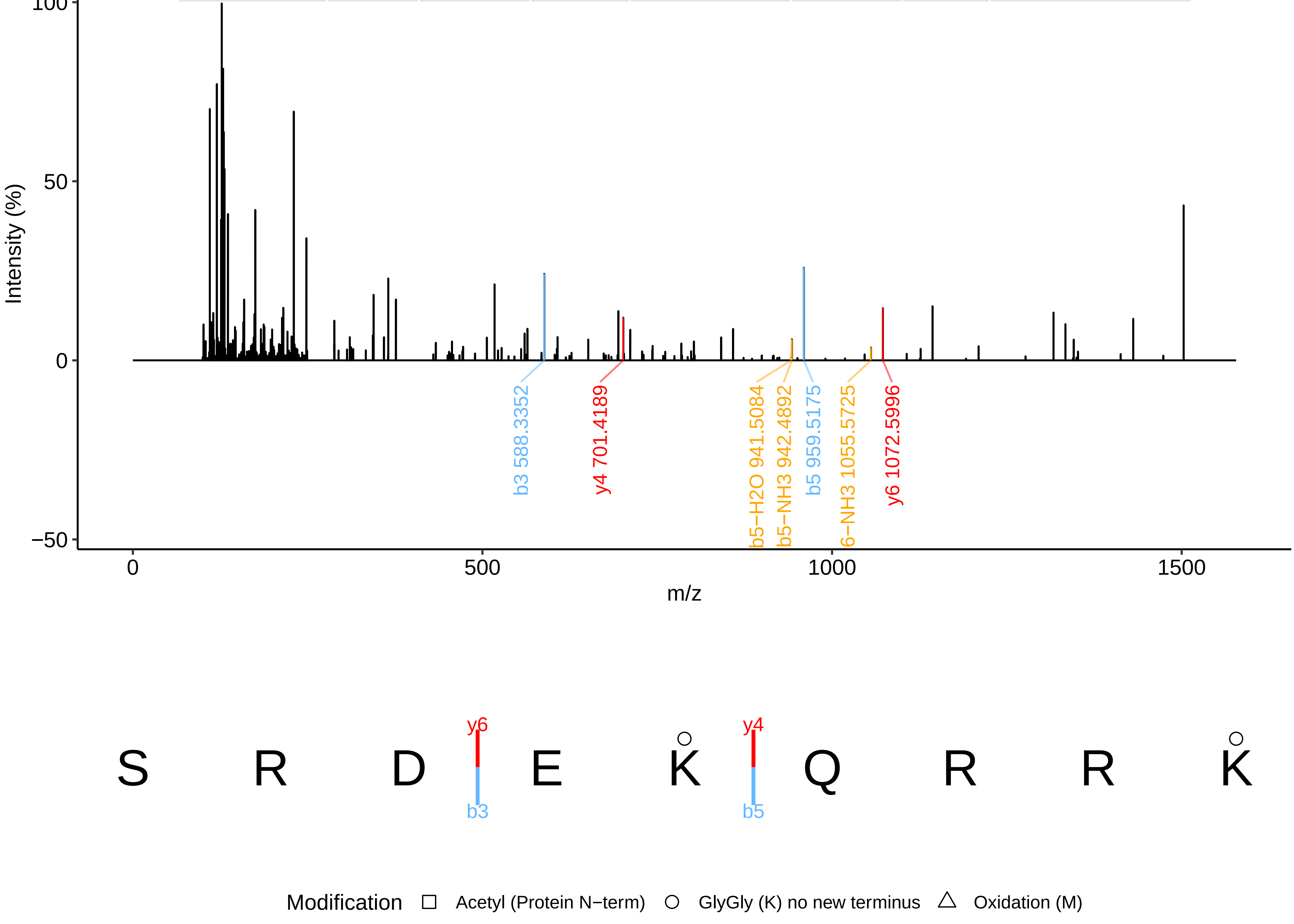

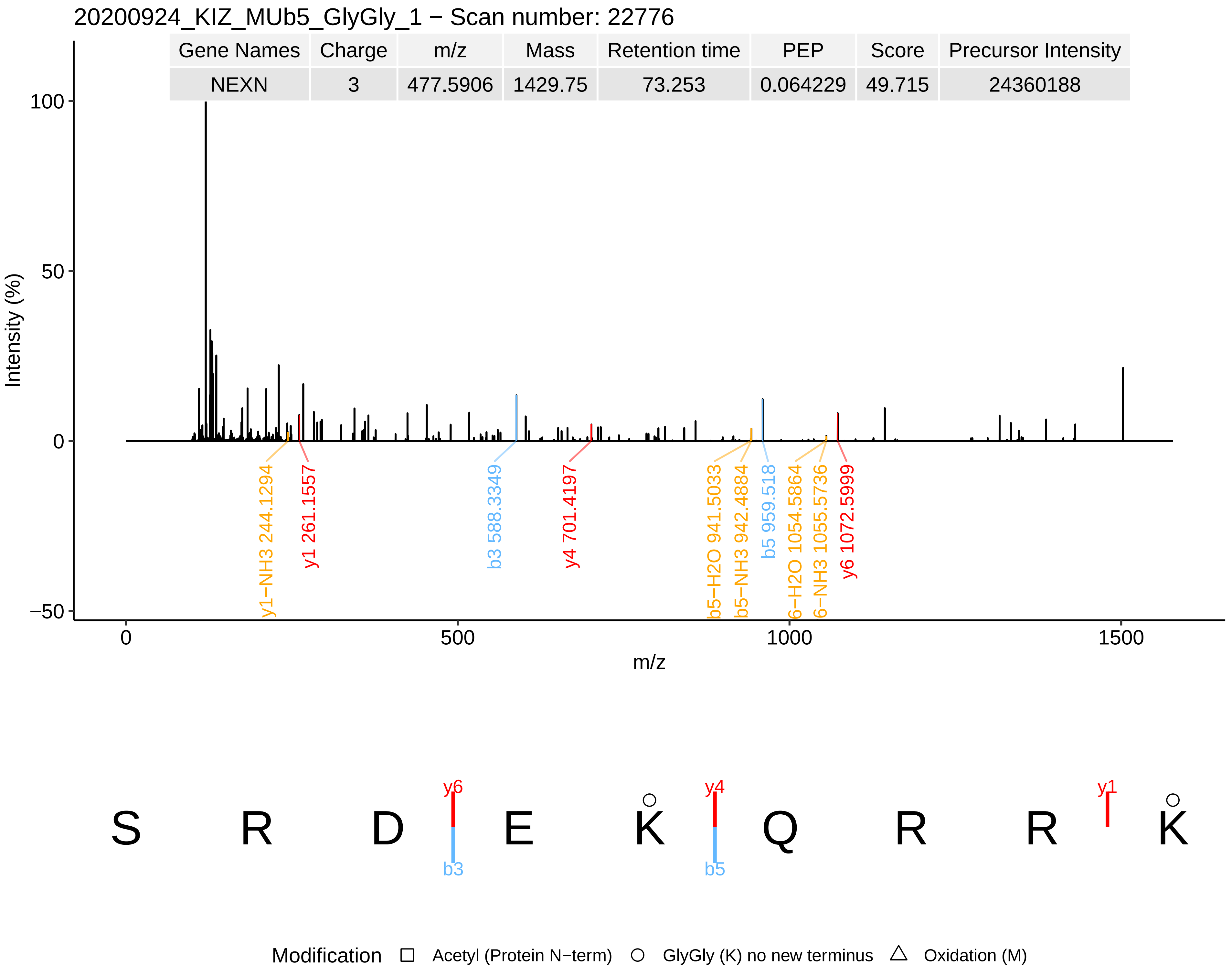

20200924_KIZ_MUb5_GlyGly_1 − Scan number: 22776
Gene Names | Charge | m/z | Mass | Retention time | PEP | Score | Precursor Intensity
NEXN | 3 | 477.5906 | 1429.75 | 73.253 | 0.064229 | 49.715 | 24360188
Intensity (%)
100
50
0
−50
0
500
1000
1500
m/z
y1−NH3 244.1294
y1 261.1557
b3 588.3349
y4 701.4197
b5−H2O 941.5033
b5−NH3 942.4884
b5 959.518
6−H2O 1054.5864
6−NH3 1055.5736
y6 1072.5999
S R D E K Q R R K
y6
y4
y1
b3
b5
Modification
Acetyl (Protein N−term)
GlyGly (K) no new terminus
Oxidation (M)

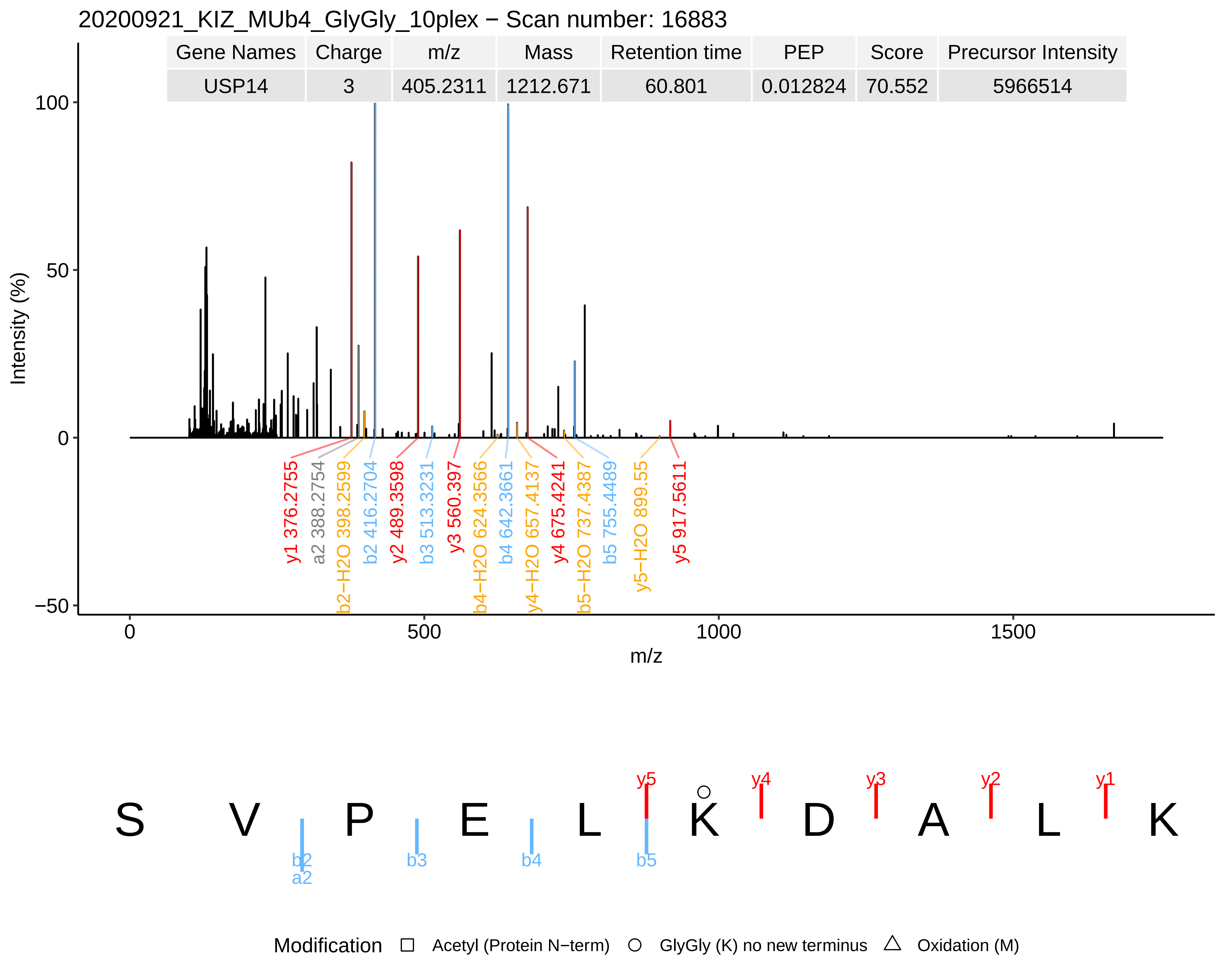
20200921_KIZ_MUb4_GlyGly_10plex − Scan number: 16883
Gene Names | Charge | m/z | Mass | Retention time | PEP | Score | Precursor Intensity
USP14 | 3 | 405.2311 | 1212.671 | 60.801 | 0.012824 | 70.552 | 5966514
Intensity (%)
m/z
y1 376.2755
a2 388.2754
b2−H2O 398.2599
b2 416.2704
y2 489.3598
b3 513.3231
y3 560.397
b4−H2O 624.3566
b4 642.3661
y4−H2O 657.4137
y4 675.4241
b5−H2O 737.4387
b5 755.4489
y5−H2O 899.55
y5 917.5611
S V P E L K D A L K
Modification
Acetyl (Protein N−term)
GlyGly (K) no new terminus
Oxidation (M)

20200921_KIZ_MUb4_GlyGly_10plex_2 − Scan number: 15709

Gene Names	Charge	m/z	Mass	Retention time	PEP	Score	Precursor Intensity
USP14	3	405.2311	1212.671	61.488	0.089344	53.751	494865.8

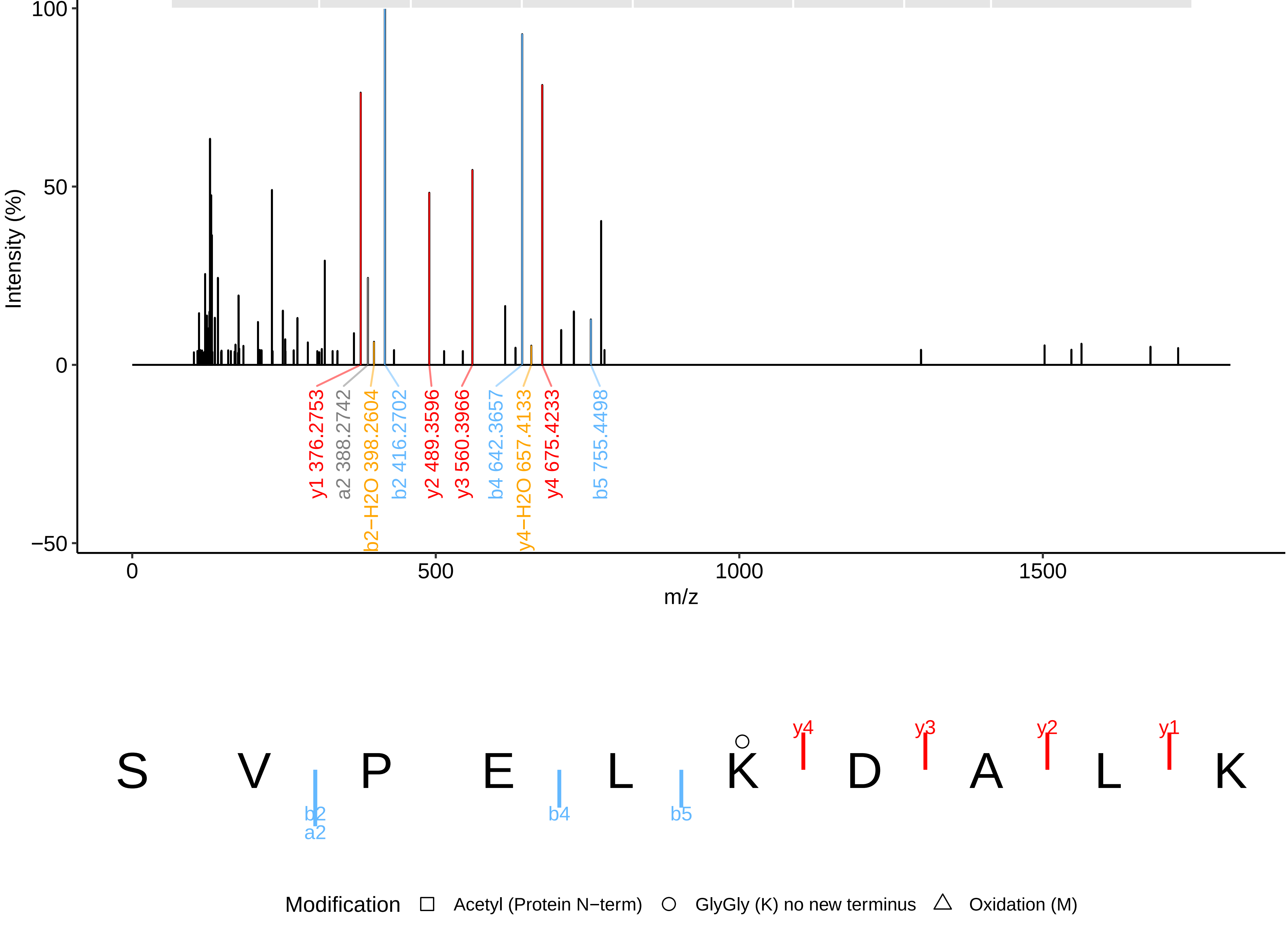

20200921_KIZ_MUb4_GlyGly_10plex − Scan number: 8651

Gene Names	Charge	m/z	Mass	Retention time	PEP	Score	Precursor Intensity
ENO1	3	413.5507	1237.63	29.427	0.041876	58.17	4883860

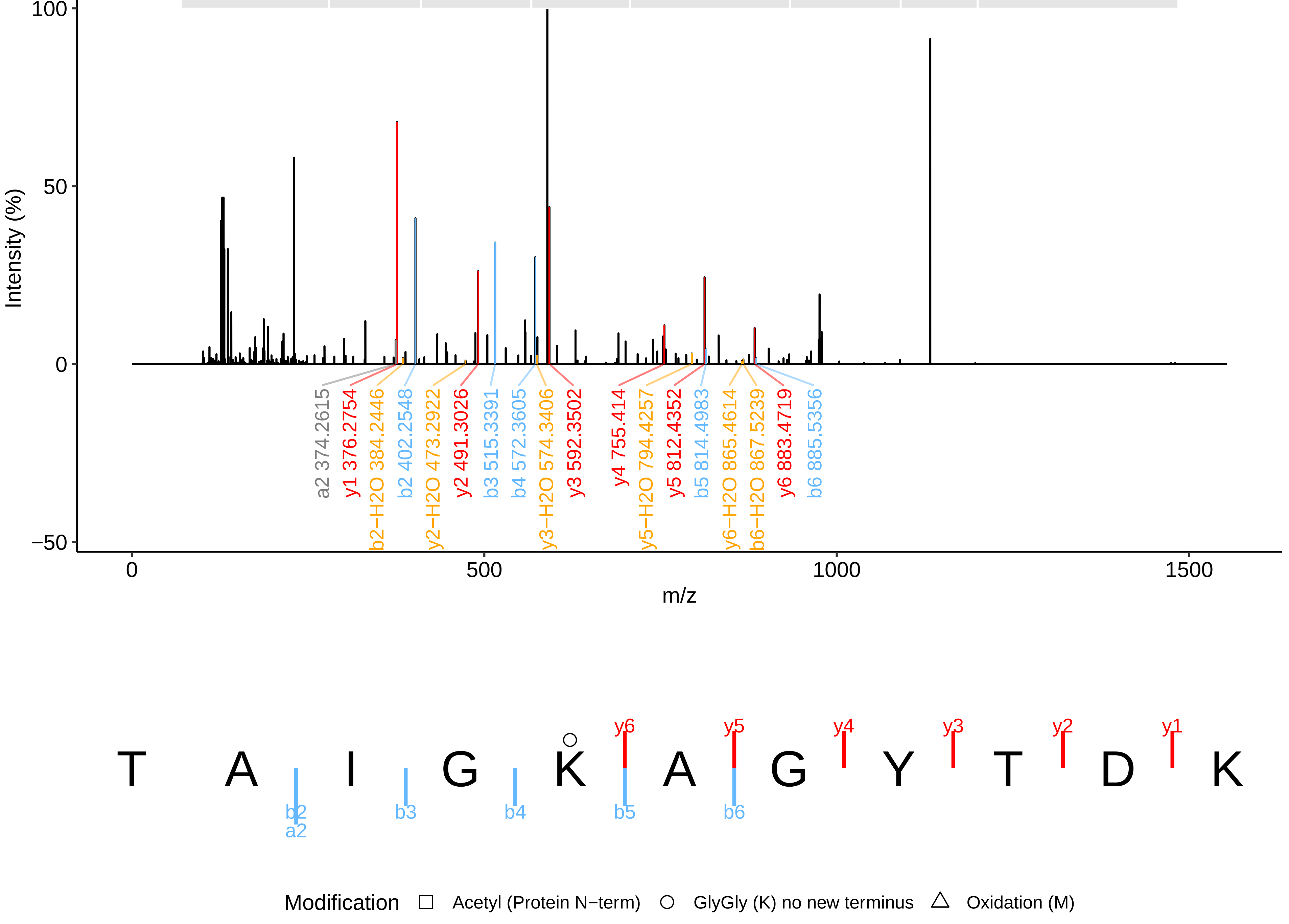

20200921_KIZ_MUb4_GlyGly_10plex_2 − Scan number: 16569

Gene Names	Charge	m/z	Mass	Retention time	PEP	Score	Precursor Intensity
CPS1	3	490.5843	1468.731	64.693	3.8831e−06	113.93	1389346

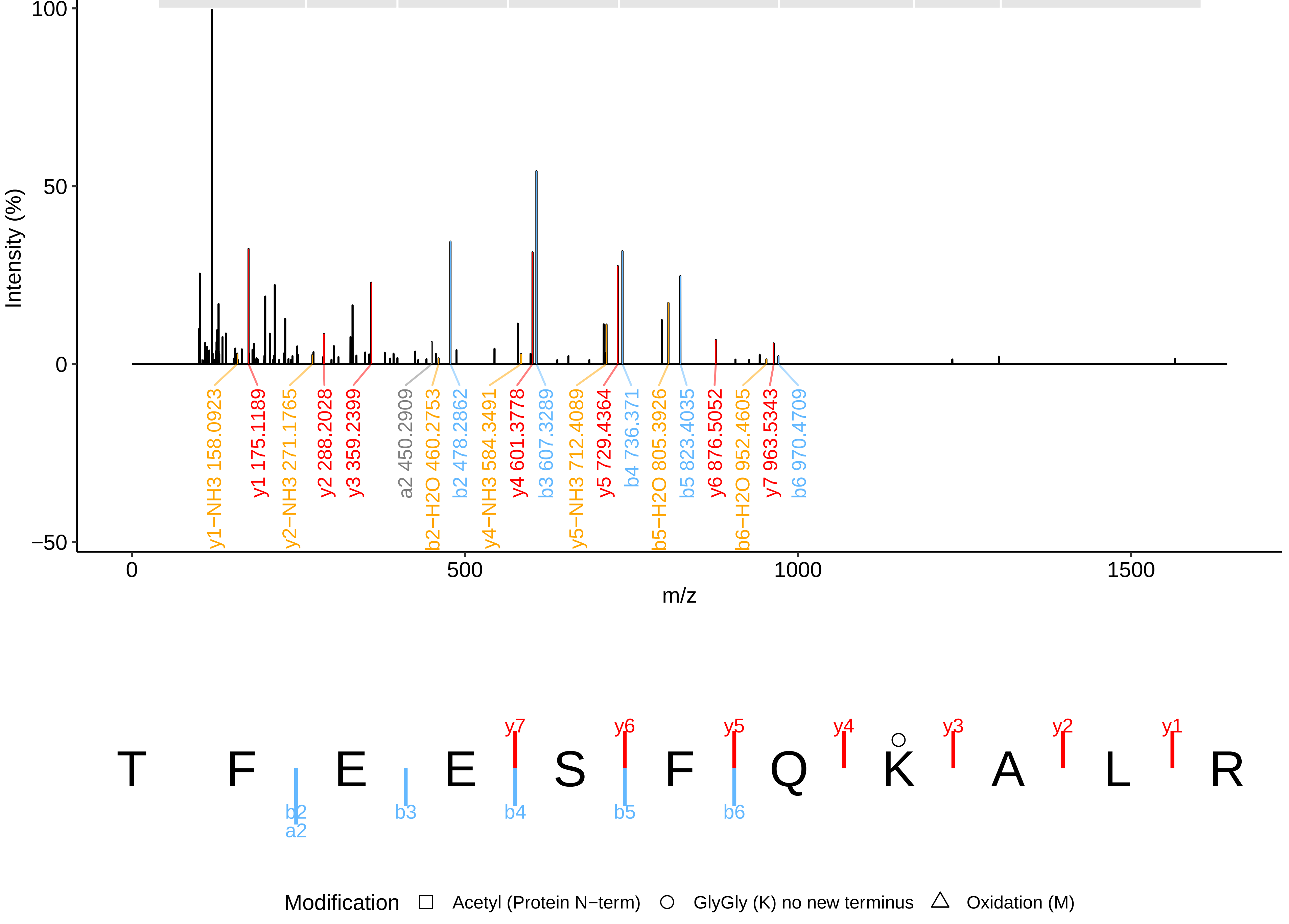

20200921_KIZ_MUb4_GlyGly_10plex − Scan number: 15256

Gene Names	Charge	m/z	Mass	Retention time	PEP	Score	Precursor Intensity
NME1−NME2;NME2;NME1	3	486.9403	1457.799	54.461	4.6475e−06	111.52	25425760

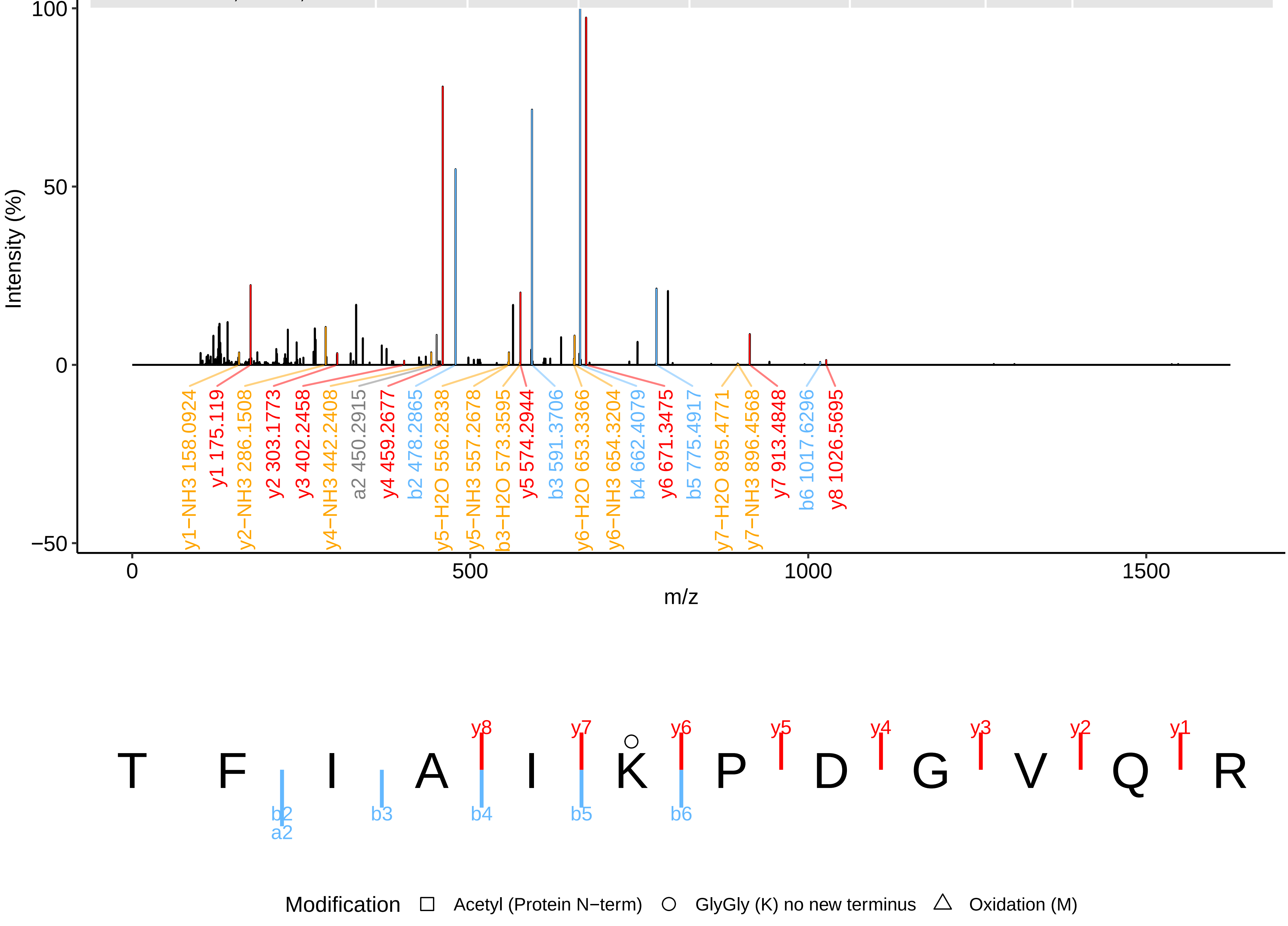

20200921_KIZ_MUb4_GlyGly_10plex_2 − Scan number: 14078

Gene Names	Charge	m/z	Mass	Retention time	PEP	Score	Precursor Intensity
NME1−NME2;NME2;NME1	3	486.9403	1457.799	54.811	0.0044059	75.819	839724.5

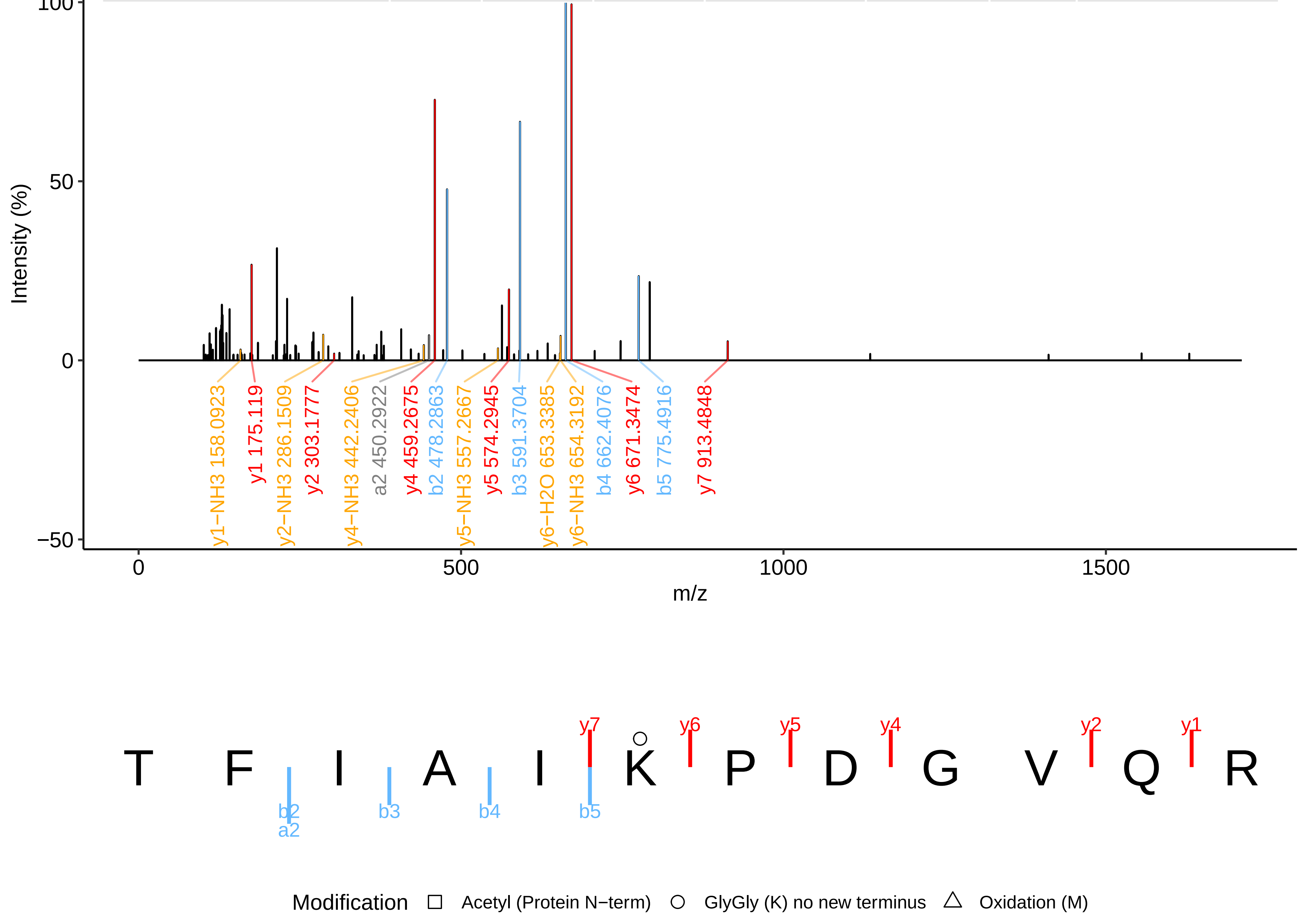

20200924_KIZ_MUb5_GlyGly_1 − Scan number: 14268

Gene Names	Charge	m/z	Mass	Retention time	PEP	Score	Precursor Intensity
NME1−NME2;NME2;NME1	3	486.9403	1457.799	46.577	0.016213	62.528	25460808

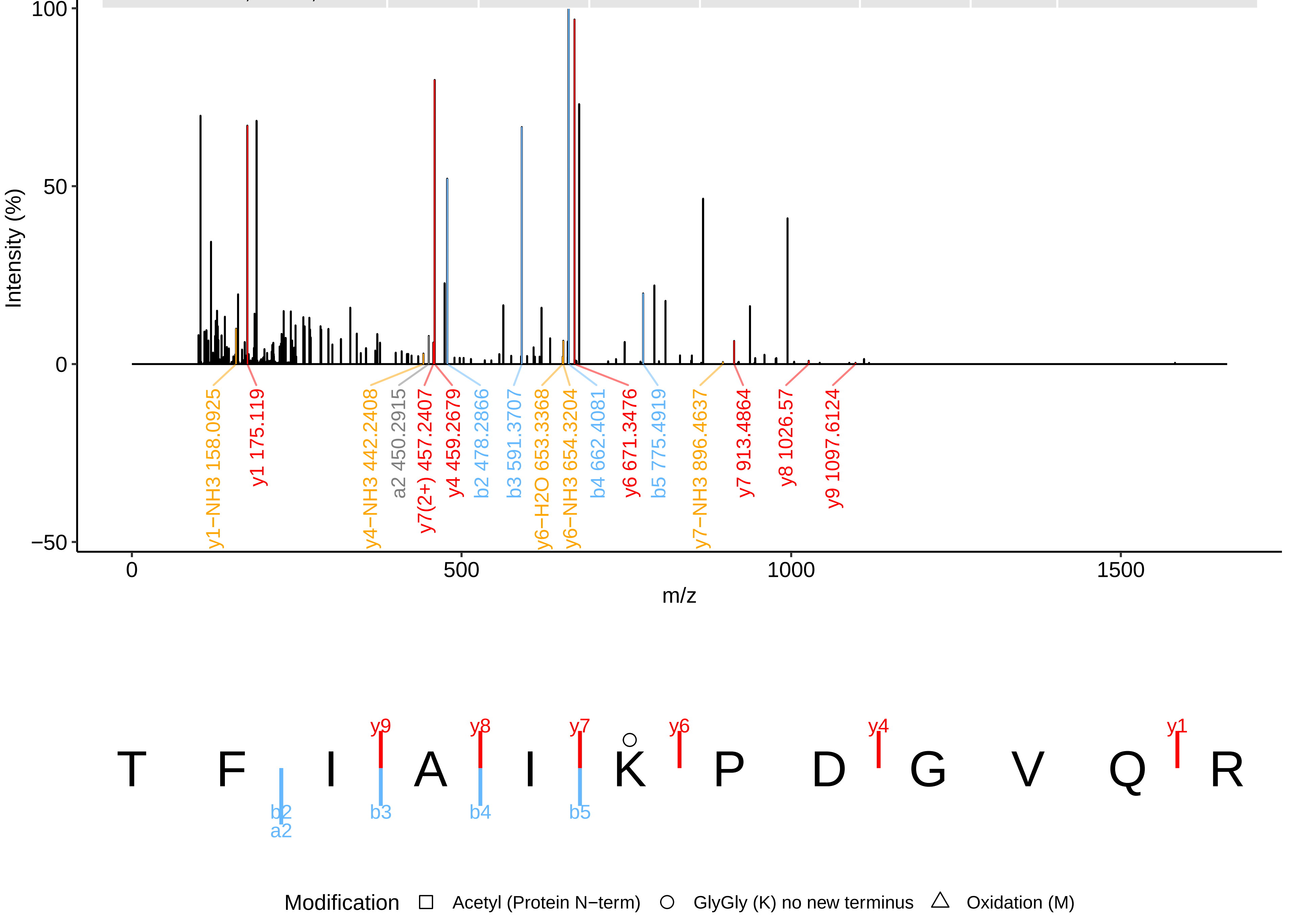

20200924_KIZ_MUb5_GlyGly_1 − Scan number: 14419

Gene Names	Charge	m/z	Mass	Retention time	PEP	Score	Precursor Intensity
NME1−NME2;NME2;NME1	3	486.9403	1457.799	47.085	0.0011895	81.002	35467320

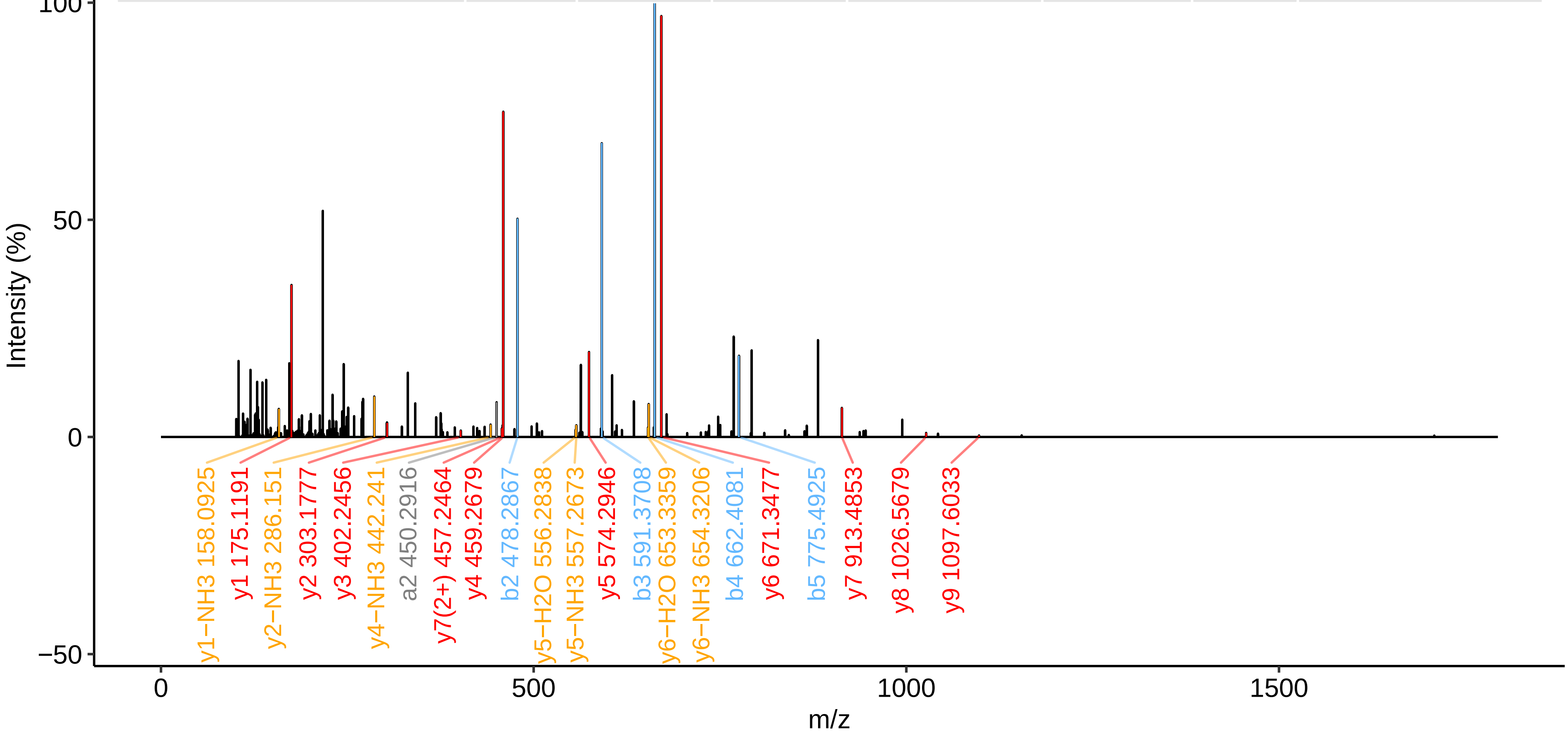

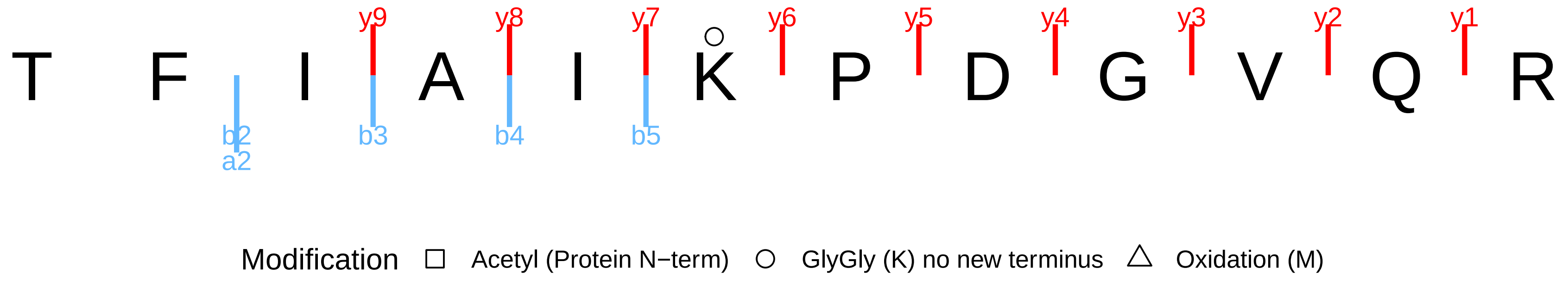

20200924_KIZ_MUb5_GlyGly_2 − Scan number: 13557

Gene Names	Charge	m/z	Mass	Retention time	PEP	Score	Precursor Intensity
NME1−NME2;NME2;NME1	3	486.9403	1457.799	50.7	0.043774	57.225	5286814

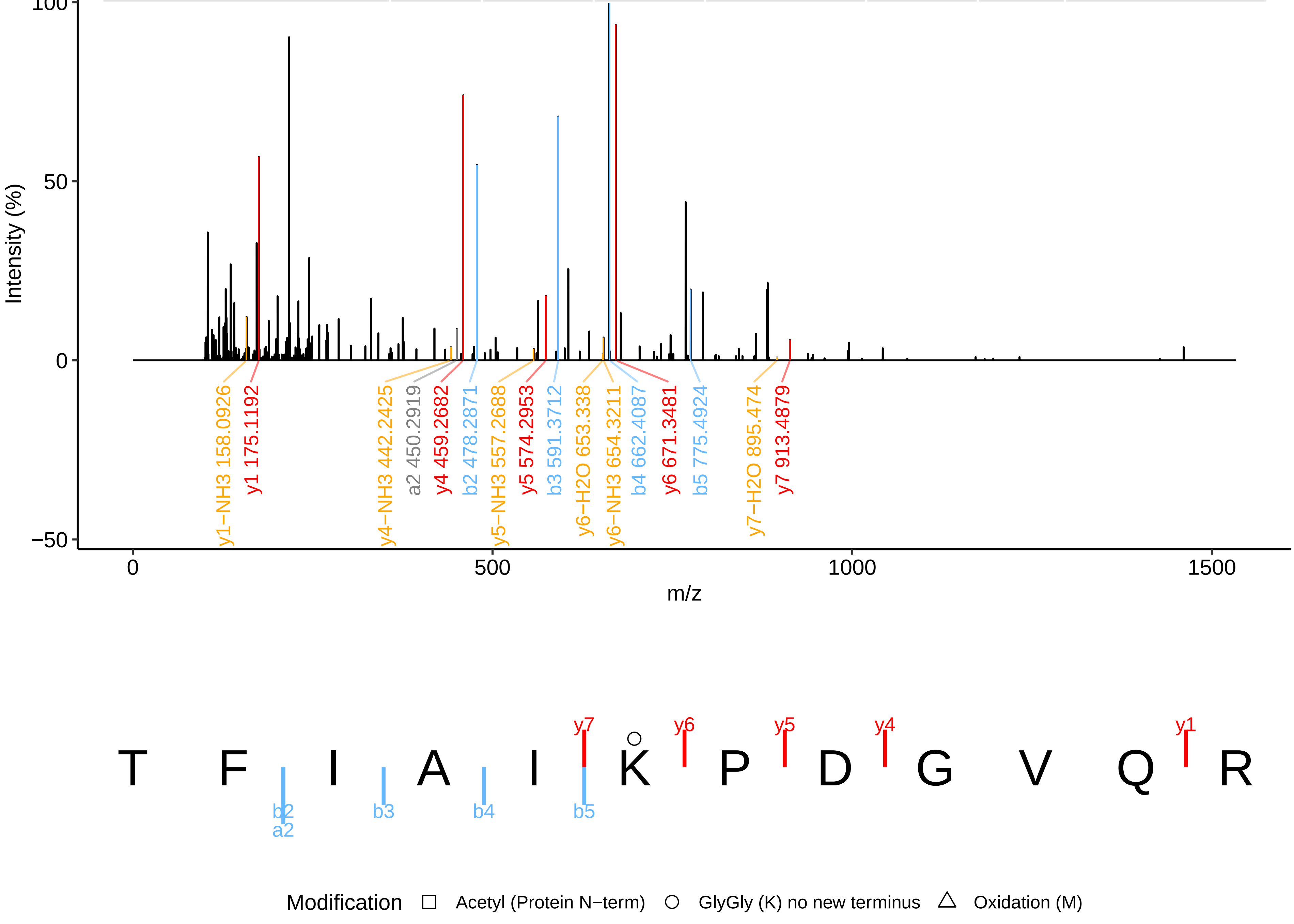

20200924_KIZ_MUb5_GlyGly_2 − Scan number: 13295

Gene Names	Charge	m/z	Mass	Retention time	PEP	Score	Precursor Intensity
NME1−NME2;NME2;NME1	3	486.9403	1457.799	49.746	0.0031664	78.692	1932832

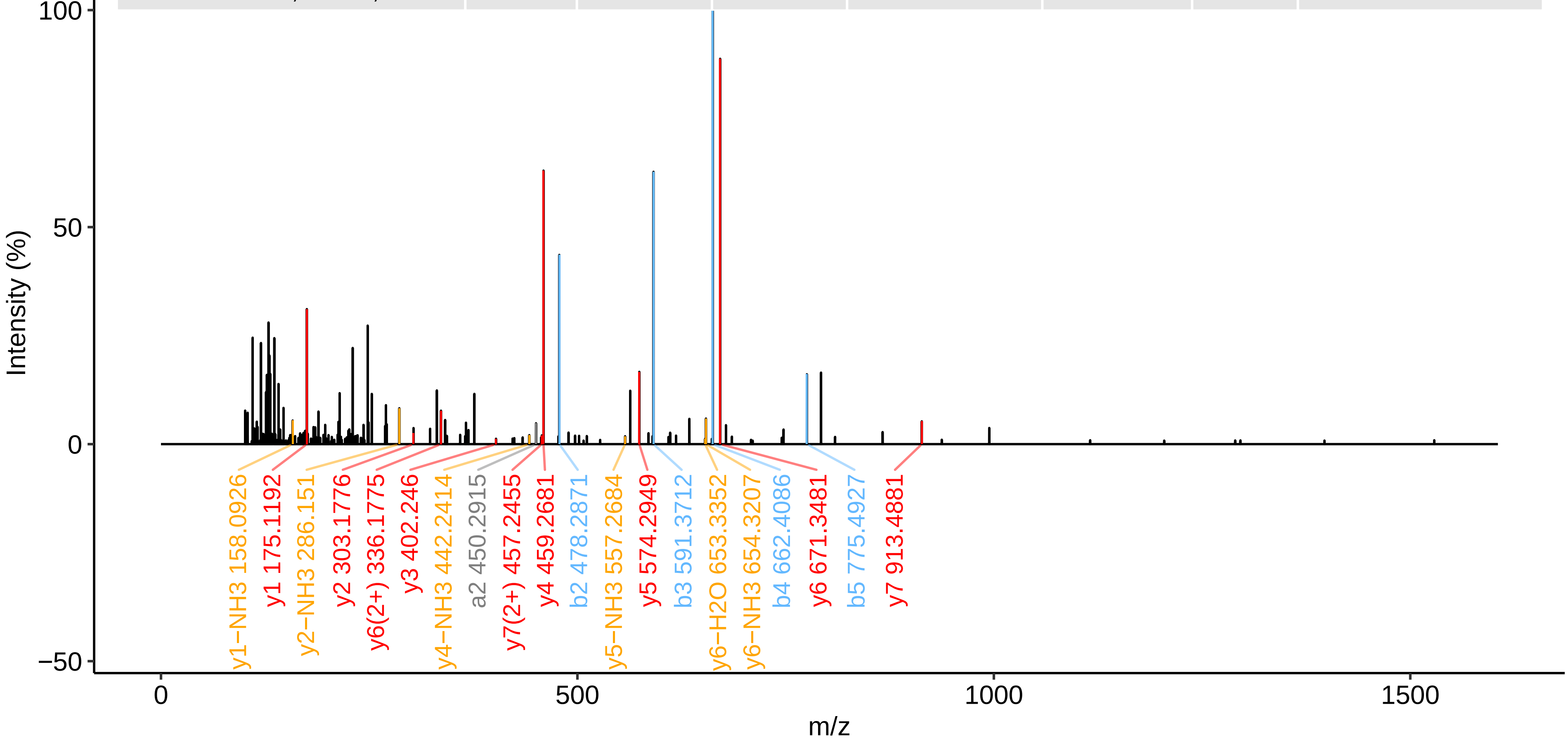

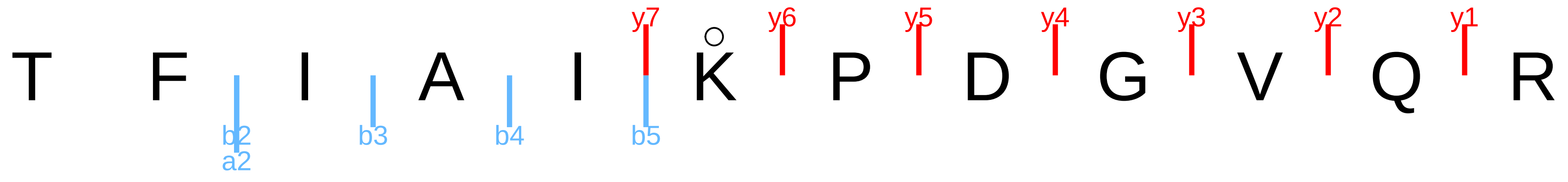

Modification □ Acetyl (Protein N−term) ○ GlyGly (K) no new terminus △ Oxidation (M)

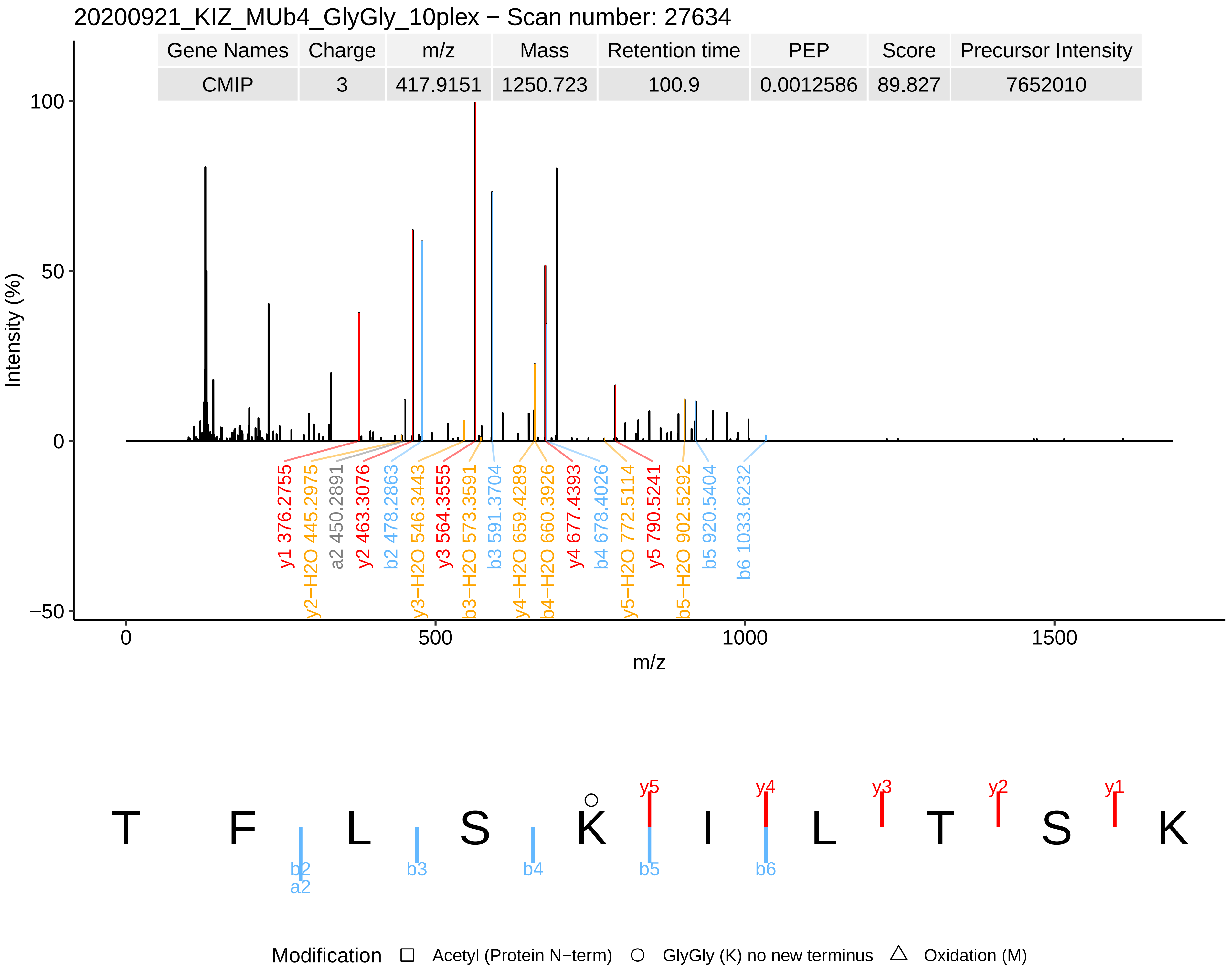
20200921_KIZ_MUb4_GlyGly_10plex − Scan number: 27634
Gene Names | Charge | m/z | Mass | Retention time | PEP | Score | Precursor Intensity
CMIP | 3 | 417.9151 | 1250.723 | 100.9 | 0.0012586 | 89.827 | 7652010
Intensity (%)
100
50
0
−50
0
500
1000
1500
m/z
y1 376.2755
y2−H2O 445.2975
a2 450.2891
y2 463.3076
b2 478.2863
y3−H2O 546.3443
y3 564.3555
b3−H2O 573.3591
b3 591.3704
y4−H2O 659.4289
b4−H2O 660.3926
y4 677.4393
b4 678.4026
y5−H2O 772.5114
y5 790.5241
b5−H2O 902.5292
b5 920.5404
b6 1033.6232
T F L S K I L T S K
y5 y4 y3 y2 y1
b2 a2 b3 b4 b5 b6
Modification
Acetyl (Protein N−term)
GlyGly (K) no new terminus
Oxidation (M)

20200921_KIZ_MUb4_GlyGly_10plex_2 − Scan number: 23460

Gene Names	Charge	m/z	Mass	Retention time	PEP	Score	Precursor Intensity
CMIP	3	417.9151	1250.723	92.594	0.0058645	77.379	942998.9

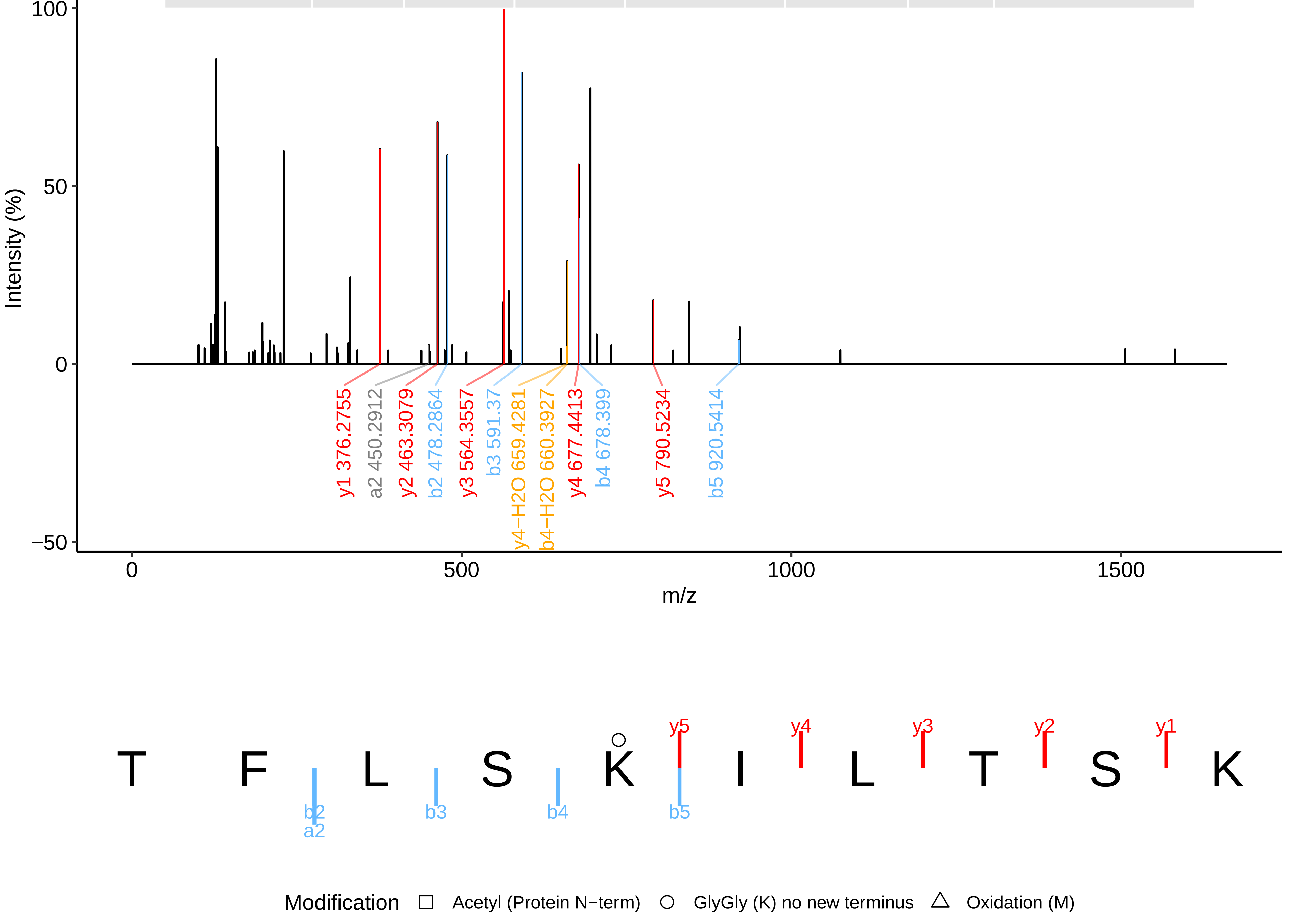

20200921_KIZ_MUb4_GlyGly_10plex − Scan number: 7548

Gene Names	Charge	m/z	Mass	Retention time	PEP	Score	Precursor Intensity
KRAS	3	487.2548	1458.743	25.366	1.1575e−08	118.77	4299802

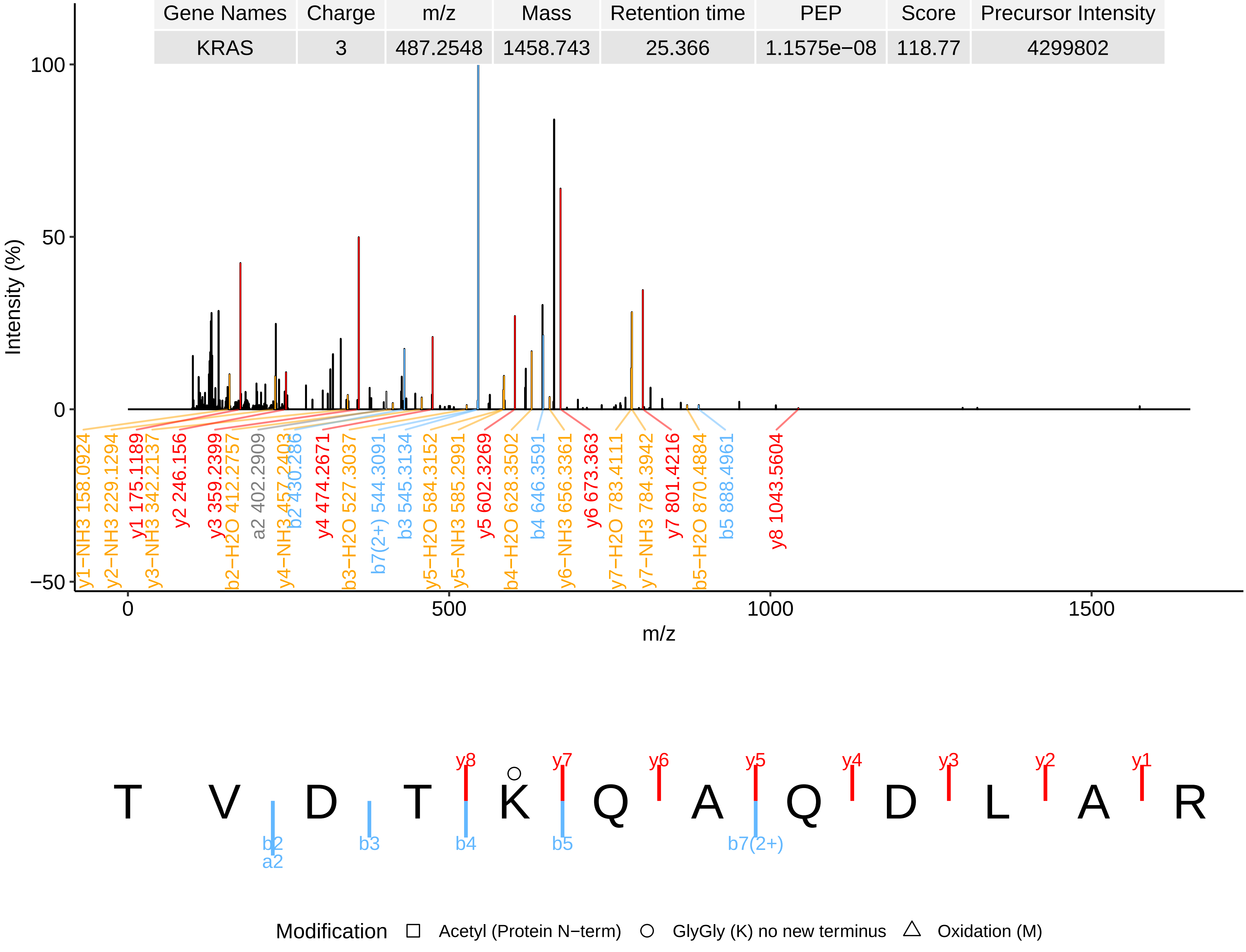

20200921_KIZ_MUb4_GlyGly_10plex_2 − Scan number: 6905

Gene Names	Charge	m/z	Mass	Retention time	PEP	Score	Precursor Intensity
KRAS	3	487.2548	1458.743	25.576	0.016213	62.528	1087386

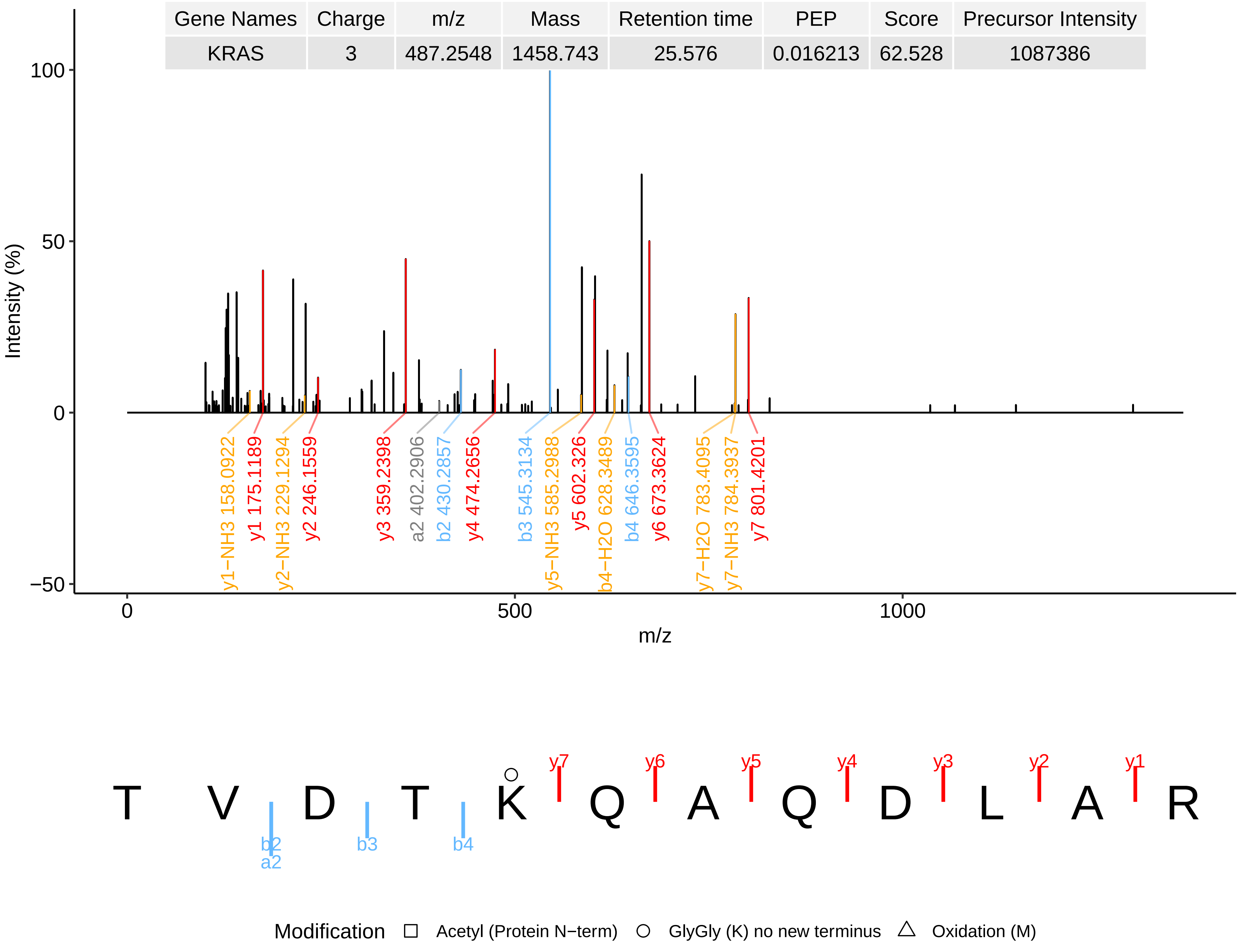

20200924_KIZ_MUb5_GlyGly_1 − Scan number: 6596

Gene Names	Charge	m/z	Mass	Retention time	PEP	Score	Precursor Intensity
KRAS	3	487.2548	1458.743	20.778	0.029	61.235	3668220

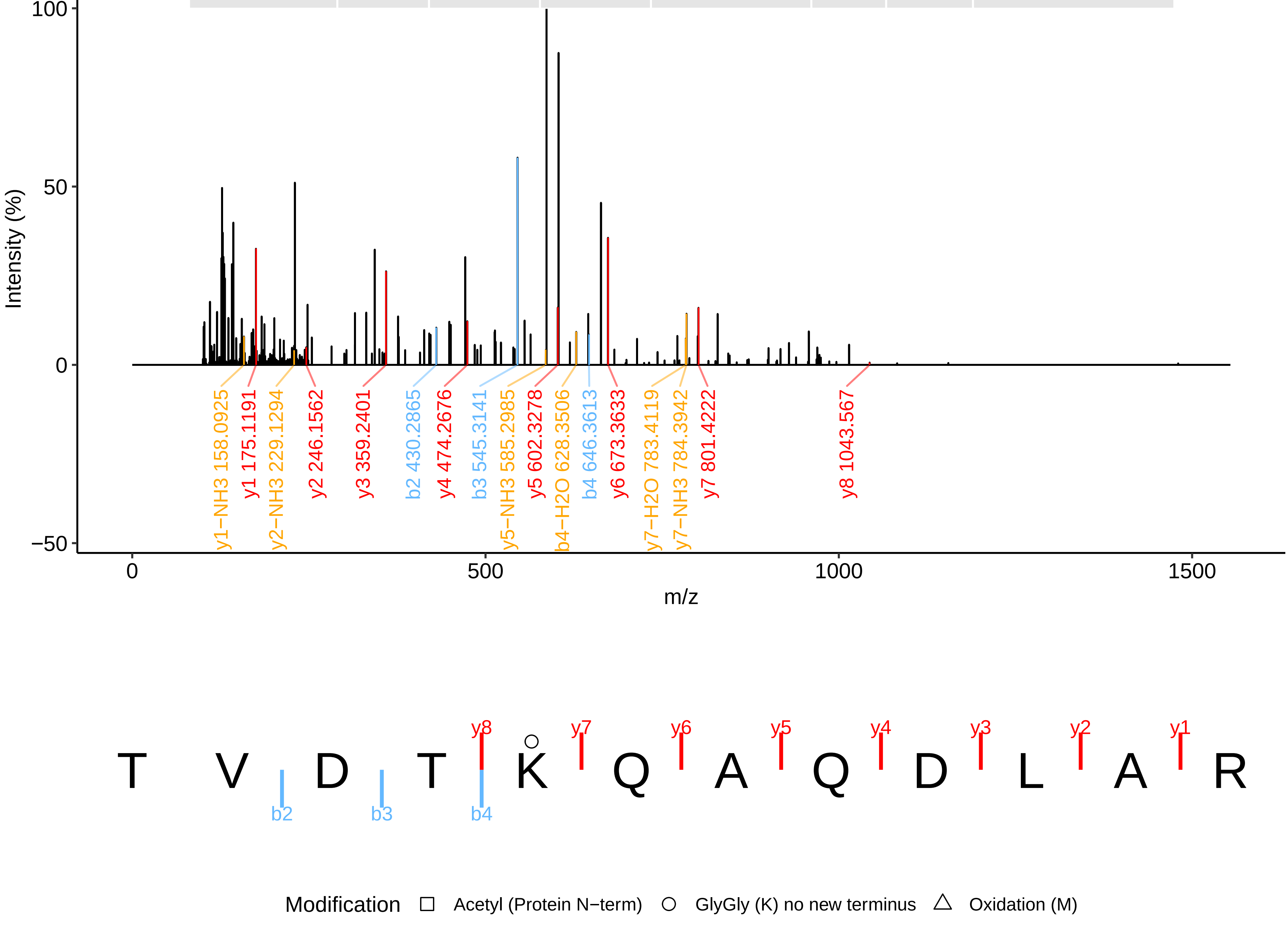

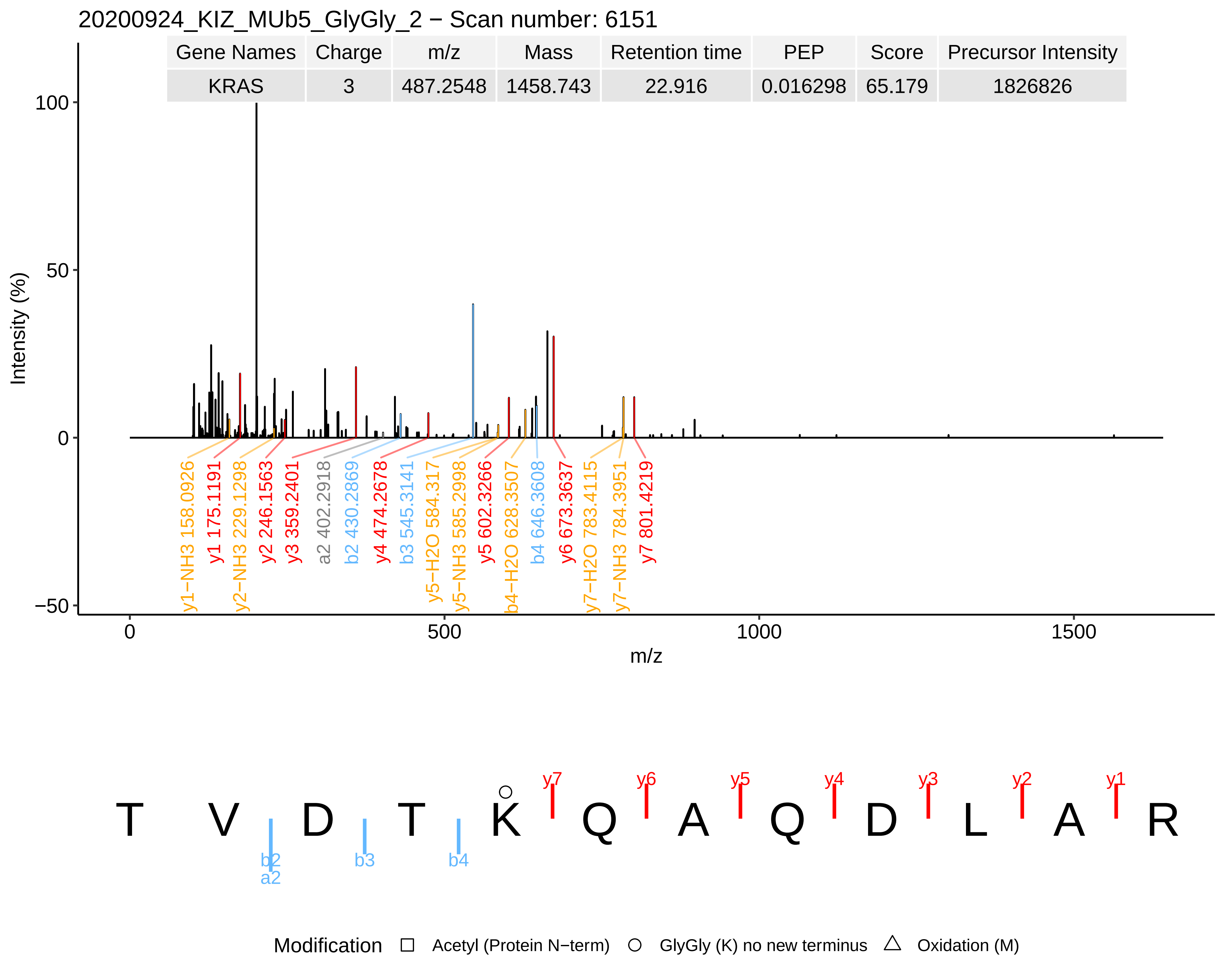
20200924_KIZ_MUb5_GlyGly_2 − Scan number: 6151
Gene Names | Charge | m/z | Mass | Retention time | PEP | Score | Precursor Intensity
KRAS | 3 | 487.2548 | 1458.743 | 22.916 | 0.016298 | 65.179 | 1826826
Intensity (%)
100
50
0
−50
y1−NH3 158.0926
y1 175.1191
y2−NH3 229.1298
y2 246.1563
y3 359.2401
a2 402.2918
b2 430.2869
y4 474.2678
b3 545.3141
y5−H2O 584.317
y5−NH3 585.2998
y5 602.3266
b4−H2O 628.3507
b4 646.3608
y6 673.3637
y7−H2O 783.4115
y7−NH3 784.3951
y7 801.4219
0
500
1000
1500
m/z
T V D T K Q A Q D L A R
y7 y6 y5 y4 y3 y2 y1
b2 a2 b3 b4
Modification
Acetyl (Protein N−term)
GlyGly (K) no new terminus
Oxidation (M)

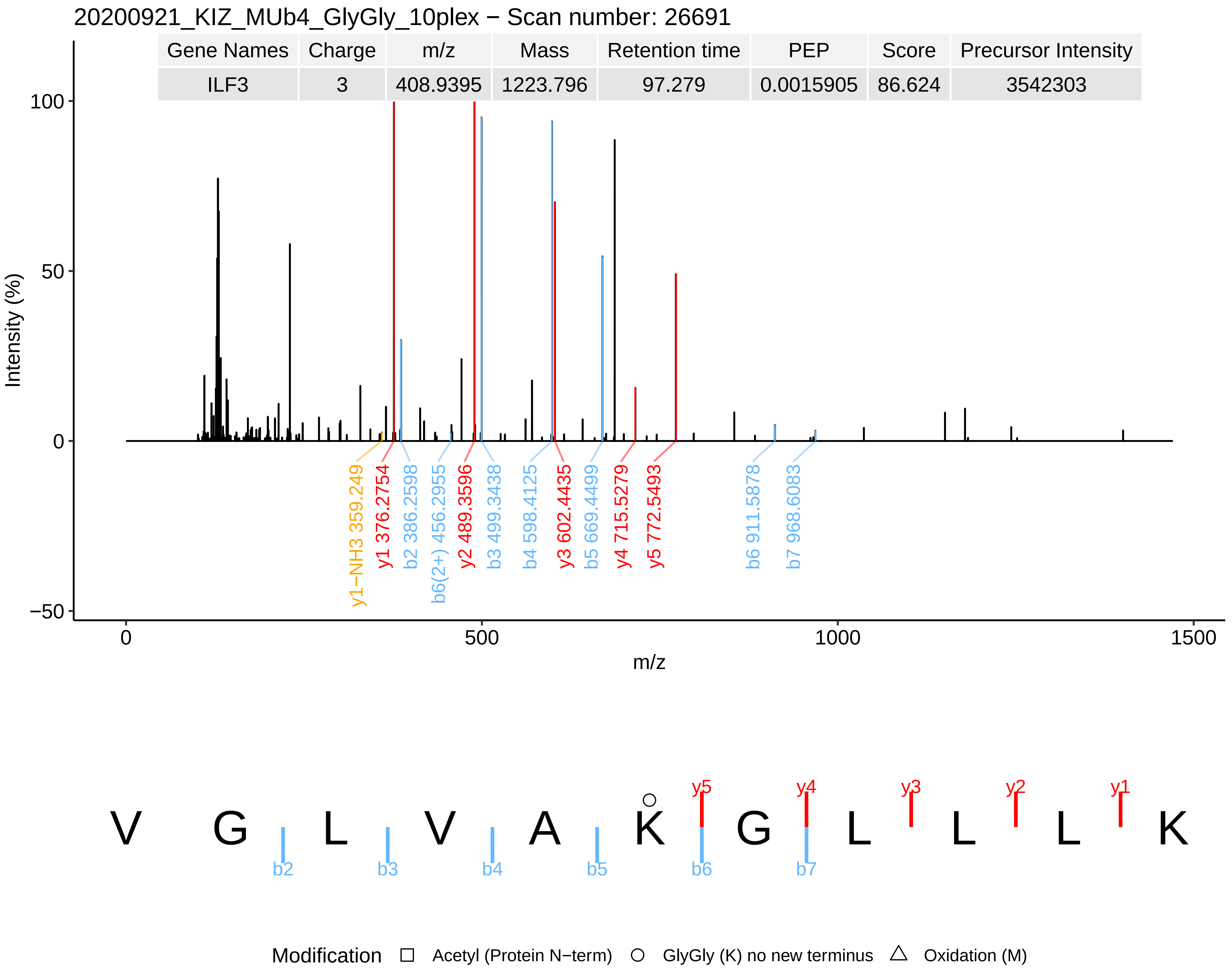

20200921_KIZ_MUb4_GlyGly_10plex − Scan number: 26691
Gene Names | Charge | m/z | Mass | Retention time | PEP | Score | Precursor Intensity
ILF3 | 3 | 408.9395 | 1223.796 | 97.279 | 0.0015905 | 86.624 | 3542303
Intensity (%)
m/z
y1−NH3 359.249
y1 376.2754
b2 386.2598
b6(2+) 456.2955
y2 489.3596
b3 499.3438
b4 598.4125
y3 602.4435
b5 669.4499
y4 715.5279
y5 772.5493
b6 911.5878
b7 968.6083
V G L V A K G L L L K
y5 y4 y3 y2 y1
b2 b3 b4 b5 b6 b7
Modification
Acetyl (Protein N−term)
GlyGly (K) no new terminus
Oxidation (M)

20200921_KIZ_MUb4_GlyGly_10plex − Scan number: 26825

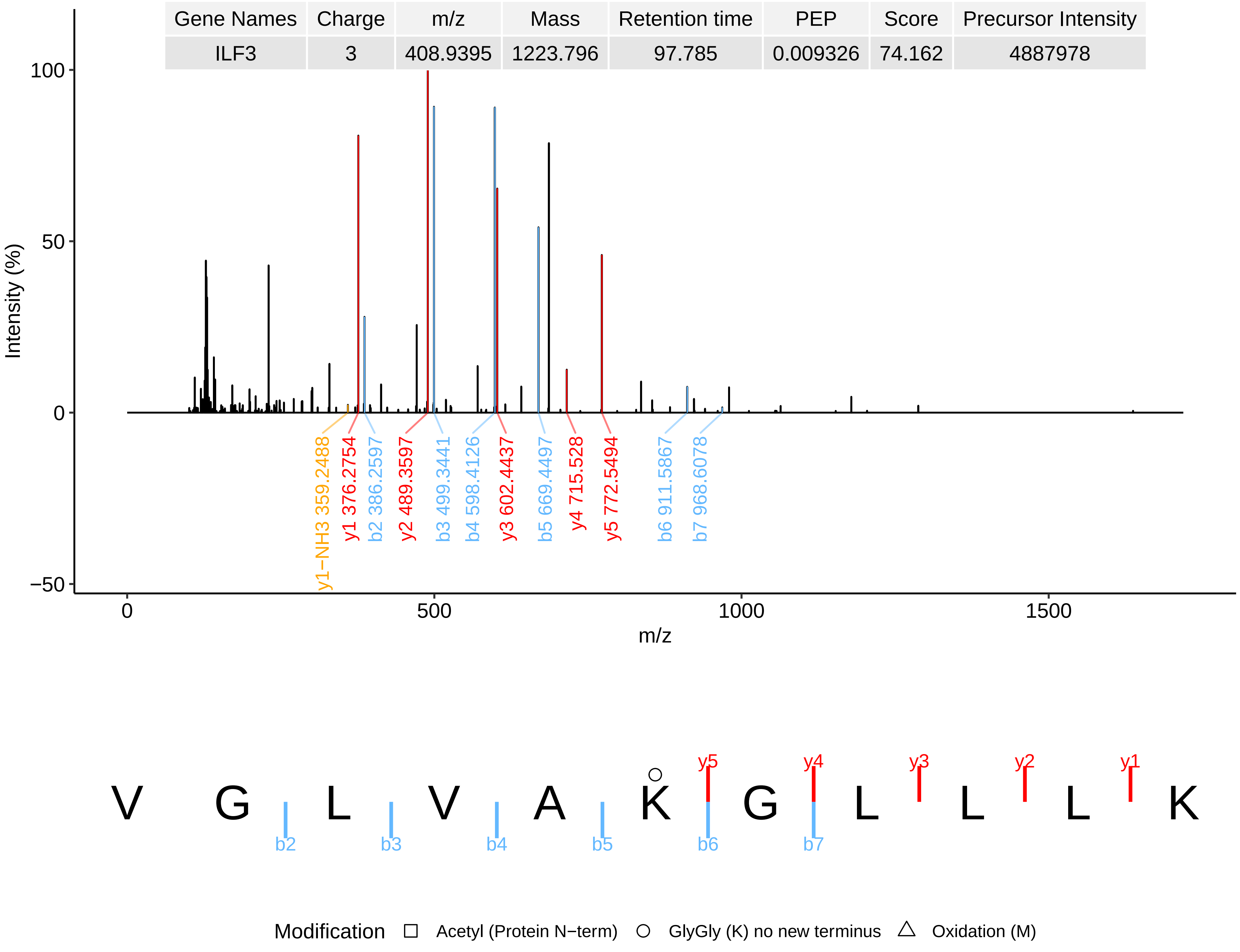

20200921_KIZ_MUb4_GlyGly_10plex_2 − Scan number: 22784

Gene Names	Charge	m/z	Mass	Retention time	PEP	Score	Precursor Intensity
ILF3	3	408.9395	1223.796	89.872	0.0021207	81.338	1021376

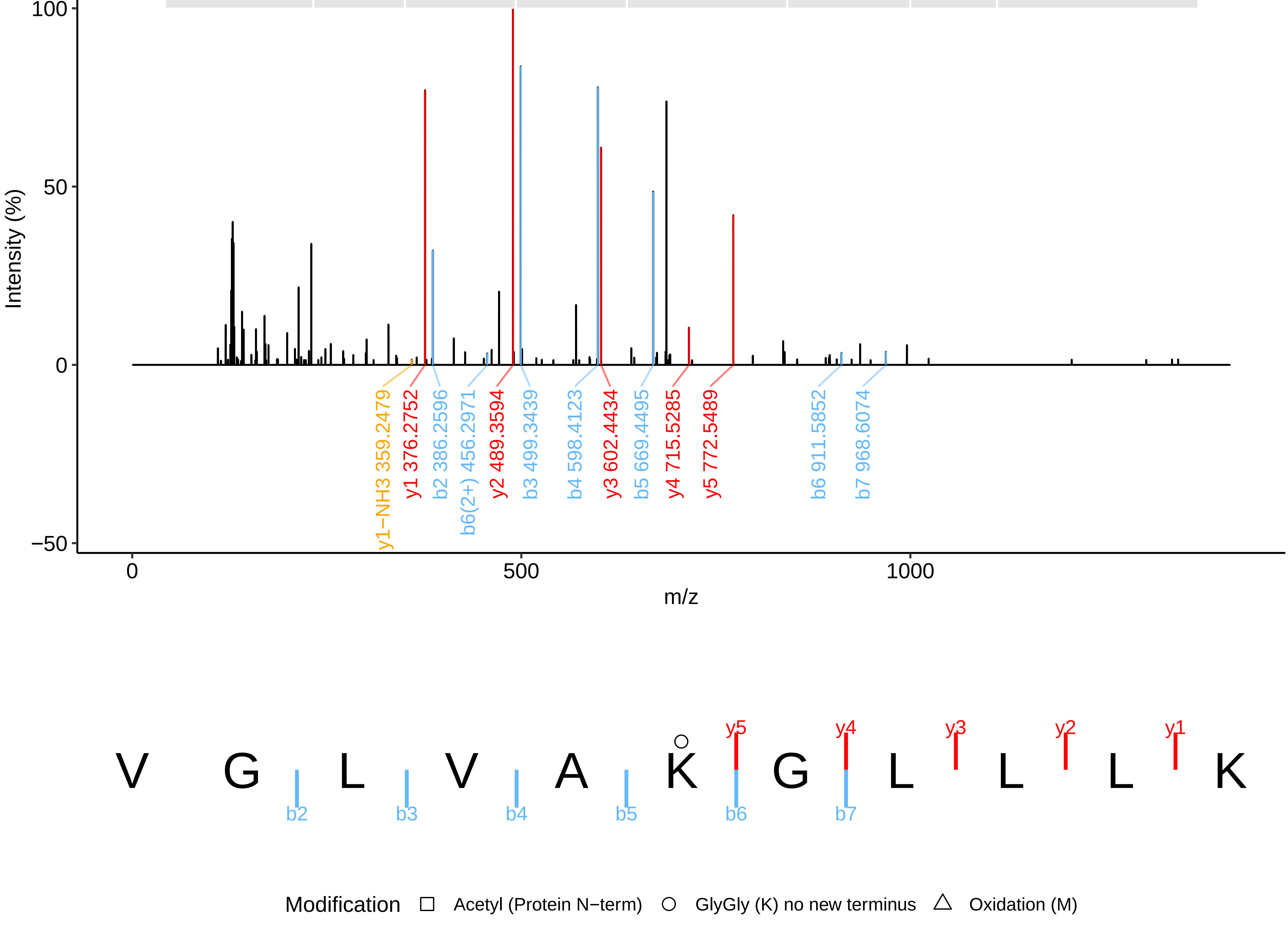

20201118_KIZ_MUb6_GlyGly_1 − Scan number: 25447

Gene Names	Charge	m/z	Mass	Retention time	PEP	Score	Precursor Intensity
ILF3	3	408.9395	1223.796	77.941	0.0024768	79.974	18558446

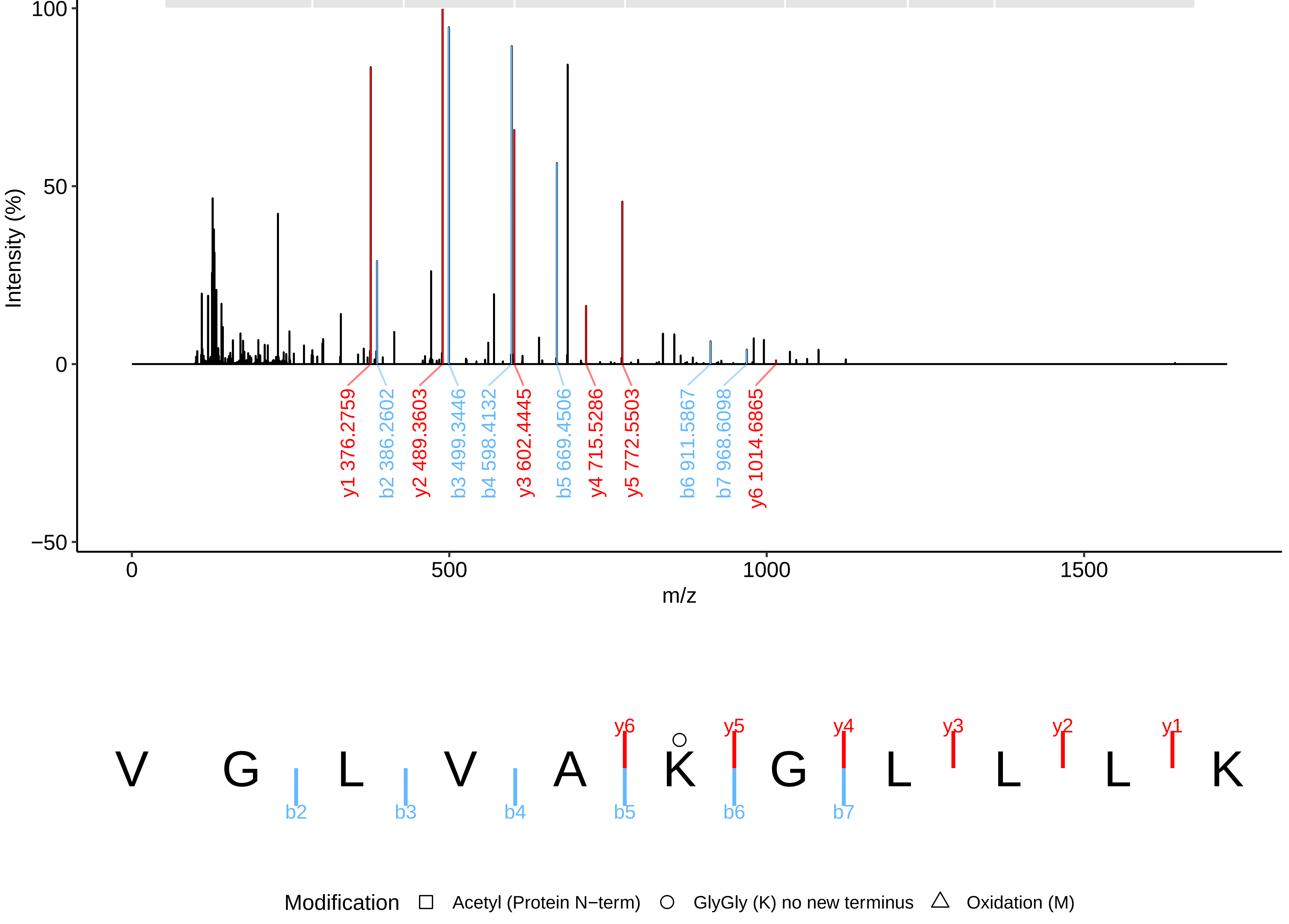

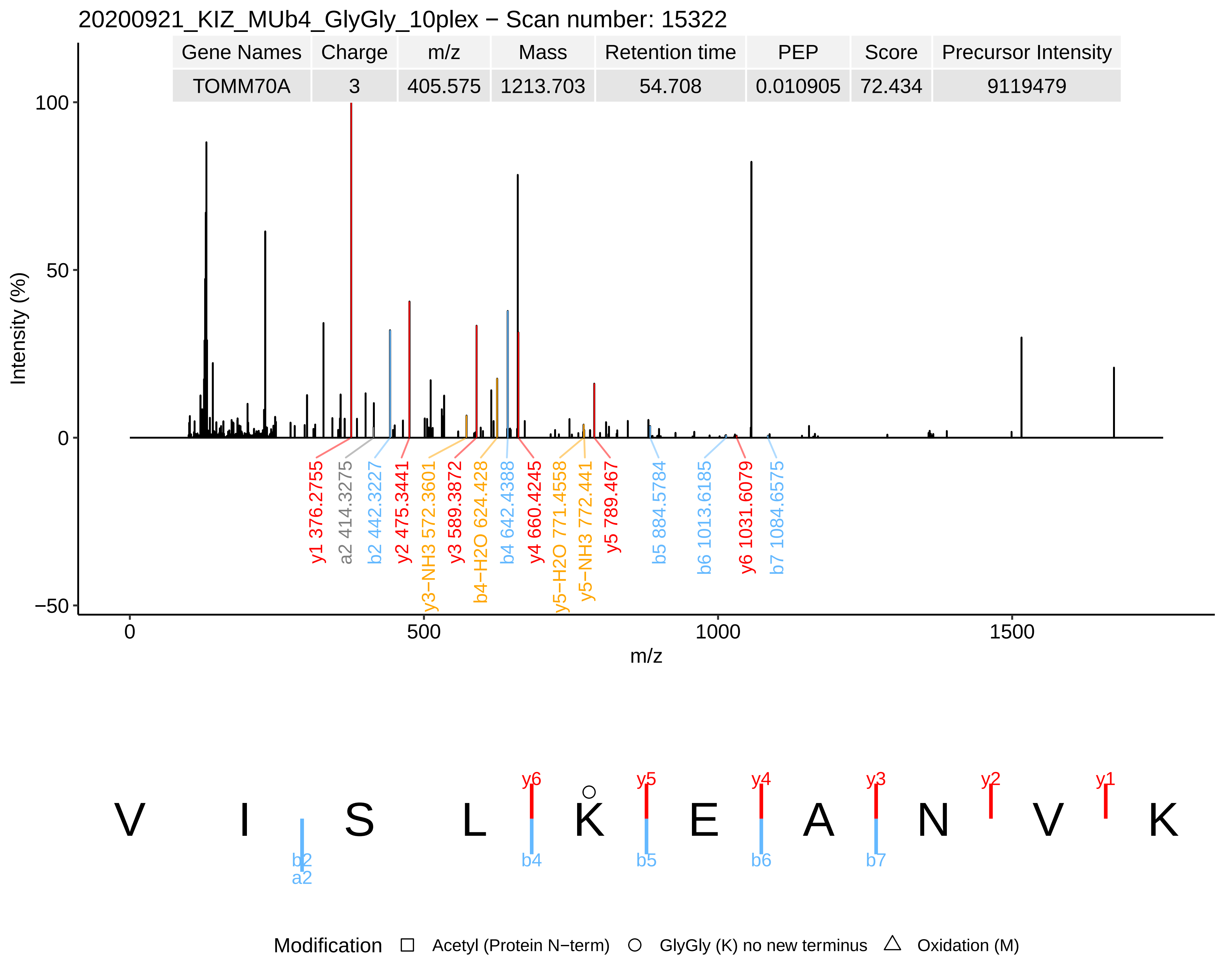
20200921_KIZ_MUb4_GlyGly_10plex − Scan number: 15322
Gene Names | Charge | m/z | Mass | Retention time | PEP | Score | Precursor Intensity
TOMM70A | 3 | 405.575 | 1213.703 | 54.708 | 0.010905 | 72.434 | 9119479
Intensity (%)
100
50
0
−50
0
500
1000
1500
m/z
y1 376.2755
a2 414.3275
b2 442.3227
y2 475.3441
y3−NH3 572.3601
y3 589.3872
b4−H2O 624.428
b4 642.4388
y4 660.4245
y5−H2O 771.4558
y5−NH3 772.441
y5 789.467
b5 884.5784
b6 1013.6185
y6 1031.6079
b7 1084.6575
V I S L K E A N V K
y6 y5 y4 y3 y2 y1
b2 a2 b4 b5 b6 b7
Modification
Acetyl (Protein N−term)
GlyGly (K) no new terminus
Oxidation (M)

20200921_KIZ_MUb4_GlyGly_10plex − Scan number: 15357

Gene Names	Charge	m/z	Mass	Retention time	PEP	Score	Precursor Intensity
TOMM70A	3	405.575	1213.703	54.838	0.00096332	91.867	15820748

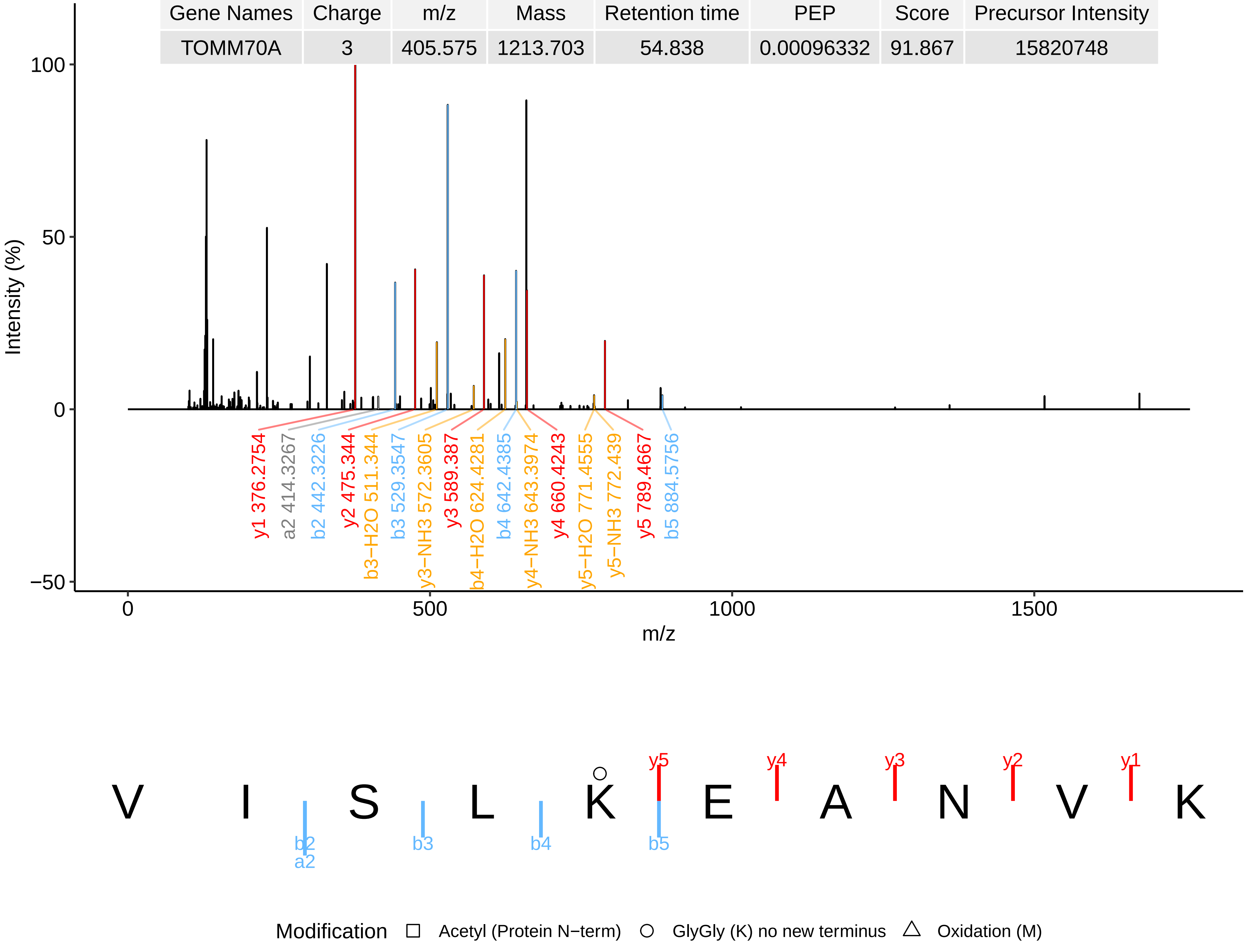

20200921_KIZ_MUb4_GlyGly_10plex_2 − Scan number: 14205

Gene Names	Charge	m/z	Mass	Retention time	PEP	Score	Precursor Intensity
TOMM70A	3	405.575	1213.703	55.337	0.0093242	73.985	1270674

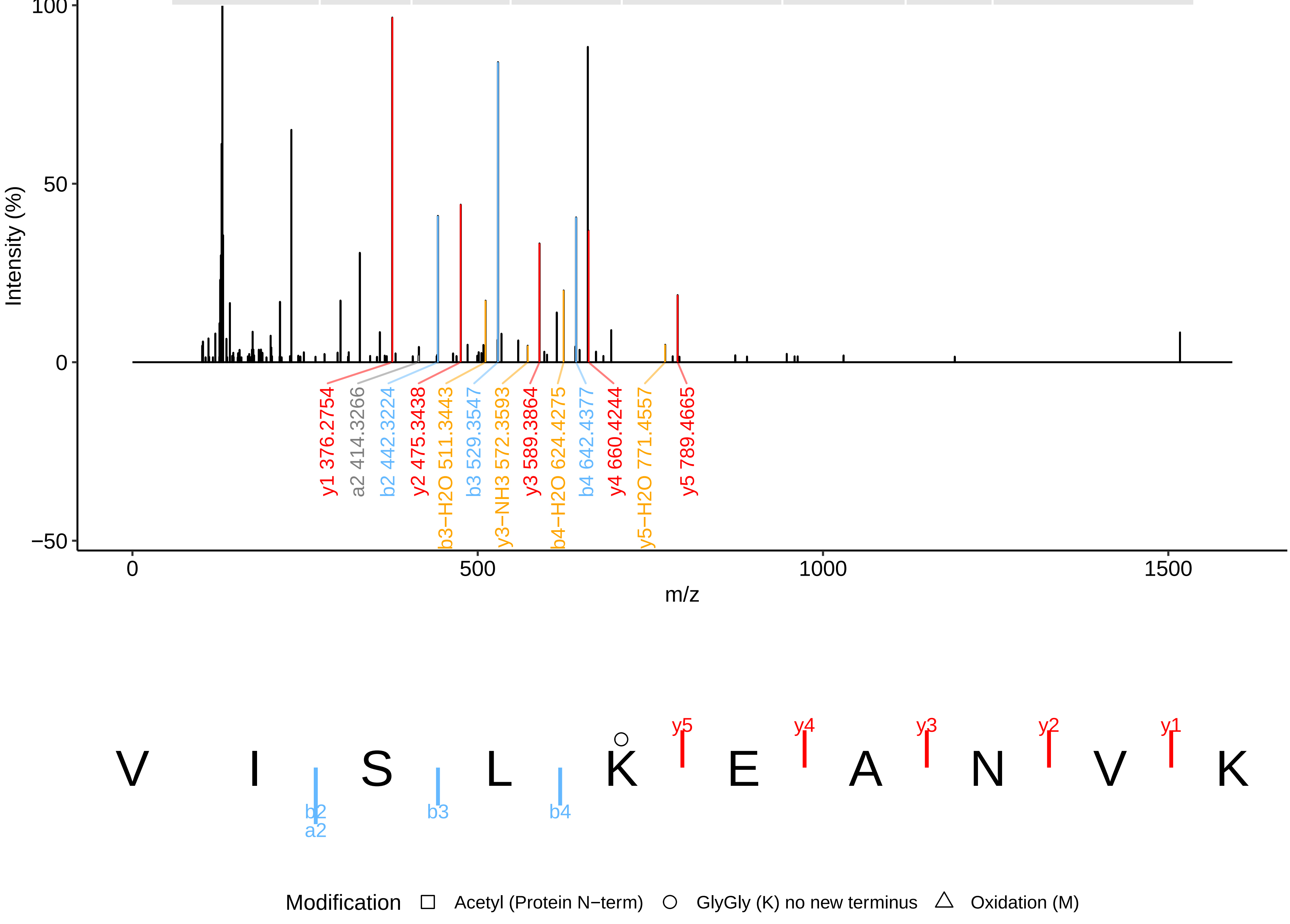

20201118_KIZ_MUb6_GlyGly_2 − Scan number: 16809

Gene Names	Charge	m/z	Mass	Retention time	PEP	Score	Precursor Intensity
TOMM70A	3	405.575	1213.703	56.821	0.0031385	87.258	7935567

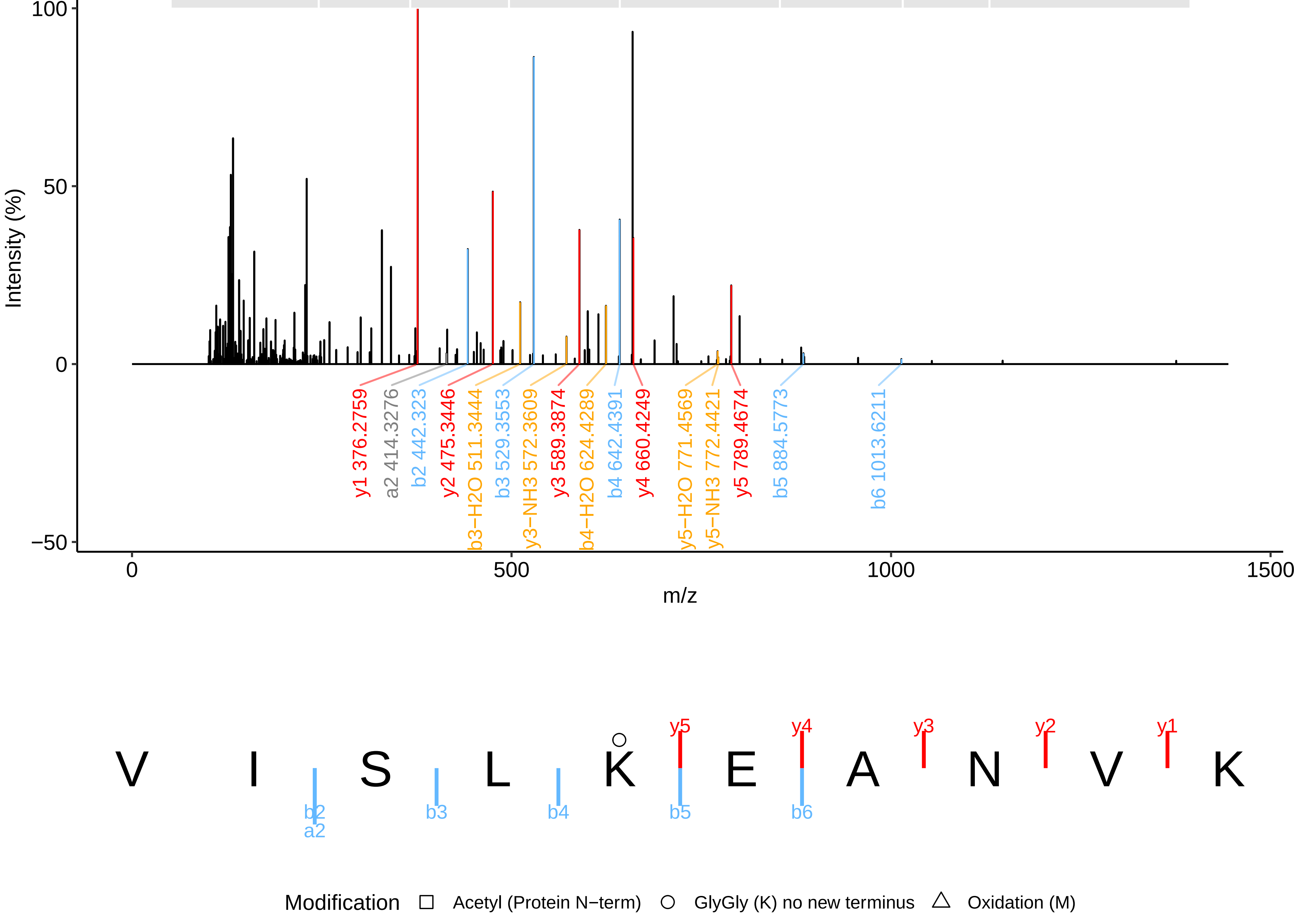

20200921_KIZ_MUb4_GlyGly_10plex − Scan number: 8181

Gene Names	Charge	m/z	Mass	Retention time	PEP	Score	Precursor Intensity
MSN	4	306.428	1221.683	27.696	0.013619	68.371	9548186

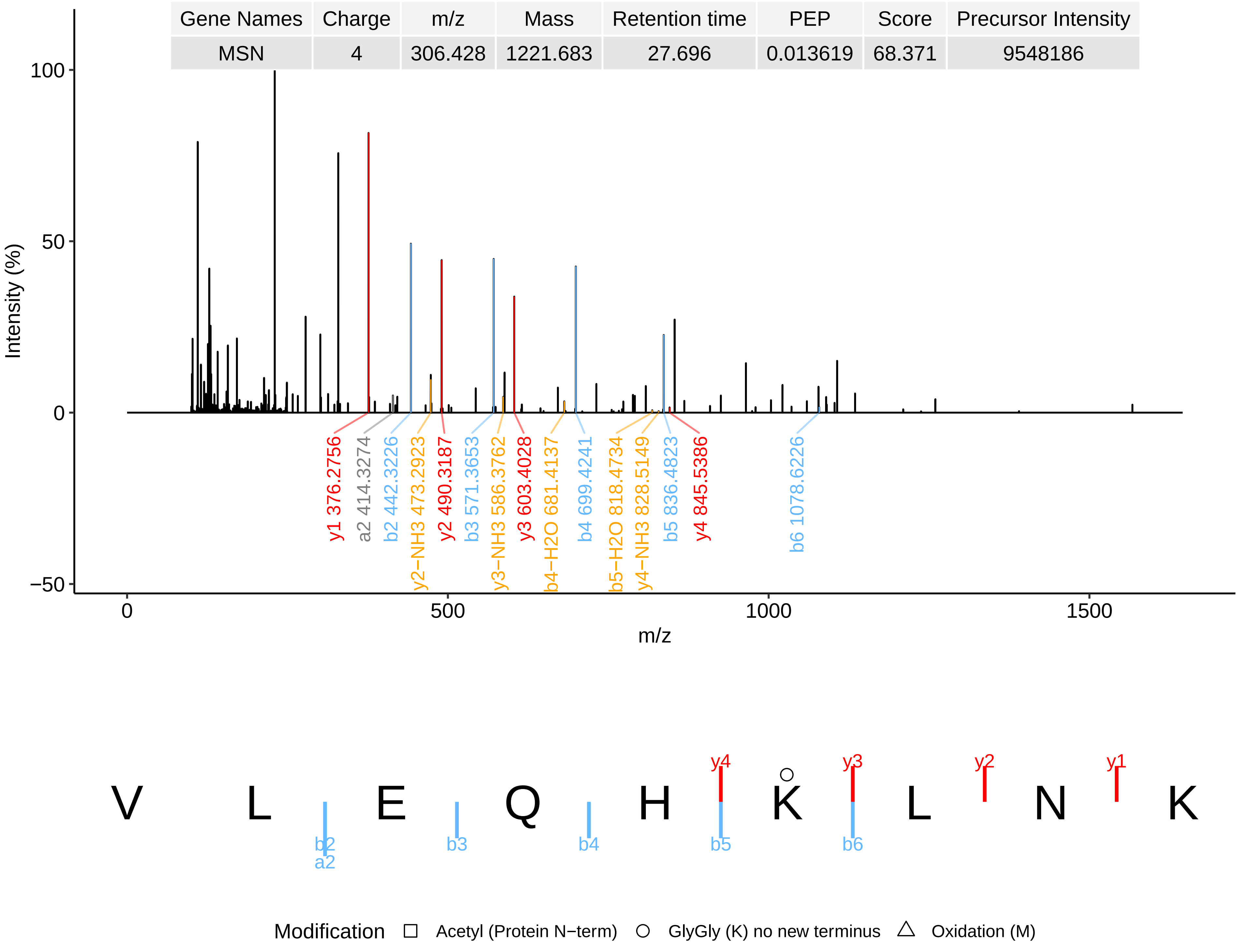

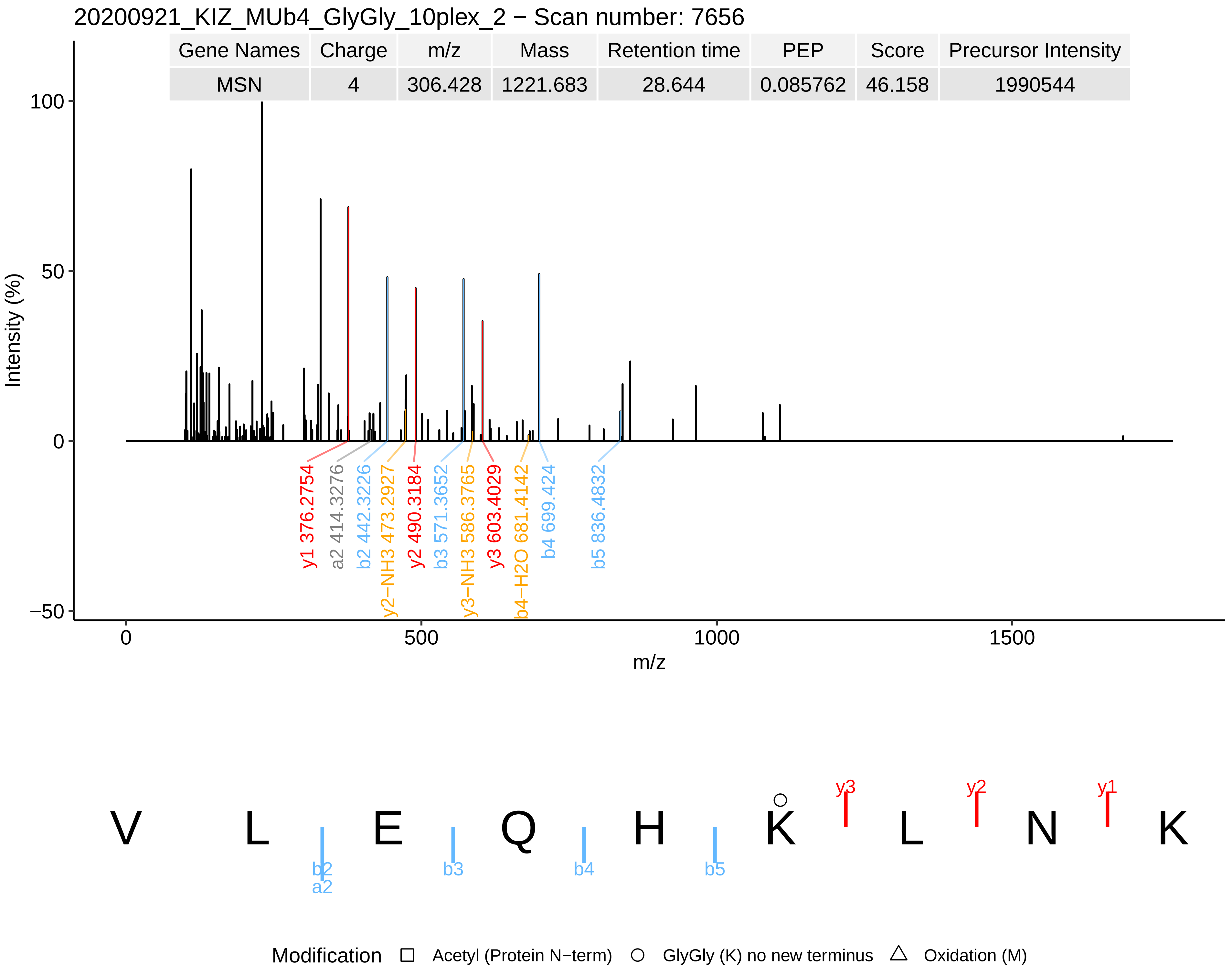
20200921_KIZ_MUb4_GlyGly_10plex_2 − Scan number: 7656
Gene Names | Charge | m/z | Mass | Retention time | PEP | Score | Precursor Intensity
MSN | 4 | 306.428 | 1221.683 | 28.644 | 0.085762 | 46.158 | 1990544
Intensity (%)
100
50
0
−50
0
500
1000
1500
m/z
y1 376.2754
a2 414.3276
b2 442.3226
y2−NH3 473.2927
y2 490.3184
b3 571.3652
y3−NH3 586.3765
y3 603.4029
b4−H2O 681.4142
b4 699.424
b5 836.4832
V L E Q H K L N K
y3 y2 y1
b2 a2 b3 b4 b5
Modification
Acetyl (Protein N−term)
GlyGly (K) no new terminus
Oxidation (M)

20200921_KIZ_MUb4_GlyGly_10plex − Scan number: 13476

Gene Names	Charge	m/z	Mass	Retention time	PEP	Score	Precursor Intensity
PSMA4	3	416.8921	1247.654	47.566	0.013743	69.65	3617497

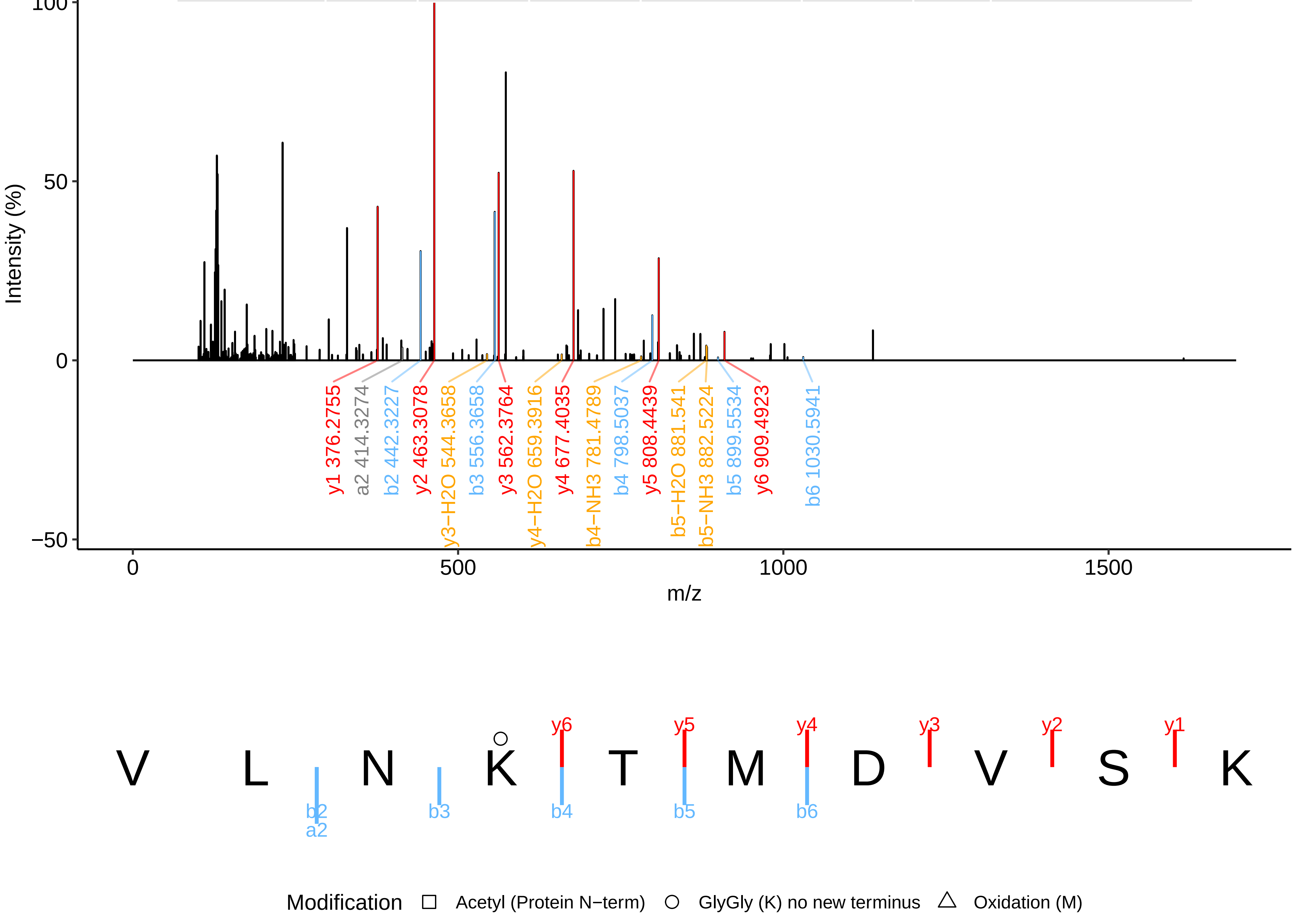

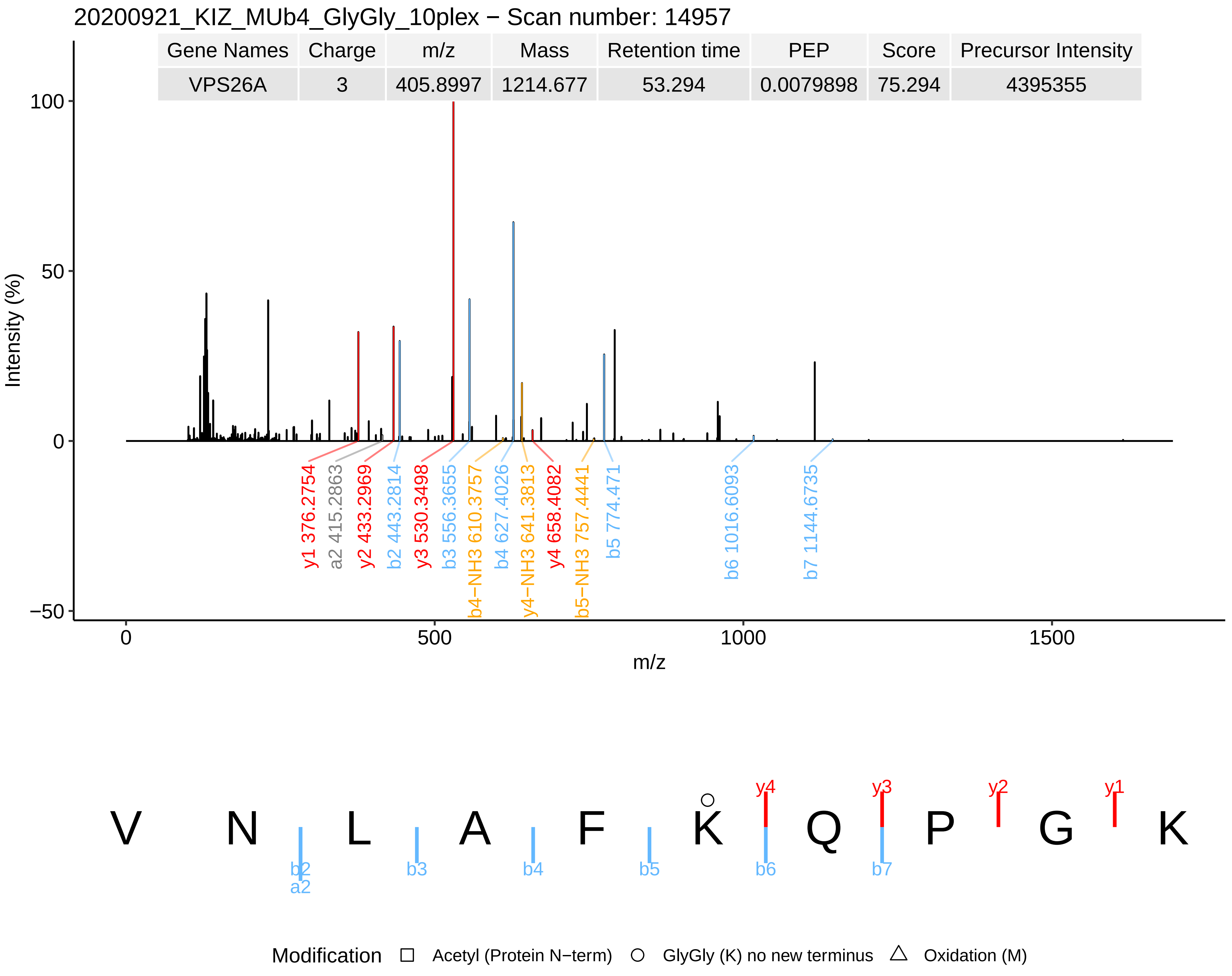

20200921_KIZ_MUb4_GlyGly_10plex − Scan number: 14957
Gene Names | Charge | m/z | Mass | Retention time | PEP | Score | Precursor Intensity
VPS26A | 3 | 405.8997 | 1214.677 | 53.294 | 0.0079898 | 75.294 | 4395355
Intensity (%)
m/z
y1 376.2754
a2 415.2863
y2 433.2969
b2 443.2814
y3 530.3498
b3 556.3655
b4−NH3 610.3757
b4 627.4026
y4−NH3 641.3813
y4 658.4082
b5−NH3 757.4441
b5 774.471
b6 1016.6093
b7 1144.6735
V N L A F K Q P G K
y4 y3 y2 y1
b2 a2 b3 b4 b5 b6 b7
Modification
Acetyl (Protein N−term)
GlyGly (K) no new terminus
Oxidation (M)

20200921_KIZ_MUb4_GlyGly_10plex_2 − Scan number: 13810

Gene Names	Charge	m/z	Mass	Retention time	PEP	Score	Precursor Intensity
VPS26A	3	405.8997	1214.677	53.702	0.022142	62.823	619641.4

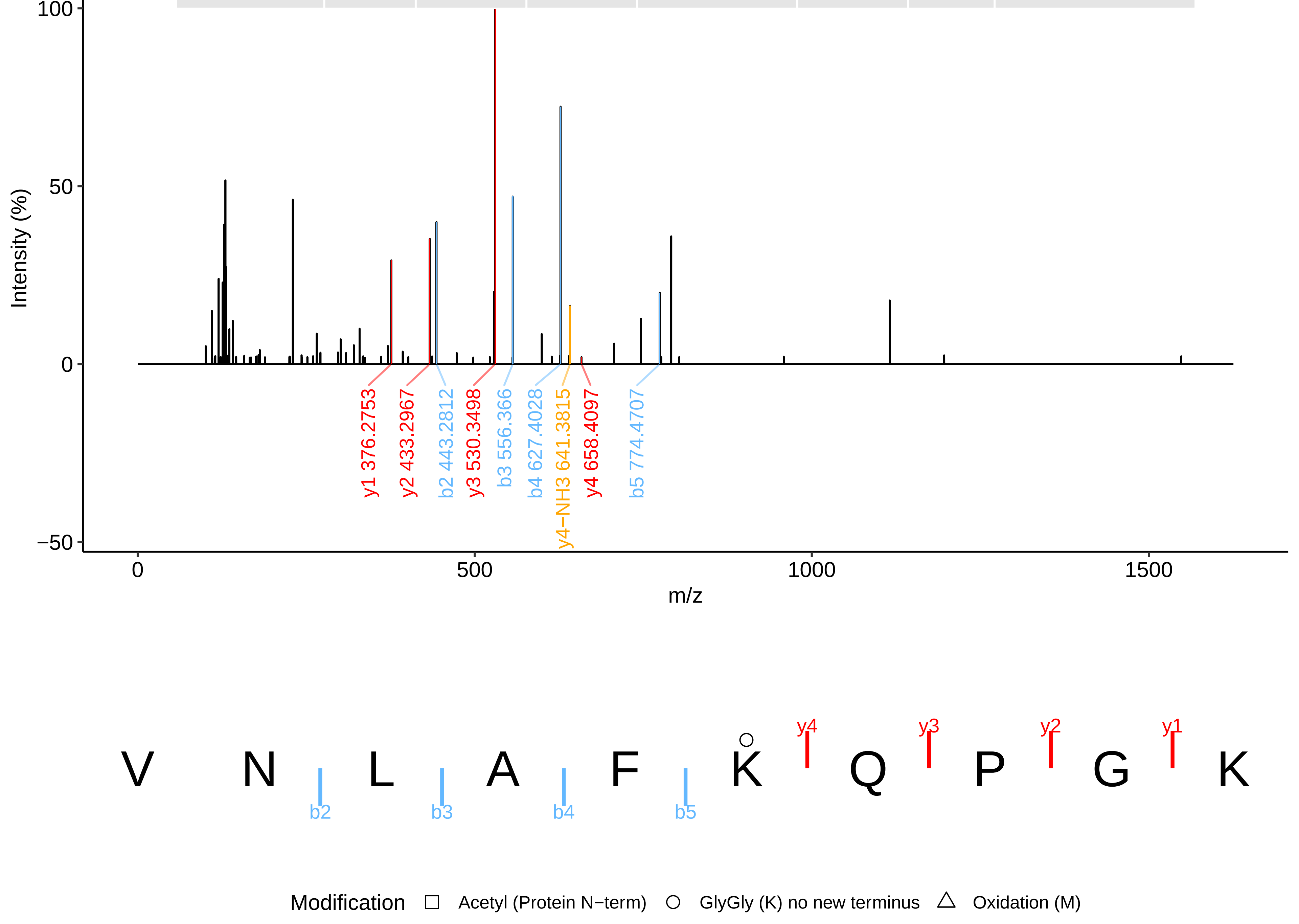

20200921_KIZ_MUb4_GlyGly_10plex − Scan number: 4068

Gene Names	Charge	m/z	Mass	Retention time	PEP	Score	Precursor Intensity
NCL	3	405.2143	1212.621	13.231	0.00060984	94.309	2549293

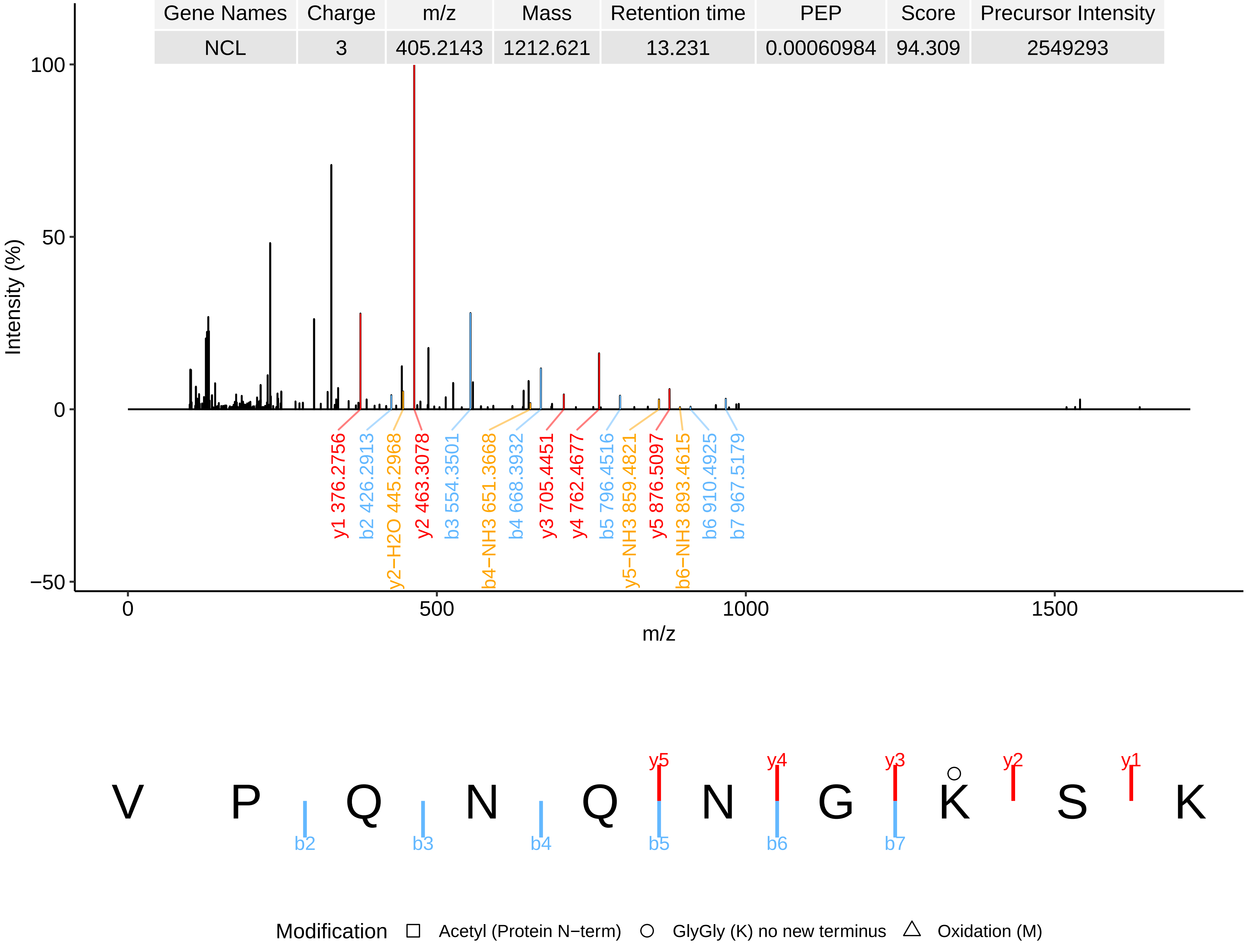

20200921_KIZ_MUb4_GlyGly_10plex − Scan number: 9546

Gene Names	Charge	m/z	Mass	Retention time	PEP	Score	Precursor Intensity
HSPA1B;HSPA1A	3	418.2226	1251.646	32.712	0.0010093	91.549	5397841

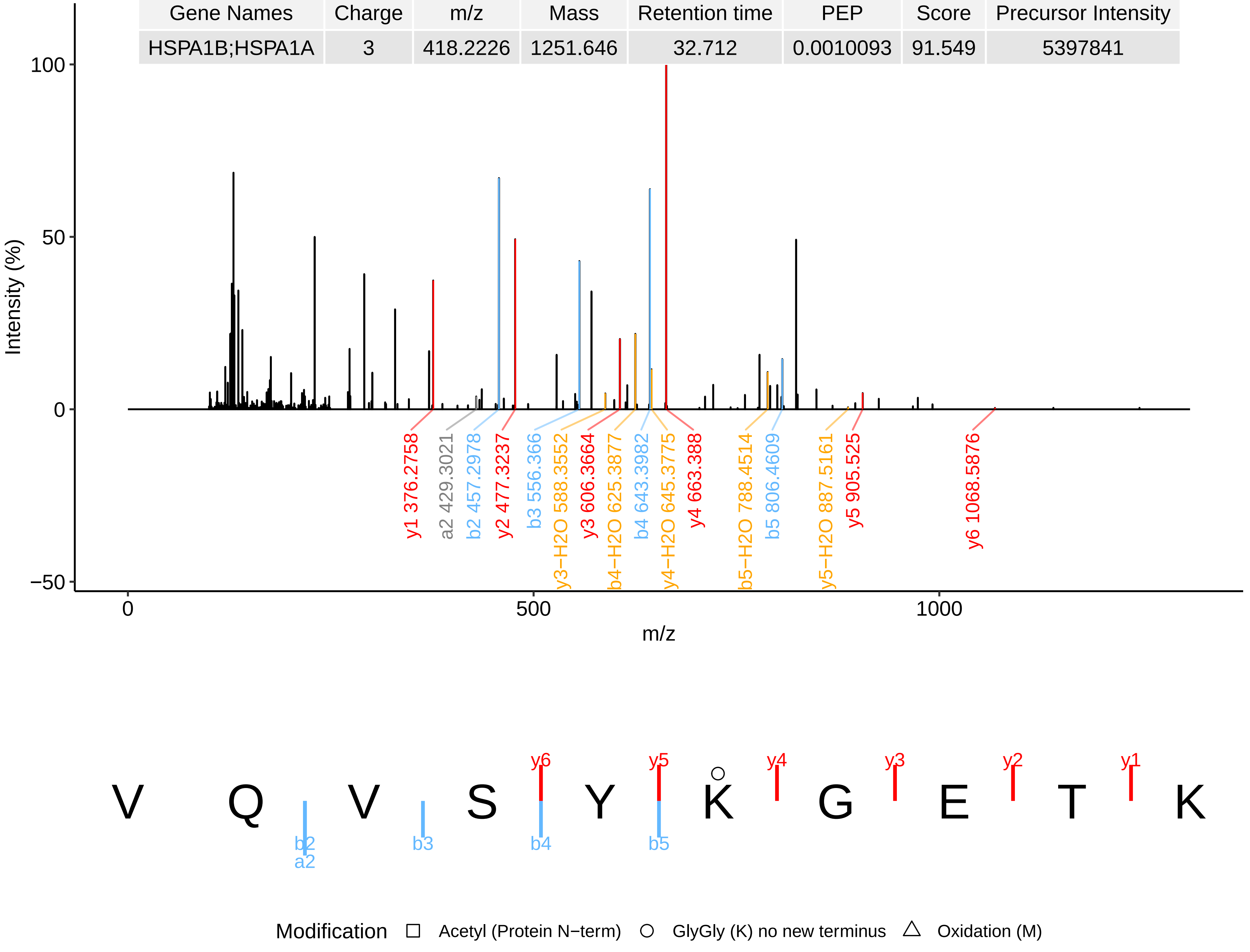

20200921_KIZ_MUb4_GlyGly_10plex − Scan number: 12203

Gene Names	Charge	m/z	Mass	Retention time	PEP	Score	Precursor Intensity
VDAC2	3	418.5627	1252.666	42.722	4.0403e−05	106.2	5616326

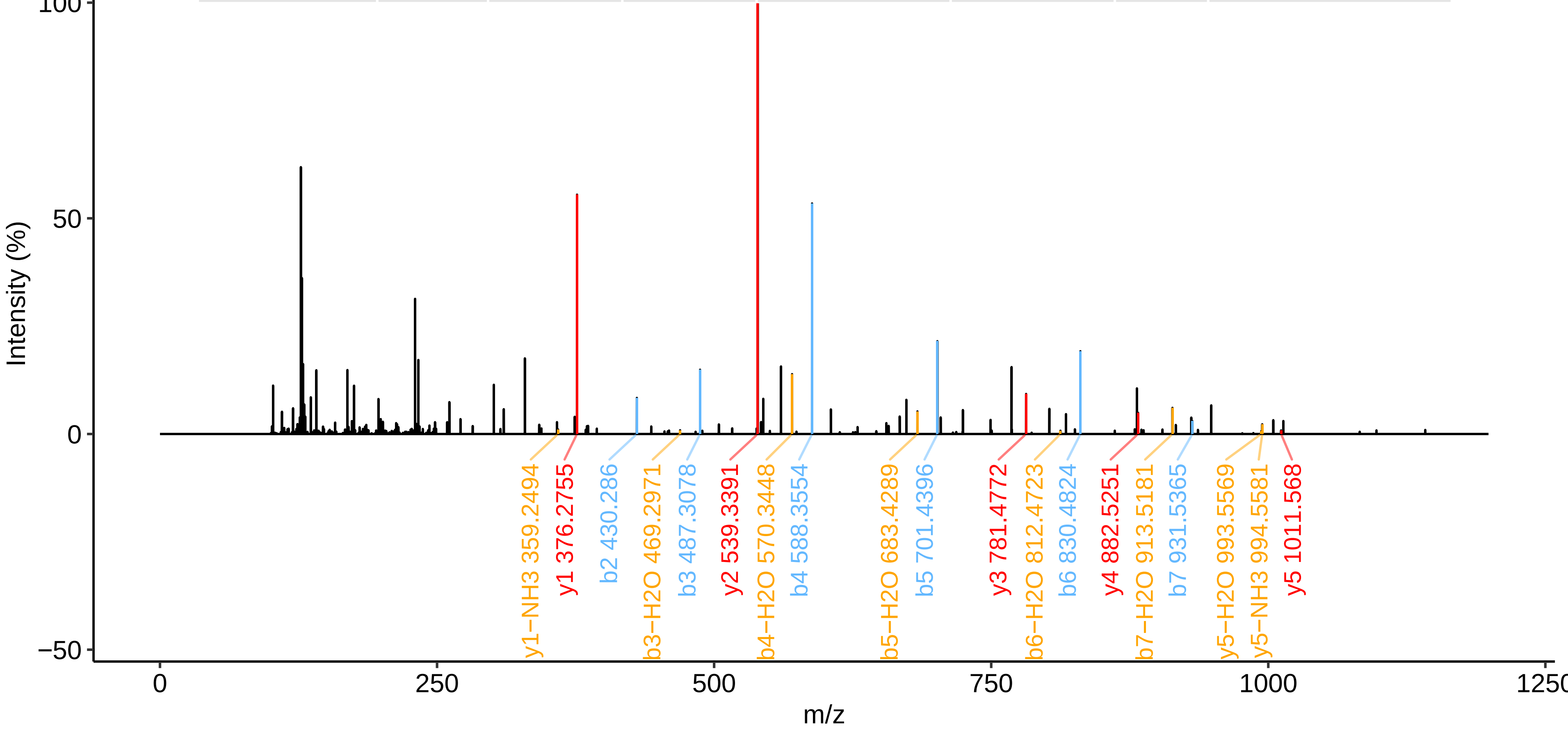

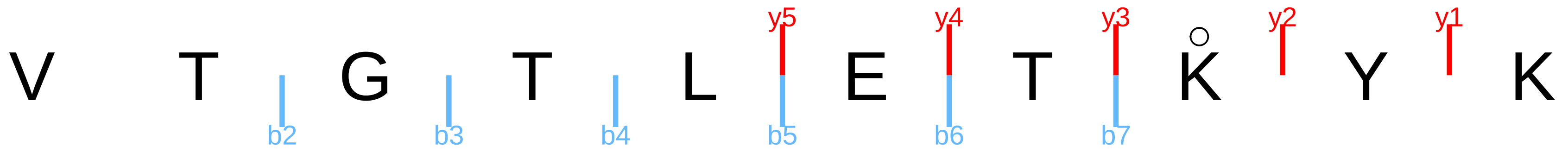

Modification □ Acetyl (Protein N−term) ○ GlyGly (K) no new terminus △ Oxidation (M)

20200921_KIZ_MUb4_GlyGly_10plex_2 − Scan number: 11251

Gene Names	Charge	m/z	Mass	Retention time	PEP	Score	Precursor Intensity
VDAC2	3	418.5627	1252.666	43.256	0.014125	69.276	1149537

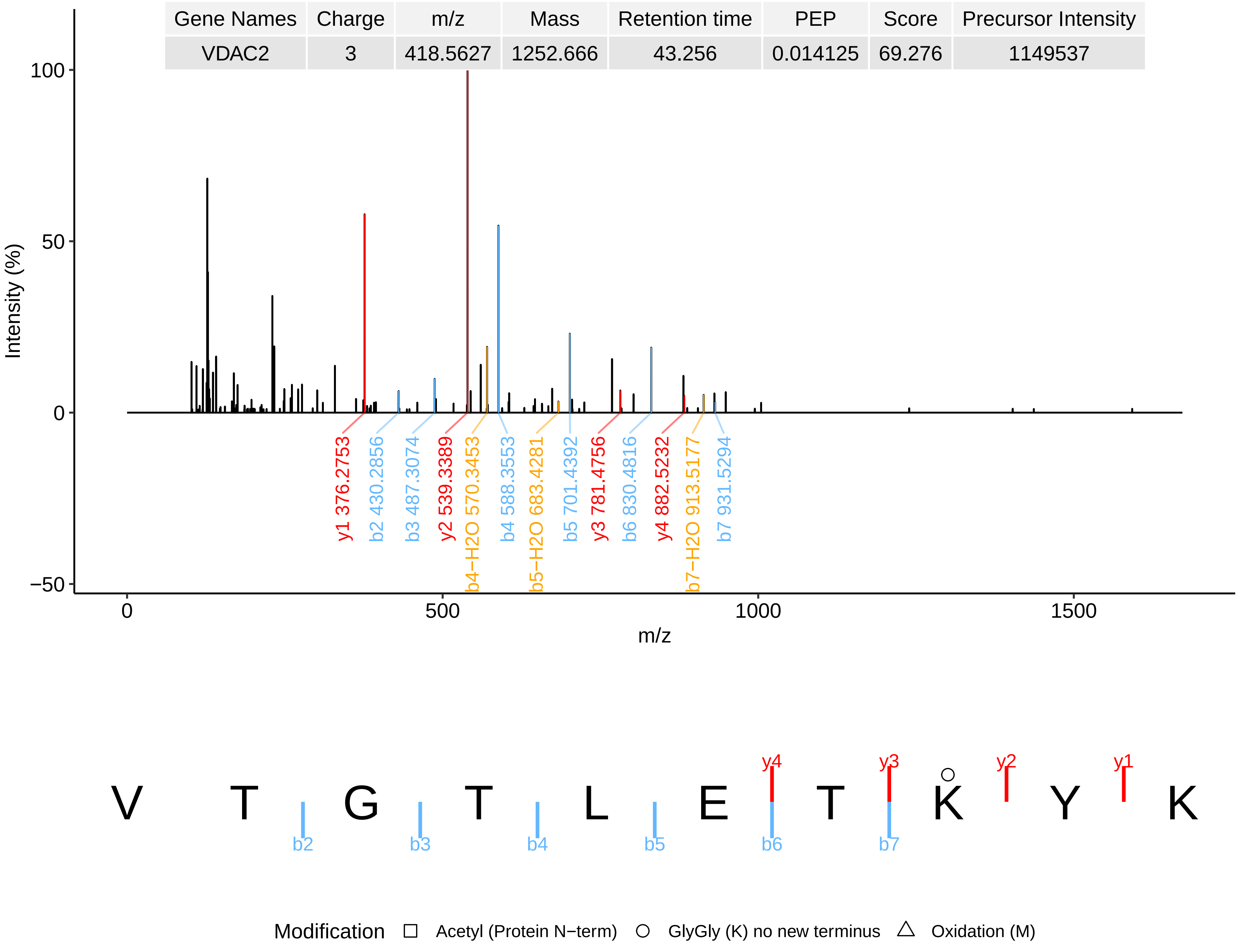

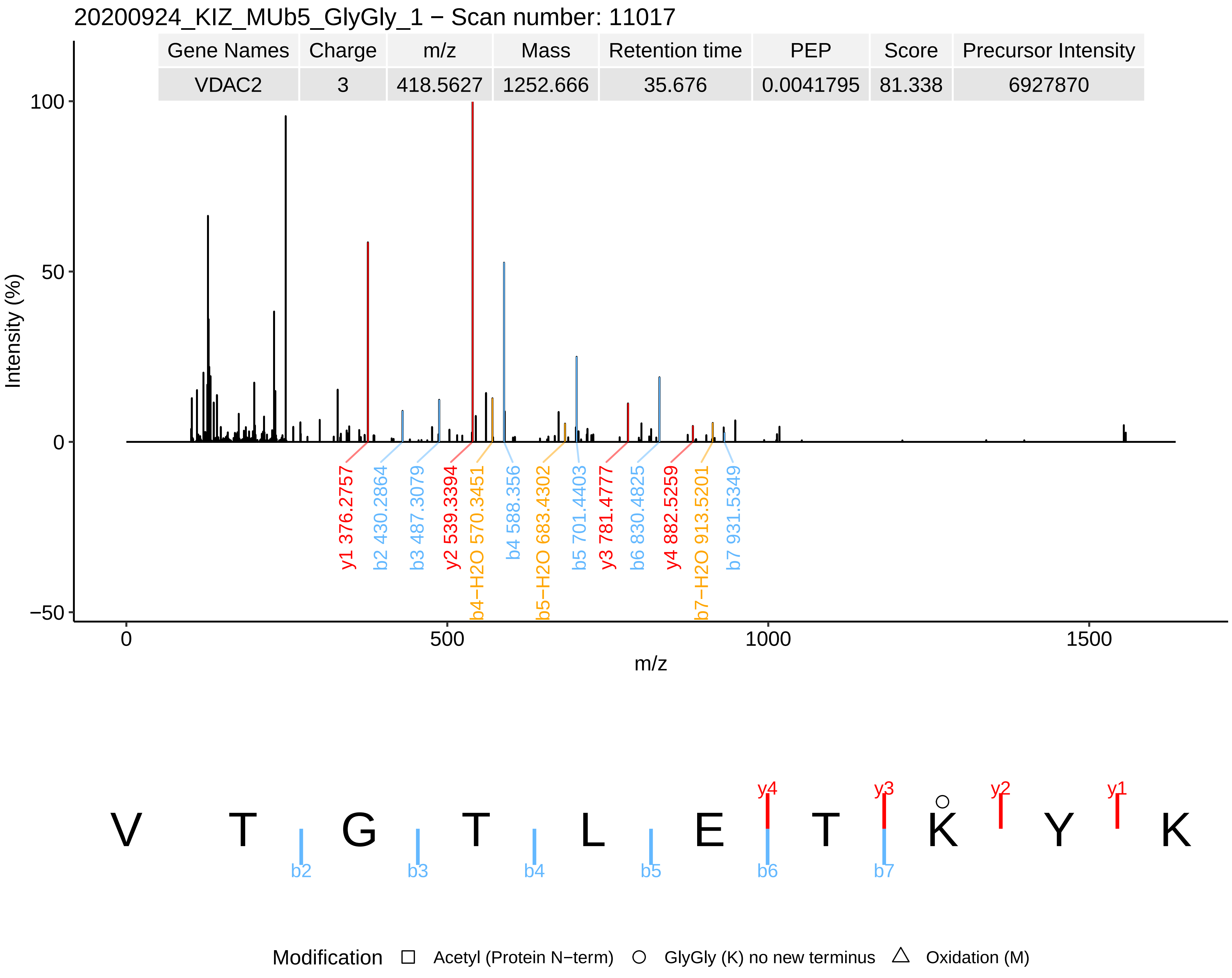

20200924_KIZ_MUb5_GlyGly_1 − Scan number: 11017
Gene Names | Charge | m/z | Mass | Retention time | PEP | Score | Precursor Intensity
VDAC2 | 3 | 418.5627 | 1252.666 | 35.676 | 0.0041795 | 81.338 | 6927870
Intensity (%)
m/z
y1 376.2757
b2 430.2864
b3 487.3079
y2 539.3394
b4−H2O 570.3451
b4 588.356
b5−H2O 683.4302
b5 701.4403
y3 781.4777
b6 830.4825
y4 882.5259
b7−H2O 913.5201
b7 931.5349
V T G T L E T K Y K
Modification
Acetyl (Protein N−term)
GlyGly (K) no new terminus
Oxidation (M)

20200924_KIZ_MUb5_GlyGly_1 − Scan number: 11278

Gene Names	Charge	m/z	Mass	Retention time	PEP	Score	Precursor Intensity
VDAC2	3	418.5627	1252.666	36.518	0.0016853	95.523	14059959

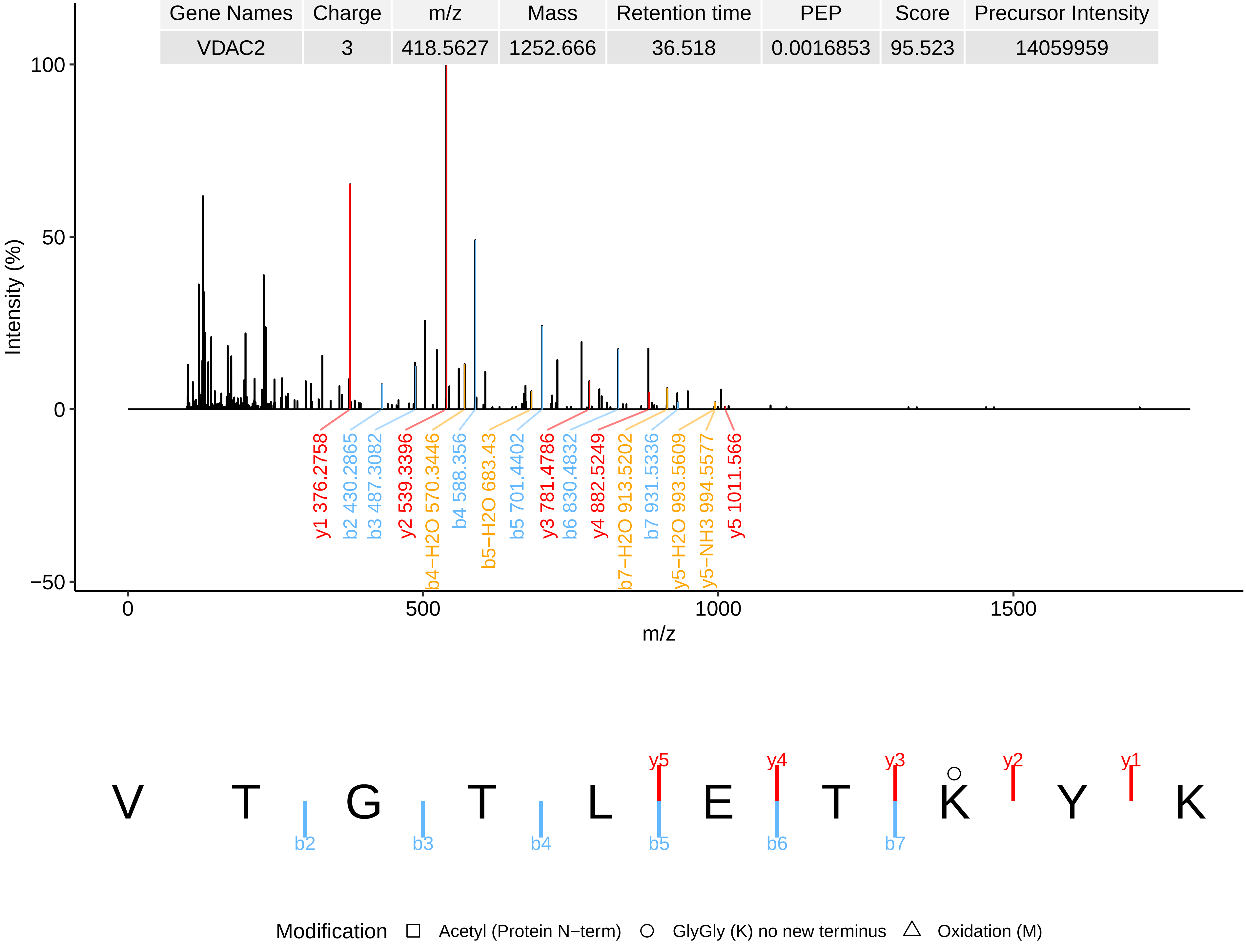

20200924_KIZ_MUb5_GlyGly_2 − Scan number: 10356

Gene Names	Charge	m/z	Mass	Retention time	PEP	Score	Precursor Intensity
VDAC2	3	418.5627	1252.666	38.667	0.014755	68.657	2908183

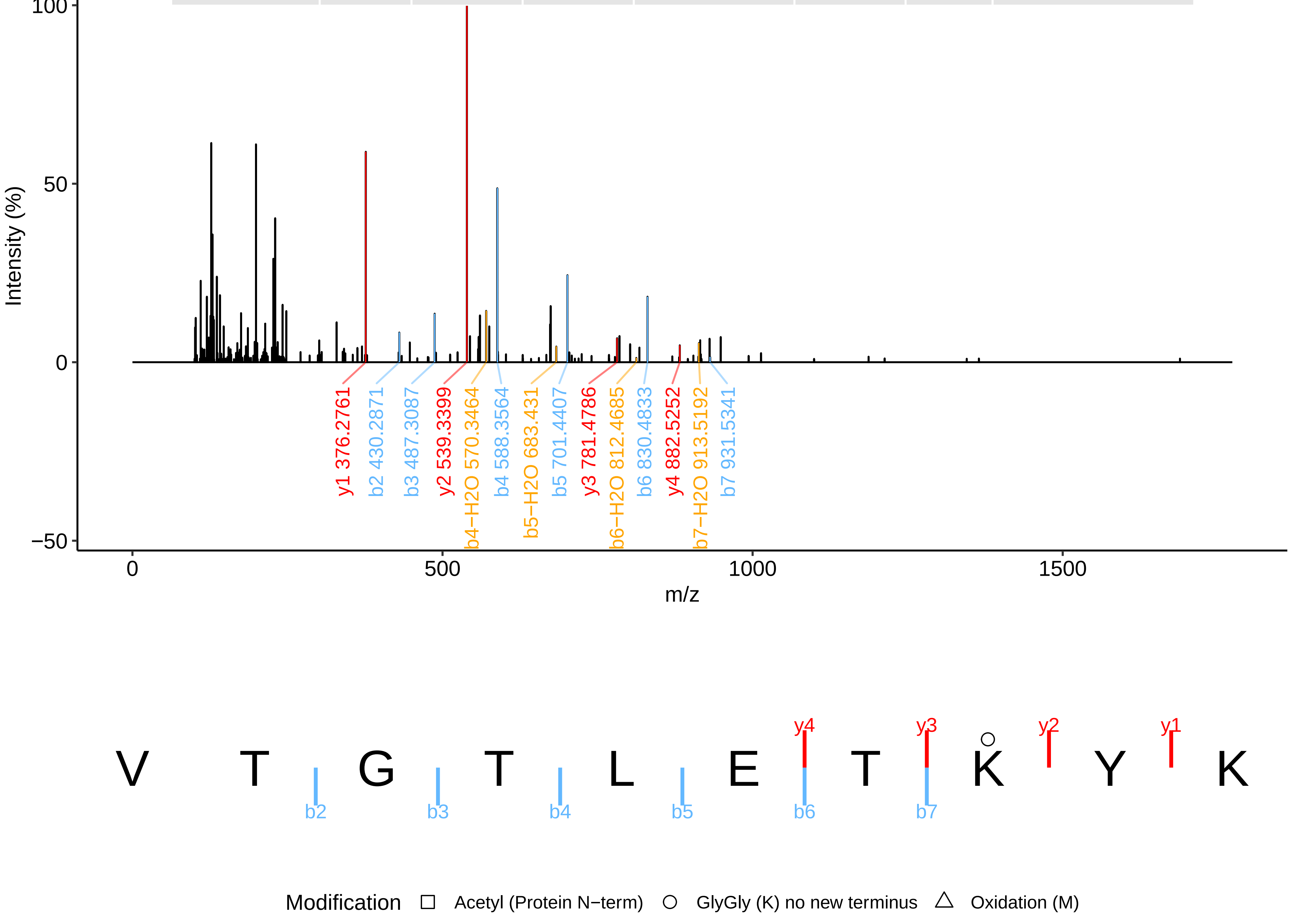

20200924_KIZ_MUb5_GlyGly_2 − Scan number: 10497

Gene Names	Charge	m/z	Mass	Retention time	PEP	Score	Precursor Intensity
VDAC2	3	418.5627	1252.666	39.179	0.0063088	76.943	6399758

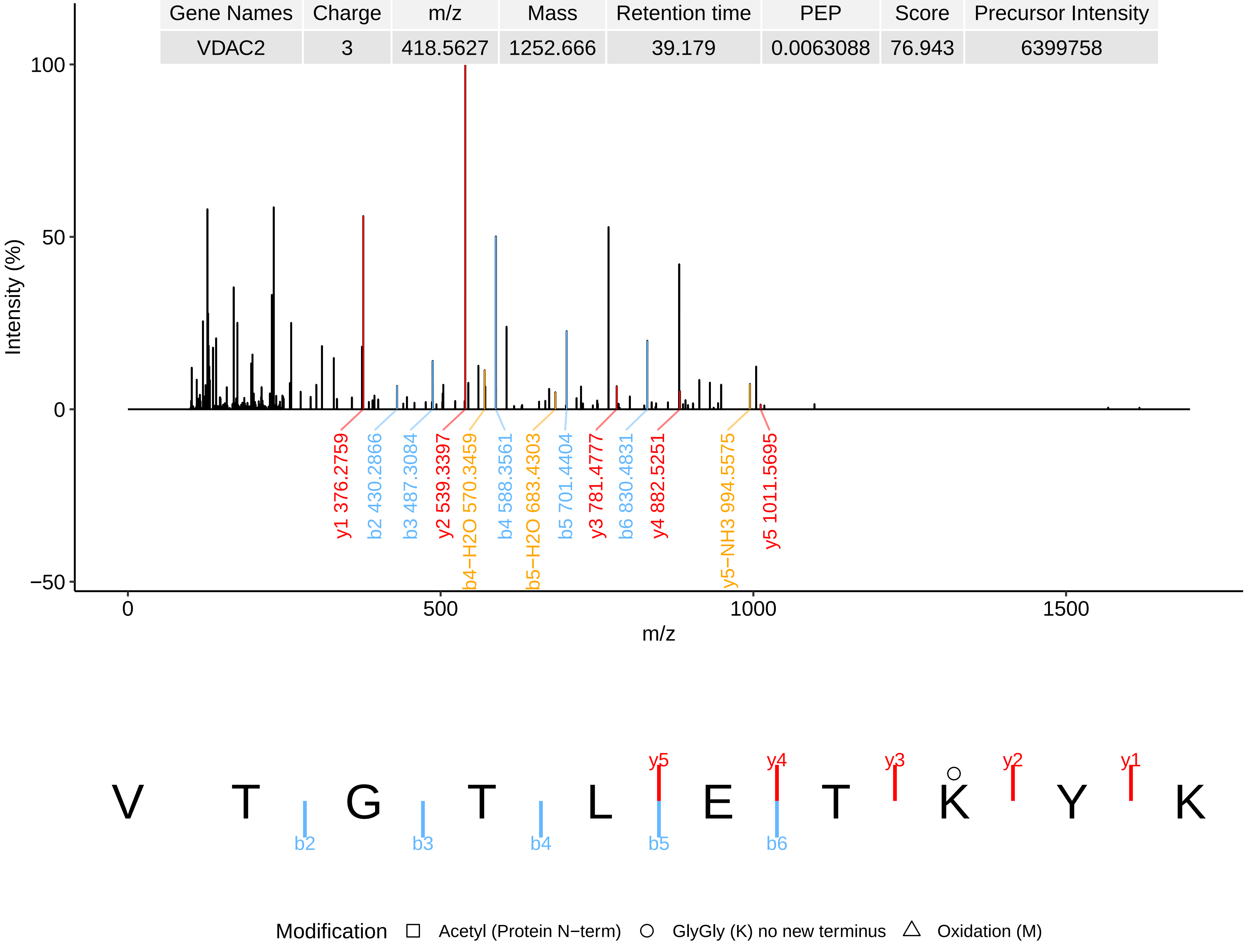

20201118_KIZ_MUb6_GlyGly_1 − Scan number: 11021

Gene Names	Charge	m/z	Mass	Retention time	PEP	Score	Precursor Intensity
VDAC2	3	418.5627	1252.666	34.79	0.01862	67.207	NA

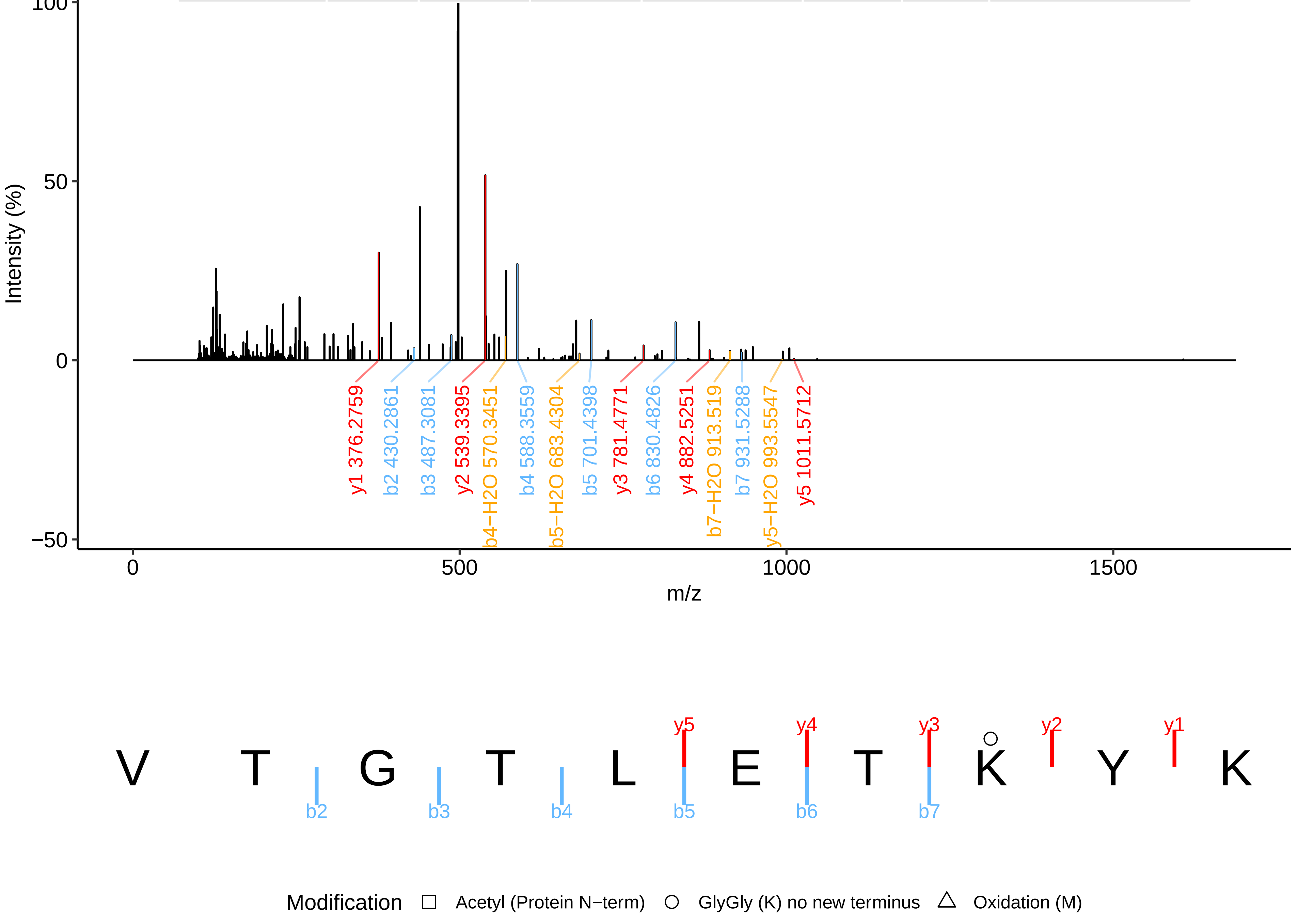

20201118_KIZ_MUb6_GlyGly_2 − Scan number: 13084

Gene Names	Charge	m/z	Mass	Retention time	PEP	Score	Precursor Intensity
VDAC2	3	418.5627	1252.666	44.076	0.0091406	74.165	3305904

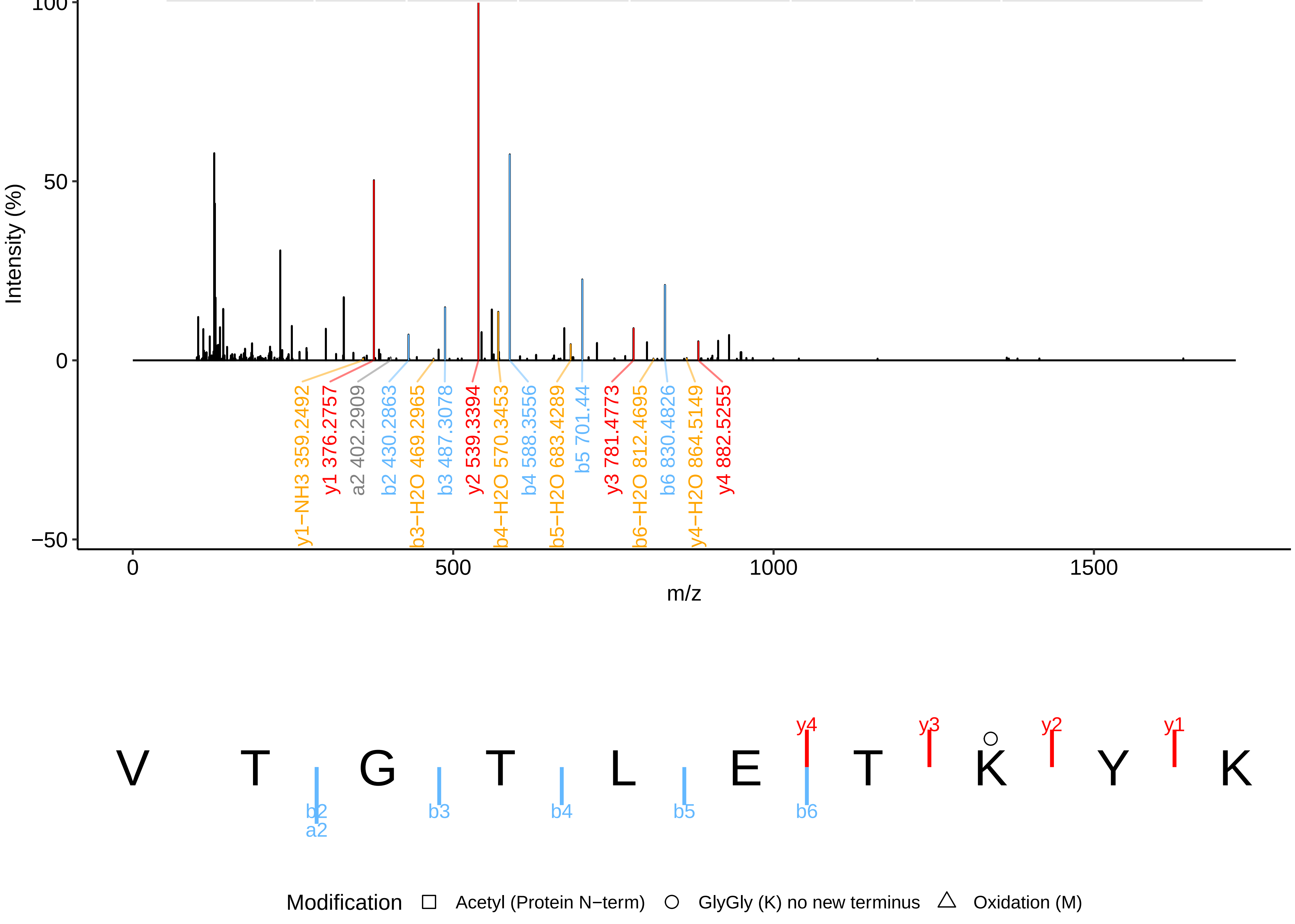

20200921_KIZ_MUb4_GlyGly_10plex − Scan number: 16629

Gene Names	Charge	m/z	Mass	Retention time	PEP	Score	Precursor Intensity
MFAP3L;MFAP3	3	491.2502	1470.729	59.793	0.001177	86.276	2624099

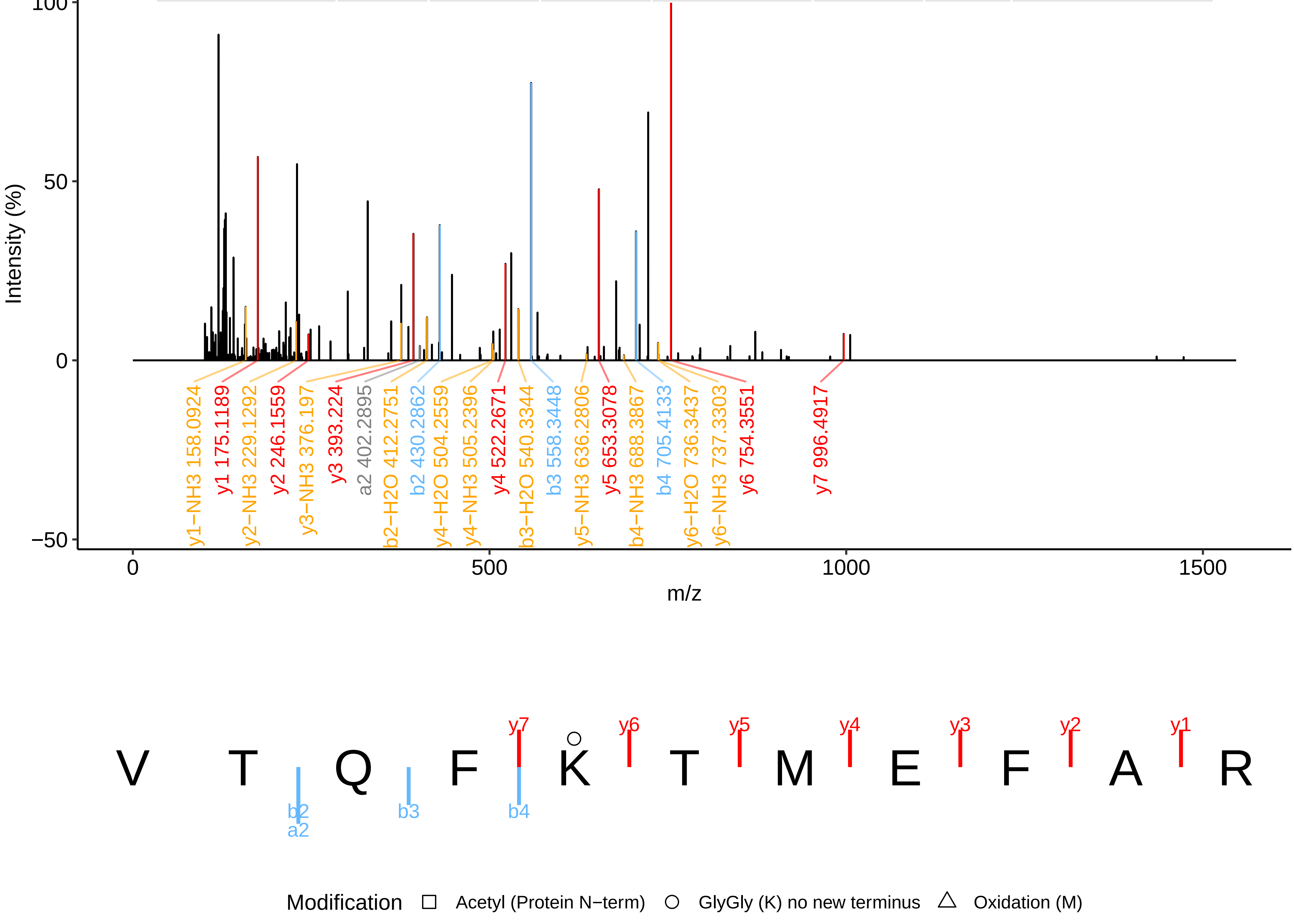

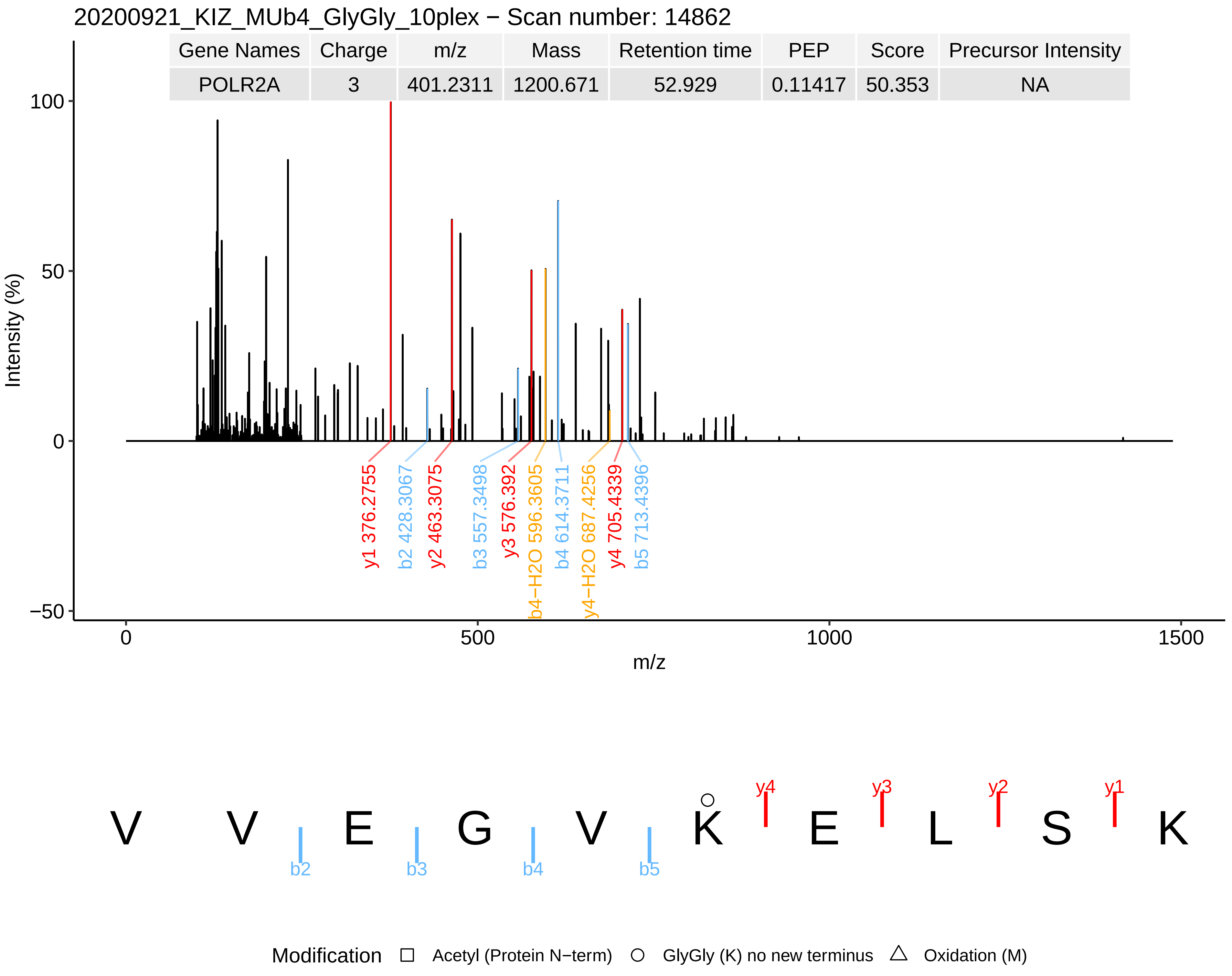

20200921_KIZ_MUb4_GlyGly_10plex − Scan number: 14862
Gene Names | Charge | m/z | Mass | Retention time | PEP | Score | Precursor Intensity
POLR2A | 3 | 401.2311 | 1200.671 | 52.929 | 0.11417 | 50.353 | NA
Intensity (%)
m/z
y1 376.2755
b2 428.3067
y2 463.3075
b3 557.3498
y3 576.392
b4−H2O 596.3605
b4 614.3711
y4−H2O 687.4256
y4 705.4339
b5 713.4396
V V E G V K E L S K
b2 b3 b4 b5
y4 y3 y2 y1
Modification
Acetyl (Protein N−term)
GlyGly (K) no new terminus
Oxidation (M)

20200921_KIZ_MUb4_GlyGly_10plex − Scan number: 9598

Gene Names	Charge	m/z	Mass	Retention time	PEP	Score	Precursor Intensity
KRT18	3	492.2668	1473.779	32.908	8.0006e−31	201.46	5318587

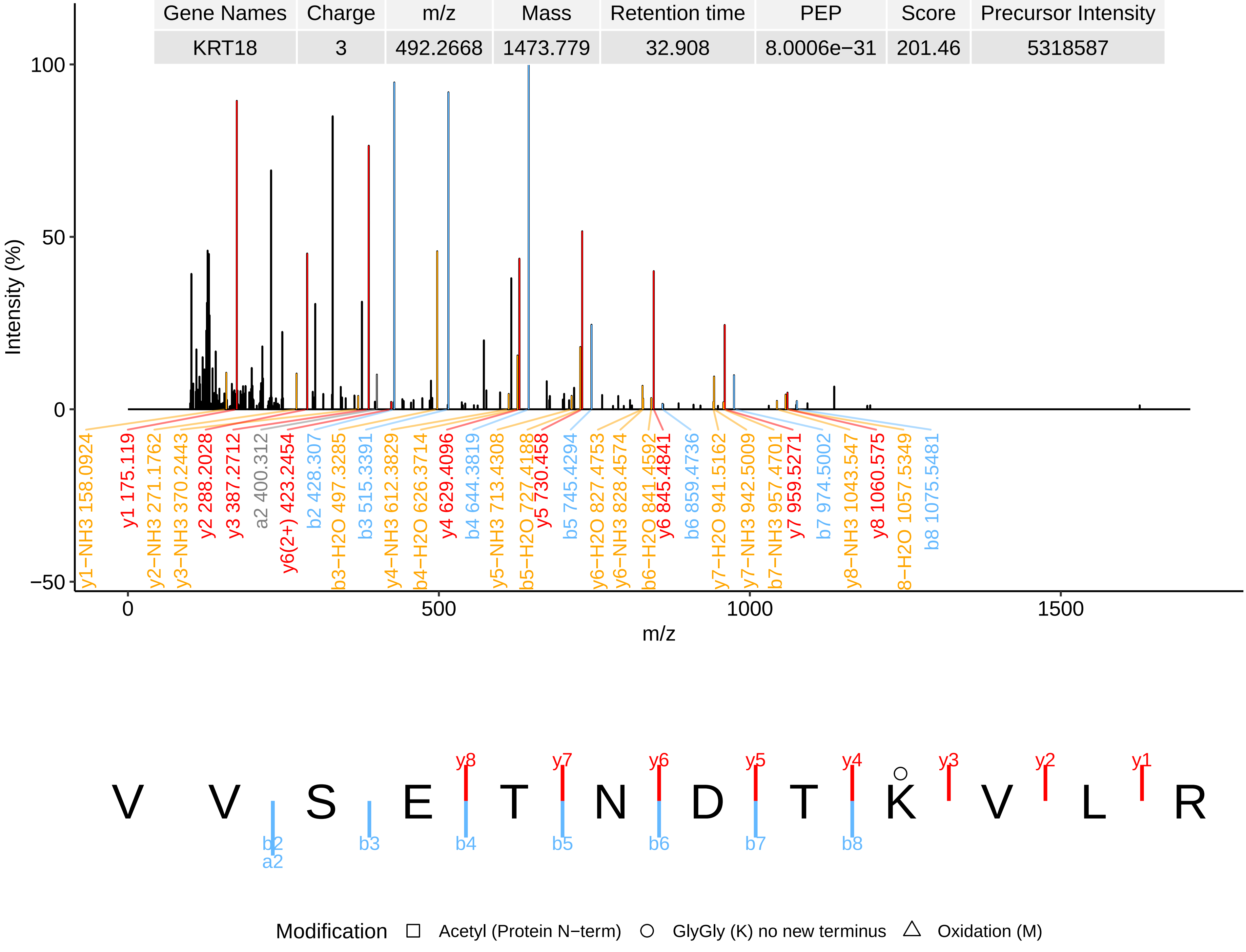

20200921_KIZ_MUb4_GlyGly_10plex − Scan number: 9387

Gene Names	Charge	m/z	Mass	Retention time	PEP	Score	Precursor Intensity
CYB5R3	4	312.9147	1247.63	32.134	0.016378	65.224	21632206

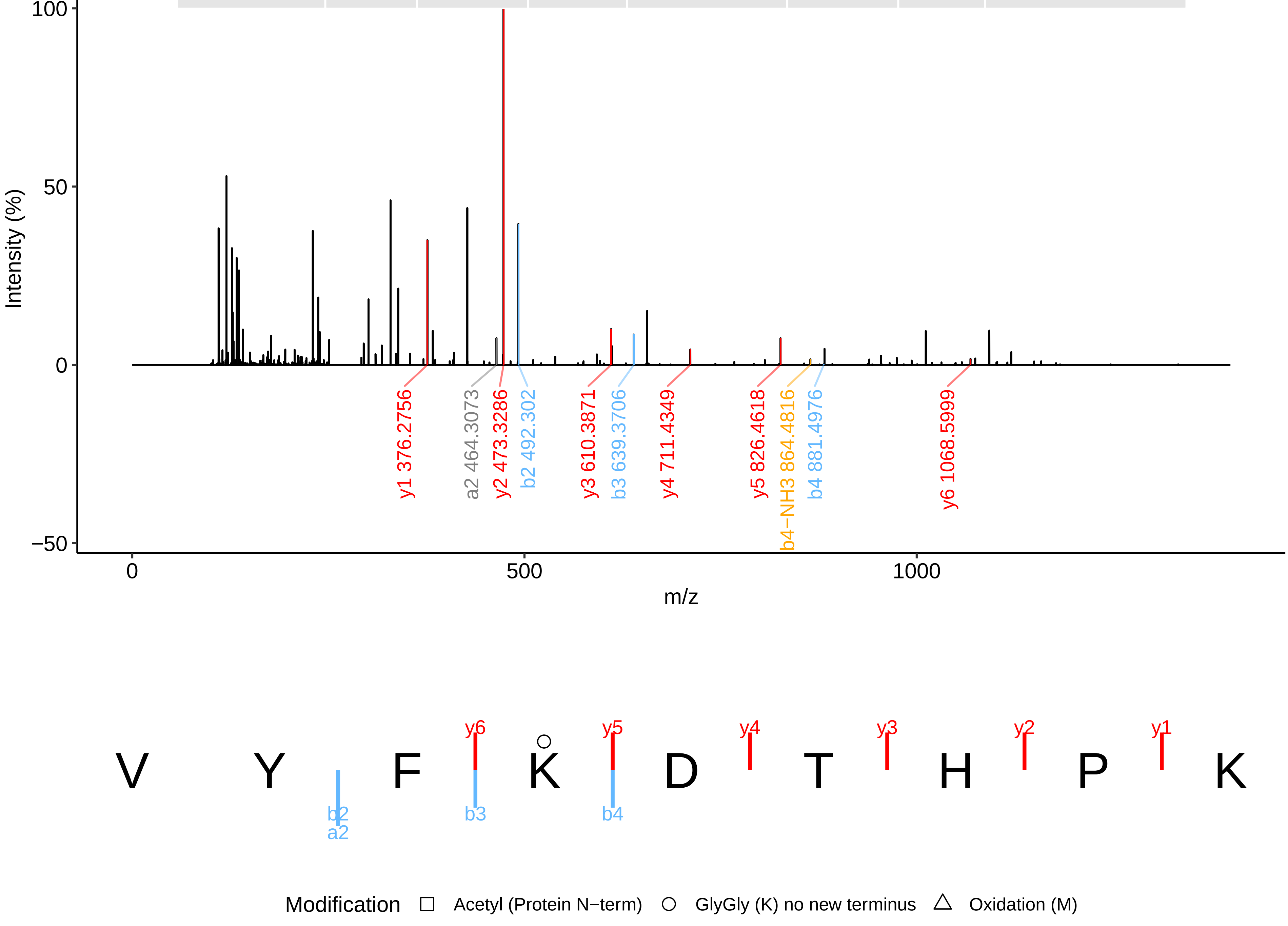

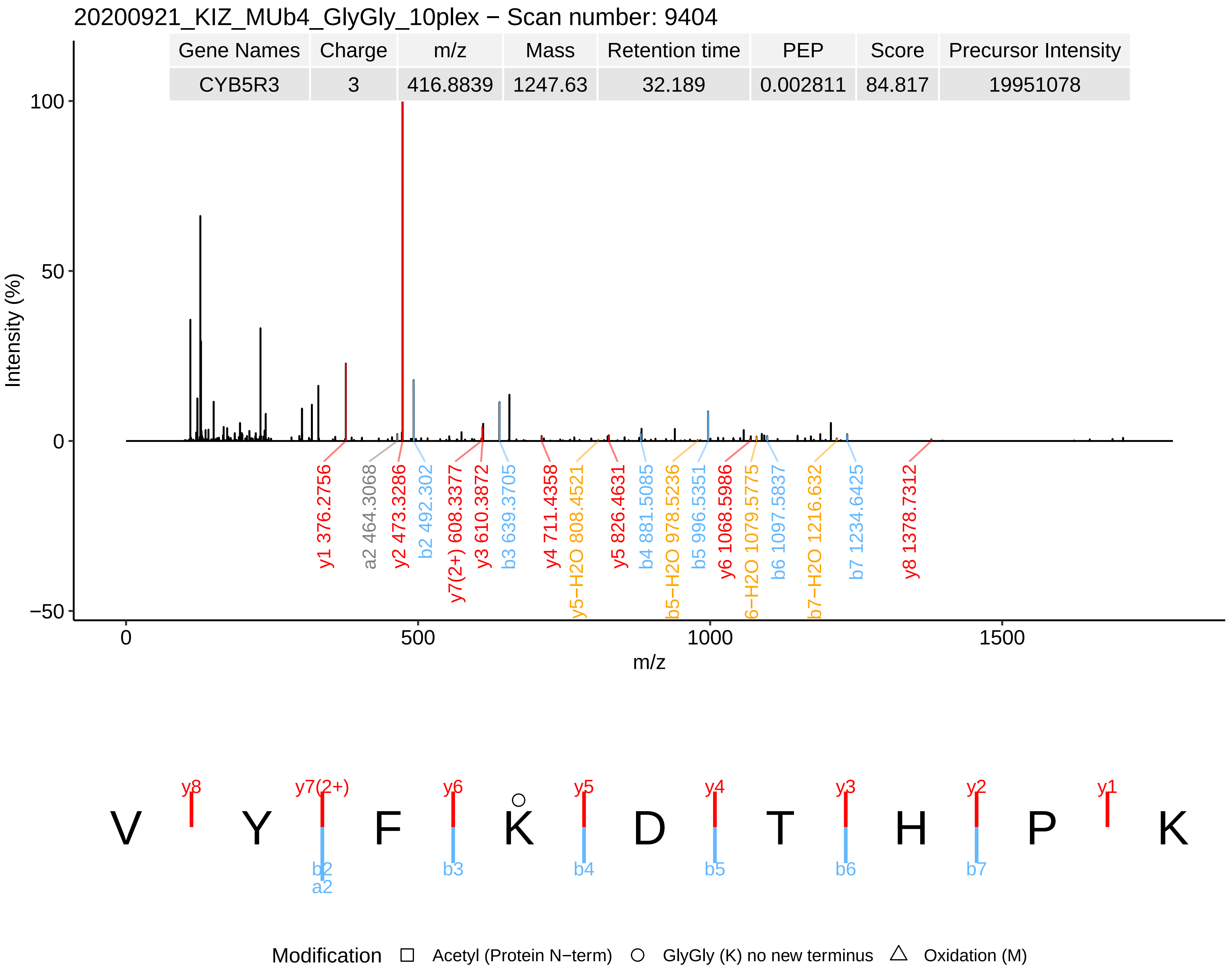
20200921_KIZ_MUb4_GlyGly_10plex − Scan number: 9404
Gene Names | Charge | m/z | Mass | Retention time | PEP | Score | Precursor Intensity
CYB5R3 | 3 | 416.8839 | 1247.63 | 32.189 | 0.002811 | 84.817 | 19951078
Intensity (%)
m/z
y1 376.2756
a2 464.3068
y2 473.3286
b2 492.302
y7(2+) 608.3377
y3 610.3872
b3 639.3705
y4 711.4358
y5−H2O 808.4521
y5 826.4631
b4 881.5085
b5−H2O 978.5236
b5 996.5351
y6 1068.5986
y6−H2O 1079.5775
b6 1097.5837
b7−H2O 1216.632
b7 1234.6425
y8 1378.7312
V Y F K D T H P K
y8 y7(2+) y6 y5 y4 y3 y2 y1
b2 a2 b3 b4 b5 b6 b7
Modification □ Acetyl (Protein N−term) ○ GlyGly (K) no new terminus △ Oxidation (M)

20200921_KIZ_MUb4_GlyGly_10plex_2 − Scan number: 8737

Gene Names	Charge	m/z	Mass	Retention time	PEP	Score	Precursor Intensity
CYB5R3	4	312.9147	1247.63	33.016	0.050387	55.064	6283659

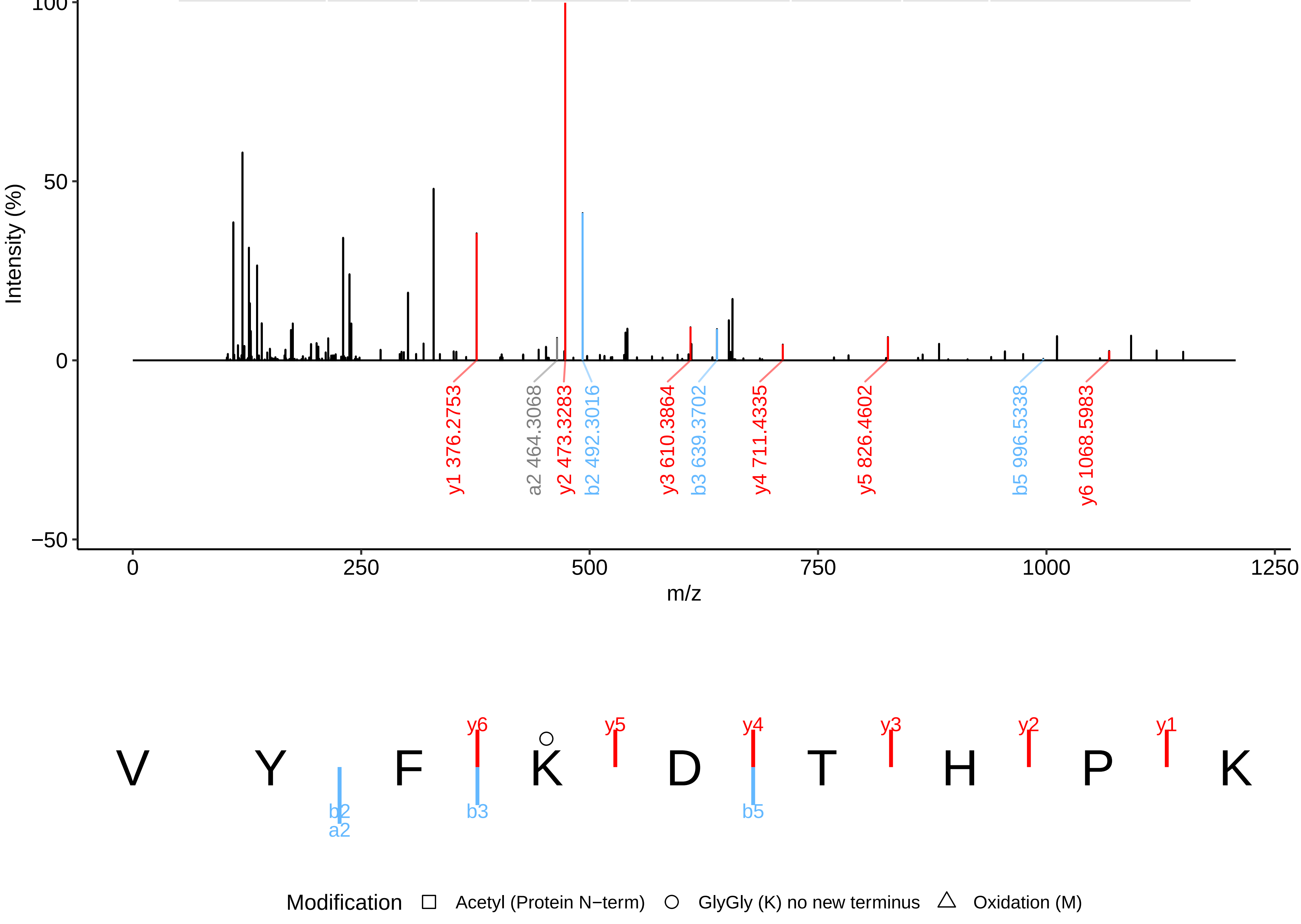

20200921_KIZ_MUb4_GlyGly_10plex_2 − Scan number: 8742

Gene Names	Charge	m/z	Mass	Retention time	PEP	Score	Precursor Intensity
CYB5R3	3	416.8839	1247.63	33.036	0.01056	73.985	1813343

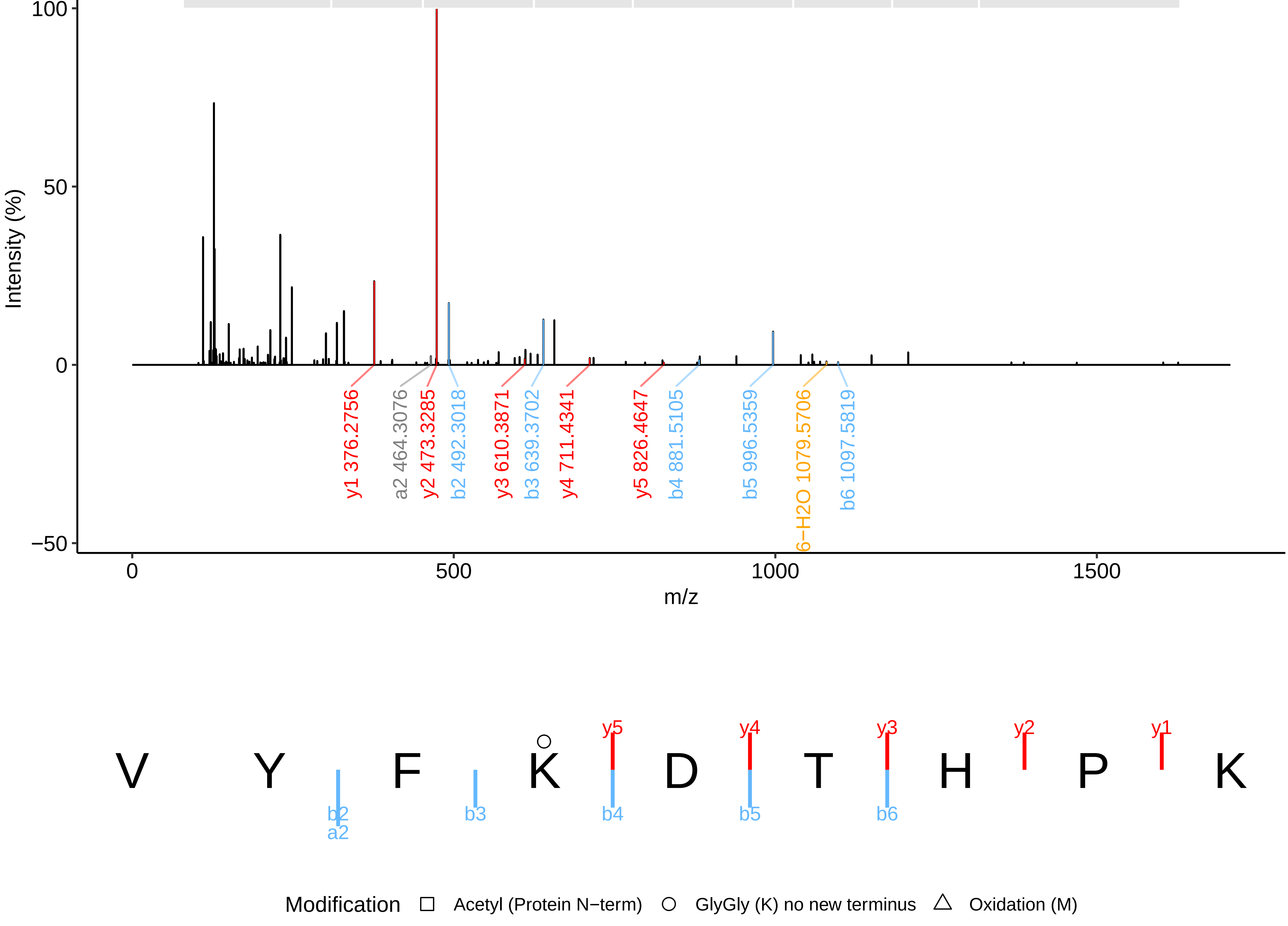

20200924_KIZ_MUb5_GlyGly_1 − Scan number: 7822

Gene Names	Charge	m/z	Mass	Retention time	PEP	Score	Precursor Intensity
CYB5R3	3	416.8839	1247.63	24.917	0.017643	67.334	6181574

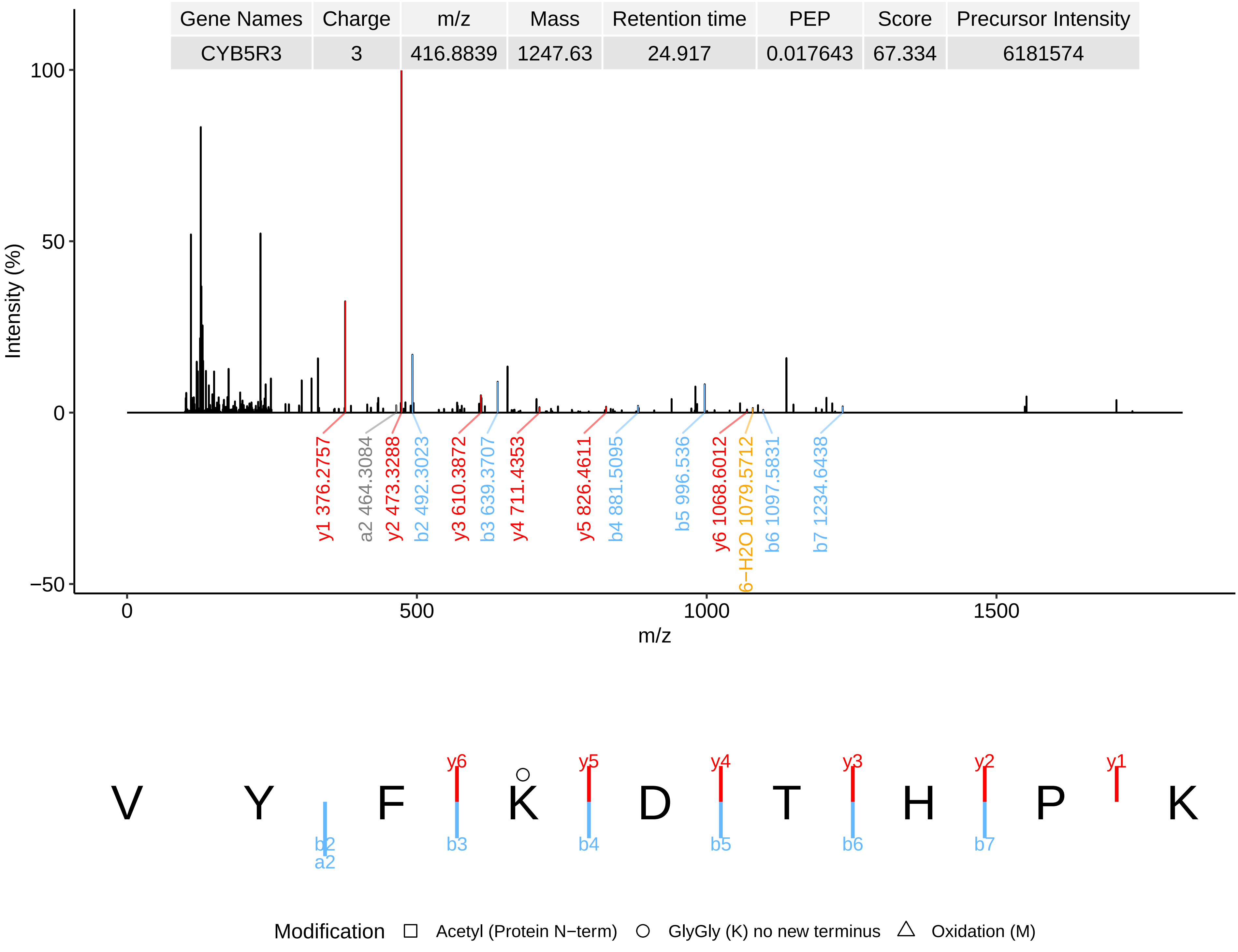

20200924_KIZ_MUb5_GlyGly_1 − Scan number: 7850

Gene Names	Charge	m/z	Mass	Retention time	PEP	Score	Precursor Intensity
CYB5R3	4	312.9147	1247.63	25.01	0.034828	58.981	20749120

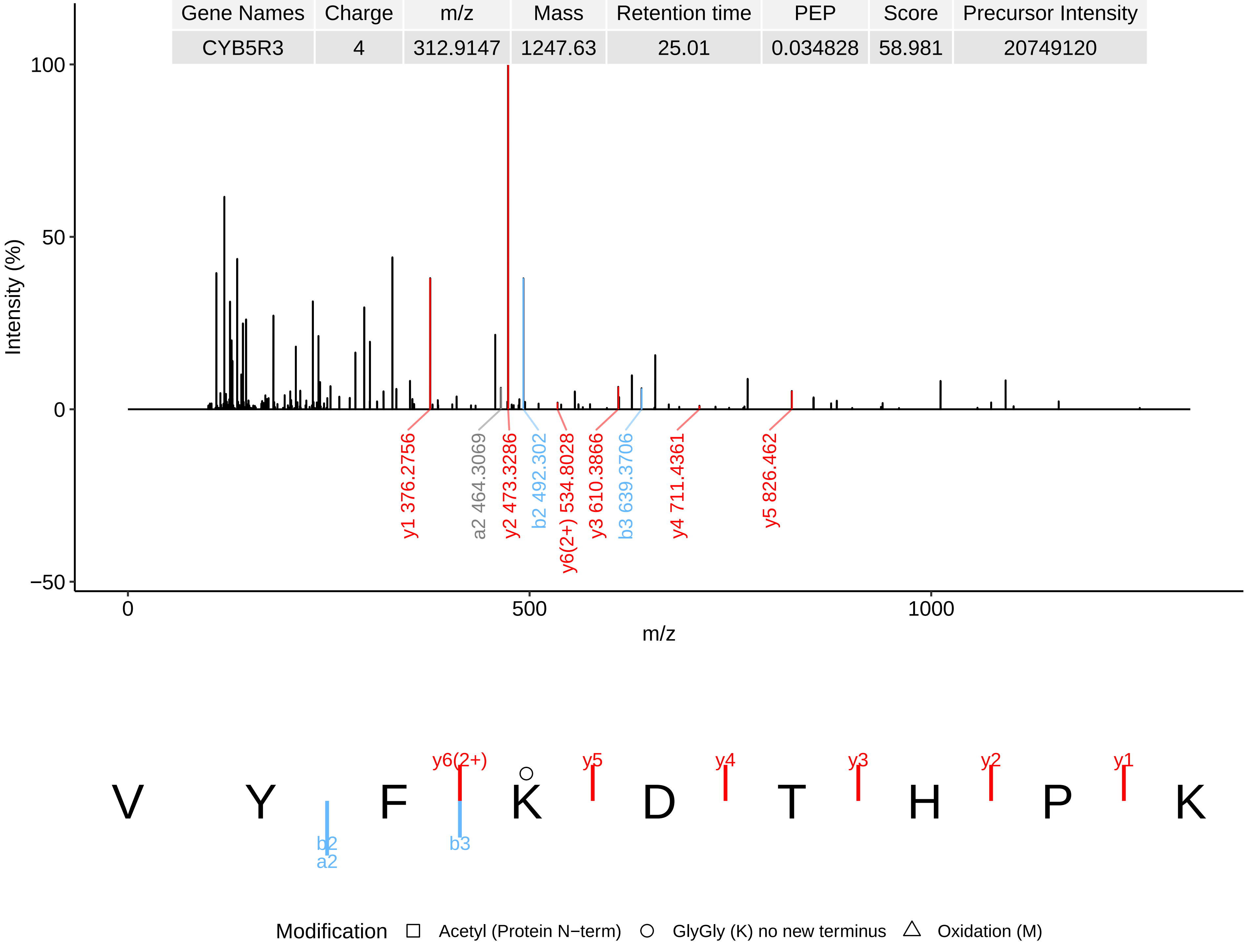

20200924_KIZ_MUb5_GlyGly_1 − Scan number: 8010

Gene Names	Charge	m/z	Mass	Retention time	PEP	Score	Precursor Intensity
CYB5R3	4	312.9147	1247.63	25.521	0.075291	48.794	39509200

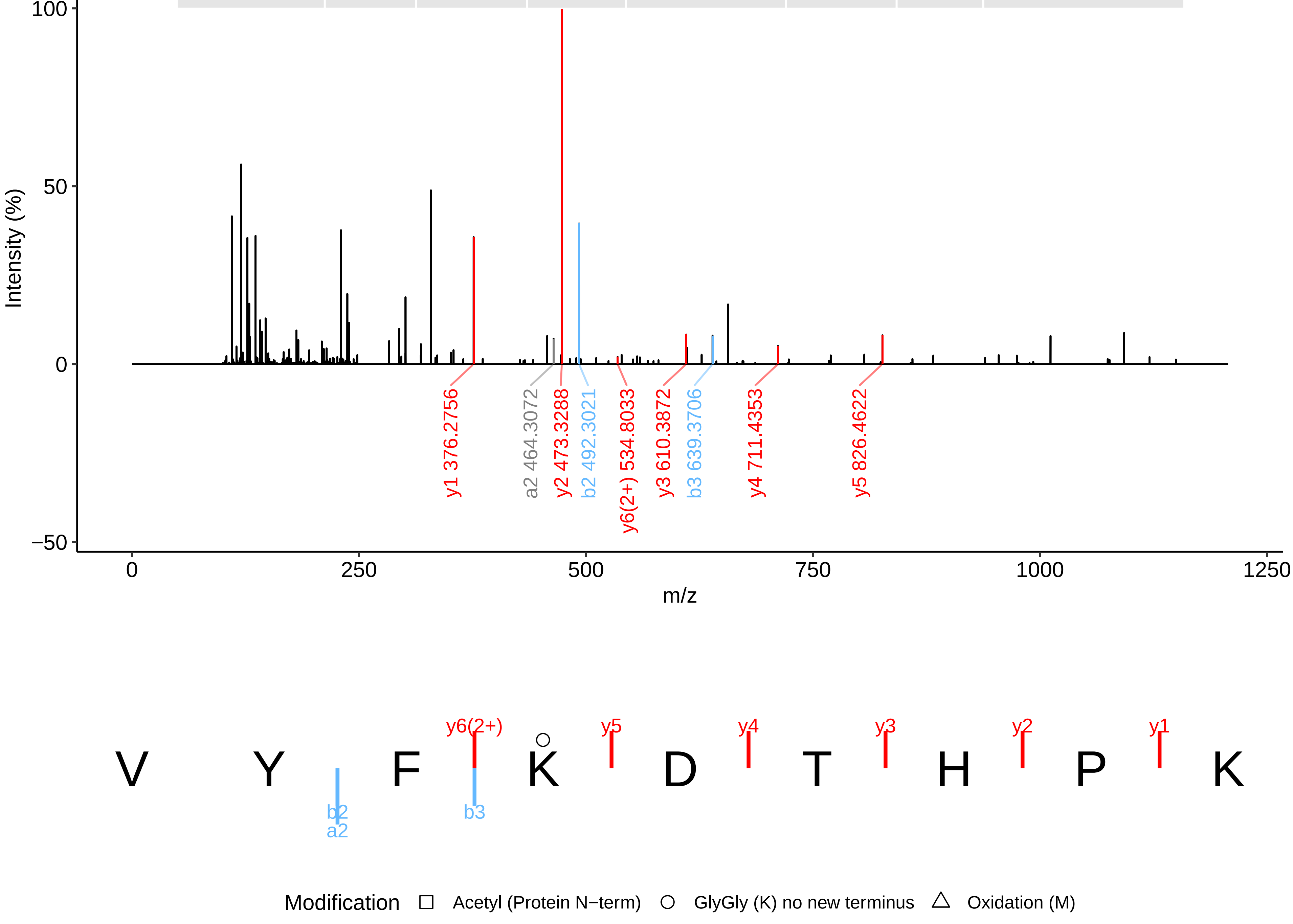

20200924_KIZ_MUb5_GlyGly_1 − Scan number: 8160

Gene Names	Charge	m/z	Mass	Retention time	PEP	Score	Precursor Intensity
CYB5R3	4	312.9147	1247.63	26.027	0.052198	54.608	88689536

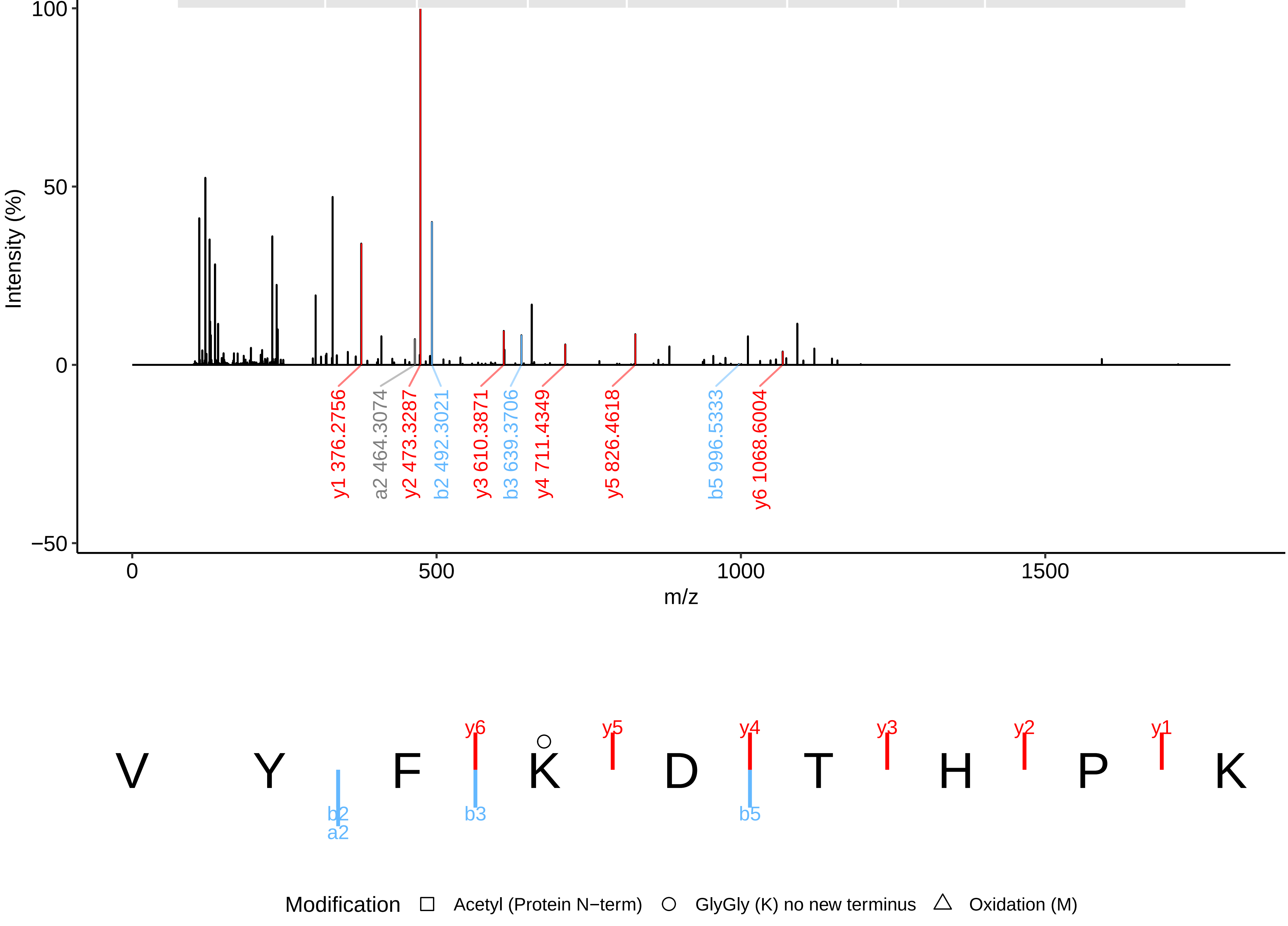

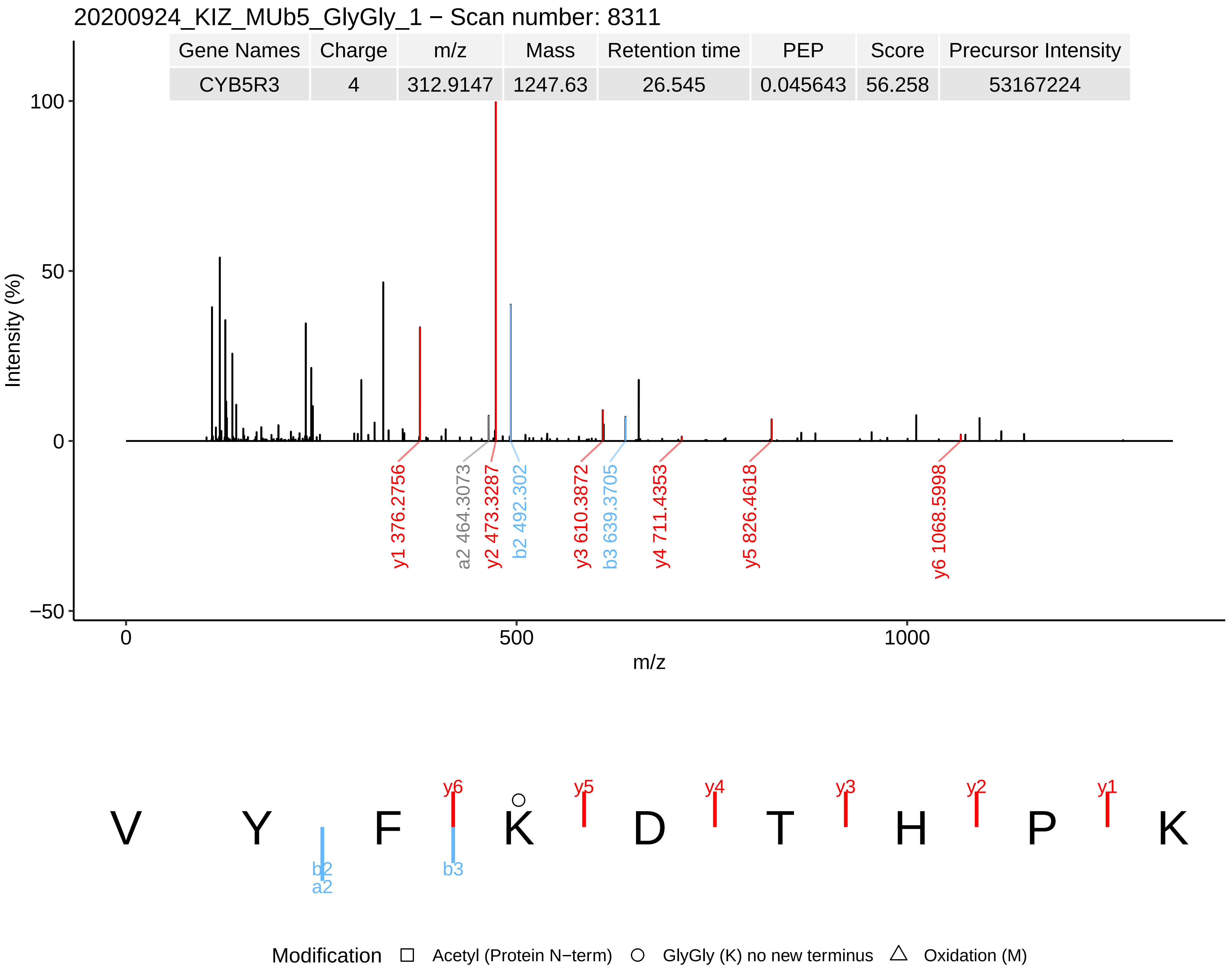

20200924_KIZ_MUb5_GlyGly_1 − Scan number: 8311
Gene Names | Charge | m/z | Mass | Retention time | PEP | Score | Precursor Intensity
CYB5R3 | 4 | 312.9147 | 1247.63 | 26.545 | 0.045643 | 56.258 | 53167224
Intensity (%)
100
50
0
−50
0
500
1000
m/z
y1 376.2756
a2 464.3073
y2 473.3287
b2 492.302
y3 610.3872
b3 639.3705
y4 711.4353
y5 826.4618
y6 1068.5998
V
Y
F
K
D
T
H
P
K
y6
y5
y4
y3
y2
y1
b2
a2
b3
Modification
Acetyl (Protein N−term)
GlyGly (K) no new terminus
Oxidation (M)

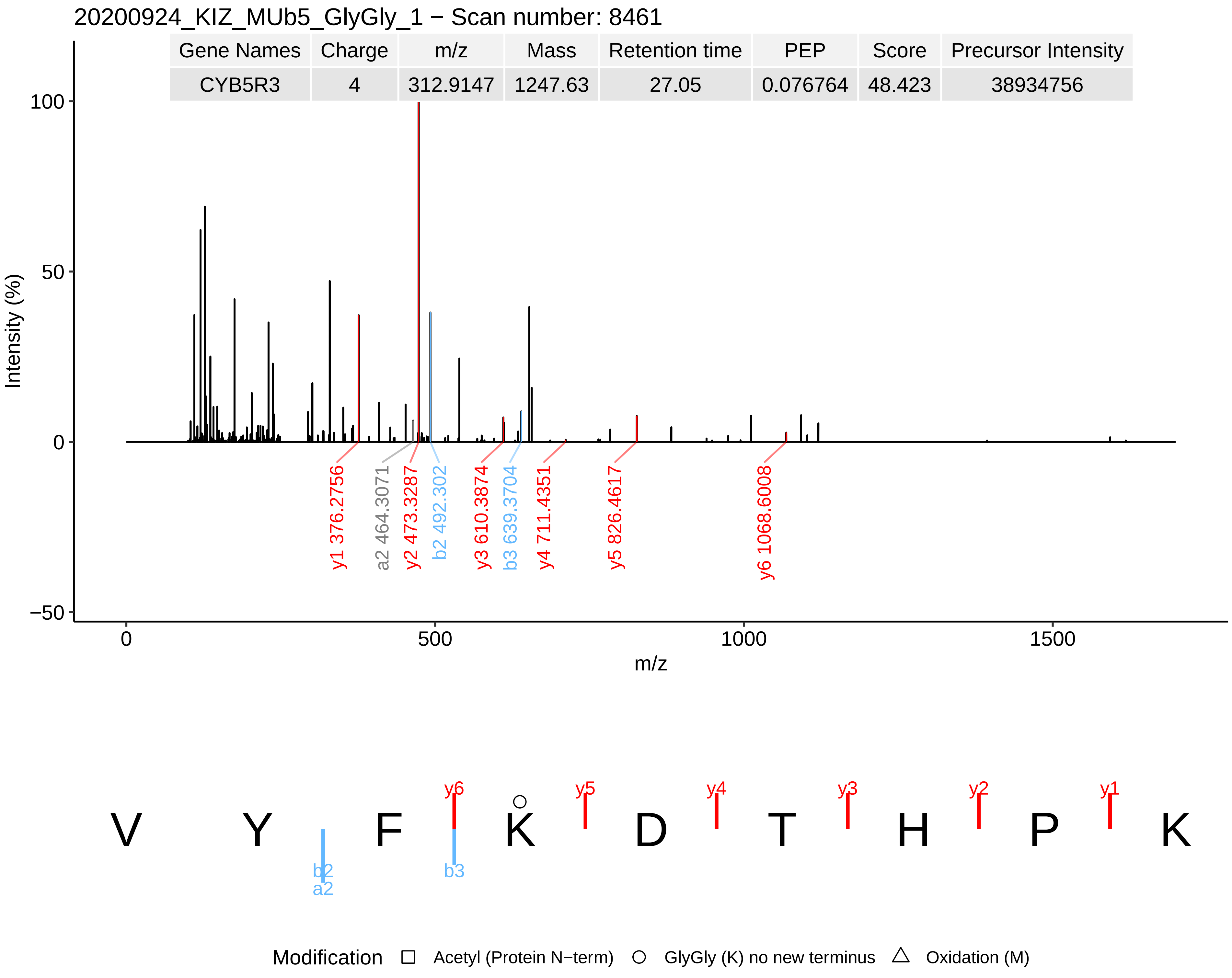
20200924_KIZ_MUb5_GlyGly_1 − Scan number: 8461
Gene Names | Charge | m/z | Mass | Retention time | PEP | Score | Precursor Intensity
CYB5R3 | 4 | 312.9147 | 1247.63 | 27.05 | 0.076764 | 48.423 | 38934756
Intensity (%)
m/z
y1 376.2756
a2 464.3071
y2 473.3287
b2 492.302
y3 610.3874
b3 639.3704
y4 711.4351
y5 826.4617
y6 1068.6008
V Y F K D T H P K
y6 y5 y4 y3 y2 y1
b2 a2 b3
Modification
Acetyl (Protein N−term)
GlyGly (K) no new terminus
Oxidation (M)

20200924_KIZ_MUb5_GlyGly_1 − Scan number: 8131

Gene Names	Charge	m/z	Mass	Retention time	PEP	Score	Precursor Intensity
CYB5R3	3	416.8839	1247.63	25.932	0.00050857	99.973	NA

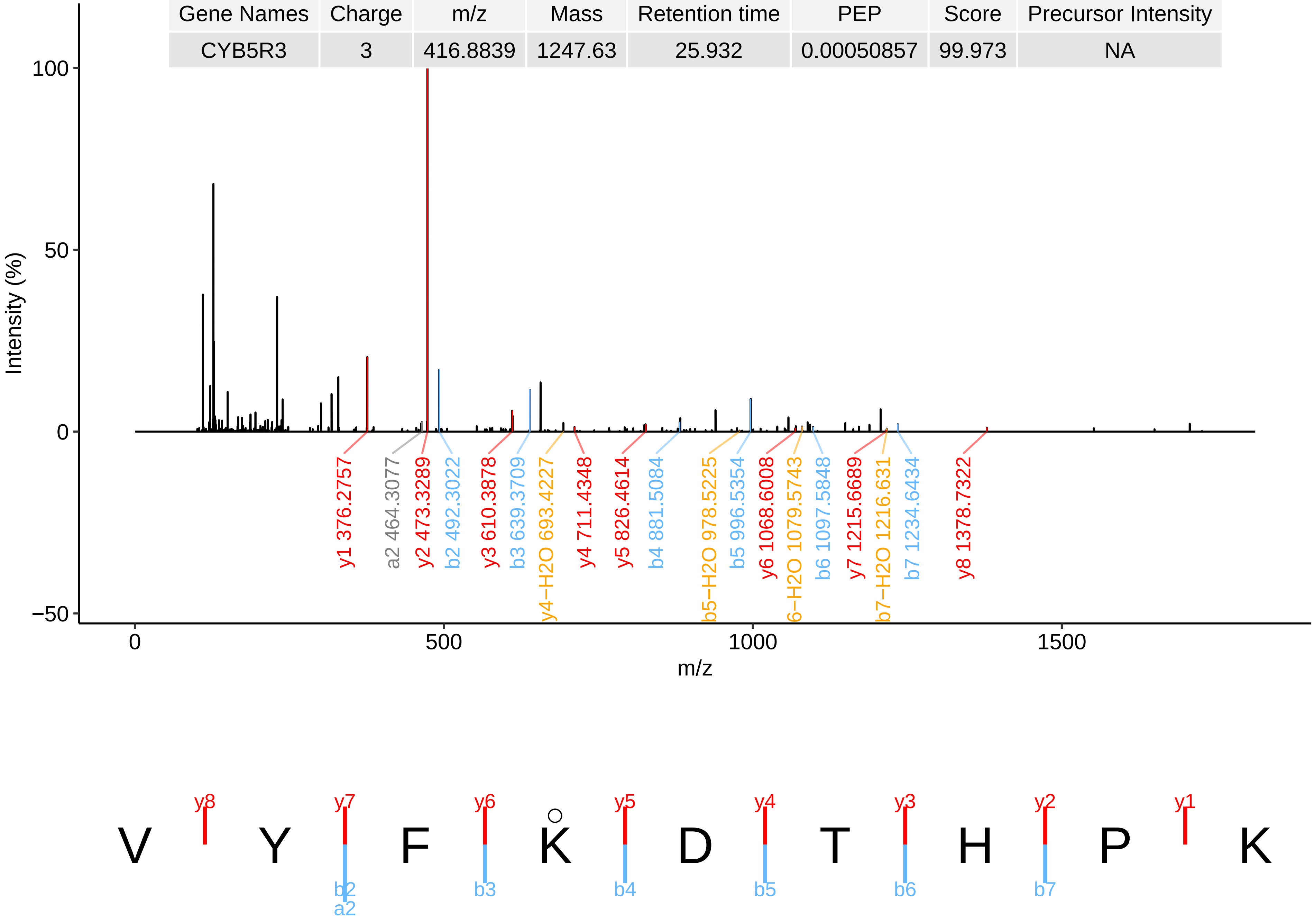

Modification □ Acetyl (Protein N−term) ○ GlyGly (K) no new terminus △ Oxidation (M)

20200924_KIZ_MUb5_GlyGly_1 − Scan number: 8281

Gene Names	Charge	m/z	Mass	Retention time	PEP	Score	Precursor Intensity
CYB5R3	3	416.8839	1247.63	26.447	0.00024127	108.43	33303104

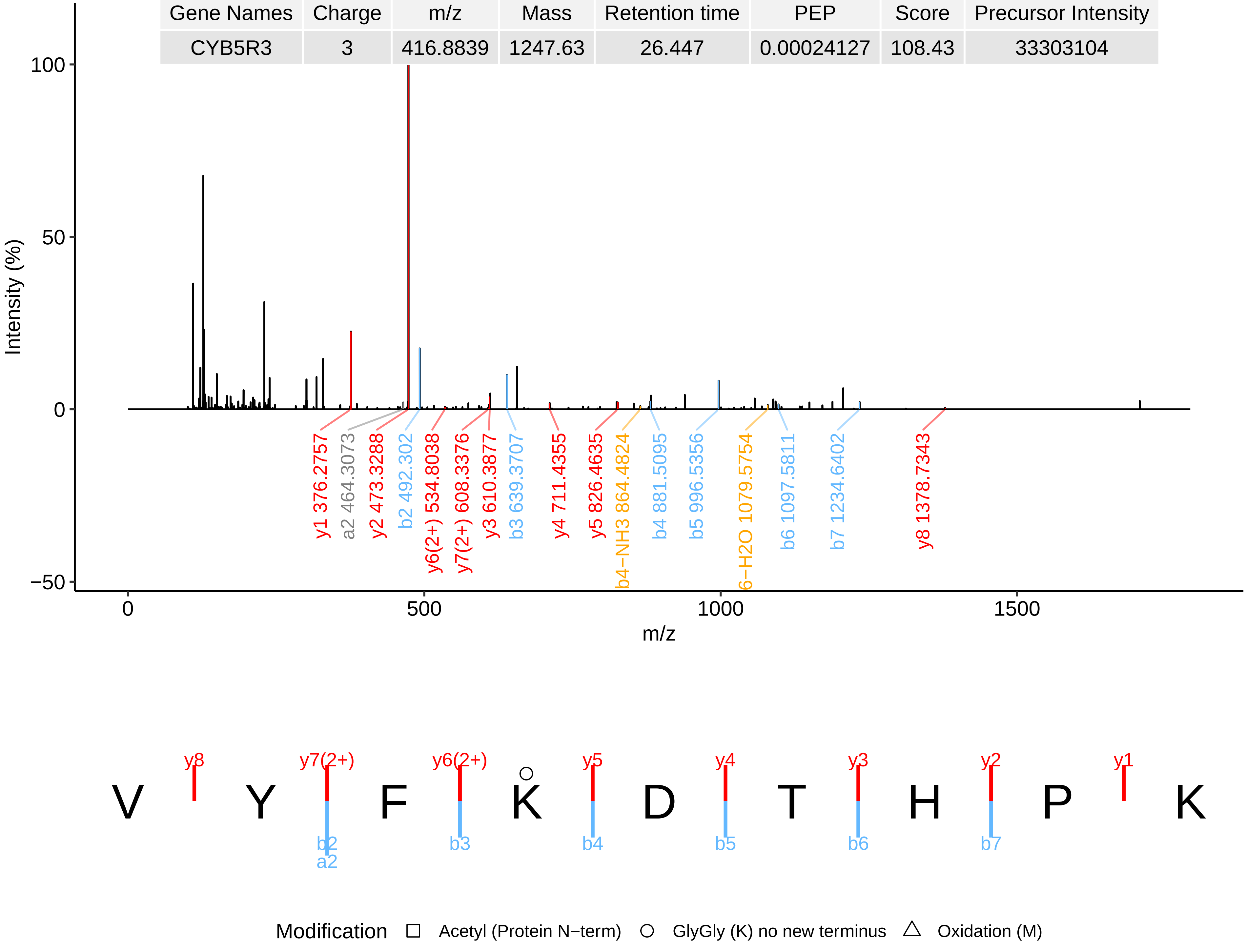

20200924_KIZ_MUb5_GlyGly_1 − Scan number: 8462

Gene Names	Charge	m/z	Mass	Retention time	PEP	Score	Precursor Intensity
CYB5R3	3	416.8839	1247.63	27.052	0.0066431	77.662	24327106

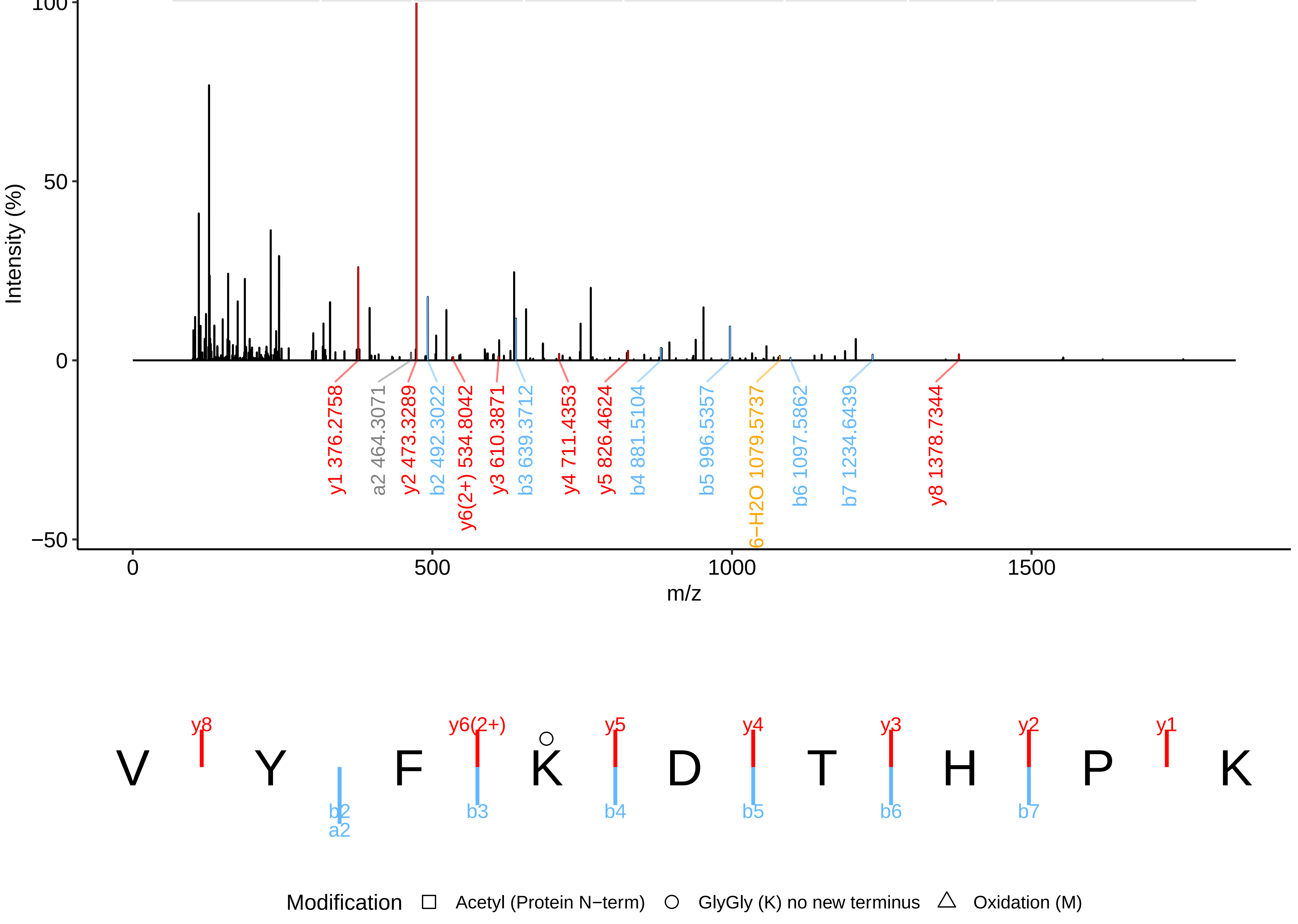

20200924_KIZ_MUb5_GlyGly_1 − Scan number: 7983

Gene Names	Charge	m/z	Mass	Retention time	PEP	Score	Precursor Intensity
CYB5R3	3	416.8839	1247.63	25.43	0.0028342	93.556	11129666

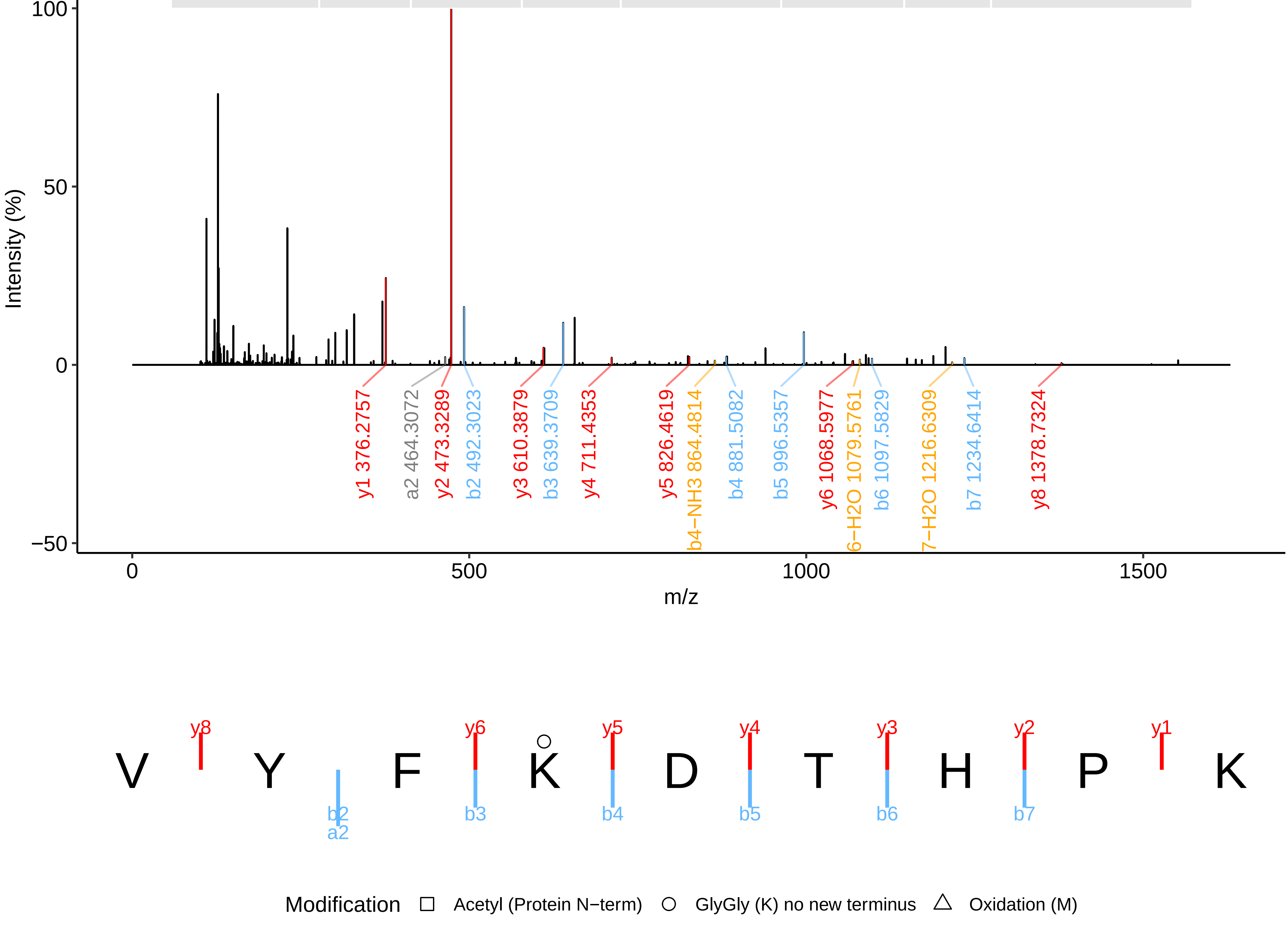

20200924_KIZ_MUb5_GlyGly_2 − Scan number: 7708

Gene Names	Charge	m/z	Mass	Retention time	PEP	Score	Precursor Intensity
CYB5R3	3	416.8839	1247.63	28.788	0.0066431	77.662	6290910

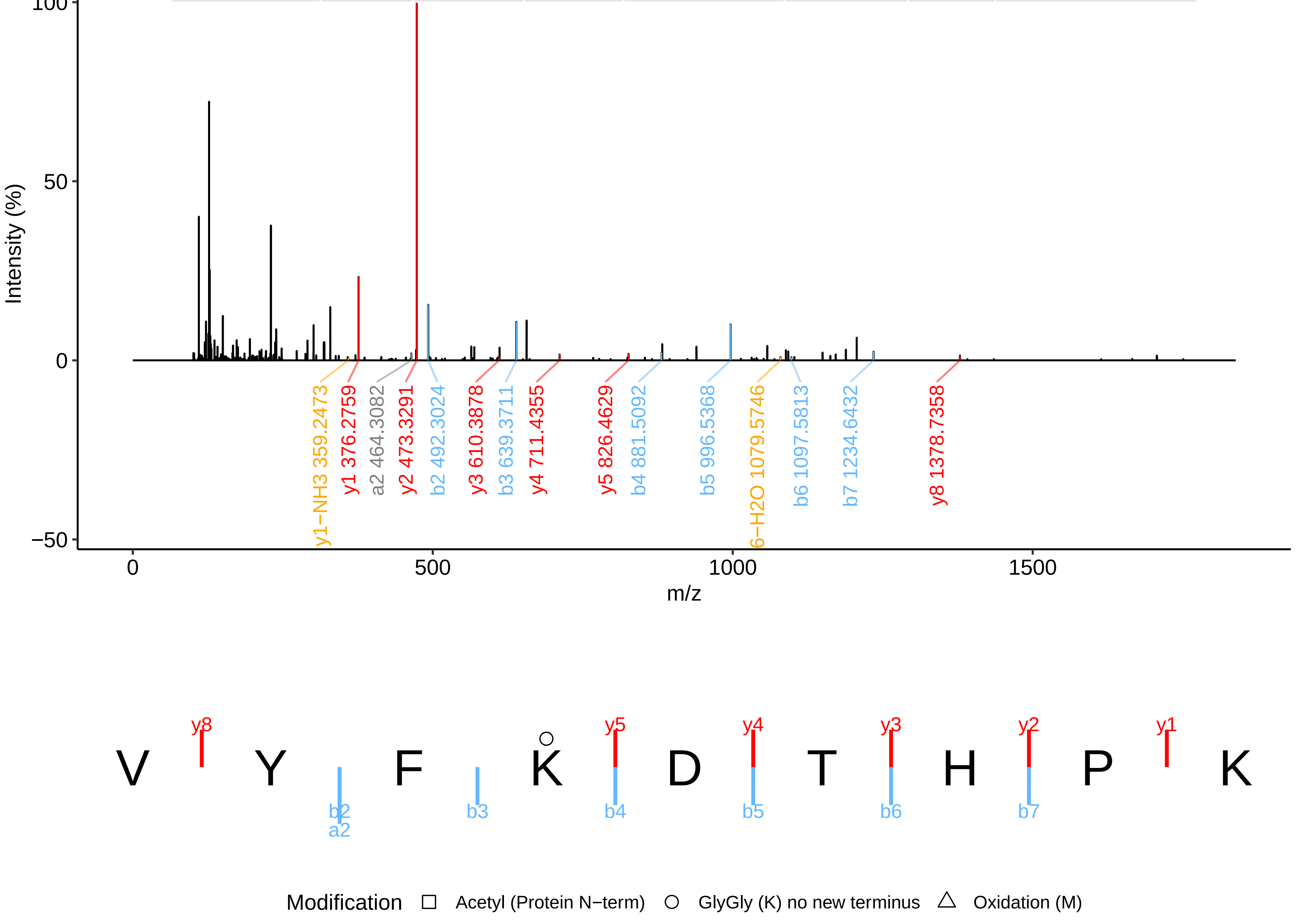

20200924_KIZ_MUb5_GlyGly_2 − Scan number: 7770

Gene Names	Charge	m/z	Mass	Retention time	PEP	Score	Precursor Intensity
CYB5R3	4	312.9147	1247.63	29.024	0.015153	66.621	21580600

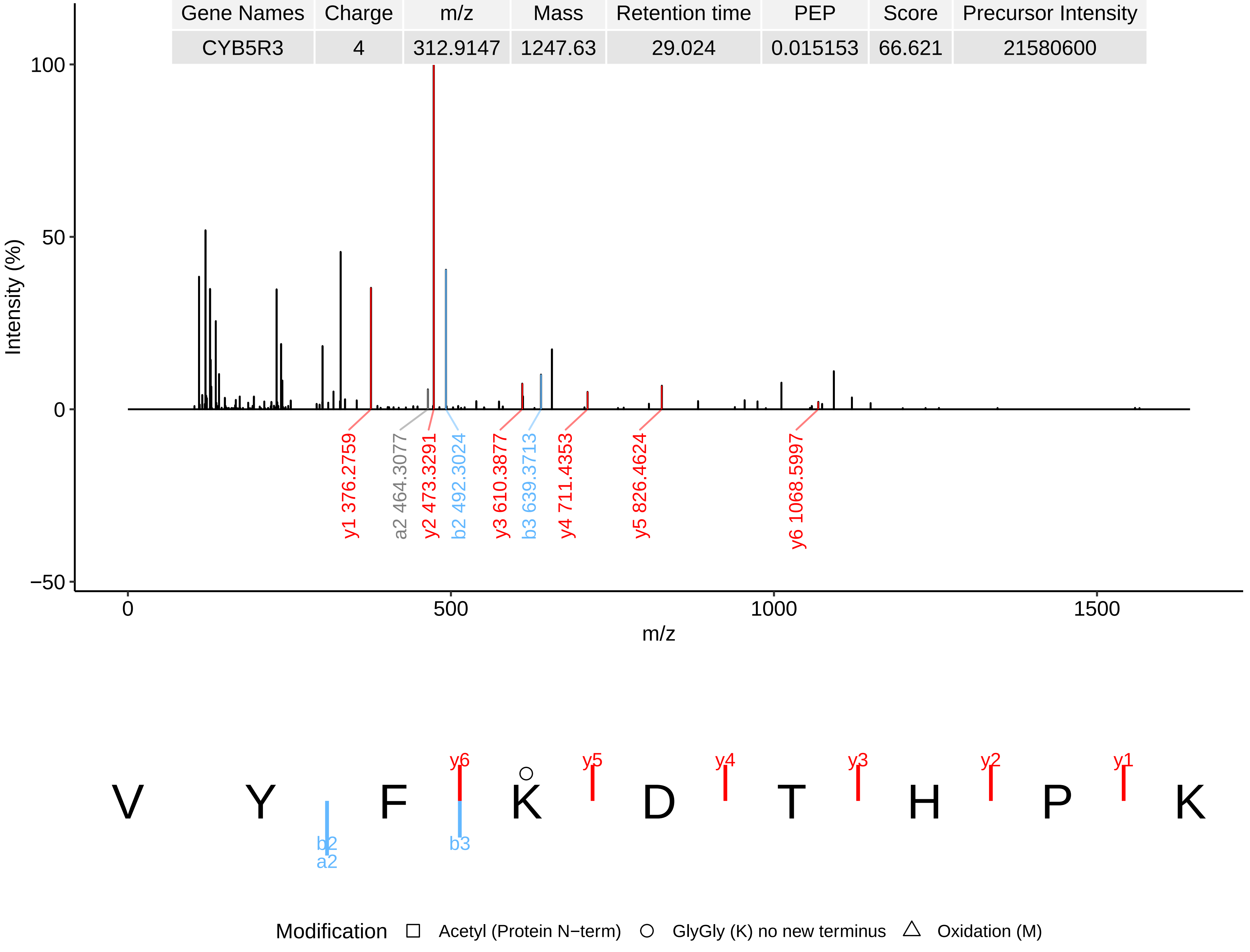

20200924_KIZ_MUb5_GlyGly_2 − Scan number: 7903

Gene Names	Charge	m/z	Mass	Retention time	PEP	Score	Precursor Intensity
CYB5R3	4	312.9147	1247.63	29.529	0.045643	56.258	24749122

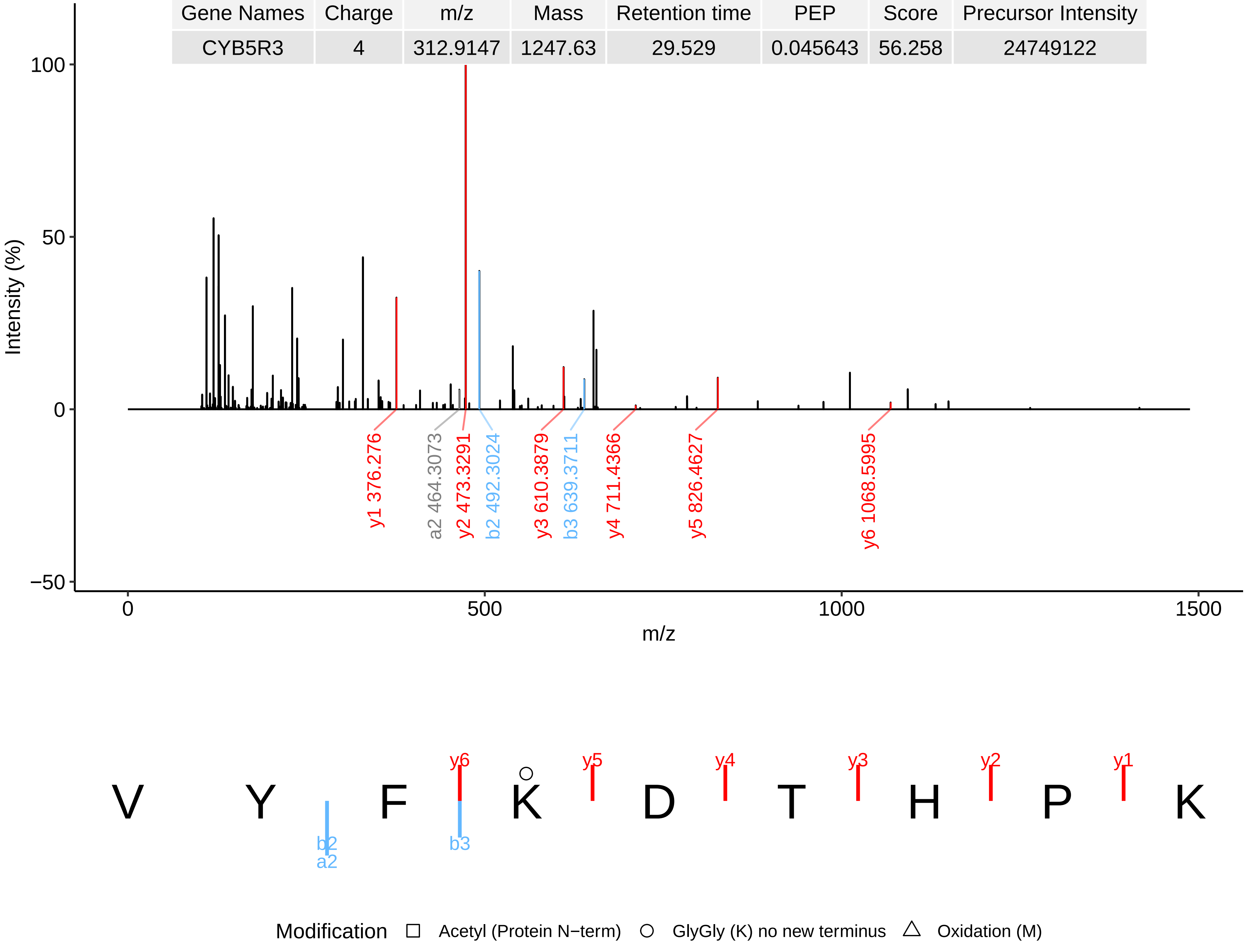

20200924_KIZ_MUb5_GlyGly_2 − Scan number: 8035

Gene Names	Charge	m/z	Mass	Retention time	PEP	Score	Precursor Intensity
CYB5R3	4	312.9147	1247.63	30.041	0.081633	47.198	8007673

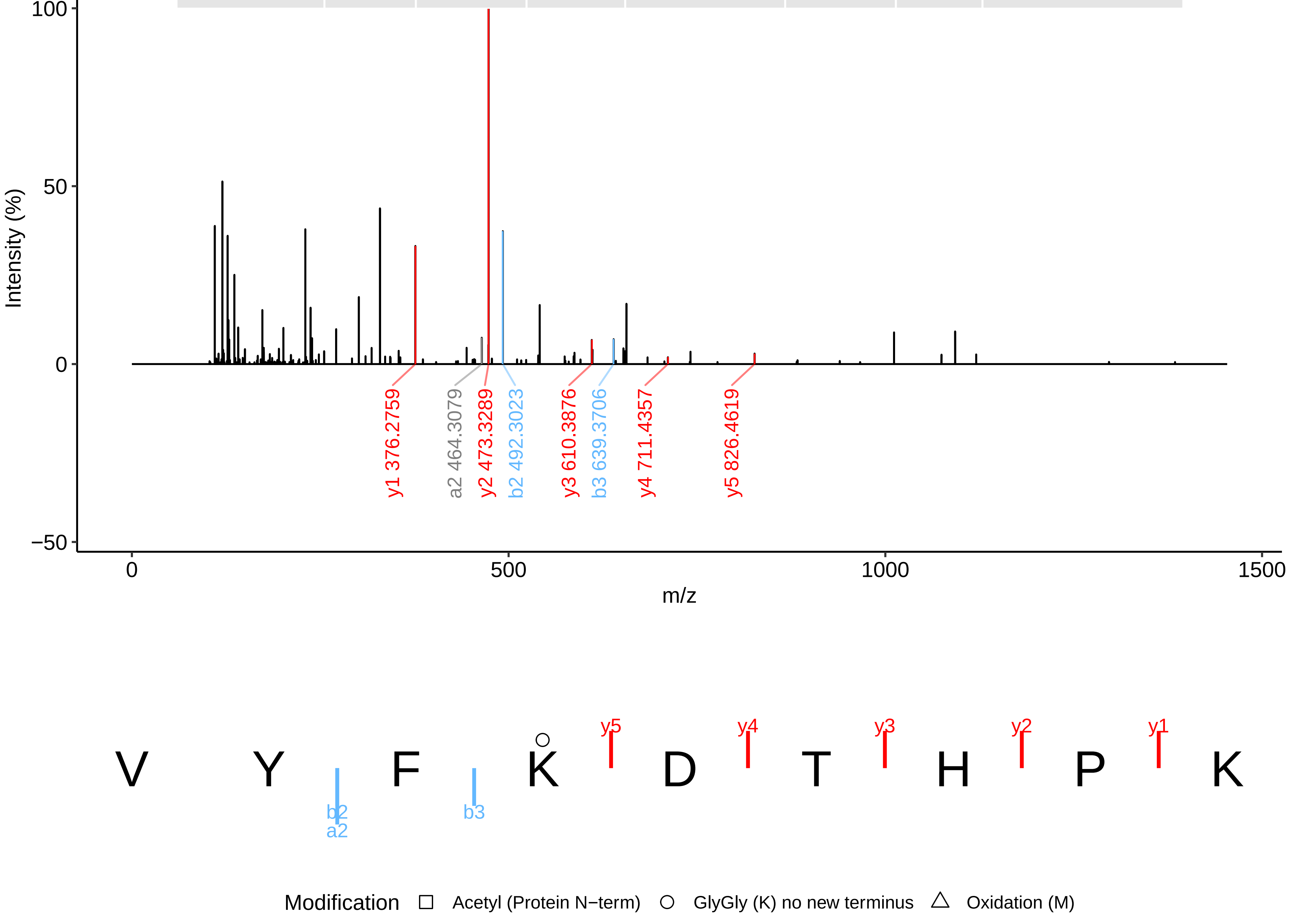

20200924_KIZ_MUb5_GlyGly_2 − Scan number: 7841

Gene Names	Charge	m/z	Mass	Retention time	PEP	Score	Precursor Intensity
CYB5R3	3	416.8839	1247.63	29.297	0.012327	72.325	4593223

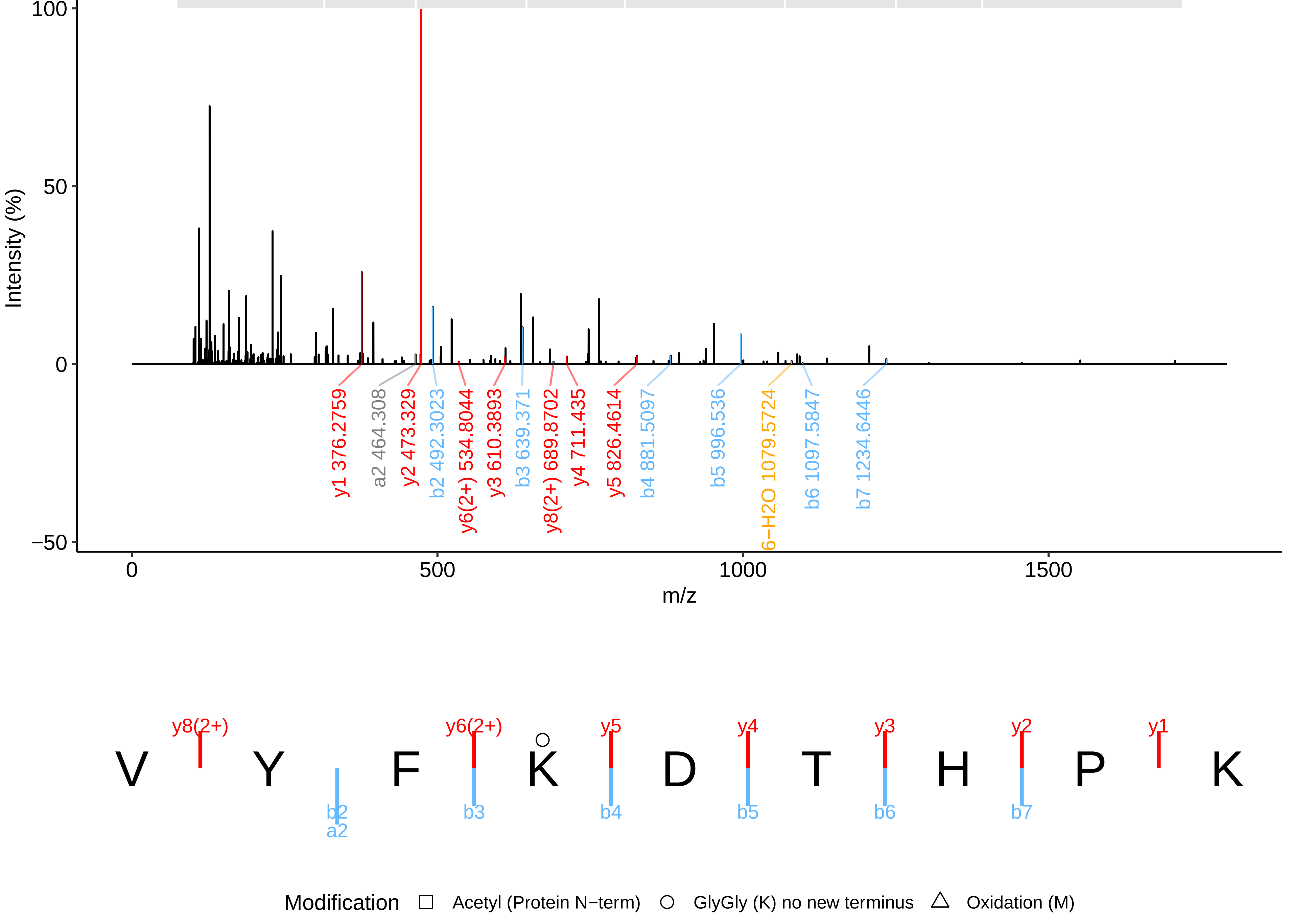

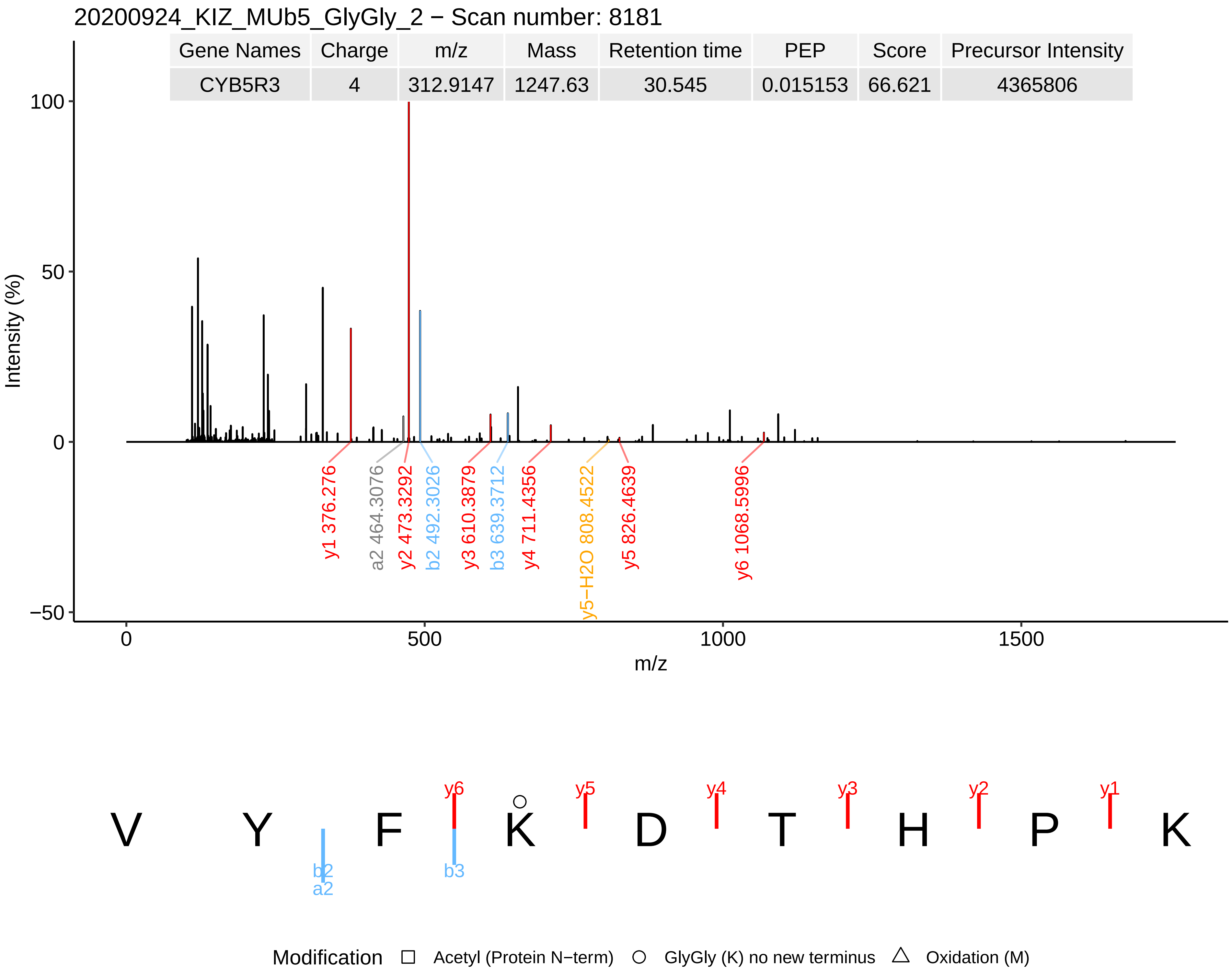
20200924_KIZ_MUb5_GlyGly_2 − Scan number: 8181
Gene Names | Charge | m/z | Mass | Retention time | PEP | Score | Precursor Intensity
CYB5R3 | 4 | 312.9147 | 1247.63 | 30.545 | 0.015153 | 66.621 | 4365806
Intensity (%)
m/z
y1 376.276
a2 464.3076
y2 473.3292
b2 492.3026
y3 610.3879
b3 639.3712
y4 711.4356
y5−H2O 808.4522
y5 826.4639
y6 1068.5996
V Y F K D T H P K
y6 y5 y4 y3 y2 y1
b2 a2 b3
Modification
Acetyl (Protein N−term)
GlyGly (K) no new terminus
Oxidation (M)

20200924_KIZ_MUb5_GlyGly_2 − Scan number: 7986

Gene Names	Charge	m/z	Mass	Retention time	PEP	Score	Precursor Intensity
CYB5R3	3	416.8839	1247.63	29.859	0.0059388	82.749	2532705

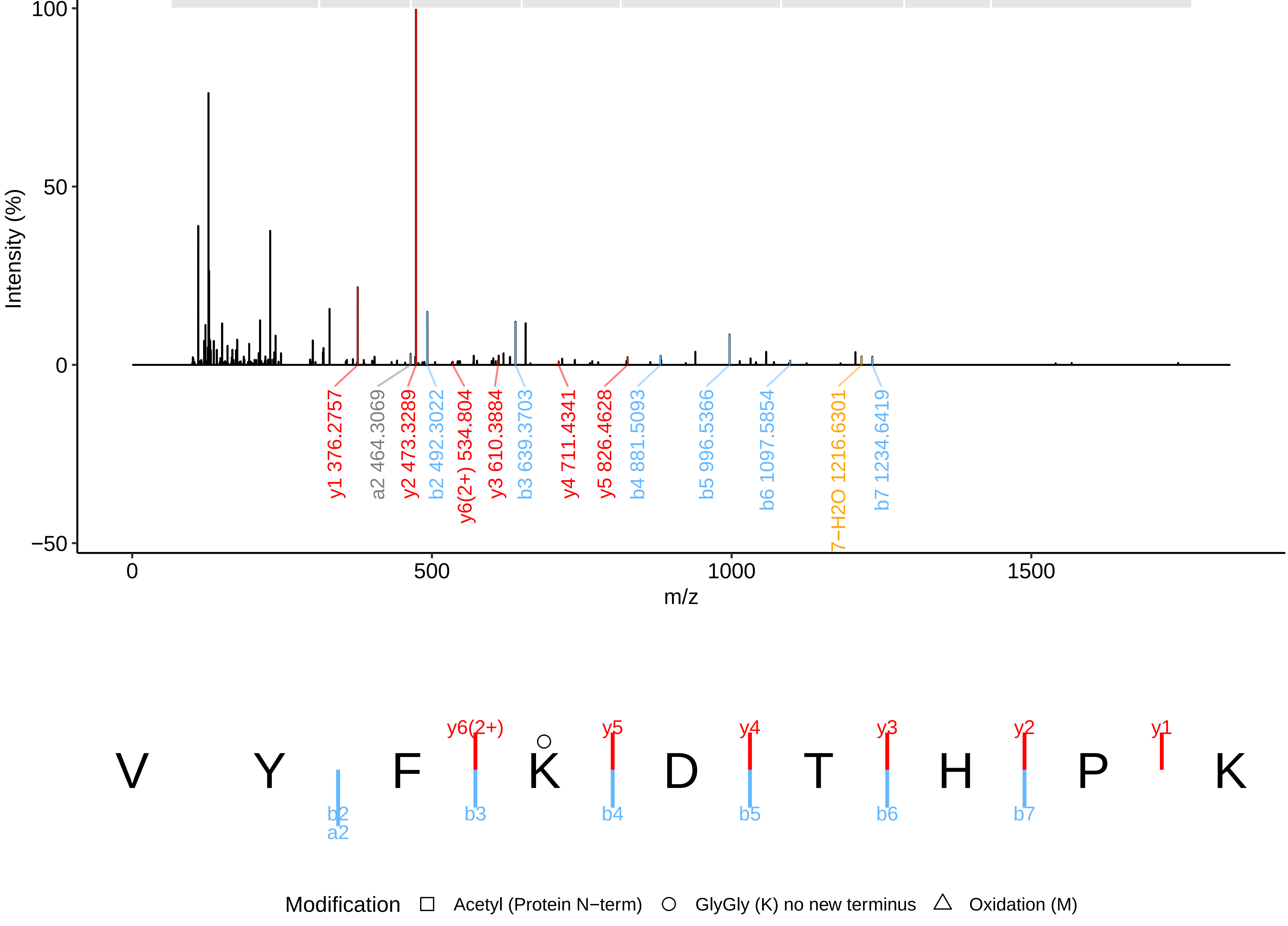

20201118_KIZ_MUb6_GlyGly_2 − Scan number: 10303

Gene Names	Charge	m/z	Mass	Retention time	PEP	Score	Precursor Intensity
CYB5R3	4	312.9147	1247.63	34.775	0.012695	69.423	106411000

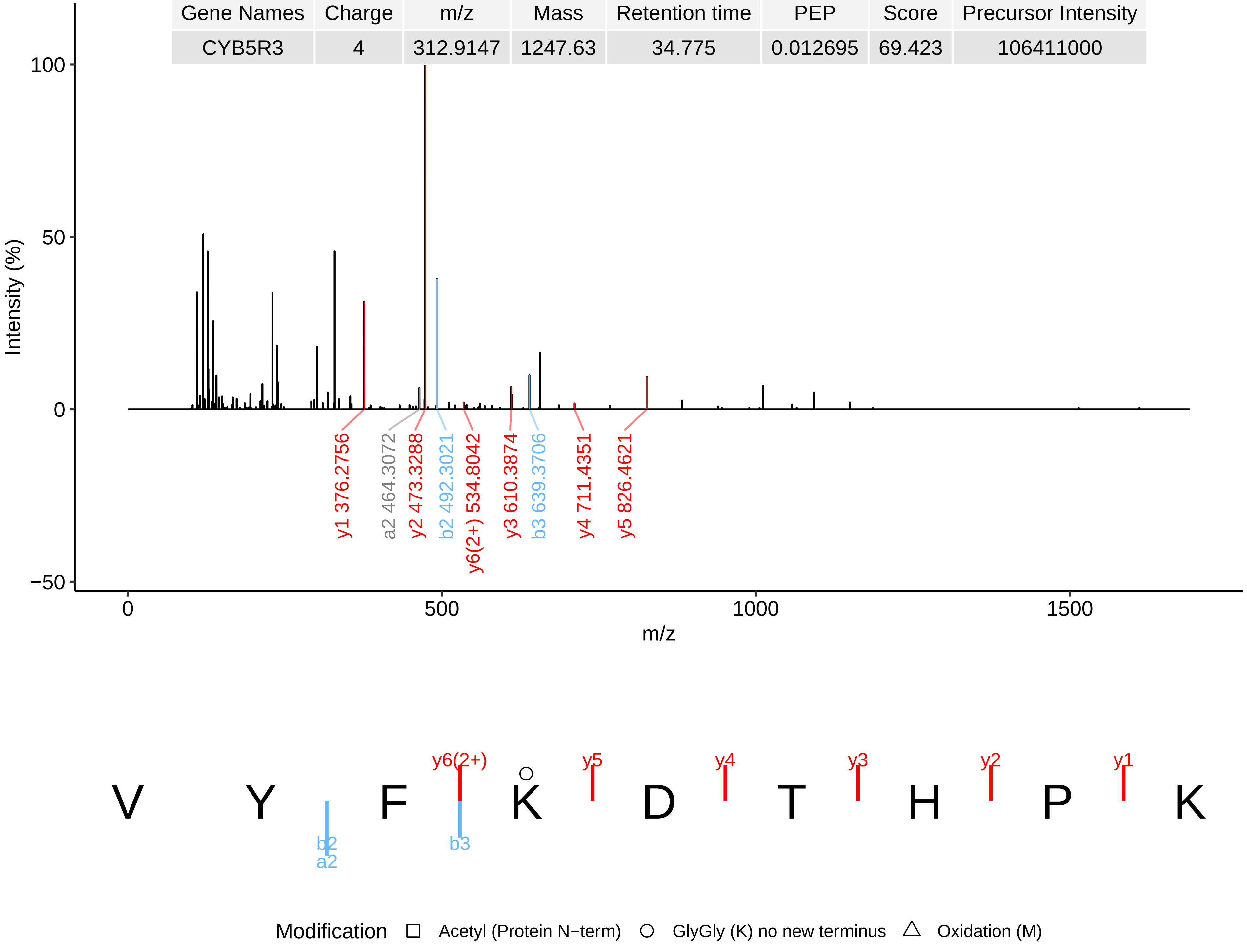

20201118_KIZ_MUb6_GlyGly_2 − Scan number: 10281

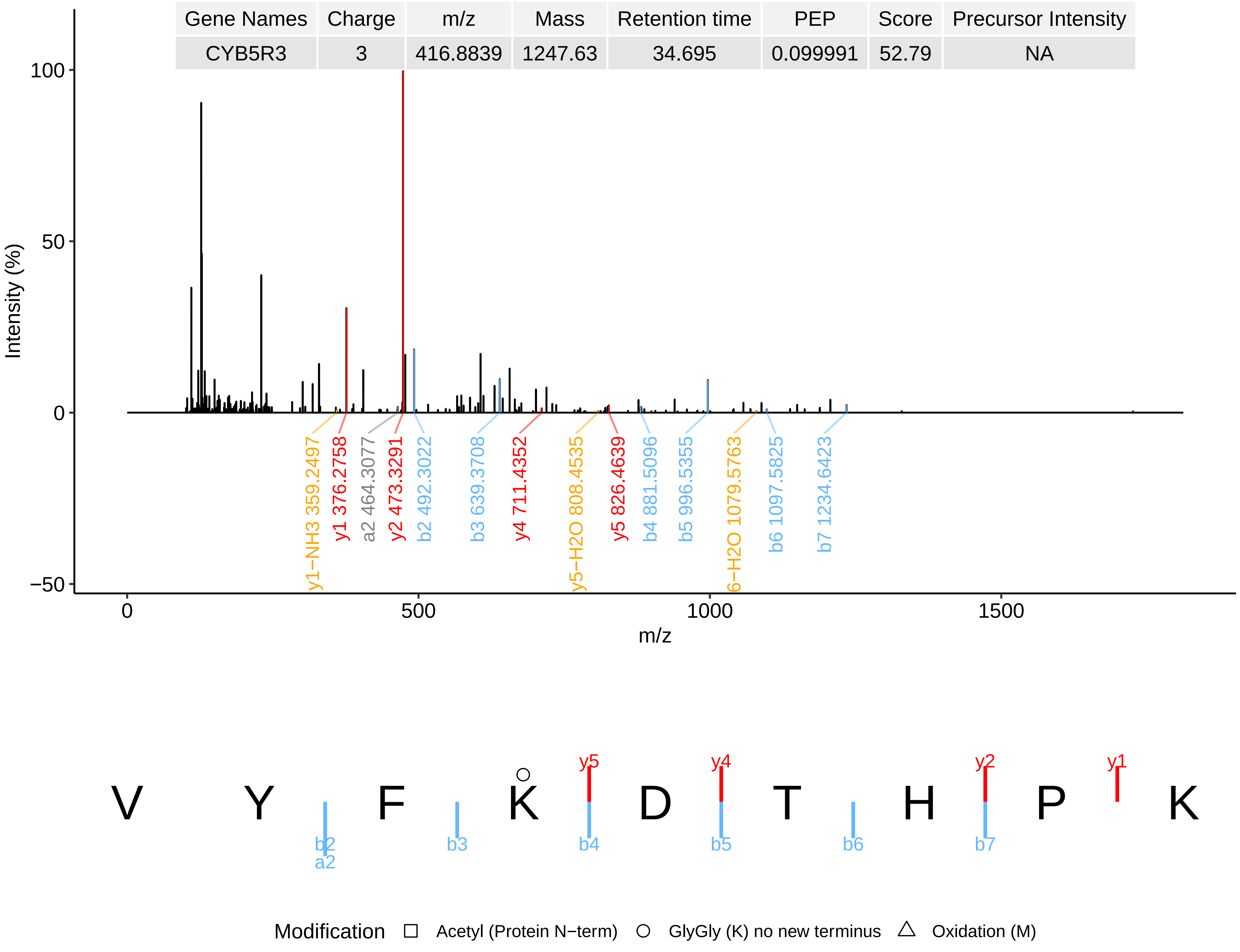

20200924_KIZ_MUb5_GlyGly_1 − Scan number: 4537

Gene Names	Charge	m/z	Mass	Retention time	PEP	Score	Precursor Intensity
SOX6	3	417.8644	1250.571	14.007	0.055801	58.981	6577348

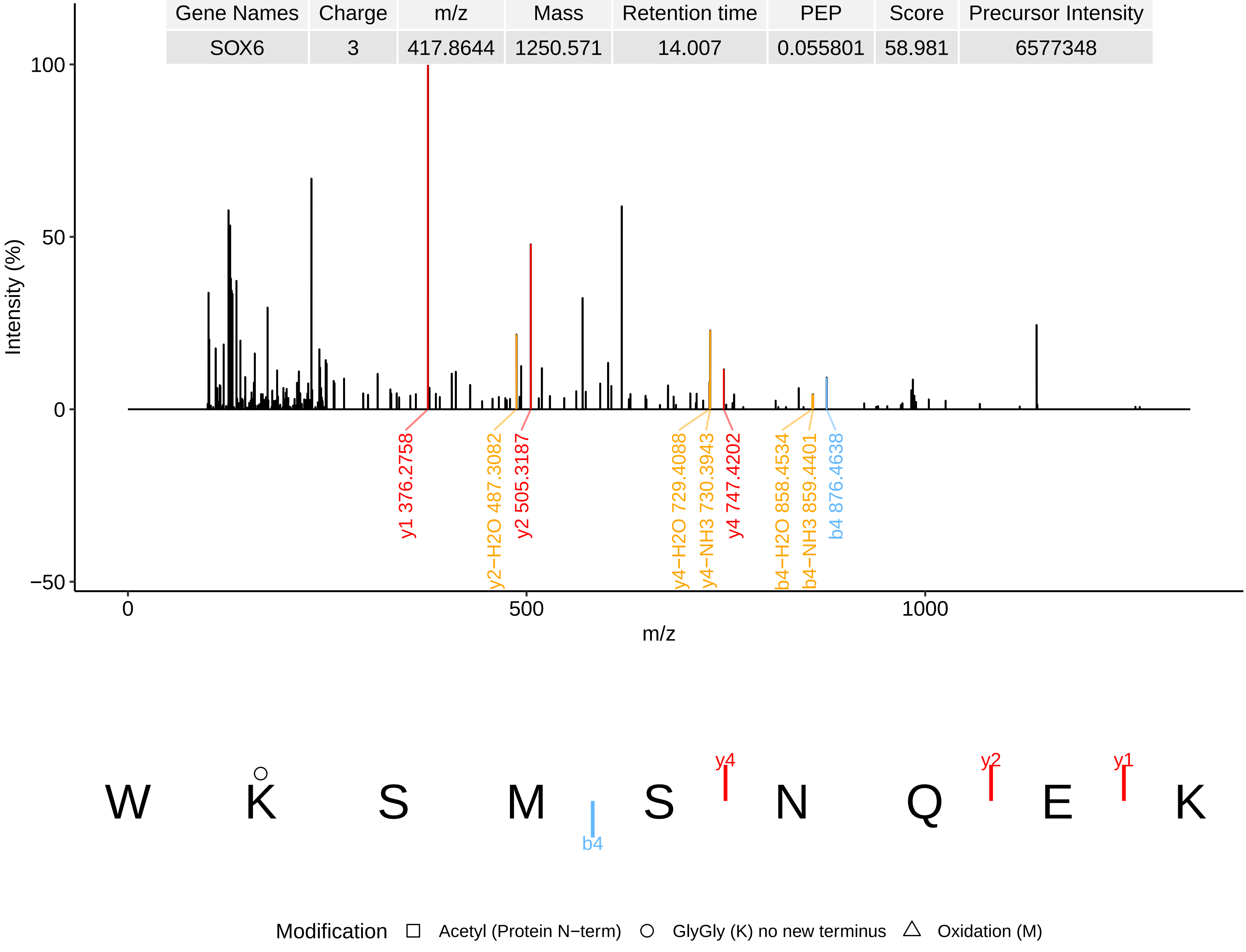

20200921_KIZ_MUb4_GlyGly_10plex − Scan number: 15176

Gene Names	Charge	m/z	Mass	Retention time	PEP	Score	Precursor Intensity
KRT8	3	491.6038	1471.79	54.145	2.7123e−10	178.67	7505263

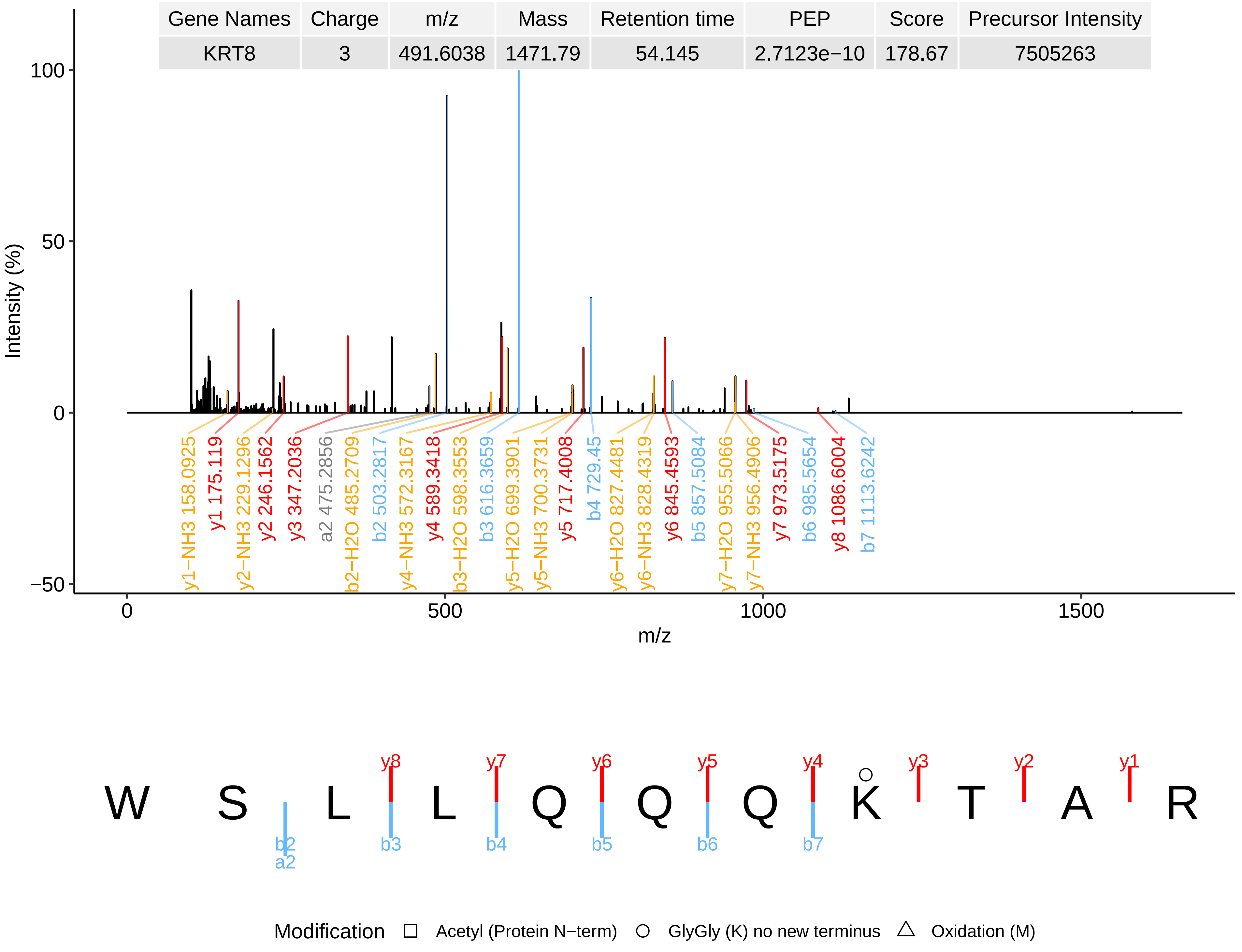

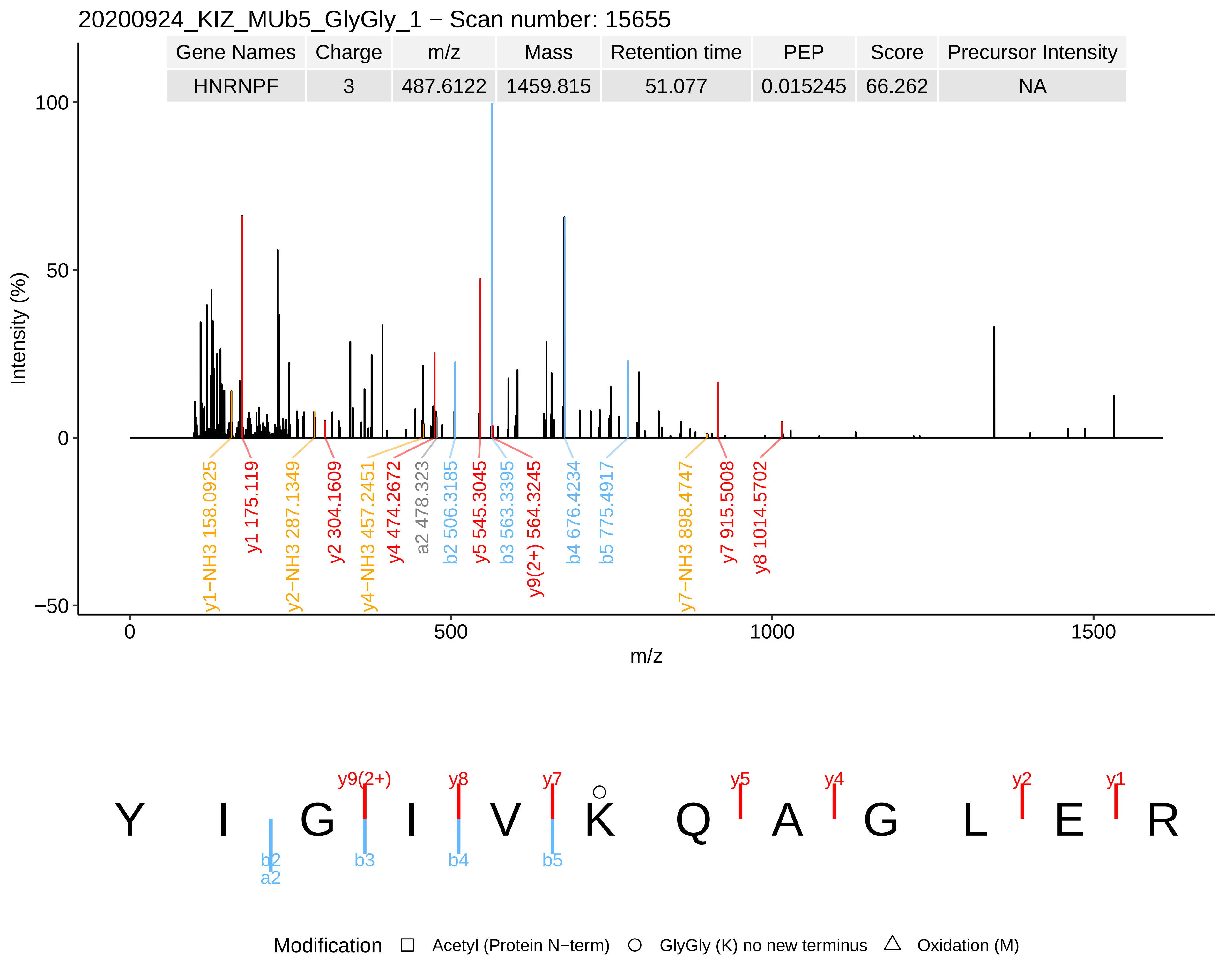

20200924_KIZ_MUb5_GlyGly_1 − Scan number: 15655
Gene Names | Charge | m/z | Mass | Retention time | PEP | Score | Precursor Intensity
HNRNPF | 3 | 487.6122 | 1459.815 | 51.077 | 0.015245 | 66.262 | NA
Intensity (%)
m/z
y1−NH3 158.0925
y1 175.119
y2−NH3 287.1349
y2 304.1609
y4−NH3 457.2451
y4 474.2672
a2 478.323
b2 506.3185
y5 545.3045
b3 563.3395
y9(2+) 564.3245
b4 676.4234
b5 775.4917
y7−NH3 898.4747
y7 915.5008
y8 1014.5702
Y I G I V K Q A G L E R
y9(2+) y8 y7 y5 y4 y2 y1
b2 a2 b3 b4 b5
Modification
Acetyl (Protein N−term)
GlyGly (K) no new terminus
Oxidation (M)

20200921_KIZ_MUb4_GlyGly_10plex − Scan number: 8522

Gene Names	Charge	m/z	Mass	Retention time	PEP	Score	Precursor Intensity
ABCB1	3	408.2069	1221.599	28.94	0.0013101	93.649	5834247

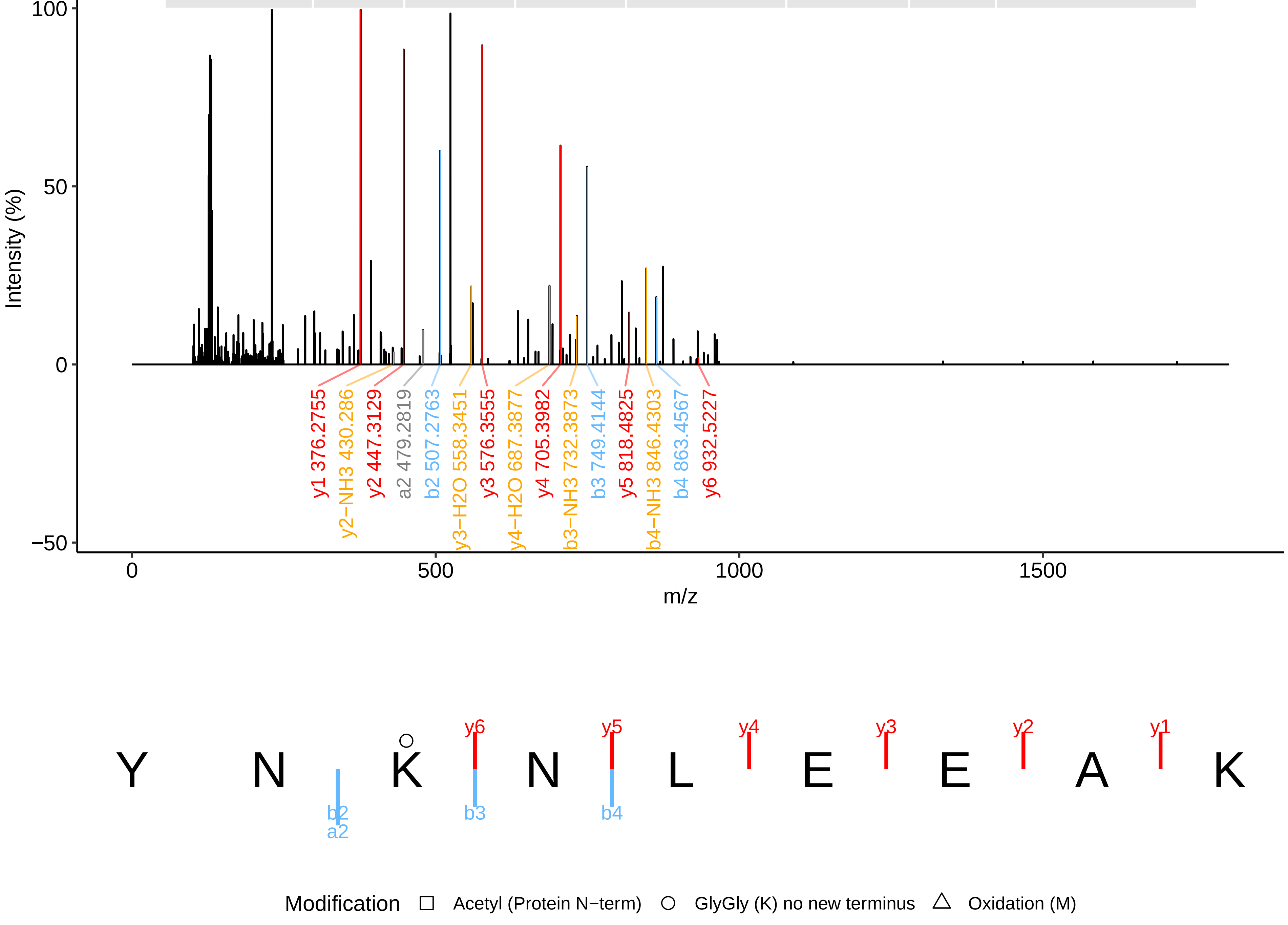

20200921_KIZ_MUb4_GlyGly_10plex_2 − Scan number: 7882

Gene Names	Charge	m/z	Mass	Retention time	PEP	Score	Precursor Intensity
ABCB1	3	408.2069	1221.599	29.556	0.0015797	92.062	1726748

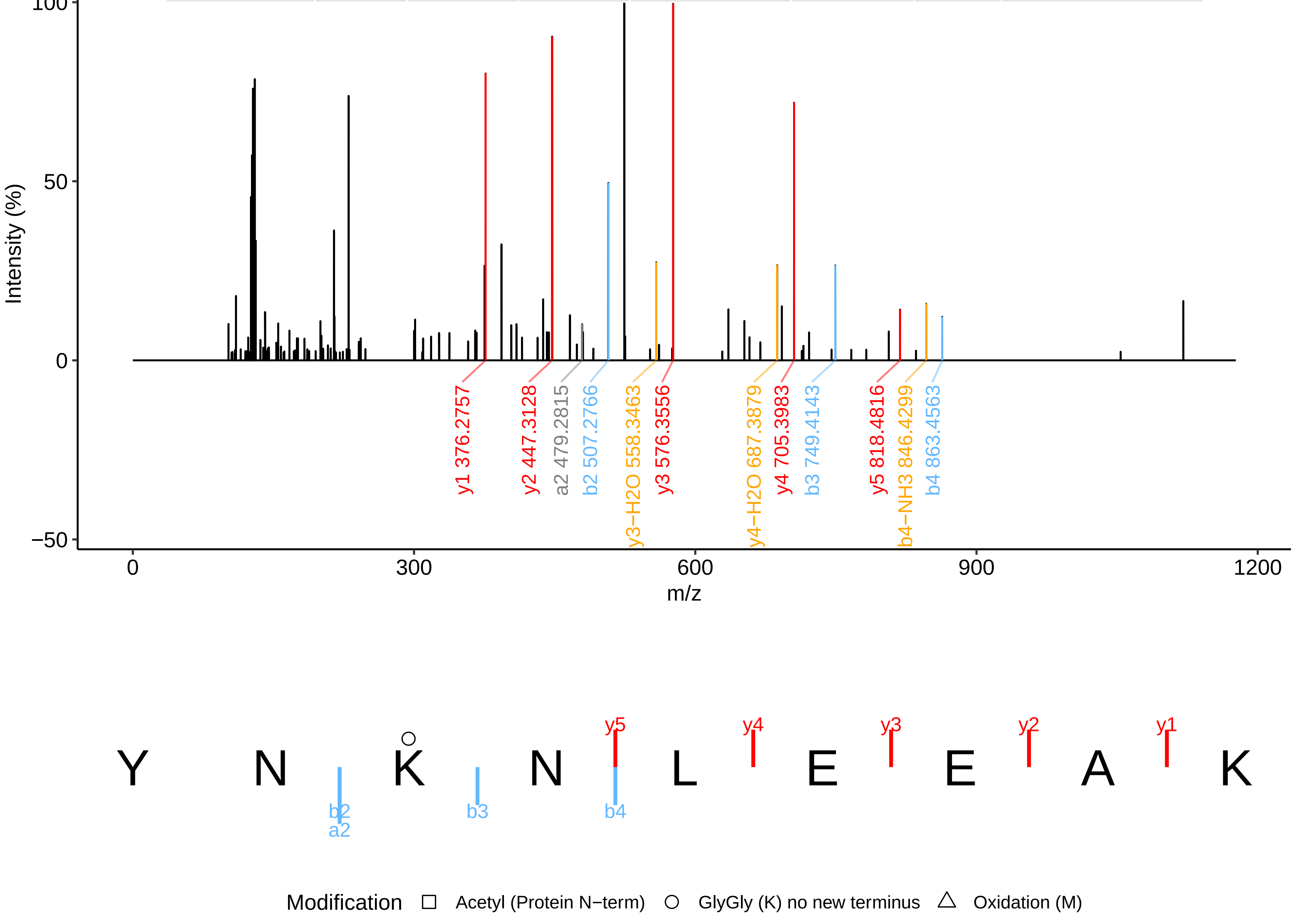

20200921_KIZ_MUb4_GlyGly_10plex − Scan number: 5470

Gene Names	Charge	m/z	Mass	Retention time	PEP	Score	Precursor Intensity
SLCO4A1	4	372.4321	1485.699	17.914	0.00076327	84.821	8050678

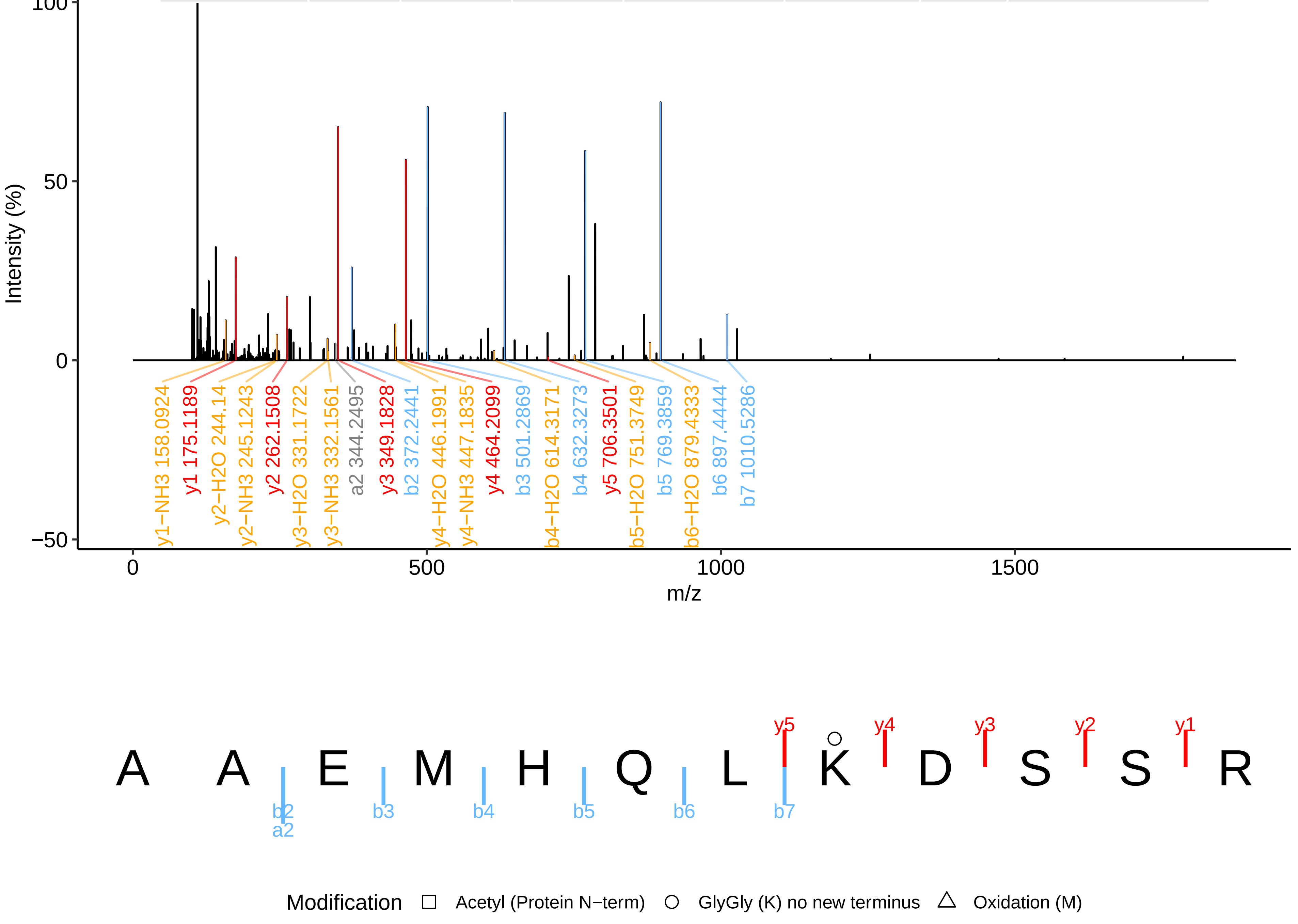

20200921_KIZ_MUb4_GlyGly_10plex_2 − Scan number: 5032

Gene Names	Charge	m/z	Mass	Retention time	PEP	Score	Precursor Intensity
SLCO4A1	4	372.4321	1485.699	17.906	0.0087682	66.486	1294359

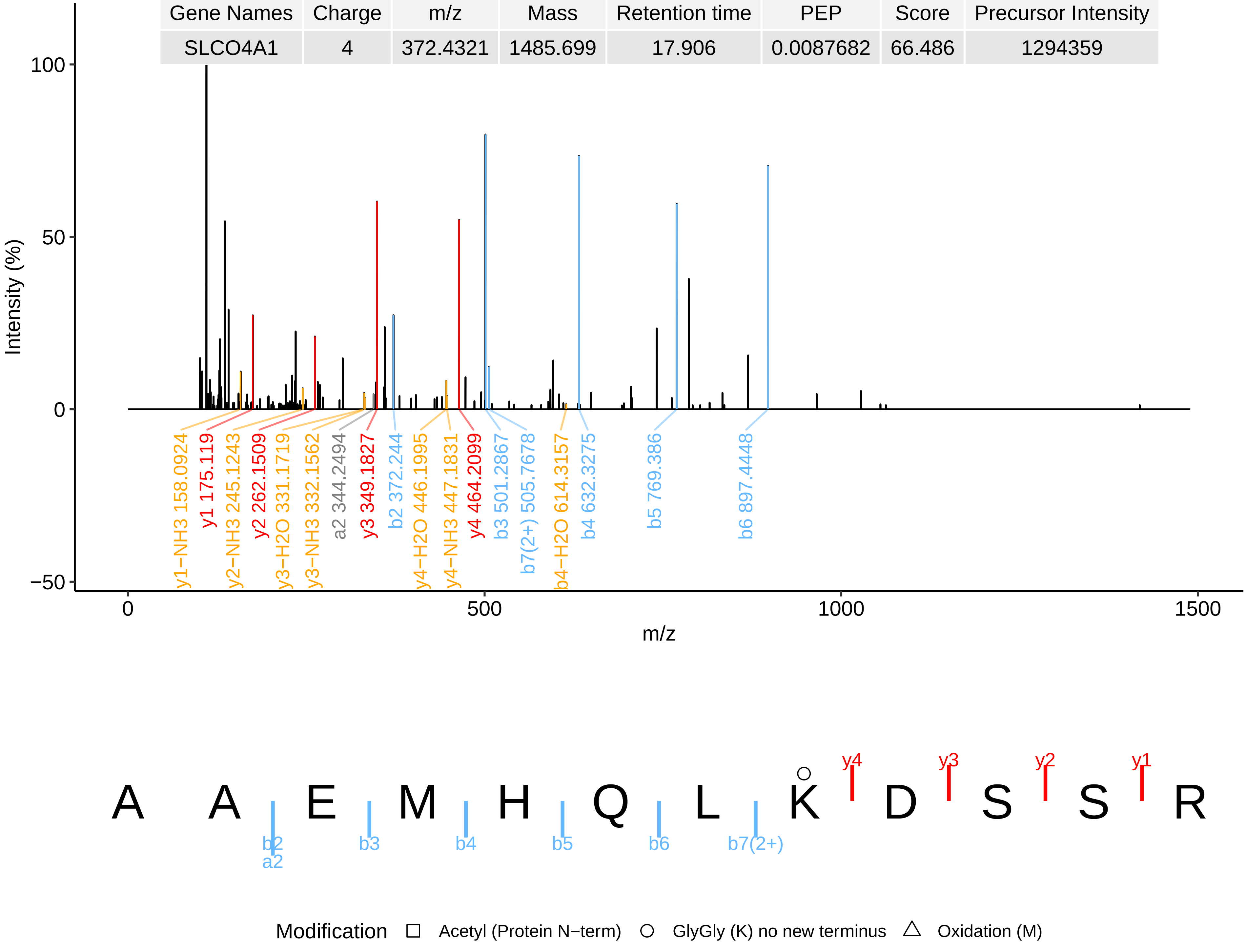

20200921_KIZ_MUb4_GlyGly_10plex_2 − Scan number: 5046

Gene Names	Charge	m/z	Mass	Retention time	PEP	Score	Precursor Intensity
SLCO4A1	3	496.2404	1485.699	17.964	0.0009504	84.479	862317.8

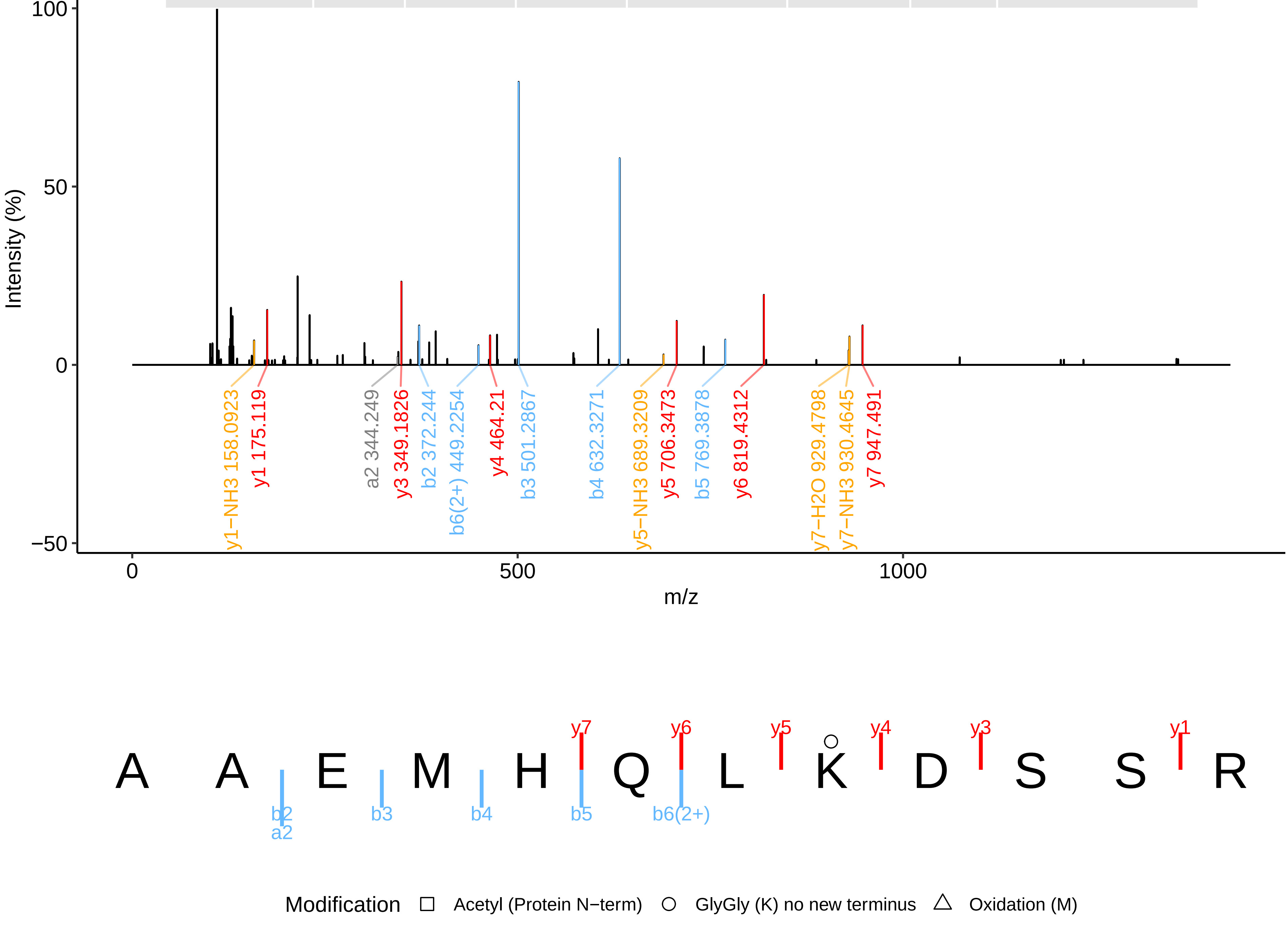

20200921_KIZ_MUb4_GlyGly_10plex − Scan number: 3772

Gene Names	Charge	m/z	Mass	Retention time	PEP	Score	Precursor Intensity
ITGA3	3	502.8999	1505.678	12.143	6.6247e−07	114.87	3307851

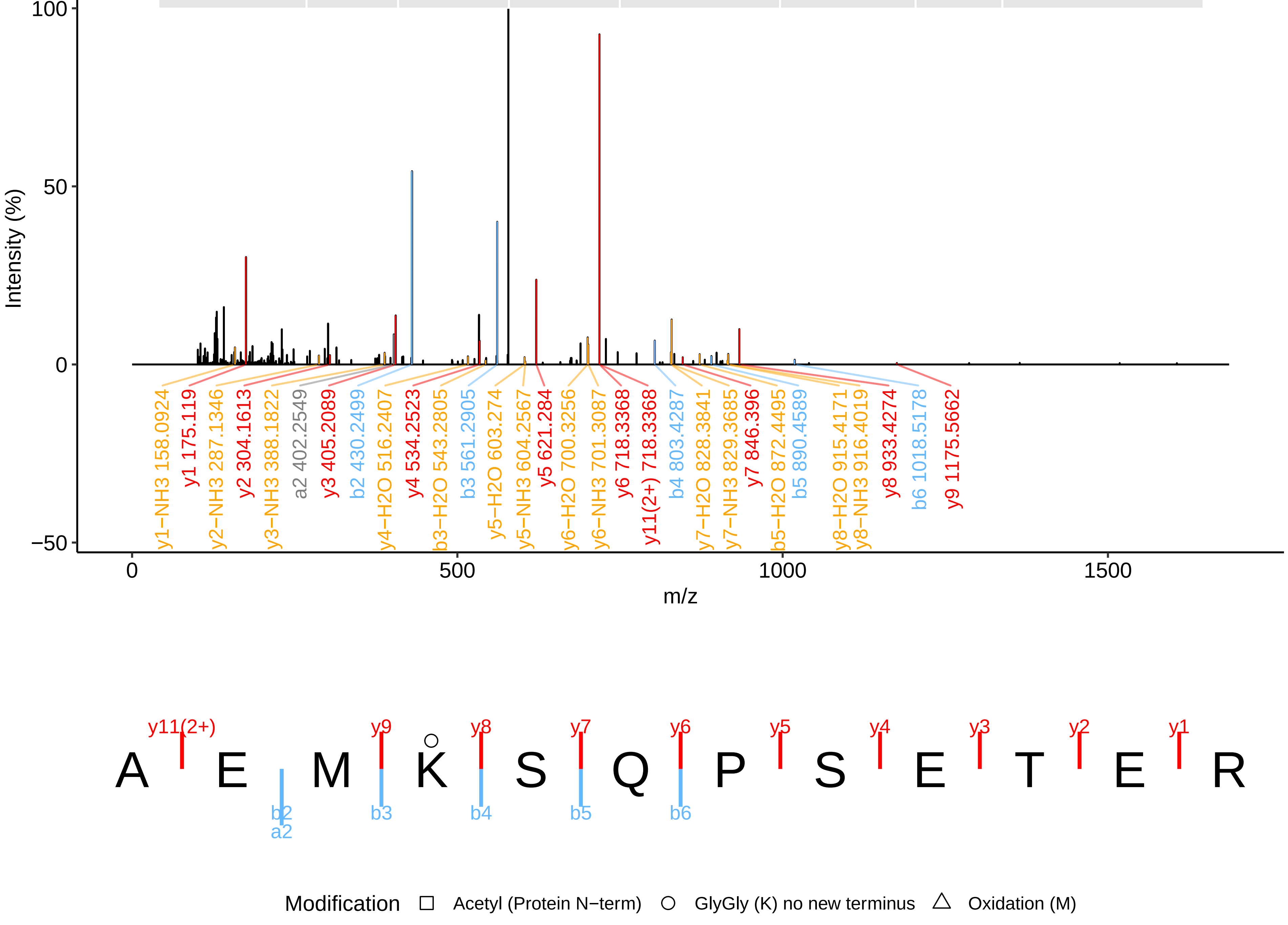

20200921_KIZ_MUb4_GlyGly_10plex − Scan number: 3805

Gene Names	Charge	m/z	Mass	Retention time	PEP	Score	Precursor Intensity
ITGA3	3	502.8999	1505.678	12.268	0.0027308	78.324	1533543

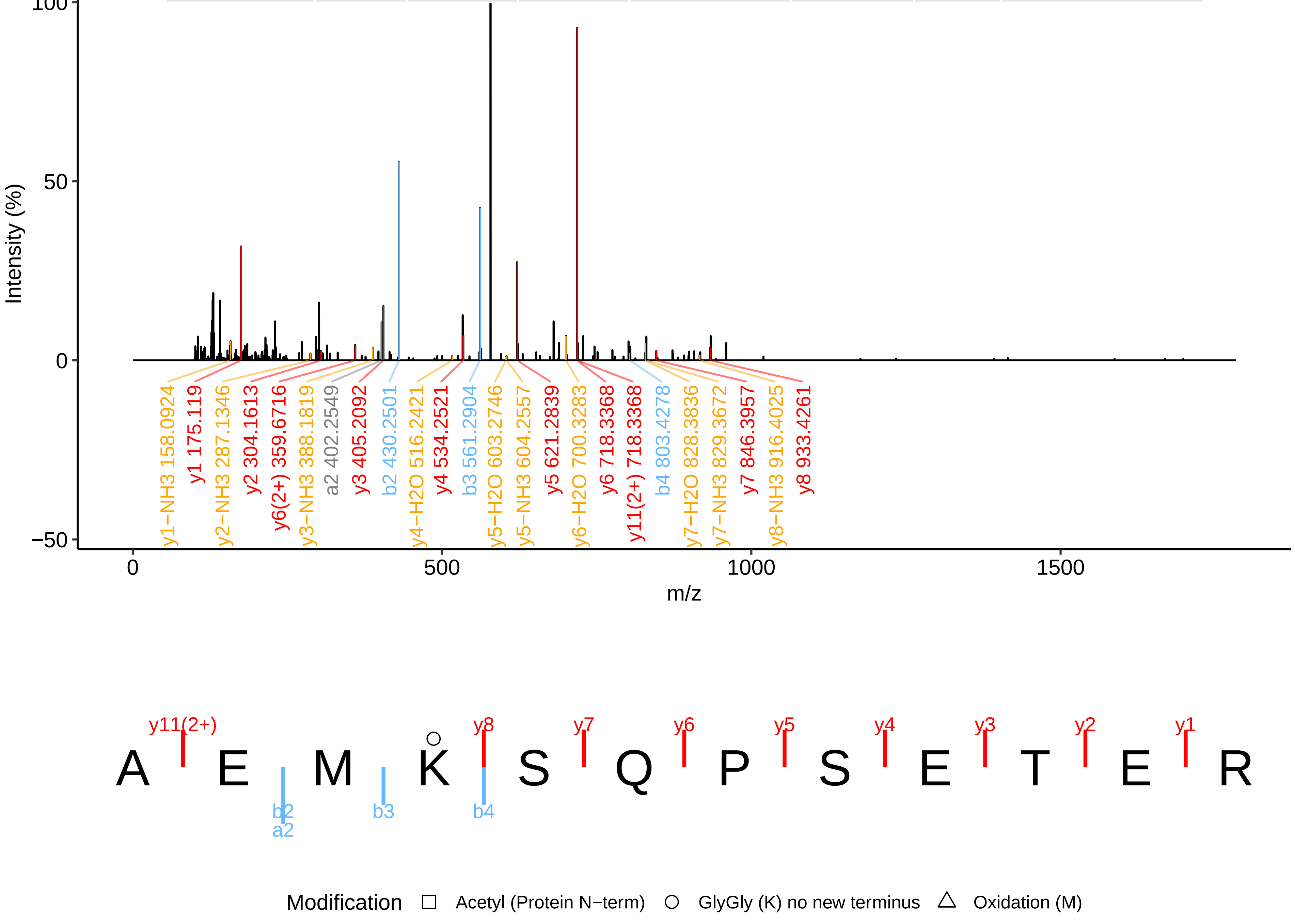

20200921_KIZ_MUb4_GlyGly_10plex_2 − Scan number: 3594

Gene Names	Charge	m/z	Mass	Retention time	PEP	Score	Precursor Intensity
ITGA3	3	502.8999	1505.678	12.159	0.060418	54.023	1627112

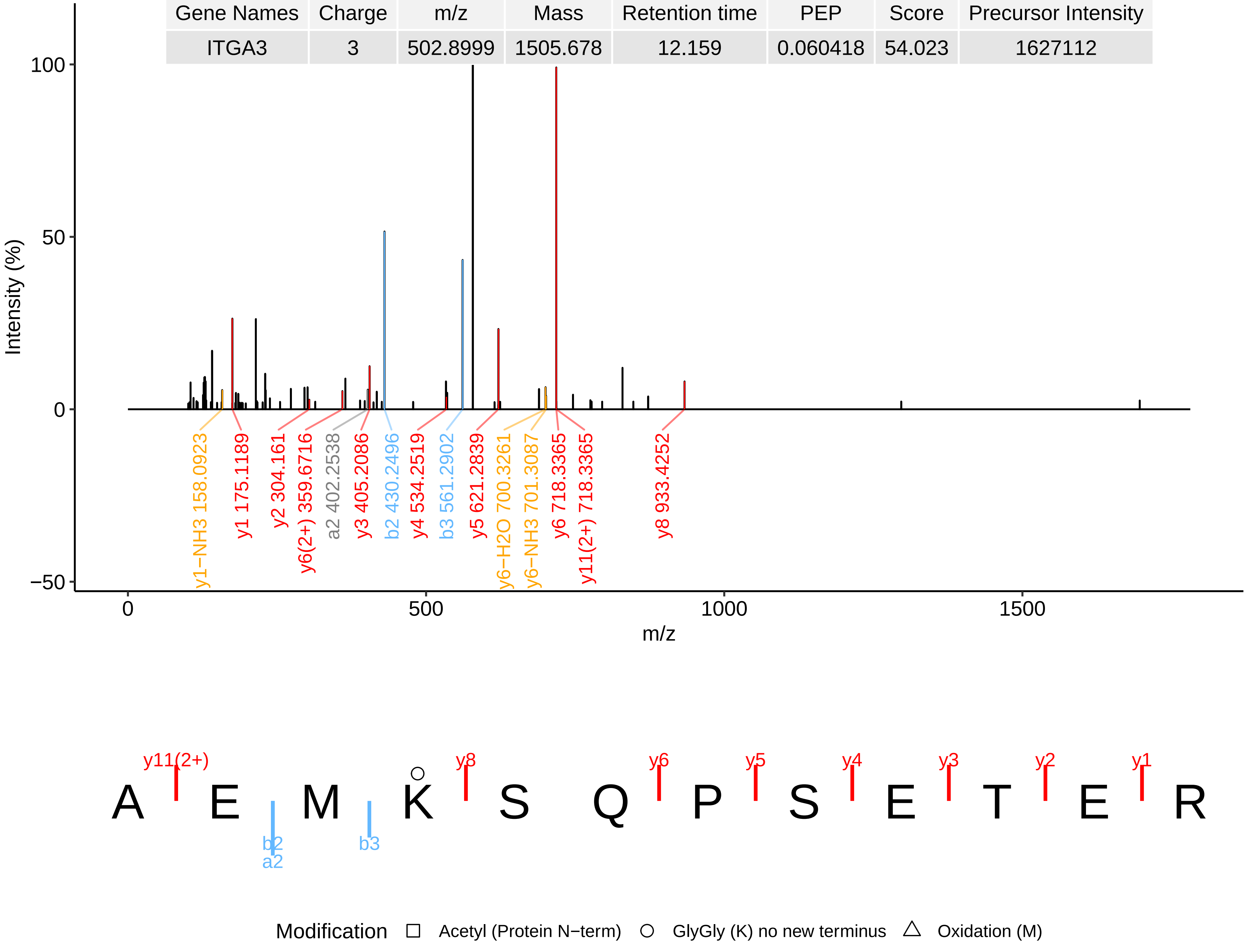

20200924_KIZ_MUb5_GlyGly_1 − Scan number: 3324

Gene Names	Charge	m/z	Mass	Retention time	PEP	Score	Precursor Intensity
ITGA3	3	502.8999	1505.678	10.205	4.3362e−09	127.56	9643271

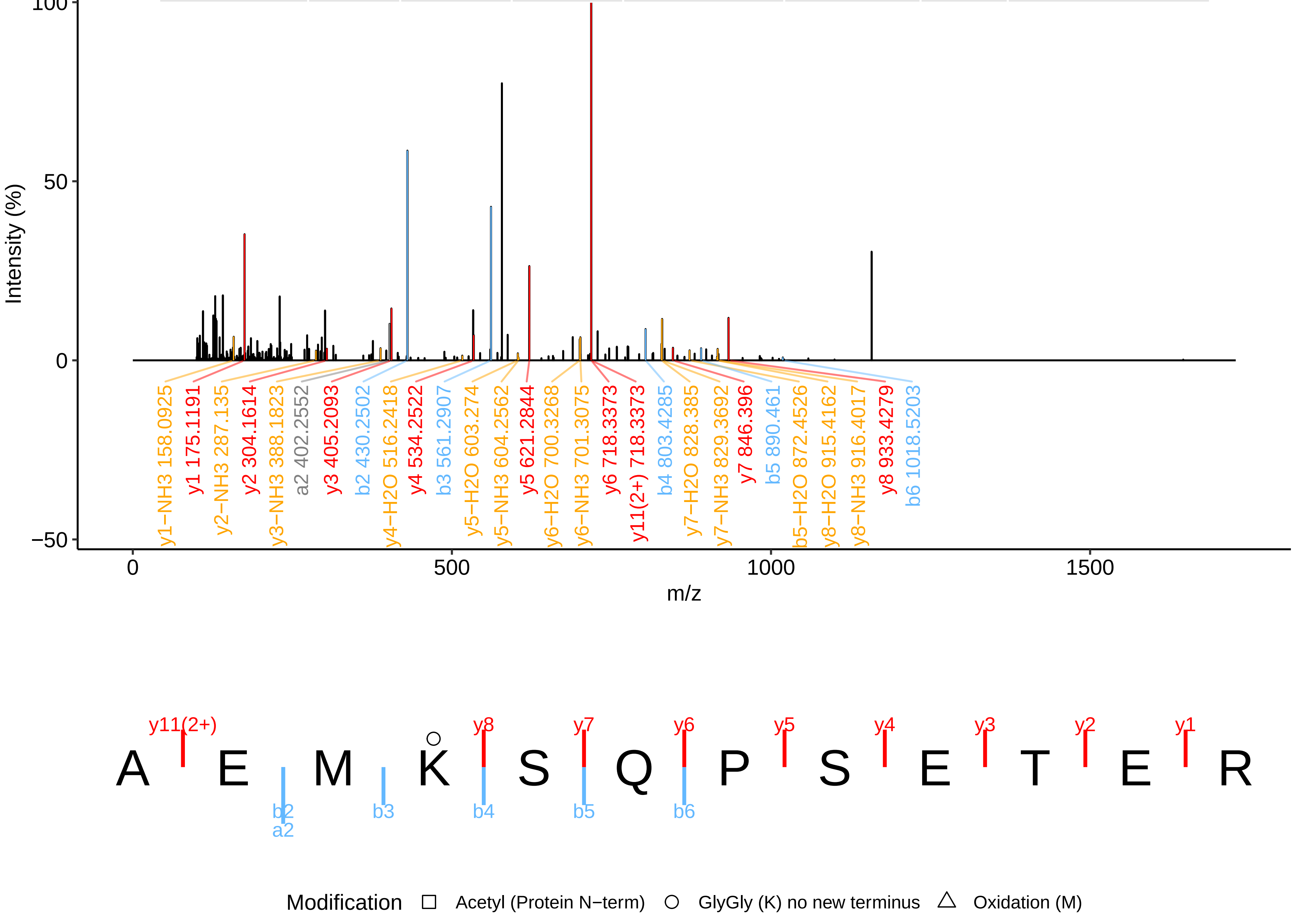

20200924_KIZ_MUb5_GlyGly_2 − Scan number: 3184

Gene Names	Charge	m/z	Mass	Retention time	PEP	Score	Precursor Intensity
ITGA3	3	502.8999	1505.678	11.456	1.579e−05	112.84	NA

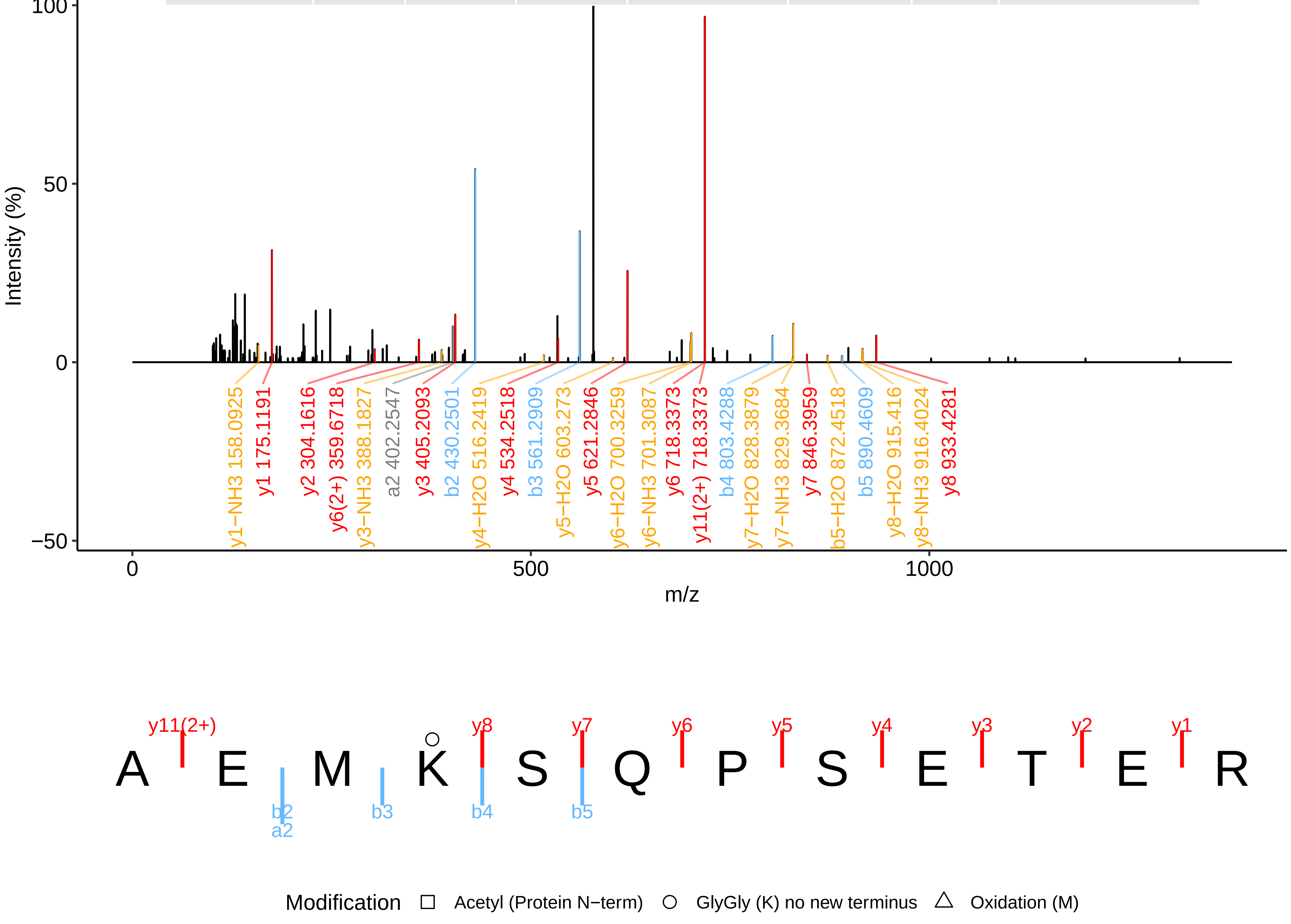

20201118_KIZ_MUb6_GlyGly_1 − Scan number: 3244

Gene Names	Charge	m/z	Mass	Retention time	PEP	Score	Precursor Intensity
ITGA3	3	502.8999	1505.678	9.9766	0.00081545	89.629	NA

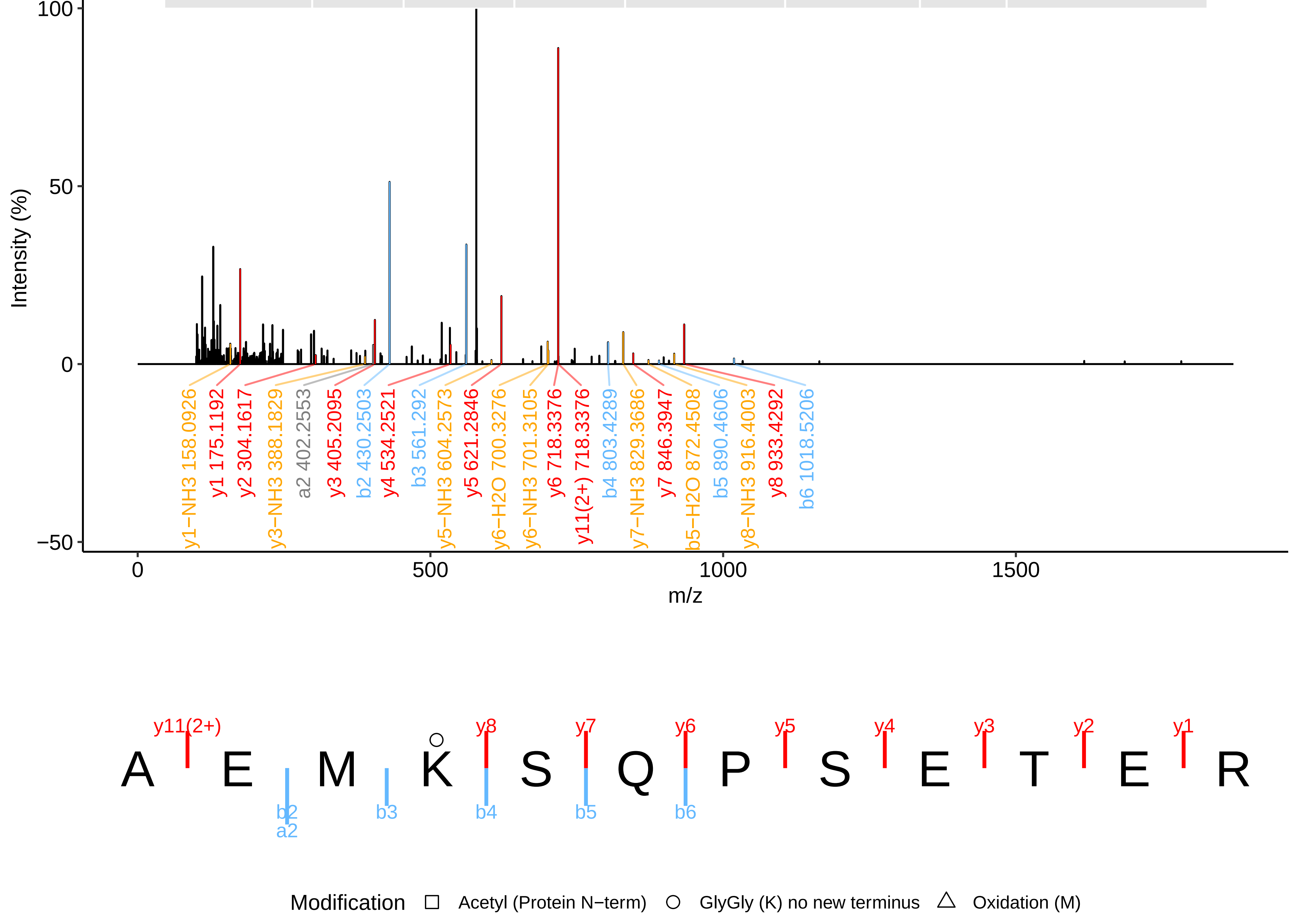

20201118_KIZ_MUb6_GlyGly_2 − Scan number: 3573

Gene Names	Charge	m/z	Mass	Retention time	PEP	Score	Precursor Intensity
ITGA3	3	502.8999	1505.678	11.878	0.00097176	84.169	3365813

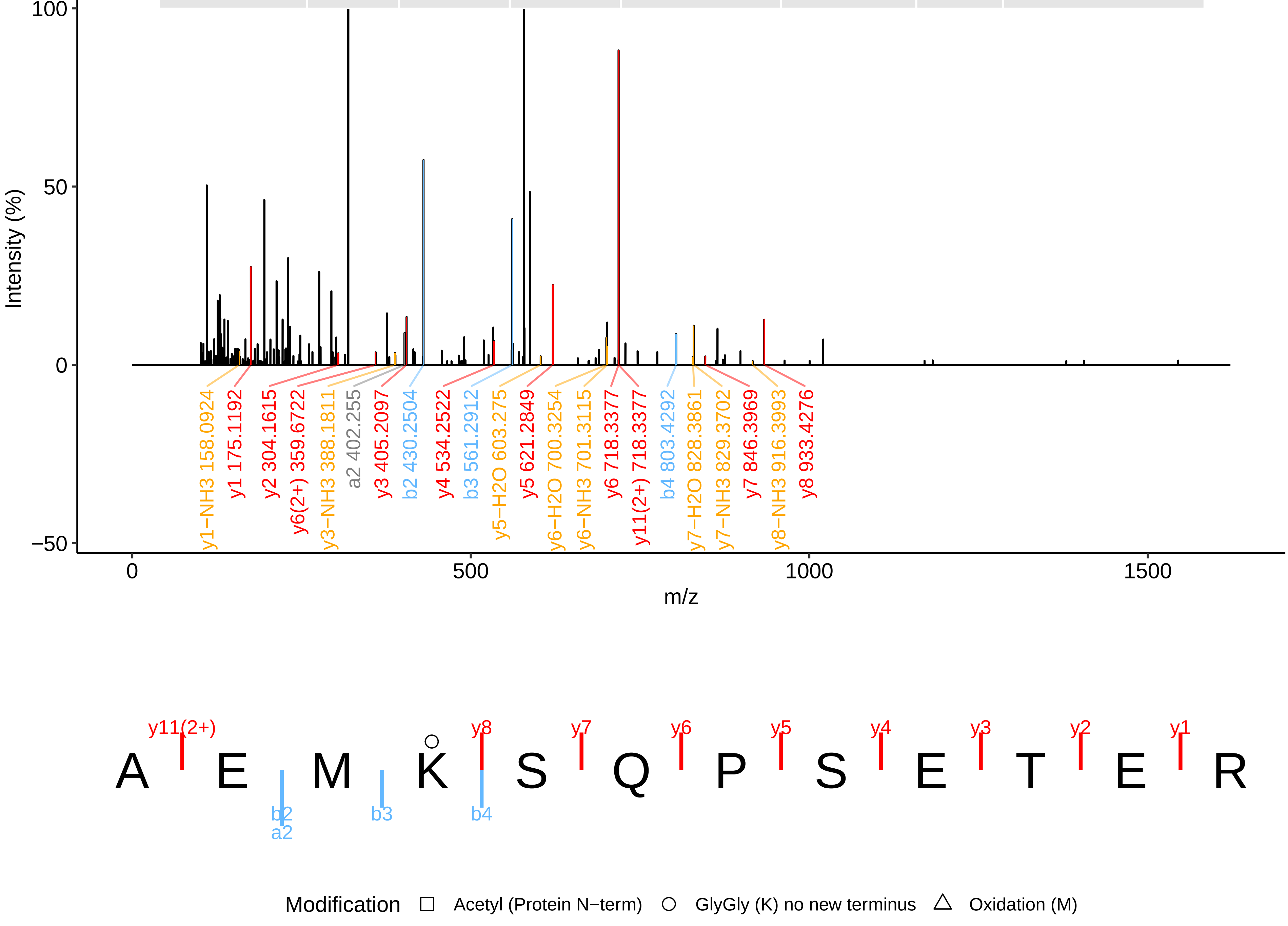

20200921_KIZ_MUb4_GlyGly_10plex − Scan number: 2640

Gene Names	Charge	m/z	Mass	Retention time	PEP	Score	Precursor Intensity
ITGA3	3	508.2316	1521.673	8.3218	0.0012923	87.641	2095743

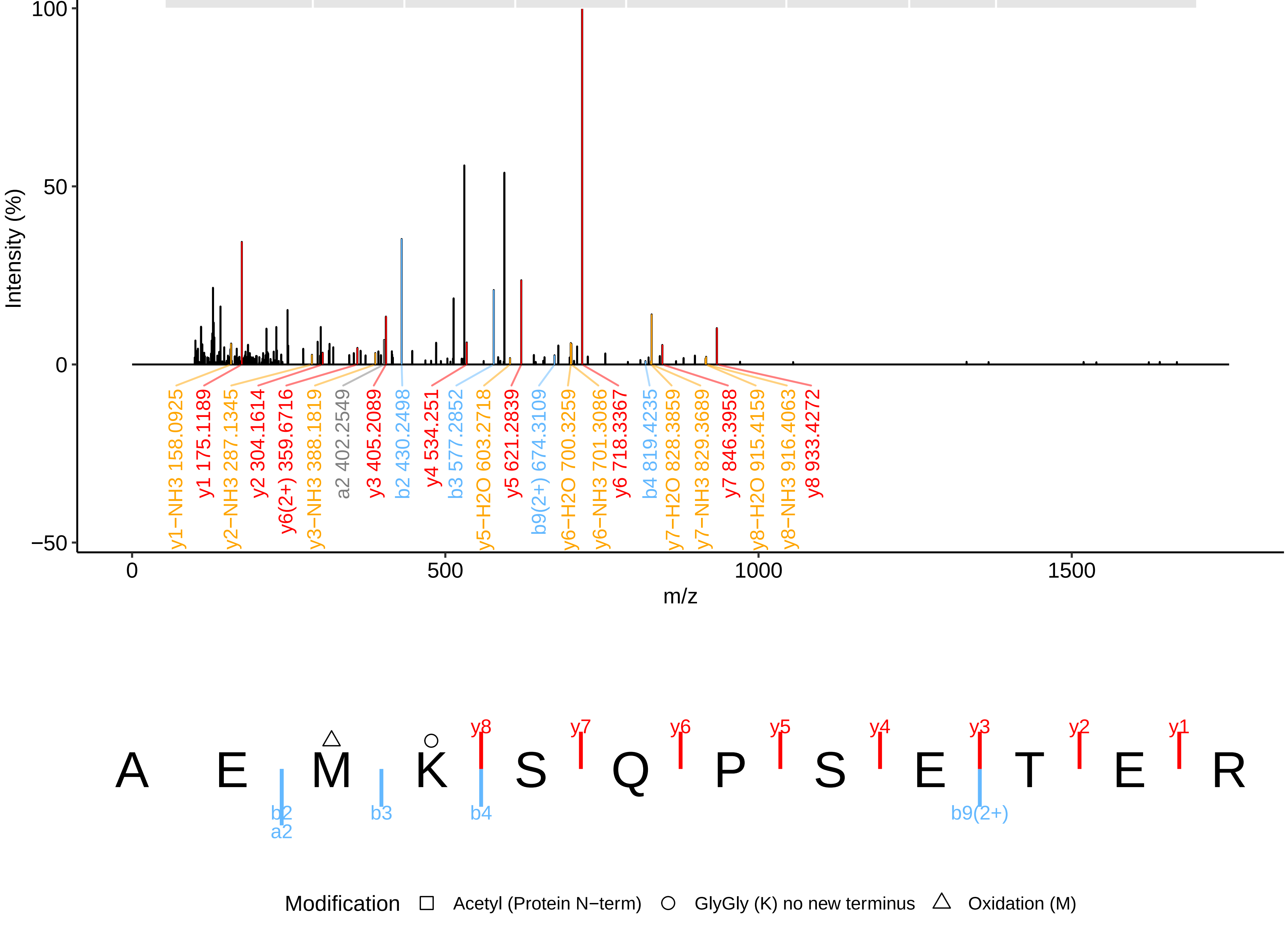

20200921_KIZ_MUb4_GlyGly_10plex − Scan number: 13797

Gene Names	Charge	m/z	Mass	Retention time	PEP	Score	Precursor Intensity
ABCB6	3	505.9402	1514.799	48.799	3.2848e−20	159.99	5684020

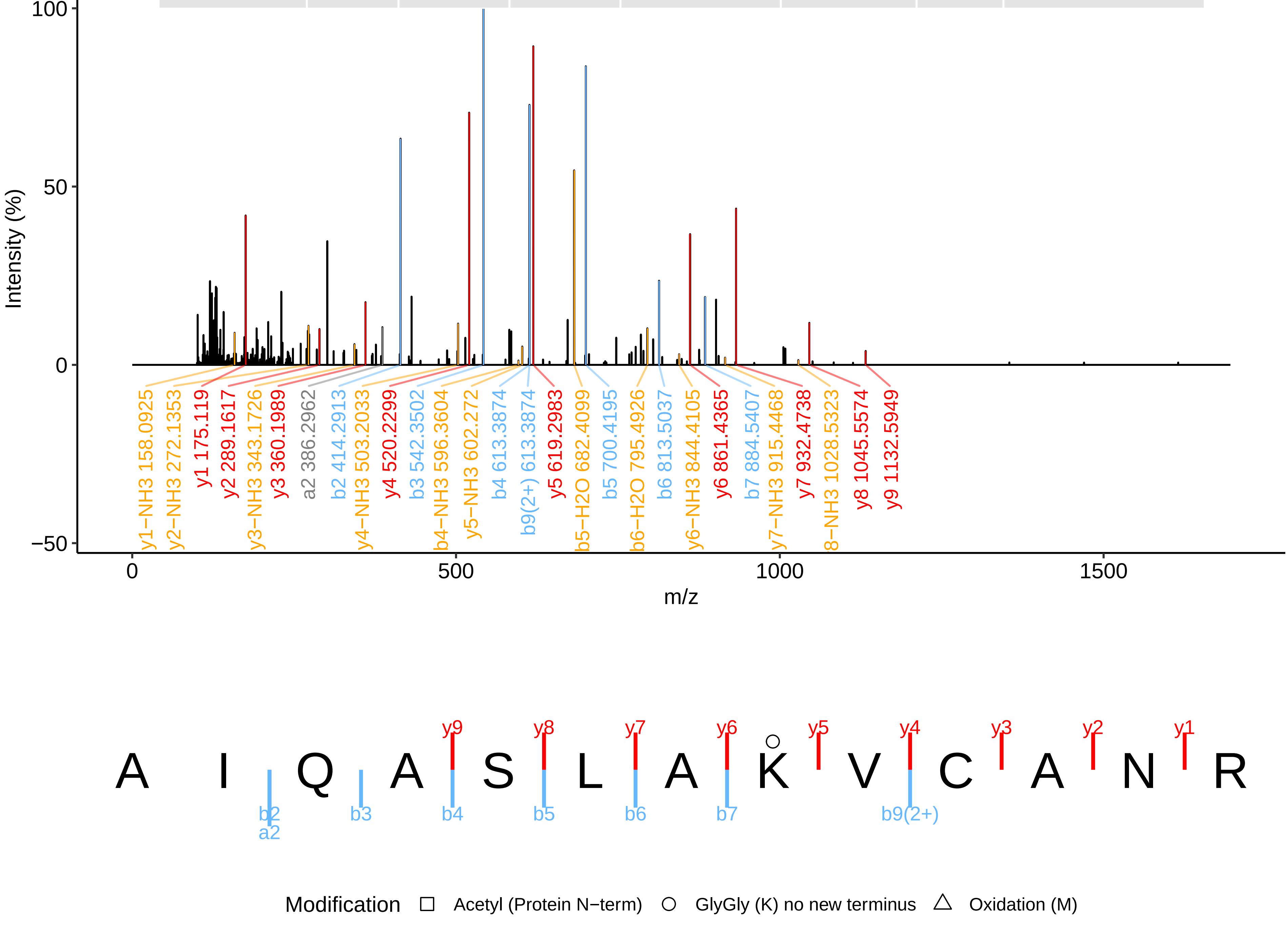

20200921_KIZ_MUb4_GlyGly_10plex_2 − Scan number: 12695

Gene Names	Charge	m/z	Mass	Retention time	PEP	Score	Precursor Intensity
ABCB6	3	505.9402	1514.799	49.17	1.1546e−08	118.87	1511544

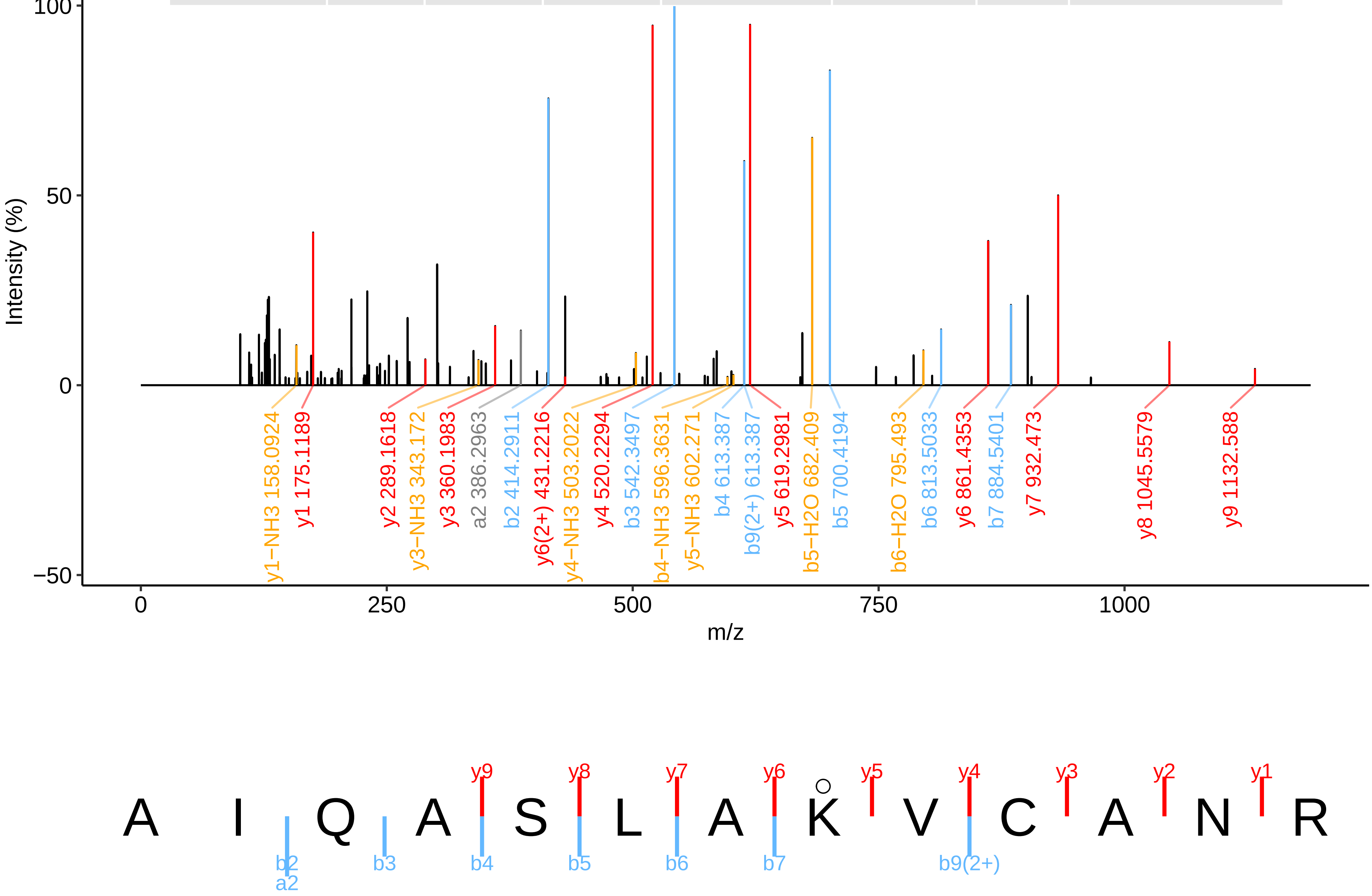

Modification □ Acetyl (Protein N−term) ○ GlyGly (K) no new terminus △ Oxidation (M)

20201118_KIZ_MUb6_GlyGly_1 − Scan number: 12646

Gene Names	Charge	m/z	Mass	Retention time	PEP	Score	Precursor Intensity
ABCB6	3	505.9402	1514.799	39.981	0.00059027	91.858	8856992

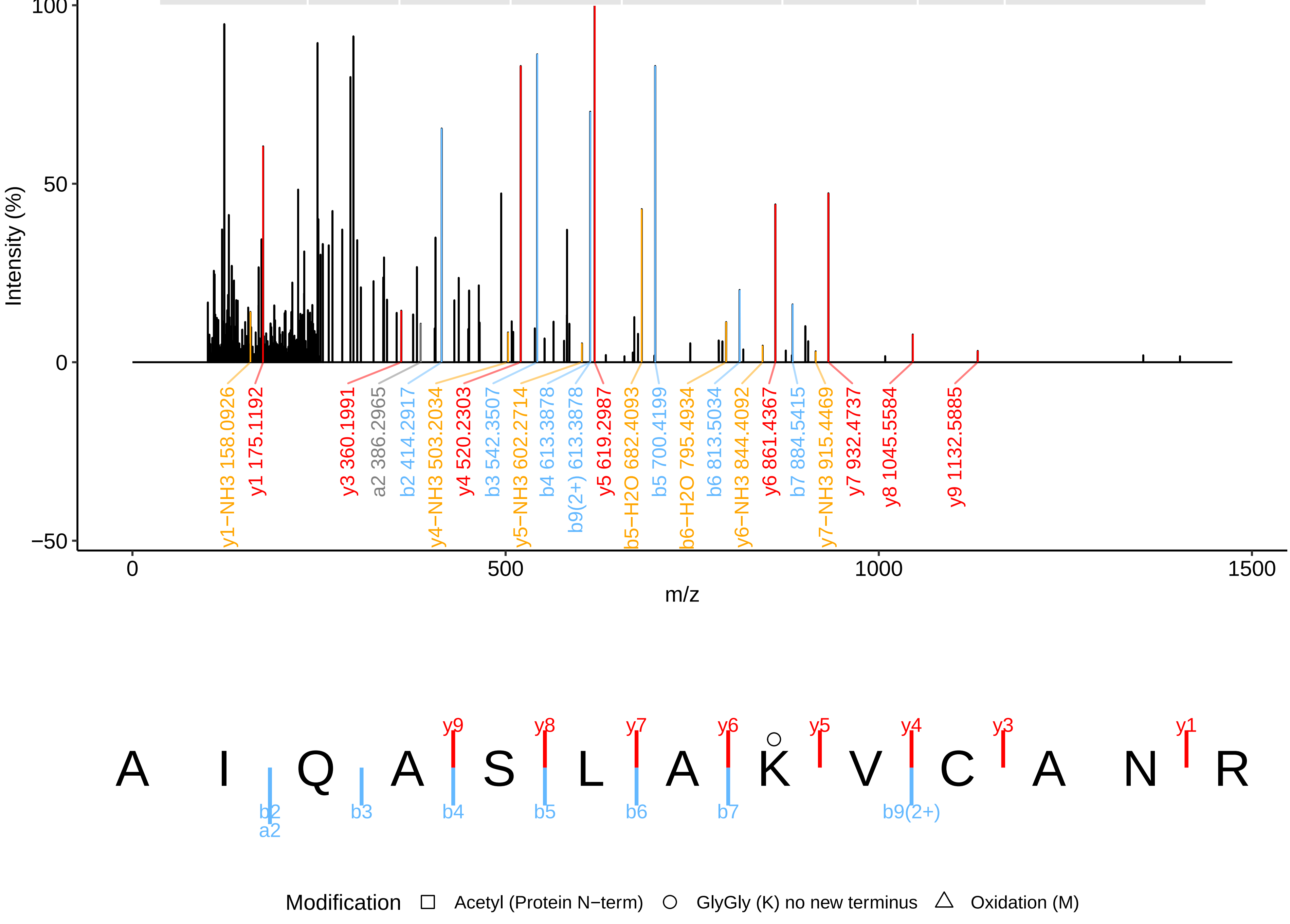

20200924_KIZ_MUb5_GlyGly_1 − Scan number: 20565

Gene Names	Charge	m/z	Mass	Retention time	PEP	Score	Precursor Intensity
RPL24	3	511.2802	1530.819	66.291	0.0014145	91.28	NA

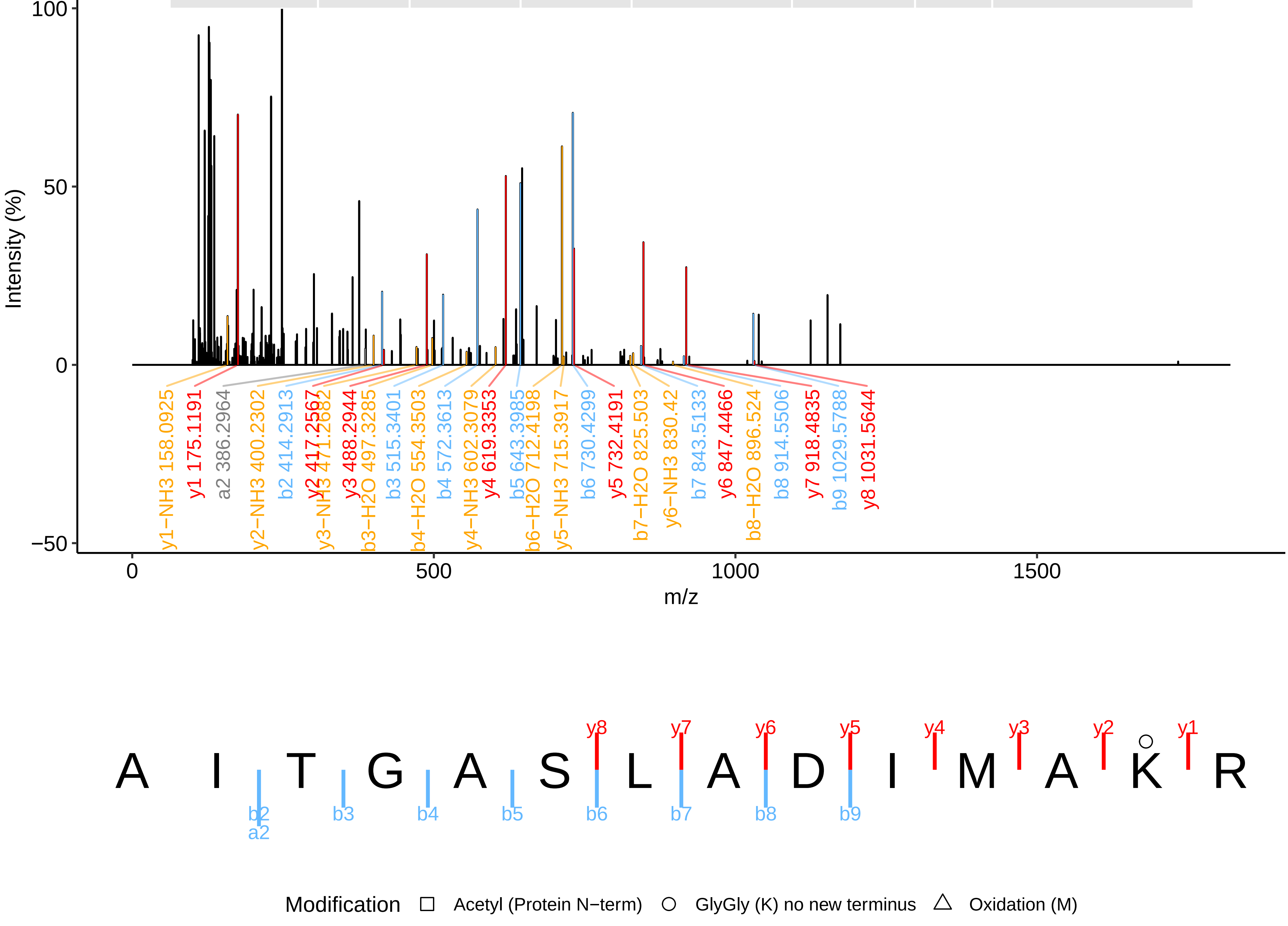

20200924_KIZ_MUb5_GlyGly_2 − Scan number: 18981

Gene Names	Charge	m/z	Mass	Retention time	PEP	Score	Precursor Intensity
RPL24	3	511.2802	1530.819	70.398	0.0019401	88.552	3368555

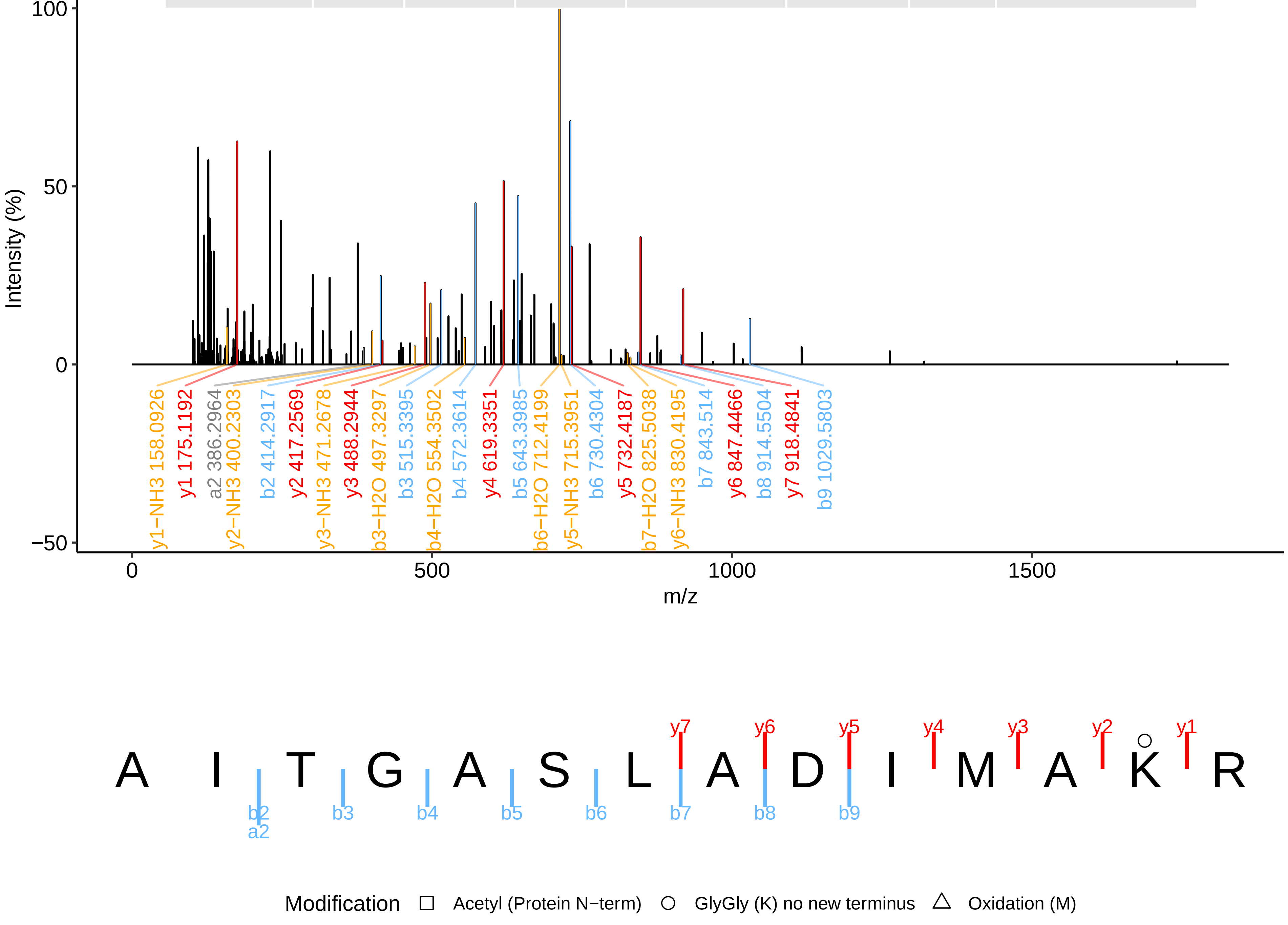

20200921_KIZ_MUb4_GlyGly_10plex − Scan number: 4784

Gene Names	Charge	m/z	Mass	Retention time	PEP	Score	Precursor Intensity
ATAD3B;ATAD3A	4	375.7036	1498.785	15.599	0.026566	76.24	8253988

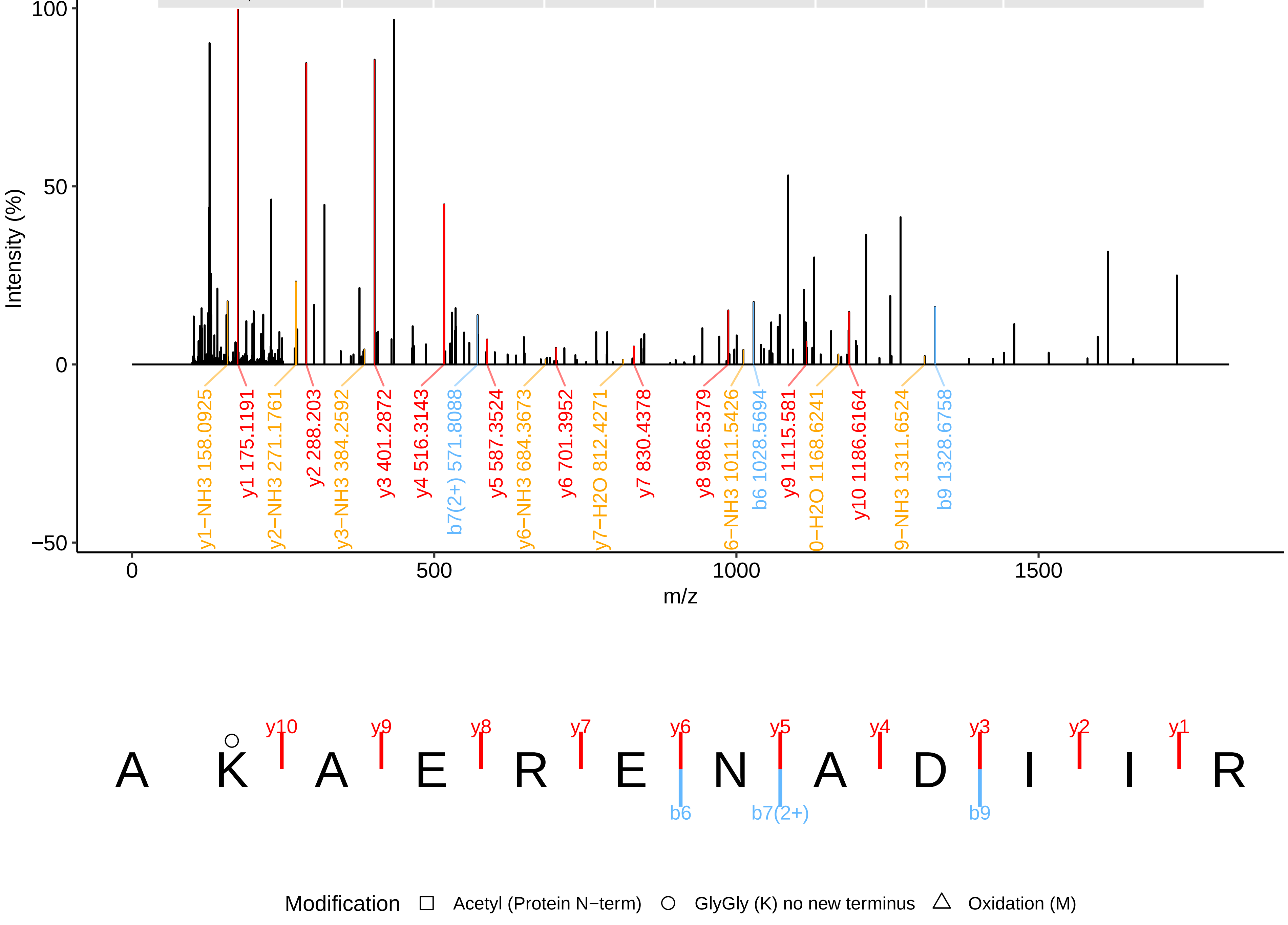

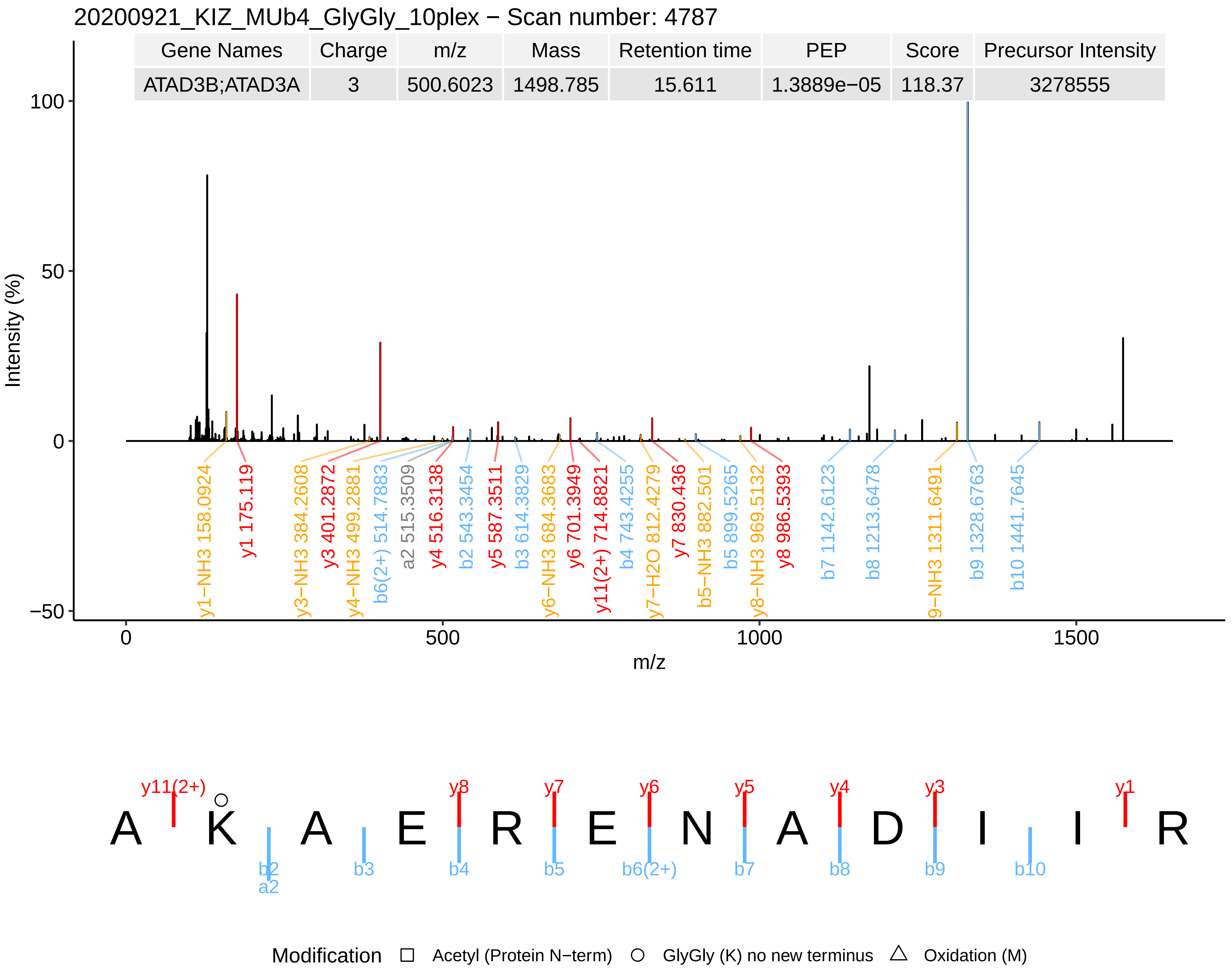

20200921_KIZ_MUb4_GlyGly_10plex − Scan number: 4787
Gene Names | Charge | m/z | Mass | Retention time | PEP | Score | Precursor Intensity
ATAD3B;ATAD3A | 3 | 500.6023 | 1498.785 | 15.611 | 1.3889e−05 | 118.37 | 3278555
Intensity (%)
100
50
0
−50
0
500
1000
1500
m/z
y1−NH3 158.0924
y1 175.119
y3−NH3 384.2608
y3 401.2872
y4−NH3 499.2881
b6(2+) 514.7883
a2 515.3509
y4 516.3138
b2 543.3454
y5 587.3511
b3 614.3829
y6−NH3 684.3683
y6 701.3949
y11(2+) 714.8821
b4 743.4255
y7−H2O 812.4279
y7 830.436
b5−NH3 882.501
b5 899.5265
y8−NH3 969.5132
y8 986.5393
b7 1142.6123
b8 1213.6478
9−NH3 1311.6491
b9 1328.6763
b10 1441.7645
y11(2+)
y8
y7
y6
y5
y4
y3
y1
A K A E R E N A D I I R
b2
a2
b3
b4
b5
b6(2+)
b7
b8
b9
b10
Modification
Acetyl (Protein N−term)
GlyGly (K) no new terminus
Oxidation (M)

20200921_KIZ_MUb4_GlyGly_10plex_2 − Scan number: 16622

Gene Names	Charge	m/z	Mass	Retention time	PEP	Score	Precursor Intensity
UBE3A	3	426.2468	1275.719	64.913	0.0079898	75.294	3264937

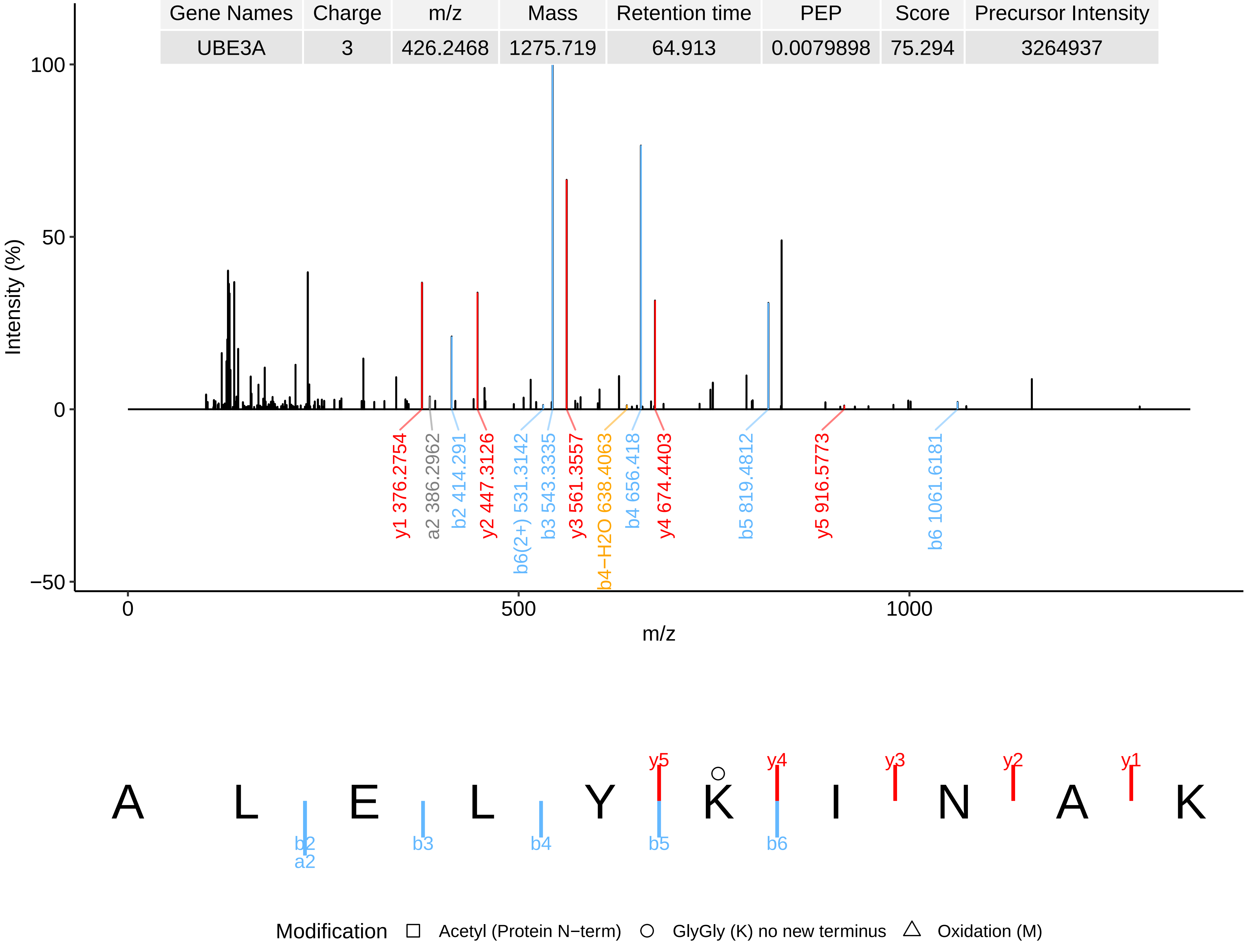

20200921_KIZ_MUb4_GlyGly_10plex − Scan number: 9474

Gene Names	Charge	m/z	Mass	Retention time	PEP	Score	Precursor Intensity
PGAM1;PGAM2	3	430.2274	1287.66	32.443	0.0015316	82.171	2414487

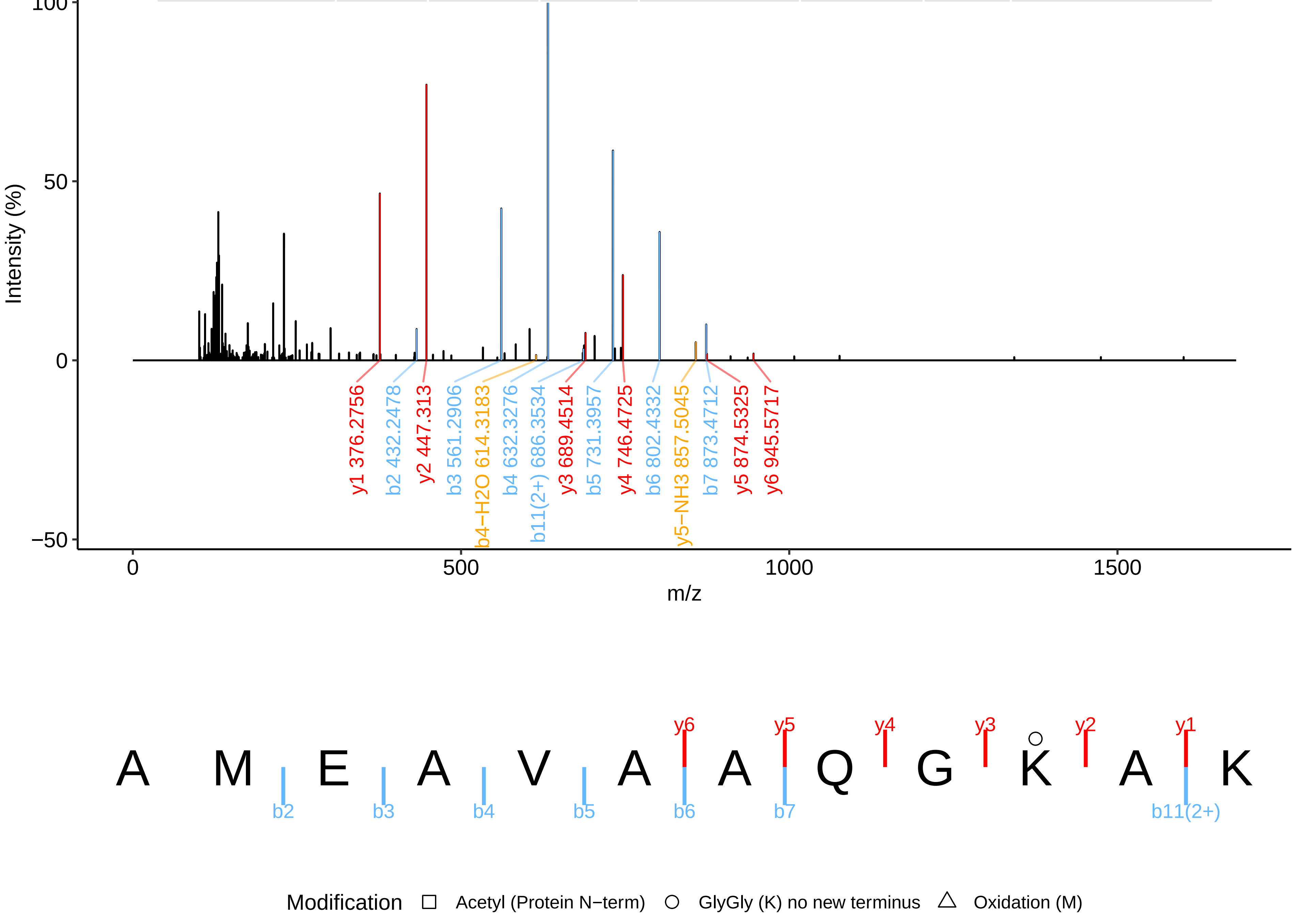

20200921_KIZ_MUb4_GlyGly_10plex − Scan number: 10986

Gene Names	Charge	m/z	Mass	Retention time	PEP	Score	Precursor Intensity
HSPBP1	3	439.2432	1314.708	38.202	0.0024871	81.338	4099888

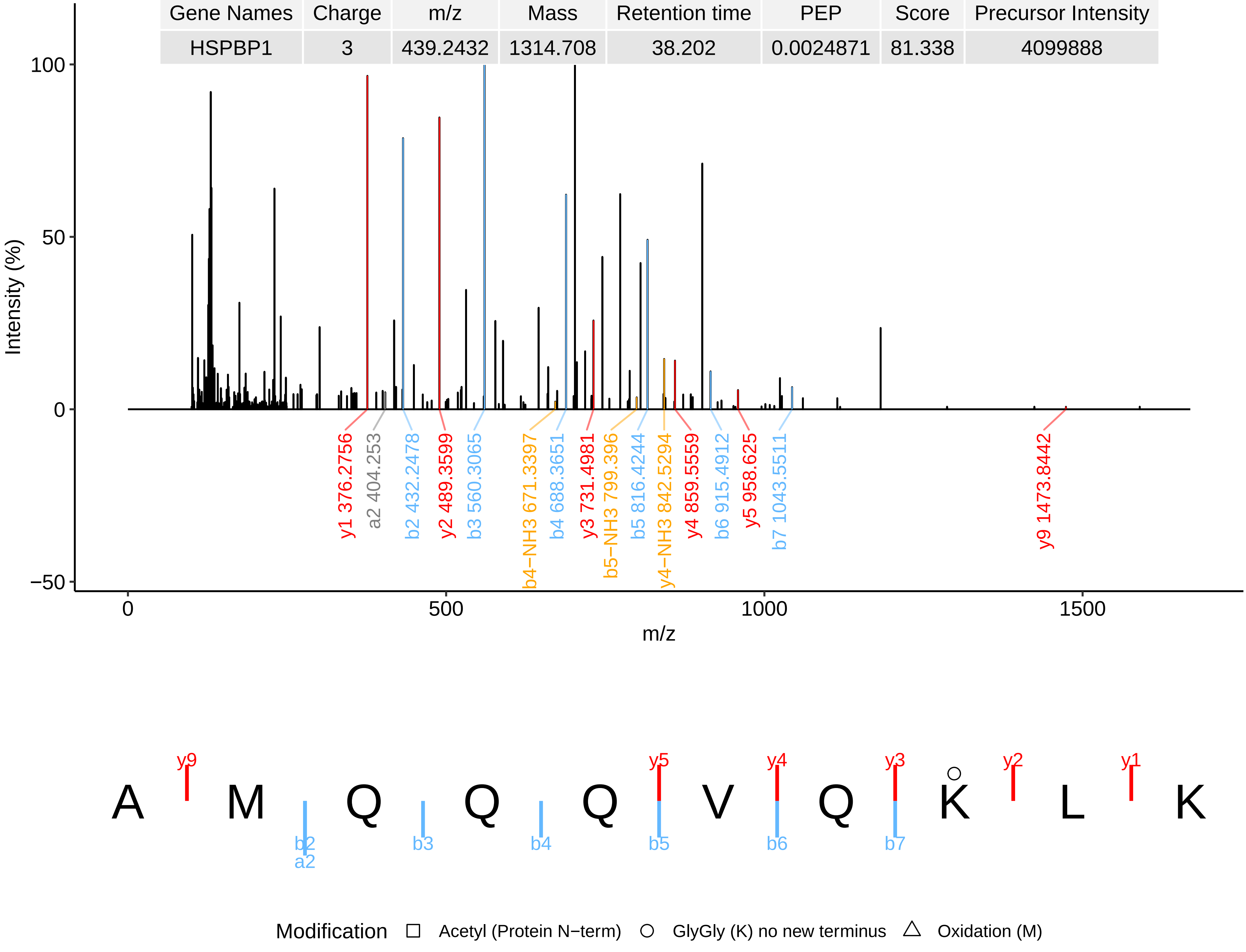

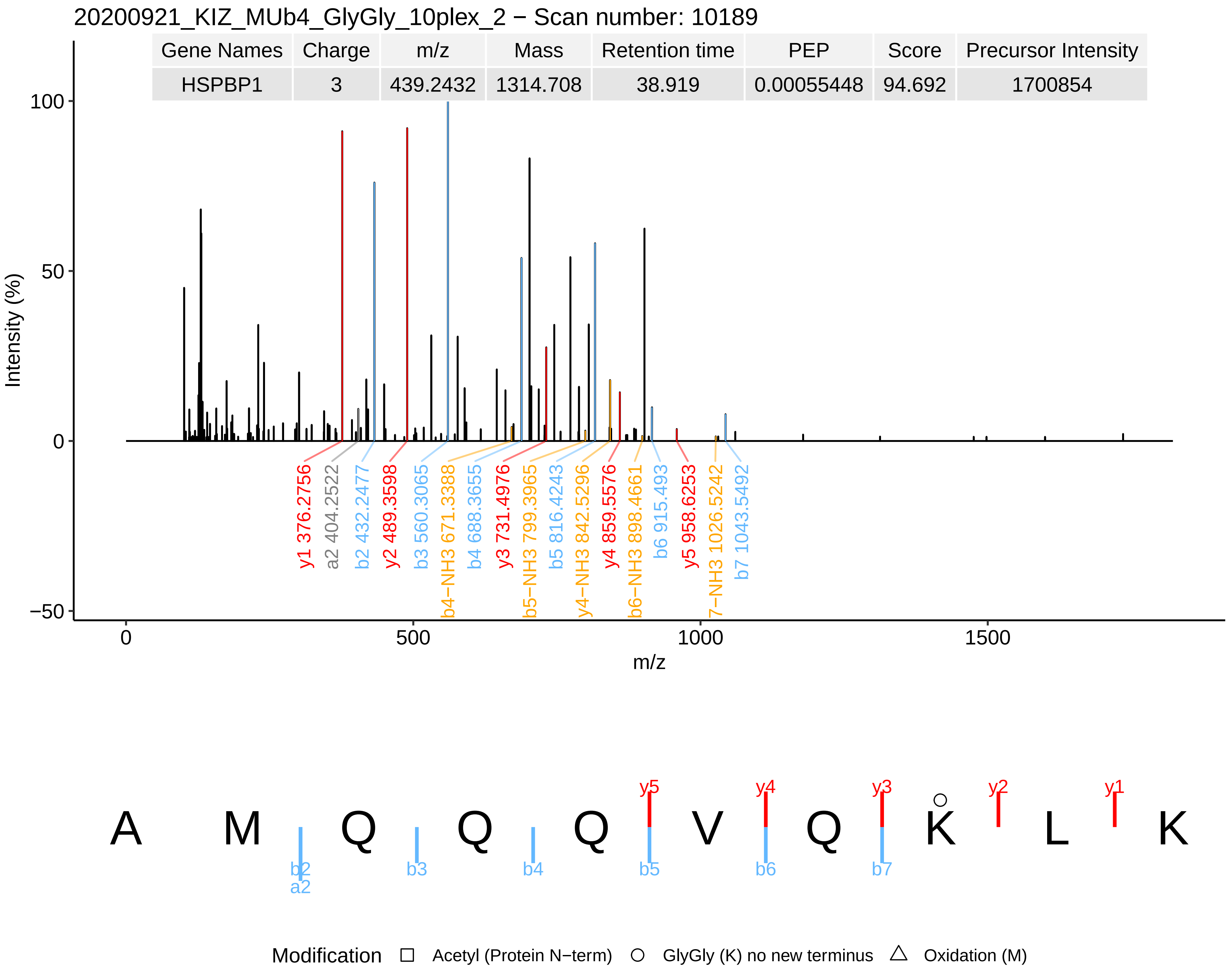
20200921_KIZ_MUb4_GlyGly_10plex_2 − Scan number: 10189
Gene Names | Charge | m/z | Mass | Retention time | PEP | Score | Precursor Intensity
HSPBP1 | 3 | 439.2432 | 1314.708 | 38.919 | 0.00055448 | 94.692 | 1700854
Intensity (%)
100
50
0
−50
0
500
1000
1500
m/z
y1 376.2756
a2 404.2522
b2 432.2477
y2 489.3598
b3 560.3065
b4−NH3 671.3388
b4 688.3655
y3 731.4976
b5−NH3 799.3965
b5 816.4243
y4−NH3 842.5296
y4 859.5576
b6−NH3 898.4661
b6 915.493
y5 958.6253
7−NH3 1026.5242
b7 1043.5492
A M Q Q Q V Q K L K
y5 y4 y3 y2 y1
b2 a2 b3 b4 b5 b6 b7
Modification
Acetyl (Protein N−term)
GlyGly (K) no new terminus
Oxidation (M)

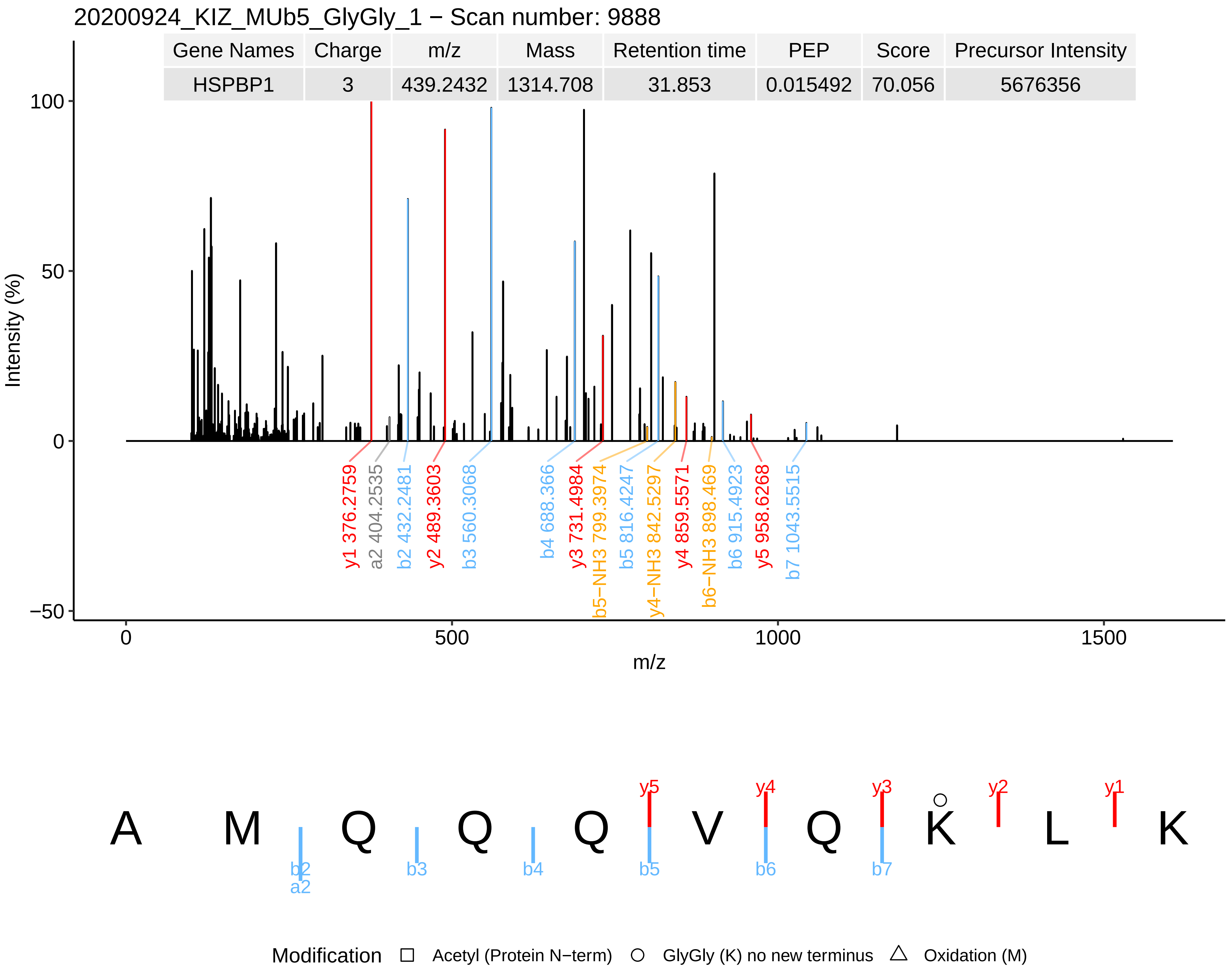

20200924_KIZ_MUb5_GlyGly_1 − Scan number: 9888
Gene Names | Charge | m/z | Mass | Retention time | PEP | Score | Precursor Intensity
HSPBP1 | 3 | 439.2432 | 1314.708 | 31.853 | 0.015492 | 70.056 | 5676356
Intensity (%)
100
50
0
−50
y1 376.2759
a2 404.2535
b2 432.2481
y2 489.3603
b3 560.3068
b4 688.366
y3 731.4984
b5−NH3 799.3974
b5 816.4247
y4−NH3 842.5297
y4 859.5571
b6−NH3 898.469
b6 915.4923
y5 958.6268
b7 1043.5515
0
500
1000
1500
m/z
A M Q Q Q V Q K L K
y5 y4 y3 y2 y1
b2 a2 b3 b4 b5 b6 b7
Modification
Acetyl (Protein N−term)
GlyGly (K) no new terminus
Oxidation (M)

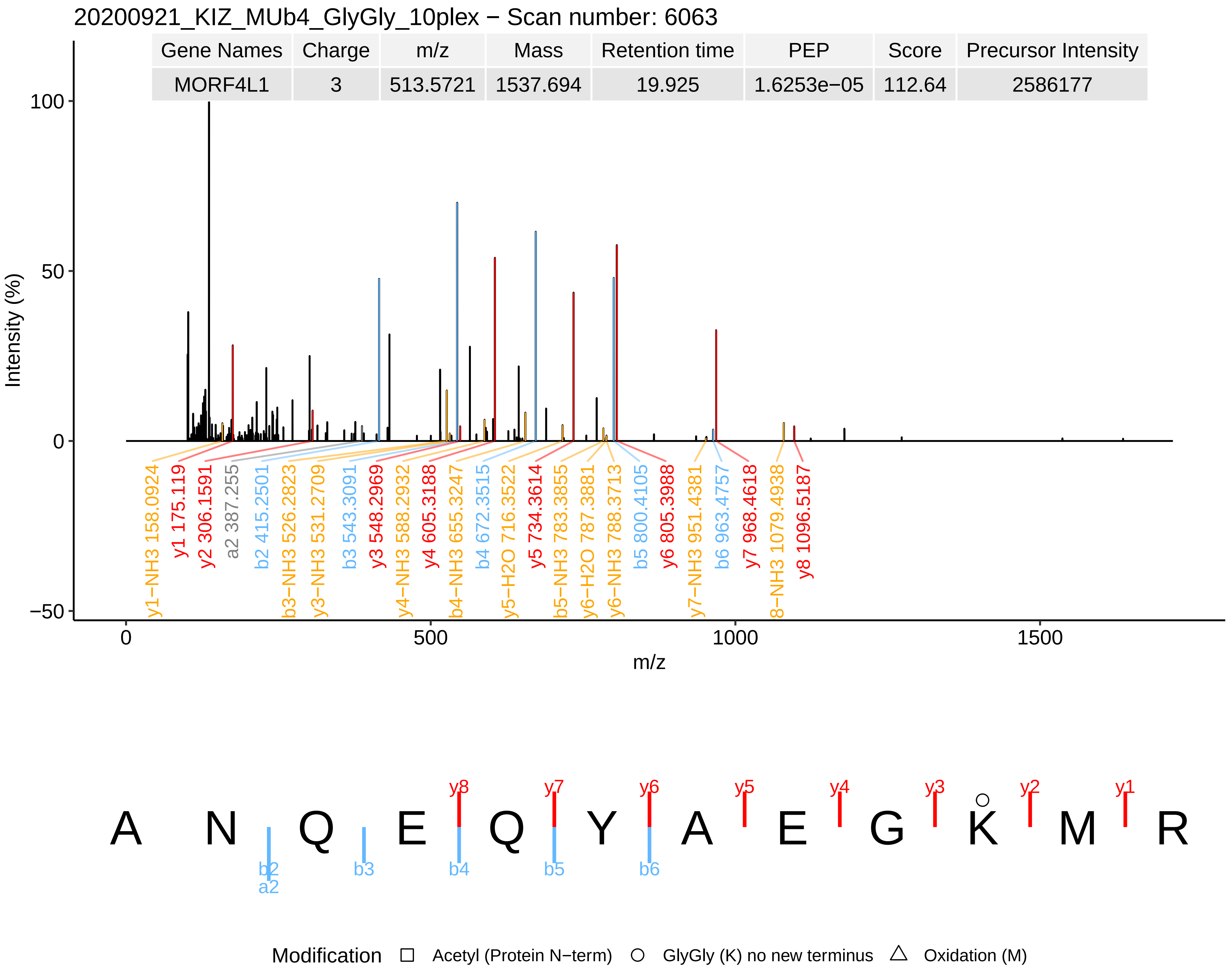
20200921_KIZ_MUb4_GlyGly_10plex − Scan number: 6063
Gene Names
MORF4L1
Charge
3
m/z
513.5721
Mass
1537.694
Retention time
19.925
PEP
1.6253e−05
Score
112.64
Precursor Intensity
2586177
Intensity (%)
100
50
0
−50
m/z
0
500
1000
1500
y1−NH3 158.0924
y1 175.119
y2 306.1591
a2 387.255
b2 415.2501
b3−NH3 526.2823
y3−NH3 531.2709
b3 543.3091
y3 548.2969
y4−NH3 588.2932
y4 605.3188
b4−NH3 655.3247
b4 672.3515
y5−H2O 716.3522
y5 734.3614
b5−NH3 783.3855
y6−H2O 787.3881
y6−NH3 788.3713
b5 800.4105
y6 805.3988
y7−NH3 951.4381
b6 963.4757
y7 968.4618
8−NH3 1079.4938
y8 1096.5187
A N Q E Q Y A E G K M R
y8 y7 y6 y5 y4 y3 y2 y1
b2 a2 b3 b4 b5 b6
Modification
Acetyl (Protein N−term)
GlyGly (K) no new terminus
Oxidation (M)

20200921_KIZ_MUb4_GlyGly_10plex_2 − Scan number: 5508

Gene Names	Charge	m/z	Mass	Retention time	PEP	Score	Precursor Intensity
MORF4L1	3	513.5721	1537.694	19.837	0.014156	62.924	401614.6

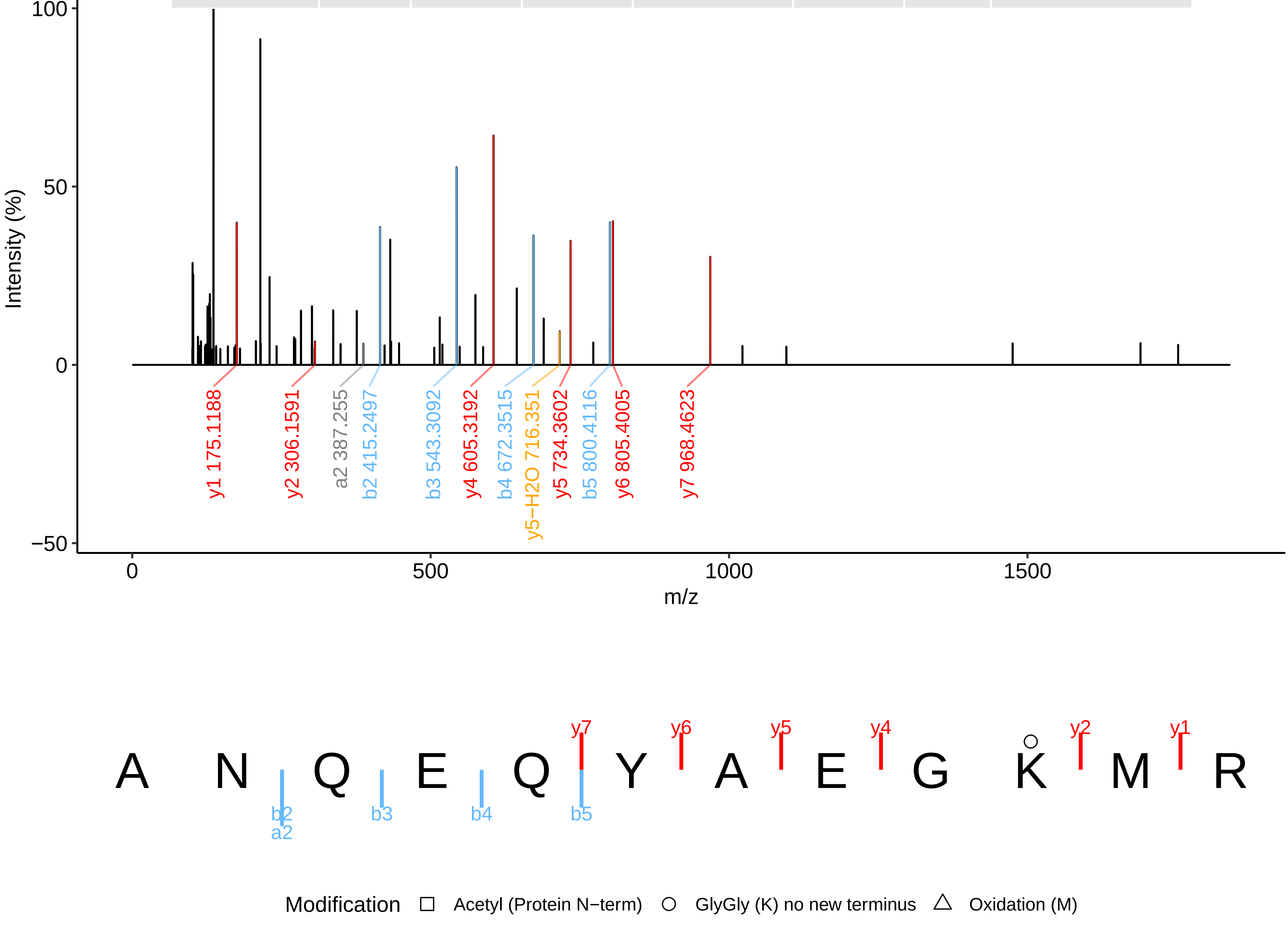

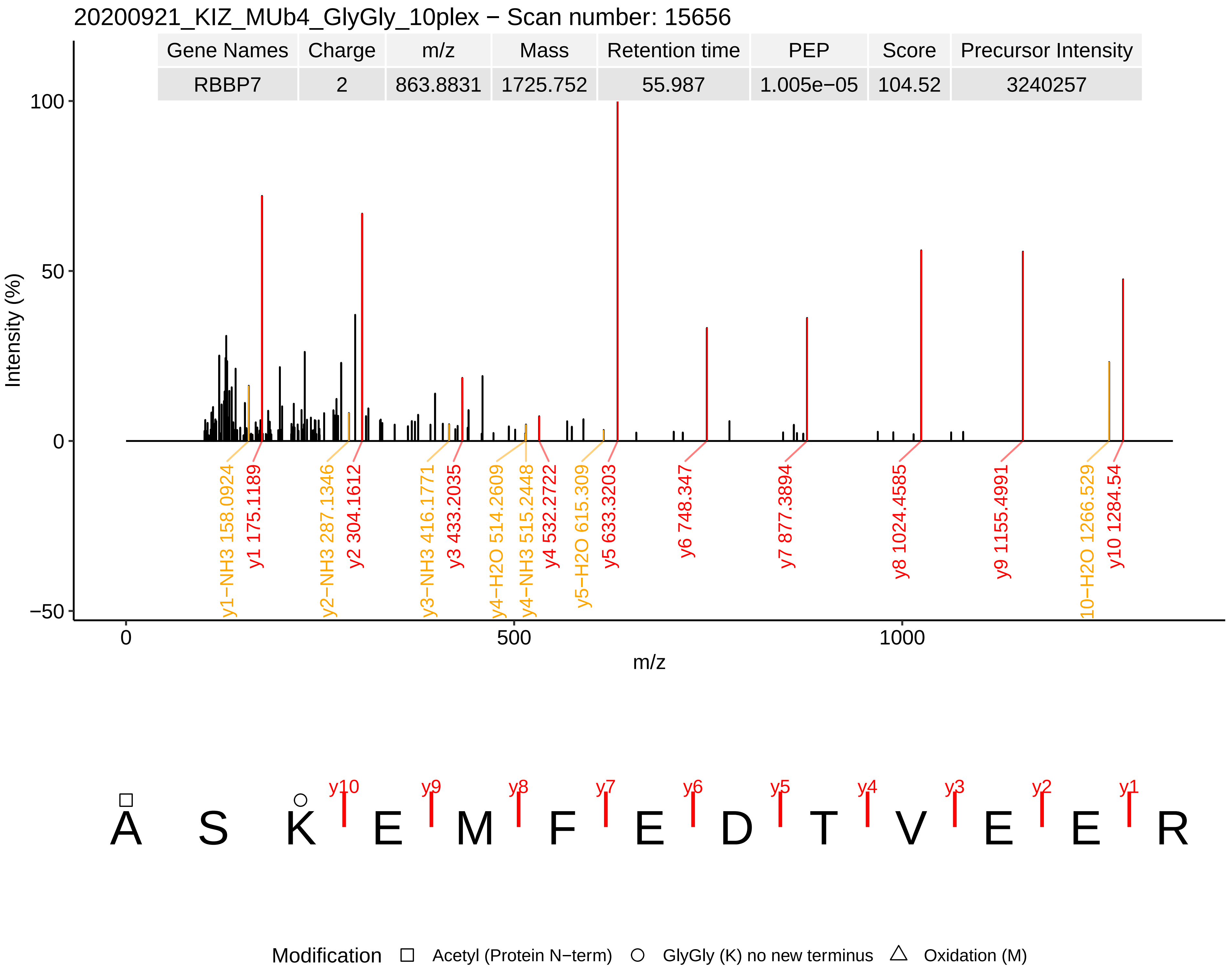

20200921_KIZ_MUb4_GlyGly_10plex − Scan number: 15656
Gene Names | Charge | m/z | Mass | Retention time | PEP | Score | Precursor Intensity
RBBP7 | 2 | 863.8831 | 1725.752 | 55.987 | 1.005e−05 | 104.52 | 3240257
Intensity (%)
100
50
0
−50
0
500
1000
m/z
y1−NH3 158.0924
y1 175.1189
y2−NH3 287.1346
y2 304.1612
y3−NH3 416.1771
y3 433.2035
y4−H2O 514.2609
y4−NH3 515.2448
y4 532.2722
y5−H2O 615.309
y5 633.3203
y6 748.347
y7 877.3894
y8 1024.4585
y9 1155.4991
10−H2O 1266.529
y10 1284.54
A S K E M F E D T V E E R
y10 y9 y8 y7 y6 y5 y4 y3 y2 y1
Modification
Acetyl (Protein N−term)
GlyGly (K) no new terminus
Oxidation (M)

20200921_KIZ_MUb4_GlyGly_10plex_2 − Scan number: 14421

Gene Names	Charge	m/z	Mass	Retention time	PEP	Score	Precursor Intensity
RBBP7	2	863.8831	1725.752	56.227	0.00078773	81.475	591719.6

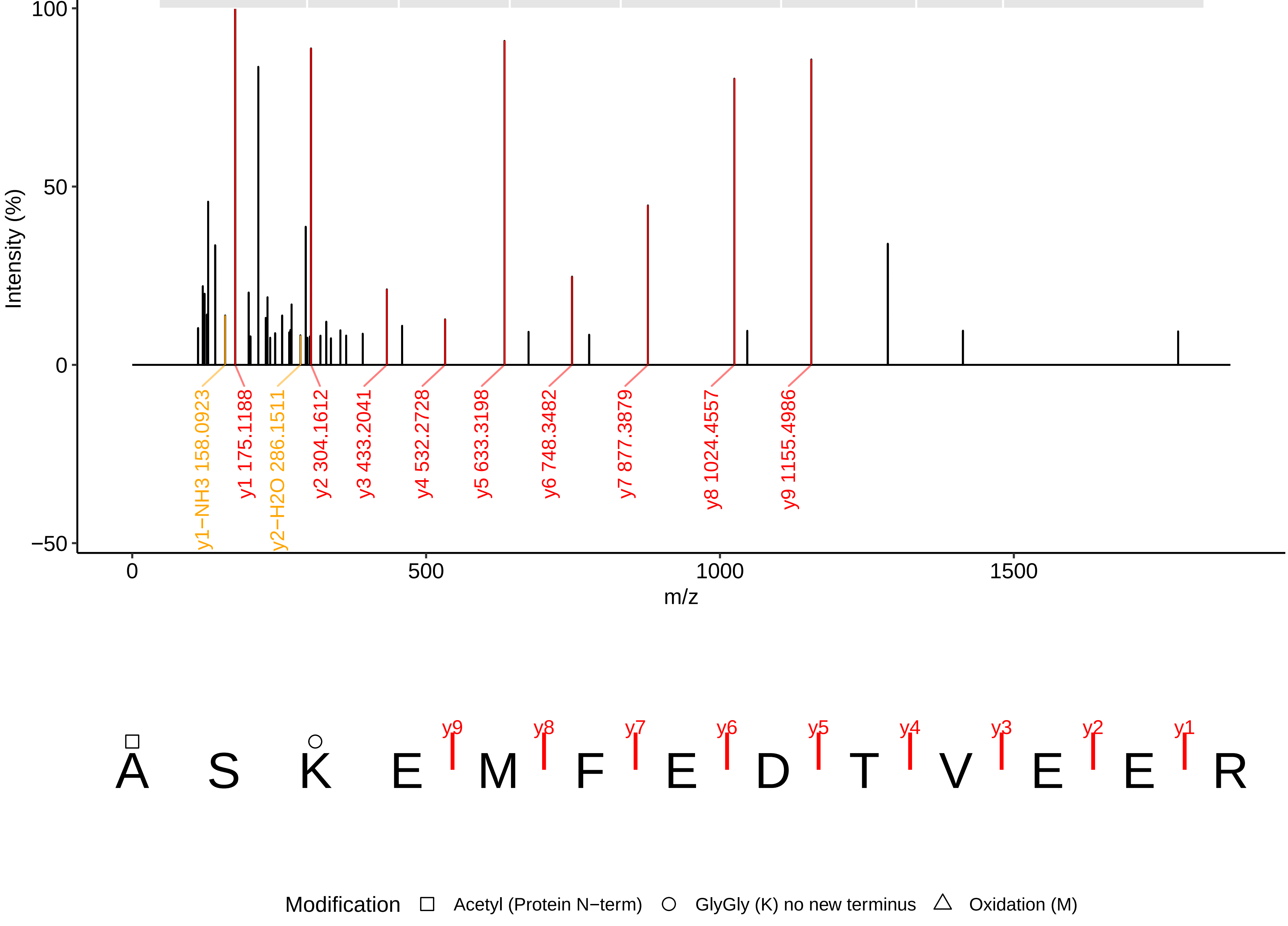

20200921_KIZ_MUb4_GlyGly_10plex − Scan number: 7824

Gene Names	Charge	m/z	Mass	Retention time	PEP	Score	Precursor Intensity
OGT	2	887.9319	1773.849	26.404	2.7496e−63	143.97	4175806

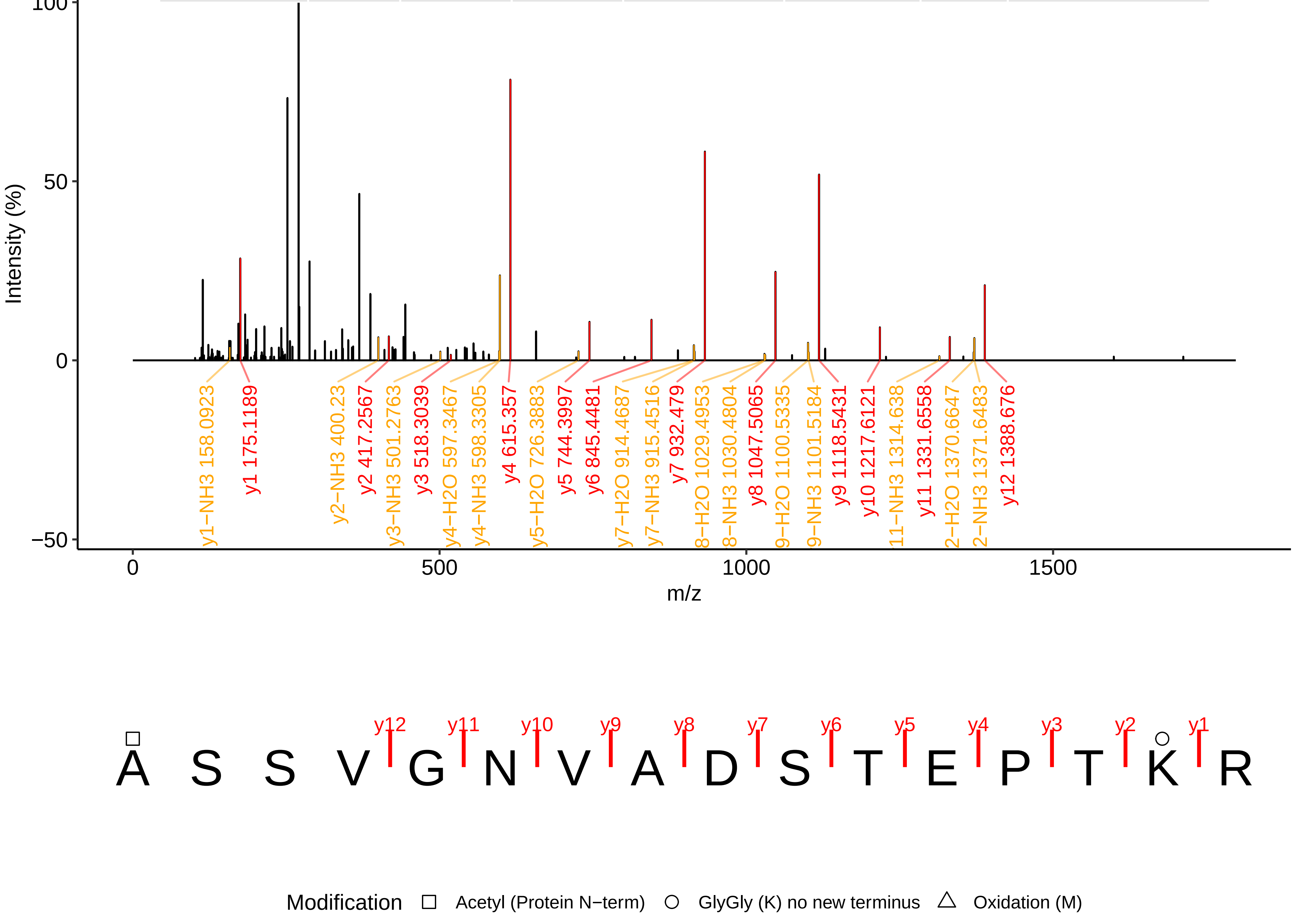

20200921_KIZ_MUb4_GlyGly_10plex_2 − Scan number: 7075

Gene Names	Charge	m/z	Mass	Retention time	PEP	Score	Precursor Intensity
OGT	2	887.9319	1773.849	26.271	1.1413e−14	85.288	564595.6

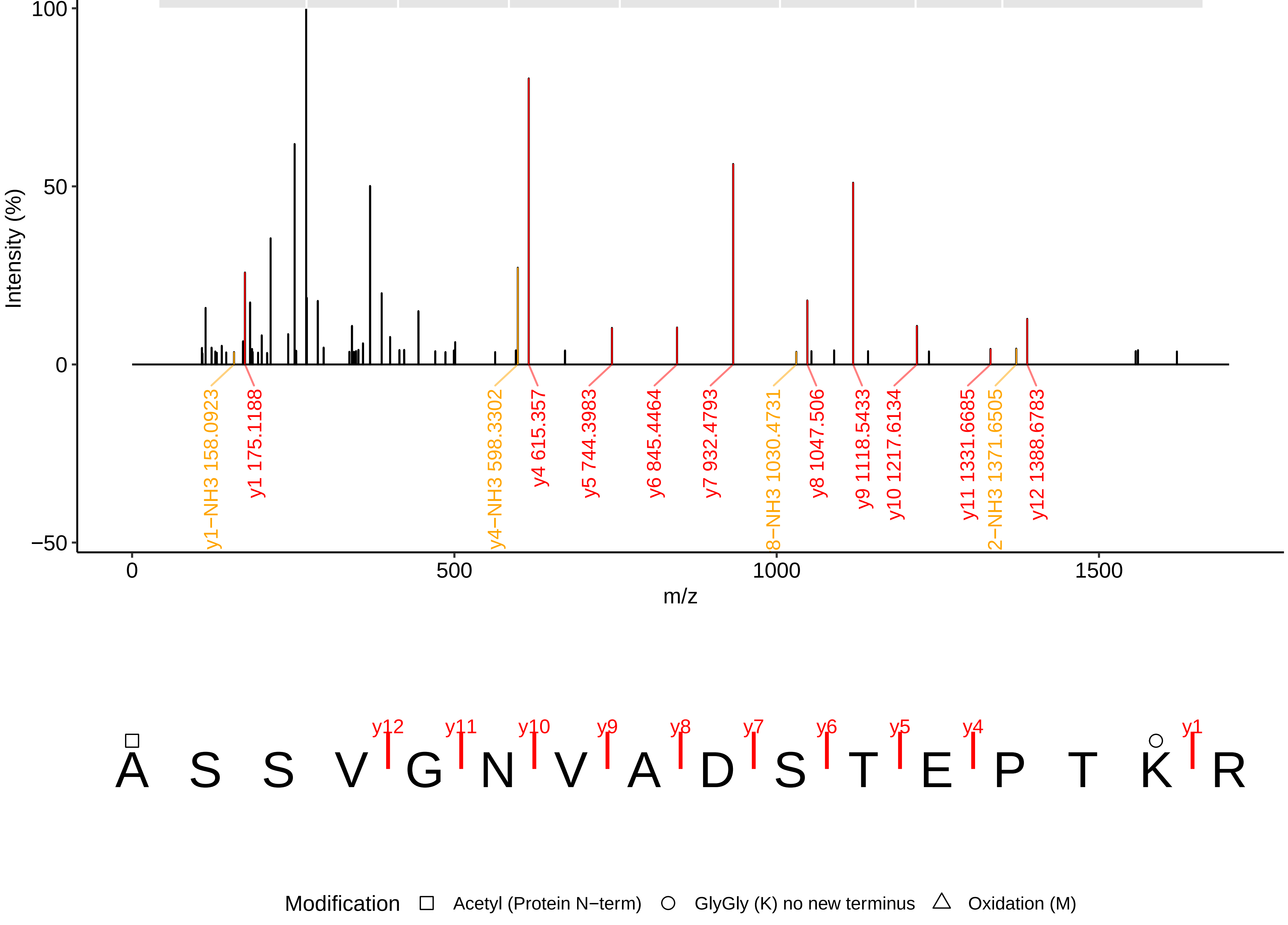

20200924_KIZ_MUb5_GlyGly_2 − Scan number: 6484

Gene Names	Charge	m/z	Mass	Retention time	PEP	Score	Precursor Intensity
OGT	2	887.9319	1773.849	24.185	1.0236e−13	84.289	1948095

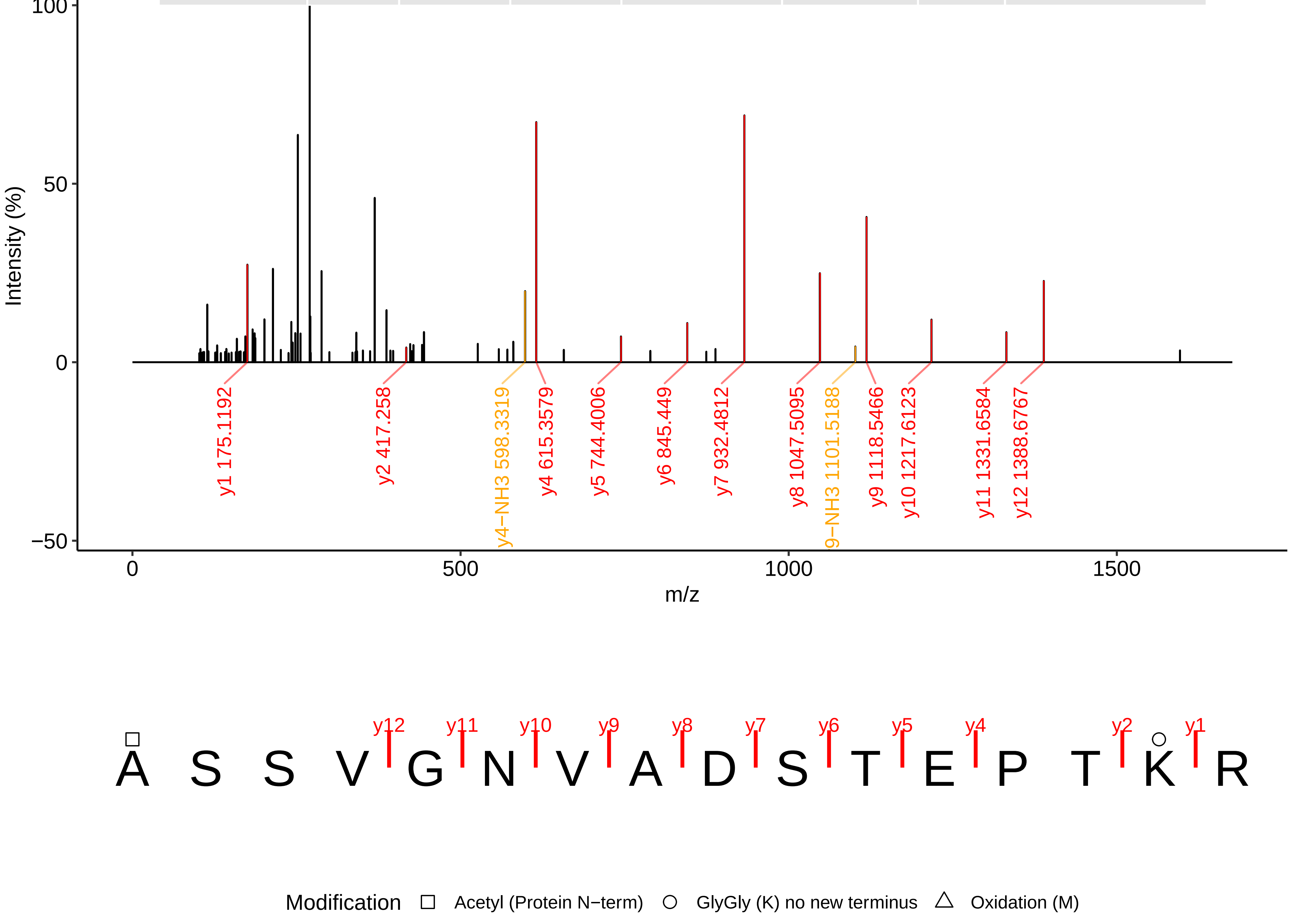

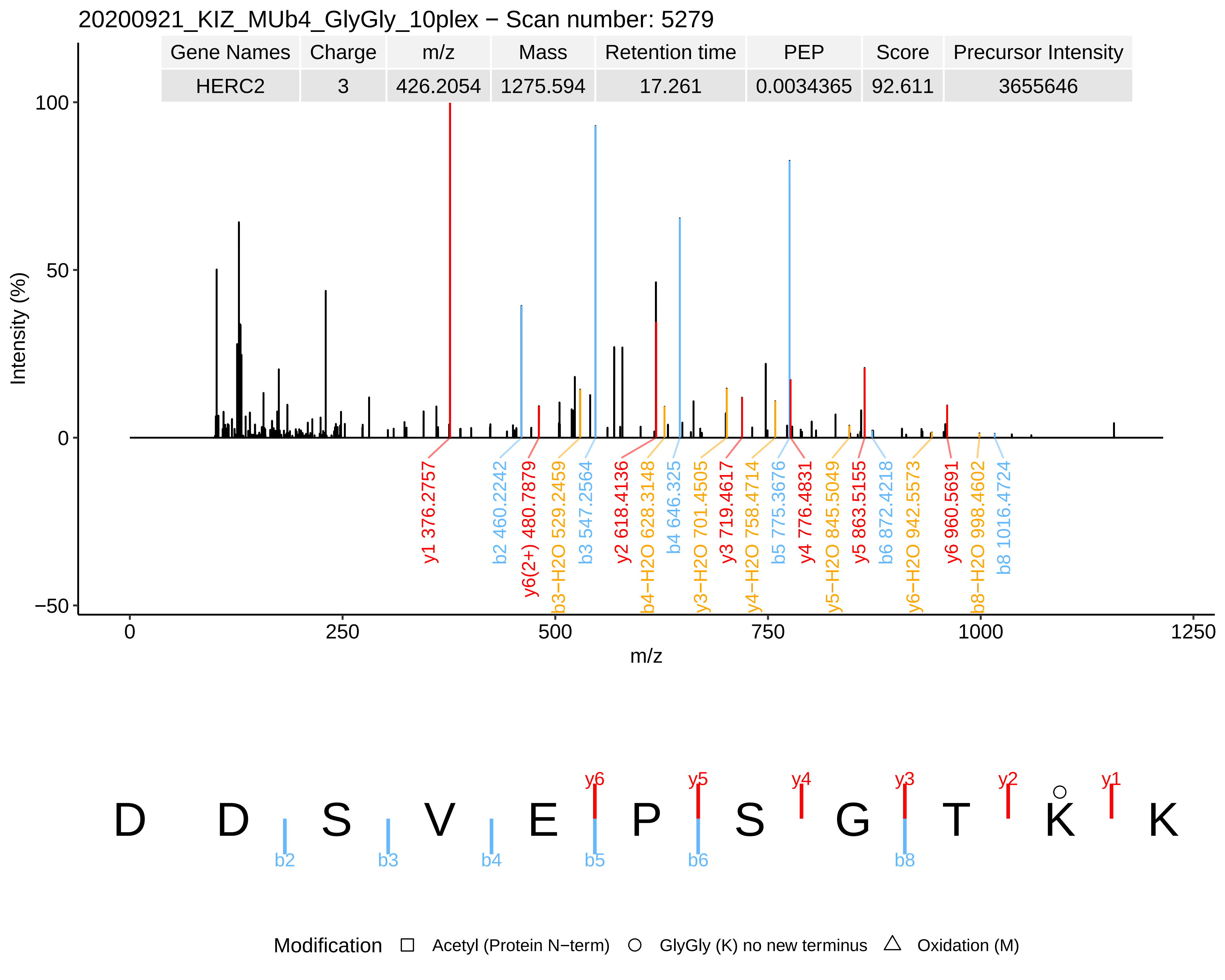

20200921_KIZ_MUb4_GlyGly_10plex − Scan number: 5279
Gene Names | Charge | m/z | Mass | Retention time | PEP | Score | Precursor Intensity
HERC2 | 3 | 426.2054 | 1275.594 | 17.261 | 0.0034365 | 92.611 | 3655646
Intensity (%)
m/z
y1 376.2757
b2 460.2242
y6(2+) 480.7879
b3−H2O 529.2459
b3 547.2564
y2 618.4136
b4−H2O 628.3148
b4 646.325
y3−H2O 701.4505
y3 719.4617
y4−H2O 758.4714
b5 775.3676
y4 776.4831
y5−H2O 845.5049
y5 863.5155
b6 872.4218
y6−H2O 942.5573
y6 960.5691
b8−H2O 998.4602
b8 1016.4724
D D S V E P S G T K K
y6 y5 y4 y3 y2 y1
b2 b3 b4 b5 b6 b8
Modification
Acetyl (Protein N−term)
GlyGly (K) no new terminus
Oxidation (M)

20200921_KIZ_MUb4_GlyGly_10plex_2 − Scan number: 4835

Gene Names	Charge	m/z	Mass	Retention time	PEP	Score	Precursor Intensity
HERC2	3	426.2054	1275.594	17.132	0.0058903	88.596	1092925

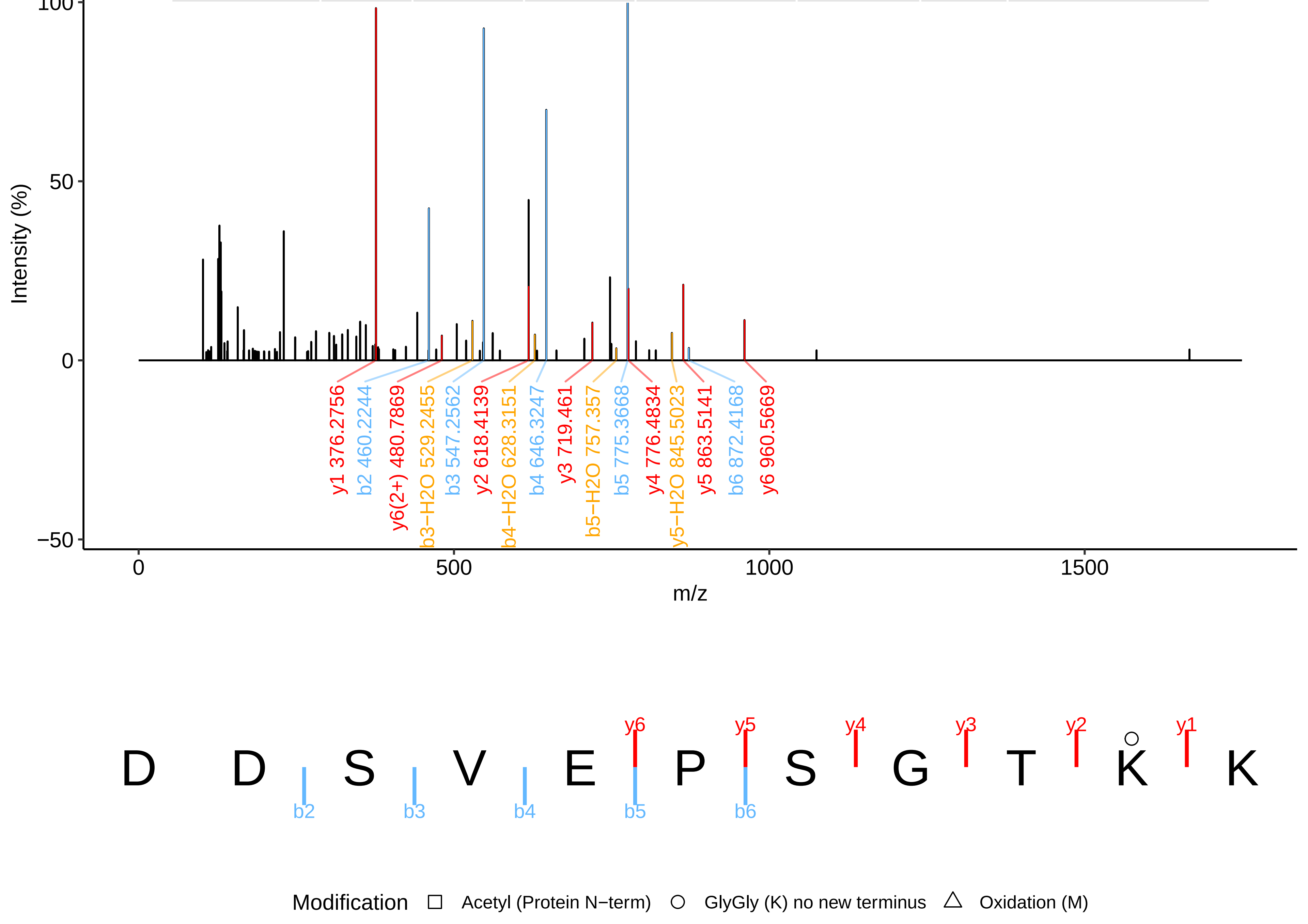

20200924_KIZ_MUb5_GlyGly_1 − Scan number: 4684

Gene Names	Charge	m/z	Mass	Retention time	PEP	Score	Precursor Intensity
HERC2	3	426.2054	1275.594	14.489	7.3202e−05	110.84	8606945

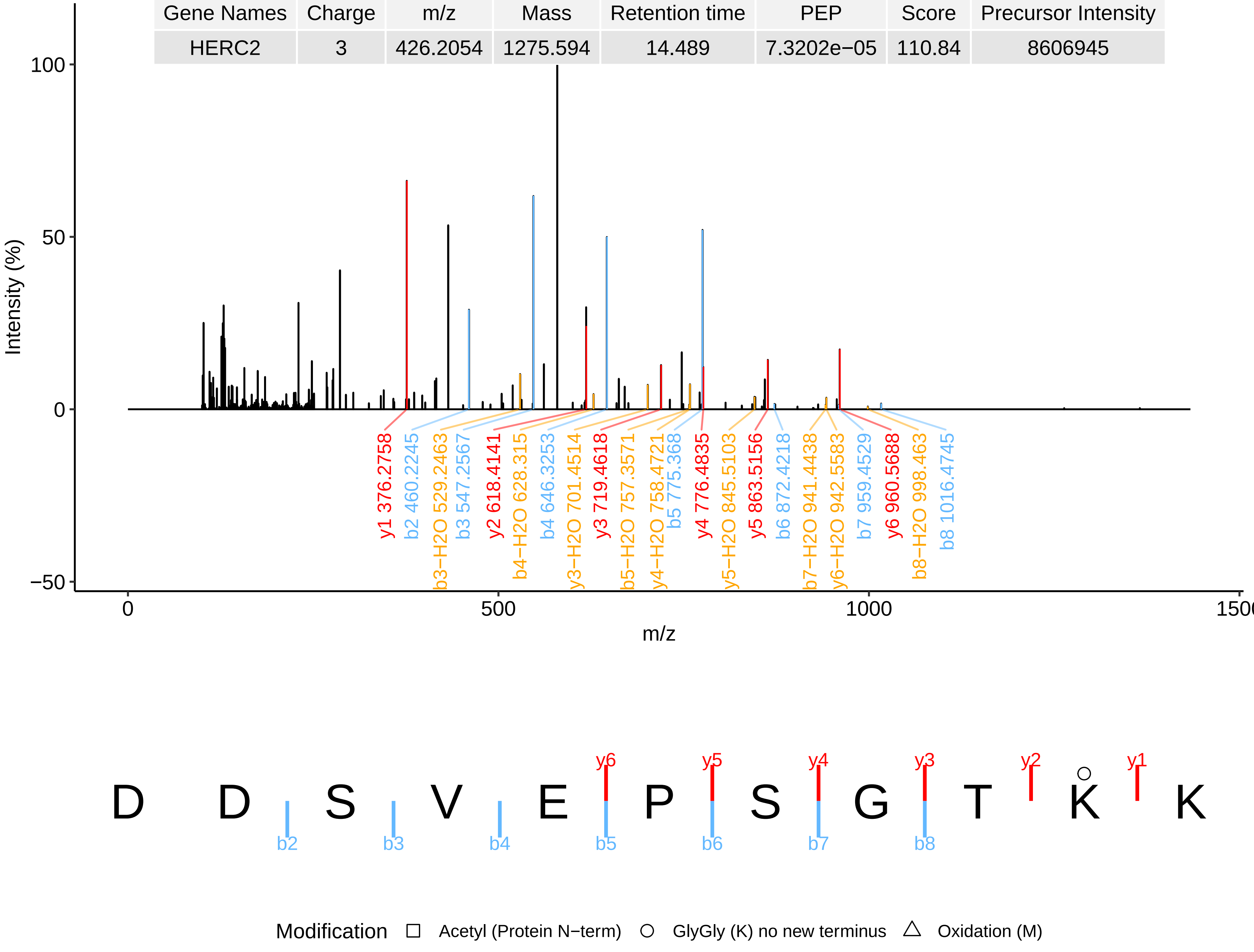

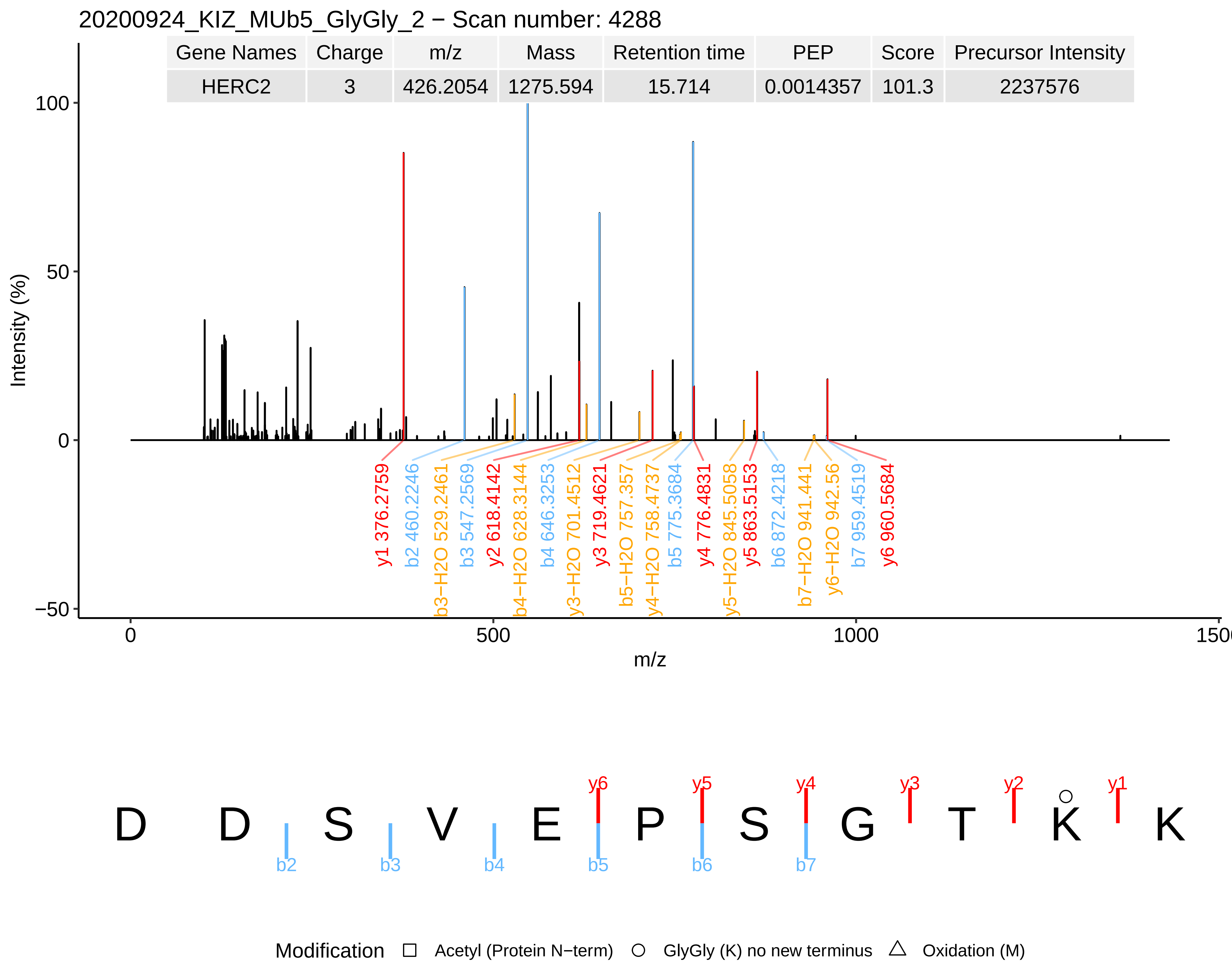
20200924_KIZ_MUb5_GlyGly_2 − Scan number: 4288
Gene Names
Charge
m/z
Mass
Retention time
PEP
Score
Precursor Intensity
HERC2
3
426.2054
1275.594
15.714
0.0014357
101.3
2237576
Intensity (%)
100
50
0
−50
0
500
1000
1500
m/z
y1 376.2759
b2 460.2246
b3−H2O 529.2461
b3 547.2569
y2 618.4142
b4−H2O 628.3144
b4 646.3253
y3−H2O 701.4512
y3 719.4621
b5−H2O 757.357
y4−H2O 758.4737
b5 775.3684
y4 776.4831
y5−H2O 845.5058
y5 863.5153
b6 872.4218
b7−H2O 941.441
y6−H2O 942.56
b7 959.4519
y6 960.5684
D D S V E P S G T K K
y6 y5 y4 y3 y2 y1
b2 b3 b4 b5 b6 b7
Modification
Acetyl (Protein N−term)
GlyGly (K) no new terminus
Oxidation (M)

20201118_KIZ_MUb6_GlyGly_2 − Scan number: 5135

Gene Names	Charge	m/z	Mass	Retention time	PEP	Score	Precursor Intensity
HERC2	3	426.2054	1275.594	17.693	0.0057327	86.014	926322.1

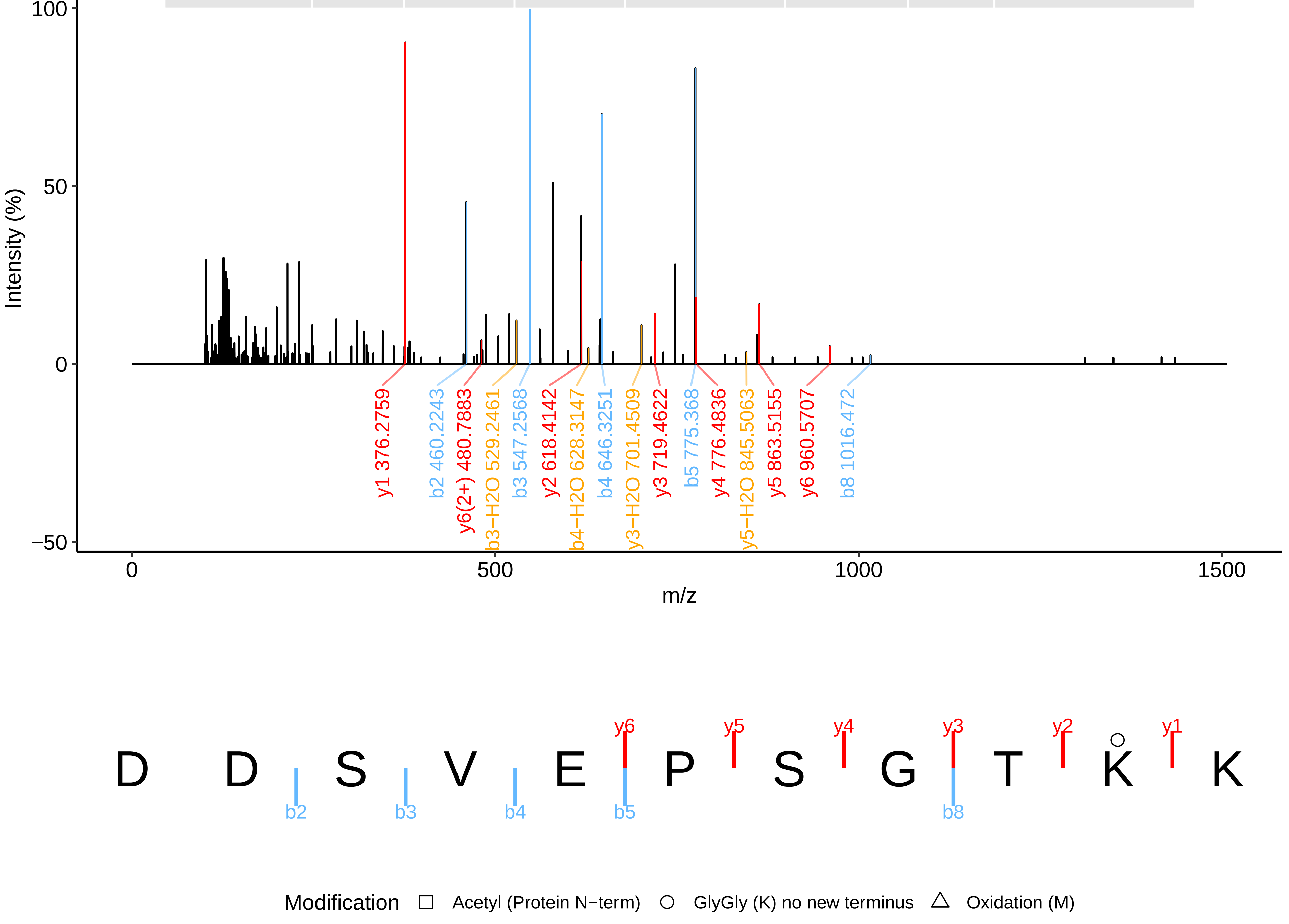

20200921_KIZ_MUb4_GlyGly_10plex_2 − Scan number: 15393

Gene Names	Charge	m/z	Mass	Retention time	PEP	Score	Precursor Intensity
CHAF1B	3	419.2346	1254.682	60.185	0.0036815	84.17	359753.5

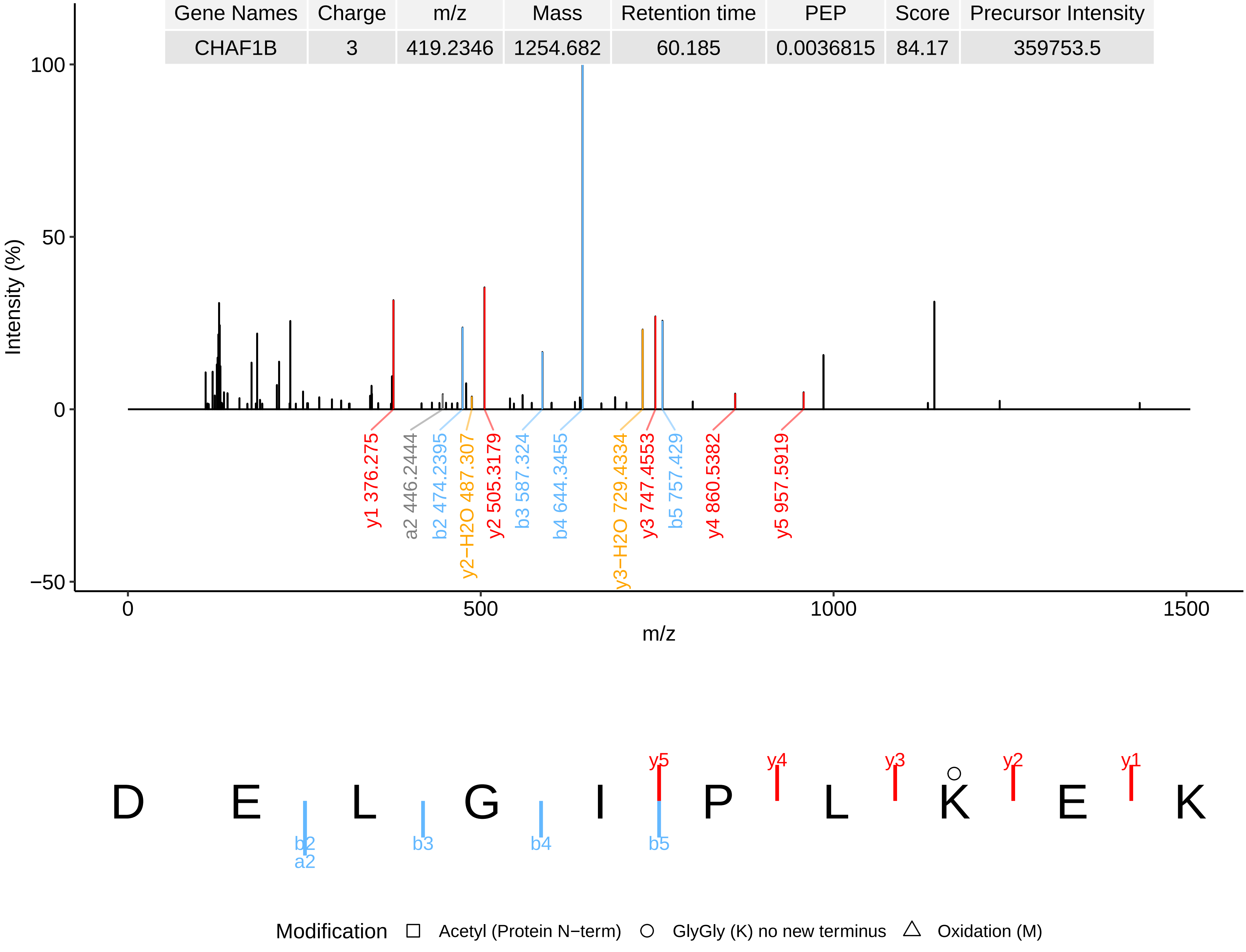

20200921_KIZ_MUb4_GlyGly_10plex − Scan number: 19591

Gene Names	Charge	m/z	Mass	Retention time	PEP	Score	Precursor Intensity
JAK1	3	423.5472	1267.62	70.69	0.0083074	76.1	NA

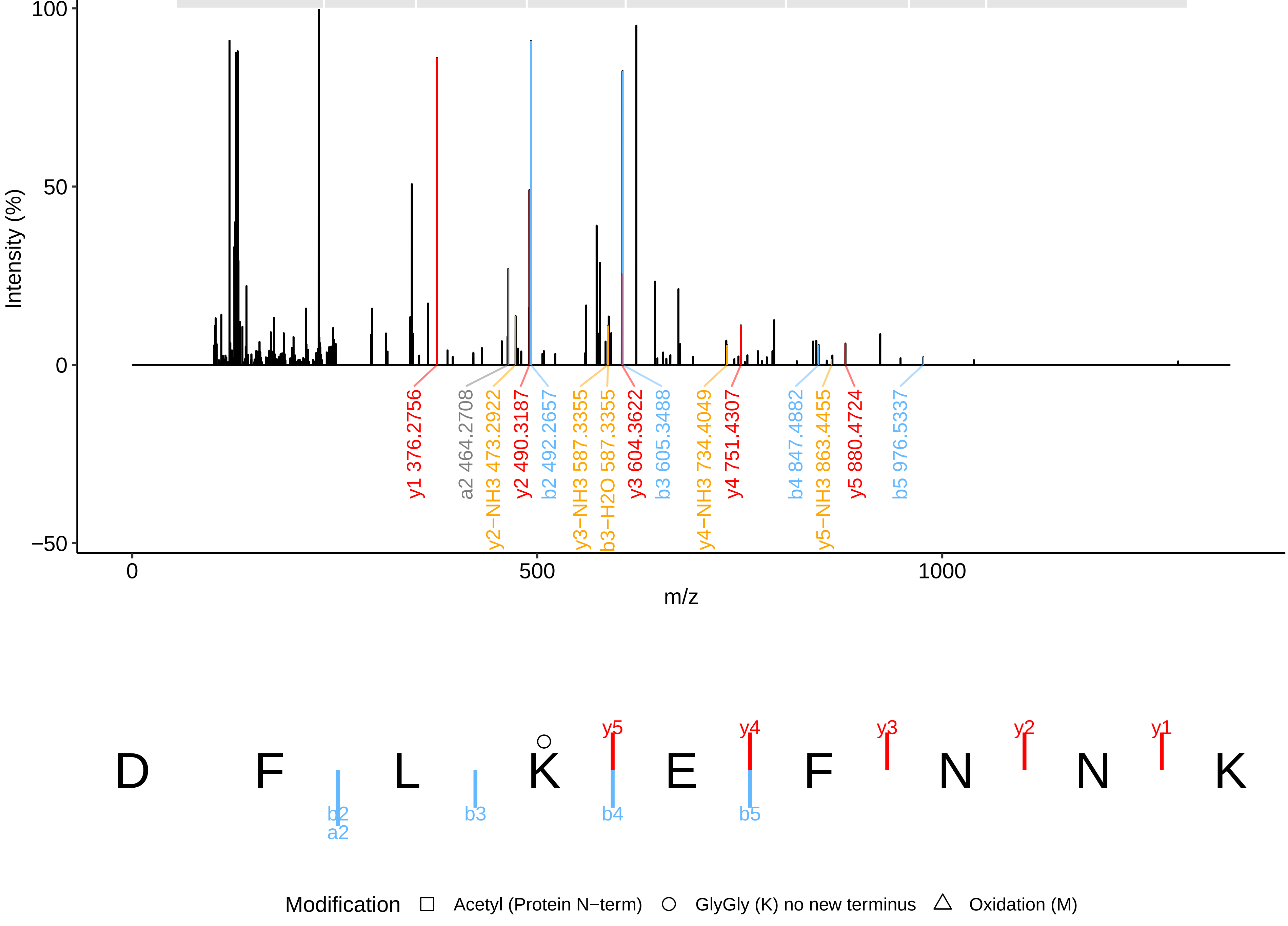

20200921_KIZ_MUb4_GlyGly_10plex − Scan number: 19726

Gene Names	Charge	m/z	Mass	Retention time	PEP	Score	Precursor Intensity
JAK1	3	423.5472	1267.62	71.195	0.00056988	98.033	102387032

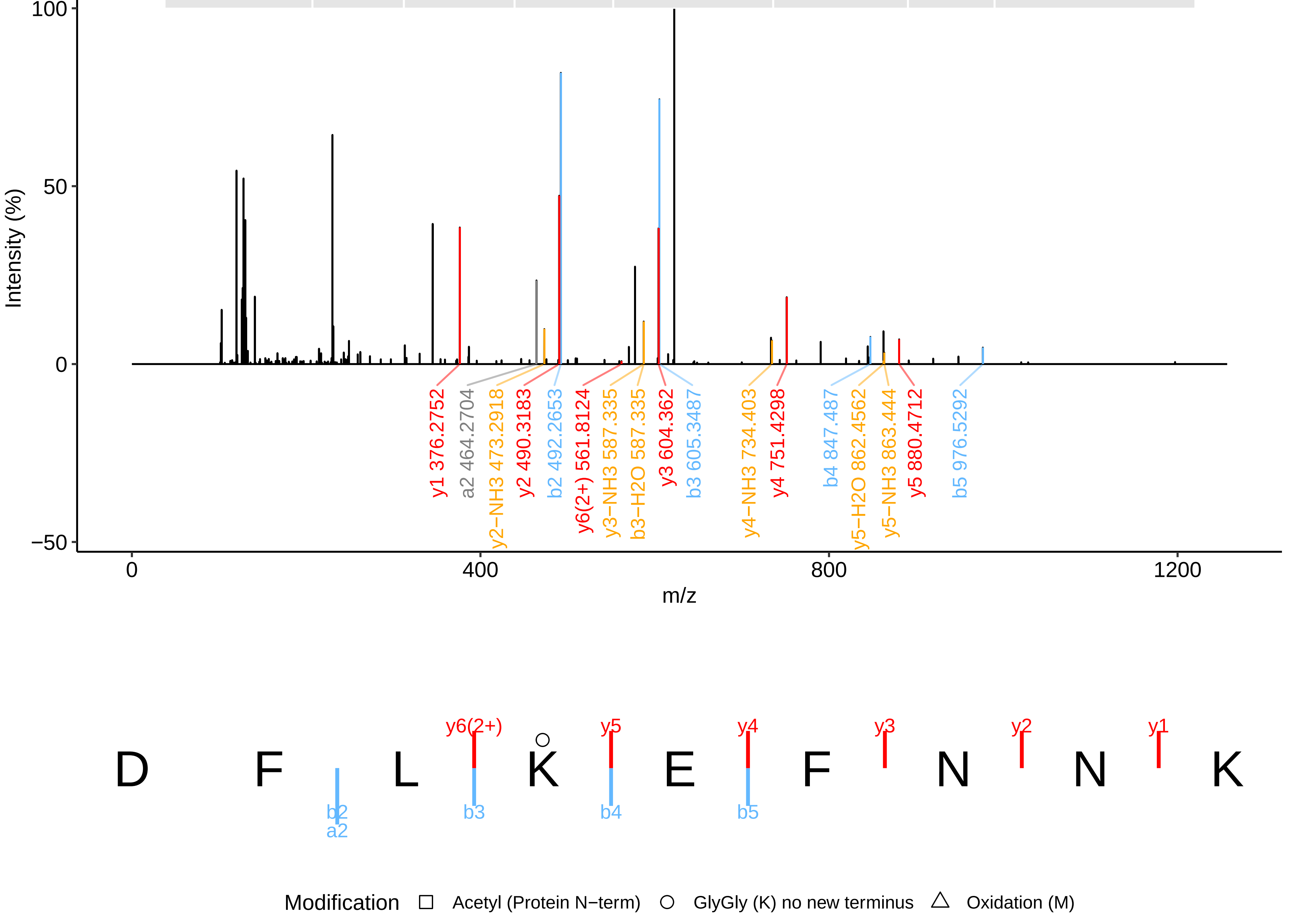

20200921_KIZ_MUb4_GlyGly_10plex − Scan number: 19863

Gene Names	Charge	m/z	Mass	Retention time	PEP	Score	Precursor Intensity
JAK1	3	423.5472	1267.62	71.703	0.0002684	107.57	73108976

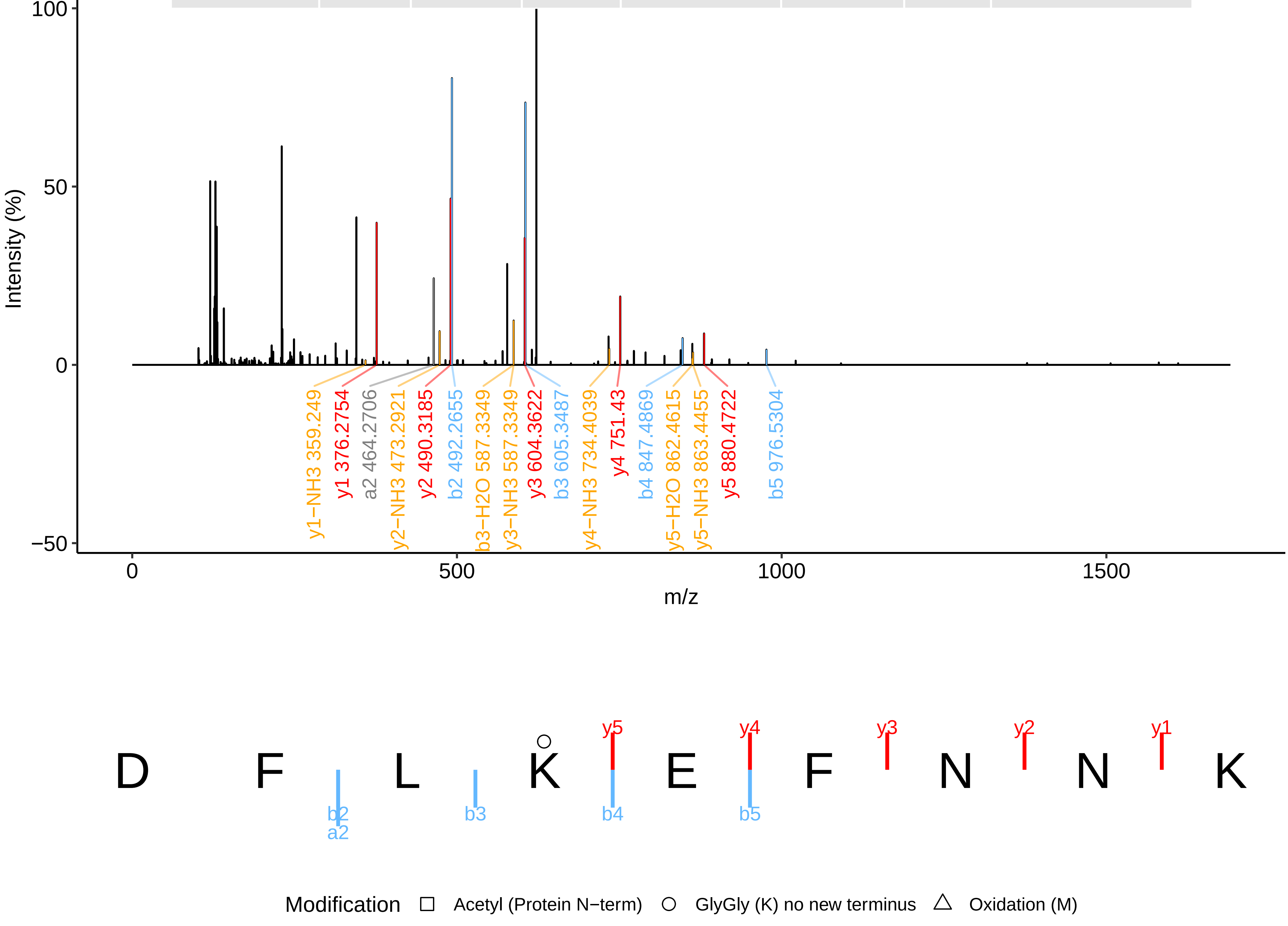

20200921_KIZ_MUb4_GlyGly_10plex − Scan number: 20002

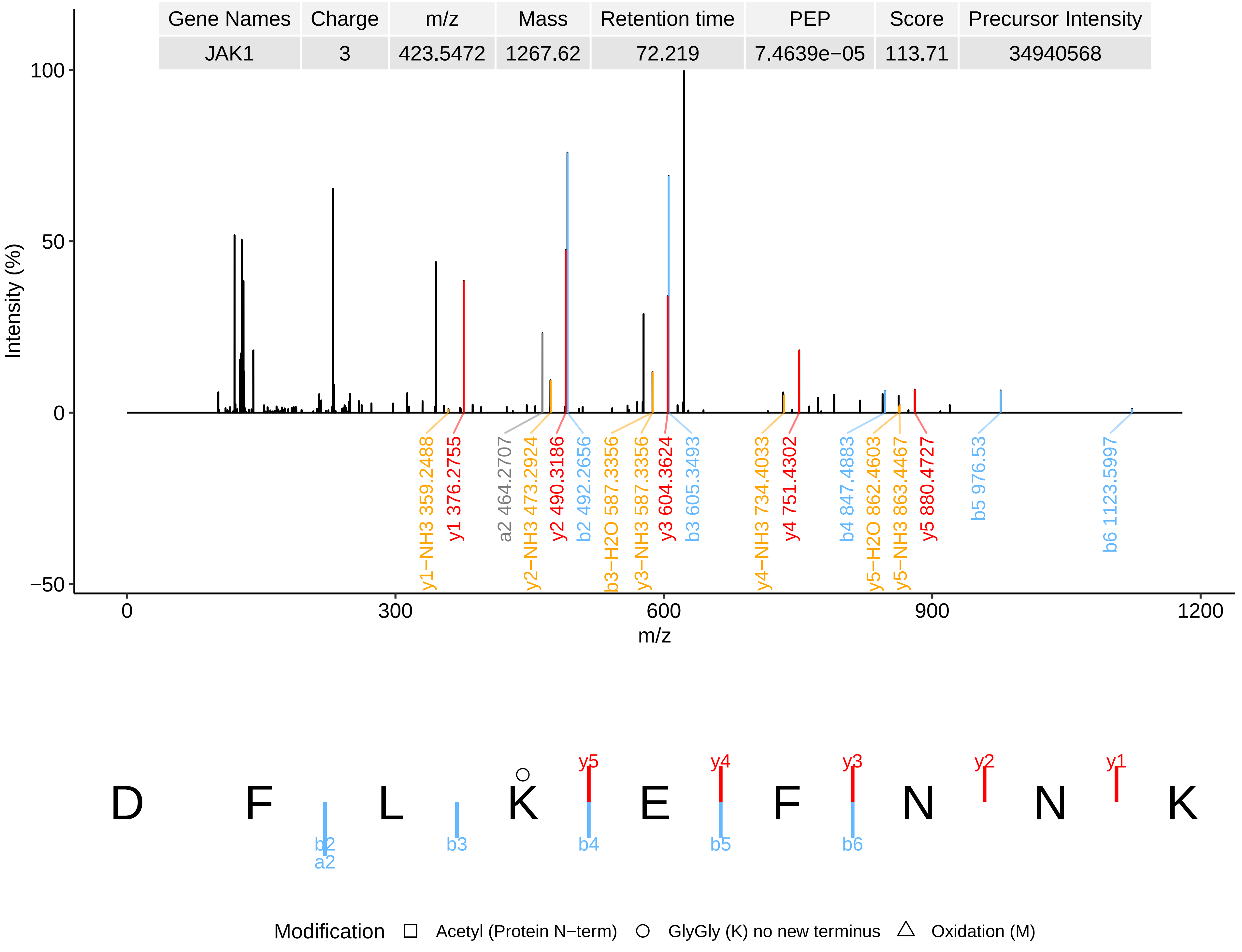

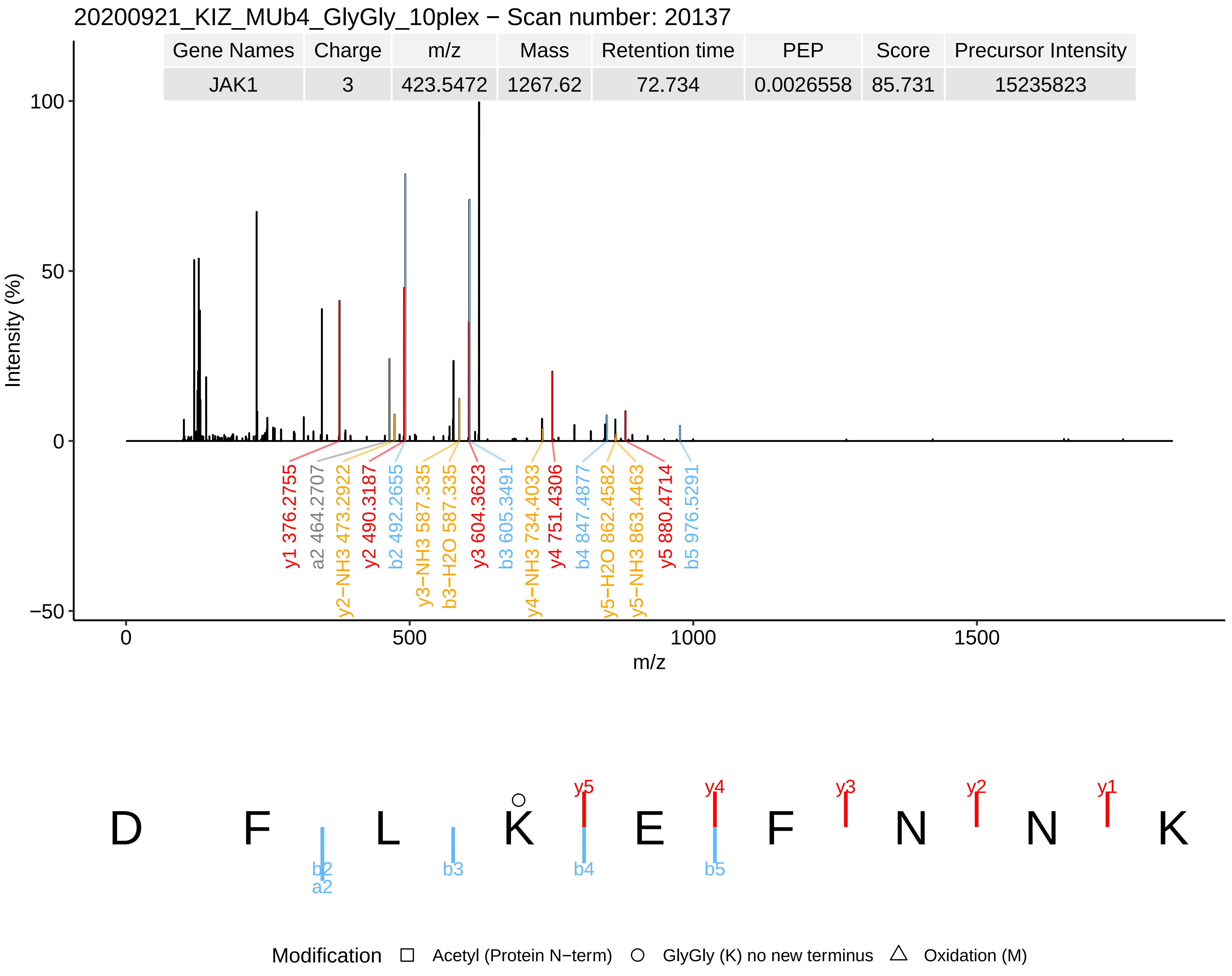

20200921_KIZ_MUb4_GlyGly_10plex − Scan number: 20137
Gene Names | Charge | m/z | Mass | Retention time | PEP | Score | Precursor Intensity
JAK1 | 3 | 423.5472 | 1267.62 | 72.734 | 0.0026558 | 85.731 | 15235823
Intensity (%)
100
50
0
−50
y1 376.2755
a2 464.2707
y2−NH3 473.2922
y2 490.3187
b2 492.2655
y3−NH3 587.335
b3−H2O 587.335
y3 604.3623
b3 605.3491
y4−NH3 734.4033
y4 751.4306
b4 847.4877
y5−H2O 862.4582
y5−NH3 863.4463
y5 880.4714
b5 976.5291
0
500
1000
1500
m/z
D F L K E F N N K
y5 y4 y3 y2 y1
b2 a2 b3 b4 b5
Modification
Acetyl (Protein N−term)
GlyGly (K) no new terminus
Oxidation (M)

20200921_KIZ_MUb4_GlyGly_10plex − Scan number: 20268

Gene Names	Charge	m/z	Mass	Retention time	PEP	Score	Precursor Intensity
JAK1	3	423.5472	1267.62	73.237	0.00028168	107.15	26944132

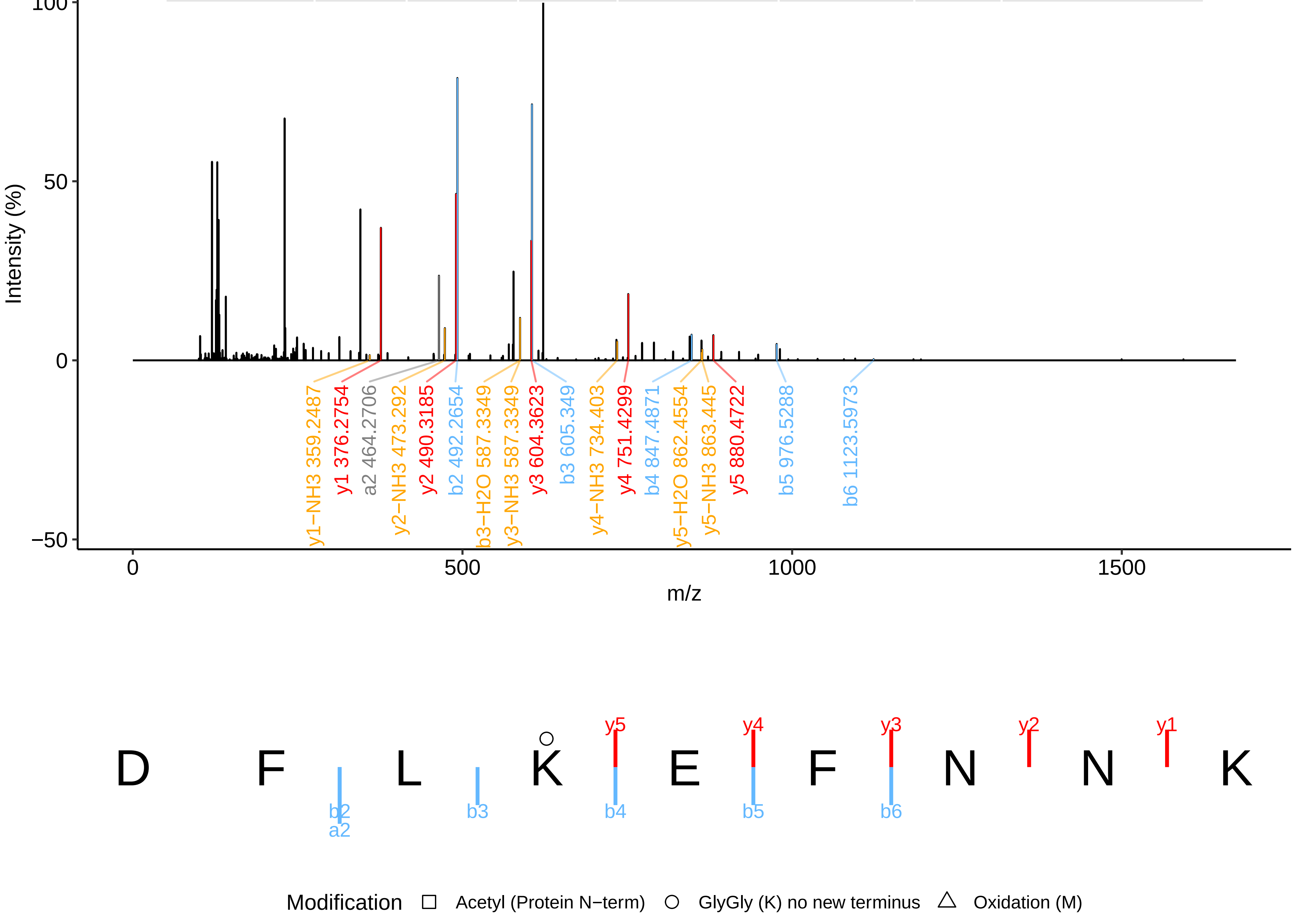

20200921_KIZ_MUb4_GlyGly_10plex − Scan number: 20548

Gene Names	Charge	m/z	Mass	Retention time	PEP	Score	Precursor Intensity
JAK1	3	423.5472	1267.62	74.288	0.01056	73.985	2182189

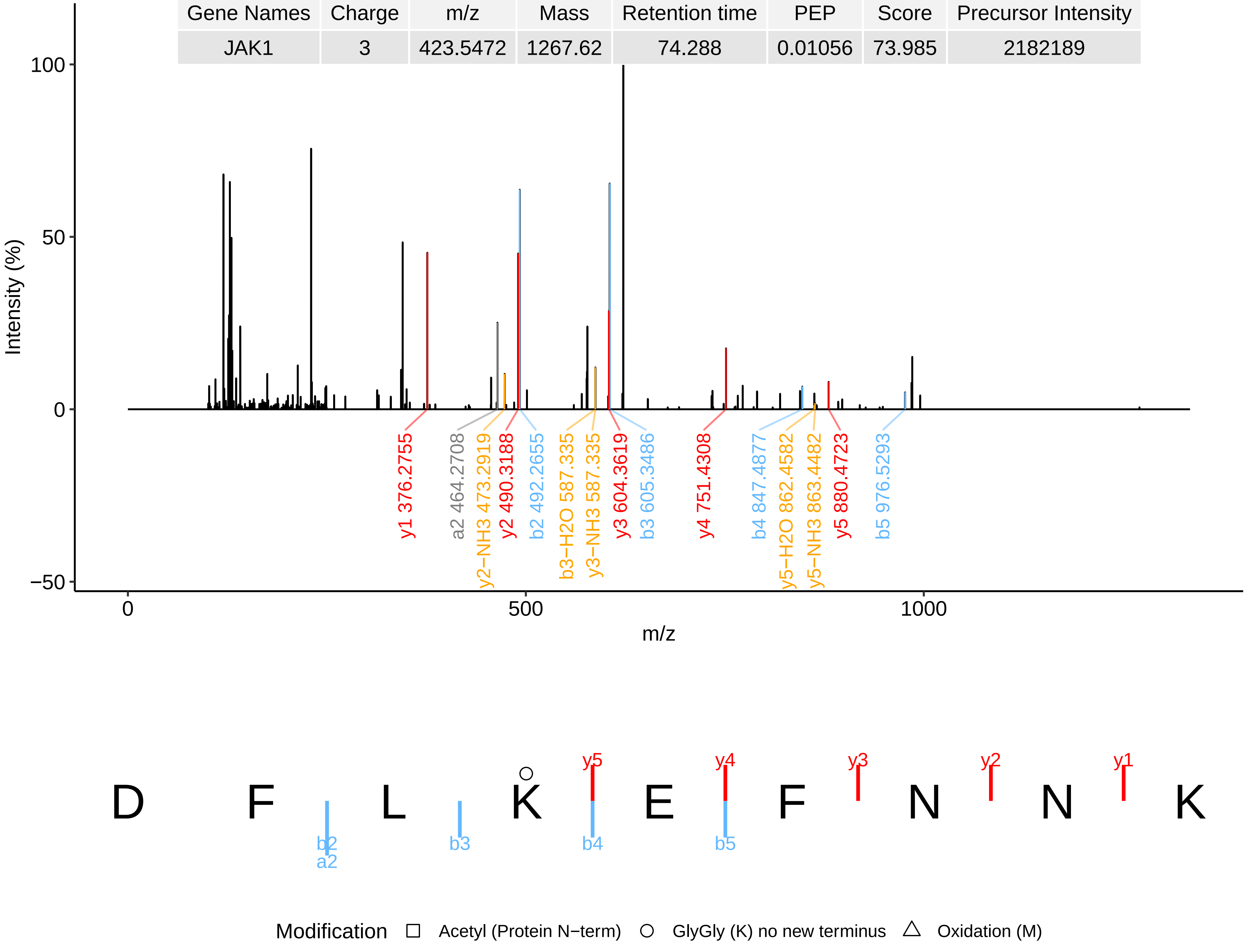

20200921_KIZ_MUb4_GlyGly_10plex − Scan number: 20956

Gene Names	Charge	m/z	Mass	Retention time	PEP	Score	Precursor Intensity
JAK1	3	423.5472	1267.62	75.818	0.059273	55.461	3489962

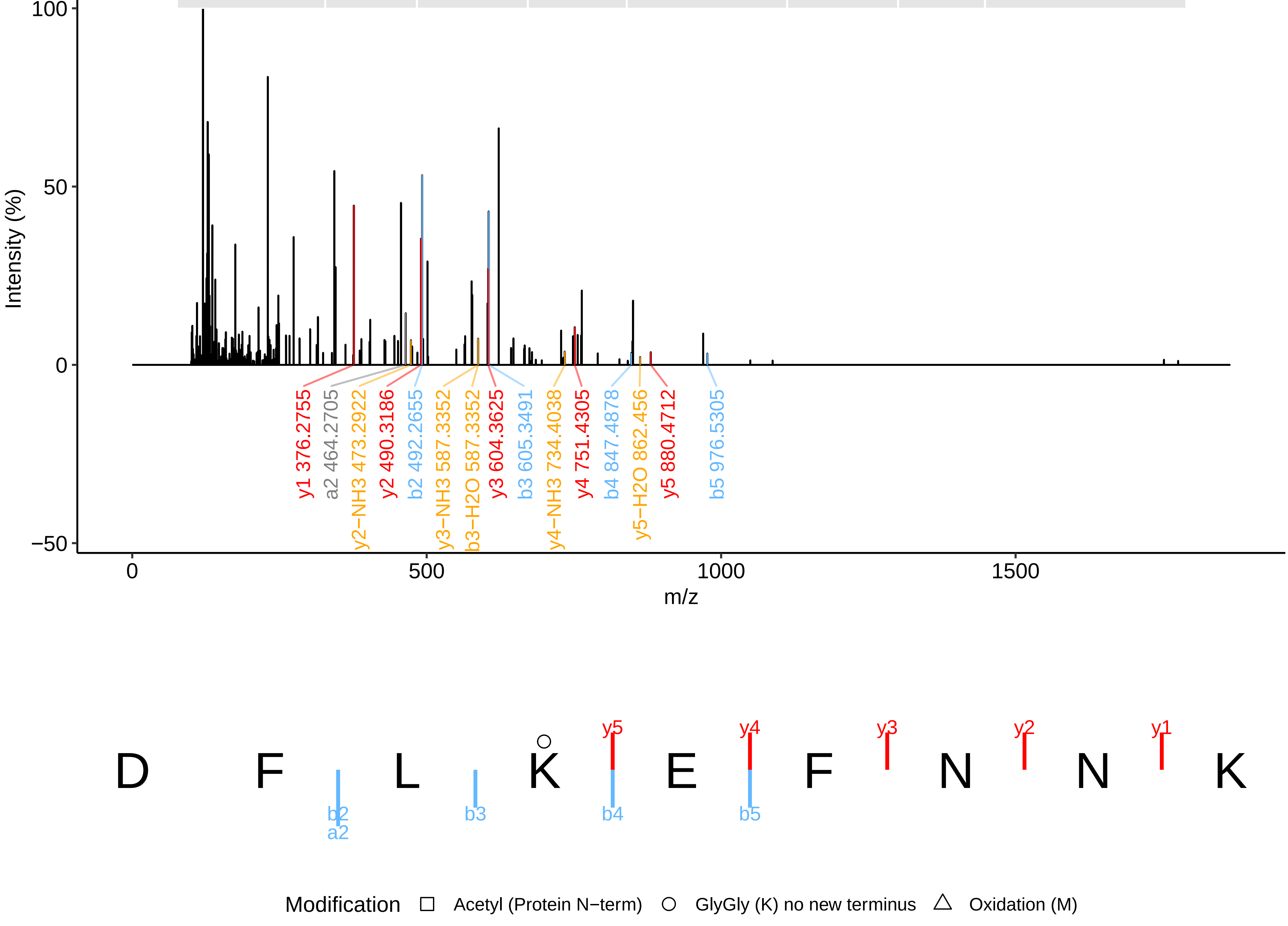

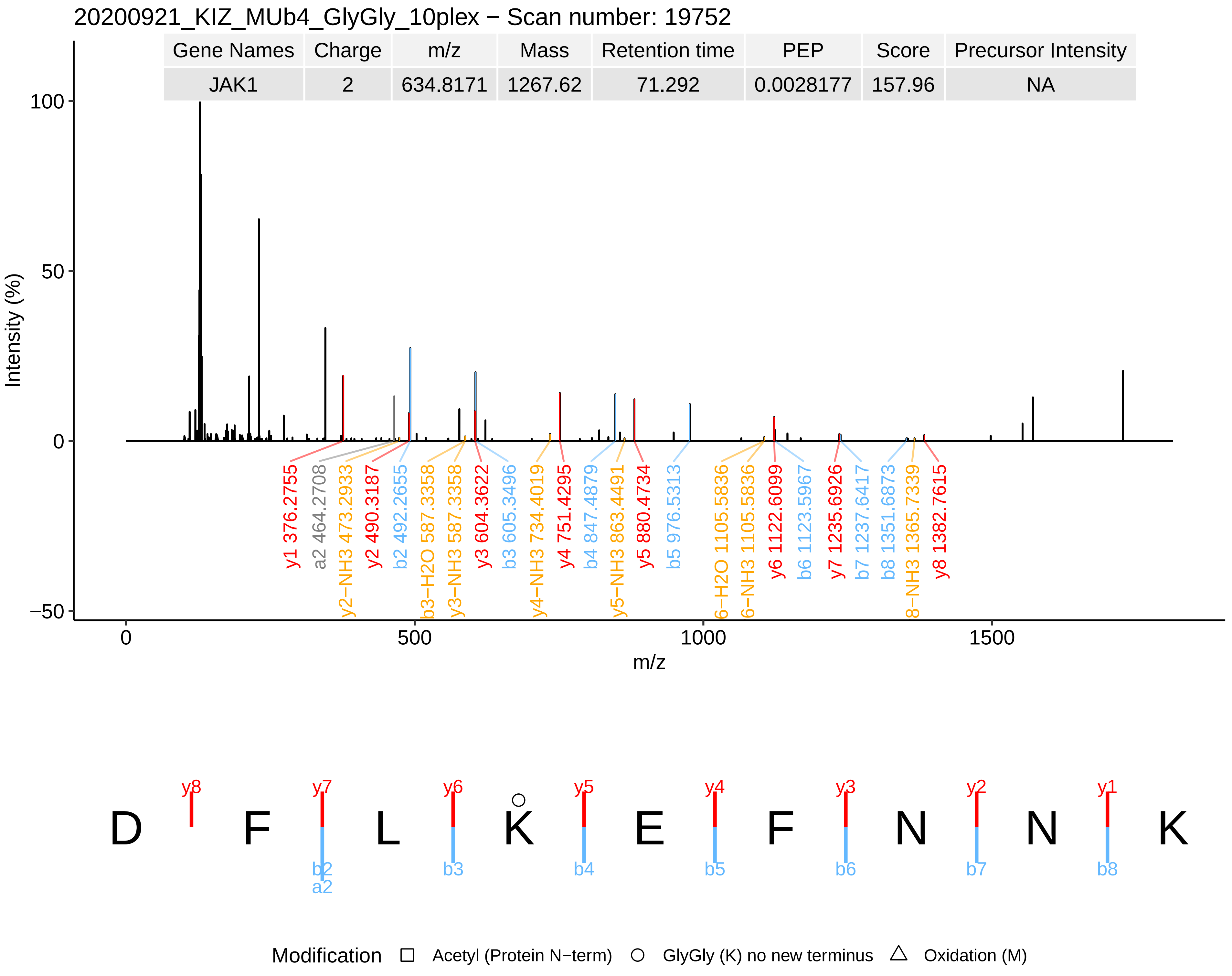
20200921_KIZ_MUb4_GlyGly_10plex − Scan number: 19752
Gene Names | Charge | m/z | Mass | Retention time | PEP | Score | Precursor Intensity
JAK1 | 2 | 634.8171 | 1267.62 | 71.292 | 0.0028177 | 157.96 | NA
Intensity (%)
100
50
0
−50
0
500
1000
1500
m/z
y1 376.2755
a2 464.2708
y2−NH3 473.2933
y2 490.3187
b2 492.2655
b3−H2O 587.3358
y3−NH3 587.3358
y3 604.3622
b3 605.3496
y4−NH3 734.4019
y4 751.4295
b4 847.4879
y5−NH3 863.4491
y5 880.4734
b5 976.5313
6−H2O 1105.5836
6−NH3 1105.5836
y6 1122.6099
b6 1123.5967
y7 1235.6926
b7 1237.6417
b8 1351.6873
8−NH3 1365.7339
y8 1382.7615
D F L K E F N N K
y8 y7 y6 y5 y4 y3 y2 y1
b2 a2 b3 b4 b5 b6 b7 b8
Modification
Acetyl (Protein N−term)
GlyGly (K) no new terminus
Oxidation (M)

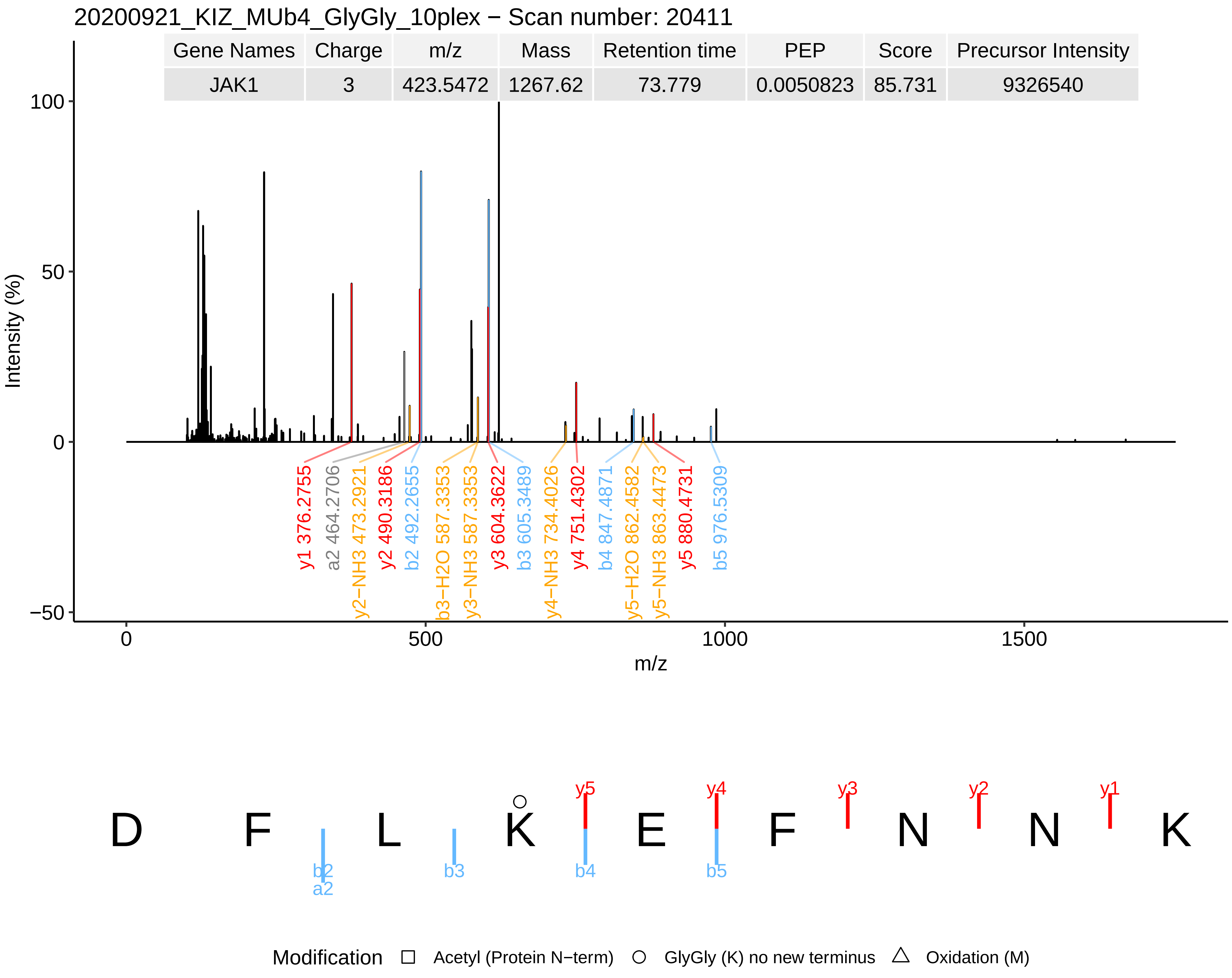
20200921_KIZ_MUb4_GlyGly_10plex − Scan number: 20411
Gene Names | Charge | m/z | Mass | Retention time | PEP | Score | Precursor Intensity
JAK1 | 3 | 423.5472 | 1267.62 | 73.779 | 0.0050823 | 85.731 | 9326540
Intensity (%)
m/z
y1 376.2755
a2 464.2706
y2−NH3 473.2921
y2 490.3186
b2 492.2655
b3−H2O 587.3353
y3−NH3 587.3353
y3 604.3622
b3 605.3489
y4−NH3 734.4026
y4 751.4302
b4 847.4871
y5−H2O 862.4582
y5−NH3 863.4473
y5 880.4731
b5 976.5309
D F L K E F N N K
y5 y4 y3 y2 y1
b2 a2 b3 b4 b5
Modification
Acetyl (Protein N−term)
GlyGly (K) no new terminus
Oxidation (M)

20200921_KIZ_MUb4_GlyGly_10plex − Scan number: 20683

Gene Names	Charge	m/z	Mass	Retention time	PEP	Score	Precursor Intensity
JAK1	3	423.5472	1267.62	74.8	0.0024625	94.85	NA

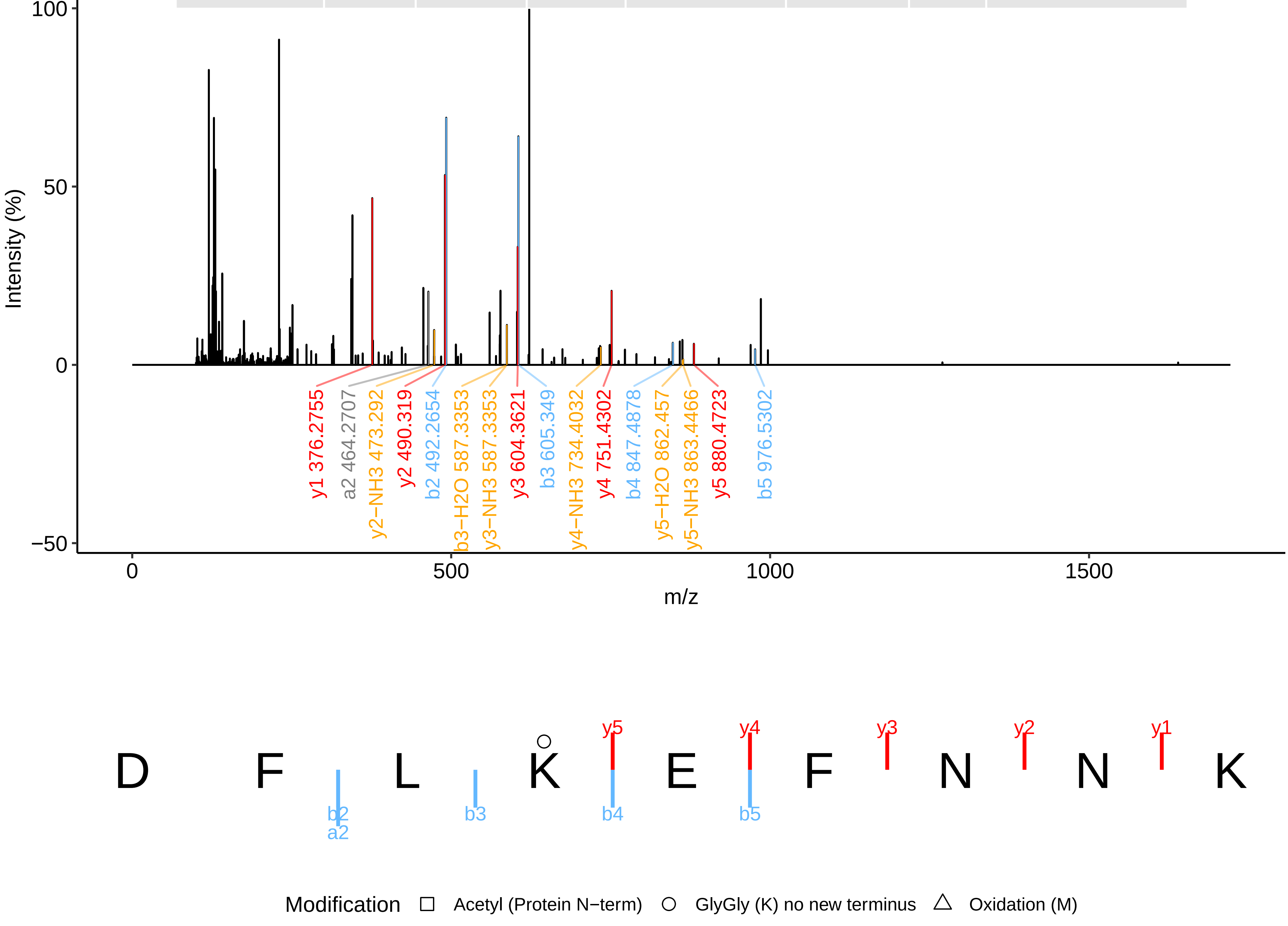

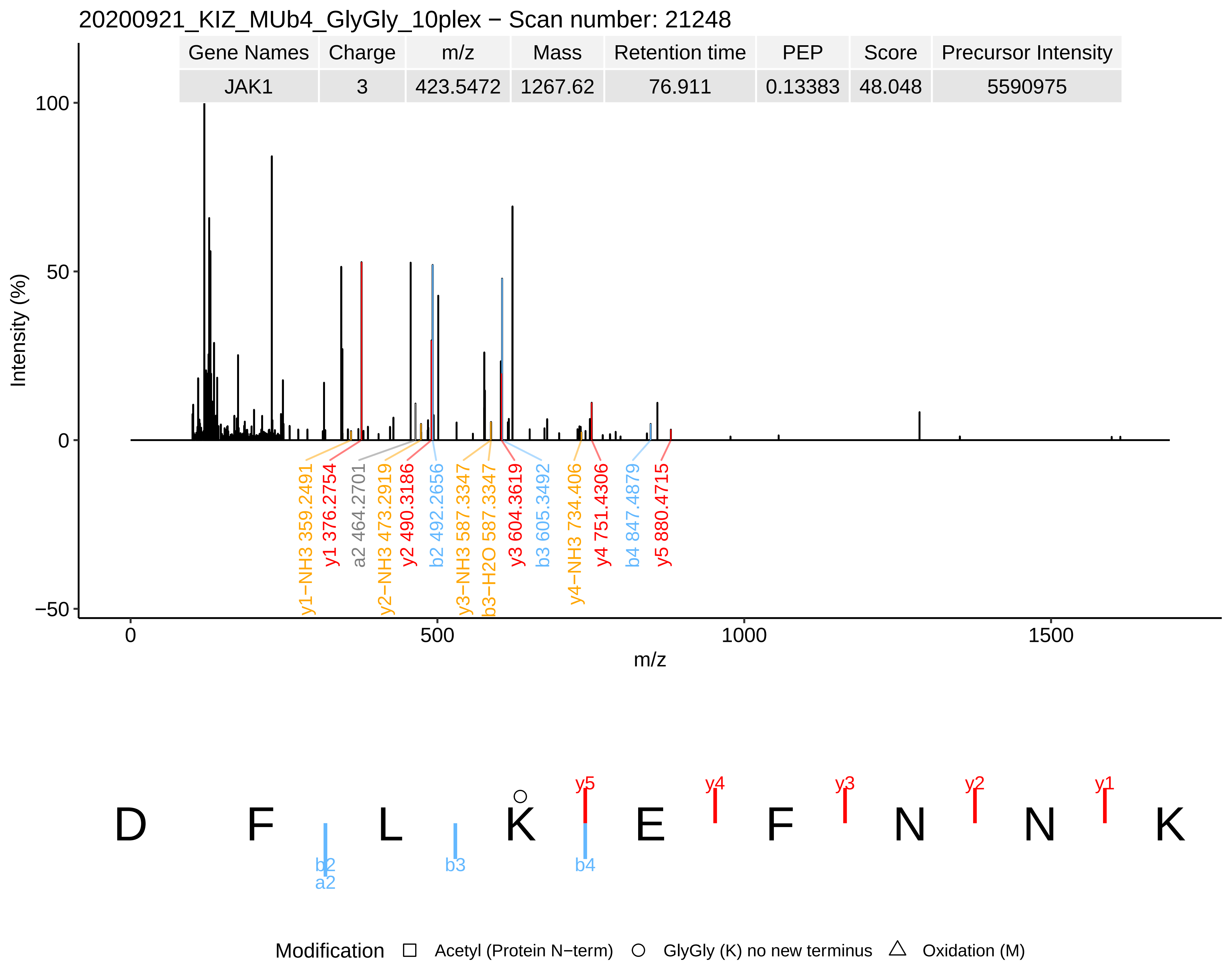

20200921_KIZ_MUb4_GlyGly_10plex − Scan number: 21248

Gene Names	Charge	m/z	Mass	Retention time	PEP	Score	Precursor Intensity
JAK1	3	423.5472	1267.62	76.911	0.13383	48.048	5590975

Intensity (%)
100
50
0
−50
0
500
1000
1500
m/z
y1−NH3 359.2491
y1 376.2754
a2 464.2701
y2−NH3 473.2919
y2 490.3186
b2 492.2656
y3−NH3 587.3347
b3−H2O 587.3347
y3 604.3619
b3 605.3492
y4−NH3 734.406
y4 751.4306
b4 847.4879
y5 880.4715
D F L K E F N N K
y5 y4 y3 y2 y1
b2 a2 b3 b4
Modification
Acetyl (Protein N−term)
GlyGly (K) no new terminus
Oxidation (M)

20200921_KIZ_MUb4_GlyGly_10plex_2 − Scan number: 18005

Gene Names	Charge	m/z	Mass	Retention time	PEP	Score	Precursor Intensity
JAK1	3	423.5472	1267.62	70.461	0.0029587	83.948	8327051

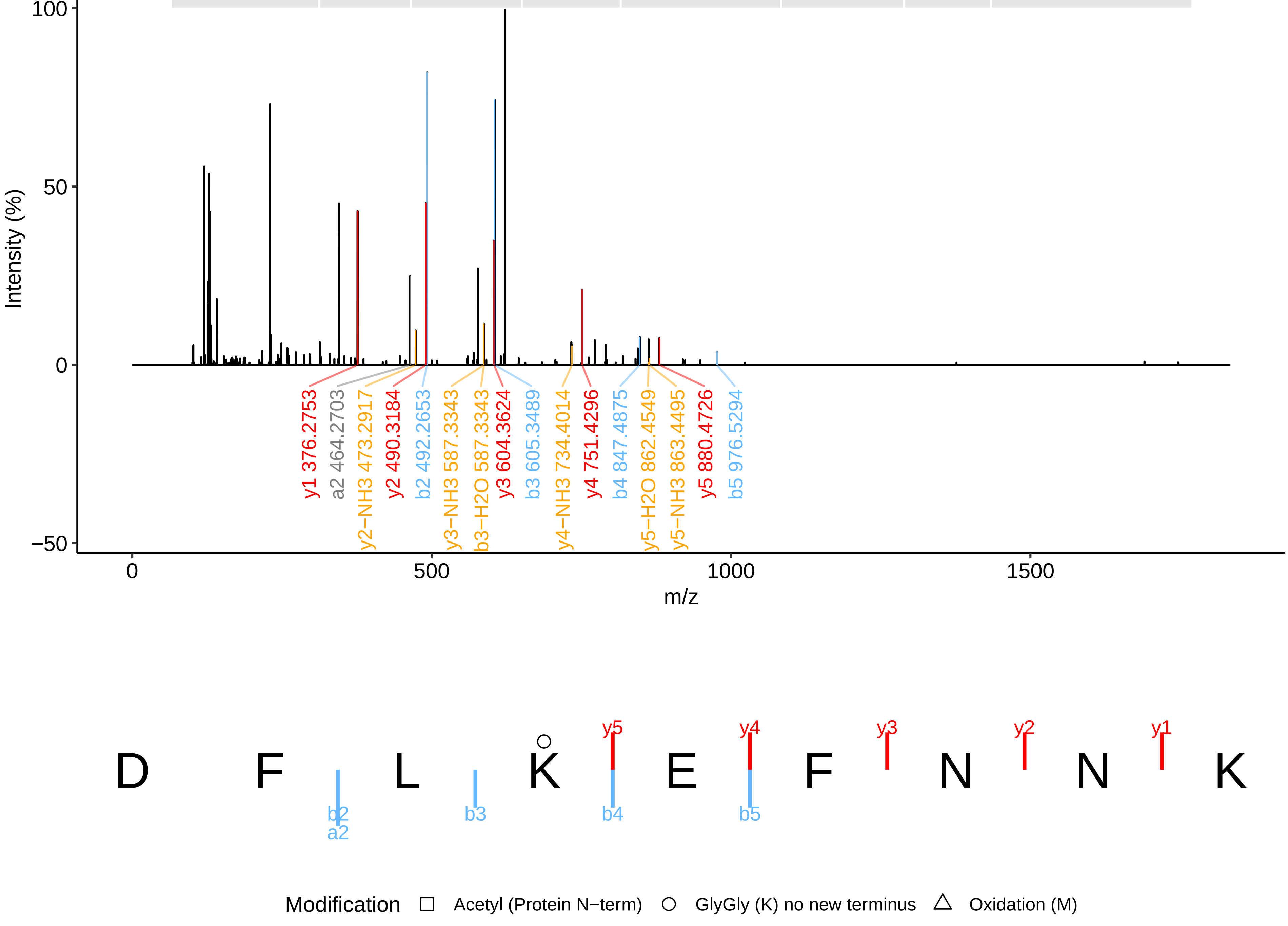

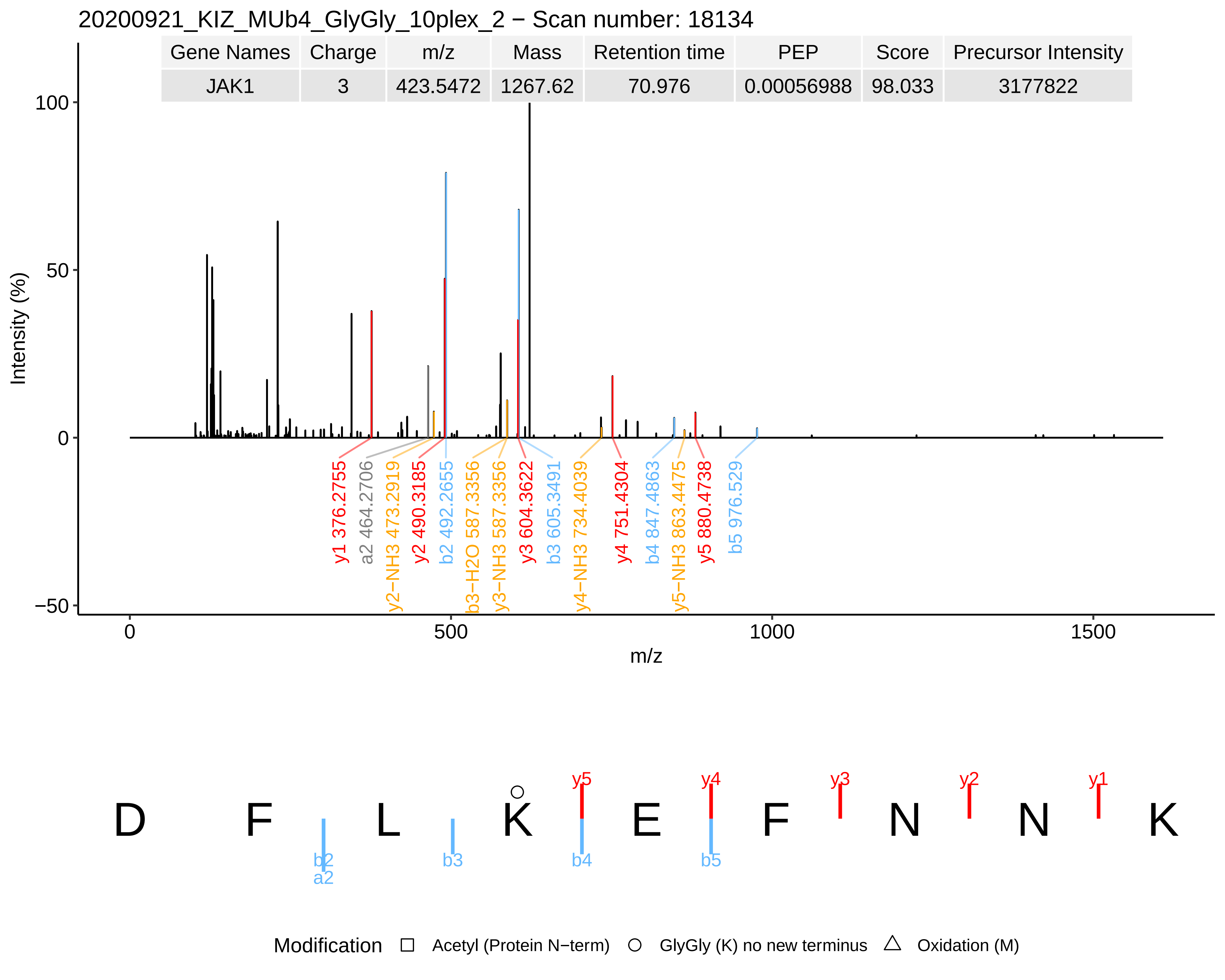
20200921_KIZ_MUb4_GlyGly_10plex_2 − Scan number: 18134
Gene Names | Charge | m/z | Mass | Retention time | PEP | Score | Precursor Intensity
JAK1 | 3 | 423.5472 | 1267.62 | 70.976 | 0.00056988 | 98.033 | 3177822
Intensity (%)
m/z
y1 376.2755
a2 464.2706
y2−NH3 473.2919
y2 490.3185
b2 492.2655
b3−H2O 587.3356
y3−NH3 587.3356
y3 604.3622
b3 605.3491
y4−NH3 734.4039
y4 751.4304
b4 847.4863
y5−NH3 863.4475
y5 880.4738
b5 976.529
D F L K E F N N K
Modification
Acetyl (Protein N−term)
GlyGly (K) no new terminus
Oxidation (M)

20200924_KIZ_MUb5_GlyGly_1 − Scan number: 18805

Gene Names	Charge	m/z	Mass	Retention time	PEP	Score	Precursor Intensity
JAK1	3	423.5472	1267.62	60.77	0.010143	74.376	11547555

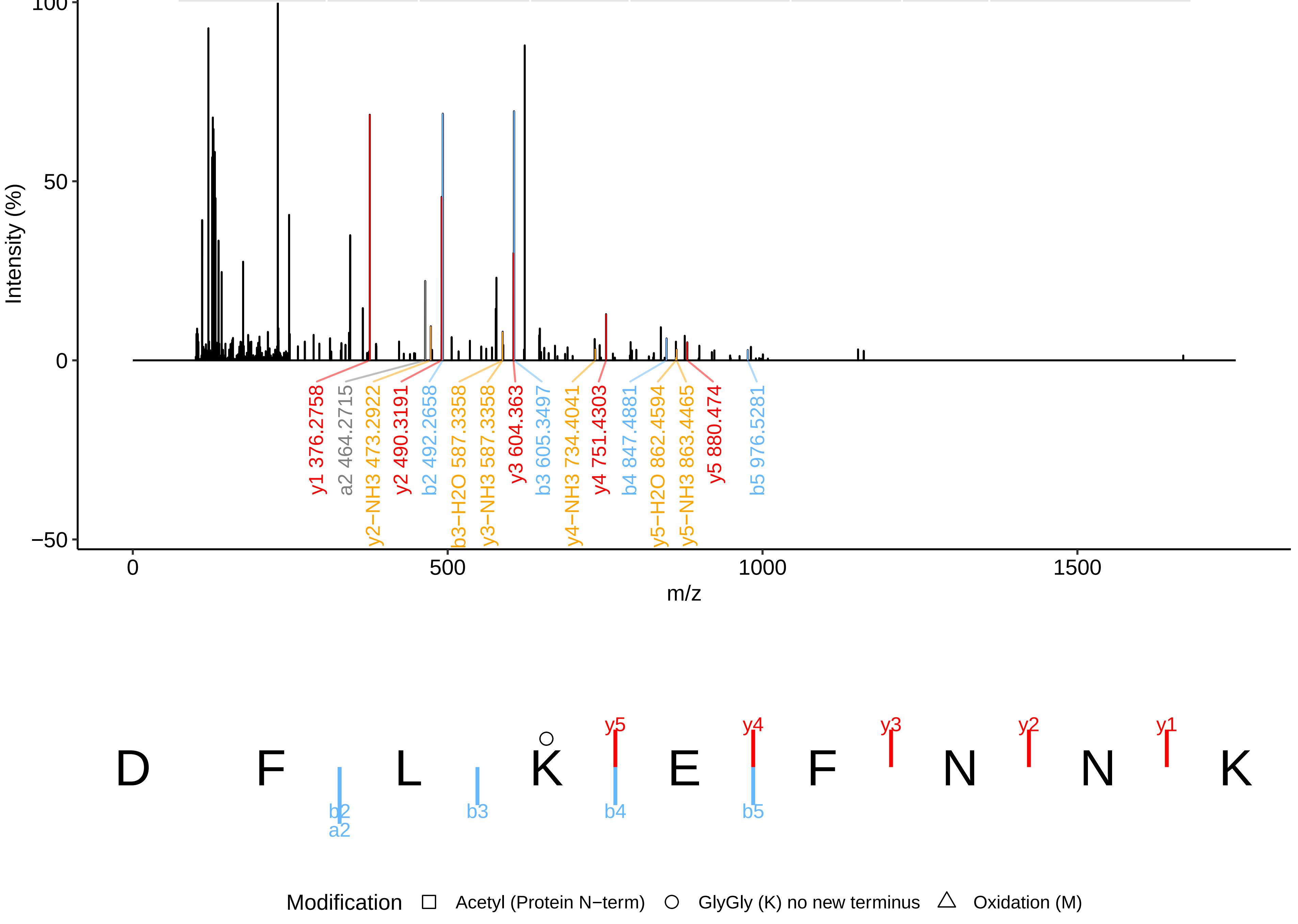

20200924_KIZ_MUb5_GlyGly_1 − Scan number: 18965

Gene Names	Charge	m/z	Mass	Retention time	PEP	Score	Precursor Intensity
JAK1	3	423.5472	1267.62	61.283	0.001613	91.867	113188000

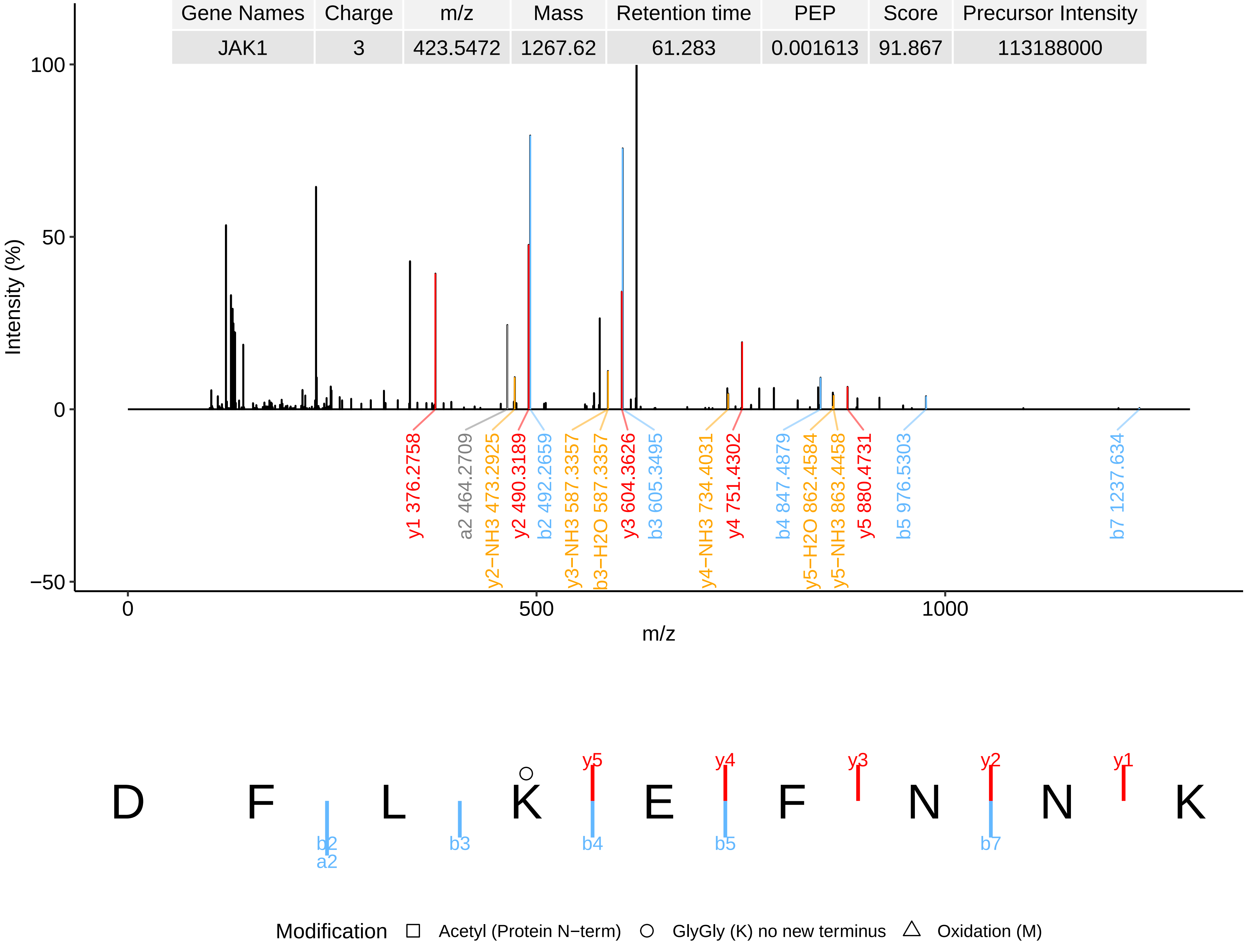

20200924_KIZ_MUb5_GlyGly_1 − Scan number: 19118

Gene Names	Charge	m/z	Mass	Retention time	PEP	Score	Precursor Intensity
JAK1	3	423.5472	1267.62	61.802	0.00030477	106.42	256976464

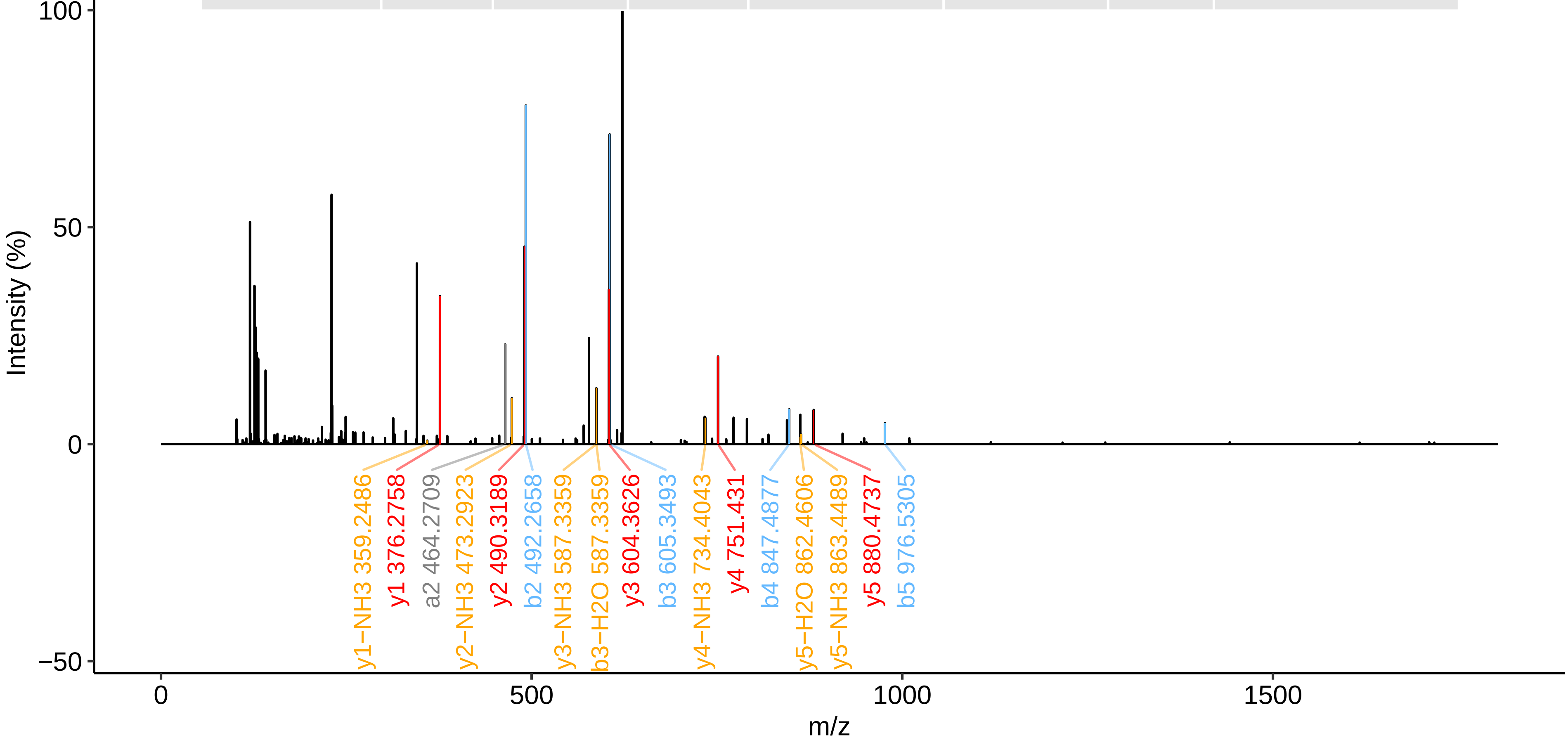

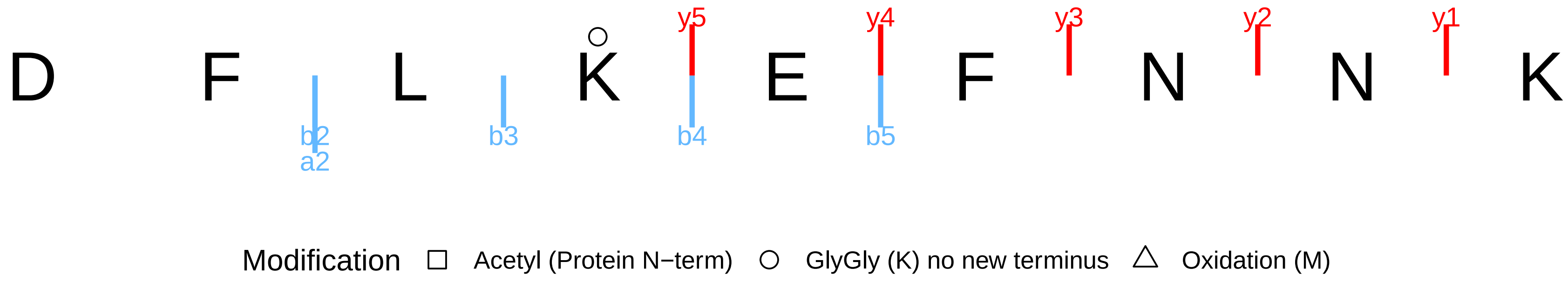

20200924_KIZ_MUb5_GlyGly_1 − Scan number: 19281

Gene Names	Charge	m/z	Mass	Retention time	PEP	Score	Precursor Intensity
JAK1	3	423.5472	1267.62	62.311	0.0011061	94.85	180663456

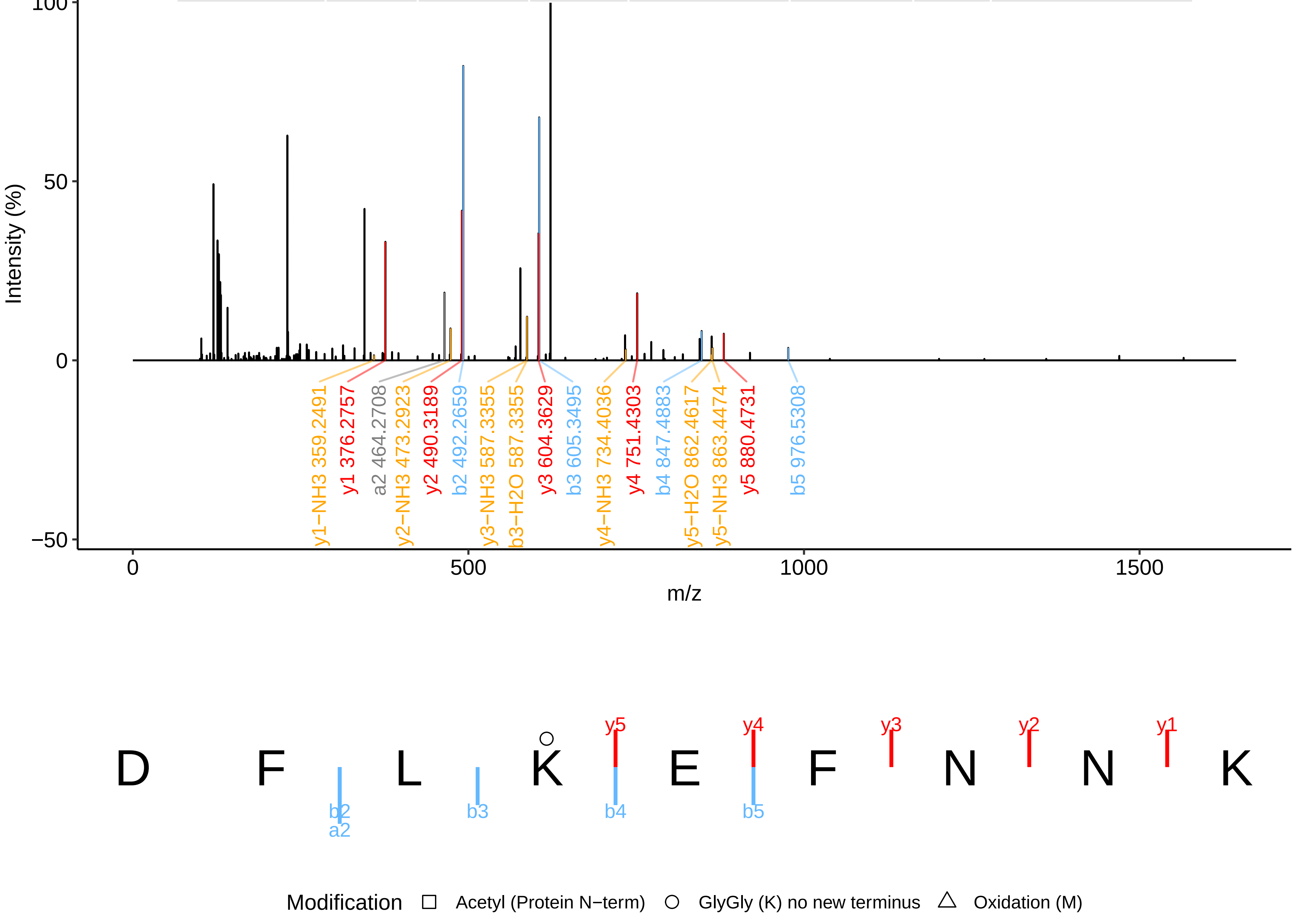

20200924_KIZ_MUb5_GlyGly_1 − Scan number: 19436

Gene Names	Charge	m/z	Mass	Retention time	PEP	Score	Precursor Intensity
JAK1	3	423.5472	1267.62	62.822	0.00028168	107.15	29492754

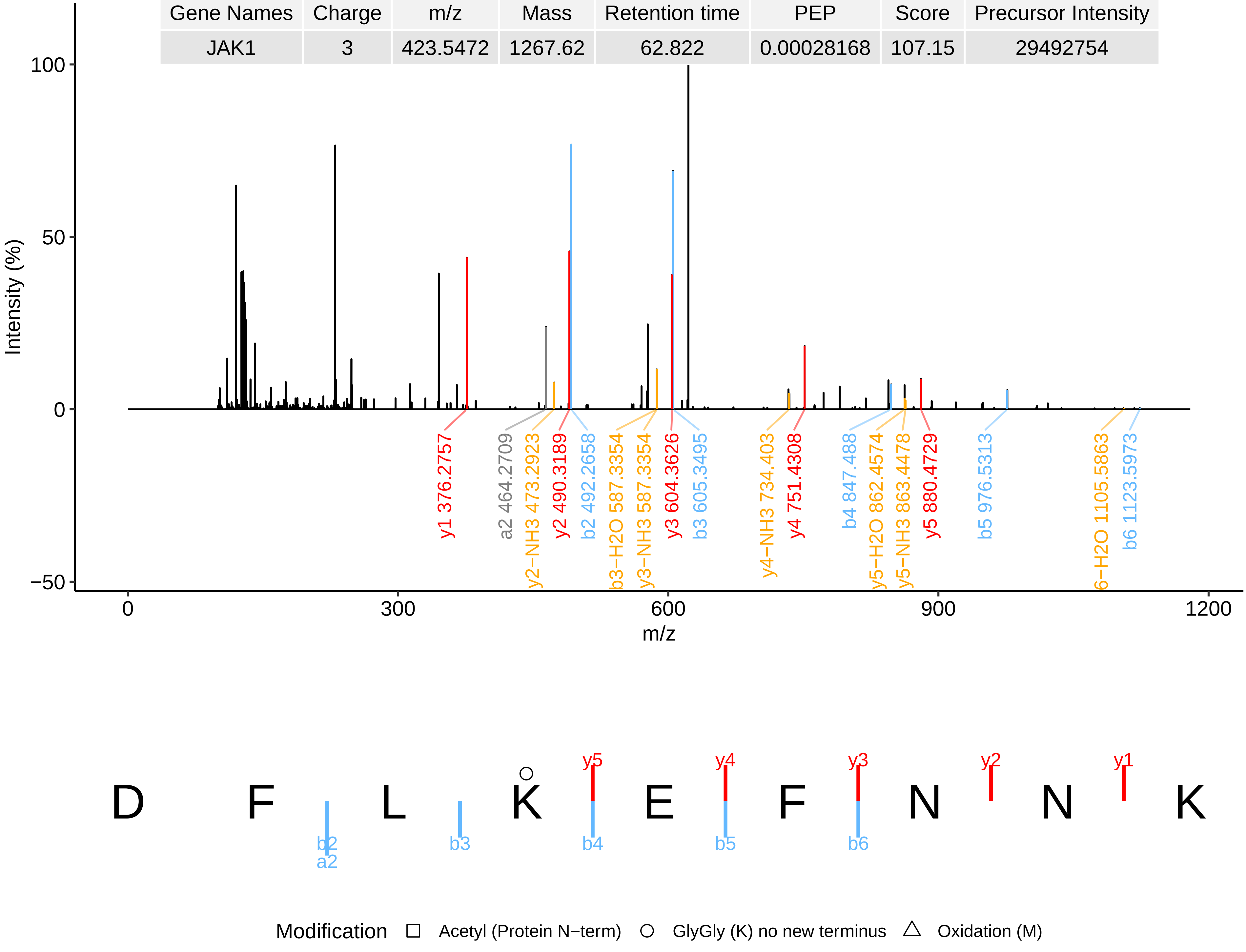

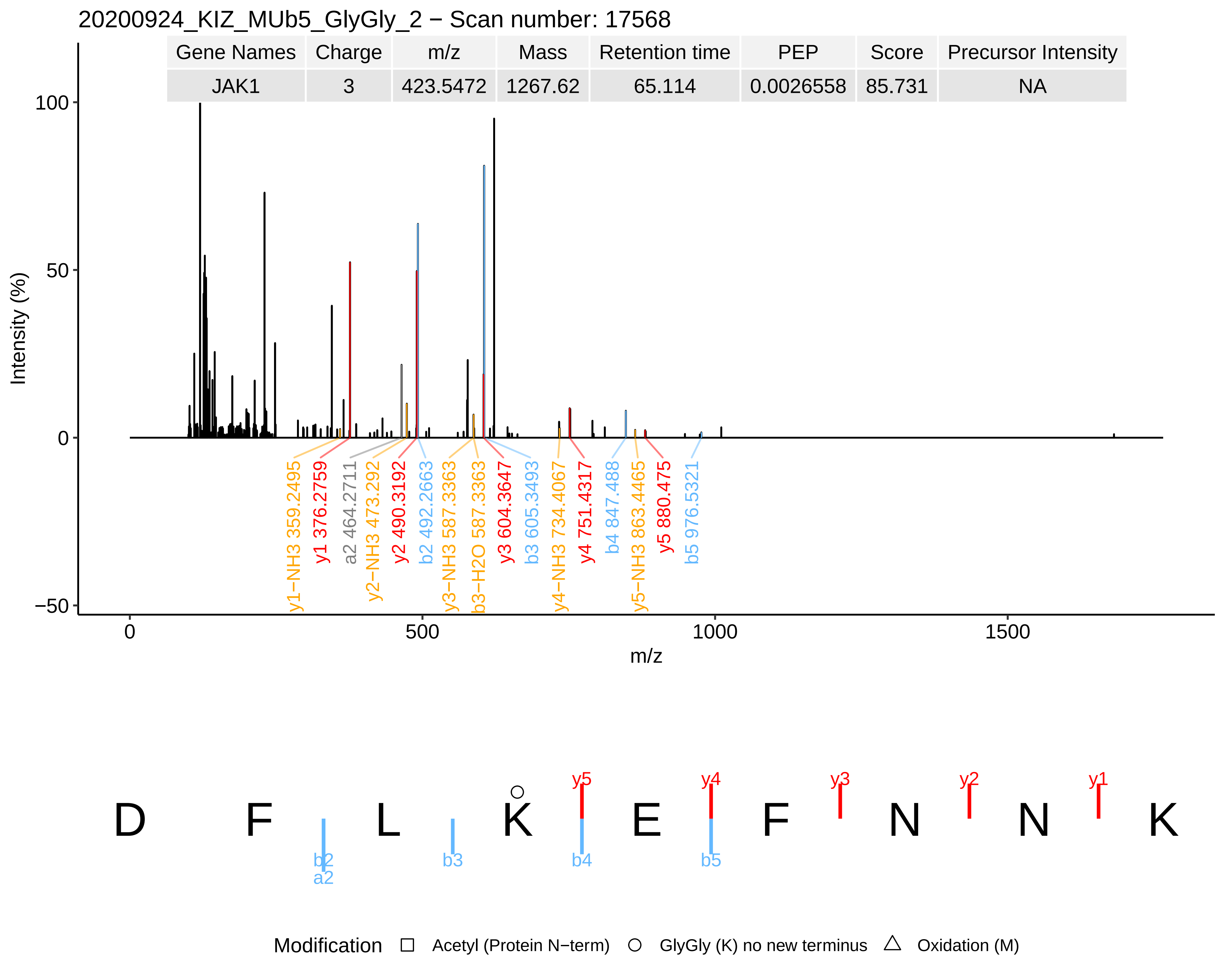
20200924_KIZ_MUb5_GlyGly_2 − Scan number: 17568
Gene Names | Charge | m/z | Mass | Retention time | PEP | Score | Precursor Intensity
JAK1 | 3 | 423.5472 | 1267.62 | 65.114 | 0.0026558 | 85.731 | NA
Intensity (%)
100
50
0
−50
0
500
1000
1500
m/z
y1−NH3 359.2495
y1 376.2759
a2 464.2711
y2−NH3 473.292
y2 490.3192
b2 492.2663
y3−NH3 587.3363
b3−H2O 587.3363
y3 604.3647
b3 605.3493
y4−NH3 734.4067
y4 751.4317
b4 847.488
y5−NH3 863.4465
y5 880.475
b5 976.5321
D F L K E F N N K
y5 y4 y3 y2 y1
b2 a2 b3 b4 b5
Modification
Acetyl (Protein N−term)
GlyGly (K) no new terminus
Oxidation (M)

20200924_KIZ_MUb5_GlyGly_2 − Scan number: 17735

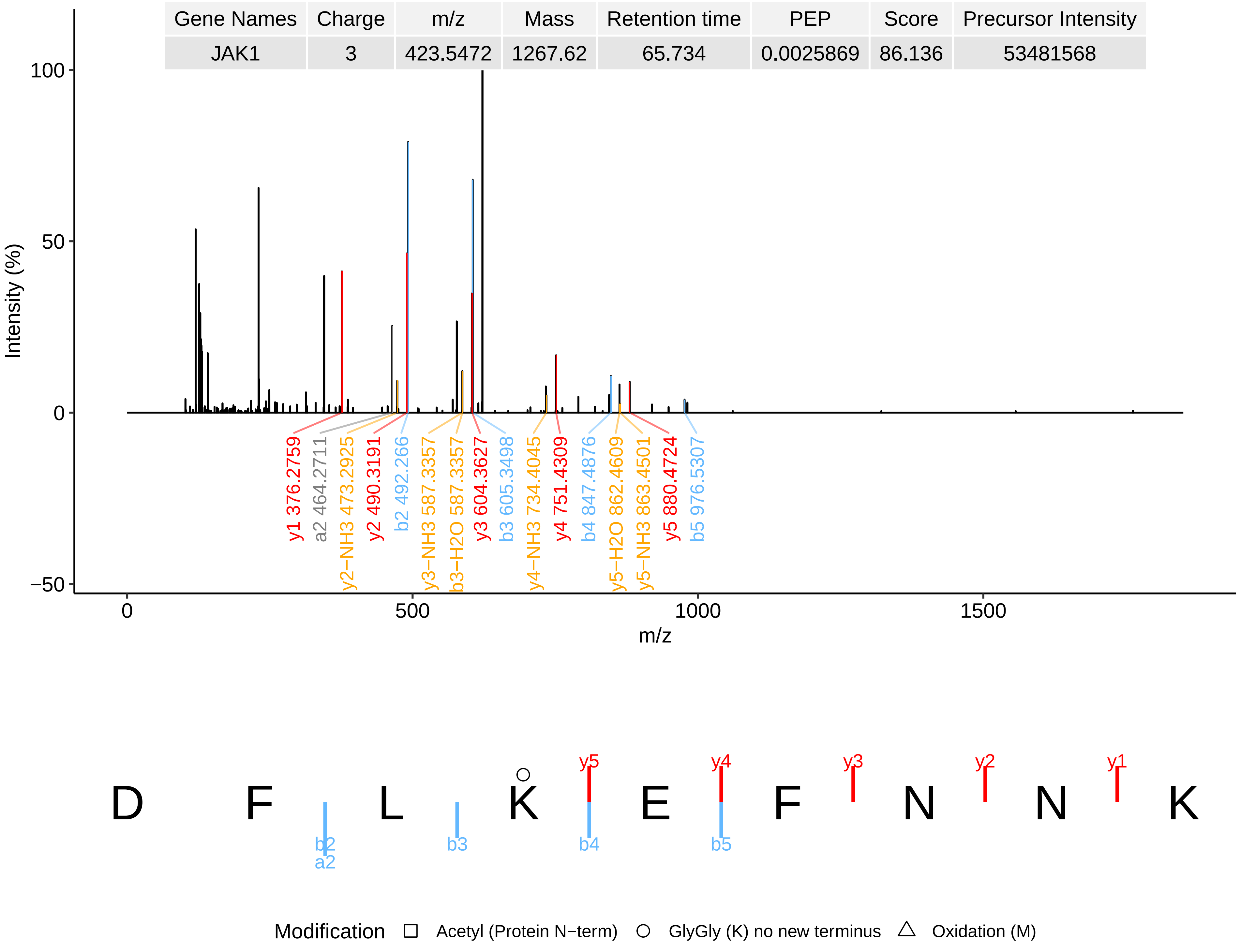

20200924_KIZ_MUb5_GlyGly_2 − Scan number: 17874

Gene Names	Charge	m/z	Mass	Retention time	PEP	Score	Precursor Intensity
JAK1	3	423.5472	1267.62	66.254	0.0011061	94.85	65097432

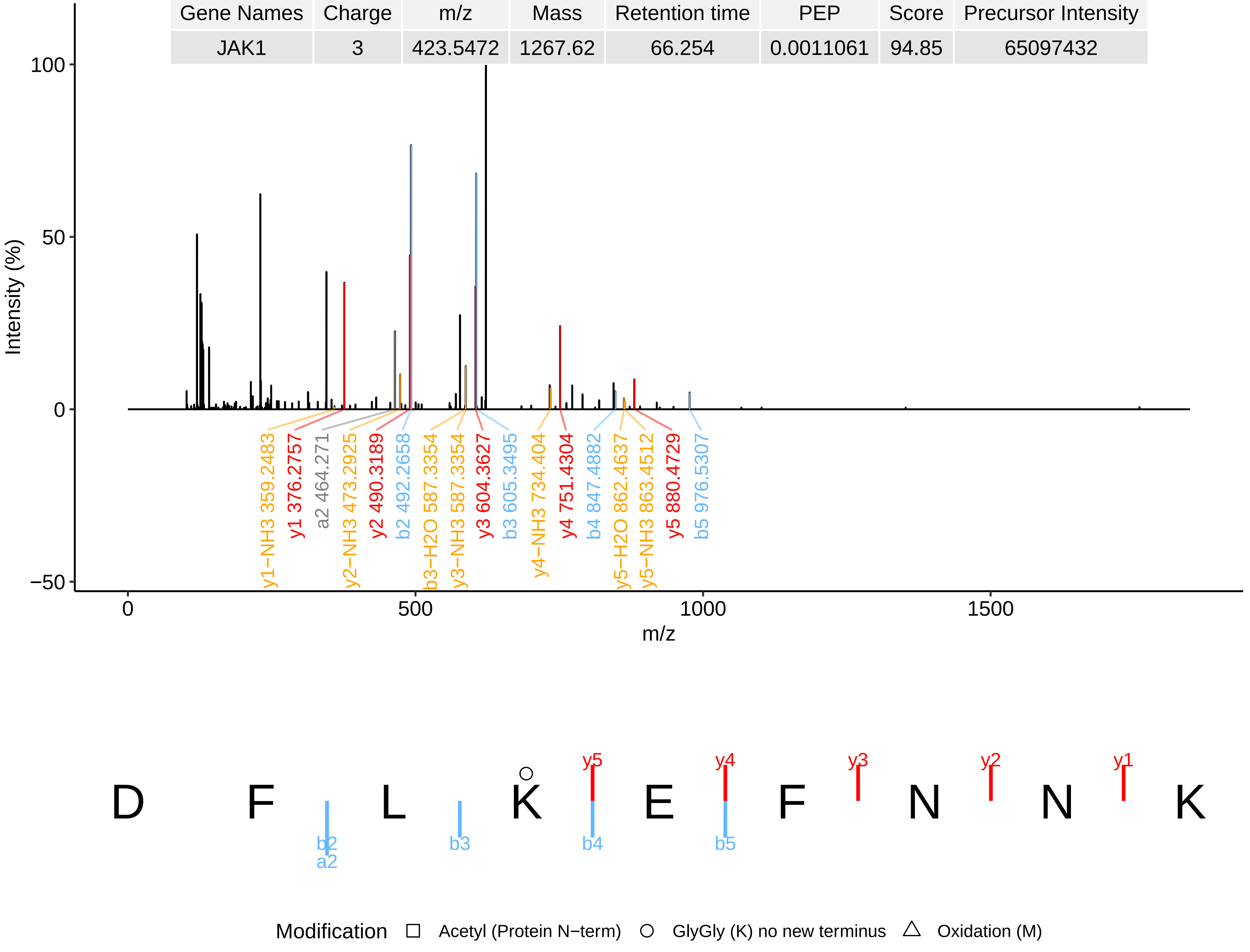

20200924_KIZ_MUb5_GlyGly_2 − Scan number: 18014

Gene Names	Charge	m/z	Mass	Retention time	PEP	Score	Precursor Intensity
JAK1	3	423.5472	1267.62	66.778	0.0026558	85.731	33315492

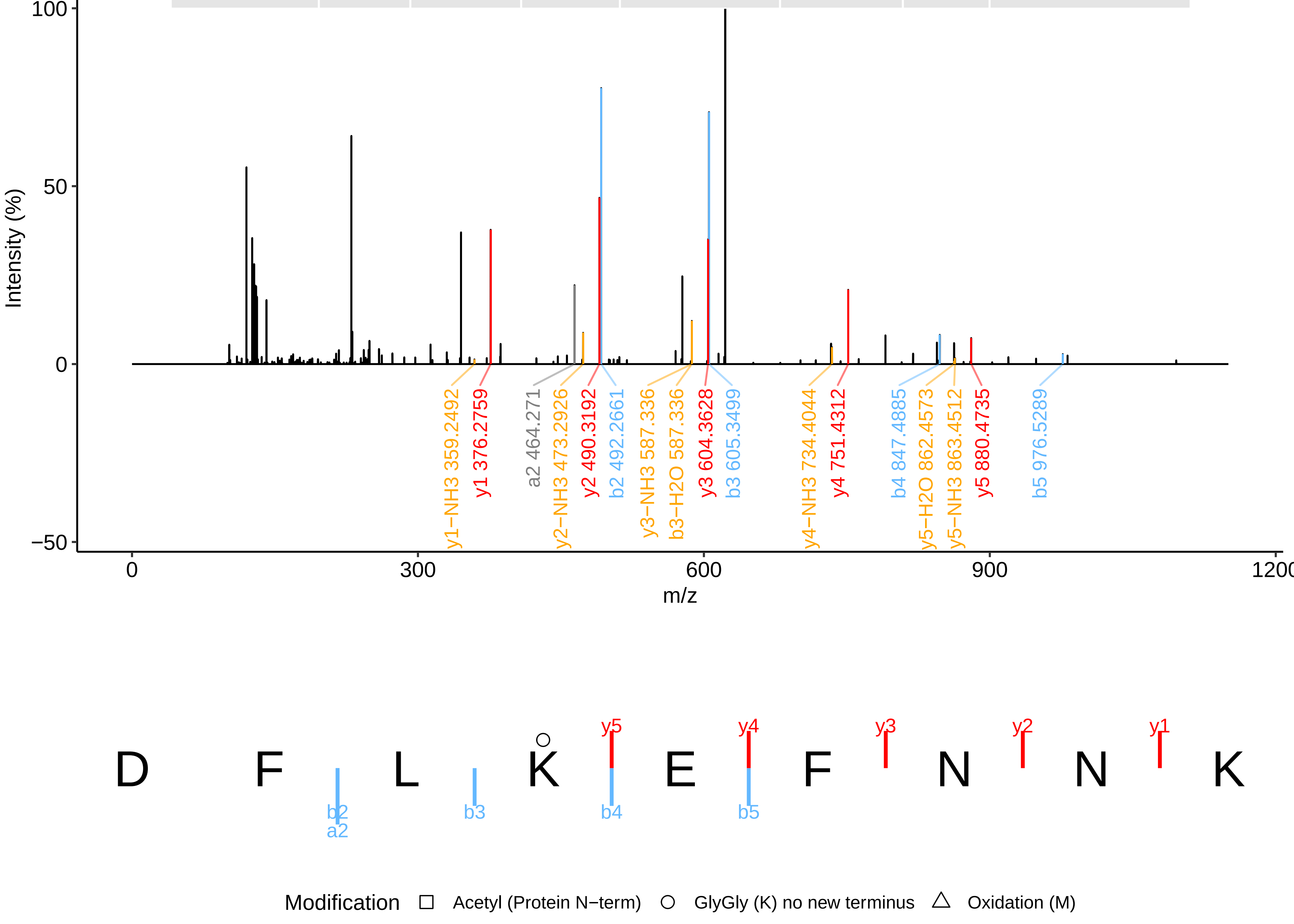

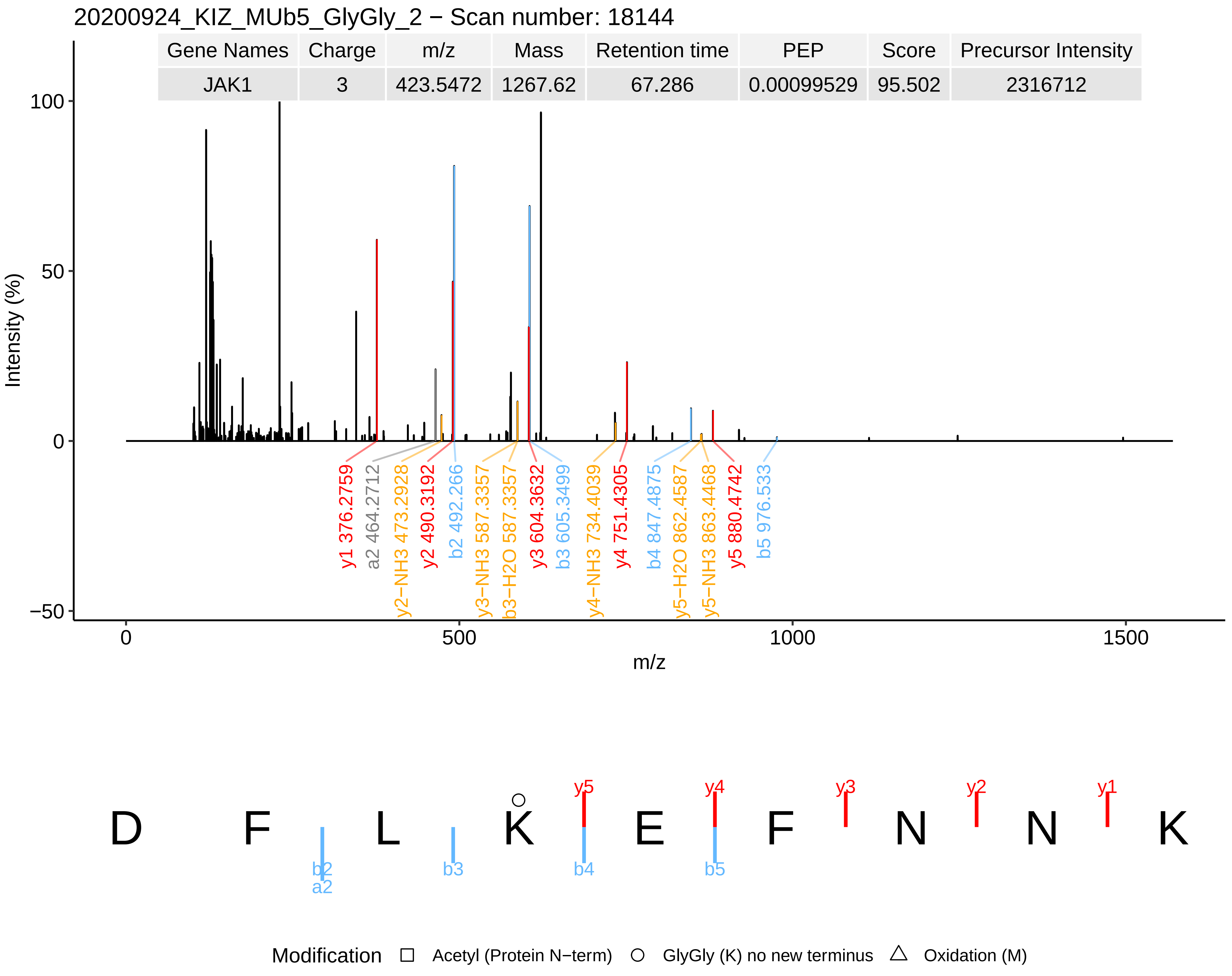
20200924_KIZ_MUb5_GlyGly_2 − Scan number: 18144
Gene Names | Charge | m/z | Mass | Retention time | PEP | Score | Precursor Intensity
JAK1 | 3 | 423.5472 | 1267.62 | 67.286 | 0.00099529 | 95.502 | 2316712
Intensity (%)
m/z
y1 376.2759
a2 464.2712
y2−NH3 473.2928
y2 490.3192
b2 492.266
y3−NH3 587.3357
b3−H2O 587.3357
y3 604.3632
b3 605.3499
y4−NH3 734.4039
y4 751.4305
b4 847.4875
y5−H2O 862.4587
y5−NH3 863.4468
y5 880.4742
b5 976.533
D F L K E F N N K
y5 y4 y3 y2 y1
b2 a2 b3 b4 b5
Modification
Acetyl (Protein N−term)
GlyGly (K) no new terminus
Oxidation (M)

20201118_KIZ_MUb6_GlyGly_1 − Scan number: 19103

Gene Names	Charge	m/z	Mass	Retention time	PEP	Score	Precursor Intensity
JAK1	3	423.5472	1267.62	59.958	0.0010197	95.358	24740544

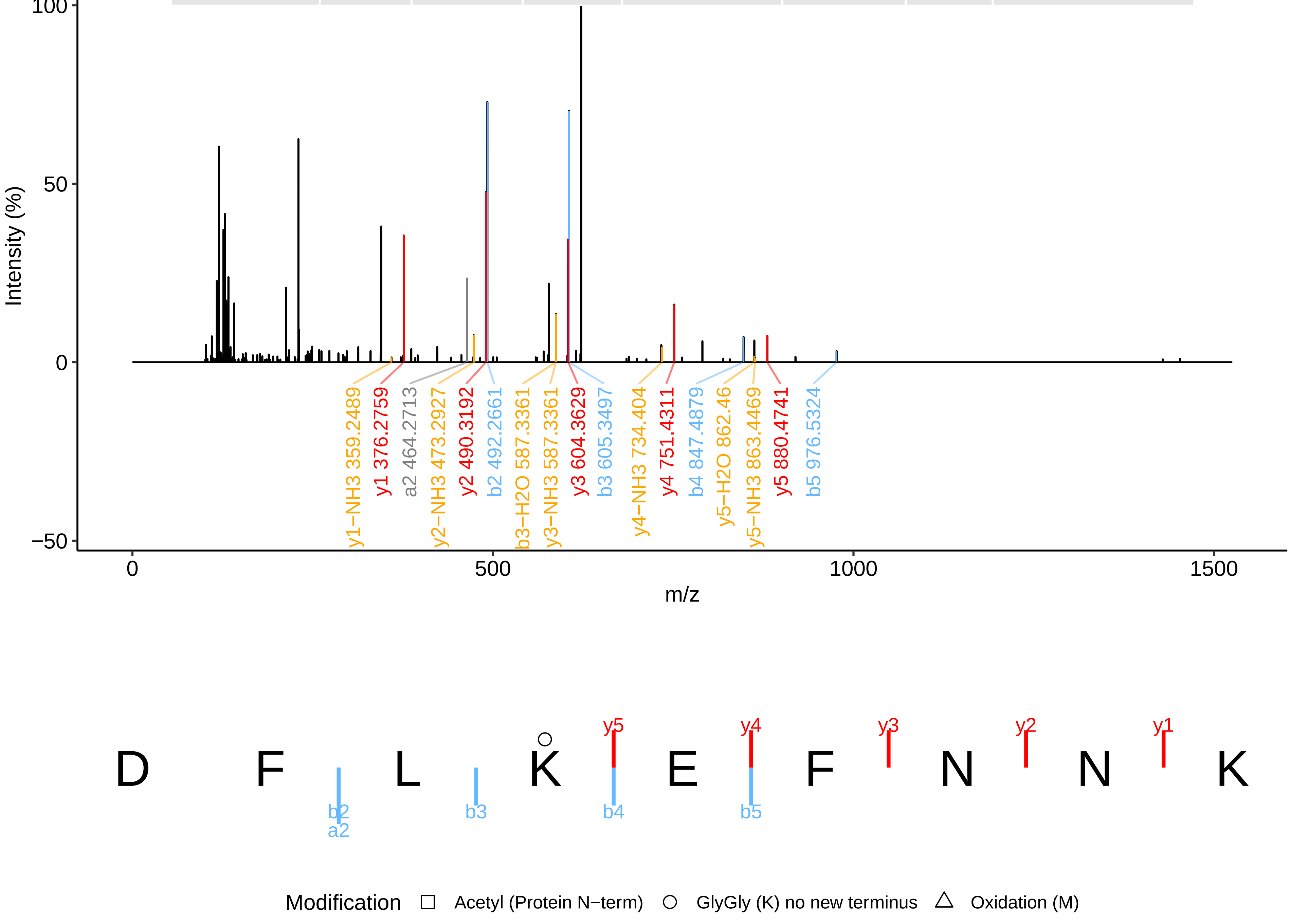

20201118_KIZ_MUb6_GlyGly_2 − Scan number: 21245

Gene Names	Charge	m/z	Mass	Retention time	PEP	Score	Precursor Intensity
JAK1	3	423.5472	1267.62	71.592	7.4639e−05	113.71	14283552

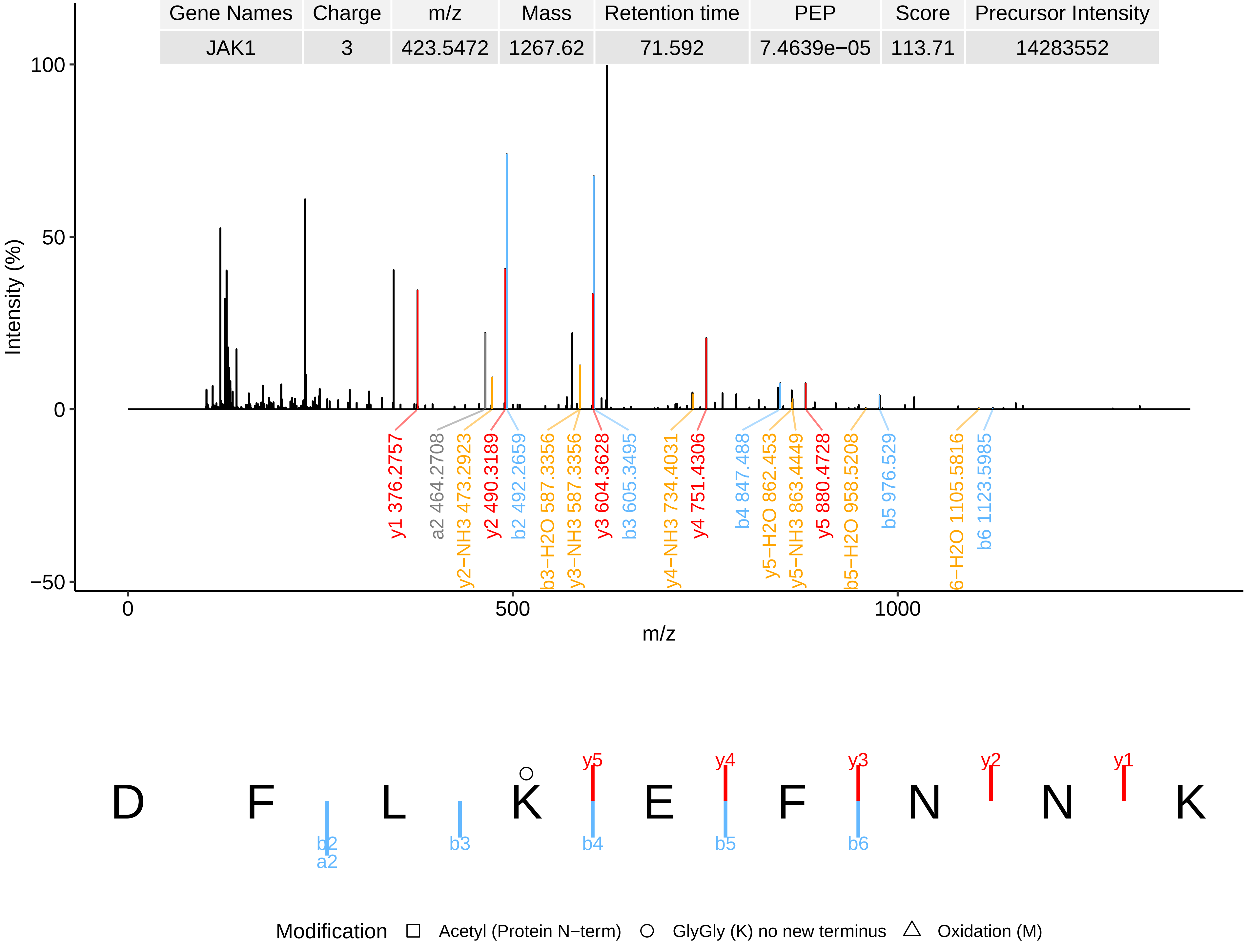

20201118_KIZ_MUb6_GlyGly_2 − Scan number: 21357

Gene Names	Charge	m/z	Mass	Retention time	PEP	Score	Precursor Intensity
JAK1	3	423.5472	1267.62	71.942	0.00016421	110.87	10828678

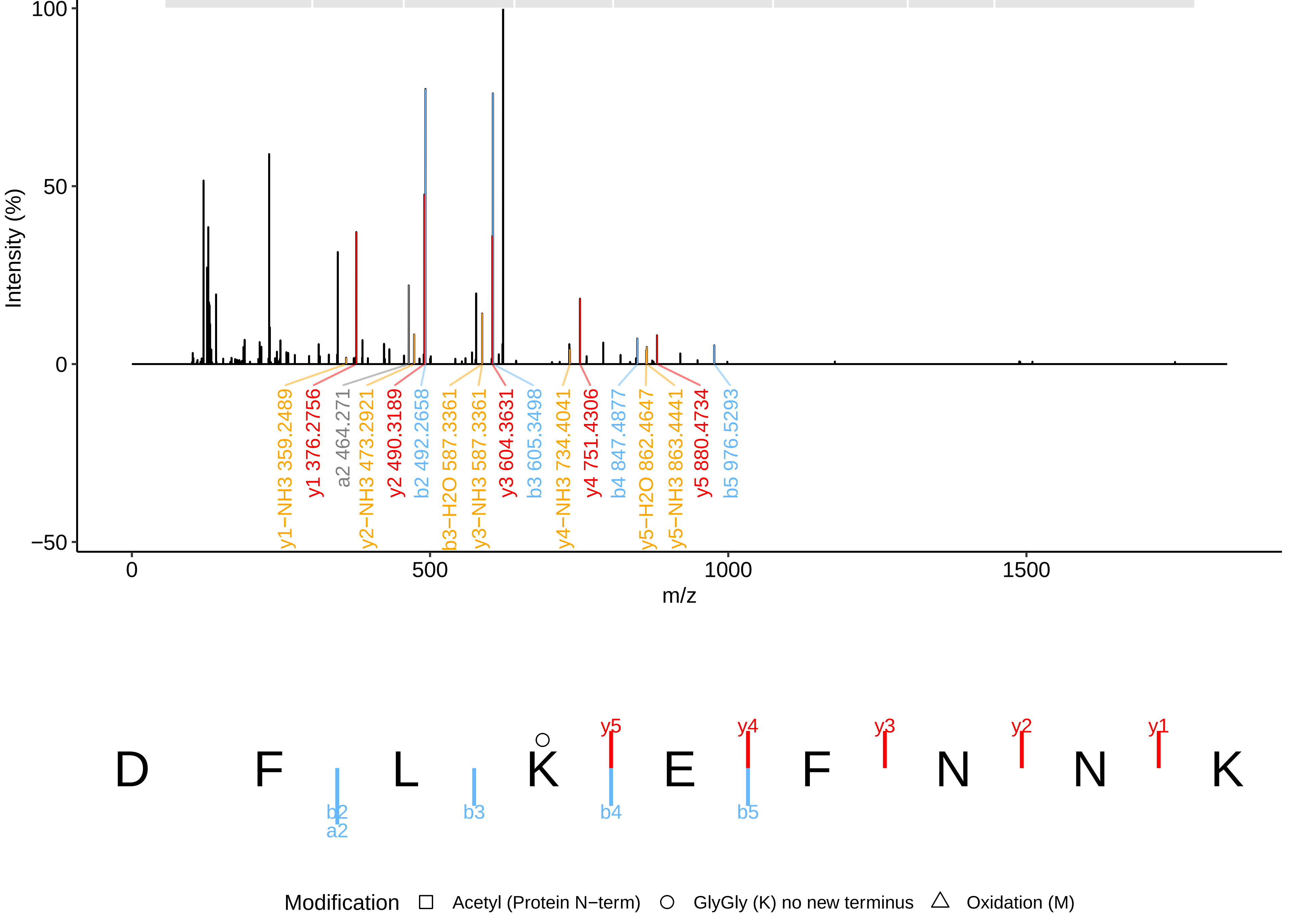

20200921_KIZ_MUb4_GlyGly_10plex − Scan number: 16428

Gene Names	Charge	m/z	Mass	Retention time	PEP	Score	Precursor Intensity
BAG1	3	515.9278	1544.762	58.992	3.7971e−09	128.21	12745653

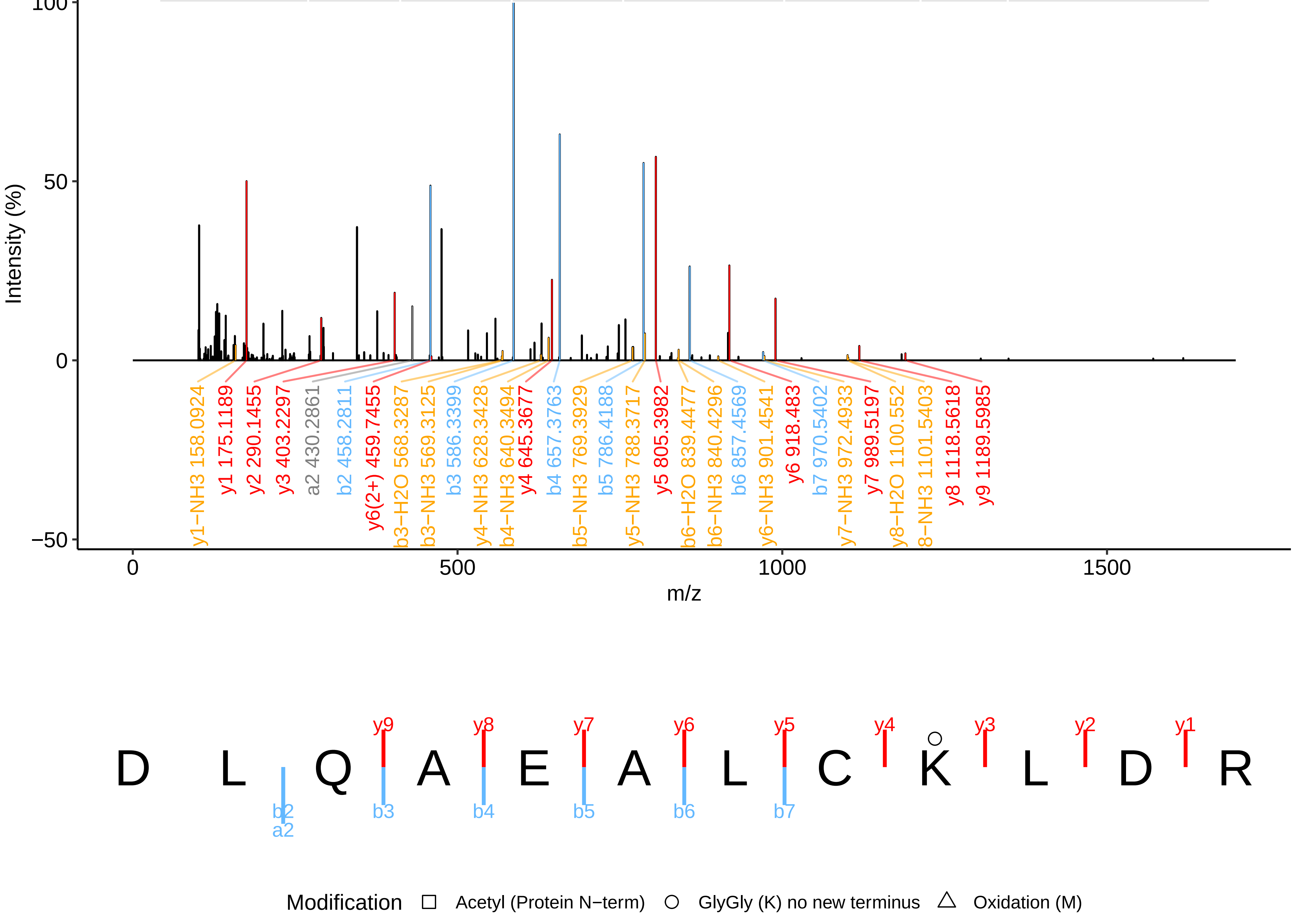

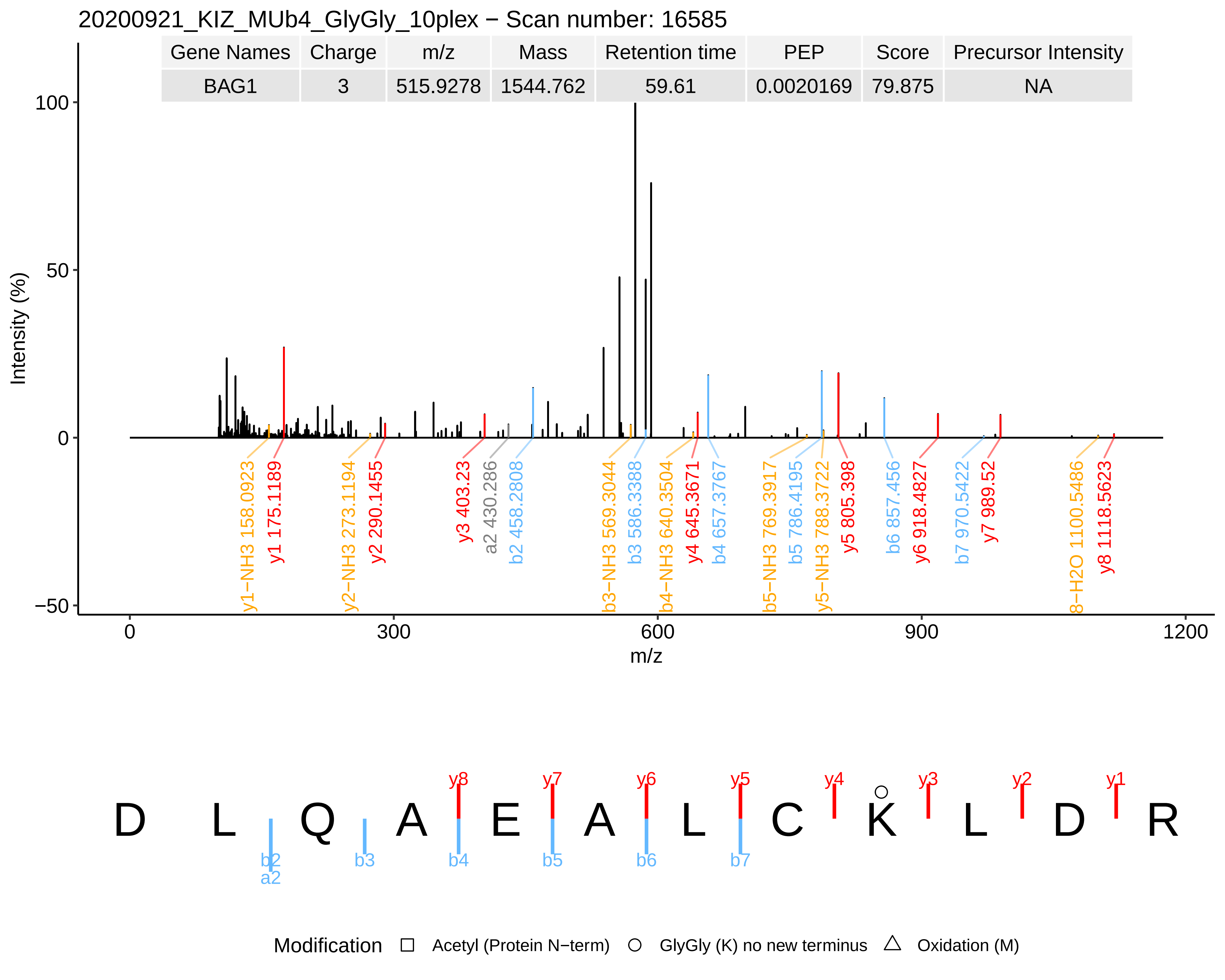
20200921_KIZ_MUb4_GlyGly_10plex − Scan number: 16585
Gene Names
Charge
m/z
Mass
Retention time
PEP
Score
Precursor Intensity
BAG1
3
515.9278
1544.762
59.61
0.0020169
79.875
NA
Intensity (%)
100
50
0
−50
0
300
600
900
1200
m/z
y1−NH3 158.0923
y1 175.1189
y2−NH3 273.1194
y2 290.1455
y3 403.23
a2 430.286
b2 458.2808
b3−NH3 569.3044
b3 586.3388
b4−NH3 640.3504
y4 645.3671
b4 657.3767
b5−NH3 769.3917
b5 786.4195
y5−NH3 788.3722
y5 805.398
b6 857.456
y6 918.4827
b7 970.5422
y7 989.52
8−H2O 1100.5486
y8 1118.5623
D L Q A E A L C K L D R
y8
y7
y6
y5
y4
y3
y2
y1
b2
a2
b3
b4
b5
b6
b7
Modification
Acetyl (Protein N−term)
GlyGly (K) no new terminus
Oxidation (M)

20200921_KIZ_MUb4_GlyGly_10plex_2 − Scan number: 15204

Gene Names	Charge	m/z	Mass	Retention time	PEP	Score	Precursor Intensity
BAG1	3	515.9278	1544.762	59.405	0.033369	59.227	1433776

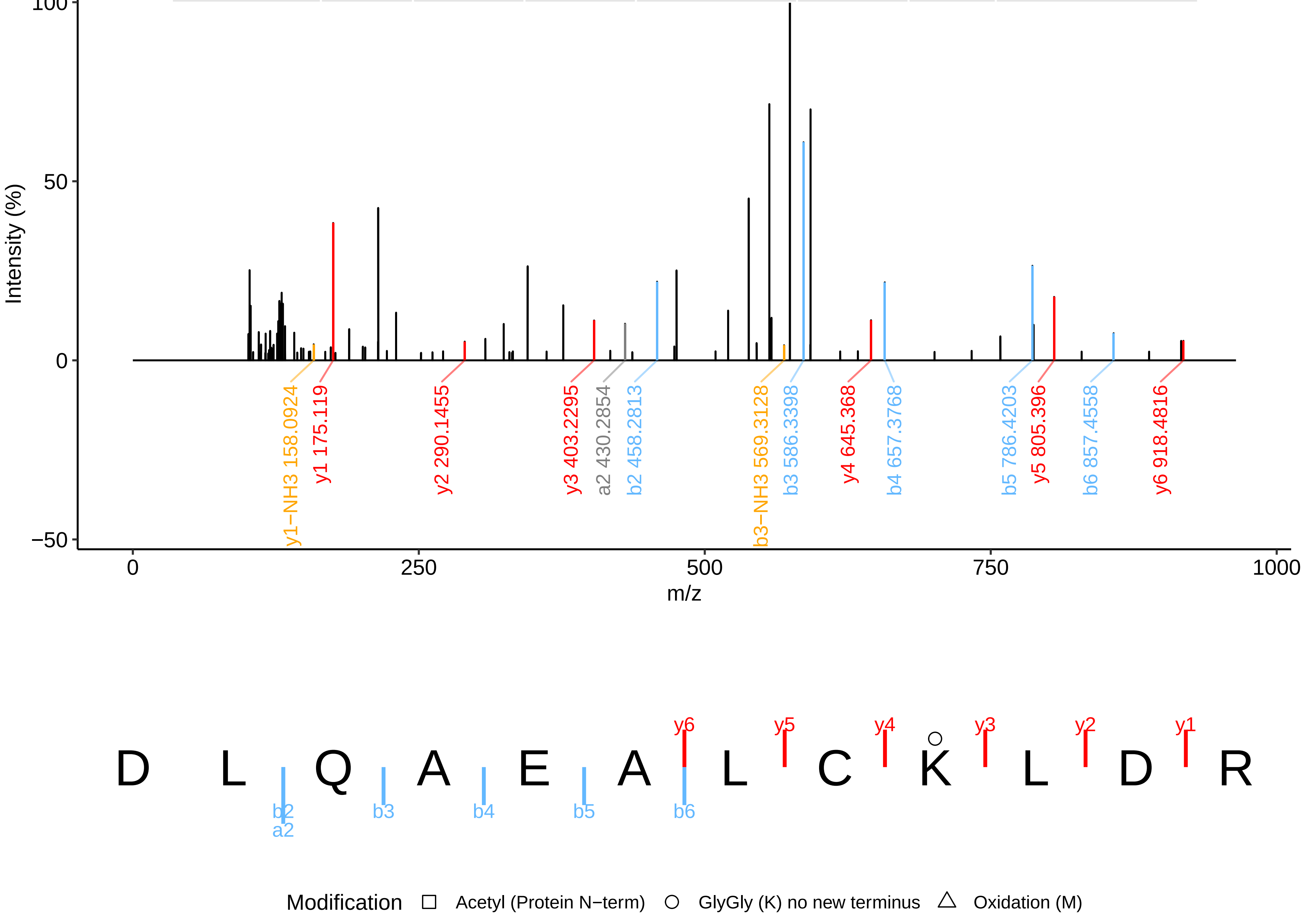

20200921_KIZ_MUb4_GlyGly_10plex_2 − Scan number: 15250

Gene Names	Charge	m/z	Mass	Retention time	PEP	Score	Precursor Intensity
BAG1	3	515.9278	1544.762	59.598	4.8389e−09	126.95	3474428

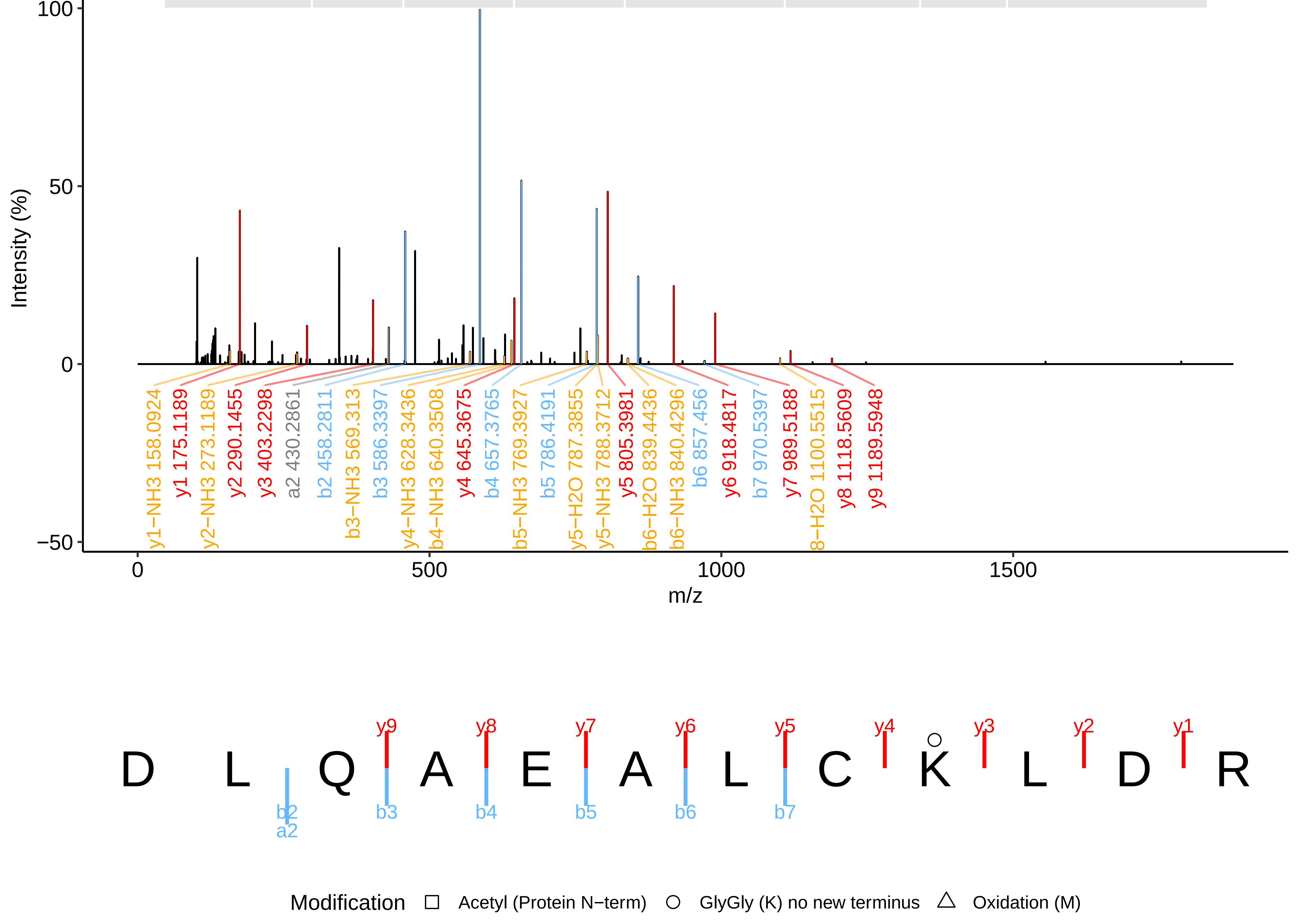

20200924_KIZ_MUb5_GlyGly_1 − Scan number: 15622

Gene Names	Charge	m/z	Mass	Retention time	PEP	Score	Precursor Intensity
BAG1	3	515.9278	1544.762	50.986	2.5226e−09	129.76	15742464

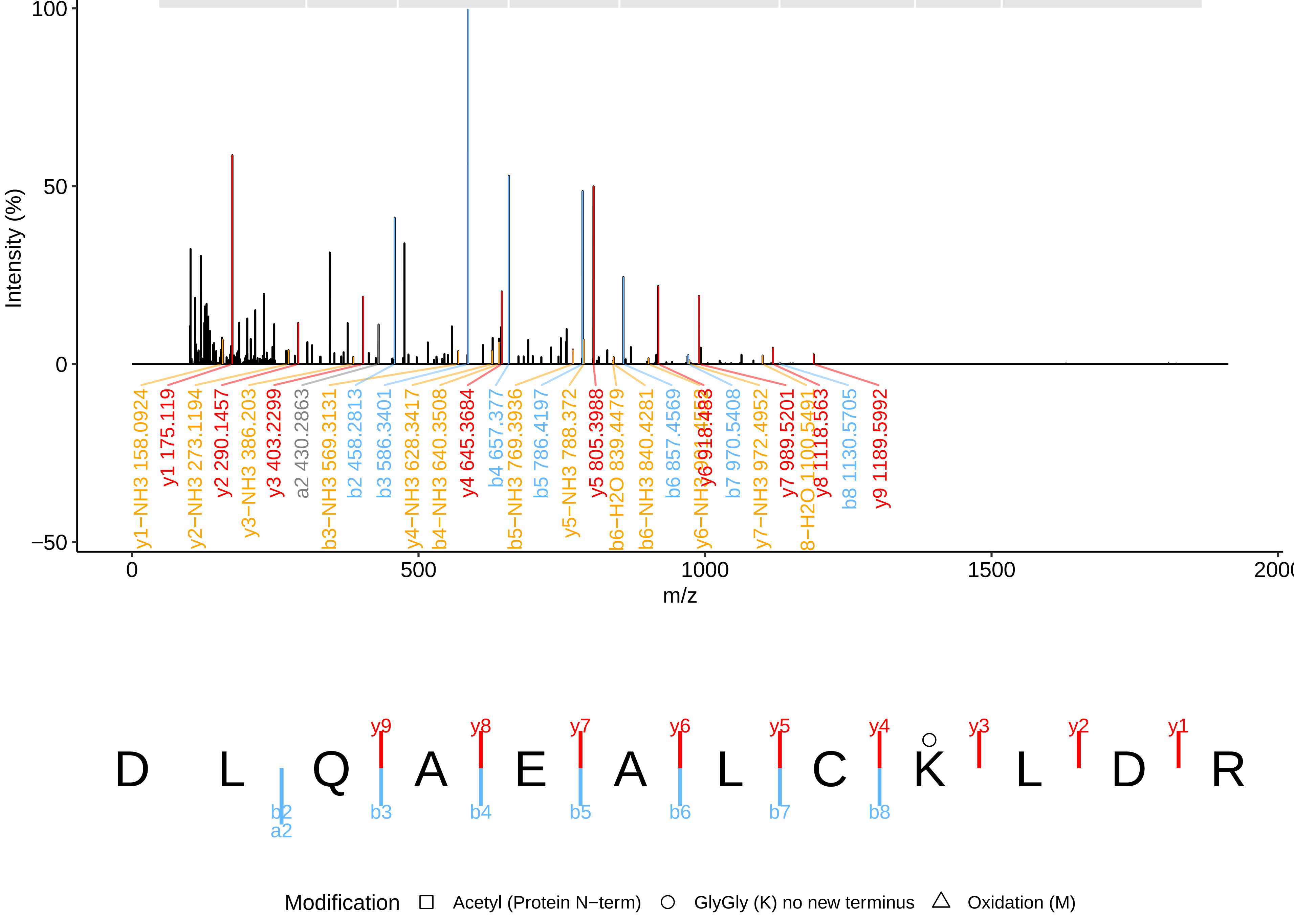

20200924_KIZ_MUb5_GlyGly_2 − Scan number: 14832

Gene Names	Charge	m/z	Mass	Retention time	PEP	Score	Precursor Intensity
BAG1	3	515.9278	1544.762	55.417	2.6267e−13	138.42	3571414

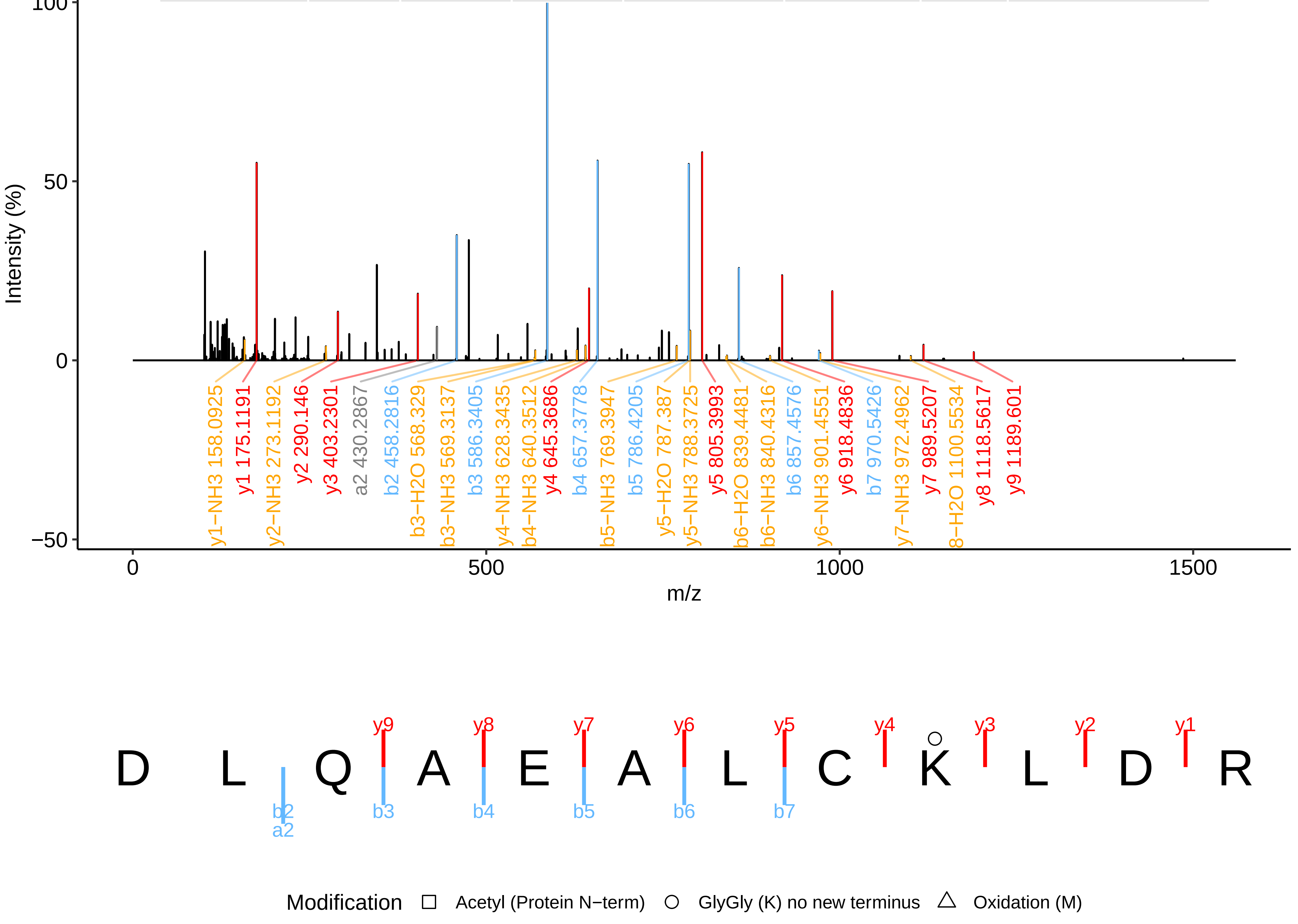

20201118_KIZ_MUb6_GlyGly_1 − Scan number: 15851

Gene Names	Charge	m/z	Mass	Retention time	PEP	Score	Precursor Intensity
BAG1	3	515.9278	1544.762	50.135	1.9906e−19	156.81	26582746

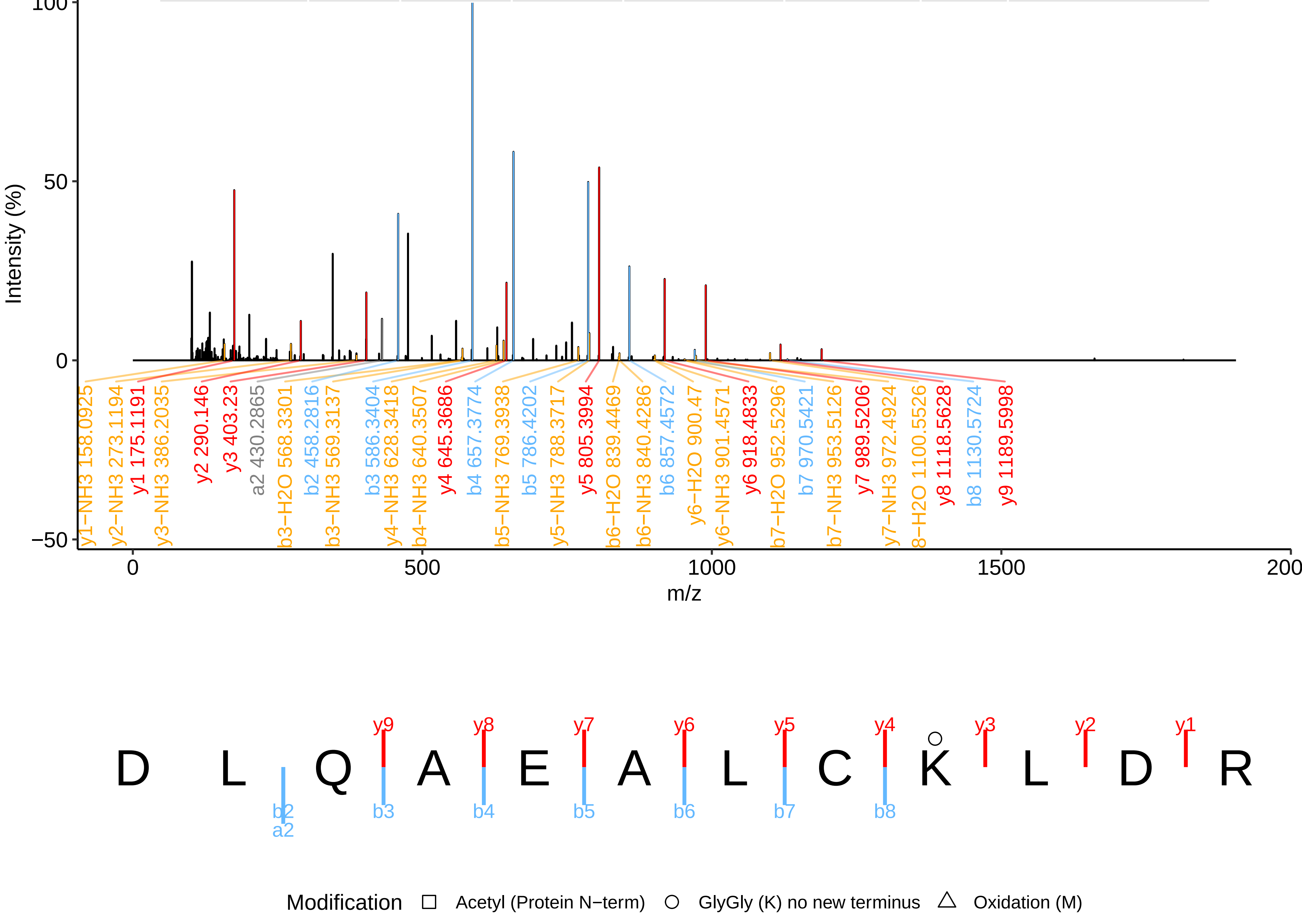

20200924_KIZ_MUb5_GlyGly_1 − Scan number: 19468

Gene Names	Charge	m/z	Mass	Retention time	PEP	Score	Precursor Intensity
GORASP2	3	430.2296	1287.667	62.933	0.051959	57.019	9033257

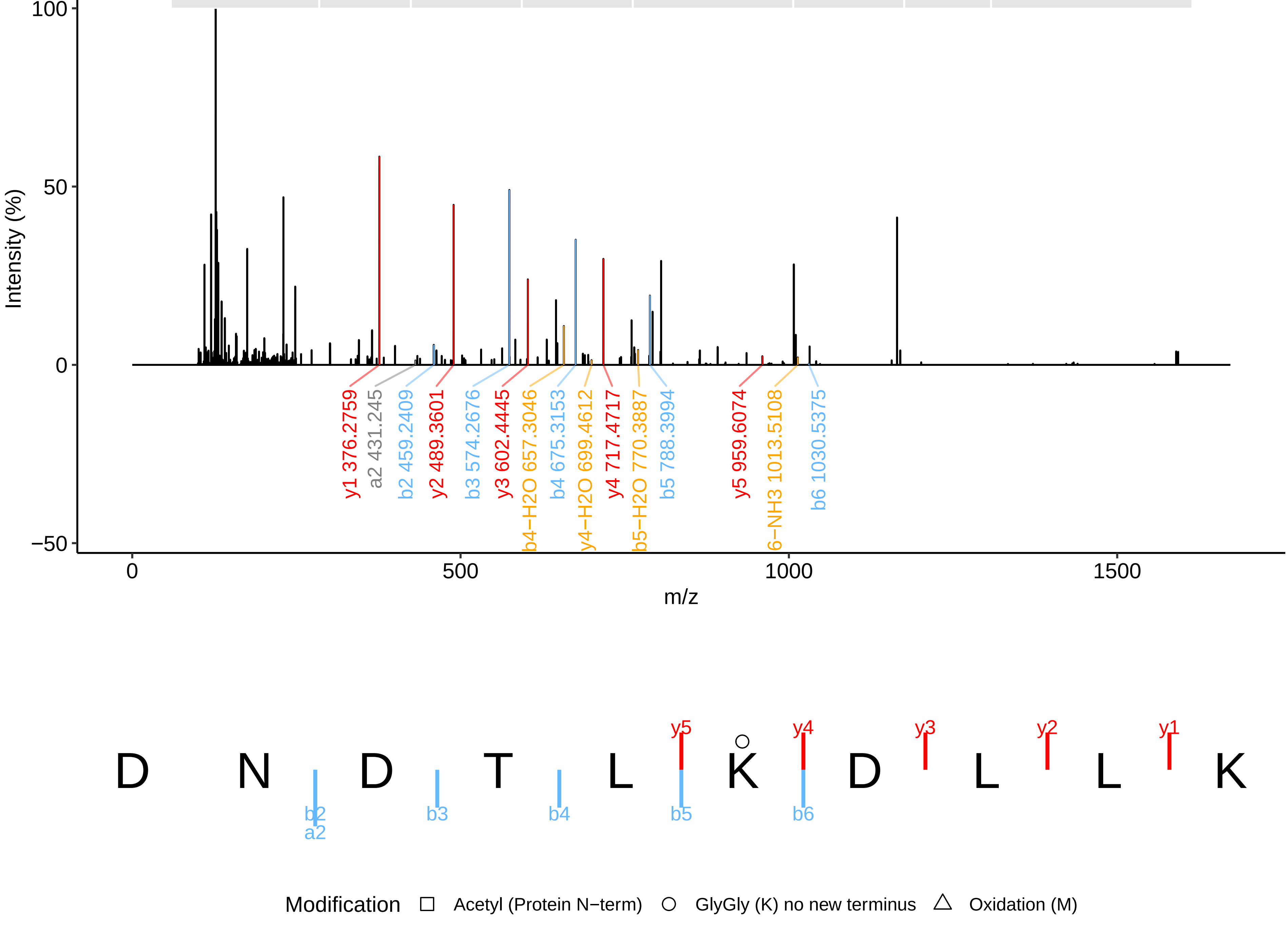

20200924_KIZ_MUb5_GlyGly_2 − Scan number: 18081

Gene Names	Charge	m/z	Mass	Retention time	PEP	Score	Precursor Intensity
GORASP2	3	430.2296	1287.667	67.042	0.077293	55.401	3717979

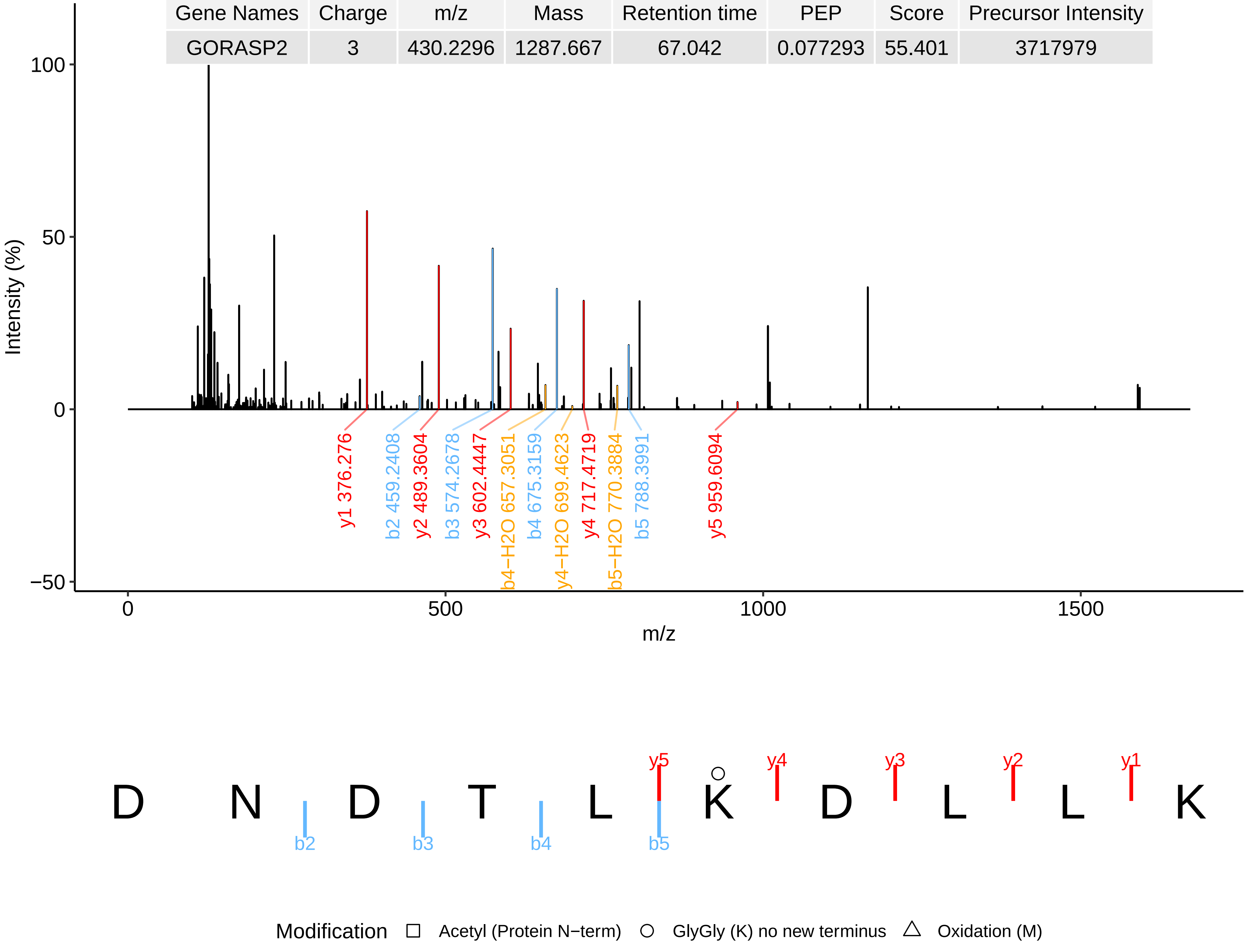

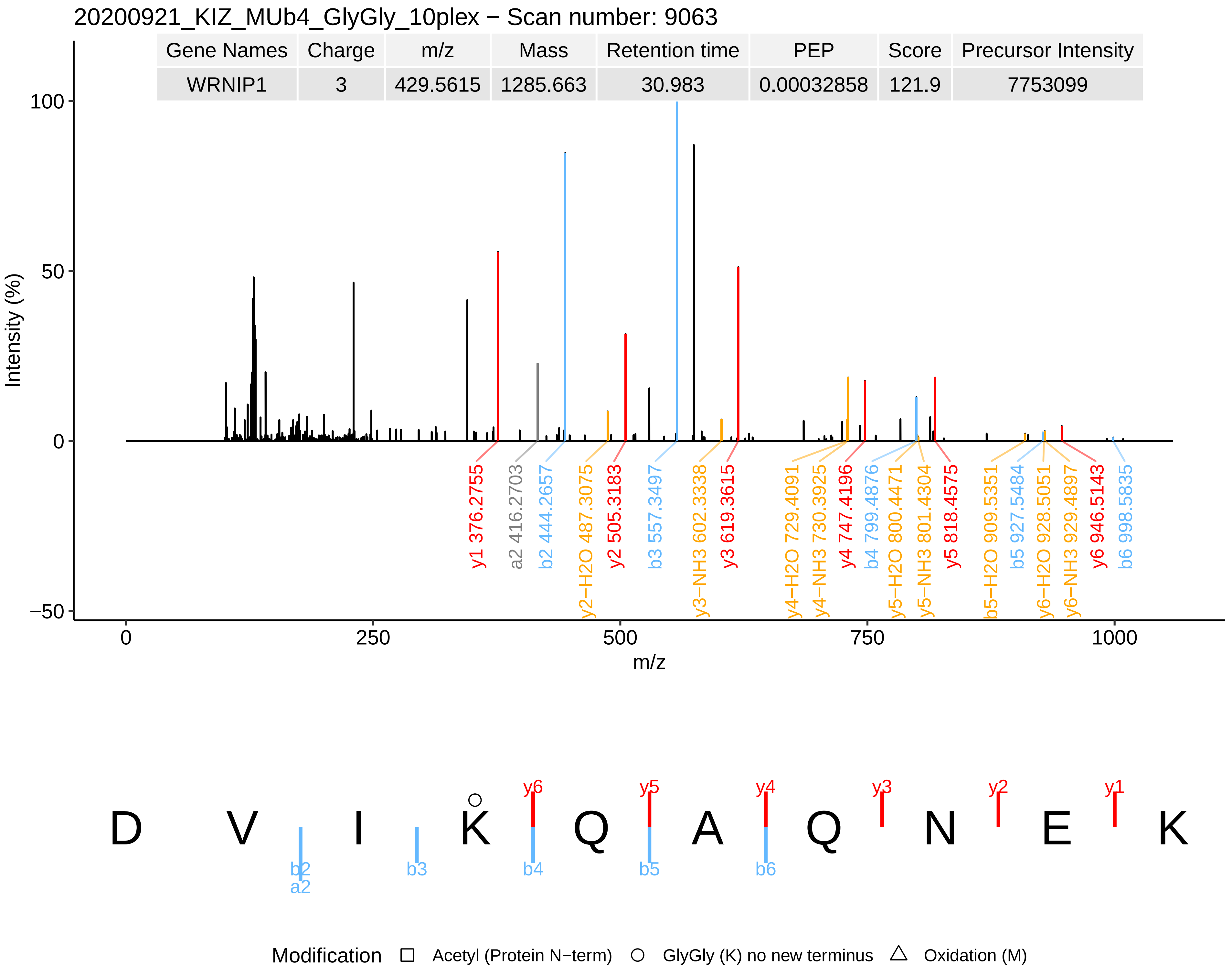

20200921_KIZ_MUb4_GlyGly_10plex − Scan number: 9063
Gene Names | Charge | m/z | Mass | Retention time | PEP | Score | Precursor Intensity
WRNIP1 | 3 | 429.5615 | 1285.663 | 30.983 | 0.00032858 | 121.9 | 7753099
Intensity (%)
m/z
y1 376.2755
a2 416.2703
b2 444.2657
y2−H2O 487.3075
y2 505.3183
b3 557.3497
y3−NH3 602.3338
y3 619.3615
y4−H2O 729.4091
y4−NH3 730.3925
y4 747.4196
b4 799.4876
y5−H2O 800.4471
y5−NH3 801.4304
y5 818.4575
b5−H2O 909.5351
b5 927.5484
y6−H2O 928.5051
y6−NH3 929.4897
y6 946.5143
b6 998.5835
D V I K Q A Q N E K
y6 y5 y4 y3 y2 y1
b2 a2 b3 b4 b5 b6
Modification
Acetyl (Protein N−term)
GlyGly (K) no new terminus
Oxidation (M)

20200921_KIZ_MUb4_GlyGly_10plex_2 − Scan number: 8352

Gene Names	Charge	m/z	Mass	Retention time	PEP	Score	Precursor Intensity
WRNIP1	3	429.5615	1285.663	31.441	0.015705	67.726	607019.1

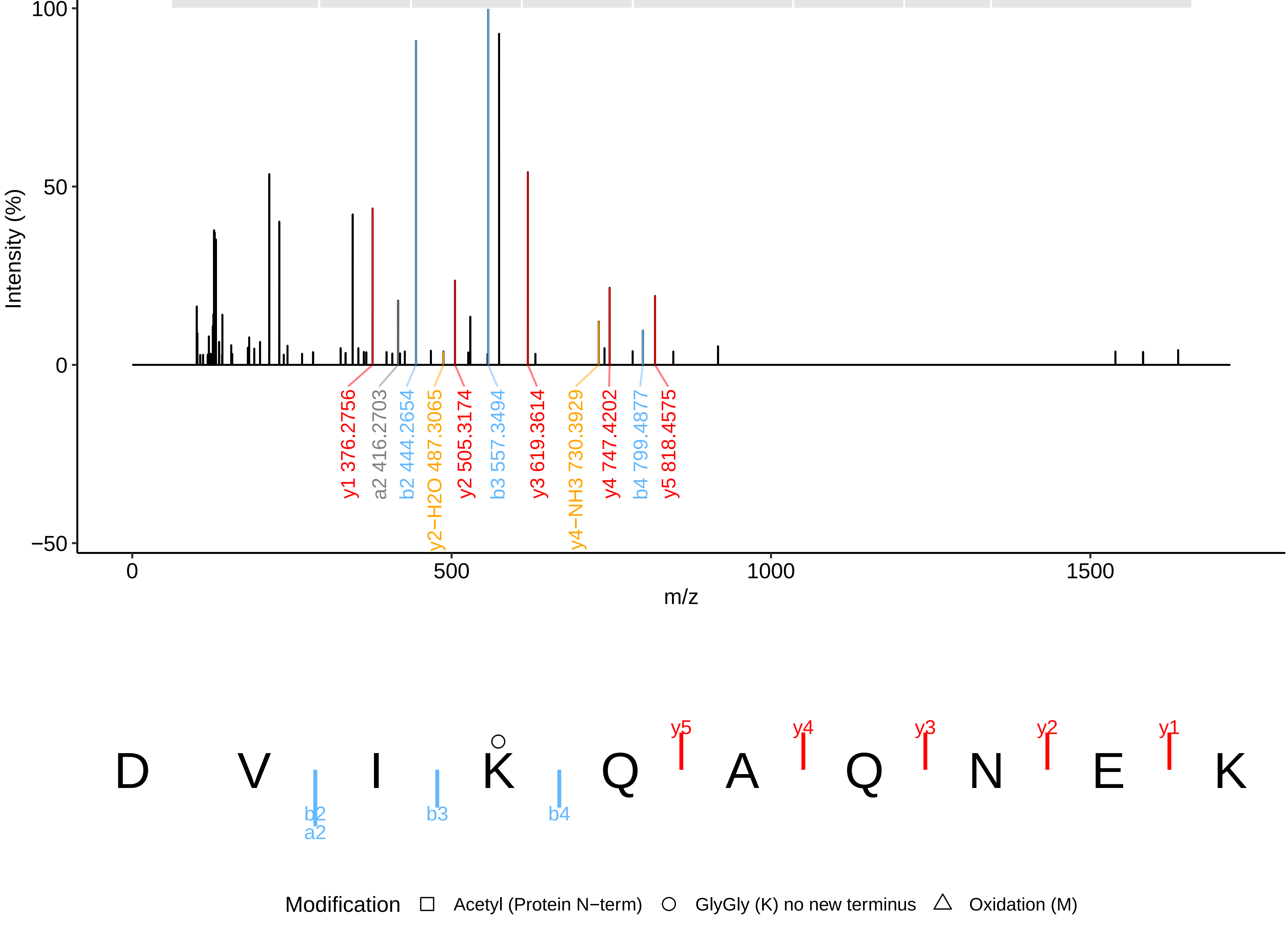

20200921_KIZ_MUb4_GlyGly_10plex − Scan number: 12756

Gene Names	Charge	m/z	Mass	Retention time	PEP	Score	Precursor Intensity
PTGES3	3	431.886	1292.636	44.833	0.0054922	77.744	3692544

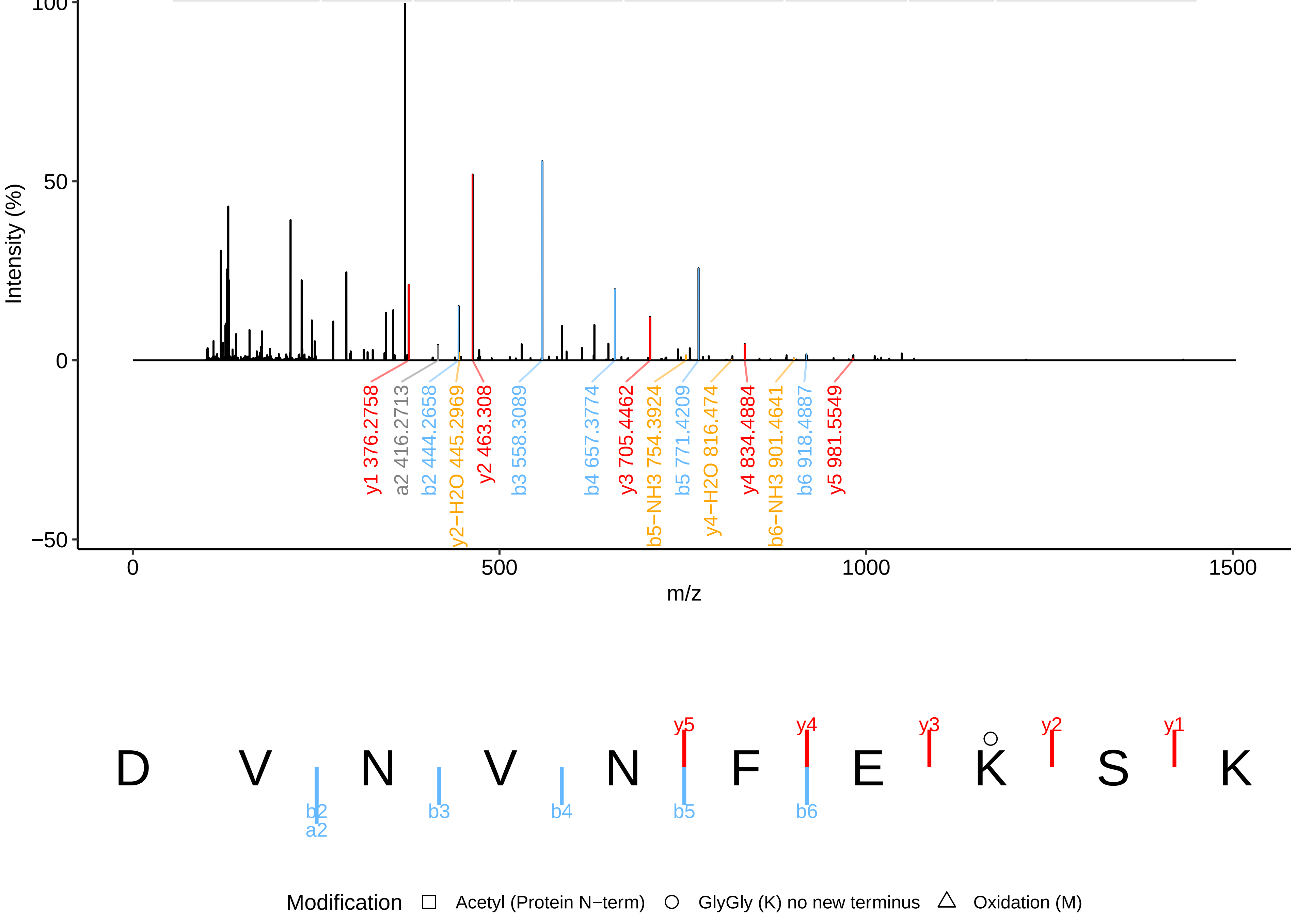

20200921_KIZ_MUb4_GlyGly_10plex_2 − Scan number: 11656

Gene Names	Charge	m/z	Mass	Retention time	PEP	Score	Precursor Intensity
PTGES3	3	431.886	1292.636	44.904	0.094492	48.741	496992.3

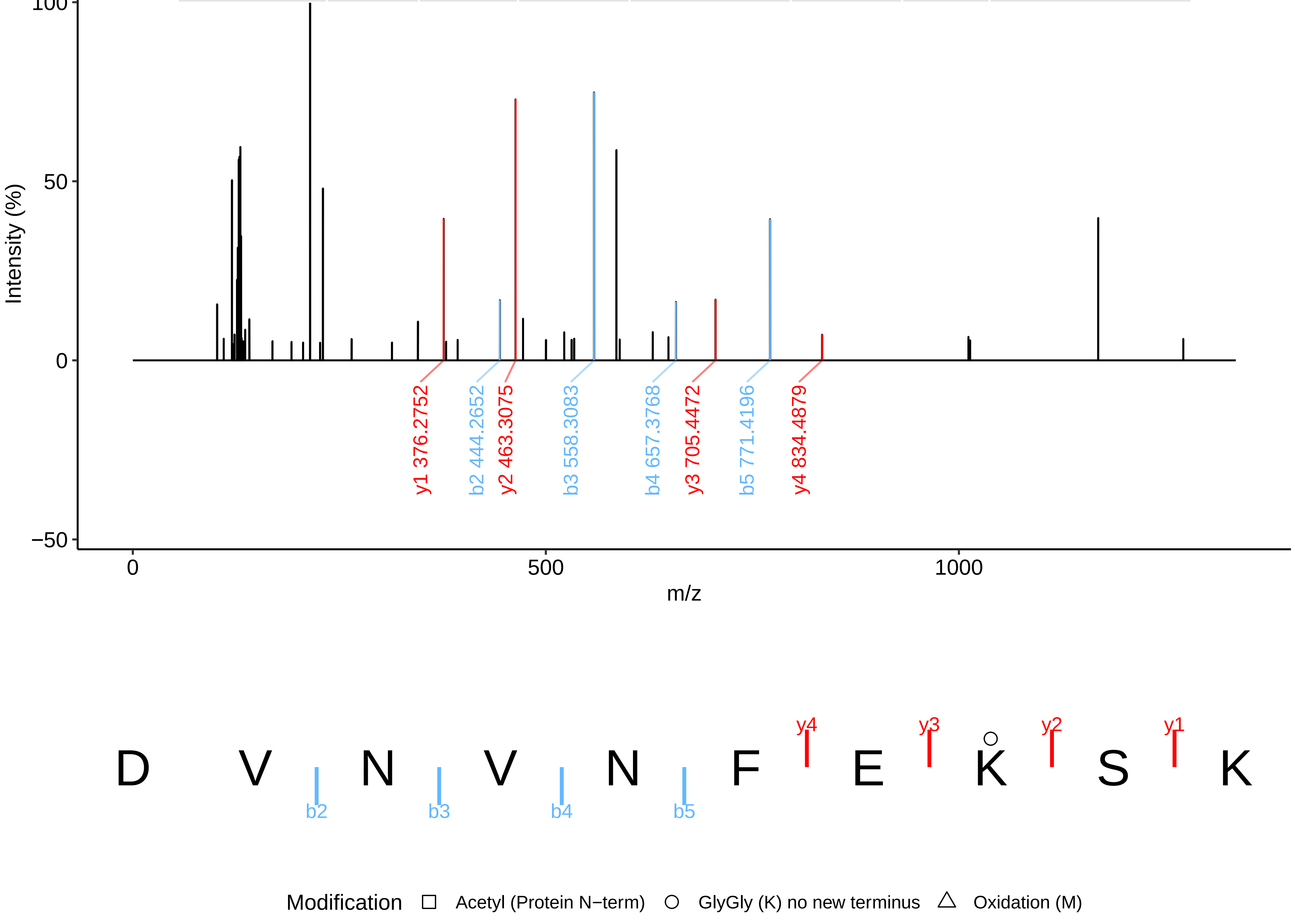

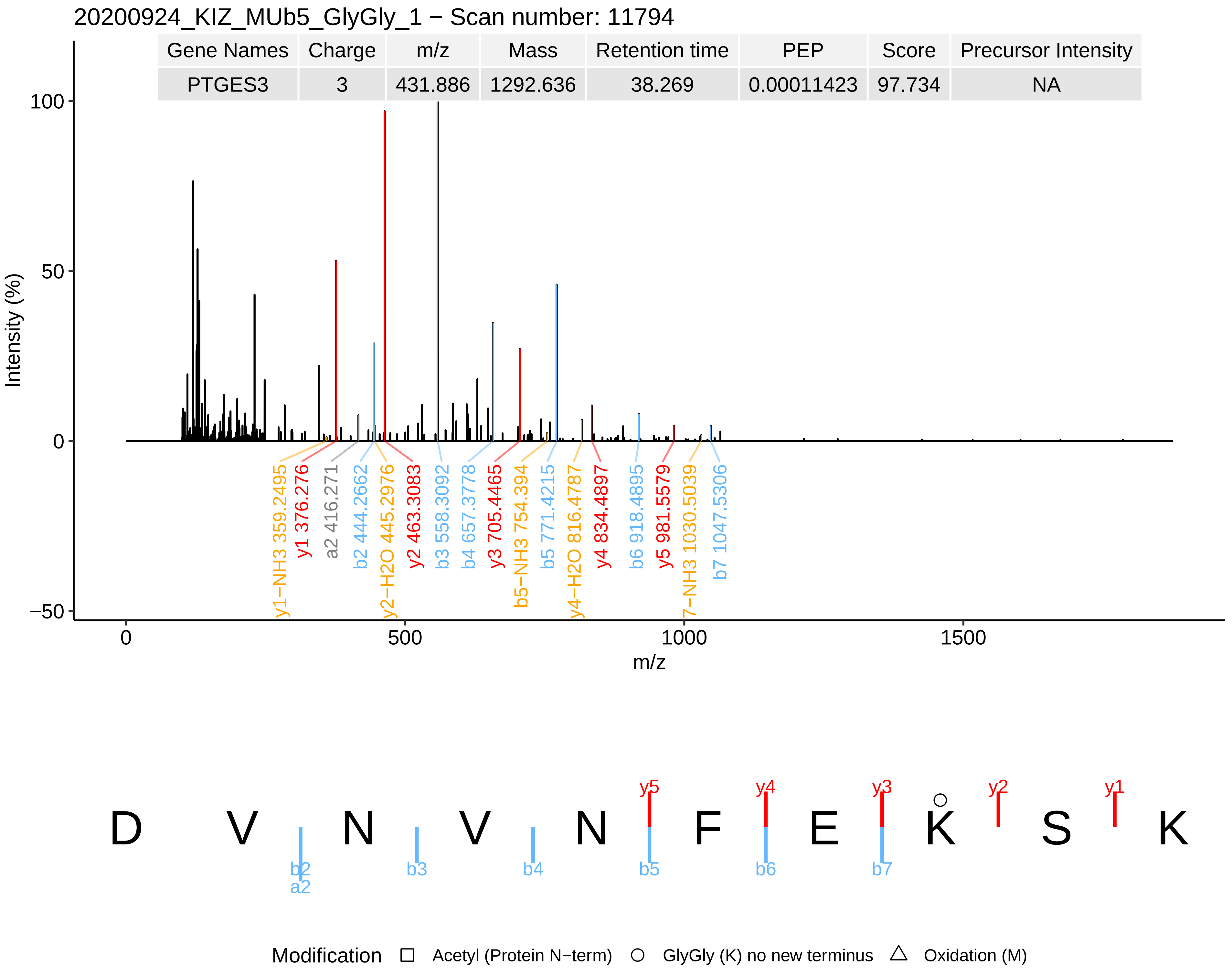
20200924_KIZ_MUb5_GlyGly_1 − Scan number: 11794
Gene Names | Charge | m/z | Mass | Retention time | PEP | Score | Precursor Intensity
PTGES3 | 3 | 431.886 | 1292.636 | 38.269 | 0.00011423 | 97.734 | NA
Intensity (%)
100
50
0
−50
0
500
1000
1500
m/z
y1−NH3 359.2495
y1 376.276
a2 416.271
b2 444.2662
y2−H2O 445.2976
y2 463.3083
b3 558.3092
b4 657.3778
y3 705.4465
b5−NH3 754.394
b5 771.4215
y4−H2O 816.4787
y4 834.4897
b6 918.4895
y5 981.5579
7−NH3 1030.5039
b7 1047.5306
D V N V N F E K S K
y5 y4 y3 y2 y1
b2 a2 b3 b4 b5 b6 b7
Modification
Acetyl (Protein N−term)
GlyGly (K) no new terminus
Oxidation (M)

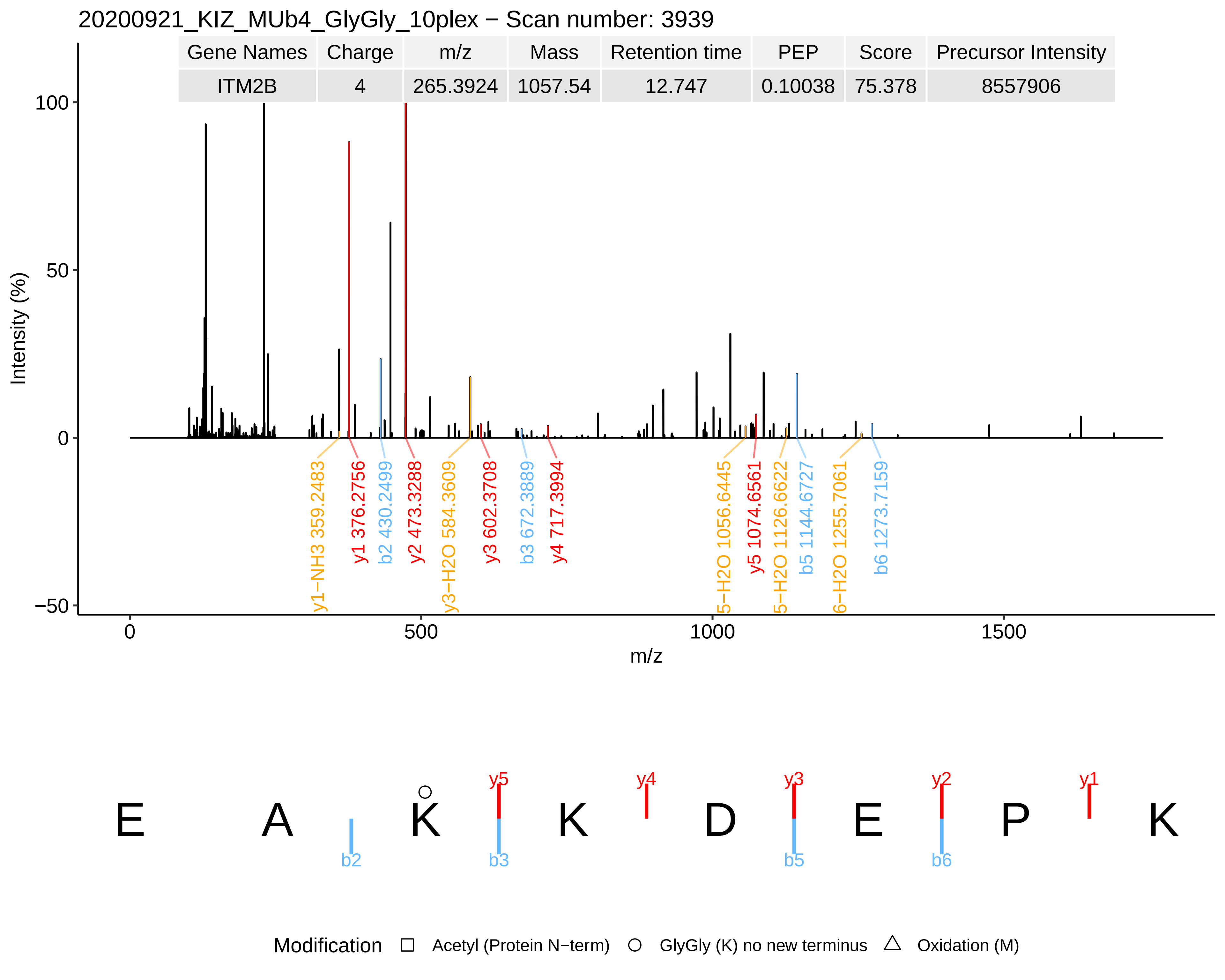

20200921_KIZ_MUb4_GlyGly_10plex − Scan number: 3939
Gene Names | Charge | m/z | Mass | Retention time | PEP | Score | Precursor Intensity
ITM2B | 4 | 265.3924 | 1057.54 | 12.747 | 0.10038 | 75.378 | 8557906
Intensity (%)
100
50
0
−50
y1−NH3 359.2483
y1 376.2756
b2 430.2499
y2 473.3288
y3−H2O 584.3609
y3 602.3708
b3 672.3889
y4 717.3994
5−H2O 1056.6445
y5 1074.6561
5−H2O 1126.6622
b5 1144.6727
6−H2O 1255.7061
b6 1273.7159
0
500
1000
1500
m/z
E A K K D E P K
y5 y4 y3 y2 y1
b2 b3 b5 b6
Modification
Acetyl (Protein N−term)
GlyGly (K) no new terminus
Oxidation (M)

20200921_KIZ_MUb4_GlyGly_10plex − Scan number: 3950

Gene Names	Charge	m/z	Mass	Retention time	PEP	Score	Precursor Intensity
ITM2B	3	353.5207	1057.54	12.79	0.0053966	98.048	3085290

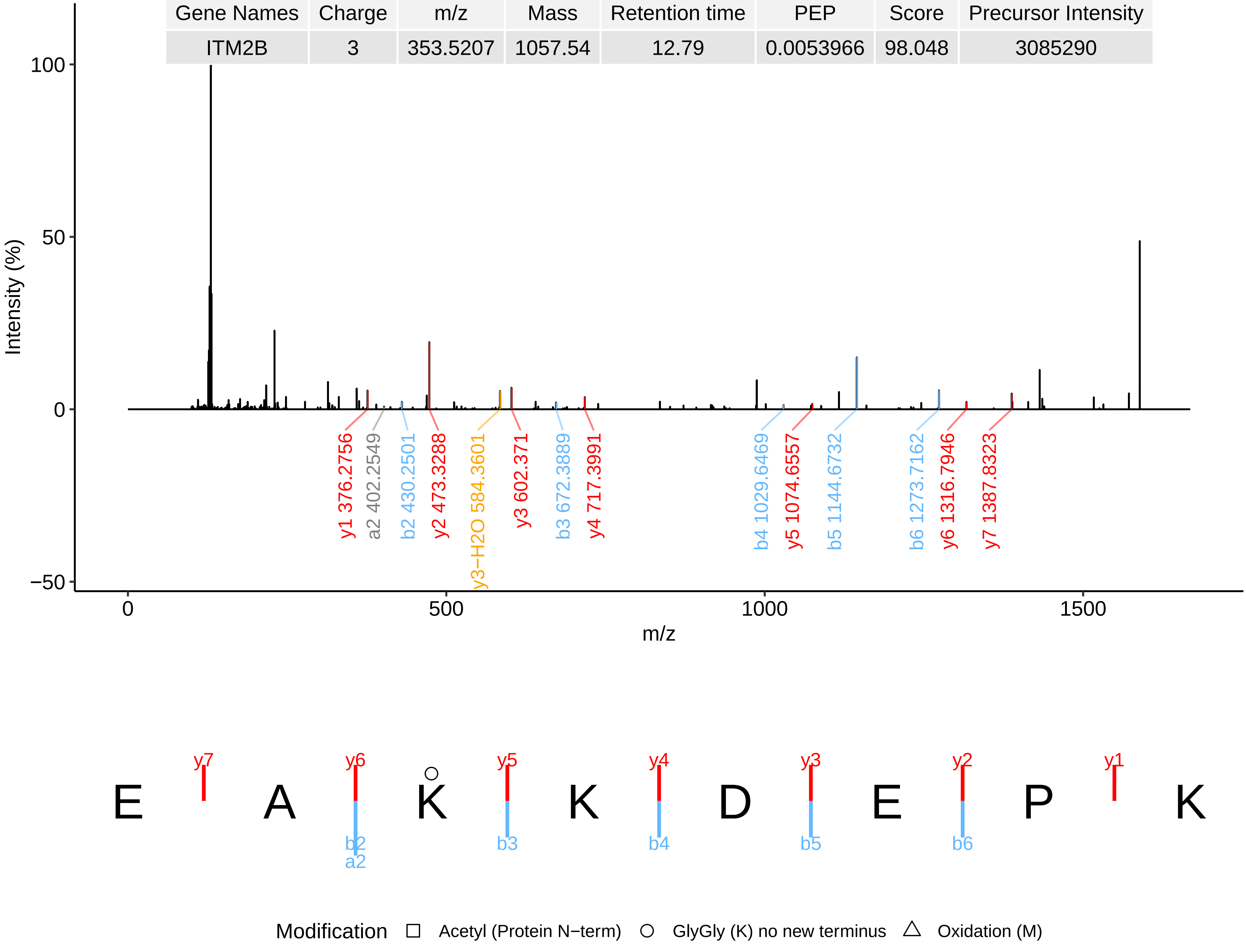

20200921_KIZ_MUb4_GlyGly_10plex_2 − Scan number: 3758

Gene Names	Charge	m/z	Mass	Retention time	PEP	Score	Precursor Intensity
ITM2B	3	353.5207	1057.54	12.829	0.017348	78.934	1276141

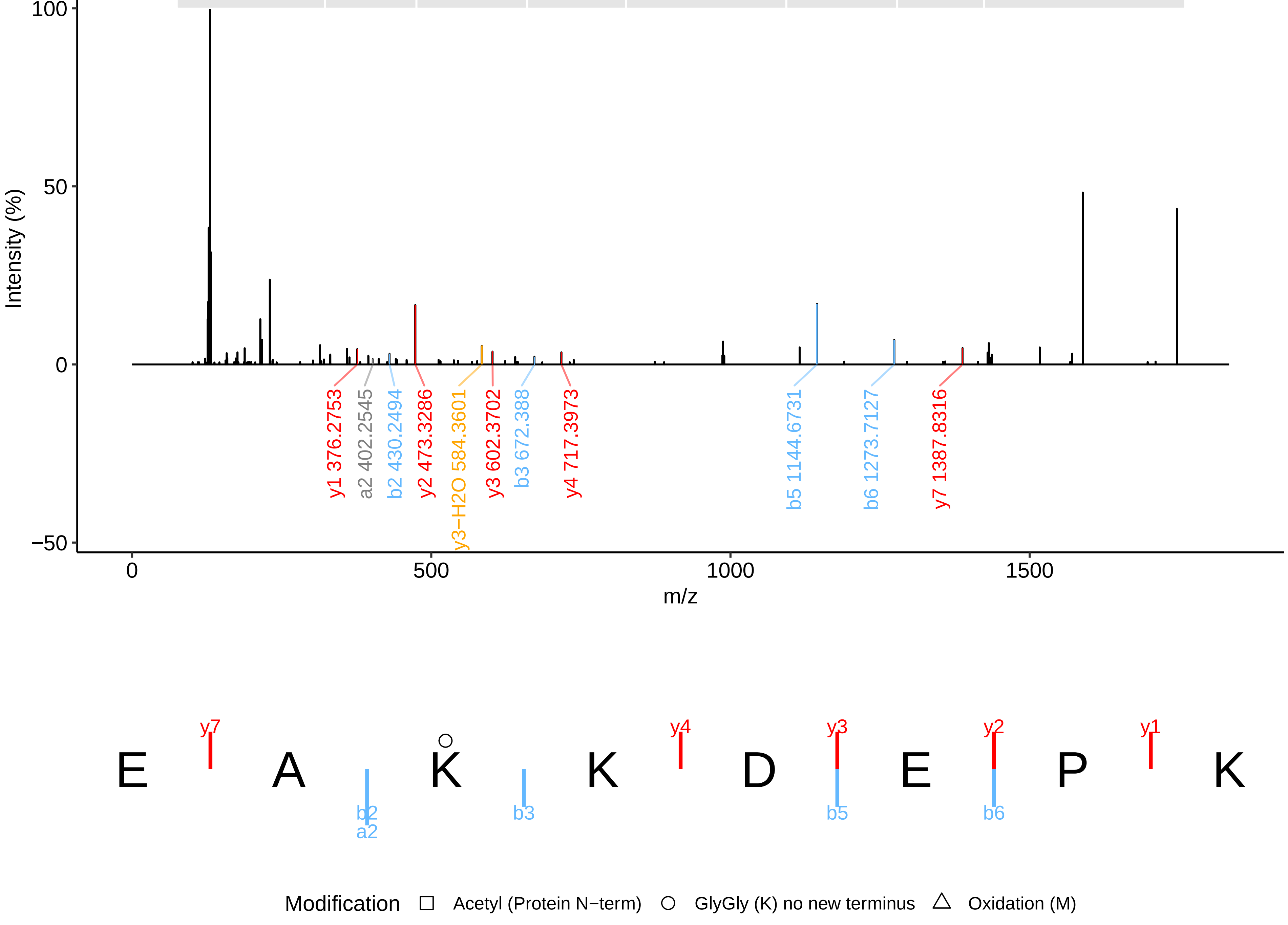

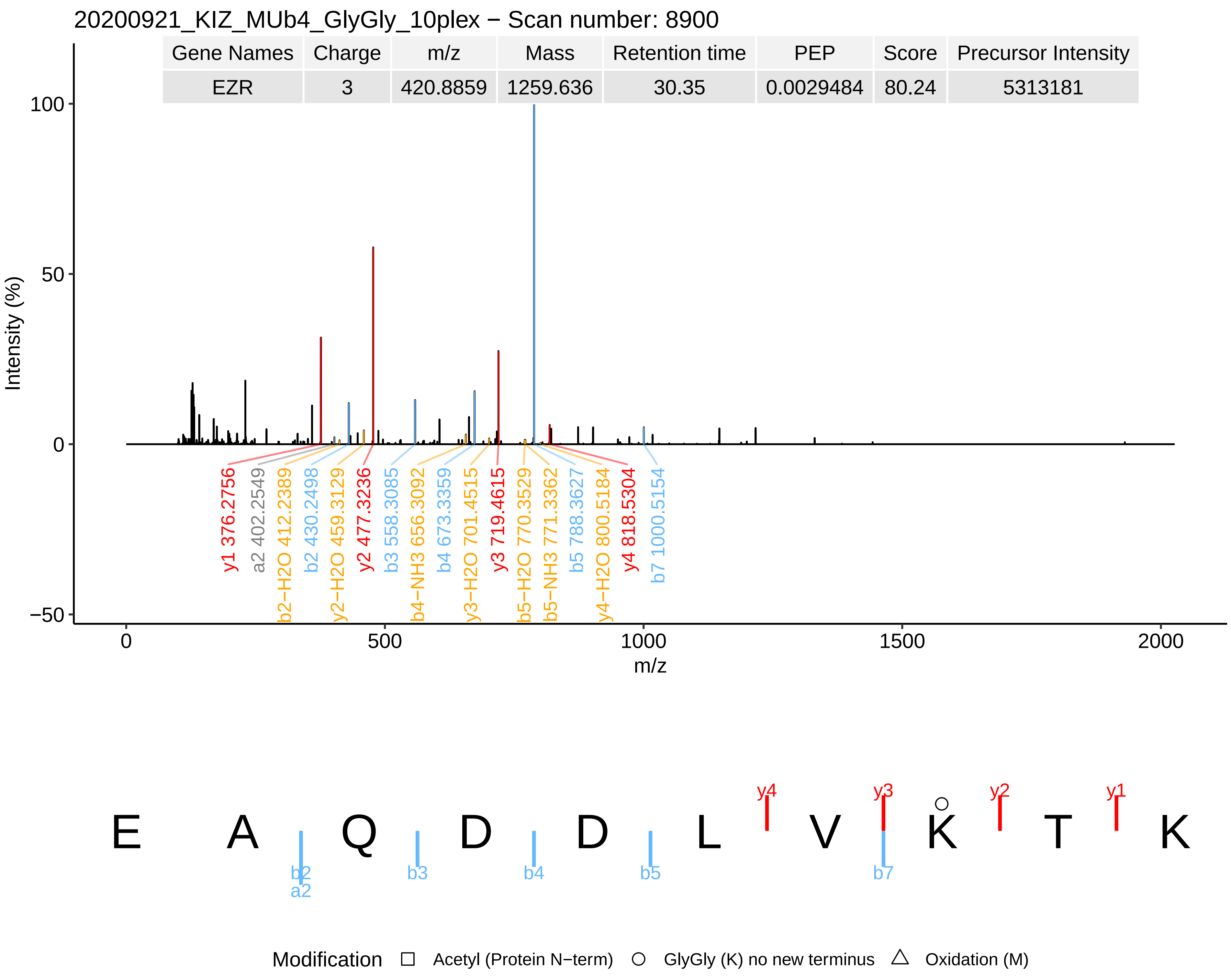

20200921_KIZ_MUb4_GlyGly_10plex − Scan number: 8900
Gene Names | Charge | m/z | Mass | Retention time | PEP | Score | Precursor Intensity
EZR | 3 | 420.8859 | 1259.636 | 30.35 | 0.0029484 | 80.24 | 5313181
Intensity (%)
m/z
y1 376.2756
a2 402.2549
b2−H2O 412.2389
b2 430.2498
y2−H2O 459.3129
y2 477.3236
b3 558.3085
b4−NH3 656.3092
b4 673.3359
y3−H2O 701.4515
y3 719.4615
b5−H2O 770.3529
b5−NH3 771.3362
b5 788.3627
y4−H2O 800.5184
y4 818.5304
b7 1000.5154
E A Q D D L V K T K
y4 y3 y2 y1
b2 a2 b3 b4 b5 b7
Modification
Acetyl (Protein N−term)
GlyGly (K) no new terminus
Oxidation (M)

20200924_KIZ_MUb5_GlyGly_1 − Scan number: 7907

Gene Names	Charge	m/z	Mass	Retention time	PEP	Score	Precursor Intensity
EZR	3	420.8859	1259.636	25.19	0.0082202	76.682	2049075

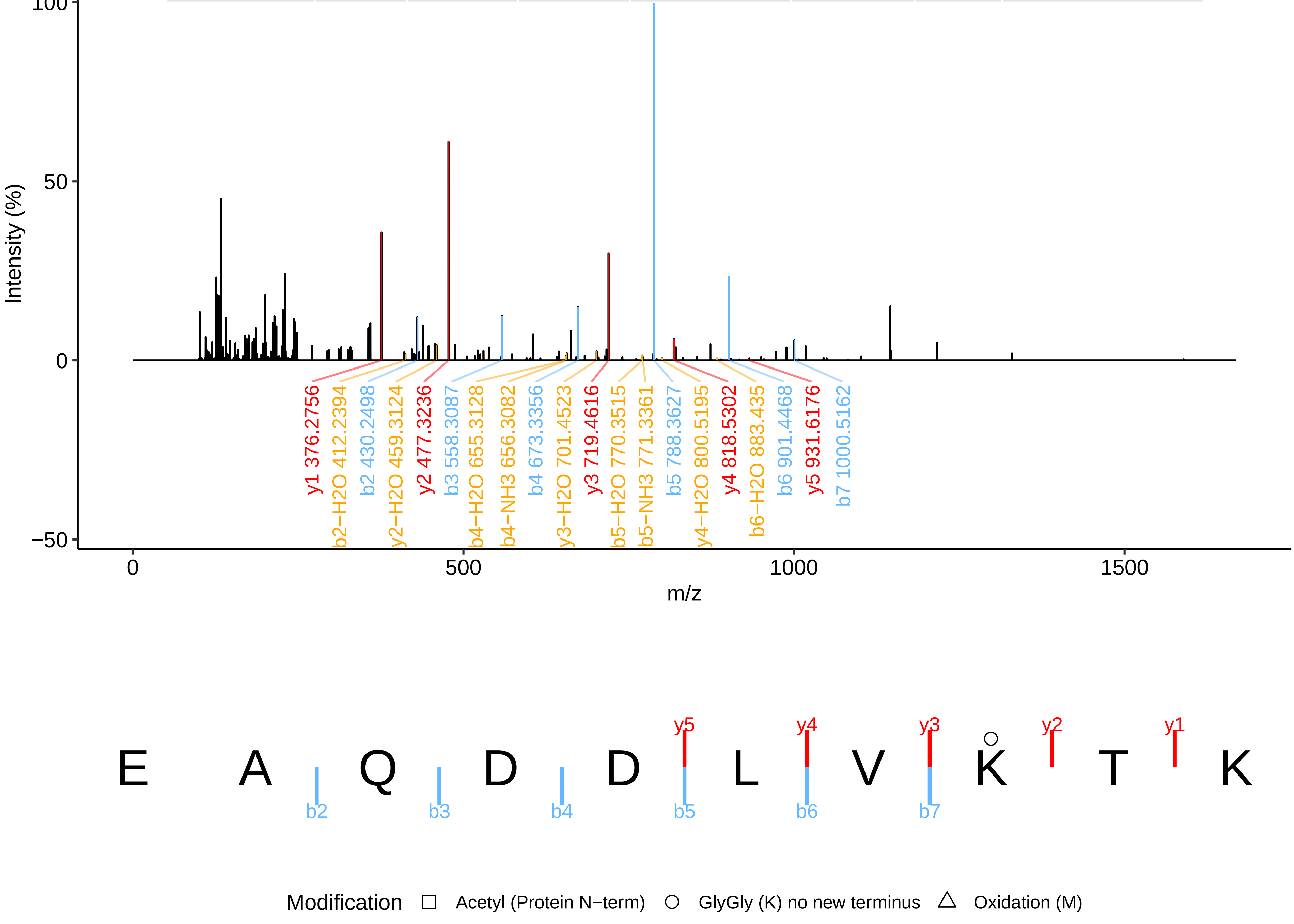

20200924_KIZ_MUb5_GlyGly_2 − Scan number: 7424

Gene Names	Charge	m/z	Mass	Retention time	PEP	Score	Precursor Intensity
EZR	3	420.8859	1259.636	27.713	0.016288	67.153	1655377

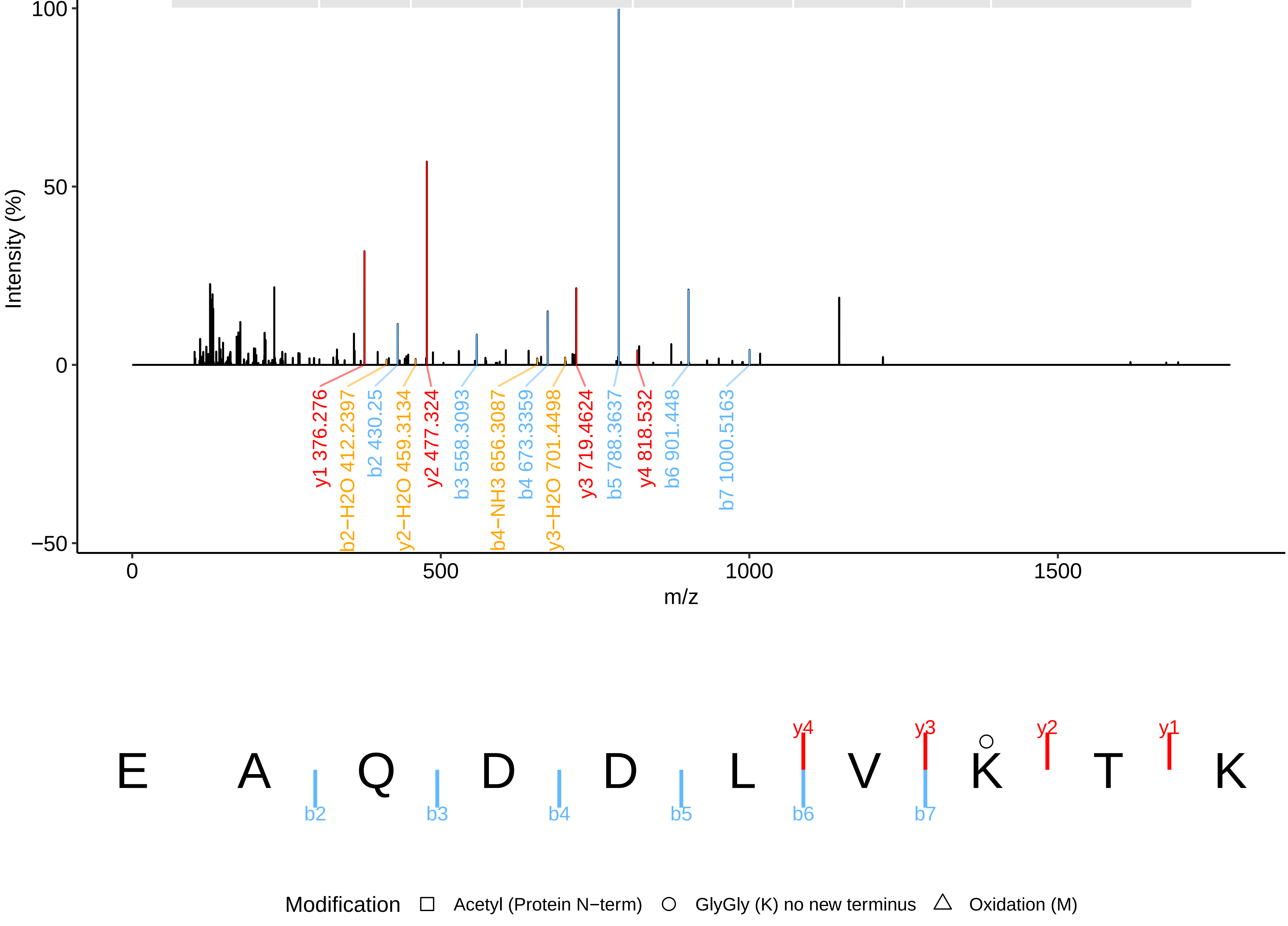

20200924_KIZ_MUb5_GlyGly_2 − Scan number: 7573

Gene Names	Charge	m/z	Mass	Retention time	PEP	Score	Precursor Intensity
EZR	3	420.8859	1259.636	28.281	0.018678	67.153	NA

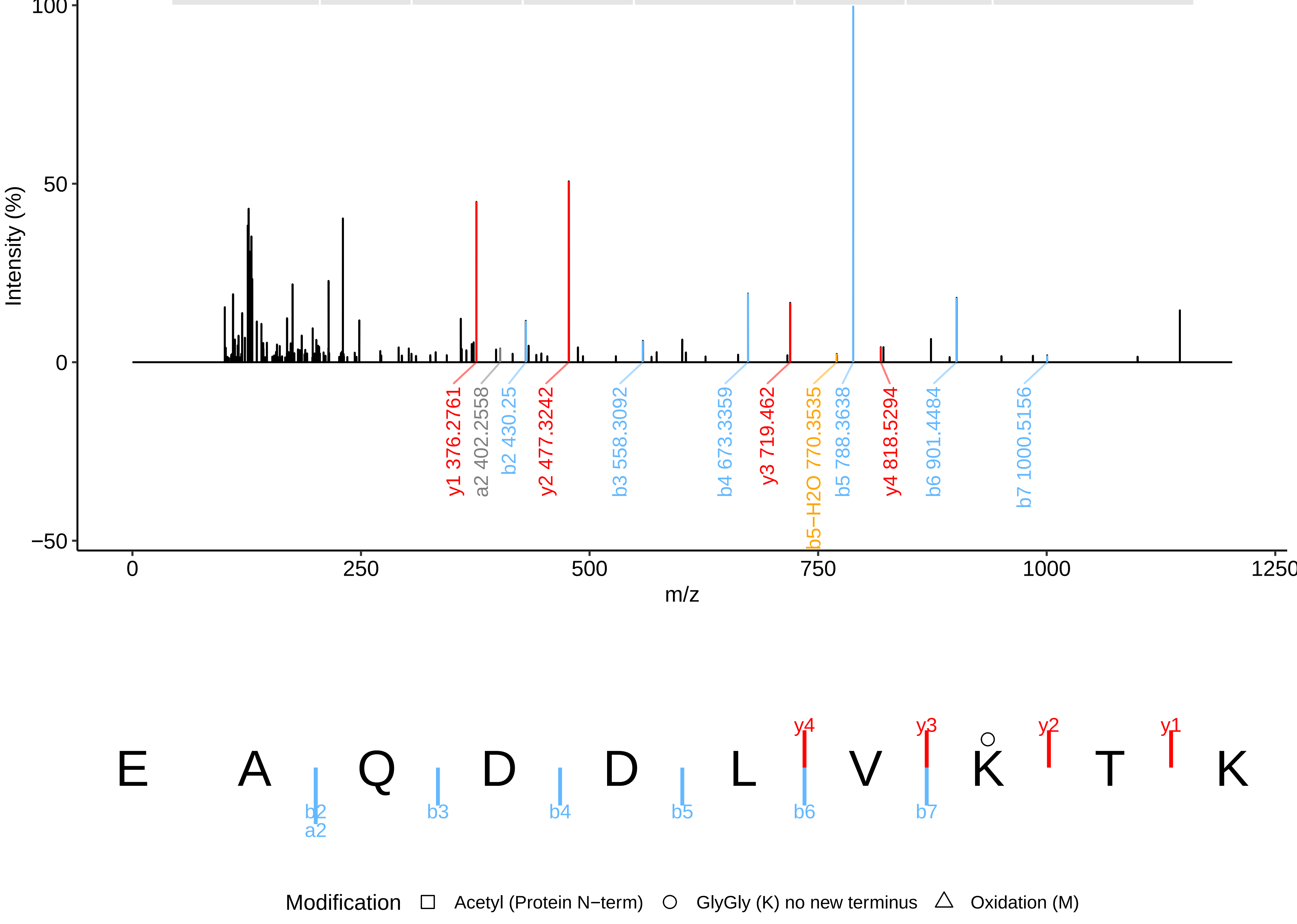

20200924_KIZ_MUb5_GlyGly_1 − Scan number: 2371

Gene Names	Charge	m/z	Mass	Retention time	PEP	Score	Precursor Intensity
SLC43A3	3	495.8903	1484.649	7.3909	5.2278e−09	131.17	21527546

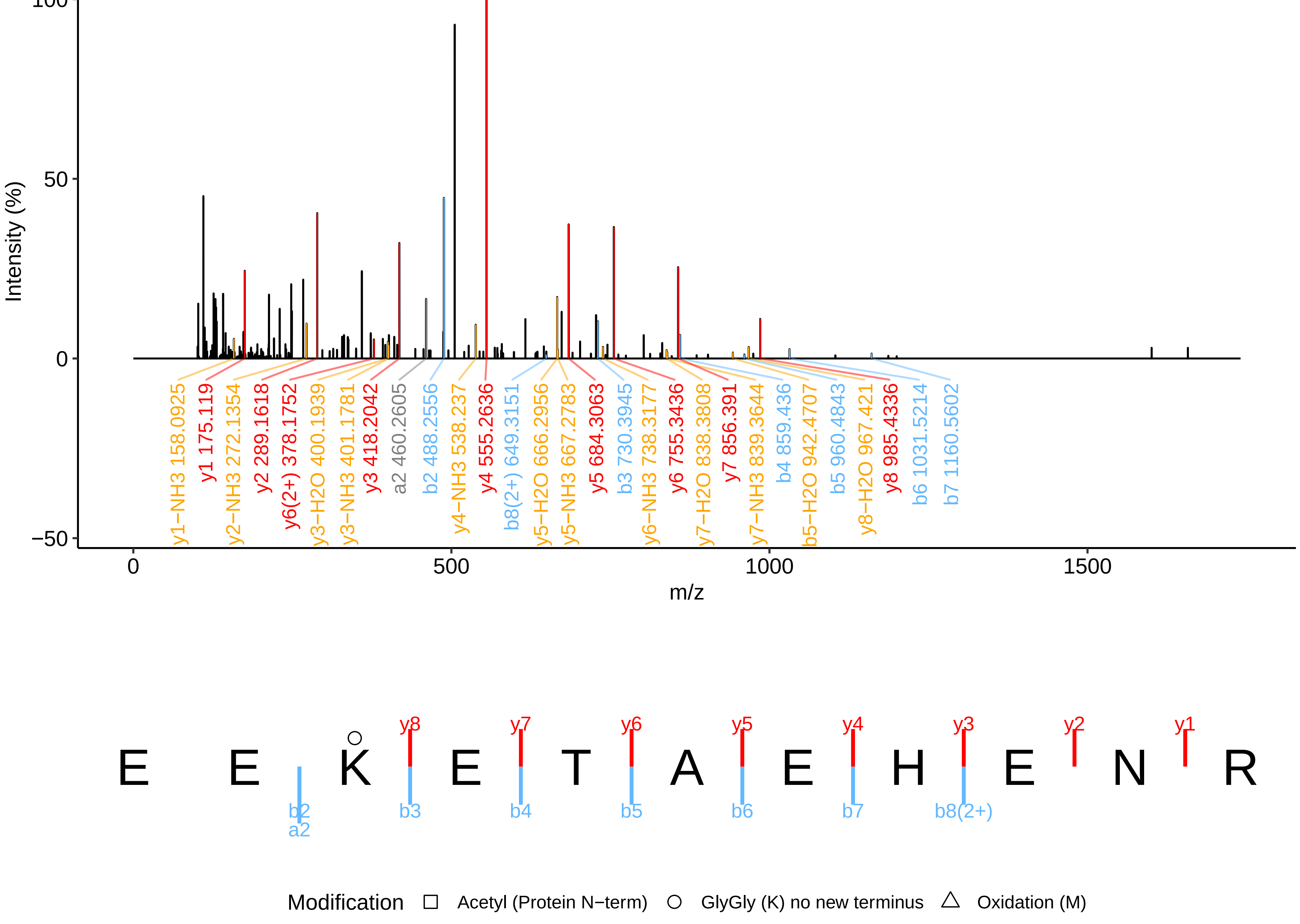

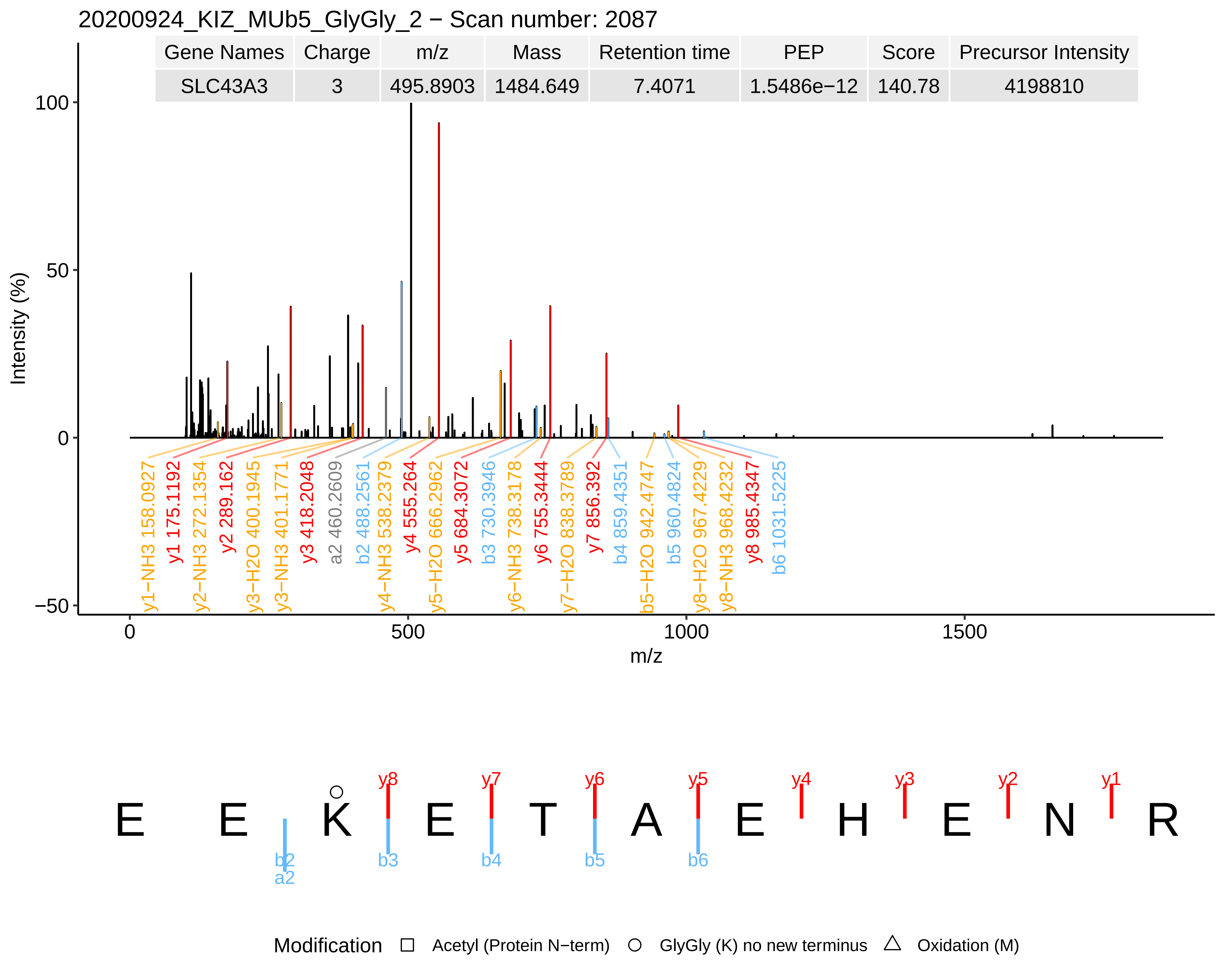
20200924_KIZ_MUb5_GlyGly_2 − Scan number: 2087
Gene Names | Charge | m/z | Mass | Retention time | PEP | Score | Precursor Intensity
SLC43A3 | 3 | 495.8903 | 1484.649 | 7.4071 | 1.5486e−12 | 140.78 | 4198810
Intensity (%)
m/z
y1−NH3 158.0927
y1 175.1192
y2−NH3 272.1354
y2 289.162
y3−H2O 400.1945
y3−NH3 401.1771
y3 418.2048
a2 460.2609
b2 488.2561
y4−NH3 538.2379
y4 555.264
y5−H2O 666.2962
y5 684.3072
b3 730.3946
y6−NH3 738.3178
y6 755.3444
y7−H2O 838.3789
y7 856.392
b4 859.4351
b5−H2O 942.4747
b5 960.4824
y8−H2O 967.4229
y8−NH3 968.4232
y8 985.4347
b6 1031.5225
E E K E T A E H E N R
y8 y7 y6 y5 y4 y3 y2 y1
b2 a2 b3 b4 b5 b6
Modification
Acetyl (Protein N−term)
GlyGly (K) no new terminus
Oxidation (M)

20201118_KIZ_MUb6_GlyGly_2 − Scan number: 2530

Gene Names	Charge	m/z	Mass	Retention time	PEP	Score	Precursor Intensity
SLC43A3	4	372.1696	1484.649	7.8891	0.00018689	96.113	10199214

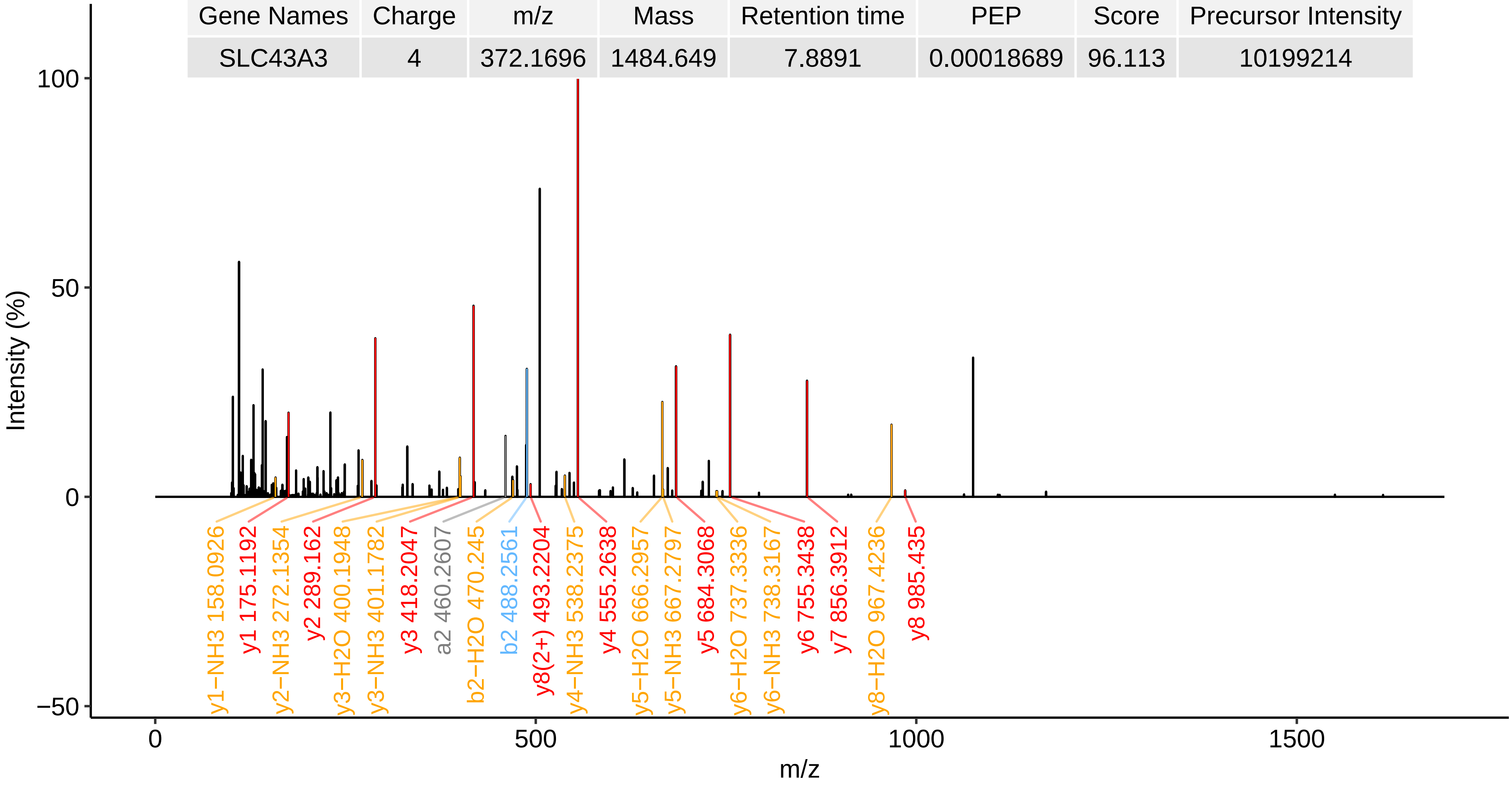

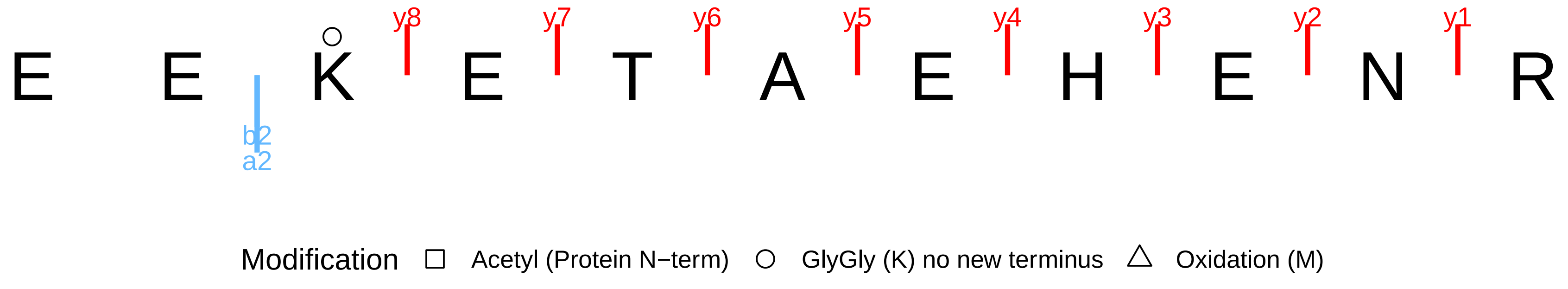

20200921_KIZ_MUb4_GlyGly_10plex − Scan number: 7057

Gene Names	Charge	m/z	Mass	Retention time	PEP	Score	Precursor Intensity
PFN1	4	317.1795	1264.689	23.573	0.0056641	72.848	20238814

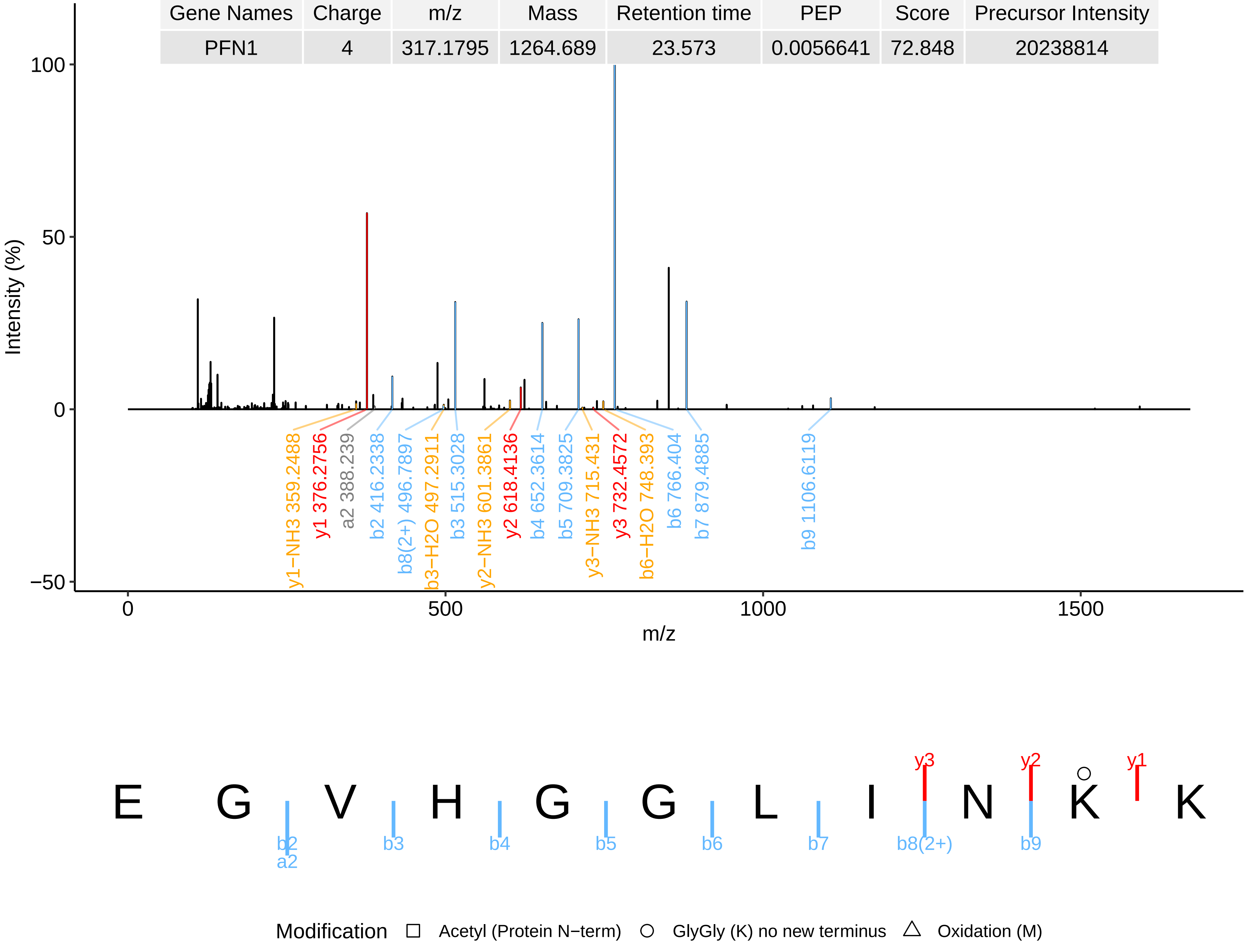

20200921_KIZ_MUb4_GlyGly_10plex_2 − Scan number: 6589

Gene Names	Charge	m/z	Mass	Retention time	PEP	Score	Precursor Intensity
PFN1	4	317.1795	1264.689	24.286	0.0090185	67.022	2936268

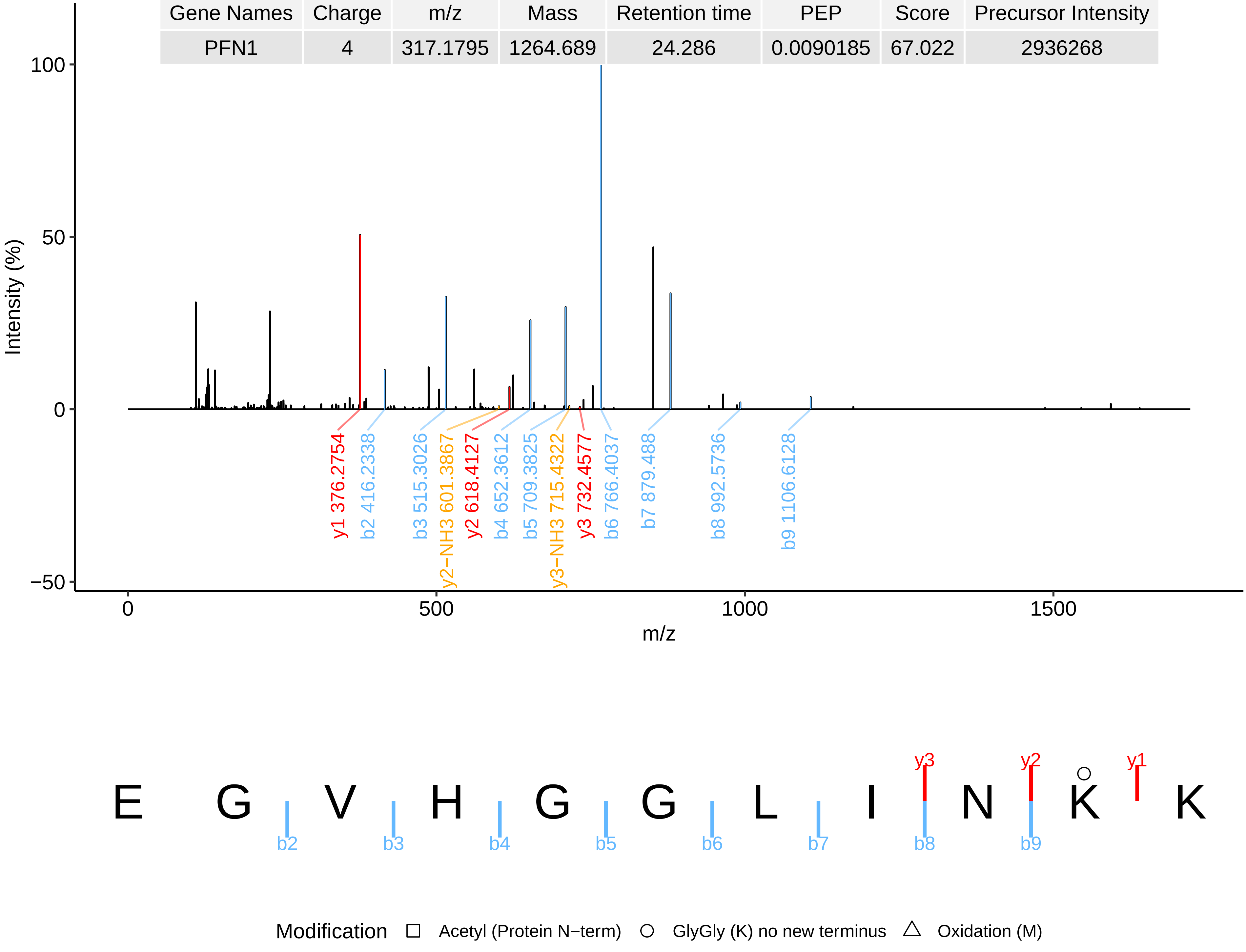

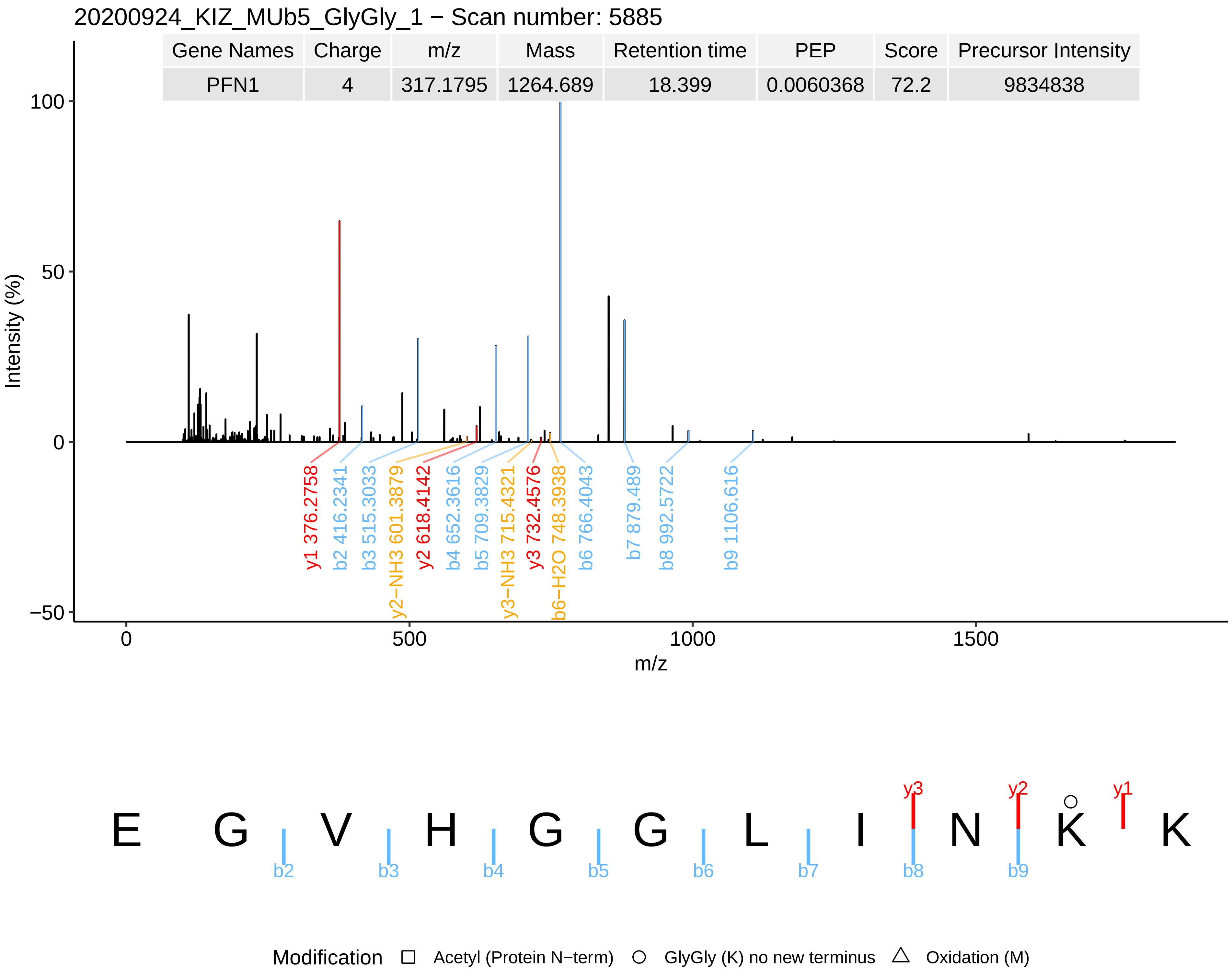

20200924_KIZ_MUb5_GlyGly_1 − Scan number: 5885

Gene Names	Charge	m/z	Mass	Retention time	PEP	Score	Precursor Intensity
PFN1	4	317.1795	1264.689	18.399	0.0060368	72.2	9834838

Intensity (%)
m/z
y1 376.2758
b2 416.2341
b3 515.3033
y2−NH3 601.3879
y2 618.4142
b4 652.3616
b5 709.3829
y3−NH3 715.4321
y3 732.4576
b6−H2O 748.3938
b6 766.4043
b7 879.489
b8 992.5722
b9 1106.616
E G V H G G L I N K K
Modification
Acetyl (Protein N−term)
GlyGly (K) no new terminus
Oxidation (M)

20200924_KIZ_MUb5_GlyGly_1 − Scan number: 5898

Gene Names	Charge	m/z	Mass	Retention time	PEP	Score	Precursor Intensity
PFN1	4	317.1795	1264.689	18.442	0.07869	44.188	5575234

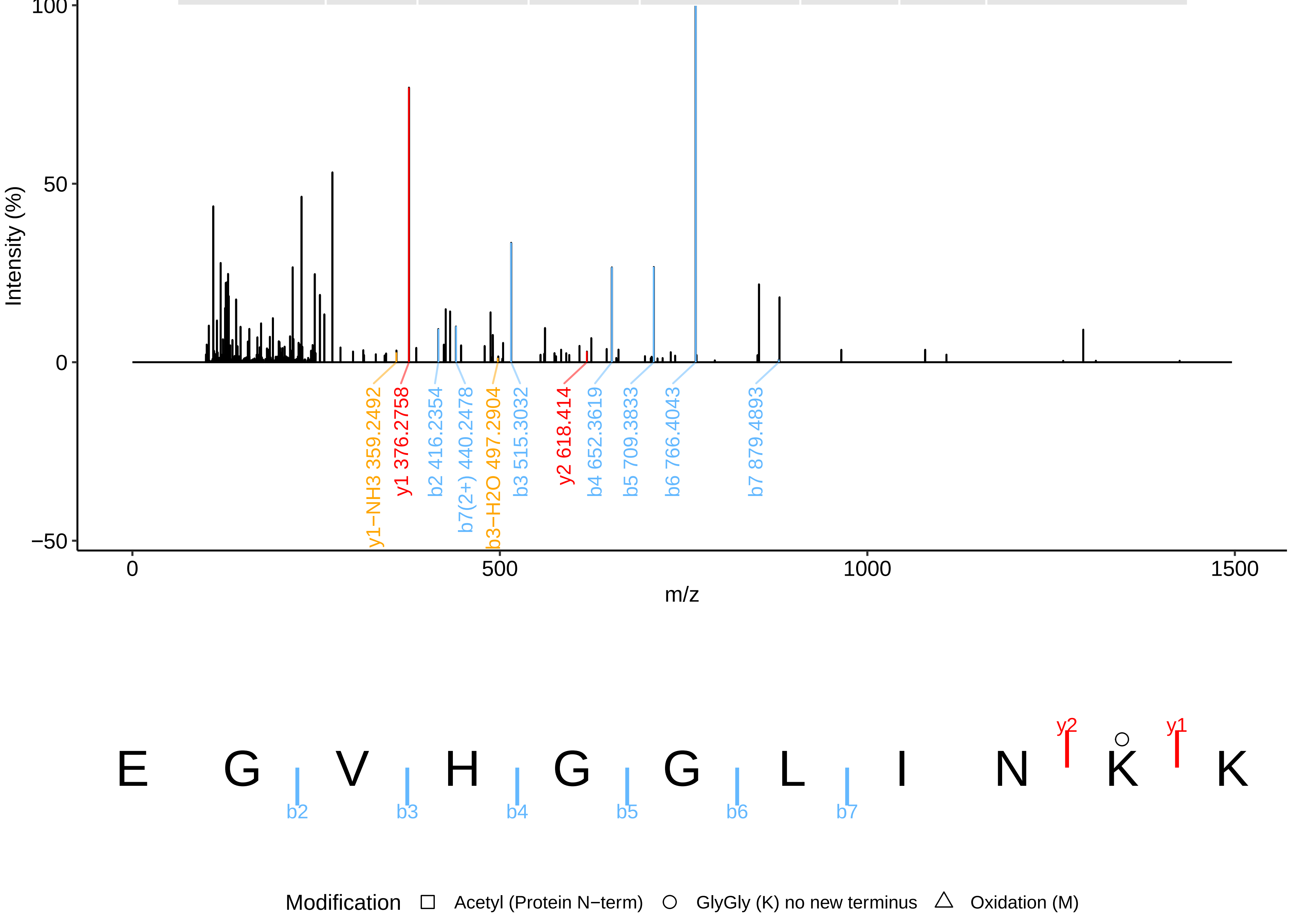

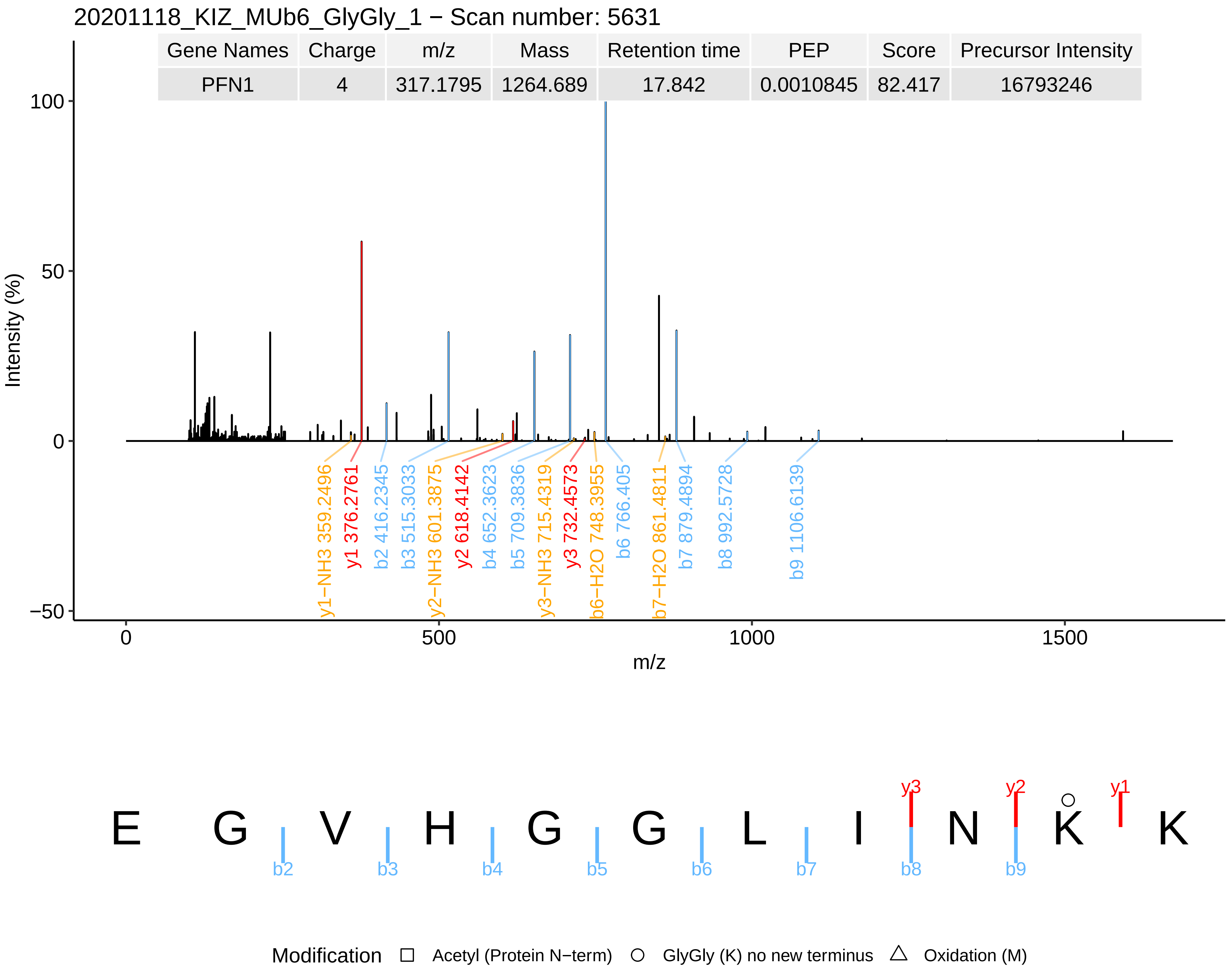

20201118_KIZ_MUb6_GlyGly_1 − Scan number: 5631
Gene Names | Charge | m/z | Mass | Retention time | PEP | Score | Precursor Intensity
PFN1 | 4 | 317.1795 | 1264.689 | 17.842 | 0.0010845 | 82.417 | 16793246
Intensity (%)
m/z
y1−NH3 359.2496
y1 376.2761
b2 416.2345
b3 515.3033
y2−NH3 601.3875
y2 618.4142
b4 652.3623
b5 709.3836
y3−NH3 715.4319
y3 732.4573
b6−H2O 748.3955
b6 766.405
b7−H2O 861.4811
b7 879.4894
b8 992.5728
b9 1106.6139
E G V H G G L I N K K
Modification
Acetyl (Protein N−term)
GlyGly (K) no new terminus
Oxidation (M)

20201118_KIZ_MUb6_GlyGly_2 − Scan number: 7712

Gene Names	Charge	m/z	Mass	Retention time	PEP	Score	Precursor Intensity
PFN1	4	317.1795	1264.689	26.326	0.0092644	66.595	6769652

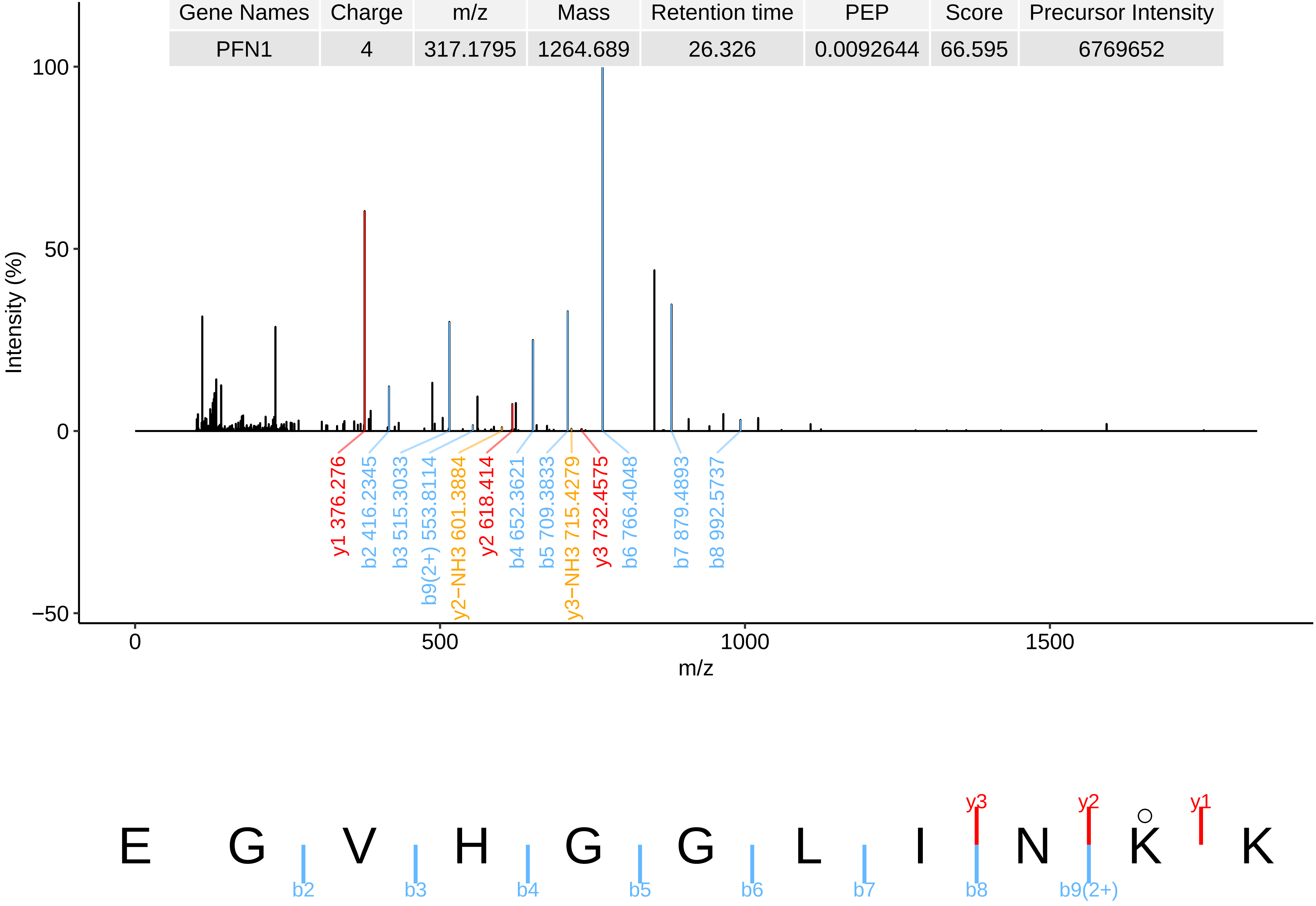

Modification □ Acetyl (Protein N−term) ○ GlyGly (K) no new terminus △ Oxidation (M)

20200921_KIZ_MUb4_GlyGly_10plex − Scan number: 10719

Gene Names	Charge	m/z	Mass	Retention time	PEP	Score	Precursor Intensity
SCAMP1	4	373.9612	1491.816	37.178	0.0013644	79.492	5976165

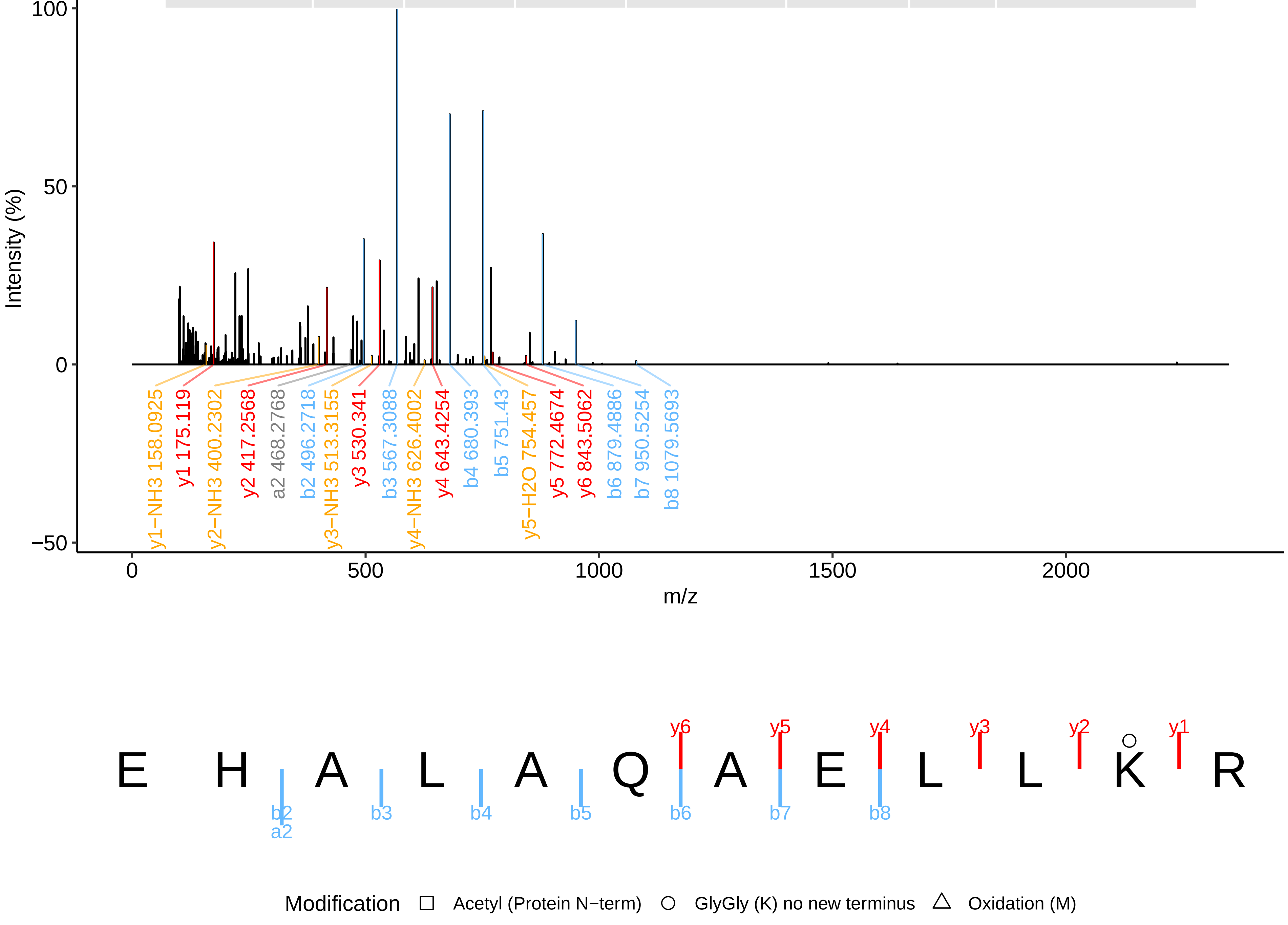

20200921_KIZ_MUb4_GlyGly_10plex − Scan number: 10735

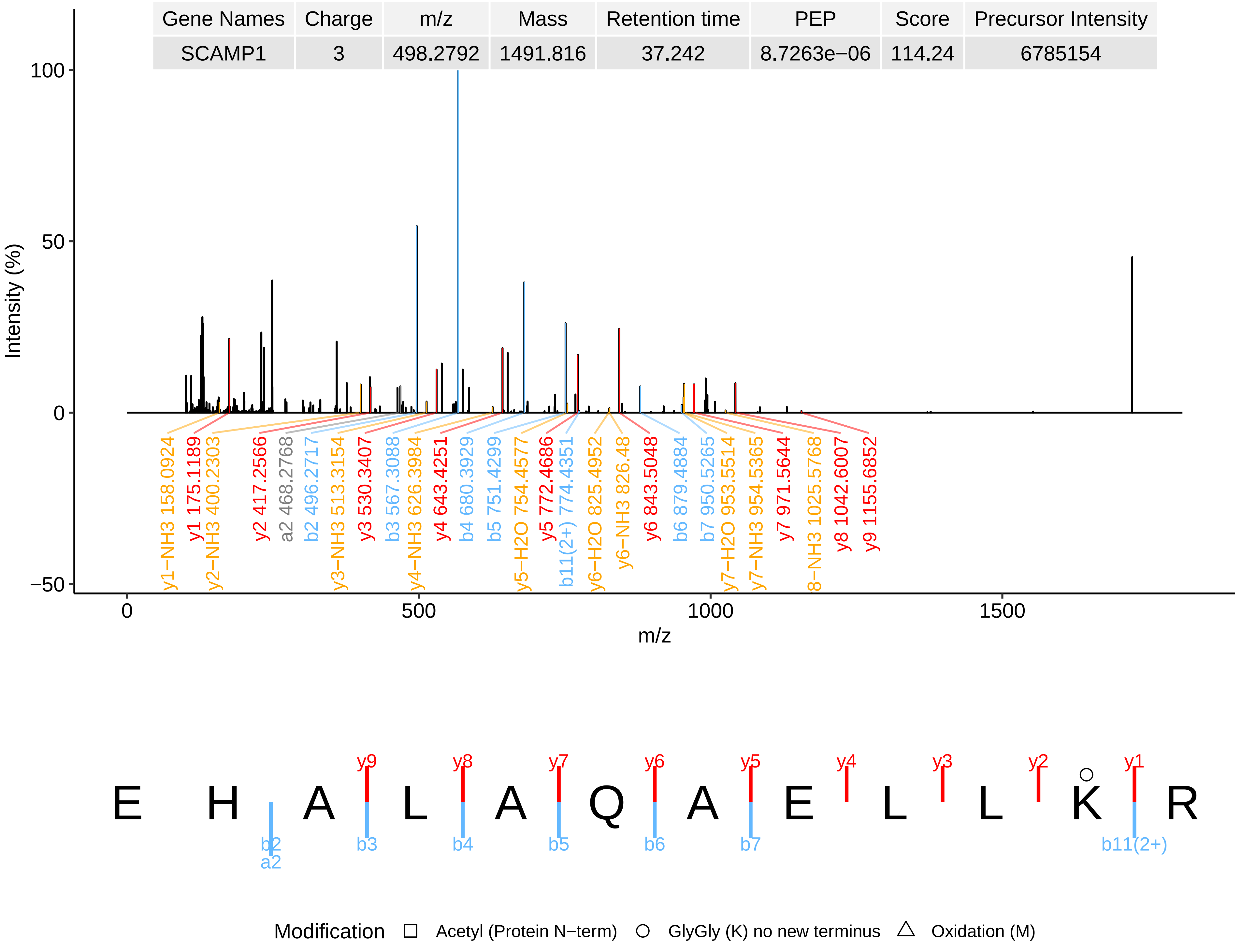

20200921_KIZ_MUb4_GlyGly_10plex_2 − Scan number: 9977

Gene Names	Charge	m/z	Mass	Retention time	PEP	Score	Precursor Intensity
SCAMP1	3	498.2792	1491.816	38.049	2.5571e−09	132.32	1290353

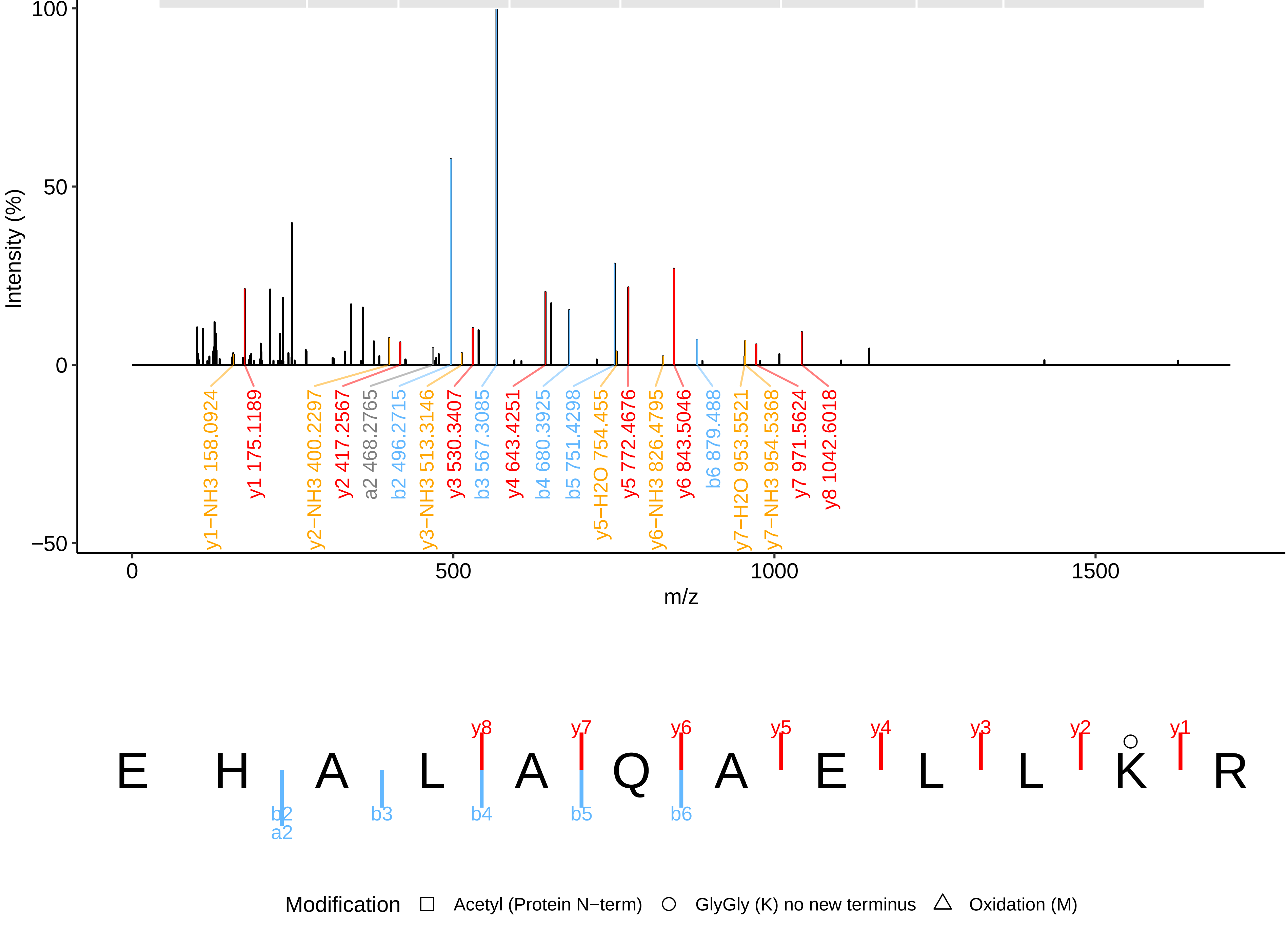

20200921_KIZ_MUb4_GlyGly_10plex_2 − Scan number: 9988

Gene Names	Charge	m/z	Mass	Retention time	PEP	Score	Precursor Intensity
SCAMP1	4	373.9612	1491.816	38.09	0.00029135	92.247	5077422

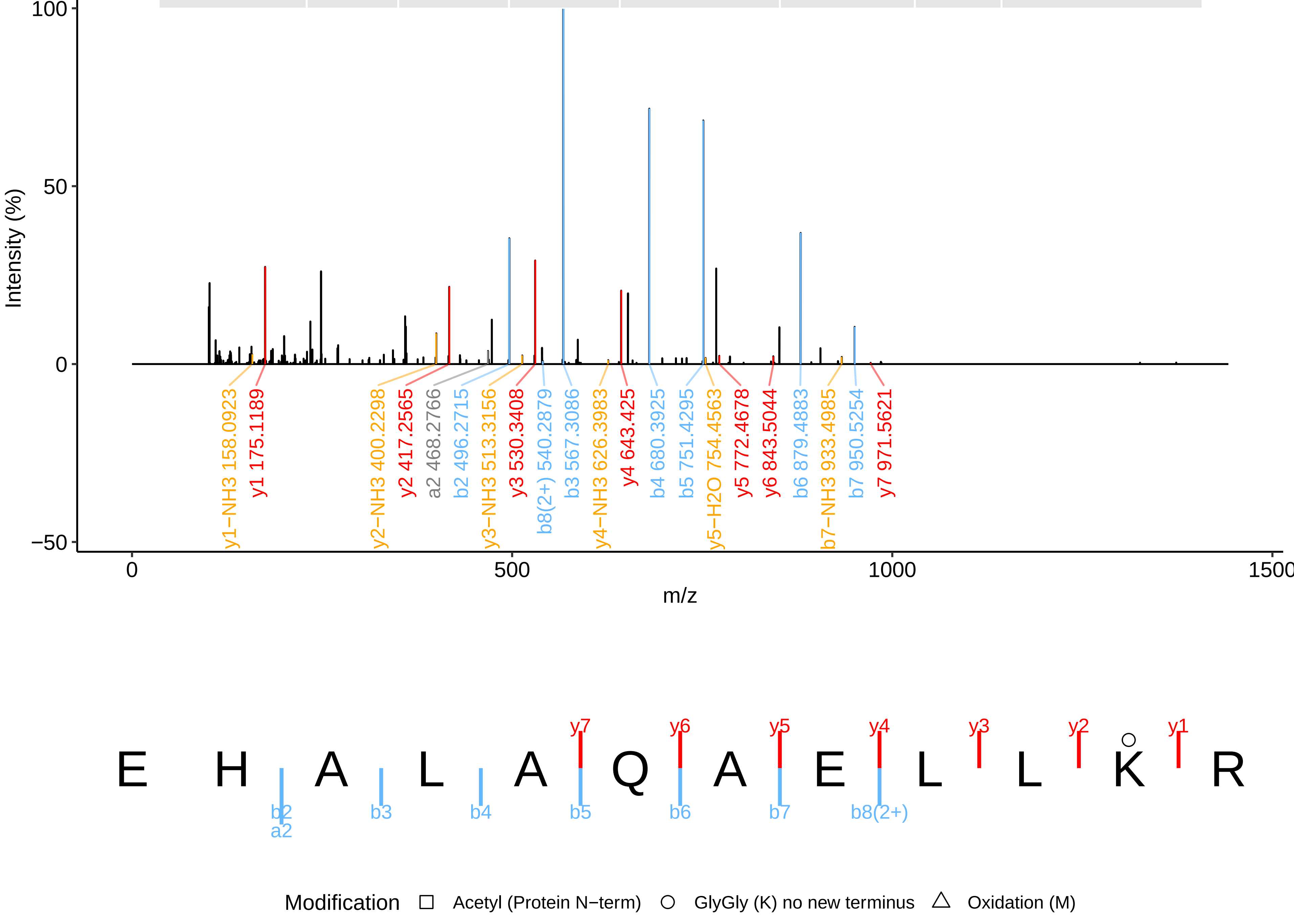

20200921_KIZ_MUb4_GlyGly_10plex − Scan number: 7411

Gene Names	Charge	m/z	Mass	Retention time	PEP	Score	Precursor Intensity
IMMT	3	420.8859	1259.636	24.866	0.0084515	74.841	5405023

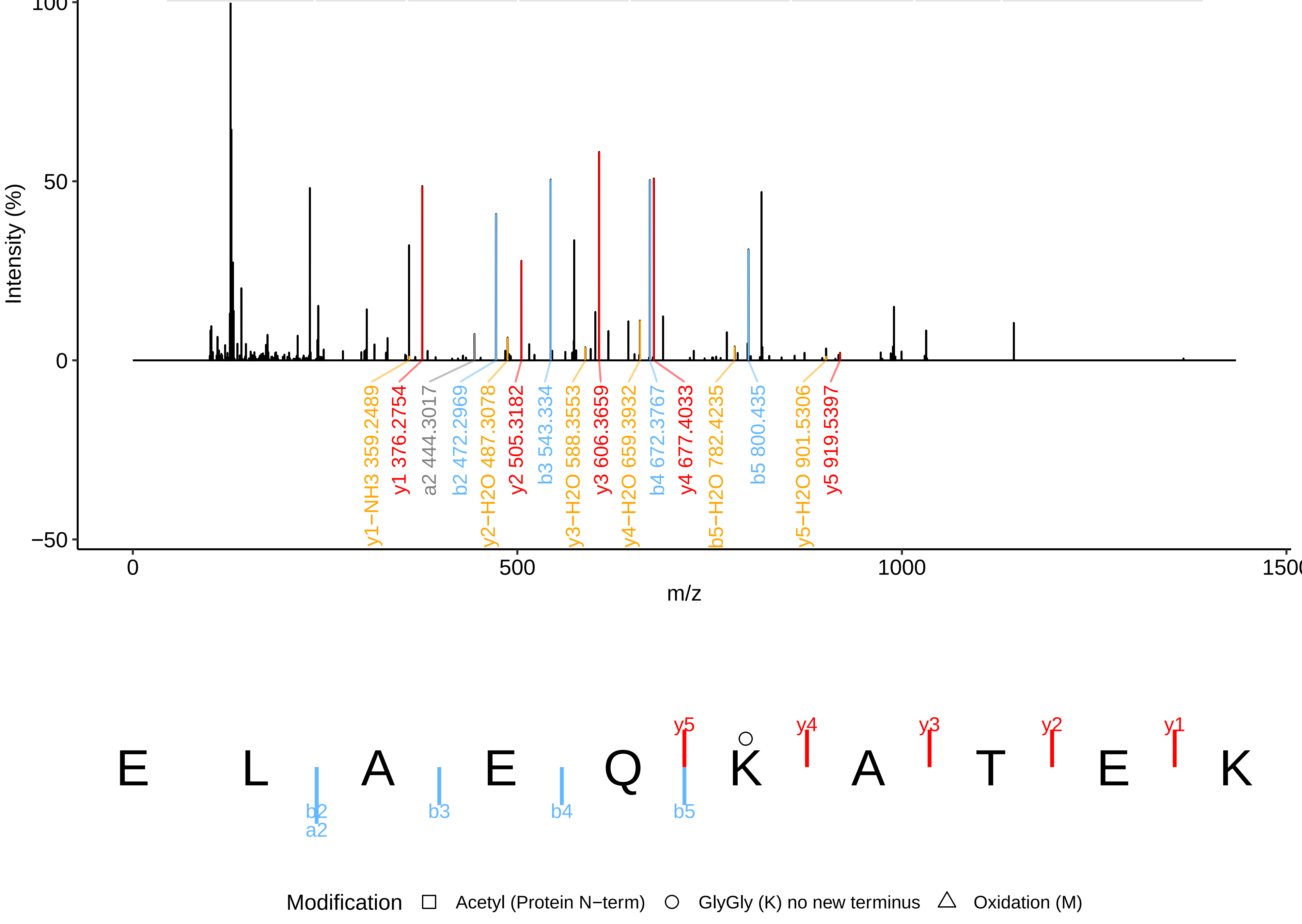

20200921_KIZ_MUb4_GlyGly_10plex_2 − Scan number: 6840

Gene Names	Charge	m/z	Mass	Retention time	PEP	Score	Precursor Intensity
IMMT	3	420.8859	1259.636	25.311	0.015727	67.704	634229.5

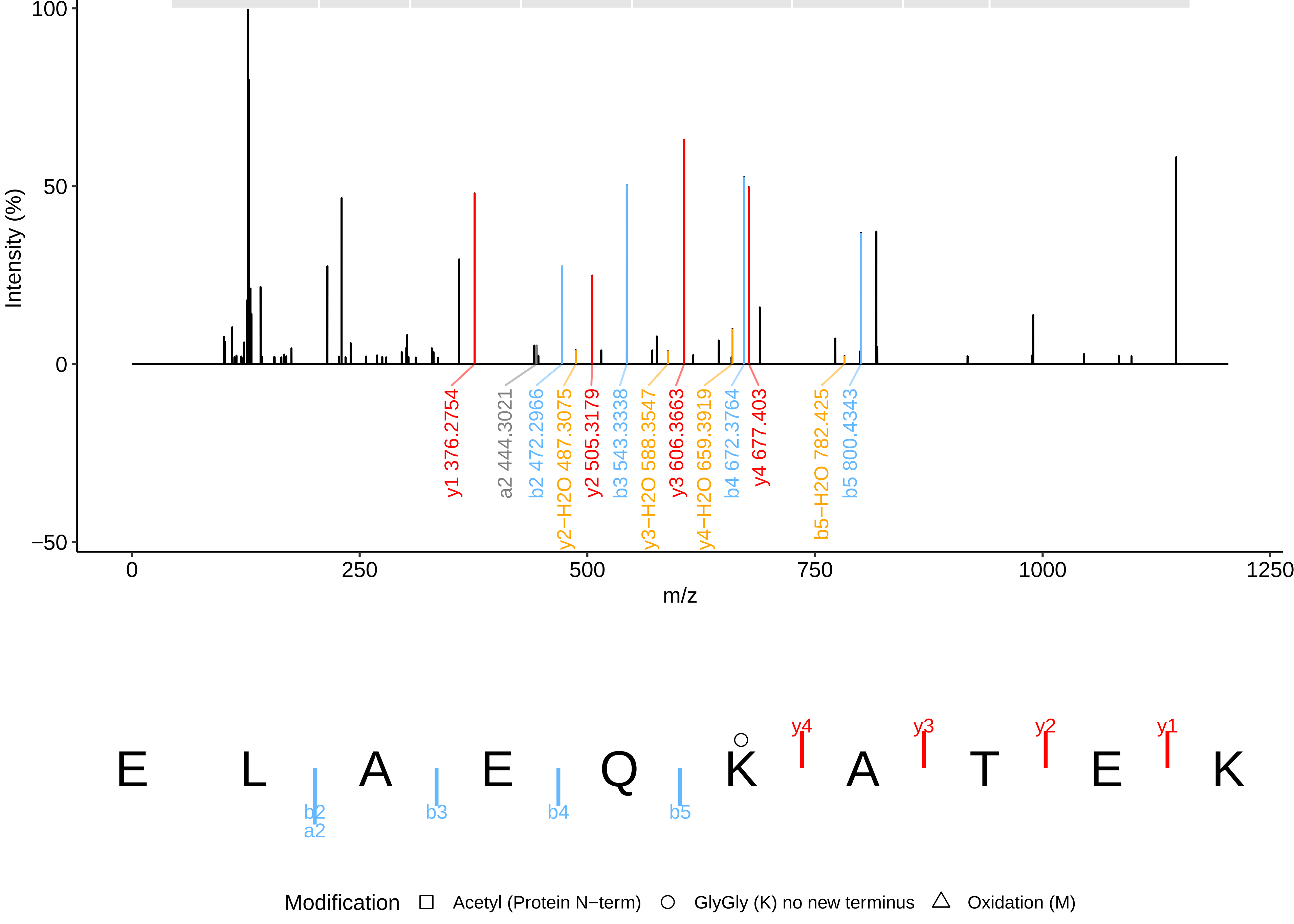

20200924_KIZ_MUb5_GlyGly_1 − Scan number: 6461

Gene Names	Charge	m/z	Mass	Retention time	PEP	Score	Precursor Intensity
IMMT	3	420.8859	1259.636	20.321	0.0087652	74.533	5665542

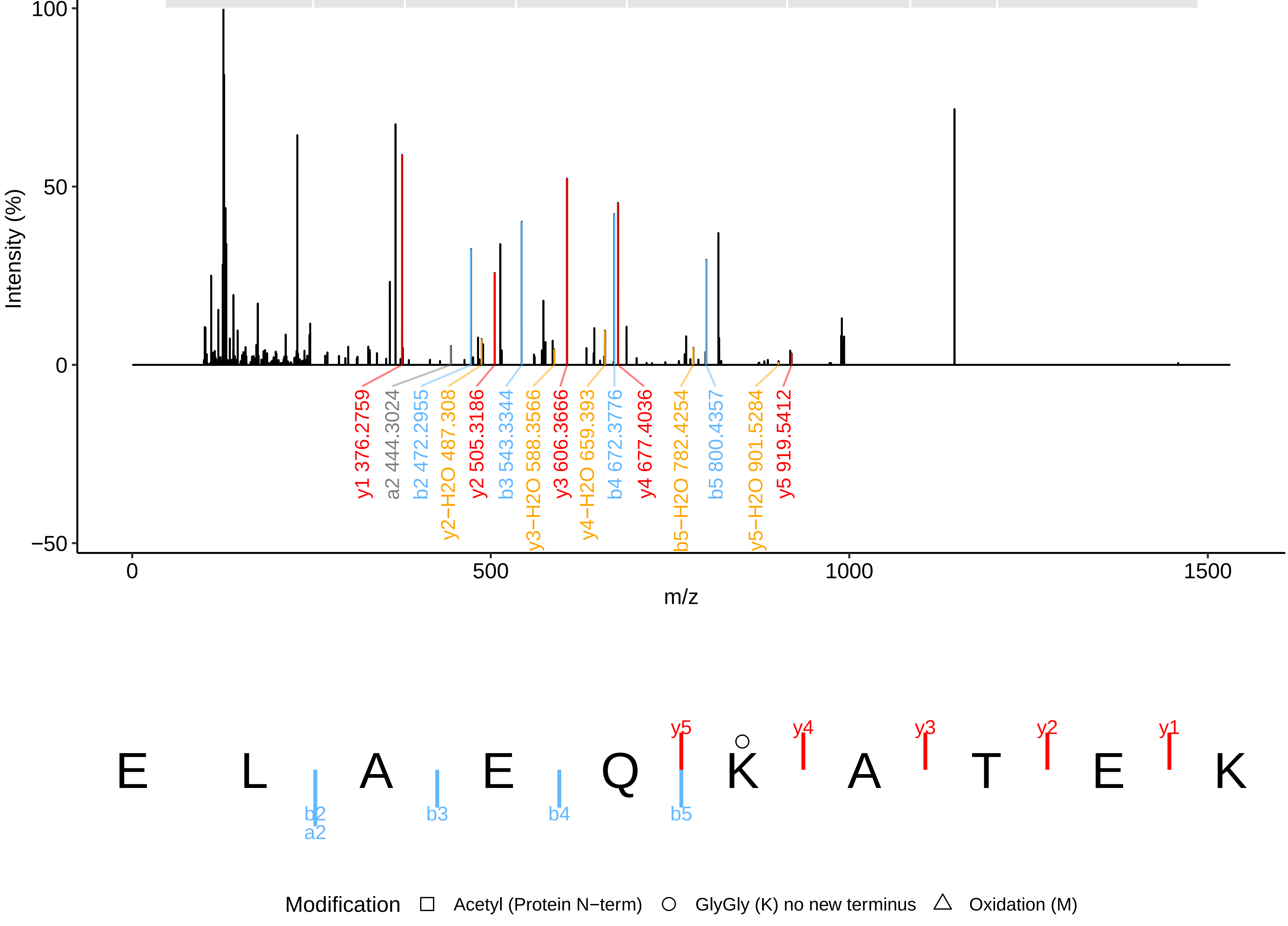

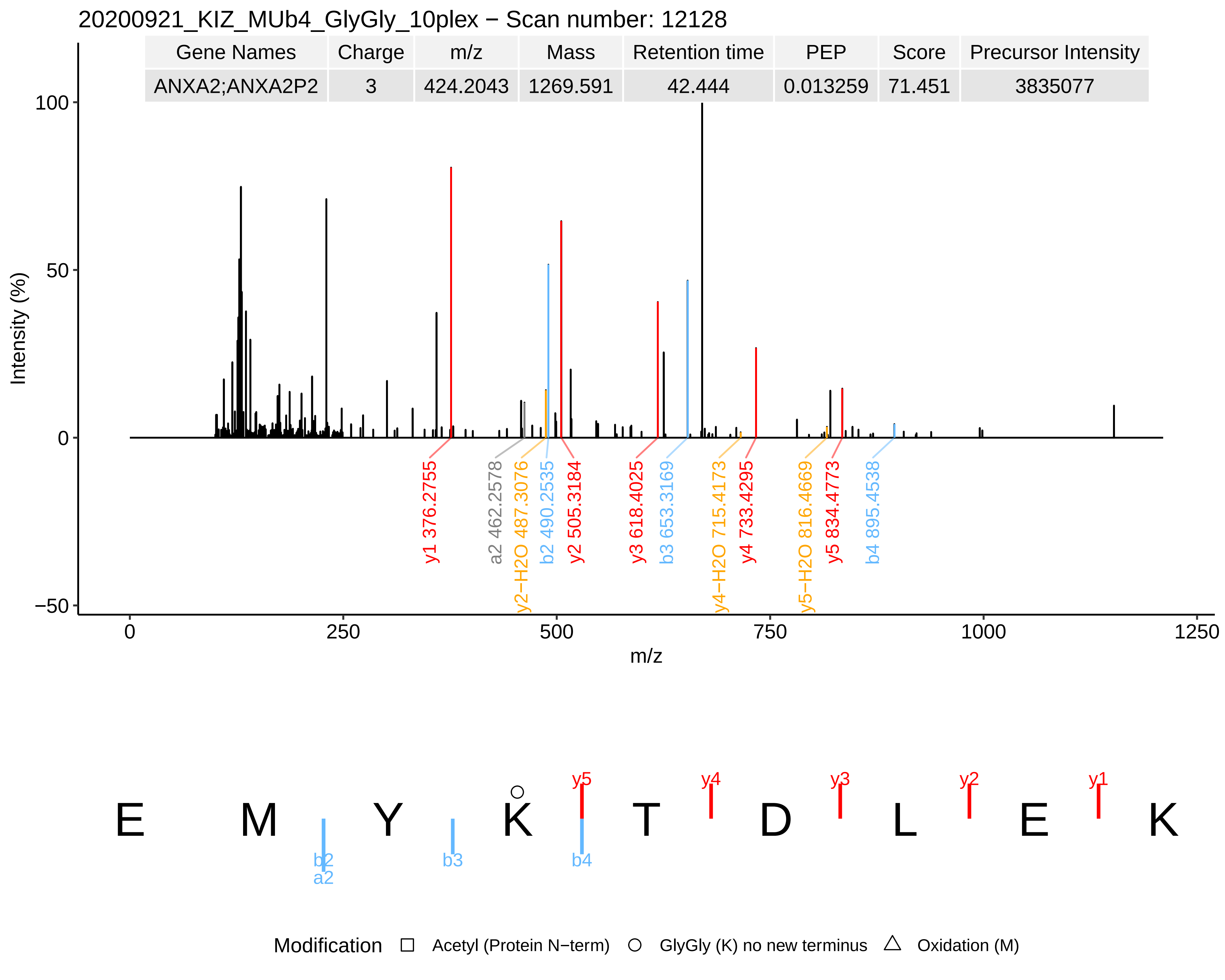
20200921_KIZ_MUb4_GlyGly_10plex − Scan number: 12128
Gene Names | Charge | m/z | Mass | Retention time | PEP | Score | Precursor Intensity
ANXA2;ANXA2P2 | 3 | 424.2043 | 1269.591 | 42.444 | 0.013259 | 71.451 | 3835077
Intensity (%)
m/z
y1 376.2755
a2 462.2578
y2−H2O 487.3076
b2 490.2535
y2 505.3184
y3 618.4025
b3 653.3169
y4−H2O 715.4173
y4 733.4295
y5−H2O 816.4669
y5 834.4773
b4 895.4538
E M Y K T D L E K
y5 y4 y3 y2 y1
b2 a2 b3 b4
Modification
Acetyl (Protein N−term)
GlyGly (K) no new terminus
Oxidation (M)

20200921_KIZ_MUb4_GlyGly_10plex − Scan number: 9107

Gene Names	Charge	m/z	Mass	Retention time	PEP	Score	Precursor Intensity
APOO	3	425.5544	1273.641	31.141	0.020252	65.719	4101424

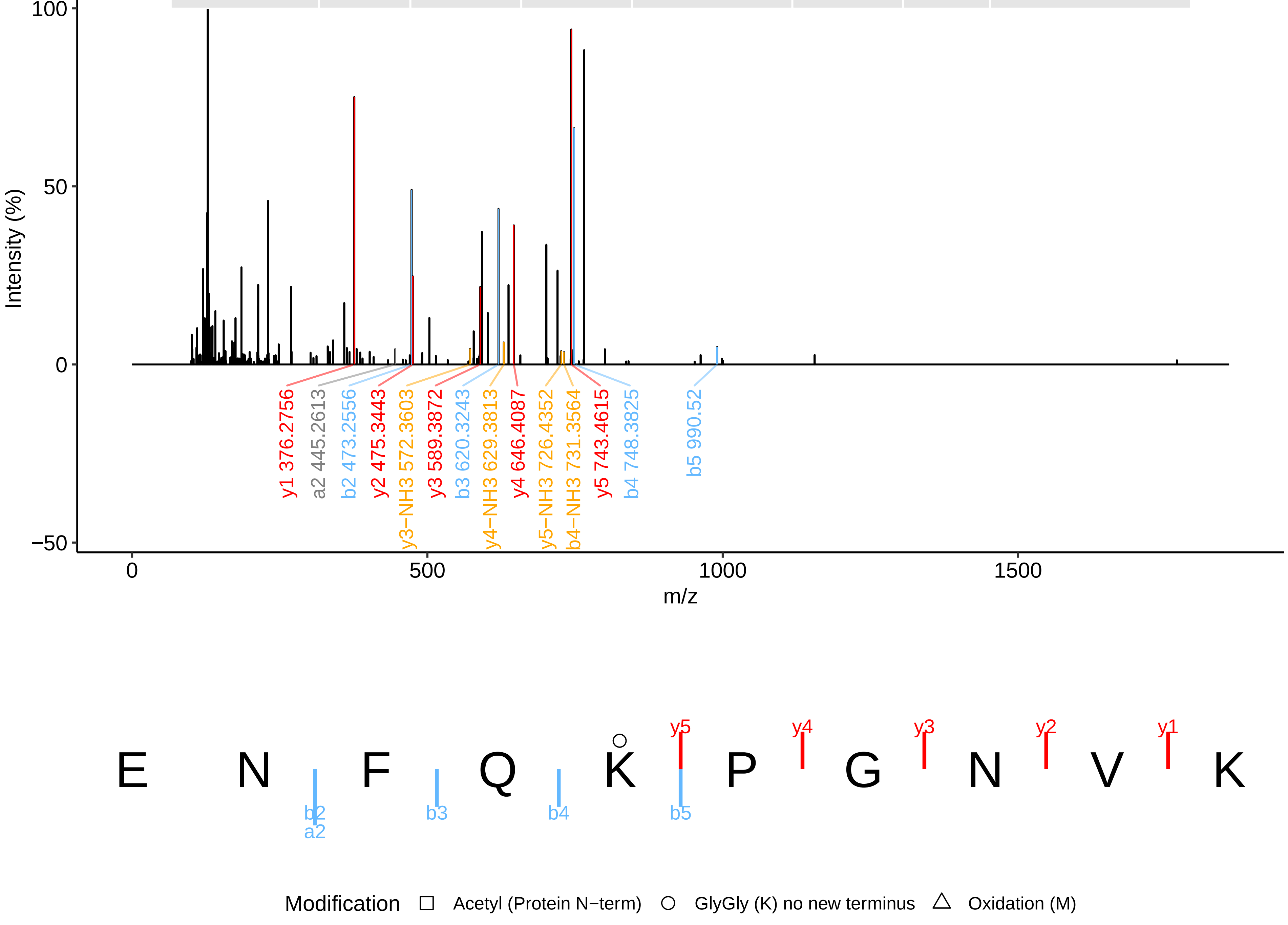

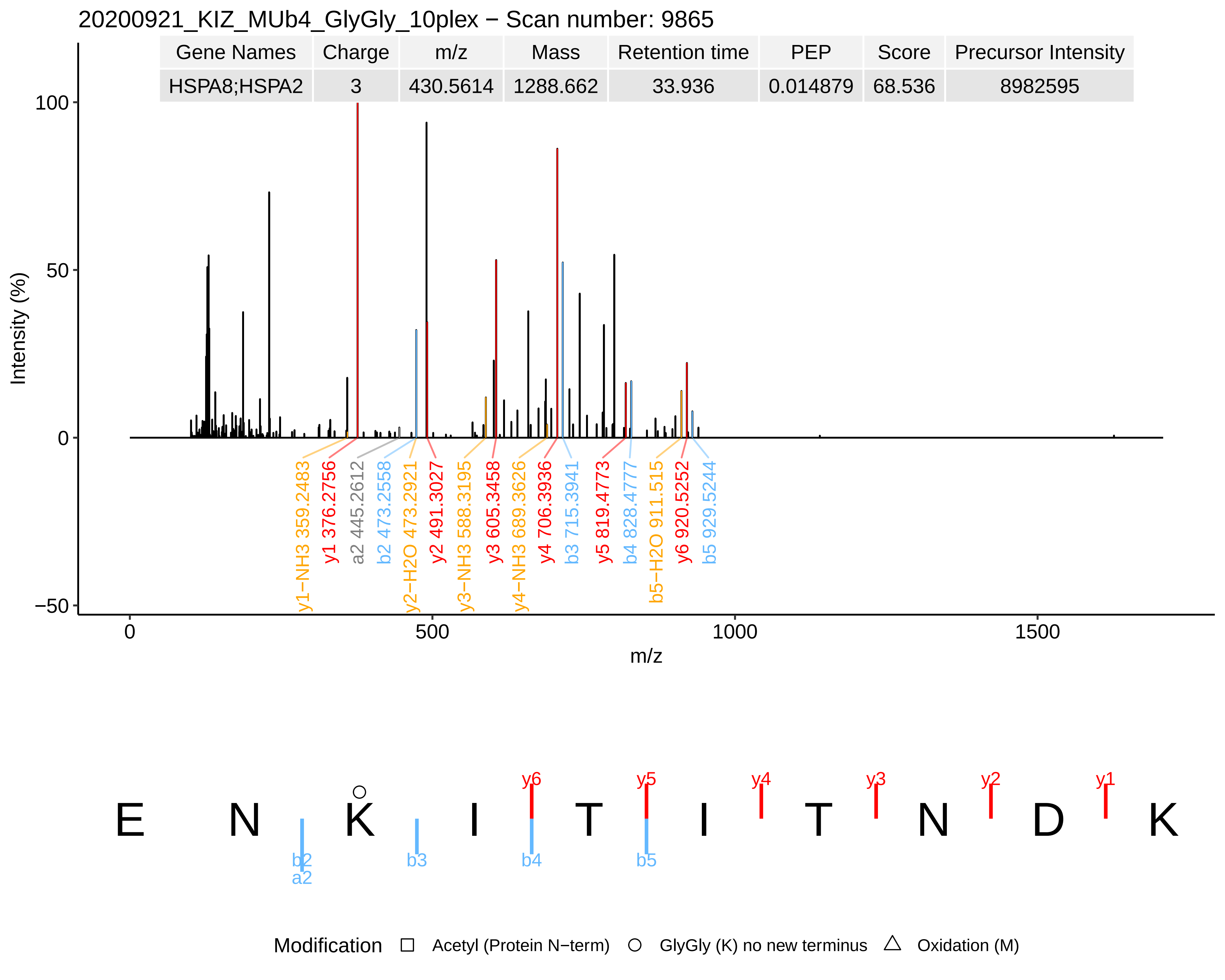
20200921_KIZ_MUb4_GlyGly_10plex − Scan number: 9865
Gene Names
Charge
m/z
Mass
Retention time
PEP
Score
Precursor Intensity
HSPA8;HSPA2
3
430.5614
1288.662
33.936
0.014879
68.536
8982595
Intensity (%)
100
50
0
−50
0
500
1000
1500
m/z
y1−NH3 359.2483
y1 376.2756
a2 445.2612
b2 473.2558
y2−H2O 473.2921
y2 491.3027
y3−NH3 588.3195
y3 605.3458
y4−NH3 689.3626
y4 706.3936
b3 715.3941
y5 819.4773
b4 828.4777
b5−H2O 911.515
y6 920.5252
b5 929.5244
E N K I T I T N D K
y6 y5 y4 y3 y2 y1
b2 a2 b3 b4 b5
Modification
Acetyl (Protein N−term)
GlyGly (K) no new terminus
Oxidation (M)

20200921_KIZ_MUb4_GlyGly_10plex_2 − Scan number: 9069

Gene Names	Charge	m/z	Mass	Retention time	PEP	Score	Precursor Intensity
HSPA8;HSPA2	3	430.5614	1288.662	34.371	0.011801	71.555	2527830

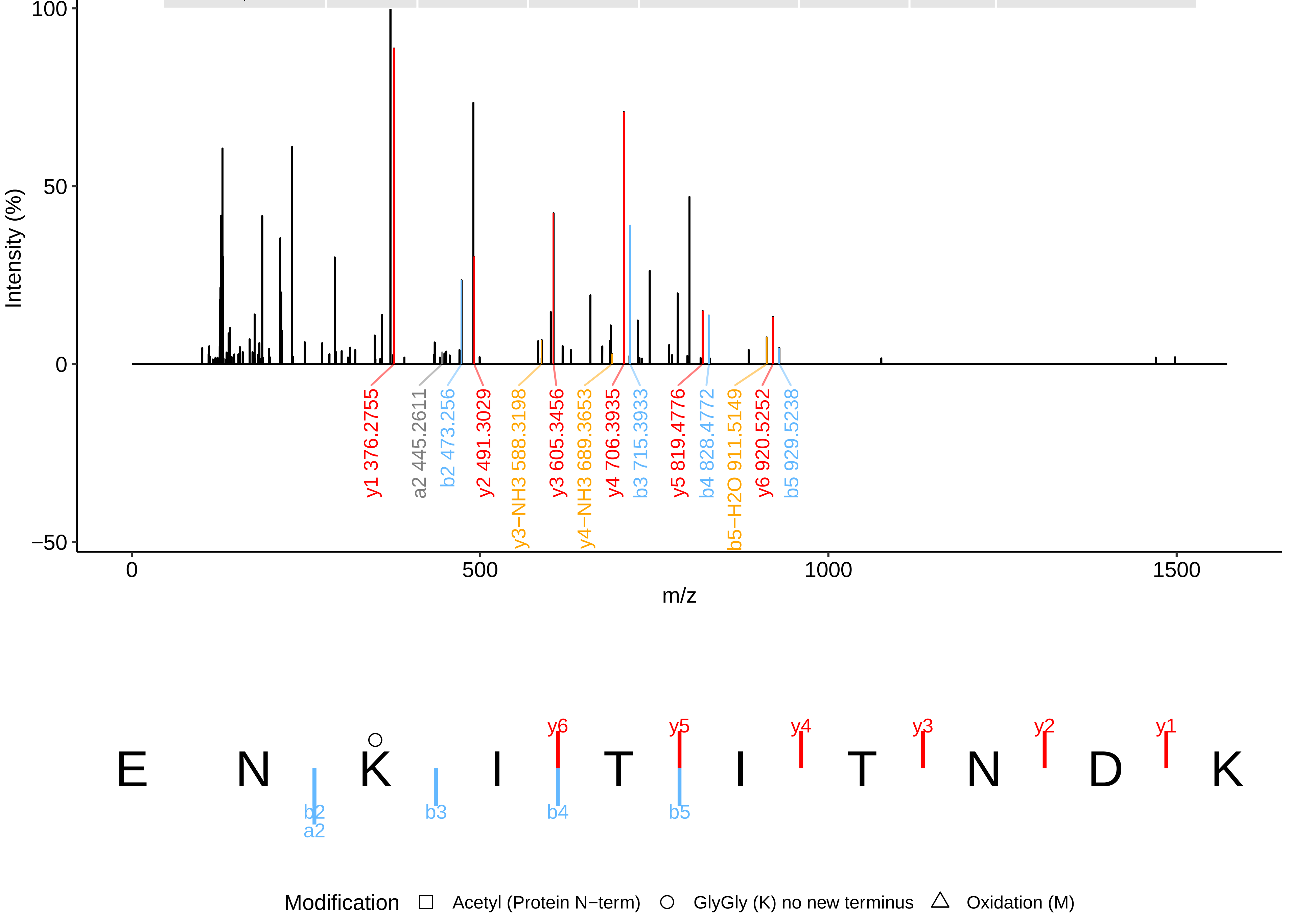

20200921_KIZ_MUb4_GlyGly_10plex − Scan number: 5412

Gene Names	Charge	m/z	Mass	Retention time	PEP	Score	Precursor Intensity
FDPS	3	437.2055	1308.595	17.726	0.012427	70.942	5312423

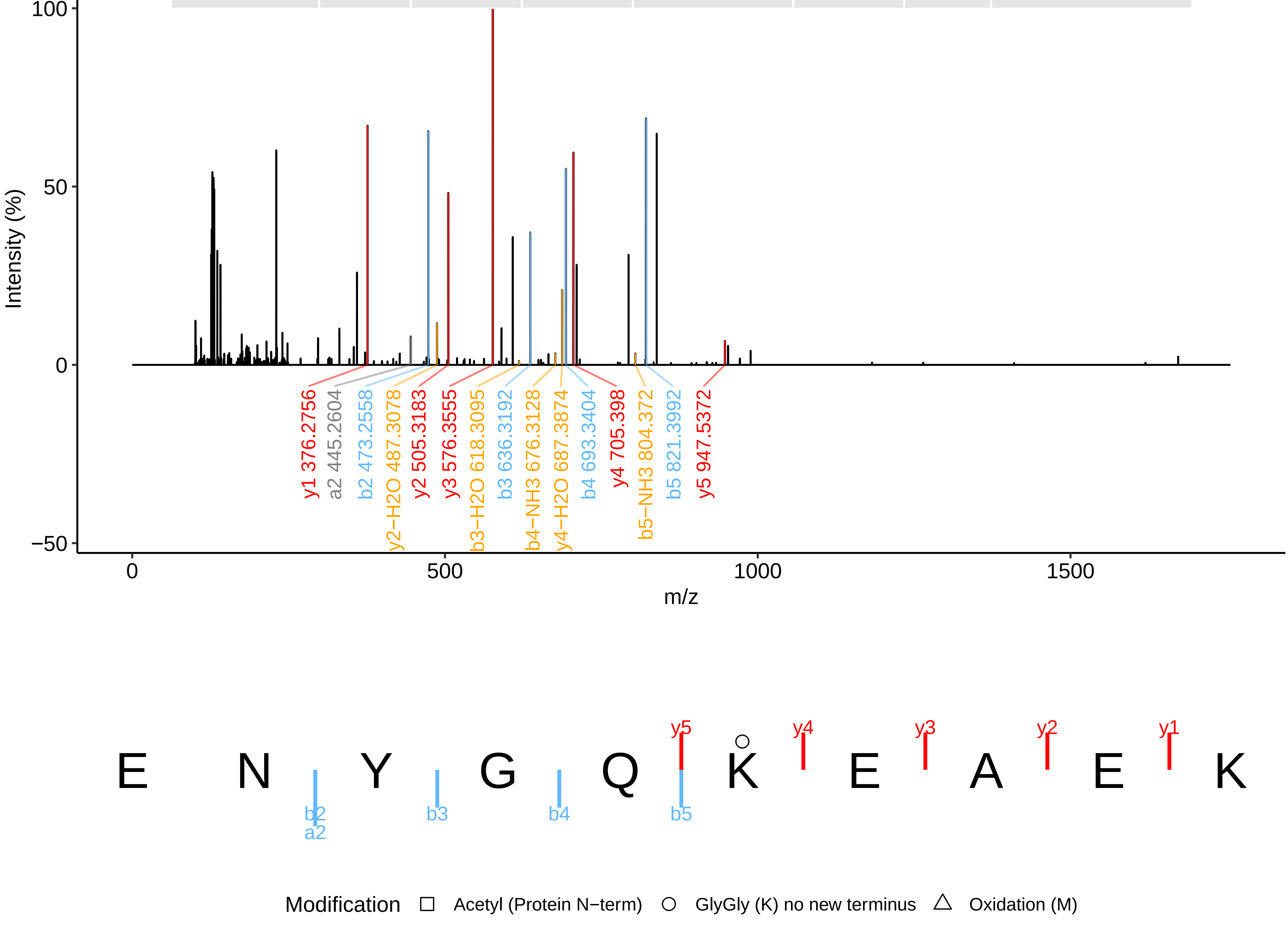

20200921_KIZ_MUb4_GlyGly_10plex_2 − Scan number: 4975

Gene Names	Charge	m/z	Mass	Retention time	PEP	Score	Precursor Intensity
FDPS	3	437.2055	1308.595	17.676	0.019885	63.624	1164180

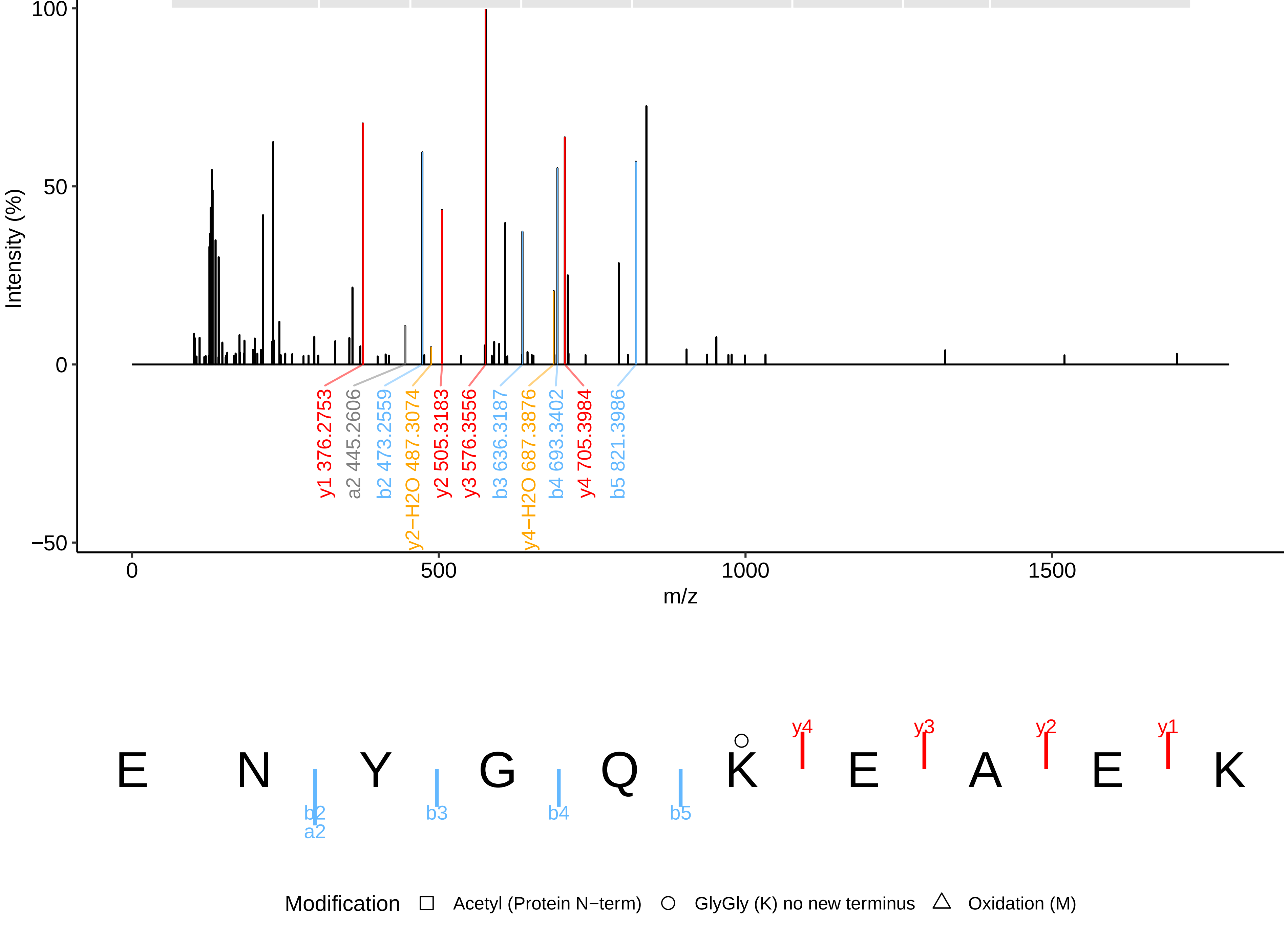

20200924_KIZ_MUb5_GlyGly_1 − Scan number: 4692

Gene Names	Charge	m/z	Mass	Retention time	PEP	Score	Precursor Intensity
FDPS	3	437.2055	1308.595	14.521	0.022789	63.408	NA

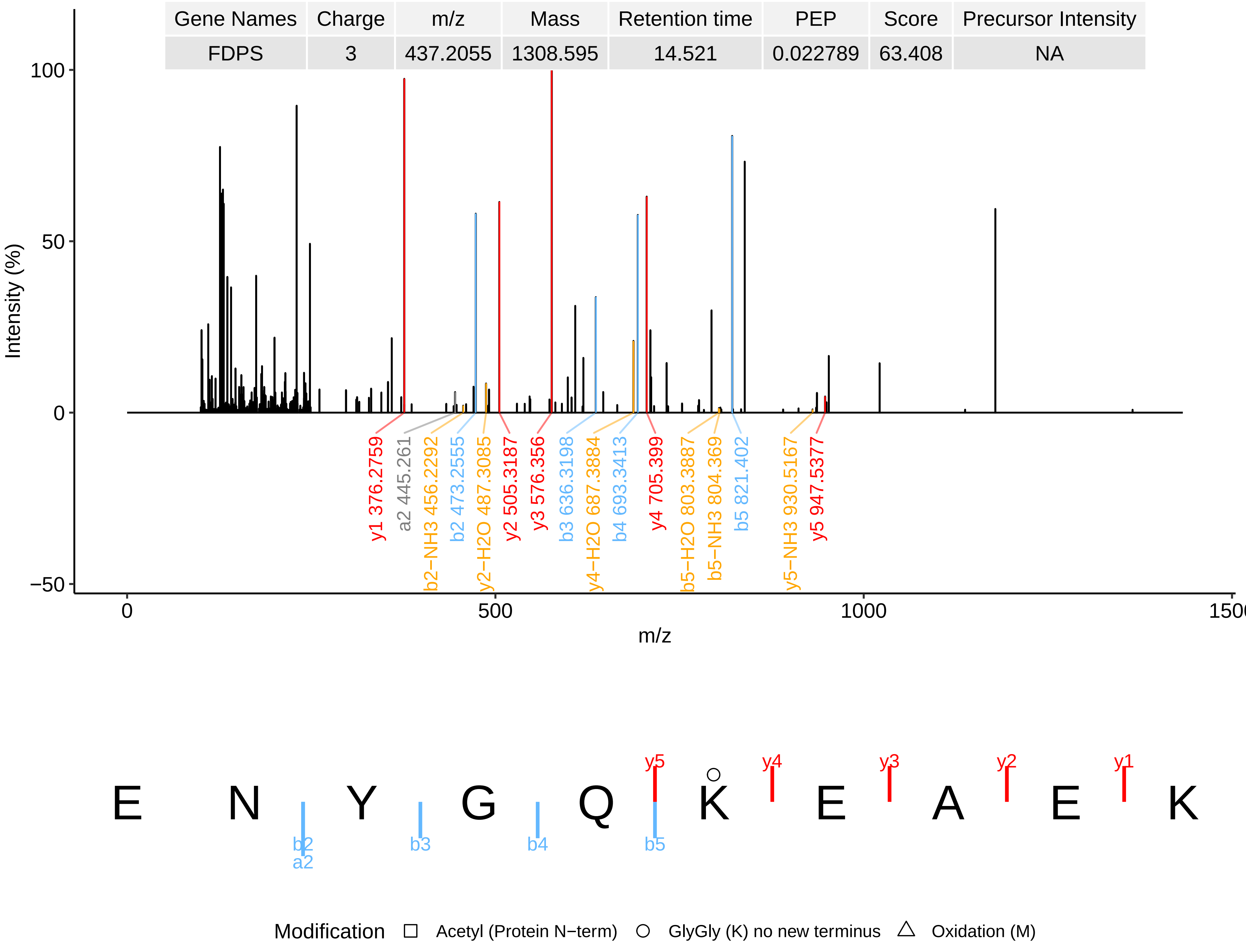

20200921_KIZ_MUb4_GlyGly_10plex − Scan number: 5036

Gene Names	Charge	m/z	Mass	Retention time	PEP	Score	Precursor Intensity
SPRTN	3	439.5529	1315.637	16.43	0.0013275	84.718	5763237

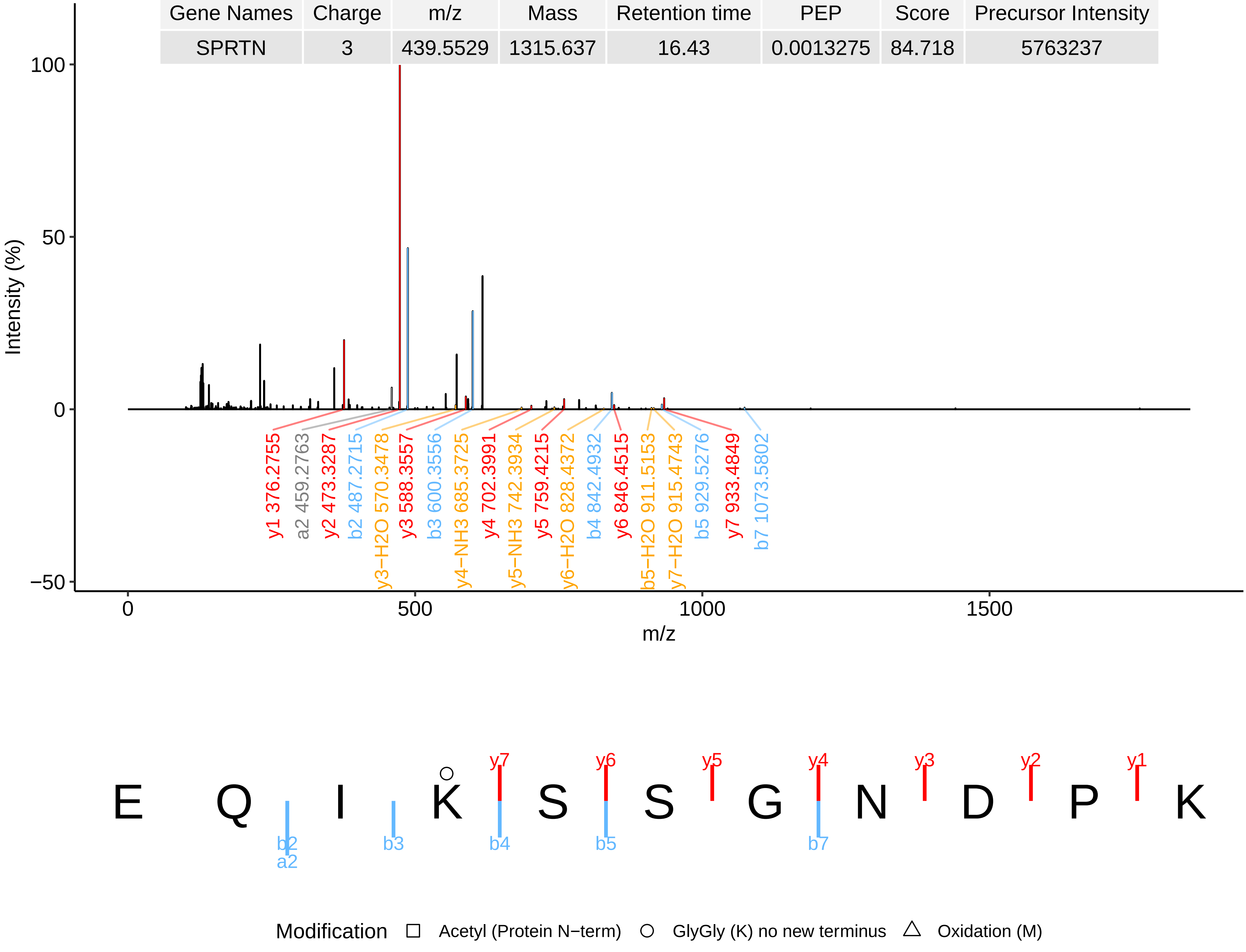

20200921_KIZ_MUb4_GlyGly_10plex_2 − Scan number: 4649

Gene Names	Charge	m/z	Mass	Retention time	PEP	Score	Precursor Intensity
SPRTN	3	439.5529	1315.637	16.39	0.067261	53.252	2141926

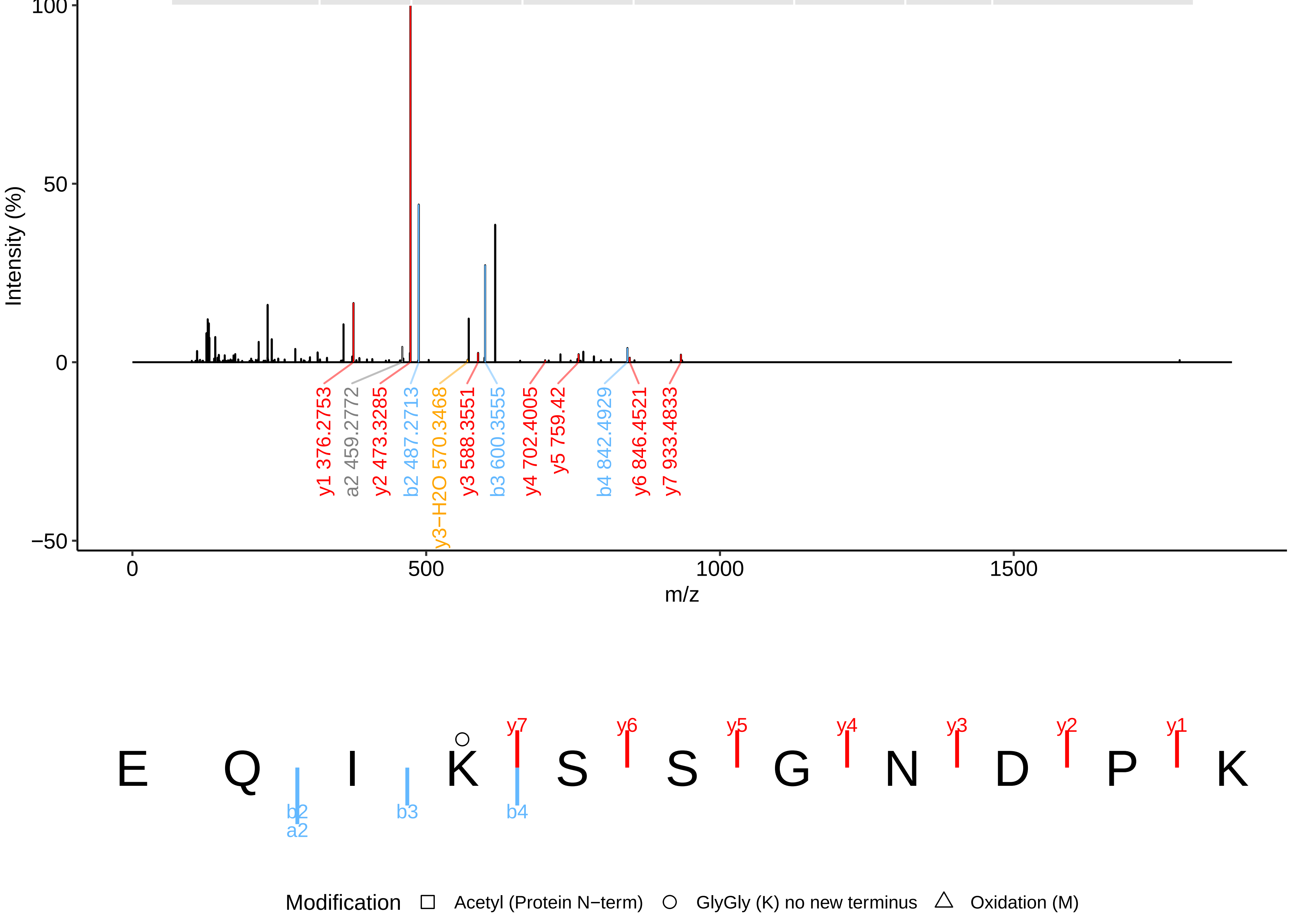

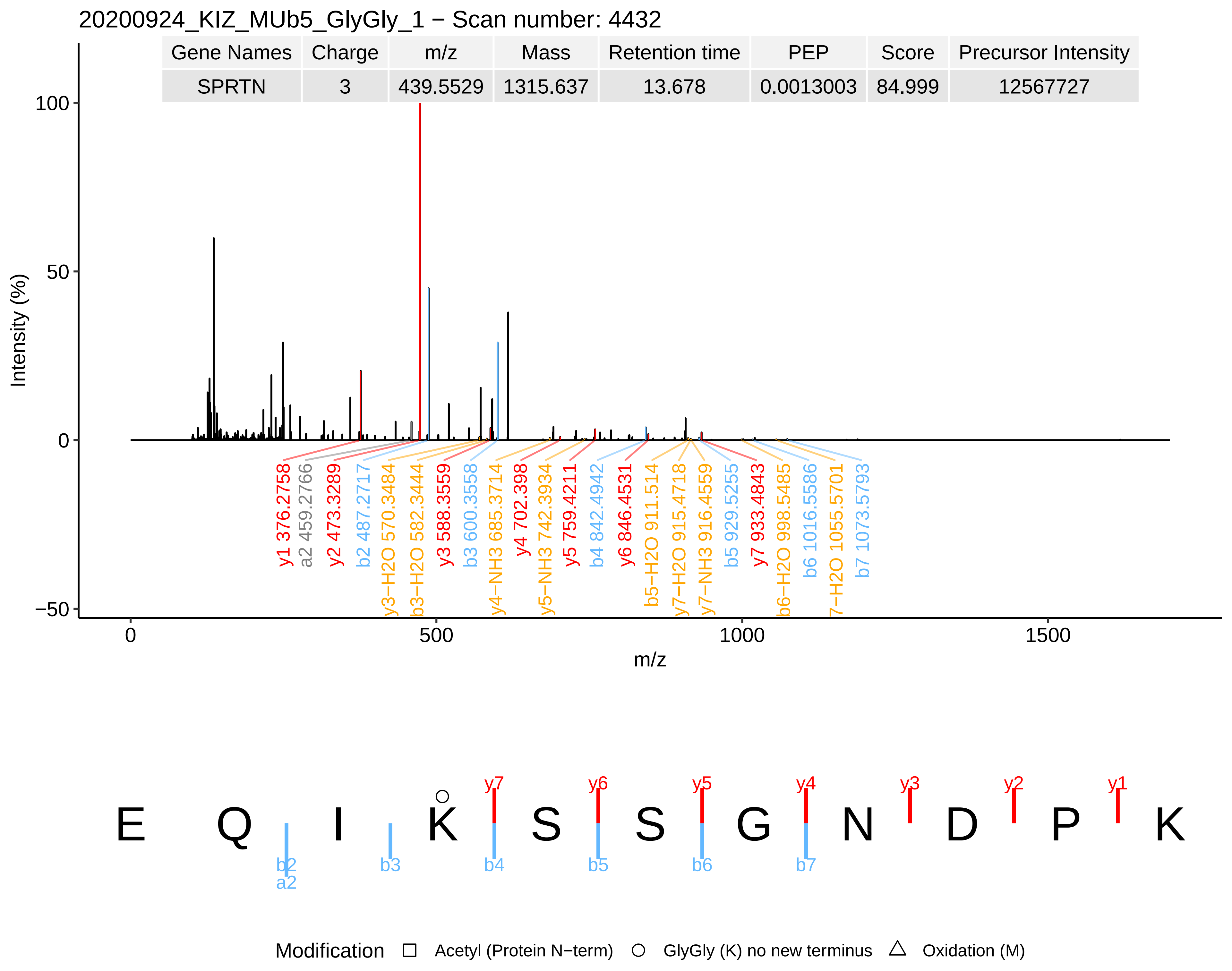
20200924_KIZ_MUb5_GlyGly_1 − Scan number: 4432
Gene Names | Charge | m/z | Mass | Retention time | PEP | Score | Precursor Intensity
SPRTN | 3 | 439.5529 | 1315.637 | 13.678 | 0.0013003 | 84.999 | 12567727
Intensity (%)
100
50
0
−50
0
500
1000
1500
m/z
y1 376.2758
a2 459.2766
y2 473.3289
b2 487.2717
y3−H2O 570.3484
b3−H2O 582.3444
y3 588.3559
b3 600.3558
y4−NH3 685.3714
y4 702.398
y5−NH3 742.3934
y5 759.4211
b4 842.4942
y6 846.4531
b5−H2O 911.514
y7−H2O 915.4718
y7−NH3 916.4559
b5 929.5255
y7 933.4843
b6−H2O 998.5485
b6 1016.5586
7−H2O 1055.5701
b7 1073.5793
E Q I K S S G N D P K
y7 y6 y5 y4 y3 y2 y1
b2 a2 b3 b4 b5 b6 b7
Modification
Acetyl (Protein N−term)
GlyGly (K) no new terminus
Oxidation (M)

20200924_KIZ_MUb5_GlyGly_2 − Scan number: 4079

Gene Names	Charge	m/z	Mass	Retention time	PEP	Score	Precursor Intensity
SPRTN	3	439.5529	1315.637	14.921	0.013318	66.498	2071873

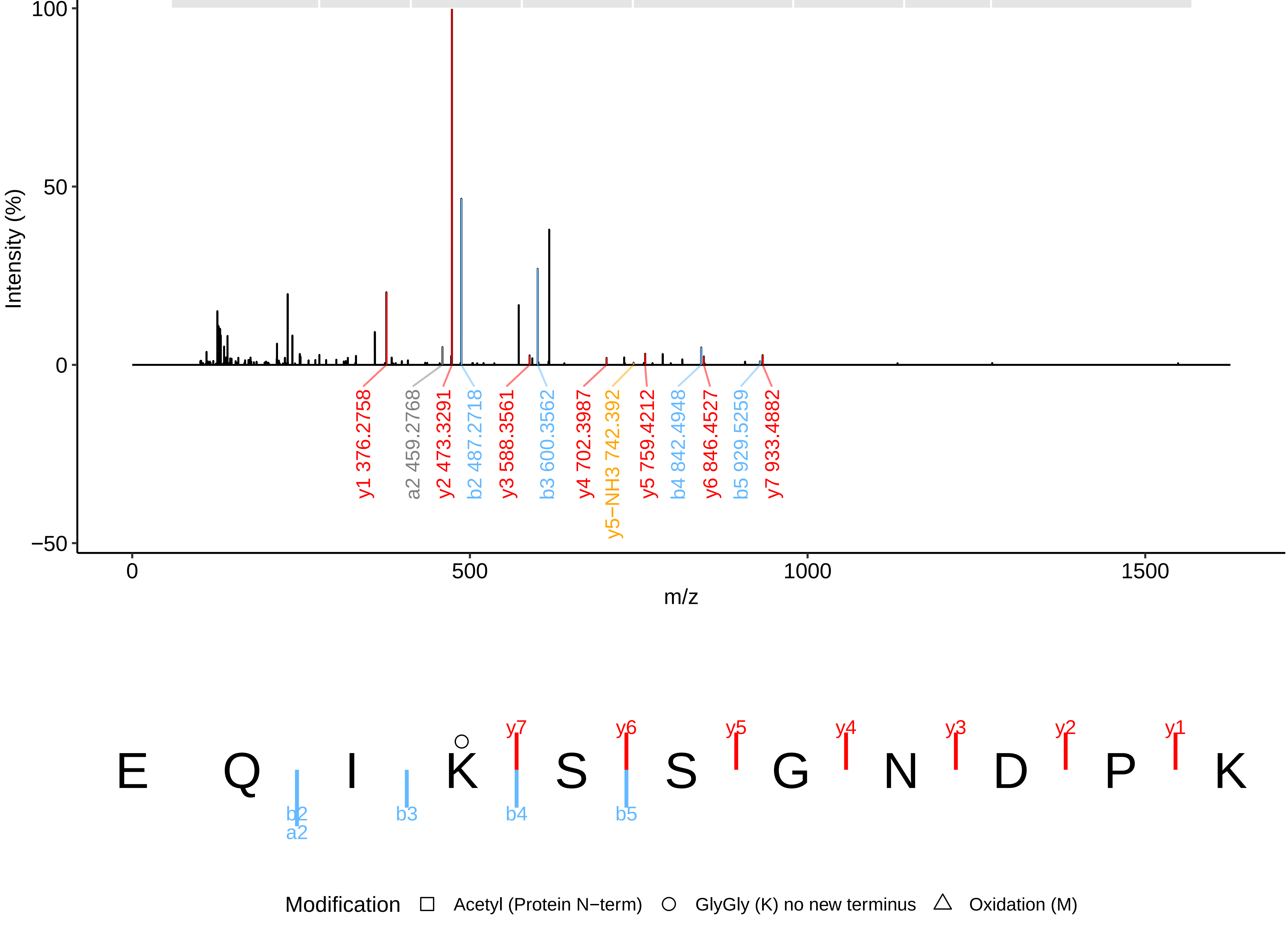

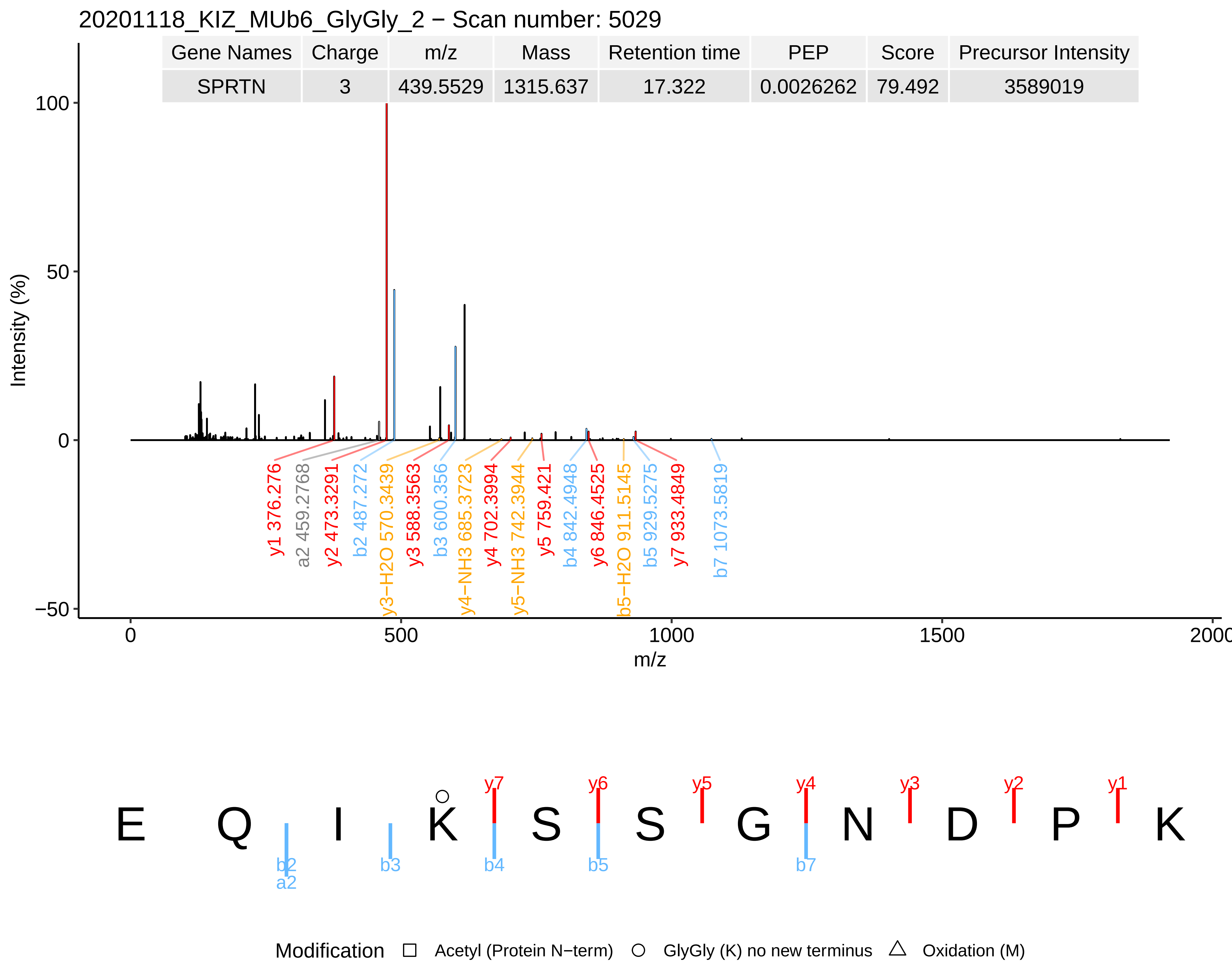

20201118_KIZ_MUb6_GlyGly_2 − Scan number: 5029
Gene Names | Charge | m/z | Mass | Retention time | PEP | Score | Precursor Intensity
SPRTN | 3 | 439.5529 | 1315.637 | 17.322 | 0.0026262 | 79.492 | 3589019
Intensity (%)
m/z
y1 376.276
a2 459.2768
y2 473.3291
b2 487.272
y3−H2O 570.3439
y3 588.3563
b3 600.356
y4−NH3 685.3723
y4 702.3994
y5−NH3 742.3944
y5 759.421
b4 842.4948
y6 846.4525
b5−H2O 911.5145
b5 929.5275
y7 933.4849
b7 1073.5819
E Q I K S S G N D P K
y7 y6 y5 y4 y3 y2 y1
b2 a2 b3 b4 b5 b7
Modification
Acetyl (Protein N−term)
GlyGly (K) no new terminus
Oxidation (M)

20200924_KIZ_MUb5_GlyGly_1 − Scan number: 3135

Gene Names	Charge	m/z	Mass	Retention time	PEP	Score	Precursor Intensity
MOSPD1	3	430.8651	1289.573	9.6778	0.10959	51.445	NA

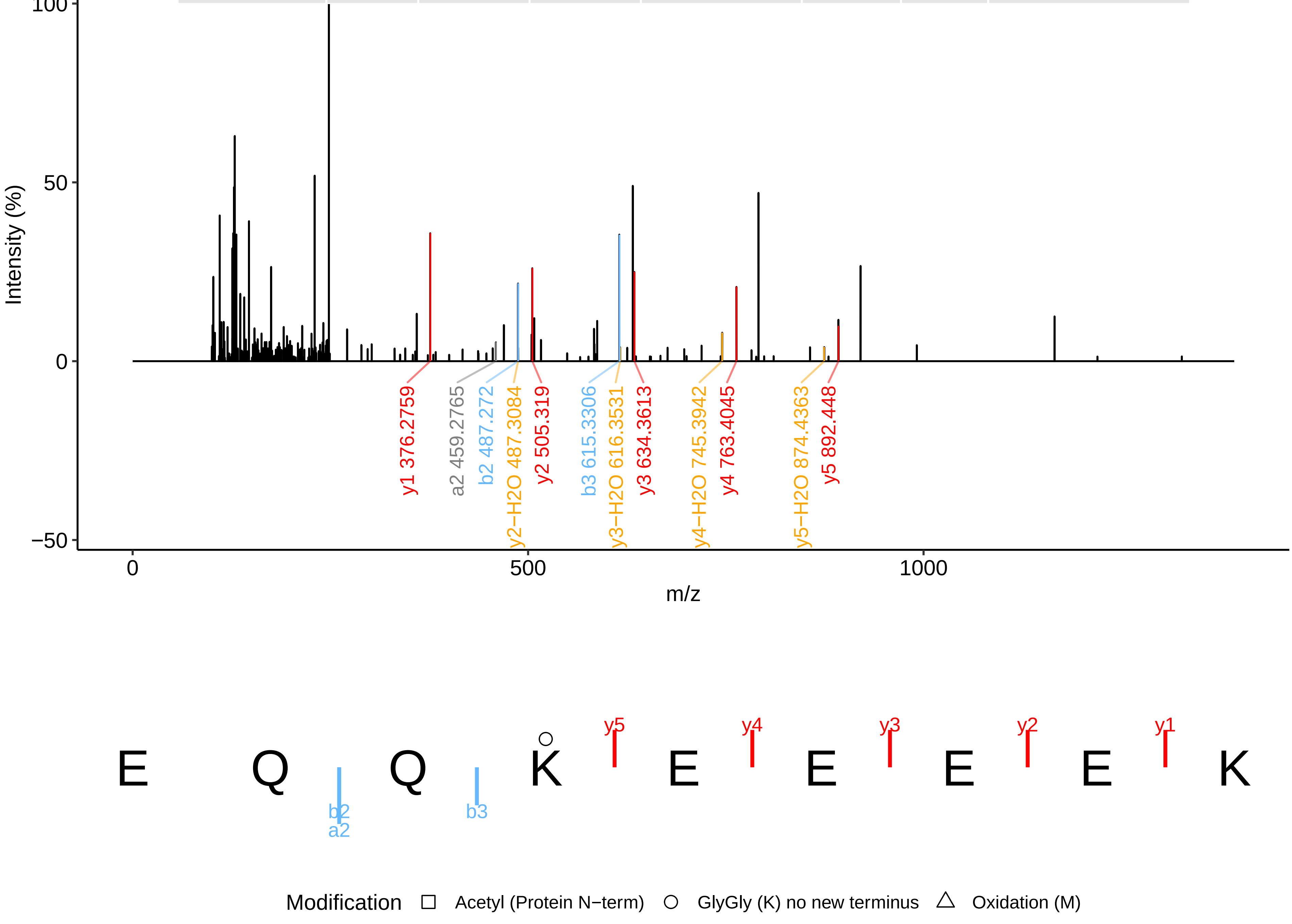

20200921_KIZ_MUb4_GlyGly_10plex_2 − Scan number: 10368

Gene Names	Charge	m/z	Mass	Retention time	PEP	Score	Precursor Intensity
TAX1BP1	3	424.57	1270.688	39.648	0.050269	57.348	627714.8

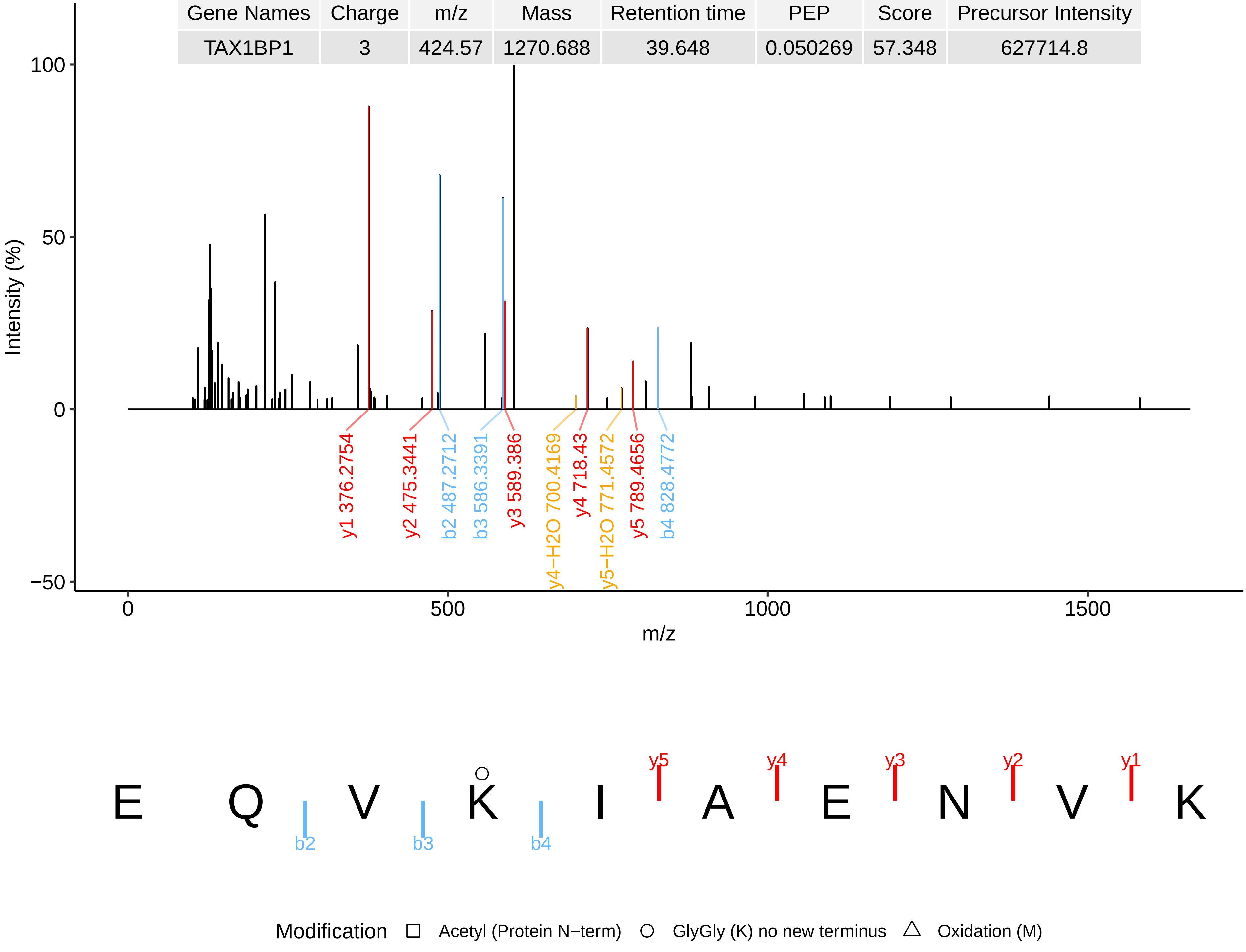

20200921_KIZ_MUb4_GlyGly_10plex_2 − Scan number: 10395

Gene Names	Charge	m/z	Mass	Retention time	PEP	Score	Precursor Intensity
TAX1BP1	3	424.57	1270.688	39.76	0.10056	47.559	335377.3

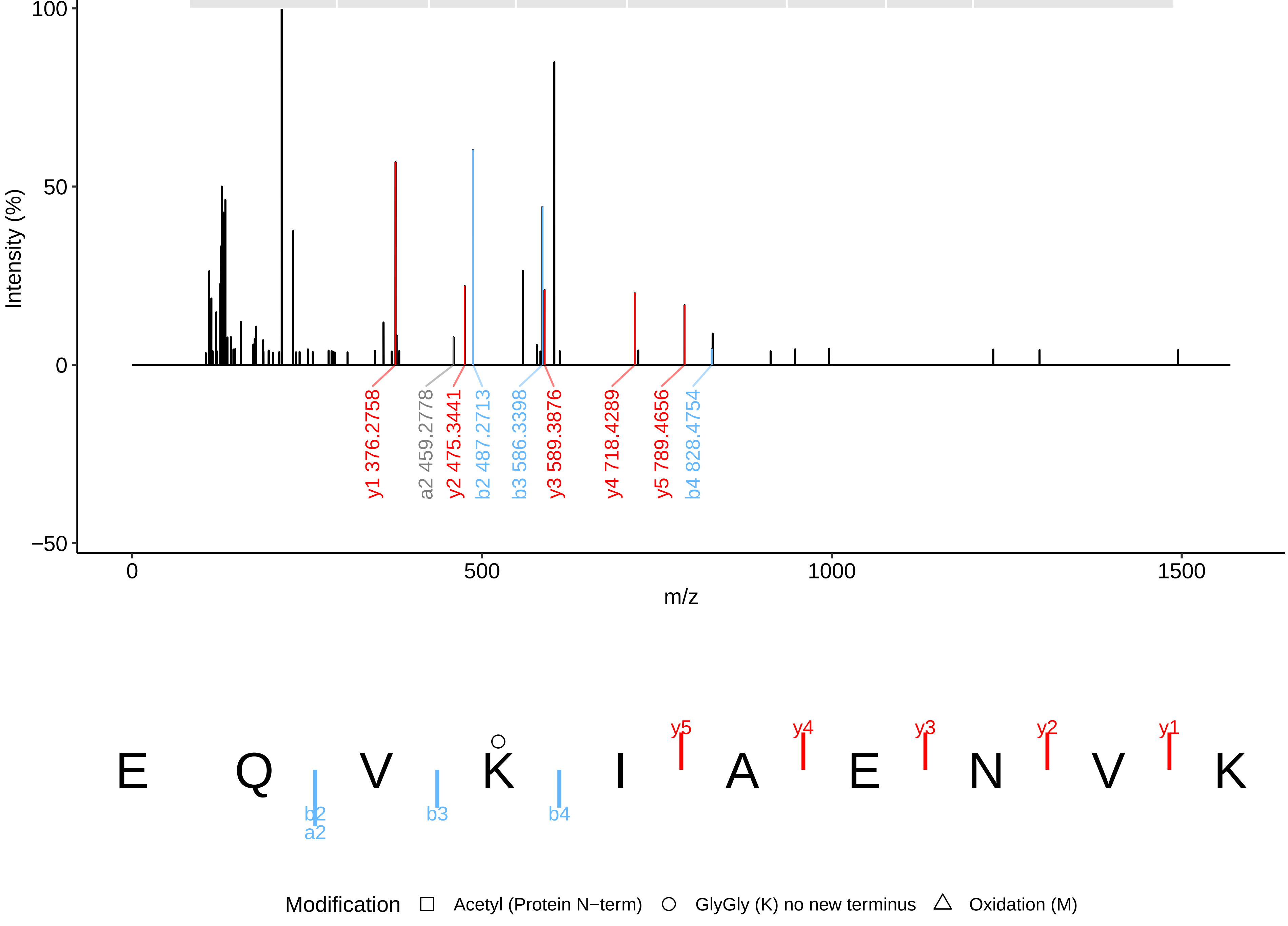

20200921_KIZ_MUb4_GlyGly_10plex − Scan number: 9639

Gene Names	Charge	m/z	Mass	Retention time	PEP	Score	Precursor Intensity
REEP5	3	430.8894	1289.646	33.069	0.0024888	79.659	NA

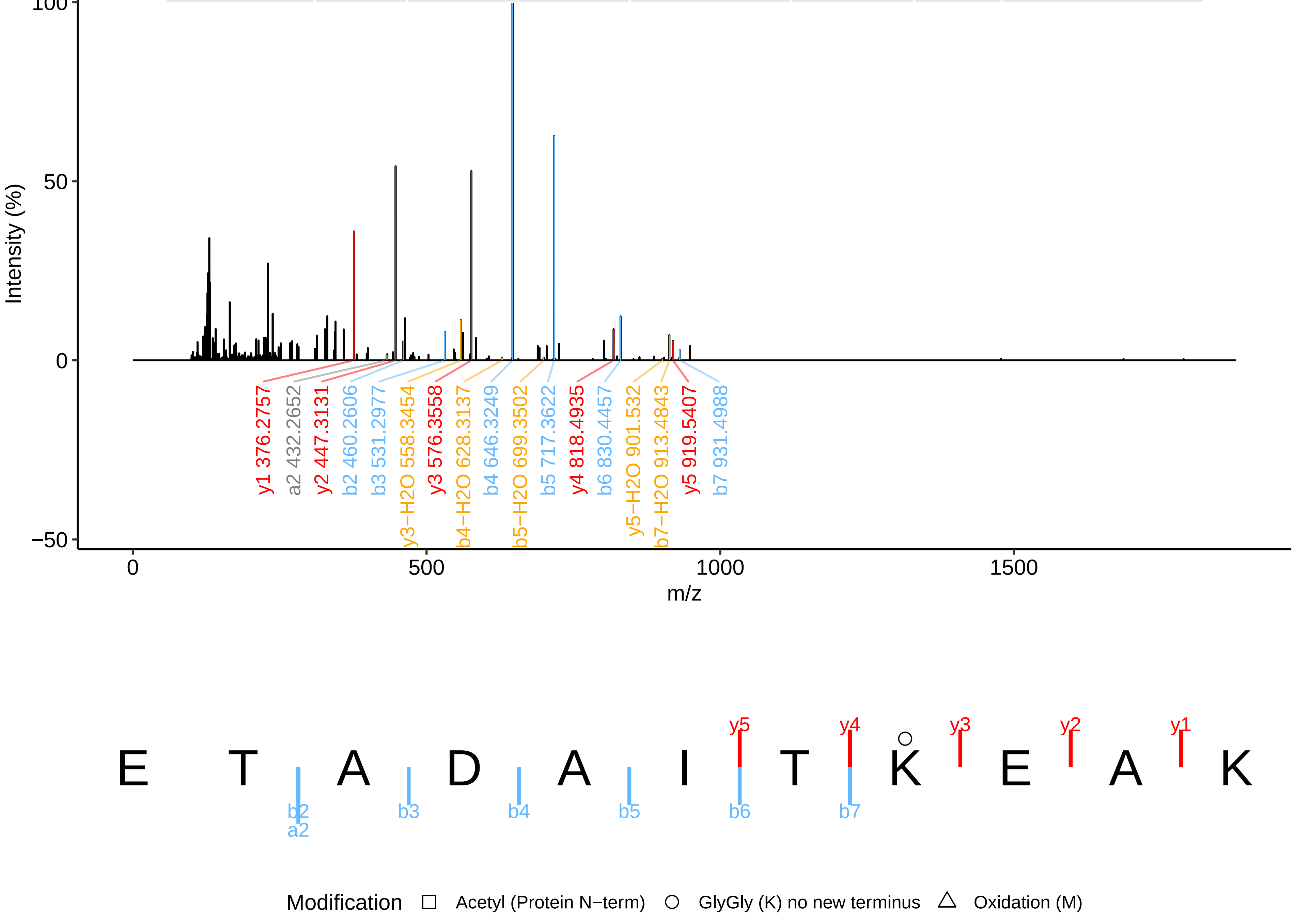

20200921_KIZ_MUb4_GlyGly_10plex_2 − Scan number: 8843

Gene Names	Charge	m/z	Mass	Retention time	PEP	Score	Precursor Intensity
REEP5	3	430.8894	1289.646	33.452	0.00081248	90.05	1173829

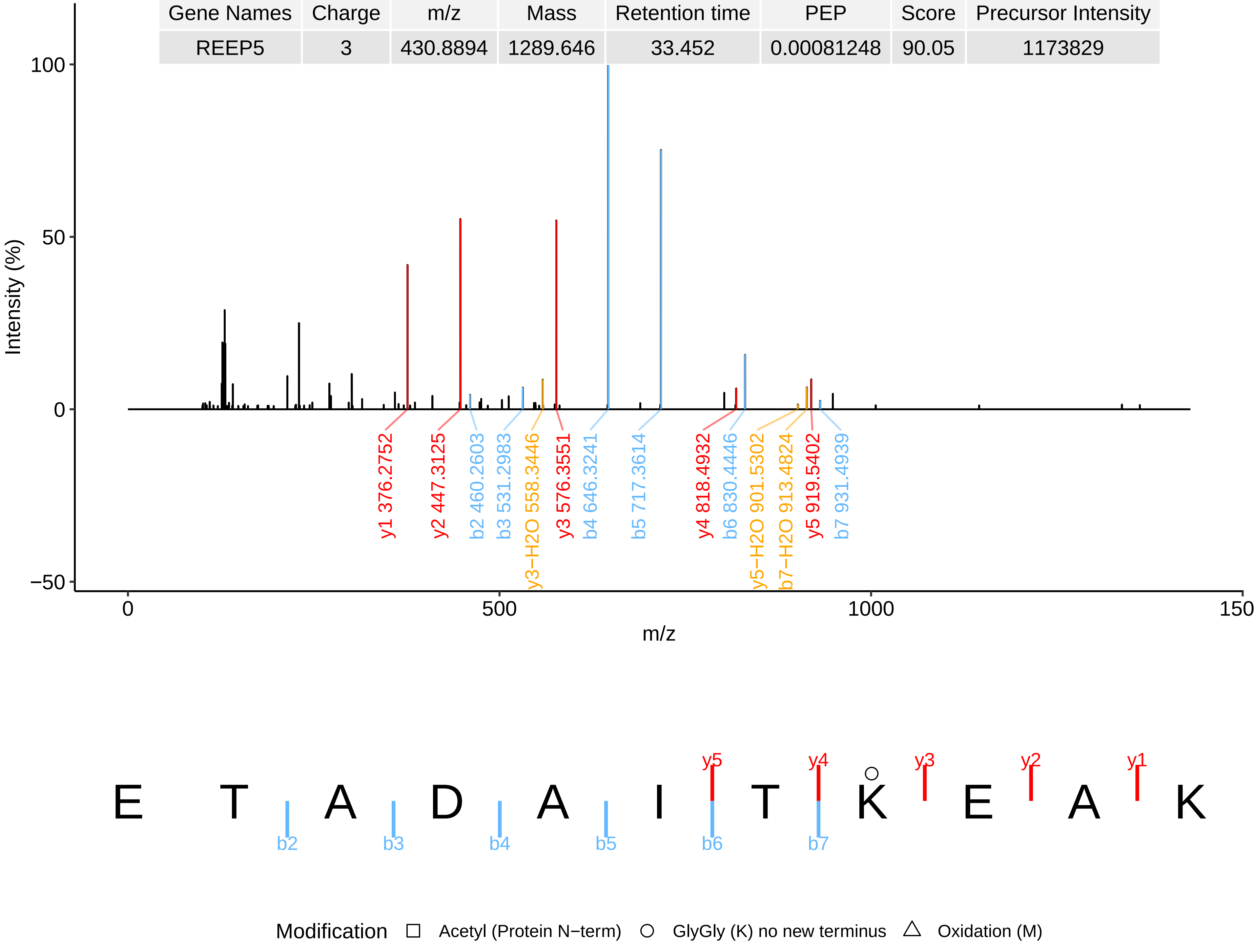

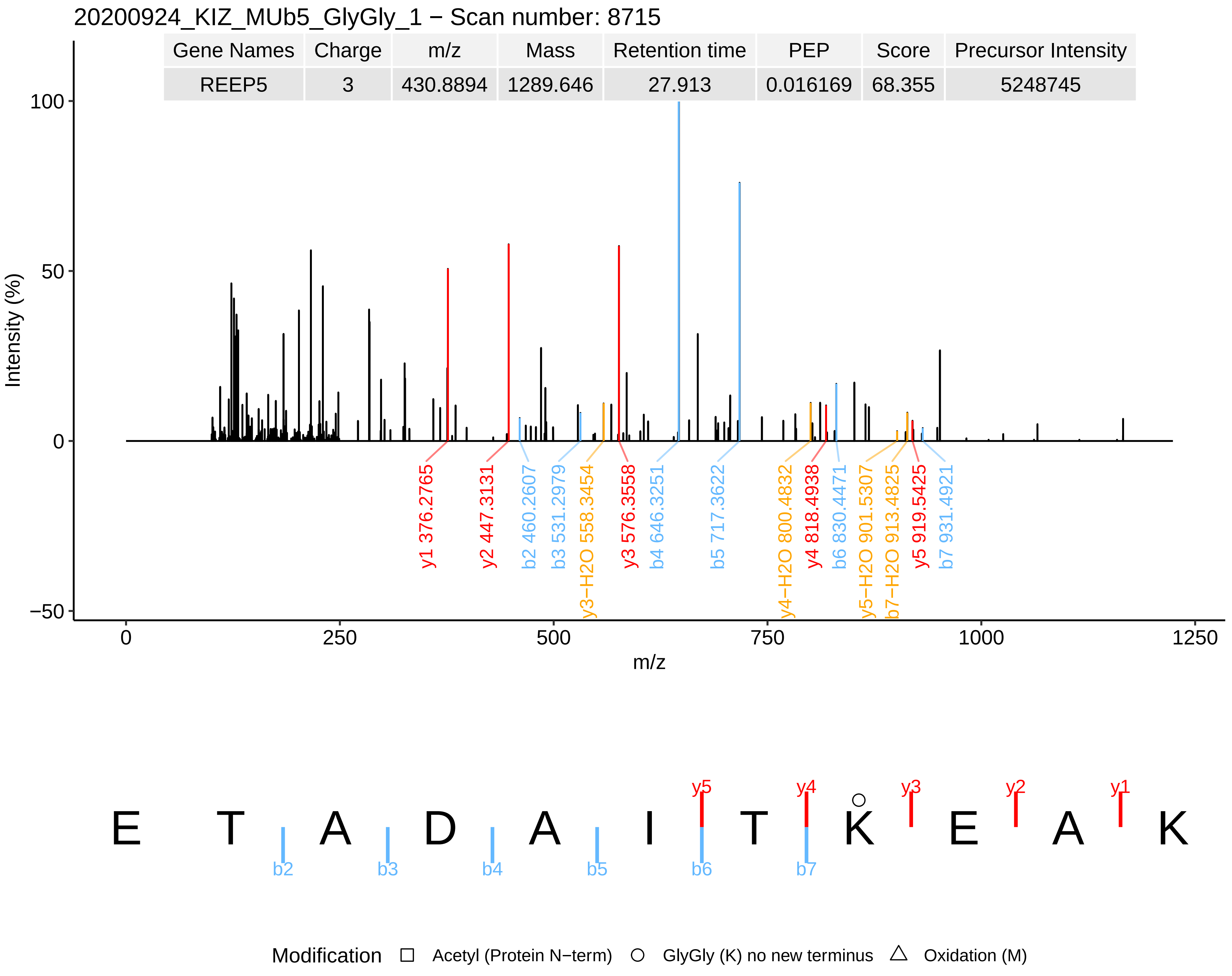
20200924_KIZ_MUb5_GlyGly_1 − Scan number: 8715
Gene Names | Charge | m/z | Mass | Retention time | PEP | Score | Precursor Intensity
REEP5 | 3 | 430.8894 | 1289.646 | 27.913 | 0.016169 | 68.355 | 5248745
Intensity (%)
m/z
y1 376.2765
y2 447.3131
b2 460.2607
b3 531.2979
y3−H2O 558.3454
y3 576.3558
b4 646.3251
b5 717.3622
y4−H2O 800.4832
y4 818.4938
b6 830.4471
y5−H2O 901.5307
b7−H2O 913.4825
y5 919.5425
b7 931.4921
E T A D A I T K E A K
Modification □ Acetyl (Protein N−term) ○ GlyGly (K) no new terminus △ Oxidation (M)

20200921_KIZ_MUb4_GlyGly_10plex − Scan number: 9882

Gene Names	Charge	m/z	Mass	Retention time	PEP	Score	Precursor Intensity
EEF2	3	515.5987	1543.774	34	0.00017793	94.287	4823114

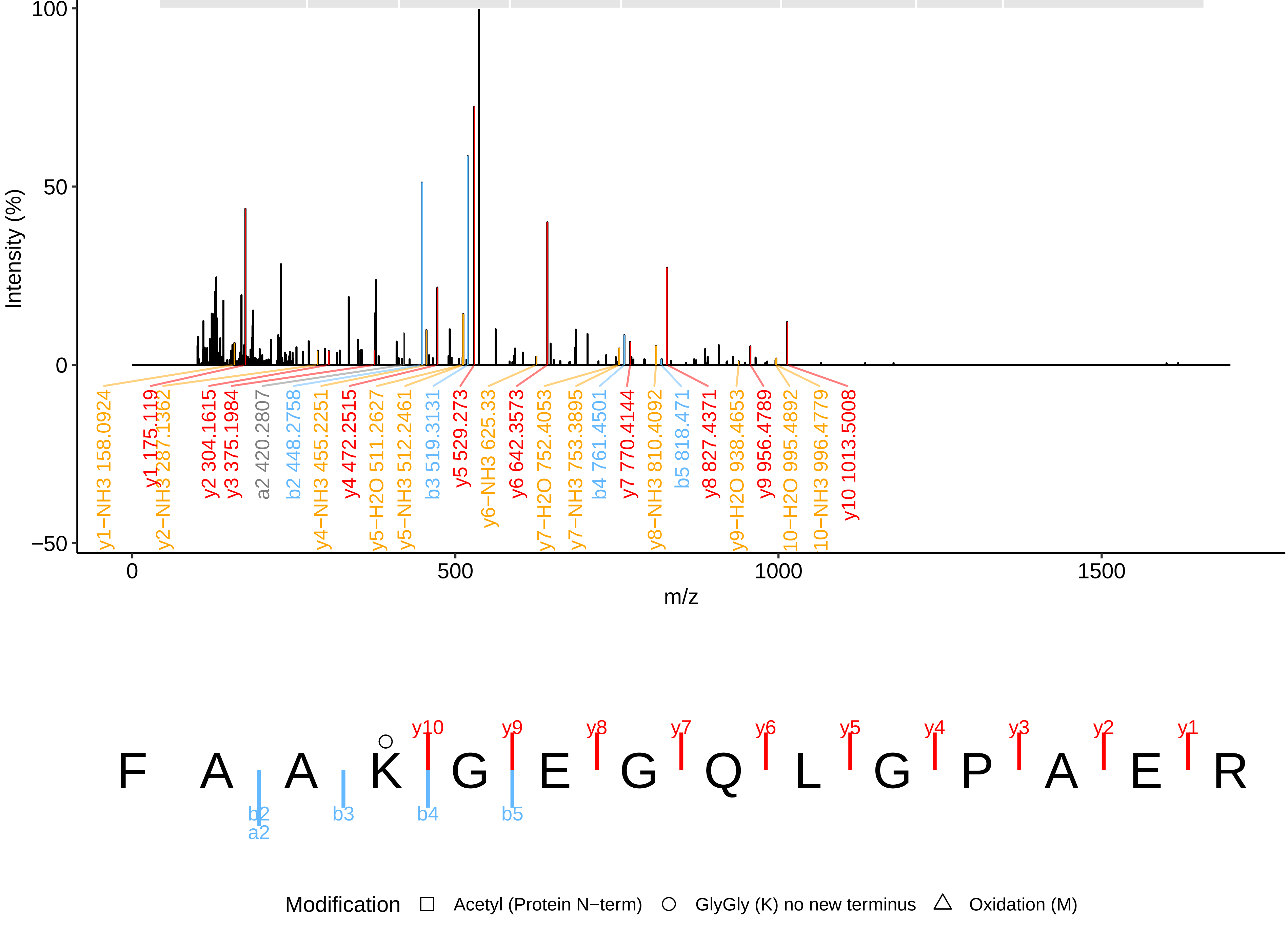

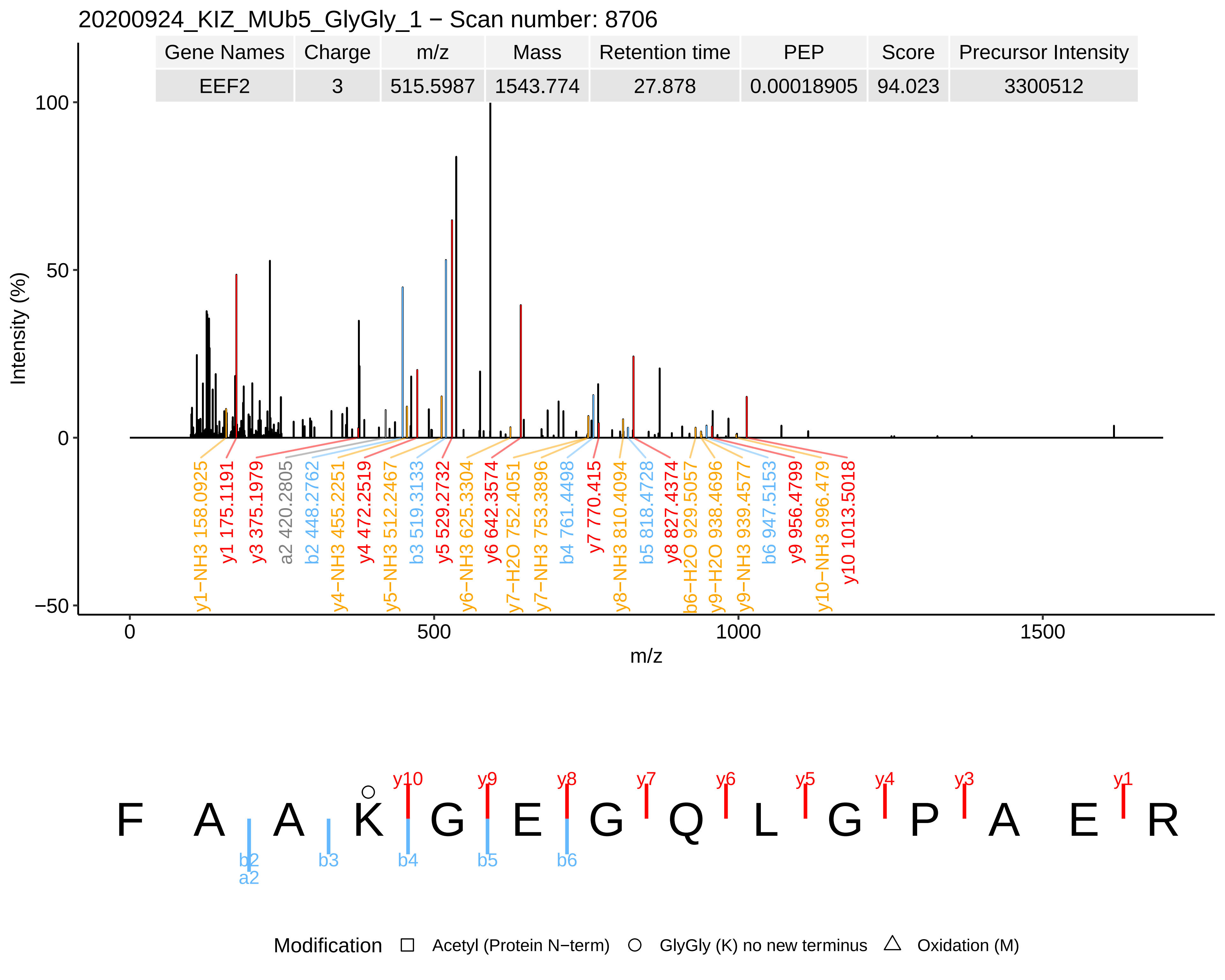

20200924_KIZ_MUb5_GlyGly_1 − Scan number: 8706
Gene Names | Charge | m/z | Mass | Retention time | PEP | Score | Precursor Intensity
EEF2 | 3 | 515.5987 | 1543.774 | 27.878 | 0.00018905 | 94.023 | 3300512
Intensity (%)
100
50
0
−50
0
500
1000
1500
m/z
y1−NH3 158.0925
y1 175.1191
y3 375.1979
a2 420.2805
b2 448.2762
y4−NH3 455.2251
y4 472.2519
y5−NH3 512.2467
b3 519.3133
y5 529.2732
y6−NH3 625.3304
y6 642.3574
y7−H2O 752.4051
y7−NH3 753.3896
b4 761.4498
y7 770.415
y8−NH3 810.4094
b5 818.4728
y8 827.4374
b6−H2O 929.5057
y9−H2O 938.4696
y9−NH3 939.4577
b6 947.5153
y9 956.4799
y10−NH3 996.479
y10 1013.5018
F A A K G E G Q L G P A E R
y10 y9 y8 y7 y6 y5 y4 y3 y1
b2 a2 b3 b4 b5 b6
Modification
Acetyl (Protein N−term)
GlyGly (K) no new terminus
Oxidation (M)

20200924_KIZ_MUb5_GlyGly_1 − Scan number: 8853

Gene Names	Charge	m/z	Mass	Retention time	PEP	Score	Precursor Intensity
EEF2	3	515.5987	1543.774	28.39	1.9591e−13	141.54	26559474

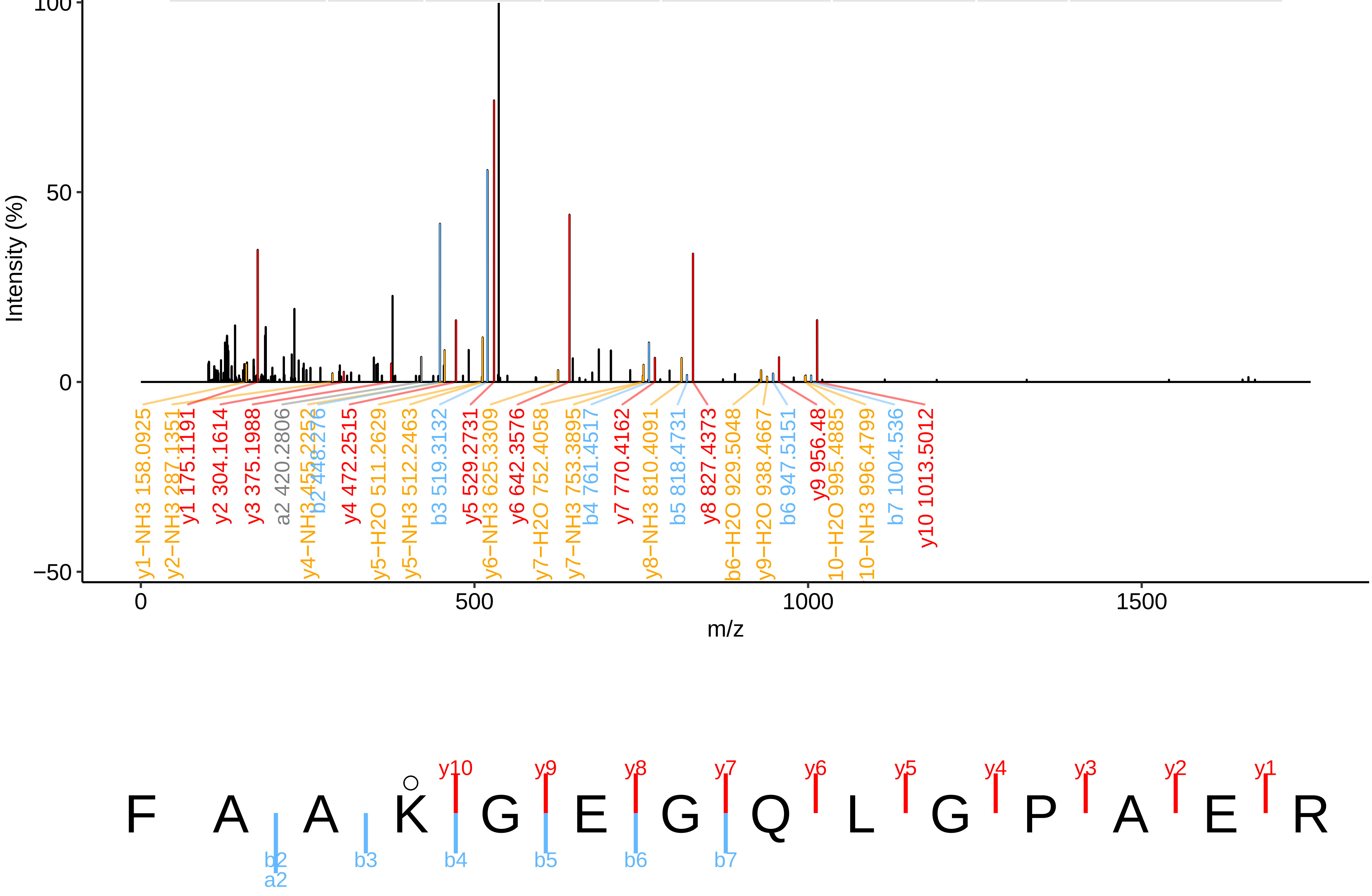

Modification □ Acetyl (Protein N−term) ○ GlyGly (K) no new terminus △ Oxidation (M)

20200924_KIZ_MUb5_GlyGly_1 − Scan number: 9001

Gene Names	Charge	m/z	Mass	Retention time	PEP	Score	Precursor Intensity
EEF2	3	515.5987	1543.774	28.904	1.1021e−05	106.55	9118797

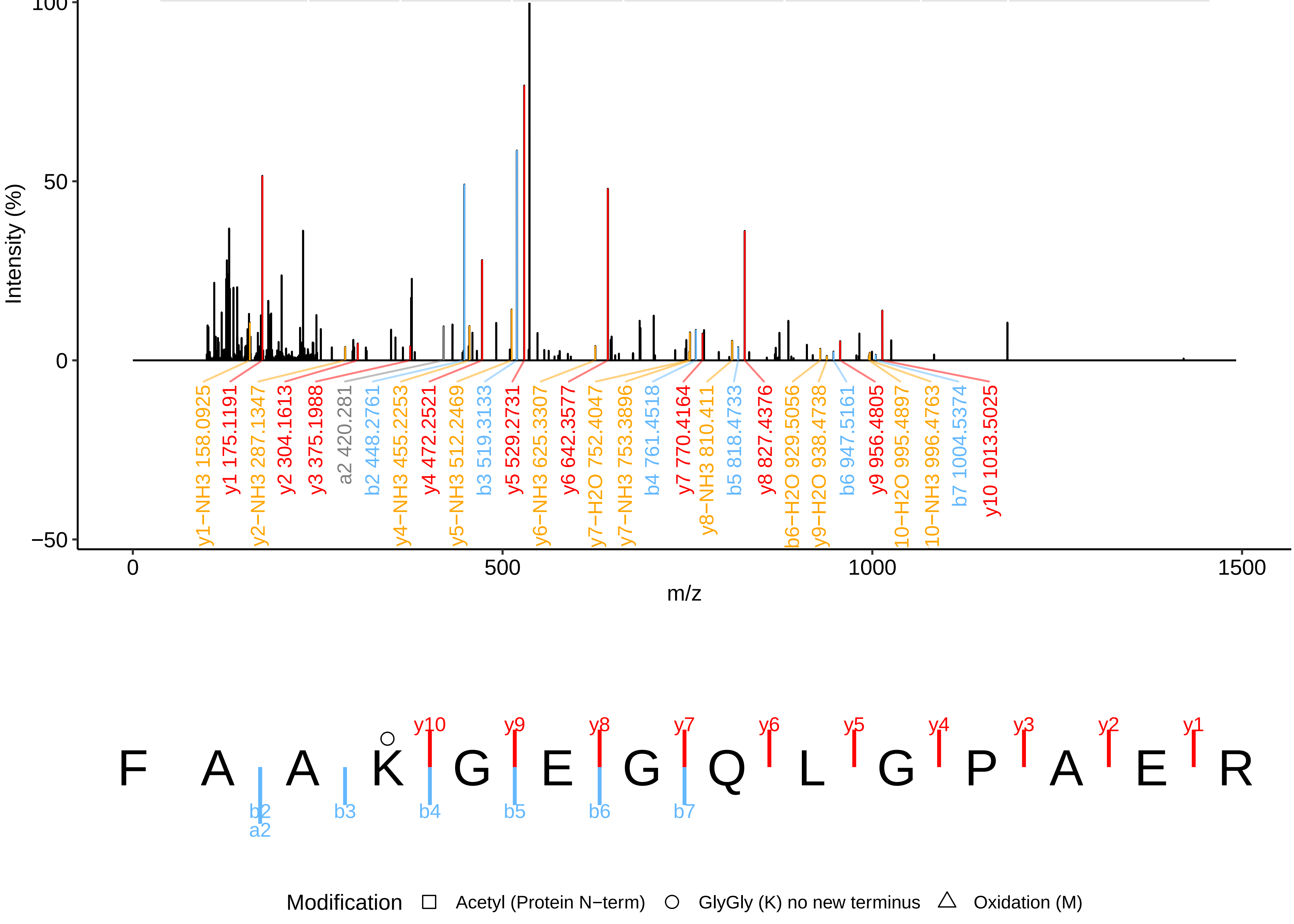

20200924_KIZ_MUb5_GlyGly_2 − Scan number: 8331

Gene Names	Charge	m/z	Mass	Retention time	PEP	Score	Precursor Intensity
EEF2	3	515.5987	1543.774	31.129	0.0066318	64.798	1171874

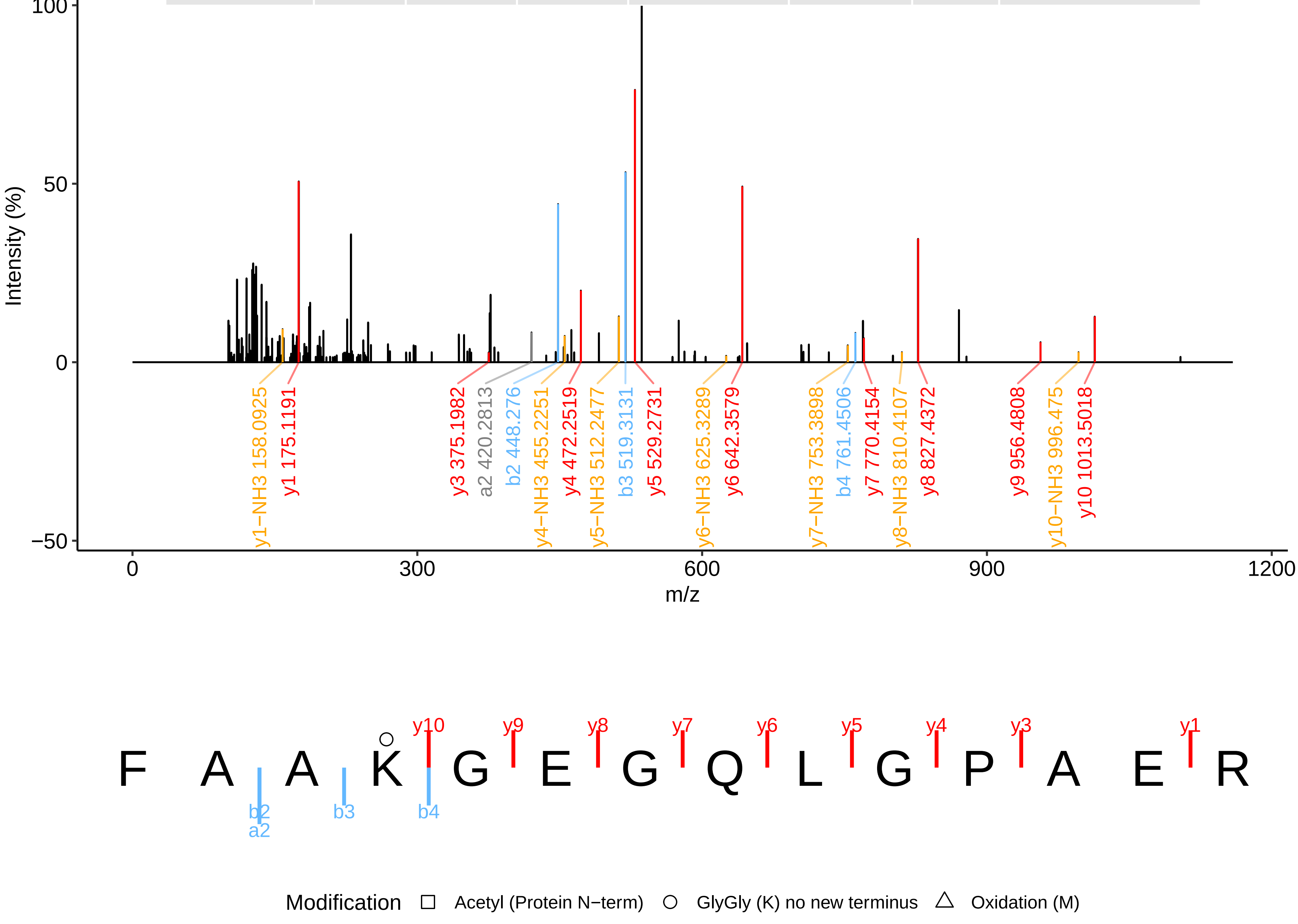

20200924_KIZ_MUb5_GlyGly_2 − Scan number: 8466

Gene Names	Charge	m/z	Mass	Retention time	PEP	Score	Precursor Intensity
EEF2	3	515.5987	1543.774	31.628	9.5822e−09	124.42	9309988

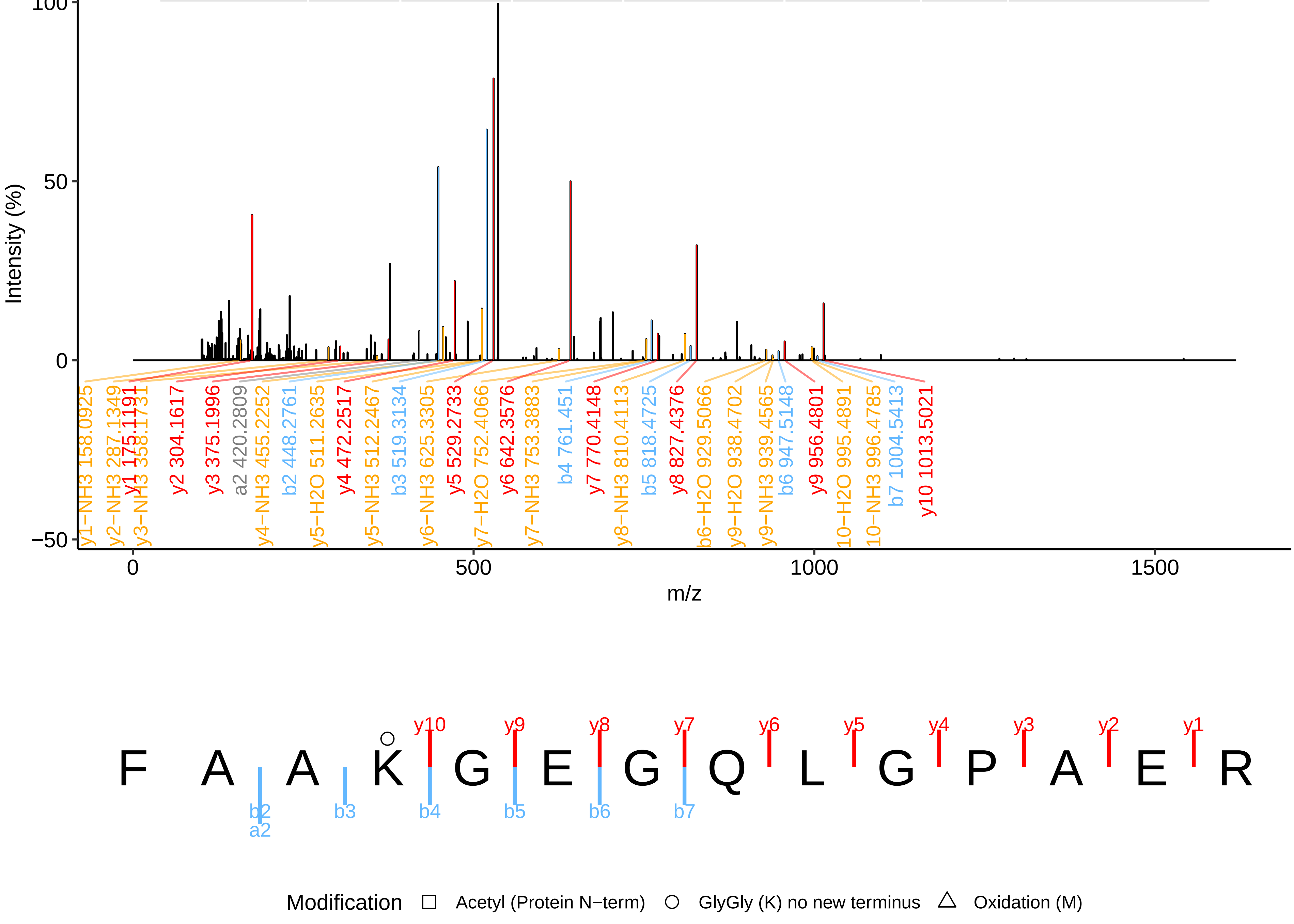

20201118_KIZ_MUb6_GlyGly_1 − Scan number: 8450

Gene Names	Charge	m/z	Mass	Retention time	PEP	Score	Precursor Intensity
EEF2	3	515.5987	1543.774	26.734	4.1256e−05	102.45	22871072

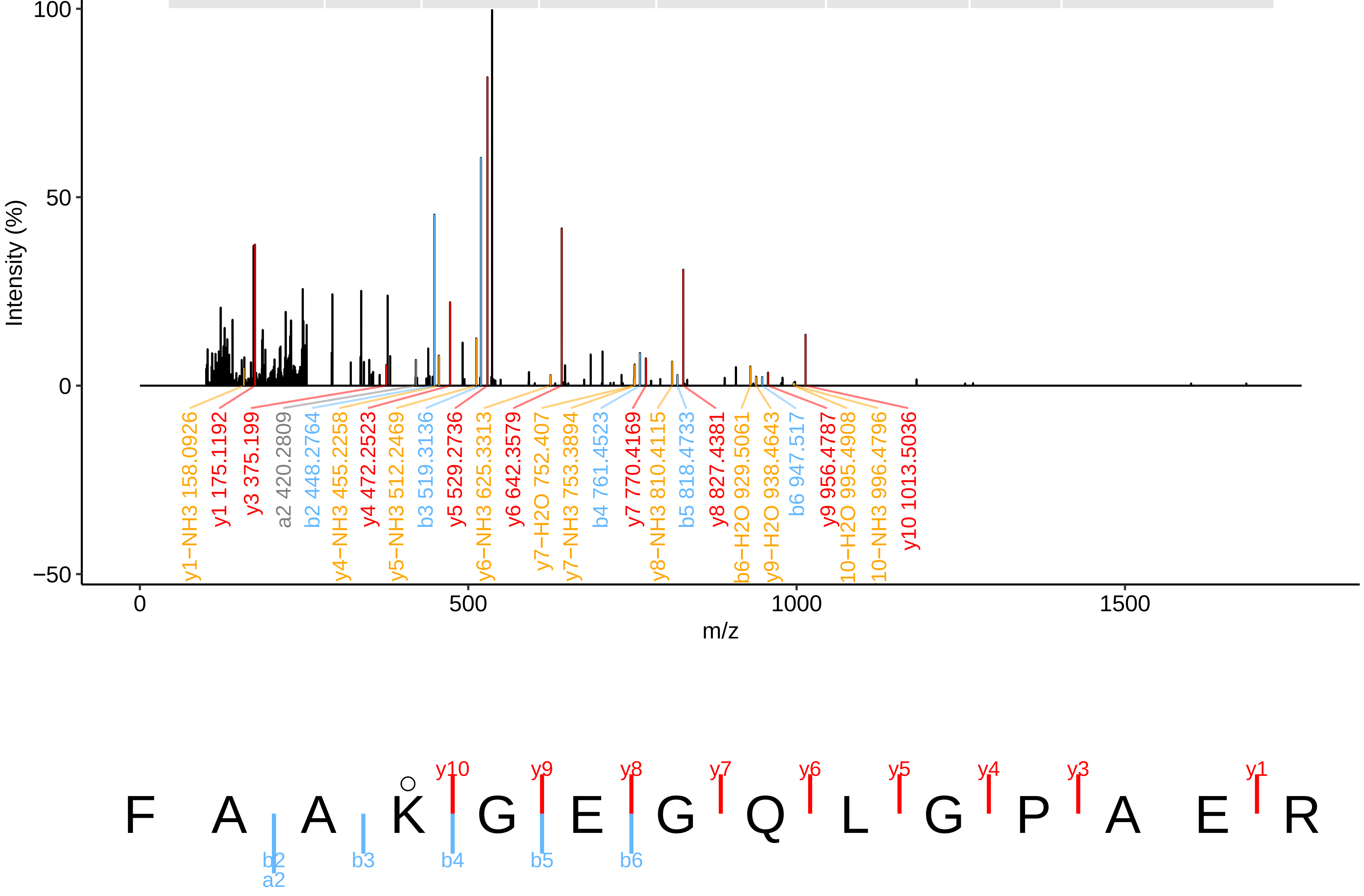

Modification □ Acetyl (Protein N−term) ○ GlyGly (K) no new terminus △ Oxidation (M)

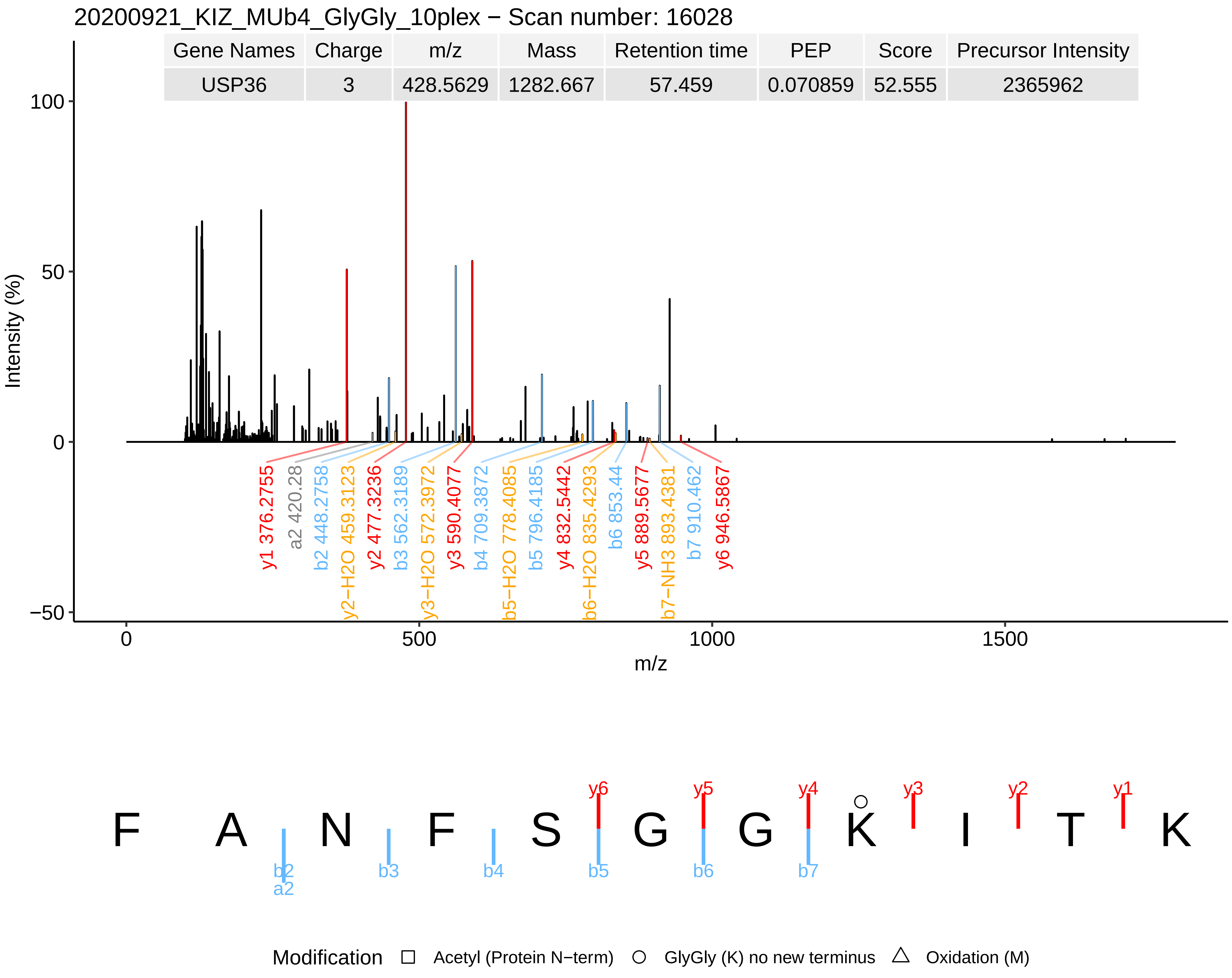

20200921_KIZ_MUb4_GlyGly_10plex − Scan number: 16028
Gene Names | Charge | m/z | Mass | Retention time | PEP | Score | Precursor Intensity
USP36 | 3 | 428.5629 | 1282.667 | 57.459 | 0.070859 | 52.555 | 2365962
Intensity (%)
m/z
y1 376.2755
a2 420.28
b2 448.2758
y2−H2O 459.3123
y2 477.3236
b3 562.3189
y3−H2O 572.3972
y3 590.4077
b4 709.3872
b5−H2O 778.4085
b5 796.4185
y4 832.5442
b6−H2O 835.4293
b6 853.44
y5 889.5677
b7−NH3 893.4381
b7 910.462
y6 946.5867
F A N F S G G K I T K
Modification
Acetyl (Protein N−term)
GlyGly (K) no new terminus
Oxidation (M)

20200921_KIZ_MUb4_GlyGly_10plex − Scan number: 16532

Gene Names	Charge	m/z	Mass	Retention time	PEP	Score	Precursor Intensity
FAM129B	3	505.5915	1513.753	59.406	9.6101e−14	145.91	3525441

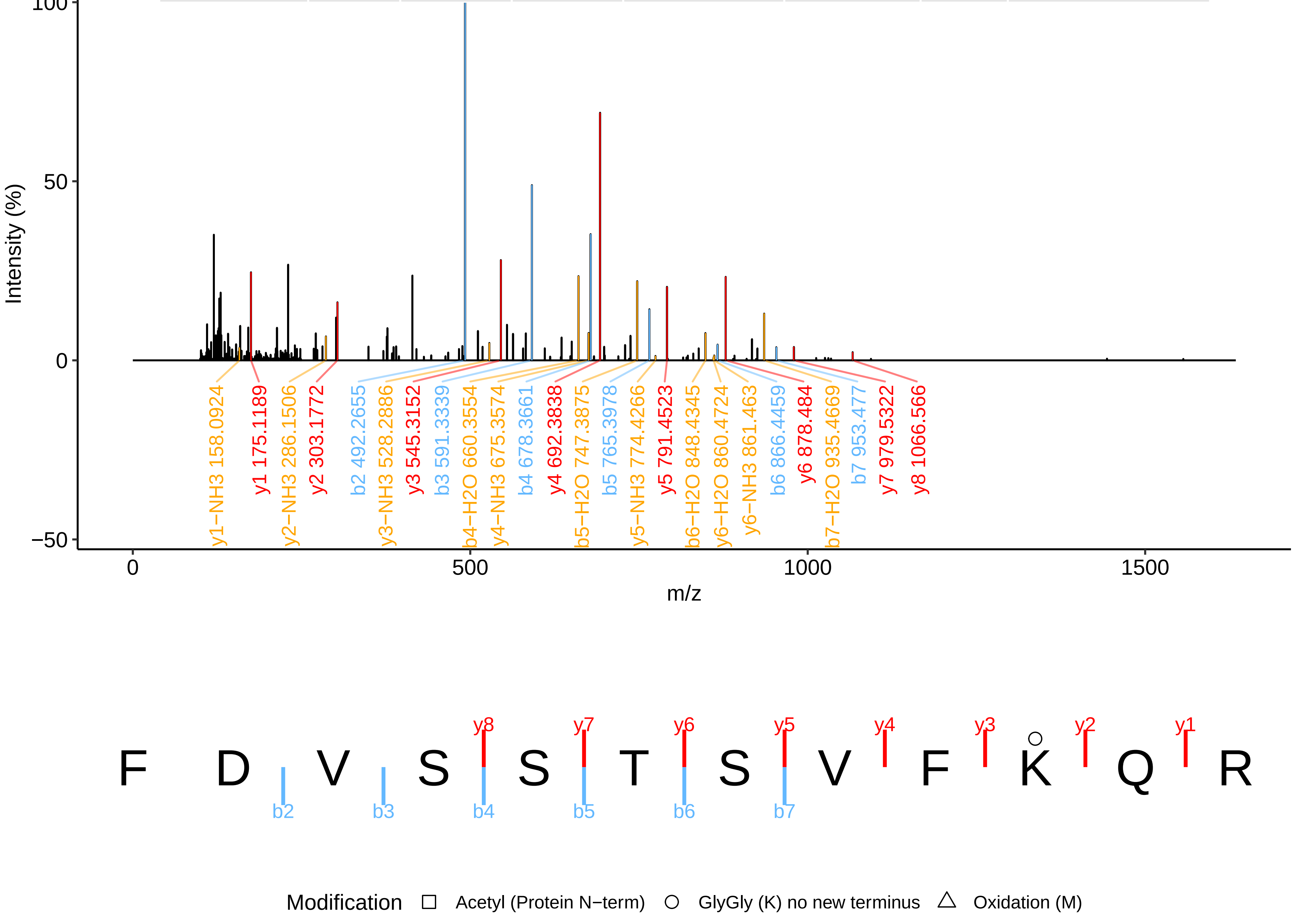

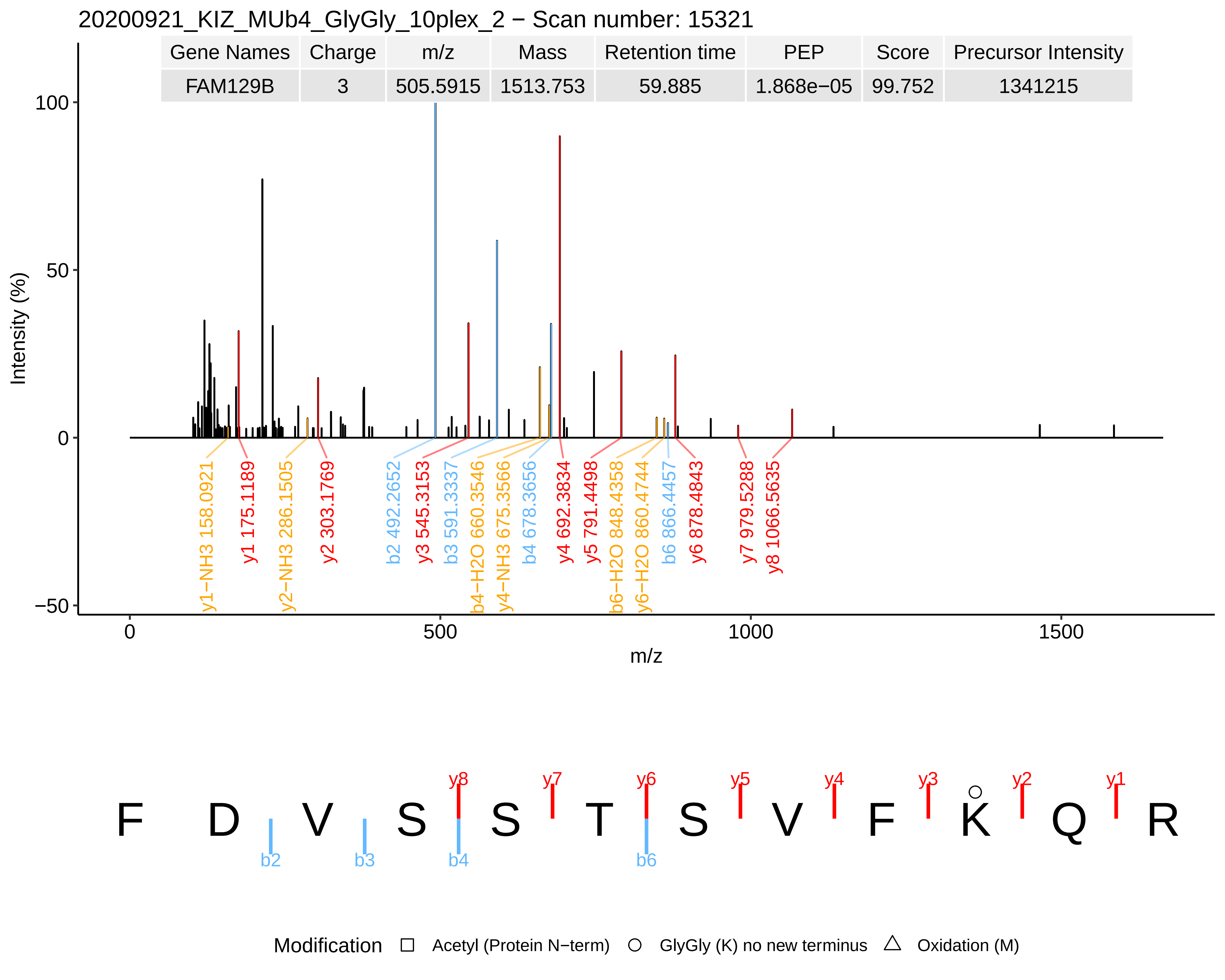
20200921_KIZ_MUb4_GlyGly_10plex_2 − Scan number: 15321
Gene Names | Charge | m/z | Mass | Retention time | PEP | Score | Precursor Intensity
FAM129B | 3 | 505.5915 | 1513.753 | 59.885 | 1.868e−05 | 99.752 | 1341215
Intensity (%)
100
50
0
−50
m/z
0
500
1000
1500
y1−NH3 158.0921
y1 175.1189
y2−NH3 286.1505
y2 303.1769
b2 492.2652
y3 545.3153
b3 591.3337
b4−H2O 660.3546
y4−NH3 675.3566
b4 678.3656
y4 692.3834
y5 791.4498
b6−H2O 848.4358
y6−H2O 860.4744
b6 866.4457
y6 878.4843
y7 979.5288
y8 1066.5635
F D V S S T S V F K Q R
y8 y7 y6 y5 y4 y3 y2 y1
b2 b3 b4 b6
Modification
Acetyl (Protein N−term)
GlyGly (K) no new terminus
Oxidation (M)

20200921_KIZ_MUb4_GlyGly_10plex − Scan number: 5704

Gene Names	Charge	m/z	Mass	Retention time	PEP	Score	Precursor Intensity
GAPDH	4	326.1704	1300.652	18.711	0.012615	64.064	8898260

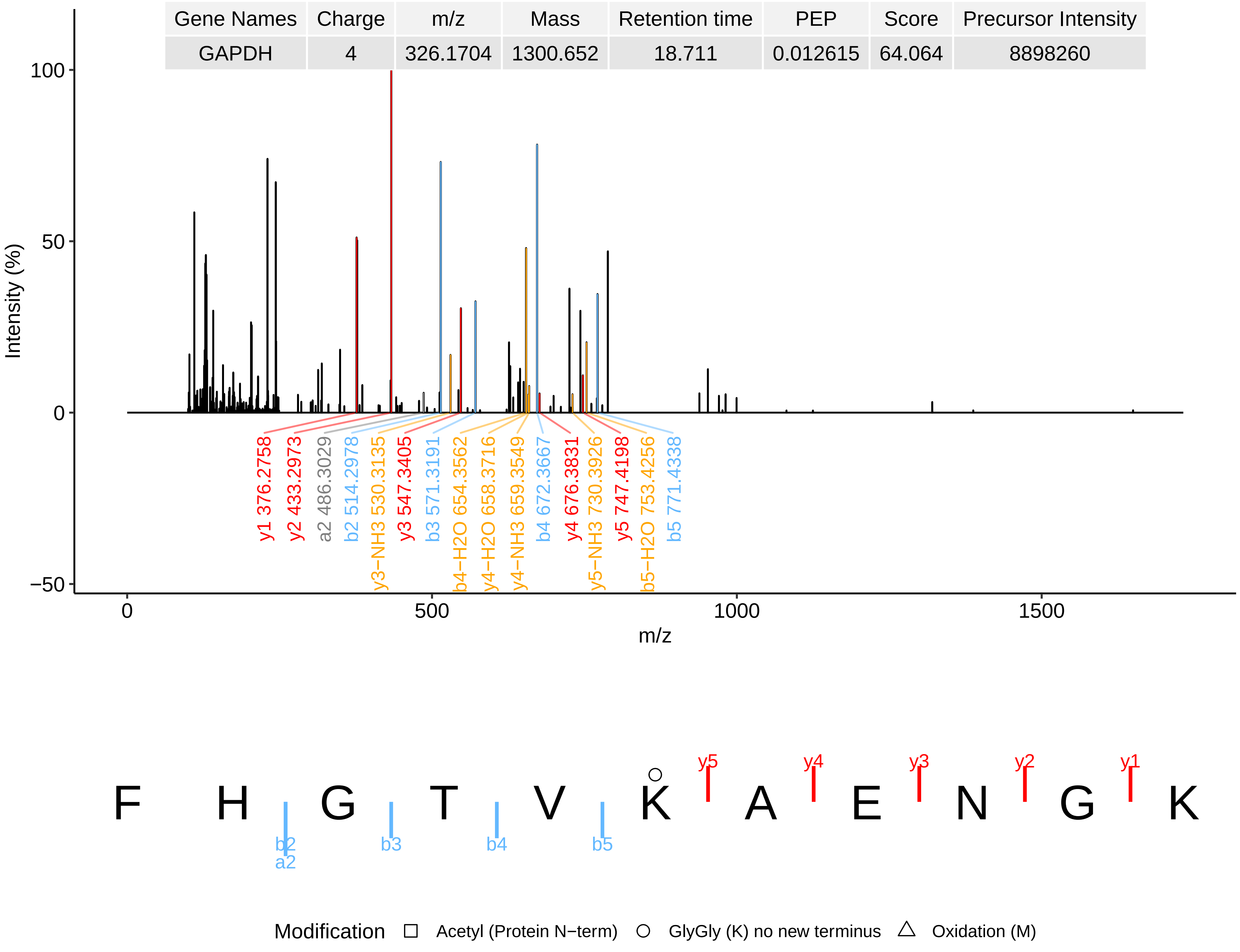

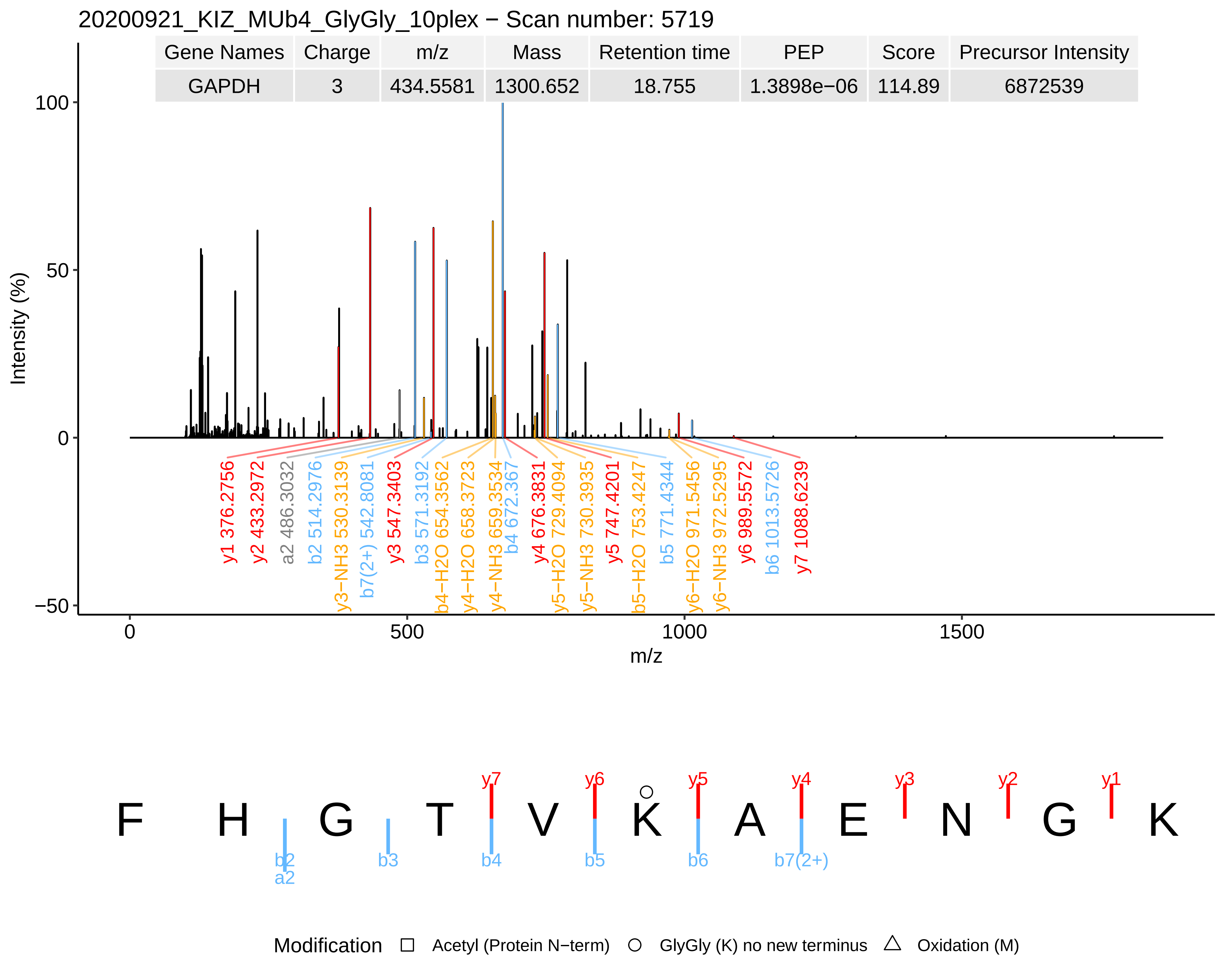

20200921_KIZ_MUb4_GlyGly_10plex − Scan number: 5719
Gene Names | Charge | m/z | Mass | Retention time | PEP | Score | Precursor Intensity
GAPDH | 3 | 434.5581 | 1300.652 | 18.755 | 1.3898e−06 | 114.89 | 6872539
Intensity (%)
100
50
0
−50
y1 376.2756
y2 433.2972
a2 486.3032
b2 514.2976
y3−NH3 530.3139
b7(2+) 542.8081
y3 547.3403
b3 571.3192
b4−H2O 654.3562
y4−H2O 658.3723
y4−NH3 659.3534
b4 672.367
y4 676.3831
y5−H2O 729.4094
y5−NH3 730.3935
y5 747.4201
b5−H2O 753.4247
b5 771.4344
y6−H2O 971.5456
y6−NH3 972.5295
y6 989.5572
b6 1013.5726
y7 1088.6239
0
500
1000
1500
m/z
F H G T V K A E N G K
y7 y6 y5 y4 y3 y2 y1
b2 a2 b3 b4 b5 b6 b7(2+)
Modification
Acetyl (Protein N−term)
GlyGly (K) no new terminus
Oxidation (M)

20200921_KIZ_MUb4_GlyGly_10plex_2 − Scan number: 5263

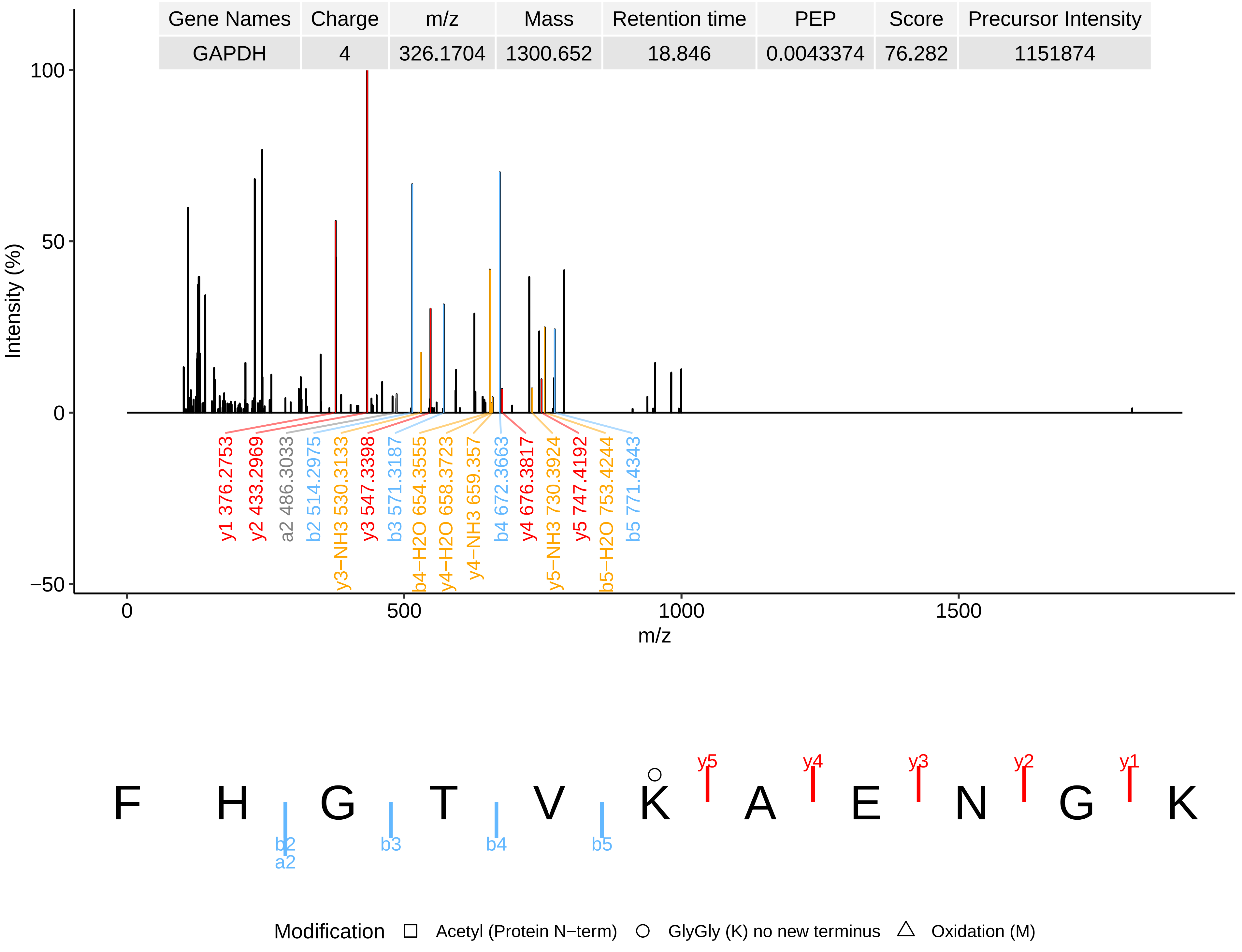

20200921_KIZ_MUb4_GlyGly_10plex_2 − Scan number: 5276

Gene Names	Charge	m/z	Mass	Retention time	PEP	Score	Precursor Intensity
GAPDH	3	434.5581	1300.652	18.896	0.005312	76.228	1412323

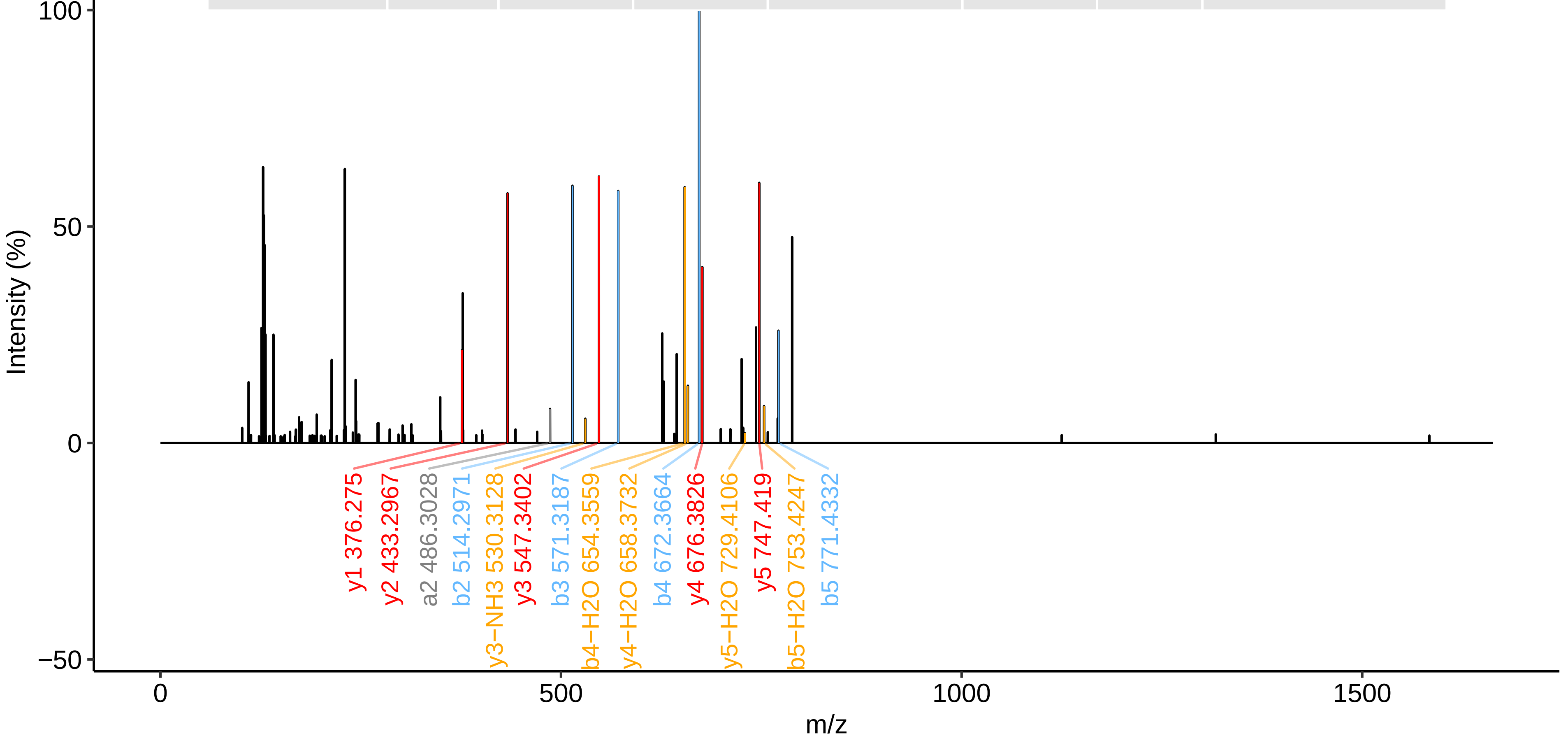

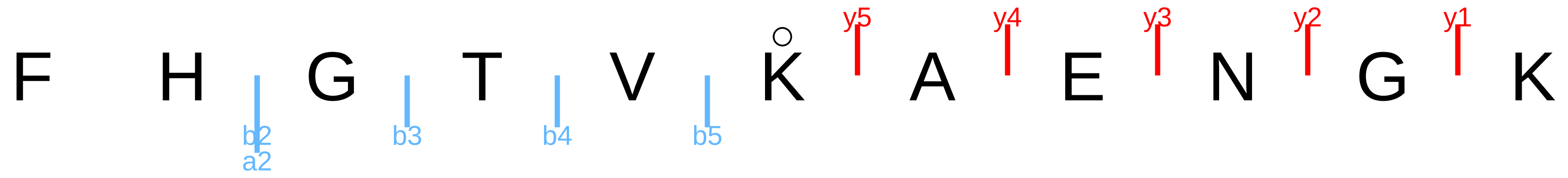

Modification □ Acetyl (Protein N−term) ○ GlyGly (K) no new terminus △ Oxidation (M)

20200924_KIZ_MUb5_GlyGly_1 − Scan number: 4738

Gene Names	Charge	m/z	Mass	Retention time	PEP	Score	Precursor Intensity
GAPDH	4	326.1704	1300.652	14.655	0.011642	65.5	9435550

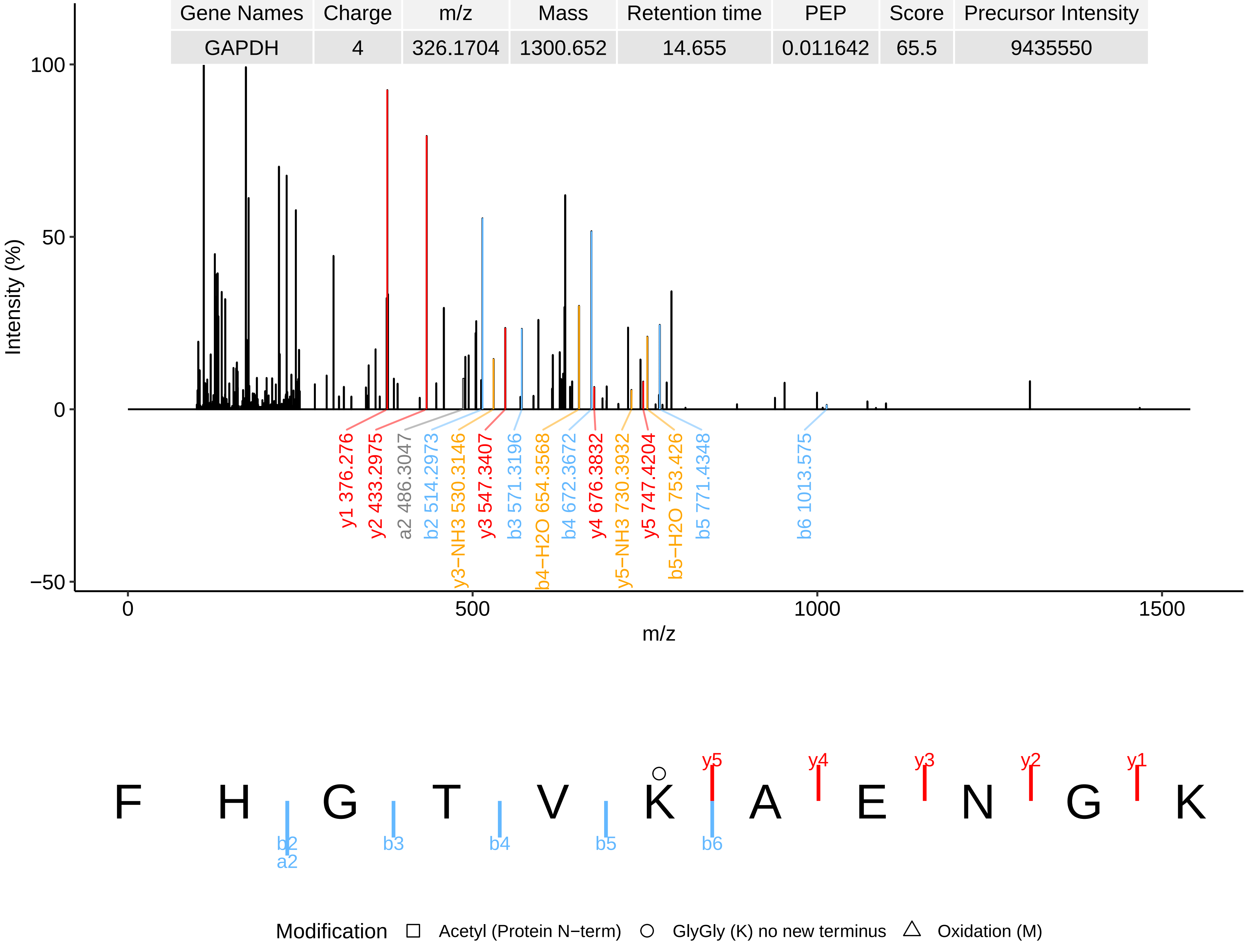

20200924_KIZ_MUb5_GlyGly_1 − Scan number: 4897

Gene Names	Charge	m/z	Mass	Retention time	PEP	Score	Precursor Intensity
GAPDH	4	326.1704	1300.652	15.168	0.0083478	70.363	32092622

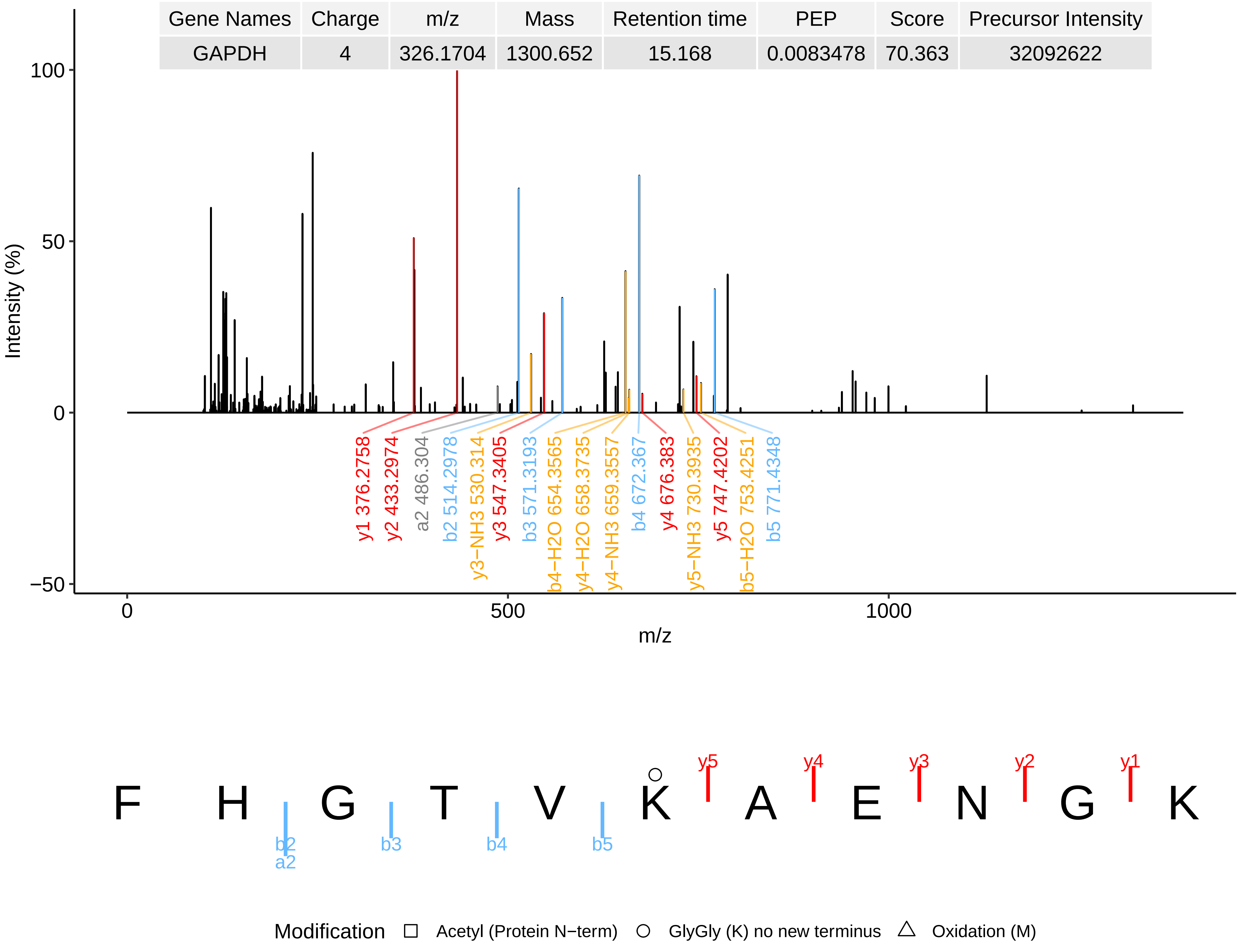

20200924_KIZ_MUb5_GlyGly_1 − Scan number: 4889

Gene Names	Charge	m/z	Mass	Retention time	PEP	Score	Precursor Intensity
GAPDH	3	434.5581	1300.652	15.142	0.0015871	82.029	11499878

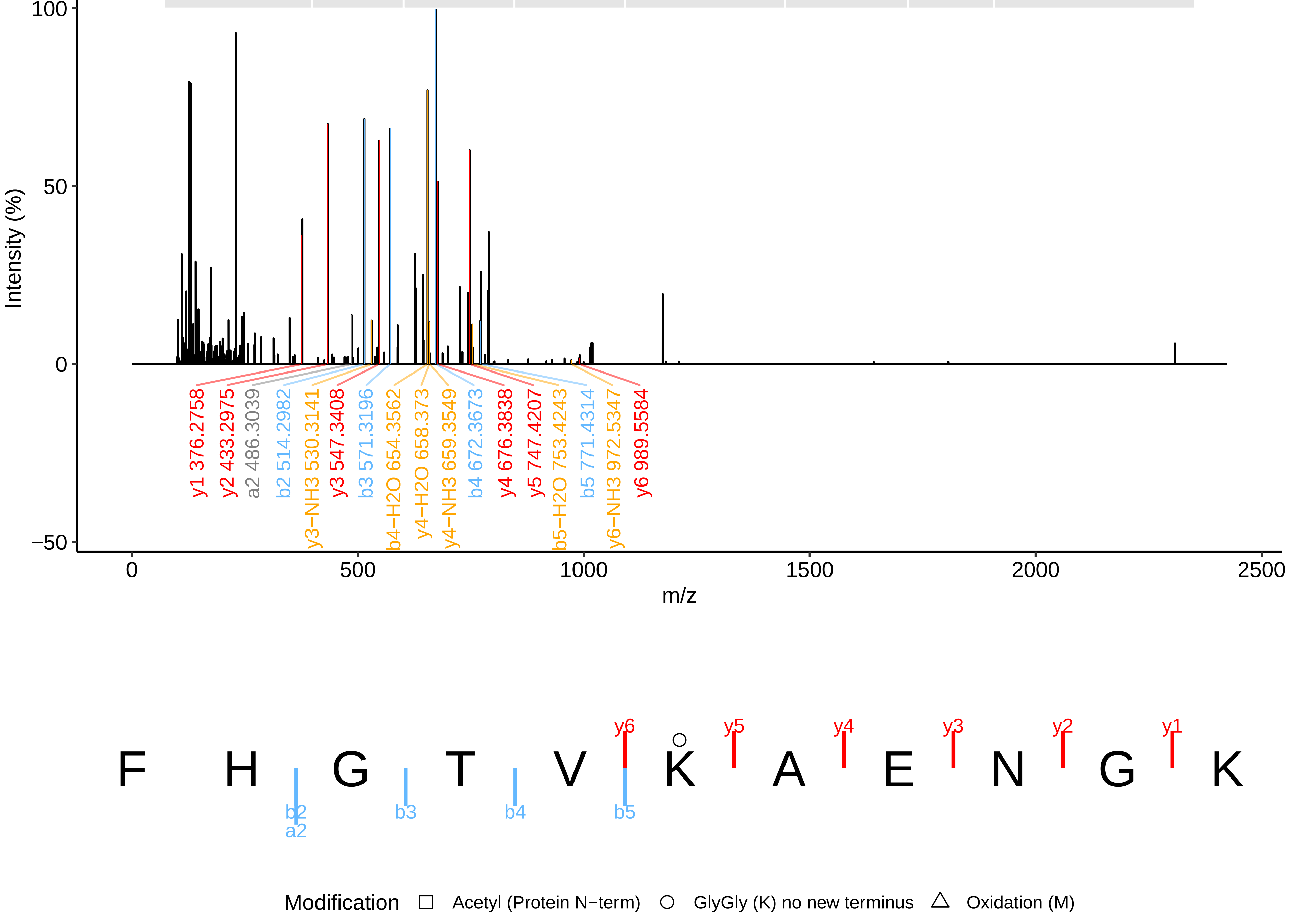

20200924_KIZ_MUb5_GlyGly_1 − Scan number: 5041

Gene Names	Charge	m/z	Mass	Retention time	PEP	Score	Precursor Intensity
GAPDH	3	434.5581	1300.652	15.648	4.4824e−05	98.04	2305399

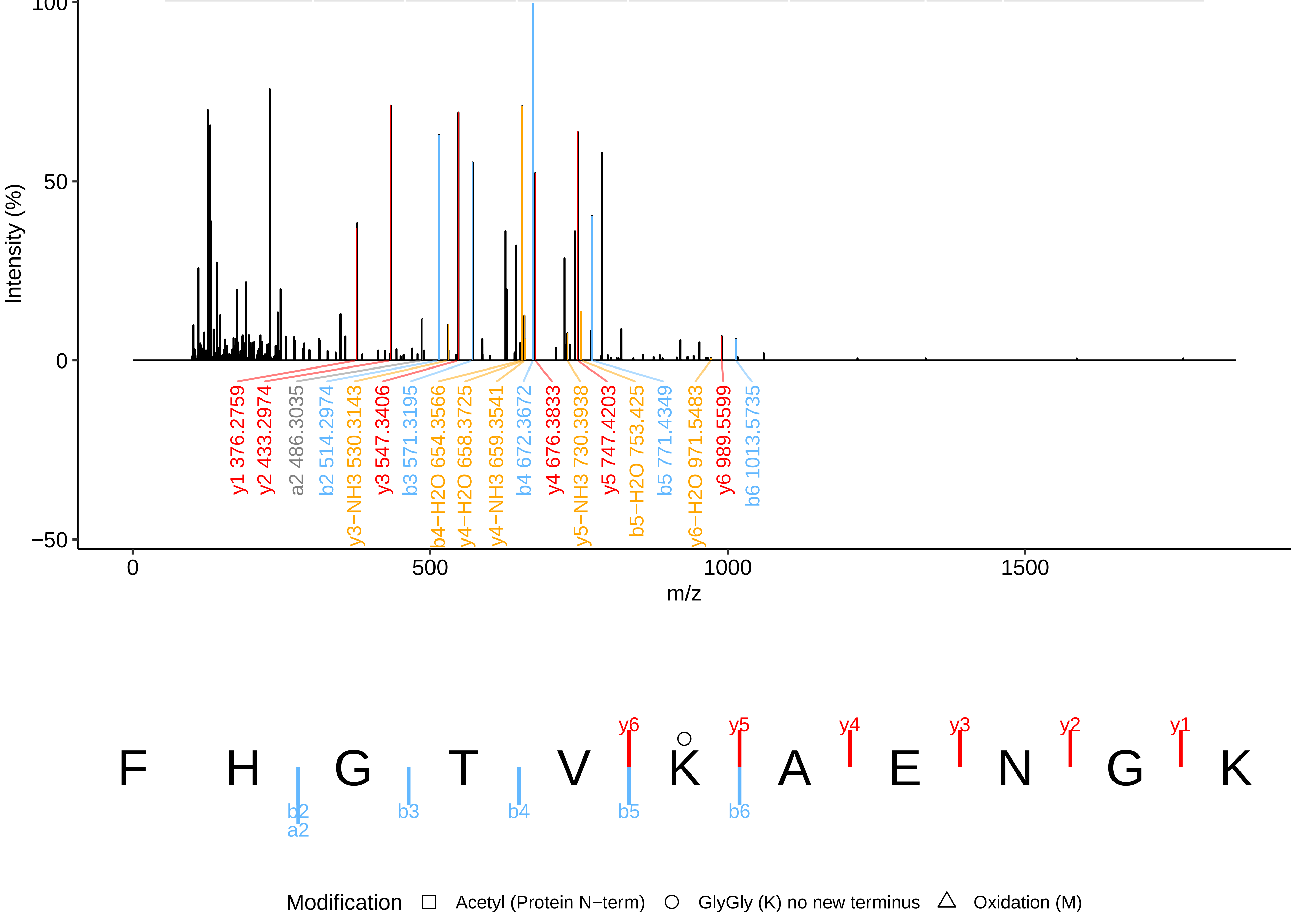

20200924_KIZ_MUb5_GlyGly_1 − Scan number: 5054

Gene Names	Charge	m/z	Mass	Retention time	PEP	Score	Precursor Intensity
GAPDH	4	326.1704	1300.652	15.688	0.0012431	82.831	19831890

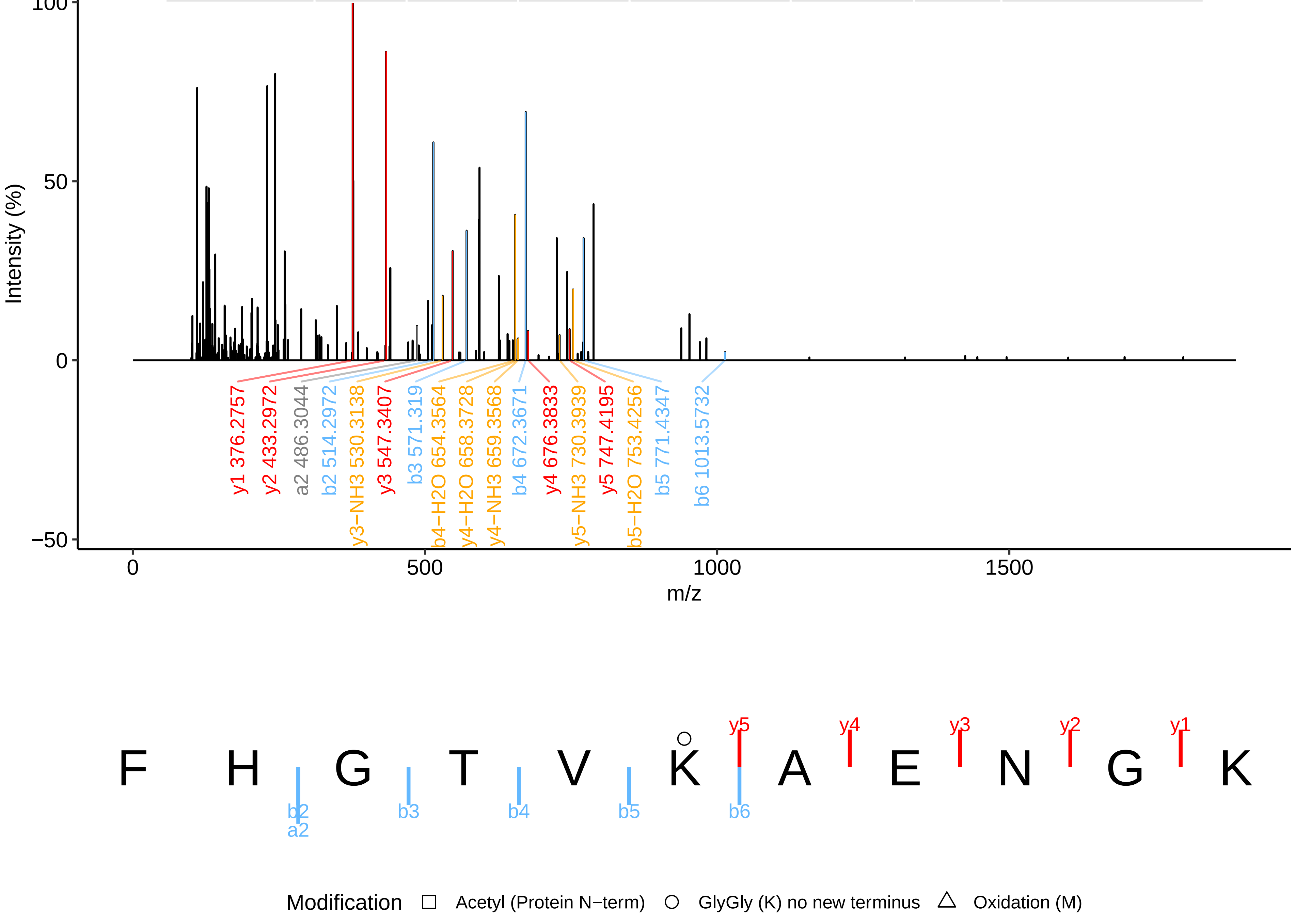

20200924_KIZ_MUb5_GlyGly_1 − Scan number: 4810

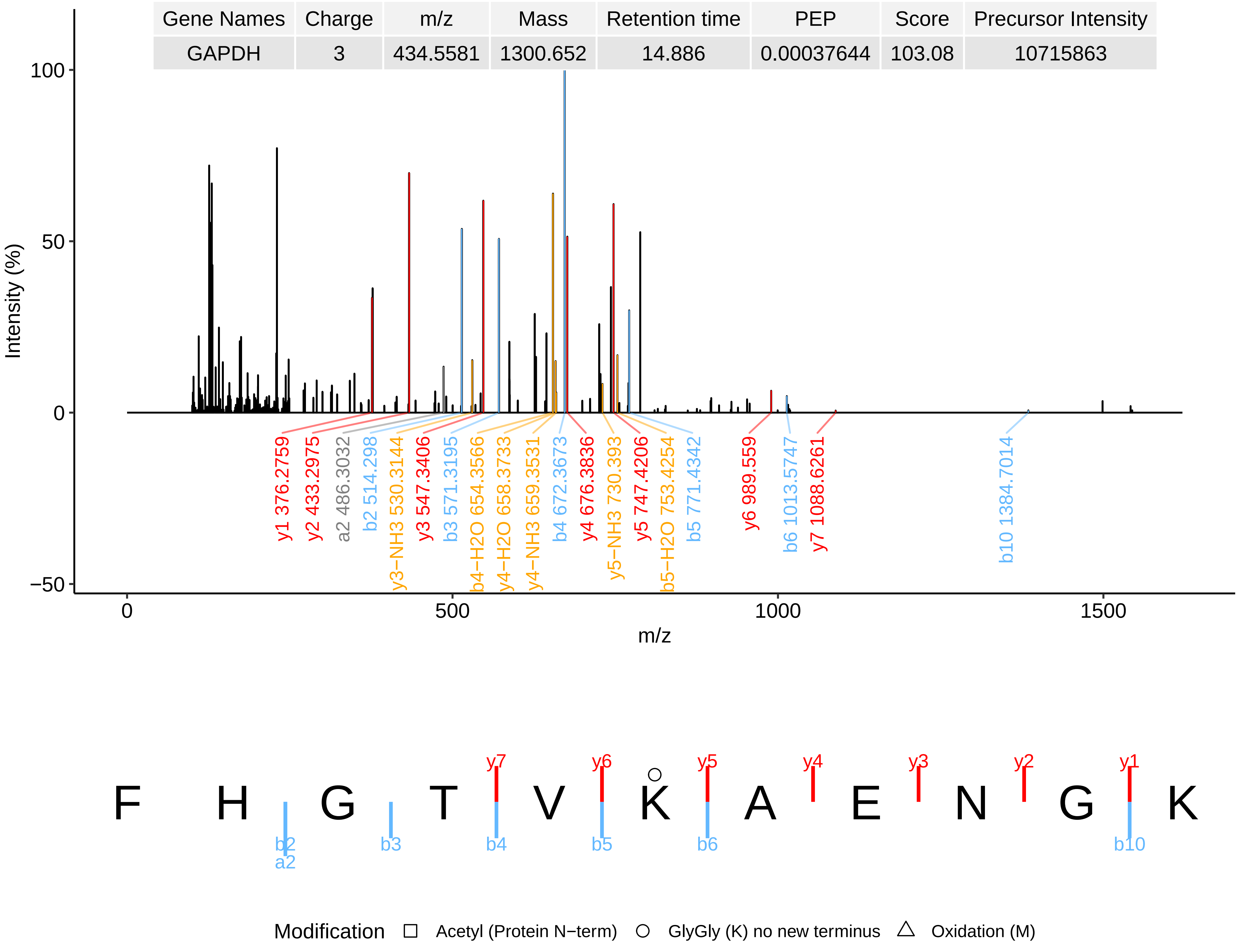

20200924_KIZ_MUb5_GlyGly_2 − Scan number: 4395

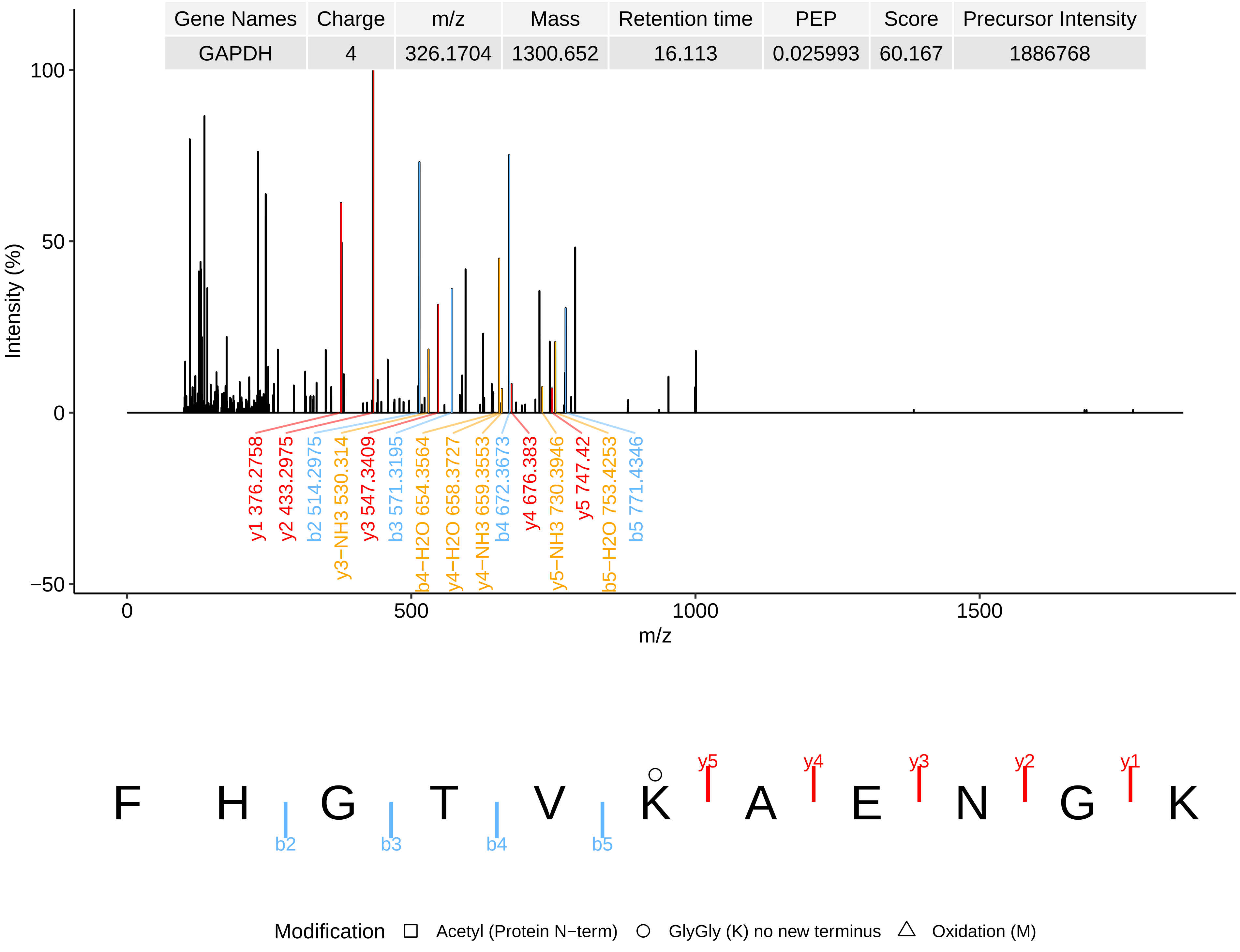

20200924_KIZ_MUb5_GlyGly_2 − Scan number: 4531

Gene Names	Charge	m/z	Mass	Retention time	PEP	Score	Precursor Intensity
GAPDH	4	326.1704	1300.652	16.643	0.0095245	68.626	3665983

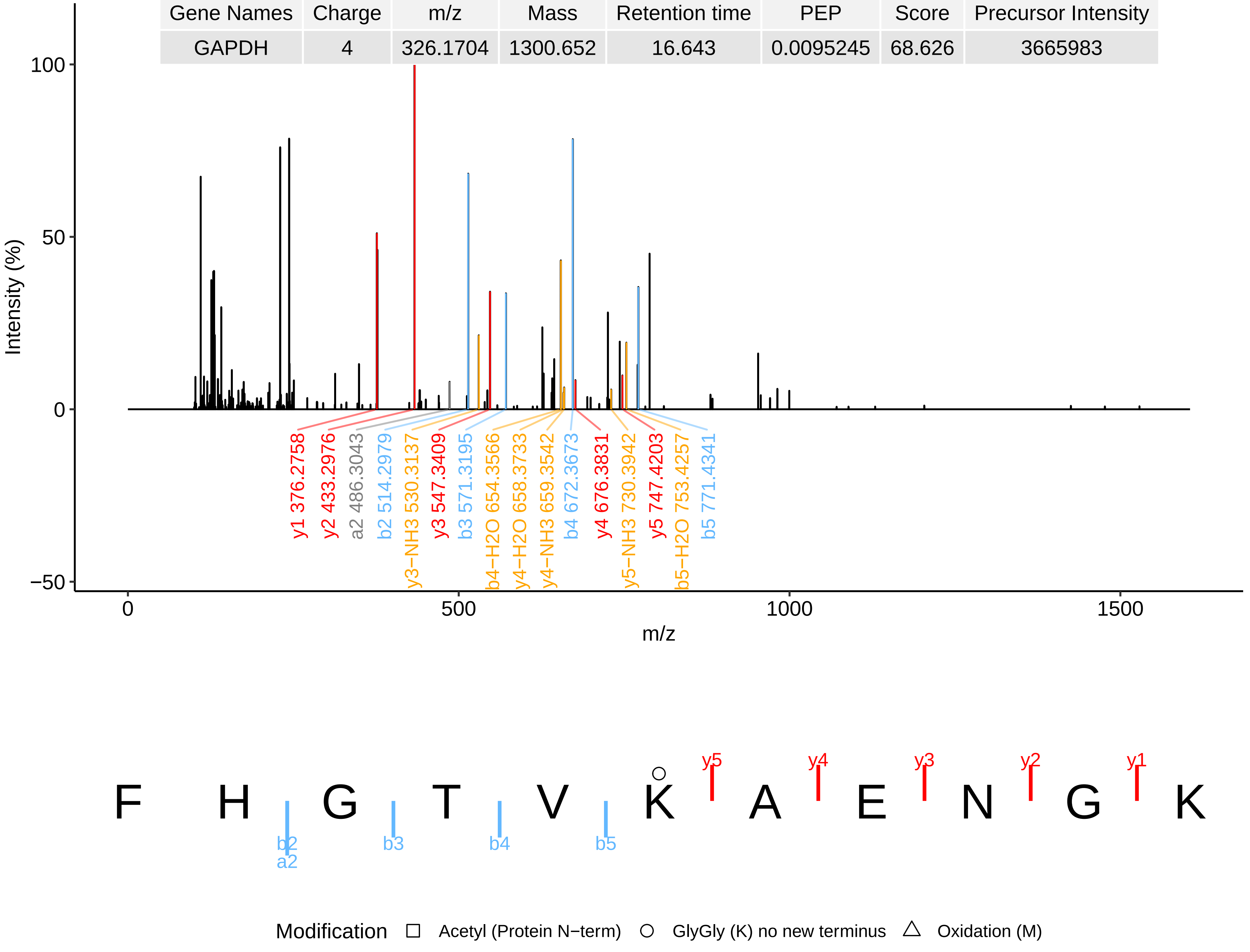

20200924_KIZ_MUb5_GlyGly_2 − Scan number: 4670

Gene Names	Charge	m/z	Mass	Retention time	PEP	Score	Precursor Intensity
GAPDH	4	326.1704	1300.652	17.185	0.01079	66.758	981339.4

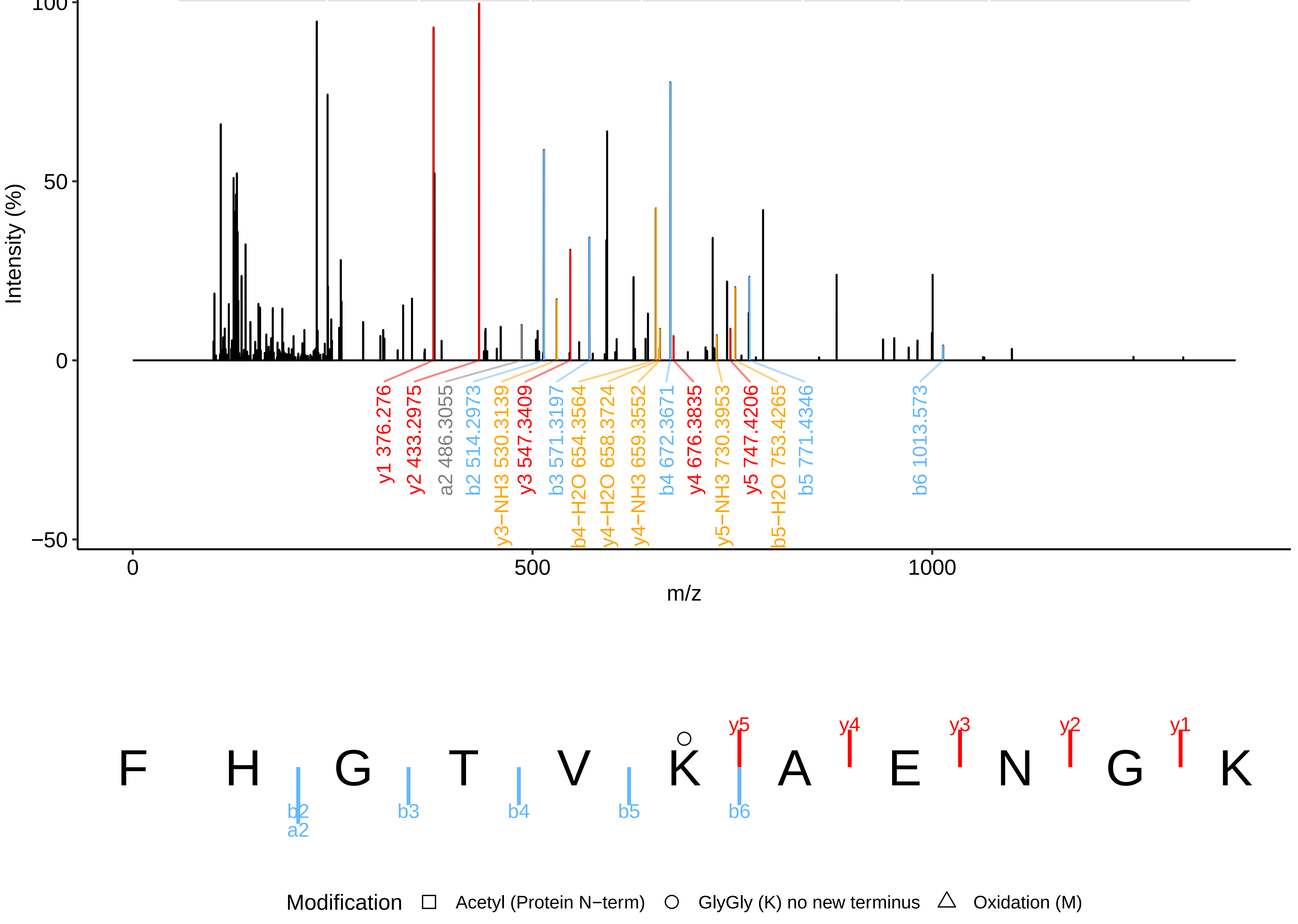

20200924_KIZ_MUb5_GlyGly_2 − Scan number: 4555

Gene Names	Charge	m/z	Mass	Retention time	PEP	Score	Precursor Intensity
GAPDH	3	434.5581	1300.652	16.734	0.00078142	94.692	837370.3

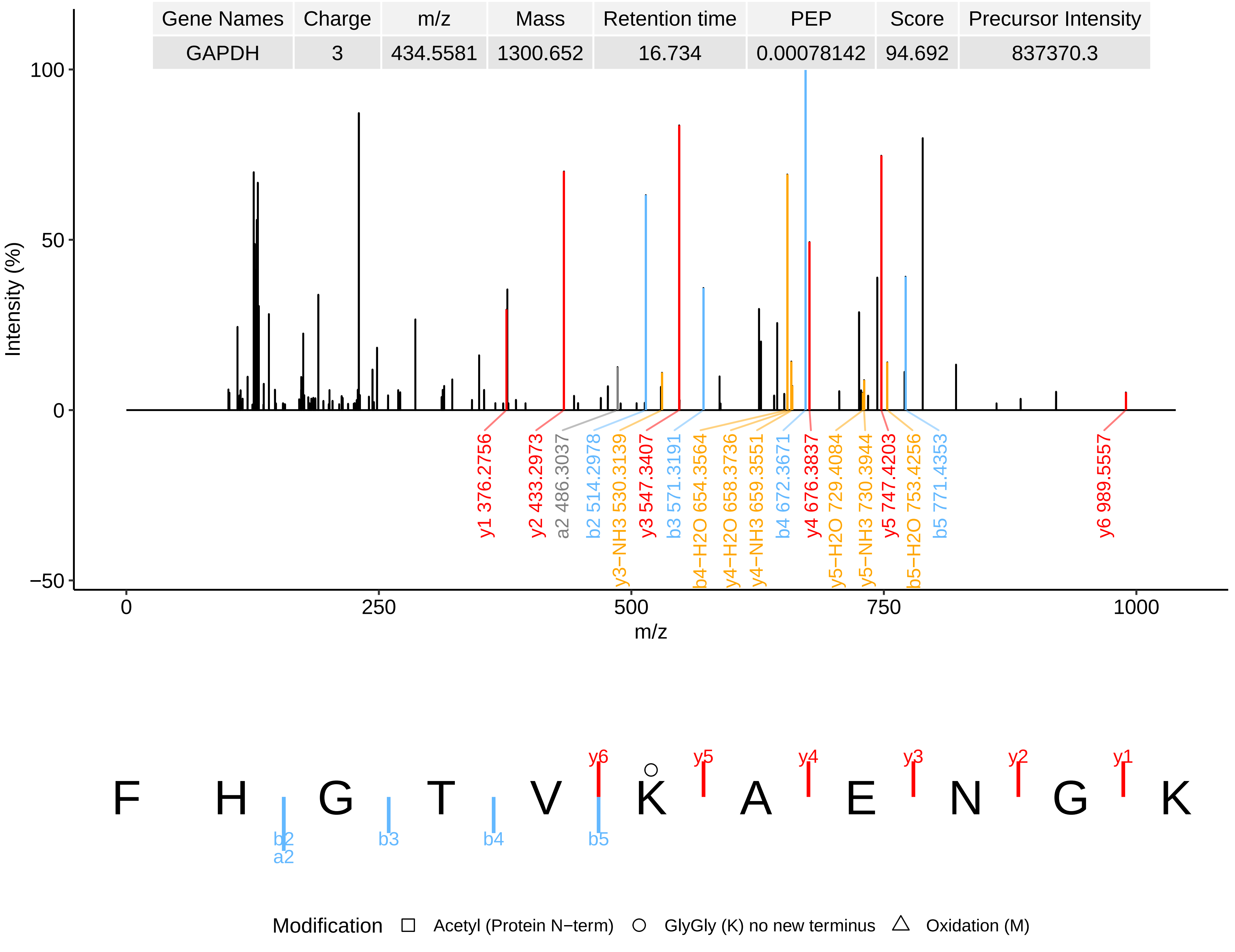

20201118_KIZ_MUb6_GlyGly_1 − Scan number: 4727

Gene Names	Charge	m/z	Mass	Retention time	PEP	Score	Precursor Intensity
GAPDH	4	326.1704	1300.652	14.886	0.004861	75.509	16640817

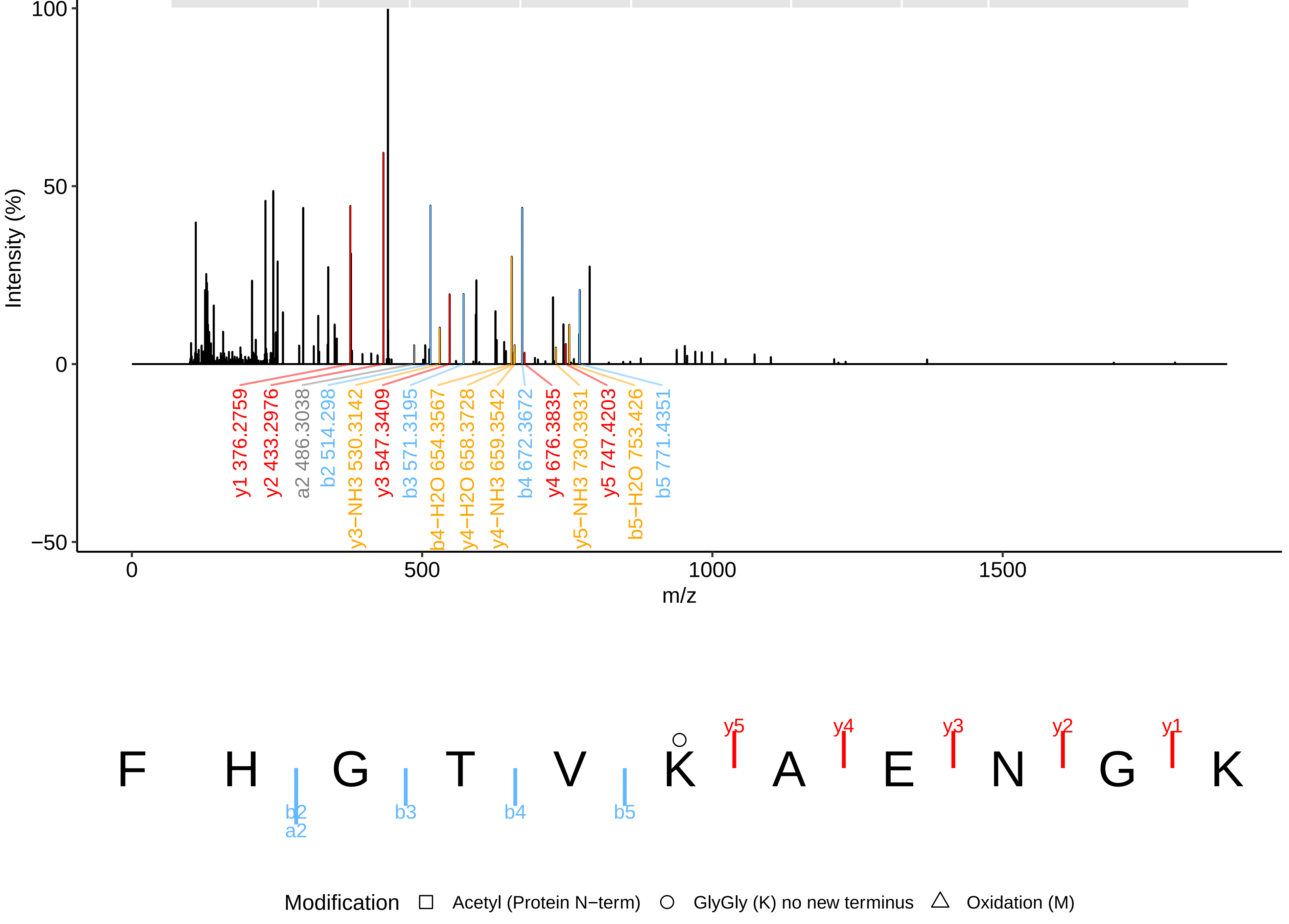

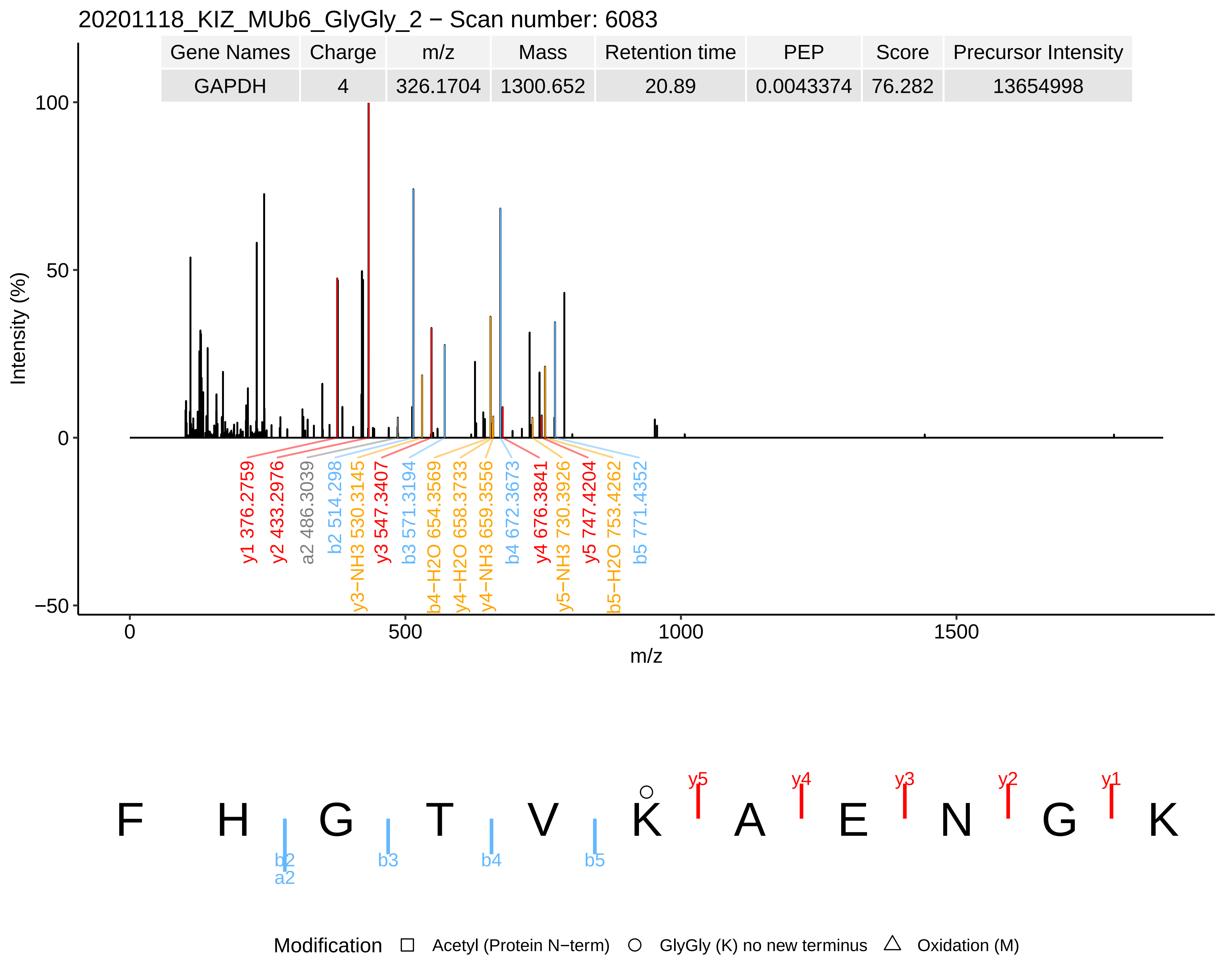
20201118_KIZ_MUb6_GlyGly_2 − Scan number: 6083
Gene Names | Charge | m/z | Mass | Retention time | PEP | Score | Precursor Intensity
GAPDH | 4 | 326.1704 | 1300.652 | 20.89 | 0.0043374 | 76.282 | 13654998
Intensity (%)
m/z
y1 376.2759
y2 433.2976
a2 486.3039
b2 514.298
y3−NH3 530.3145
y3 547.3407
b3 571.3194
b4−H2O 654.3569
y4−H2O 658.3733
y4−NH3 659.3556
b4 672.3673
y4 676.3841
y5−NH3 730.3926
y5 747.4204
b5−H2O 753.4262
b5 771.4352
F H G T V K A E N G K
y5 y4 y3 y2 y1
b2 a2 b3 b4 b5
Modification
Acetyl (Protein N−term)
GlyGly (K) no new terminus
Oxidation (M)

20200921_KIZ_MUb4_GlyGly_10plex_2 − Scan number: 16397

Gene Names	Charge	m/z	Mass	Retention time	PEP	Score	Precursor Intensity
WIZ	3	495.5941	1483.761	64.015	4.7836e−08	117.7	2714322

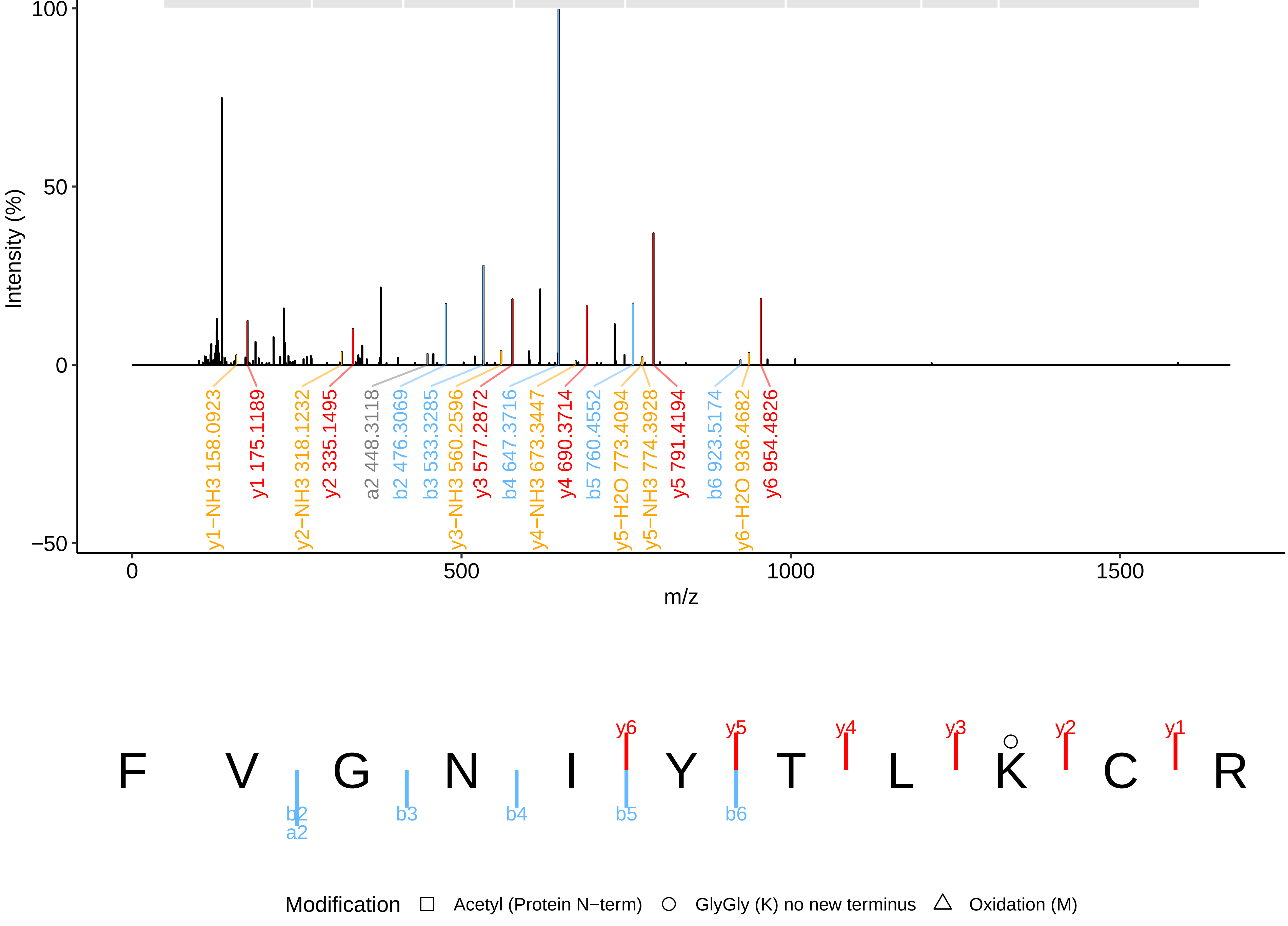

20200921_KIZ_MUb4_GlyGly_10plex_2 − Scan number: 16434

Gene Names	Charge	m/z	Mass	Retention time	PEP	Score	Precursor Intensity
WIZ	3	495.5941	1483.761	64.158	0.00040121	94.309	2498493

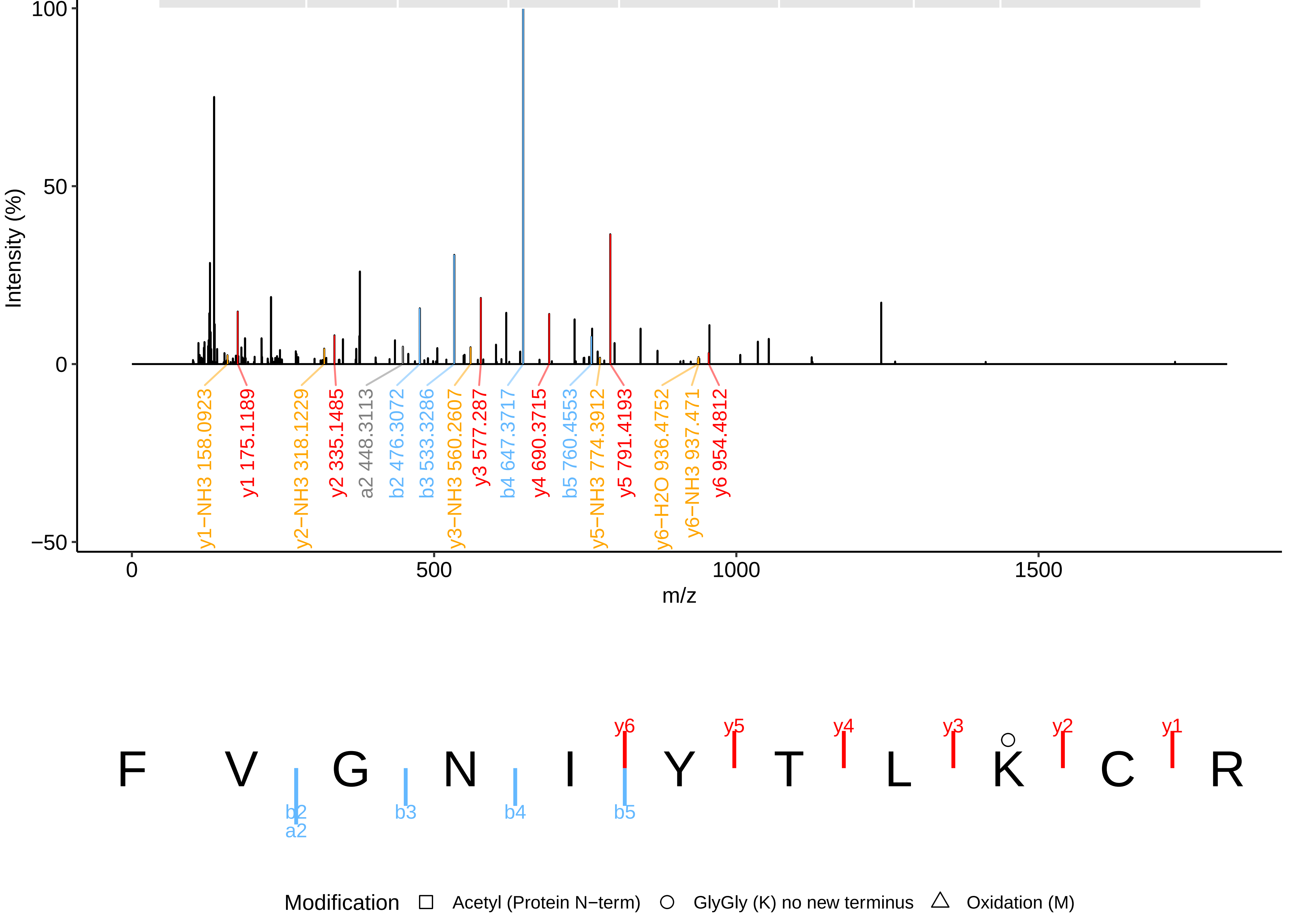

20200924_KIZ_MUb5_GlyGly_2 − Scan number: 16106

Gene Names	Charge	m/z	Mass	Retention time	PEP	Score	Precursor Intensity
WIZ	3	495.5941	1483.761	59.795	0.00080297	90.149	3761575

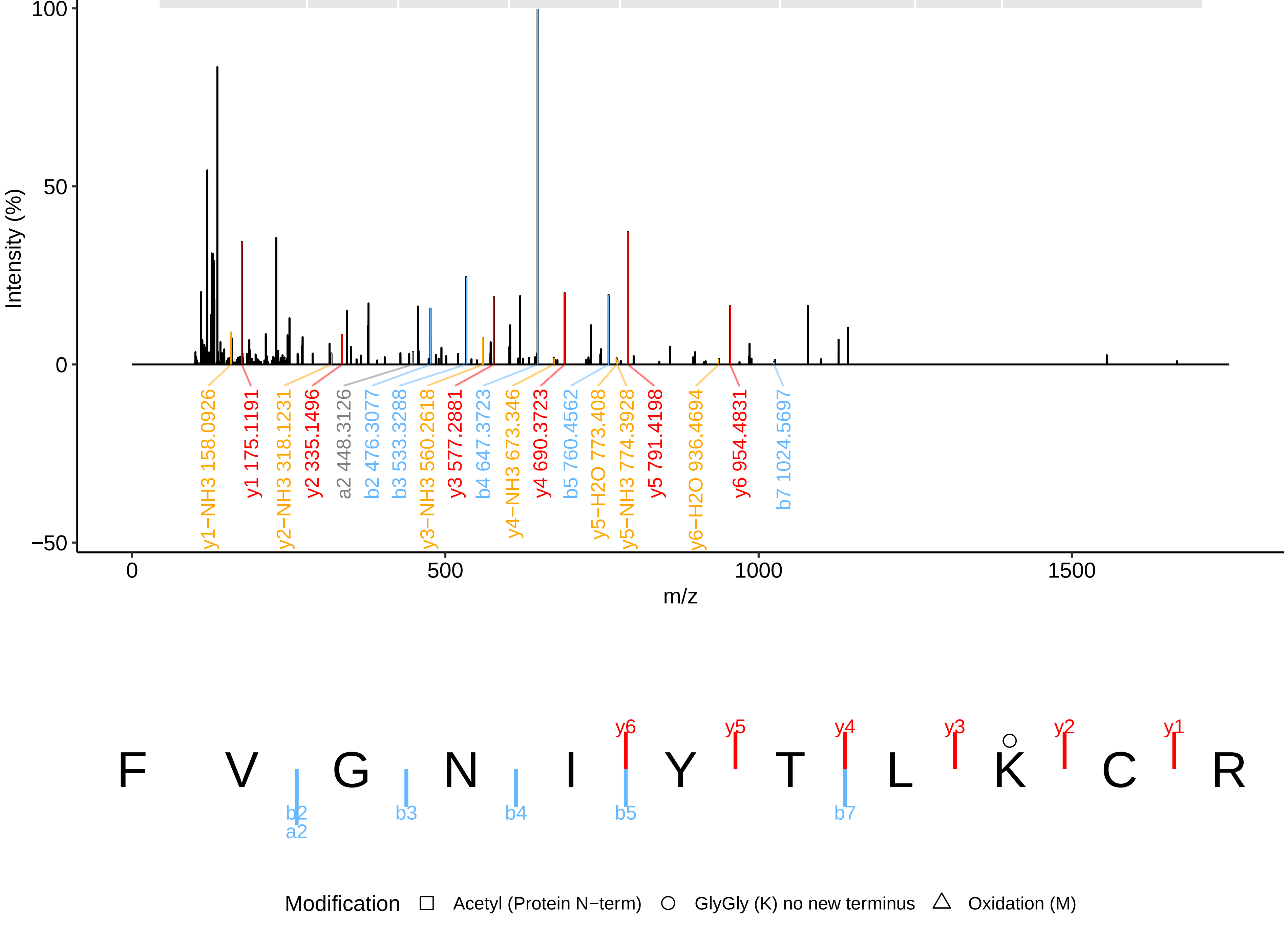

20201118_KIZ_MUb6_GlyGly_1 − Scan number: 16927

Gene Names	Charge	m/z	Mass	Retention time	PEP	Score	Precursor Intensity
WIZ	3	495.5941	1483.761	53.312	2.1744e−12	136.96	15757289

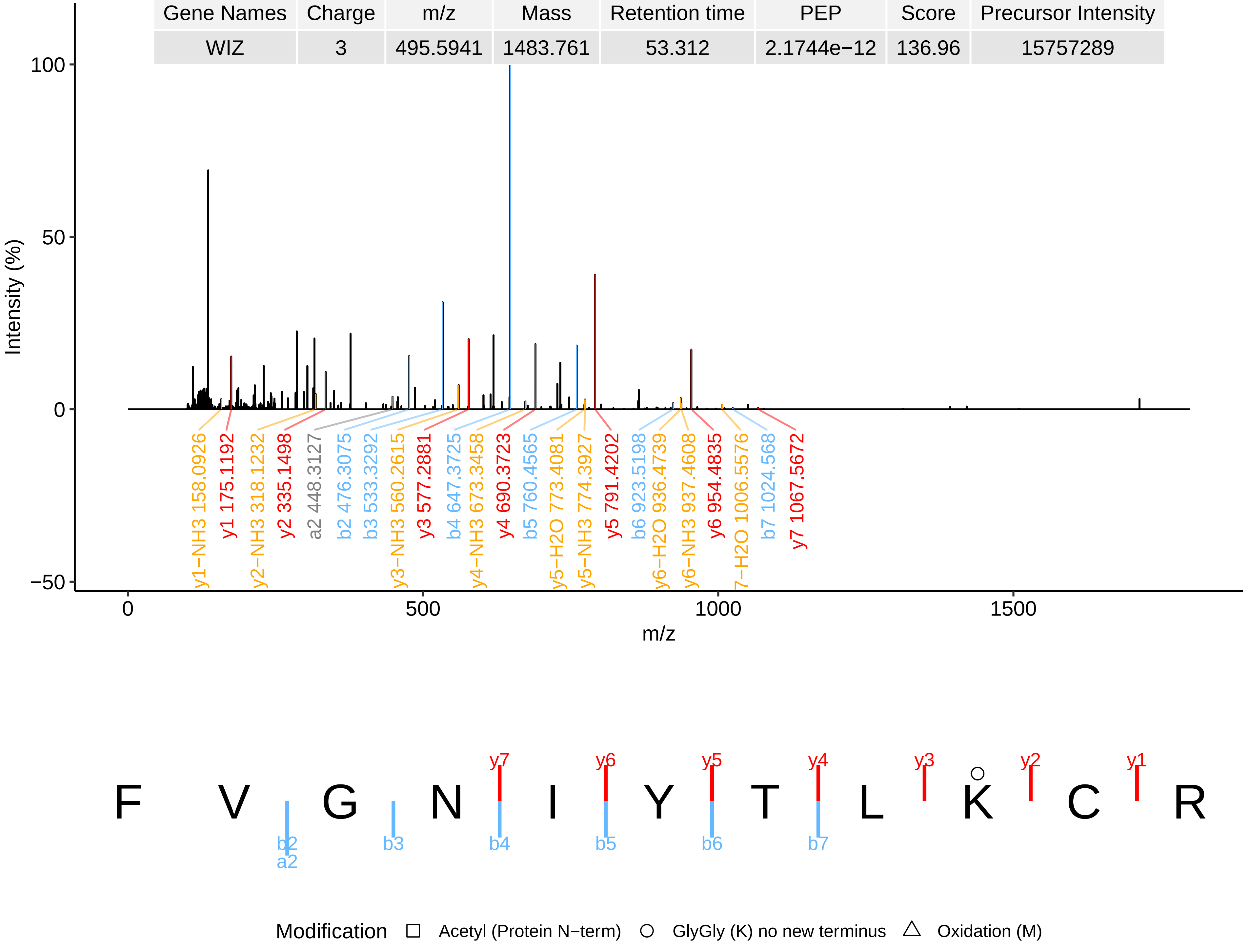

20201118_KIZ_MUb6_GlyGly_2 − Scan number: 19308

Gene Names	Charge	m/z	Mass	Retention time	PEP	Score	Precursor Intensity
WIZ	3	495.5941	1483.761	65.155	4.7836e−08	117.7	9473148

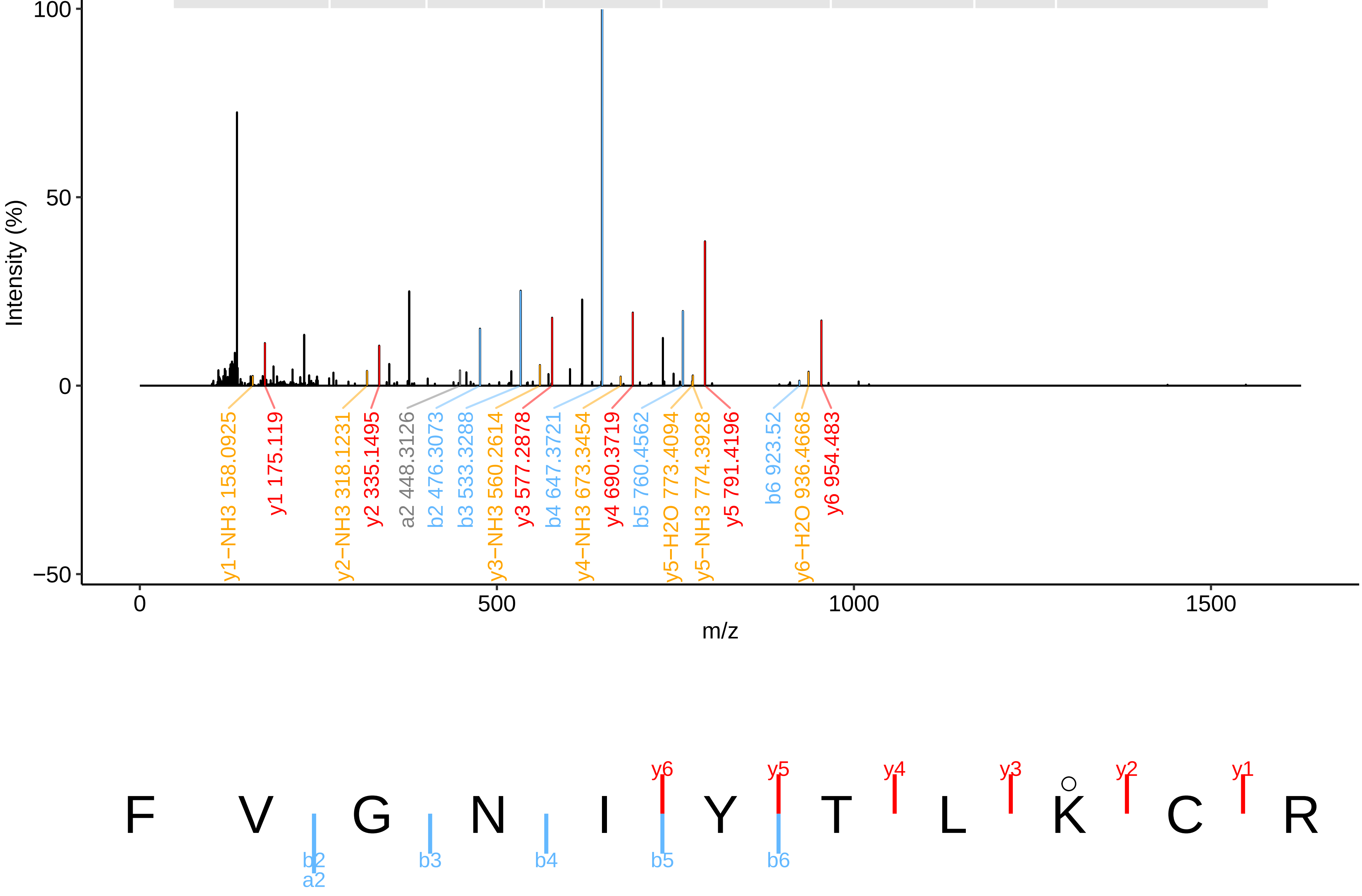

Modification □ Acetyl (Protein N−term) ○ GlyGly (K) no new terminus △ Oxidation (M)

20200921_KIZ_MUb4_GlyGly_10plex_2 − Scan number: 7955

Gene Names	Charge	m/z	Mass	Retention time	PEP	Score	Precursor Intensity
SLCO4A1	3	502.5829	1504.727	29.855	0.0061594	72.496	282316.1

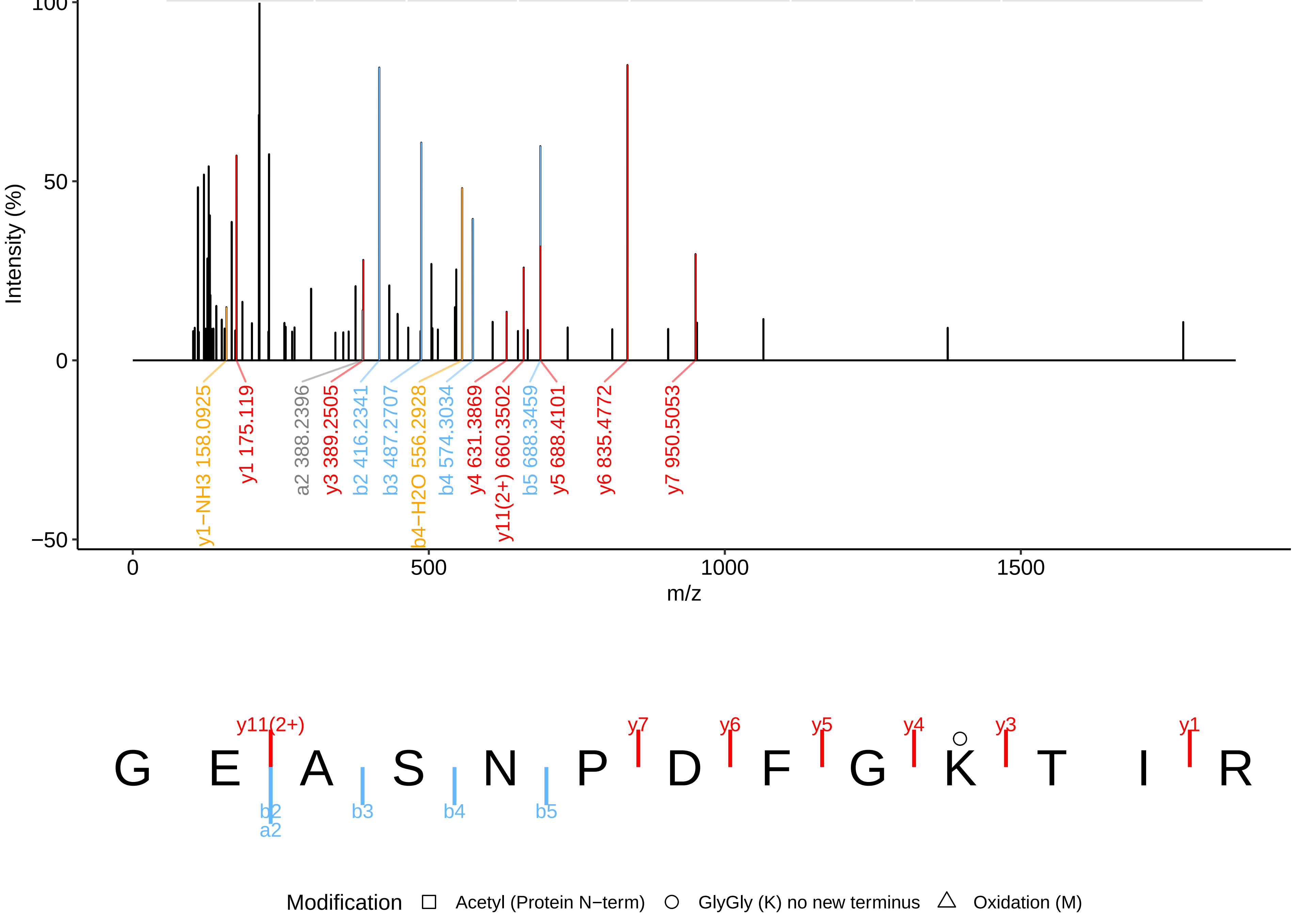

20200921_KIZ_MUb4_GlyGly_10plex − Scan number: 4331

Gene Names	Charge	m/z	Mass	Retention time	PEP	Score	Precursor Intensity
GPR56	3	496.2463	1485.717	14.113	3.4569e−05	104.49	6053405

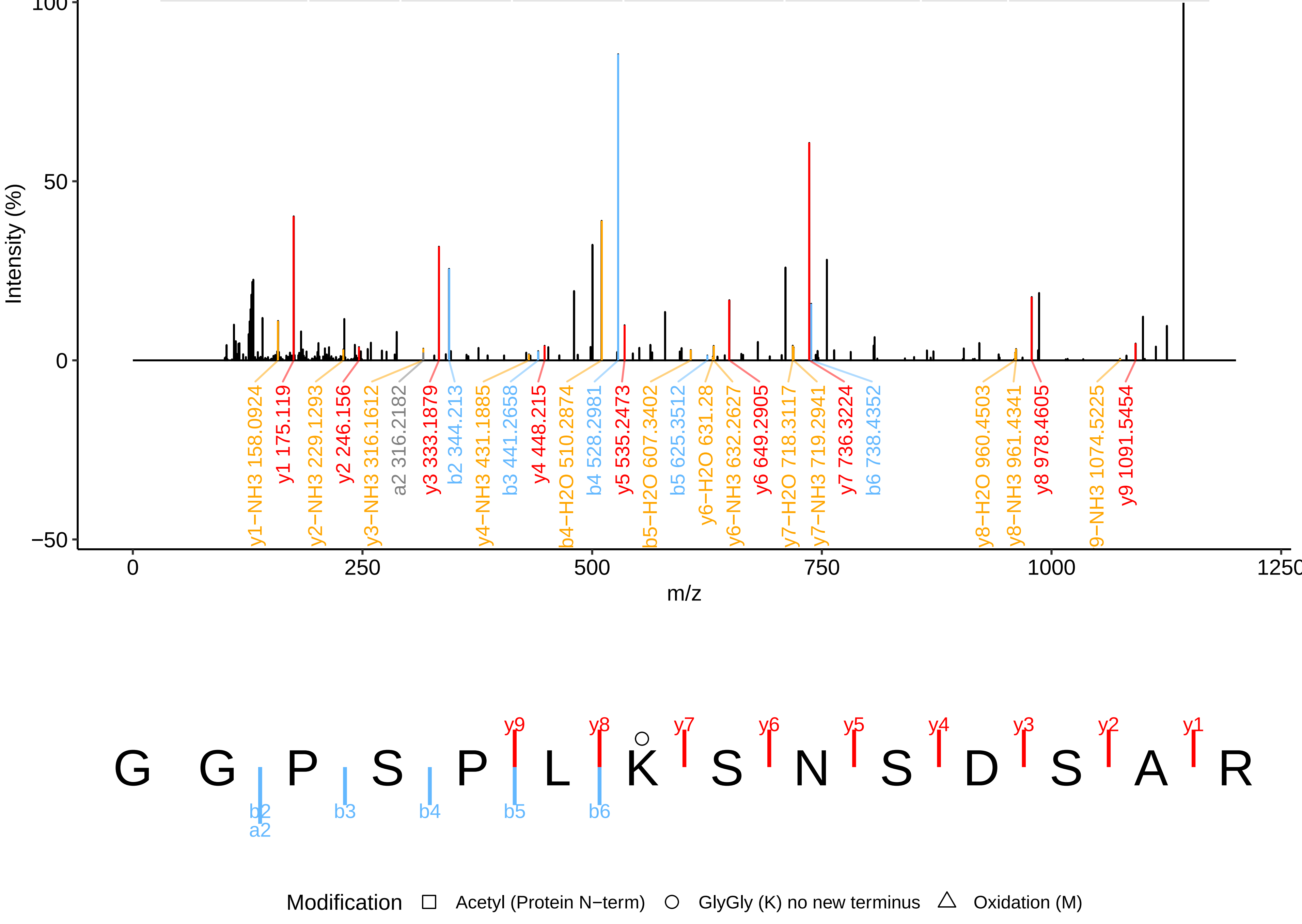

20200921_KIZ_MUb4_GlyGly_10plex − Scan number: 7759

Gene Names	Charge	m/z	Mass	Retention time	PEP	Score	Precursor Intensity
CYB5R1	4	324.9256	1295.674	26.157	0.0029948	78.934	10071317

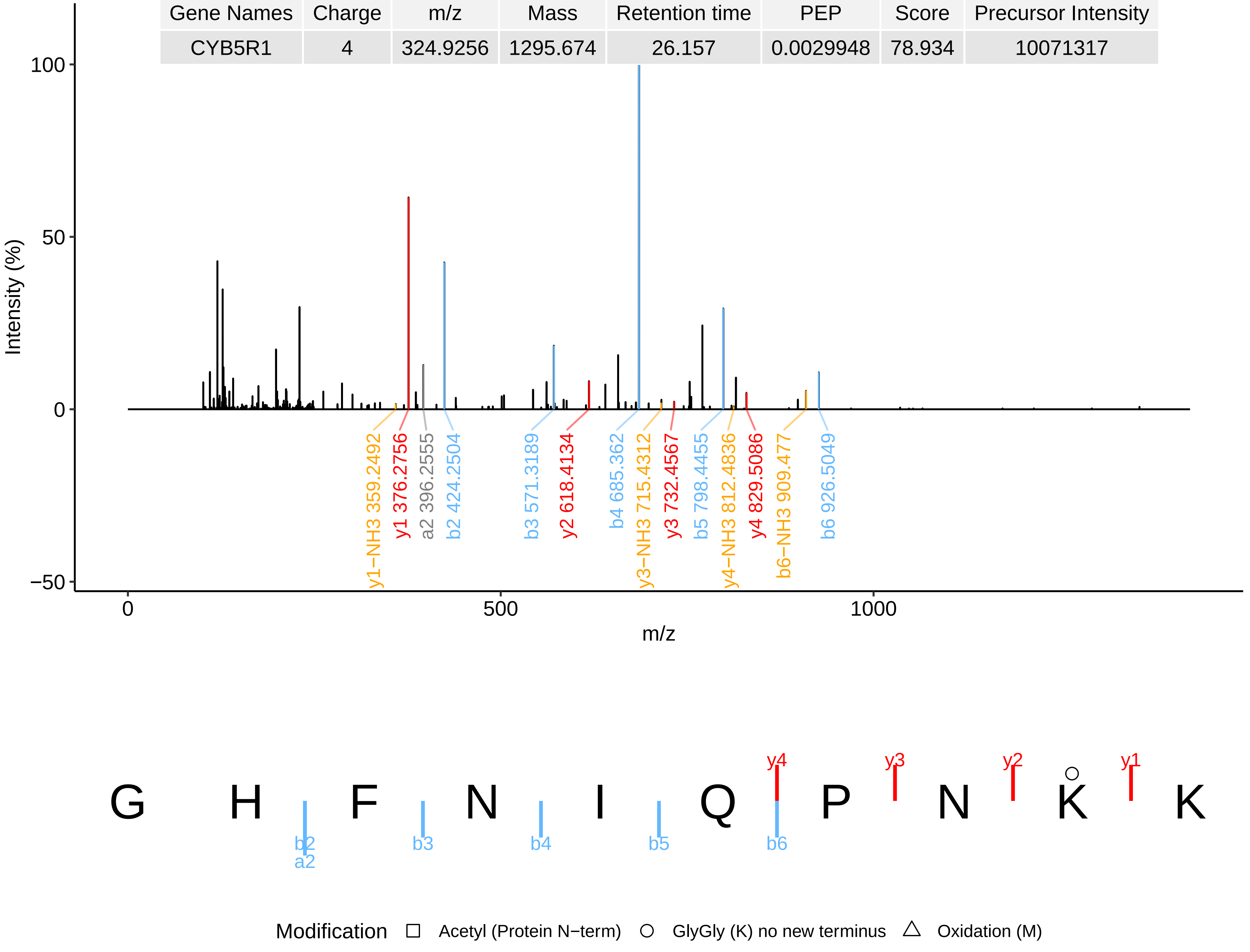

20200921_KIZ_MUb4_GlyGly_10plex_2 − Scan number: 7219

Gene Names	Charge	m/z	Mass	Retention time	PEP	Score	Precursor Intensity
CYB5R1	4	324.9256	1295.674	26.856	0.020621	61.398	808455.6

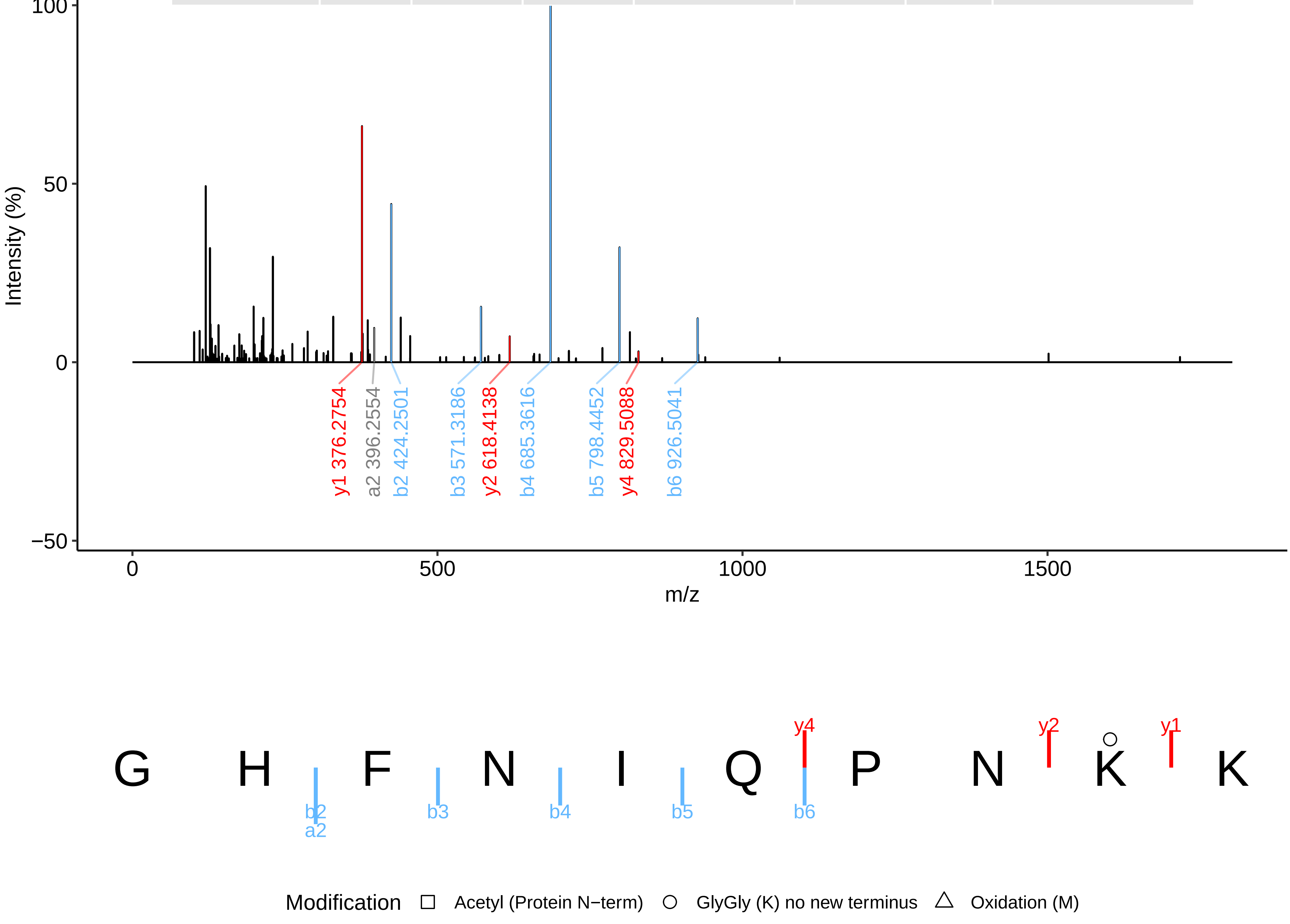

20200921_KIZ_MUb4_GlyGly_10plex − Scan number: 2432

Gene Names	Charge	m/z	Mass	Retention time	PEP	Score	Precursor Intensity
EIF1AX;EIF1AY	4	326.9102	1303.612	7.6678	9.4841e−05	112.13	5969929

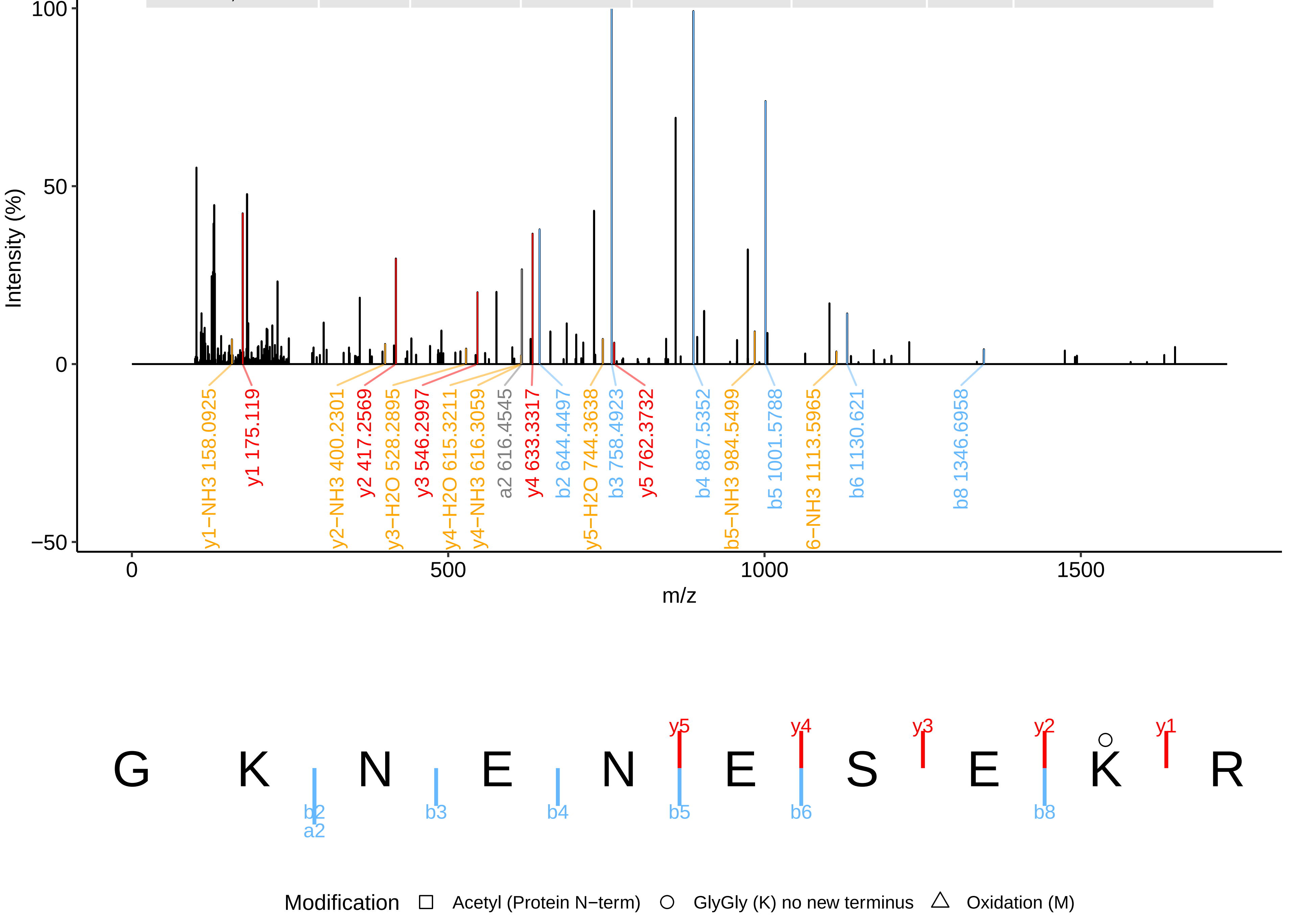

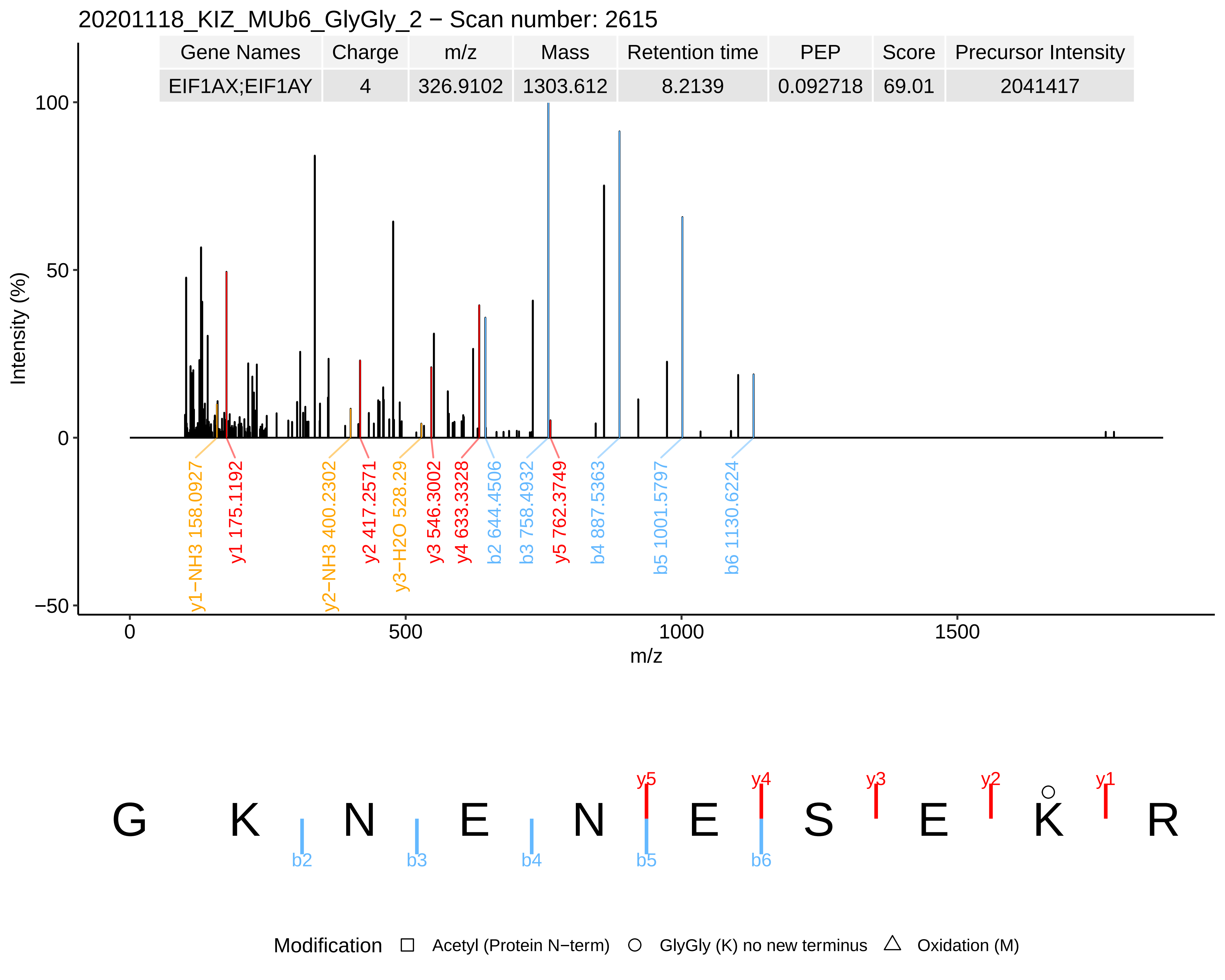

Gene Names	Charge	m/z	Mass	Retention time	PEP	Score	Precursor Intensity
EIF1AX;EIF1AY	4	326.9102	1303.612	8.2139	0.092718	69.01	2041417

20200921_KIZ_MUb4_GlyGly_10plex − Scan number: 18263

Gene Names	Charge	m/z	Mass	Retention time	PEP	Score	Precursor Intensity
LDHB	3	438.9295	1313.767	65.628	2.3544e−12	135.86	6860418

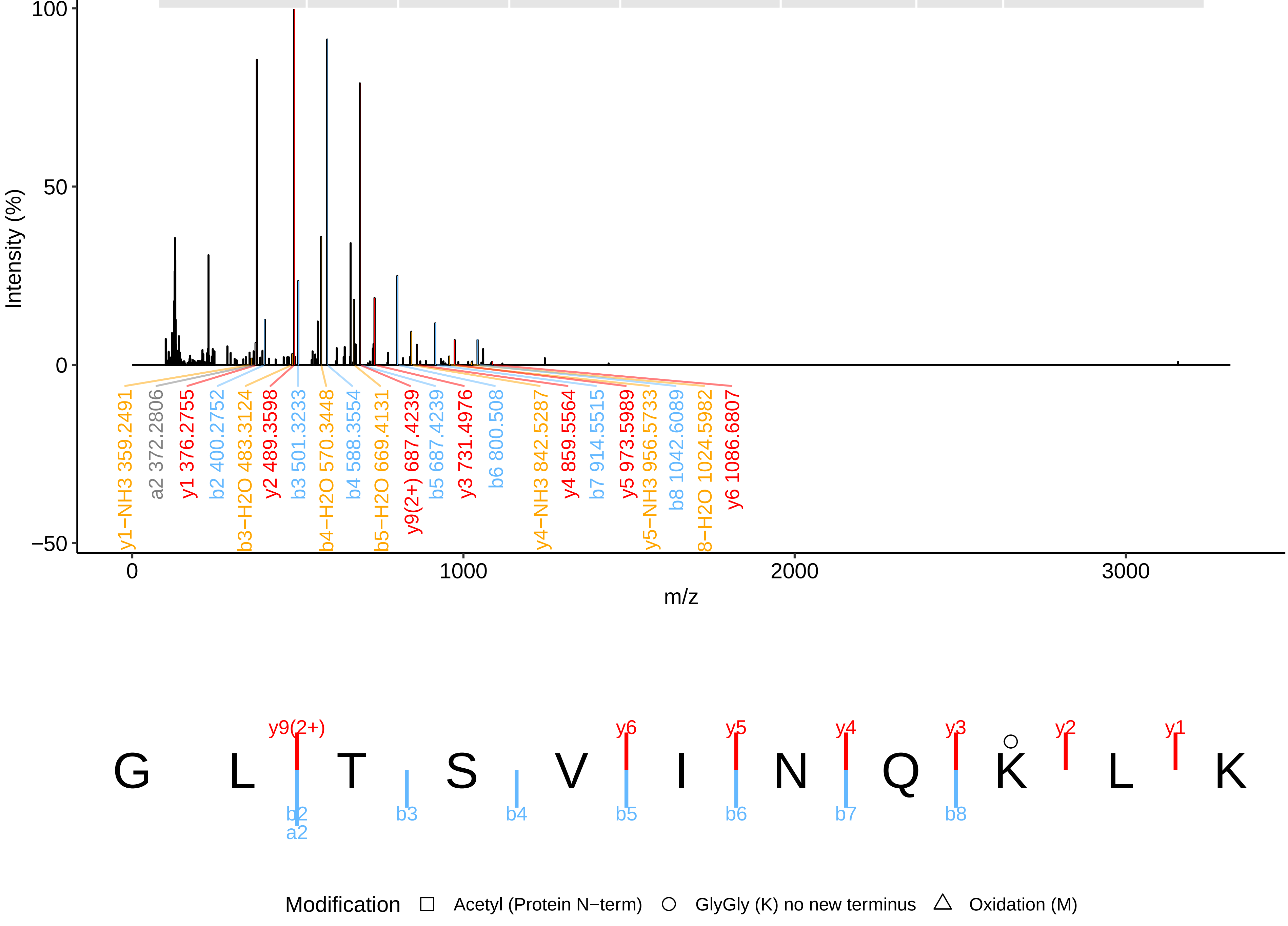

20200921_KIZ_MUb4_GlyGly_10plex − Scan number: 18395

Gene Names	Charge	m/z	Mass	Retention time	PEP	Score	Precursor Intensity
LDHB	3	438.9295	1313.767	66.139	2.0077e−12	137.98	77390136

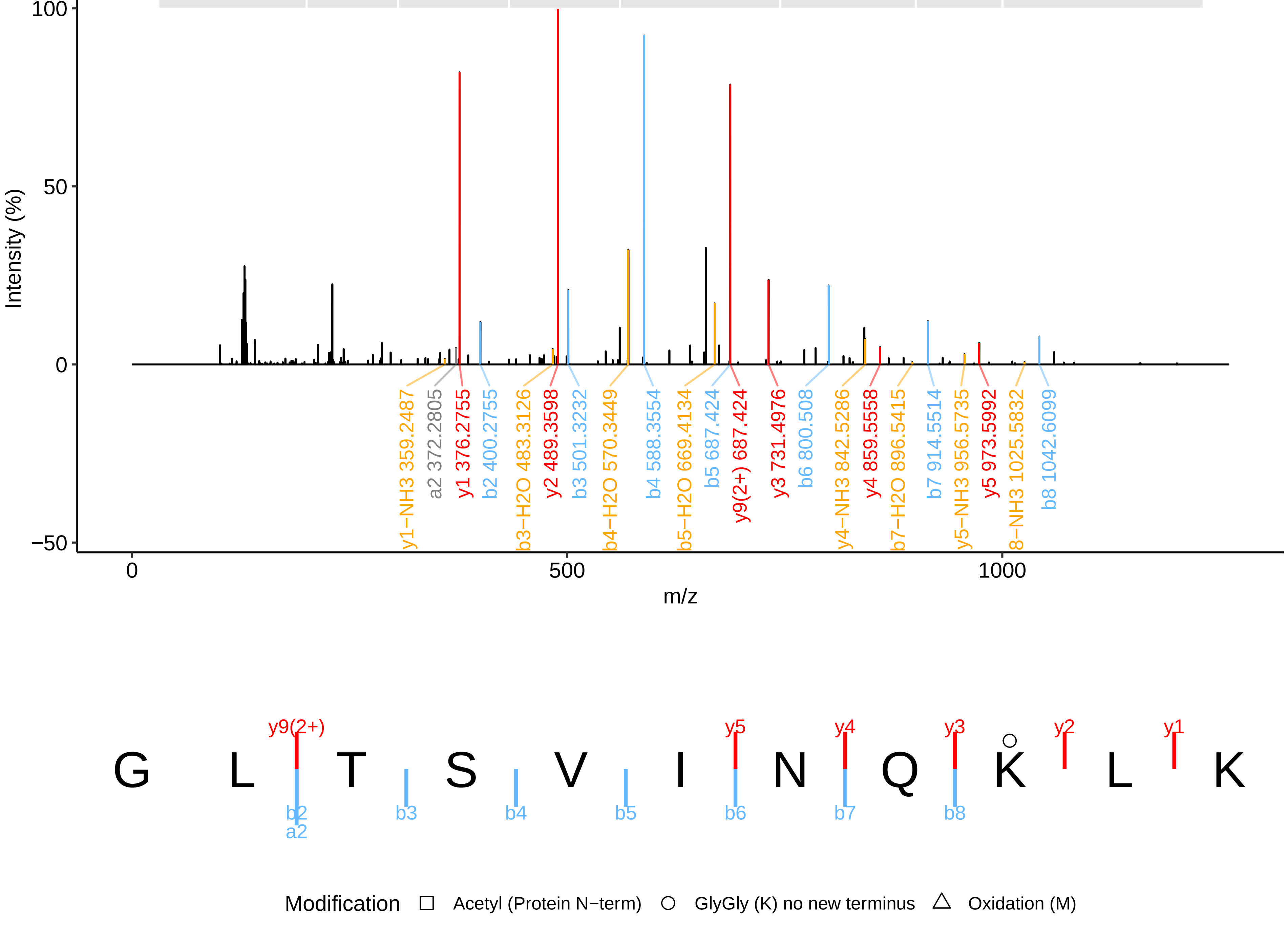

20200921_KIZ_MUb4_GlyGly_10plex − Scan number: 18525

Gene Names	Charge	m/z	Mass	Retention time	PEP	Score	Precursor Intensity
LDHB	3	438.9295	1313.767	66.654	7.1263e−13	145.89	10911179

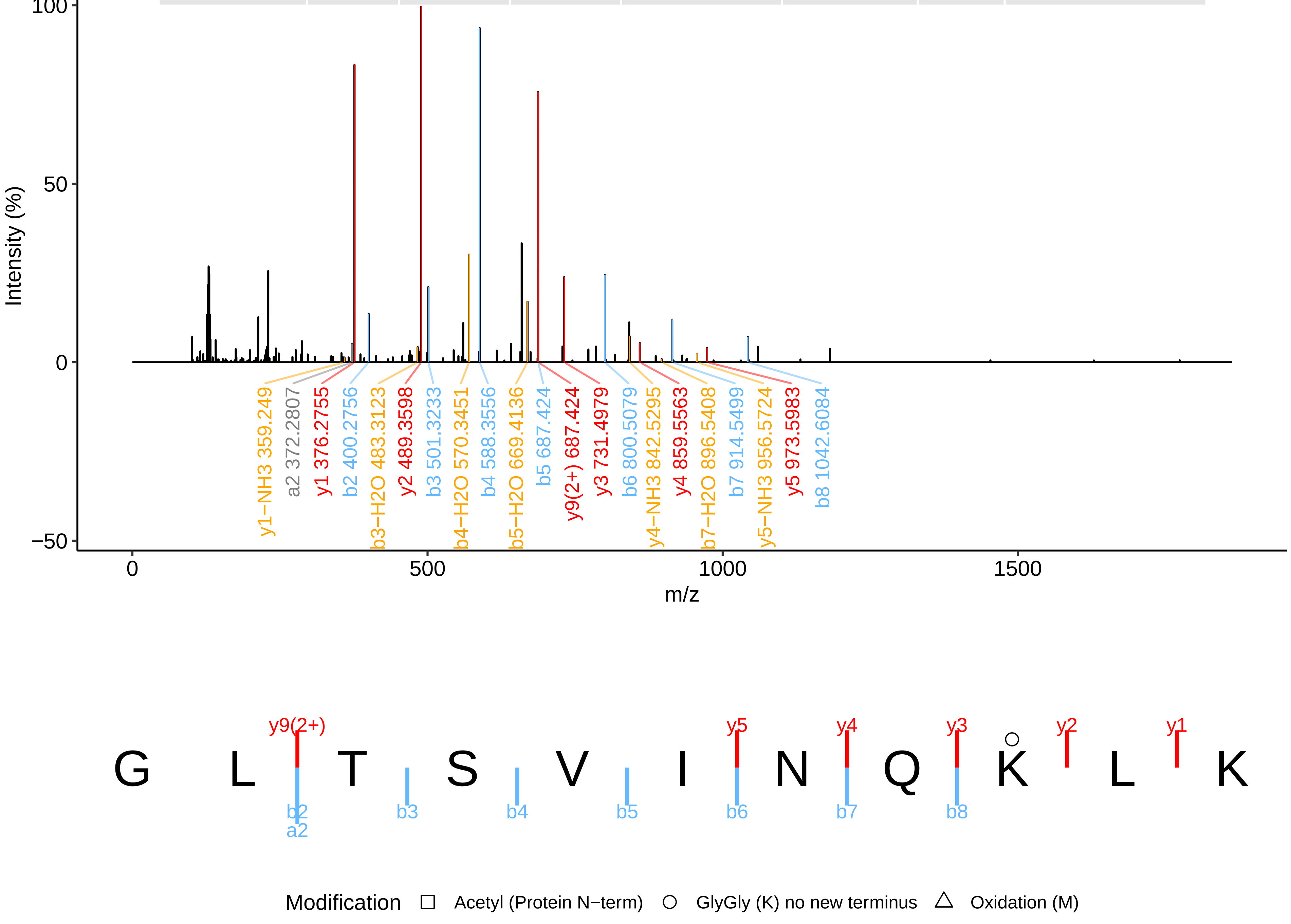

20200921_KIZ_MUb4_GlyGly_10plex_2 − Scan number: 16781

Gene Names	Charge	m/z	Mass	Retention time	PEP	Score	Precursor Intensity
LDHB	3	438.9295	1313.767	65.548	2.0077e−12	137.98	944664.1

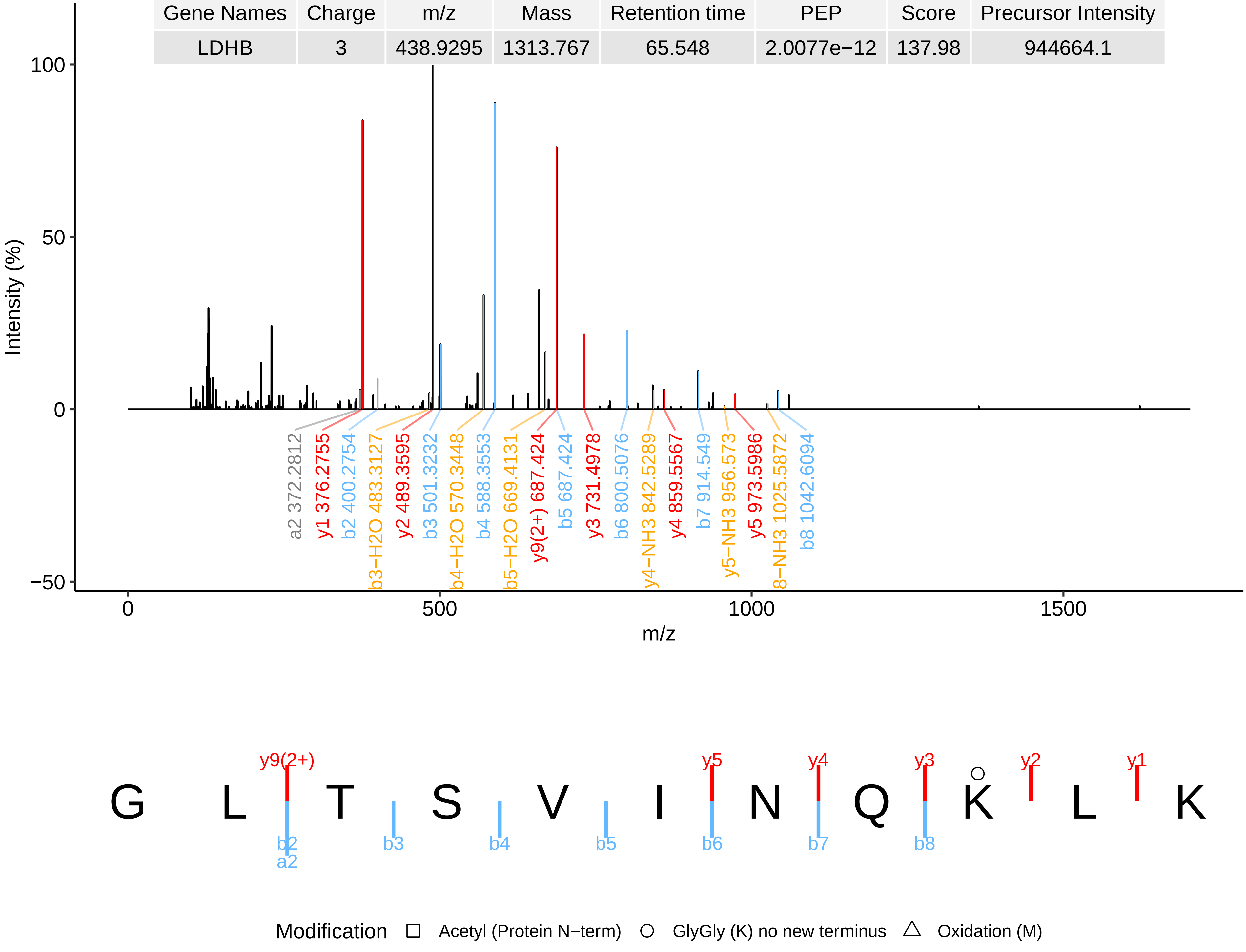

20200924_KIZ_MUb5_GlyGly_1 − Scan number: 17305

Gene Names	Charge	m/z	Mass	Retention time	PEP	Score	Precursor Intensity
LDHB	3	438.9295	1313.767	55.871	2.2385e−08	125.75	40958908

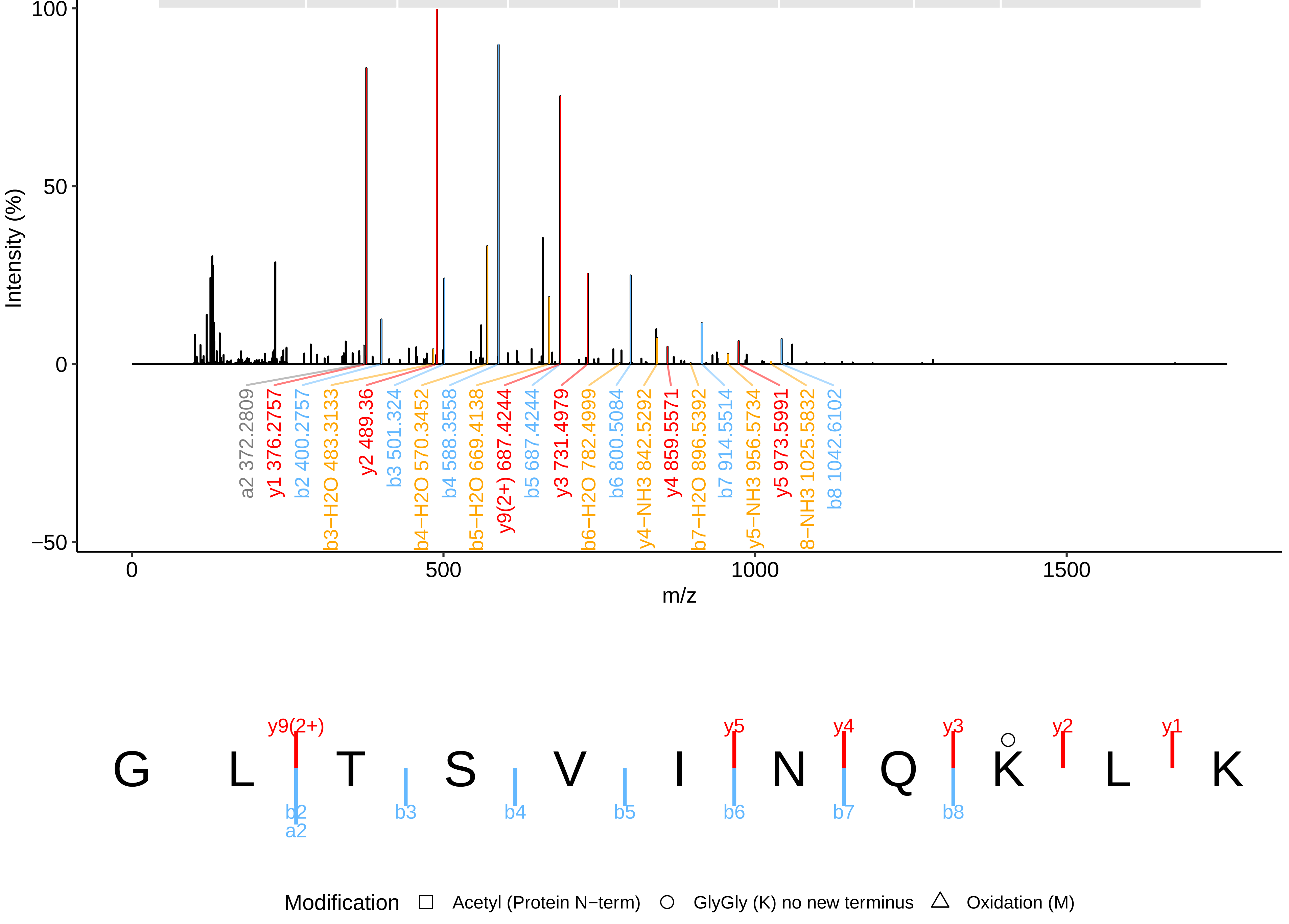

20200924_KIZ_MUb5_GlyGly_1 − Scan number: 17465

Gene Names	Charge	m/z	Mass	Retention time	PEP	Score	Precursor Intensity
LDHB	3	438.9295	1313.767	56.383	3.1403e−13	148.32	65164336

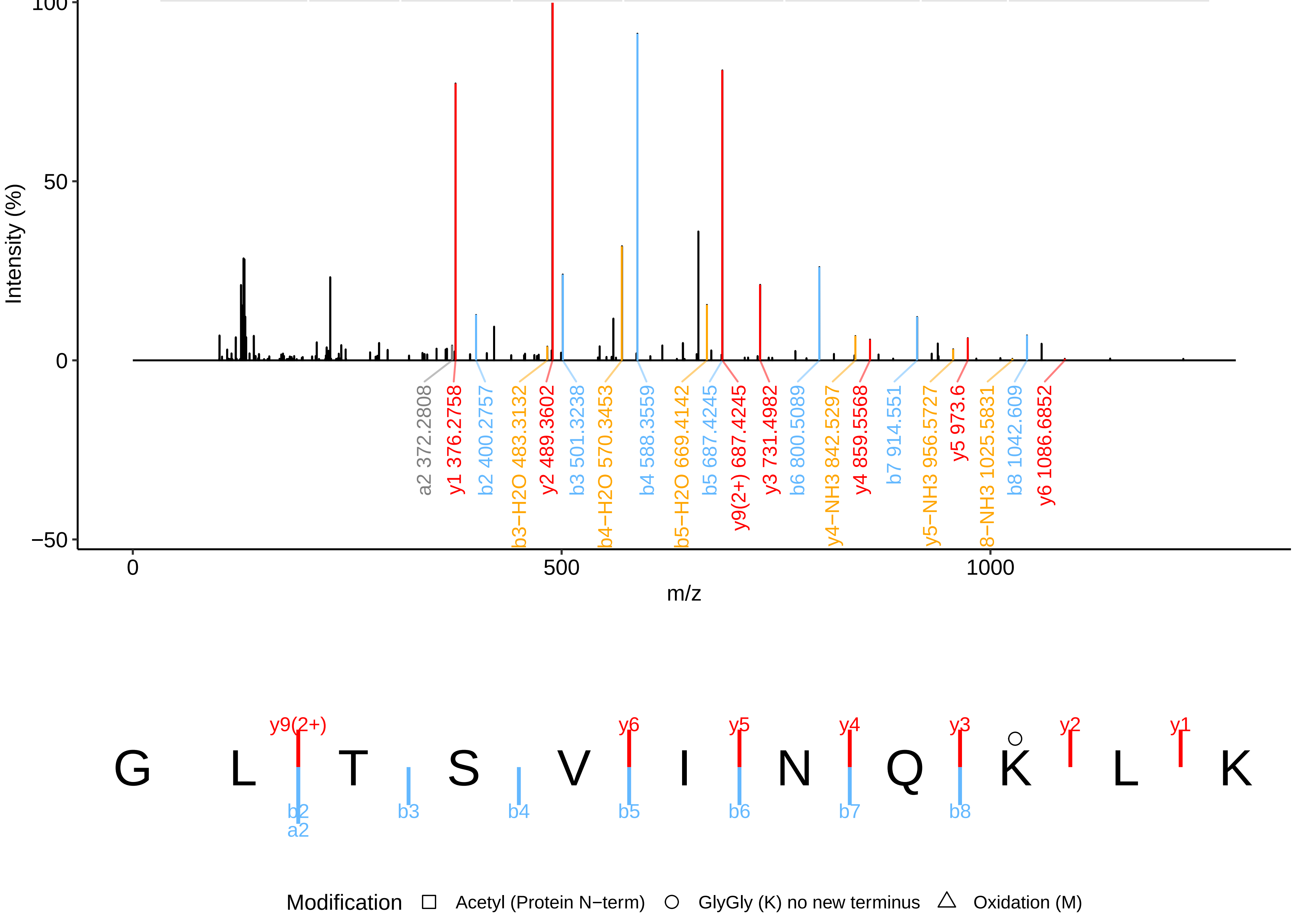

20200924_KIZ_MUb5_GlyGly_1 − Scan number: 17616

Gene Names	Charge	m/z	Mass	Retention time	PEP	Score	Precursor Intensity
LDHB	3	438.9295	1313.767	56.893	3.1403e−13	148.32	62276944

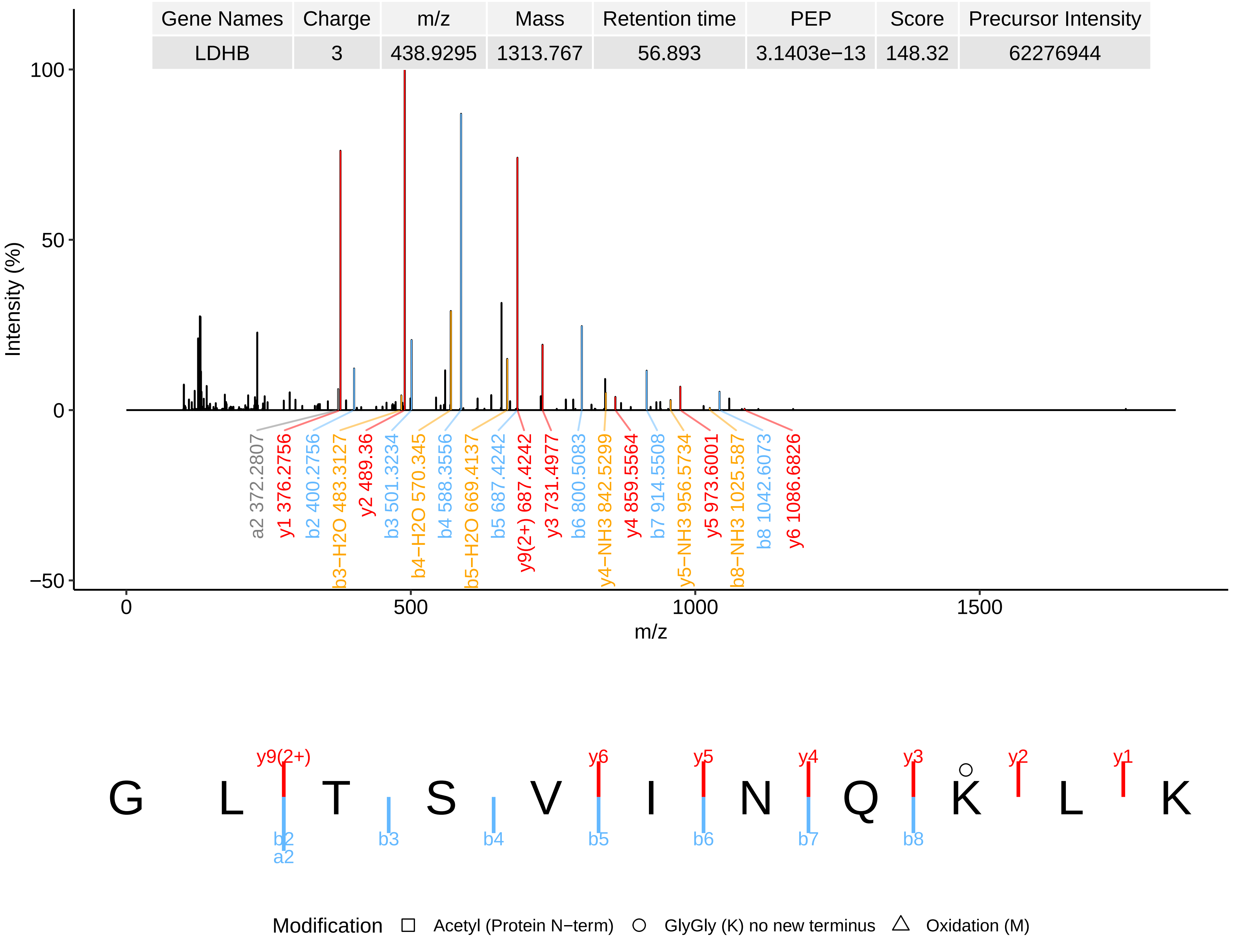

20200924_KIZ_MUb5_GlyGly_1 − Scan number: 17768

Gene Names	Charge	m/z	Mass	Retention time	PEP	Score	Precursor Intensity
LDHB	3	438.9295	1313.767	57.402	3.1403e−13	148.32	57968596

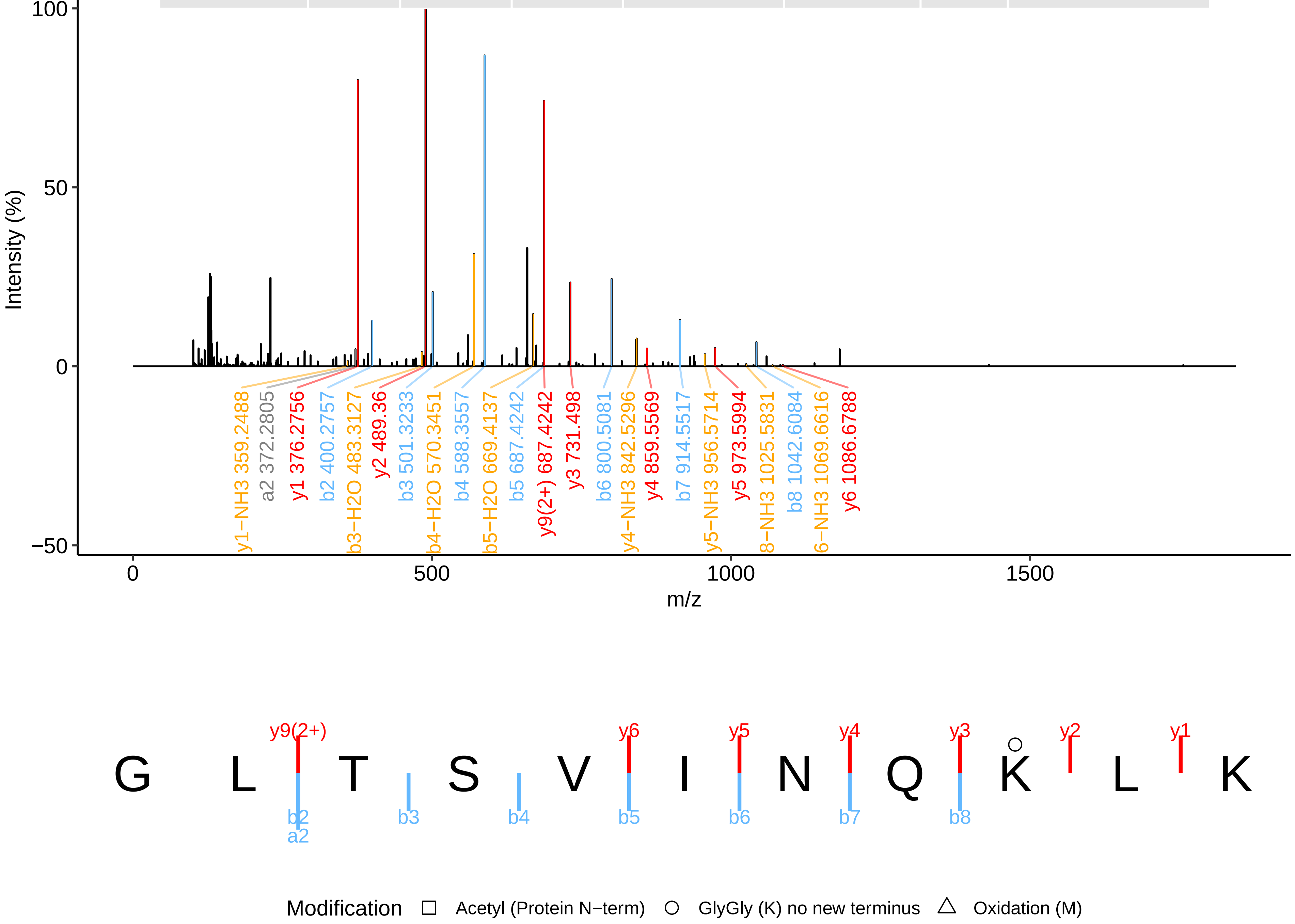

20200924_KIZ_MUb5_GlyGly_1 − Scan number: 17919

Gene Names	Charge	m/z	Mass	Retention time	PEP	Score	Precursor Intensity
LDHB	3	438.9295	1313.767	57.913	6.8572e−17	161.17	17004722

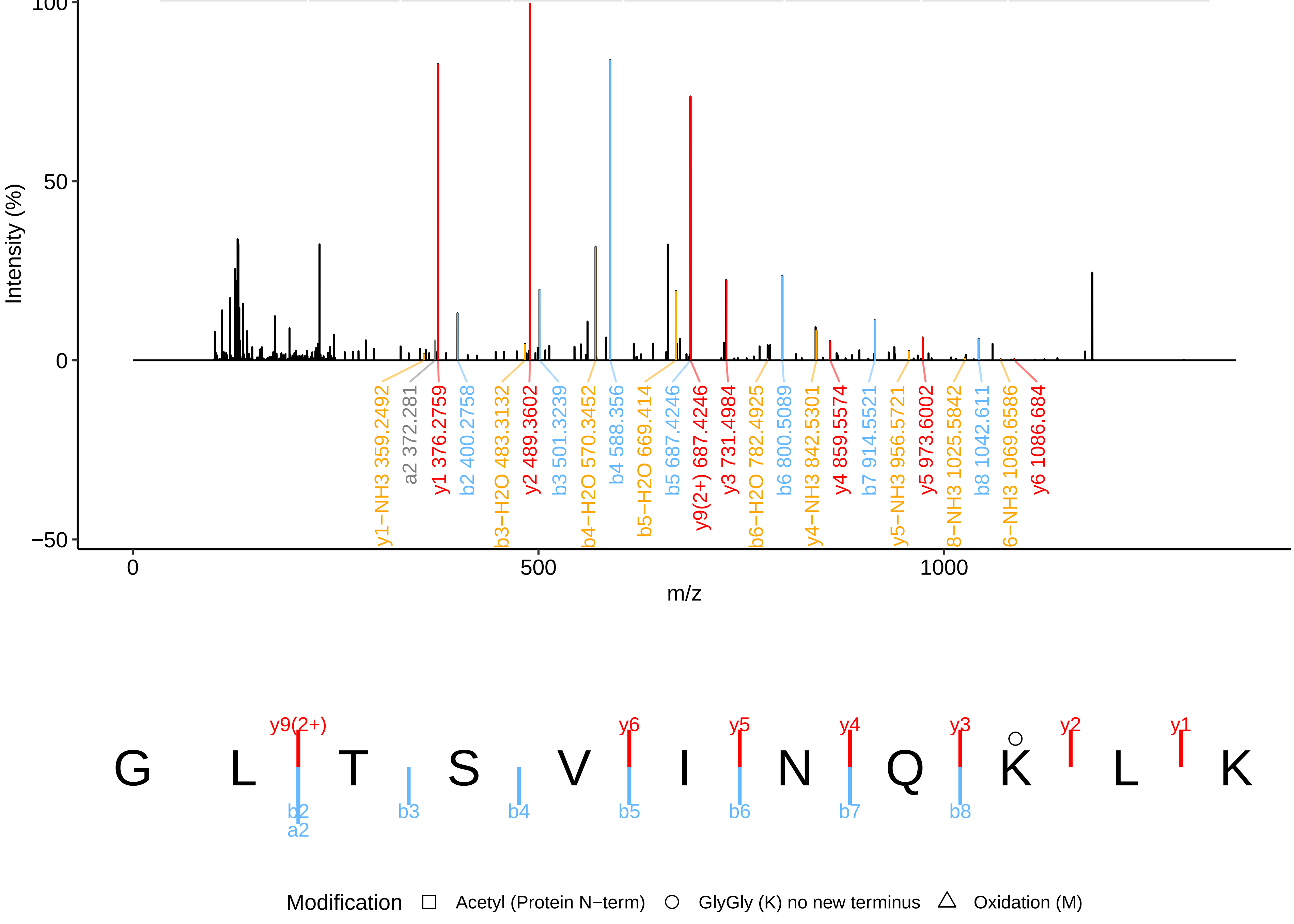

20200924_KIZ_MUb5_GlyGly_1 − Scan number: 17140

Gene Names	Charge	m/z	Mass	Retention time	PEP	Score	Precursor Intensity
LDHB	3	438.9295	1313.767	55.361	9.5566e−05	119.53	16822570

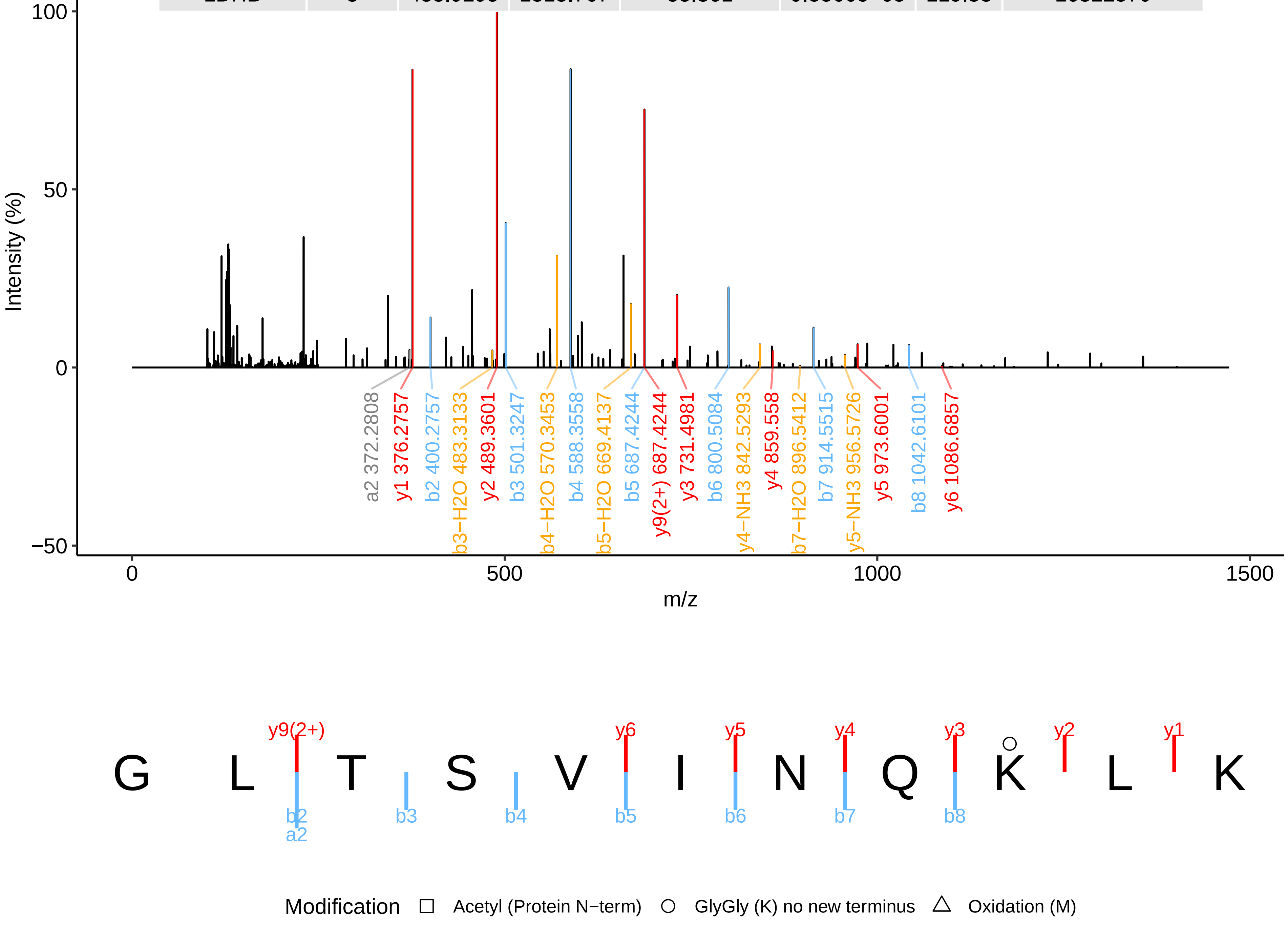

20200924_KIZ_MUb5_GlyGly_2 − Scan number: 16089

Gene Names	Charge	m/z	Mass	Retention time	PEP	Score	Precursor Intensity
LDHB	3	438.9295	1313.767	59.734	0.00015075	113.93	4921618

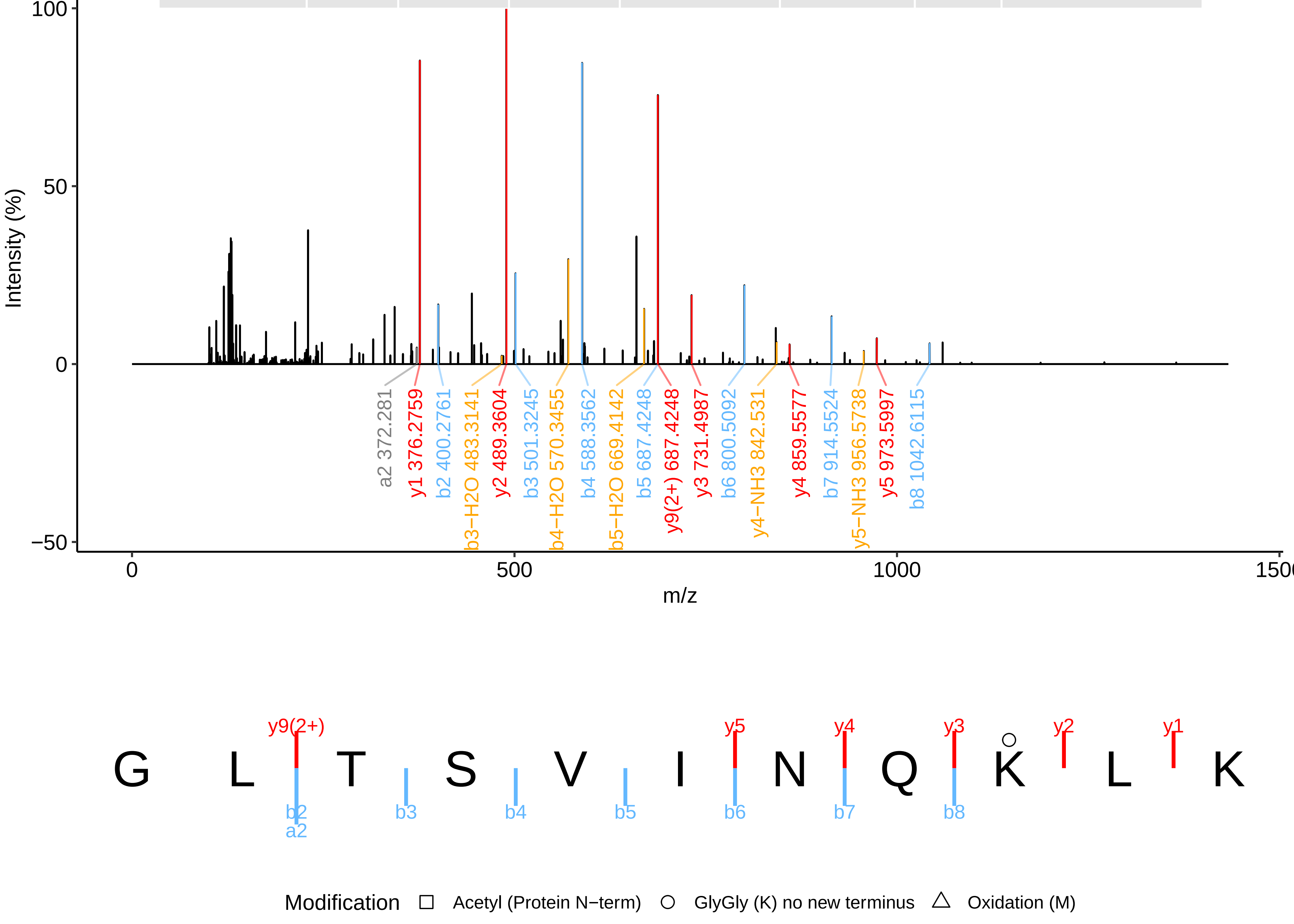

20200924_KIZ_MUb5_GlyGly_2 − Scan number: 16372

Gene Names	Charge	m/z	Mass	Retention time	PEP	Score	Precursor Intensity
LDHB	3	438.9295	1313.767	60.746	1.5385e−06	137.98	27207270

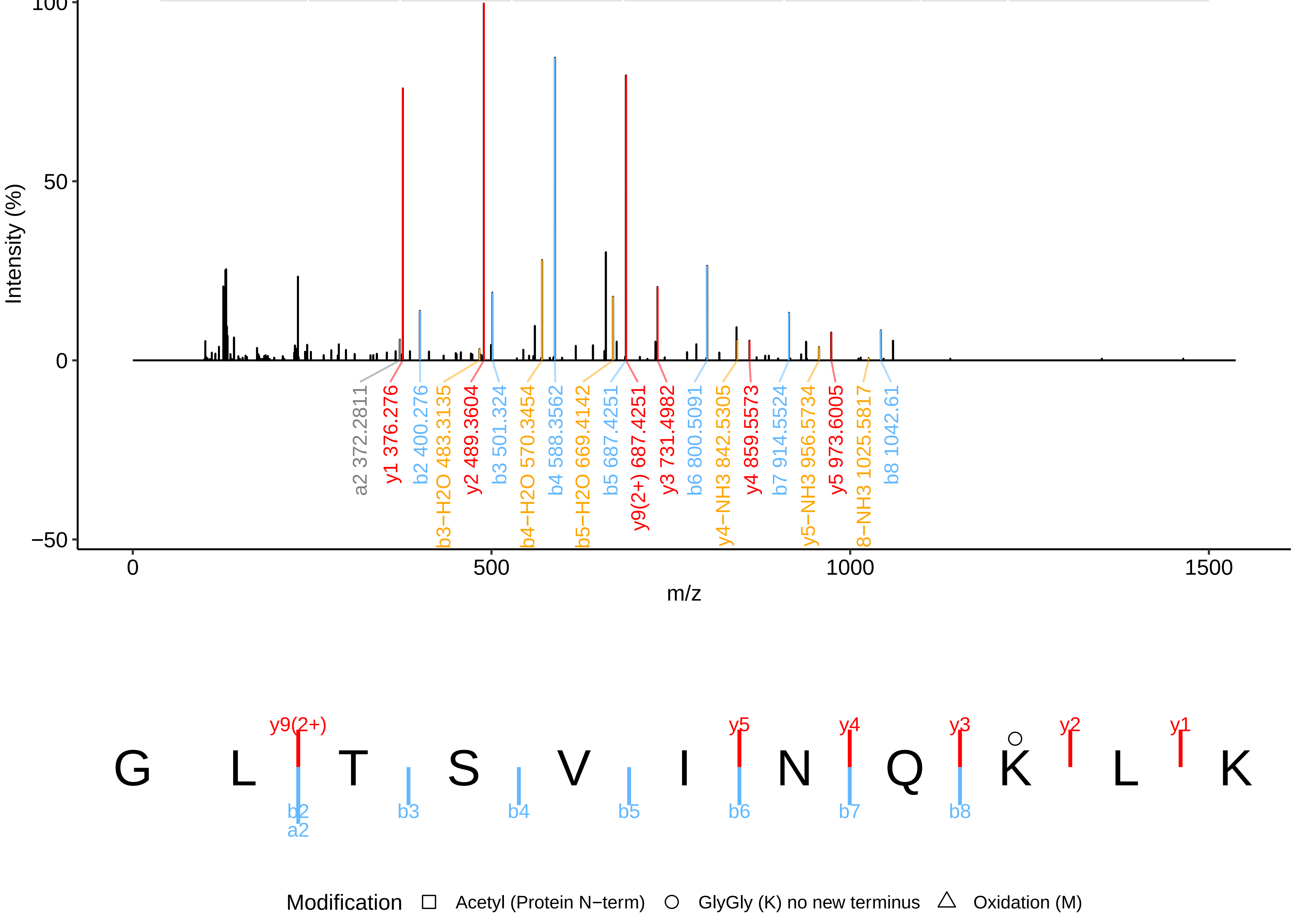

20200924_KIZ_MUb5_GlyGly_2 − Scan number: 16672

Gene Names	Charge	m/z	Mass	Retention time	PEP	Score	Precursor Intensity
LDHB	3	438.9295	1313.767	61.849	1.8124e−06	135.86	4624586

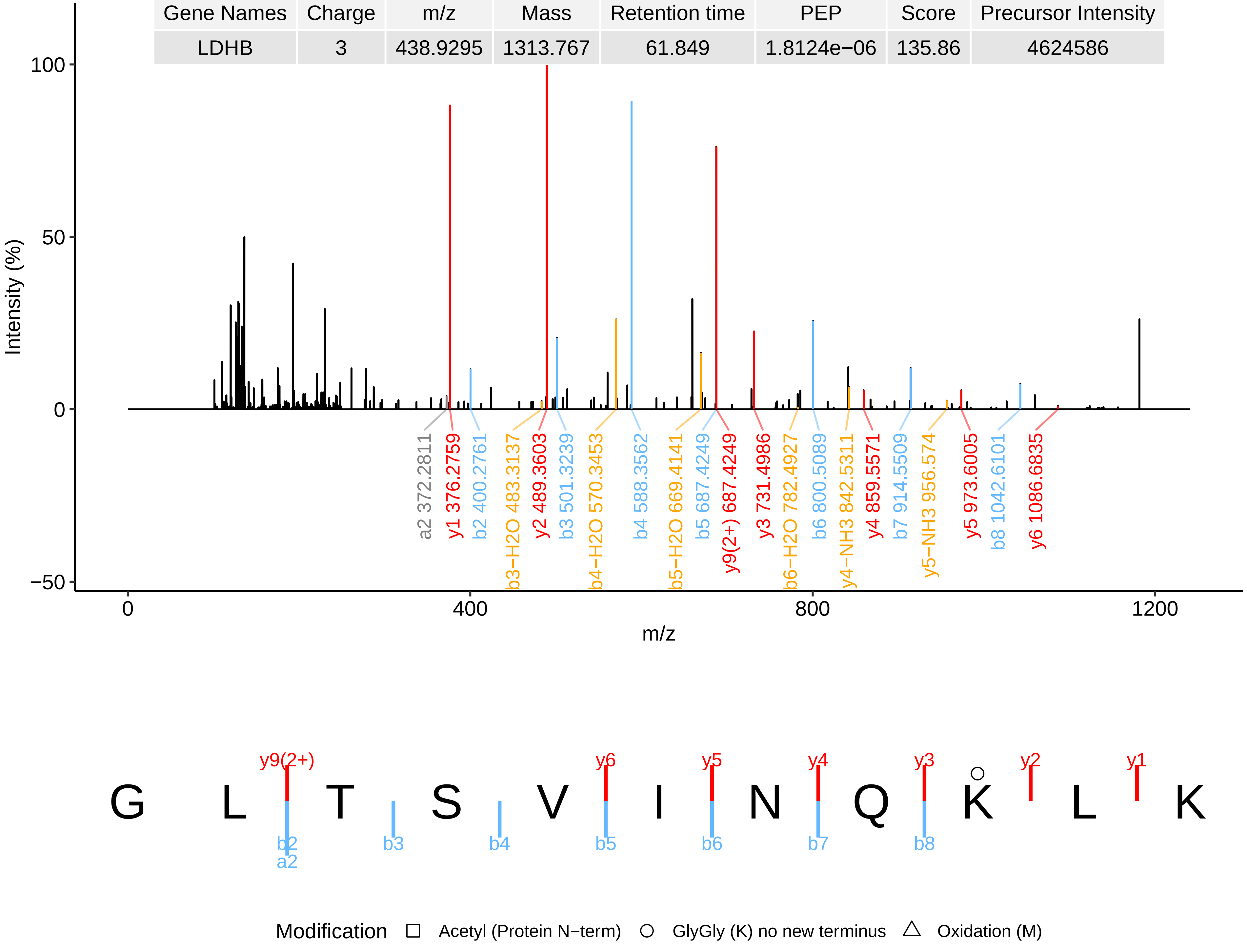

20200921_KIZ_MUb4_GlyGly_10plex − Scan number: 11016

Gene Names	Charge	m/z	Mass	Retention time	PEP	Score	Precursor Intensity
SLC6A9	3	515.5998	1543.778	38.313	0.041602	64.534	11338081

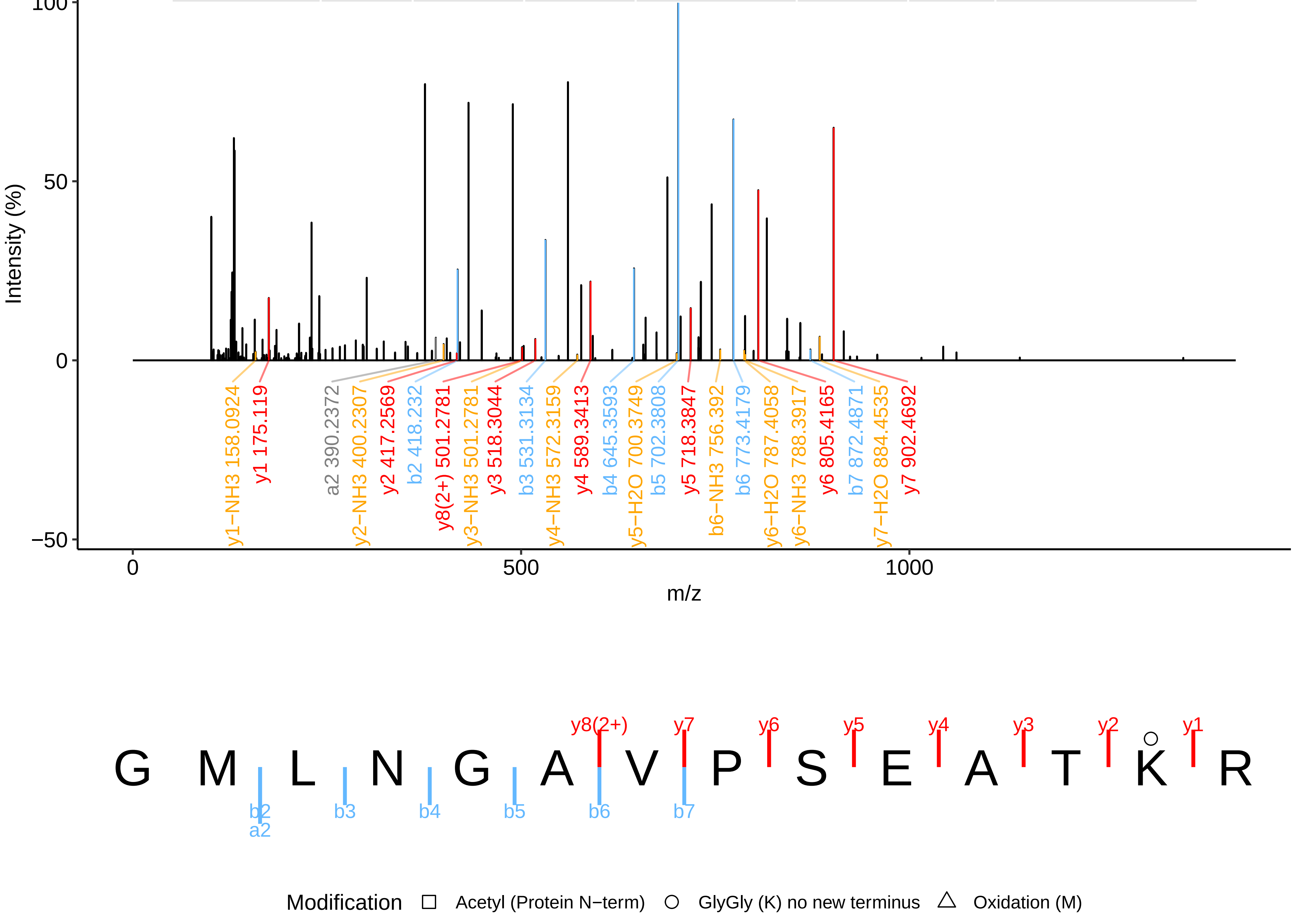

20200921_KIZ_MUb4_GlyGly_10plex_2 − Scan number: 10038

Gene Names	Charge	m/z	Mass	Retention time	PEP	Score	Precursor
IST1H1E;HIST1H1D;HIST1H1A;HIST1H1B;HIST1H1C	3	427.558	1279.652	38.296	0.041307	59.418	8913

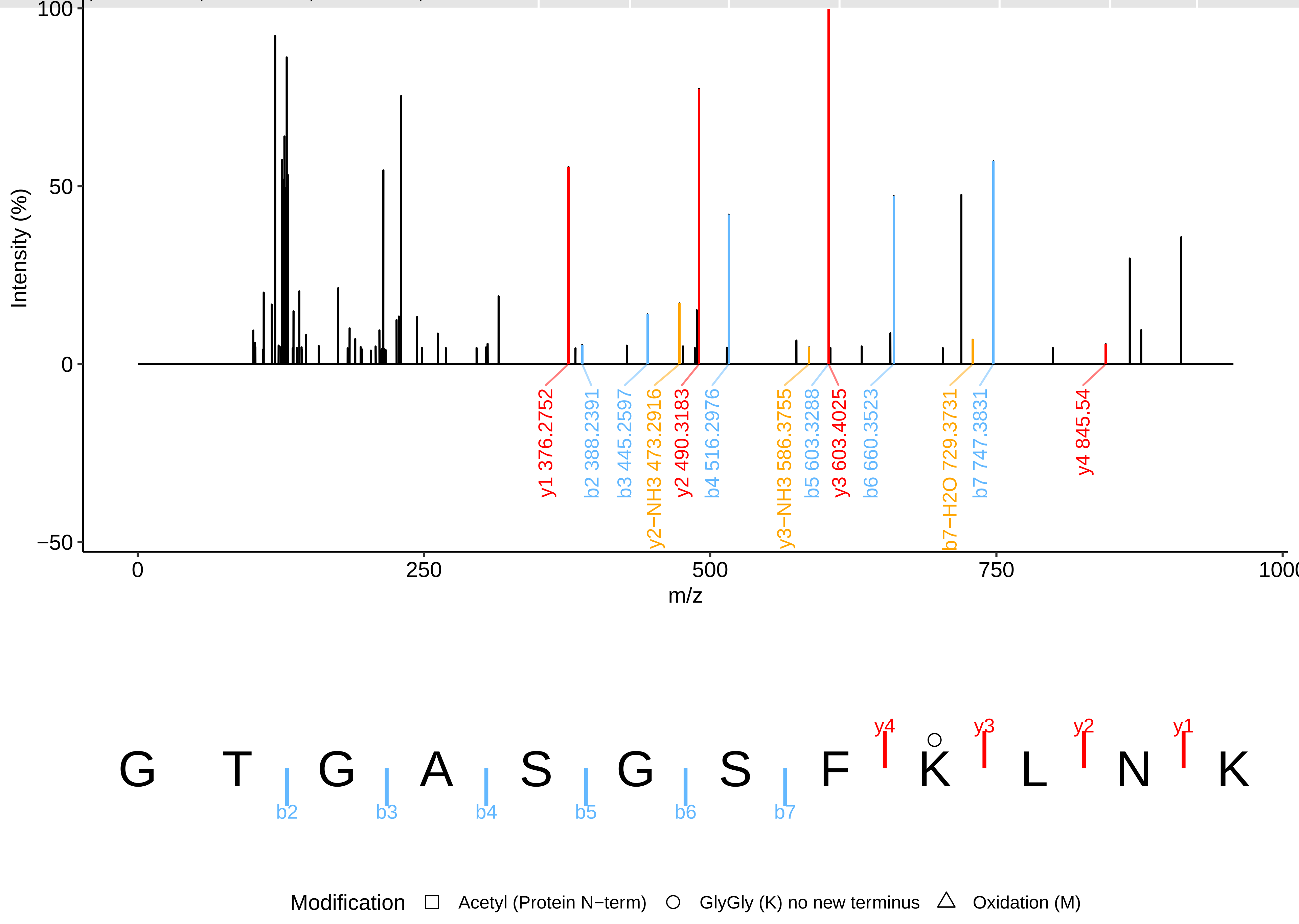

20200921_KIZ_MUb4_GlyGly_10plex − Scan number: 8090

Gene Names	Charge	m/z	Mass	Retention time	PEP	Score	Precursor Intensity
ORMDL1;ORMDL2;ORMDL3	3	506.5829	1516.727	27.373	1.3805e−08	116.13	5760826

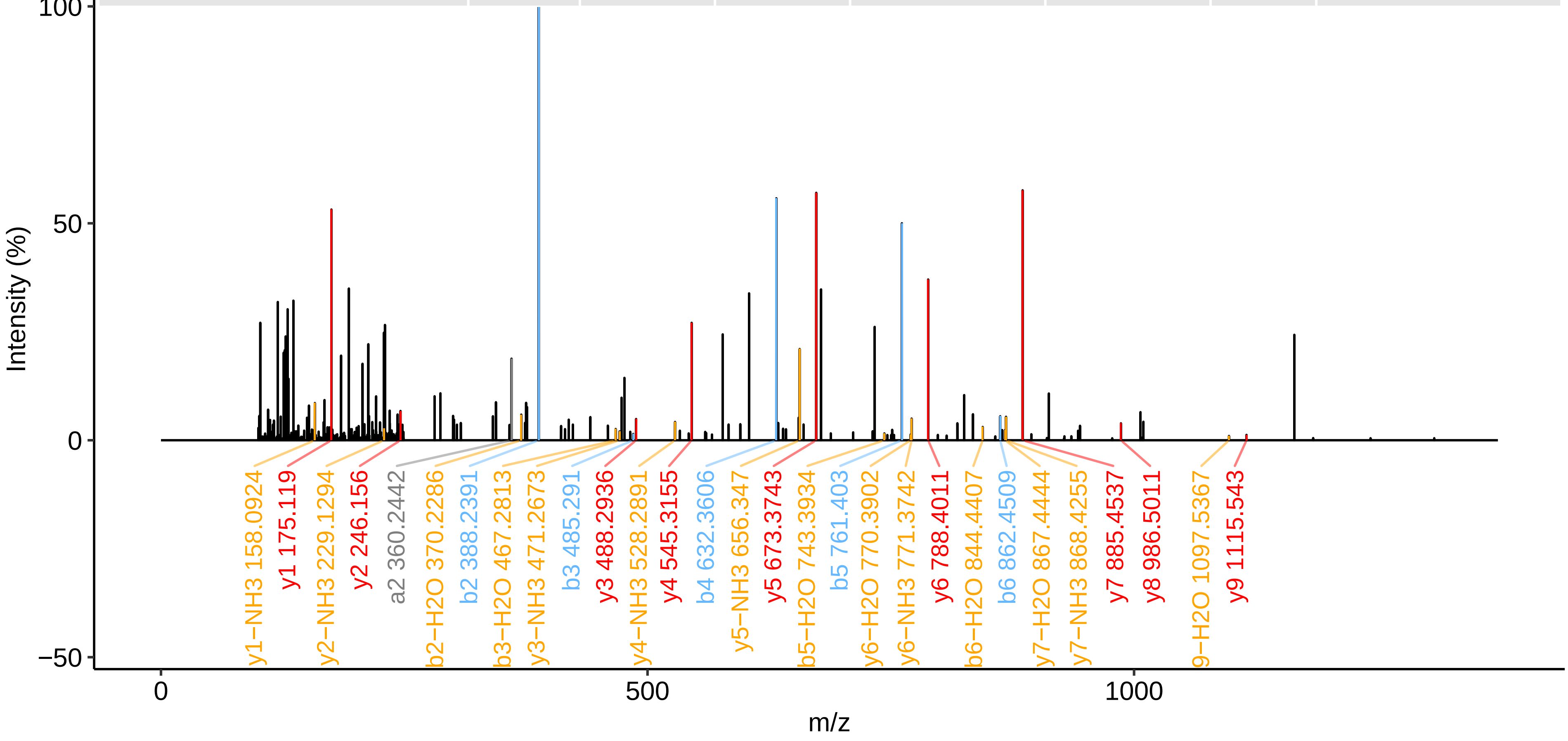

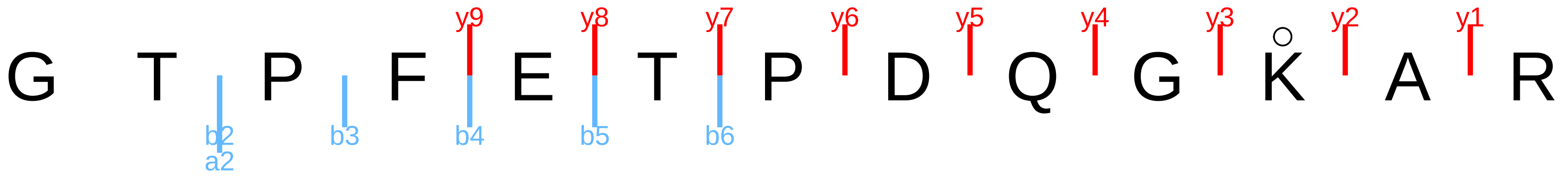

Modification □ Acetyl (Protein N−term) ○ GlyGly (K) no new terminus △ Oxidation (M)

20200921_KIZ_MUb4_GlyGly_10plex_2 − Scan number: 7432

Gene Names	Charge	m/z	Mass	Retention time	PEP	Score	Precursor Intensity
ORMDL1;ORMDL2;ORMDL3	3	506.5829	1516.727	27.734	0.035517	57.802	1166972

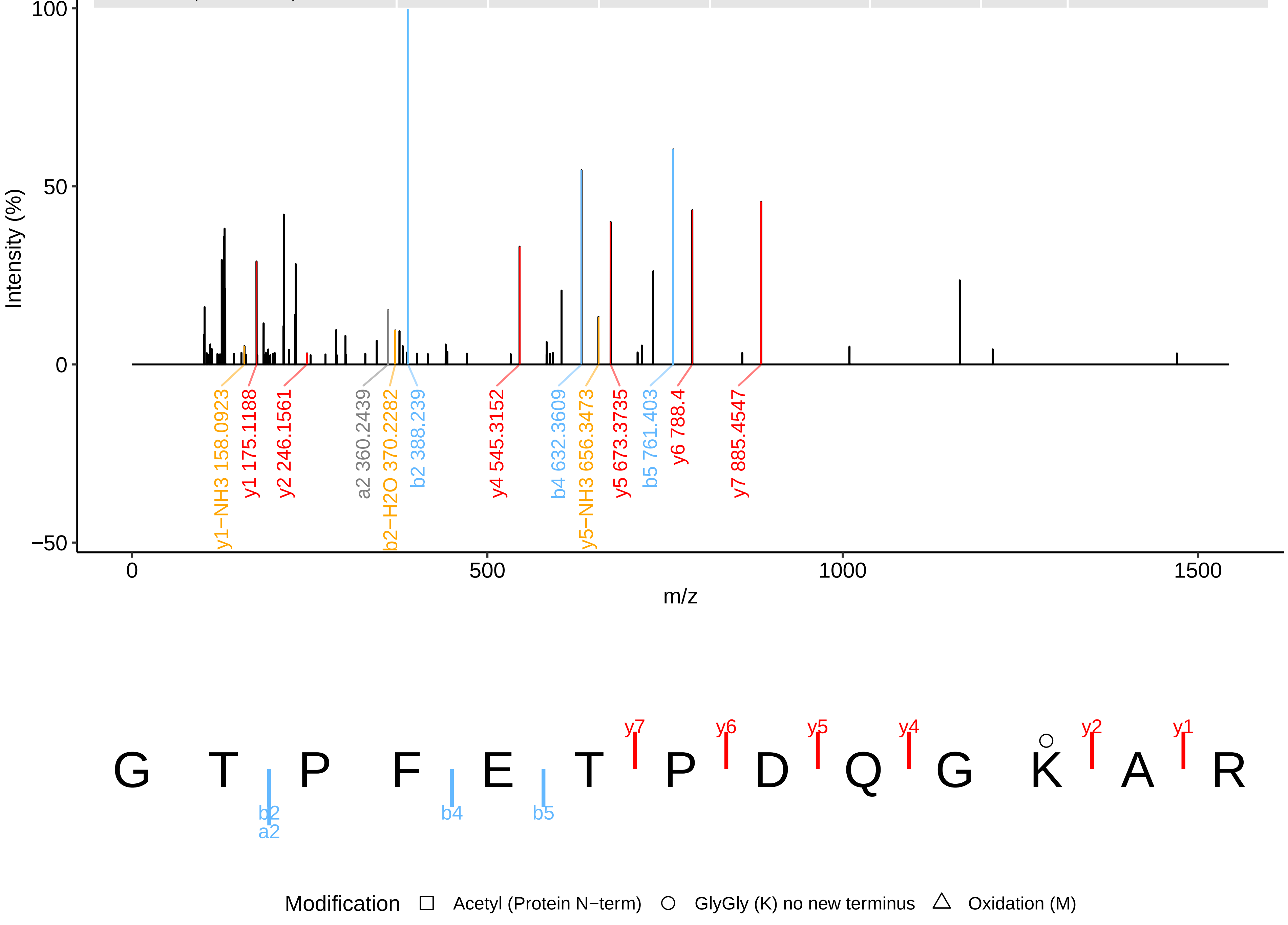

20200921_KIZ_MUb4_GlyGly_10plex − Scan number: 15116

Gene Names	Charge	m/z	Mass	Retention time	PEP	Score	Precursor Intensity
PRDX6	3	426.2347	1275.682	53.918	0.00095291	88.596	4714966

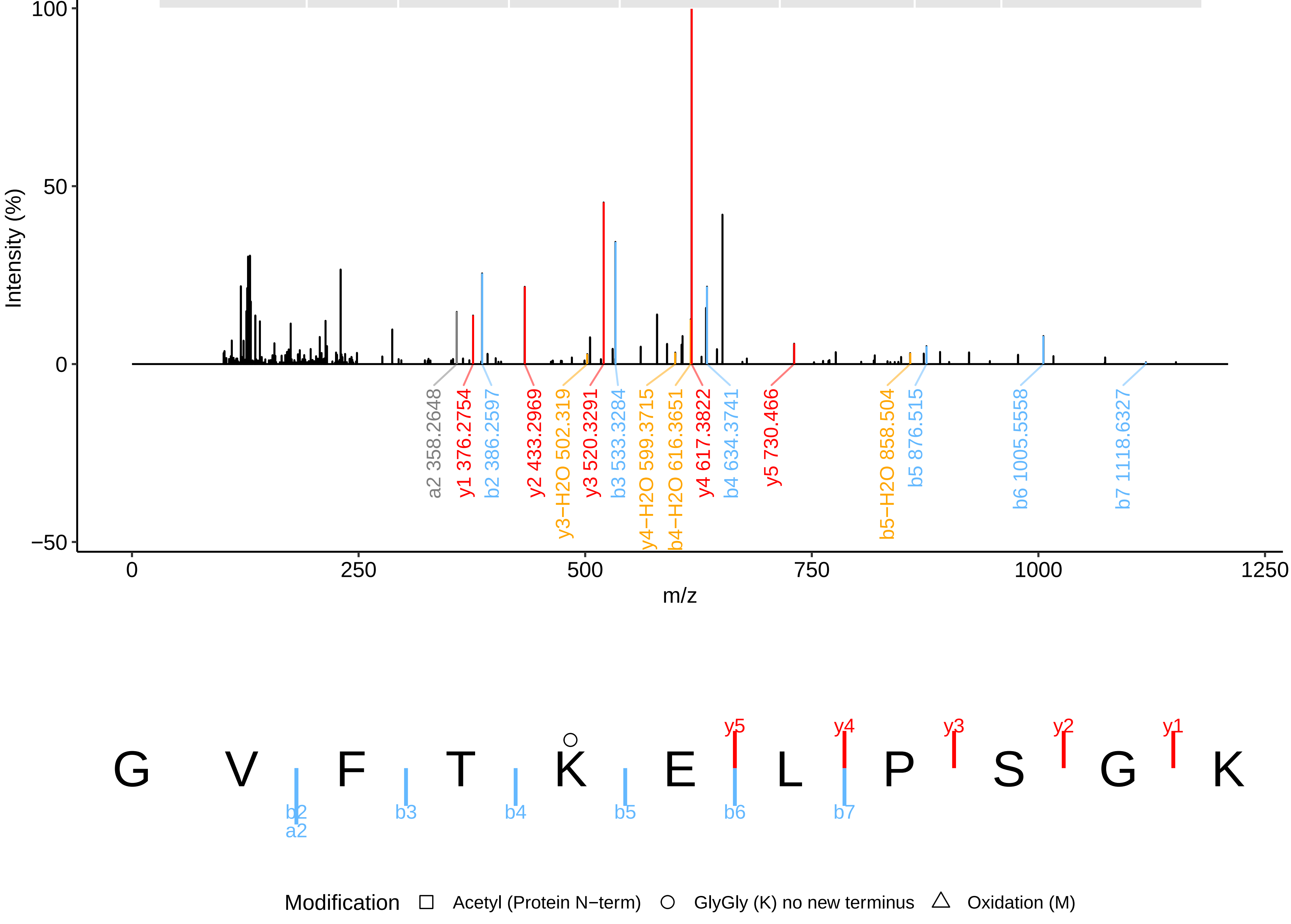

20200921_KIZ_MUb4_GlyGly_10plex_2 − Scan number: 7803

Gene Names	Charge	m/z	Mass	Retention time	PEP	Score	Precursor Intensity
CYB5R1	4	317.4202	1265.652	29.235	0.024499	60.518	677706.1

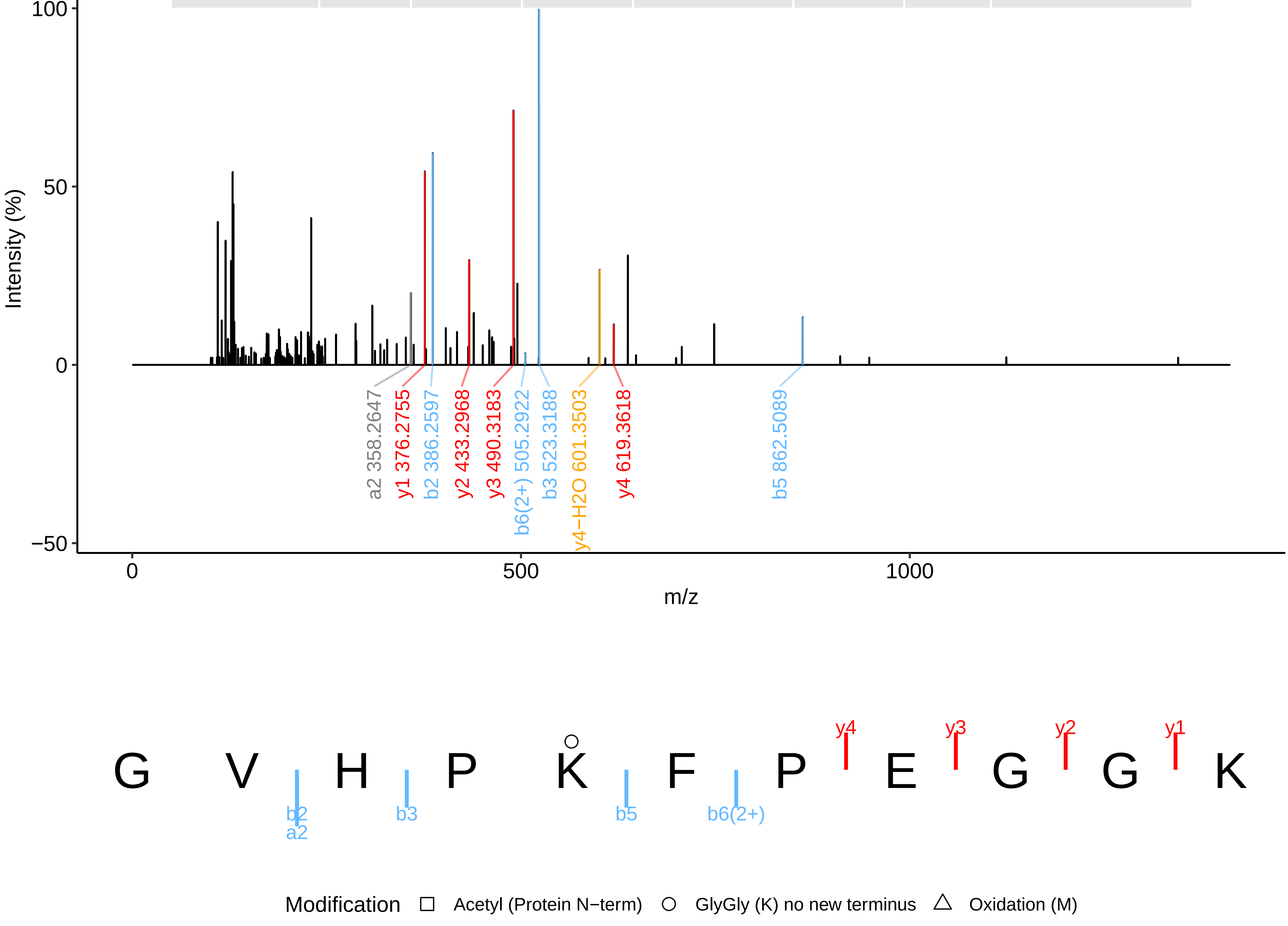

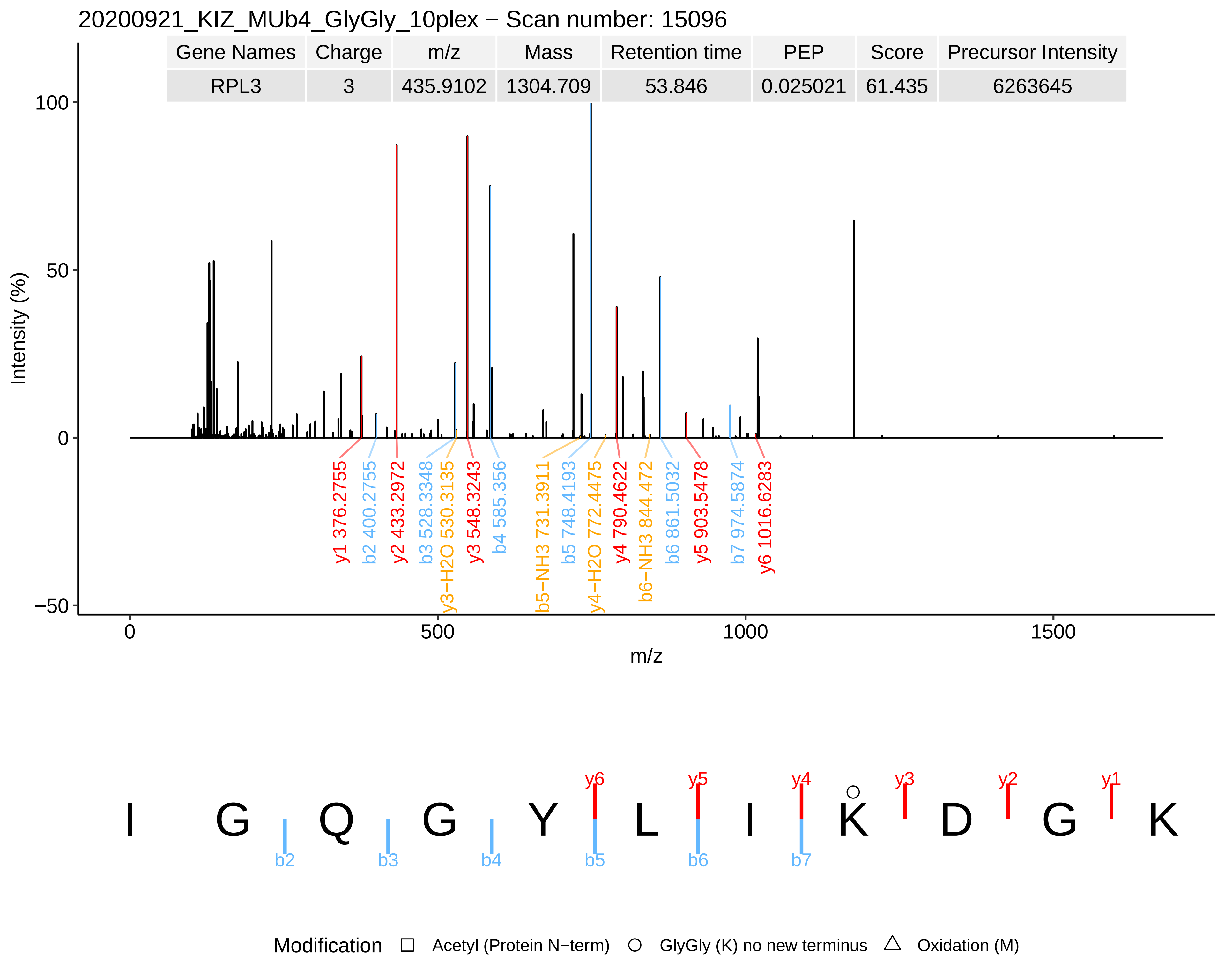
20200921_KIZ_MUb4_GlyGly_10plex − Scan number: 15096
Gene Names | Charge | m/z | Mass | Retention time | PEP | Score | Precursor Intensity
RPL3 | 3 | 435.9102 | 1304.709 | 53.846 | 0.025021 | 61.435 | 6263645
Intensity (%)
100
50
0
−50
0
500
1000
1500
m/z
y1 376.2755
b2 400.2755
y2 433.2972
b3 528.3348
y3−H2O 530.3135
y3 548.3243
b4 585.356
b5−NH3 731.3911
b5 748.4193
y4−H2O 772.4475
y4 790.4622
b6−NH3 844.472
b6 861.5032
y5 903.5478
b7 974.5874
y6 1016.6283
I G Q G Y L I K D G K
y6 y5 y4 y3 y2 y1
b2 b3 b4 b5 b6 b7
Modification
Acetyl (Protein N−term)
GlyGly (K) no new terminus
Oxidation (M)

20200921_KIZ_MUb4_GlyGly_10plex_2 − Scan number: 13976

Gene Names	Charge	m/z	Mass	Retention time	PEP	Score	Precursor Intensity
RPL3	3	435.9102	1304.709	54.386	0.034794	59.542	1084351

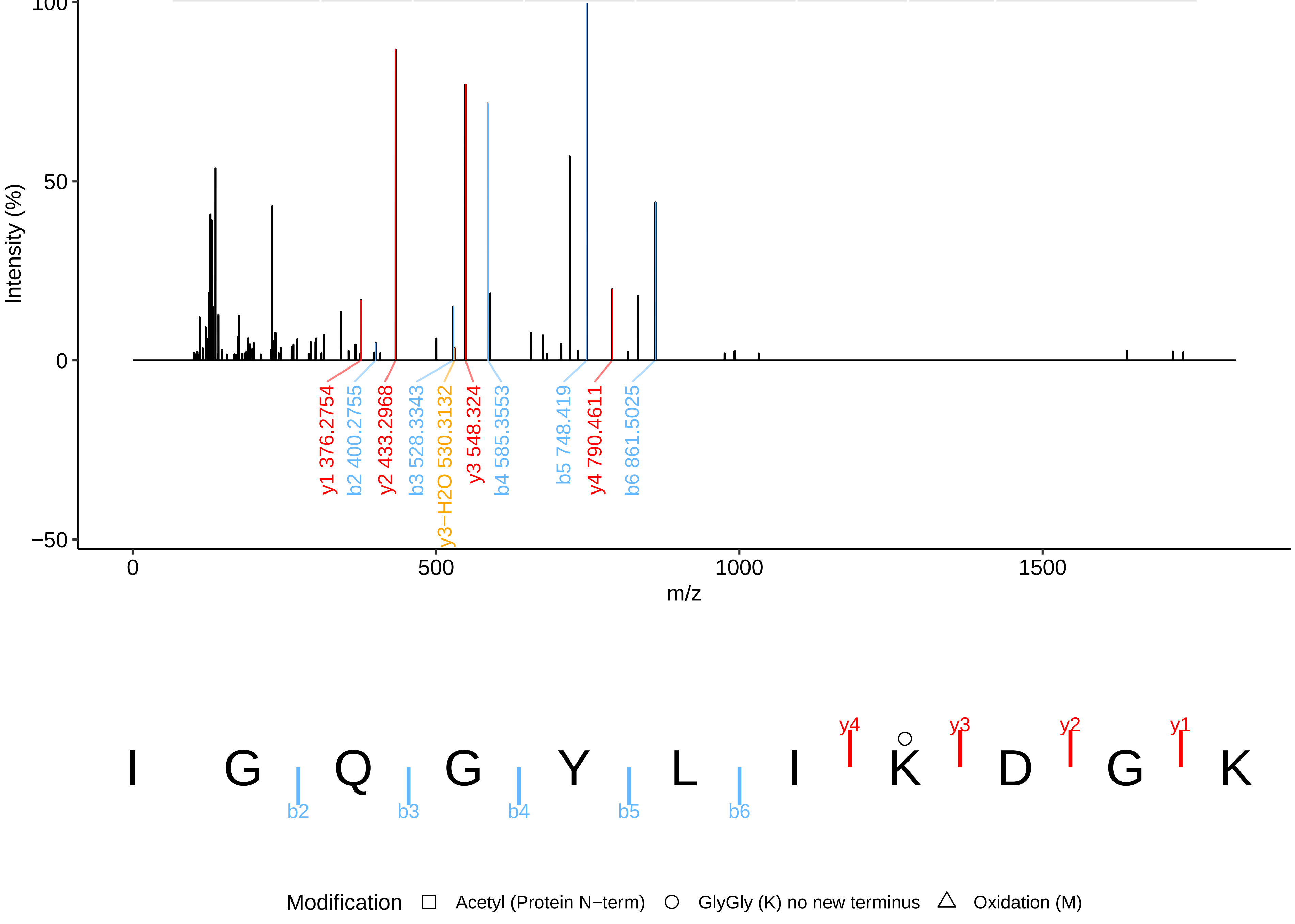

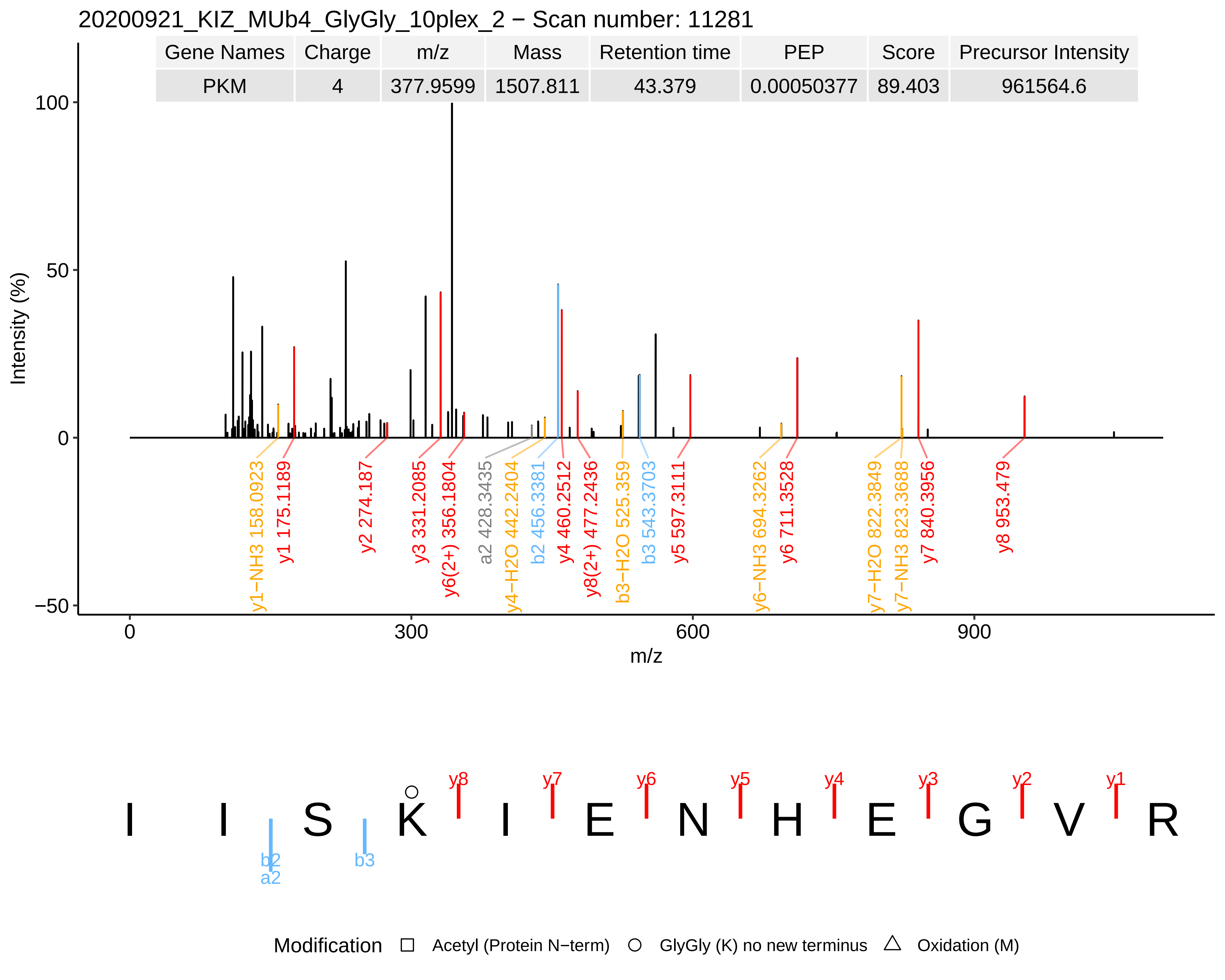

20200921_KIZ_MUb4_GlyGly_10plex_2 − Scan number: 11281
Gene Names | Charge | m/z | Mass | Retention time | PEP | Score | Precursor Intensity
PKM | 4 | 377.9599 | 1507.811 | 43.379 | 0.00050377 | 89.403 | 961564.6
Intensity (%)
100
50
0
−50
0
300
600
900
m/z
y1−NH3 158.0923
y1 175.1189
y2 274.187
y3 331.2085
y6(2+) 356.1804
a2 428.3435
y4−H2O 442.2404
b2 456.3381
y4 460.2512
y8(2+) 477.2436
b3−H2O 525.359
b3 543.3703
y5 597.3111
y6−NH3 694.3262
y6 711.3528
y7−H2O 822.3849
y7−NH3 823.3688
y7 840.3956
y8 953.479
I I S K I E N H E G V R
y8 y7 y6 y5 y4 y3 y2 y1
b2
a2
b3
Modification
Acetyl (Protein N−term)
GlyGly (K) no new terminus
Oxidation (M)

20200921_KIZ_MUb4_GlyGly_10plex_2 − Scan number: 11302

Gene Names	Charge	m/z	Mass	Retention time	PEP	Score	Precursor Intensity
PKM	3	503.6108	1507.811	43.465	0.0003774	94.191	818783.9

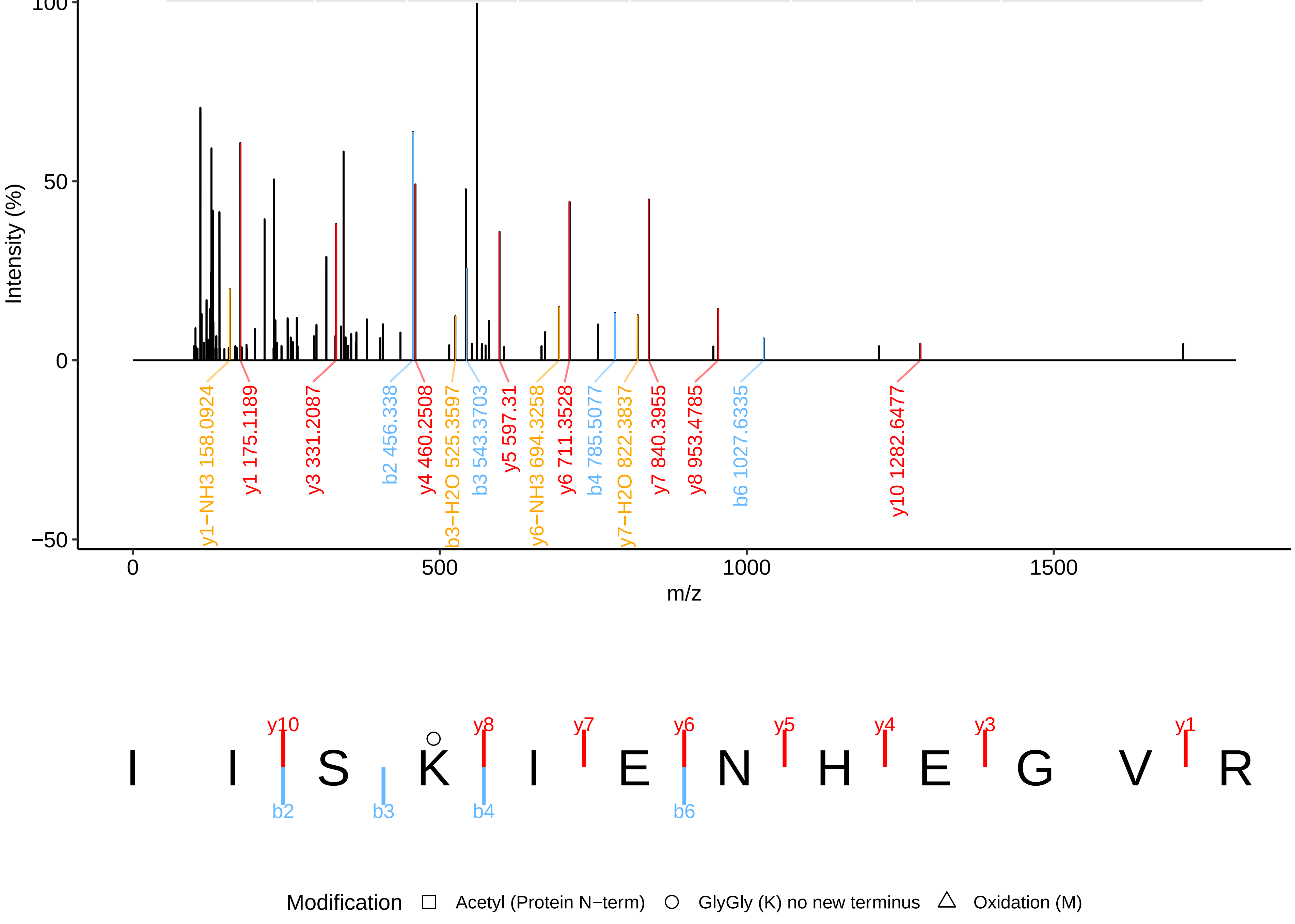

20200921_KIZ_MUb4_GlyGly_10plex_2 − Scan number: 19795

Gene Names	Charge	m/z	Mass	Retention time	PEP	Score	Precursor Intensity
RPL6	3	505.2998	1512.878	77.739	0.01349	68.069	NA

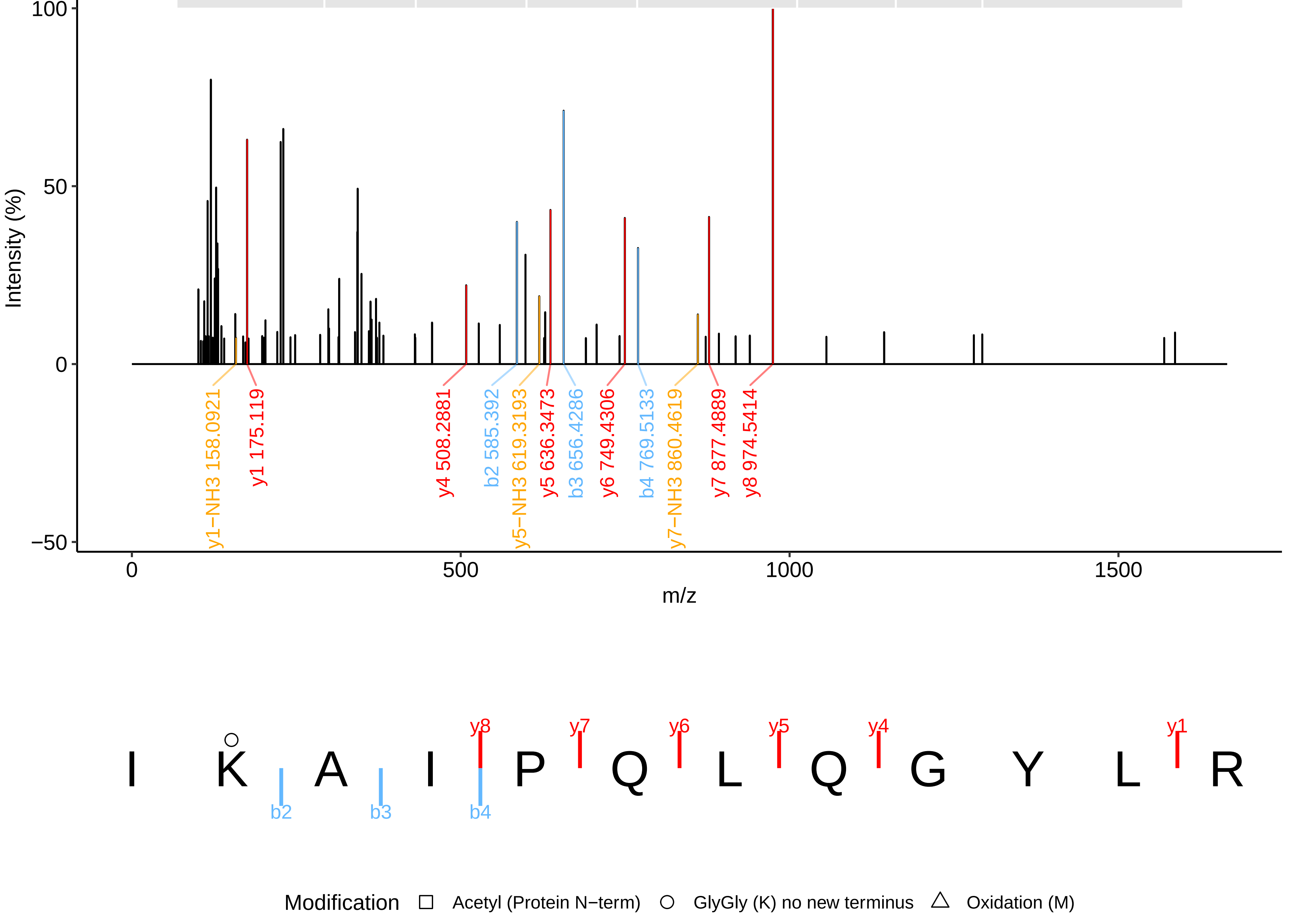

20200921_KIZ_MUb4_GlyGly_10plex − Scan number: 14639

Gene Names	Charge	m/z	Mass	Retention time	PEP	Score	Precursor Intensity
GNA11	3	438.2347	1311.682	52.073	0.00021657	109.21	28650944

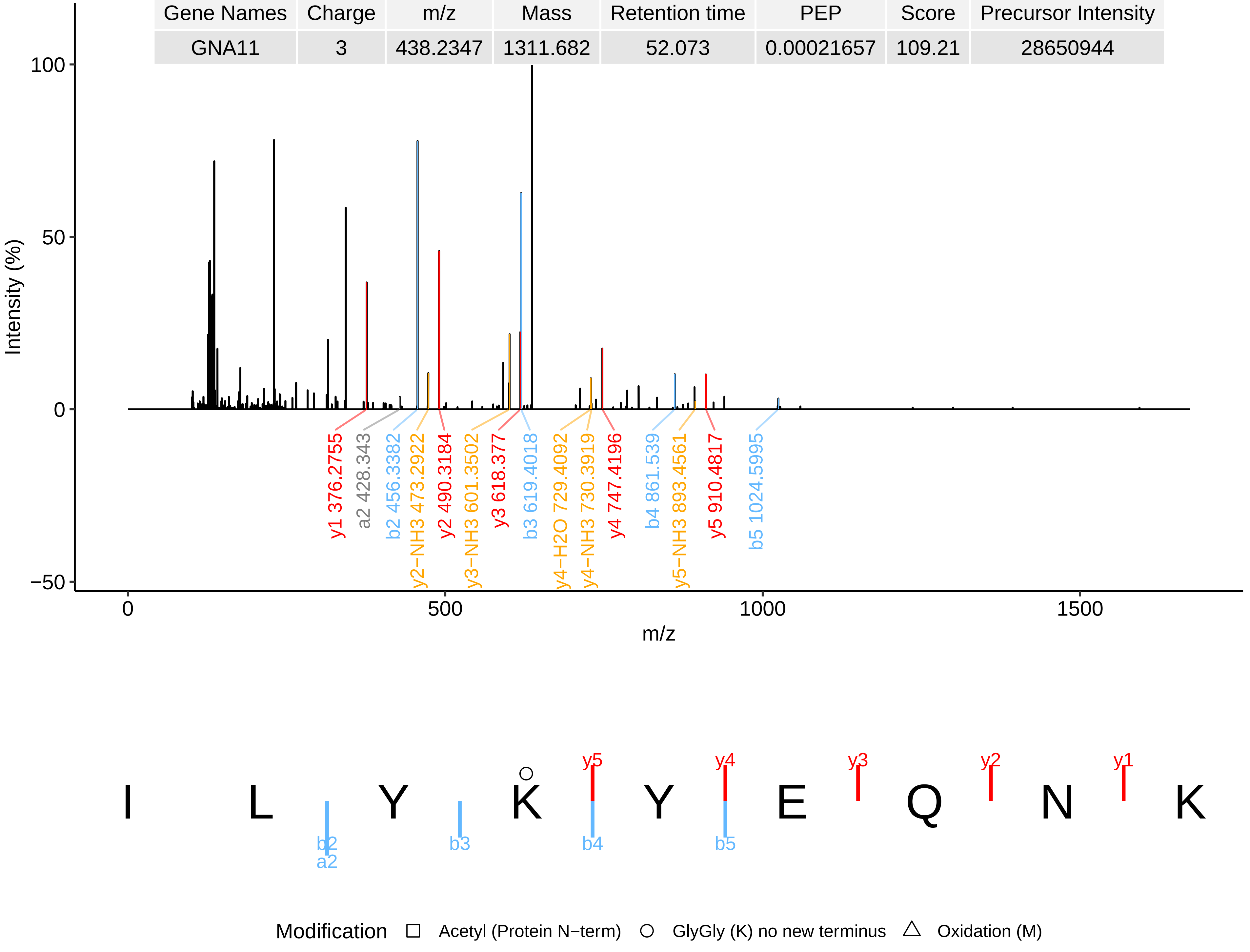

20200921_KIZ_MUb4_GlyGly_10plex − Scan number: 14626

Gene Names	Charge	m/z	Mass	Retention time	PEP	Score	Precursor Intensity
GNA11	3	438.2347	1311.682	52.033	0.0028708	93.429	NA

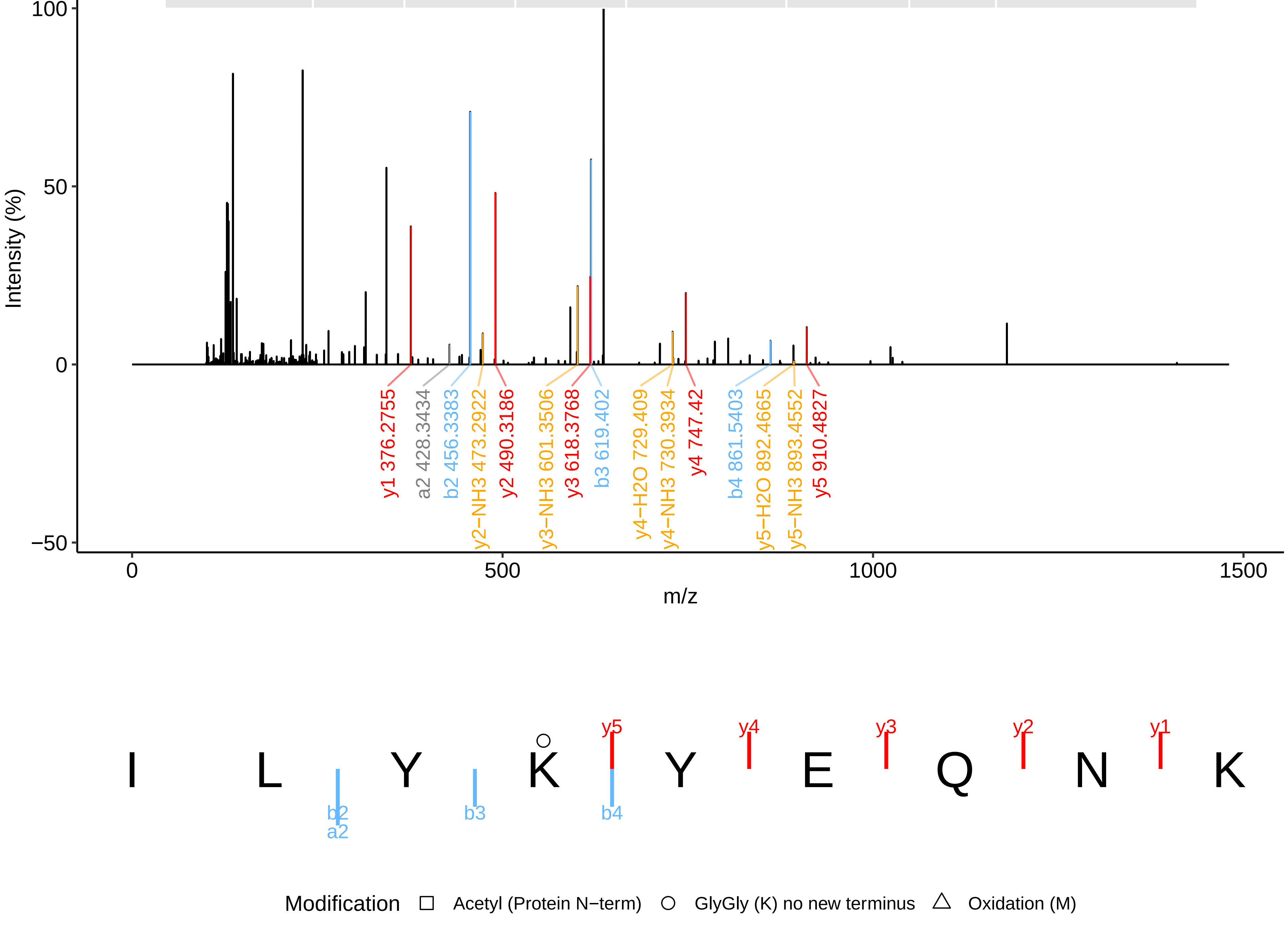

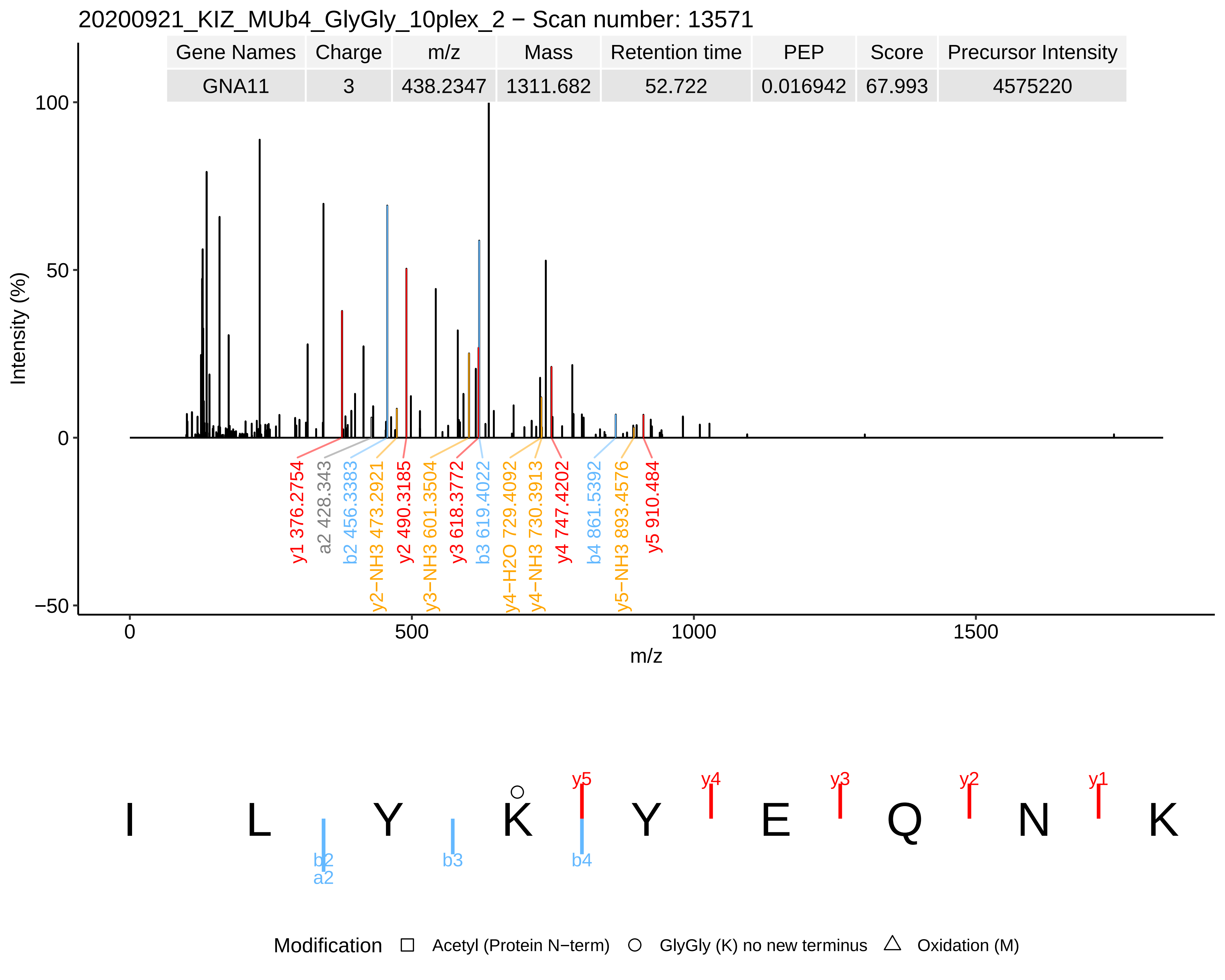

20200921_KIZ_MUb4_GlyGly_10plex_2 − Scan number: 13571
Gene Names | Charge | m/z | Mass | Retention time | PEP | Score | Precursor Intensity
GNA11 | 3 | 438.2347 | 1311.682 | 52.722 | 0.016942 | 67.993 | 4575220
Intensity (%)
m/z
y1 376.2754
a2 428.343
b2 456.3383
y2−NH3 473.2921
y2 490.3185
y3−NH3 601.3504
y3 618.3772
b3 619.4022
y4−H2O 729.4092
y4−NH3 730.3913
y4 747.4202
b4 861.5392
y5−NH3 893.4576
y5 910.484
I L Y K Y E Q N K
y5 y4 y3 y2 y1
b2 a2 b3 b4
Modification
Acetyl (Protein N−term)
GlyGly (K) no new terminus
Oxidation (M)

20200924_KIZ_MUb5_GlyGly_1 − Scan number: 13273

Gene Names	Charge	m/z	Mass	Retention time	PEP	Score	Precursor Intensity
GNA11	3	438.2347	1311.682	43.254	0.00010196	112.84	18381362

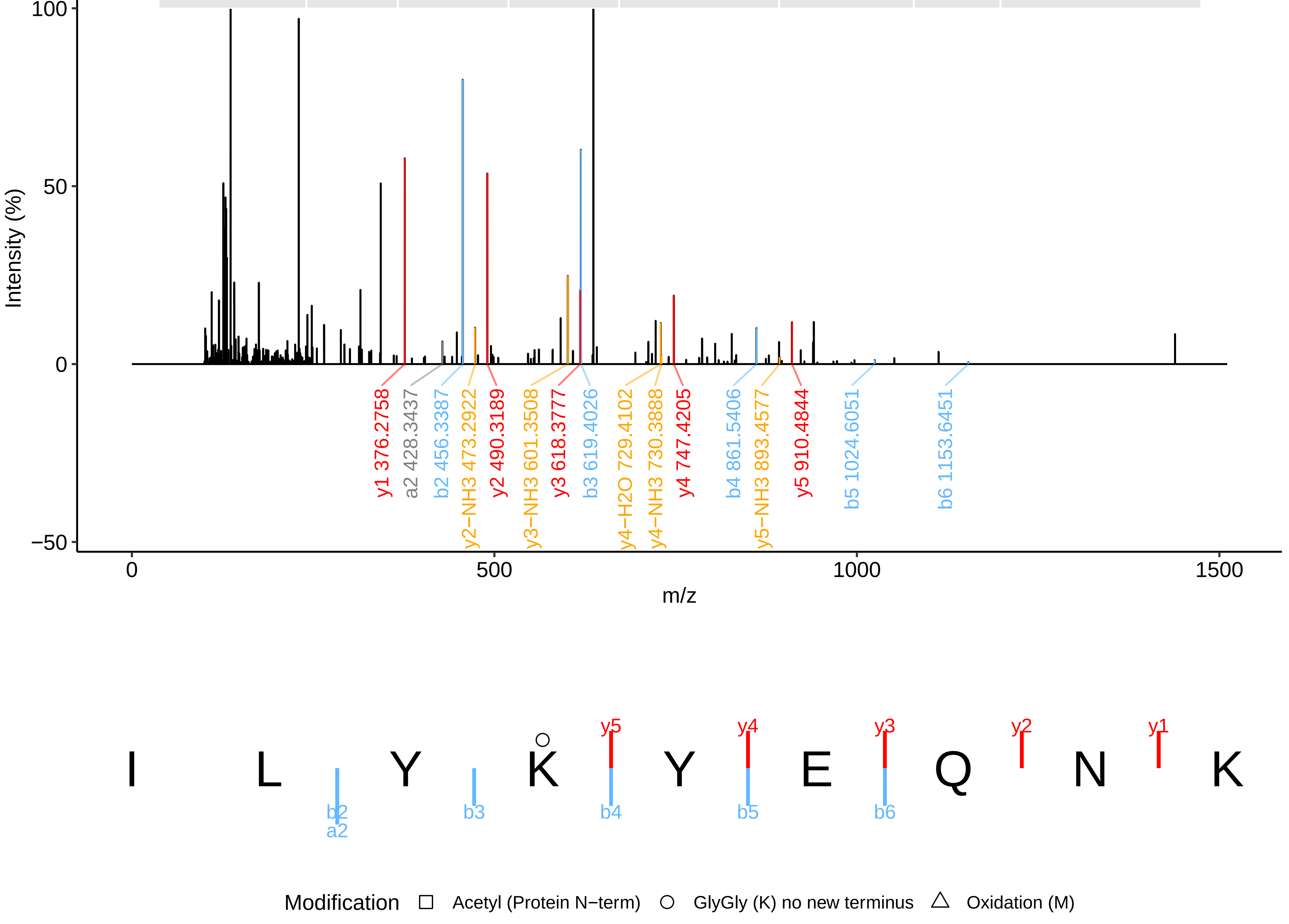

20200924_KIZ_MUb5_GlyGly_1 − Scan number: 13307

Gene Names	Charge	m/z	Mass	Retention time	PEP	Score	Precursor Intensity
GNA11	3	438.2347	1311.682	43.366	0.0015157	92.439	NA

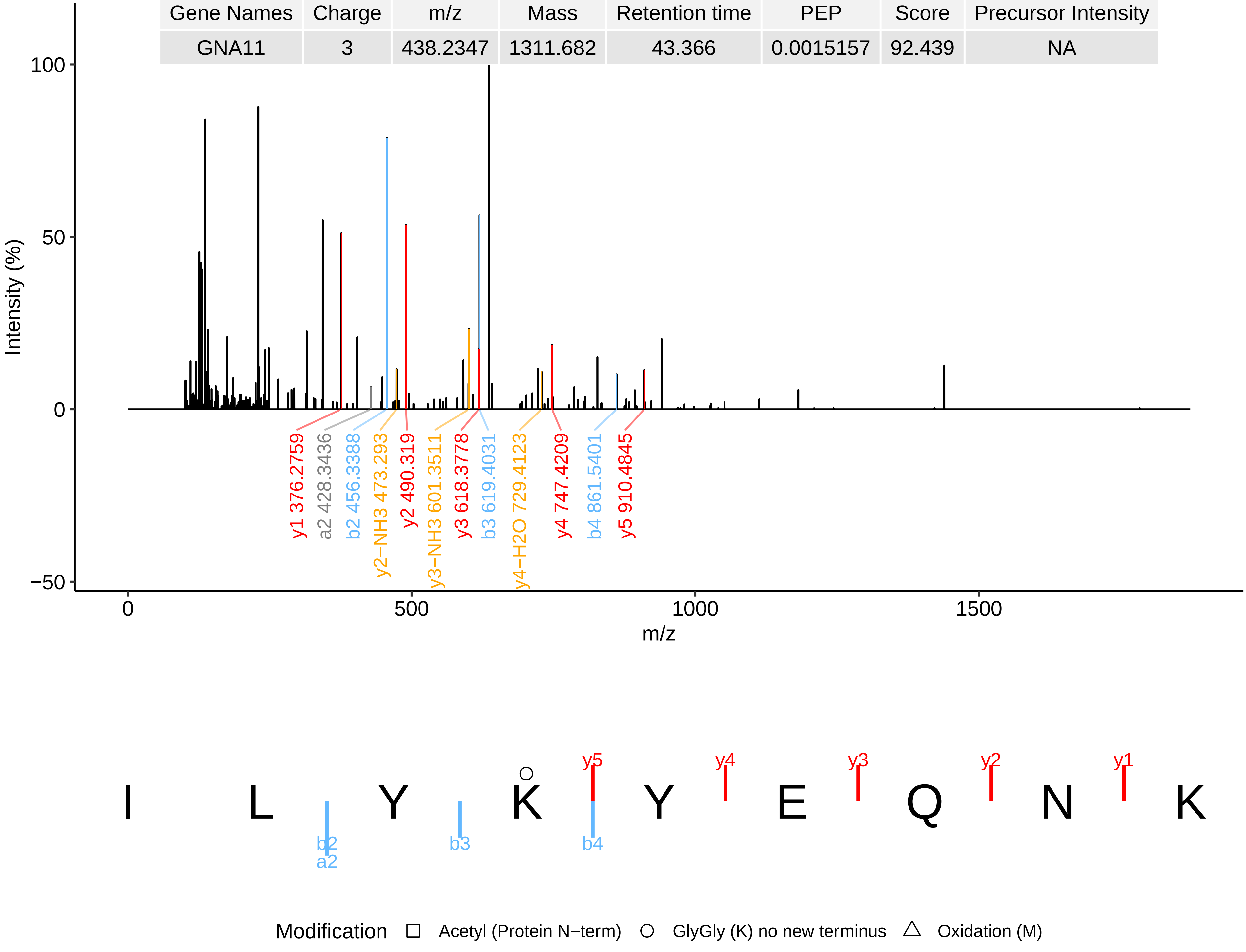

20200924_KIZ_MUb5_GlyGly_1 − Scan number: 13455

Gene Names	Charge	m/z	Mass	Retention time	PEP	Score	Precursor Intensity
GNA11	3	438.2347	1311.682	43.875	0.00039514	103.56	43998756

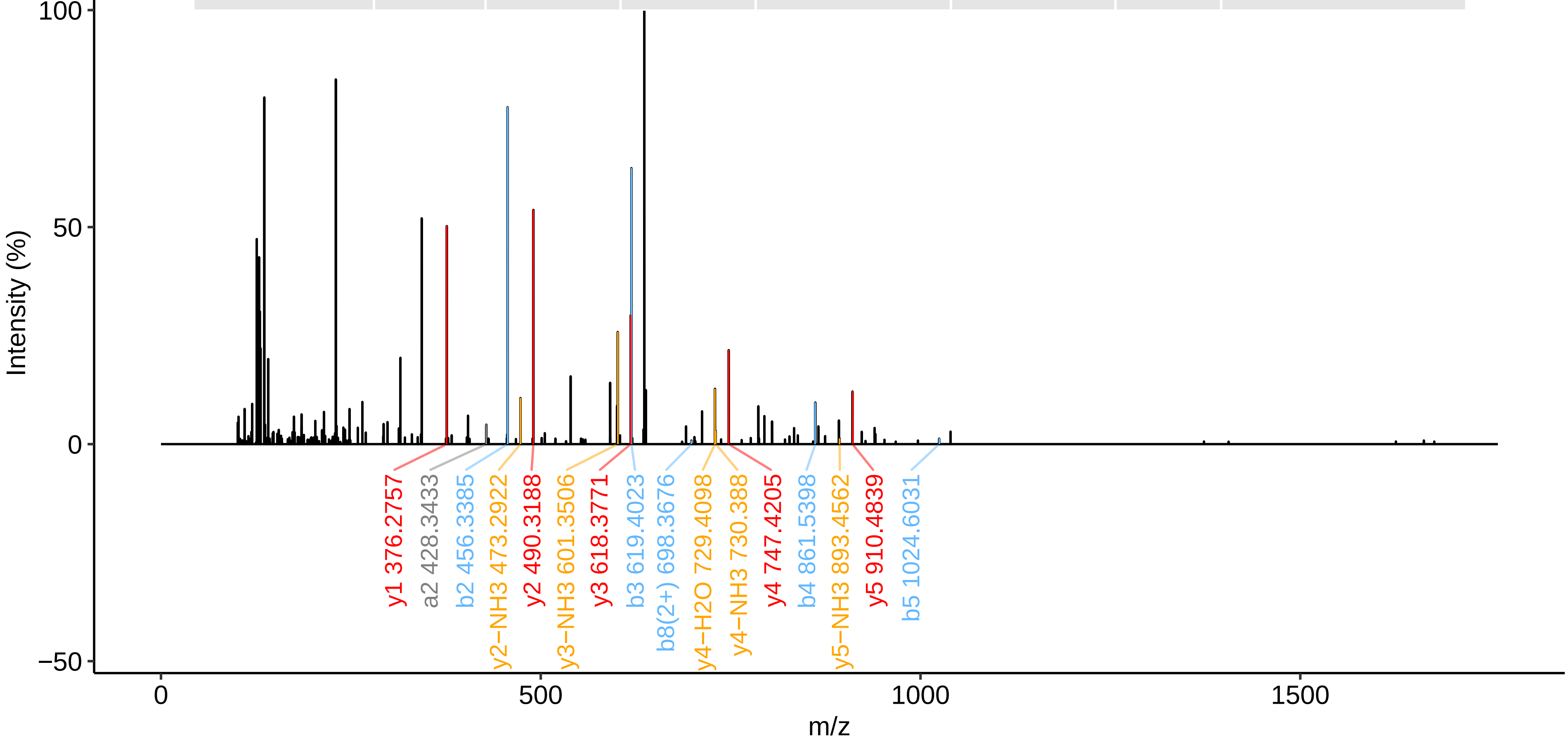

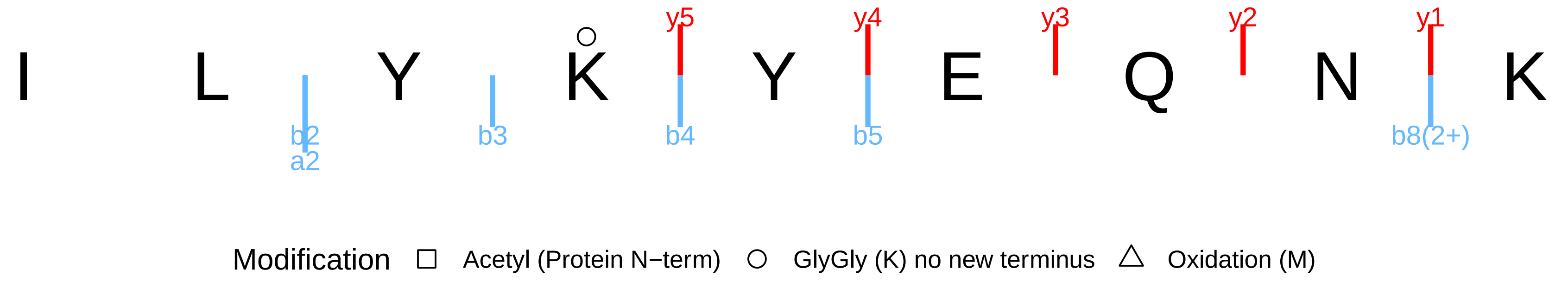

20200924_KIZ_MUb5_GlyGly_2 − Scan number: 12651

Gene Names	Charge	m/z	Mass	Retention time	PEP	Score	Precursor Intensity
GNA11	3	438.2347	1311.682	47.302	0.00045901	101.54	3236130

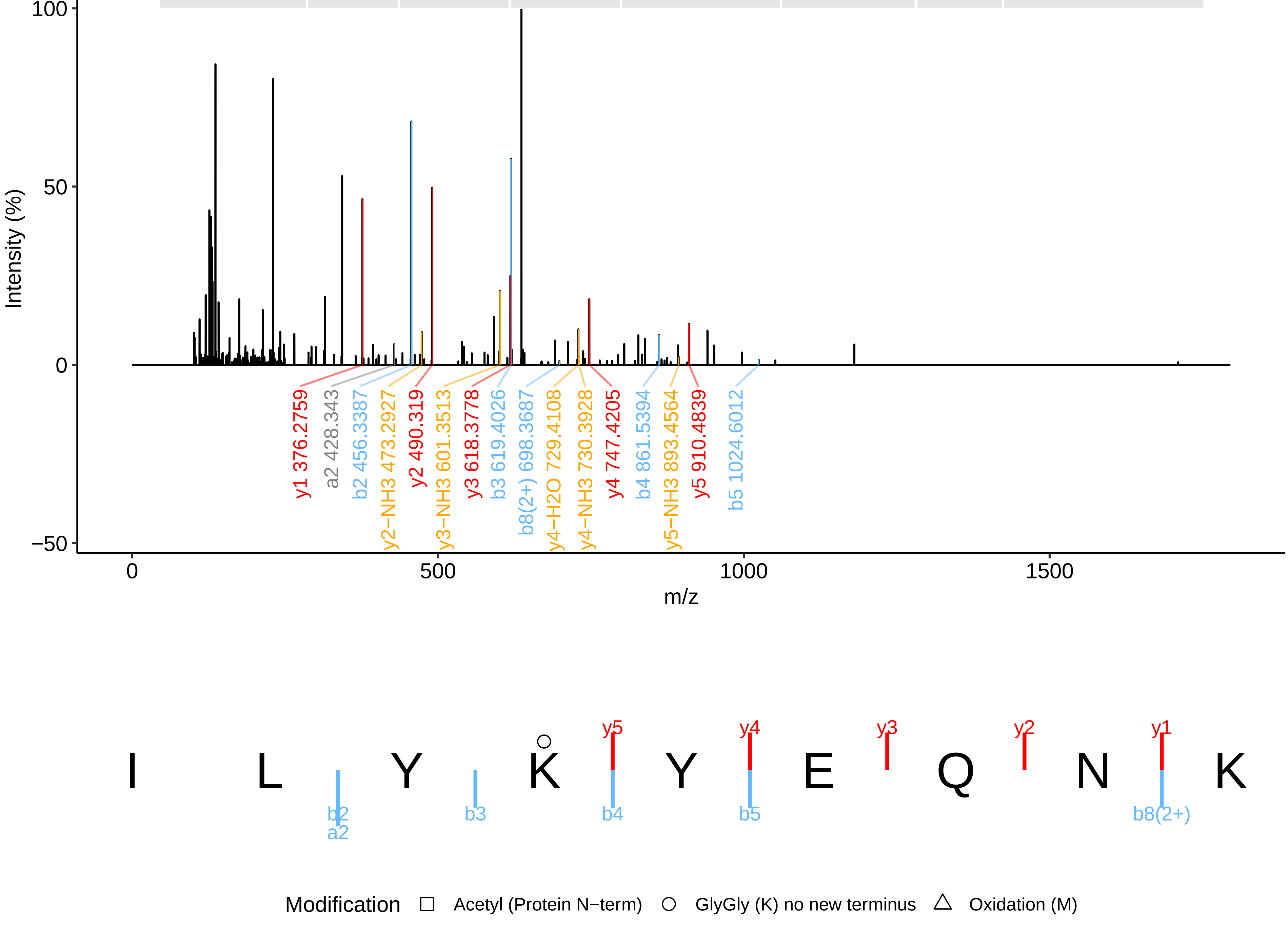

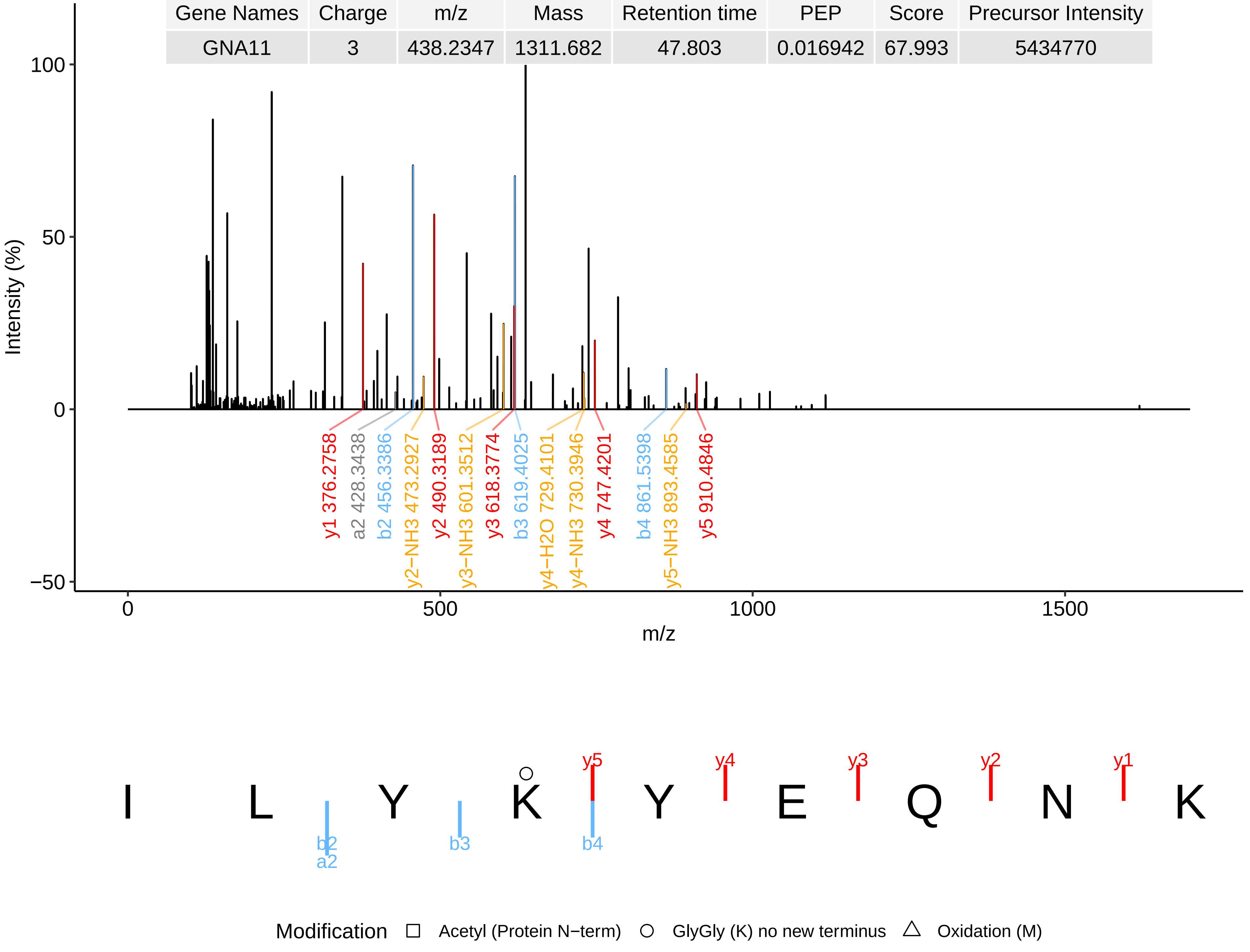

Gene Names	Charge	m/z	Mass	Retention time	PEP	Score	Precursor Intensity
GNA11	3	438.2347	1311.682	47.803	0.016942	67.993	5434770

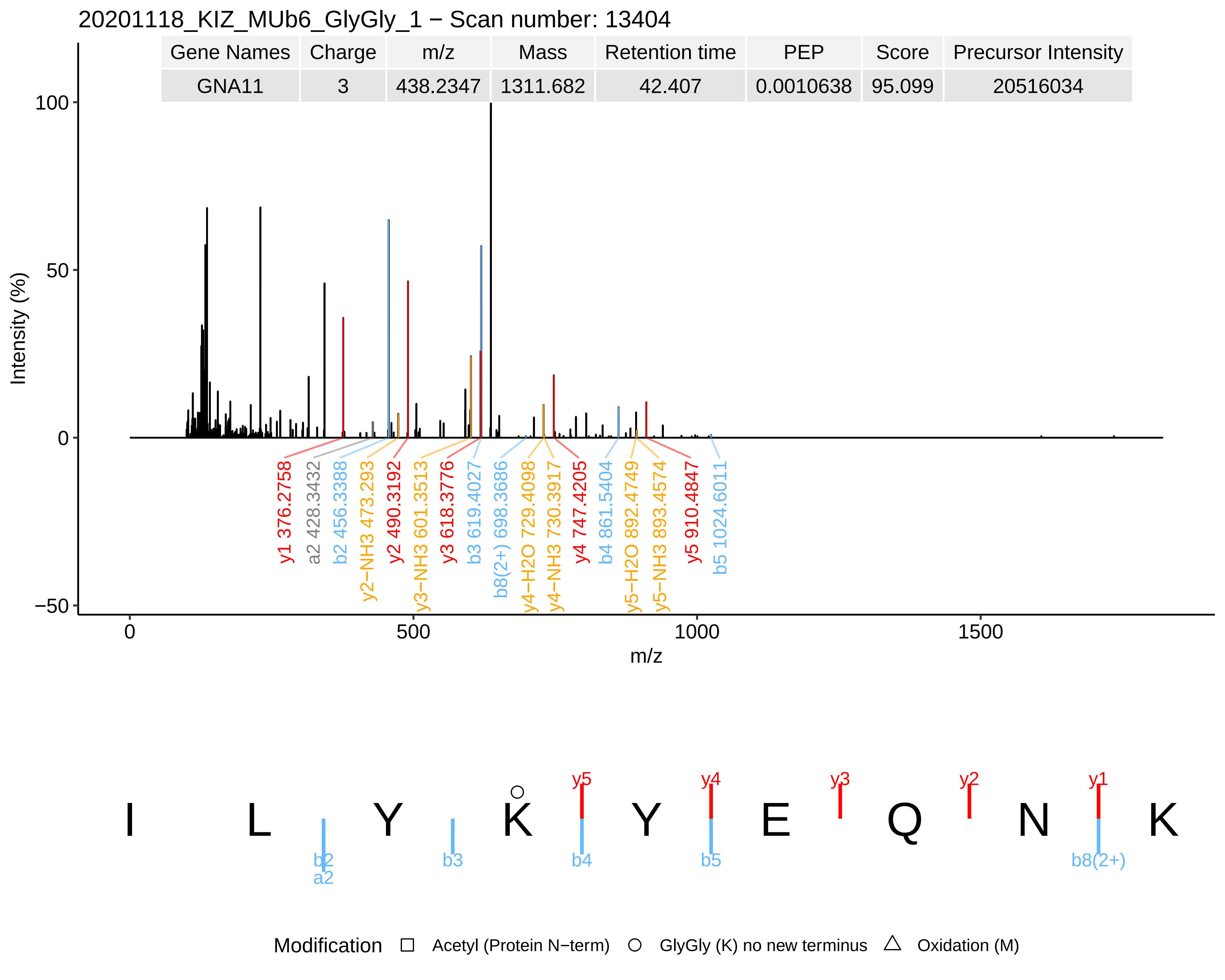
20201118_KIZ_MUb6_GlyGly_1 − Scan number: 13404
Gene Names
Charge
m/z
Mass
Retention time
PEP
Score
Precursor Intensity
GNA11
3
438.2347
1311.682
42.407
0.0010638
95.099
20516034
Intensity (%)
100
50
0
−50
0
500
1000
1500
m/z
y1 376.2758
a2 428.3432
b2 456.3388
y2−NH3 473.293
y2 490.3192
y3−NH3 601.3513
y3 618.3776
b3 619.4027
b8(2+) 698.3686
y4−H2O 729.4098
y4−NH3 730.3917
y4 747.4205
b4 861.5404
y5−H2O 892.4749
y5−NH3 893.4574
y5 910.4847
b5 1024.6011
I
L
Y
K
Y
E
Q
N
K
y5
y4
y3
y2
y1
b2
a2
b3
b4
b5
b8(2+)
Modification
Acetyl (Protein N−term)
GlyGly (K) no new terminus
Oxidation (M)

20201118_KIZ_MUb6_GlyGly_2 − Scan number: 16020

Gene Names	Charge	m/z	Mass	Retention time	PEP	Score	Precursor Intensity
GNA11	3	438.2347	1311.682	54.071	5.6479e−05	114.28	5236257

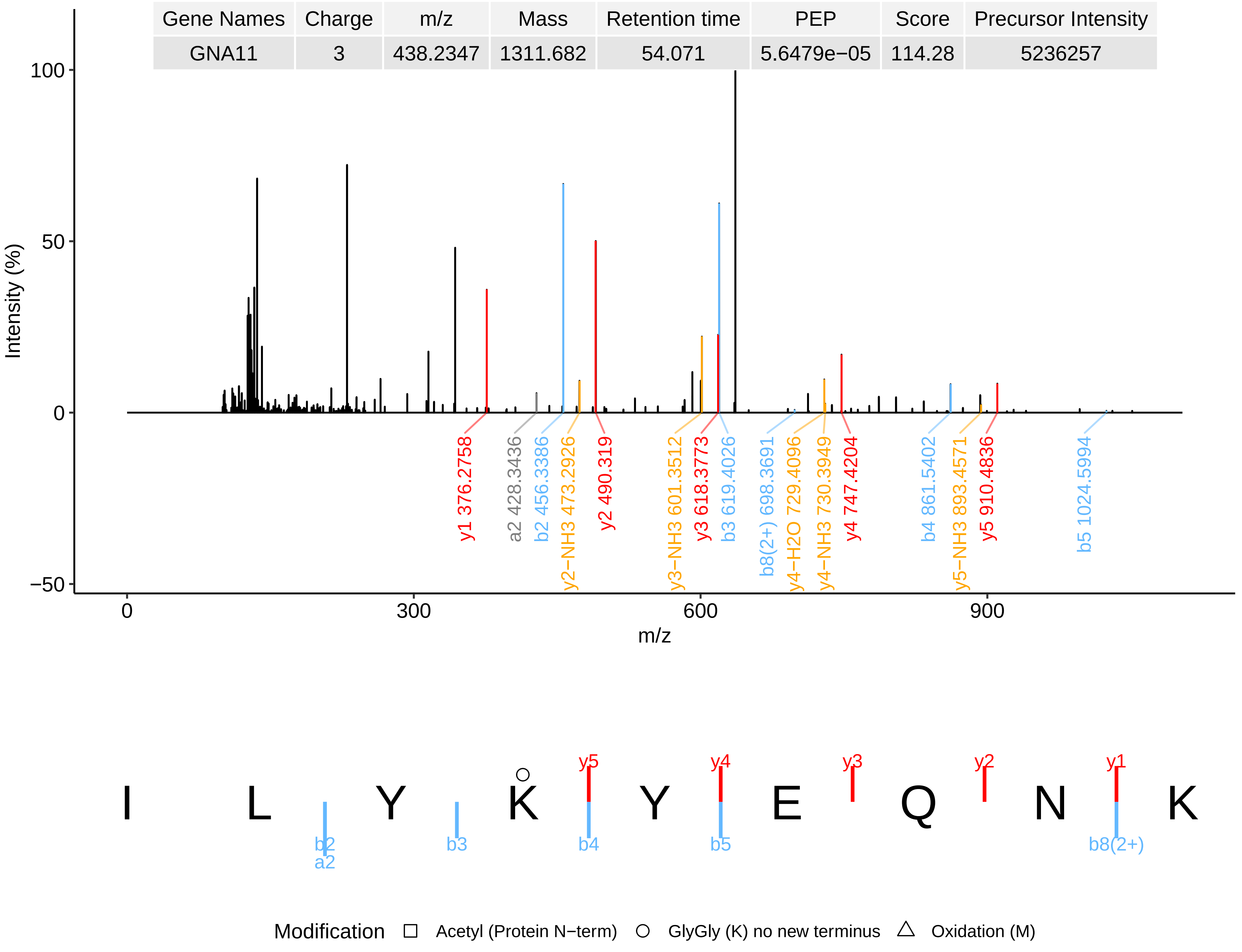

20200921_KIZ_MUb4_GlyGly_10plex − Scan number: 14168

Gene Names	Charge	m/z	Mass	Retention time	PEP	Score	Precursor Intensity
POLR2F	3	506.2537	1515.739	50.248	0.0014338	88.187	4177150

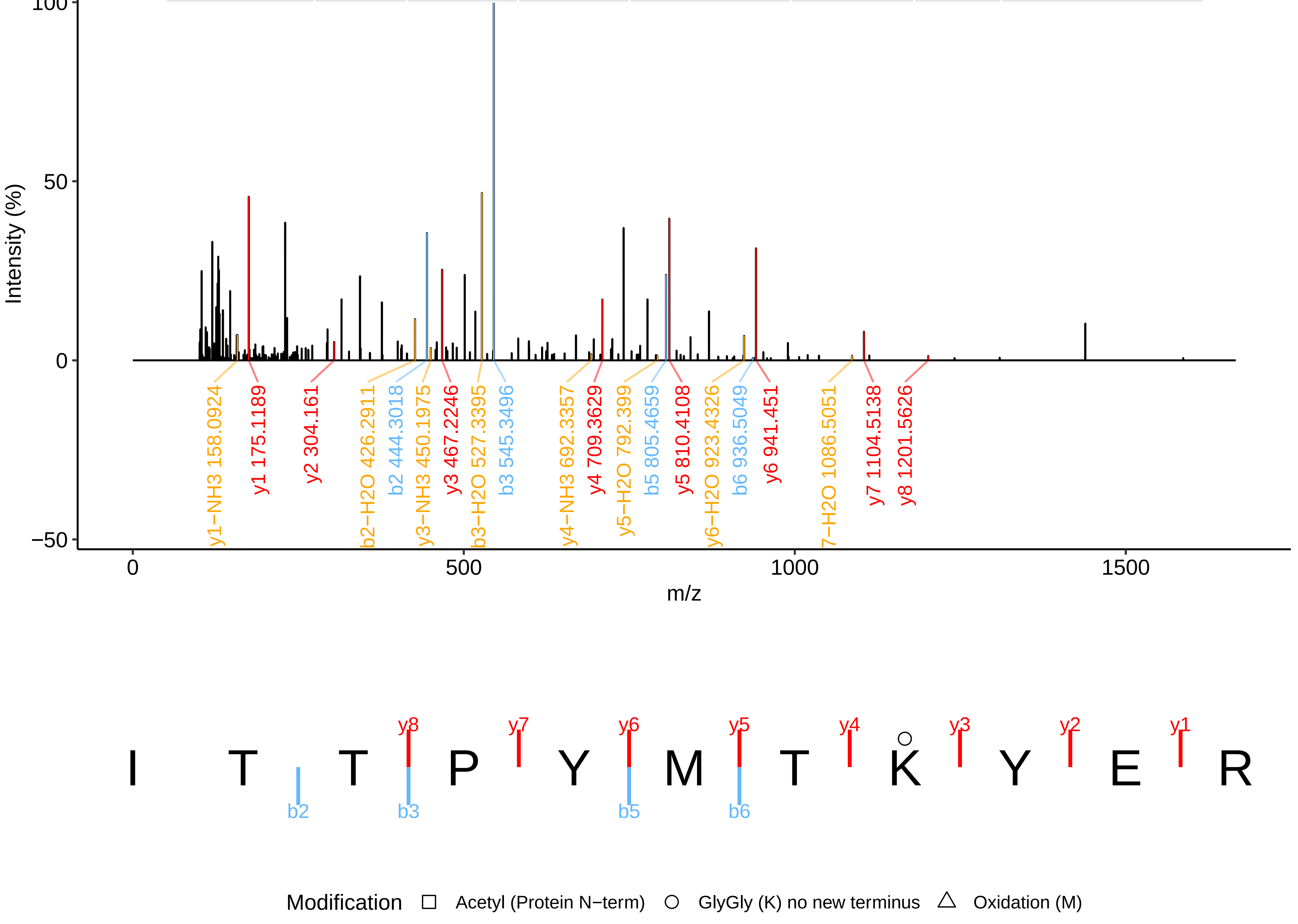

20200921_KIZ_MUb4_GlyGly_10plex_2 − Scan number: 14967

Gene Names	Charge	m/z	Mass	Retention time	PEP	Score	Precursor Intensity
PLA2G4A	3	435.9065	1304.698	58.437	0.0032194	79.974	1032983

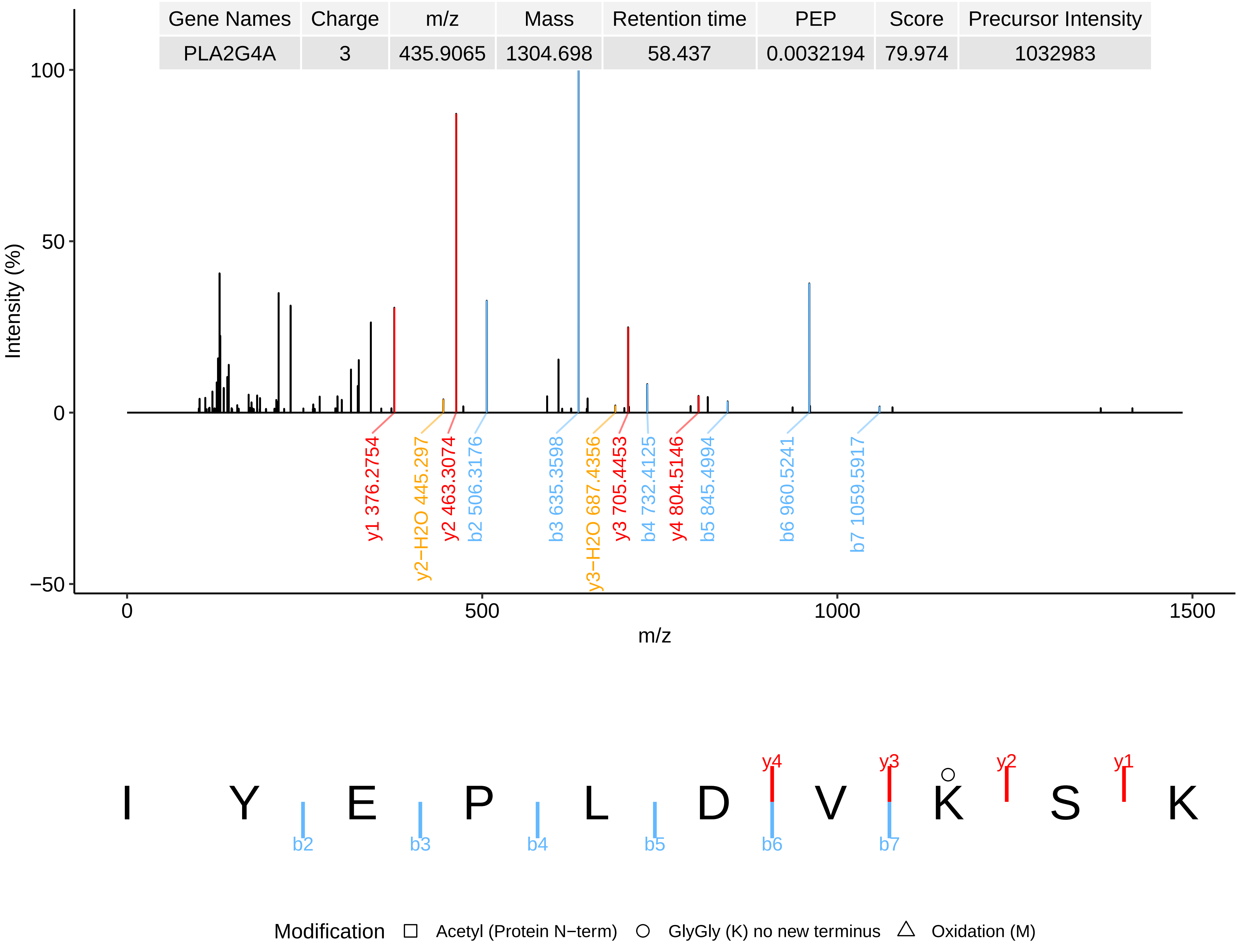

20200921_KIZ_MUb4_GlyGly_10plex − Scan number: 10190

Gene Names	Charge	m/z	Mass	Retention time	PEP	Score	Precursor Intensity
PHB	3	435.2333	1302.678	35.183	0.063744	66.989	8242848

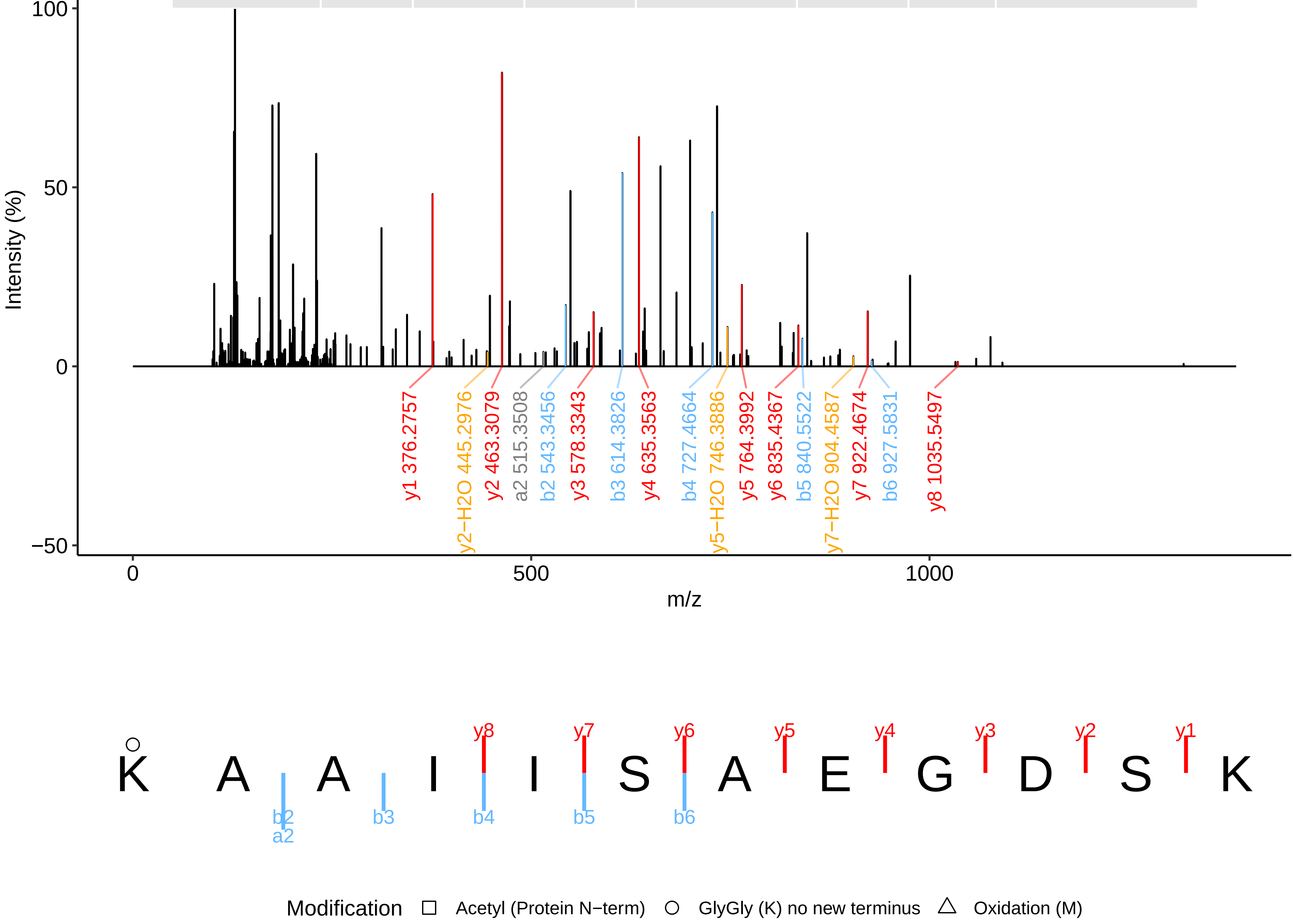

20201118_KIZ_MUb6_GlyGly_2 − Scan number: 10890

Gene Names	Charge	m/z	Mass	Retention time	PEP	Score	Precursor Intensity
PHB	3	435.2333	1302.678	36.712	0.026187	76.073	3844608

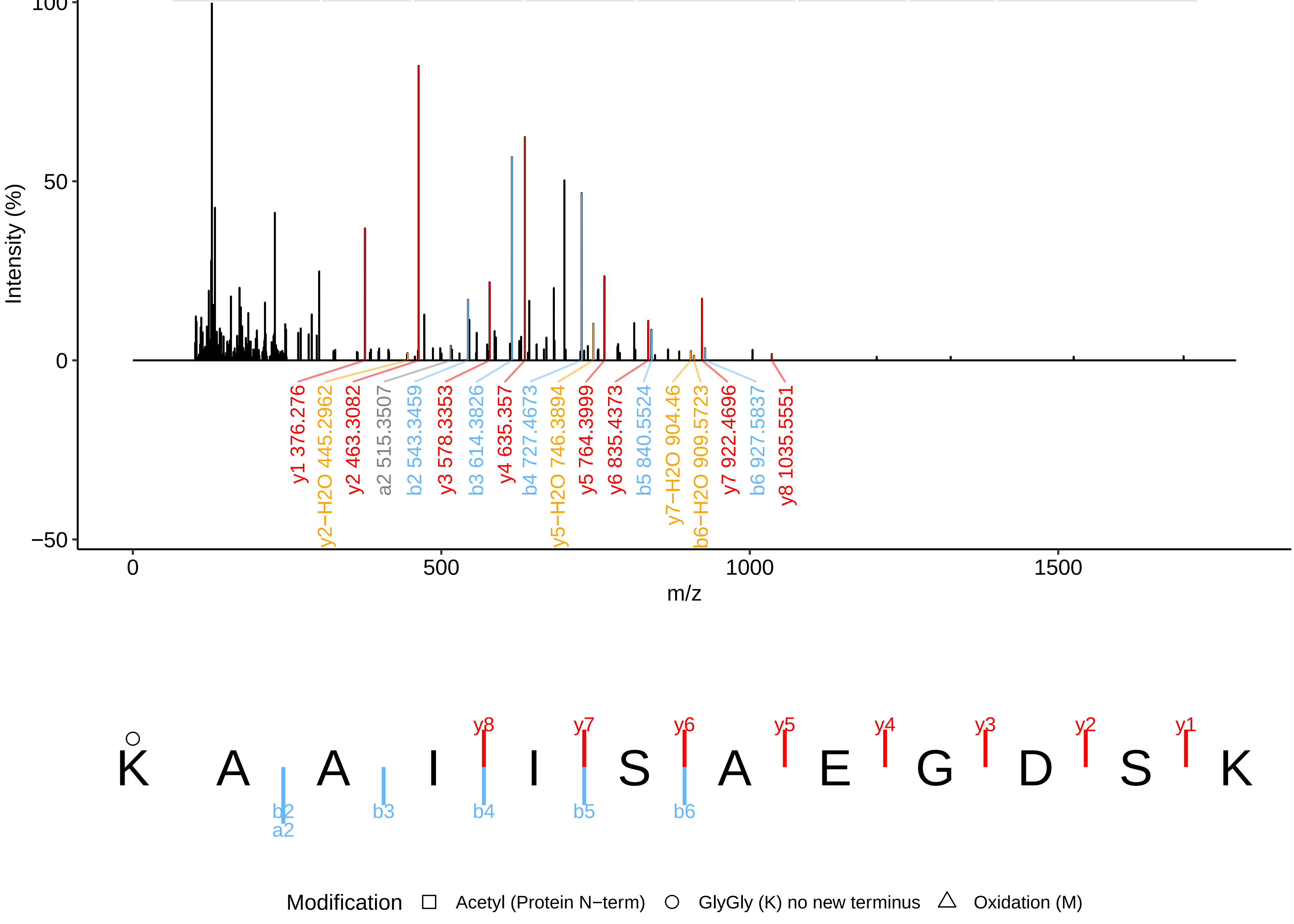

20200924_KIZ_MUb5_GlyGly_2 − Scan number: 19928

Gene Names	Charge	m/z	Mass	Retention time	PEP	Score	Precursor Intensity
CCAR1	3	515.274	1542.8	73.878	0.068402	46.73	15070323

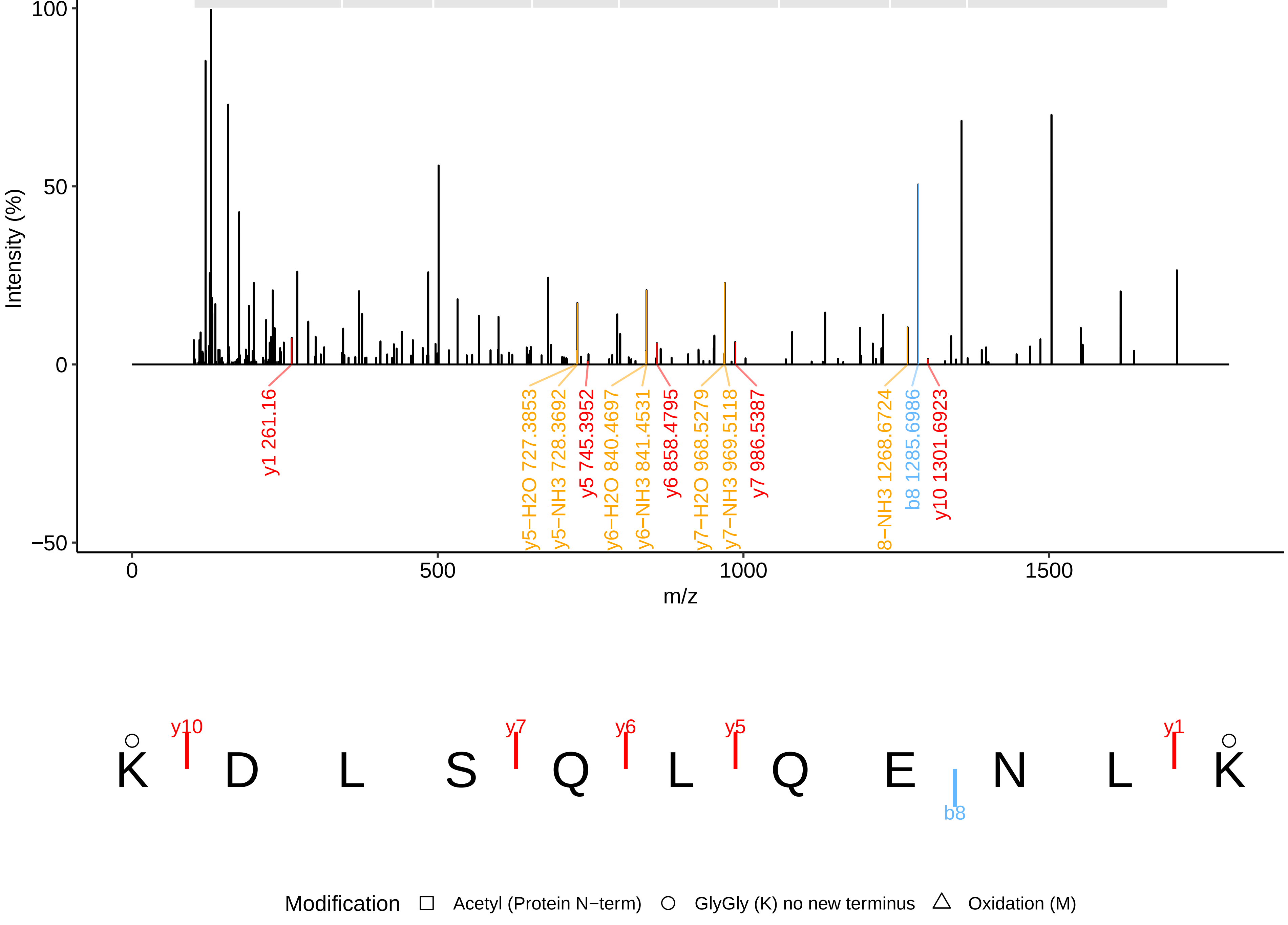

20200921_KIZ_MUb4_GlyGly_10plex − Scan number: 7610

Gene Names	Charge	m/z	Mass	Retention time	PEP	Score	Precursor Intensity
SLC39A14	3	436.203	1305.587	25.596	0.0088213	90.653	NA

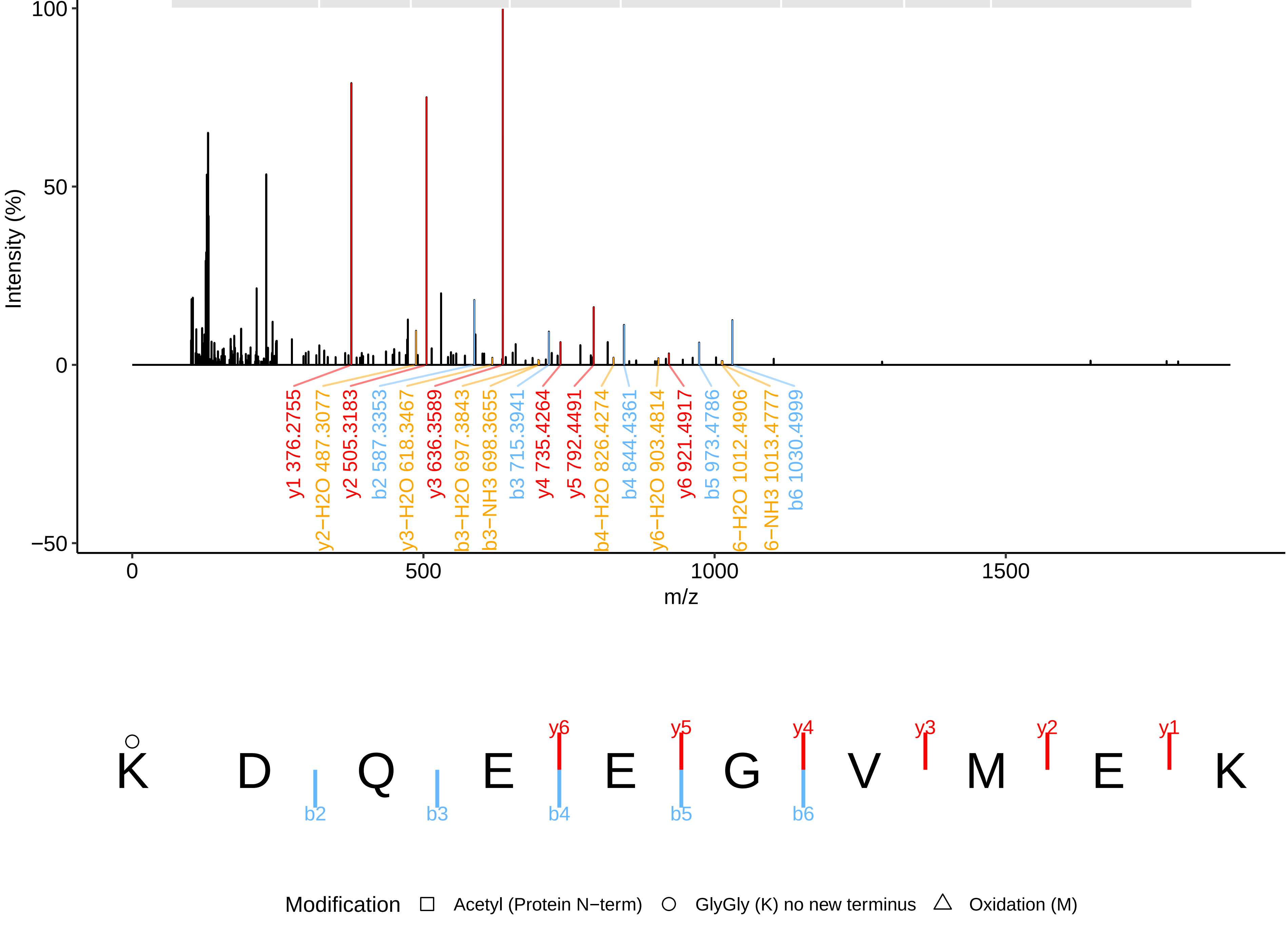

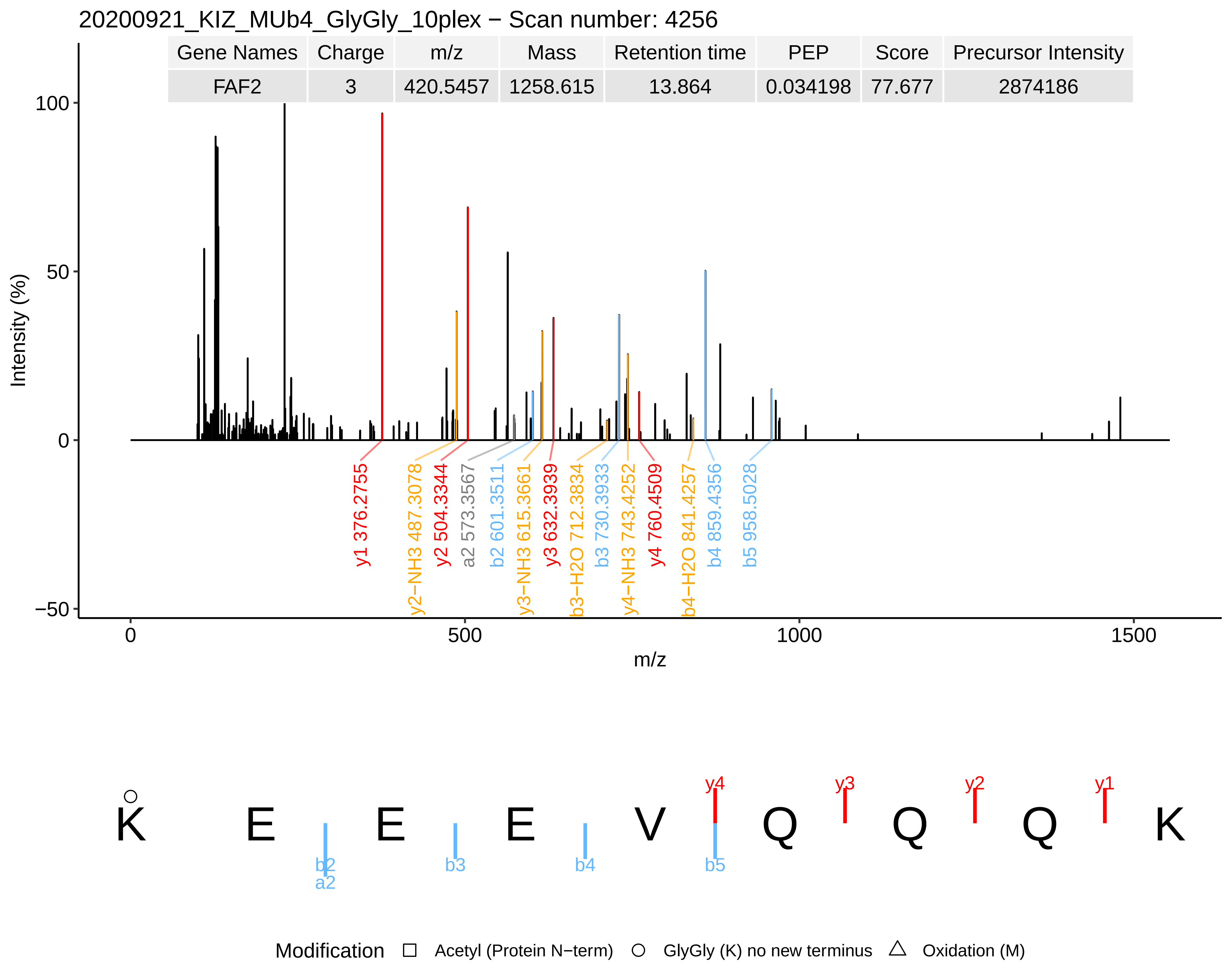
20200921_KIZ_MUb4_GlyGly_10plex − Scan number: 4256
Gene Names | Charge | m/z | Mass | Retention time | PEP | Score | Precursor Intensity
FAF2 | 3 | 420.5457 | 1258.615 | 13.864 | 0.034198 | 77.677 | 2874186
Intensity (%)
m/z
y1 376.2755
y2−NH3 487.3078
y2 504.3344
a2 573.3567
b2 601.3511
y3−NH3 615.3661
y3 632.3939
b3−H2O 712.3834
b3 730.3933
y4−NH3 743.4252
y4 760.4509
b4−H2O 841.4257
b4 859.4356
b5 958.5028
K E E E V Q Q Q K
y4 y3 y2 y1
b2 a2 b3 b4 b5
Modification
Acetyl (Protein N−term)
GlyGly (K) no new terminus
Oxidation (M)

20200921_KIZ_MUb4_GlyGly_10plex − Scan number: 28845

Gene Names	Charge	m/z	Mass	Retention time	PEP	Score	Precursor Intensity
	2	644.367	1286.719	105.59	0.14873	71.223	3333153

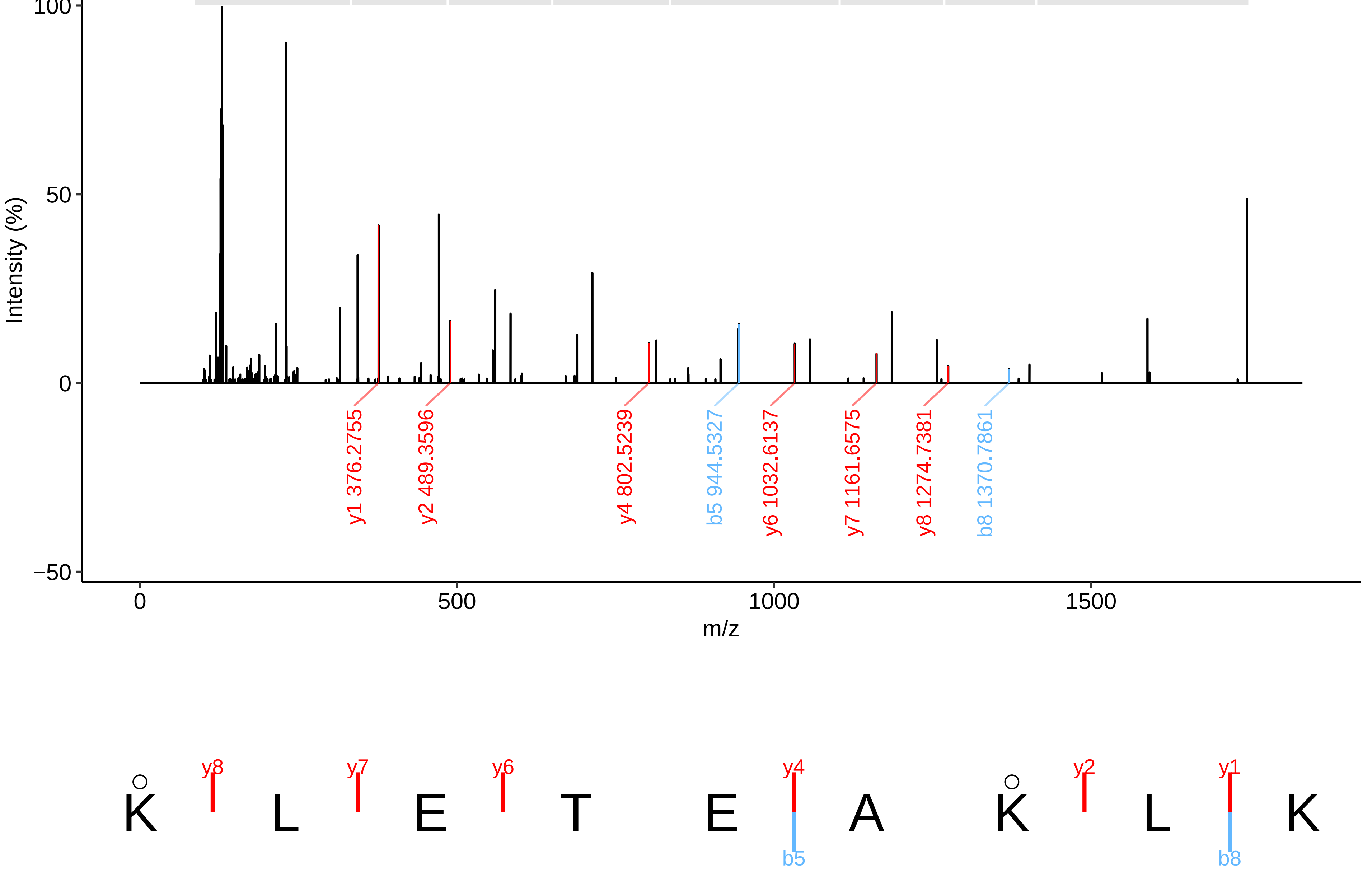

Modification □ Acetyl (Protein N−term) ○ GlyGly (K) no new terminus △ Oxidation (M)

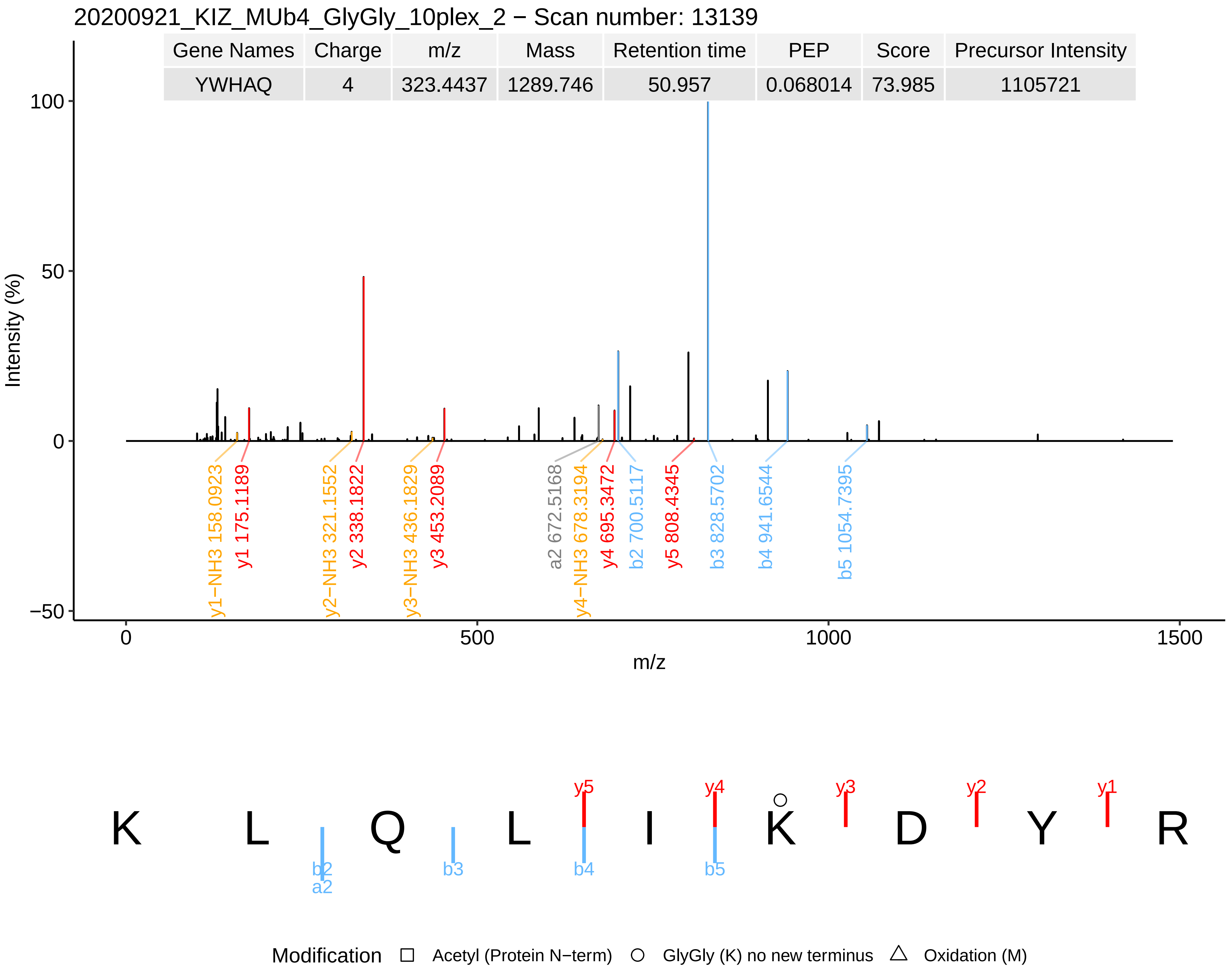

20200921_KIZ_MUb4_GlyGly_10plex_2 − Scan number: 13139
Gene Names | Charge | m/z | Mass | Retention time | PEP | Score | Precursor Intensity
YWHAQ | 4 | 323.4437 | 1289.746 | 50.957 | 0.068014 | 73.985 | 1105721
Intensity (%)
m/z
y1−NH3 158.0923
y1 175.1189
y2−NH3 321.1552
y2 338.1822
y3−NH3 436.1829
y3 453.2089
a2 672.5168
y4−NH3 678.3194
y4 695.3472
b2 700.5117
y5 808.4345
b3 828.5702
b4 941.6544
b5 1054.7395
K L Q L I K D Y R
y5 y4 y3 y2 y1
b2 a2 b3 b4 b5
Modification
Acetyl (Protein N−term)
GlyGly (K) no new terminus
Oxidation (M)

20200921_KIZ_MUb4_GlyGly_10plex − Scan number: 5324

Gene Names	Charge	m/z	Mass	Retention time	PEP	Score	Precursor Intensity
ITFG3	3	439.2249	1314.653	17.418	0.044584	71.176	NA

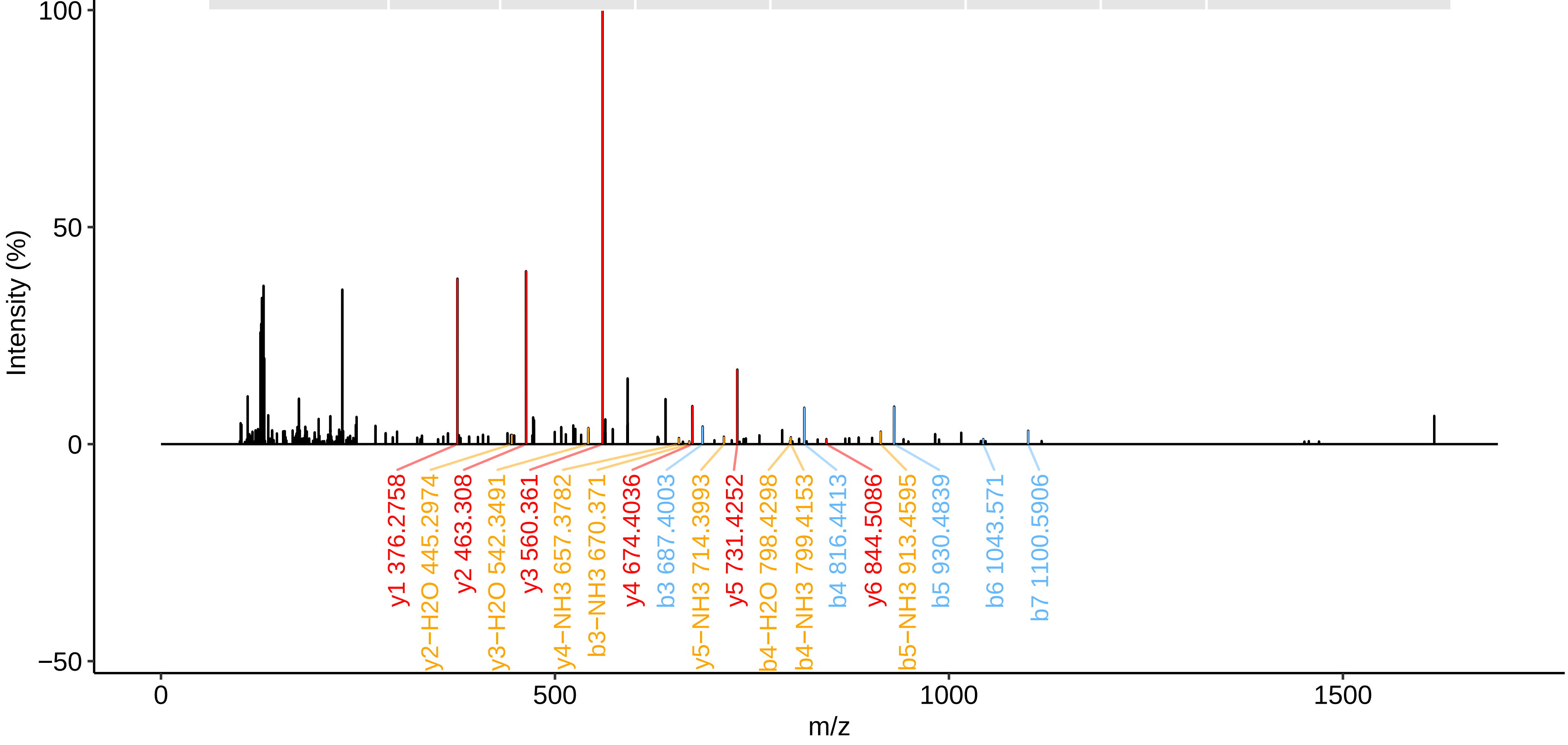

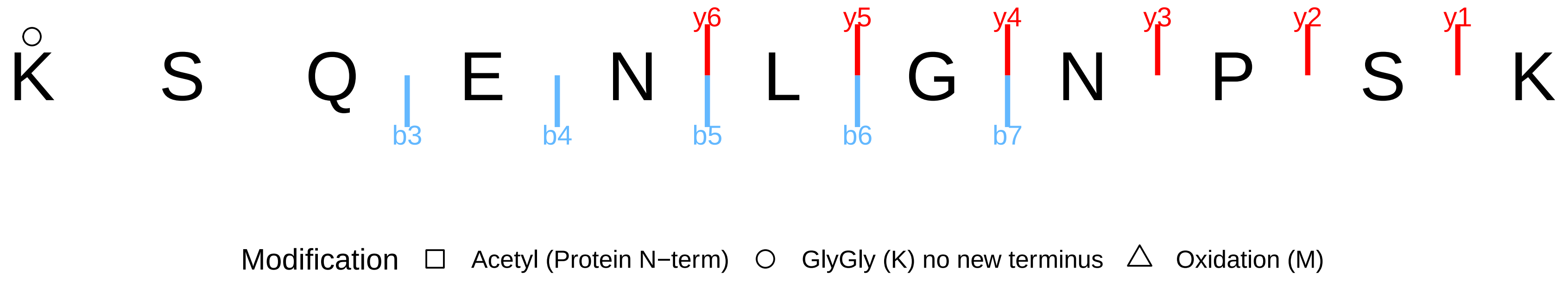

20200921_KIZ_MUb4_GlyGly_10plex − Scan number: 13184

Gene Names	Charge	m/z	Mass	Retention time	PEP	Score	Precursor Intensity
CLTA	3	437.2117	1308.613	46.426	0.020492	65.5	6006465

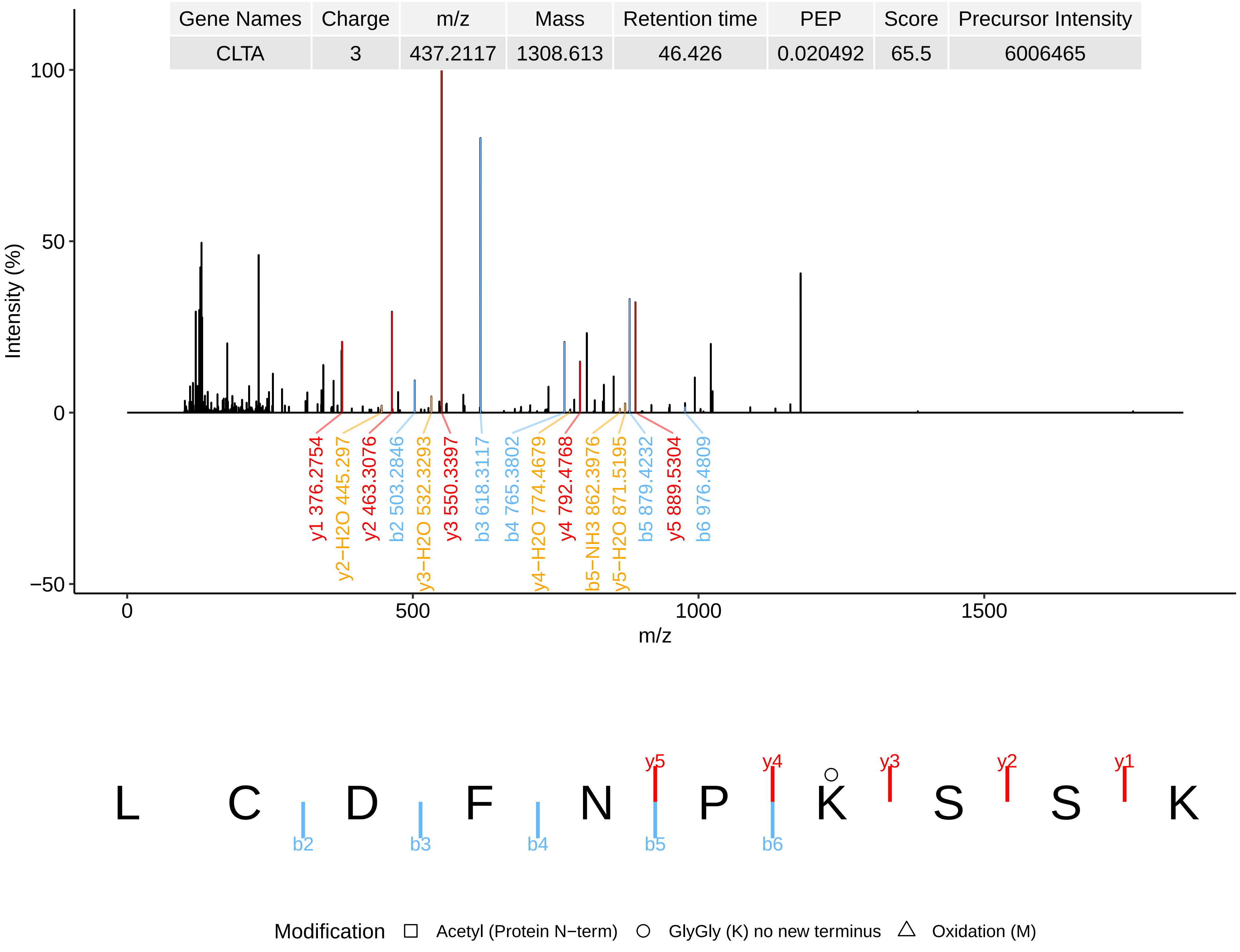

20200921_KIZ_MUb4_GlyGly_10plex − Scan number: 7300

Gene Names	Charge	m/z	Mass	Retention time	PEP	Score	Precursor Intensity
HGS	3	501.5963	1501.767	24.464	4.1622e−05	99.283	2349501

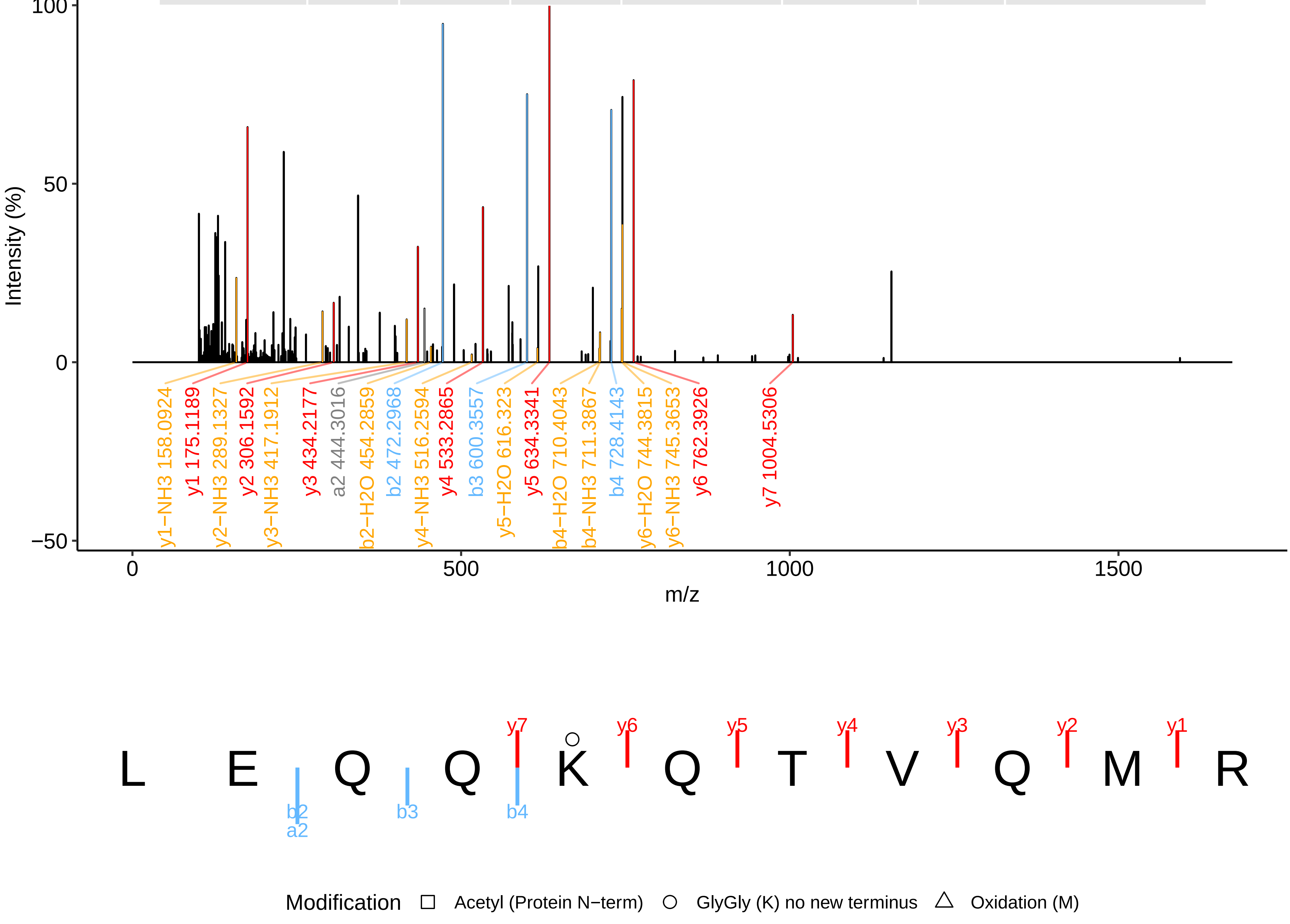

20200921_KIZ_MUb4_GlyGly_10plex_2 − Scan number: 6723

Gene Names	Charge	m/z	Mass	Retention time	PEP	Score	Precursor Intensity
HGS	3	501.5963	1501.767	24.839	0.00095291	88.596	592591.8

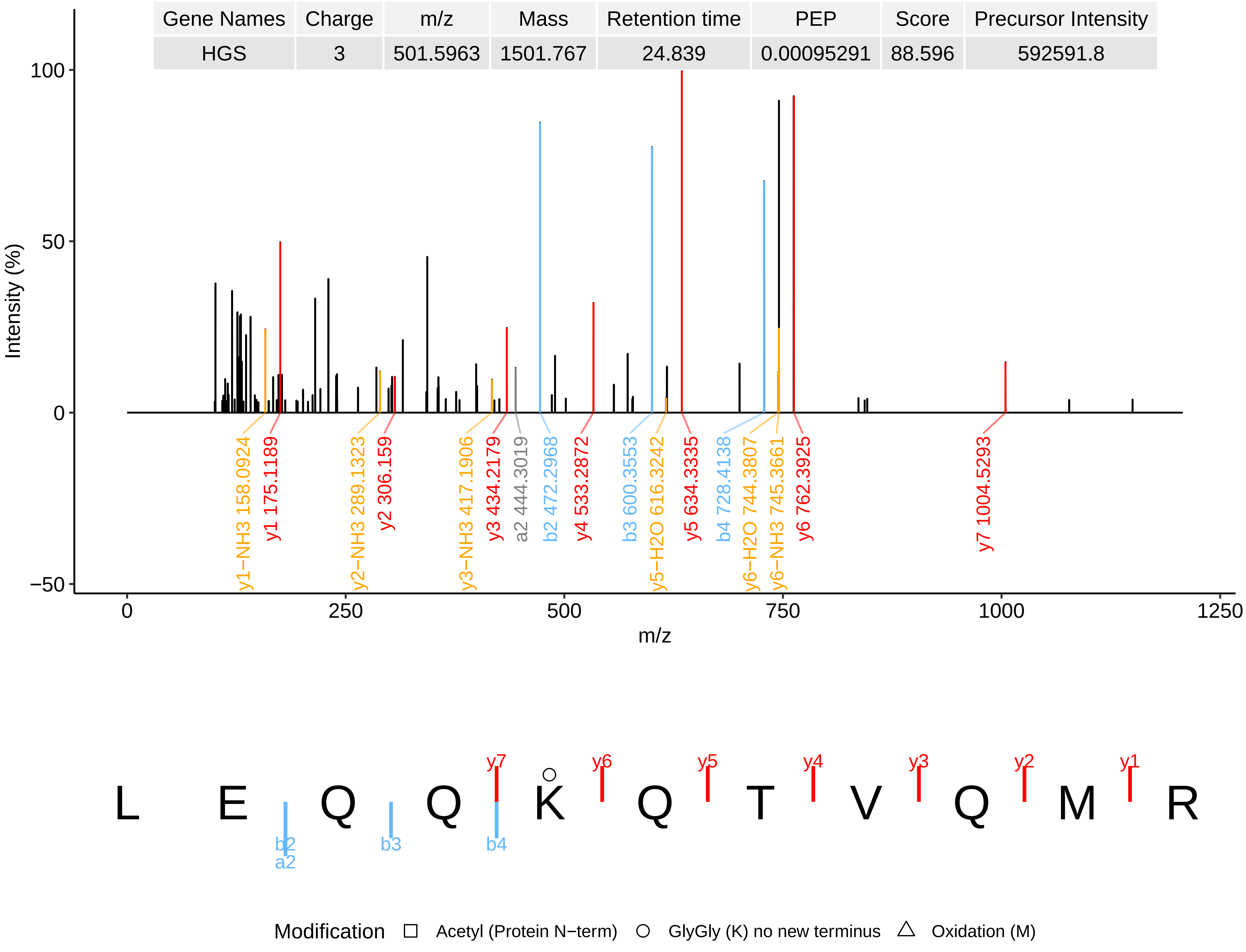

20200924_KIZ_MUb5_GlyGly_2 − Scan number: 5945

Gene Names	Charge	m/z	Mass	Retention time	PEP	Score	Precursor Intensity
HGS	3	501.5963	1501.767	22.122	0.0018339	84.198	NA

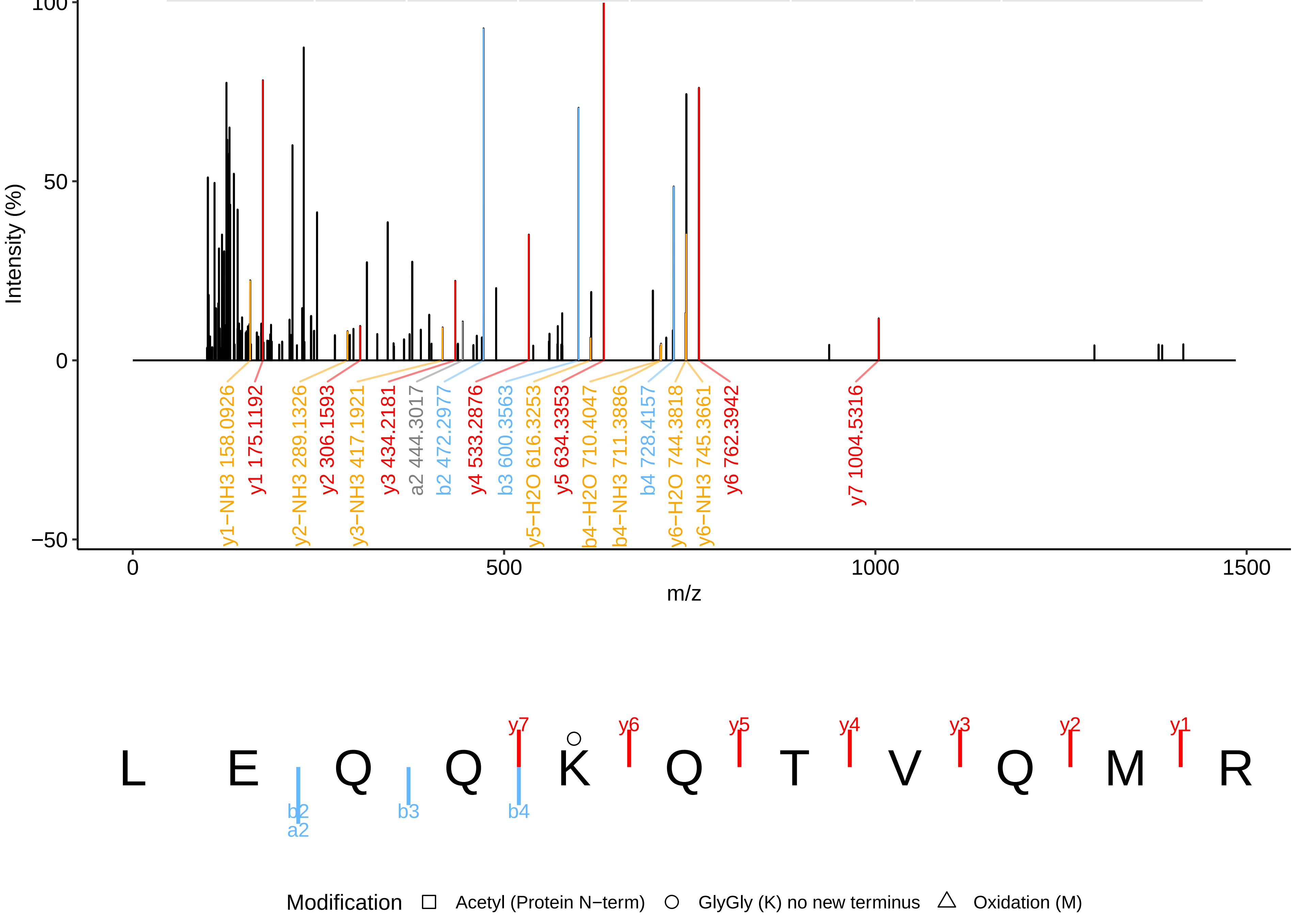

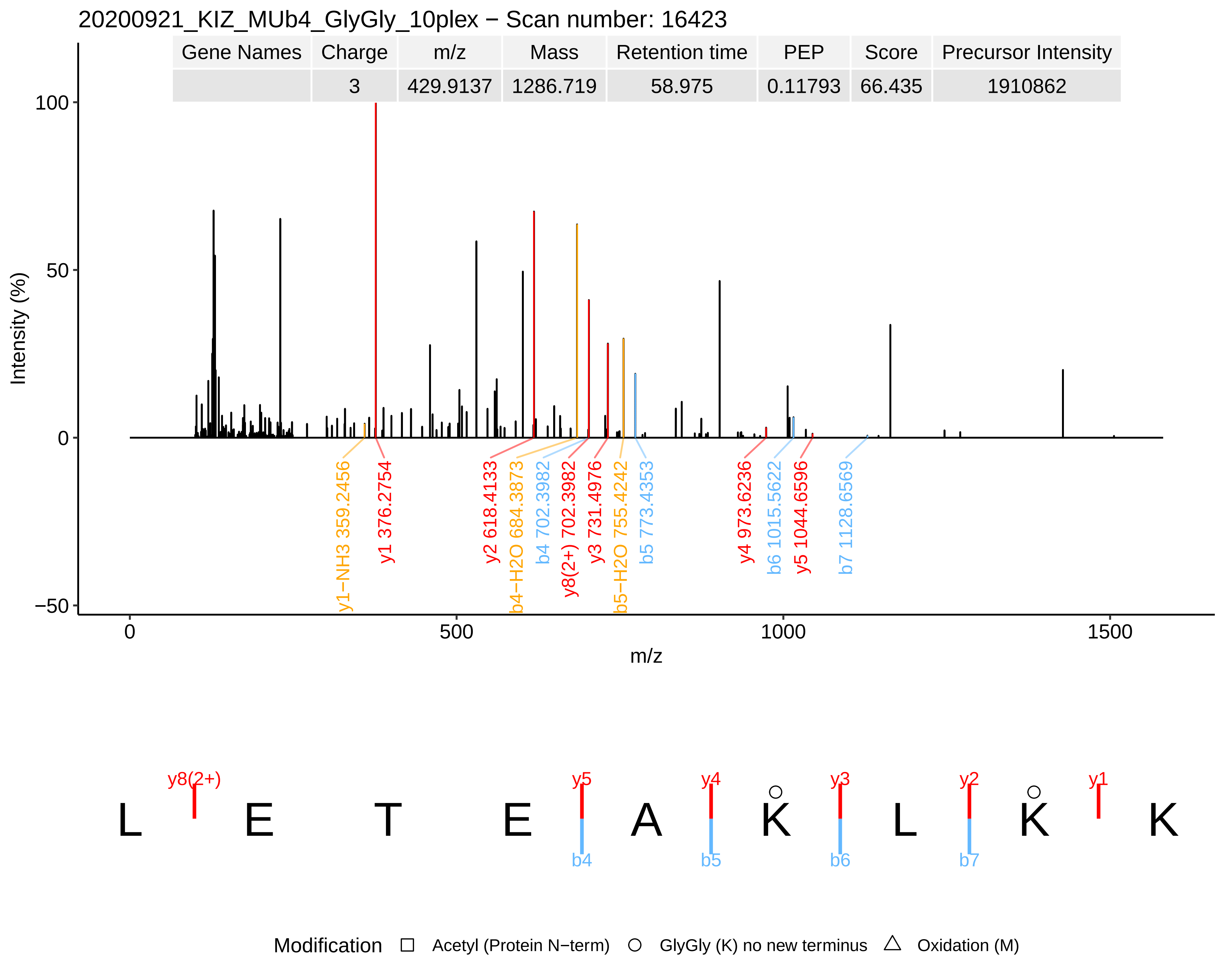
20200921_KIZ_MUb4_GlyGly_10plex − Scan number: 16423
Gene Names
Charge
m/z
Mass
Retention time
PEP
Score
Precursor Intensity
3
429.9137
1286.719
58.975
0.11793
66.435
1910862
Intensity (%)
m/z
y1−NH3 359.2456
y1 376.2754
y2 618.4133
b4−H2O 684.3873
b4 702.3982
y8(2+) 702.3982
y3 731.4976
b5−H2O 755.4242
b5 773.4353
y4 973.6236
b6 1015.5622
y5 1044.6596
b7 1128.6569
L E T E A K L K K
y8(2+)
y5
y4
y3
y2
y1
b4
b5
b6
b7
Modification
Acetyl (Protein N−term)
GlyGly (K) no new terminus
Oxidation (M)

20200921_KIZ_MUb4_GlyGly_10plex − Scan number: 14194

Gene Names	Charge	m/z	Mass	Retention time	PEP	Score	Precursor Intensity
STX4	3	424.57	1270.688	50.351	0.0079332	76.943	NA

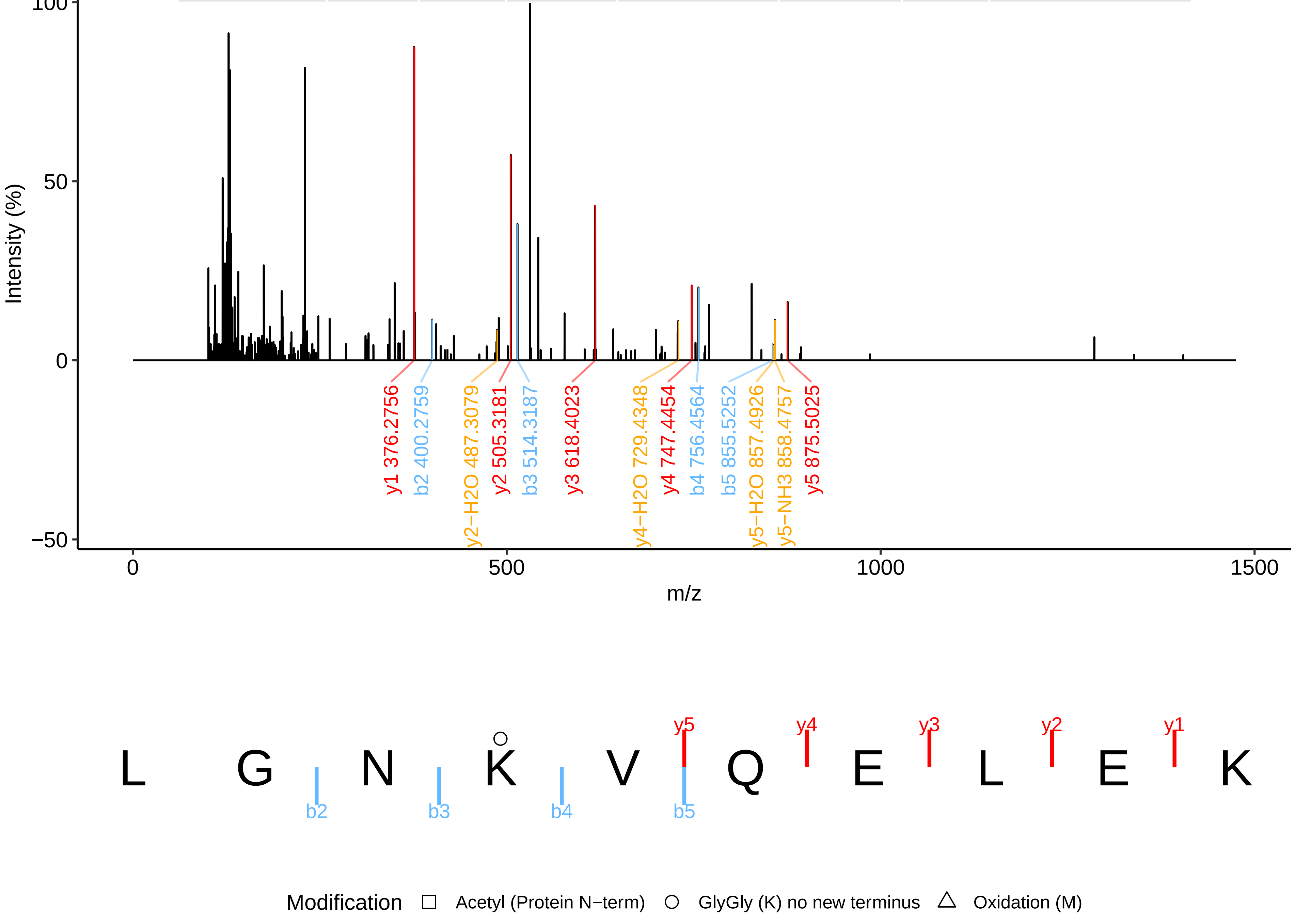

20200921_KIZ_MUb4_GlyGly_10plex − Scan number: 8282

Gene Names	Charge	m/z	Mass	Retention time	PEP	Score	Precursor Intensity
SLC16A3	4	380.456	1517.795	28.057	0.00076332	84.169	11728500

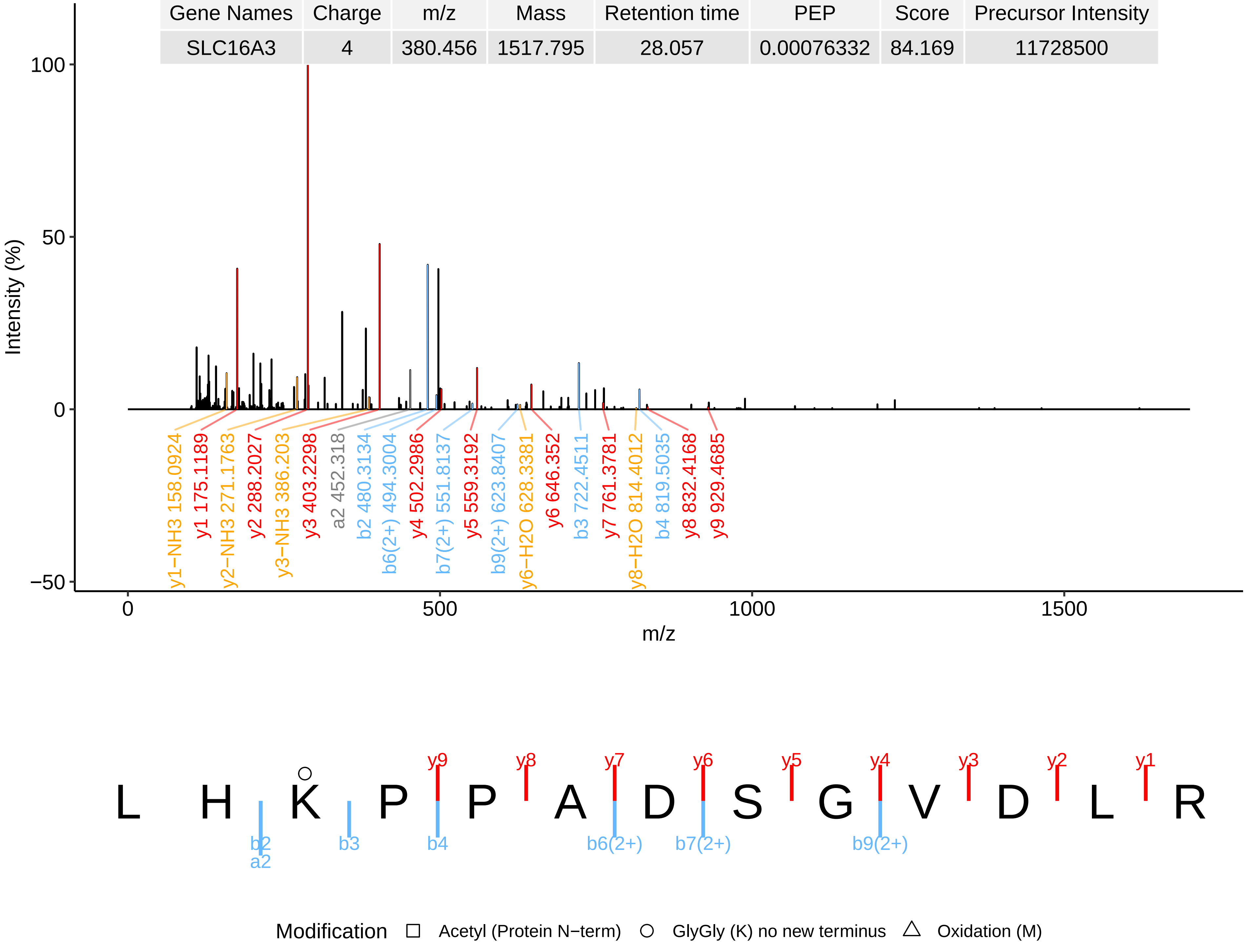

20200921_KIZ_MUb4_GlyGly_10plex − Scan number: 8287

Gene Names	Charge	m/z	Mass	Retention time	PEP	Score	Precursor Intensity
SLC16A3	3	506.939	1517.795	28.079	1.812e−05	101.39	4210503

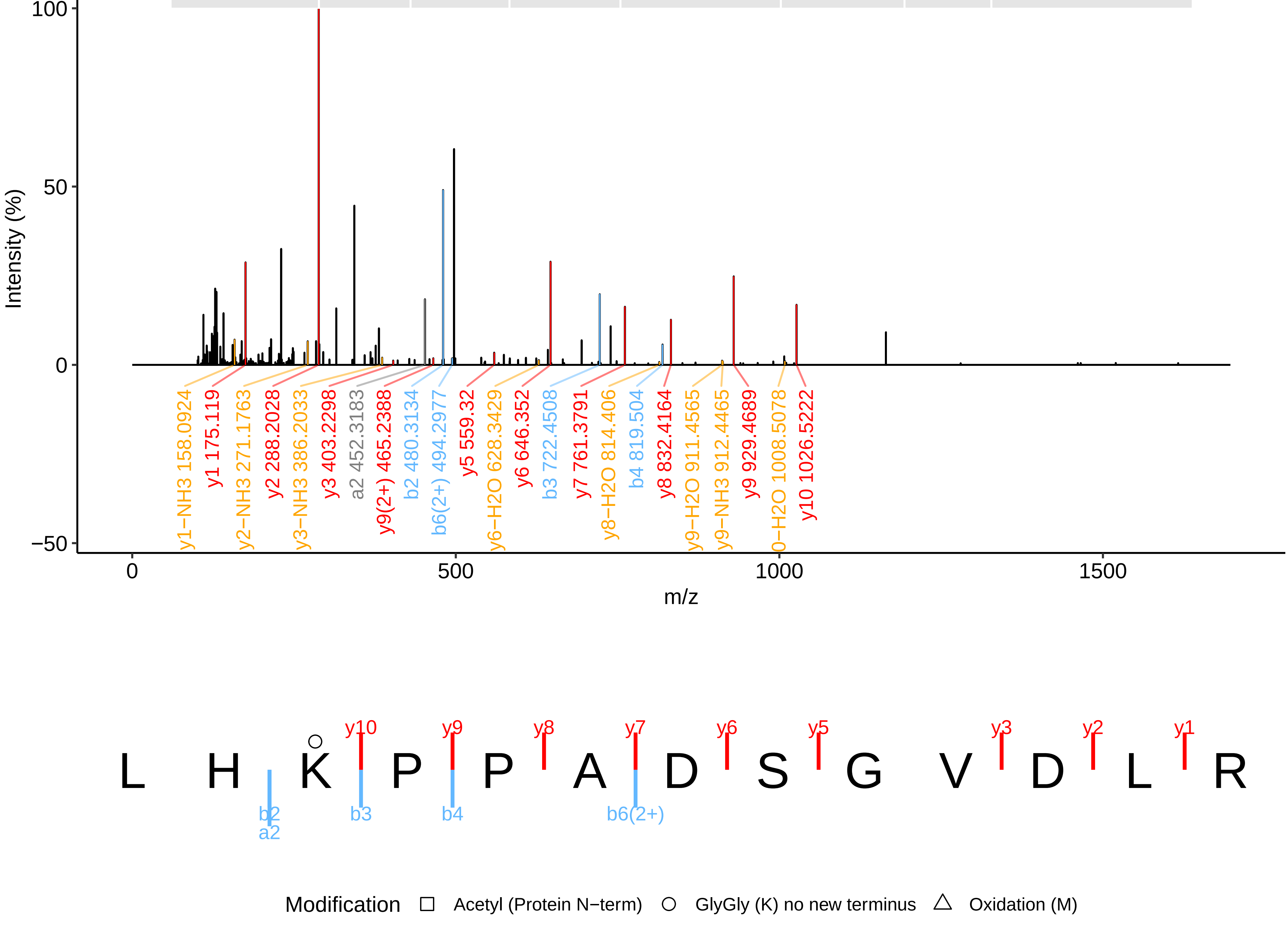

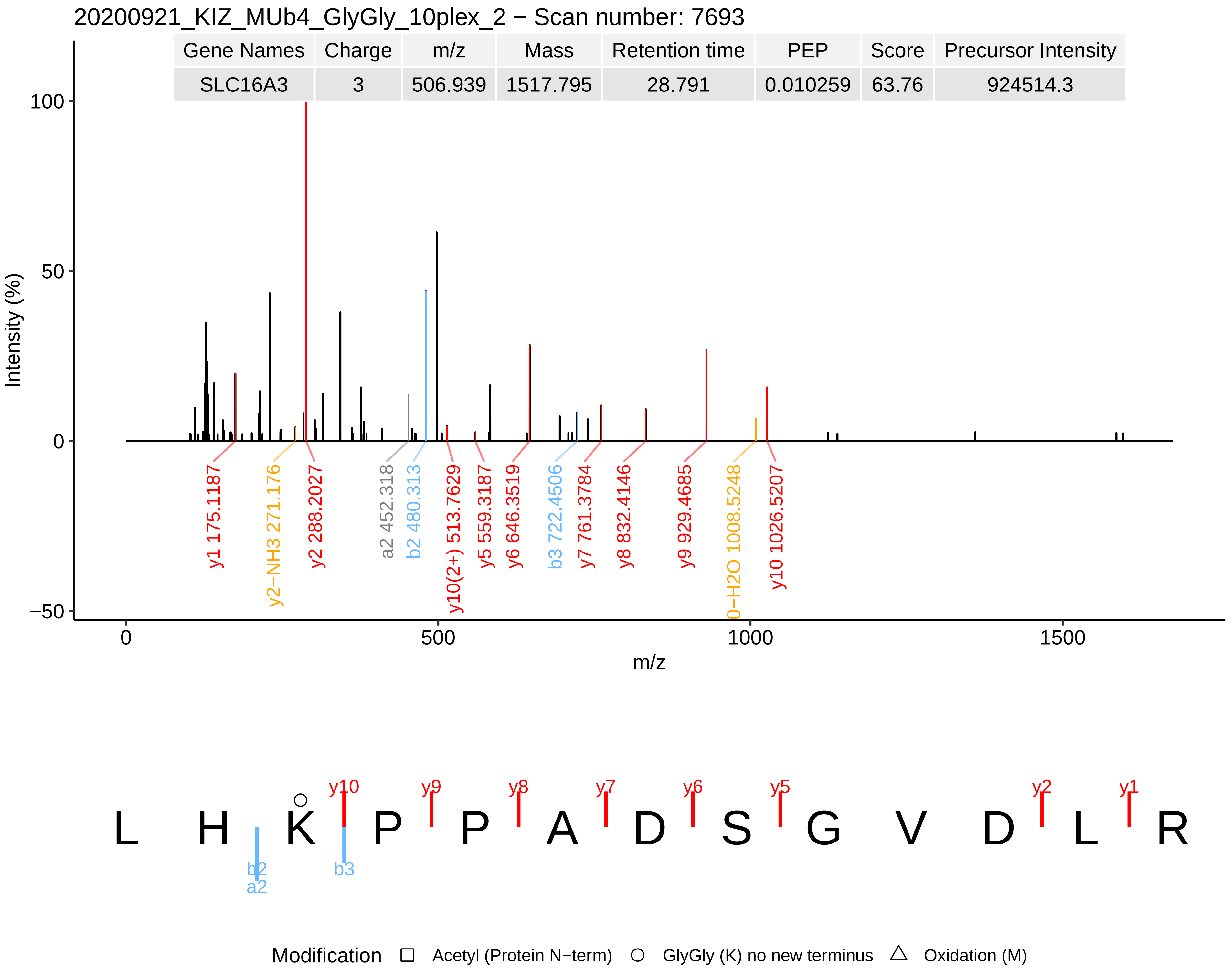
20200921_KIZ_MUb4_GlyGly_10plex_2 − Scan number: 7693
Gene Names | Charge | m/z | Mass | Retention time | PEP | Score | Precursor Intensity
SLC16A3 | 3 | 506.939 | 1517.795 | 28.791 | 0.010259 | 63.76 | 924514.3
Intensity (%)
m/z
y1 175.1187
y2−NH3 271.176
y2 288.2027
a2 452.318
b2 480.313
y10(2+) 513.7629
y5 559.3187
y6 646.3519
b3 722.4506
y7 761.3784
y8 832.4146
y9 929.4685
0−H2O 1008.5248
y10 1026.5207
L H K P P A D S G V D L R
Modification
Acetyl (Protein N−term)
GlyGly (K) no new terminus
Oxidation (M)

20200921_KIZ_MUb4_GlyGly_10plex − Scan number: 16242

Gene Names	Charge	m/z	Mass	Retention time	PEP	Score	Precursor Intensity
TMUB1	3	511.9388	1532.795	58.28	0.00011416	96.64	6509784

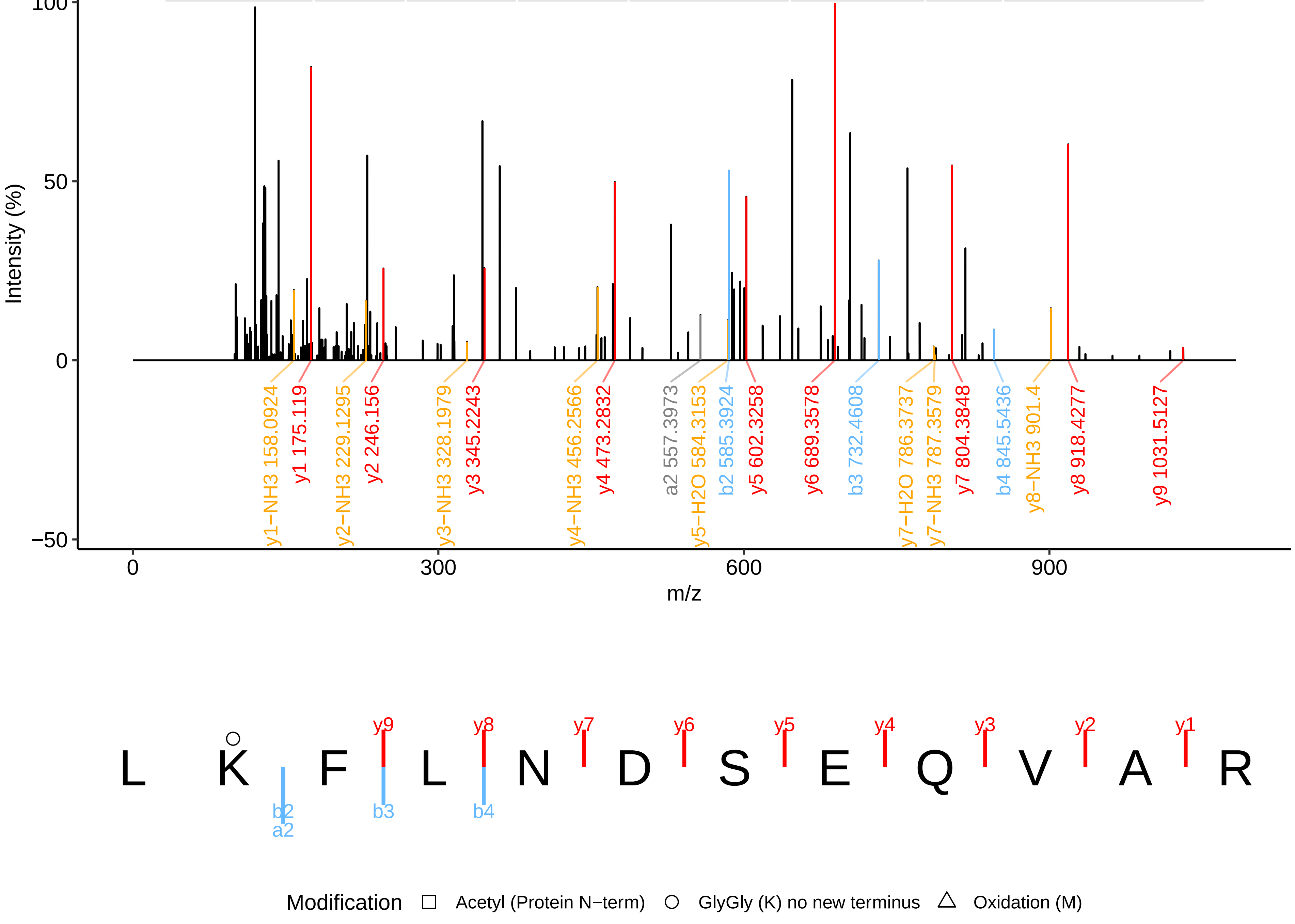

20200921_KIZ_MUb4_GlyGly_10plex_2 − Scan number: 15066

Gene Names	Charge	m/z	Mass	Retention time	PEP	Score	Precursor Intensity
TMUB1	3	511.9388	1532.795	58.842	0.012664	63.473	1651584

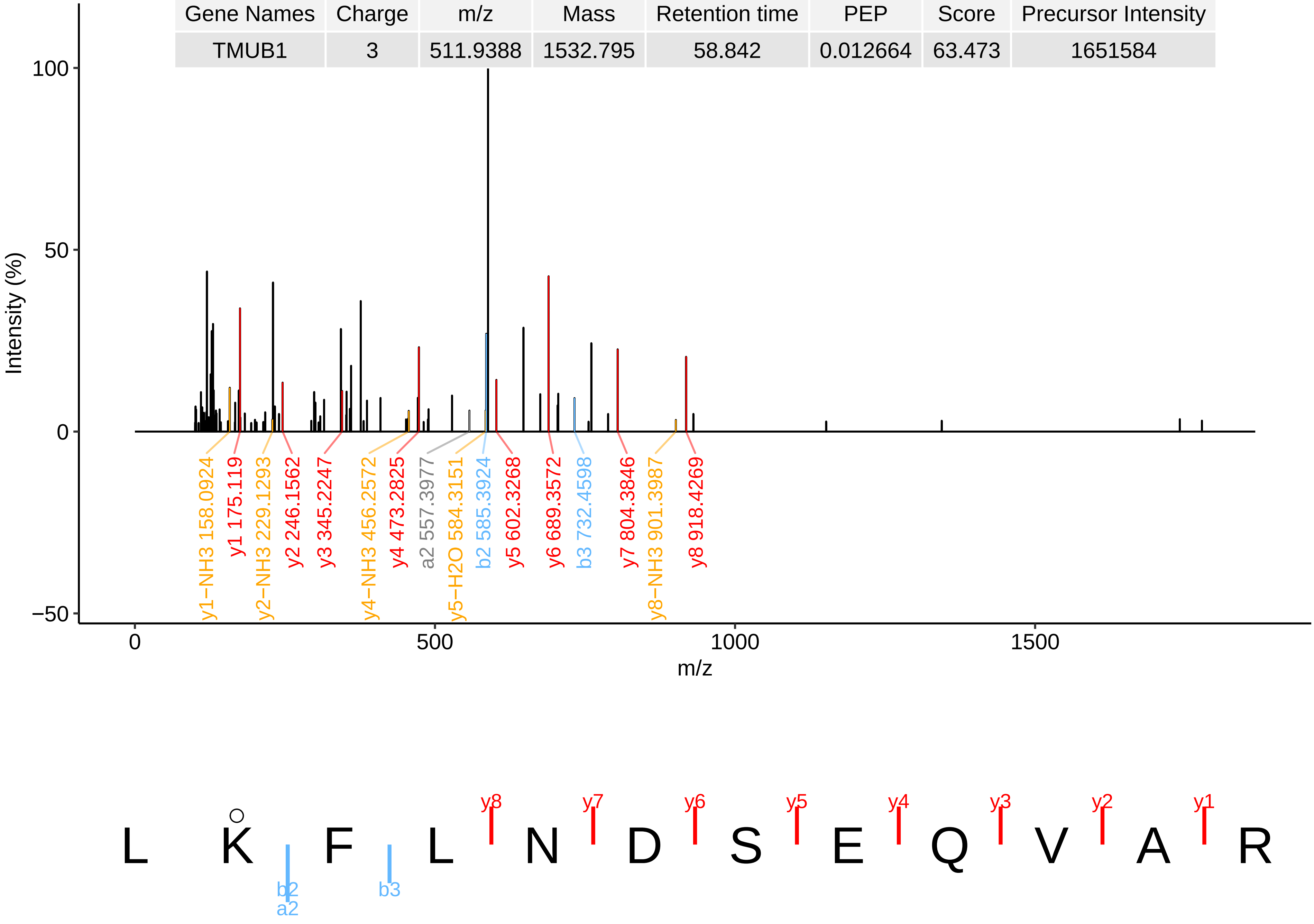

Modification □ Acetyl (Protein N−term) ○ GlyGly (K) no new terminus △ Oxidation (M)

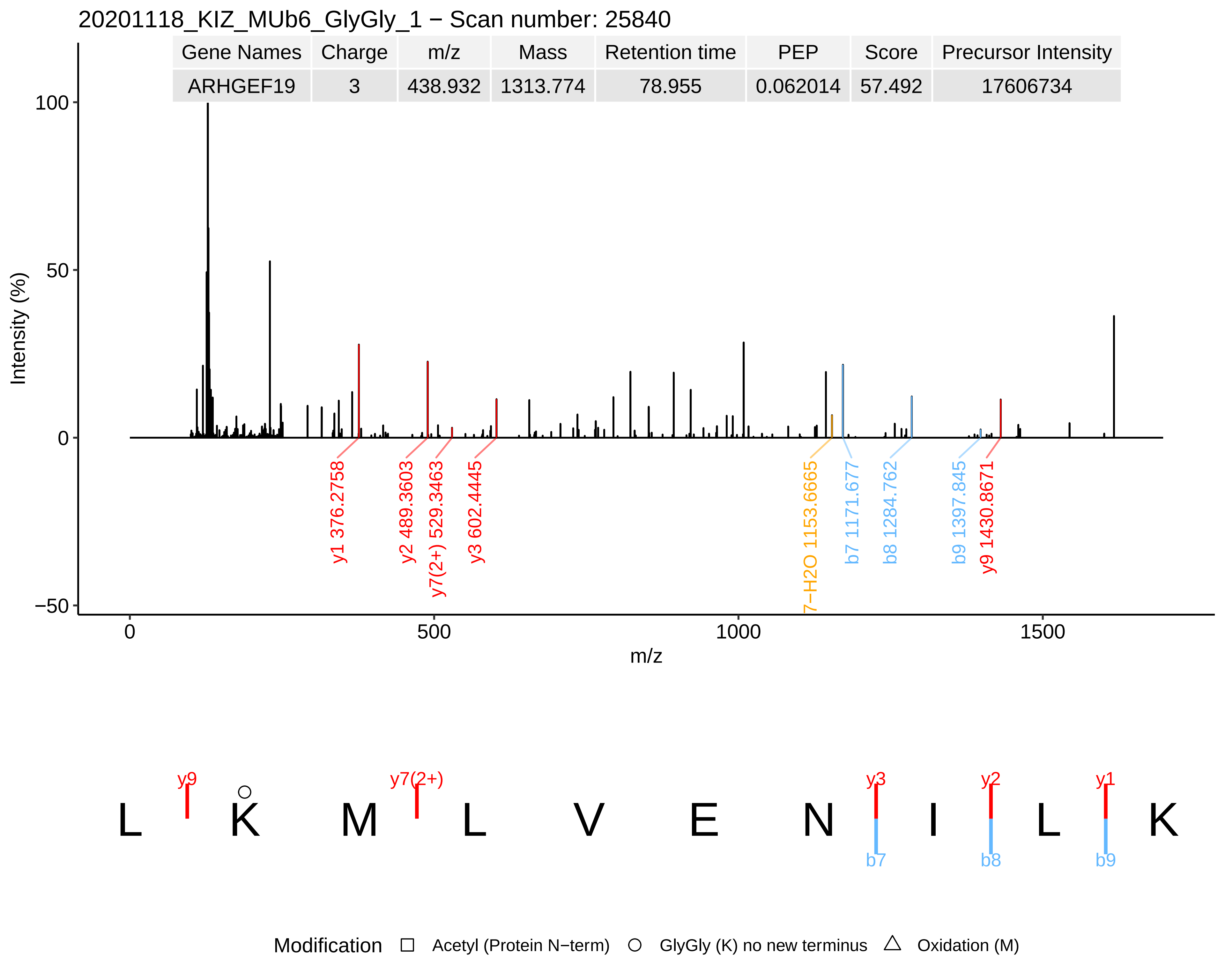
20201118_KIZ_MUb6_GlyGly_1 − Scan number: 25840
Gene Names | Charge | m/z | Mass | Retention time | PEP | Score | Precursor Intensity
ARHGEF19 | 3 | 438.932 | 1313.774 | 78.955 | 0.062014 | 57.492 | 17606734
Intensity (%)
m/z
y1 376.2758
y2 489.3603
y7(2+) 529.3463
y3 602.4445
7−H2O 1153.6665
b7 1171.677
b8 1284.762
b9 1397.845
y9 1430.8671
L K M L V E N I L K
y9
y7(2+)
y3
y2
y1
b7
b8
b9
Modification
Acetyl (Protein N−term)
GlyGly (K) no new terminus
Oxidation (M)

20200921_KIZ_MUb4_GlyGly_10plex_2 − Scan number: 13209

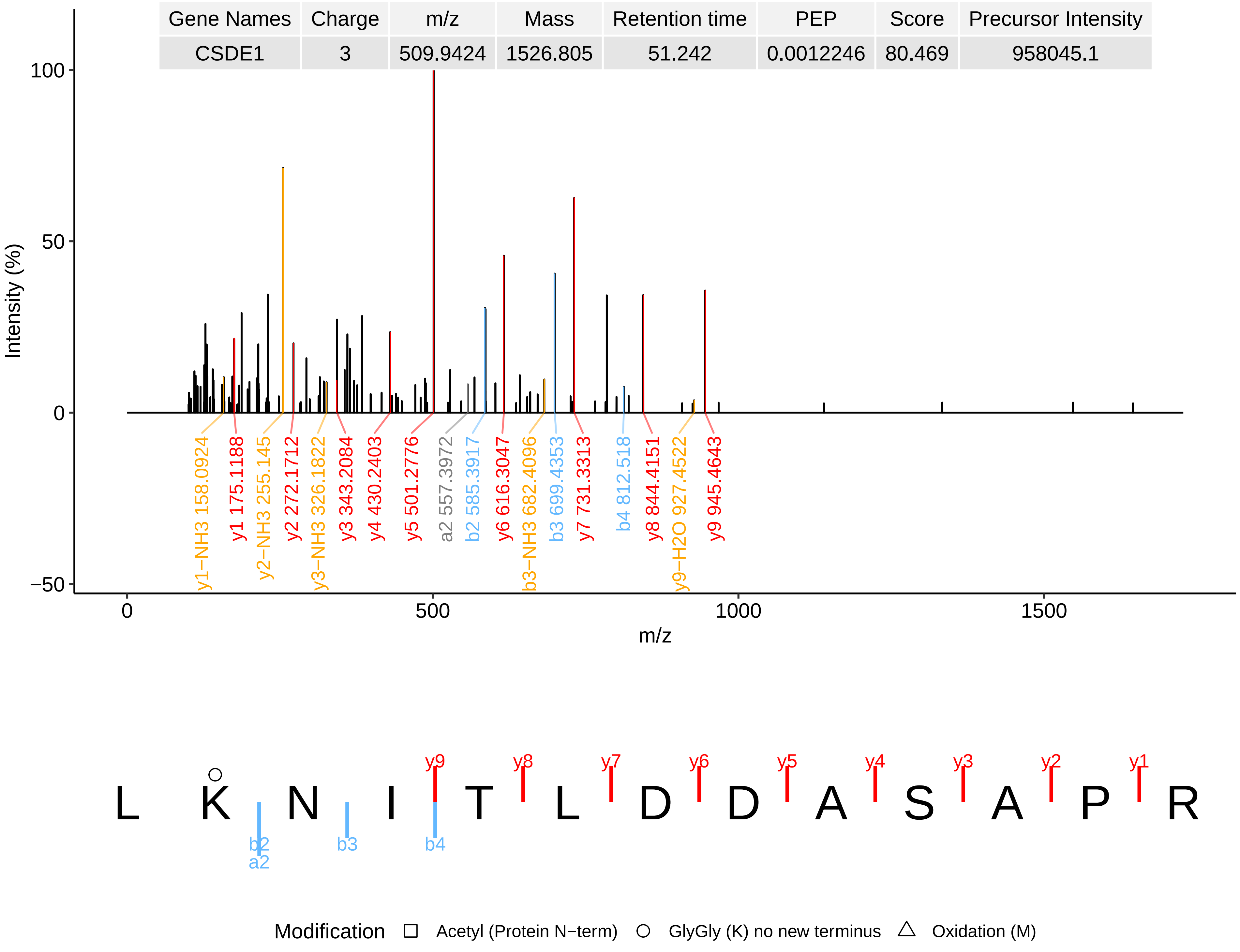

20200924_KIZ_MUb5_GlyGly_1 − Scan number: 13426

Gene Names	Charge	m/z	Mass	Retention time	PEP	Score	Precursor Intensity
CSDE1	3	509.9424	1526.805	43.776	0.00027533	95.183	8204262

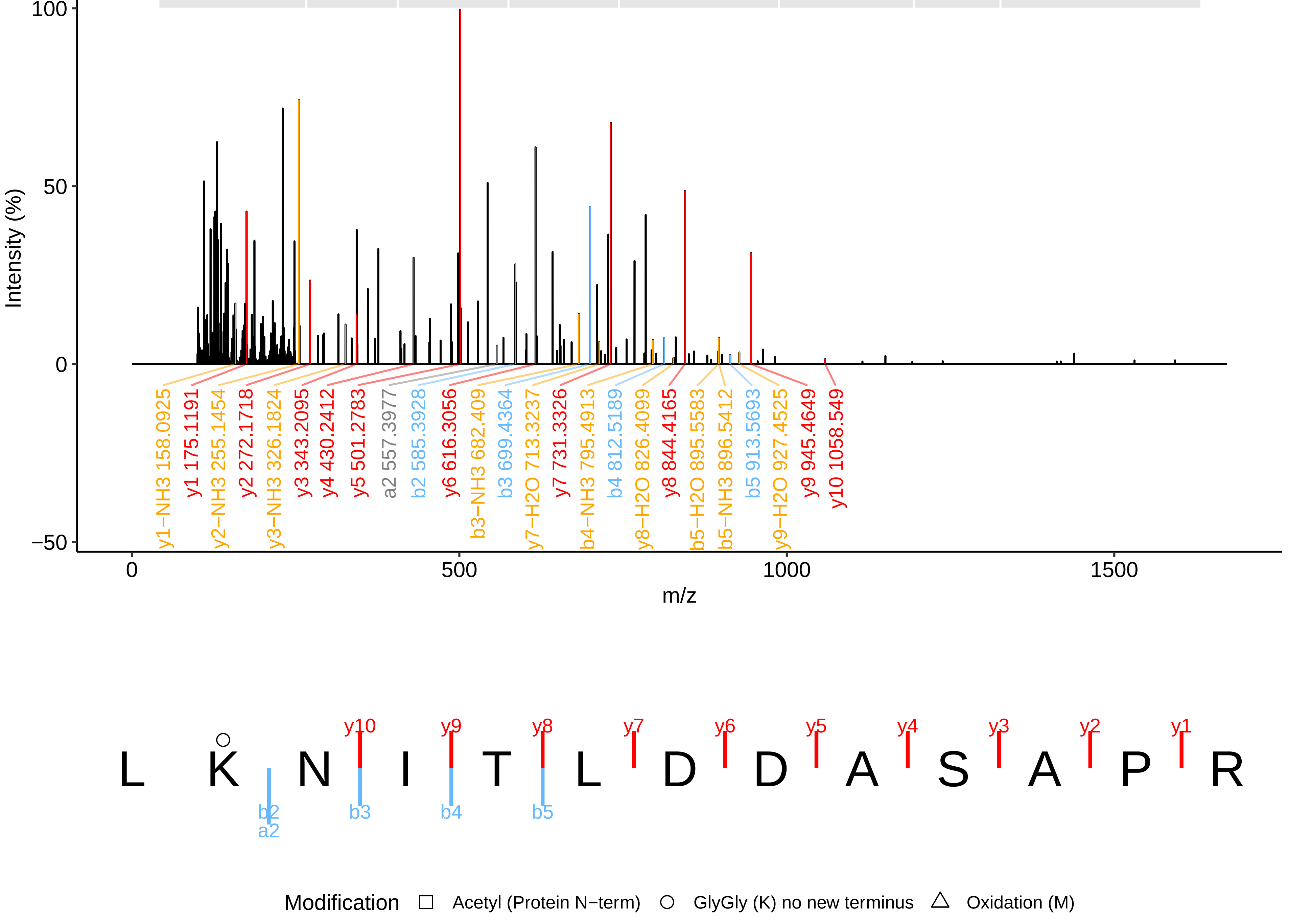

20200921_KIZ_MUb4_GlyGly_10plex − Scan number: 23614

Gene Names	Charge	m/z	Mass	Retention time	PEP	Score	Precursor Intensity
PSMC3	3	435.2479	1302.722	85.732	0.020661	65.347	NA

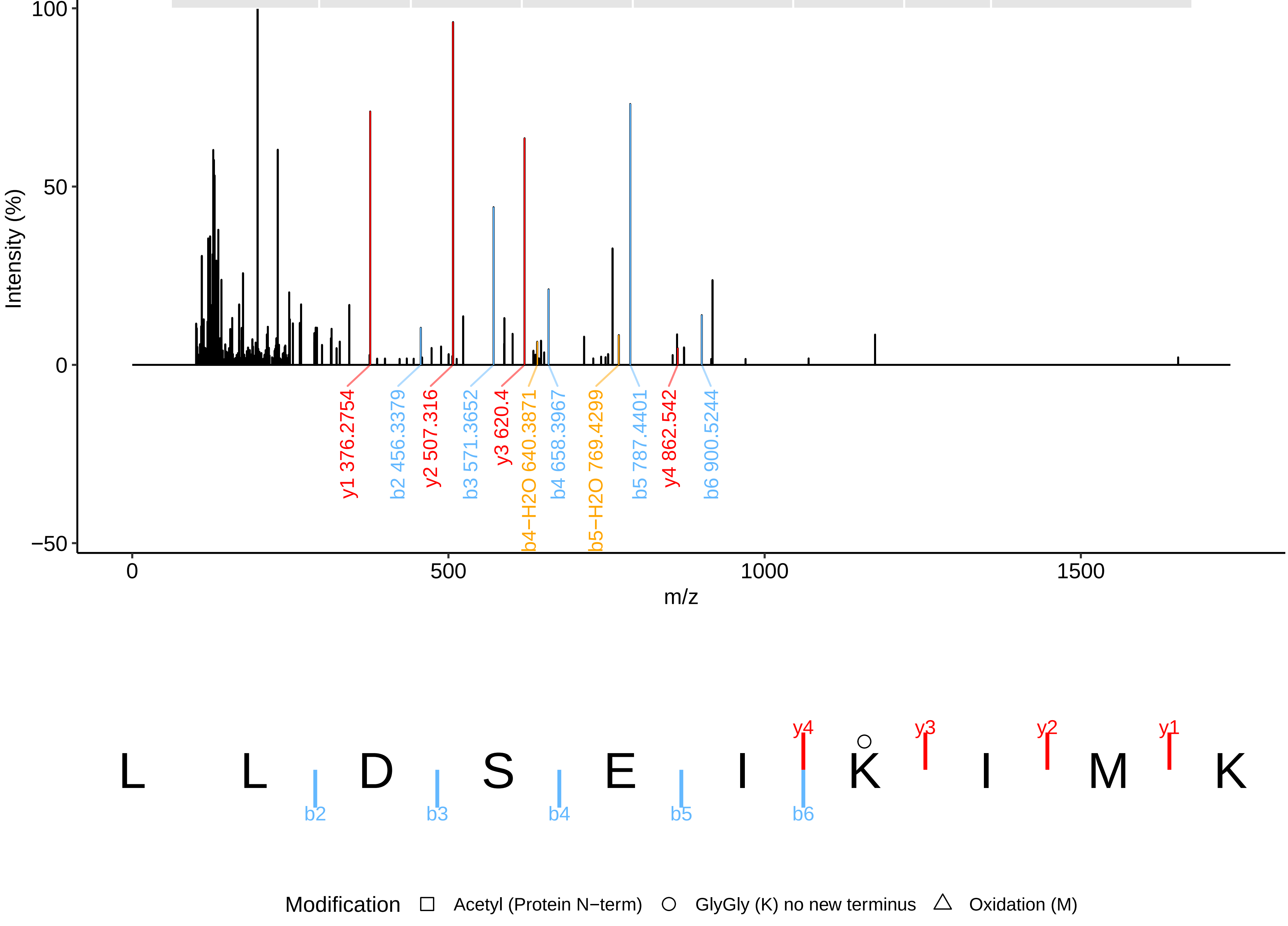

20200921_KIZ_MUb4_GlyGly_10plex − Scan number: 9635

Gene Names	Charge	m/z	Mass	Retention time	PEP	Score	Precursor Intensity
PTRF	3	426.9031	1277.687	33.054	0.099886	43.282	13786564

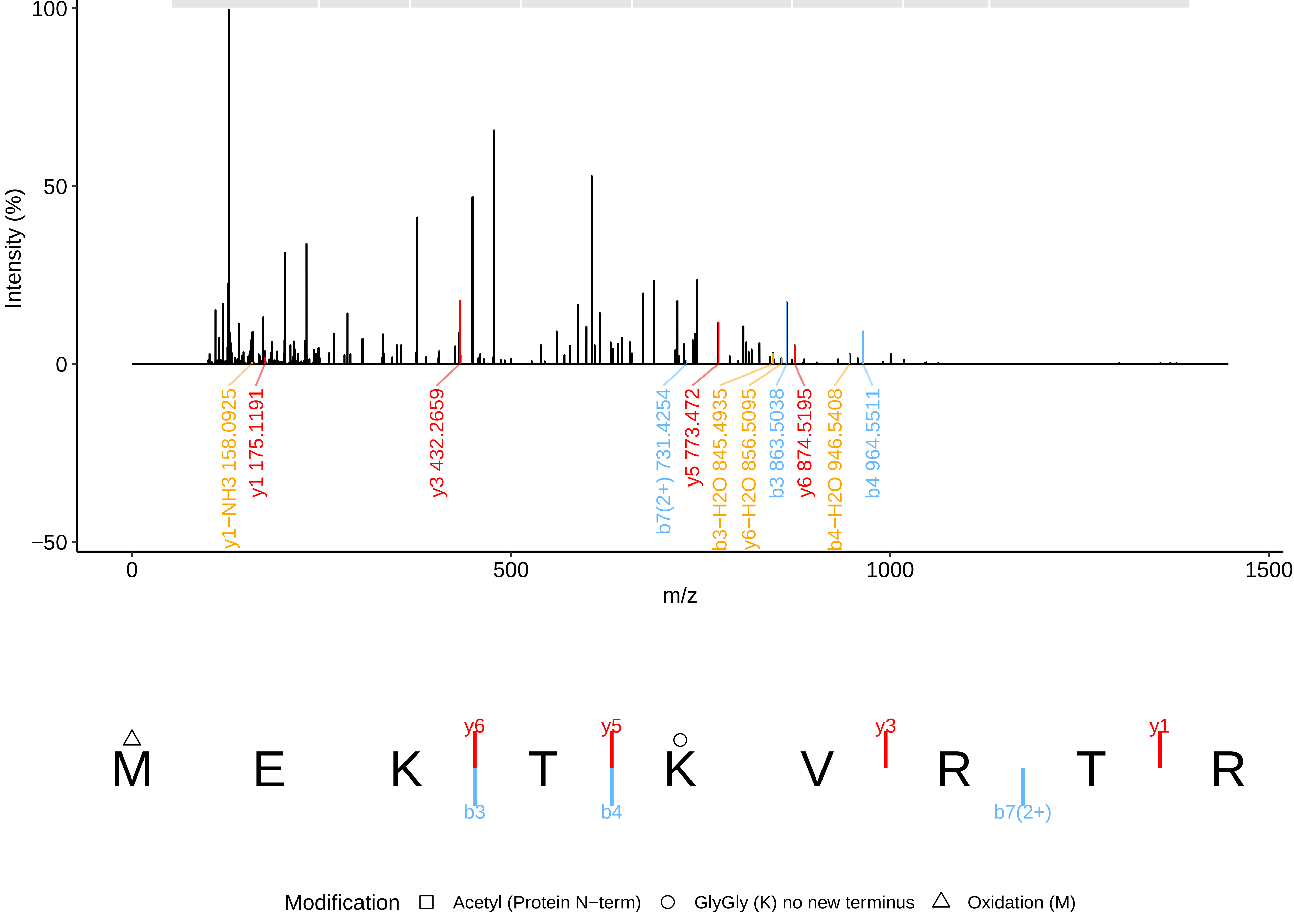

20200921_KIZ_MUb4_GlyGly_10plex − Scan number: 3674

Gene Names	Charge	m/z	Mass	Retention time	PEP	Score	Precursor Intensity
RHOB	3	515.9195	1544.736	11.777	0.010309	72.496	5349799

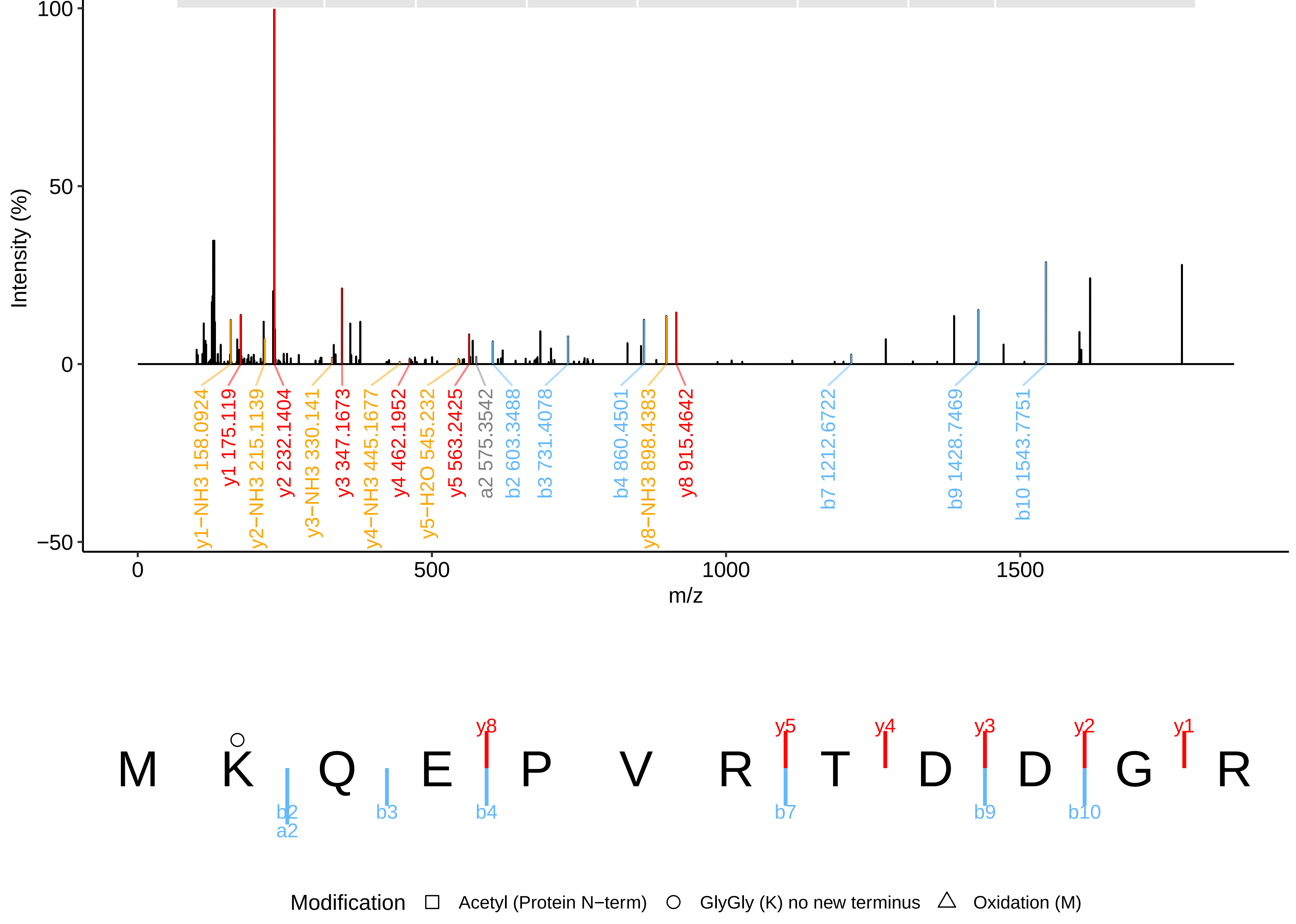

20200924_KIZ_MUb5_GlyGly_1 − Scan number: 3268

Gene Names	Charge	m/z	Mass	Retention time	PEP	Score	Precursor Intensity
RHOB	4	387.1914	1544.736	10.052	0.048383	71.176	20264756

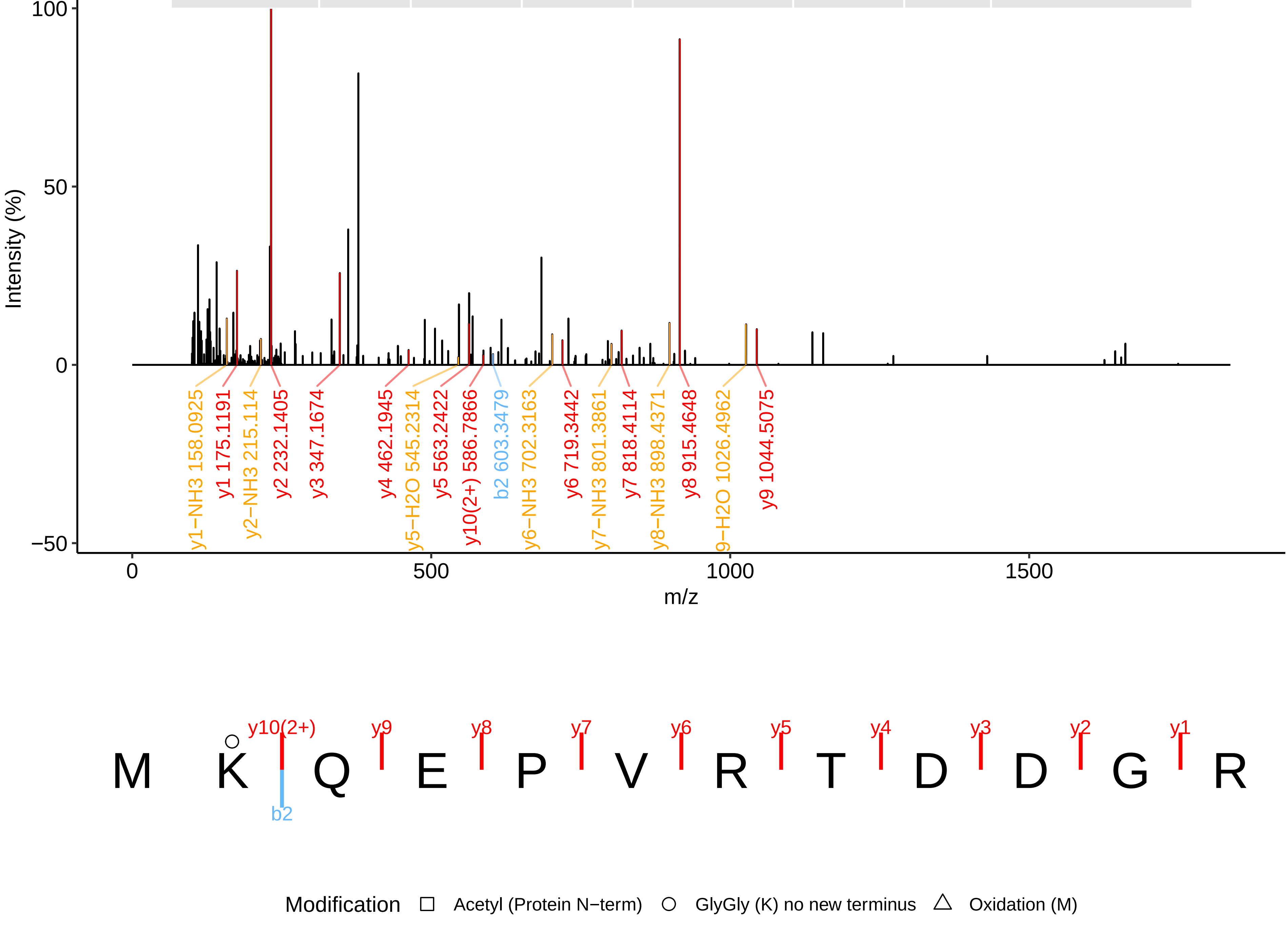

20200924_KIZ_MUb5_GlyGly_1 − Scan number: 3270

Gene Names	Charge	m/z	Mass	Retention time	PEP	Score	Precursor Intensity
RHOB	3	515.9195	1544.736	10.058	1.4185e−07	115.35	18845668

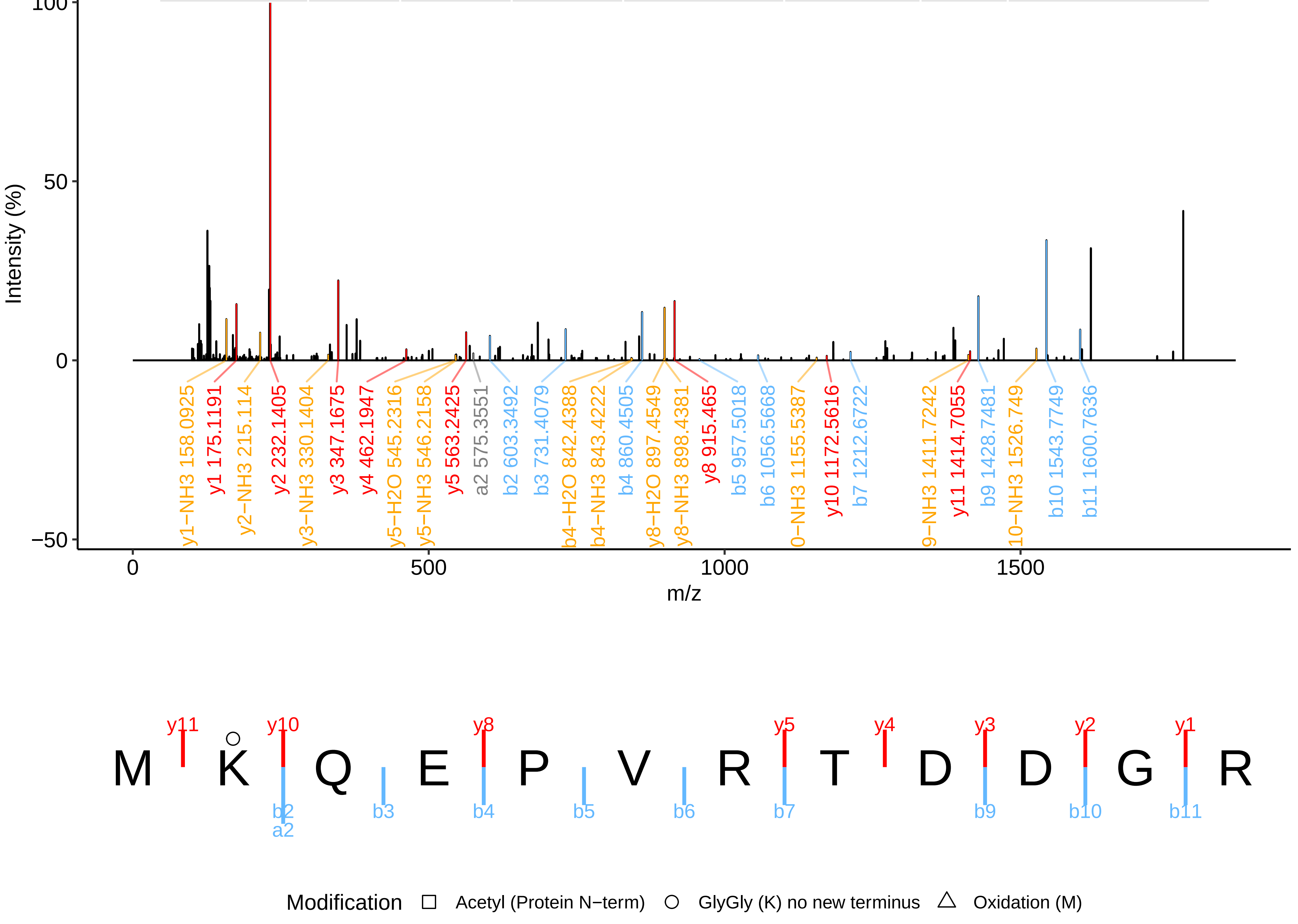

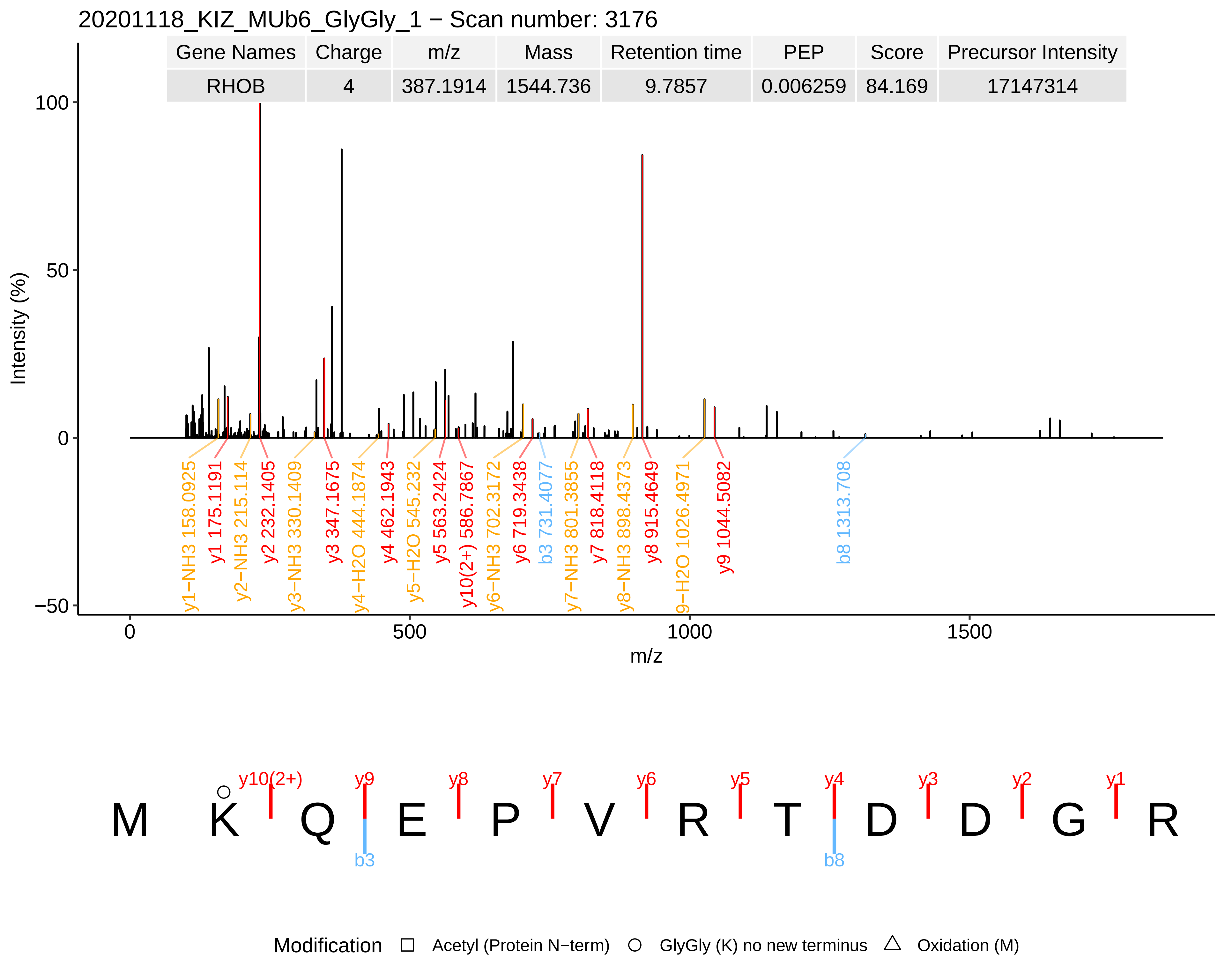

20201118_KIZ_MUb6_GlyGly_1 − Scan number: 3176
Gene Names | Charge | m/z | Mass | Retention time | PEP | Score | Precursor Intensity
RHOB | 4 | 387.1914 | 1544.736 | 9.7857 | 0.006259 | 84.169 | 17147314
Intensity (%)
m/z
y1−NH3 158.0925
y1 175.1191
y2−NH3 215.114
y2 232.1405
y3−NH3 330.1409
y3 347.1675
y4−H2O 444.1874
y4 462.1943
y5−H2O 545.232
y5 563.2424
y10(2+) 586.7867
y6−NH3 702.3172
y6 719.3438
b3 731.4077
y7−NH3 801.3855
y7 818.4118
y8−NH3 898.4373
y8 915.4649
y9−H2O 1026.4971
y9 1044.5082
b8 1313.708
M K Q E P V R T D D G R
y10(2+) y9 y8 y7 y6 y5 y4 y3 y2 y1
b3 b8
Modification
Acetyl (Protein N−term)
GlyGly (K) no new terminus
Oxidation (M)

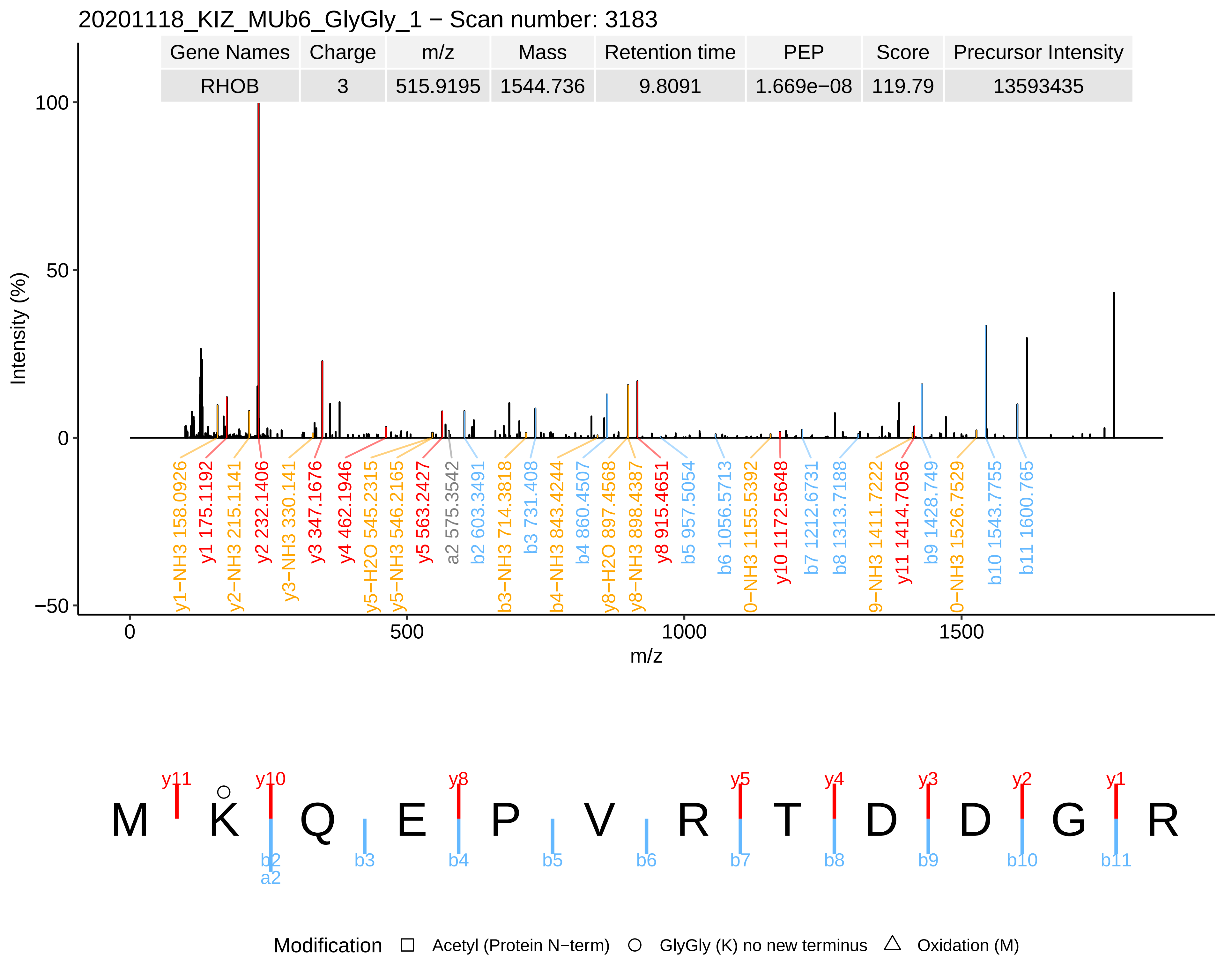

20201118_KIZ_MUb6_GlyGly_1 − Scan number: 3183
Gene Names | Charge | m/z | Mass | Retention time | PEP | Score | Precursor Intensity
RHOB | 3 | 515.9195 | 1544.736 | 9.8091 | 1.669e−08 | 119.79 | 13593435
Intensity (%)
m/z
y1−NH3 158.0926
y1 175.1192
y2−NH3 215.1141
y2 232.1406
y3−NH3 330.141
y3 347.1676
y4 462.1946
y5−H2O 545.2315
y5−NH3 546.2165
y5 563.2427
a2 575.3542
b2 603.3491
b3−NH3 714.3818
b3 731.408
b4−NH3 843.4244
b4 860.4507
y8−H2O 897.4568
y8−NH3 898.4387
y8 915.4651
b5 957.5054
b6 1056.5713
0−NH3 1155.5392
y10 1172.5648
b7 1212.6731
b8 1313.7188
9−NH3 1411.7222
y11 1414.7056
b9 1428.749
0−NH3 1526.7529
b10 1543.7755
b11 1600.765
M K Q E P V R T D D G R
y11 y10 y8 y5 y4 y3 y2 y1
b2 a2 b3 b4 b5 b6 b7 b8 b9 b10 b11
Modification
Acetyl (Protein N−term)
GlyGly (K) no new terminus
Oxidation (M)

20201118_KIZ_MUb6_GlyGly_2 − Scan number: 3560

Gene Names	Charge	m/z	Mass	Retention time	PEP	Score	Precursor Intensity
RHOB	4	387.1914	1544.736	11.825	0.006979	82.543	3450685

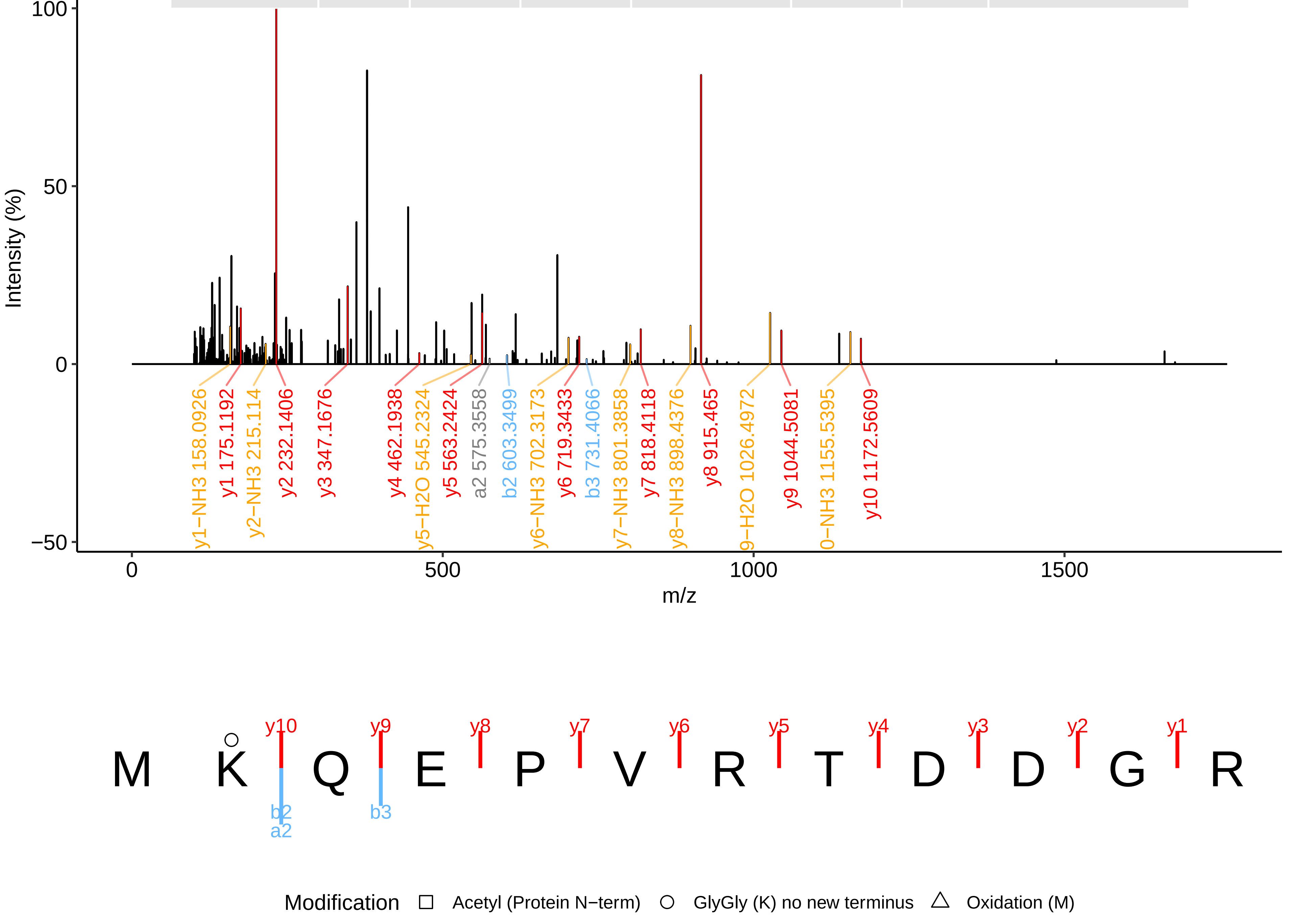

20201118_KIZ_MUb6_GlyGly_2 − Scan number: 3570

Gene Names	Charge	m/z	Mass	Retention time	PEP	Score	Precursor Intensity
RHOB	3	515.9195	1544.736	11.868	0.0019603	80.522	2778188

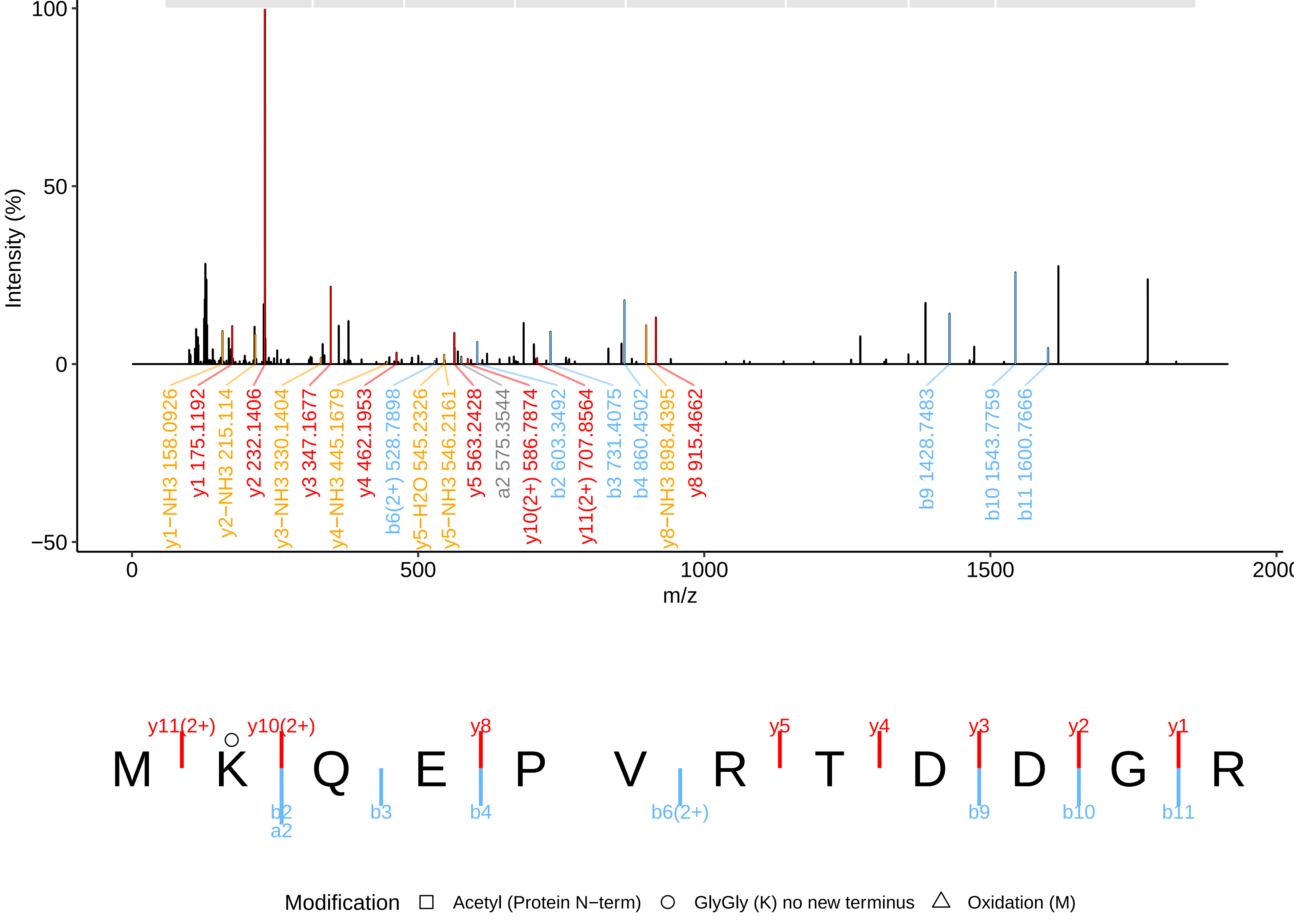

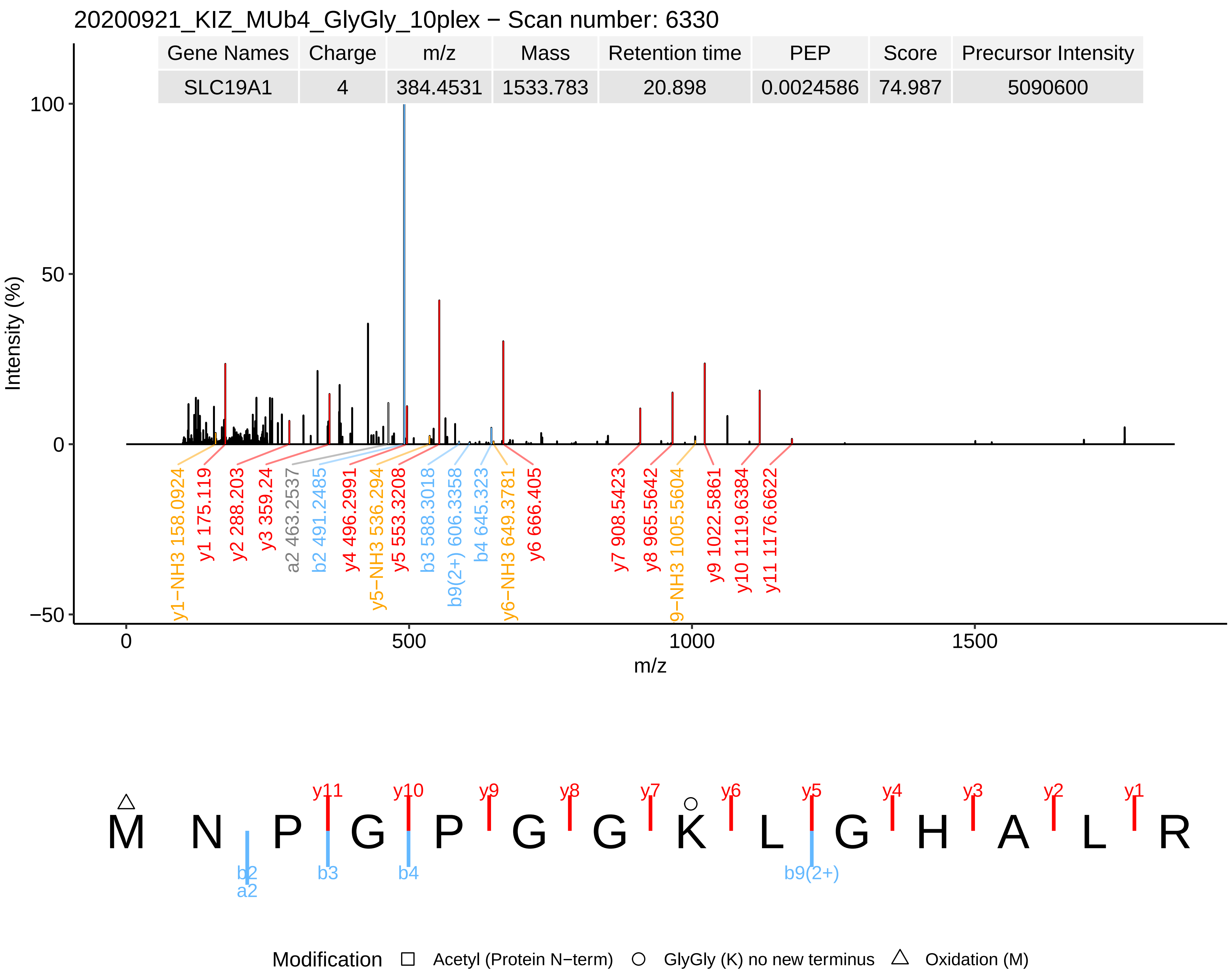

20200921_KIZ_MUb4_GlyGly_10plex − Scan number: 6330
Gene Names | Charge | m/z | Mass | Retention time | PEP | Score | Precursor Intensity
SLC19A1 | 4 | 384.4531 | 1533.783 | 20.898 | 0.0024586 | 74.987 | 5090600
Intensity (%)
m/z
y1−NH3 158.0924
y1 175.119
y2 288.203
y3 359.24
a2 463.2537
b2 491.2485
y4 496.2991
y5−NH3 536.294
y5 553.3208
b3 588.3018
b9(2+) 606.3358
b4 645.323
y6−NH3 649.3781
y6 666.405
y7 908.5423
y8 965.5642
9−NH3 1005.5604
y9 1022.5861
y10 1119.6384
y11 1176.6622
M N P G P G G K L G H A L R
Modification
Acetyl (Protein N−term)
GlyGly (K) no new terminus
Oxidation (M)

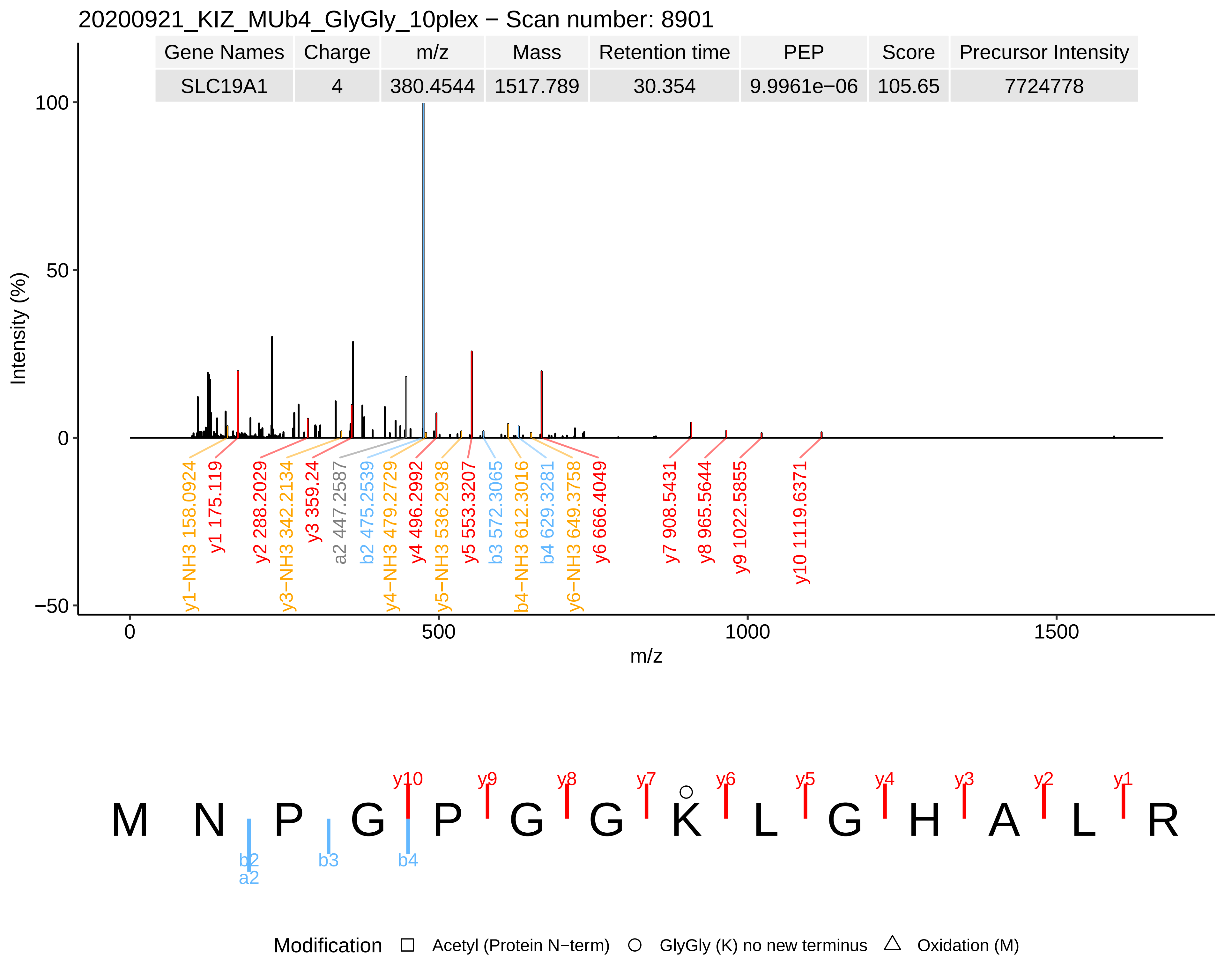
20200921_KIZ_MUb4_GlyGly_10plex − Scan number: 8901
Gene Names | Charge | m/z | Mass | Retention time | PEP | Score | Precursor Intensity
SLC19A1 | 4 | 380.4544 | 1517.789 | 30.354 | 9.9961e−06 | 105.65 | 7724778
Intensity (%)
m/z
y1−NH3 158.0924
y1 175.119
y2 288.2029
y3−NH3 342.2134
y3 359.24
a2 447.2587
b2 475.2539
y4−NH3 479.2729
y4 496.2992
y5−NH3 536.2938
y5 553.3207
b3 572.3065
b4−NH3 612.3016
b4 629.3281
y6−NH3 649.3758
y6 666.4049
y7 908.5431
y8 965.5644
y9 1022.5855
y10 1119.6371
M N P G P G G K L G H A L R
Modification
Acetyl (Protein N−term)
GlyGly (K) no new terminus
Oxidation (M)

20200921_KIZ_MUb4_GlyGly_10plex − Scan number: 8903

Gene Names	Charge	m/z	Mass	Retention time	PEP	Score	Precursor Intensity
SLC19A1	4	380.4544	1517.789	30.362	7.2258e−07	114.72	8192918

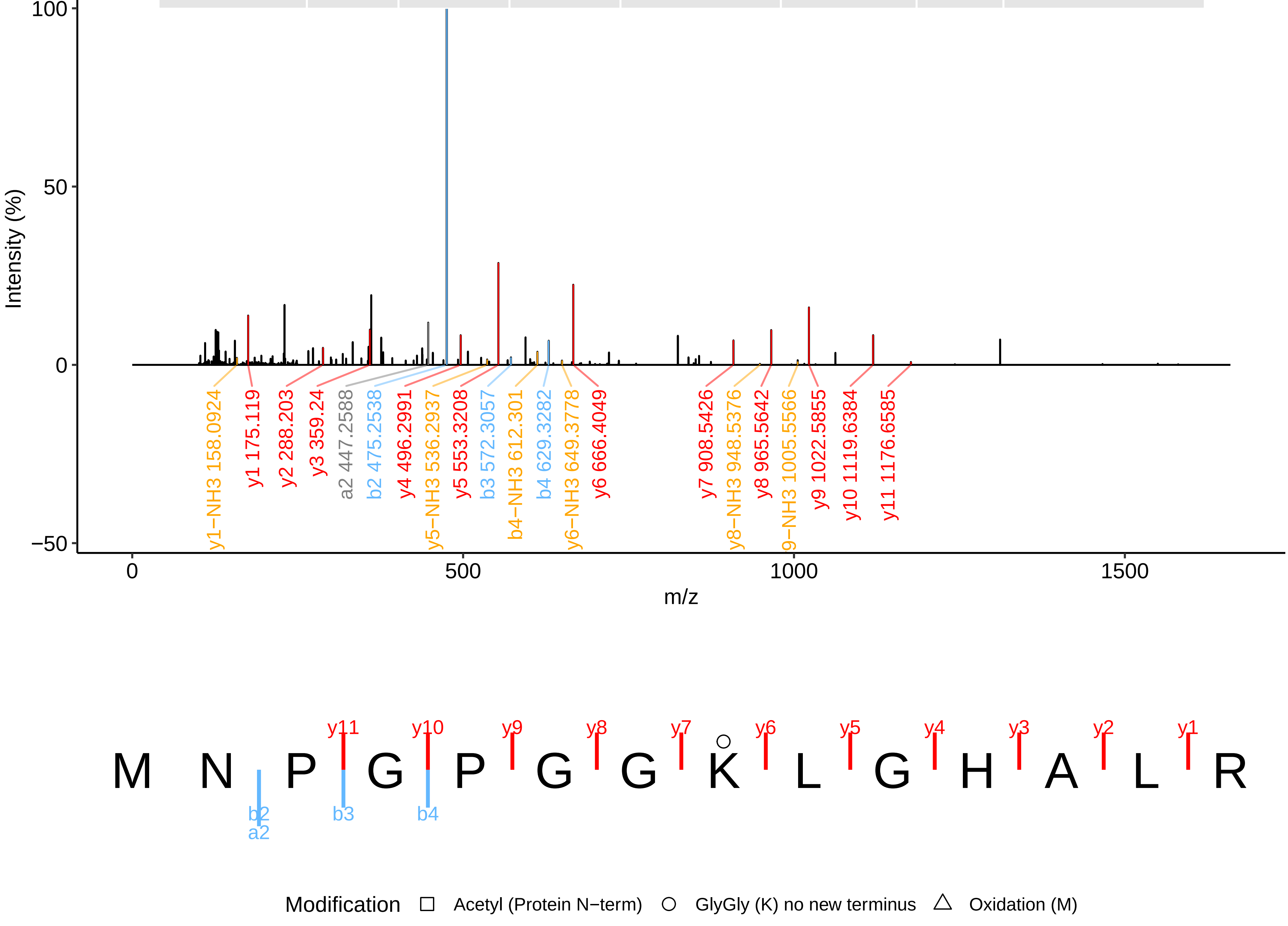

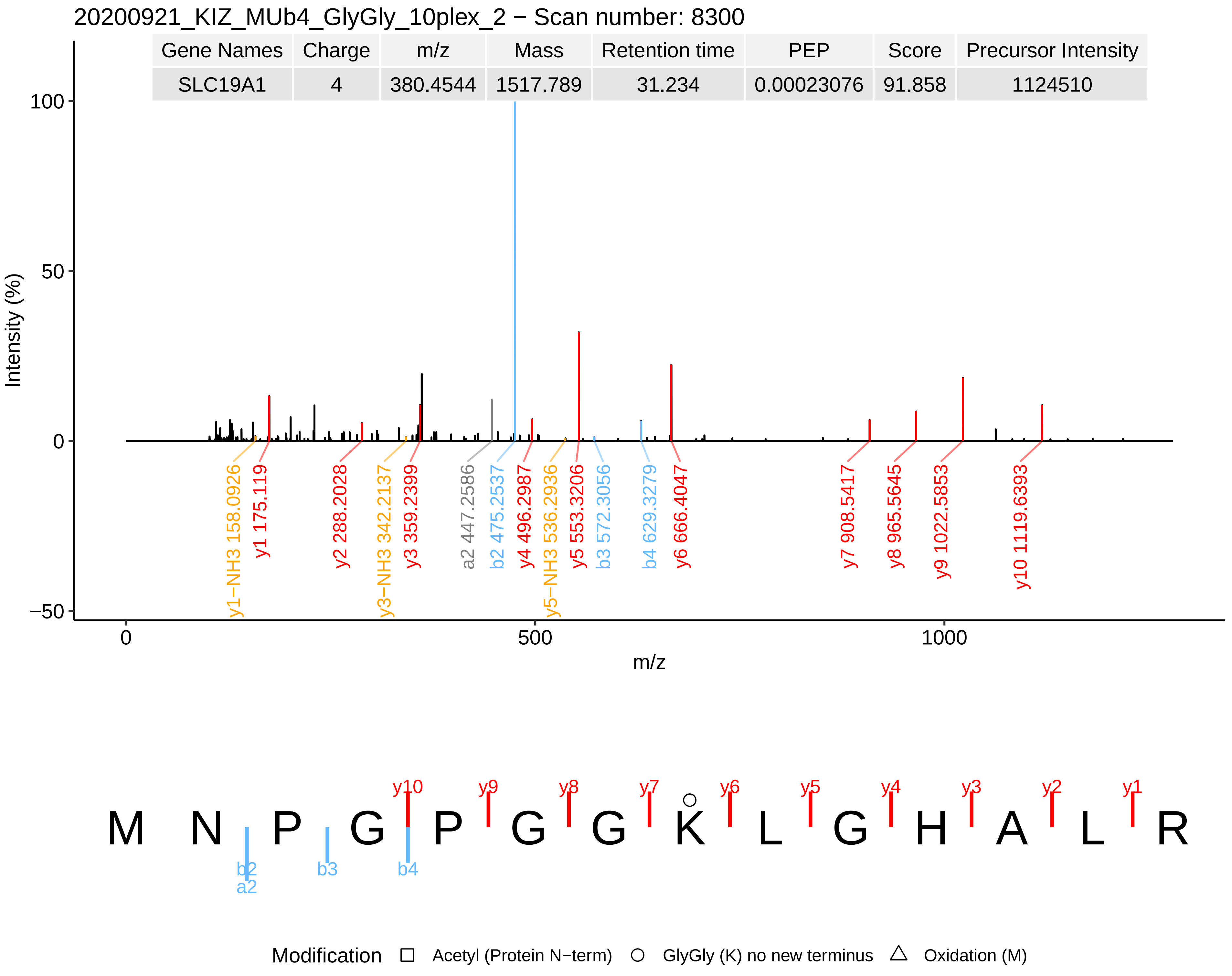
20200921_KIZ_MUb4_GlyGly_10plex_2 − Scan number: 8300
Gene Names | Charge | m/z | Mass | Retention time | PEP | Score | Precursor Intensity
SLC19A1 | 4 | 380.4544 | 1517.789 | 31.234 | 0.00023076 | 91.858 | 1124510
Intensity (%)
m/z
y1−NH3 158.0926
y1 175.119
y2 288.2028
y3−NH3 342.2137
y3 359.2399
a2 447.2586
b2 475.2537
y4 496.2987
y5−NH3 536.2936
y5 553.3206
b3 572.3056
b4 629.3279
y6 666.4047
y7 908.5417
y8 965.5645
y9 1022.5853
y10 1119.6393
M N P G P G G K L G H A L R
Modification
Acetyl (Protein N−term)
GlyGly (K) no new terminus
Oxidation (M)

20200921_KIZ_MUb4_GlyGly_10plex − Scan number: 15689

Gene Names	Charge	m/z	Mass	Retention time	PEP	Score	Precursor Intensity
AHNAK	3	424.8861	1271.637	56.11	0.020233	63.283	2900751

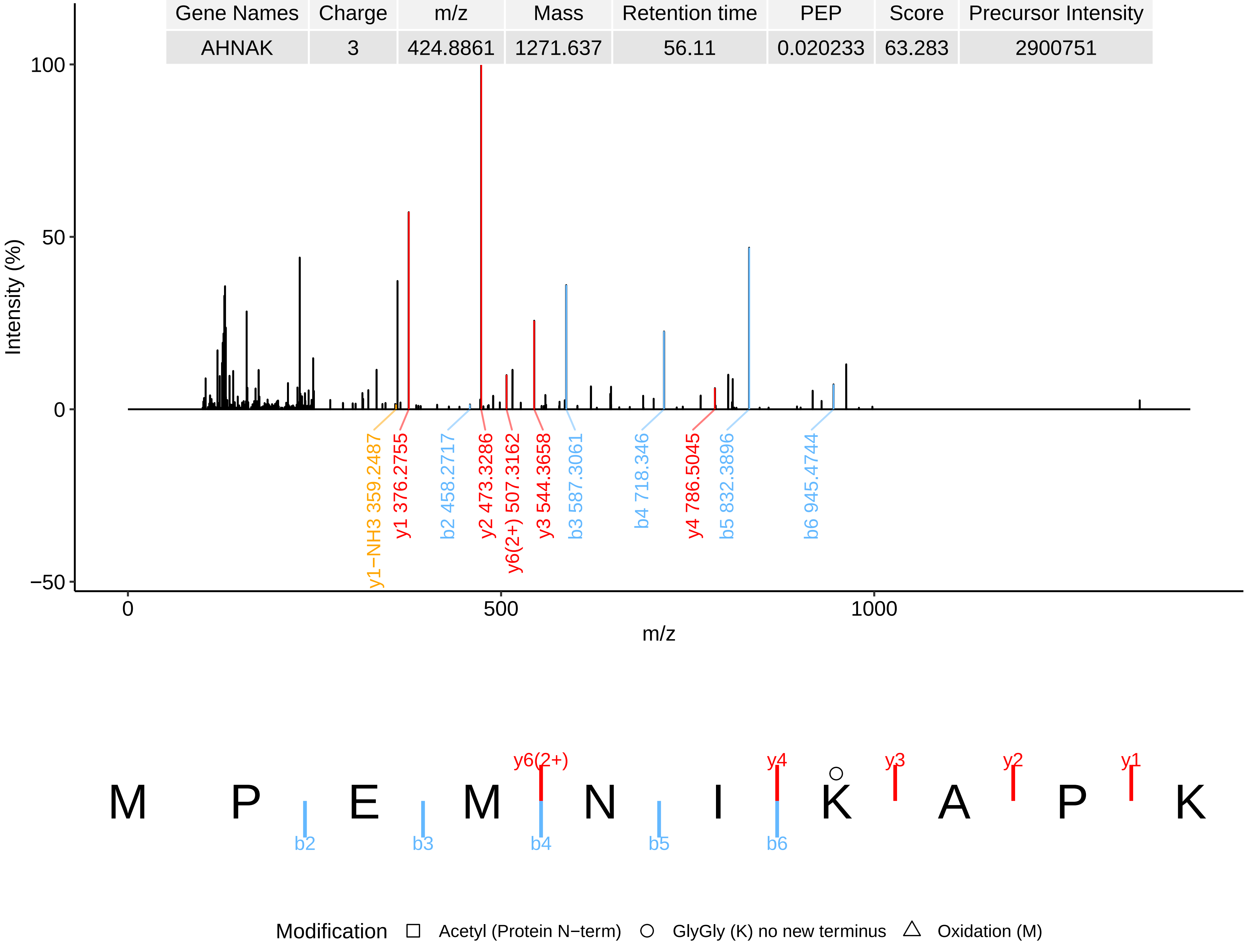

20200921_KIZ_MUb4_GlyGly_10plex − Scan number: 15700

Gene Names	Charge	m/z	Mass	Retention time	PEP	Score	Precursor Intensity
AHNAK	3	424.8861	1271.637	56.155	0.07369	52.79	3205363

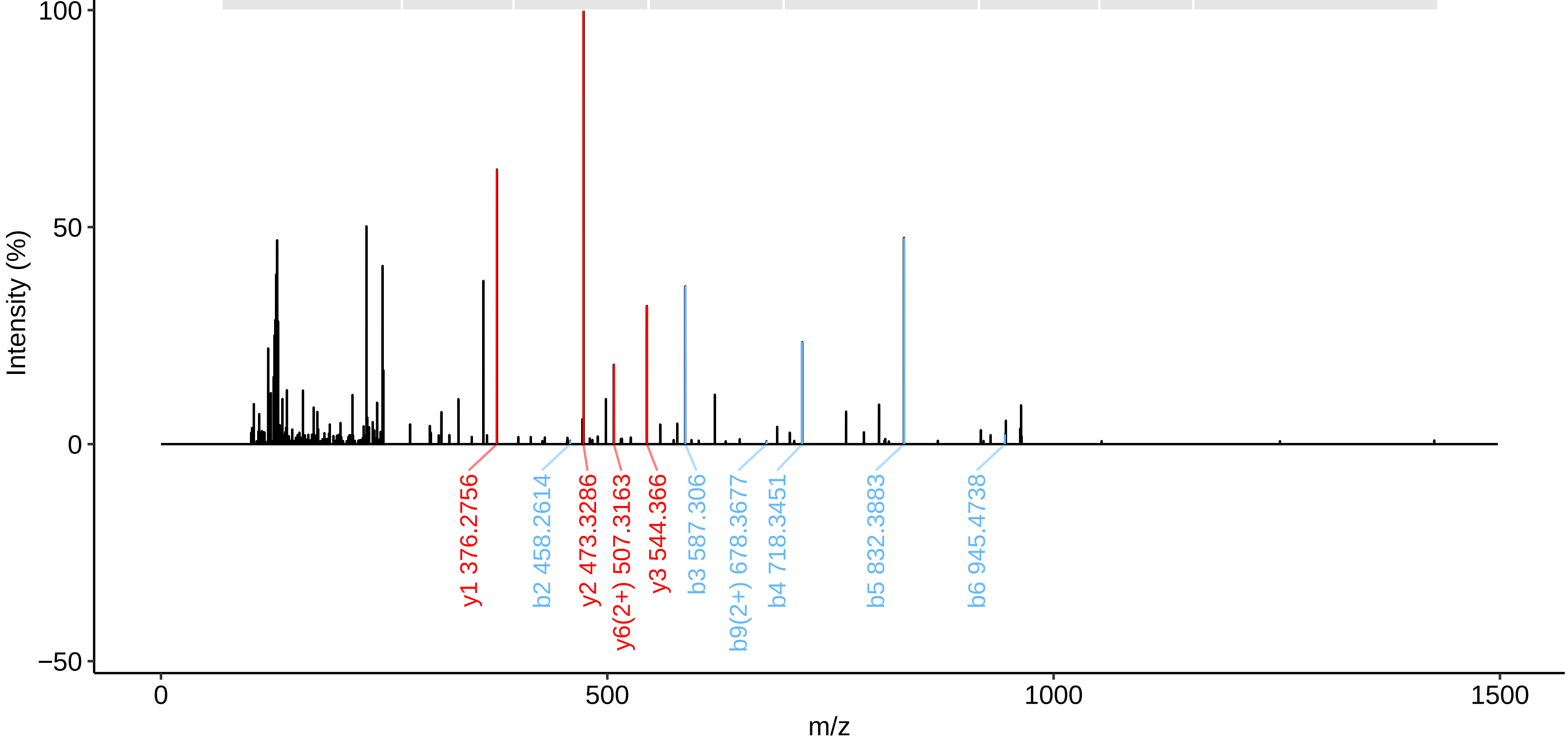

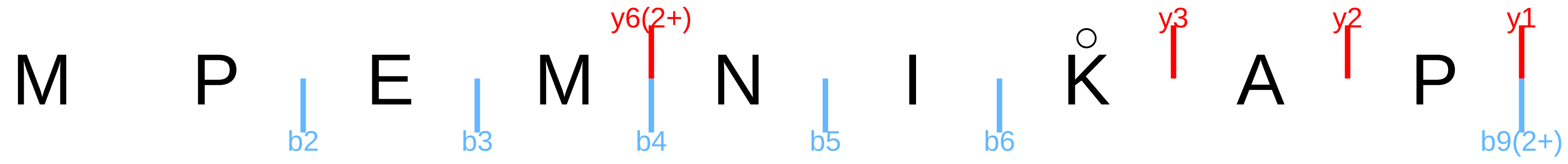

Modification □ Acetyl (Protein N−term) ○ GlyGly (K) no new terminus △ Oxidation (M)

20200924_KIZ_MUb5_GlyGly_1 − Scan number: 14832

Gene Names	Charge	m/z	Mass	Retention time	PEP	Score	Precursor Intensity
AHNAK	3	424.8861	1271.637	48.439	0.11468	50.284	NA

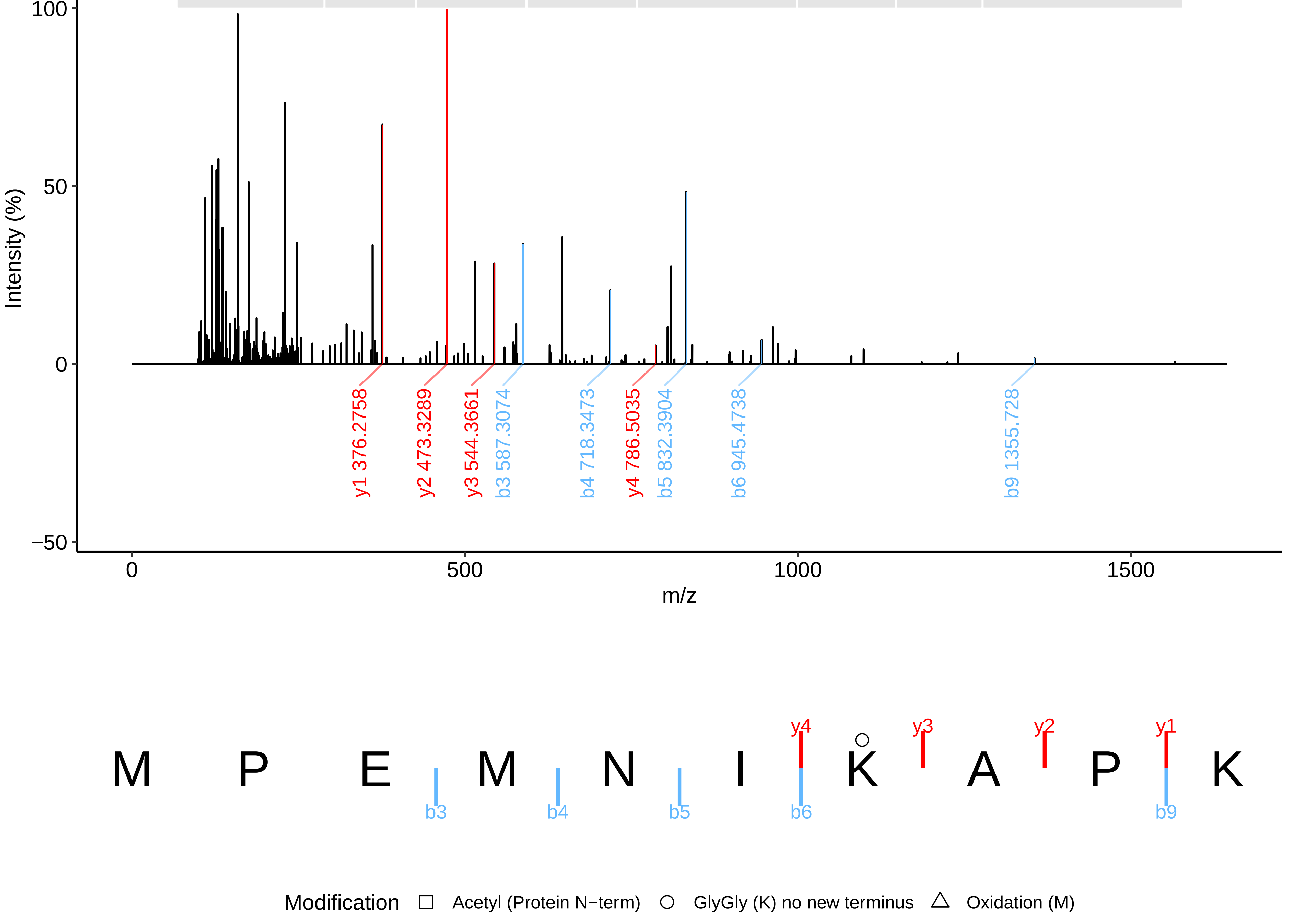

20200921_KIZ_MUb4_GlyGly_10plex − Scan number: 8135

Gene Names	Charge	m/z	Mass	Retention time	PEP	Score	Precursor Intensity
HSPA8;HSPA1B;HSPA1A	3	419.2044	1254.591	27.526	0.0059186	76.943	2516333

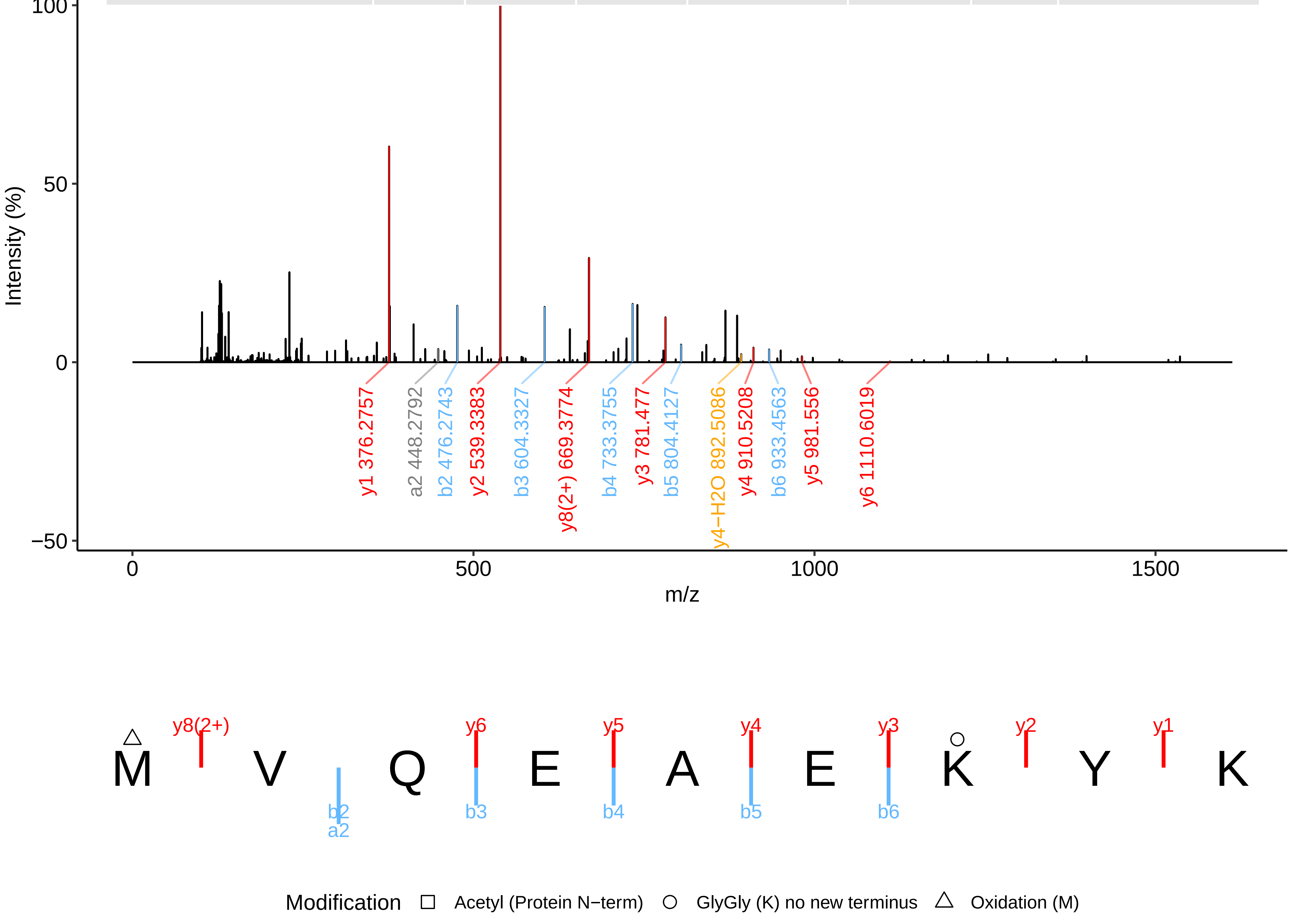

20200921_KIZ_MUb4_GlyGly_10plex_2 − Scan number: 7533

Gene Names	Charge	m/z	Mass	Retention time	PEP	Score	Precursor Intensity
HSPA8;HSPA1B;HSPA1A	3	419.2044	1254.591	28.14	0.0093344	72.928	1131887

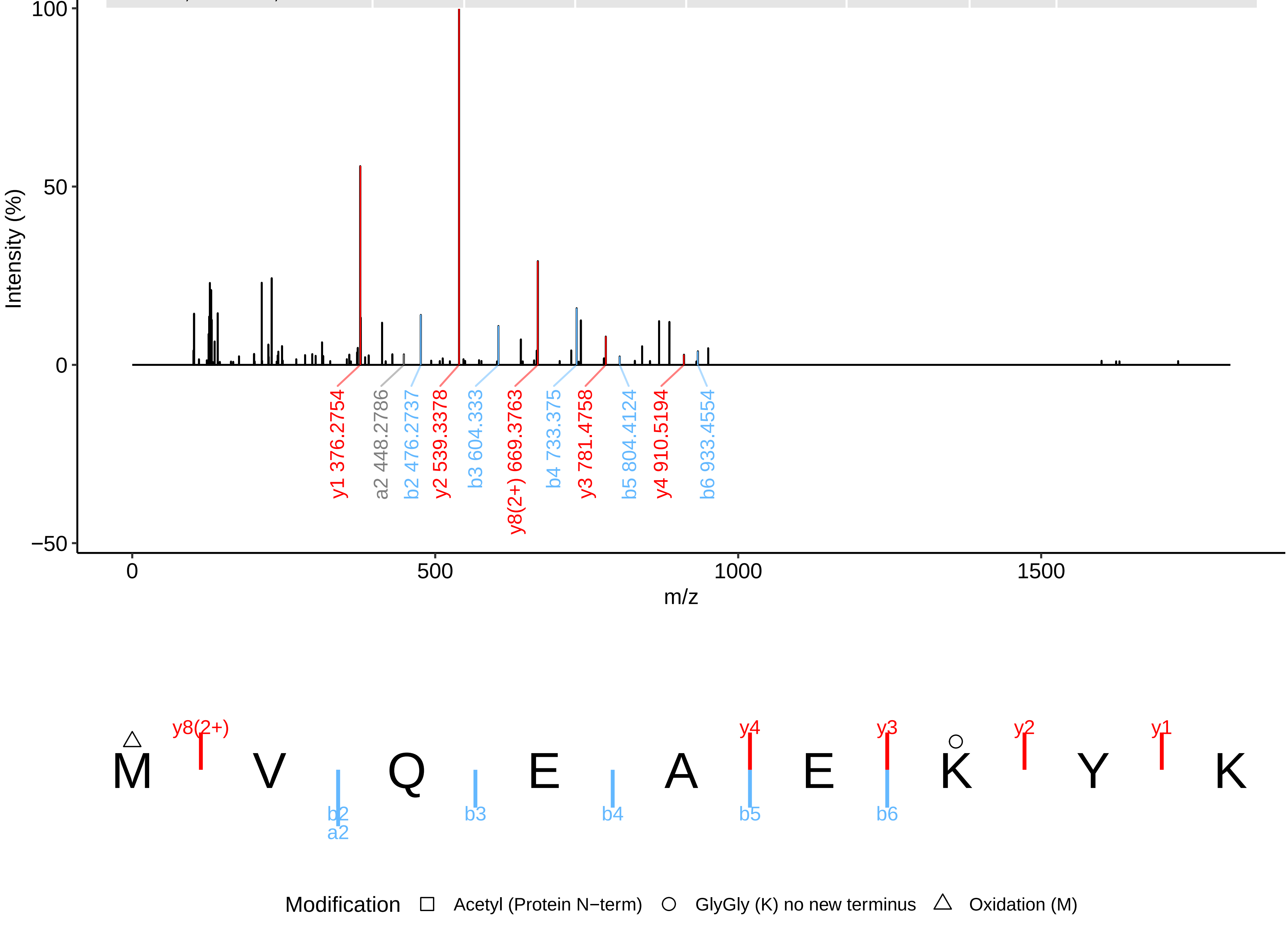

20200924_KIZ_MUb5_GlyGly_1 − Scan number: 7104

Gene Names	Charge	m/z	Mass	Retention time	PEP	Score	Precursor Intensity
HSPA8;HSPA1B;HSPA1A	3	419.2044	1254.591	22.497	0.011777	70.056	8898452

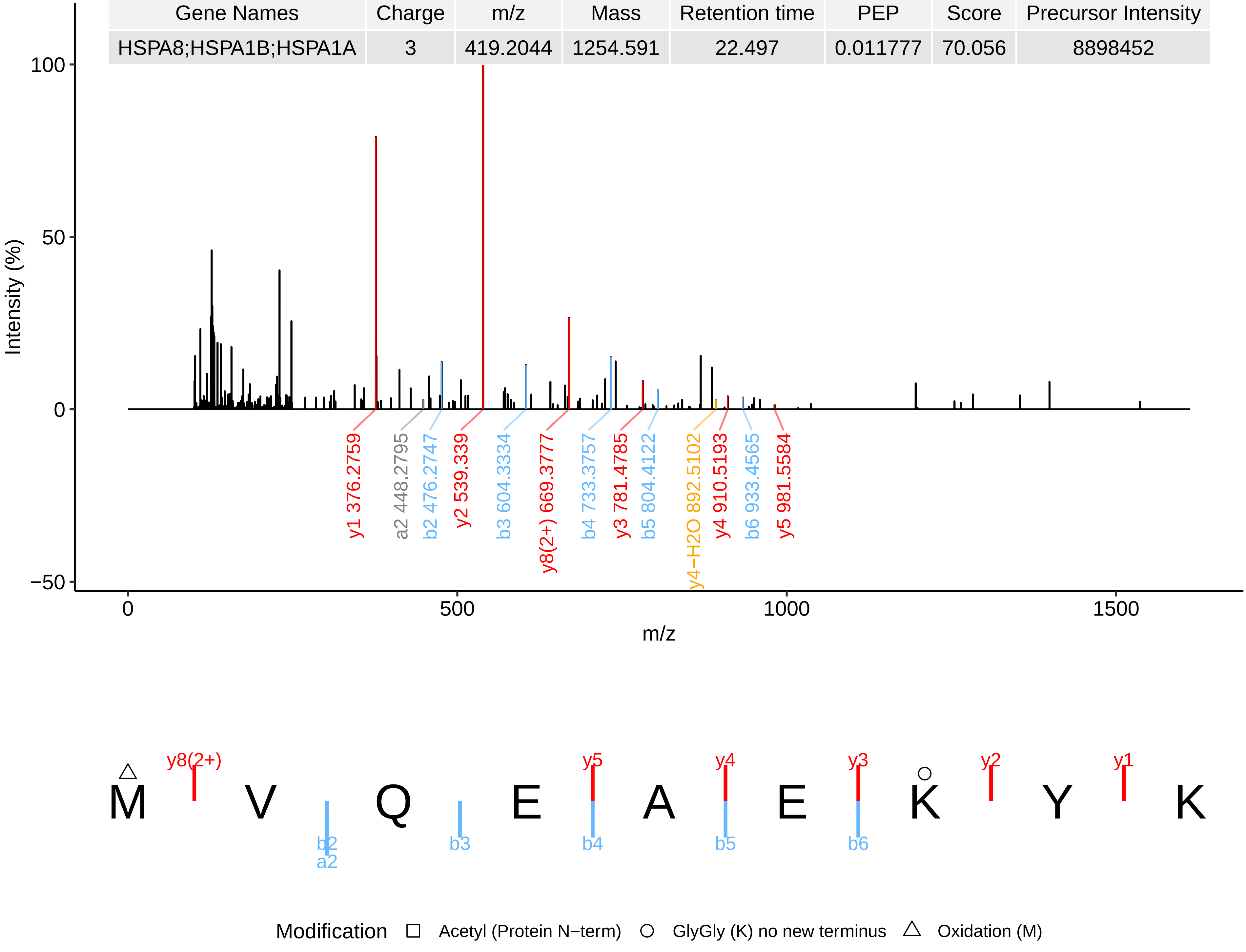

20200924_KIZ_MUb5_GlyGly_1 − Scan number: 7258

Gene Names	Charge	m/z	Mass	Retention time	PEP	Score	Precursor Intensity
HSPA8;HSPA1B;HSPA1A	3	419.2044	1254.591	23.002	0.057556	60.518	NA

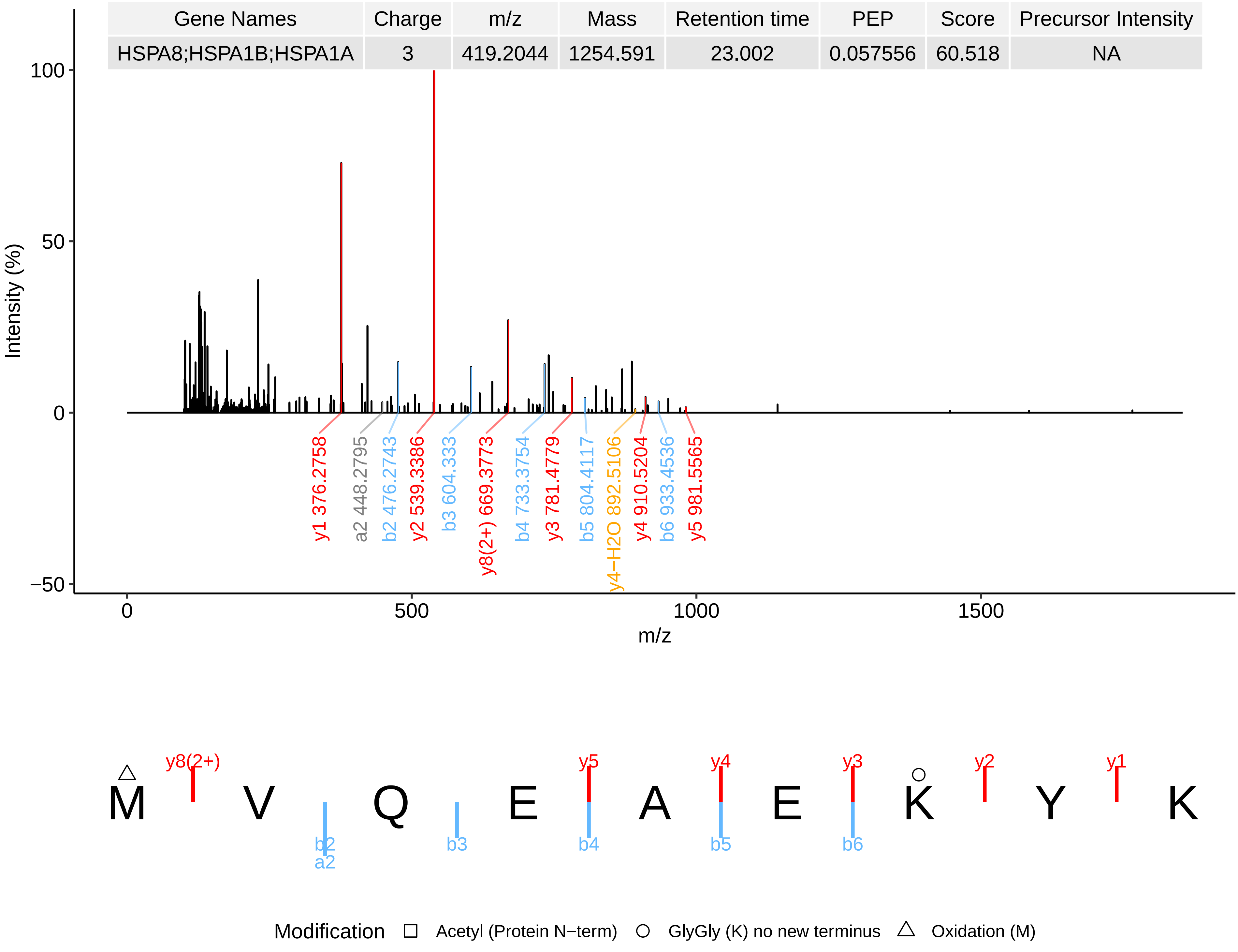

20200924_KIZ_MUb5_GlyGly_2 − Scan number: 6744

Gene Names	Charge	m/z	Mass	Retention time	PEP	Score	Precursor Intensity
HSPA8;HSPA1B;HSPA1A	3	419.2044	1254.591	25.176	0.022972	71.349	4183460

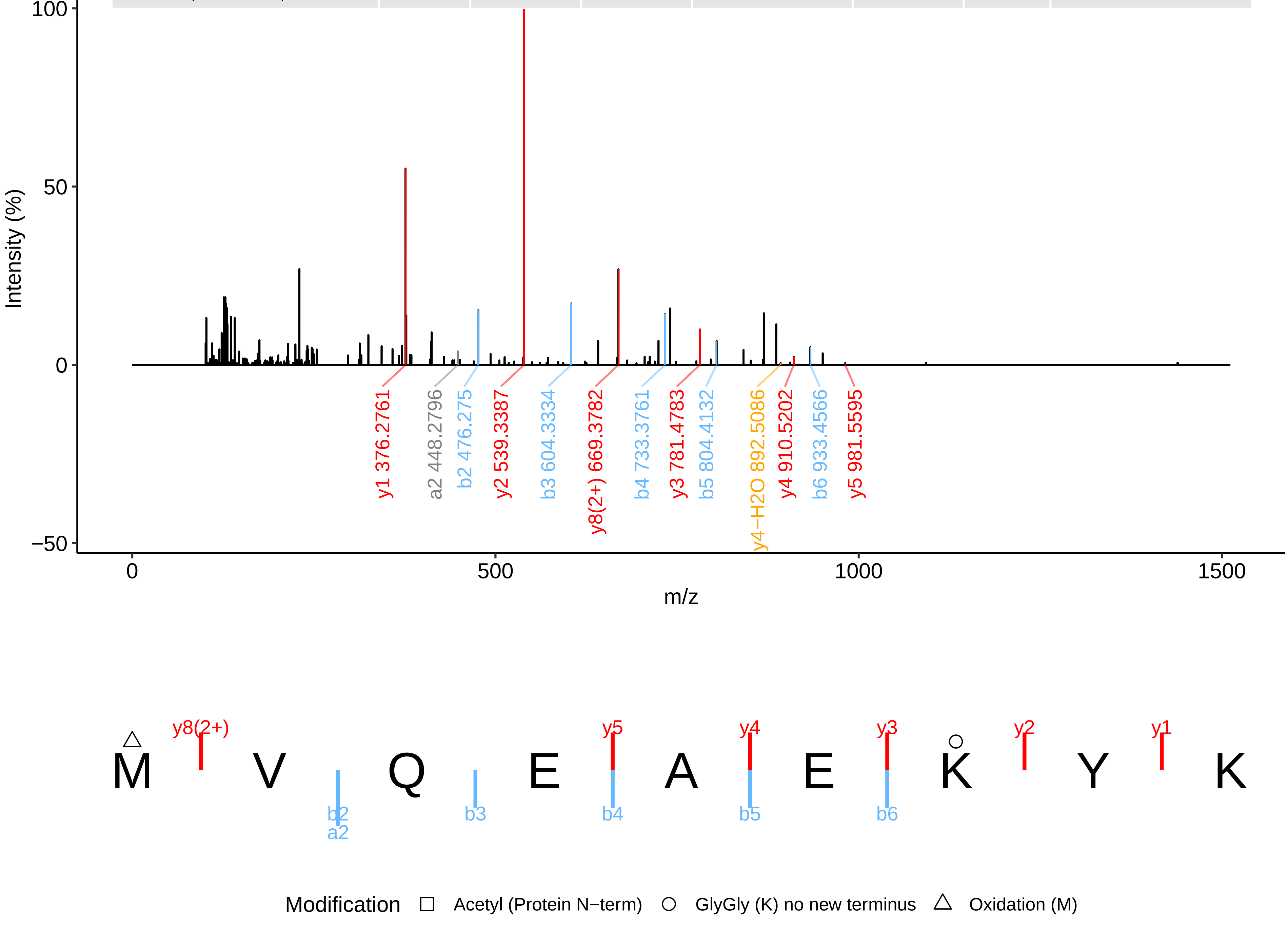

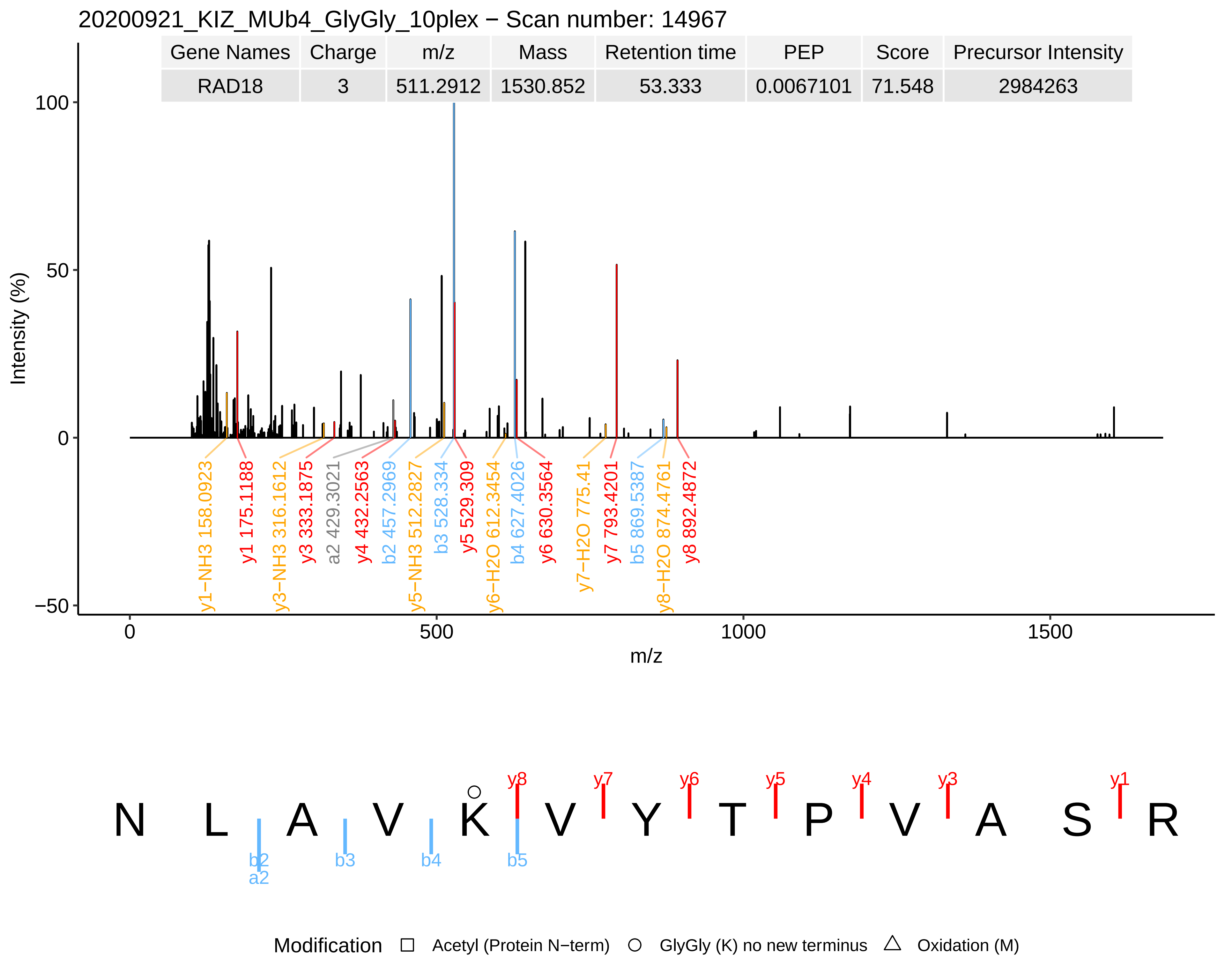
20200921_KIZ_MUb4_GlyGly_10plex − Scan number: 14967
Gene Names | Charge | m/z | Mass | Retention time | PEP | Score | Precursor Intensity
RAD18 | 3 | 511.2912 | 1530.852 | 53.333 | 0.0067101 | 71.548 | 2984263
Intensity (%)
m/z
y1−NH3 158.0923
y1 175.1188
y3−NH3 316.1612
y3 333.1875
a2 429.3021
y4 432.2563
b2 457.2969
y5−NH3 512.2827
b3 528.334
y5 529.309
y6−H2O 612.3454
b4 627.4026
y6 630.3564
y7−H2O 775.41
y7 793.4201
b5 869.5387
y8−H2O 874.4761
y8 892.4872
N L A V K V Y T P V A S R
y8 y7 y6 y5 y4 y3 y1
b2 a2 b3 b4 b5
Modification
Acetyl (Protein N−term)
GlyGly (K) no new terminus
Oxidation (M)

20200921_KIZ_MUb4_GlyGly_10plex − Scan number: 2487

Gene Names	Charge	m/z	Mass	Retention time	PEP	Score	Precursor Intensity
VIM	4	378.1906	1508.733	7.8278	0.0002416	100.88	21975540

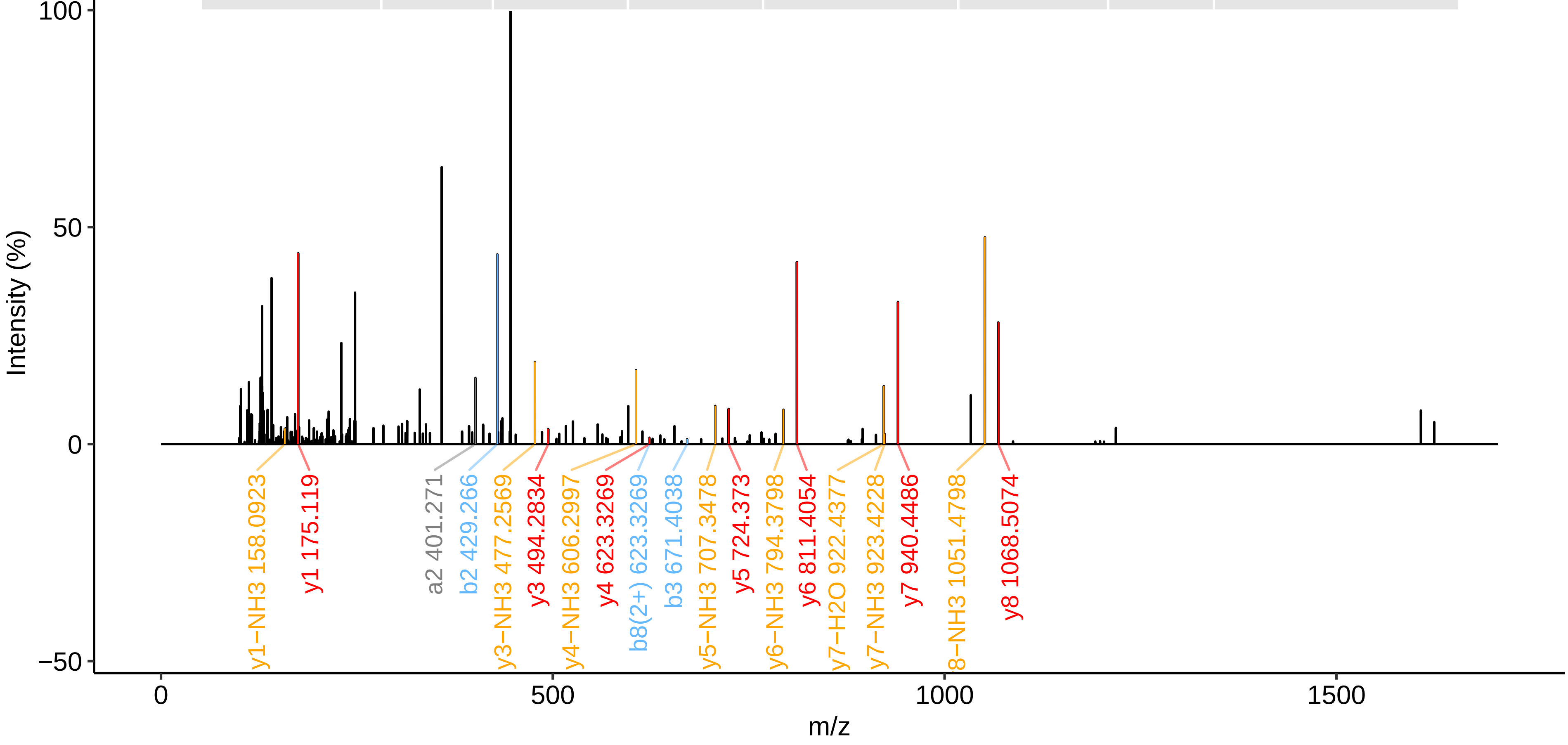

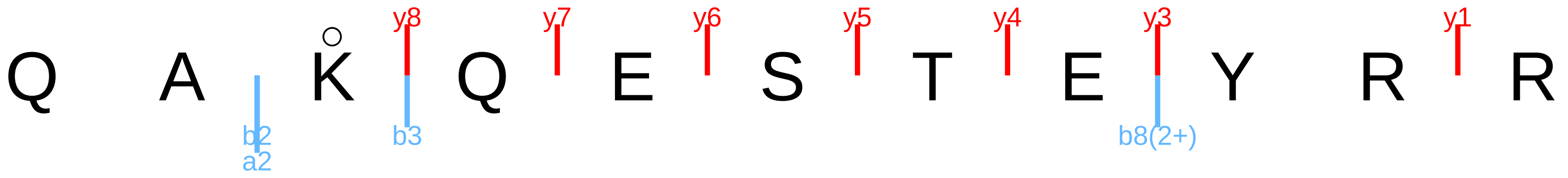

Modification □ Acetyl (Protein N−term) ○ GlyGly (K) no new terminus △ Oxidation (M)

20200921_KIZ_MUb4_GlyGly_10plex − Scan number: 2492

Gene Names	Charge	m/z	Mass	Retention time	PEP	Score	Precursor Intensity
VIM	3	503.9183	1508.733	7.8455	7.7379e−08	117.09	10115902

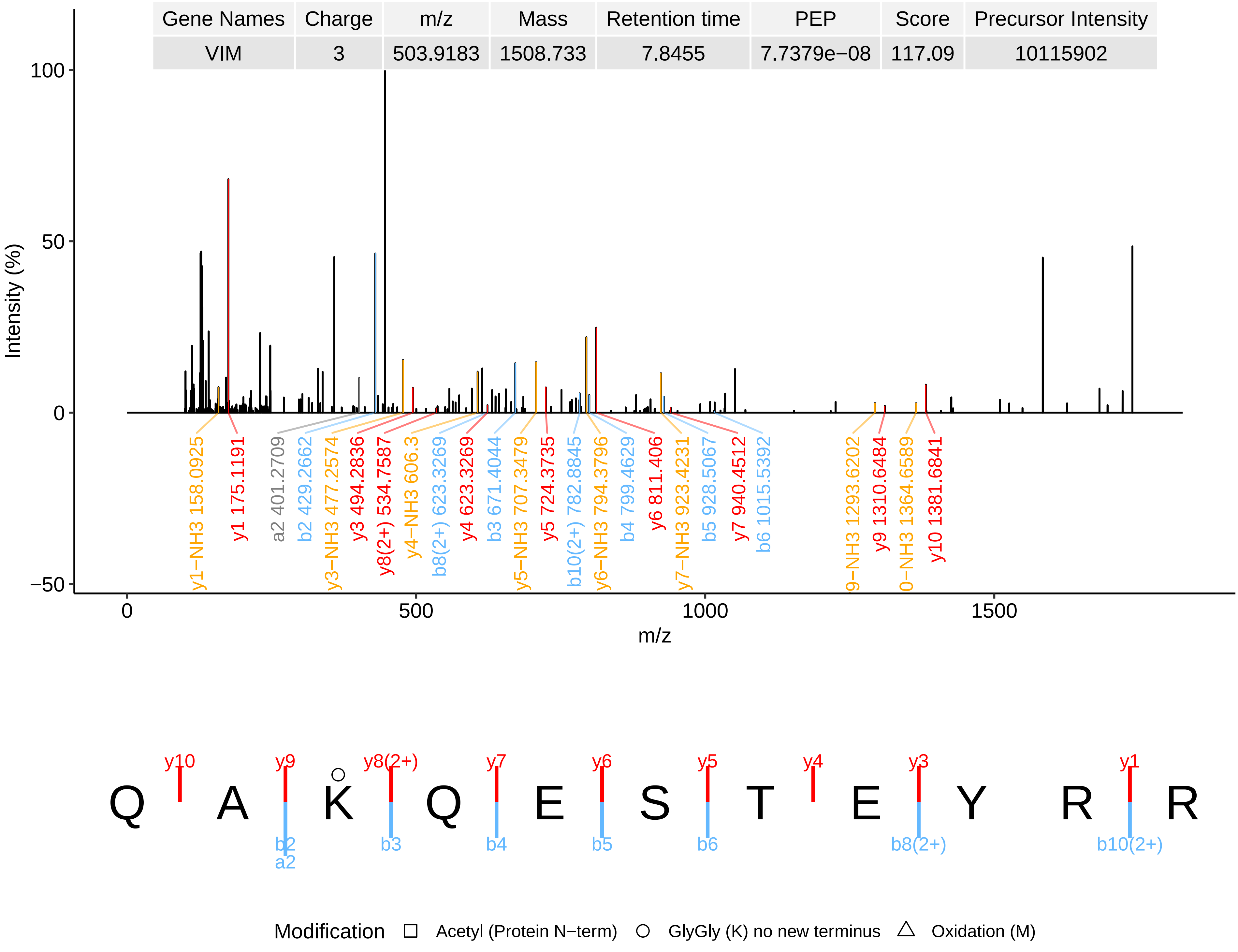

20200921_KIZ_MUb4_GlyGly_10plex_2 − Scan number: 2479

Gene Names	Charge	m/z	Mass	Retention time	PEP	Score	Precursor Intensity
VIM	4	378.1906	1508.733	7.8295	0.0077784	85.958	2319945

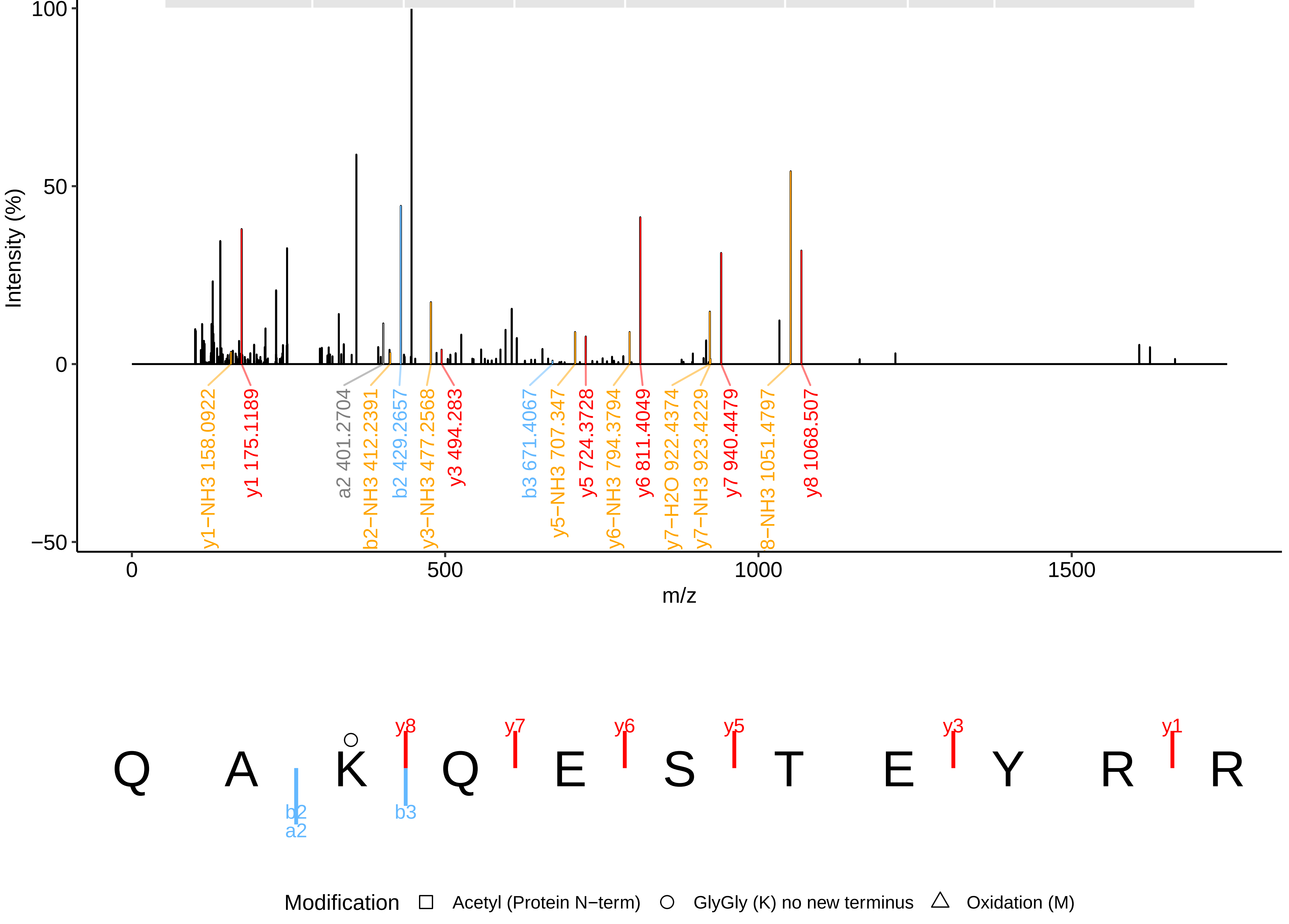

20200921_KIZ_MUb4_GlyGly_10plex_2 − Scan number: 11262

Gene Names	Charge	m/z	Mass	Retention time	PEP	Score	Precursor Intensity
TANK	3	425.2348	1272.683	43.301	0.029692	61.353	645101.4

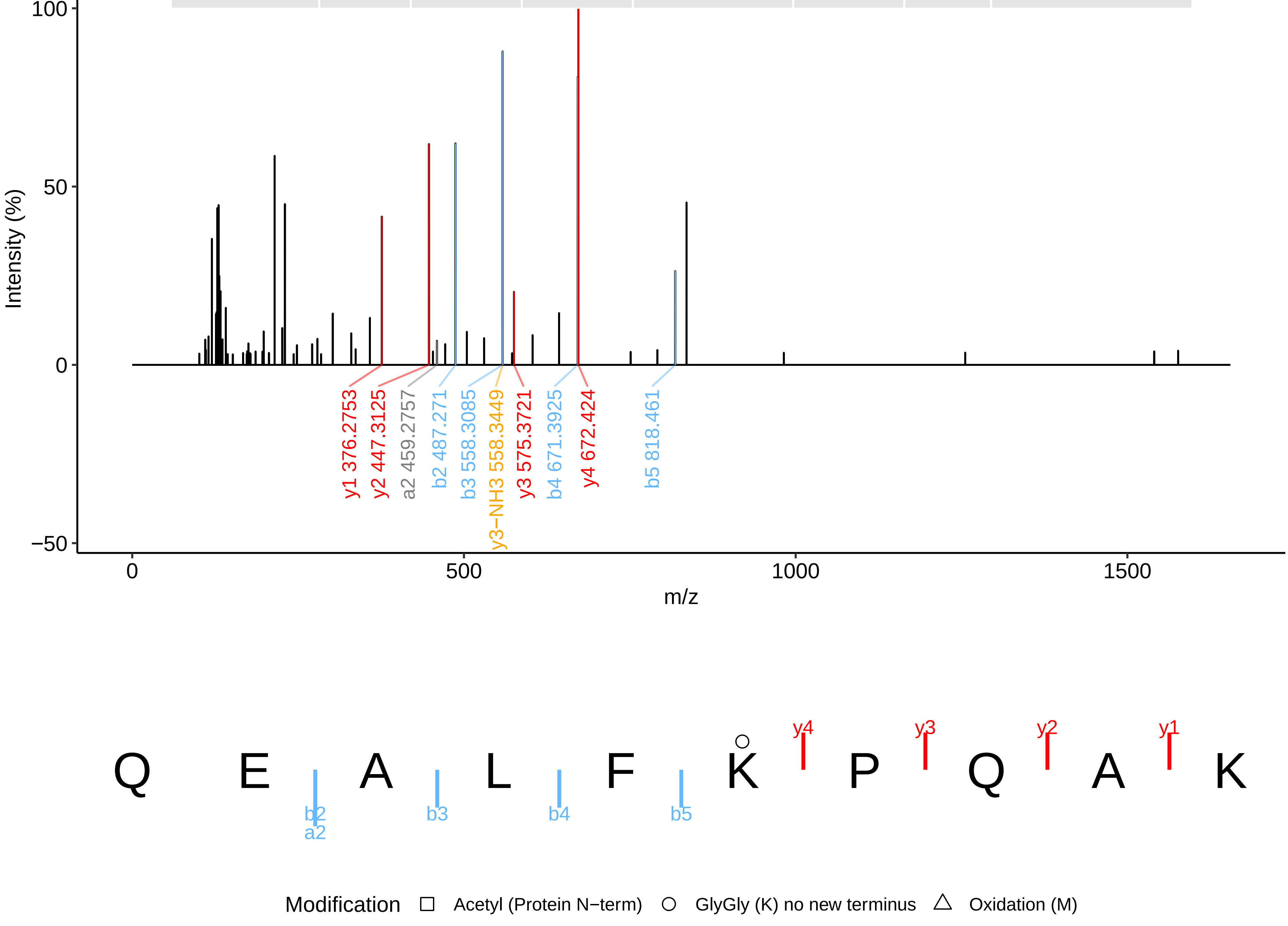

20200921_KIZ_MUb4_GlyGly_10plex − Scan number: 6027

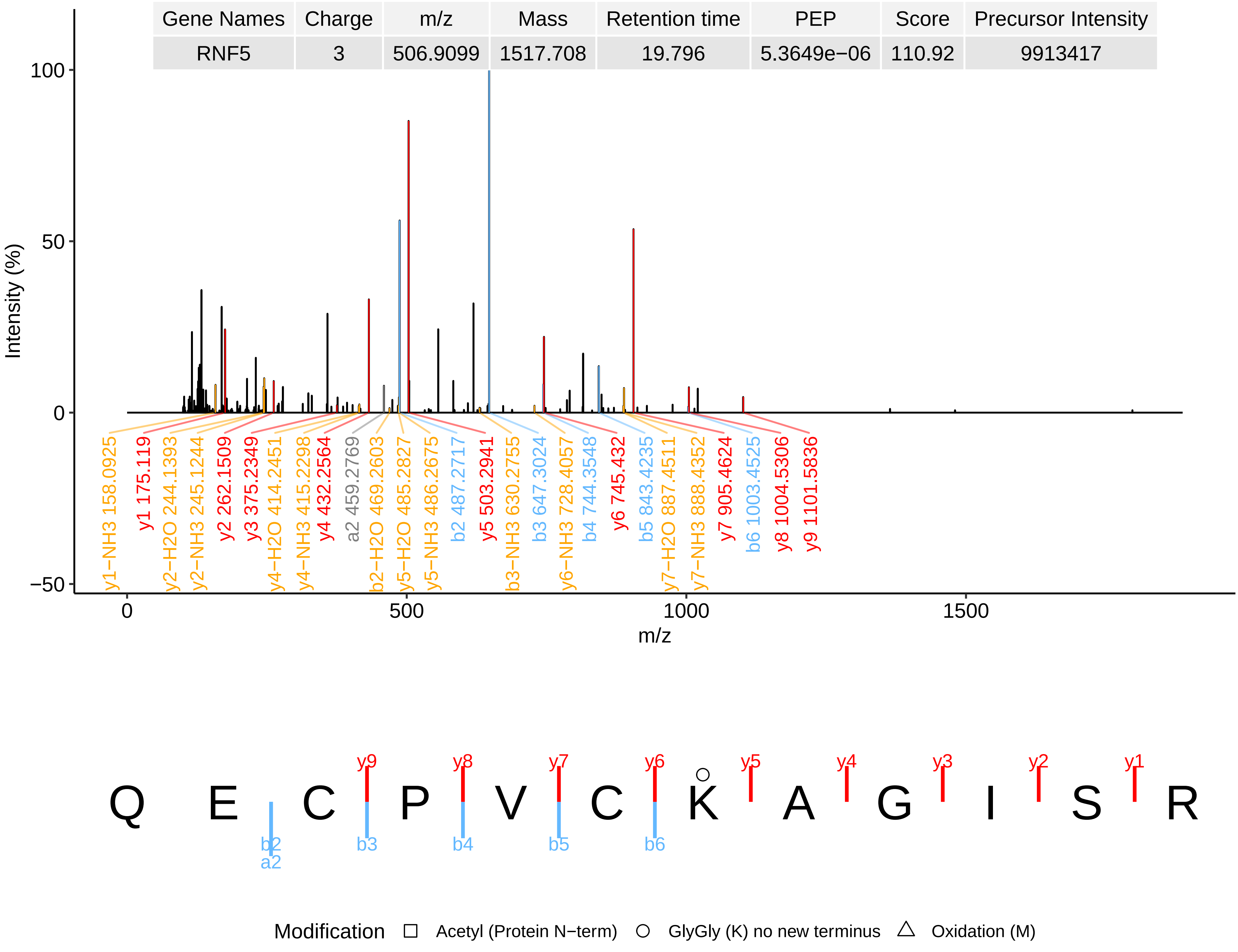

20200921_KIZ_MUb4_GlyGly_10plex_2 − Scan number: 5464

Gene Names	Charge	m/z	Mass	Retention time	PEP	Score	Precursor Intensity
RNF5	3	506.9099	1517.708	19.656	0.006489	72.705	487117.8

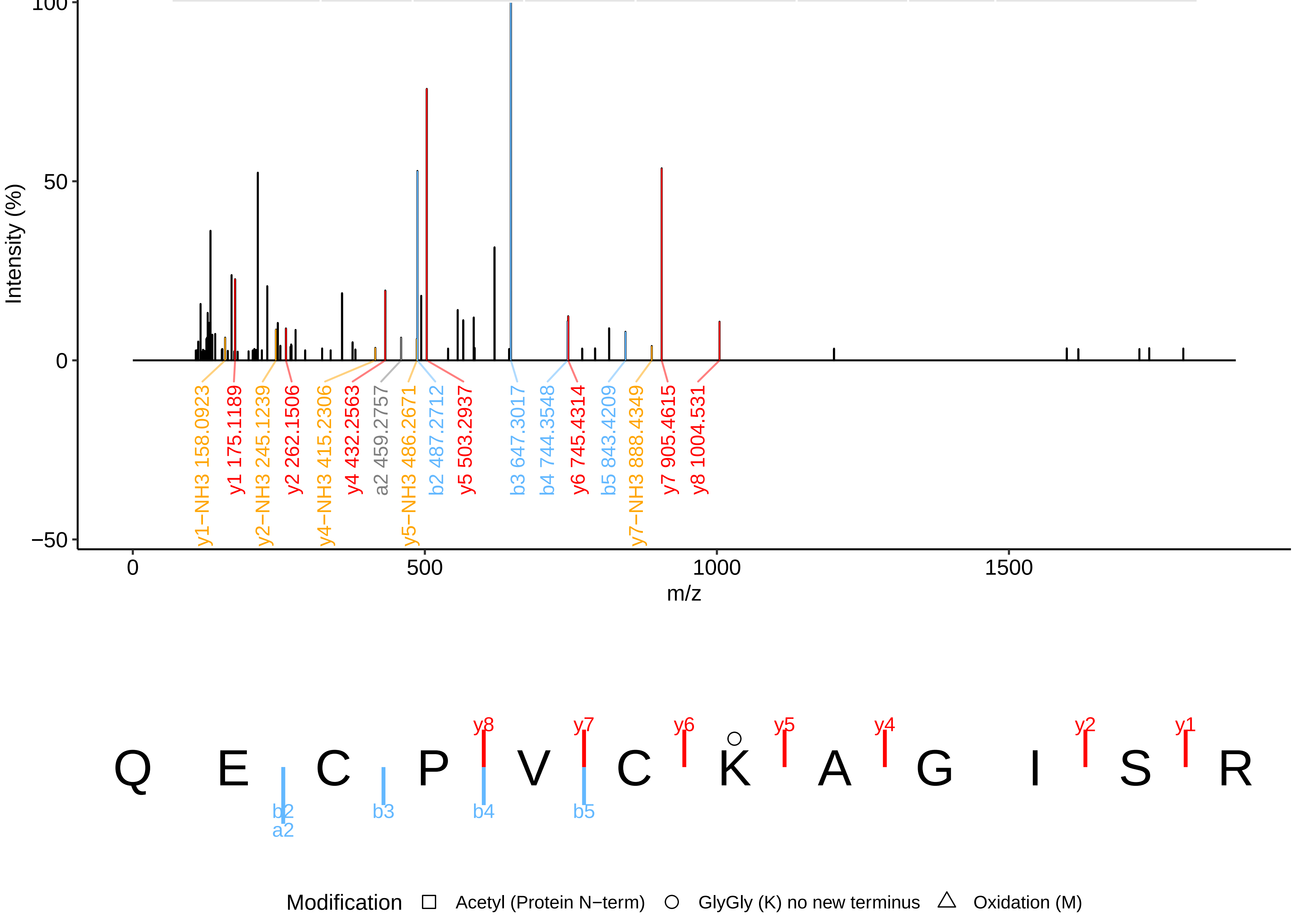

20200924_KIZ_MUb5_GlyGly_2 − Scan number: 4864

Gene Names	Charge	m/z	Mass	Retention time	PEP	Score	Precursor Intensity
RNF5	3	506.9099	1517.708	17.948	0.10449	50.088	2173050

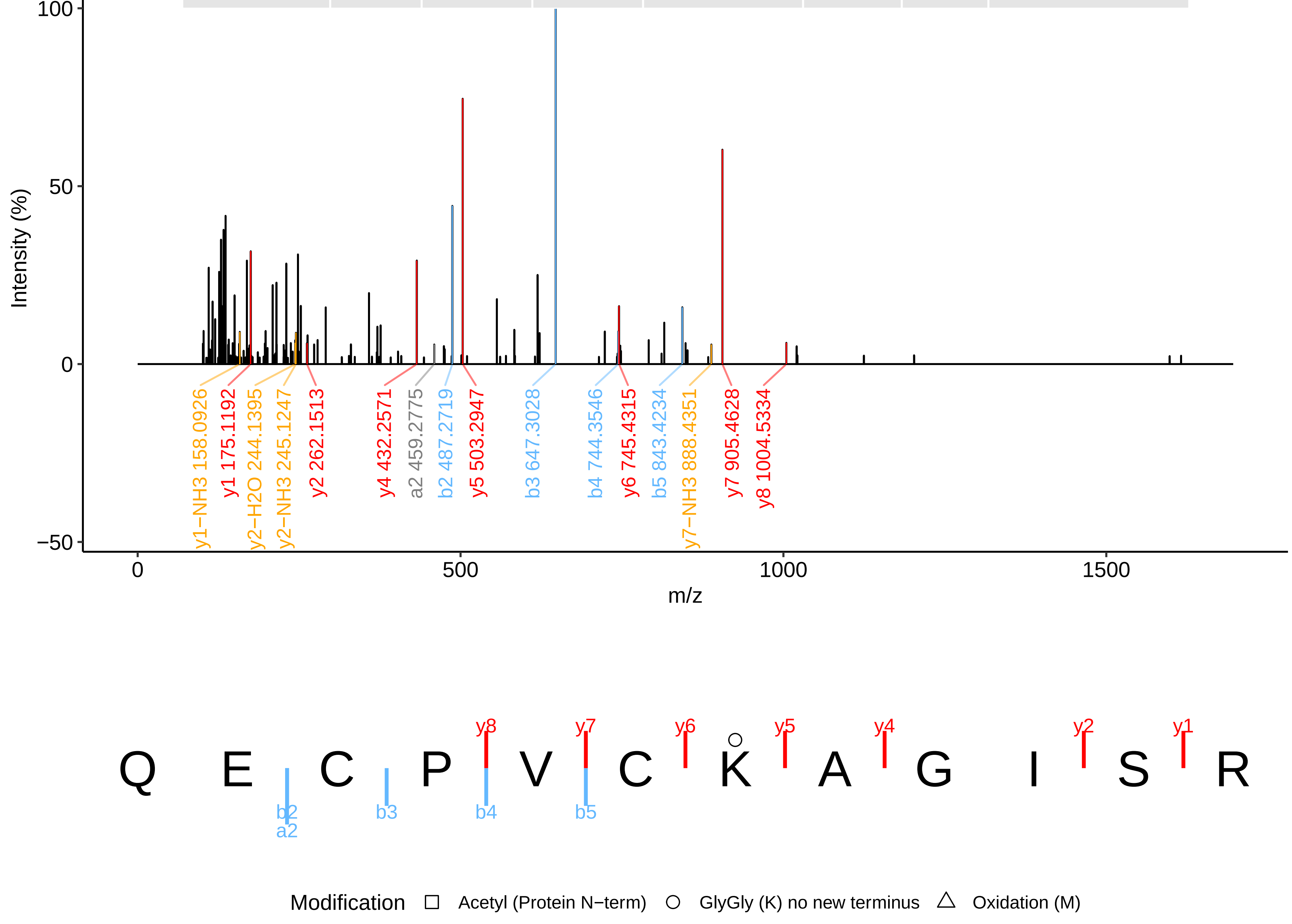

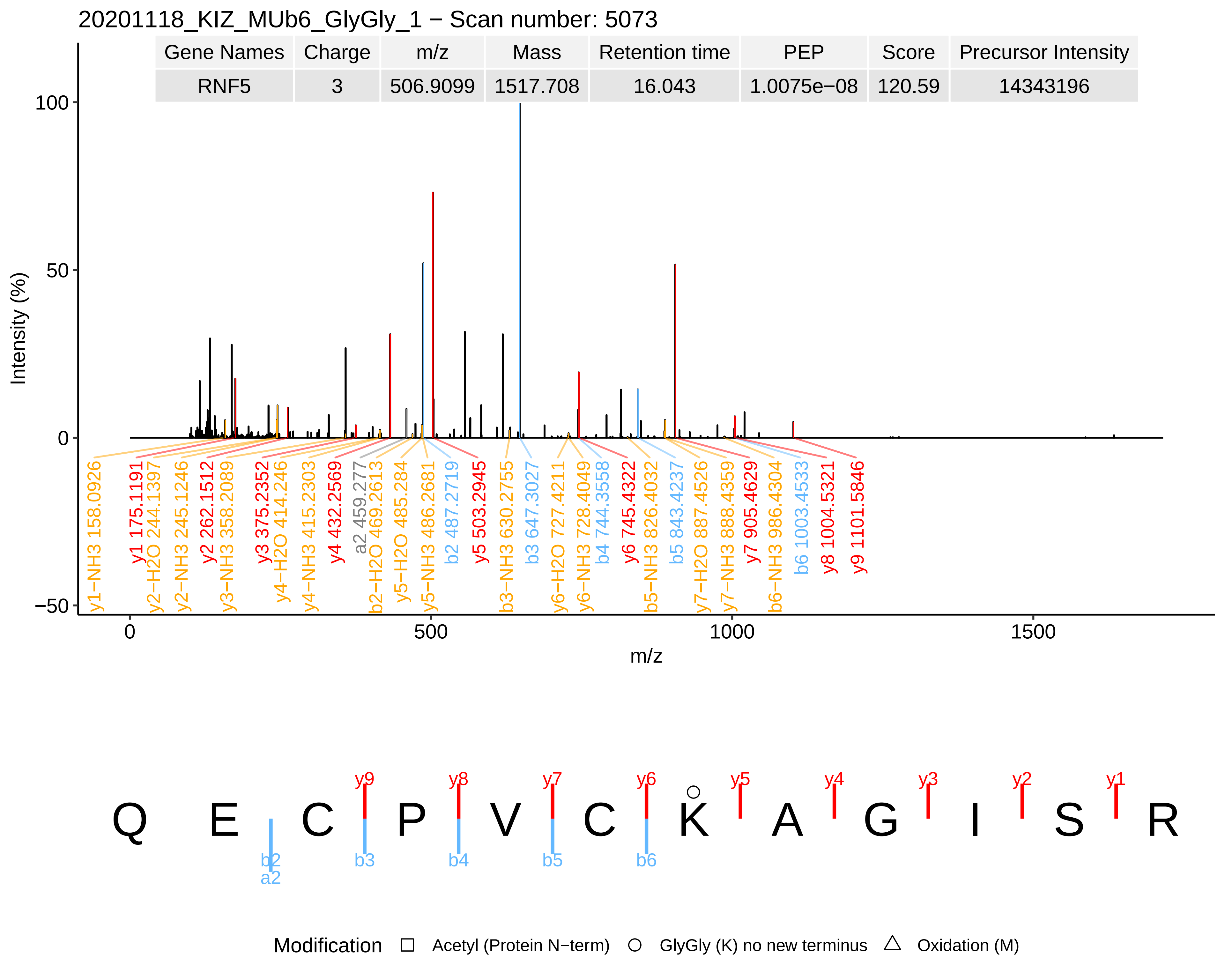

20201118_KIZ_MUb6_GlyGly_1 − Scan number: 5073
Gene Names | Charge | m/z | Mass | Retention time | PEP | Score | Precursor Intensity
RNF5 | 3 | 506.9099 | 1517.708 | 16.043 | 1.0075e−08 | 120.59 | 14343196
Intensity (%)
m/z
y1−NH3 158.0926
y1 175.1191
y2−H2O 244.1397
y2−NH3 245.1246
y2 262.1512
y3−NH3 358.2089
y3 375.2352
y4−H2O 414.246
y4−NH3 415.2303
y4 432.2569
a2 459.277
b2−H2O 469.2613
y5−H2O 485.284
y5−NH3 486.2681
b2 487.2719
y5 503.2945
b3−NH3 630.2755
b3 647.3027
y6−H2O 727.4211
y6−NH3 728.4049
b4 744.3558
y6 745.4322
b5−NH3 826.4032
b5 843.4237
y7−H2O 887.4526
y7−NH3 888.4359
y7 905.4629
b6−NH3 986.4304
b6 1003.4533
y8 1004.5321
y9 1101.5846
Q E C P V C K A G I S R
y9 y8 y7 y6 y5 y4 y3 y2 y1
b2 a2 b3 b4 b5 b6
Modification
Acetyl (Protein N−term)
GlyGly (K) no new terminus
Oxidation (M)

20201118_KIZ_MUb6_GlyGly_2 − Scan number: 6005

Gene Names	Charge	m/z	Mass	Retention time	PEP	Score	Precursor Intensity
RNF5	3	506.9099	1517.708	20.632	1.681e−05	101.32	2846129

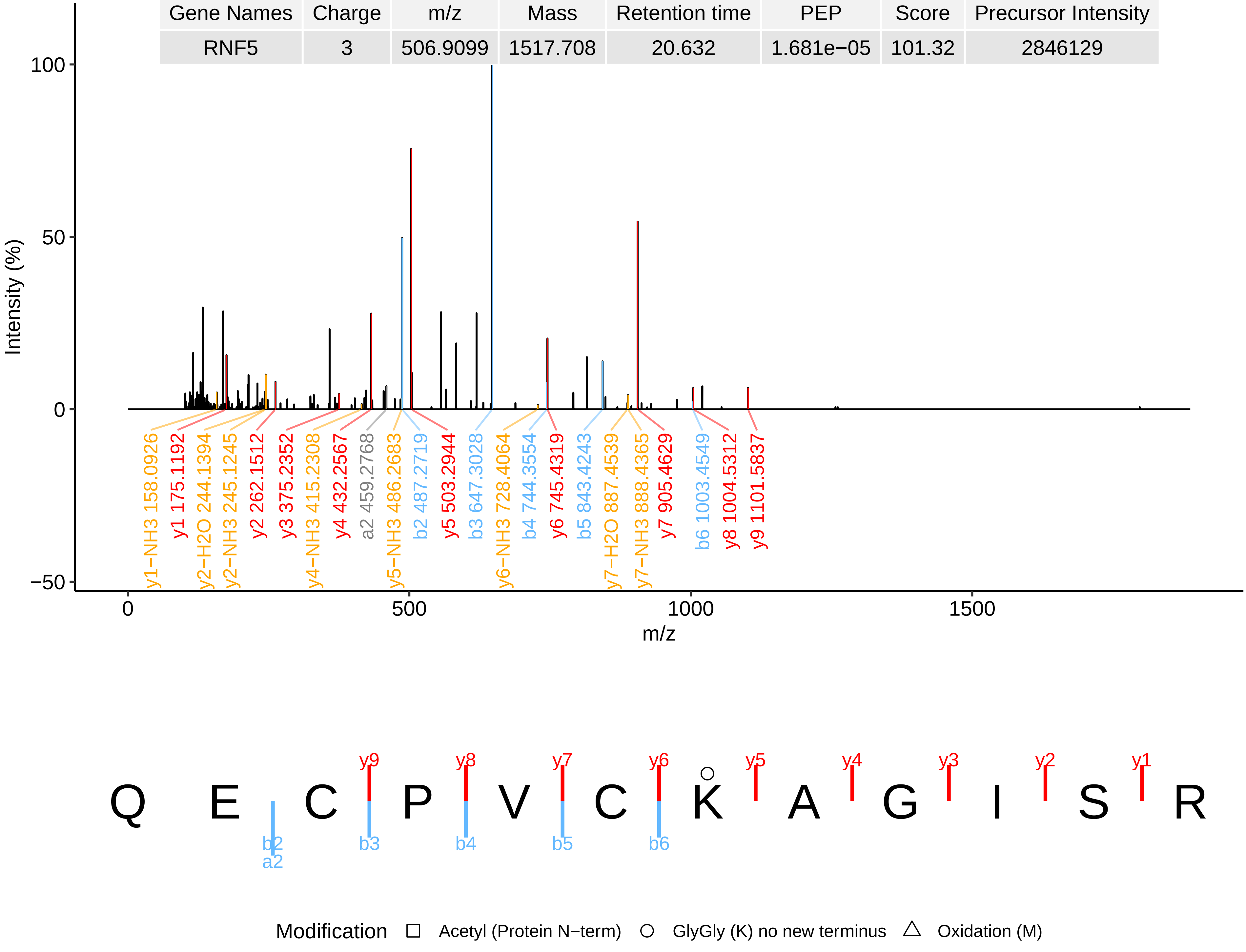

20200921_KIZ_MUb4_GlyGly_10plex − Scan number: 5532

Gene Names	Charge	m/z	Mass	Retention time	PEP	Score	Precursor Intensity
MFSD1	4	380.189	1516.727	18.13	0.012952	80.24	6856276

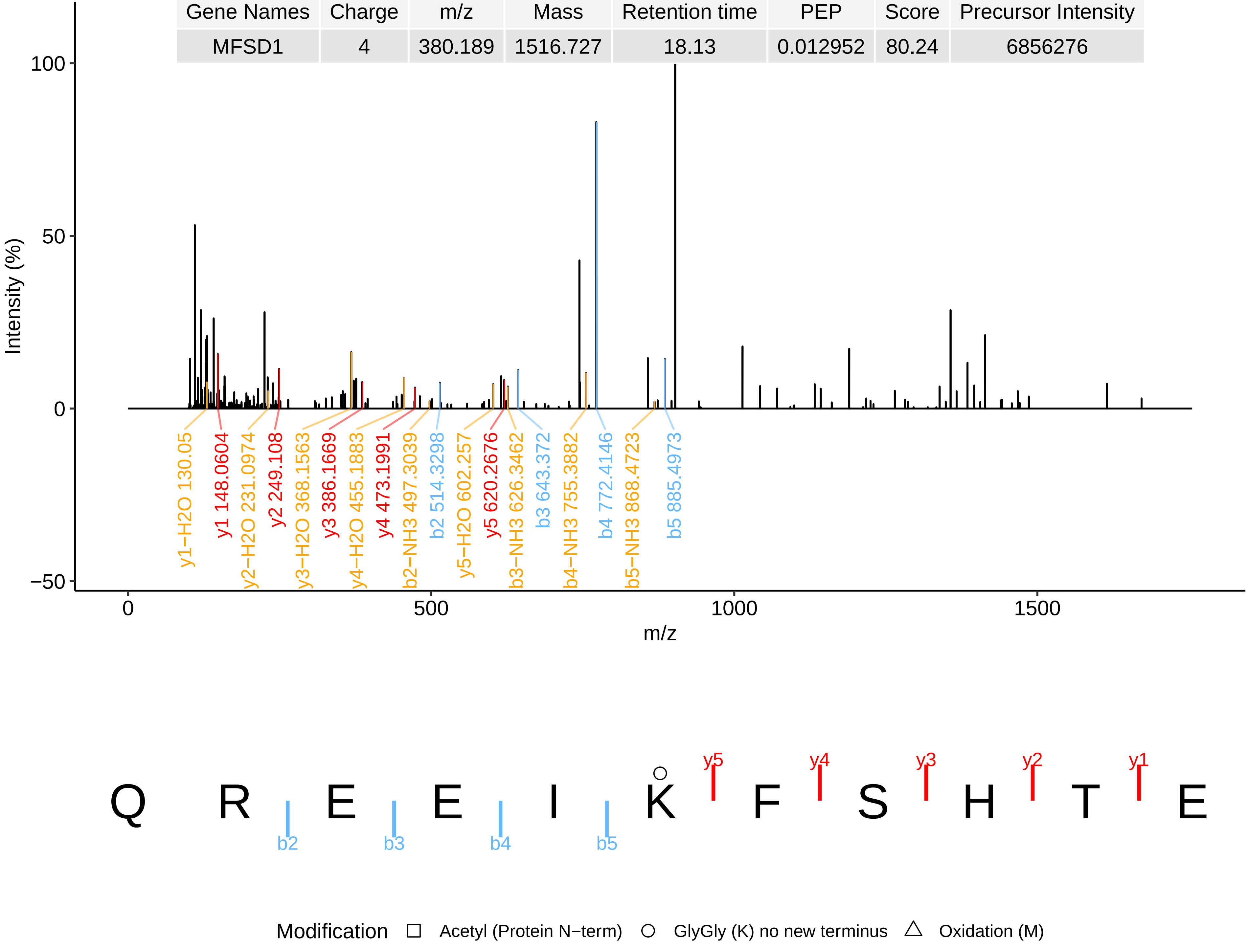

20200921_KIZ_MUb4_GlyGly_10plex − Scan number: 5534

Gene Names	Charge	m/z	Mass	Retention time	PEP	Score	Precursor Intensity
MFSD1	3	506.5829	1516.727	18.137	2.4837e−05	109.24	4534792

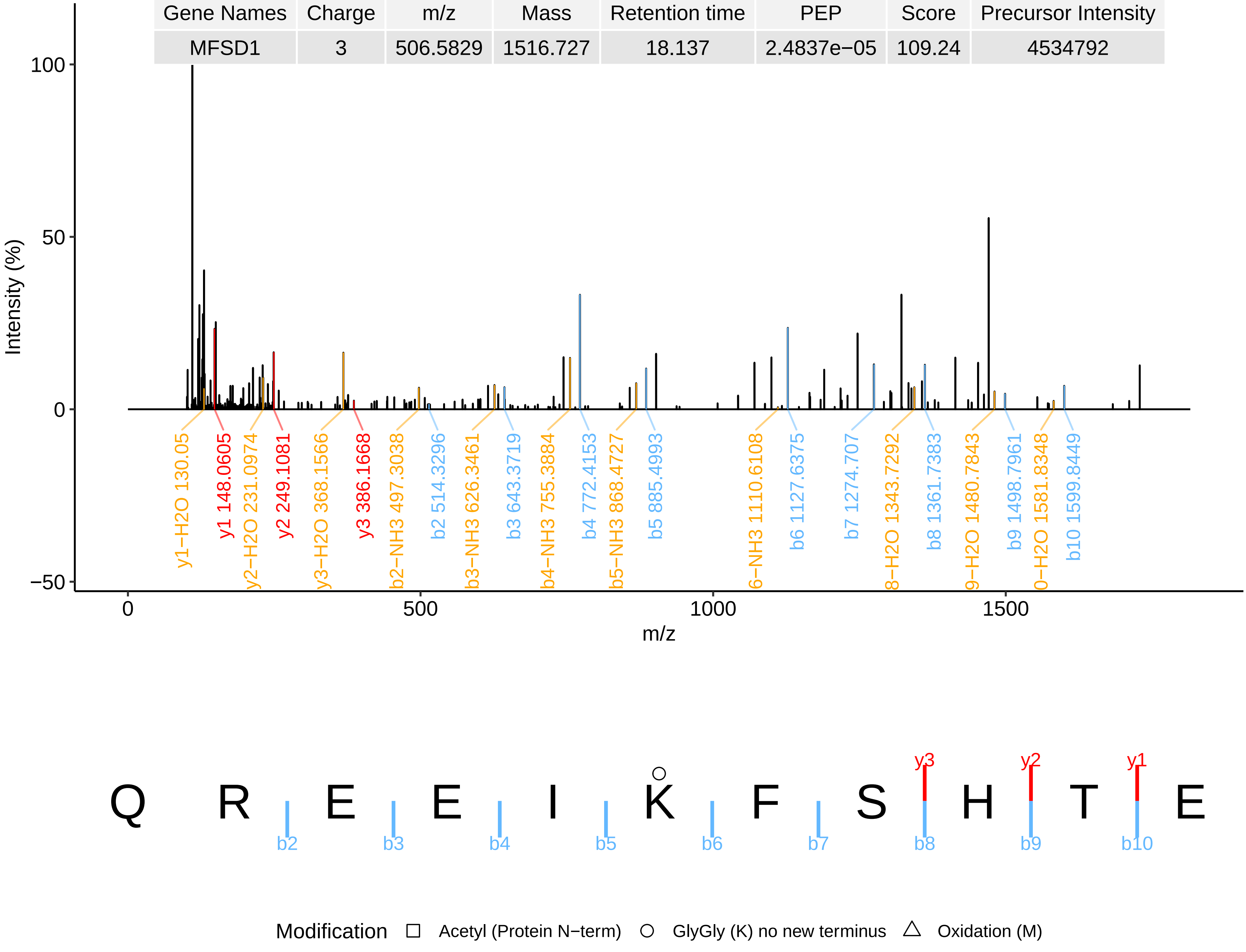

20200921_KIZ_MUb4_GlyGly_10plex_2 − Scan number: 5102

Gene Names	Charge	m/z	Mass	Retention time	PEP	Score	Precursor Intensity
MFSD1	4	380.189	1516.727	18.188	0.0090823	83.862	1914549

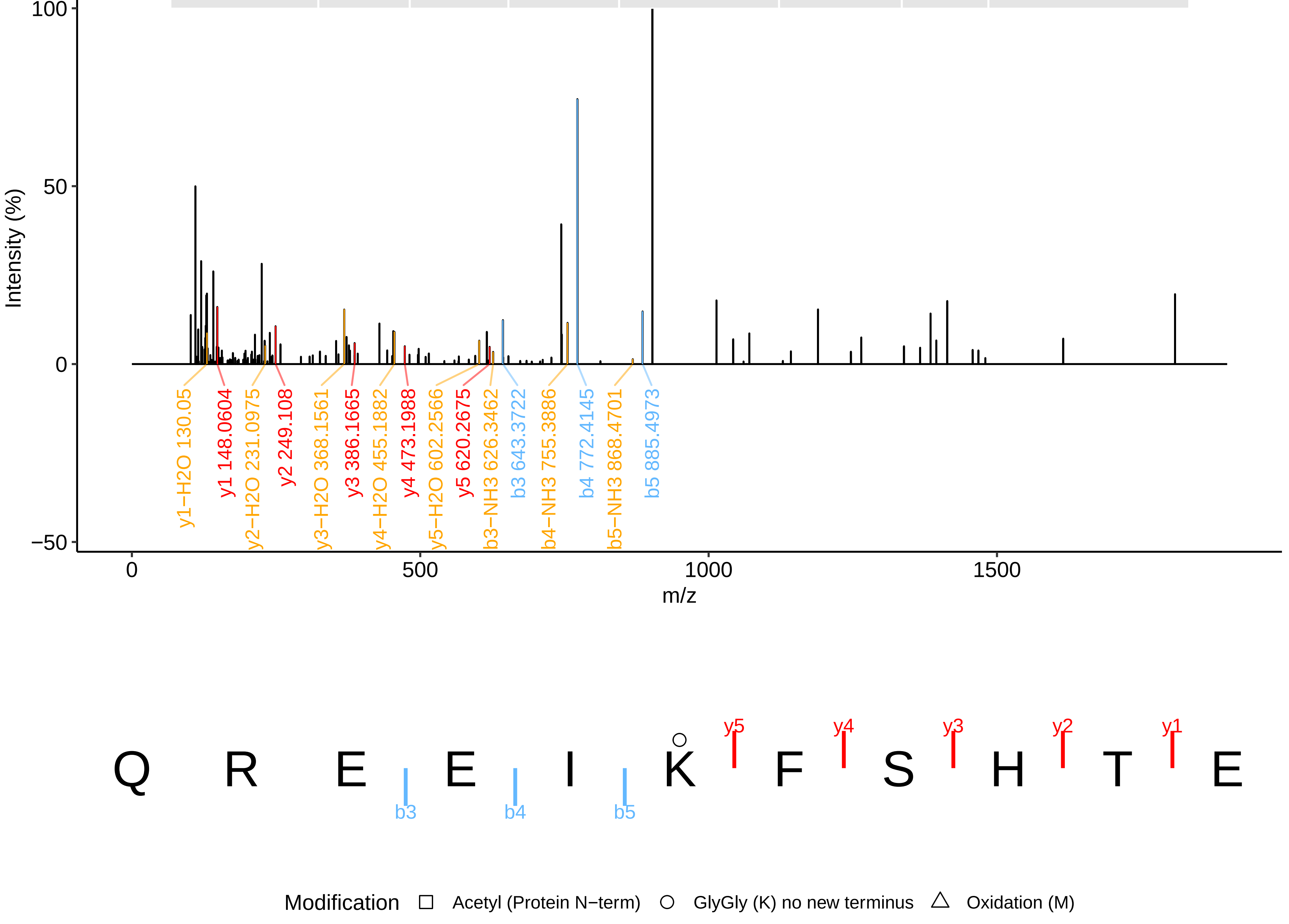

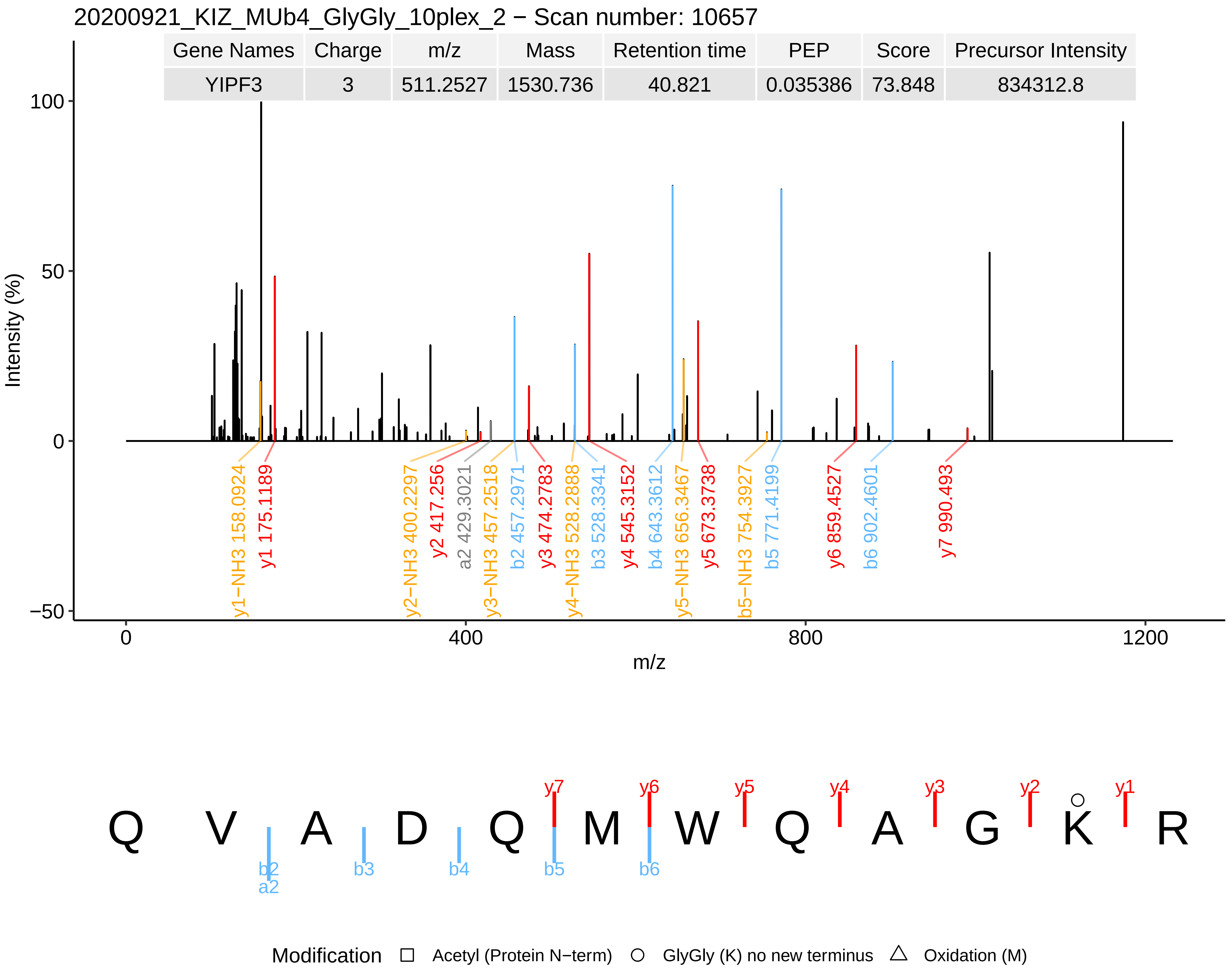
20200921_KIZ_MUb4_GlyGly_10plex_2 − Scan number: 10657
Gene Names | Charge | m/z | Mass | Retention time | PEP | Score | Precursor Intensity
YIPF3 | 3 | 511.2527 | 1530.736 | 40.821 | 0.035386 | 73.848 | 834312.8
Intensity (%)
100
50
0
−50
0
400
800
1200
m/z
y1−NH3 158.0924
y1 175.1189
y2−NH3 400.2297
y2 417.256
a2 429.3021
y3−NH3 457.2518
b2 457.2971
y3 474.2783
y4−NH3 528.2888
b3 528.3341
y4 545.3152
b4 643.3612
y5−NH3 656.3467
y5 673.3738
b5−NH3 754.3927
b5 771.4199
y6 859.4527
b6 902.4601
y7 990.493
Q V A D Q M W Q A G K R
y7 y6 y5 y4 y3 y2 y1
b2 a2 b3 b4 b5 b6
Modification
Acetyl (Protein N−term)
GlyGly (K) no new terminus
Oxidation (M)

20200921_KIZ_MUb4_GlyGly_10plex − Scan number: 7823

Gene Names	Charge	m/z	Mass	Retention time	PEP	Score	Precursor Intensity
RNF185	3	496.9185	1487.734	26.399	0.0010676	82.774	4602714

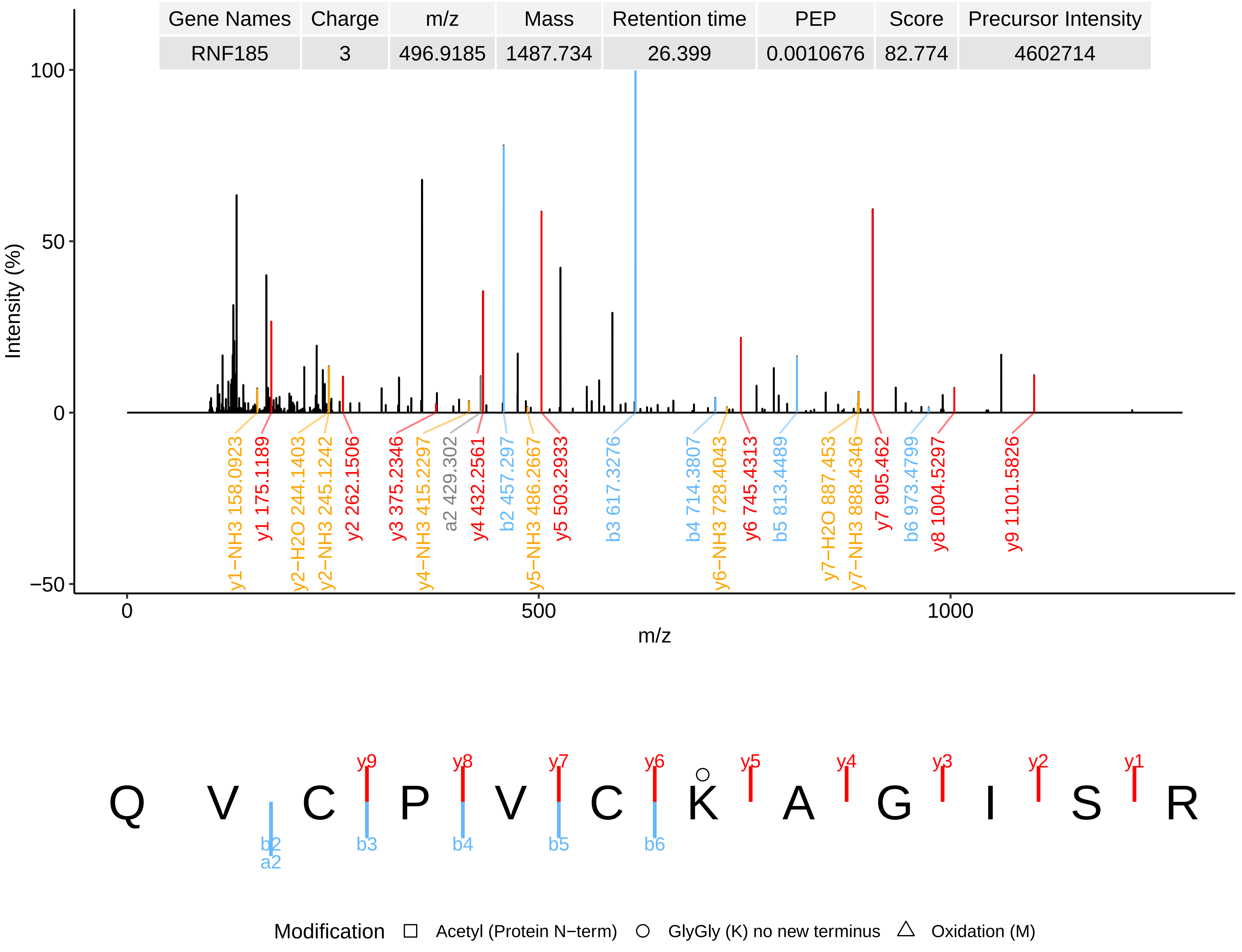

20200921_KIZ_MUb4_GlyGly_10plex_2 − Scan number: 7197

Gene Names	Charge	m/z	Mass	Retention time	PEP	Score	Precursor Intensity
RNF185	3	496.9185	1487.734	26.766	0.060182	54.068	1856493

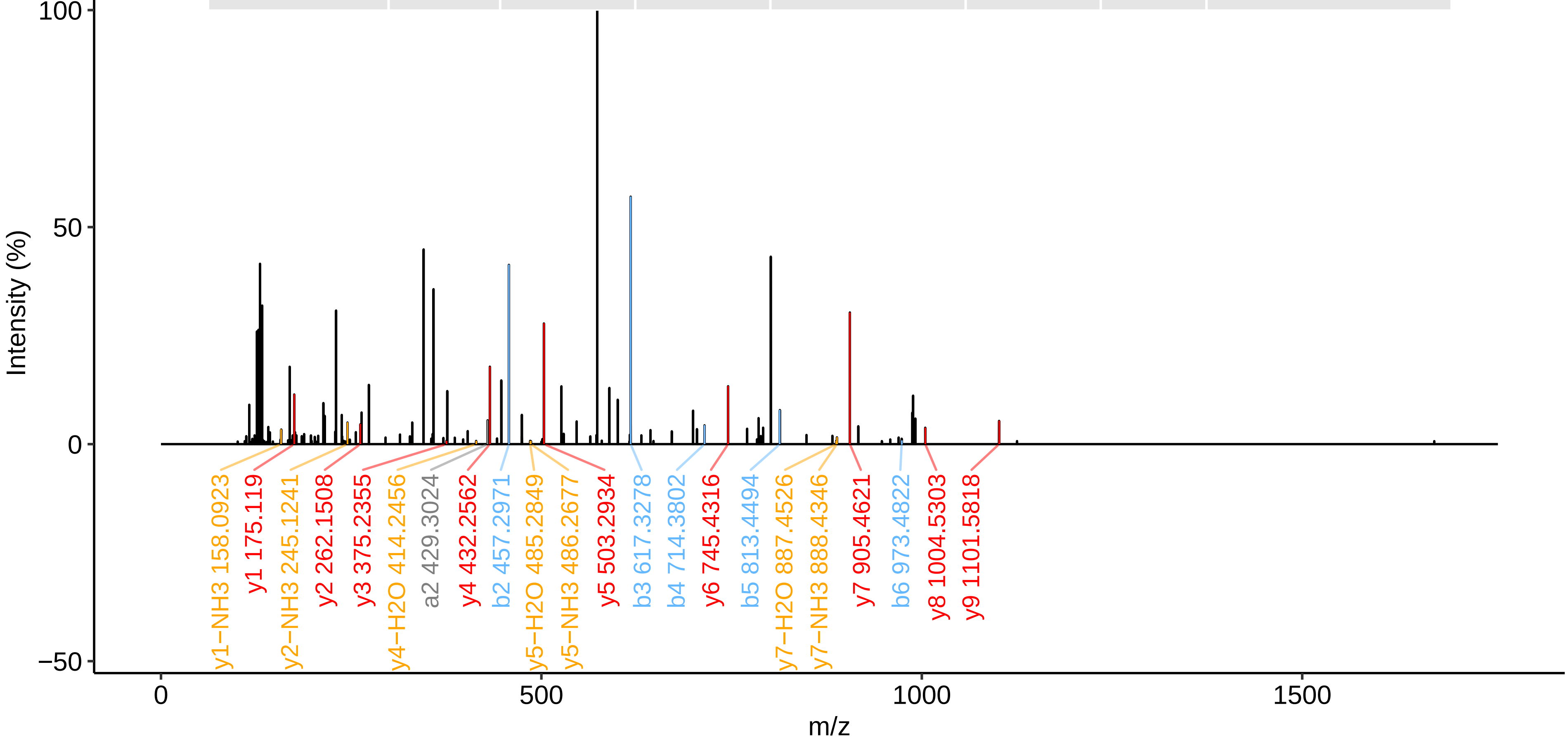

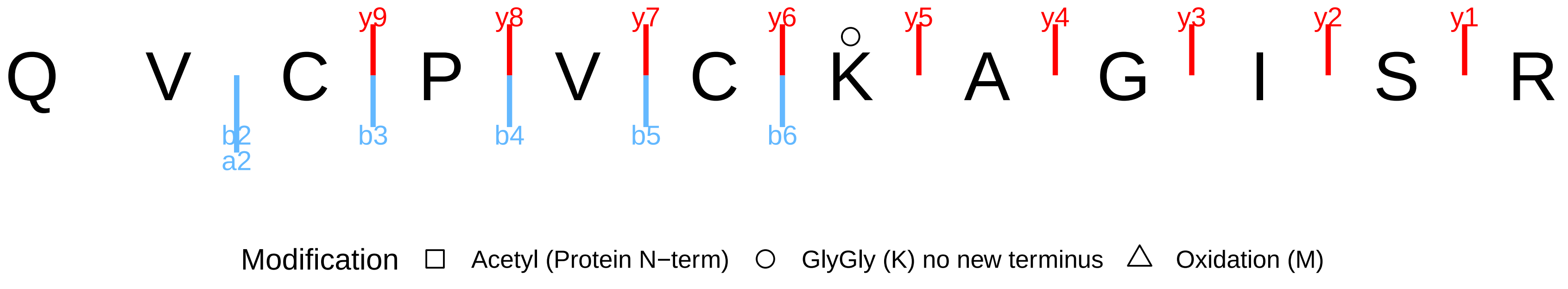

20201118_KIZ_MUb6_GlyGly_1 − Scan number: 6520

Gene Names	Charge	m/z	Mass	Retention time	PEP	Score	Precursor Intensity
RNF185	3	496.9185	1487.734	20.644	0.00060996	89.43	22783634

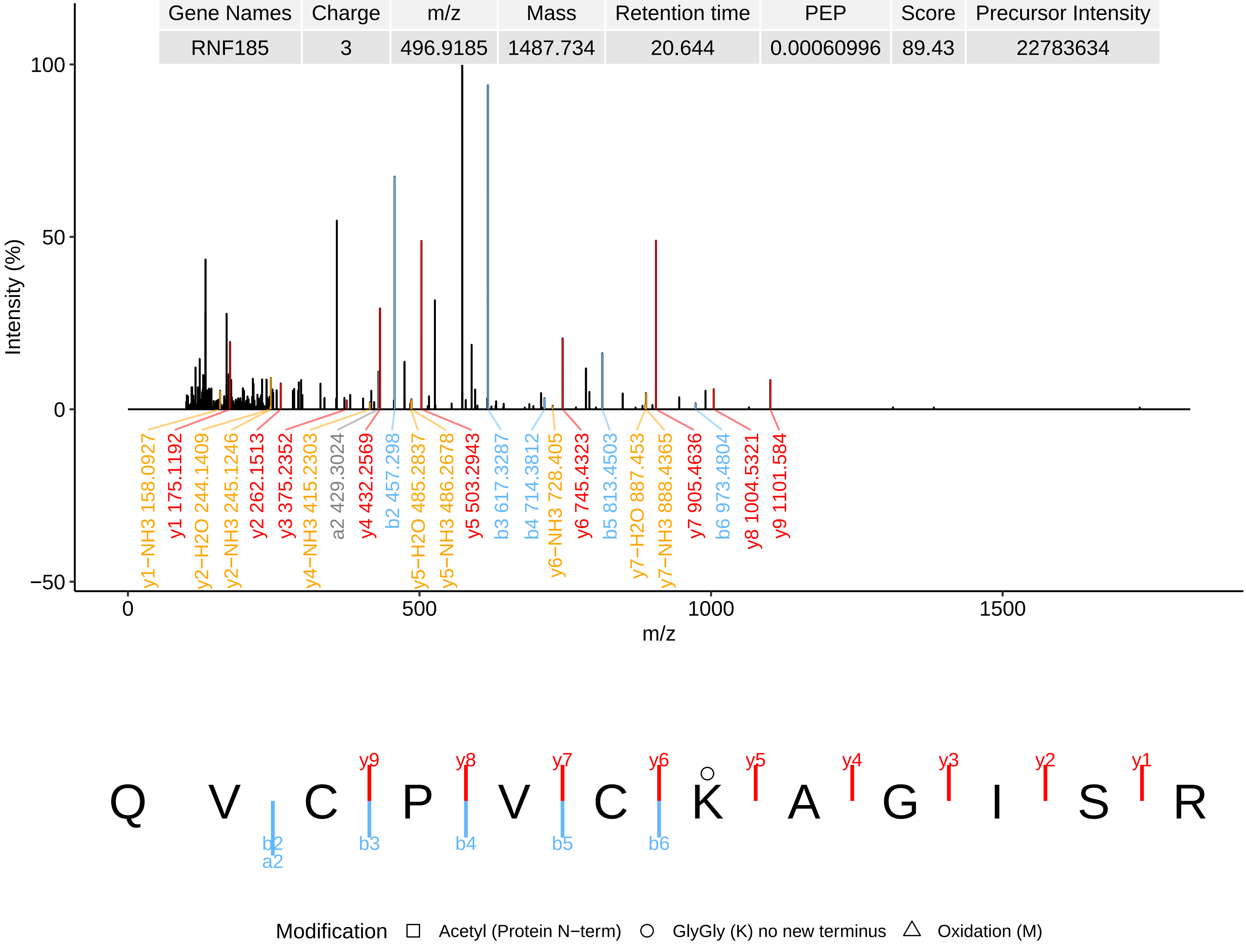

20200921_KIZ_MUb4_GlyGly_10plex − Scan number: 10290

Gene Names	Charge	m/z	Mass	Retention time	PEP	Score	Precursor Intensity
ATIC	3	503.261	1506.761	35.561	4.6613e−05	99.5	2421782

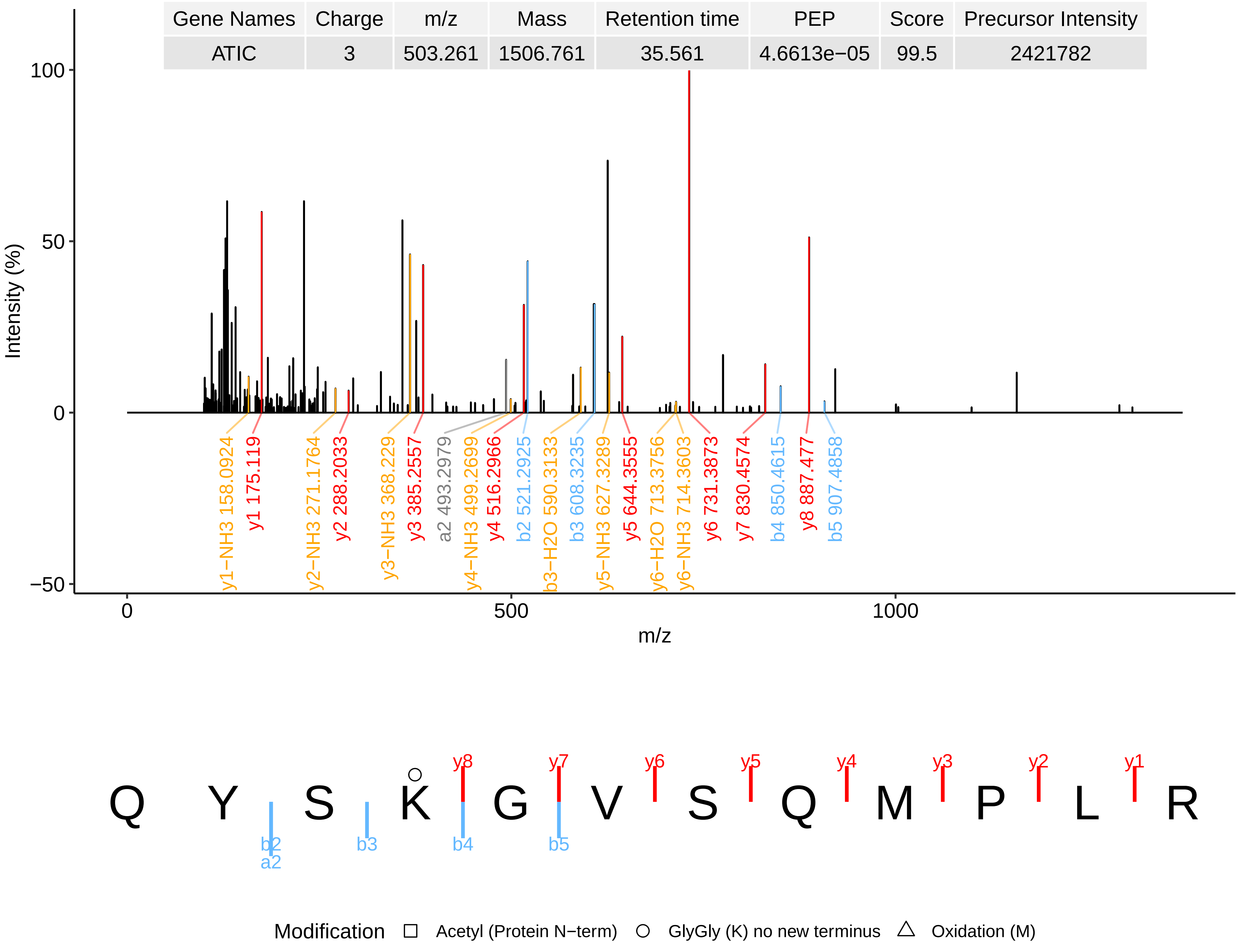

20201118_KIZ_MUb6_GlyGly_2 − Scan number: 33943

Gene Names	Charge	m/z	Mass	Retention time	PEP	Score	Precursor Intensity
CRYBB2	3	496.2827	1485.826	110.62	0.068485	57.175	31407076

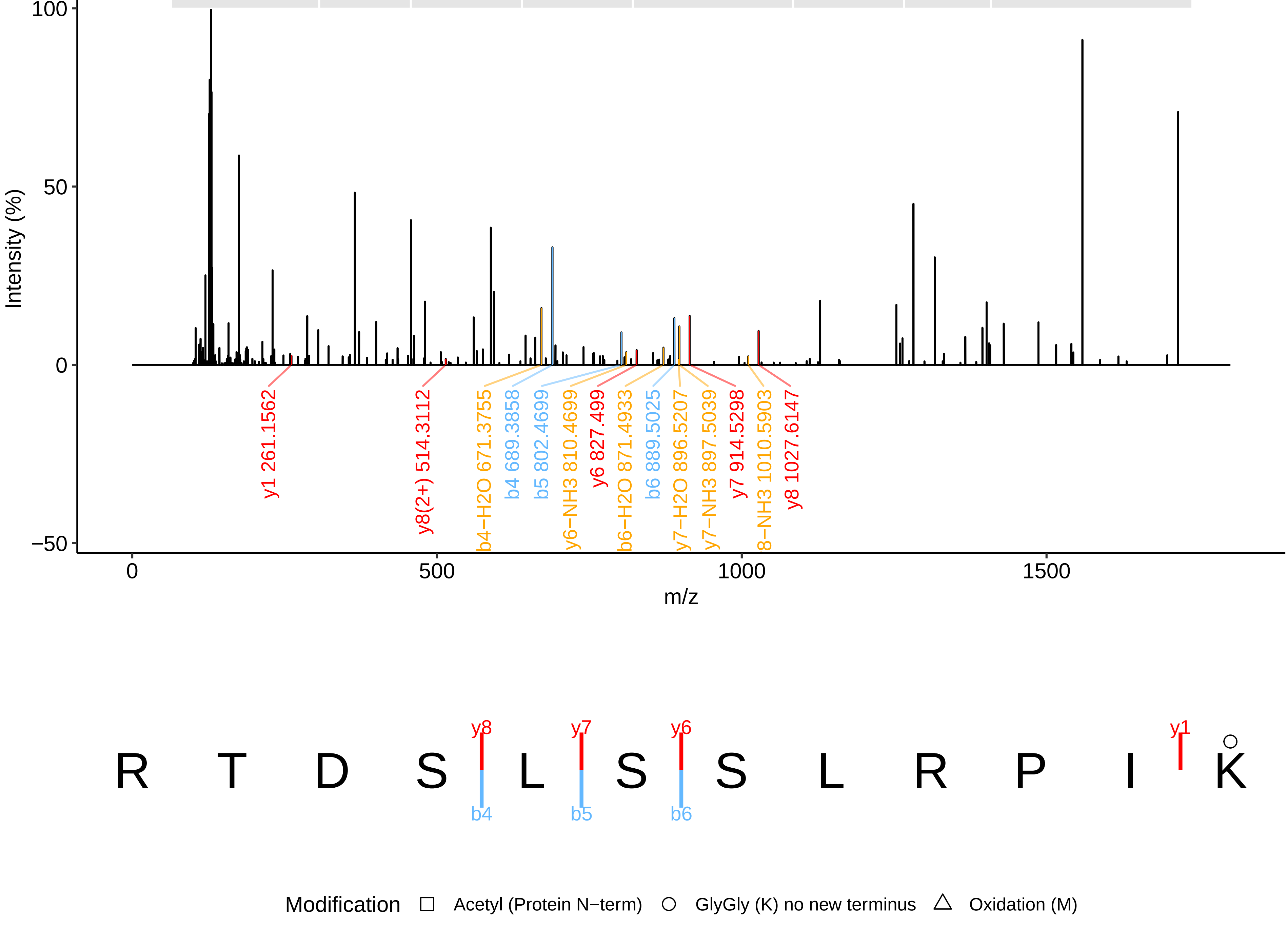

20200921_KIZ_MUb4_GlyGly_10plex − Scan number: 7290

Gene Names	Charge	m/z	Mass	Retention time	PEP	Score	Precursor Intensity
IFNGR1	3	435.5613	1303.662	24.422	5.4554e−08	115.57	6739937

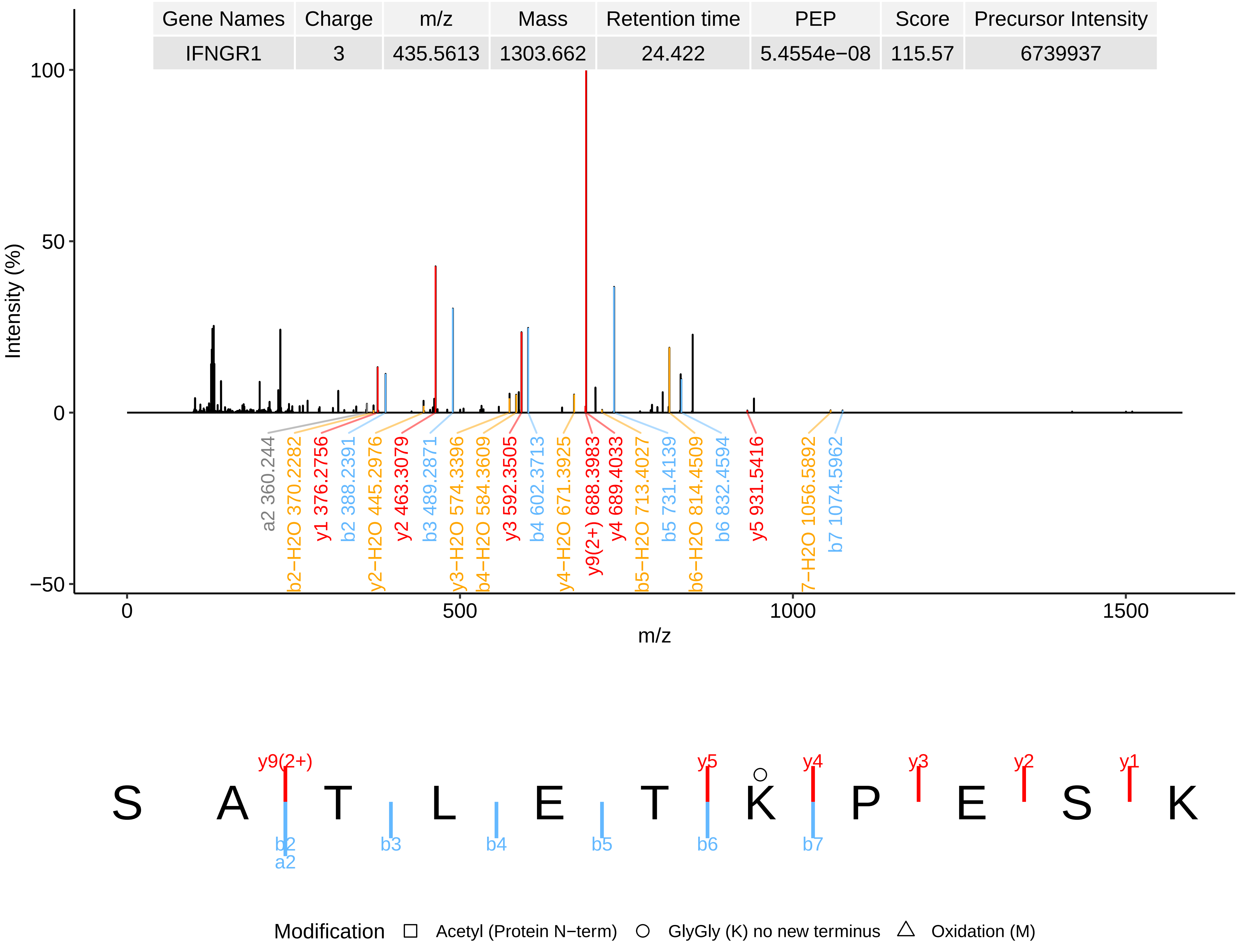

20200921_KIZ_MUb4_GlyGly_10plex_2 − Scan number: 6712

Gene Names	Charge	m/z	Mass	Retention time	PEP	Score	Precursor Intensity
IFNGR1	3	435.5613	1303.662	24.796	0.00073748	90.827	976720.5

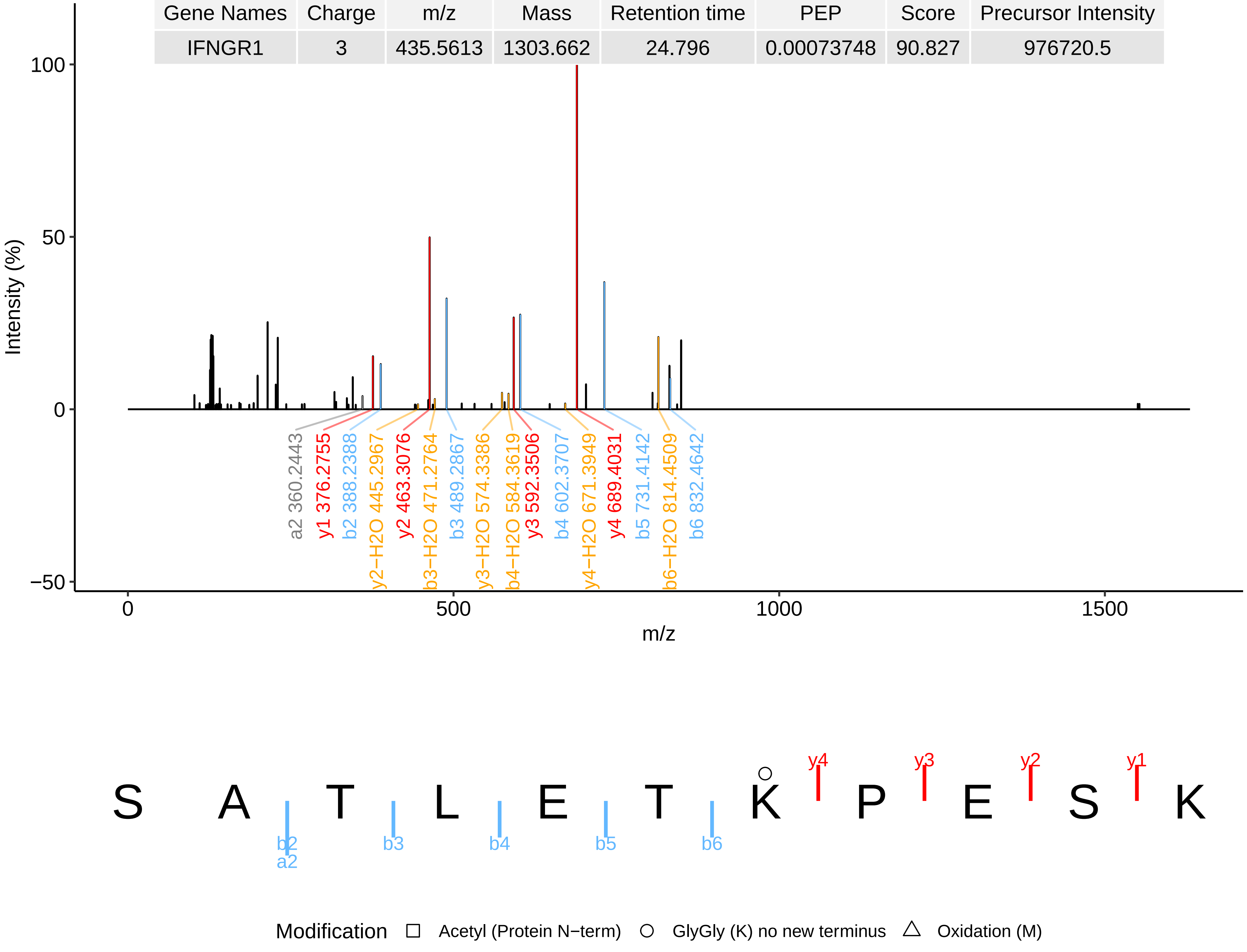

20200924_KIZ_MUb5_GlyGly_1 − Scan number: 6393

Gene Names	Charge	m/z	Mass	Retention time	PEP	Score	Precursor Intensity
IFNGR1	3	435.5613	1303.662	20.089	0.0049605	76.655	NA

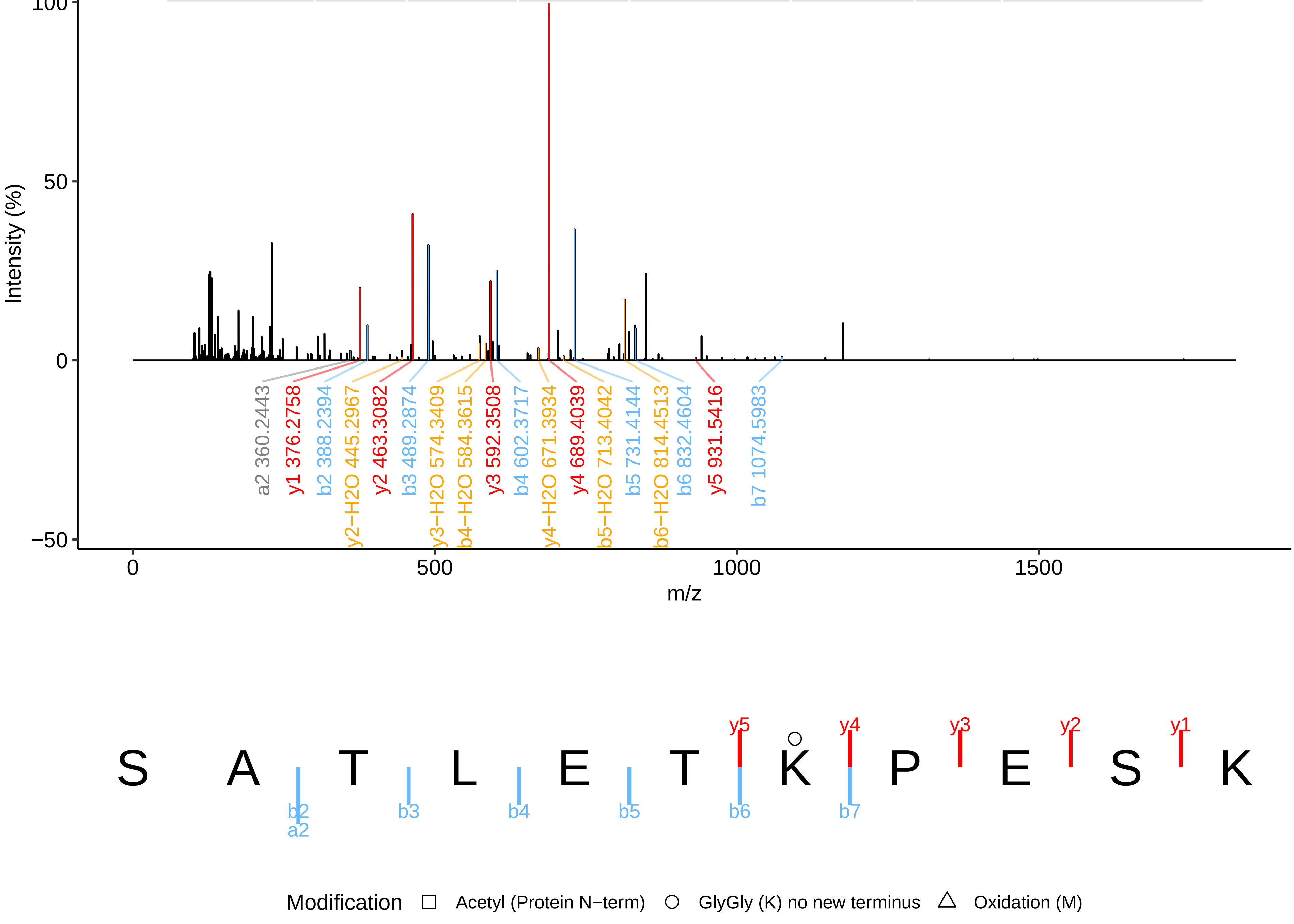

20200924_KIZ_MUb5_GlyGly_1 − Scan number: 6530

Gene Names	Charge	m/z	Mass	Retention time	PEP	Score	Precursor Intensity
IFNGR1	3	435.5613	1303.662	20.544	0.012779	67.153	4662596

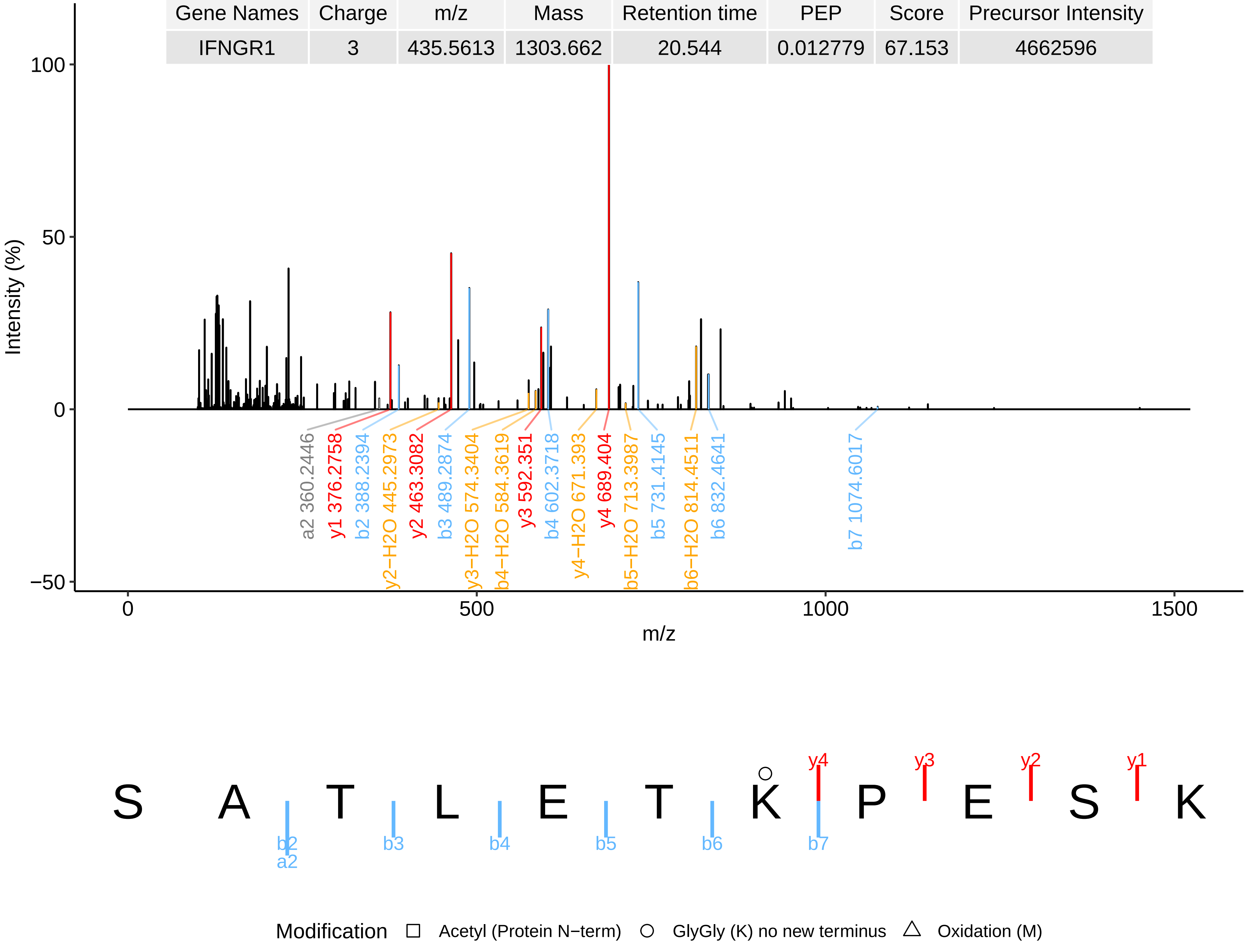

20200924_KIZ_MUb5_GlyGly_1 − Scan number: 6548

Gene Names	Charge	m/z	Mass	Retention time	PEP	Score	Precursor Intensity
IFNGR1	3	435.5613	1303.662	20.606	0.020002	62.408	9175455

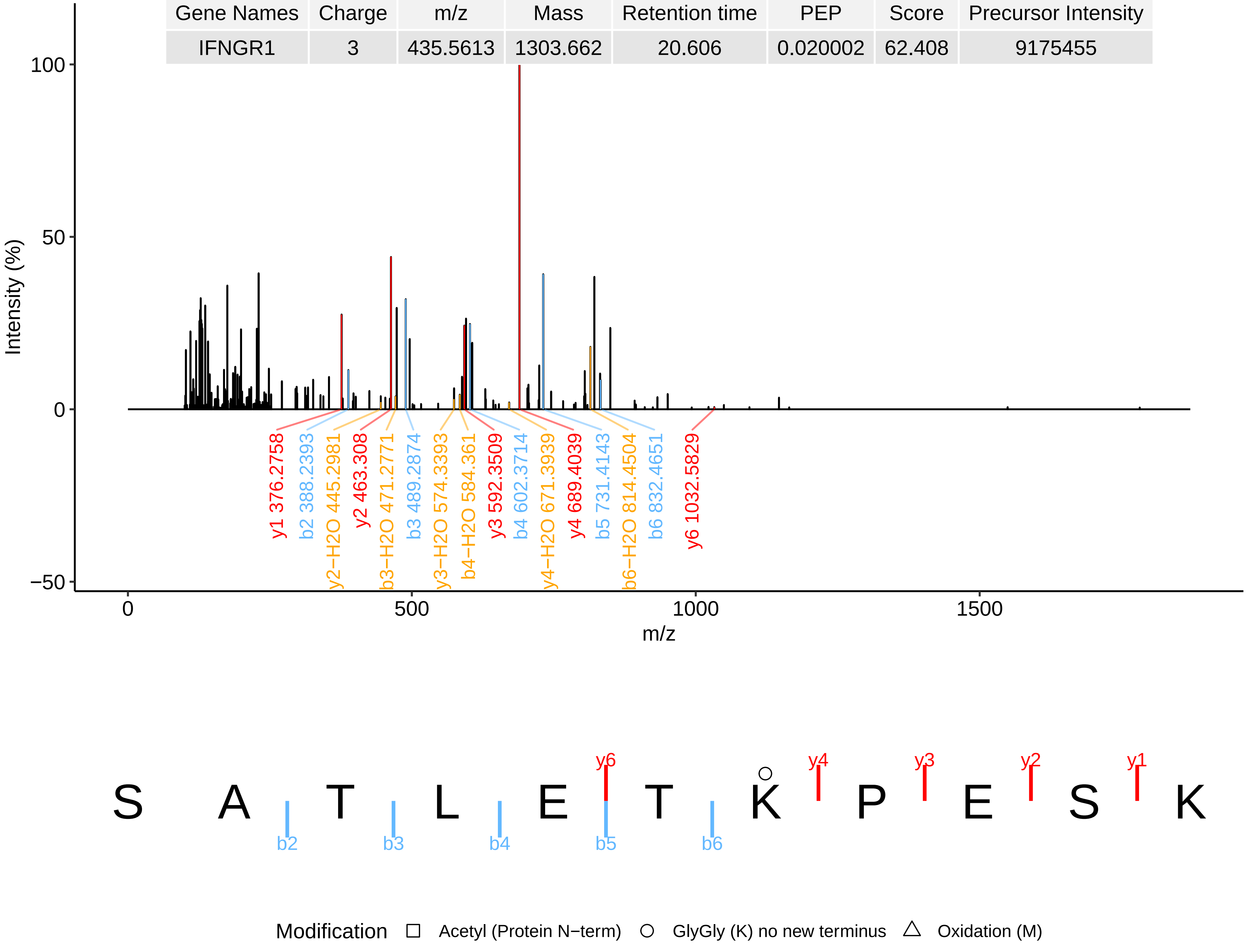

20200924_KIZ_MUb5_GlyGly_2 − Scan number: 5911

Gene Names	Charge	m/z	Mass	Retention time	PEP	Score	Precursor Intensity
IFNGR1	3	435.5613	1303.662	21.995	0.024794	62.466	1821015

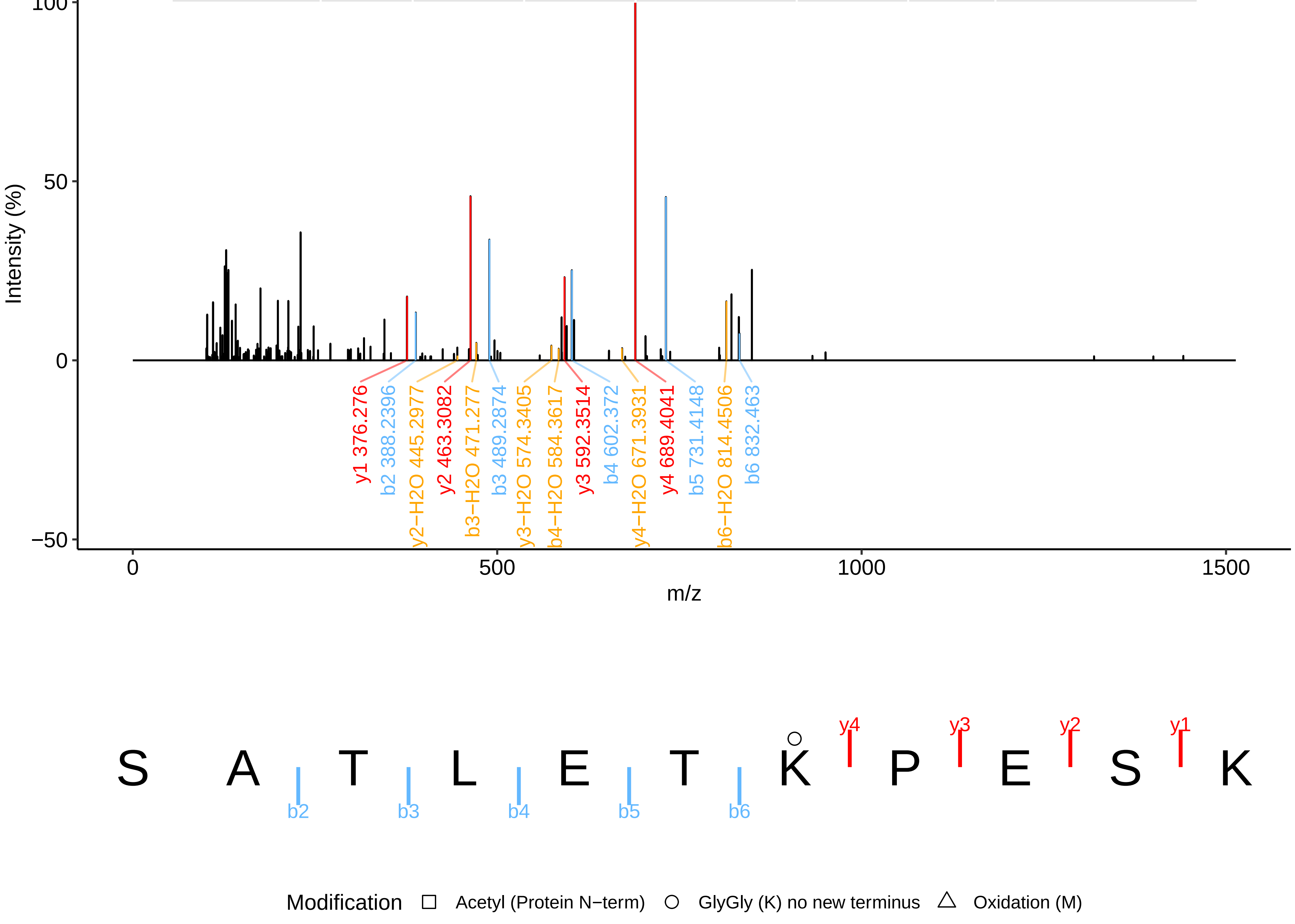

20201118_KIZ_MUb6_GlyGly_1 − Scan number: 6004

Gene Names	Charge	m/z	Mass	Retention time	PEP	Score	Precursor Intensity
IFNGR1	3	435.5613	1303.662	18.999	1.736e−05	108.7	10113168

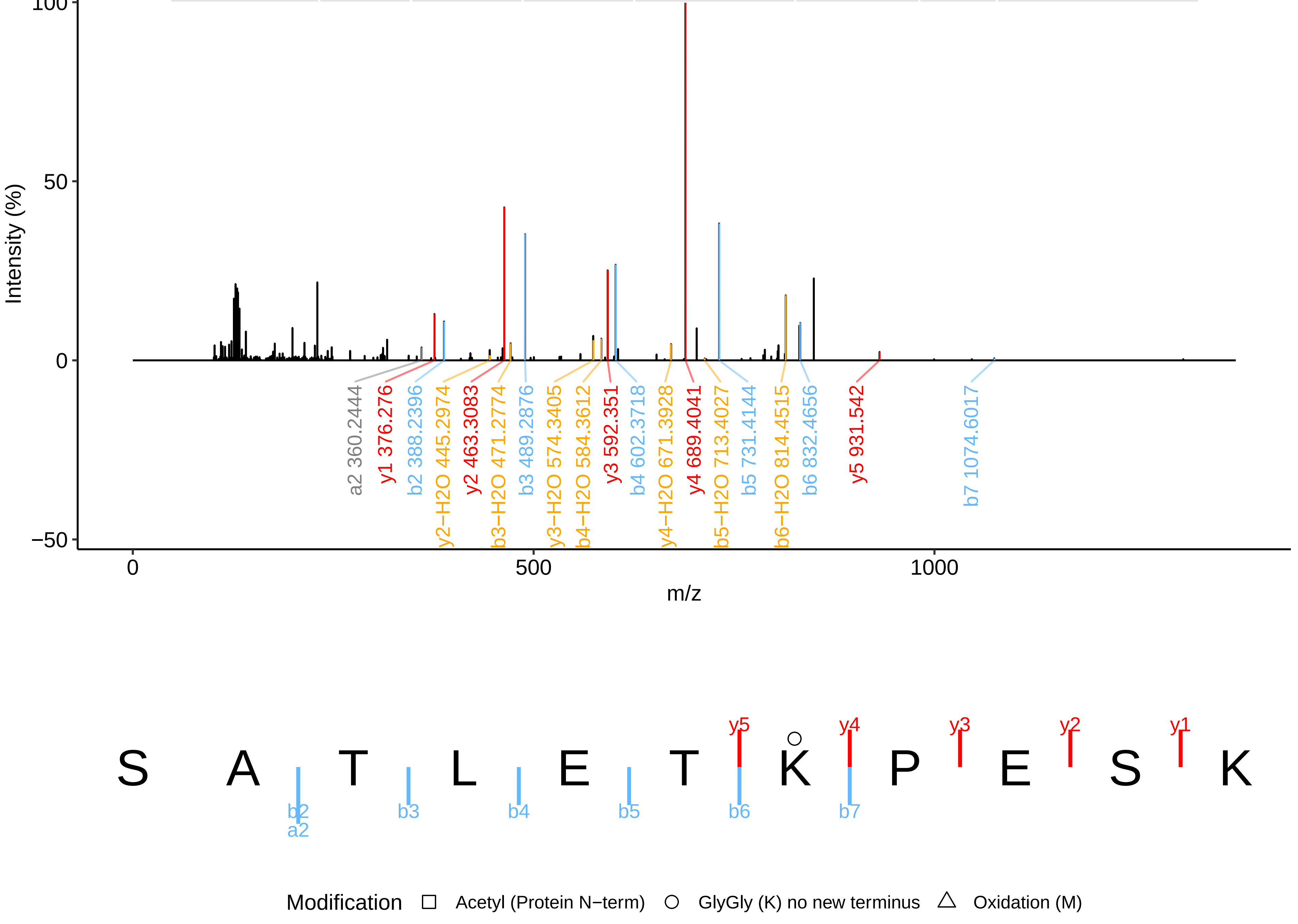

20200921_KIZ_MUb4_GlyGly_10plex − Scan number: 12628

Gene Names	Charge	m/z	Mass	Retention time	PEP	Score	Precursor Intensity
SHMT2	3	508.9266	1523.758	44.355	0.091056	52.071	NA

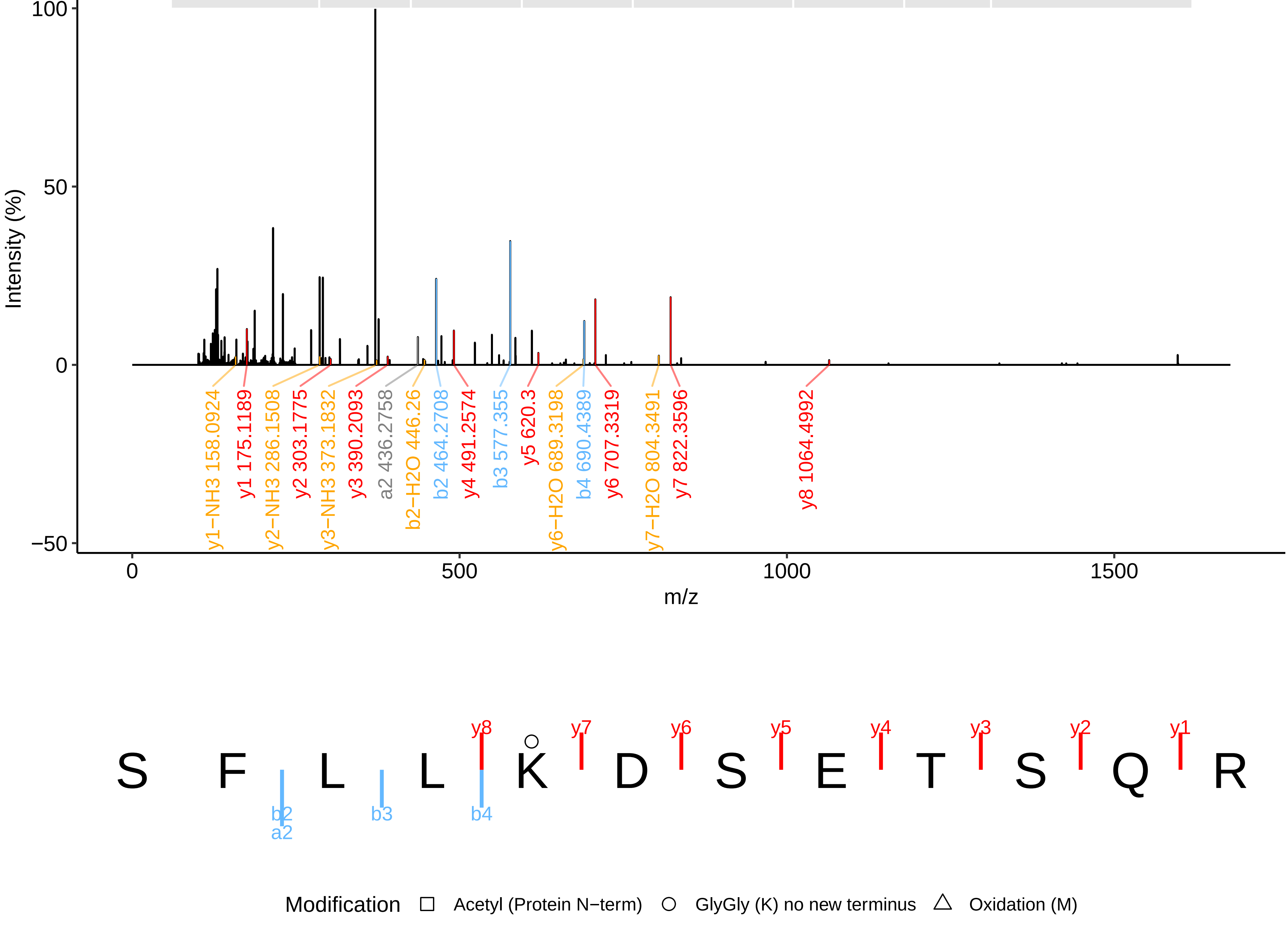

20200924_KIZ_MUb5_GlyGly_2 − Scan number: 11635

Gene Names	Charge	m/z	Mass	Retention time	PEP	Score	Precursor Intensity
BARD1	3	501.278	1500.812	43.497	0.058501	48.561	6099672

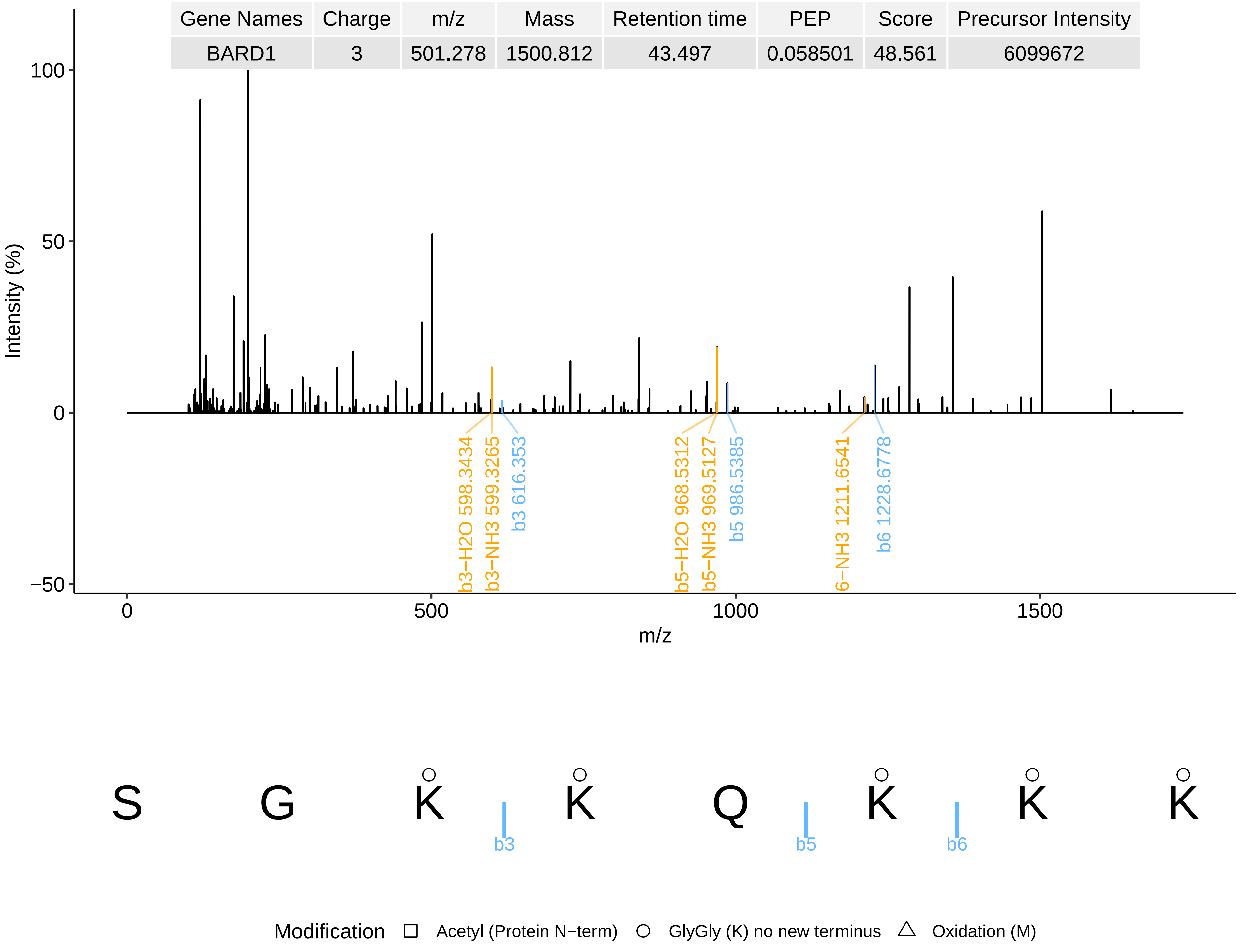

20200921_KIZ_MUb4_GlyGly_10plex − Scan number: 27348

Gene Names	Charge	m/z	Mass	Retention time	PEP	Score	Precursor Intensity
IFNGR1	3	513.6591	1537.956	99.804	0.0016836	80.312	5142565

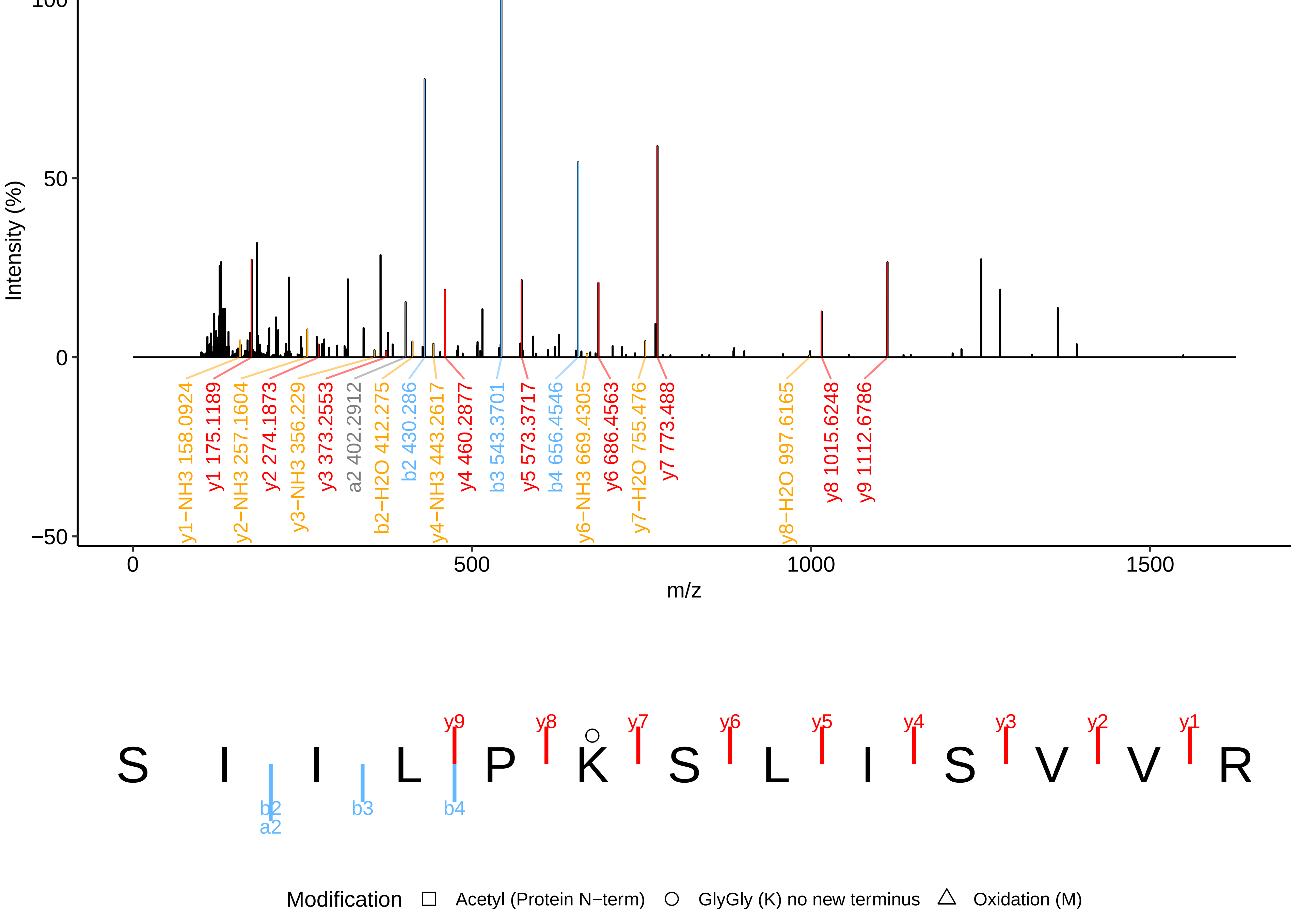

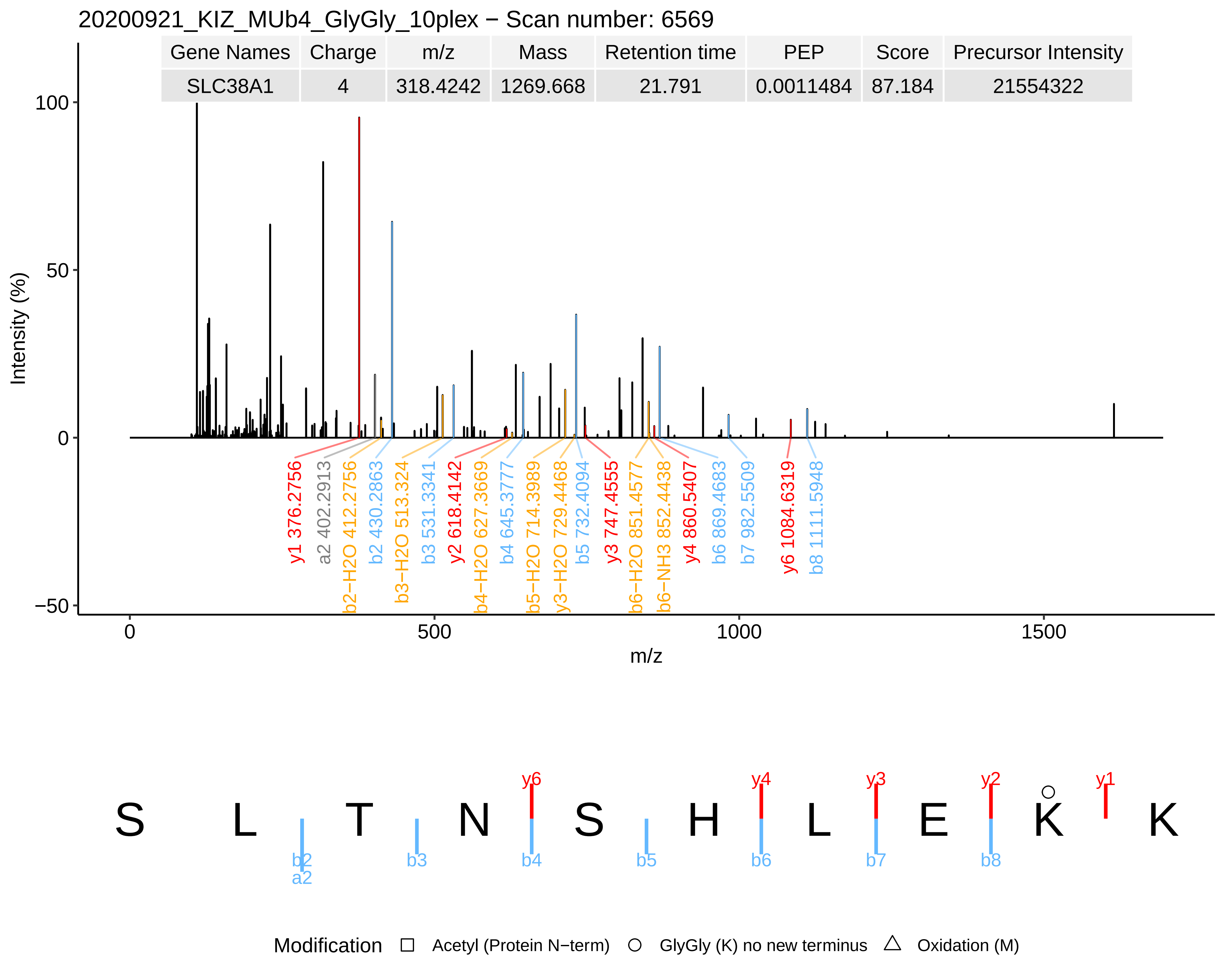

20200921_KIZ_MUb4_GlyGly_10plex − Scan number: 6569
Gene Names | Charge | m/z | Mass | Retention time | PEP | Score | Precursor Intensity
SLC38A1 | 4 | 318.4242 | 1269.668 | 21.791 | 0.0011484 | 87.184 | 21554322
Intensity (%)
m/z
y1 376.2756
a2 402.2913
b2−H2O 412.2756
b2 430.2863
b3−H2O 513.324
b3 531.3341
y2 618.4142
b4−H2O 627.3669
b4 645.3777
b5−H2O 714.3989
y3−H2O 729.4468
b5 732.4094
y3 747.4555
b6−H2O 851.4577
b6−NH3 852.4438
y4 860.5407
b6 869.4683
b7 982.5509
y6 1084.6319
b8 1111.5948
S L T N S H L E K K
y6 y4 y3 y2 y1
b2 a2 b3 b4 b5 b6 b7 b8
Modification
Acetyl (Protein N−term)
GlyGly (K) no new terminus
Oxidation (M)

20200921_KIZ_MUb4_GlyGly_10plex − Scan number: 6577

Gene Names	Charge	m/z	Mass	Retention time	PEP	Score	Precursor Intensity
SLC38A1	3	424.2298	1269.668	21.817	0.00063487	97.813	5126790

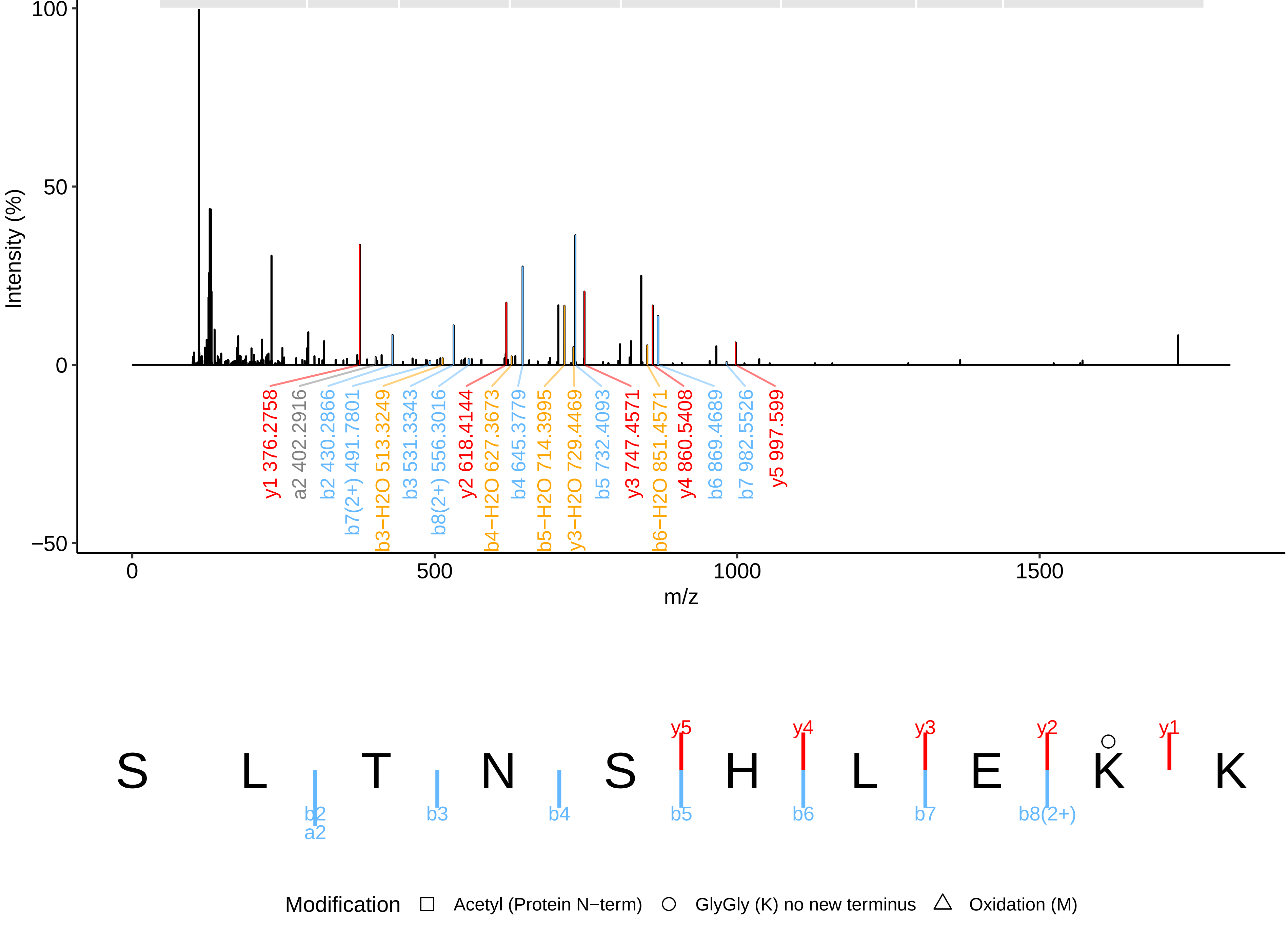

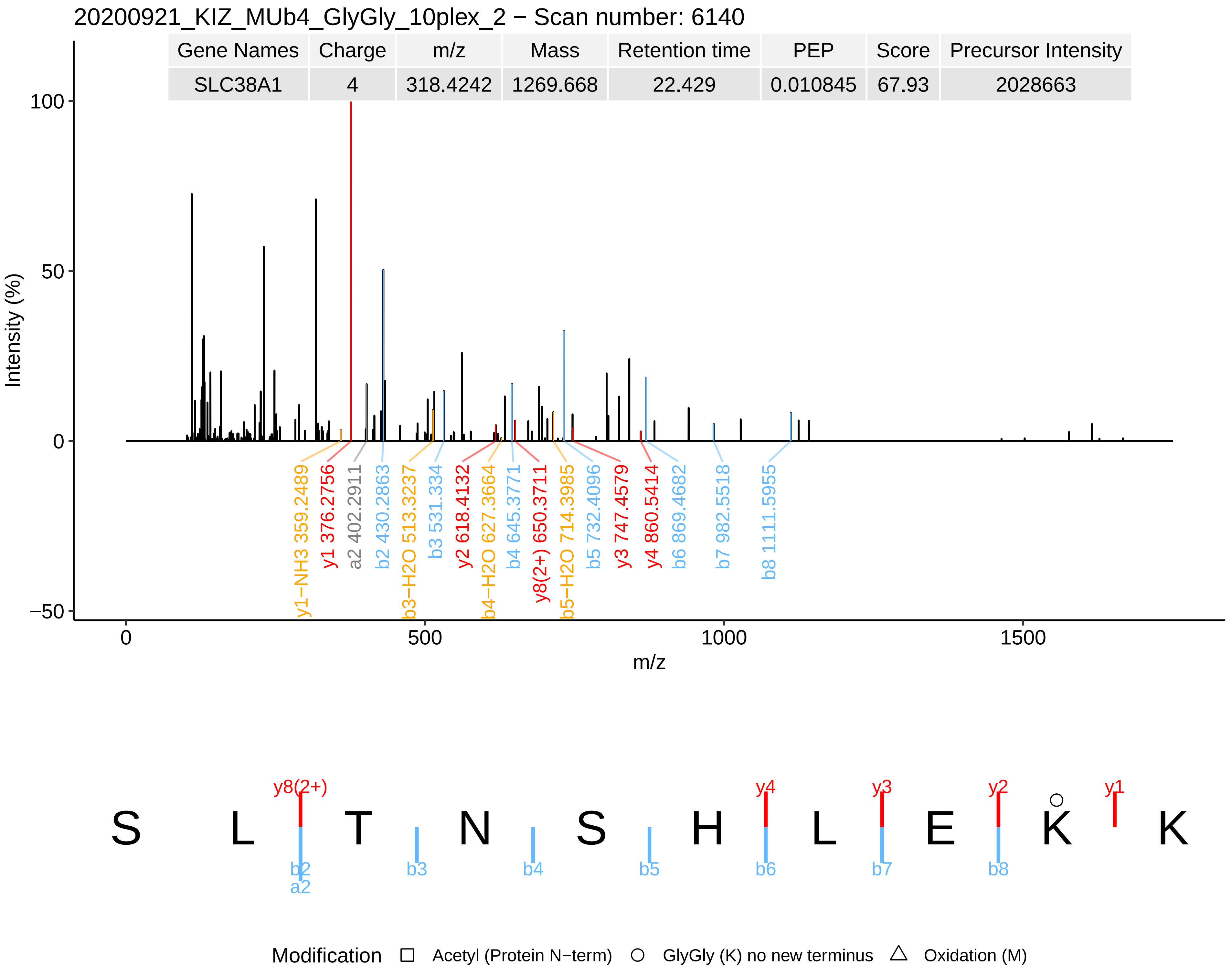

20200921_KIZ_MUb4_GlyGly_10plex_2 − Scan number: 6140
Gene Names | Charge | m/z | Mass | Retention time | PEP | Score | Precursor Intensity
SLC38A1 | 4 | 318.4242 | 1269.668 | 22.429 | 0.010845 | 67.93 | 2028663
Intensity (%)
100
50
0
−50
0
500
1000
1500
m/z
y1−NH3 359.2489
y1 376.2756
a2 402.2911
b2 430.2863
b3−H2O 513.3237
b3 531.334
y2 618.4132
b4−H2O 627.3664
b4 645.3771
y8(2+) 650.3711
b5−H2O 714.3985
b5 732.4096
y3 747.4579
y4 860.5414
b6 869.4682
b7 982.5518
b8 1111.5955
S L T N S H L E K K
y8(2+) y4 y3 y2 y1
b2 a2 b3 b4 b5 b6 b7 b8
Modification
Acetyl (Protein N−term)
GlyGly (K) no new terminus
Oxidation (M)

20200921_KIZ_MUb4_GlyGly_10plex − Scan number: 5883

Gene Names	Charge	m/z	Mass	Retention time	PEP	Score	Precursor Intensity
PLGRKT	3	437.5324	1309.575	19.298	0.0057072	77.533	12576838

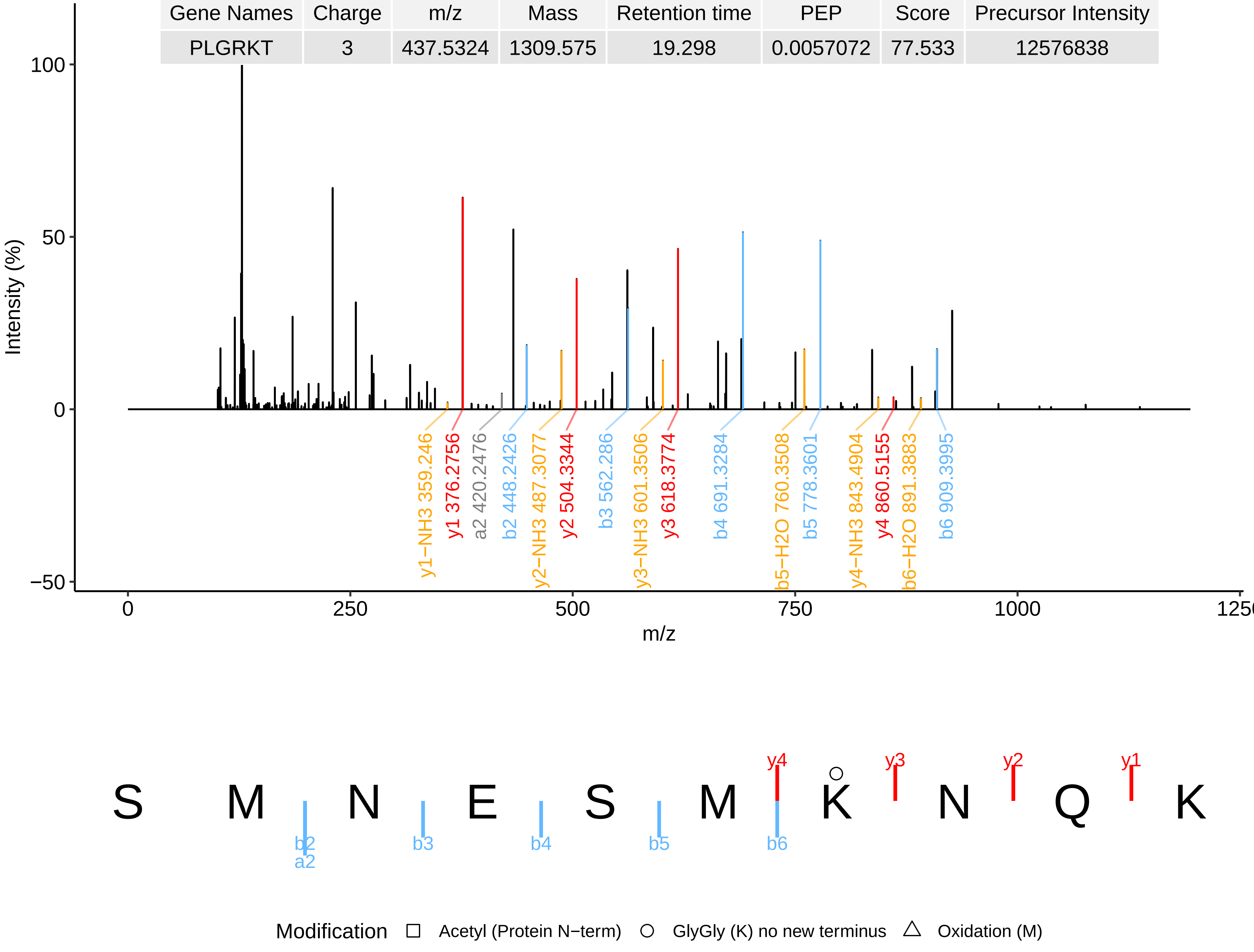

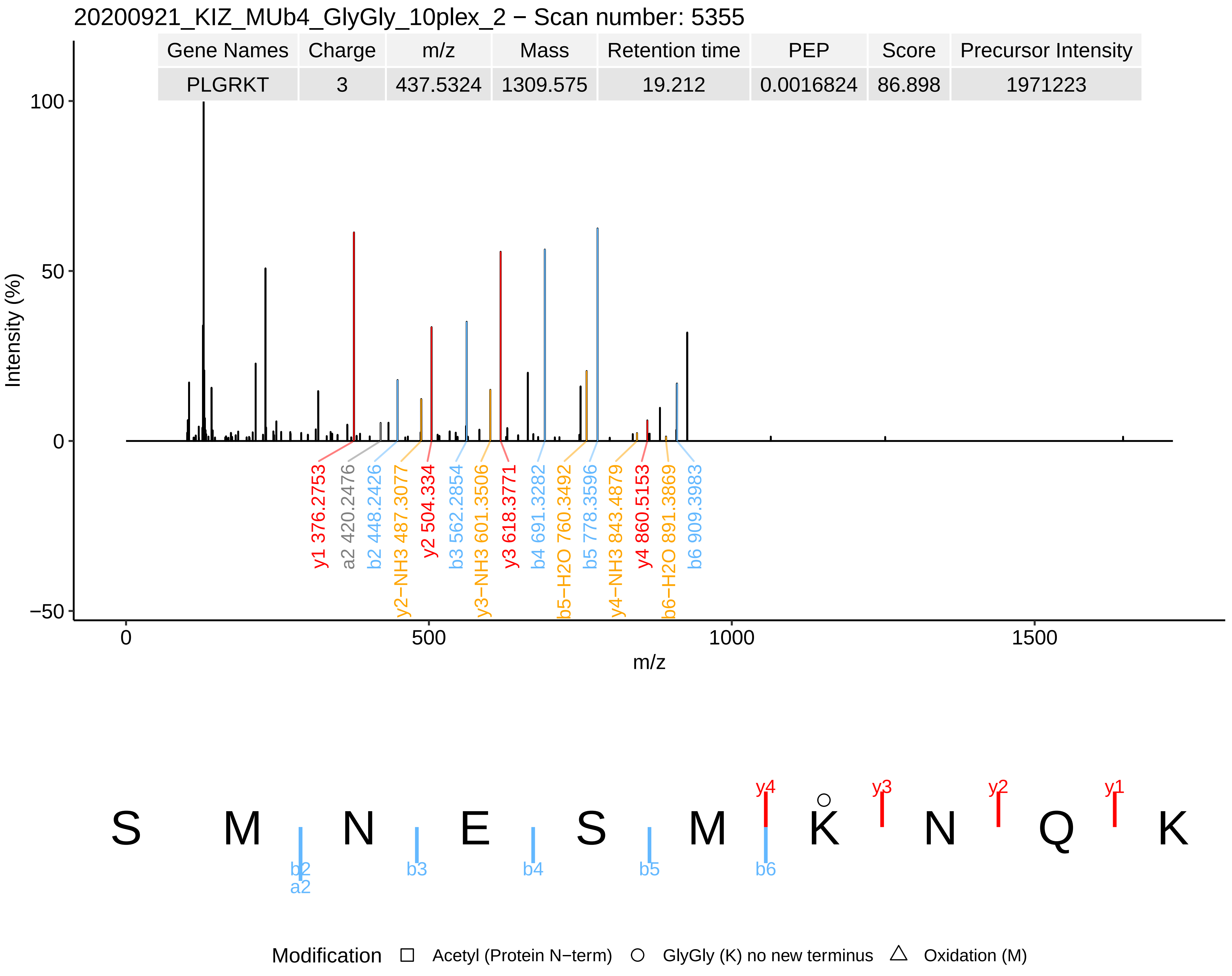
20200921_KIZ_MUb4_GlyGly_10plex_2 − Scan number: 5355

Gene Names	Charge	m/z	Mass	Retention time	PEP	Score	Precursor Intensity
PLGRKT	3	437.5324	1309.575	19.212	0.0016824	86.898	1971223

Intensity (%)
m/z
y1 376.2753
a2 420.2476
b2 448.2426
y2−NH3 487.3077
y2 504.334
b3 562.2854
y3−NH3 601.3506
y3 618.3771
b4 691.3282
b5−H2O 760.3492
b5 778.3596
y4−NH3 843.4879
y4 860.5153
b6−H2O 891.3869
b6 909.3983
S M N E S M K N Q K
Modification
Acetyl (Protein N−term)
GlyGly (K) no new terminus
Oxidation (M)

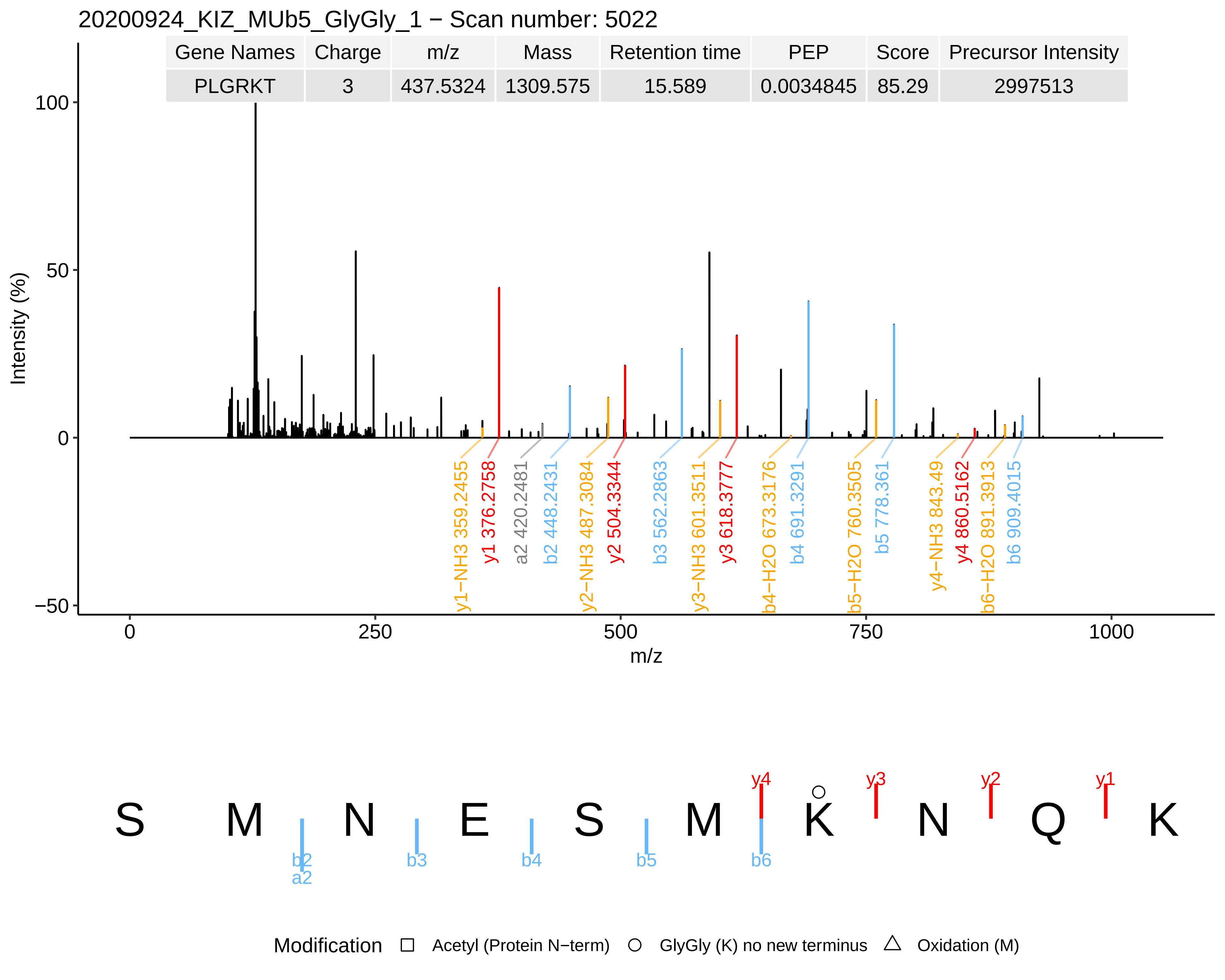
20200924_KIZ_MUb5_GlyGly_1 − Scan number: 5022
Gene Names | Charge | m/z | Mass | Retention time | PEP | Score | Precursor Intensity
PLGRKT | 3 | 437.5324 | 1309.575 | 15.589 | 0.0034845 | 85.29 | 2997513
Intensity (%)
m/z
y1−NH3 359.2455
y1 376.2758
a2 420.2481
b2 448.2431
y2−NH3 487.3084
y2 504.3344
b3 562.2863
y3−NH3 601.3511
y3 618.3777
b4−H2O 673.3176
b4 691.3291
b5−H2O 760.3505
b5 778.361
y4−NH3 843.49
y4 860.5162
b6−H2O 891.3913
b6 909.4015
S M N E S M K N Q K
y4 y3 y2 y1
b2 a2 b3 b4 b5 b6
Modification
Acetyl (Protein N−term)
GlyGly (K) no new terminus
Oxidation (M)

20200924_KIZ_MUb5_GlyGly_2 − Scan number: 4798

Gene Names	Charge	m/z	Mass	Retention time	PEP	Score	Precursor Intensity
PLGRKT	3	437.5324	1309.575	17.688	0.0086589	76.282	NA

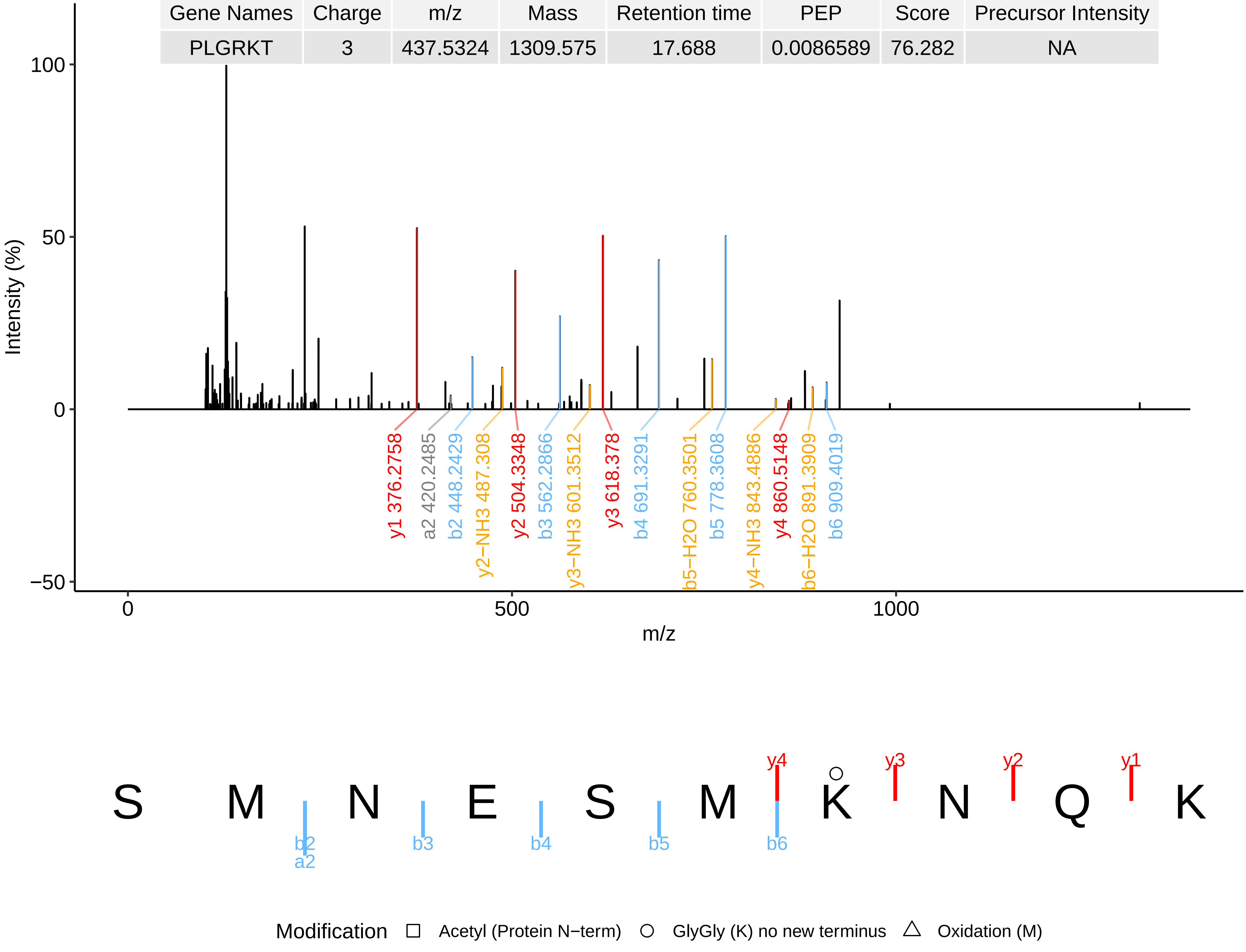

20200921_KIZ_MUb4_GlyGly_10plex − Scan number: 9074

Gene Names	Charge	m/z	Mass	Retention time	PEP	Score	Precursor Intensity
RAB1A	3	438.9174	1313.73	31.022	0.009469	71.176	6467472

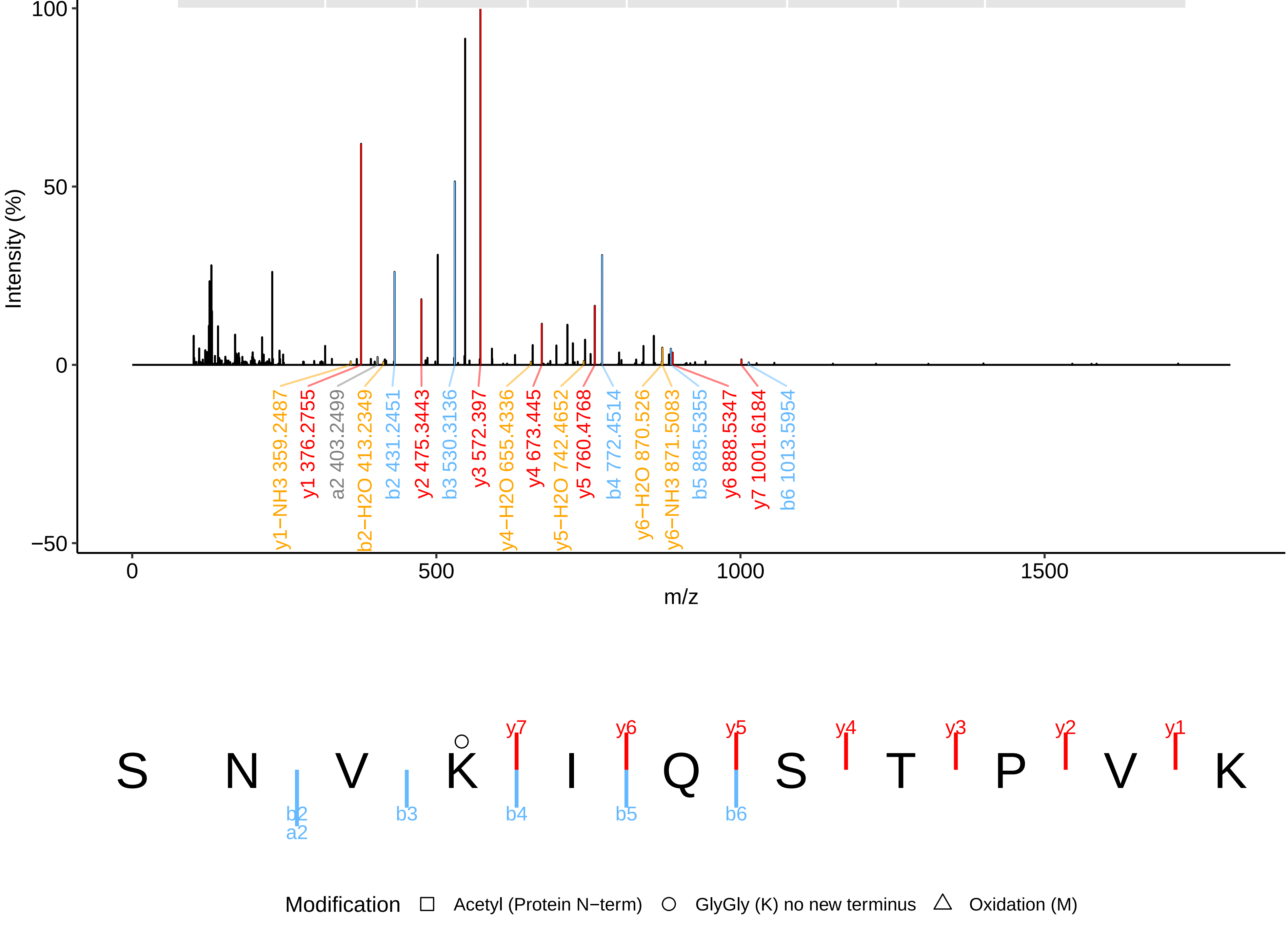

20200921_KIZ_MUb4_GlyGly_10plex_2 − Scan number: 8390

Gene Names	Charge	m/z	Mass	Retention time	PEP	Score	Precursor Intensity
RAB1A	3	438.9174	1313.73	31.594	0.10513	45.915	1559761

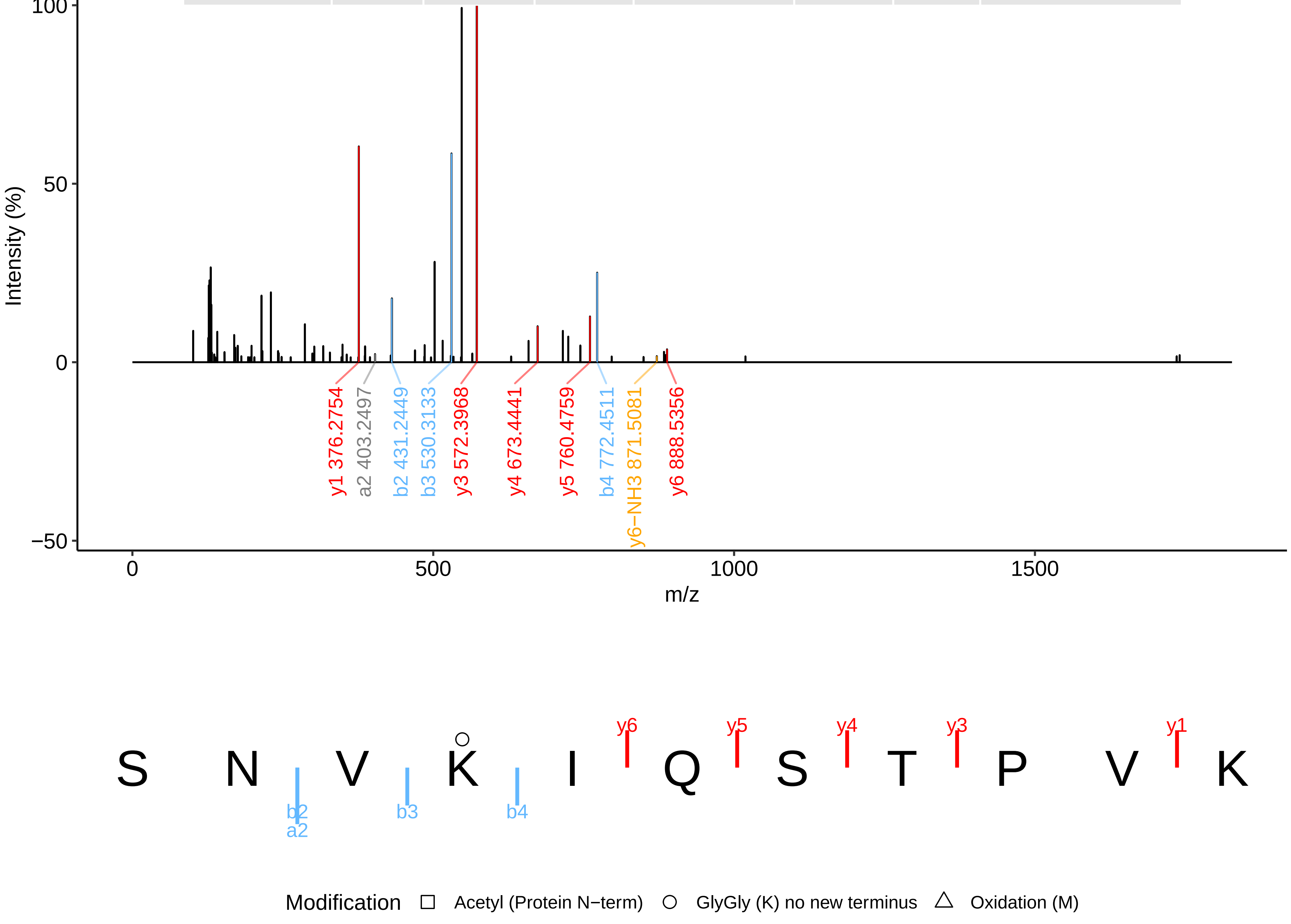

20200921_KIZ_MUb4_GlyGly_10plex − Scan number: 7646

Gene Names	Charge	m/z	Mass	Retention time	PEP	Score	Precursor Intensity
IGF2R	3	426.2104	1275.61	25.725	0.07139	53.237	4144347

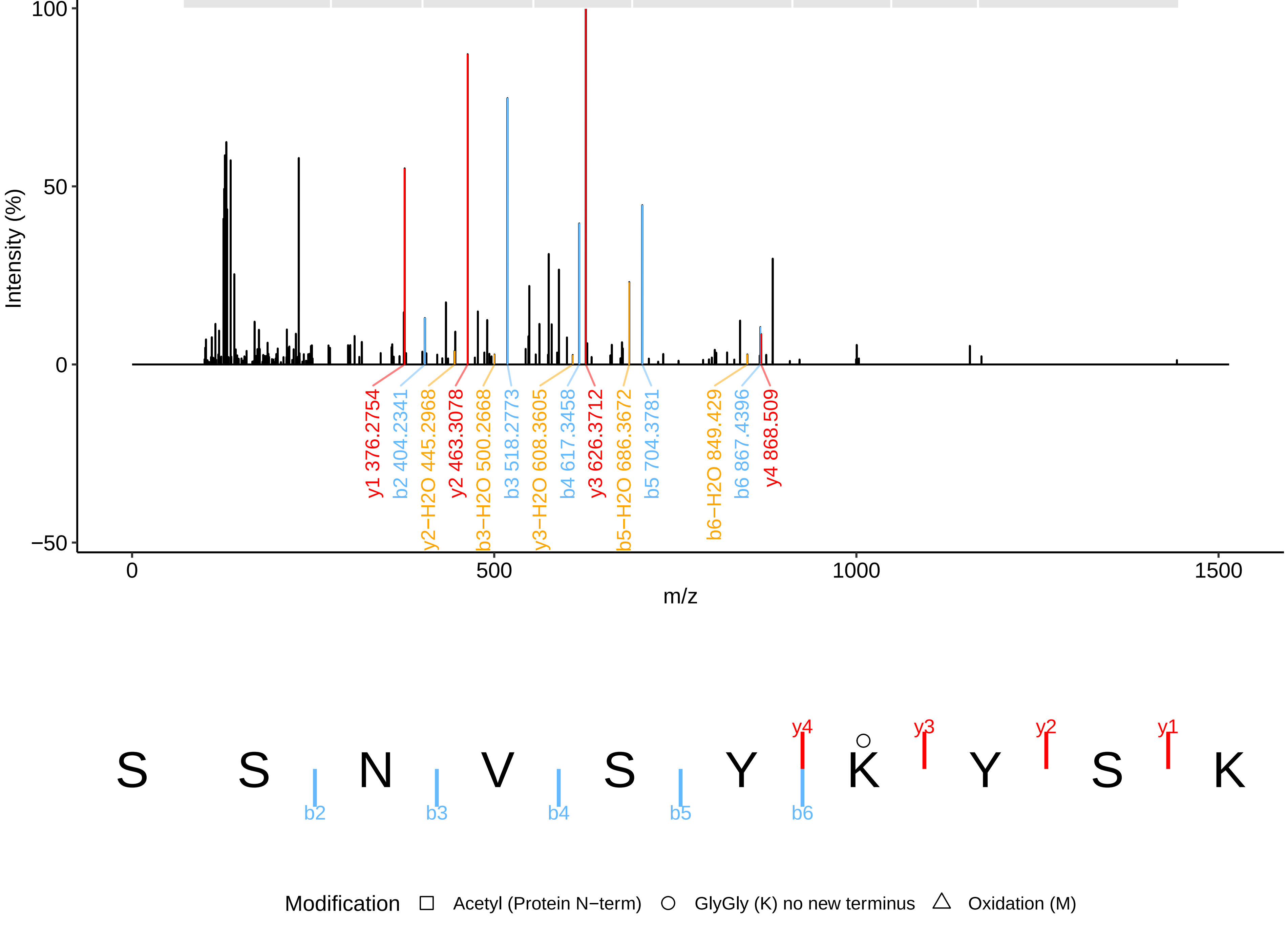

20200921_KIZ_MUb4_GlyGly_10plex_2 − Scan number: 7046

Gene Names	Charge	m/z	Mass	Retention time	PEP	Score	Precursor Intensity
IGF2R	3	426.2104	1275.61	26.156	0.023974	62.466	907424.4

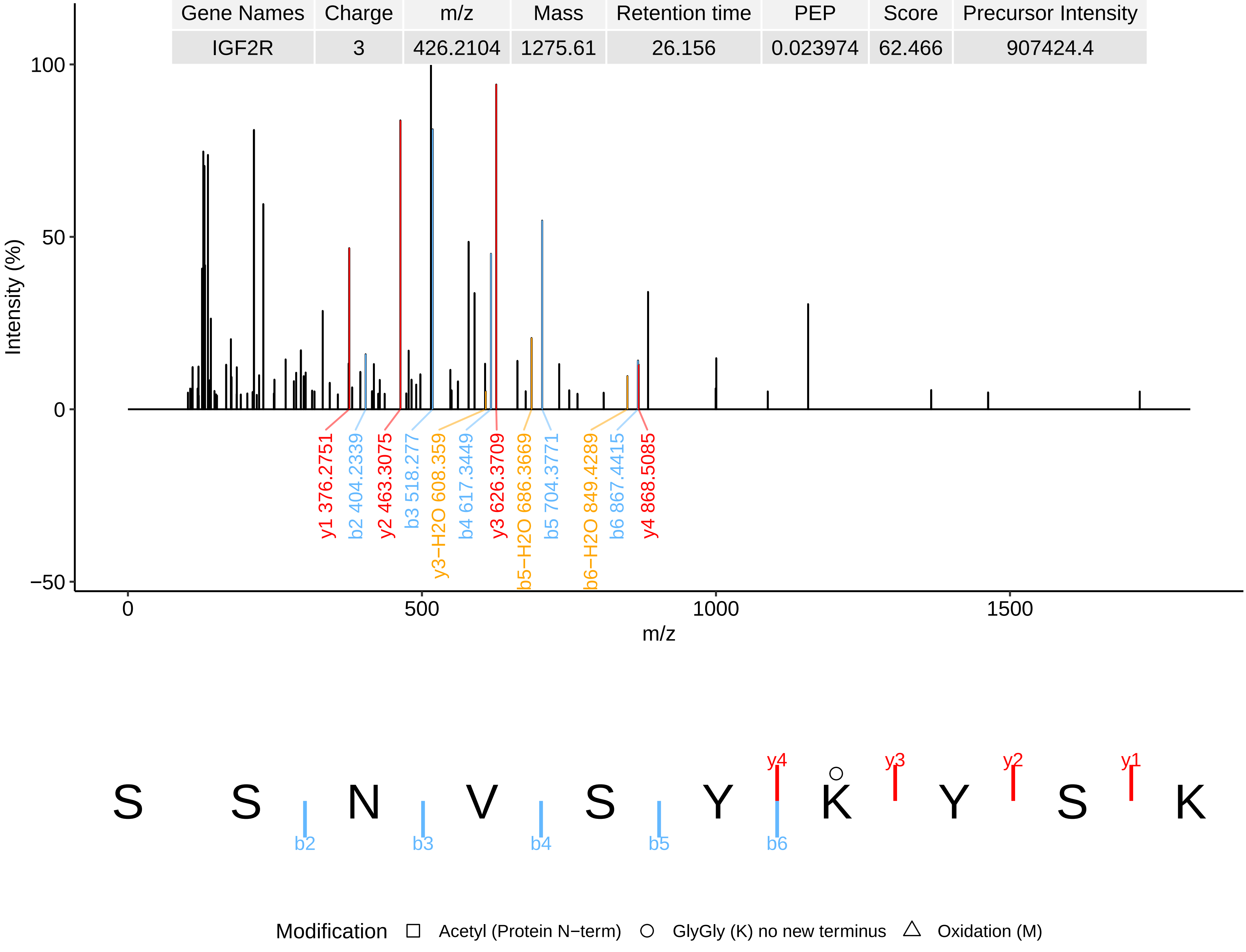

20200921_KIZ_MUb4_GlyGly_10plex_2 − Scan number: 7061

Gene Names	Charge	m/z	Mass	Retention time	PEP	Score	Precursor Intensity
IGF2R	3	426.2104	1275.61	26.216	0.094492	48.741	1488560

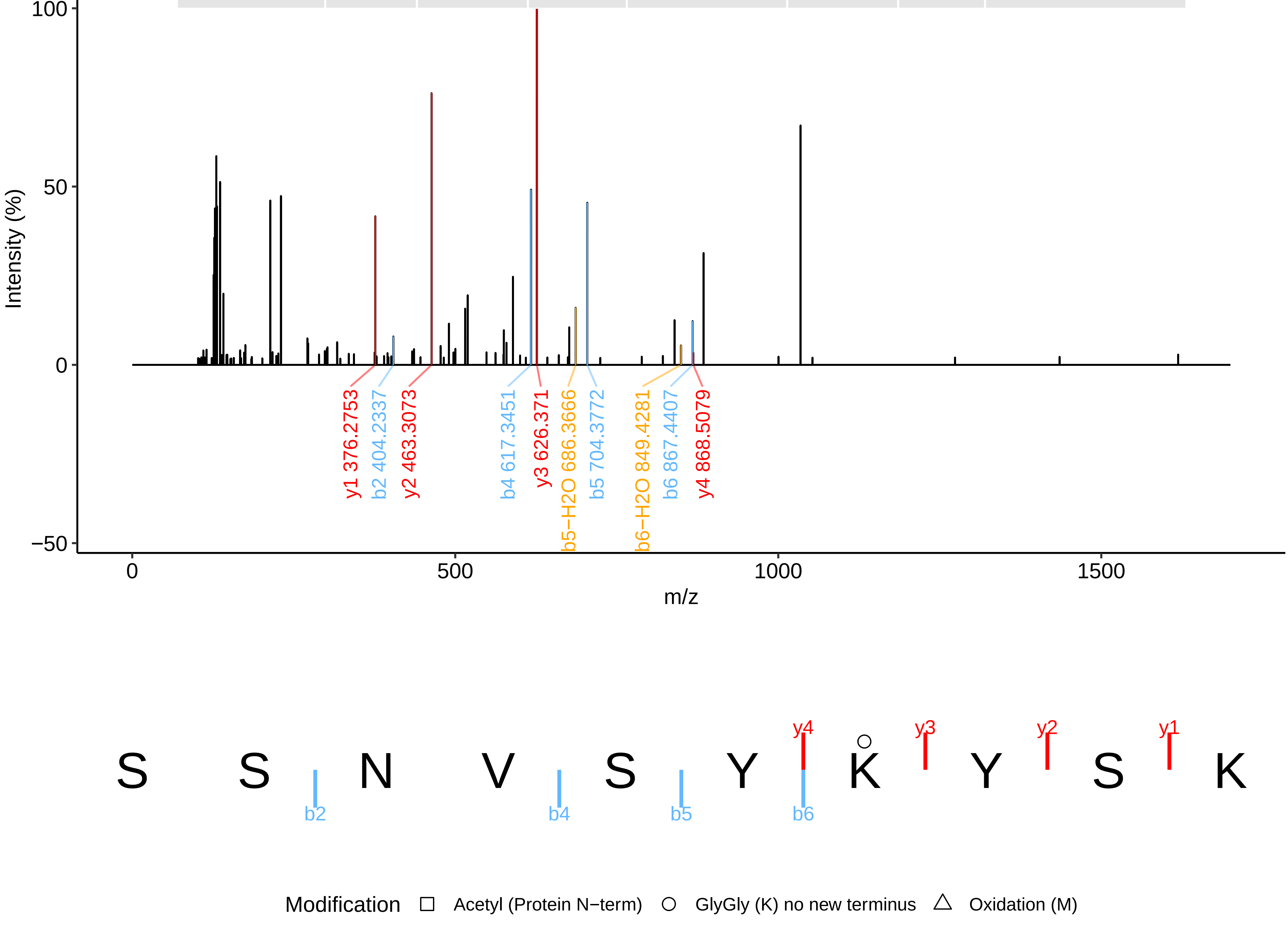

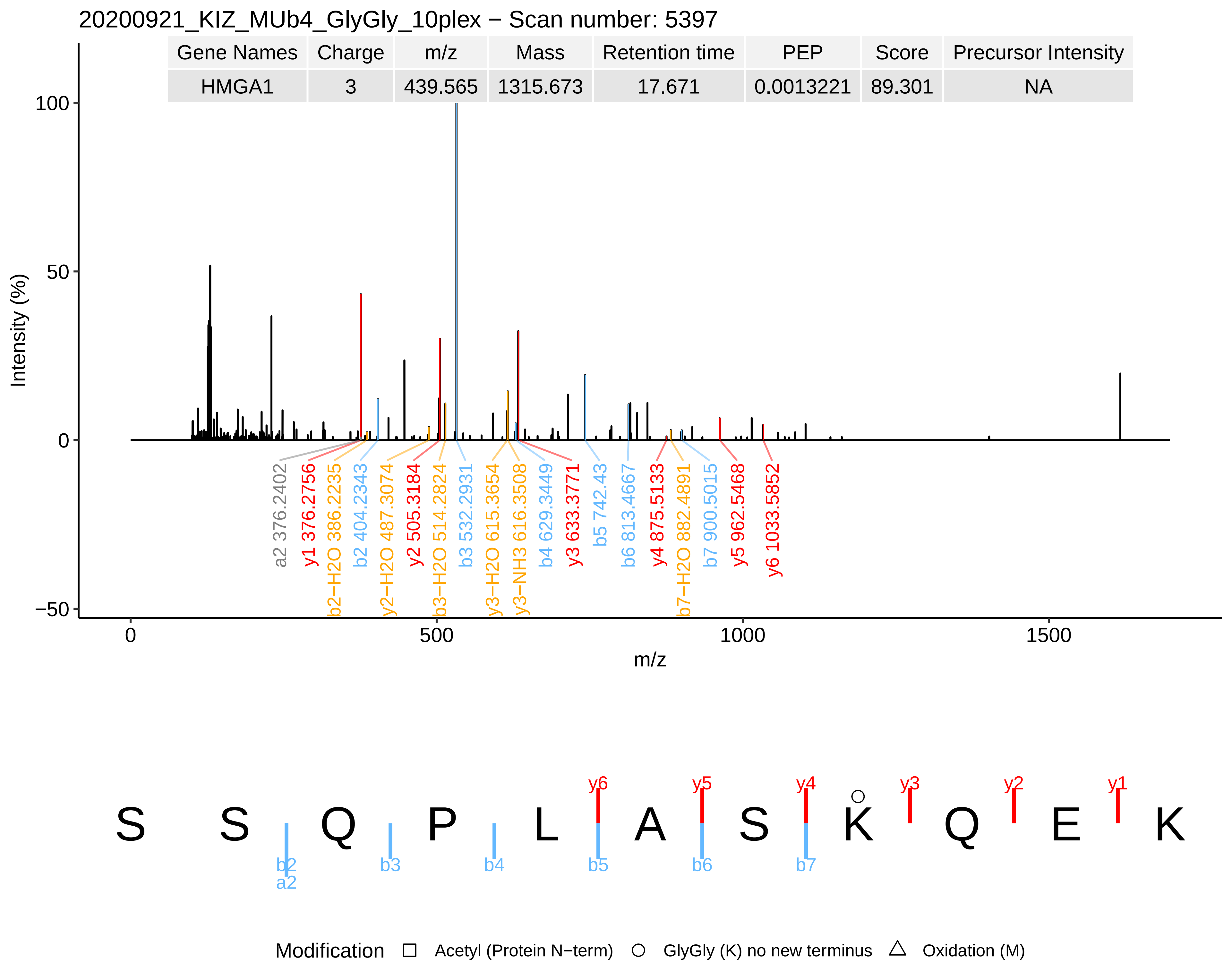
20200921_KIZ_MUb4_GlyGly_10plex − Scan number: 5397
Gene Names
Charge
m/z
Mass
Retention time
PEP
Score
Precursor Intensity
HMGA1
3
439.565
1315.673
17.671
0.0013221
89.301
NA
Intensity (%)
m/z
a2 376.2402
y1 376.2756
b2−H2O 386.2235
b2 404.2343
y2−H2O 487.3074
y2 505.3184
b3−H2O 514.2824
b3 532.2931
y3−H2O 615.3654
y3−NH3 616.3508
b4 629.3449
y3 633.3771
b5 742.43
b6 813.4667
y4 875.5133
b7−H2O 882.4891
b7 900.5015
y5 962.5468
y6 1033.5852
S S Q P L A S K Q E K
Modification
Acetyl (Protein N−term)
GlyGly (K) no new terminus
Oxidation (M)

20201118_KIZ_MUb6_GlyGly_1 − Scan number: 2837

Gene Names	Charge	m/z	Mass	Retention time	PEP	Score	Precursor Intensity
TMEM87A	3	435.2128	1302.616	8.686	0.00091082	89.032	3671074

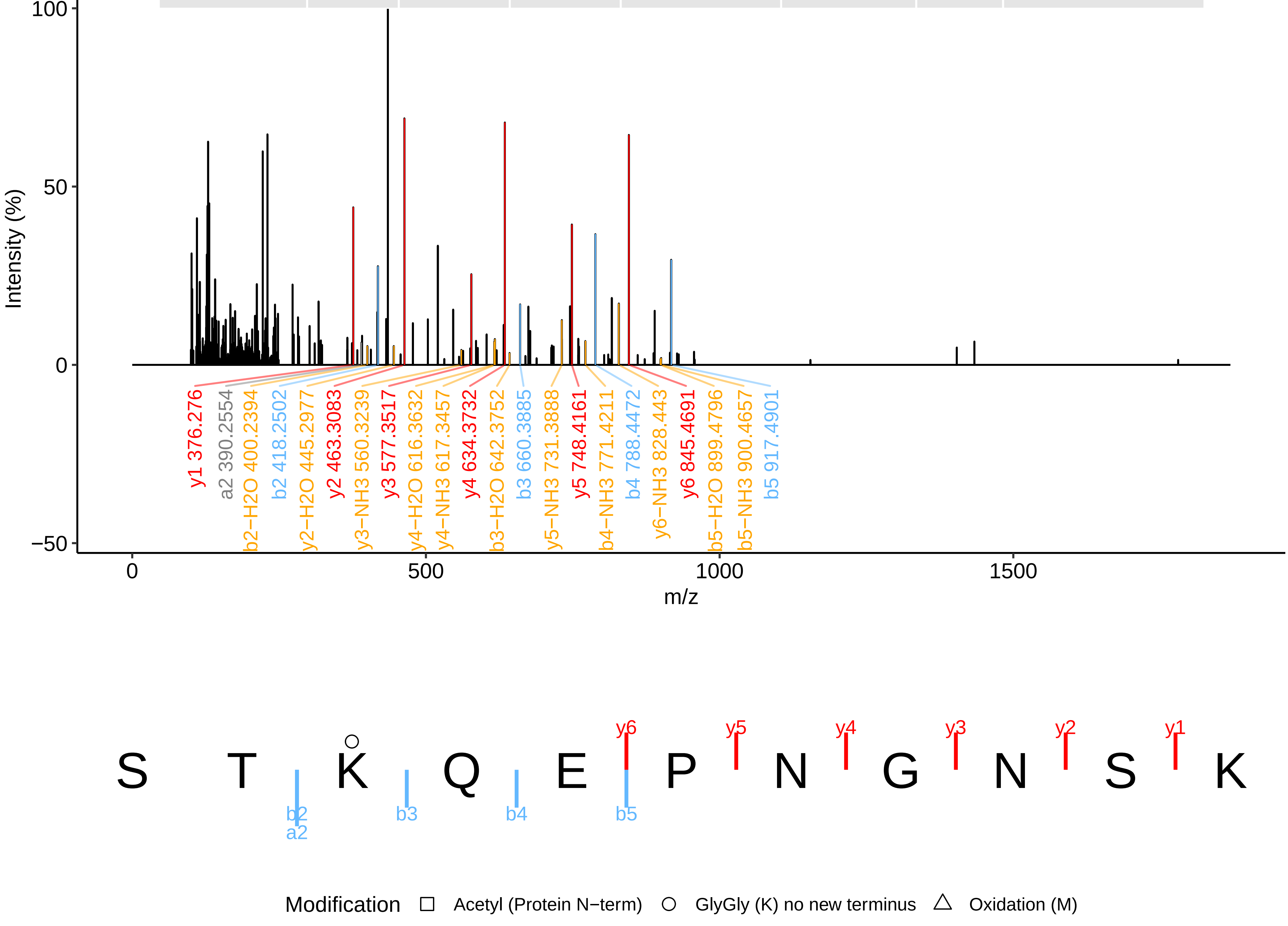

20200921_KIZ_MUb4_GlyGly_10plex_2 − Scan number: 15979

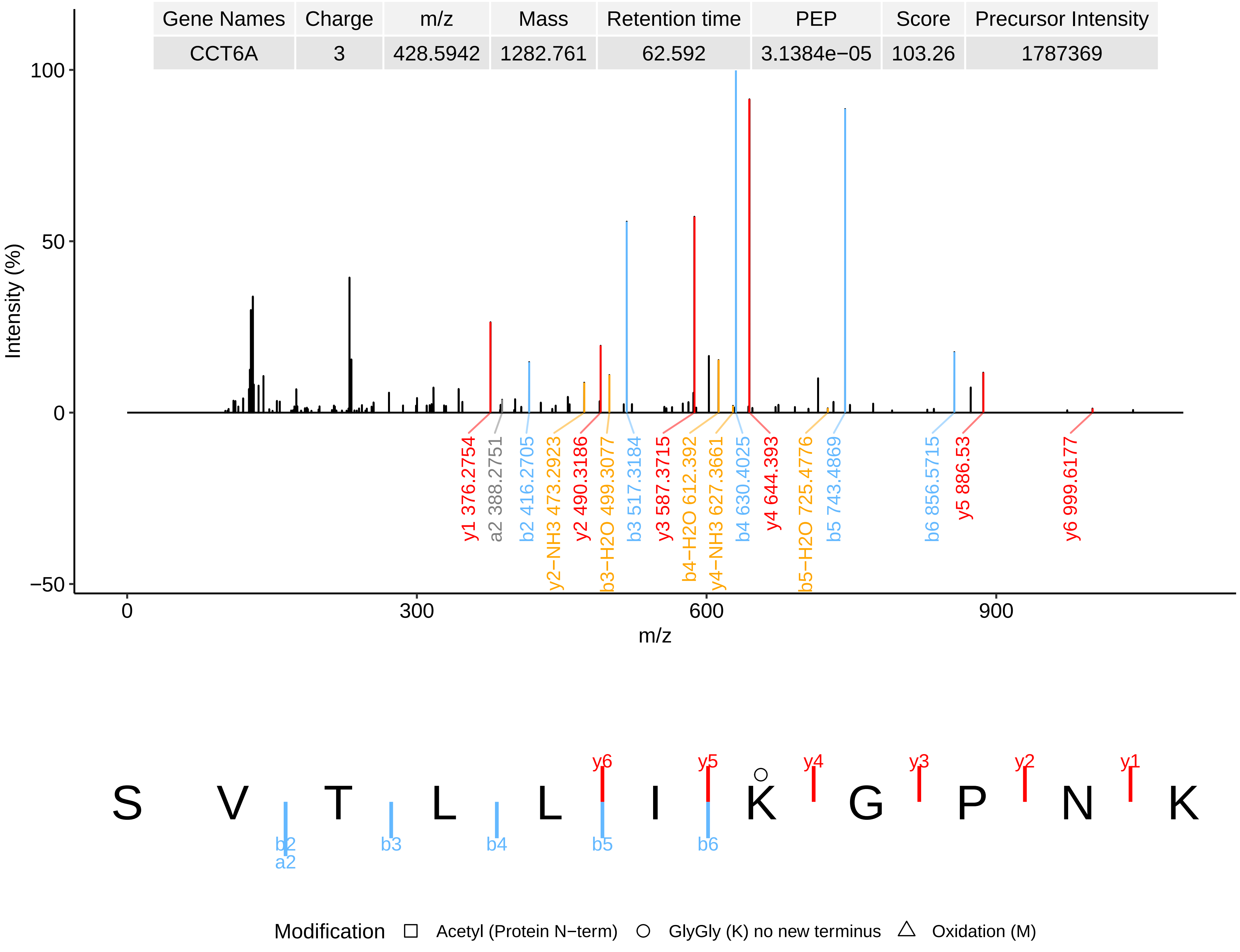

20200921_KIZ_MUb4_GlyGly_10plex − Scan number: 12728

Gene Names	Charge	m/z	Mass	Retention time	PEP	Score	Precursor Intensity
CCT3	3	422.8716	1265.593	44.725	0.024815	62.303	4125818

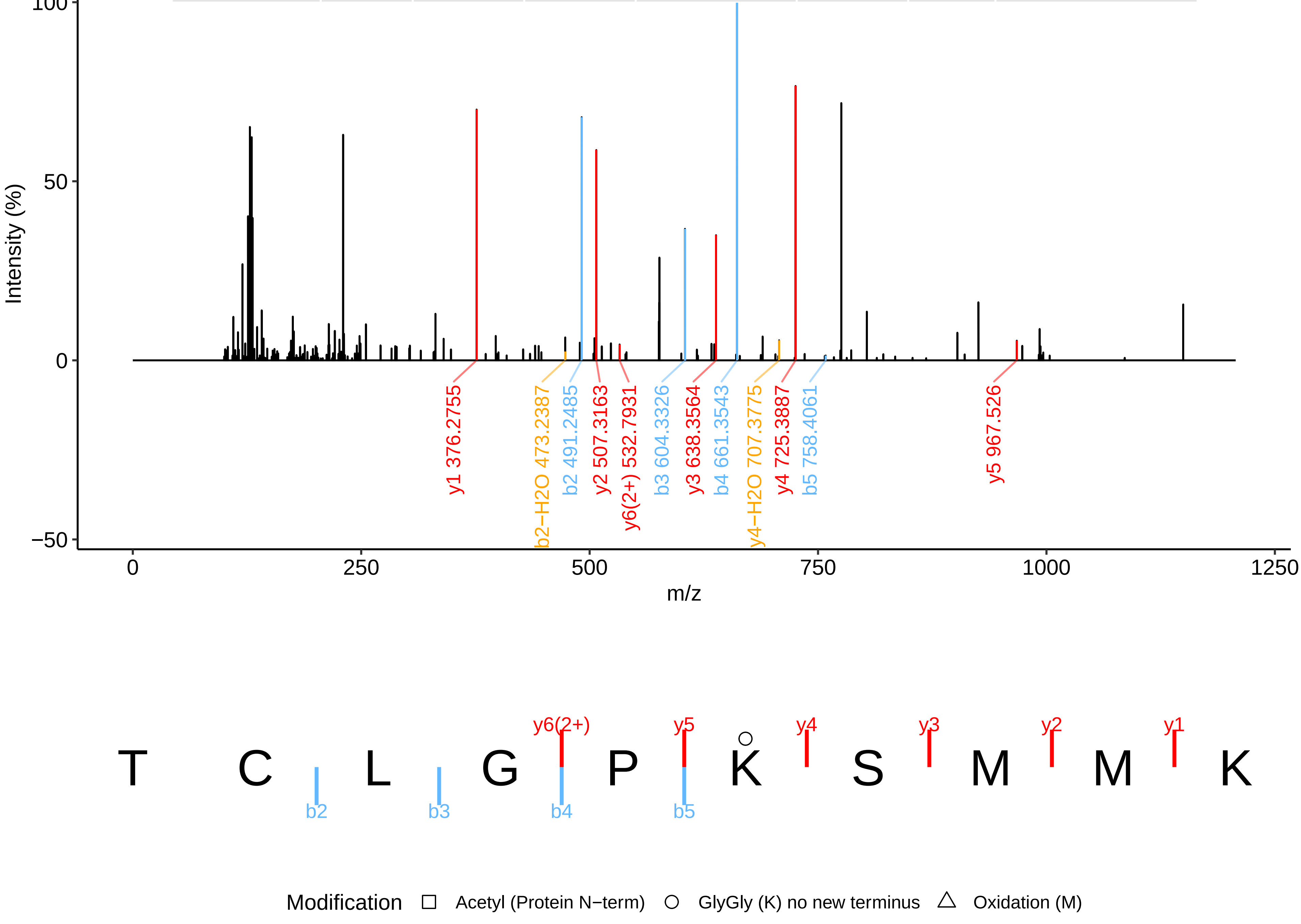

20200924_KIZ_MUb5_GlyGly_1 − Scan number: 11664

Gene Names	Charge	m/z	Mass	Retention time	PEP	Score	Precursor Intensity
CCT3	3	422.8716	1265.593	37.833	0.012188	73.067	16607212

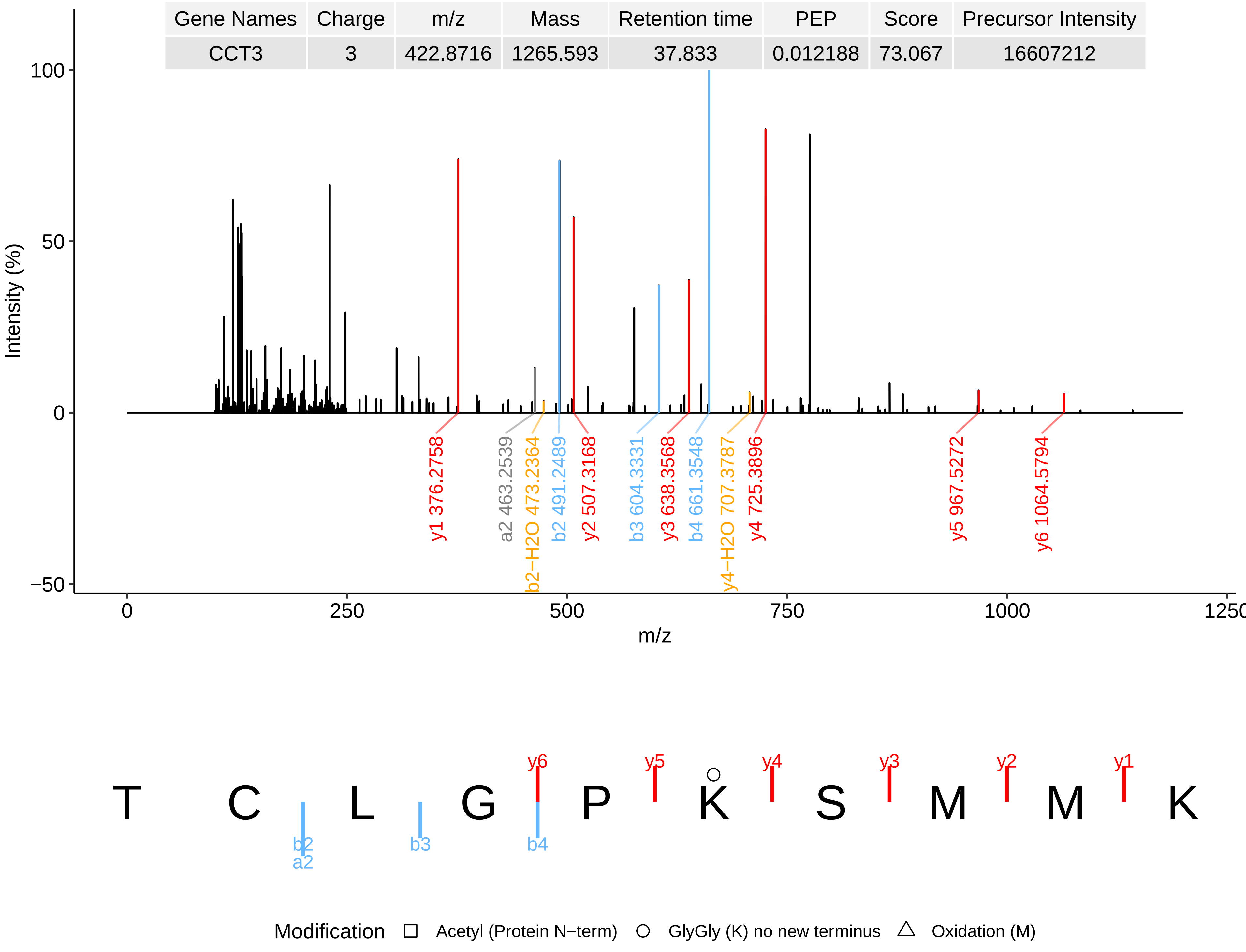

20200921_KIZ_MUb4_GlyGly_10plex_2 − Scan number: 4107

Gene Names	Charge	m/z	Mass	Retention time	PEP	Score	Precursor Intensity
RNF167	4	378.1908	1508.734	14.223	0.0013953	80.916	3143615

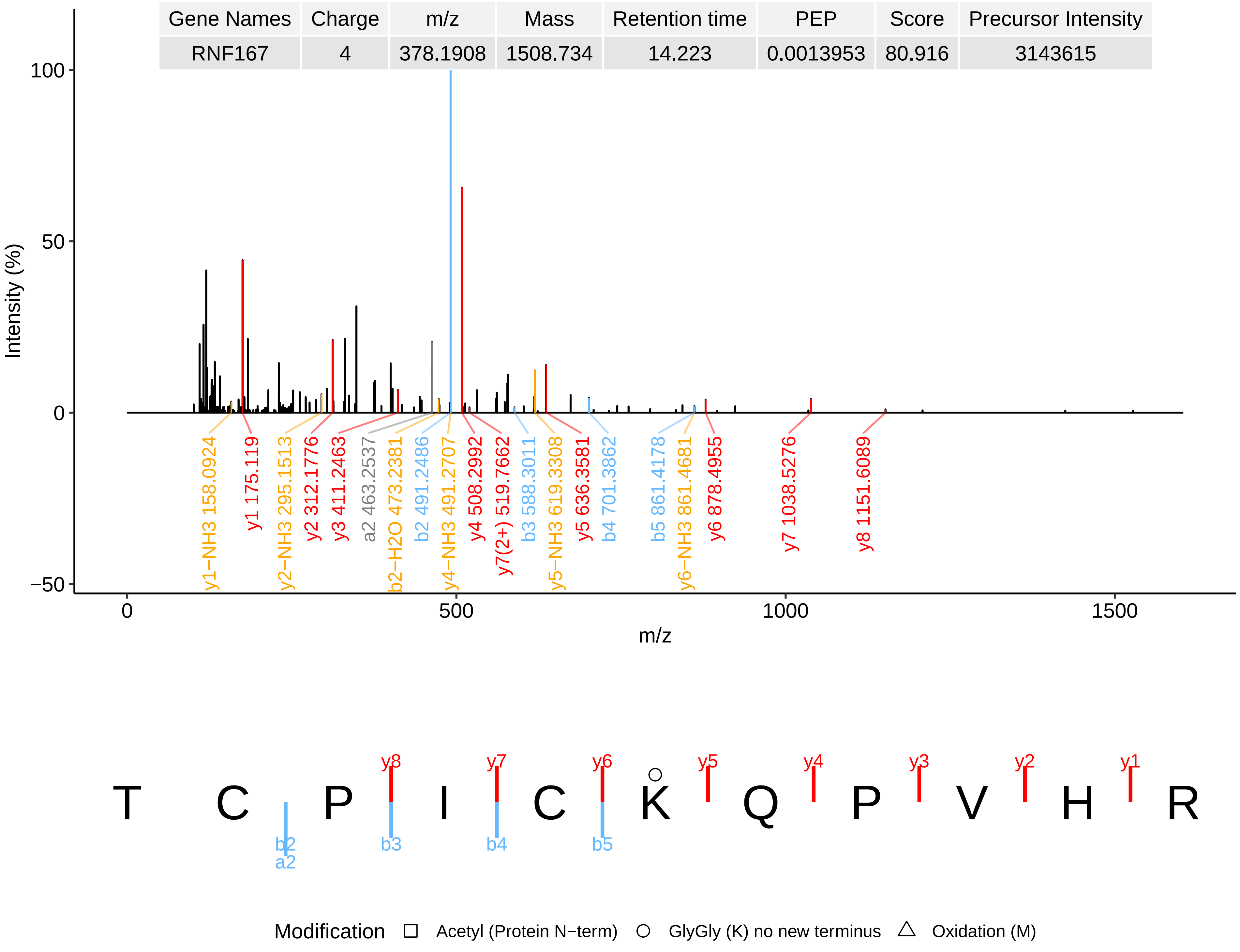

20201118_KIZ_MUb6_GlyGly_1 − Scan number: 3526

Gene Names	Charge	m/z	Mass	Retention time	PEP	Score	Precursor Intensity
RNF167	4	378.1908	1508.734	10.818	0.00040865	93.325	27478642

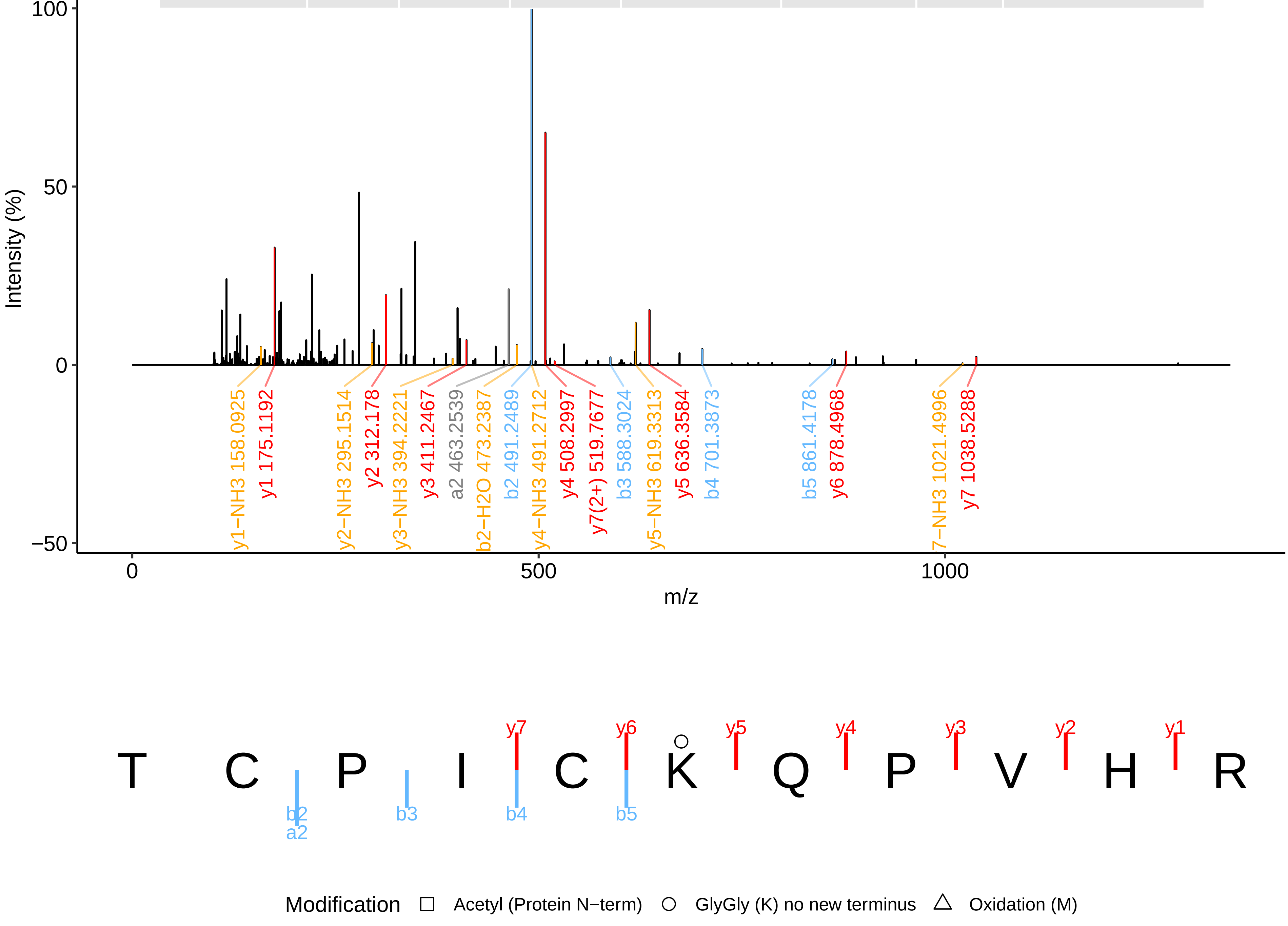

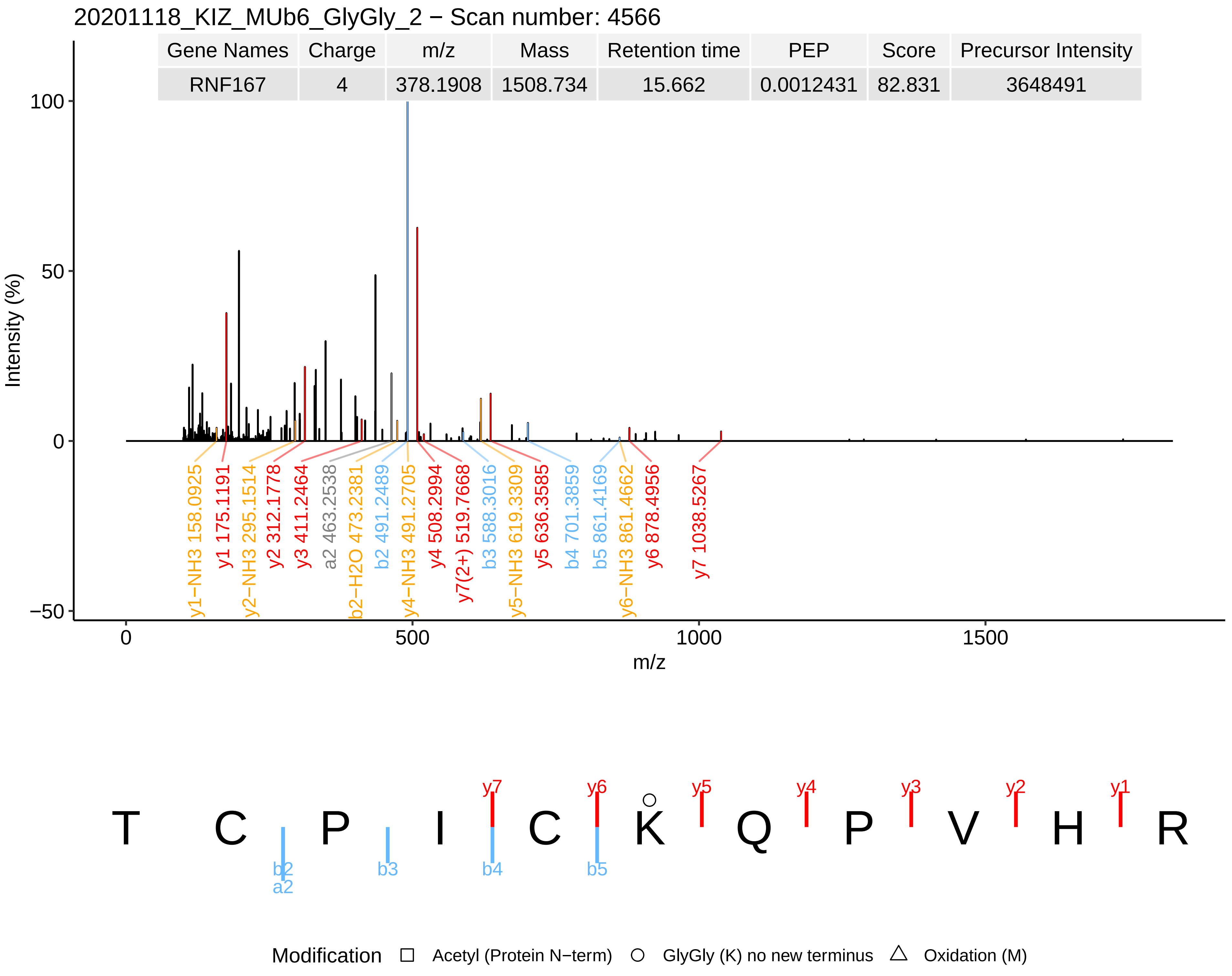

20201118_KIZ_MUb6_GlyGly_2 − Scan number: 4566
Gene Names
Charge
m/z
Mass
Retention time
PEP
Score
Precursor Intensity
RNF167
4
378.1908
1508.734
15.662
0.0012431
82.831
3648491
Intensity (%)
m/z
y1−NH3 158.0925
y1 175.1191
y2−NH3 295.1514
y2 312.1778
y3 411.2464
a2 463.2538
b2−H2O 473.2381
b2 491.2489
y4−NH3 491.2705
y4 508.2994
y7(2+) 519.7668
b3 588.3016
y5−NH3 619.3309
y5 636.3585
b4 701.3859
b5 861.4169
y6−NH3 861.4662
y6 878.4956
y7 1038.5267
T C P I C K Q P V H R
Modification
Acetyl (Protein N−term)
GlyGly (K) no new terminus
Oxidation (M)

20201118_KIZ_MUb6_GlyGly_1 − Scan number: 4387

Gene Names	Charge	m/z	Mass	Retention time	PEP	Score	Precursor Intensity
PAGE1	3	507.2317	1518.673	13.715	1.7712e−07	120.84	3663349

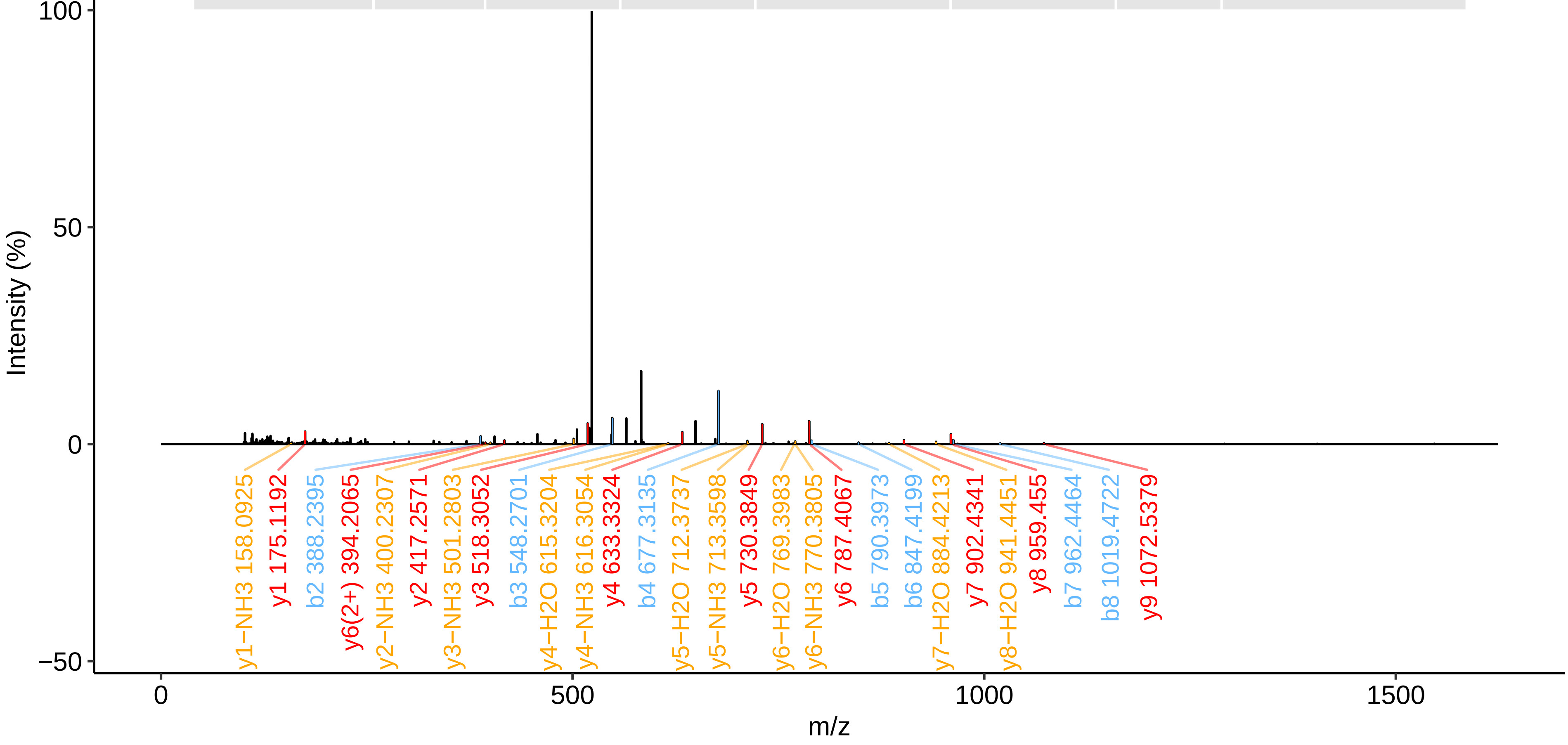

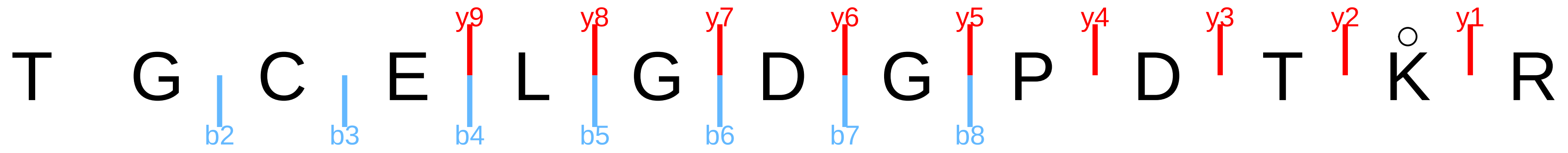

Modification □ Acetyl (Protein N−term) ○ GlyGly (K) no new terminus △ Oxidation (M)

20200921_KIZ_MUb4_GlyGly_10plex_2 − Scan number: 15649

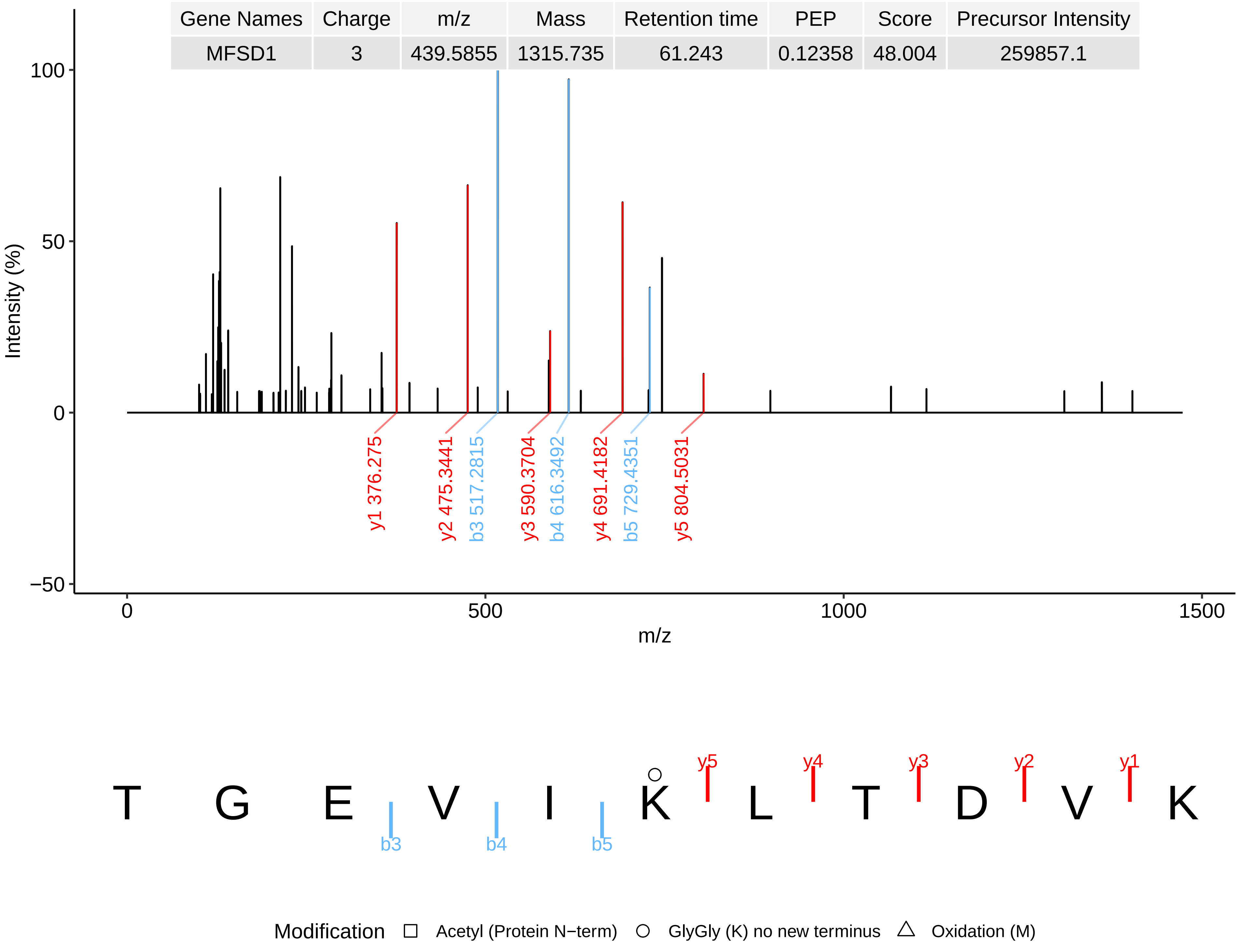

20200921_KIZ_MUb4_GlyGly_10plex − Scan number: 13985

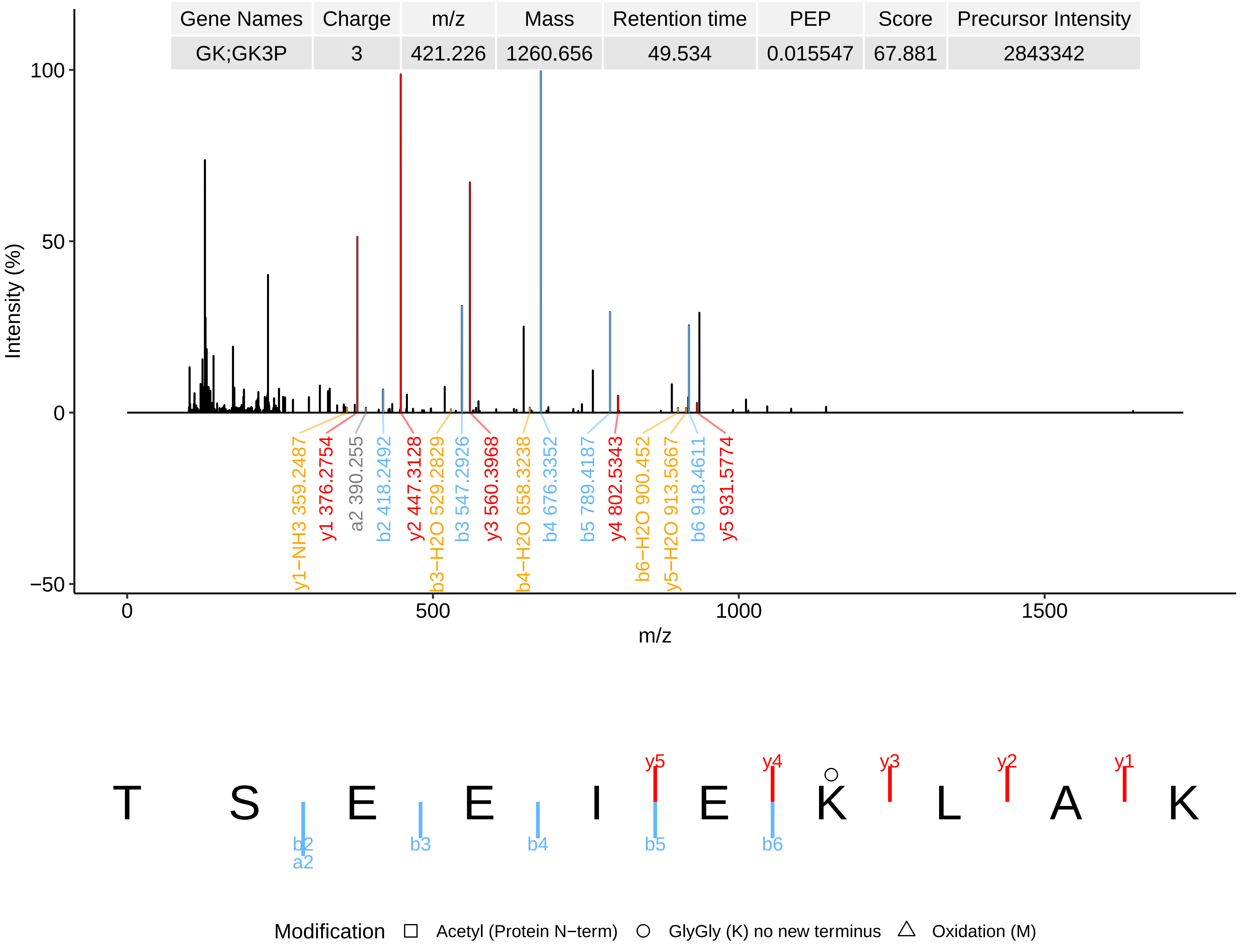

20200921_KIZ_MUb4_GlyGly_10plex_2 − Scan number: 12877

Gene Names	Charge	m/z	Mass	Retention time	PEP	Score	Precursor Intensity
GK;GK3P	3	421.226	1260.656	49.927	0.019199	64.297	998209.9

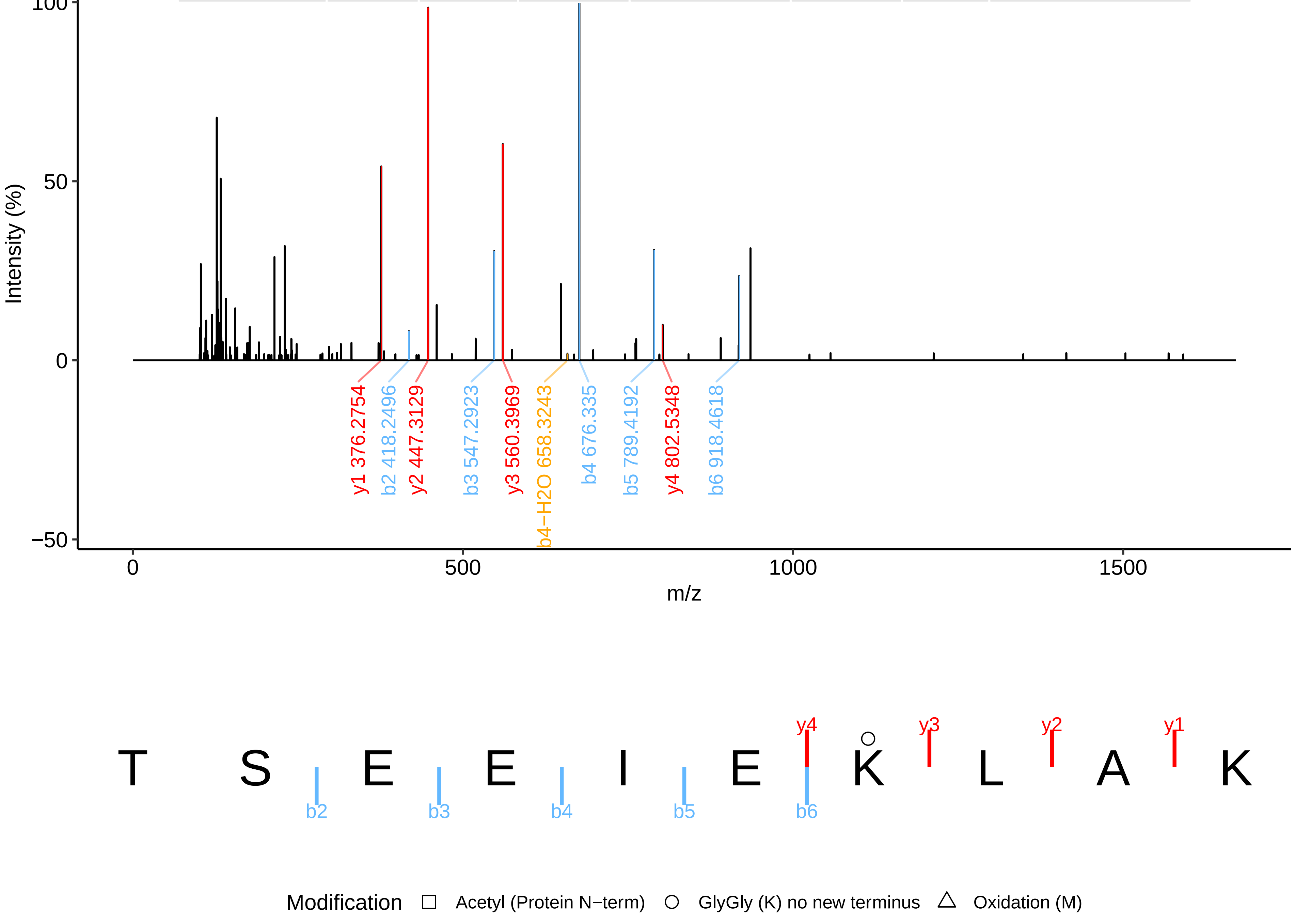

20200921_KIZ_MUb4_GlyGly_10plex − Scan number: 16725

Gene Names	Charge	m/z	Mass	Retention time	PEP	Score	Precursor Intensity
STIP1	3	424.8909	1271.651	60.17	0.0010043	91.584	7803855

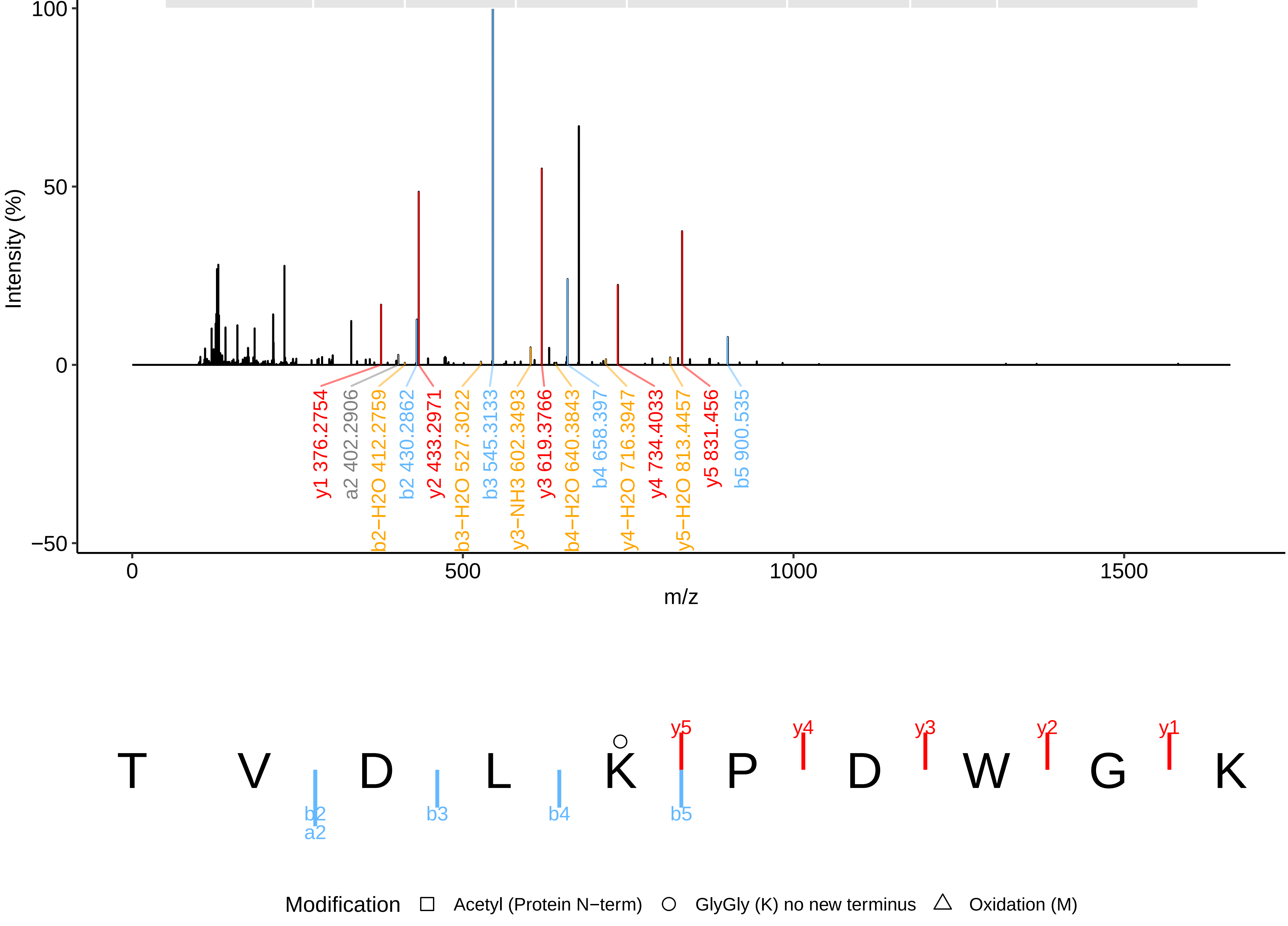

20200924_KIZ_MUb5_GlyGly_2 − Scan number: 15057

Gene Names	Charge	m/z	Mass	Retention time	PEP	Score	Precursor Intensity
STIP1	3	424.8909	1271.651	56.269	0.014515	68.893	NA

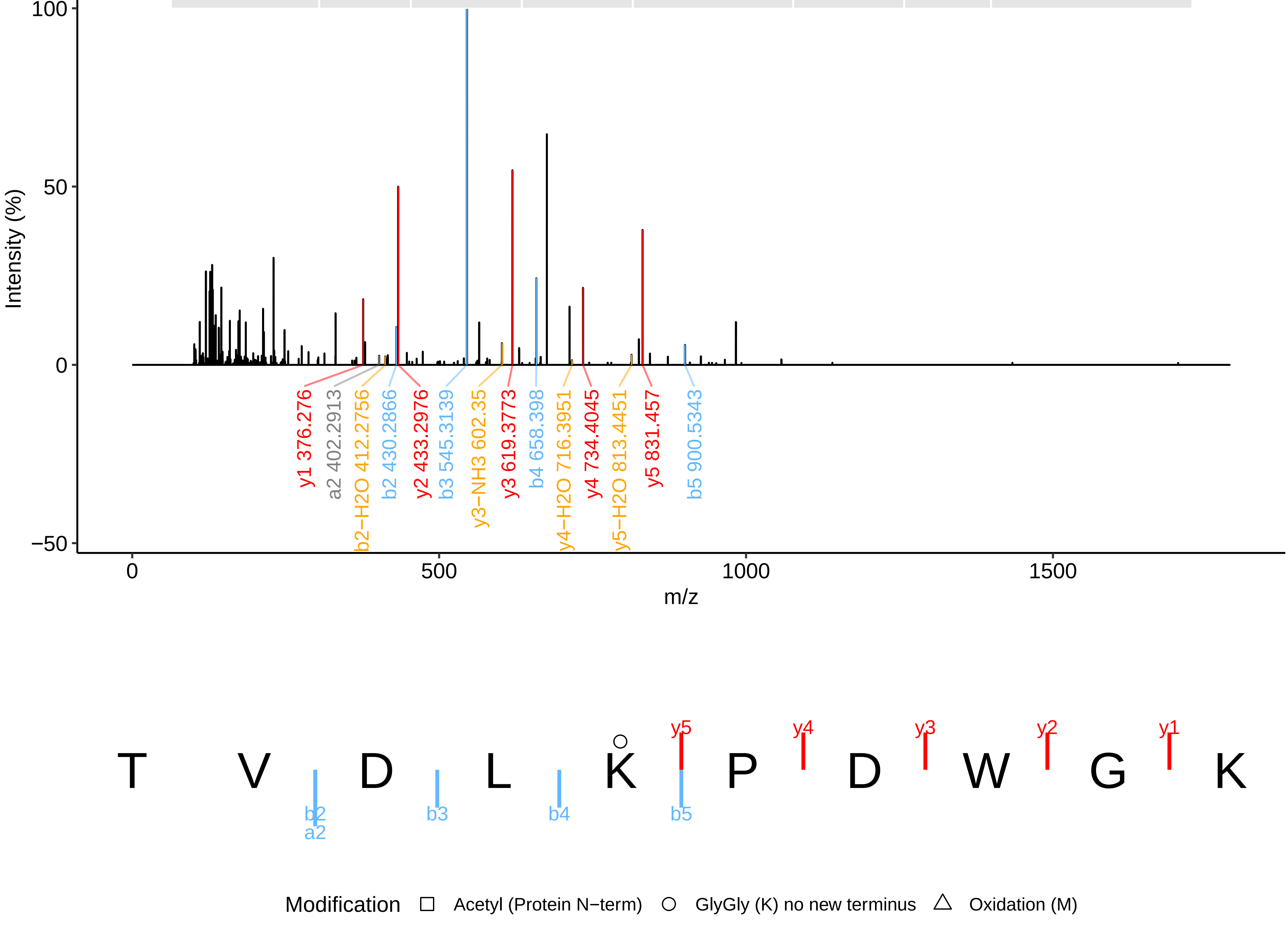

20200921_KIZ_MUb4_GlyGly_10plex − Scan number: 7149

Gene Names	Charge	m/z	Mass	Retention time	PEP	Score	Precursor Intensity
ABCC1	4	327.4198	1305.65	23.901	0.077807	48.741	11604642

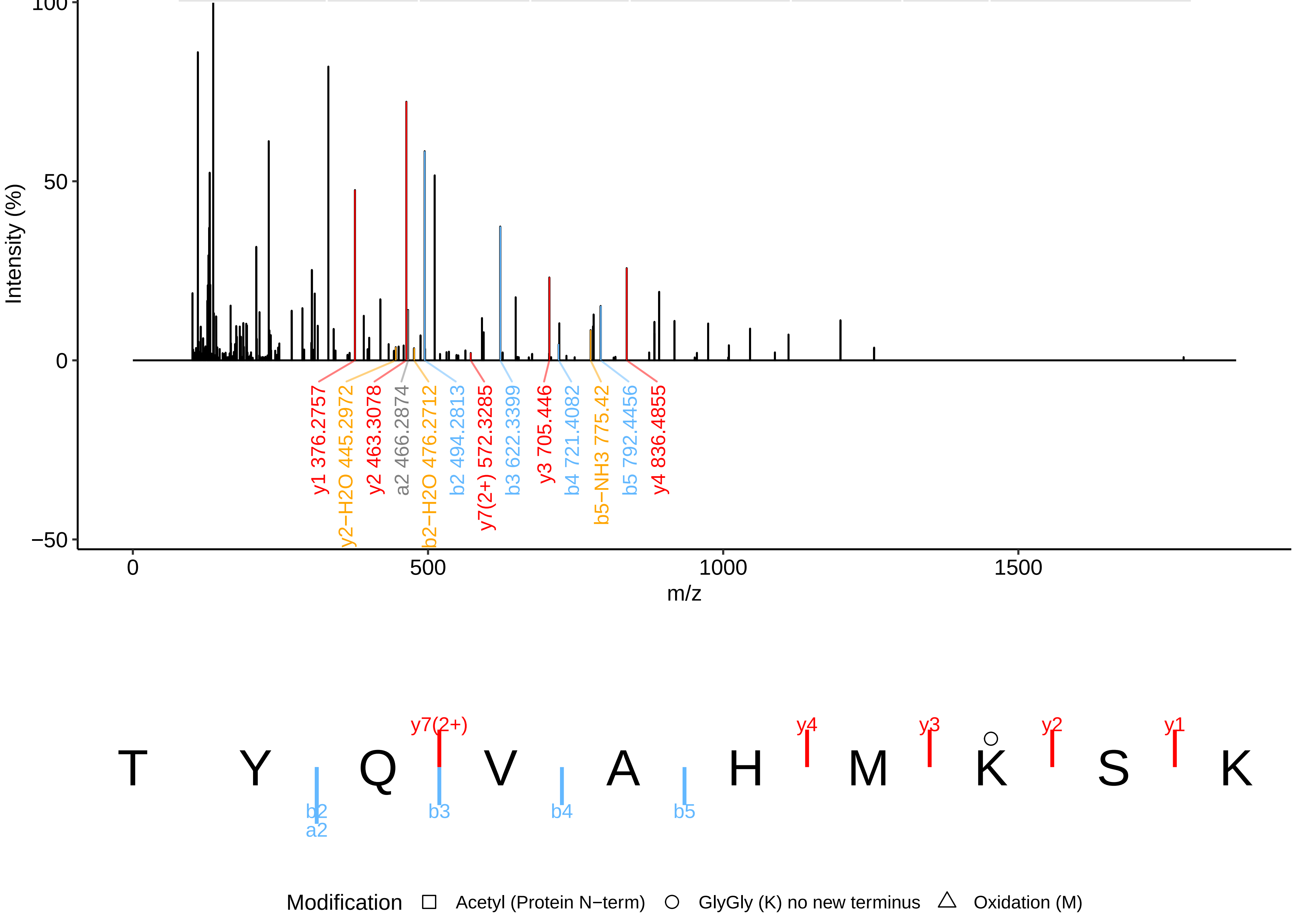

20200921_KIZ_MUb4_GlyGly_10plex_2 − Scan number: 6663

Gene Names	Charge	m/z	Mass	Retention time	PEP	Score	Precursor Intensity
ABCC1	4	327.4198	1305.65	24.589	0.013412	67.153	1774510

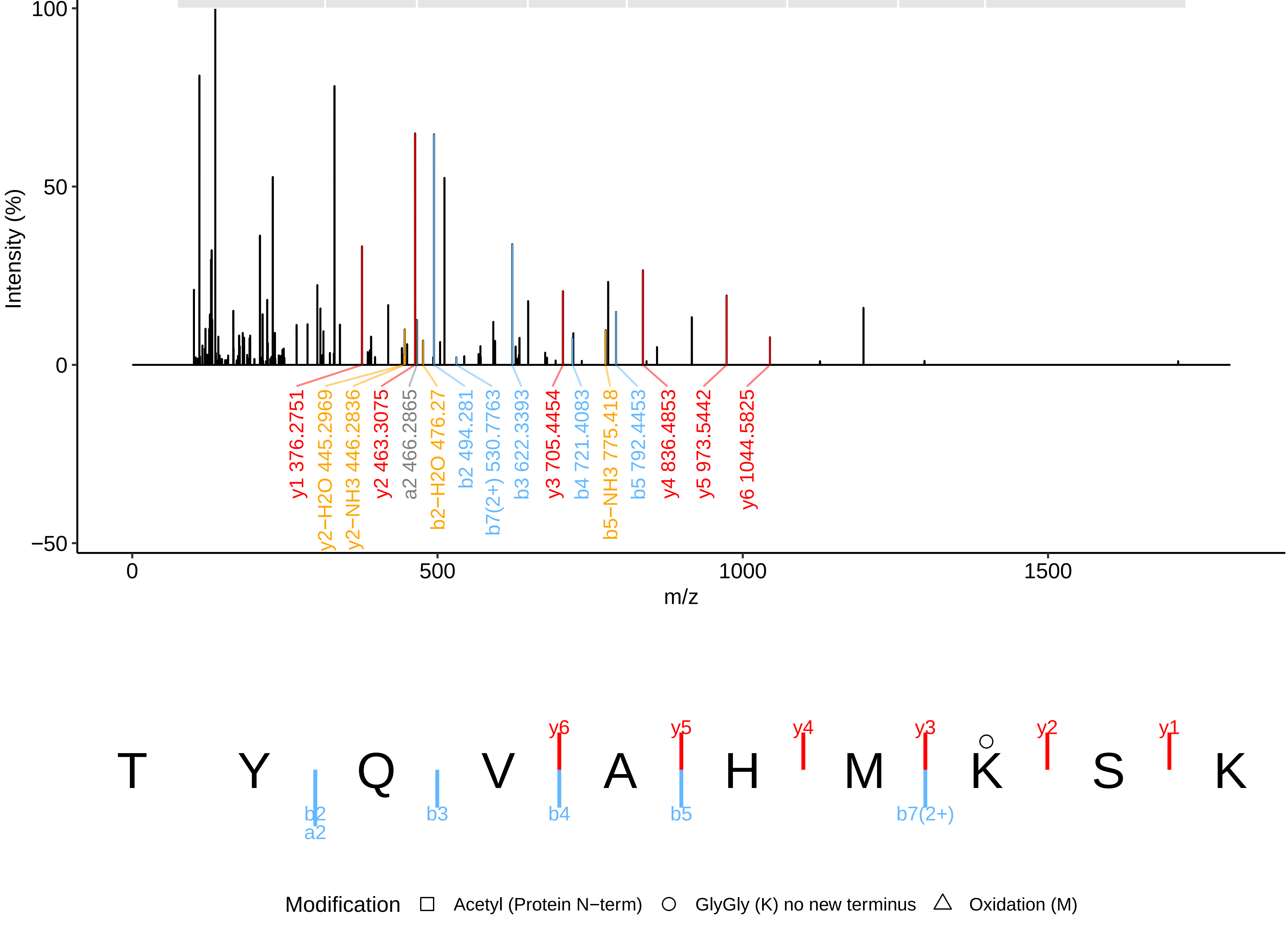

20200921_KIZ_MUb4_GlyGly_10plex_2 − Scan number: 6679

Gene Names	Charge	m/z	Mass	Retention time	PEP	Score	Precursor Intensity
ABCC1	3	436.2239	1305.65	24.656	0.0014591	88.441	887044.2

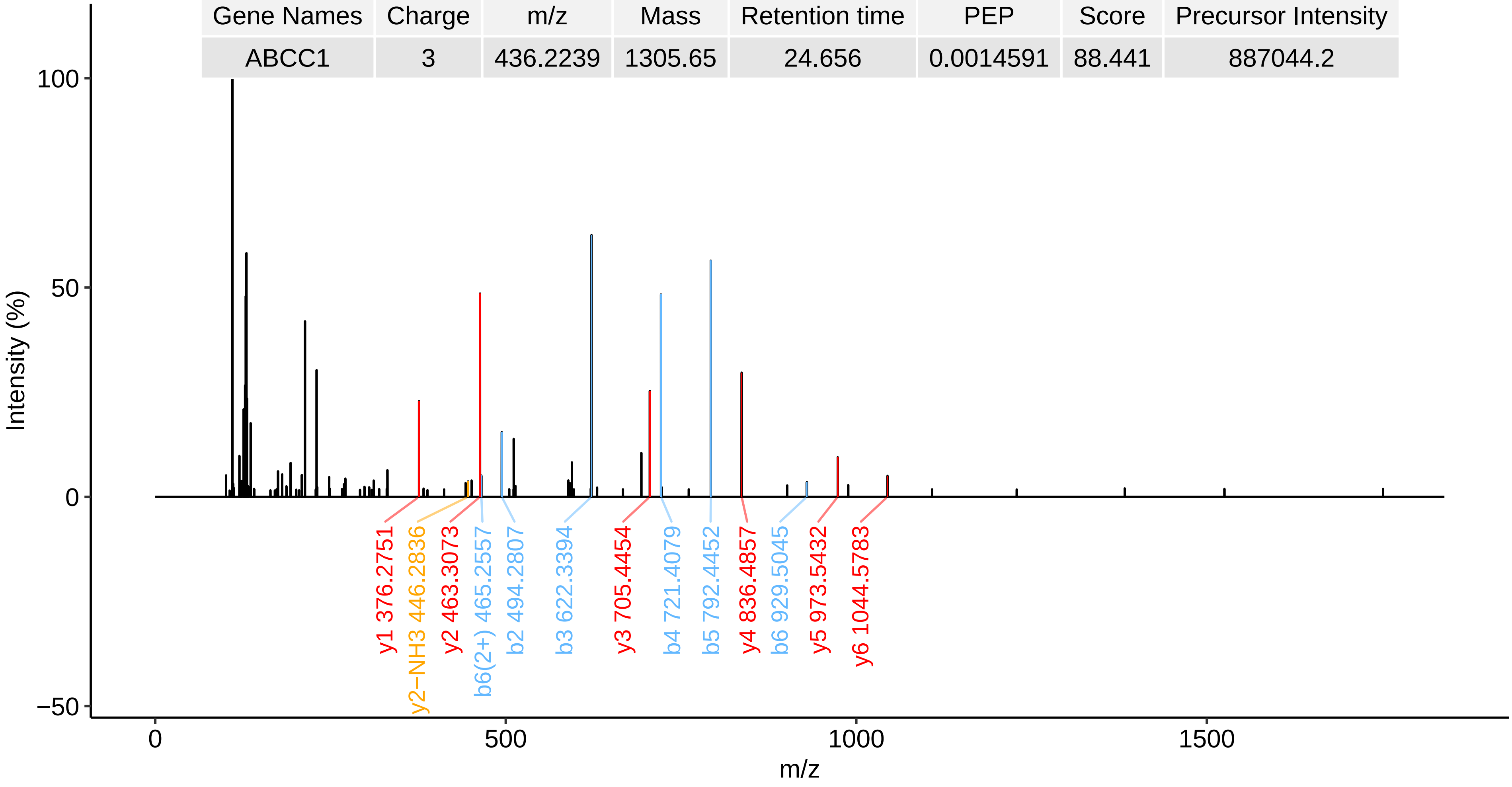

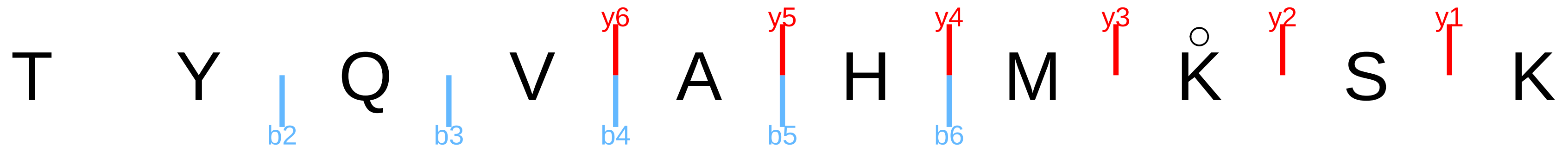

Modification □ Acetyl (Protein N−term) ○ GlyGly (K) no new terminus △ Oxidation (M)

20201118_KIZ_MUb6_GlyGly_1 − Scan number: 5734

Gene Names	Charge	m/z	Mass	Retention time	PEP	Score	Precursor Intensity
ABCC1	4	327.4198	1305.65	18.161	0.0054366	76.655	26018790

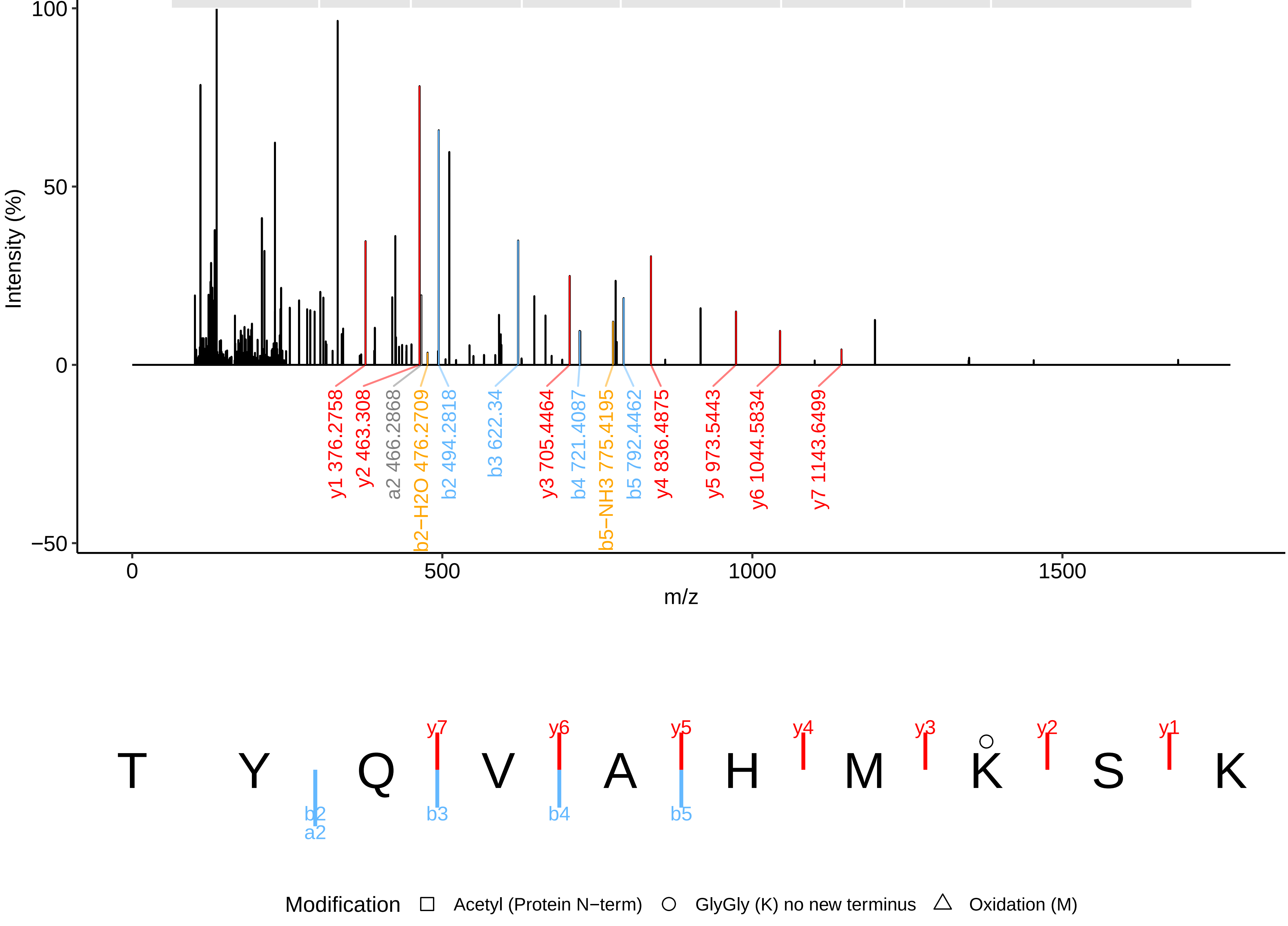

20201118_KIZ_MUb6_GlyGly_2 − Scan number: 7628

Gene Names	Charge	m/z	Mass	Retention time	PEP	Score	Precursor Intensity
ABCC1	4	327.4198	1305.65	26.022	0.011257	69.721	9429717

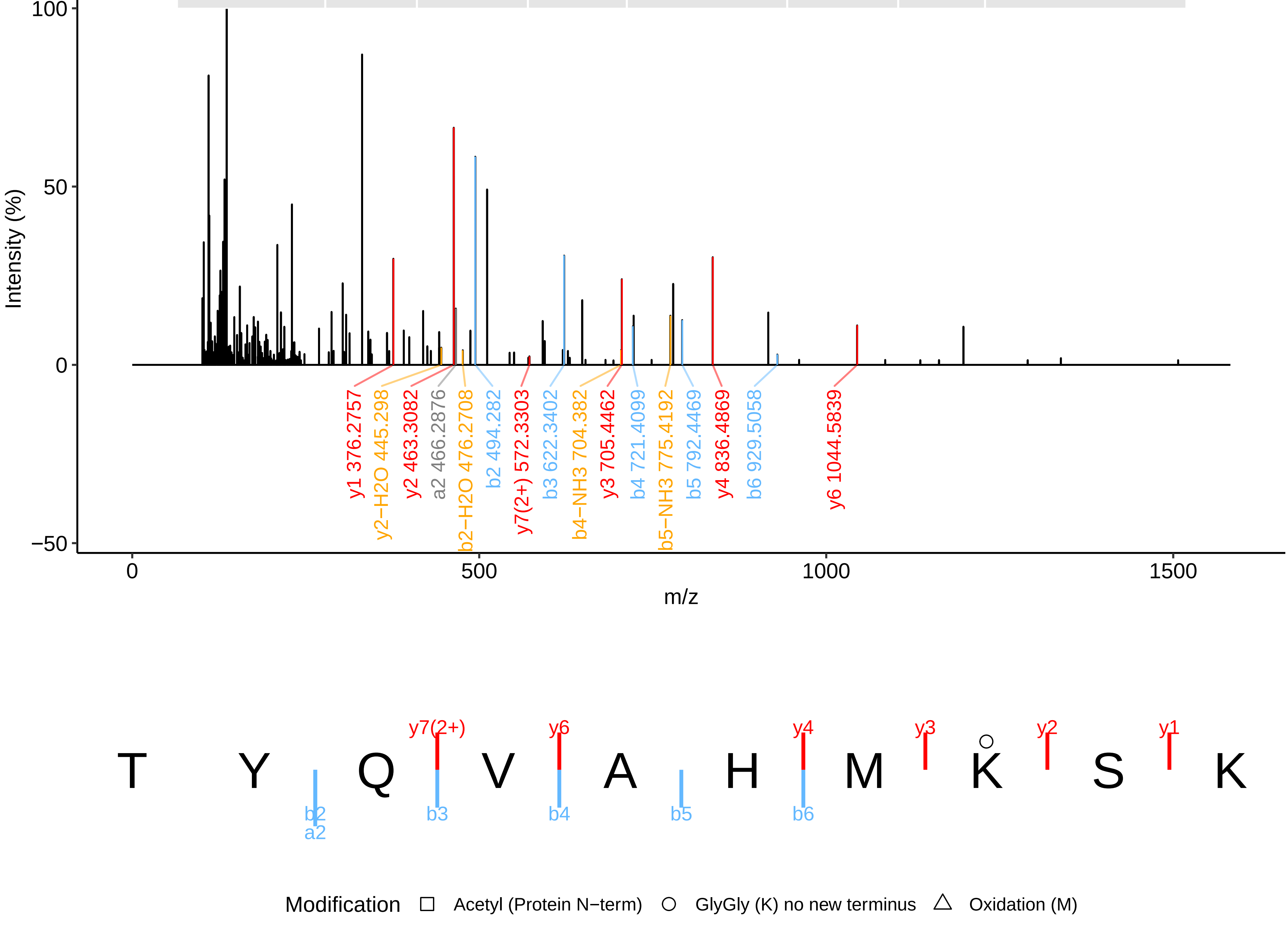

20200921_KIZ_MUb4_GlyGly_10plex_2 − Scan number: 12737

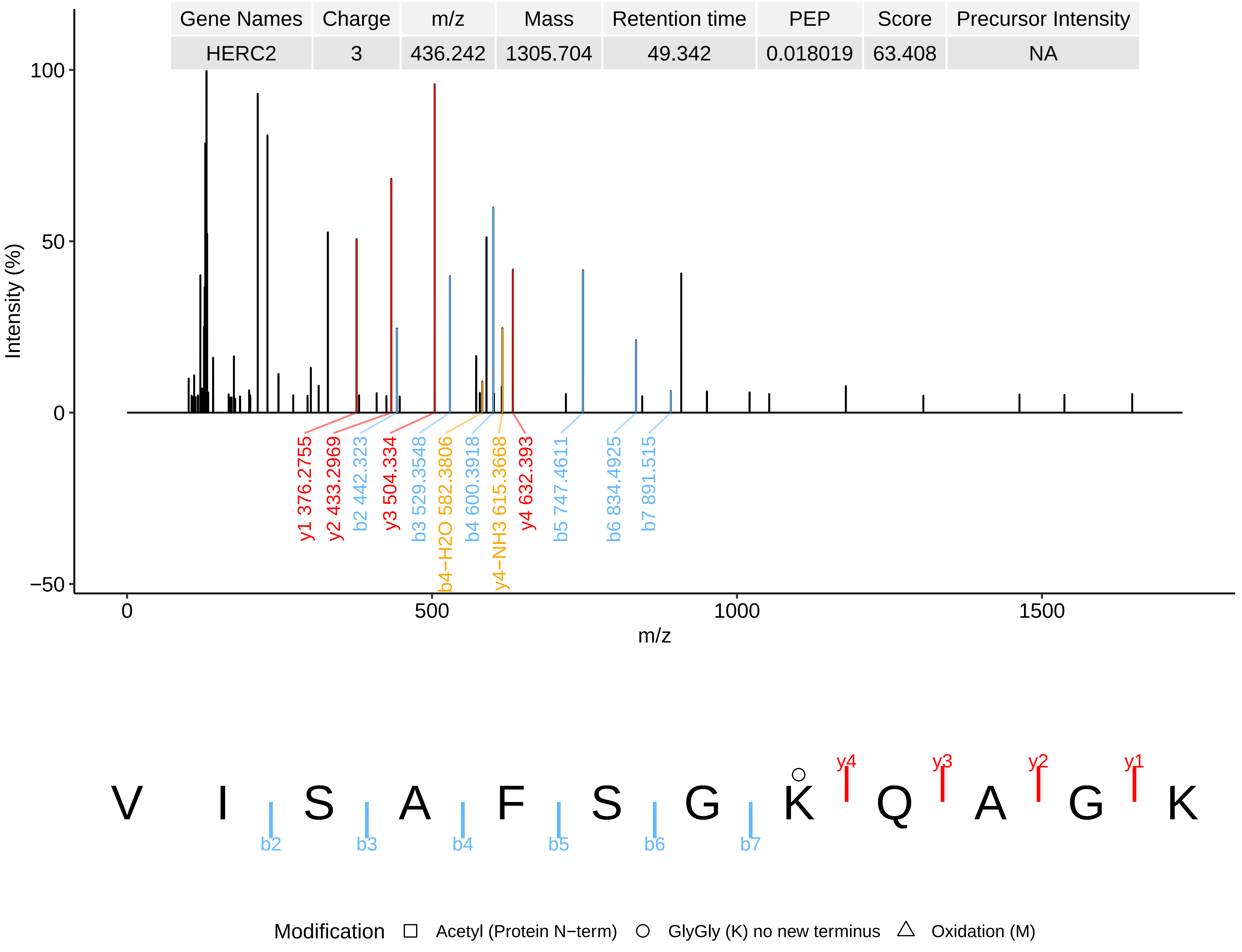

20200921_KIZ_MUb4_GlyGly_10plex_2 − Scan number: 10834

Gene Names	Charge	m/z	Mass	Retention time	PEP	Score	Precursor Intensity
CKB	3	436.2251	1305.653	41.545	0.1206	48.44	215533.1

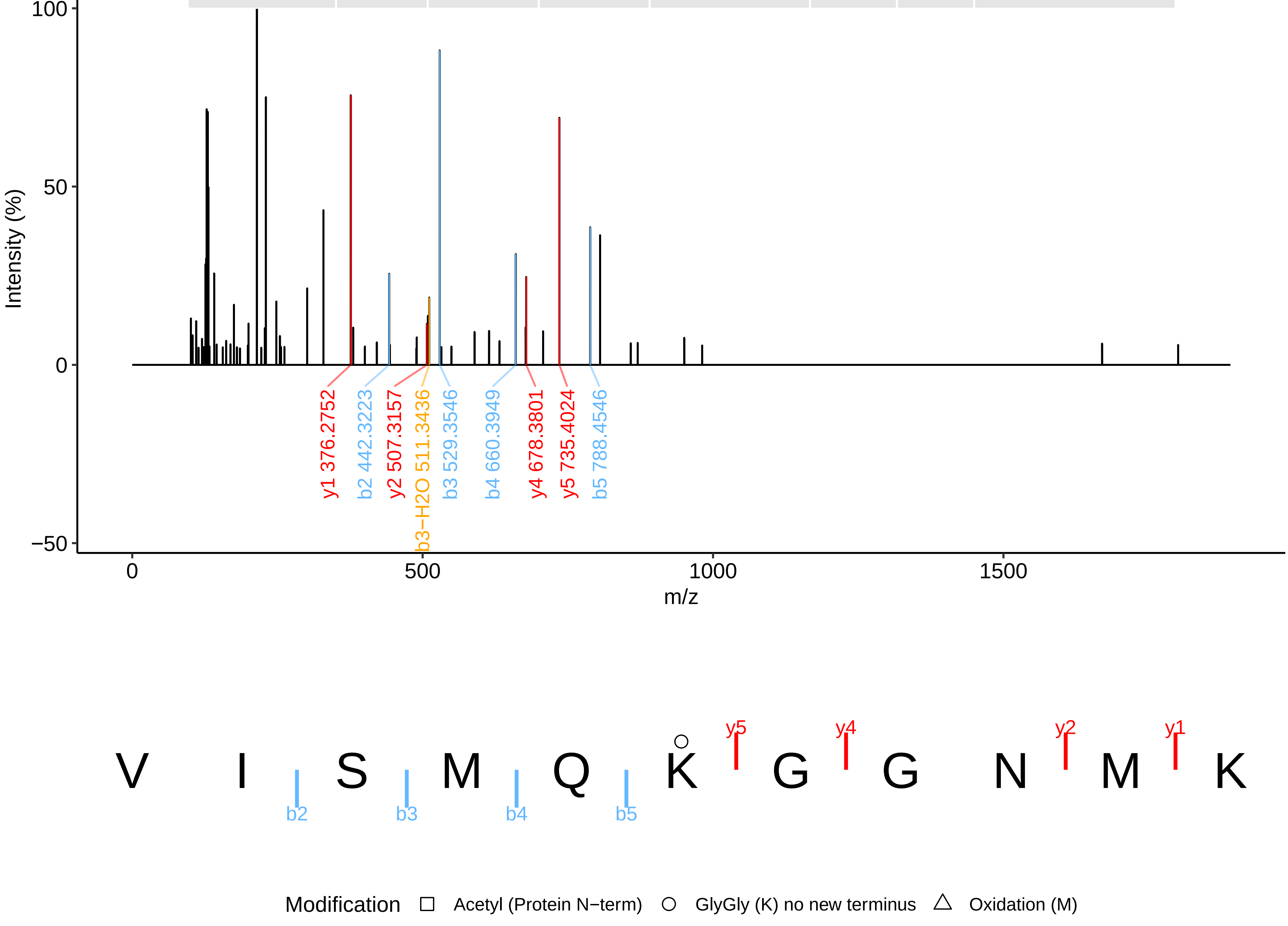

20200924_KIZ_MUb5_GlyGly_1 − Scan number: 10569

Gene Names	Charge	m/z	Mass	Retention time	PEP	Score	Precursor Intensity
CKB	3	436.2251	1305.653	34.143	0.0075698	75.652	5446443

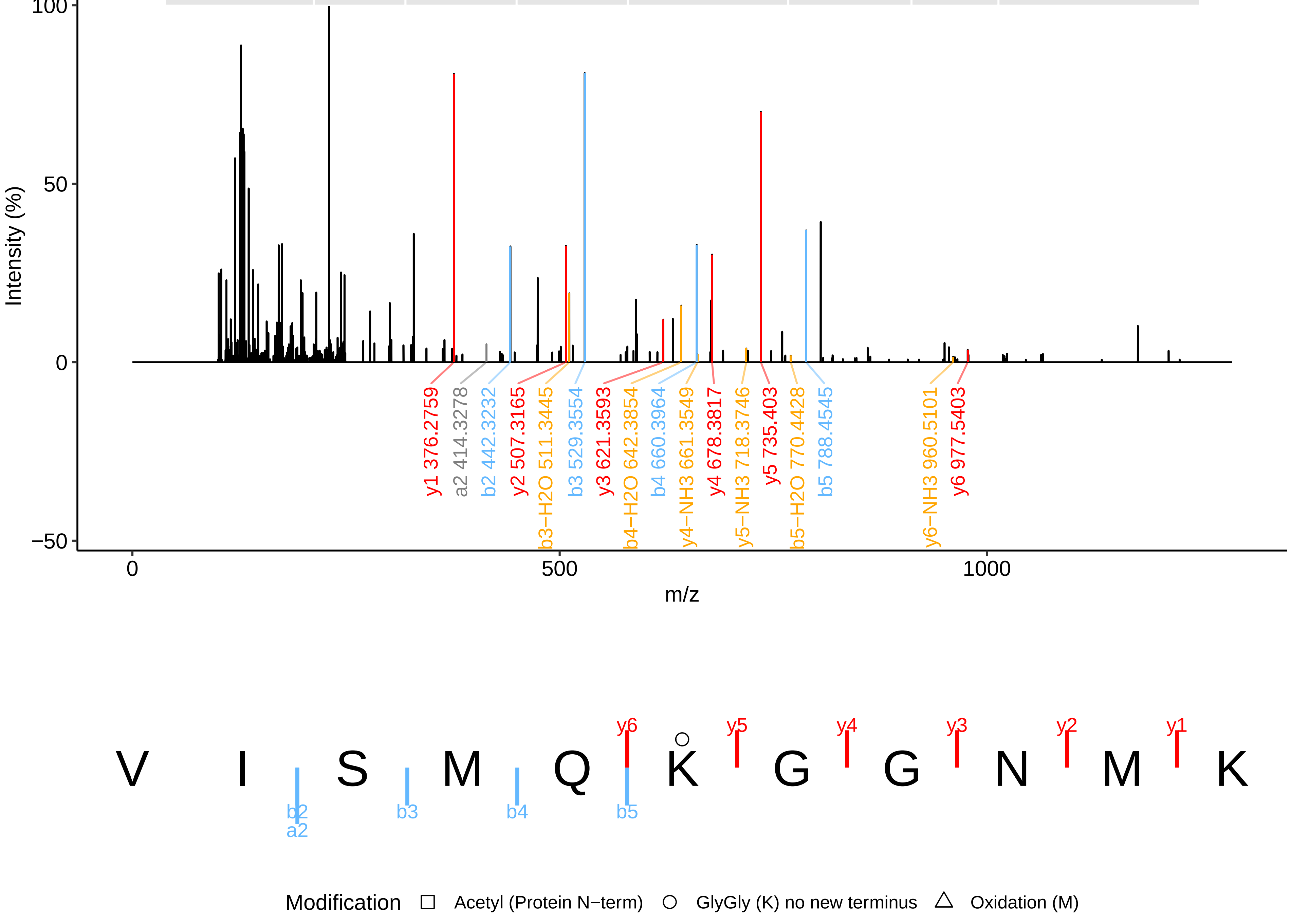

20200921_KIZ_MUb4_GlyGly_10plex − Scan number: 8021

Gene Names	Charge	m/z	Mass	Retention time	PEP	Score	Precursor Intensity
TMEM87A	3	425.2176	1272.631	27.105	0.01727	66.19	5922569

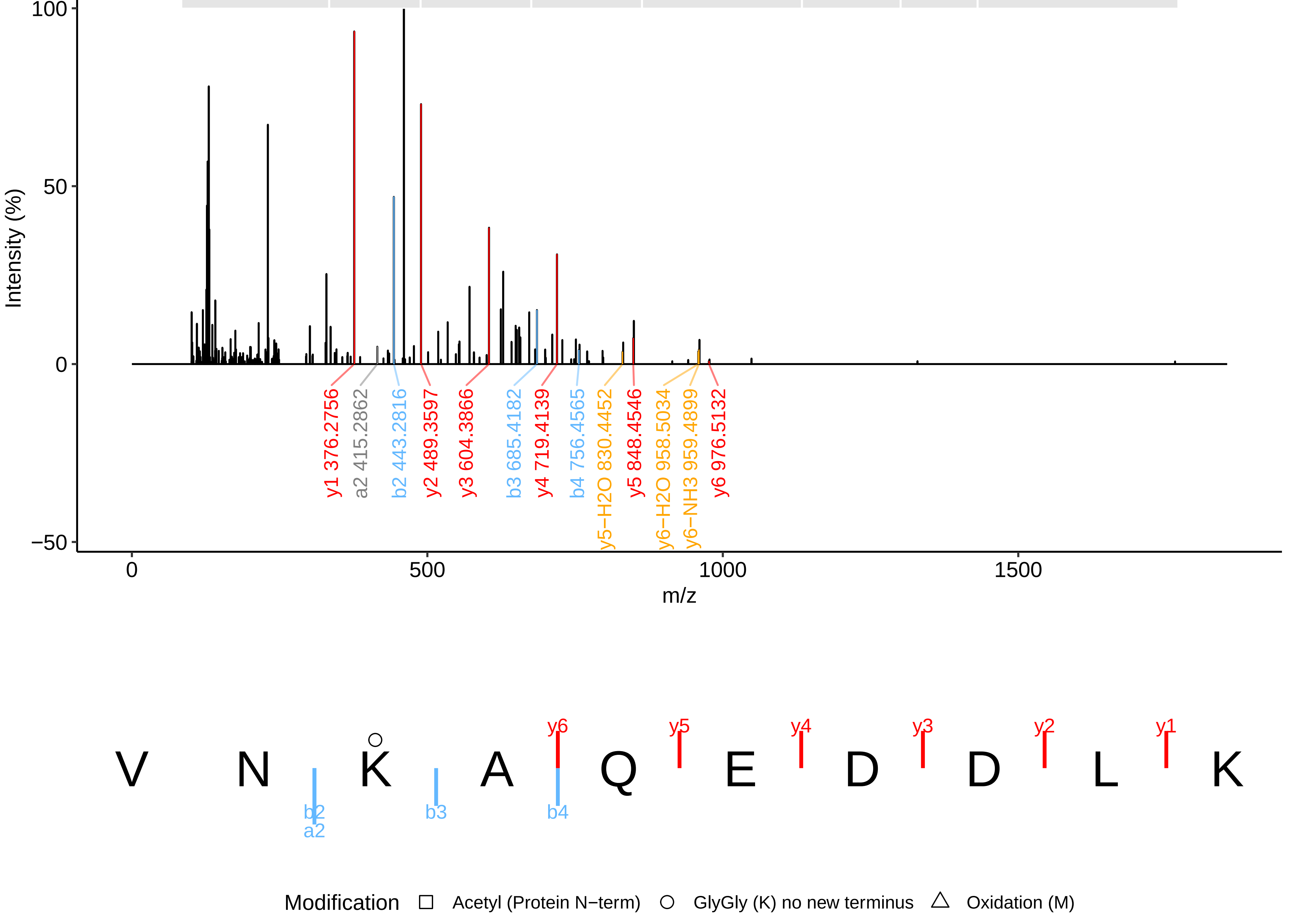

20200921_KIZ_MUb4_GlyGly_10plex − Scan number: 8009

Gene Names	Charge	m/z	Mass	Retention time	PEP	Score	Precursor Intensity
CSDE1	3	512.6023	1534.785	27.06	0.00034872	93.011	11583807

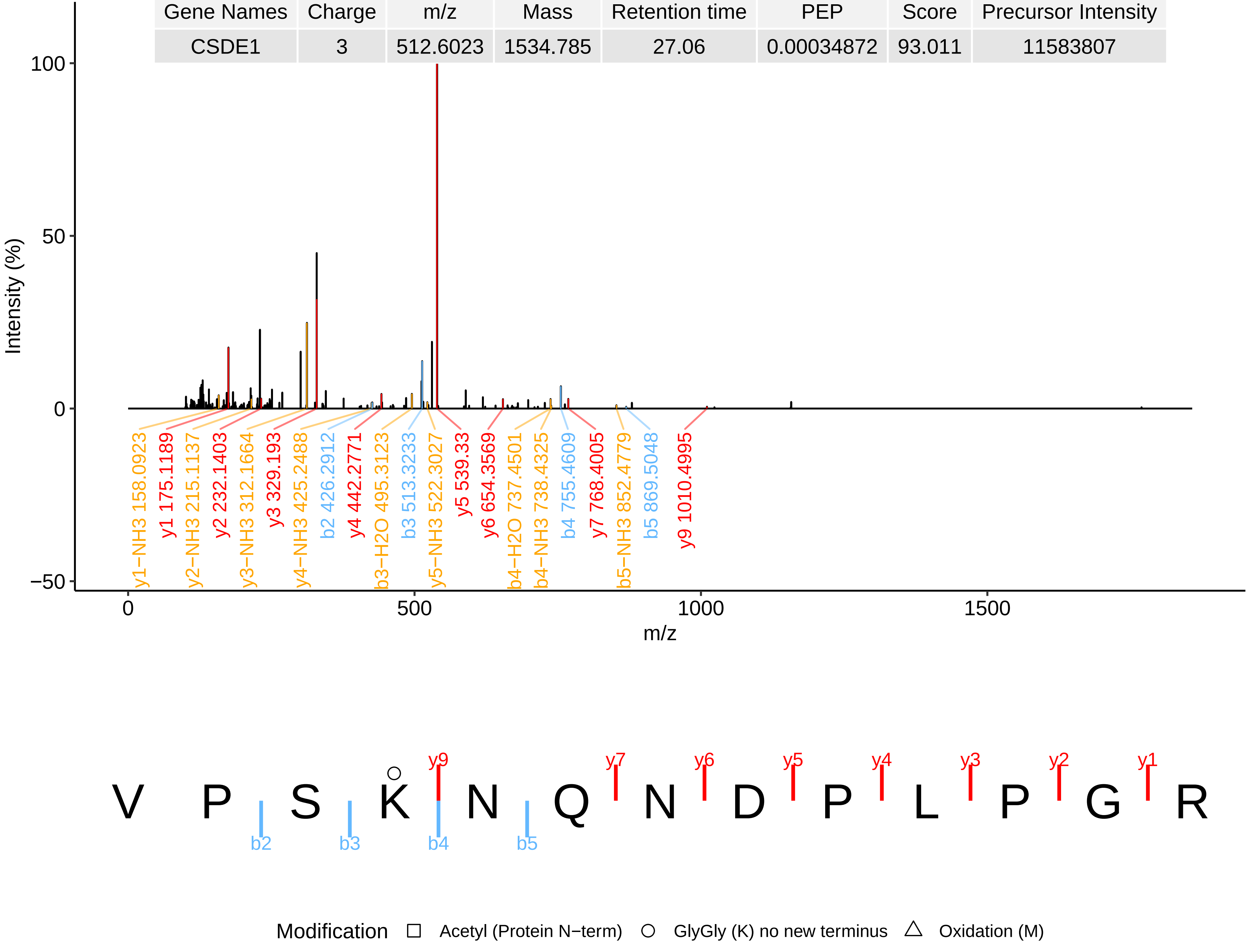

20200924_KIZ_MUb5_GlyGly_1 − Scan number: 7039

Gene Names	Charge	m/z	Mass	Retention time	PEP	Score	Precursor Intensity
CSDE1	3	512.6023	1534.785	22.284	0.0030004	77.185	5794775

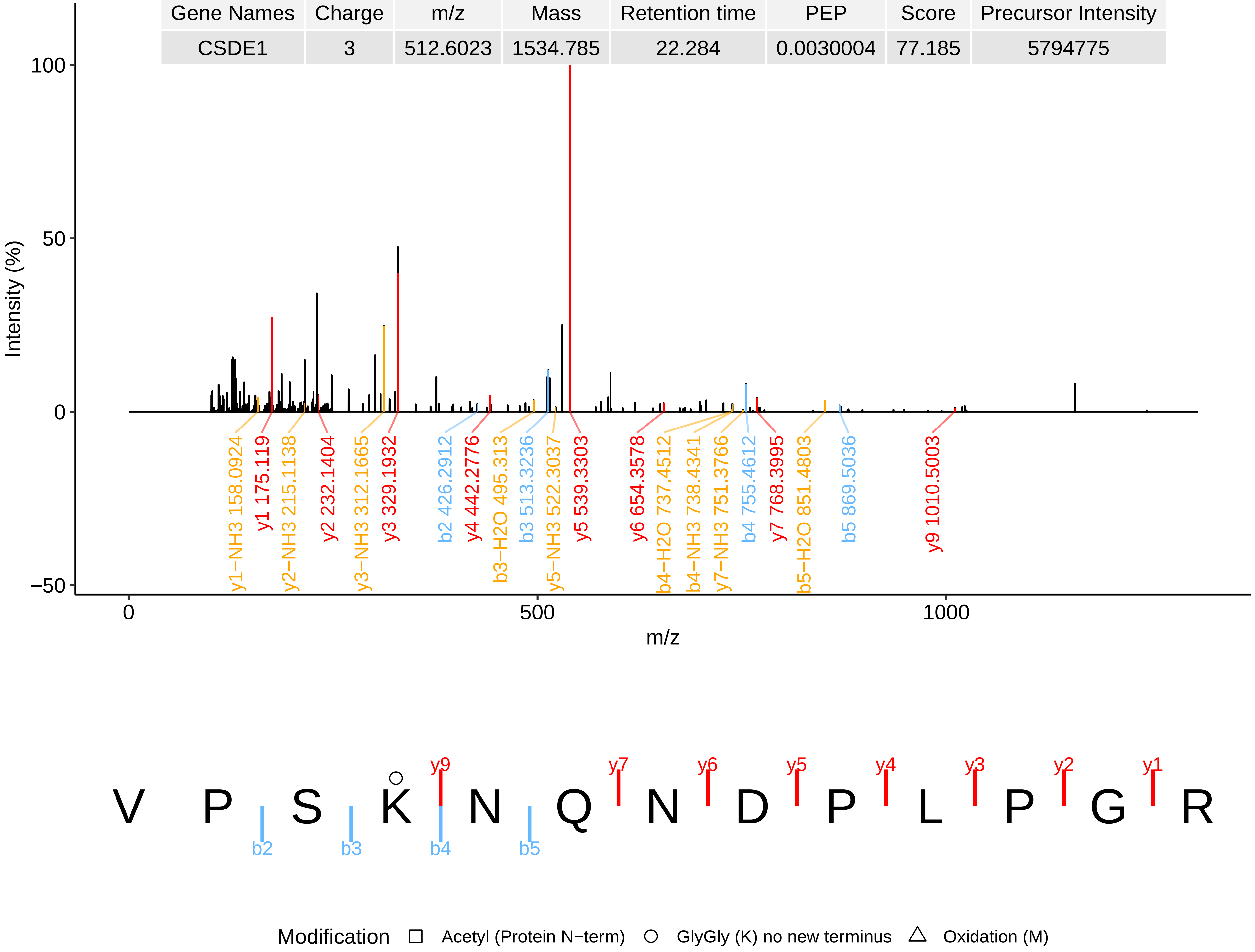

20200924_KIZ_MUb5_GlyGly_1 − Scan number: 7051

Gene Names	Charge	m/z	Mass	Retention time	PEP	Score	Precursor Intensity
CSDE1	3	512.6023	1534.785	22.324	8.66e−06	108.72	5620881

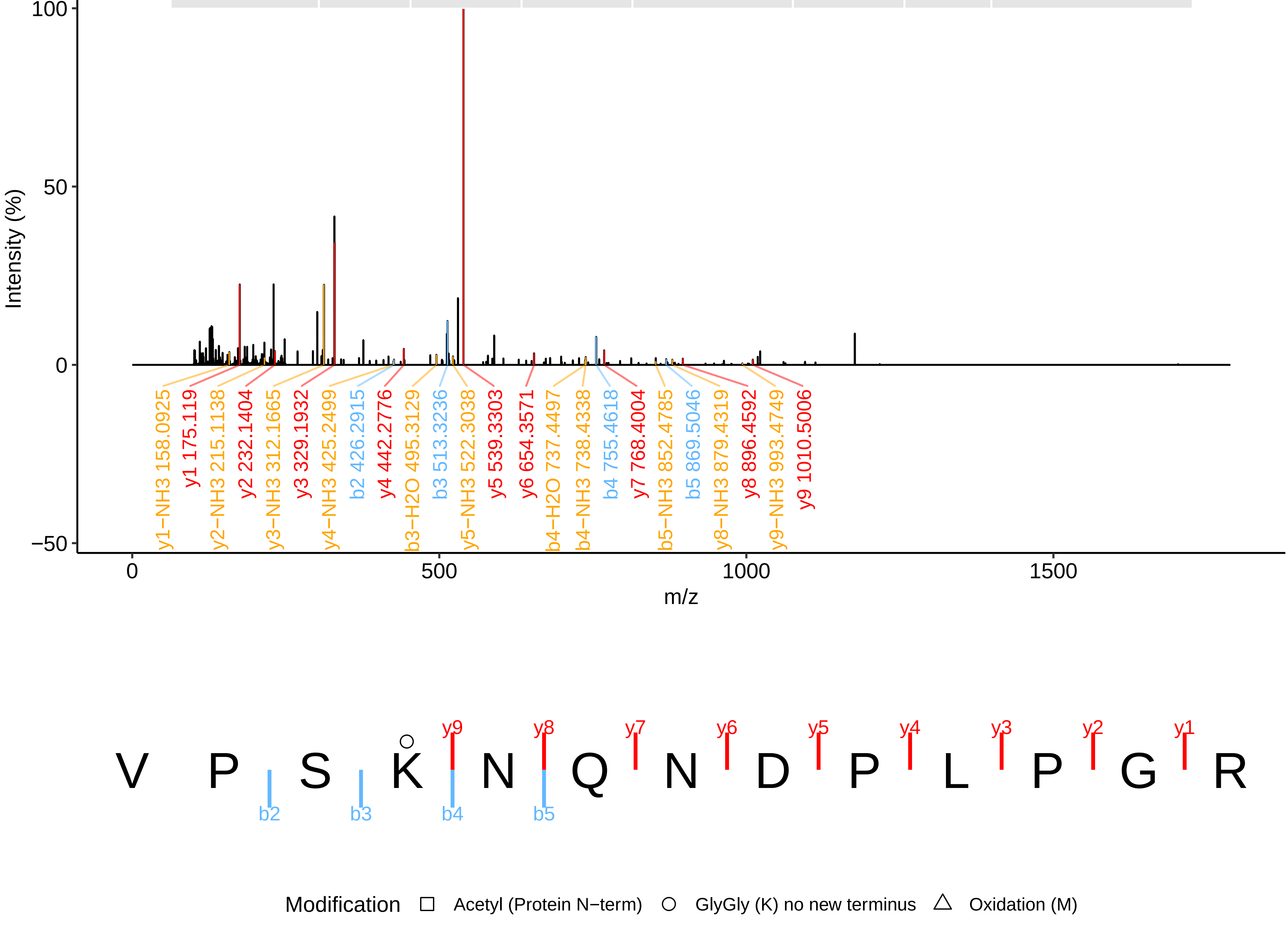

20200921_KIZ_MUb4_GlyGly_10plex − Scan number: 9800

Gene Names	Charge	m/z	Mass	Retention time	PEP	Score	Precursor Intensity
HSPA8;HSPA2	3	432.2261	1293.657	33.684	0.0010093	91.549	5625883

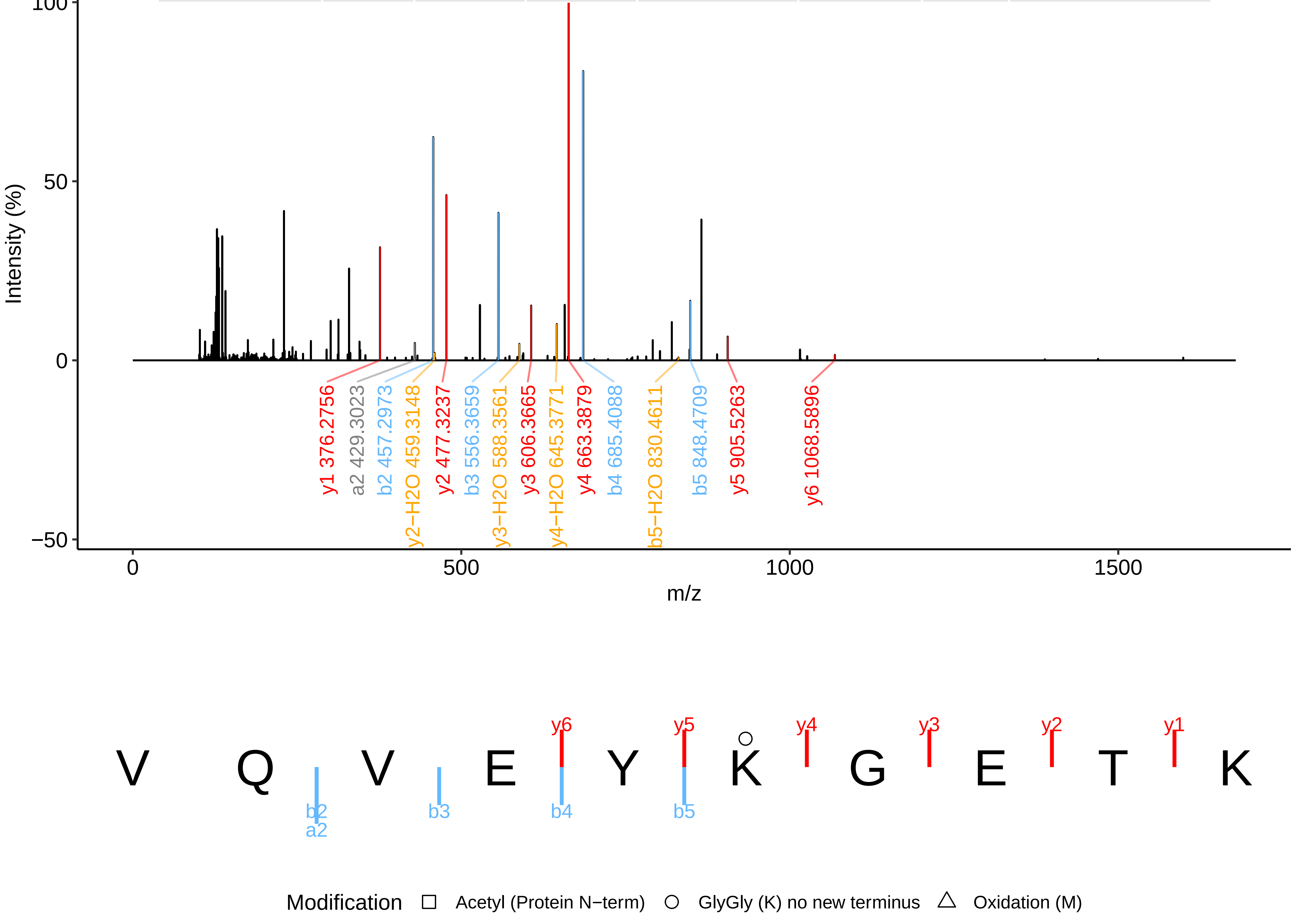

20200921_KIZ_MUb4_GlyGly_10plex_2 − Scan number: 9006

Gene Names	Charge	m/z	Mass	Retention time	PEP	Score	Precursor Intensity
HSPA8;HSPA2	3	432.2261	1293.657	34.113	0.034417	60.434	788382.5

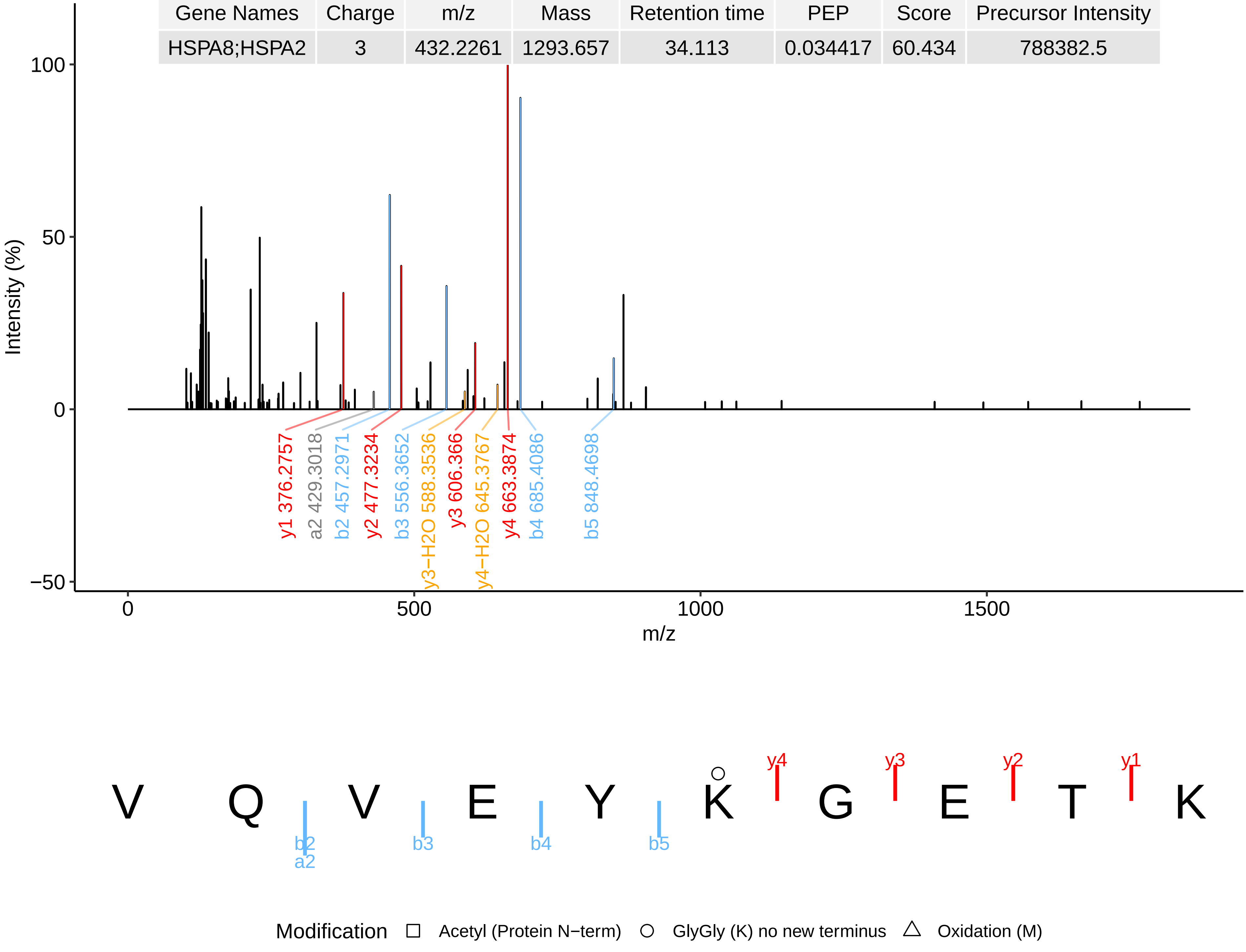

20200924_KIZ_MUb5_GlyGly_1 − Scan number: 8793

Gene Names	Charge	m/z	Mass	Retention time	PEP	Score	Precursor Intensity
HSPA8;HSPA2	3	432.2261	1293.657	28.17	0.0018103	86.014	16255158

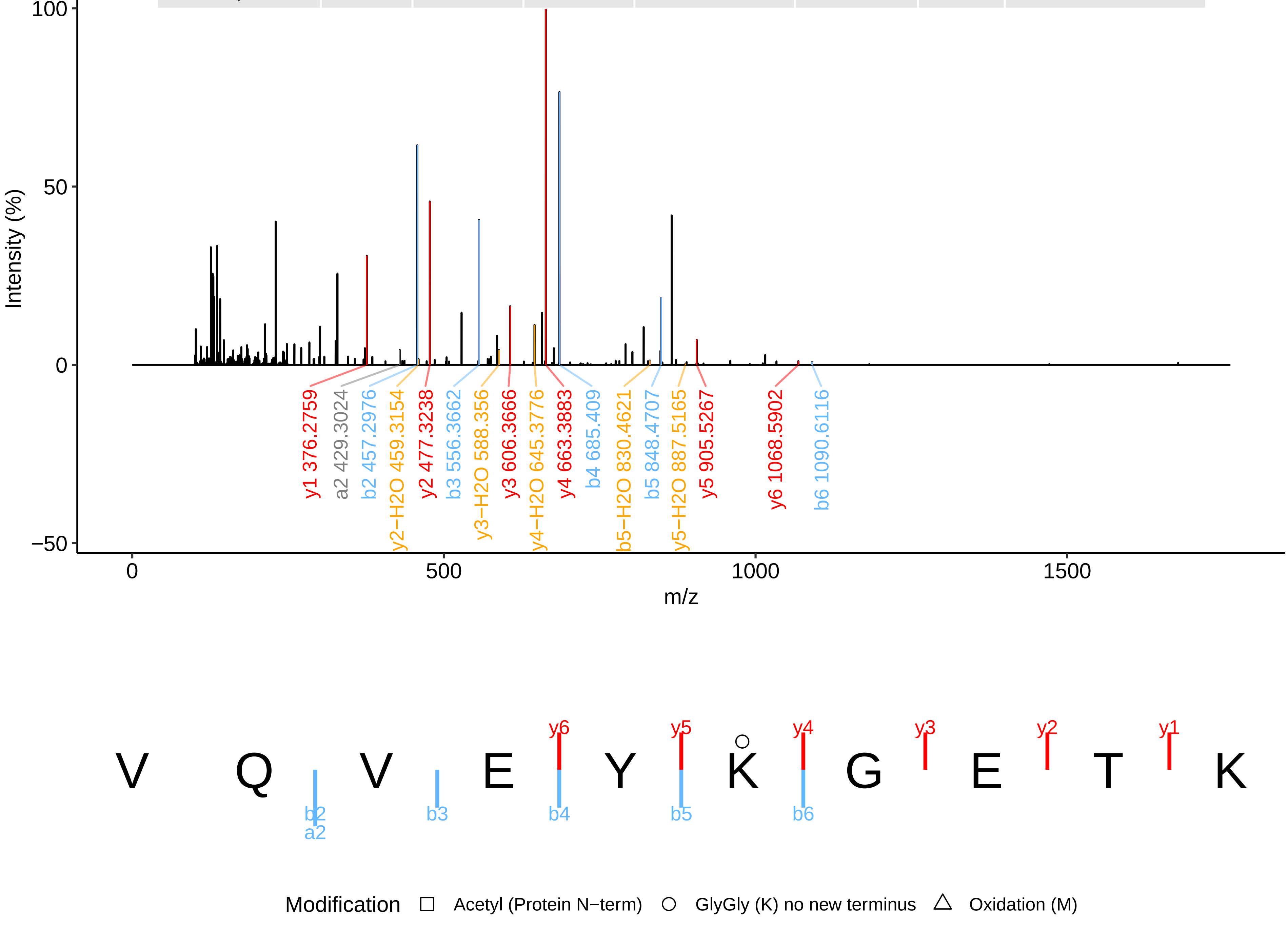

20200924_KIZ_MUb5_GlyGly_1 − Scan number: 8807

Gene Names	Charge	m/z	Mass	Retention time	PEP	Score	Precursor Intensity
HSPA8;HSPA2	3	432.2261	1293.657	28.224	0.10553	46.592	13643407

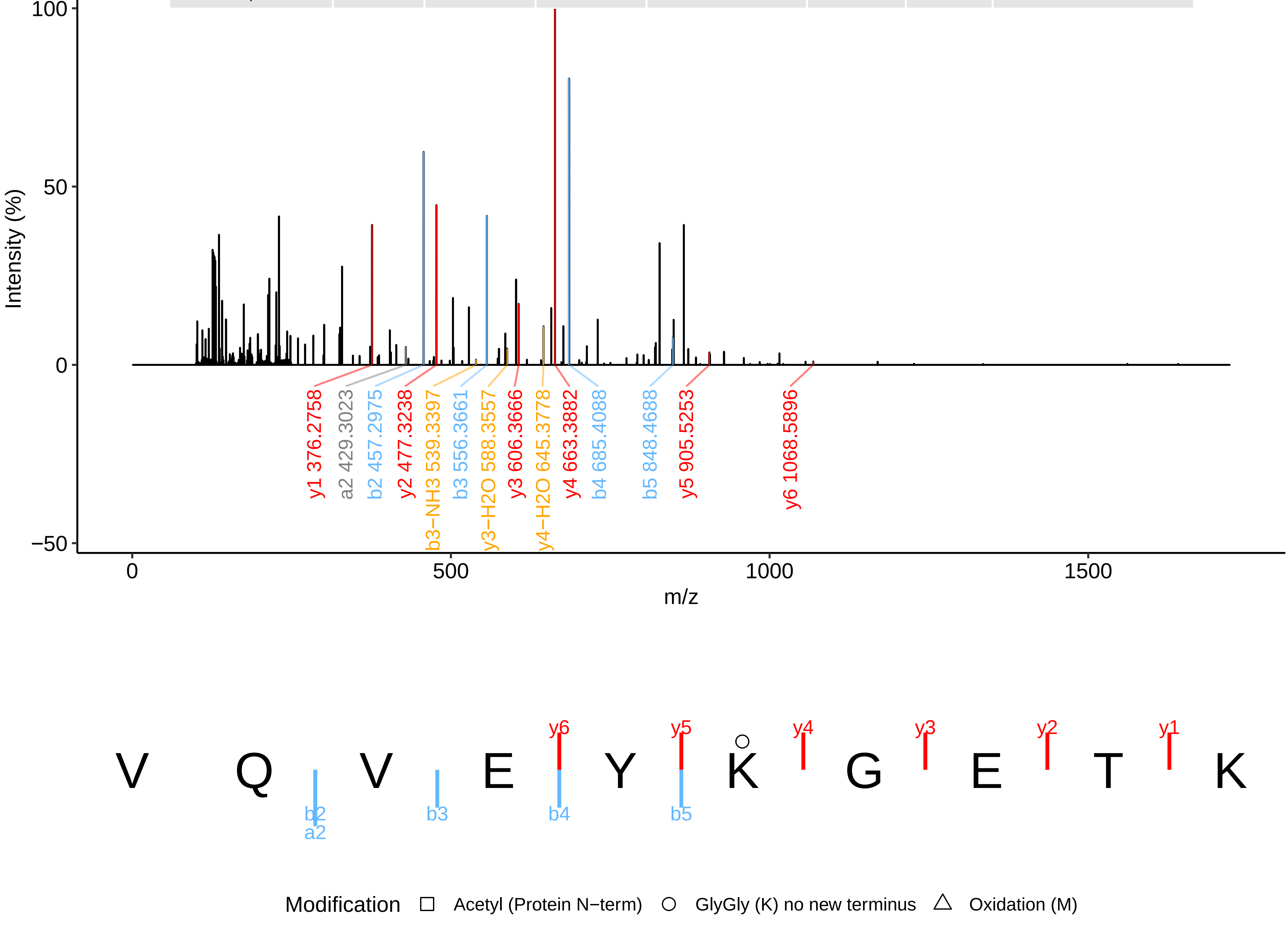

20200924_KIZ_MUb5_GlyGly_2 − Scan number: 8420

Gene Names	Charge	m/z	Mass	Retention time	PEP	Score	Precursor Intensity
HSPA8;HSPA2	3	432.2261	1293.657	31.46	0.017973	65.5	3295031

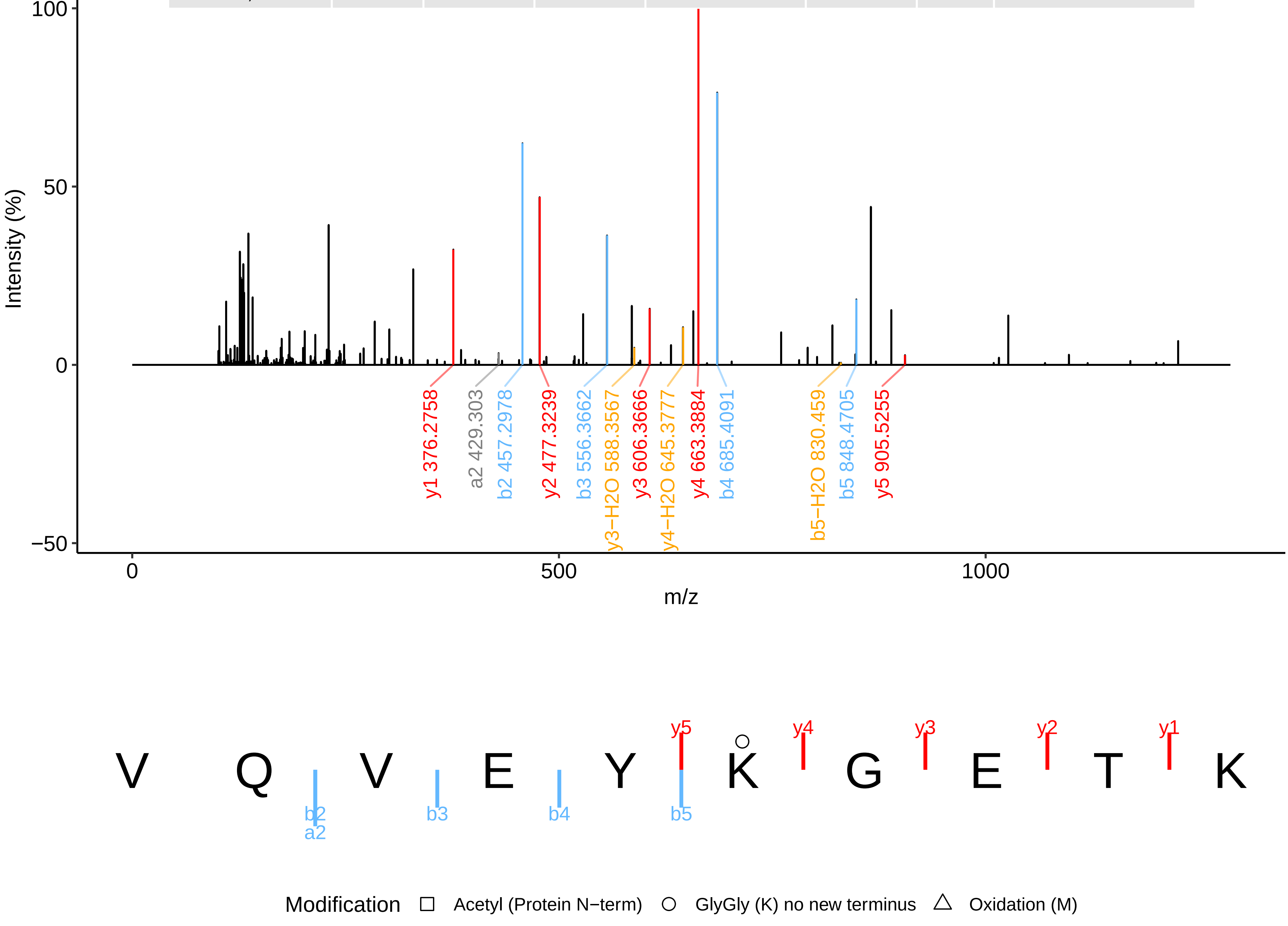

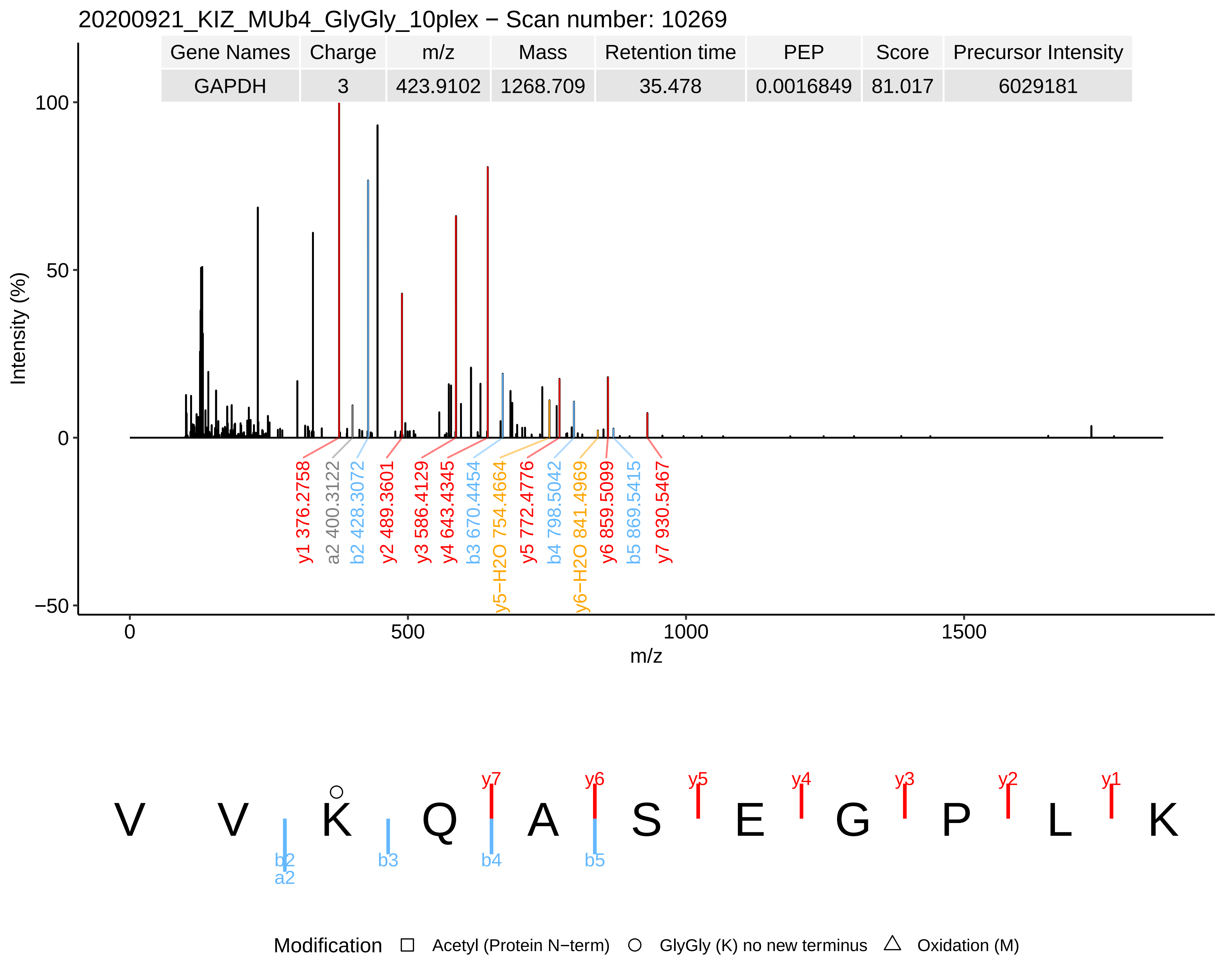
20200921_KIZ_MUb4_GlyGly_10plex − Scan number: 10269
Gene Names
Charge
m/z
Mass
Retention time
PEP
Score
Precursor Intensity
GAPDH
3
423.9102
1268.709
35.478
0.0016849
81.017
6029181
Intensity (%)
100
50
0
−50
0
500
1000
1500
m/z
y1 376.2758
a2 400.3122
b2 428.3072
y2 489.3601
y3 586.4129
y4 643.4345
b3 670.4454
y5−H2O 754.4664
y5 772.4776
b4 798.5042
y6−H2O 841.4969
y6 859.5099
b5 869.5415
y7 930.5467
V V K Q A S E G P L K
y7 y6 y5 y4 y3 y2 y1
b2 a2 b3 b4 b5
Modification
Acetyl (Protein N−term)
GlyGly (K) no new terminus
Oxidation (M)

20200921_KIZ_MUb4_GlyGly_10plex_2 − Scan number: 9541

Gene Names	Charge	m/z	Mass	Retention time	PEP	Score	Precursor Intensity
GAPDH	3	423.9102	1268.709	36.3	0.00095291	88.596	13790134

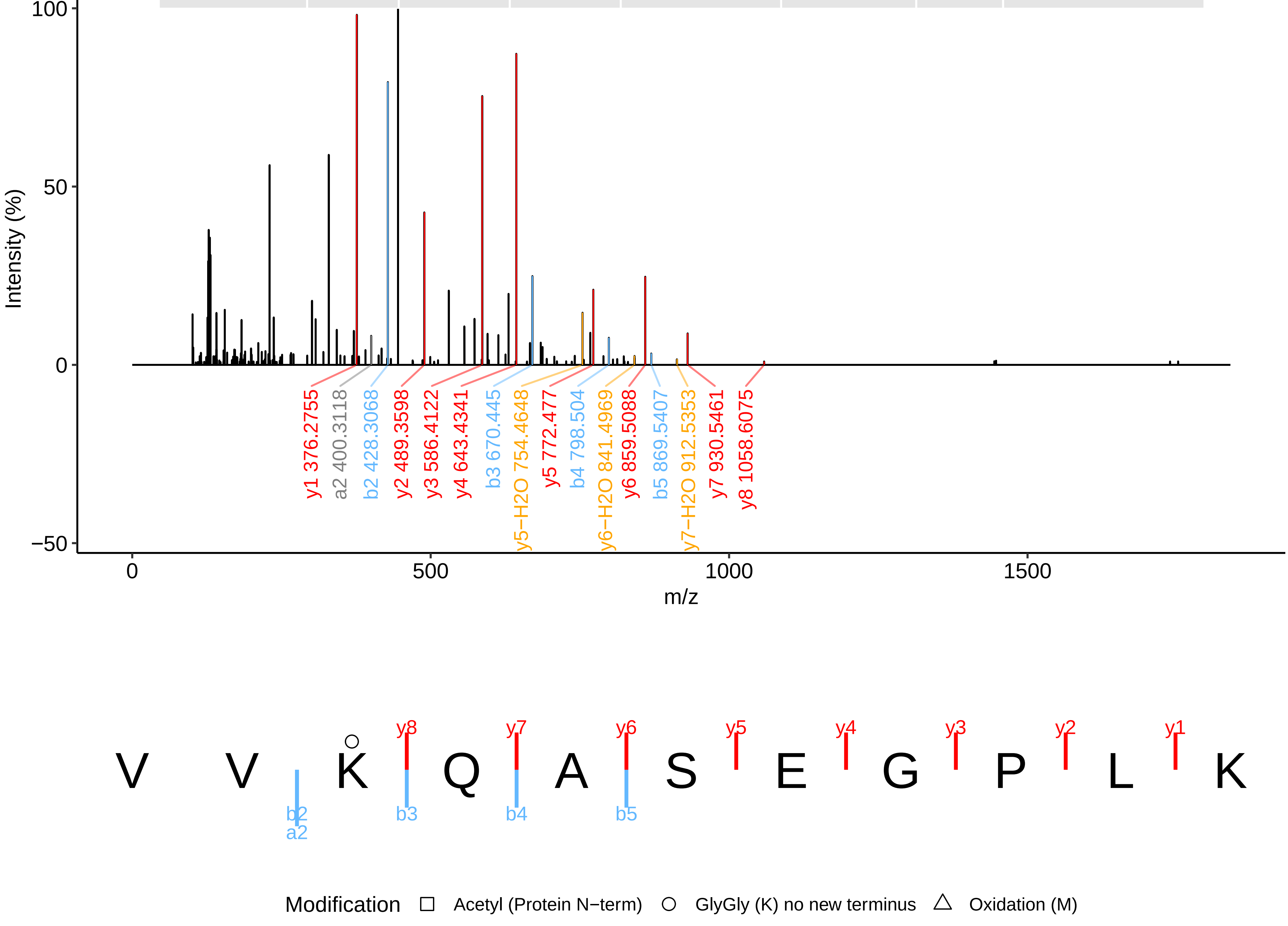

20200924_KIZ_MUb5_GlyGly_1 − Scan number: 9293

Gene Names	Charge	m/z	Mass	Retention time	PEP	Score	Precursor Intensity
GAPDH	3	423.9102	1268.709	29.864	0.00021577	96.229	45631660

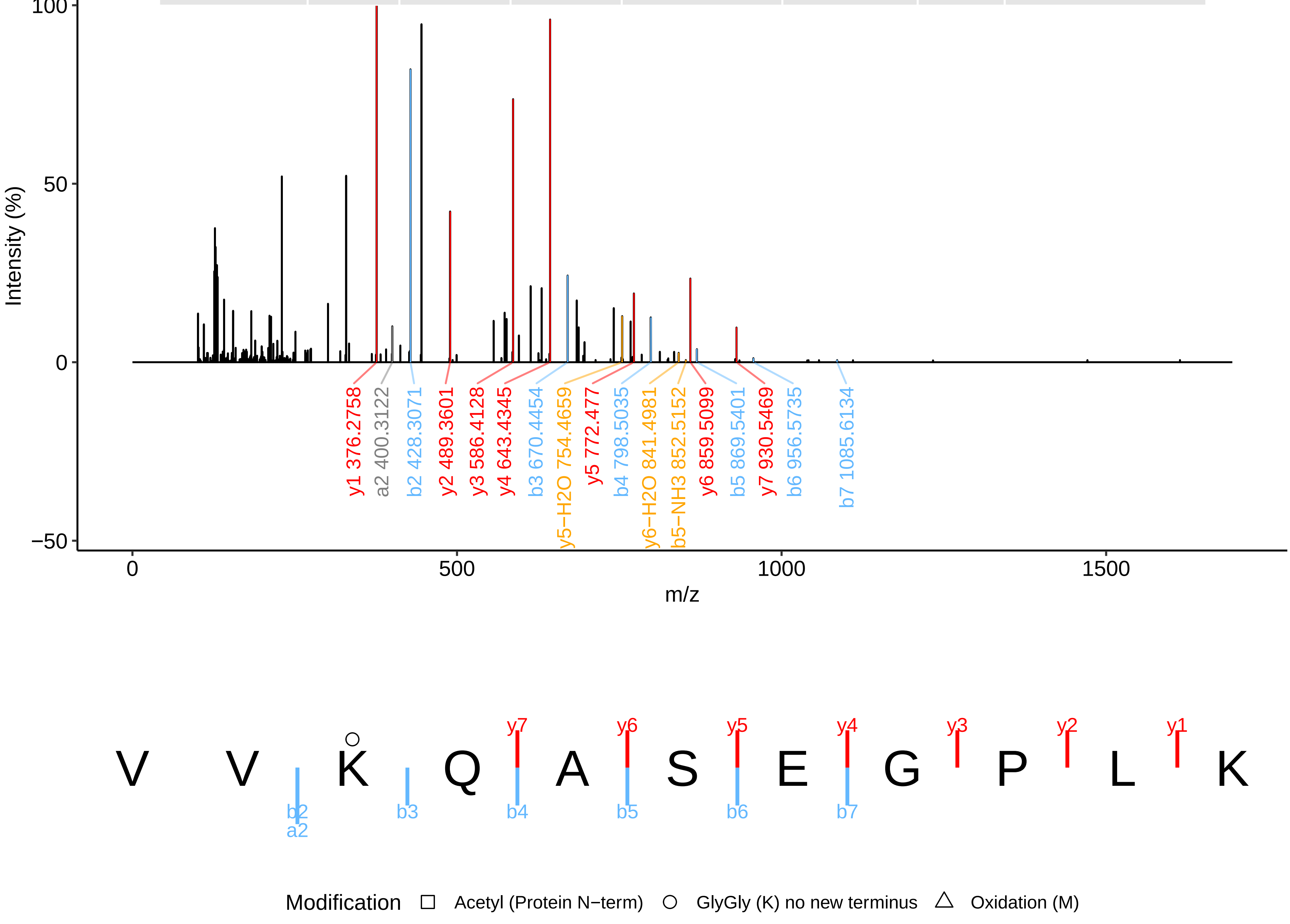

20200924_KIZ_MUb5_GlyGly_1 − Scan number: 9011

Gene Names	Charge	m/z	Mass	Retention time	PEP	Score	Precursor Intensity
GAPDH	3	423.9102	1268.709	28.938	0.0021529	81.017	NA

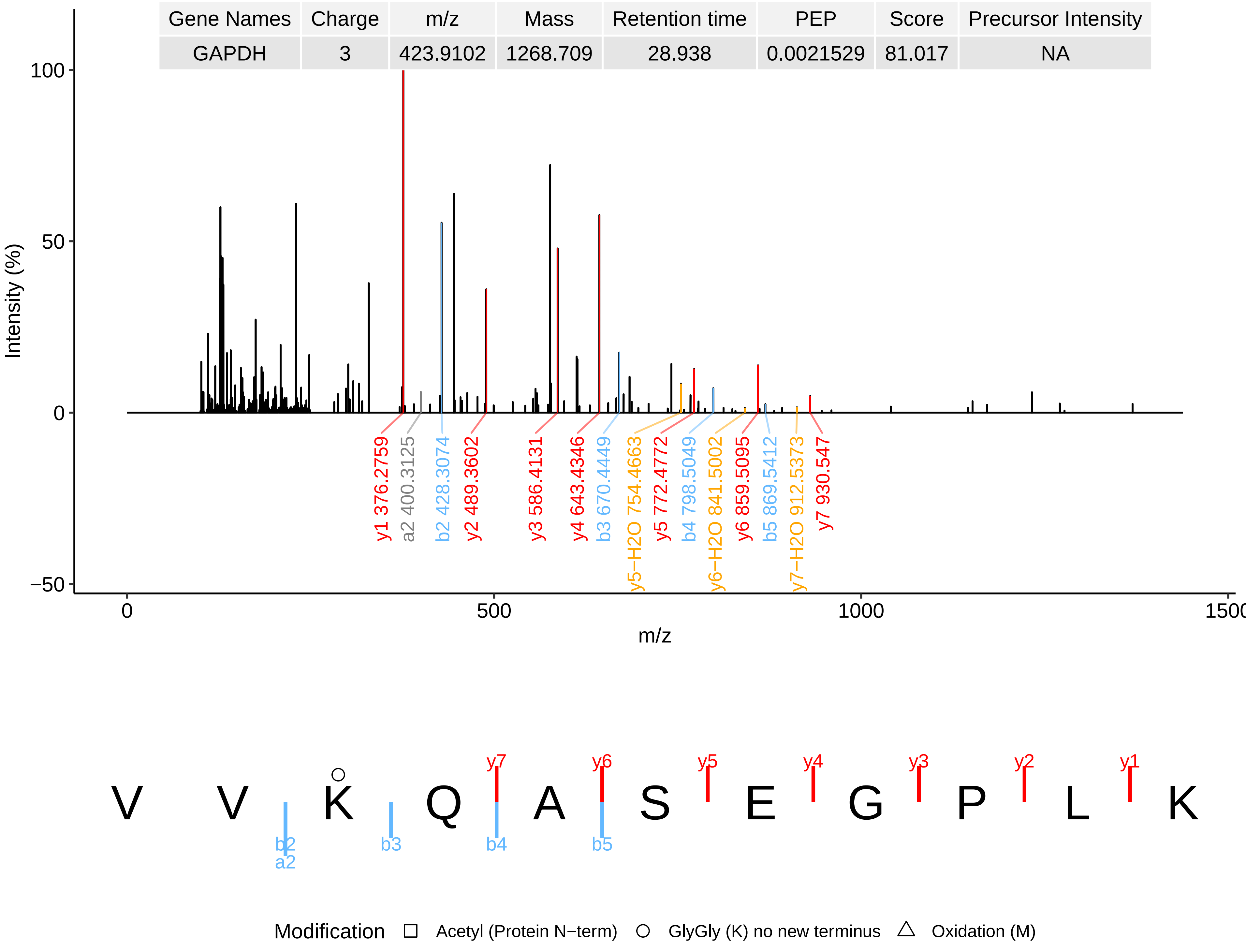

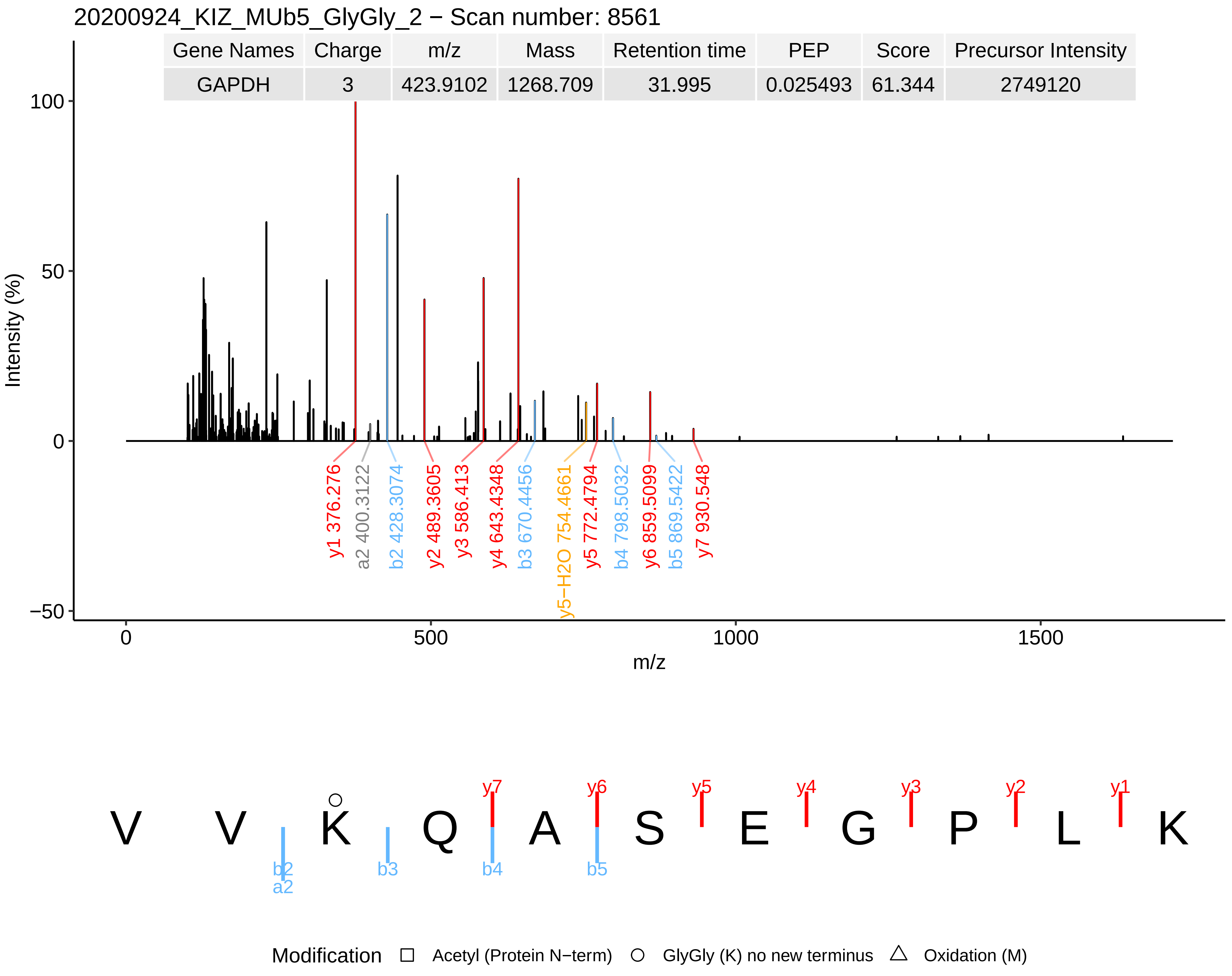
20200924_KIZ_MUb5_GlyGly_2 − Scan number: 8561
Gene Names | Charge | m/z | Mass | Retention time | PEP | Score | Precursor Intensity
GAPDH | 3 | 423.9102 | 1268.709 | 31.995 | 0.025493 | 61.344 | 2749120
Intensity (%)
100
50
0
−50
0
500
1000
1500
m/z
y1 376.276
a2 400.3122
b2 428.3074
y2 489.3605
y3 586.413
y4 643.4348
b3 670.4456
y5−H2O 754.4661
y5 772.4794
b4 798.5032
y6 859.5099
b5 869.5422
y7 930.548
V V K Q A S E G P L K
y7 y6 y5 y4 y3 y2 y1
b2 a2 b3 b4 b5
Modification
Acetyl (Protein N−term)
GlyGly (K) no new terminus
Oxidation (M)

20200924_KIZ_MUb5_GlyGly_2 − Scan number: 8694

Gene Names	Charge	m/z	Mass	Retention time	PEP	Score	Precursor Intensity
GAPDH	3	423.9102	1268.709	32.499	2.1591e−05	107.06	10265401

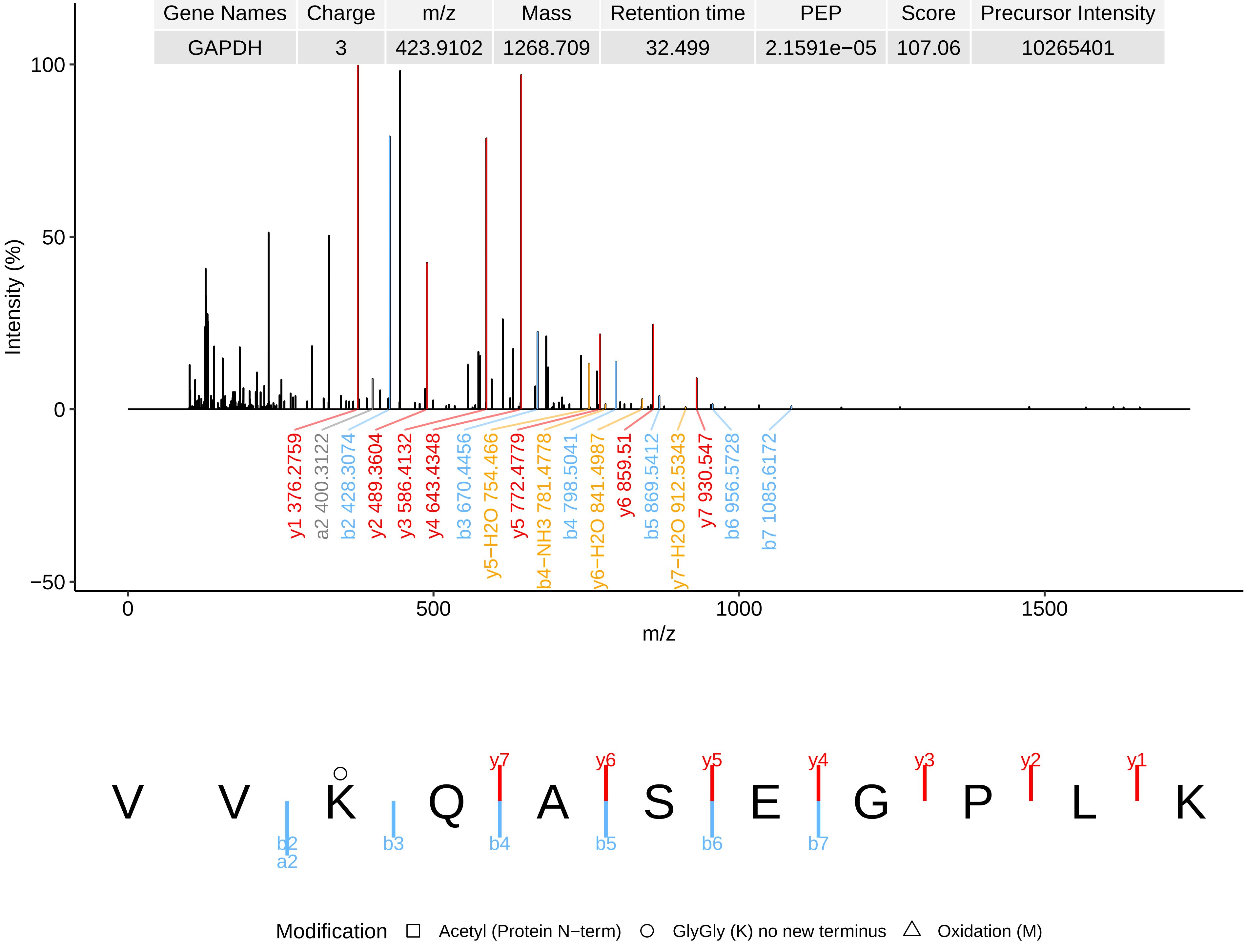

20201118_KIZ_MUb6_GlyGly_1 − Scan number: 8844

Gene Names	Charge	m/z	Mass	Retention time	PEP	Score	Precursor Intensity
GAPDH	3	423.9102	1268.709	27.963	3.7374e−05	100.93	13682369

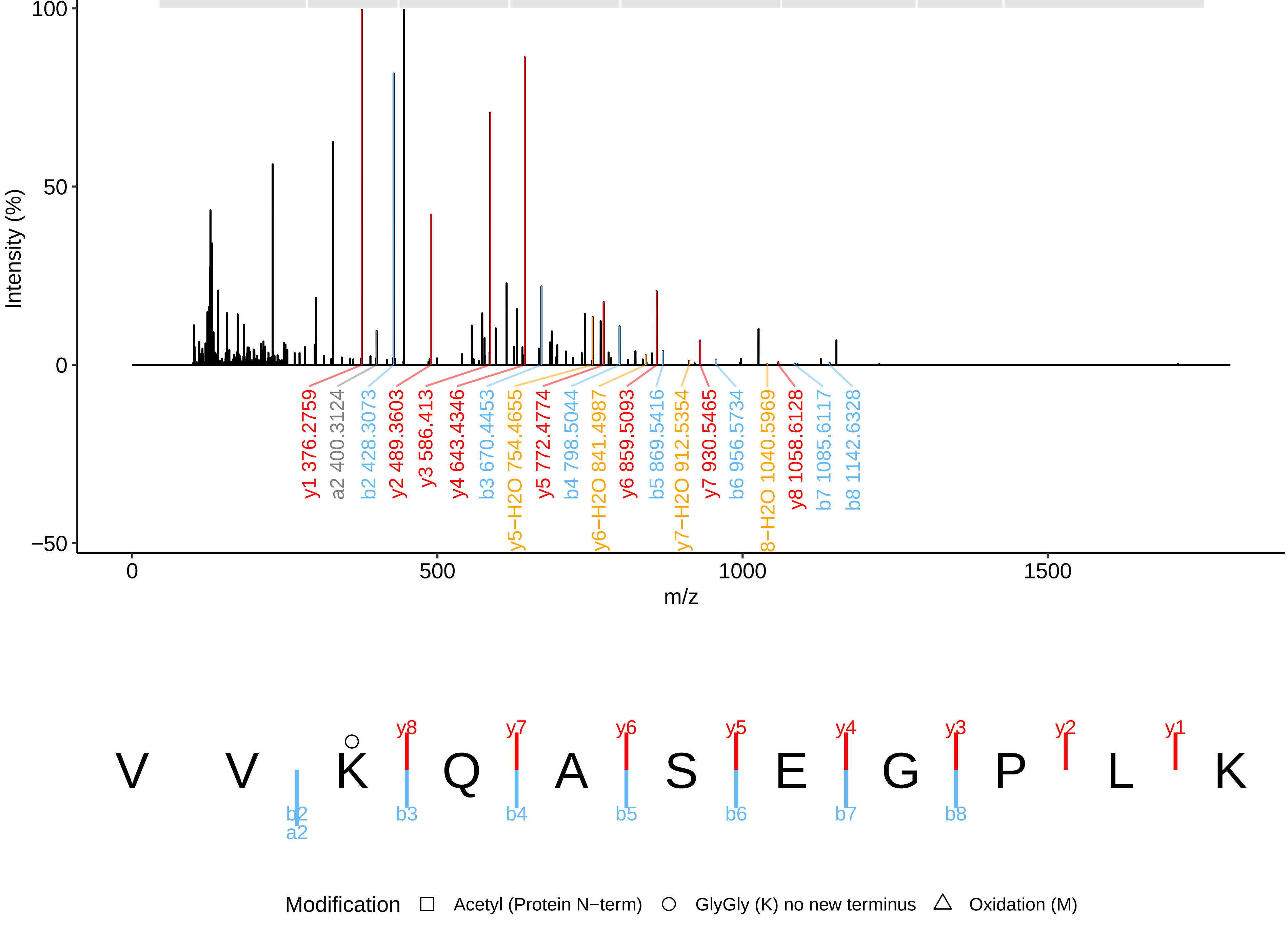

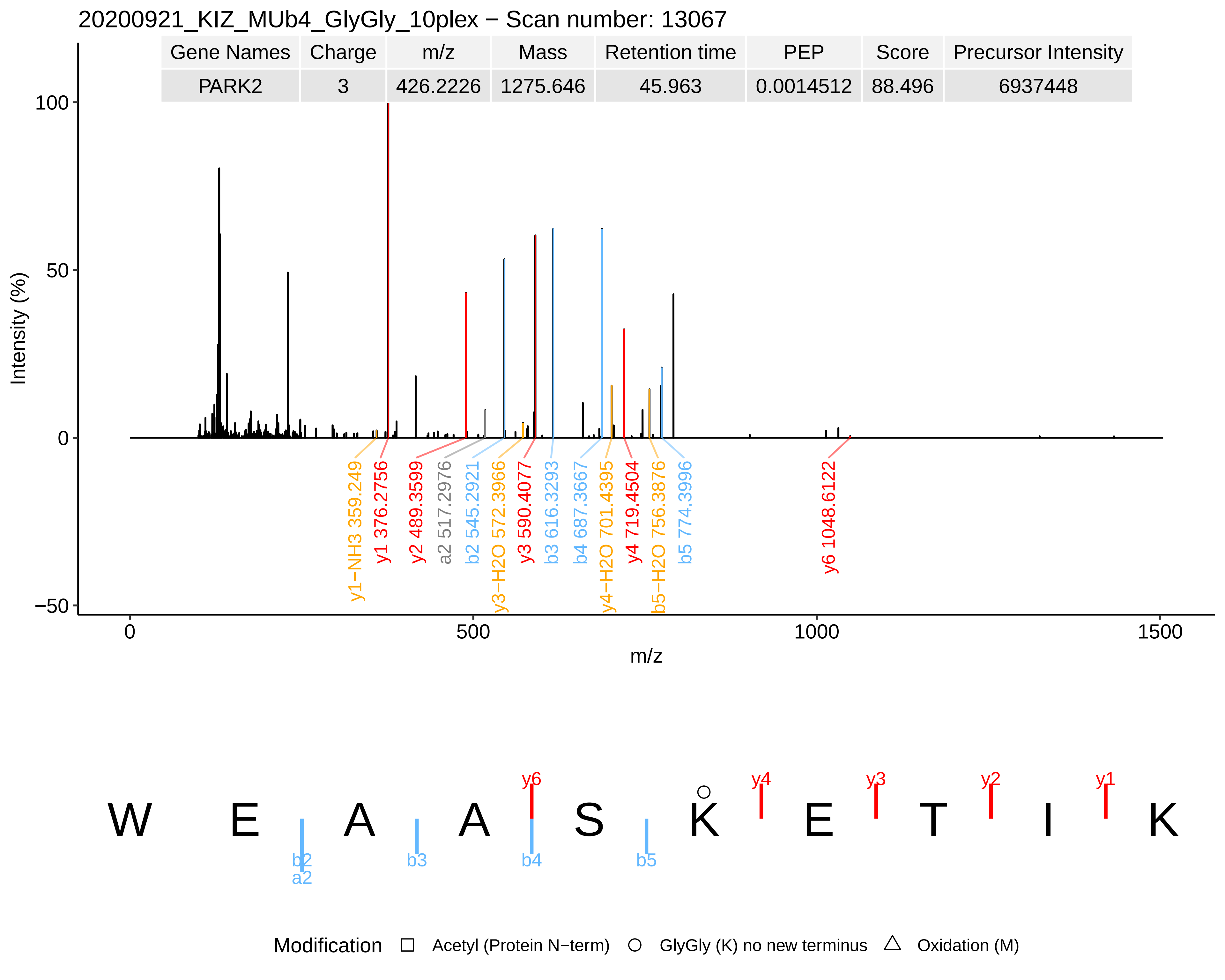
20200921_KIZ_MUb4_GlyGly_10plex − Scan number: 13067
Gene Names
Charge
m/z
Mass
Retention time
PEP
Score
Precursor Intensity
PARK2
3
426.2226
1275.646
45.963
0.0014512
88.496
6937448
Intensity (%)
100
50
0
−50
m/z
0
500
1000
1500
y1−NH3 359.249
y1 376.2756
y2 489.3599
a2 517.2976
b2 545.2921
y3−H2O 572.3966
y3 590.4077
b3 616.3293
b4 687.3667
y4−H2O 701.4395
y4 719.4504
b5−H2O 756.3876
b5 774.3996
y6 1048.6122
W E A A S K E T I K
y6
y4
y3
y2
y1
b2
a2
b3
b4
b5
Modification
Acetyl (Protein N−term)
GlyGly (K) no new terminus
Oxidation (M)

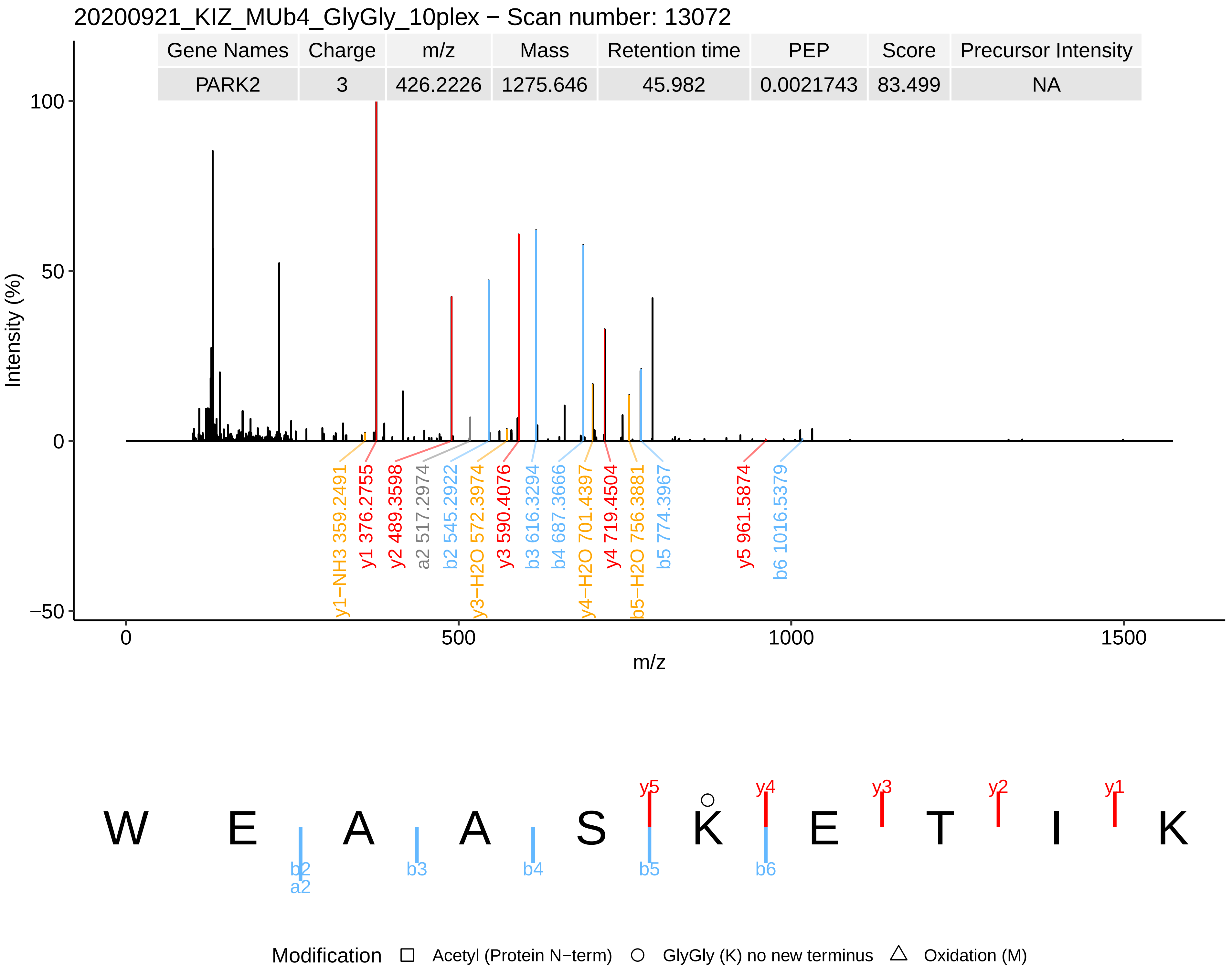

20200921_KIZ_MUb4_GlyGly_10plex − Scan number: 13072
Gene Names | Charge | m/z | Mass | Retention time | PEP | Score | Precursor Intensity
PARK2 | 3 | 426.2226 | 1275.646 | 45.982 | 0.0021743 | 83.499 | NA
Intensity (%)
100
50
0
−50
0
500
1000
1500
m/z
y1−NH3 359.2491
y1 376.2755
y2 489.3598
a2 517.2974
b2 545.2922
y3−H2O 572.3974
y3 590.4076
b3 616.3294
b4 687.3666
y4−H2O 701.4397
y4 719.4504
b5−H2O 756.3881
b5 774.3967
y5 961.5874
b6 1016.5379
W E A A S K E T I K
y5 y4 y3 y2 y1
b2 a2 b3 b4 b5 b6
Modification
Acetyl (Protein N−term)
GlyGly (K) no new terminus
Oxidation (M)

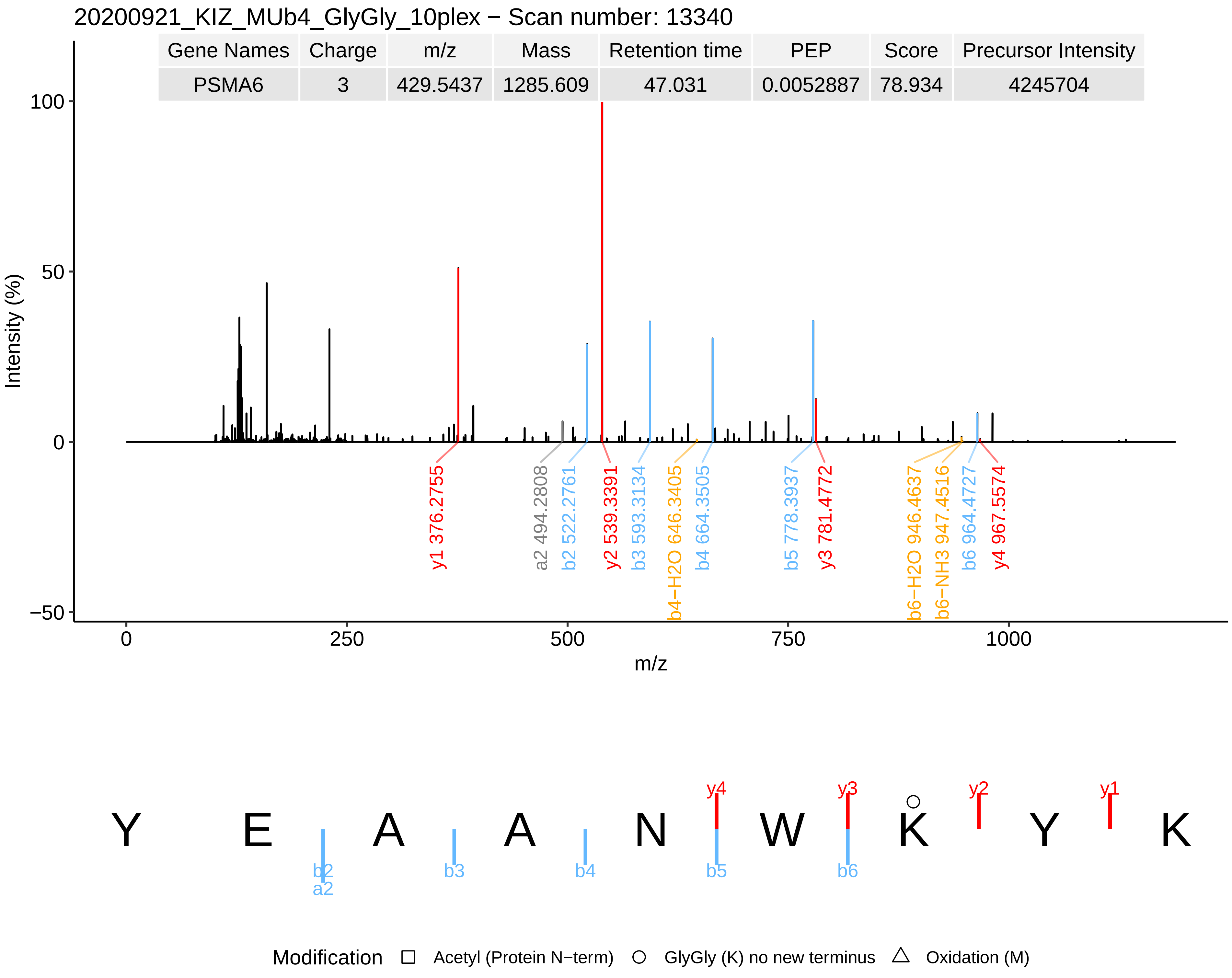
20200921_KIZ_MUb4_GlyGly_10plex − Scan number: 13340
Gene Names | Charge | m/z | Mass | Retention time | PEP | Score | Precursor Intensity
PSMA6 | 3 | 429.5437 | 1285.609 | 47.031 | 0.0052887 | 78.934 | 4245704
Intensity (%)
100
50
0
−50
0
250
500
750
1000
m/z
y1 376.2755
a2 494.2808
b2 522.2761
y2 539.3391
b3 593.3134
b4−H2O 646.3405
b4 664.3505
b5 778.3937
y3 781.4772
b6−H2O 946.4637
b6−NH3 947.4516
b6 964.4727
y4 967.5574
Y E A A N W K Y K
y4 y3 y2 y1
b2 a2 b3 b4 b5 b6
Modification
Acetyl (Protein N−term)
GlyGly (K) no new terminus
Oxidation (M)

20200921_KIZ_MUb4_GlyGly_10plex_2 − Scan number: 12310

Gene Names	Charge	m/z	Mass	Retention time	PEP	Score	Precursor Intensity
PSMA6	3	429.5437	1285.609	47.599	0.017399	67.563	1391046

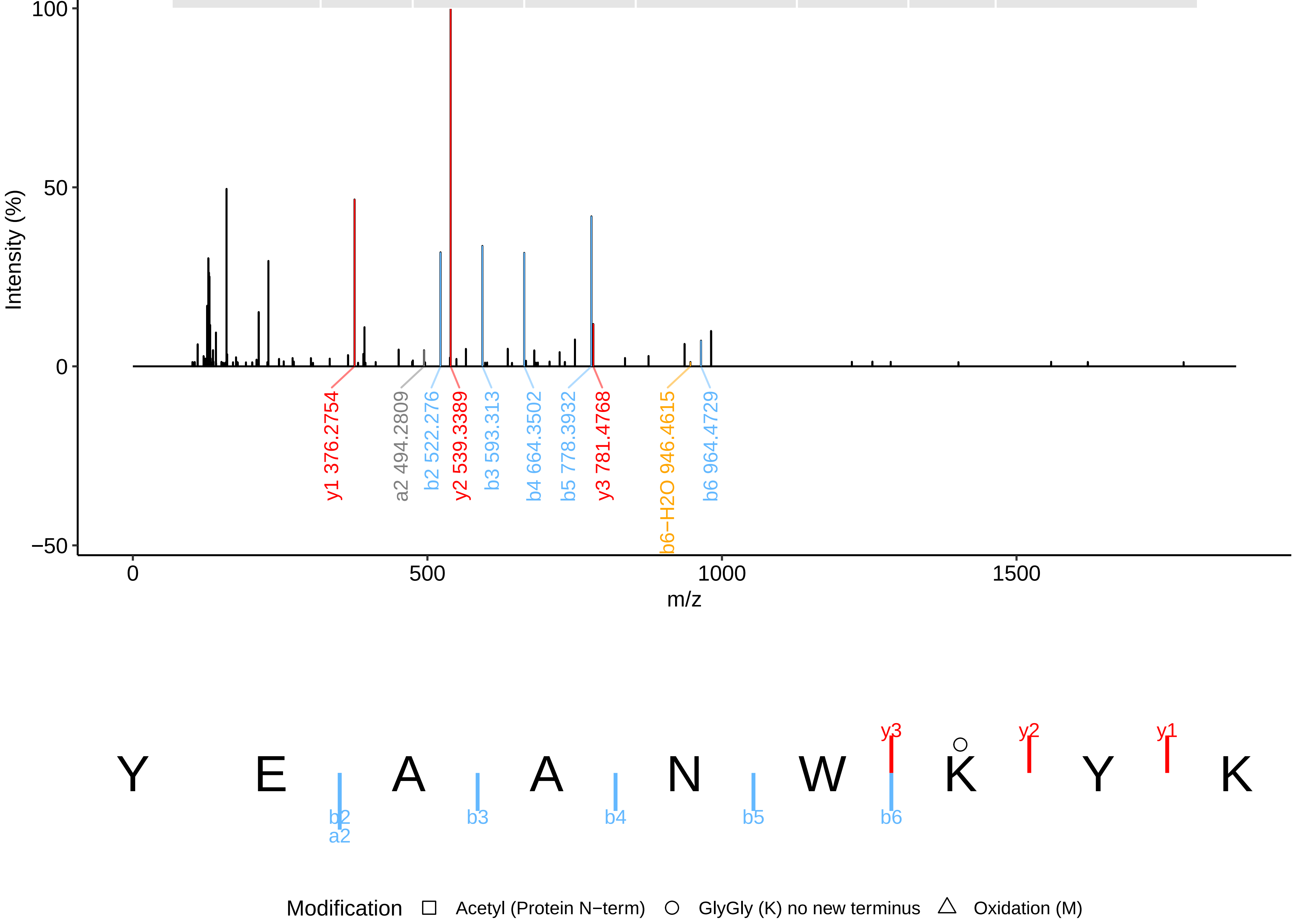

20200924_KIZ_MUb5_GlyGly_1 − Scan number: 12218

Gene Names	Charge	m/z	Mass	Retention time	PEP	Score	Precursor Intensity
PSMA6	3	429.5437	1285.609	39.71	0.0061636	78.113	22737830

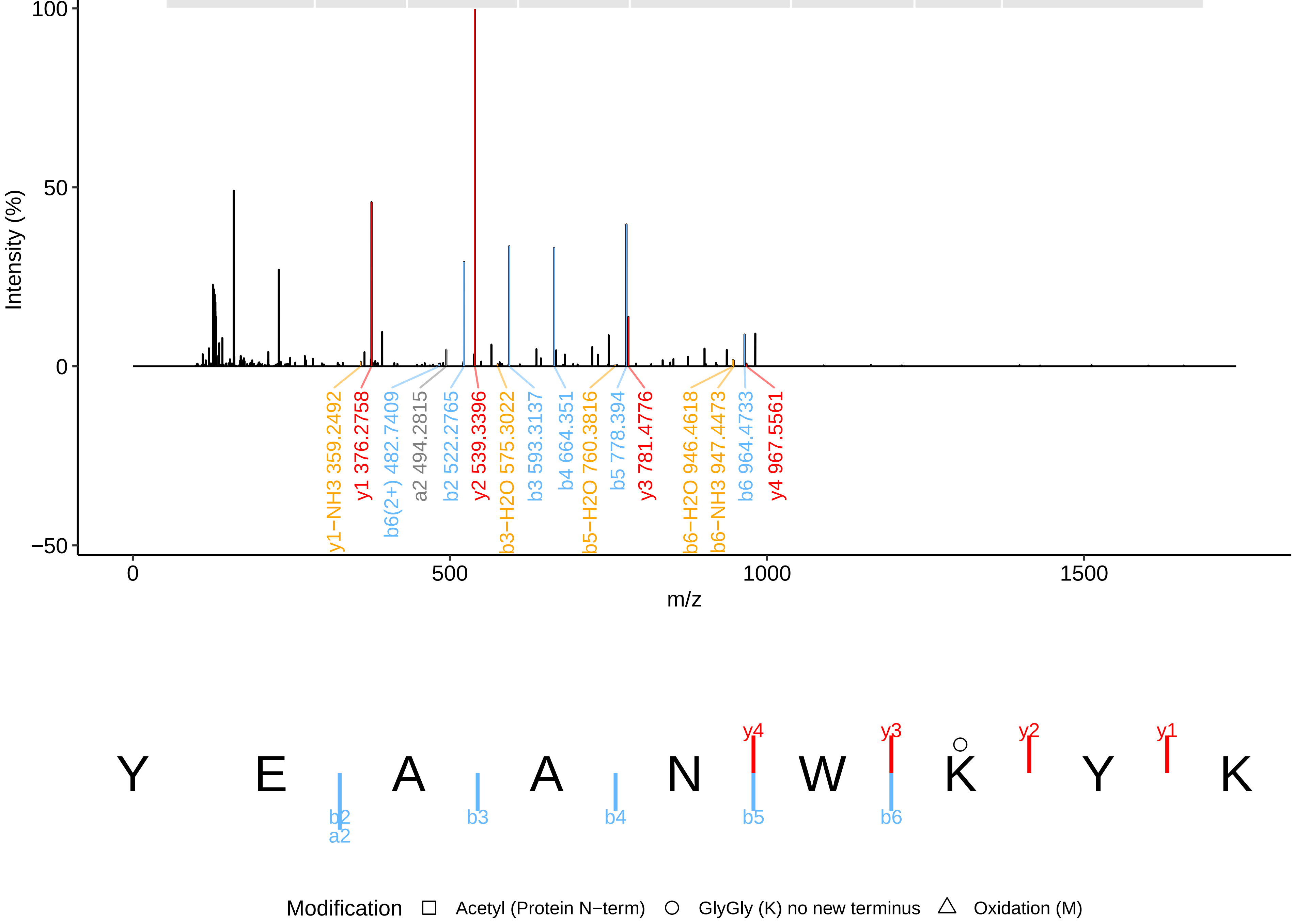

20200924_KIZ_MUb5_GlyGly_1 − Scan number: 12367

Gene Names	Charge	m/z	Mass	Retention time	PEP	Score	Precursor Intensity
PSMA6	3	429.5437	1285.609	40.228	0.0090758	75.378	10488540

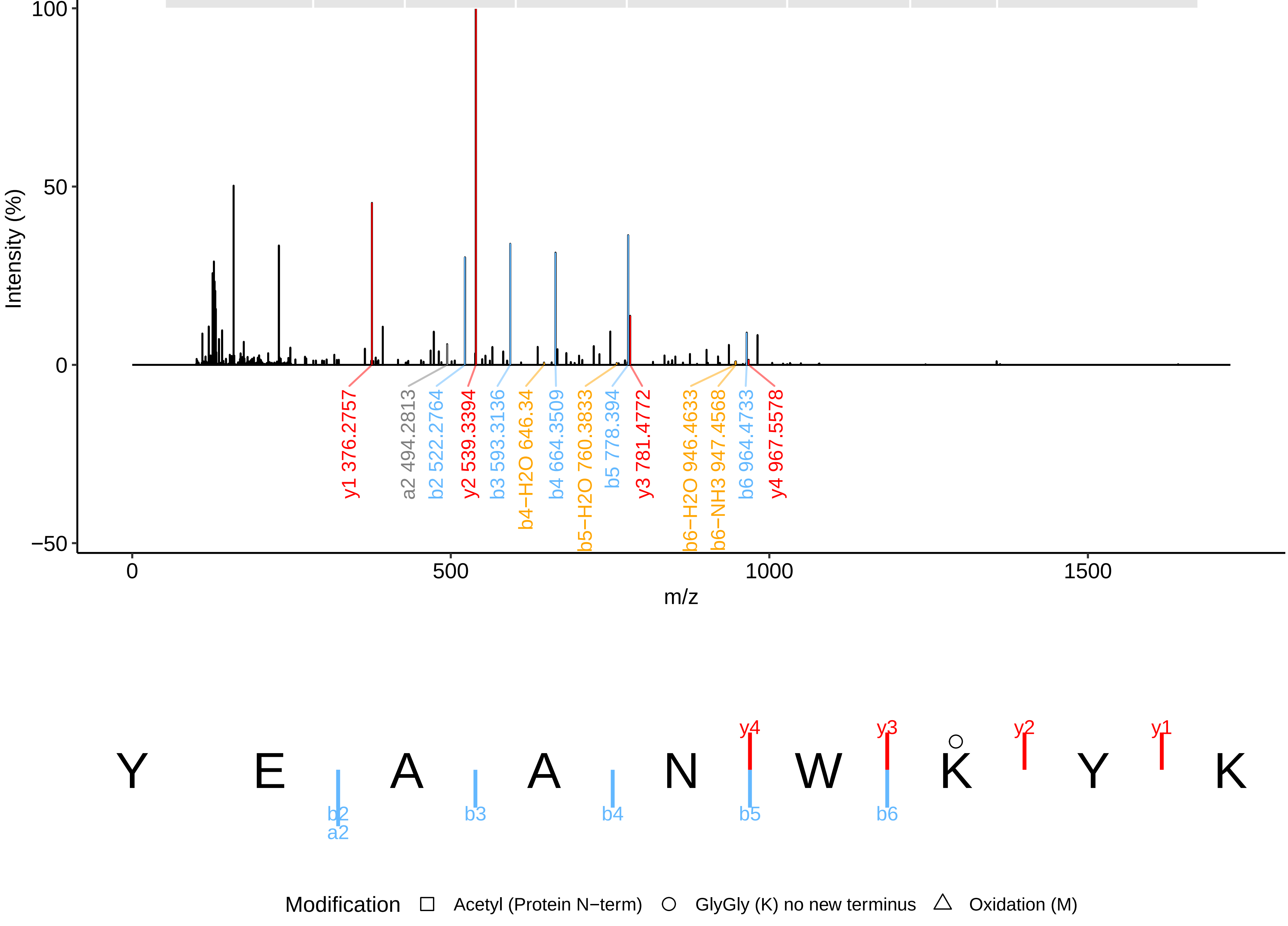

20200924_KIZ_MUb5_GlyGly_1 − Scan number: 12068

Gene Names	Charge	m/z	Mass	Retention time	PEP	Score	Precursor Intensity
PSMA6	3	429.5437	1285.609	39.202	0.024133	66.435	NA

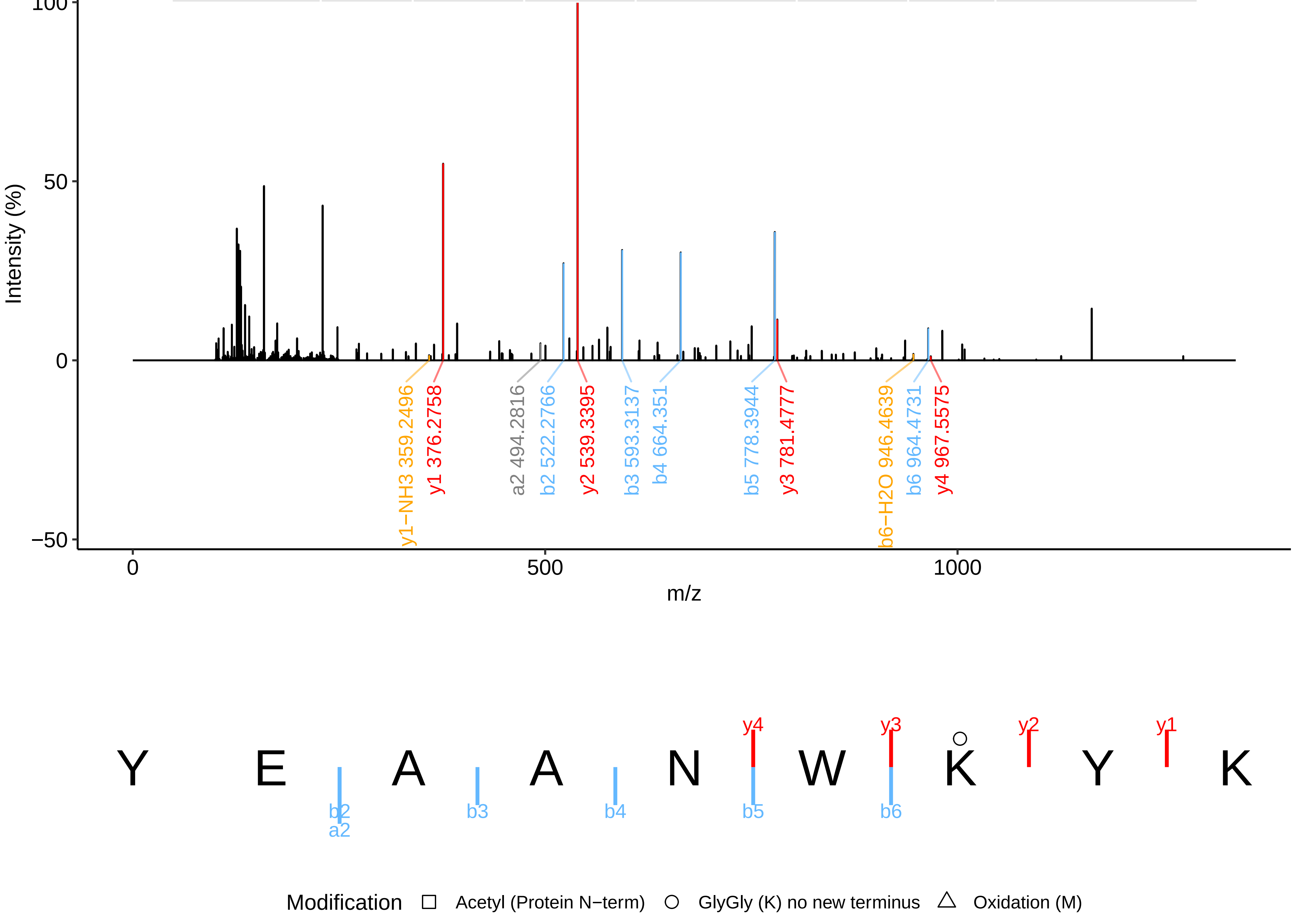

20200924_KIZ_MUb5_GlyGly_2 − Scan number: 11563

Gene Names	Charge	m/z	Mass	Retention time	PEP	Score	Precursor Intensity
PSMA6	3	429.5437	1285.609	43.234	0.011702	76.221	6894819

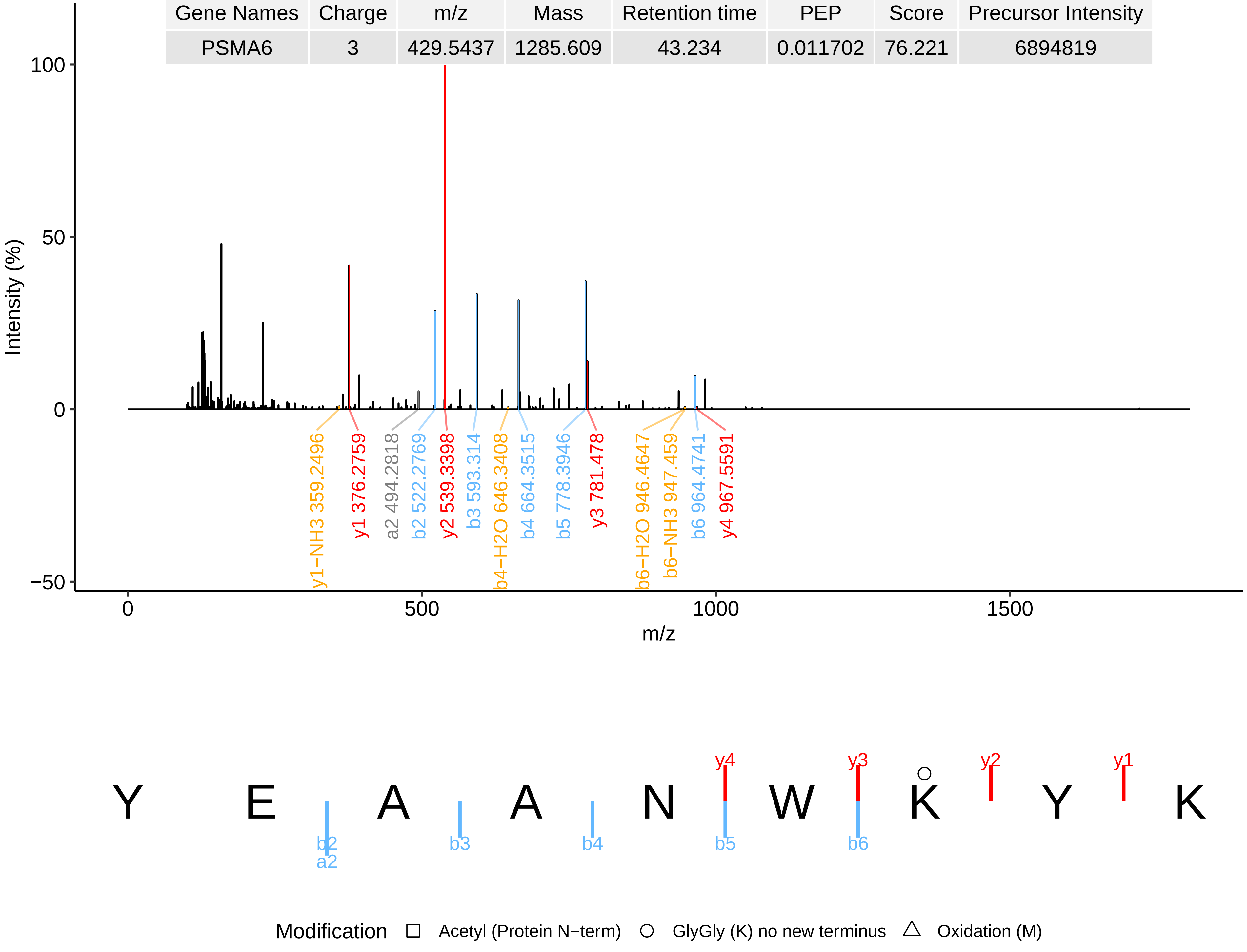

20201118_KIZ_MUb6_GlyGly_2 − Scan number: 14192

Gene Names	Charge	m/z	Mass	Retention time	PEP	Score	Precursor Intensity
PSMA6	3	429.5437	1285.609	47.803	0.017399	67.563	6087083

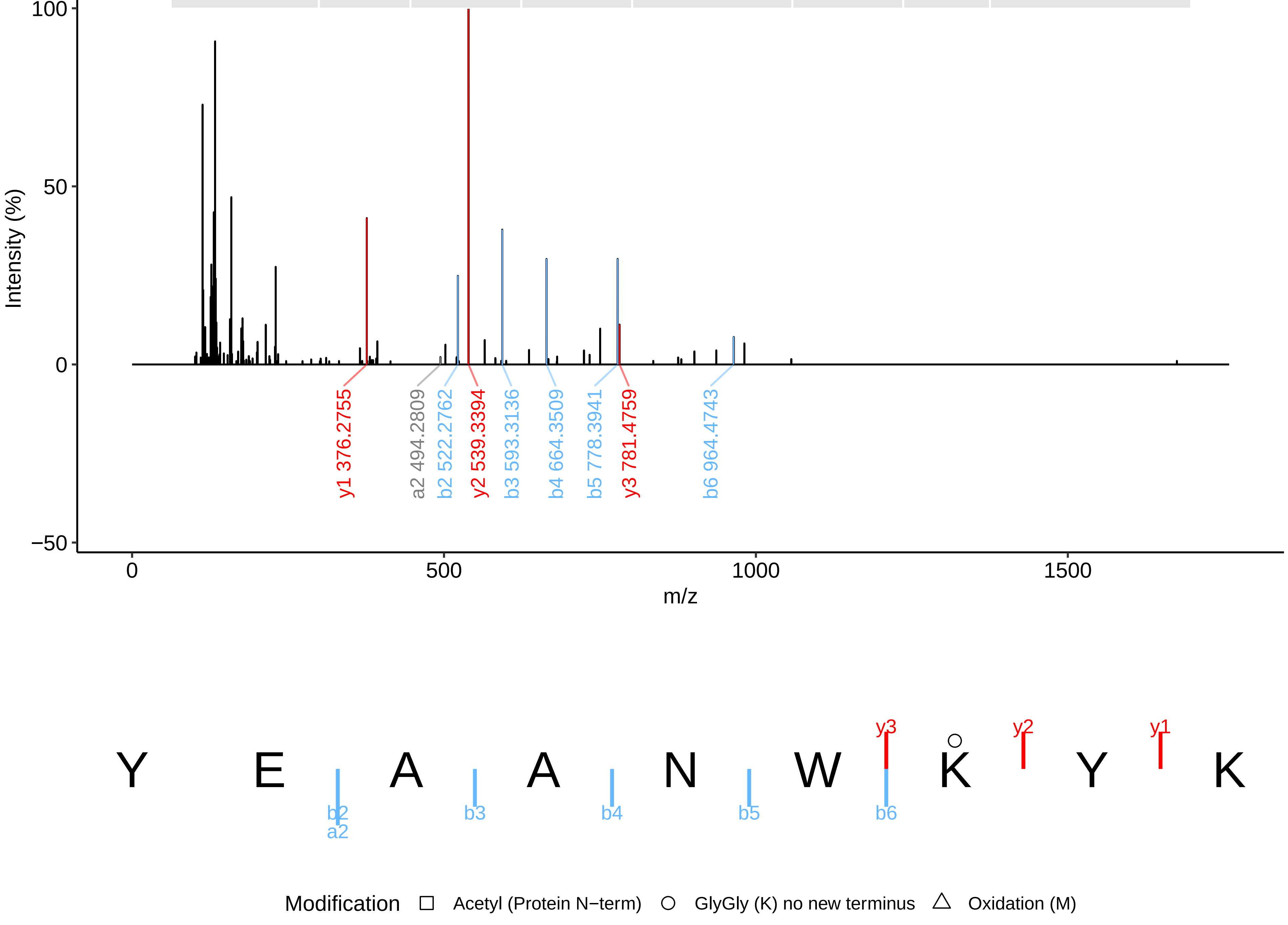

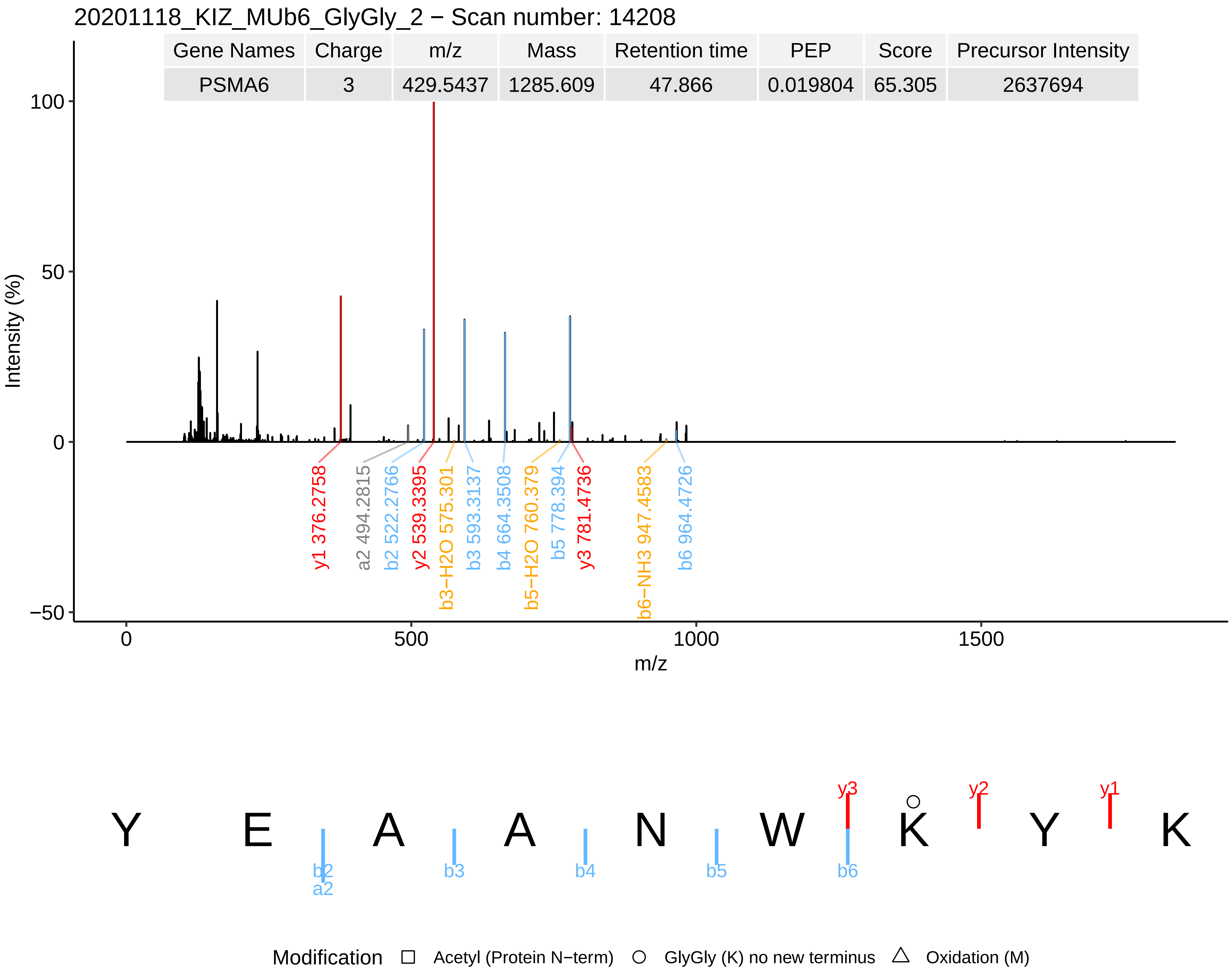
20201118_KIZ_MUb6_GlyGly_2 − Scan number: 14208
Gene Names
Charge
m/z
Mass
Retention time
PEP
Score
Precursor Intensity
PSMA6
3
429.5437
1285.609
47.866
0.019804
65.305
2637694
Intensity (%)
100
50
0
−50
0
500
1000
1500
m/z
y1 376.2758
a2 494.2815
b2 522.2766
y2 539.3395
b3−H2O 575.301
b3 593.3137
b4 664.3508
b5−H2O 760.379
b5 778.394
y3 781.4736
b6−NH3 947.4583
b6 964.4726
Y
E
A
A
N
W
K
Y
K
y3
y2
y1
b2
a2
b3
b4
b5
b6
Modification
Acetyl (Protein N−term)
GlyGly (K) no new terminus
Oxidation (M)

20200921_KIZ_MUb4_GlyGly_10plex − Scan number: 9948

Gene Names	Charge	m/z	Mass	Retention time	PEP	Score	Precursor Intensity
UBR2	3	495.5758	1483.706	34.259	2.0971e−08	126.19	4903545

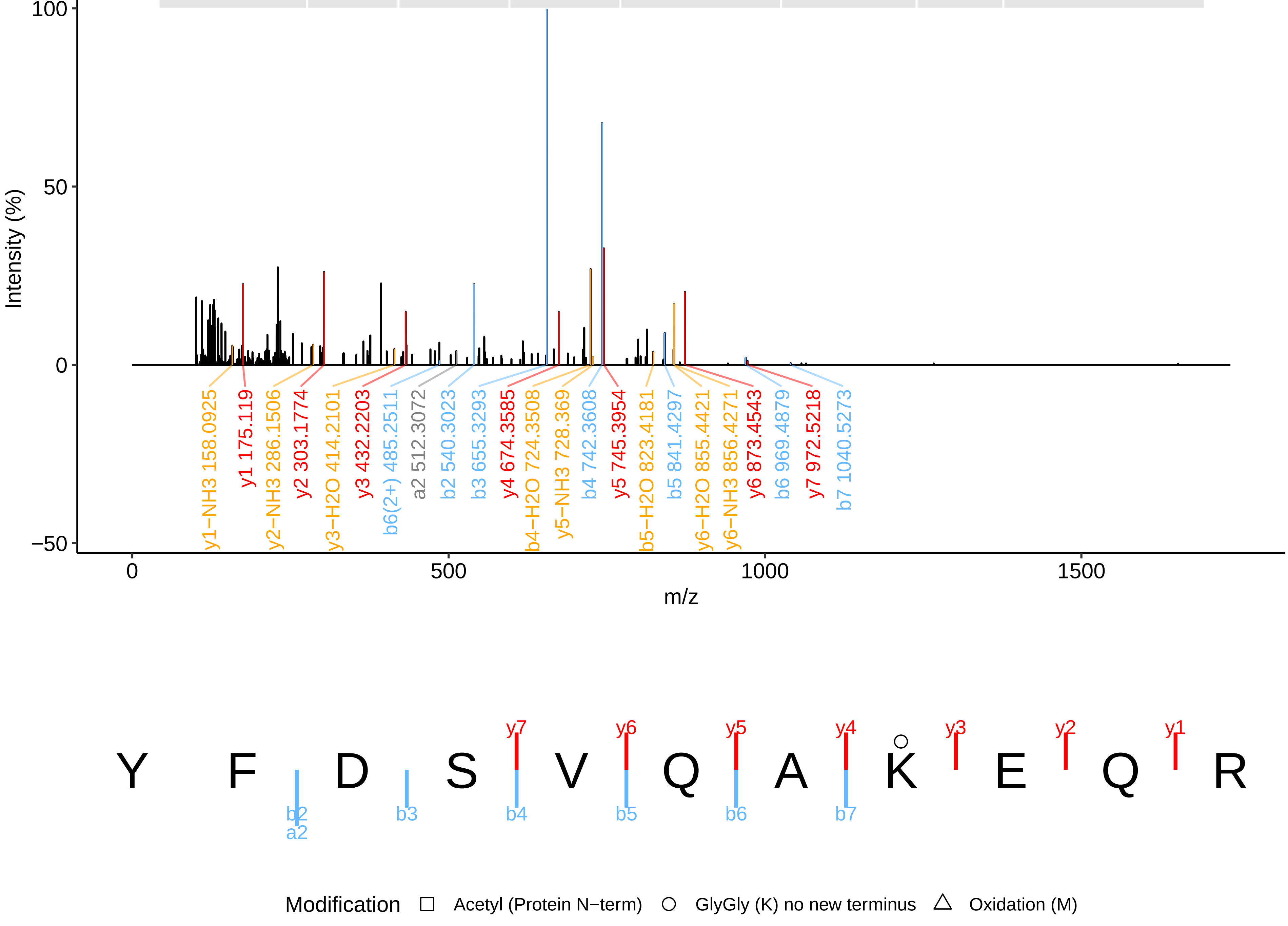

20200921_KIZ_MUb4_GlyGly_10plex_2 − Scan number: 9139

Gene Names	Charge	m/z	Mass	Retention time	PEP	Score	Precursor Intensity
UBR2	3	495.5758	1483.706	34.655	3.3269e−05	102.52	374321

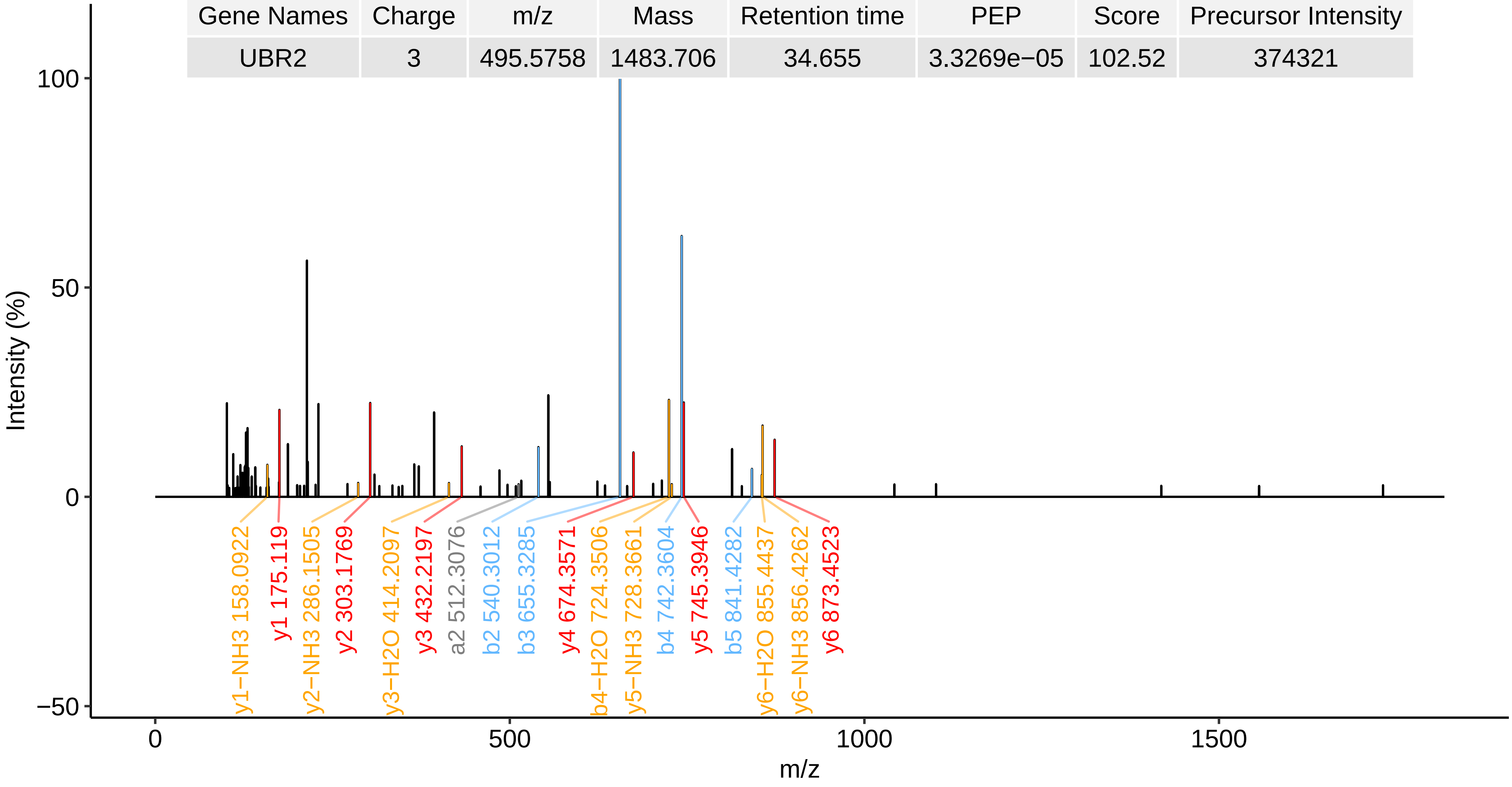

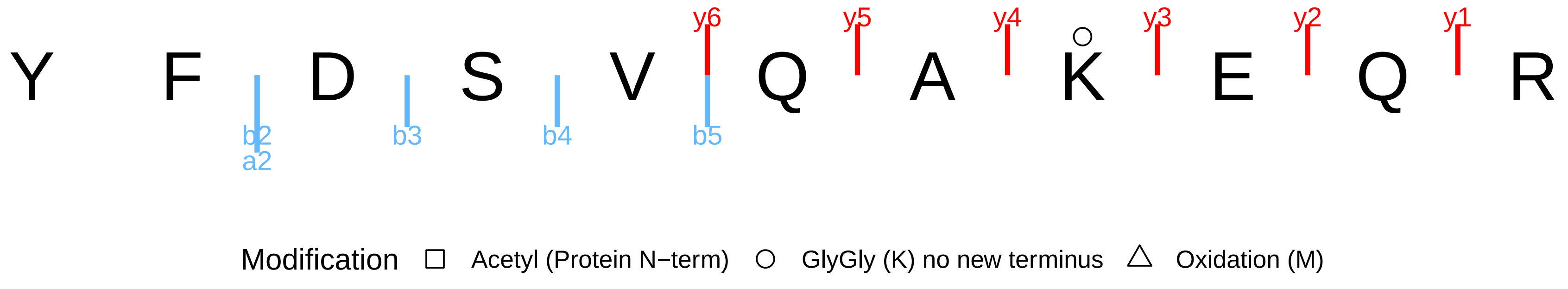

20200921_KIZ_MUb4_GlyGly_10plex − Scan number: 3409

Gene Names	Charge	m/z	Mass	Retention time	PEP	Score	Precursor Intensity
HSPA8	4	320.9112	1279.616	10.782	0.0090277	93.111	5915708

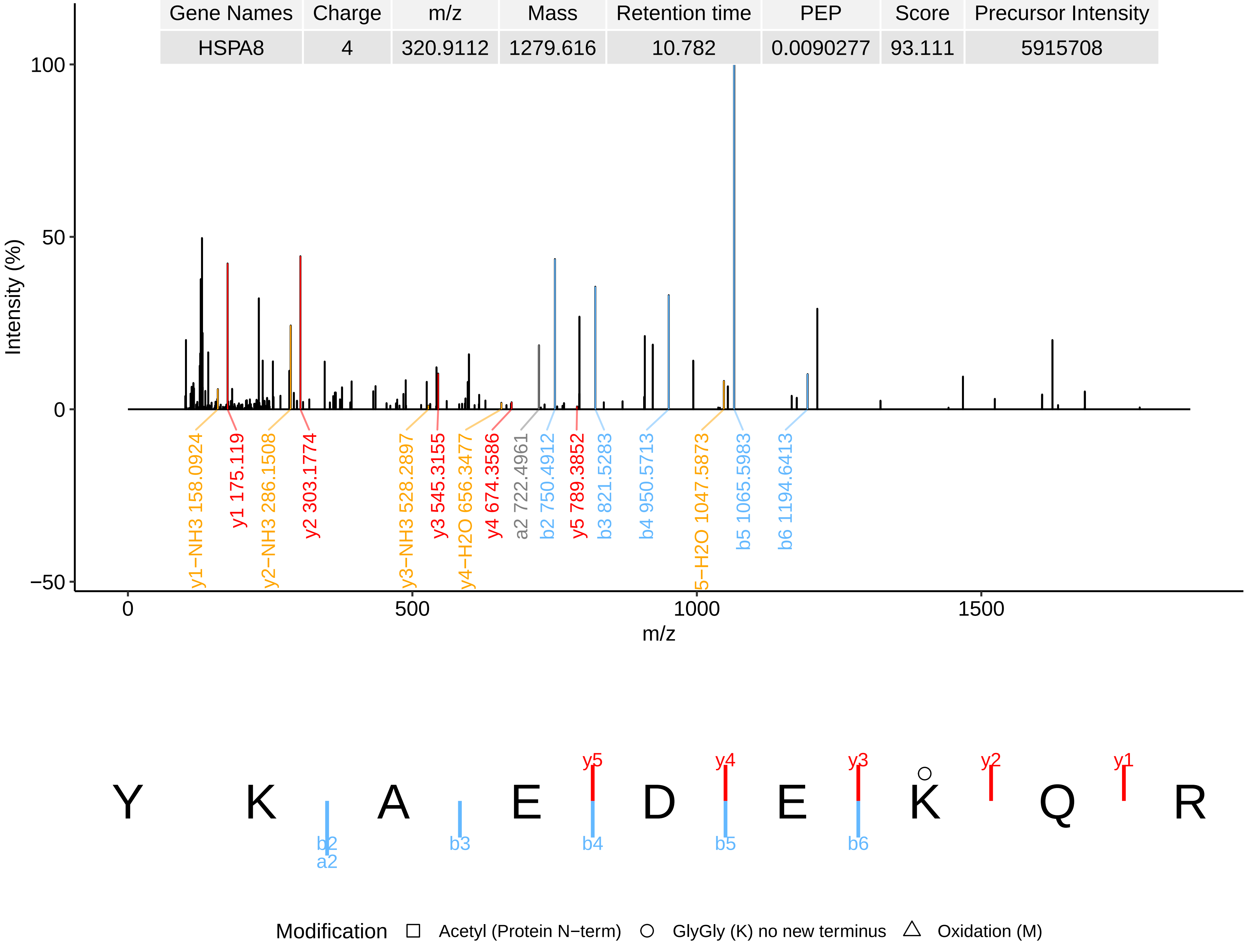

20200921_KIZ_MUb4_GlyGly_10plex − Scan number: 5753

Gene Names	Charge	m/z	Mass	Retention time	PEP	Score	Precursor Intensity
ATP5A1	3	454.888	1361.642	18.868	7.9948e−06	108.72	4853543

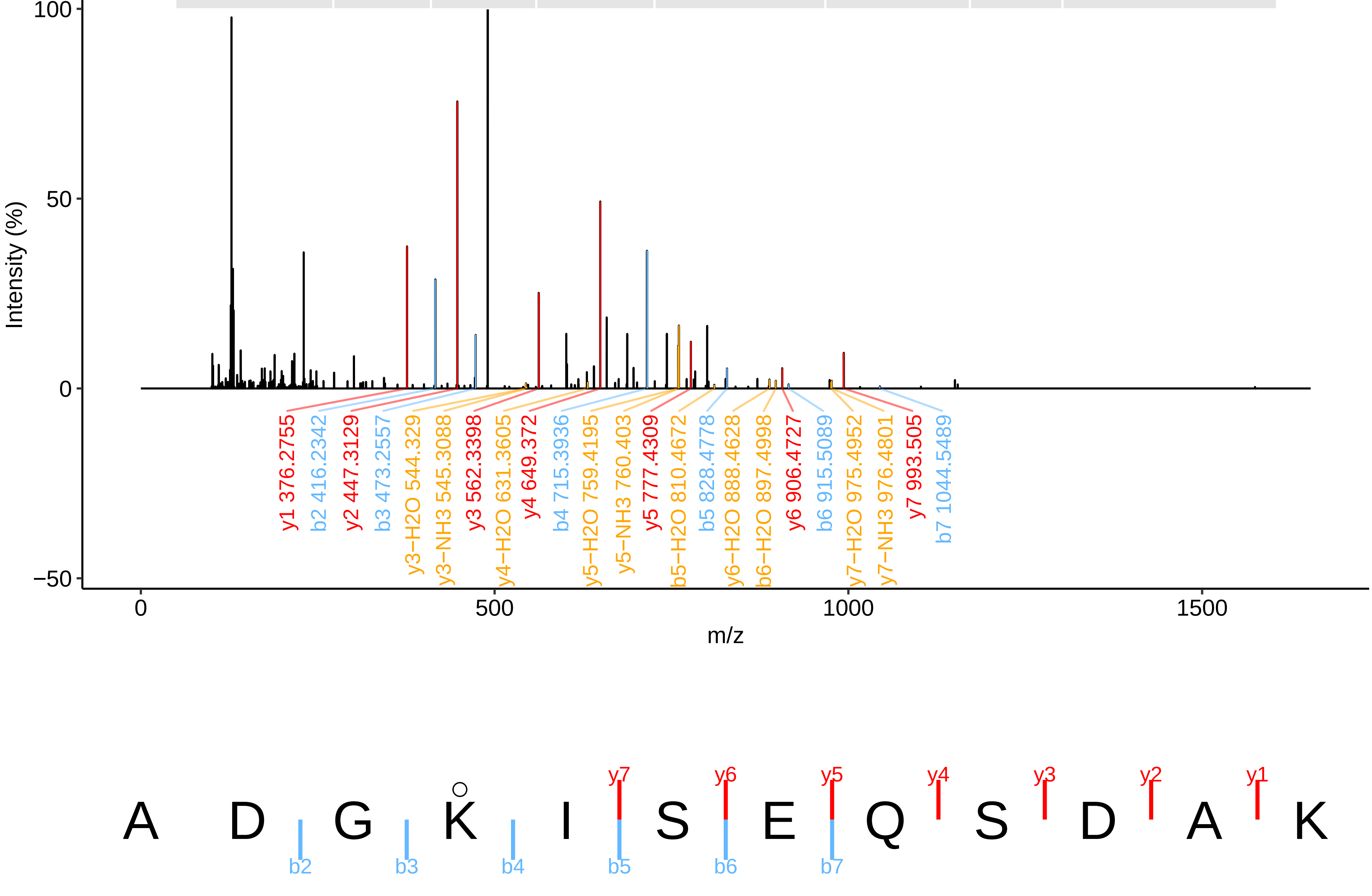

Modification □ Acetyl (Protein N−term) ○ GlyGly (K) no new terminus △ Oxidation (M)

20200921_KIZ_MUb4_GlyGly_10plex_2 − Scan number: 5234

Gene Names	Charge	m/z	Mass	Retention time	PEP	Score	Precursor Intensity
ATP5A1	3	454.888	1361.642	18.727	0.064426	53.252	779082.4

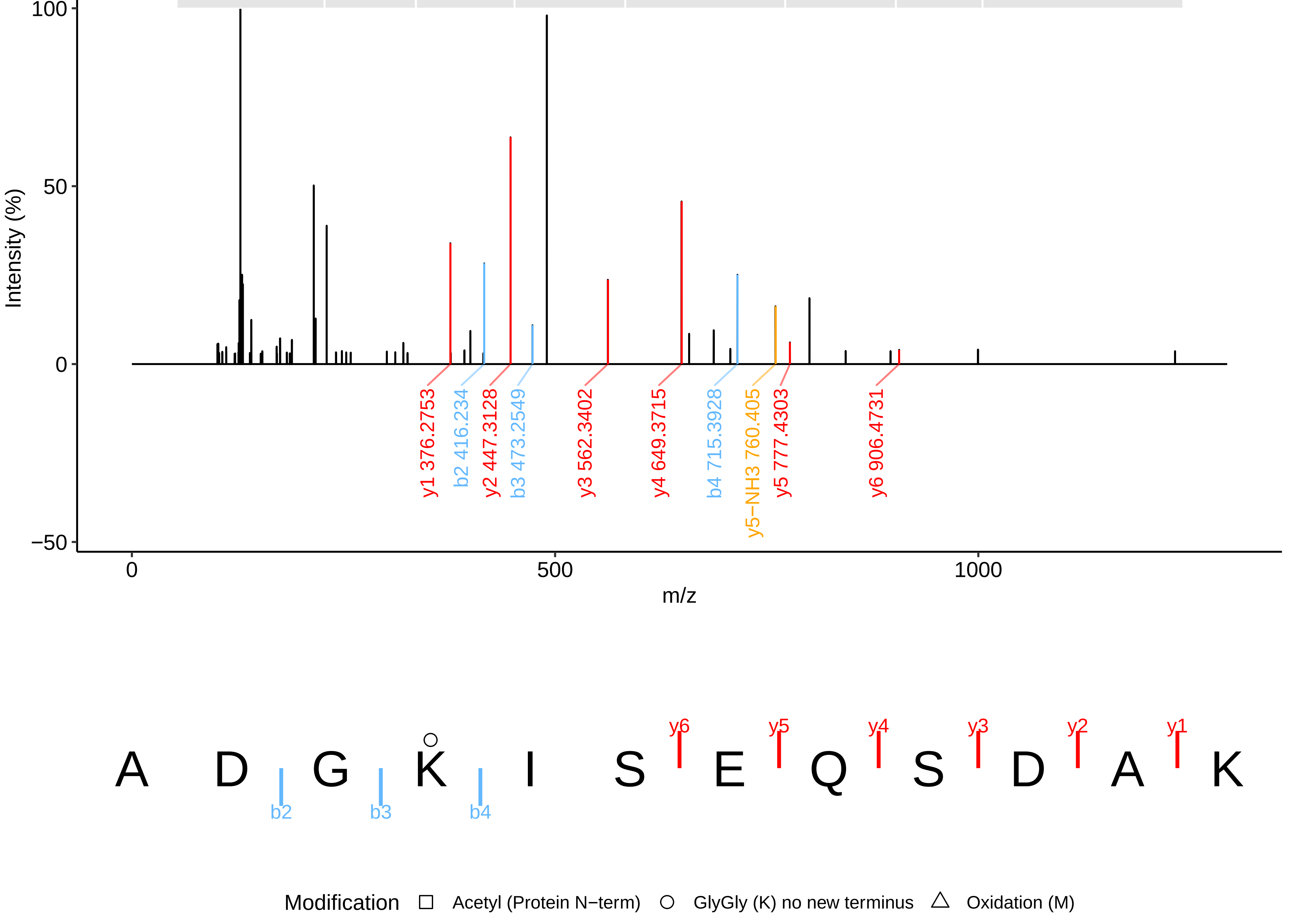

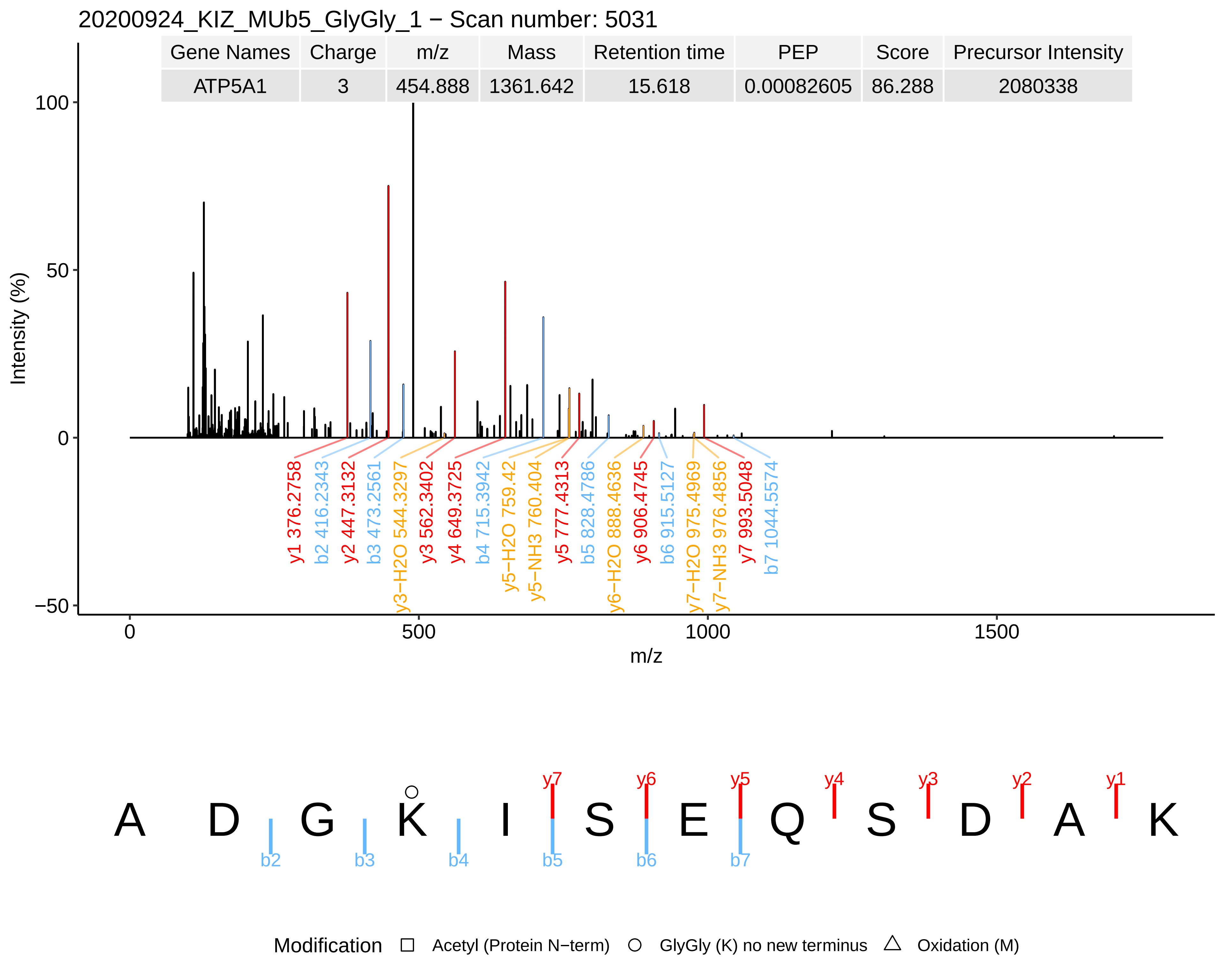
20200924_KIZ_MUb5_GlyGly_1 − Scan number: 5031
Gene Names
Charge
m/z
Mass
Retention time
PEP
Score
Precursor Intensity
ATP5A1
3
454.888
1361.642
15.618
0.00082605
86.288
2080338
Intensity (%)
100
50
0
−50
0
500
1000
1500
m/z
y1 376.2758
b2 416.2343
y2 447.3132
b3 473.2561
y3−H2O 544.3297
y3 562.3402
y4 649.3725
b4 715.3942
y5−H2O 759.42
y5−NH3 760.404
y5 777.4313
b5 828.4786
y6−H2O 888.4636
y6 906.4745
b6 915.5127
y7−H2O 975.4969
y7−NH3 976.4856
y7 993.5048
b7 1044.5574
A D G K I S E Q S D A K
y7 y6 y5 y4 y3 y2 y1
b2 b3 b4 b5 b6 b7
Modification
Acetyl (Protein N−term)
GlyGly (K) no new terminus
Oxidation (M)

20200921_KIZ_MUb4_GlyGly_10plex_2 − Scan number: 17037

Gene Names	Charge	m/z	Mass	Retention time	PEP	Score	Precursor Intensity
RDH11	3	442.2347	1323.682	66.571	0.062062	54.259	930022

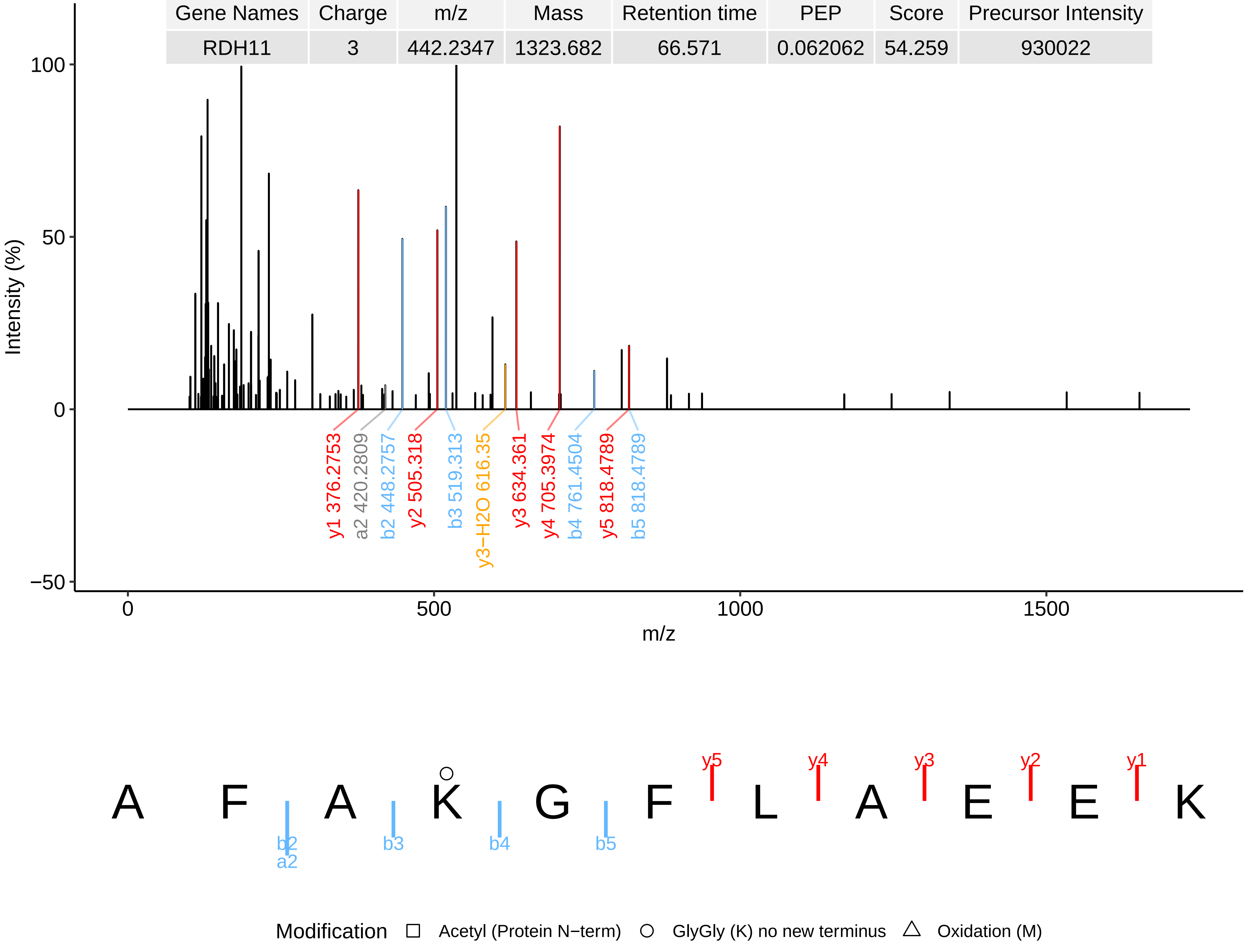

20200921_KIZ_MUb4_GlyGly_10plex − Scan number: 13353

Gene Names	Charge	m/z	Mass	Retention time	PEP	Score	Precursor Intensity
ARMC6	3	524.2776	1569.811	47.082	0.00012133	95.631	3018195

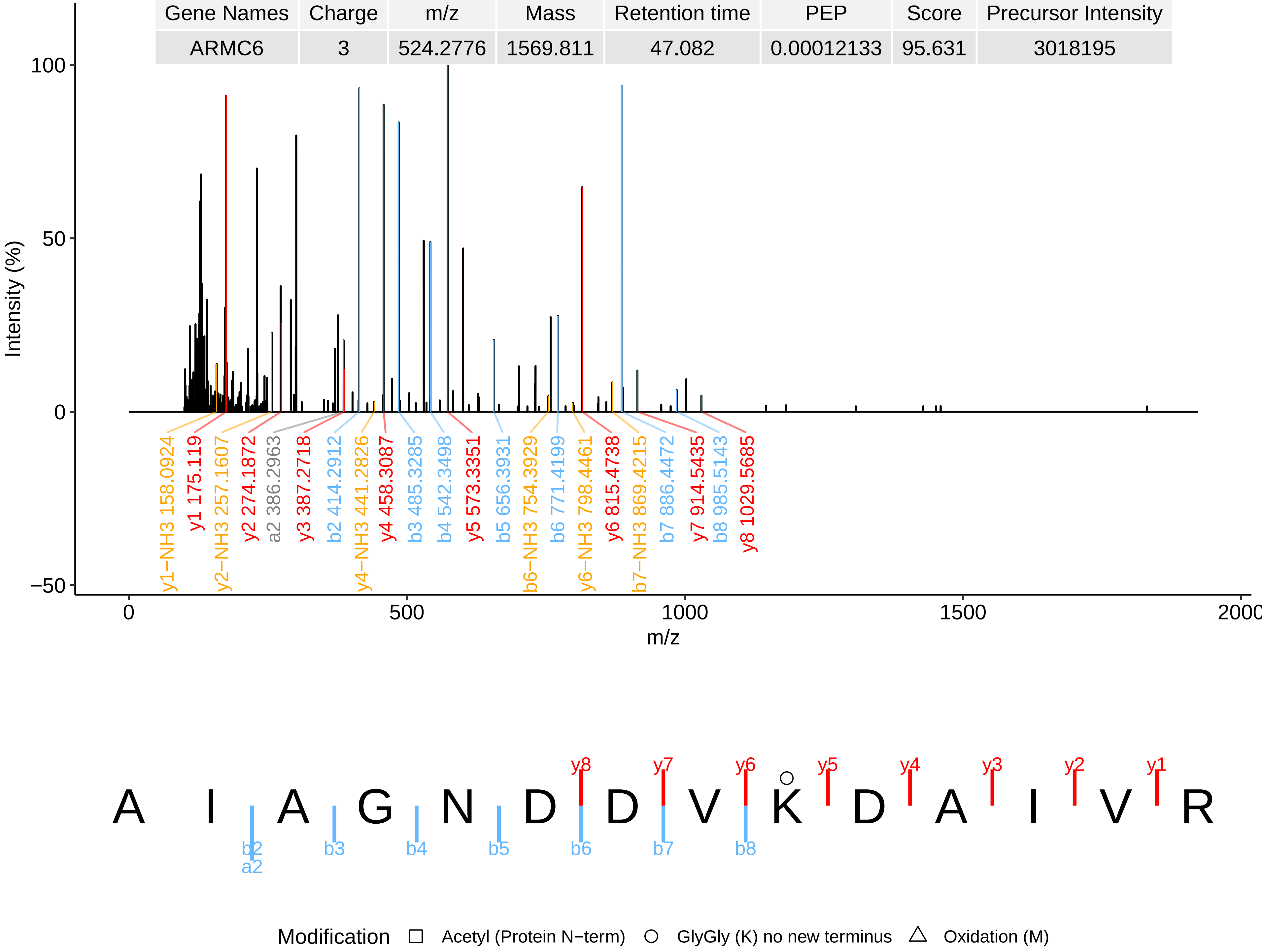

20200921_KIZ_MUb4_GlyGly_10plex_2 − Scan number: 14212

Gene Names	Charge	m/z	Mass	Retention time	PEP	Score	Precursor Intensity
RPL24	3	516.6119	1546.814	55.365	0.09077	40.066	367192.1

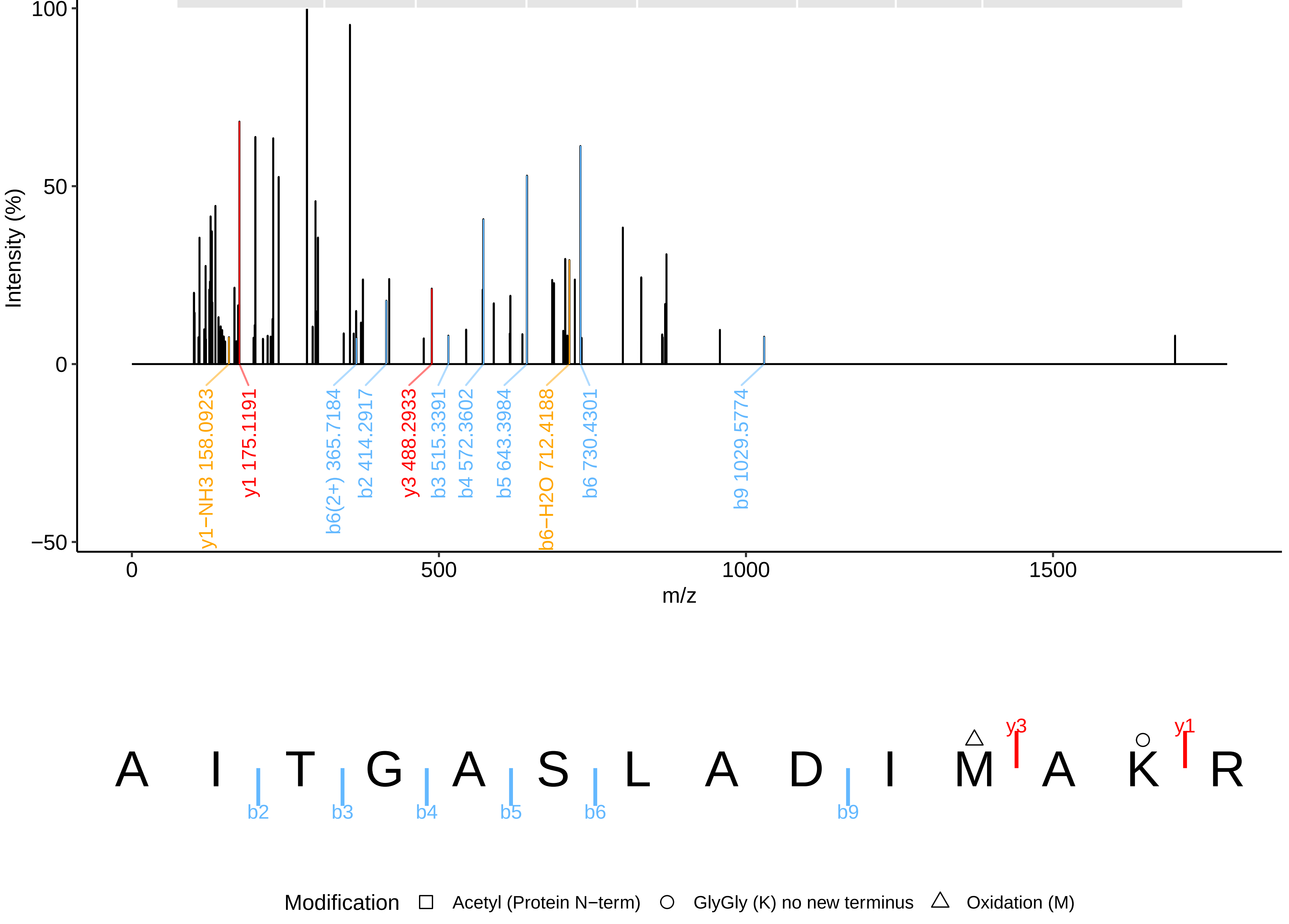

20200921_KIZ_MUb4_GlyGly_10plex − Scan number: 15855

Gene Names	Charge	m/z	Mass	Retention time	PEP	Score	Precursor Intensity
PABPC1;PABPC4	3	442.9066	1325.698	56.777	0.051165	57.174	3486531

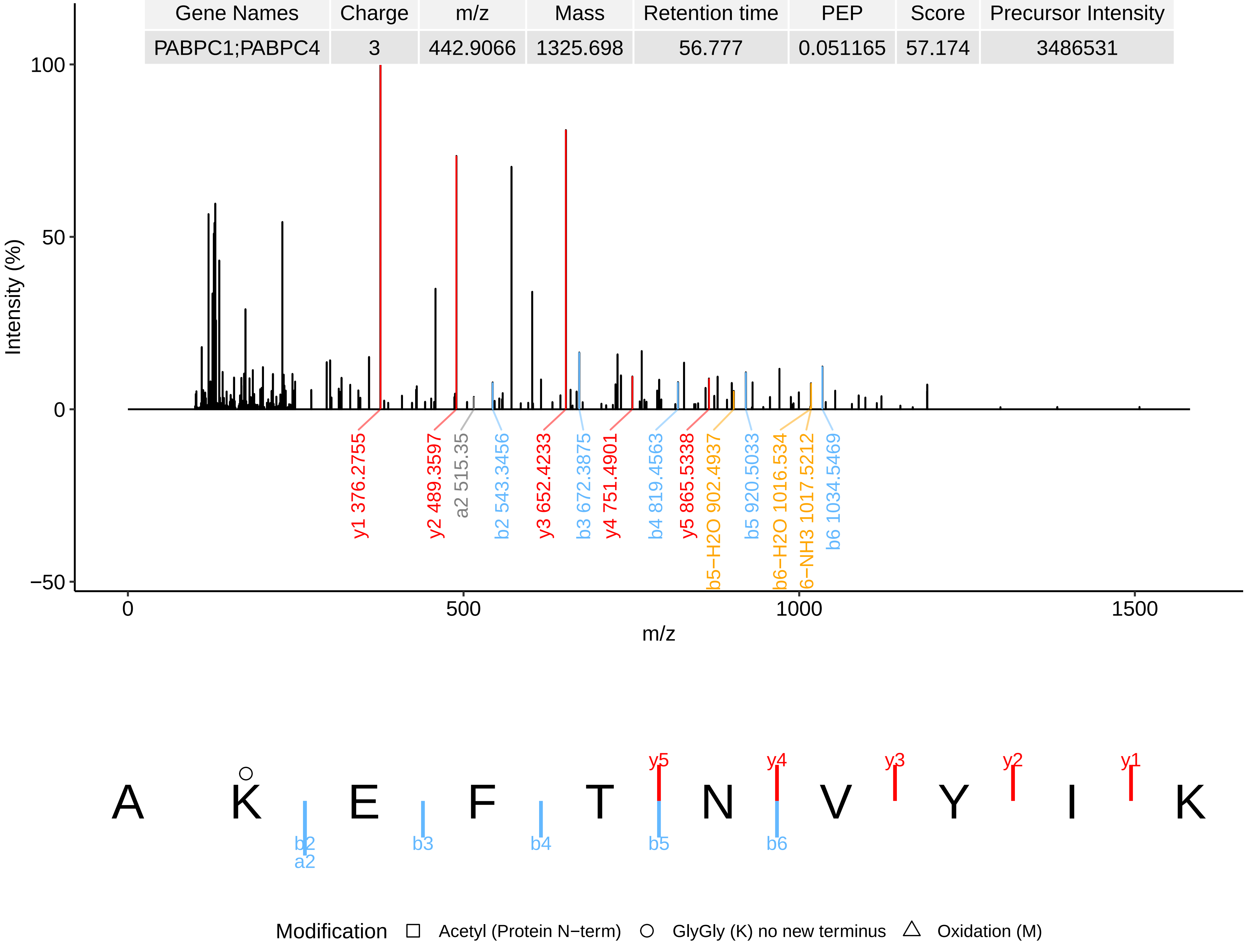

20200921_KIZ_MUb4_GlyGly_10plex − Scan number: 7832

Gene Names	Charge	m/z	Mass	Retention time	PEP	Score	Precursor Intensity
SLC7A5	3	523.9338	1568.779	26.43	2.3306e−13	139.89	5486702

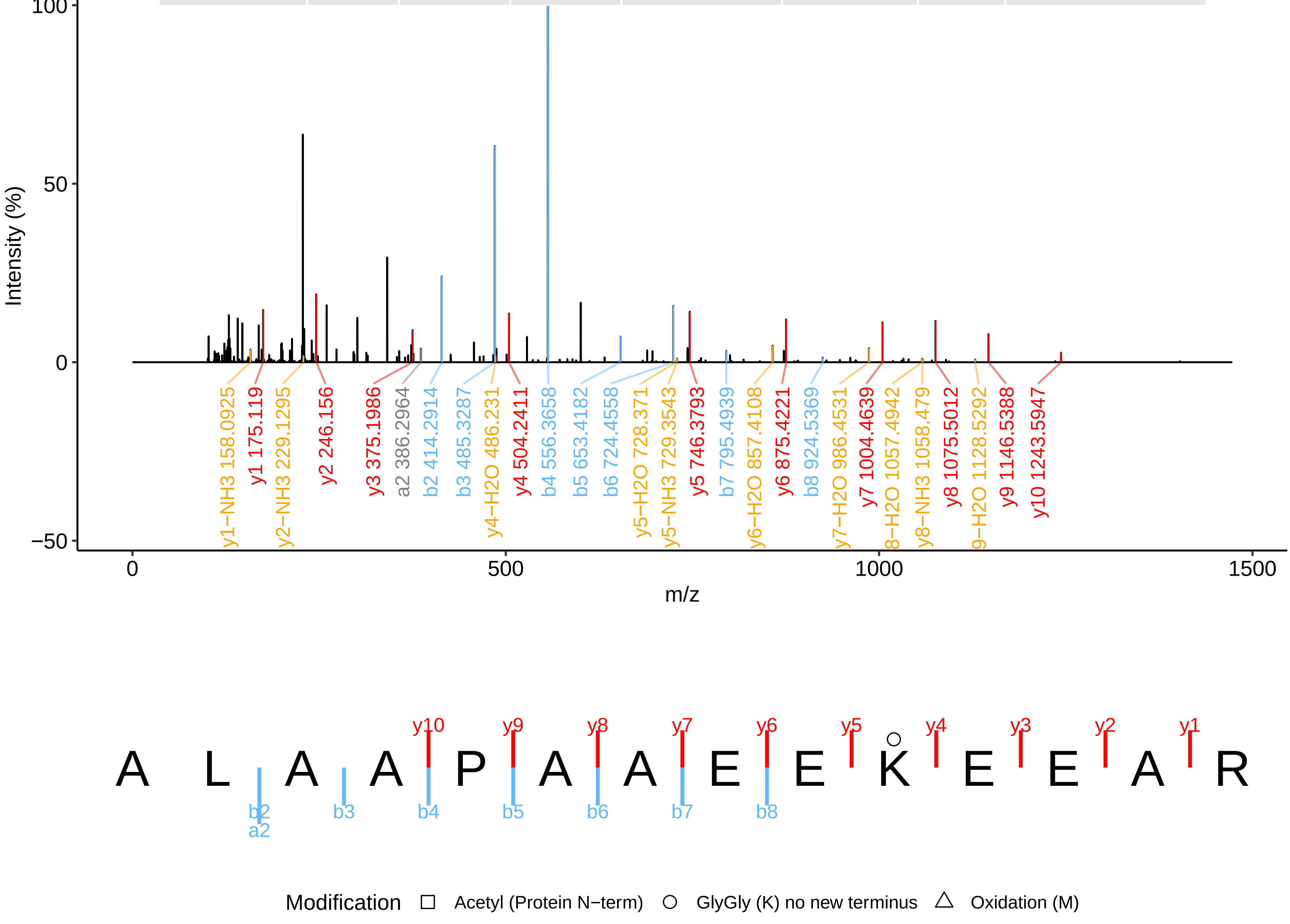

20200921_KIZ_MUb4_GlyGly_10plex_2 − Scan number: 7198

Gene Names	Charge	m/z	Mass	Retention time	PEP	Score	Precursor Intensity
SLC7A5	3	523.9338	1568.779	26.771	6.9308e−06	109.84	1125762

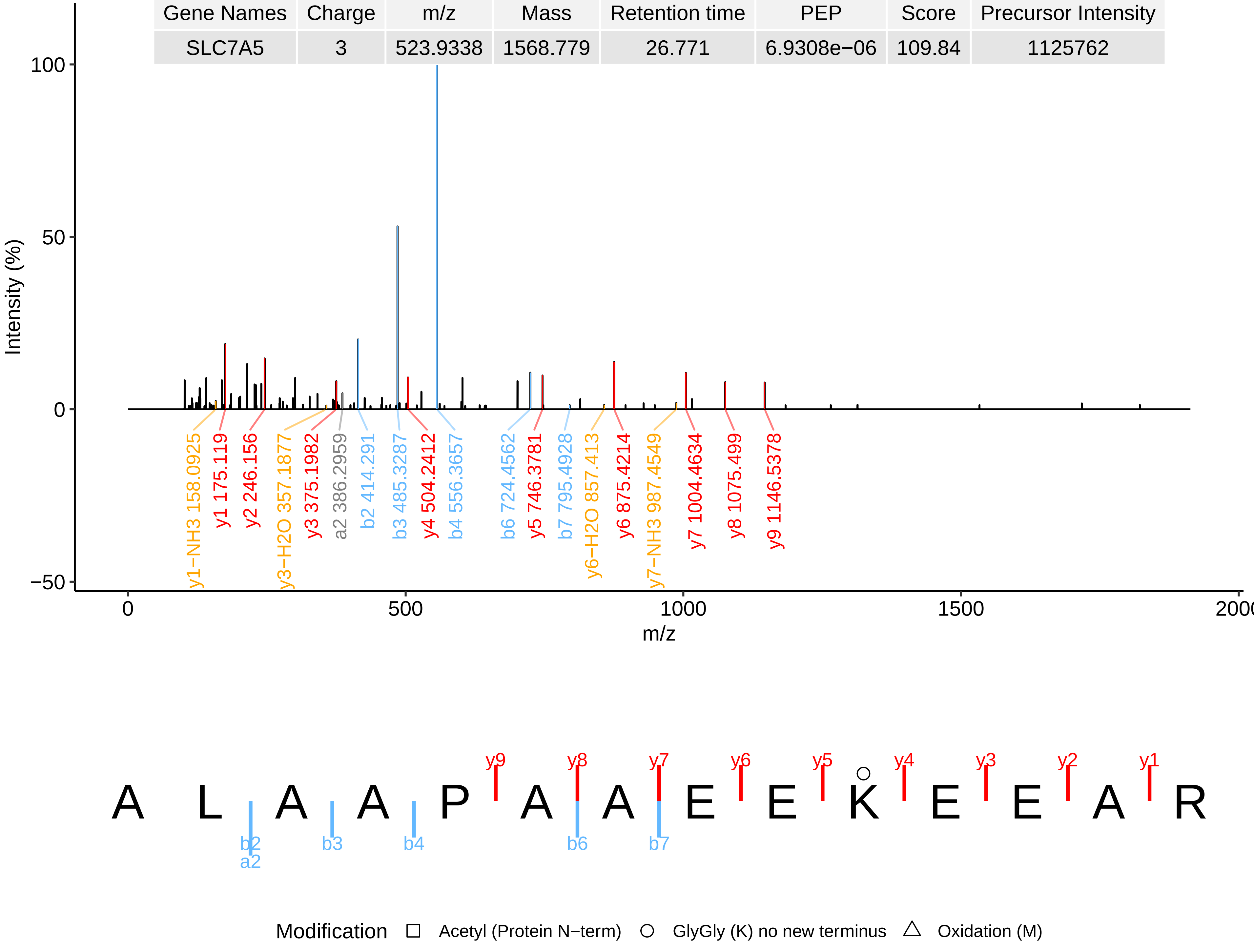

20200921_KIZ_MUb4_GlyGly_10plex_2 − Scan number: 7323

Gene Names	Charge	m/z	Mass	Retention time	PEP	Score	Precursor Intensity
SLC7A5	3	523.9338	1568.779	27.282	0.0020605	77.078	351107.6

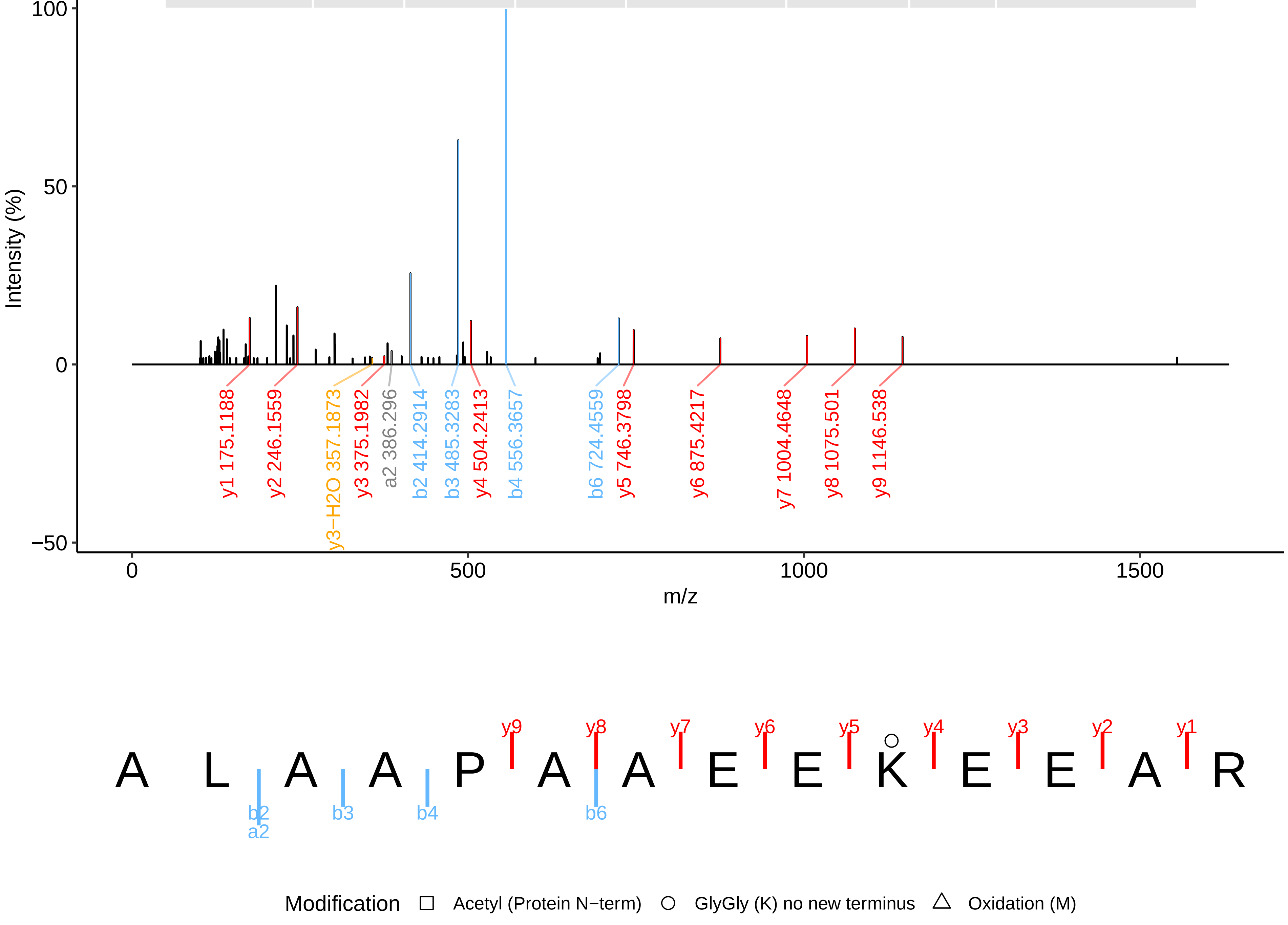

20200924_KIZ_MUb5_GlyGly_1 − Scan number: 6794

Gene Names	Charge	m/z	Mass	Retention time	PEP	Score	Precursor Intensity
SLC7A5	3	523.9338	1568.779	21.458	1.312e−19	152.4	NA

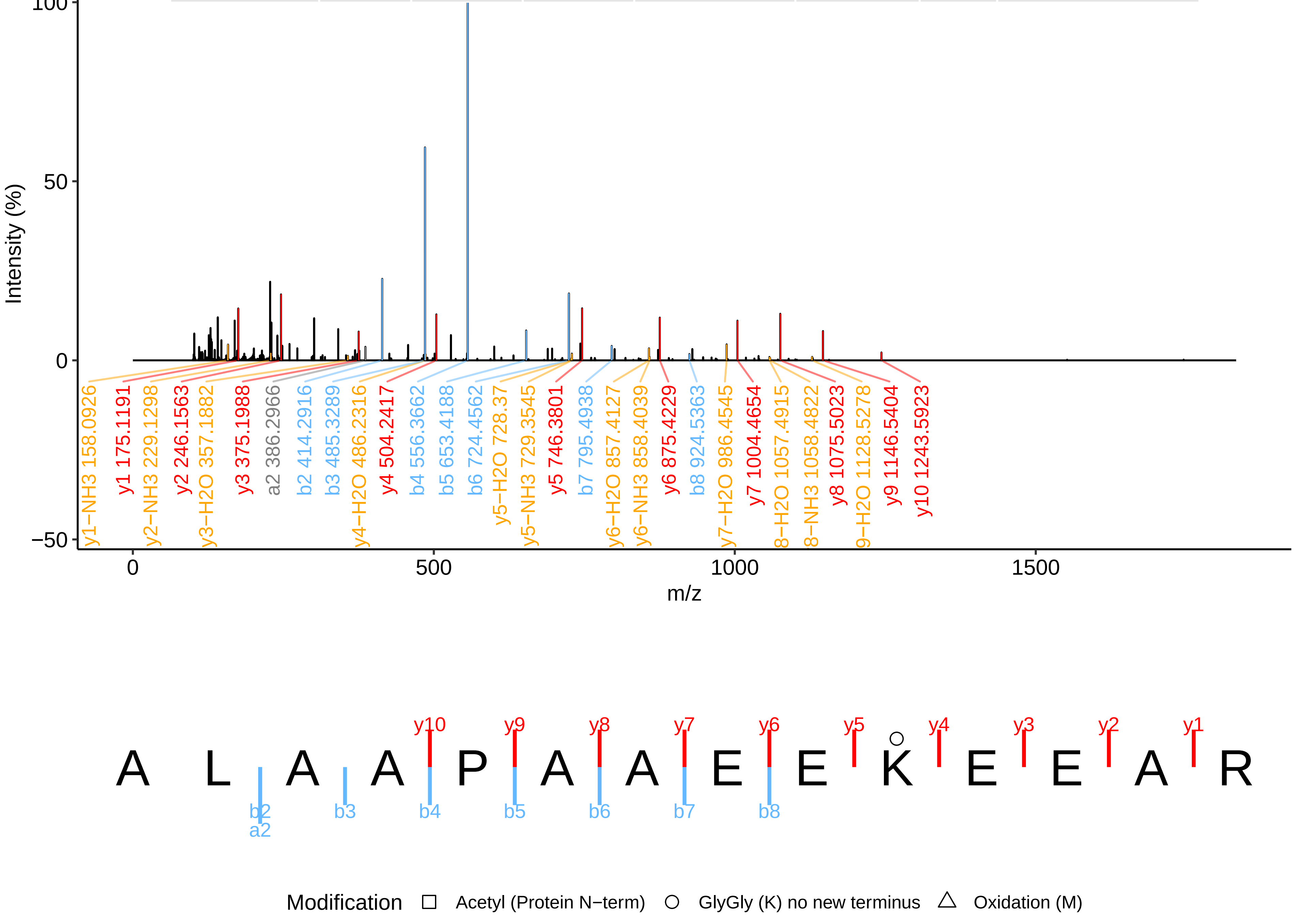

20200924_KIZ_MUb5_GlyGly_1 − Scan number: 6943

Gene Names	Charge	m/z	Mass	Retention time	PEP	Score	Precursor Intensity
SLC7A5	3	523.9338	1568.779	21.96	6.2705e−28	174.73	43432352

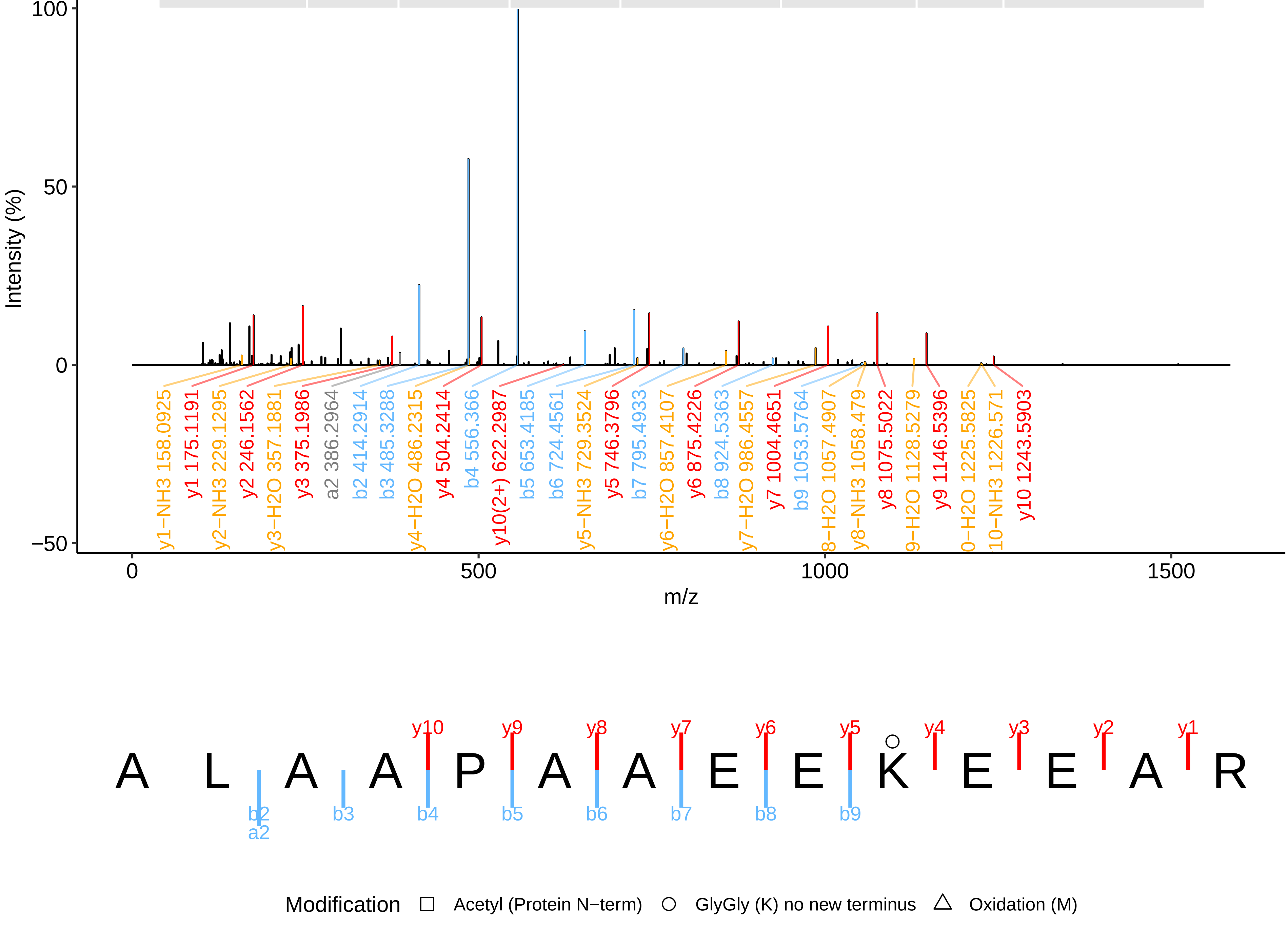

20200924_KIZ_MUb5_GlyGly_2 − Scan number: 6321

Gene Names	Charge	m/z	Mass	Retention time	PEP	Score	Precursor Intensity
SLC7A5	3	523.9338	1568.779	23.569	5.1422e−09	128.31	NA

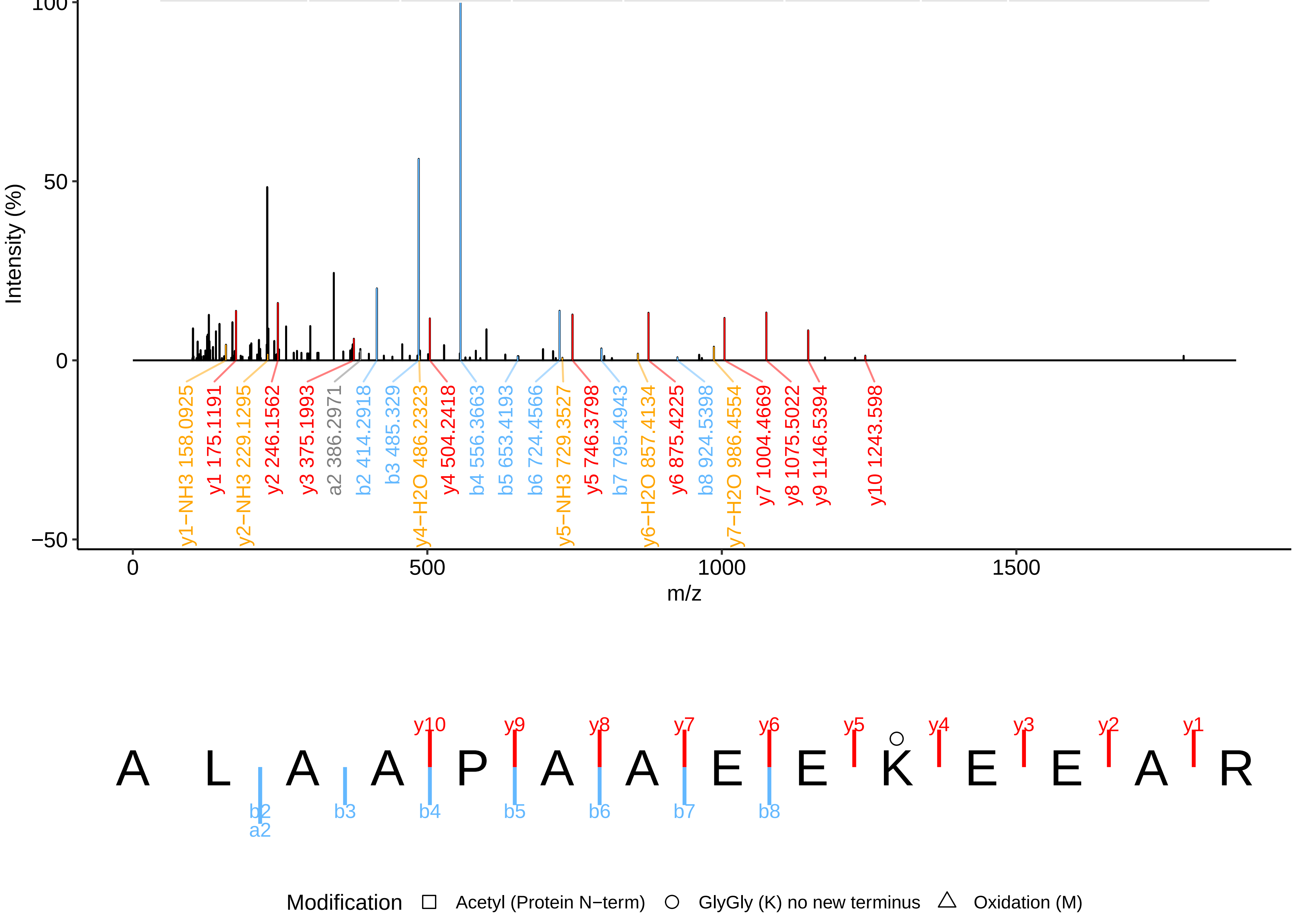

20200924_KIZ_MUb5_GlyGly_2 − Scan number: 6458

Gene Names	Charge	m/z	Mass	Retention time	PEP	Score	Precursor Intensity
SLC7A5	3	523.9338	1568.779	24.082	7.2543e−14	147.02	5100051

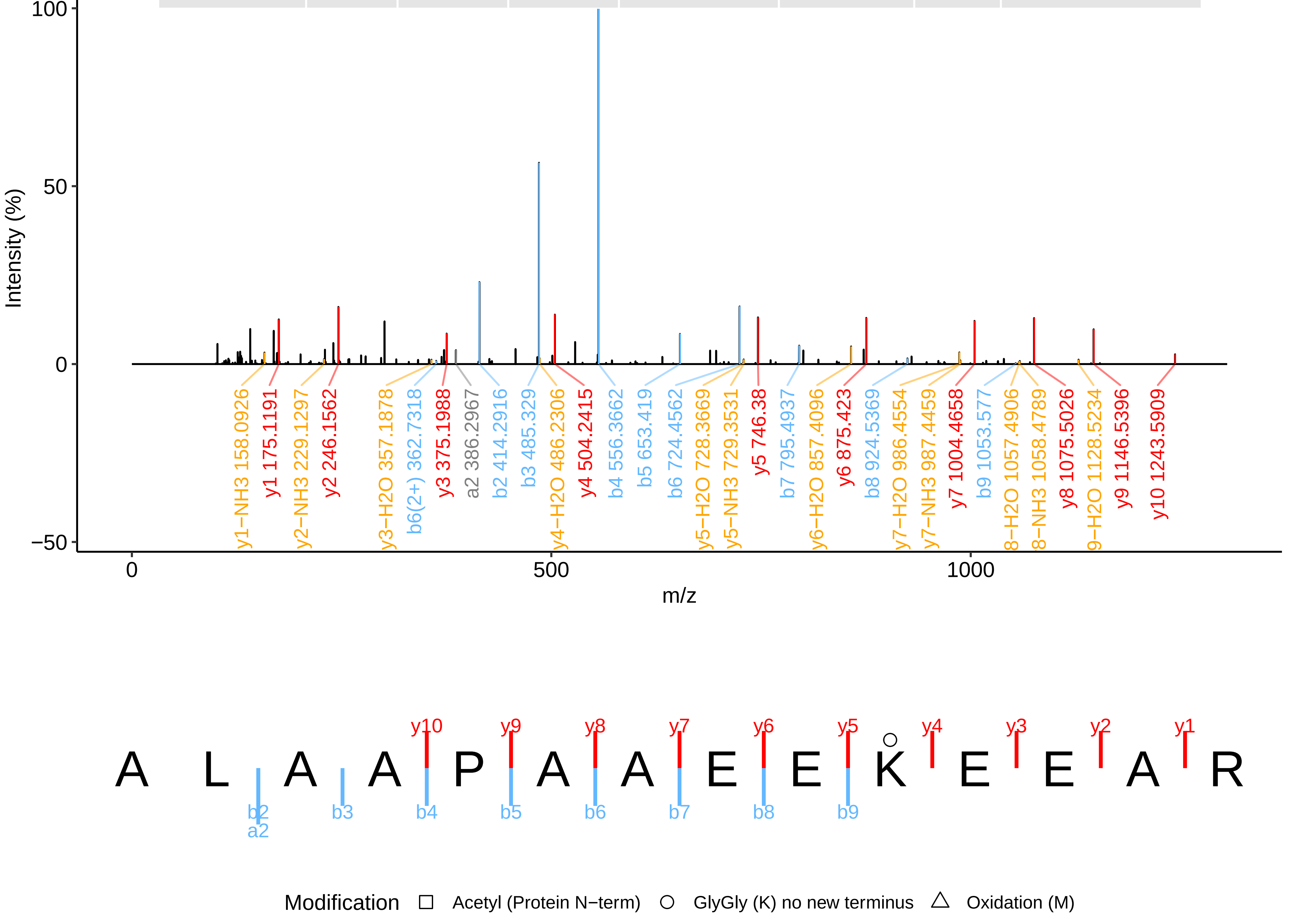

20201118_KIZ_MUb6_GlyGly_2 − Scan number: 8015

Gene Names	Charge	m/z	Mass	Retention time	PEP	Score	Precursor Intensity
SLC7A5	3	523.9338	1568.779	27.317	1.104e−12	138.42	4193642

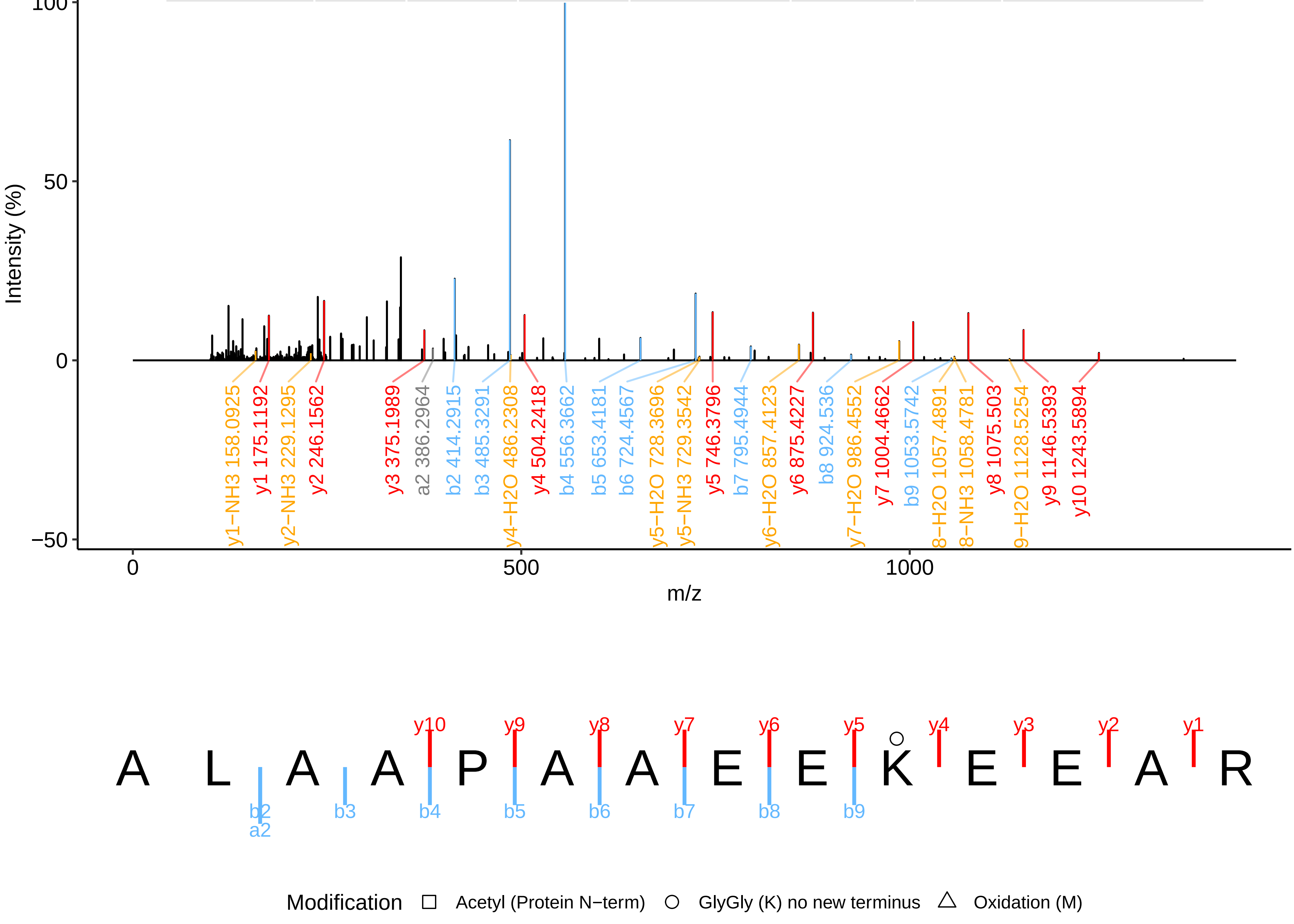

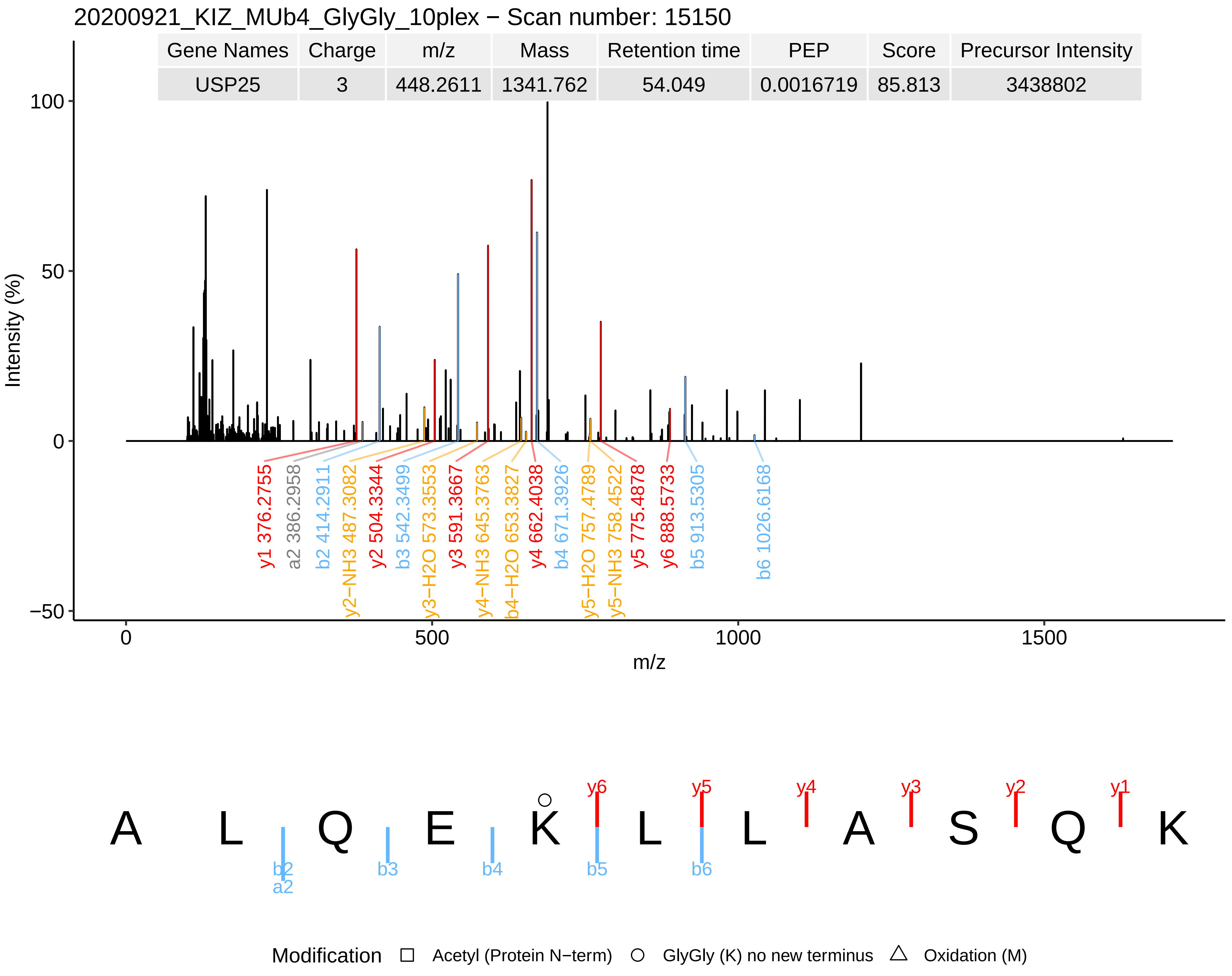
20200921_KIZ_MUb4_GlyGly_10plex − Scan number: 15150
Gene Names | Charge | m/z | Mass | Retention time | PEP | Score | Precursor Intensity
USP25 | 3 | 448.2611 | 1341.762 | 54.049 | 0.0016719 | 85.813 | 3438802
Intensity (%)
100
50
0
−50
0
500
1000
1500
m/z
y1 376.2755
a2 386.2958
b2 414.2911
y2−NH3 487.3082
y2 504.3344
b3 542.3499
y3−H2O 573.3553
y3 591.3667
y4−NH3 645.3763
b4−H2O 653.3827
y4 662.4038
b4 671.3926
y5−H2O 757.4789
y5−NH3 758.4522
y5 775.4878
y6 888.5733
b5 913.5305
b6 1026.6168
A L Q E K L L A S Q K
y6 y5 y4 y3 y2 y1
b2 a2 b3 b4 b5 b6
Modification
Acetyl (Protein N−term)
GlyGly (K) no new terminus
Oxidation (M)

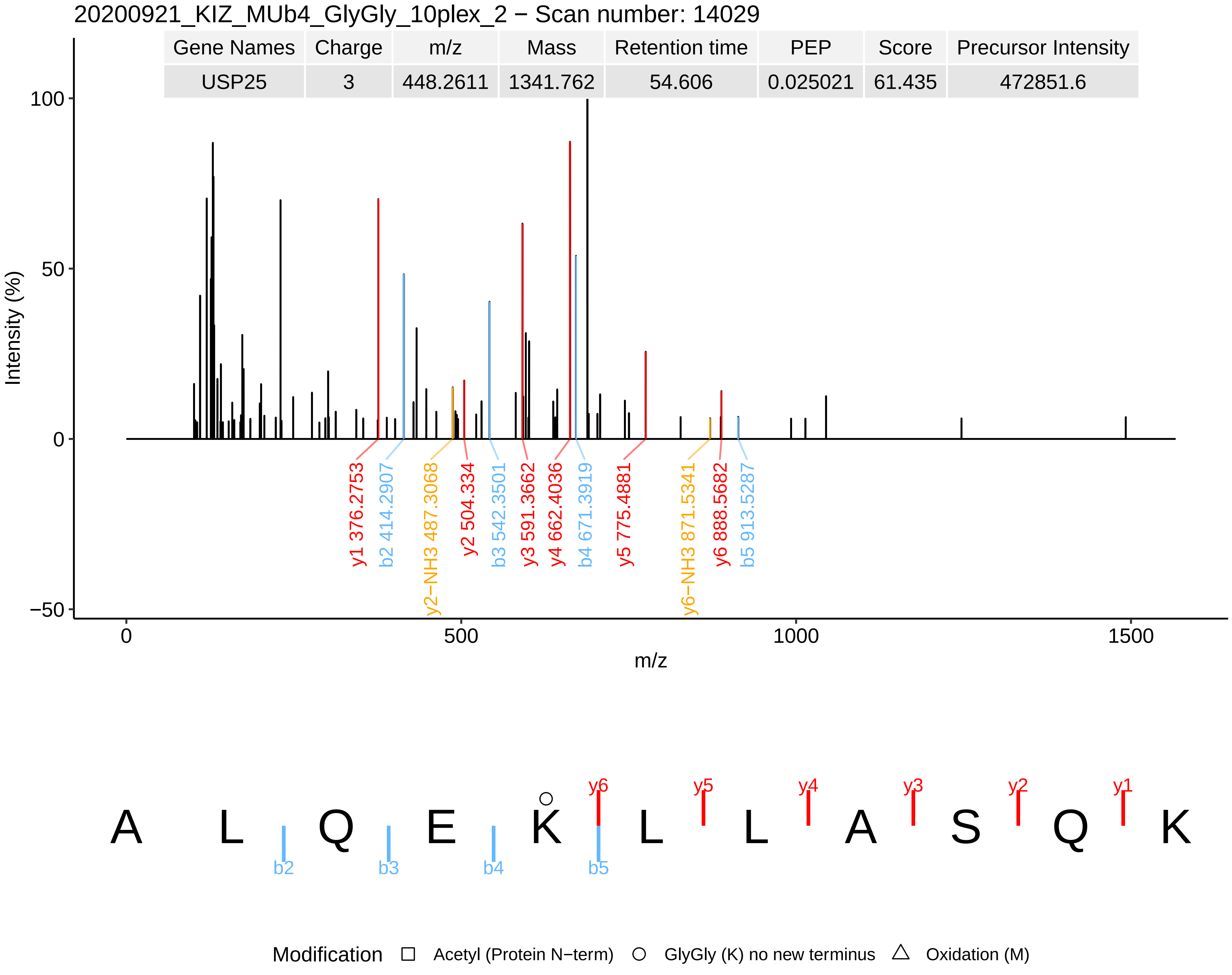
20200921_KIZ_MUb4_GlyGly_10plex_2 − Scan number: 14029
Gene Names | Charge | m/z | Mass | Retention time | PEP | Score | Precursor Intensity
USP25 | 3 | 448.2611 | 1341.762 | 54.606 | 0.025021 | 61.435 | 472851.6
Intensity (%)
m/z
y1 376.2753
b2 414.2907
y2−NH3 487.3068
y2 504.334
b3 542.3501
y3 591.3662
y4 662.4036
b4 671.3919
y5 775.4881
y6−NH3 871.5341
y6 888.5682
b5 913.5287
A L Q E K L L A S Q K
Modification
Acetyl (Protein N−term)
GlyGly (K) no new terminus
Oxidation (M)

20200921_KIZ_MUb4_GlyGly_10plex − Scan number: 13587

Gene Names	Charge	m/z	Mass	Retention time	PEP	Score	Precursor Intensity
PPIA	3	457.9016	1370.683	47.984	8.0181e−06	108.7	6054794

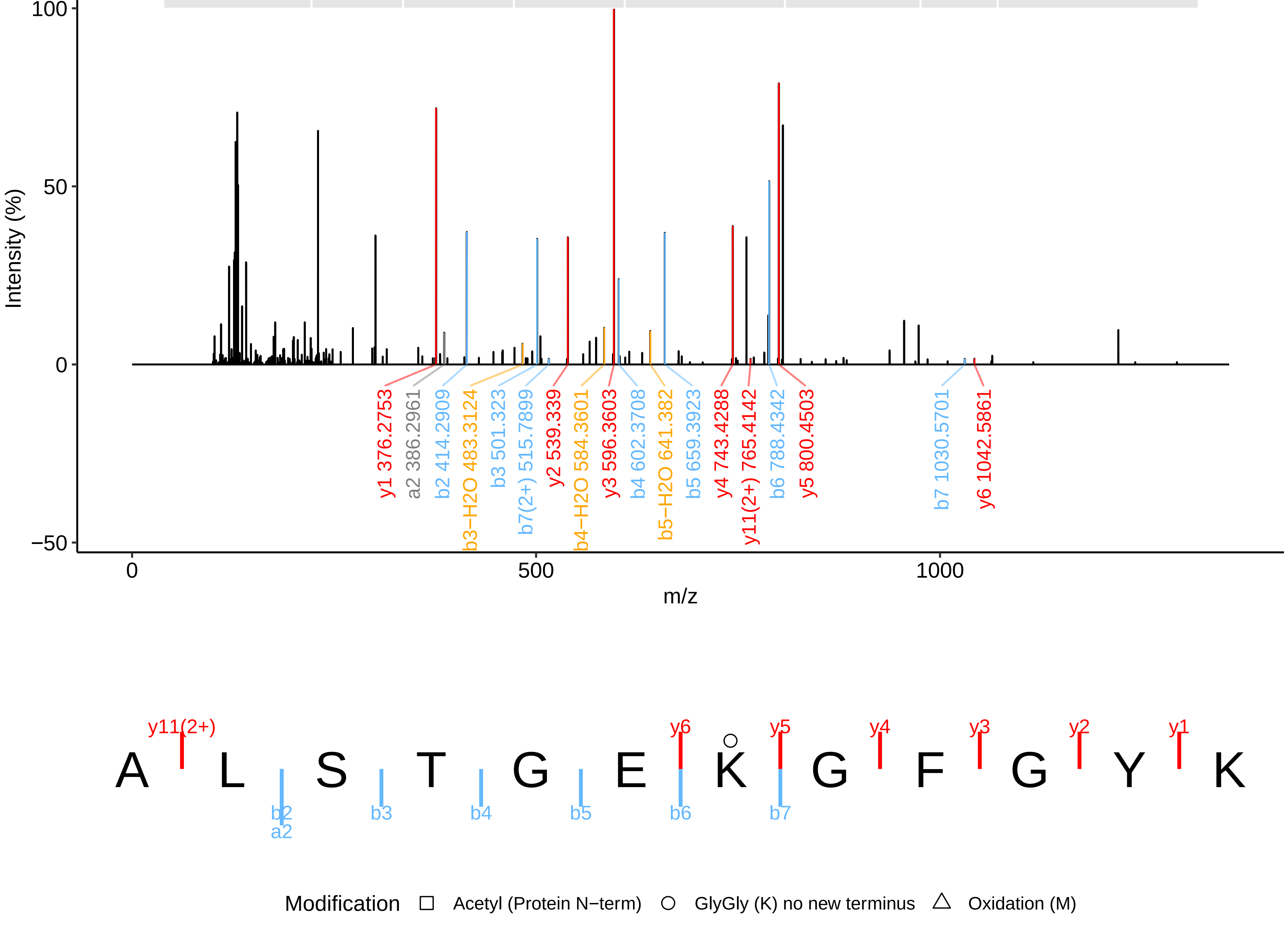

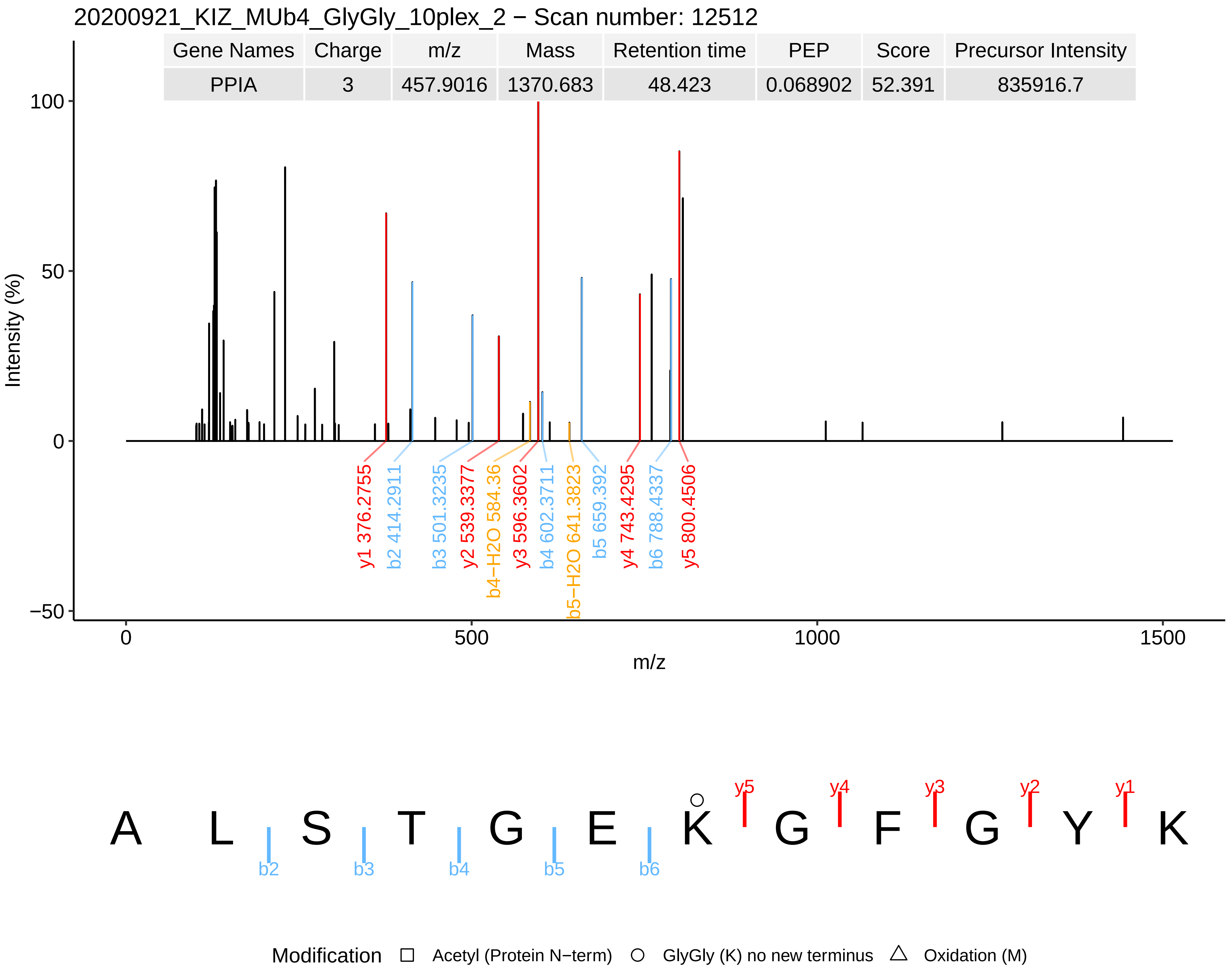
20200921_KIZ_MUb4_GlyGly_10plex_2 − Scan number: 12512
Gene Names | Charge | m/z | Mass | Retention time | PEP | Score | Precursor Intensity
PPIA | 3 | 457.9016 | 1370.683 | 48.423 | 0.068902 | 52.391 | 835916.7
Intensity (%)
100
50
0
−50
0
500
1000
1500
m/z
y1 376.2755
b2 414.2911
b3 501.3235
y2 539.3377
b4−H2O 584.36
y3 596.3602
b4 602.3711
b5−H2O 641.3823
b5 659.392
y4 743.4295
b6 788.4337
y5 800.4506
A L S T G E K G F G Y K
b2 b3 b4 b5 b6
y5 y4 y3 y2 y1
Modification
Acetyl (Protein N−term)
GlyGly (K) no new terminus
Oxidation (M)

20200921_KIZ_MUb4_GlyGly_10plex − Scan number: 4621

Gene Names	Charge	m/z	Mass	Retention time	PEP	Score	Precursor Intensity
ADRM1	3	458.2261	1371.657	15.06	0.0033871	79.201	NA

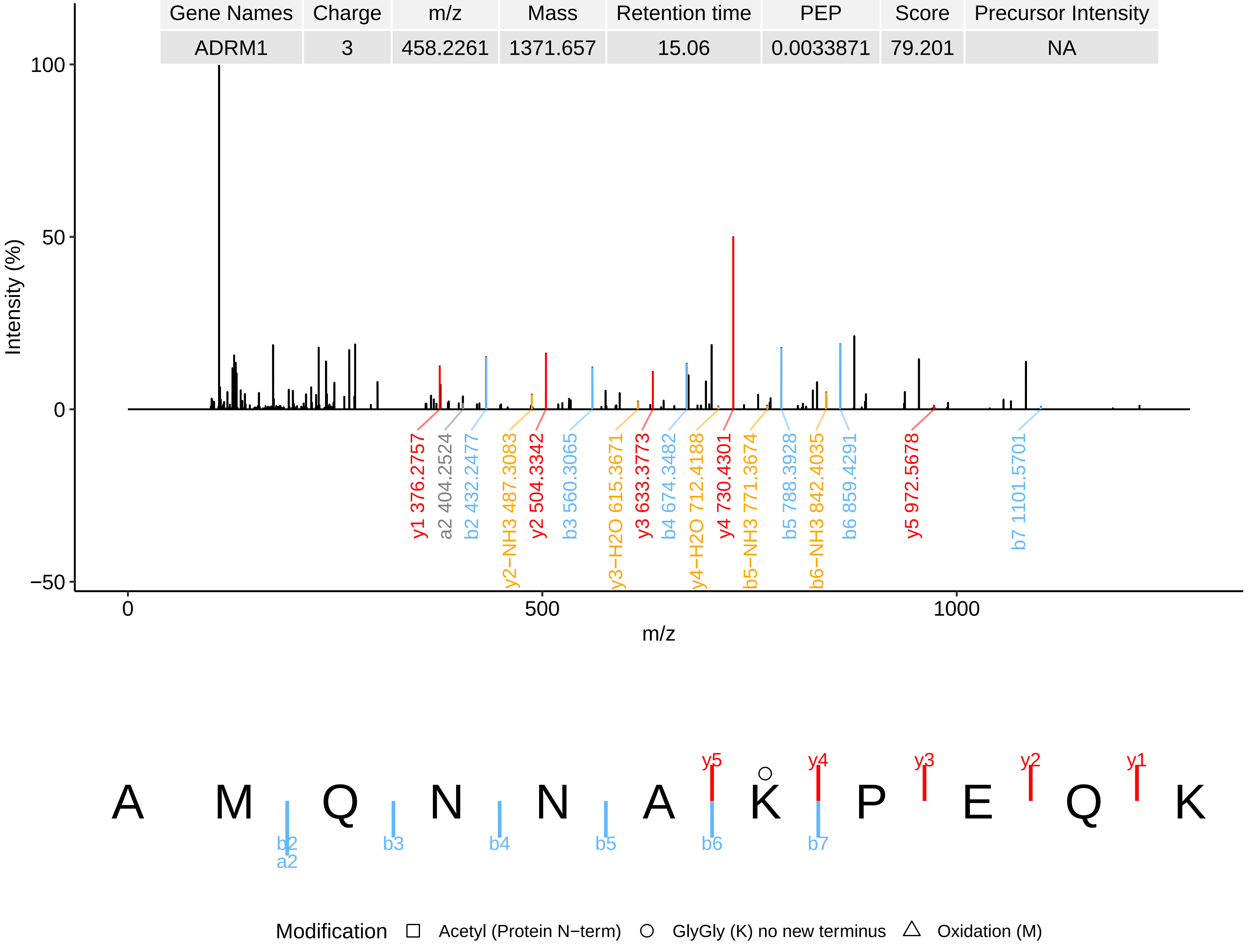

20200921_KIZ_MUb4_GlyGly_10plex_2 − Scan number: 4283

Gene Names	Charge	m/z	Mass	Retention time	PEP	Score	Precursor Intensity
ADRM1	3	458.2261	1371.657	14.946	0.0012659	85.355	689784

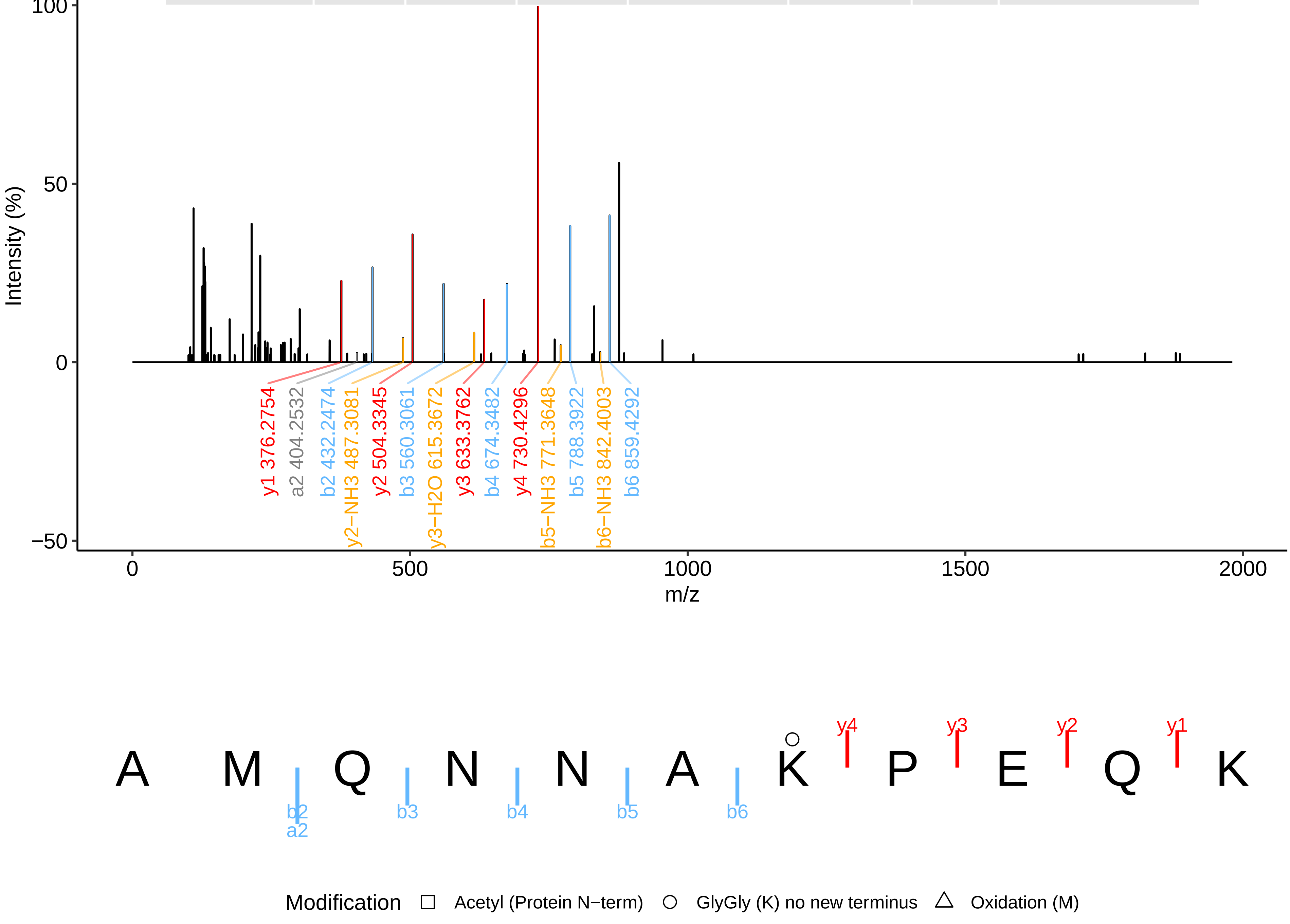

20200924_KIZ_MUb5_GlyGly_2 − Scan number: 3858

Gene Names	Charge	m/z	Mass	Retention time	PEP	Score	Precursor Intensity
ADRM1	3	458.2261	1371.657	14.081	0.044768	59.542	NA

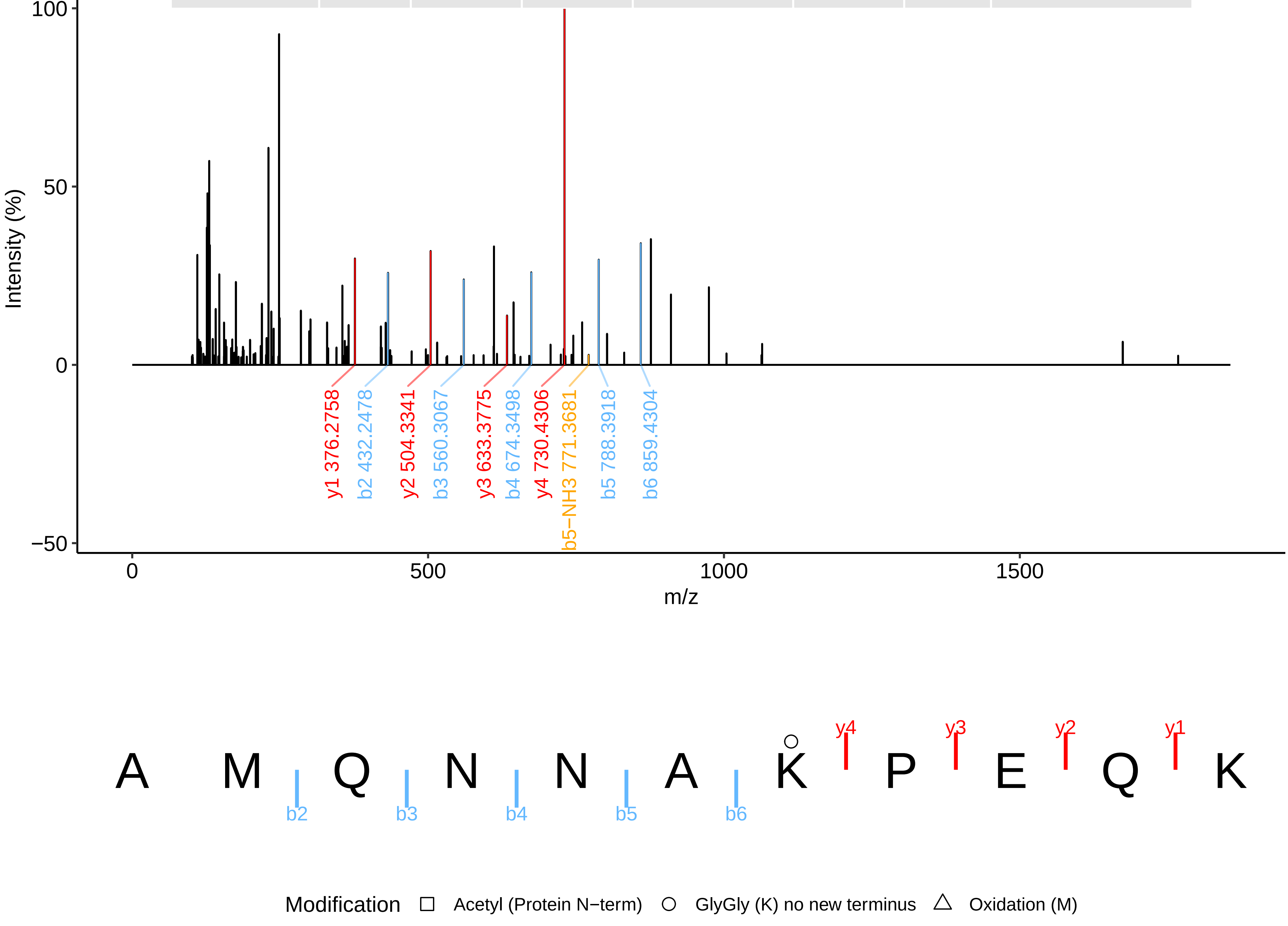

20200921_KIZ_MUb4_GlyGly_10plex − Scan number: 13087

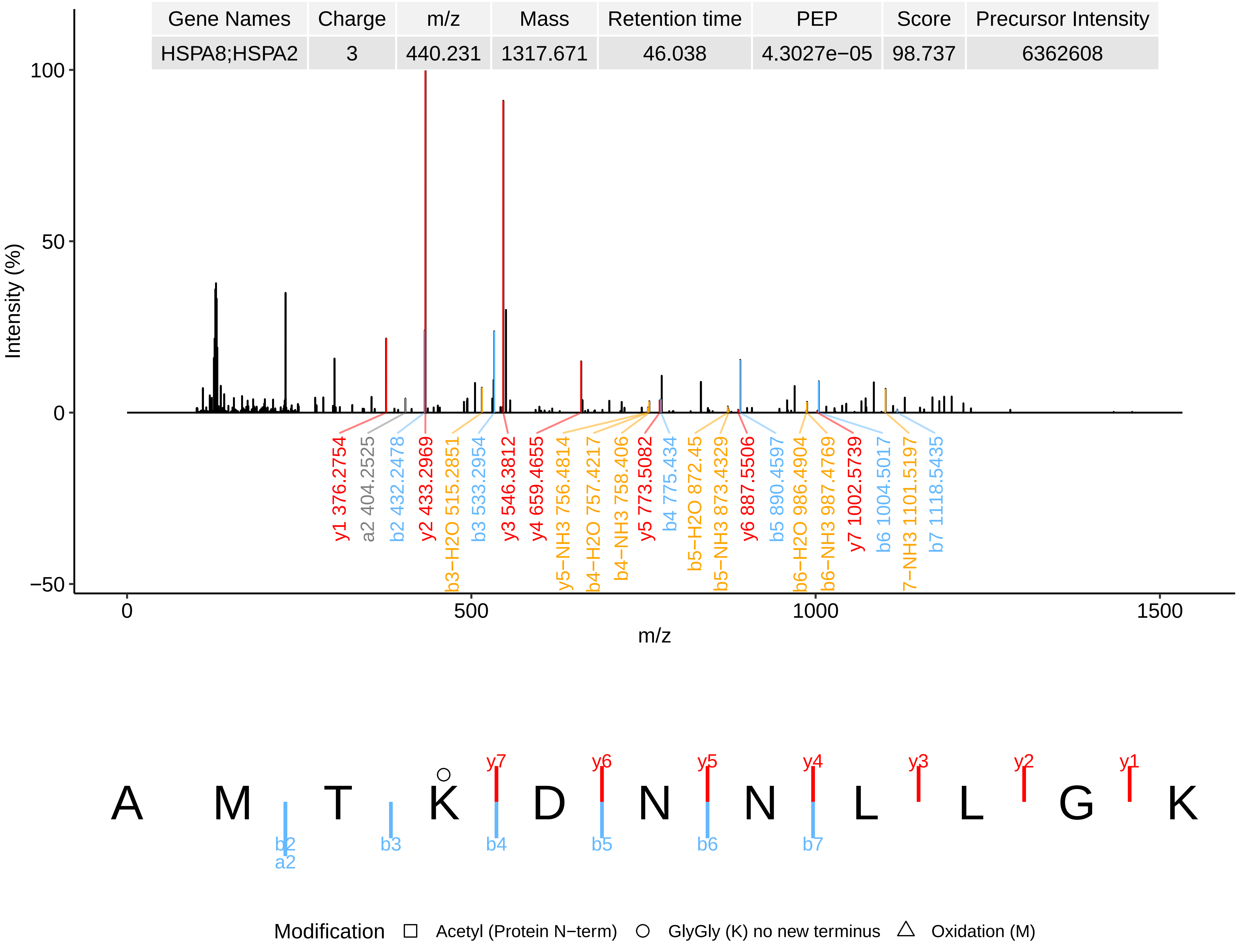

20200921_KIZ_MUb4_GlyGly_10plex_2 − Scan number: 12028

Gene Names	Charge	m/z	Mass	Retention time	PEP	Score	Precursor Intensity
HSPA8;HSPA2	3	440.231	1317.671	46.445	0.092099	48.44	613090.4

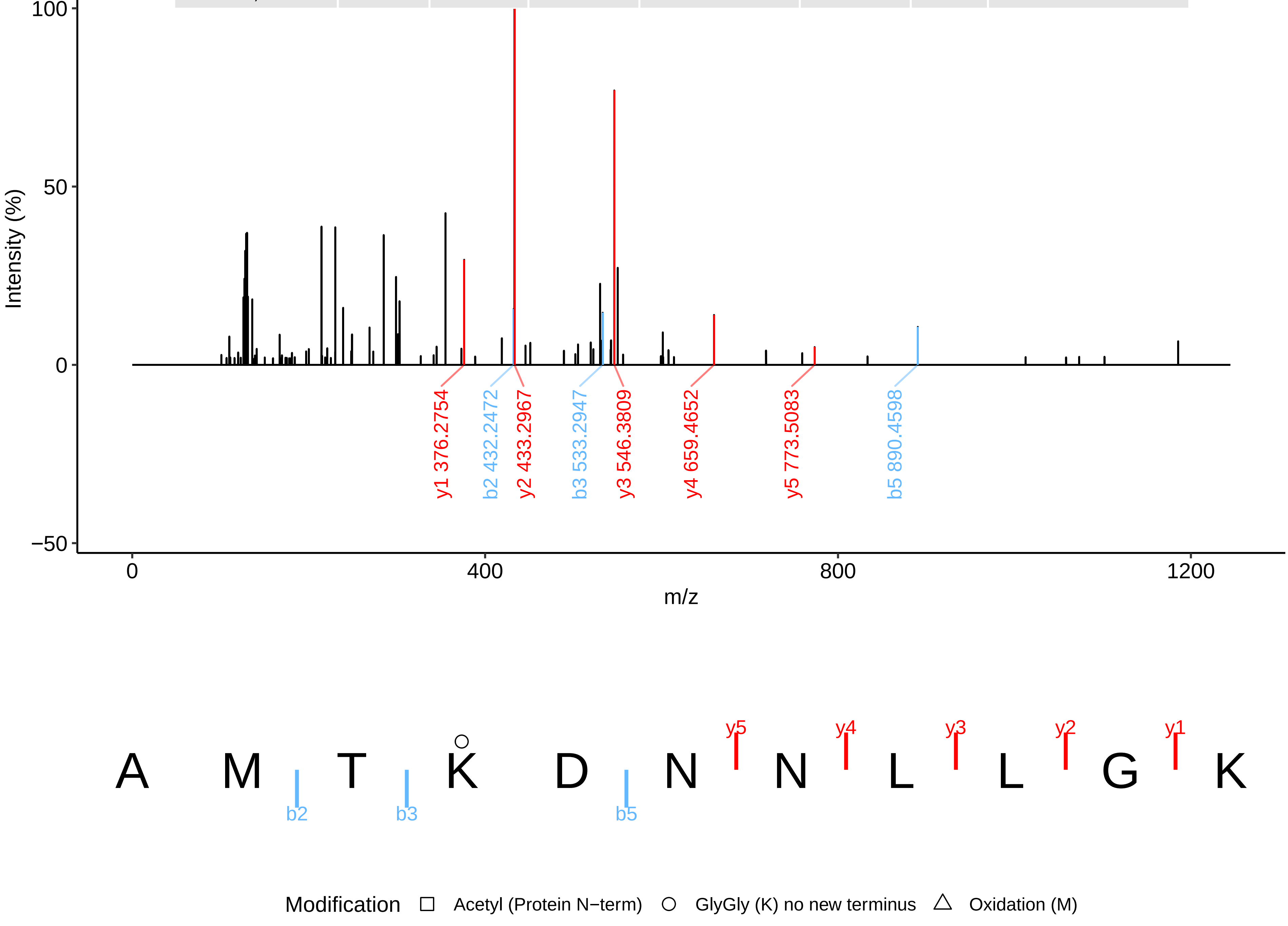

20200924_KIZ_MUb5_GlyGly_1 − Scan number: 11982

Gene Names	Charge	m/z	Mass	Retention time	PEP	Score	Precursor Intensity
HSPA8;HSPA2	3	440.231	1317.671	38.923	0.0013332	84.658	8354920

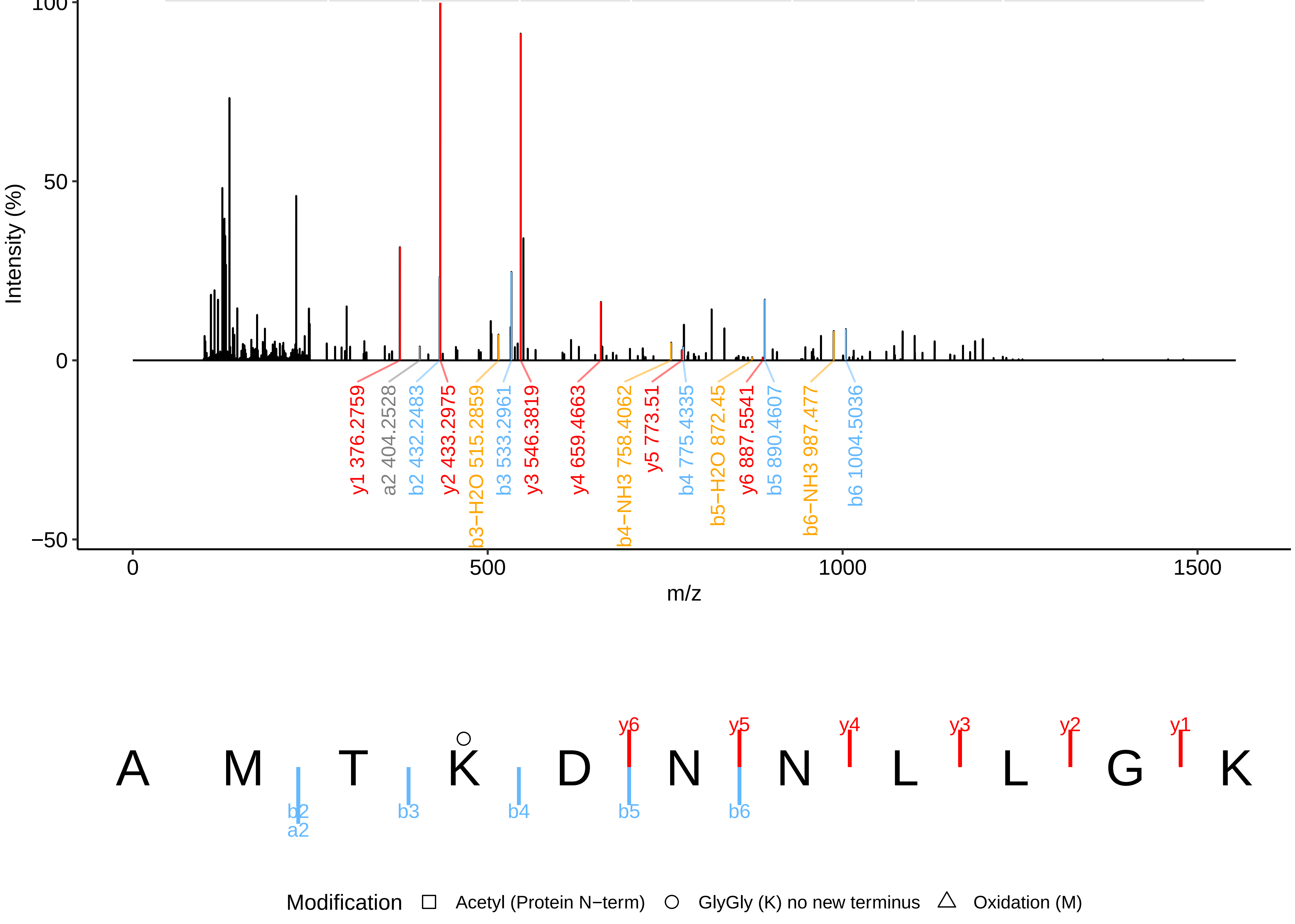

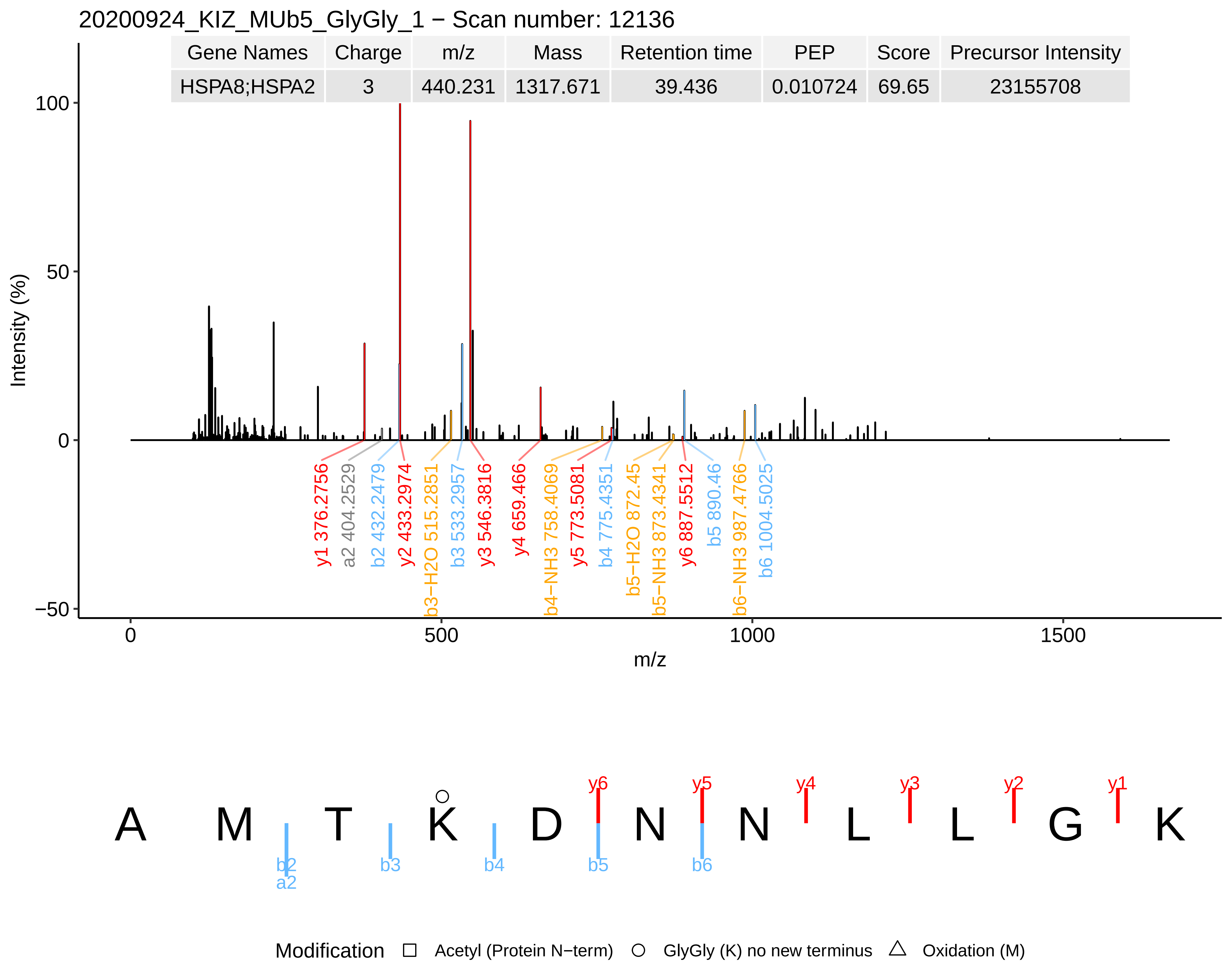
20200924_KIZ_MUb5_GlyGly_1 − Scan number: 12136
Gene Names | Charge | m/z | Mass | Retention time | PEP | Score | Precursor Intensity
HSPA8;HSPA2 | 3 | 440.231 | 1317.671 | 39.436 | 0.010724 | 69.65 | 23155708
Intensity (%)
100
50
0
−50
0
500
1000
1500
m/z
y1 376.2756
a2 404.2529
b2 432.2479
y2 433.2974
b3−H2O 515.2851
b3 533.2957
y3 546.3816
y4 659.466
b4−NH3 758.4069
y5 773.5081
b4 775.4351
b5−H2O 872.45
b5−NH3 873.4341
y6 887.5512
b5 890.46
b6−NH3 987.4766
b6 1004.5025
A M T K D N N L L G K
y6 y5 y4 y3 y2 y1
b2 a2 b3 b4 b5 b6
Modification
Acetyl (Protein N−term)
GlyGly (K) no new terminus
Oxidation (M)

20200924_KIZ_MUb5_GlyGly_2 − Scan number: 11217

Gene Names	Charge	m/z	Mass	Retention time	PEP	Score	Precursor Intensity
HSPA8;HSPA2	3	440.231	1317.671	41.899	0.023058	61.815	3493167

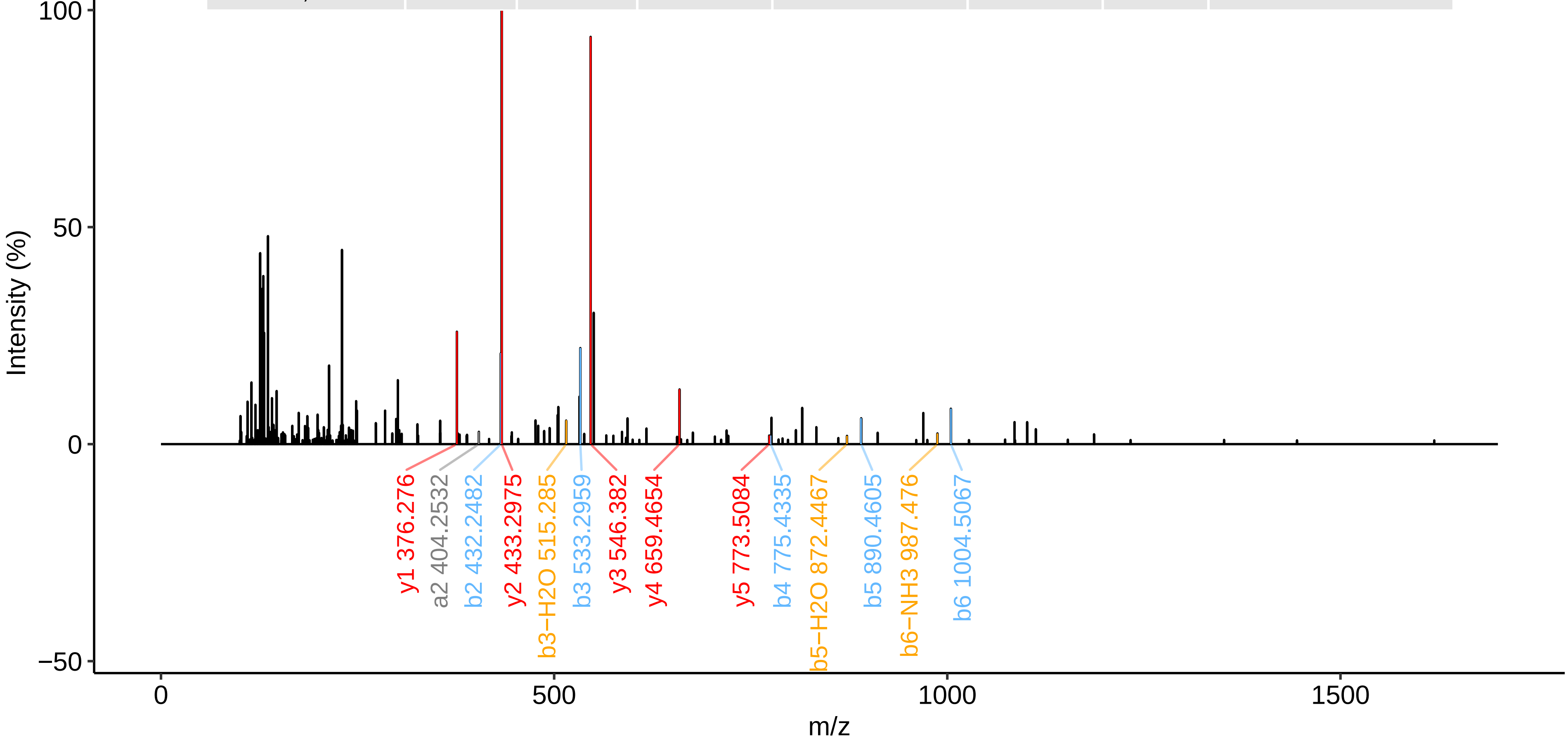

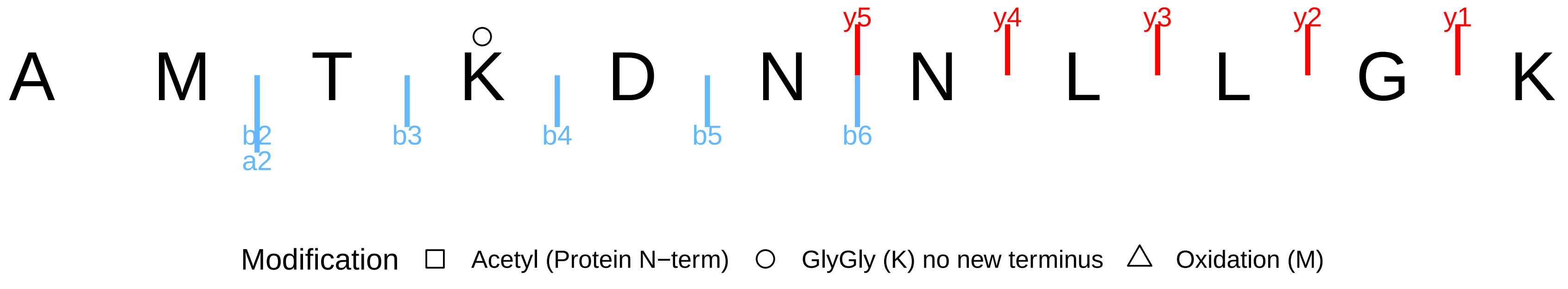

20200924_KIZ_MUb5_GlyGly_2 − Scan number: 11379

Gene Names	Charge	m/z	Mass	Retention time	PEP	Score	Precursor Intensity
HSPA8;HSPA2	3	440.231	1317.671	42.526	0.0017149	80.706	1924121

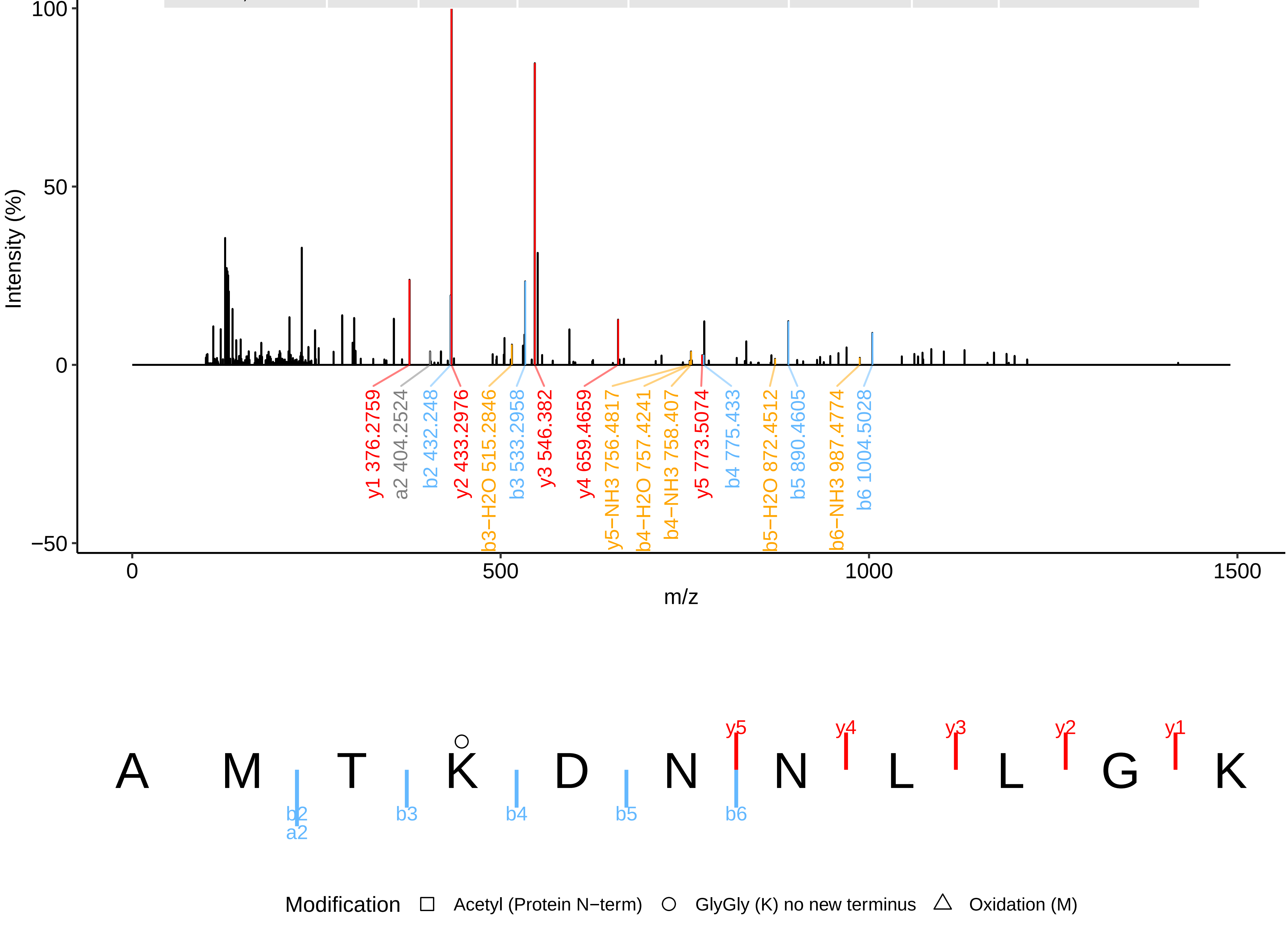

20200921_KIZ_MUb4_GlyGly_10plex − Scan number: 7532

Gene Names	Charge	m/z	Mass	Retention time	PEP	Score	Precursor Intensity
HIP1	3	516.9193	1547.736	25.312	1.493e−26	179.94	8233884

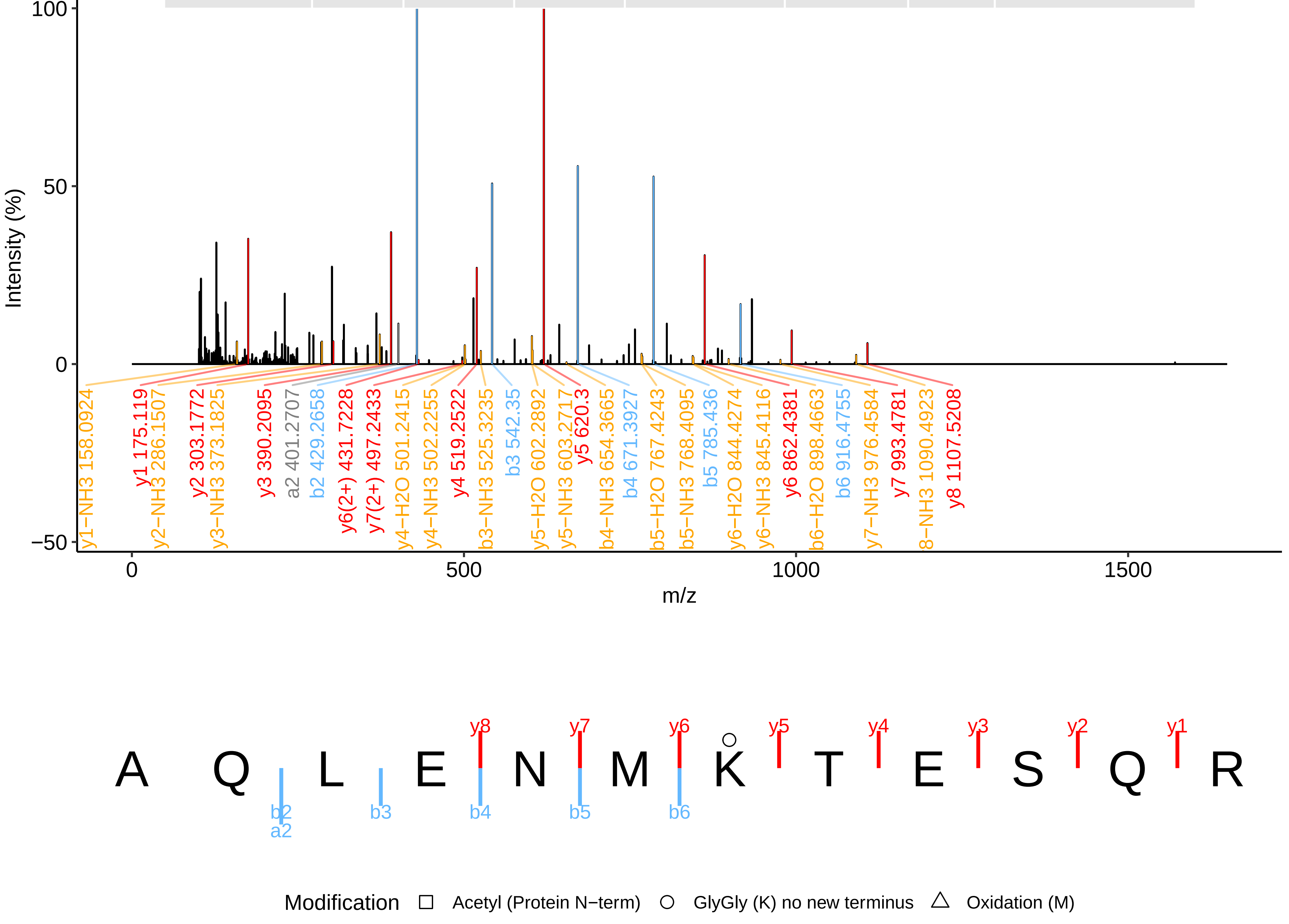

20200921_KIZ_MUb4_GlyGly_10plex_2 − Scan number: 6939

Gene Names	Charge	m/z	Mass	Retention time	PEP	Score	Precursor Intensity
HIP1	3	516.9193	1547.736	25.713	1.6727e−05	101.39	1136292

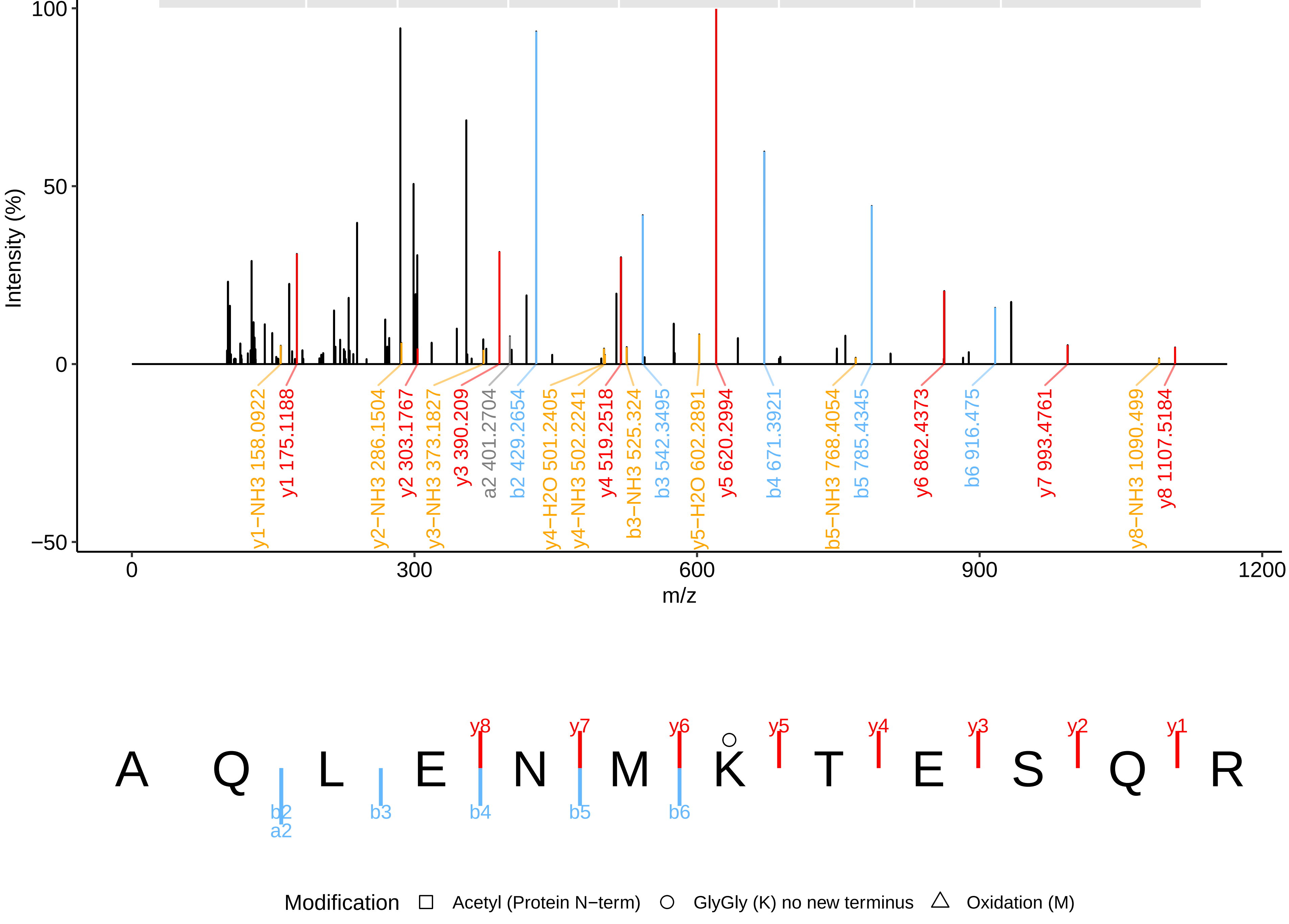

20200924_KIZ_MUb5_GlyGly_1 − Scan number: 6451

Gene Names	Charge	m/z	Mass	Retention time	PEP	Score	Precursor Intensity
HIP1	3	516.9193	1547.736	20.288	1.0106e−08	120.55	5912038

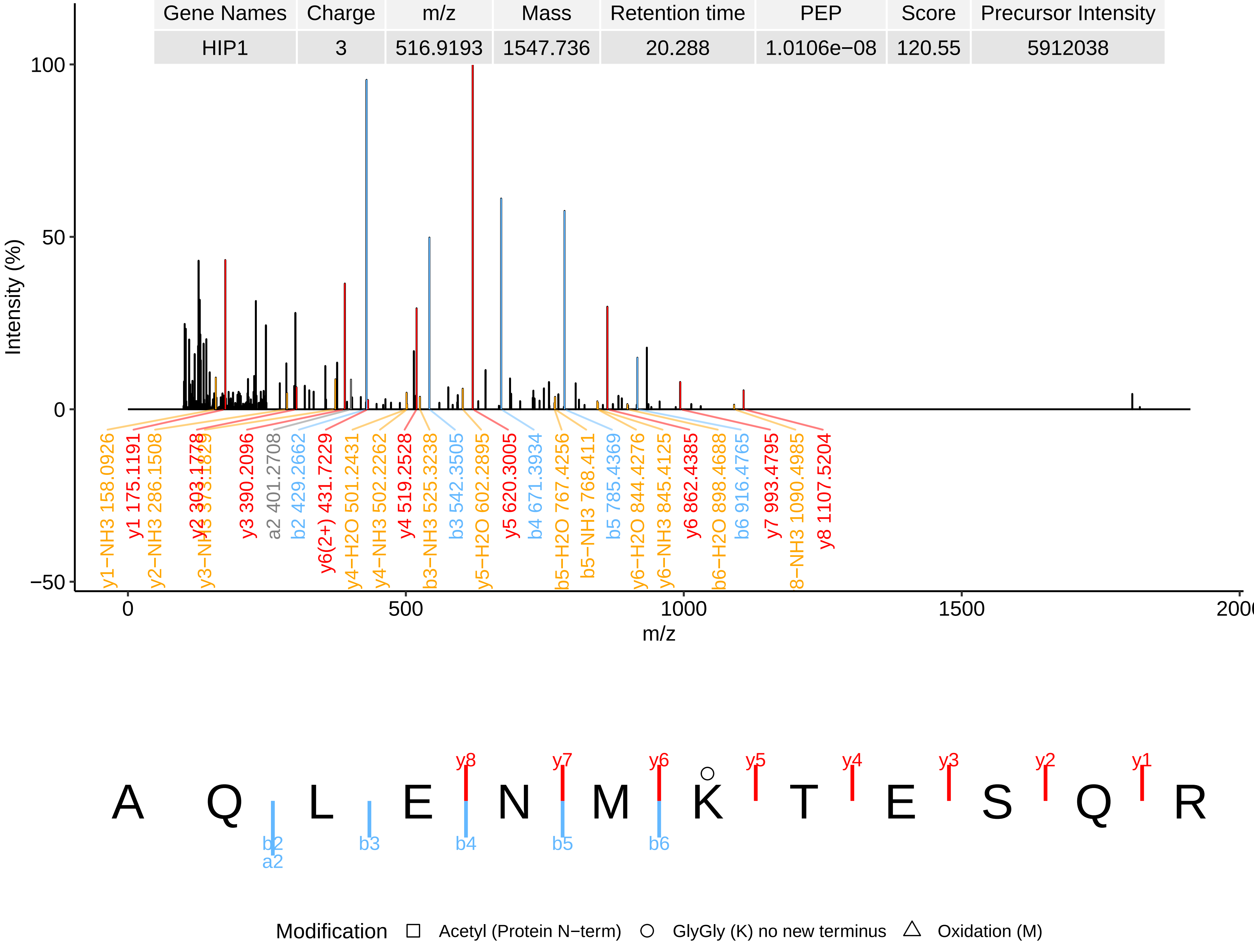

20200924_KIZ_MUb5_GlyGly_1 − Scan number: 6482

Gene Names	Charge	m/z	Mass	Retention time	PEP	Score	Precursor Intensity
HIP1	3	516.9193	1547.736	20.388	1.1617e−13	145.01	5093337

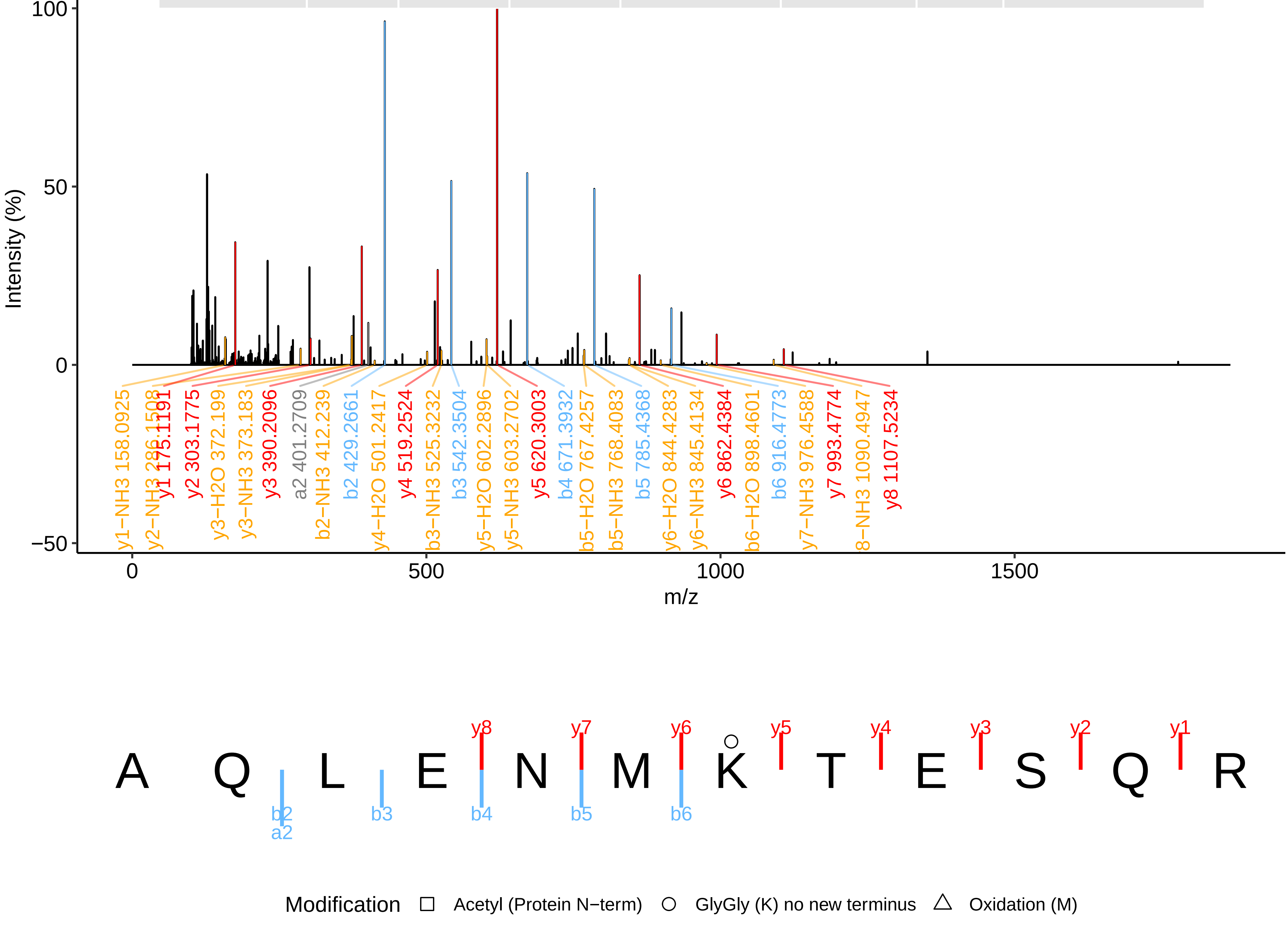

20201118_KIZ_MUb6_GlyGly_2 − Scan number: 7593

Gene Names	Charge	m/z	Mass	Retention time	PEP	Score	Precursor Intensity
HIP1	3	516.9193	1547.736	25.899	0.0011363	86.288	4201172

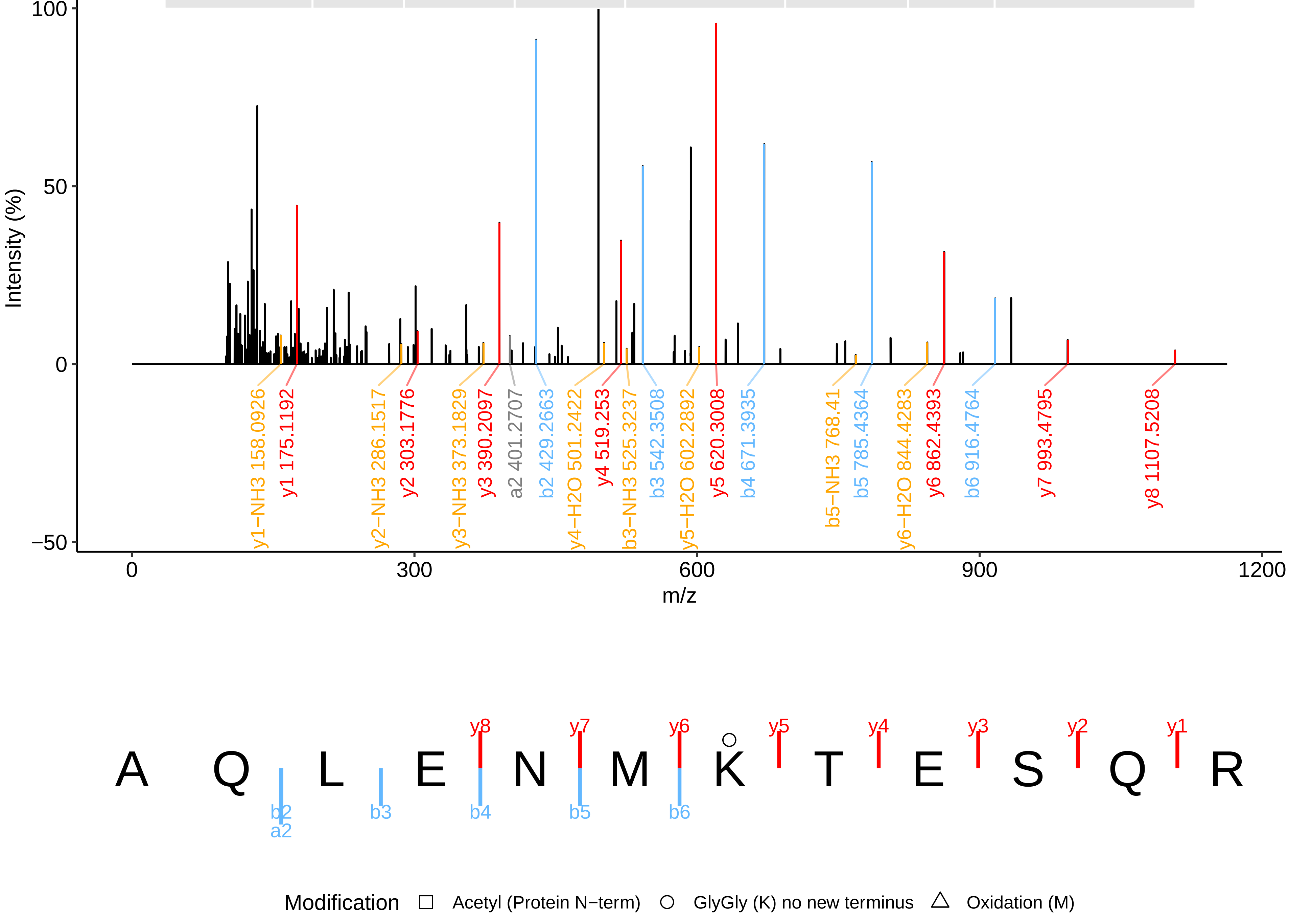

20200921_KIZ_MUb4_GlyGly_10plex_2 − Scan number: 17724

Gene Names	Charge	m/z	Mass	Retention time	PEP	Score	Precursor Intensity
PHB2	3	611.6745	1832.002	69.337	1.1379e−05	73.887	1321123

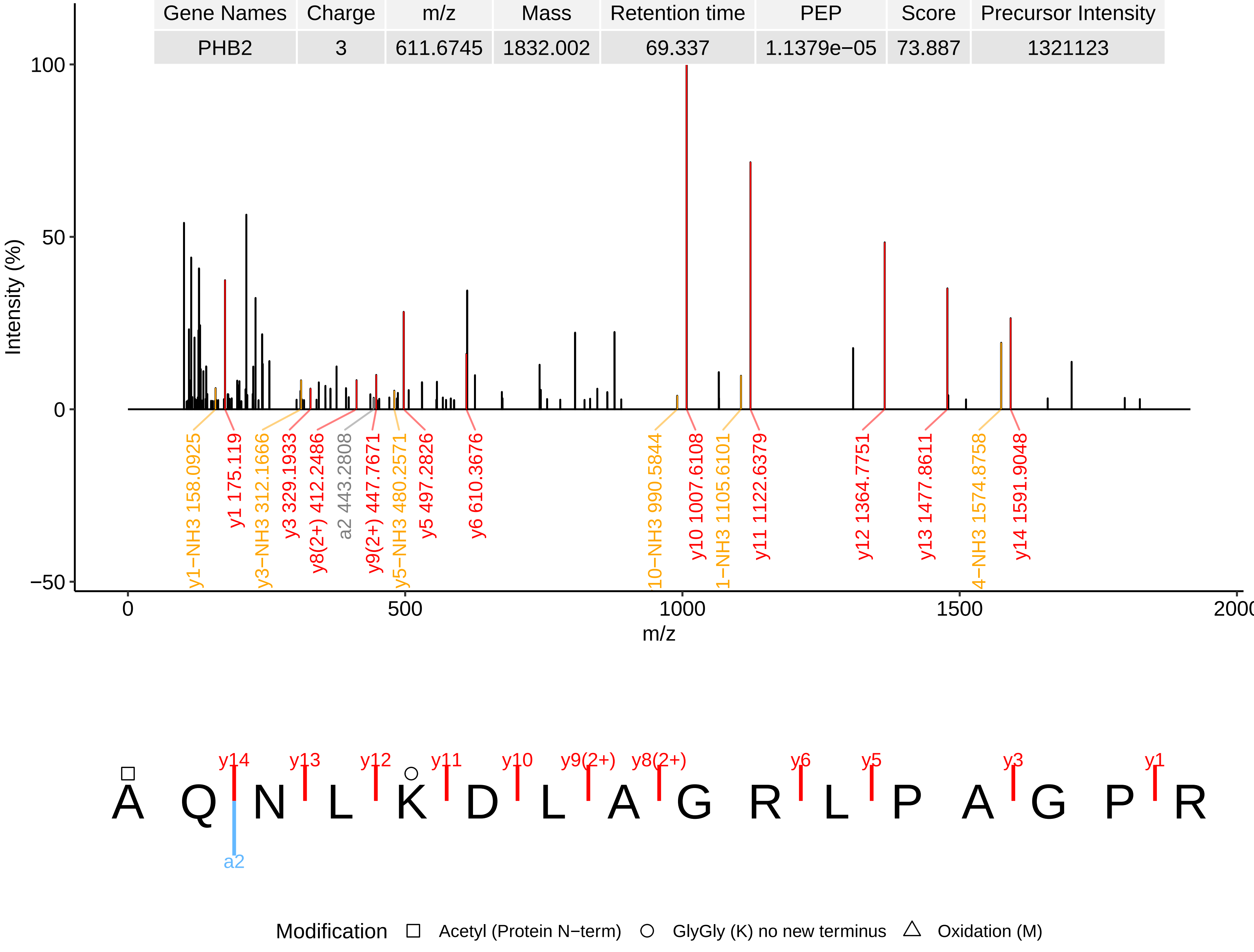

20200921_KIZ_MUb4_GlyGly_10plex − Scan number: 16878

Gene Names	Charge	m/z	Mass	Retention time	PEP	Score	Precursor Intensity
GAPDH	3	446.9295	1337.767	60.784	0.0027189	78.342	3009290

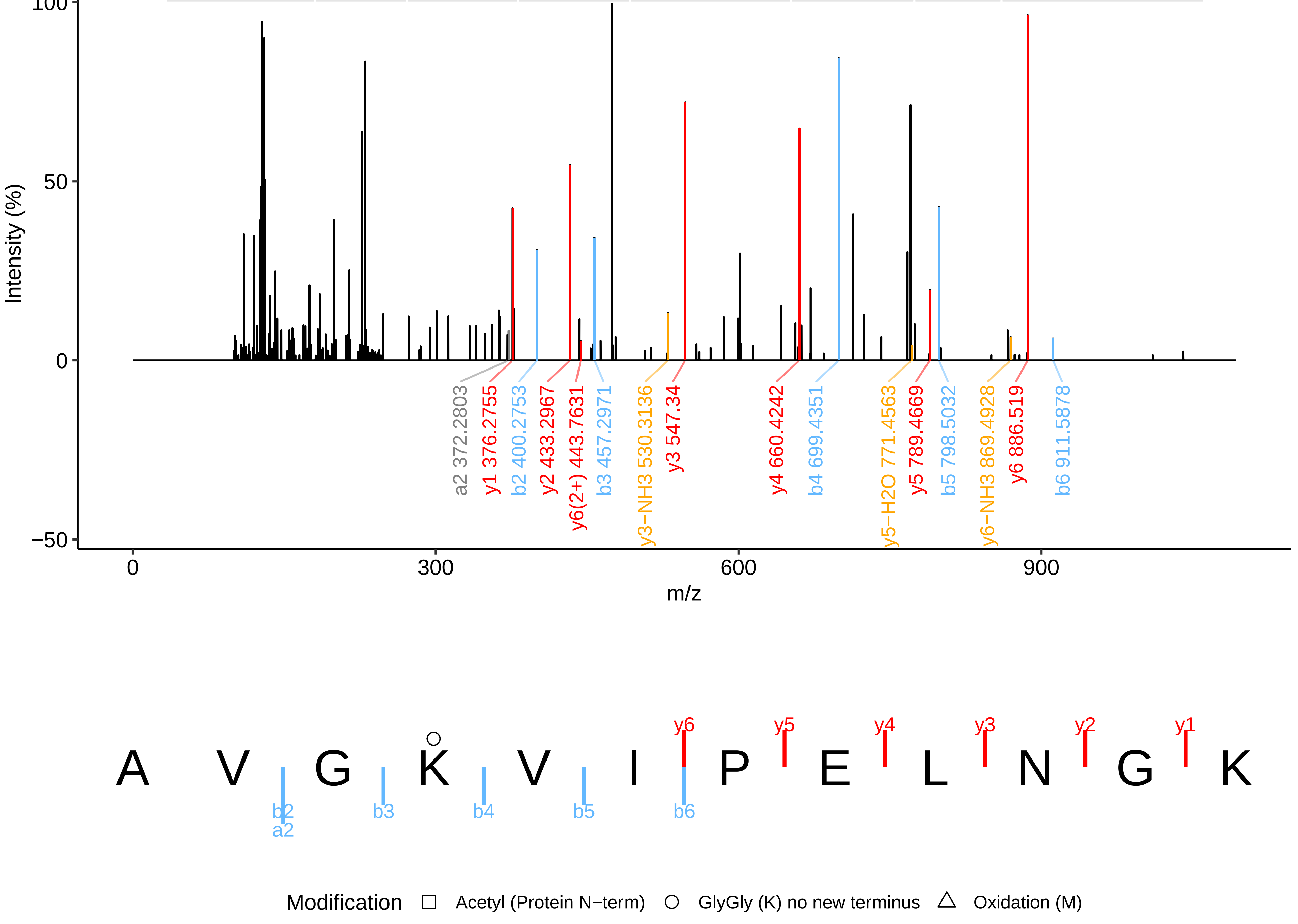

20200921_KIZ_MUb4_GlyGly_10plex_2 − Scan number: 9396

Gene Names	Charge	m/z	Mass	Retention time	PEP	Score	Precursor Intensity
EMC8	3	529.2691	1584.786	35.701	0.10661	46.964	NA

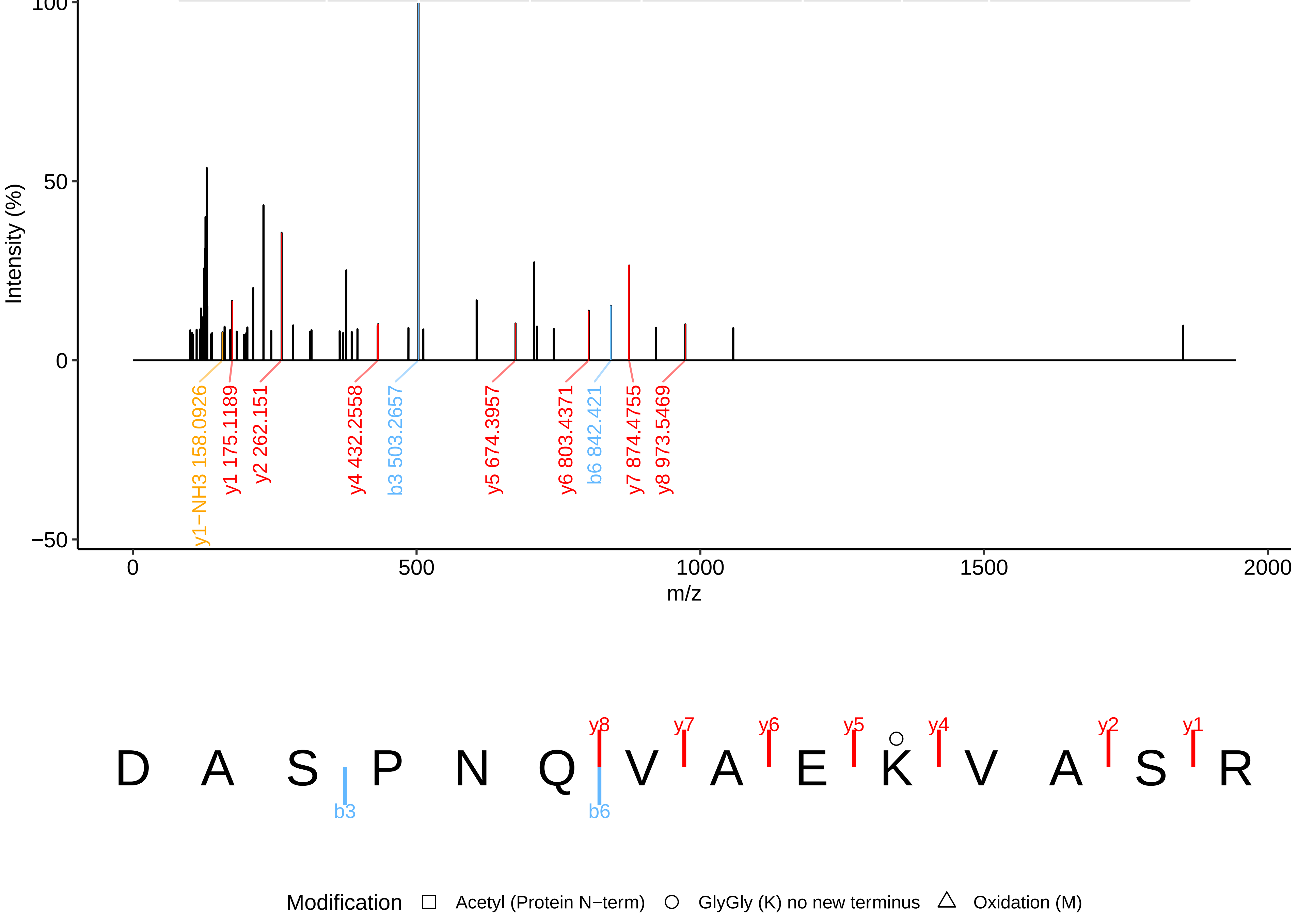

20200921_KIZ_MUb4_GlyGly_10plex_2 − Scan number: 3870

Gene Names	Charge	m/z	Mass	Retention time	PEP	Score	Precursor Intensity
LEO1	3	535.5764	1603.707	13.278	0.11035	41.644	284244.5

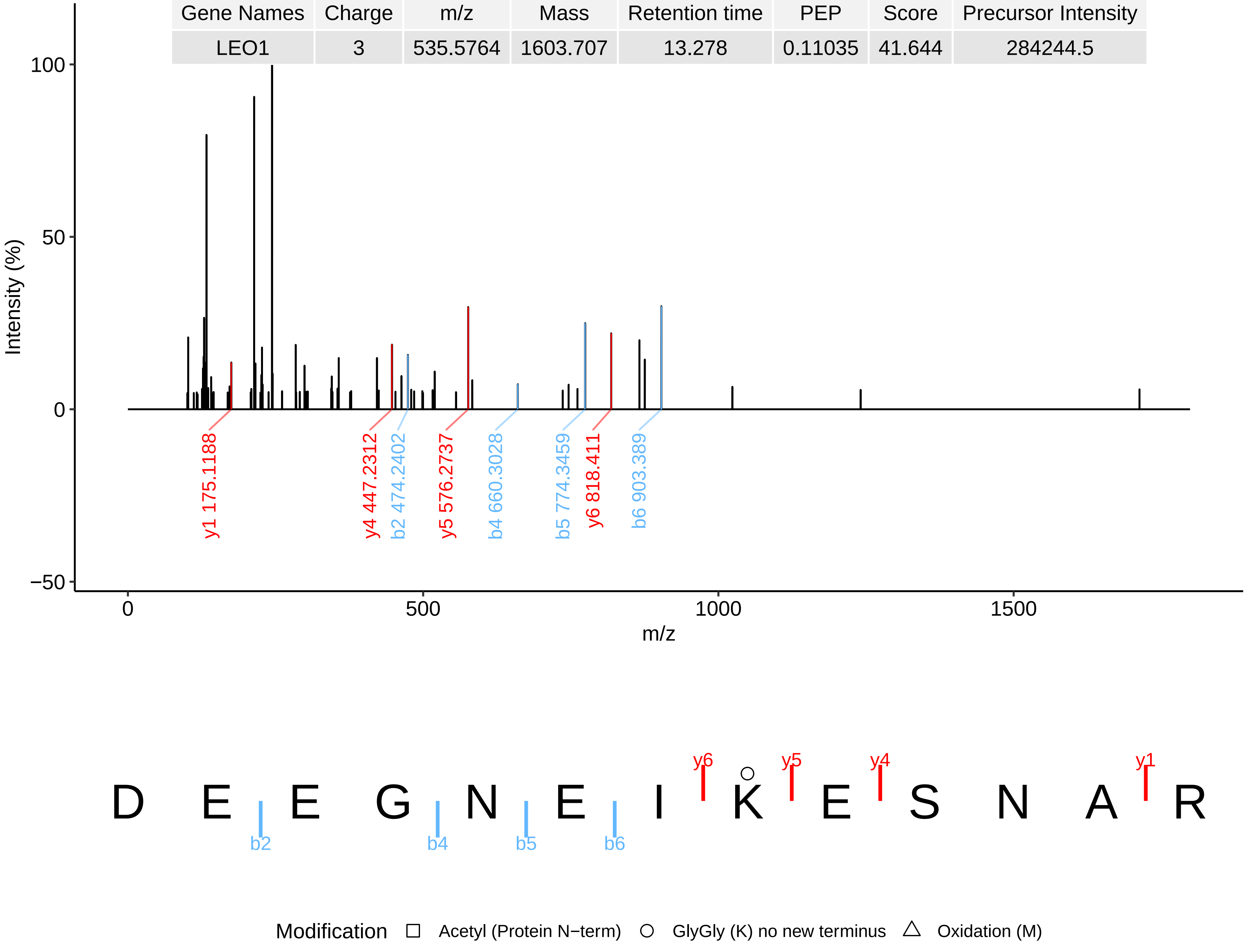

20200921_KIZ_MUb4_GlyGly_10plex_2 − Scan number: 15098

Gene Names	Charge	m/z	Mass	Retention time	PEP	Score	Precursor Intensity
HSPD1	3	453.9208	1358.741	58.976	0.00173	79.82	614501.2

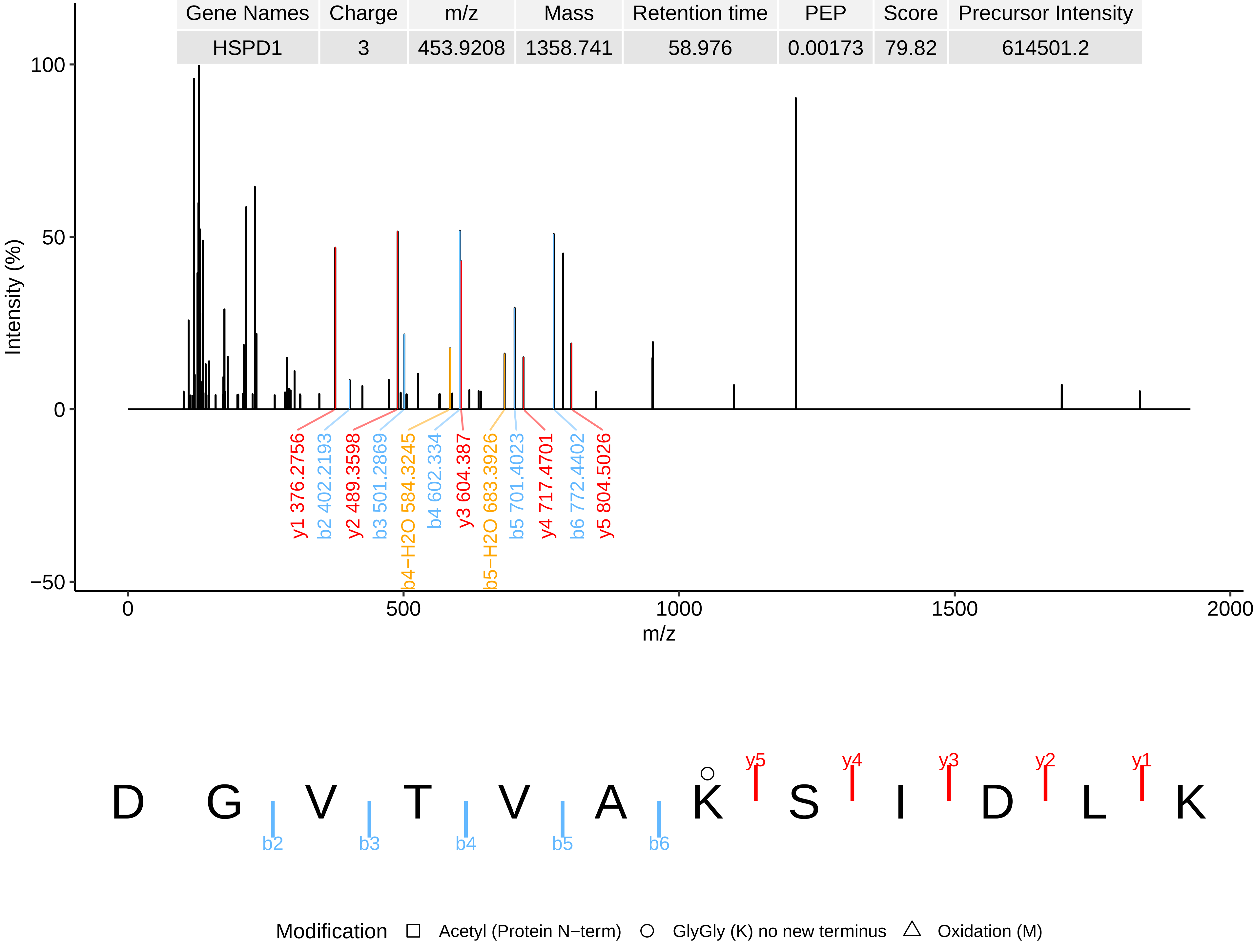

20200921_KIZ_MUb4_GlyGly_10plex_2 − Scan number: 11108

Gene Names	Charge	m/z	Mass	Retention time	PEP	Score	Precursor Intensity
PSMD7	3	516.6056	1546.795	42.673	0.064565	51.546	1087583

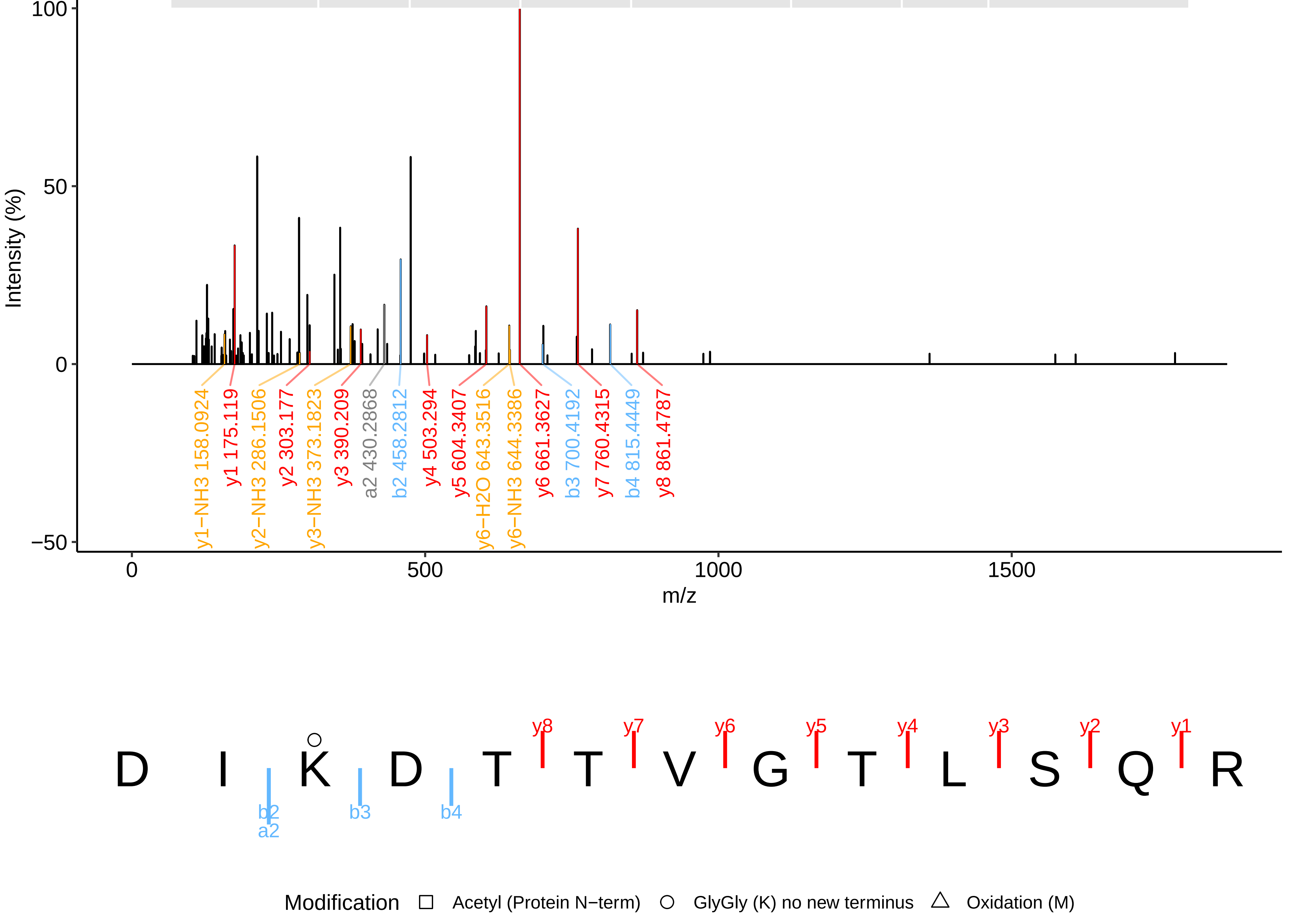

20200921_KIZ_MUb4_GlyGly_10plex − Scan number: 8479

Gene Names	Charge	m/z	Mass	Retention time	PEP	Score	Precursor Intensity
CLPTM1	3	458.2245	1371.652	28.781	0.0014757	83.182	6034893

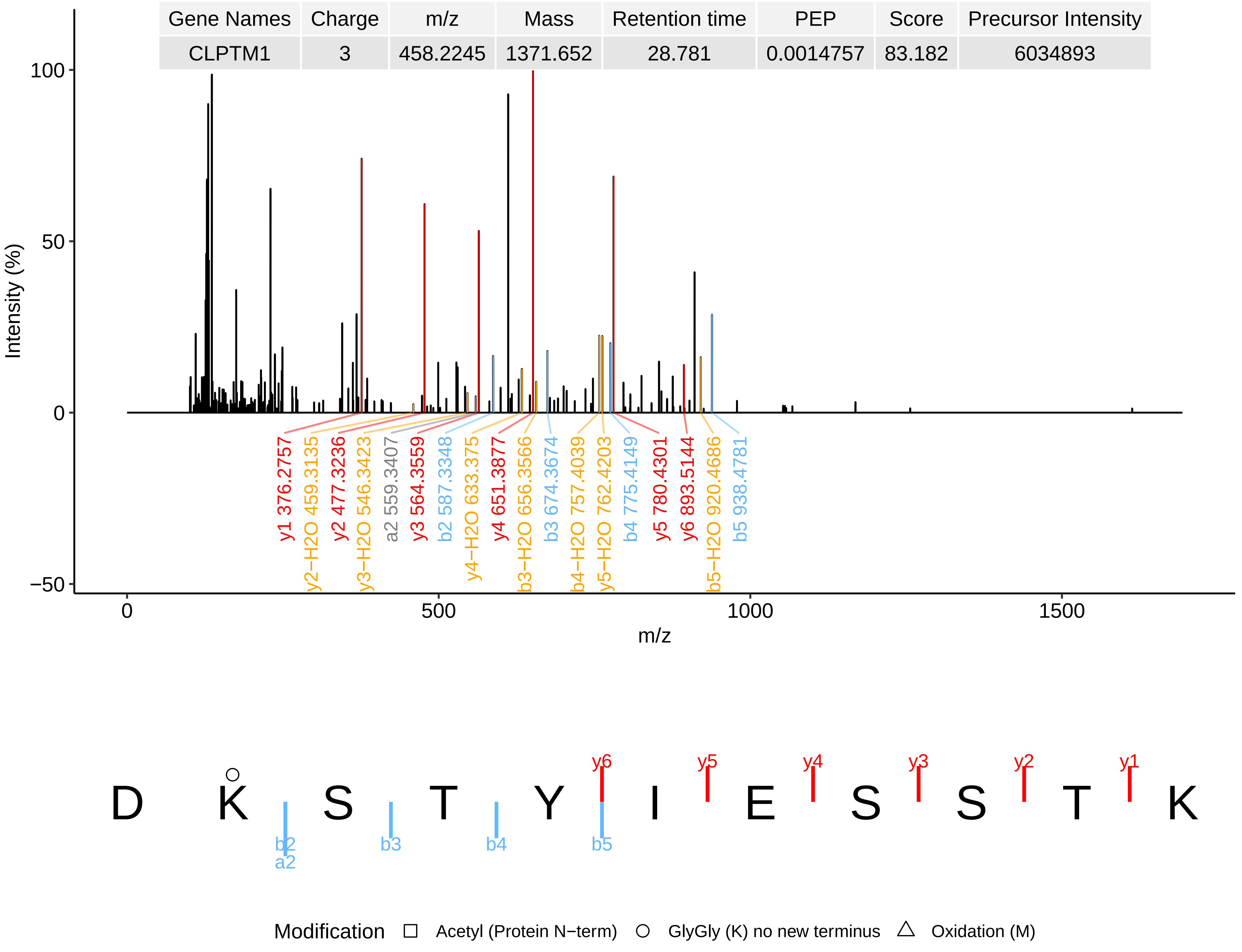

20200921_KIZ_MUb4_GlyGly_10plex_2 − Scan number: 7797

Gene Names	Charge	m/z	Mass	Retention time	PEP	Score	Precursor Intensity
CLPTM1	3	458.2245	1371.652	29.213	0.0076099	73.435	939321.1

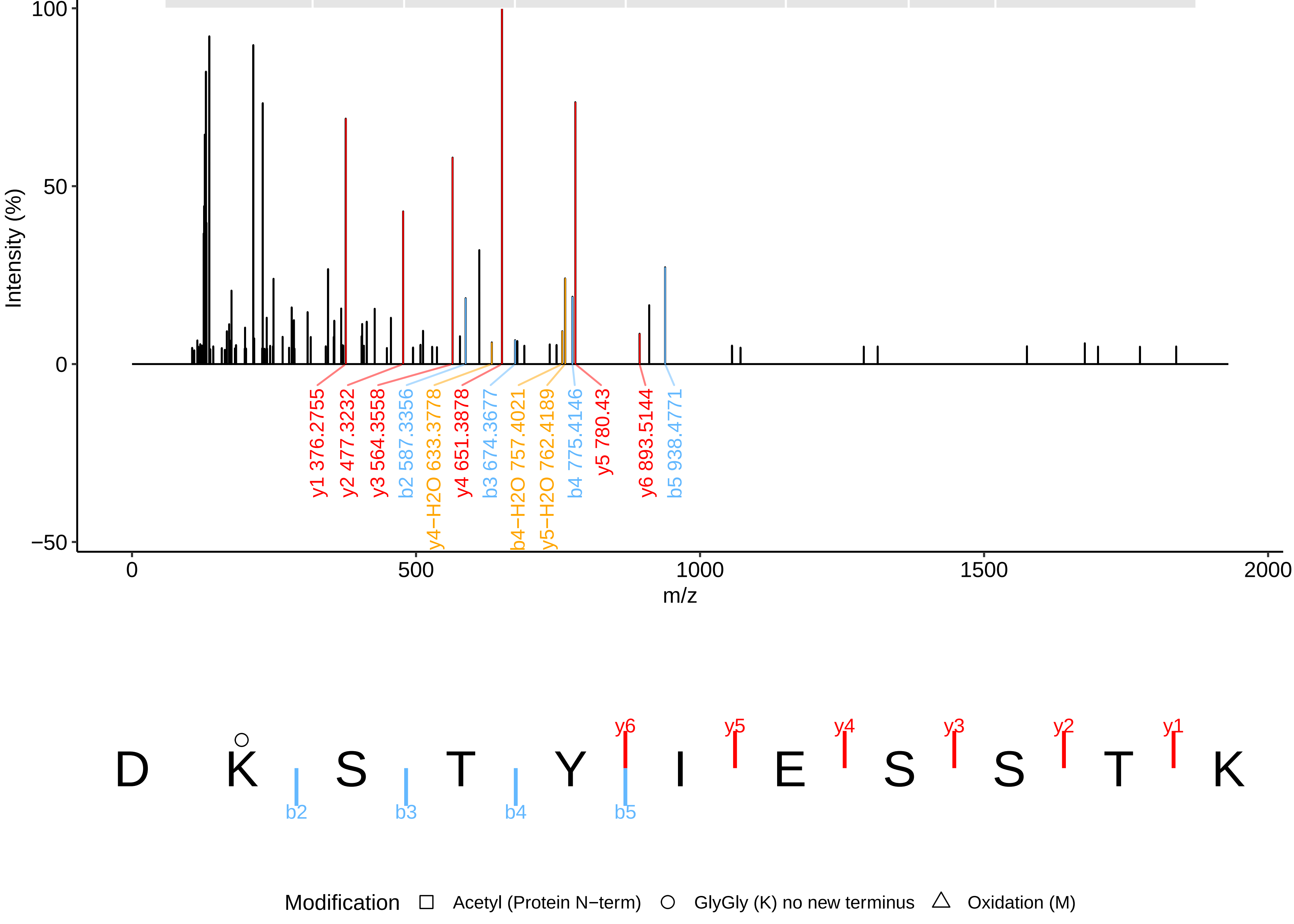

20200921_KIZ_MUb4_GlyGly_10plex − Scan number: 10172

Gene Names	Charge	m/z	Mass	Retention time	PEP	Score	Precursor Intensity
XPO1	3	533.9192	1598.736	35.114	0.0085062	66.486	8391580

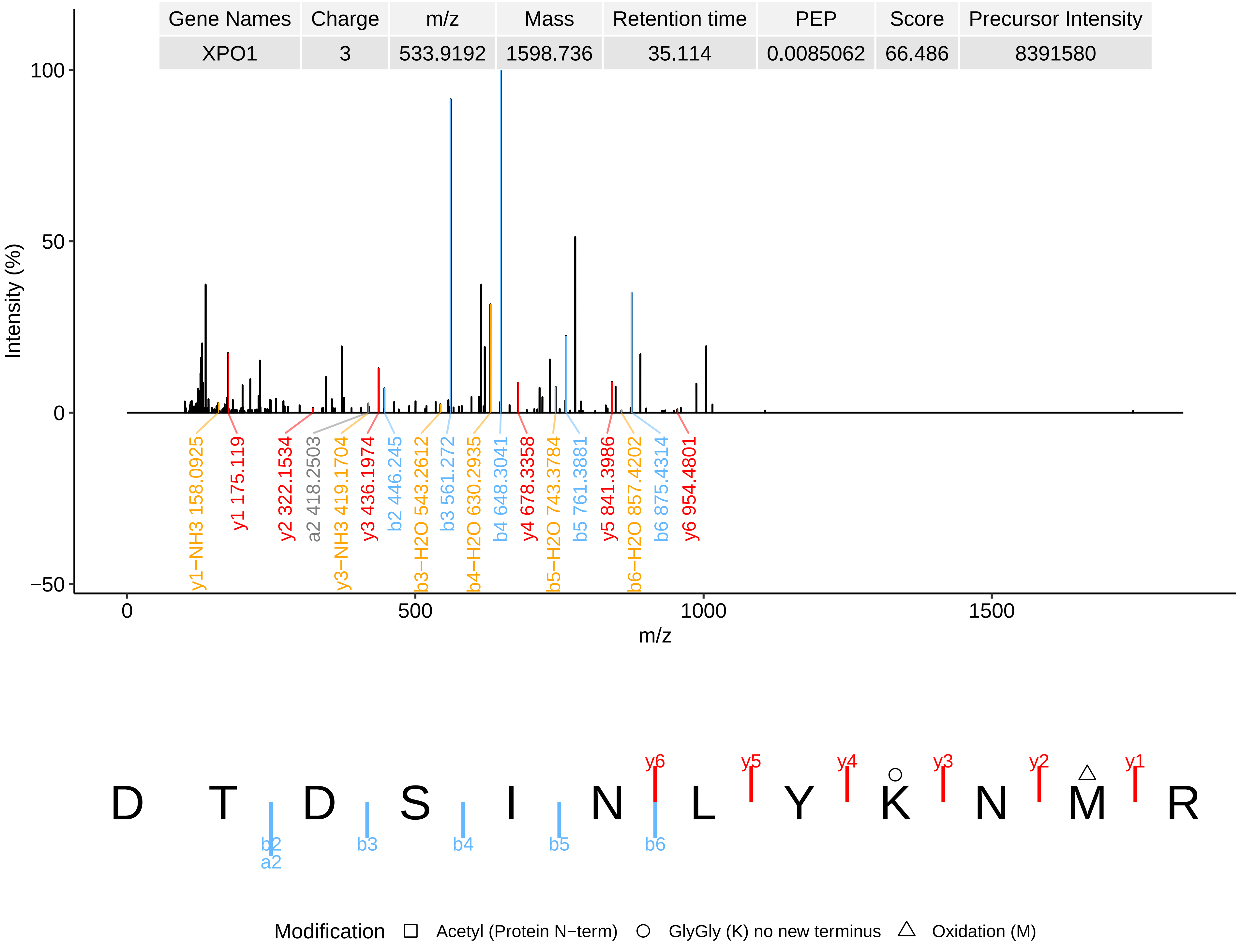

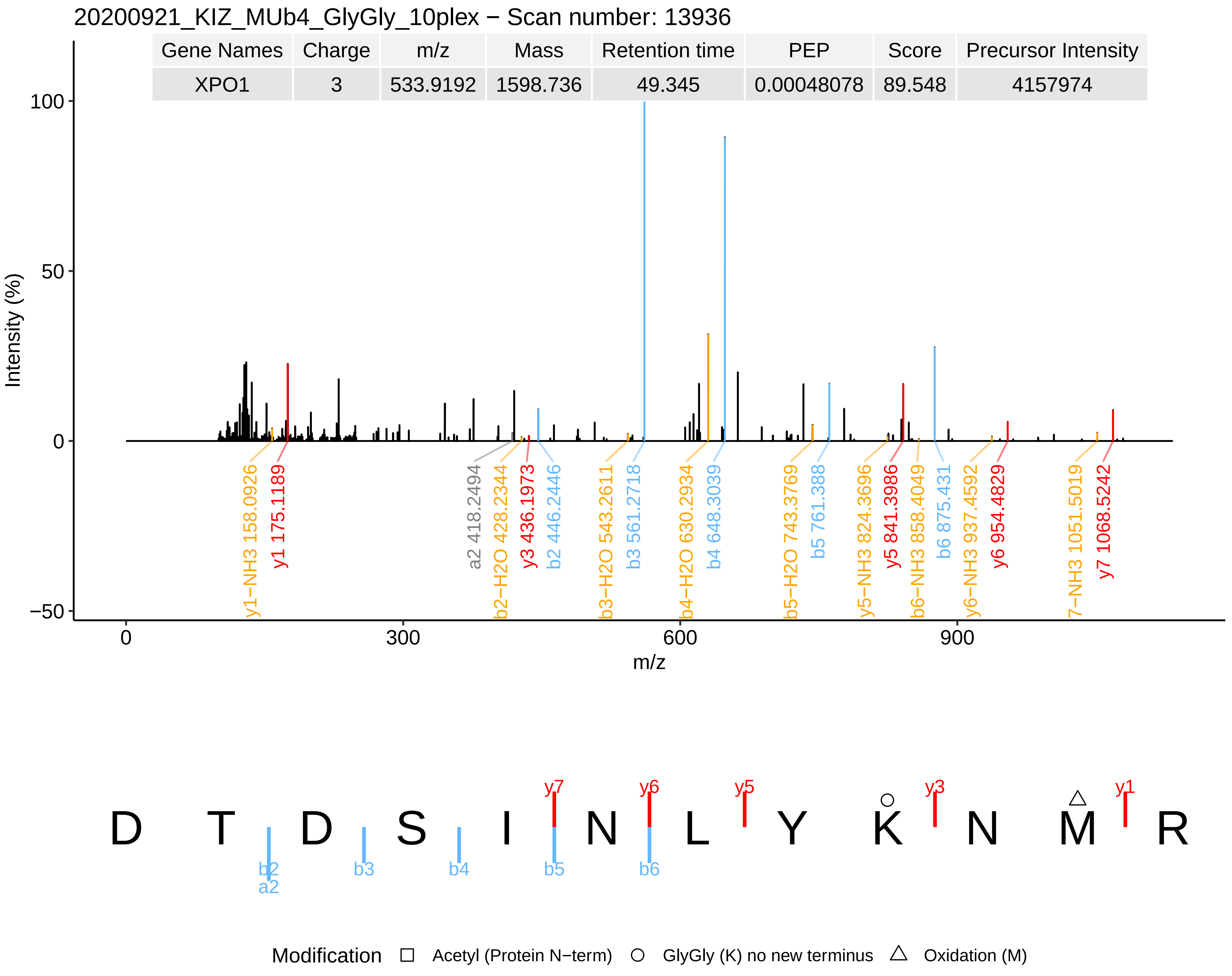

20200921_KIZ_MUb4_GlyGly_10plex − Scan number: 13936
Gene Names | Charge | m/z | Mass | Retention time | PEP | Score | Precursor Intensity
XPO1 | 3 | 533.9192 | 1598.736 | 49.345 | 0.00048078 | 89.548 | 4157974
Intensity (%)
m/z
y1−NH3 158.0926
y1 175.1189
a2 418.2494
b2−H2O 428.2344
y3 436.1973
b2 446.2446
b3−H2O 543.2611
b3 561.2718
b4−H2O 630.2934
b4 648.3039
b5−H2O 743.3769
b5 761.388
y5−NH3 824.3696
y5 841.3986
b6−NH3 858.4049
b6 875.431
y6−NH3 937.4592
y6 954.4829
y7−NH3 1051.5019
y7 1068.5242
D T D S I N L Y K N M R
y7 y6 y5 y3 y1
b2 a2 b3 b4 b5 b6
Modification
Acetyl (Protein N−term)
GlyGly (K) no new terminus
Oxidation (M)

20200921_KIZ_MUb4_GlyGly_10plex − Scan number: 13942

Gene Names	Charge	m/z	Mass	Retention time	PEP	Score	Precursor Intensity
XPO1	3	533.9192	1598.736	49.366	0.0046883	73.632	1139304

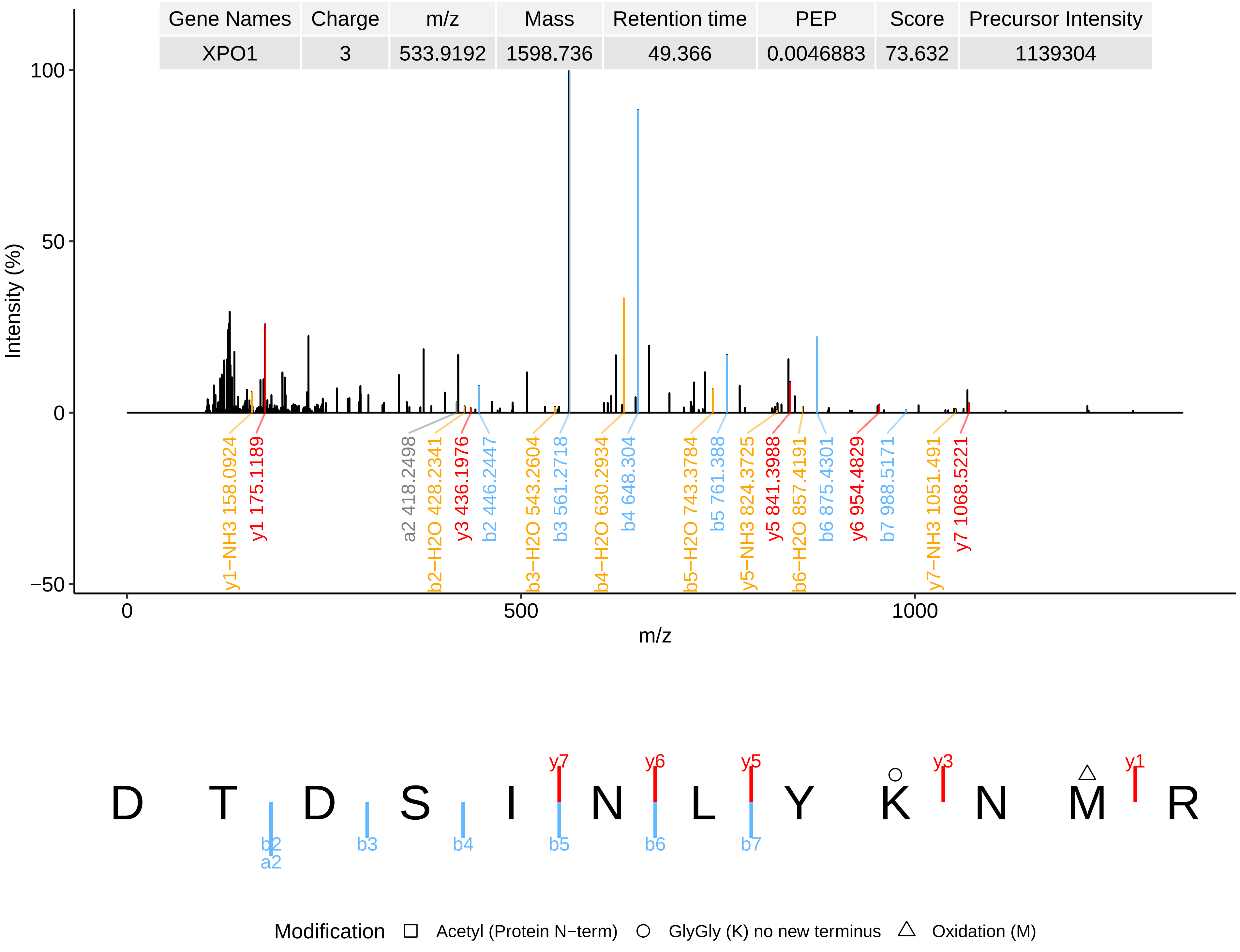

20200921_KIZ_MUb4_GlyGly_10plex_2 − Scan number: 9309

Gene Names	Charge	m/z	Mass	Retention time	PEP	Score	Precursor Intensity
XPO1	3	533.9192	1598.736	35.354	0.031037	58.172	1033337

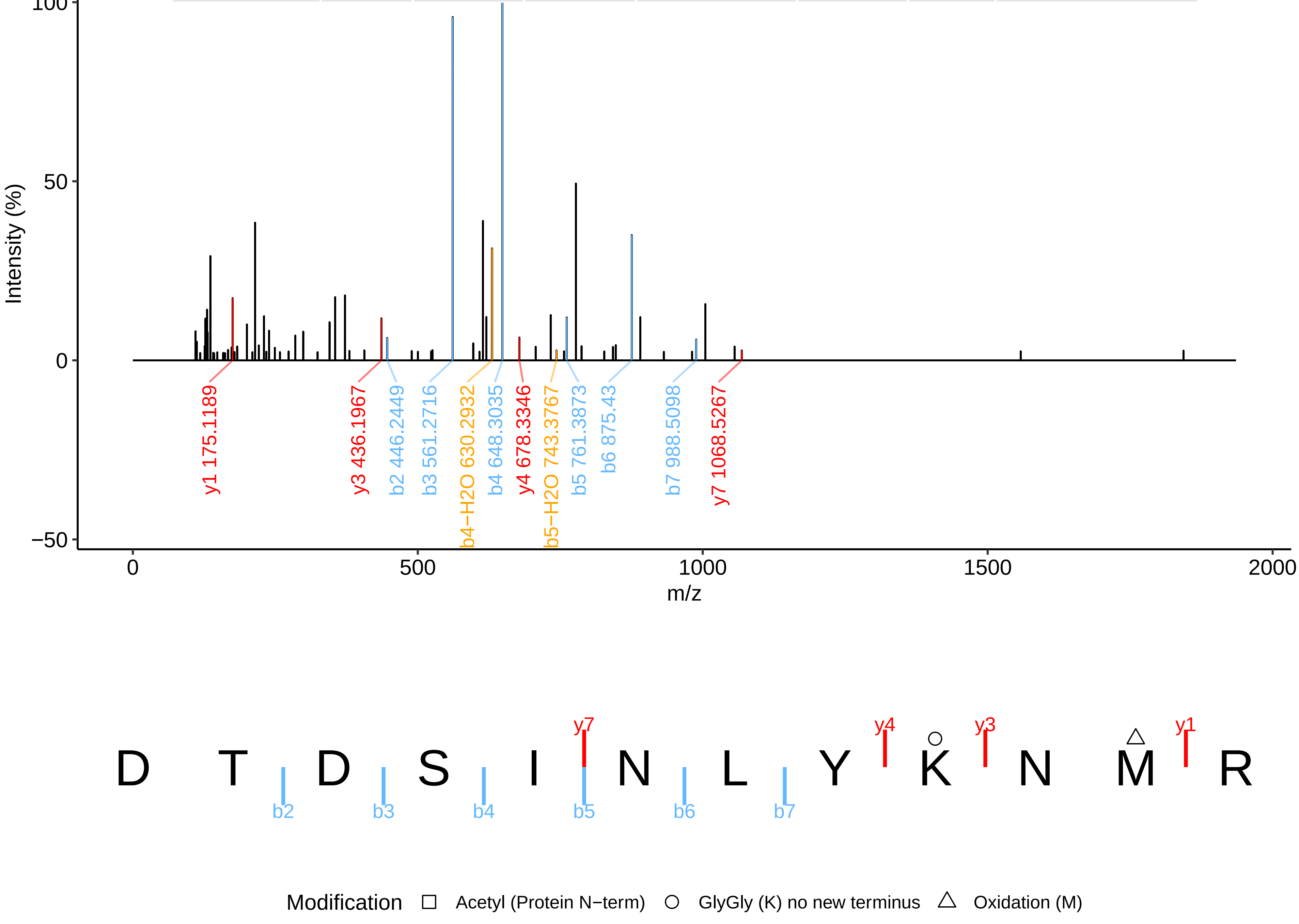

20200921_KIZ_MUb4_GlyGly_10plex − Scan number: 13903

Gene Names	Charge	m/z	Mass	Retention time	PEP	Score	Precursor Intensity
XPO1	3	528.5876	1582.741	49.212	1.1031e−13	145.28	4863962

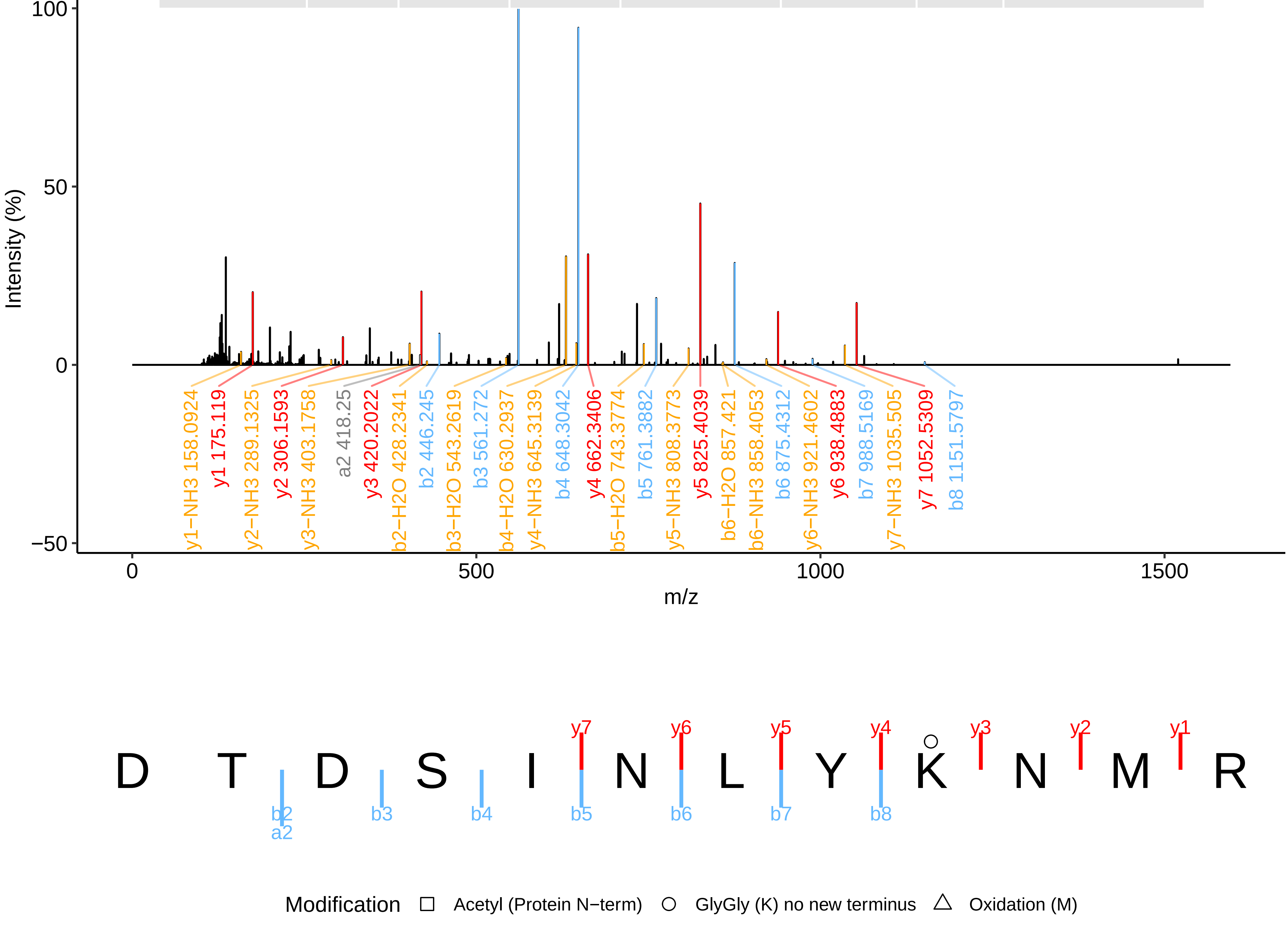

20200921_KIZ_MUb4_GlyGly_10plex_2 − Scan number: 12746

Gene Names	Charge	m/z	Mass	Retention time	PEP	Score	Precursor Intensity
XPO1	3	528.5876	1582.741	49.378	0.00067798	88.441	870759.7

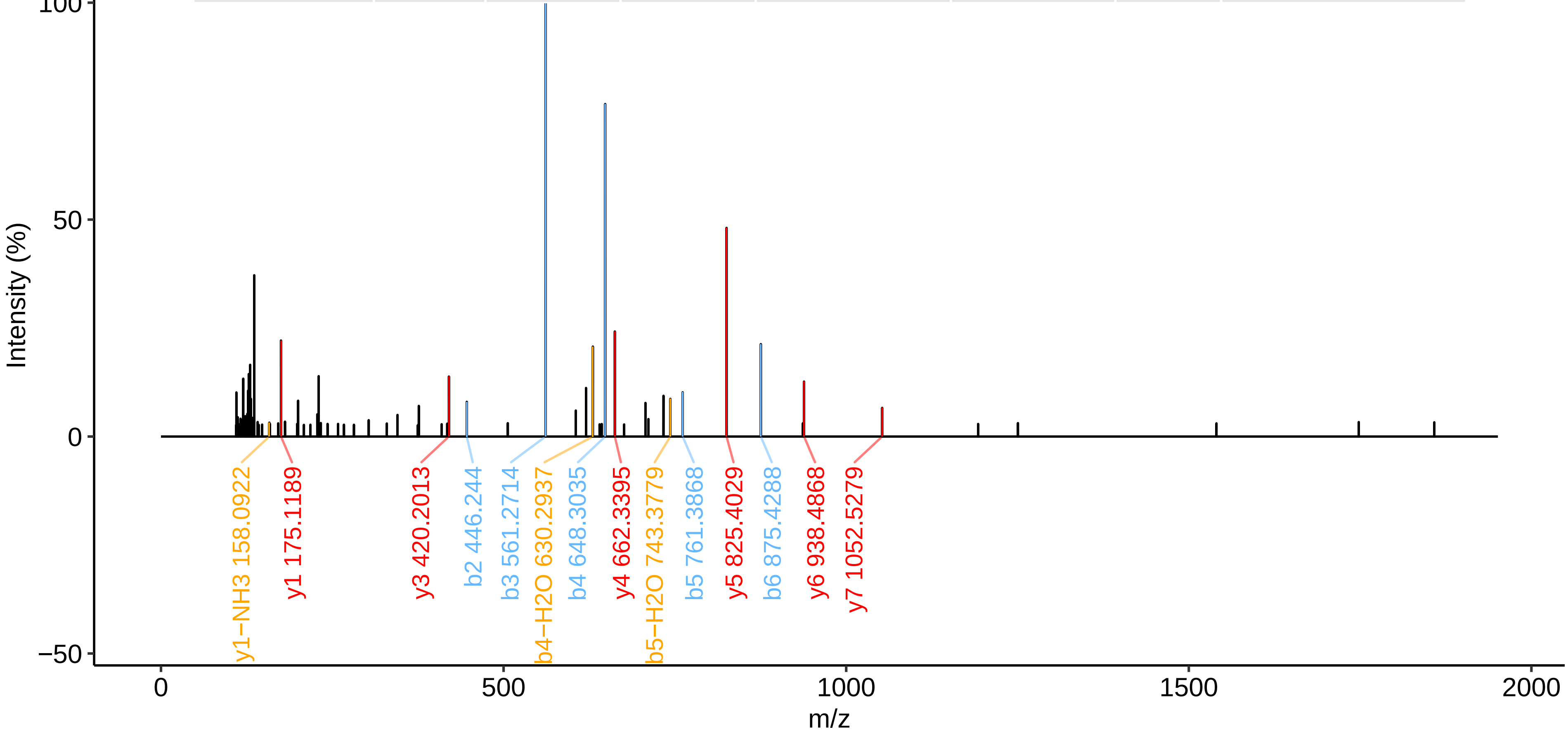

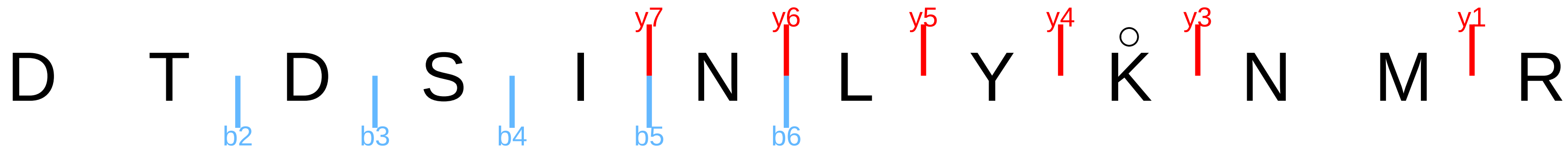

Modification □ Acetyl (Protein N−term) ○ GlyGly (K) no new terminus △ Oxidation (M)

20200924_KIZ_MUb5_GlyGly_1 − Scan number: 12871

Gene Names	Charge	m/z	Mass	Retention time	PEP	Score	Precursor Intensity
XPO1	3	528.5876	1582.741	41.94	2.0897e−06	113.67	13448631

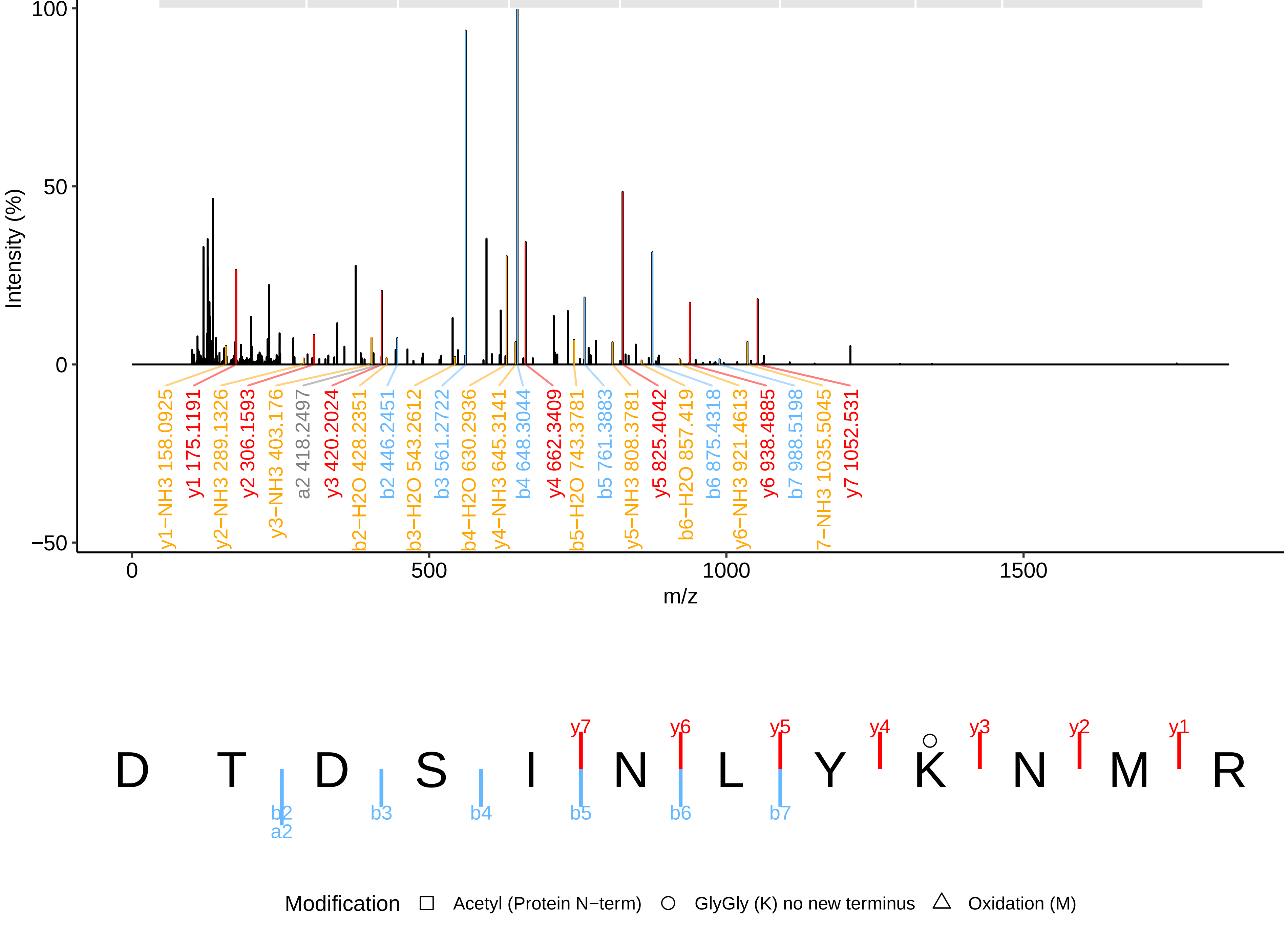

20200924_KIZ_MUb5_GlyGly_1 − Scan number: 13019

Gene Names	Charge	m/z	Mass	Retention time	PEP	Score	Precursor Intensity
XPO1	3	528.5876	1582.741	42.45	1.556e−13	143.24	22976242

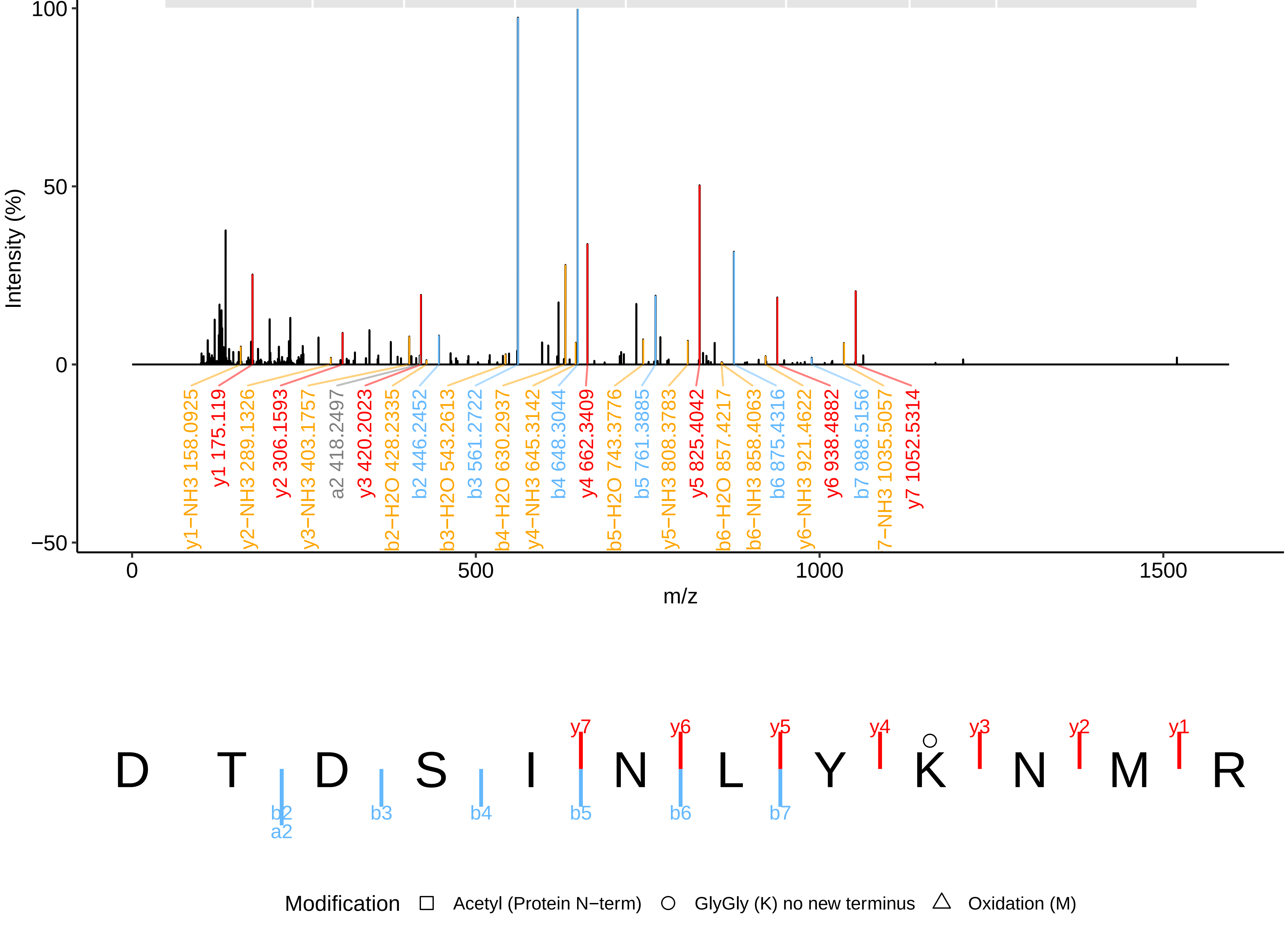

20200924_KIZ_MUb5_GlyGly_2 − Scan number: 12188

Gene Names	Charge	m/z	Mass	Retention time	PEP	Score	Precursor Intensity
XPO1	3	528.5876	1582.741	45.568	1.556e−13	143.24	10541162

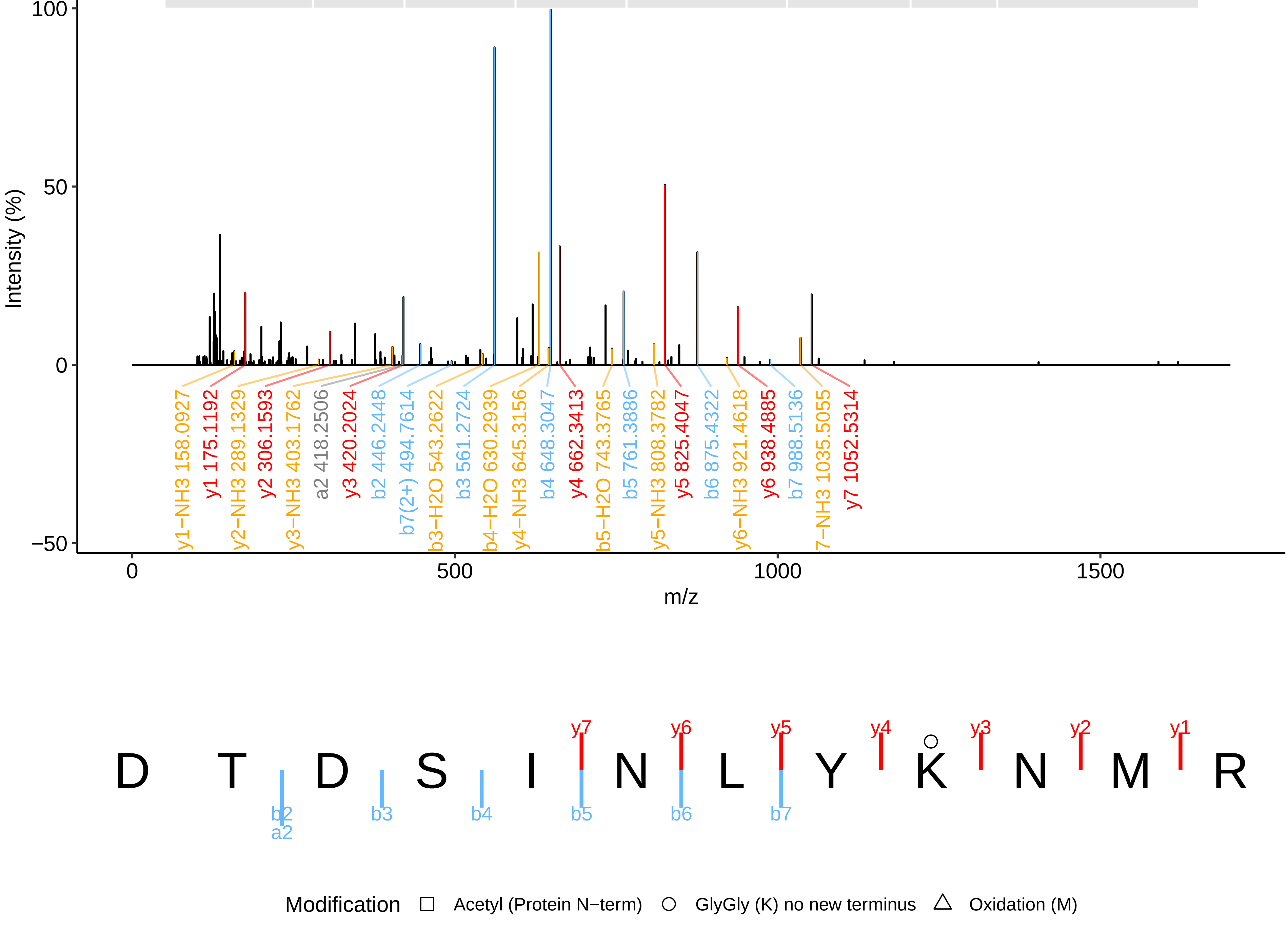

20200924_KIZ_MUb5_GlyGly_2 − Scan number: 12331

Gene Names	Charge	m/z	Mass	Retention time	PEP	Score	Precursor Intensity
XPO1	3	528.5876	1582.741	46.094	4.1121e−09	127.83	2468849

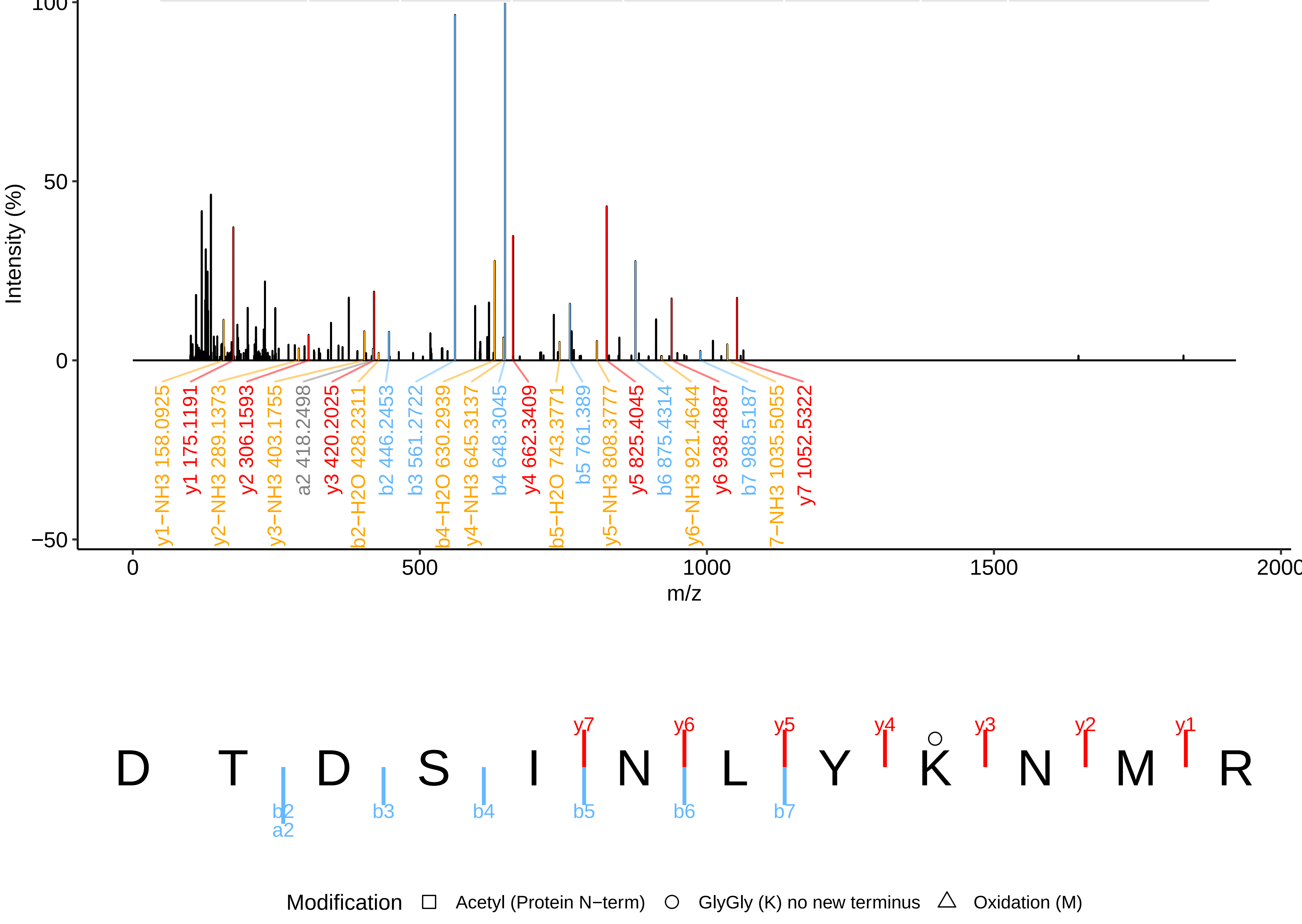

20201118_KIZ_MUb6_GlyGly_2 − Scan number: 14566

Gene Names	Charge	m/z	Mass	Retention time	PEP	Score	Precursor Intensity
XPO1	3	528.5876	1582.741	49.147	2.5508e−13	138.76	14237986

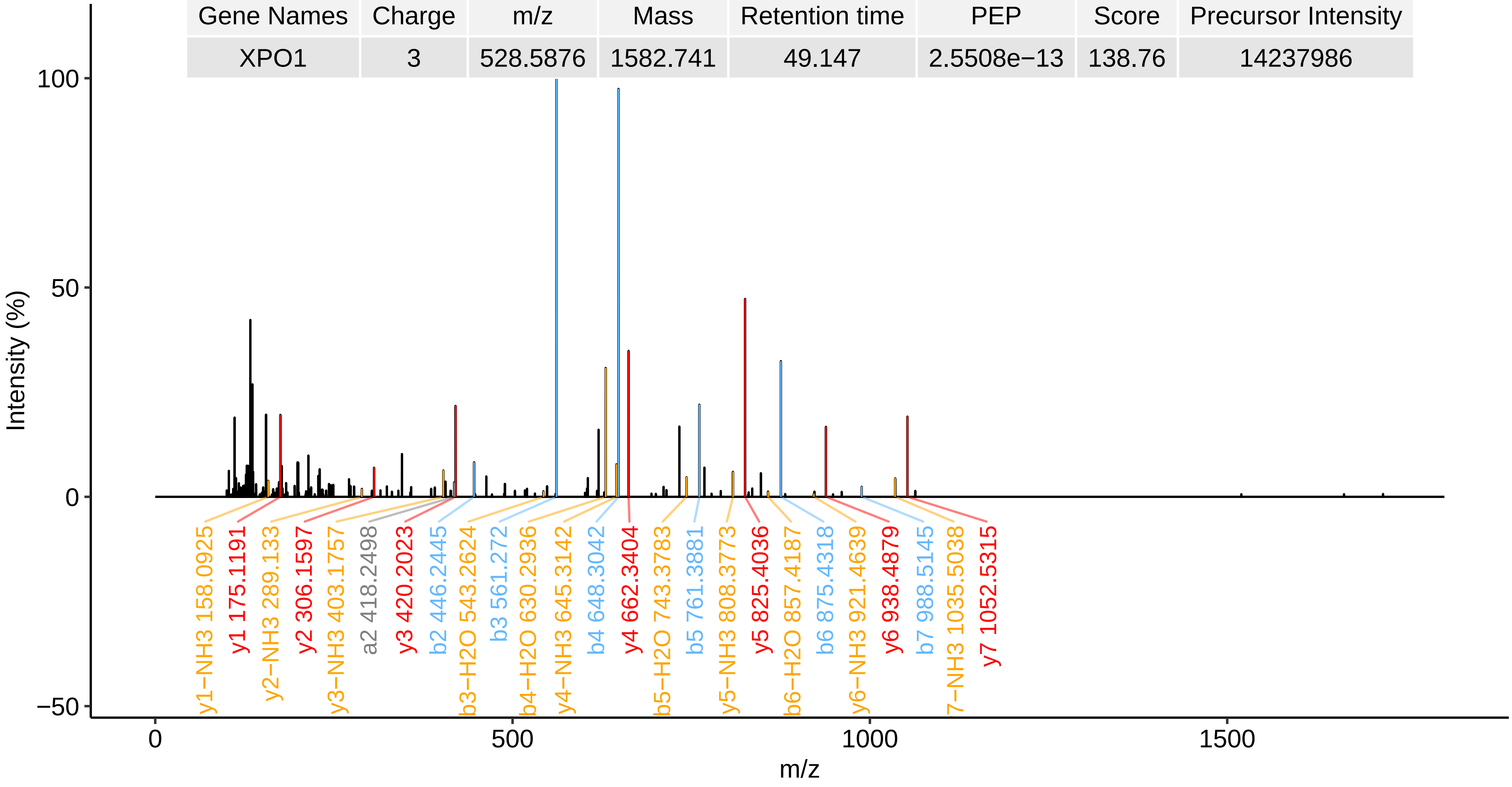

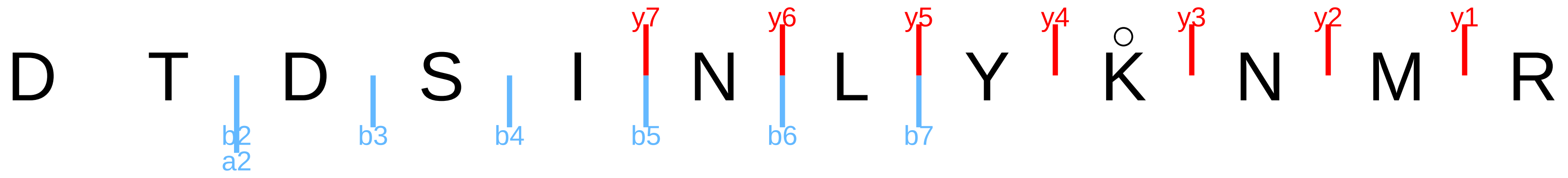

Modification □ Acetyl (Protein N−term) ○ GlyGly (K) no new terminus △ Oxidation (M)

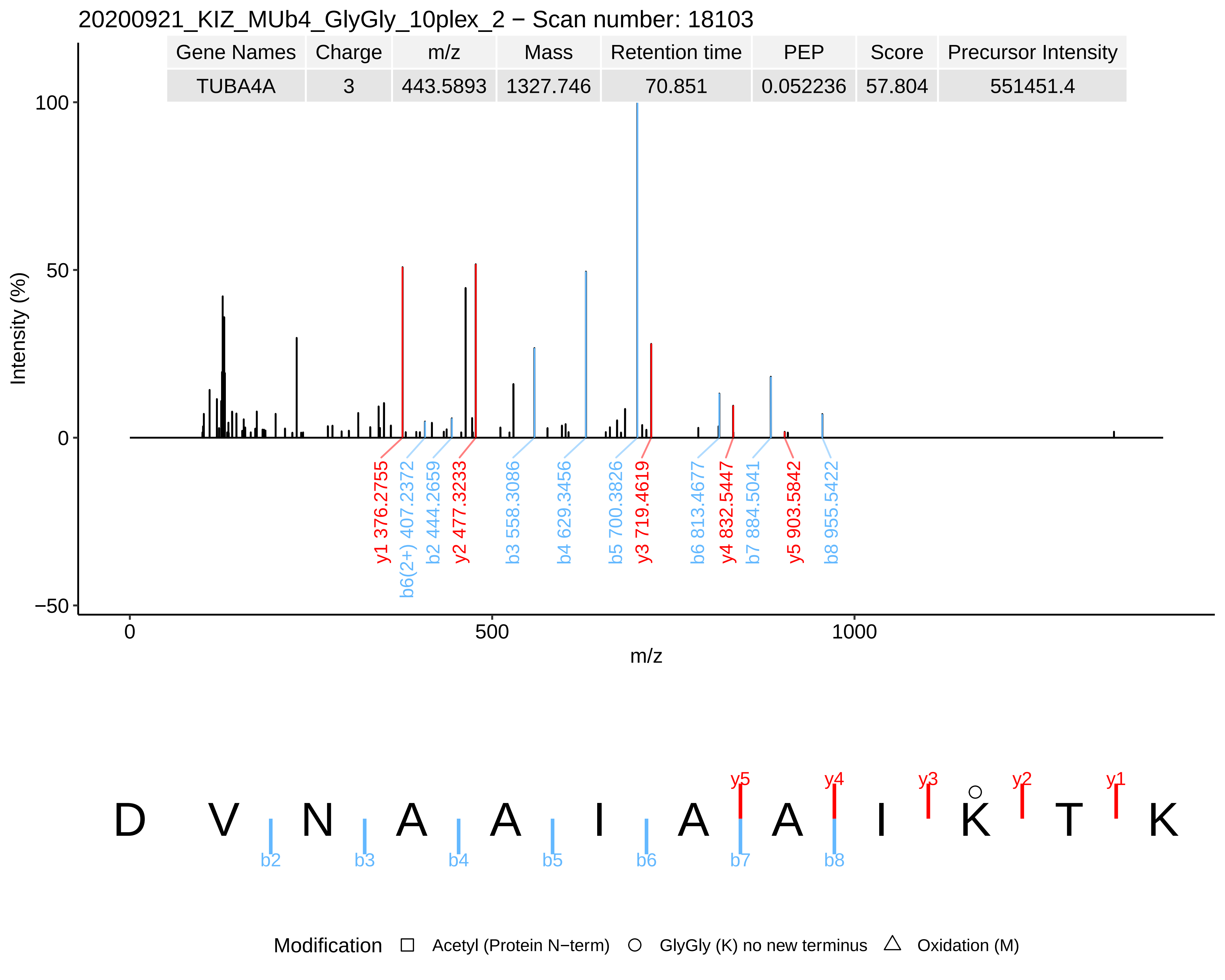
20200921_KIZ_MUb4_GlyGly_10plex_2 − Scan number: 18103
Gene Names | Charge | m/z | Mass | Retention time | PEP | Score | Precursor Intensity
TUBA4A | 3 | 443.5893 | 1327.746 | 70.851 | 0.052236 | 57.804 | 551451.4
Intensity (%)
m/z
y1 376.2755
b6(2+) 407.2372
b2 444.2659
y2 477.3233
b3 558.3086
b4 629.3456
b5 700.3826
y3 719.4619
b6 813.4677
y4 832.5447
b7 884.5041
y5 903.5842
b8 955.5422
D V N A A I A A I K T K
Modification
Acetyl (Protein N−term)
GlyGly (K) no new terminus
Oxidation (M)

20200921_KIZ_MUb4_GlyGly_10plex − Scan number: 17929

Gene Names	Charge	m/z	Mass	Retention time	PEP	Score	Precursor Intensity
TUBA1C;TUBA1B	3	453.5928	1357.756	64.394	1.8943e−05	99.531	10632264

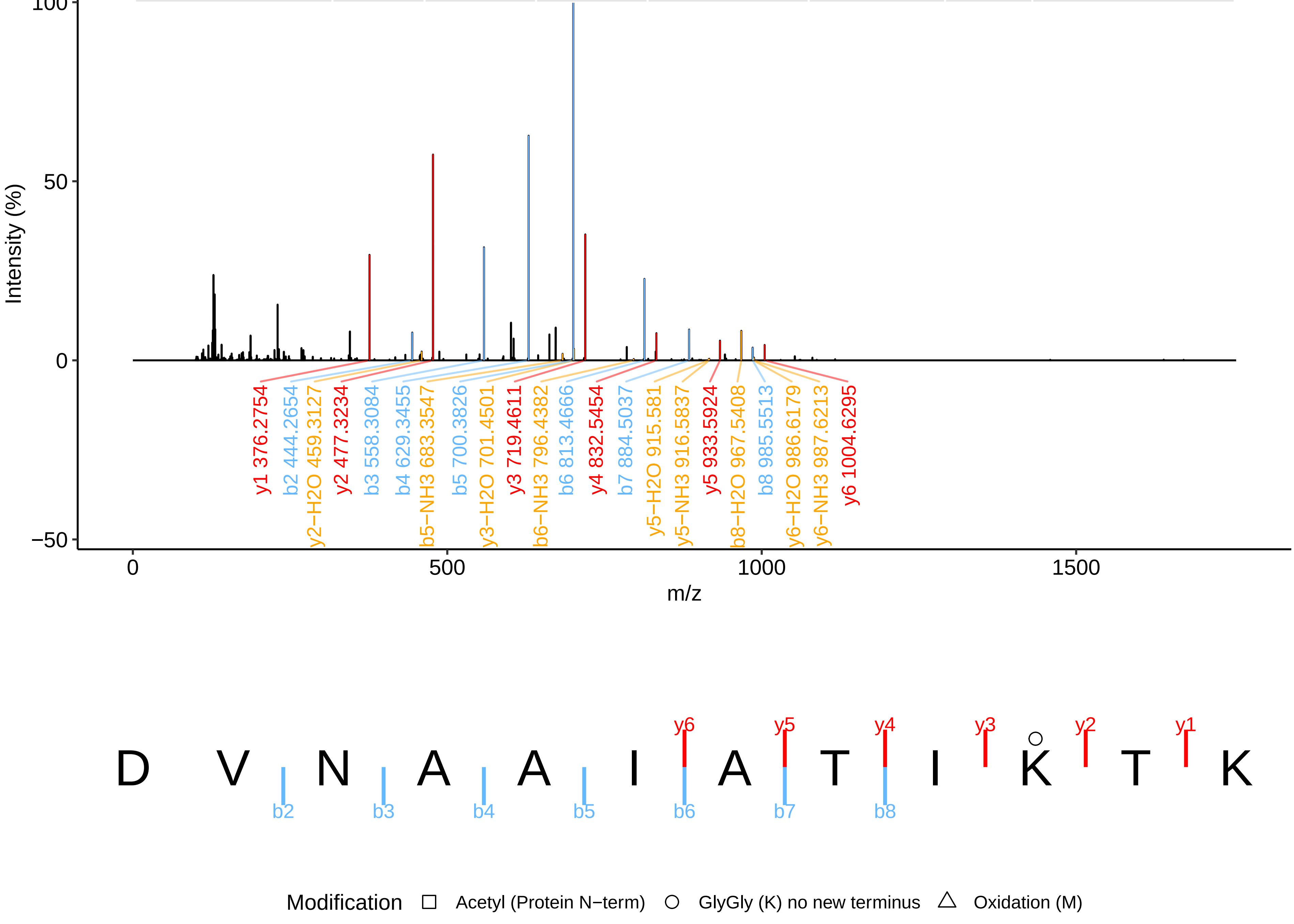

20200921_KIZ_MUb4_GlyGly_10plex − Scan number: 18067

Gene Names	Charge	m/z	Mass	Retention time	PEP	Score	Precursor Intensity
TUBA1C;TUBA1B	3	453.5928	1357.756	64.894	9.5797e−06	107.39	21598246

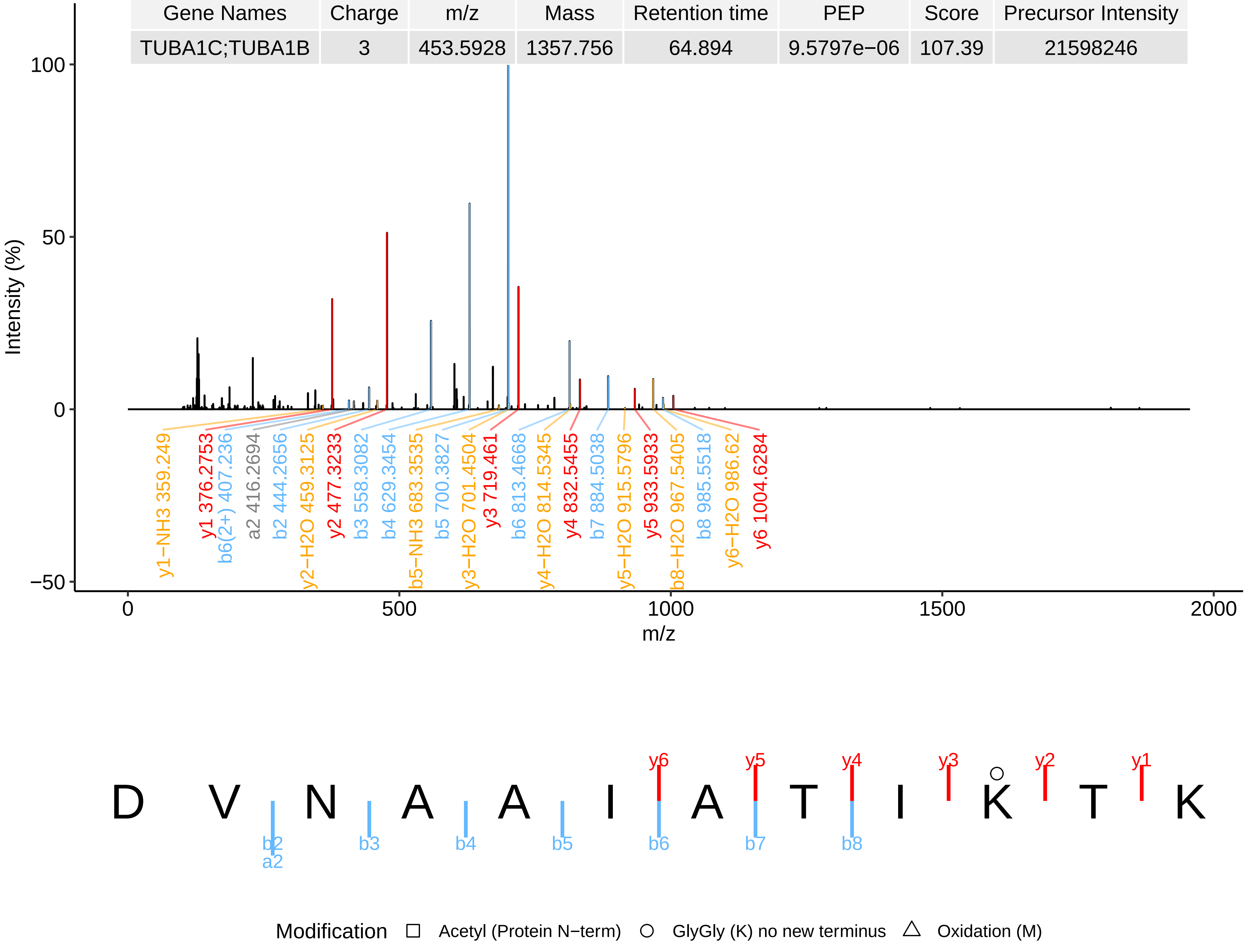

20200921_KIZ_MUb4_GlyGly_10plex_2 − Scan number: 16498

Gene Names	Charge	m/z	Mass	Retention time	PEP	Score	Precursor Intensity
TUBA1C;TUBA1B	3	453.5928	1357.756	64.407	1.6558e−05	101.53	2243574

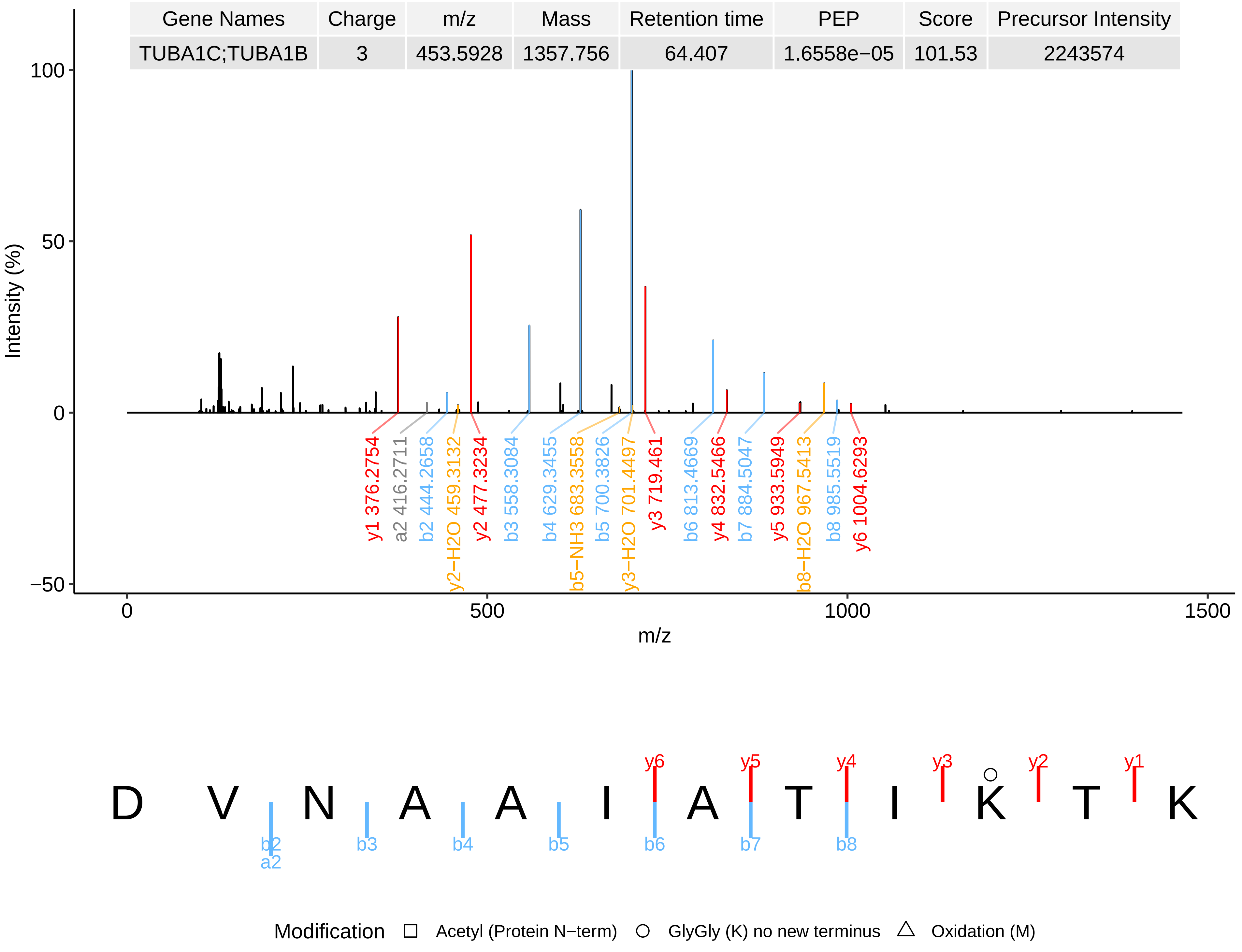

20200924_KIZ_MUb5_GlyGly_1 − Scan number: 17387

Gene Names	Charge	m/z	Mass	Retention time	PEP	Score	Precursor Intensity
TUBA1C;TUBA1B	3	453.5928	1357.756	56.131	0.00060791	89.46	11313401

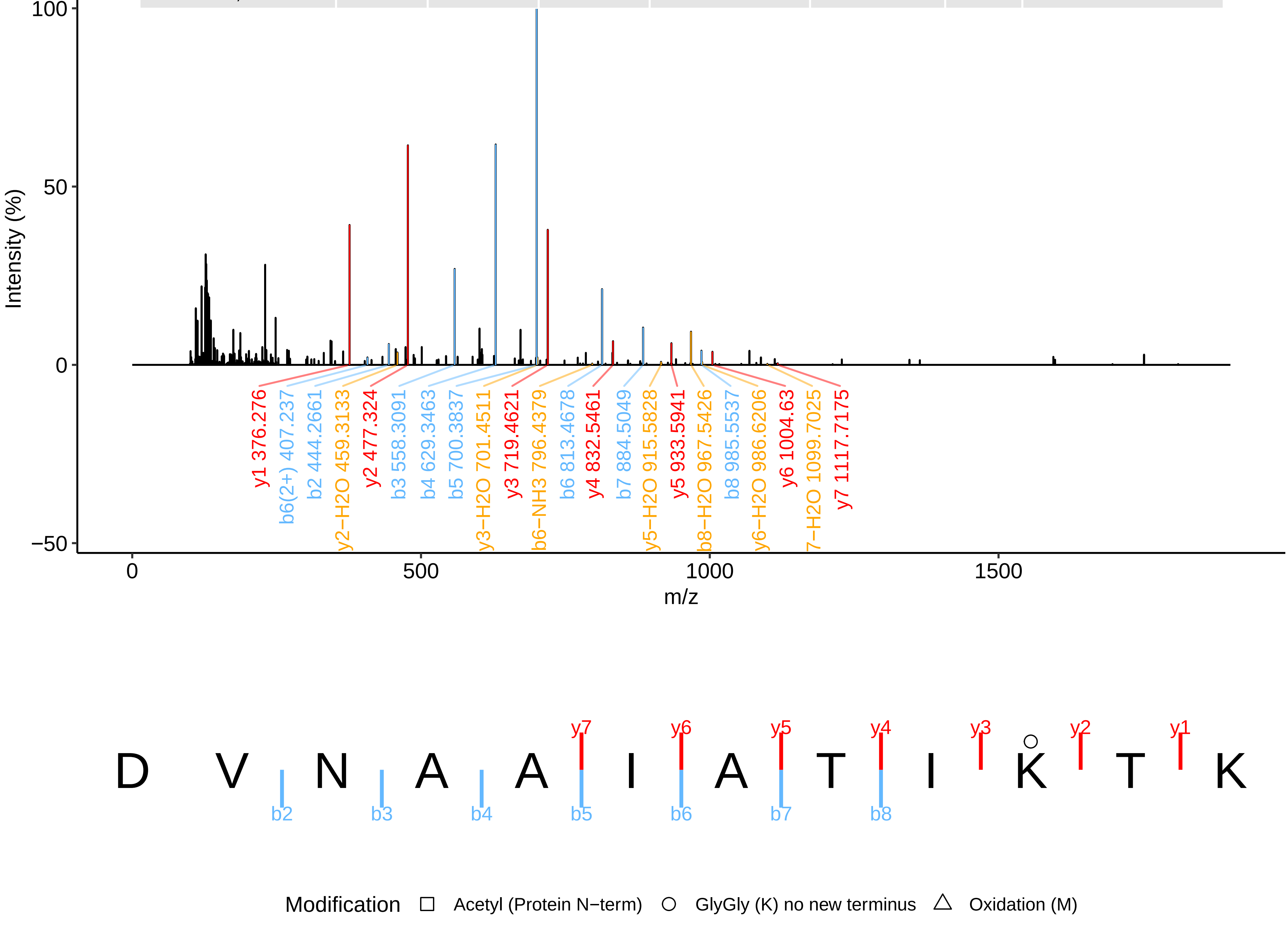

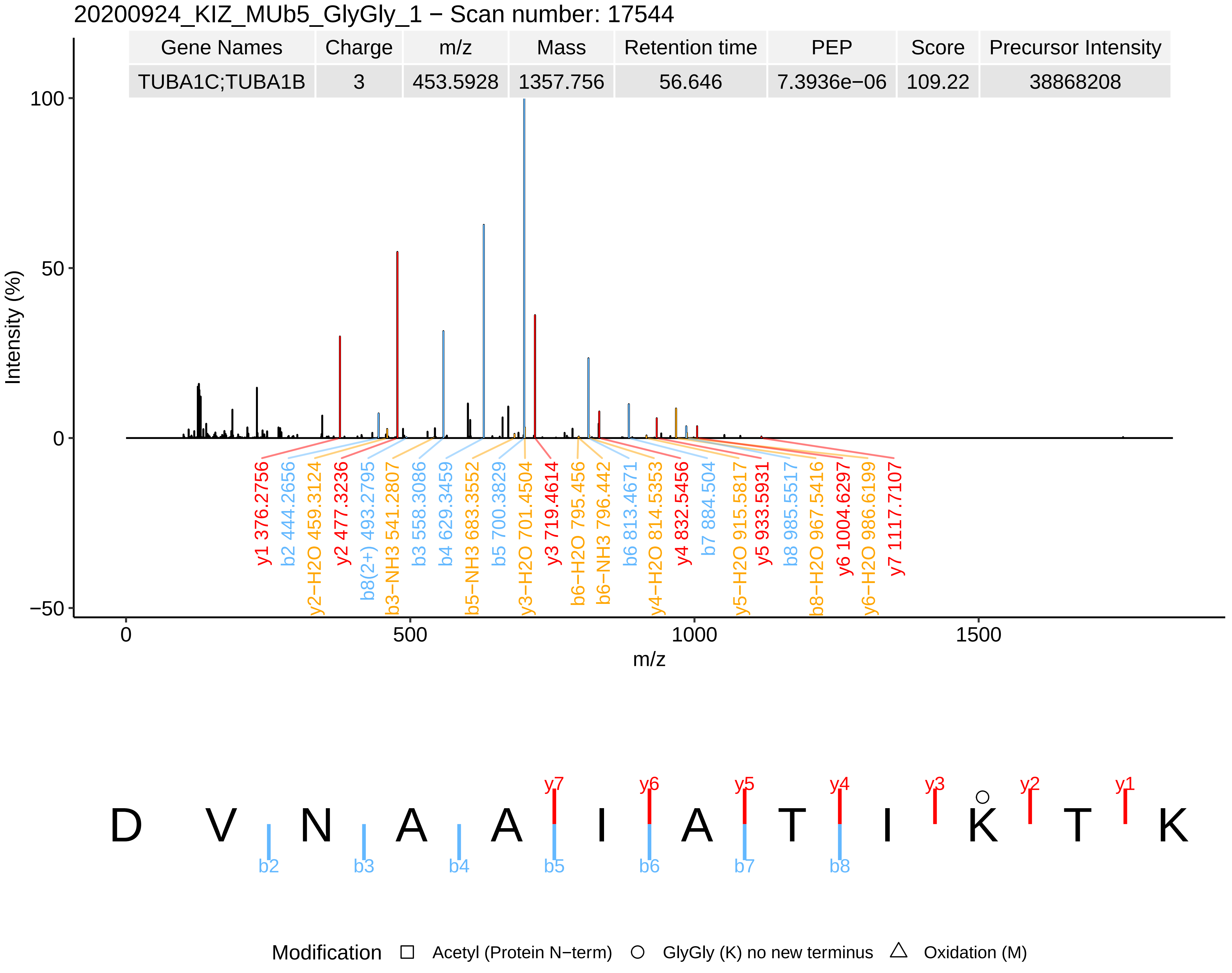
20200924_KIZ_MUb5_GlyGly_1 − Scan number: 17544
Gene Names | Charge | m/z | Mass | Retention time | PEP | Score | Precursor Intensity
TUBA1C;TUBA1B | 3 | 453.5928 | 1357.756 | 56.646 | 7.3936e−06 | 109.22 | 38868208
Intensity (%)
100
50
0
−50
0
500
1000
1500
m/z
y1 376.2756
b2 444.2656
y2−H2O 459.3124
y2 477.3236
b8(2+) 493.2795
b3−NH3 541.2807
b3 558.3086
b4 629.3459
b5−NH3 683.3552
b5 700.3829
y3−H2O 701.4504
y3 719.4614
b6−H2O 795.456
b6−NH3 796.442
b6 813.4671
y4−H2O 814.5353
y4 832.5456
b7 884.504
y5−H2O 915.5817
y5 933.5931
b8 985.5517
b8−H2O 967.5416
y6 1004.6297
y6−H2O 986.6199
y7 1117.7107
D V N A A I A T I K T K
y7 y6 y5 y4 y3 y2 y1
b2 b3 b4 b5 b6 b7 b8
Modification
Acetyl (Protein N−term)
GlyGly (K) no new terminus
Oxidation (M)

20200924_KIZ_MUb5_GlyGly_1 − Scan number: 17692

Gene Names	Charge	m/z	Mass	Retention time	PEP	Score	Precursor Intensity
TUBA1C;TUBA1B	3	453.5928	1357.756	57.154	0.00045936	91.62	74893088

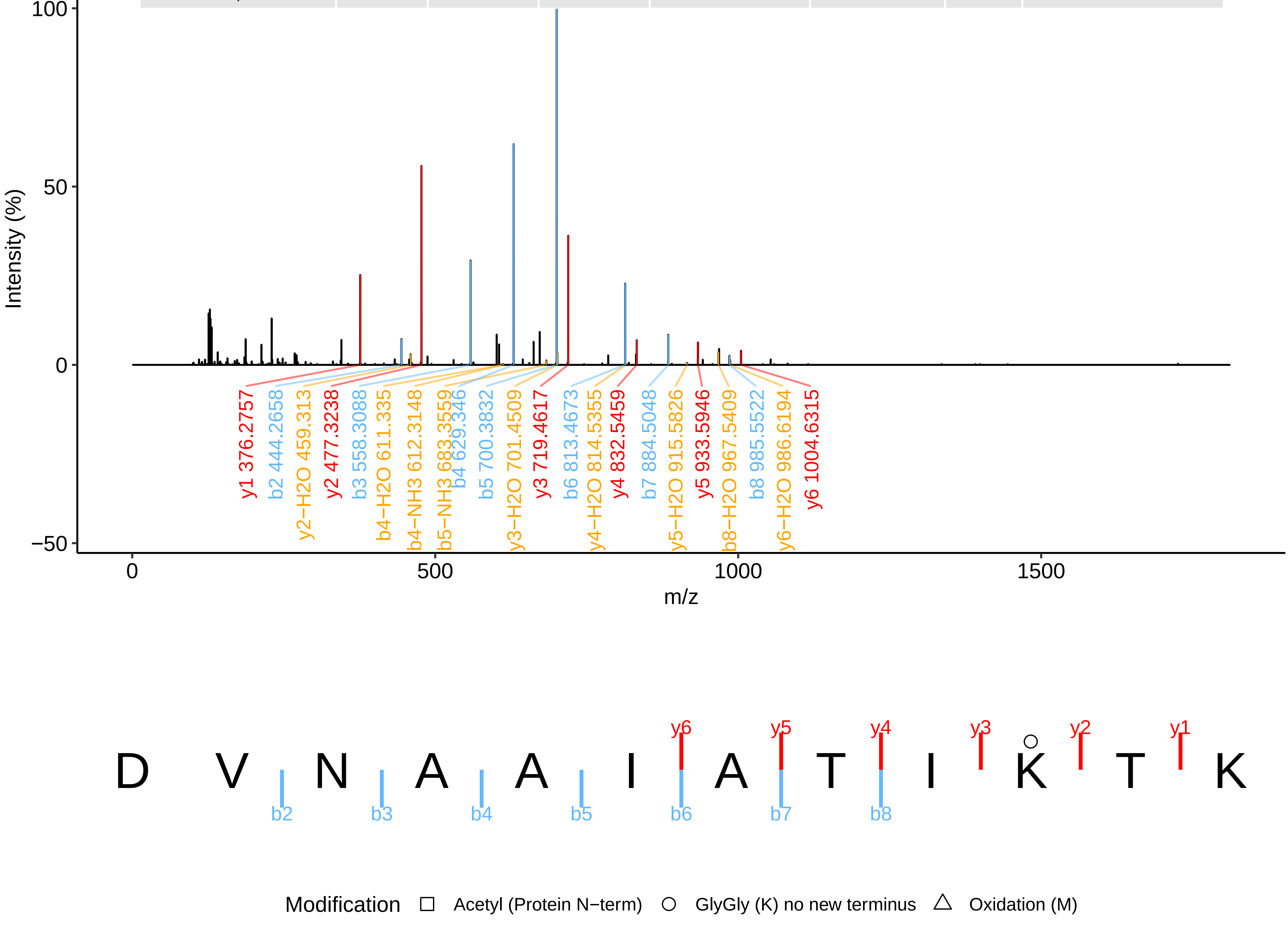

20200924_KIZ_MUb5_GlyGly_1 − Scan number: 17845

Gene Names	Charge	m/z	Mass	Retention time	PEP	Score	Precursor Intensity
TUBA1C;TUBA1B	3	453.5928	1357.756	57.662	0.00045936	91.62	27997070

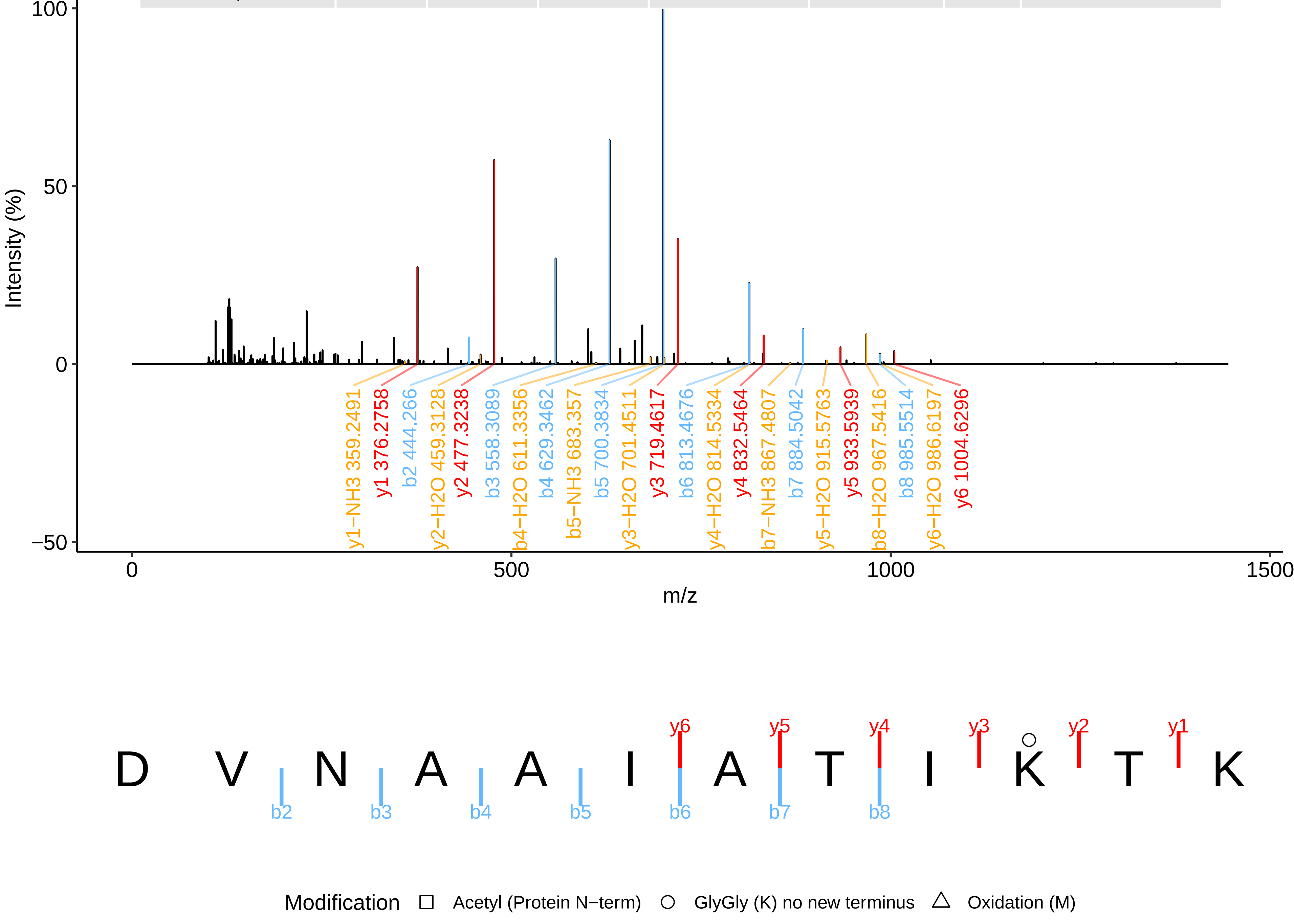

20200924_KIZ_MUb5_GlyGly_2 − Scan number: 16090

Gene Names	Charge	m/z	Mass	Retention time	PEP	Score	Precursor Intensity
TUBA1C;TUBA1B	3	453.5928	1357.756	59.738	0.00070235	88.087	4400291

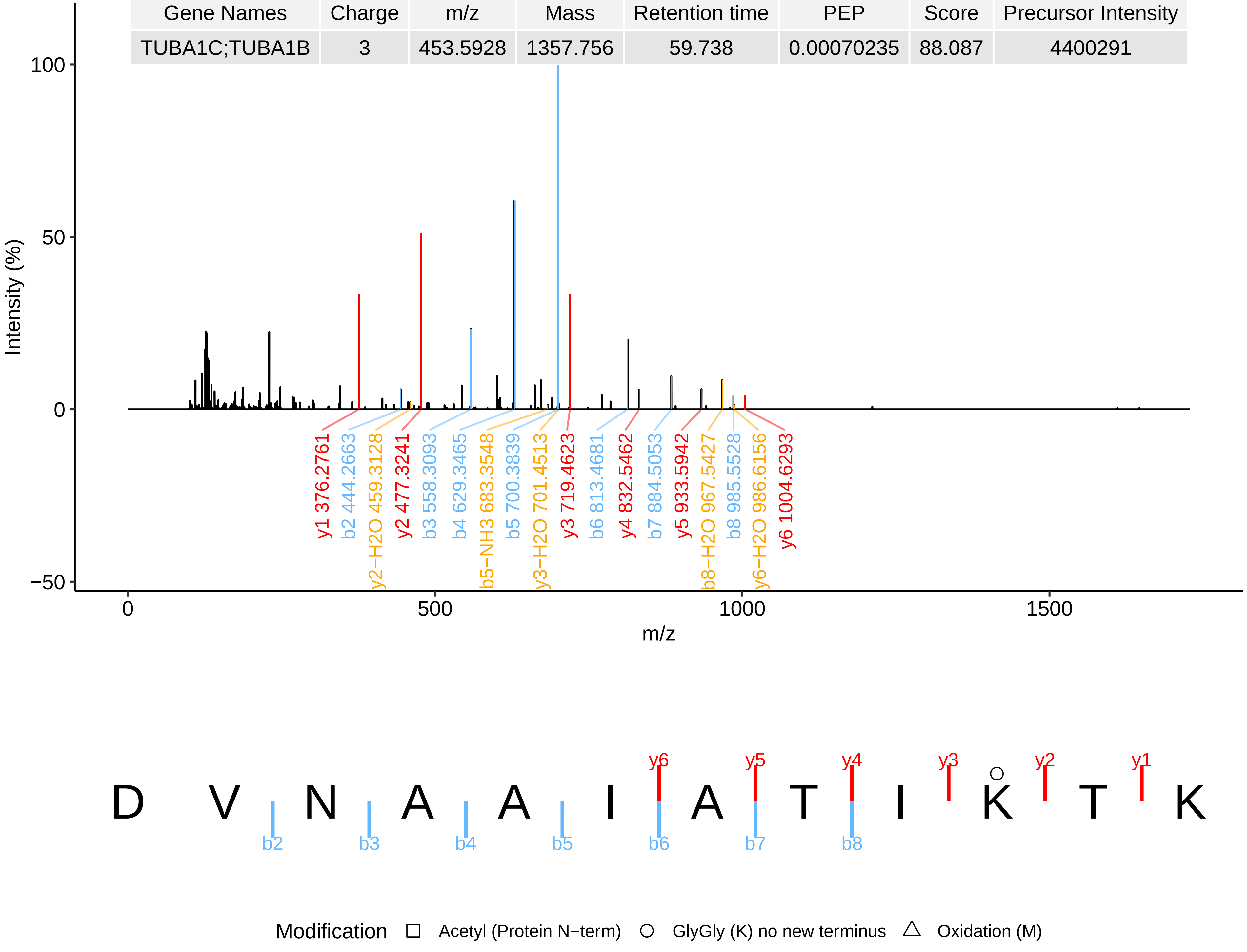

20200924_KIZ_MUb5_GlyGly_2 − Scan number: 16234

Gene Names	Charge	m/z	Mass	Retention time	PEP	Score	Precursor Intensity
TUBA1C;TUBA1B	3	453.5928	1357.756	60.243	9.7027e−09	121.04	22972972

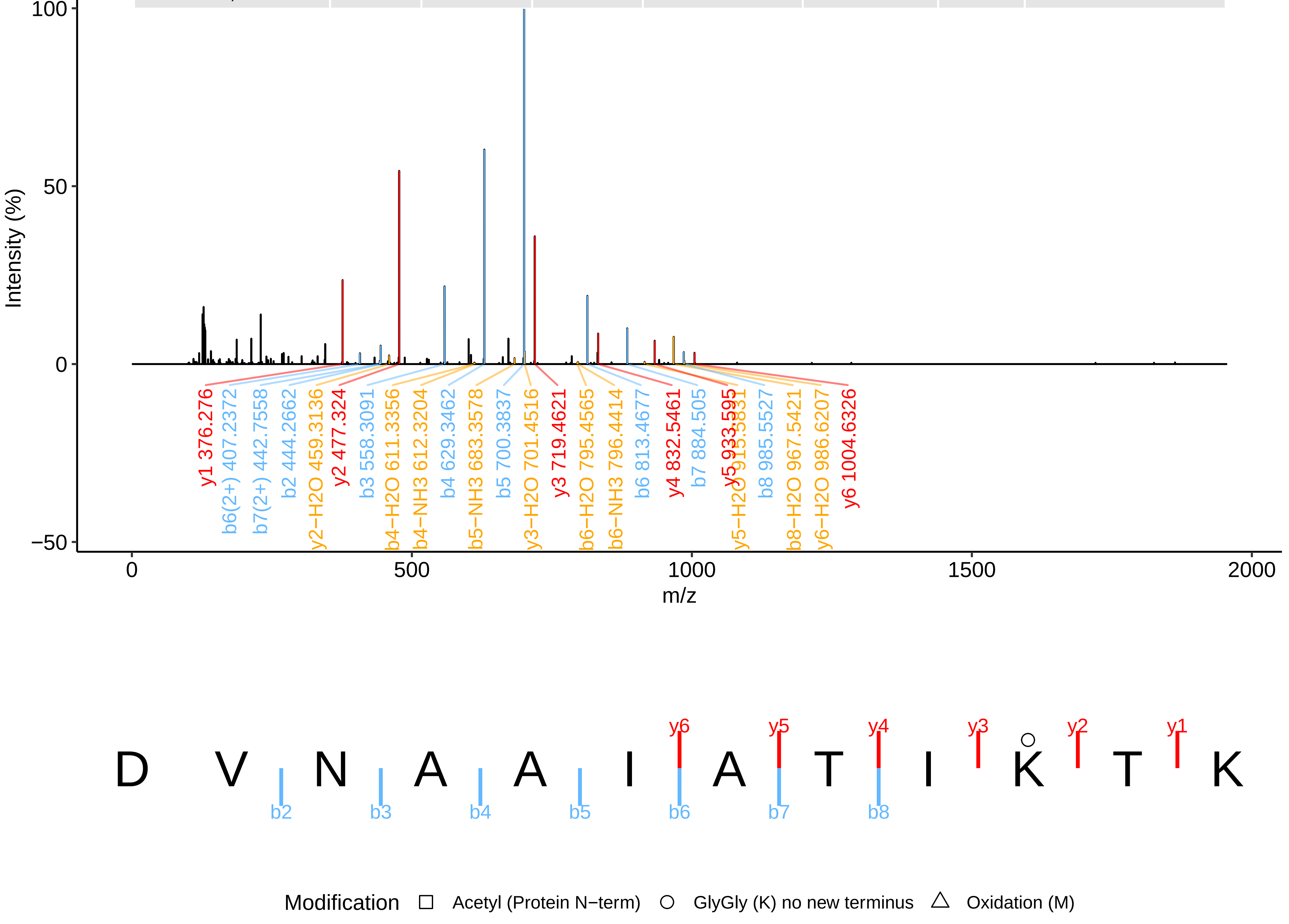

20200924_KIZ_MUb5_GlyGly_2 − Scan number: 16378

Gene Names	Charge	m/z	Mass	Retention time	PEP	Score	Precursor Intensity
TUBA1C;TUBA1B	3	453.5928	1357.756	60.768	1.1647e−05	105.65	24832070

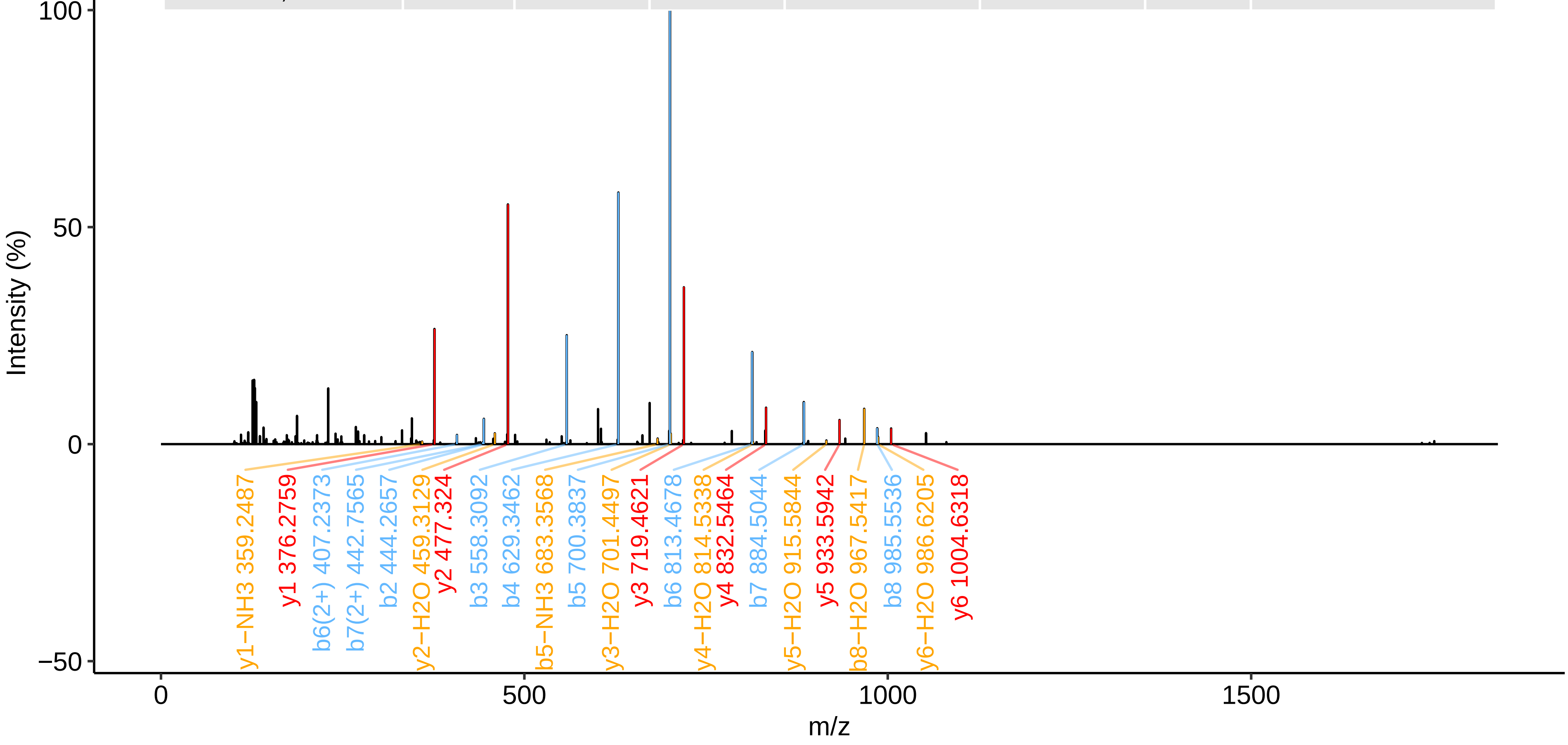

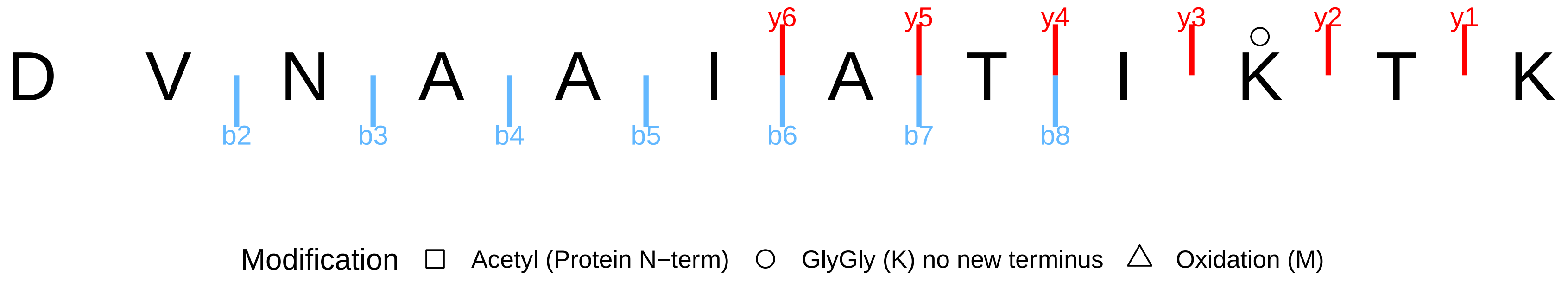

20201118_KIZ_MUb6_GlyGly_1 − Scan number: 17932

Gene Names	Charge	m/z	Mass	Retention time	PEP	Score	Precursor Intensity
TUBA1C;TUBA1B	3	453.5928	1357.756	56.4	1.9563e−05	99.011	58531160

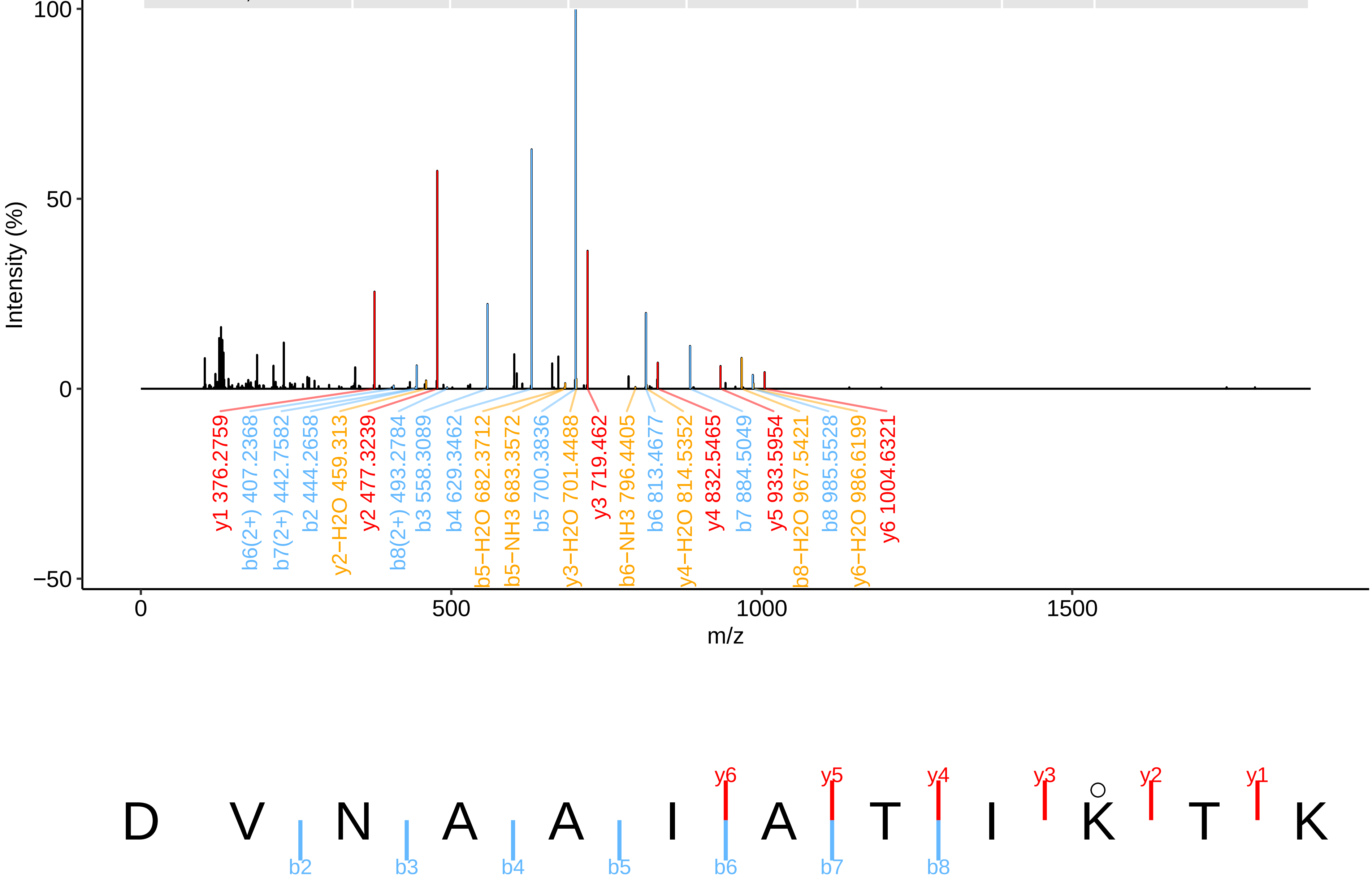

Modification □ Acetyl (Protein N−term) ○ GlyGly (K) no new terminus △ Oxidation (M)

20201118_KIZ_MUb6_GlyGly_2 − Scan number: 19405

Gene Names	Charge	m/z	Mass	Retention time	PEP	Score	Precursor Intensity
TUBA1C;TUBA1B	3	453.5928	1357.756	65.487	2.0366e−05	98.337	11230882

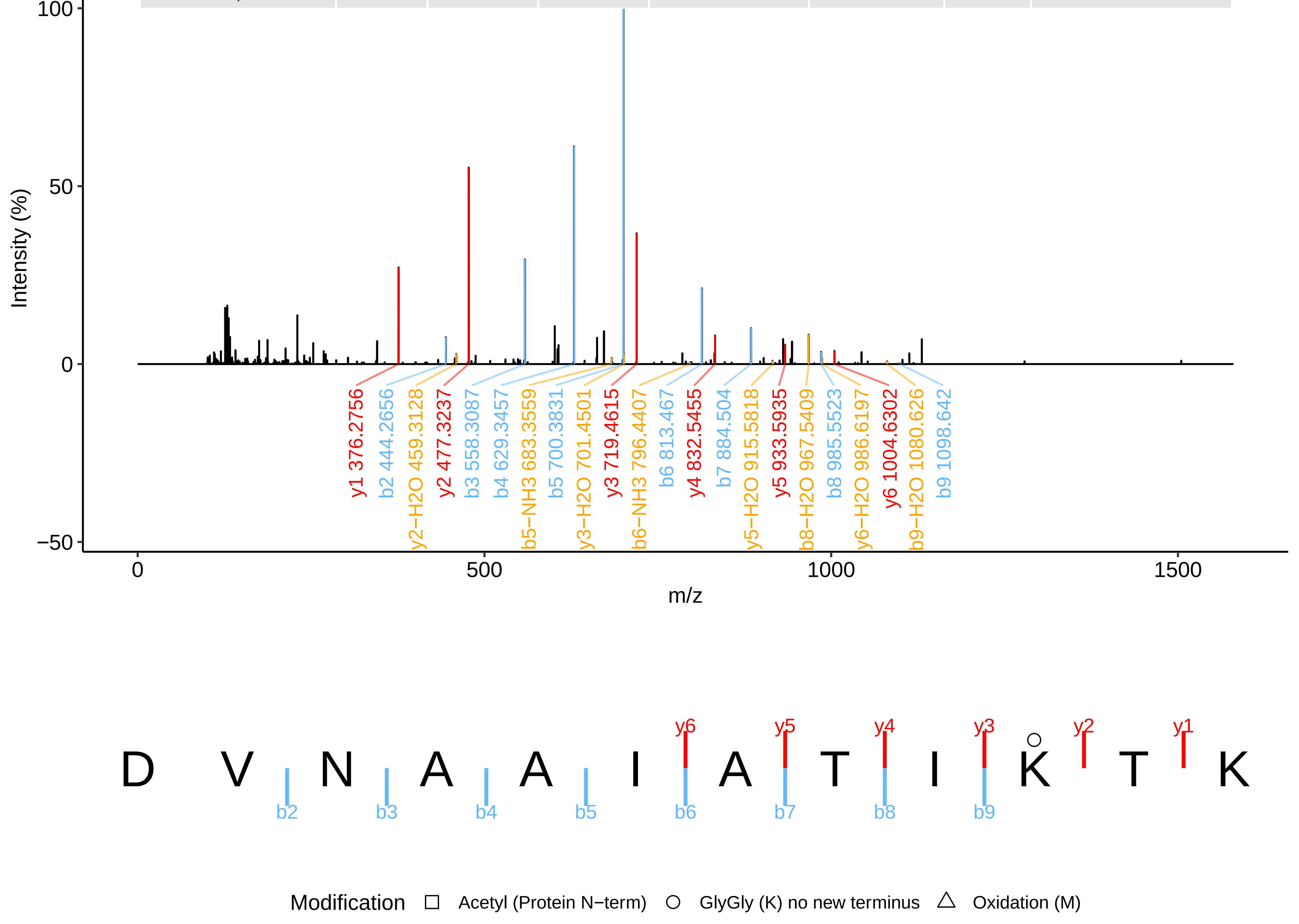

20200921_KIZ_MUb4_GlyGly_10plex − Scan number: 7155

Gene Names	Charge	m/z	Mass	Retention time	PEP	Score	Precursor Intensity
SF3B2	3	449.2246	1344.652	23.921	2.6669e−07	123.79	14005465

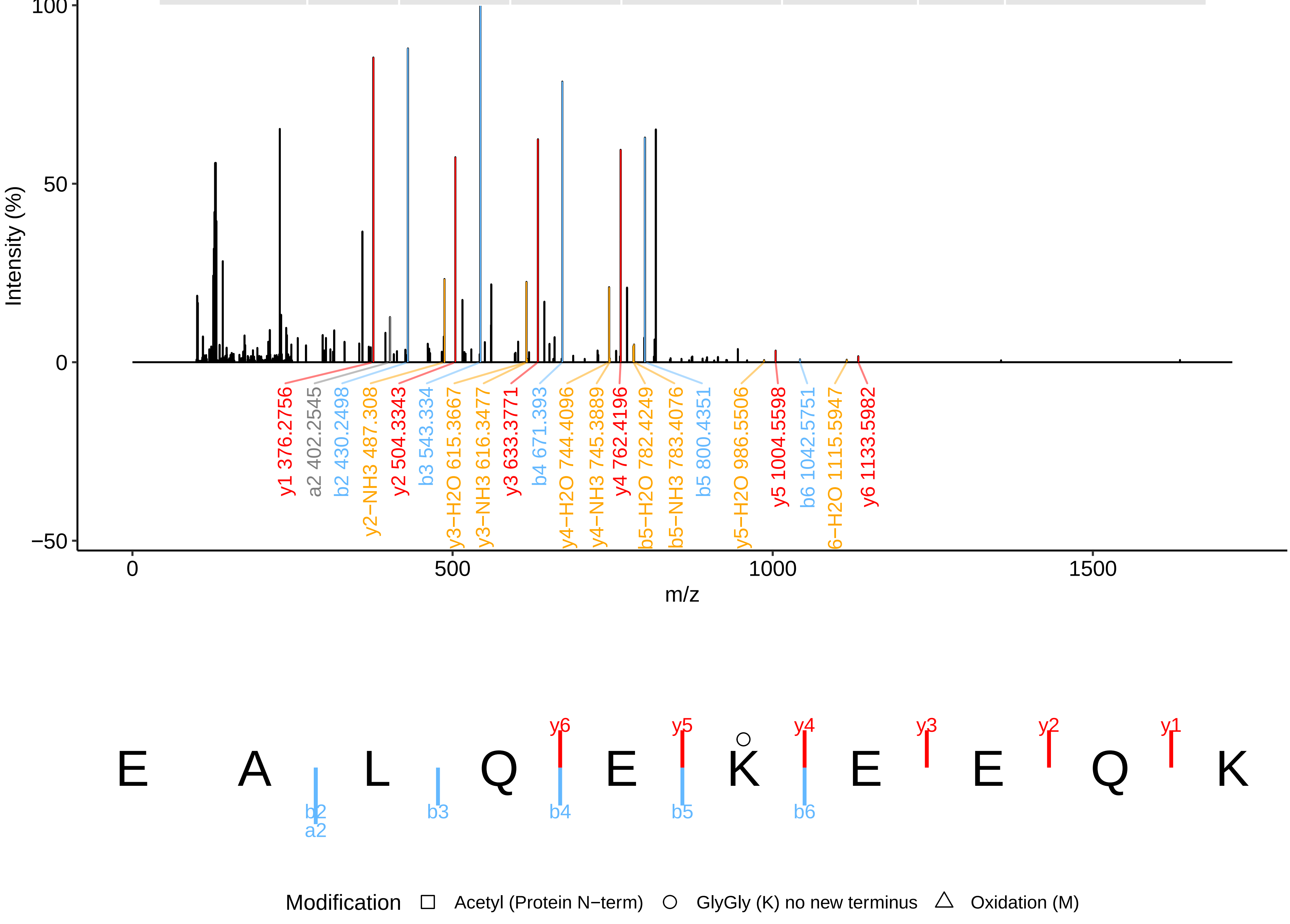

20200921_KIZ_MUb4_GlyGly_10plex − Scan number: 7165

Gene Names	Charge	m/z	Mass	Retention time	PEP	Score	Precursor Intensity
SF3B2	3	449.2246	1344.652	23.958	0.0032194	79.974	8207658

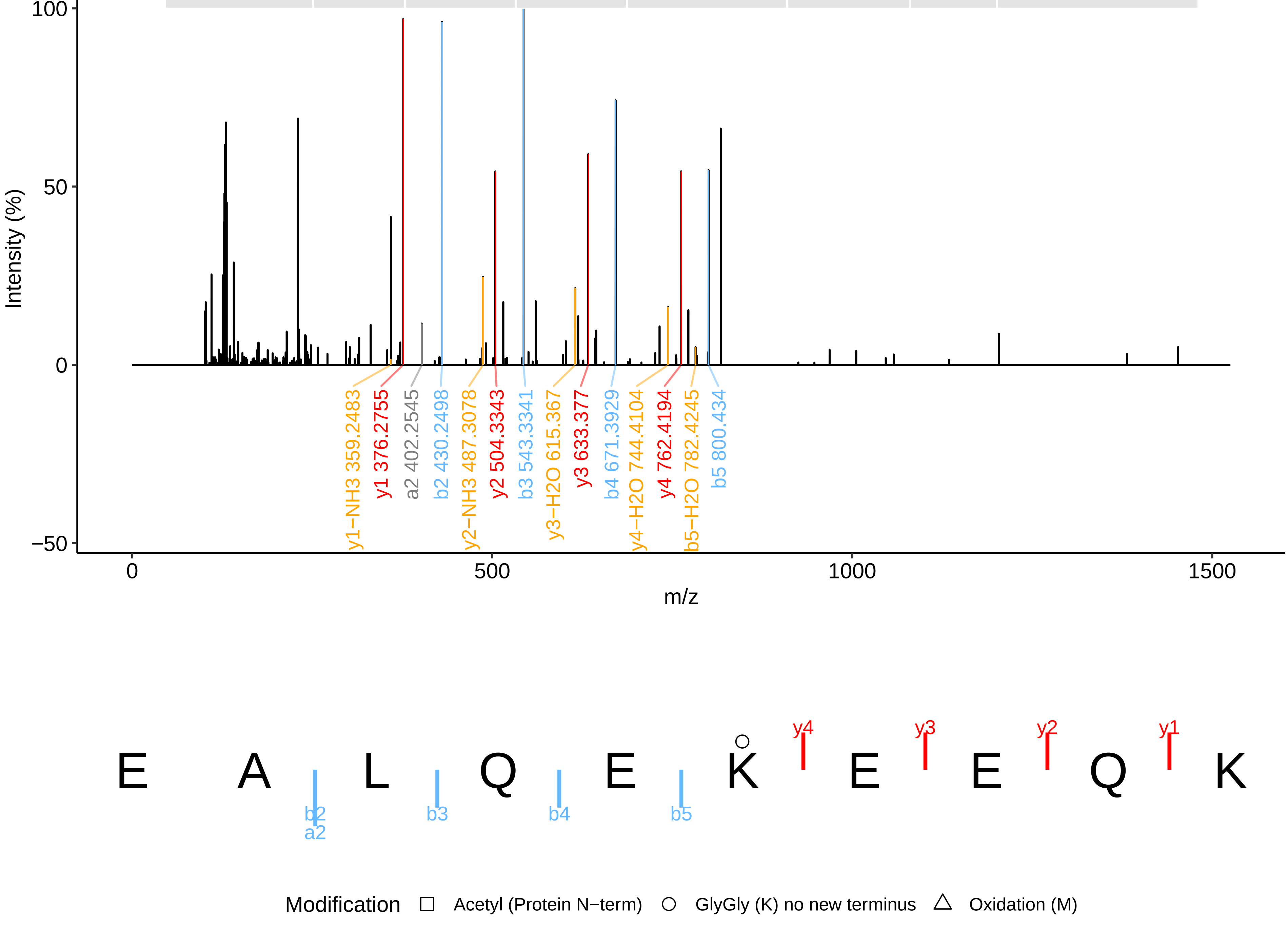

20200921_KIZ_MUb4_GlyGly_10plex_2 − Scan number: 6630

Gene Names	Charge	m/z	Mass	Retention time	PEP	Score	Precursor Intensity
SF3B2	3	449.2246	1344.652	24.453	0.018074	67.704	2007715

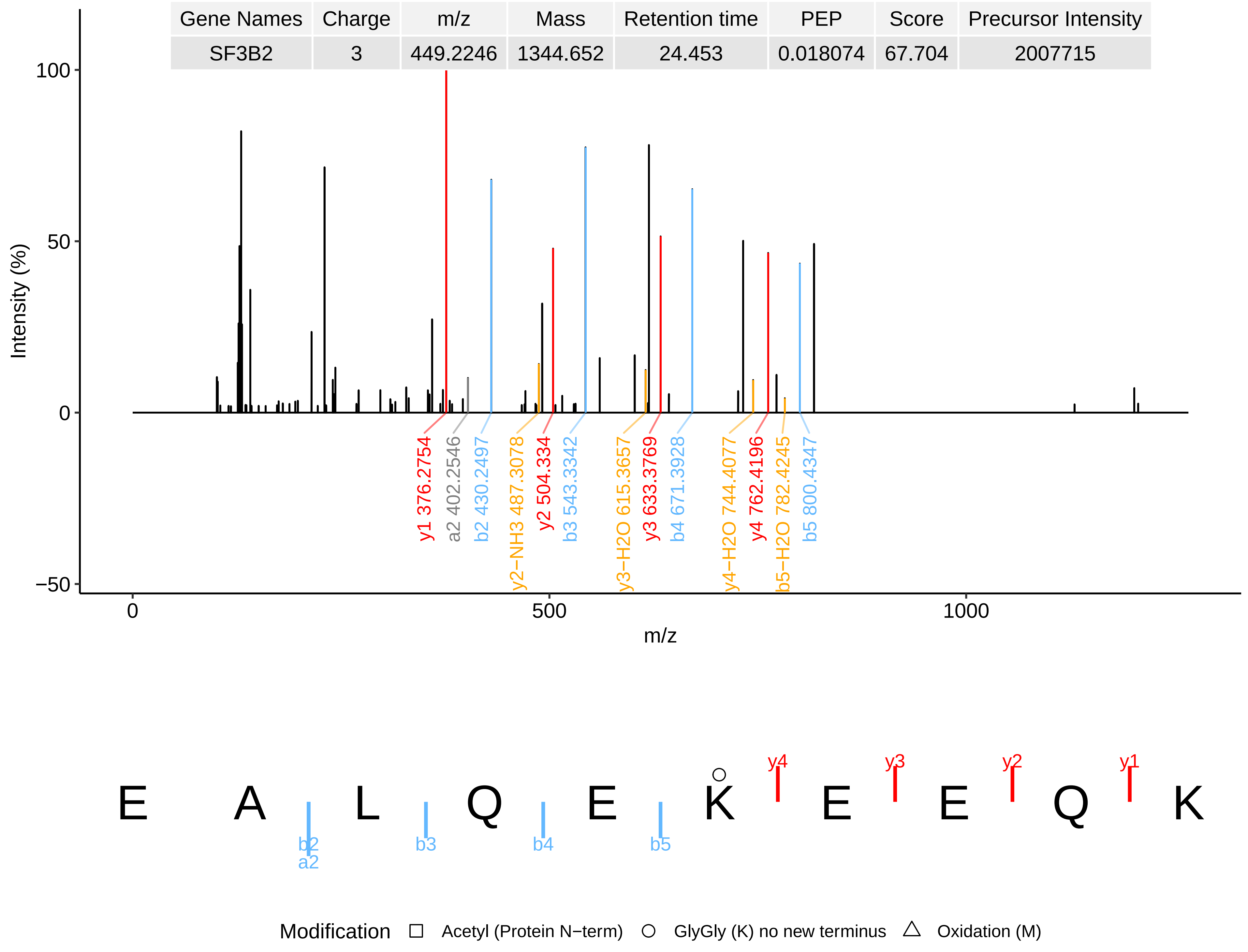

20200924_KIZ_MUb5_GlyGly_1 − Scan number: 6219

Gene Names	Charge	m/z	Mass	Retention time	PEP	Score	Precursor Intensity
SF3B2	3	449.2246	1344.652	19.516	0.0021121	93.096	10358194

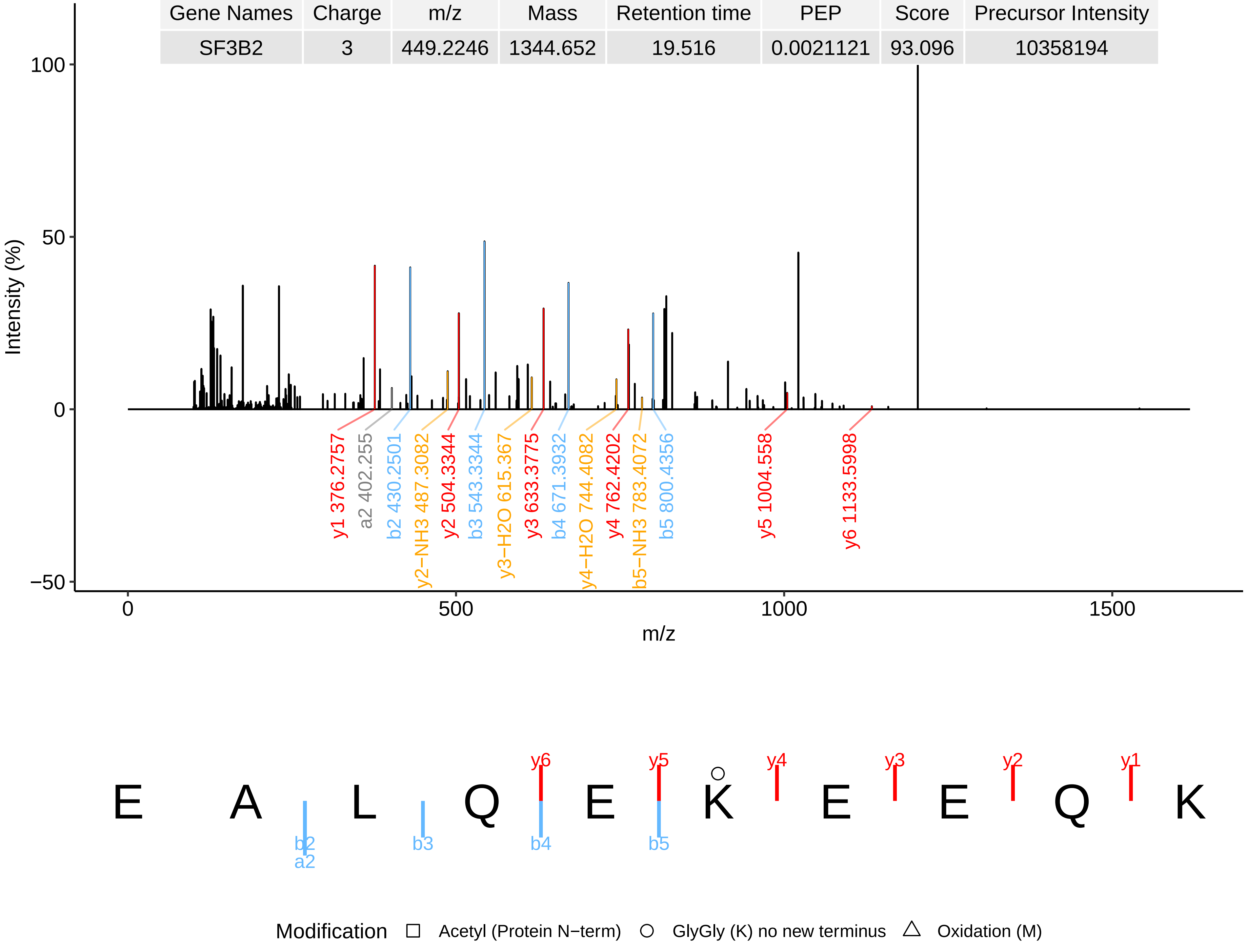

20200924_KIZ_MUb5_GlyGly_2 − Scan number: 5847

Gene Names	Charge	m/z	Mass	Retention time	PEP	Score	Precursor Intensity
SF3B2	3	449.2246	1344.652	21.752	0.0055755	77.662	2081669

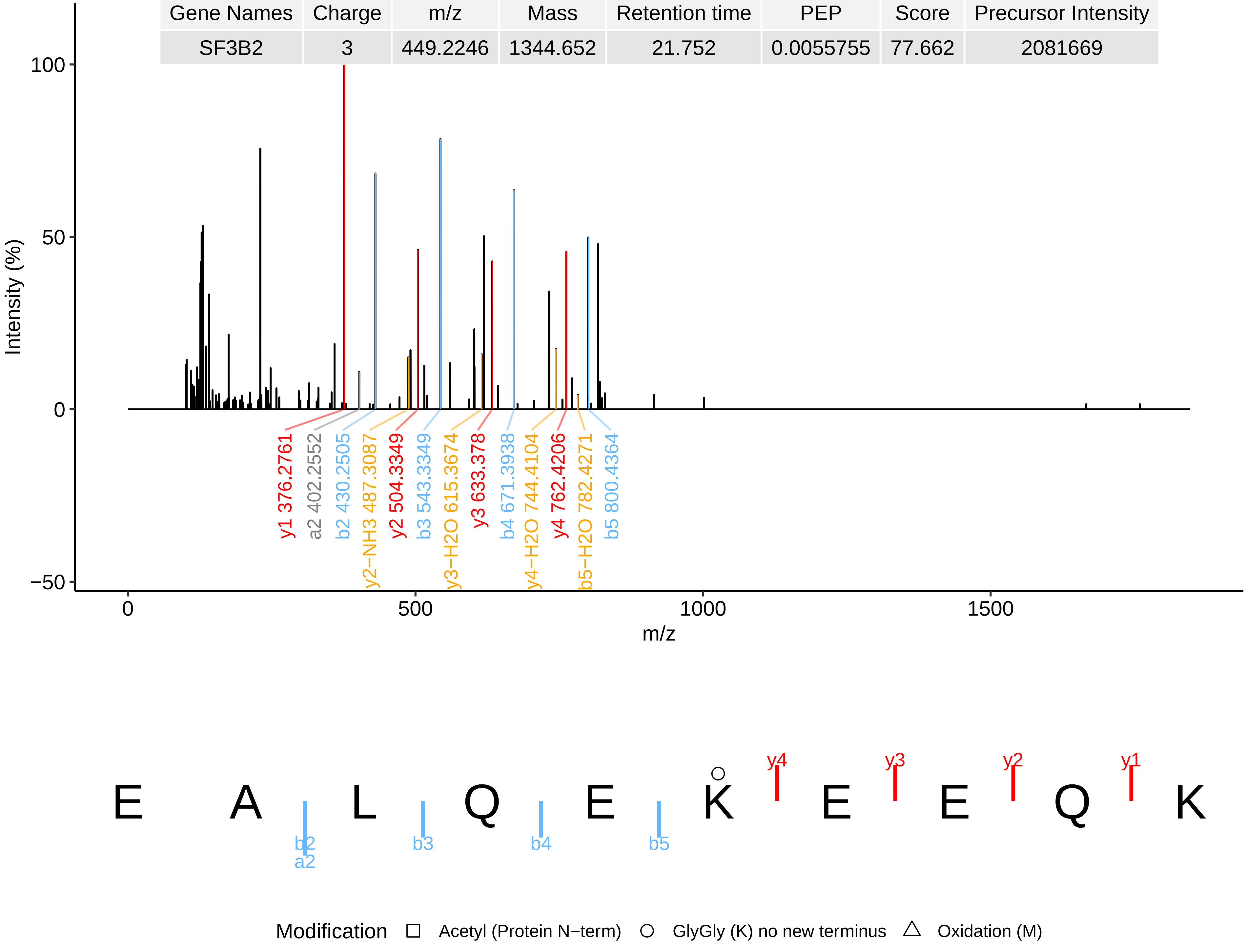

20200924_KIZ_MUb5_GlyGly_2 − Scan number: 5983

Gene Names	Charge	m/z	Mass	Retention time	PEP	Score	Precursor Intensity
SF3B2	3	449.2246	1344.652	22.266	0.016769	68.893	NA

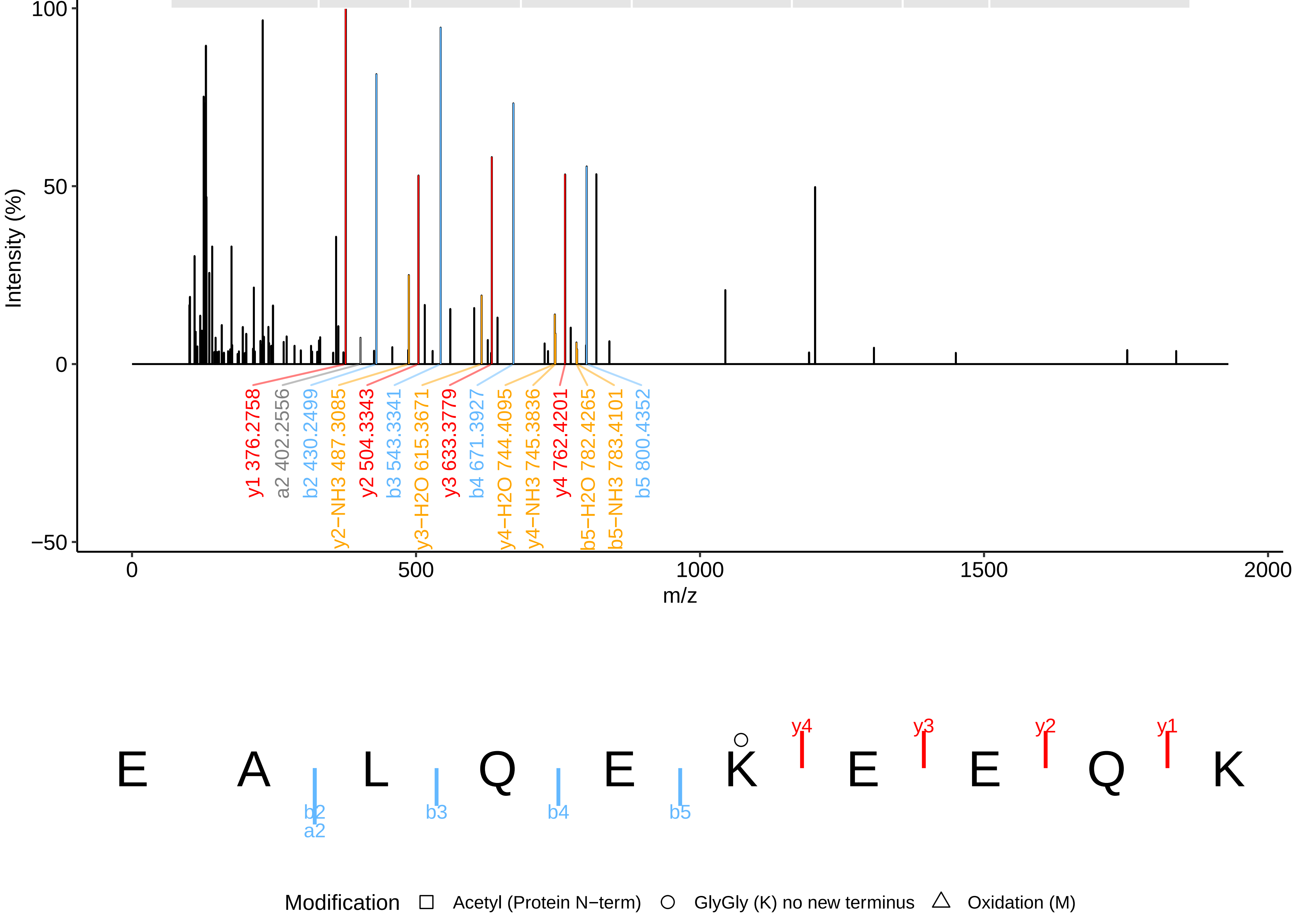

20200921_KIZ_MUb4_GlyGly_10plex_2 − Scan number: 15738

Gene Names	Charge	m/z	Mass	Retention time	PEP	Score	Precursor Intensity
CCT2	3	518.2645	1551.772	61.601	0.063039	51.875	752269.1

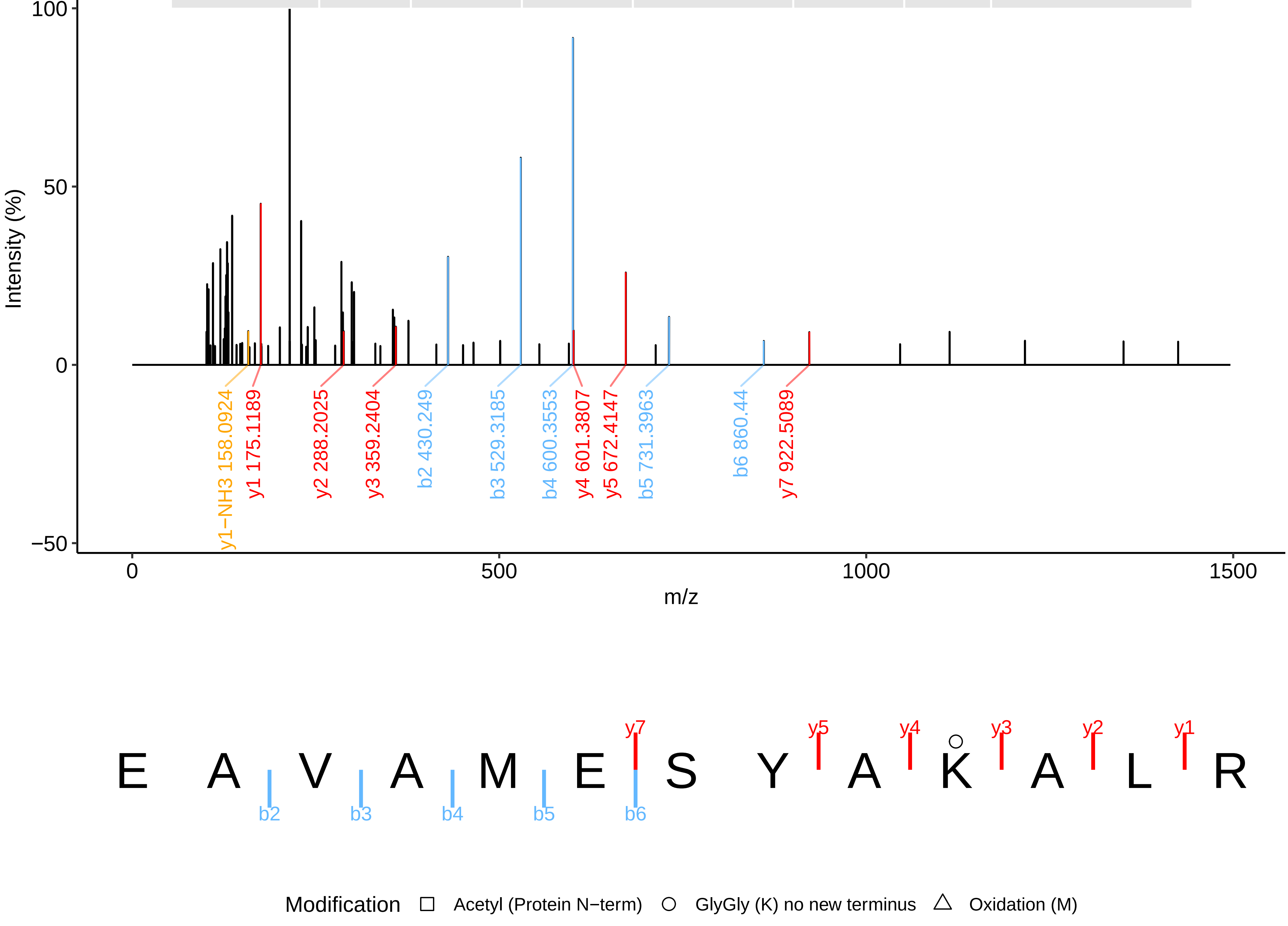

20200921_KIZ_MUb4_GlyGly_10plex − Scan number: 4698

Gene Names	Charge	m/z	Mass	Retention time	PEP	Score	Precursor Intensity
SLC43A3	3	519.5936	1555.759	15.313	3.2016e−20	160.18	6390317

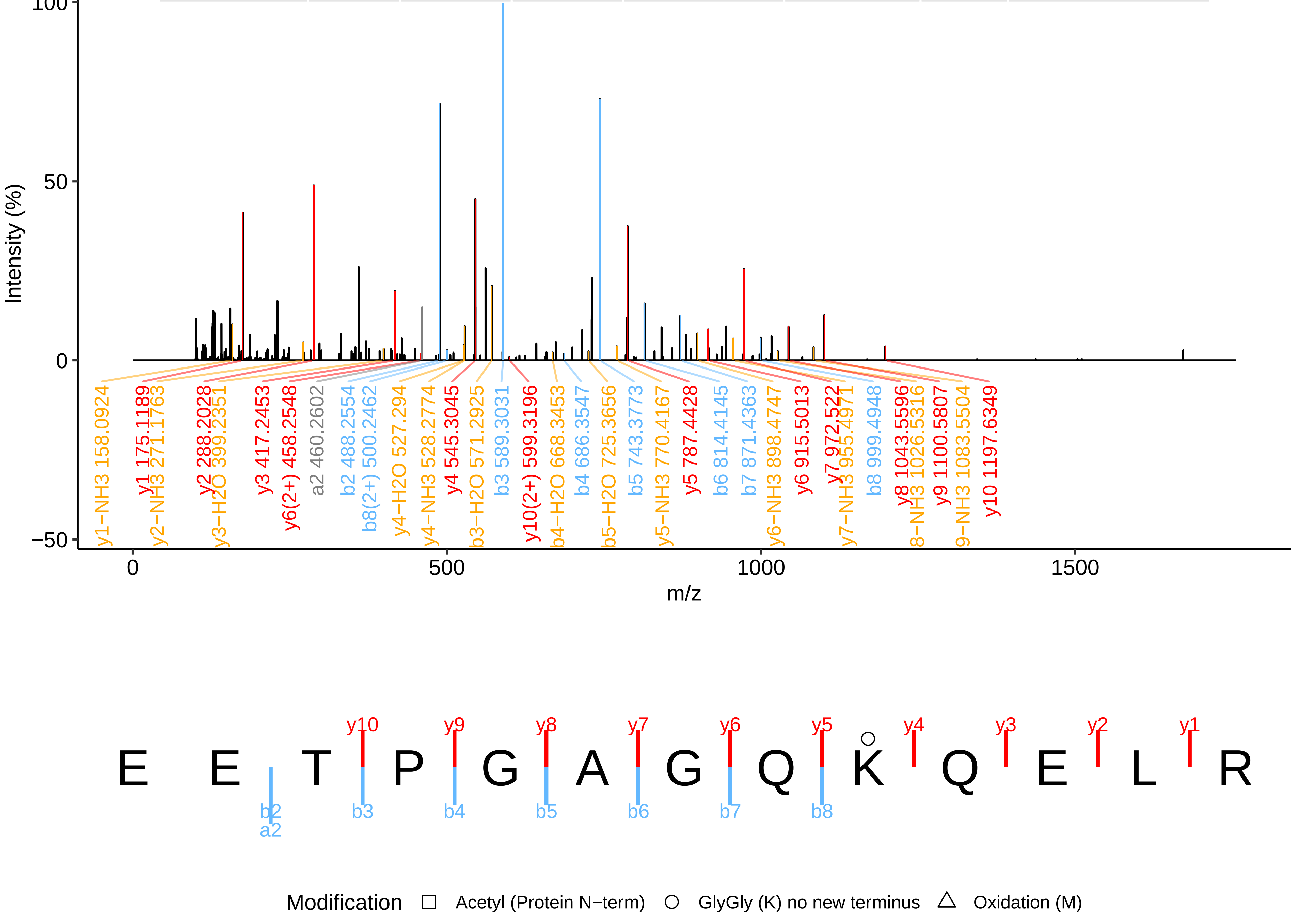

20200921_KIZ_MUb4_GlyGly_10plex − Scan number: 4727

Gene Names	Charge	m/z	Mass	Retention time	PEP	Score	Precursor Intensity
SLC43A3	2	778.8868	1555.759	15.416	6.1981e−49	231.17	5012640

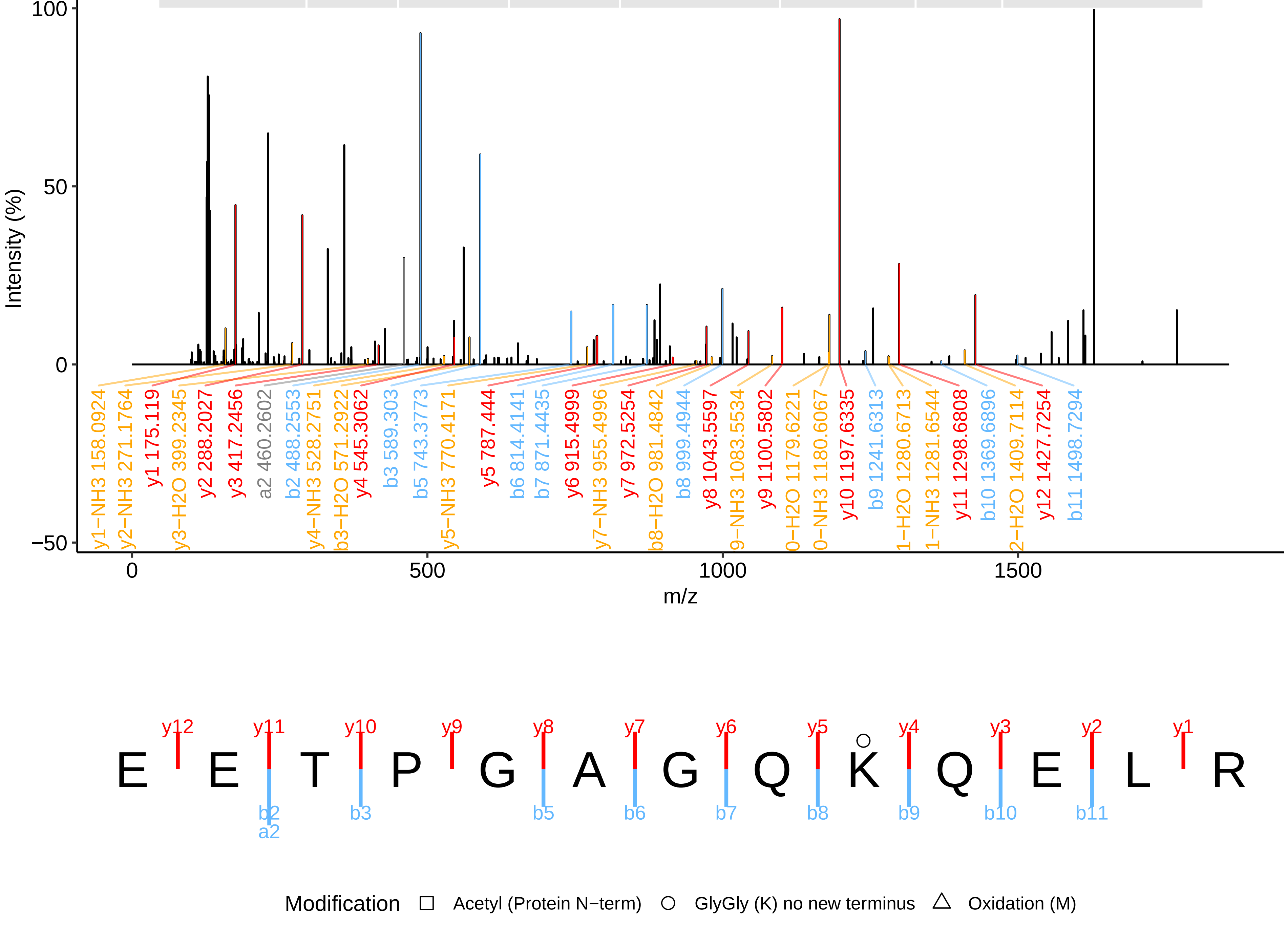

20200921_KIZ_MUb4_GlyGly_10plex_2 − Scan number: 4358

Gene Names	Charge	m/z	Mass	Retention time	PEP	Score	Precursor Intensity
SLC43A3	3	519.5936	1555.759	15.251	1.5454e−13	137.84	764682

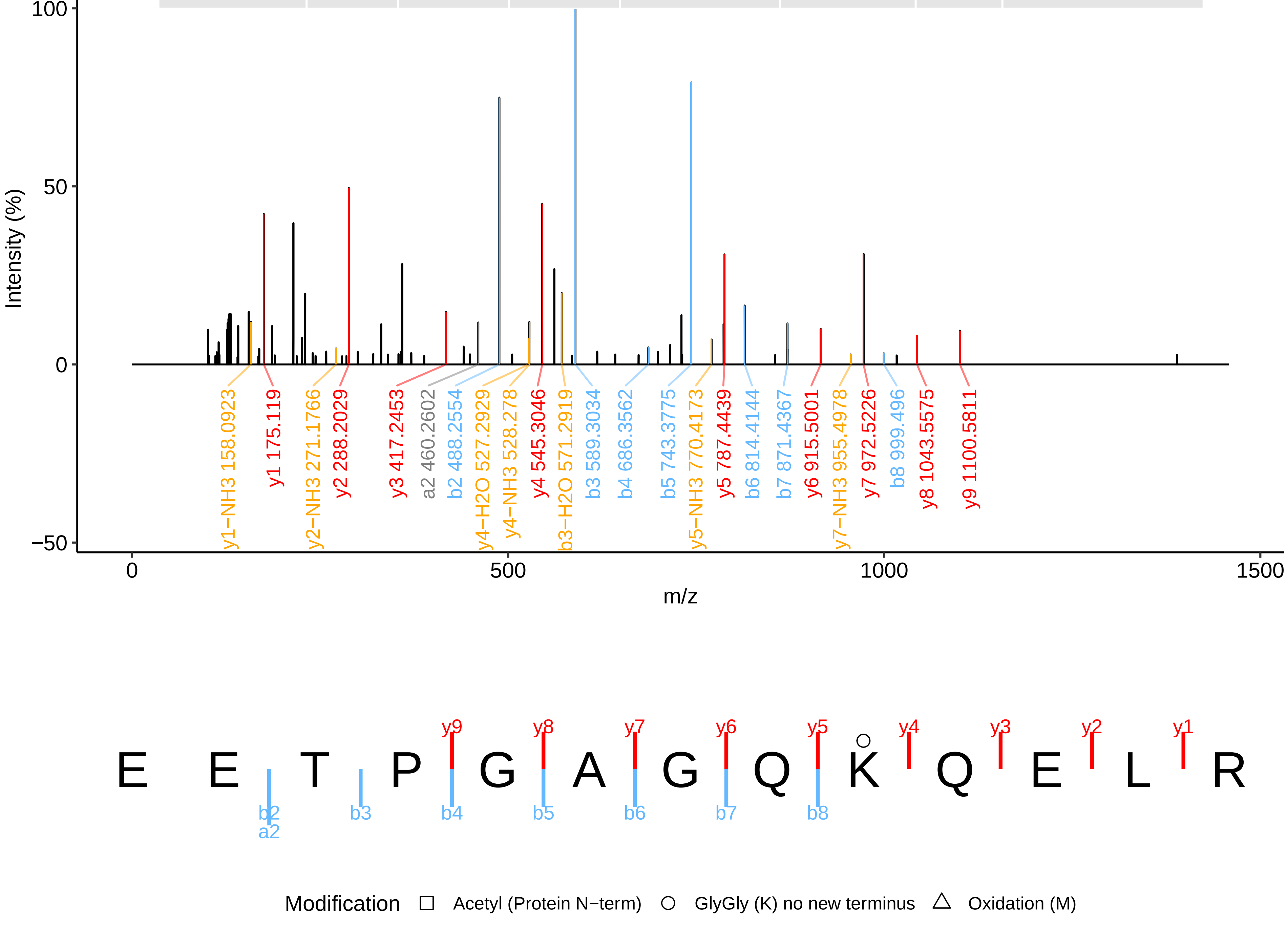

20200921_KIZ_MUb4_GlyGly_10plex_2 − Scan number: 4387

Gene Names	Charge	m/z	Mass	Retention time	PEP	Score	Precursor Intensity
SLC43A3	2	778.8868	1555.759	15.368	2.4766e−19	151.15	1617887

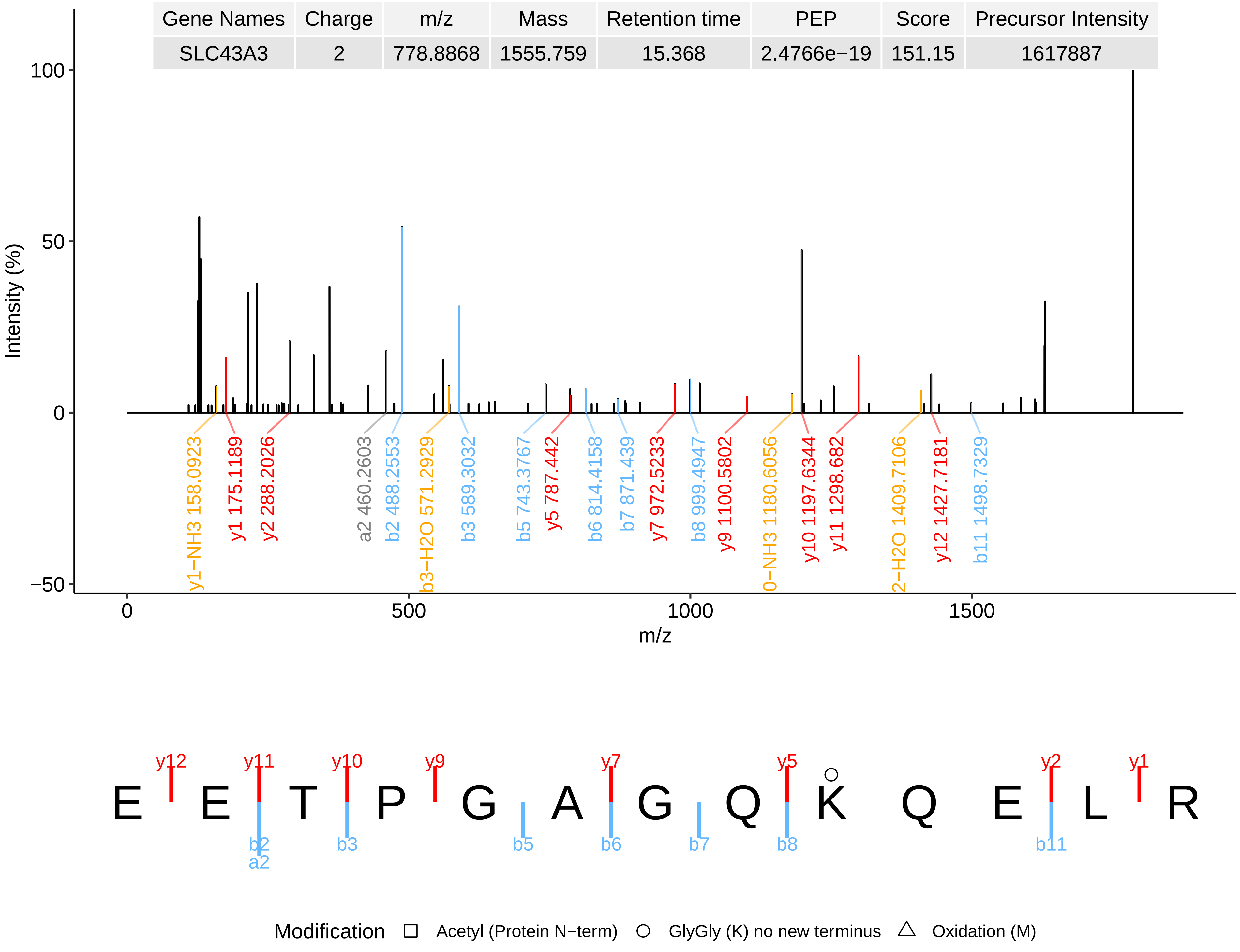

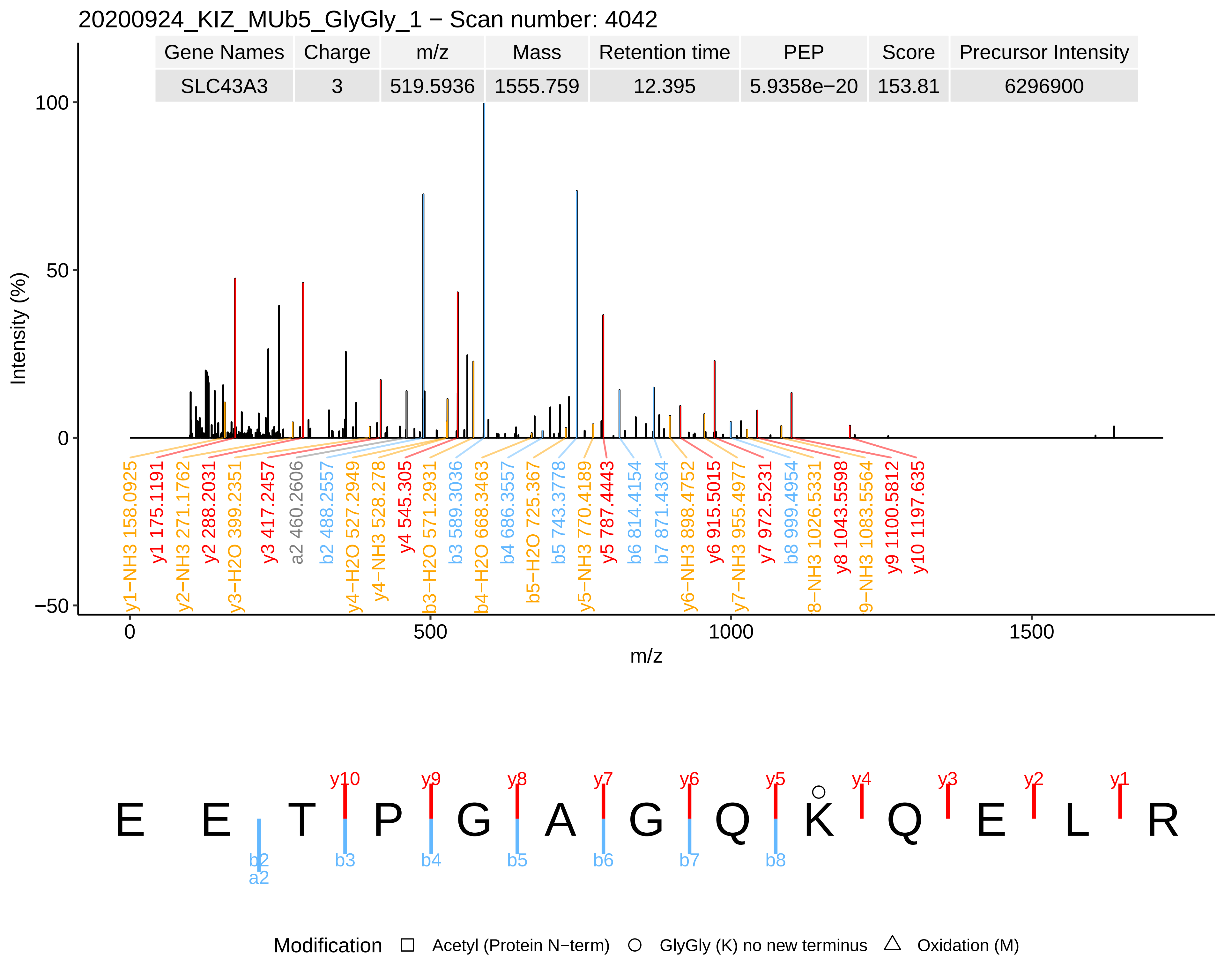

20200924_KIZ_MUb5_GlyGly_1 − Scan number: 4042
Gene Names
Charge
m/z
Mass
Retention time
PEP
Score
Precursor Intensity
SLC43A3
3
519.5936
1555.759
12.395
5.9358e−20
153.81
6296900
Intensity (%)
m/z
y1−NH3 158.0925
y1 175.1191
y2−NH3 271.1762
y2 288.2031
y3−H2O 399.2351
y3 417.2457
a2 460.2606
b2 488.2557
y4−H2O 527.2949
y4−NH3 528.278
y4 545.305
b3−H2O 571.2931
b3 589.3036
b4−H2O 668.3463
b4 686.3557
b5−H2O 725.367
b5 743.3778
y5−NH3 770.4189
y5 787.4443
b6 814.4154
b7 871.4364
y6−NH3 898.4752
y6 915.5015
y7−NH3 955.4977
y7 972.5231
b8 999.4954
y8−NH3 1026.5331
y8 1043.5598
y9−NH3 1083.5564
y9 1100.5812
y10 1197.635
E E T P G A G Q K Q E L R
Modification
Acetyl (Protein N−term)
GlyGly (K) no new terminus
Oxidation (M)

20200924_KIZ_MUb5_GlyGly_1 − Scan number: 4053

Gene Names	Charge	m/z	Mass	Retention time	PEP	Score	Precursor Intensity
SLC43A3	3	519.5936	1555.759	12.437	0.00068753	87.831	3213846

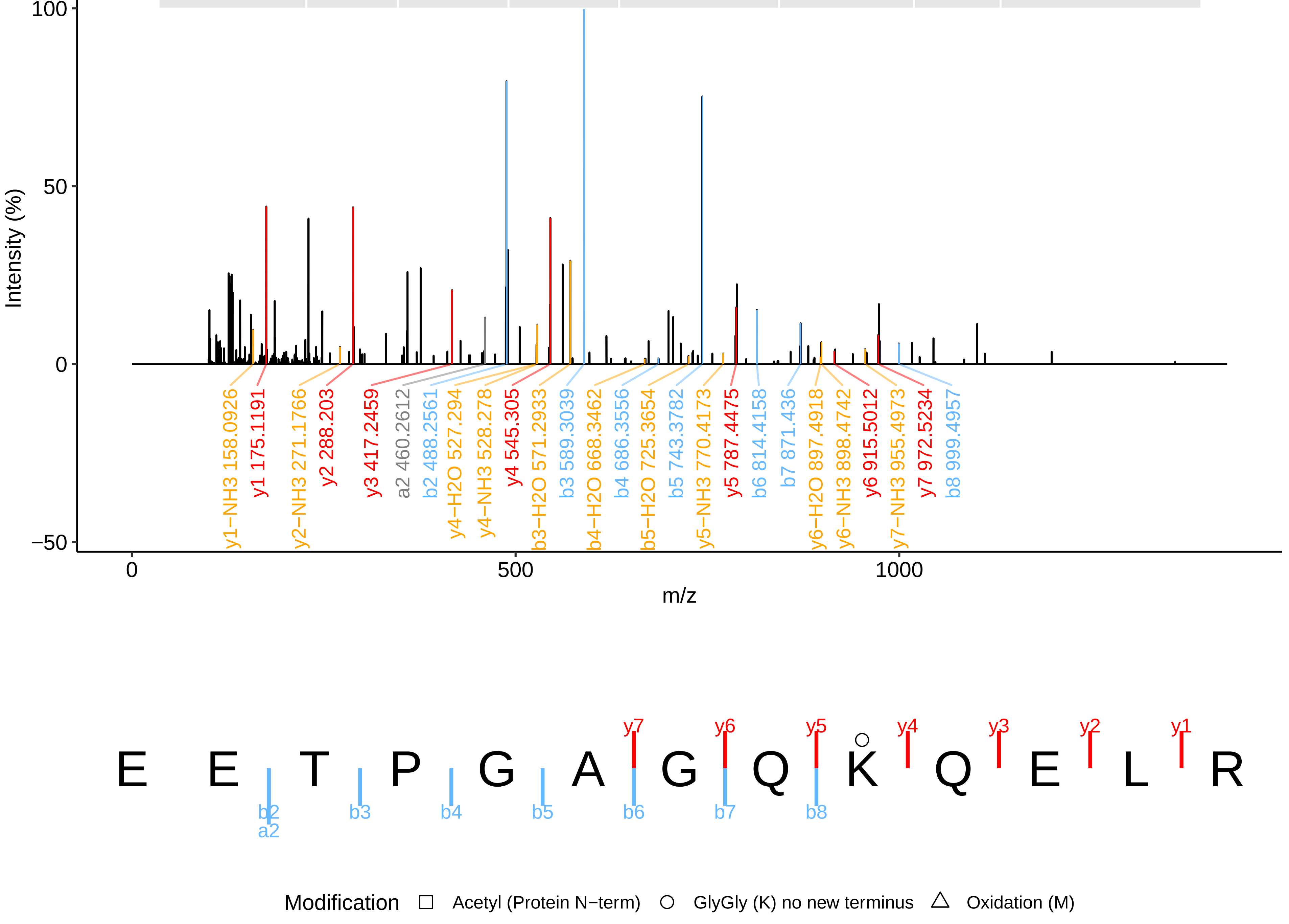

20200924_KIZ_MUb5_GlyGly_1 − Scan number: 4208

Gene Names	Charge	m/z	Mass	Retention time	PEP	Score	Precursor Intensity
SLC43A3	3	519.5936	1555.759	12.952	1.388e−28	181.44	96931944

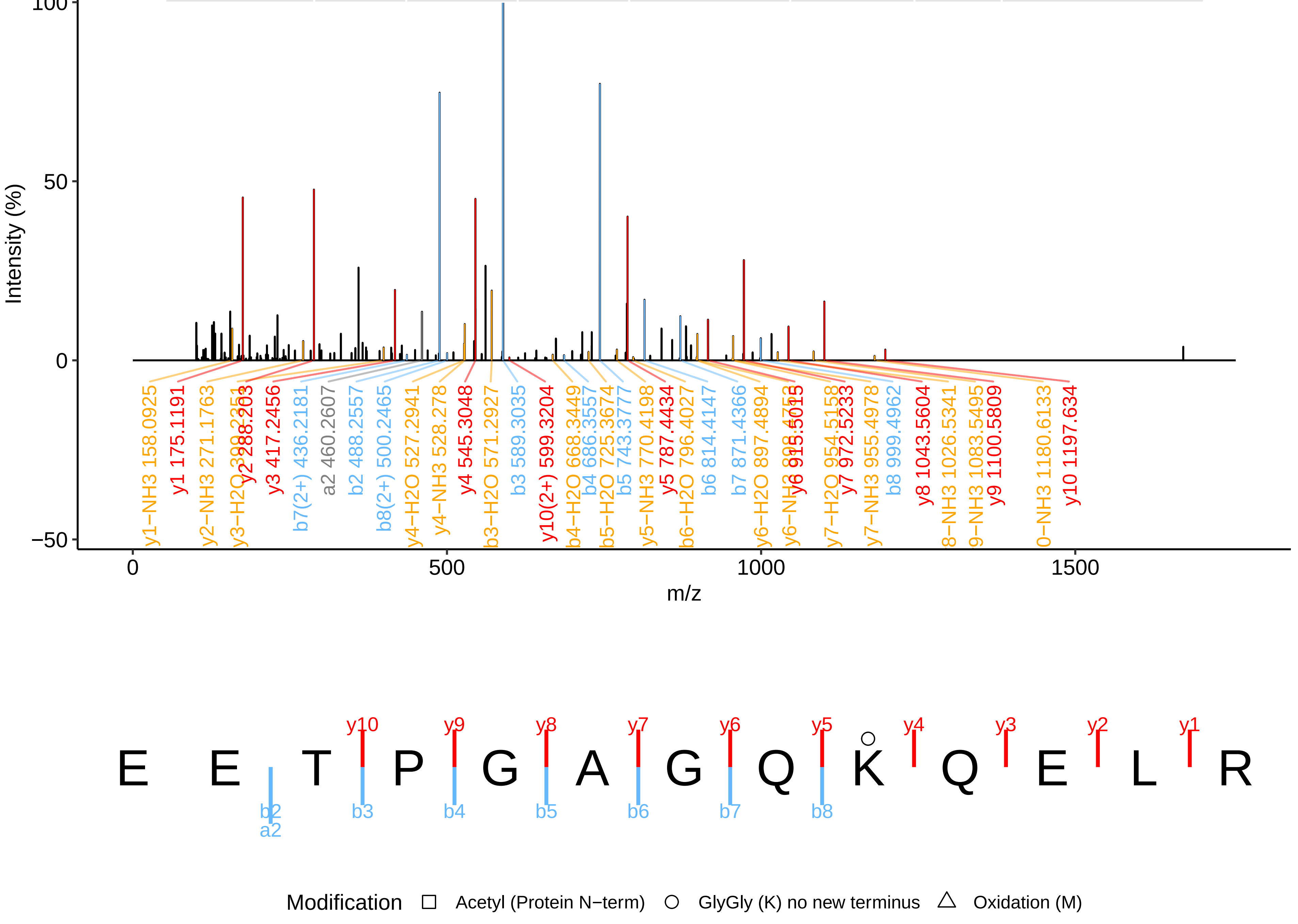

20200924_KIZ_MUb5_GlyGly_2 − Scan number: 3830

Gene Names	Charge	m/z	Mass	Retention time	PEP	Score	Precursor Intensity
SLC43A3	3	519.5936	1555.759	13.967	3.7709e−20	158.86	3556379

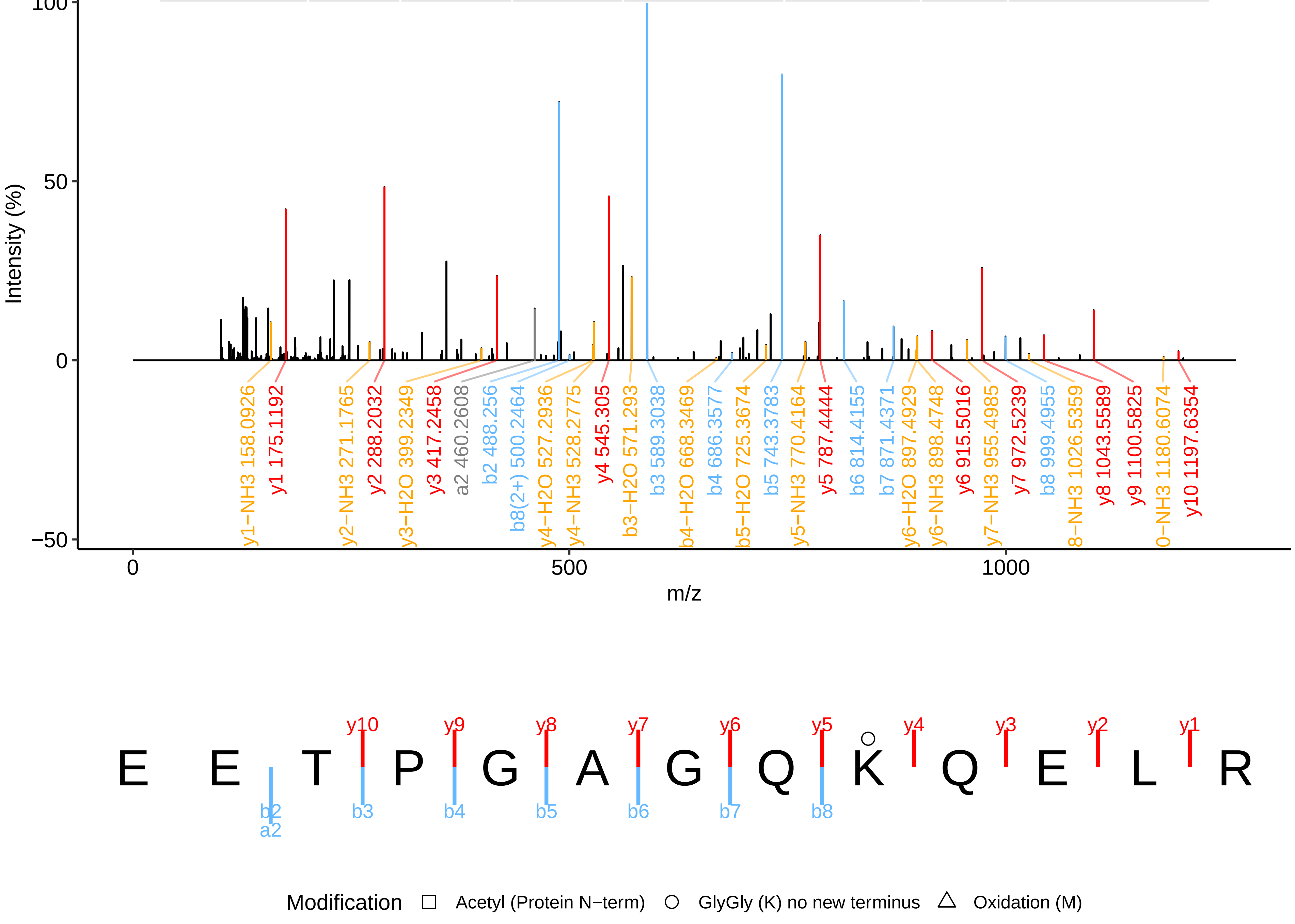

20200924_KIZ_MUb5_GlyGly_2 − Scan number: 3959

Gene Names	Charge	m/z	Mass	Retention time	PEP	Score	Precursor Intensity
SLC43A3	3	519.5936	1555.759	14.469	1.1084e−13	141.34	19735002

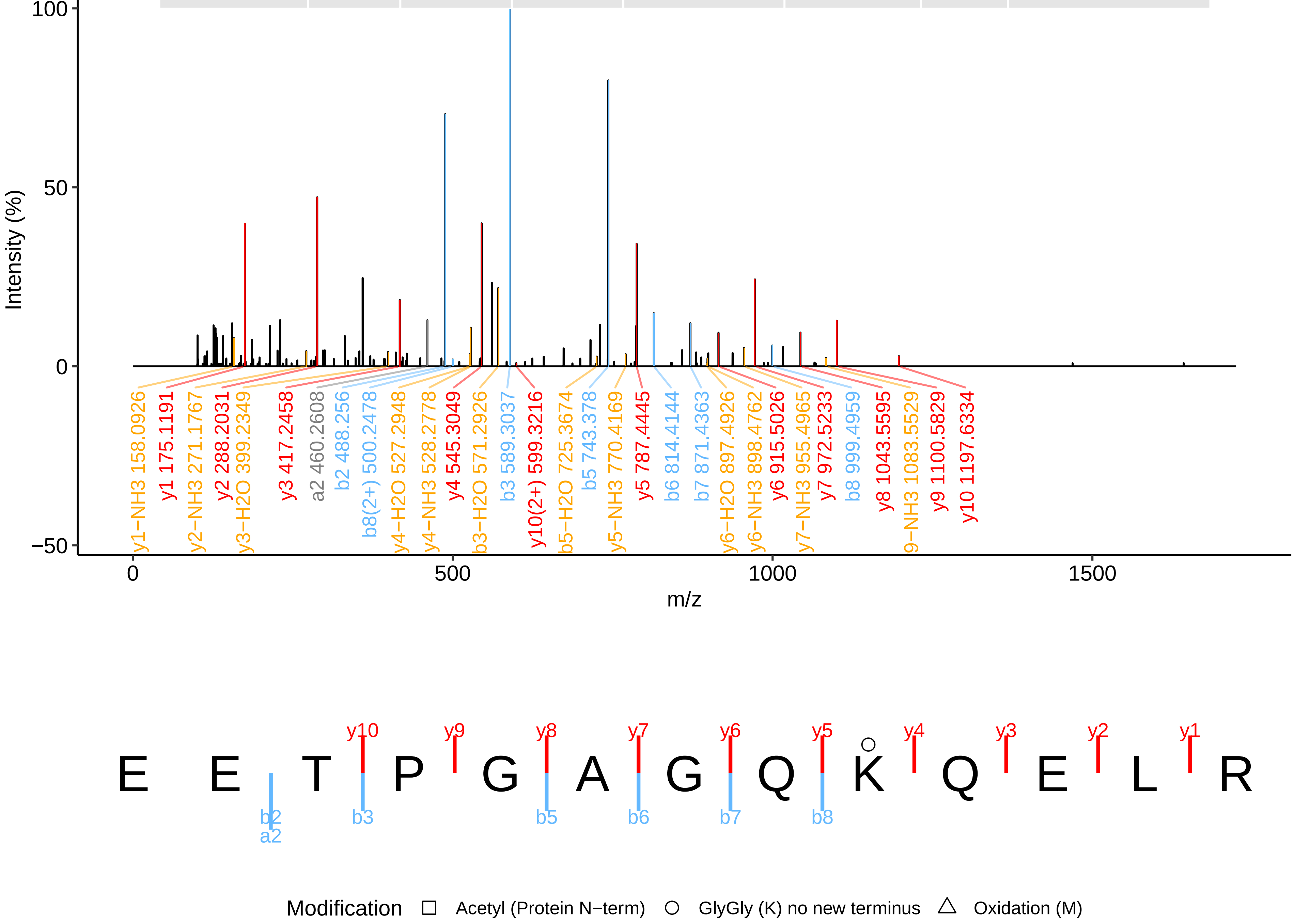

20201118_KIZ_MUb6_GlyGly_1 − Scan number: 3721

Gene Names	Charge	m/z	Mass	Retention time	PEP	Score	Precursor Intensity
SLC43A3	3	519.5936	1555.759	11.463	5.3078e−28	171.2	4688123

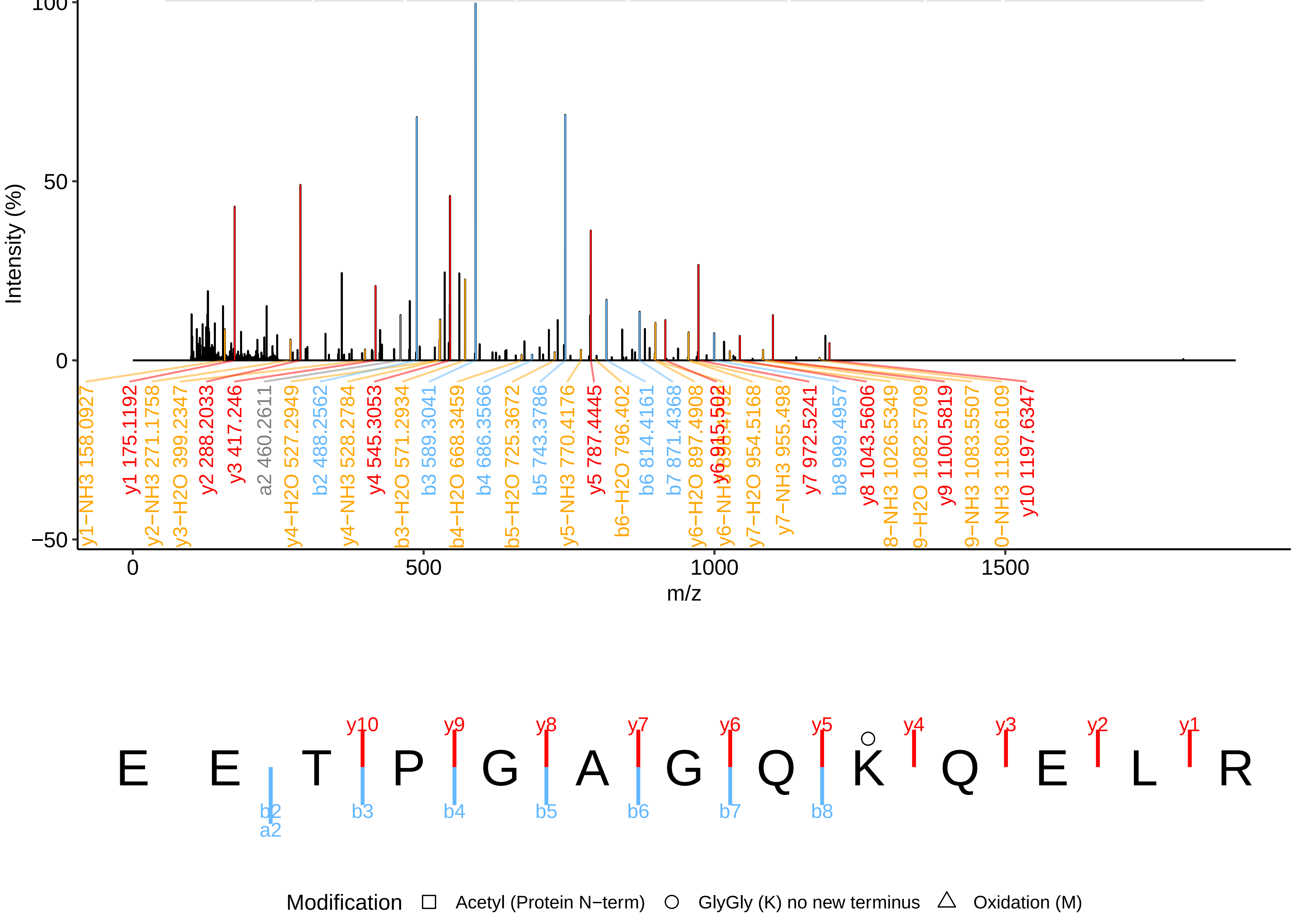

20201118_KIZ_MUb6_GlyGly_2 − Scan number: 4659

Gene Names	Charge	m/z	Mass	Retention time	PEP	Score	Precursor Intensity
SLC43A3	3	519.5936	1555.759	15.996	4.9942e−20	156	3475033

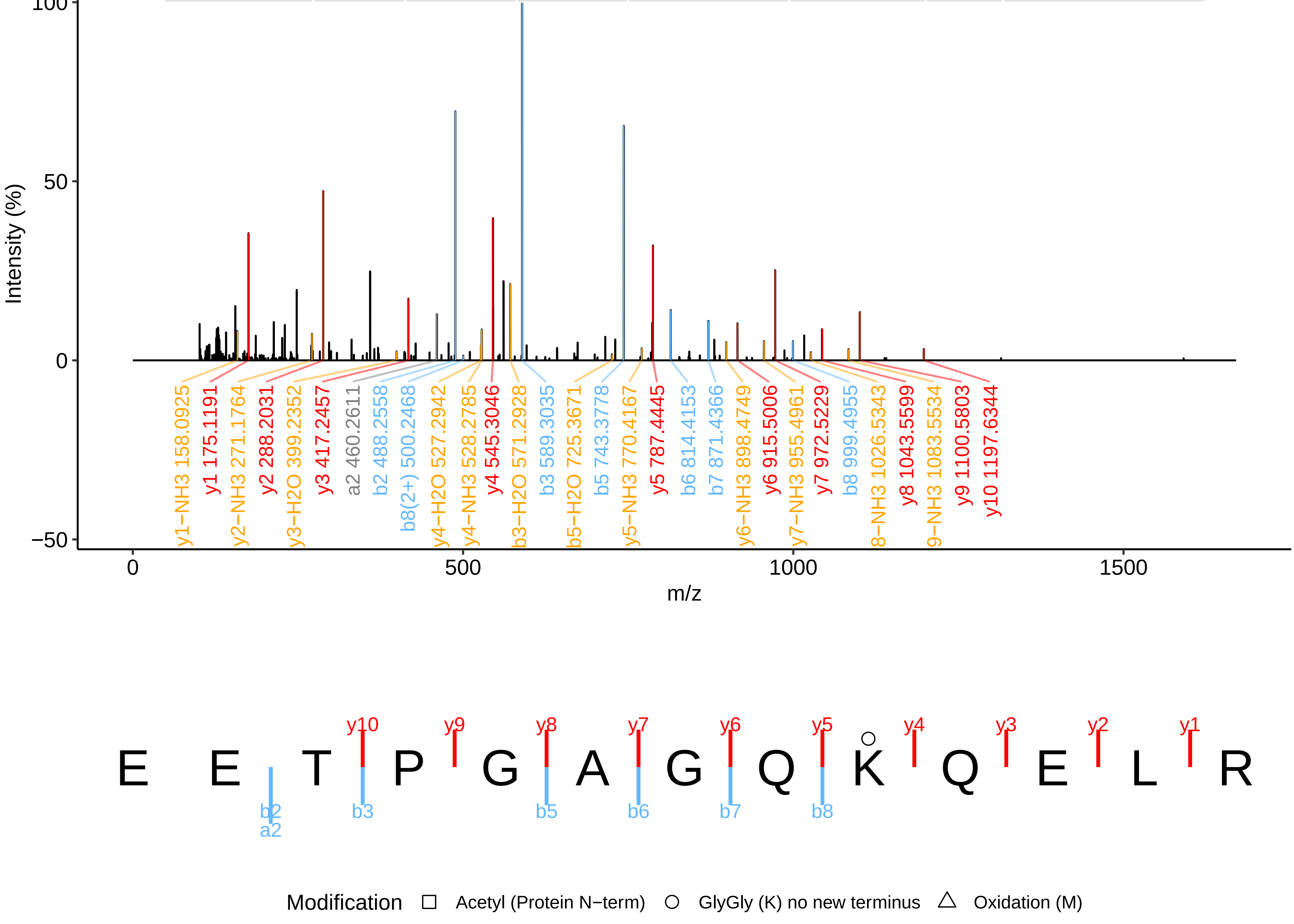

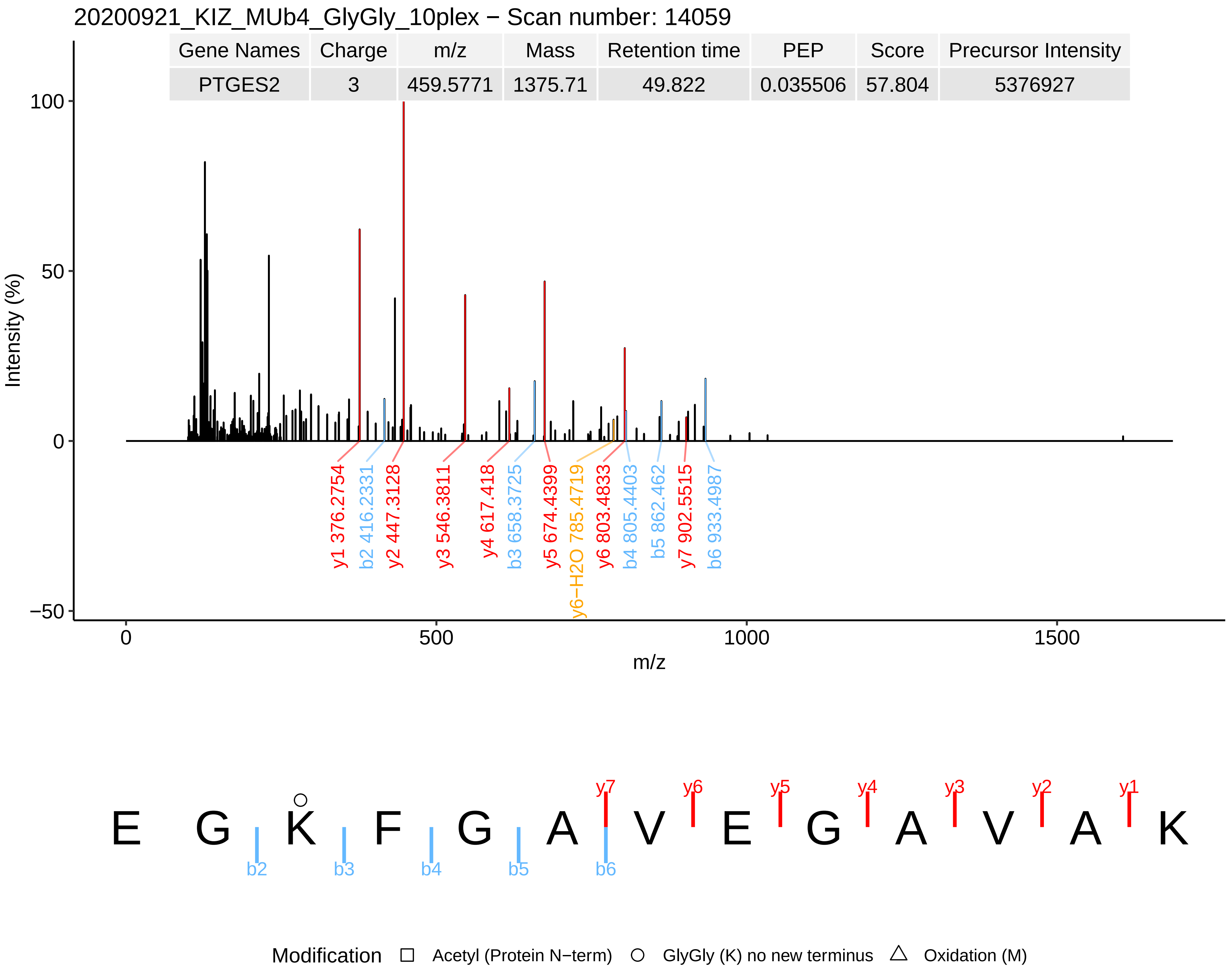

20200921_KIZ_MUb4_GlyGly_10plex − Scan number: 14059
Gene Names
Charge
m/z
Mass
Retention time
PEP
Score
Precursor Intensity
PTGES2
3
459.5771
1375.71
49.822
0.035506
57.804
5376927
Intensity (%)
m/z
y1 376.2754
b2 416.2331
y2 447.3128
y3 546.3811
y4 617.418
b3 658.3725
y5 674.4399
y6−H2O 785.4719
y6 803.4833
b4 805.4403
b5 862.462
y7 902.5515
b6 933.4987
E G K F G A V E G A V A K
Modification
Acetyl (Protein N−term)
GlyGly (K) no new terminus
Oxidation (M)

20200921_KIZ_MUb4_GlyGly_10plex − Scan number: 14520

Gene Names	Charge	m/z	Mass	Retention time	PEP	Score	Precursor Intensity
CPS1	3	455.9051	1364.694	51.635	0.00037157	94.616	4107339

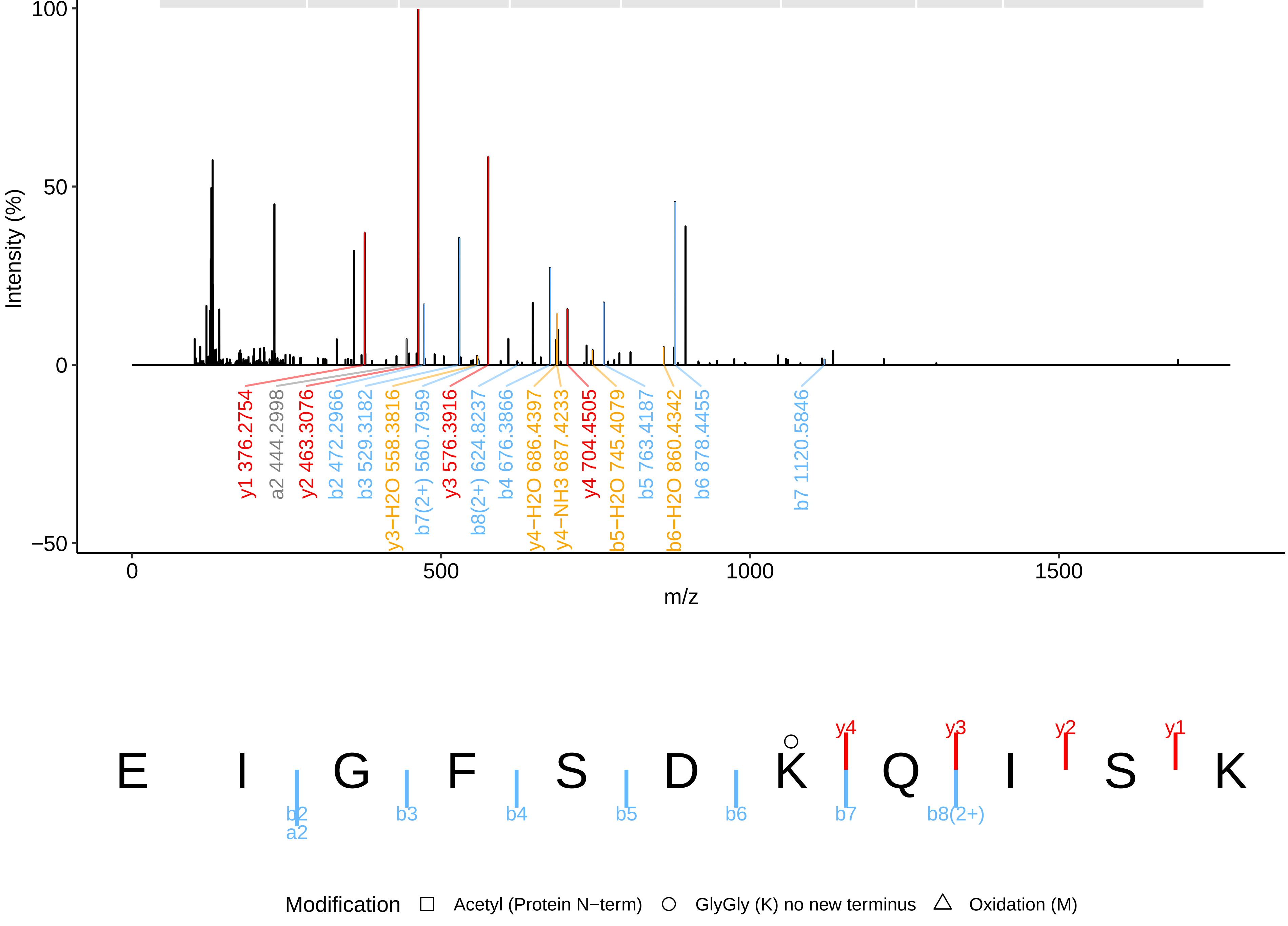

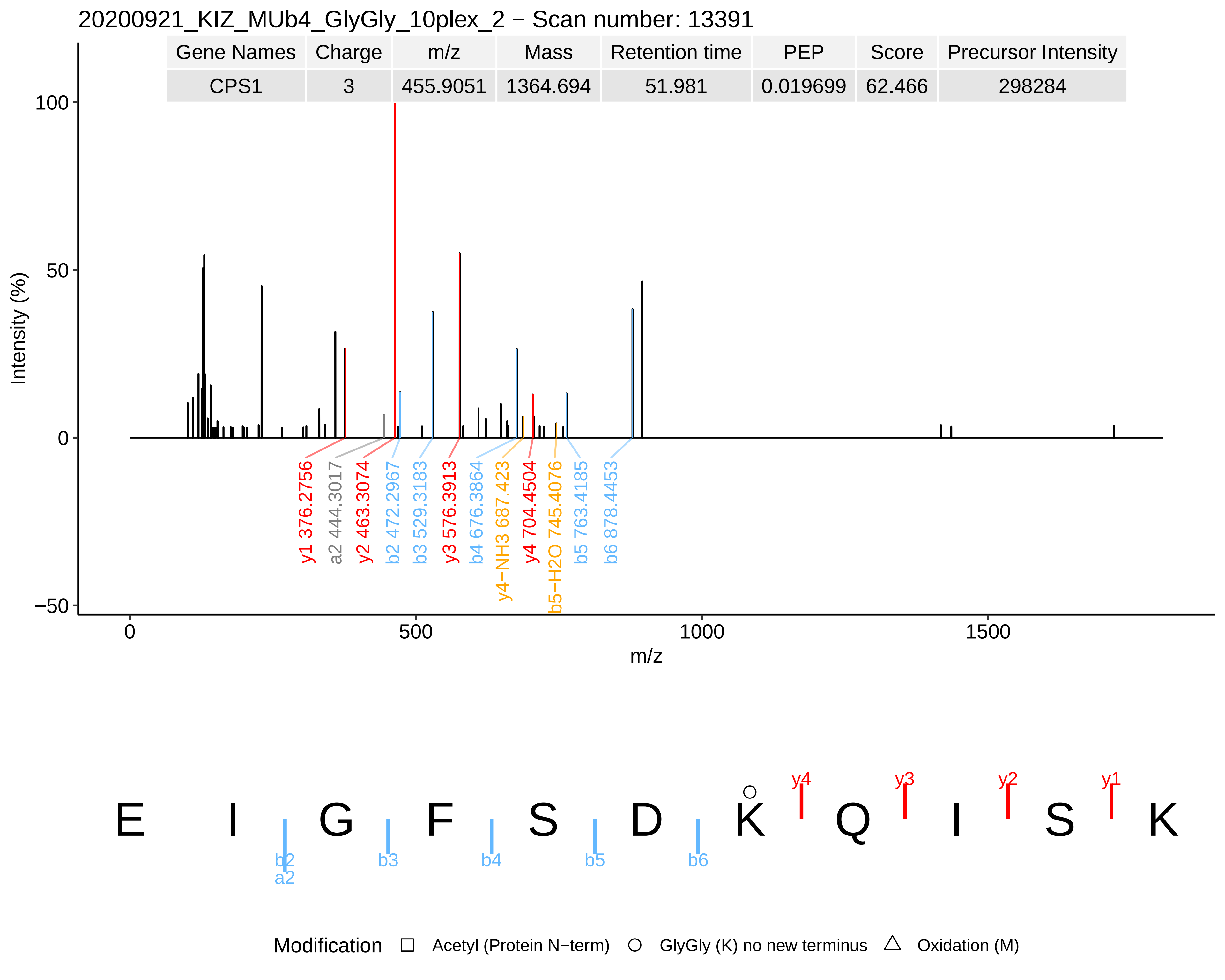
20200921_KIZ_MUb4_GlyGly_10plex_2 − Scan number: 13391
Gene Names | Charge | m/z | Mass | Retention time | PEP | Score | Precursor Intensity
CPS1 | 3 | 455.9051 | 1364.694 | 51.981 | 0.019699 | 62.466 | 298284
Intensity (%)
m/z
y1 376.2756
a2 444.3017
y2 463.3074
b2 472.2967
b3 529.3183
y3 576.3913
b4 676.3864
y4−NH3 687.423
y4 704.4504
b5−H2O 745.4076
b5 763.4185
b6 878.4453
E I G F S D K Q I S K
y4 y3 y2 y1
b2 a2 b3 b4 b5 b6
Modification
Acetyl (Protein N−term)
GlyGly (K) no new terminus
Oxidation (M)

20200921_KIZ_MUb4_GlyGly_10plex_2 − Scan number: 16575

Gene Names	Charge	m/z	Mass	Retention time	PEP	Score	Precursor Intensity
DEGS1	3	439.9186	1316.734	64.72	0.015705	67.726	662242.9

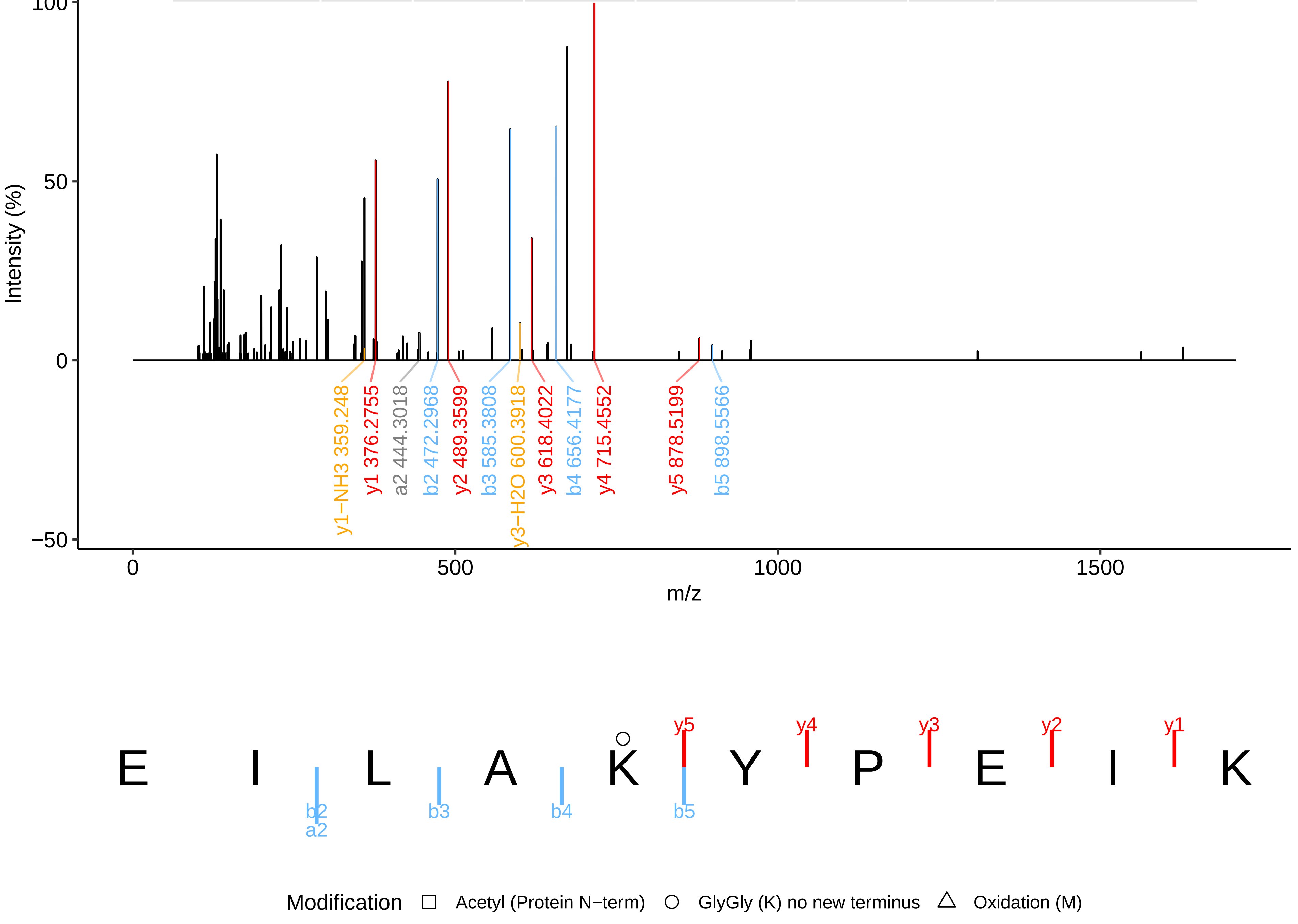

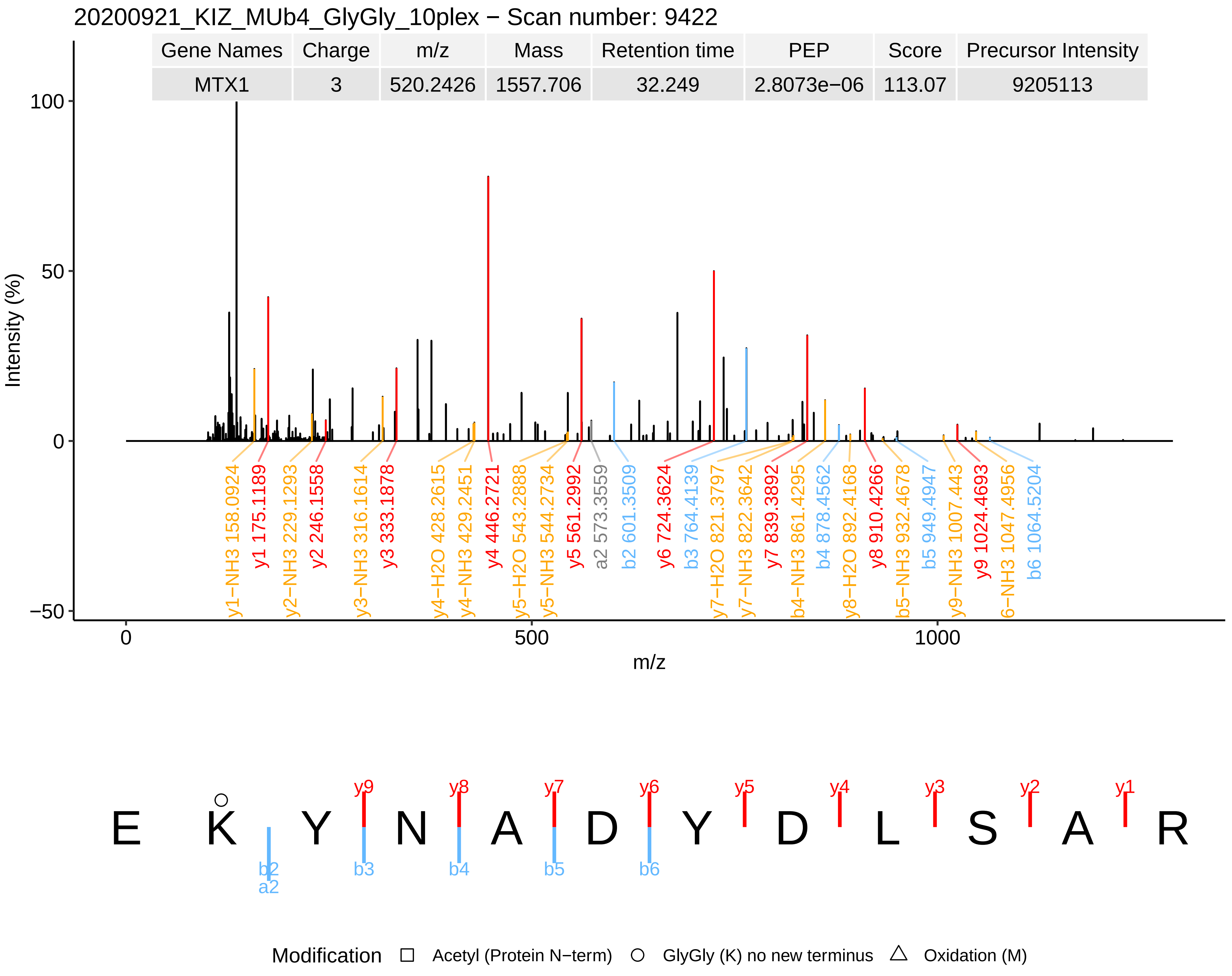
20200921_KIZ_MUb4_GlyGly_10plex − Scan number: 9422
Gene Names | Charge | m/z | Mass | Retention time | PEP | Score | Precursor Intensity
MTX1 | 3 | 520.2426 | 1557.706 | 32.249 | 2.8073e−06 | 113.07 | 9205113
Intensity (%)
100
50
0
−50
0
500
1000
m/z
y1−NH3 158.0924
y1 175.1189
y2−NH3 229.1293
y2 246.1558
y3−NH3 316.1614
y3 333.1878
y4−H2O 428.2615
y4−NH3 429.2451
y4 446.2721
y5−H2O 543.2888
y5−NH3 544.2734
y5 561.2992
a2 573.3559
b2 601.3509
y6 724.3624
b3 764.4139
y7−H2O 821.3797
y7−NH3 822.3642
y7 839.3892
b4−NH3 861.4295
b4 878.4562
y8−H2O 892.4168
y8 910.4266
b5−NH3 932.4678
b5 949.4947
y9−NH3 1007.443
y9 1024.4693
6−NH3 1047.4956
b6 1064.5204
E K Y N A D Y D L S A R
y9 y8 y7 y6 y5 y4 y3 y2 y1
b2 a2 b3 b4 b5 b6
Modification
Acetyl (Protein N−term)
GlyGly (K) no new terminus
Oxidation (M)

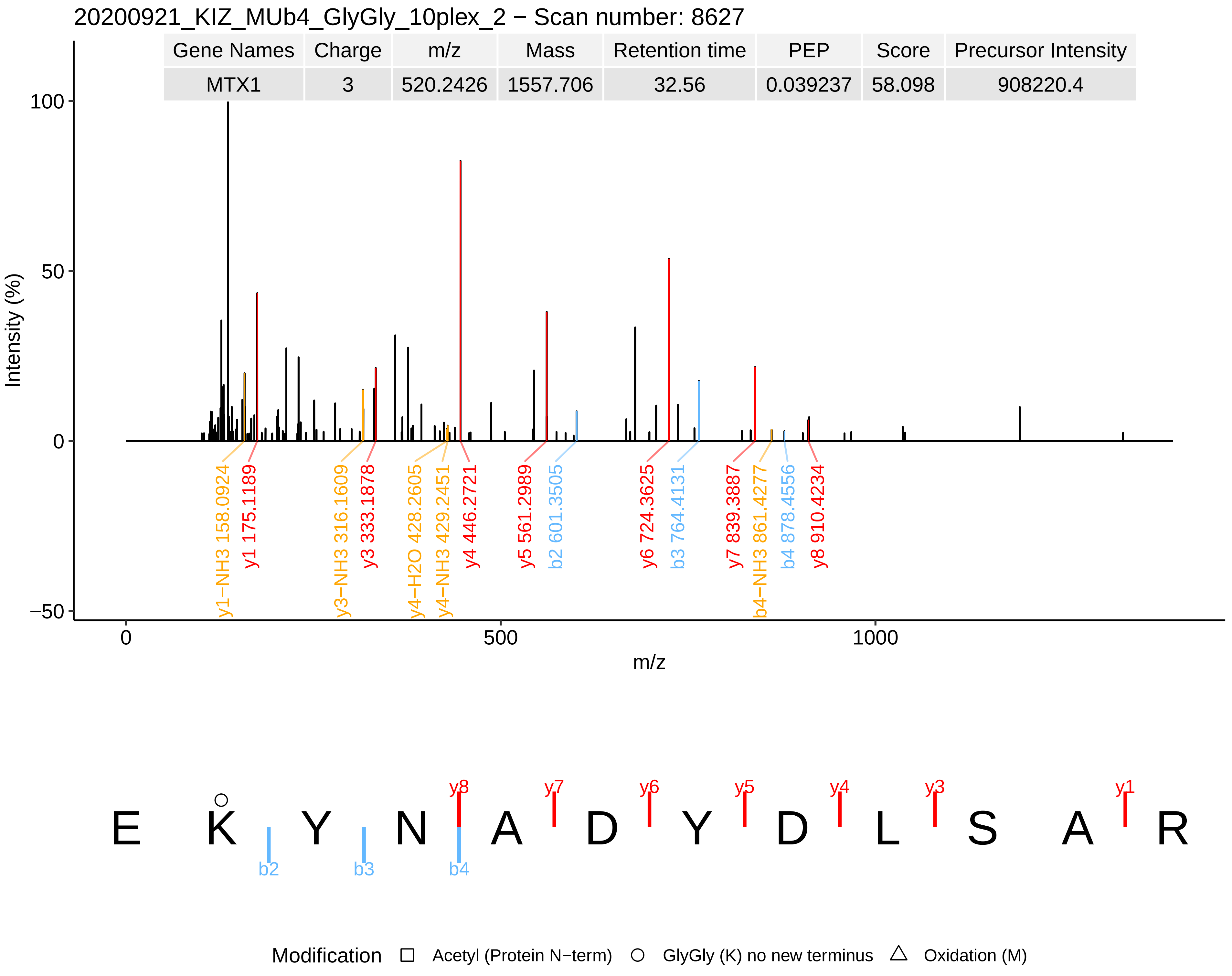
20200921_KIZ_MUb4_GlyGly_10plex_2 − Scan number: 8627
Gene Names | Charge | m/z | Mass | Retention time | PEP | Score | Precursor Intensity
MTX1 | 3 | 520.2426 | 1557.706 | 32.56 | 0.039237 | 58.098 | 908220.4
Intensity (%)
m/z
y1−NH3 158.0924
y1 175.1189
y3−NH3 316.1609
y3 333.1878
y4−H2O 428.2605
y4−NH3 429.2451
y4 446.2721
y5 561.2989
b2 601.3505
y6 724.3625
b3 764.4131
y7 839.3887
b4−NH3 861.4277
b4 878.4556
y8 910.4234
E K Y N A D Y D L S A R
y8 y7 y6 y5 y4 y3 y1
b2 b3 b4
Modification
Acetyl (Protein N−term)
GlyGly (K) no new terminus
Oxidation (M)

20200921_KIZ_MUb4_GlyGly_10plex − Scan number: 11836

Gene Names	Charge	m/z	Mass	Retention time	PEP	Score	Precursor Intensity
HSP90AA1;HSP90AB1;HSP90AB3P	3	449.9149	1346.723	41.348	0.083268	46.844	2190609

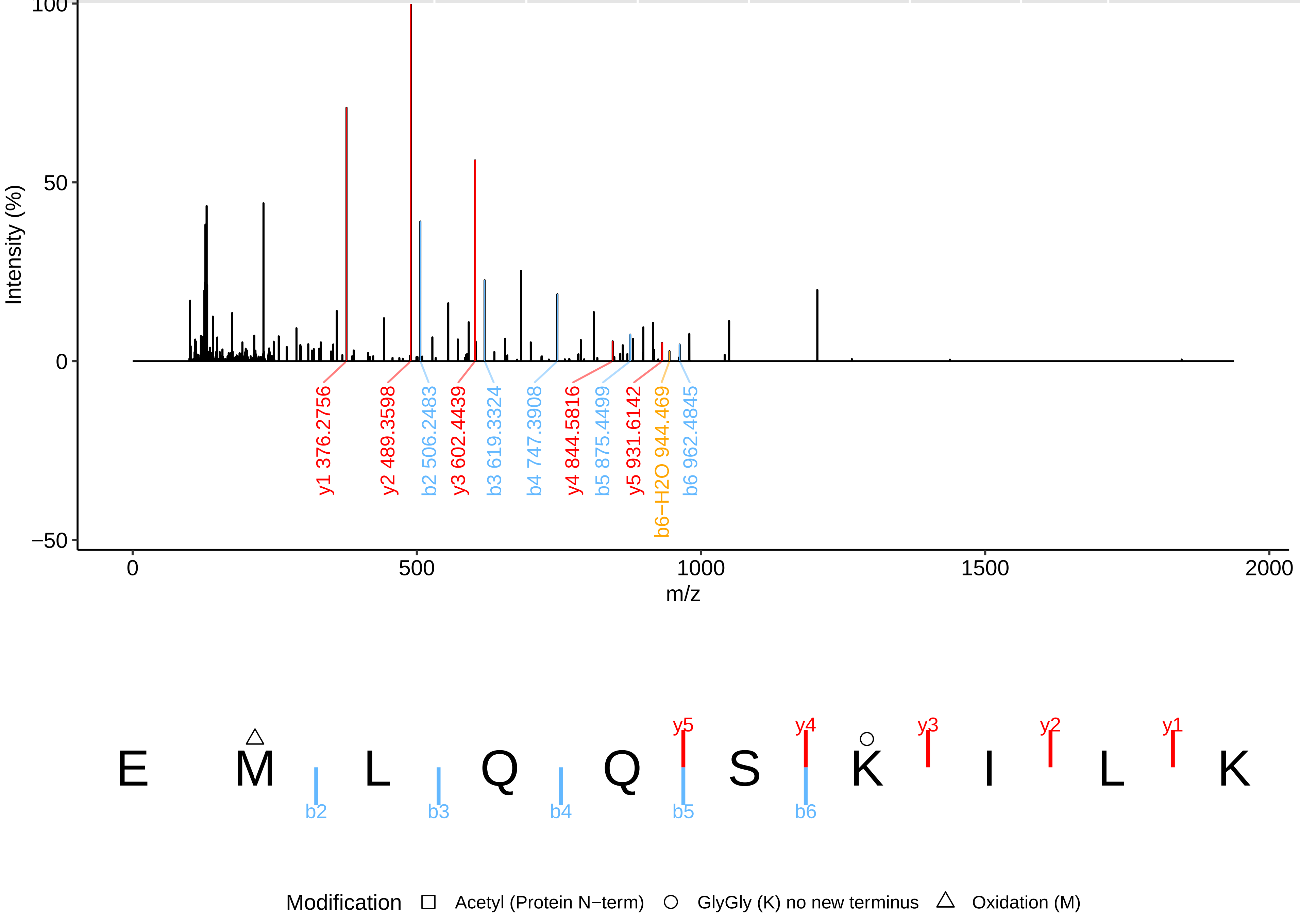

20200921_KIZ_MUb4_GlyGly_10plex_2 − Scan number: 10897

Gene Names	Charge	m/z	Mass	Retention time	PEP	Score	Precursor Intensity
HSP90AA1;HSP90AB1;HSP90AB3P	3	449.9149	1346.723	41.811	0.13967	51.092	684594.2

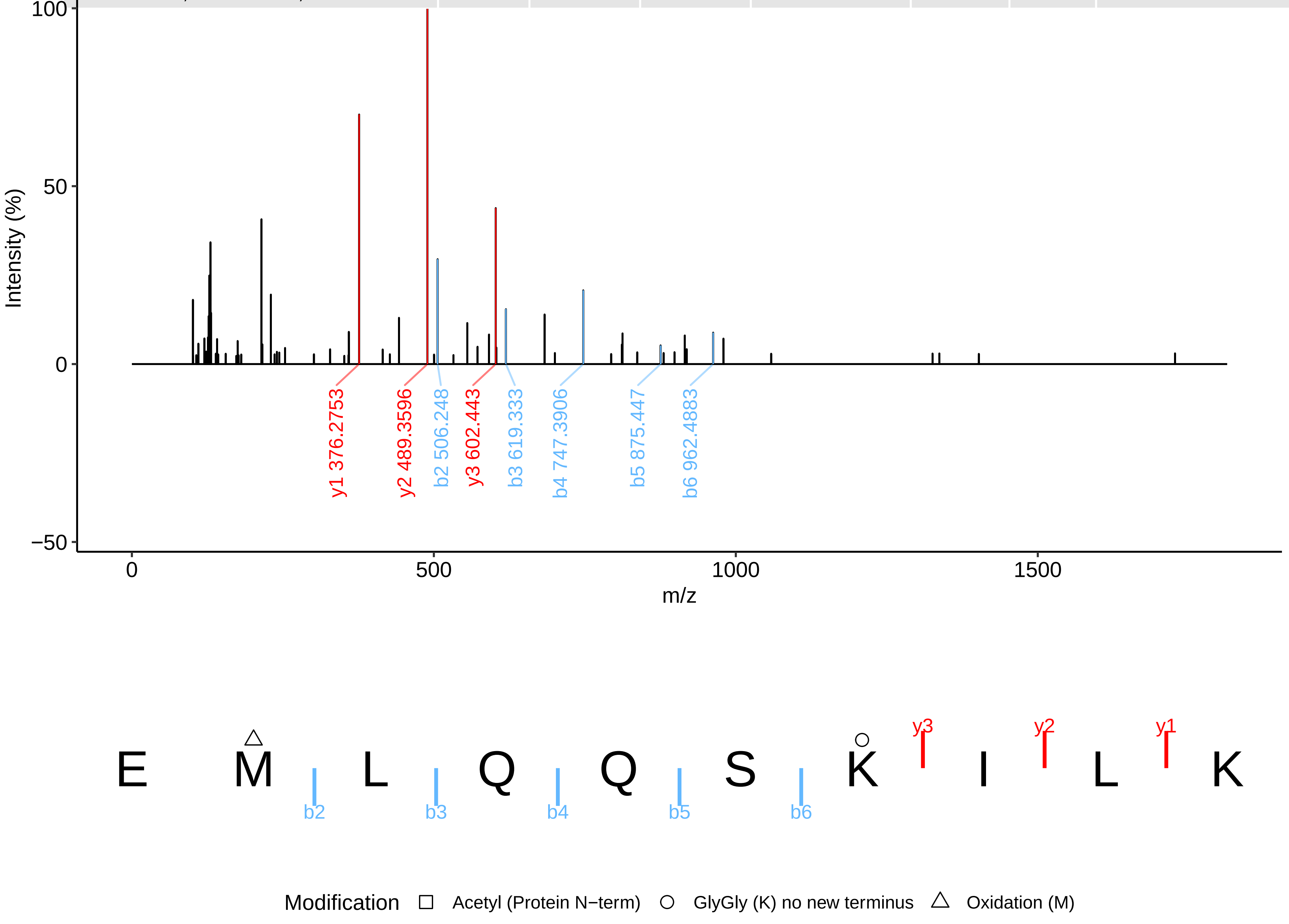

20200921_KIZ_MUb4_GlyGly_10plex − Scan number: 15175

Gene Names	Charge	m/z	Mass	Retention time	PEP	Score	Precursor Intensity
HSP90AA1;HSP90AB1;HSP90AB3P	3	444.5832	1330.728	54.14	0.0087652	74.533	18980880

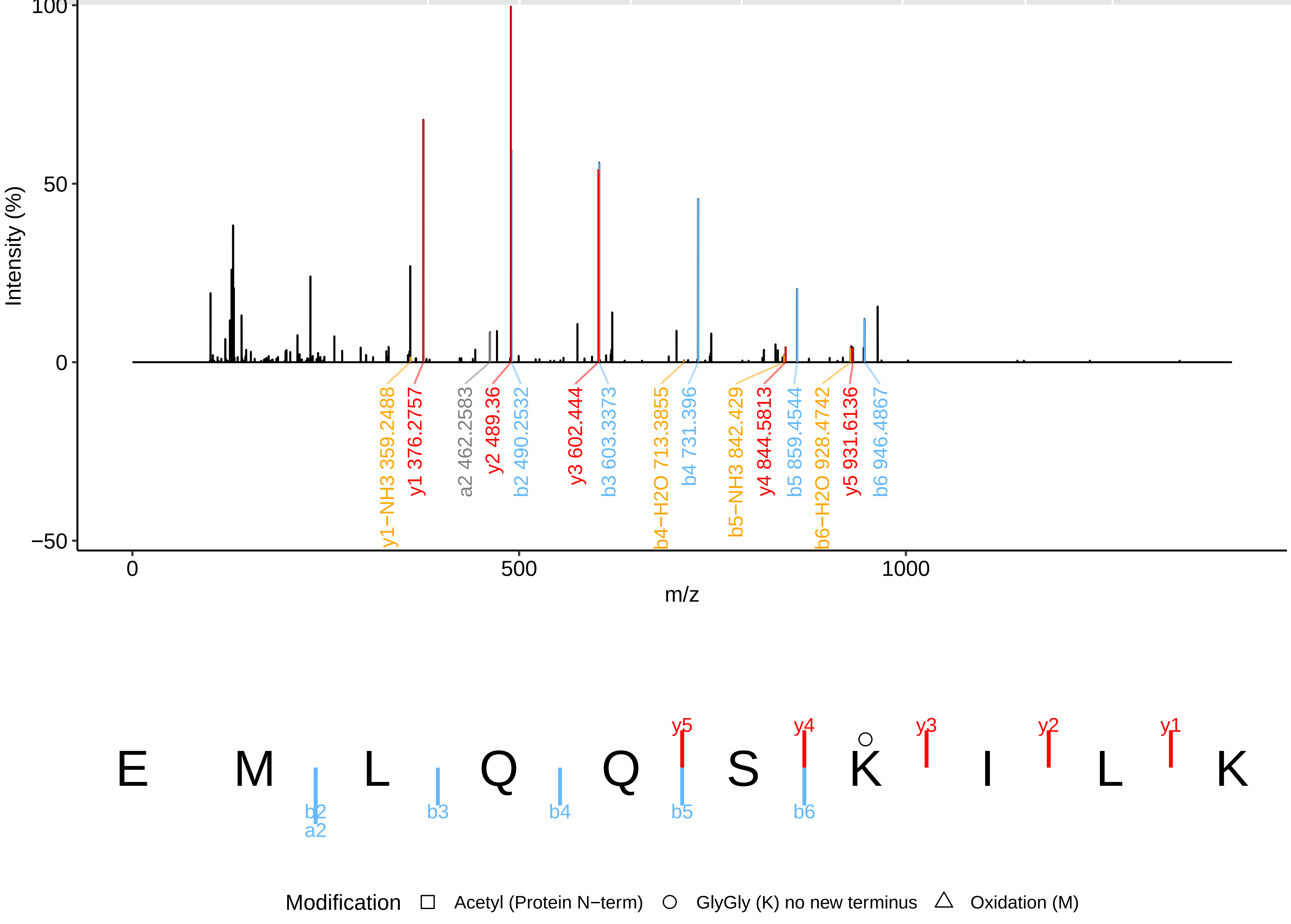

20200921_KIZ_MUb4_GlyGly_10plex_2 − Scan number: 14021

Gene Names	Charge	m/z	Mass	Retention time	PEP	Score	Precursor Intensity
HSP90AA1;HSP90AB1;HSP90AB3P	3	444.5832	1330.728	54.574	0.034417	60.434	738483

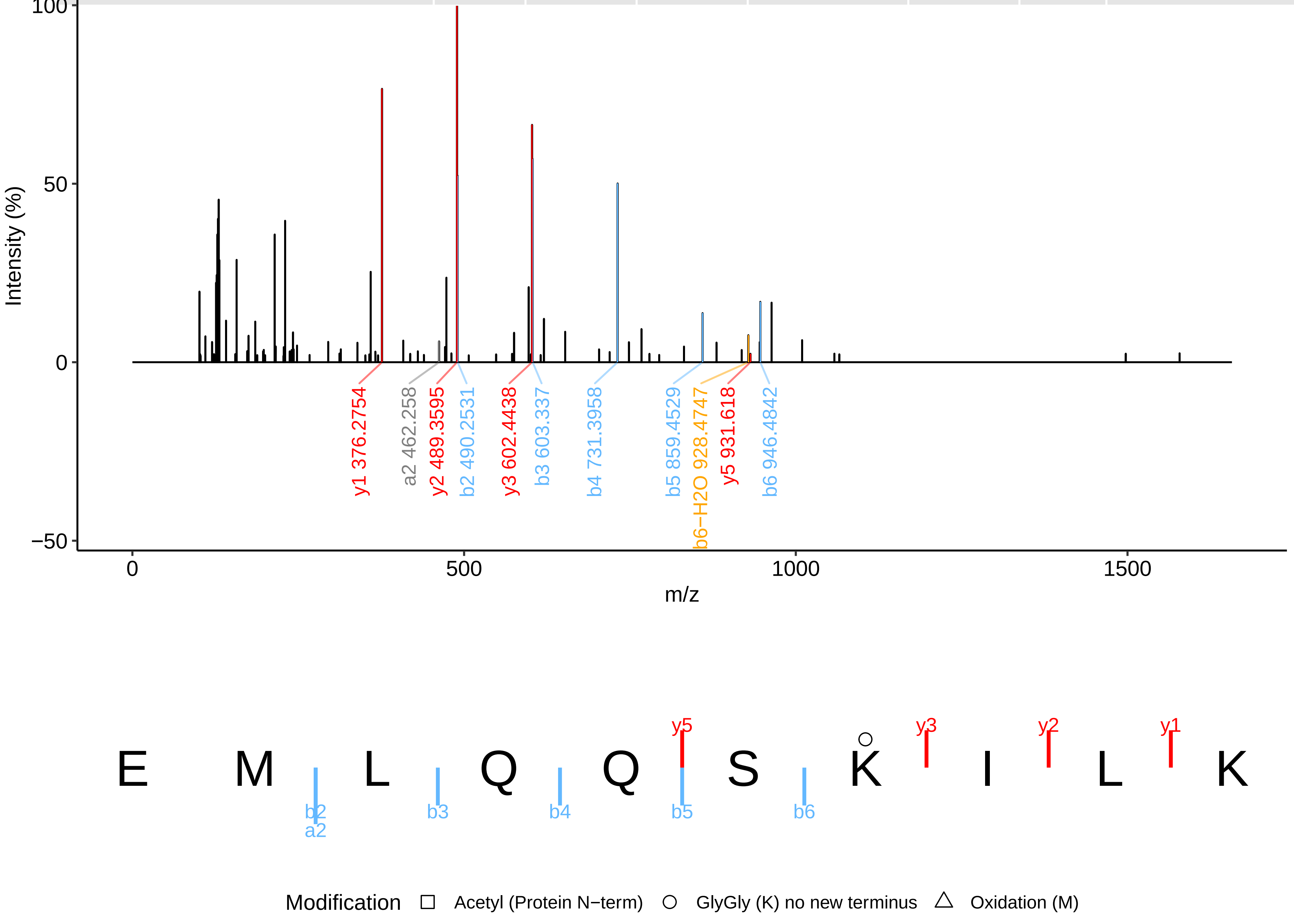

20200924_KIZ_MUb5_GlyGly_1 − Scan number: 14204

Gene Names	Charge	m/z	Mass	Retention time	PEP	Score	Precursor Intensity
HSP90AA1;HSP90AB1;HSP90AB3P	3	444.5832	1330.728	46.361	0.094492	48.741	6847480

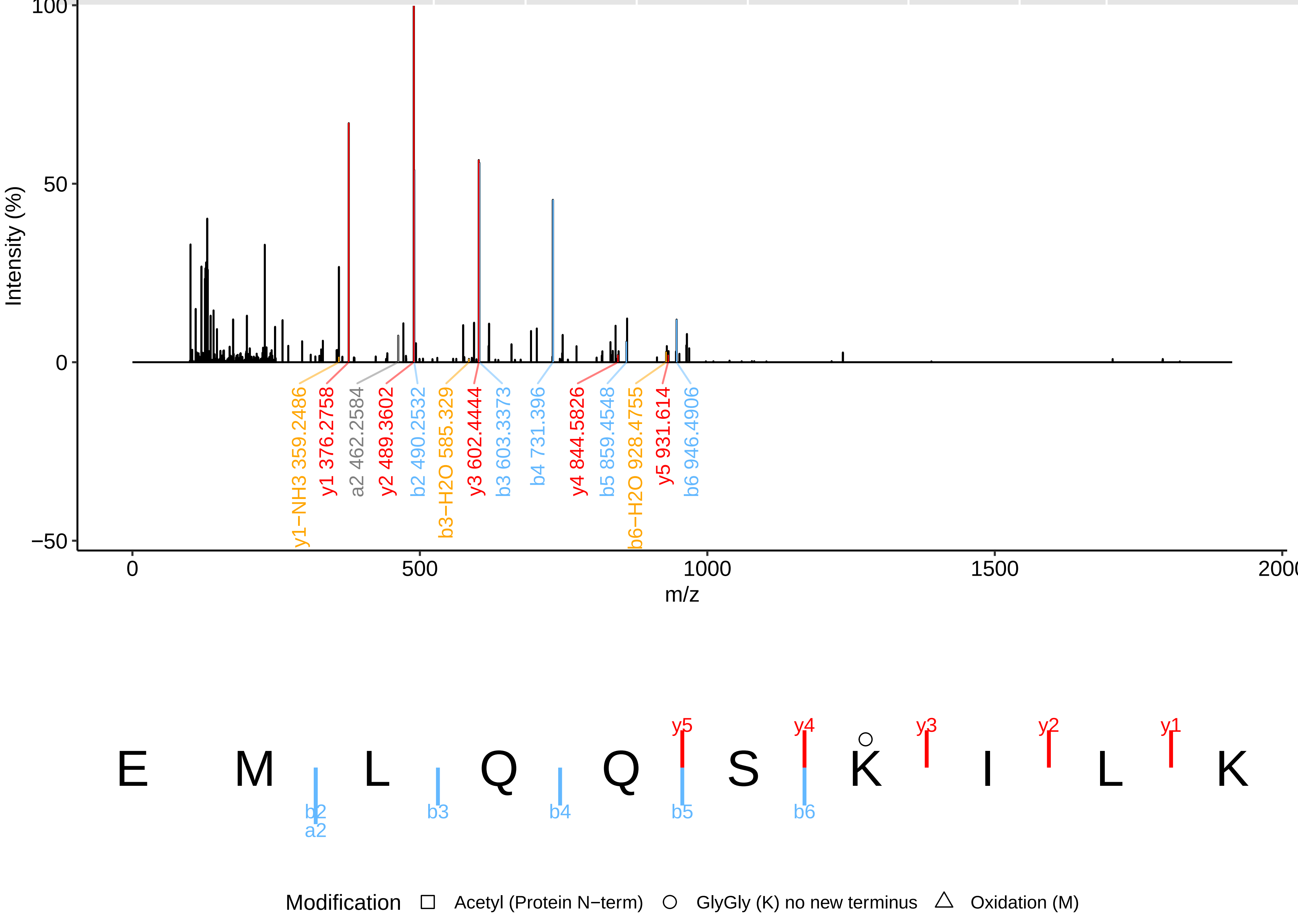

20200924_KIZ_MUb5_GlyGly_1 − Scan number: 14357

Gene Names	Charge	m/z	Mass	Retention time	PEP	Score	Precursor Intensity
HSP90AA1;HSP90AB1;HSP90AB3P	3	444.5832	1330.728	46.878	0.0013454	97.456	7073716

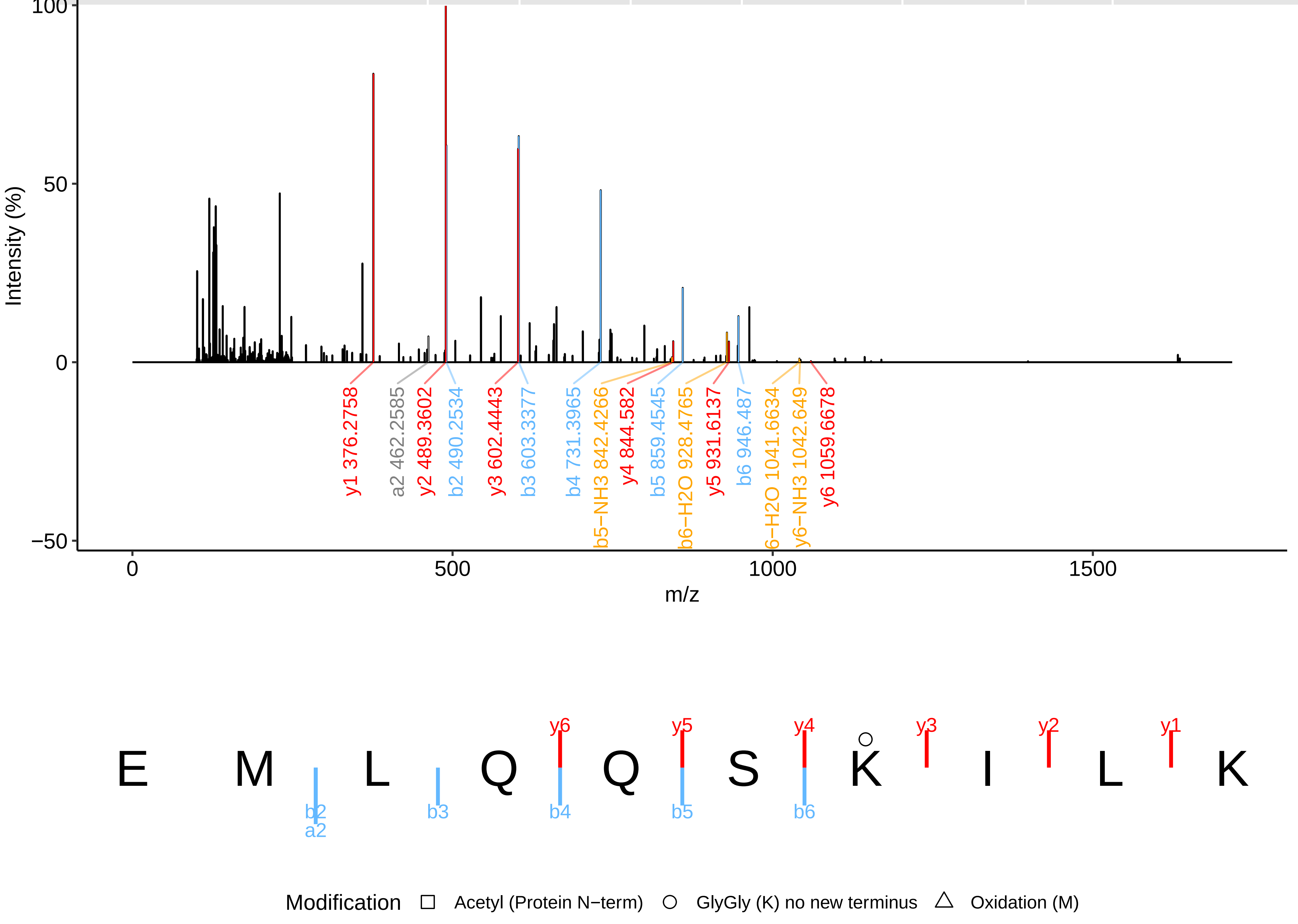

20201118_KIZ_MUb6_GlyGly_1 − Scan number: 14309

Gene Names	Charge	m/z	Mass	Retention time	PEP	Score	Precursor Intensity
HSP90AA1;HSP90AB1;HSP90AB3P	3	444.5832	1330.728	45.26	0.0021121	93.096	13265288

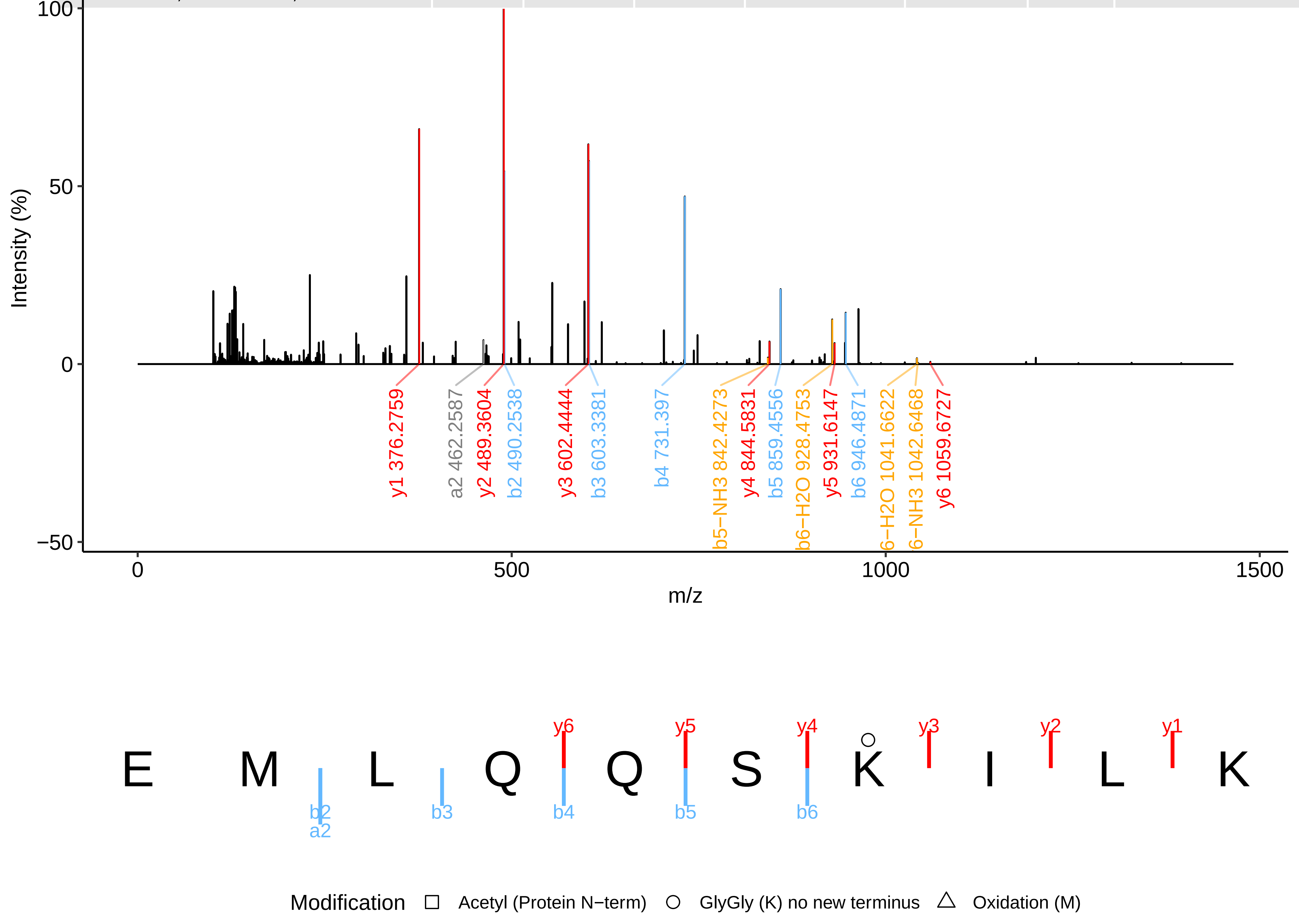

20201118_KIZ_MUb6_GlyGly_2 − Scan number: 16566

Gene Names	Charge	m/z	Mass	Retention time	PEP	Score	Precursor Intensity
HSP90AA1;HSP90AB1;HSP90AB3P	3	444.5832	1330.728	55.963	0.0058645	77.379	5727050

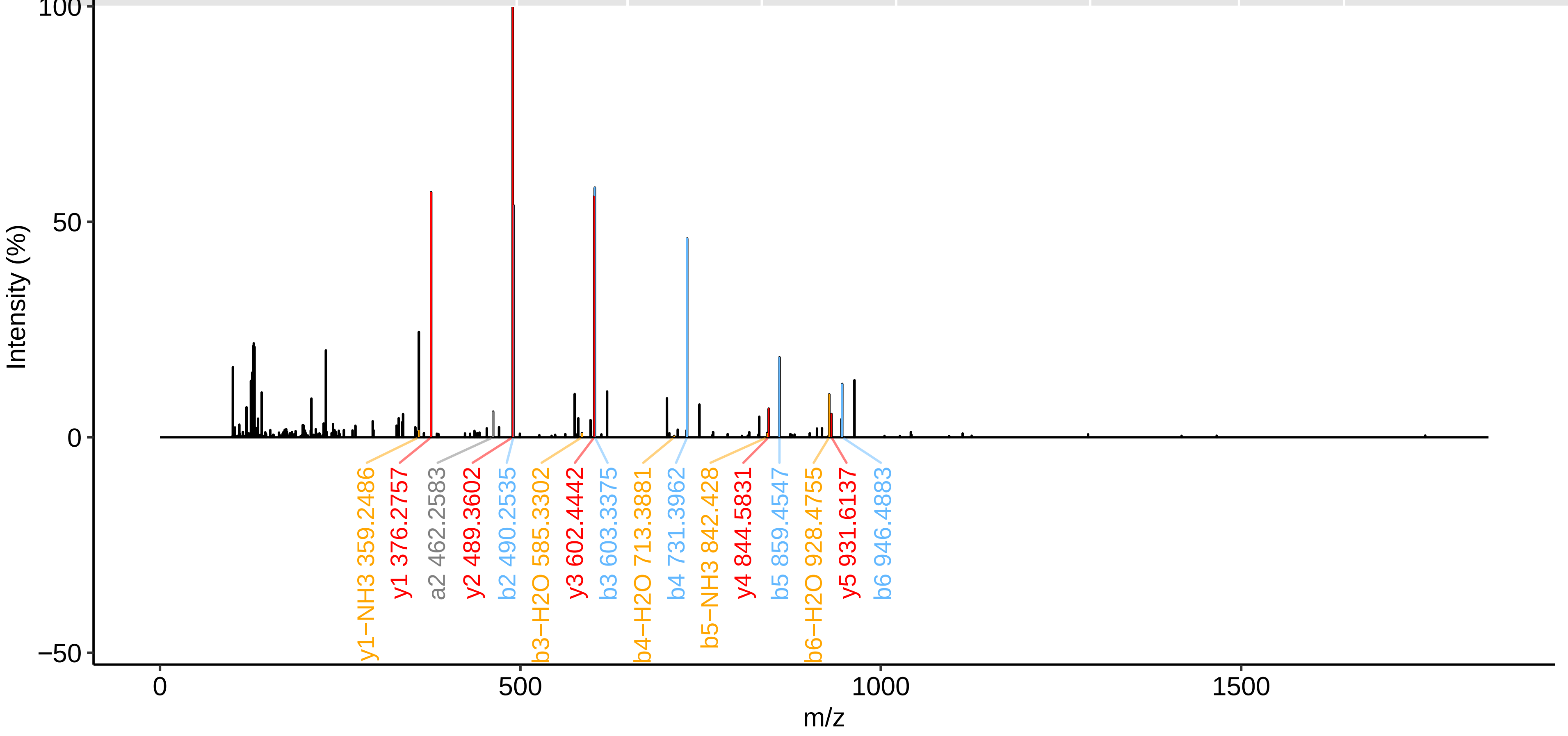

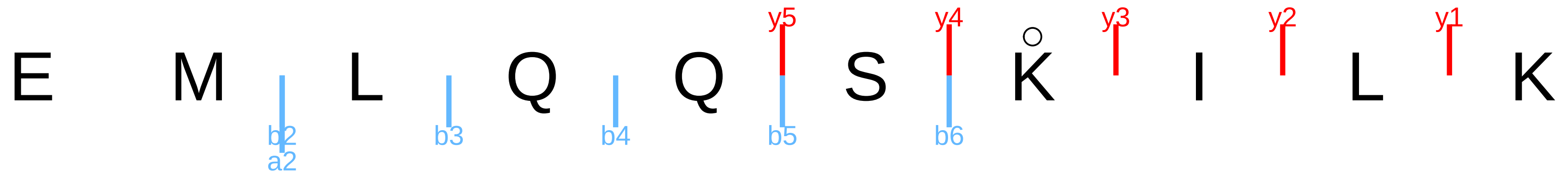

Modification □ Acetyl (Protein N−term) ○ GlyGly (K) no new terminus △ Oxidation (M)

20201118_KIZ_MUb6_GlyGly_2 − Scan number: 16574

Gene Names	Charge	m/z	Mass	Retention time	PEP	Score	Precursor Intensity
HSP90AA1;HSP90AB1;HSP90AB3P	3	444.5832	1330.728	55.995	0.038999	59.542	5341307

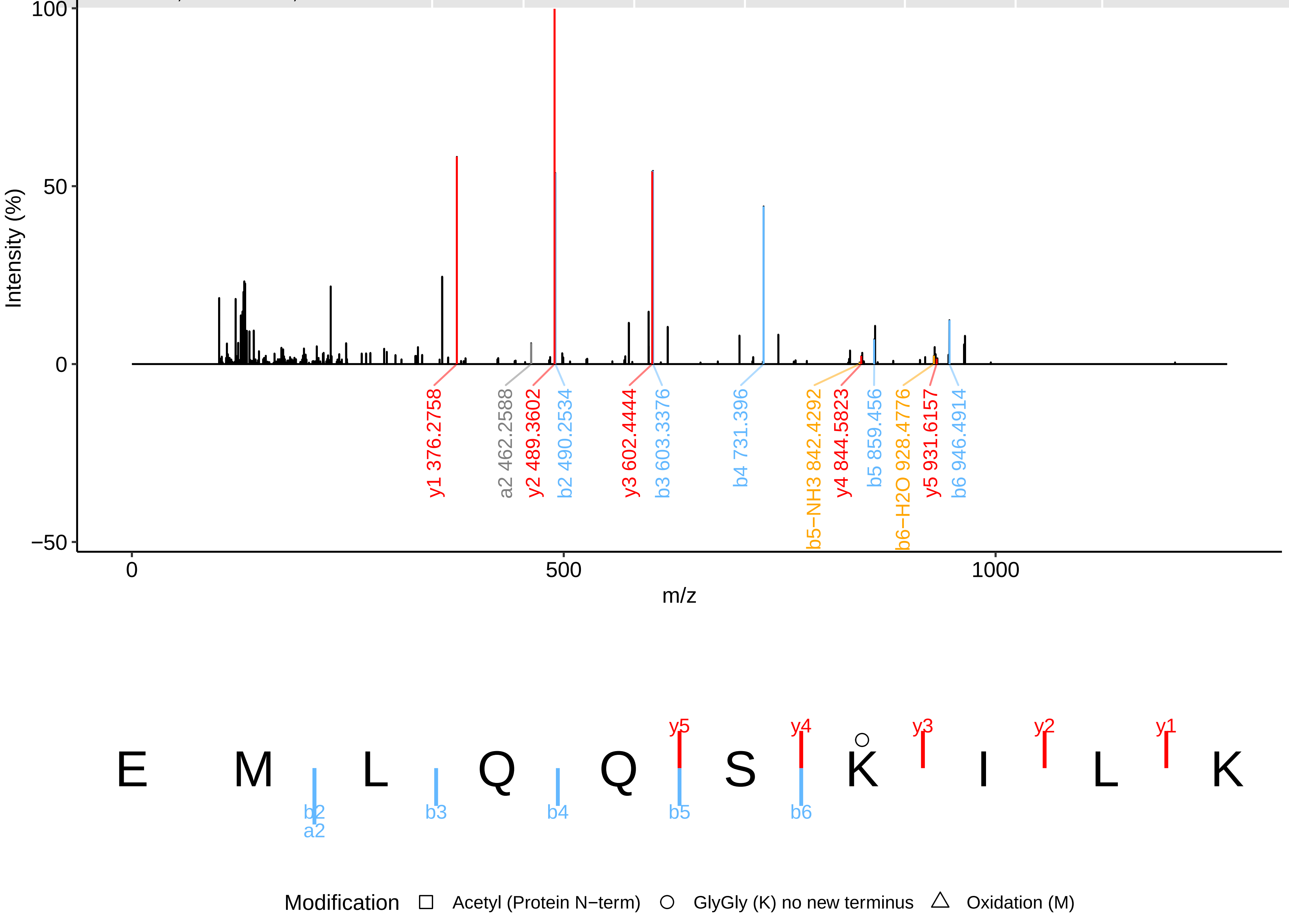

20200921_KIZ_MUb4_GlyGly_10plex − Scan number: 20734

Gene Names	Charge	m/z	Mass	Retention time	PEP	Score	Precursor Intensity
CD320	2	779.9198	1557.825	74.995	0.0010968	93.551	5914713

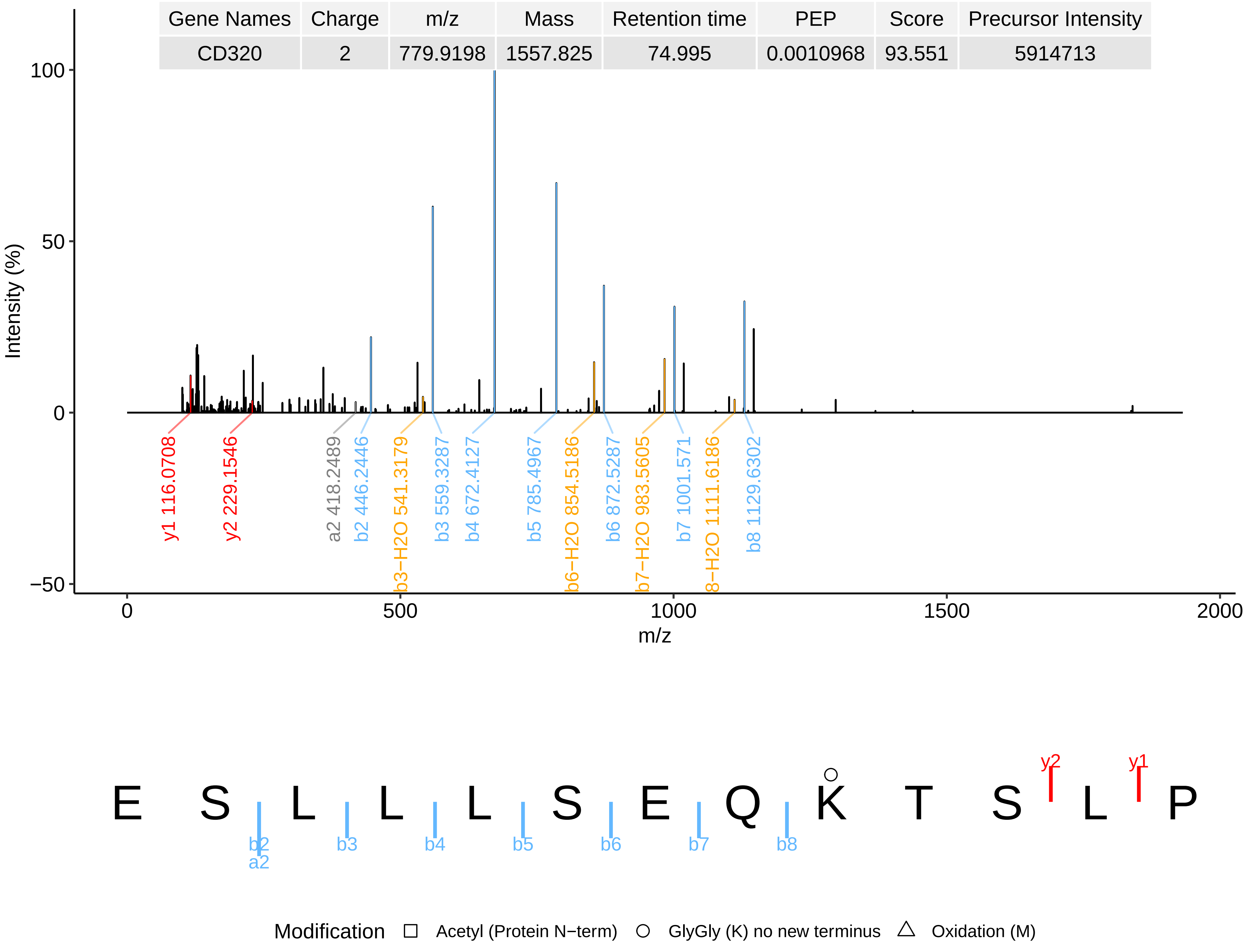

20200921_KIZ_MUb4_GlyGly_10plex − Scan number: 20871

Gene Names	Charge	m/z	Mass	Retention time	PEP	Score	Precursor Intensity
CD320	2	779.9198	1557.825	75.496	0.0024499	87.639	9248051

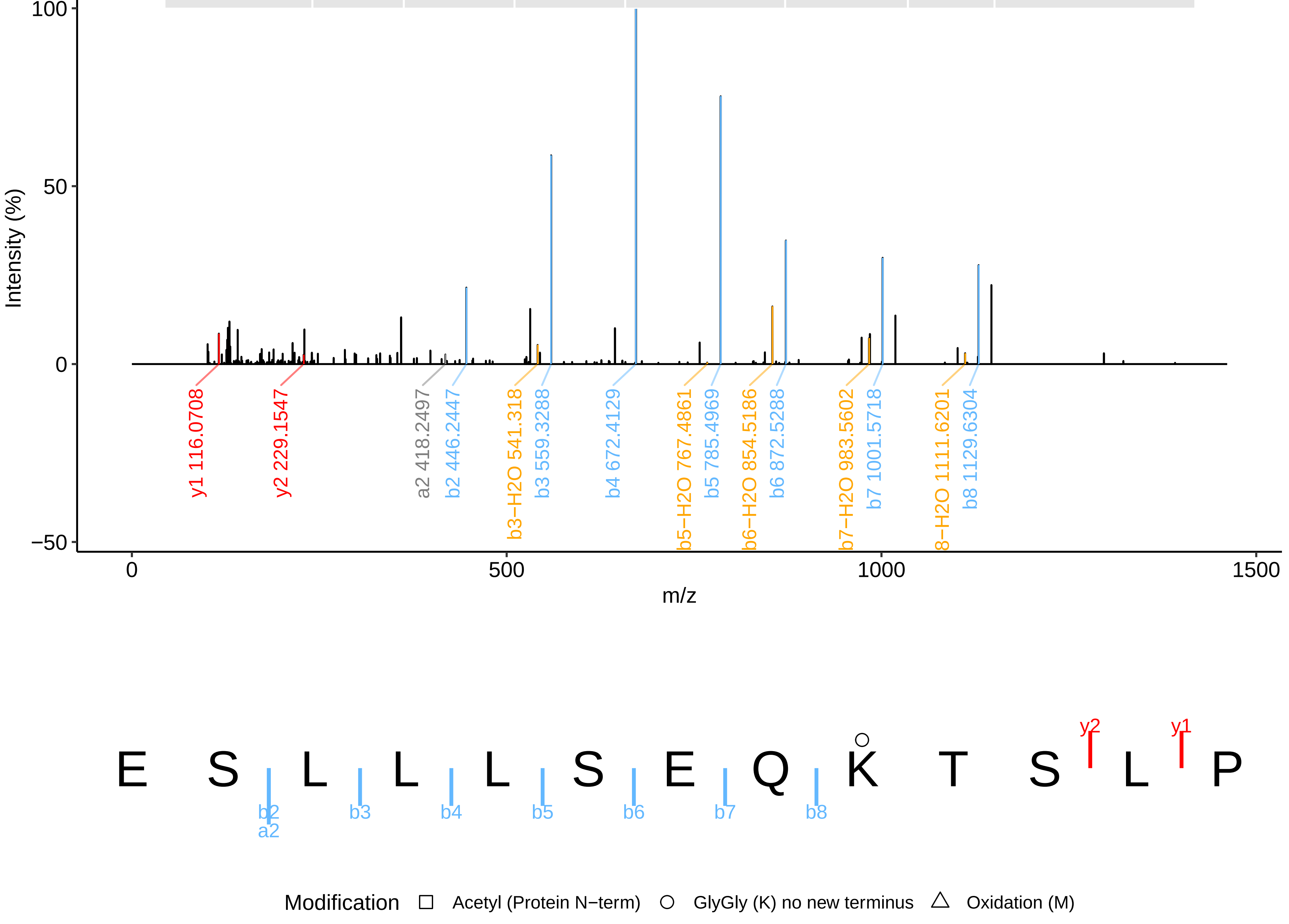

20200921_KIZ_MUb4_GlyGly_10plex − Scan number: 20789

Gene Names	Charge	m/z	Mass	Retention time	PEP	Score	Precursor Intensity
CD320	3	520.2823	1557.825	75.19	3.1968e−07	115.18	17957044

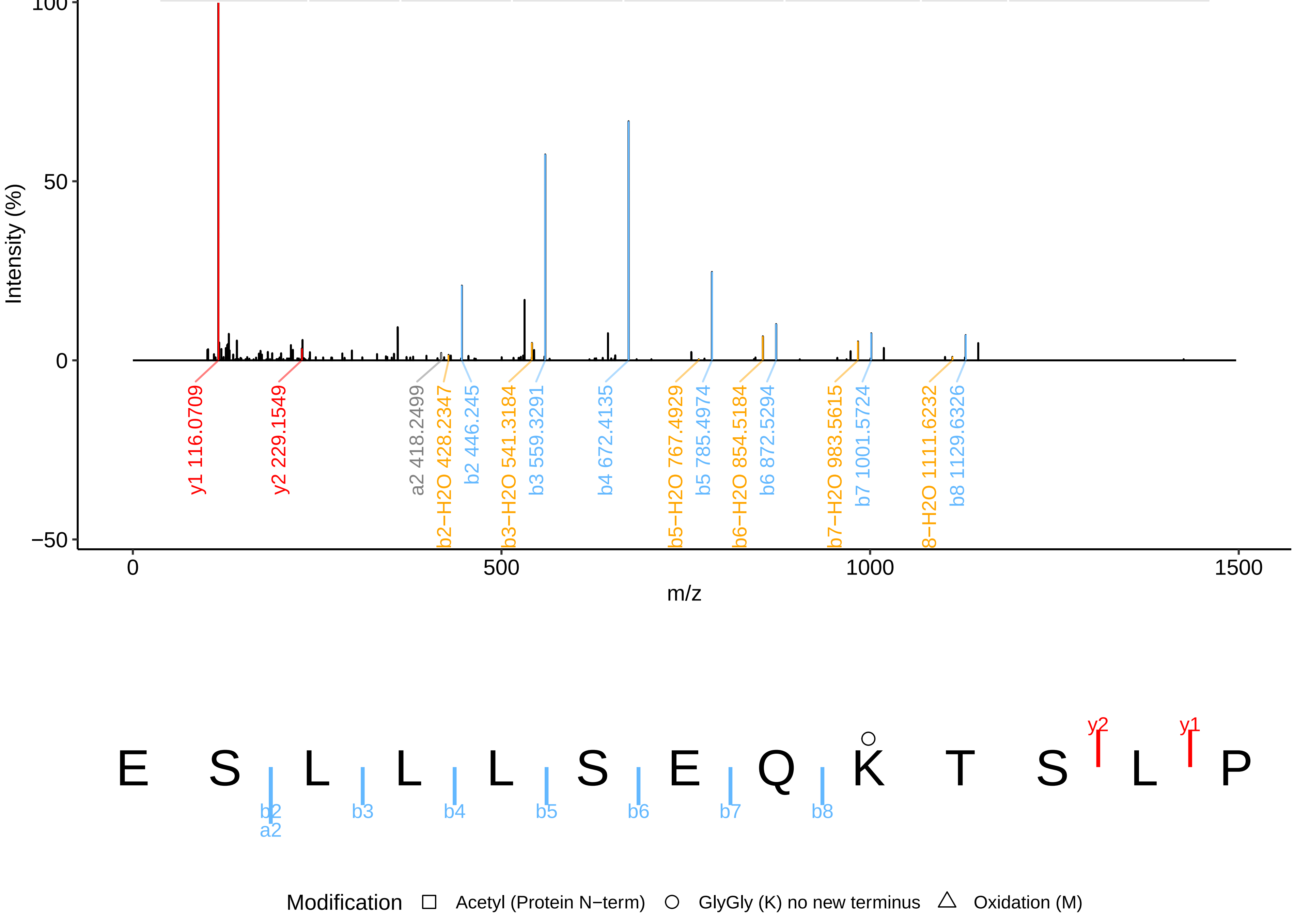

20200921_KIZ_MUb4_GlyGly_10plex − Scan number: 20925

Gene Names	Charge	m/z	Mass	Retention time	PEP	Score	Precursor Intensity
CD320	3	520.2823	1557.825	75.695	0.00025359	94.465	5330018

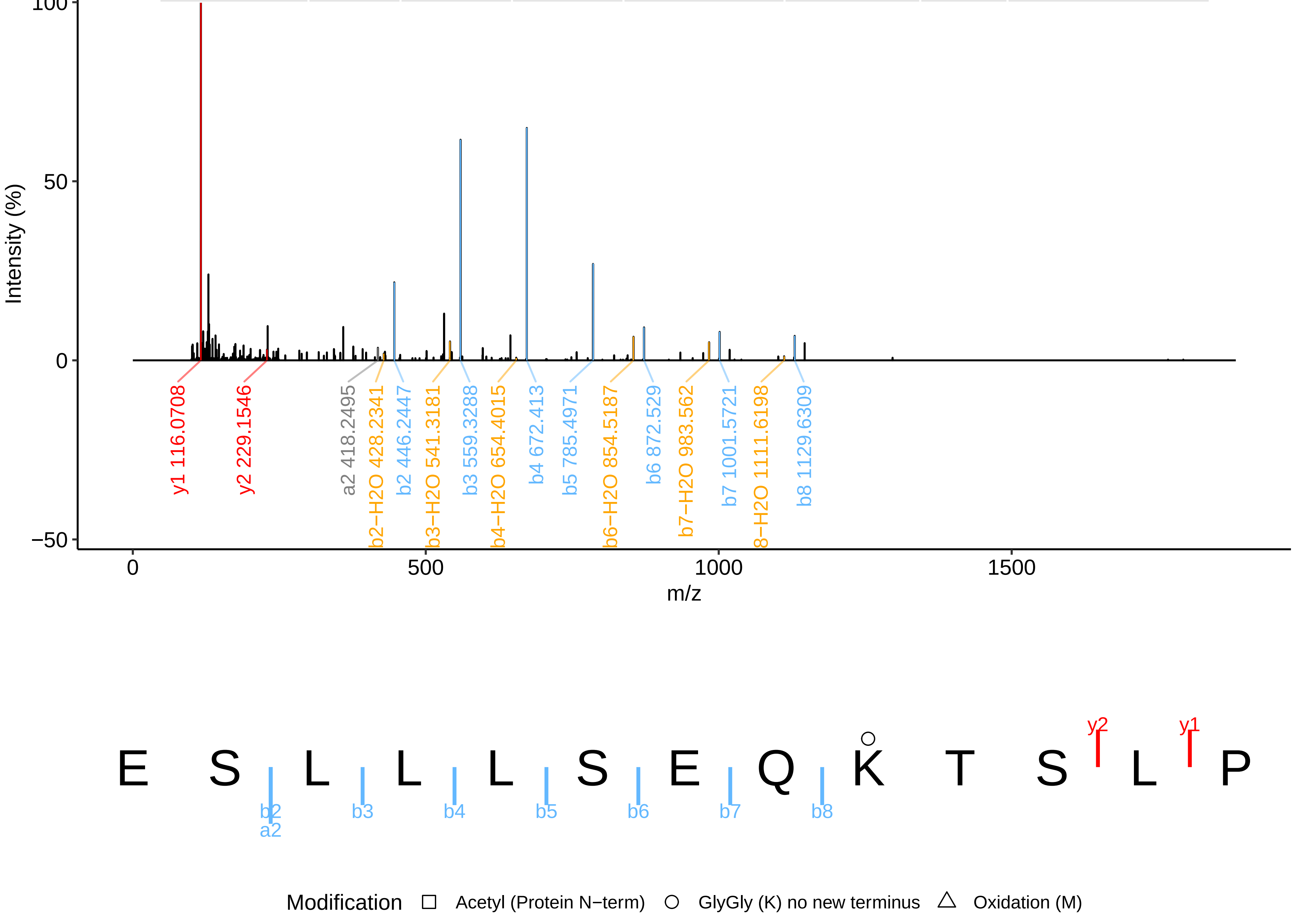

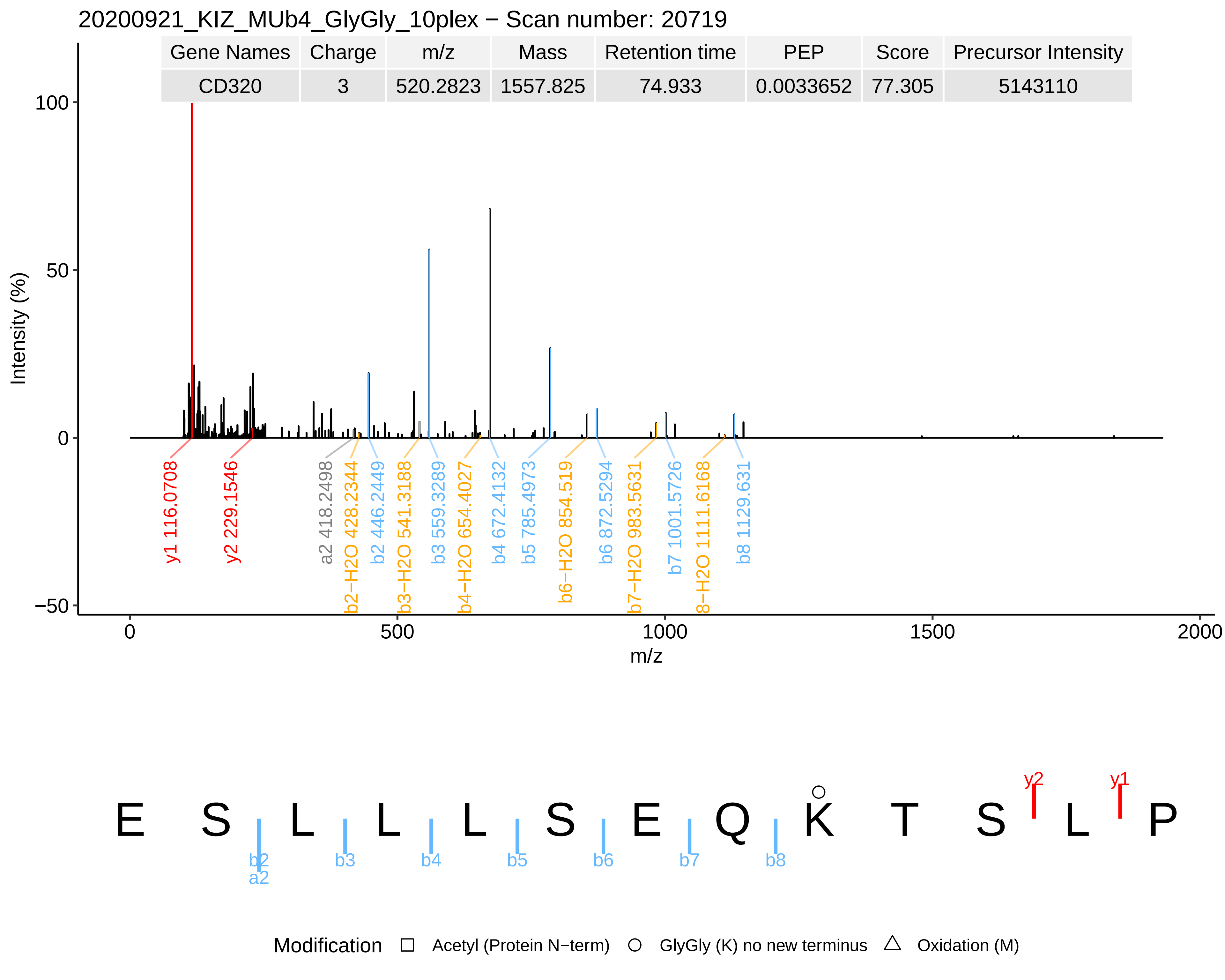
20200921_KIZ_MUb4_GlyGly_10plex − Scan number: 20719
Gene Names | Charge | m/z | Mass | Retention time | PEP | Score | Precursor Intensity
CD320 | 3 | 520.2823 | 1557.825 | 74.933 | 0.0033652 | 77.305 | 5143110
Intensity (%)
100
50
0
−50
0
500
1000
1500
2000
m/z
y1 116.0708
y2 229.1546
a2 418.2498
b2−H2O 428.2344
b2 446.2449
b3−H2O 541.3188
b3 559.3289
b4−H2O 654.4027
b4 672.4132
b5 785.4973
b6−H2O 854.519
b6 872.5294
b7−H2O 983.5631
b7 1001.5726
8−H2O 1111.6168
b8 1129.631
E S L L L S E Q K T S L P
y2
y1
b2
a2
b3
b4
b5
b6
b7
b8
Modification
Acetyl (Protein N−term)
GlyGly (K) no new terminus
Oxidation (M)

20200921_KIZ_MUb4_GlyGly_10plex_2 − Scan number: 19104

Gene Names	Charge	m/z	Mass	Retention time	PEP	Score	Precursor Intensity
CD320	2	779.9198	1557.825	74.932	0.028709	67.563	903161.8

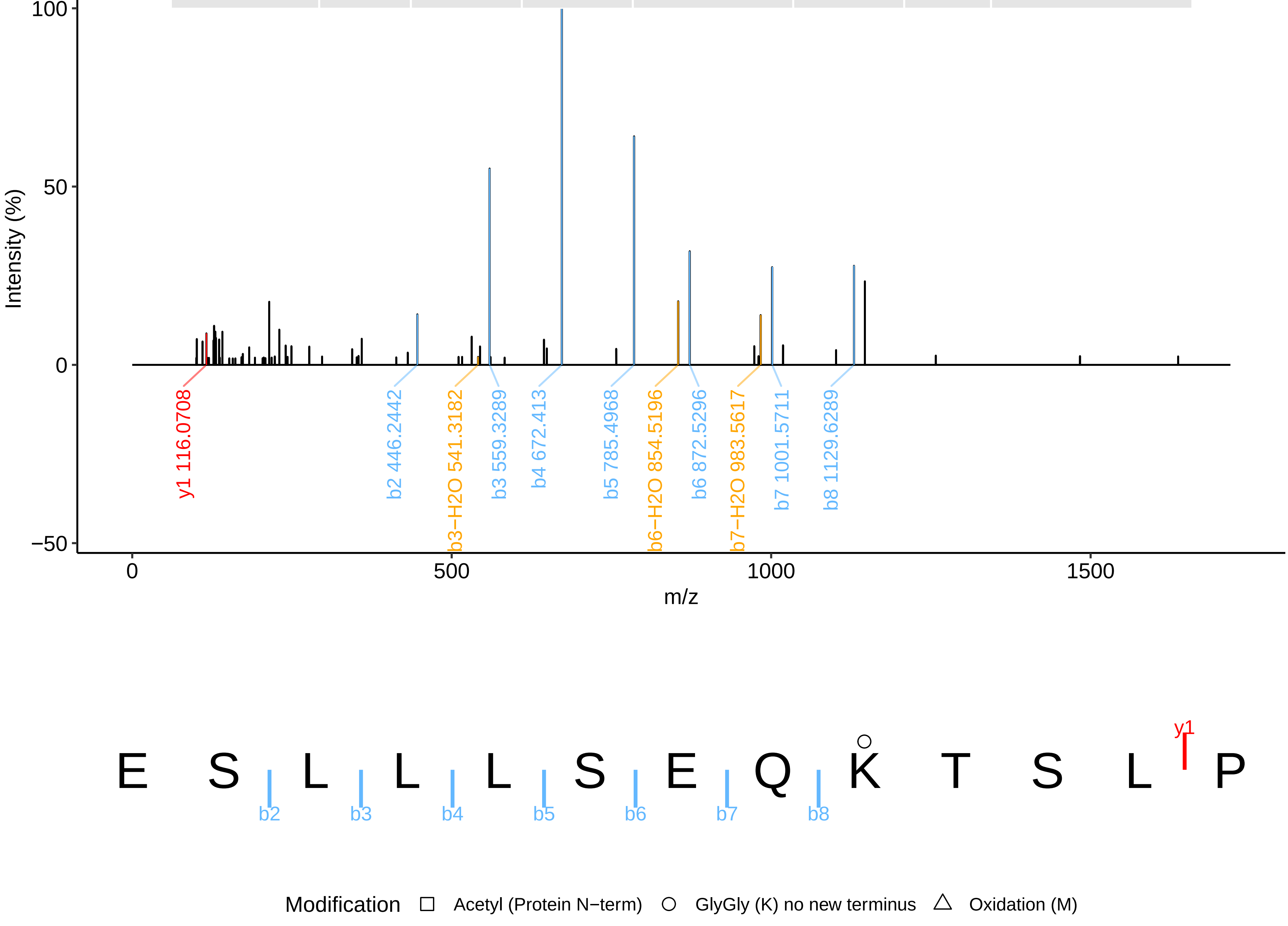

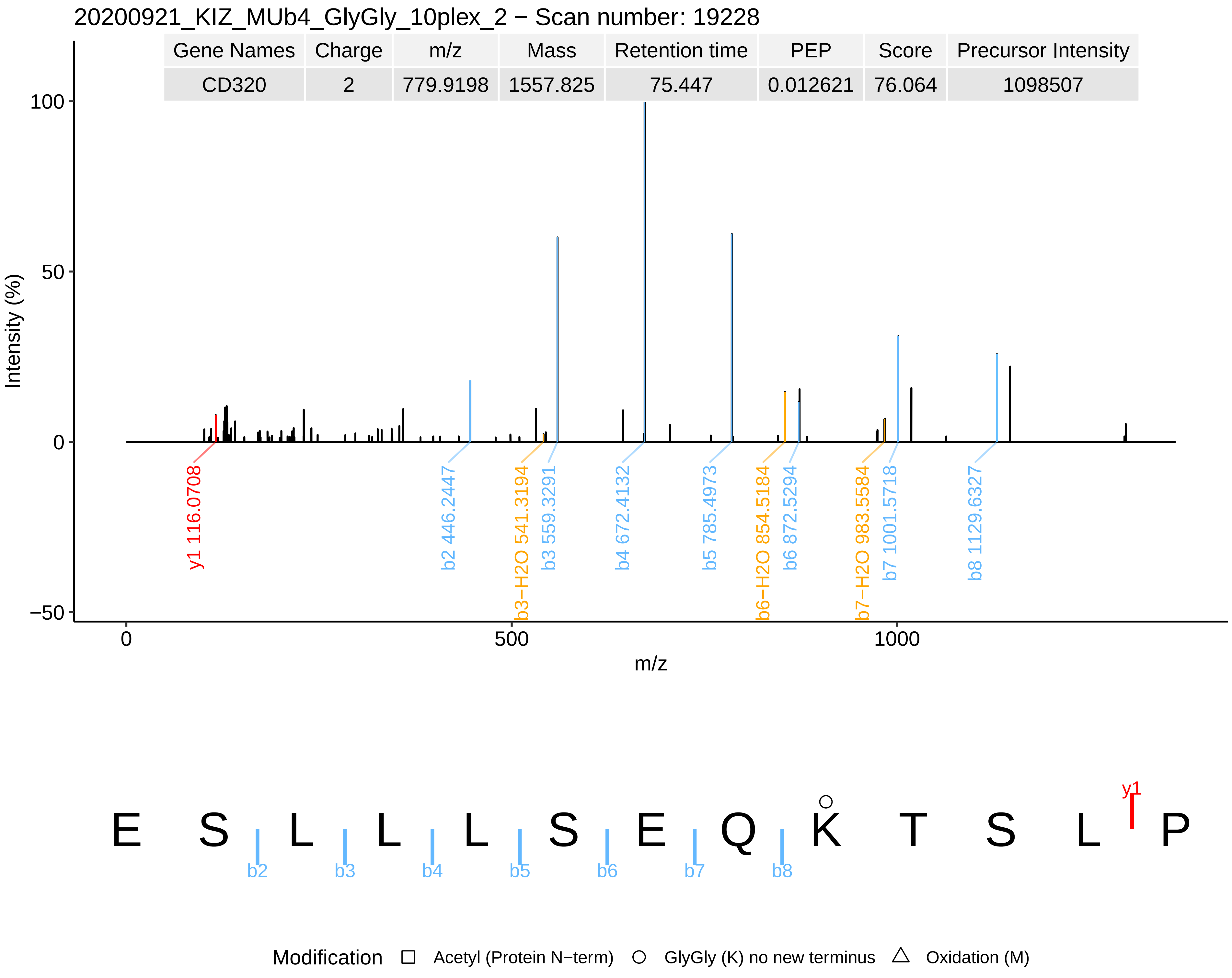
20200921_KIZ_MUb4_GlyGly_10plex_2 − Scan number: 19228
Gene Names | Charge | m/z | Mass | Retention time | PEP | Score | Precursor Intensity
CD320 | 2 | 779.9198 | 1557.825 | 75.447 | 0.012621 | 76.064 | 1098507
Intensity (%)
100
50
0
−50
0
500
1000
m/z
y1 116.0708
b2 446.2447
b3−H2O 541.3194
b3 559.3291
b4 672.4132
b5 785.4973
b6−H2O 854.5184
b6 872.5294
b7−H2O 983.5584
b7 1001.5718
b8 1129.6327
E S L L L S E Q K T S L P
b2 b3 b4 b5 b6 b7 b8
y1
Modification
Acetyl (Protein N−term)
GlyGly (K) no new terminus
Oxidation (M)

20200921_KIZ_MUb4_GlyGly_10plex_2 − Scan number: 19114

Gene Names	Charge	m/z	Mass	Retention time	PEP	Score	Precursor Intensity
CD320	3	520.2823	1557.825	74.974	0.023035	60.49	1071856

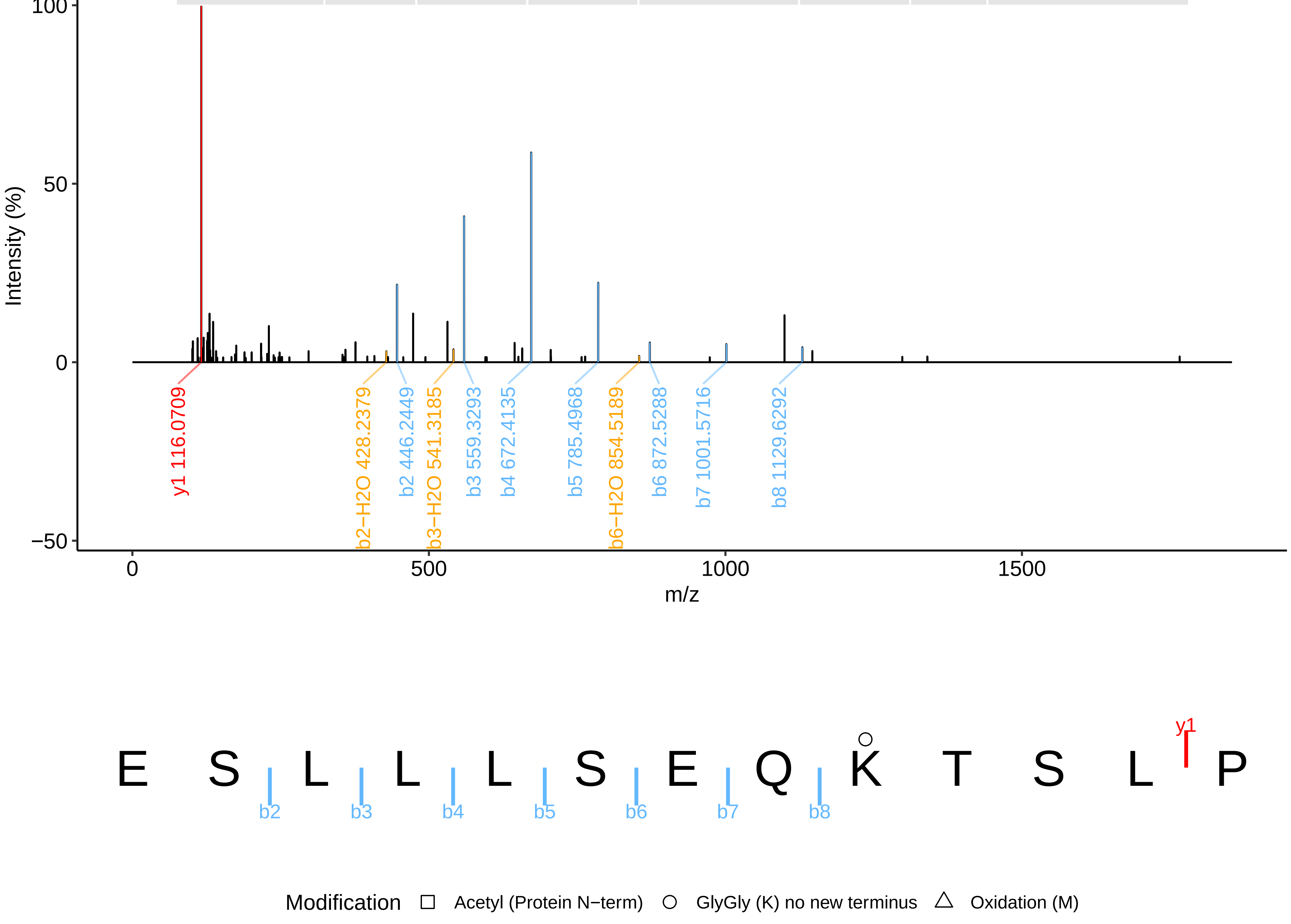

20200921_KIZ_MUb4_GlyGly_10plex_2 − Scan number: 19241

Gene Names	Charge	m/z	Mass	Retention time	PEP	Score	Precursor Intensity
CD320	3	520.2823	1557.825	75.499	0.00041893	91.937	1787768

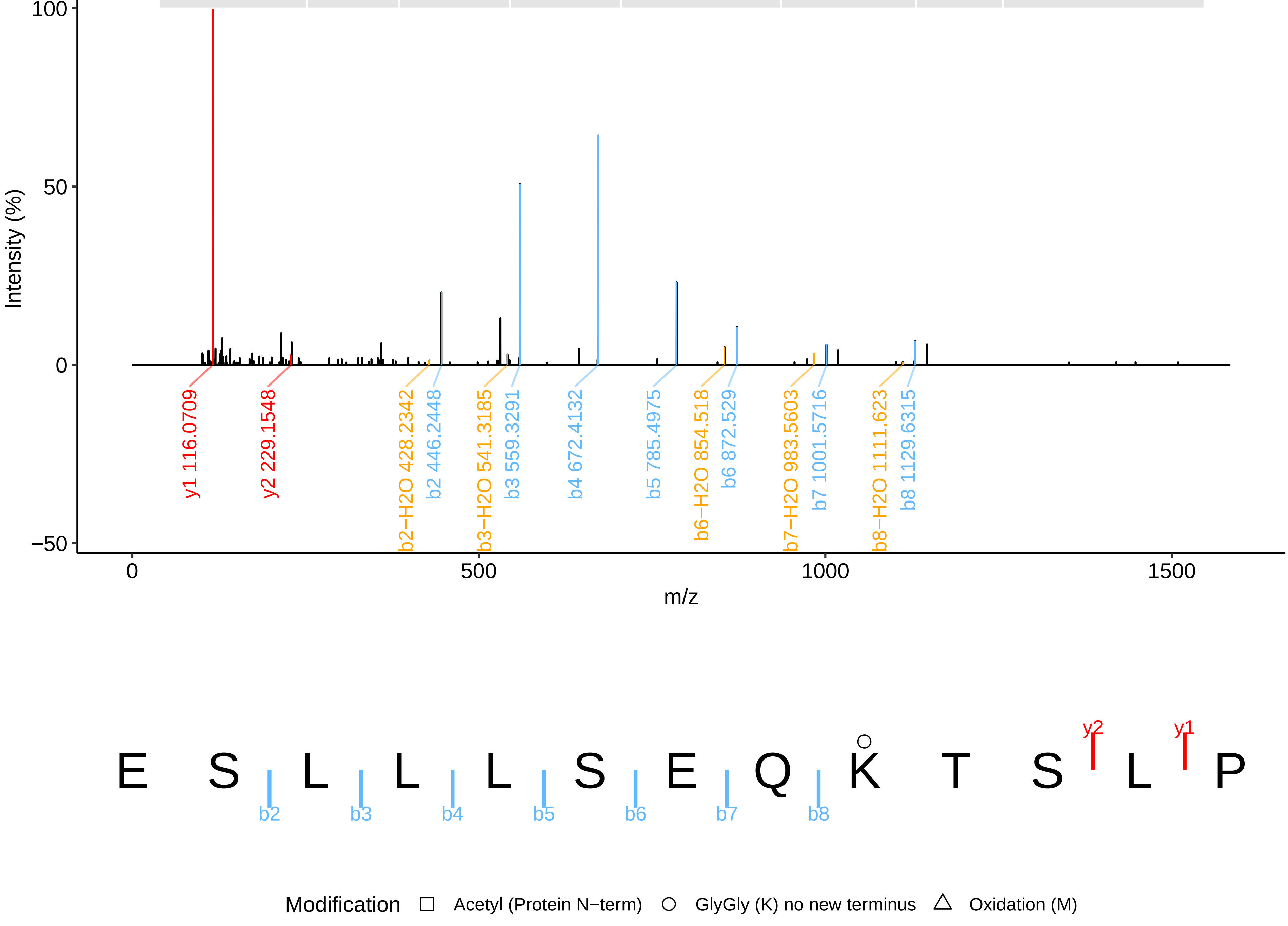

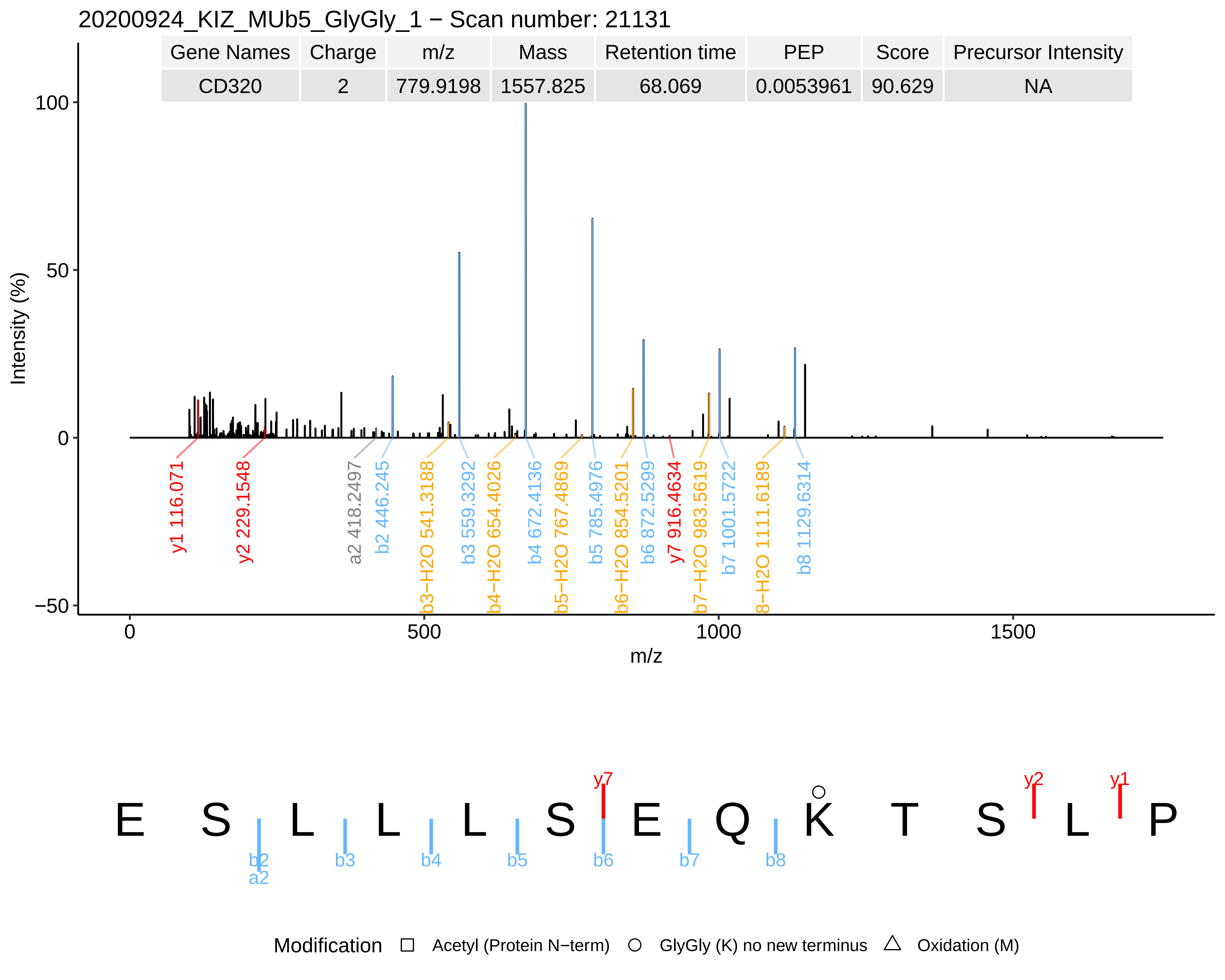
20200924_KIZ_MUb5_GlyGly_1 − Scan number: 21131
Gene Names | Charge | m/z | Mass | Retention time | PEP | Score | Precursor Intensity
CD320 | 2 | 779.9198 | 1557.825 | 68.069 | 0.0053961 | 90.629 | NA
Intensity (%)
100
50
0
−50
0
500
1000
1500
m/z
y1 116.071
y2 229.1548
a2 418.2497
b2 446.245
b3−H2O 541.3188
b3 559.3292
b4−H2O 654.4026
b4 672.4136
b5−H2O 767.4869
b5 785.4976
b6−H2O 854.5201
b6 872.5299
y7 916.4634
b7−H2O 983.5619
b7 1001.5722
8−H2O 1111.6189
b8 1129.6314
E S L L L S E Q K T S L P
y7
y2
y1
b2
a2
b3
b4
b5
b6
b7
b8
Modification
Acetyl (Protein N−term)
GlyGly (K) no new terminus
Oxidation (M)

20200924_KIZ_MUb5_GlyGly_2 − Scan number: 19255

Gene Names	Charge	m/z	Mass	Retention time	PEP	Score	Precursor Intensity
CD320	2	779.9198	1557.825	71.389	6.227e−05	101.64	3520204

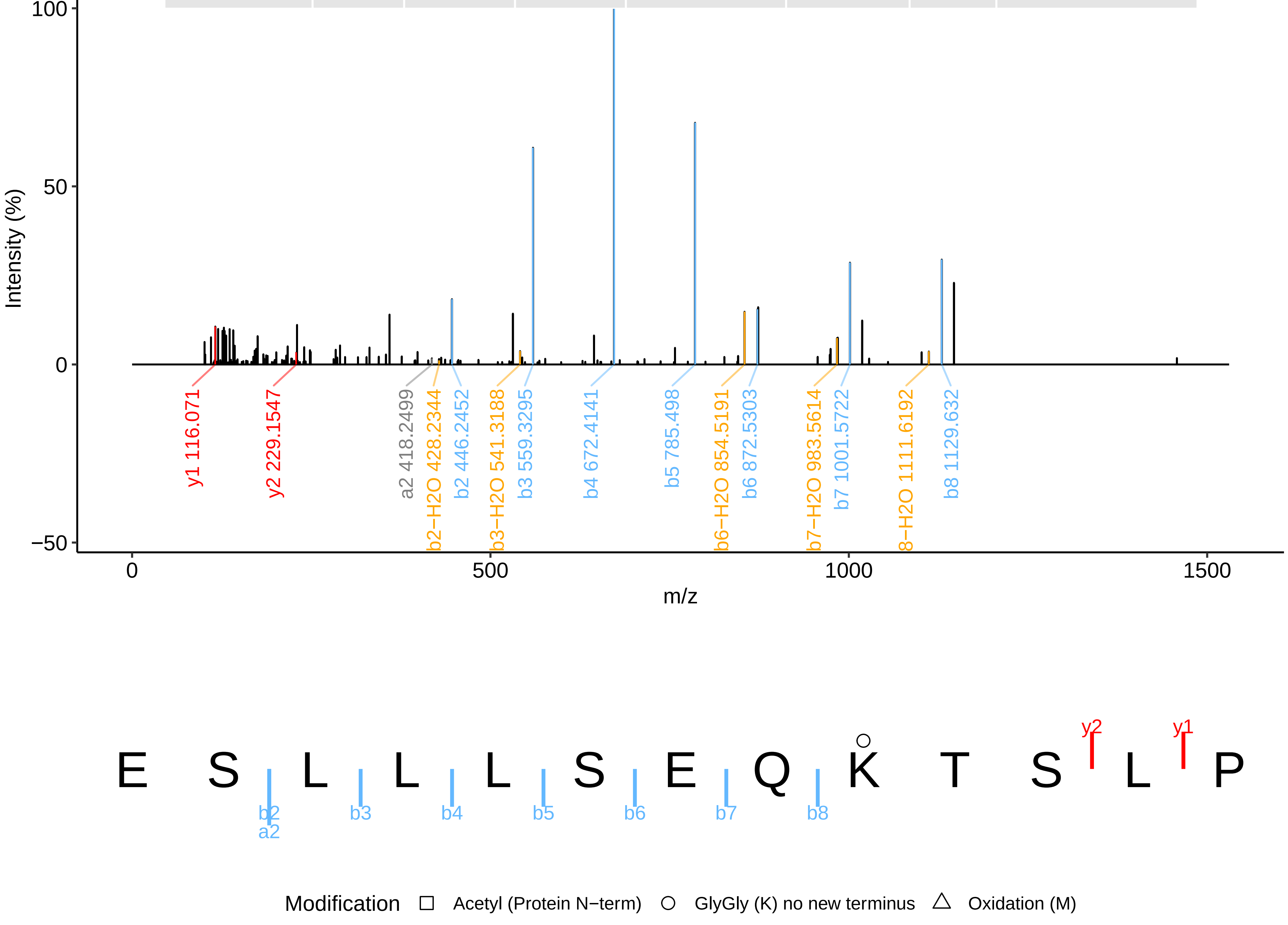

20200924_KIZ_MUb5_GlyGly_2 − Scan number: 19256

Gene Names	Charge	m/z	Mass	Retention time	PEP	Score	Precursor Intensity
CD320	2	779.9198	1557.825	71.394	0.0024499	87.639	3328446

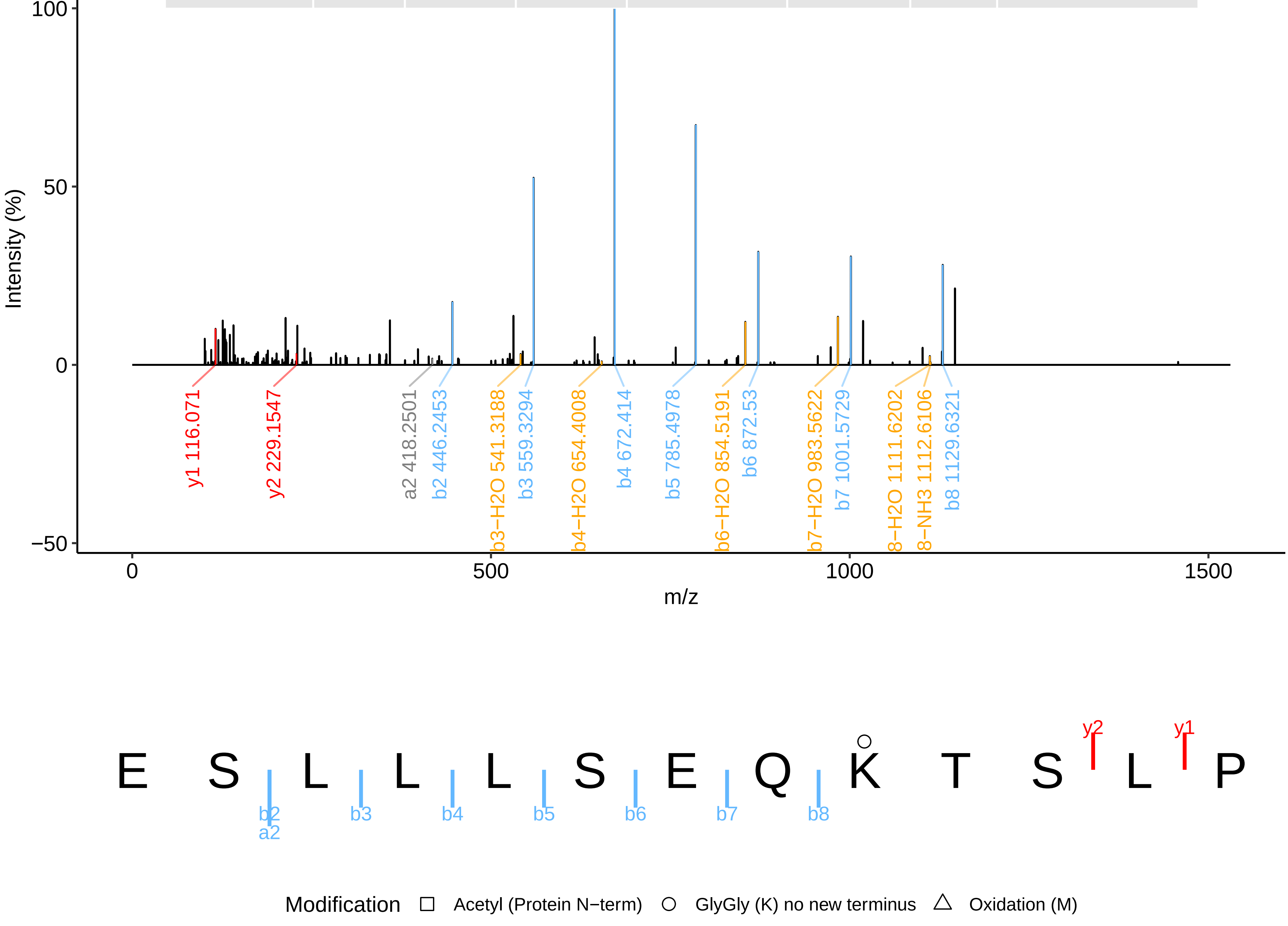

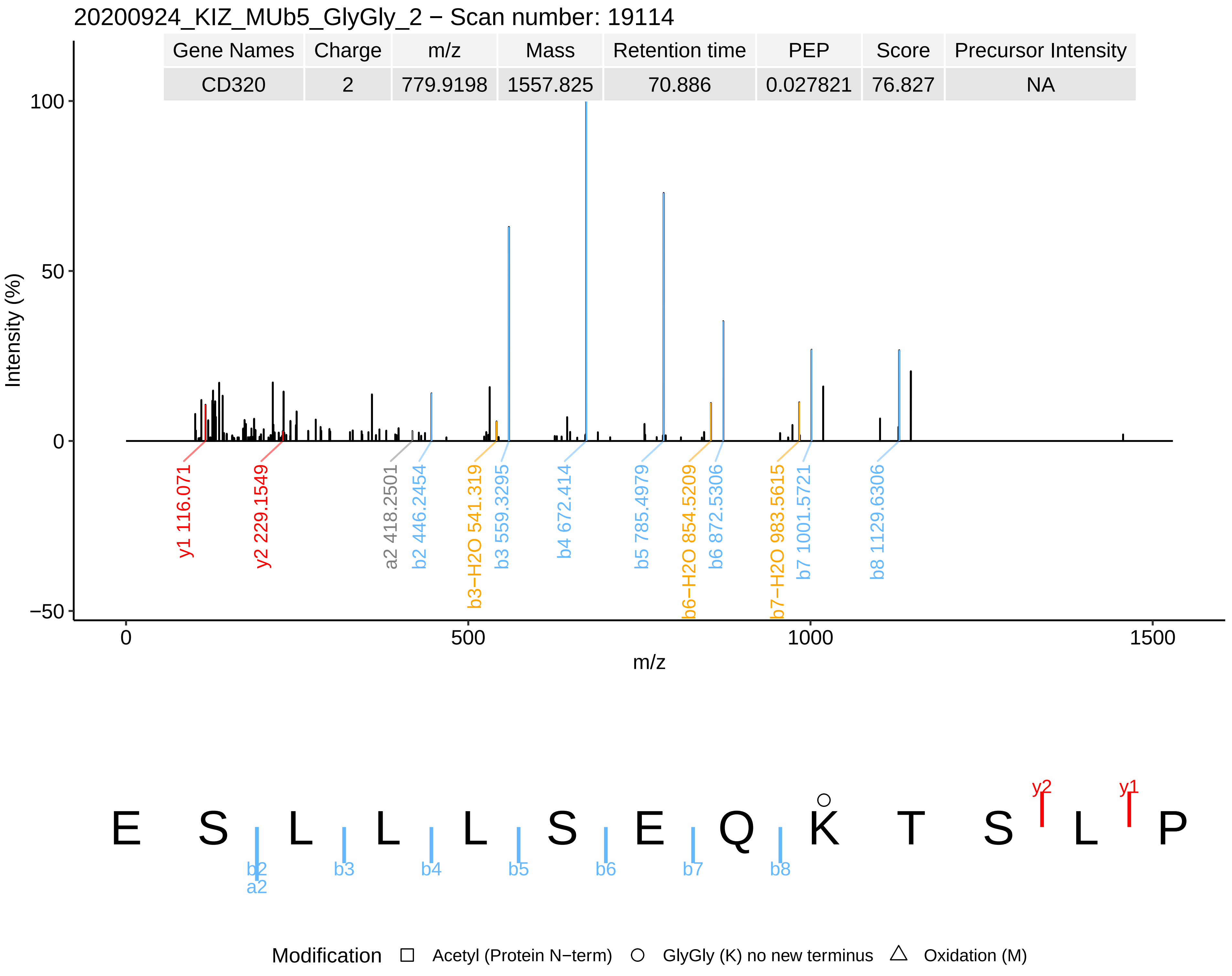
20200924_KIZ_MUb5_GlyGly_2 − Scan number: 19114
Gene Names
Charge
m/z
Mass
Retention time
PEP
Score
Precursor Intensity
CD320
2
779.9198
1557.825
70.886
0.027821
76.827
NA
Intensity (%)
m/z
y1 116.071
y2 229.1549
a2 418.2501
b2 446.2454
b3−H2O 541.319
b3 559.3295
b4 672.414
b5 785.4979
b6−H2O 854.5209
b6 872.5306
b7−H2O 983.5615
b7 1001.5721
b8 1129.6306
E S L L L S E Q K T S L P
b2
a2
b3
b4
b5
b6
b7
b8
y2
y1
Modification
Acetyl (Protein N−term)
GlyGly (K) no new terminus
Oxidation (M)

20200921_KIZ_MUb4_GlyGly_10plex_2 − Scan number: 9013

Gene Names	Charge	m/z	Mass	Retention time	PEP	Score	Precursor Intensity
HPD	4	335.1938	1336.746	34.14	0.00091967	86.898	1160674

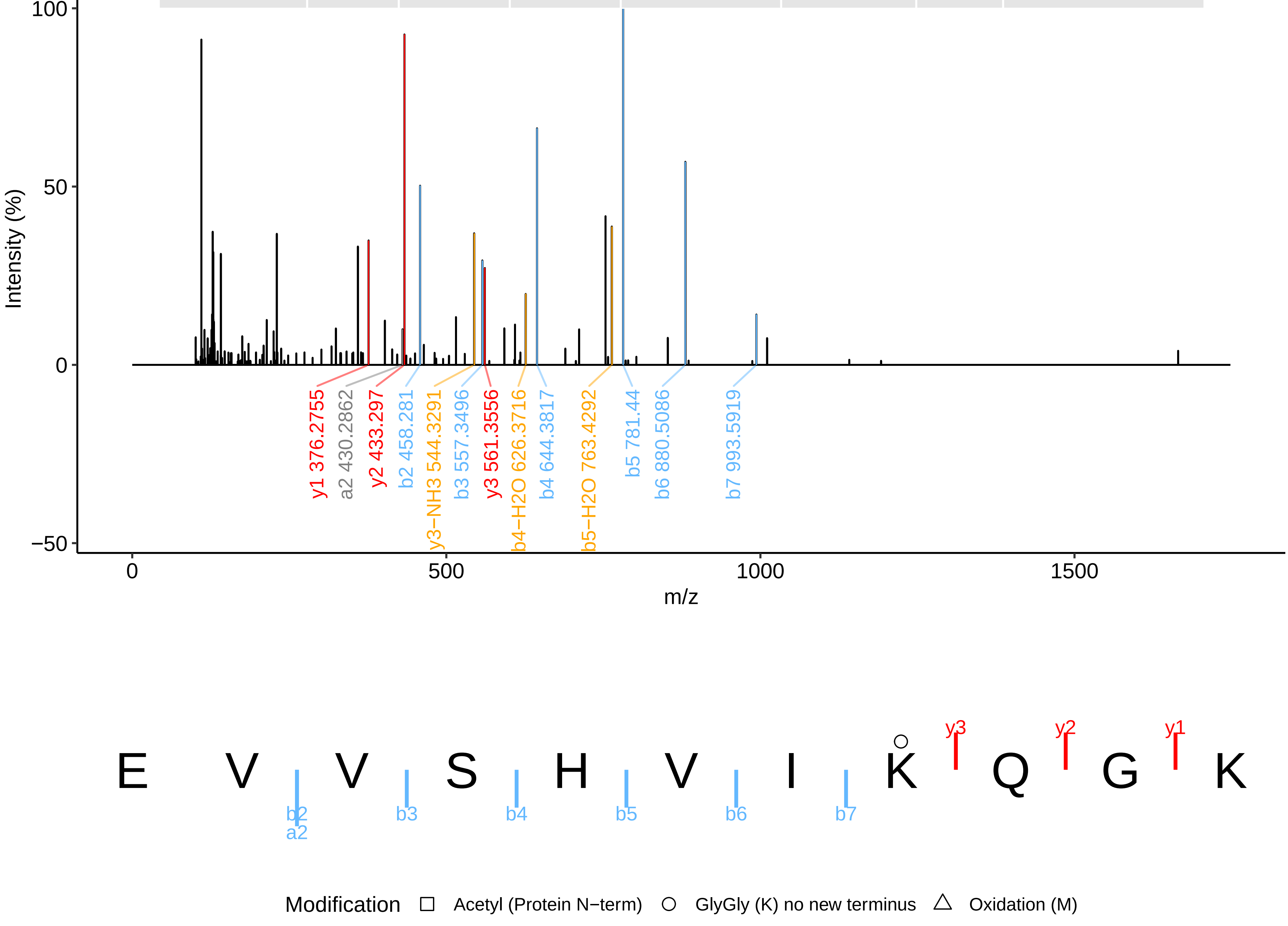

20200921_KIZ_MUb4_GlyGly_10plex − Scan number: 21504

Gene Names	Charge	m/z	Mass	Retention time	PEP	Score	Precursor Intensity
RPS15A	3	450.2418	1347.703	77.891	0.015577	69.979	NA

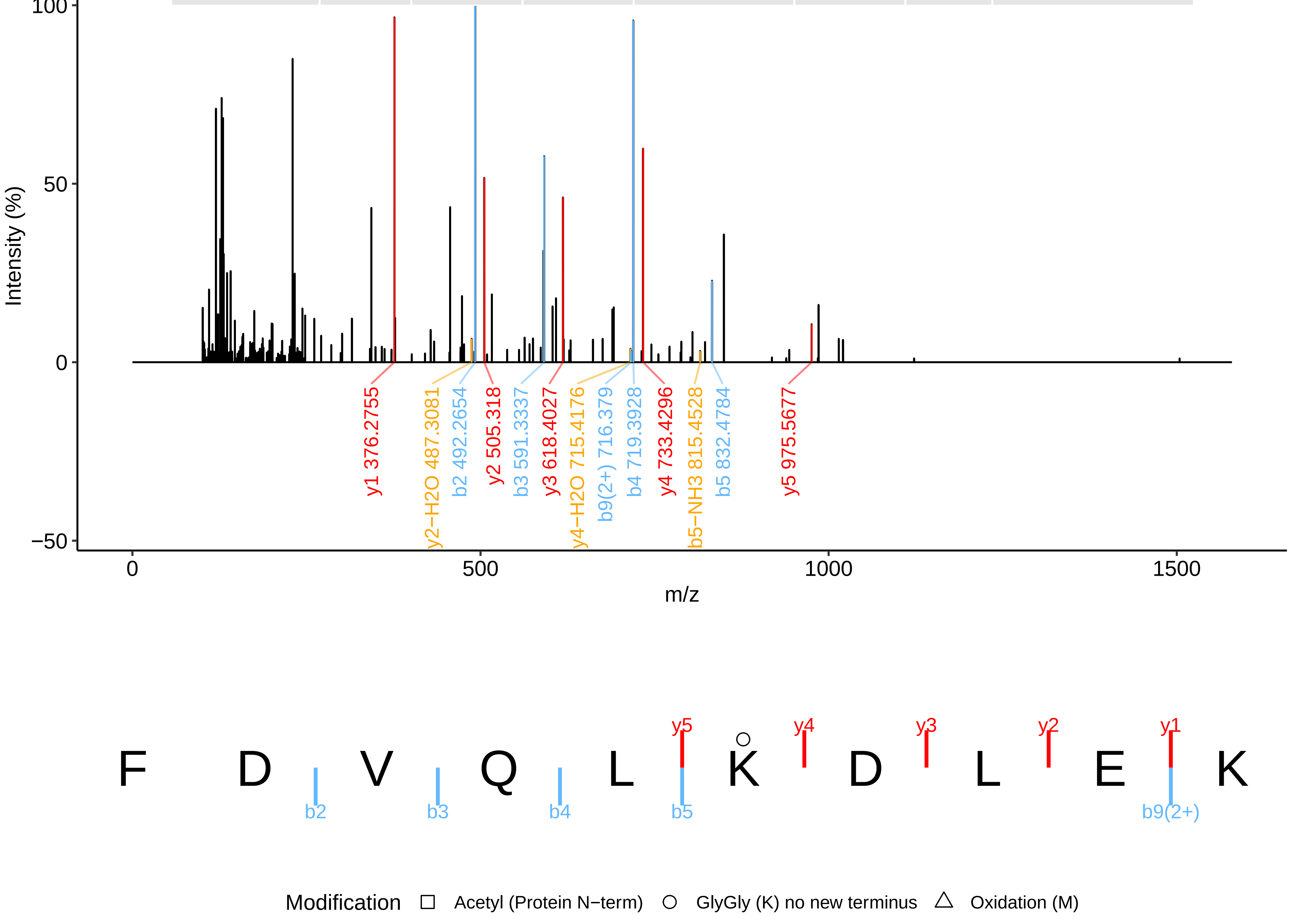

20200921_KIZ_MUb4_GlyGly_10plex_2 − Scan number: 19672

Gene Names	Charge	m/z	Mass	Retention time	PEP	Score	Precursor Intensity
RPS15A	3	450.2418	1347.703	77.239	0.034417	60.434	1391210

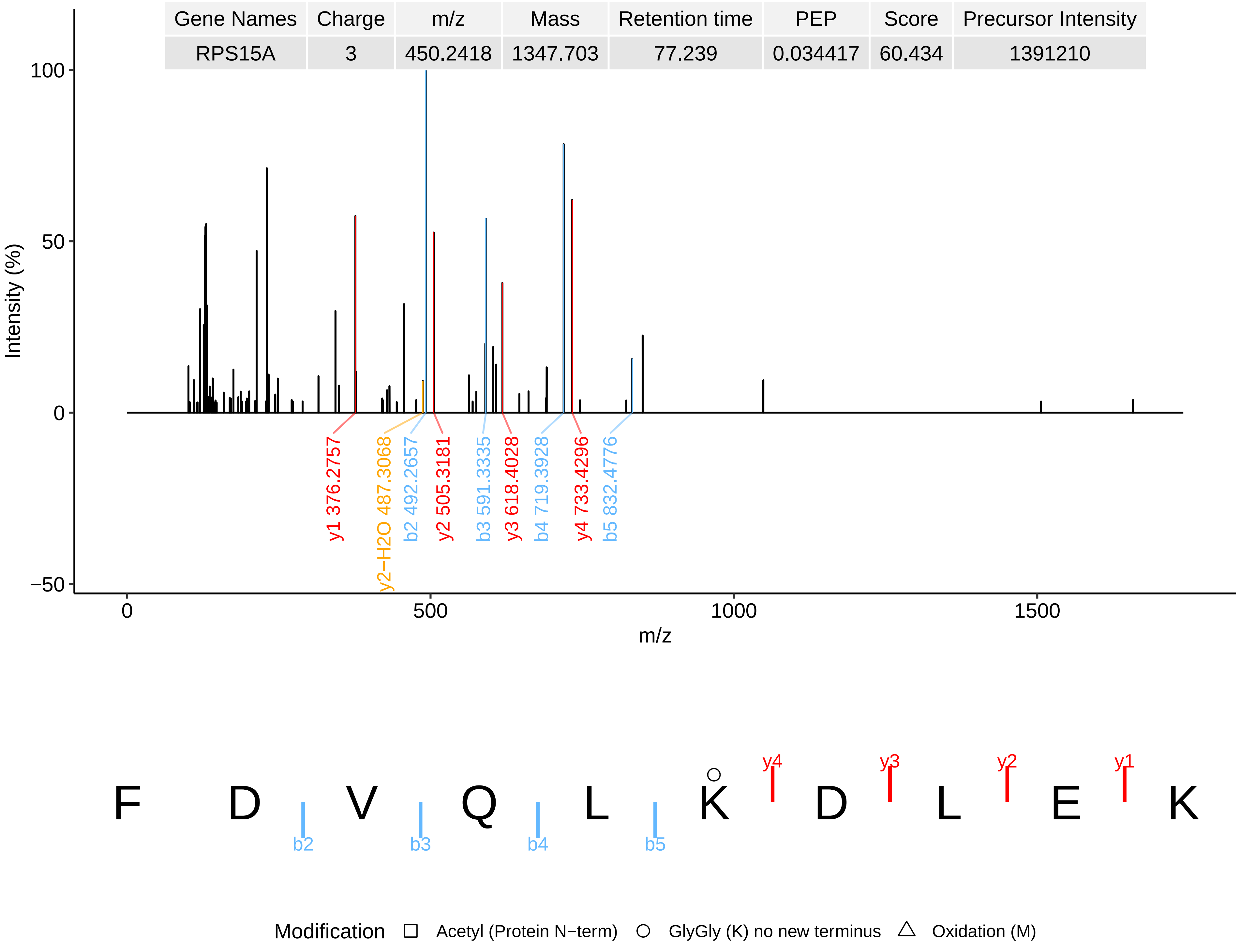

20200921_KIZ_MUb4_GlyGly_10plex − Scan number: 9009

Gene Names	Charge	m/z	Mass	Retention time	PEP	Score	Precursor Intensity
FIS1	3	451.565	1351.673	30.771	0.010971	66.004	5388517

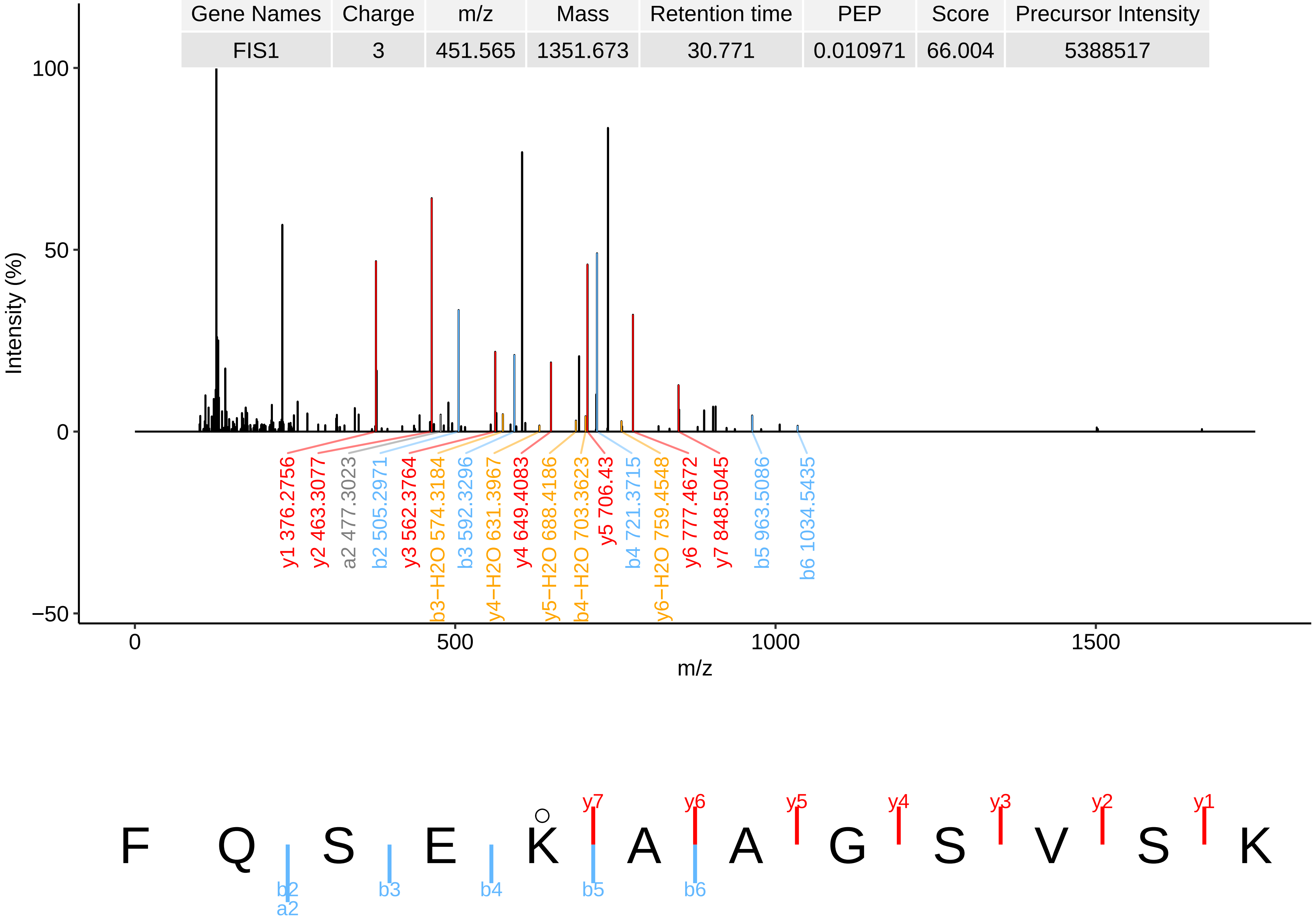

Modification □ Acetyl (Protein N−term) ○ GlyGly (K) no new terminus △ Oxidation (M)

20200921_KIZ_MUb4_GlyGly_10plex_2 − Scan number: 8349

Gene Names	Charge	m/z	Mass	Retention time	PEP	Score	Precursor Intensity
FIS1	3	451.565	1351.673	31.428	0.00097173	84.169	2900674

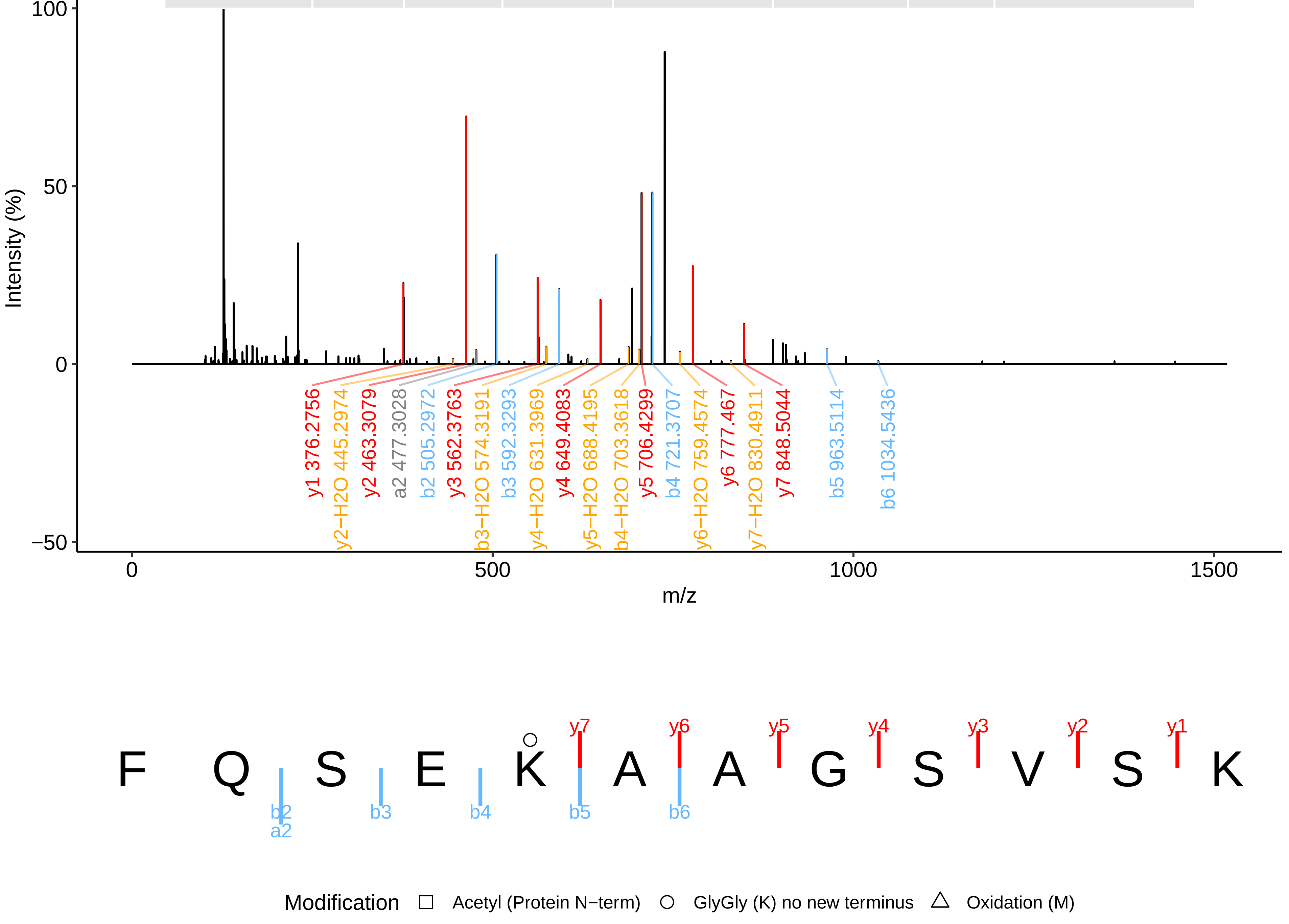

20200924_KIZ_MUb5_GlyGly_1 − Scan number: 7912

Gene Names	Charge	m/z	Mass	Retention time	PEP	Score	Precursor Intensity
FIS1	3	451.565	1351.673	25.206	0.0078824	70.622	3906729

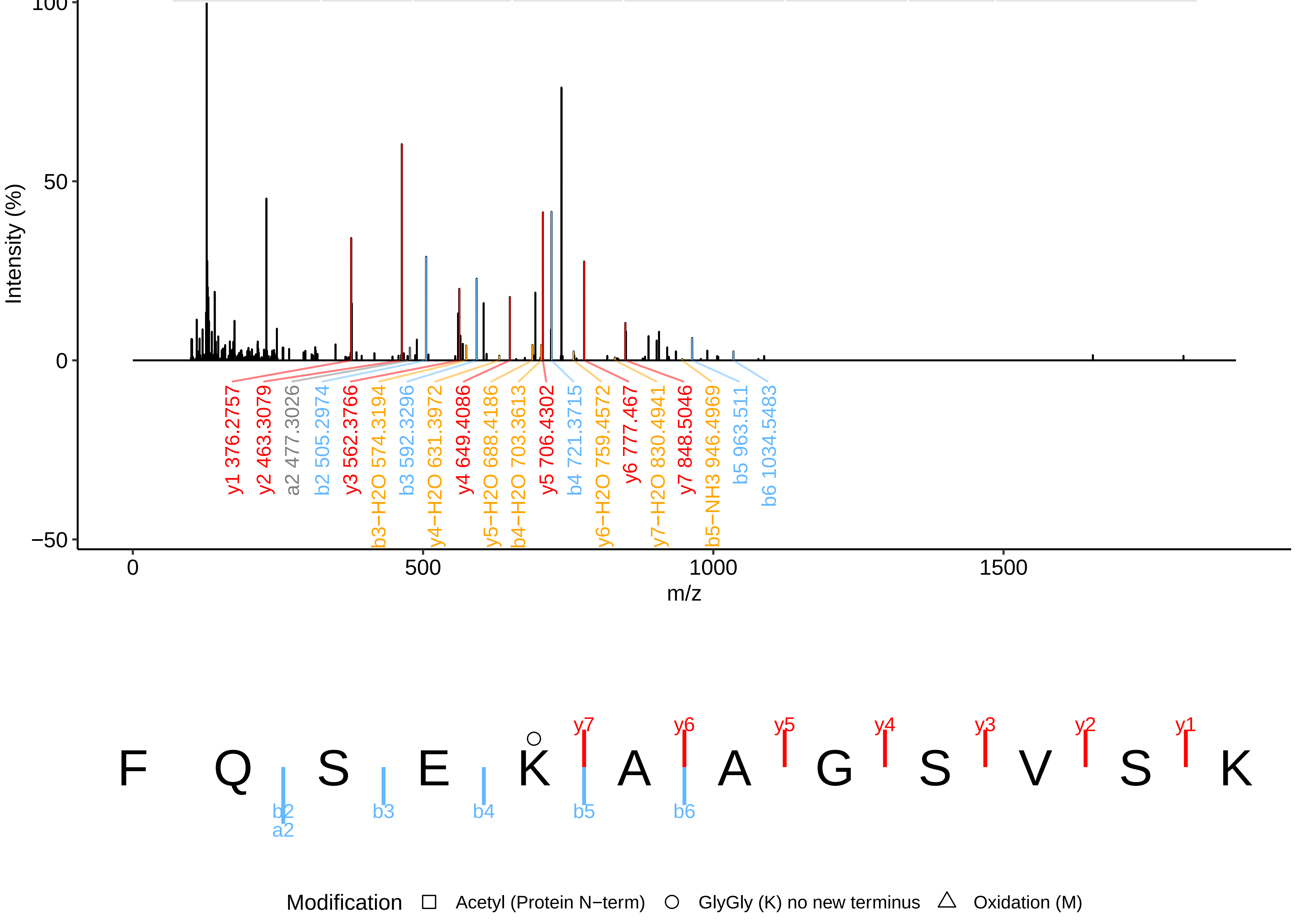

20200924_KIZ_MUb5_GlyGly_1 − Scan number: 8020

Gene Names	Charge	m/z	Mass	Retention time	PEP	Score	Precursor Intensity
FIS1	3	451.565	1351.673	25.553	0.061221	53.869	10924753

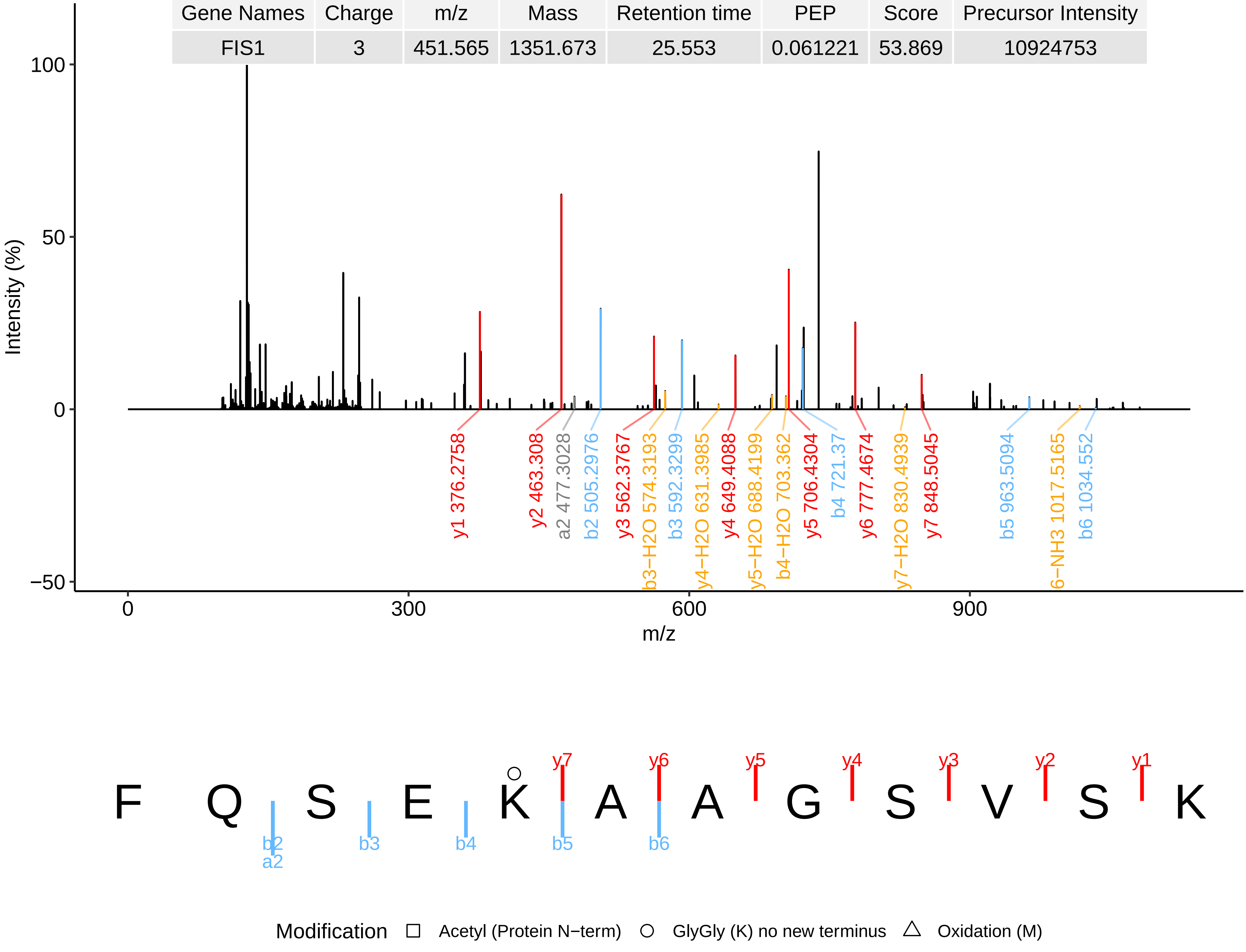

20200924_KIZ_MUb5_GlyGly_1 − Scan number: 8170

Gene Names	Charge	m/z	Mass	Retention time	PEP	Score	Precursor Intensity
FIS1	3	451.565	1351.673	26.062	0.00083458	89.43	22125538

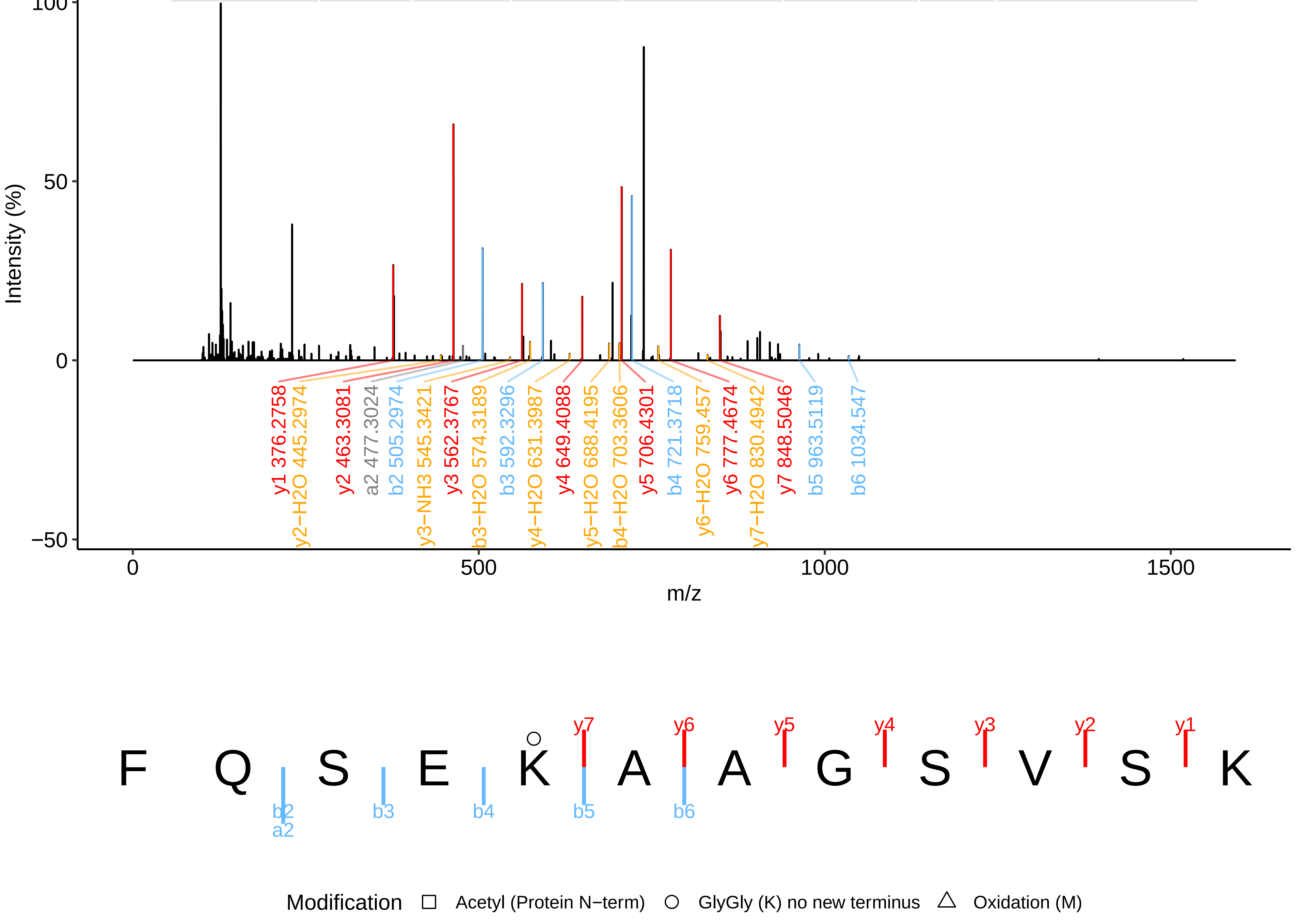

20200924_KIZ_MUb5_GlyGly_2 − Scan number: 7497

Gene Names	Charge	m/z	Mass	Retention time	PEP	Score	Precursor Intensity
FIS1	3	451.565	1351.673	27.987	0.061817	53.754	1619266

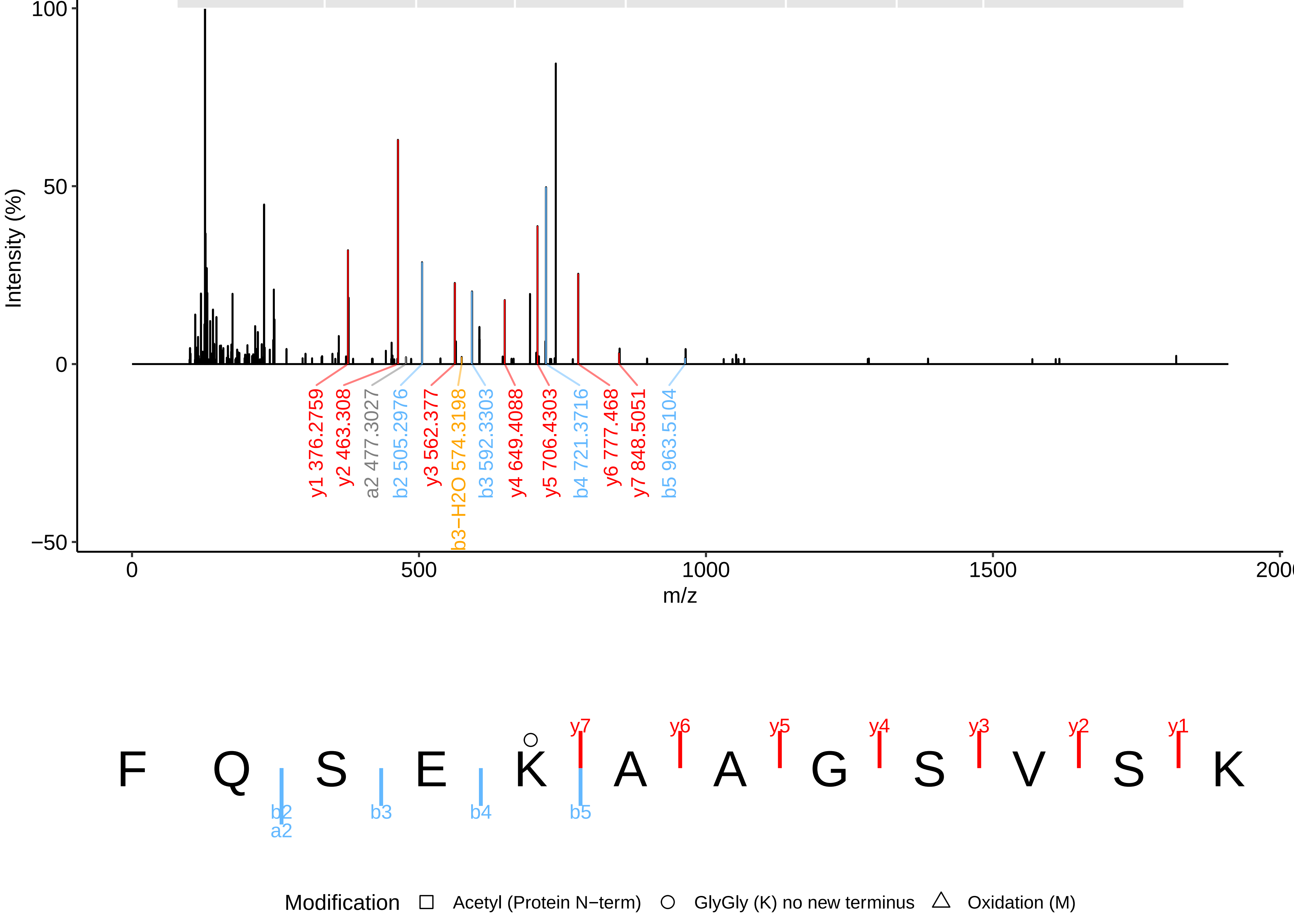

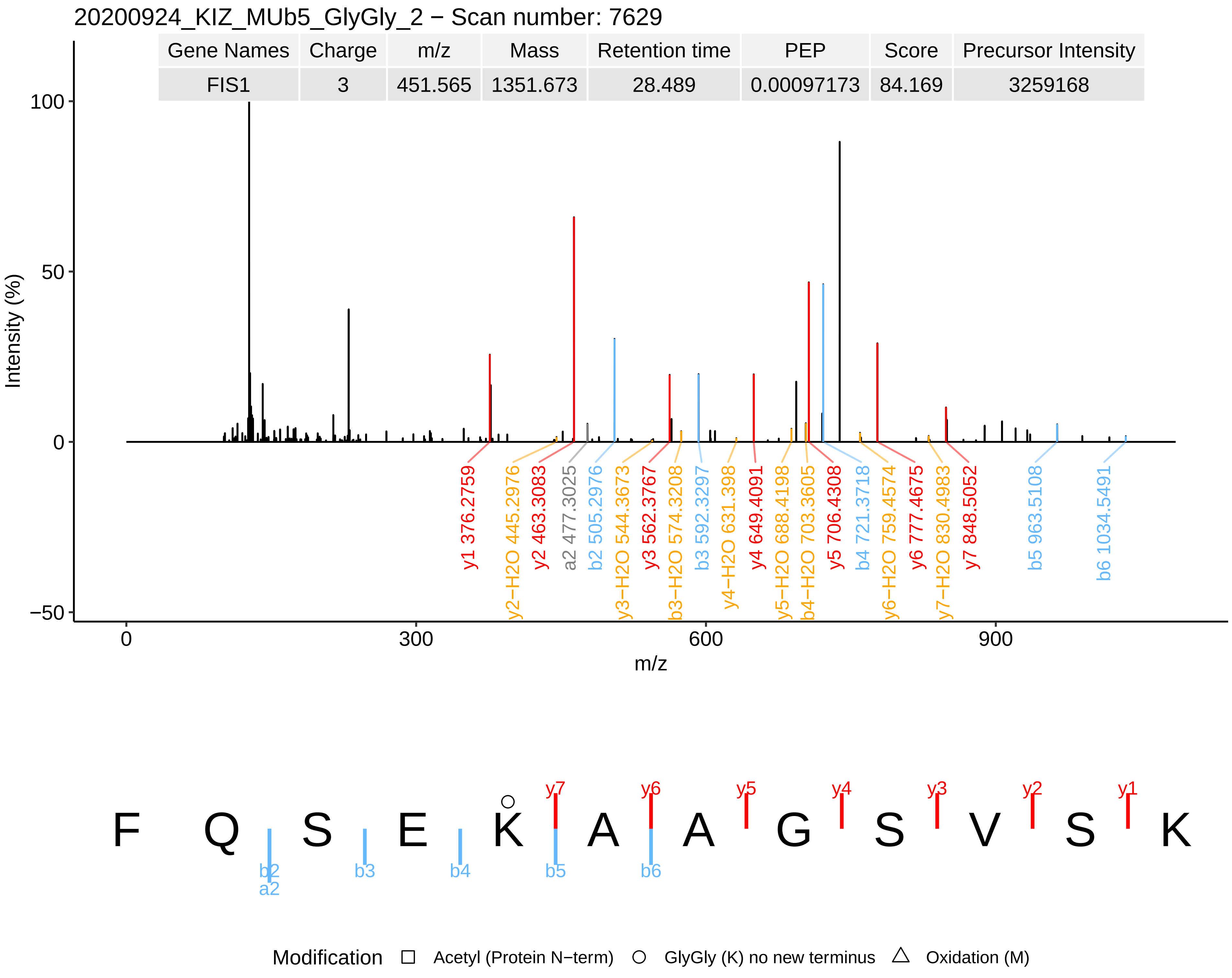
20200924_KIZ_MUb5_GlyGly_2 − Scan number: 7629
Gene Names | Charge | m/z | Mass | Retention time | PEP | Score | Precursor Intensity
FIS1 | 3 | 451.565 | 1351.673 | 28.489 | 0.00097173 | 84.169 | 3259168
Intensity (%)
100
50
0
−50
0
300
600
900
m/z
y1 376.2759
y2−H2O 445.2976
y2 463.3083
a2 477.3025
b2 505.2976
y3−H2O 544.3673
y3 562.3767
b3−H2O 574.3208
b3 592.3297
y4−H2O 631.398
y4 649.4091
y5−H2O 688.4198
b4−H2O 703.3605
y5 706.4308
b4 721.3718
y6−H2O 759.4574
y6 777.4675
y7−H2O 830.4983
y7 848.5052
b5 963.5108
b6 1034.5491
F Q S E K A A G S V S K
y7 y6 y5 y4 y3 y2 y1
b2 a2 b3 b4 b5 b6
Modification
Acetyl (Protein N−term)
GlyGly (K) no new terminus
Oxidation (M)

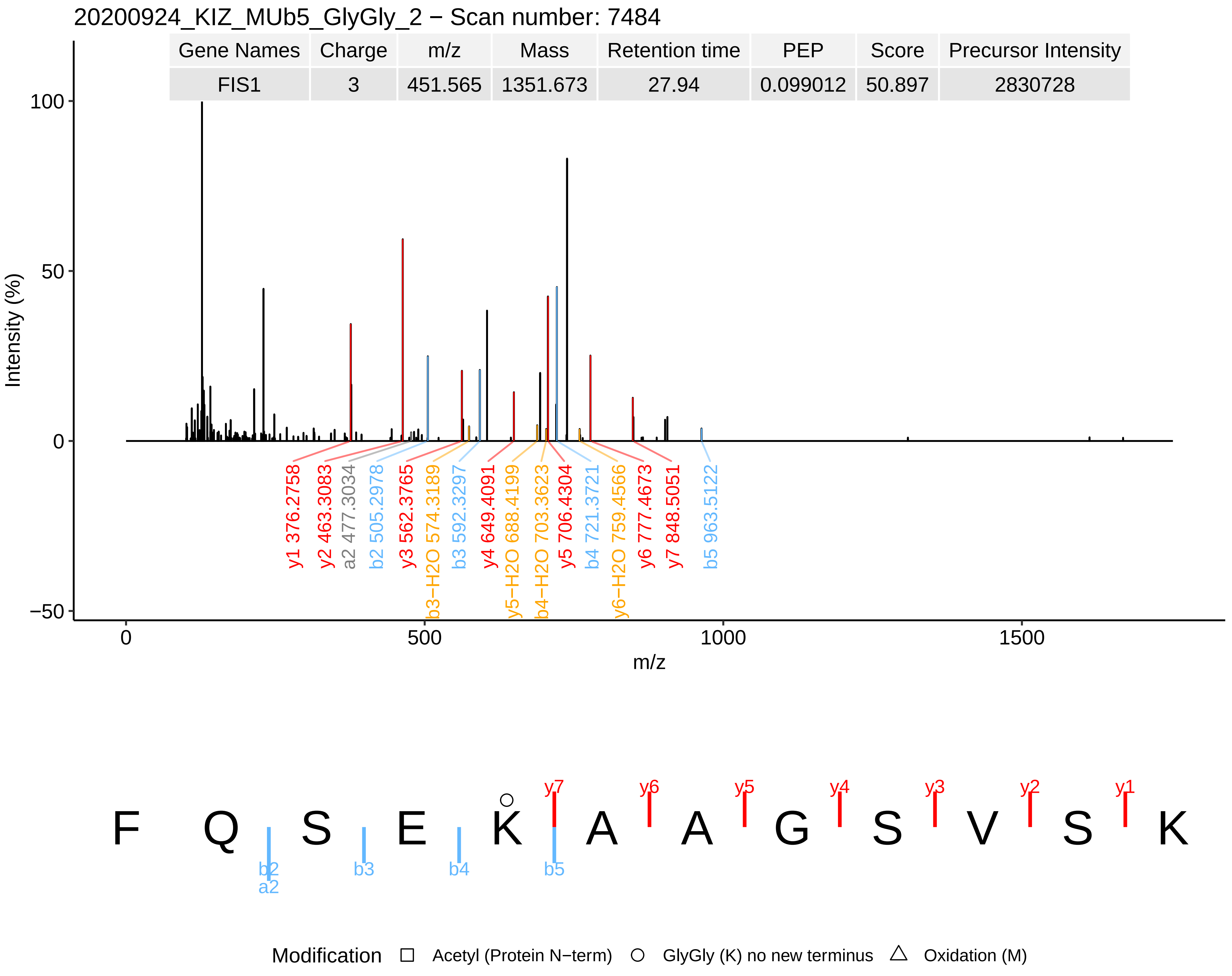
20200924_KIZ_MUb5_GlyGly_2 − Scan number: 7484
Gene Names | Charge | m/z | Mass | Retention time | PEP | Score | Precursor Intensity
FIS1 | 3 | 451.565 | 1351.673 | 27.94 | 0.099012 | 50.897 | 2830728
Intensity (%)
100
50
0
−50
m/z
0
500
1000
1500
y1 376.2758
y2 463.3083
a2 477.3034
b2 505.2978
y3 562.3765
b3−H2O 574.3189
b3 592.3297
y4 649.4091
y5−H2O 688.4199
b4−H2O 703.3623
y5 706.4304
b4 721.3721
y6−H2O 759.4566
y6 777.4673
y7 848.5051
b5 963.5122
F Q S E K A A G S V S K
y7 y6 y5 y4 y3 y2 y1
b2 a2 b3 b4 b5
Modification
Acetyl (Protein N−term)
GlyGly (K) no new terminus
Oxidation (M)

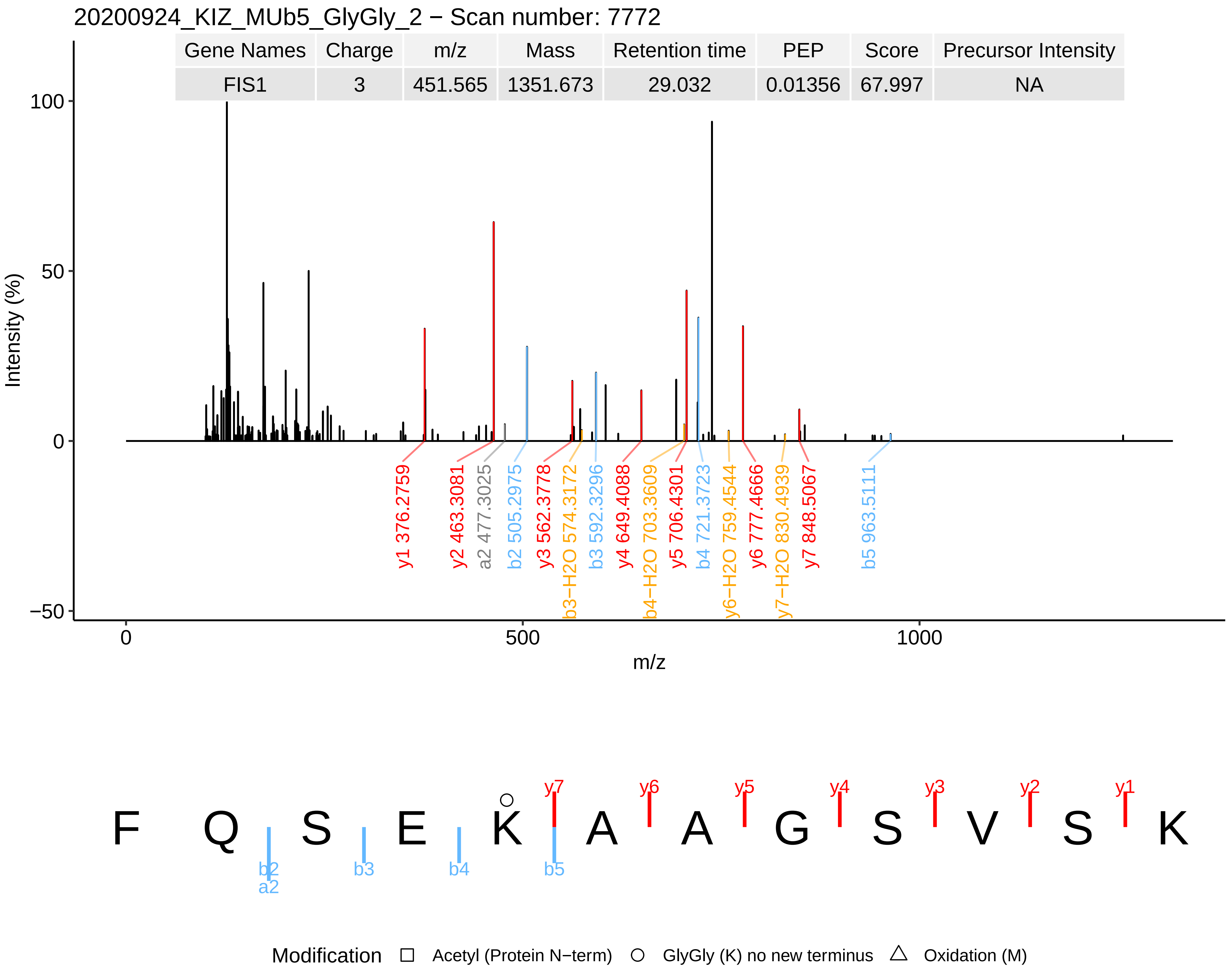
20200924_KIZ_MUb5_GlyGly_2 − Scan number: 7772
Gene Names | Charge | m/z | Mass | Retention time | PEP | Score | Precursor Intensity
FIS1 | 3 | 451.565 | 1351.673 | 29.032 | 0.01356 | 67.997 | NA
Intensity (%)
m/z
y1 376.2759
y2 463.3081
a2 477.3025
b2 505.2975
y3 562.3778
b3−H2O 574.3172
b3 592.3296
y4 649.4088
b4−H2O 703.3609
y5 706.4301
b4 721.3723
y6−H2O 759.4544
y6 777.4666
y7−H2O 830.4939
y7 848.5067
b5 963.5111
F Q S E K A A G S V S K
Modification □ Acetyl (Protein N−term) ○ GlyGly (K) no new terminus △ Oxidation (M)

20201118_KIZ_MUb6_GlyGly_1 − Scan number: 7705

Gene Names	Charge	m/z	Mass	Retention time	PEP	Score	Precursor Intensity
FIS1	3	451.565	1351.673	24.414	0.0033667	78.486	16298153

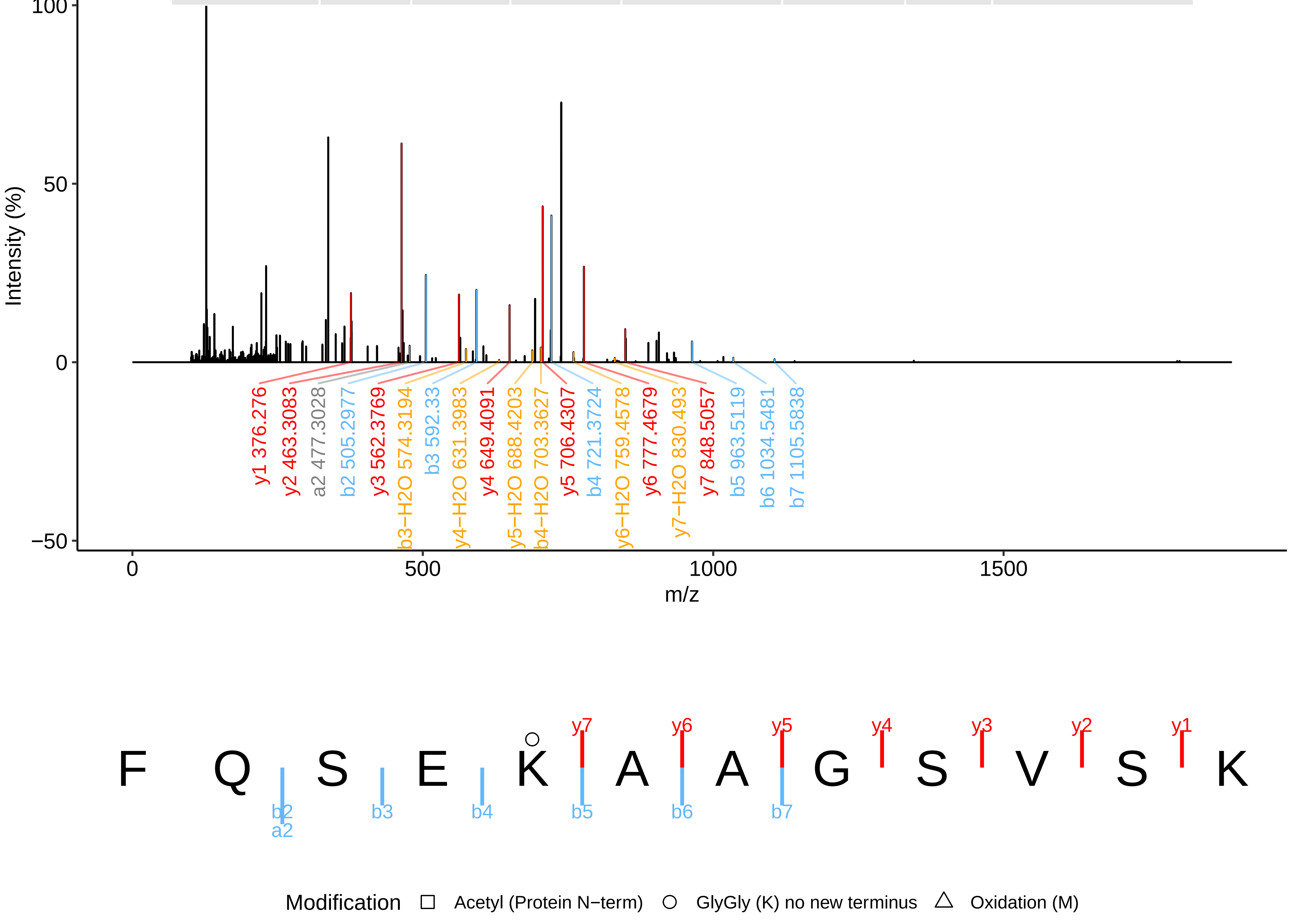

20200921_KIZ_MUb4_GlyGly_10plex − Scan number: 15048

Gene Names	Charge	m/z	Mass	Retention time	PEP	Score	Precursor Intensity
CD97	3	441.5518	1321.634	53.663	0.01311	83.869	20073748

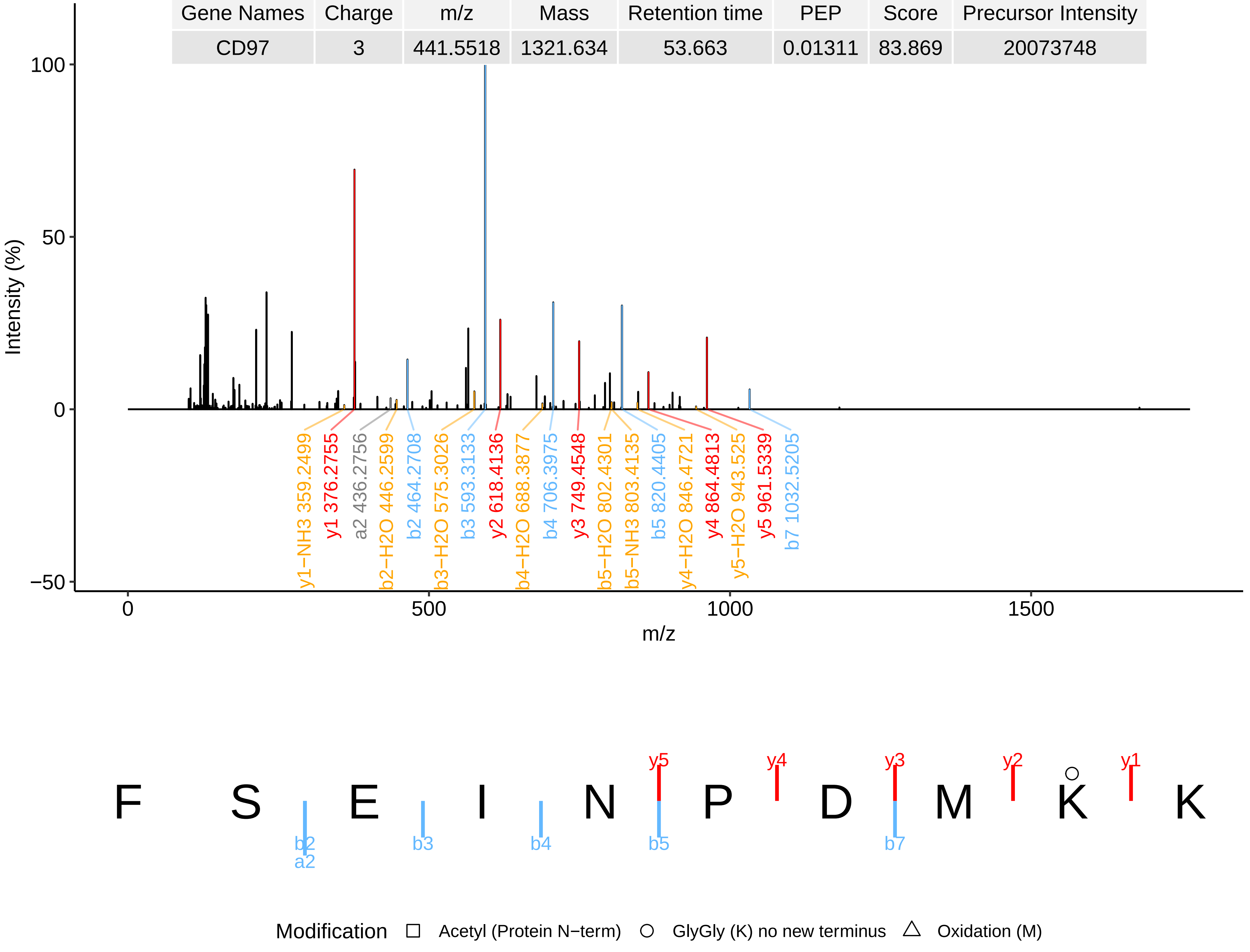

20200921_KIZ_MUb4_GlyGly_10plex_2 − Scan number: 13888

Gene Names	Charge	m/z	Mass	Retention time	PEP	Score	Precursor Intensity
CD97	3	441.5518	1321.634	54.021	0.063423	73.067	655037

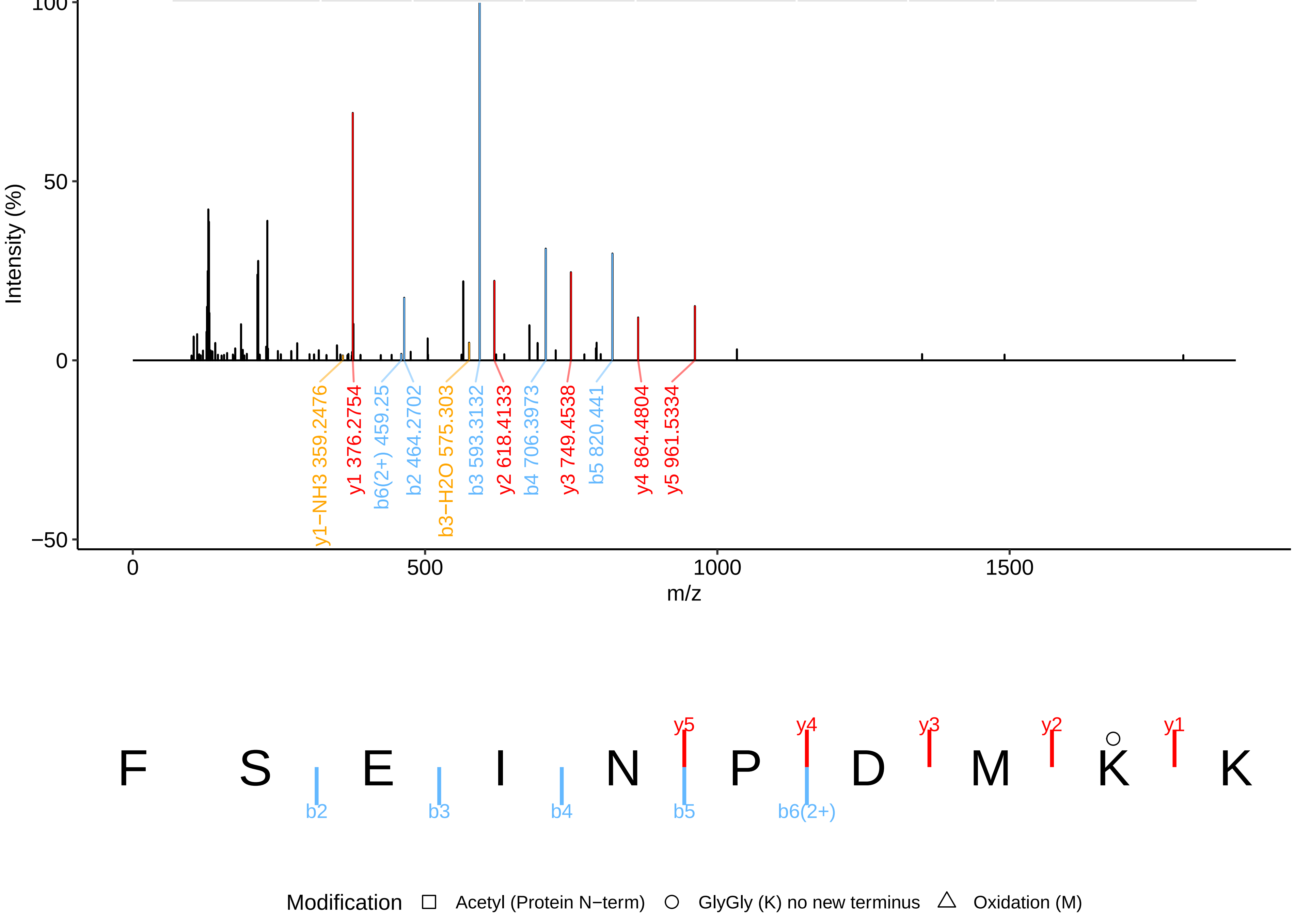

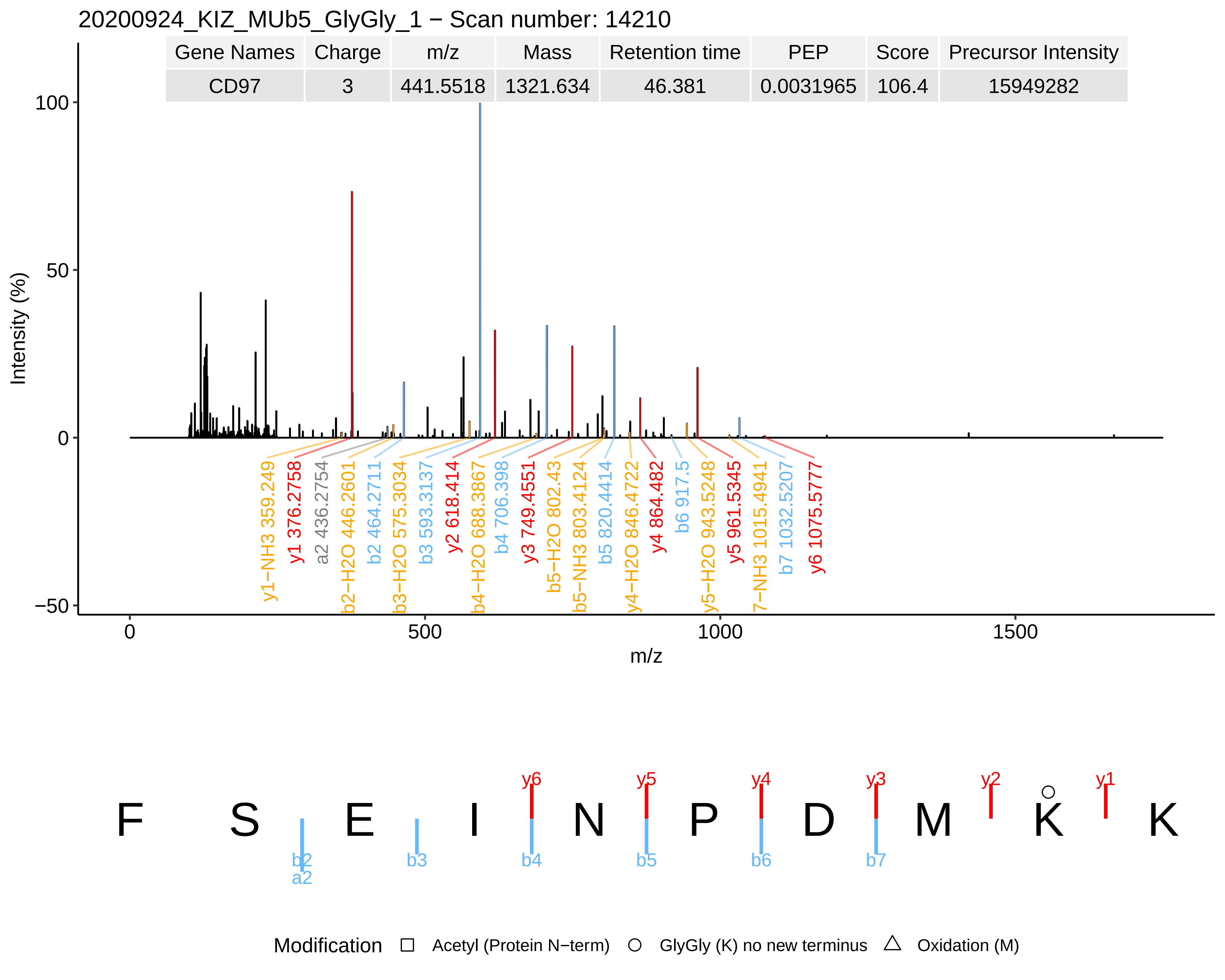
20200924_KIZ_MUb5_GlyGly_1 − Scan number: 14210
Gene Names
CD97
Charge
3
m/z
441.5518
Mass
1321.634
Retention time
46.381
PEP
0.0031965
Score
106.4
Precursor Intensity
15949282
Intensity (%)
100
50
0
−50
0
500
1000
1500
m/z
y1−NH3 359.249
y1 376.2758
a2 436.2754
b2−H2O 446.2601
b2 464.2711
b3−H2O 575.3034
b3 593.3137
y2 618.414
b4−H2O 688.3867
b4 706.398
y3 749.4551
b5−H2O 802.43
b5−NH3 803.4124
b5 820.4414
y4−H2O 846.4722
y4 864.482
b6 917.5
y5−H2O 943.5248
y5 961.5345
7−NH3 1015.4941
b7 1032.5207
y6 1075.5777
F S E I N P D M K K
y6
y5
y4
y3
y2
y1
b2
a2
b3
b4
b5
b6
b7
Modification
Acetyl (Protein N−term)
GlyGly (K) no new terminus
Oxidation (M)

20200924_KIZ_MUb5_GlyGly_1 − Scan number: 8416

Gene Names	Charge	m/z	Mass	Retention time	PEP	Score	Precursor Intensity
RAD18	3	458.9053	1373.694	26.906	0.0014959	82.543	6945803

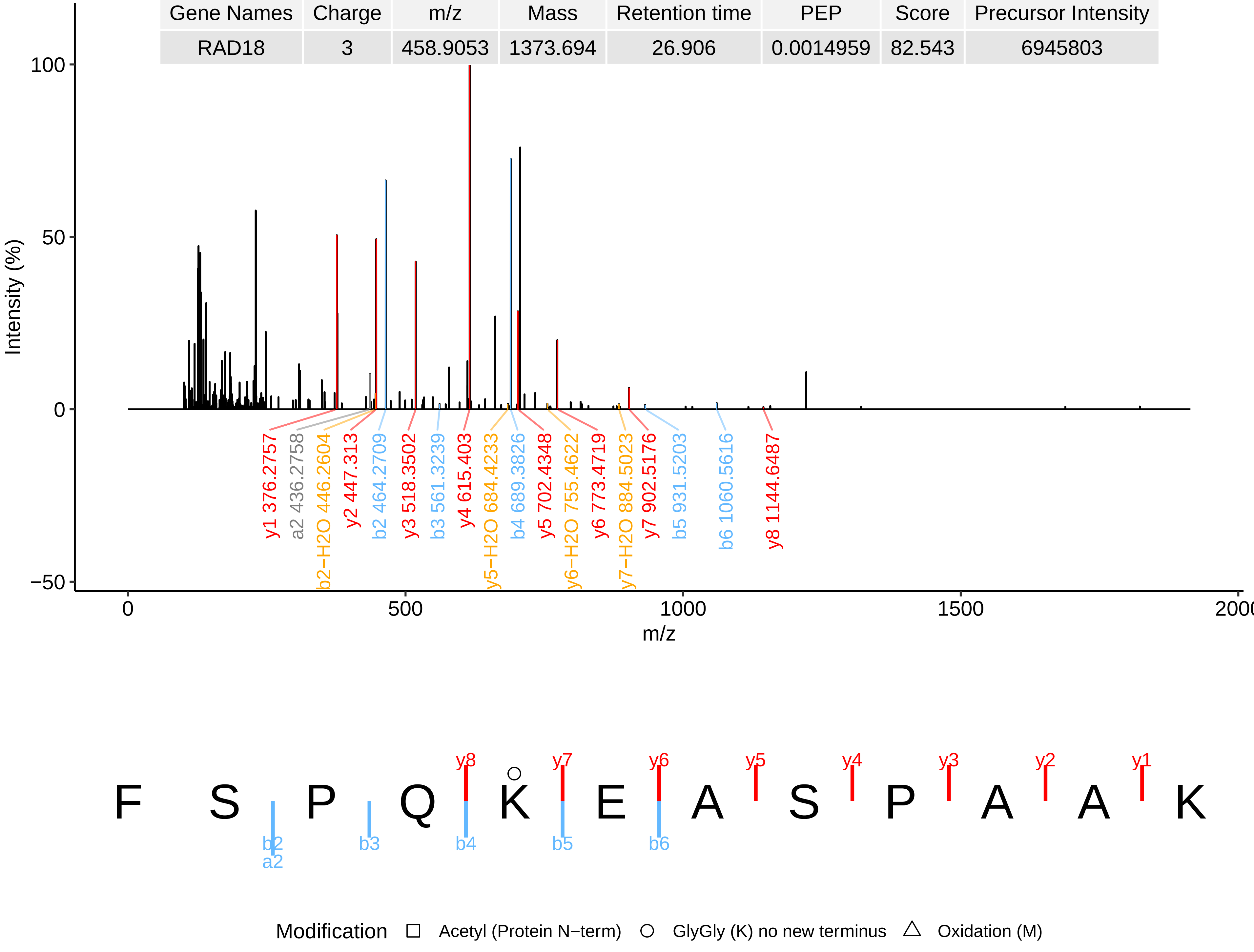

20200921_KIZ_MUb4_GlyGly_10plex − Scan number: 20355

Gene Names	Charge	m/z	Mass	Retention time	PEP	Score	Precursor Intensity
SLC3A2	3	460.2574	1377.75	73.571	0.00066773	91.549	6906878

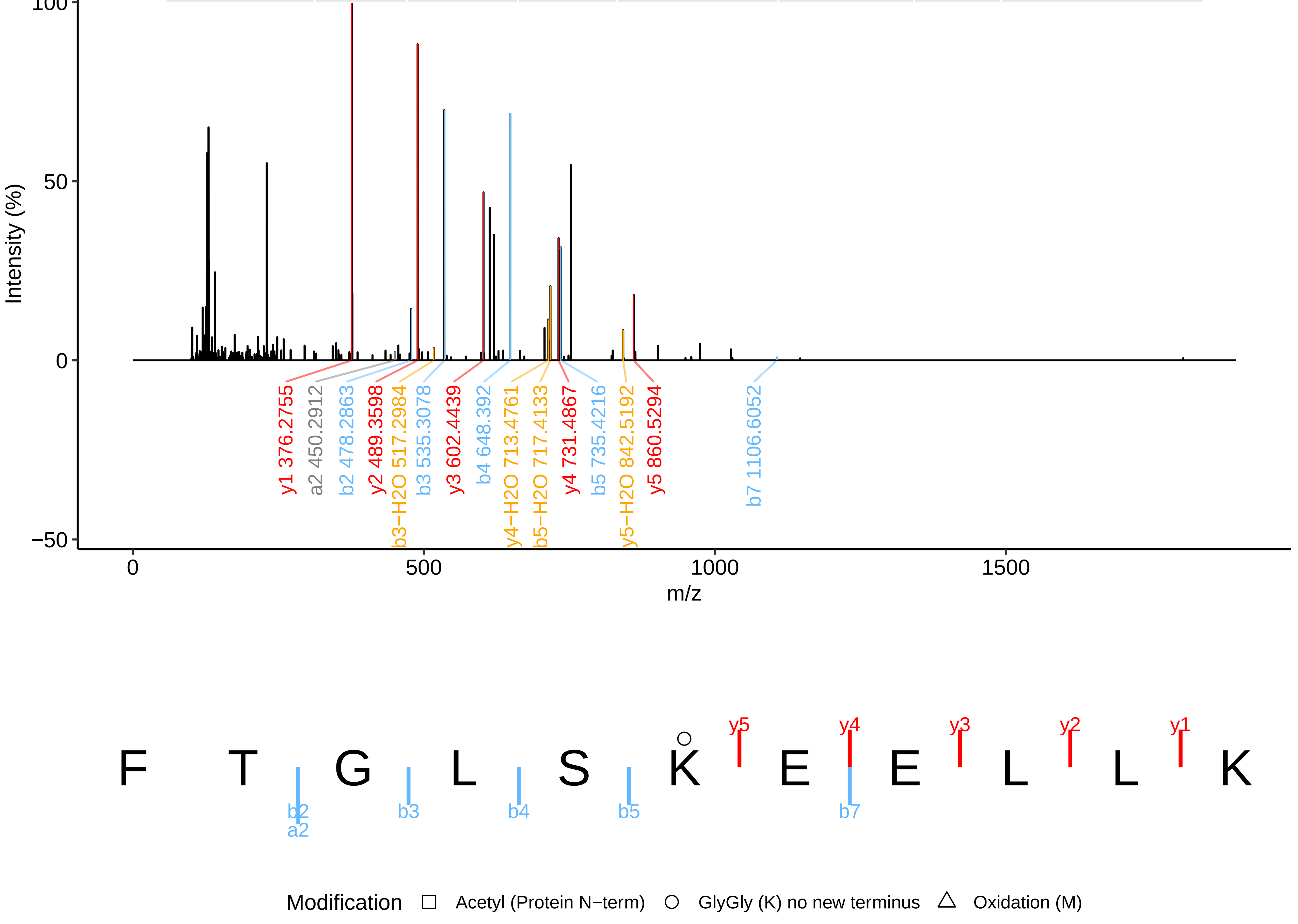

20200921_KIZ_MUb4_GlyGly_10plex − Scan number: 20493

Gene Names	Charge	m/z	Mass	Retention time	PEP	Score	Precursor Intensity
SLC3A2	3	460.2574	1377.75	74.076	0.00075427	90.653	15512029

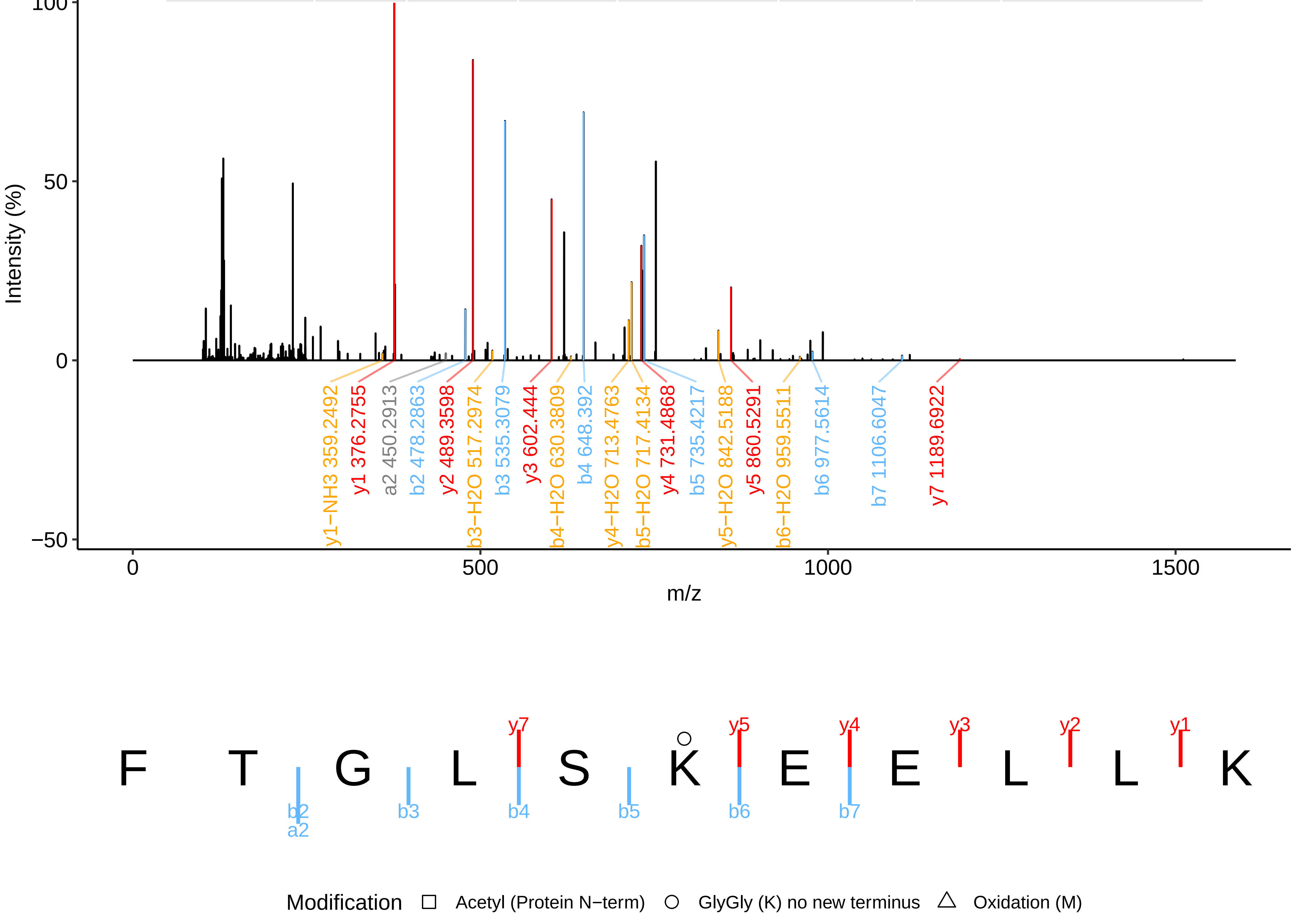

20200921_KIZ_MUb4_GlyGly_10plex − Scan number: 20628

Gene Names	Charge	m/z	Mass	Retention time	PEP	Score	Precursor Intensity
SLC3A2	3	460.2574	1377.75	74.586	0.0017877	84.658	6951082

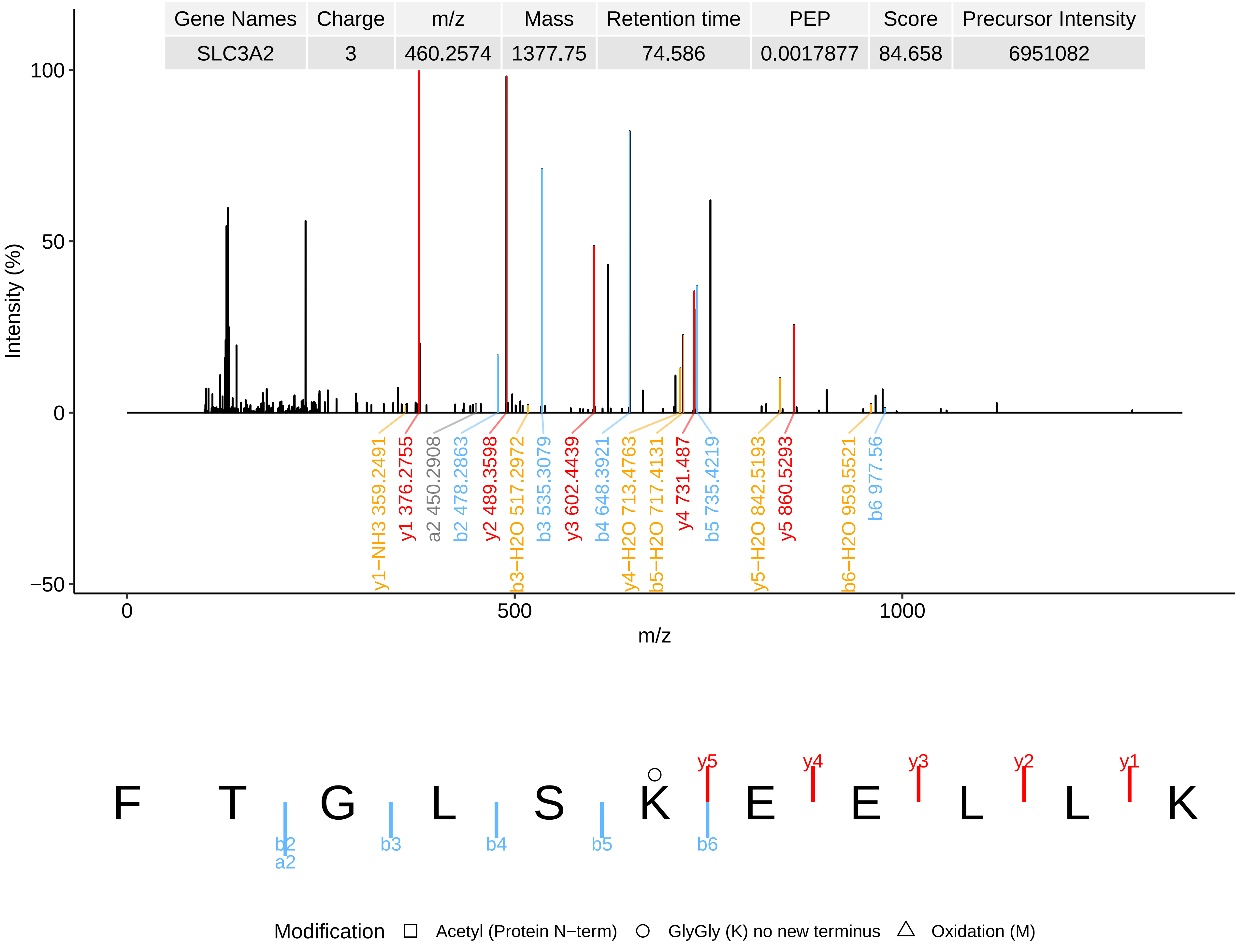

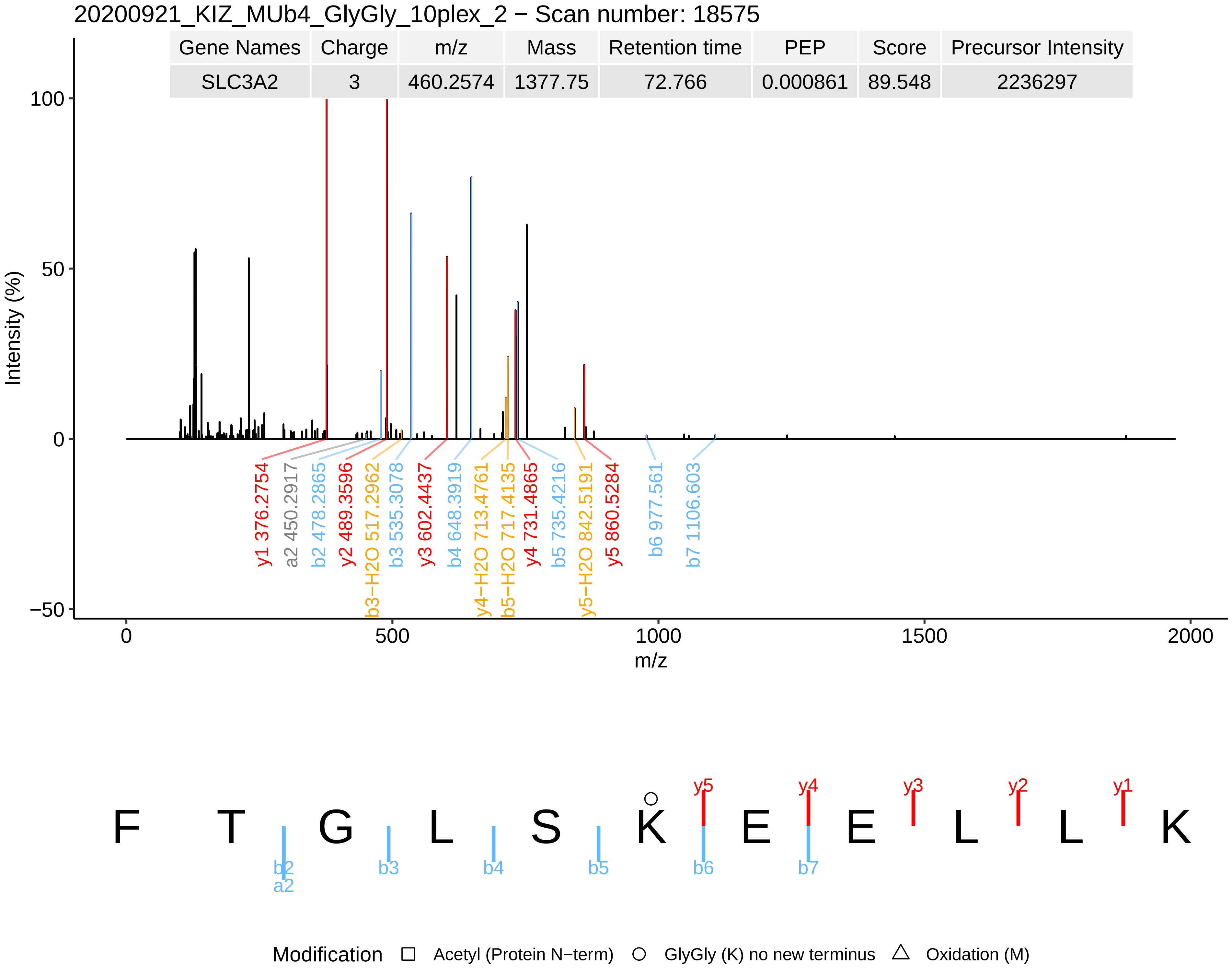
20200921_KIZ_MUb4_GlyGly_10plex_2 − Scan number: 18575
Gene Names | Charge | m/z | Mass | Retention time | PEP | Score | Precursor Intensity
SLC3A2 | 3 | 460.2574 | 1377.75 | 72.766 | 0.000861 | 89.548 | 2236297
Intensity (%)
m/z
y1 376.2754
a2 450.2917
b2 478.2865
y2 489.3596
b3−H2O 517.2962
b3 535.3078
y3 602.4437
b4 648.3919
y4−H2O 713.4761
b5−H2O 717.4135
y4 731.4865
b5 735.4216
y5−H2O 842.5191
y5 860.5284
b6 977.561
b7 1106.603
F T G L S K E E L L K
Modification
Acetyl (Protein N−term)
GlyGly (K) no new terminus
Oxidation (M)

20200924_KIZ_MUb5_GlyGly_1 − Scan number: 19633

Gene Names	Charge	m/z	Mass	Retention time	PEP	Score	Precursor Intensity
SLC3A2	3	460.2574	1377.75	63.46	0.014878	69.451	13273223

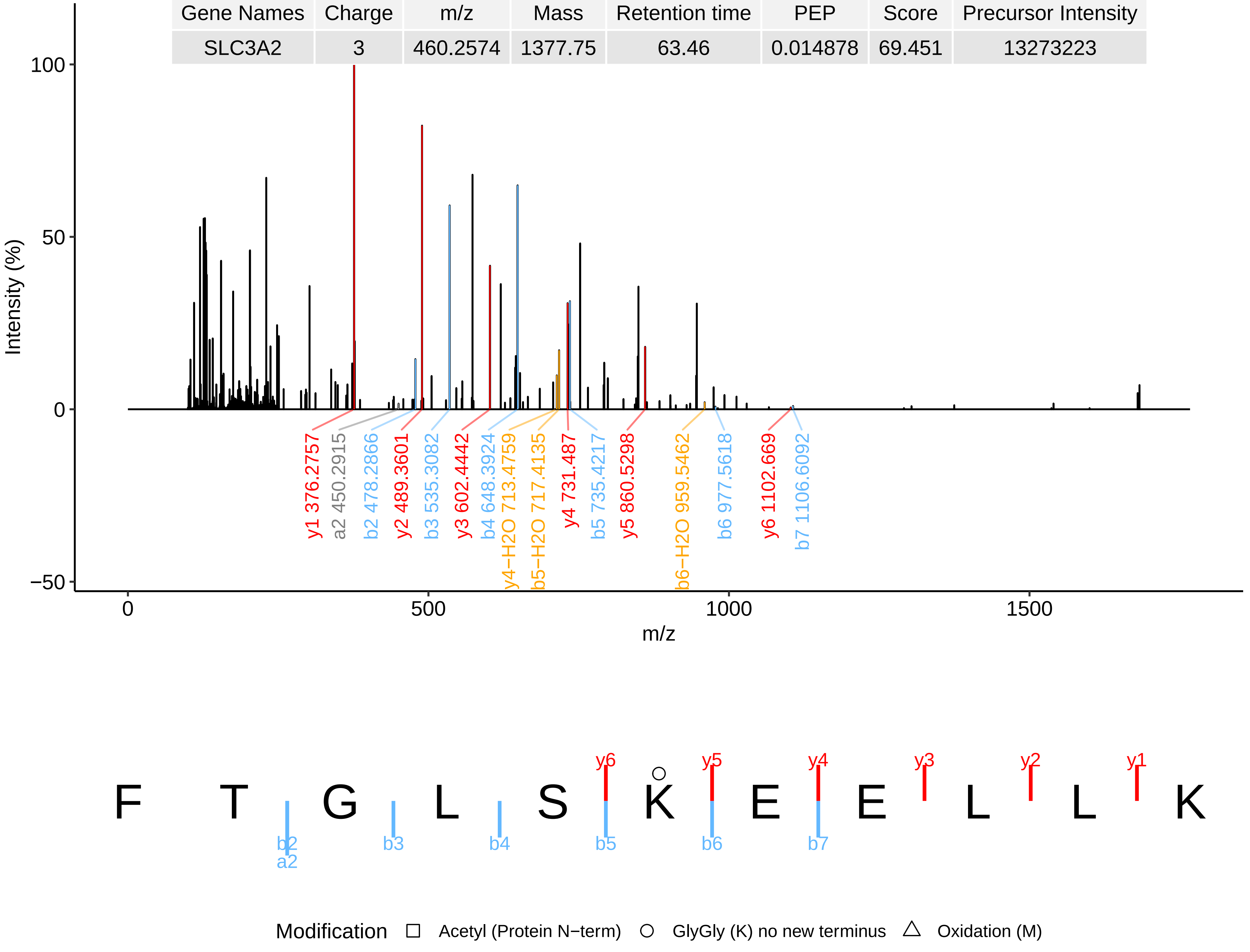

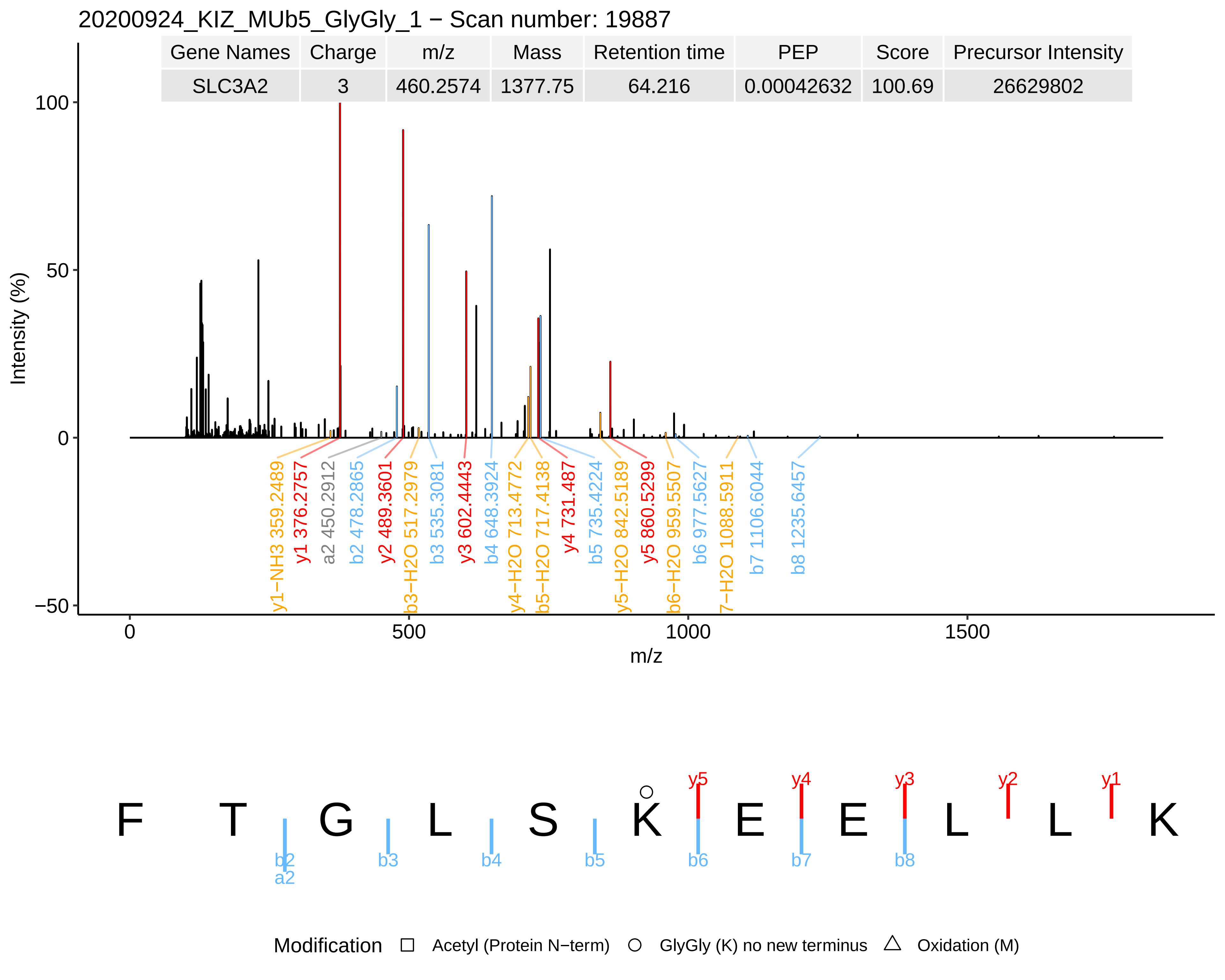
20200924_KIZ_MUb5_GlyGly_1 − Scan number: 19887
Gene Names
Charge
m/z
Mass
Retention time
PEP
Score
Precursor Intensity
SLC3A2
3
460.2574
1377.75
64.216
0.00042632
100.69
26629802
Intensity (%)
100
50
0
−50
0
500
1000
1500
m/z
y1−NH3 359.2489
y1 376.2757
a2 450.2912
b2 478.2865
y2 489.3601
b3−H2O 517.2979
b3 535.3081
y3 602.4443
b4 648.3924
y4−H2O 713.4772
b5−H2O 717.4138
y4 731.487
b5 735.4224
y5−H2O 842.5189
y5 860.5299
b6−H2O 959.5507
b6 977.5627
y7−H2O 1088.5911
b7 1106.6044
b8 1235.6457
F T G L S K E E L L K
y5
y4
y3
y2
y1
b2
a2
b3
b4
b5
b6
b7
b8
Modification
Acetyl (Protein N−term)
GlyGly (K) no new terminus
Oxidation (M)

20200924_KIZ_MUb5_GlyGly_2 − Scan number: 18270

Gene Names	Charge	m/z	Mass	Retention time	PEP	Score	Precursor Intensity
SLC3A2	3	460.2574	1377.75	67.758	0.00095291	88.596	5033257

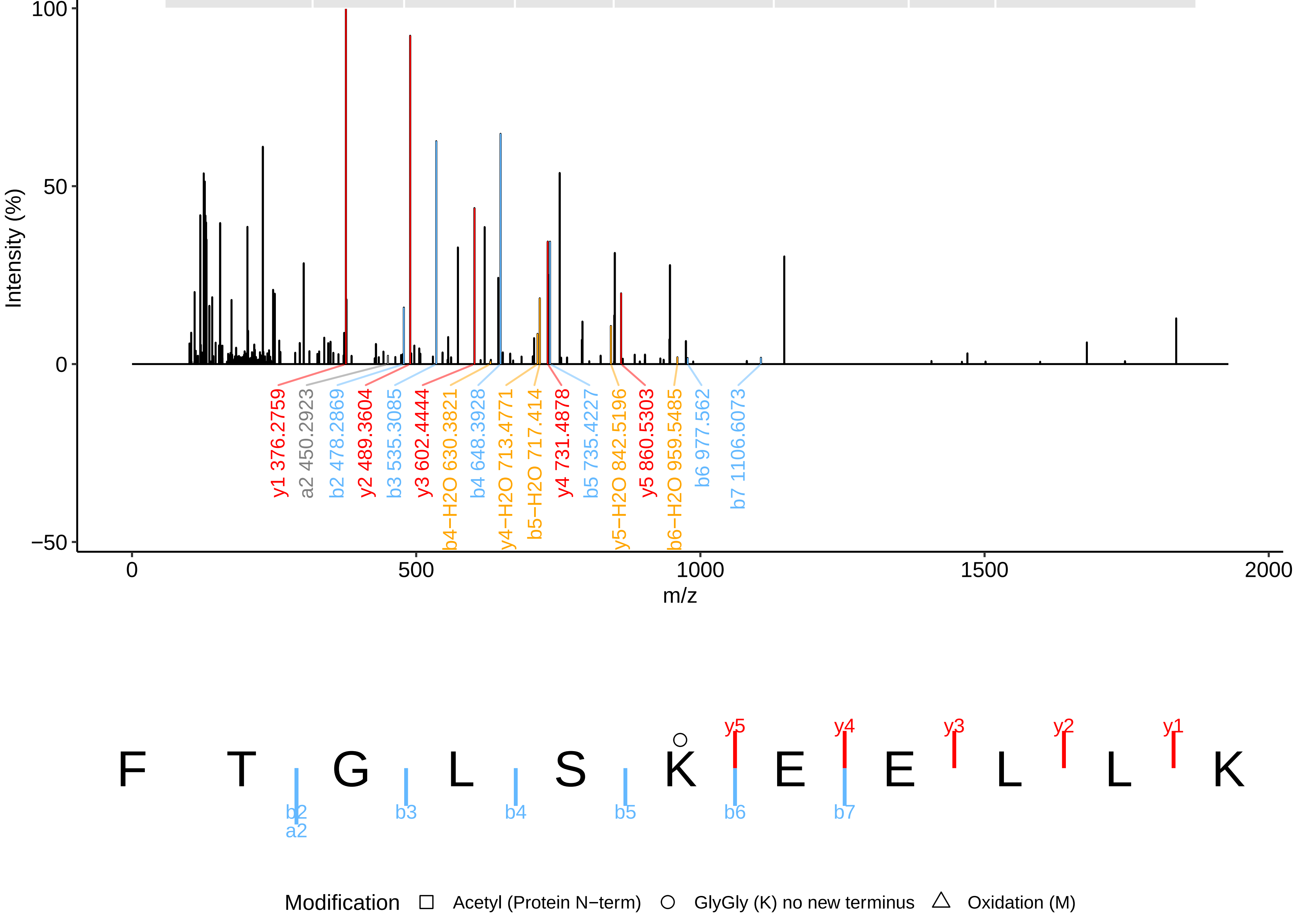

20201118_KIZ_MUb6_GlyGly_1 − Scan number: 20282

Gene Names	Charge	m/z	Mass	Retention time	PEP	Score	Precursor Intensity
SLC3A2	3	460.2574	1377.75	63.469	0.012025	68.069	17919764

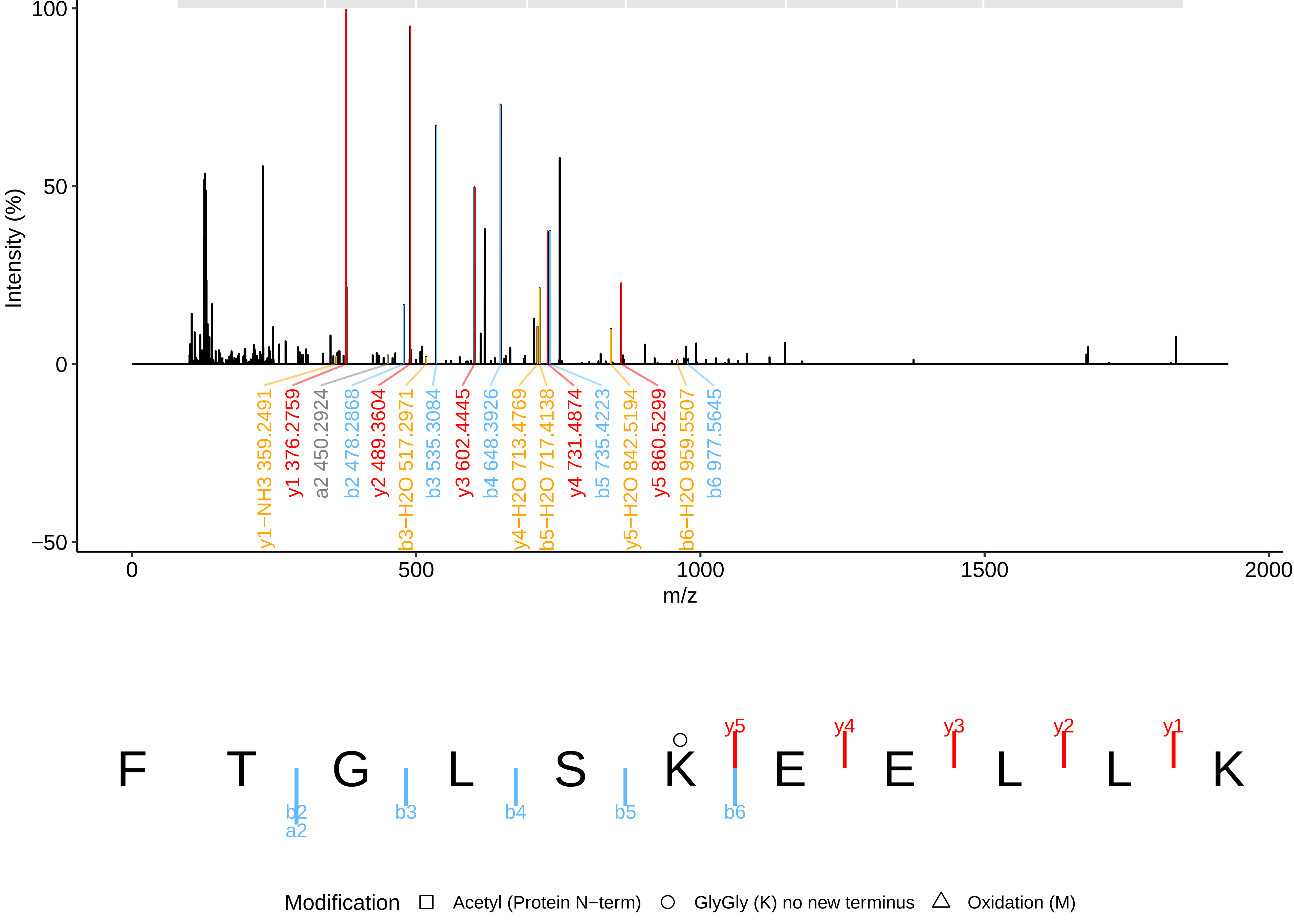

20201118_KIZ_MUb6_GlyGly_1 − Scan number: 20325

Gene Names	Charge	m/z	Mass	Retention time	PEP	Score	Precursor Intensity
SLC3A2	3	460.2574	1377.75	63.594	0.011032	69.276	35041828

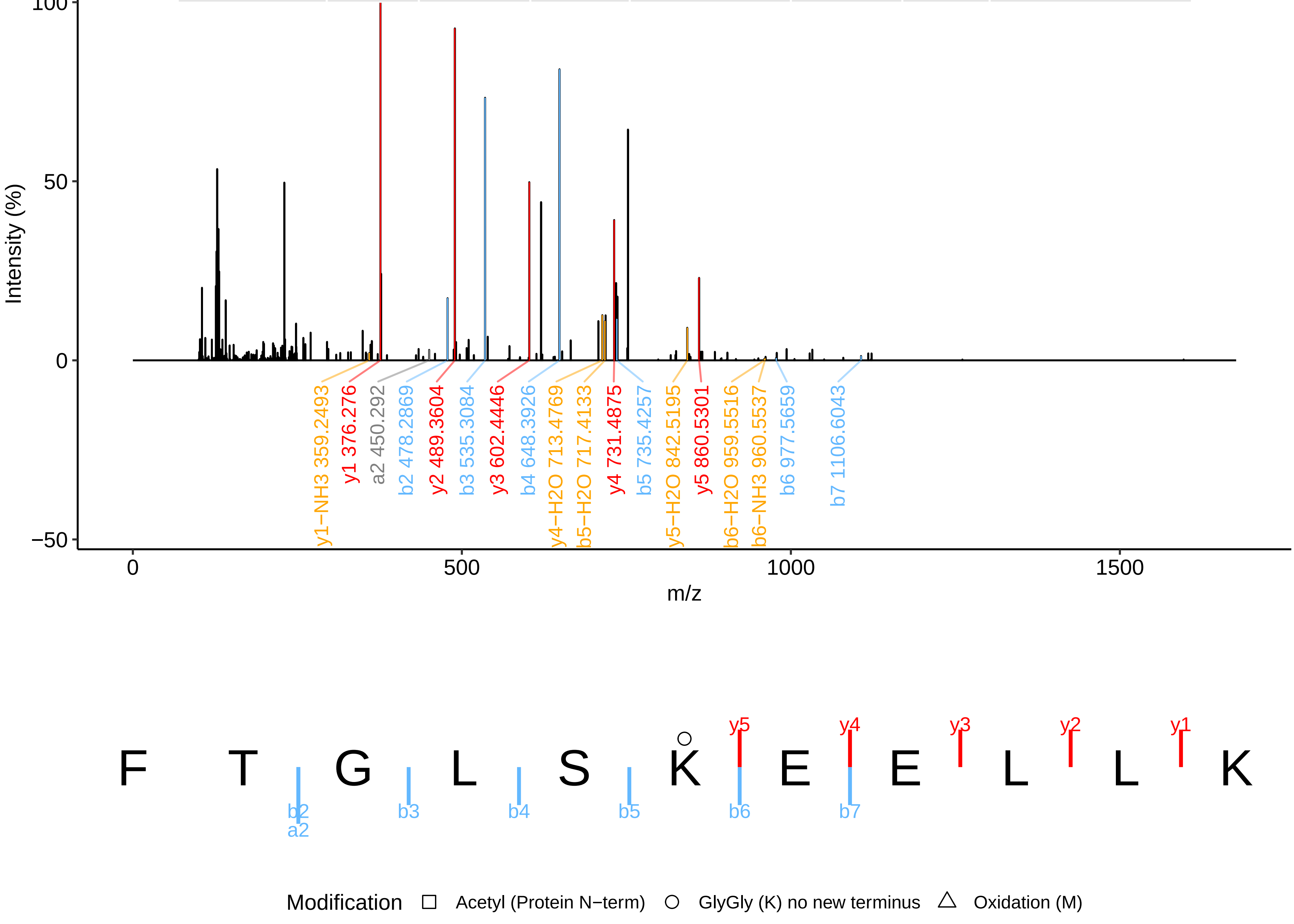

20201118_KIZ_MUb6_GlyGly_2 − Scan number: 22343

Gene Names	Charge	m/z	Mass	Retention time	PEP	Score	Precursor Intensity
SLC3A2	3	460.2574	1377.75	75.122	0.0091567	71.555	7471517

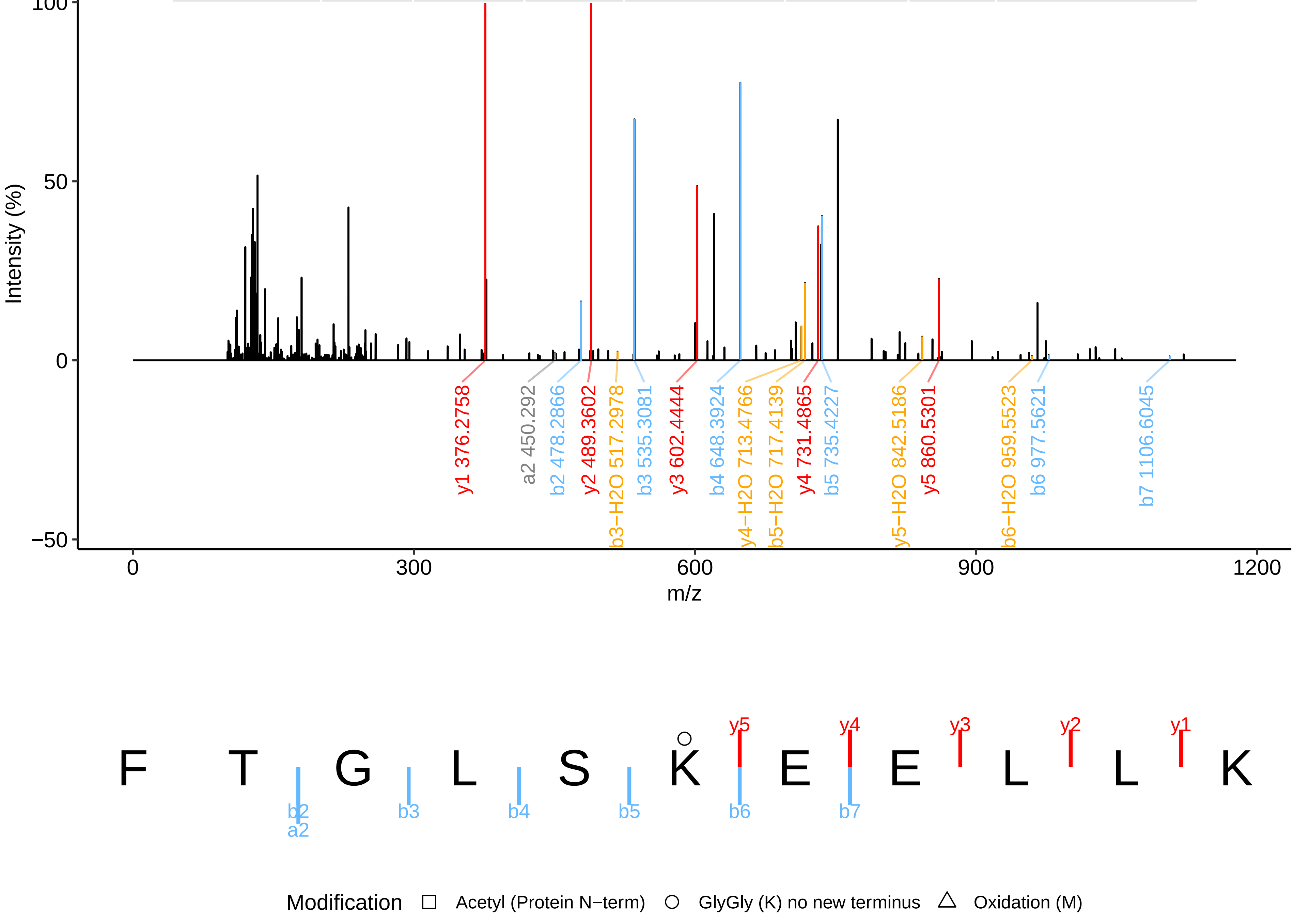

20200921_KIZ_MUb4_GlyGly_10plex − Scan number: 12844

Gene Names	Charge	m/z	Mass	Retention time	PEP	Score	Precursor Intensity
PHB	3	440.57	1318.688	45.178	0.00096332	91.867	3877883

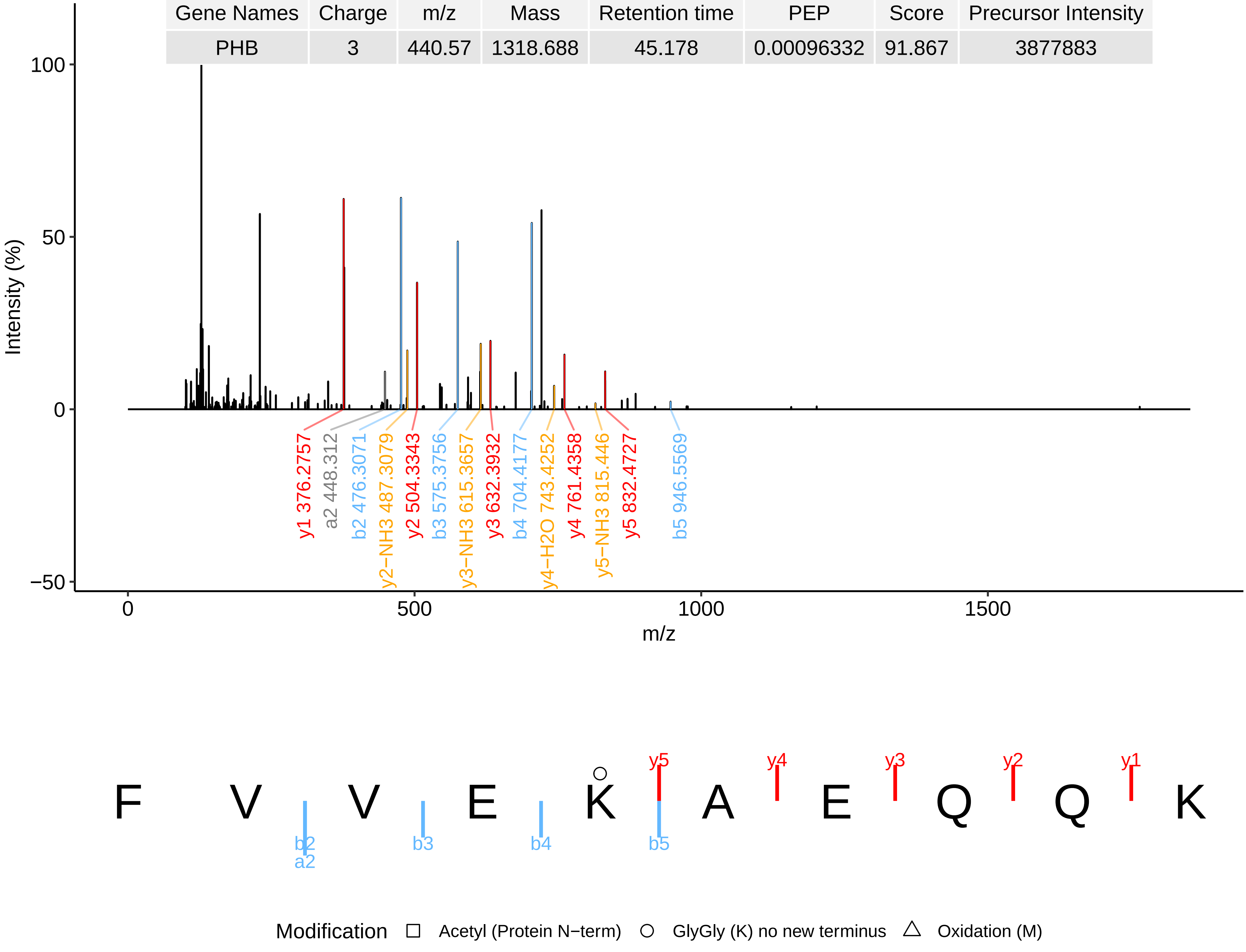

20200921_KIZ_MUb4_GlyGly_10plex_2 − Scan number: 11812

Gene Names	Charge	m/z	Mass	Retention time	PEP	Score	Precursor Intensity
PHB	3	440.57	1318.688	45.554	0.0018513	85.731	256220.3

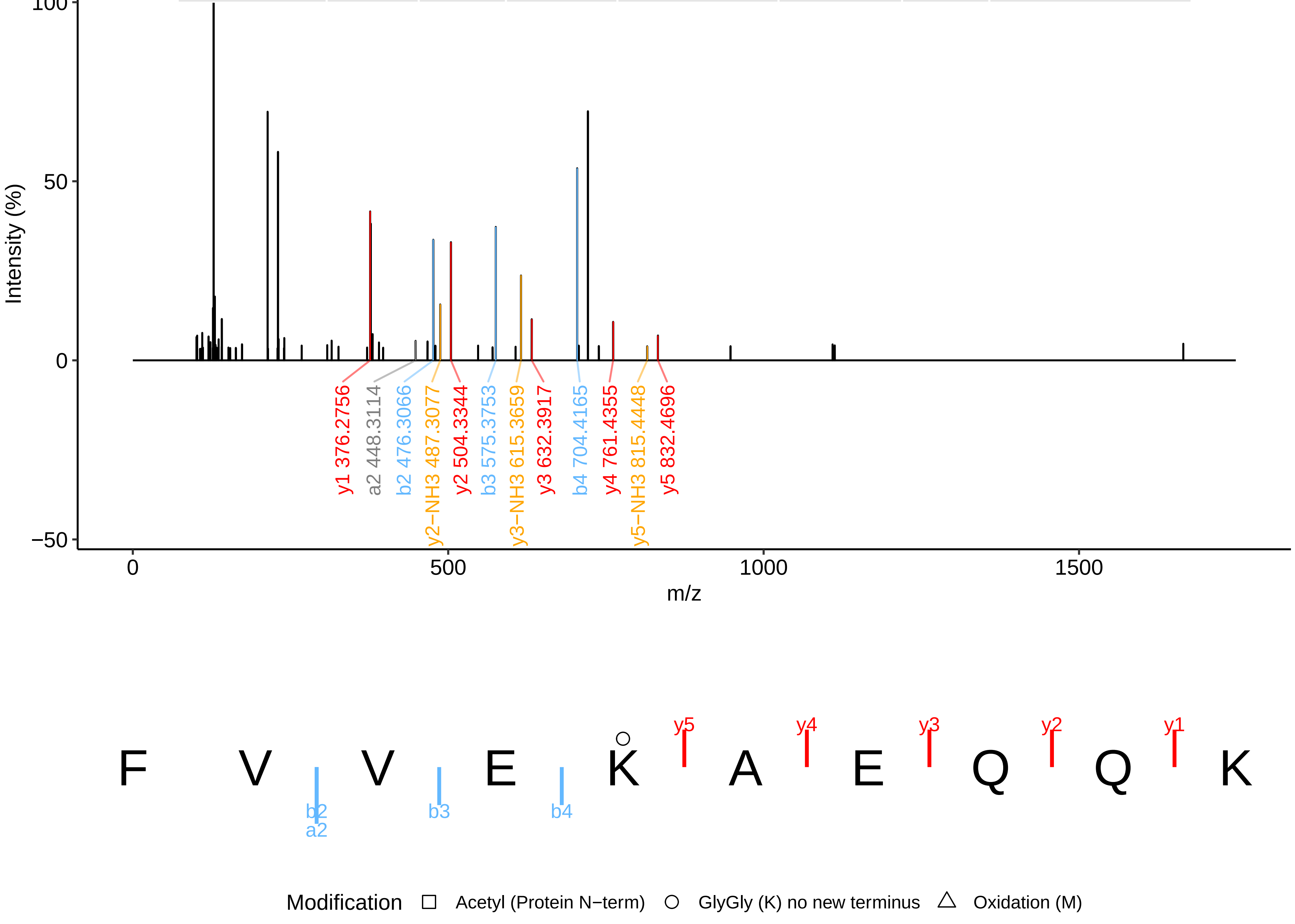

20200921_KIZ_MUb4_GlyGly_10plex − Scan number: 21088

Gene Names	Charge	m/z	Mass	Retention time	PEP	Score	Precursor Intensity
HSP90AB1	3	454.2347	1359.682	76.312	0.072816	56.013	NA

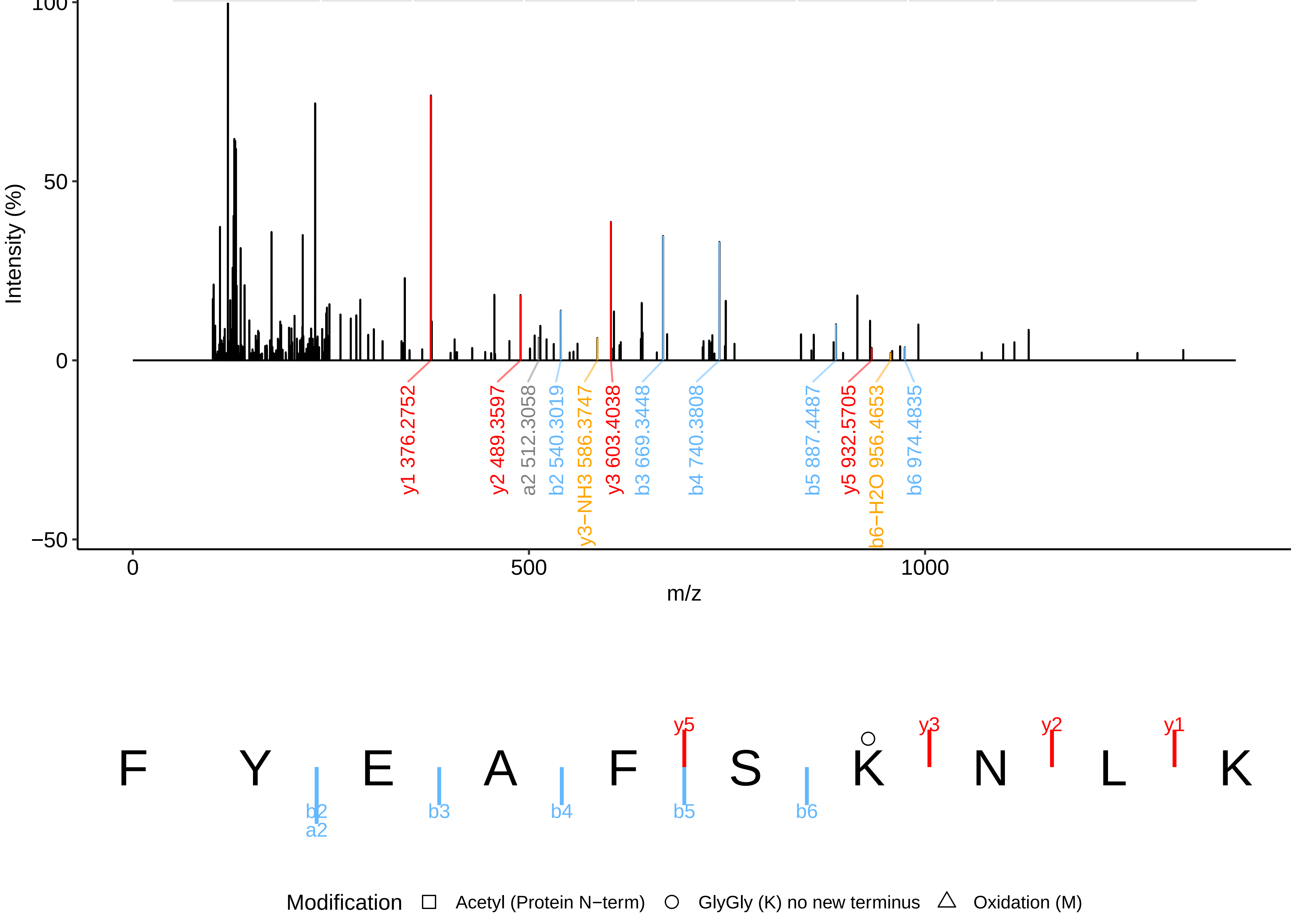

20200921_KIZ_MUb4_GlyGly_10plex_2 − Scan number: 18853

Gene Names	Charge	m/z	Mass	Retention time	PEP	Score	Precursor Intensity
HSP90AB1	3	454.2347	1359.682	73.916	0.013329	70.056	1180666

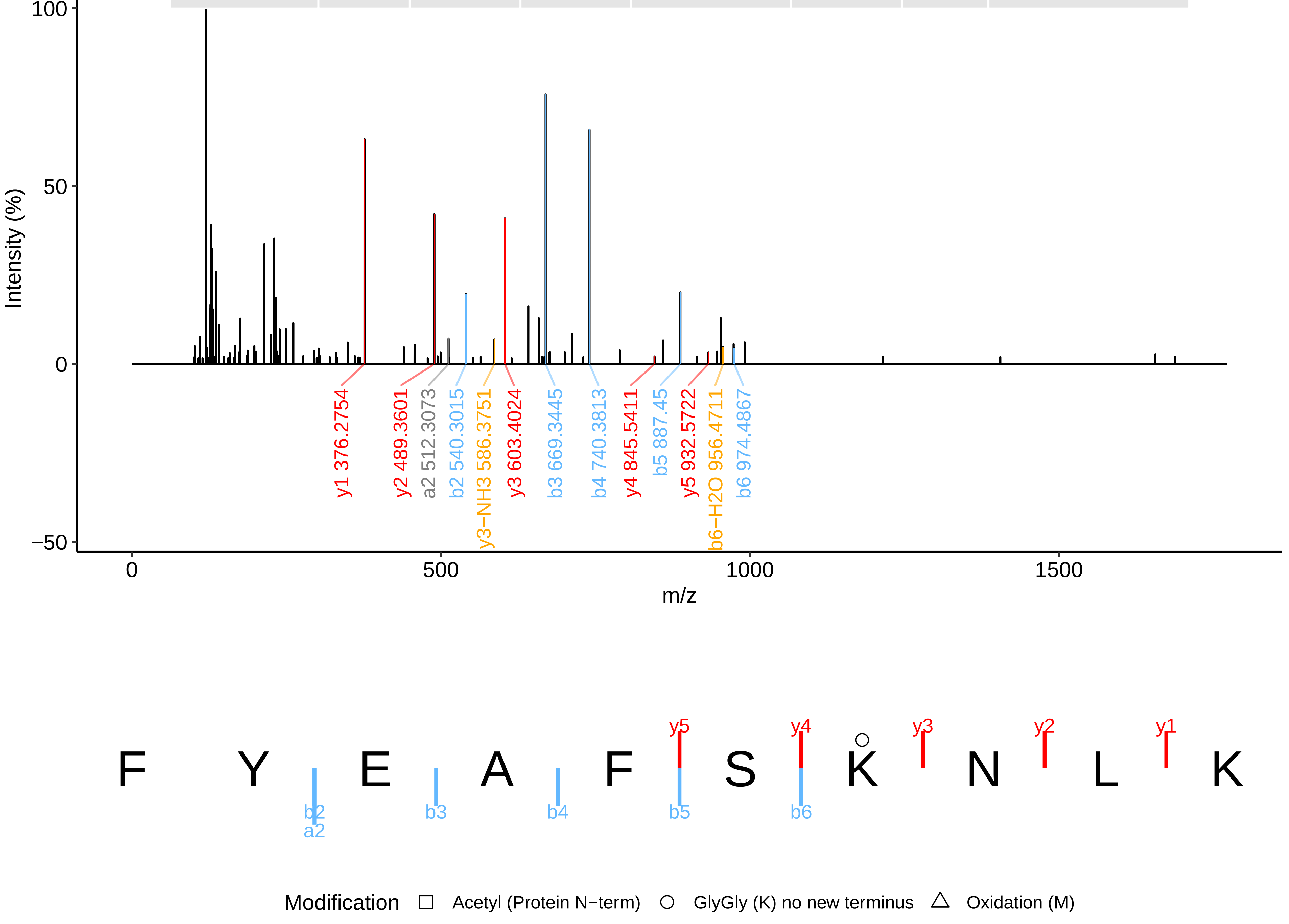

20200921_KIZ_MUb4_GlyGly_10plex − Scan number: 3831

Gene Names	Charge	m/z	Mass	Retention time	PEP	Score	Precursor Intensity
BAG2	3	520.929	1559.765	12.364	5.6557e−28	175.74	7580666

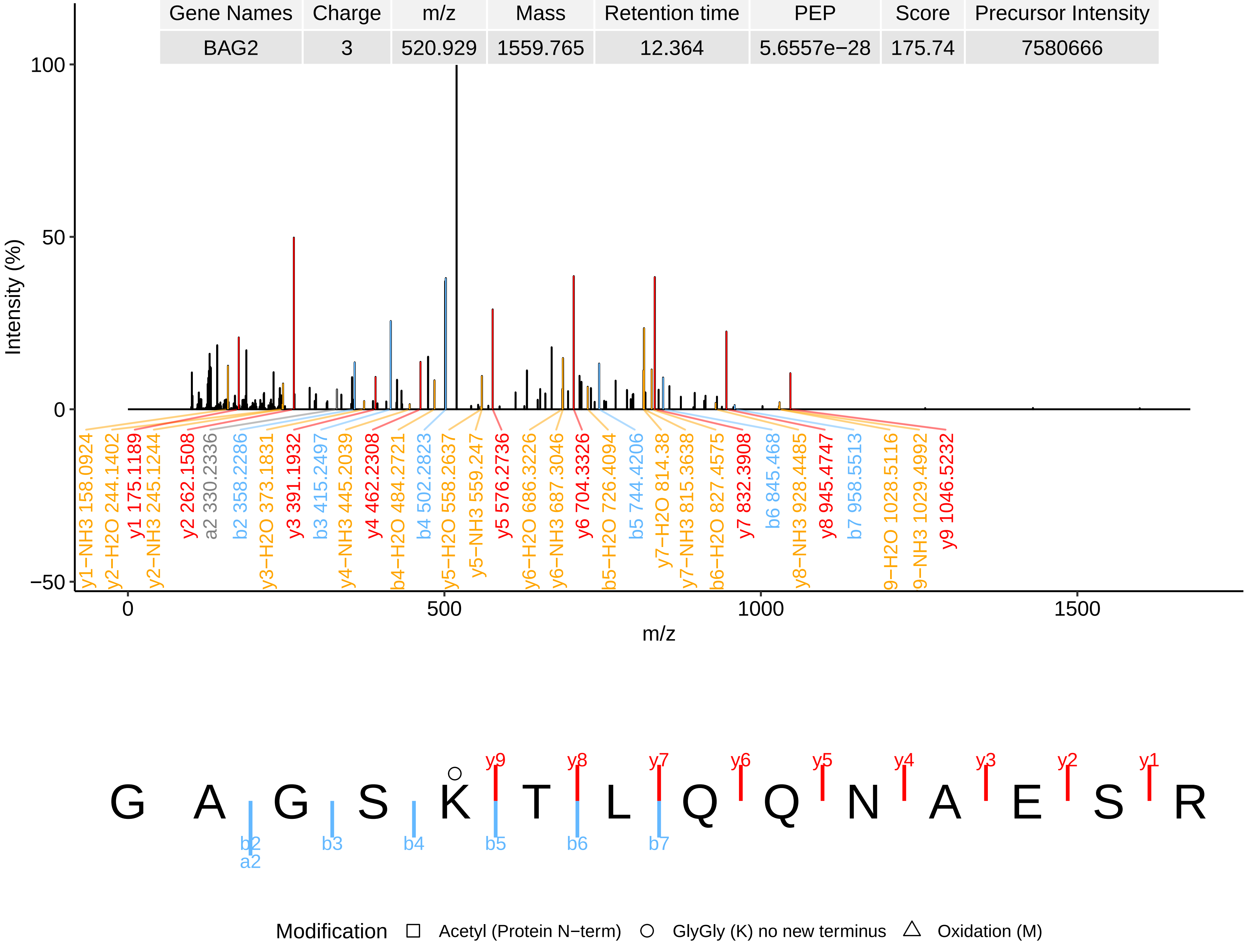

20200921_KIZ_MUb4_GlyGly_10plex_2 − Scan number: 3653

Gene Names	Charge	m/z	Mass	Retention time	PEP	Score	Precursor Intensity
BAG2	3	520.929	1559.765	12.404	3.7466e−09	129.54	1472424

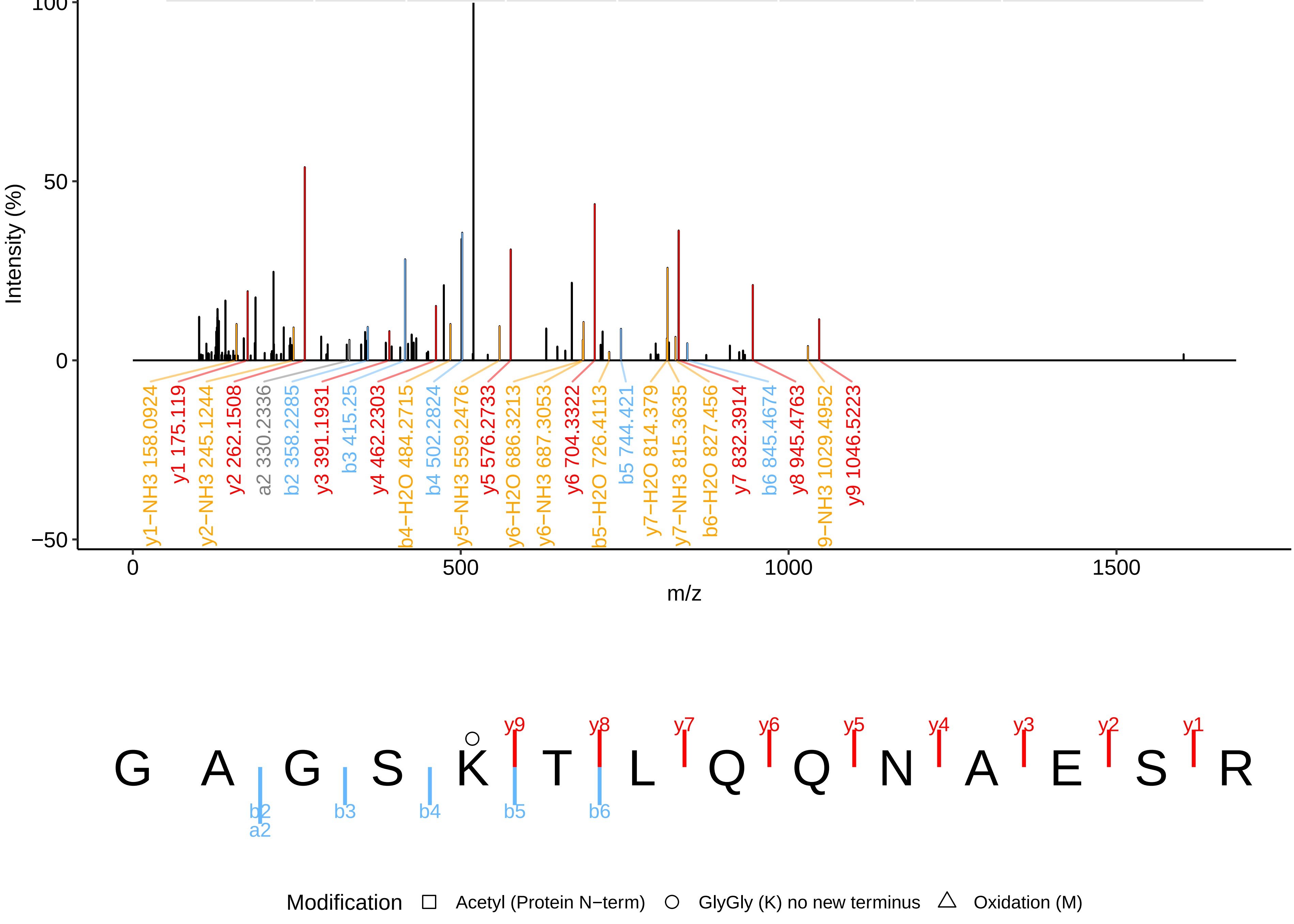

20200924_KIZ_MUb5_GlyGly_1 − Scan number: 3329

Gene Names	Charge	m/z	Mass	Retention time	PEP	Score	Precursor Intensity
BAG2	3	520.929	1559.765	10.22	9.5972e−38	186.51	30420590

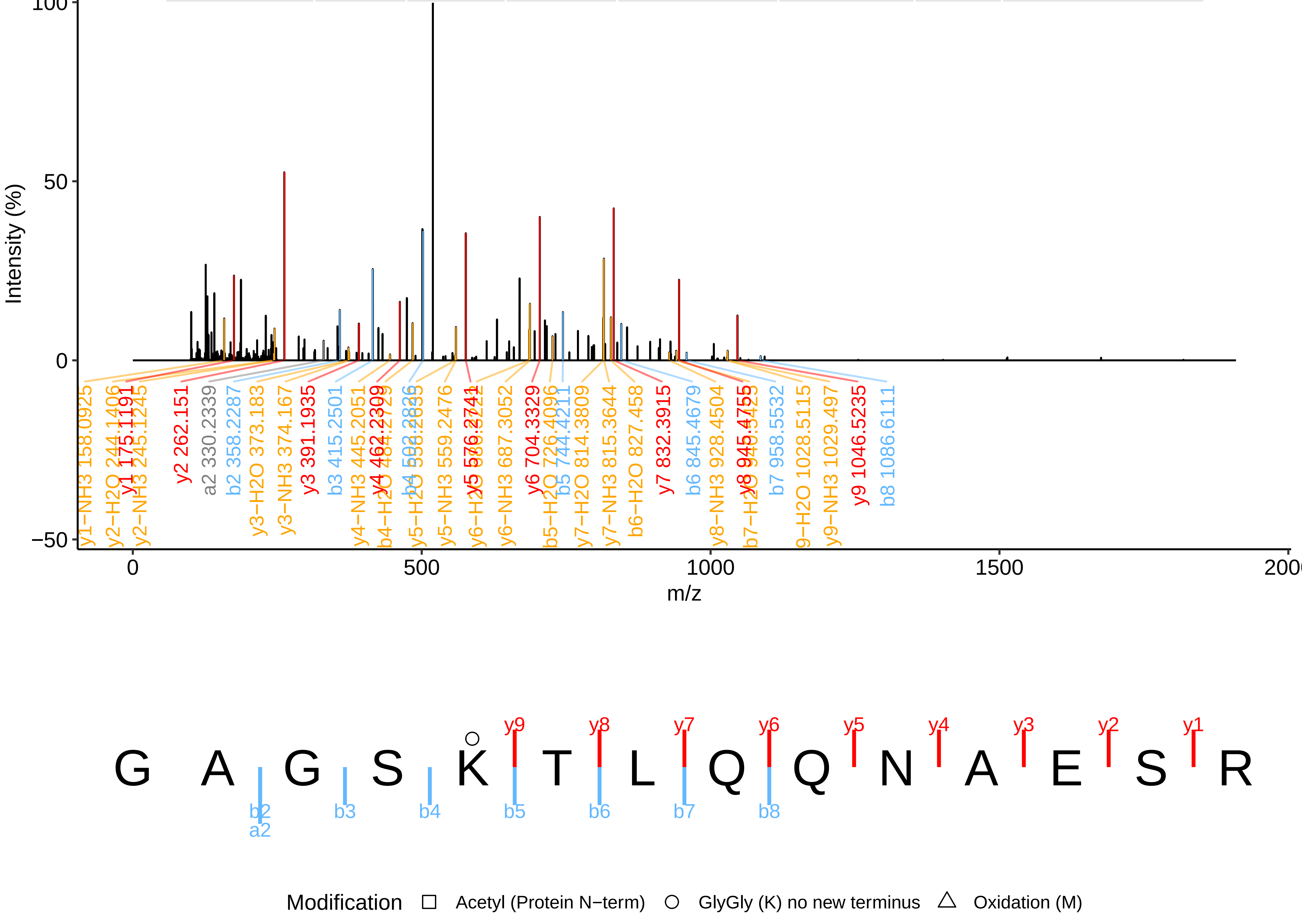

20200924_KIZ_MUb5_GlyGly_2 − Scan number: 3289

Gene Names	Charge	m/z	Mass	Retention time	PEP	Score	Precursor Intensity
BAG2	3	520.929	1559.765	11.867	1.3595e−08	120.9	799674.1

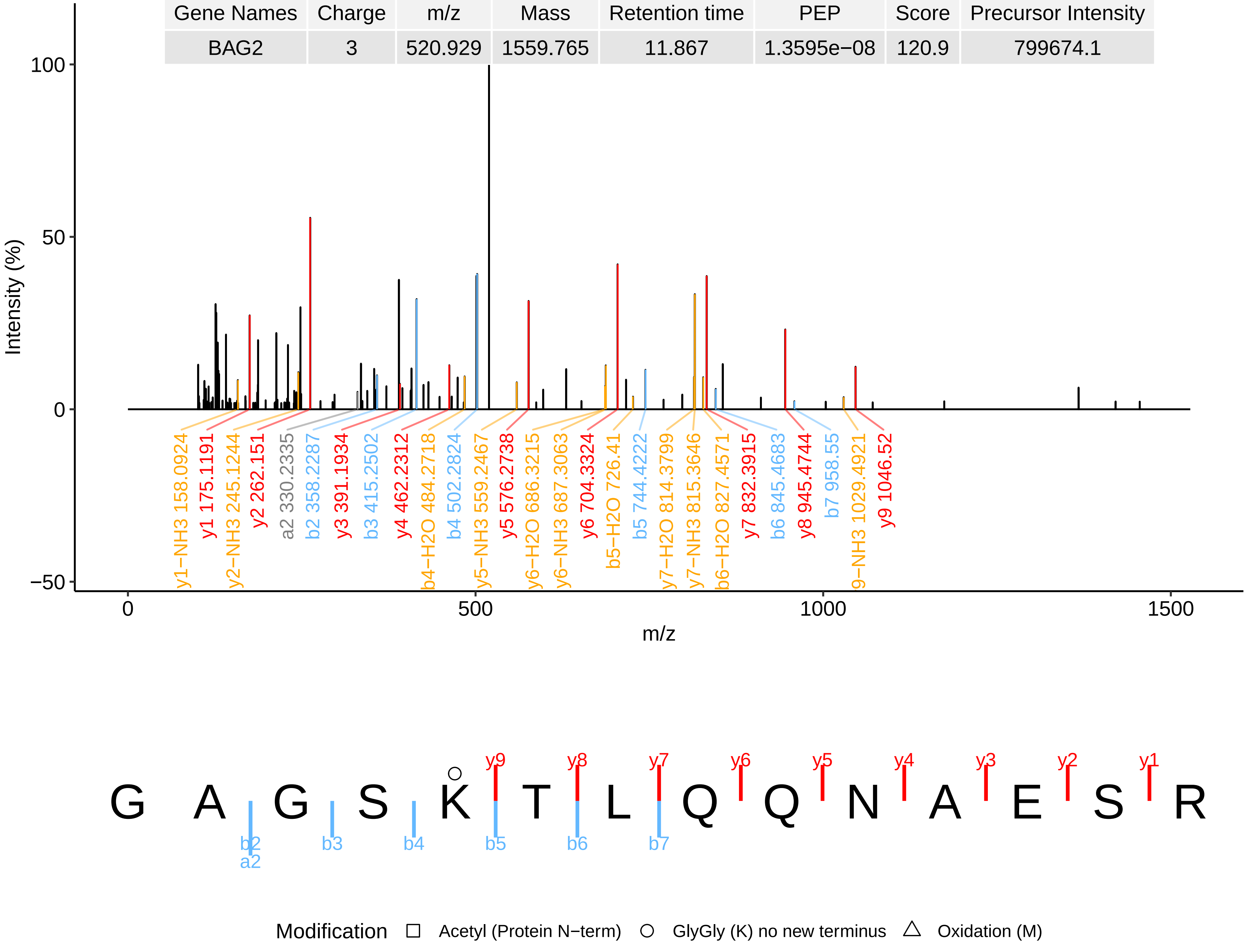

20201118_KIZ_MUb6_GlyGly_1 − Scan number: 3242

Gene Names	Charge	m/z	Mass	Retention time	PEP	Score	Precursor Intensity
BAG2	3	520.929	1559.765	9.9718	1.1542e−19	154.23	51021652

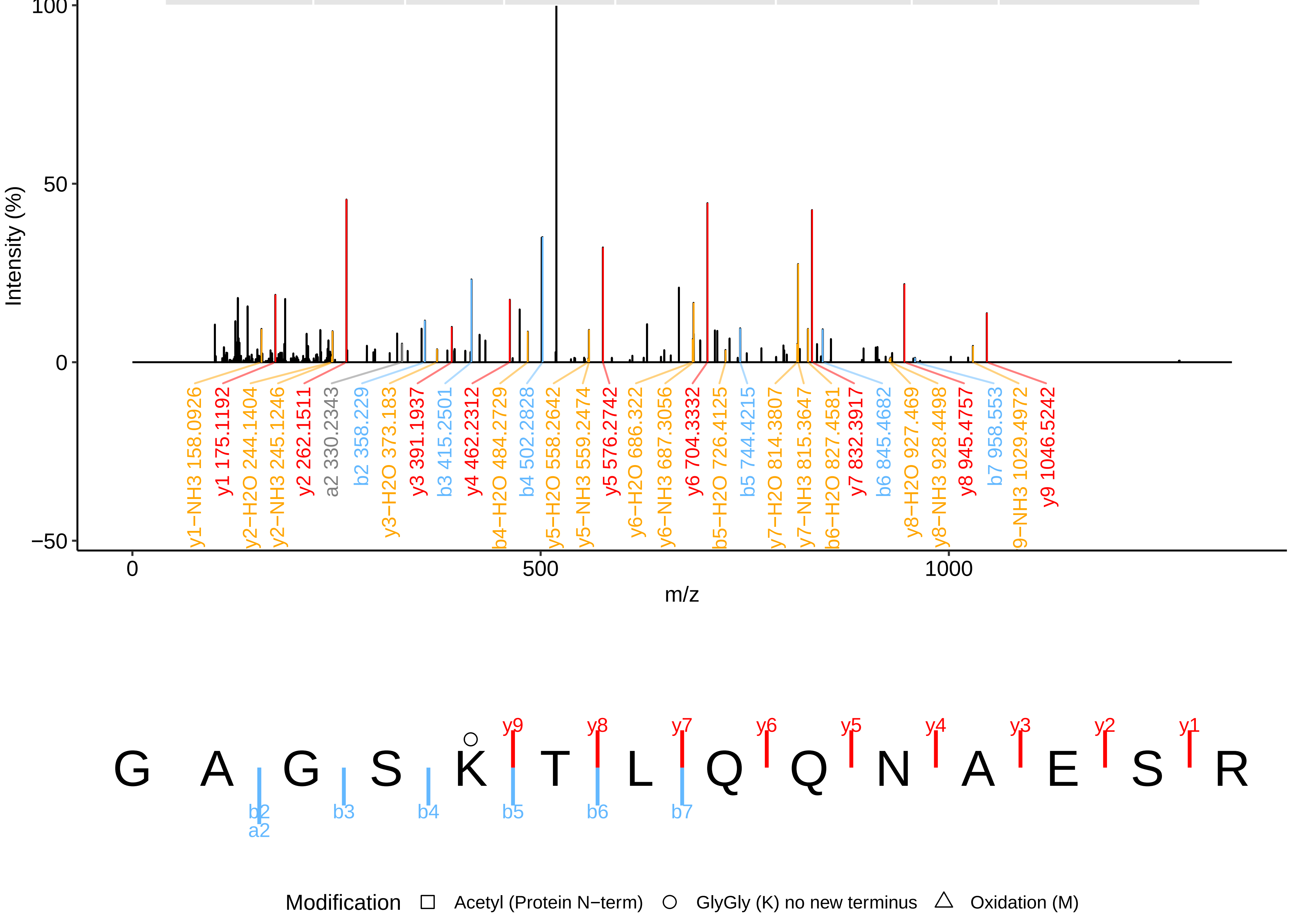

20201118_KIZ_MUb6_GlyGly_2 − Scan number: 3653

Gene Names	Charge	m/z	Mass	Retention time	PEP	Score	Precursor Intensity
BAG2	3	520.929	1559.765	12.197	3.7466e−09	129.54	3809214

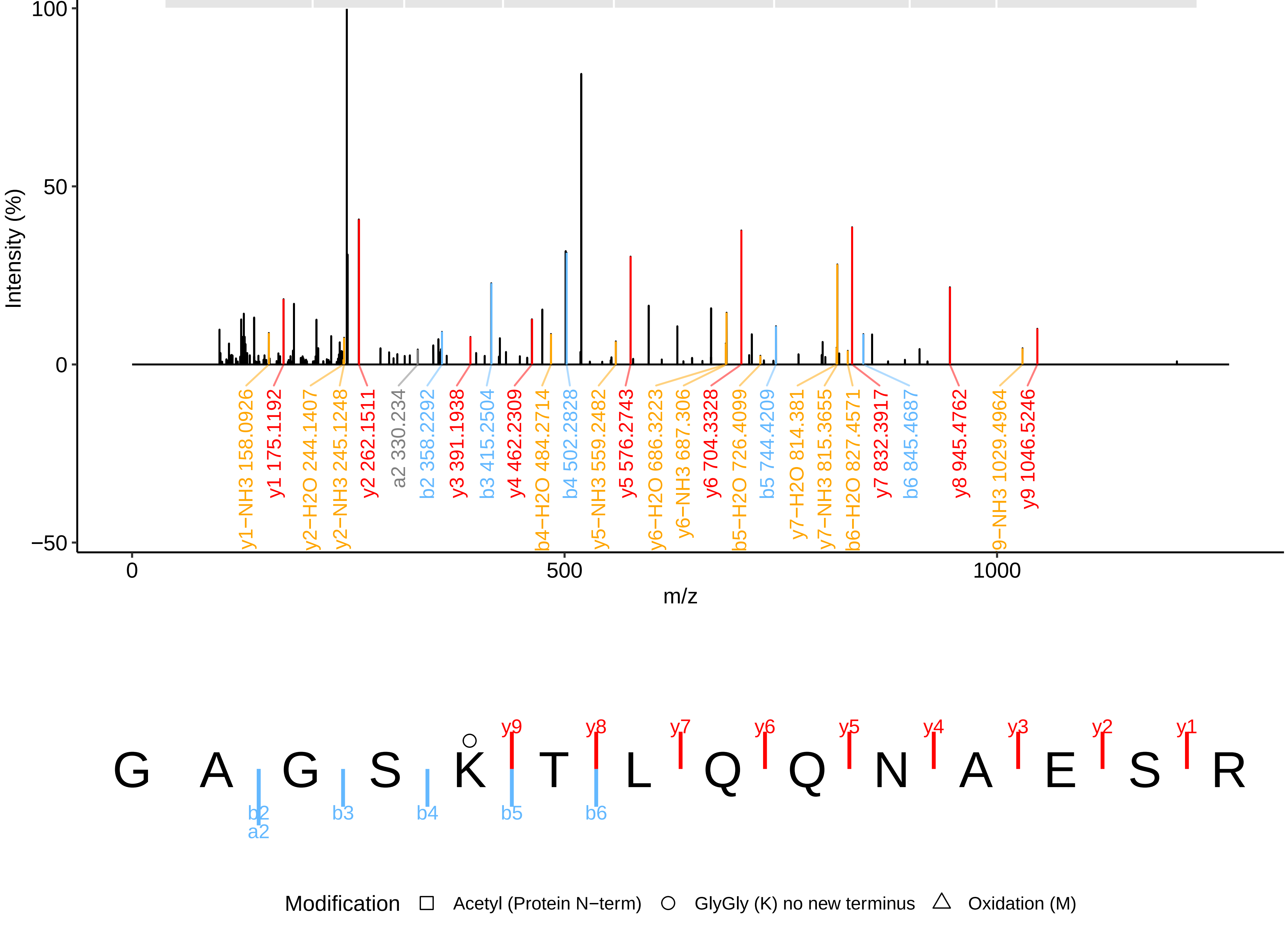

20200924_KIZ_MUb5_GlyGly_1 − Scan number: 2525

Gene Names	Charge	m/z	Mass	Retention time	PEP	Score	Precursor Intensity
ZC3H14	3	520.9169	1559.729	7.8541	1.4603e−09	131.06	9278104

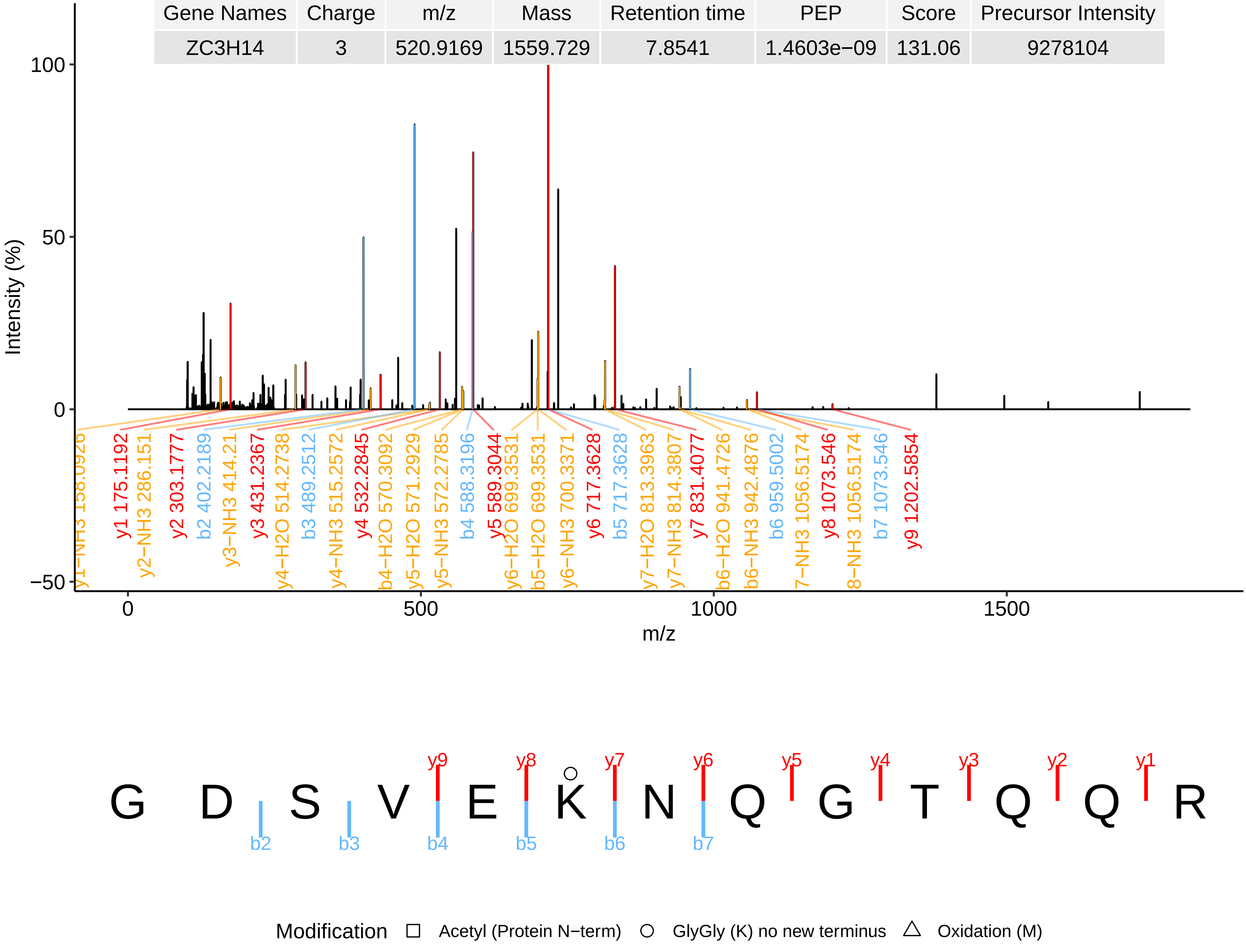

20200921_KIZ_MUb4_GlyGly_10plex − Scan number: 12570

Gene Names	Charge	m/z	Mass	Retention time	PEP	Score	Precursor Intensity
HSPE1;HSPE1−MOB4	3	453.5747	1357.702	44.126	0.00059385	91.937	2156359

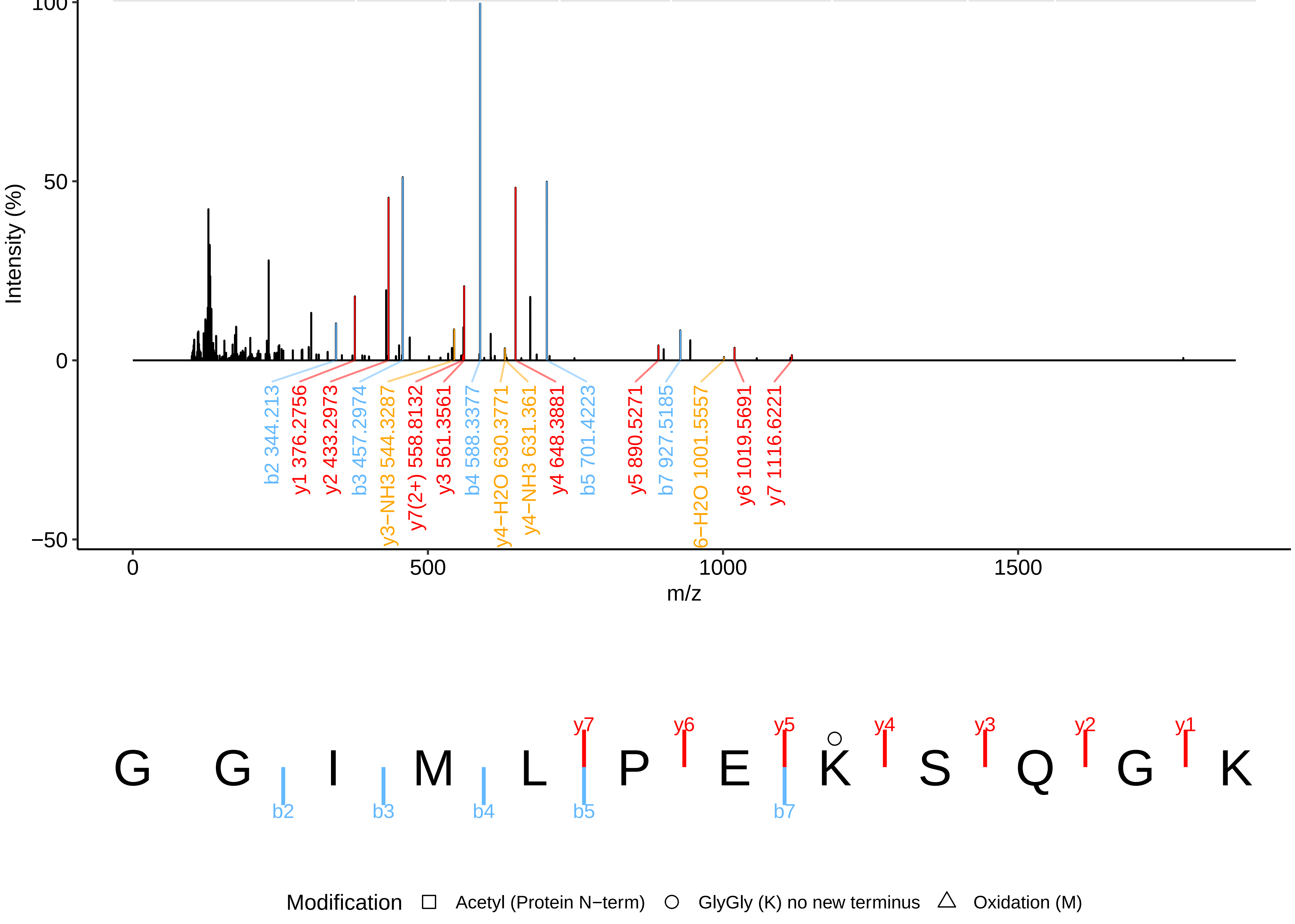

20200921_KIZ_MUb4_GlyGly_10plex − Scan number: 12085

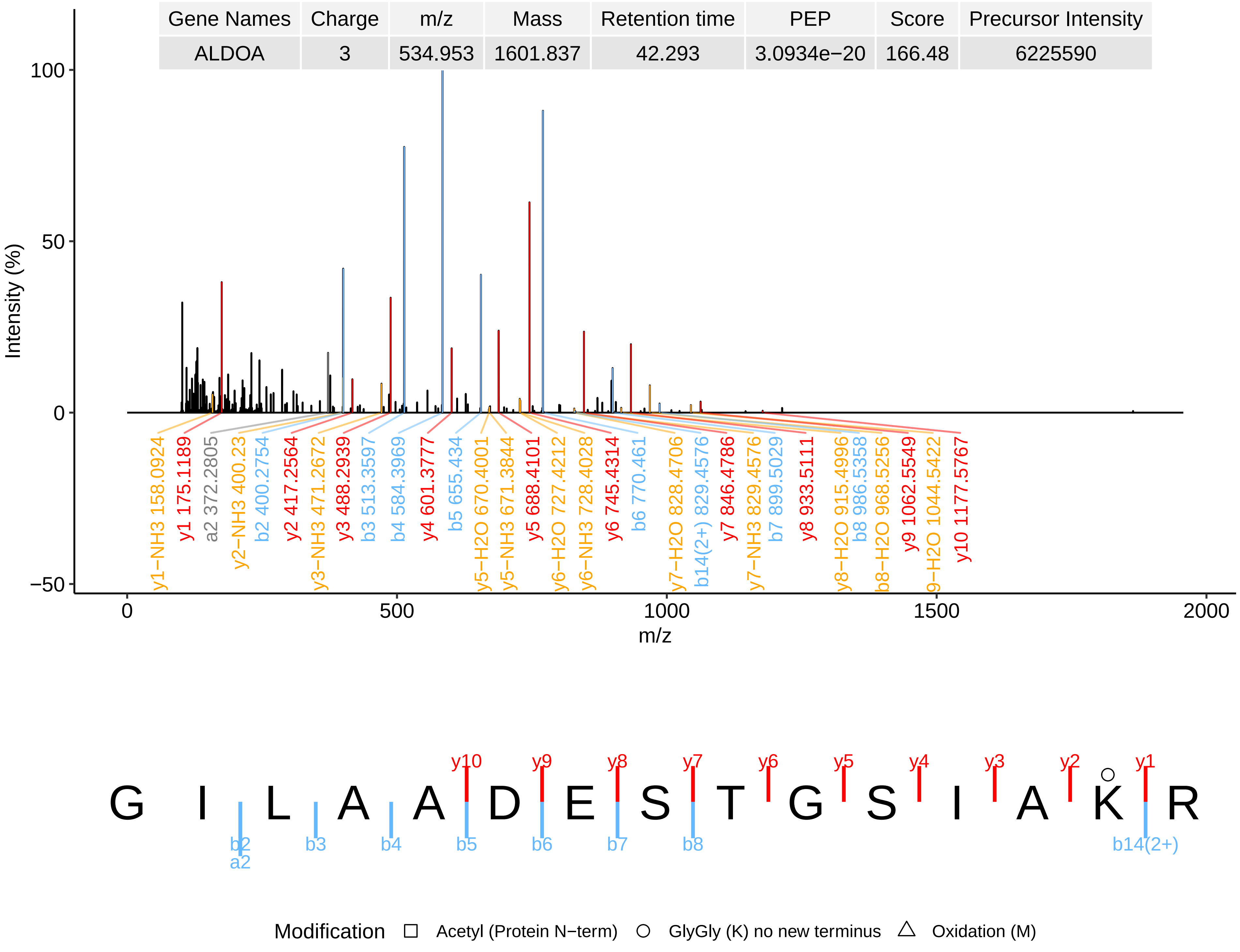

20200921_KIZ_MUb4_GlyGly_10plex_2 − Scan number: 11131

Gene Names	Charge	m/z	Mass	Retention time	PEP	Score	Precursor Intensity
ALDOA	3	534.953	1601.837	42.77	1.1492e−05	109	605189.4

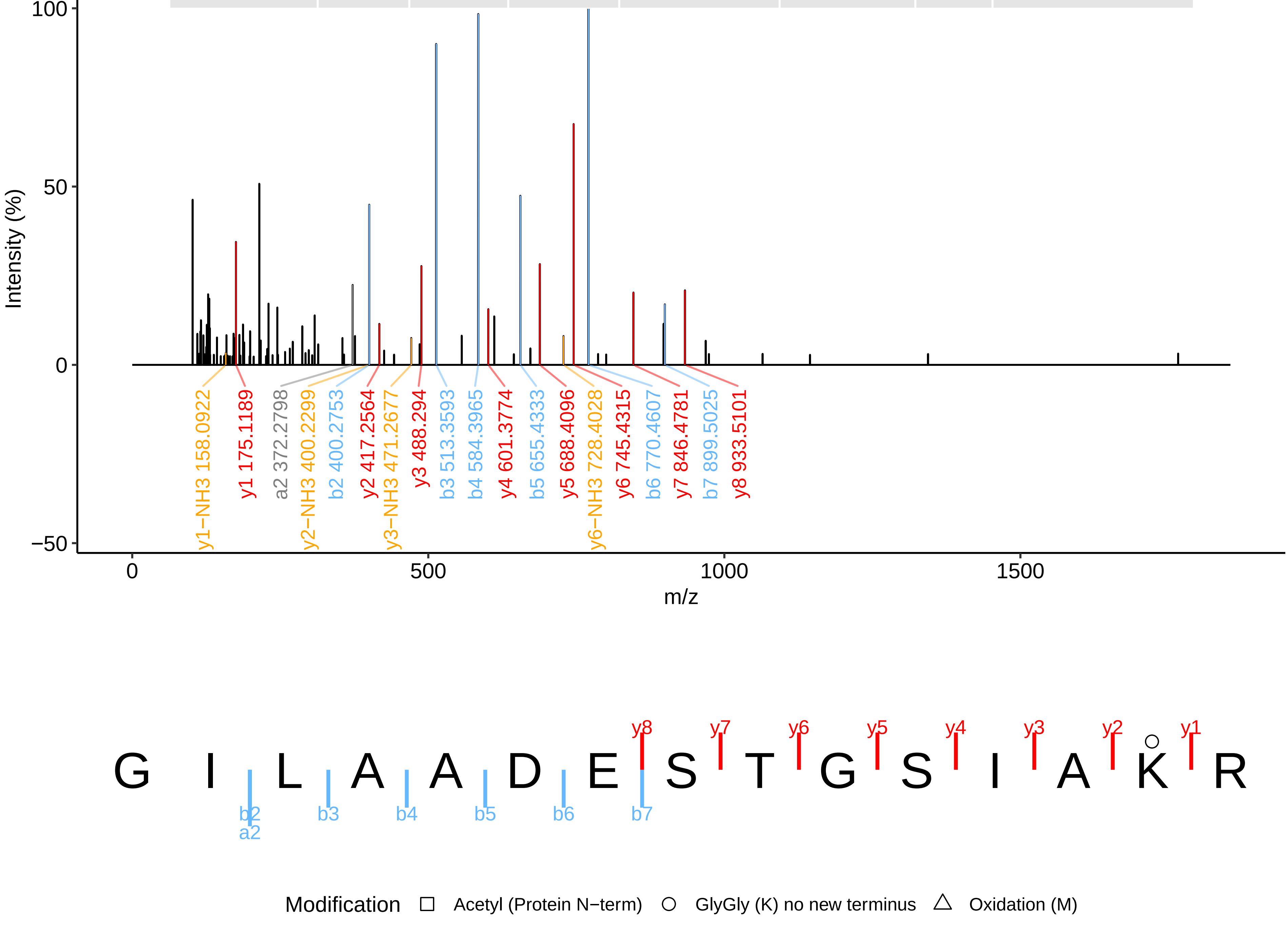

20200921_KIZ_MUb4_GlyGly_10plex − Scan number: 15626

Gene Names	Charge	m/z	Mass	Retention time	PEP	Score	Precursor Intensity
CPS1	3	443.9294	1328.766	55.867	0.12892	46.481	2544313

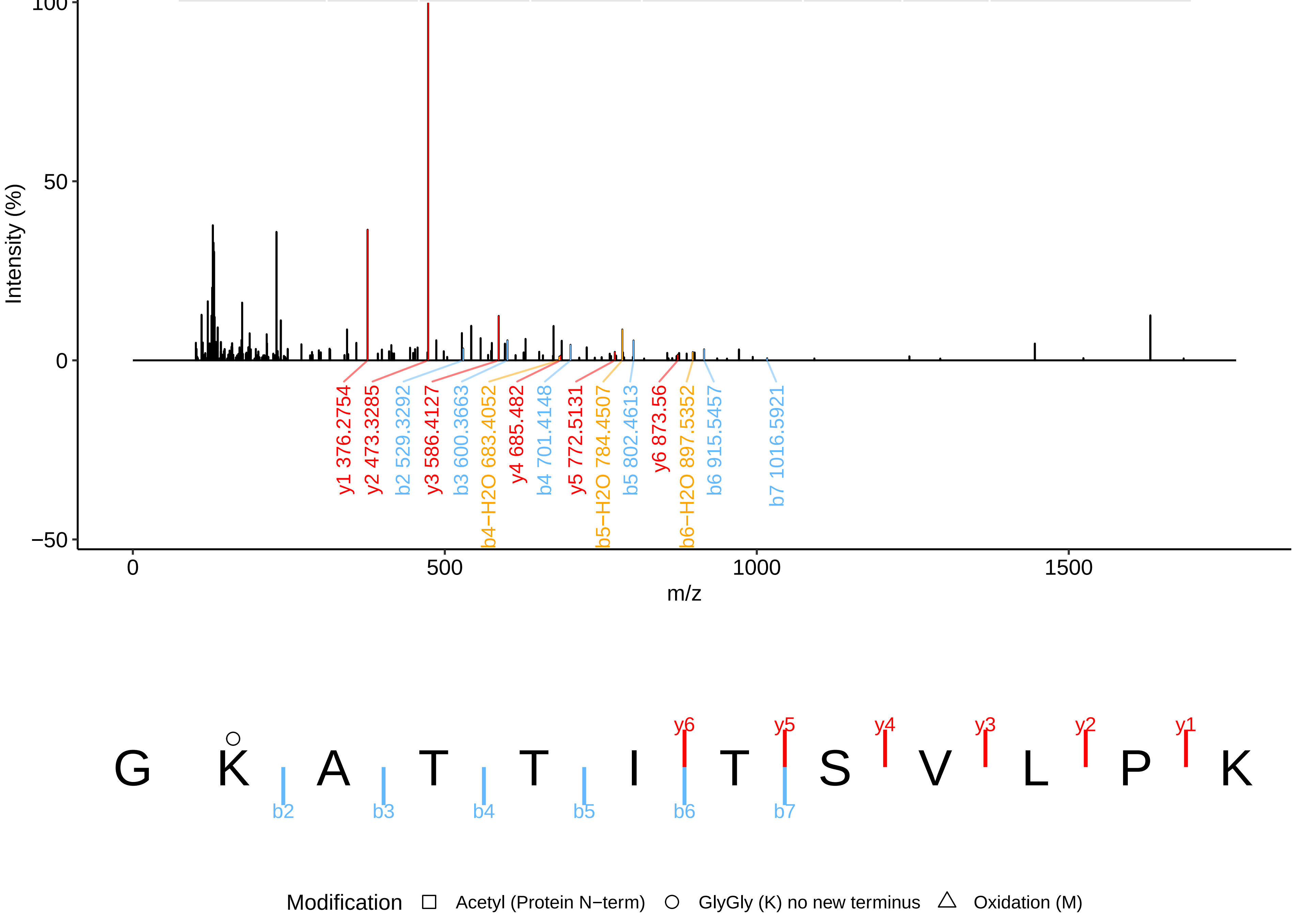

20200921_KIZ_MUb4_GlyGly_10plex − Scan number: 18644

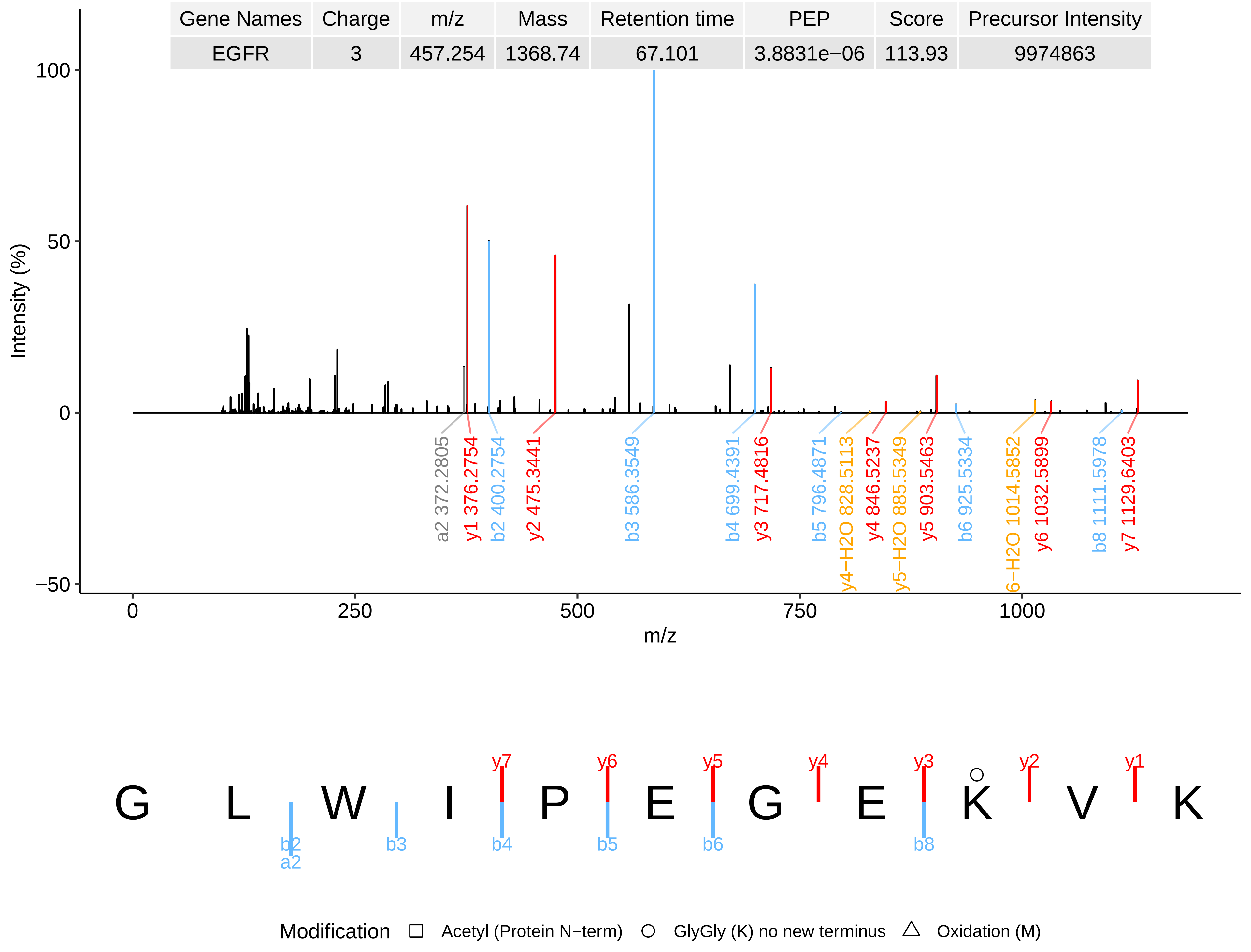

20200921_KIZ_MUb4_GlyGly_10plex_2 − Scan number: 17166

Gene Names	Charge	m/z	Mass	Retention time	PEP	Score	Precursor Intensity
EGFR	3	457.254	1368.74	67.09	0.0015497	82.417	2155913

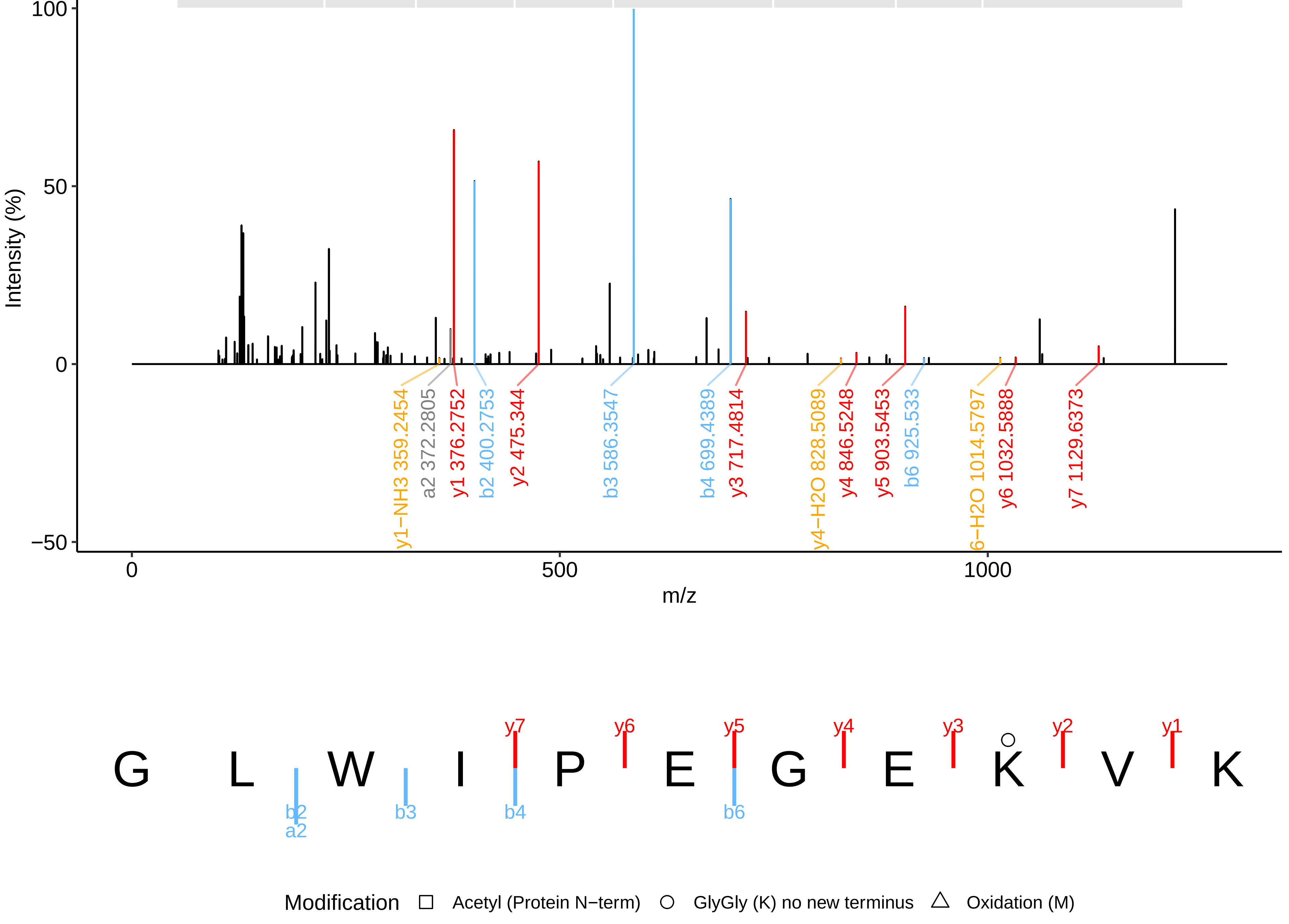

20200924_KIZ_MUb5_GlyGly_2 − Scan number: 16758

Gene Names	Charge	m/z	Mass	Retention time	PEP	Score	Precursor Intensity
EGFR	3	457.254	1368.74	62.17	0.0084042	74.944	1671873

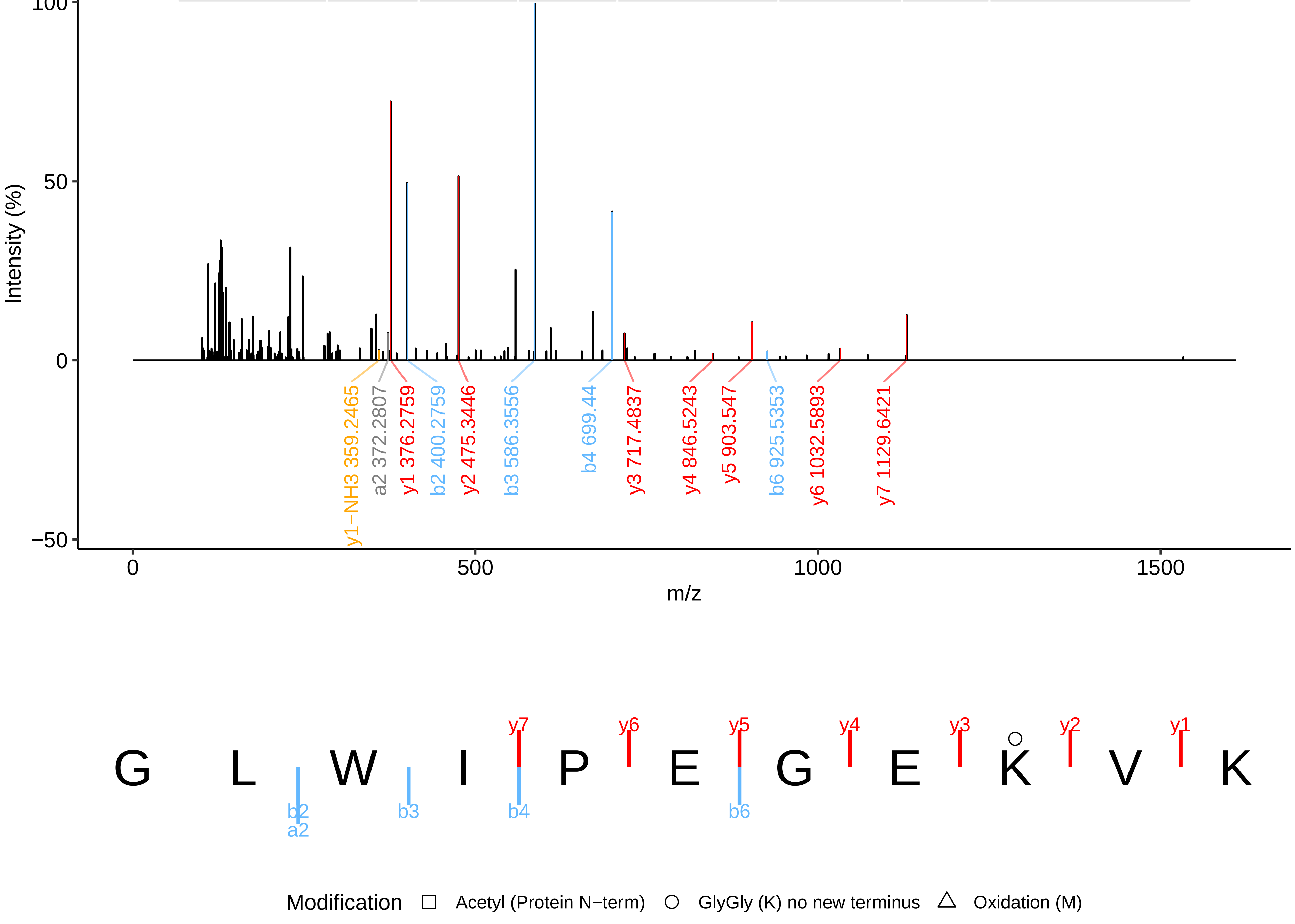

20200921_KIZ_MUb4_GlyGly_10plex − Scan number: 11515

Gene Names	Charge	m/z	Mass	Retention time	PEP	Score	Precursor Intensity
ATP5B	3	442.9174	1325.73	40.151	0.012472	63.76	5805203

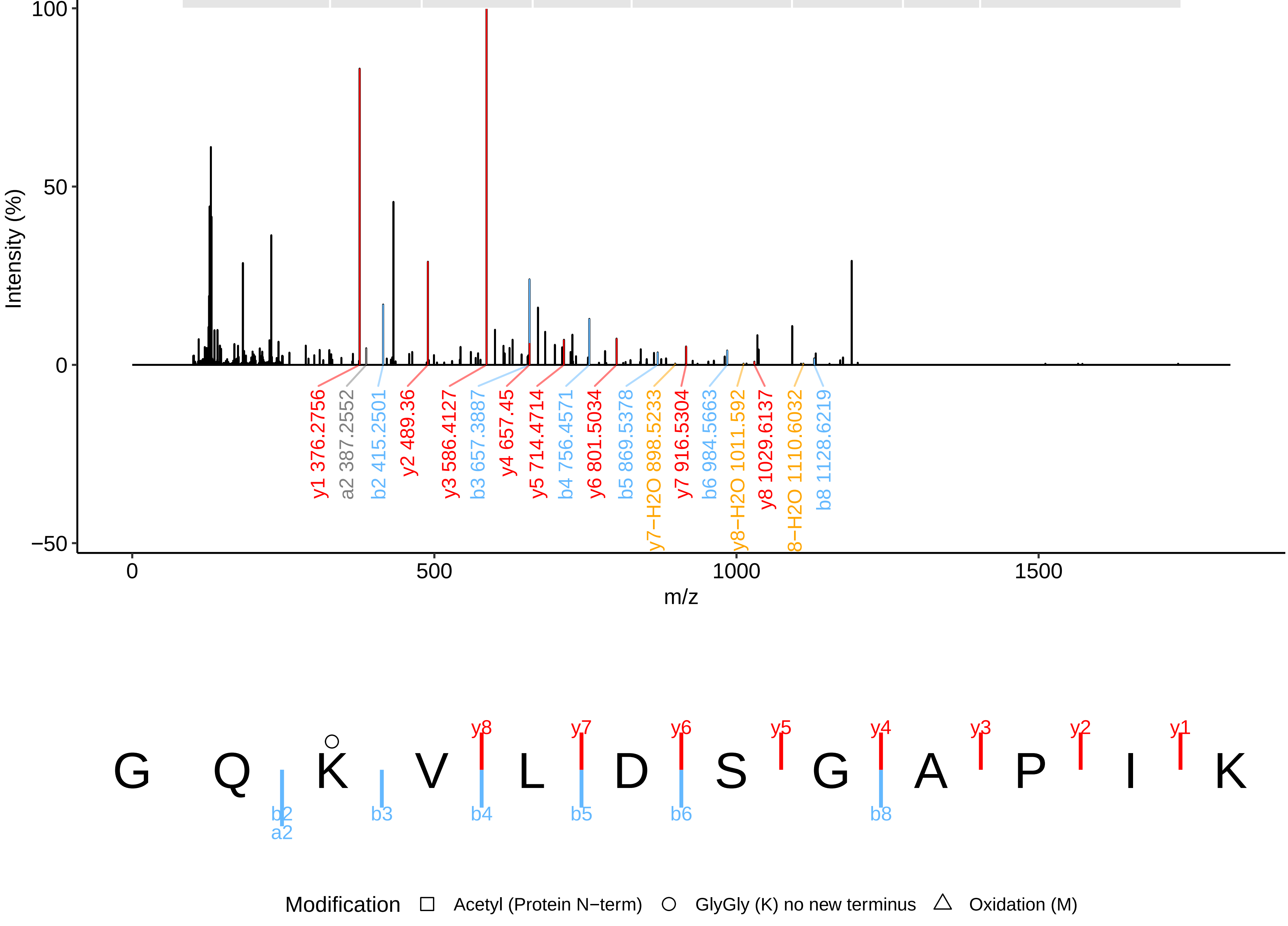

20200921_KIZ_MUb4_GlyGly_10plex_2 − Scan number: 10626

Gene Names	Charge	m/z	Mass	Retention time	PEP	Score	Precursor Intensity
ATP5B	3	442.9174	1325.73	40.689	0.060711	53.967	1428286

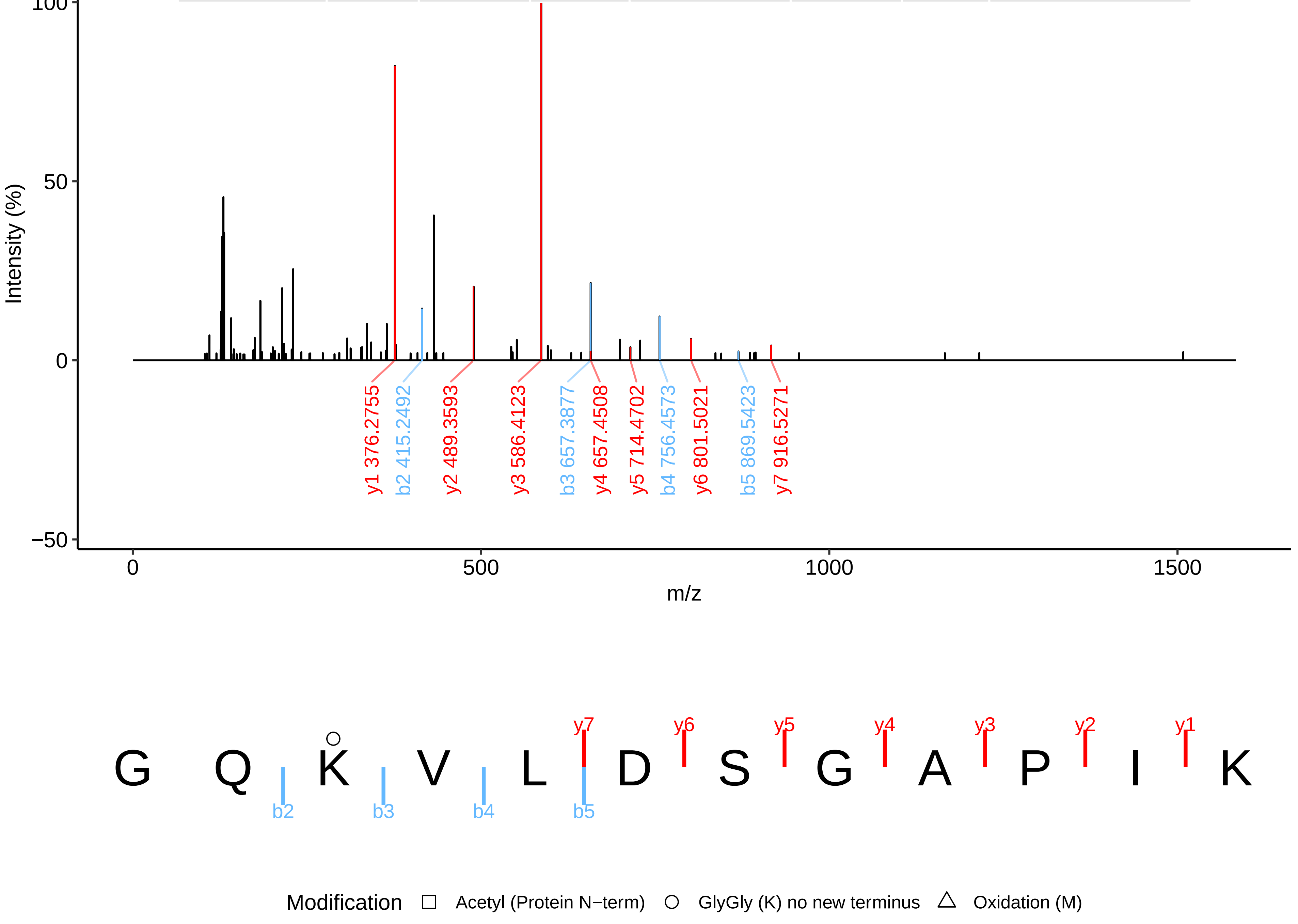

20200921_KIZ_MUb4_GlyGly_10plex − Scan number: 8862

Gene Names	Charge	m/z	Mass	Retention time	PEP	Score	Precursor Intensity
PHB2	4	287.4174	1145.64	30.208	0.073817	71.889	3976677

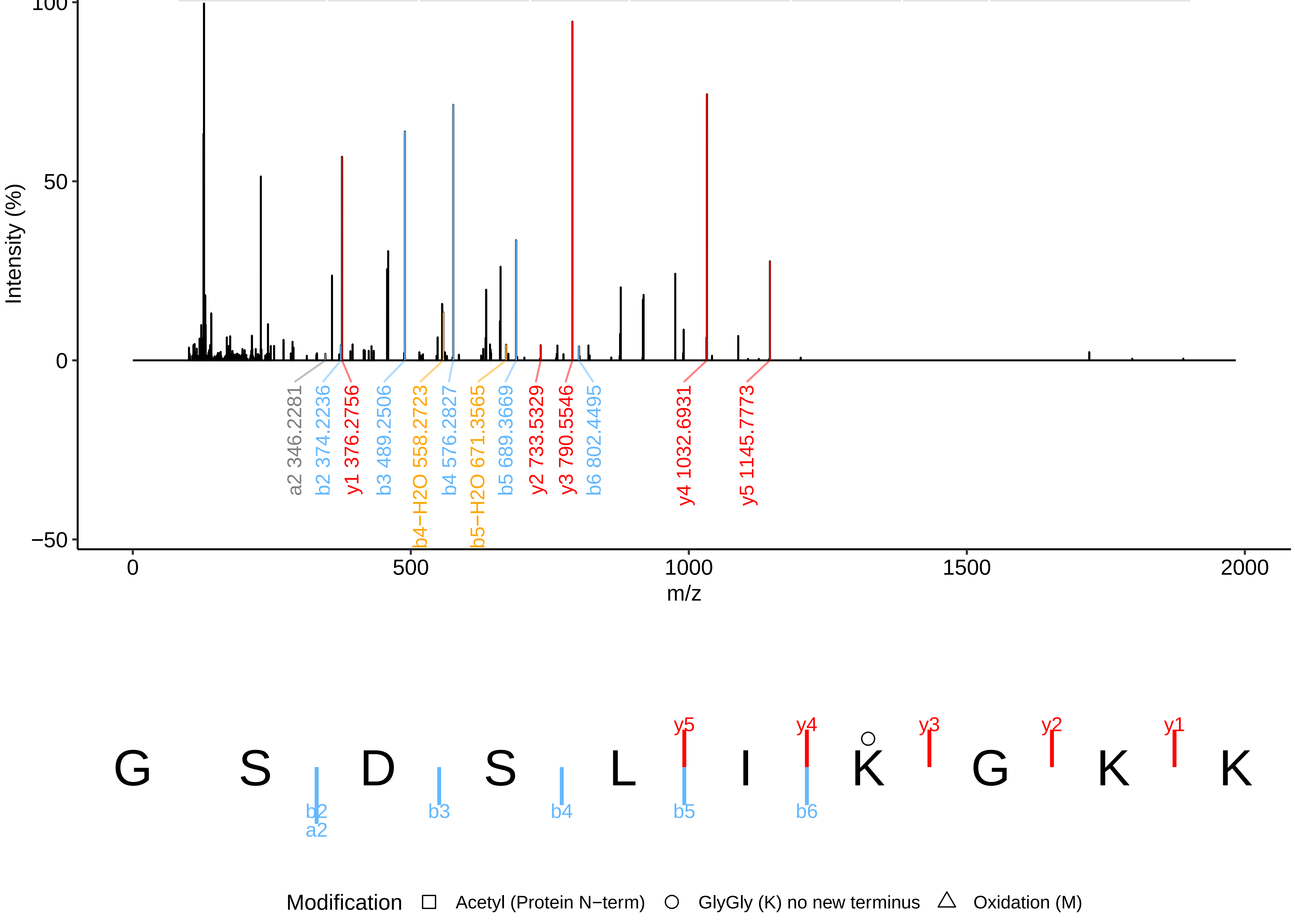

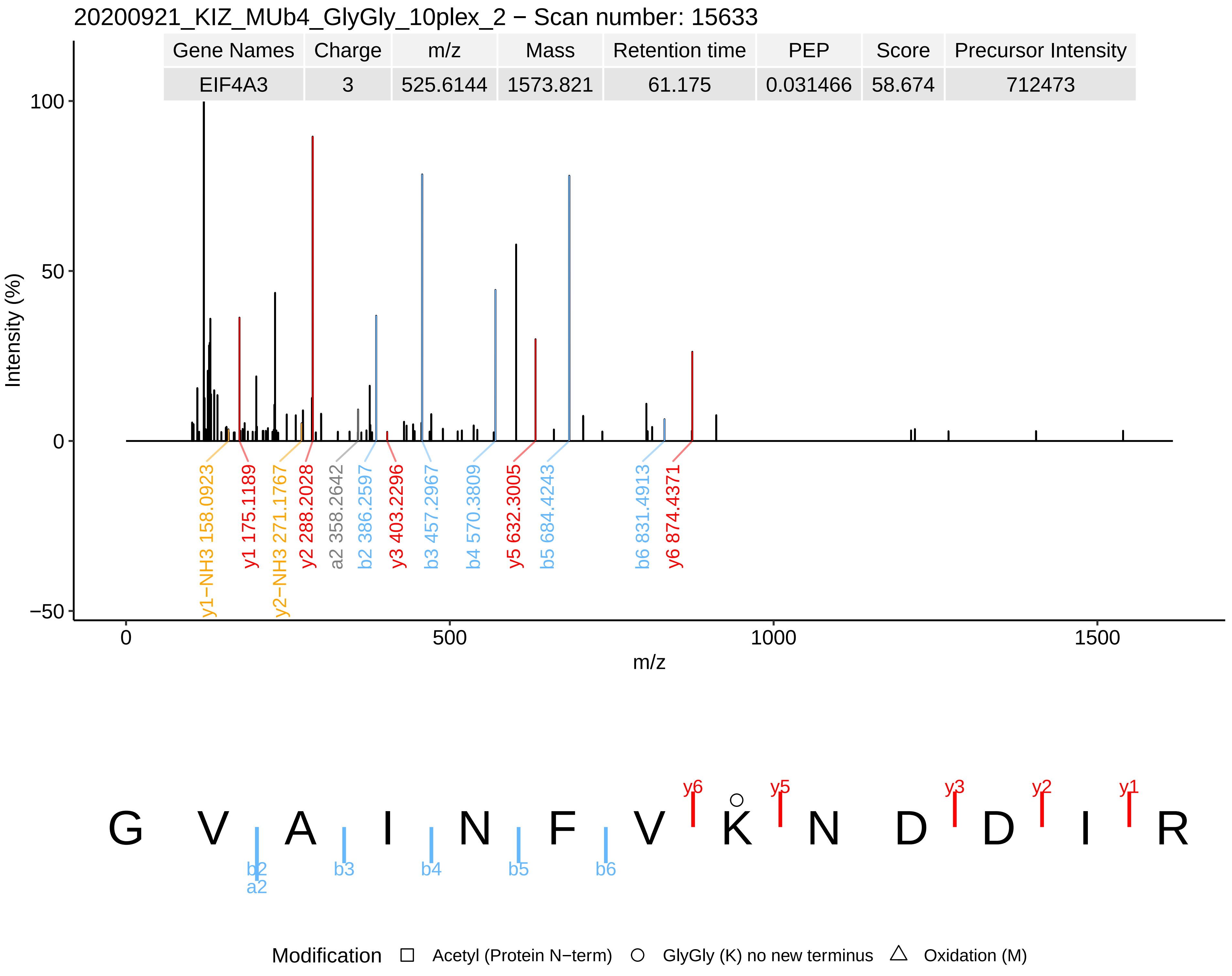
20200921_KIZ_MUb4_GlyGly_10plex_2 − Scan number: 15633
Gene Names | Charge | m/z | Mass | Retention time | PEP | Score | Precursor Intensity
EIF4A3 | 3 | 525.6144 | 1573.821 | 61.175 | 0.031466 | 58.674 | 712473
Intensity (%)
100
50
0
−50
0
500
1000
1500
m/z
y1−NH3 158.0923
y1 175.1189
y2−NH3 271.1767
y2 288.2028
a2 358.2642
b2 386.2597
y3 403.2296
b3 457.2967
b4 570.3809
y5 632.3005
b5 684.4243
b6 831.4913
y6 874.4371
G V A I N F V K N D D I R
y6 y5 y3 y2 y1
b2 a2 b3 b4 b5 b6
Modification
Acetyl (Protein N−term)
GlyGly (K) no new terminus
Oxidation (M)

20200921_KIZ_MUb4_GlyGly_10plex − Scan number: 4917

Gene Names	Charge	m
BC;HIST1H2BD;HIST1H2BL;HIST1H2BM;HIST2H2BF;HIST2H2BE;HIST1H2BO;HIST1H2BB;HIST3H2BB;HIST1H2BA	4	336.

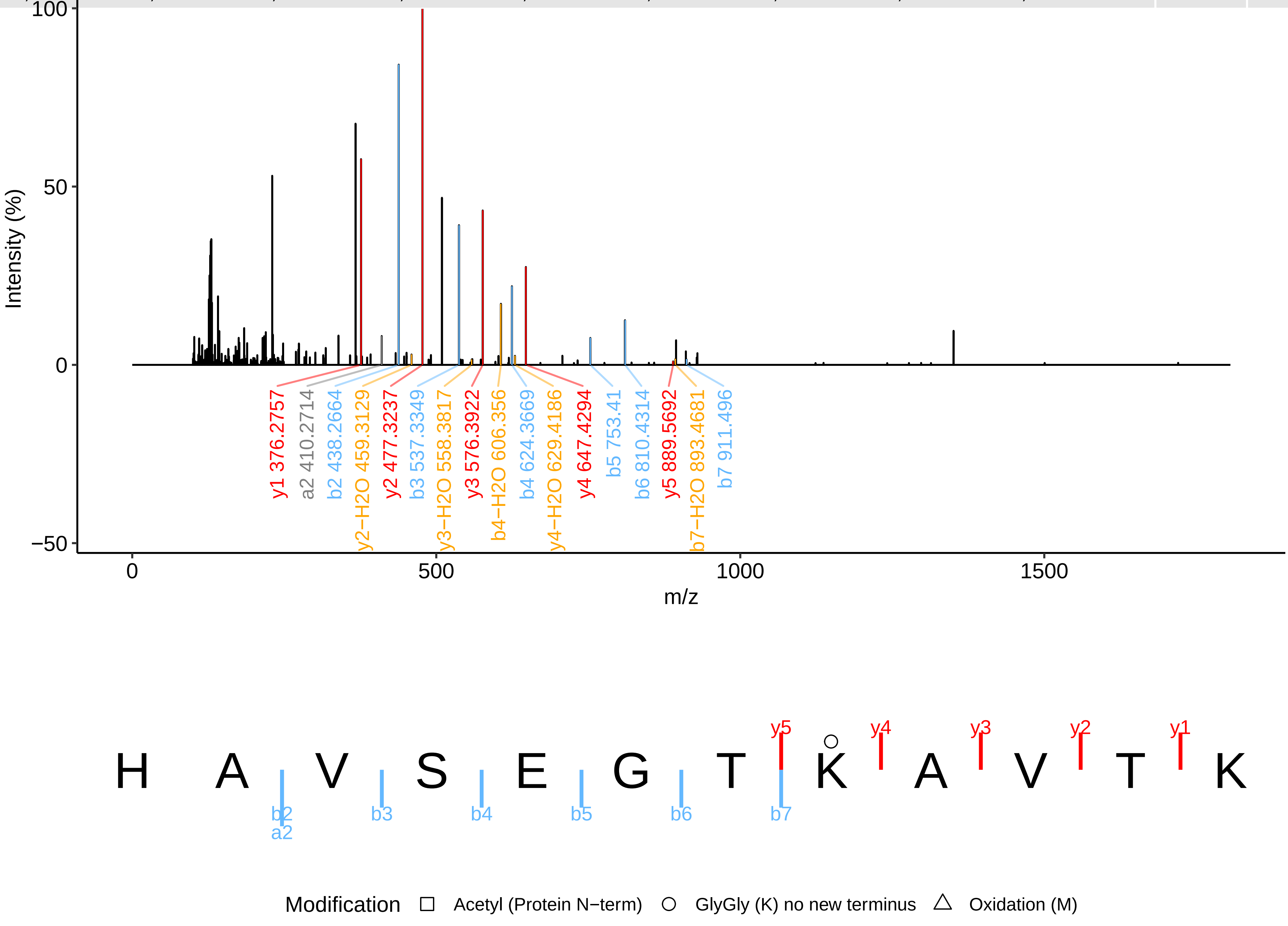

20200921_KIZ_MUb4_GlyGly_10plex_2 − Scan number: 4595

Gene Names	Charge	m
BC;HIST1H2BD;HIST1H2BL;HIST1H2BM;HIST2H2BF;HIST2H2BE;HIST1H2BO;HIST1H2BB;HIST3H2BB;HIST1H2BA	4	336.

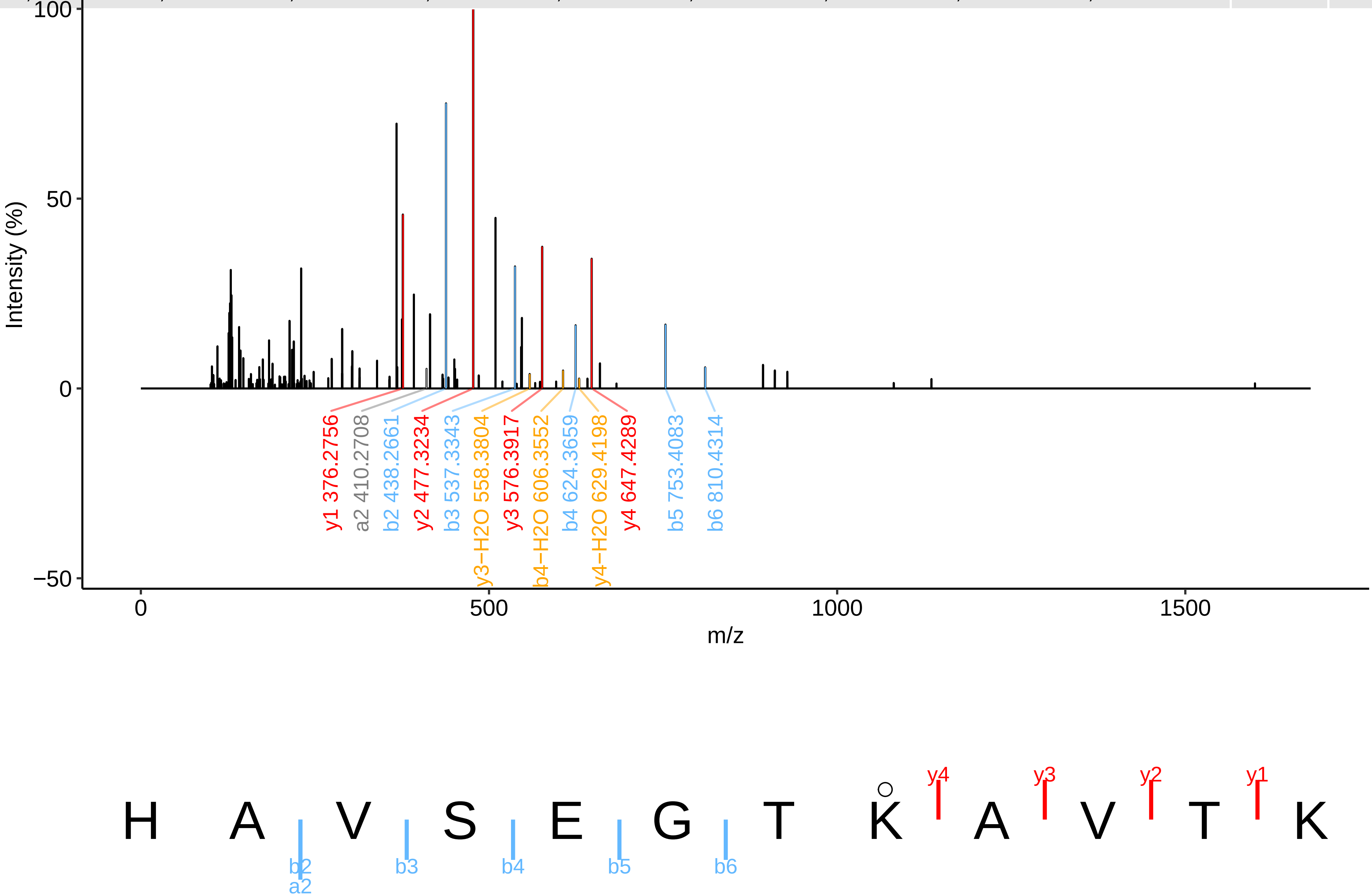

Modification □ Acetyl (Protein N−term) ○ GlyGly (K) no new terminus △ Oxidation (M)

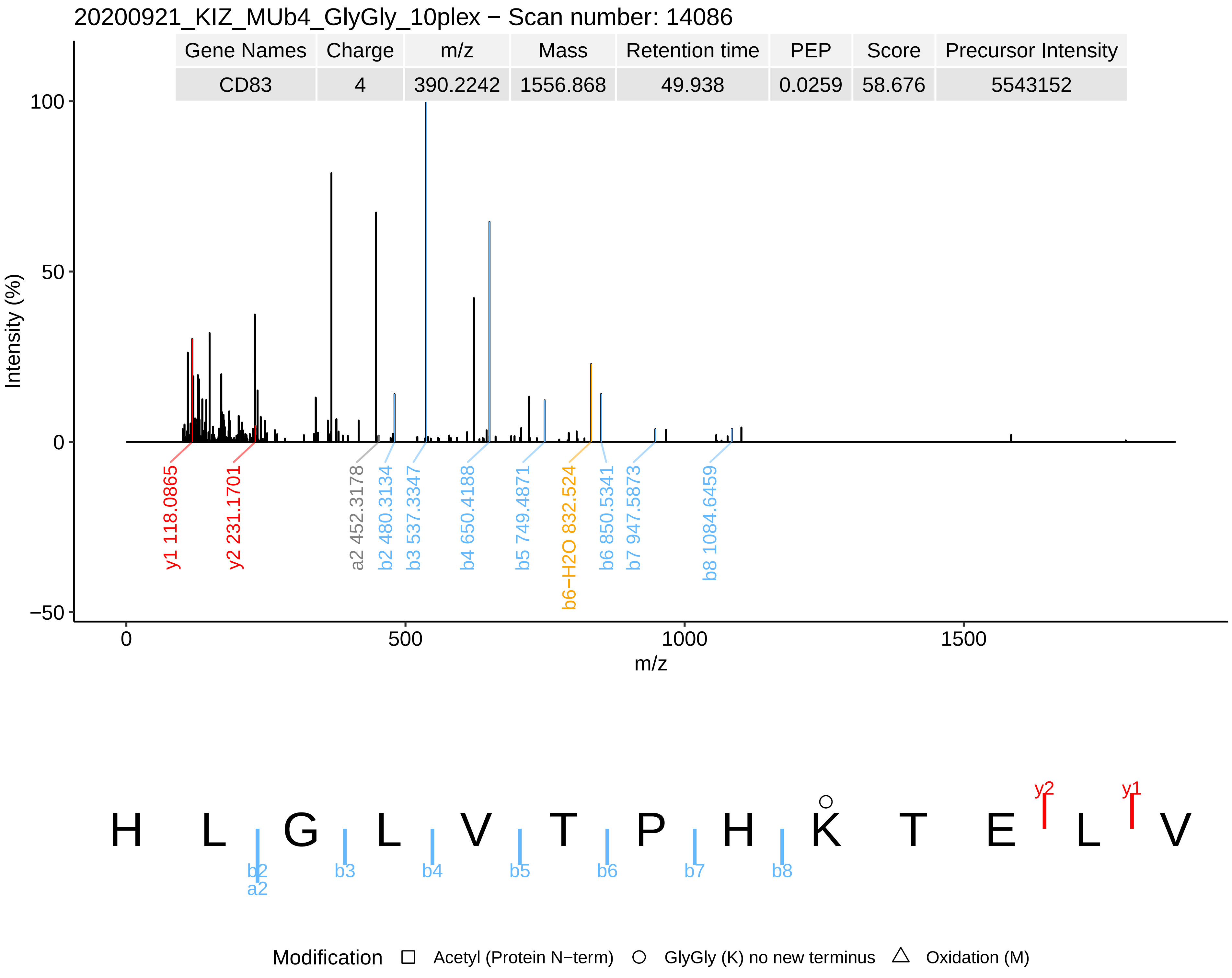
20200921_KIZ_MUb4_GlyGly_10plex − Scan number: 14086
Gene Names
Charge
m/z
Mass
Retention time
PEP
Score
Precursor Intensity
CD83
4
390.2242
1556.868
49.938
0.0259
58.676
5543152
Intensity (%)
100
50
0
−50
0
500
1000
1500
m/z
y1 118.0865
y2 231.1701
a2 452.3178
b2 480.3134
b3 537.3347
b4 650.4188
b5 749.4871
b6−H2O 832.524
b6 850.5341
b7 947.5873
b8 1084.6459
H L G L V T P H K T E L V
b2
a2
b3
b4
b5
b6
b7
b8
y2
y1
Modification
Acetyl (Protein N−term)
GlyGly (K) no new terminus
Oxidation (M)

20200921_KIZ_MUb4_GlyGly_10plex_2 − Scan number: 14899

Gene Names	Charge	m/z	Mass	Retention time	PEP	Score	Precursor Intensity
MFN1	3	458.5806	1372.72	58.161	0.0023561	79.82	499449.3

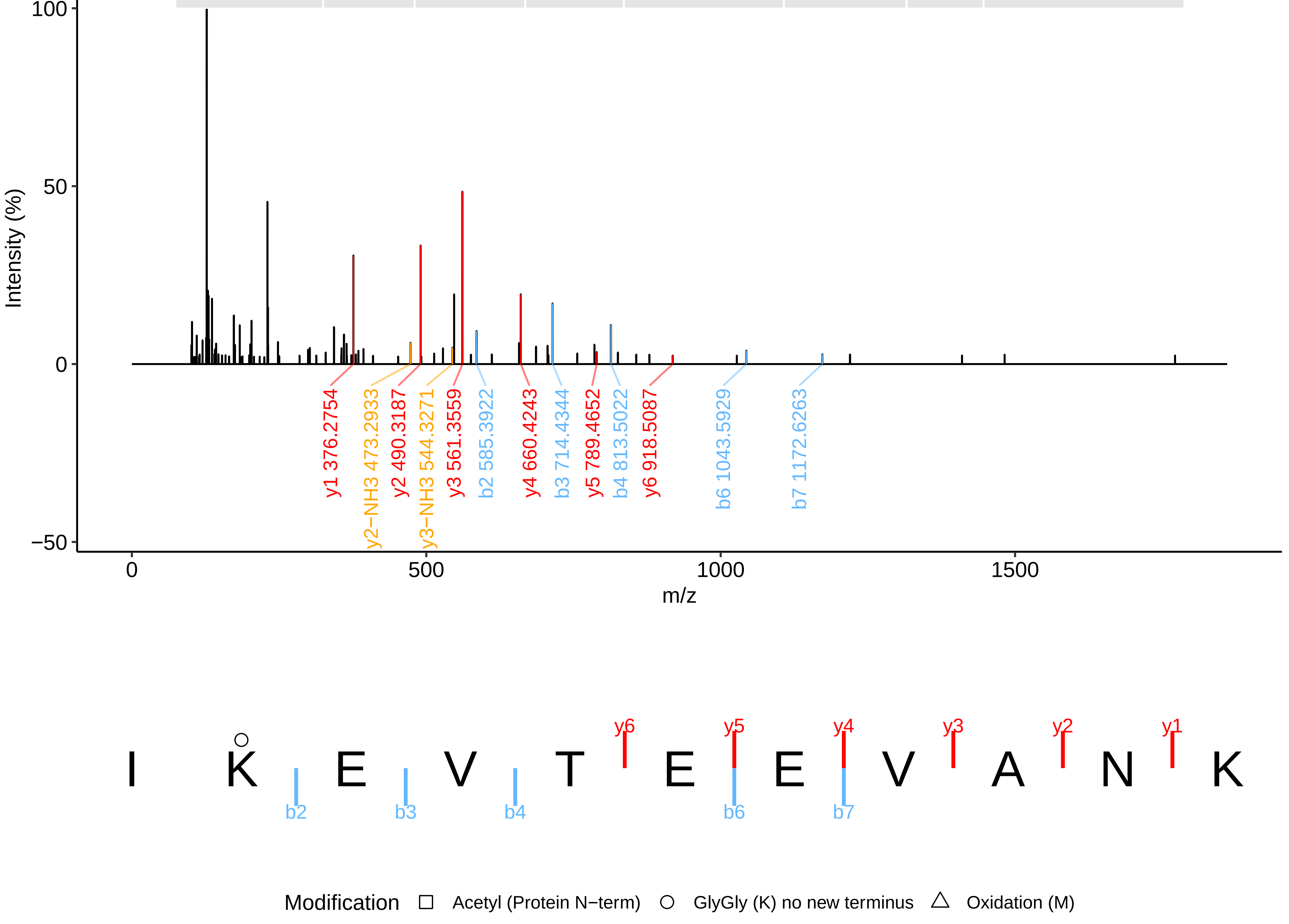

20201118_KIZ_MUb6_GlyGly_1 − Scan number: 36005

Gene Names	Charge	m/z	Mass	Retention time	PEP	Score	Precursor Intensity
	2	783.4836	1564.953	104.76	0.030068	84.083	NA

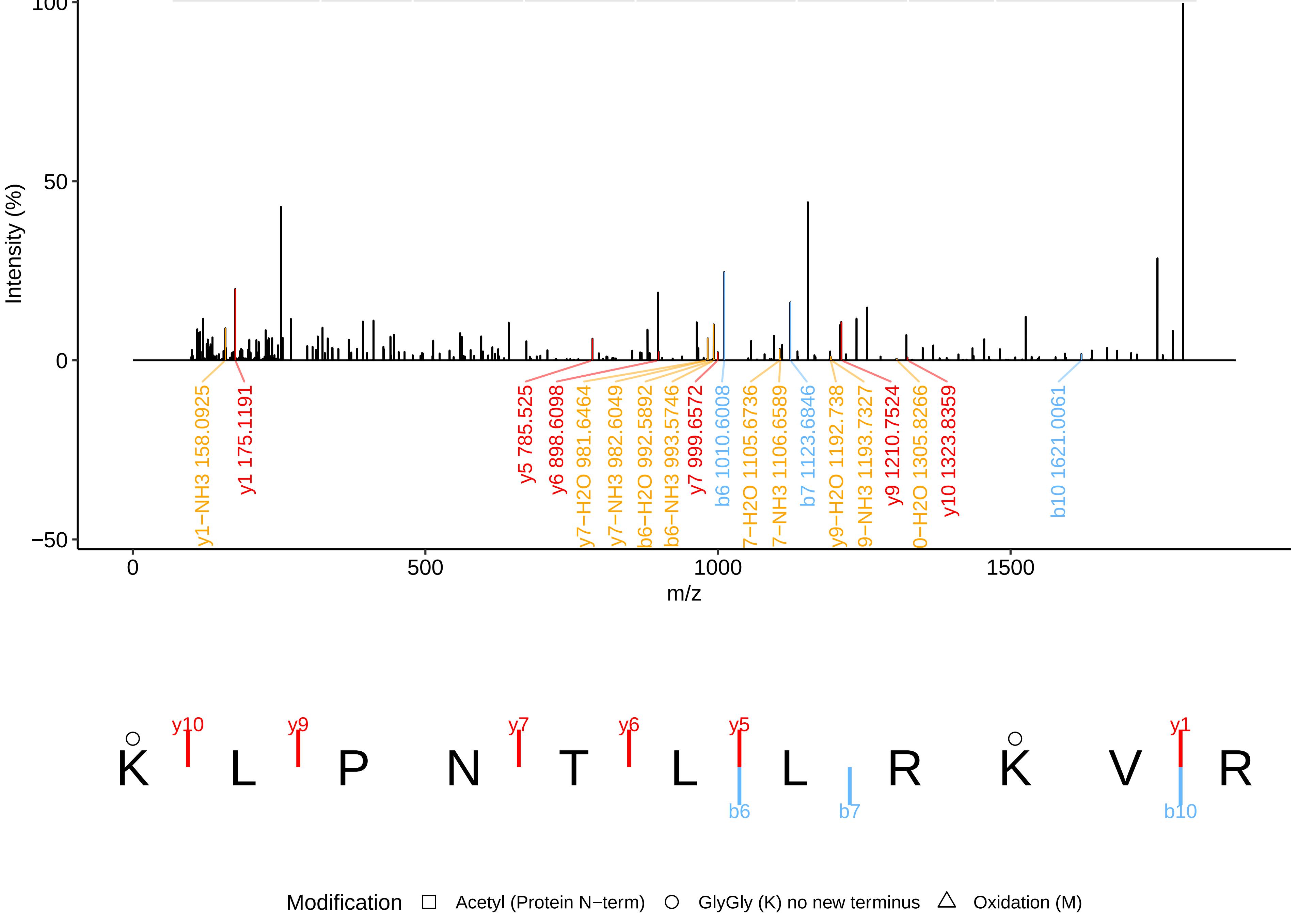

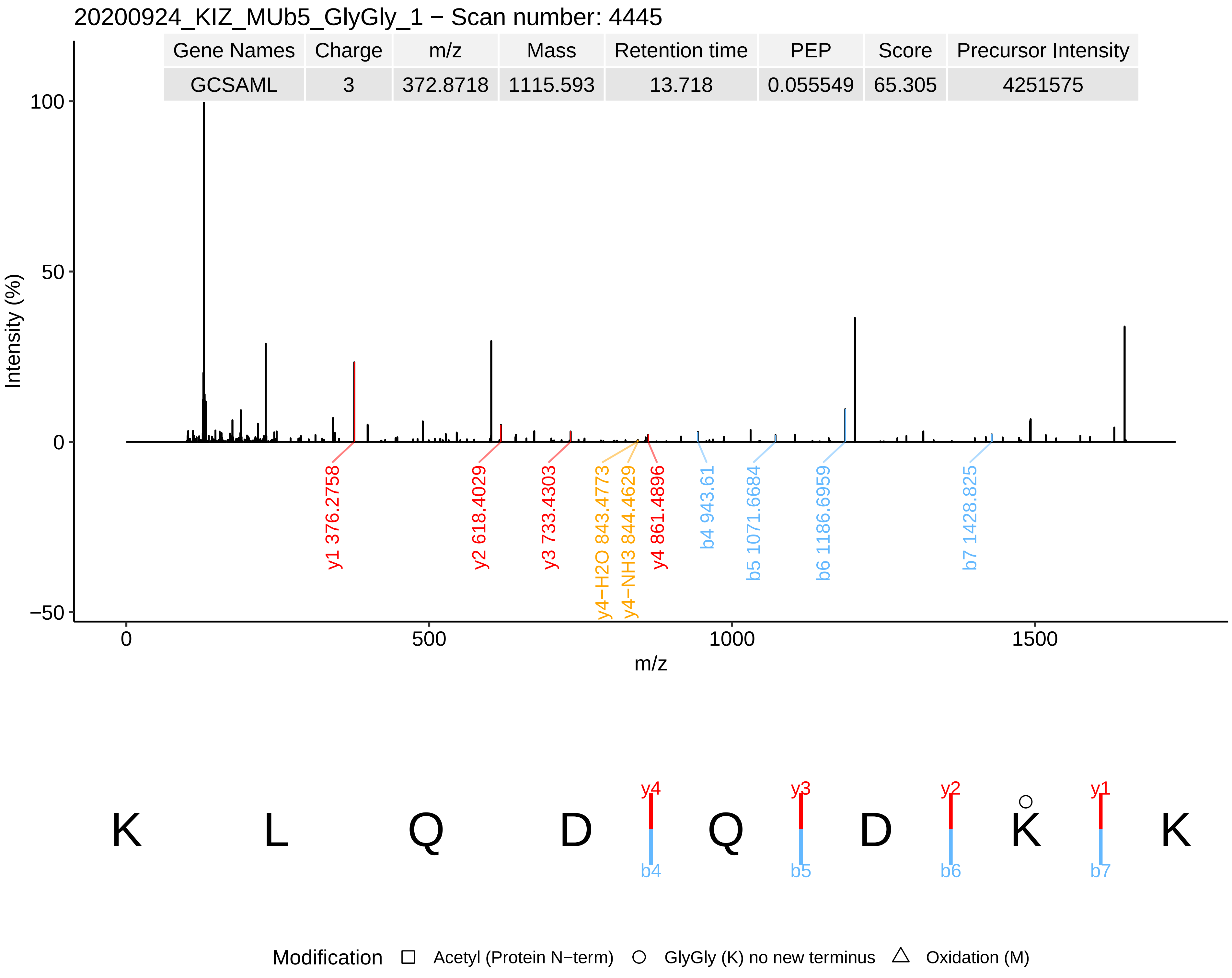
20200924_KIZ_MUb5_GlyGly_1 − Scan number: 4445
Gene Names | Charge | m/z | Mass | Retention time | PEP | Score | Precursor Intensity
GCSAML | 3 | 372.8718 | 1115.593 | 13.718 | 0.055549 | 65.305 | 4251575
Intensity (%)
m/z
y1 376.2758
y2 618.4029
y3 733.4303
y4−H2O 843.4773
y4−NH3 844.4629
y4 861.4896
b4 943.61
b5 1071.6684
b6 1186.6959
b7 1428.825
K L Q D Q D K K
y4 y3 y2 y1
b4 b5 b6 b7
Modification
Acetyl (Protein N−term)
GlyGly (K) no new terminus
Oxidation (M)

20200921_KIZ_MUb4_GlyGly_10plex − Scan number: 24690

Gene Names	Charge	m/z	Mass	Retention time	PEP	Score	Precursor Intensity
	3	534.2779	1599.812	89.806	0.0028582	79.659	12976037

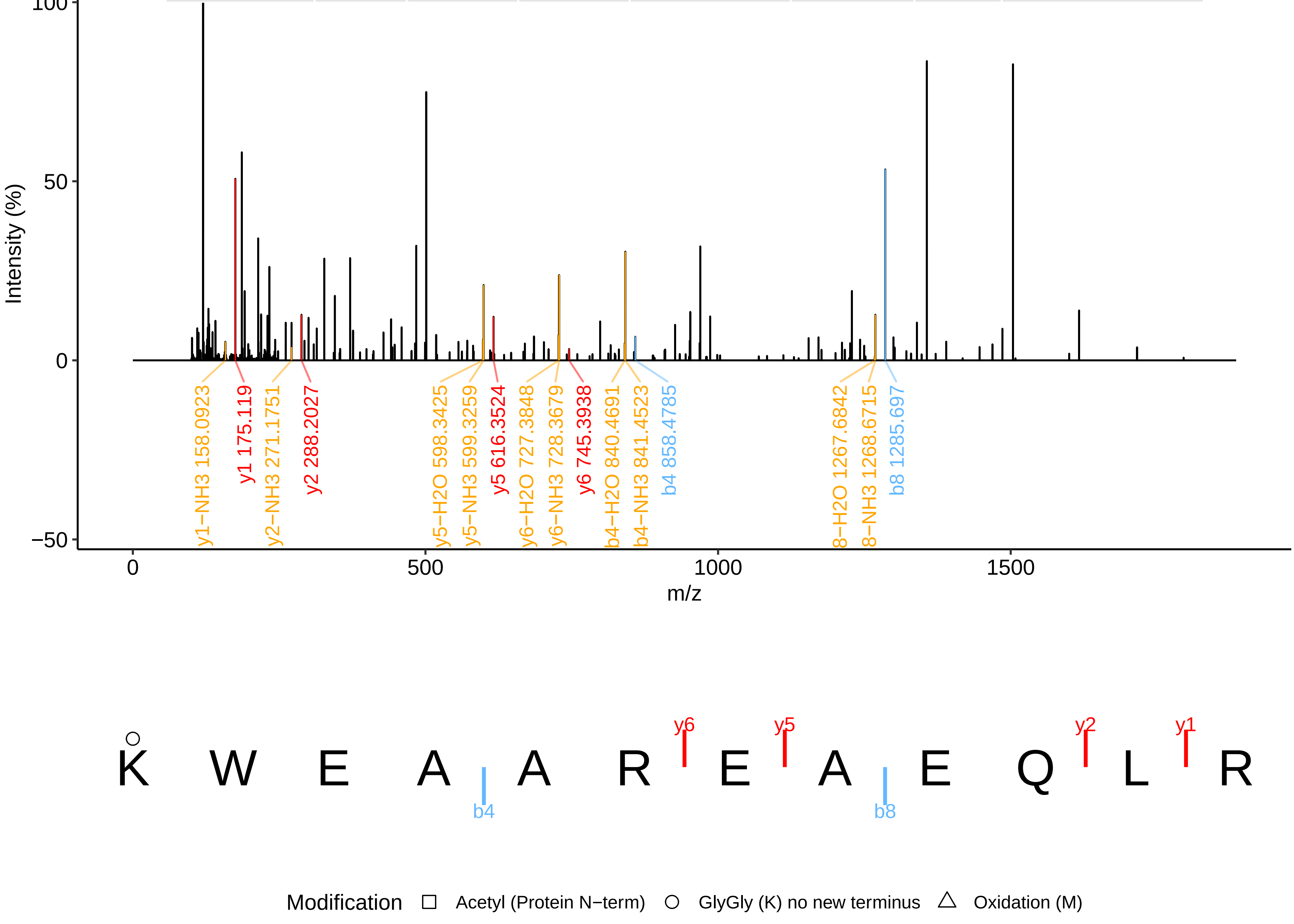

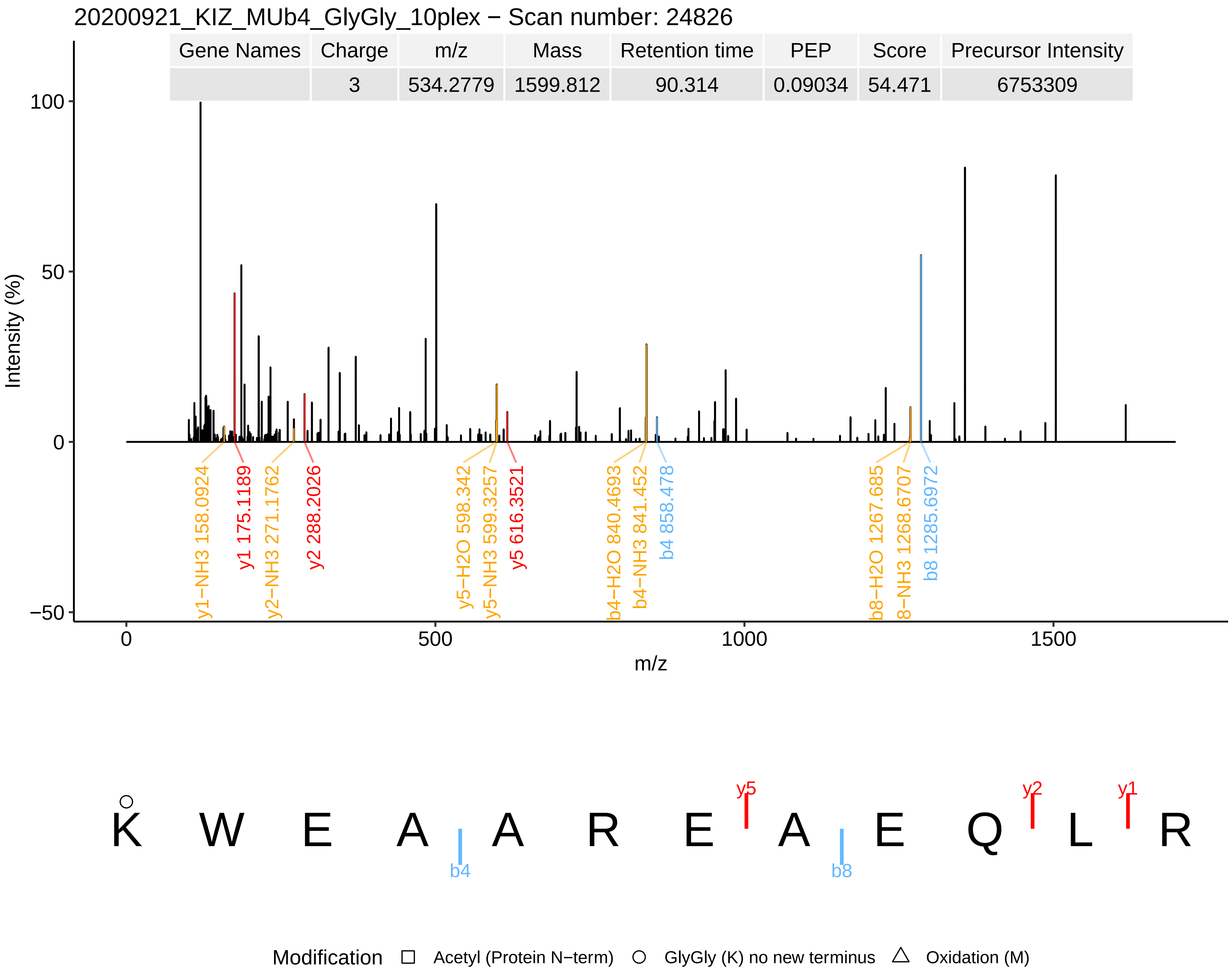
20200921_KIZ_MUb4_GlyGly_10plex − Scan number: 24826
Gene Names
Charge
m/z
Mass
Retention time
PEP
Score
Precursor Intensity
3
534.2779
1599.812
90.314
0.09034
54.471
6753309
Intensity (%)
100
50
0
−50
0
500
1000
1500
m/z
y1−NH3 158.0924
y1 175.1189
y2−NH3 271.1762
y2 288.2026
y5−H2O 598.342
y5−NH3 599.3257
y5 616.3521
b4−H2O 840.4693
b4−NH3 841.452
b4 858.478
b8−H2O 1267.685
b8−NH3 1268.6707
b8 1285.6972
K W E A A R E A E Q L R
y5
y2
y1
b4
b8
Modification
Acetyl (Protein N−term)
GlyGly (K) no new terminus
Oxidation (M)

20201118_KIZ_MUb6_GlyGly_1 − Scan number: 23554

Gene Names	Charge	m/z	Mass	Retention time	PEP	Score	Precursor Intensity
	3	534.2779	1599.812	72.809	0.10031	53.237	44697528

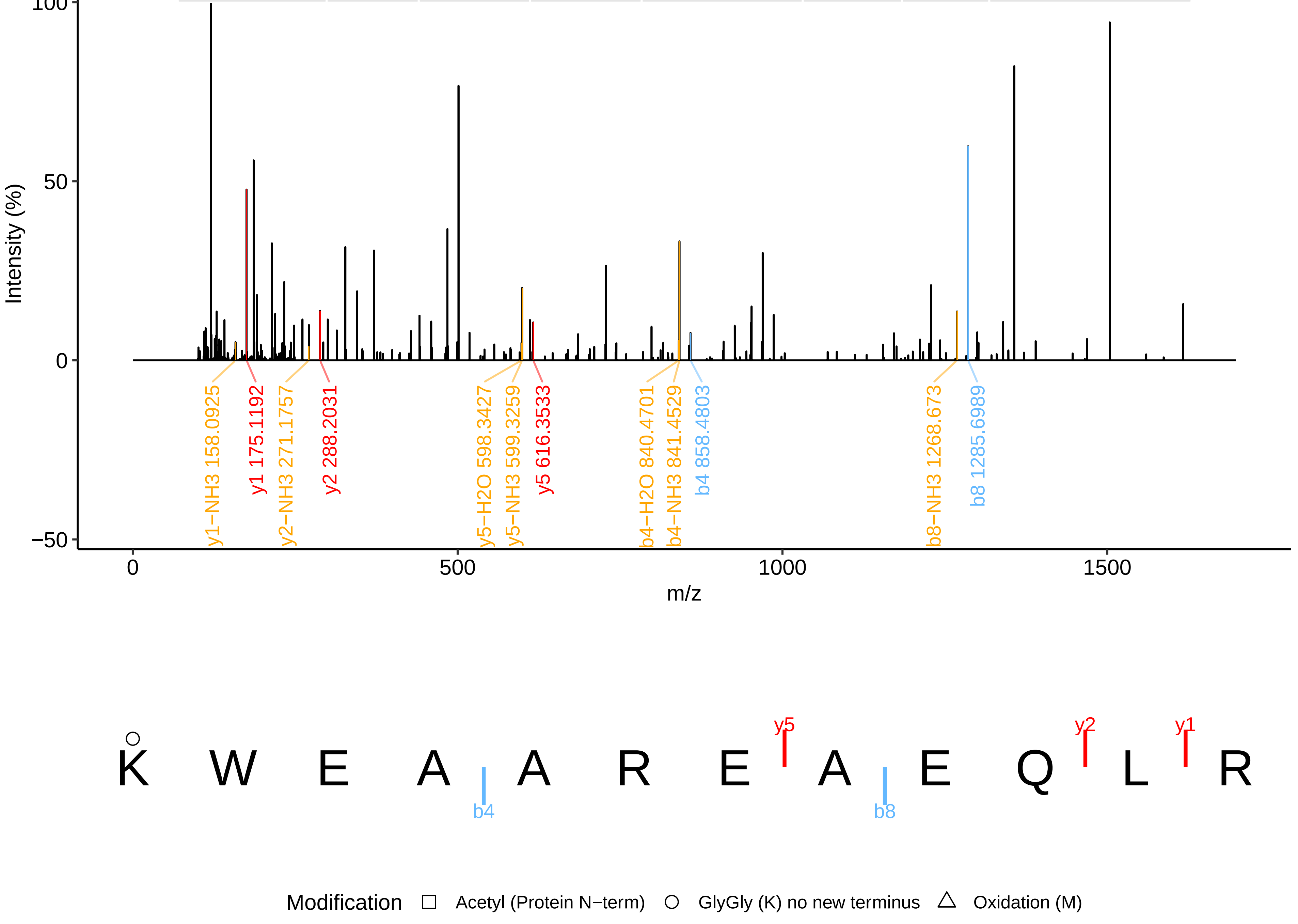

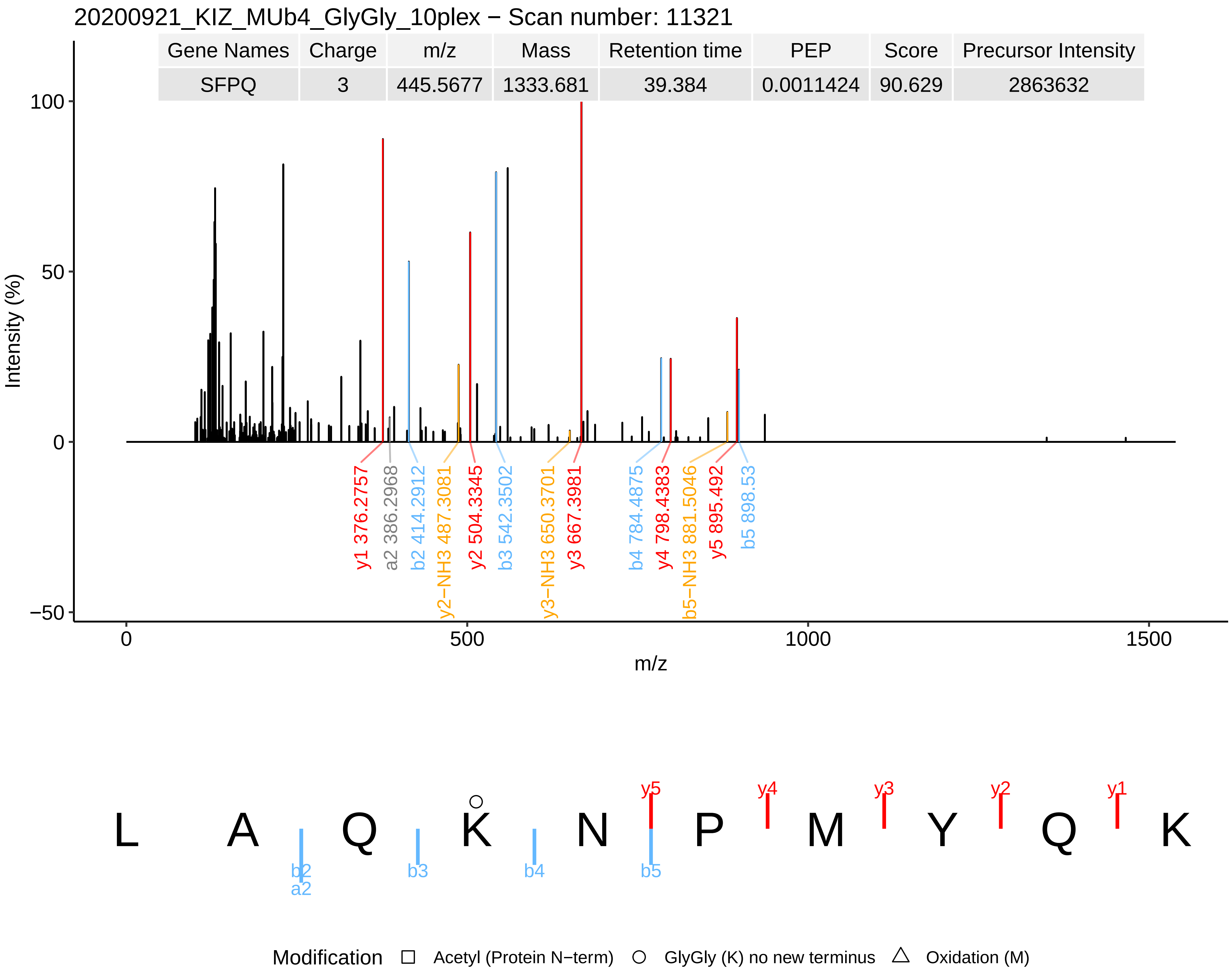
20200921_KIZ_MUb4_GlyGly_10plex − Scan number: 11321
Gene Names
Charge
m/z
Mass
Retention time
PEP
Score
Precursor Intensity
SFPQ
3
445.5677
1333.681
39.384
0.0011424
90.629
2863632
Intensity (%)
100
50
0
−50
0
500
1000
1500
m/z
y1 376.2757
a2 386.2968
b2 414.2912
y2−NH3 487.3081
y2 504.3345
b3 542.3502
y3−NH3 650.3701
y3 667.3981
b4 784.4875
y4 798.4383
b5−NH3 881.5046
y5 895.492
b5 898.53
L
A
Q
K
N
P
M
Y
Q
K
y5
y4
y3
y2
y1
b2
a2
b3
b4
b5
Modification
Acetyl (Protein N−term)
GlyGly (K) no new terminus
Oxidation (M)

20200921_KIZ_MUb4_GlyGly_10plex_2 − Scan number: 10460

Gene Names	Charge	m/z	Mass	Retention time	PEP	Score	Precursor Intensity
SFPQ	3	445.5677	1333.681	40.021	0.0034071	85.731	1396585

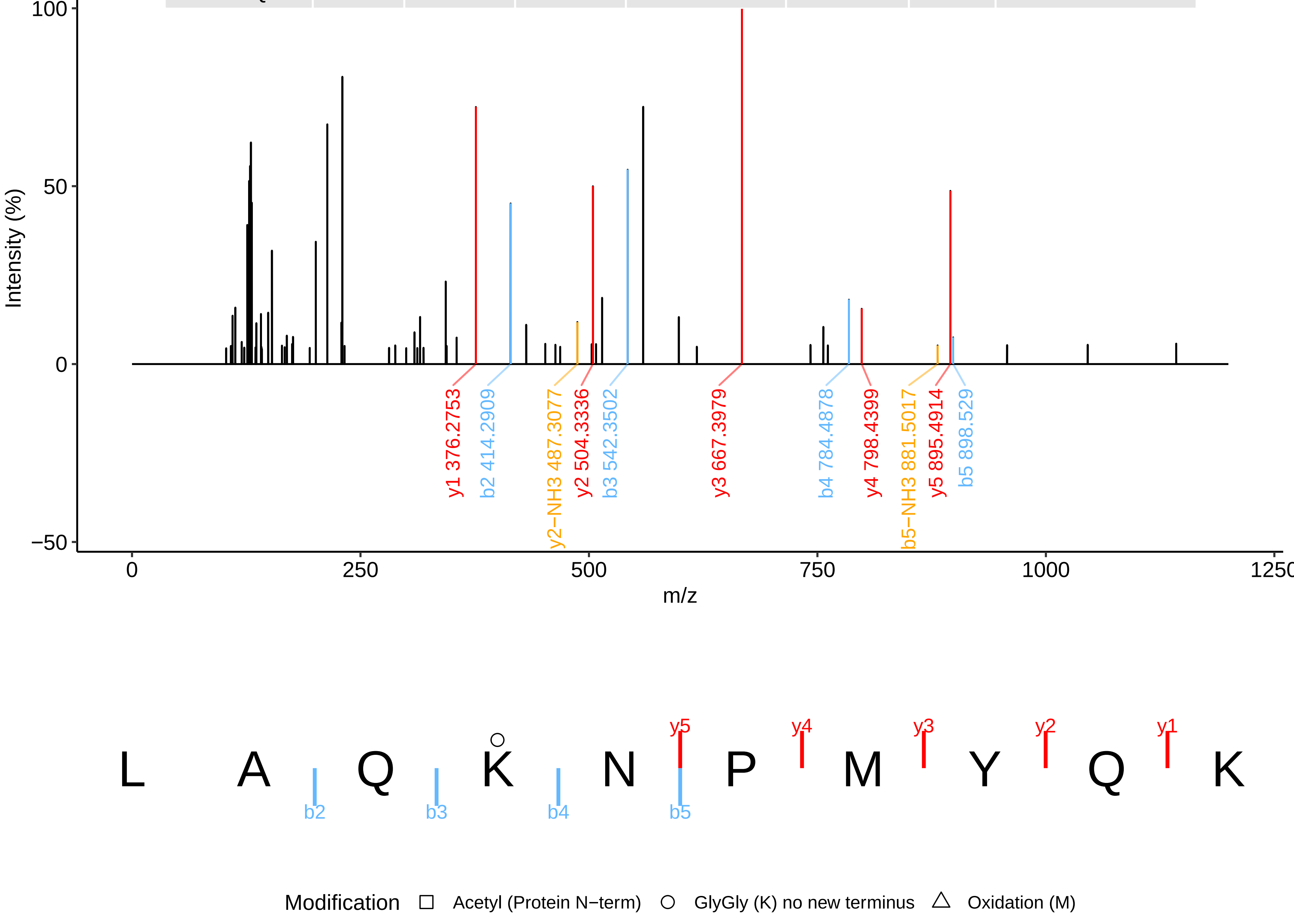

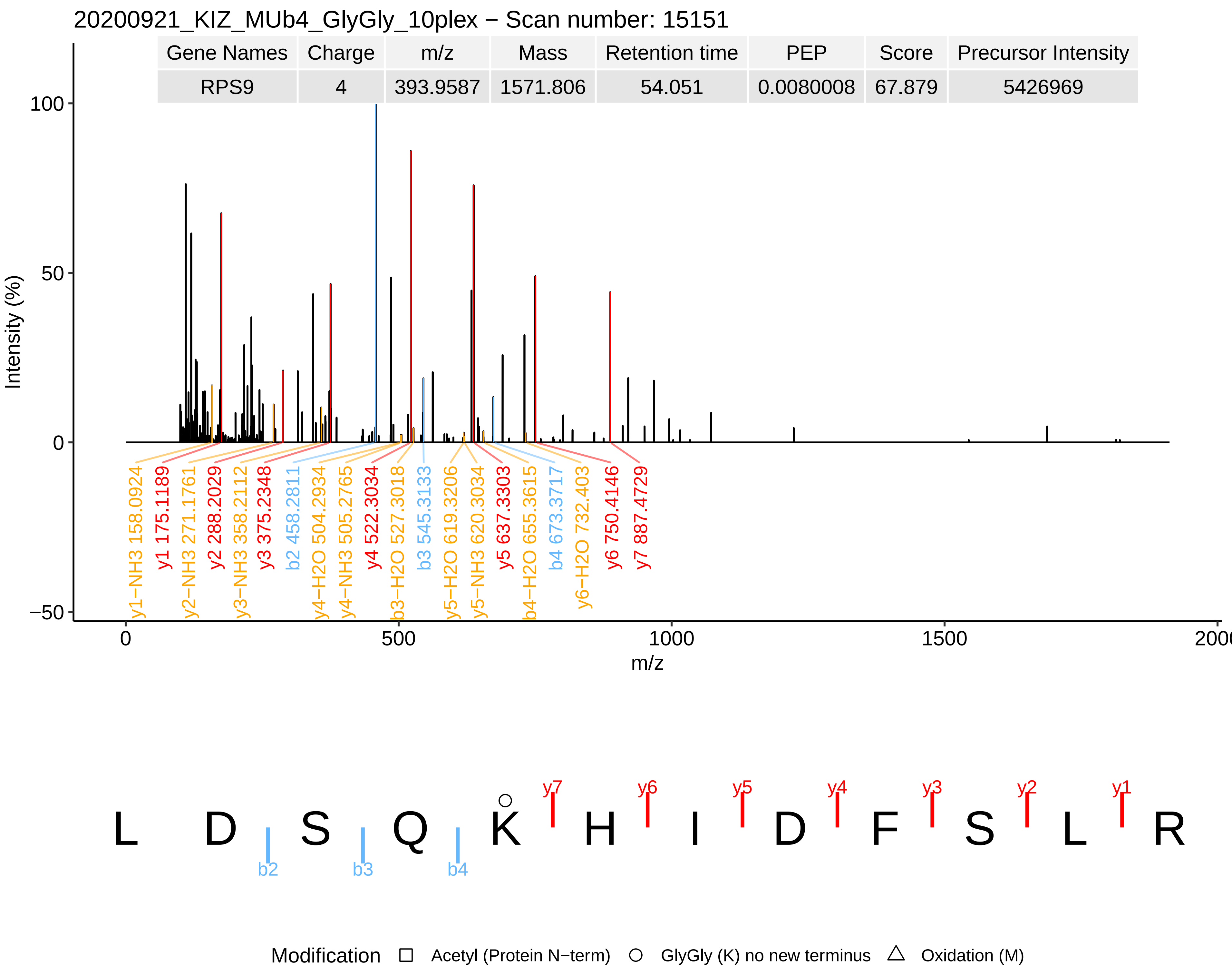
20200921_KIZ_MUb4_GlyGly_10plex − Scan number: 15151
Gene Names | Charge | m/z | Mass | Retention time | PEP | Score | Precursor Intensity
RPS9 | 4 | 393.9587 | 1571.806 | 54.051 | 0.0080008 | 67.879 | 5426969
Intensity (%)
m/z
y1−NH3 158.0924
y1 175.1189
y2−NH3 271.1761
y2 288.2029
y3−NH3 358.2112
y3 375.2348
b2 458.2811
y4−H2O 504.2934
y4−NH3 505.2765
y4 522.3034
b3−H2O 527.3018
b3 545.3133
y5−H2O 619.3206
y5−NH3 620.3034
y5 637.3303
b4−H2O 655.3615
b4 673.3717
y6−H2O 732.403
y6 750.4146
y7 887.4729
L D S Q K H I D F S L R
y7 y6 y5 y4 y3 y2 y1
b2 b3 b4
Modification
Acetyl (Protein N−term)
GlyGly (K) no new terminus
Oxidation (M)

20200921_KIZ_MUb4_GlyGly_10plex_2 − Scan number: 17534

Gene Names	Charge	m/z	Mass	Retention time	PEP	Score	Precursor Intensity
PSMC3	3	440.5795	1318.717	68.572	0.08337	46.819	1557731

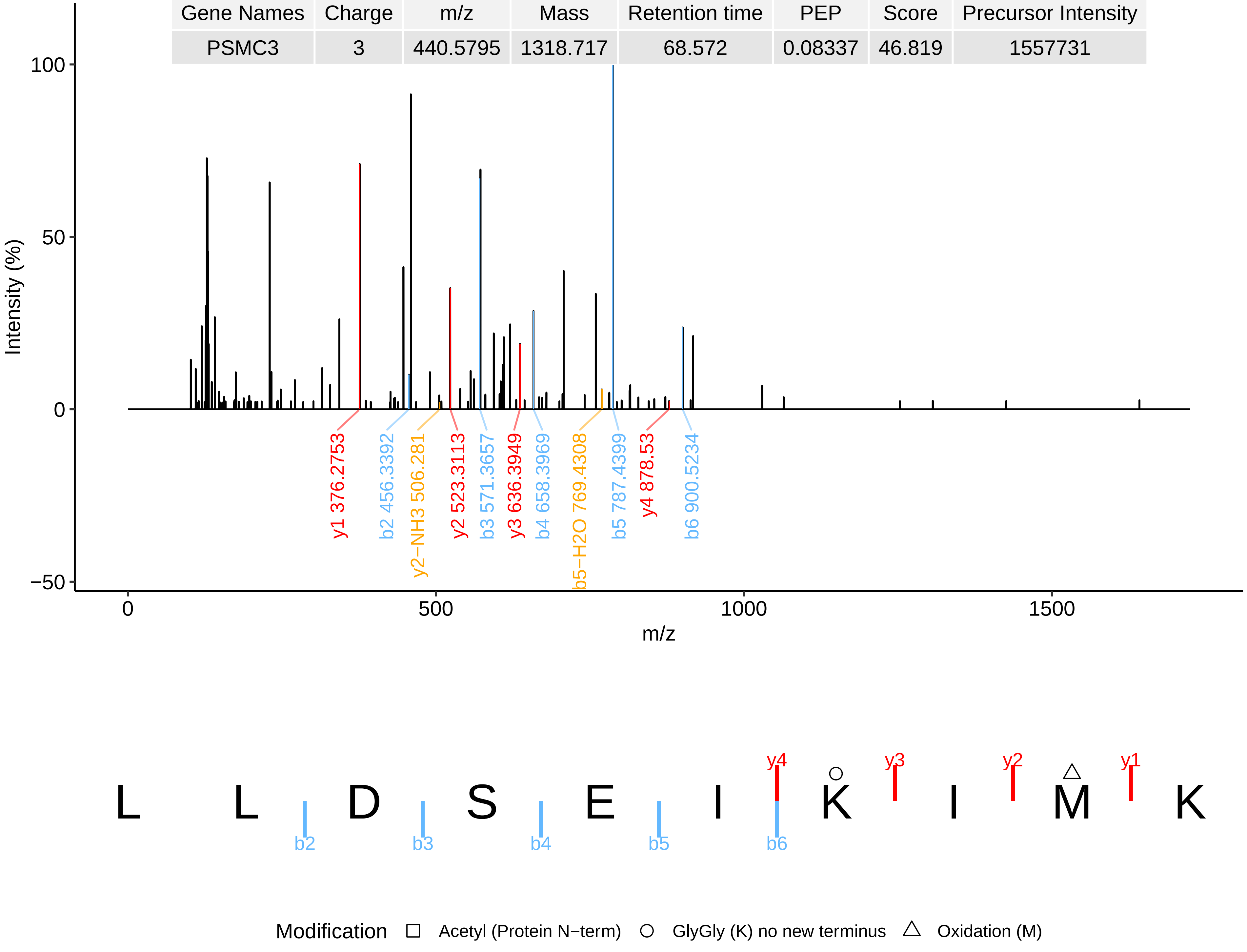

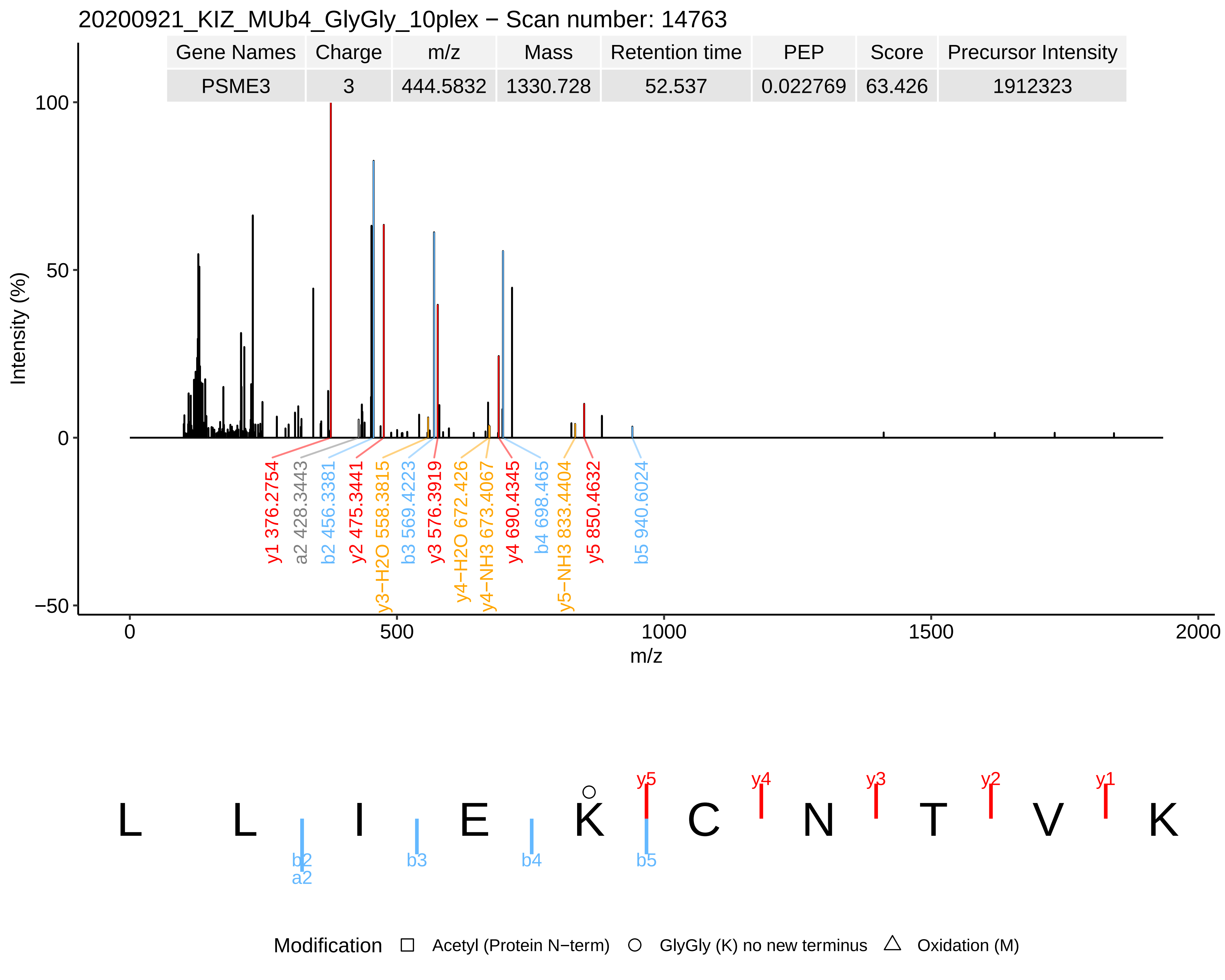
20200921_KIZ_MUb4_GlyGly_10plex − Scan number: 14763
Gene Names | Charge | m/z | Mass | Retention time | PEP | Score | Precursor Intensity
PSME3 | 3 | 444.5832 | 1330.728 | 52.537 | 0.022769 | 63.426 | 1912323
Intensity (%)
100
50
0
−50
0
500
1000
1500
2000
m/z
y1 376.2754
a2 428.3443
b2 456.3381
y2 475.3441
y3−H2O 558.3815
b3 569.4223
y3 576.3919
y4−H2O 672.426
y4−NH3 673.4067
y4 690.4345
b4 698.465
y5−NH3 833.4404
y5 850.4632
b5 940.6024
L L I E K C N T V K
y5 y4 y3 y2 y1
b2 a2 b3 b4 b5
Modification
Acetyl (Protein N−term)
GlyGly (K) no new terminus
Oxidation (M)

20201118_KIZ_MUb6_GlyGly_1 − Scan number: 20353

Gene Names	Charge	m/z	Mass	Retention time	PEP	Score	Precursor Intensity
ARL2;ARL2−SNX15	3	533.9582	1598.853	63.677	6.2729e−10	134.61	8147396

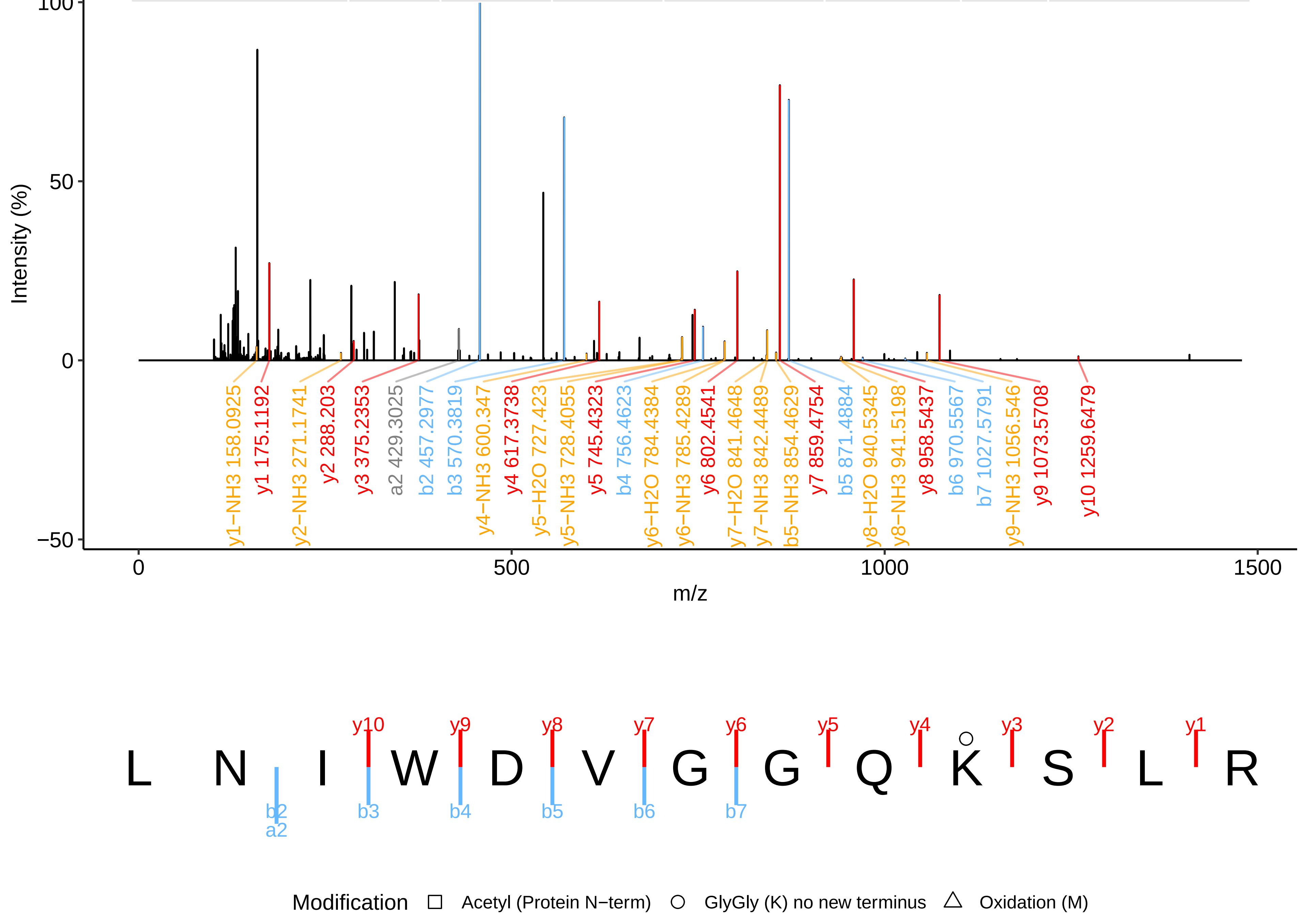

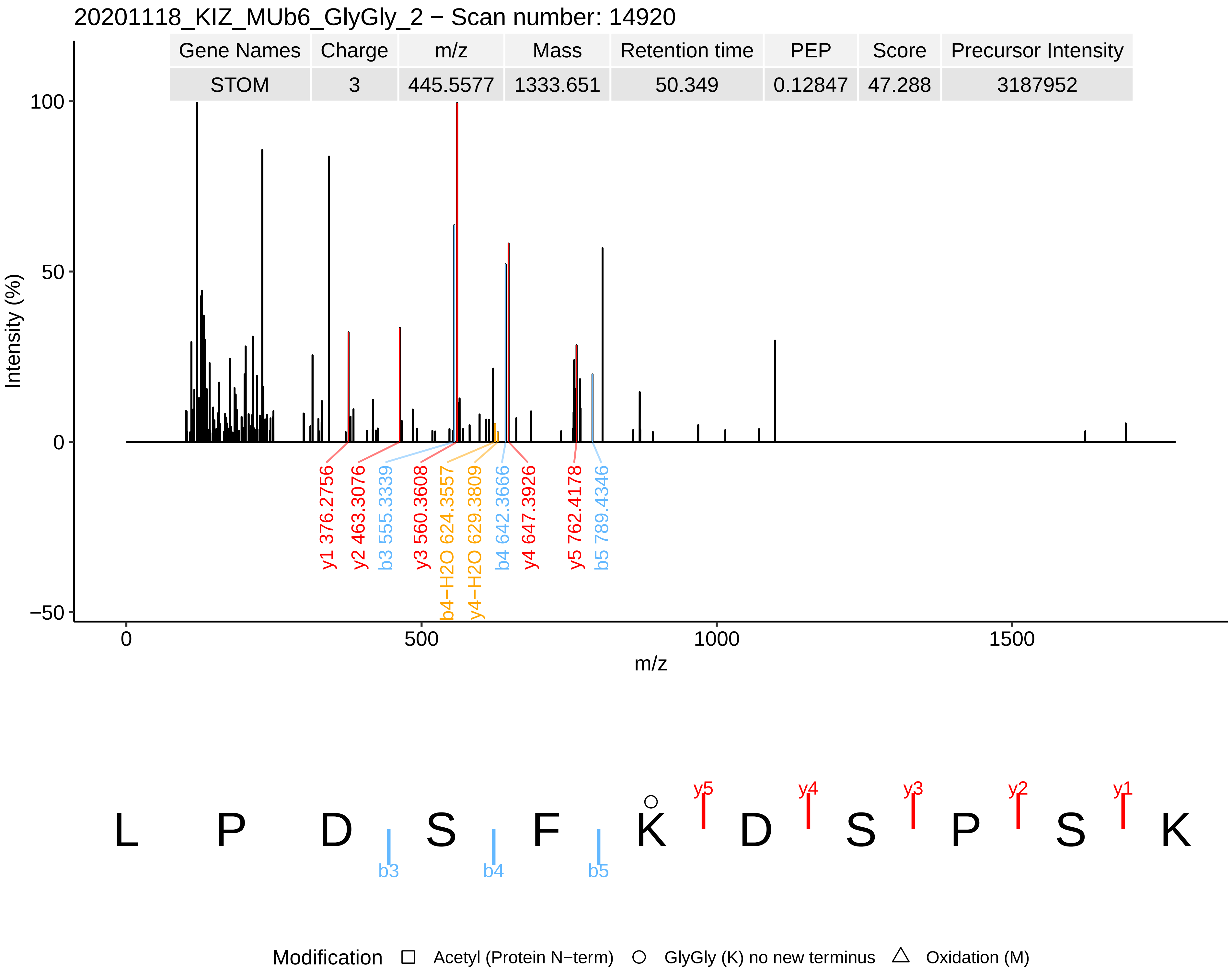
20201118_KIZ_MUb6_GlyGly_2 − Scan number: 14920
Gene Names | Charge | m/z | Mass | Retention time | PEP | Score | Precursor Intensity
STOM | 3 | 445.5577 | 1333.651 | 50.349 | 0.12847 | 47.288 | 3187952
Intensity (%)
100
50
0
−50
0
500
1000
1500
m/z
y1 376.2756
y2 463.3076
b3 555.3339
y3 560.3608
b4−H2O 624.3557
y4−H2O 629.3809
b4 642.3666
y4 647.3926
y5 762.4178
b5 789.4346
L P D S F K D S P S K
y5 y4 y3 y2 y1
b3 b4 b5
Modification
Acetyl (Protein N−term)
GlyGly (K) no new terminus
Oxidation (M)

20200921_KIZ_MUb4_GlyGly_10plex_2 − Scan number: 15011

Gene Names	Charge	m/z	Mass	Retention time	PEP	Score	Precursor Intensity
KRT7	3	519.2777	1554.811	58.617	0.0004163	92.247	441020.3

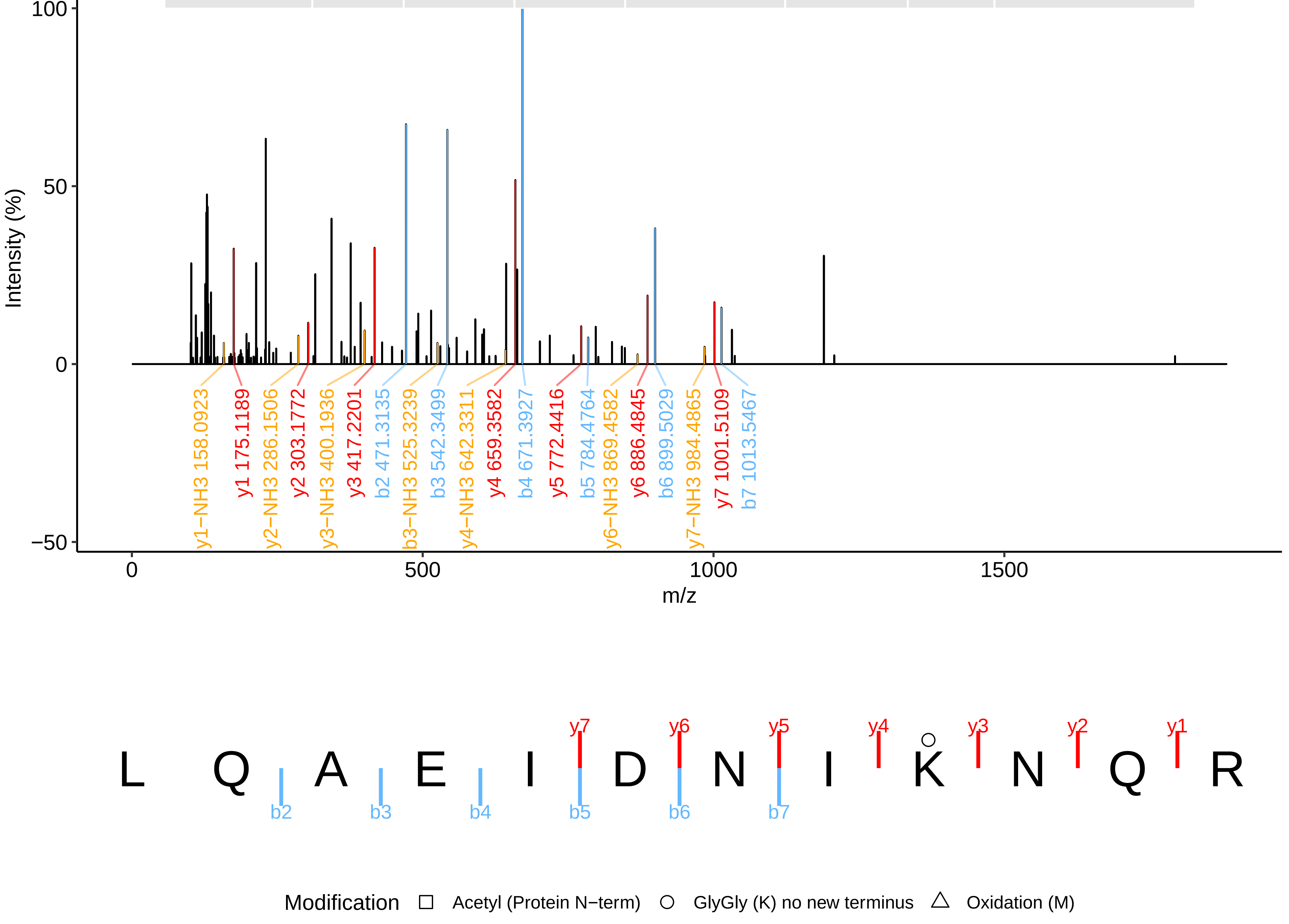

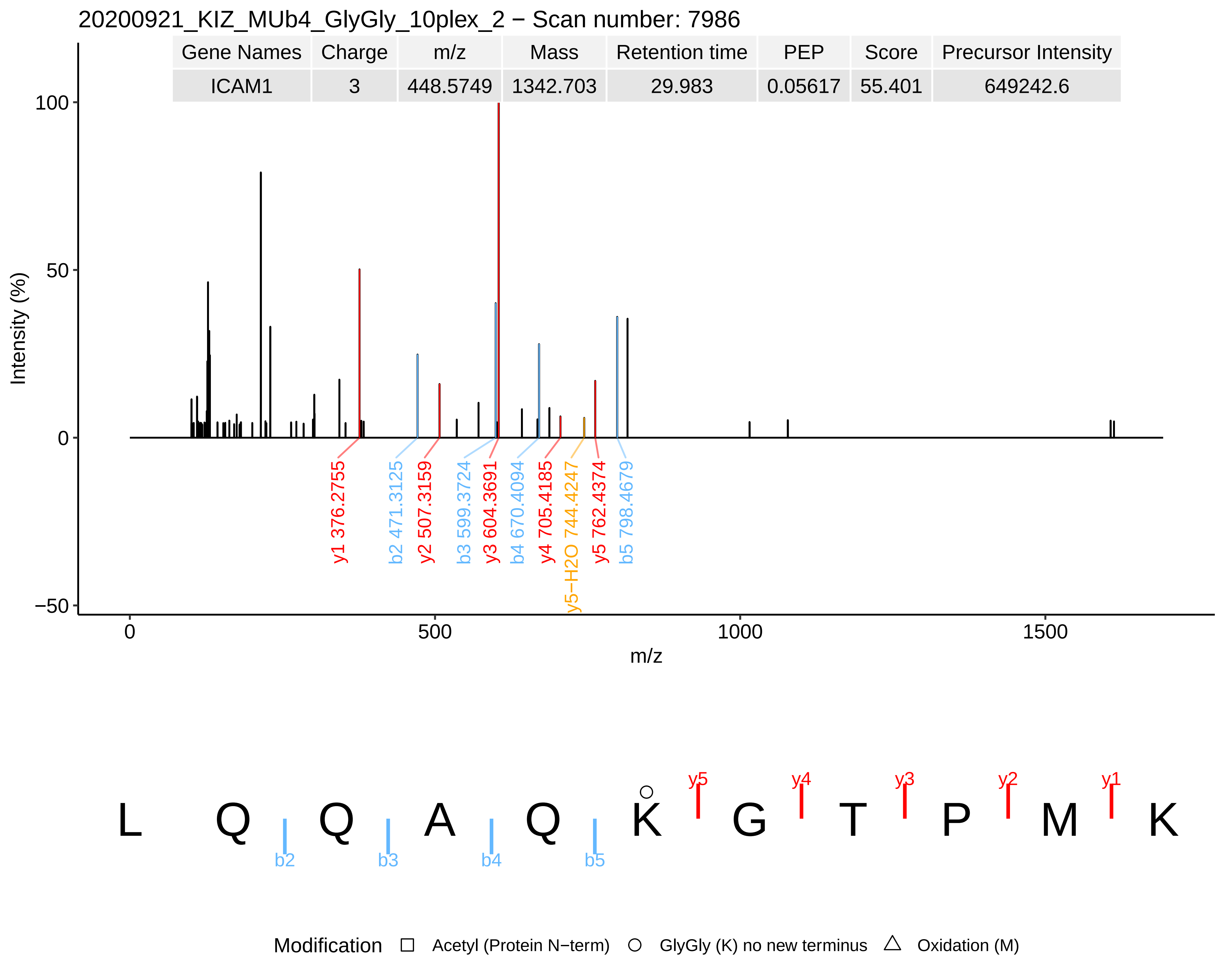
20200921_KIZ_MUb4_GlyGly_10plex_2 − Scan number: 7986
Gene Names | Charge | m/z | Mass | Retention time | PEP | Score | Precursor Intensity
ICAM1 | 3 | 448.5749 | 1342.703 | 29.983 | 0.05617 | 55.401 | 649242.6
Intensity (%)
100
50
0
−50
0
500
1000
1500
m/z
y1 376.2755
b2 471.3125
y2 507.3159
b3 599.3724
y3 604.3691
b4 670.4094
y4 705.4185
y5−H2O 744.4247
y5 762.4374
b5 798.4679
L Q Q A Q K G T P M K
b2 b3 b4 b5
y5 y4 y3 y2 y1
Modification
Acetyl (Protein N−term)
GlyGly (K) no new terminus
Oxidation (M)

20200921_KIZ_MUb4_GlyGly_10plex − Scan number: 5499

Gene Names	Charge	m/z	Mass	Retention time	PEP	Score	Precursor Intensity
VTI1A	4	390.9577	1559.802	18.017	0.0042884	88.163	8365965

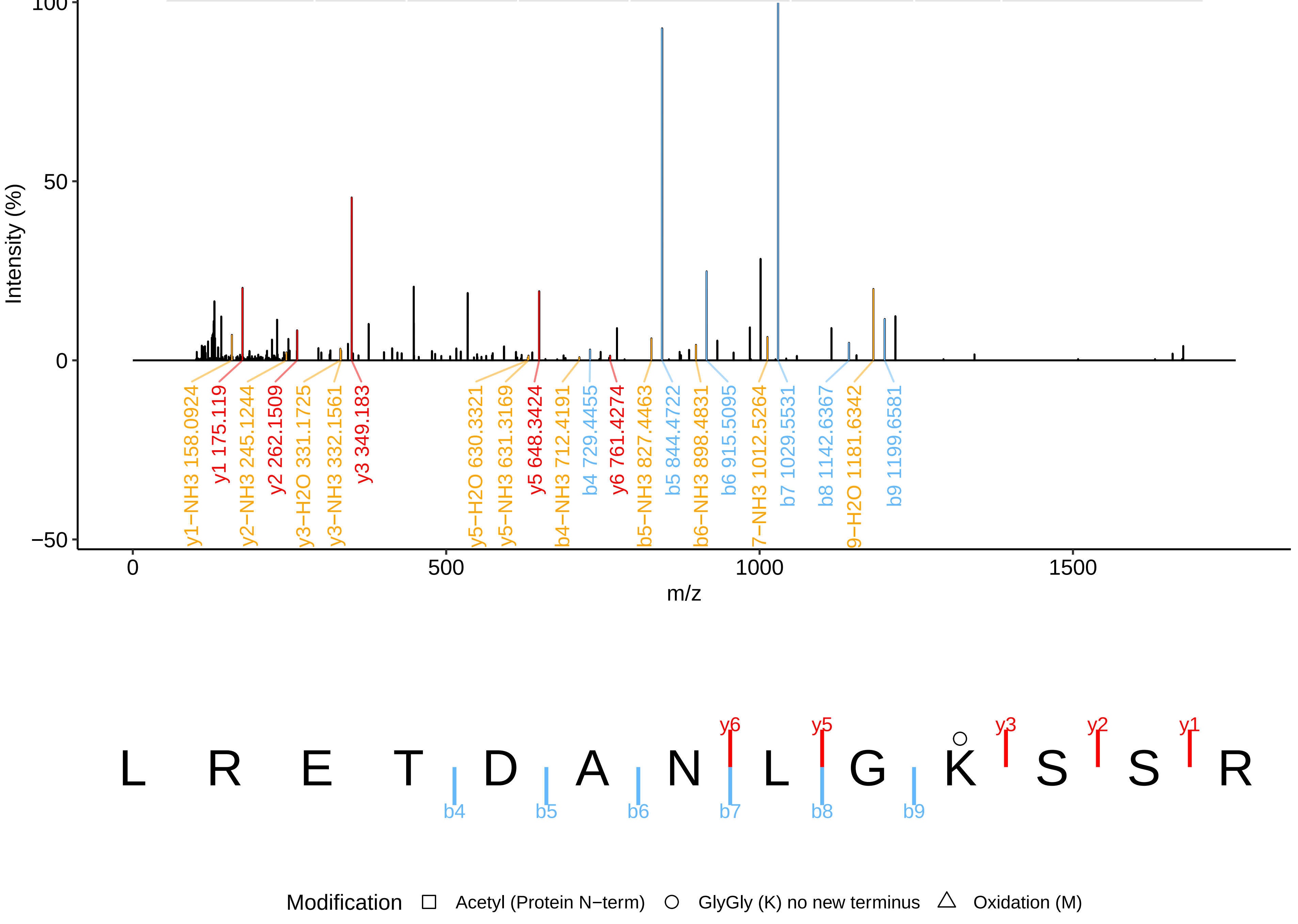

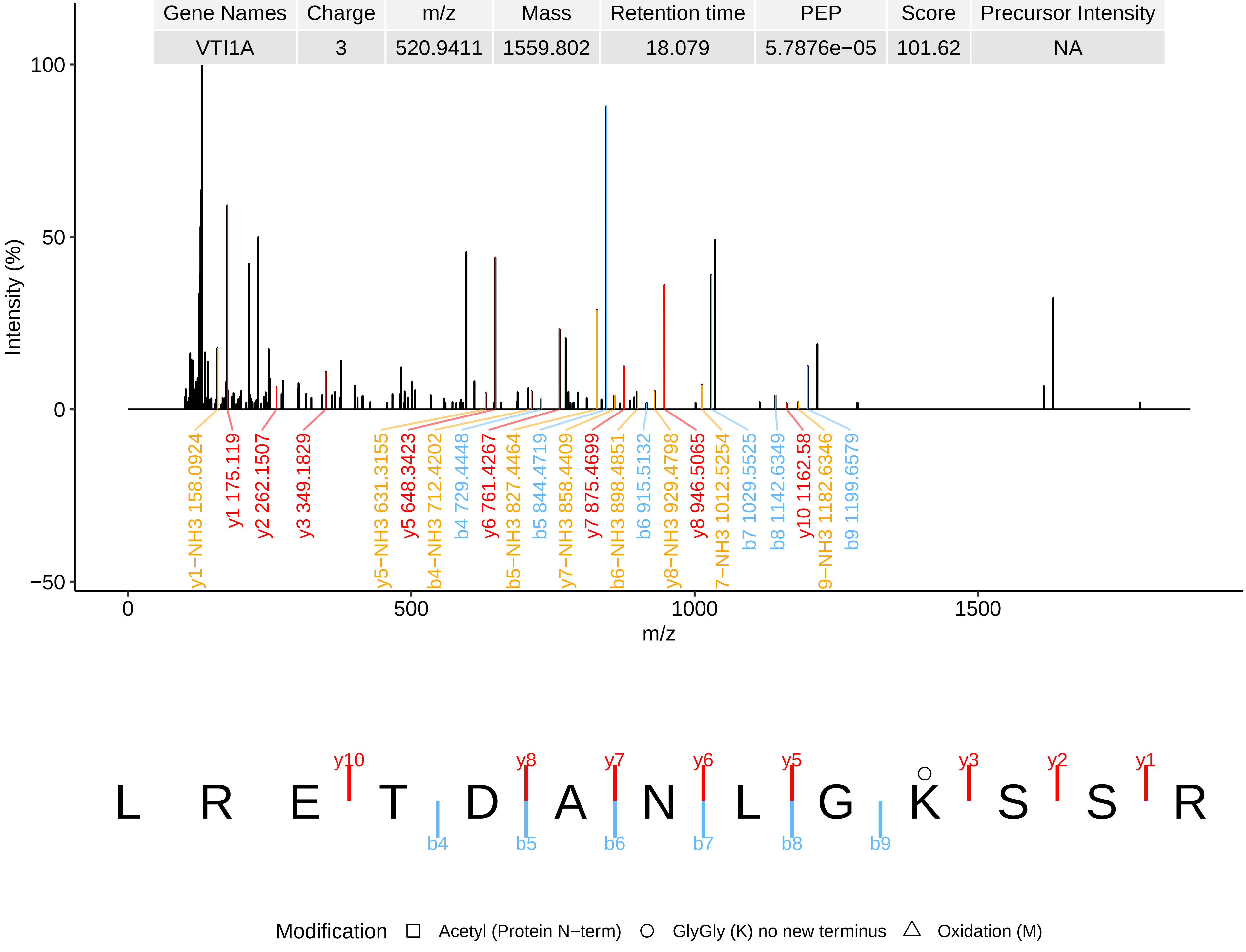

Gene Names	Charge	m/z	Mass	Retention time	PEP	Score	Precursor Intensity
VTI1A	3	520.9411	1559.802	18.079	5.7876e−05	101.62	NA

20200921_KIZ_MUb4_GlyGly_10plex_2 − Scan number: 5045

Gene Names	Charge	m/z	Mass	Retention time	PEP	Score	Precursor Intensity
VTI1A	4	390.9577	1559.802	17.96	0.0070977	81.494	727347.8

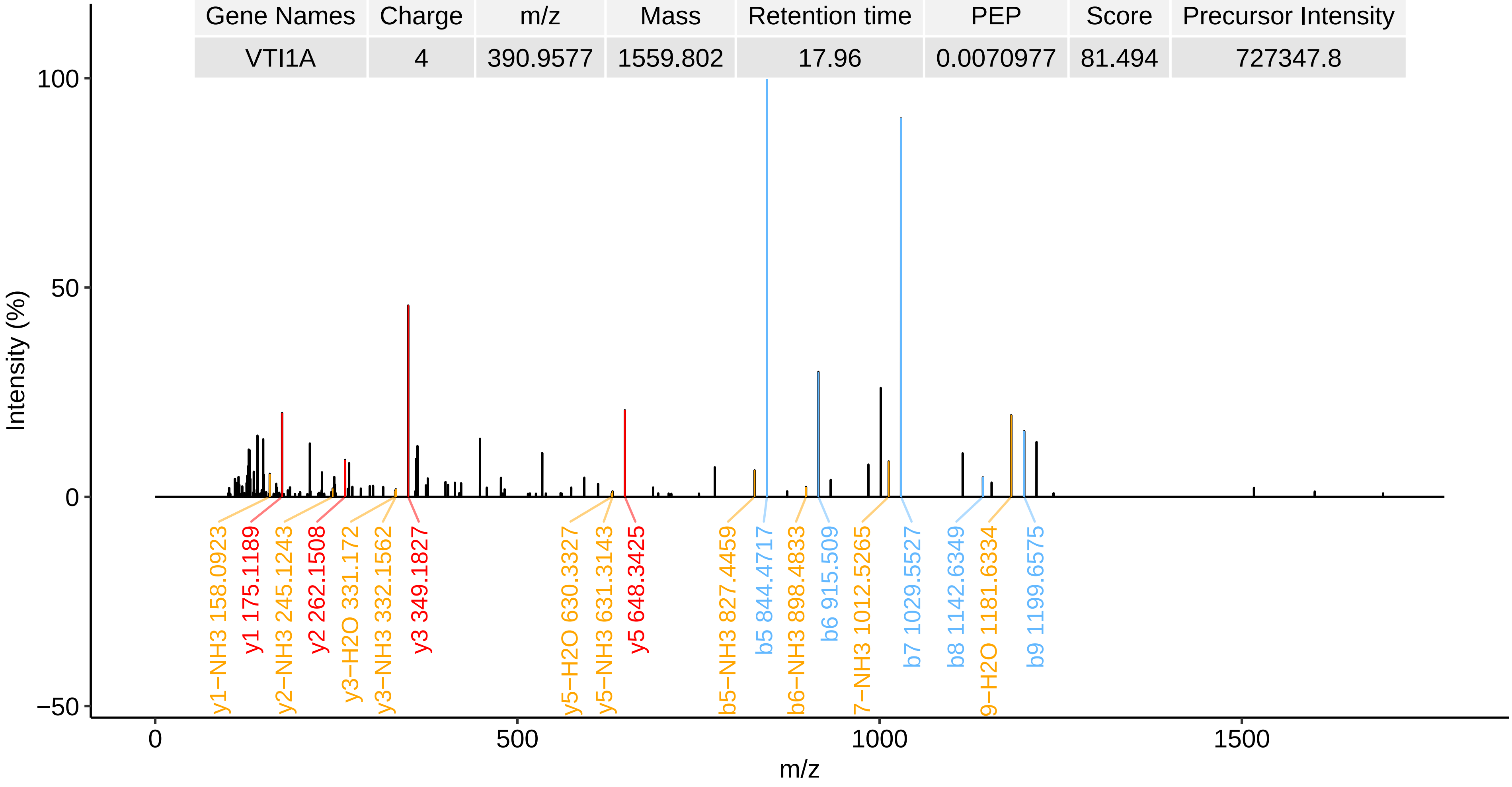

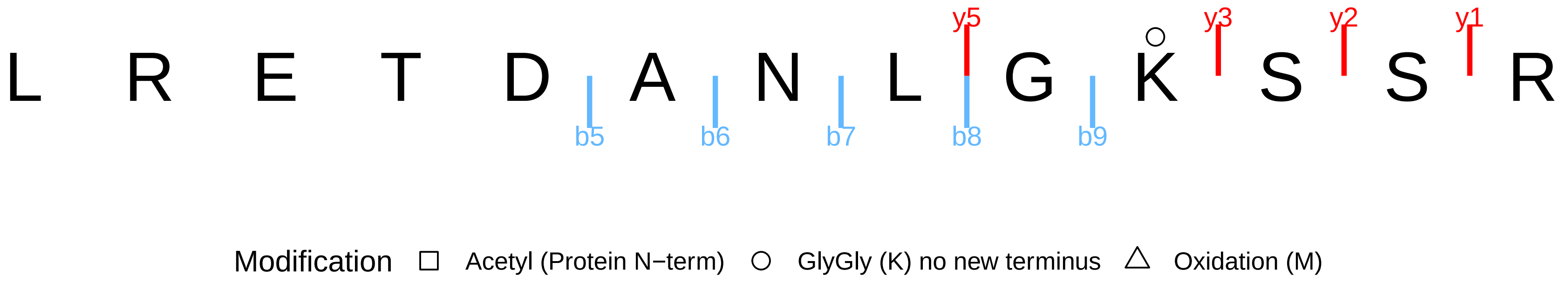

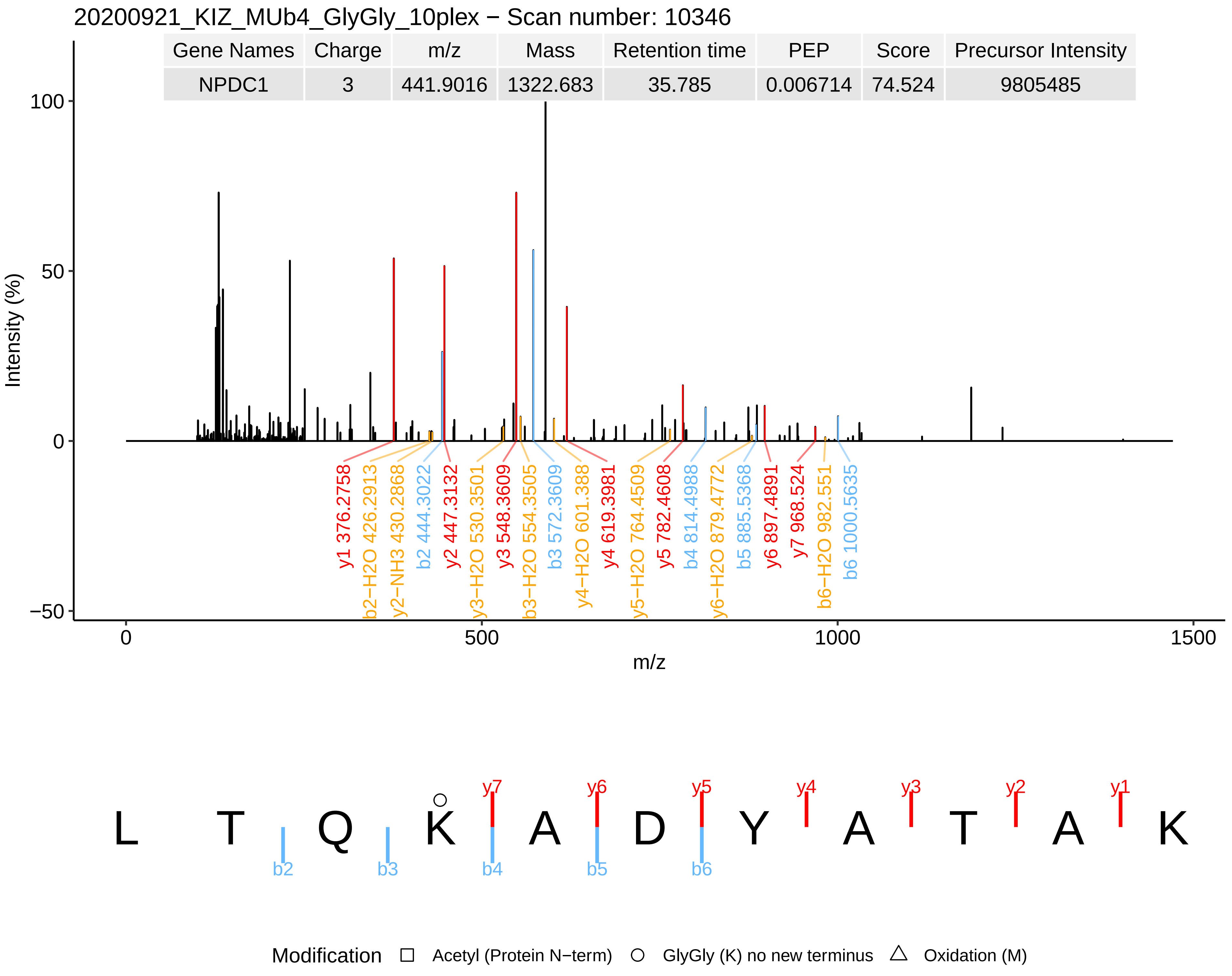
20200921_KIZ_MUb4_GlyGly_10plex − Scan number: 10346
Gene Names | Charge | m/z | Mass | Retention time | PEP | Score | Precursor Intensity
NPDC1 | 3 | 441.9016 | 1322.683 | 35.785 | 0.006714 | 74.524 | 9805485
Intensity (%)
m/z
y1 376.2758
b2−H2O 426.2913
y2−NH3 430.2868
b2 444.3022
y2 447.3132
y3−H2O 530.3501
y3 548.3609
b3−H2O 554.3505
b3 572.3609
y4−H2O 601.388
y4 619.3981
y5−H2O 764.4509
y5 782.4608
b4 814.4988
y6−H2O 879.4772
b5 885.5368
y6 897.4891
y7 968.524
b6−H2O 982.551
b6 1000.5635
L T Q K A D Y A T A K
y7 y6 y5 y4 y3 y2 y1
b2 b3 b4 b5 b6
Modification
Acetyl (Protein N−term)
GlyGly (K) no new terminus
Oxidation (M)

20200921_KIZ_MUb4_GlyGly_10plex_2 − Scan number: 9532

Gene Names	Charge	m/z	Mass	Retention time	PEP	Score	Precursor Intensity
NPDC1	3	441.9016	1322.683	36.265	0.10801	45.257	928809.9

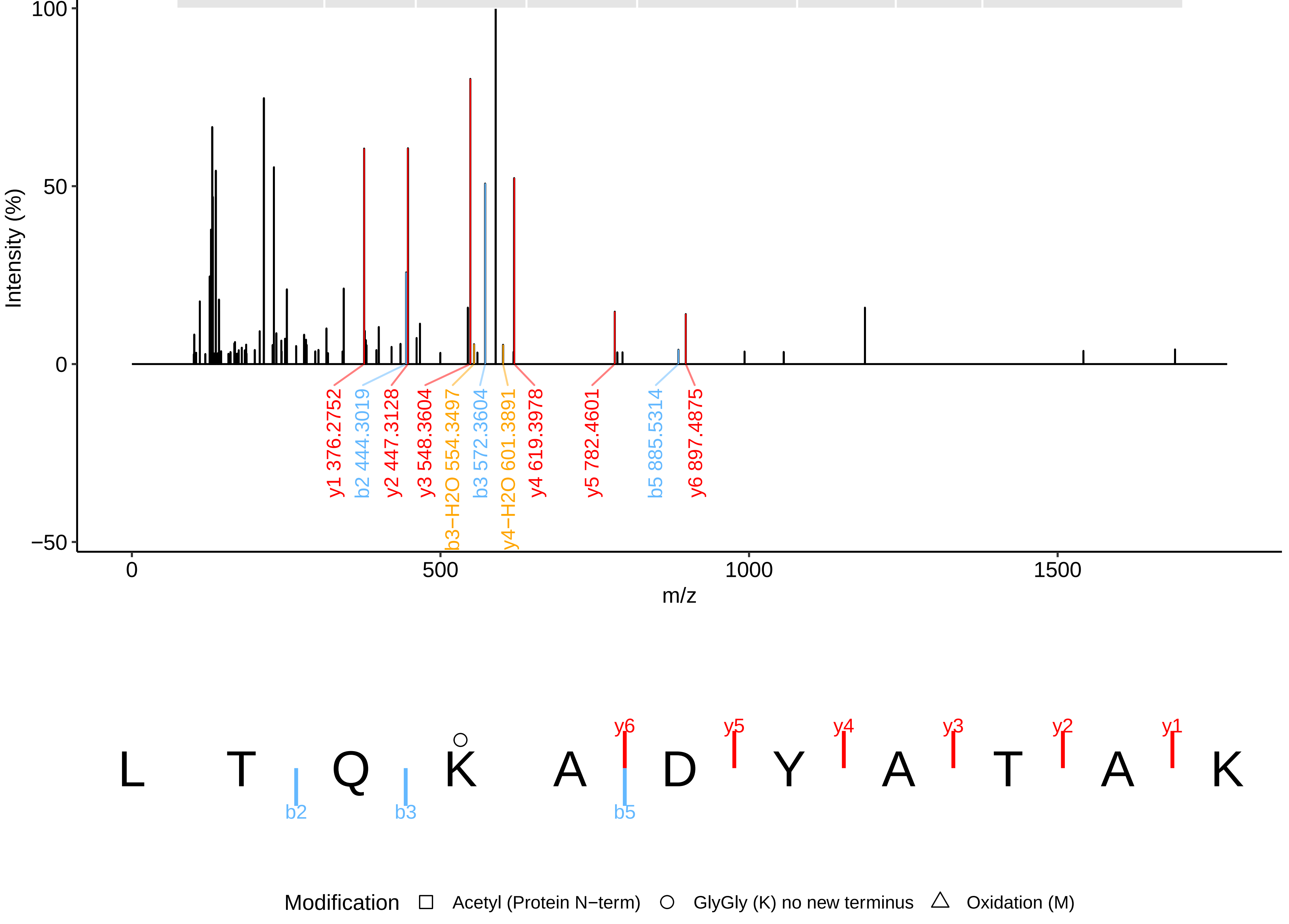

20200921_KIZ_MUb4_GlyGly_10plex − Scan number: 7019

Gene Names	Charge	m/z	Mass	Retention time	PEP	Score	Precursor Intensity
EEF1A1;EEF1A1P5	3	518.2402	1551.699	23.434	0.0037322	86.923	3503792

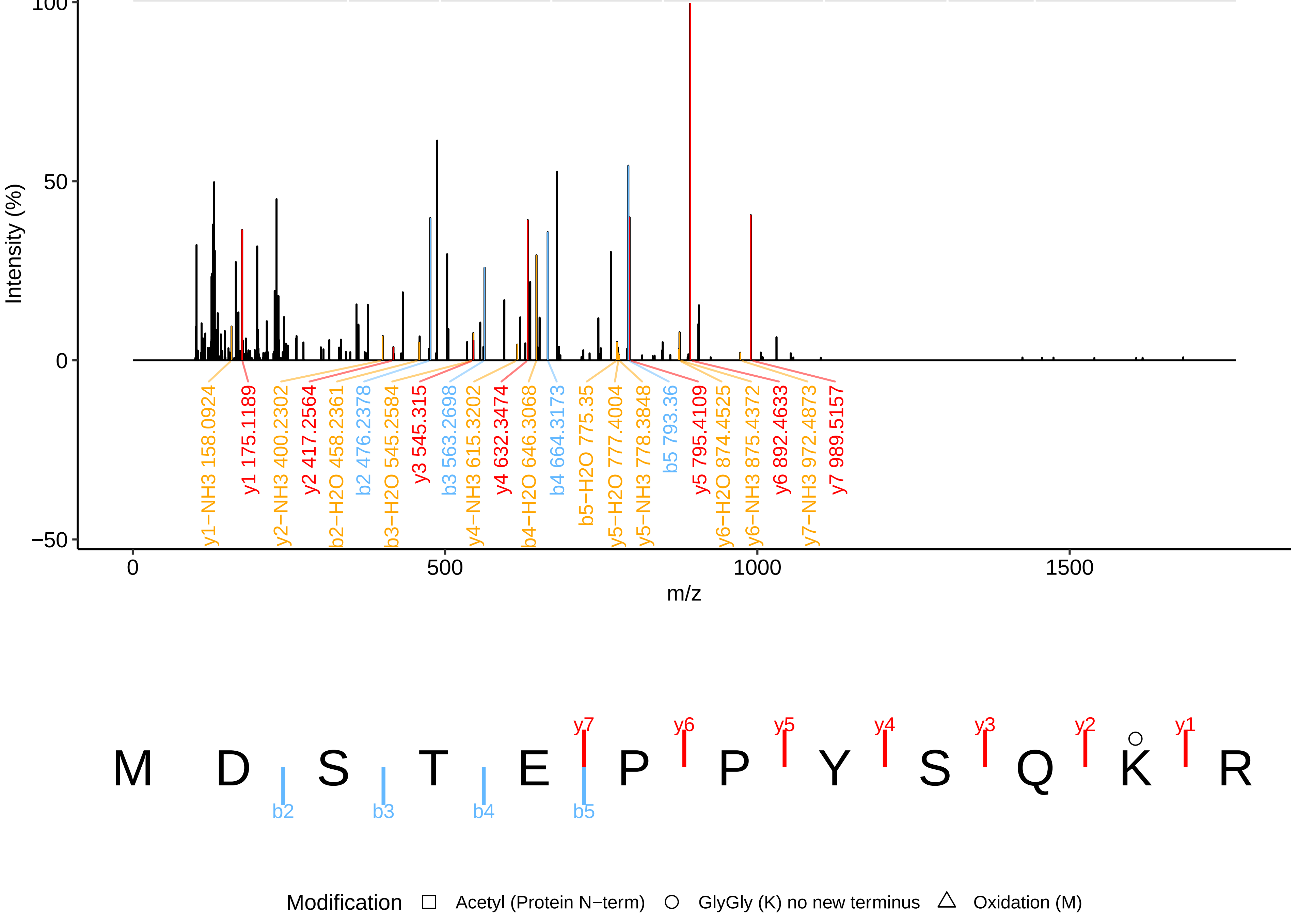

20200921_KIZ_MUb4_GlyGly_10plex − Scan number: 15220

Gene Names	Charge	m/z	Mass	Retention time	PEP	Score	Precursor Intensity
PLA2G4A	3	455.9063	1364.697	54.321	1.7247e−06	114.76	6701795

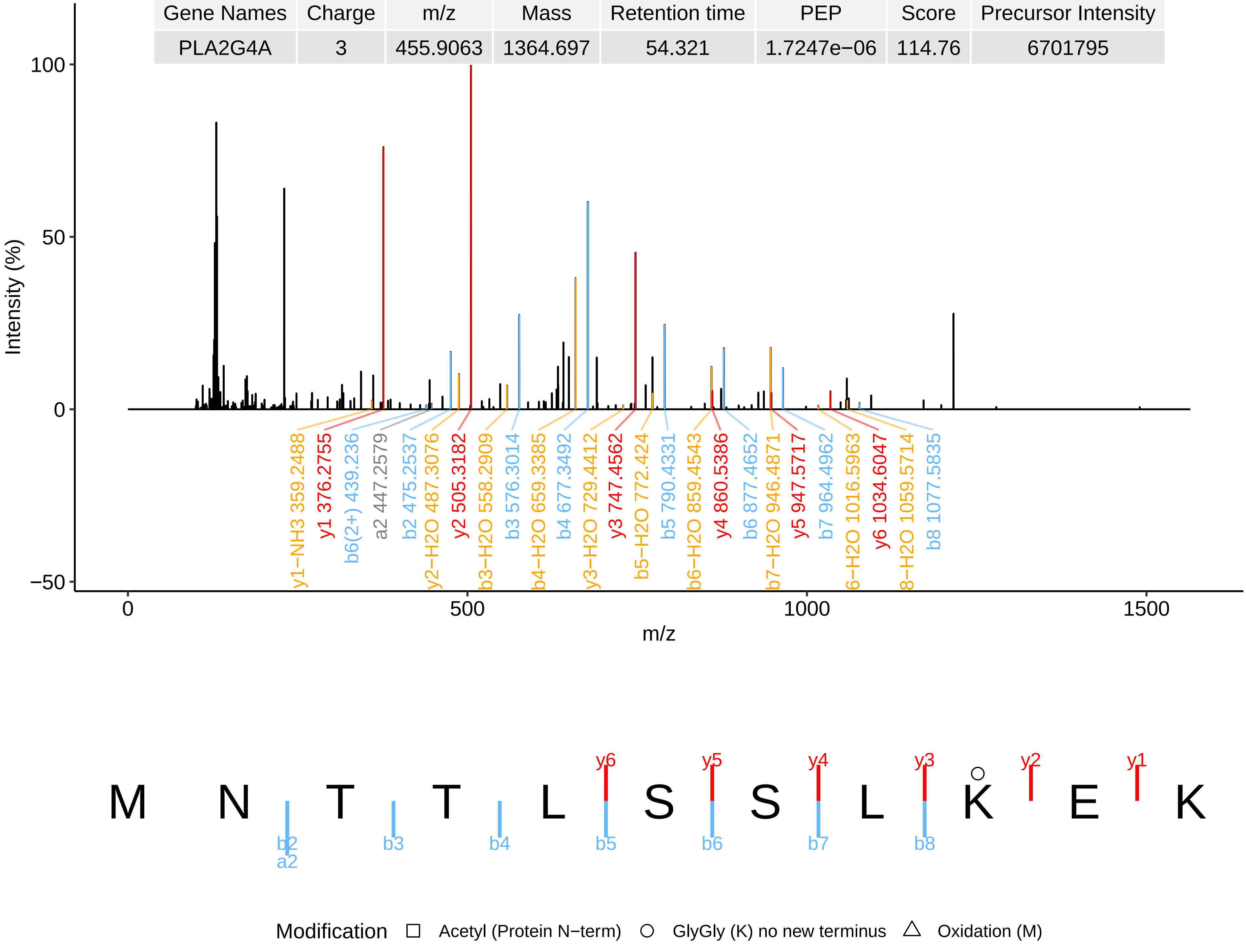

20200921_KIZ_MUb4_GlyGly_10plex − Scan number: 17979

Gene Names	Charge	m/z	Mass	Retention time	PEP	Score	Precursor Intensity
LAPTM4A	2	790.3949	1578.775	64.567	0.10518	58.978	4418452

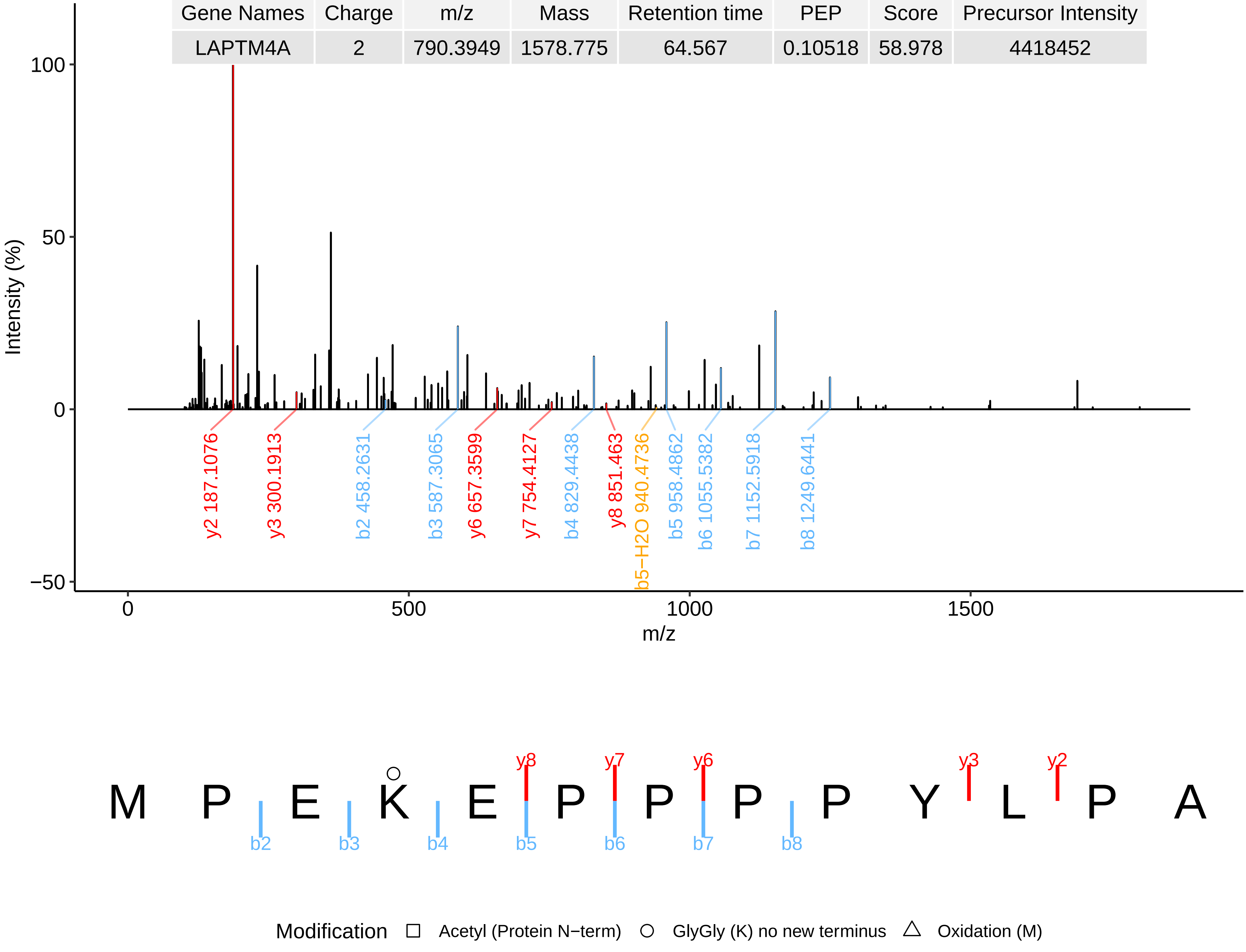

20200921_KIZ_MUb4_GlyGly_10plex − Scan number: 18116

Gene Names	Charge	m/z	Mass	Retention time	PEP	Score	Precursor Intensity
LAPTM4A	2	790.3949	1578.775	65.073	0.10518	58.978	30609768

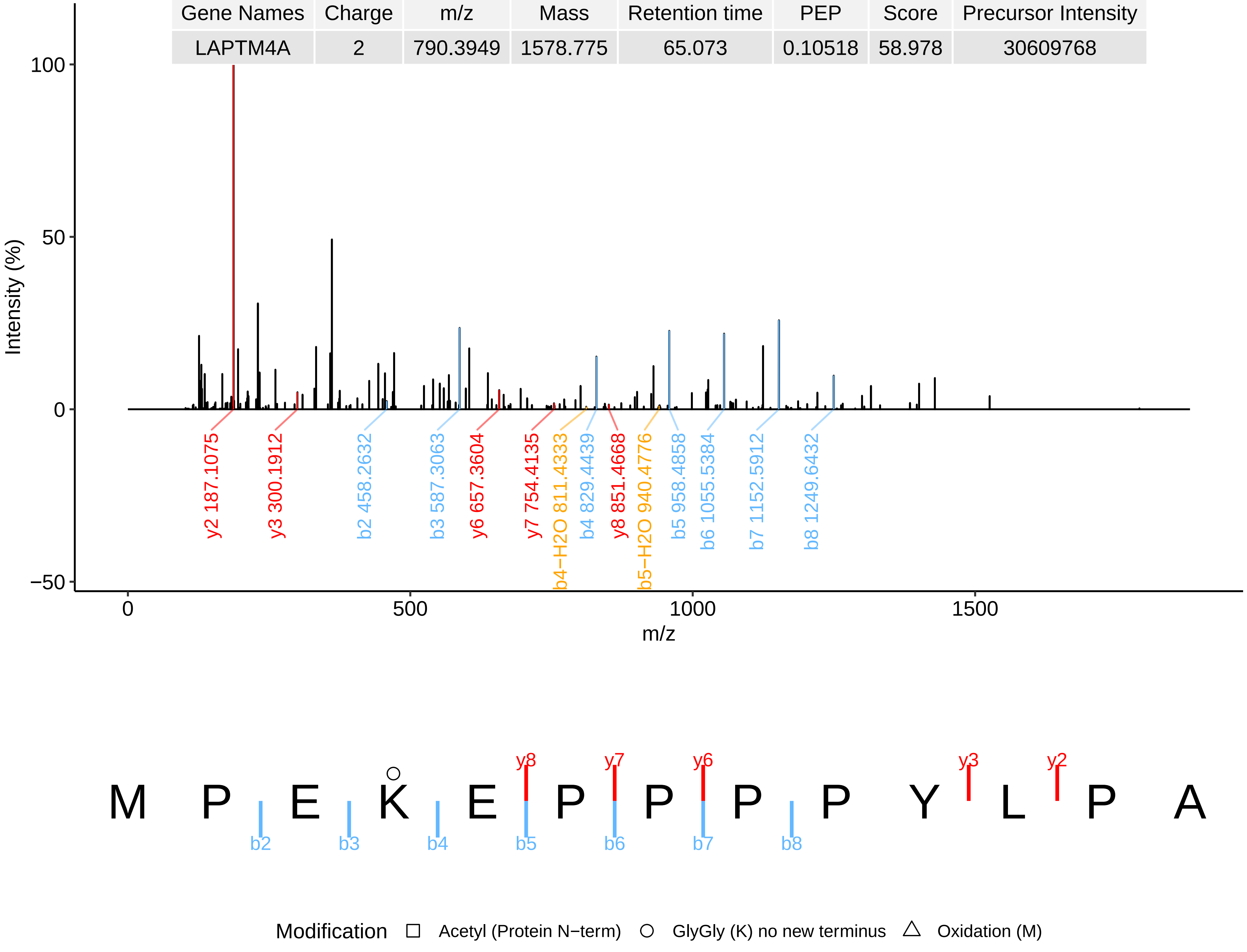

20200921_KIZ_MUb4_GlyGly_10plex_2 − Scan number: 16771

Gene Names	Charge	m/z	Mass	Retention time	PEP	Score	Precursor Intensity
LAPTM4A	2	790.3949	1578.775	65.509	0.079417	60.563	2052580

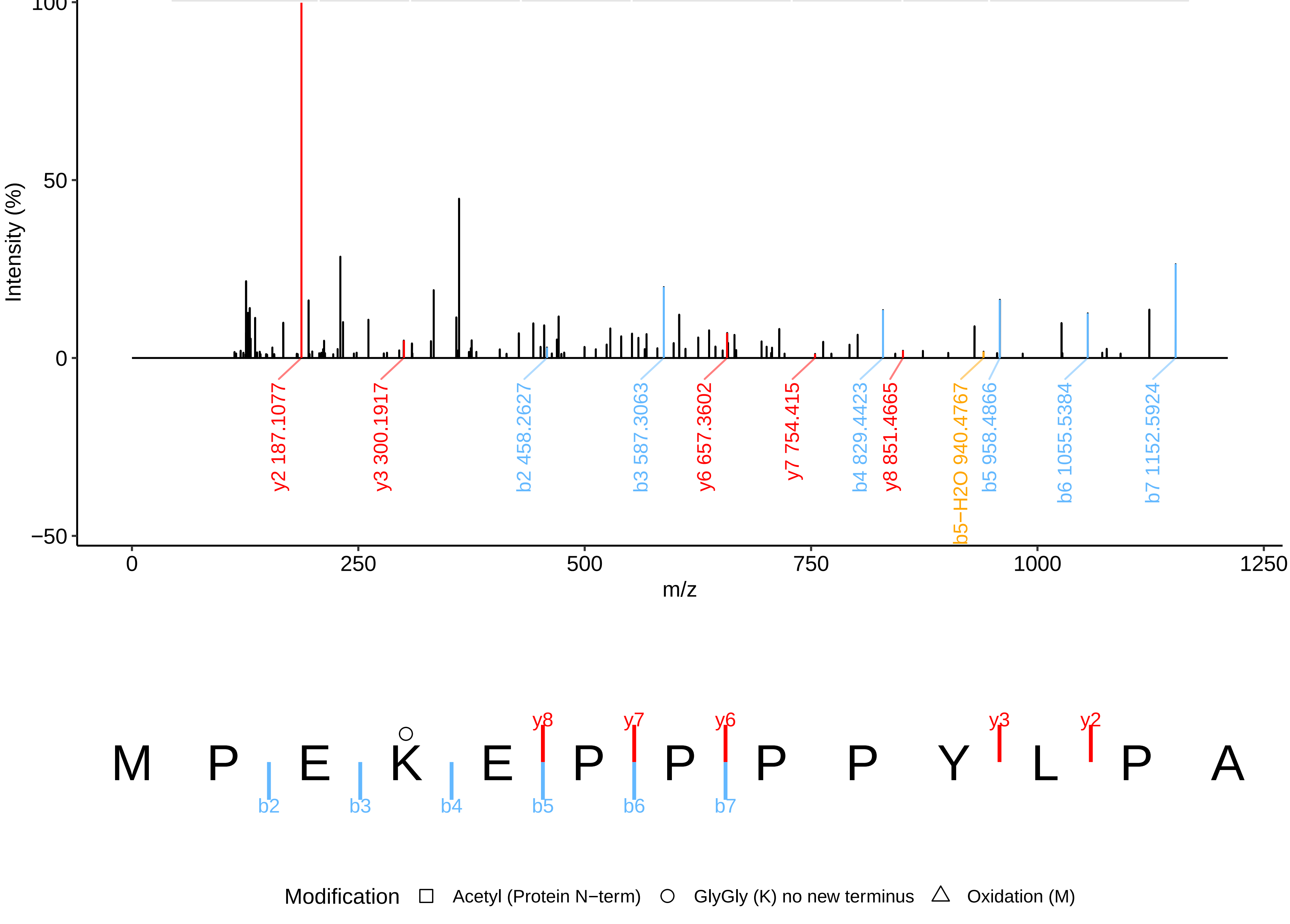

20200924_KIZ_MUb5_GlyGly_1 − Scan number: 18164

Gene Names	Charge	m/z	Mass	Retention time	PEP	Score	Precursor Intensity
LAPTM4A	2	790.3949	1578.775	58.734	0.026638	68.657	27239056

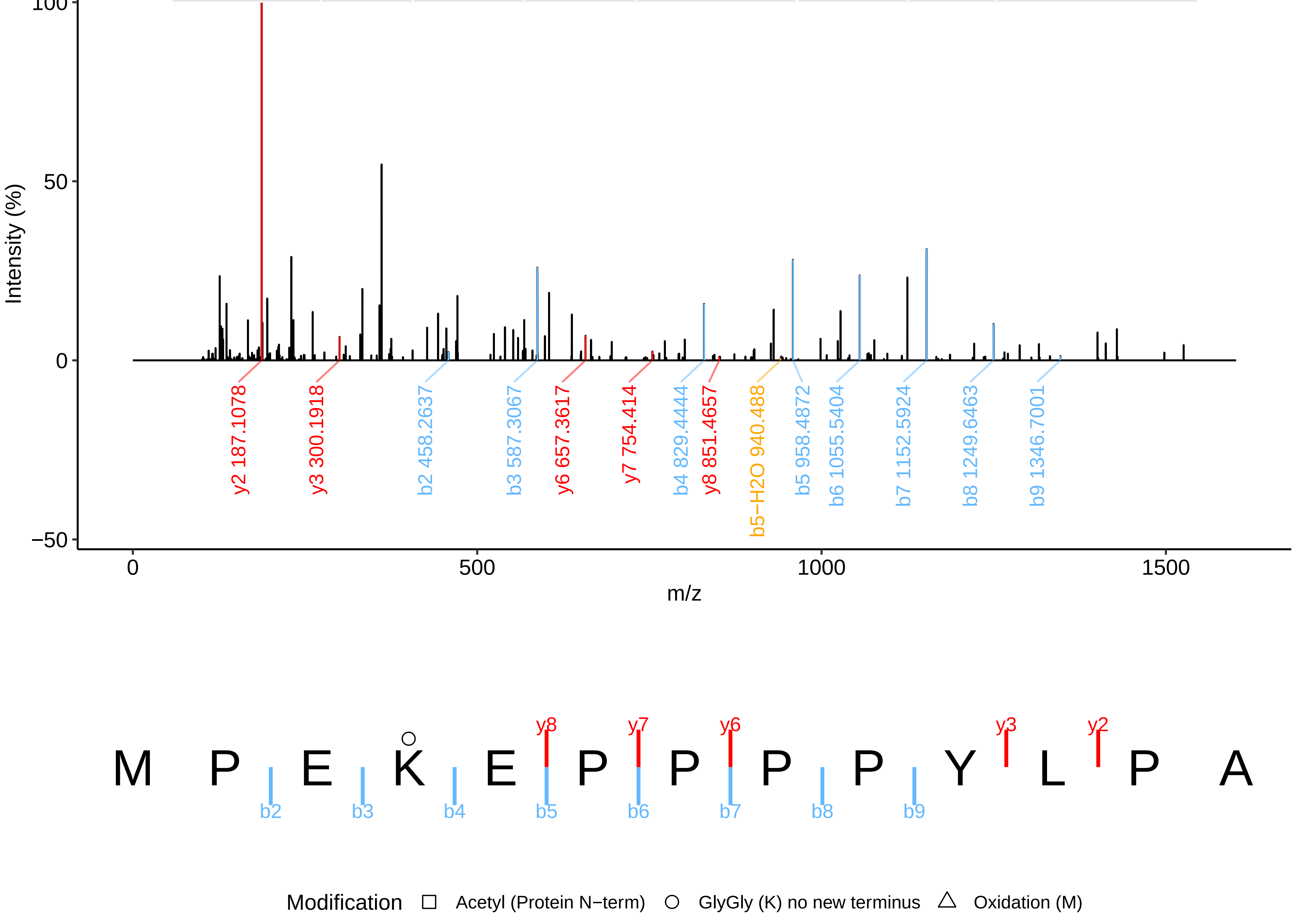

20200924_KIZ_MUb5_GlyGly_1 − Scan number: 18011

Gene Names	Charge	m/z	Mass	Retention time	PEP	Score	Precursor Intensity
LAPTM4A	2	790.3949	1578.775	58.217	0.039644	74.162	NA

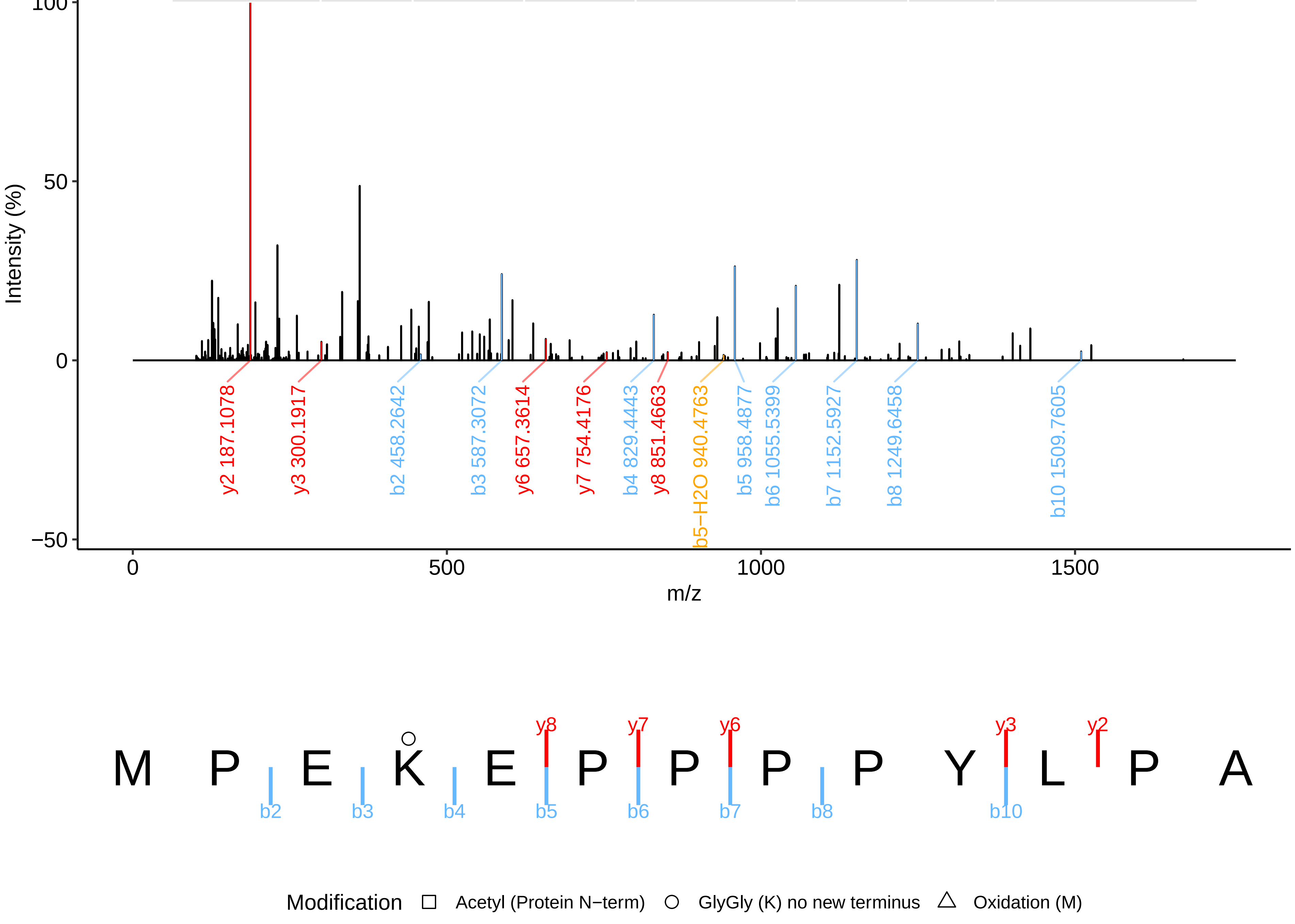

20200924_KIZ_MUb5_GlyGly_1 − Scan number: 18022

Gene Names	Charge	m/z	Mass	Retention time	PEP	Score	Precursor Intensity
LAPTM4A	3	527.2657	1578.775	58.254	0.023494	61.038	15429173

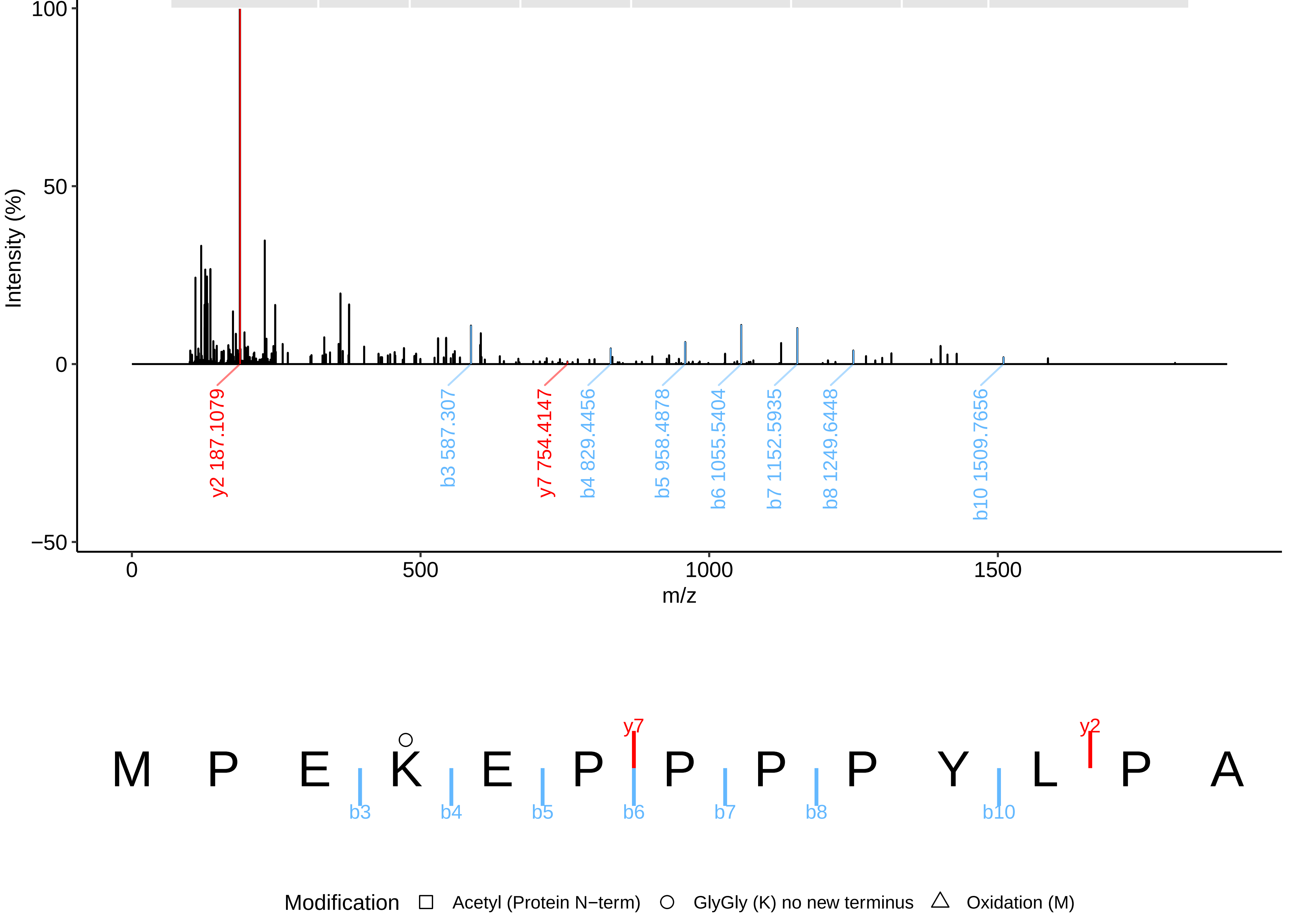

20200924_KIZ_MUb5_GlyGly_2 − Scan number: 16537

Gene Names	Charge	m/z	Mass	Retention time	PEP	Score	Precursor Intensity
LAPTM4A	3	527.2657	1578.775	61.353	0.071071	50.145	3851203

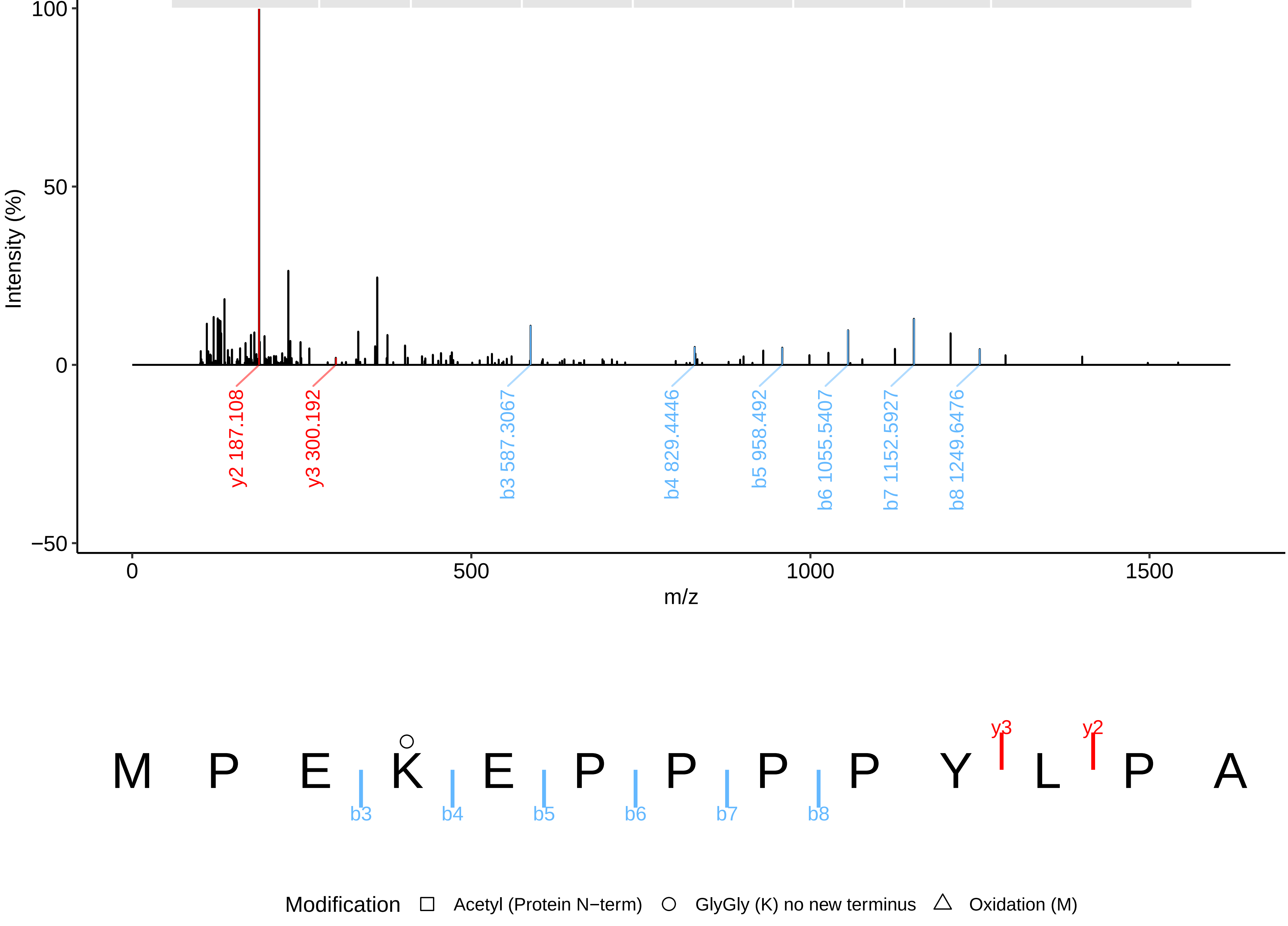

20200924_KIZ_MUb5_GlyGly_2 − Scan number: 16696

Gene Names	Charge	m/z	Mass	Retention time	PEP	Score	Precursor Intensity
LAPTM4A	3	527.2657	1578.775	61.944	0.10509	42.789	998281.8

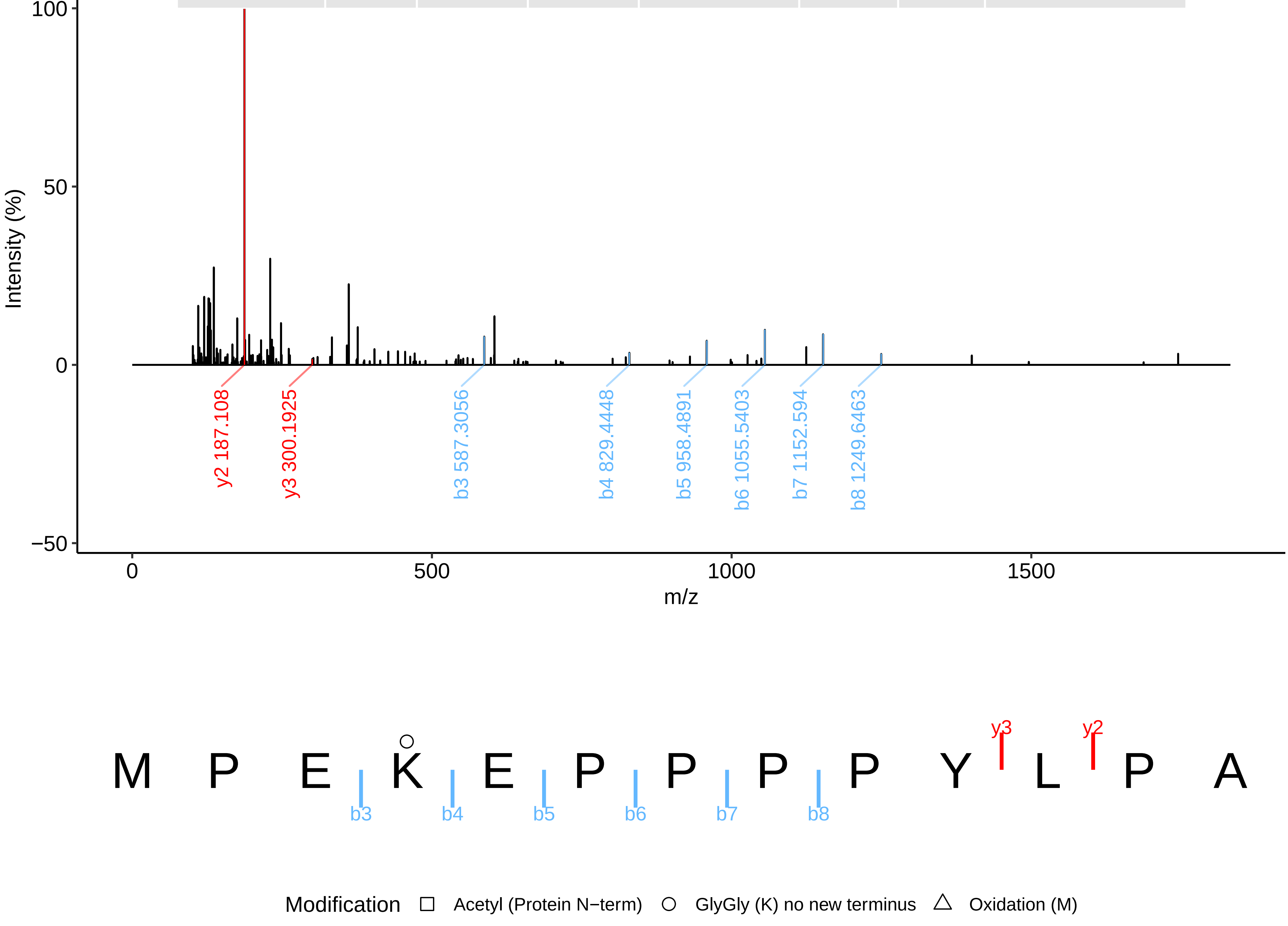

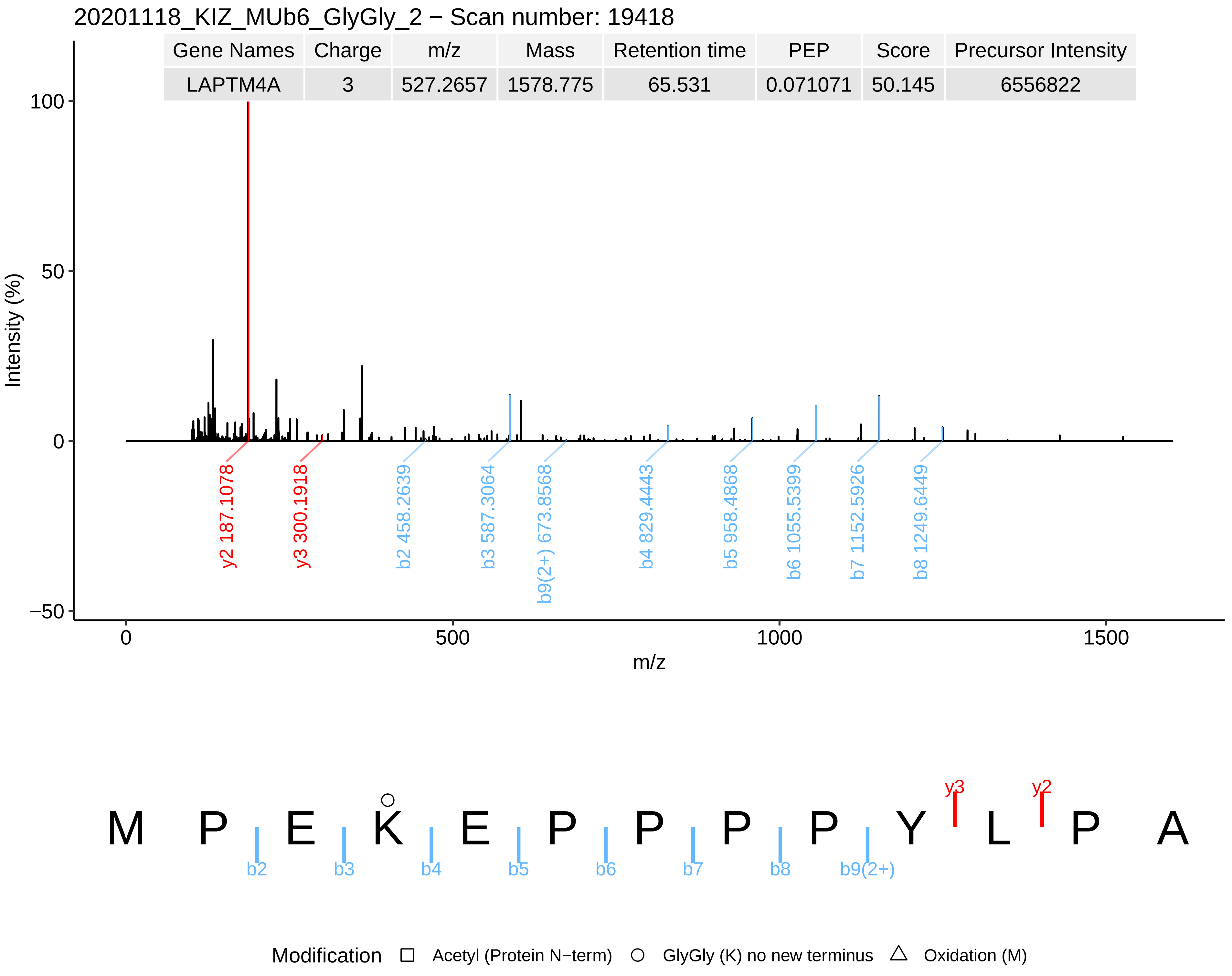
20201118_KIZ_MUb6_GlyGly_2 − Scan number: 19418
Gene Names | Charge | m/z | Mass | Retention time | PEP | Score | Precursor Intensity
LAPTM4A | 3 | 527.2657 | 1578.775 | 65.531 | 0.071071 | 50.145 | 6556822
Intensity (%)
100
50
0
−50
m/z
0
500
1000
1500
y2 187.1078
y3 300.1918
b2 458.2639
b3 587.3064
b9(2+) 673.8568
b4 829.4443
b5 958.4868
b6 1055.5399
b7 1152.5926
b8 1249.6449
M P E K E P P P P Y L P A
b2
b3
b4
b5
b6
b7
b8
b9(2+)
y3
y2
Modification
Acetyl (Protein N−term)
GlyGly (K) no new terminus
Oxidation (M)

20200921_KIZ_MUb4_GlyGly_10plex − Scan number: 28380

Gene Names	Charge	m/z	Mass	Retention time	PEP	Score	Precursor Intensity
RPS27A;UBA52;UBC;UBB	3	460.5954	1378.764	103.83	0.001455	83.397	2269168

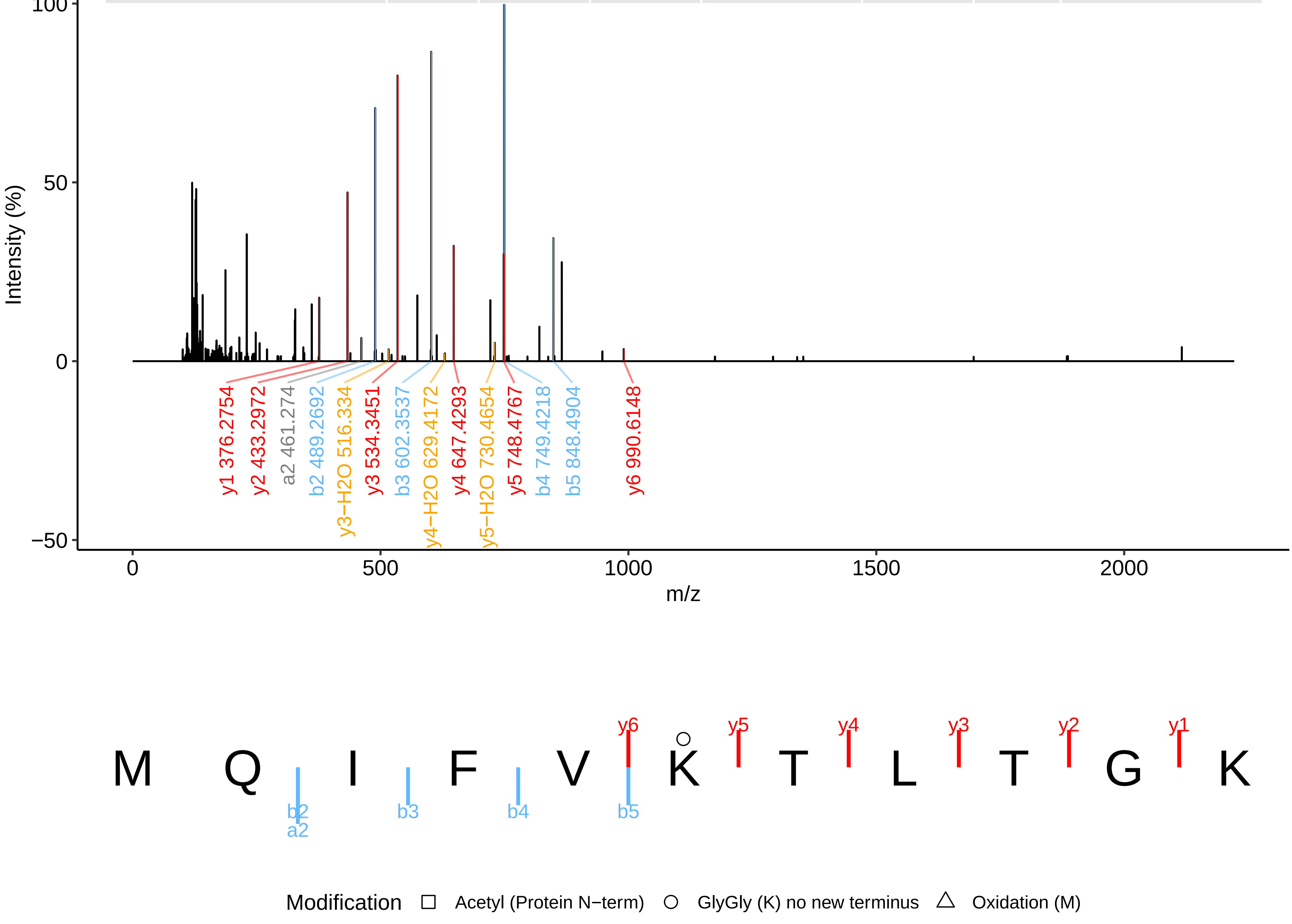

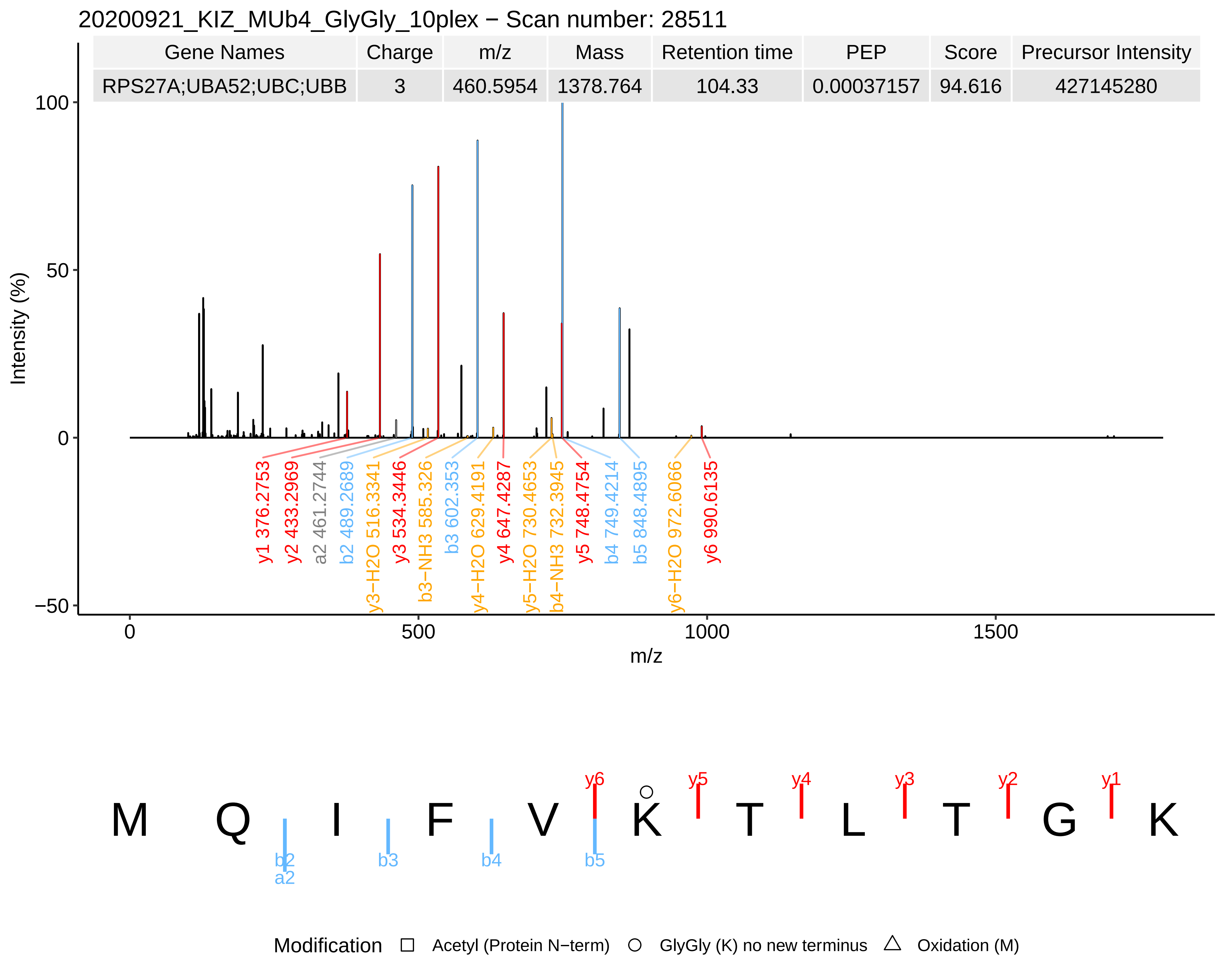

Gene Names	Charge	m/z	Mass	Retention time	PEP	Score	Precursor Intensity
RPS27A;UBA52;UBC;UBB	3	460.5954	1378.764	104.33	0.00037157	94.616	427145280

20200921_KIZ_MUb4_GlyGly_10plex − Scan number: 28514

Gene Names	Charge	m/z	Mass	Retention time	PEP	Score	Precursor Intensity
RPS27A;UBA52;UBC;UBB	3	460.5954	1378.764	104.34	4.6487e−06	113.63	29651378

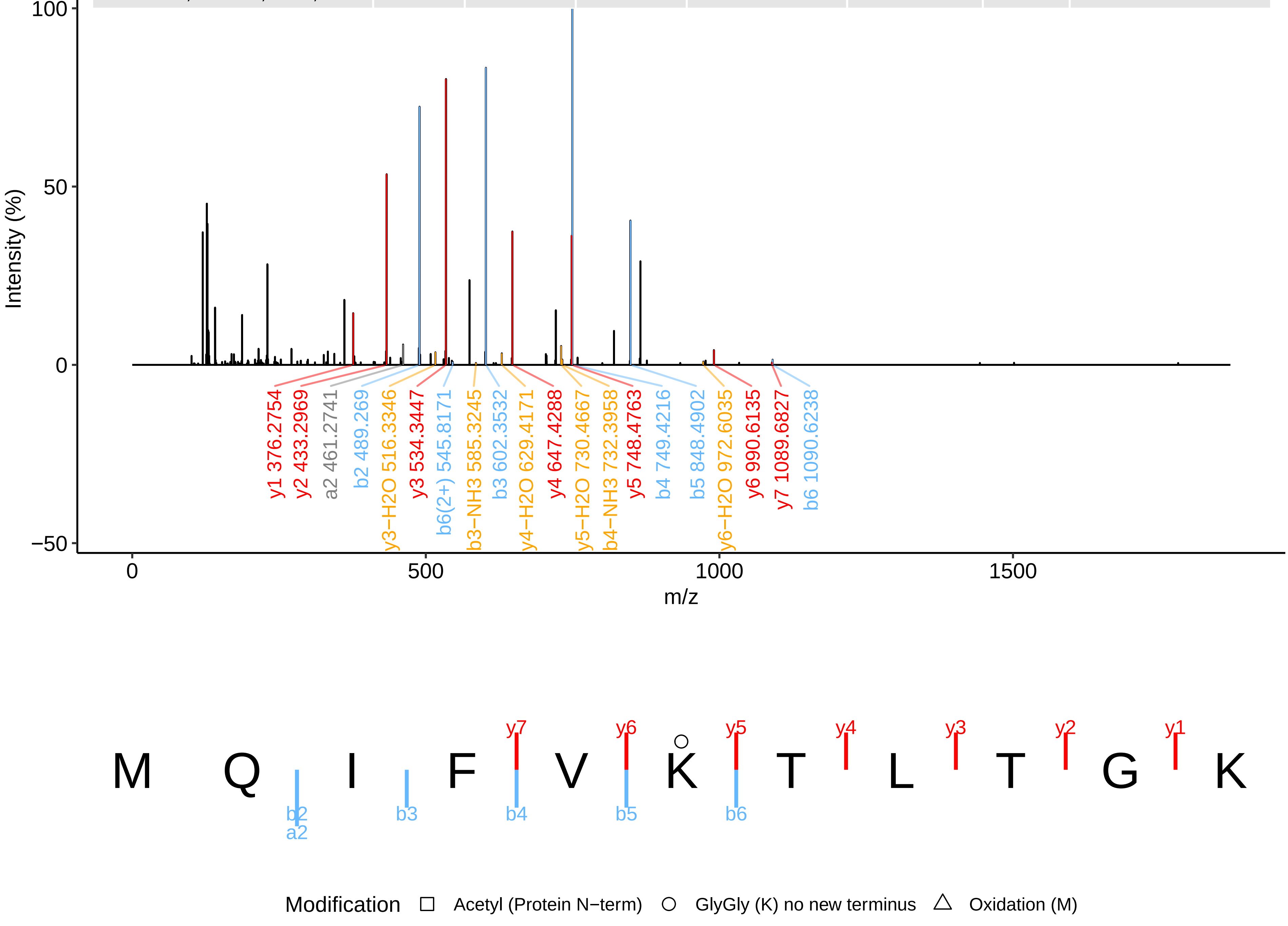

20200921_KIZ_MUb4_GlyGly_10plex − Scan number: 28652

Gene Names	Charge	m/z	Mass	Retention time	PEP	Score	Precursor Intensity
RPS27A;UBA52;UBC;UBB	3	460.5954	1378.764	104.85	0.00050276	93.258	617416960

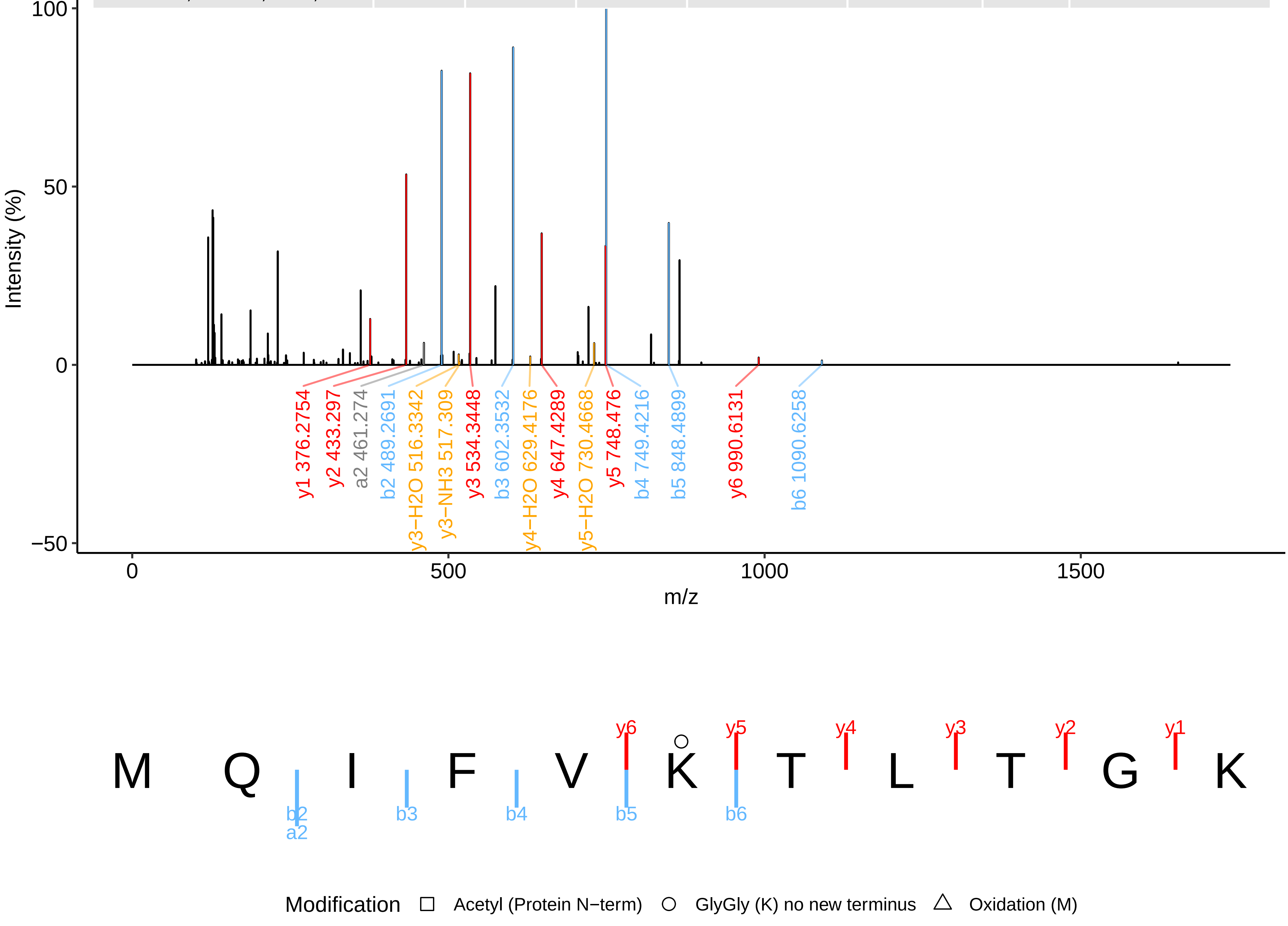

20200921_KIZ_MUb4_GlyGly_10plex − Scan number: 28433

Gene Names	Charge	m/z	Mass	Retention time	PEP	Score	Precursor Intensity
RPS27A;UBA52;UBC;UBB	2	690.3894	1378.764	104.04	6.8678e−28	206.48	2576483

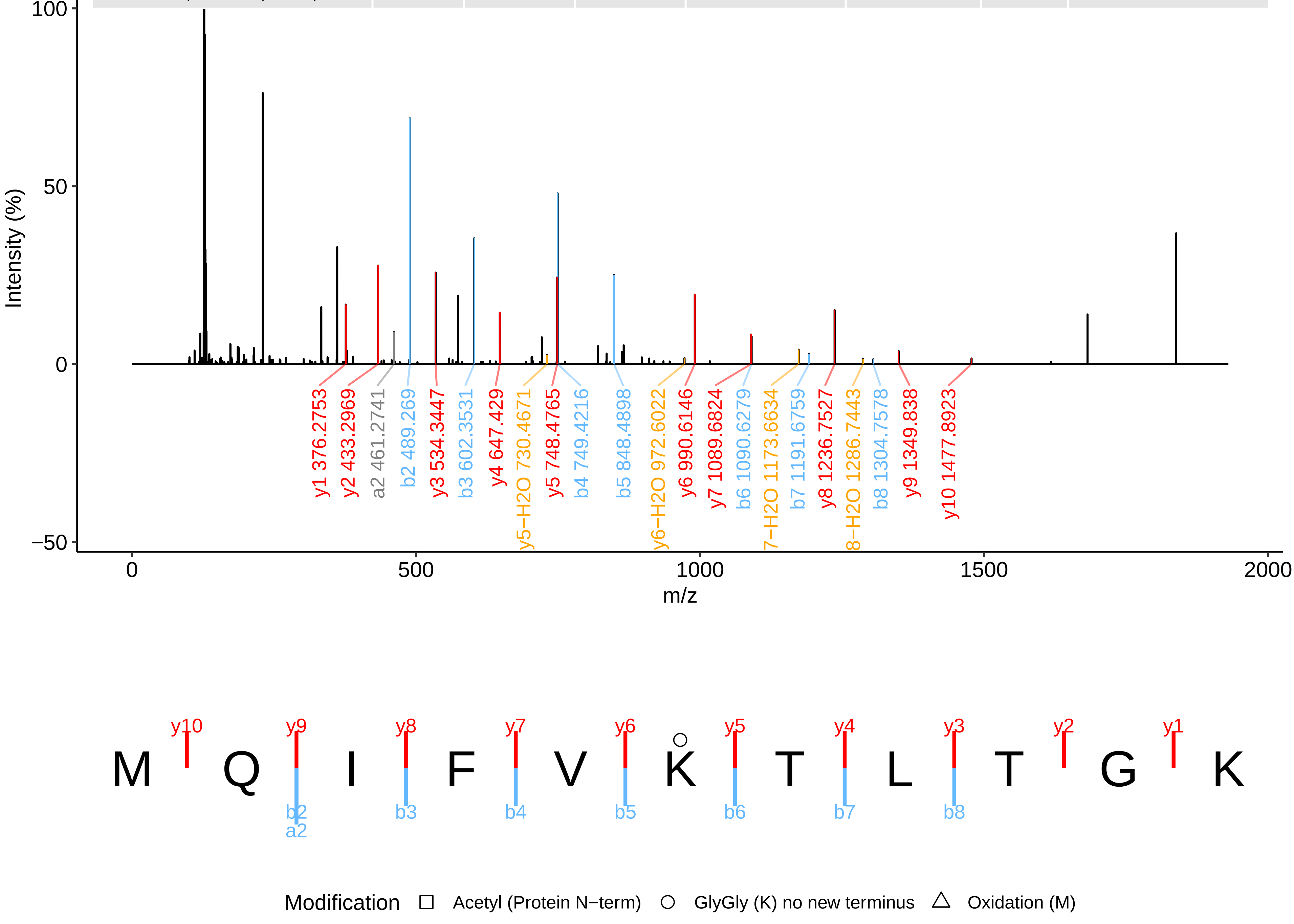

20200921_KIZ_MUb4_GlyGly_10plex − Scan number: 28568

Gene Names	Charge	m/z	Mass	Retention time	PEP	Score	Precursor Intensity
RPS27A;UBA52;UBC;UBB	2	690.3894	1378.764	104.55	2.4647e−54	259.1	17576842

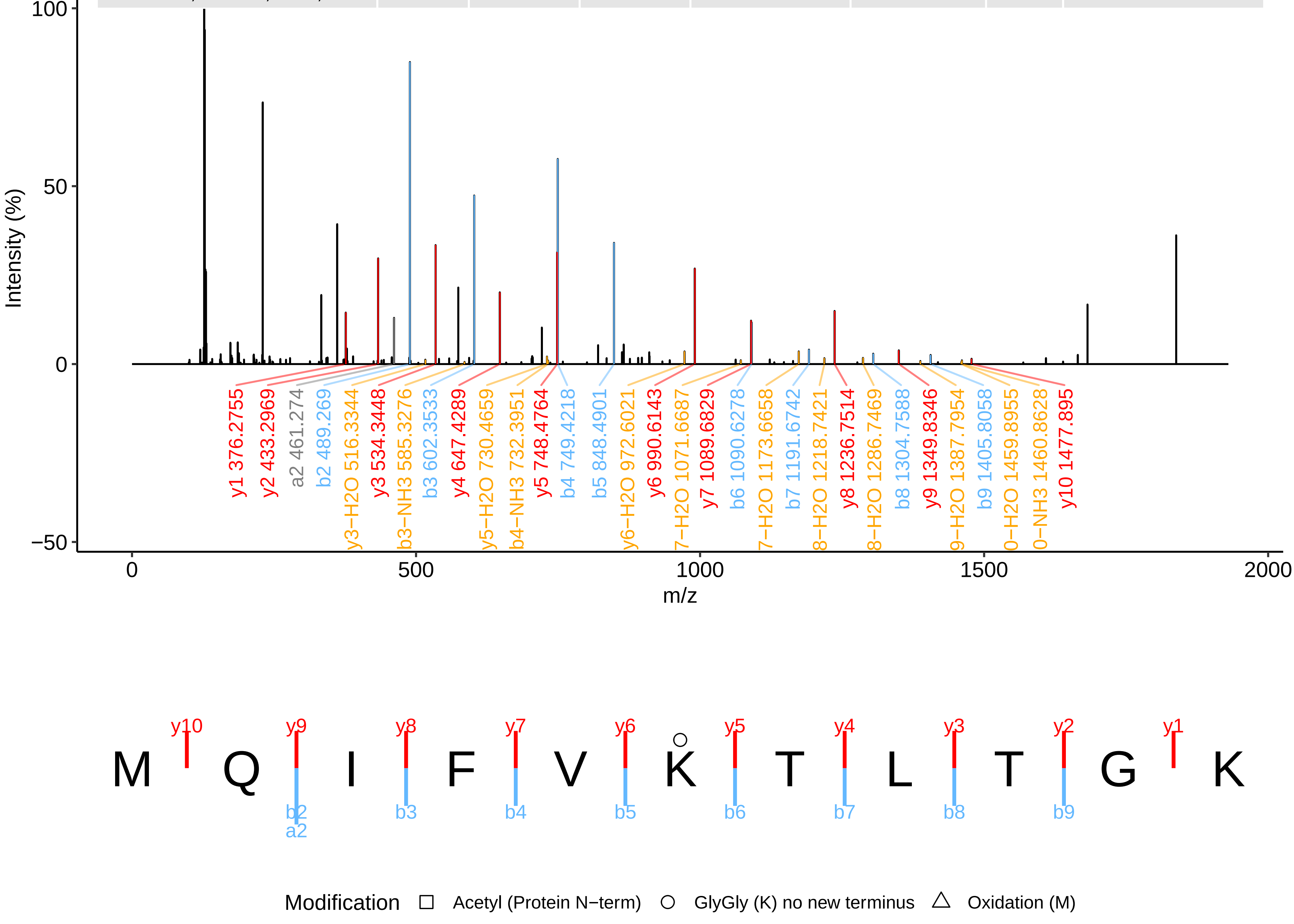

20200921_KIZ_MUb4_GlyGly_10plex − Scan number: 28675

Gene Names	Charge	m/z	Mass	Retention time	PEP	Score	Precursor Intensity
RPS27A;UBA52;UBC;UBB	3	460.5954	1378.764	104.93	0.023058	61.815	40228684

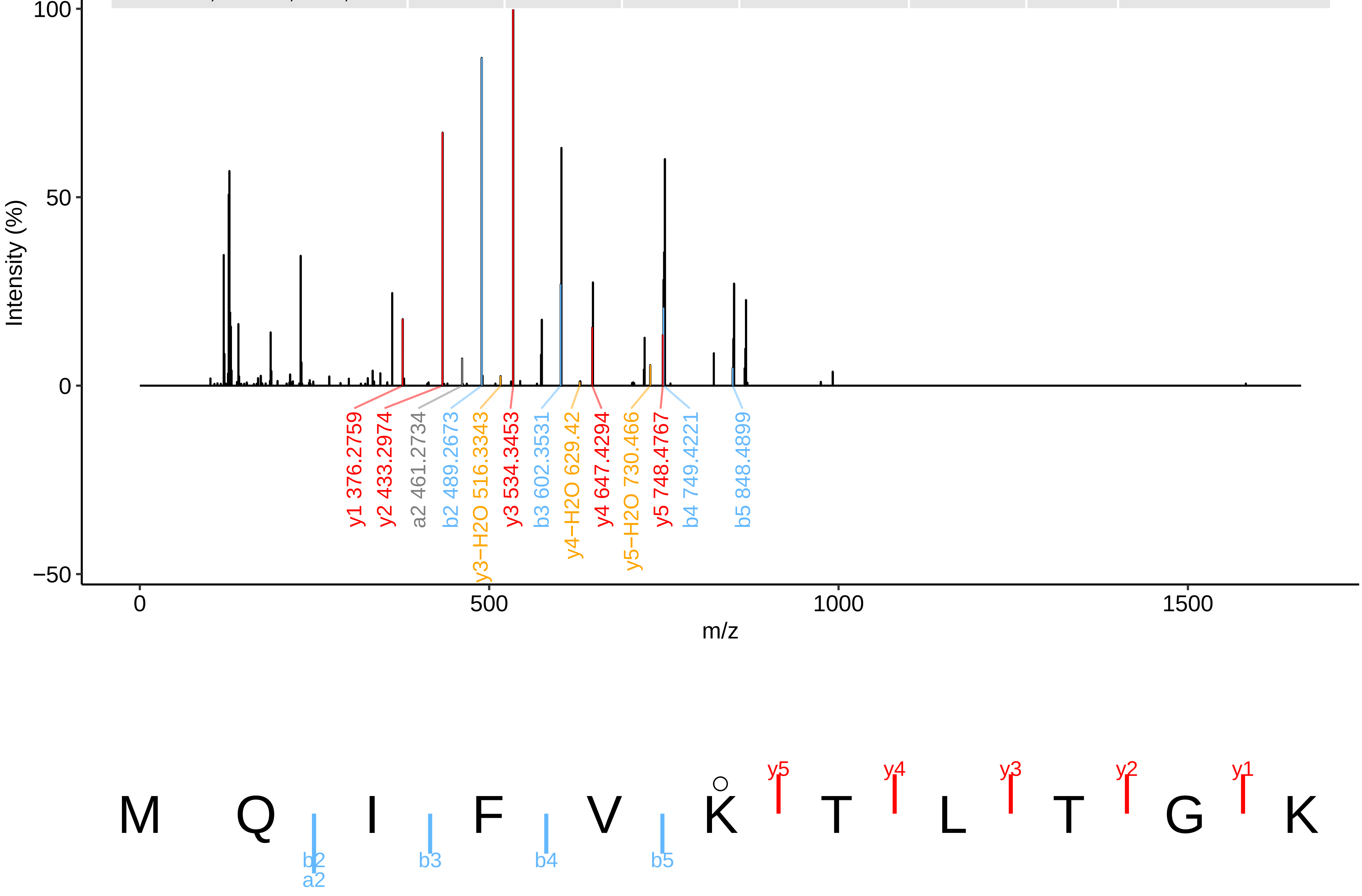

Modification □ Acetyl (Protein N−term) ○ GlyGly (K) no new terminus △ Oxidation (M)

20200921_KIZ_MUb4_GlyGly_10plex − Scan number: 28783

Gene Names	Charge	m/z	Mass	Retention time	PEP	Score	Precursor Intensity
RPS27A;UBA52;UBC;UBB	3	460.5954	1378.764	105.35	1.3004e−05	110.39	879336960

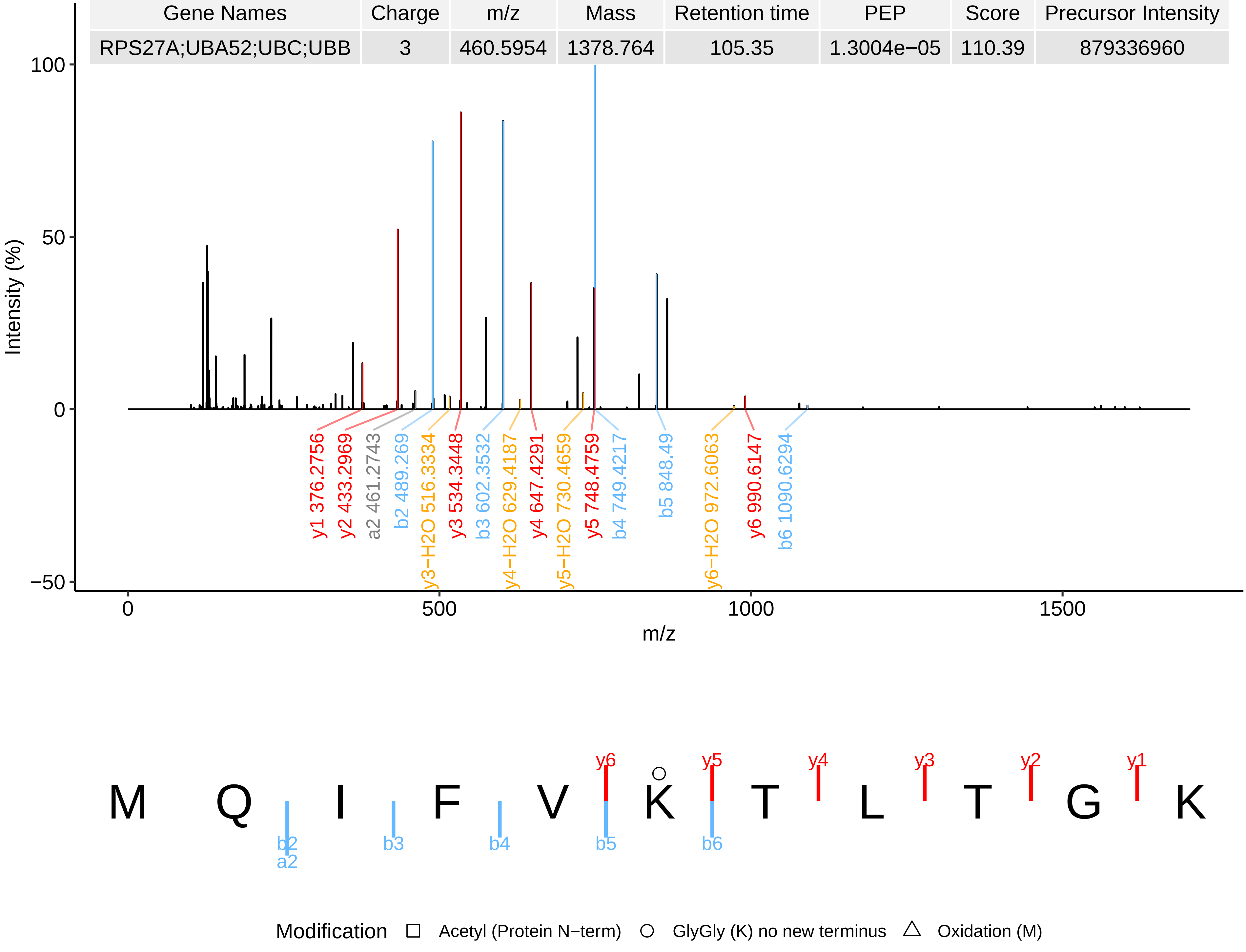

20200921_KIZ_MUb4_GlyGly_10plex − Scan number: 28883

Gene Names	Charge	m/z	Mass	Retention time	PEP	Score	Precursor Intensity
RPS27A;UBA52;UBC;UBB	3	460.5954	1378.764	105.74	0.019699	62.466	29233348

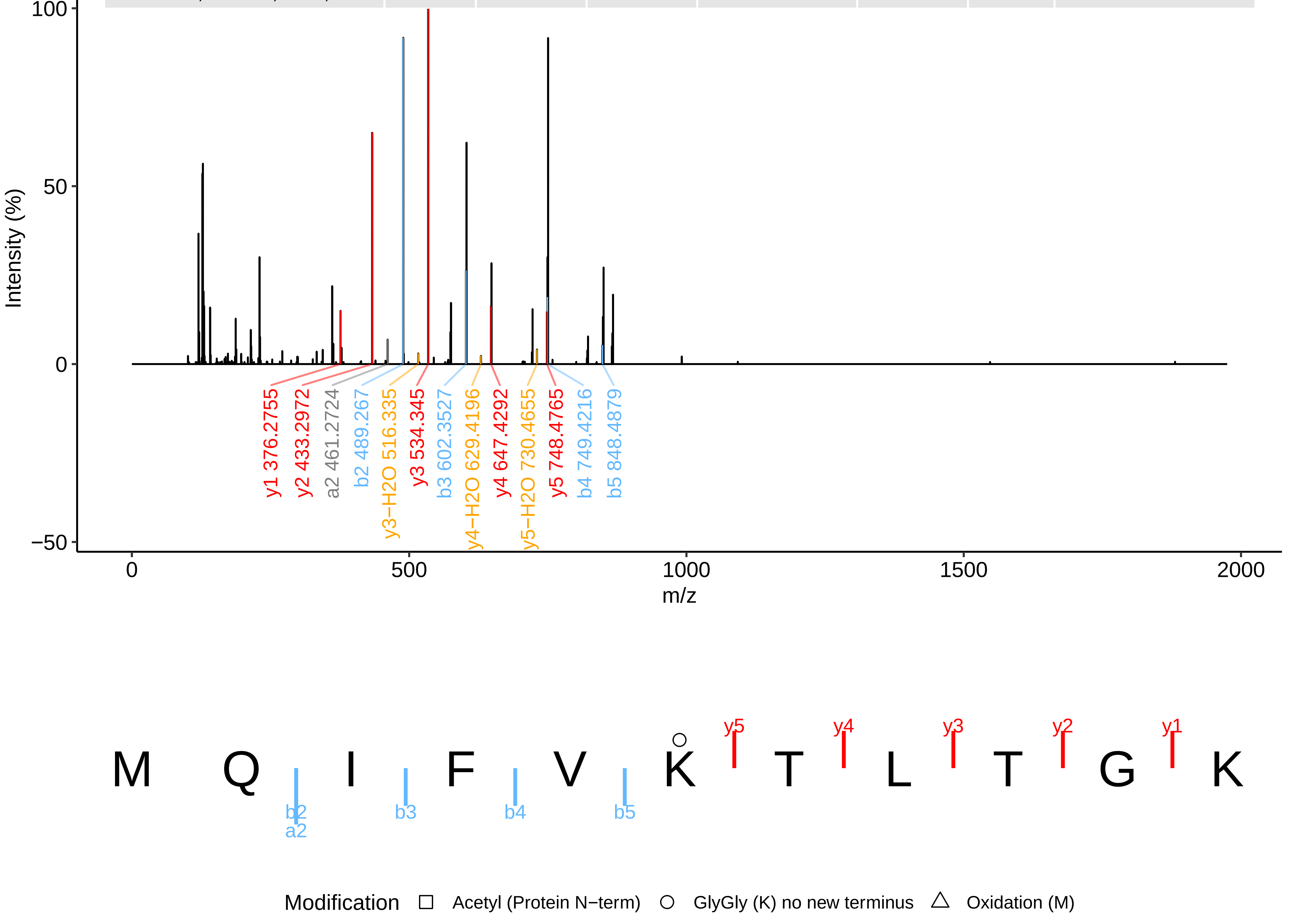

20200921_KIZ_MUb4_GlyGly_10plex − Scan number: 28914

Gene Names	Charge	m/z	Mass	Retention time	PEP	Score	Precursor Intensity
RPS27A;UBA52;UBC;UBB	3	460.5954	1378.764	105.86	0.00050276	93.258	271240512

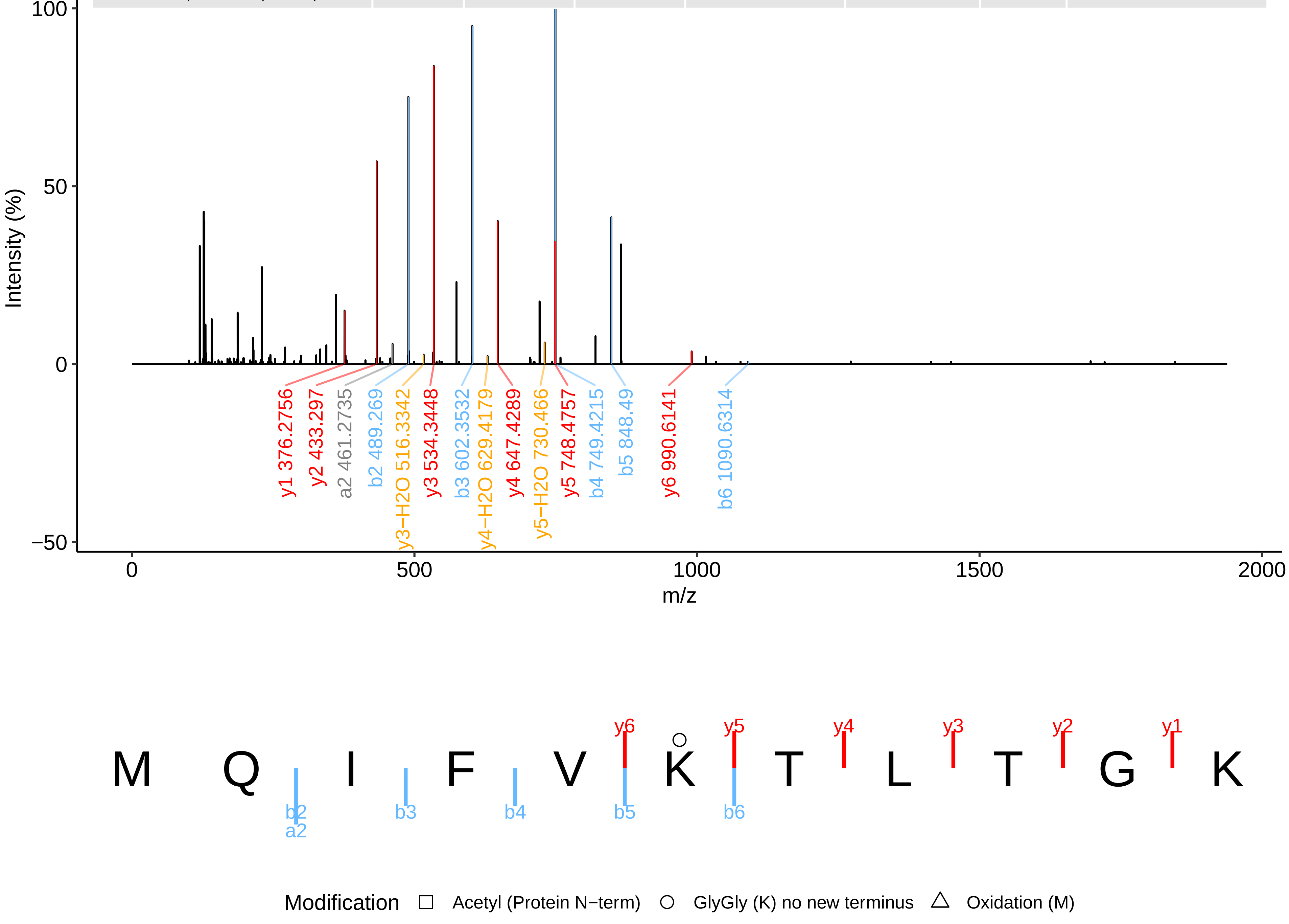

20200921_KIZ_MUb4_GlyGly_10plex − Scan number: 29044

Gene Names	Charge	m/z	Mass	Retention time	PEP	Score	Precursor Intensity
RPS27A;UBA52;UBC;UBB	3	460.5954	1378.764	106.38	0.0033169	78.653	2327380

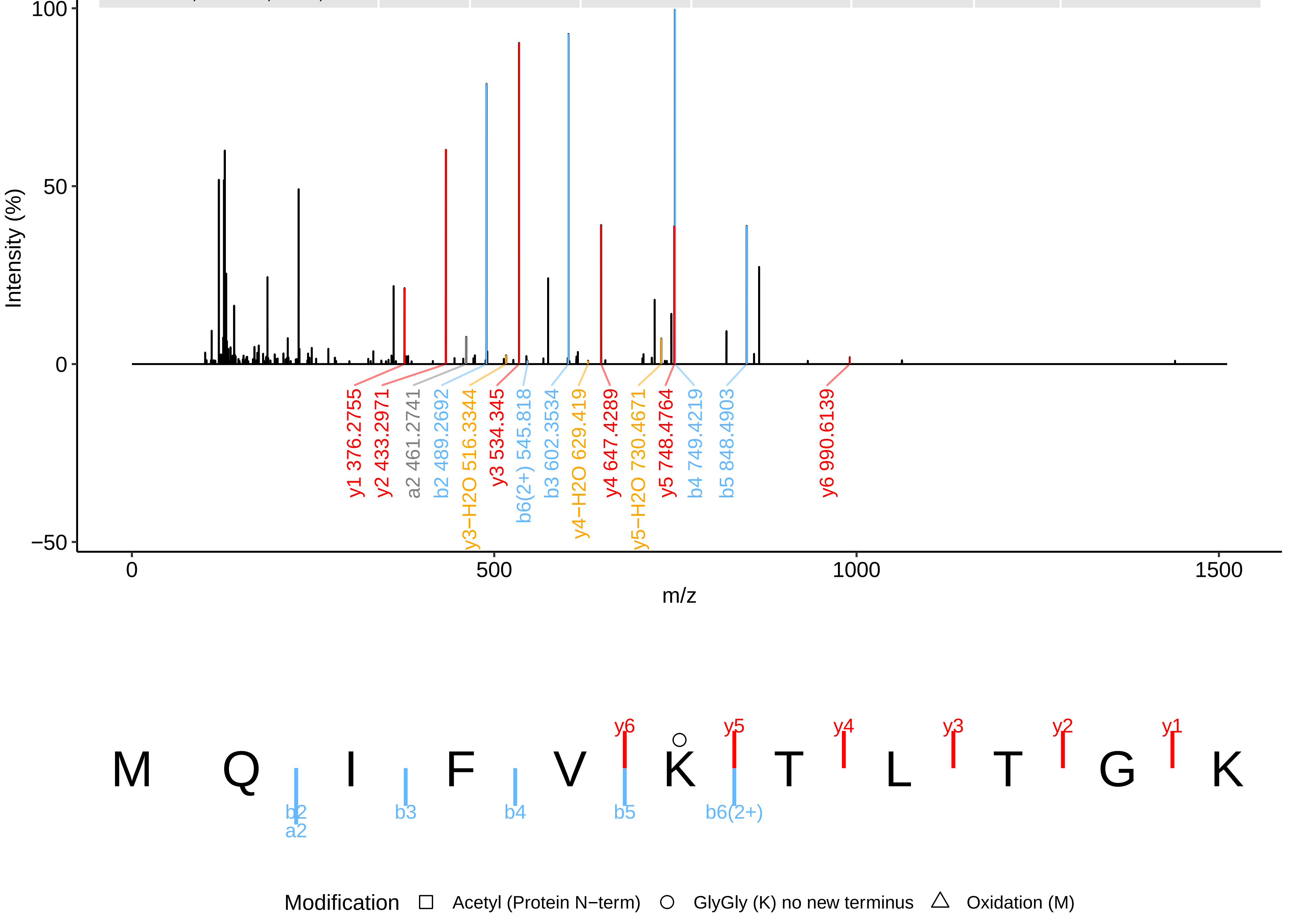

20200921_KIZ_MUb4_GlyGly_10plex − Scan number: 28708

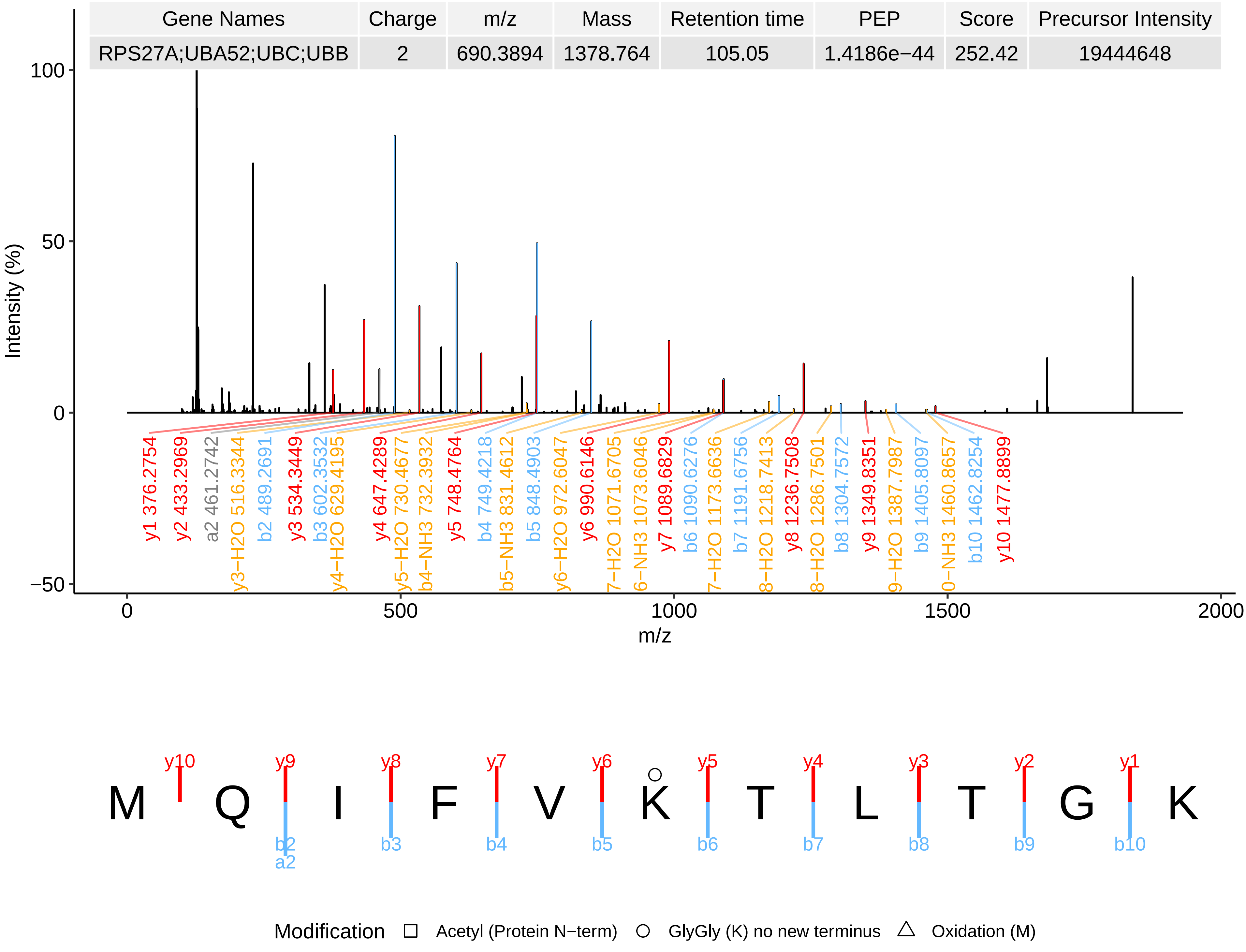

20200921_KIZ_MUb4_GlyGly_10plex − Scan number: 28838

Gene Names	Charge	m/z	Mass	Retention time	PEP	Score	Precursor Intensity
RPS27A;UBA52;UBC;UBB	2	690.3894	1378.764	105.56	6.4209e−55	268.73	11708684

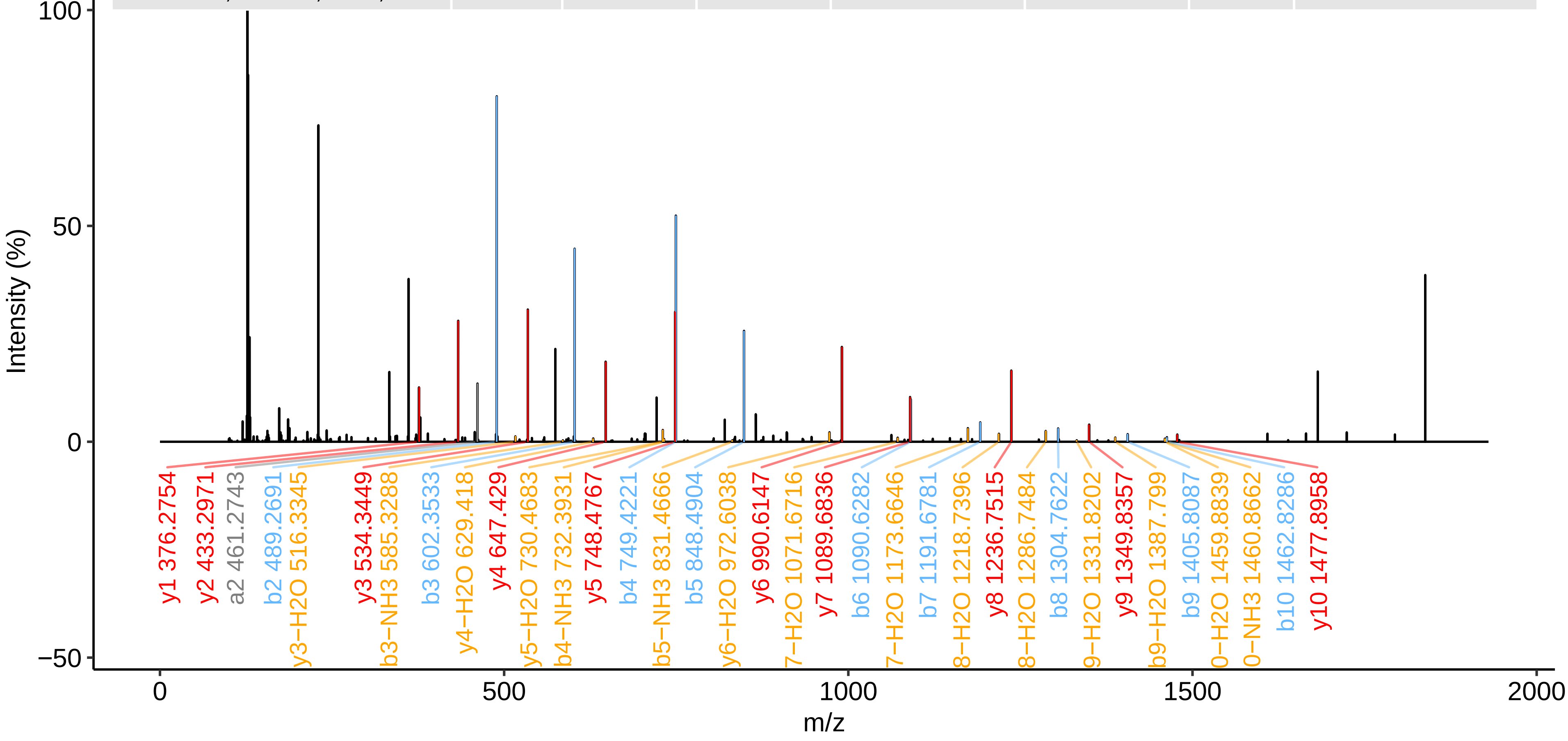

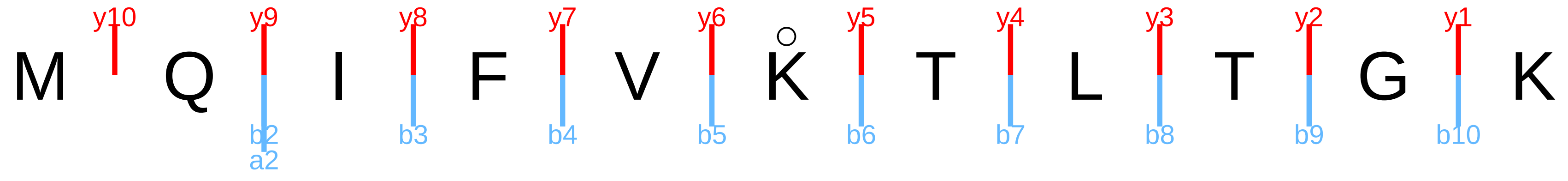

Modification □ Acetyl (Protein N−term) ○ GlyGly (K) no new terminus △ Oxidation (M)

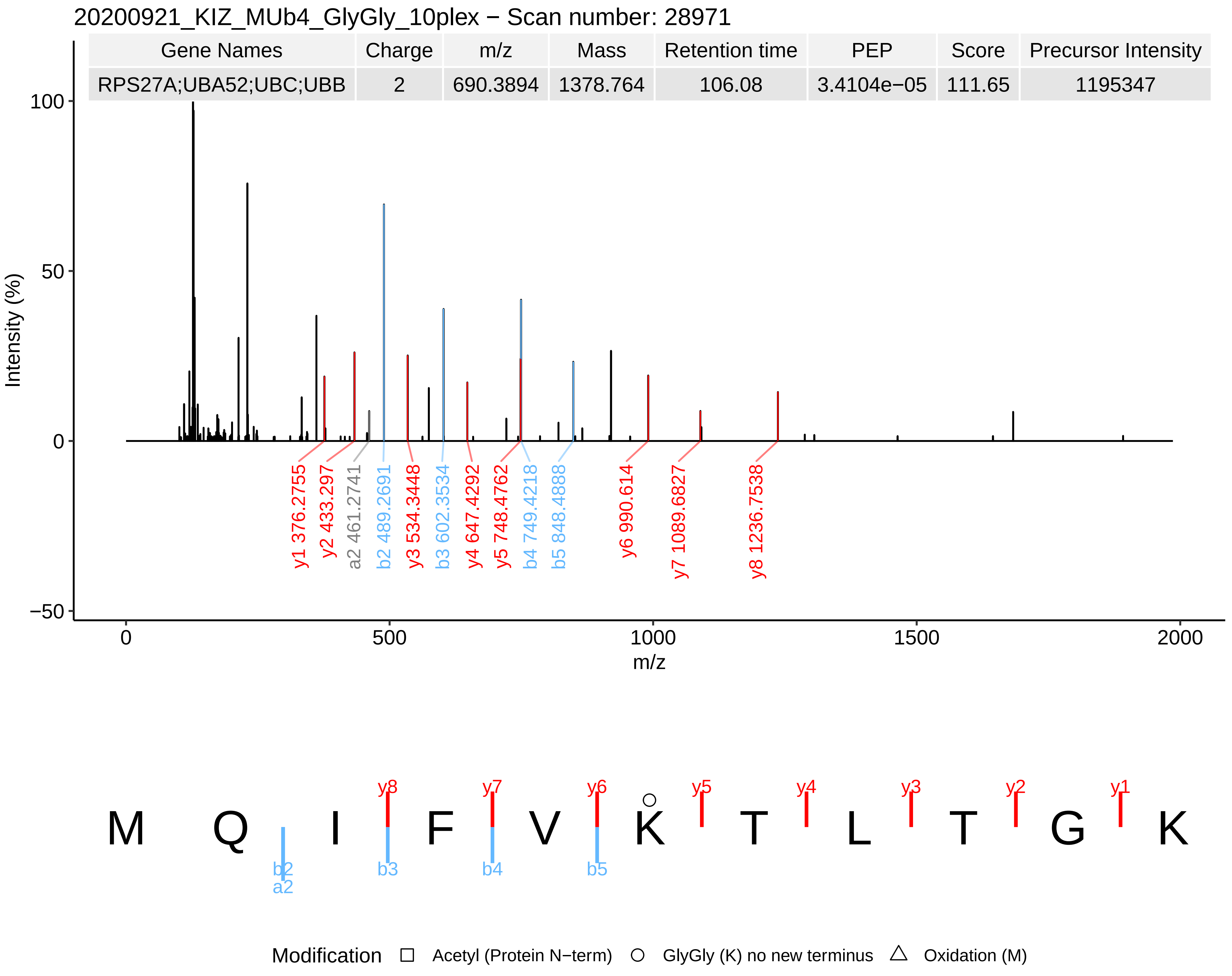

20200921_KIZ_MUb4_GlyGly_10plex − Scan number: 28971
Gene Names | Charge | m/z | Mass | Retention time | PEP | Score | Precursor Intensity
RPS27A;UBA52;UBC;UBB | 2 | 690.3894 | 1378.764 | 106.08 | 3.4104e−05 | 111.65 | 1195347
Intensity (%)
100
50
0
−50
y1 376.2755
y2 433.297
a2 461.2741
b2 489.2691
y3 534.3448
b3 602.3534
y4 647.4292
y5 748.4762
b4 749.4218
b5 848.4888
y6 990.614
y7 1089.6827
y8 1236.7538
0
500
1000
1500
2000
m/z
M Q I F V K T L T G K
y8 y7 y6 y5 y4 y3 y2 y1
b2 a2 b3 b4 b5
Modification
Acetyl (Protein N−term)
GlyGly (K) no new terminus
Oxidation (M)

20200921_KIZ_MUb4_GlyGly_10plex − Scan number: 35375

Gene Names	Charge	m/z	Mass	Retention time	PEP	Score	Precursor Intensity
RPS27A;UBA52;UBC;UBB	3	460.5954	1378.764	129.65	0.011251	69.01	2444031

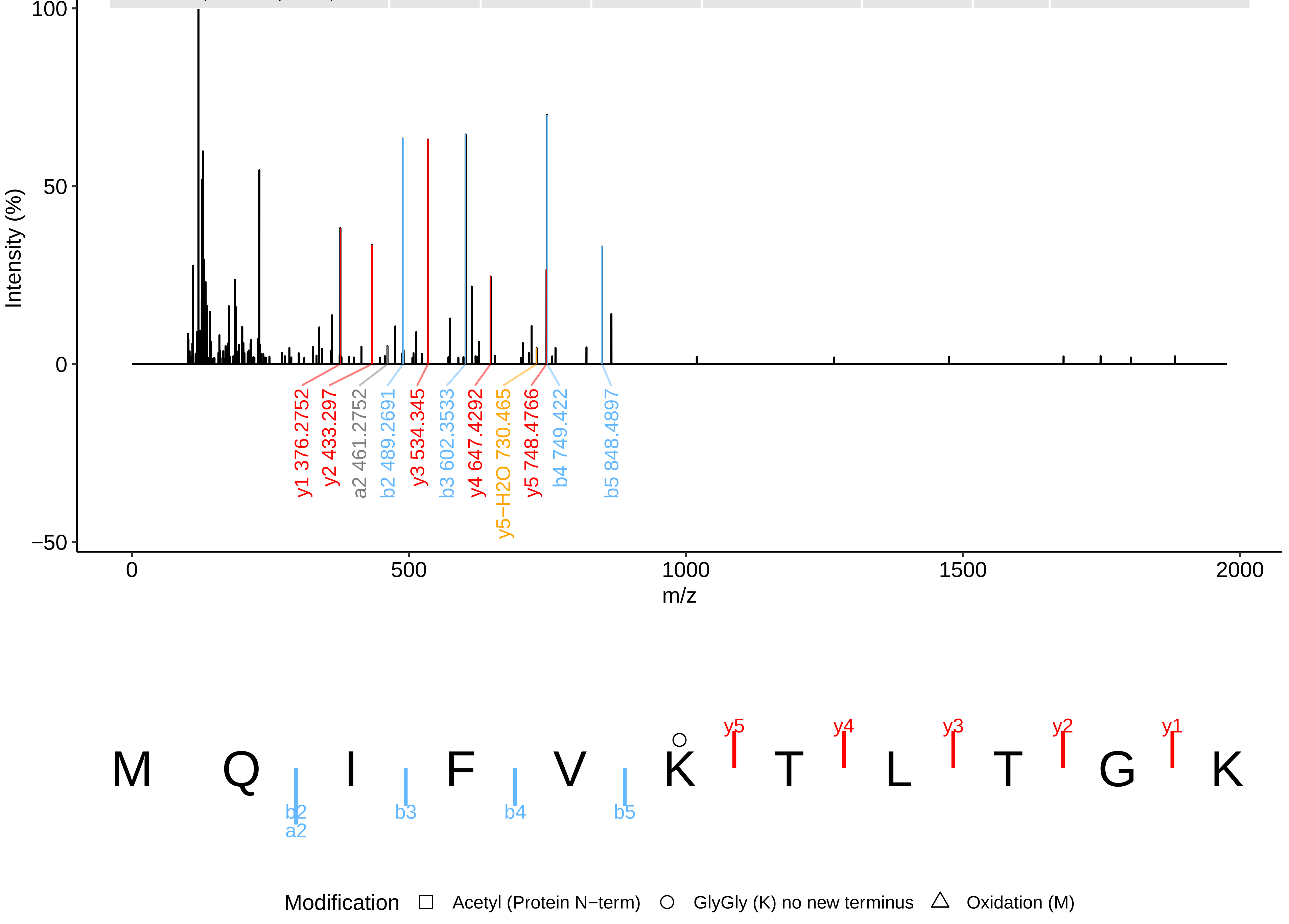

20200921_KIZ_MUb4_GlyGly_10plex_2 − Scan number: 24549

Gene Names	Charge	m/z	Mass	Retention time	PEP	Score	Precursor Intensity
RPS27A;UBA52;UBC;UBB	3	460.5954	1378.764	97.011	0.001455	83.397	2002725

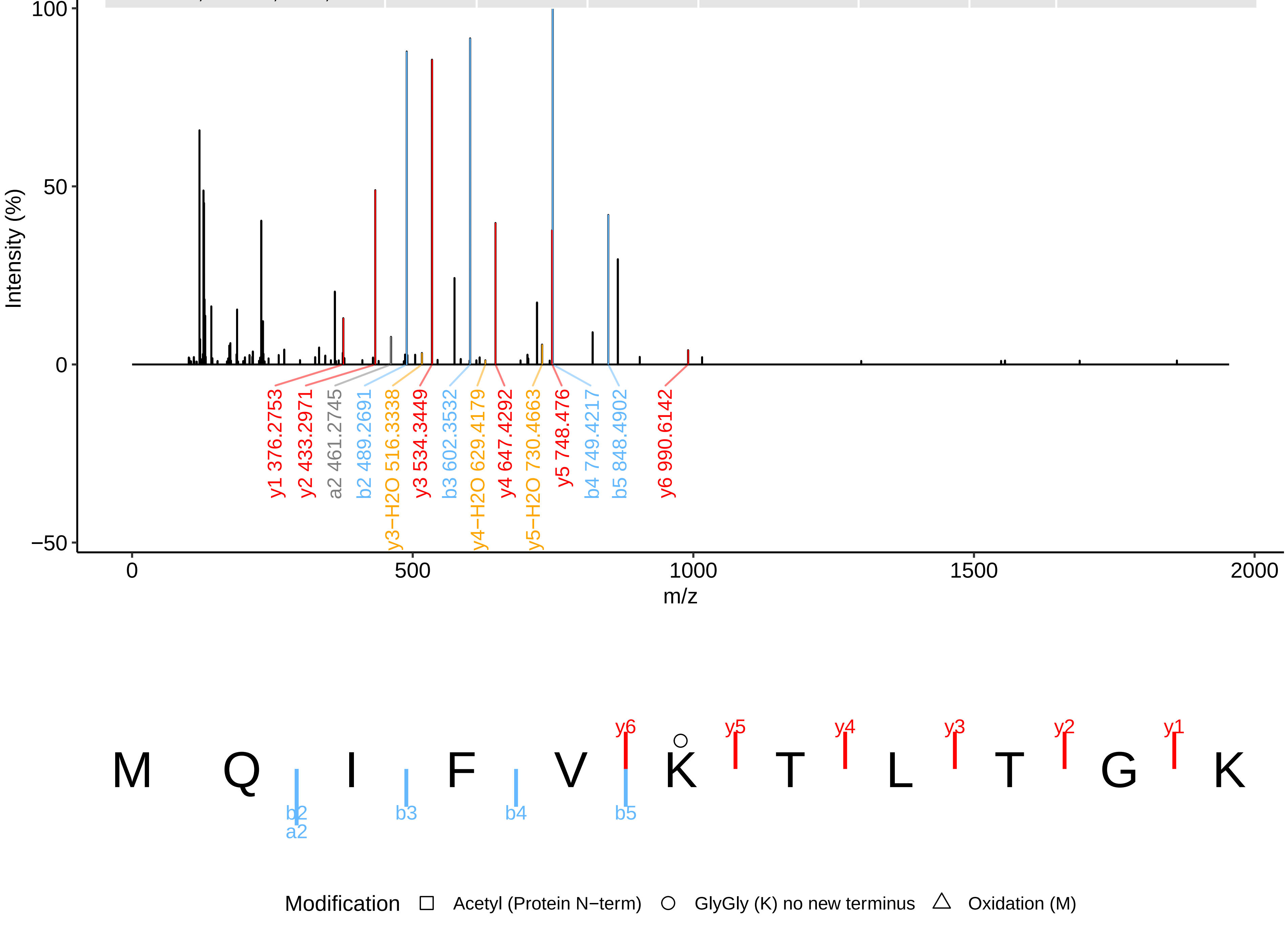

20200921_KIZ_MUb4_GlyGly_10plex_2 − Scan number: 24678

Gene Names	Charge	m/z	Mass	Retention time	PEP	Score	Precursor Intensity
RPS27A;UBA52;UBC;UBB	3	460.5954	1378.764	97.518	0.00050276	93.258	182355360

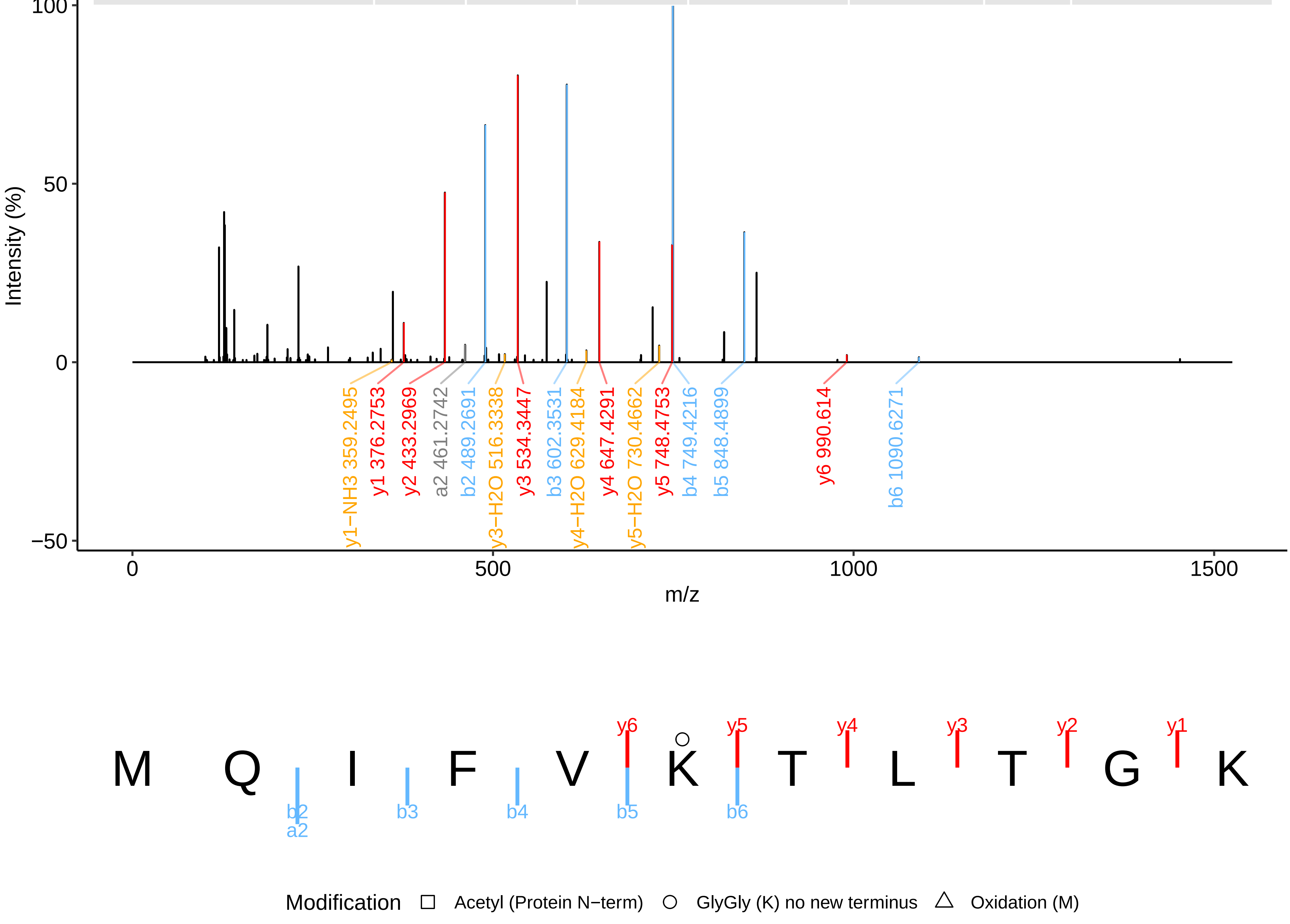

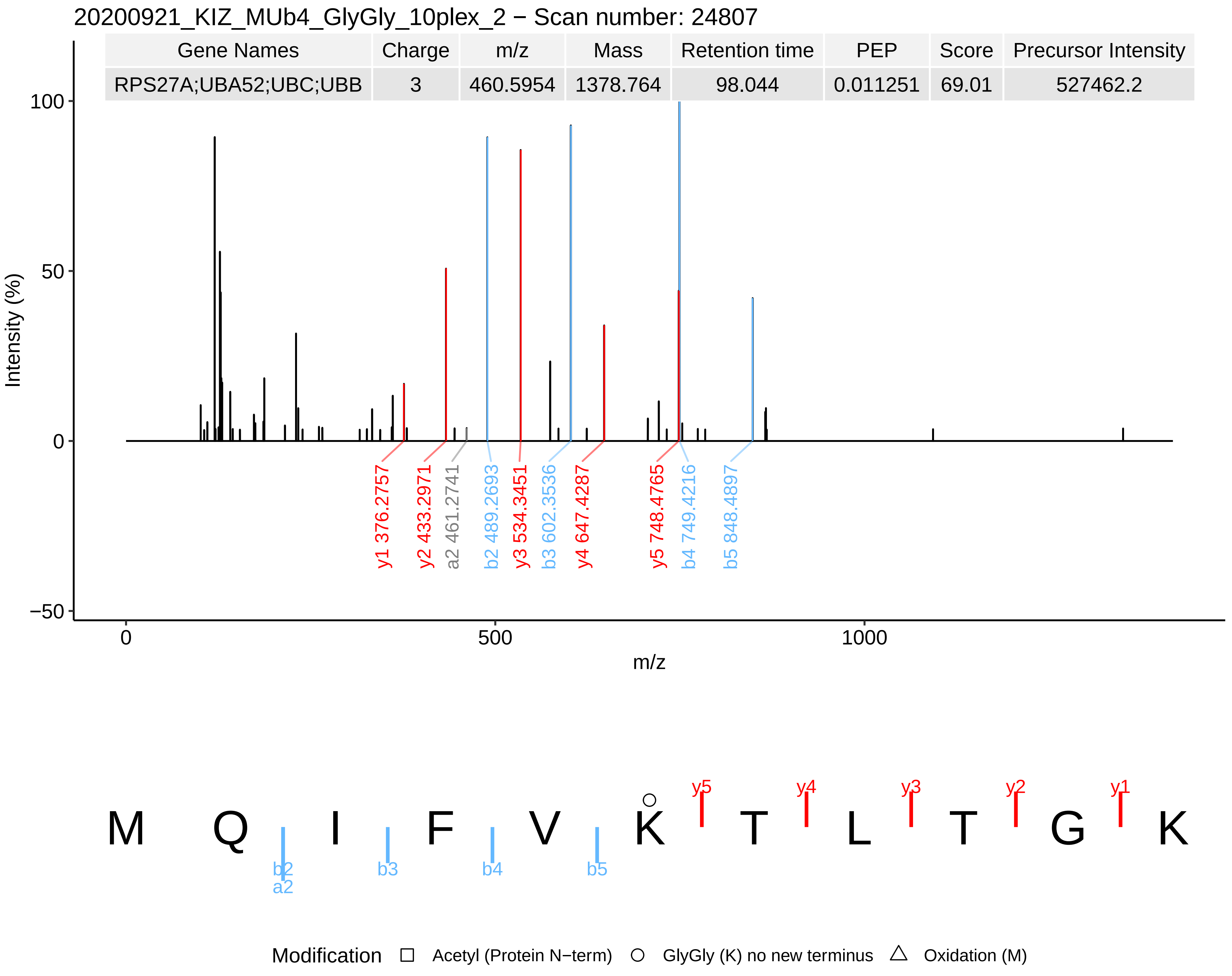

20200921_KIZ_MUb4_GlyGly_10plex_2 − Scan number: 24807
Gene Names
Charge
m/z
Mass
Retention time
PEP
Score
Precursor Intensity
RPS27A;UBA52;UBC;UBB
3
460.5954
1378.764
98.044
0.011251
69.01
527462.2
Intensity (%)
m/z
y1 376.2757
y2 433.2971
a2 461.2741
b2 489.2693
y3 534.3451
b3 602.3536
y4 647.4287
y5 748.4765
b4 749.4216
b5 848.4897
M Q I F V K T L T G K
Modification
Acetyl (Protein N−term)
GlyGly (K) no new terminus
Oxidation (M)

20200921_KIZ_MUb4_GlyGly_10plex_2 − Scan number: 24583

Gene Names	Charge	m/z	Mass	Retention time	PEP	Score	Precursor Intensity
RPS27A;UBA52;UBC;UBB	2	690.3894	1378.764	97.147	0.0035657	87.913	1100326

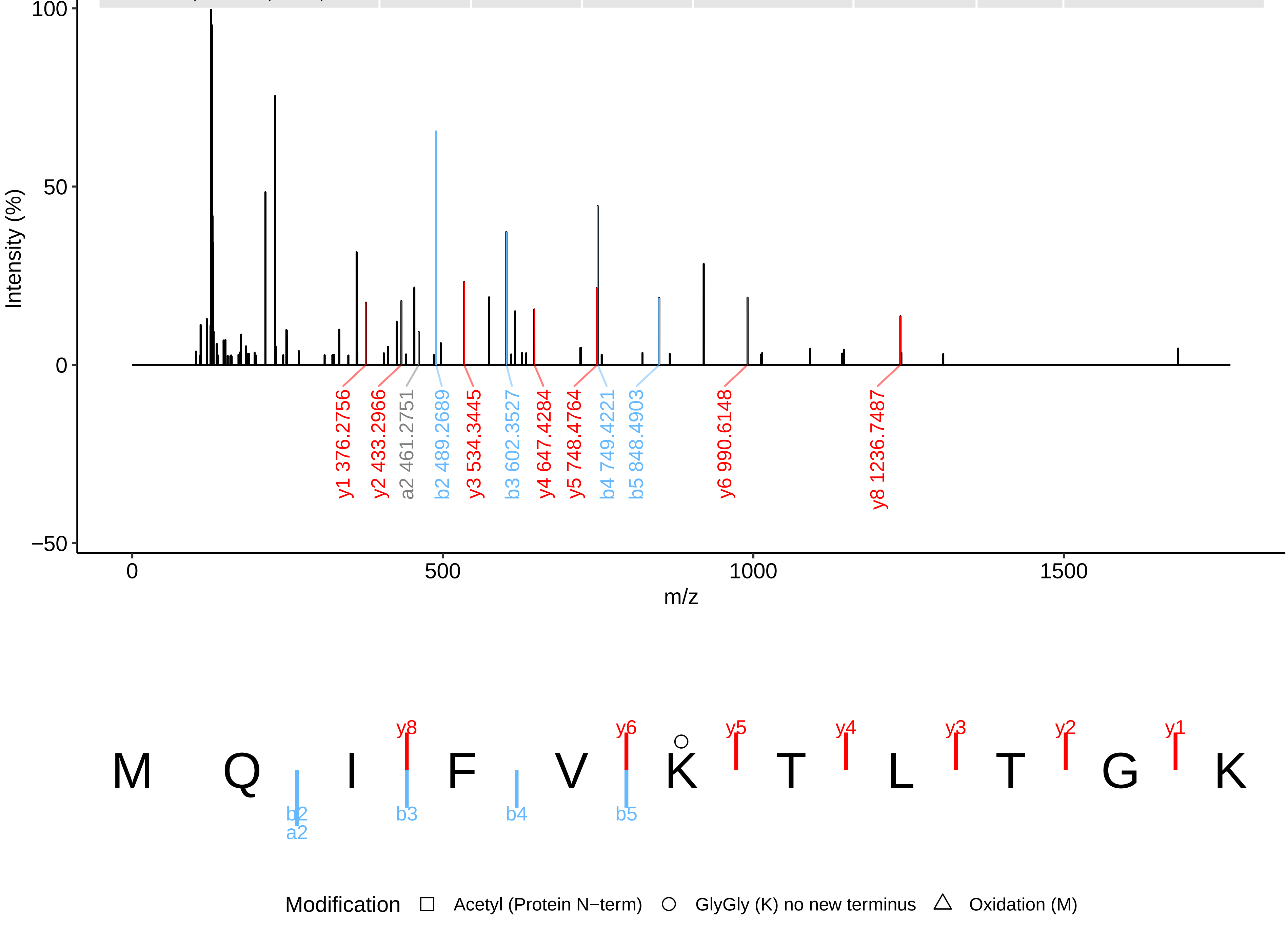

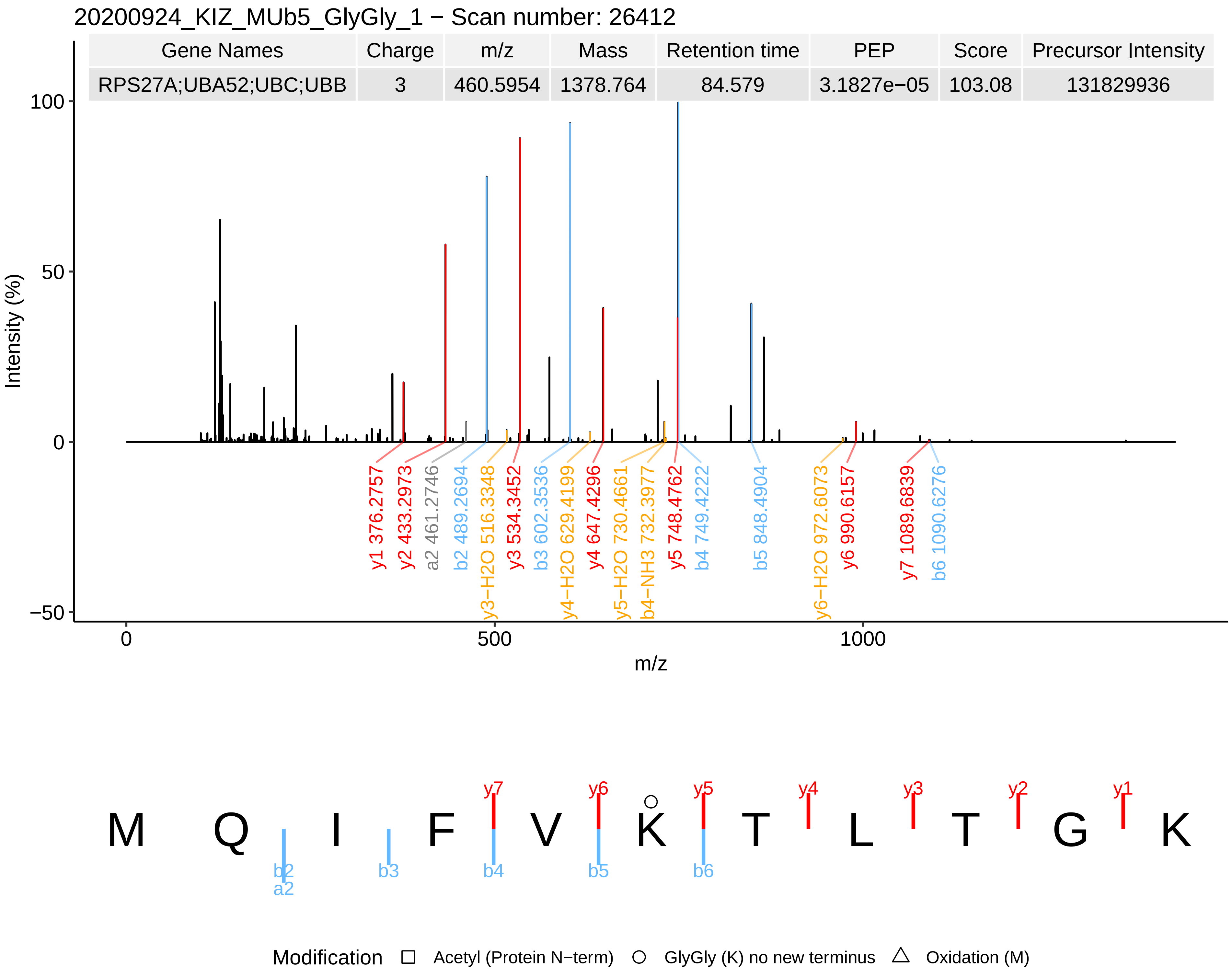
20200924_KIZ_MUb5_GlyGly_1 − Scan number: 26412
Gene Names | Charge | m/z | Mass | Retention time | PEP | Score | Precursor Intensity
RPS27A;UBA52;UBC;UBB | 3 | 460.5954 | 1378.764 | 84.579 | 3.1827e−05 | 103.08 | 131829936
Intensity (%)
100
50
0
−50
0
500
1000
m/z
y1 376.2757
y2 433.2973
a2 461.2746
b2 489.2694
y3−H2O 516.3348
y3 534.3452
b3 602.3536
y4−H2O 629.4199
y4 647.4296
y5−H2O 730.4661
b4−NH3 732.3977
y5 748.4762
b4 749.4222
b5 848.4904
y6−H2O 972.6073
y6 990.6157
y7 1089.6839
b6 1090.6276
M Q I F V K T L T G K
y7 y6 y5 y4 y3 y2 y1
b2 a2 b3 b4 b5 b6
Modification
Acetyl (Protein N−term)
GlyGly (K) no new terminus
Oxidation (M)

20200924_KIZ_MUb5_GlyGly_1 − Scan number: 26580

Gene Names	Charge	m/z	Mass	Retention time	PEP	Score	Precursor Intensity
RPS27A;UBA52;UBC;UBB	3	460.5954	1378.764	85.085	8.4678e−06	112.15	559493824

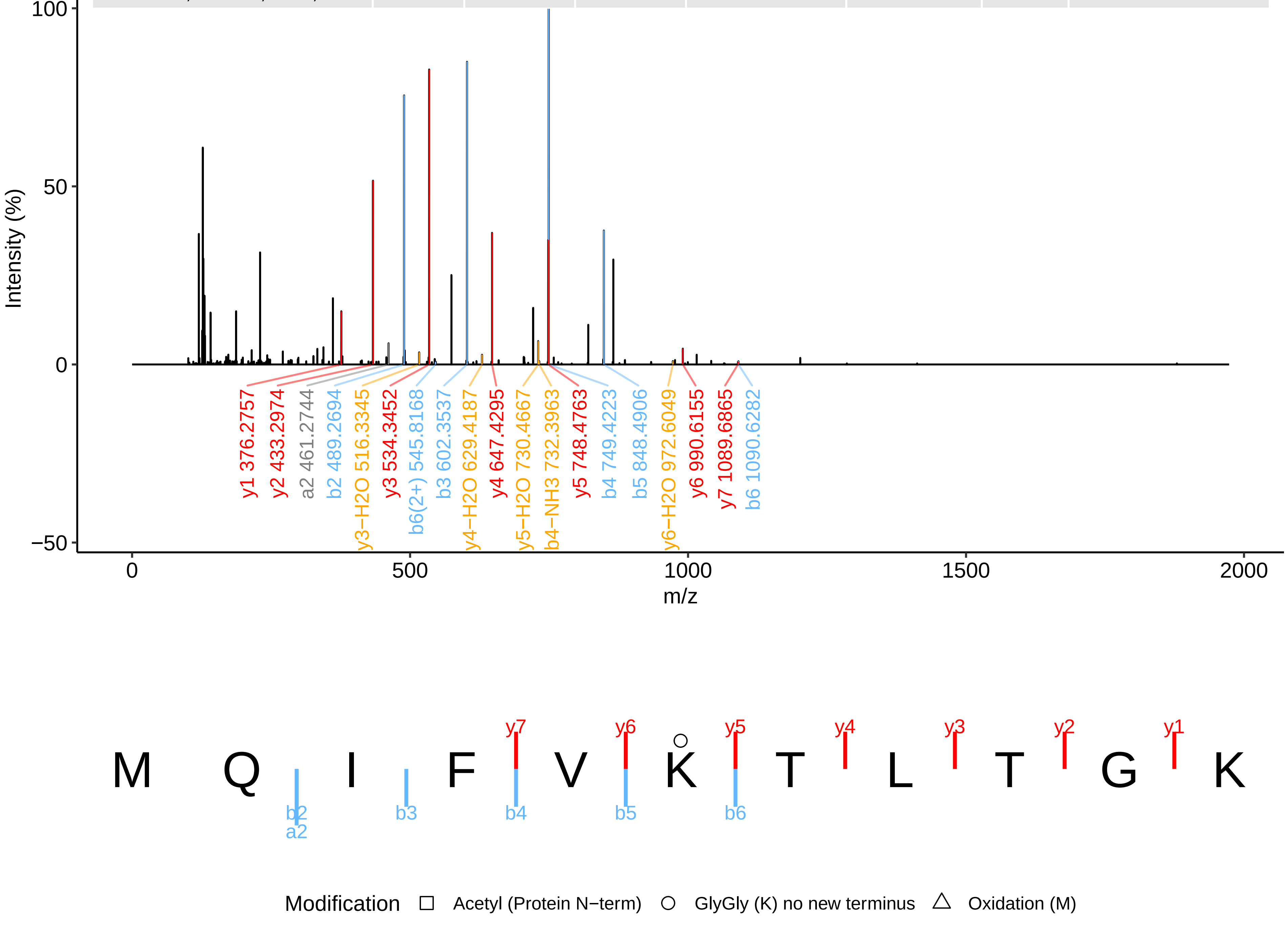

20200924_KIZ_MUb5_GlyGly_1 − Scan number: 26754

Gene Names	Charge	m/z	Mass	Retention time	PEP	Score	Precursor Intensity
RPS27A;UBA52;UBC;UBB	3	460.5954	1378.764	85.595	3.4803e−05	101.93	525956736

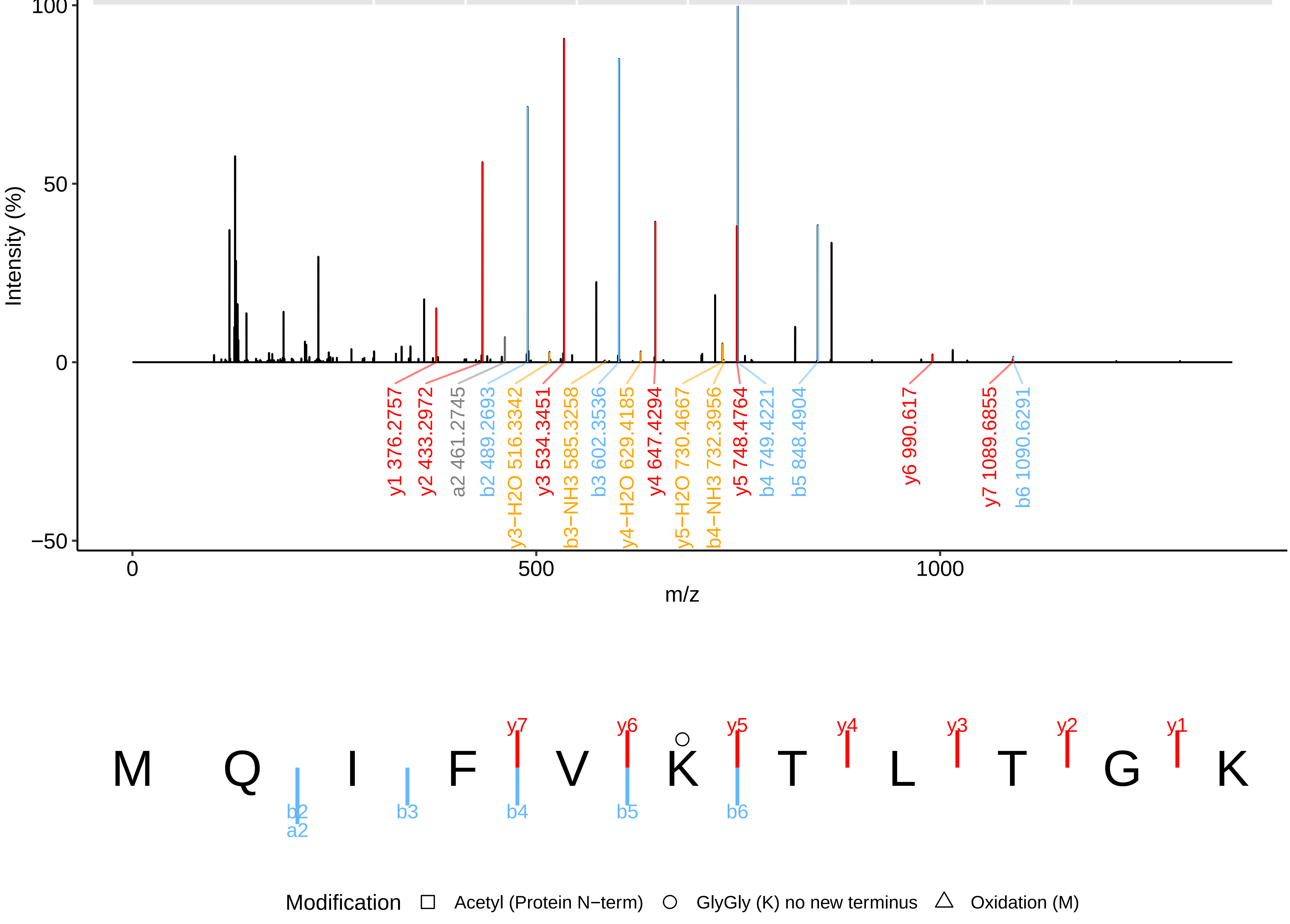

20200924_KIZ_MUb5_GlyGly_1 − Scan number: 26921

Gene Names	Charge	m/z	Mass	Retention time	PEP	Score	Precursor Intensity
RPS27A;UBA52;UBC;UBB	3	460.5954	1378.764	86.104	0.00022697	96.113	397807456

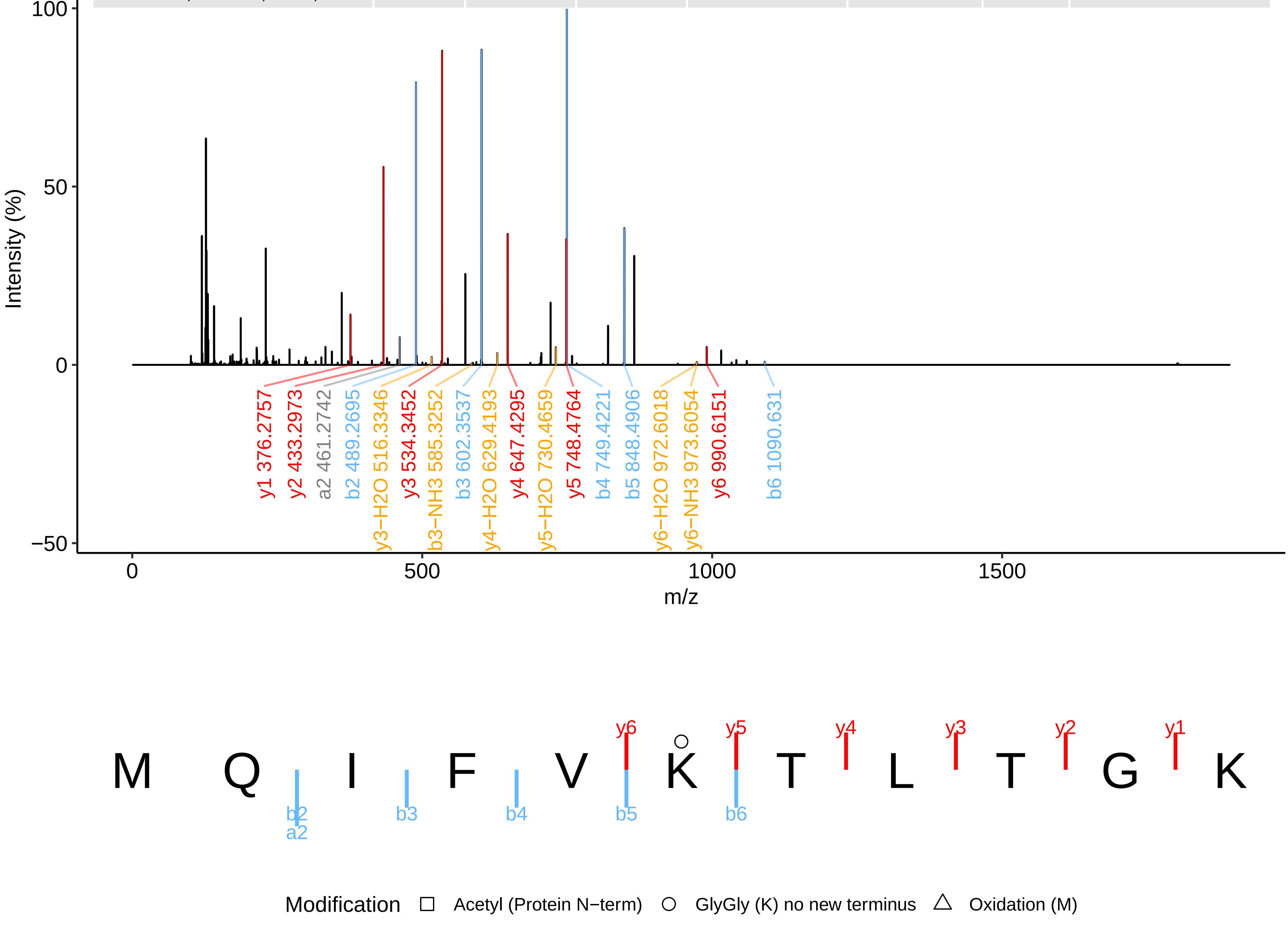

20200924_KIZ_MUb5_GlyGly_1 − Scan number: 27084

Gene Names	Charge	m/z	Mass	Retention time	PEP	Score	Precursor Intensity
RPS27A;UBA52;UBC;UBB	3	460.5954	1378.764	86.61	1.3004e−05	110.39	270714688

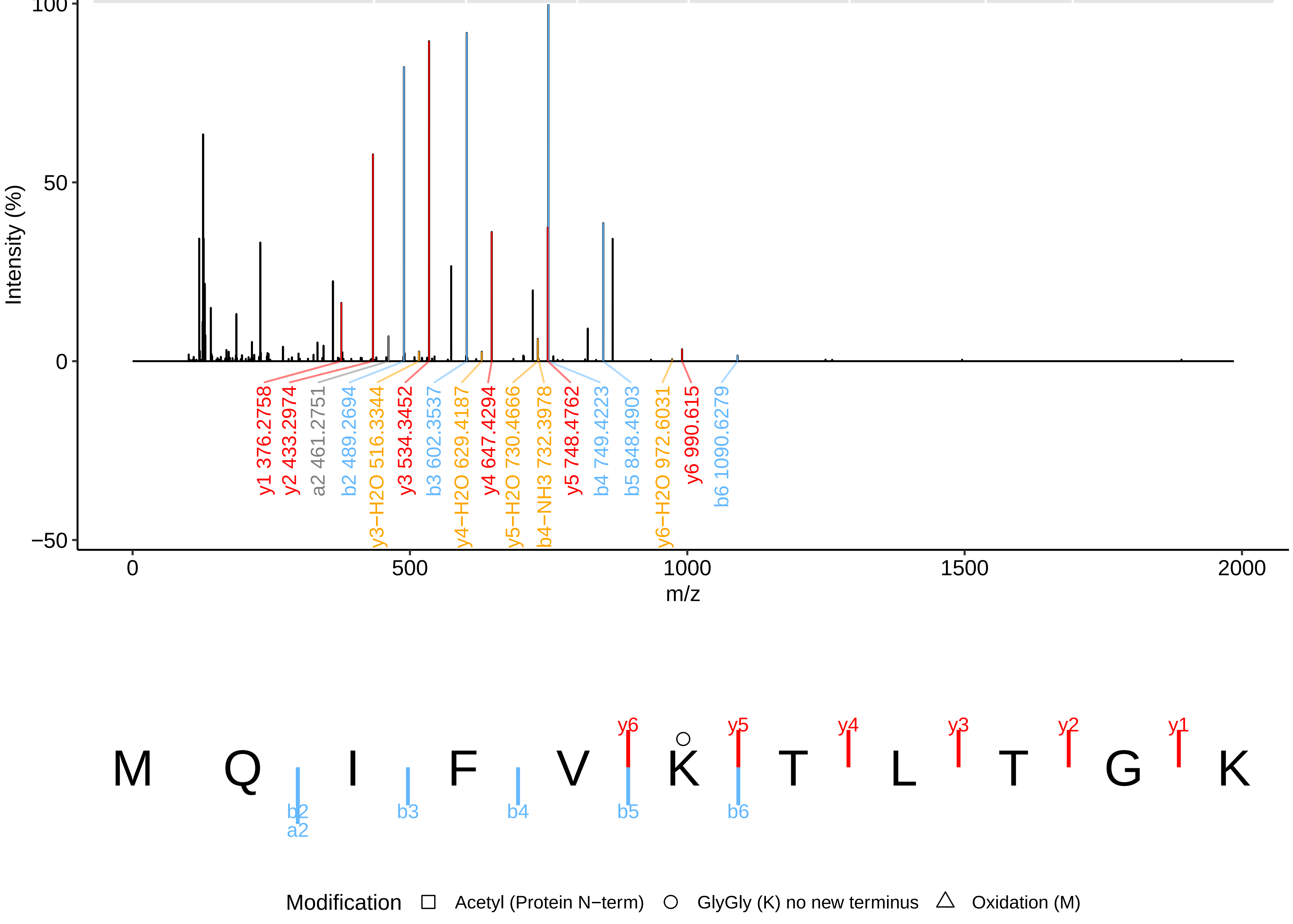

20200924_KIZ_MUb5_GlyGly_1 − Scan number: 27248

Gene Names	Charge	m/z	Mass	Retention time	PEP	Score	Precursor Intensity
RPS27A;UBA52;UBC;UBB	3	460.5954	1378.764	87.12	2.7416e−05	104.79	88876232

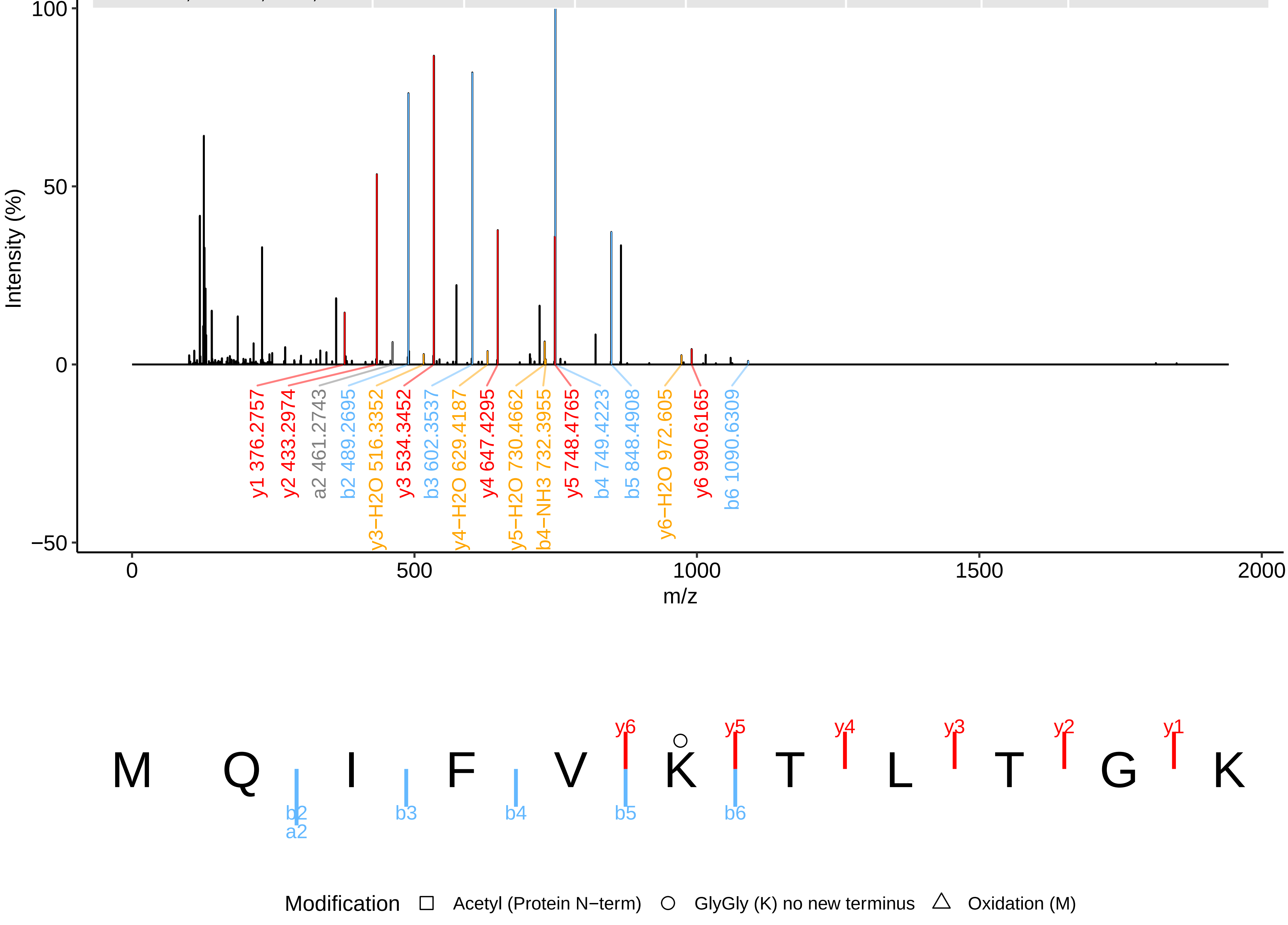

20200924_KIZ_MUb5_GlyGly_1 − Scan number: 26565

Gene Names	Charge	m/z	Mass	Retention time	PEP	Score	Precursor Intensity
RPS27A;UBA52;UBC;UBB	2	690.3894	1378.764	85.04	3.7777e−15	210.87	11605820

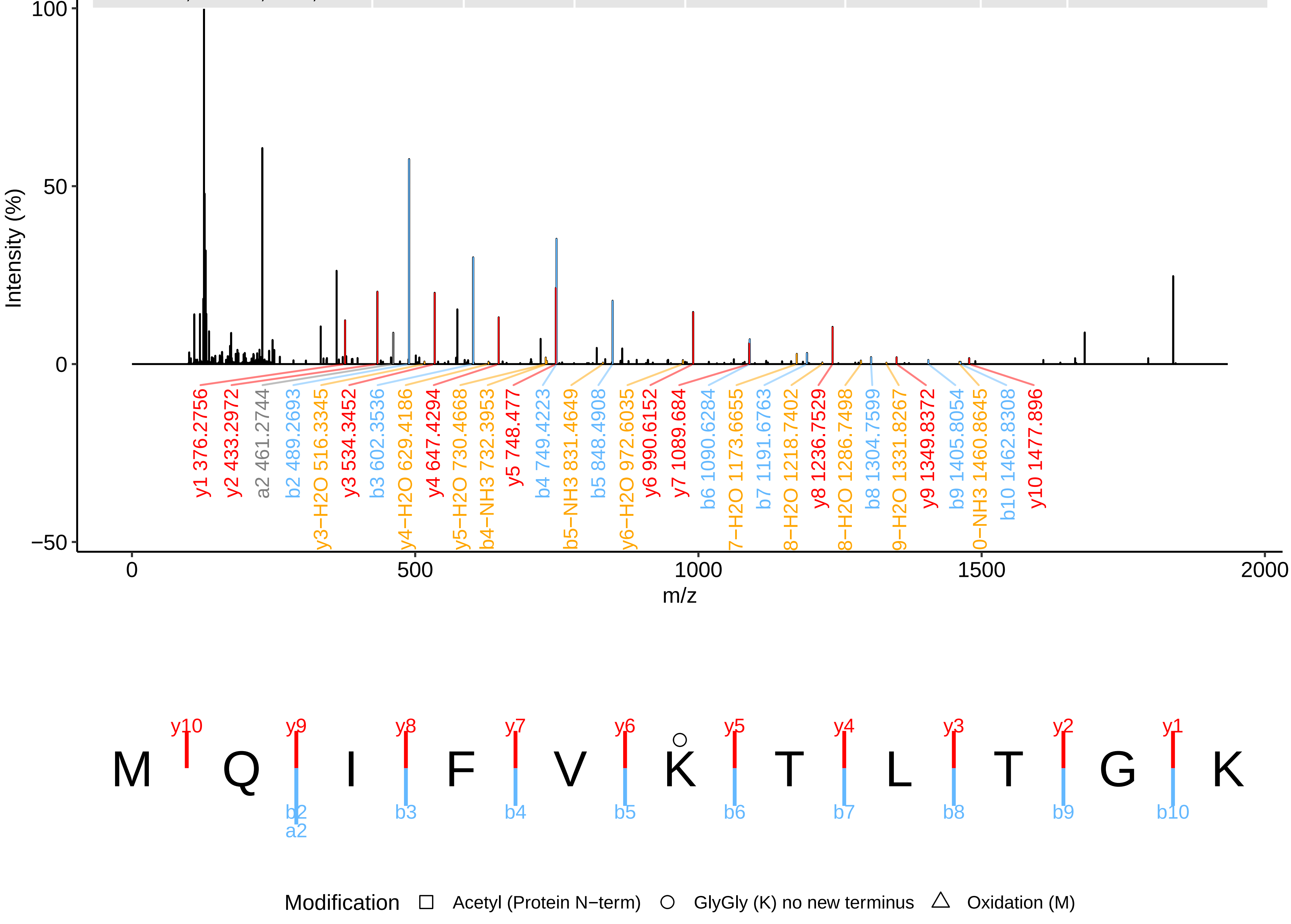

20200924_KIZ_MUb5_GlyGly_1 − Scan number: 26812

Gene Names	Charge	m/z	Mass	Retention time	PEP	Score	Precursor Intensity
RPS27A;UBA52;UBC;UBB	2	690.3894	1378.764	85.774	1.3579e−37	267.24	15088600

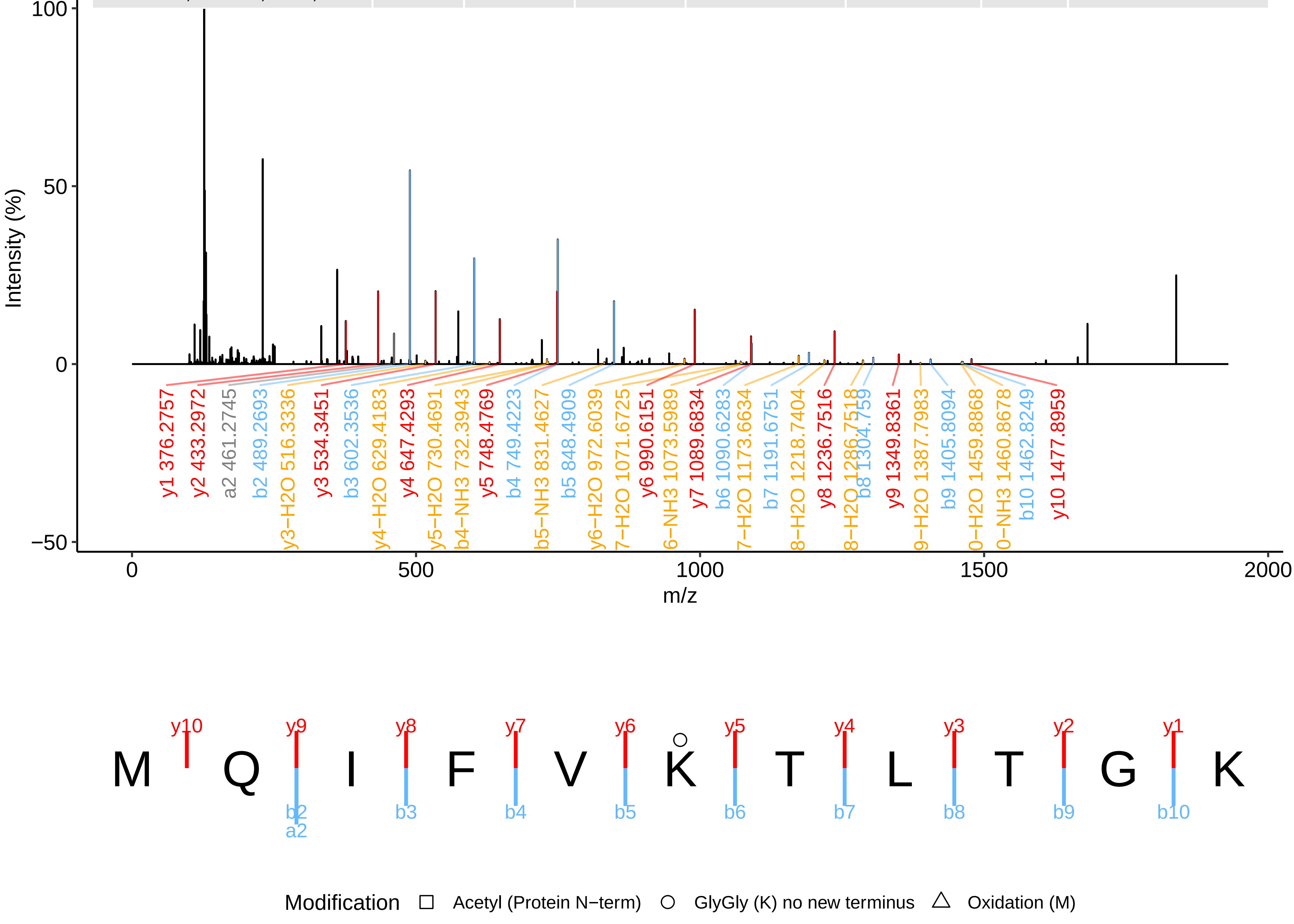

20200924_KIZ_MUb5_GlyGly_1 − Scan number: 27415

Gene Names	Charge	m/z	Mass	Retention time	PEP	Score	Precursor Intensity
RPS27A;UBA52;UBC;UBB	3	460.5954	1378.764	87.626	0.00096454	92.866	41048036

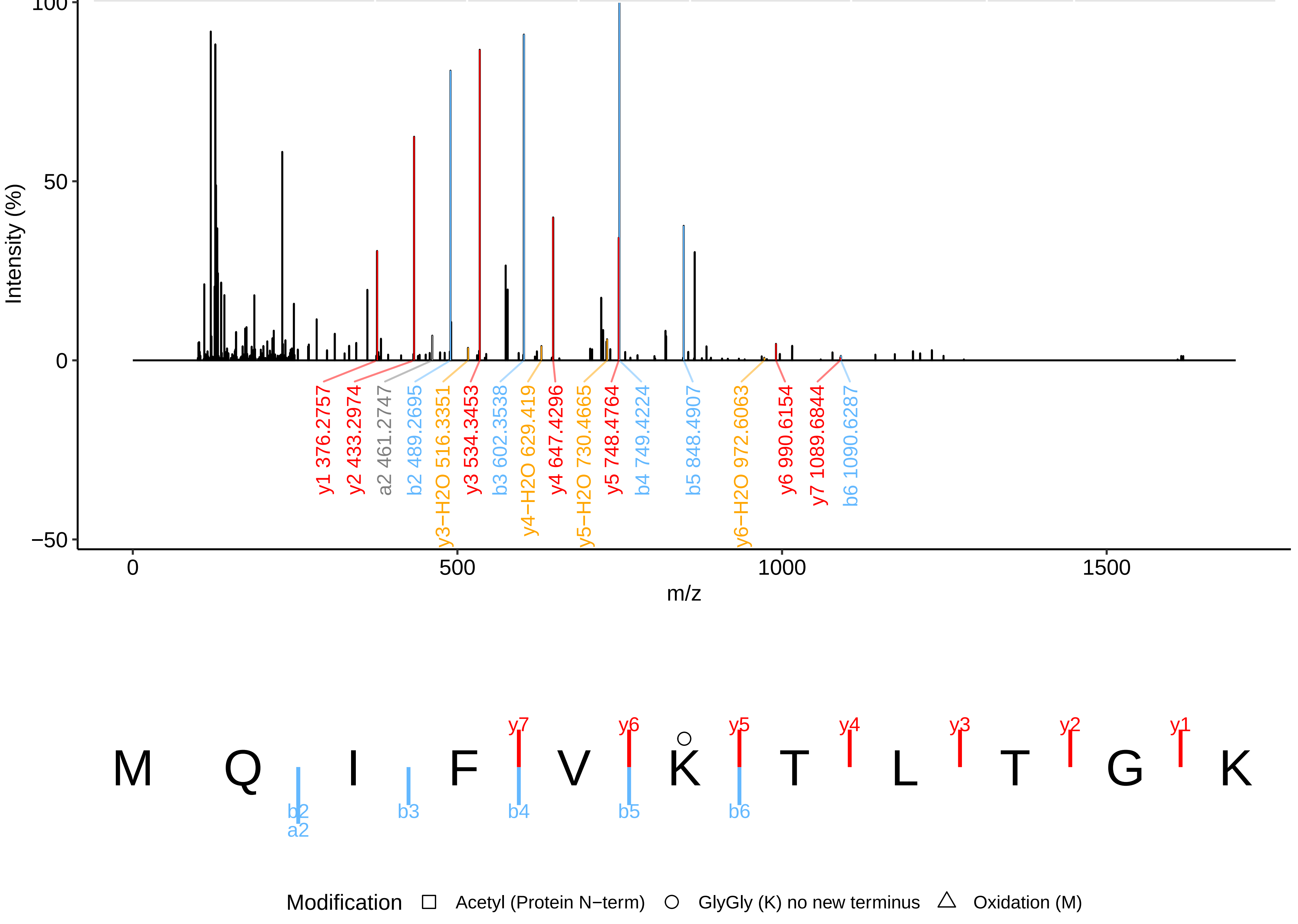

20200924_KIZ_MUb5_GlyGly_2 − Scan number: 23951

Gene Names	Charge	m/z	Mass	Retention time	PEP	Score	Precursor Intensity
RPS27A;UBA52;UBC;UBB	3	460.5954	1378.764	88.736	2.7416e−05	104.79	31647010

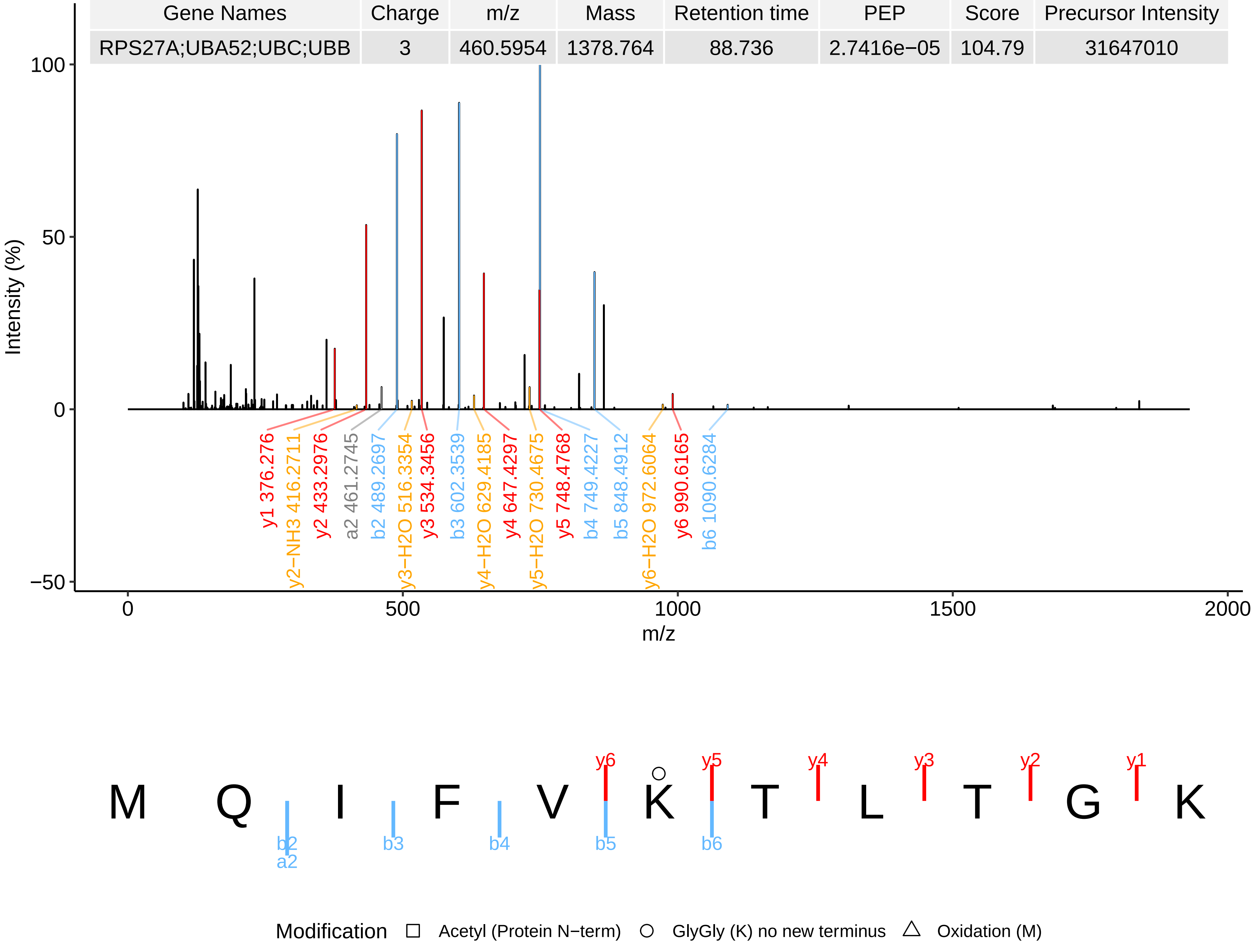

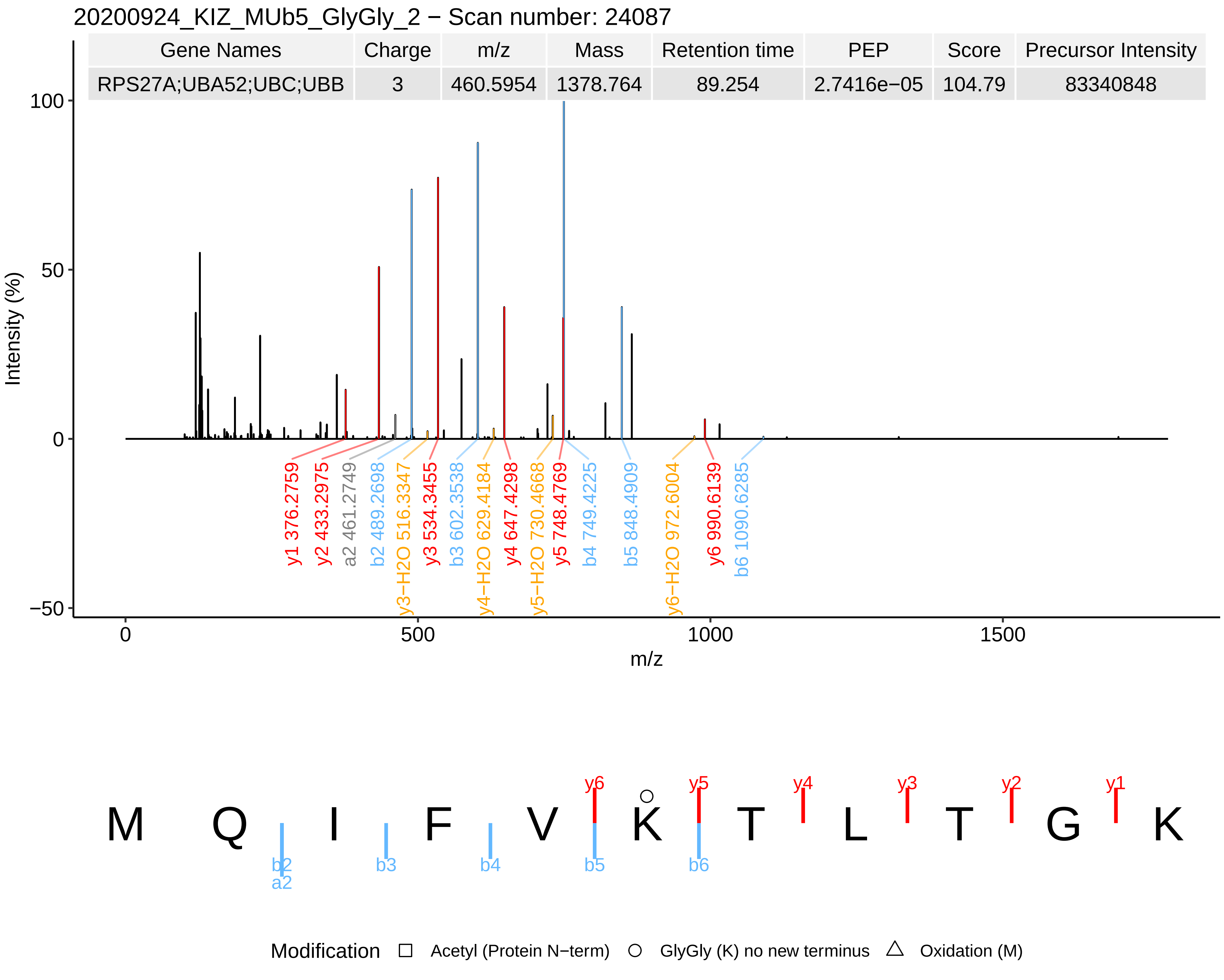

20200924_KIZ_MUb5_GlyGly_2 − Scan number: 24087
Gene Names | Charge | m/z | Mass | Retention time | PEP | Score | Precursor Intensity
RPS27A;UBA52;UBC;UBB | 3 | 460.5954 | 1378.764 | 89.254 | 2.7416e−05 | 104.79 | 83340848
Intensity (%)
m/z
y1 376.2759
y2 433.2975
a2 461.2749
b2 489.2698
y3−H2O 516.3347
y3 534.3455
b3 602.3538
y4−H2O 629.4184
y4 647.4298
y5−H2O 730.4668
y5 748.4769
b4 749.4225
b5 848.4909
y6−H2O 972.6004
y6 990.6139
b6 1090.6285
M Q I F V K T L T G K
Modification
Acetyl (Protein N−term)
GlyGly (K) no new terminus
Oxidation (M)

20200924_KIZ_MUb5_GlyGly_2 − Scan number: 24226

Gene Names	Charge	m/z	Mass	Retention time	PEP	Score	Precursor Intensity
RPS27A;UBA52;UBC;UBB	3	460.5954	1378.764	89.766	0.00050276	93.258	138405408

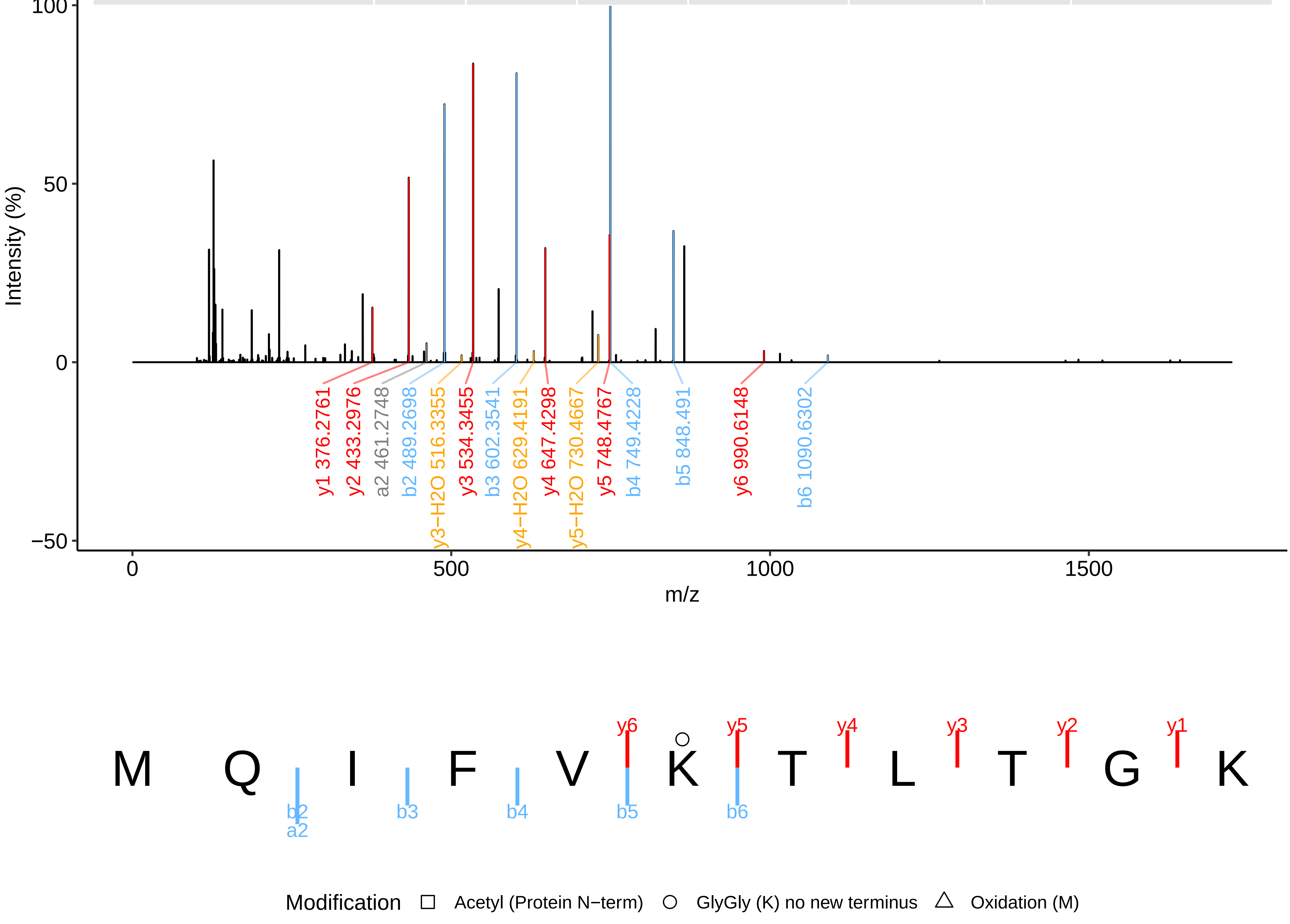

20200924_KIZ_MUb5_GlyGly_2 − Scan number: 24365

Gene Names	Charge	m/z	Mass	Retention time	PEP	Score	Precursor Intensity
RPS27A;UBA52;UBC;UBB	3	460.5954	1378.764	90.273	3.751e−05	100.88	118384352

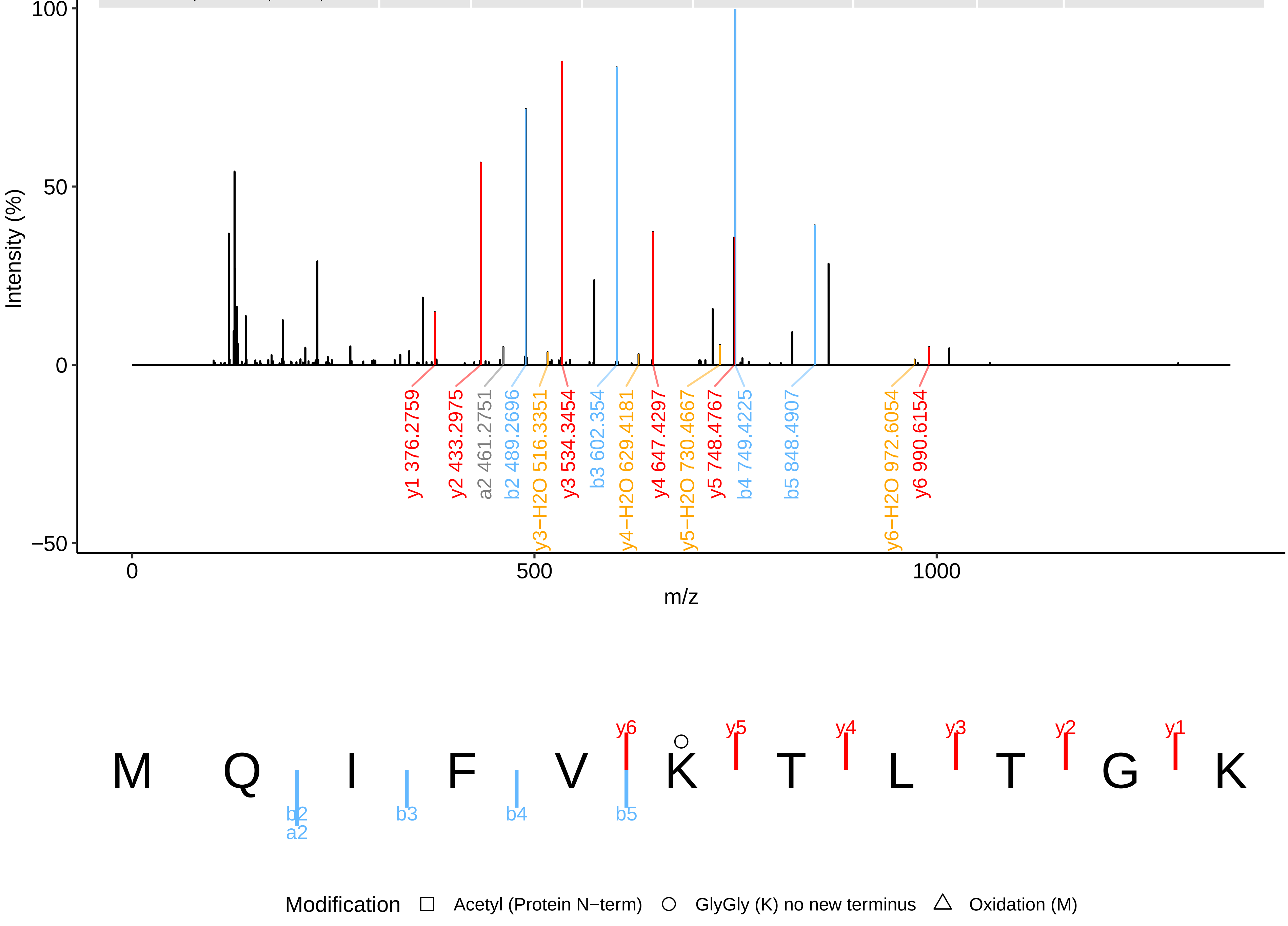

20200924_KIZ_MUb5_GlyGly_2 − Scan number: 24504

Gene Names	Charge	m/z	Mass	Retention time	PEP	Score	Precursor Intensity
RPS27A;UBA52;UBC;UBB	3	460.5954	1378.764	90.787	0.000861	89.548	85833128

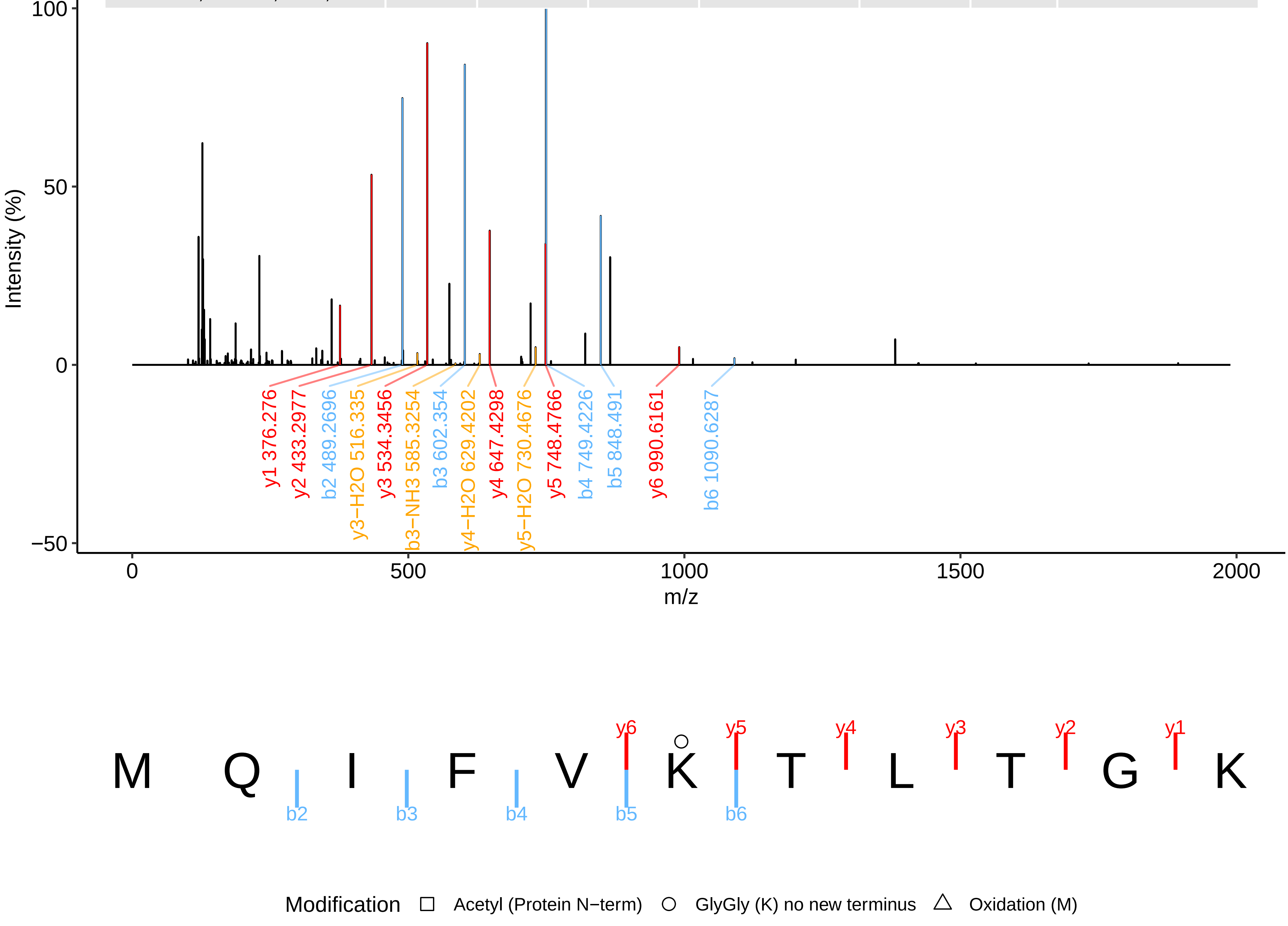

20200924_KIZ_MUb5_GlyGly_2 − Scan number: 24648

Gene Names	Charge	m/z	Mass	Retention time	PEP	Score	Precursor Intensity
RPS27A;UBA52;UBC;UBB	3	460.5954	1378.764	91.294	3.4737e−05	101.95	11350696

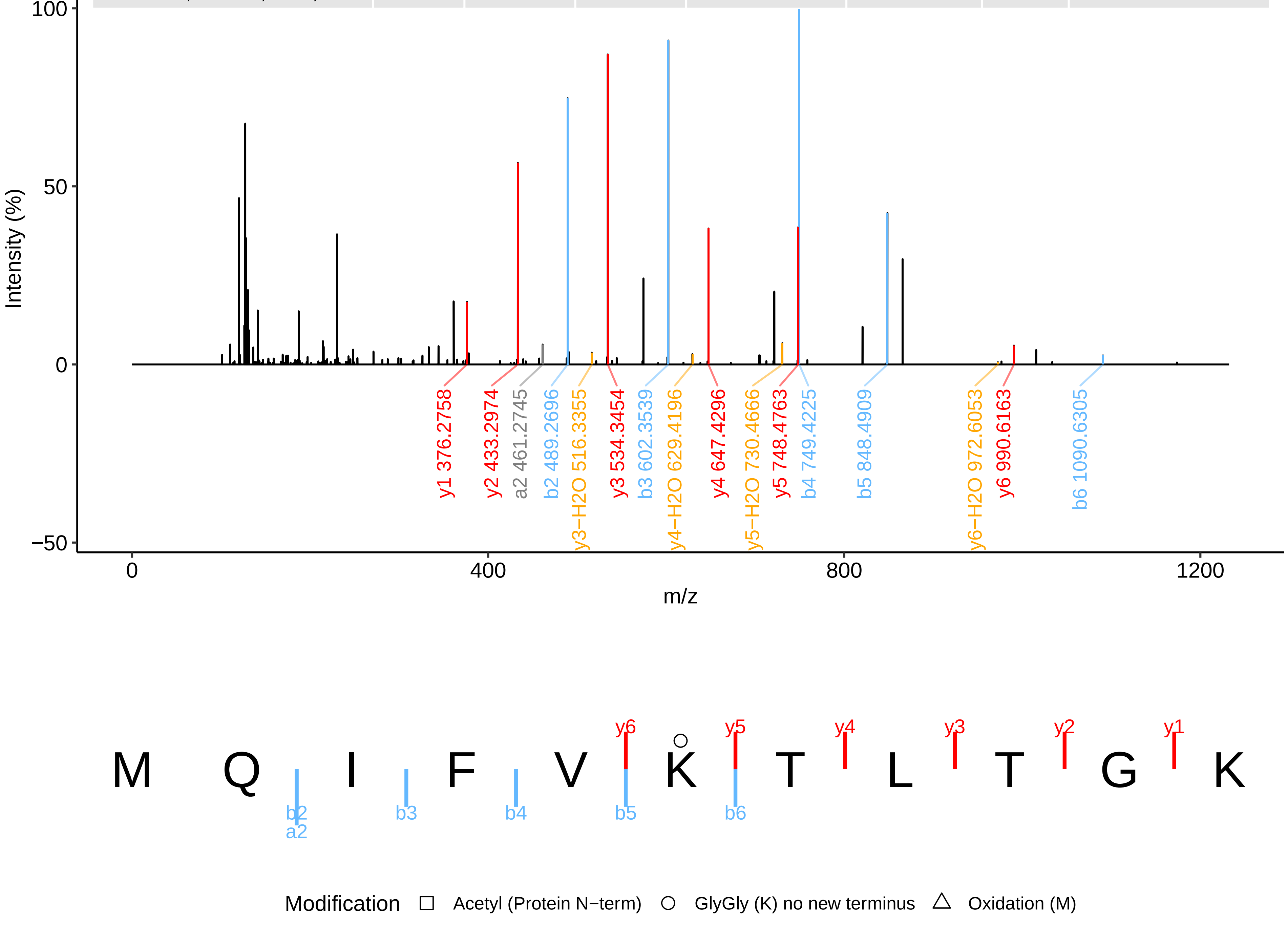

20200924_KIZ_MUb5_GlyGly_2 − Scan number: 23813

Gene Names	Charge	m/z	Mass	Retention time	PEP	Score	Precursor Intensity
RPS27A;UBA52;UBC;UBB	3	460.5954	1378.764	88.222	0.0040339	78.653	5796239

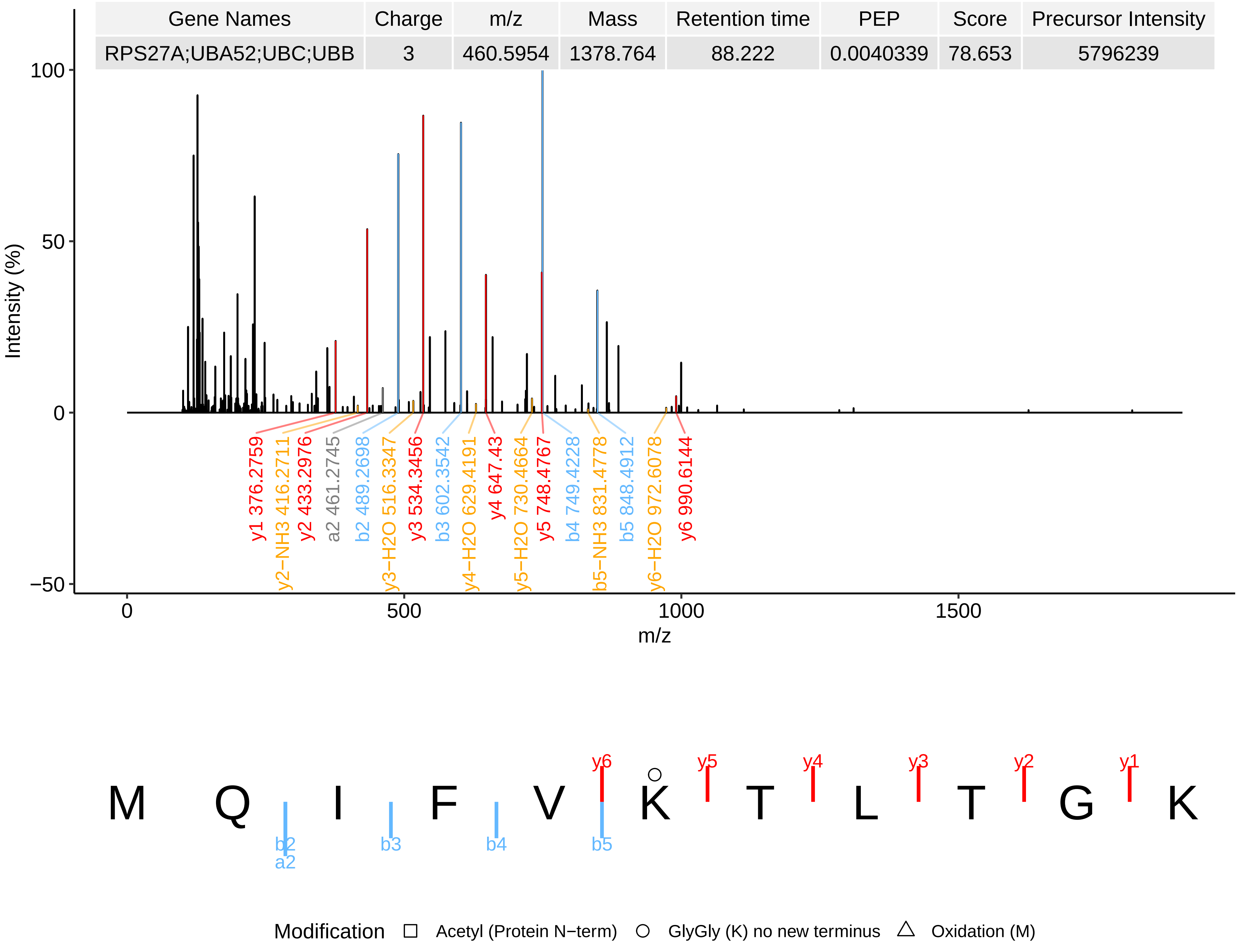

20200924_KIZ_MUb5_GlyGly_2 − Scan number: 24223

Gene Names	Charge	m/z	Mass	Retention time	PEP	Score	Precursor Intensity
RPS27A;UBA52;UBC;UBB	2	690.3894	1378.764	89.757	1.3672e−08	163.34	3961381

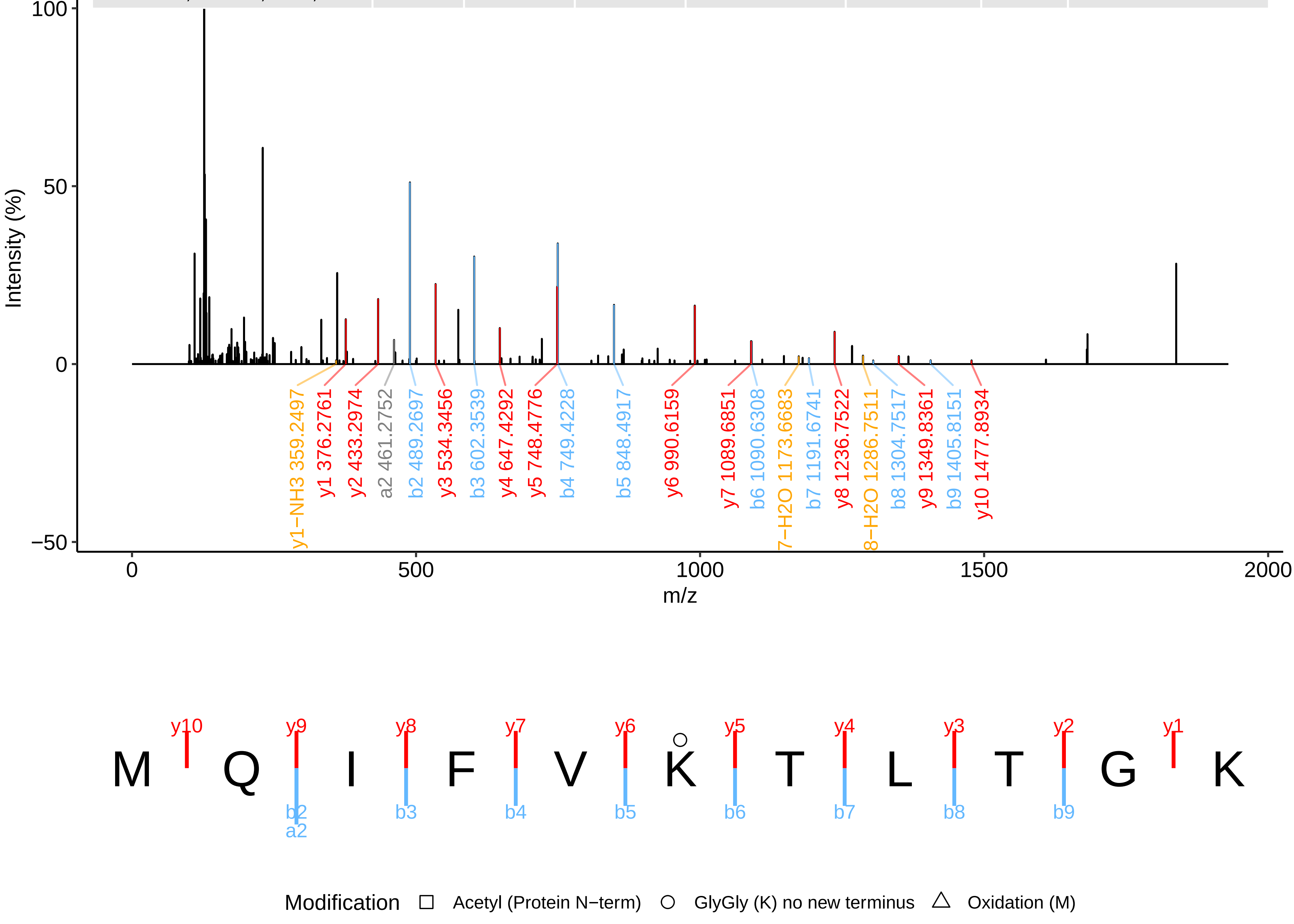

20200924_KIZ_MUb5_GlyGly_2 − Scan number: 24396

Gene Names	Charge	m/z	Mass	Retention time	PEP	Score	Precursor Intensity
RPS27A;UBA52;UBC;UBB	2	690.3894	1378.764	90.388	3.7897e−08	151.15	NA

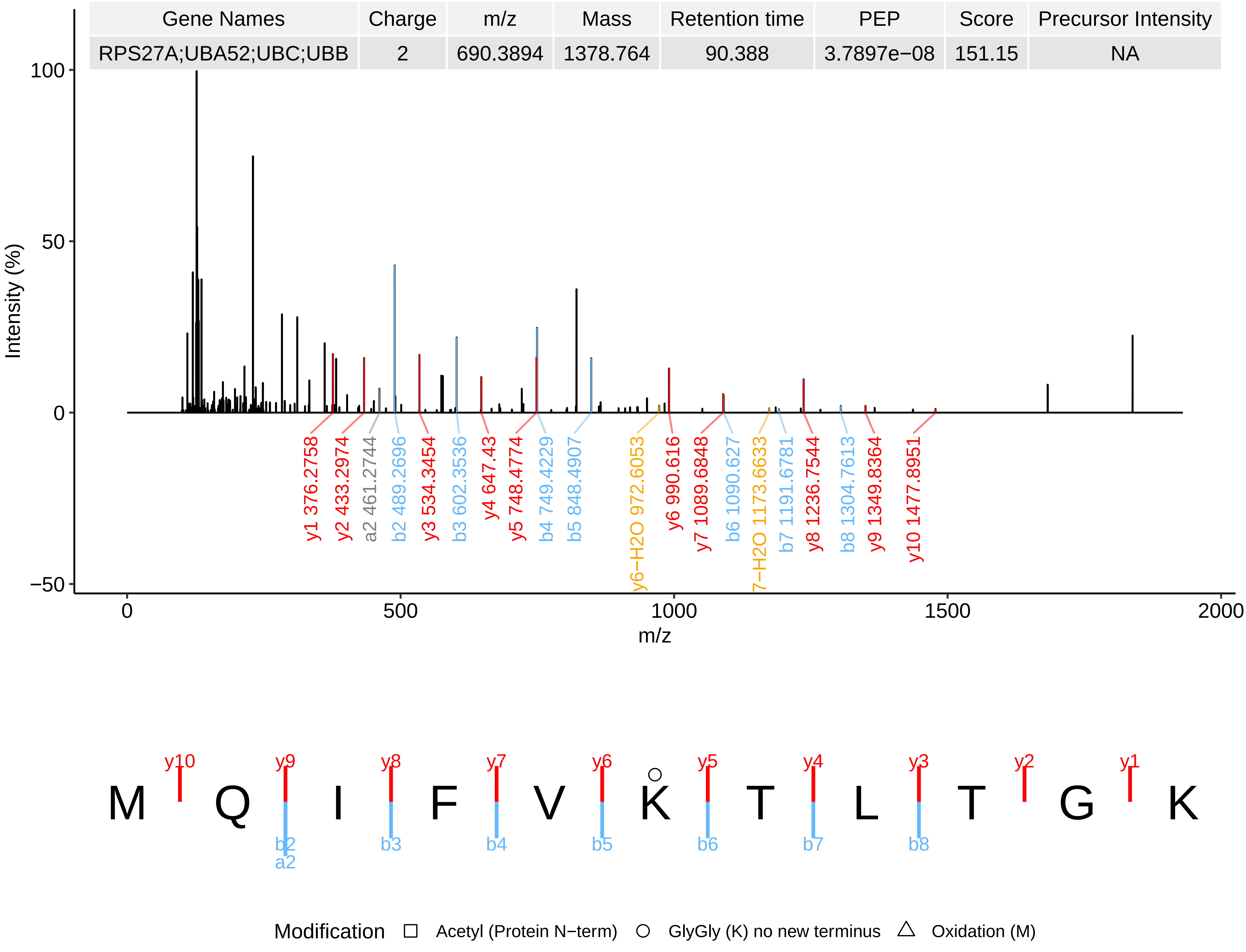

20201118_KIZ_MUb6_GlyGly_1 − Scan number: 27464

Gene Names	Charge	m/z	Mass	Retention time	PEP	Score	Precursor Intensity
RPS27A;UBA52;UBC;UBB	3	460.5954	1378.764	83.158	3.1827e−05	103.08	44960876

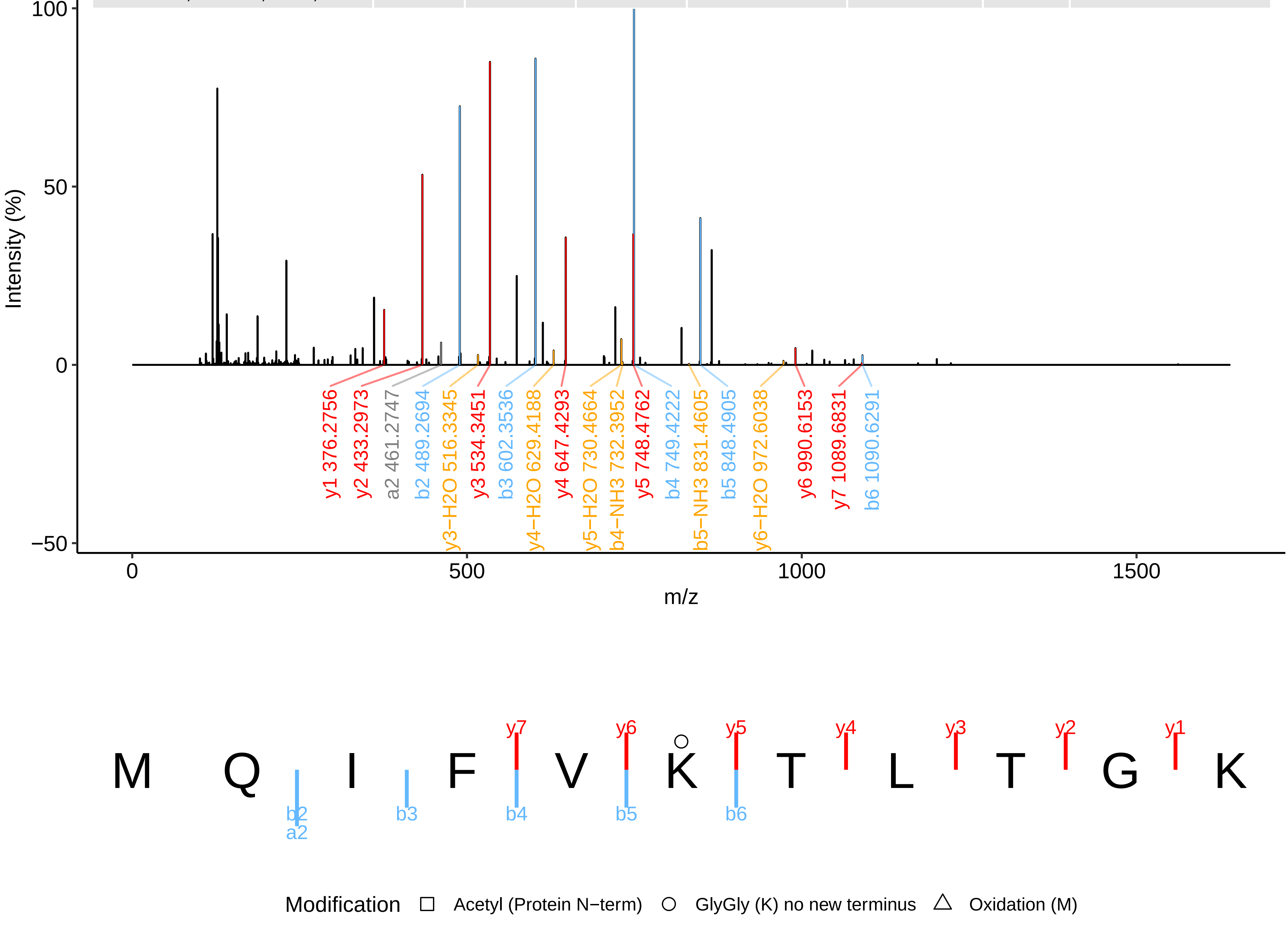

20201118_KIZ_MUb6_GlyGly_1 − Scan number: 27642

Gene Names	Charge	m/z	Mass	Retention time	PEP	Score	Precursor Intensity
RPS27A;UBA52;UBC;UBB	3	460.5954	1378.764	83.669	0.00037157	94.616	421984064

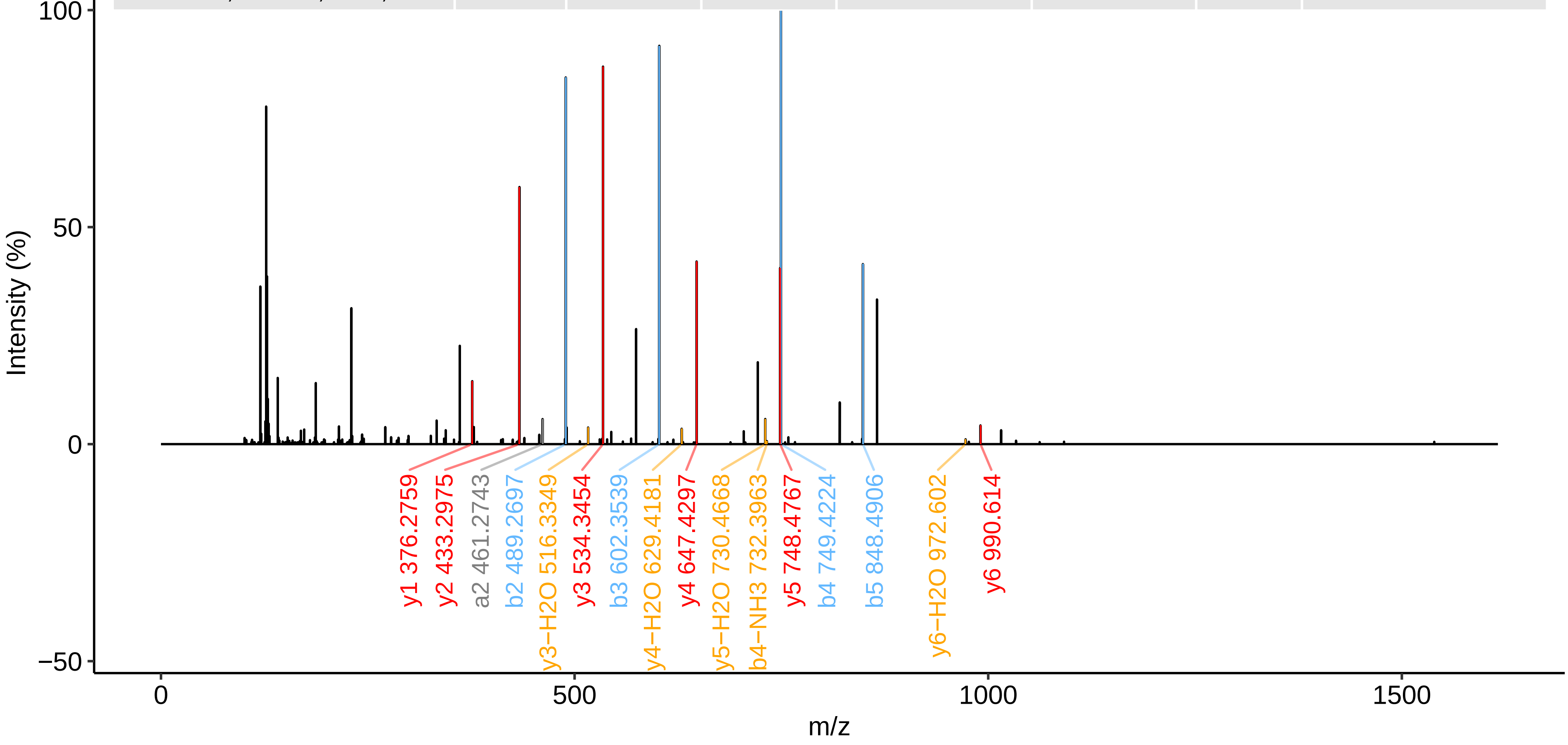

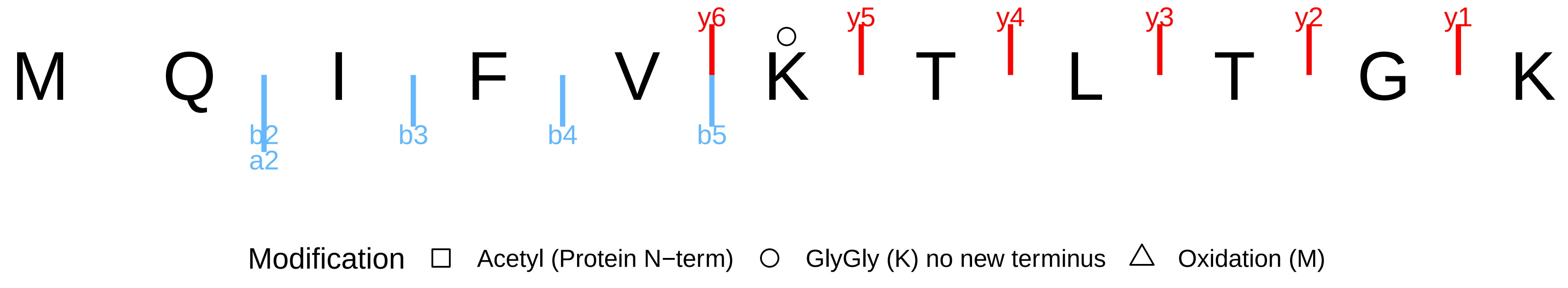

20201118_KIZ_MUb6_GlyGly_2 − Scan number: 30310

Gene Names	Charge	m/z	Mass	Retention time	PEP	Score	Precursor Intensity
RPS27A;UBA52;UBC;UBB	3	460.5954	1378.764	100.06	2.1591e−05	107.06	130006672

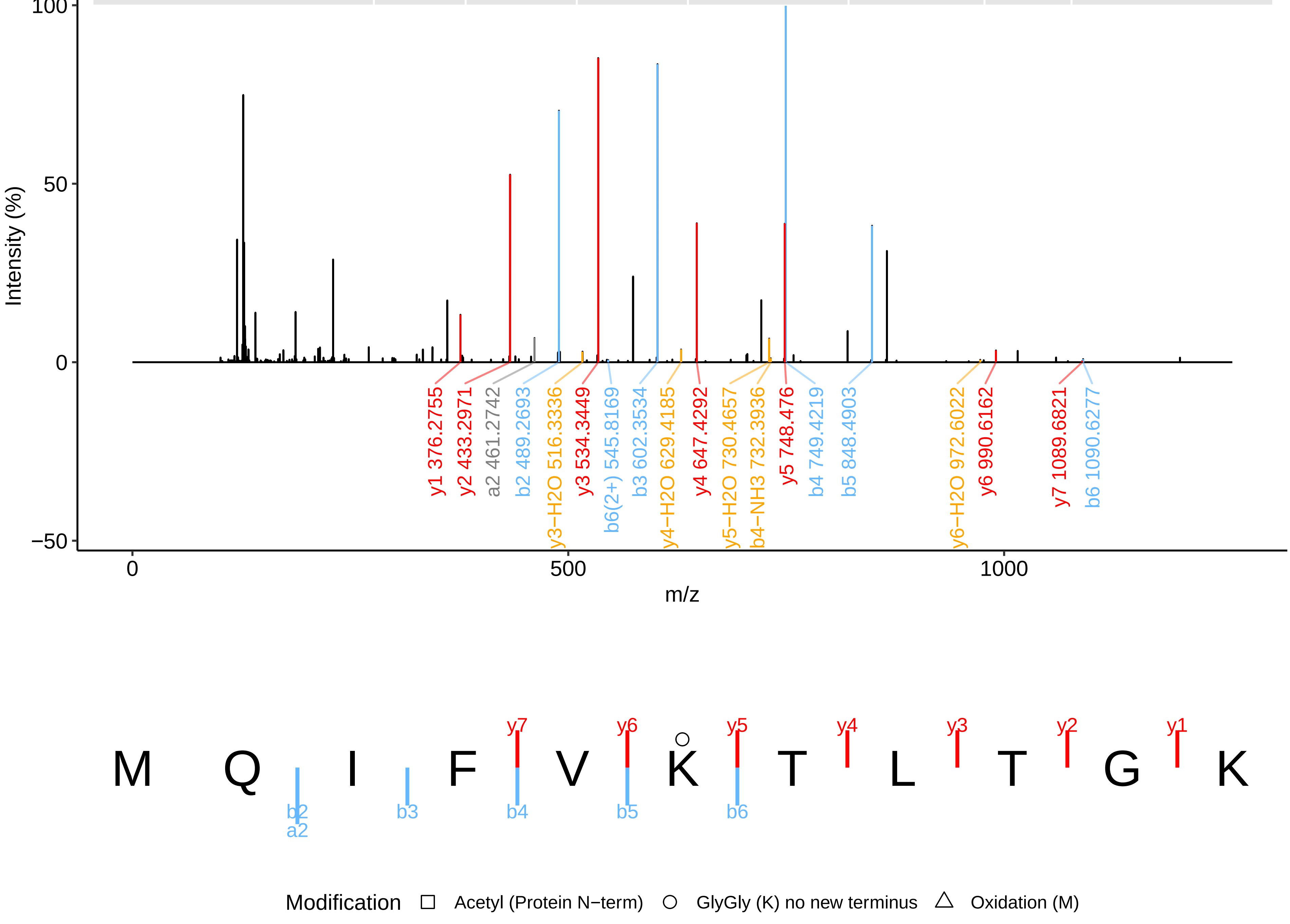

20201118_KIZ_MUb6_GlyGly_2 − Scan number: 30330

Gene Names	Charge	m/z	Mass	Retention time	PEP	Score	Precursor Intensity
RPS27A;UBA52;UBC;UBB	2	690.3894	1378.764	100.12	4.0551e−23	199.11	21803204

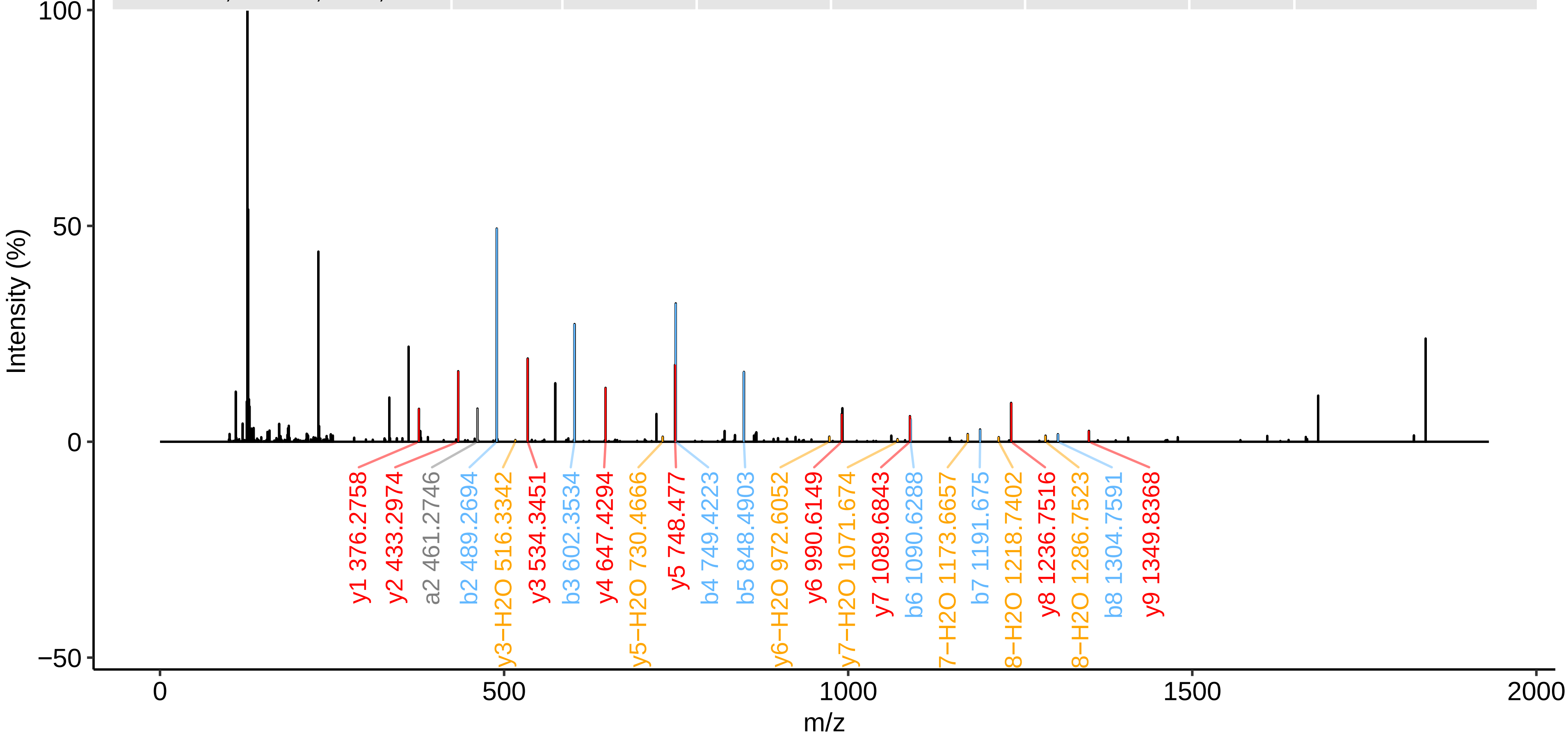

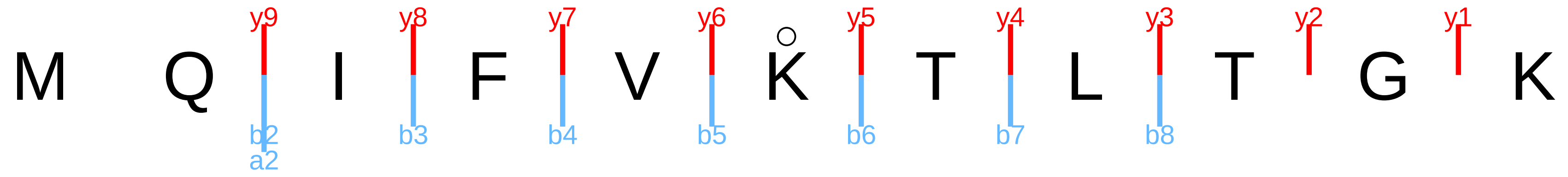

Modification □ Acetyl (Protein N−term) ○ GlyGly (K) no new terminus △ Oxidation (M)

20200921_KIZ_MUb4_GlyGly_10plex − Scan number: 24359

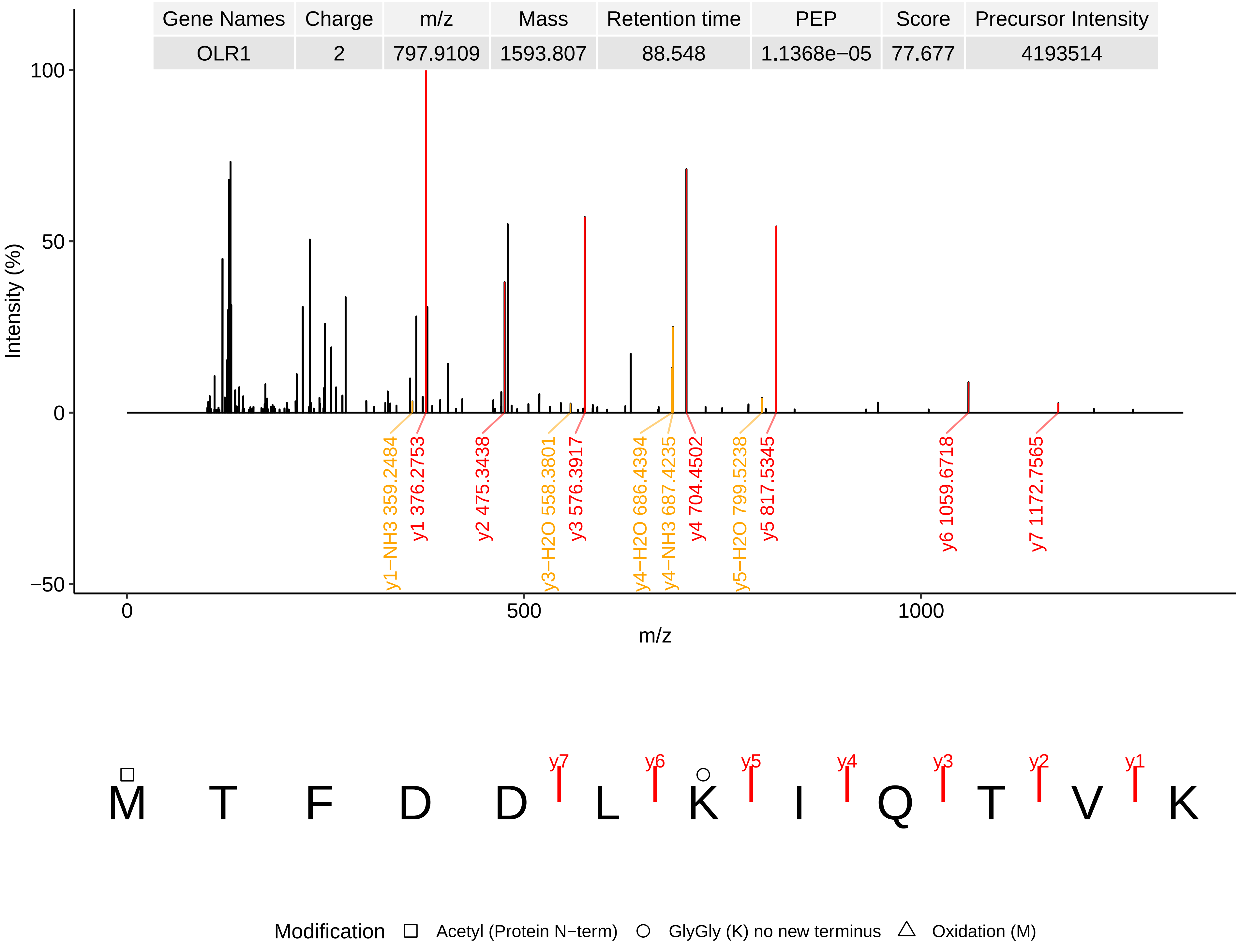

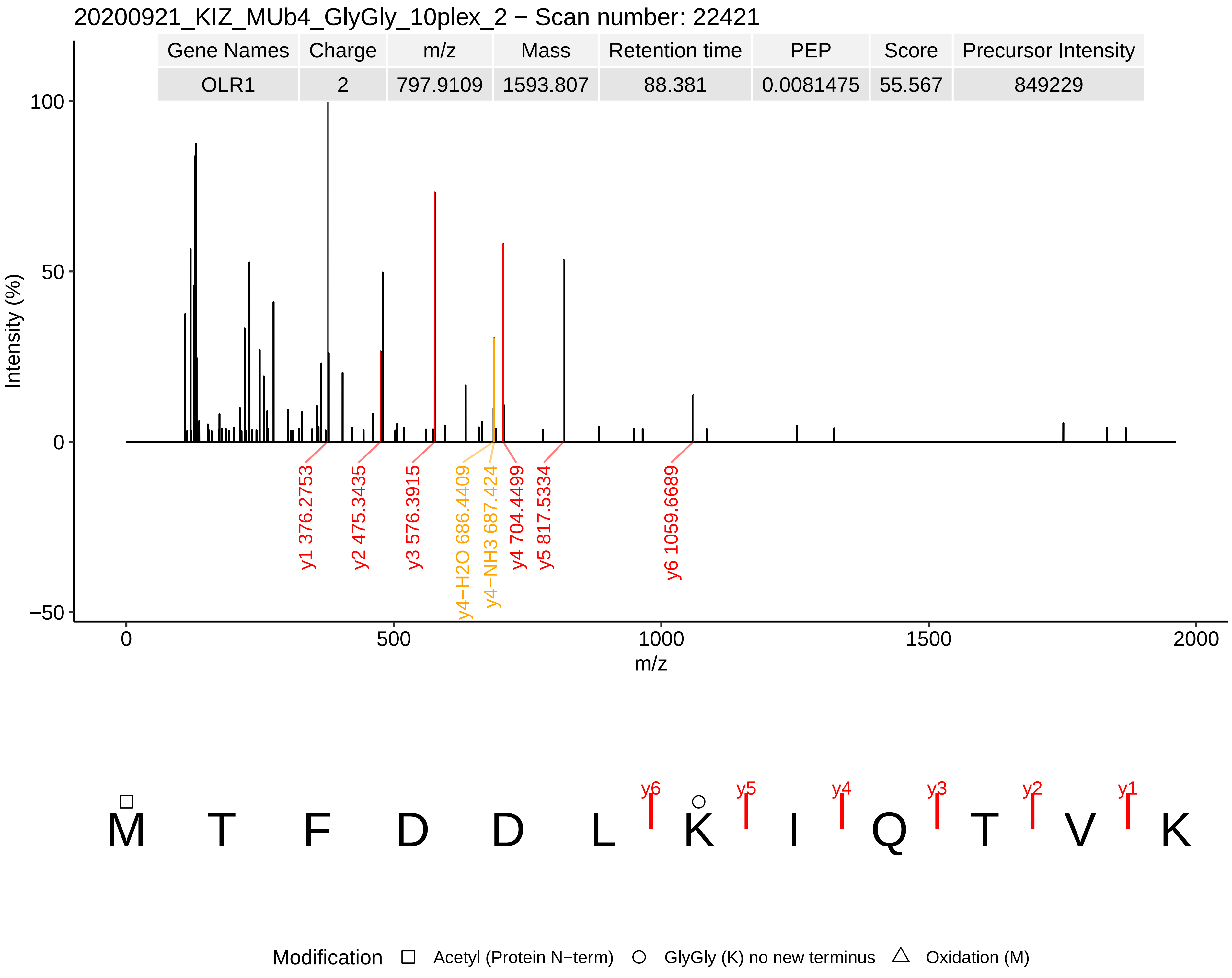
20200921_KIZ_MUb4_GlyGly_10plex_2 − Scan number: 22421
Gene Names | Charge | m/z | Mass | Retention time | PEP | Score | Precursor Intensity
OLR1 | 2 | 797.9109 | 1593.807 | 88.381 | 0.0081475 | 55.567 | 849229
Intensity (%)
100
50
0
−50
0
500
1000
1500
2000
m/z
y1 376.2753
y2 475.3435
y3 576.3915
y4−H2O 686.4409
y4−NH3 687.424
y4 704.4499
y5 817.5334
y6 1059.6689
M T F D D L K I Q T V K
y6 y5 y4 y3 y2 y1
Modification
Acetyl (Protein N−term)
GlyGly (K) no new terminus
Oxidation (M)

20200921_KIZ_MUb4_GlyGly_10plex − Scan number: 8565

Gene Names	Charge	m/z	Mass	Retention time	PEP	Score	Precursor Intensity
YWHAG	4	397.4662	1585.836	29.1	0.026196	76.326	NA

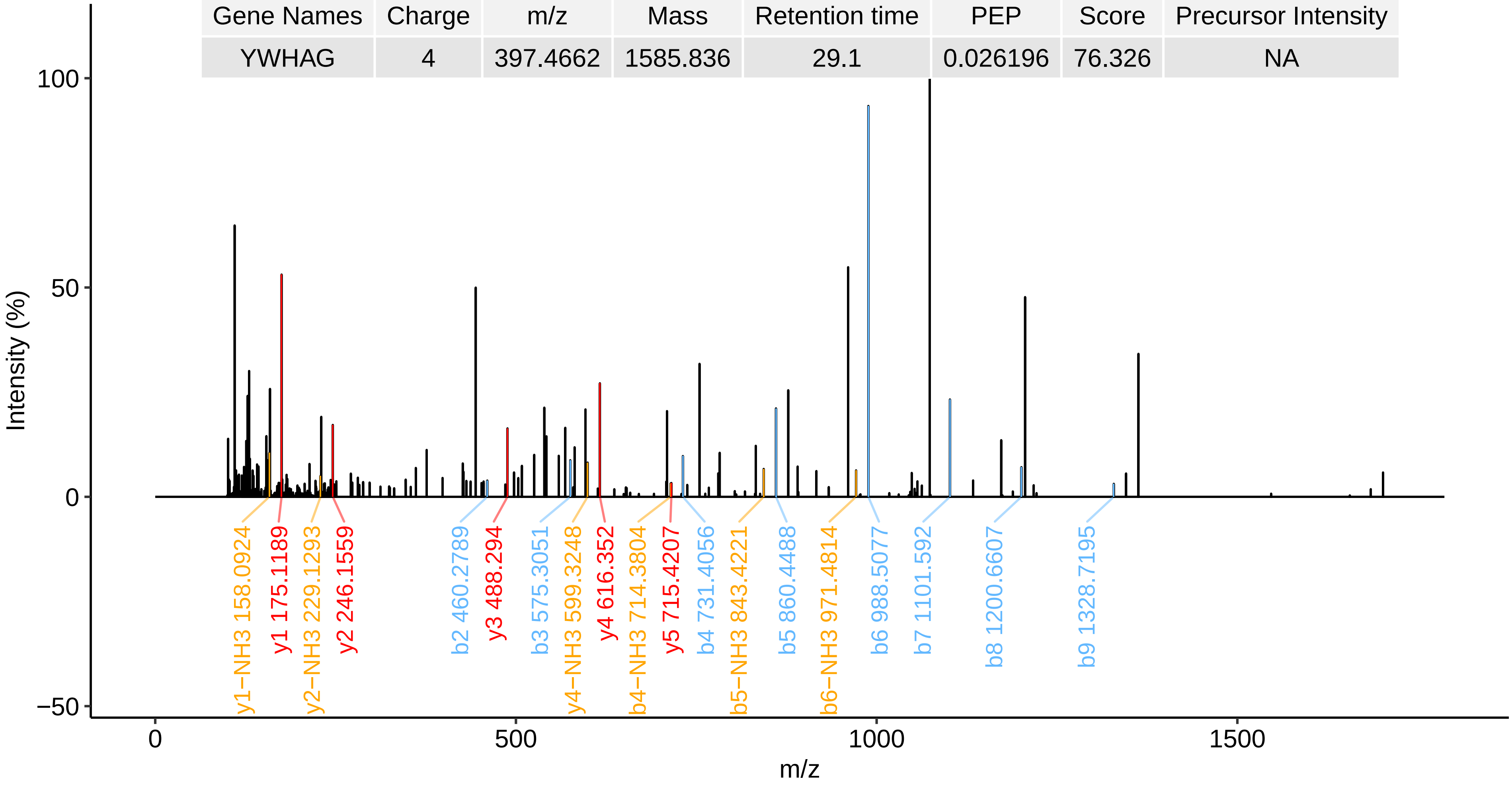

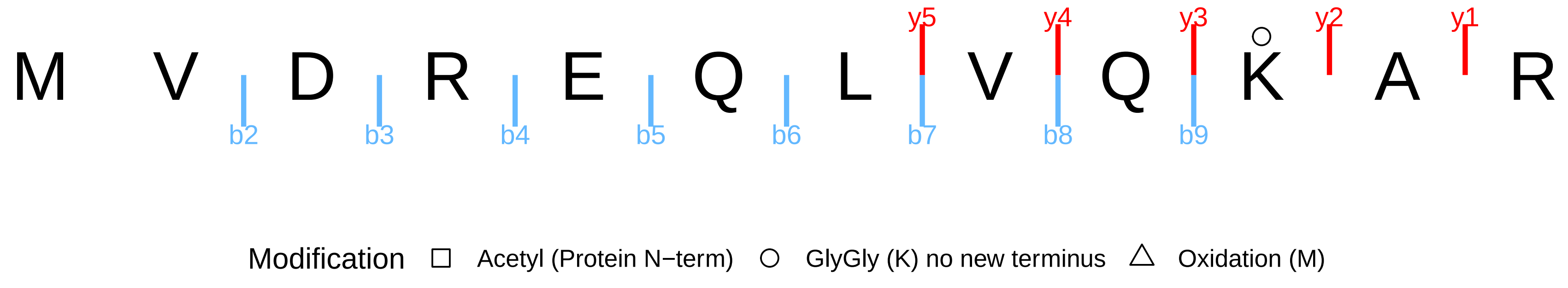

20200921_KIZ_MUb4_GlyGly_10plex_2 − Scan number: 11287

Gene Names	Charge	m/z	Mass	Retention time	PEP	Score	Precursor Intensity
ATP2B4	3	454.9015	1361.683	43.403	0.11468	50.284	781958.1

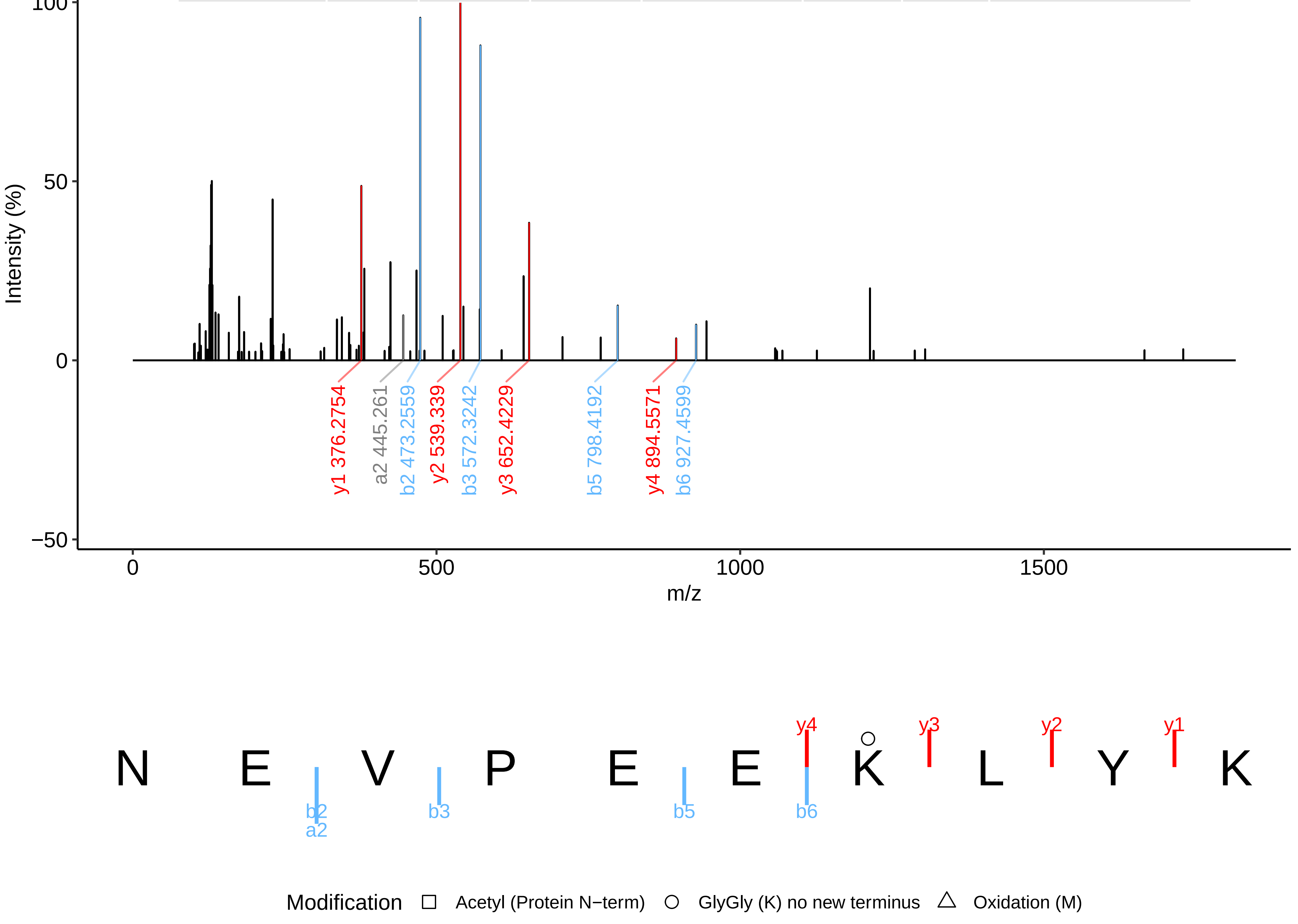

20200921_KIZ_MUb4_GlyGly_10plex − Scan number: 16548

Gene Names	Charge	m/z	Mass	Retention time	PEP	Score	Precursor Intensity
AKR1B1	3	522.6385	1564.894	59.468	0.00048936	90.861	4196061

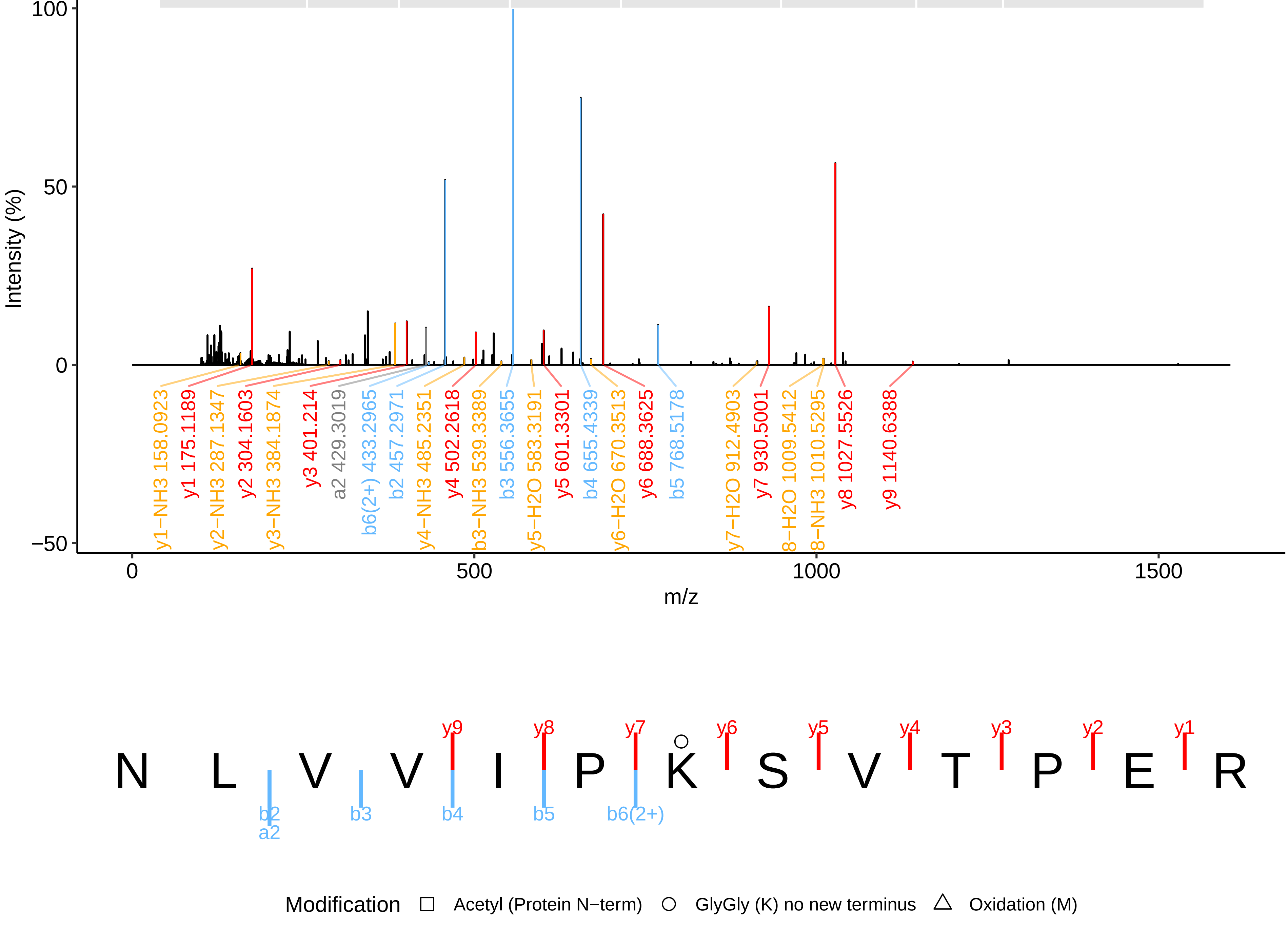

20200921_KIZ_MUb4_GlyGly_10plex_2 − Scan number: 15296

Gene Names	Charge	m/z	Mass	Retention time	PEP	Score	Precursor Intensity
AKR1B1	3	522.6385	1564.894	59.785	0.032336	58.487	557227.3

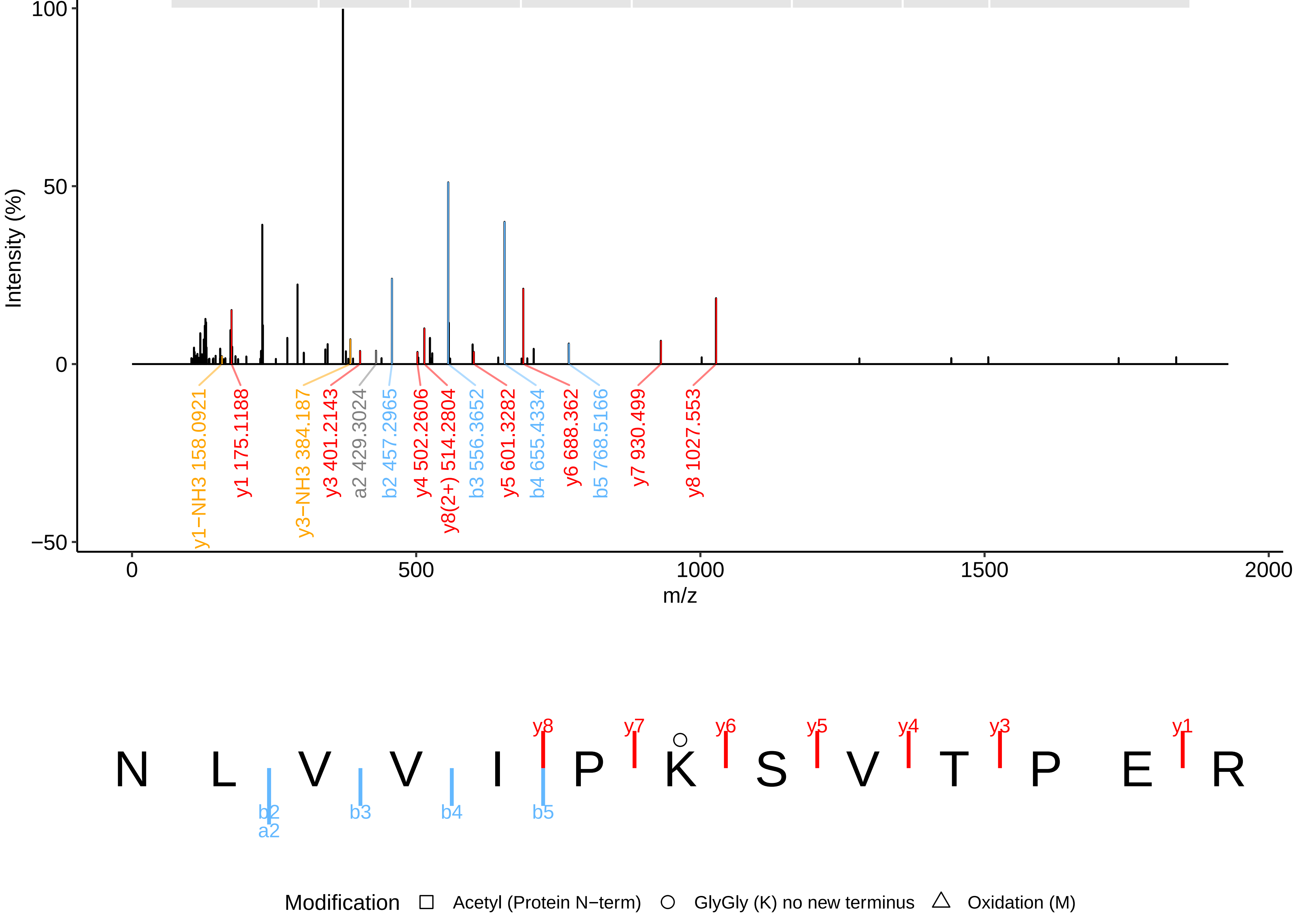

20200921_KIZ_MUb4_GlyGly_10plex − Scan number: 5294

Gene Names	Charge	m/z	Mass	Retention time	PEP	Score	Precursor Intensity
SDC1	3	459.5688	1375.684	17.315	7.7721e−06	108.9	4000428

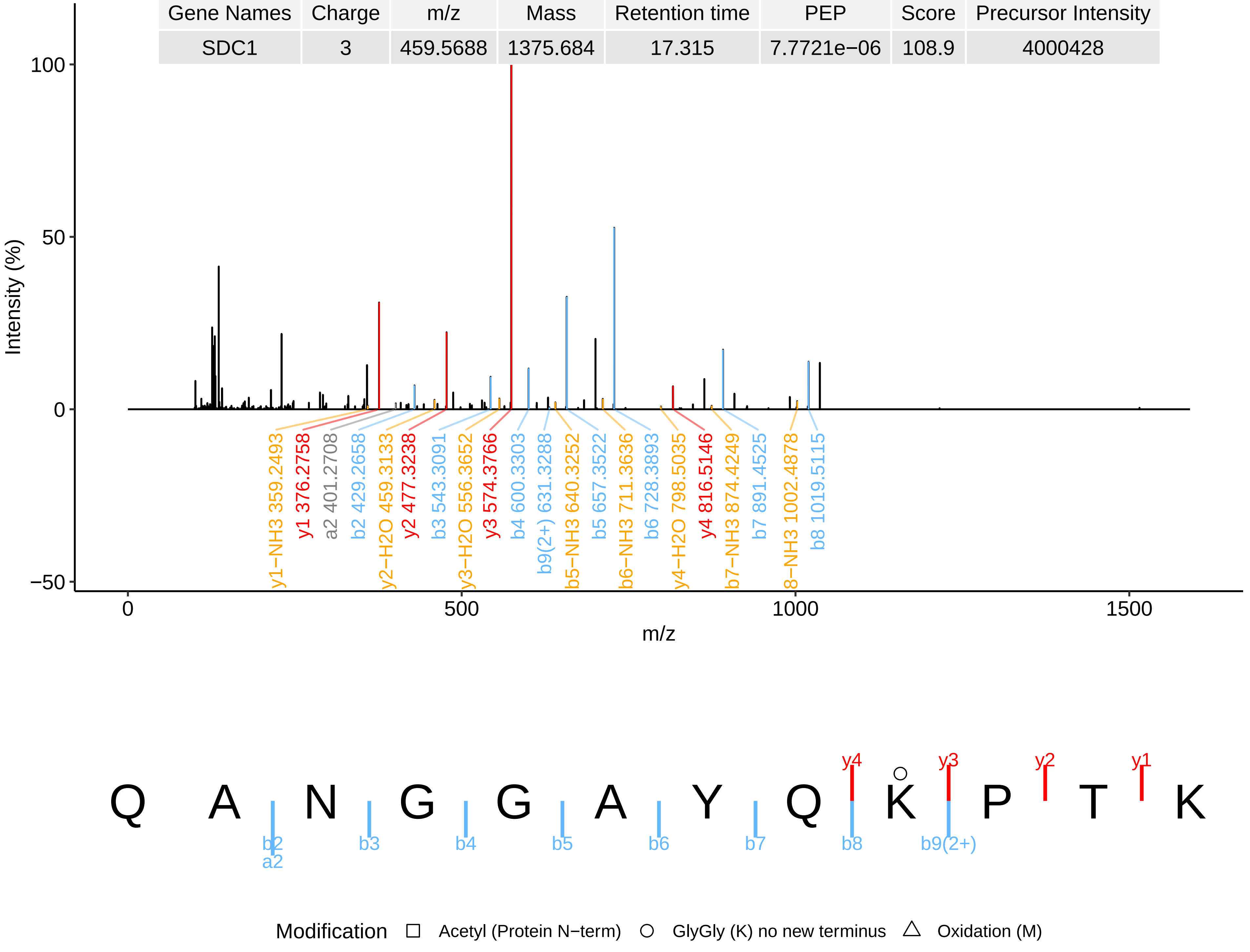

20200921_KIZ_MUb4_GlyGly_10plex − Scan number: 5323

Gene Names	Charge	m/z	Mass	Retention time	PEP	Score	Precursor Intensity
SDC1	3	459.5688	1375.684	17.413	0.0051145	74.76	4667064

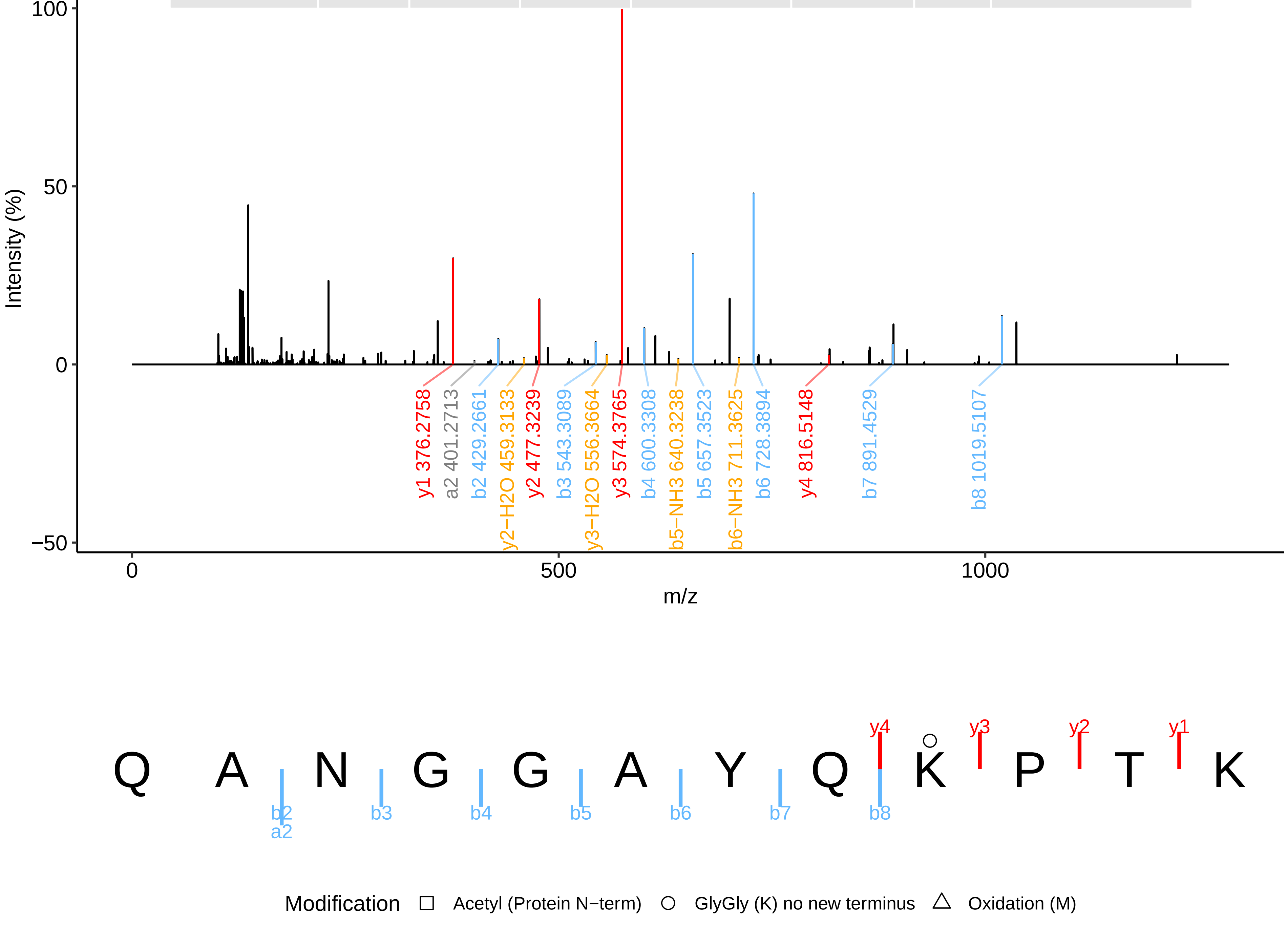

20200921_KIZ_MUb4_GlyGly_10plex_2 − Scan number: 4879

Gene Names	Charge	m/z	Mass	Retention time	PEP	Score	Precursor Intensity
SDC1	3	459.5688	1375.684	17.302	0.011523	65.179	1367549

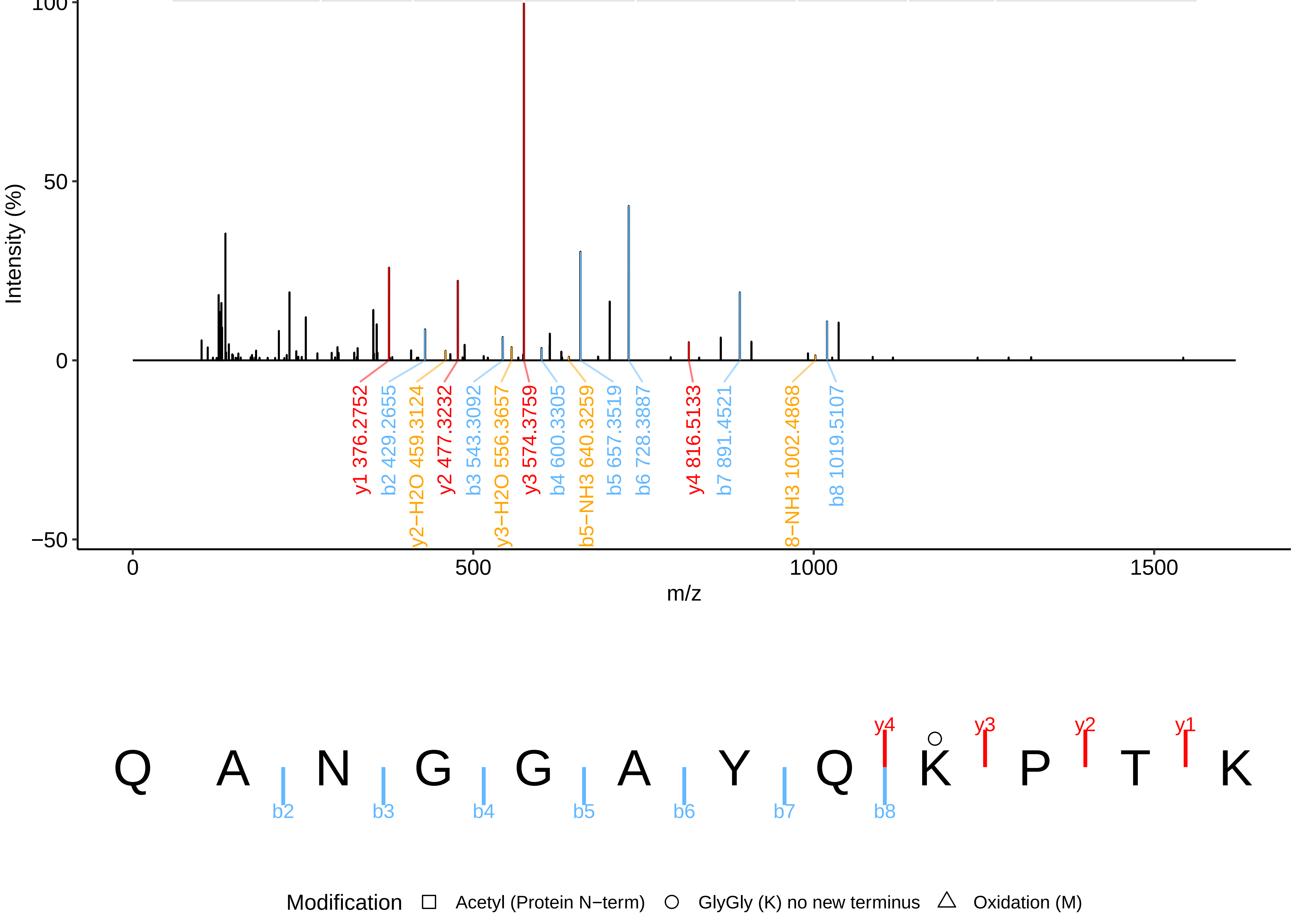

20200924_KIZ_MUb5_GlyGly_1 − Scan number: 4665

Gene Names	Charge	m/z	Mass	Retention time	PEP	Score	Precursor Intensity
SDC1	3	459.5688	1375.684	14.429	0.0051145	74.76	8931730

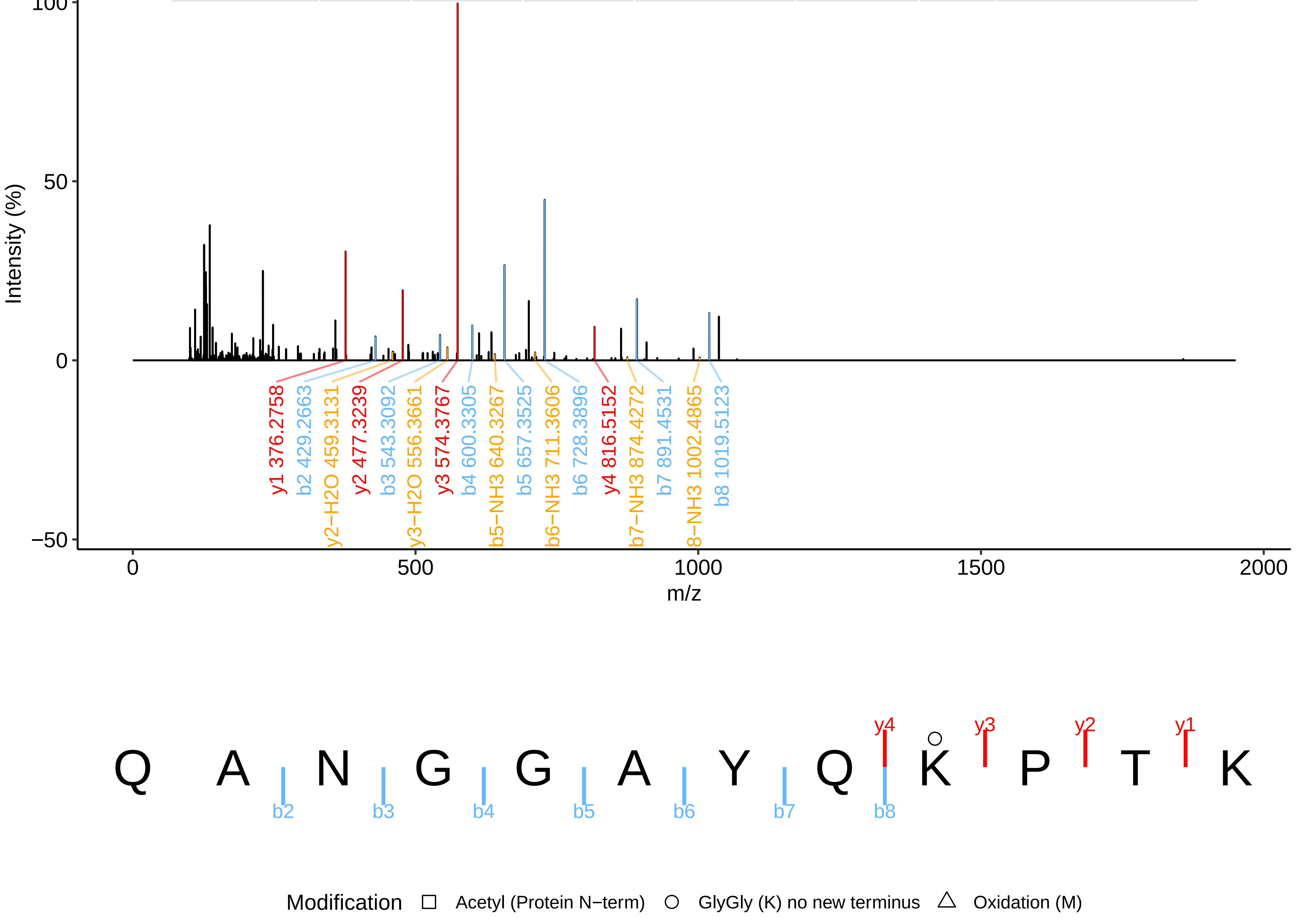

20200924_KIZ_MUb5_GlyGly_1 − Scan number: 4674

Gene Names	Charge	m/z	Mass	Retention time	PEP	Score	Precursor Intensity
SDC1	3	459.5688	1375.684	14.455	0.0093413	68.44	2343651

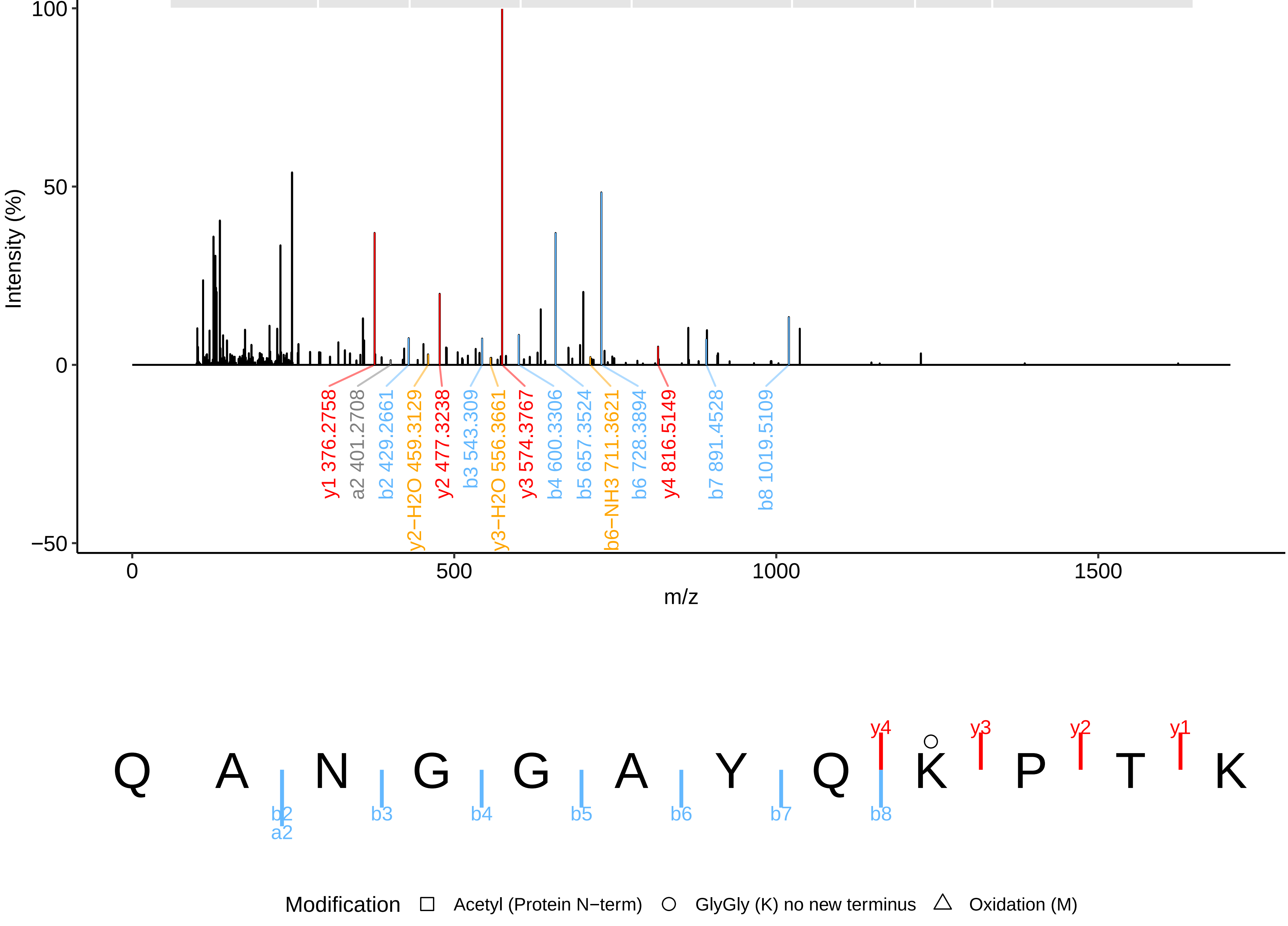

20200921_KIZ_MUb4_GlyGly_10plex − Scan number: 4095

Gene Names	Charge	m/z	Mass	Retention time	PEP	Score	Precursor Intensity
TMEM87A	3	443.5566	1327.648	13.325	0.0013505	84.479	5934916

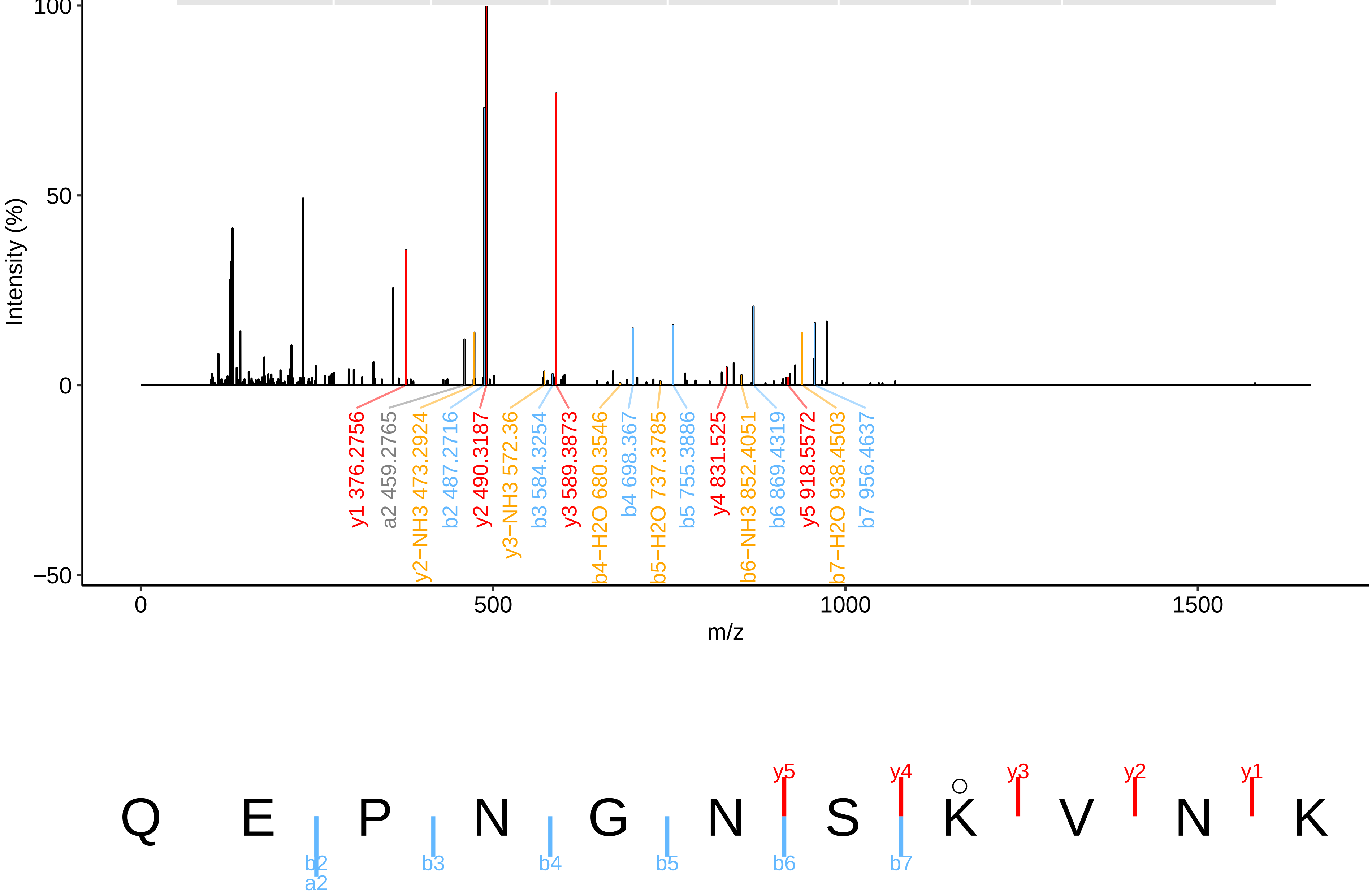

Modification □ Acetyl (Protein N−term) ○ GlyGly (K) no new terminus △ Oxidation (M)

20200921_KIZ_MUb4_GlyGly_10plex_2 − Scan number: 3838

Gene Names	Charge	m/z	Mass	Retention time	PEP	Score	Precursor Intensity
TMEM87A	3	443.5566	1327.648	13.154	0.010034	70.488	1992032

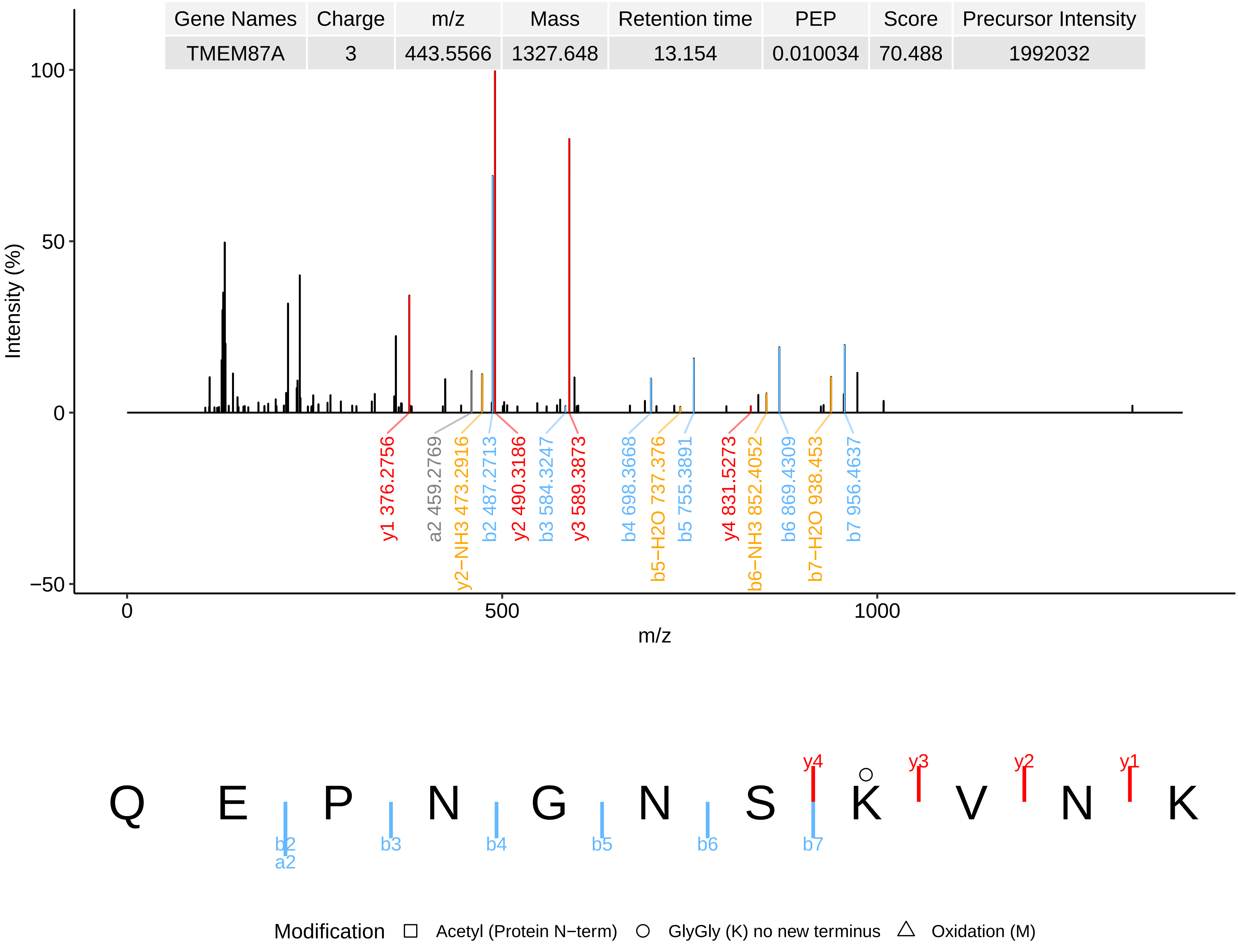

20200924_KIZ_MUb5_GlyGly_1 − Scan number: 3548

Gene Names	Charge	m/z	Mass	Retention time	PEP	Score	Precursor Intensity
TMEM87A	3	443.5566	1327.648	10.856	0.0016416	86.114	6178169

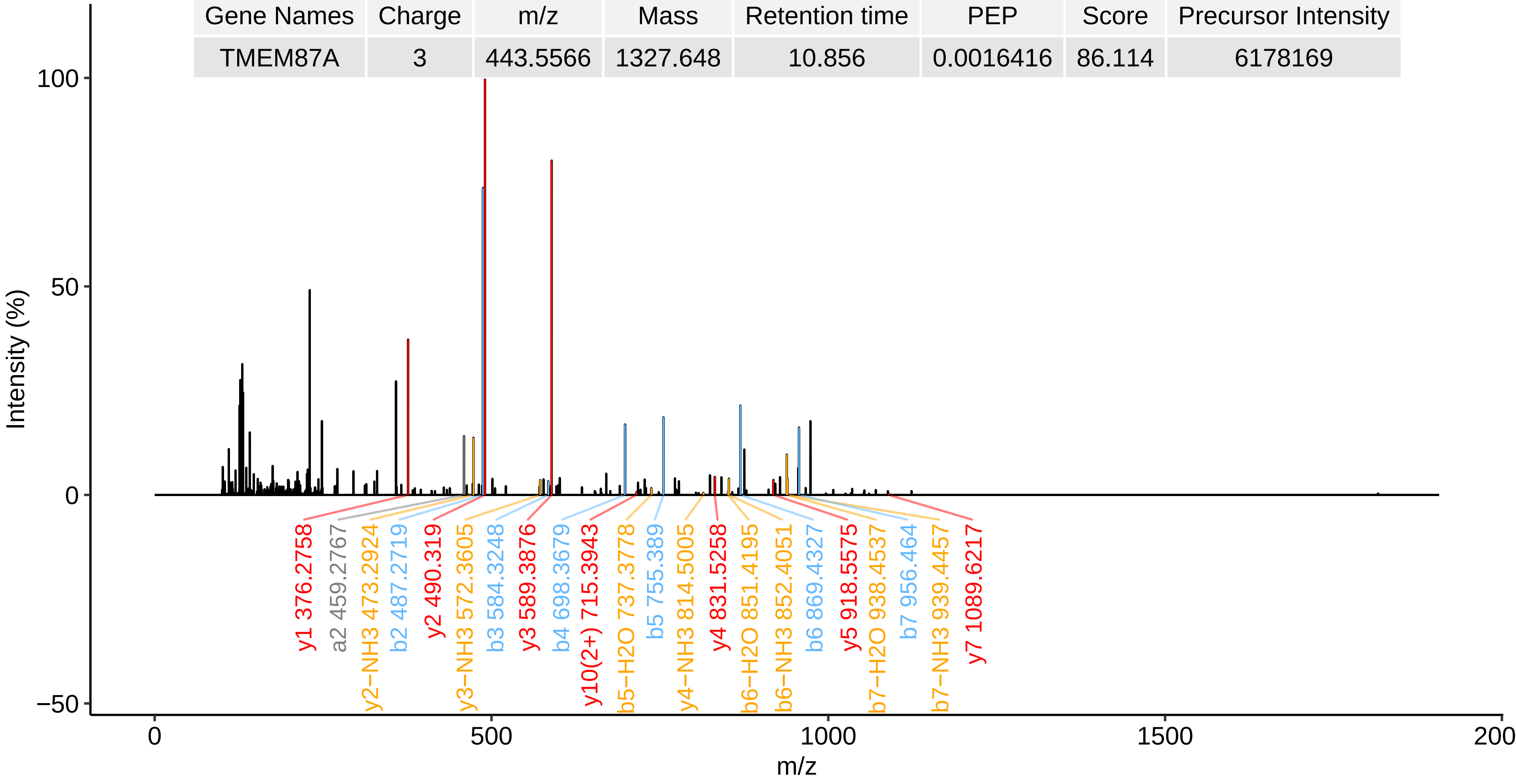

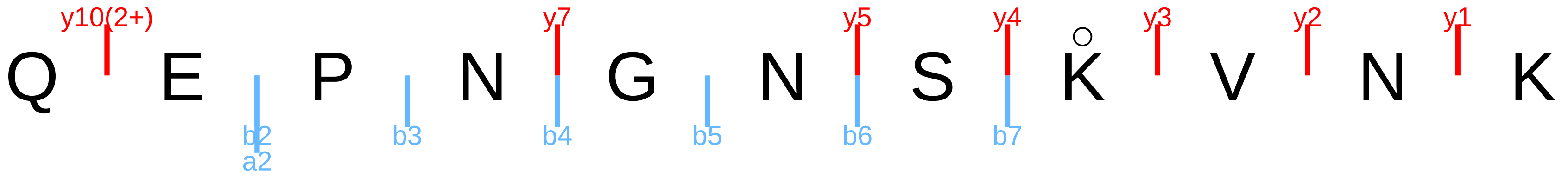

Modification □ Acetyl (Protein N−term) ○ GlyGly (K) no new terminus △ Oxidation (M)

20200921_KIZ_MUb4_GlyGly_10plex_2 − Scan number: 19108

Gene Names	Charge	m/z	Mass	Retention time	PEP	Score	Precursor Intensity
MFN2	3	535.2983	1602.873	74.95	7.2356e−07	114.82	2333155

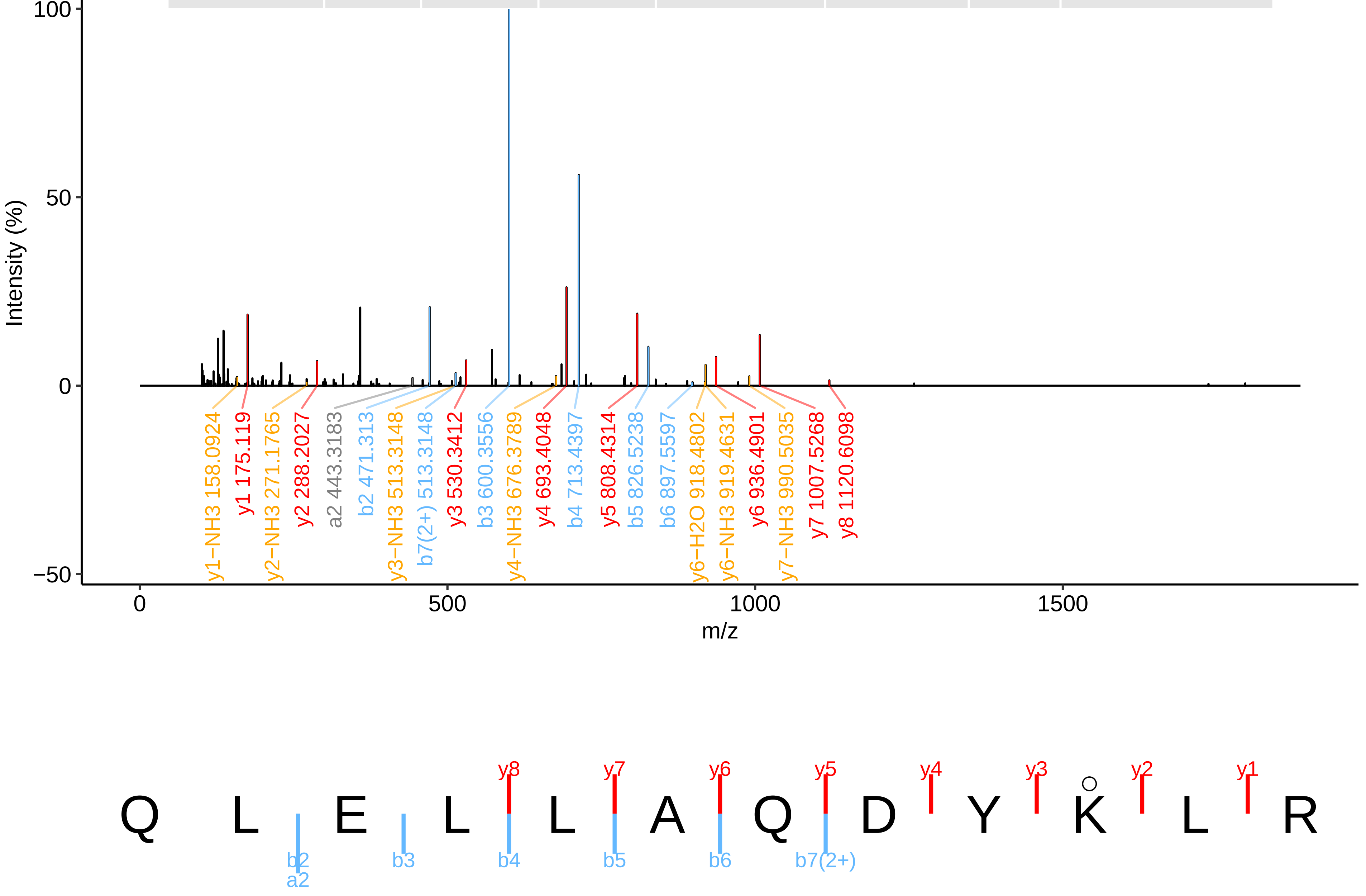

Modification □ Acetyl (Protein N−term) ○ GlyGly (K) no new terminus △ Oxidation (M)

20200924_KIZ_MUb5_GlyGly_1 − Scan number: 20507

Gene Names	Charge	m/z	Mass	Retention time	PEP	Score	Precursor Intensity
MFN2	3	535.2983	1602.873	66.103	1.2756e−07	132.5	NA

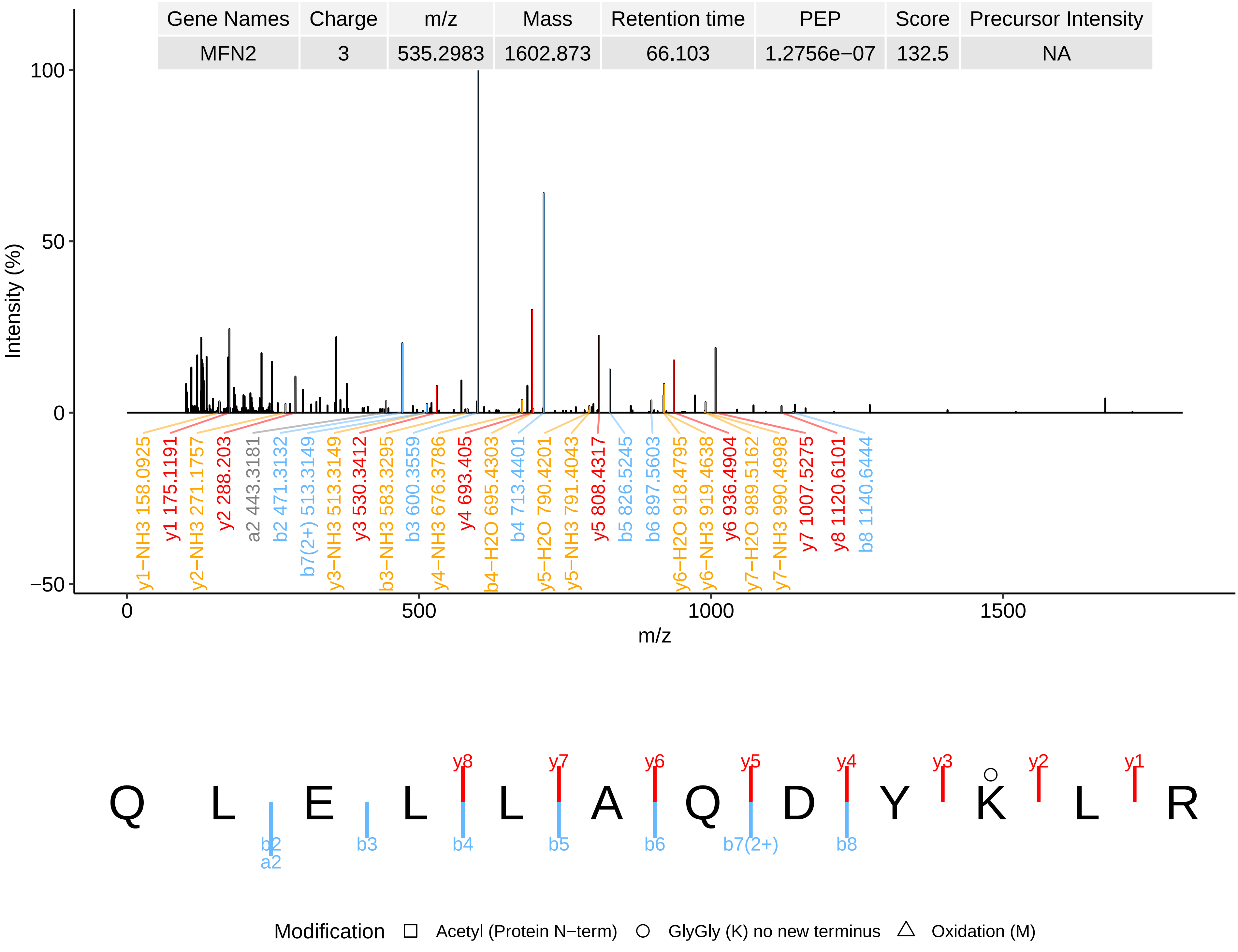

20201118_KIZ_MUb6_GlyGly_1 − Scan number: 20904

Gene Names	Charge	m/z	Mass	Retention time	PEP	Score	Precursor Intensity
MFN2	3	535.2983	1602.873	65.326	7.3088e−09	123.95	19509710

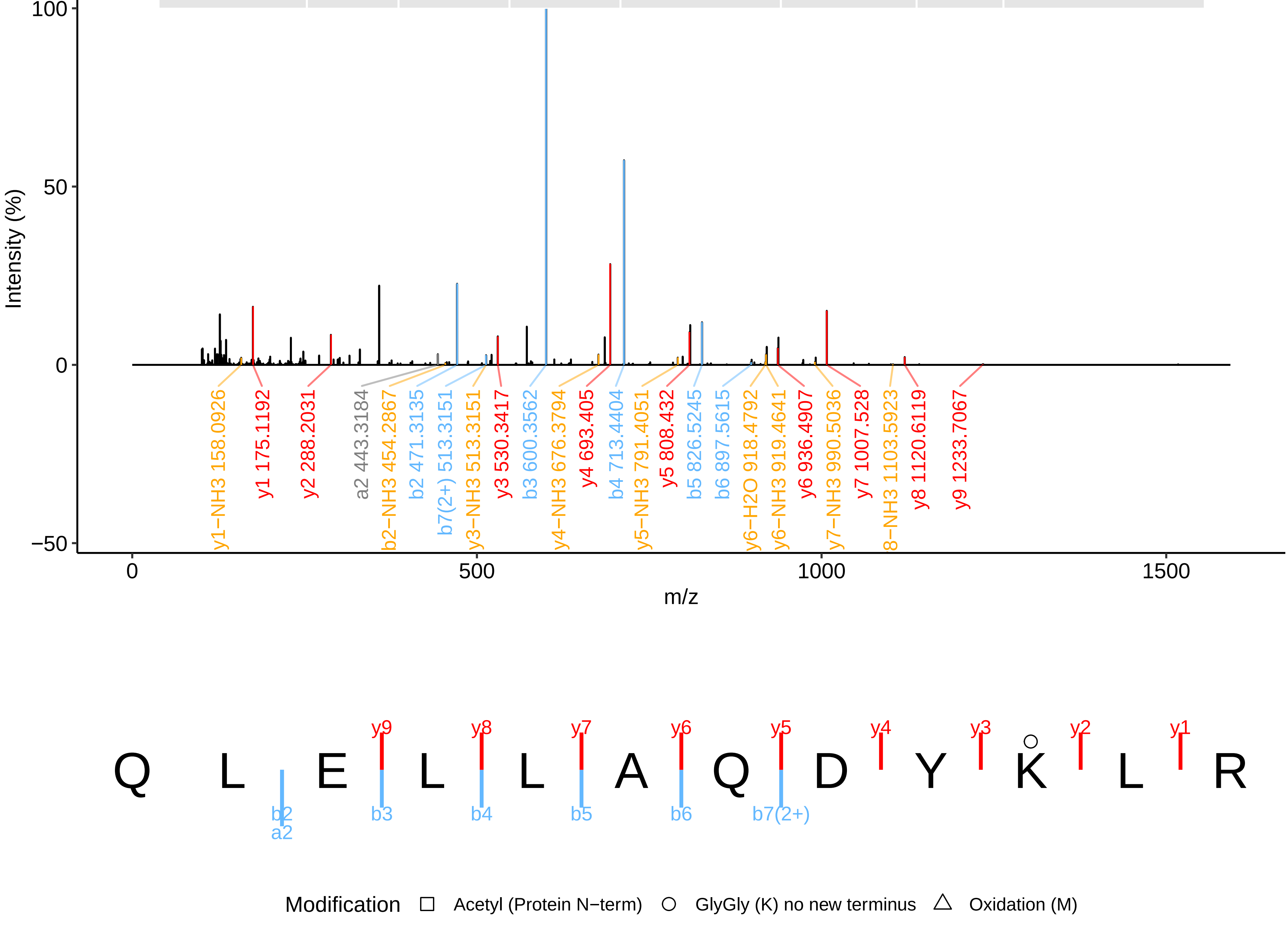

20201118_KIZ_MUb6_GlyGly_1 − Scan number: 20877

Gene Names	Charge	m/z	Mass	Retention time	PEP	Score	Precursor Intensity
MFN2	3	535.2983	1602.873	65.25	3.1067e−06	127.56	NA

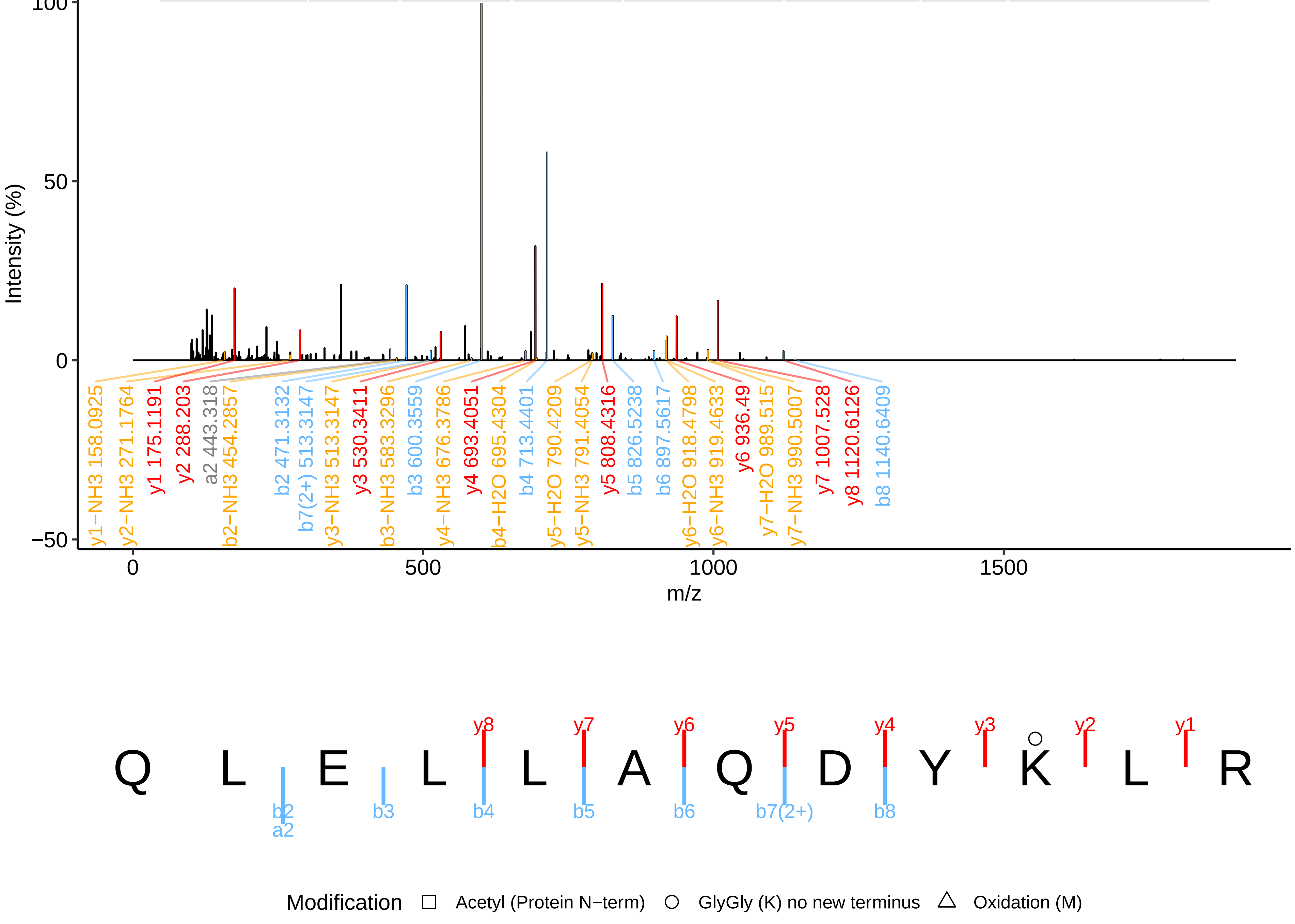

20201118_KIZ_MUb6_GlyGly_2 − Scan number: 22788

Gene Names	Charge	m/z	Mass	Retention time	PEP	Score	Precursor Intensity
MFN2	3	535.2983	1602.873	76.58	2.0643e−05	98.105	8297052

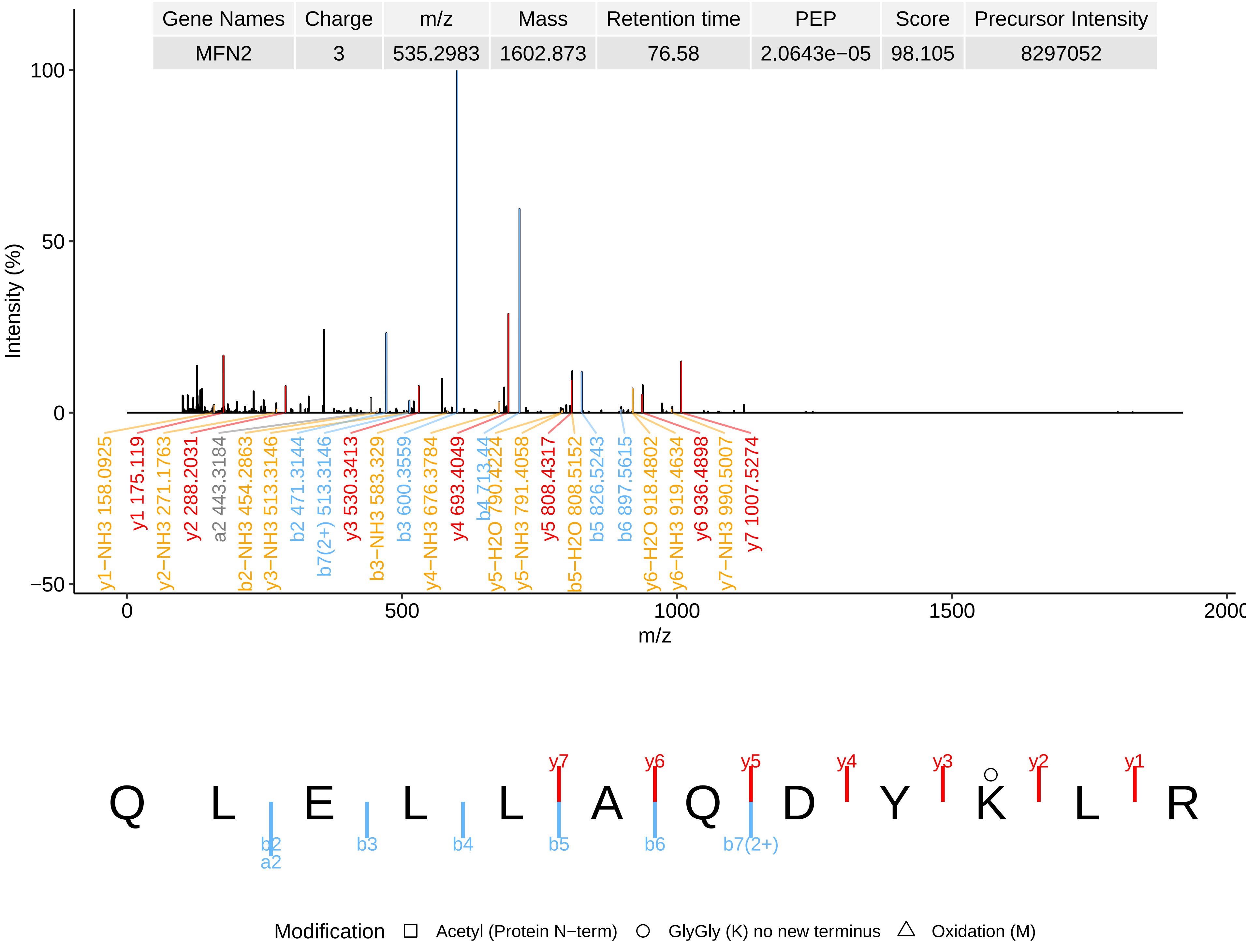

20201118_KIZ_MUb6_GlyGly_2 − Scan number: 22790

Gene Names	Charge	m/z	Mass	Retention time	PEP	Score	Precursor Intensity
MFN2	3	535.2983	1602.873	76.589	2.3833e−19	154.67	11493432

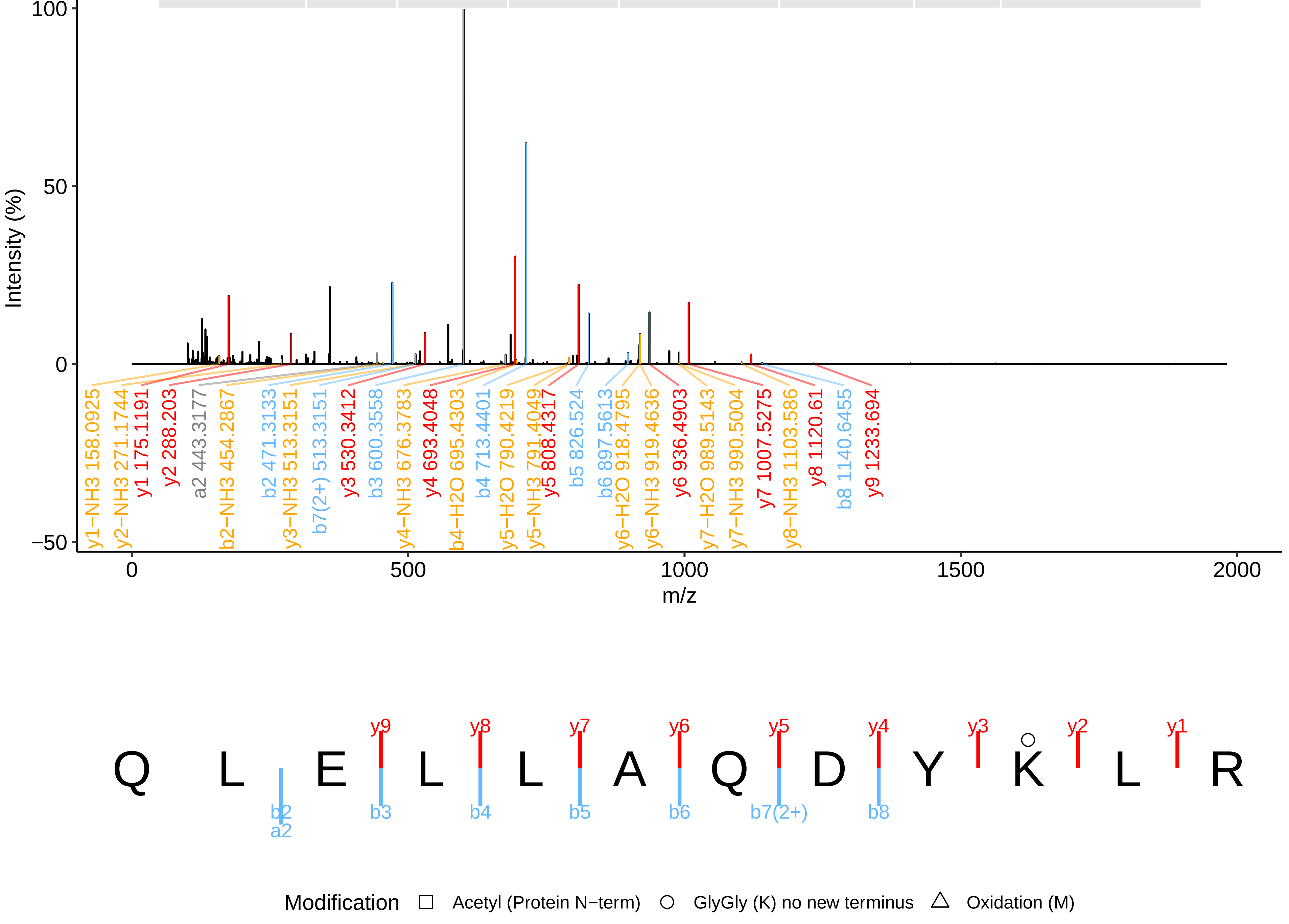

20200921_KIZ_MUb4_GlyGly_10plex − Scan number: 10522

Gene Names	Charge	m/z	Mass	Retention time	PEP	Score	Precursor Intensity
BRCC3	3	536.2437	1605.709	36.441	0.0015456	79.875	2214793

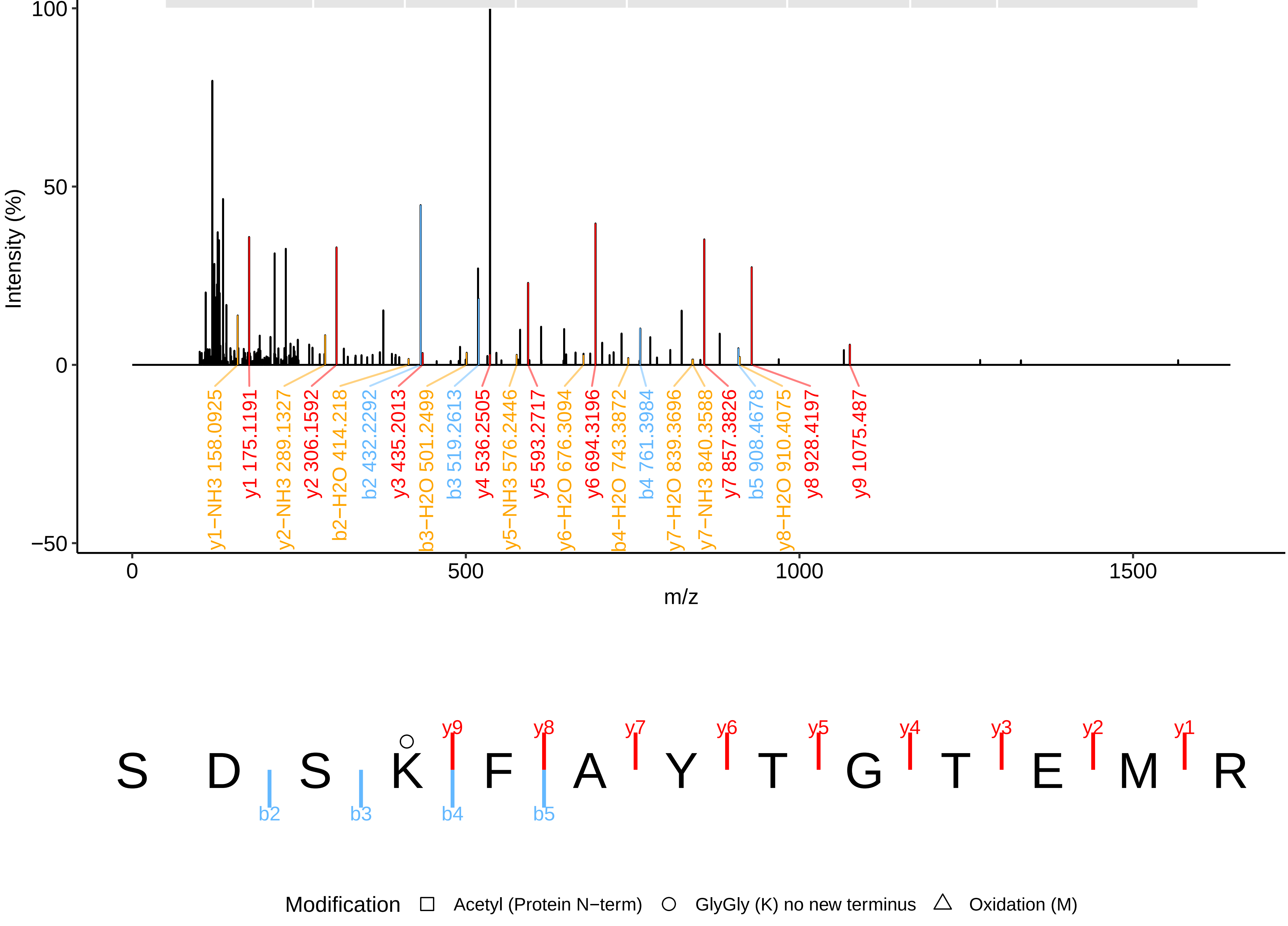

20200921_KIZ_MUb4_GlyGly_10plex_2 − Scan number: 10952

Gene Names	Charge	m/z	Mass	Retention time	PEP	Score	Precursor Intensity
ABCC4	3	443.2262	1326.657	42.038	0.12358	48.004	NA

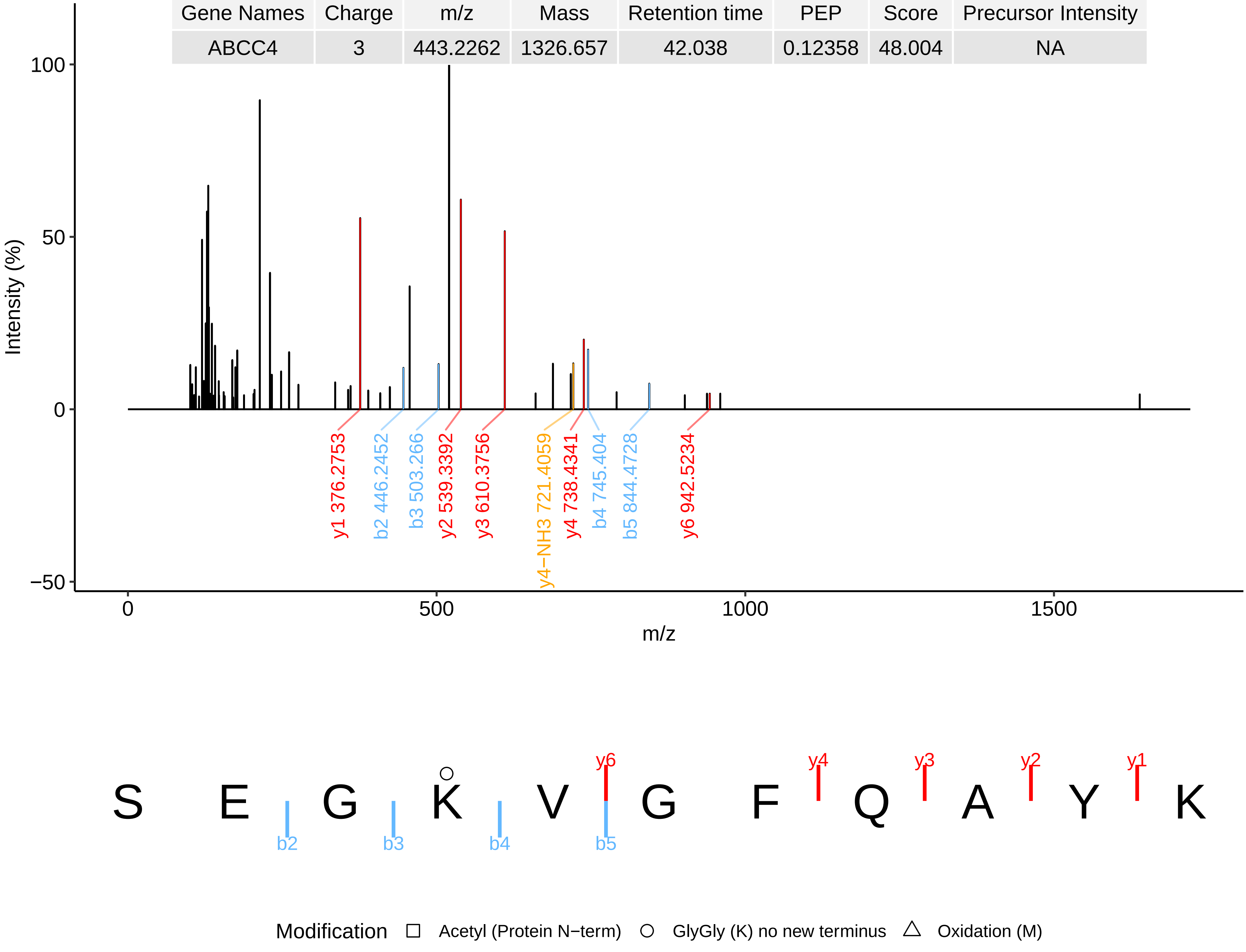

20200921_KIZ_MUb4_GlyGly_10plex_2 − Scan number: 20745

Gene Names	Charge	m/z	Mass	Retention time	PEP	Score	Precursor Intensity
FASN	3	453.2732	1356.798	81.586	0.0003025	97.214	1350684

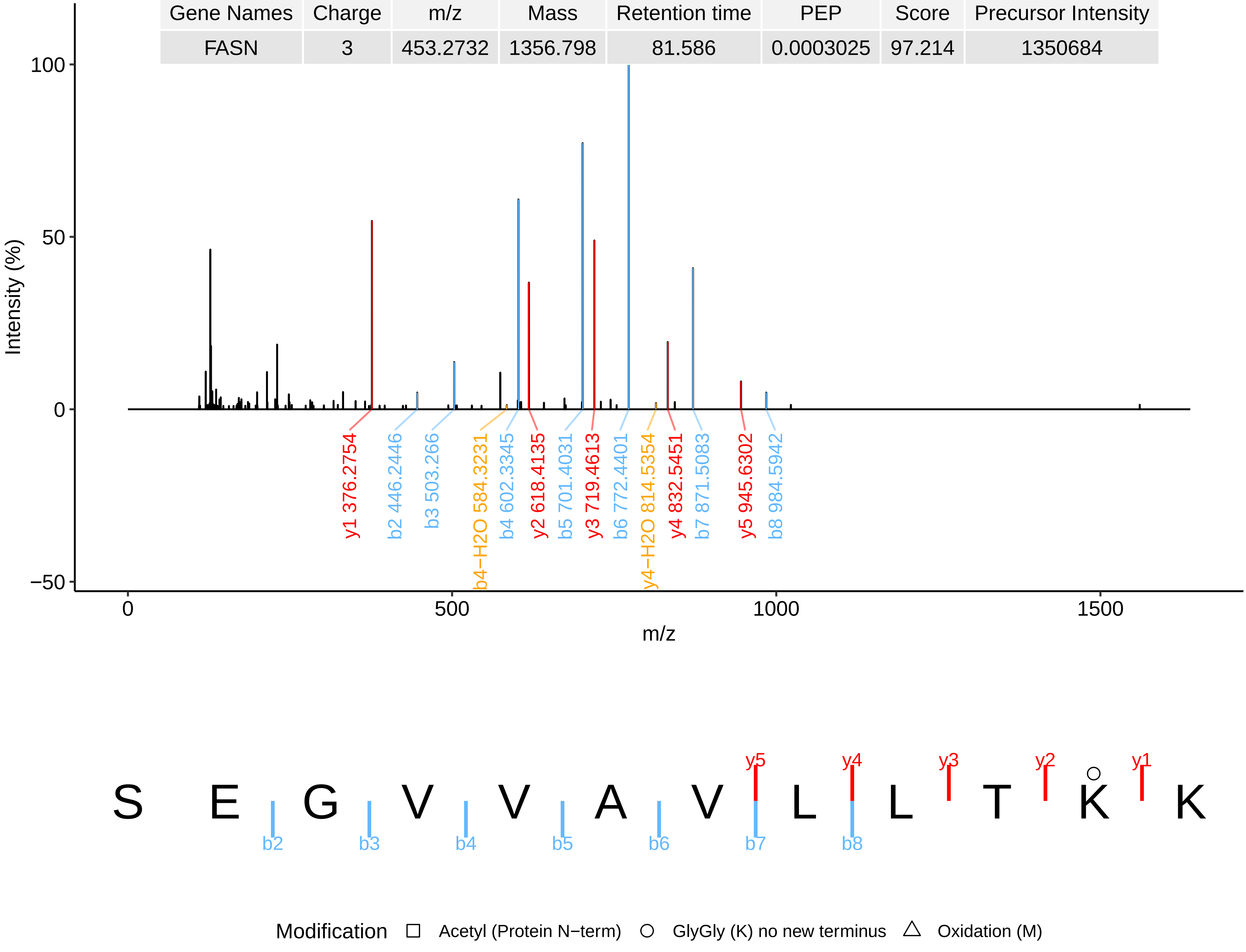

20200921_KIZ_MUb4_GlyGly_10plex_2 − Scan number: 6521

Gene Names	Charge	m/z	Mass	Retention time	PEP	Score	Precursor Intensity
TOMM40	3	455.2381	1362.693	24.008	0.010888	69.451	2007165

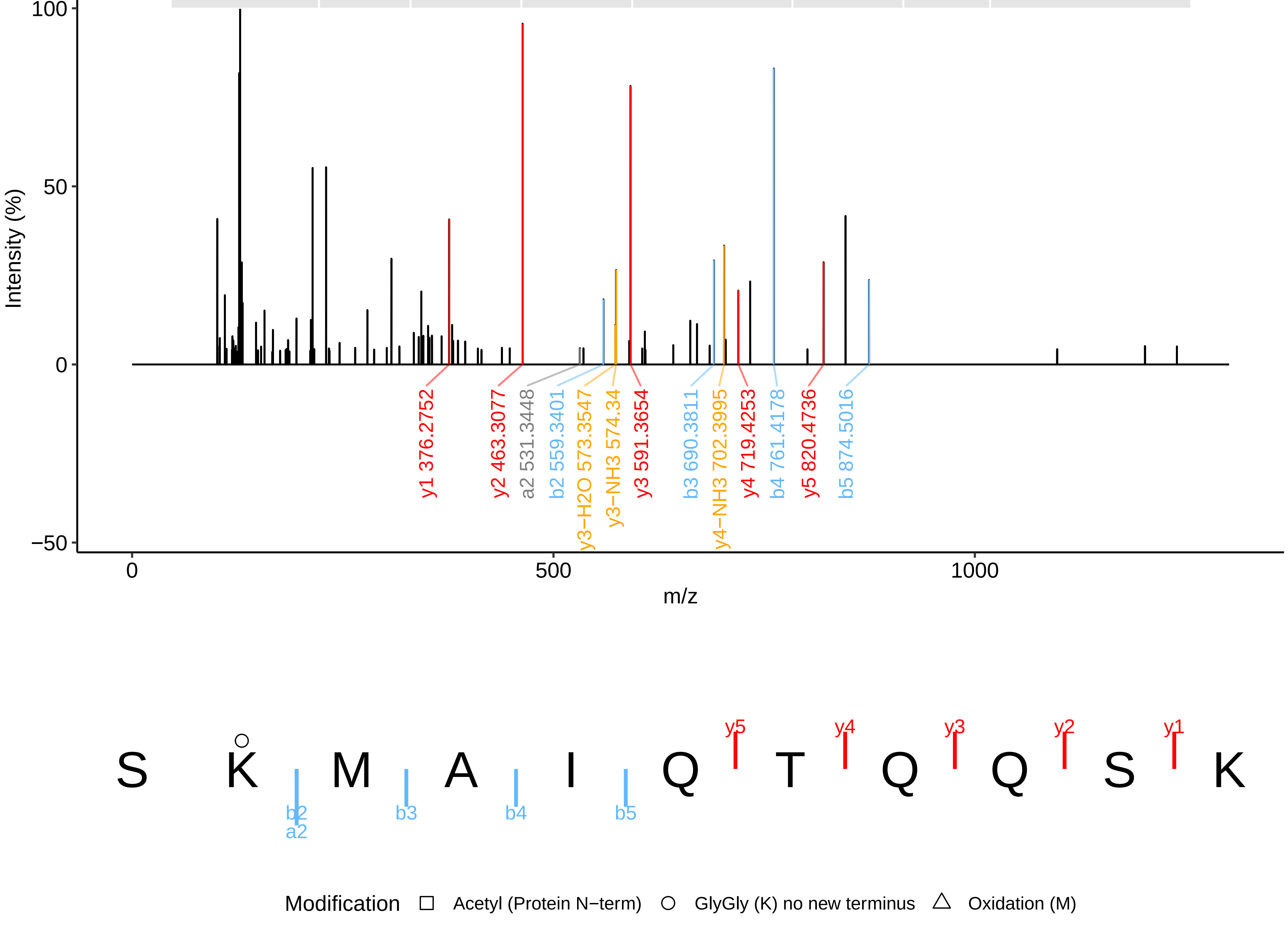

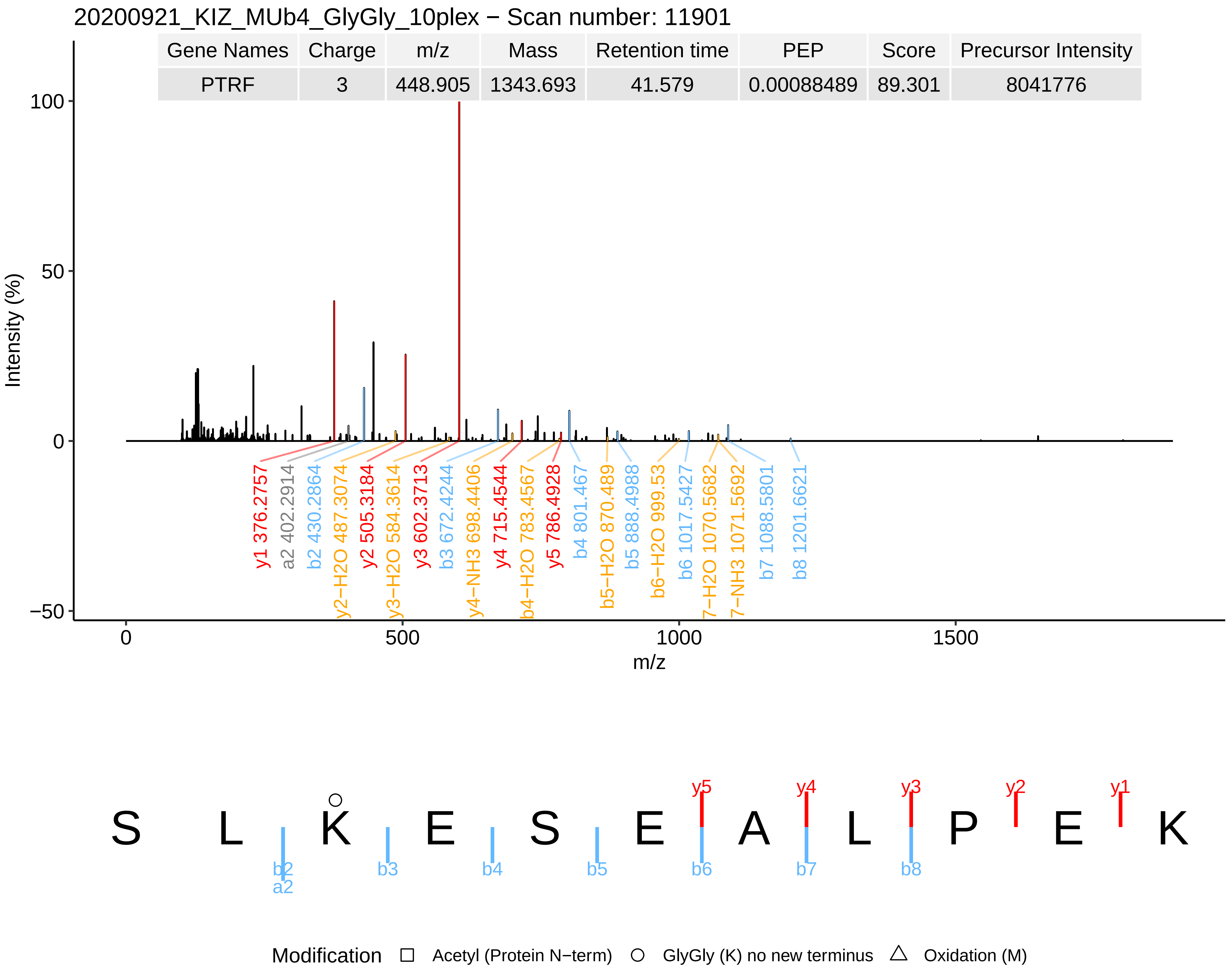
20200921_KIZ_MUb4_GlyGly_10plex − Scan number: 11901
Gene Names | Charge | m/z | Mass | Retention time | PEP | Score | Precursor Intensity
PTRF | 3 | 448.905 | 1343.693 | 41.579 | 0.00088489 | 89.301 | 8041776
Intensity (%)
m/z
y1 376.2757
a2 402.2914
b2 430.2864
y2−H2O 487.3074
y2 505.3184
y3−H2O 584.3614
y3 602.3713
b3 672.4244
y4−NH3 698.4406
y4 715.4544
b4−H2O 783.4567
y5 786.4928
b4 801.467
b5−H2O 870.489
b5 888.4988
b6−H2O 999.53
b6 1017.5427
7−H2O 1070.5682
7−NH3 1071.5692
b7 1088.5801
b8 1201.6621
S L K E S E A L P E K
Modification
Acetyl (Protein N−term)
GlyGly (K) no new terminus
Oxidation (M)

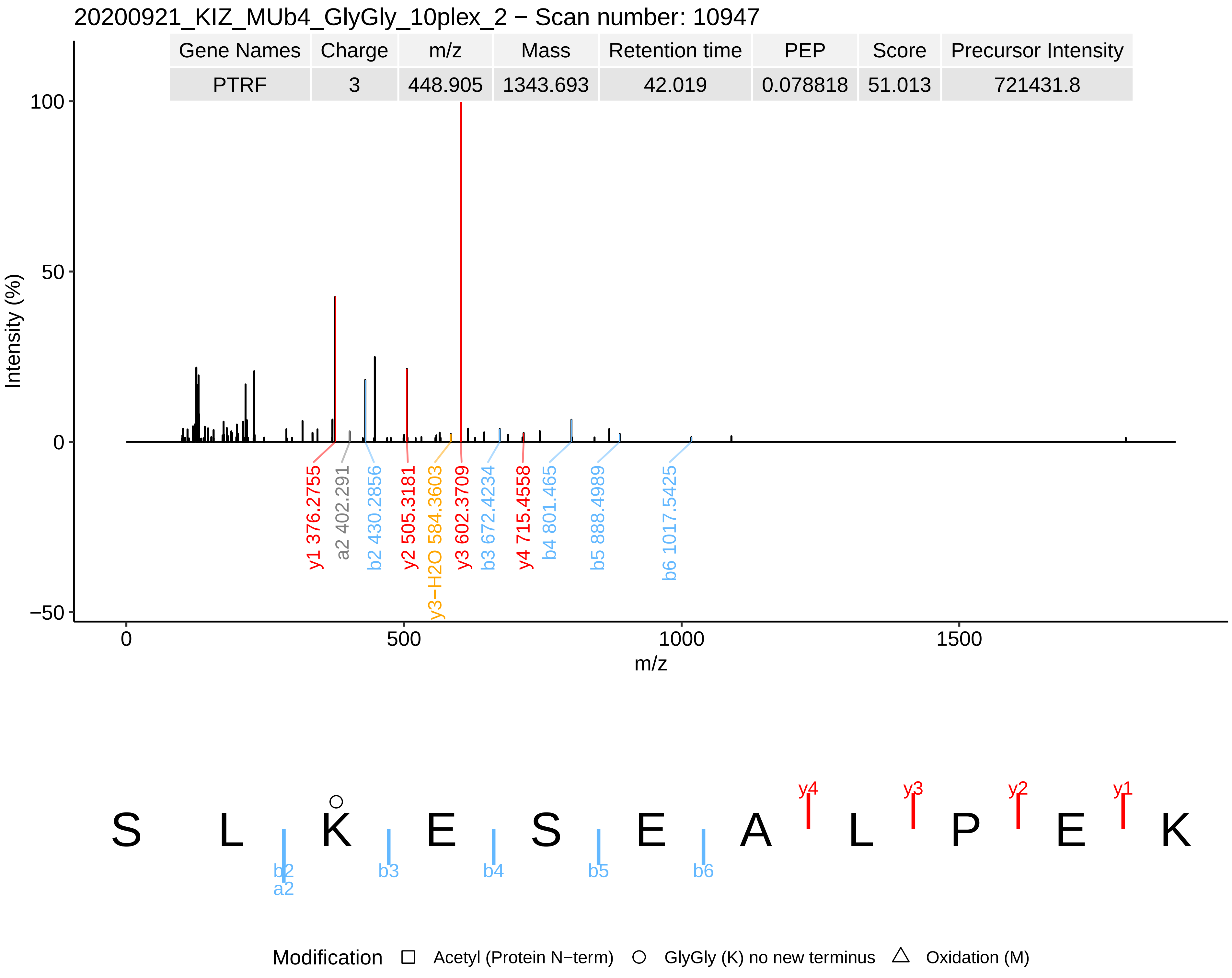
20200921_KIZ_MUb4_GlyGly_10plex_2 − Scan number: 10947
Gene Names | Charge | m/z | Mass | Retention time | PEP | Score | Precursor Intensity
PTRF | 3 | 448.905 | 1343.693 | 42.019 | 0.078818 | 51.013 | 721431.8
Intensity (%)
100
50
0
−50
y1 376.2755
a2 402.291
b2 430.2856
y2 505.3181
y3−H2O 584.3603
y3 602.3709
b3 672.4234
y4 715.4558
b4 801.465
b5 888.4989
b6 1017.5425
0
500
1000
1500
m/z
S L K E S E A L P E K
y4 y3 y2 y1
b2 a2 b3 b4 b5 b6
Modification
Acetyl (Protein N−term)
GlyGly (K) no new terminus
Oxidation (M)

20200924_KIZ_MUb5_GlyGly_1 − Scan number: 10908

Gene Names	Charge	m/z	Mass	Retention time	PEP	Score	Precursor Intensity
PTRF	3	448.905	1343.693	35.3	0.0068221	74.392	5375627

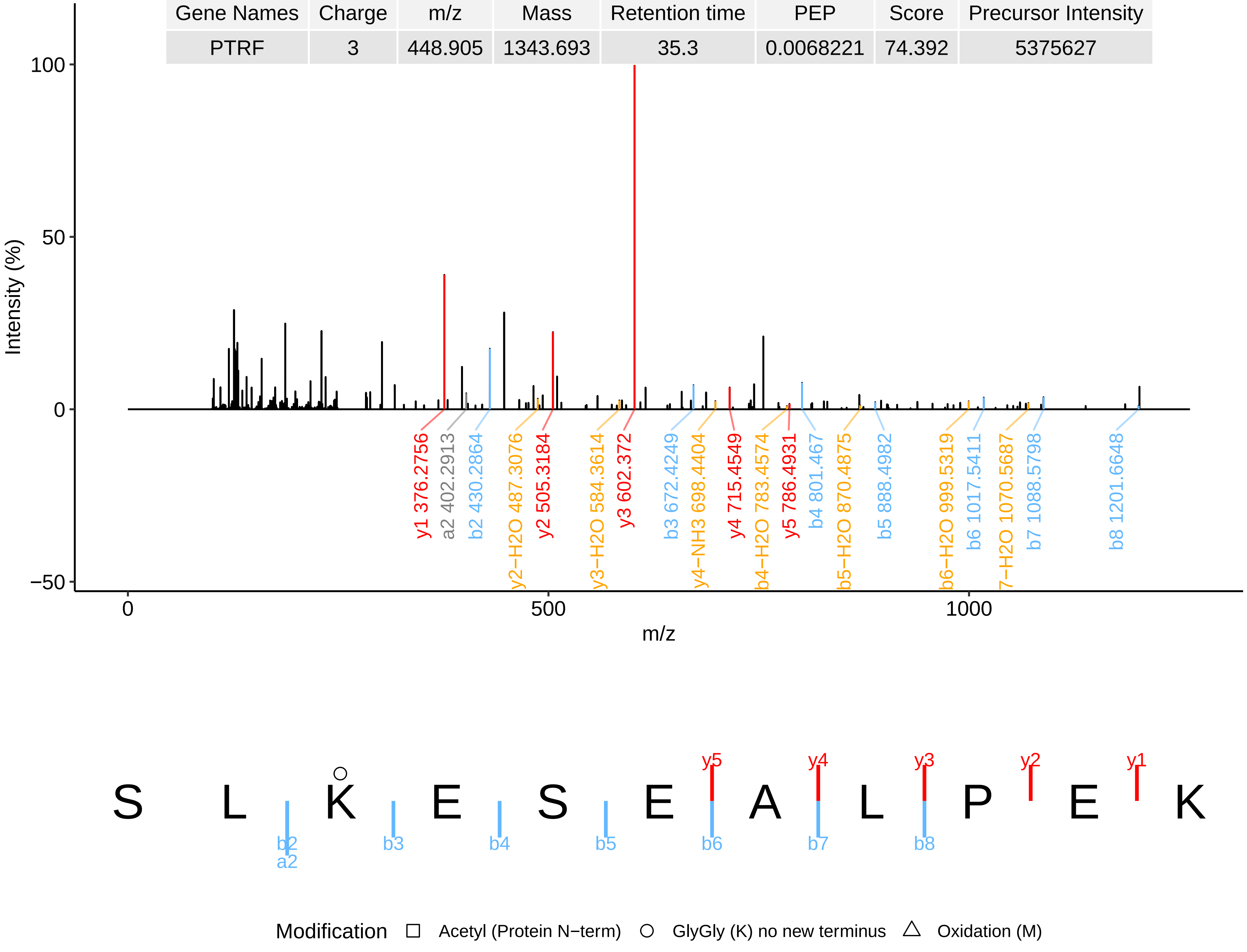

20200924_KIZ_MUb5_GlyGly_2 − Scan number: 10222

Gene Names	Charge	m/z	Mass	Retention time	PEP	Score	Precursor Intensity
PTRF	3	448.905	1343.693	38.165	0.067423	56.225	1910265

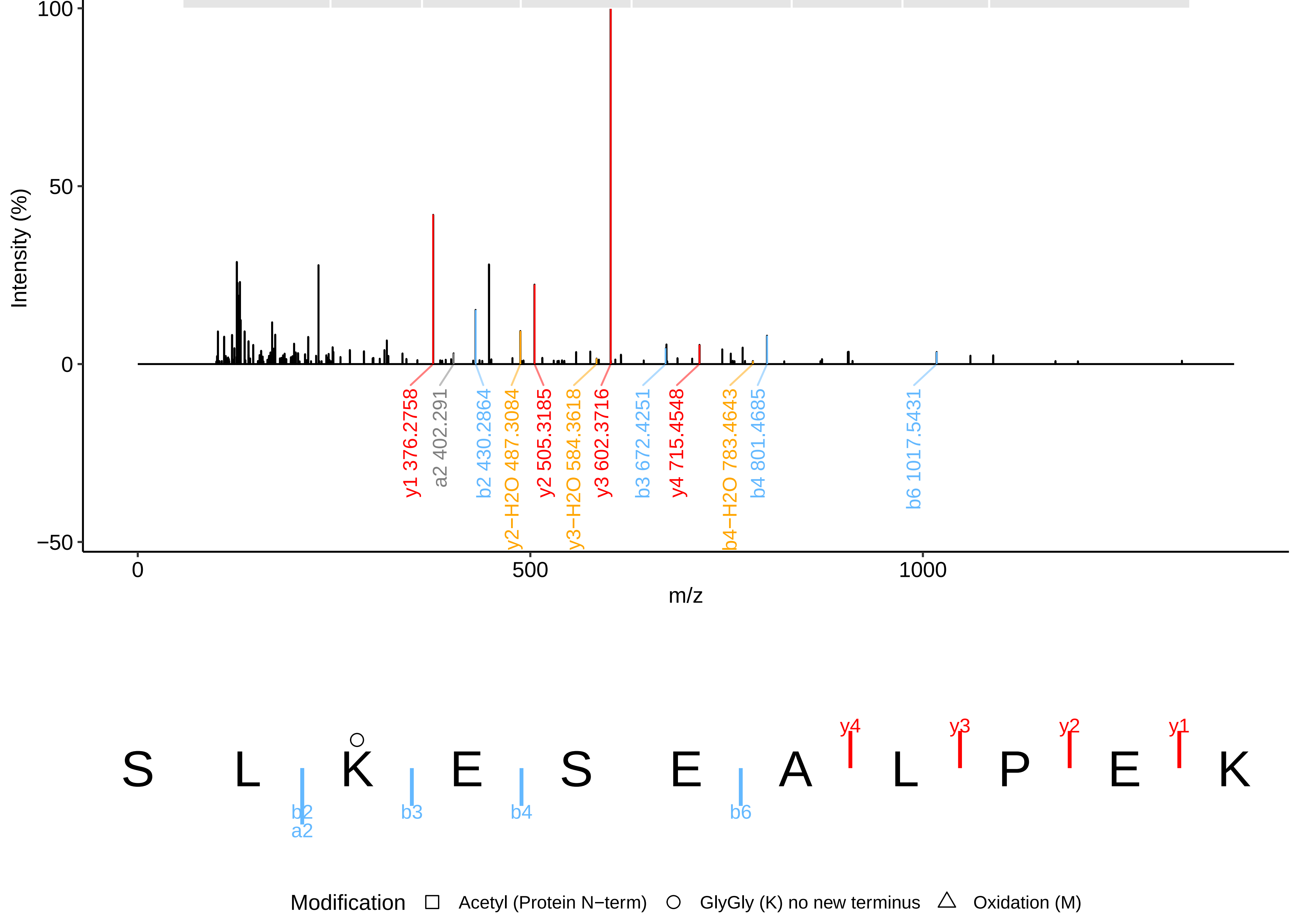

20200921_KIZ_MUb4_GlyGly_10plex − Scan number: 8300

Gene Names	Charge	m/z	Mass	Retention time	PEP	Score	Precursor Intensity
EPS15L1	3	535.2727	1602.796	28.125	0.00055881	92.19	2559391

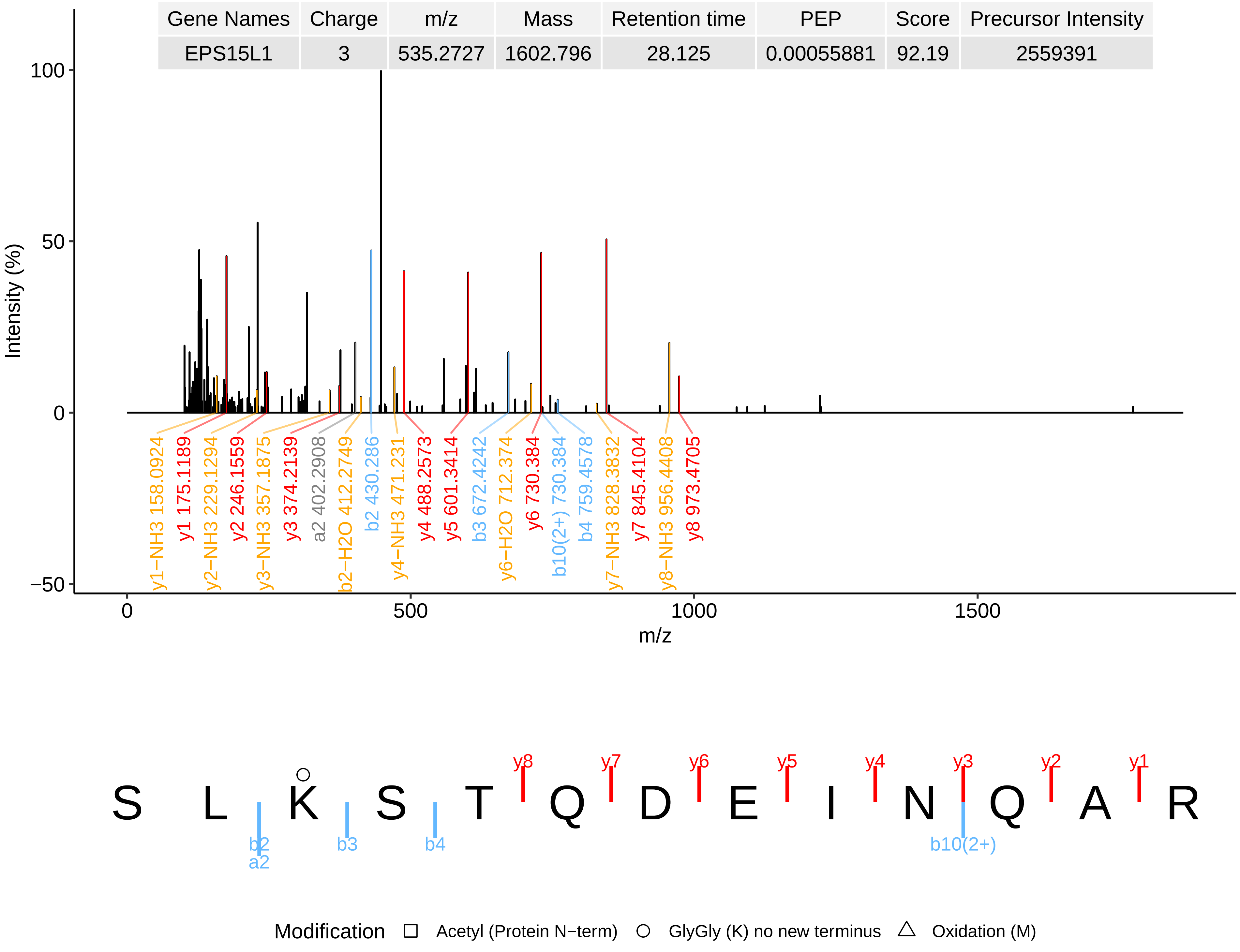

20200921_KIZ_MUb4_GlyGly_10plex − Scan number: 8311

Gene Names	Charge	m/z	Mass	Retention time	PEP	Score	Precursor Intensity
EPS15L1	3	535.2727	1602.796	28.165	5.7341e−08	119.39	1557435

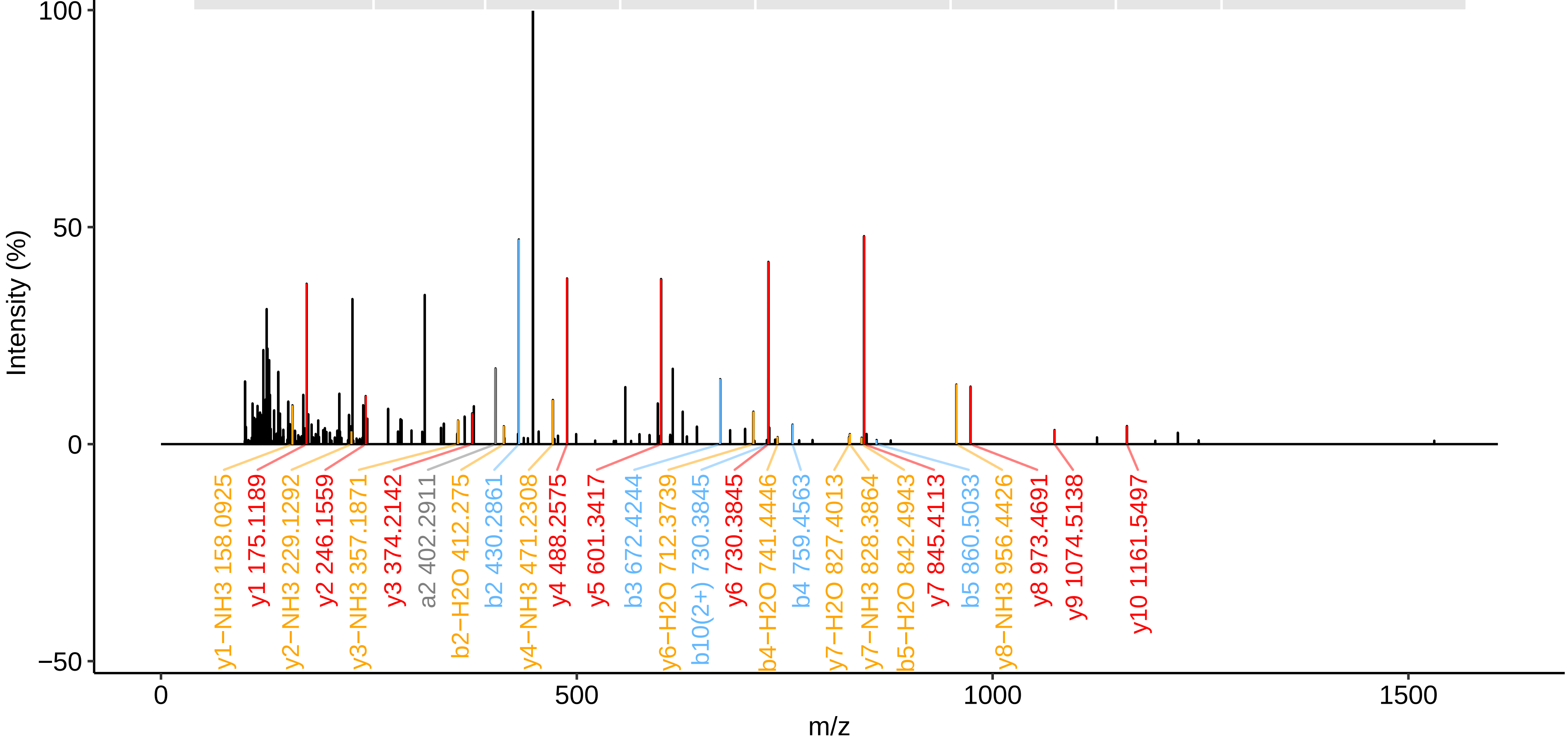

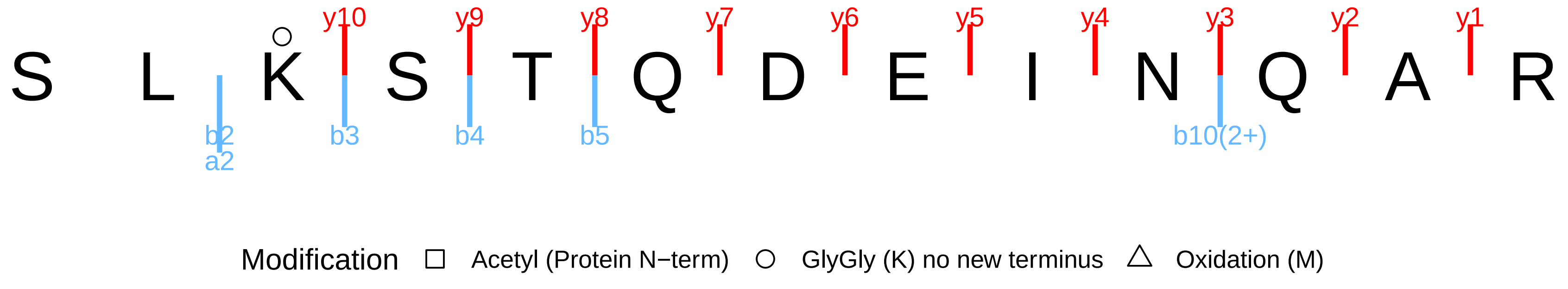

20200921_KIZ_MUb4_GlyGly_10plex_2 − Scan number: 7670

Gene Names	Charge	m/z	Mass	Retention time	PEP	Score	Precursor Intensity
EPS15L1	3	535.2727	1602.796	28.698	0.010954	63.091	957031.8

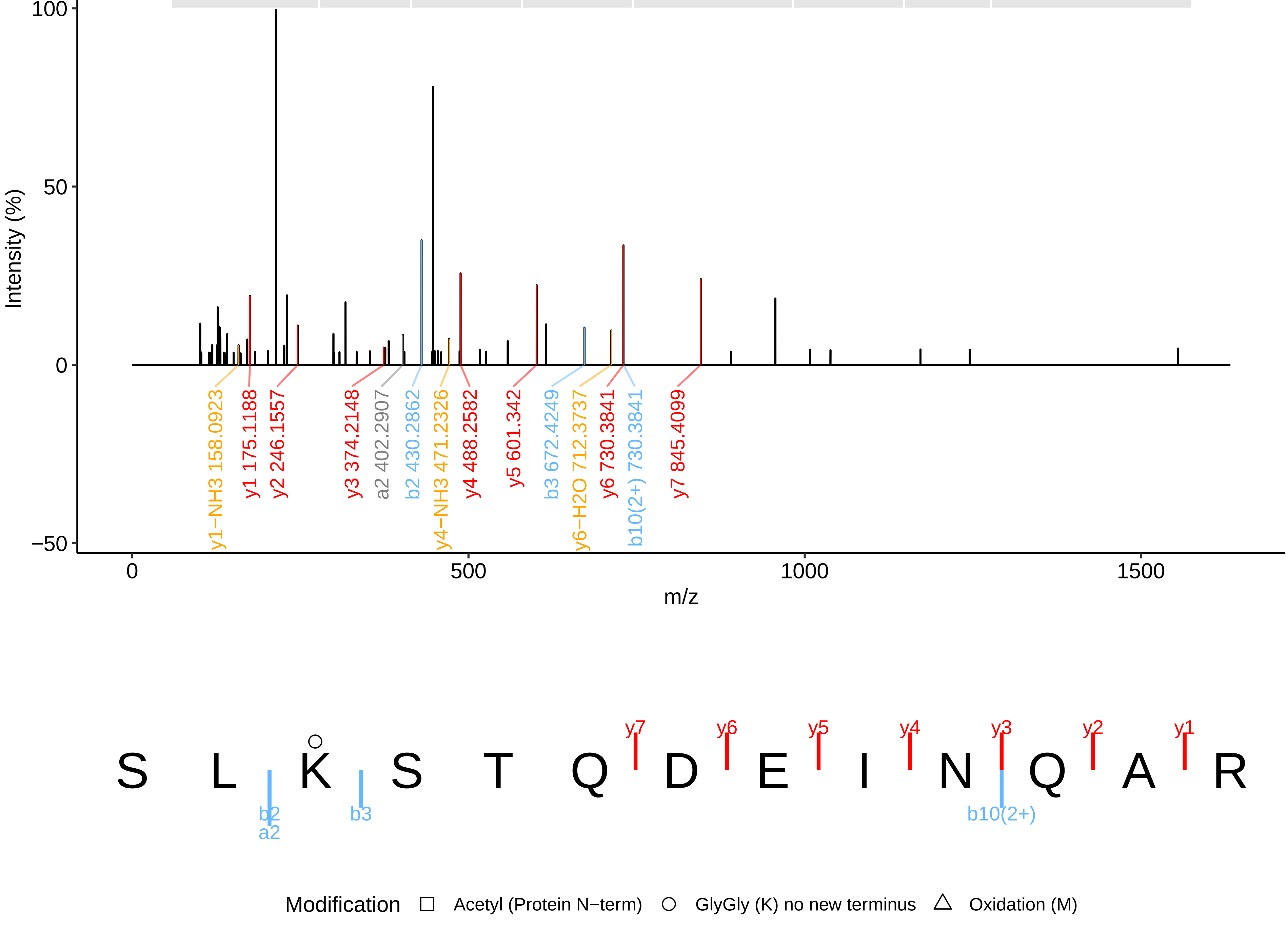

20200921_KIZ_MUb4_GlyGly_10plex − Scan number: 13625

Gene Names	Charge	m/z	Mass	Retention time	PEP	Score	Precurs
ST1H1E;HIST1H1D;HIST1H1A;HIST1H1B;HIST1H1C	3	458.9366	1373.788	48.132	0.00028736	94.122	78

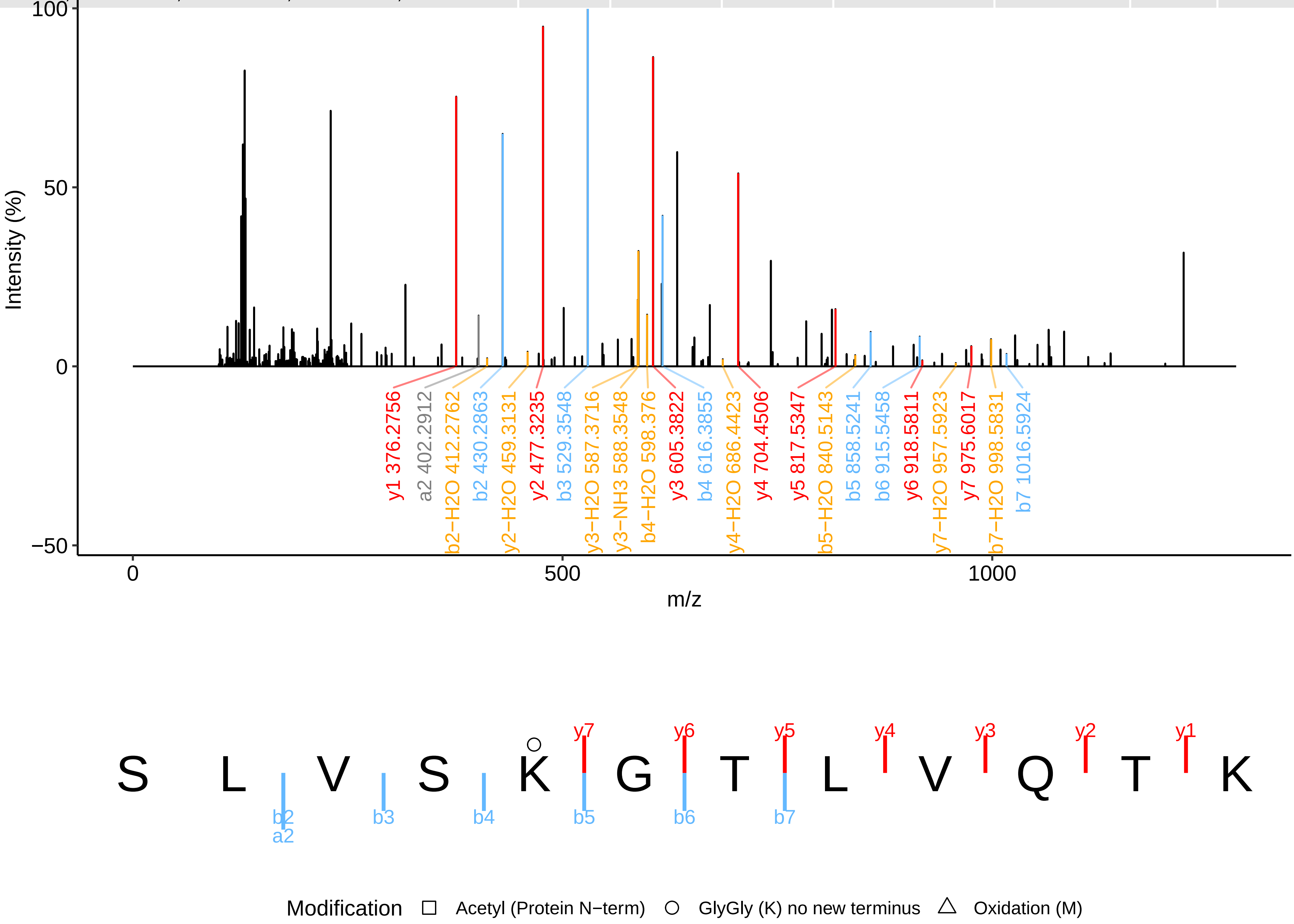

20200921_KIZ_MUb4_GlyGly_10plex_2 − Scan number: 12537

Gene Names	Charge	m/z	Mass	Retention time	PEP	Score	Precurso
ST1H1E;HIST1H1D;HIST1H1A;HIST1H1B;HIST1H1C	3	458.9366	1373.788	48.526	0.084325	49.423	114

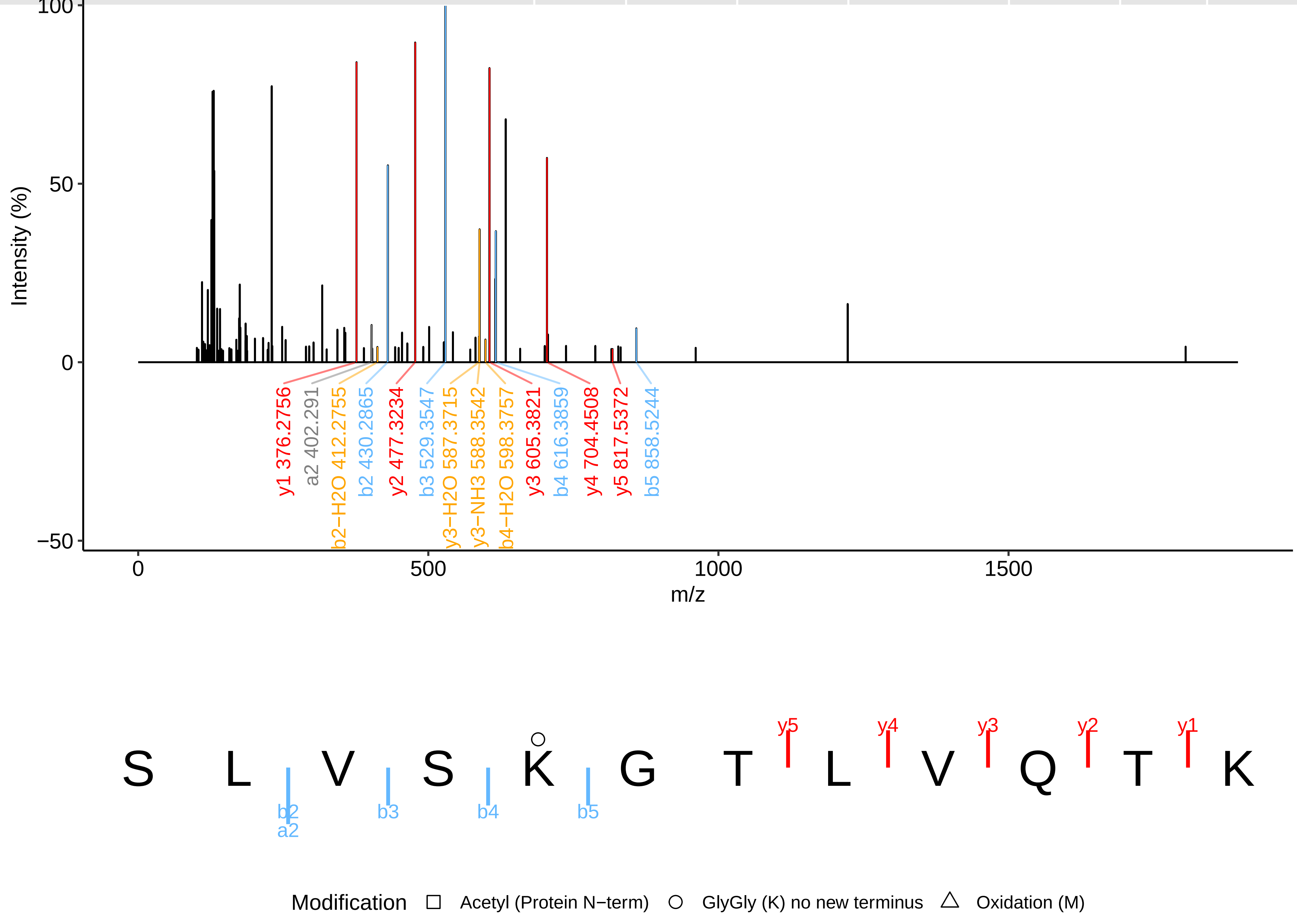

20200921_KIZ_MUb4_GlyGly_10plex − Scan number: 9083

Gene Names	Charge	m/z	Mass	Retention time	PEP	Score	Precursor Intensity
PSMD8	3	520.9193	1559.736	31.055	0.0015046	82.202	6309025

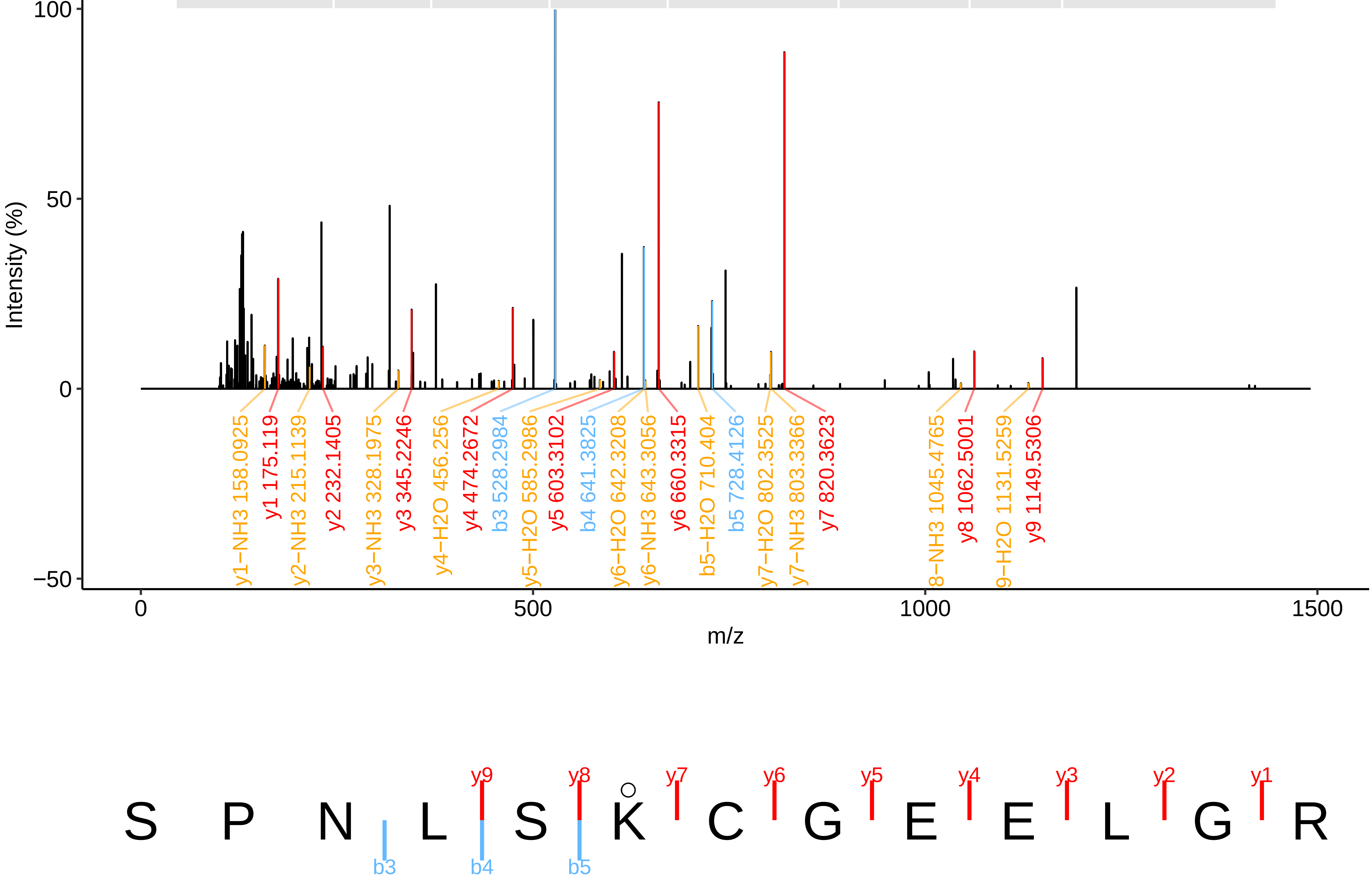

Modification □ Acetyl (Protein N−term) ○ GlyGly (K) no new terminus △ Oxidation (M)

20200921_KIZ_MUb4_GlyGly_10plex_2 − Scan number: 8355

Gene Names	Charge	m/z	Mass	Retention time	PEP	Score	Precursor Intensity
PSMD8	3	520.9193	1559.736	31.452	0.0066485	70.438	467284.2

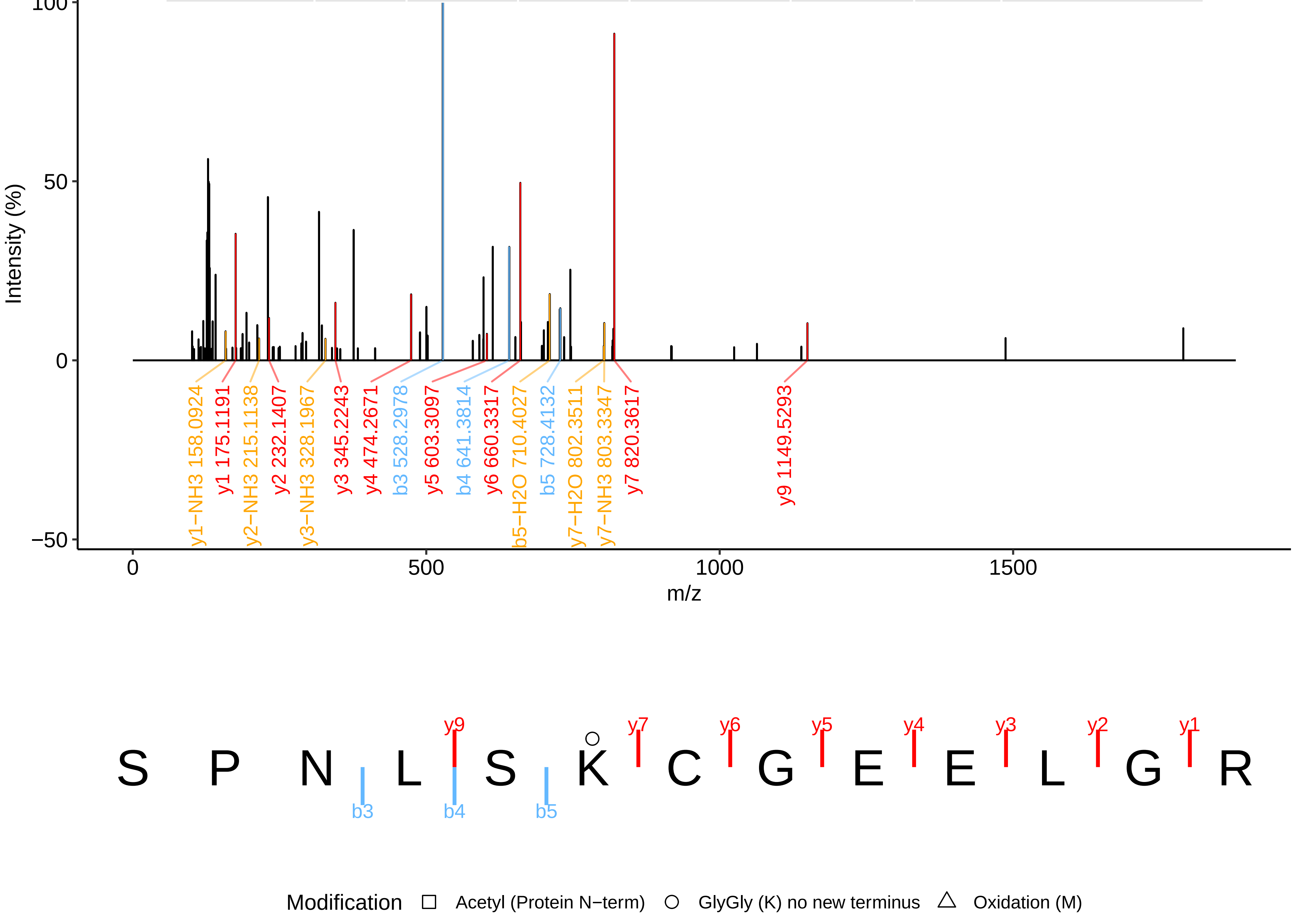

20200921_KIZ_MUb4_GlyGly_10plex_2 − Scan number: 20952

Gene Names	Charge	m/z	Mass	Retention time	PEP	Score	Precursor Intensity
HUWE1	3	449.9329	1346.777	82.43	0.0031652	77.674	2091129

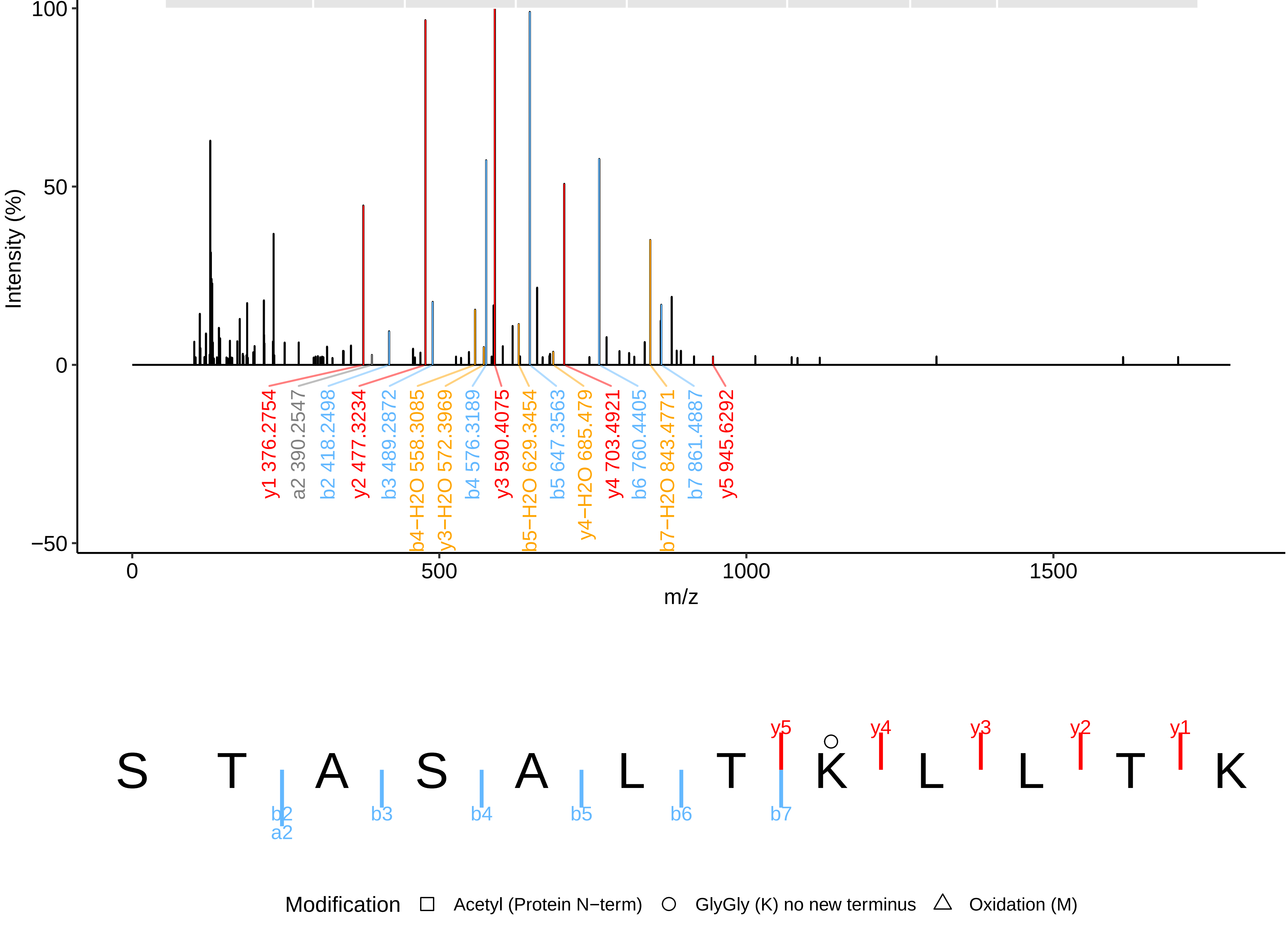

20200921_KIZ_MUb4_GlyGly_10plex − Scan number: 14617

Gene Names	Charge	m/z	Mass	Retention time	PEP	Score	Precursor Intensity
SFPQ	3	446.5735	1336.699	52.005	0.0012086	85.536	9041989

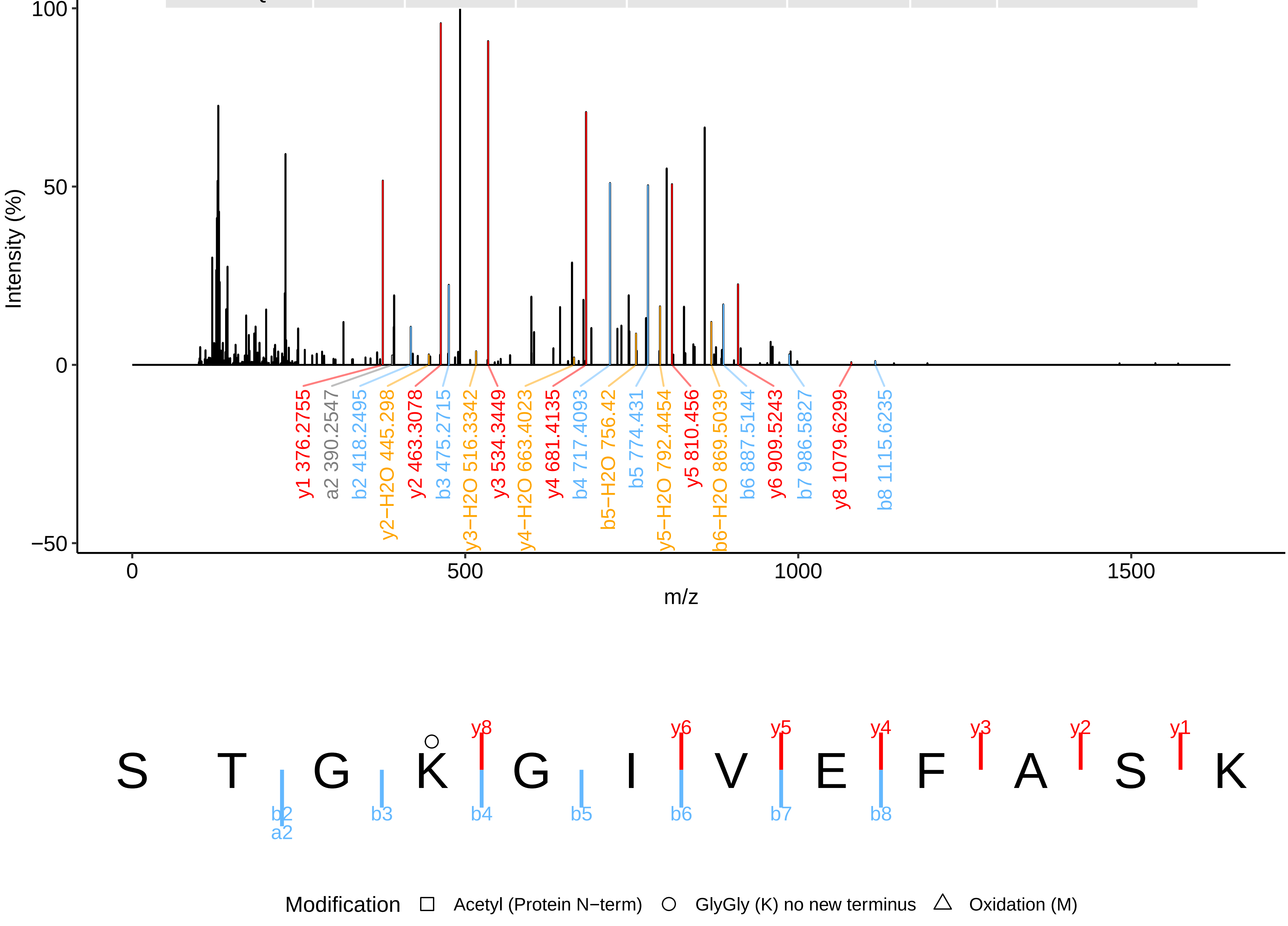

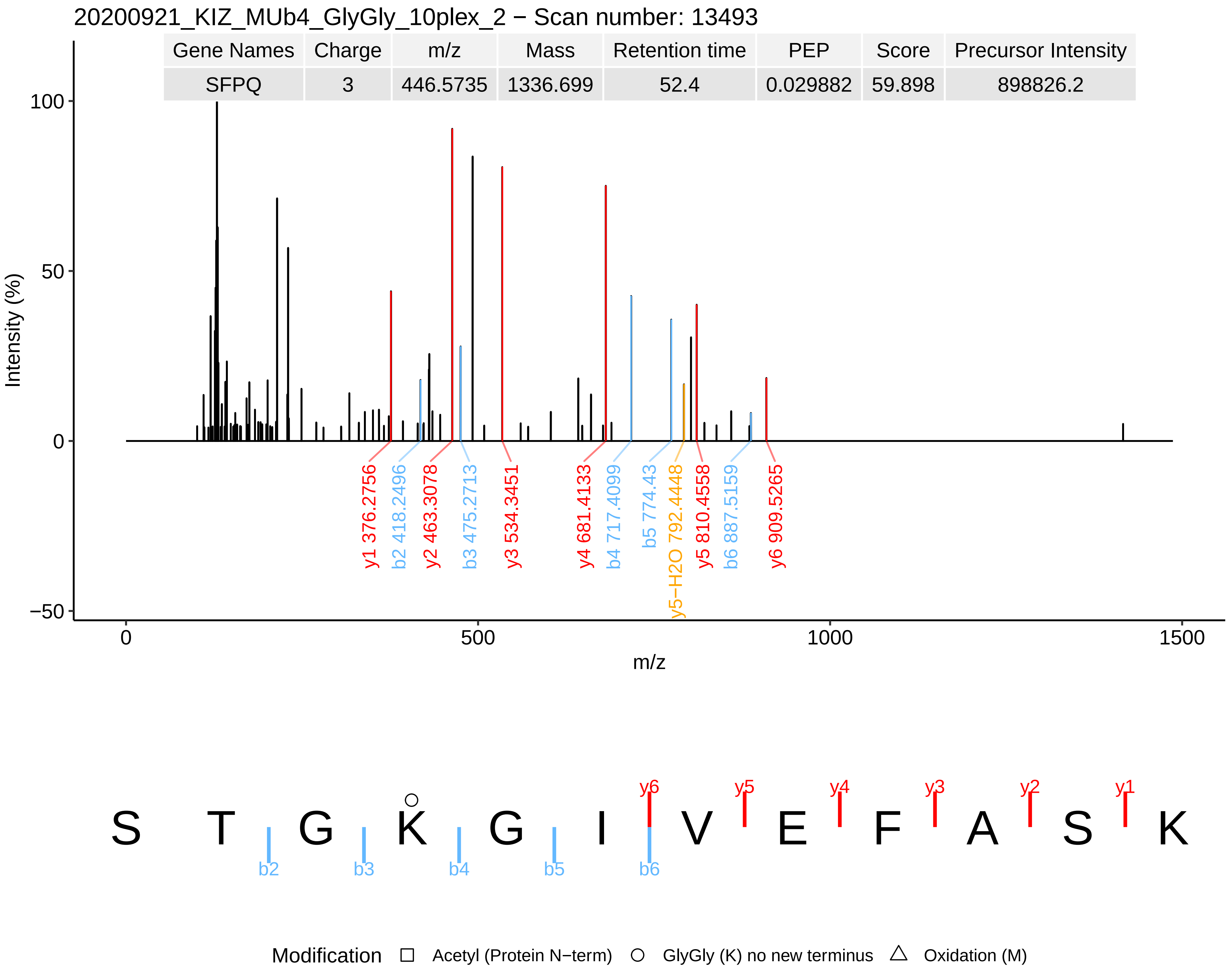
20200921_KIZ_MUb4_GlyGly_10plex_2 − Scan number: 13493
Gene Names | Charge | m/z | Mass | Retention time | PEP | Score | Precursor Intensity
SFPQ | 3 | 446.5735 | 1336.699 | 52.4 | 0.029882 | 59.898 | 898826.2
Intensity (%)
m/z
y1 376.2756
b2 418.2496
y2 463.3078
b3 475.2713
y3 534.3451
y4 681.4133
b4 717.4099
b5 774.43
y5−H2O 792.4448
y5 810.4558
b6 887.5159
y6 909.5265
S T G K G I V E F A S K
Modification
Acetyl (Protein N−term)
GlyGly (K) no new terminus
Oxidation (M)

20200921_KIZ_MUb4_GlyGly_10plex_2 − Scan number: 19291

Gene Names	Charge	m/z	Mass	Retention time	PEP	Score	Precursor Intensity
ACSL4	3	454.5748	1360.703	75.701	0.11461	50.292	NA

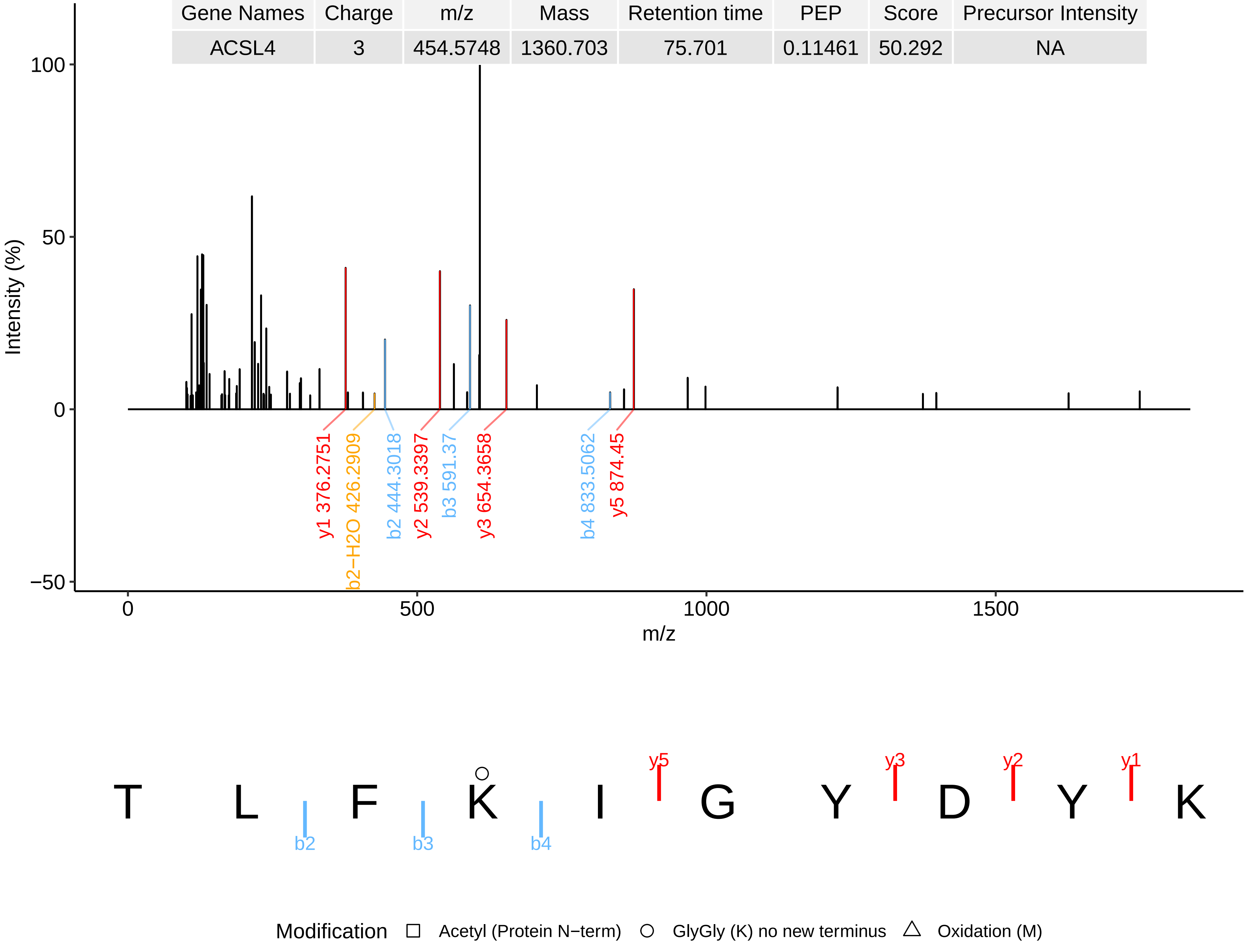

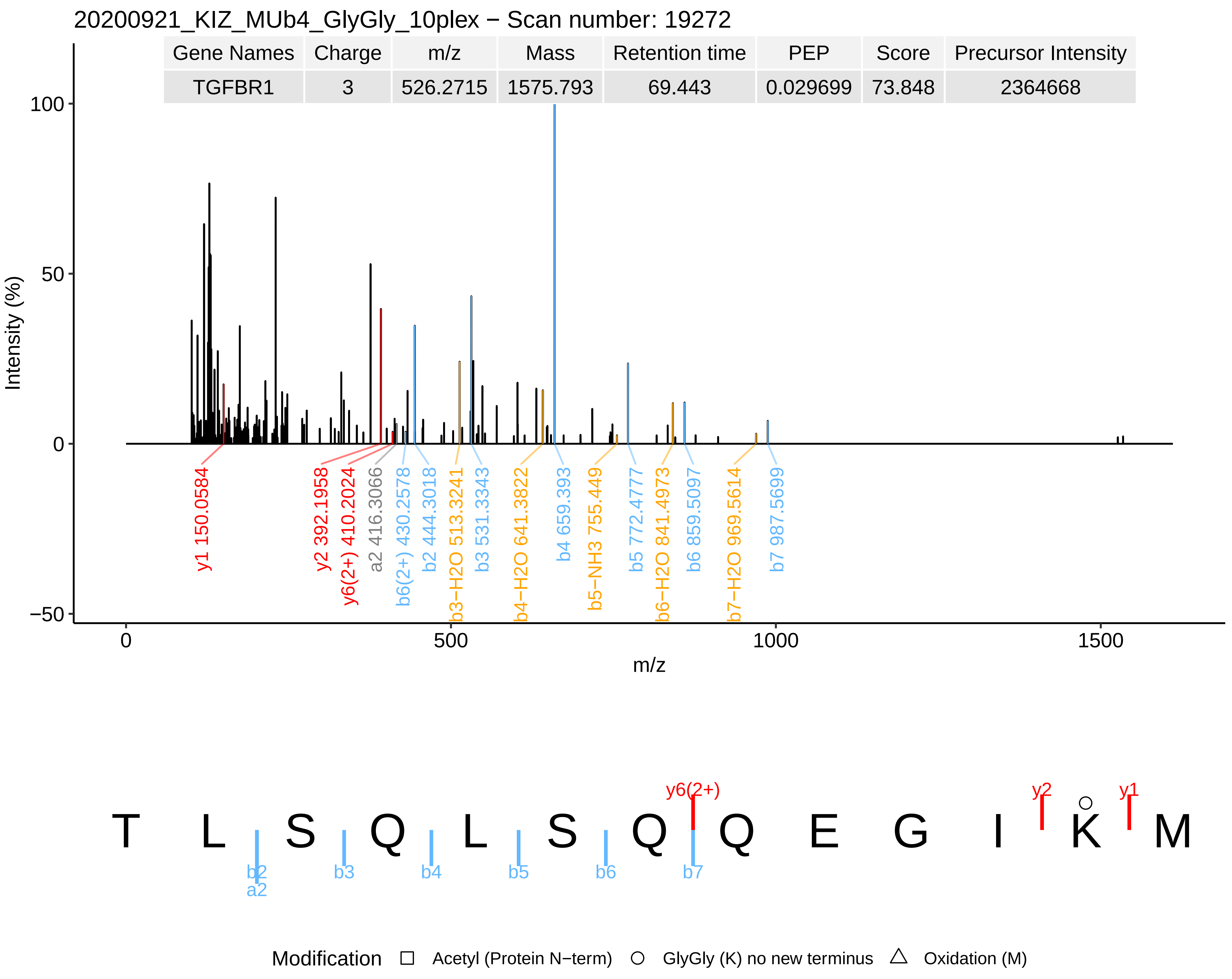
20200921_KIZ_MUb4_GlyGly_10plex − Scan number: 19272
Gene Names | Charge | m/z | Mass | Retention time | PEP | Score | Precursor Intensity
TGFBR1 | 3 | 526.2715 | 1575.793 | 69.443 | 0.029699 | 73.848 | 2364668
Intensity (%)
m/z
y1 150.0584
y2 392.1958
y6(2+) 410.2024
a2 416.3066
b6(2+) 430.2578
b2 444.3018
b3−H2O 513.3241
b3 531.3343
b4−H2O 641.3822
b4 659.393
b5−NH3 755.449
b5 772.4777
b6−H2O 841.4973
b6 859.5097
b7−H2O 969.5614
b7 987.5699
T L S Q L S Q Q E G I K M
Modification
Acetyl (Protein N−term)
GlyGly (K) no new terminus
Oxidation (M)

20200921_KIZ_MUb4_GlyGly_10plex − Scan number: 24621

Gene Names	Charge	m/z	Mass	Retention time	PEP	Score	Precursor Intensity
CUL5	3	525.9497	1574.827	89.548	1.3844e−08	116.01	6841350

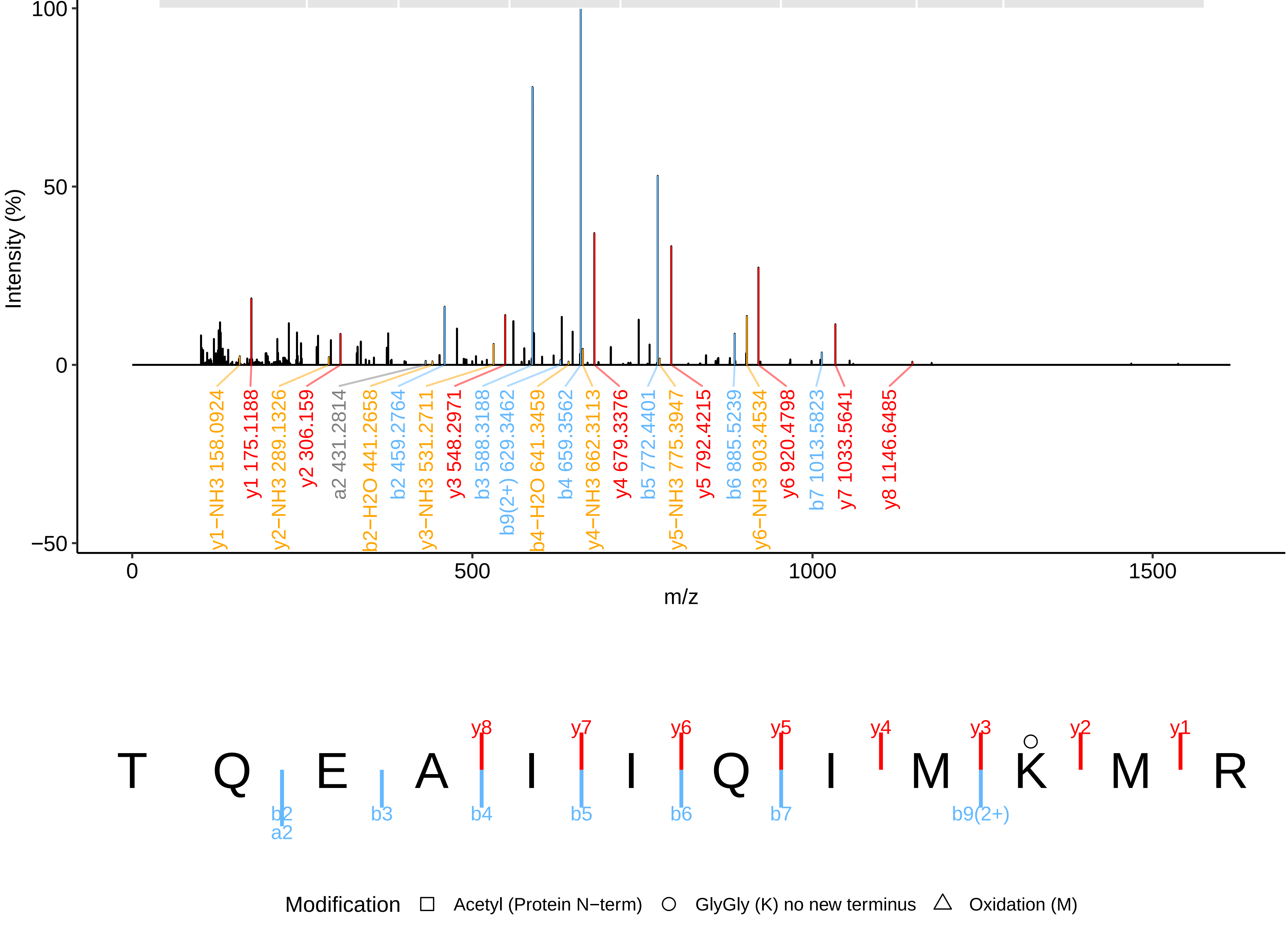

20200921_KIZ_MUb4_GlyGly_10plex − Scan number: 24756

Gene Names	Charge	m/z	Mass	Retention time	PEP	Score	Precursor Intensity
CUL5	3	525.9497	1574.827	90.059	0.00079273	86.772	1858616

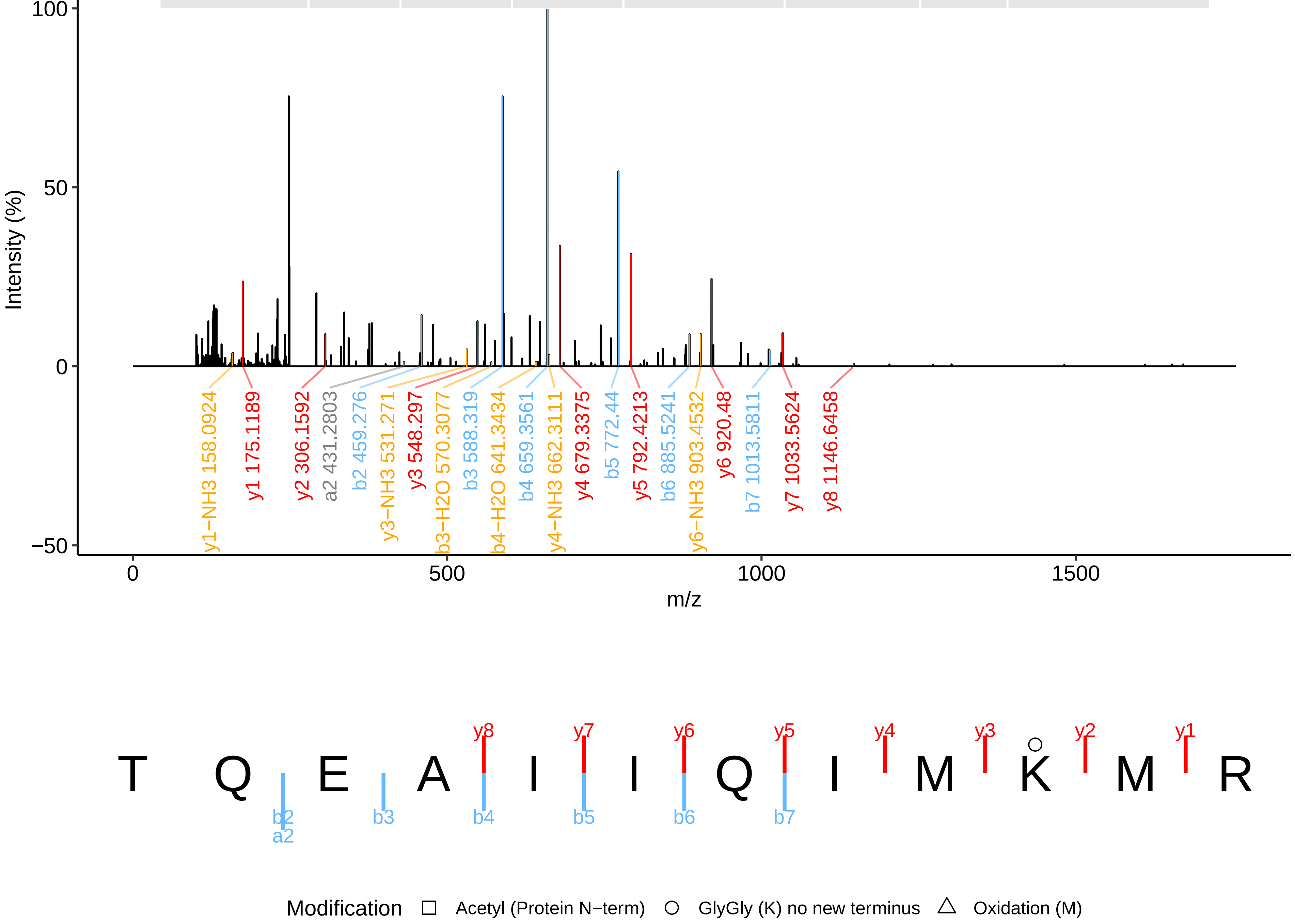

20200921_KIZ_MUb4_GlyGly_10plex_2 − Scan number: 20895

Gene Names	Charge	m/z	Mass	Retention time	PEP	Score	Precursor Intensity
CUL5	3	525.9497	1574.827	82.203	0.0008565	85.845	681355.1

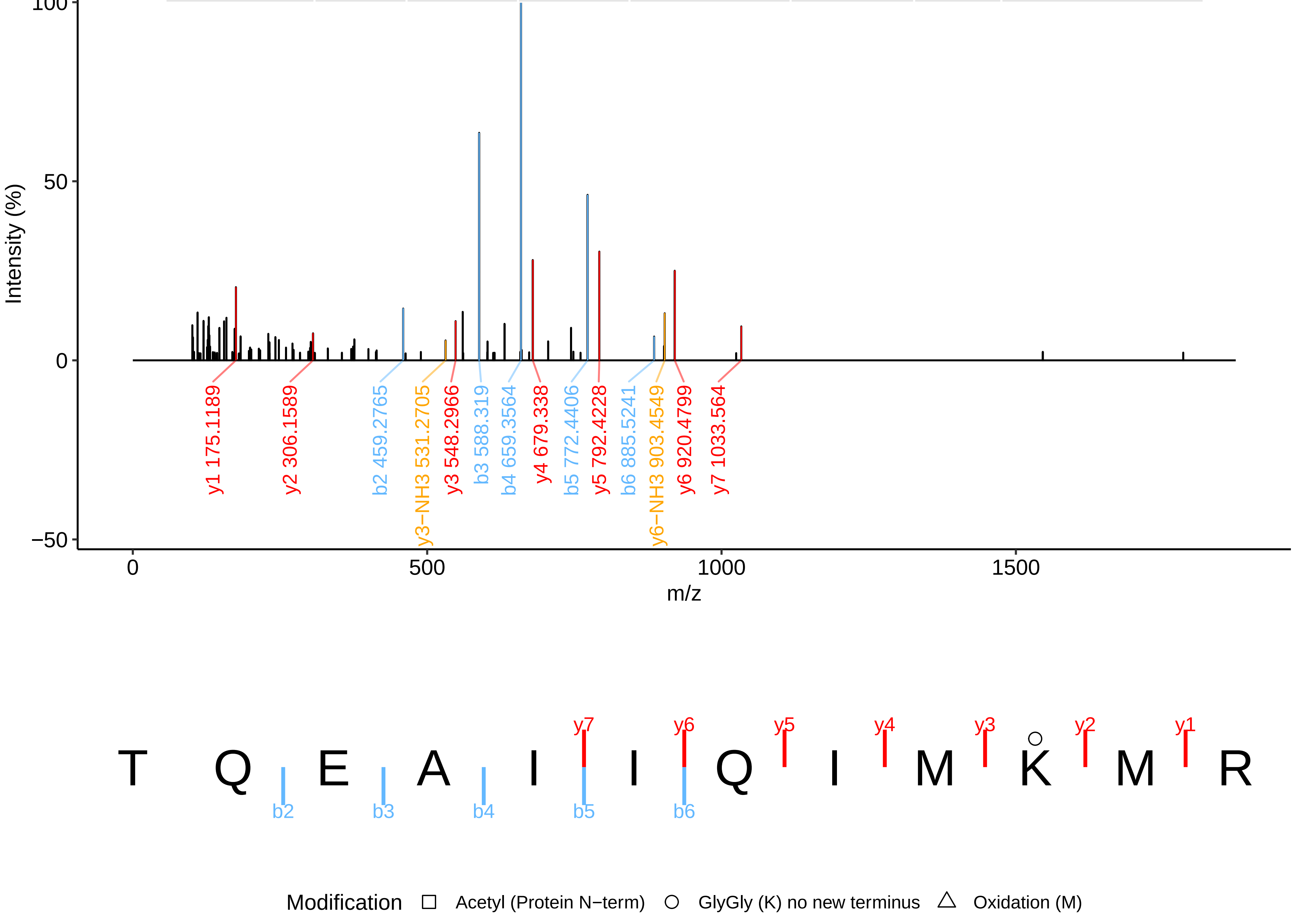

20200921_KIZ_MUb4_GlyGly_10plex_2 − Scan number: 17776

Gene Names	Charge	m/z	Mass	Retention time	PEP	Score	Precursor Intensity
CUL5	3	531.2813	1590.822	69.544	0.070532	48.659	943667.5

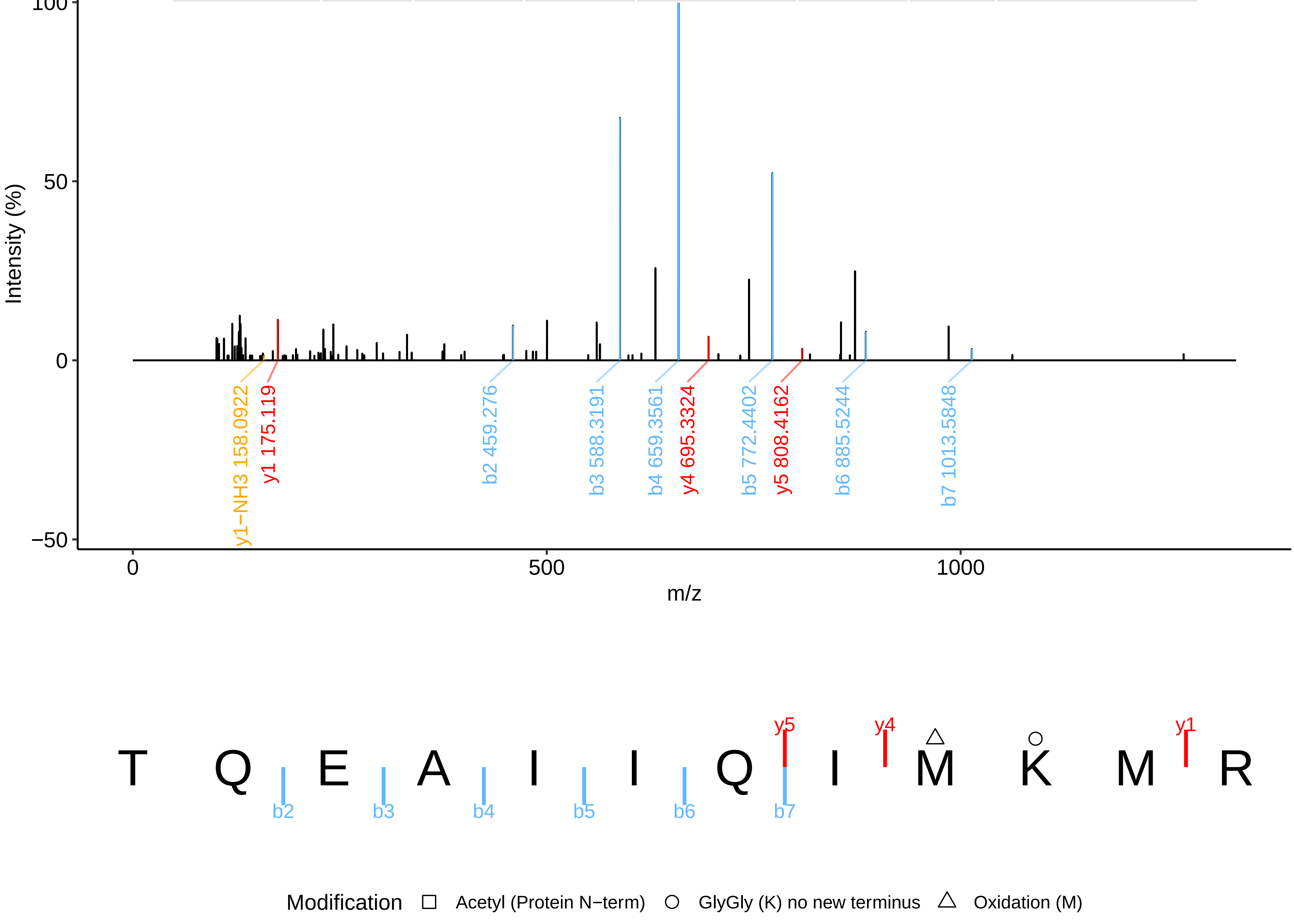

20200921_KIZ_MUb4_GlyGly_10plex_2 − Scan number: 17797

Gene Names	Charge	m/z	Mass	Retention time	PEP	Score	Precursor Intensity
CUL5	3	531.2813	1590.822	69.629	0.027116	59.116	3700788

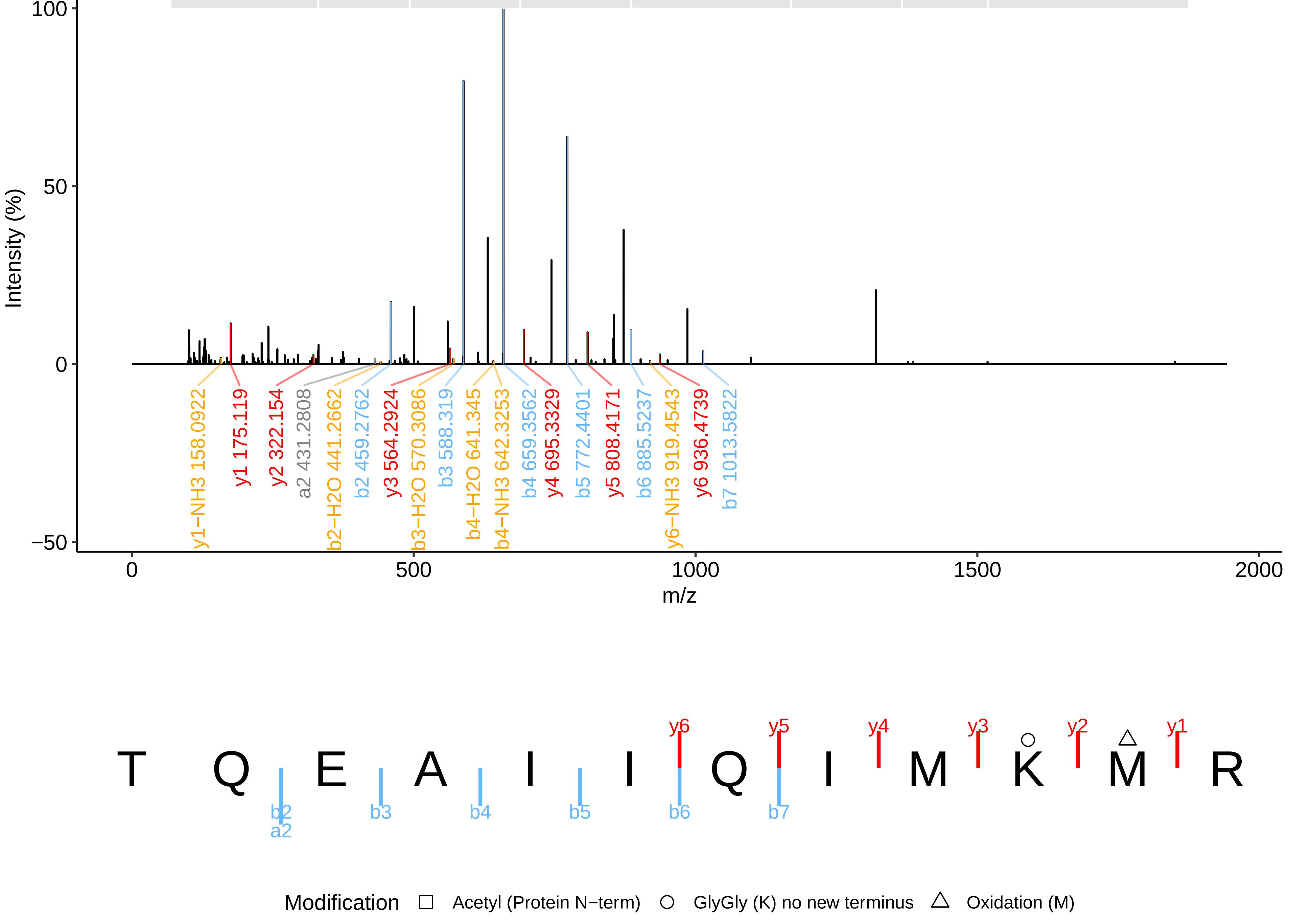

20200921_KIZ_MUb4_GlyGly_10plex − Scan number: 26945

Gene Names	Charge	m/z	Mass	Retention time	PEP	Score	Precursor Intensity
SPRTN	3	458.2468	1371.719	98.25	0.070108	72.006	875979.7

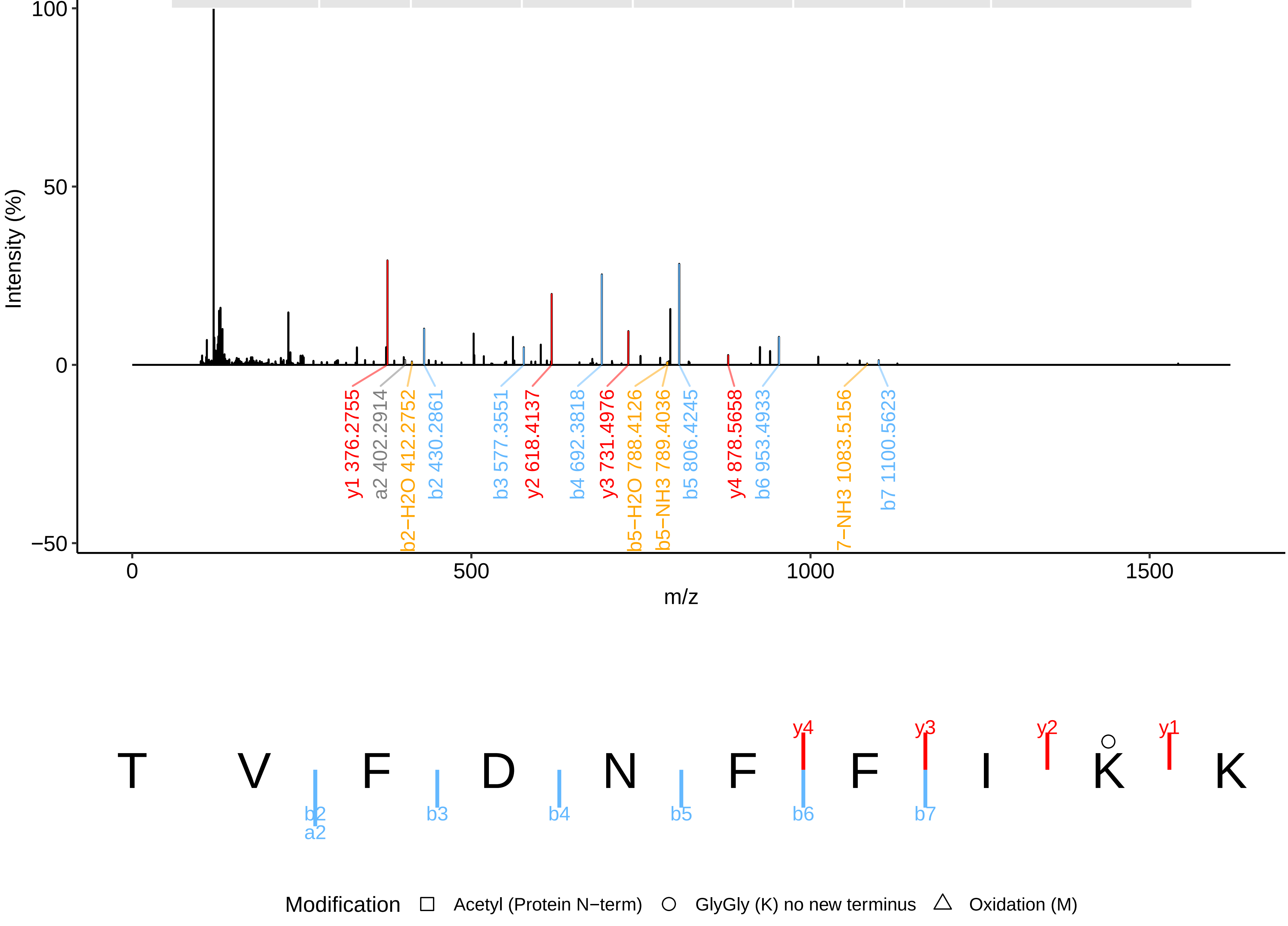

20200921_KIZ_MUb4_GlyGly_10plex − Scan number: 18557

Gene Names	Charge	m/z	Mass	Retention time	PEP	Score	Precursor Intensity
XRCC5	3	459.2575	1374.751	66.774	0.0015343	87.184	NA

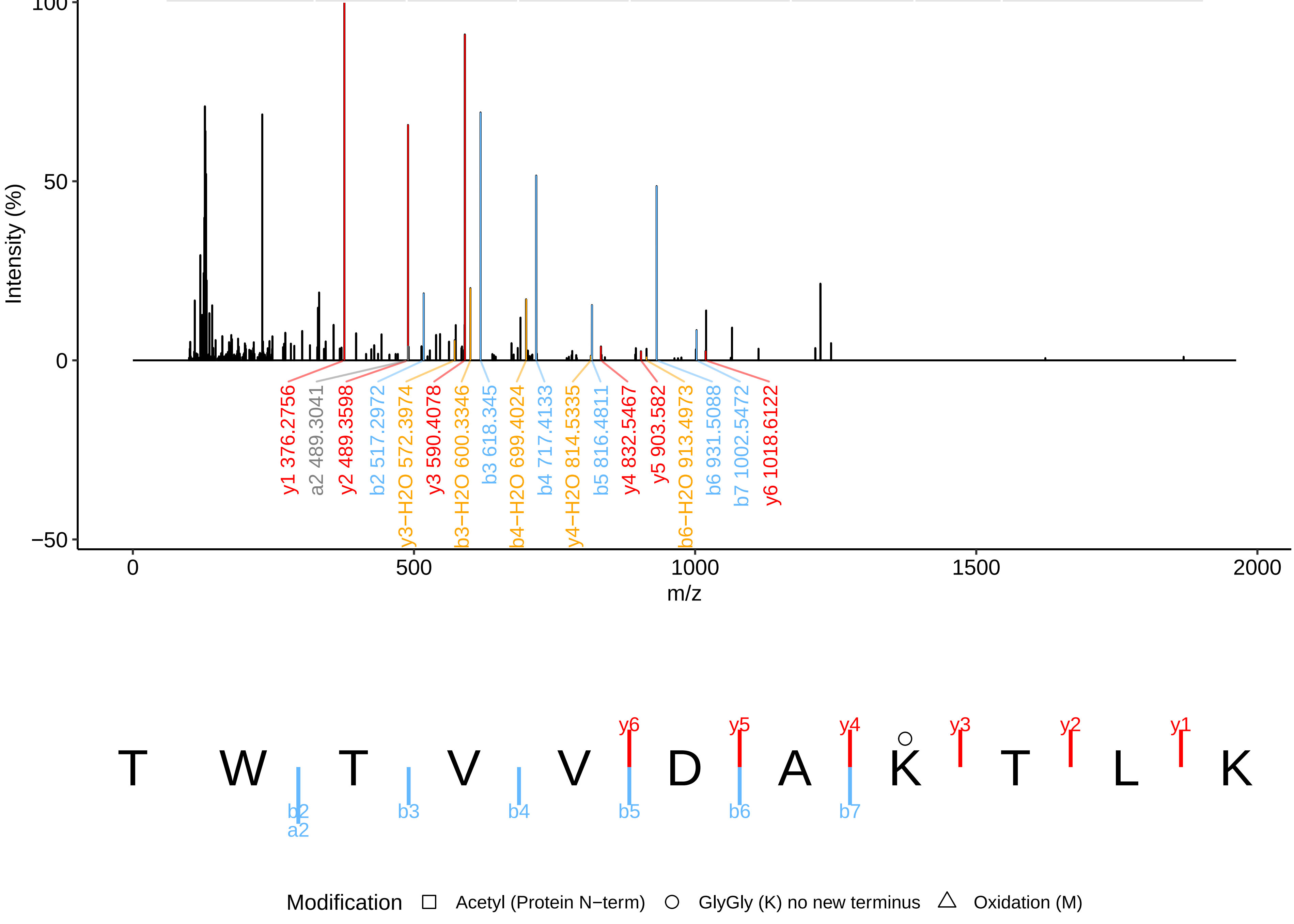

20200921_KIZ_MUb4_GlyGly_10plex_2 − Scan number: 17054

Gene Names	Charge	m/z	Mass	Retention time	PEP	Score	Precursor Intensity
XRCC5	3	459.2575	1374.751	66.638	0.044768	59.542	779336.2

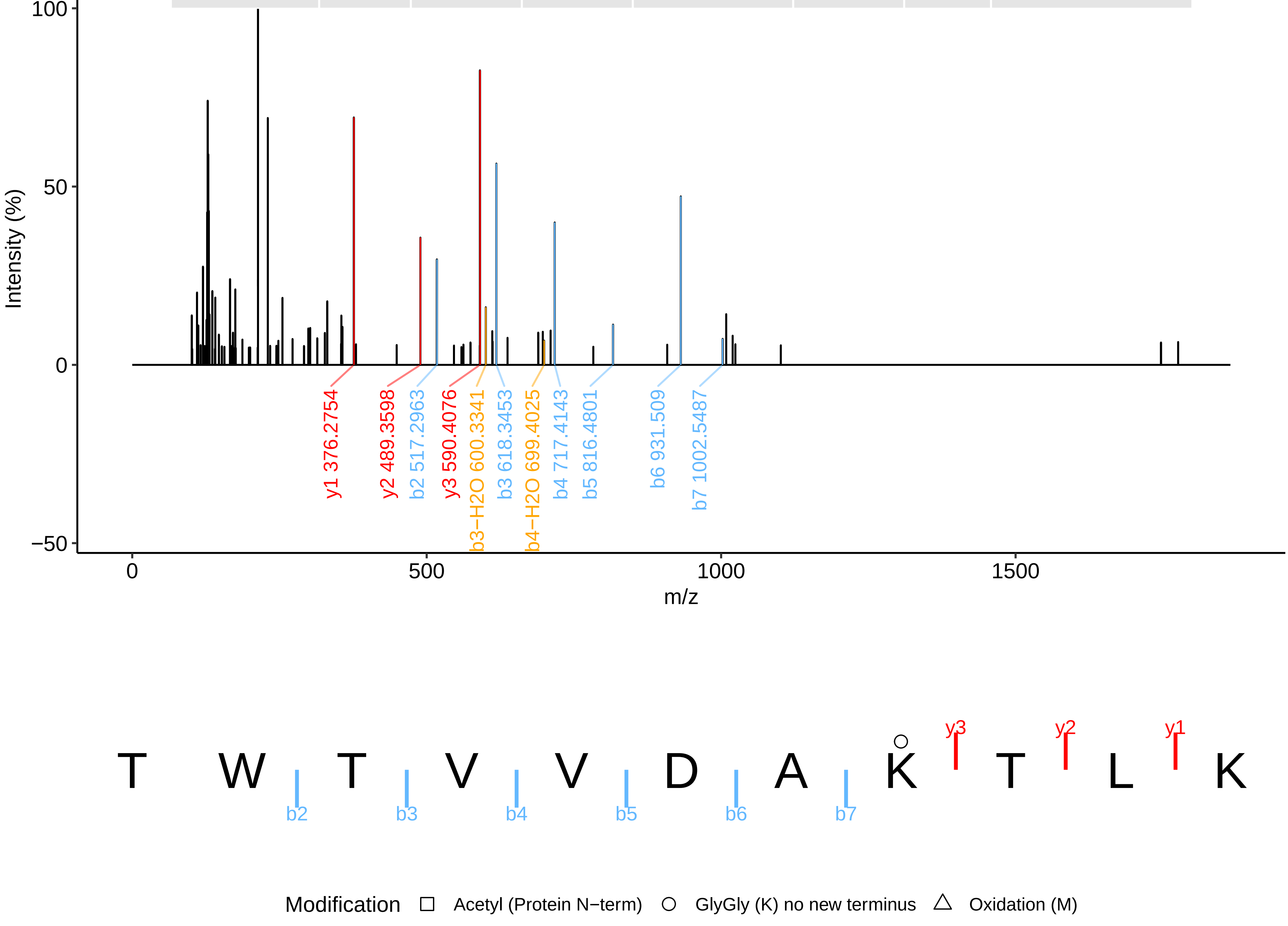

20200921_KIZ_MUb4_GlyGly_10plex − Scan number: 5273

Gene Names	Charge	m/z	Mass	Retention time	PEP	Score	Precursor Intensity
DMD;UTRN	4	335.4336	1337.705	17.241	0.00020862	94.616	10780190

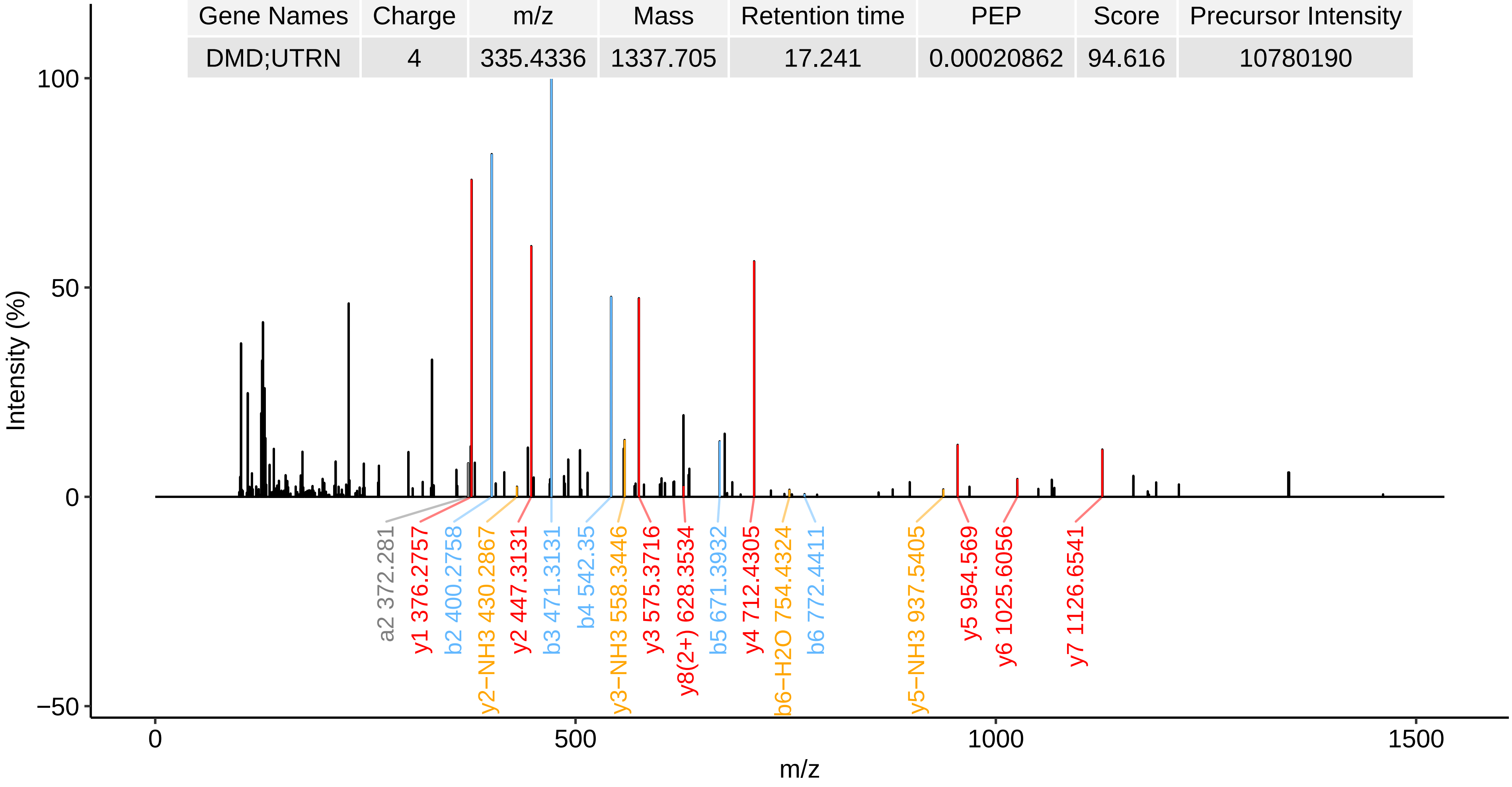

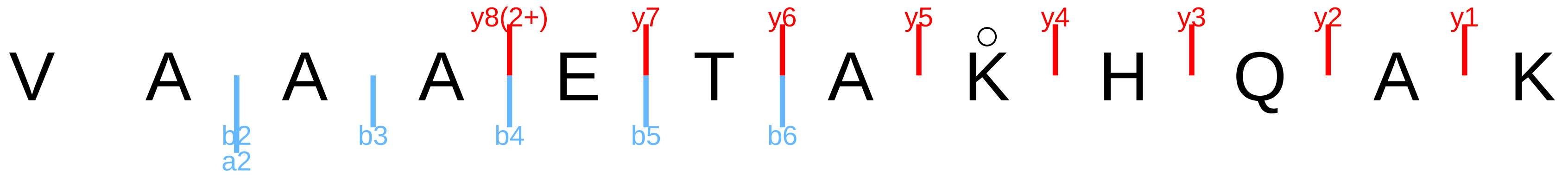

Modification □ Acetyl (Protein N−term) ○ GlyGly (K) no new terminus △ Oxidation (M)

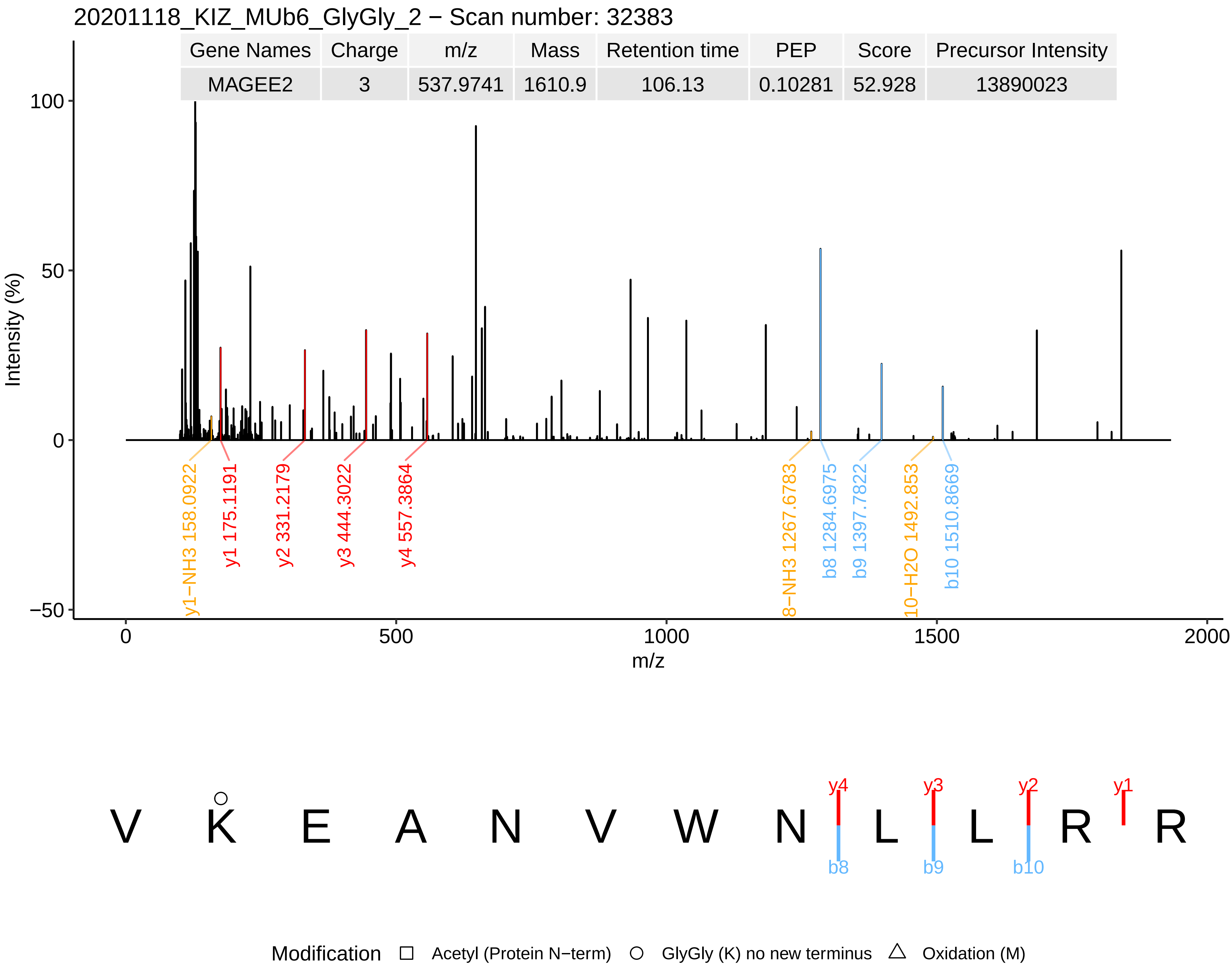
20201118_KIZ_MUb6_GlyGly_2 − Scan number: 32383
Gene Names | Charge | m/z | Mass | Retention time | PEP | Score | Precursor Intensity
MAGEE2 | 3 | 537.9741 | 1610.9 | 106.13 | 0.10281 | 52.928 | 13890023
Intensity (%)
100
50
0
−50
m/z
0
500
1000
1500
2000
y1−NH3 158.0922
y1 175.1191
y2 331.2179
y3 444.3022
y4 557.3864
8−NH3 1267.6783
b8 1284.6975
b9 1397.7822
10−H2O 1492.853
b10 1510.8669
V K E A N V W N L L R R
y4
y3
y2
y1
b8
b9
b10
Modification
Acetyl (Protein N−term)
GlyGly (K) no new terminus
Oxidation (M)

20200921_KIZ_MUb4_GlyGly_10plex_2 − Scan number: 17948

Gene Names	Charge	m/z	Mass	Retention time	PEP	Score	Precursor Intensity
MTX2	3	533.6084	1597.803	70.231	0.00173	79.82	1633096

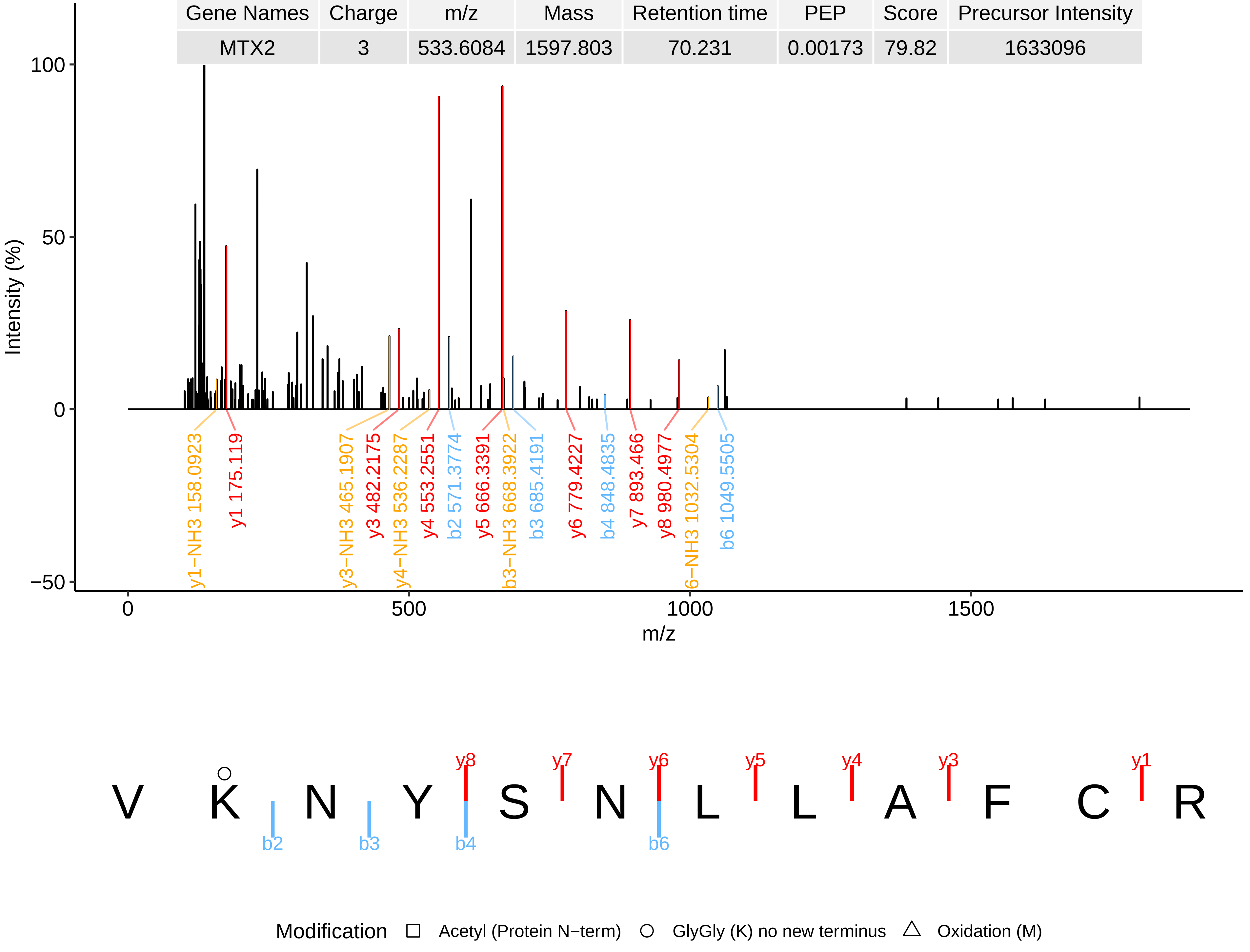

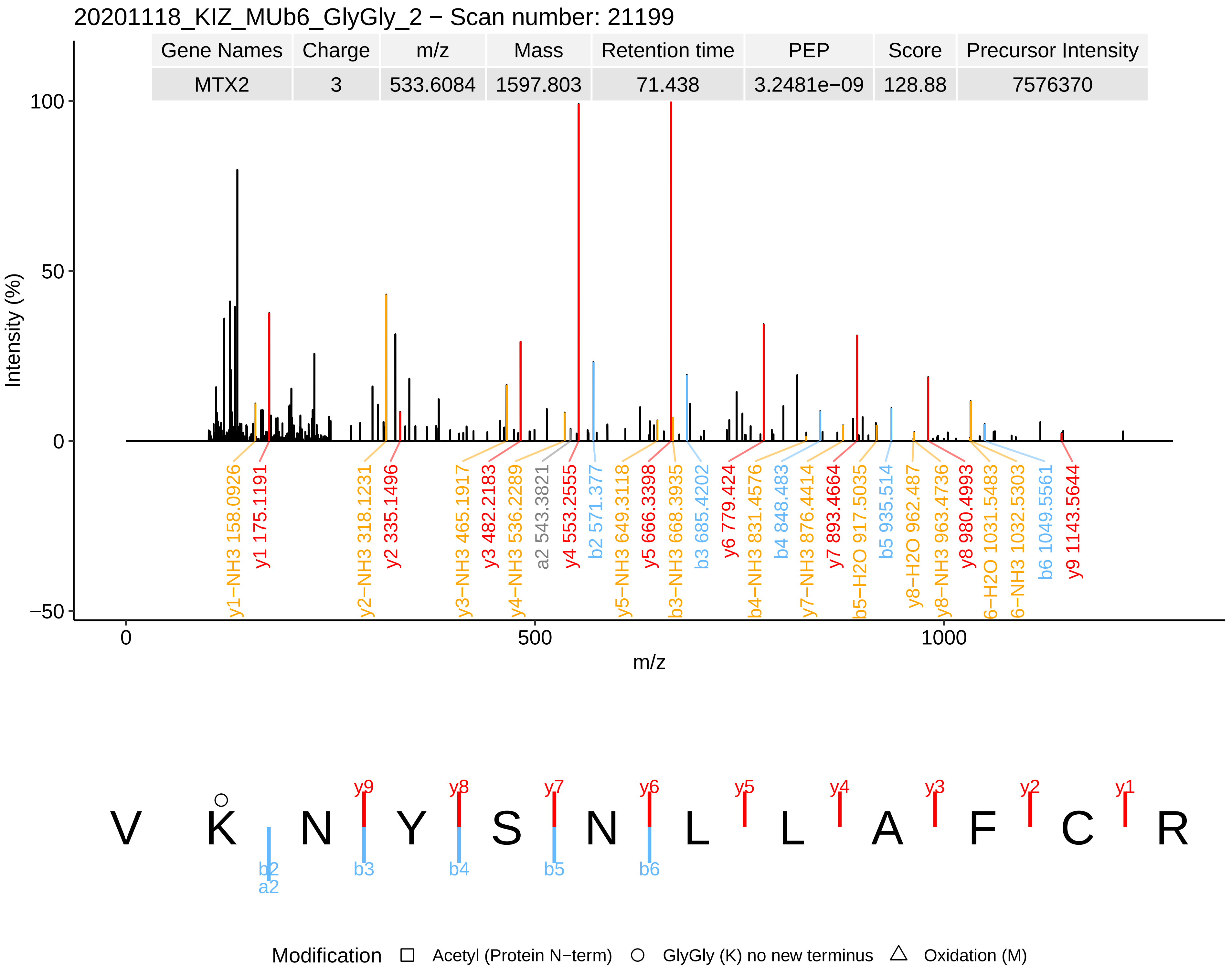

Gene Names	Charge	m/z	Mass	Retention time	PEP	Score	Precursor Intensity
MTX2	3	533.6084	1597.803	71.438	3.2481e−09	128.88	7576370

20201118_KIZ_MUb6_GlyGly_2 − Scan number: 21200

Gene Names	Charge	m/z	Mass	Retention time	PEP	Score	Precursor Intensity
MTX2	3	533.6084	1597.803	71.443	8.4334e−06	108.35	6935219

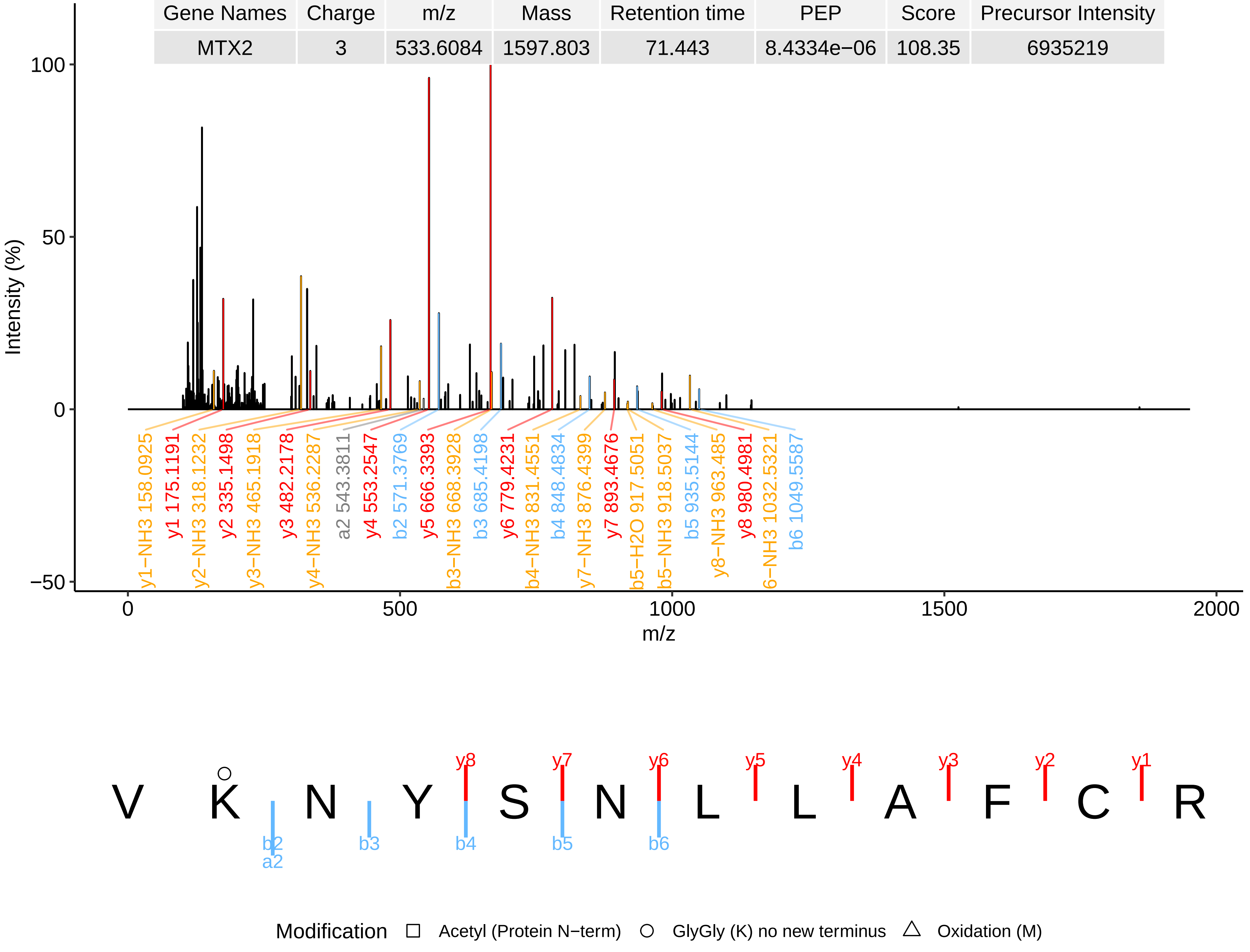

20200921_KIZ_MUb4_GlyGly_10plex − Scan number: 15389

Gene Names	Charge	m/z	Mass	Retention time	PEP	Score	Precursor Intensity
DDI2	3	443.914	1328.72	54.961	0.0007644	87.184	4568224

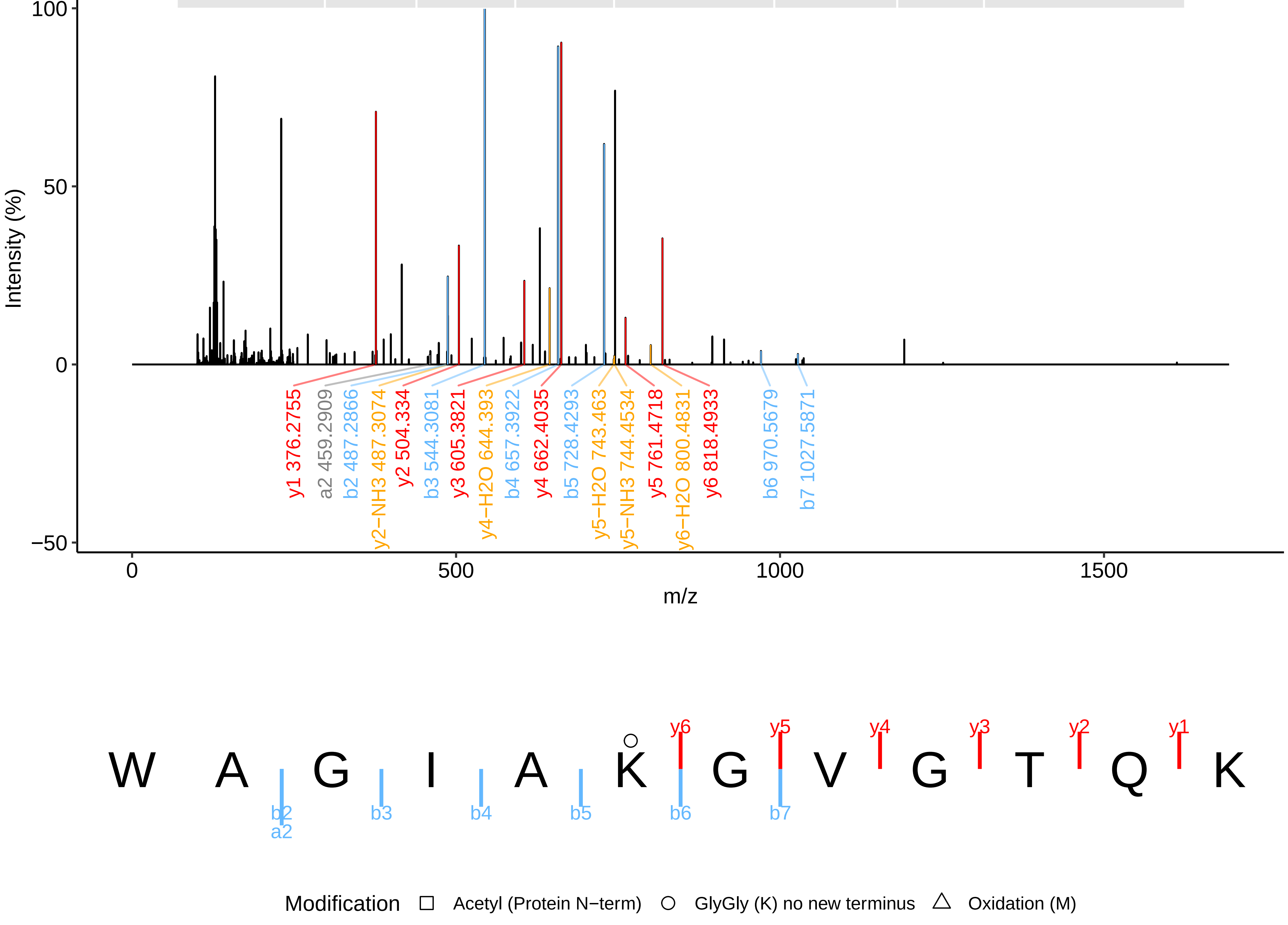

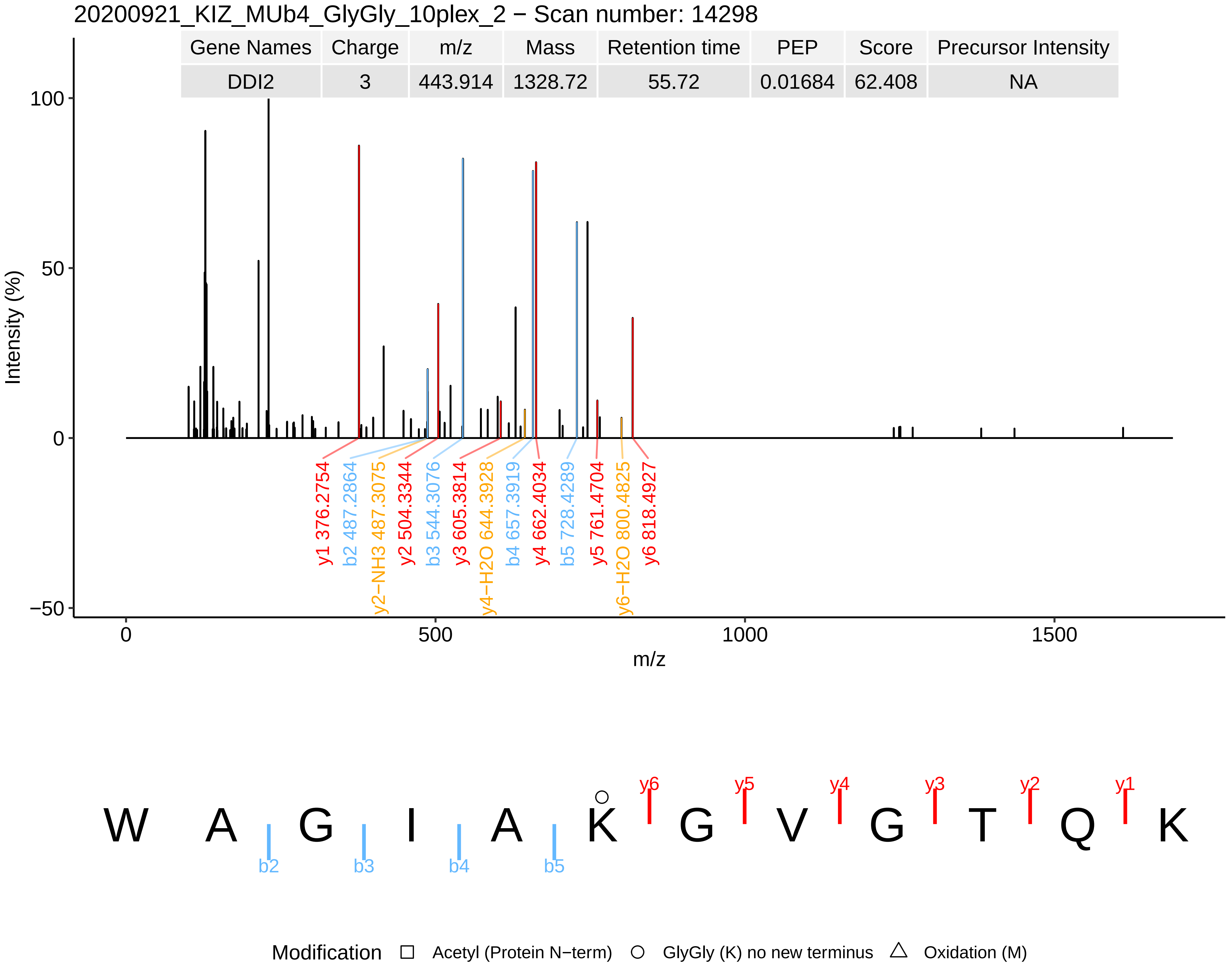
20200921_KIZ_MUb4_GlyGly_10plex_2 − Scan number: 14298
Gene Names | Charge | m/z | Mass | Retention time | PEP | Score | Precursor Intensity
DDI2 | 3 | 443.914 | 1328.72 | 55.72 | 0.01684 | 62.408 | NA
Intensity (%)
m/z
y1 376.2754
b2 487.2864
y2−NH3 487.3075
y2 504.3344
b3 544.3076
y3 605.3814
y4−H2O 644.3928
b4 657.3919
y4 662.4034
b5 728.4289
y5 761.4704
y6−H2O 800.4825
y6 818.4927
W A G I A K G V G T Q K
b2 b3 b4 b5
y6 y5 y4 y3 y2 y1
Modification
Acetyl (Protein N−term)
GlyGly (K) no new terminus
Oxidation (M)

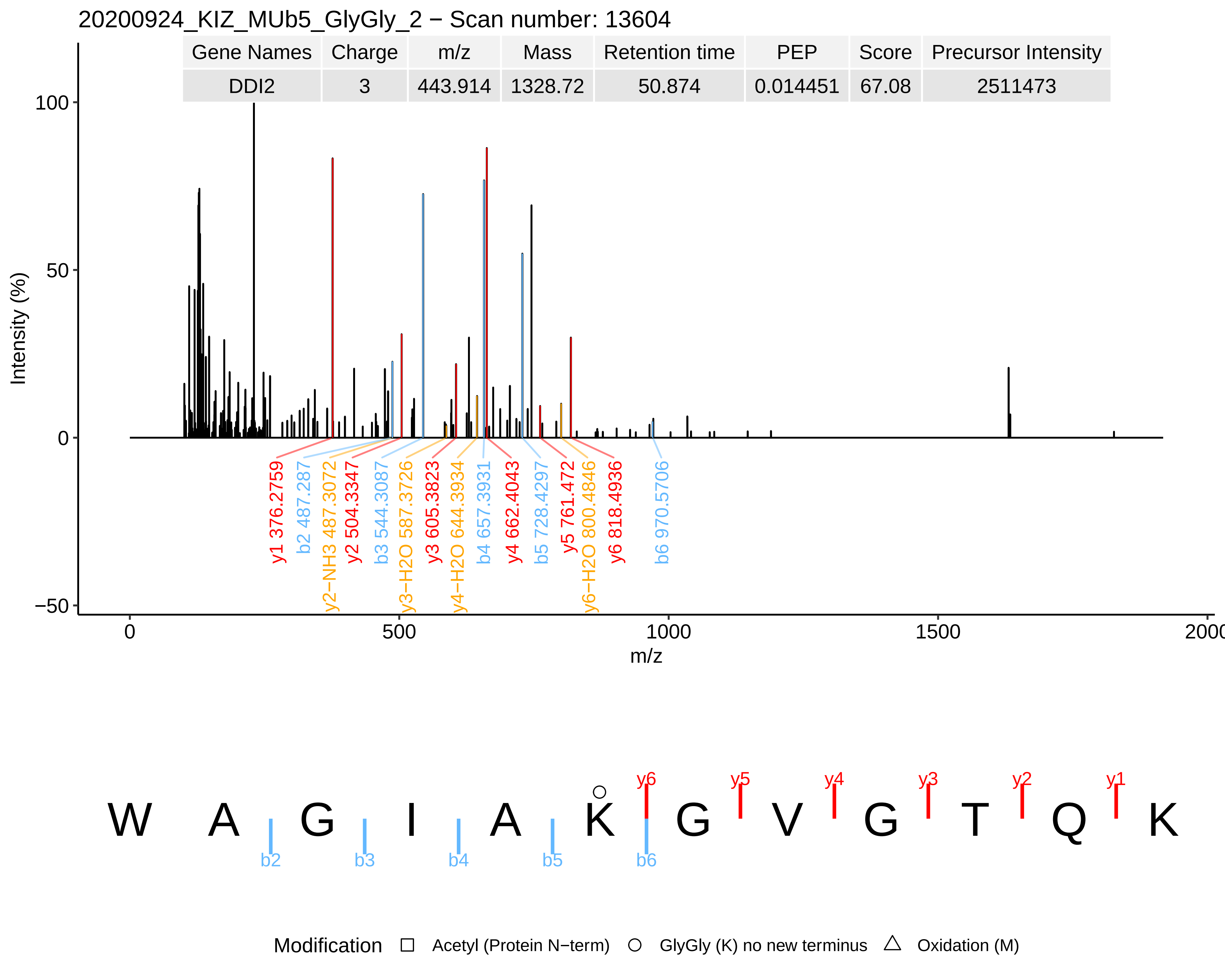

20200924_KIZ_MUb5_GlyGly_2 − Scan number: 13604
Gene Names
DDI2
Charge
3
m/z
443.914
Mass
1328.72
Retention time
50.874
PEP
0.014451
Score
67.08
Precursor Intensity
2511473
Intensity (%)
100
50
0
−50
0
500
1000
1500
2000
m/z
y1 376.2759
b2 487.287
y2−NH3 487.3072
y2 504.3347
b3 544.3087
y3−H2O 587.3726
y3 605.3823
y4−H2O 644.3934
b4 657.3931
y4 662.4043
b5 728.4297
y5 761.472
y6−H2O 800.4846
y6 818.4936
b6 970.5706
W A G I A K G V G T Q K
y6
y5
y4
y3
y2
y1
b2
b3
b4
b5
b6
Modification
Acetyl (Protein N−term)
GlyGly (K) no new terminus
Oxidation (M)

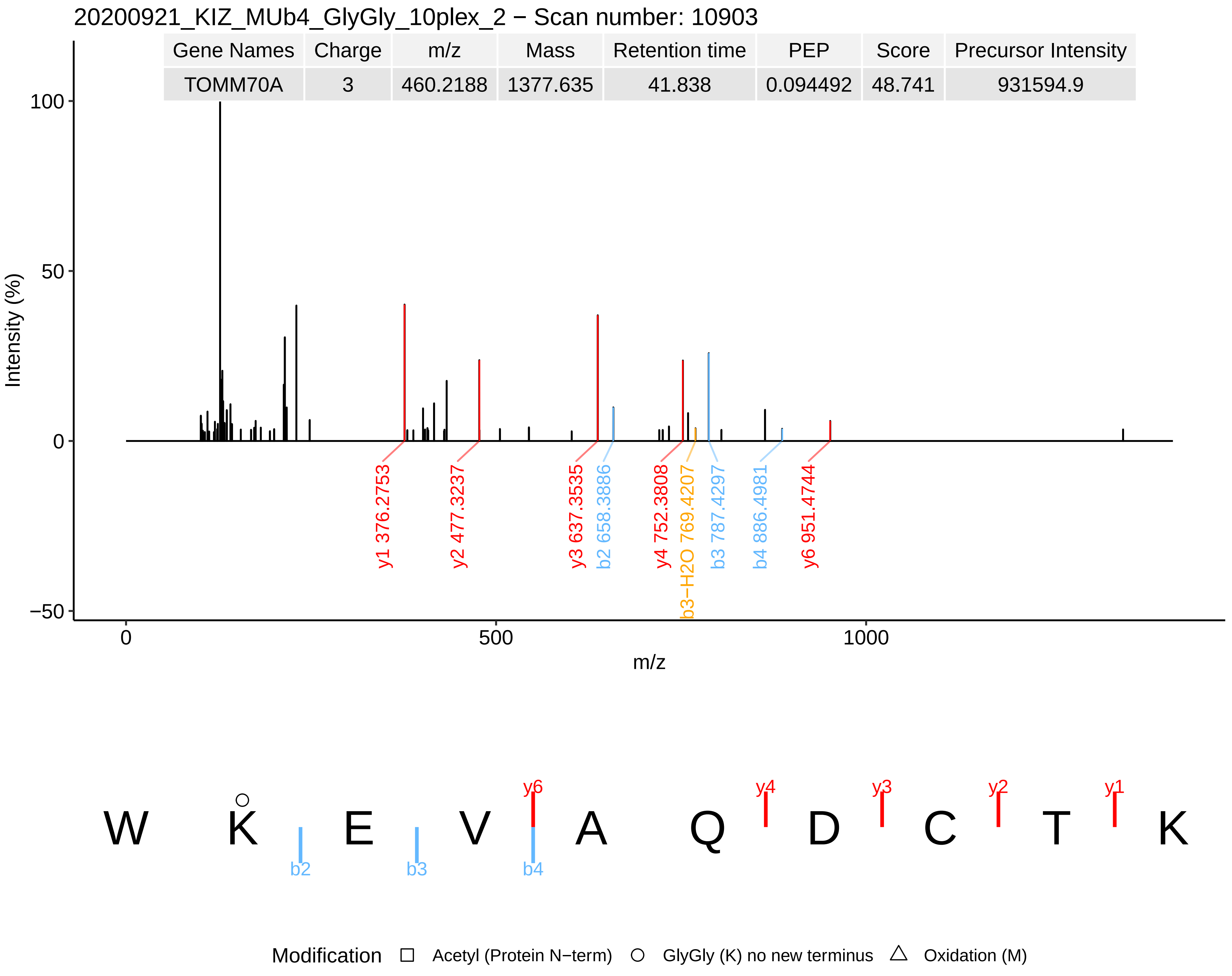
20200921_KIZ_MUb4_GlyGly_10plex_2 − Scan number: 10903
Gene Names
Charge
m/z
Mass
Retention time
PEP
Score
Precursor Intensity
TOMM70A
3
460.2188
1377.635
41.838
0.094492
48.741
931594.9
Intensity (%)
100
50
0
−50
0
500
1000
m/z
y1 376.2753
y2 477.3237
y3 637.3535
b2 658.3886
y4 752.3808
b3−H2O 769.4207
b3 787.4297
b4 886.4981
y6 951.4744
W
K
E
V
A
Q
D
C
T
K
y6
y4
y3
y2
y1
b2
b3
b4
Modification
Acetyl (Protein N−term)
GlyGly (K) no new terminus
Oxidation (M)

20201118_KIZ_MUb6_GlyGly_2 − Scan number: 12538

Gene Names	Charge	m/z	Mass	Retention time	PEP	Score	Precursor Intensity
TOMM70A	3	460.2188	1377.635	42.21	0.020777	65.241	1124582

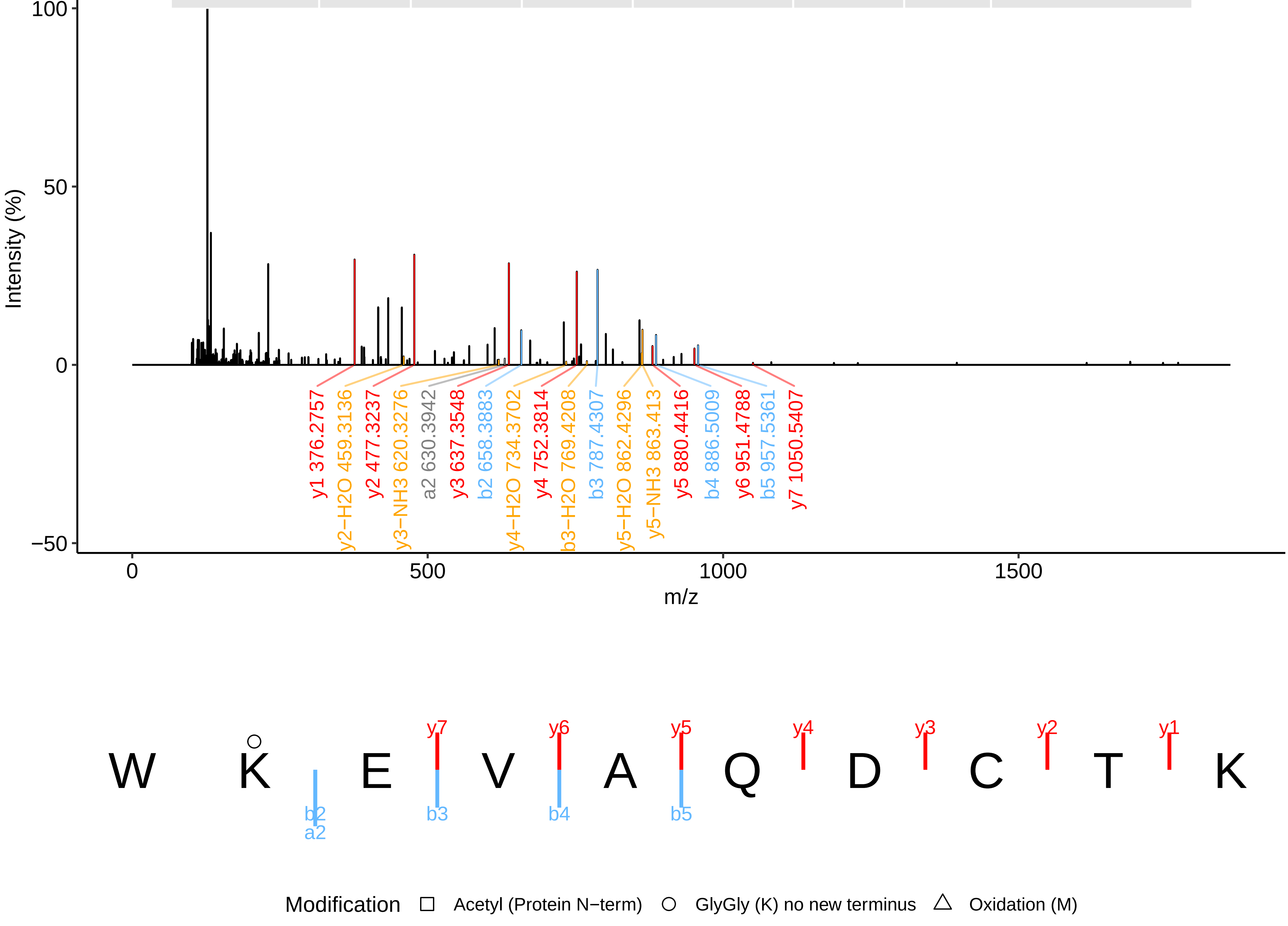

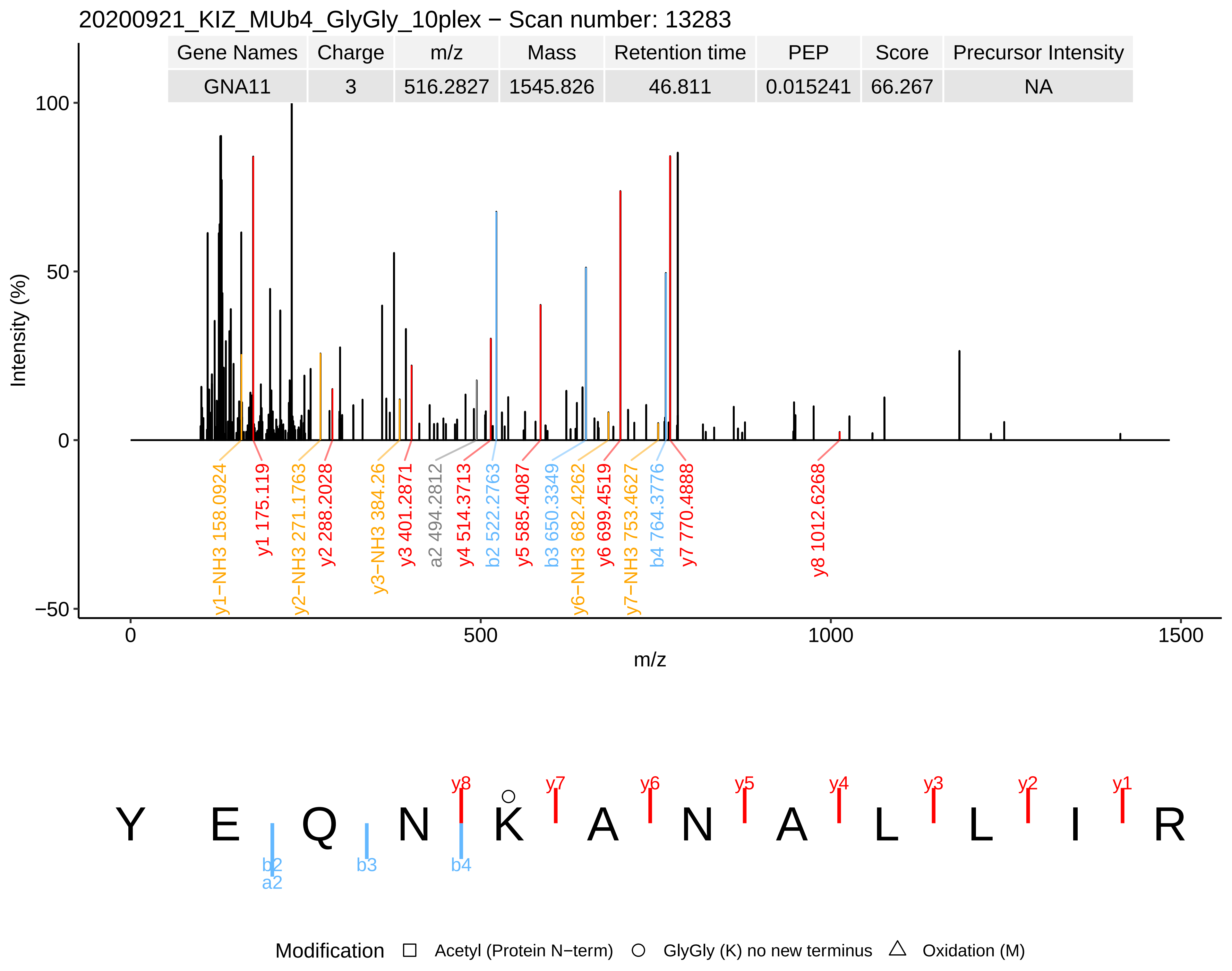
20200921_KIZ_MUb4_GlyGly_10plex − Scan number: 13283
Gene Names | Charge | m/z | Mass | Retention time | PEP | Score | Precursor Intensity
GNA11 | 3 | 516.2827 | 1545.826 | 46.811 | 0.015241 | 66.267 | NA
Intensity (%)
100
50
0
−50
0
500
1000
1500
m/z
y1−NH3 158.0924
y1 175.119
y2−NH3 271.1763
y2 288.2028
y3−NH3 384.26
y3 401.2871
a2 494.2812
y4 514.3713
b2 522.2763
y5 585.4087
b3 650.3349
y6−NH3 682.4262
y6 699.4519
y7−NH3 753.4627
b4 764.3776
y7 770.4888
y8 1012.6268
Y E Q N K A N A L L I R
y8 y7 y6 y5 y4 y3 y2 y1
b2 a2 b3 b4
Modification
Acetyl (Protein N−term)
GlyGly (K) no new terminus
Oxidation (M)

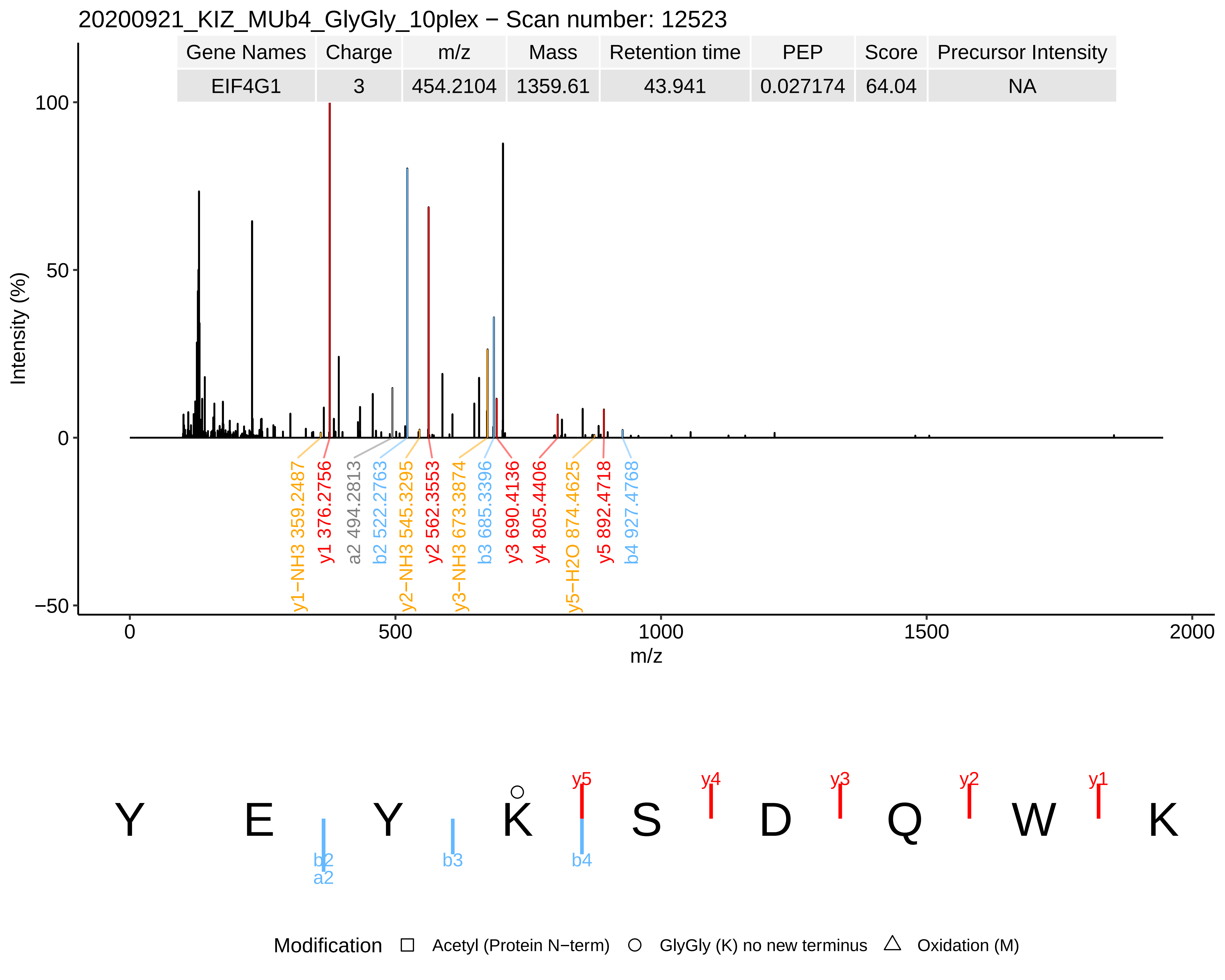

20200921_KIZ_MUb4_GlyGly_10plex − Scan number: 12523
Gene Names | Charge | m/z | Mass | Retention time | PEP | Score | Precursor Intensity
EIF4G1 | 3 | 454.2104 | 1359.61 | 43.941 | 0.027174 | 64.04 | NA
Intensity (%)
100
50
0
−50
0
500
1000
1500
2000
m/z
y1−NH3 359.2487
y1 376.2756
a2 494.2813
b2 522.2763
y2−NH3 545.3295
y2 562.3553
y3−NH3 673.3874
b3 685.3396
y3 690.4136
y4 805.4406
y5−H2O 874.4625
y5 892.4718
b4 927.4768
Y E Y K S D Q W K
y5
y4
y3
y2
y1
b2
a2
b3
b4
Modification
Acetyl (Protein N−term)
GlyGly (K) no new terminus
Oxidation (M)

20200921_KIZ_MUb4_GlyGly_10plex − Scan number: 12535

Gene Names	Charge	m/z	Mass	Retention time	PEP	Score	Precursor Intensity
EIF4G1	3	454.2104	1359.61	43.989	0.018904	70.552	5068668

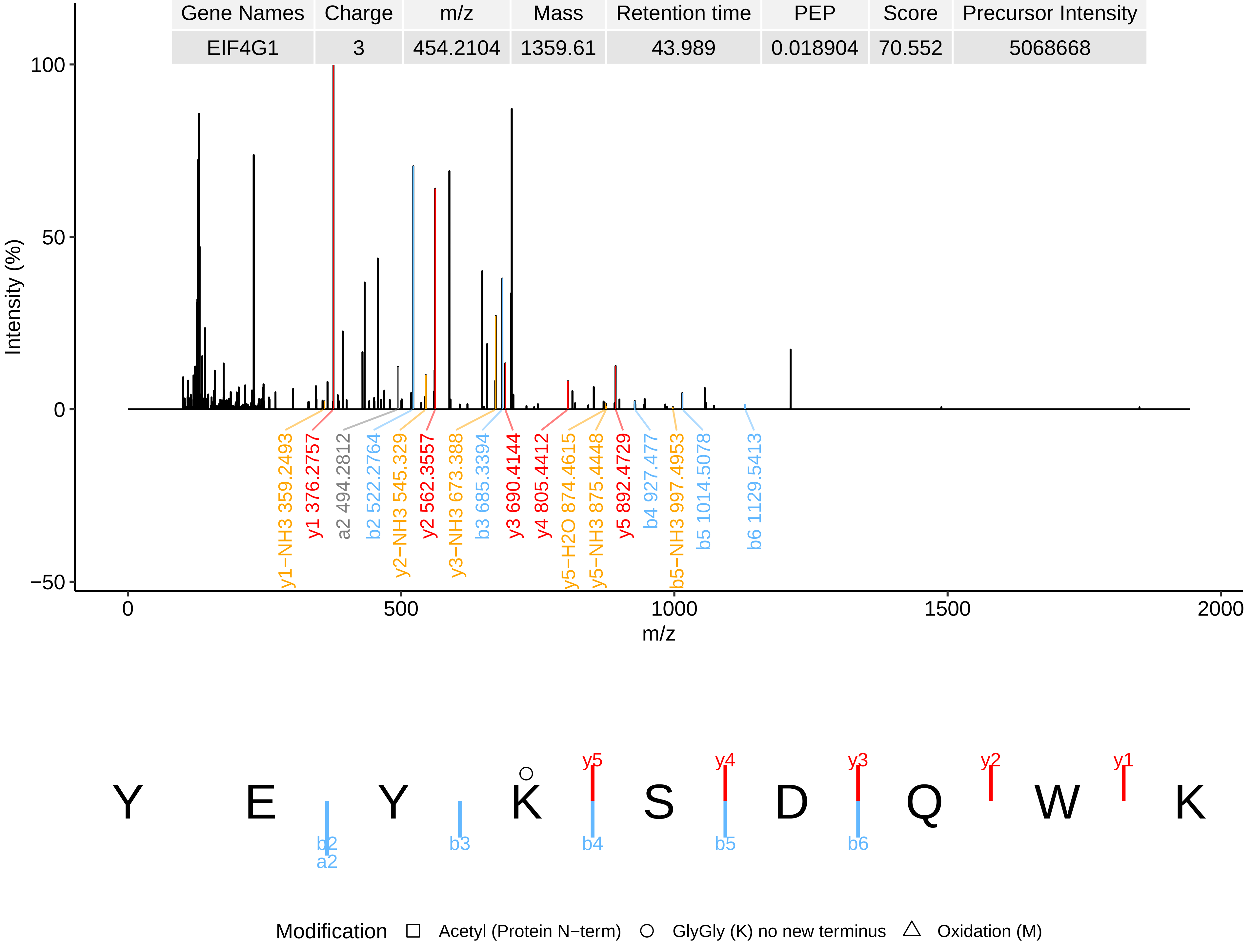

20200921_KIZ_MUb4_GlyGly_10plex_2 − Scan number: 15261

Gene Names	Charge	m/z	Mass	Retention time	PEP	Score	Precursor Intensity
PTTG1IP	3	534.2633	1599.768	59.641	0.058753	54.343	837754.8

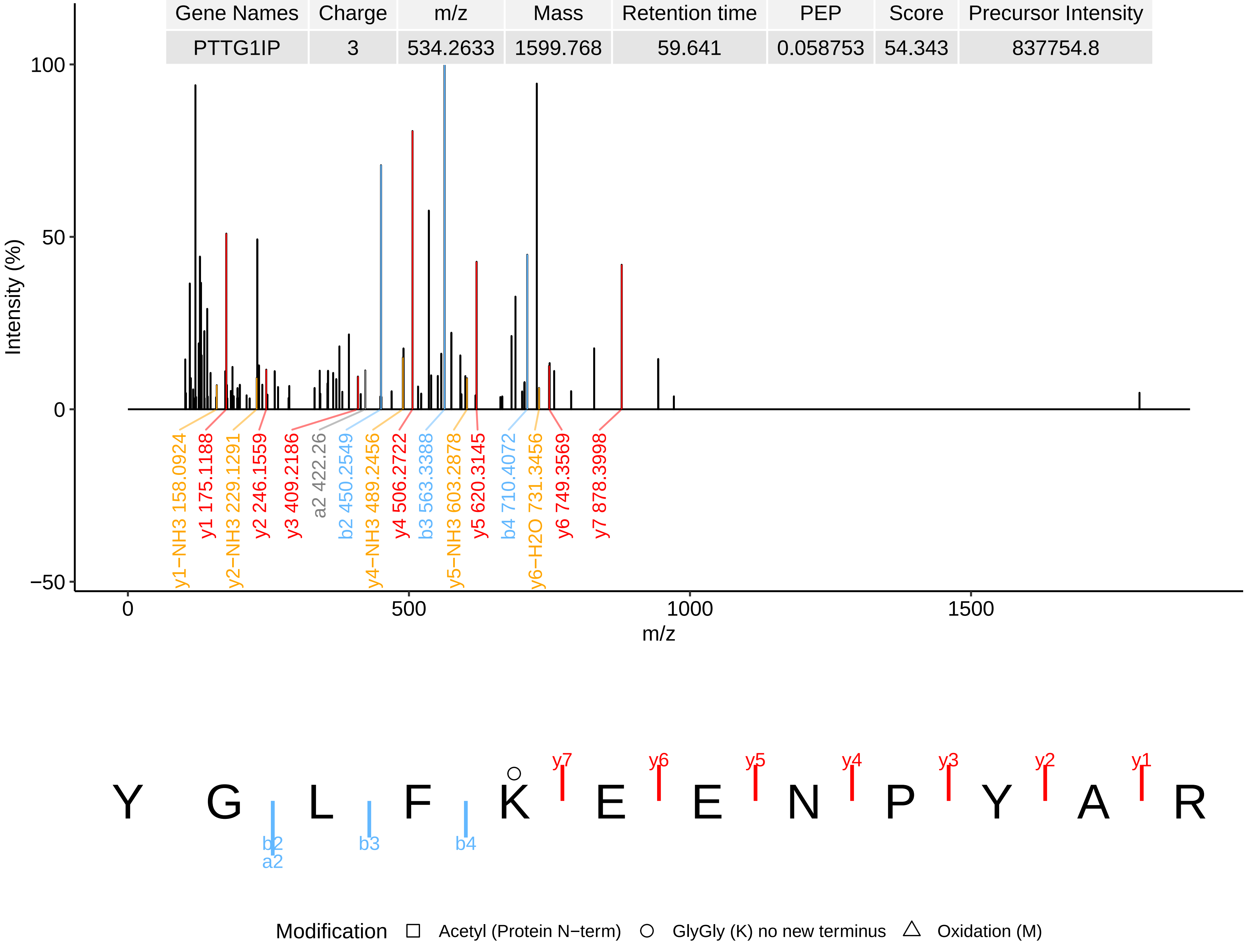

20200921_KIZ_MUb4_GlyGly_10plex_2 − Scan number: 10801

Gene Names	Charge	m/z	Mass	Retention time	PEP	Score	Precursor Intensity
ABCF2	3	520.9546	1559.842	41.405	0.0004231	93.623	NA

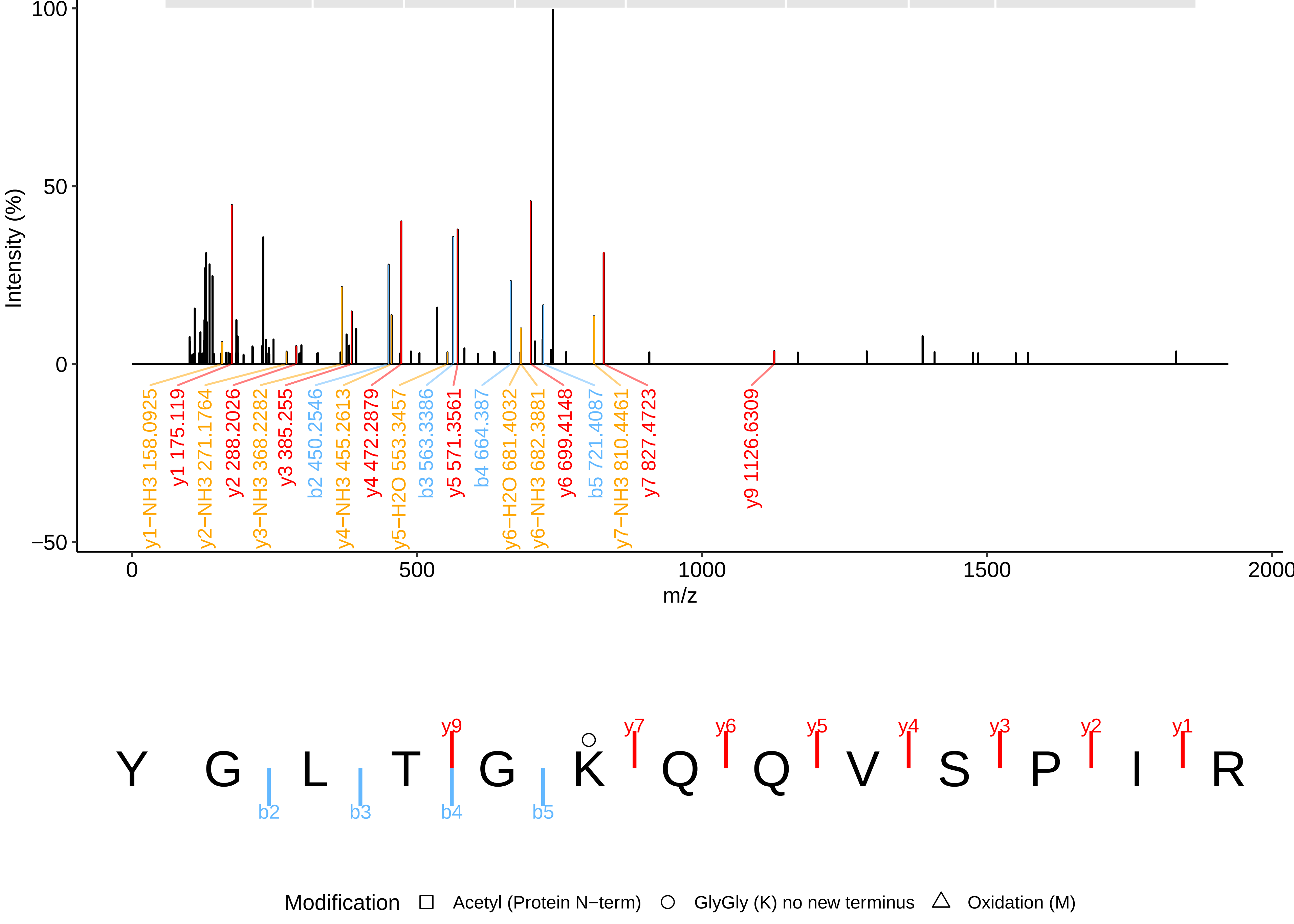

20200921_KIZ_MUb4_GlyGly_10plex − Scan number: 21227

Gene Names	Charge	m/z	Mass	Retention time	PEP	Score	Precursor Intensity
PSMC2	3	446.5907	1336.75	76.829	0.0016231	87.308	6017801

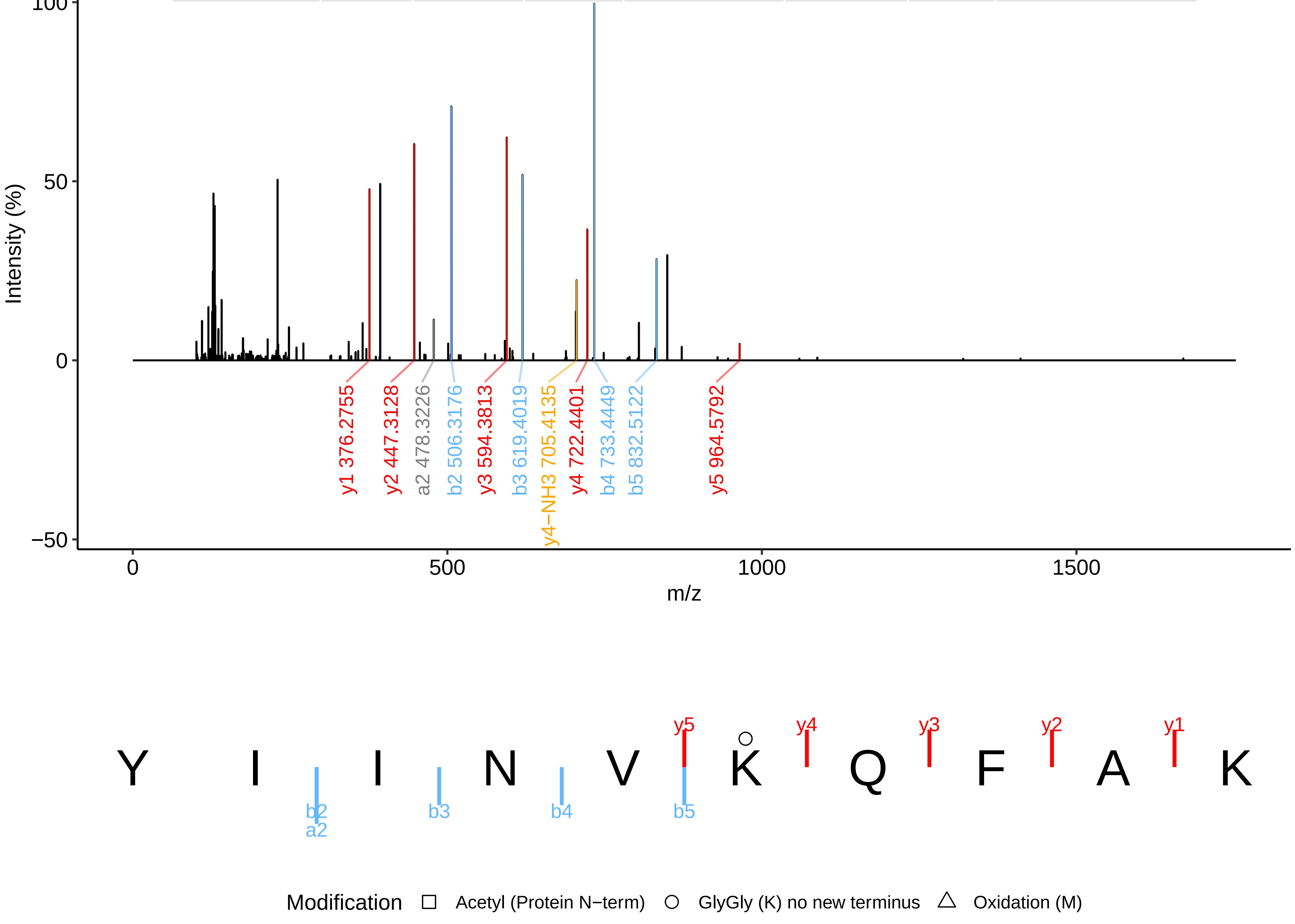

20200921_KIZ_MUb4_GlyGly_10plex_2 − Scan number: 19379

Gene Names	Charge	m/z	Mass	Retention time	PEP	Score	Precursor Intensity
PSMC2	3	446.5907	1336.75	76.06	0.0016231	87.308	751919.4

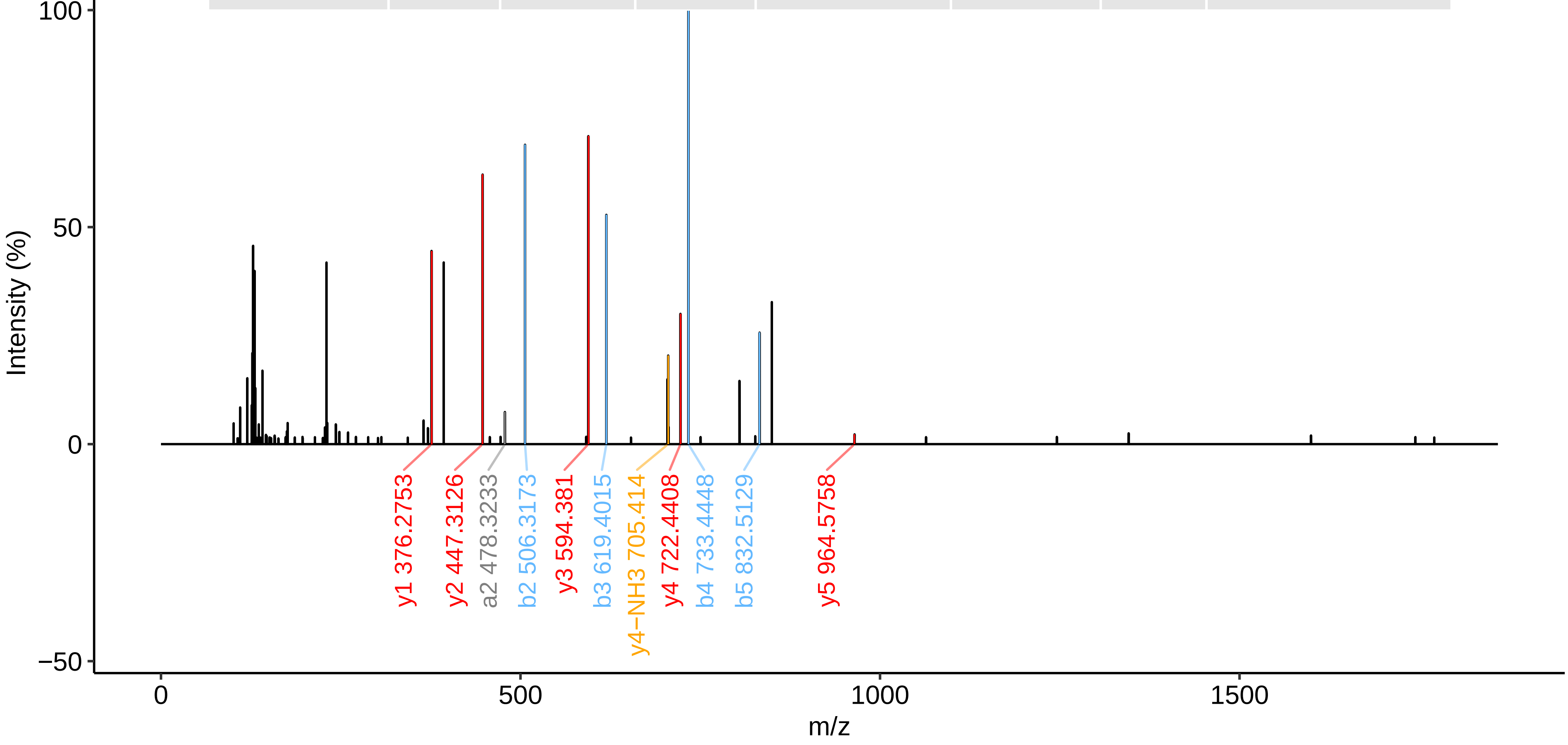

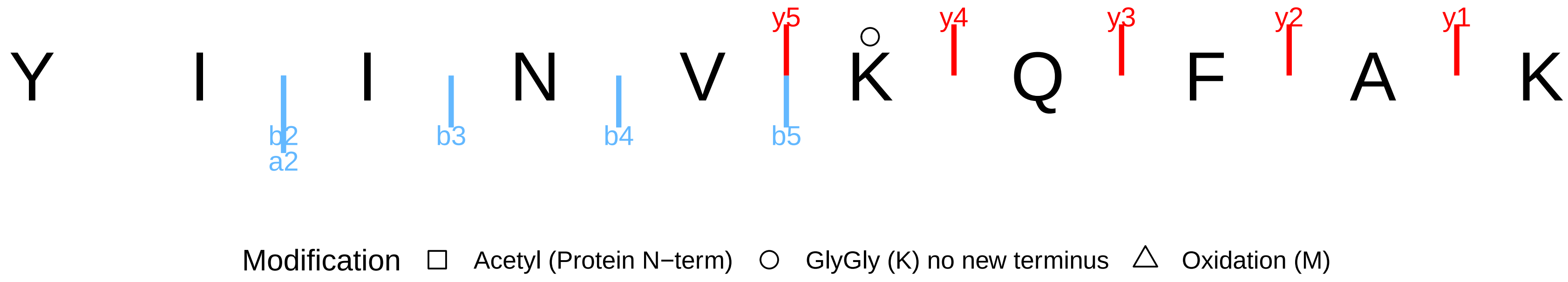

20200921_KIZ_MUb4_GlyGly_10plex_2 − Scan number: 12722

Gene Names	Charge	m/z	Mass	Retention time	PEP	Score	Precursor Intensity
NUDT21	3	535.2983	1602.873	49.283	0.010795	66.267	705055.6

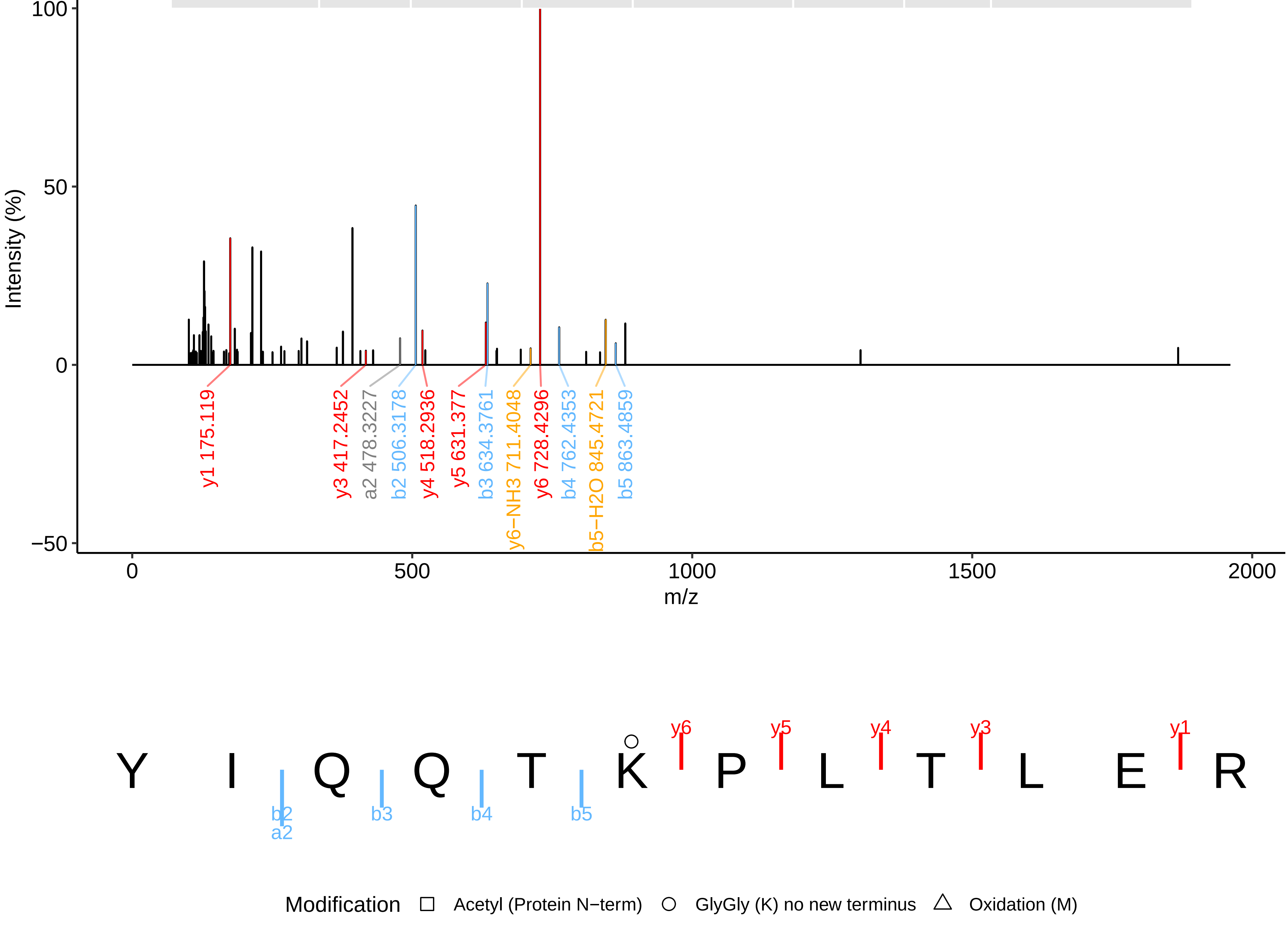

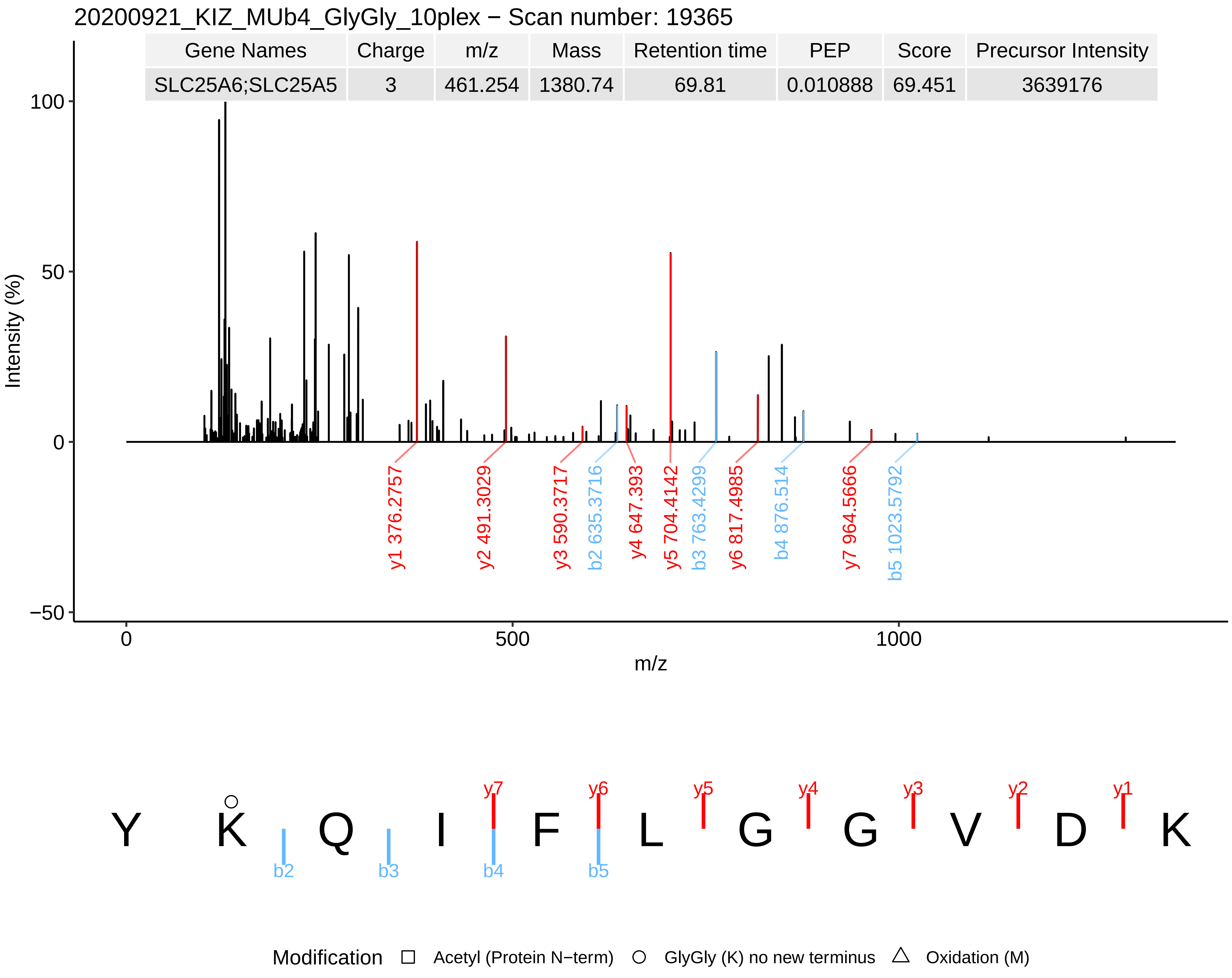
20200921_KIZ_MUb4_GlyGly_10plex − Scan number: 19365
Gene Names | Charge | m/z | Mass | Retention time | PEP | Score | Precursor Intensity
SLC25A6;SLC25A5 | 3 | 461.254 | 1380.74 | 69.81 | 0.010888 | 69.451 | 3639176
Intensity (%)
100
50
0
−50
0
500
1000
m/z
y1 376.2757
y2 491.3029
y3 590.3717
b2 635.3716
y4 647.393
y5 704.4142
b3 763.4299
y6 817.4985
b4 876.514
y7 964.5666
b5 1023.5792
Y K Q I F L G G V D K
y7 y6 y5 y4 y3 y2 y1
b2 b3 b4 b5
Modification
Acetyl (Protein N−term)
GlyGly (K) no new terminus
Oxidation (M)

20200921_KIZ_MUb4_GlyGly_10plex − Scan number: 16094

Gene Names	Charge	m/z	Mass	Retention time	PEP	Score	Precursor Intensity
RPL30	3	442.9187	1325.734	57.727	0.00031722	96.331	5352966

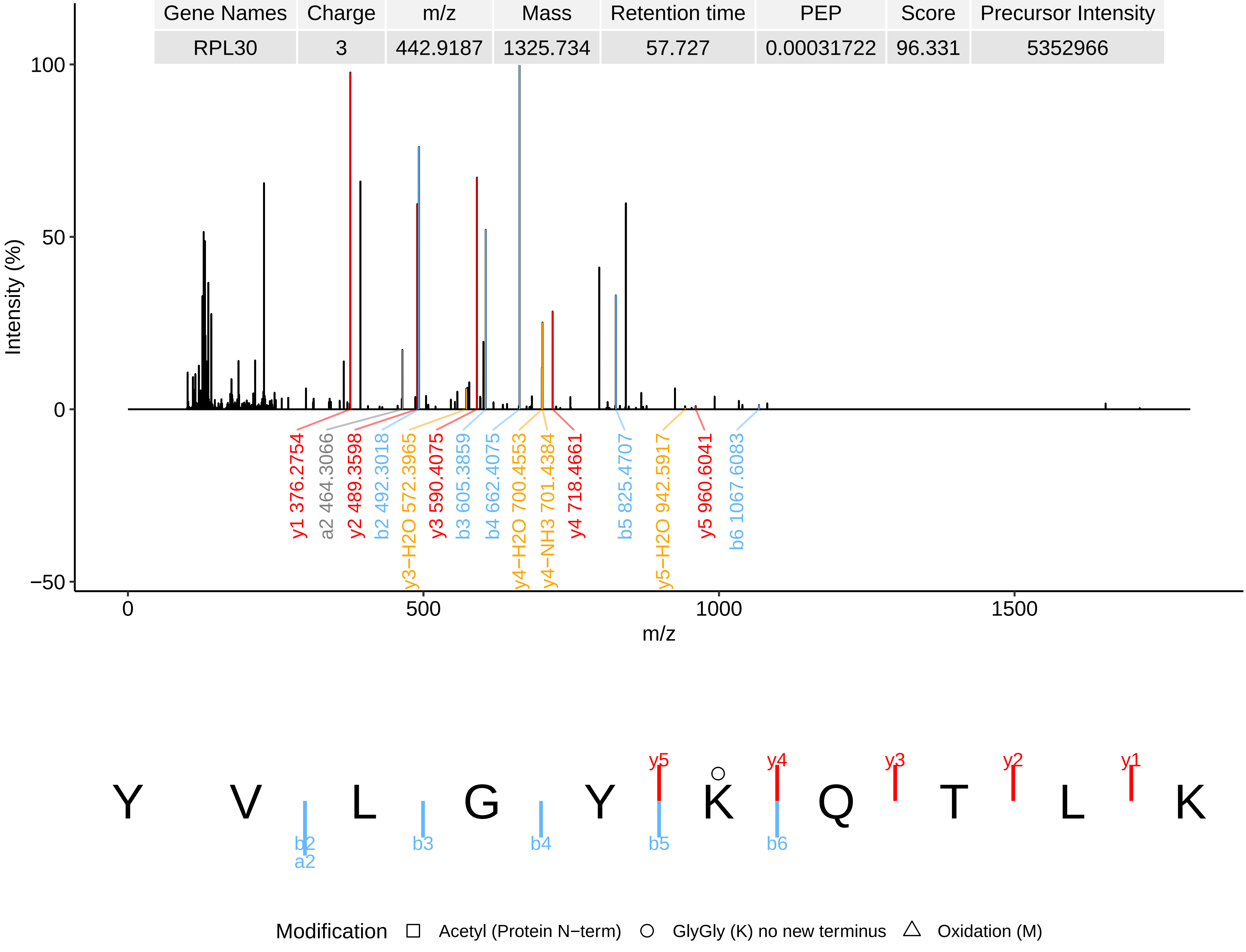

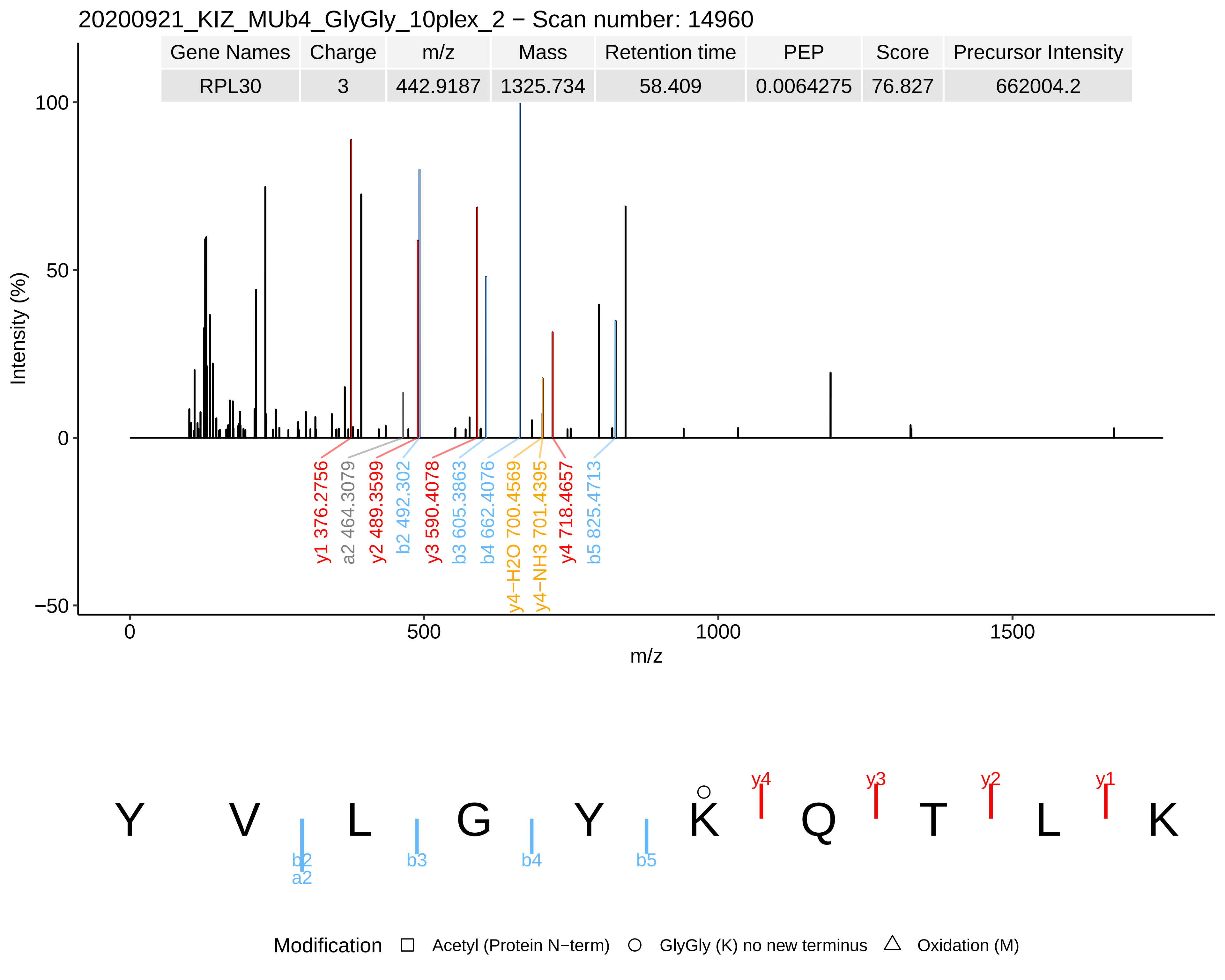

20200921_KIZ_MUb4_GlyGly_10plex_2 − Scan number: 14960
Gene Names | Charge | m/z | Mass | Retention time | PEP | Score | Precursor Intensity
RPL30 | 3 | 442.9187 | 1325.734 | 58.409 | 0.0064275 | 76.827 | 662004.2
Intensity (%)
100
50
0
−50
0
500
1000
1500
m/z
y1 376.2756
a2 464.3079
y2 489.3599
b2 492.302
y3 590.4078
b3 605.3863
b4 662.4076
y4−H2O 700.4569
y4−NH3 701.4395
y4 718.4657
b5 825.4713
Y V L G Y K Q T L K
y4 y3 y2 y1
b2 a2 b3 b4 b5
Modification
Acetyl (Protein N−term)
GlyGly (K) no new terminus
Oxidation (M)

20200921_KIZ_MUb4_GlyGly_10plex_2 − Scan number: 14998

Gene Names	Charge	m/z	Mass	Retention time	PEP	Score	Precursor Intensity
RPL30	3	442.9187	1325.734	58.565	0.062428	54.982	1479093

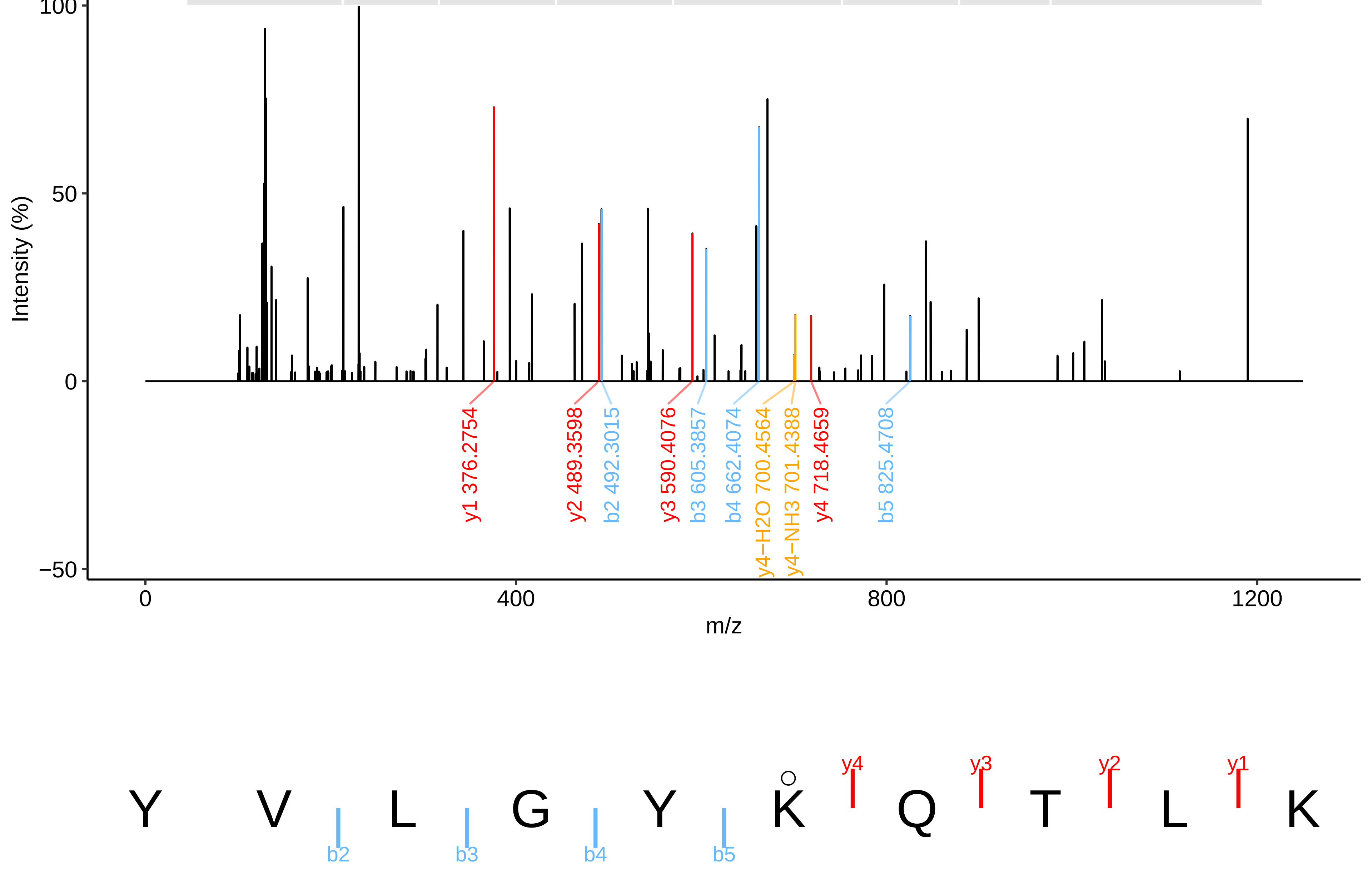

Modification □ Acetyl (Protein N−term) ○ GlyGly (K) no new terminus △ Oxidation (M)

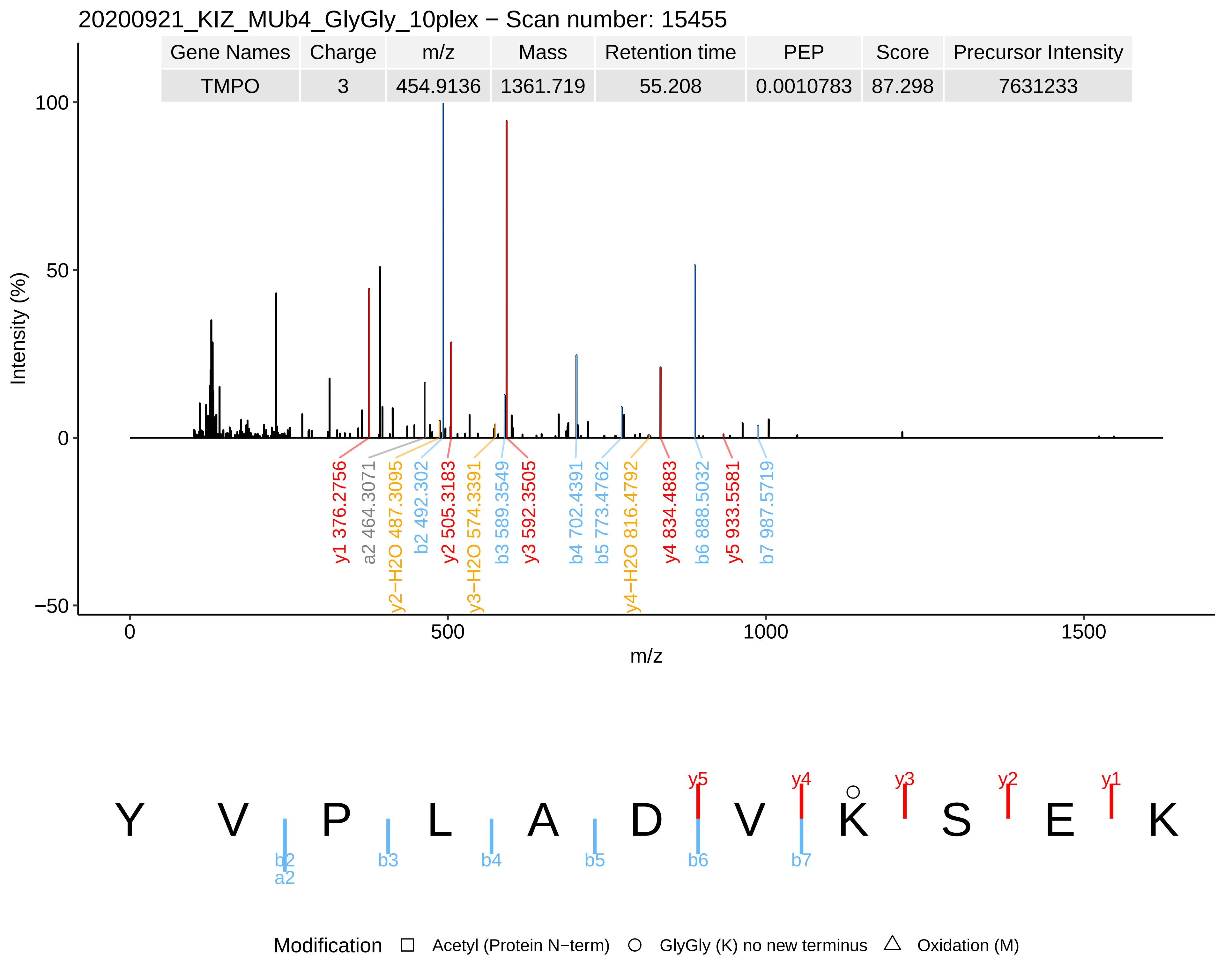

20200921_KIZ_MUb4_GlyGly_10plex − Scan number: 15455
Gene Names | Charge | m/z | Mass | Retention time | PEP | Score | Precursor Intensity
TMPO | 3 | 454.9136 | 1361.719 | 55.208 | 0.0010783 | 87.298 | 7631233
Intensity (%)
m/z
y1 376.2756
a2 464.3071
y2−H2O 487.3095
b2 492.302
y2 505.3183
y3−H2O 574.3391
b3 589.3549
y3 592.3505
b4 702.4391
b5 773.4762
y4−H2O 816.4792
y4 834.4883
b6 888.5032
y5 933.5581
b7 987.5719
Y V P L A D V K S E K
Modification
Acetyl (Protein N−term)
GlyGly (K) no new terminus
Oxidation (M)

20200921_KIZ_MUb4_GlyGly_10plex − Scan number: 15465

Gene Names	Charge	m/z	Mass	Retention time	PEP	Score	Precursor Intensity
TMPO	3	454.9136	1361.719	55.245	2.2717e−05	106.62	10290163

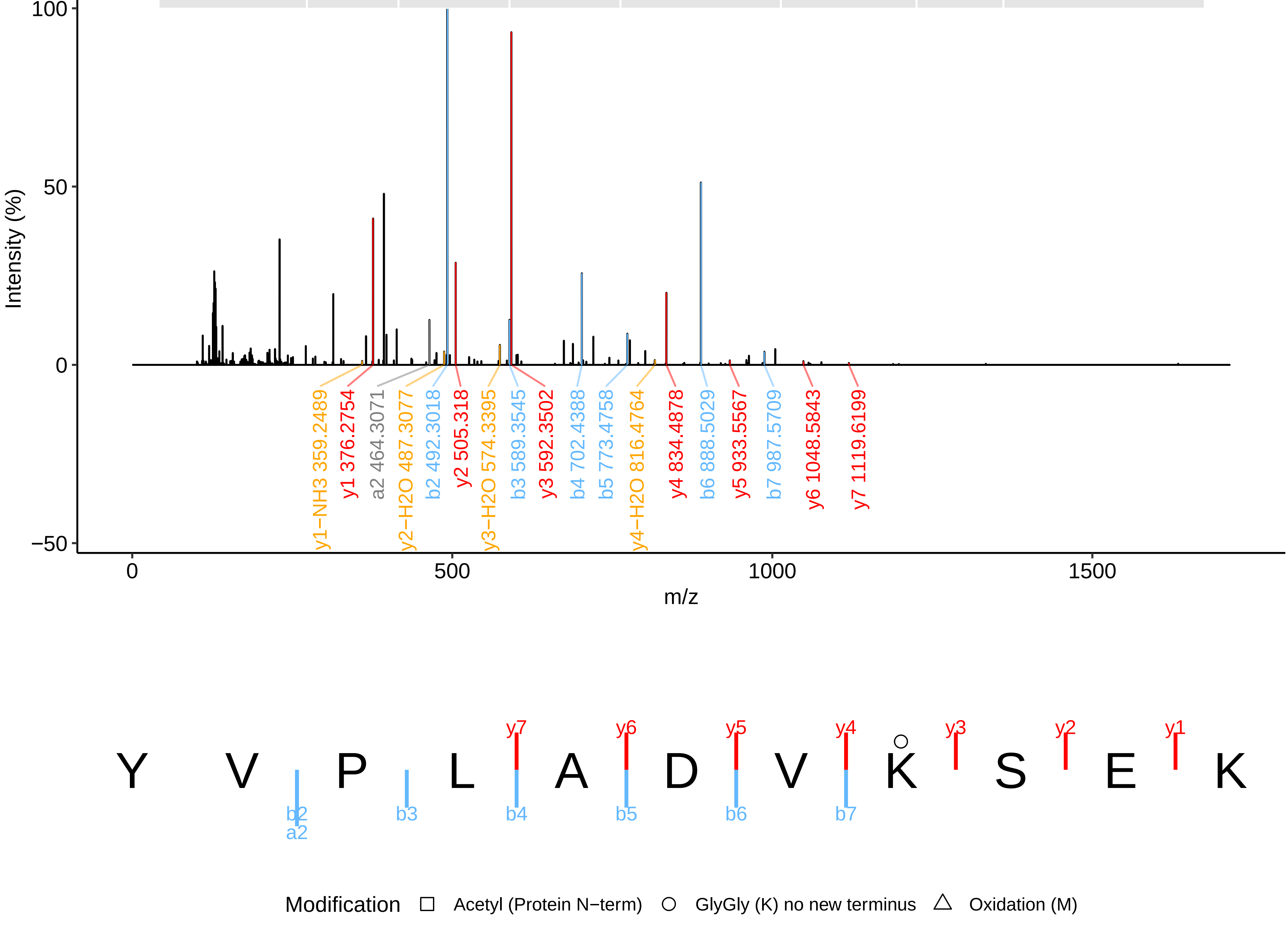

20200921_KIZ_MUb4_GlyGly_10plex − Scan number: 21378

Gene Names	Charge	m/z	Mass	Retention time	PEP	Score	Precursor Intensity
TOMM70A	3	478.5894	1432.746	77.408	2.8894e−05	104.22	4532534

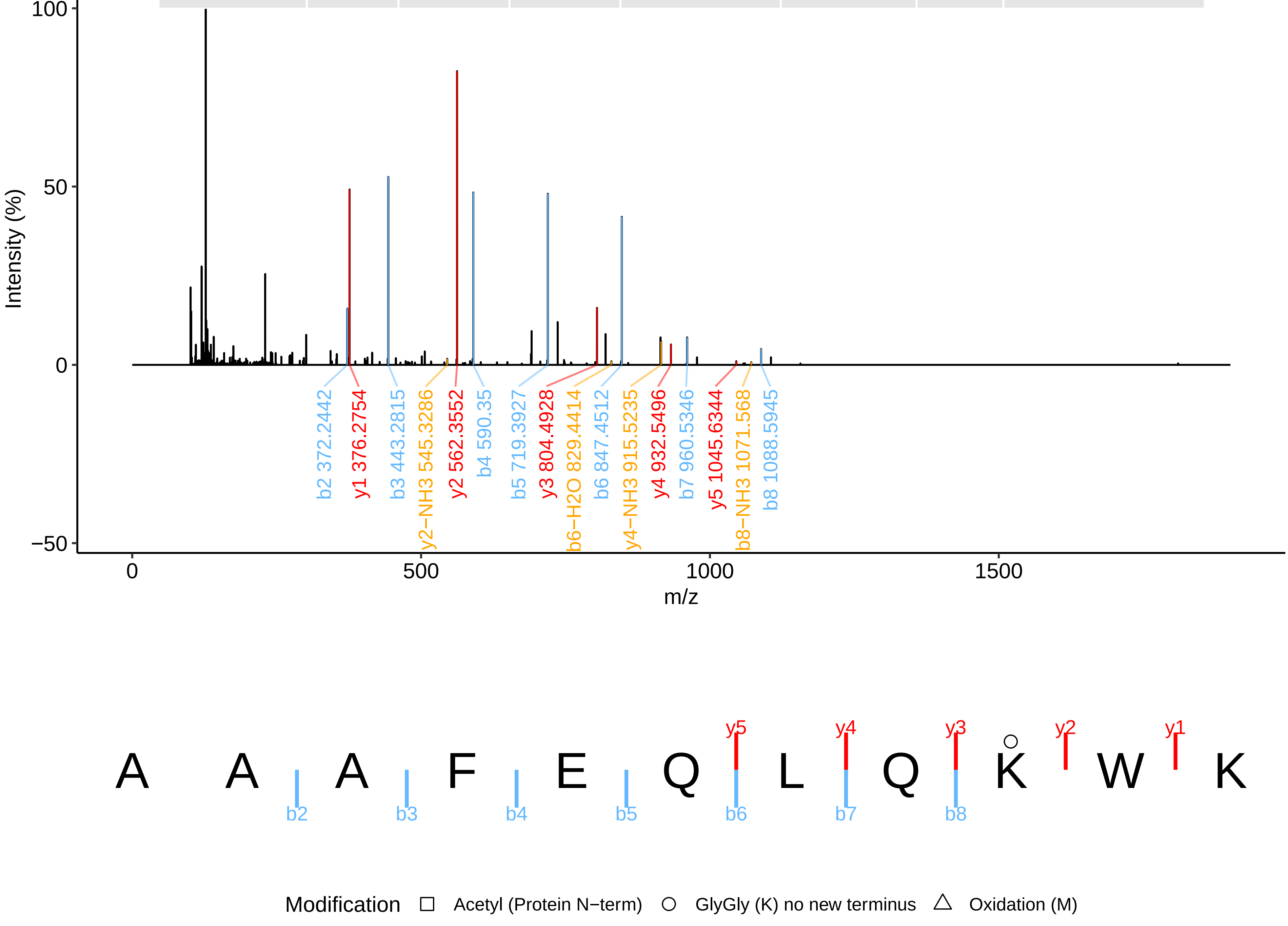

20200921_KIZ_MUb4_GlyGly_10plex − Scan number: 21427

Gene Names	Charge	m/z	Mass	Retention time	PEP	Score	Precursor Intensity
TOMM70A	3	478.5894	1432.746	77.604	0.00020492	96.342	5705077

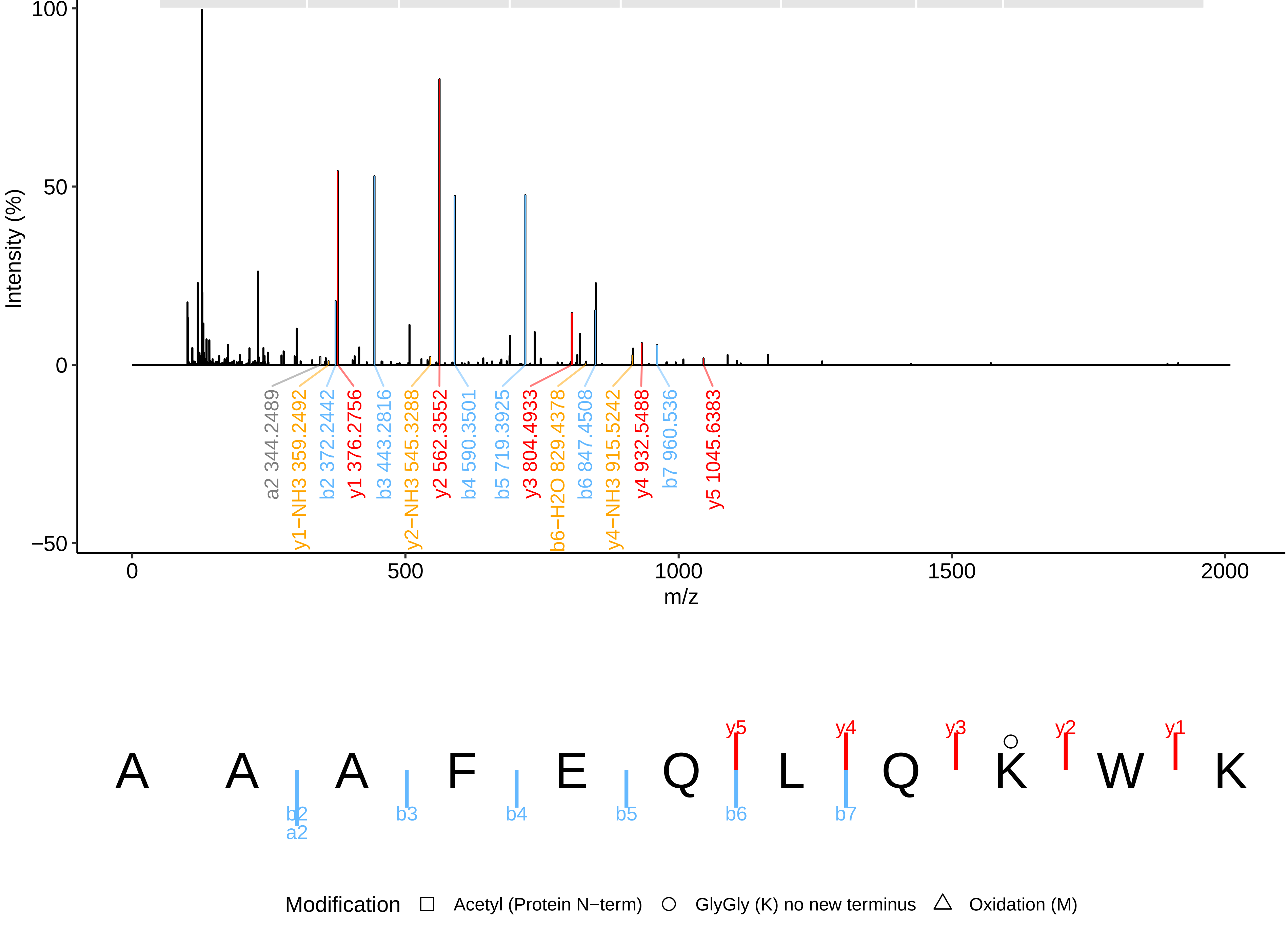

20200921_KIZ_MUb4_GlyGly_10plex − Scan number: 21566

Gene Names	Charge	m/z	Mass	Retention time	PEP	Score	Precursor Intensity
TOMM70A	3	478.5894	1432.746	78.121	5.8287e−09	130.98	3840642

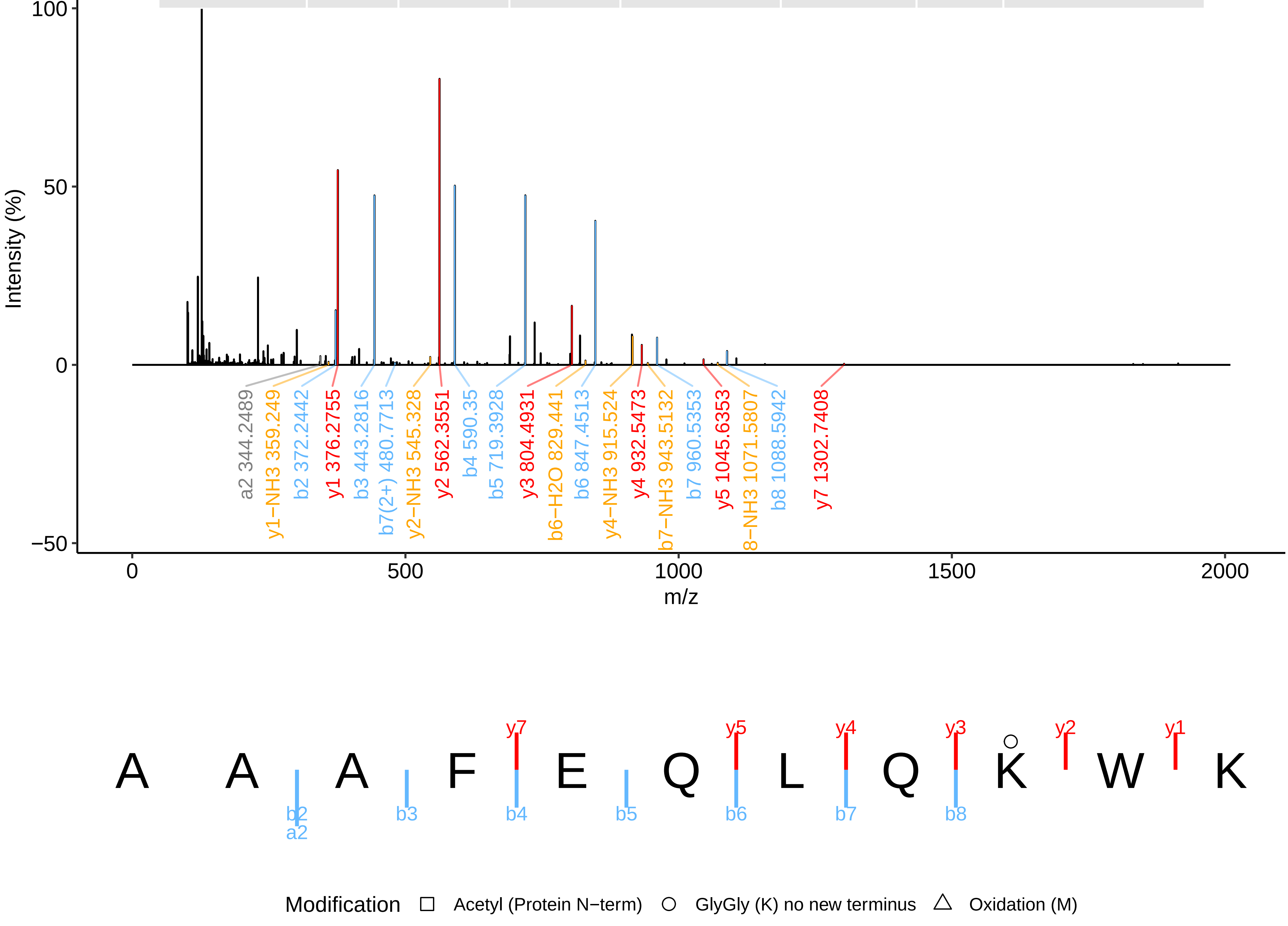

20200921_KIZ_MUb4_GlyGly_10plex − Scan number: 21700

Gene Names	Charge	m/z	Mass	Retention time	PEP	Score	Precursor Intensity
TOMM70A	3	478.5894	1432.746	78.631	3.5807e−08	121.5	7441038

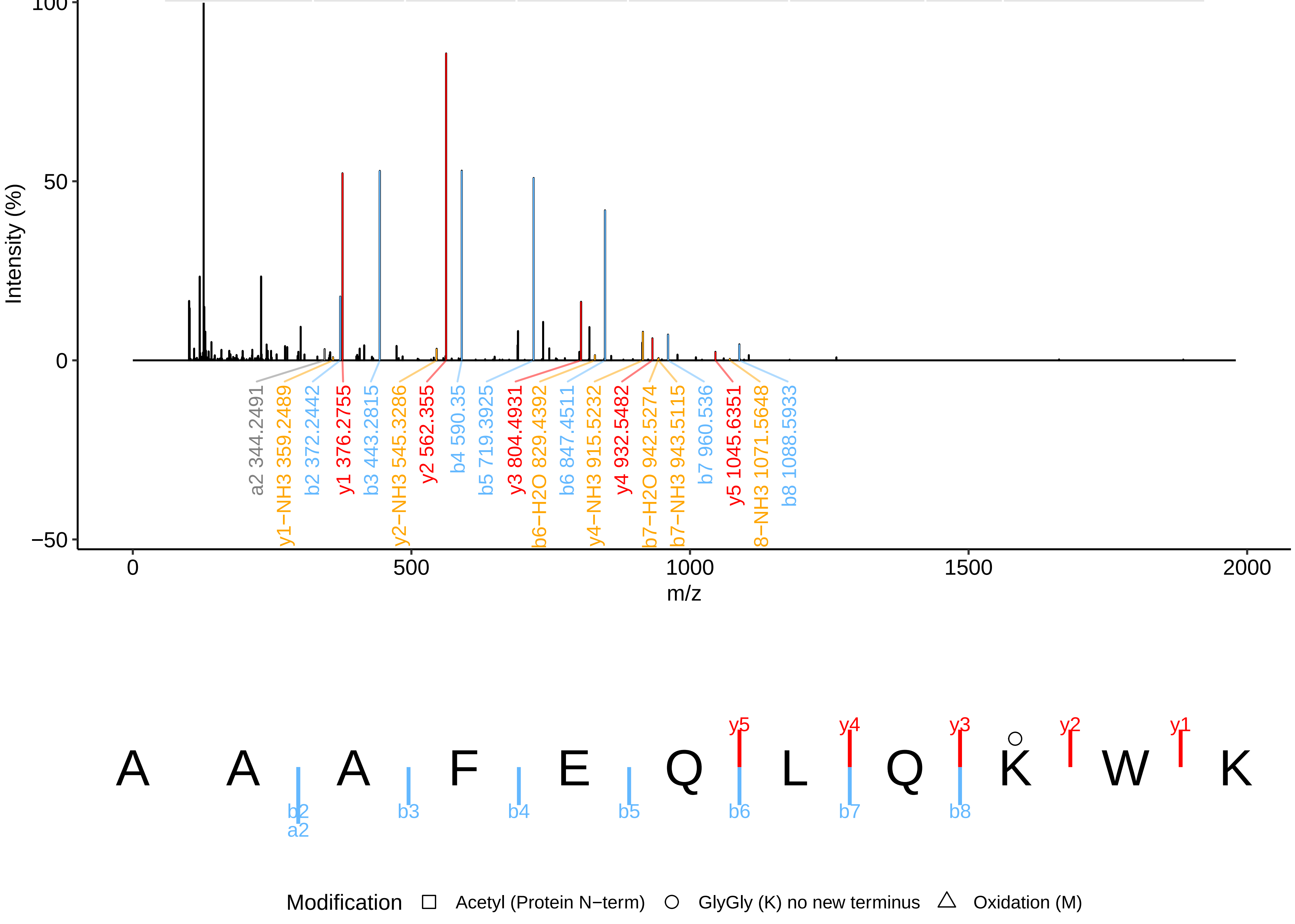

20200921_KIZ_MUb4_GlyGly_10plex − Scan number: 21834

Gene Names	Charge	m/z	Mass	Retention time	PEP	Score	Precursor Intensity
TOMM70A	3	478.5894	1432.746	79.135	1.5499e−05	109.42	11441544

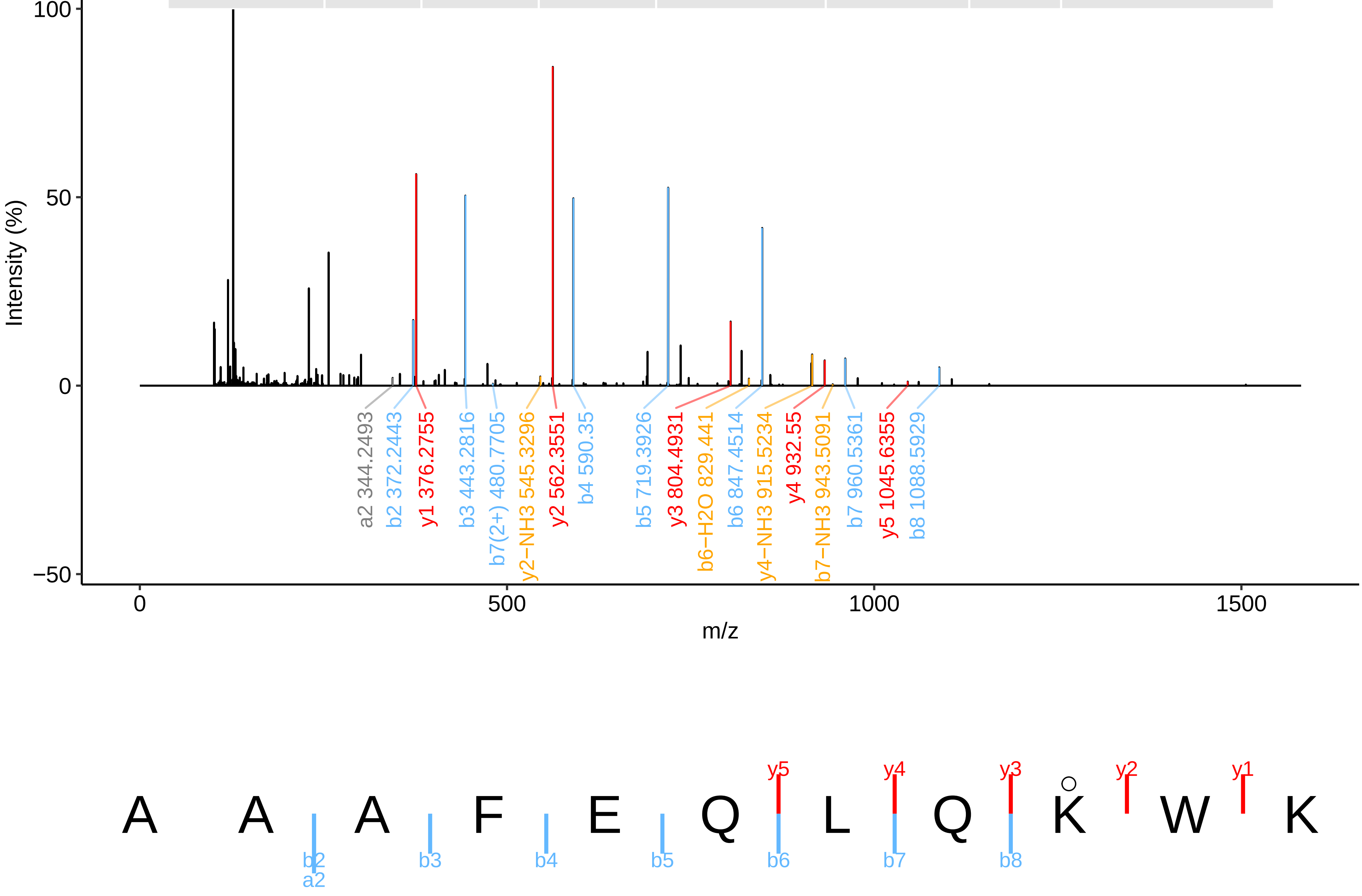

Modification □ Acetyl (Protein N−term) ○ GlyGly (K) no new terminus △ Oxidation (M)

20200921_KIZ_MUb4_GlyGly_10plex − Scan number: 21968

Gene Names	Charge	m/z	Mass	Retention time	PEP	Score	Precursor Intensity
TOMM70A	3	478.5894	1432.746	79.642	1.5499e−05	109.42	8437961

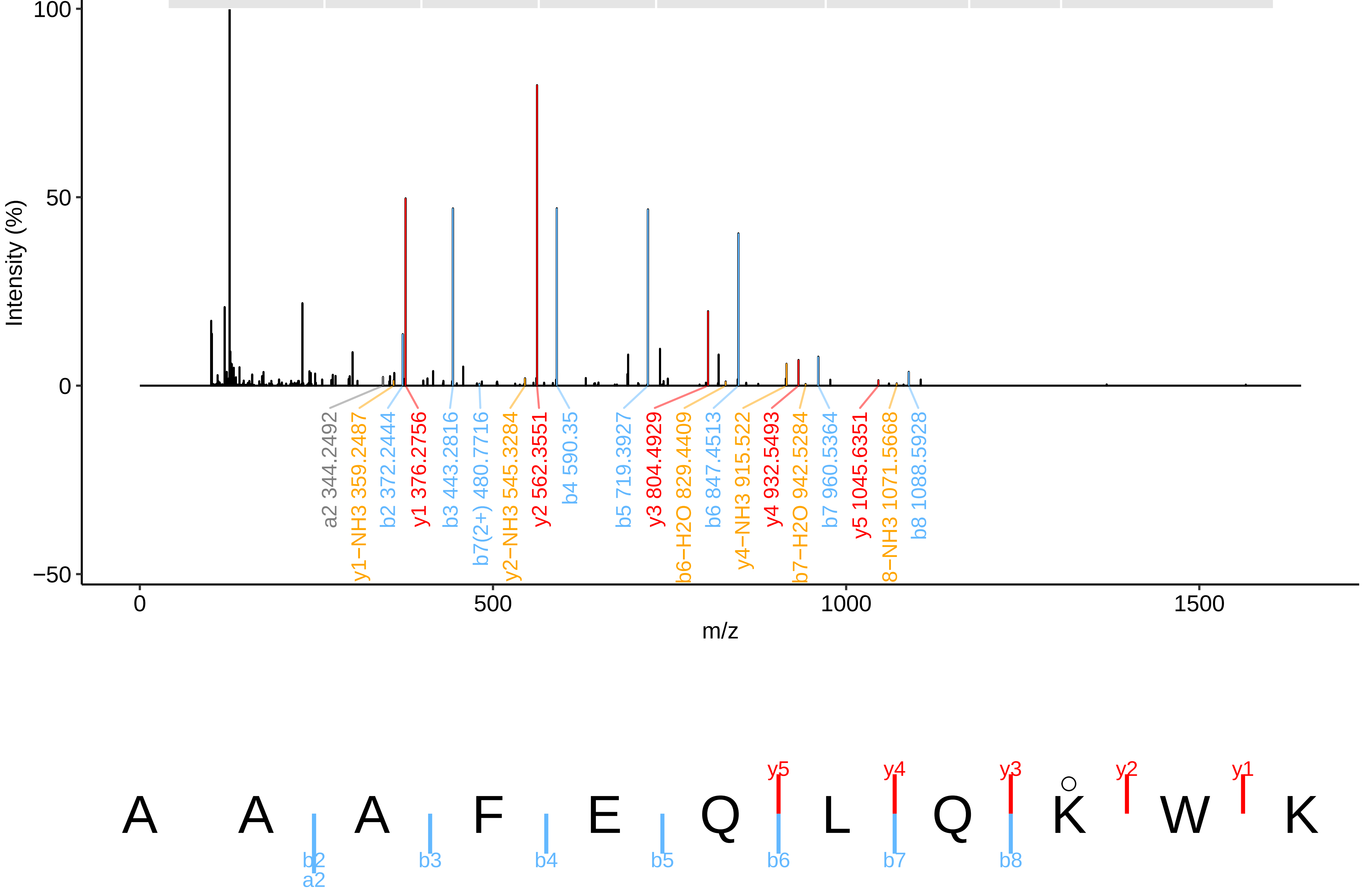

Modification □ Acetyl (Protein N−term) ○ GlyGly (K) no new terminus △ Oxidation (M)

20200921_KIZ_MUb4_GlyGly_10plex − Scan number: 22101

Gene Names	Charge	m/z	Mass	Retention time	PEP	Score	Precursor Intensity
TOMM70A	3	478.5894	1432.746	80.147	1.5499e−05	109.42	10298500

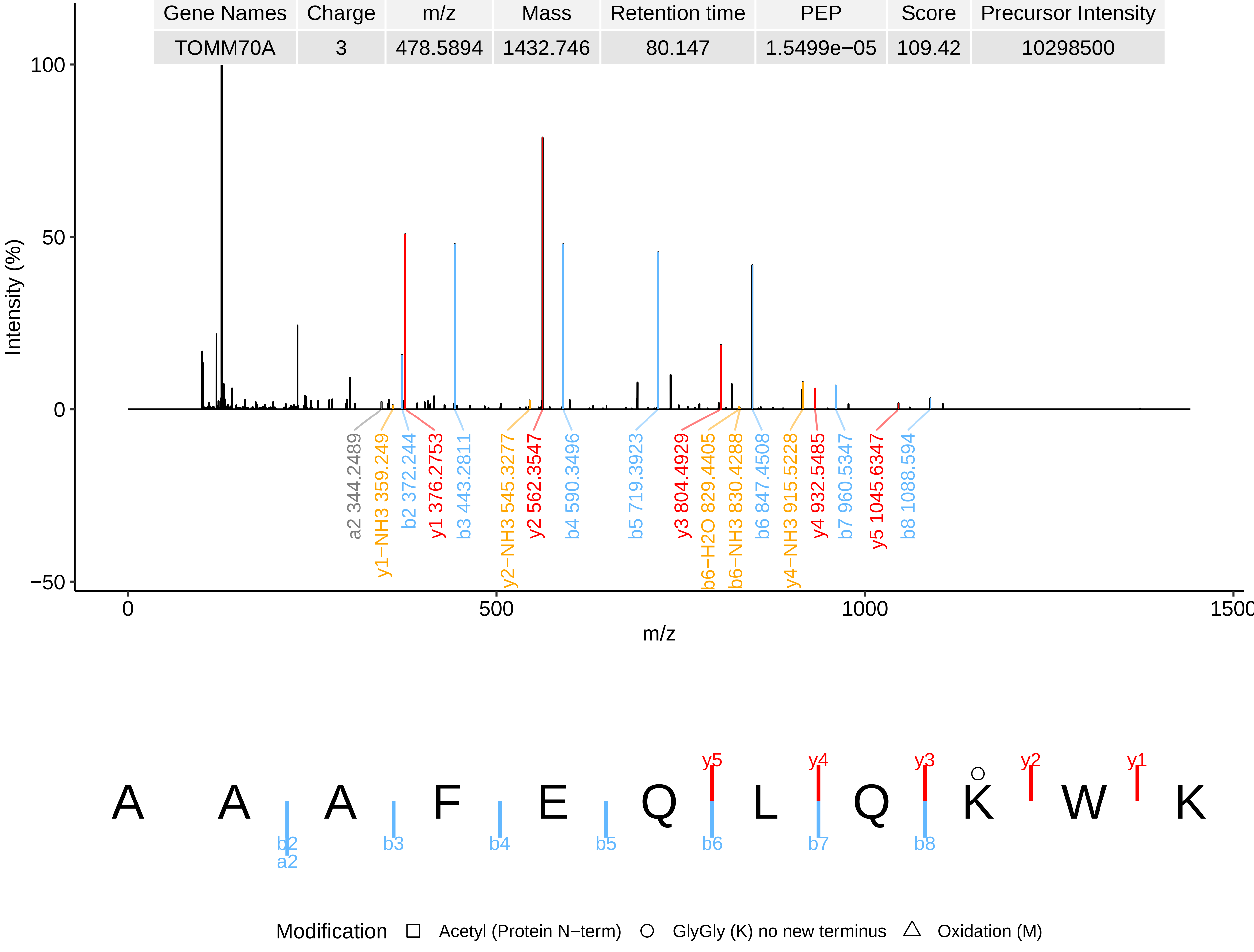

20200921_KIZ_MUb4_GlyGly_10plex − Scan number: 22241

Gene Names	Charge	m/z	Mass	Retention time	PEP	Score	Precursor Intensity
TOMM70A	3	478.5894	1432.746	80.65	3.5807e−08	121.5	2860400

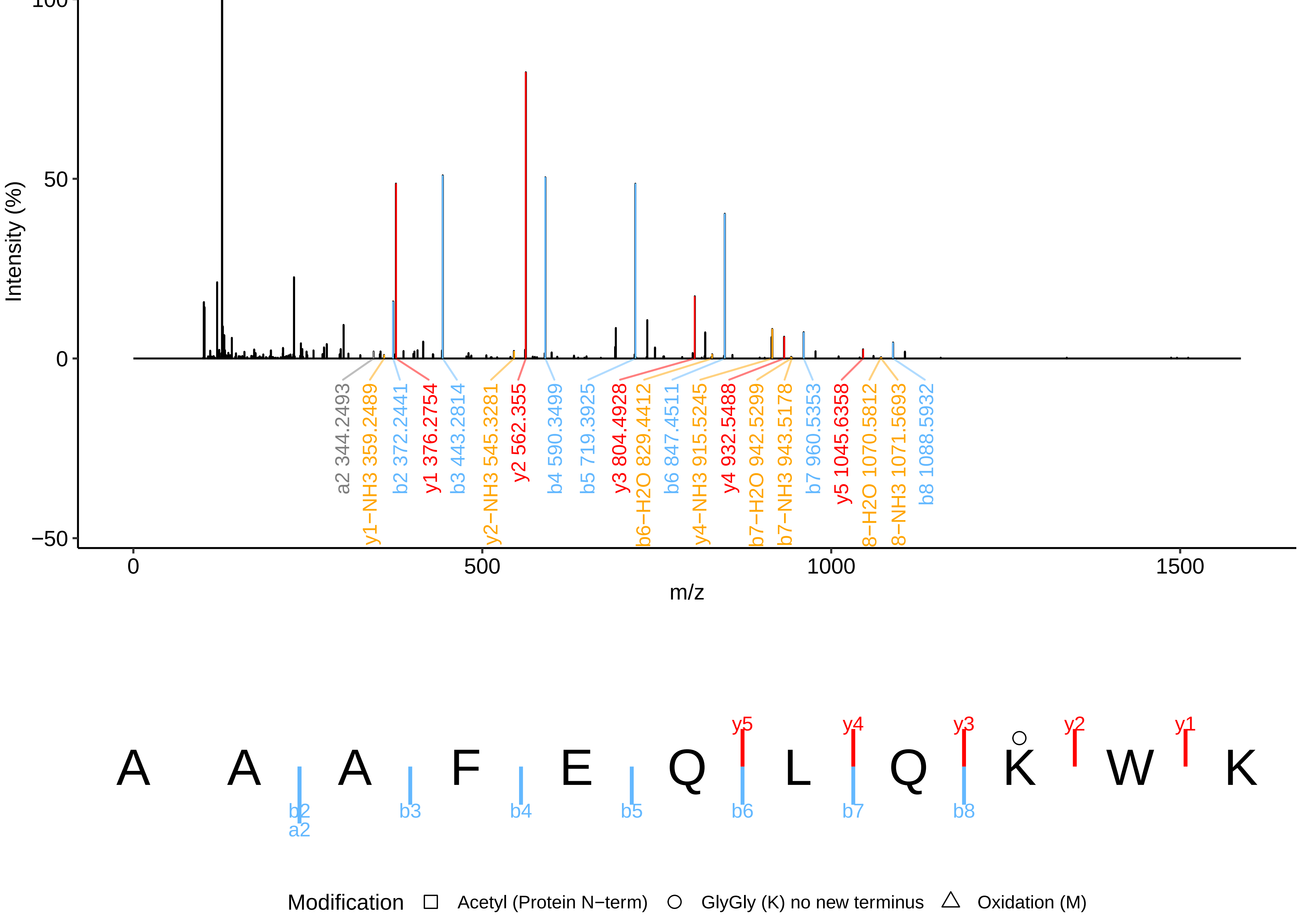

20200921_KIZ_MUb4_GlyGly_10plex − Scan number: 22515

Gene Names	Charge	m/z	Mass	Retention time	PEP	Score	Precursor Intensity
TOMM70A	3	478.5894	1432.746	81.668	4.0272e−08	120.09	10653069

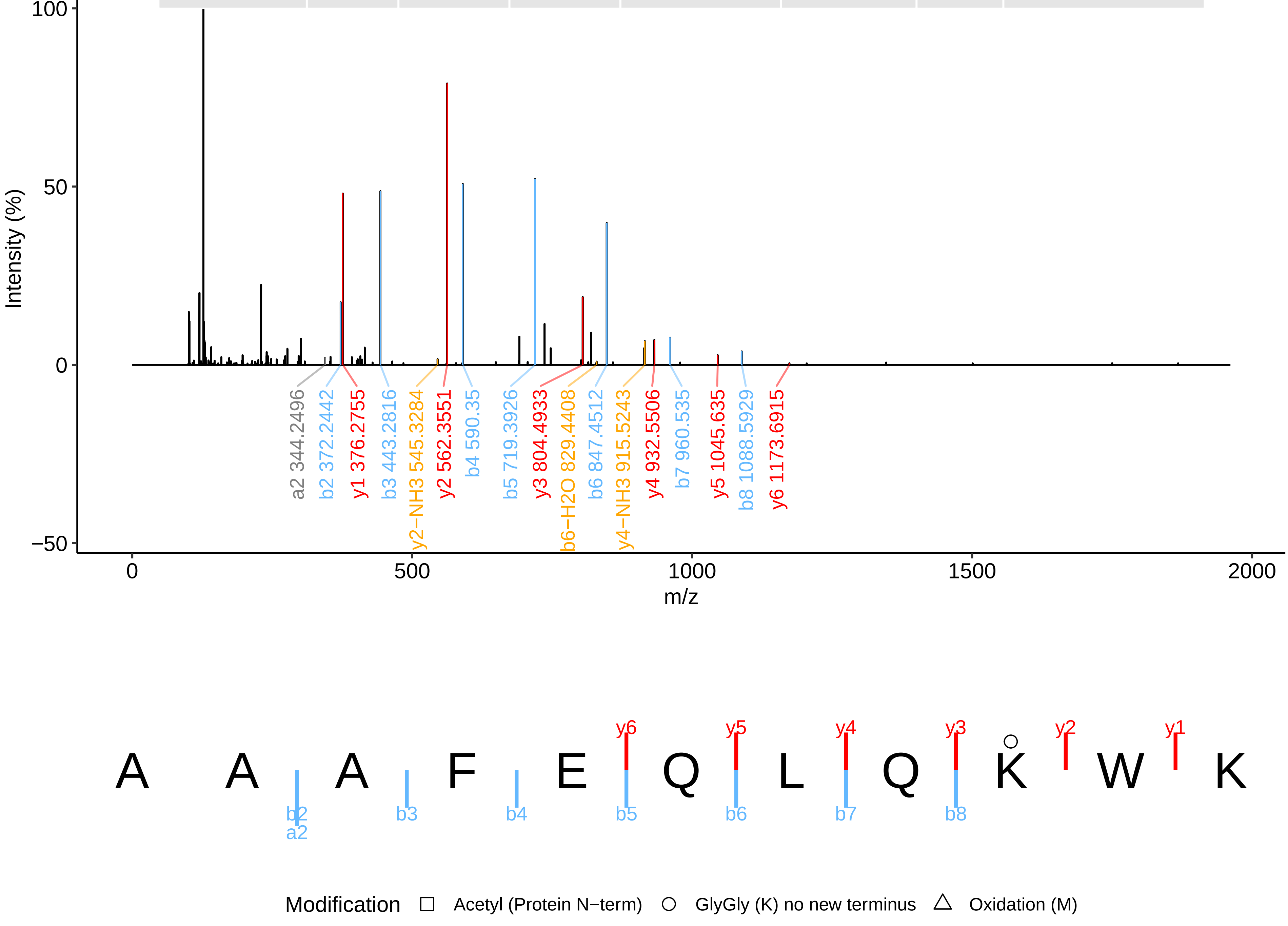

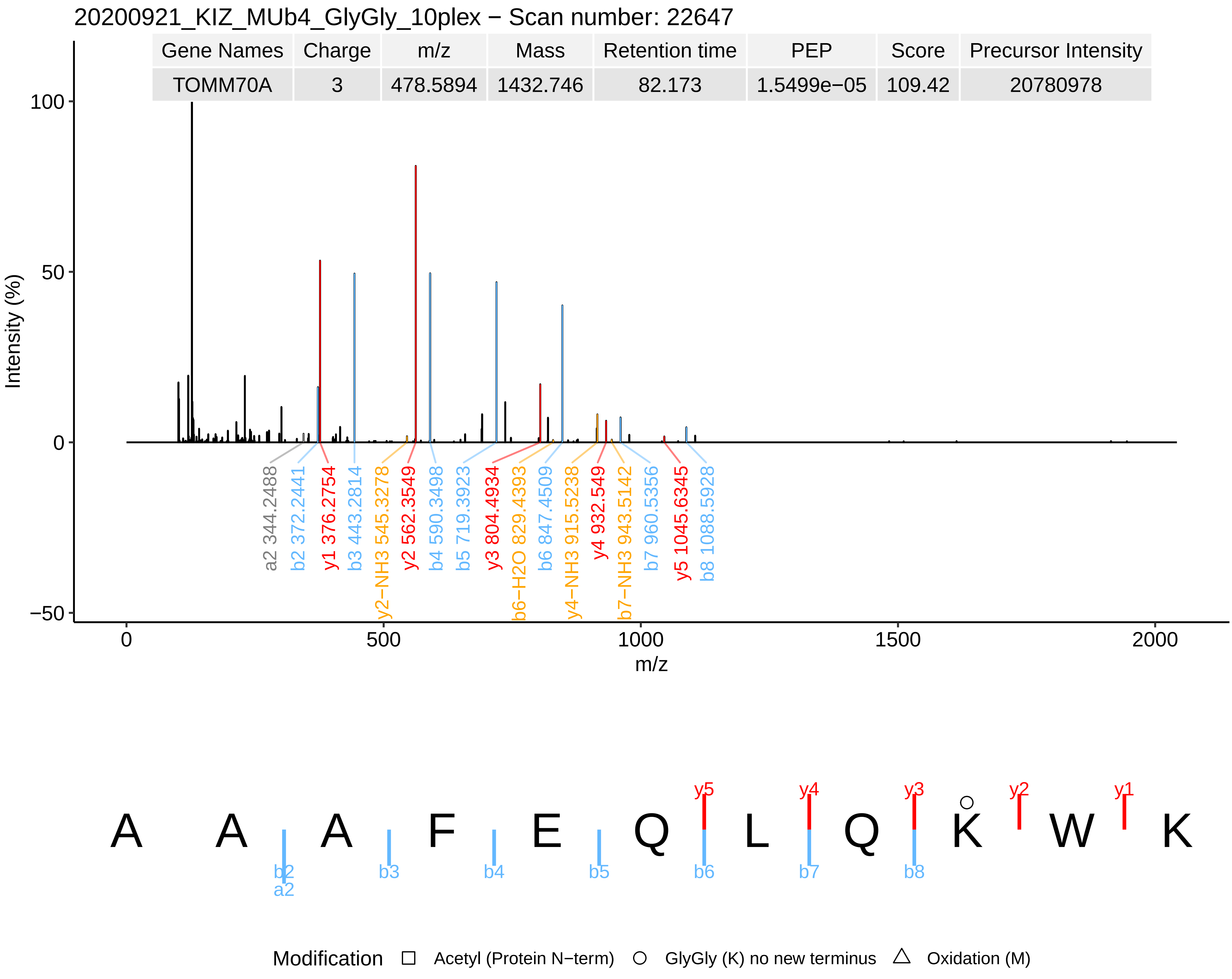

20200921_KIZ_MUb4_GlyGly_10plex − Scan number: 22647
Gene Names | Charge | m/z | Mass | Retention time | PEP | Score | Precursor Intensity
TOMM70A | 3 | 478.5894 | 1432.746 | 82.173 | 1.5499e−05 | 109.42 | 20780978
Intensity (%)
m/z
a2 344.2488
b2 372.2441
y1 376.2754
b3 443.2814
y2−NH3 545.3278
y2 562.3549
b4 590.3498
b5 719.3923
y3 804.4934
b6−H2O 829.4393
b6 847.4509
y4−NH3 915.5238
y4 932.549
b7−NH3 943.5142
b7 960.5356
y5 1045.6345
b8 1088.5928
A A A F E Q L Q K W K
y5 y4 y3 y2 y1
b2 a2 b3 b4 b5 b6 b7 b8
Modification
Acetyl (Protein N−term)
GlyGly (K) no new terminus
Oxidation (M)

20200921_KIZ_MUb4_GlyGly_10plex − Scan number: 22782

Gene Names	Charge	m/z	Mass	Retention time	PEP	Score	Precursor Intensity
TOMM70A	3	478.5894	1432.746	82.68	3.5807e−08	121.5	9768820

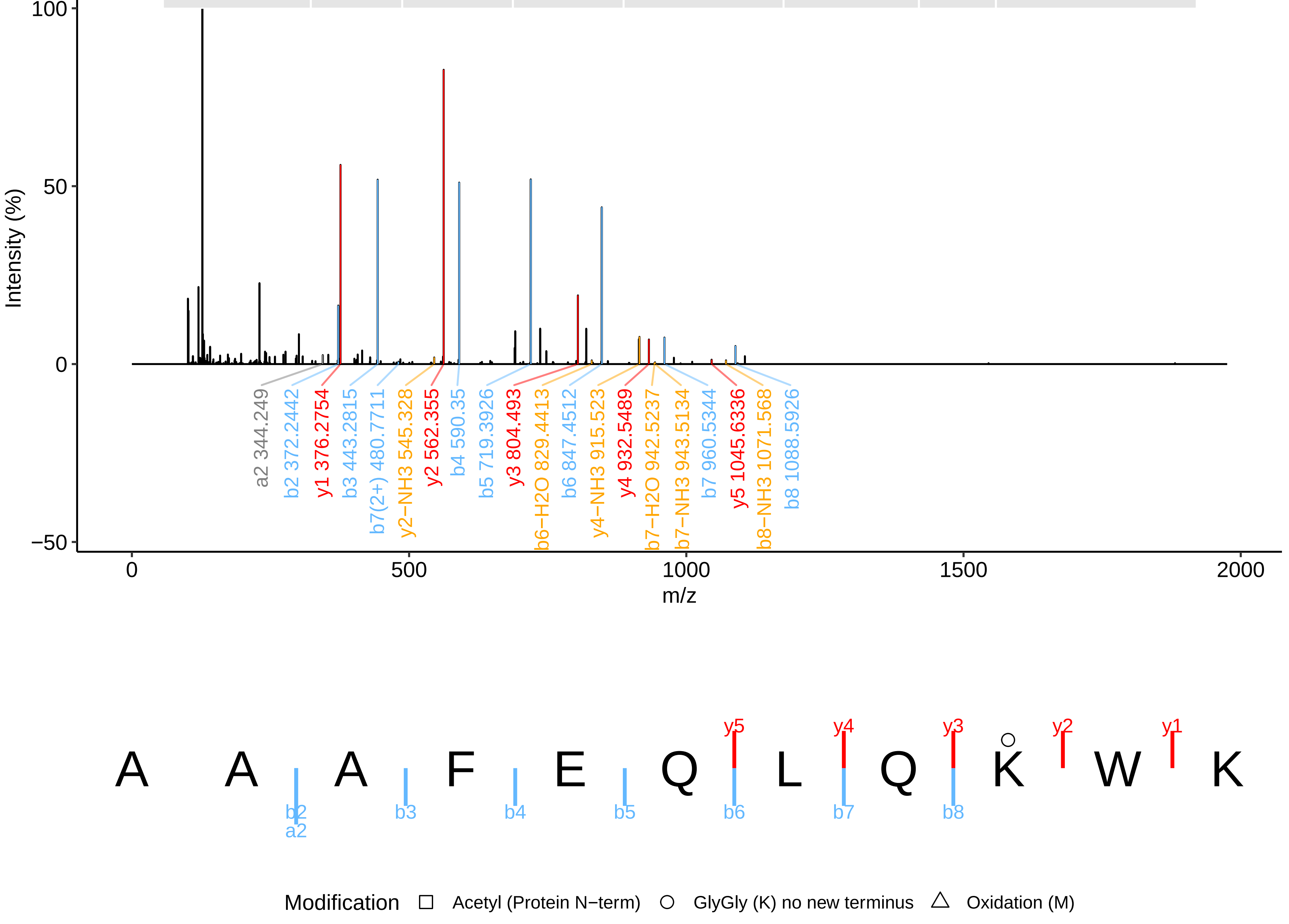

20200921_KIZ_MUb4_GlyGly_10plex − Scan number: 22939

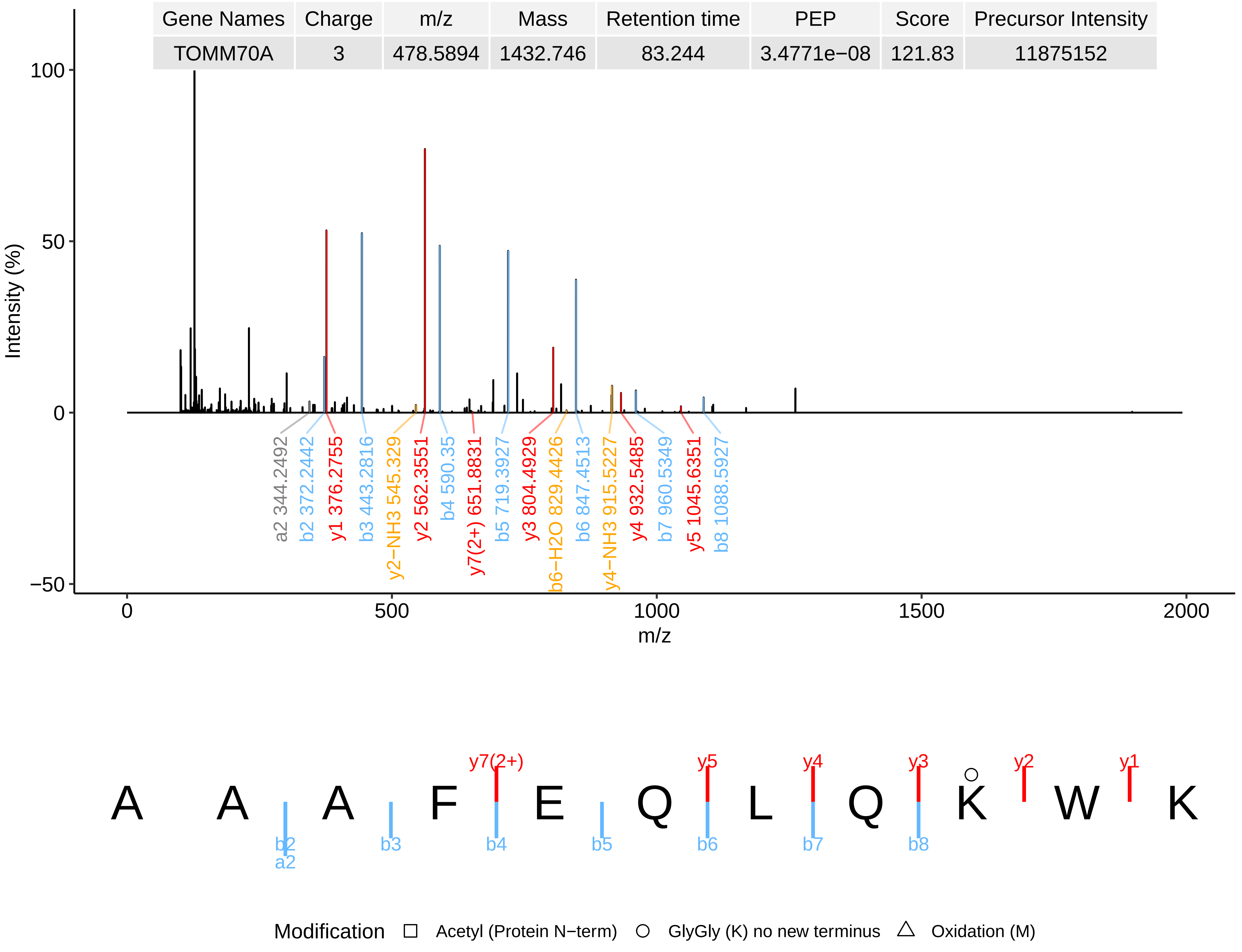

20200921_KIZ_MUb4_GlyGly_10plex_2 − Scan number: 19462

Gene Names	Charge	m/z	Mass	Retention time	PEP	Score	Precursor Intensity
TOMM70A	3	478.5894	1432.746	76.392	1.5499e−05	109.42	1882614

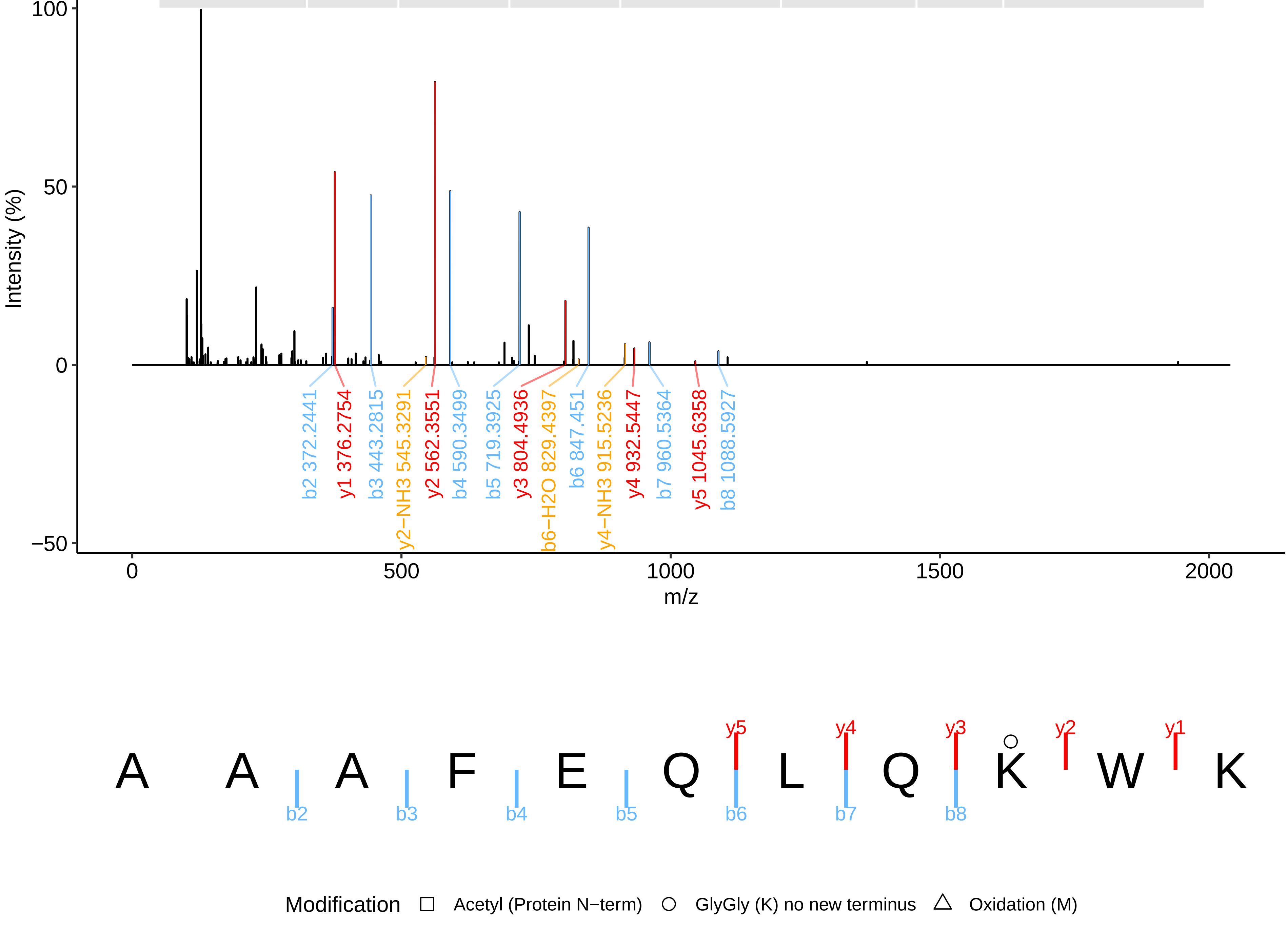

20200924_KIZ_MUb5_GlyGly_1 − Scan number: 20819

Gene Names	Charge	m/z	Mass	Retention time	PEP	Score	Precursor Intensity
TOMM70A	3	478.5894	1432.746	67.102	5.3897e−08	115.78	36451368

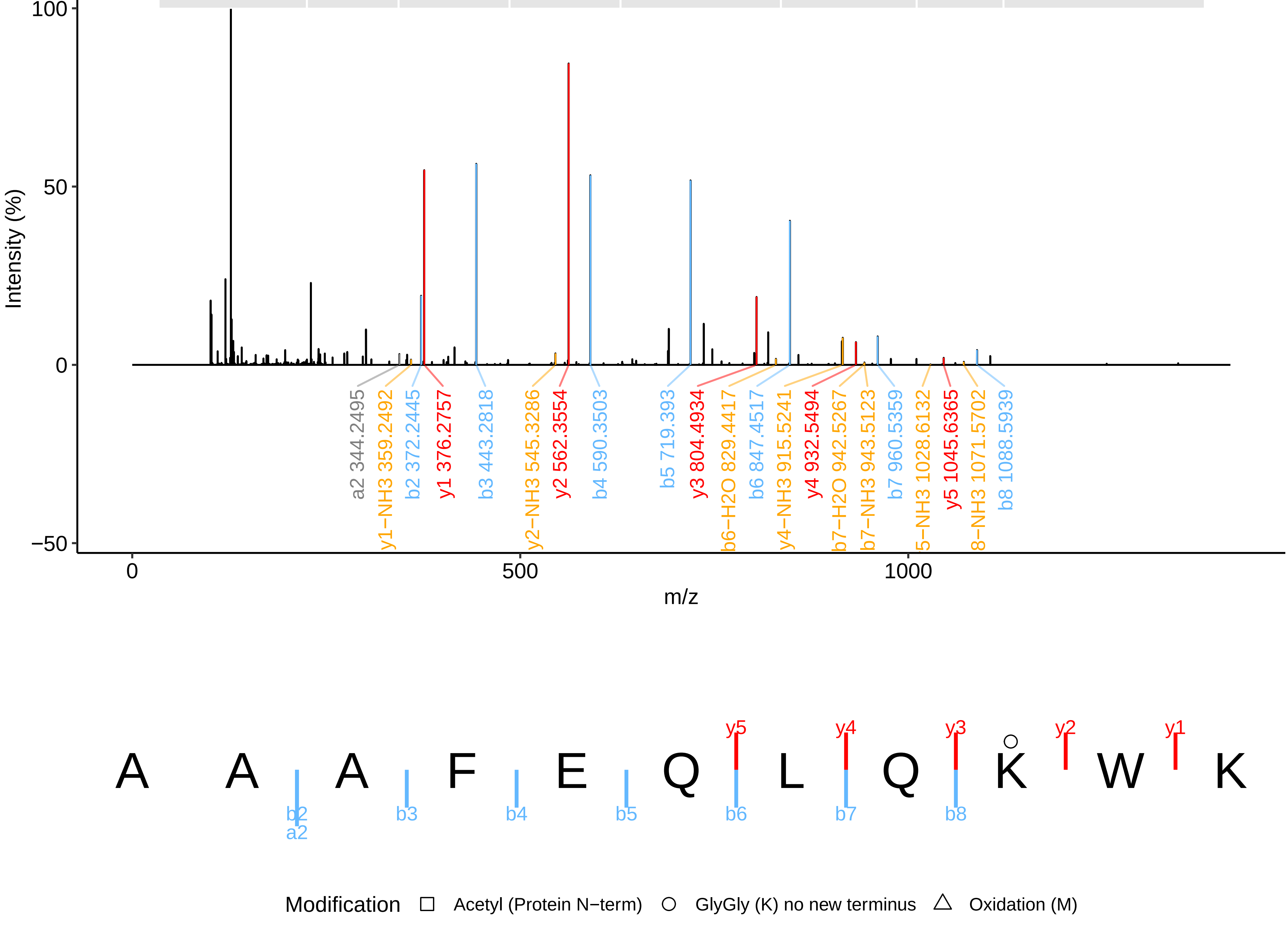

20200924_KIZ_MUb5_GlyGly_1 − Scan number: 20981

Gene Names	Charge	m/z	Mass	Retention time	PEP	Score	Precursor Intensity
TOMM70A	3	478.5894	1432.746	67.607	5.3897e−08	115.78	24849014

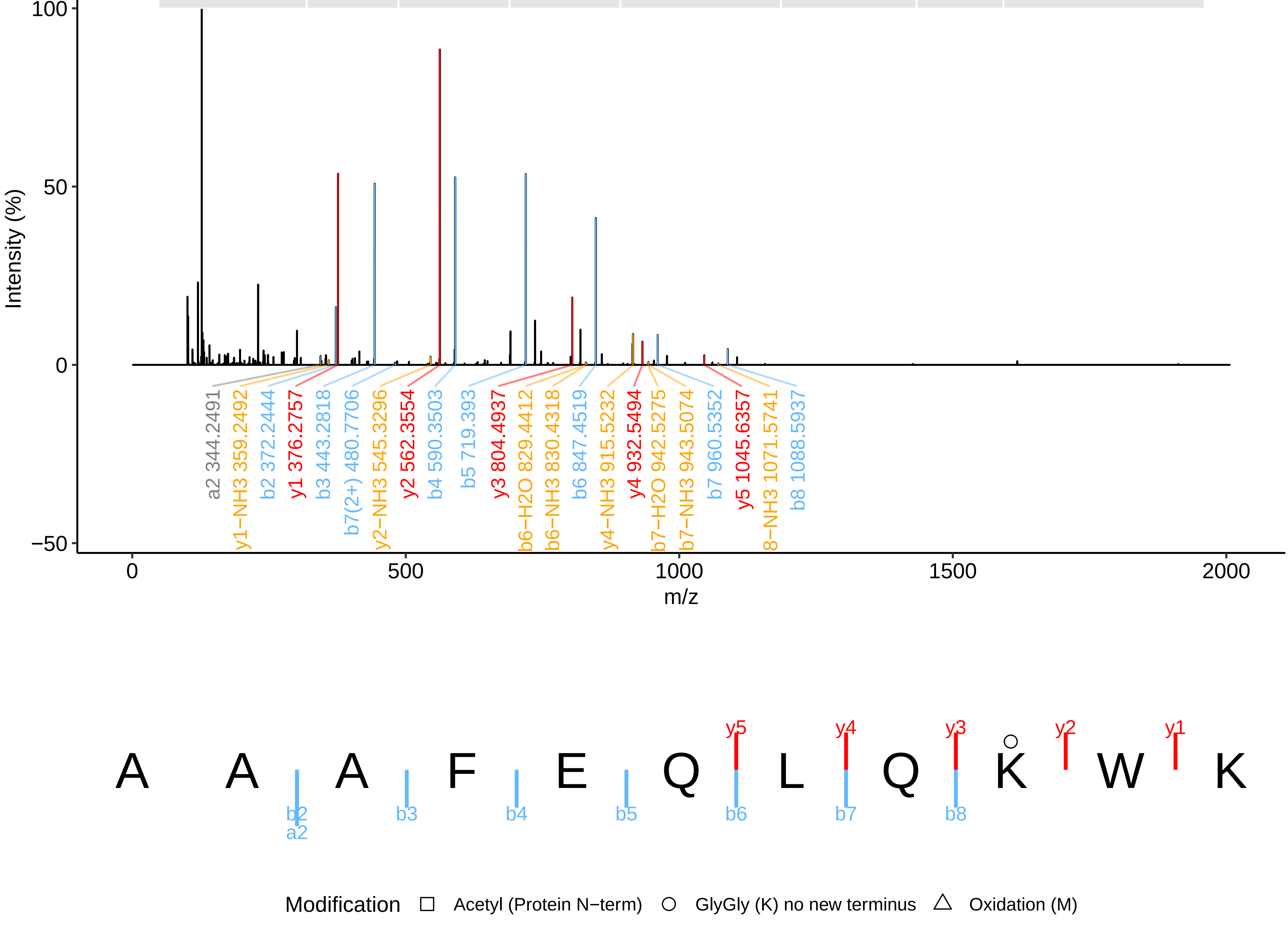

20200924_KIZ_MUb5_GlyGly_1 − Scan number: 21302

Gene Names	Charge	m/z	Mass	Retention time	PEP	Score	Precursor Intensity
TOMM70A	3	478.5894	1432.746	68.629	1.2627e−05	110.53	16907652

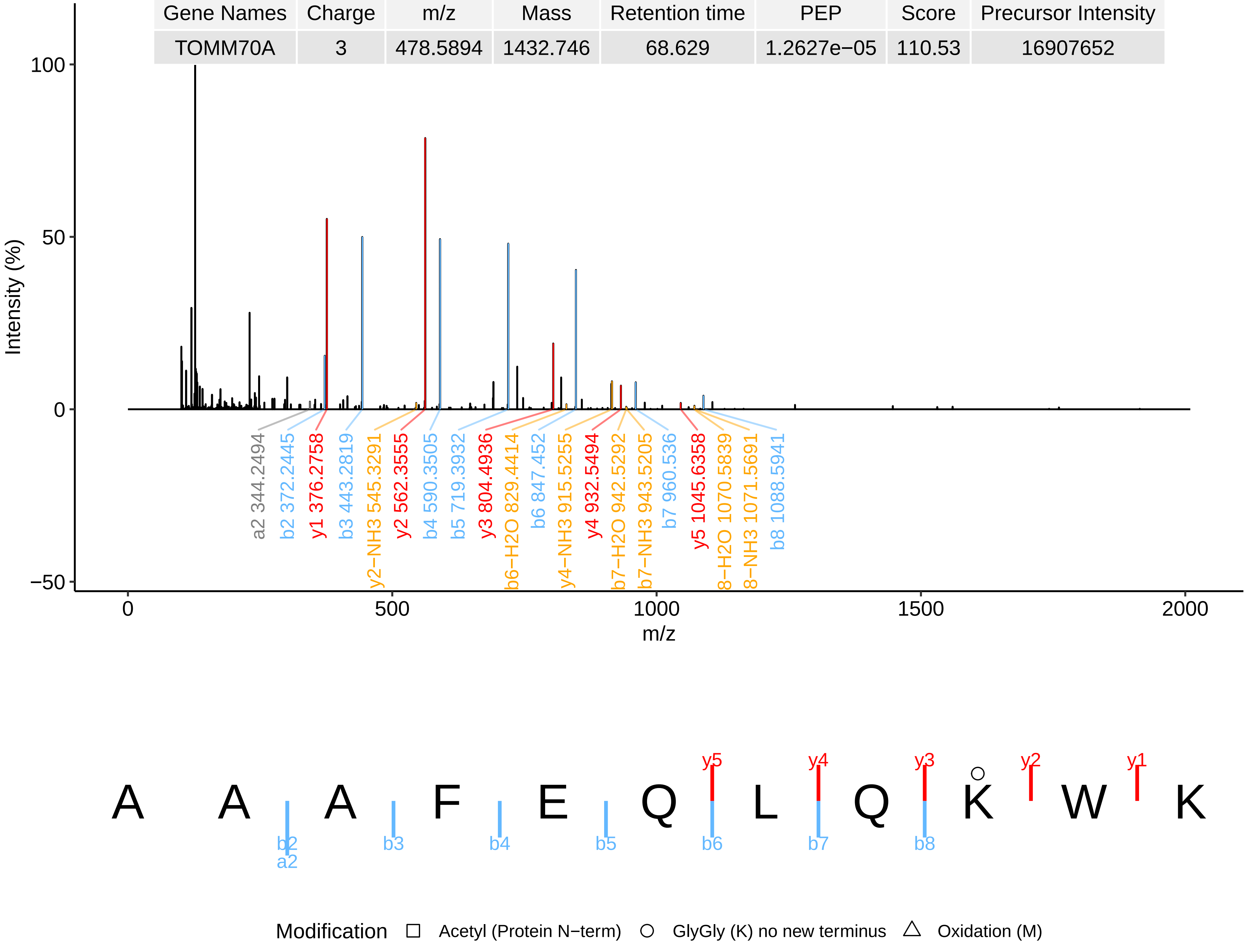

20200924_KIZ_MUb5_GlyGly_1 − Scan number: 20509

Gene Names	Charge	m/z	Mass	Retention time	PEP	Score	Precursor Intensity
TOMM70A	3	478.5894	1432.746	66.108	0.00024456	109.42	11127714

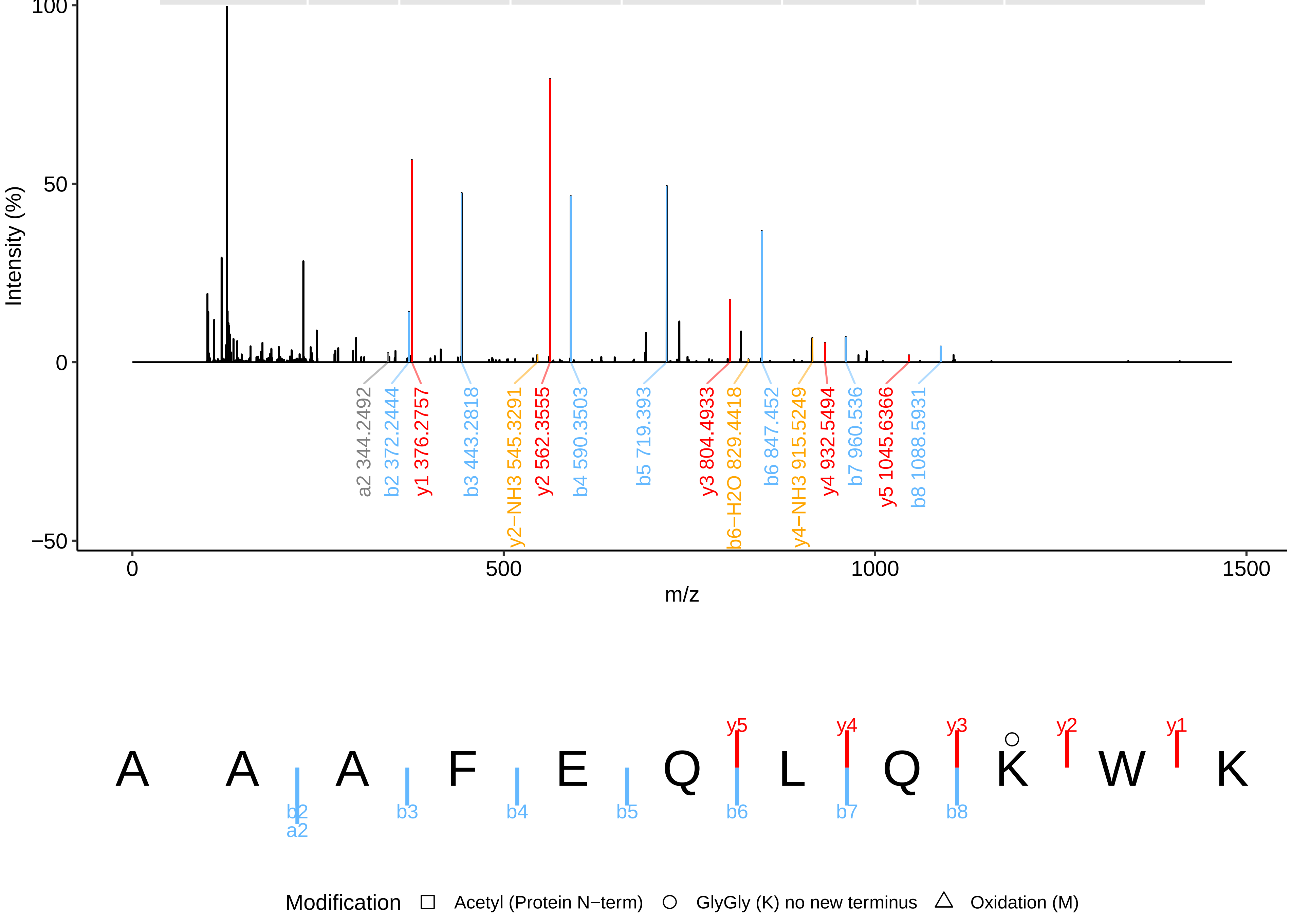

20200924_KIZ_MUb5_GlyGly_2 − Scan number: 19090

Gene Names	Charge	m/z	Mass	Retention time	PEP	Score	Precursor Intensity
TOMM70A	3	478.5894	1432.746	70.794	4.3229e−08	119.16	9018562

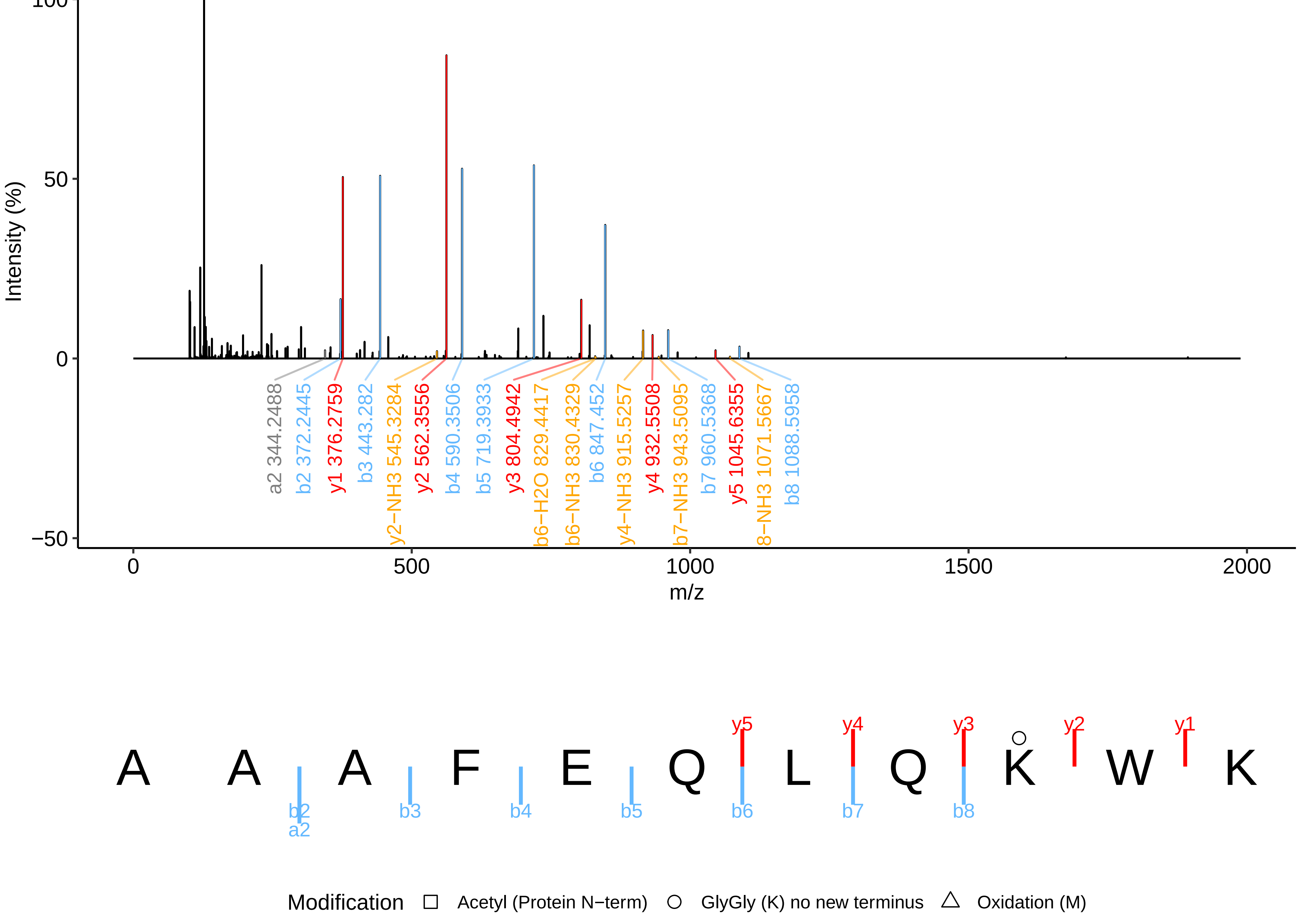

20200924_KIZ_MUb5_GlyGly_2 − Scan number: 19233

Gene Names	Charge	m/z	Mass	Retention time	PEP	Score	Precursor Intensity
TOMM70A	3	478.5894	1432.746	71.305	5.3897e−08	115.78	17638444

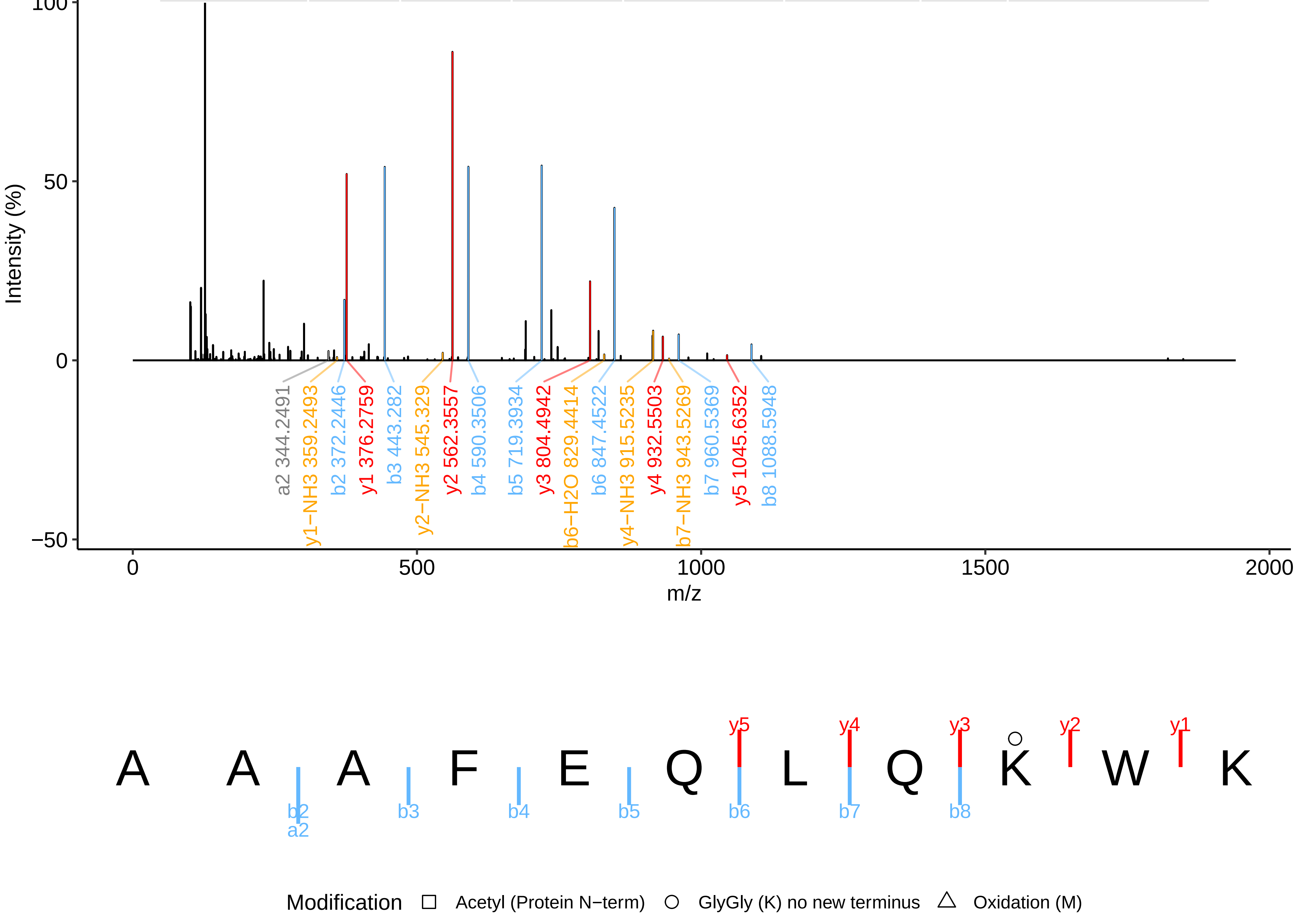

20200924_KIZ_MUb5_GlyGly_2 − Scan number: 19374

Gene Names	Charge	m/z	Mass	Retention time	PEP	Score	Precursor Intensity
TOMM70A	3	478.5894	1432.746	71.817	1.5499e−05	109.42	21322726

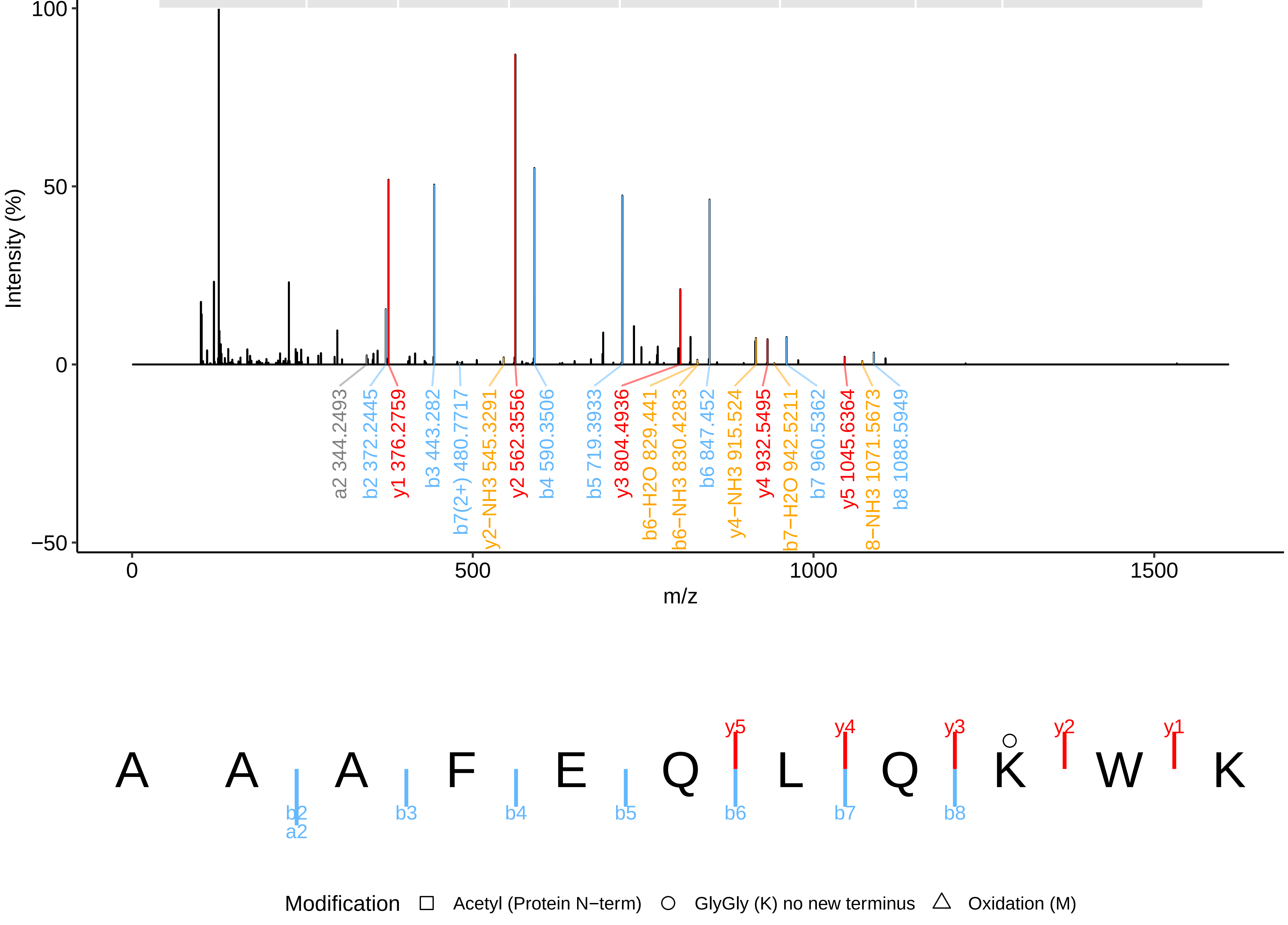

20200924_KIZ_MUb5_GlyGly_2 − Scan number: 19511

Gene Names	Charge	m/z	Mass	Retention time	PEP	Score	Precursor Intensity
TOMM70A	3	478.5894	1432.746	72.325	3.4771e−08	121.83	8257395

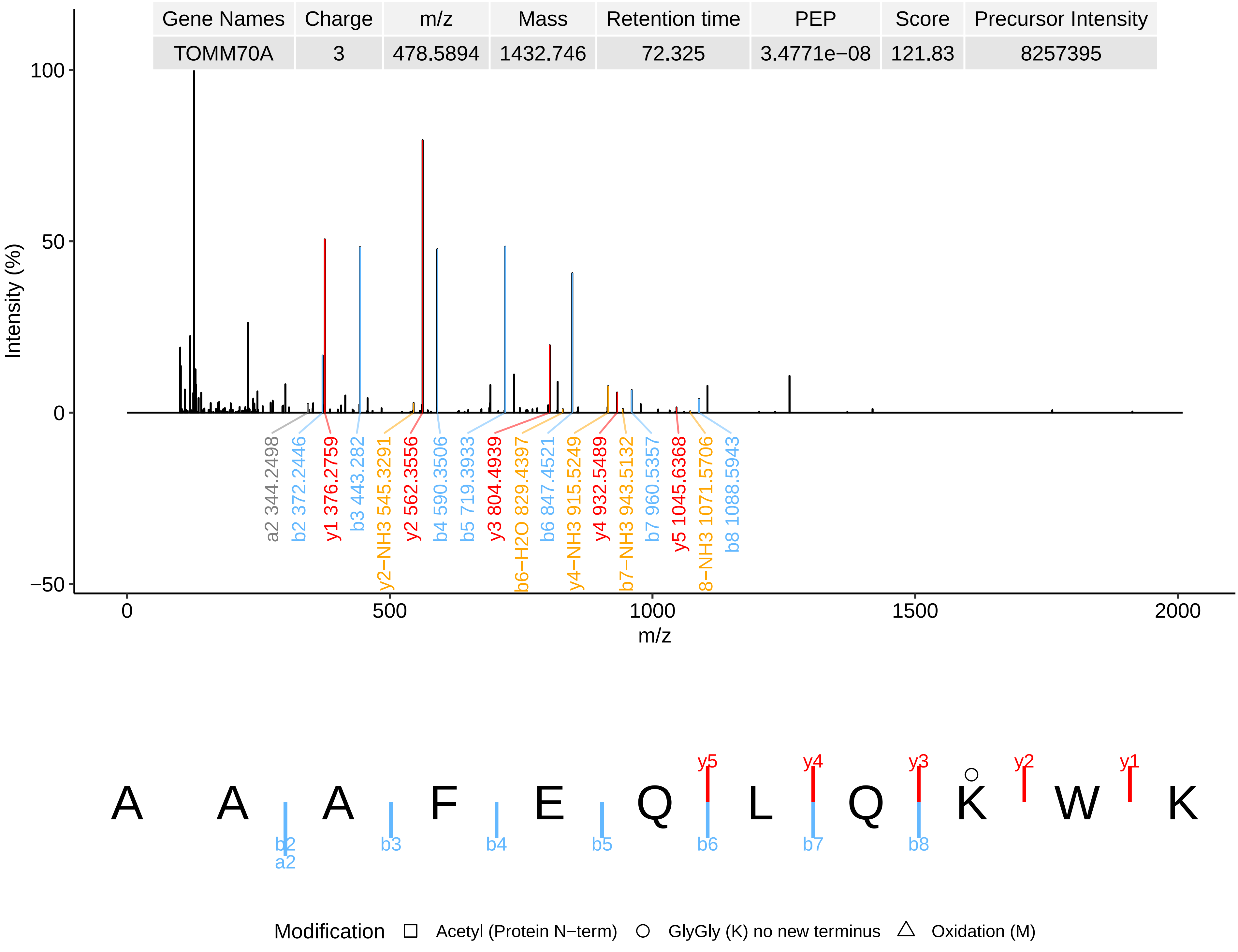

20200924_KIZ_MUb5_GlyGly_2 − Scan number: 18932

Gene Names	Charge	m/z	Mass	Retention time	PEP	Score	Precursor Intensity
TOMM70A	3	478.5894	1432.746	70.207	0.018879	66.056	2378322

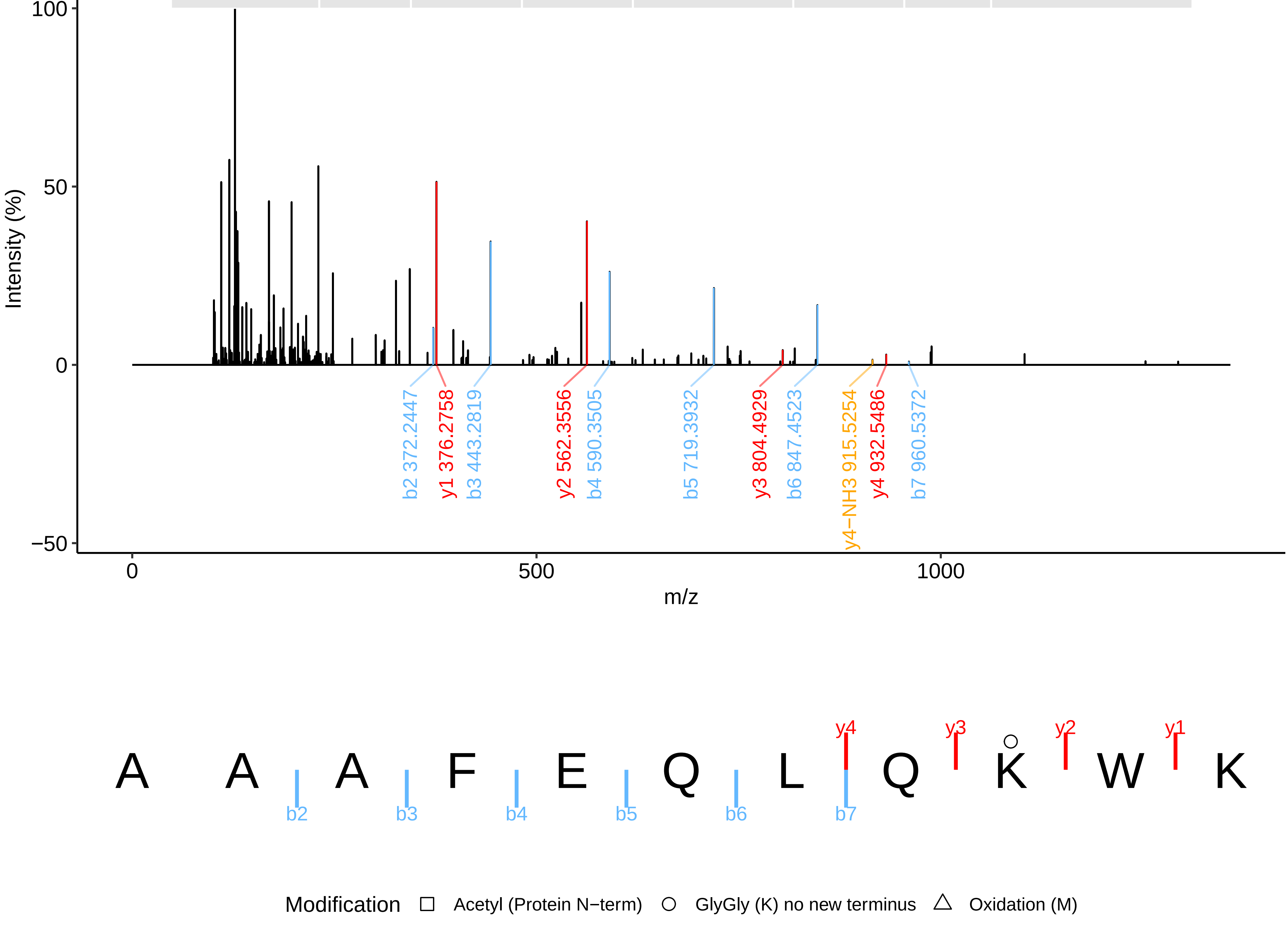

20201118_KIZ_MUb6_GlyGly_1 − Scan number: 21338

Gene Names	Charge	m/z	Mass	Retention time	PEP	Score	Precursor Intensity
TOMM70A	3	478.5894	1432.746	66.594	3.5807e−08	121.5	23699540

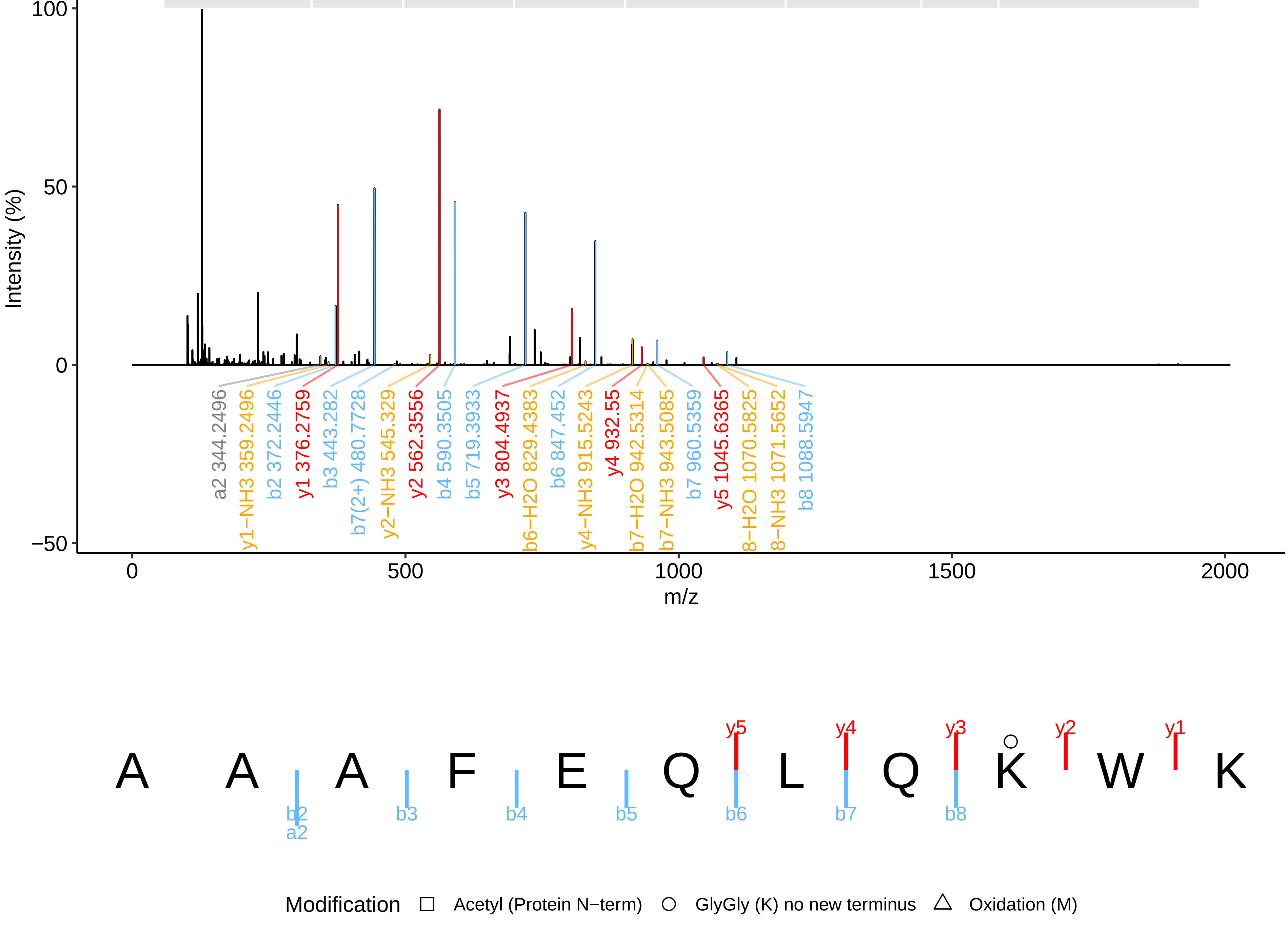

20201118_KIZ_MUb6_GlyGly_1 − Scan number: 21509

Gene Names	Charge	m/z	Mass	Retention time	PEP	Score	Precursor Intensity
TOMM70A	3	478.5894	1432.746	67.103	0.00031857	105.86	NA

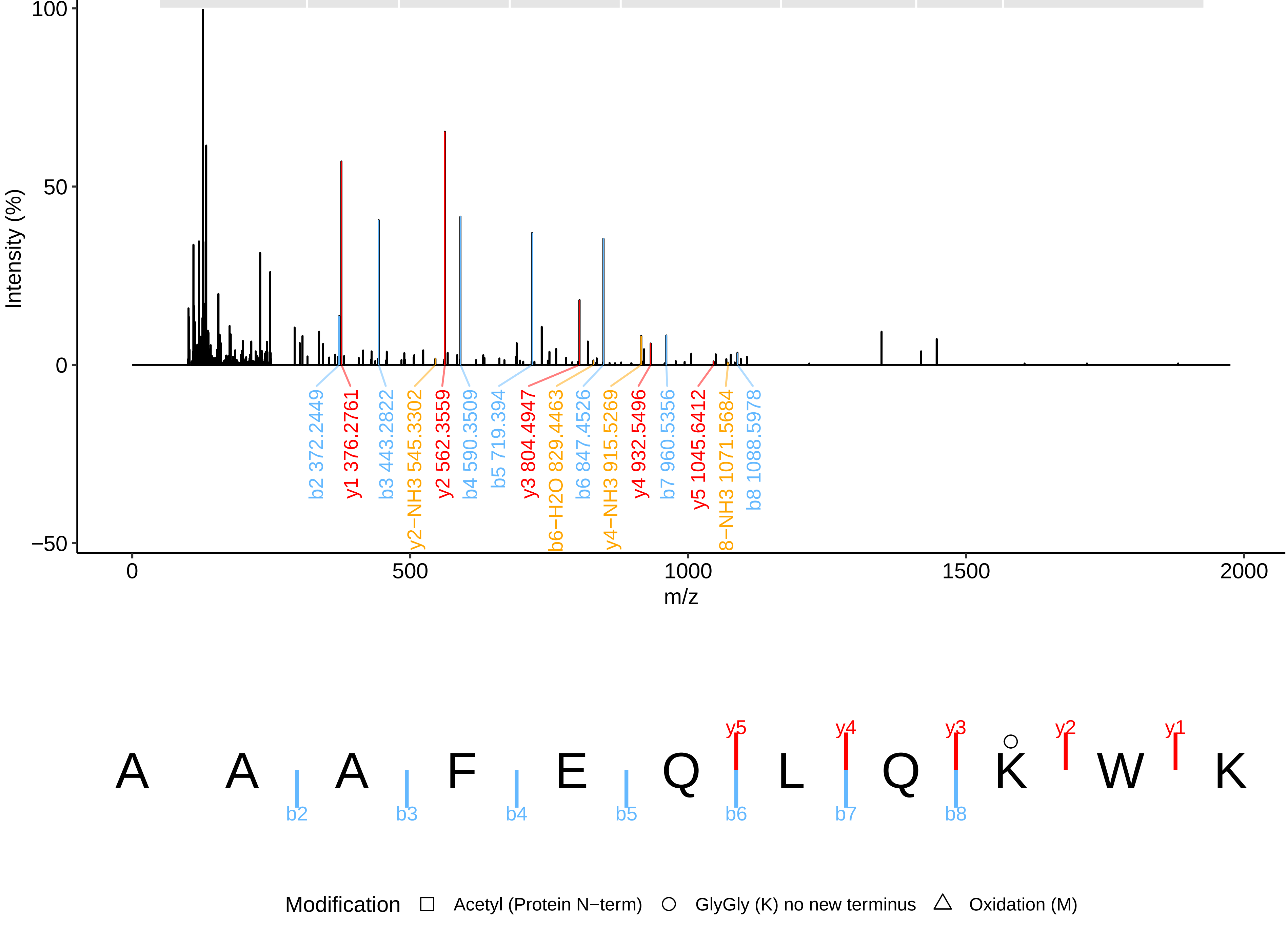

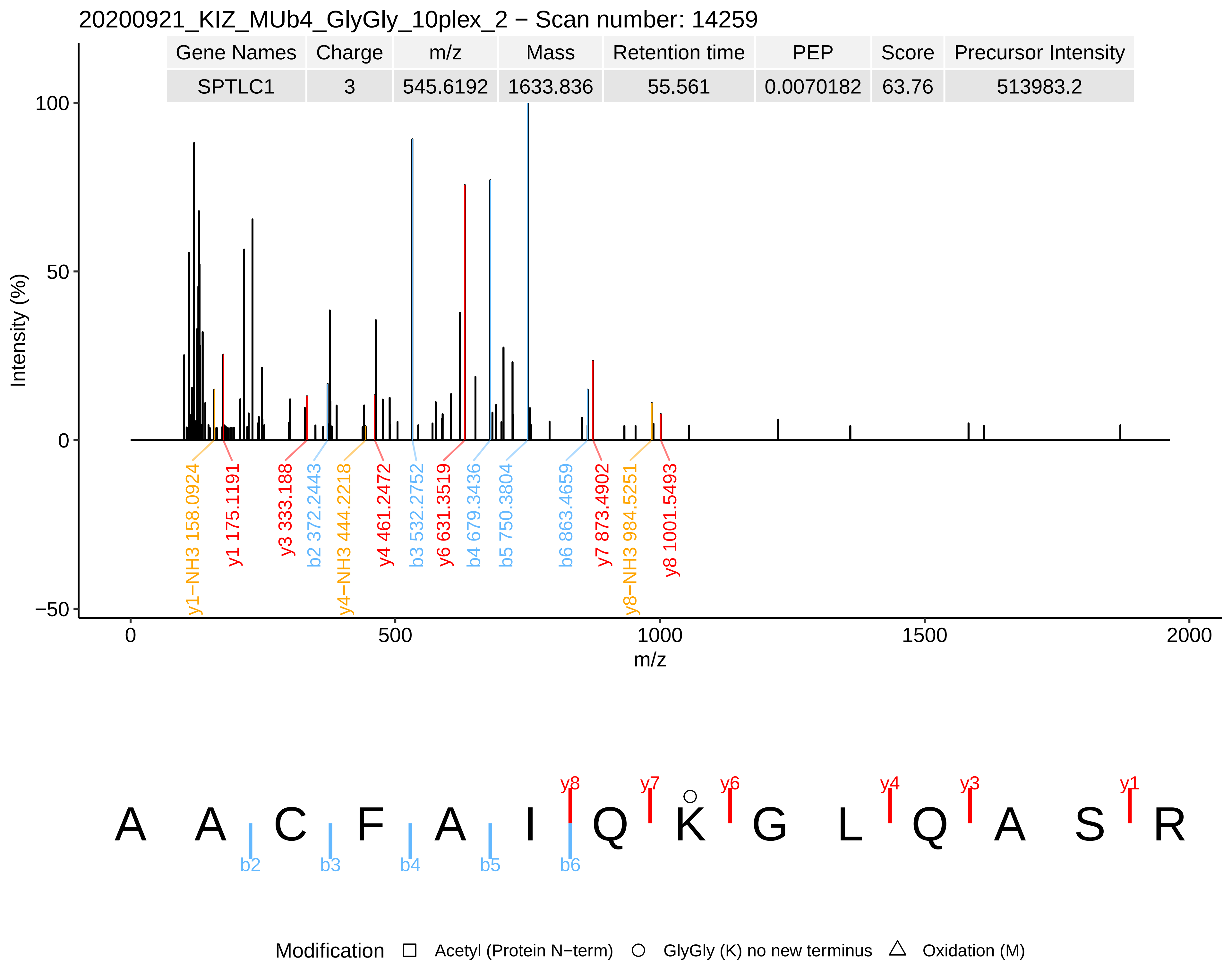
20200921_KIZ_MUb4_GlyGly_10plex_2 − Scan number: 14259
Gene Names | Charge | m/z | Mass | Retention time | PEP | Score | Precursor Intensity
SPTLC1 | 3 | 545.6192 | 1633.836 | 55.561 | 0.0070182 | 63.76 | 513983.2
Intensity (%)
m/z
y1−NH3 158.0924
y1 175.1191
y3 333.188
b2 372.2443
y4−NH3 444.2218
y4 461.2472
b3 532.2752
y6 631.3519
b4 679.3436
b5 750.3804
b6 863.4659
y7 873.4902
y8−NH3 984.5251
y8 1001.5493
A A C F A I Q K G L Q A S R
y8 y7 y6 y4 y3 y1
b2 b3 b4 b5 b6
Modification
Acetyl (Protein N−term)
GlyGly (K) no new terminus
Oxidation (M)

20200921_KIZ_MUb4_GlyGly_10plex − Scan number: 13061

Gene Names	Charge	m/z	Mass	Retention time	PEP	Score	Precursor Intensity
PSMC3	3	475.2502	1422.729	45.939	0.00094847	84.507	15259176

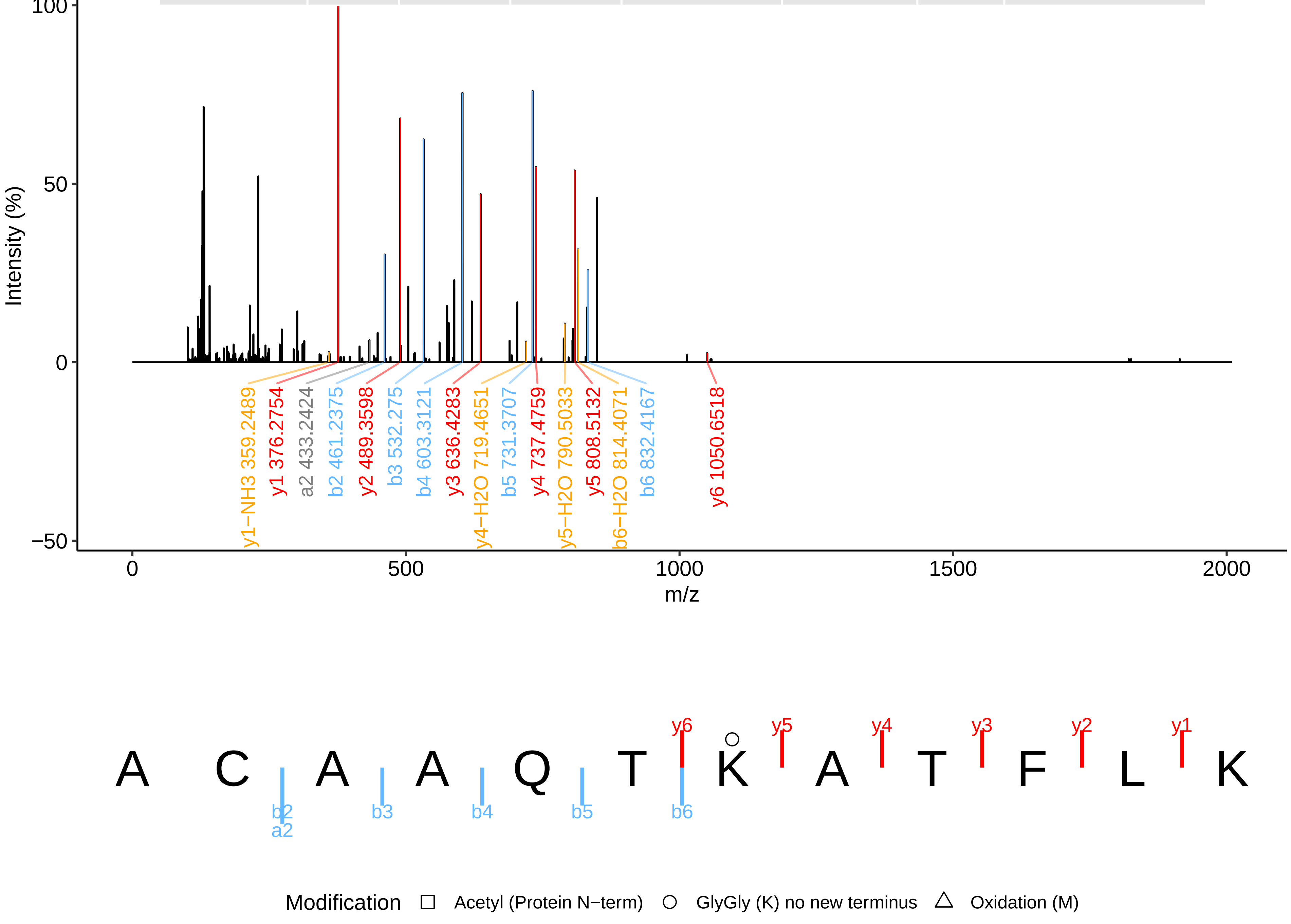

20200921_KIZ_MUb4_GlyGly_10plex_2 − Scan number: 12012

Gene Names	Charge	m/z	Mass	Retention time	PEP	Score	Precursor Intensity
PSMC3	3	475.2502	1422.729	46.381	0.0018378	79.659	1182917

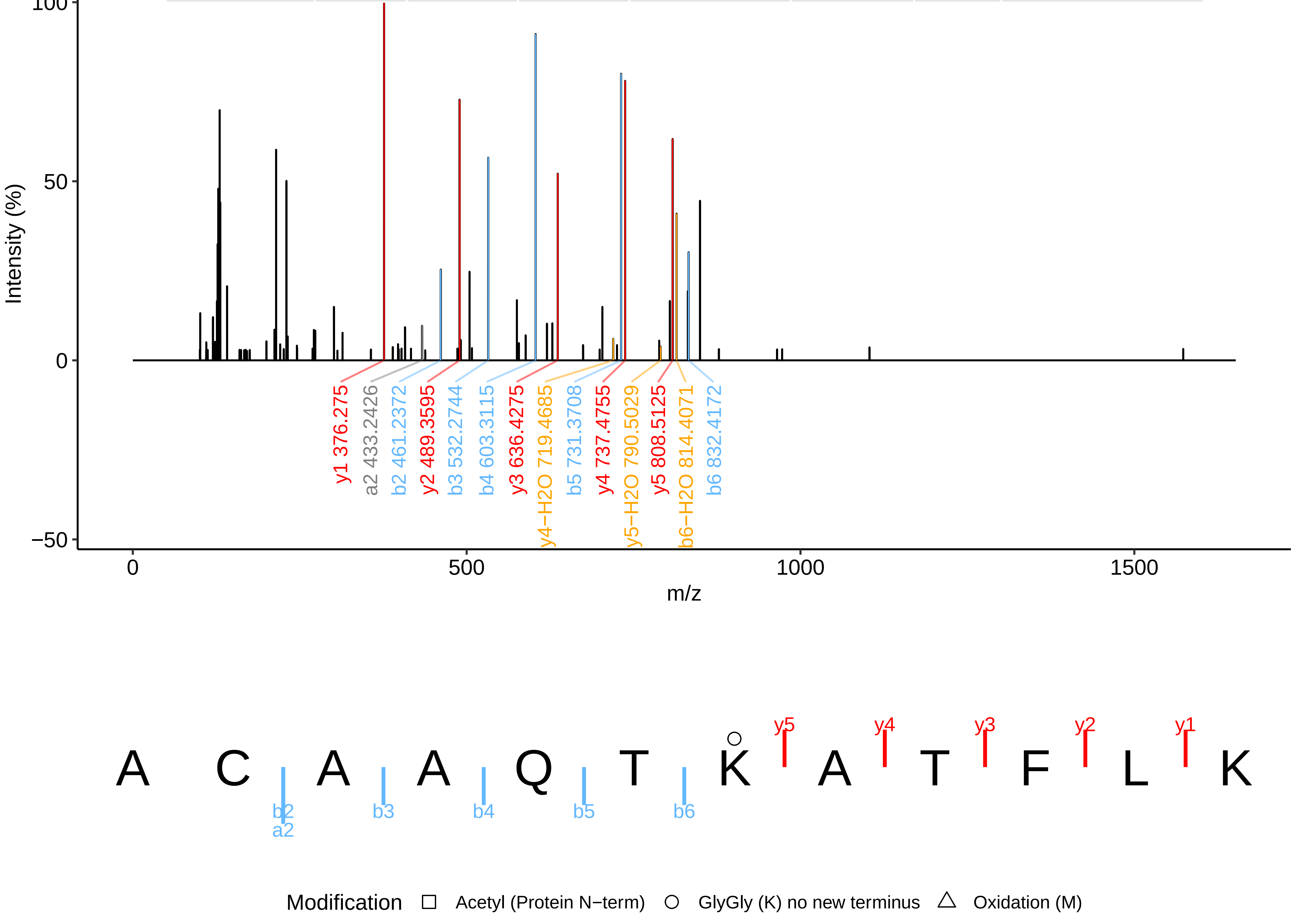

20200924_KIZ_MUb5_GlyGly_1 − Scan number: 12097

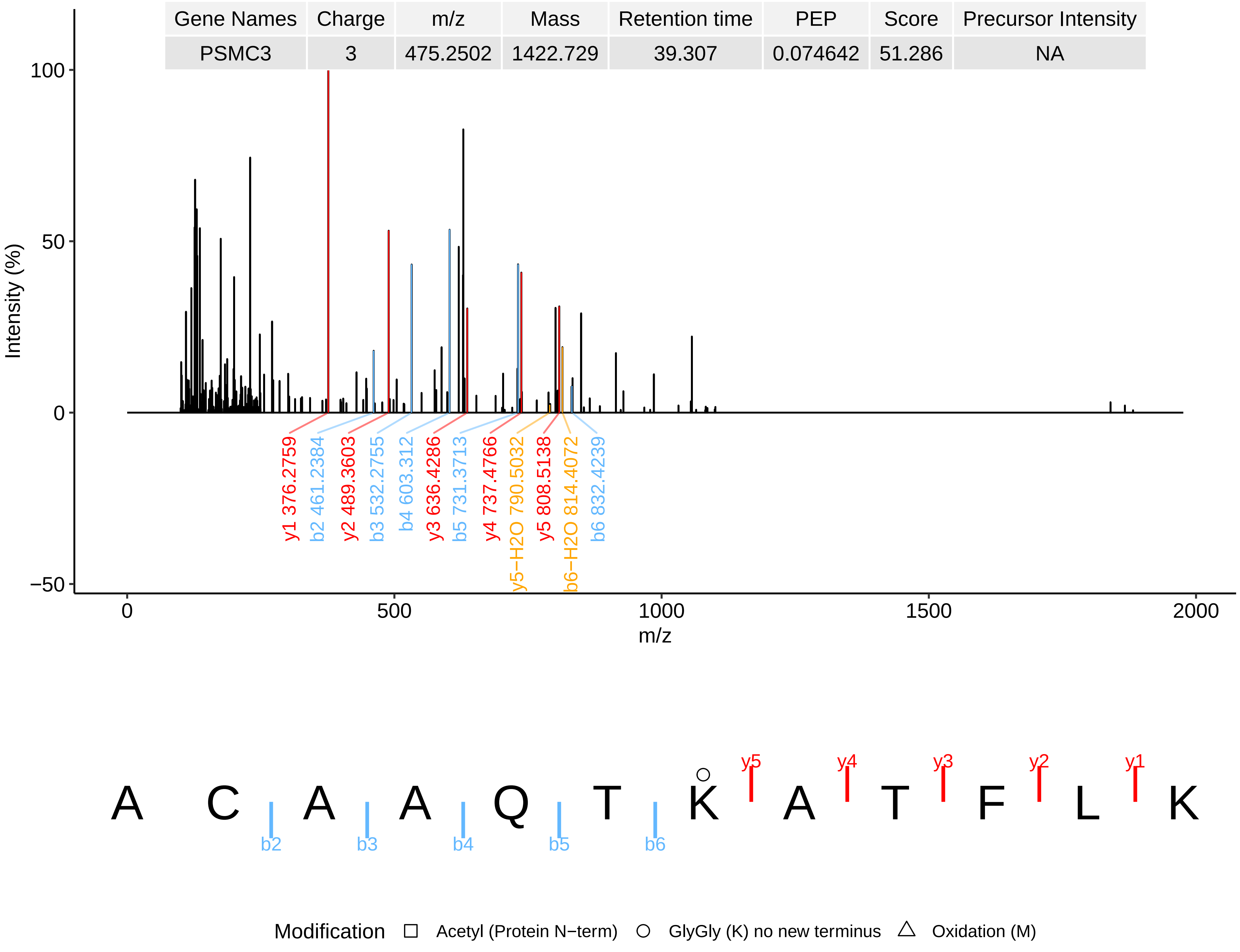

20201118_KIZ_MUb6_GlyGly_2 − Scan number: 14085

Gene Names	Charge	m/z	Mass	Retention time	PEP	Score	Precursor Intensity
PSMC3	3	475.2502	1422.729	47.422	0.00082434	86.313	3467385

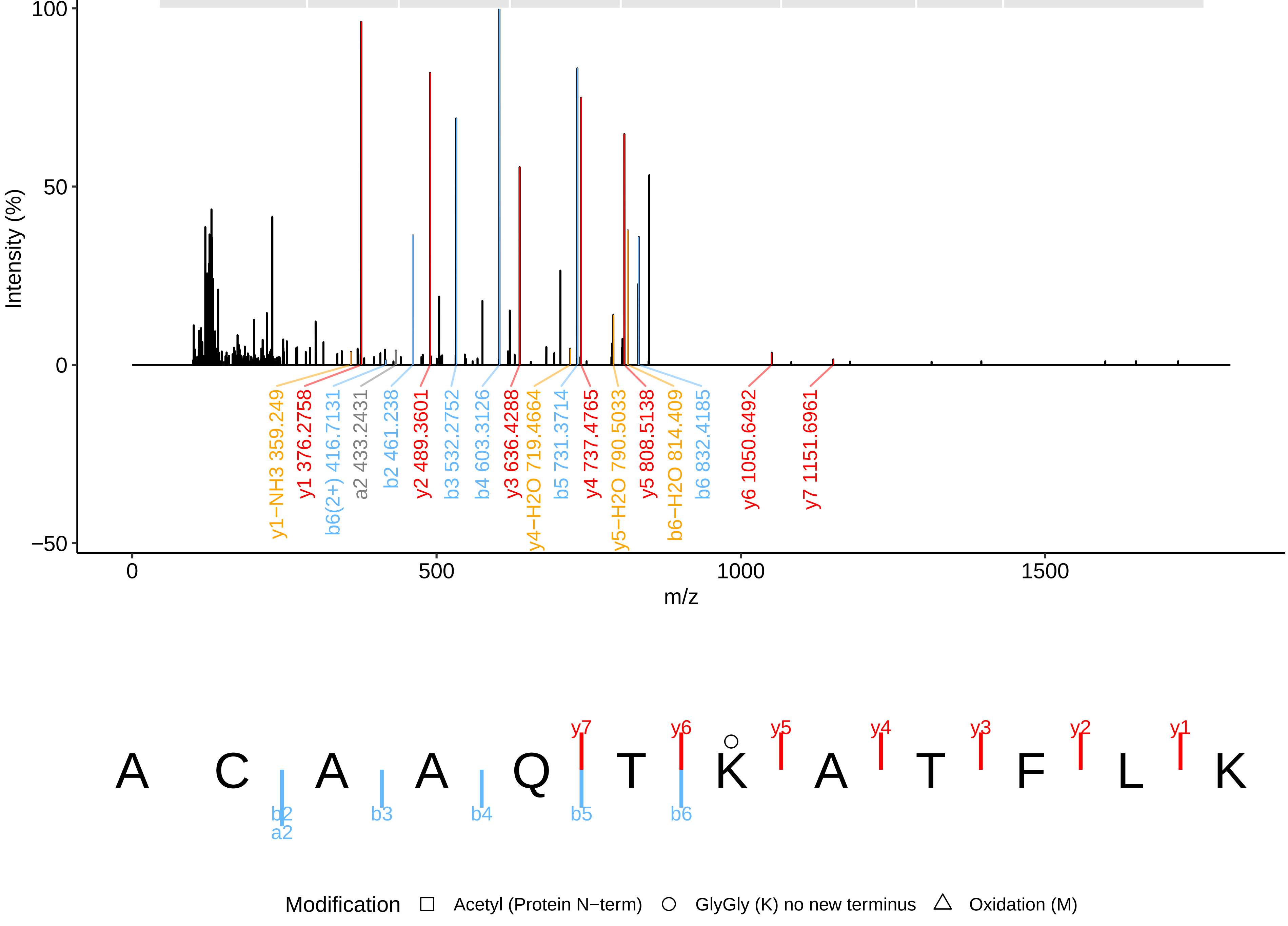

20200921_KIZ_MUb4_GlyGly_10plex − Scan number: 15327

Gene Names	Charge	m/z	Mass	Retention time	PEP	Score	Precursor Intensity
RPS2	3	483.2344	1446.681	54.725	0.0059285	75.479	66046068

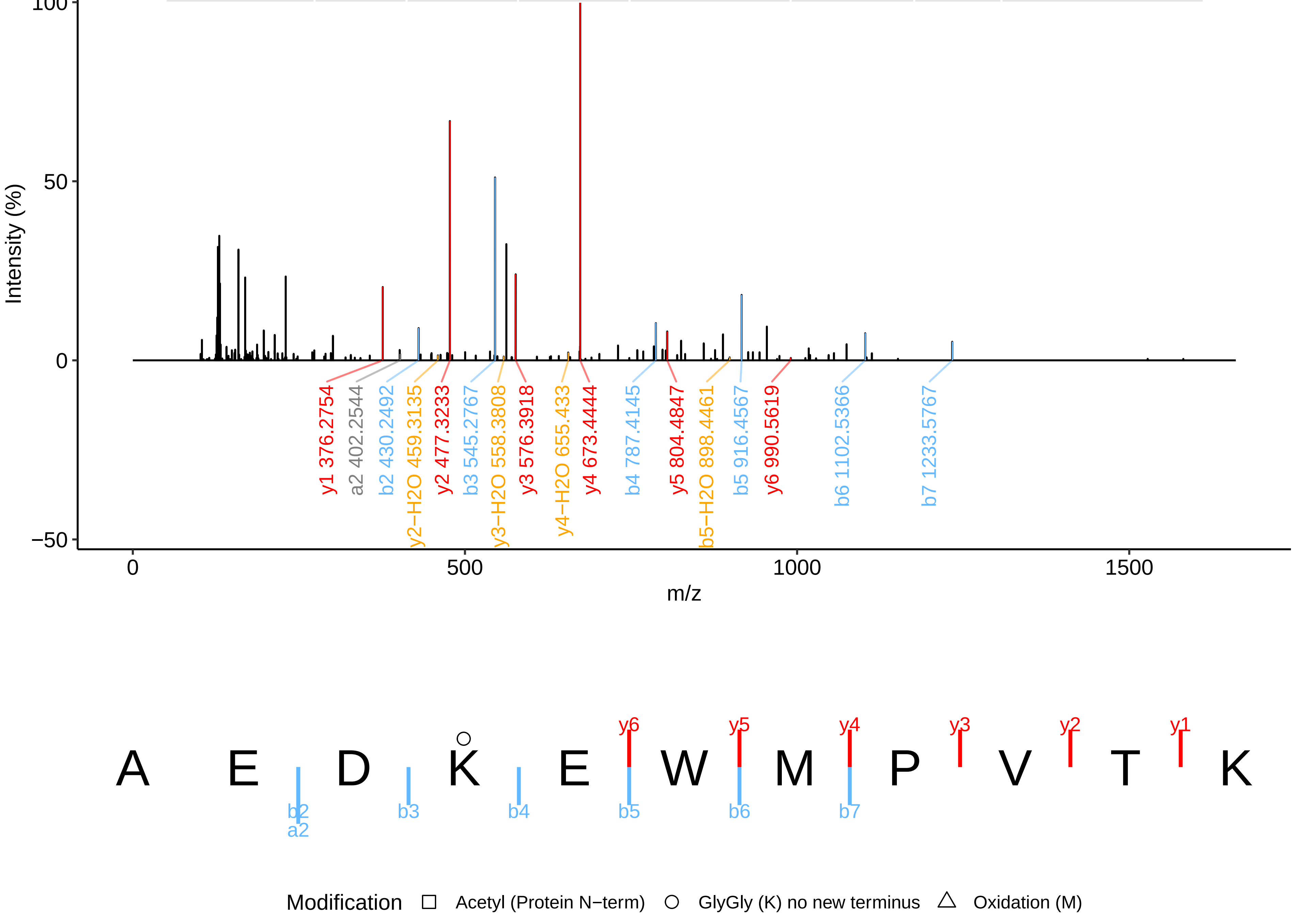

20200921_KIZ_MUb4_GlyGly_10plex_2 − Scan number: 14151

Gene Names	Charge	m/z	Mass	Retention time	PEP	Score	Precursor Intensity
RPS2	3	483.2344	1446.681	55.108	0.00083622	89.805	3680084

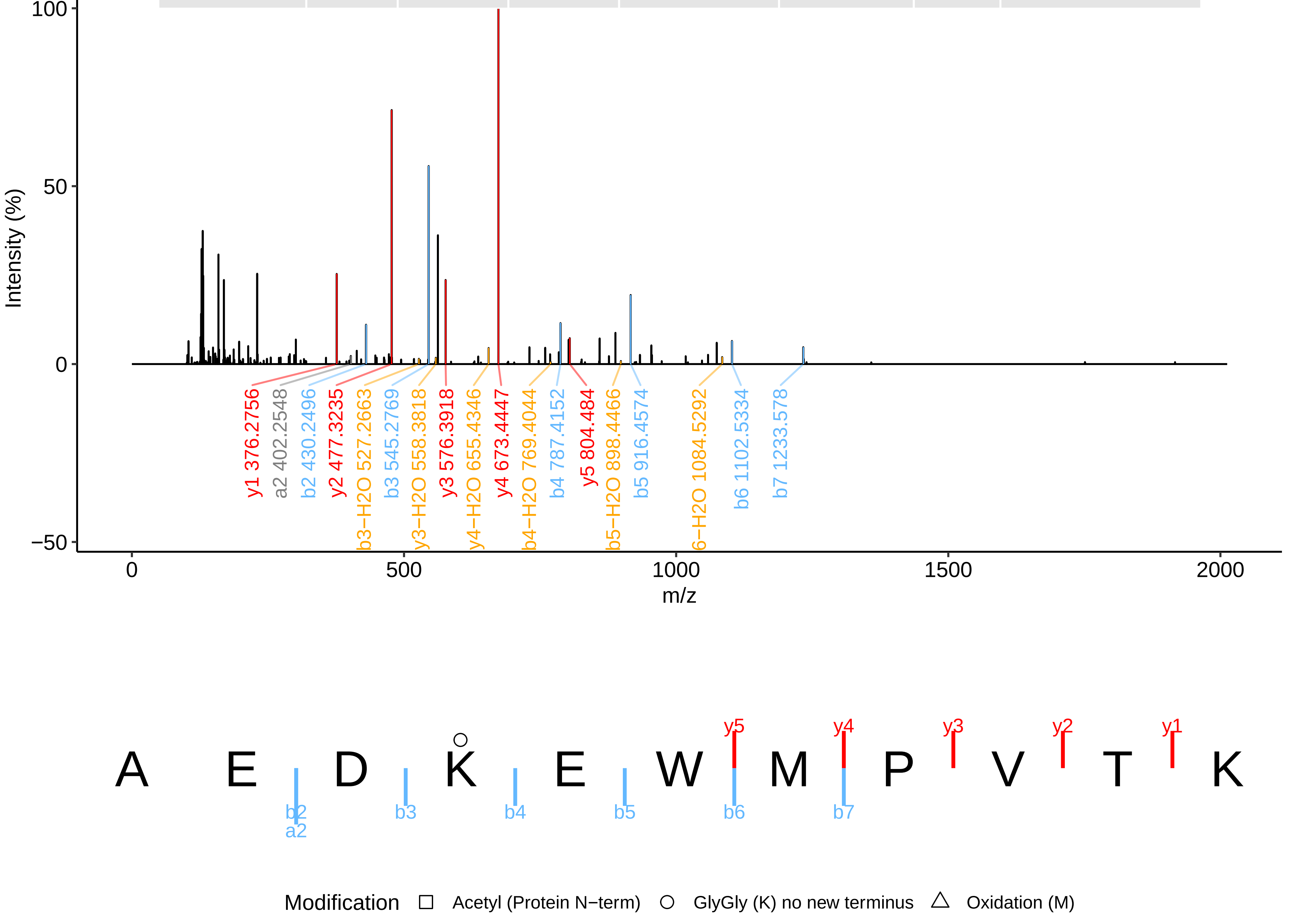

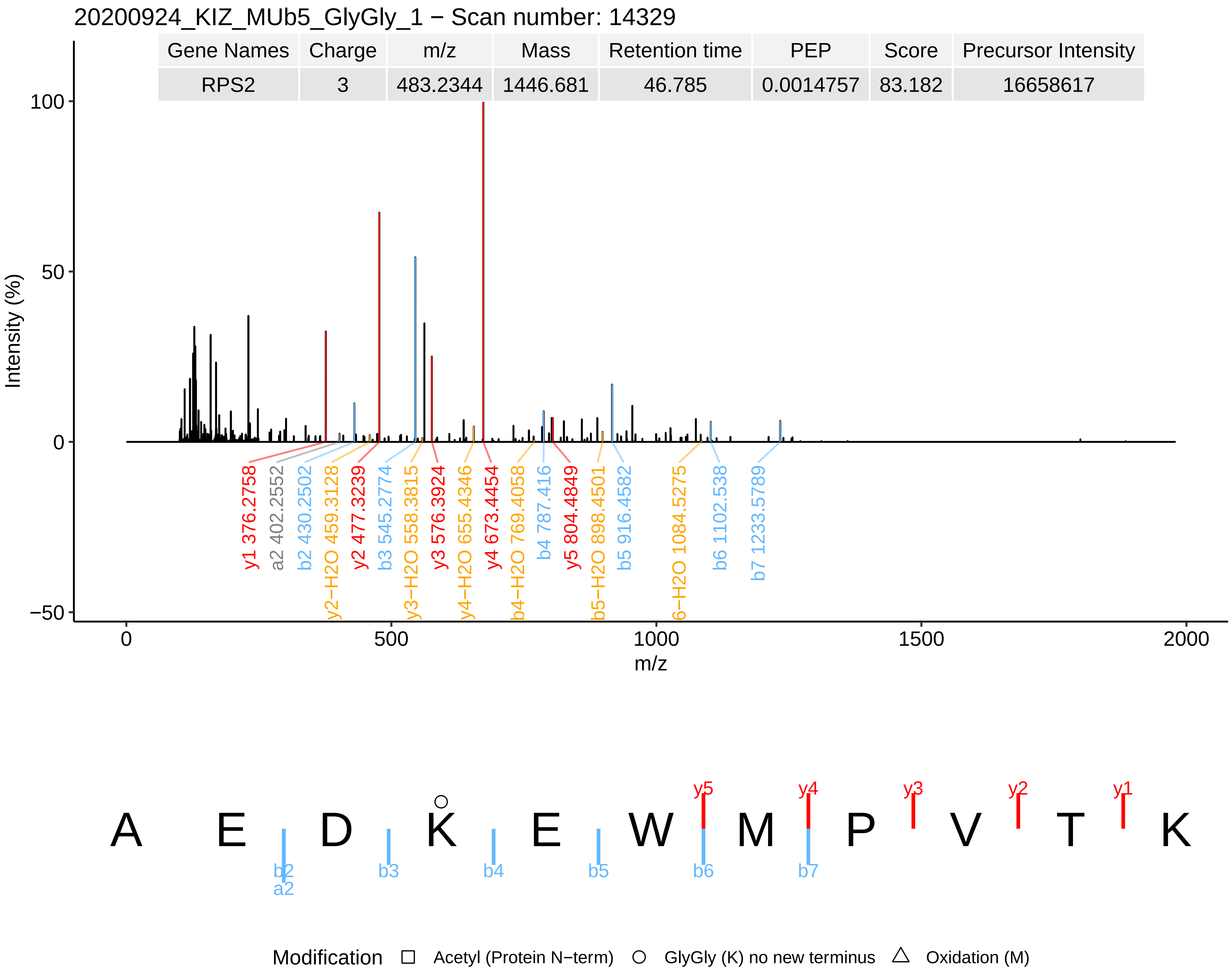
20200924_KIZ_MUb5_GlyGly_1 − Scan number: 14329
Gene Names
Charge
m/z
Mass
Retention time
PEP
Score
Precursor Intensity
RPS2
3
483.2344
1446.681
46.785
0.0014757
83.182
16658617
Intensity (%)
100
50
0
−50
0
500
1000
1500
2000
m/z
y1 376.2758
a2 402.2552
b2 430.2502
y2−H2O 459.3128
y2 477.3239
b3 545.2774
y3−H2O 558.3815
y3 576.3924
y4−H2O 655.4346
y4 673.4454
b4−H2O 769.4058
b4 787.416
y5 804.4849
b5−H2O 898.4501
b5 916.4582
6−H2O 1084.5275
b6 1102.538
b7 1233.5789
A E D K E W M P V T K
y5
y4
y3
y2
y1
b2
a2
b3
b4
b5
b6
b7
Modification
Acetyl (Protein N−term)
GlyGly (K) no new terminus
Oxidation (M)

20200924_KIZ_MUb5_GlyGly_1 − Scan number: 14484

Gene Names	Charge	m/z	Mass	Retention time	PEP	Score	Precursor Intensity
RPS2	3	483.2344	1446.681	47.294	0.0012186	85.845	47842368

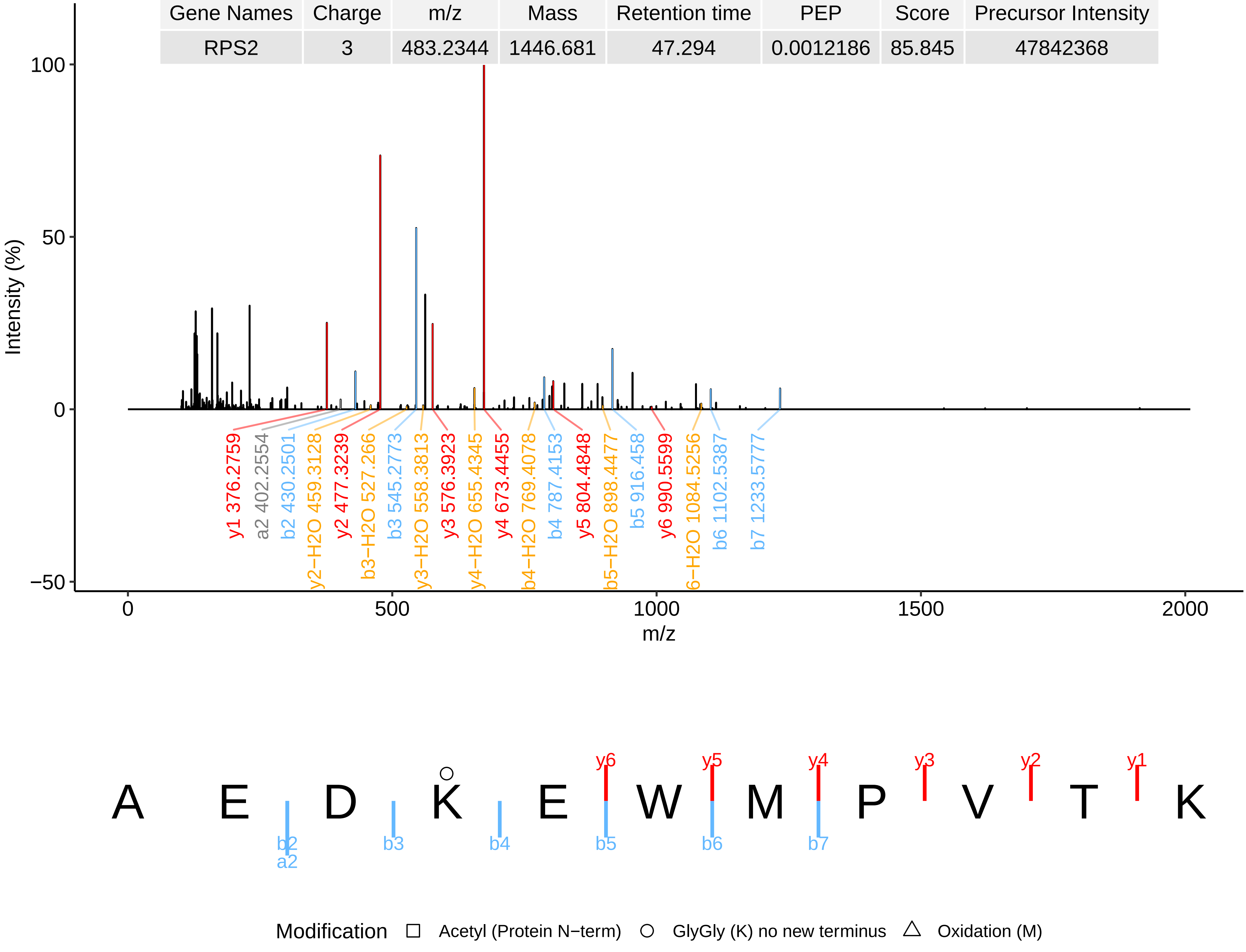

20200924_KIZ_MUb5_GlyGly_2 − Scan number: 13598

Gene Names	Charge	m/z	Mass	Retention time	PEP	Score	Precursor Intensity
RPS2	3	483.2344	1446.681	50.851	0.0087173	72.089	12527341

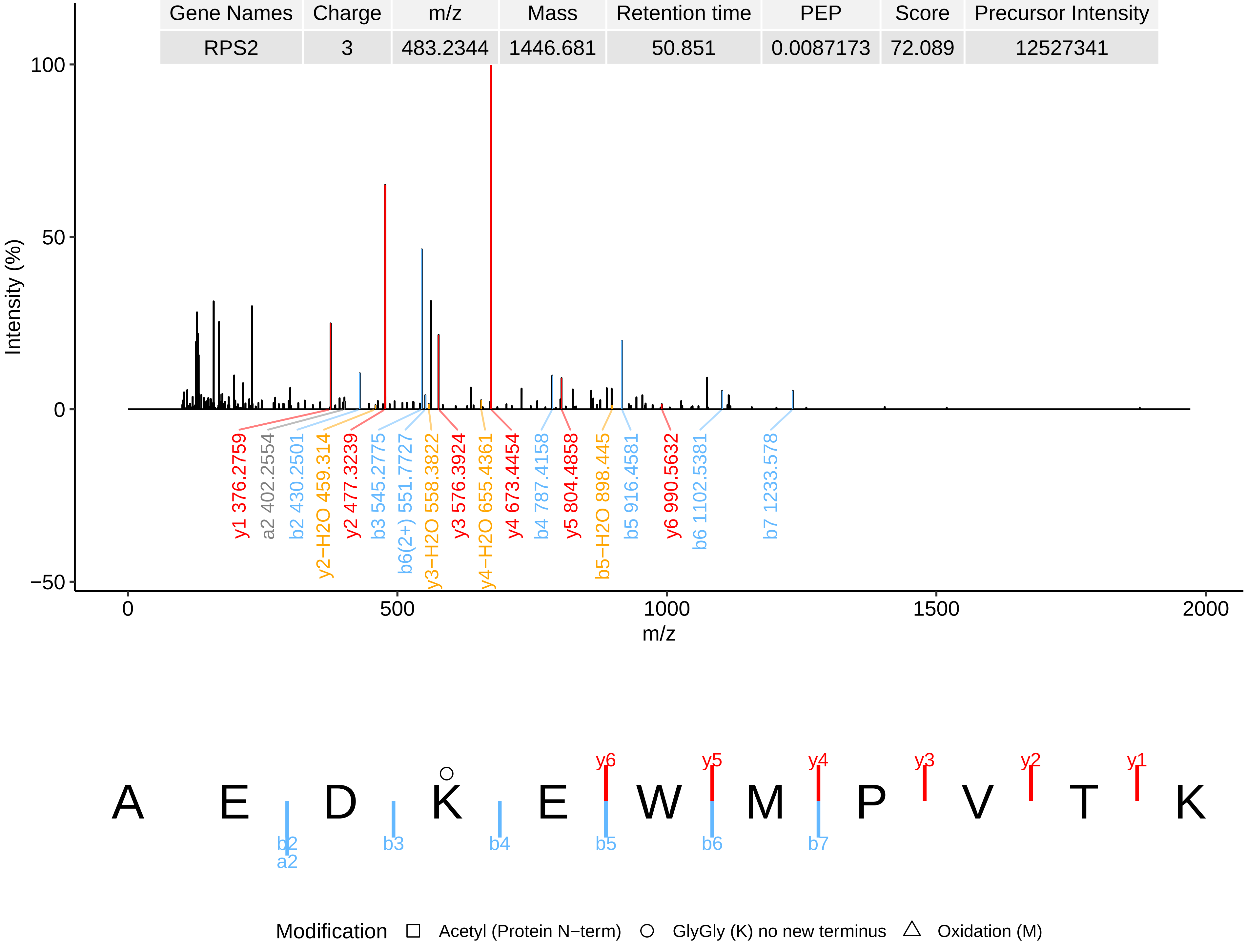

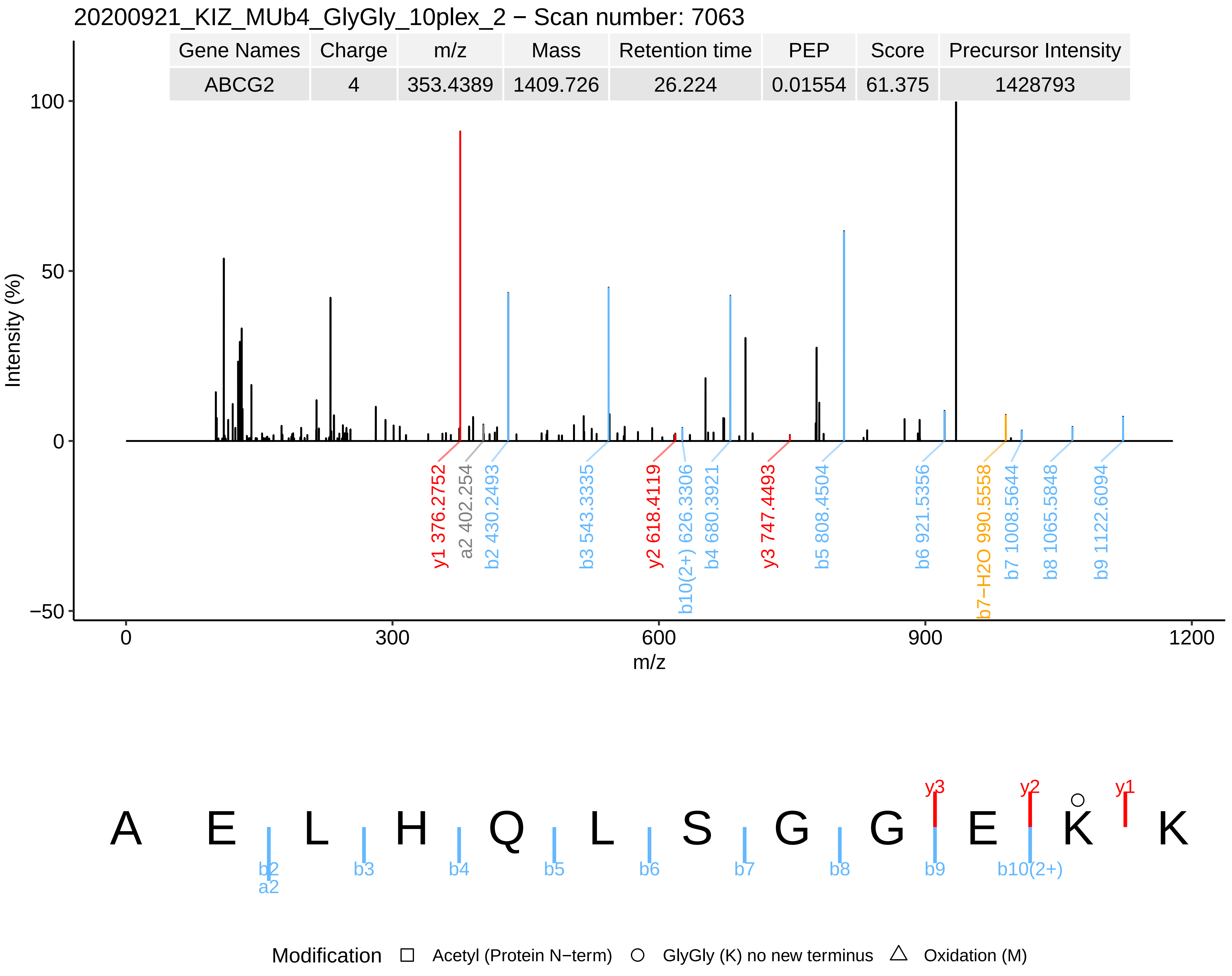
20200921_KIZ_MUb4_GlyGly_10plex_2 − Scan number: 7063
Gene Names
Charge
m/z
Mass
Retention time
PEP
Score
Precursor Intensity
ABCG2
4
353.4389
1409.726
26.224
0.01554
61.375
1428793
Intensity (%)
m/z
y1 376.2752
a2 402.254
b2 430.2493
b3 543.3335
y2 618.4119
b10(2+) 626.3306
b4 680.3921
y3 747.4493
b5 808.4504
b6 921.5356
b7−H2O 990.5558
b7 1008.5644
b8 1065.5848
b9 1122.6094
A E L H Q L S G G E K K
Modification
Acetyl (Protein N−term)
GlyGly (K) no new terminus
Oxidation (M)

20200921_KIZ_MUb4_GlyGly_10plex − Scan number: 5622

Gene Names	Charge	m/z	Mass	Retention time	PEP	Score	Precursor Intensity
BASP1	3	482.2353	1443.684	18.445	0.00080127	89.629	6767018

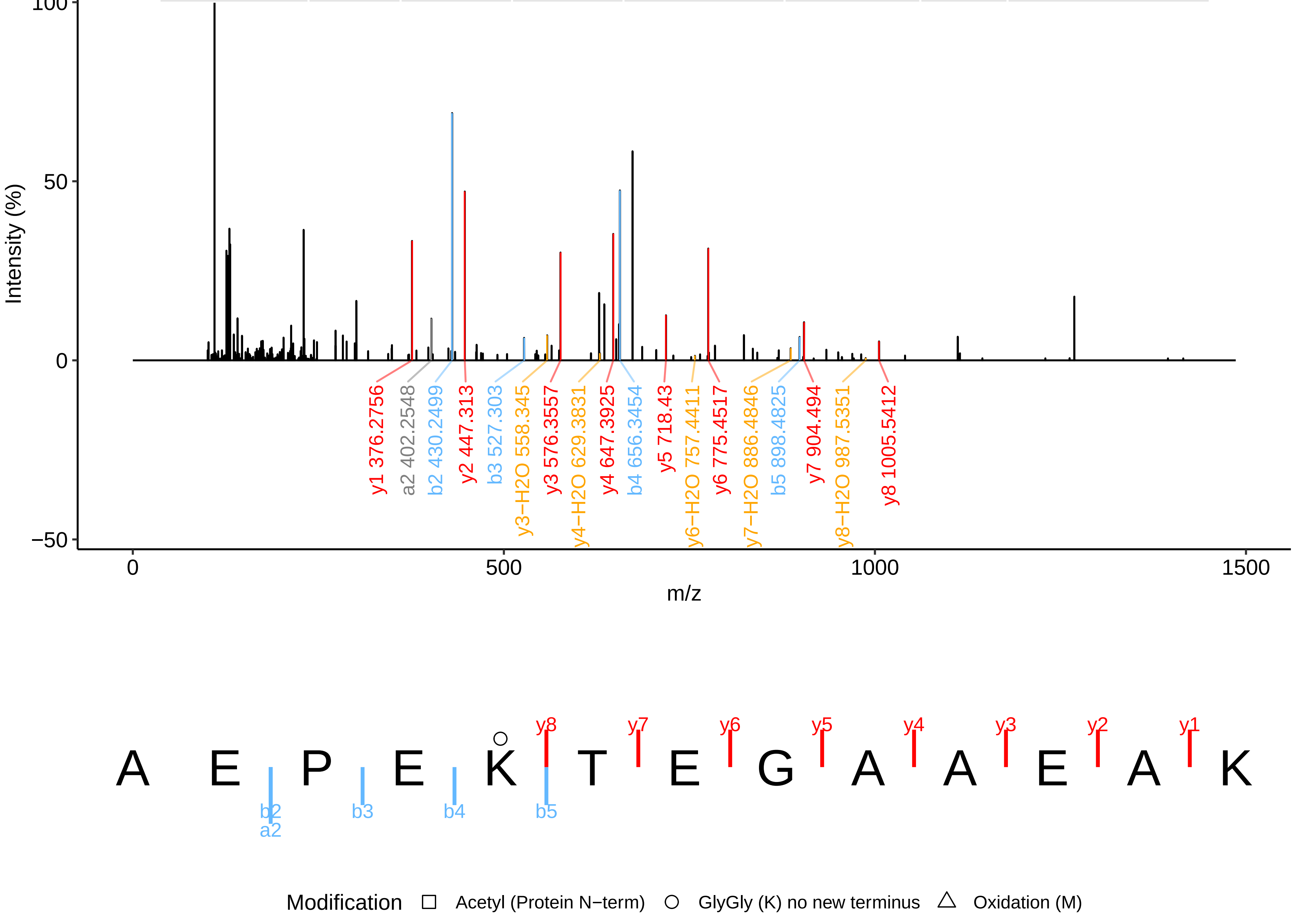

20200921_KIZ_MUb4_GlyGly_10plex_2 − Scan number: 5117

Gene Names	Charge	m/z	Mass	Retention time	PEP	Score	Precursor Intensity
BASP1	3	482.2353	1443.684	18.25	0.0060482	71.548	746874.2

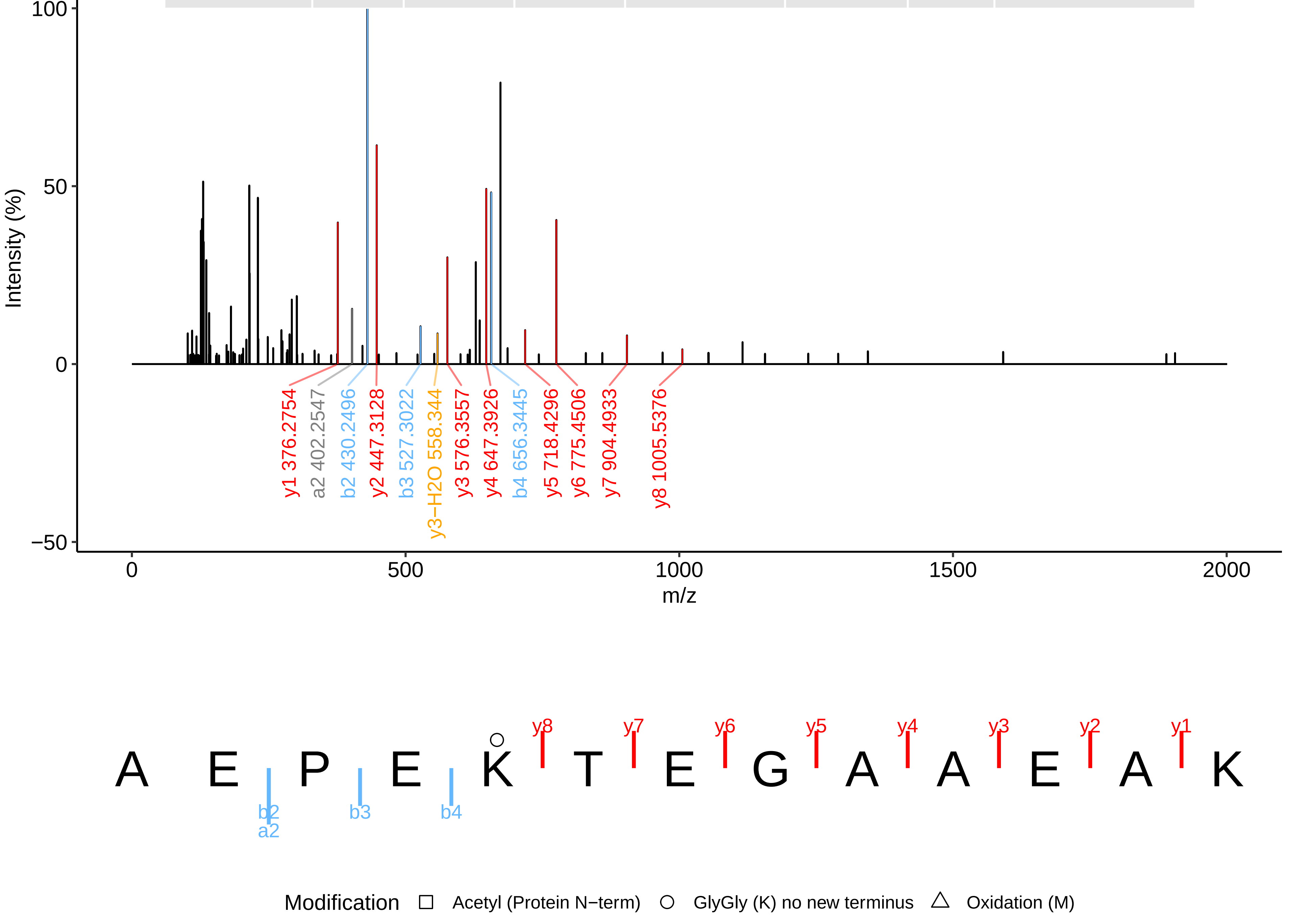

20200924_KIZ_MUb5_GlyGly_1 − Scan number: 4902

Gene Names	Charge	m/z	Mass	Retention time	PEP	Score	Precursor Intensity
BASP1	3	482.2353	1443.684	15.186	0.0058694	71.879	7975837

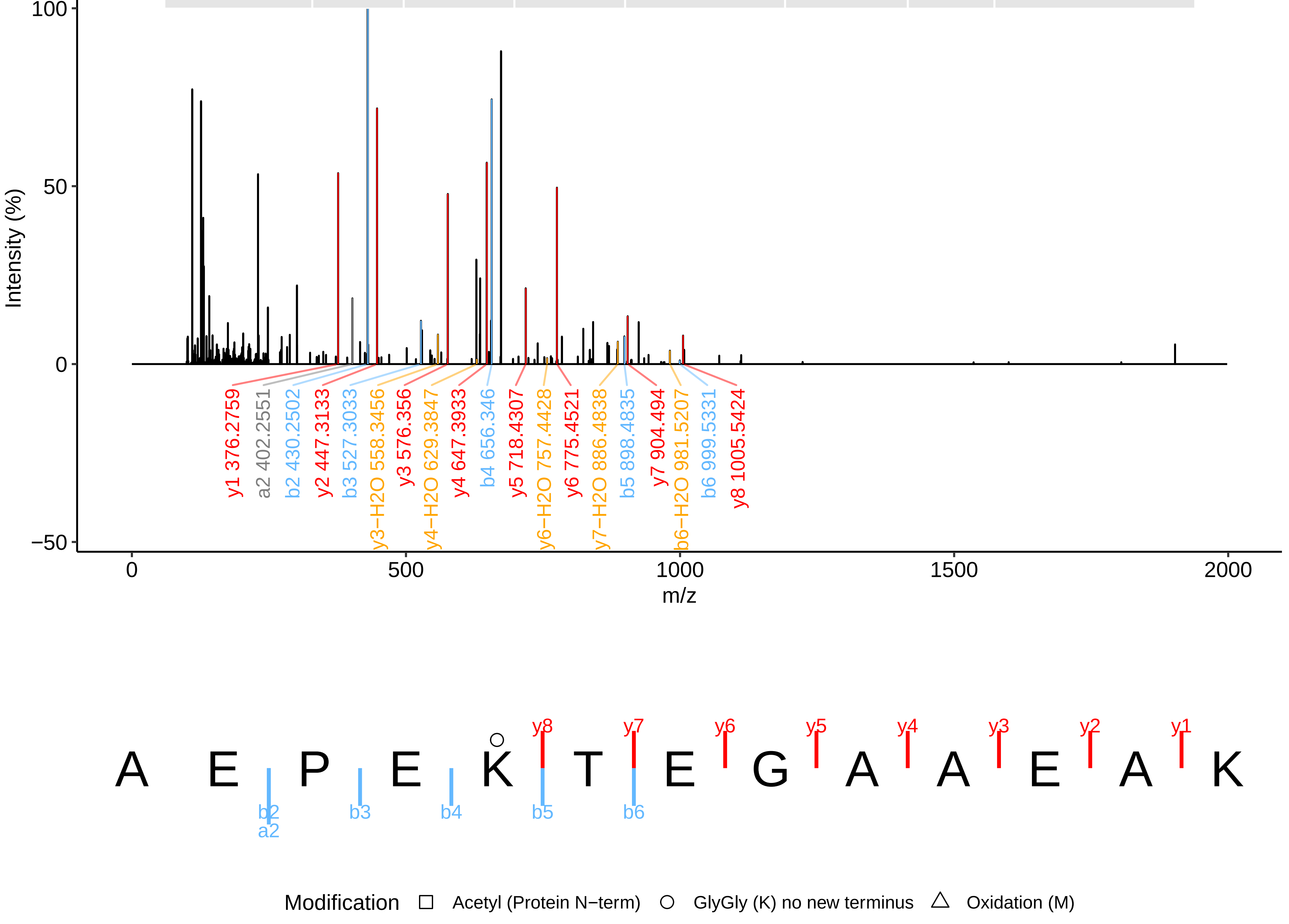

20200924_KIZ_MUb5_GlyGly_2 − Scan number: 4548

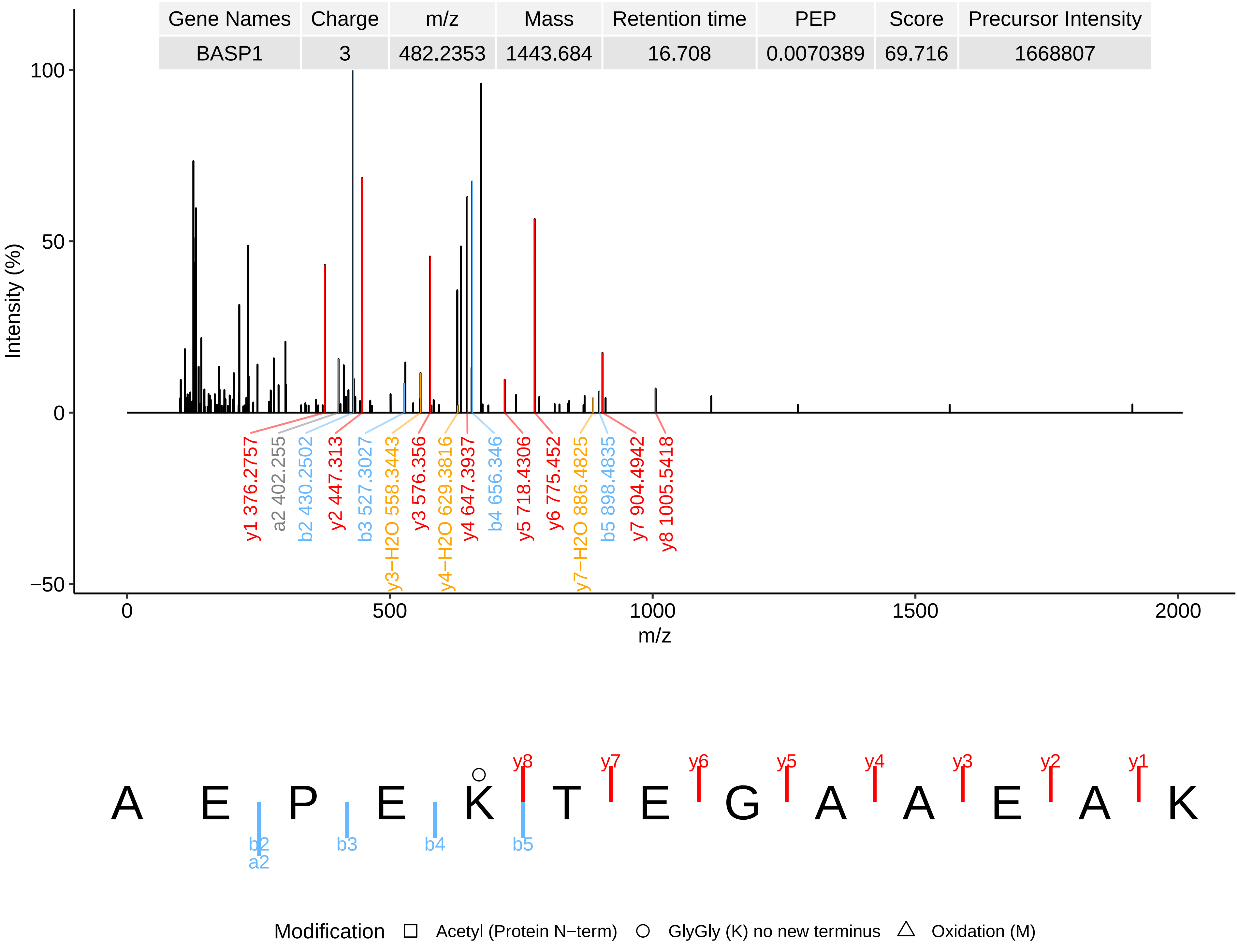

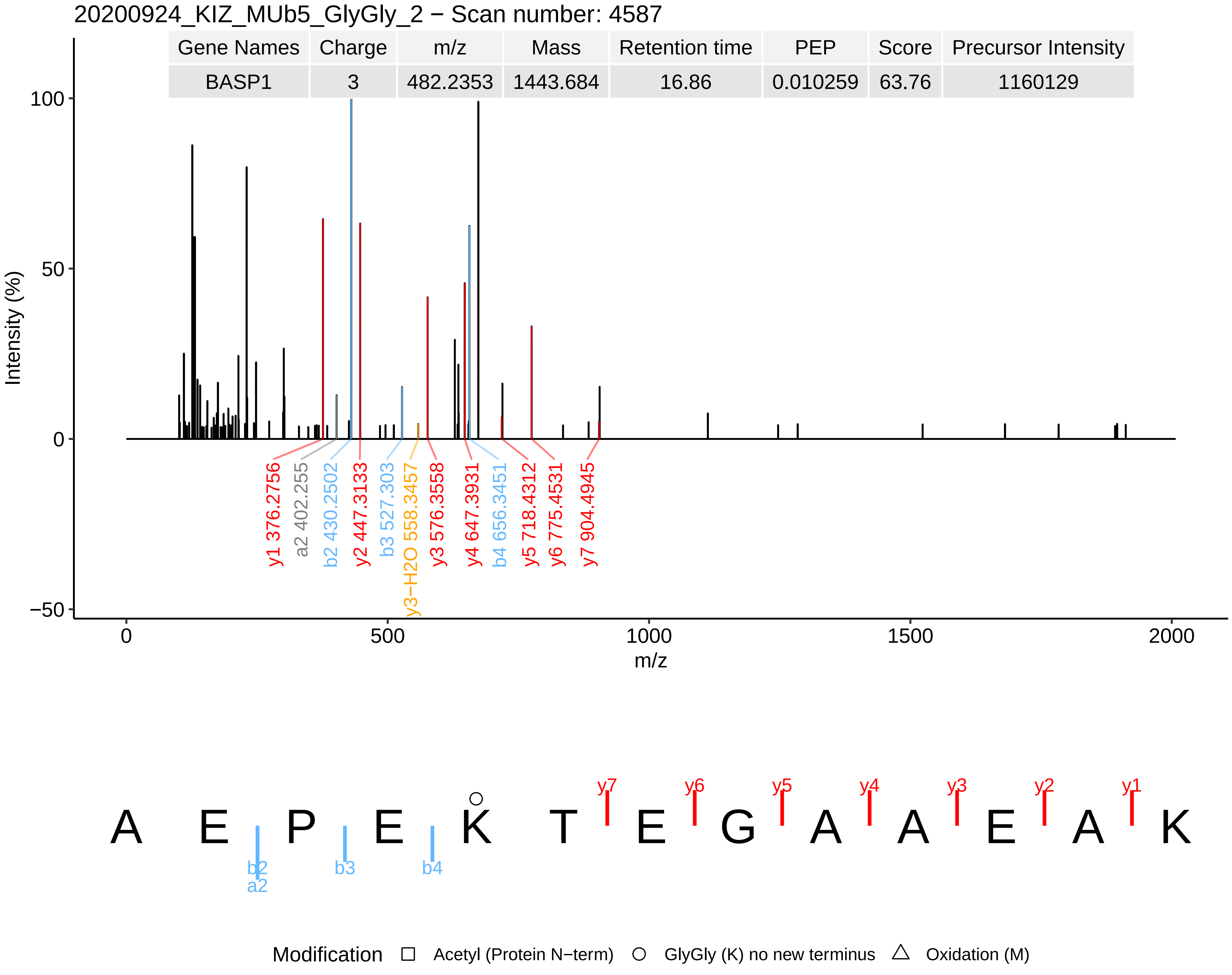
20200924_KIZ_MUb5_GlyGly_2 − Scan number: 4587
Gene Names | Charge | m/z | Mass | Retention time | PEP | Score | Precursor Intensity
BASP1 | 3 | 482.2353 | 1443.684 | 16.86 | 0.010259 | 63.76 | 1160129
Intensity (%)
100
50
0
−50
0
500
1000
1500
2000
m/z
y1 376.2756
a2 402.255
b2 430.2502
y2 447.3133
b3 527.303
y3−H2O 558.3457
y3 576.3558
y4 647.3931
b4 656.3451
y5 718.4312
y6 775.4531
y7 904.4945
A E P E K T E G A A E A K
y7 y6 y5 y4 y3 y2 y1
b2 a2 b3 b4
Modification
Acetyl (Protein N−term)
GlyGly (K) no new terminus
Oxidation (M)

20200921_KIZ_MUb4_GlyGly_10plex − Scan number: 8597

Gene Names	Charge	m/z	Mass	Retention time	PEP	Score	Precursor Intensity
NDFIP2	3	550.2739	1647.8	29.229	0.064667	46.812	4588840

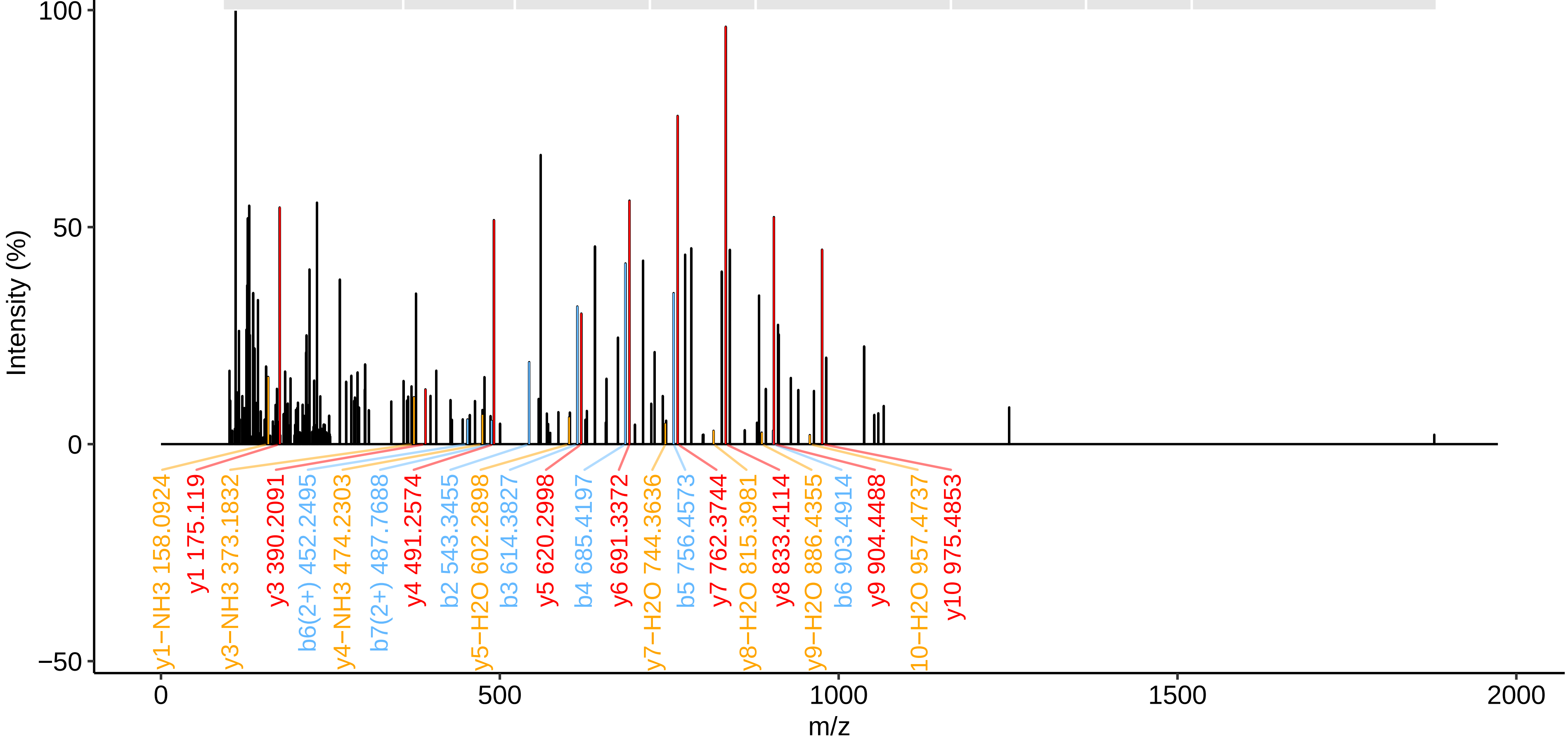

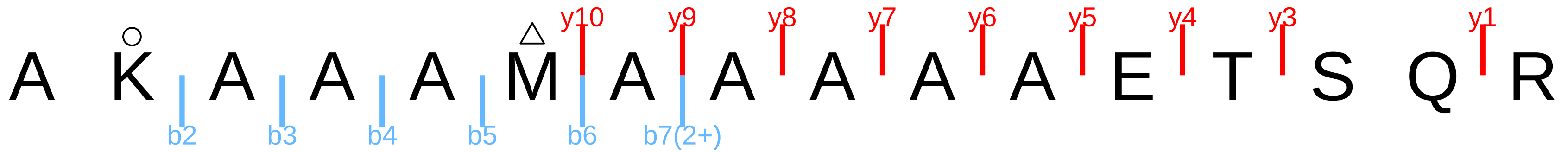

Modification □ Acetyl (Protein N−term) ○ GlyGly (K) no new terminus △ Oxidation (M)

20200921_KIZ_MUb4_GlyGly_10plex_2 − Scan number: 8029

Gene Names	Charge	m/z	Mass	Retention time	PEP	Score	Precursor Intensity
NDFIP2	3	550.2739	1647.8	30.161	0.07161	52.522	NA

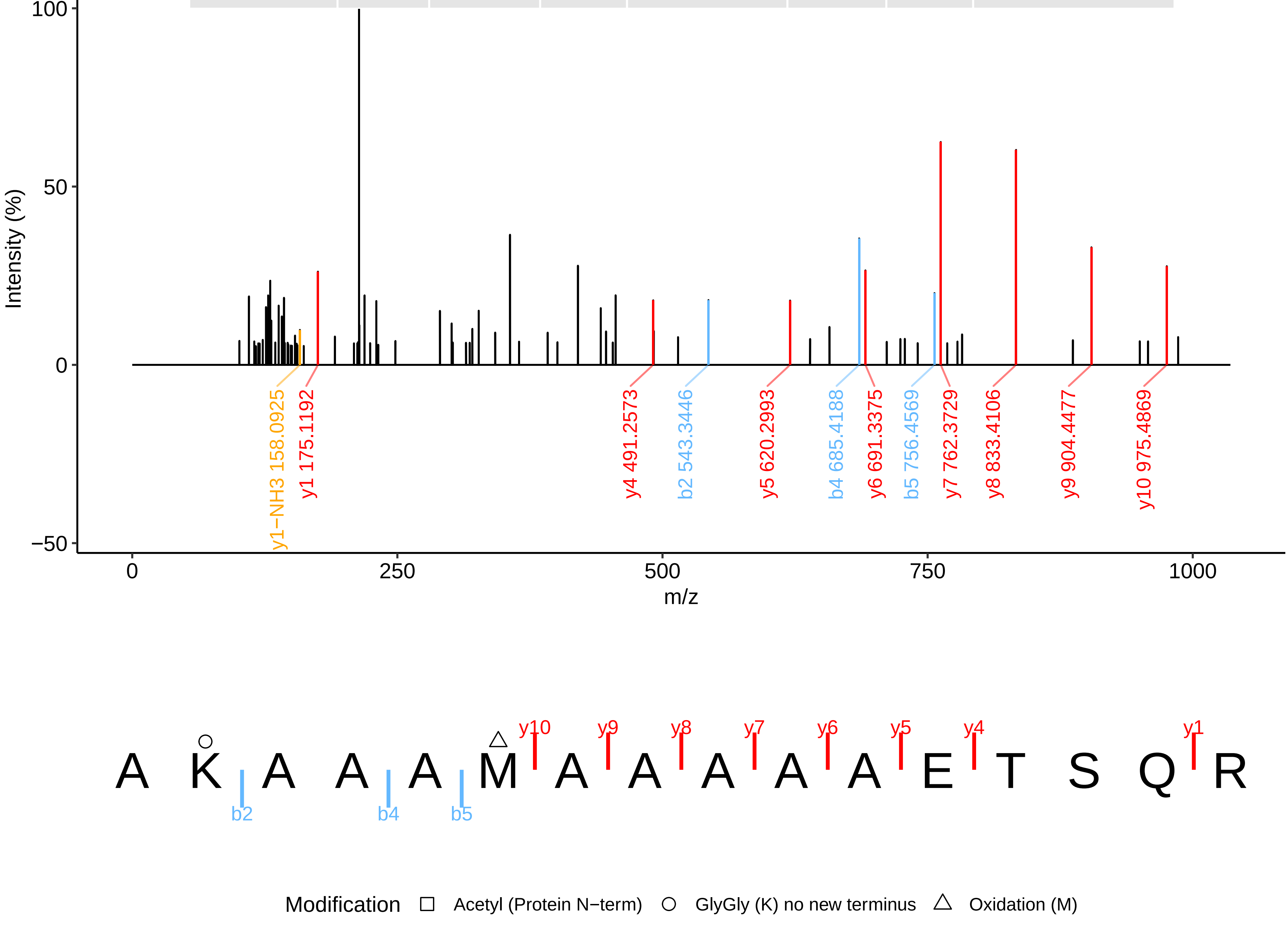

20200921_KIZ_MUb4_GlyGly_10plex − Scan number: 12324

Gene Names	Charge	m/z	Mass	Retention time	PEP	Score	Precursor Intensity
NDFIP2	3	544.9423	1631.805	43.167	0.00079403	76.158	8103181

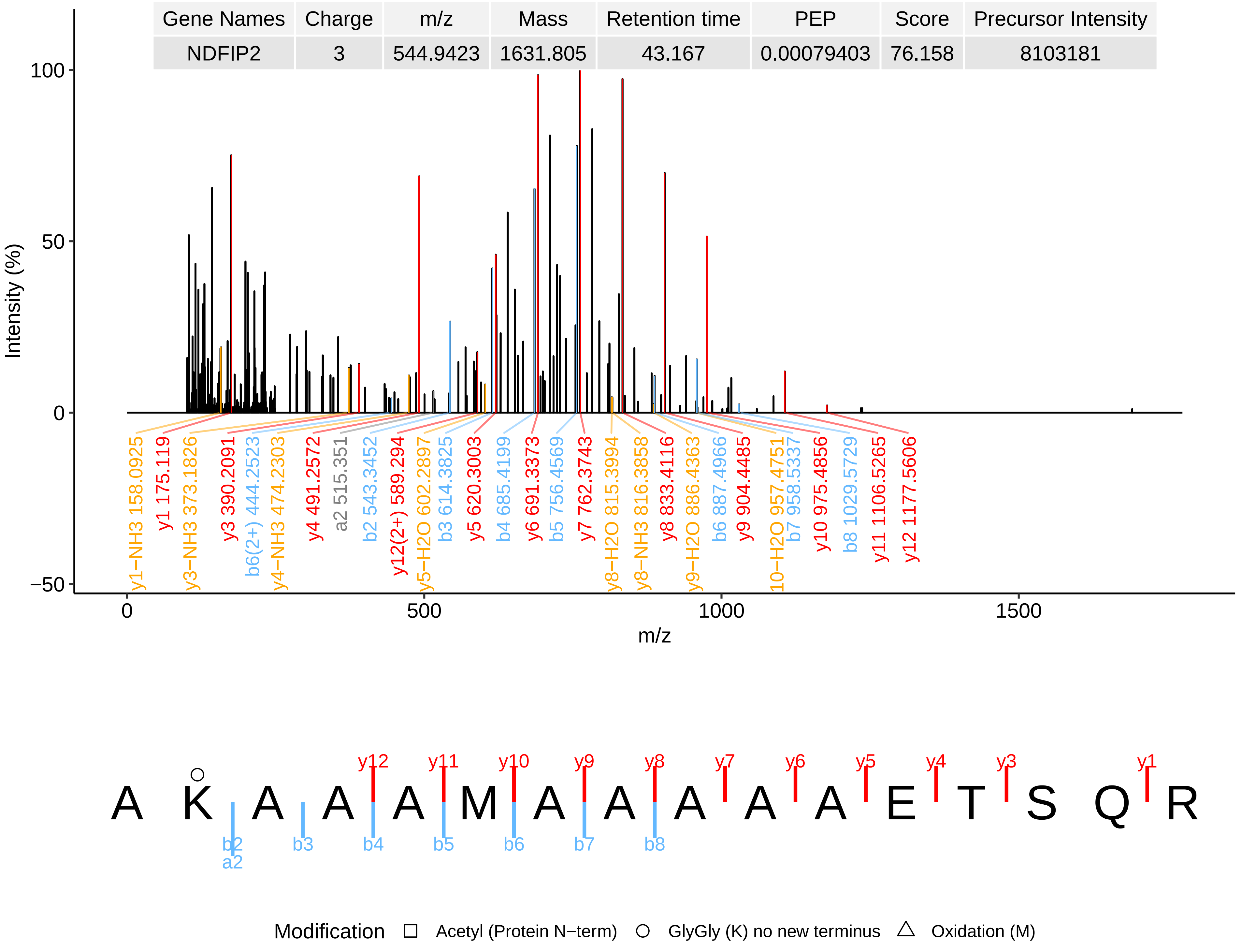

20200921_KIZ_MUb4_GlyGly_10plex_2 − Scan number: 11354

Gene Names	Charge	m/z	Mass	Retention time	PEP	Score	Precursor Intensity
NDFIP2	3	544.9423	1631.805	43.683	1.7302e−05	81.346	3200174

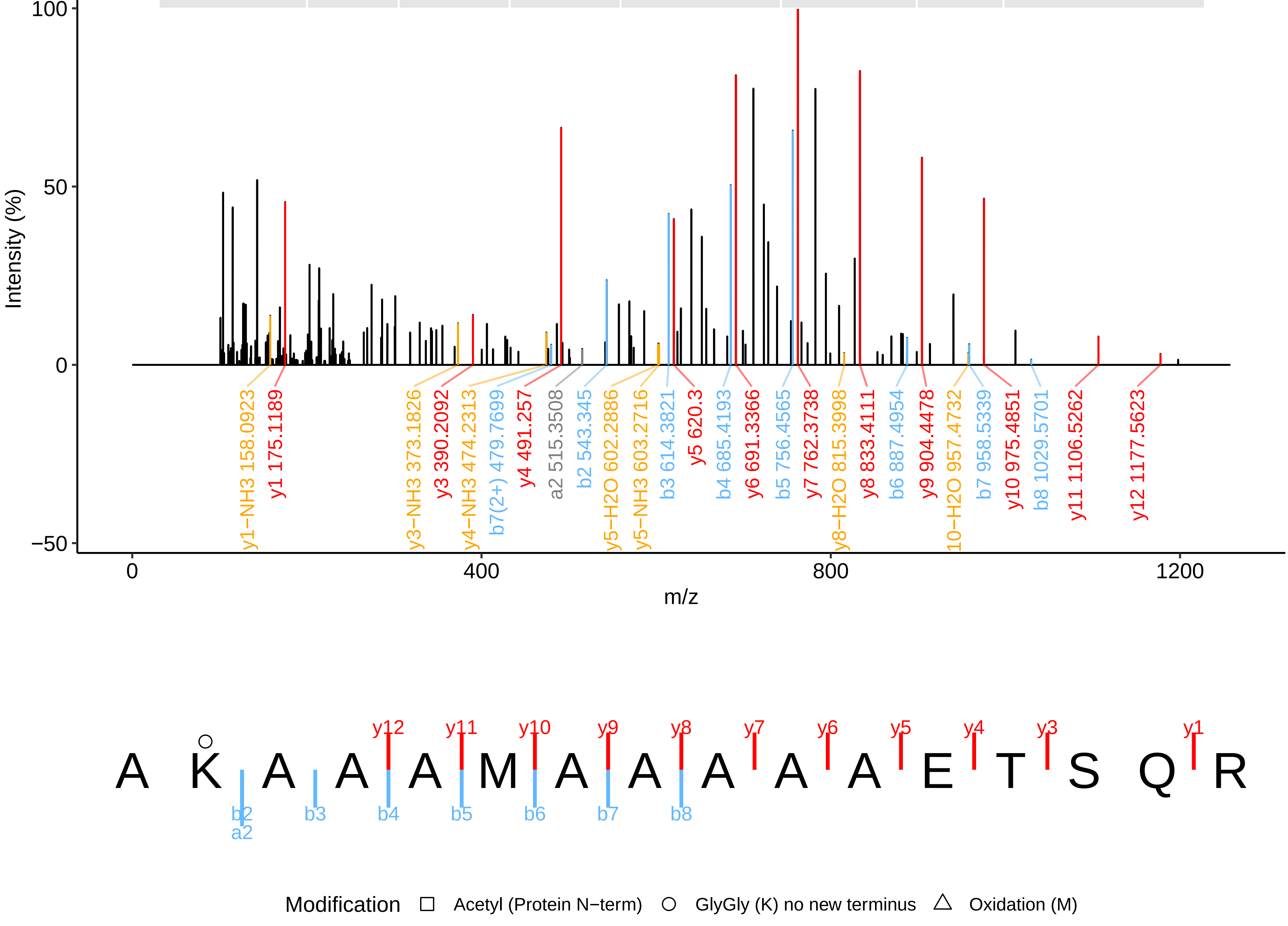

20200924_KIZ_MUb5_GlyGly_1 − Scan number: 10928

Gene Names	Charge	m/z	Mass	Retention time	PEP	Score	Precursor Intensity
NDFIP2	3	544.9423	1631.805	35.374	0.0034757	73.093	3020268

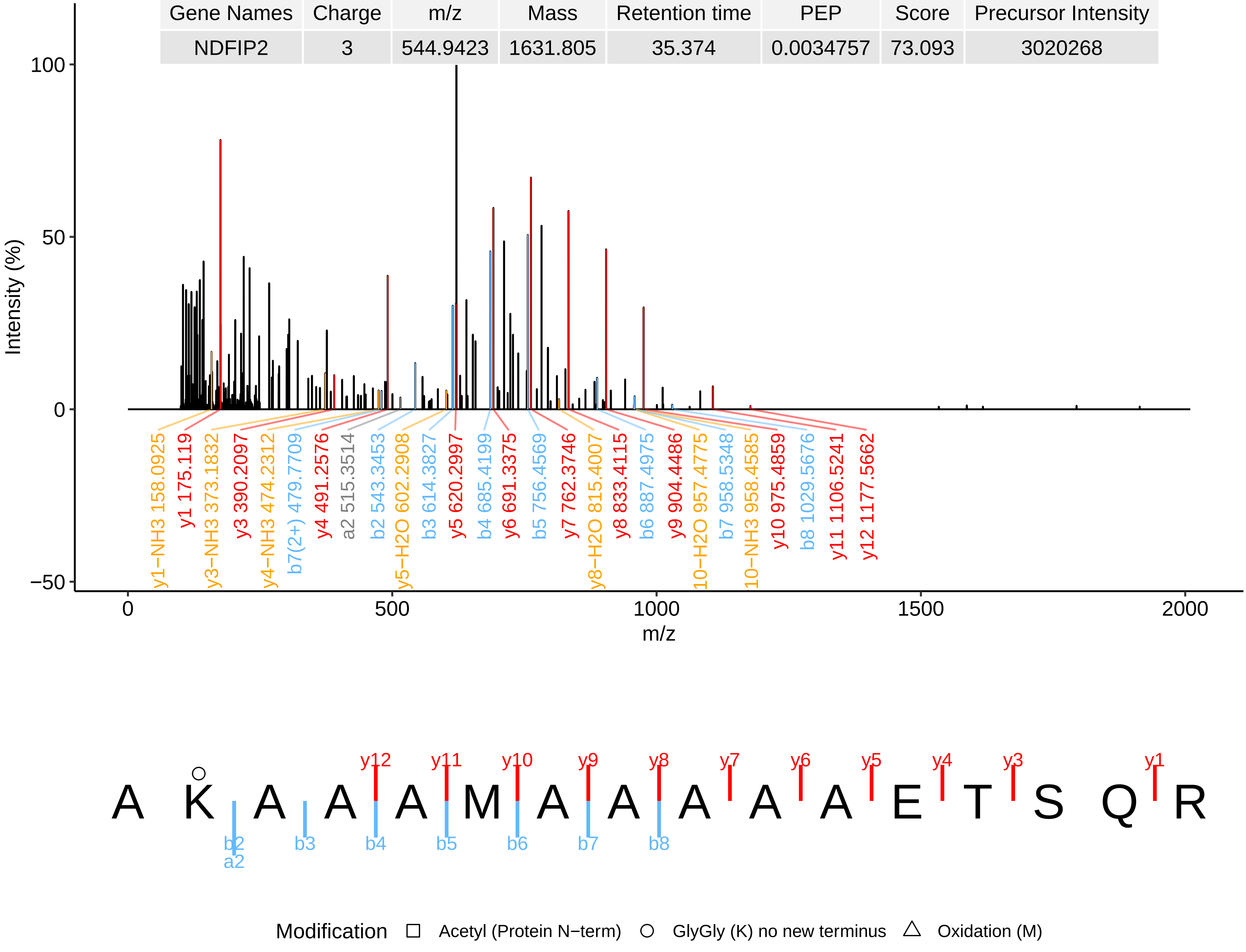

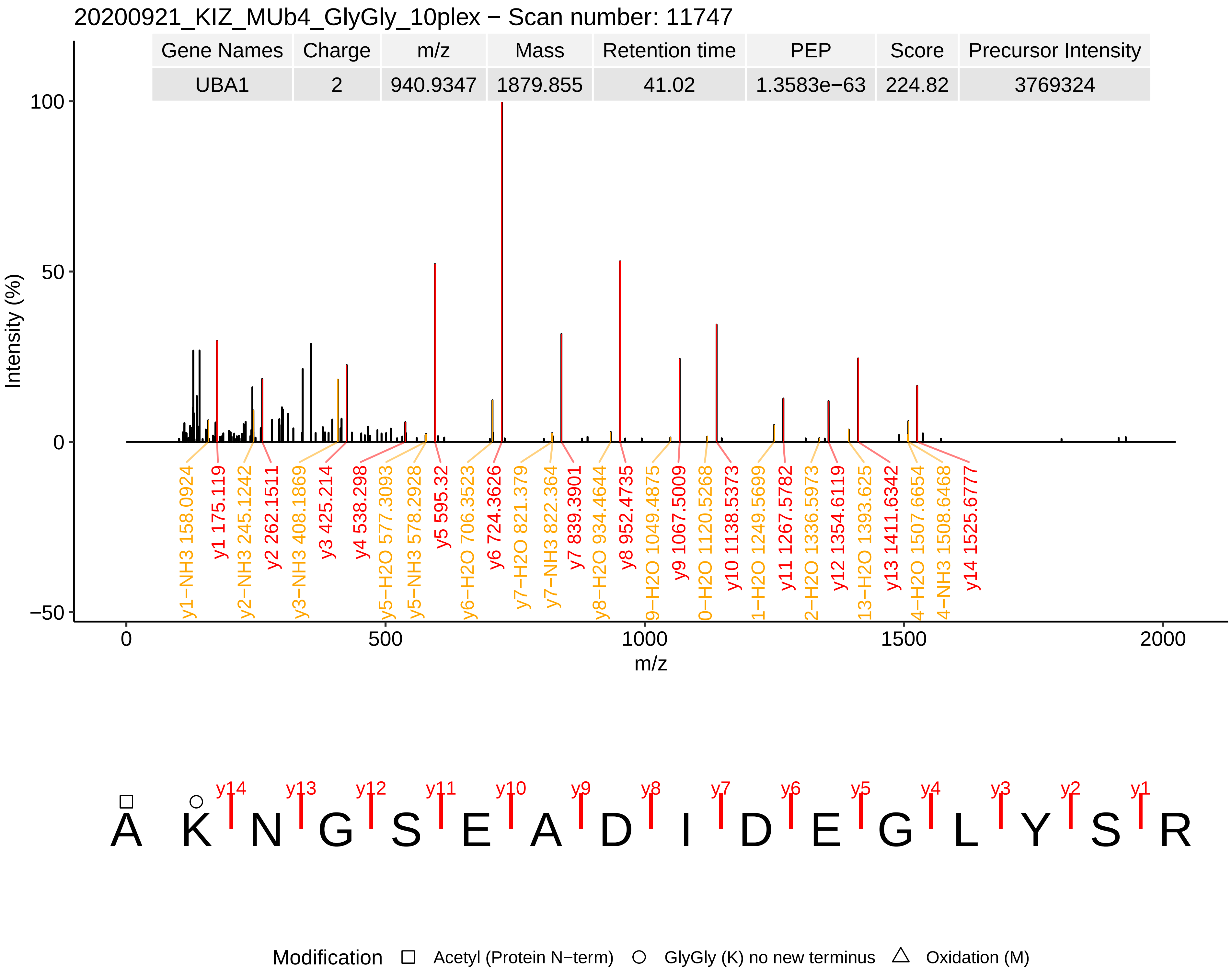
20200921_KIZ_MUb4_GlyGly_10plex − Scan number: 11747
Gene Names | Charge | m/z | Mass | Retention time | PEP | Score | Precursor Intensity
UBA1 | 2 | 940.9347 | 1879.855 | 41.02 | 1.3583e−63 | 224.82 | 3769324
Intensity (%)
100
50
0
−50
0
500
1000
1500
2000
m/z
y1−NH3 158.0924
y1 175.119
y2−NH3 245.1242
y2 262.1511
y3−NH3 408.1869
y3 425.214
y4 538.298
y5−H2O 577.3093
y5−NH3 578.2928
y5 595.32
y6−H2O 706.3523
y6 724.3626
y7−H2O 821.379
y7−NH3 822.364
y7 839.3901
y8−H2O 934.4644
y8 952.4735
9−H2O 1049.4875
y9 1067.5009
0−H2O 1120.5268
y10 1138.5373
1−H2O 1249.5699
y11 1267.5782
2−H2O 1336.5973
y12 1354.6119
13−H2O 1393.625
y13 1411.6342
4−H2O 1507.6654
4−NH3 1508.6468
y14 1525.6777
A K N G S E A D I D E G L Y S R
y14 y13 y12 y11 y10 y9 y8 y7 y6 y5 y4 y3 y2 y1
Modification
Acetyl (Protein N−term)
GlyGly (K) no new terminus
Oxidation (M)

20200921_KIZ_MUb4_GlyGly_10plex_2 − Scan number: 10690

Gene Names	Charge	m/z	Mass	Retention time	PEP	Score	Precursor Intensity
UBA1	2	940.9347	1879.855	40.953	0.0017759	67.456	625992.2

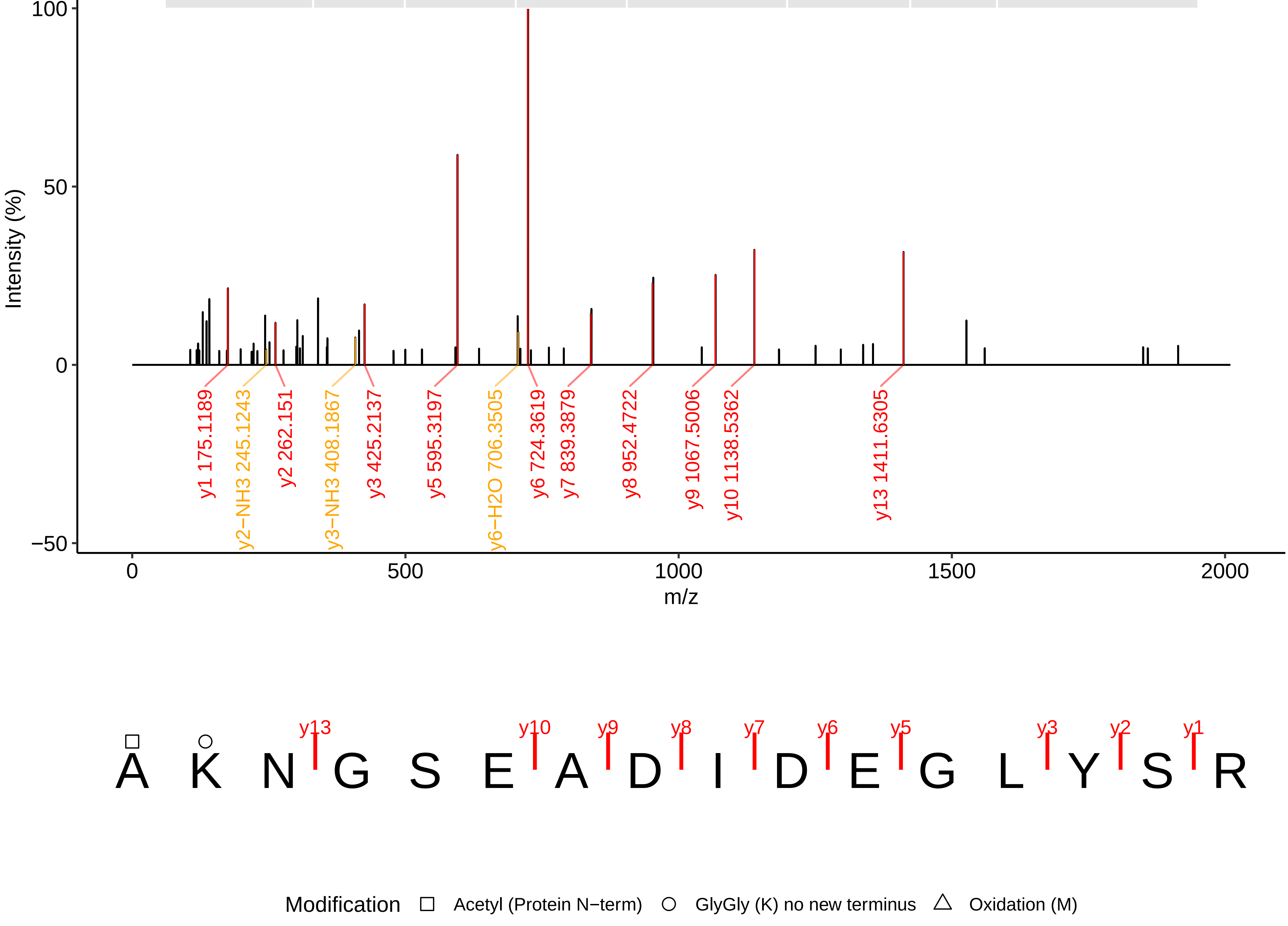

20200924_KIZ_MUb5_GlyGly_1 − Scan number: 10849

Gene Names	Charge	m/z	Mass	Retention time	PEP	Score	Precursor Intensity
UBA1	2	940.9347	1879.855	35.082	1.9537e−87	239.02	8133857

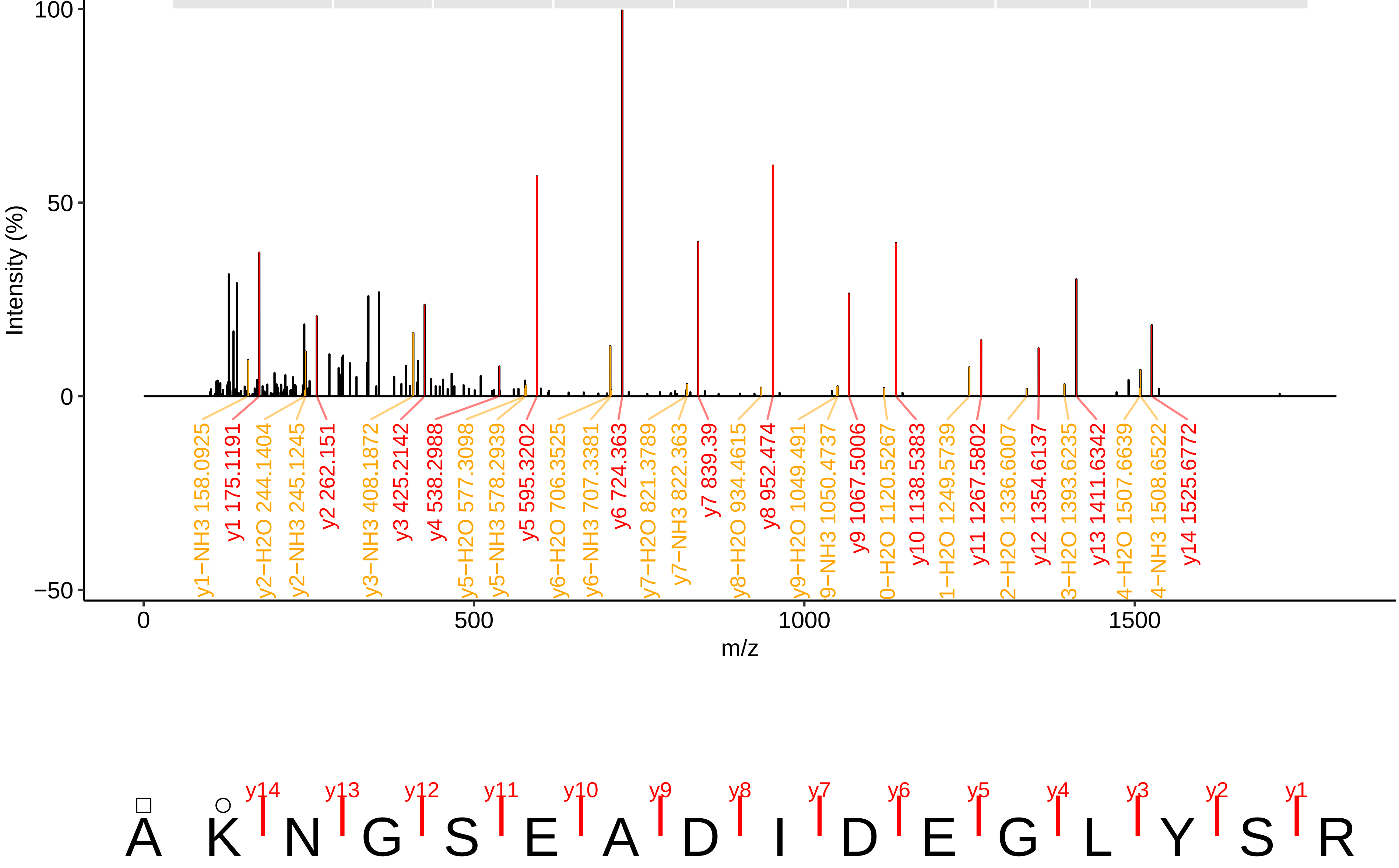

Modification □ Acetyl (Protein N−term) ○ GlyGly (K) no new terminus △ Oxidation (M)

20200921_KIZ_MUb4_GlyGly_10plex − Scan number: 9771

Gene Names	Charge	m/z	Mass	Retention time	PEP	Score	Precursor Intensity
USP5	4	410.968	1639.843	33.571	1.063e−05	105.03	14099225

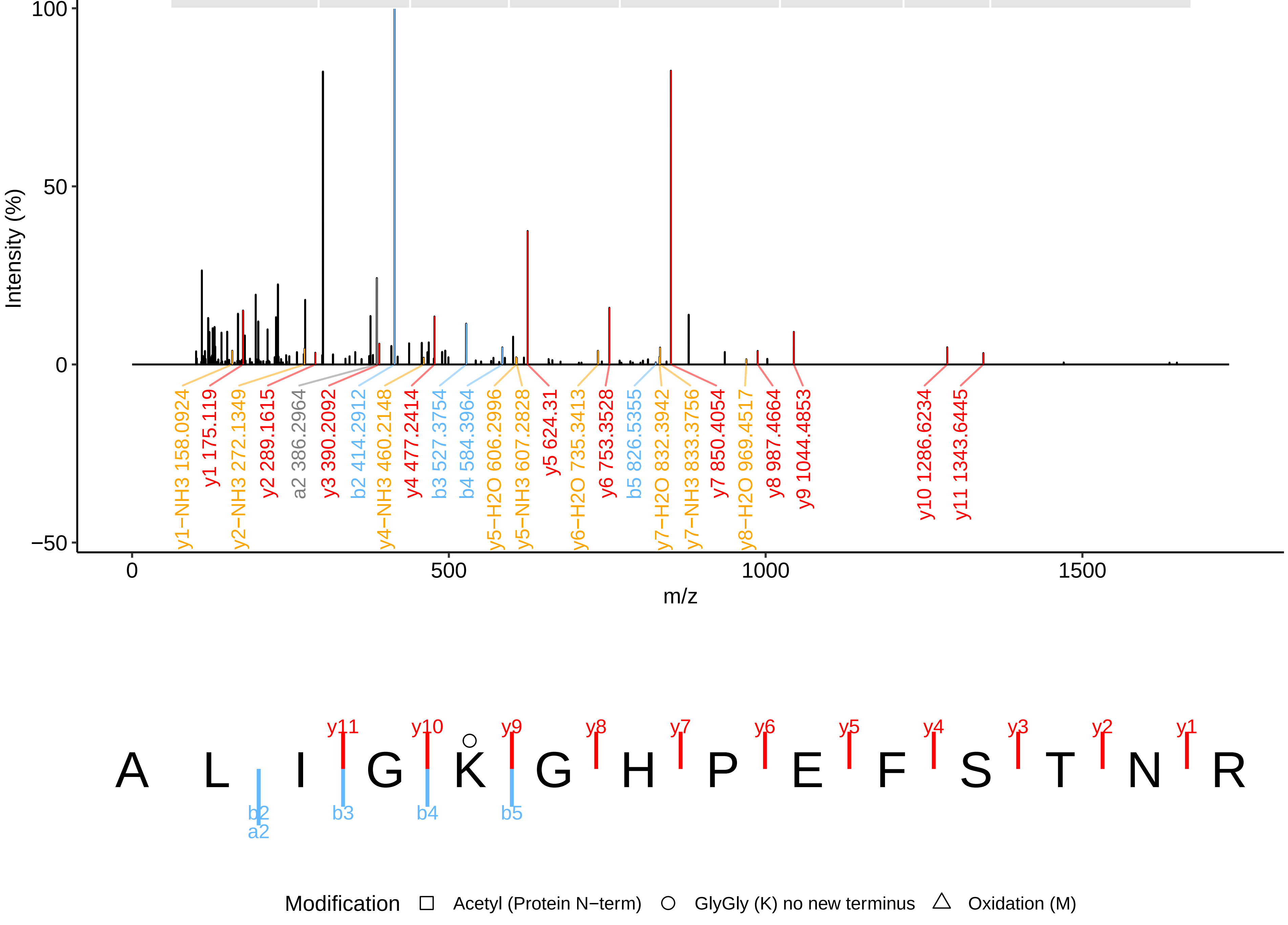

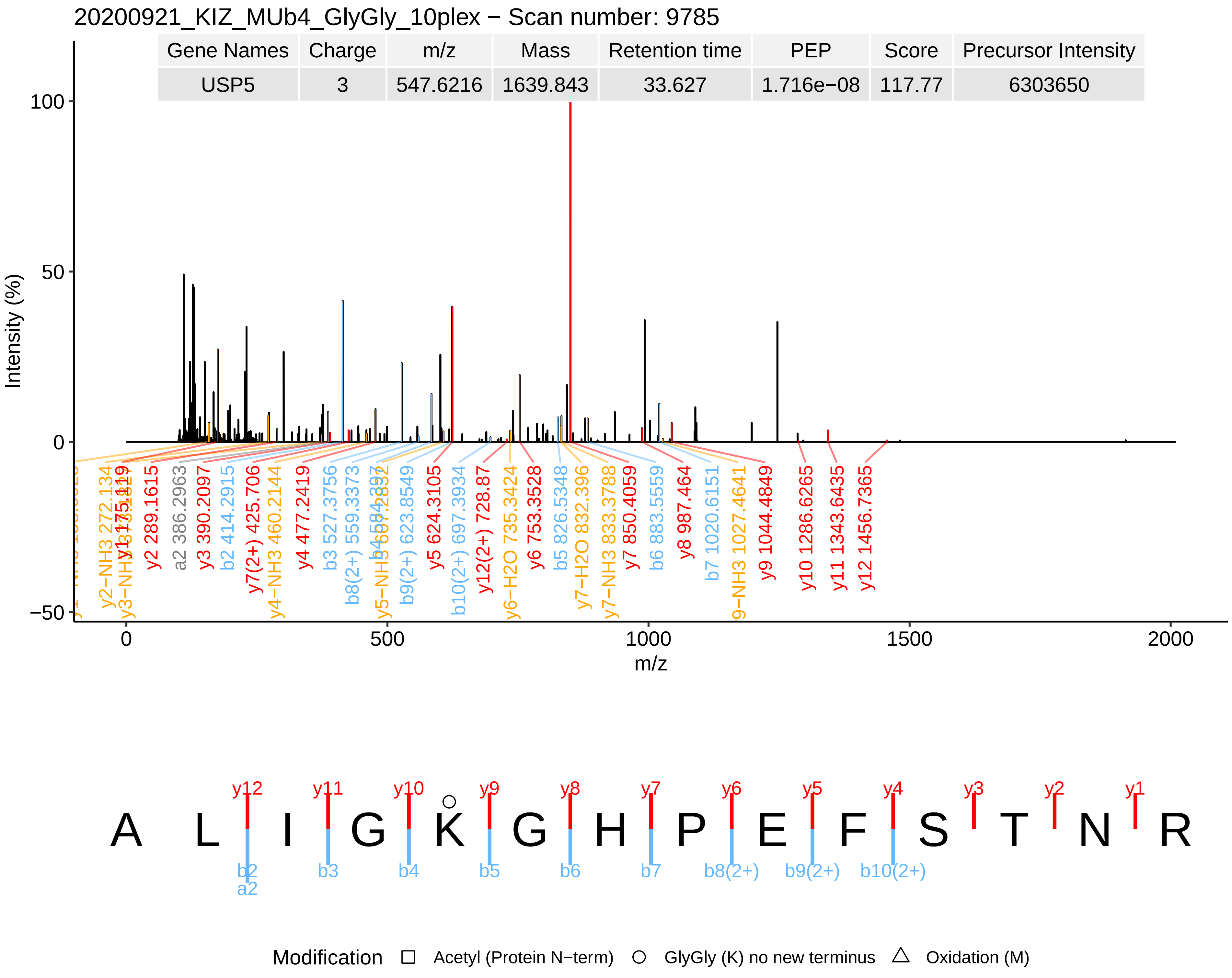
20200921_KIZ_MUb4_GlyGly_10plex − Scan number: 9785
Gene Names
Charge
m/z
Mass
Retention time
PEP
Score
Precursor Intensity
USP5
3
547.6216
1639.843
33.627
1.716e−08
117.77
6303650
Intensity (%)
100
50
0
−50
0
500
1000
1500
2000
m/z
y2−NH3 272.134
y2 289.1615
a2 386.2963
y3 390.2097
b2 414.2915
y7(2+) 425.706
y4−NH3 460.2144
y4 477.2419
b3 527.3756
b8(2+) 559.3373
b9(2+) 623.8549
y5 624.3105
b10(2+) 697.3934
y12(2+) 728.87
y6−H2O 735.3424
y6 753.3528
b5 826.5348
y7−H2O 832.396
y7−NH3 833.3788
y7 850.4059
b6 883.5559
y8 987.464
b7 1020.6151
y9 1044.4849
y10 1286.6265
y11 1343.6435
y12 1456.7365
A L I G K G H P E F S T N R
y12 y11 y10 y9 y8 y7 y6 y5 y4 y3 y2 y1
b2 b3 b4 b5 b6 b7 b8(2+) b9(2+) b10(2+)
a2
Modification
Acetyl (Protein N−term)
GlyGly (K) no new terminus
Oxidation (M)

20200921_KIZ_MUb4_GlyGly_10plex_2 − Scan number: 9044

Gene Names	Charge	m/z	Mass	Retention time	PEP	Score	Precursor Intensity
USP5	4	410.968	1639.843	34.266	0.0044803	67.997	1305388

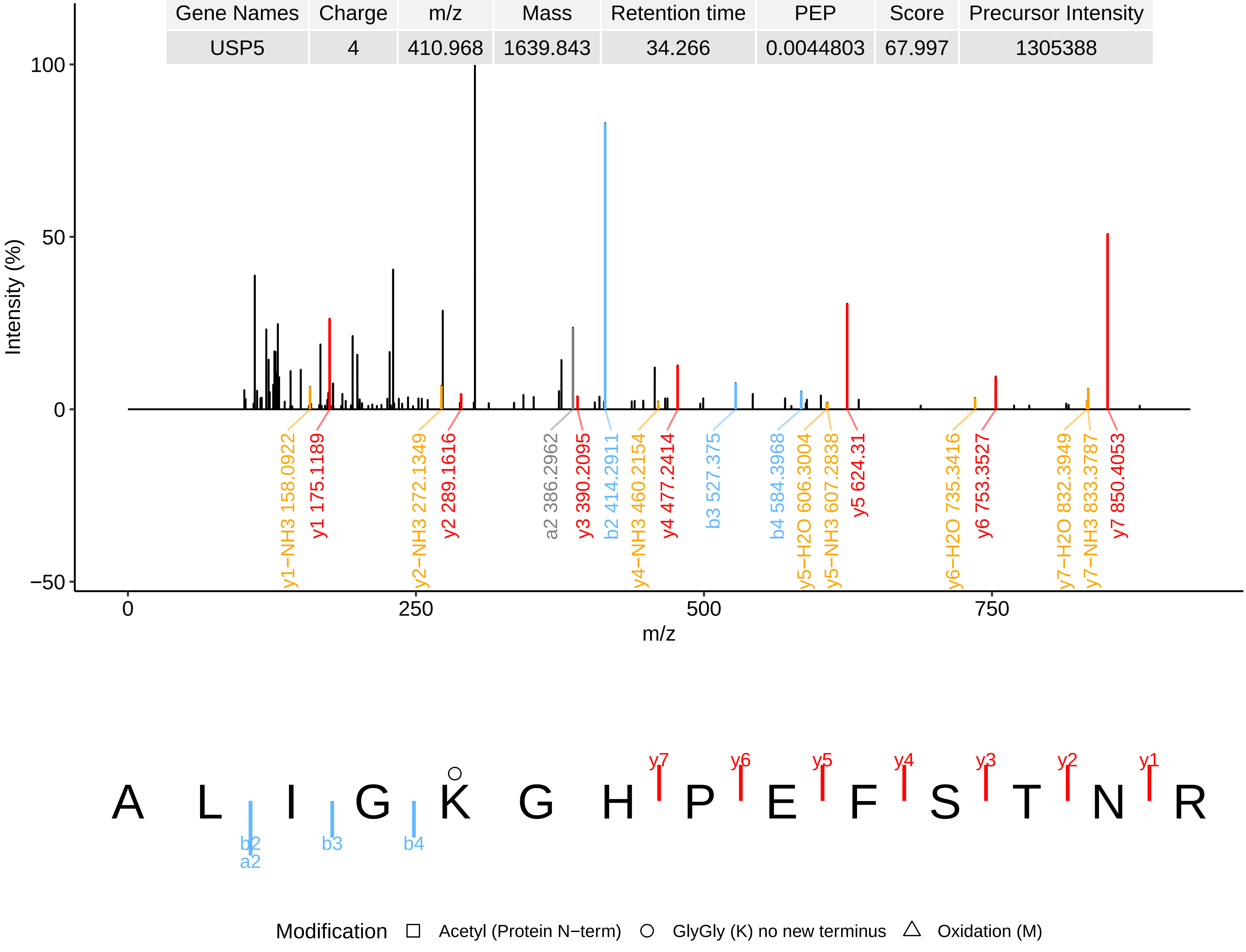

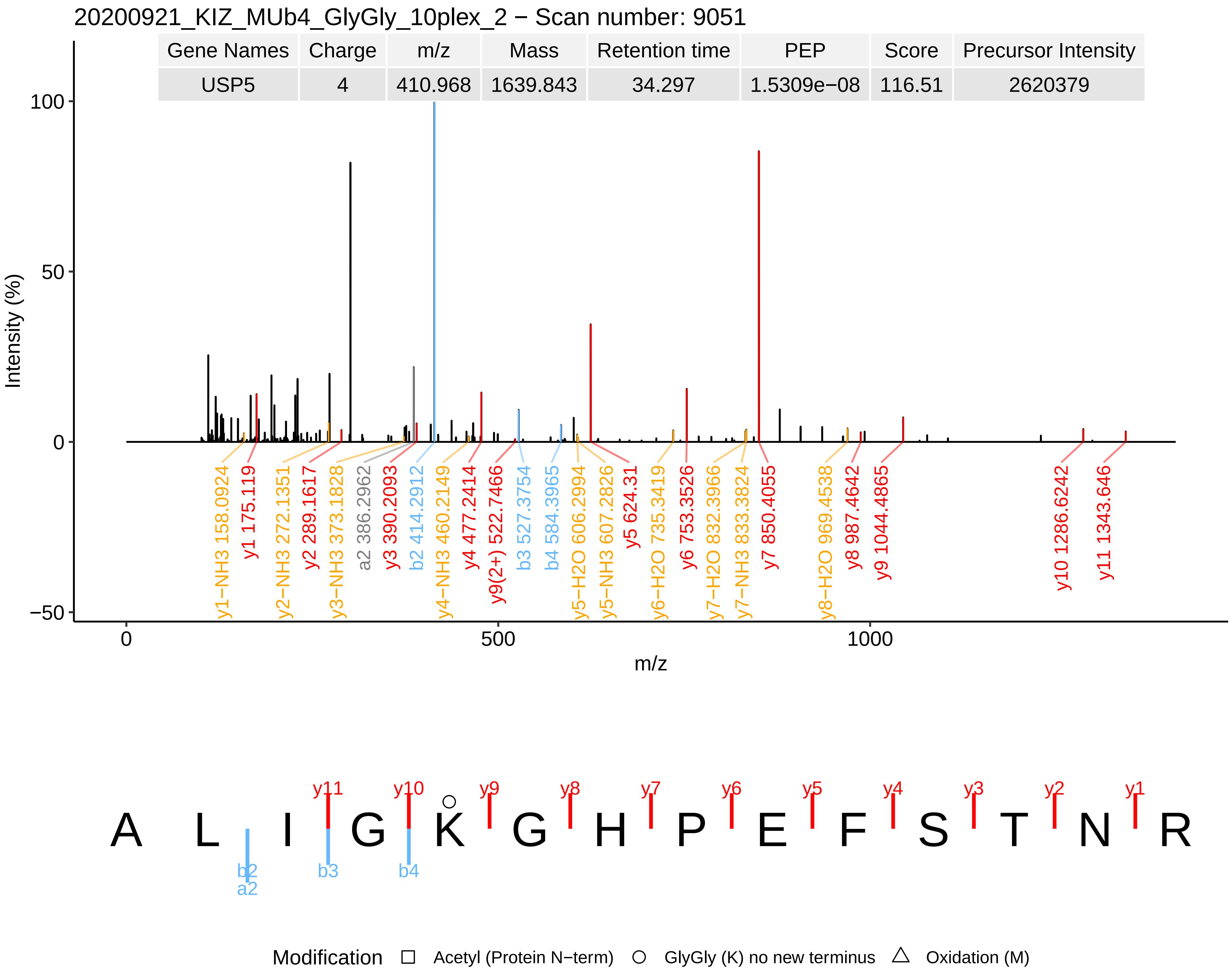
20200921_KIZ_MUb4_GlyGly_10plex_2 − Scan number: 9051
Gene Names | Charge | m/z | Mass | Retention time | PEP | Score | Precursor Intensity
USP5 | 4 | 410.968 | 1639.843 | 34.297 | 1.5309e−08 | 116.51 | 2620379
Intensity (%)
m/z
y1−NH3 158.0924
y1 175.119
y2−NH3 272.1351
y2 289.1617
y3−NH3 373.1828
a2 386.2962
y3 390.2093
b2 414.2912
y4−NH3 460.2149
y4 477.2414
y9(2+) 522.7466
b3 527.3754
b4 584.3965
y5−H2O 606.2994
y5−NH3 607.2826
y5 624.31
y6−H2O 735.3419
y6 753.3526
y7−H2O 832.3966
y7−NH3 833.3824
y7 850.4055
y8−H2O 969.4538
y8 987.4642
y9 1044.4865
y10 1286.6242
y11 1343.646
A L I G K G H P E F S T N R
y11 y10 y9 y8 y7 y6 y5 y4 y3 y2 y1
b2 a2 b3 b4
Modification
Acetyl (Protein N−term)
GlyGly (K) no new terminus
Oxidation (M)

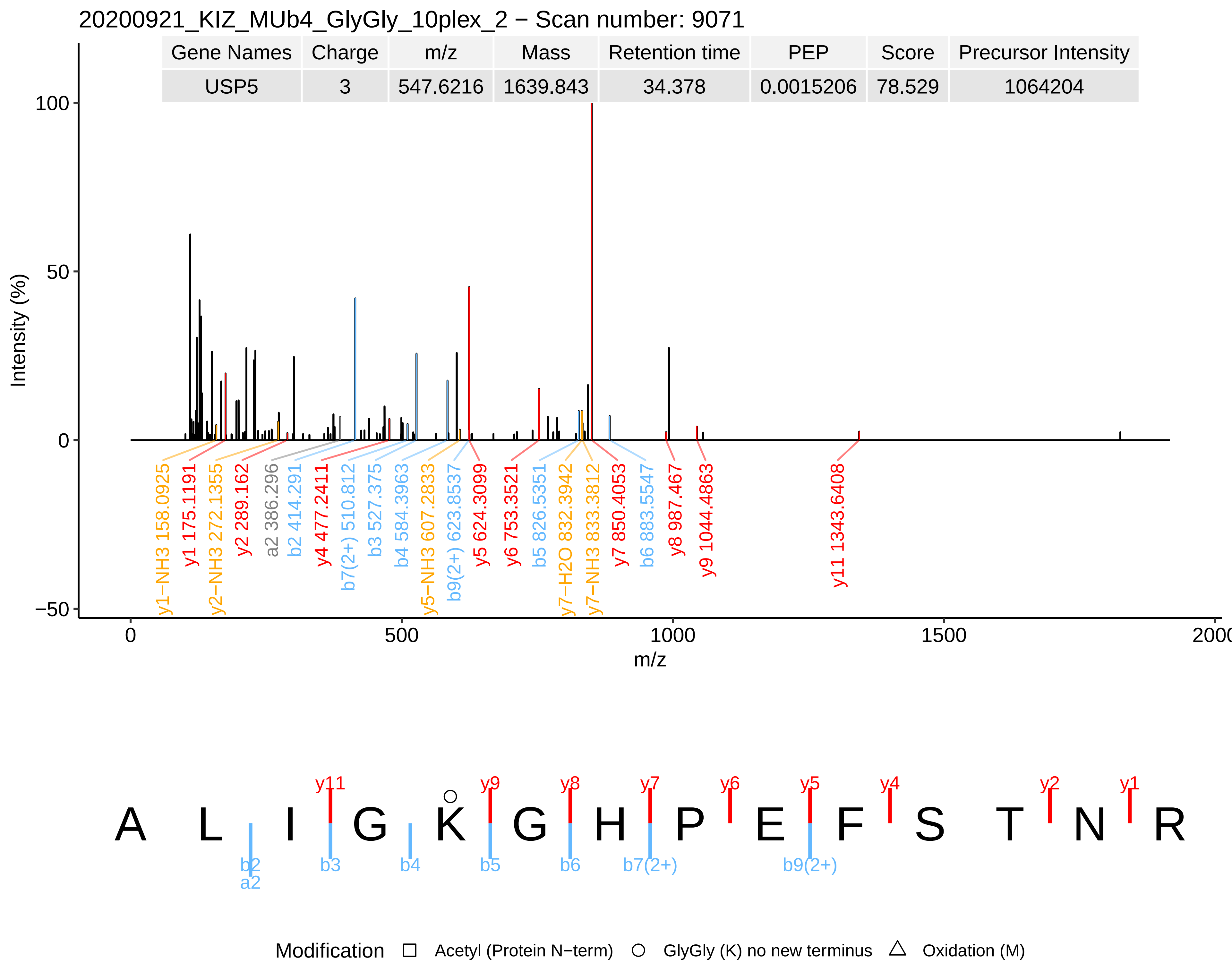
20200921_KIZ_MUb4_GlyGly_10plex_2 − Scan number: 9071
Gene Names
Charge
m/z
Mass
Retention time
PEP
Score
Precursor Intensity
USP5
3
547.6216
1639.843
34.378
0.0015206
78.529
1064204
Intensity (%)
m/z
y1−NH3 158.0925
y1 175.1191
y2−NH3 272.1355
y2 289.162
a2 386.296
b2 414.291
y4 477.2411
b7(2+) 510.812
b3 527.375
b4 584.3963
y5−NH3 607.2833
b9(2+) 623.8537
y5 624.3099
y6 753.3521
b5 826.5351
y7−H2O 832.3942
y7−NH3 833.3812
y7 850.4053
b6 883.5547
y8 987.467
y9 1044.4863
y11 1343.6408
A L I G K G H P E F S T N R
y11 y9 y8 y7 y6 y5 y4 y2 y1
b2 a2 b3 b4 b5 b6 b7(2+) b9(2+)
Modification
Acetyl (Protein N−term)
GlyGly (K) no new terminus
Oxidation (M)

20200924_KIZ_MUb5_GlyGly_1 − Scan number: 8314

Gene Names	Charge	m/z	Mass	Retention time	PEP	Score	Precursor Intensity
USP5	4	410.968	1639.843	26.557	0.0028797	73.219	4577780

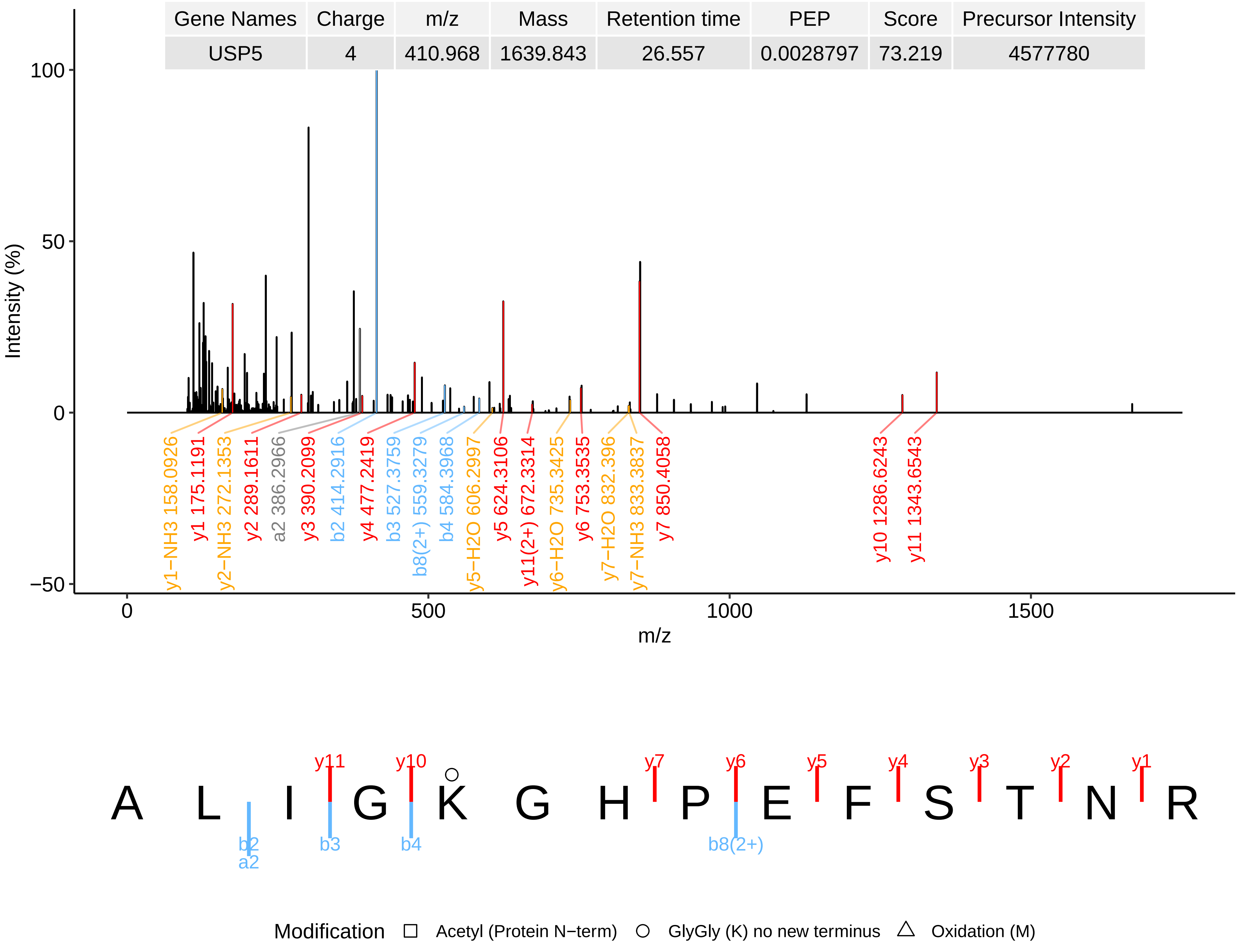

20200924_KIZ_MUb5_GlyGly_1 − Scan number: 8429

Gene Names	Charge	m/z	Mass	Retention time	PEP	Score	Precursor Intensity
USP5	3	547.6216	1639.843	26.945	0.062683	48.608	11497488

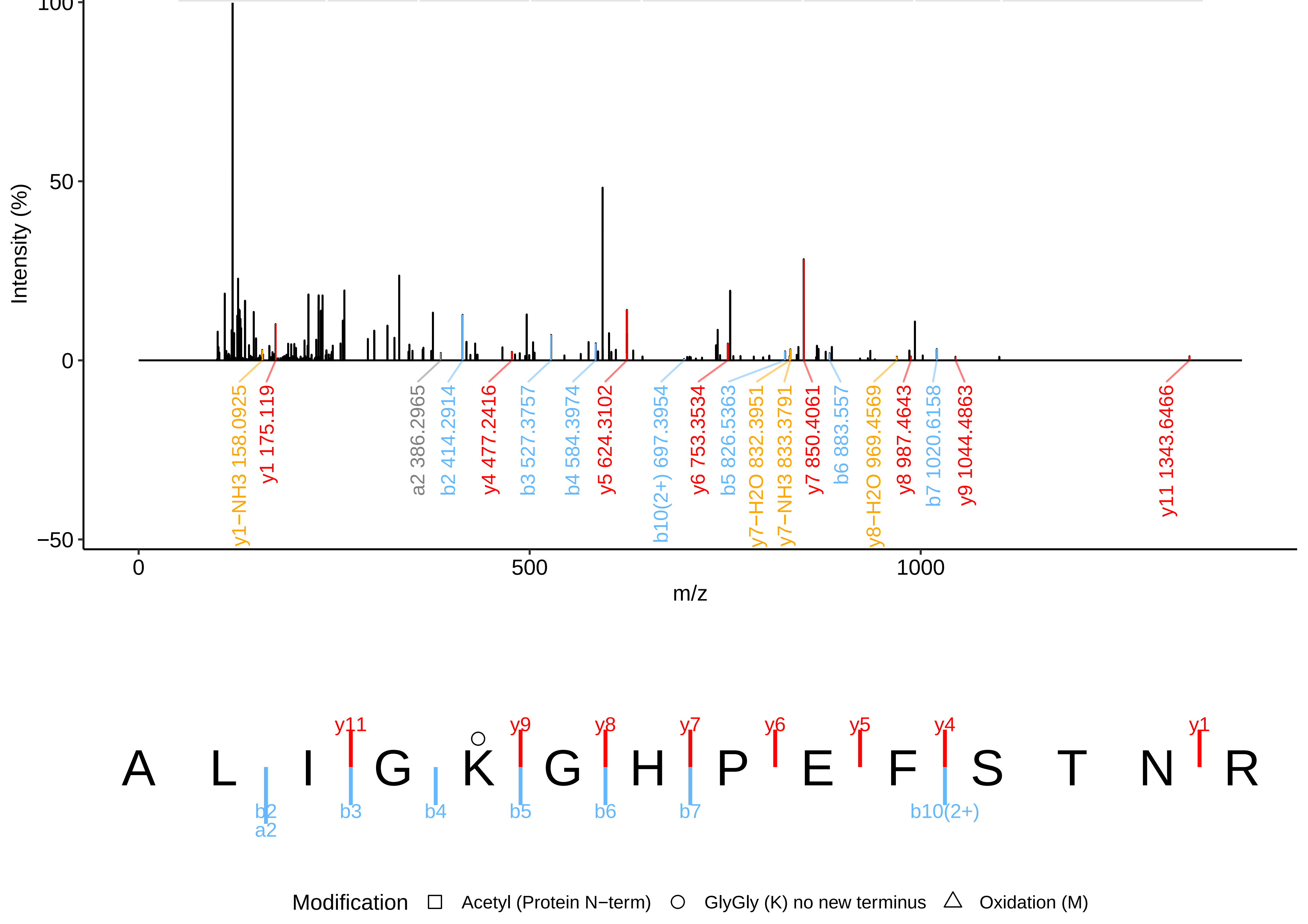

20200924_KIZ_MUb5_GlyGly_1 − Scan number: 8522

Gene Names	Charge	m/z	Mass	Retention time	PEP	Score	Precursor Intensity
USP5	4	410.968	1639.843	27.254	0.0016409	77.26	10799405

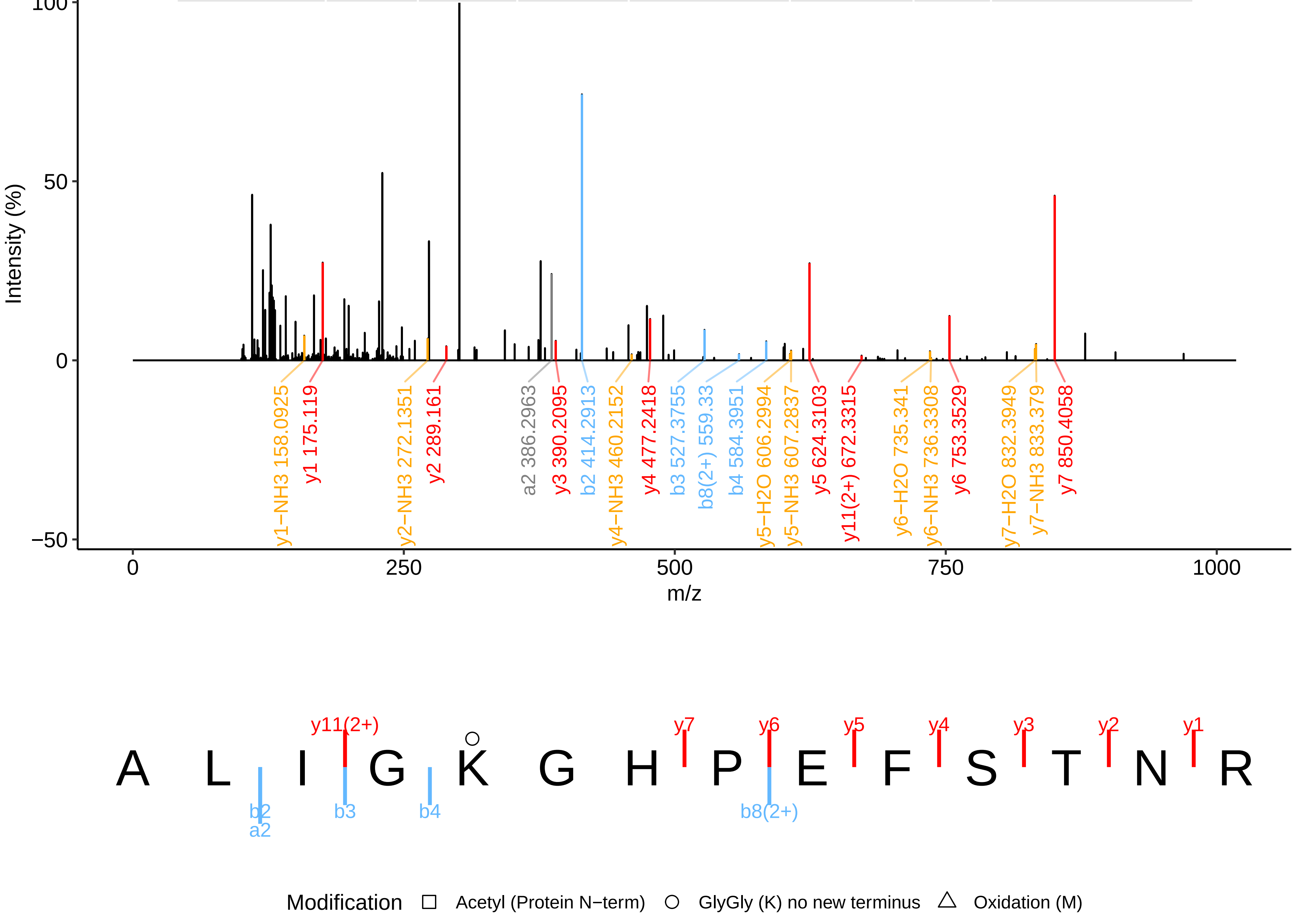

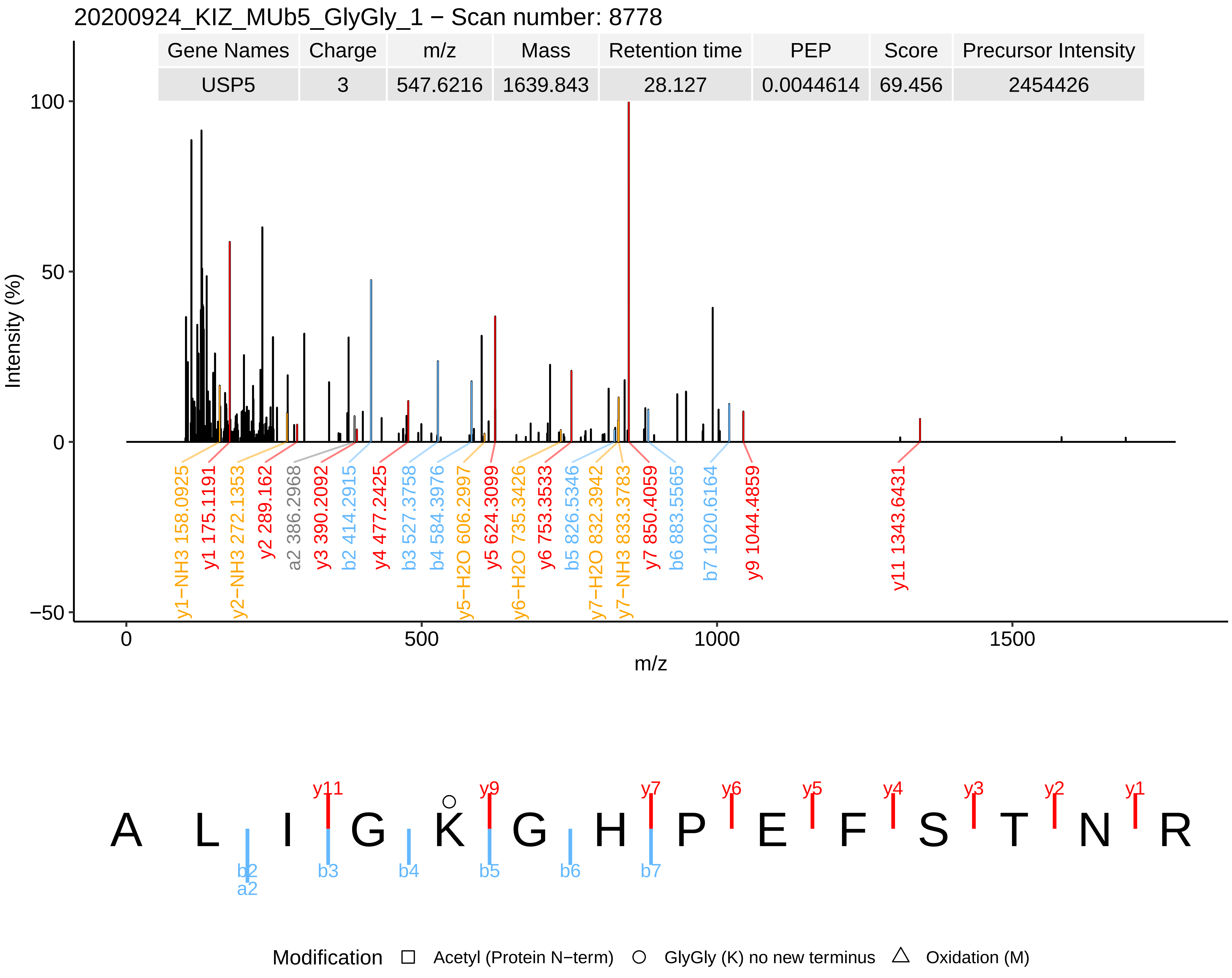
20200924_KIZ_MUb5_GlyGly_1 − Scan number: 8778
Gene Names
Charge
m/z
Mass
Retention time
PEP
Score
Precursor Intensity
USP5
3
547.6216
1639.843
28.127
0.0044614
69.456
2454426
Intensity (%)
m/z
y1−NH3 158.0925
y1 175.1191
y2−NH3 272.1353
y2 289.162
a2 386.2968
y3 390.2092
b2 414.2915
y4 477.2425
b3 527.3758
b4 584.3976
y5−H2O 606.2997
y5 624.3099
y6−H2O 735.3426
y6 753.3533
b5 826.5346
y7−H2O 832.3942
y7−NH3 833.3783
y7 850.4059
b6 883.5565
b7 1020.6164
y9 1044.4859
y11 1343.6431
A L I G K G H P E F S T N R
y11
y9
y7
y6
y5
y4
y3
y2
y1
b2
a2
b3
b4
b5
b6
b7
Modification
Acetyl (Protein N−term)
GlyGly (K) no new terminus
Oxidation (M)

20201118_KIZ_MUb6_GlyGly_1 − Scan number: 8247

Gene Names	Charge	m/z	Mass	Retention time	PEP	Score	Precursor Intensity
USP5	4	410.968	1639.843	26.095	1.4286e−05	101.45	14528310

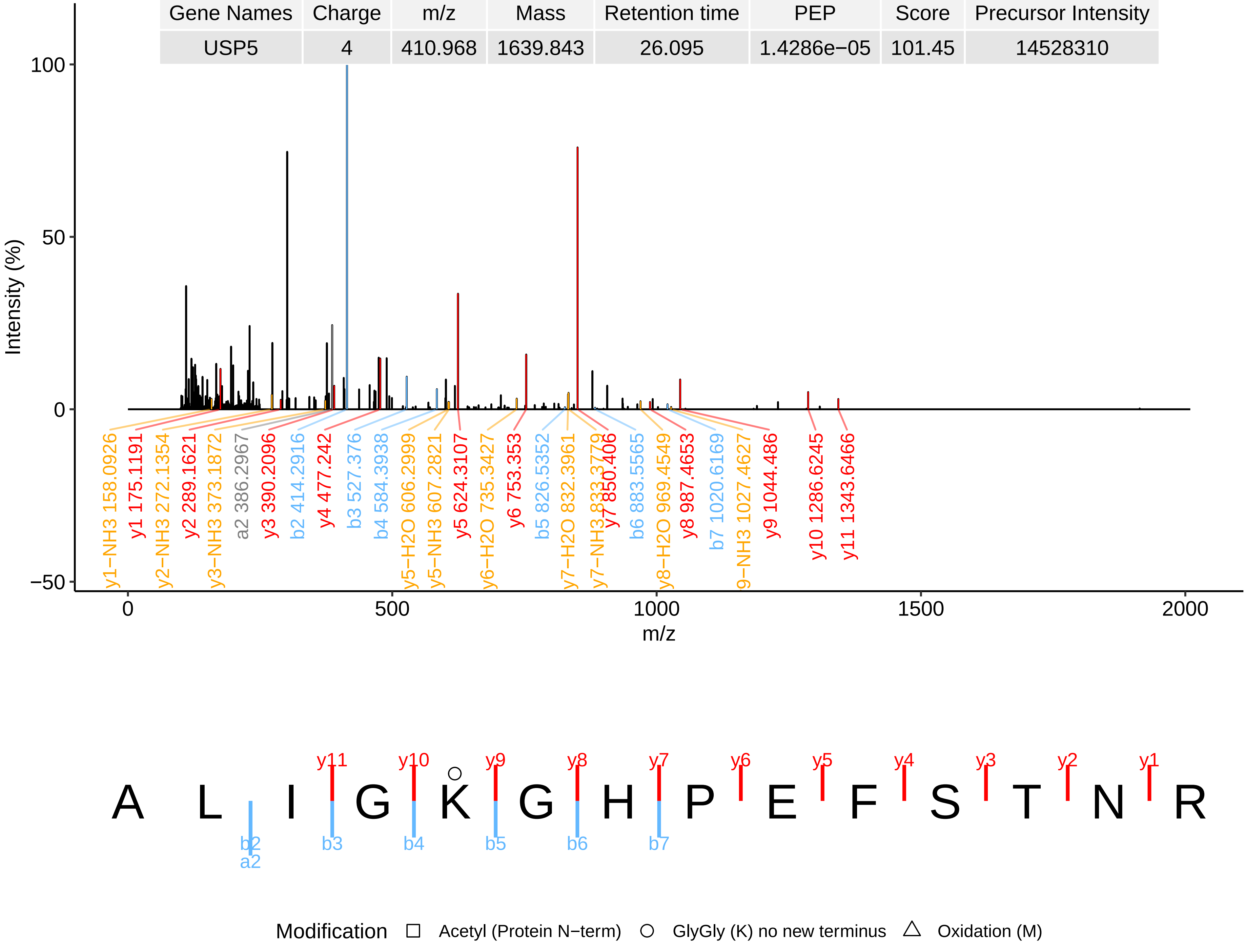

20201118_KIZ_MUb6_GlyGly_2 − Scan number: 10603

Gene Names	Charge	m/z	Mass	Retention time	PEP	Score	Precursor Intensity
USP5	4	410.968	1639.843	35.777	0.00022448	92.039	5084154

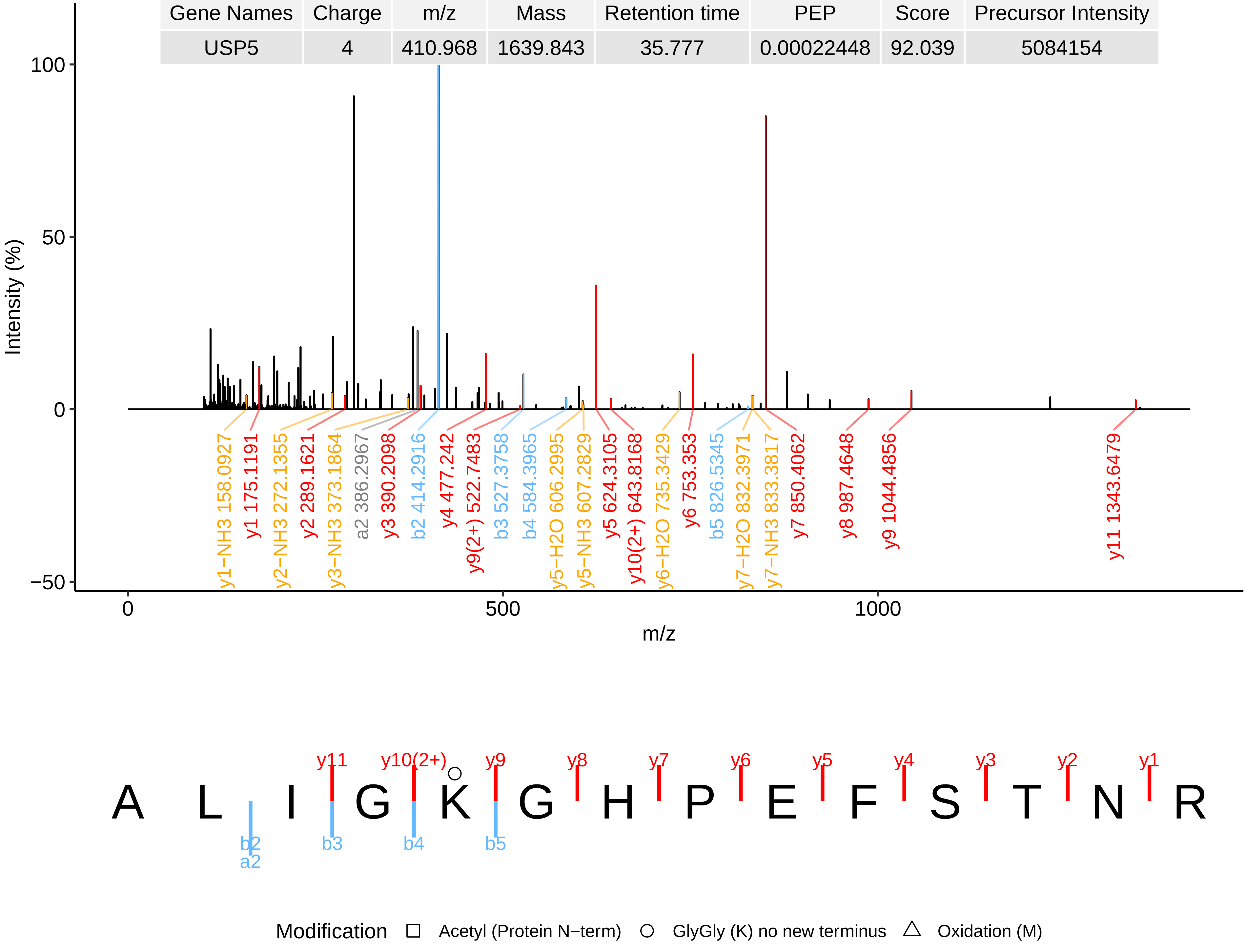

20200921_KIZ_MUb4_GlyGly_10plex − Scan number: 15535

Gene Names	Charge	m/z	Mass	Retention time	PEP	Score	Precursor Intensity
DCUN1D1	3	476.922	1427.744	55.515	0.0016849	81.017	5653367

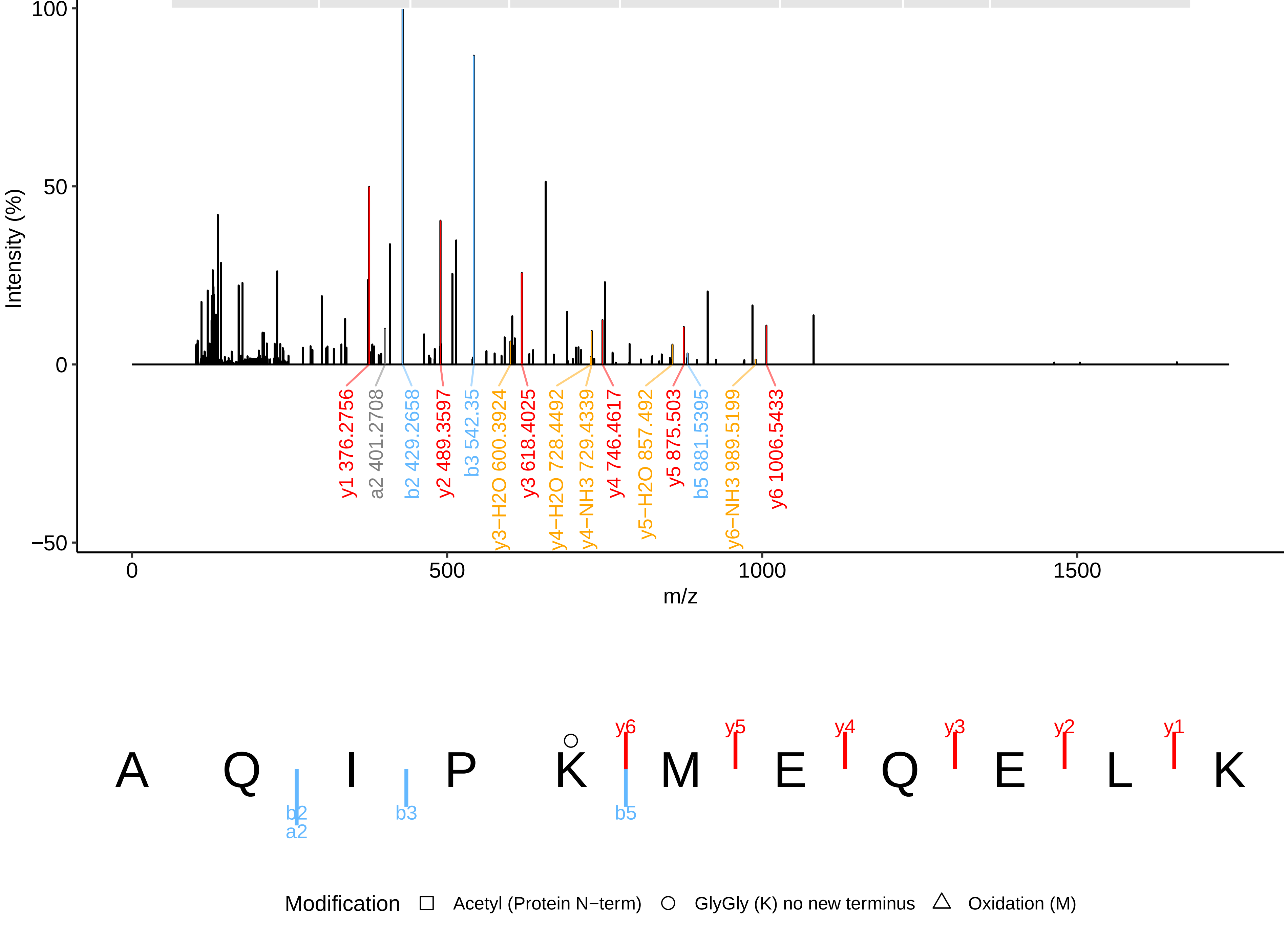

20200921_KIZ_MUb4_GlyGly_10plex_2 − Scan number: 14396

Gene Names	Charge	m/z	Mass	Retention time	PEP	Score	Precursor Intensity
DCUN1D1	3	476.922	1427.744	56.125	0.10876	45.077	757060.6

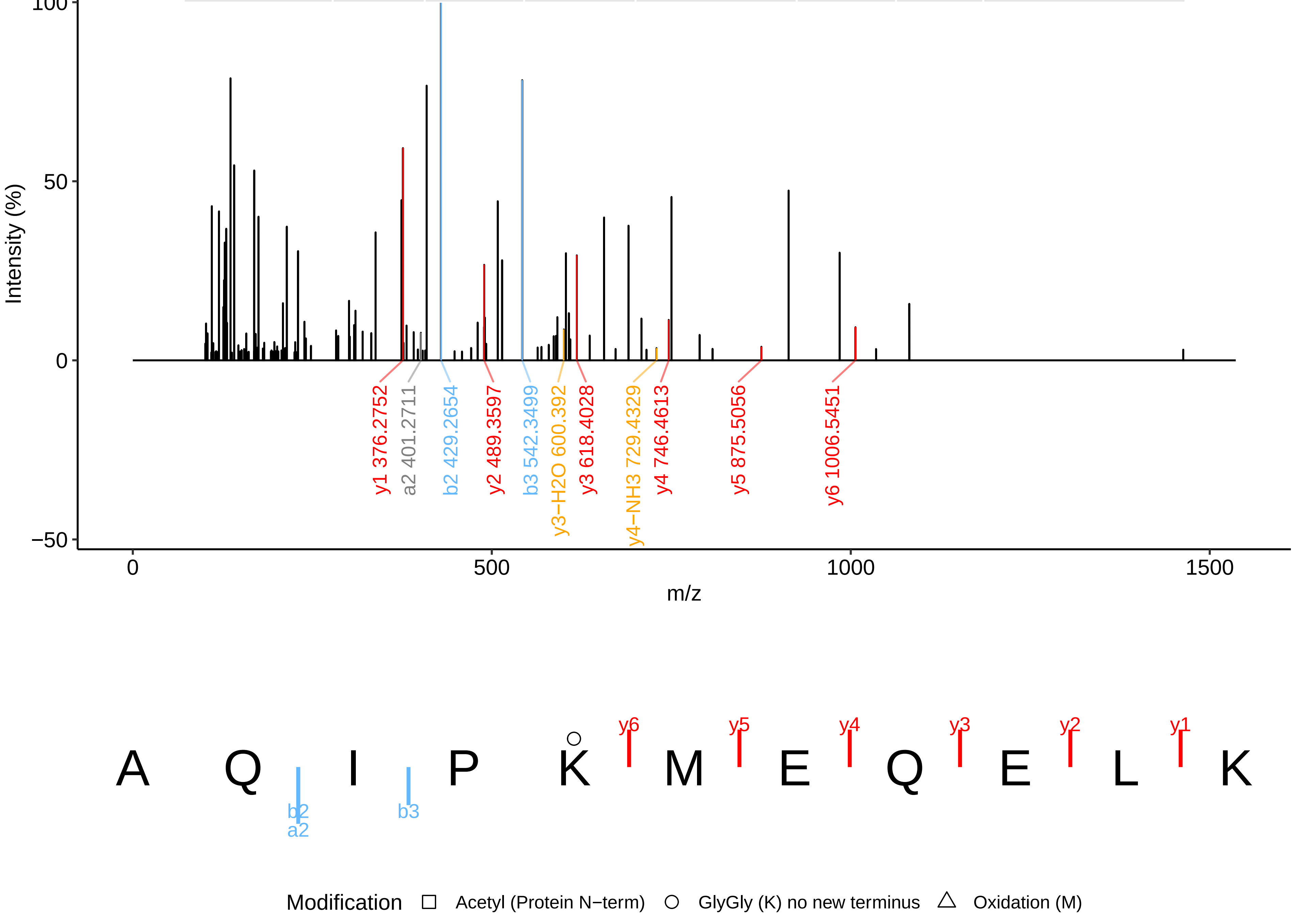

20200924_KIZ_MUb5_GlyGly_1 − Scan number: 14742

Gene Names	Charge	m/z	Mass	Retention time	PEP	Score	Precursor Intensity
DCUN1D1	3	476.922	1427.744	48.155	0.0010522	87.568	30038780

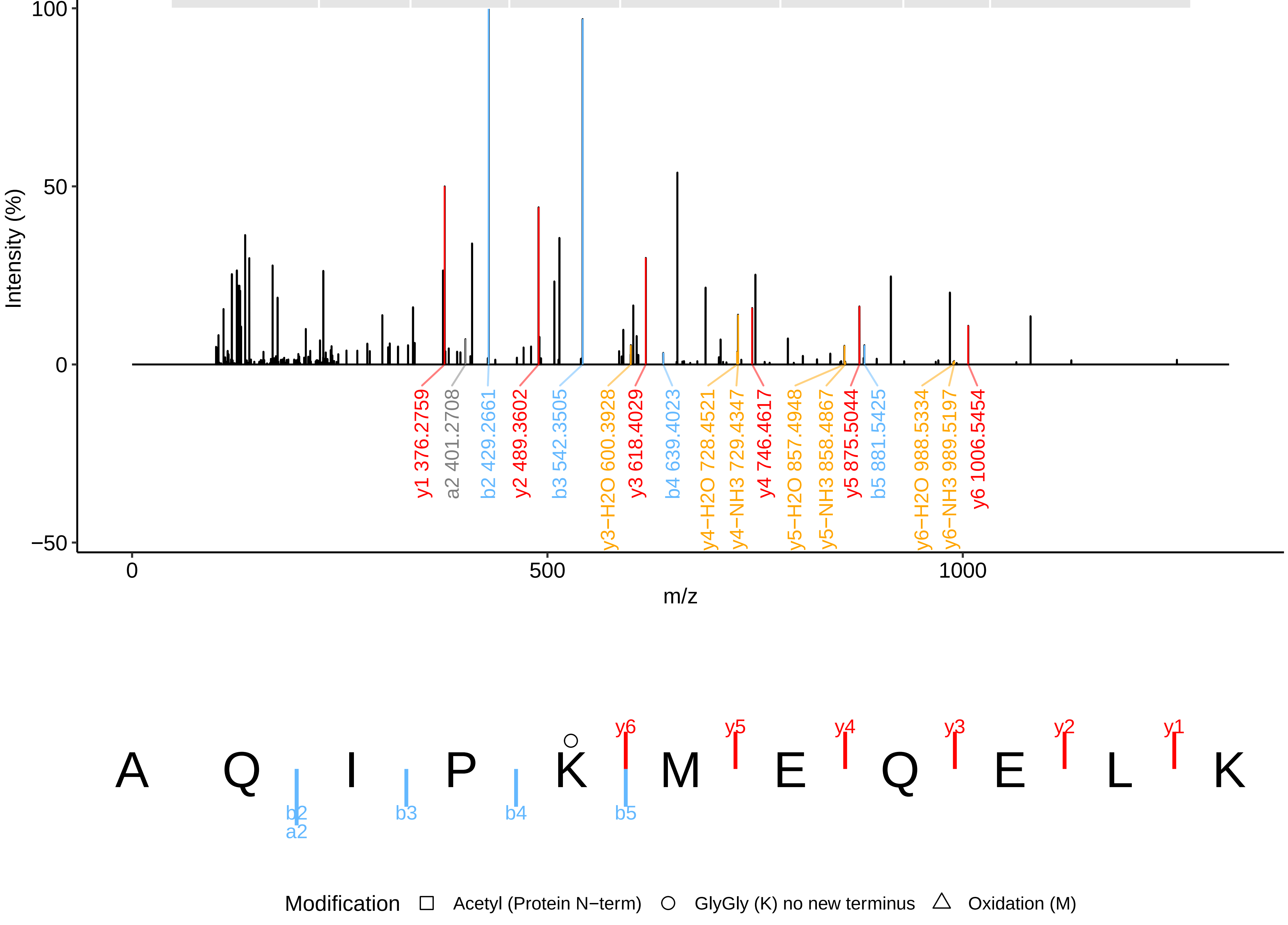

20200924_KIZ_MUb5_GlyGly_2 − Scan number: 13918

Gene Names	Charge	m/z	Mass	Retention time	PEP	Score	Precursor Intensity
DCUN1D1	3	476.922	1427.744	52.057	0.010666	69.721	2945085

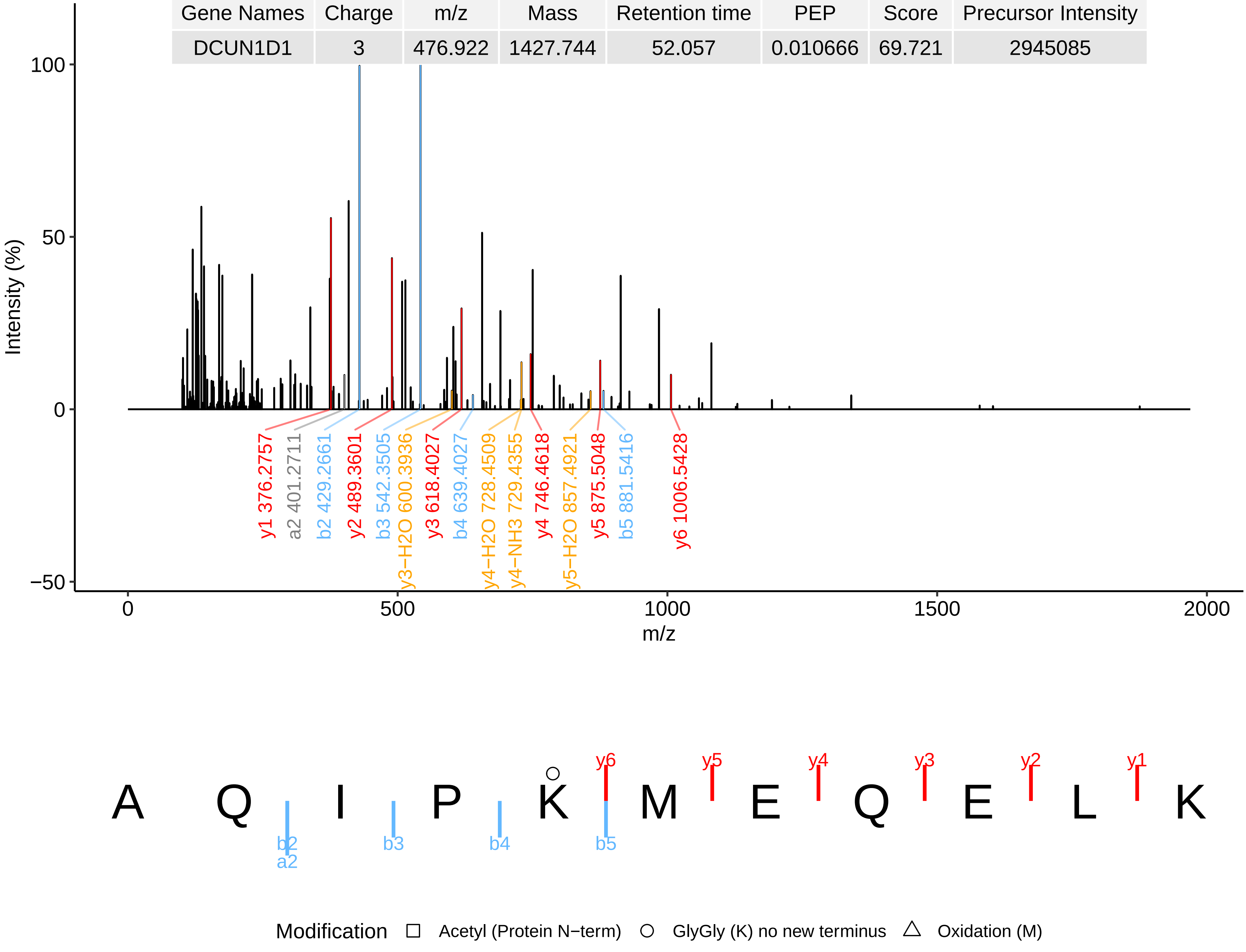

20200921_KIZ_MUb4_GlyGly_10plex_2 − Scan number: 17559

Gene Names	Charge	m/z	Mass	Retention time	PEP	Score	Precursor Intensity
CCT8	3	550.2933	1647.858	68.673	1.1543e−05	106.13	1029580

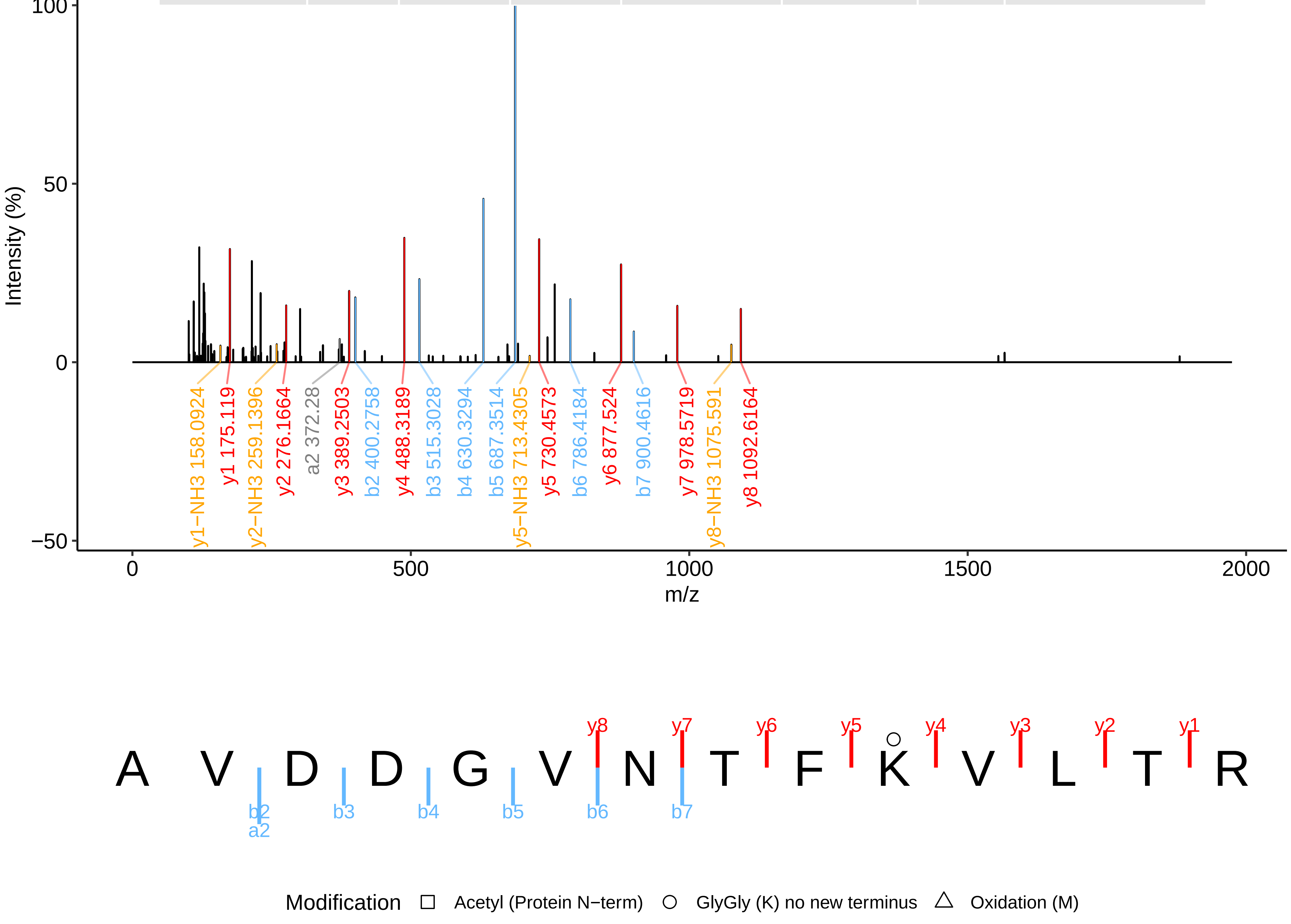

20200924_KIZ_MUb5_GlyGly_1 − Scan number: 8932

Gene Names	Charge	m/z	Mass	Retention time	PEP	Score	Precursor Intensity
MIEF2	3	475.2039	1422.59	28.656	0.079299	54.486	NA

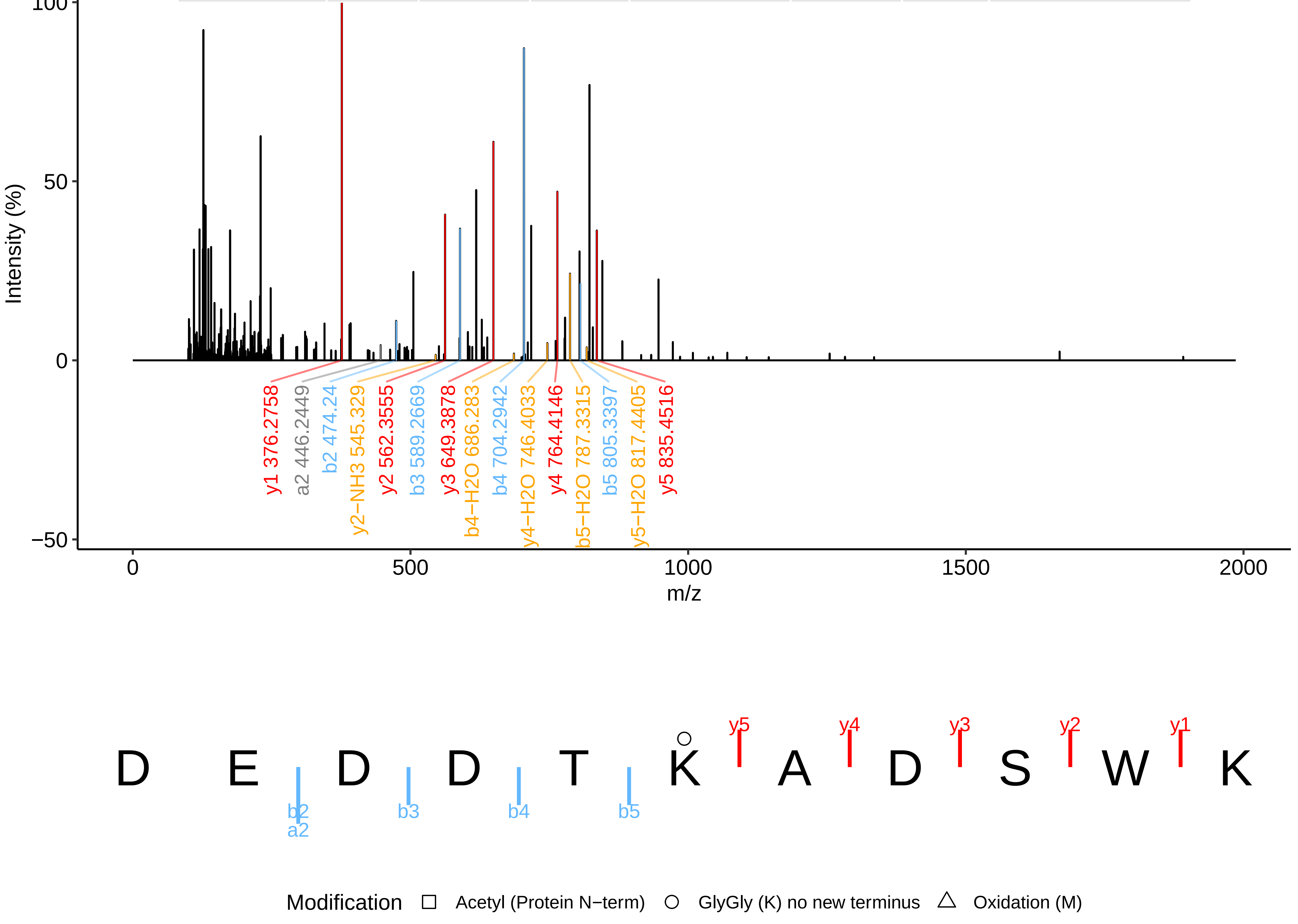

20200924_KIZ_MUb5_GlyGly_1 − Scan number: 33150

Gene Names	Charge	m/z	Mass	Retention time	PEP	Score	Precursor Intensity
ITPR1;ITPR3	2	832.4021	1662.79	105.67	0.0099664	78.149	12221089

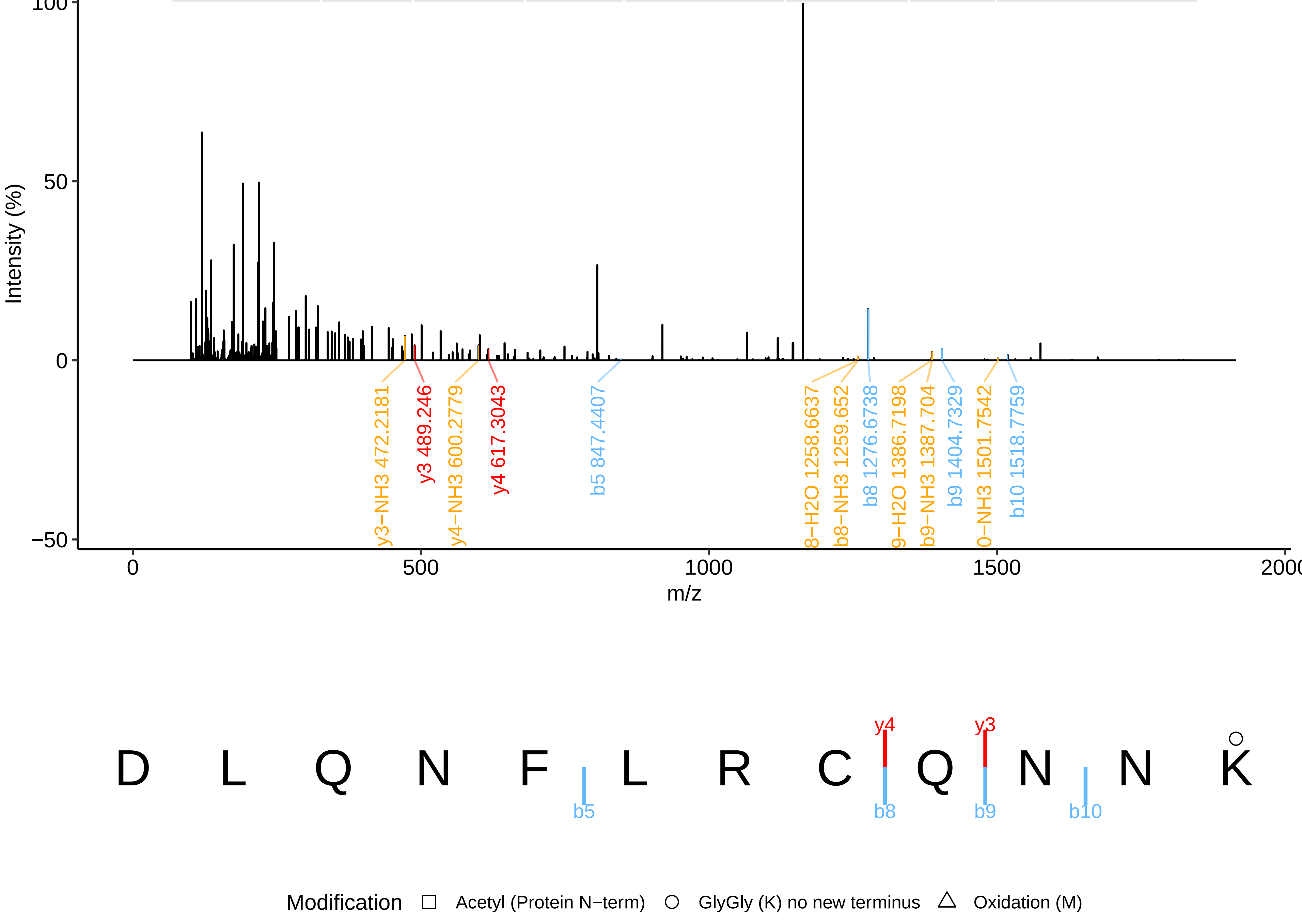

20200924_KIZ_MUb5_GlyGly_1 − Scan number: 33313

Gene Names	Charge	m/z	Mass	Retention time	PEP	Score	Precursor Intensity
ITPR1;ITPR3	2	832.4021	1662.79	106.18	0.029982	69.598	25952642

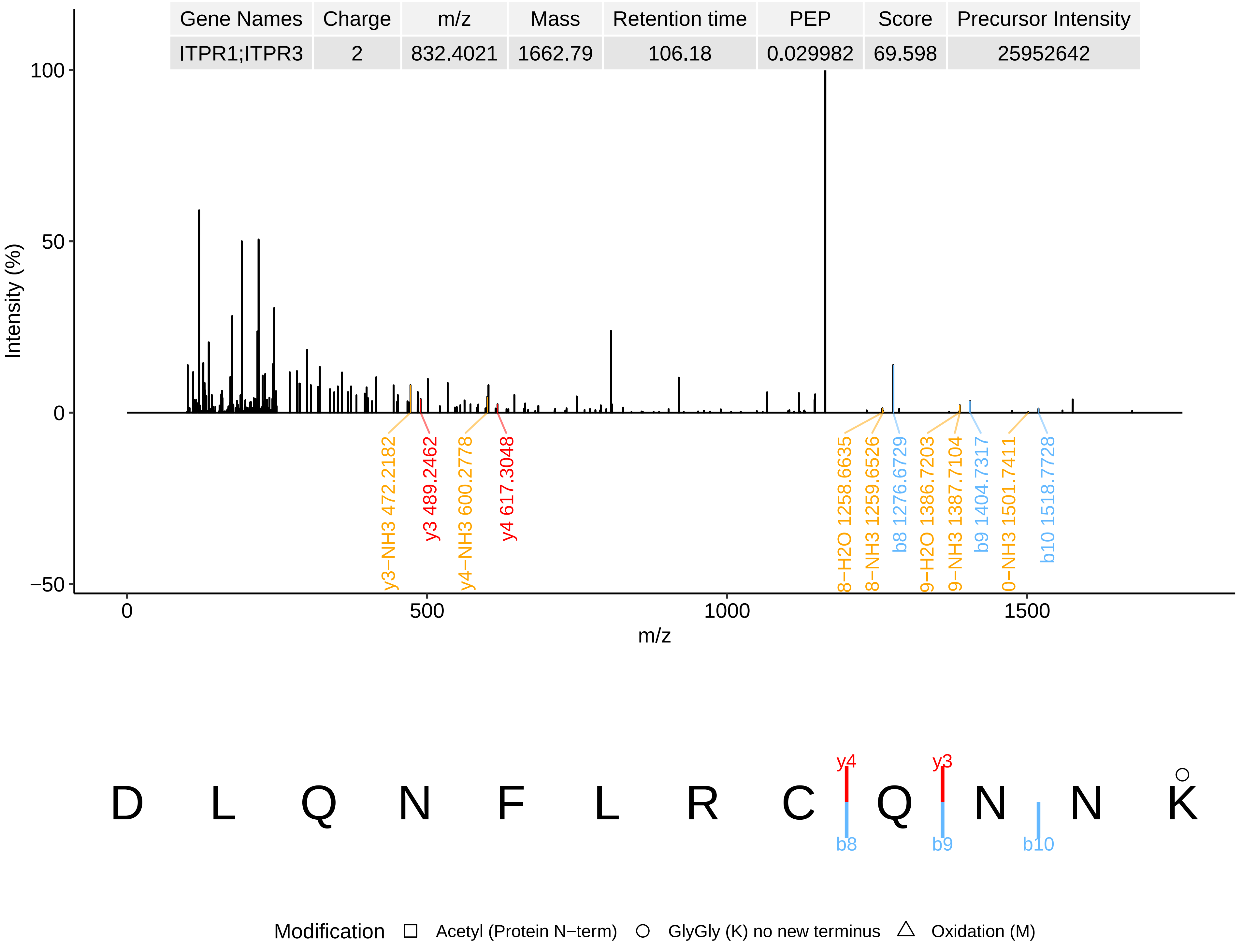

20200924_KIZ_MUb5_GlyGly_1 − Scan number: 12015

Gene Names	Charge	m/z	Mass	Retention time	PEP	Score	Precursor Intensity
EPS15L1	3	482.2525	1443.736	39.028	0.02919	60.196	11967113

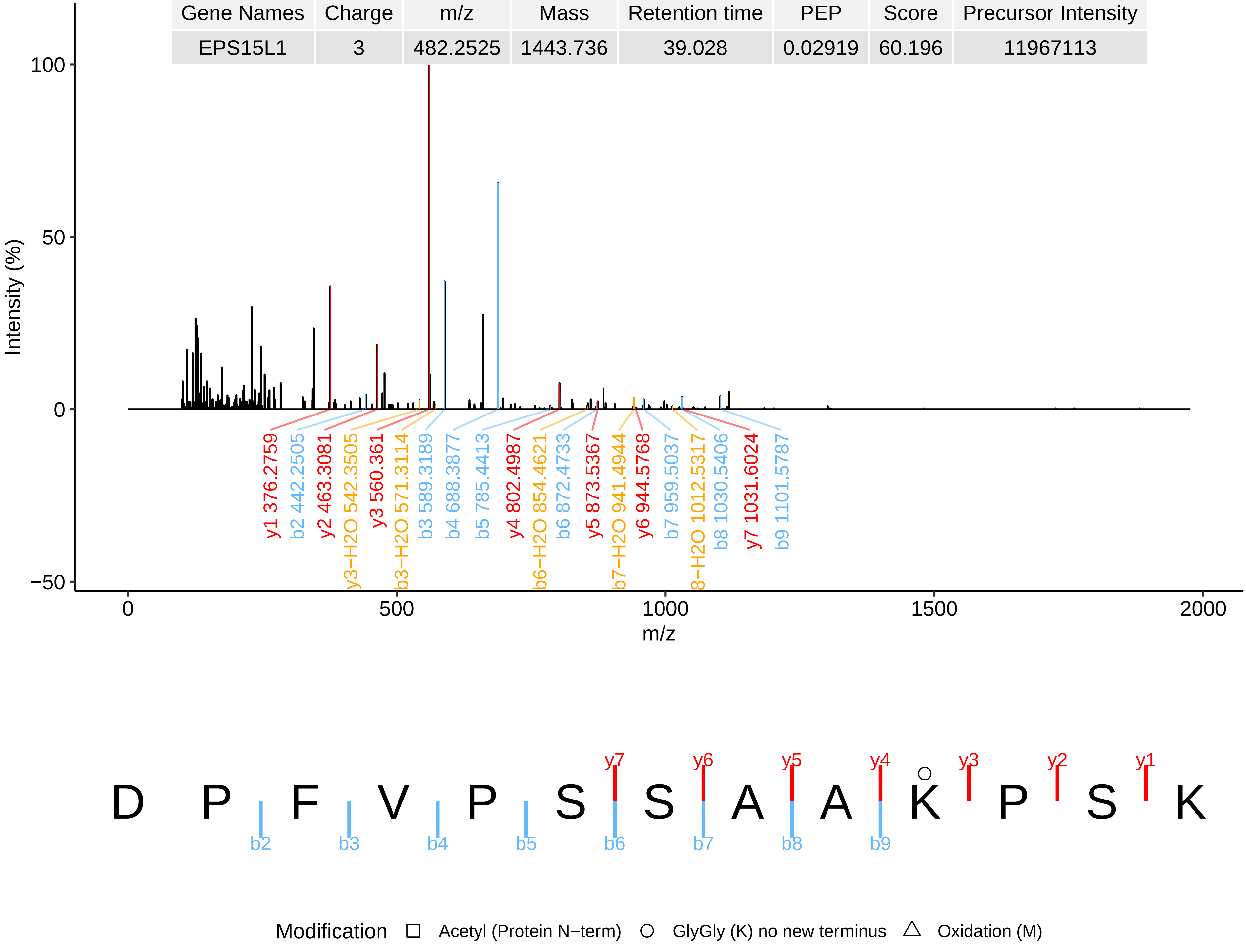

20200921_KIZ_MUb4_GlyGly_10plex_2 − Scan number: 16574

Gene Names	Charge	m/z	Mass	Retention time	PEP	Score	Precursor Intensity
SLC20A1	4	354.9425	1415.741	64.715	0.012361	64.439	1201627

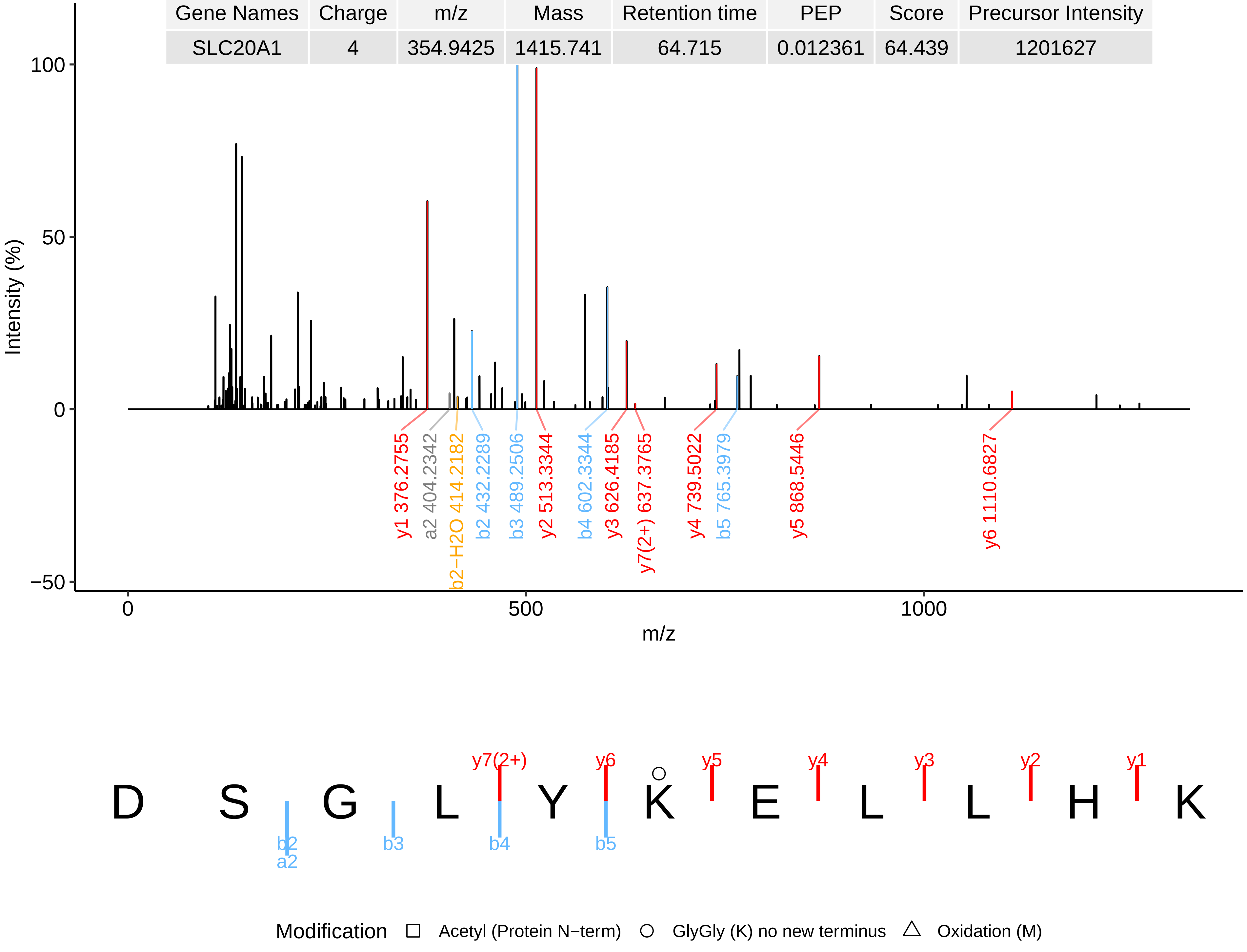

20200921_KIZ_MUb4_GlyGly_10plex_2 − Scan number: 19805

Gene Names	Charge	m/z	Mass	Retention time	PEP	Score	Precursor Intensity
RPS12	3	482.2308	1443.67	77.782	0.064349	54.608	536654.2

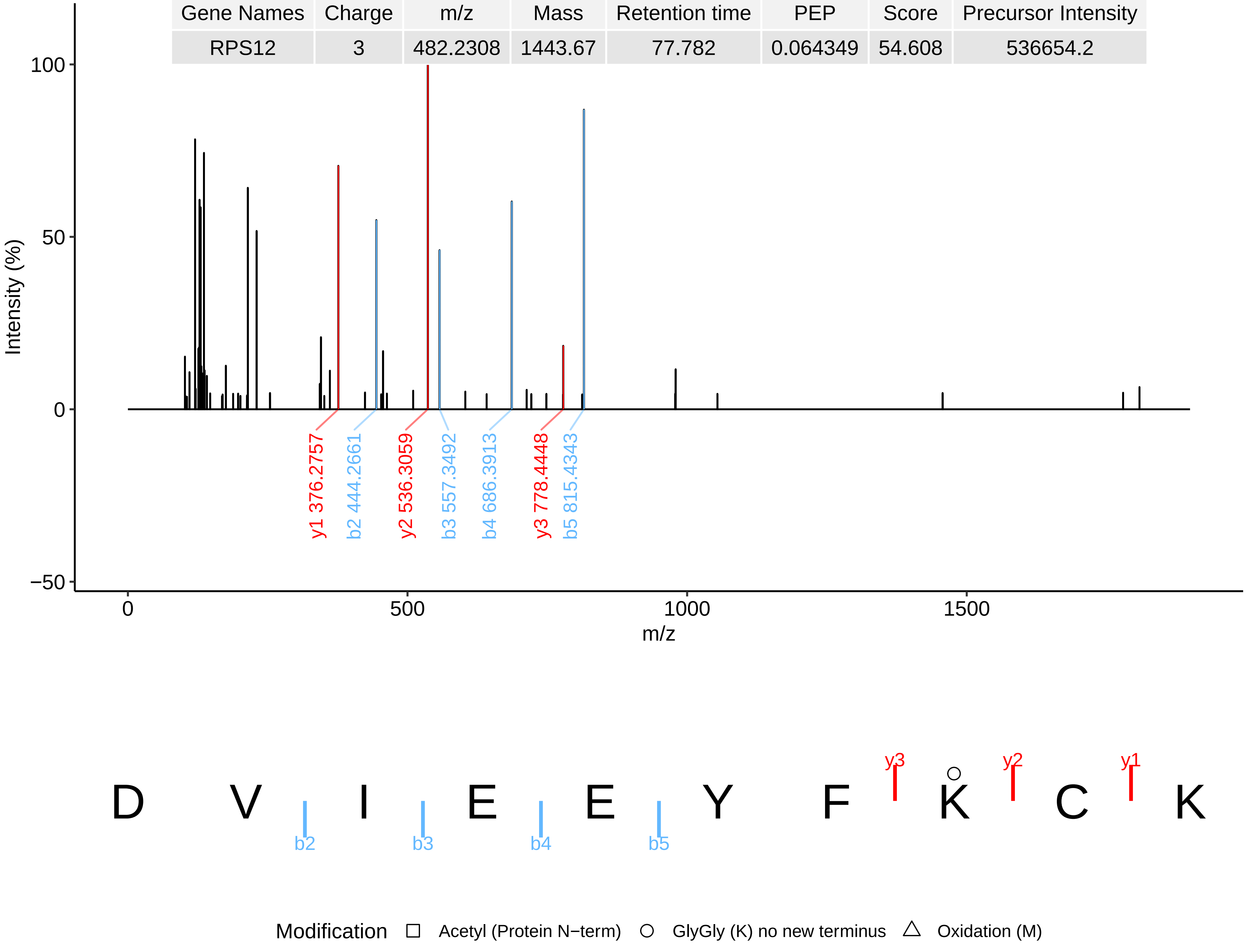

20200921_KIZ_MUb4_GlyGly_10plex − Scan number: 10242

Gene Names	Charge	m/z	Mass	Retention time	PEP	Score	Precursor Intensity
SLC16A6	3	542.5911	1624.752	35.373	8.7425e−06	118.37	2600617

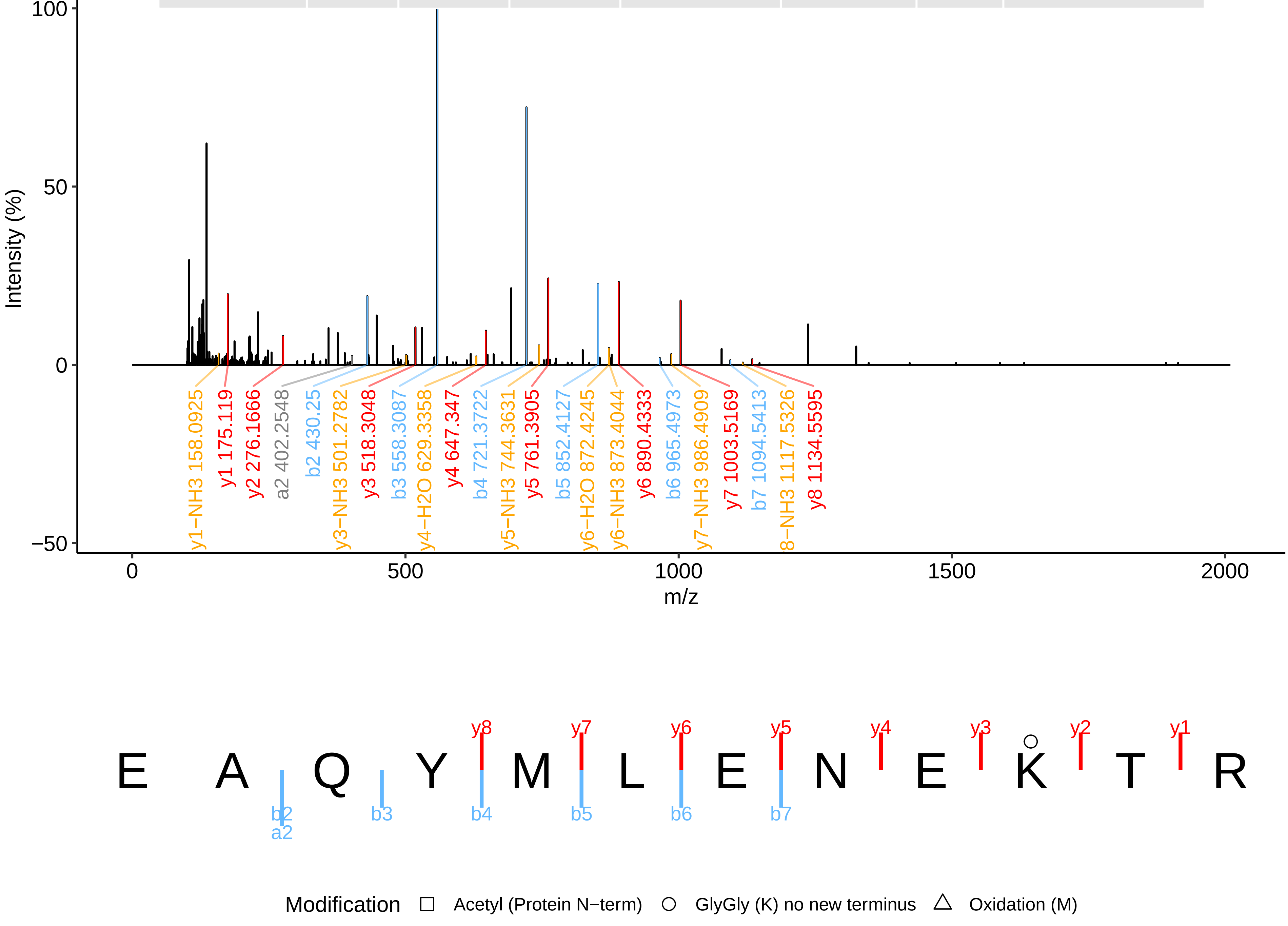

20200921_KIZ_MUb4_GlyGly_10plex − Scan number: 9919

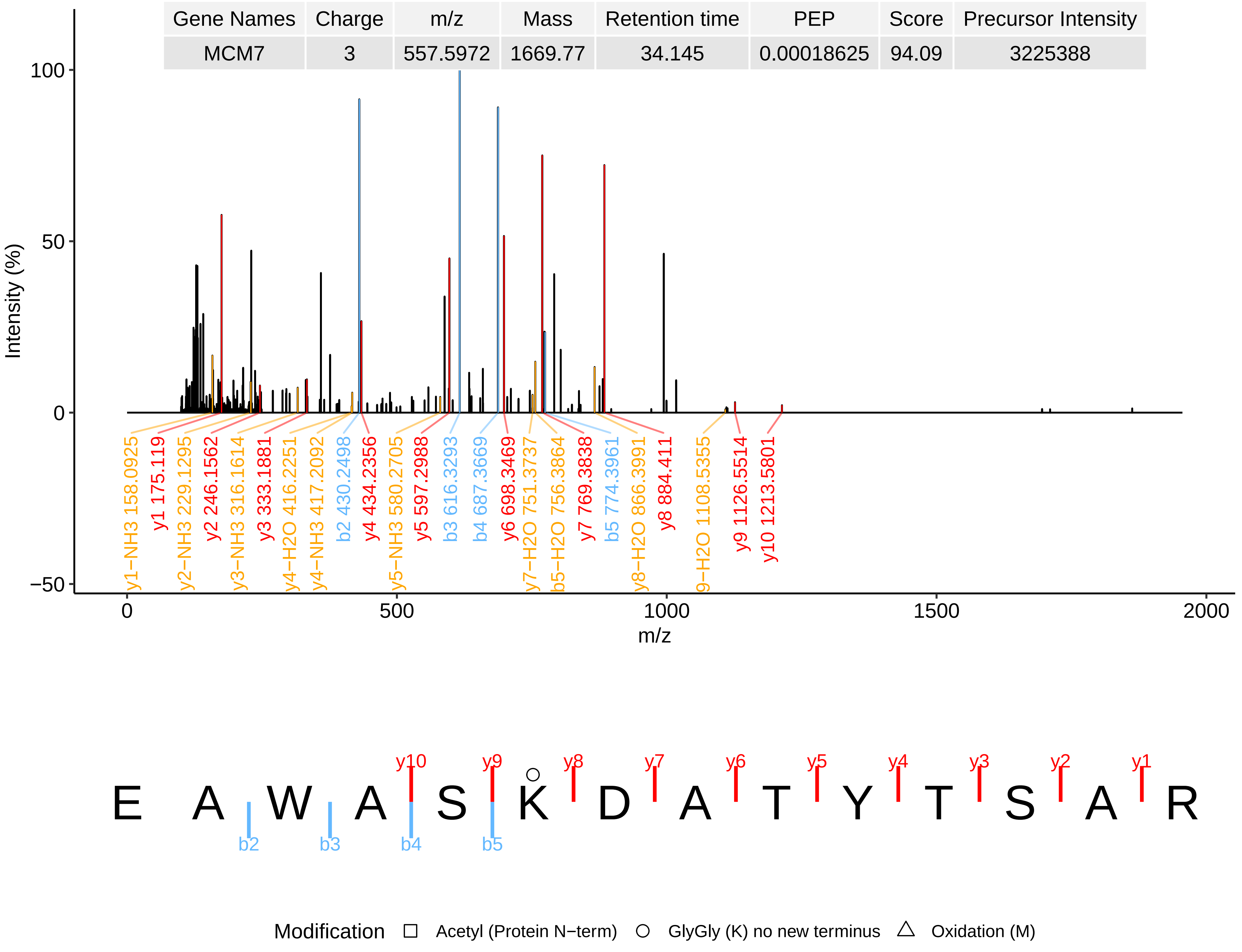

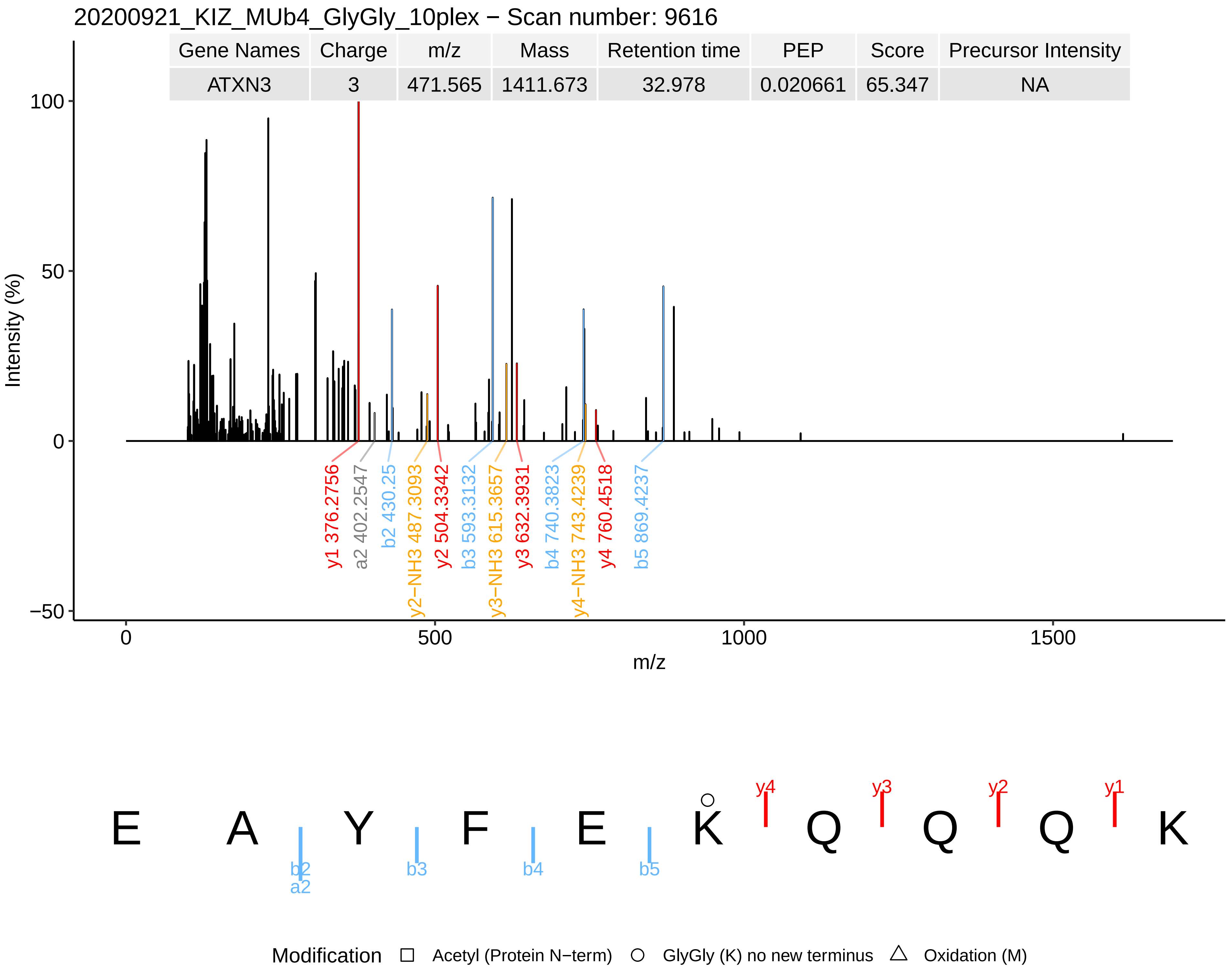
20200921_KIZ_MUb4_GlyGly_10plex − Scan number: 9616
Gene Names | Charge | m/z | Mass | Retention time | PEP | Score | Precursor Intensity
ATXN3 | 3 | 471.565 | 1411.673 | 32.978 | 0.020661 | 65.347 | NA
Intensity (%)
100
50
0
−50
0
500
1000
1500
m/z
y1 376.2756
a2 402.2547
b2 430.25
y2−NH3 487.3093
y2 504.3342
b3 593.3132
y3−NH3 615.3657
y3 632.3931
b4 740.3823
y4−NH3 743.4239
y4 760.4518
b5 869.4237
E A Y F E K Q Q Q K
y4 y3 y2 y1
b2 a2 b3 b4 b5
Modification
Acetyl (Protein N−term)
GlyGly (K) no new terminus
Oxidation (M)

20200921_KIZ_MUb4_GlyGly_10plex_2 − Scan number: 8819

Gene Names	Charge	m/z	Mass	Retention time	PEP	Score	Precursor Intensity
ATXN3	3	471.565	1411.673	33.354	0.0093242	73.985	627766.8

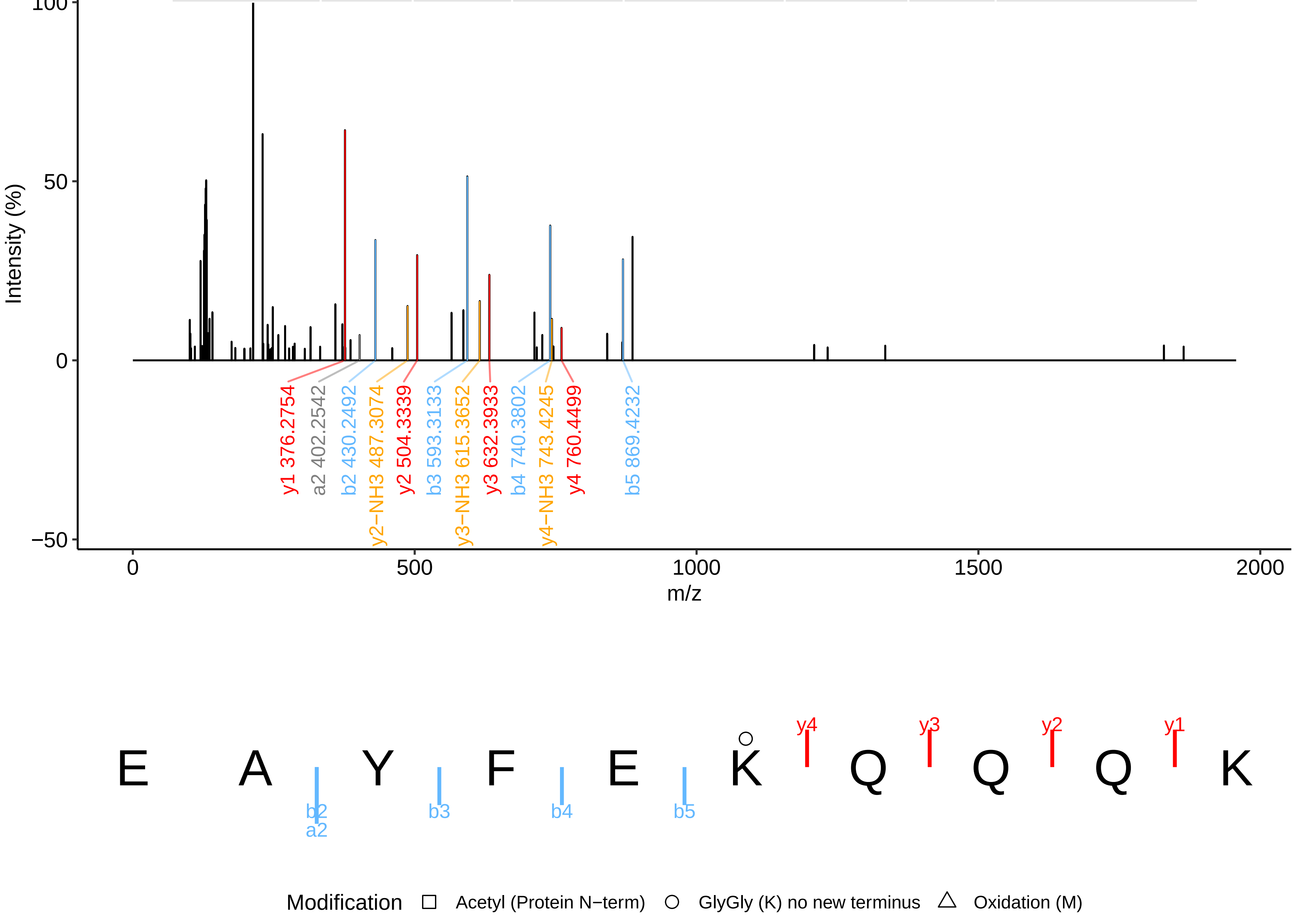

20200921_KIZ_MUb4_GlyGly_10plex_2 − Scan number: 18969

Gene Names	Charge	m/z	Mass	Retention time	PEP	Score	Precursor Intensity
SLC3A2	3	552.3019	1653.884	74.383	0.10615	47.037	2151748

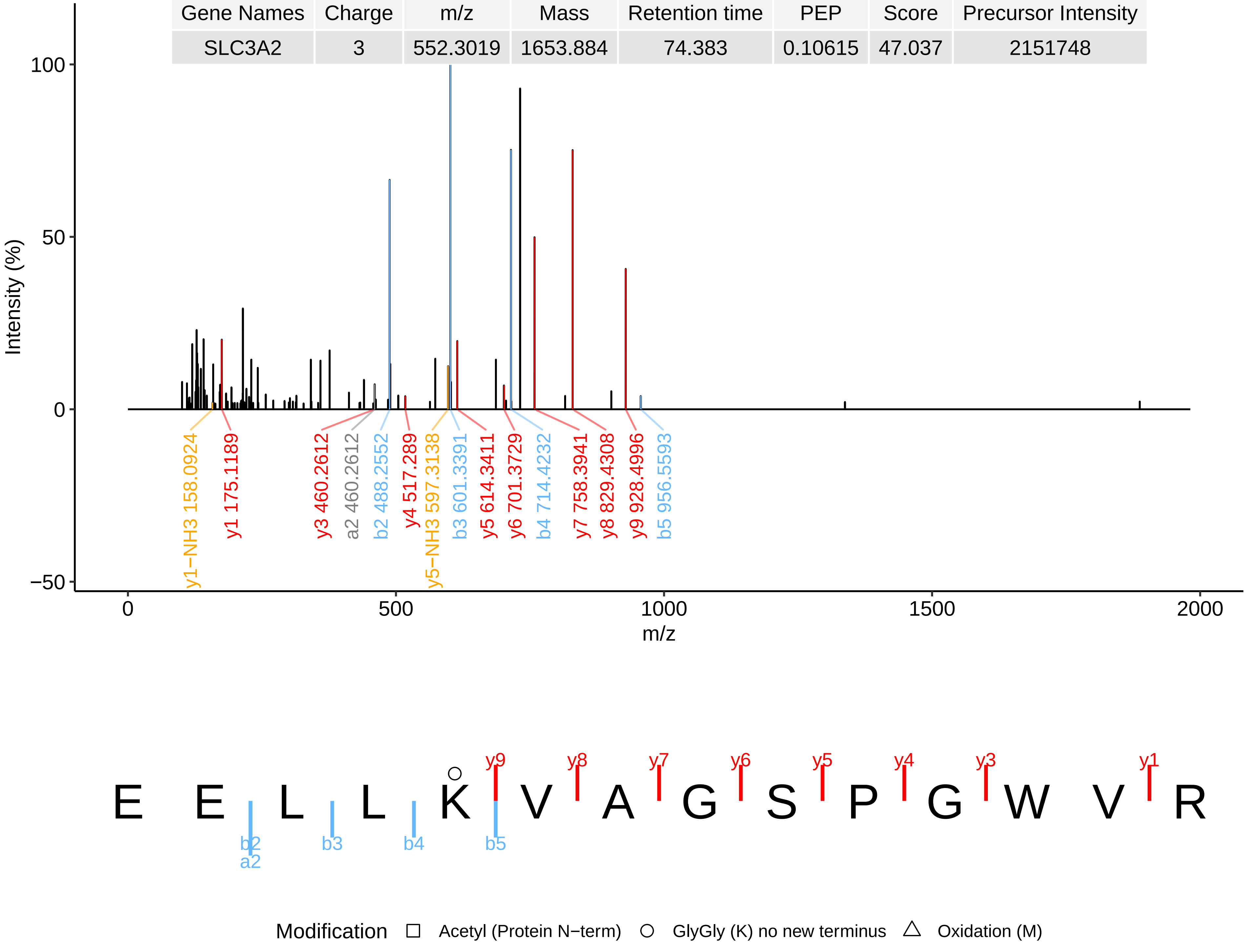

20200921_KIZ_MUb4_GlyGly_10plex_2 − Scan number: 8023

Gene Names	Charge	m/z	Mass	Retention time	PEP	Score	Precursor Intensity
RPSA	3	463.572	1387.694	30.138	0.0691	55.314	420497.6

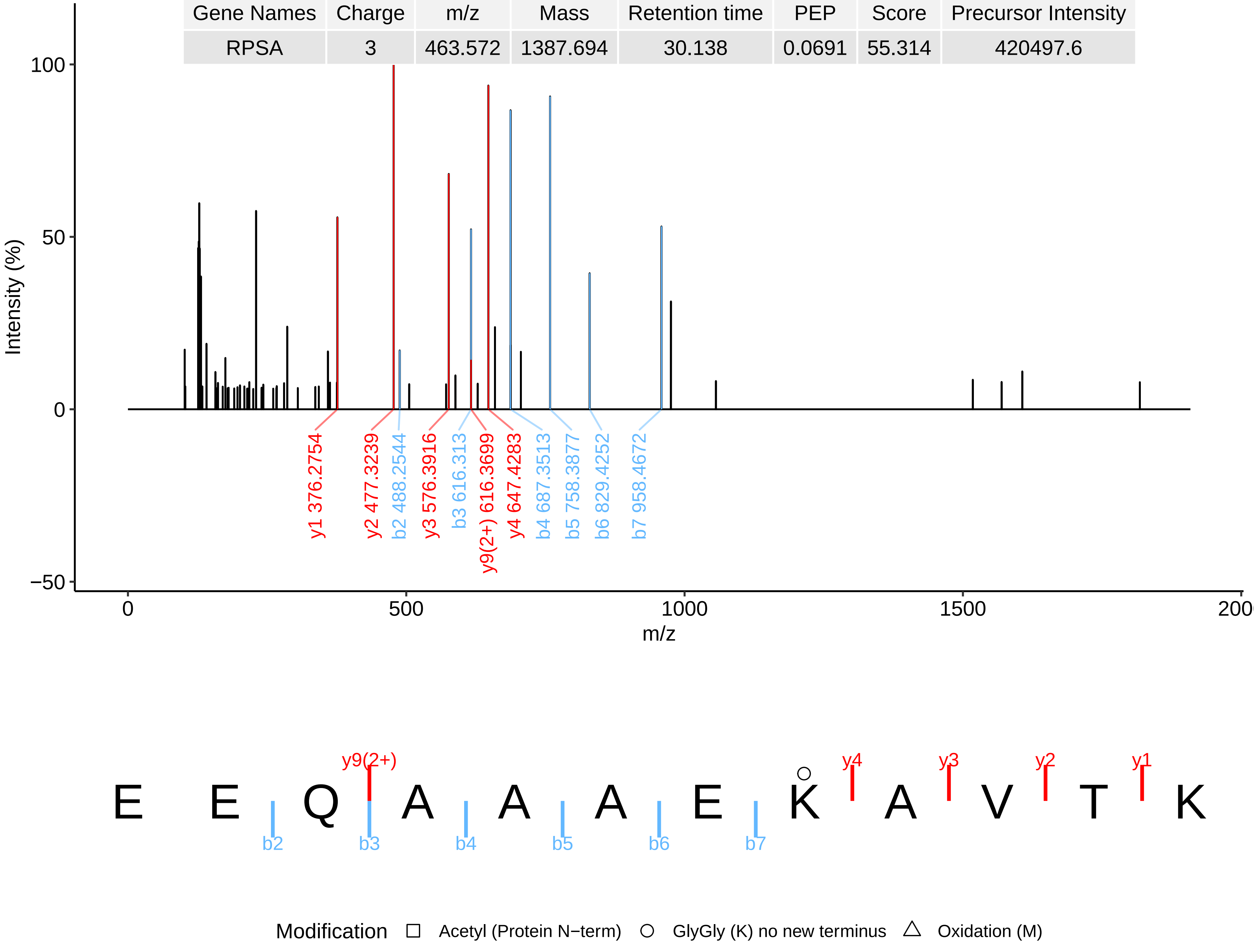

20200921_KIZ_MUb4_GlyGly_10plex_2 − Scan number: 5120

Gene Names	Charge	m/z	Mass	Retention time	PEP	Score	Precursor Intensity
SLC20A1	3	469.8734	1406.598	18.264	0.025021	61.435	NA

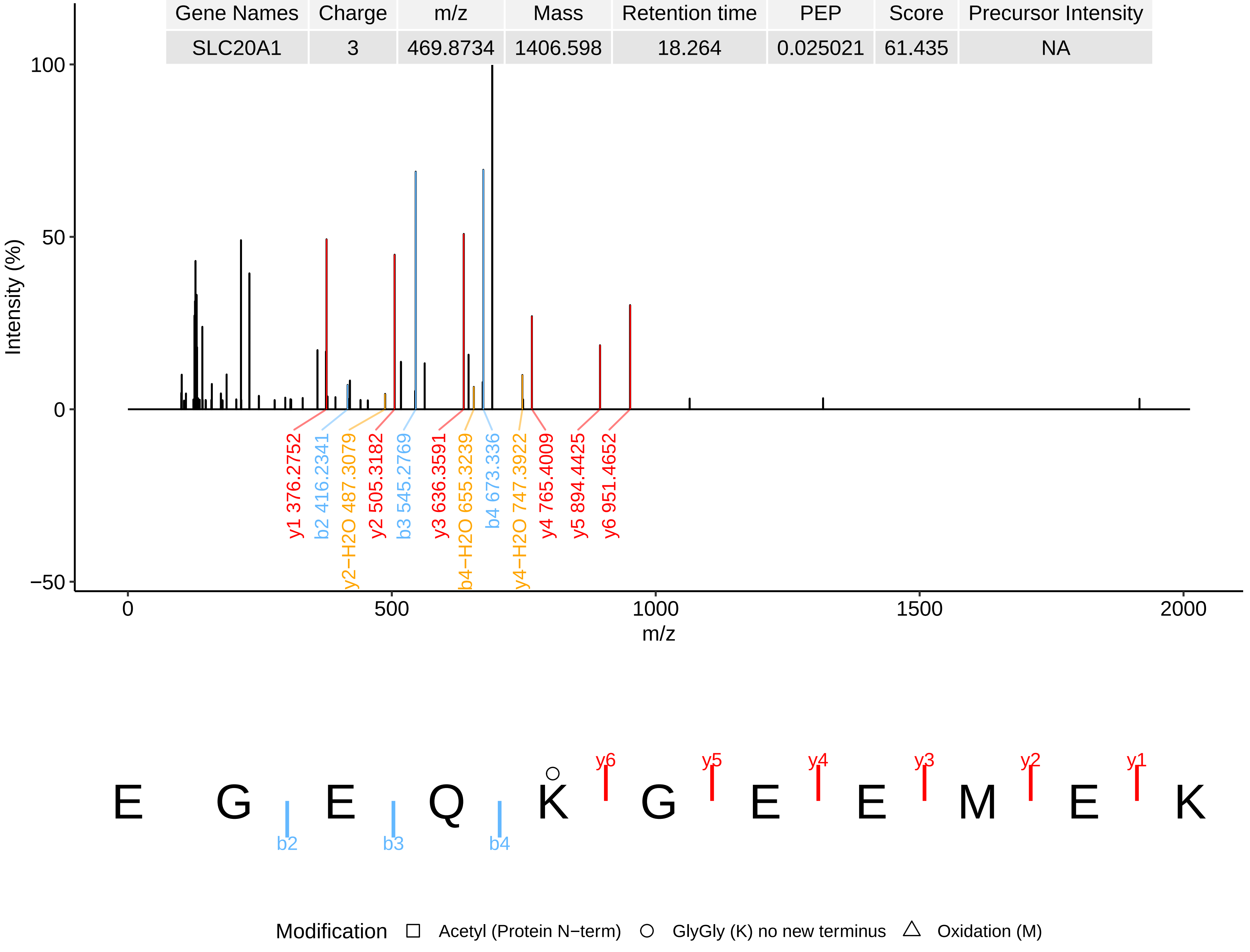

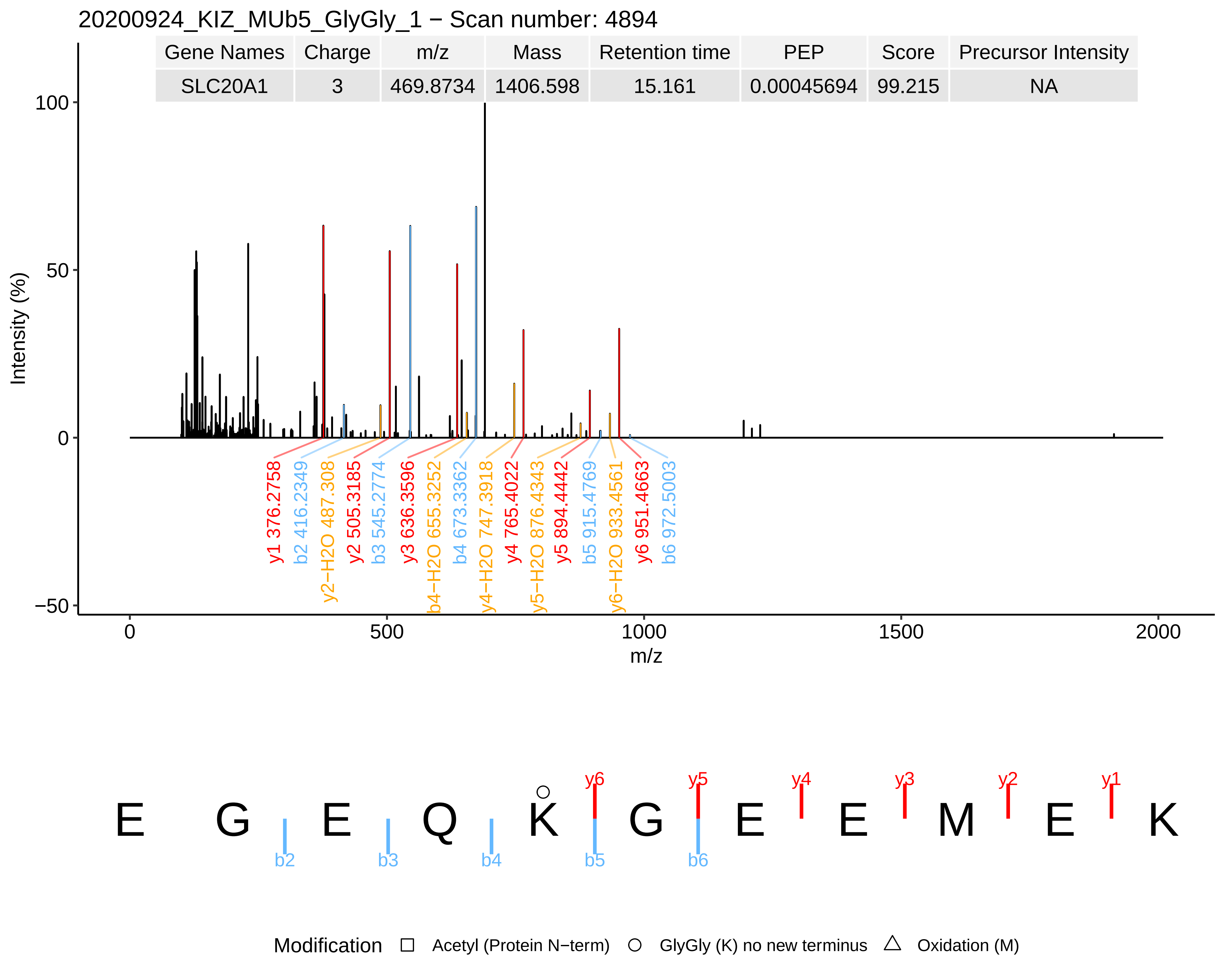

20200924_KIZ_MUb5_GlyGly_1 − Scan number: 4894
Gene Names | Charge | m/z | Mass | Retention time | PEP | Score | Precursor Intensity
SLC20A1 | 3 | 469.8734 | 1406.598 | 15.161 | 0.00045694 | 99.215 | NA
Intensity (%)
m/z
y1 376.2758
b2 416.2349
y2−H2O 487.308
y2 505.3185
b3 545.2774
y3 636.3596
b4−H2O 655.3252
b4 673.3362
y4−H2O 747.3918
y4 765.4022
y5−H2O 876.4343
y5 894.4442
b5 915.4769
y6−H2O 933.4561
y6 951.4663
b6 972.5003
E G E Q K G E E M E K
Modification
Acetyl (Protein N−term)
GlyGly (K) no new terminus
Oxidation (M)

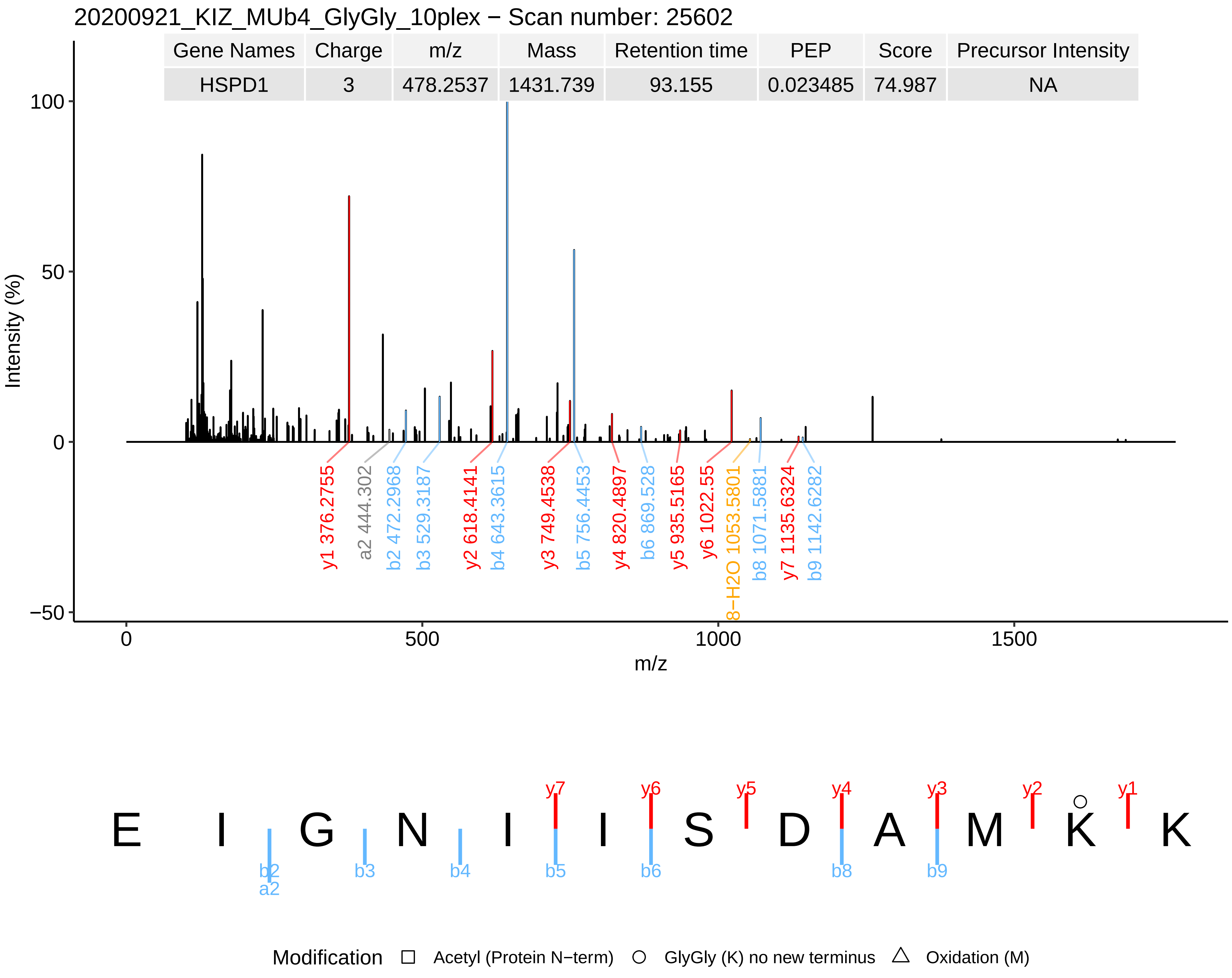

20200921_KIZ_MUb4_GlyGly_10plex − Scan number: 25602
Gene Names
Charge
m/z
Mass
Retention time
PEP
Score
Precursor Intensity
HSPD1
3
478.2537
1431.739
93.155
0.023485
74.987
NA
Intensity (%)
100
50
0
−50
0
500
1000
1500
m/z
y1 376.2755
a2 444.302
b2 472.2968
b3 529.3187
y2 618.4141
b4 643.3615
y3 749.4538
b5 756.4453
y4 820.4897
b6 869.528
y5 935.5165
y6 1022.55
8−H2O 1053.5801
b8 1071.5881
y7 1135.6324
b9 1142.6282
E I G N I I S D A M K K
y7 y6 y5 y4 y3 y2 y1
b2 a2 b3 b4 b5 b6 b8 b9
Modification
Acetyl (Protein N−term)
GlyGly (K) no new terminus
Oxidation (M)

20201118_KIZ_MUb6_GlyGly_1 − Scan number: 23956

Gene Names	Charge	m/z	Mass	Retention time	PEP	Score	Precursor Intensity
HSPD1	3	478.2537	1431.739	73.907	0.00014549	101.53	23683792

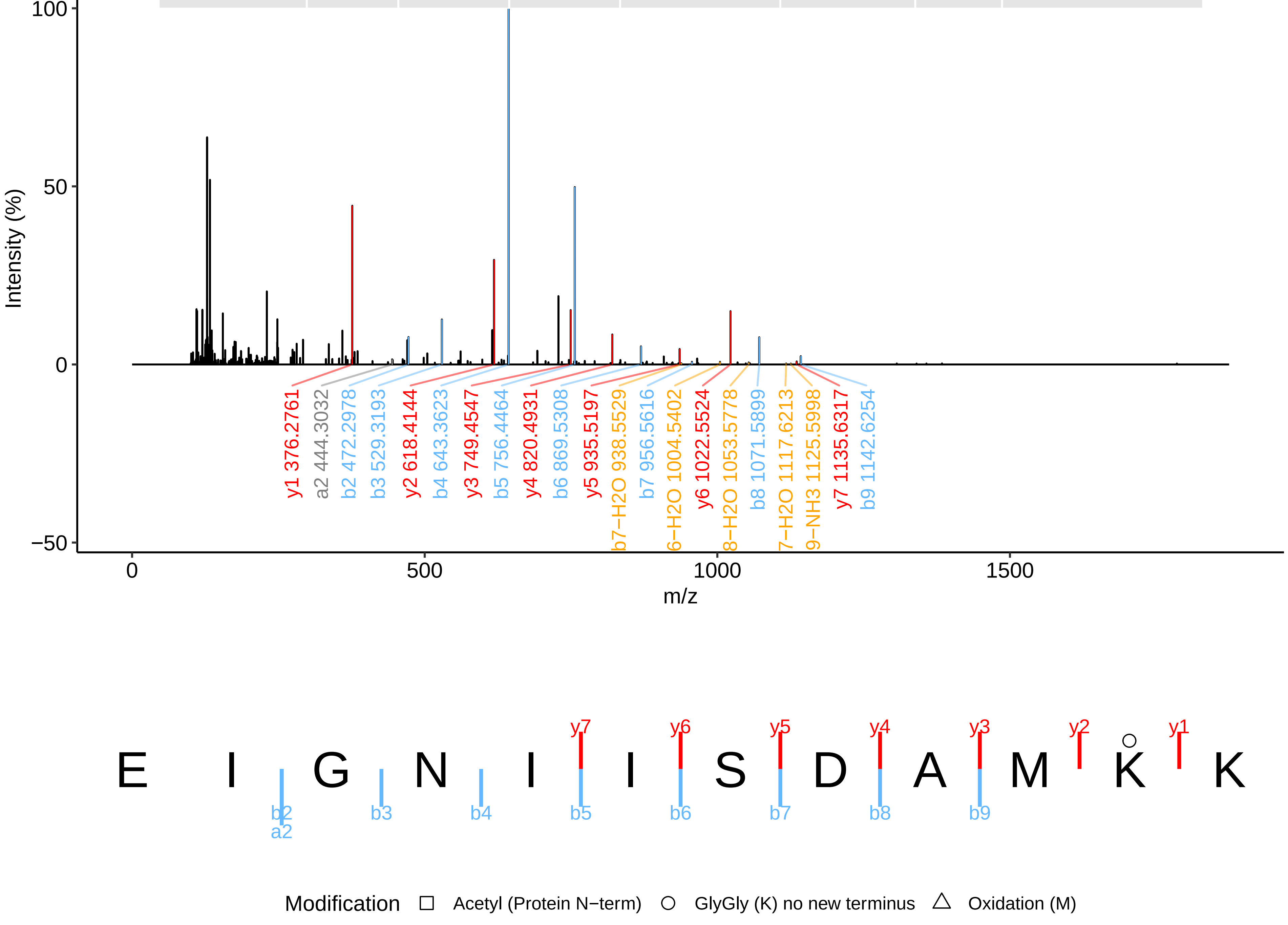

20200921_KIZ_MUb4_GlyGly_10plex_2 − Scan number: 16788

Gene Names	Charge	m/z	Mass	Retention time	PEP	Score	Precursor Intensity
HSPD1	3	483.5853	1447.734	65.575	0.012118	62.14	846133.6

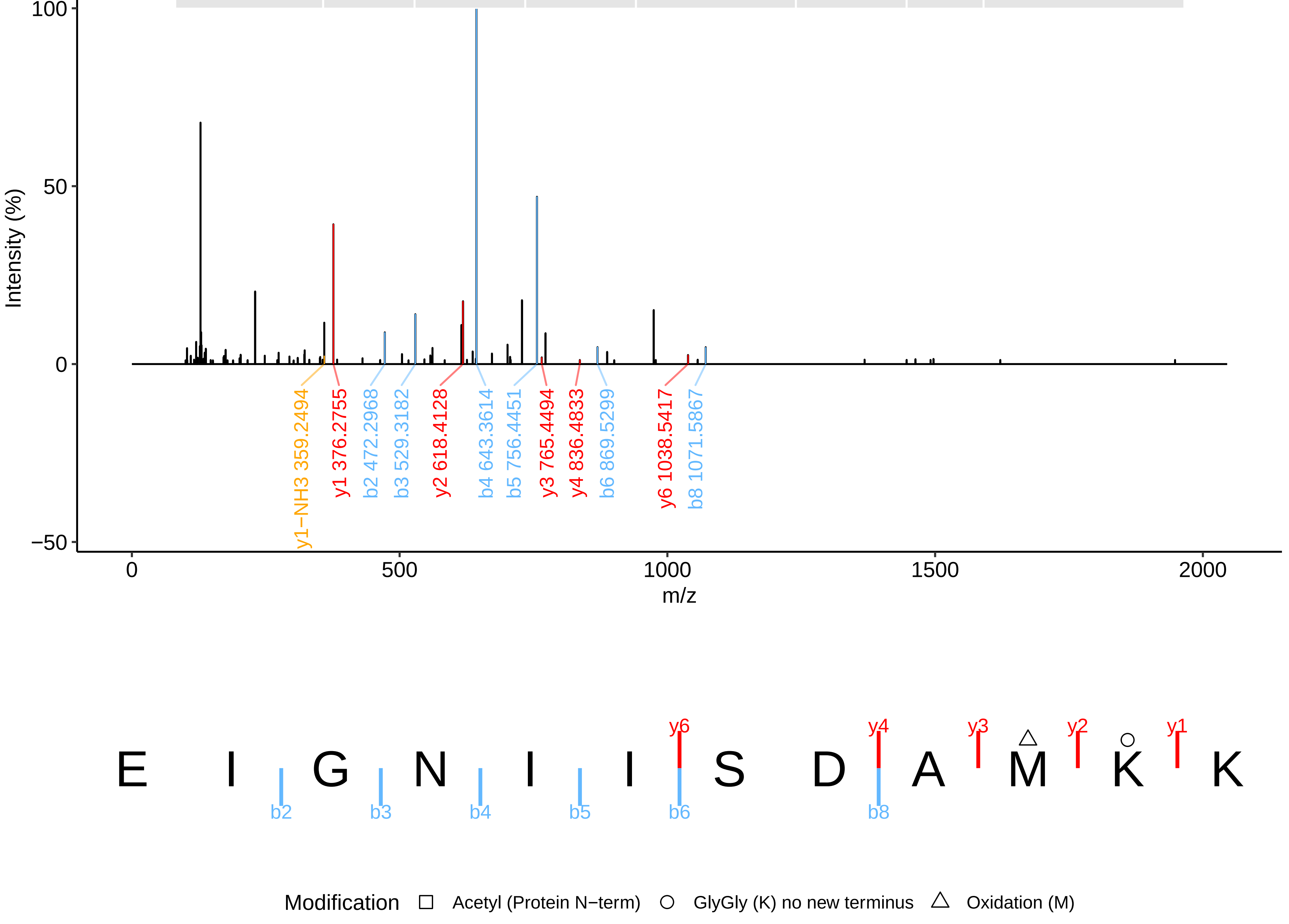

20200921_KIZ_MUb4_GlyGly_10plex_2 − Scan number: 16935

Gene Names	Charge	m/z	Mass	Retention time	PEP	Score	Precursor Intensity
HSPD1	3	483.5853	1447.734	66.162	0.071995	44.616	769186.8

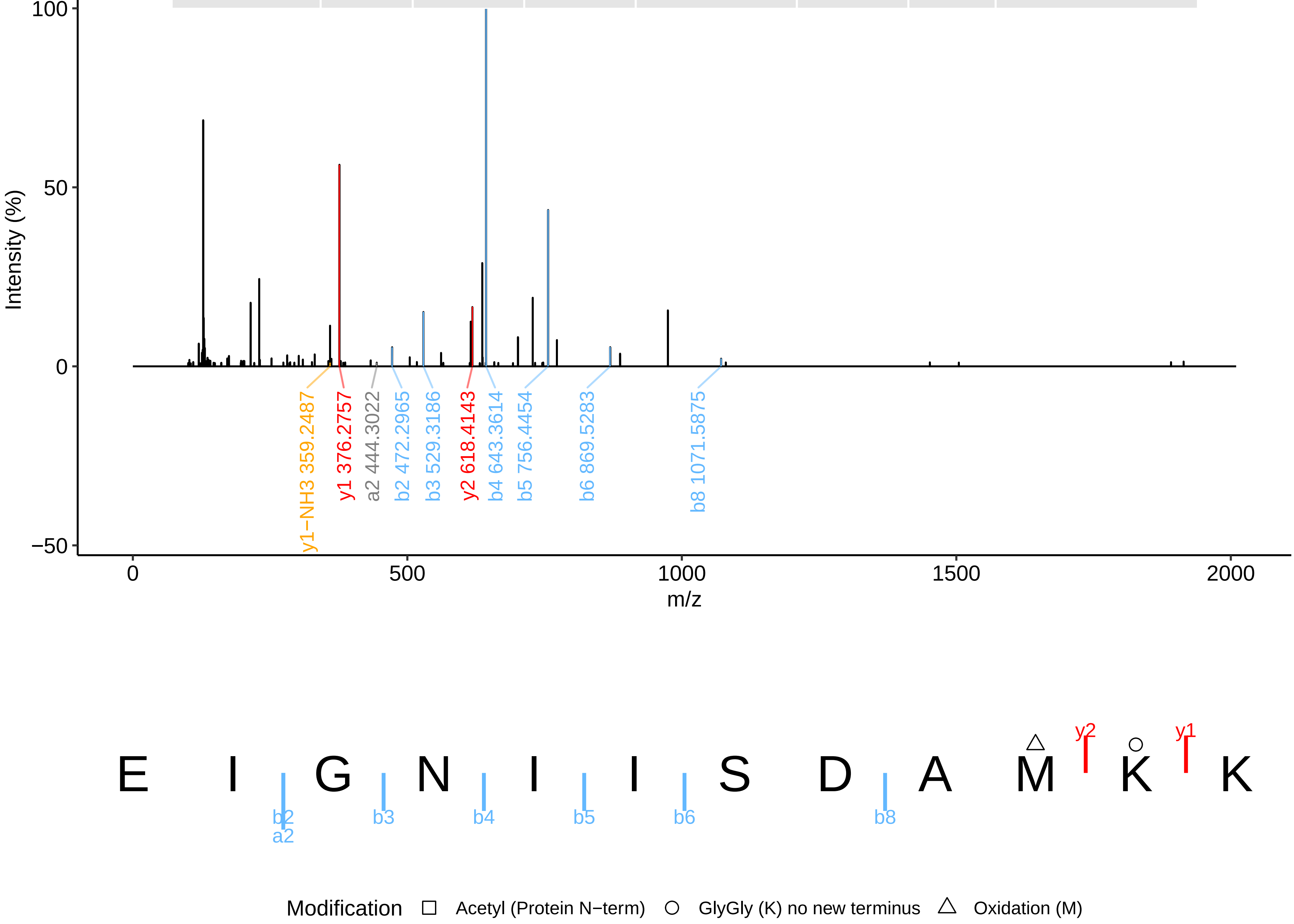

20200921_KIZ_MUb4_GlyGly_10plex − Scan number: 11358

Gene Names	Charge	m/z	Mass	Retention time	PEP	Score	Precursor Intensity
PPP2CB;PPP2CA	3	553.6285	1657.863	39.535	2.0215e−14	148.62	6170100

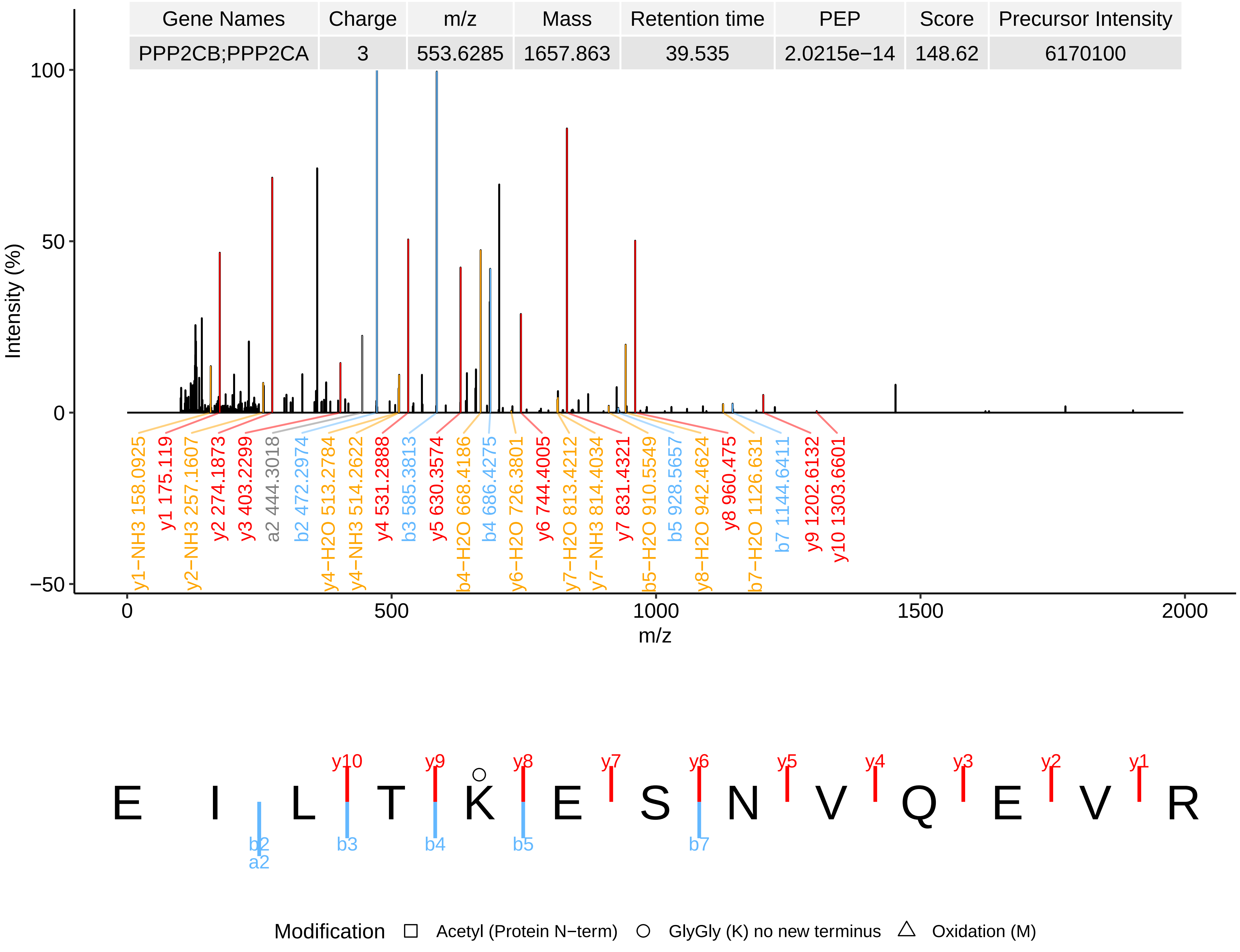

20200921_KIZ_MUb4_GlyGly_10plex_2 − Scan number: 10443

Gene Names	Charge	m/z	Mass	Retention time	PEP	Score	Precursor Intensity
PPP2CB;PPP2CA	3	553.6285	1657.863	39.955	1.9203e−05	100.55	1112611

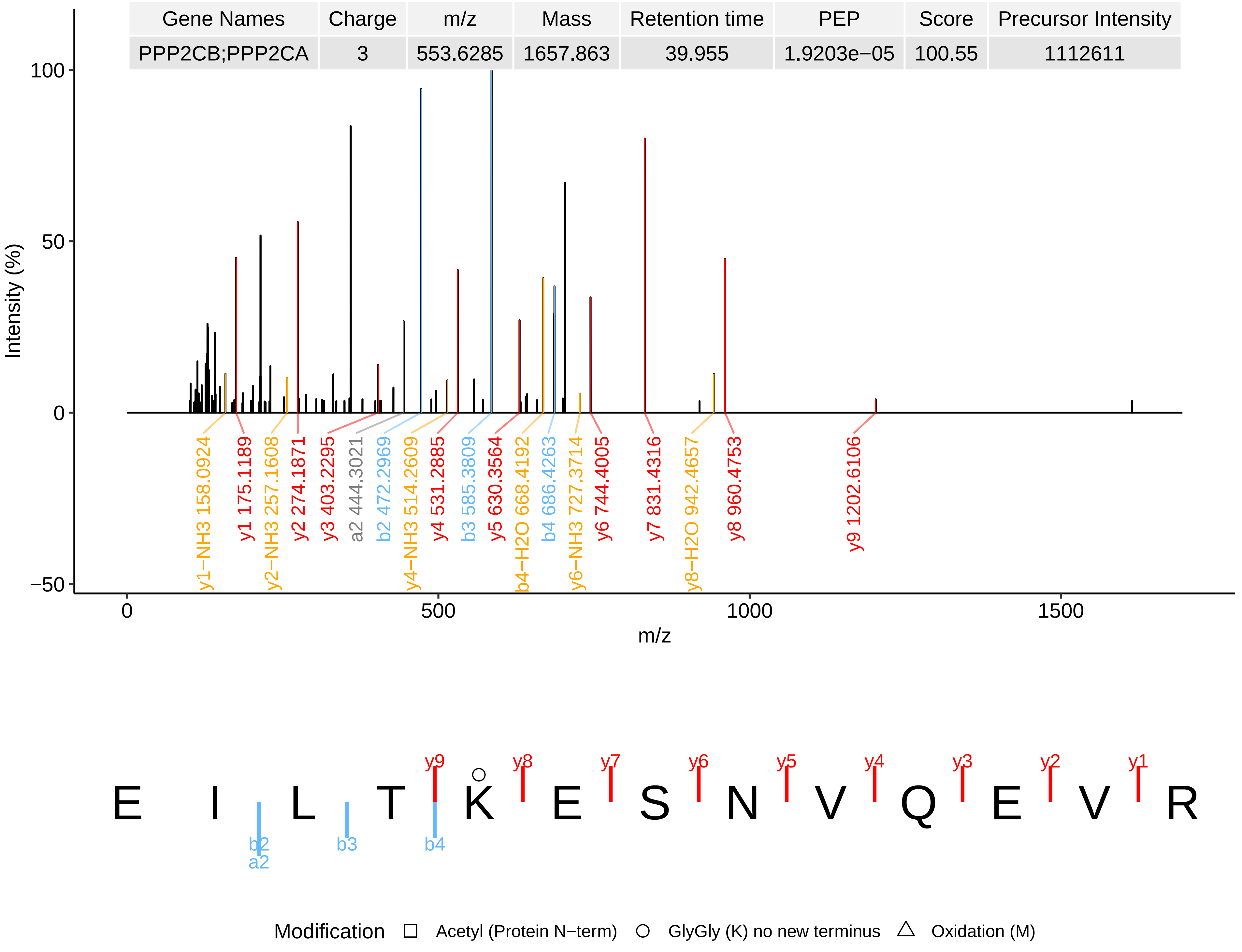

20200924_KIZ_MUb5_GlyGly_1 − Scan number: 10321

Gene Names	Charge	m/z	Mass	Retention time	PEP	Score	Precursor Intensity
PPP2CB;PPP2CA	3	553.6285	1657.863	33.285	0.0011464	80.815	11928381

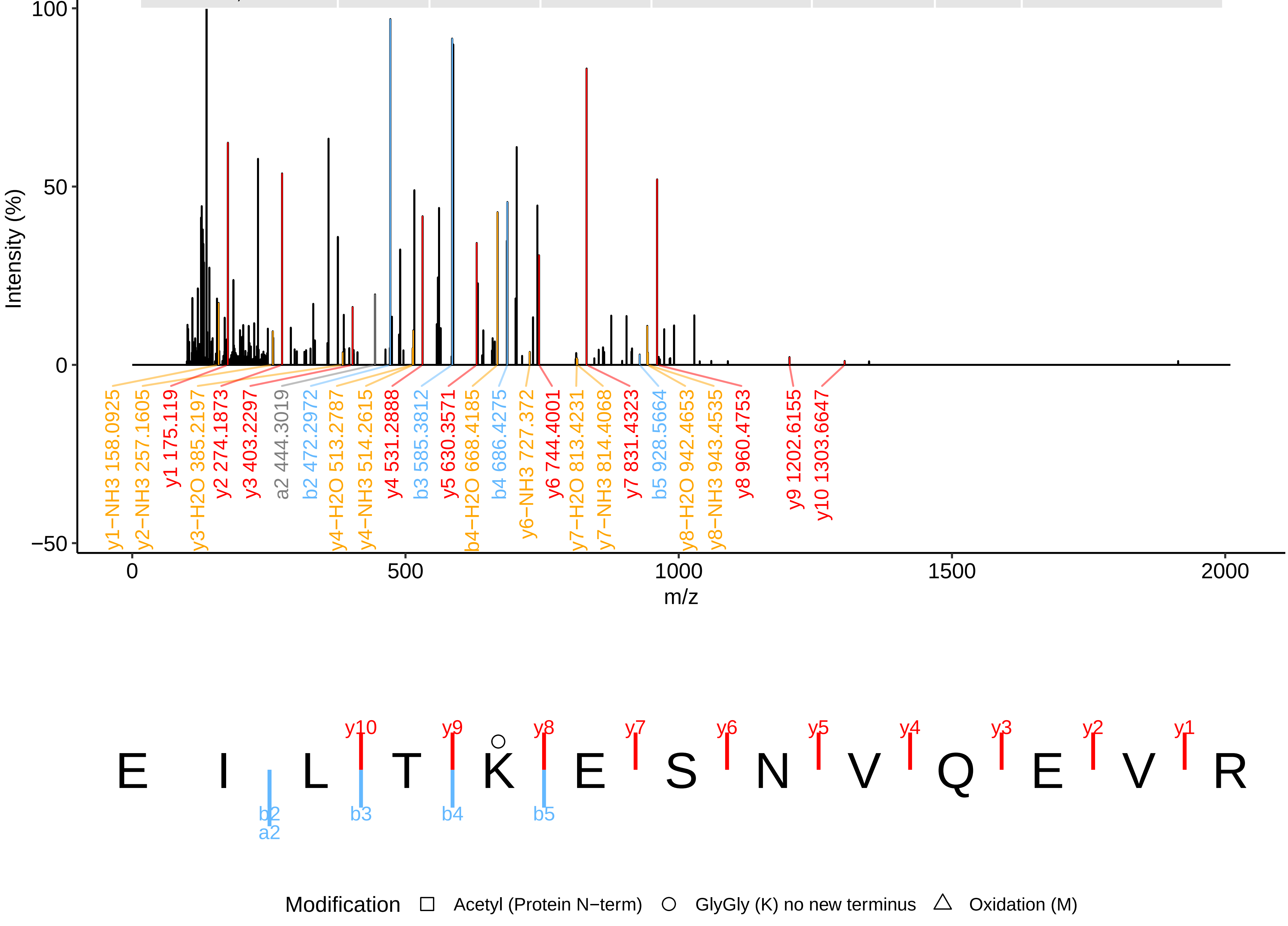

20200924_KIZ_MUb5_GlyGly_2 − Scan number: 9789

Gene Names	Charge	m/z	Mass	Retention time	PEP	Score	Precursor Intensity
PPP2CB;PPP2CA	3	553.6285	1657.863	36.583	0.0040812	76.073	1133771

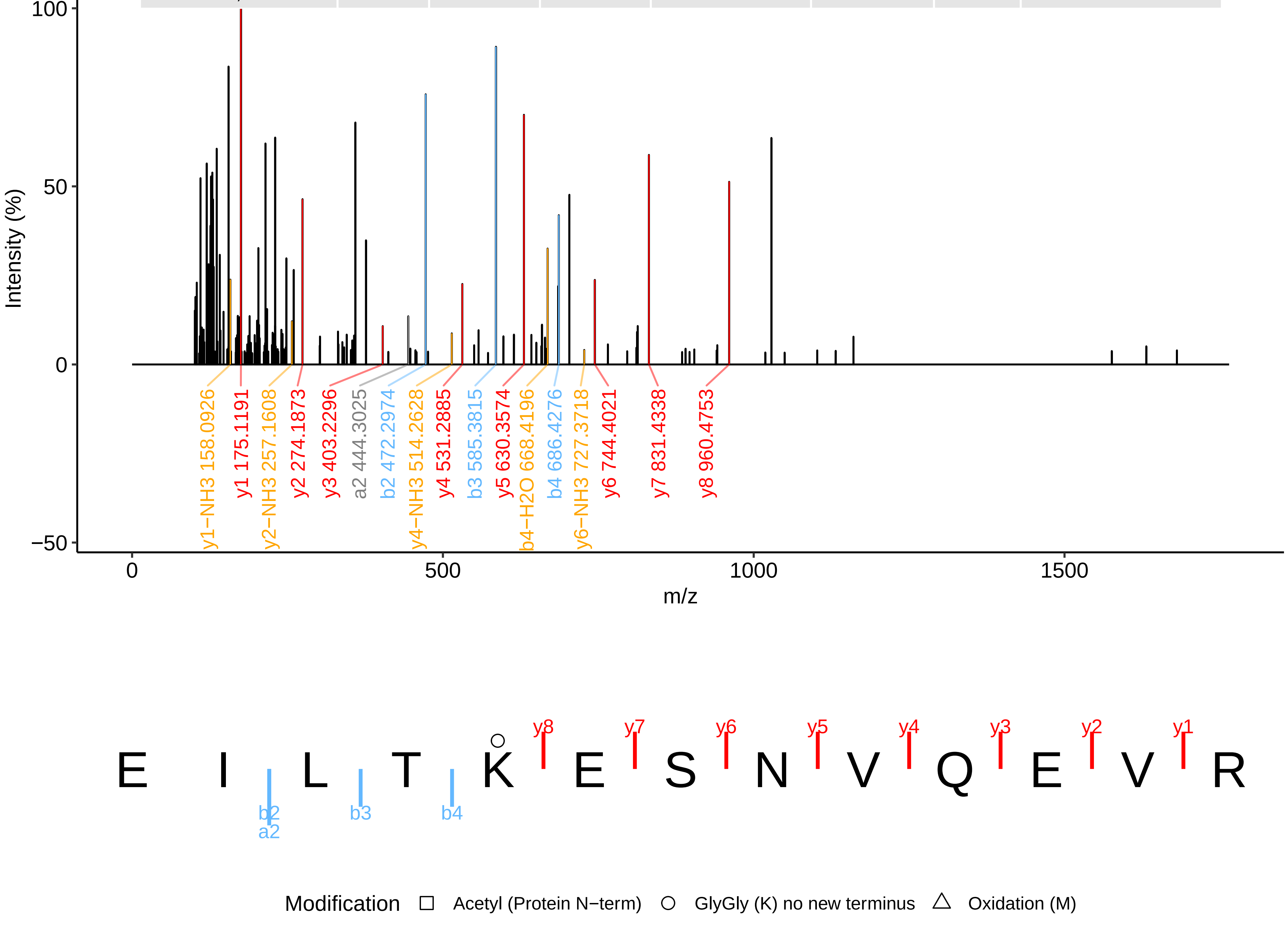

20200921_KIZ_MUb4_GlyGly_10plex − Scan number: 15399

Gene Names	Charge	m/z	Mass	Retention time	PEP	Score	Precursor Intensity
SLC43A3	3	465.2489	1392.725	54.995	0.0010426	87.667	5406487

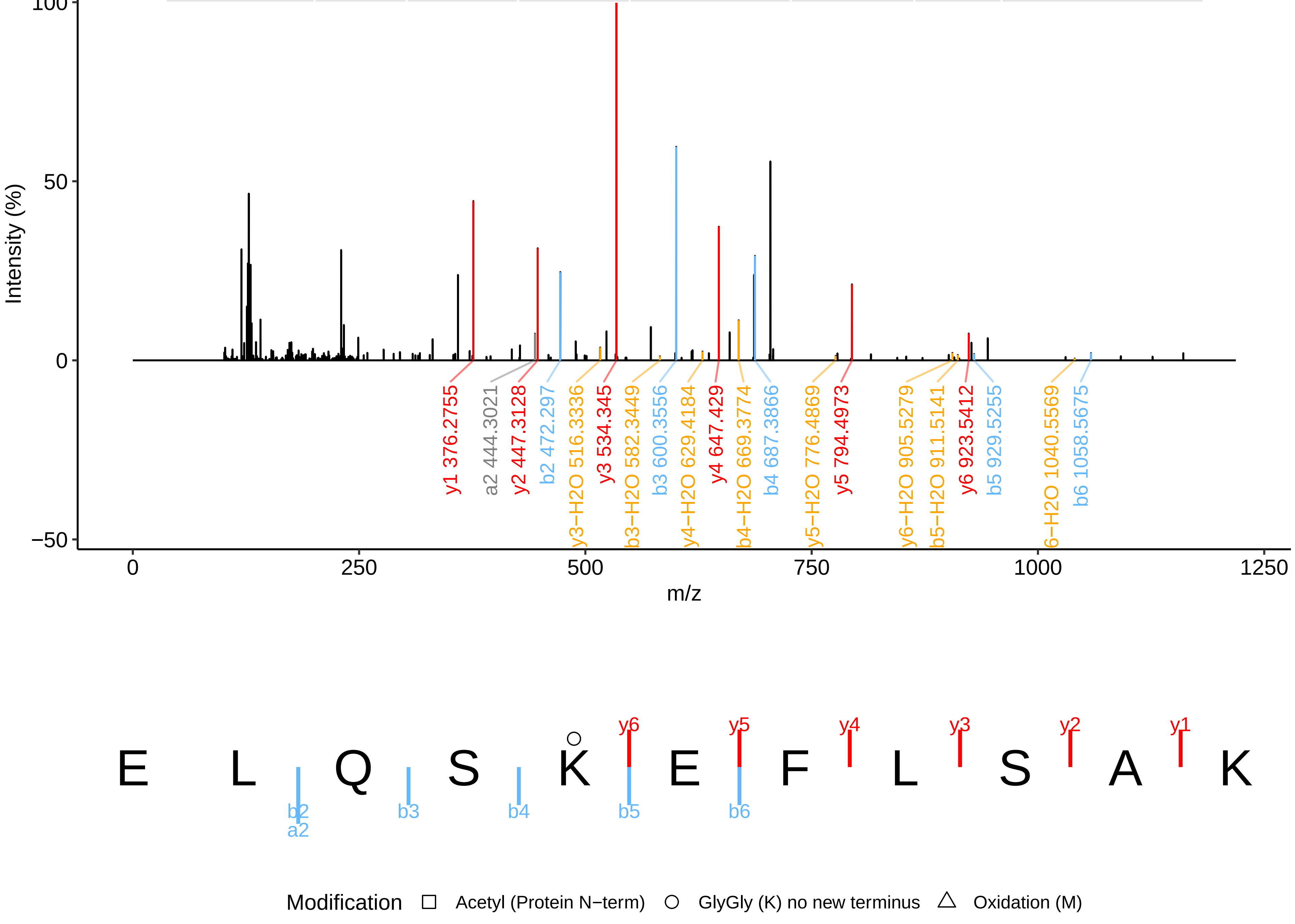

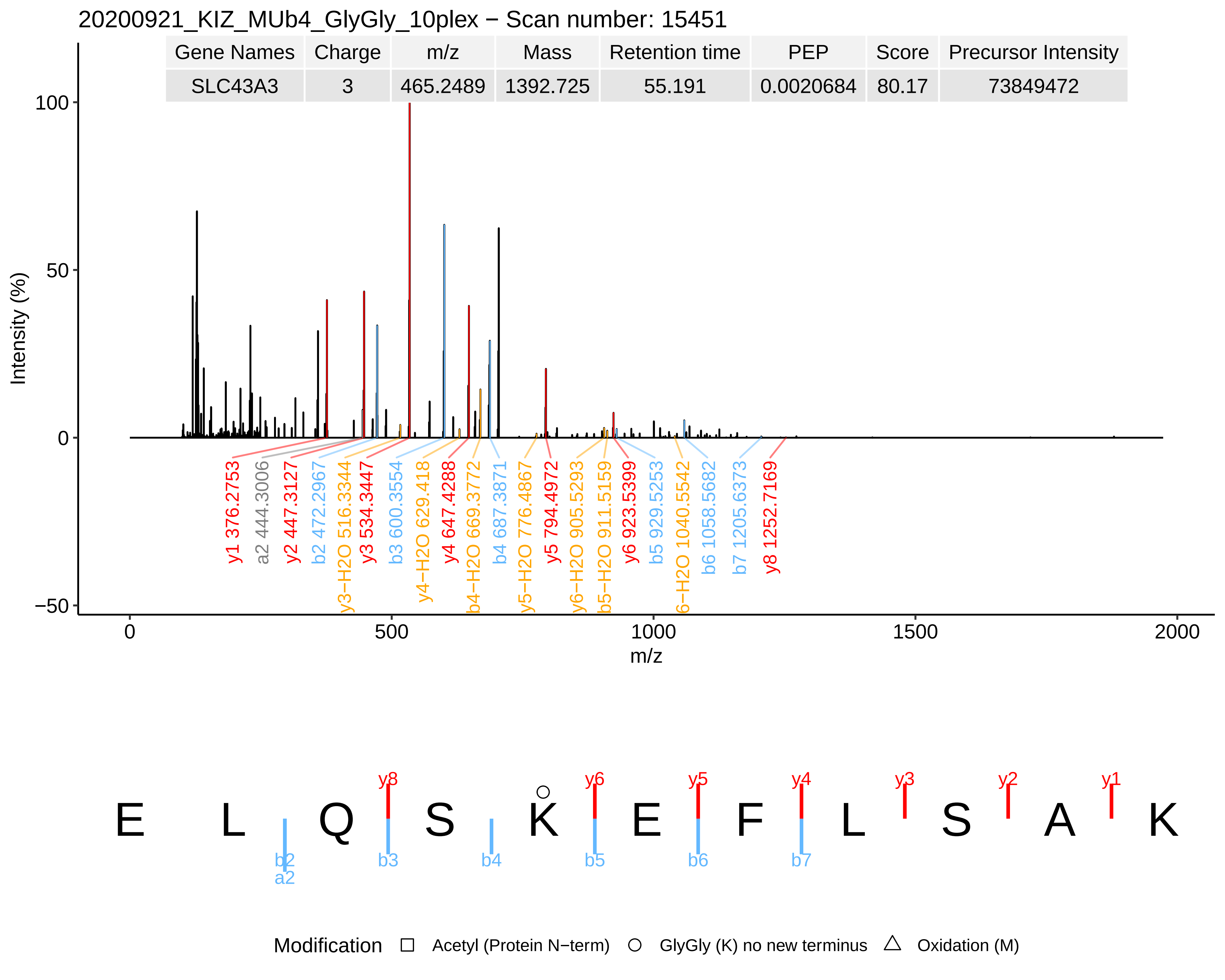

20200921_KIZ_MUb4_GlyGly_10plex − Scan number: 15451
Gene Names | Charge | m/z | Mass | Retention time | PEP | Score | Precursor Intensity
SLC43A3 | 3 | 465.2489 | 1392.725 | 55.191 | 0.0020684 | 80.17 | 73849472
Intensity (%)
m/z
y1 376.2753
a2 444.3006
y2 447.3127
b2 472.2967
y3−H2O 516.3344
y3 534.3447
b3 600.3554
y4−H2O 629.418
y4 647.4288
b4−H2O 669.3772
b4 687.3871
y5−H2O 776.4867
y5 794.4972
y6−H2O 905.5293
b5−H2O 911.5159
y6 923.5399
b5 929.5253
b6−H2O 1040.5542
b6 1058.5682
b7 1205.6373
y8 1252.7169
E L Q S K E F L S A K
Modification
Acetyl (Protein N−term)
GlyGly (K) no new terminus
Oxidation (M)

20200921_KIZ_MUb4_GlyGly_10plex − Scan number: 15587

Gene Names	Charge	m/z	Mass	Retention time	PEP	Score	Precursor Intensity
SLC43A3	3	465.2489	1392.725	55.714	0.00083622	89.805	12839591

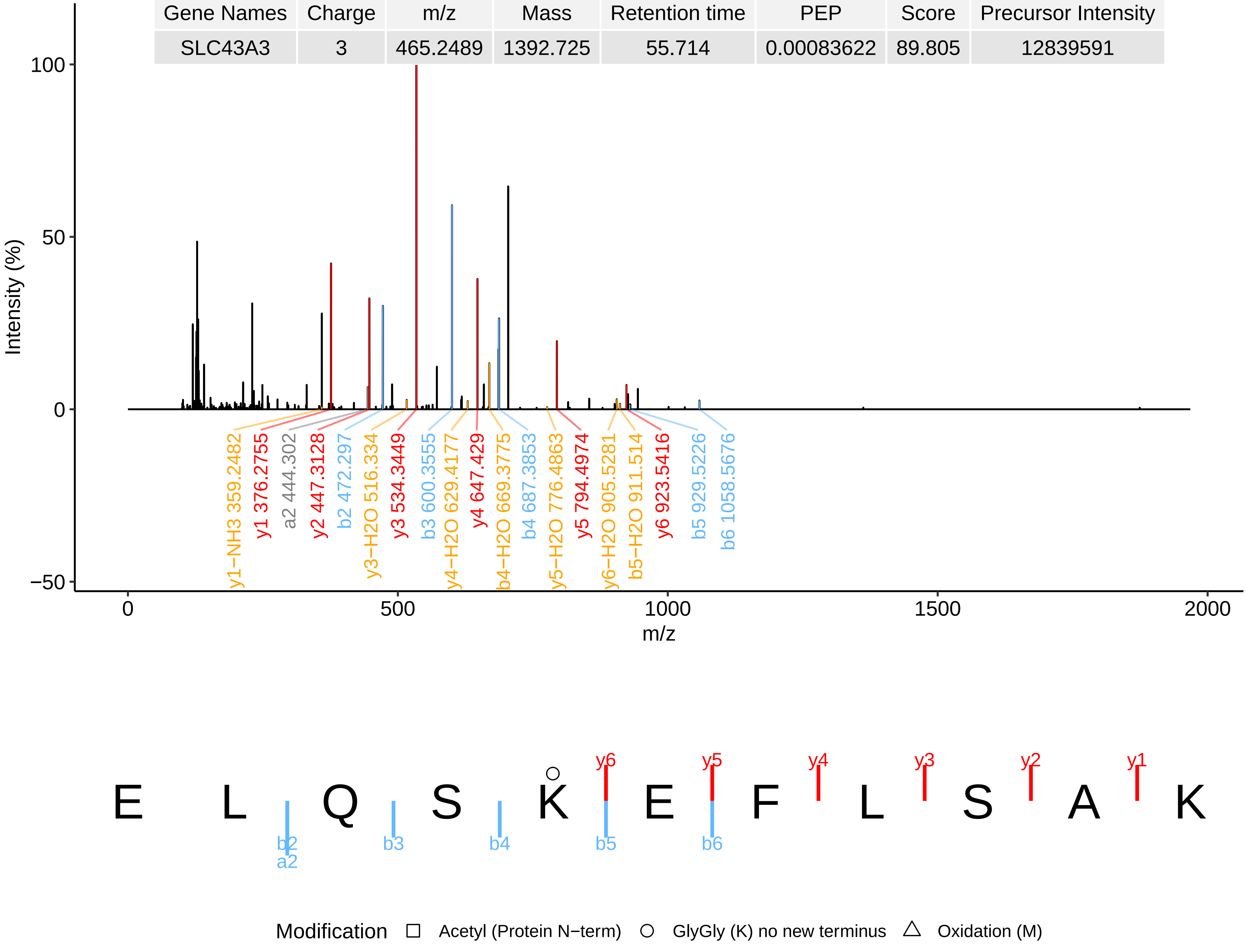

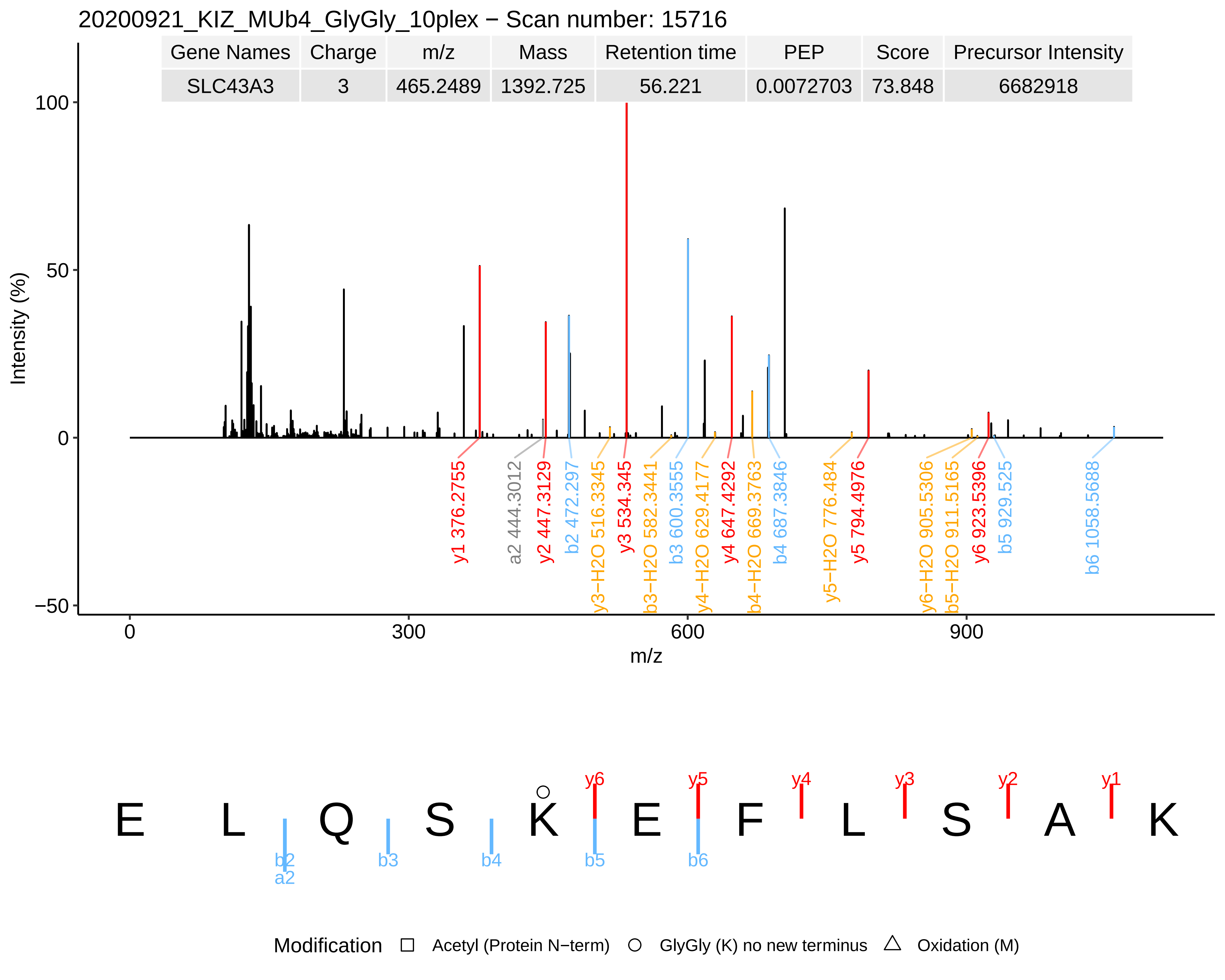
20200921_KIZ_MUb4_GlyGly_10plex − Scan number: 15716
Gene Names
Charge
m/z
Mass
Retention time
PEP
Score
Precursor Intensity
SLC43A3
3
465.2489
1392.725
56.221
0.0072703
73.848
6682918
Intensity (%)
100
50
0
−50
0
300
600
900
m/z
y1 376.2755
a2 444.3012
y2 447.3129
b2 472.297
y3−H2O 516.3345
y3 534.345
b3−H2O 582.3441
b3 600.3555
y4−H2O 629.4177
y4 647.4292
b4−H2O 669.3763
b4 687.3846
y5−H2O 776.484
y5 794.4976
y6−H2O 905.5306
b5−H2O 911.5165
y6 923.5396
b5 929.525
b6 1058.5688
E L Q S K E F L S A K
y6 y5 y4 y3 y2 y1
b2 a2 b3 b4 b5 b6
Modification
Acetyl (Protein N−term)
GlyGly (K) no new terminus
Oxidation (M)

20200921_KIZ_MUb4_GlyGly_10plex − Scan number: 15439

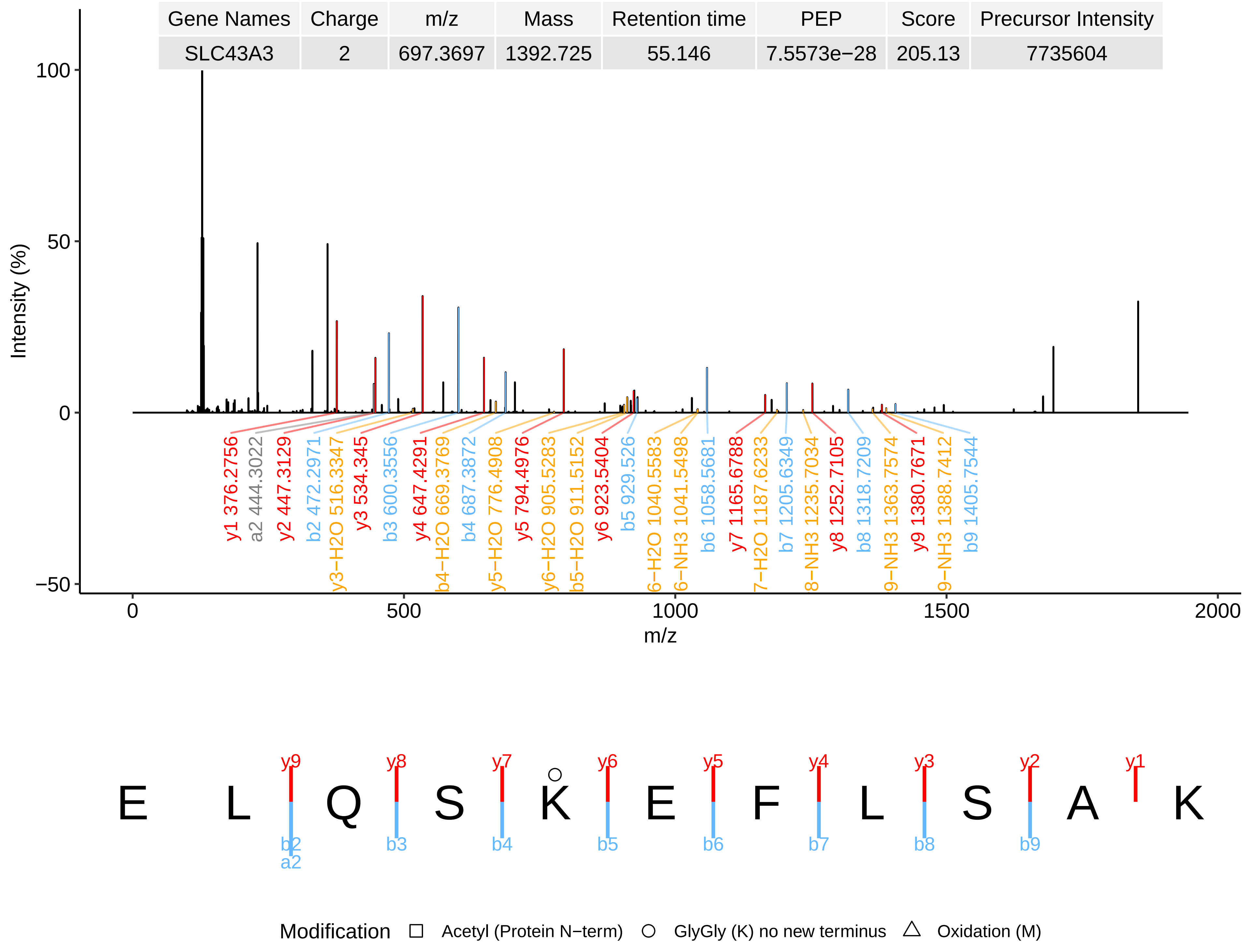

20200921_KIZ_MUb4_GlyGly_10plex_2 − Scan number: 14297

Gene Names	Charge	m/z	Mass	Retention time	PEP	Score	Precursor Intensity
SLC43A3	3	465.2489	1392.725	55.716	0.0041885	77.593	1914960

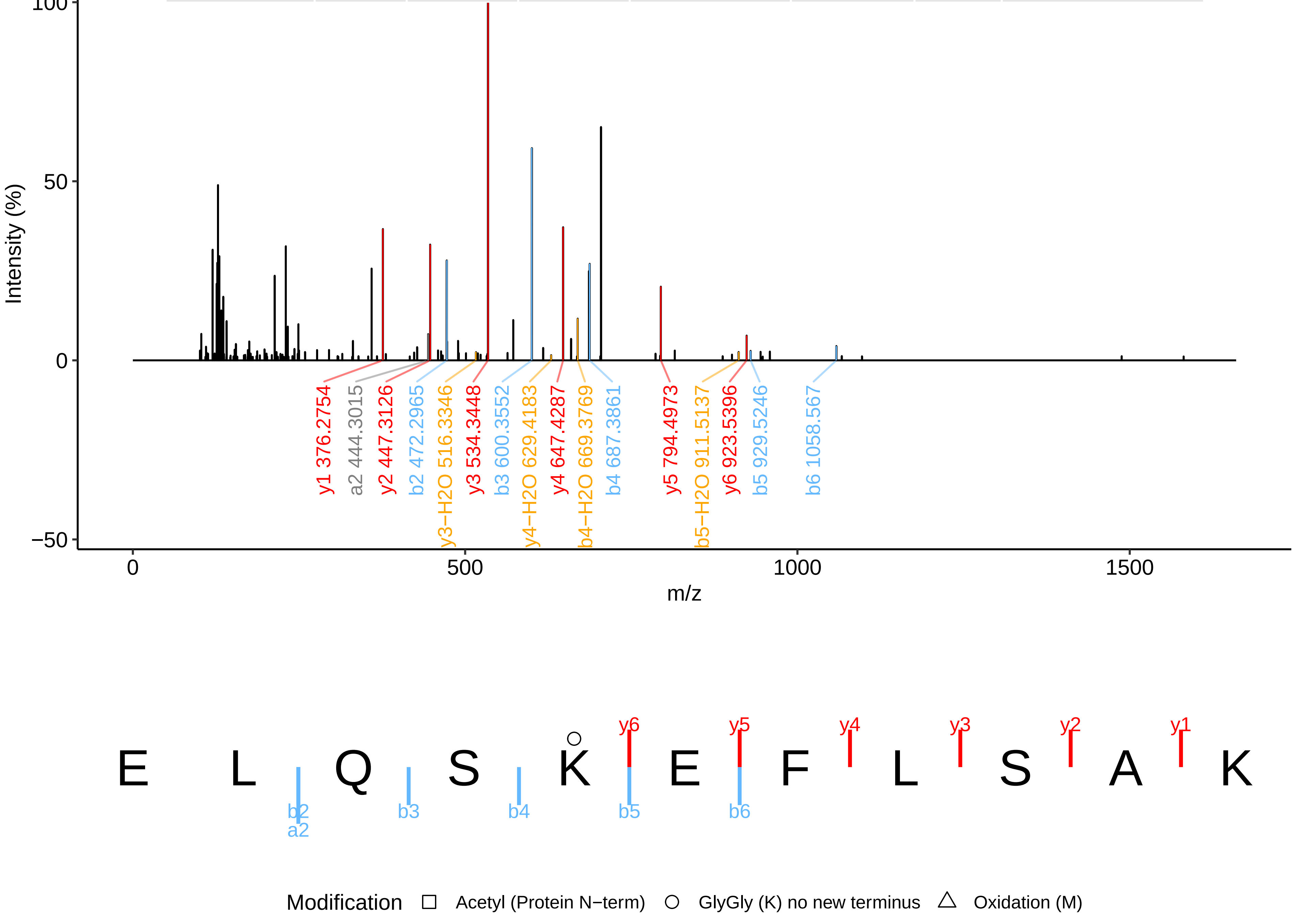

20200921_KIZ_MUb4_GlyGly_10plex_2 − Scan number: 14464

Gene Names	Charge	m/z	Mass	Retention time	PEP	Score	Precursor Intensity
SLC43A3	3	465.2489	1392.725	56.397	0.047012	57.175	1498941

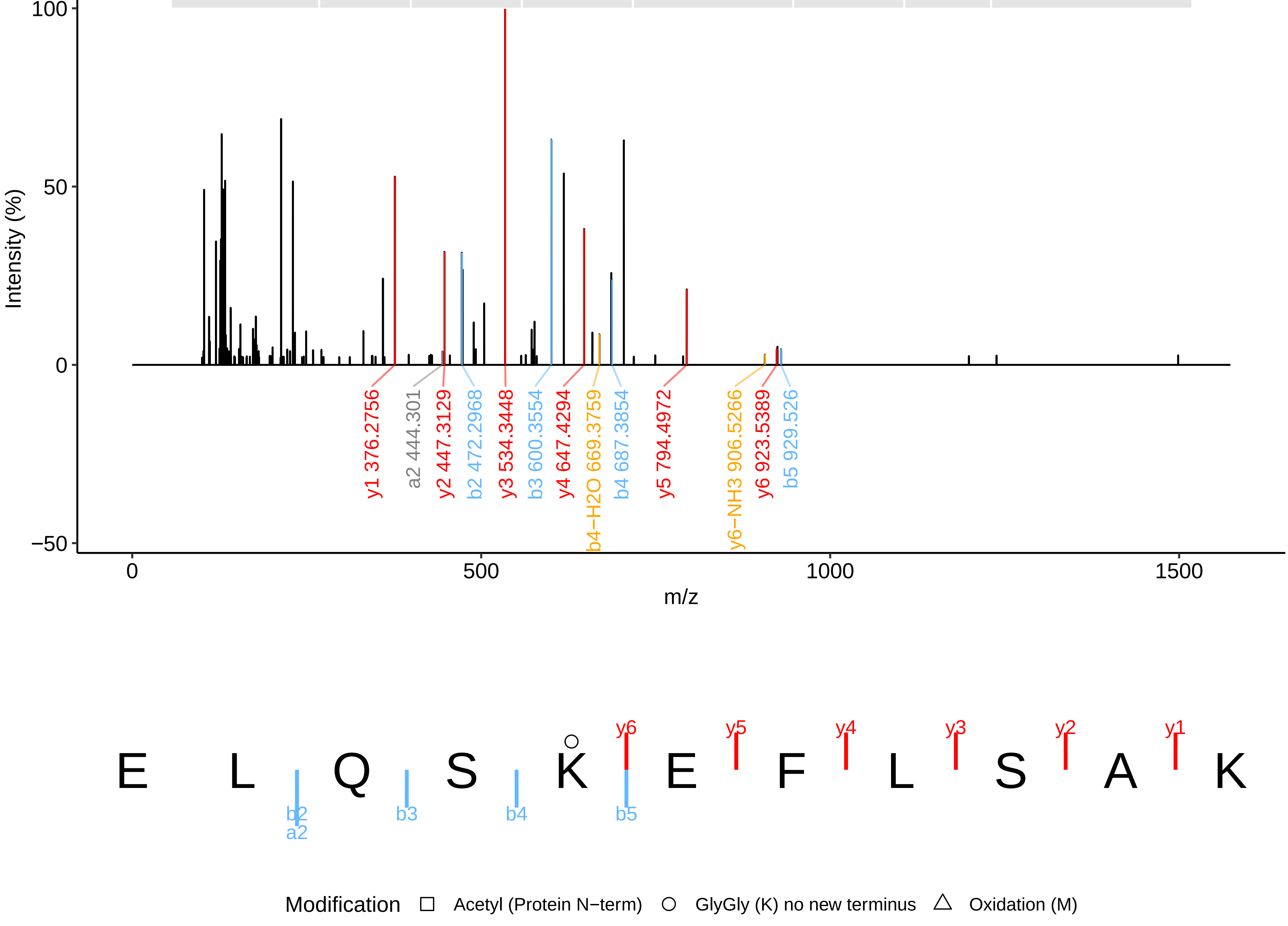

20200921_KIZ_MUb4_GlyGly_10plex_2 − Scan number: 14338

Gene Names	Charge	m/z	Mass	Retention time	PEP	Score	Precursor Intensity
SLC43A3	2	697.3697	1392.725	55.885	3.4614e−16	158.3	2561179

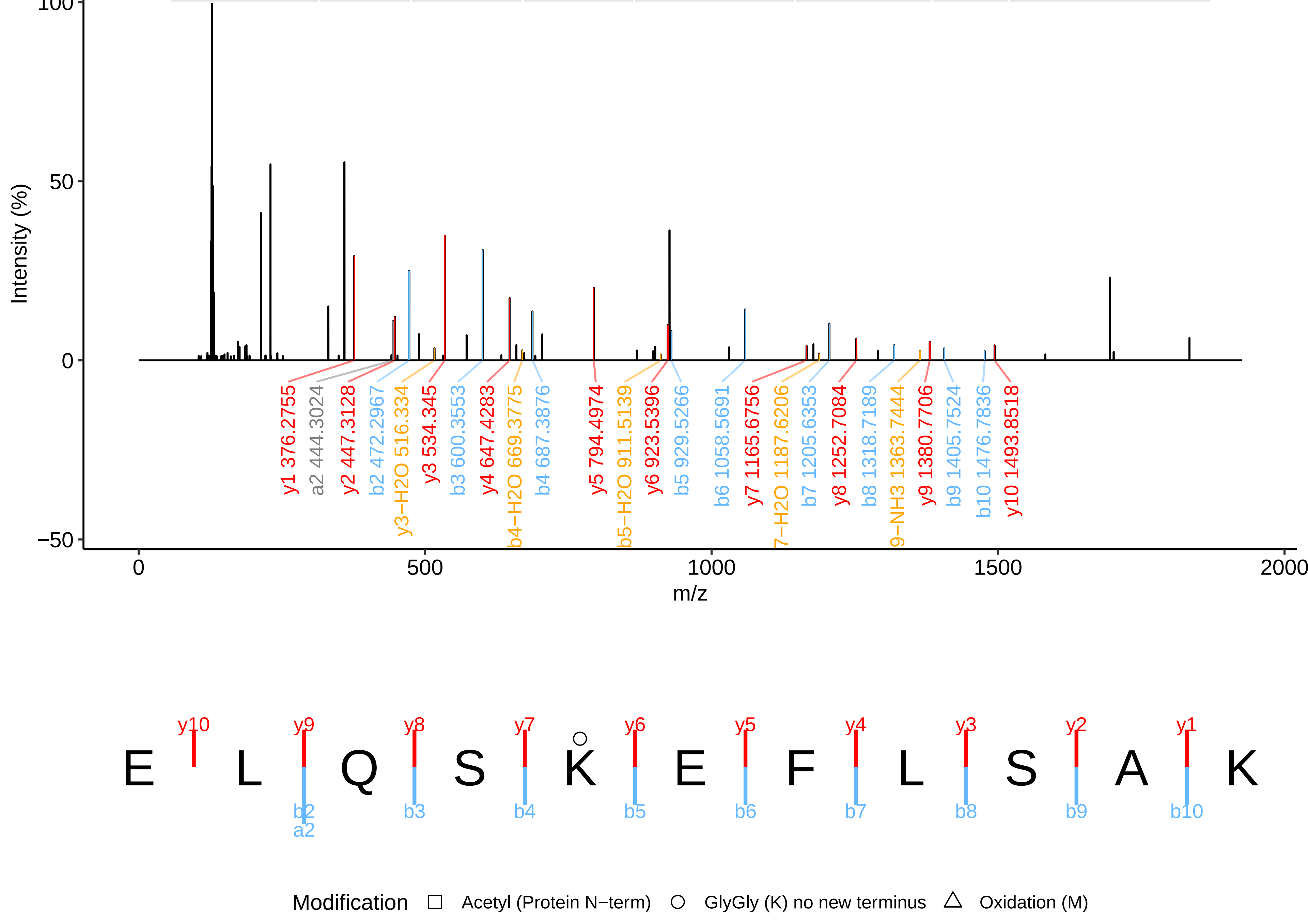

20200924_KIZ_MUb5_GlyGly_1 − Scan number: 14359

Gene Names	Charge	m/z	Mass	Retention time	PEP	Score	Precursor Intensity
SLC43A3	3	465.2489	1392.725	46.885	0.00044659	93.839	10502877

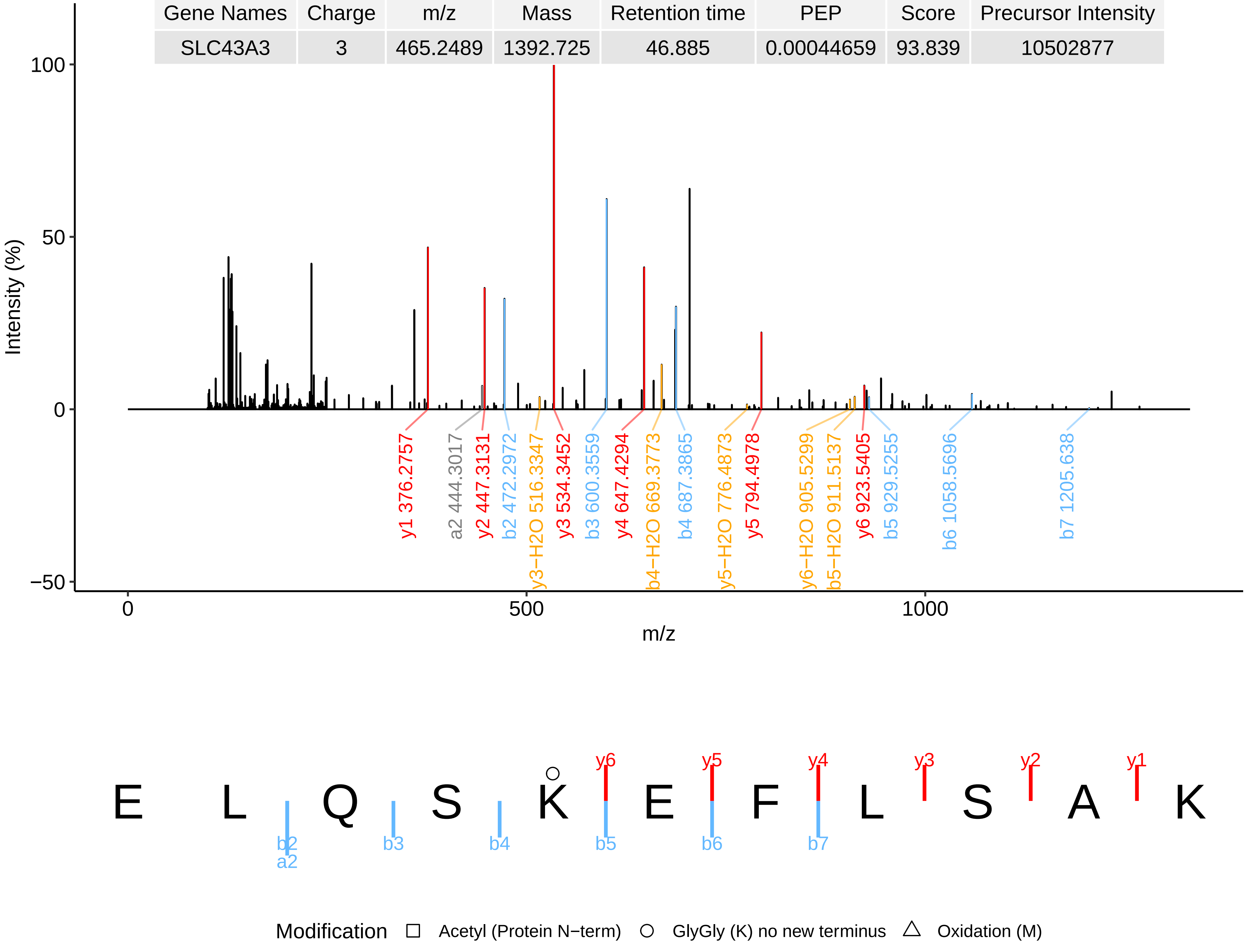

20200924_KIZ_MUb5_GlyGly_1 − Scan number: 14513

Gene Names	Charge	m/z	Mass	Retention time	PEP	Score	Precursor Intensity
SLC43A3	3	465.2489	1392.725	47.386	2.6543e−05	105.13	368758848

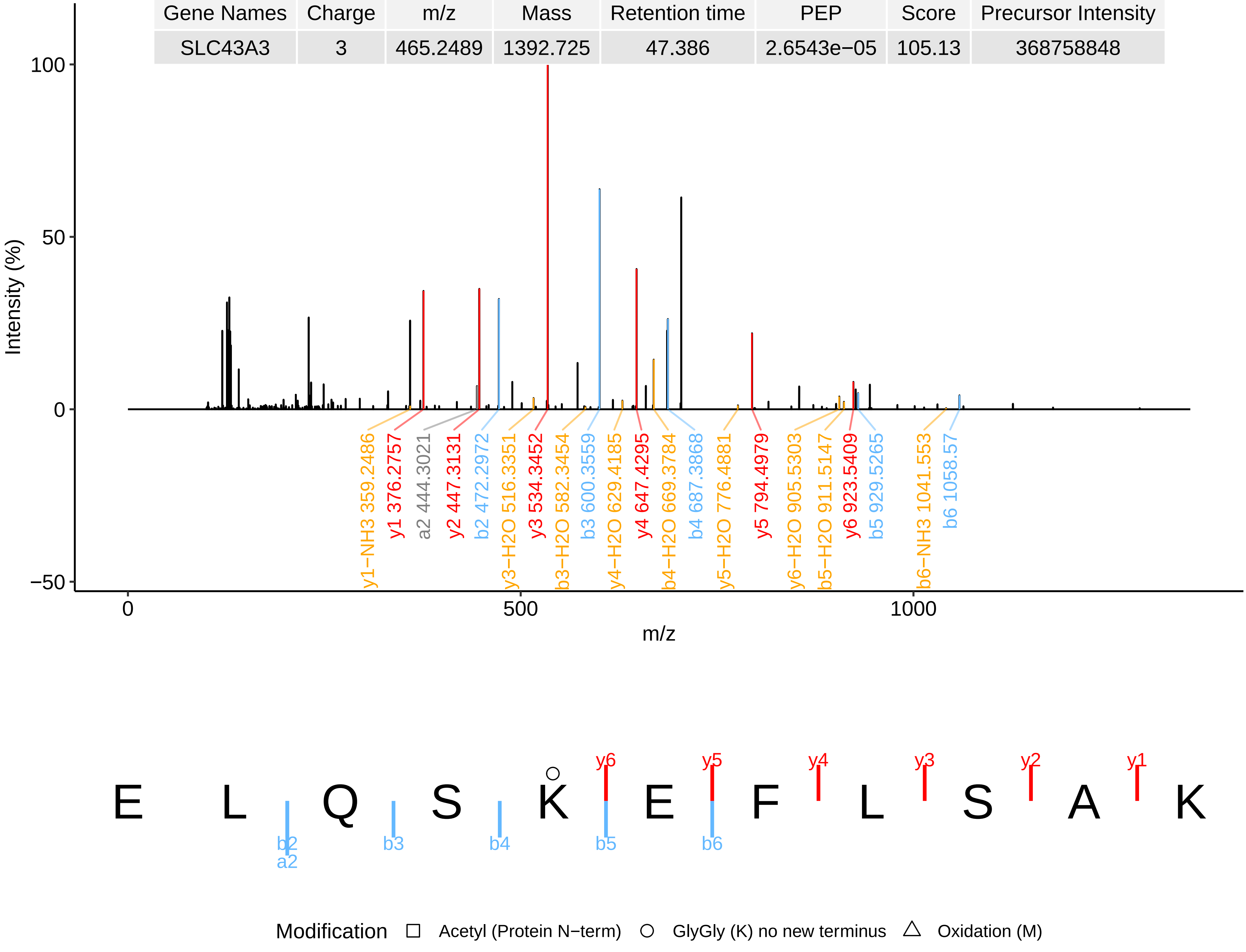

20200924_KIZ_MUb5_GlyGly_1 − Scan number: 14665

Gene Names	Charge	m/z	Mass	Retention time	PEP	Score	Precursor Intensity
SLC43A3	3	465.2489	1392.725	47.895	2.259e−05	106.67	226060768

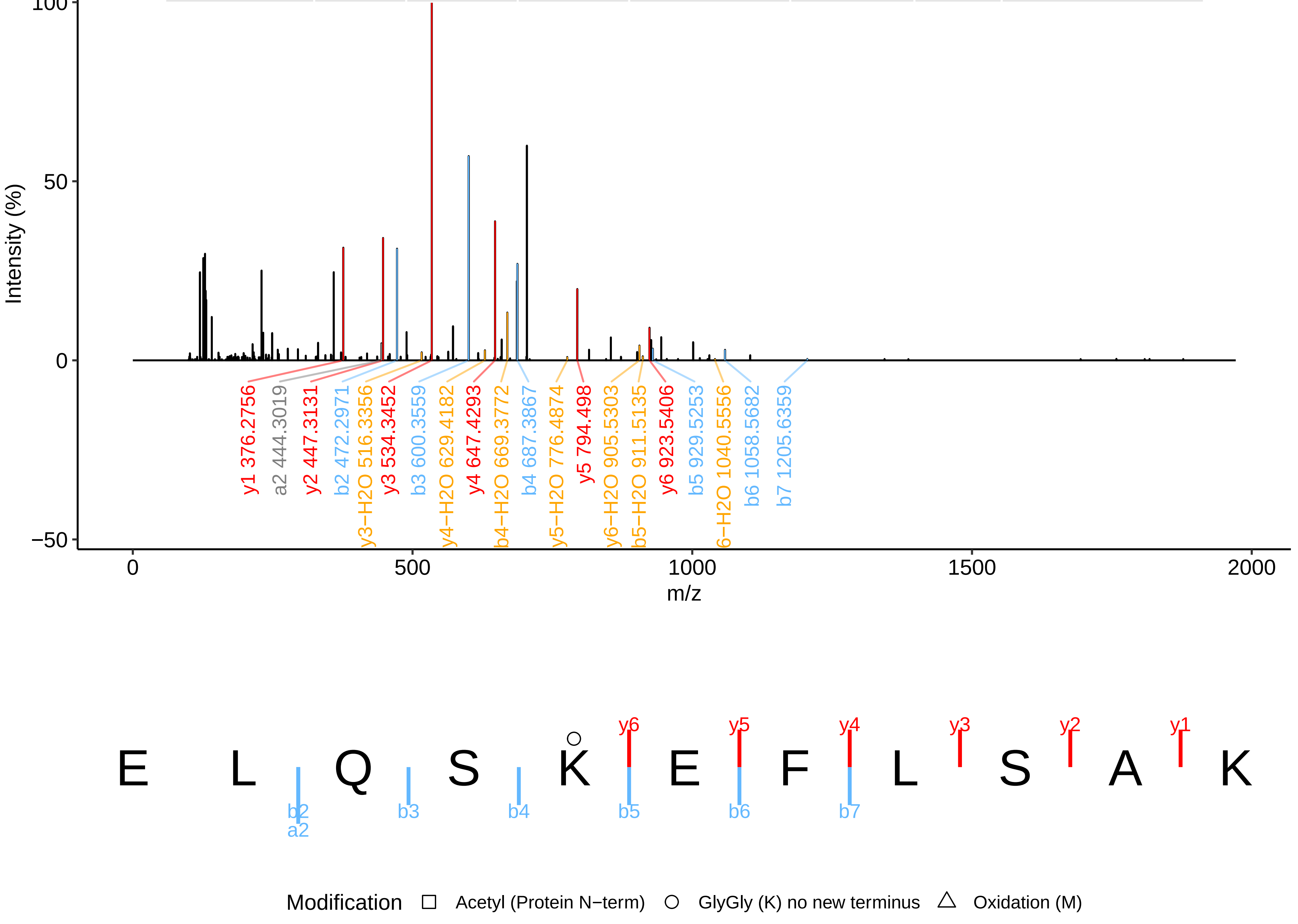

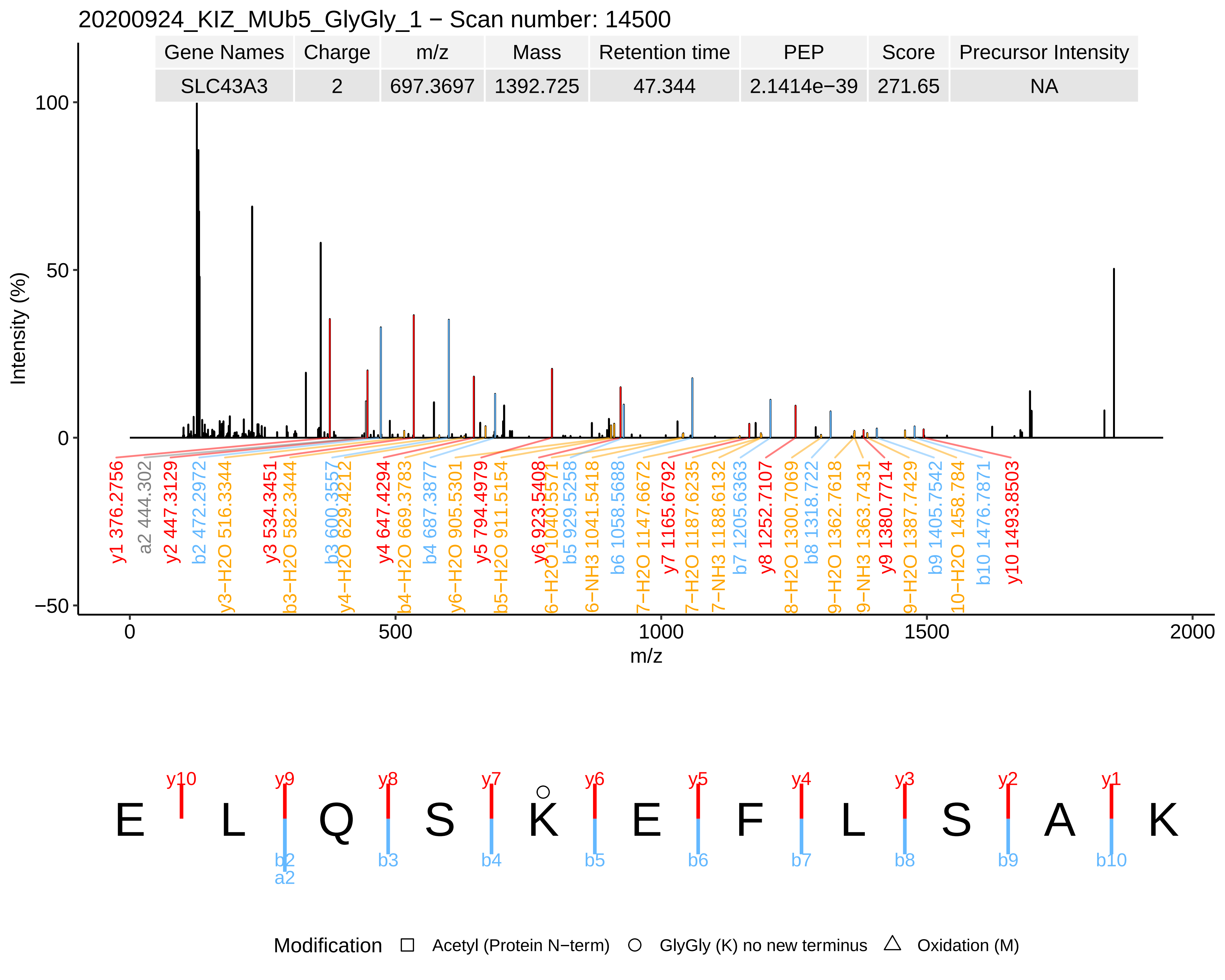

20200924_KIZ_MUb5_GlyGly_1 − Scan number: 14500
Gene Names | Charge | m/z | Mass | Retention time | PEP | Score | Precursor Intensity
SLC43A3 | 2 | 697.3697 | 1392.725 | 47.344 | 2.1414e−39 | 271.65 | NA
Intensity (%)
m/z
y1 376.2756
a2 444.302
y2 447.3129
b2 472.2972
y3−H2O 516.3344
y3 534.3451
b3−H2O 582.3444
b3 600.3557
y4−H2O 629.4212
y4 647.4294
b4−H2O 669.3783
b4 687.3877
y6−H2O 905.5301
y5 794.4979
b5−H2O 911.5154
y6 923.5408
b5 929.5258
b6 1058.5688
y7 1165.6792
b7 1205.6363
y8 1252.7107
b8 1318.722
y9 1380.7714
b9 1405.7542
b10 1476.7871
y10 1493.8503
E L Q S K E F L S A K
Modification
Acetyl (Protein N−term)
GlyGly (K) no new terminus
Oxidation (M)

20200924_KIZ_MUb5_GlyGly_2 − Scan number: 13509

Gene Names	Charge	m/z	Mass	Retention time	PEP	Score	Precursor Intensity
SLC43A3	3	465.2489	1392.725	50.519	4.0412e−05	99.752	10563270

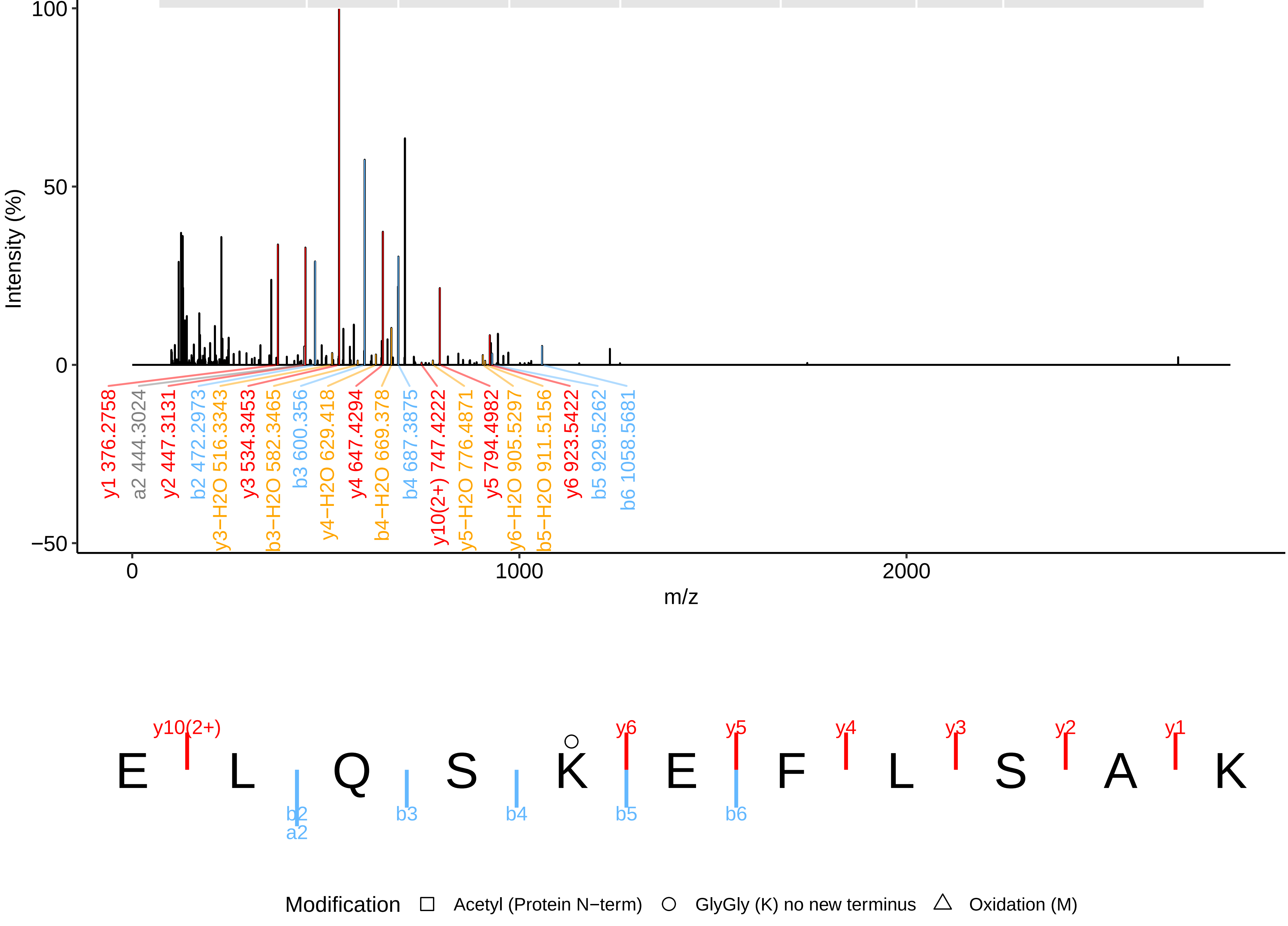

20200924_KIZ_MUb5_GlyGly_2 − Scan number: 13647

Gene Names	Charge	m/z	Mass	Retention time	PEP	Score	Precursor Intensity
SLC43A3	3	465.2489	1392.725	51.024	0.0041217	77.674	86165656

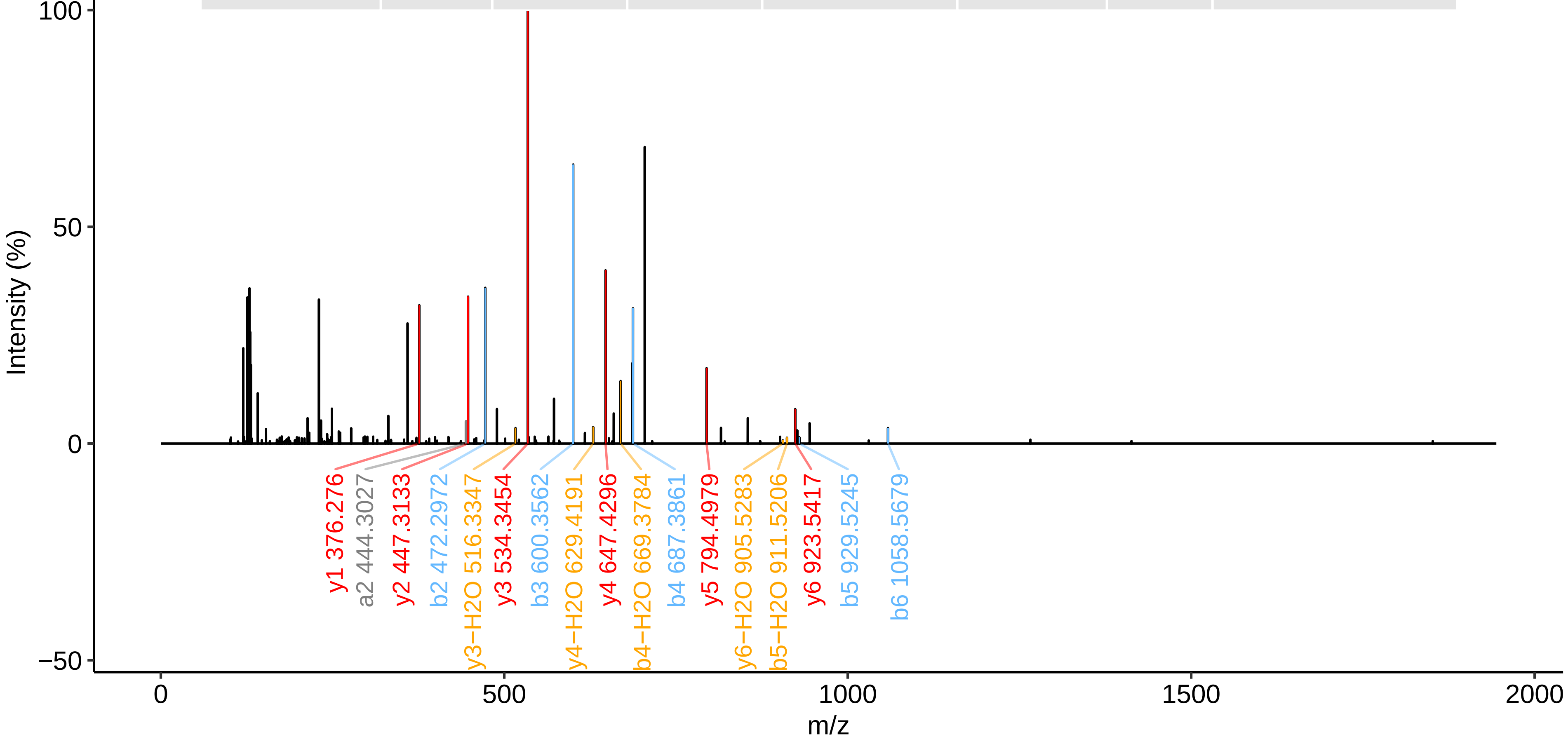

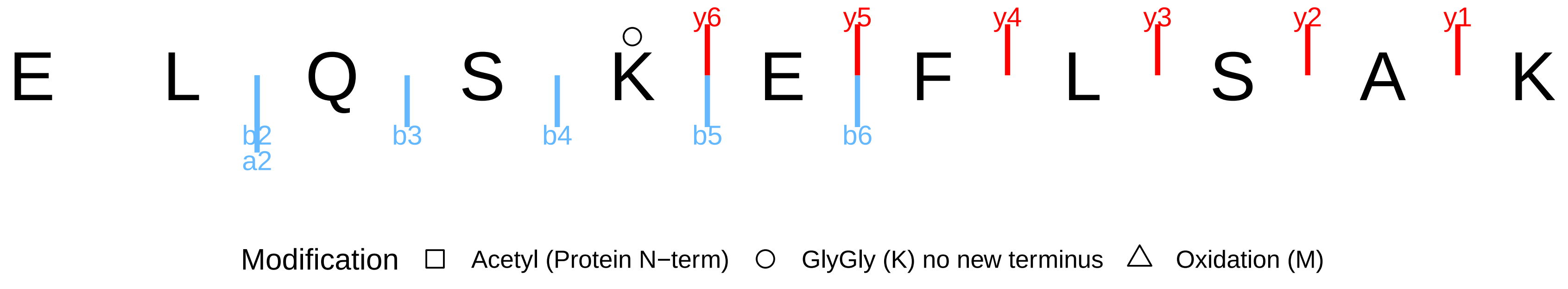

20200924_KIZ_MUb5_GlyGly_2 − Scan number: 13778

Gene Names	Charge	m/z	Mass	Retention time	PEP	Score	Precursor Intensity
SLC43A3	3	465.2489	1392.725	51.532	0.00083622	89.805	91299400

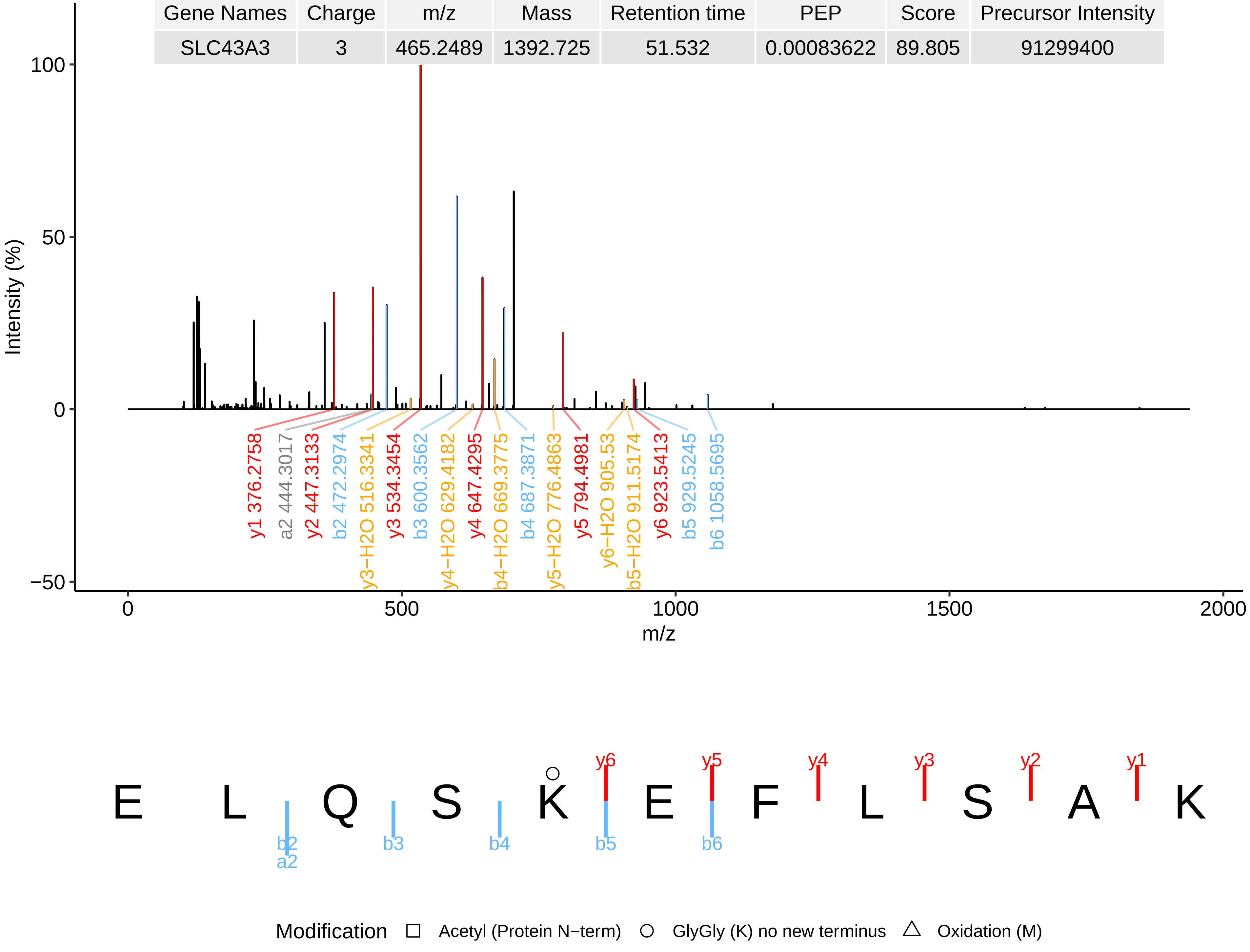

20200924_KIZ_MUb5_GlyGly_2 − Scan number: 13914

Gene Names	Charge	m/z	Mass	Retention time	PEP	Score	Precursor Intensity
SLC43A3	3	465.2489	1392.725	52.04	0.0095439	71.085	9572694

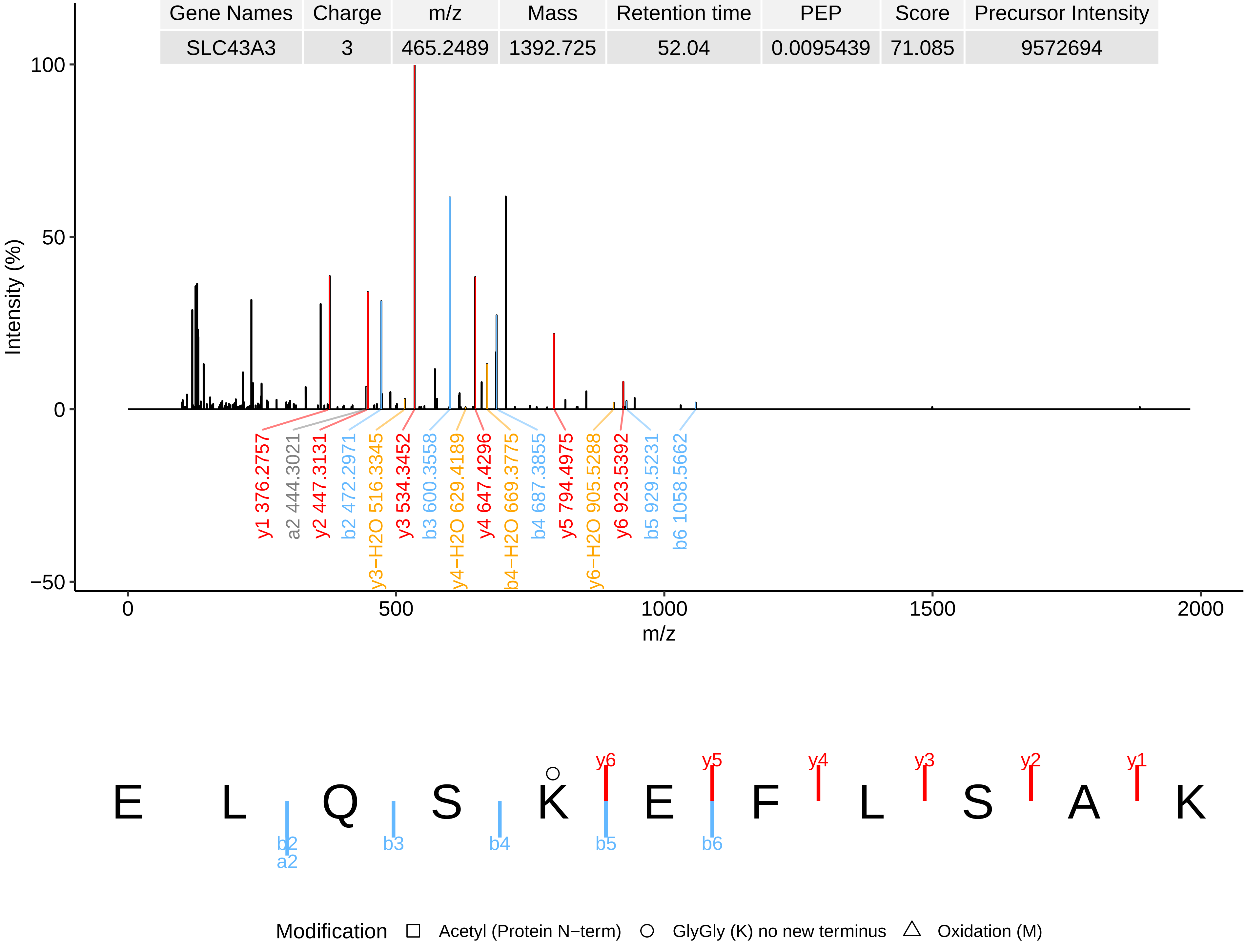

20201118_KIZ_MUb6_GlyGly_1 − Scan number: 14626

Gene Names	Charge	m/z	Mass	Retention time	PEP	Score	Precursor Intensity
SLC43A3	3	465.2489	1392.725	46.286	0.0010426	87.667	NA

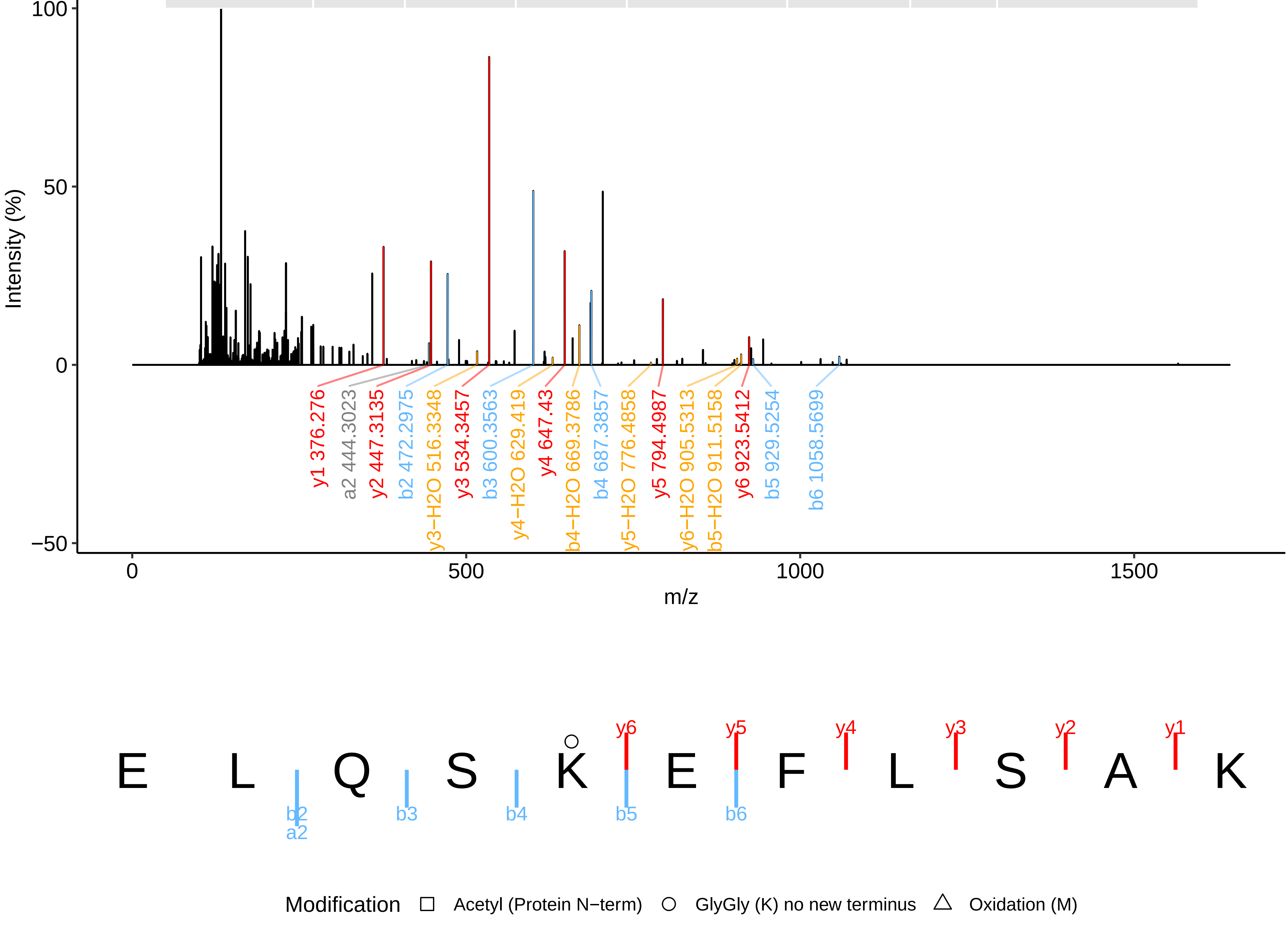

20200921_KIZ_MUb4_GlyGly_10plex − Scan number: 9079

Gene Names	Charge	m/z	Mass	Retention time	PEP	Score	Precursor Intensity
SLC12A4	4	412.9746	1647.869	31.04	7.9283e−06	107.97	11968557

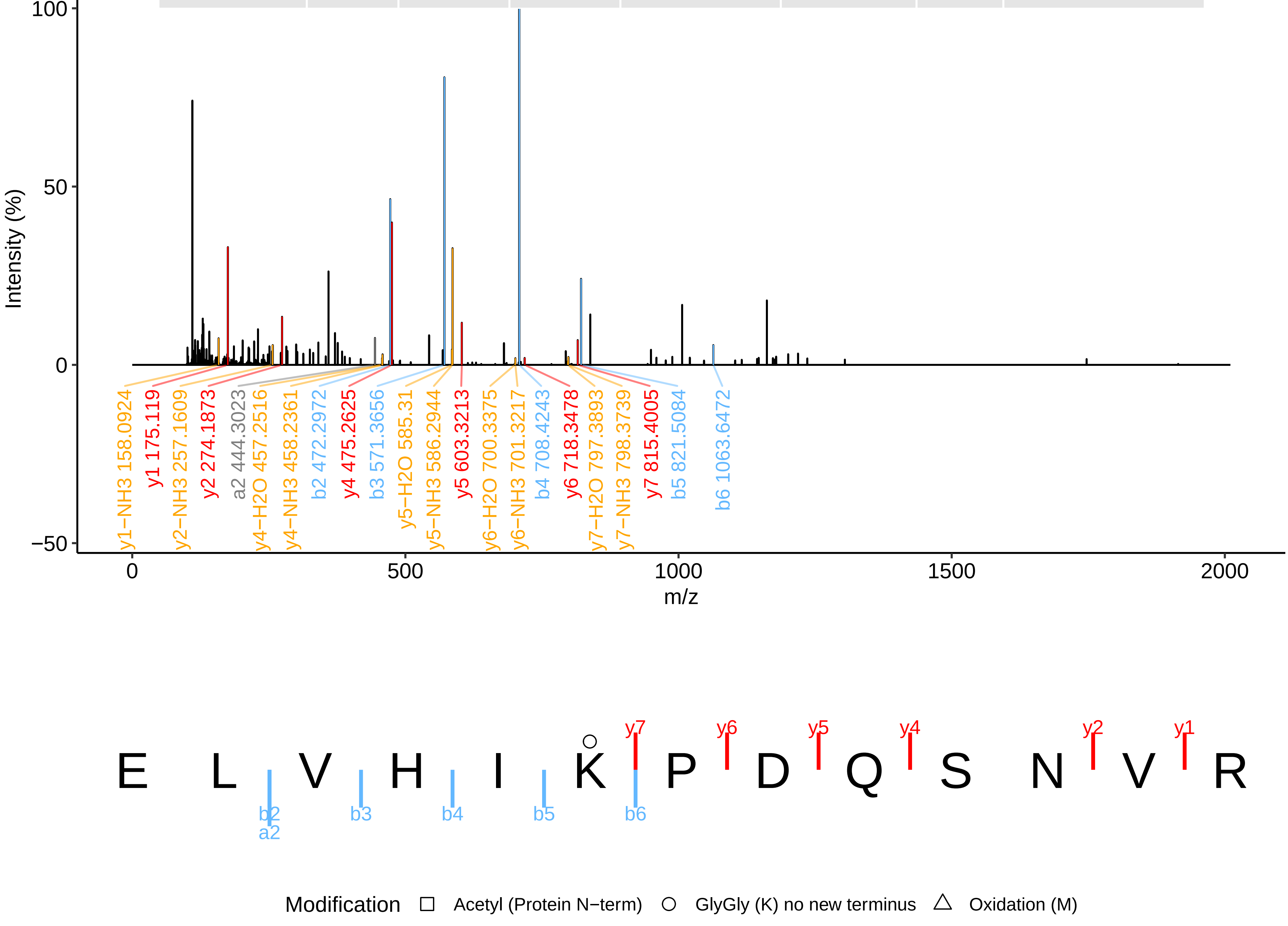

20200921_KIZ_MUb4_GlyGly_10plex − Scan number: 9119

Gene Names	Charge	m/z	Mass	Retention time	PEP	Score	Precursor Intensity
SLC12A4	3	550.297	1647.869	31.188	0.0011177	86.288	NA

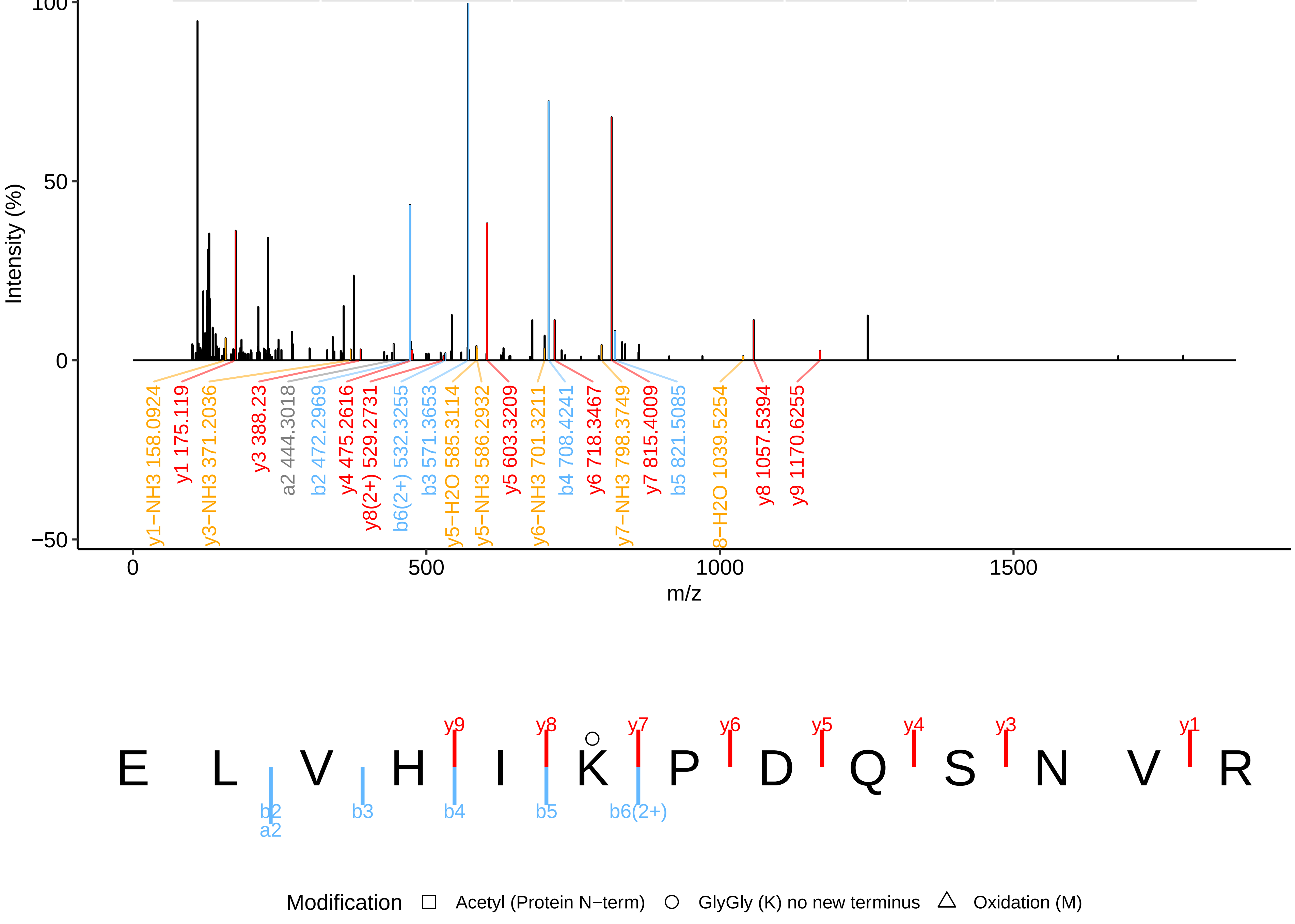

20200921_KIZ_MUb4_GlyGly_10plex_2 − Scan number: 8434

Gene Names	Charge	m/z	Mass	Retention time	PEP	Score	Precursor Intensity
SLC12A4	4	412.9746	1647.869	31.773	0.00089581	81.709	1613425

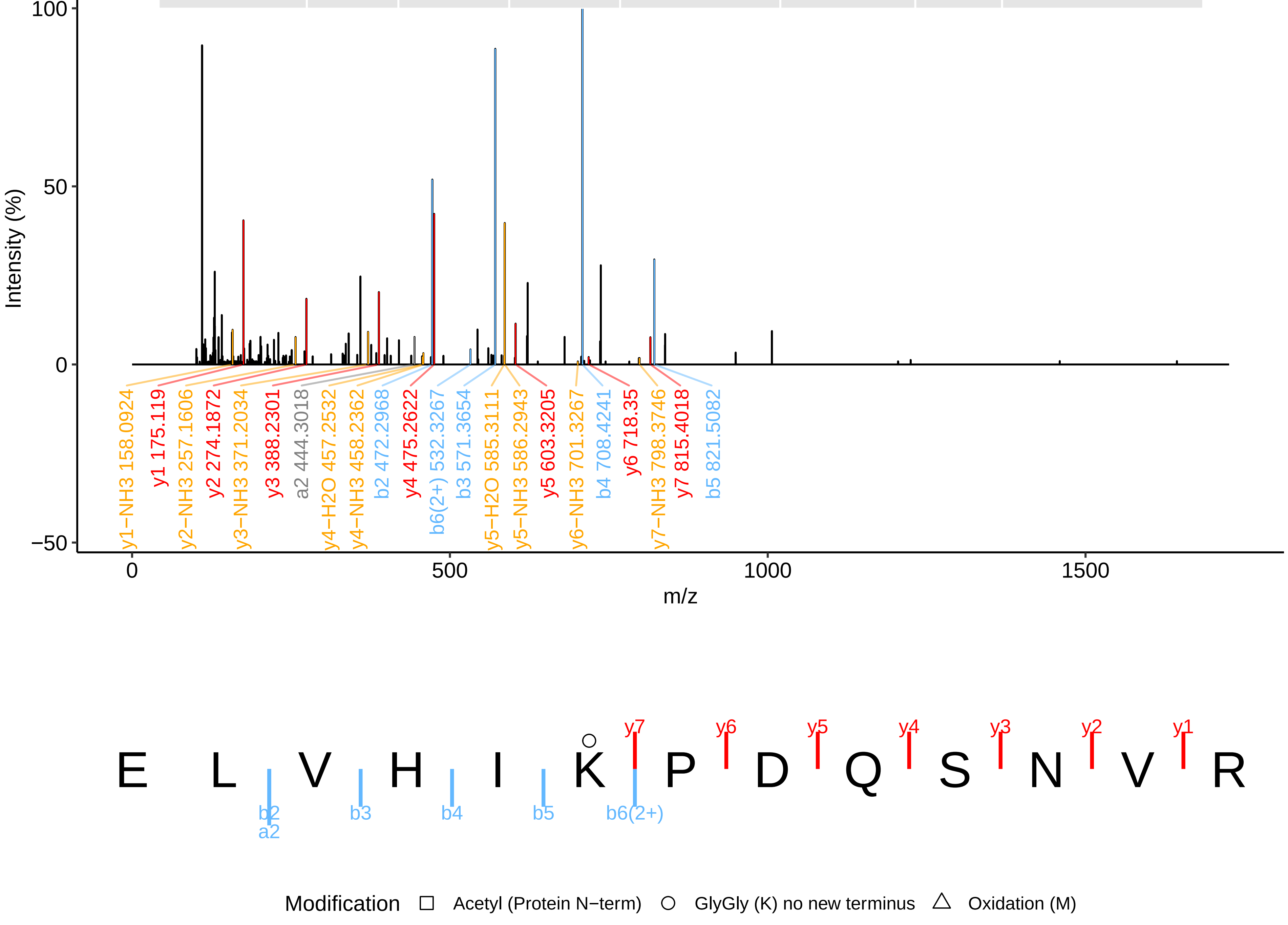

20200921_KIZ_MUb4_GlyGly_10plex − Scan number: 7023

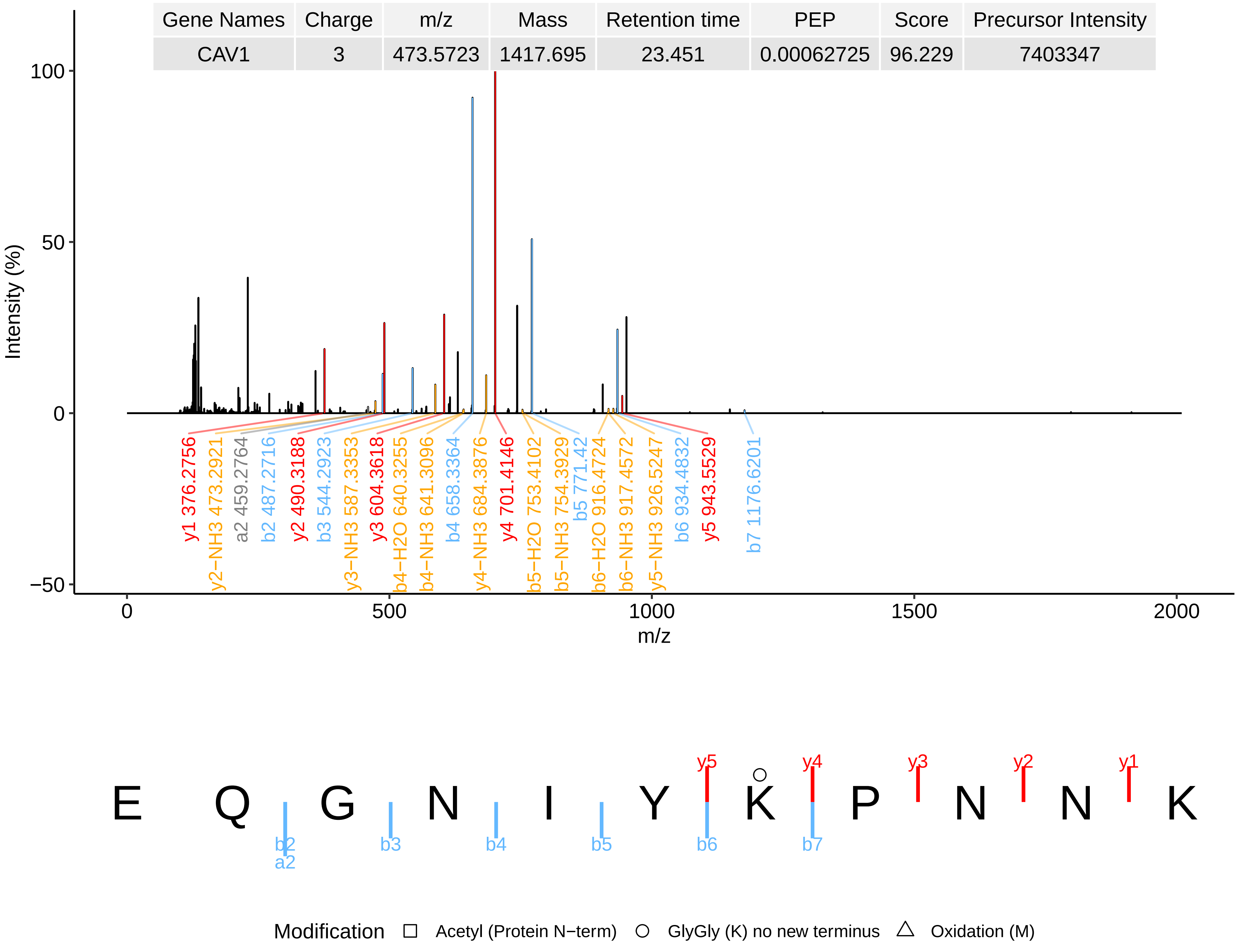

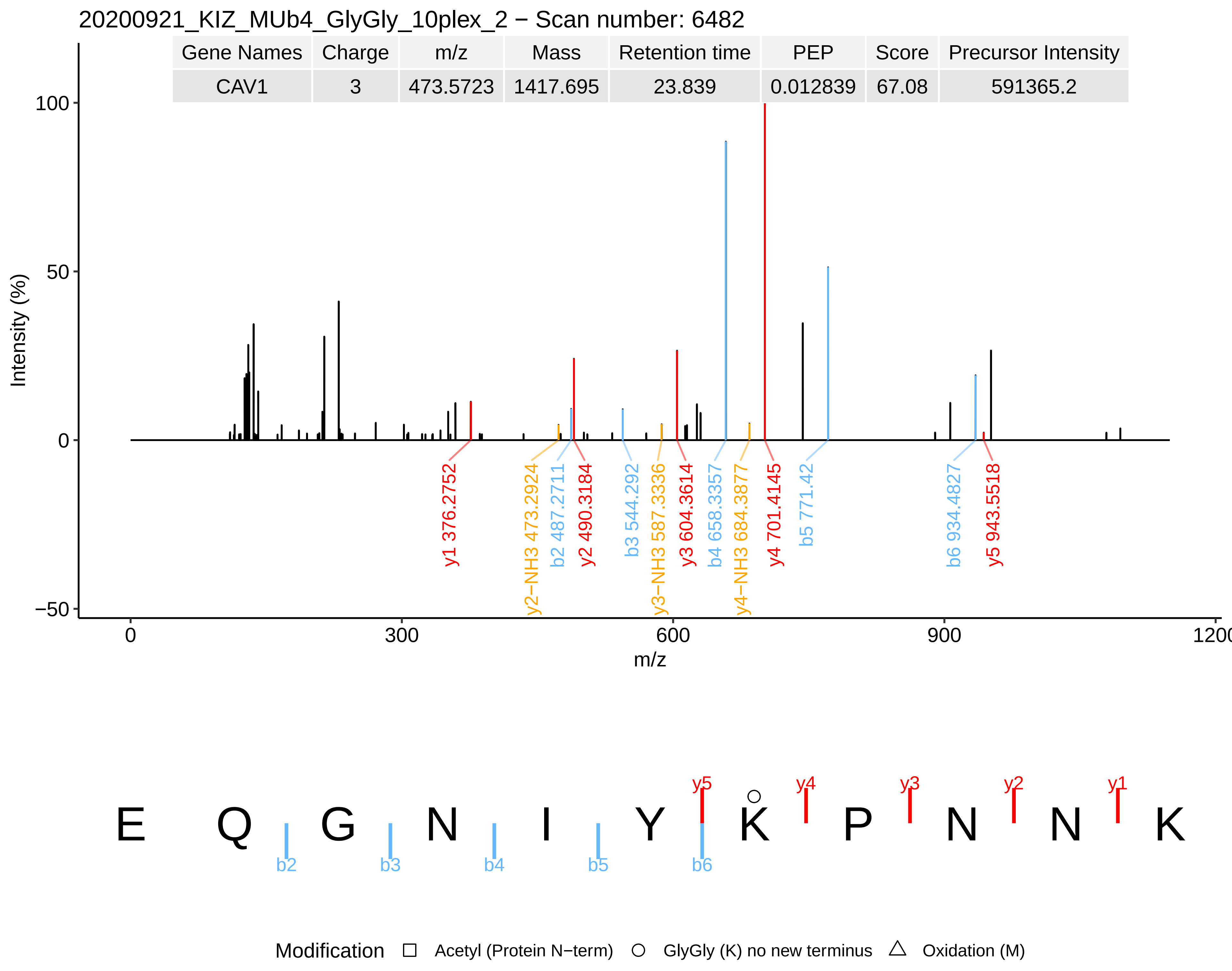

20200921_KIZ_MUb4_GlyGly_10plex_2 − Scan number: 6482
Gene Names | Charge | m/z | Mass | Retention time | PEP | Score | Precursor Intensity
CAV1 | 3 | 473.5723 | 1417.695 | 23.839 | 0.012839 | 67.08 | 591365.2
Intensity (%)
m/z
y1 376.2752
y2−NH3 473.2924
b2 487.2711
y2 490.3184
b3 544.292
y3−NH3 587.3336
y3 604.3614
b4 658.3357
y4−NH3 684.3877
y4 701.4145
b5 771.42
b6 934.4827
y5 943.5518
E Q G N I Y K P N N K
b2 b3 b4 b5 b6
y5 y4 y3 y2 y1
Modification
Acetyl (Protein N−term)
GlyGly (K) no new terminus
Oxidation (M)

20200921_KIZ_MUb4_GlyGly_10plex_2 − Scan number: 6493

Gene Names	Charge	m/z	Mass	Retention time	PEP	Score	Precursor Intensity
CAV1	3	473.5723	1417.695	23.887	0.011641	68.536	1221510

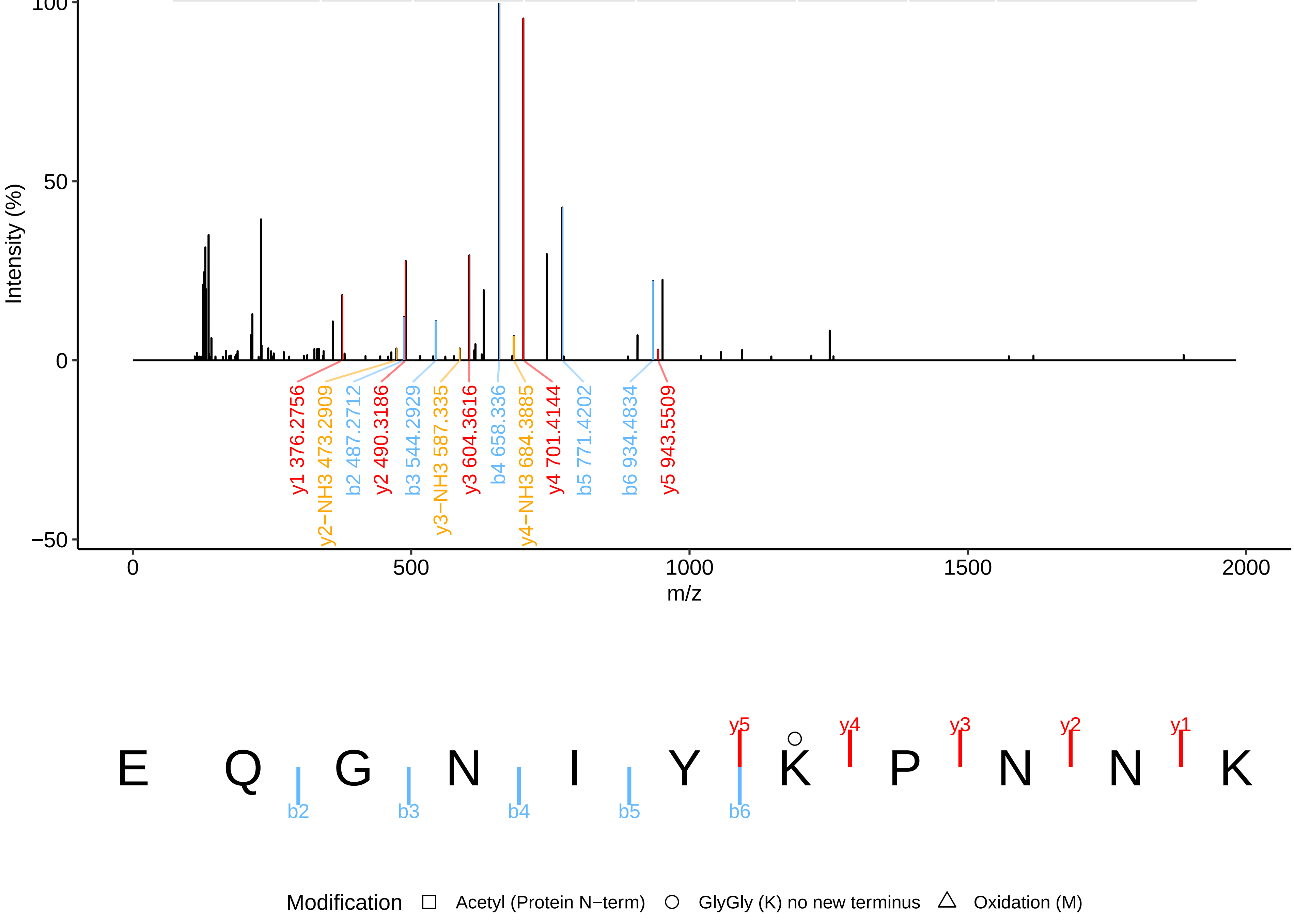

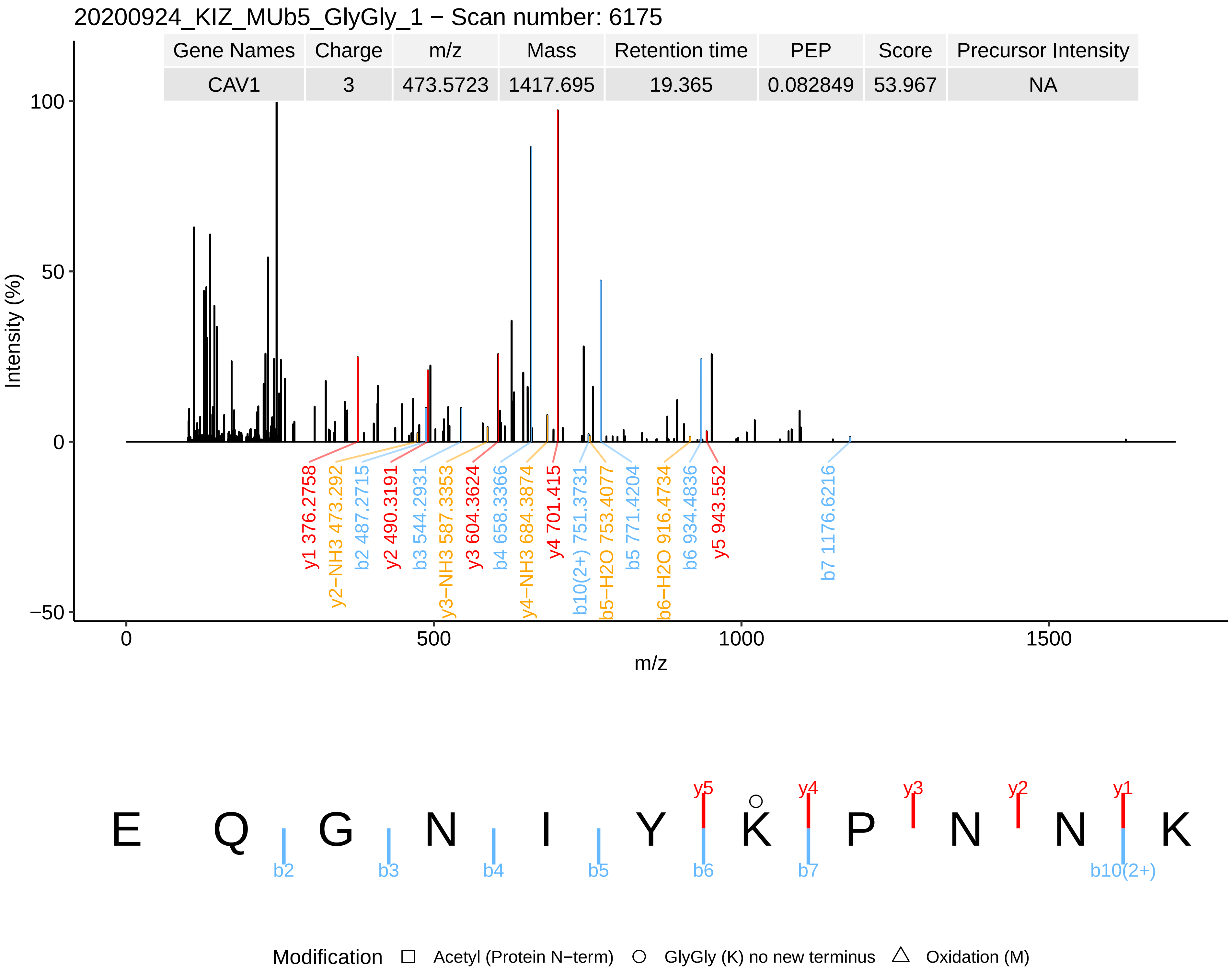
20200924_KIZ_MUb5_GlyGly_1 − Scan number: 6175
Gene Names | Charge | m/z | Mass | Retention time | PEP | Score | Precursor Intensity
CAV1 | 3 | 473.5723 | 1417.695 | 19.365 | 0.082849 | 53.967 | NA
Intensity (%)
m/z
y1 376.2758
y2−NH3 473.292
b2 487.2715
y2 490.3191
b3 544.2931
y3−NH3 587.3353
y3 604.3624
b4 658.3366
y4−NH3 684.3874
y4 701.415
b10(2+) 751.3731
b5−H2O 753.4077
b5 771.4204
b6−H2O 916.4734
b6 934.4836
y5 943.552
b7 1176.6216
E Q G N I Y K P N N K
Modification
Acetyl (Protein N−term)
GlyGly (K) no new terminus
Oxidation (M)

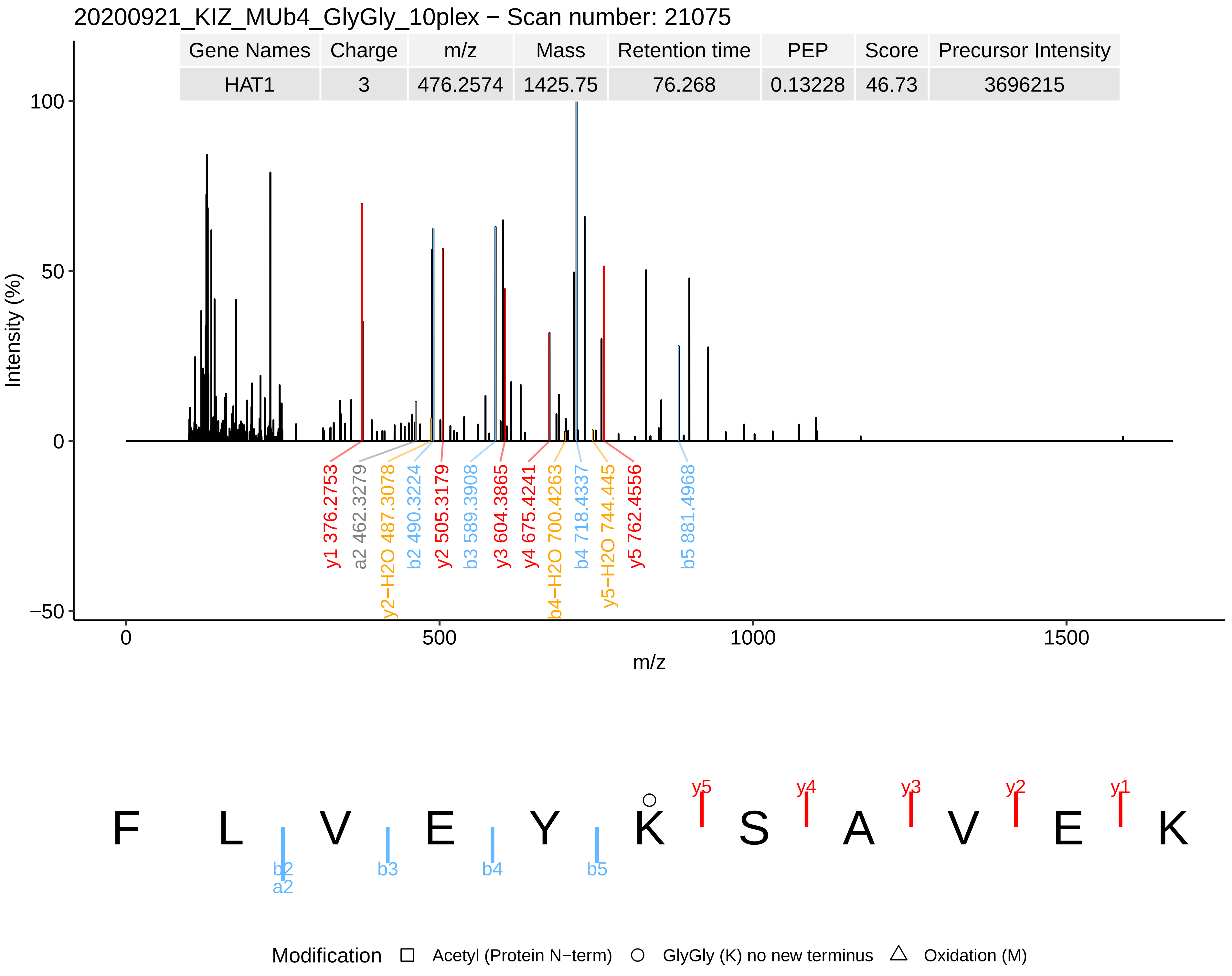

20200921_KIZ_MUb4_GlyGly_10plex − Scan number: 21075
Gene Names
Charge
m/z
Mass
Retention time
PEP
Score
Precursor Intensity
HAT1
3
476.2574
1425.75
76.268
0.13228
46.73
3696215
Intensity (%)
m/z
y1 376.2753
a2 462.3279
y2−H2O 487.3078
b2 490.3224
y2 505.3179
b3 589.3908
y3 604.3865
y4 675.4241
b4−H2O 700.4263
b4 718.4337
y5−H2O 744.445
y5 762.4556
b5 881.4968
F L V E Y K S A V E K
Modification
Acetyl (Protein N−term)
GlyGly (K) no new terminus
Oxidation (M)

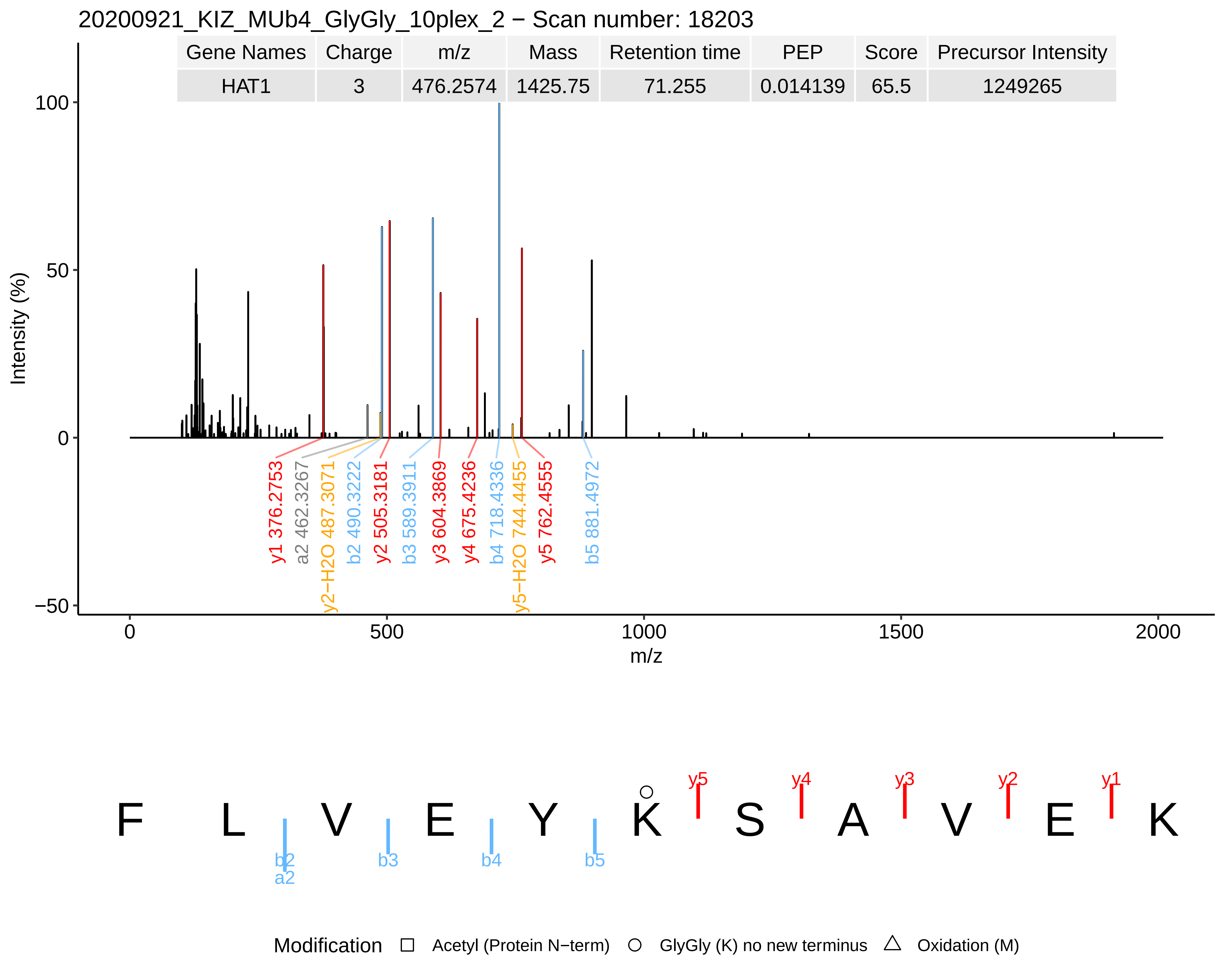

20200921_KIZ_MUb4_GlyGly_10plex_2 − Scan number: 18203
Gene Names
Charge
m/z
Mass
Retention time
PEP
Score
Precursor Intensity
HAT1
3
476.2574
1425.75
71.255
0.014139
65.5
1249265
Intensity (%)
100
50
0
−50
y1 376.2753
a2 462.3267
y2−H2O 487.3071
b2 490.3222
y2 505.3181
b3 589.3911
y3 604.3869
y4 675.4236
b4 718.4336
y5−H2O 744.4455
y5 762.4555
b5 881.4972
0
500
1000
1500
2000
m/z
F L V E Y K S A V E K
y5
y4
y3
y2
y1
b2
a2
b3
b4
b5
Modification
Acetyl (Protein N−term)
GlyGly (K) no new terminus
Oxidation (M)

20200924_KIZ_MUb5_GlyGly_2 − Scan number: 17963

Gene Names	Charge	m/z	Mass	Retention time	PEP	Score	Precursor Intensity
HAT1	3	476.2574	1425.75	66.583	0.0075569	73.499	1250727

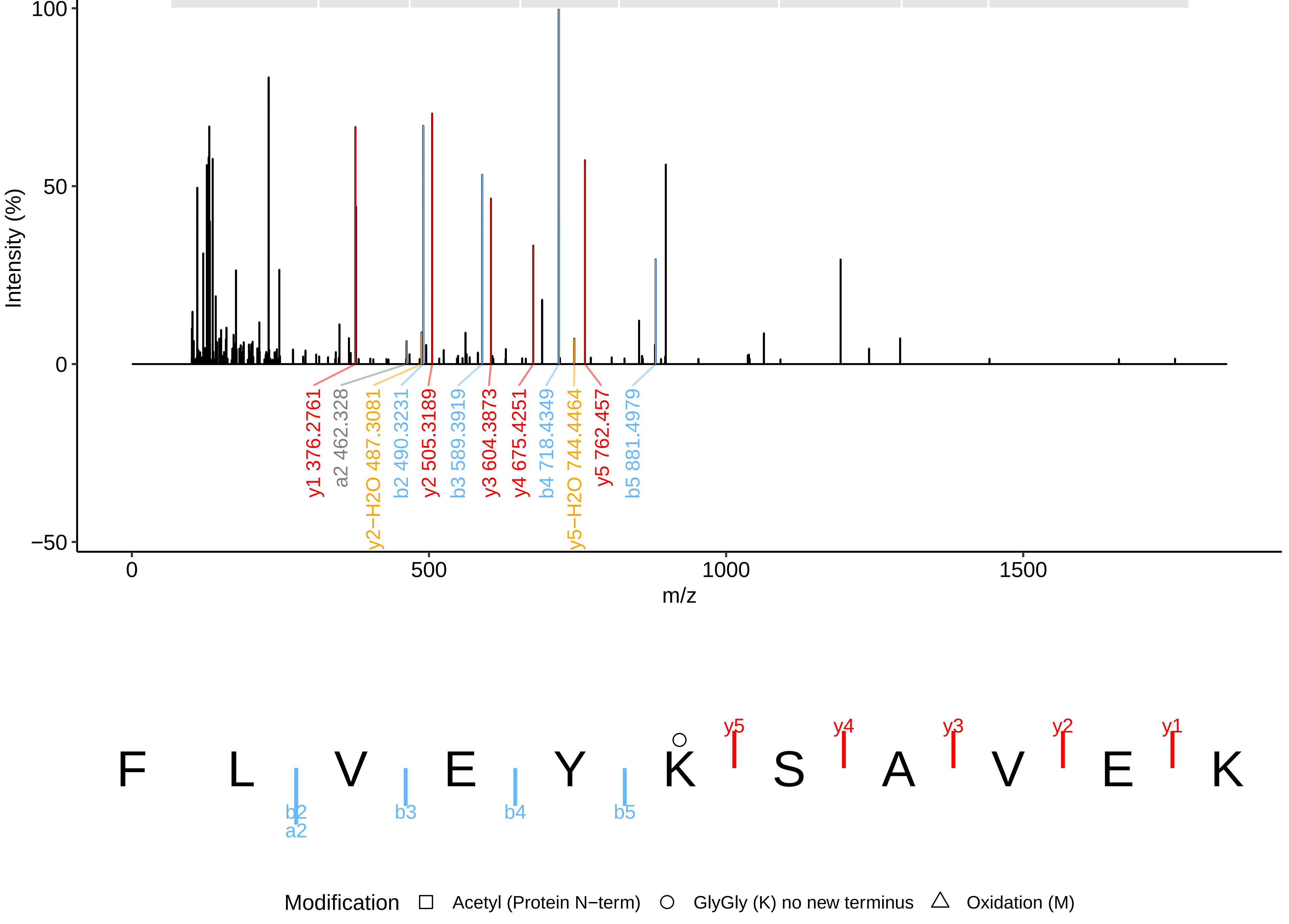

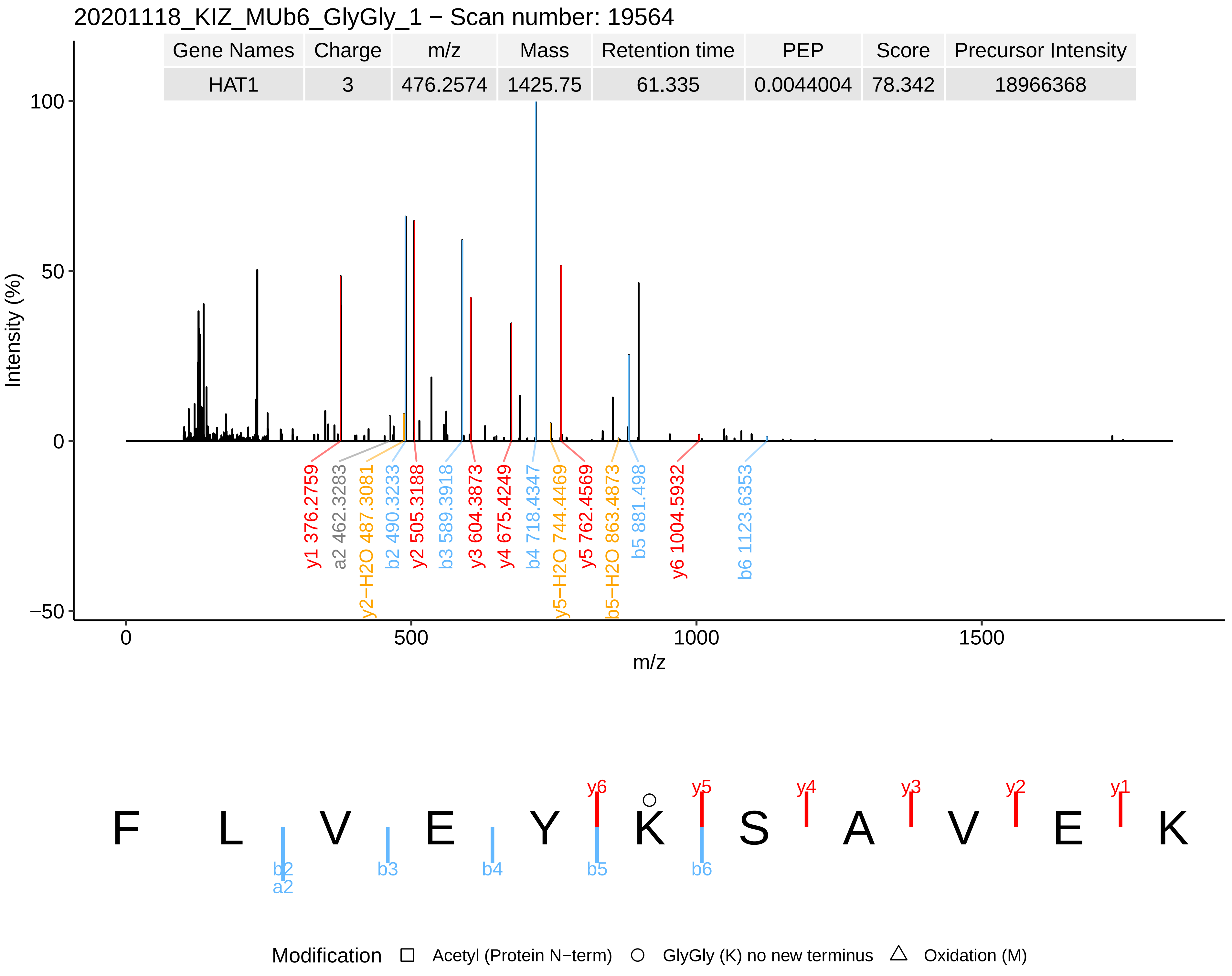
20201118_KIZ_MUb6_GlyGly_1 − Scan number: 19564
Gene Names
Charge
m/z
Mass
Retention time
PEP
Score
Precursor Intensity
HAT1
3
476.2574
1425.75
61.335
0.0044004
78.342
18966368
Intensity (%)
100
50
0
−50
0
500
1000
1500
m/z
y1 376.2759
a2 462.3283
y2−H2O 487.3081
b2 490.3233
y2 505.3188
b3 589.3918
y3 604.3873
y4 675.4249
b4 718.4347
y5−H2O 744.4469
y5 762.4569
b5−H2O 863.4873
b5 881.498
y6 1004.5932
b6 1123.6353
F L V E Y K S A V E K
y6
y5
y4
y3
y2
y1
b2
a2
b3
b4
b5
b6
Modification
Acetyl (Protein N−term)
GlyGly (K) no new terminus
Oxidation (M)

20200921_KIZ_MUb4_GlyGly_10plex_2 − Scan number: 17157

Gene Names	Charge	m/z	Mass	Retention time	PEP	Score	Precursor Intensity
HSP90AA1	3	473.2419	1416.704	67.053	0.0026374	90.108	1867702

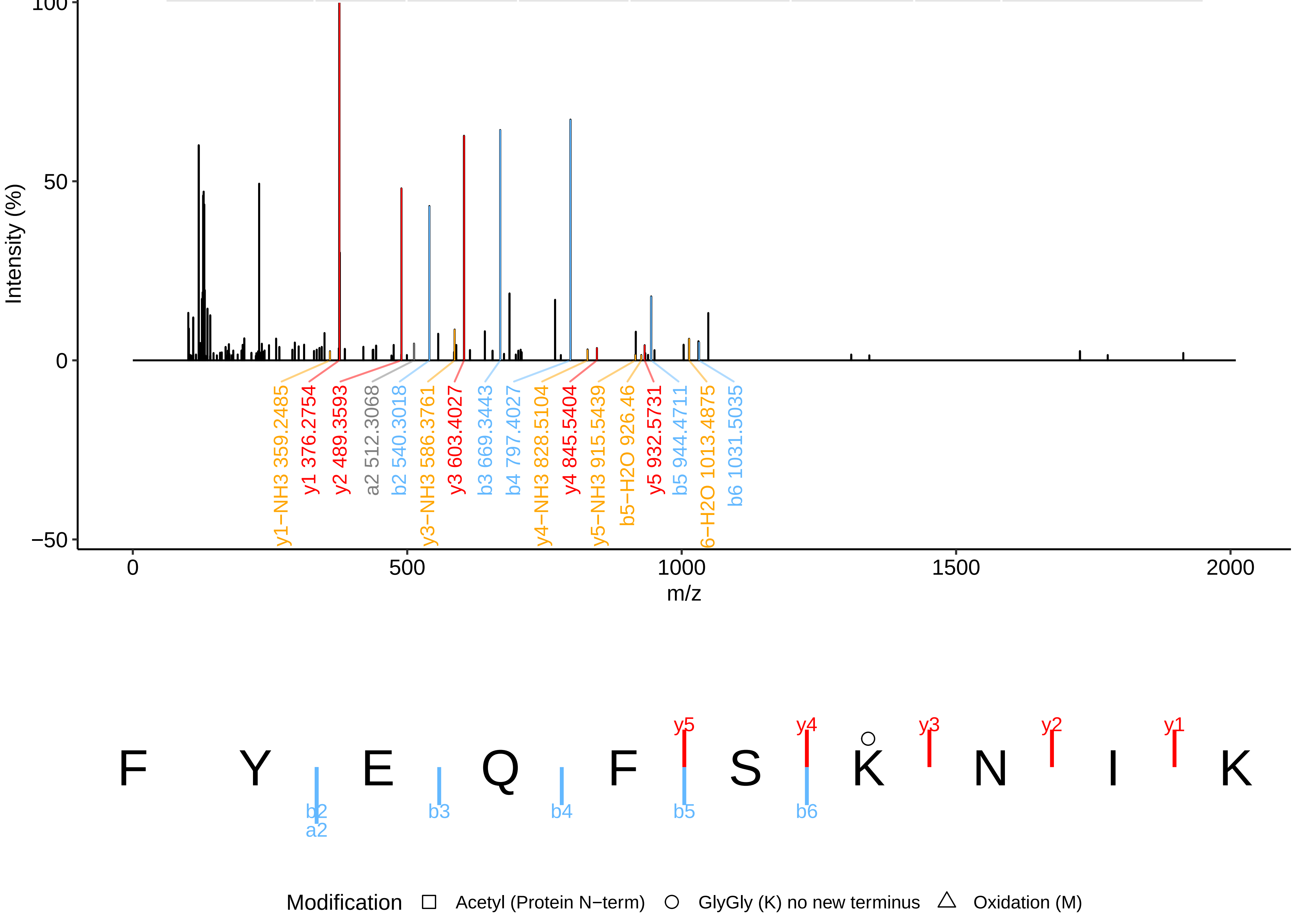

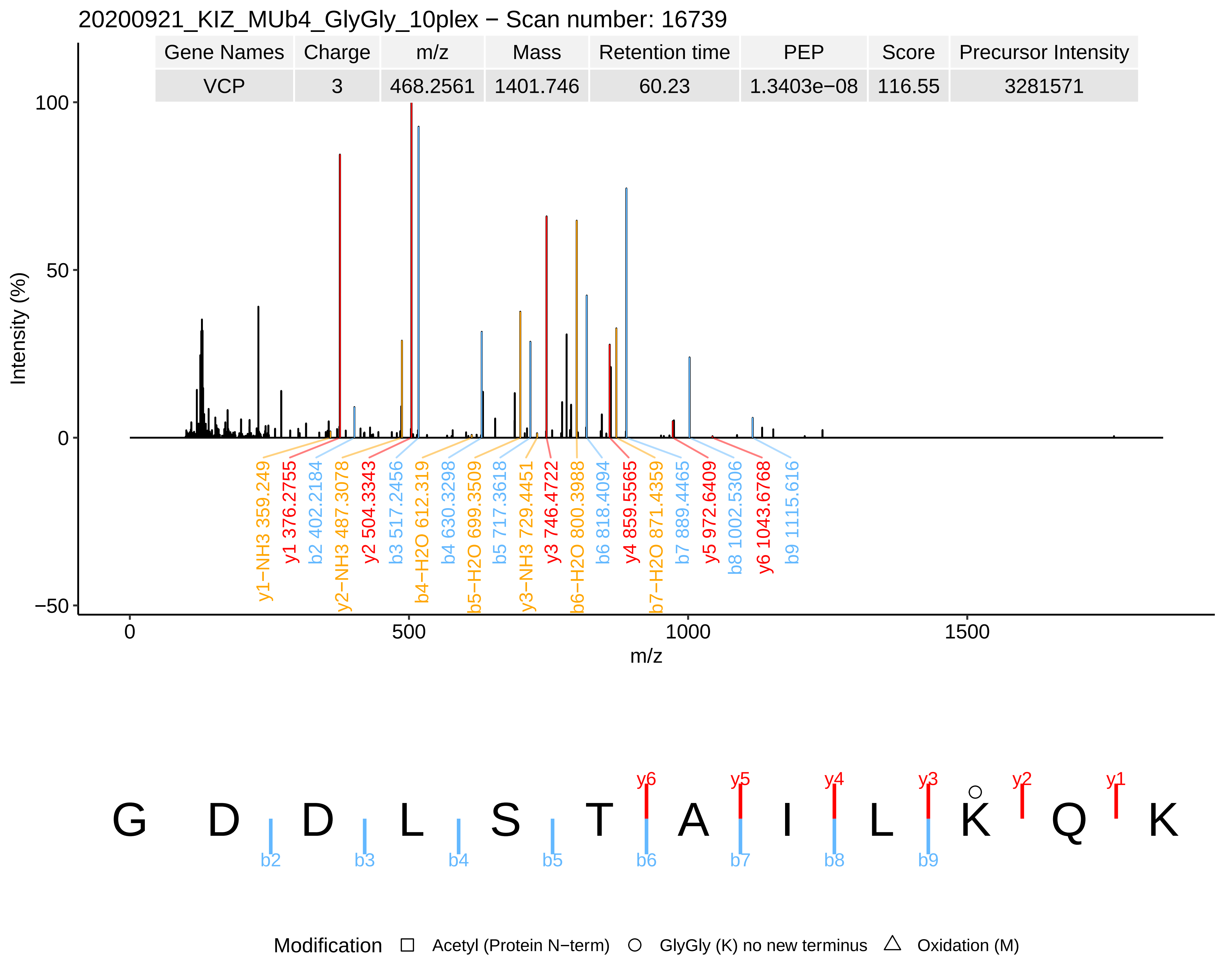
20200921_KIZ_MUb4_GlyGly_10plex − Scan number: 16739
Gene Names | Charge | m/z | Mass | Retention time | PEP | Score | Precursor Intensity
VCP | 3 | 468.2561 | 1401.746 | 60.23 | 1.3403e−08 | 116.55 | 3281571
Intensity (%)
100
50
0
−50
0
500
1000
1500
m/z
y1−NH3 359.249
y1 376.2755
b2 402.2184
y2−NH3 487.3078
y2 504.3343
b3 517.2456
b4−H2O 612.319
b4 630.3298
b5−H2O 699.3509
b5 717.3618
y3−NH3 729.4451
y3 746.4722
b6−H2O 800.3988
b6 818.4094
y4 859.5565
b7−H2O 871.4359
b7 889.4465
y5 972.6409
b8 1002.5306
y6 1043.6768
b9 1115.616
G D D L S T A I L K Q K
y6 y5 y4 y3 y2 y1
b2 b3 b4 b5 b6 b7 b8 b9
Modification
Acetyl (Protein N−term)
GlyGly (K) no new terminus
Oxidation (M)

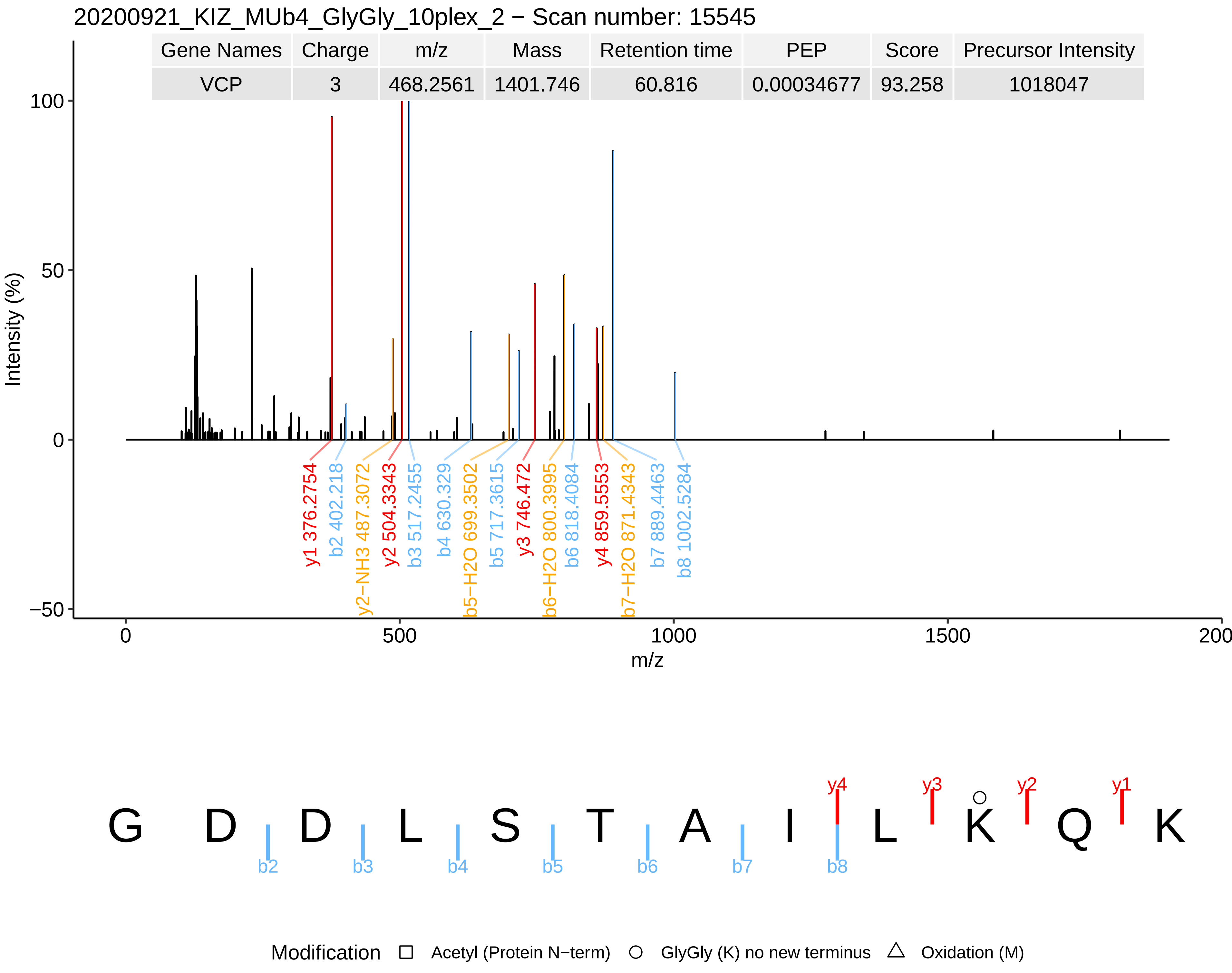
20200921_KIZ_MUb4_GlyGly_10plex_2 − Scan number: 15545
Gene Names
Charge
m/z
Mass
Retention time
PEP
Score
Precursor Intensity
VCP
3
468.2561
1401.746
60.816
0.00034677
93.258
1018047
Intensity (%)
m/z
y1 376.2754
b2 402.218
y2−NH3 487.3072
y2 504.3343
b3 517.2455
b4 630.329
b5−H2O 699.3502
b5 717.3615
y3 746.472
b6−H2O 800.3995
b6 818.4084
y4 859.5553
b7−H2O 871.4343
b7 889.4463
b8 1002.5284
G D D L S T A I L K Q K
b2 b3 b4 b5 b6 b7 b8
y4 y3 y2 y1
Modification
Acetyl (Protein N−term)
GlyGly (K) no new terminus
Oxidation (M)

20200924_KIZ_MUb5_GlyGly_1 − Scan number: 16351

Gene Names	Charge	m/z	Mass	Retention time	PEP	Score	Precursor Intensity
VCP	3	468.2561	1401.746	53.01	1.1977e−08	118.28	21455896

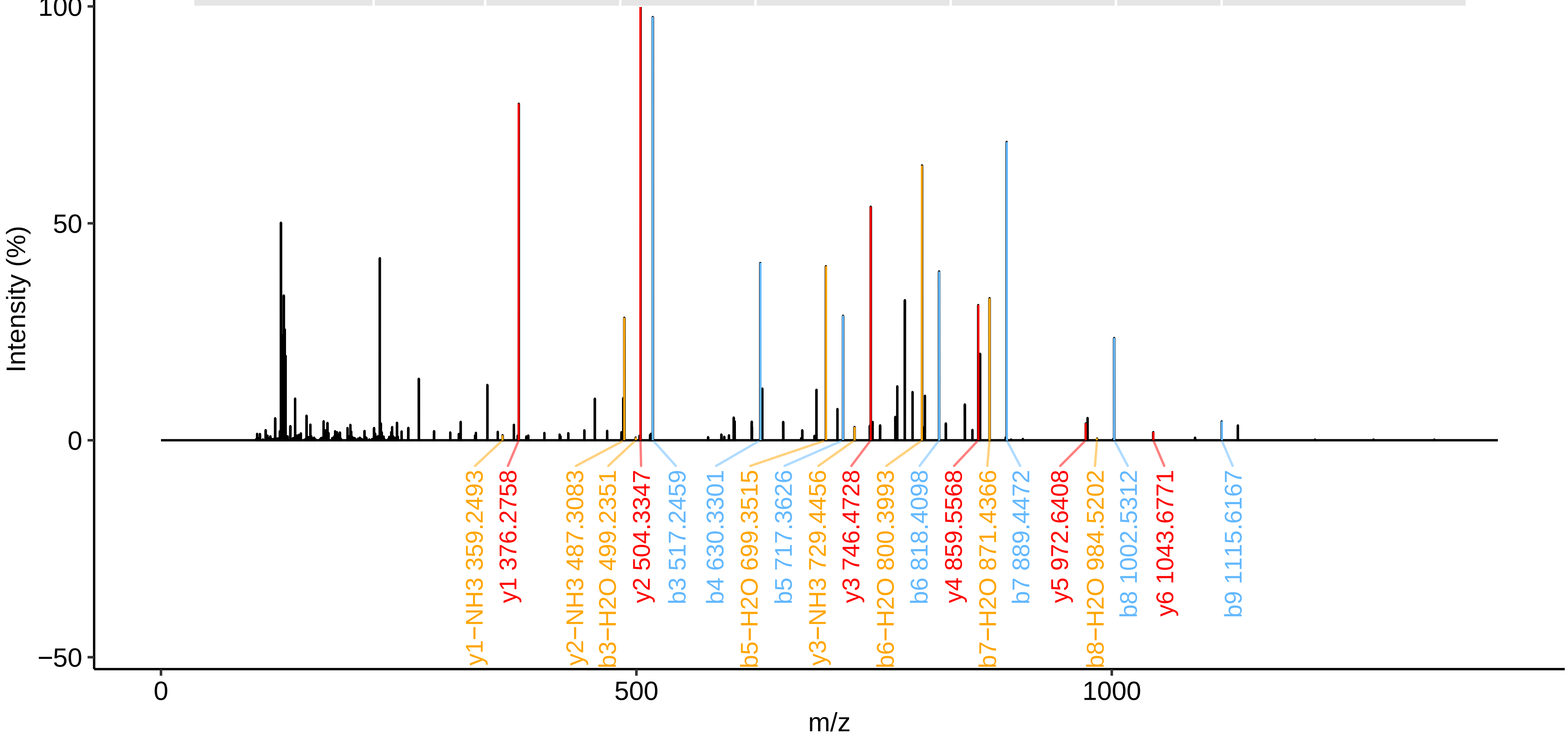

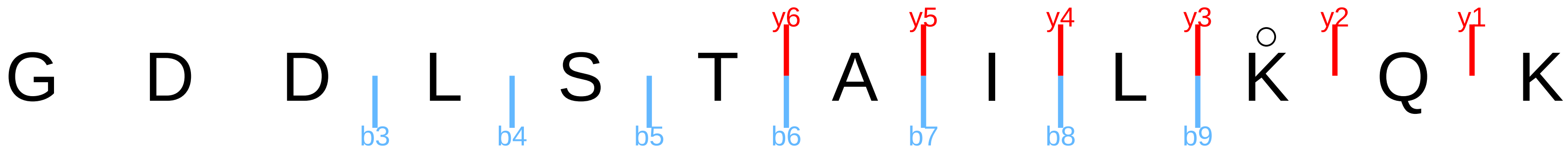

Modification □ Acetyl (Protein N−term) ○ GlyGly (K) no new terminus △ Oxidation (M)

20200924_KIZ_MUb5_GlyGly_1 − Scan number: 16447

Gene Names	Charge	m/z	Mass	Retention time	PEP	Score	Precursor Intensity
VCP	3	468.2561	1401.746	53.285	0.0011091	82.171	27316154

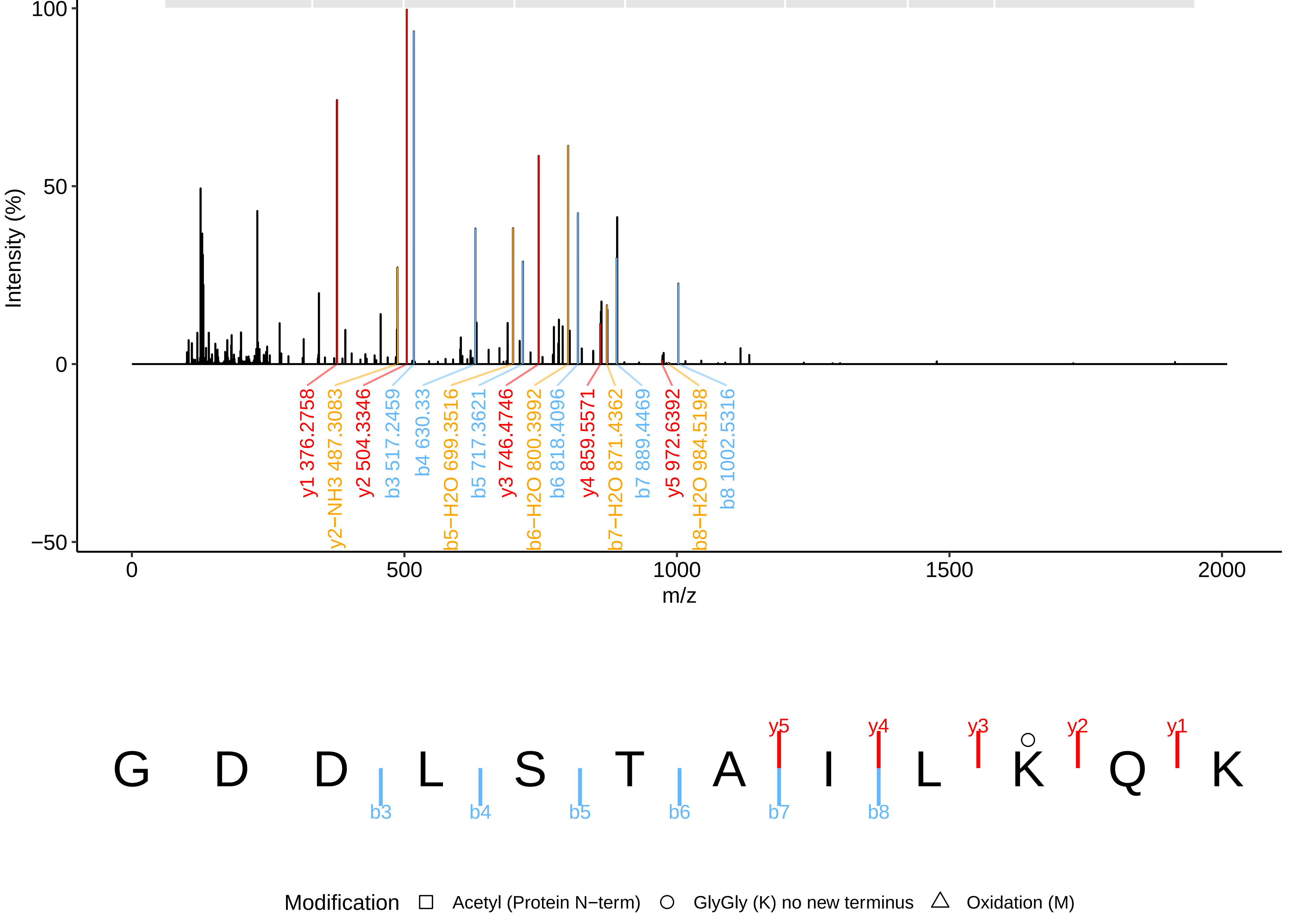

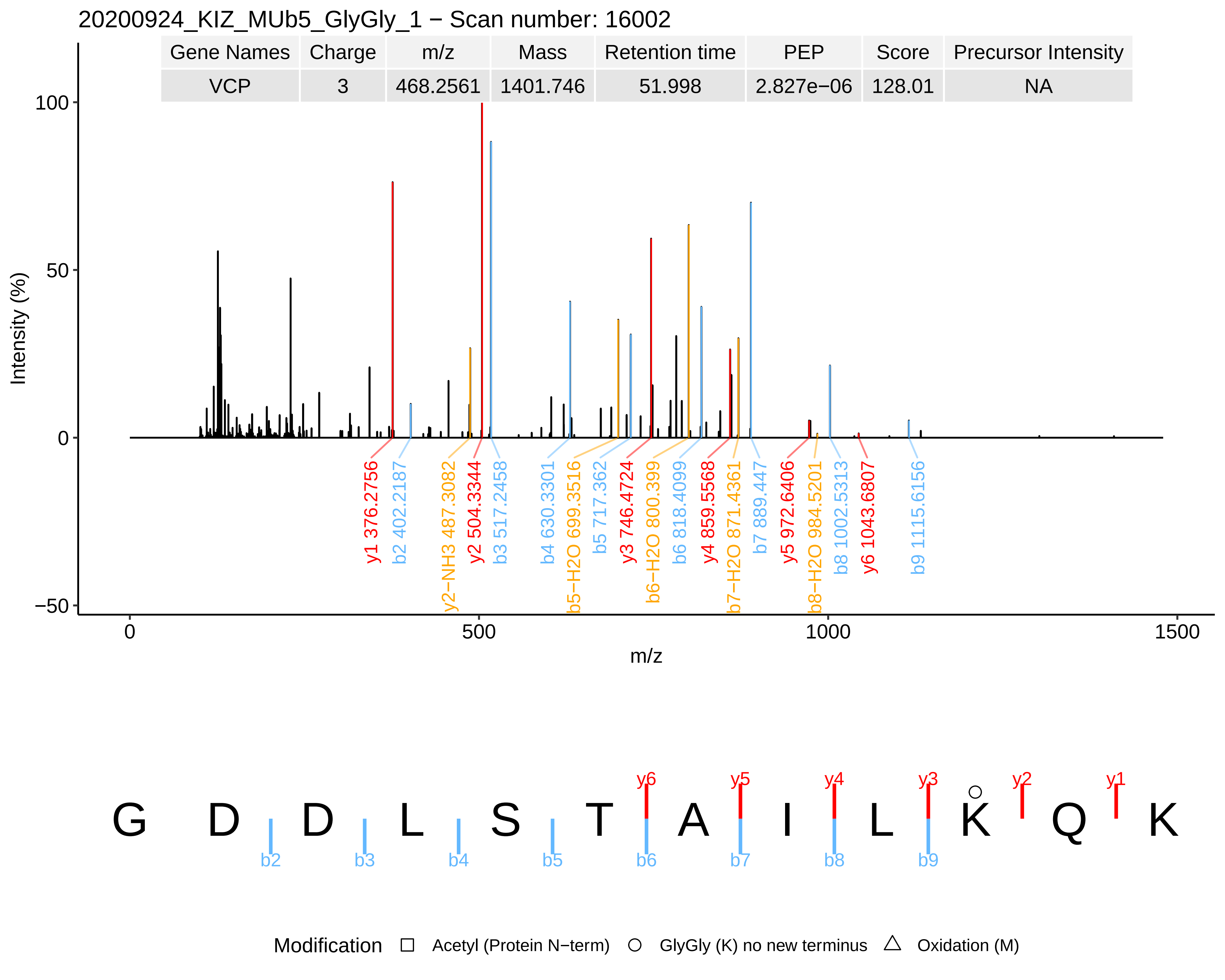

20200924_KIZ_MUb5_GlyGly_1 − Scan number: 16002
Gene Names | Charge | m/z | Mass | Retention time | PEP | Score | Precursor Intensity
VCP | 3 | 468.2561 | 1401.746 | 51.998 | 2.827e−06 | 128.01 | NA
Intensity (%)
100
50
0
−50
0
500
1000
1500
m/z
y1 376.2756
b2 402.2187
y2−NH3 487.3082
y2 504.3344
b3 517.2458
b4 630.3301
b5−H2O 699.3516
b5 717.362
y3 746.4724
b6−H2O 800.399
b6 818.4099
y4 859.5568
b7−H2O 871.4361
b7 889.447
y5 972.6406
b8−H2O 984.5201
b8 1002.5313
y6 1043.6807
b9 1115.6156
G D D L S T A I L K Q K
y6 y5 y4 y3 y2 y1
b2 b3 b4 b5 b6 b7 b8 b9
Modification
Acetyl (Protein N−term)
GlyGly (K) no new terminus
Oxidation (M)

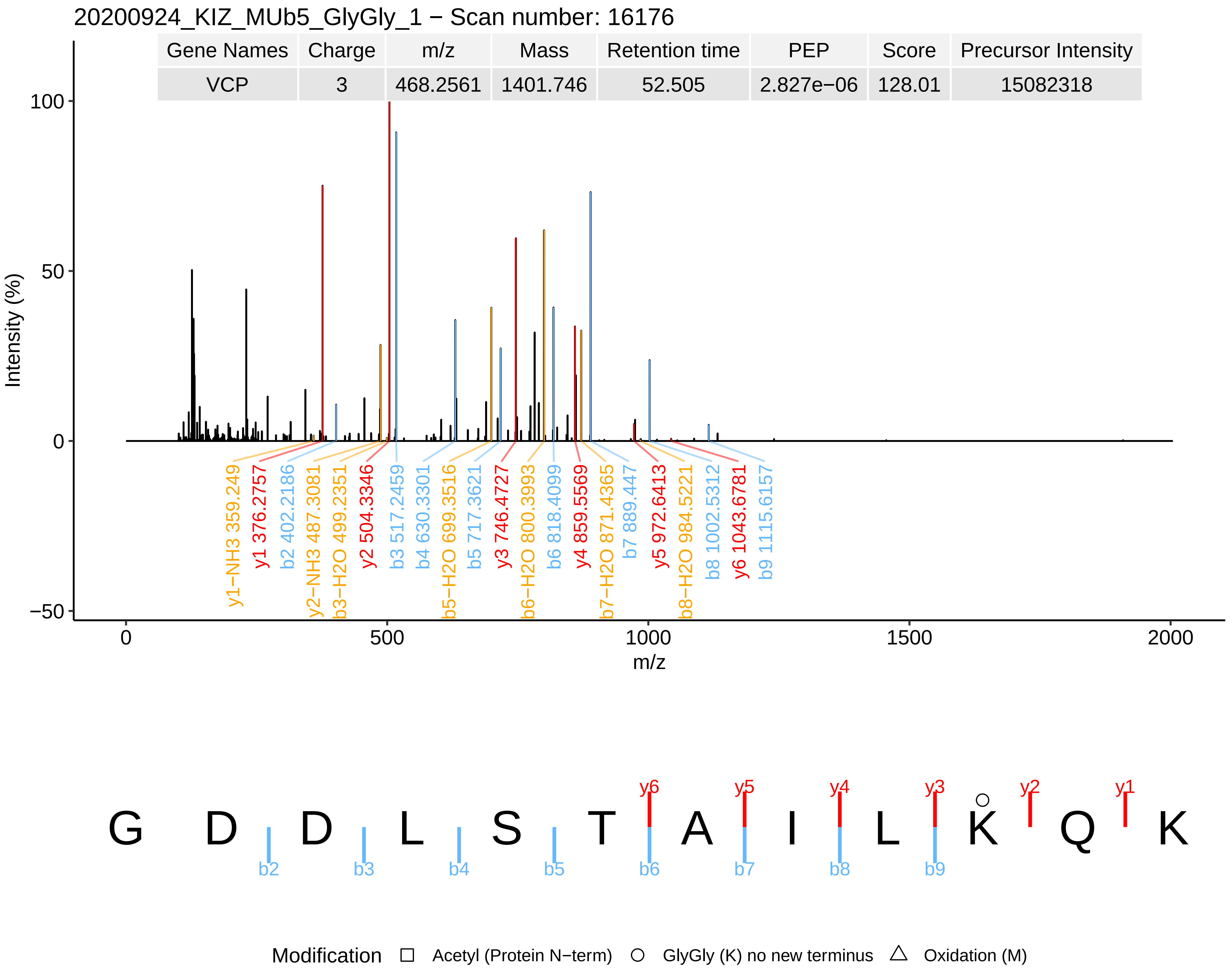
20200924_KIZ_MUb5_GlyGly_1 − Scan number: 16176
Gene Names
Charge
m/z
Mass
Retention time
PEP
Score
Precursor Intensity
VCP
3
468.2561
1401.746
52.505
2.827e−06
128.01
15082318
Intensity (%)
m/z
y1−NH3 359.249
y1 376.2757
b2 402.2186
y2−NH3 487.3081
b3−H2O 499.2351
y2 504.3346
b3 517.2459
b4 630.3301
b5−H2O 699.3516
b5 717.3621
y3 746.4727
b6−H2O 800.3993
b6 818.4099
y4 859.5569
b7−H2O 871.4365
b7 889.447
y5 972.6413
b8−H2O 984.5221
b8 1002.5312
y6 1043.6781
b9 1115.6157
G D D L S T A I L K Q K
y6 y5 y4 y3 y2 y1
b2 b3 b4 b5 b6 b7 b8 b9
Modification
Acetyl (Protein N−term)
GlyGly (K) no new terminus
Oxidation (M)

20200924_KIZ_MUb5_GlyGly_2 − Scan number: 15002

Gene Names	Charge	m/z	Mass	Retention time	PEP	Score	Precursor Intensity
VCP	3	468.2561	1401.746	56.062	8.0181e−06	108.7	4605240

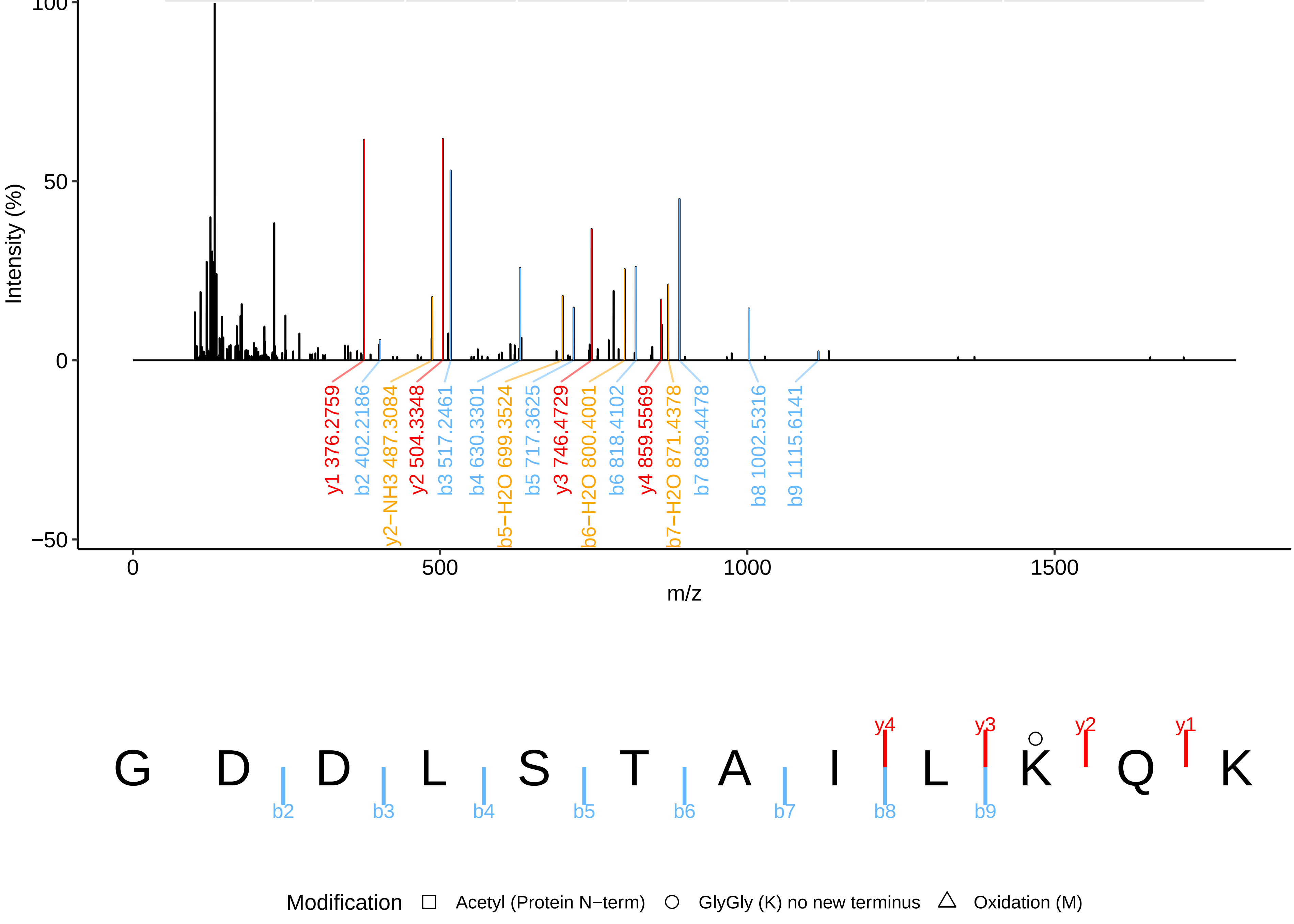

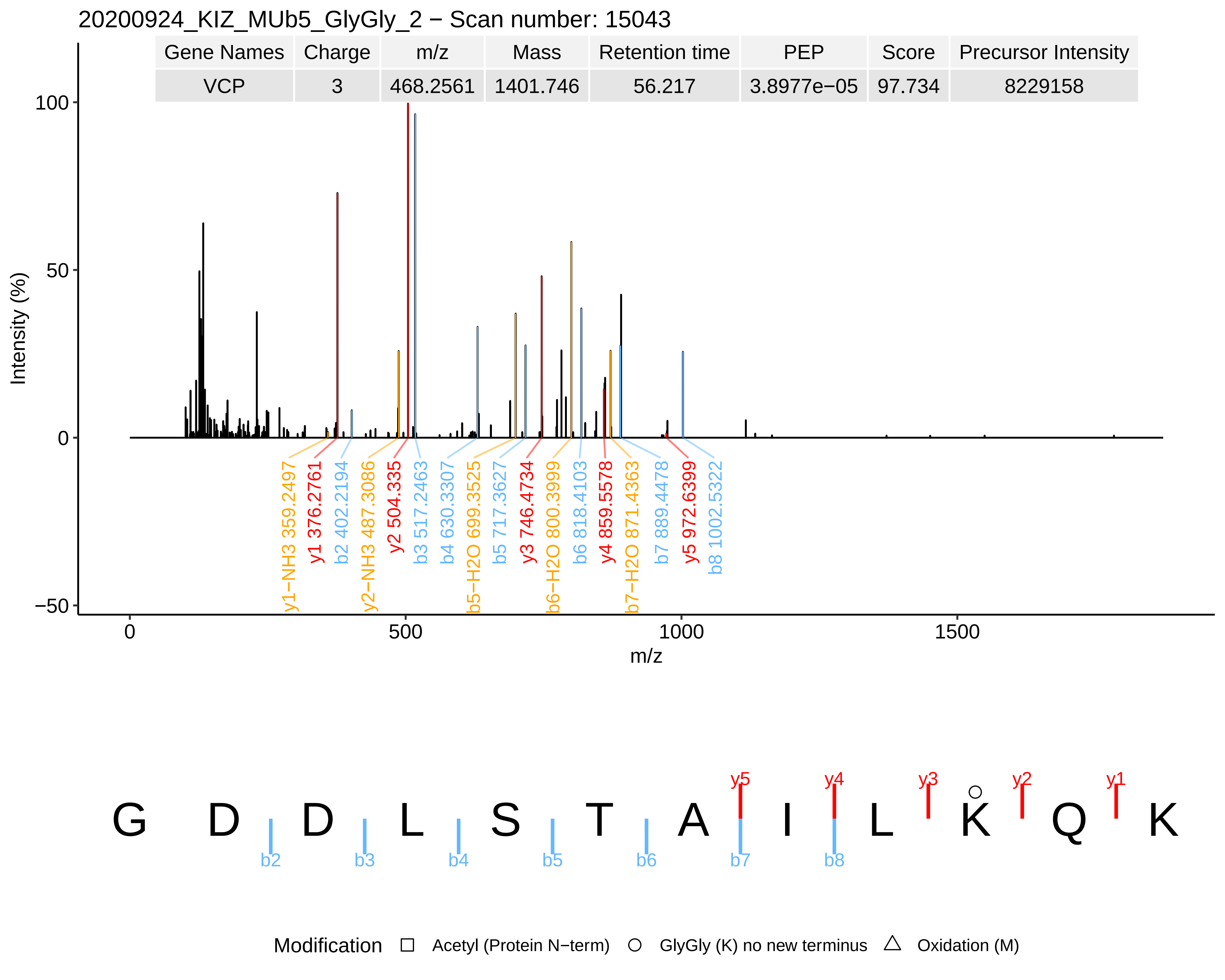
20200924_KIZ_MUb5_GlyGly_2 − Scan number: 15043
Gene Names | Charge | m/z | Mass | Retention time | PEP | Score | Precursor Intensity
VCP | 3 | 468.2561 | 1401.746 | 56.217 | 3.8977e−05 | 97.734 | 8229158
Intensity (%)
100
50
0
−50
0
500
1000
1500
m/z
y1−NH3 359.2497
y1 376.2761
b2 402.2194
y2−NH3 487.3086
y2 504.335
b3 517.2463
b4 630.3307
b5−H2O 699.3525
b5 717.3627
y3 746.4734
b6−H2O 800.3999
b6 818.4103
y4 859.5578
b7−H2O 871.4363
b7 889.4478
y5 972.6399
b8 1002.5322
G D D L S T A I L K Q K
y5 y4 y3 y2 y1
b2 b3 b4 b5 b6 b7 b8
Modification
Acetyl (Protein N−term)
GlyGly (K) no new terminus
Oxidation (M)

20200924_KIZ_MUb5_GlyGly_2 − Scan number: 15177

Gene Names	Charge	m/z	Mass	Retention time	PEP	Score	Precursor Intensity
VCP	3	468.2561	1401.746	56.728	1.3403e−08	116.55	18504264

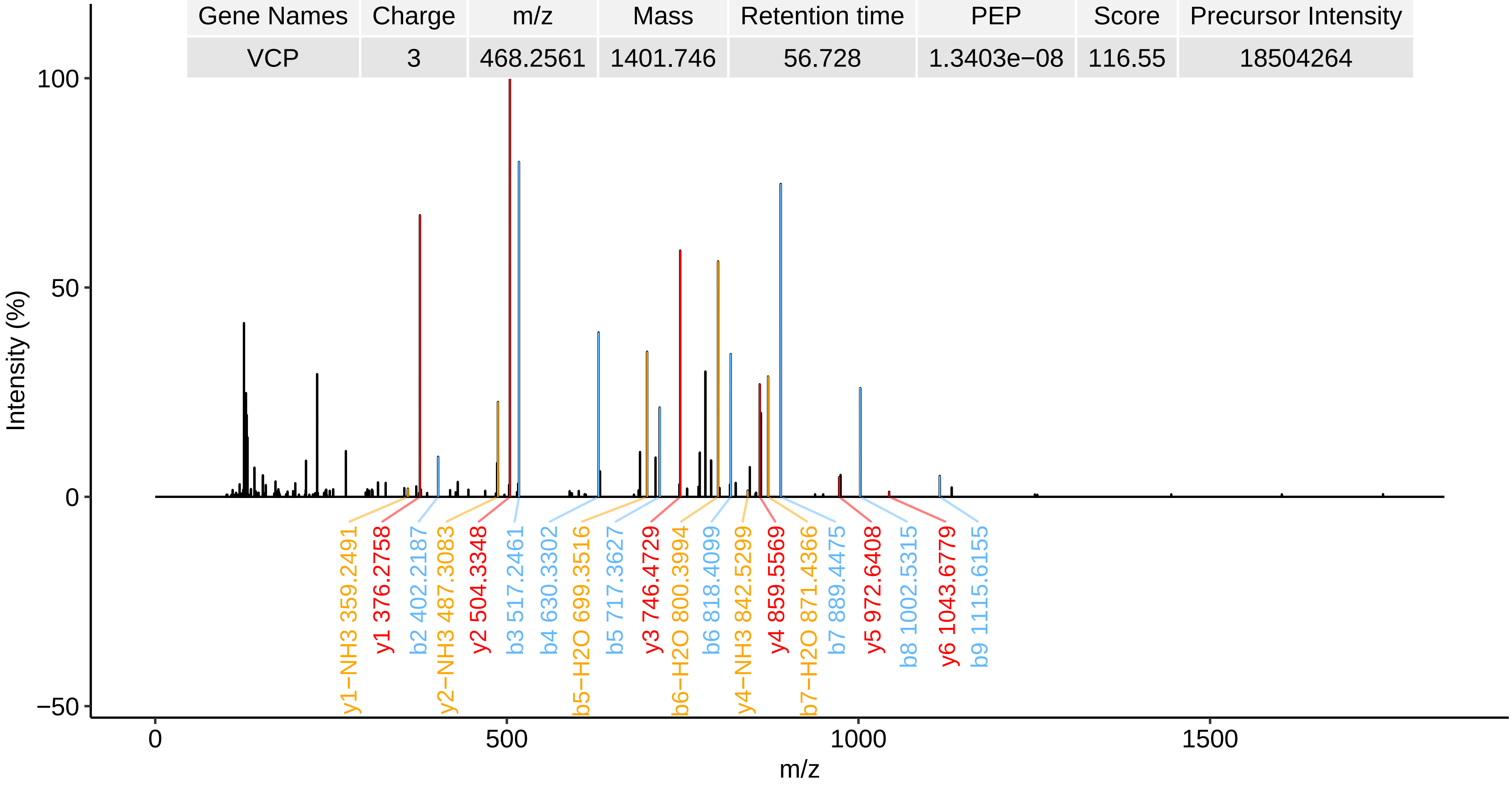

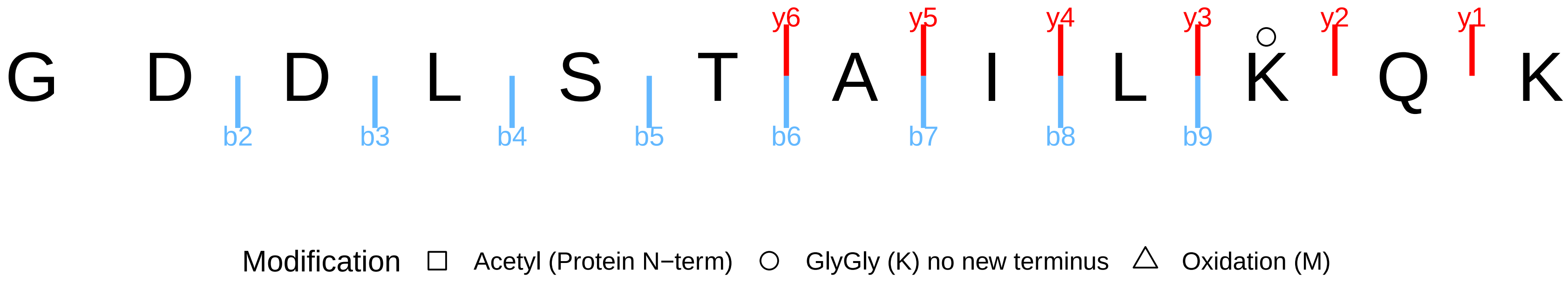

20201118_KIZ_MUb6_GlyGly_1 − Scan number: 16621

Gene Names	Charge	m/z	Mass	Retention time	PEP	Score	Precursor Intensity
VCP	3	468.2561	1401.746	52.387	2.3073e−13	139.86	33772268

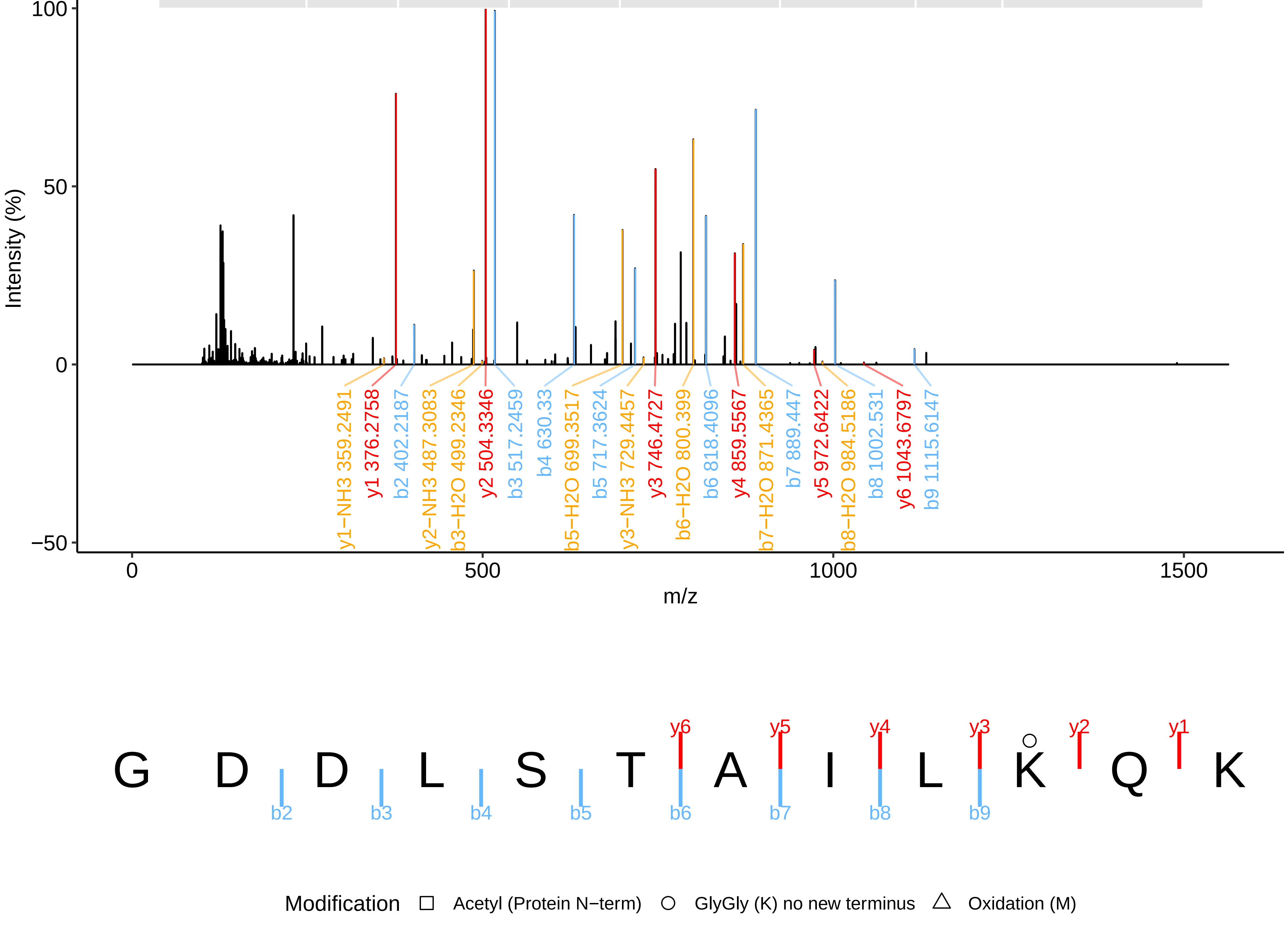

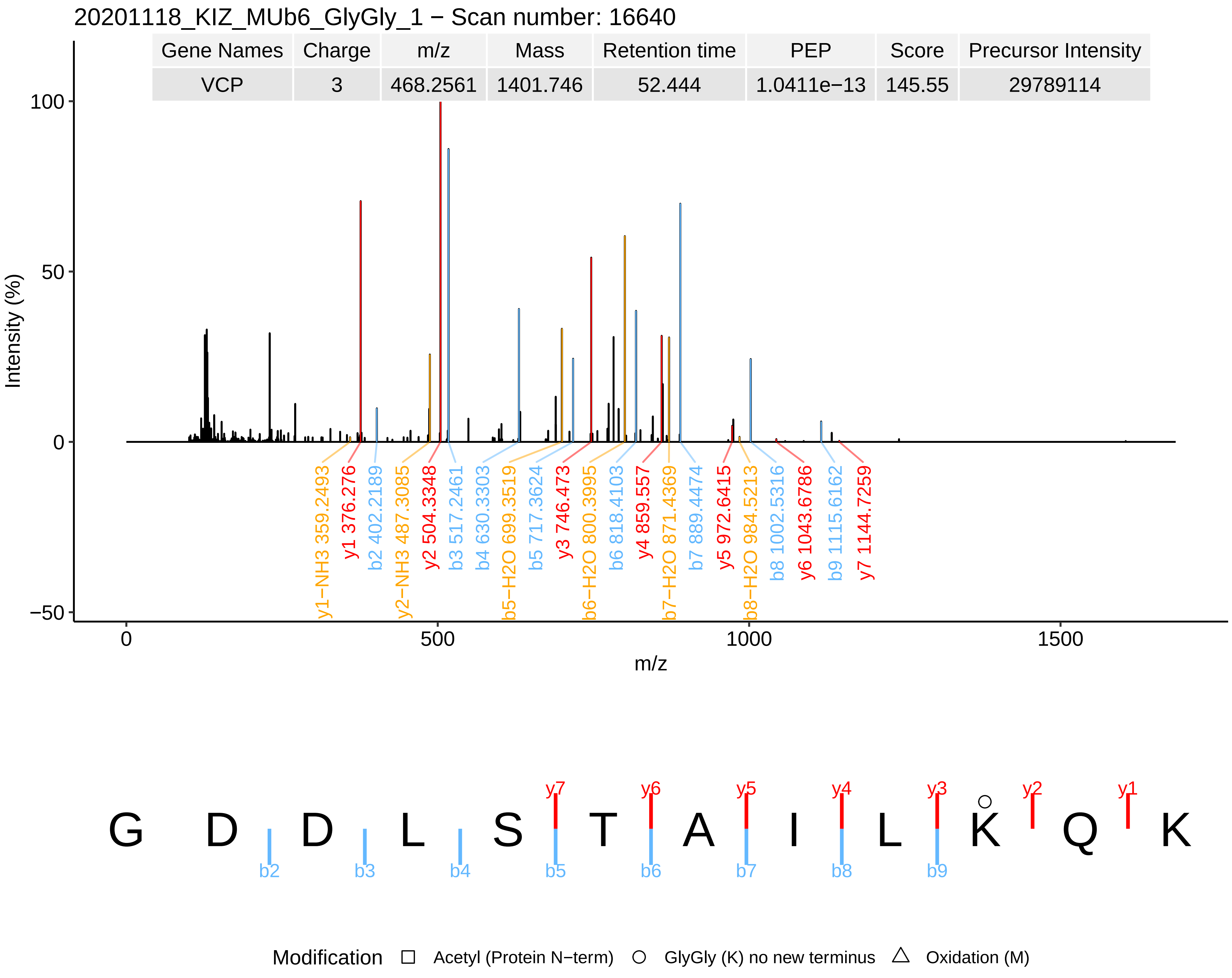
20201118_KIZ_MUb6_GlyGly_1 − Scan number: 16640
Gene Names
Charge
m/z
Mass
Retention time
PEP
Score
Precursor Intensity
VCP
3
468.2561
1401.746
52.444
1.0411e−13
145.55
29789114
Intensity (%)
100
50
0
−50
m/z
0
500
1000
1500
y1−NH3 359.2493
y1 376.276
b2 402.2189
y2−NH3 487.3085
y2 504.3348
b3 517.2461
b4 630.3303
b5−H2O 699.3519
b5 717.3624
y3 746.473
b6−H2O 800.3995
b6 818.4103
y4 859.557
b7−H2O 871.4369
b7 889.4474
y5 972.6415
b8−H2O 984.5213
b8 1002.5316
y6 1043.6786
b9 1115.6162
y7 1144.7259
G D D L S T A I L K Q K
y7 y6 y5 y4 y3 y2 y1
b2 b3 b4 b5 b6 b7 b8 b9
Modification
Acetyl (Protein N−term)
GlyGly (K) no new terminus
Oxidation (M)

20201118_KIZ_MUb6_GlyGly_2 − Scan number: 18251

Gene Names	Charge	m/z	Mass	Retention time	PEP	Score	Precursor Intensity
VCP	3	468.2561	1401.746	61.726	3.0592e−13	136.48	33061902

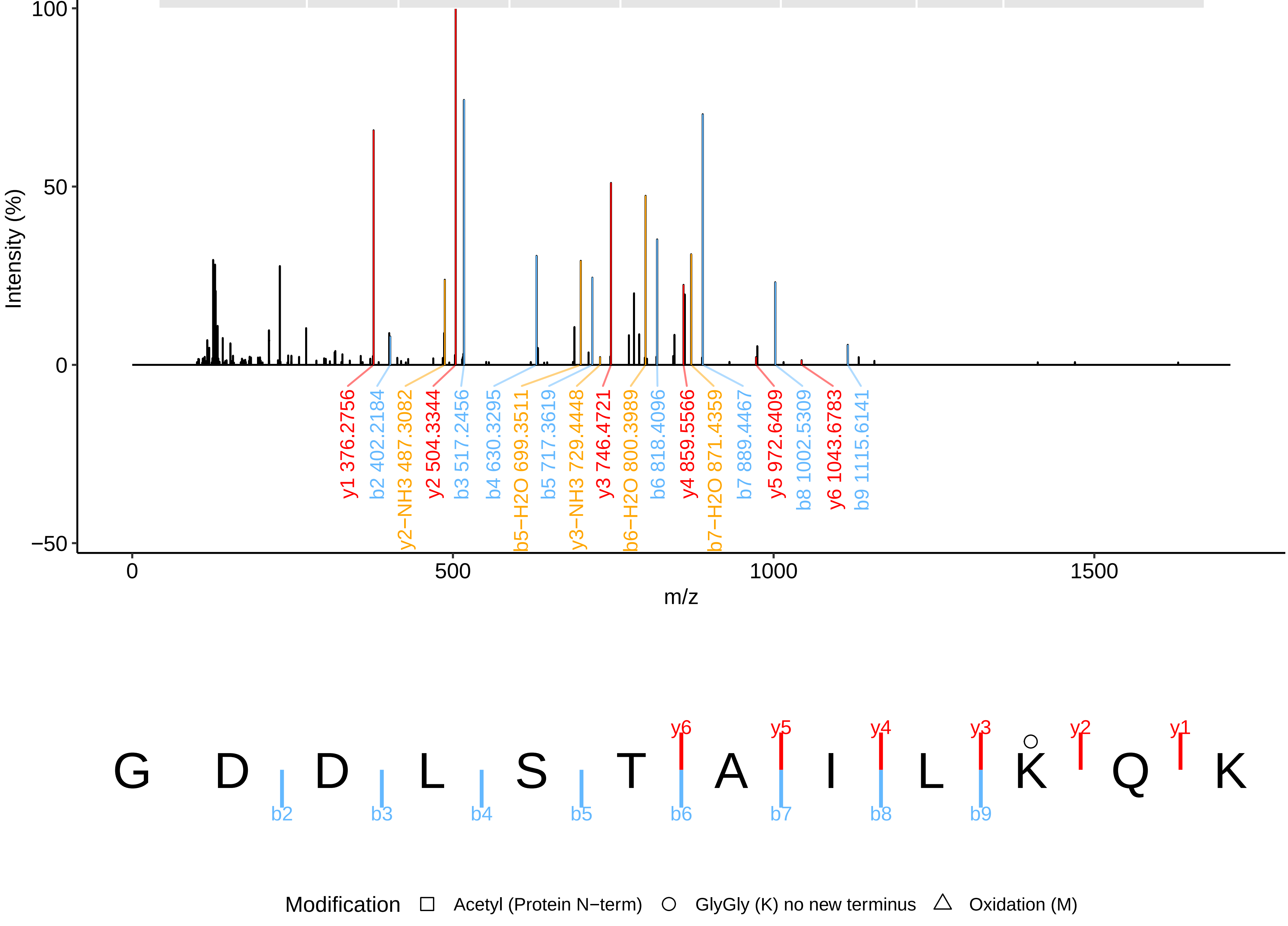

20200921_KIZ_MUb4_GlyGly_10plex − Scan number: 15789

Gene Names	Charge	m/z	Mass	Retention time	PEP	Score	Precursor Intensity
CNNM3	3	545.6093	1633.806	56.51	2.475e−06	111.72	2773310

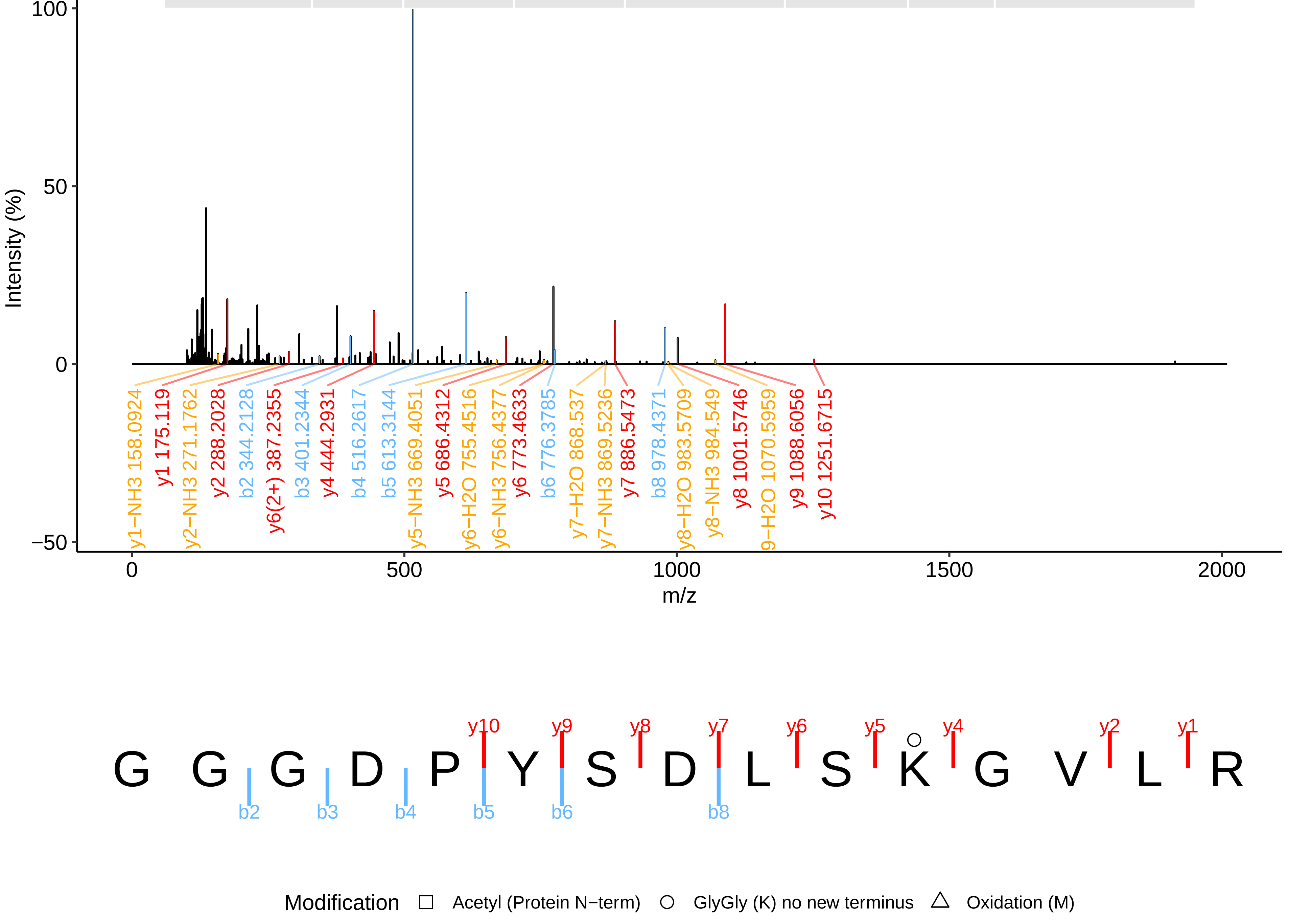

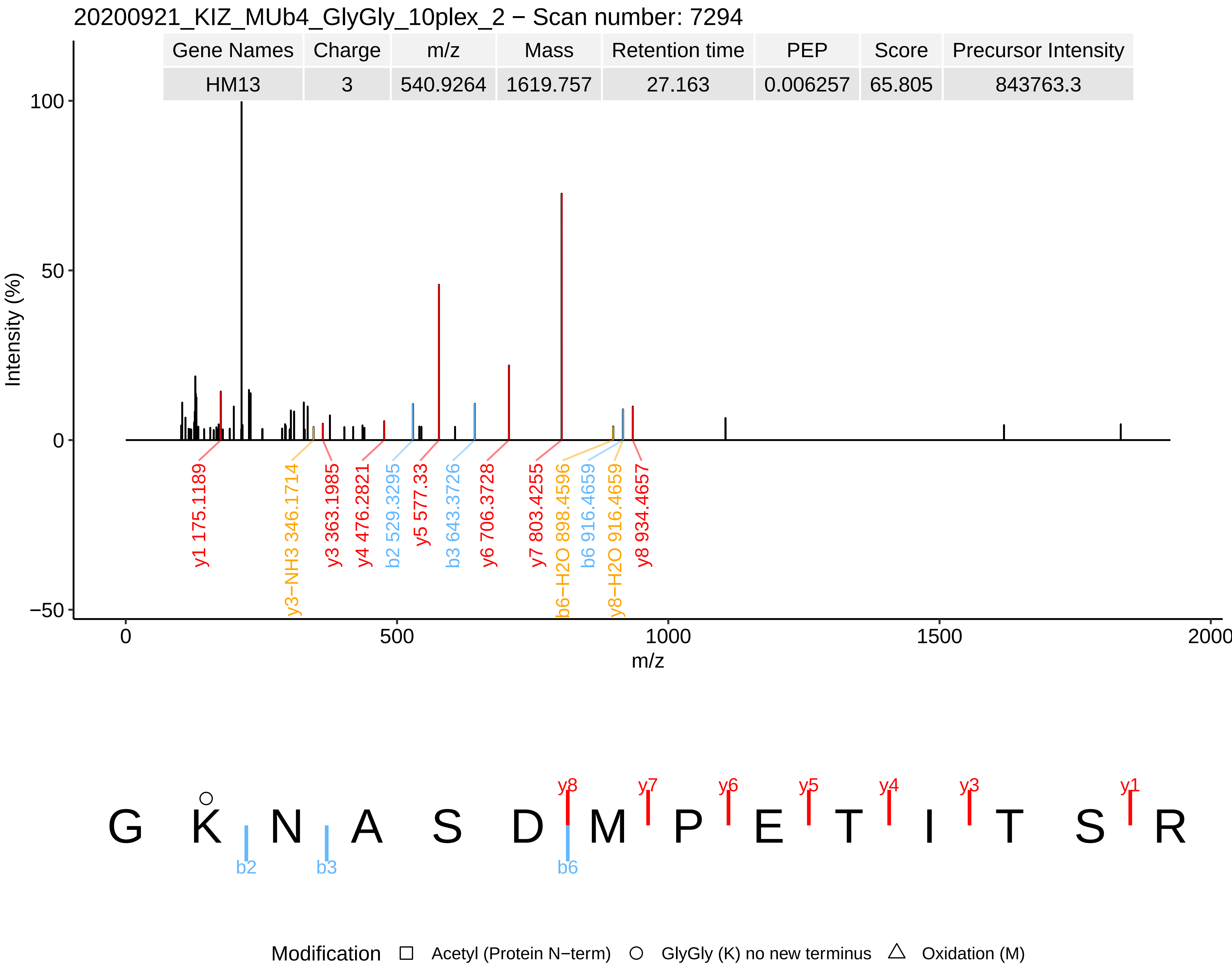
20200921_KIZ_MUb4_GlyGly_10plex_2 − Scan number: 7294
Gene Names
Charge
m/z
Mass
Retention time
PEP
Score
Precursor Intensity
HM13
3
540.9264
1619.757
27.163
0.006257
65.805
843763.3
Intensity (%)
m/z
y1 175.1189
y3−NH3 346.1714
y3 363.1985
y4 476.2821
b2 529.3295
y5 577.33
b3 643.3726
y6 706.3728
y7 803.4255
b6−H2O 898.4596
b6 916.4659
y8−H2O 916.4659
y8 934.4657
G K N A S D M P E T I T S R
y8
y7
y6
y5
y4
y3
y1
b2
b3
b6
Modification
Acetyl (Protein N−term)
GlyGly (K) no new terminus
Oxidation (M)

20200924_KIZ_MUb5_GlyGly_1 − Scan number: 6930

Gene Names	Charge	m/z	Mass	Retention time	PEP	Score	Precursor Intensity
HM13	3	540.9264	1619.757	21.92	0.00052793	85.976	3895101

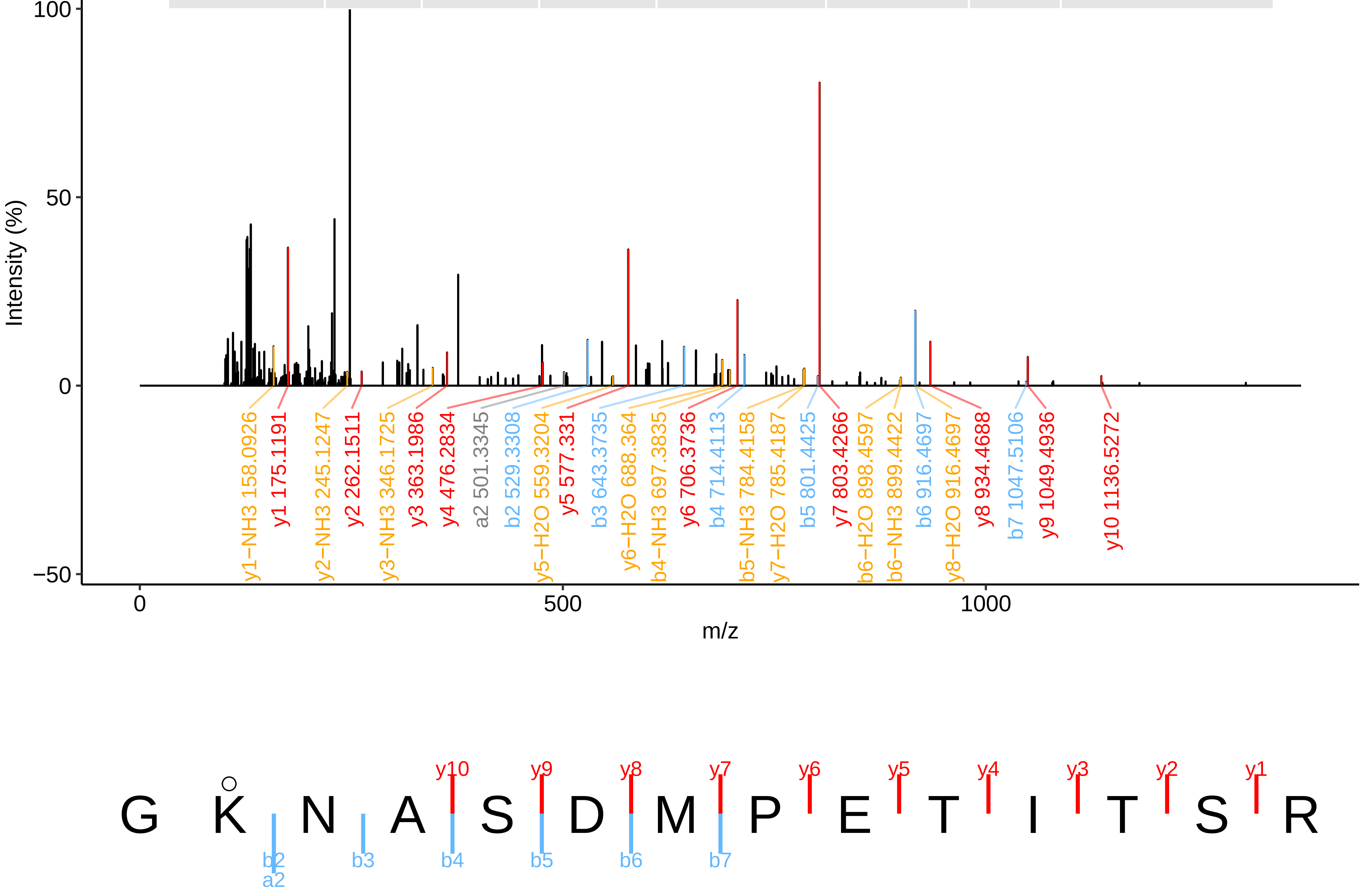

Modification □ Acetyl (Protein N−term) ○ GlyGly (K) no new terminus △ Oxidation (M)

20200921_KIZ_MUb4_GlyGly_10plex − Scan number: 13146

Gene Names	Charge	m/z	Mass	Retention time	PEP	Score	Precursor Intensity
SLC1A5	2	831.8856	1661.757	46.276	2.4252e−05	91.584	3291189

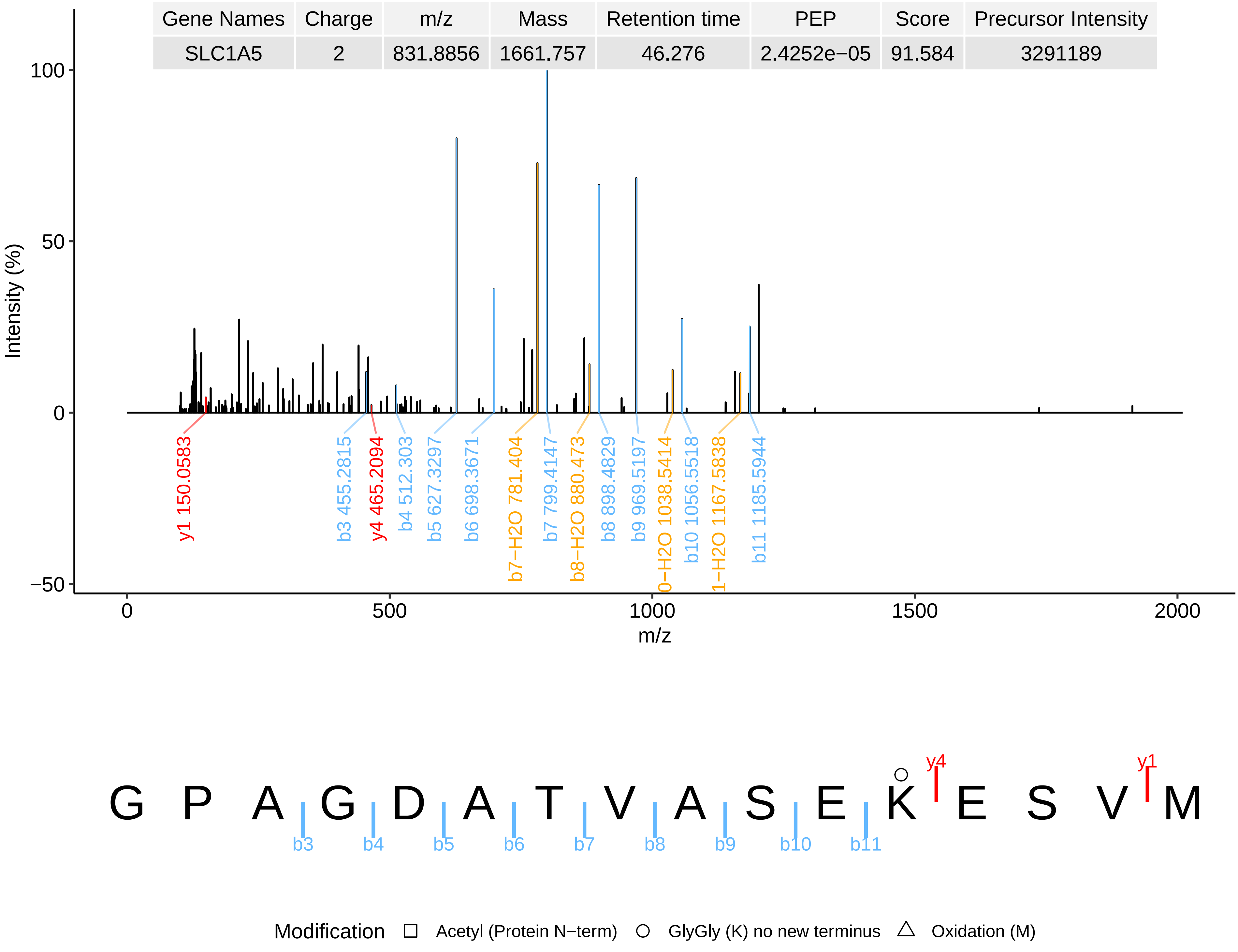

20200921_KIZ_MUb4_GlyGly_10plex − Scan number: 14806

Gene Names	Charge	m/z	Mass	Retention time	PEP	Score	Precursor Intensity
CYB5R3	3	473.2648	1416.773	52.706	1.3632e−05	104.87	1551458

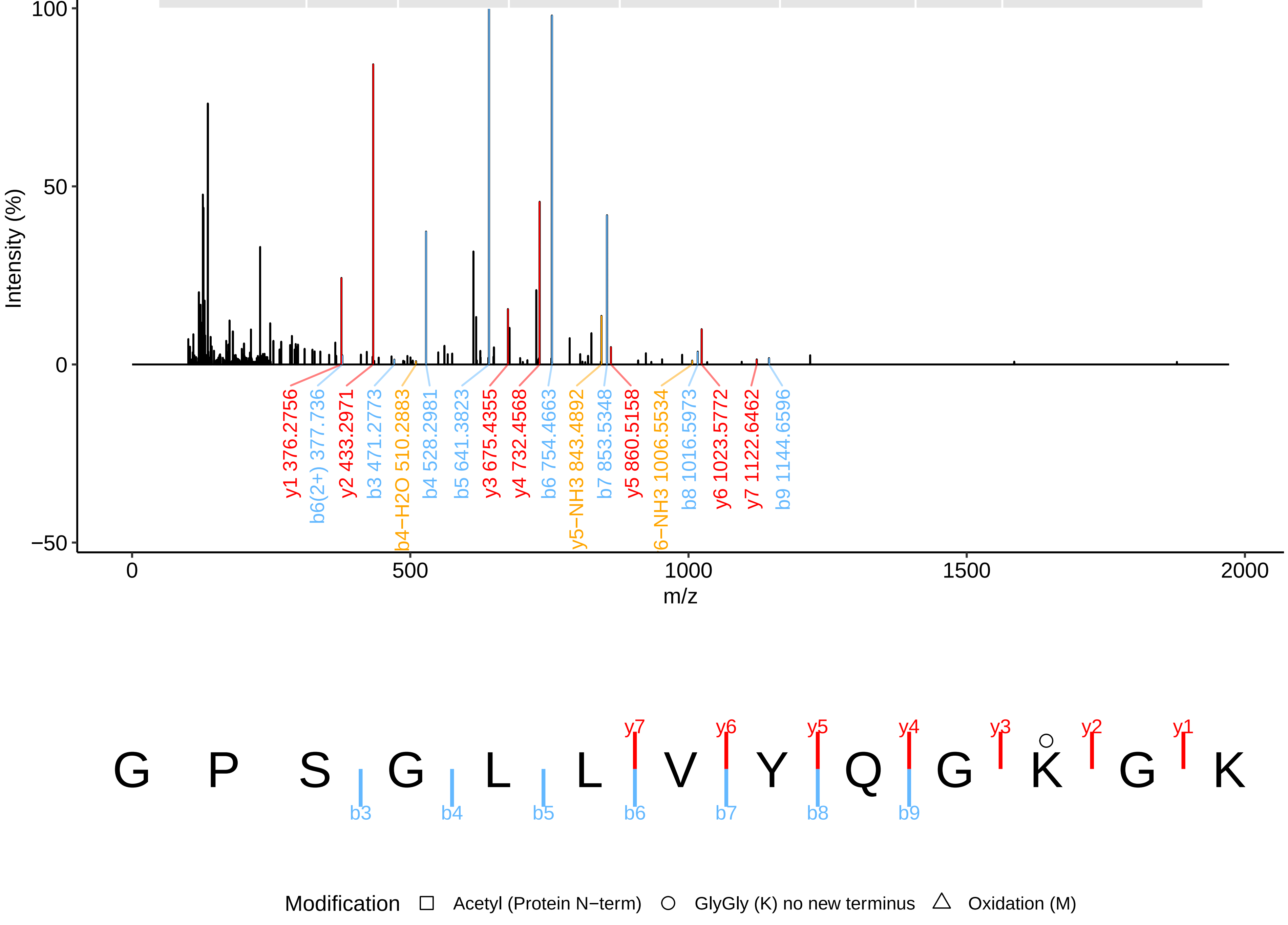

20200921_KIZ_MUb4_GlyGly_10plex − Scan number: 15879

Gene Names	Charge	m/z	Mass	Retention time	PEP	Score	Precursor Intensity
BIRC6	3	479.5963	1435.767	56.868	0.00116	81.431	7684929

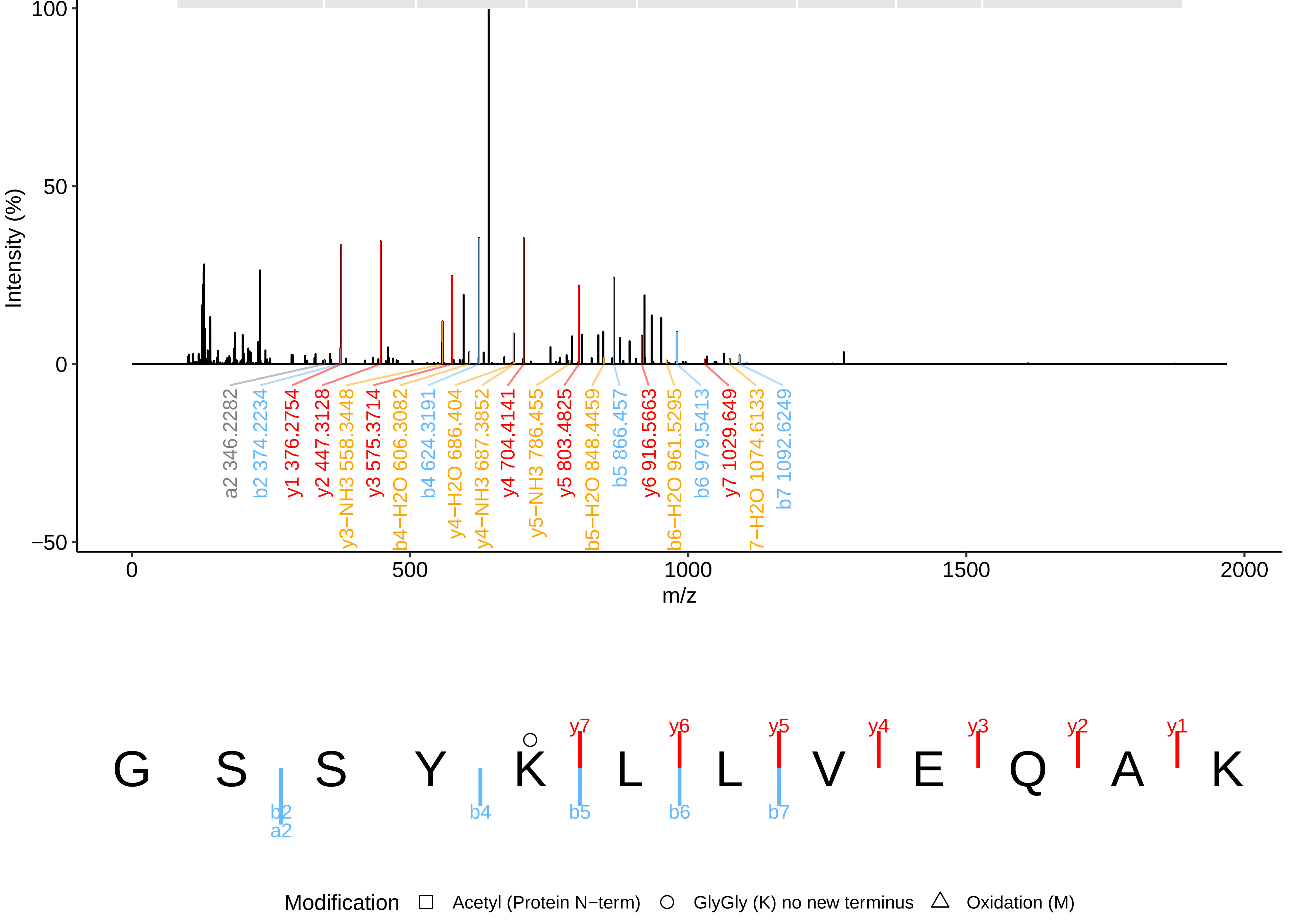

20200921_KIZ_MUb4_GlyGly_10plex − Scan number: 16011

Gene Names	Charge	m/z	Mass	Retention time	PEP	Score	Precursor Intensity
BIRC6	3	479.5963	1435.767	57.391	0.00093402	84.718	2431489

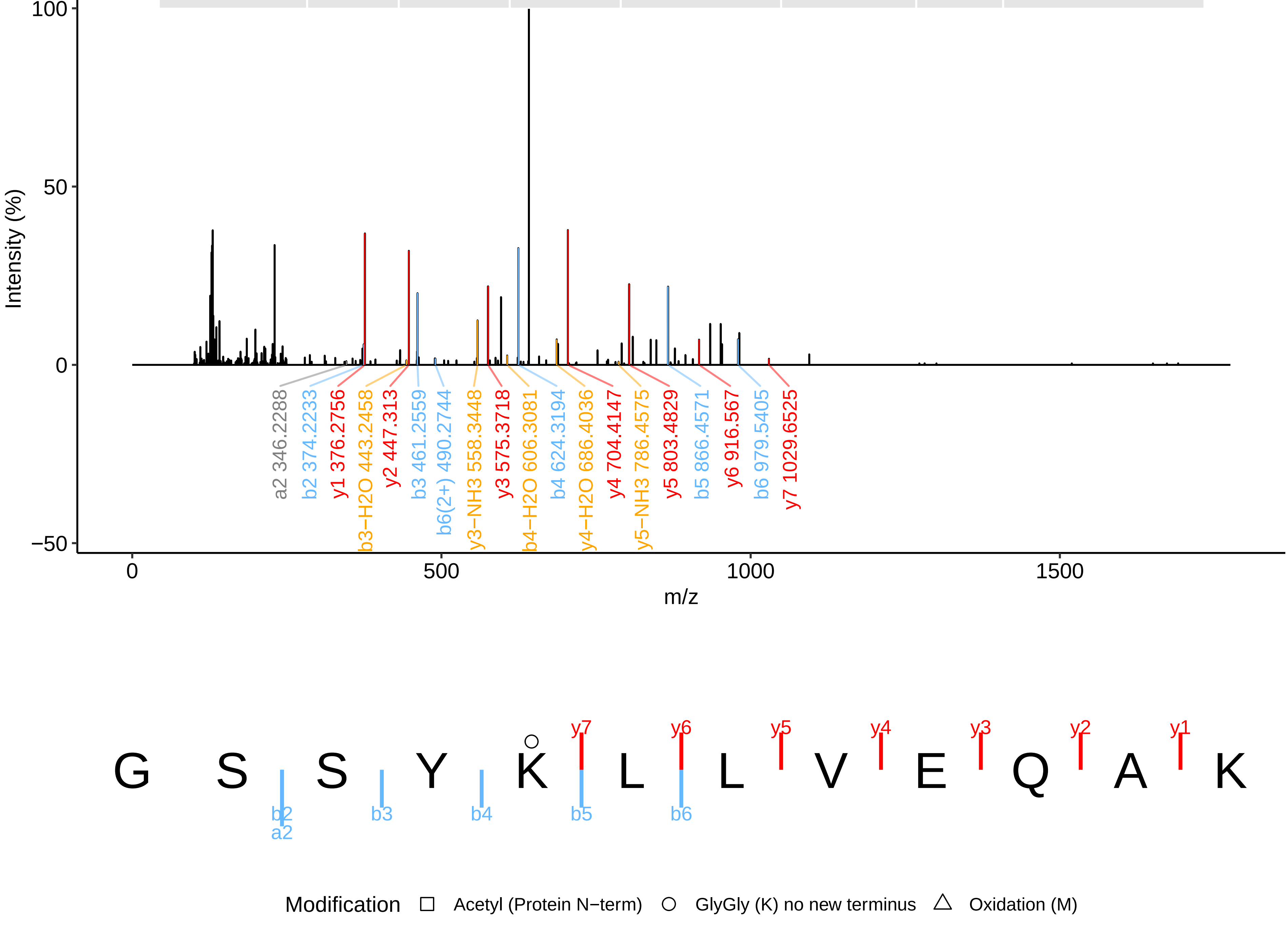

20200921_KIZ_MUb4_GlyGly_10plex_2 − Scan number: 14738

Gene Names	Charge	m/z	Mass	Retention time	PEP	Score	Precursor Intensity
BIRC6	3	479.5963	1435.767	57.509	0.018501	62.088	952314.2

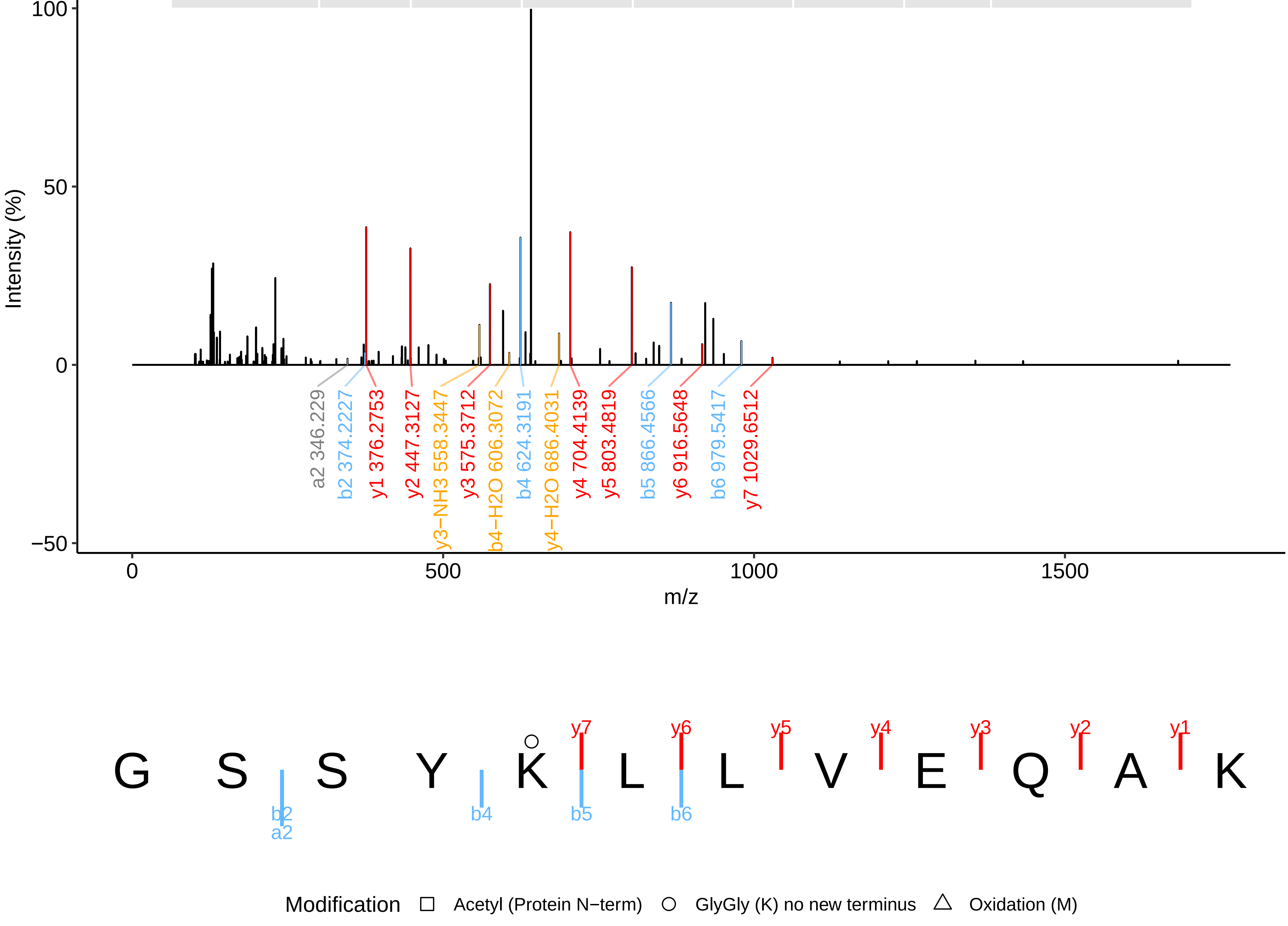

20200924_KIZ_MUb5_GlyGly_1 − Scan number: 14891

Gene Names	Charge	m/z	Mass	Retention time	PEP	Score	Precursor Intensity
BIRC6	3	479.5963	1435.767	48.644	0.00116	81.431	15763511

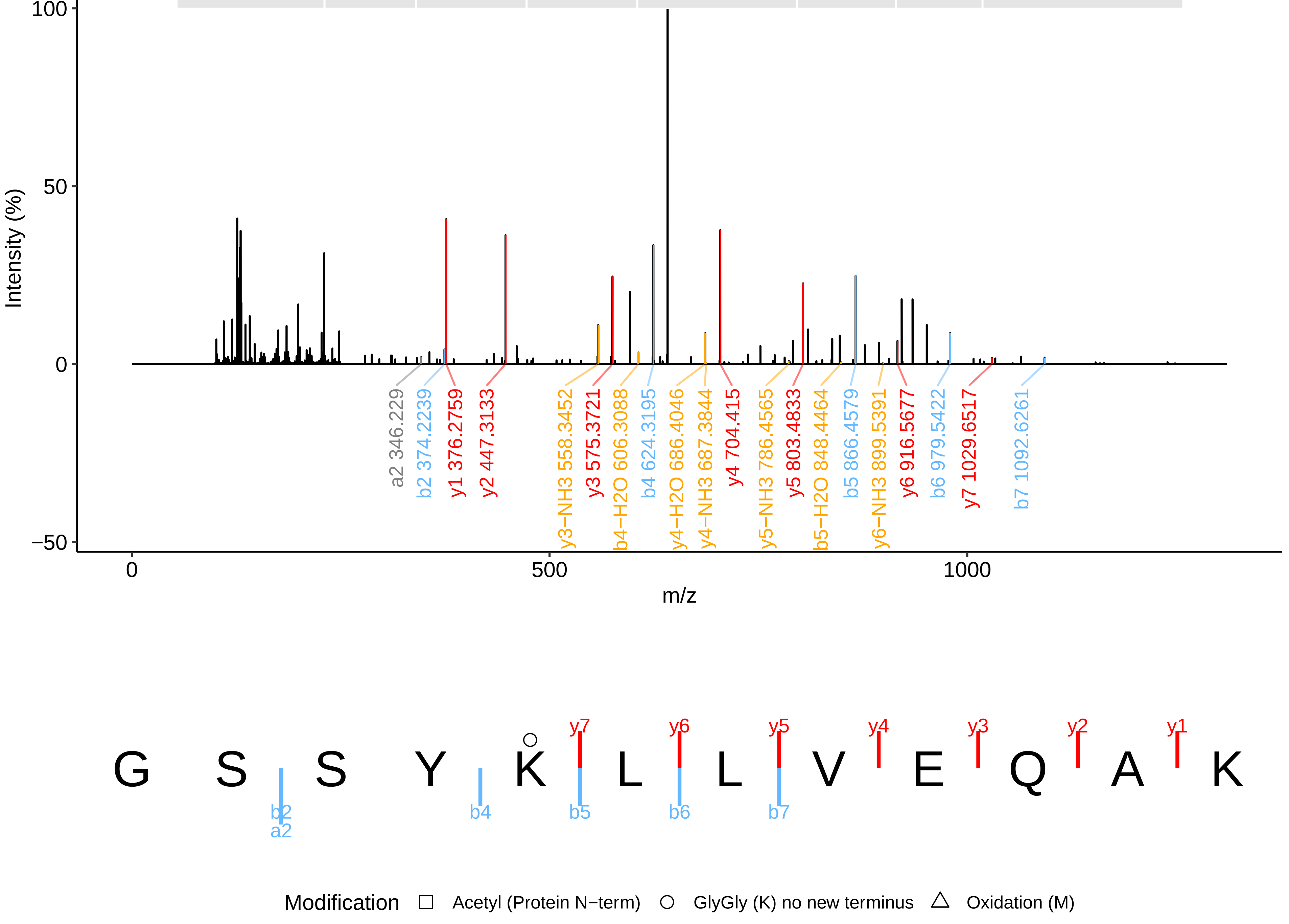

20200924_KIZ_MUb5_GlyGly_2 − Scan number: 14006

Gene Names	Charge	m/z	Mass	Retention time	PEP	Score	Precursor Intensity
BIRC6	3	479.5963	1435.767	52.37	0.011746	69.864	4400048

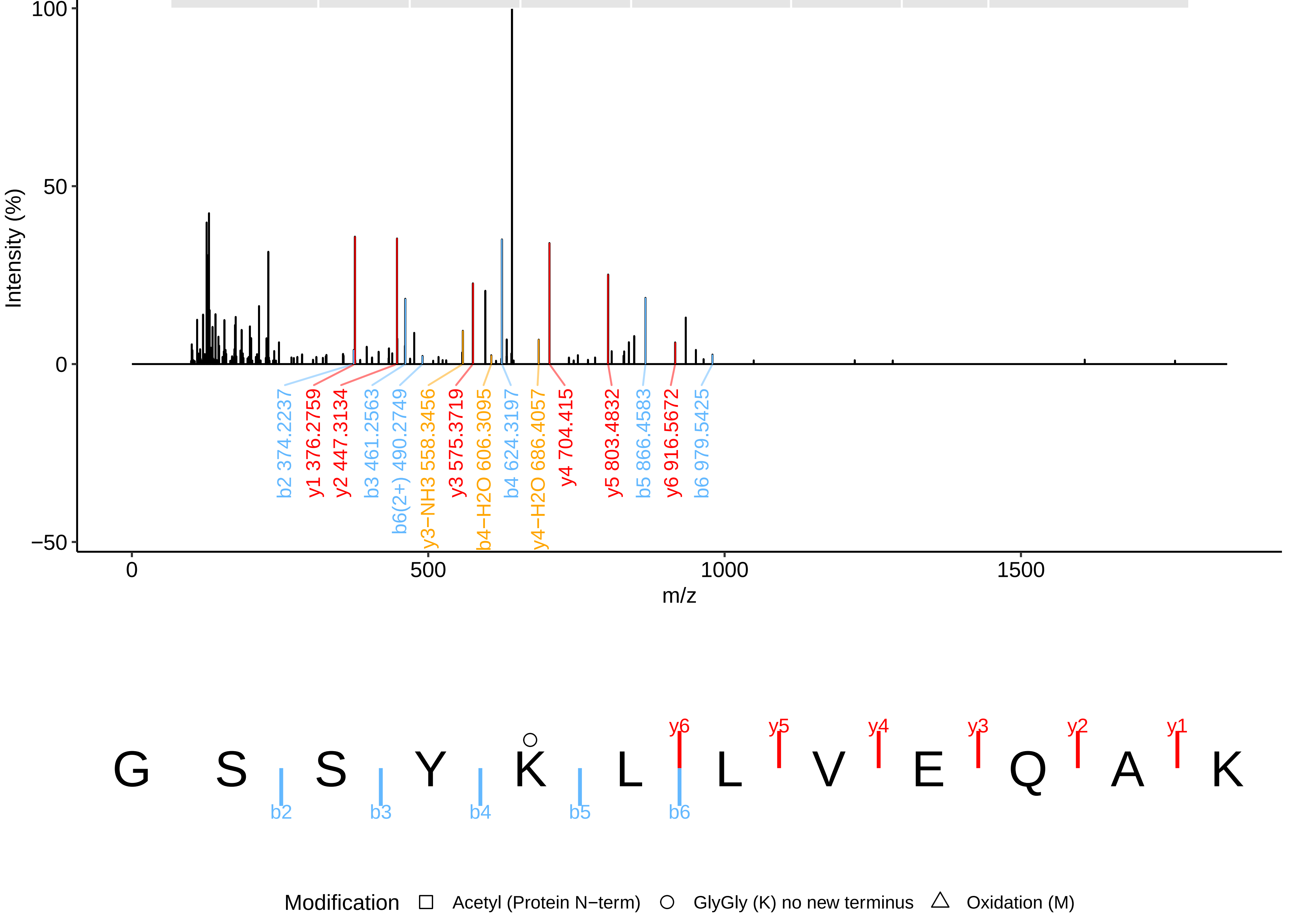

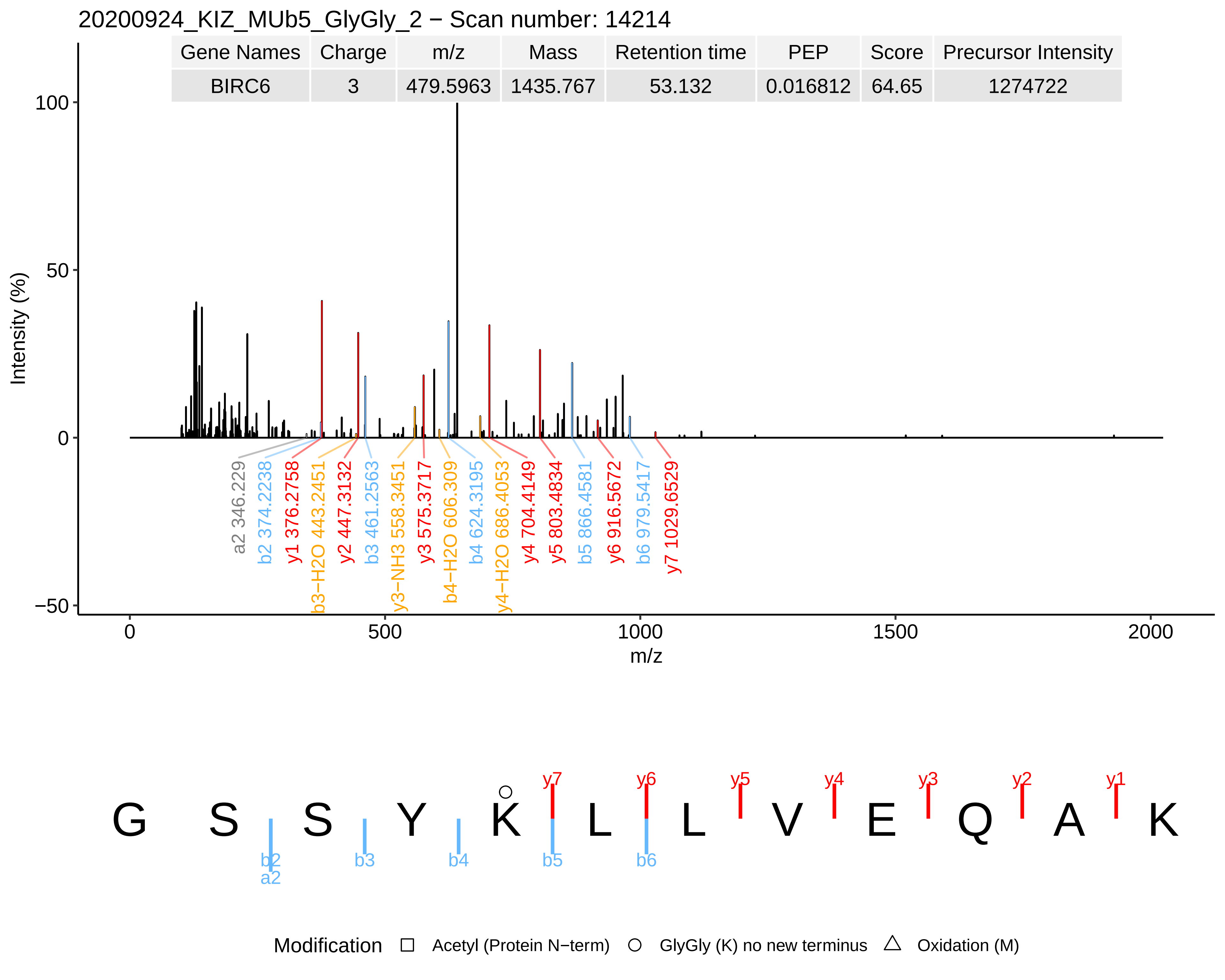
20200924_KIZ_MUb5_GlyGly_2 − Scan number: 14214
Gene Names | Charge | m/z | Mass | Retention time | PEP | Score | Precursor Intensity
BIRC6 | 3 | 479.5963 | 1435.767 | 53.132 | 0.016812 | 64.65 | 1274722
Intensity (%)
100
50
0
−50
a2 346.229
b2 374.2238
y1 376.2758
b3−H2O 443.2451
y2 447.3132
b3 461.2563
y3−NH3 558.3451
y3 575.3717
b4−H2O 606.309
b4 624.3195
y4−H2O 686.4053
y4 704.4149
y5 803.4834
b5 866.4581
y6 916.5672
b6 979.5417
y7 1029.6529
0
500
1000
1500
2000
m/z
G S S Y K L L V E Q A K
y7 y6 y5 y4 y3 y2 y1
b2 a2 b3 b4 b5 b6
Modification
Acetyl (Protein N−term)
GlyGly (K) no new terminus
Oxidation (M)

20200921_KIZ_MUb4_GlyGly_10plex − Scan number: 26007

Gene Names	Charge	m/z	Mass	Retention time	PEP	Score	Precursor Intensity
UBE2T	3	470.611	1408.811	94.675	0.0013803	84.17	4185040

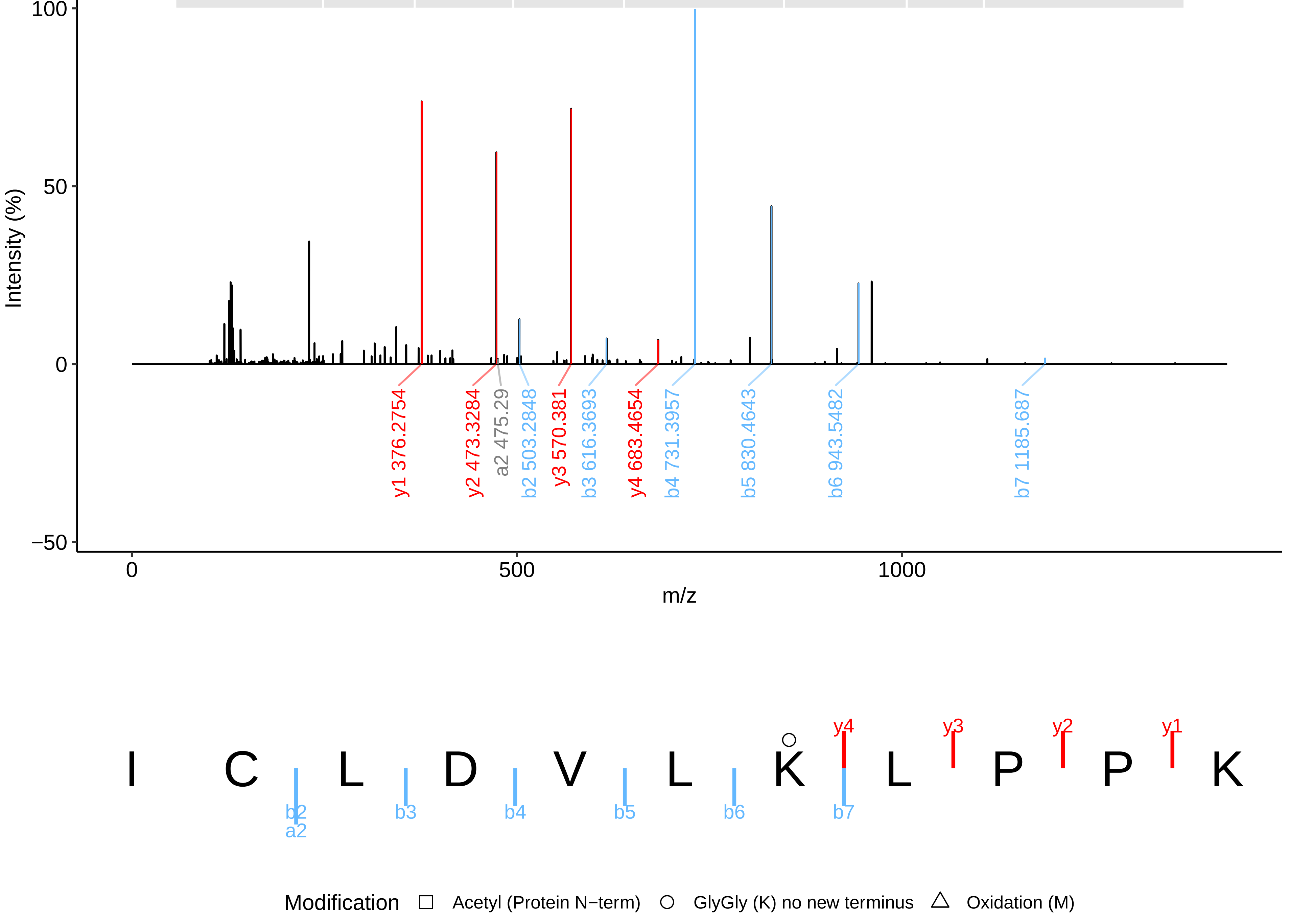

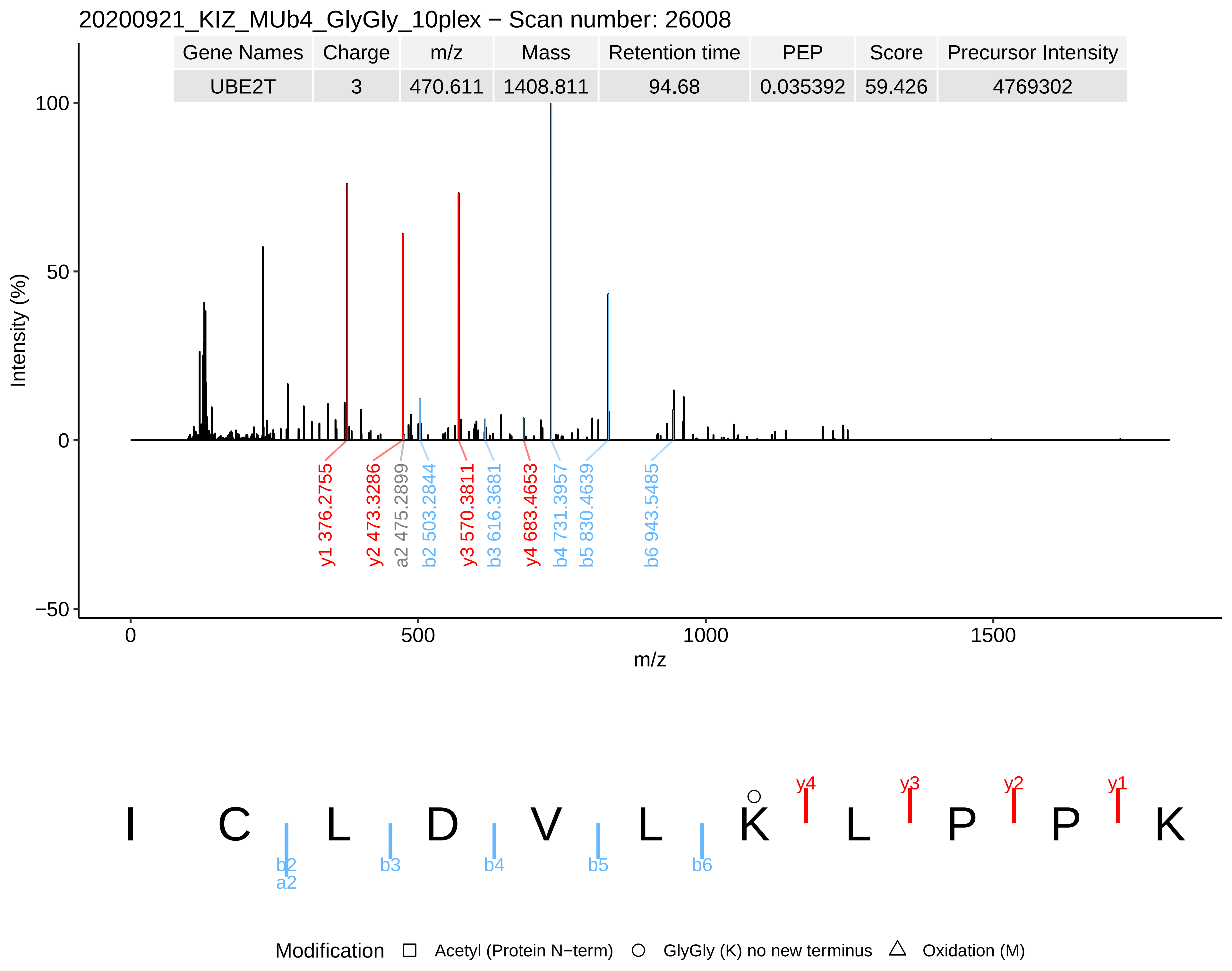
20200921_KIZ_MUb4_GlyGly_10plex − Scan number: 26008
Gene Names
Charge
m/z
Mass
Retention time
PEP
Score
Precursor Intensity
UBE2T
3
470.611
1408.811
94.68
0.035392
59.426
4769302
Intensity (%)
100
50
0
−50
0
500
1000
1500
m/z
y1 376.2755
y2 473.3286
a2 475.2899
b2 503.2844
y3 570.3811
b3 616.3681
y4 683.4653
b4 731.3957
b5 830.4639
b6 943.5485
I
C
L
D
V
L
K
L
P
P
K
y4
y3
y2
y1
b2
a2
b3
b4
b5
b6
Modification
Acetyl (Protein N−term)
GlyGly (K) no new terminus
Oxidation (M)

20200921_KIZ_MUb4_GlyGly_10plex − Scan number: 26144

Gene Names	Charge	m/z	Mass	Retention time	PEP	Score	Precursor Intensity
UBE2T	3	470.611	1408.811	95.194	0.0013803	84.17	3044096

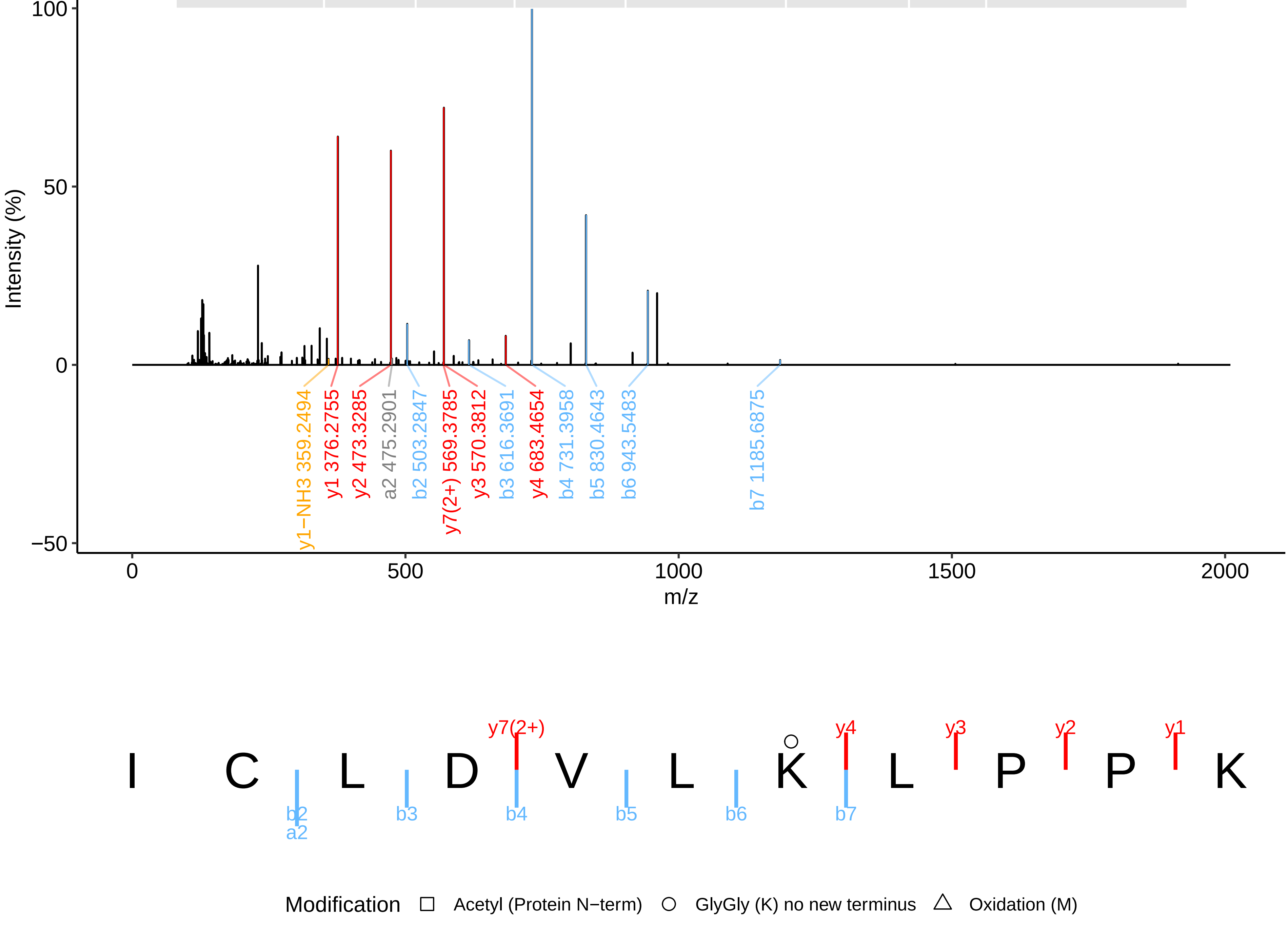

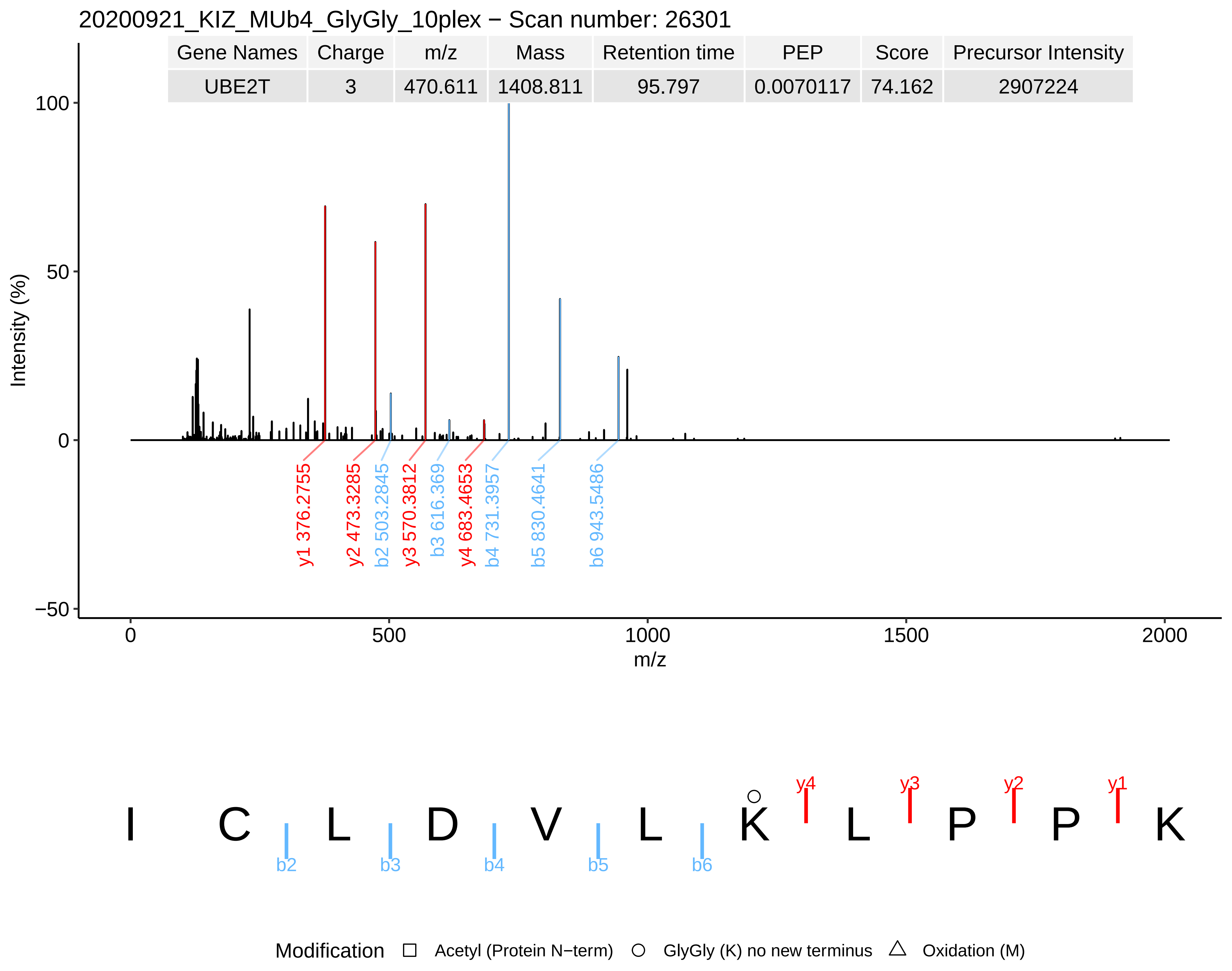

20200921_KIZ_MUb4_GlyGly_10plex − Scan number: 26301
Gene Names | Charge | m/z | Mass | Retention time | PEP | Score | Precursor Intensity
UBE2T | 3 | 470.611 | 1408.811 | 95.797 | 0.0070117 | 74.162 | 2907224
Intensity (%)
m/z
y1 376.2755
y2 473.3285
b2 503.2845
y3 570.3812
b3 616.369
y4 683.4653
b4 731.3957
b5 830.4641
b6 943.5486
I C L D V L K L P P K
Modification
Acetyl (Protein N−term)
GlyGly (K) no new terminus
Oxidation (M)

20200921_KIZ_MUb4_GlyGly_10plex − Scan number: 25862

Gene Names	Charge	m/z	Mass	Retention time	PEP	Score	Precursor Intensity
UBE2T	3	470.611	1408.811	94.151	0.011867	72.006	NA

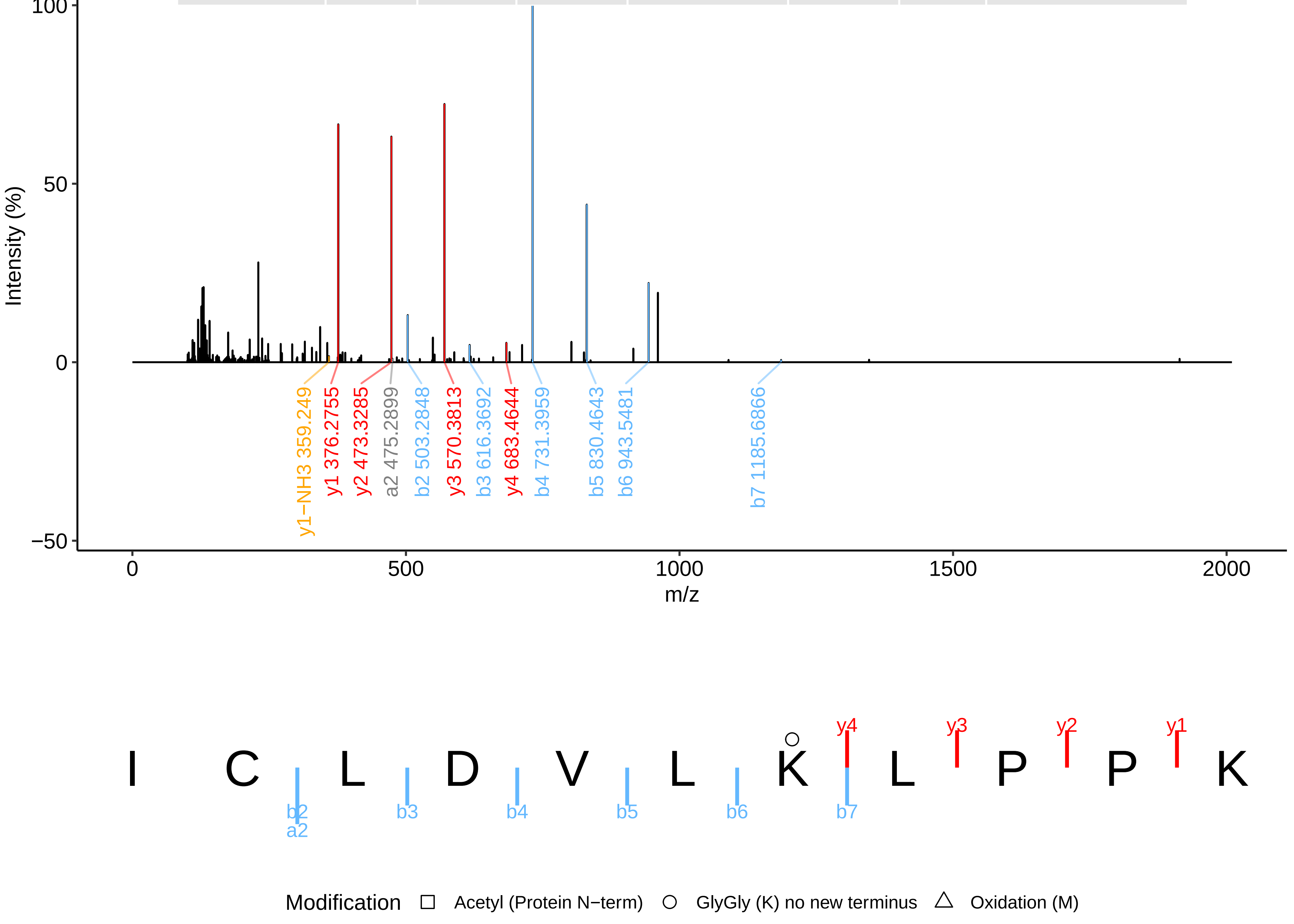

20200921_KIZ_MUb4_GlyGly_10plex_2 − Scan number: 22867

Gene Names	Charge	m/z	Mass	Retention time	PEP	Score	Precursor Intensity
UBE2T	3	470.611	1408.811	90.202	0.025911	62.303	945142.6

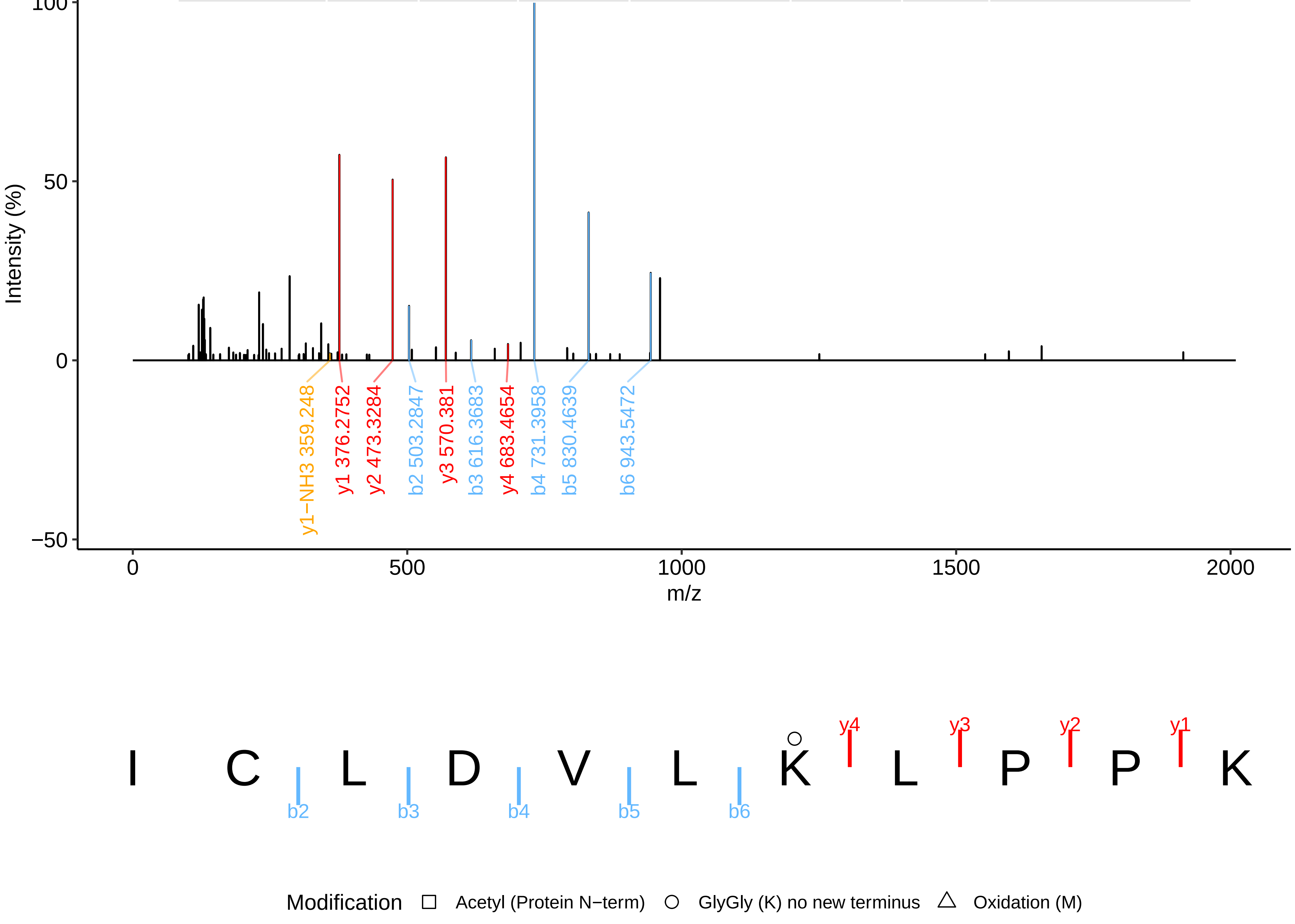

20200921_KIZ_MUb4_GlyGly_10plex_2 − Scan number: 23272

Gene Names	Charge	m/z	Mass	Retention time	PEP	Score	Precursor Intensity
UBE2T	3	470.611	1408.811	91.84	0.025911	62.303	520426

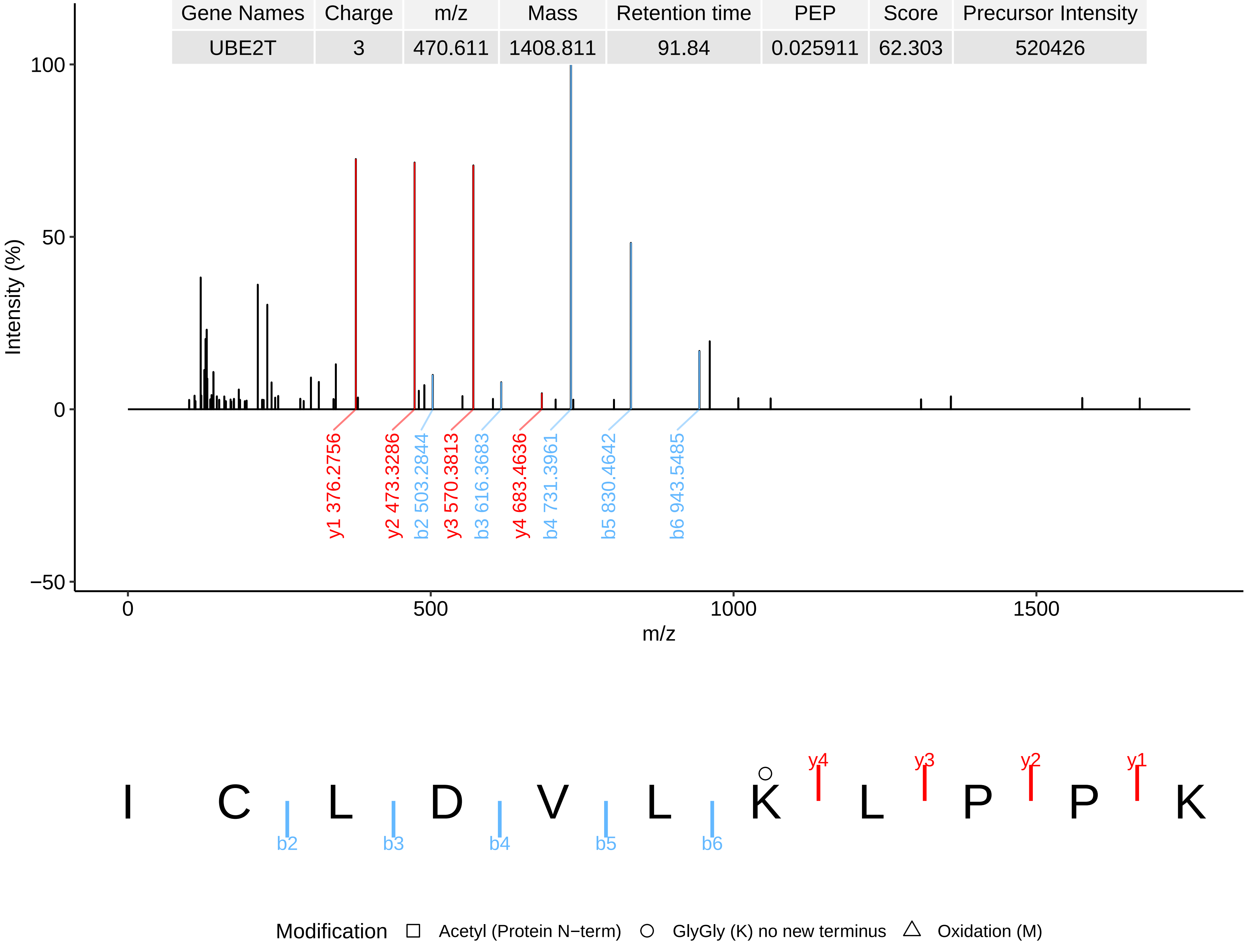

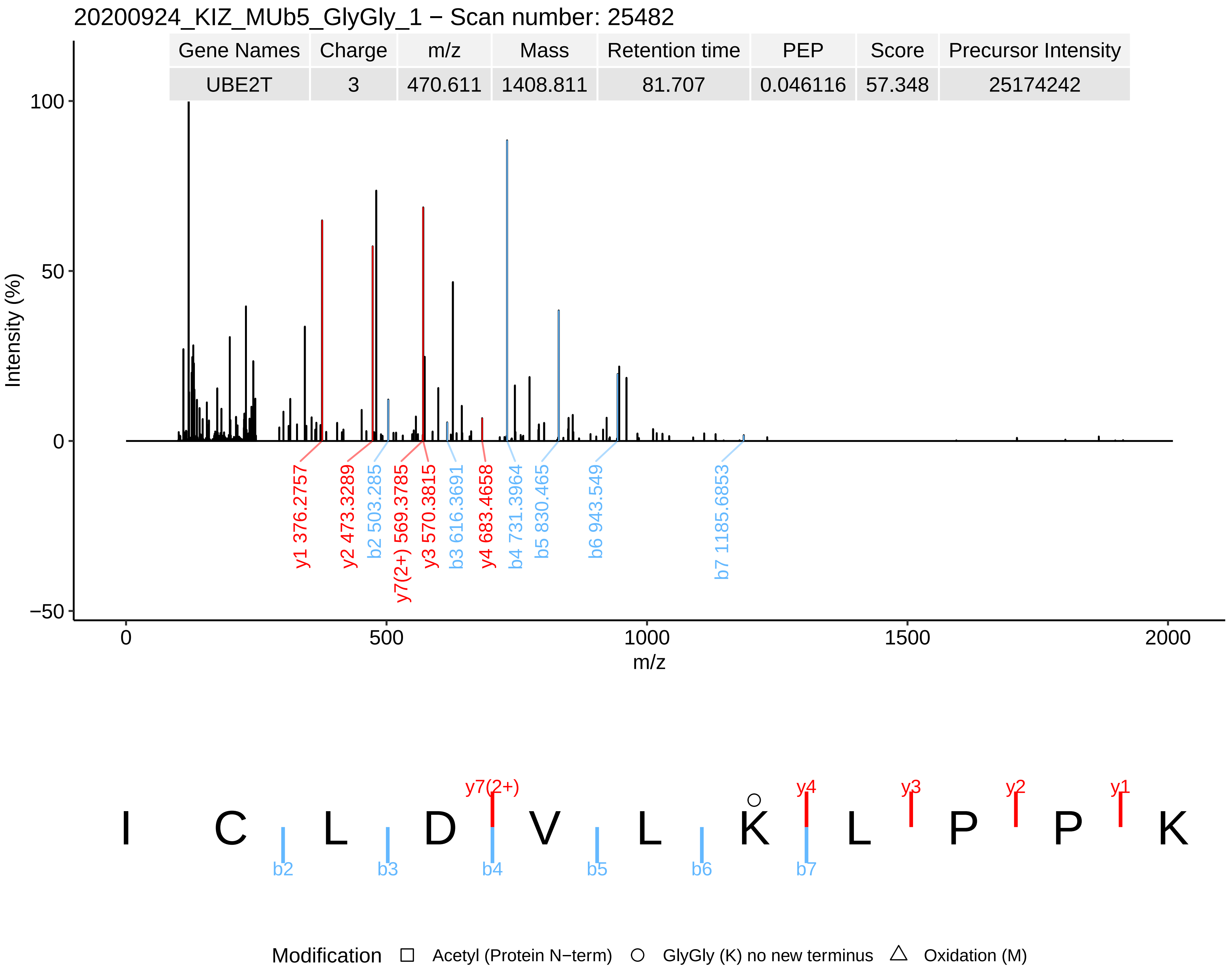

20200924_KIZ_MUb5_GlyGly_1 − Scan number: 25482
Gene Names | Charge | m/z | Mass | Retention time | PEP | Score | Precursor Intensity
UBE2T | 3 | 470.611 | 1408.811 | 81.707 | 0.046116 | 57.348 | 25174242
Intensity (%)
100
50
0
−50
0
500
1000
1500
2000
m/z
y1 376.2757
y2 473.3289
b2 503.285
y7(2+) 569.3785
y3 570.3815
b3 616.3691
y4 683.4658
b4 731.3964
b5 830.465
b6 943.549
b7 1185.6853
I C L D V L K L P P K
y7(2+) y4 y3 y2 y1
b2 b3 b4 b5 b6 b7
Modification
Acetyl (Protein N−term)
GlyGly (K) no new terminus
Oxidation (M)

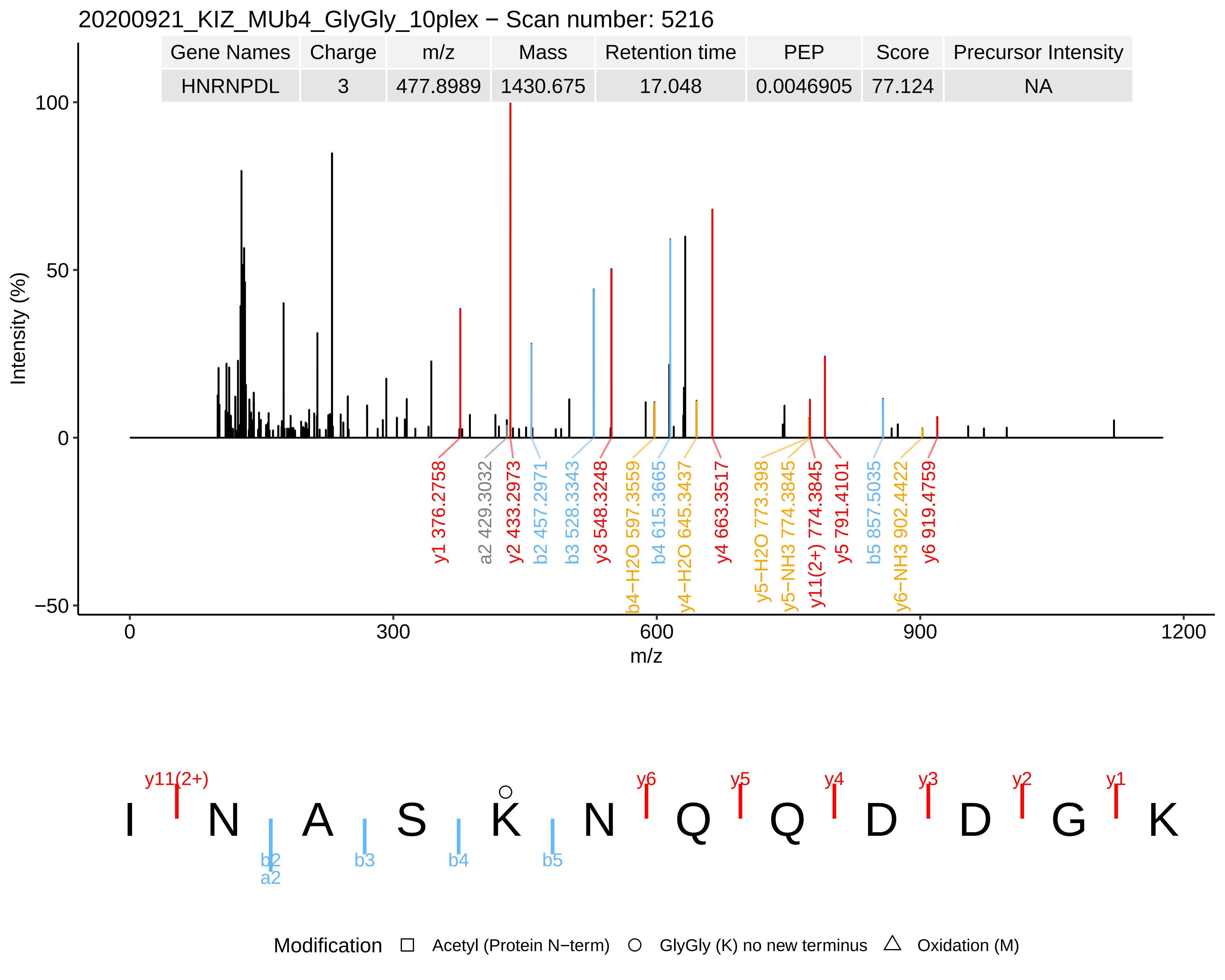
20200921_KIZ_MUb4_GlyGly_10plex − Scan number: 5216
Gene Names | Charge | m/z | Mass | Retention time | PEP | Score | Precursor Intensity
HNRNPDL | 3 | 477.8989 | 1430.675 | 17.048 | 0.0046905 | 77.124 | NA
Intensity (%)
100
50
0
−50
0
300
600
900
1200
m/z
y1 376.2758
a2 429.3032
y2 433.2973
b2 457.2971
b3 528.3343
y3 548.3248
b4−H2O 597.3559
b4 615.3665
y4−H2O 645.3437
y4 663.3517
y5−H2O 773.398
y5−NH3 774.3845
y11(2+) 774.3845
y5 791.4101
b5 857.5035
y6−NH3 902.4422
y6 919.4759
y11(2+)
y6
y5
y4
y3
y2
y1
I N A S K N Q Q D D G K
b2
a2
b3
b4
b5
Modification
Acetyl (Protein N−term)
GlyGly (K) no new terminus
Oxidation (M)

20200921_KIZ_MUb4_GlyGly_10plex − Scan number: 7207

Gene Names	Charge	m/z	Mass	Retention time	PEP	Score	Precursor Intensity
RPS27A;UBA52;UBC;UBB	3	546.6129	1636.817	24.108	0.00055491	89.858	5598568

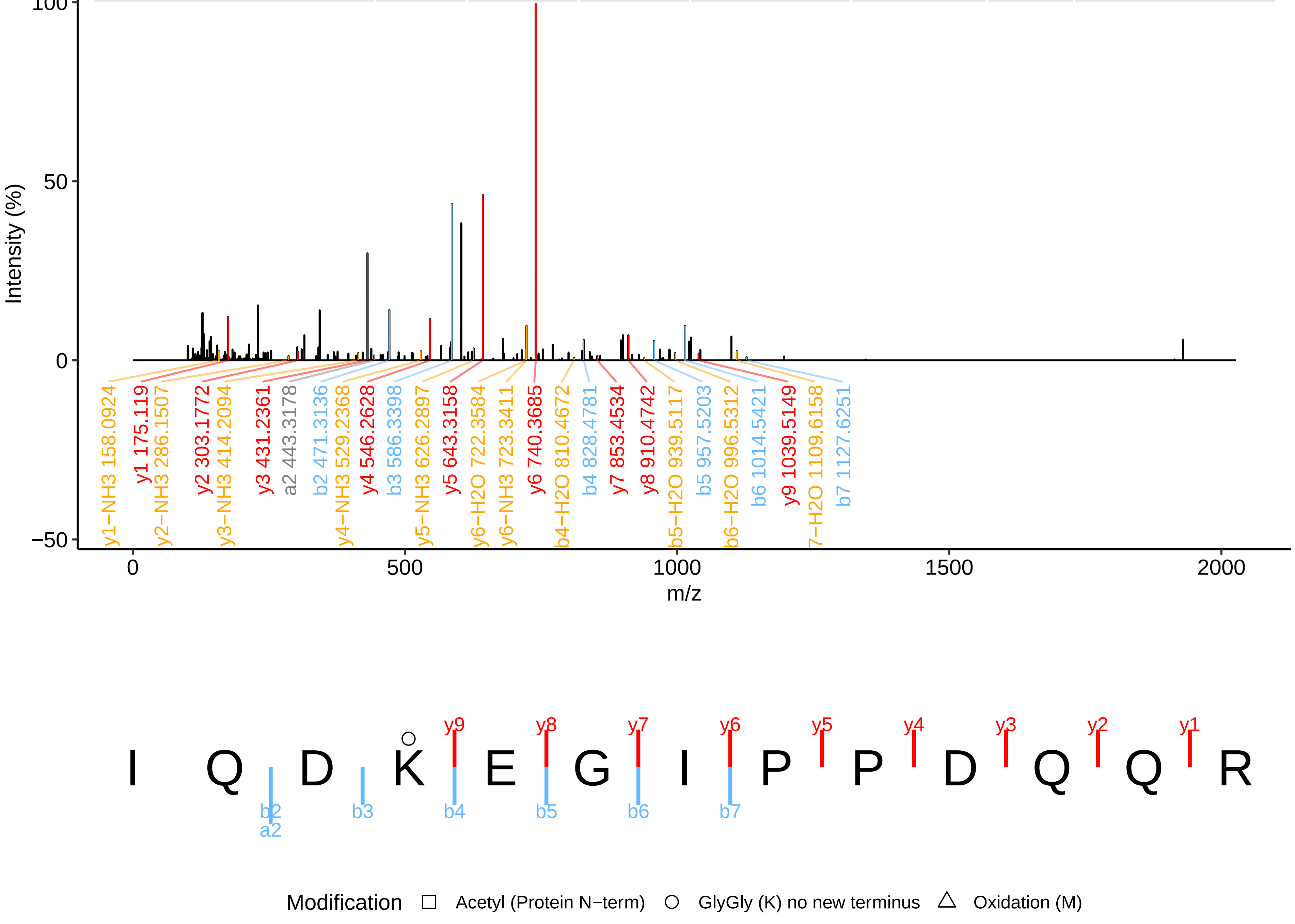

20200921_KIZ_MUb4_GlyGly_10plex − Scan number: 7481

Gene Names	Charge	m/z	Mass	Retention time	PEP	Score	Precursor Intensity
RPS27A;UBA52;UBC;UBB	3	546.6129	1636.817	25.118	0.0010095	82.908	33032552

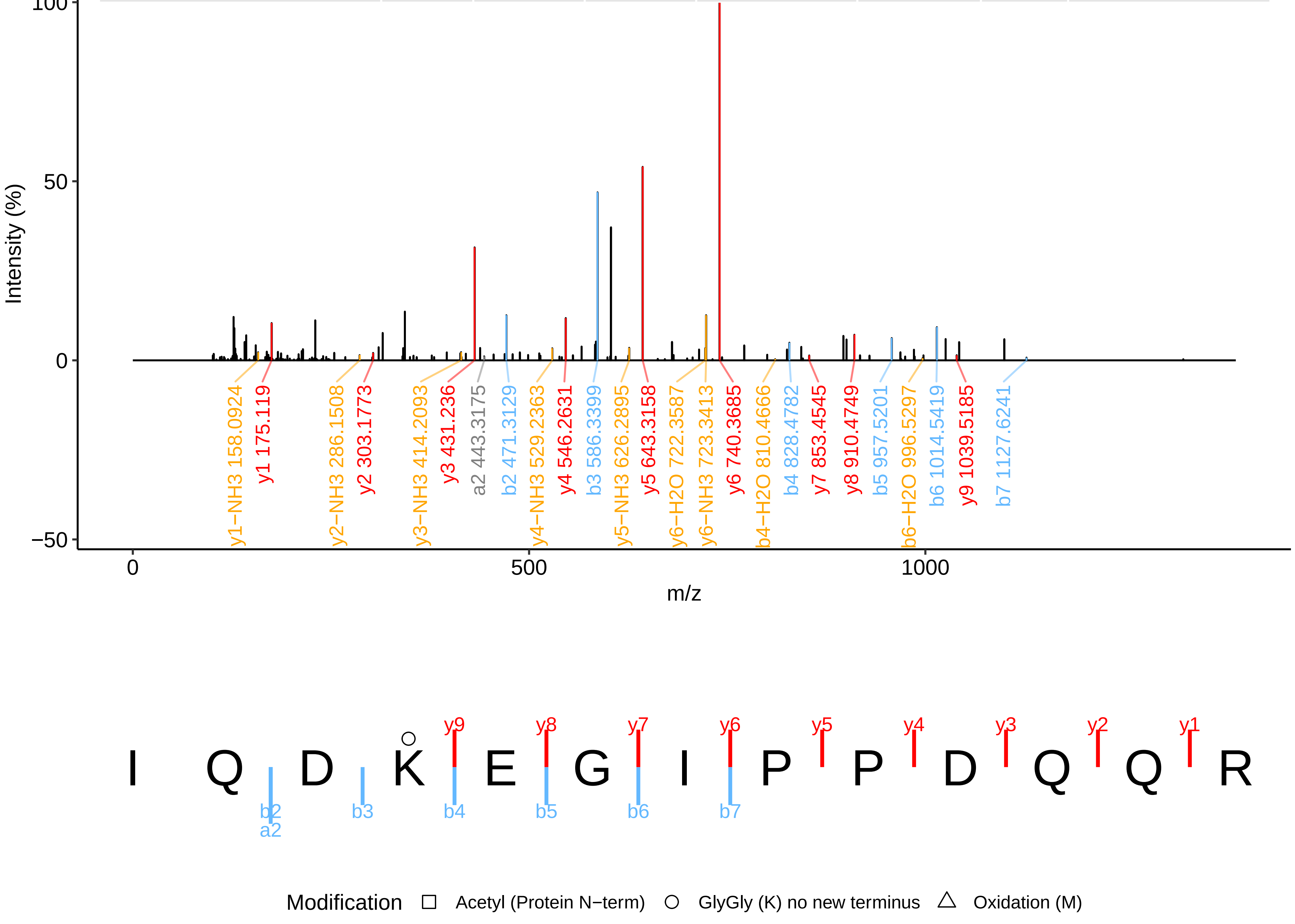

20200921_KIZ_MUb4_GlyGly_10plex − Scan number: 7623

Gene Names	Charge	m/z	Mass	Retention time	PEP	Score	Precursor Intensity
RPS27A;UBA52;UBC;UBB	3	546.6129	1636.817	25.638	0.00037233	92.65	38608940

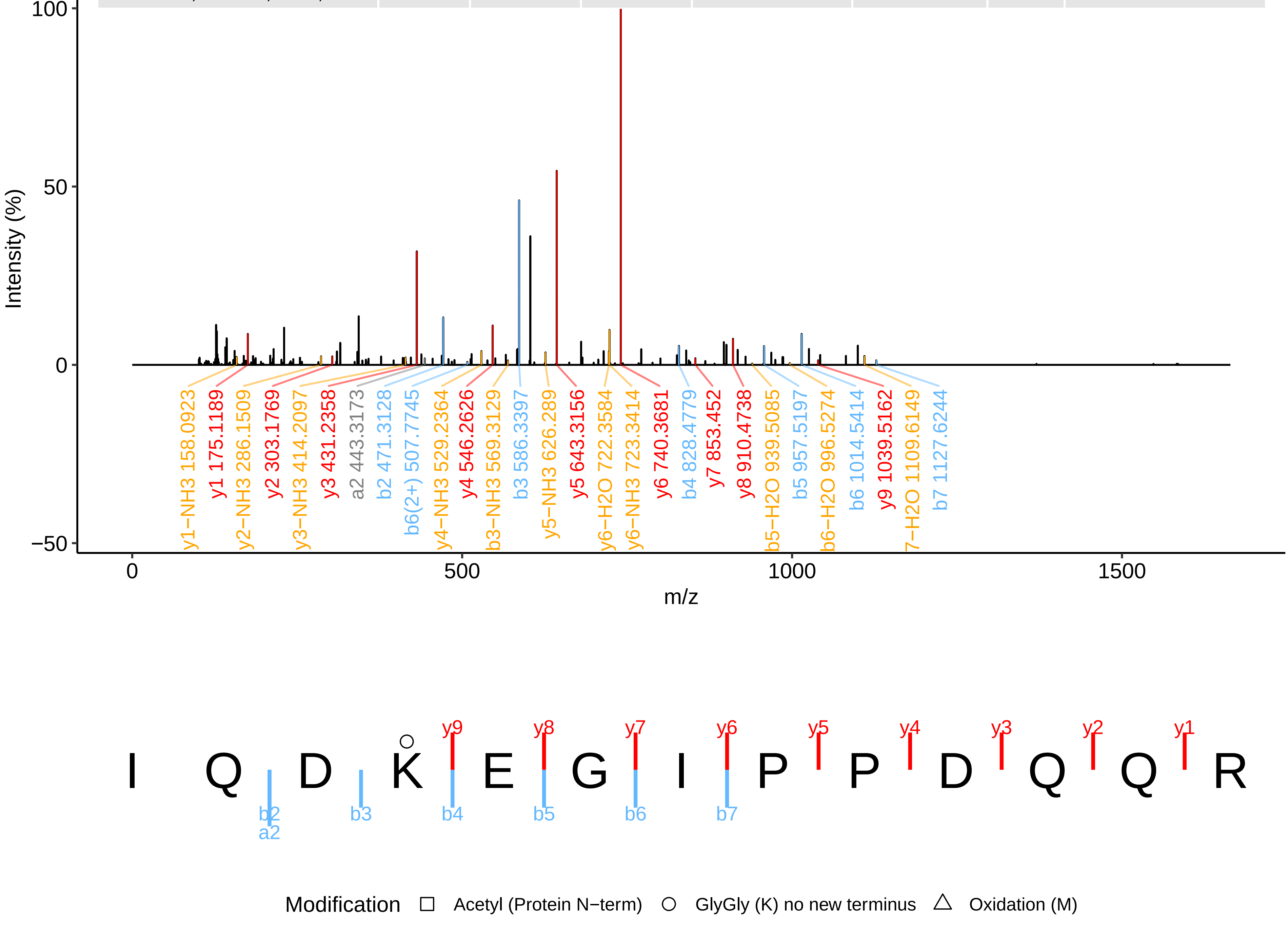

20200921_KIZ_MUb4_GlyGly_10plex − Scan number: 7758

Gene Names	Charge	m/z	Mass	Retention time	PEP	Score	Precursor Intensity
RPS27A;UBA52;UBC;UBB	3	546.6129	1636.817	26.154	0.00059407	89.26	52197064

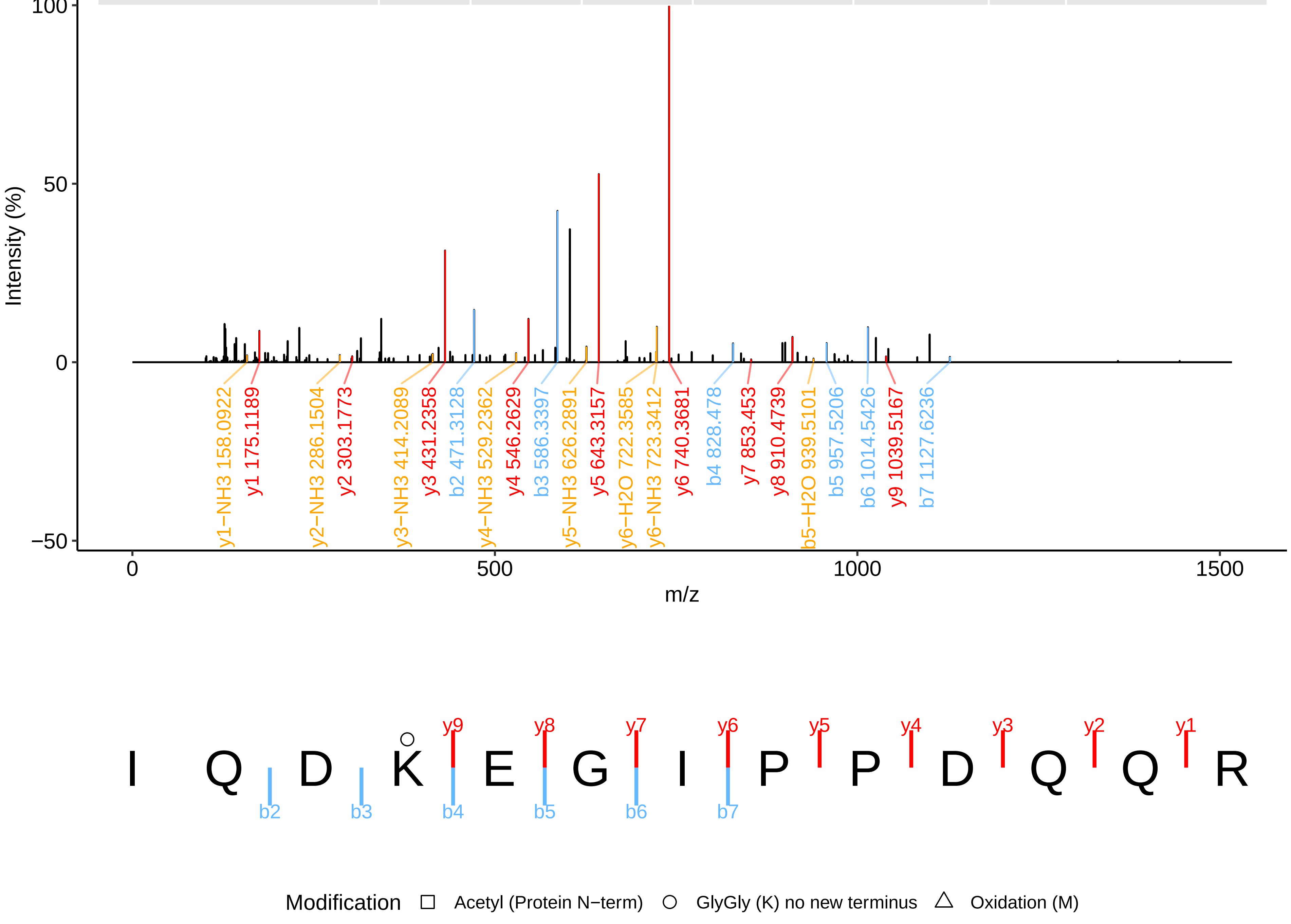

20200921_KIZ_MUb4_GlyGly_10plex − Scan number: 7895

Gene Names	Charge	m/z	Mass	Retention time	PEP	Score	Precursor Intensity
RPS27A;UBA52;UBC;UBB	3	546.6129	1636.817	26.656	0.00075898	86.738	53433404

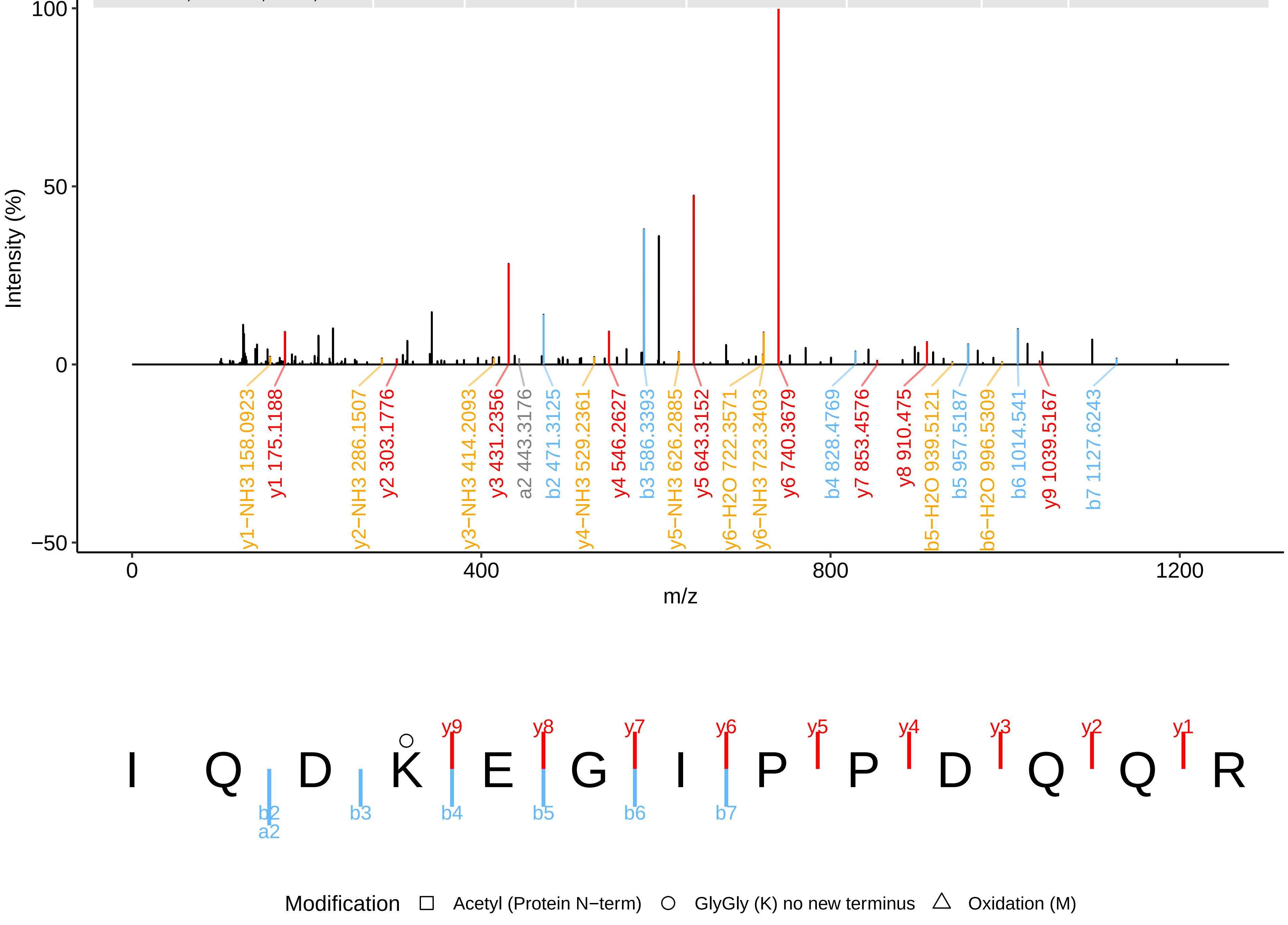

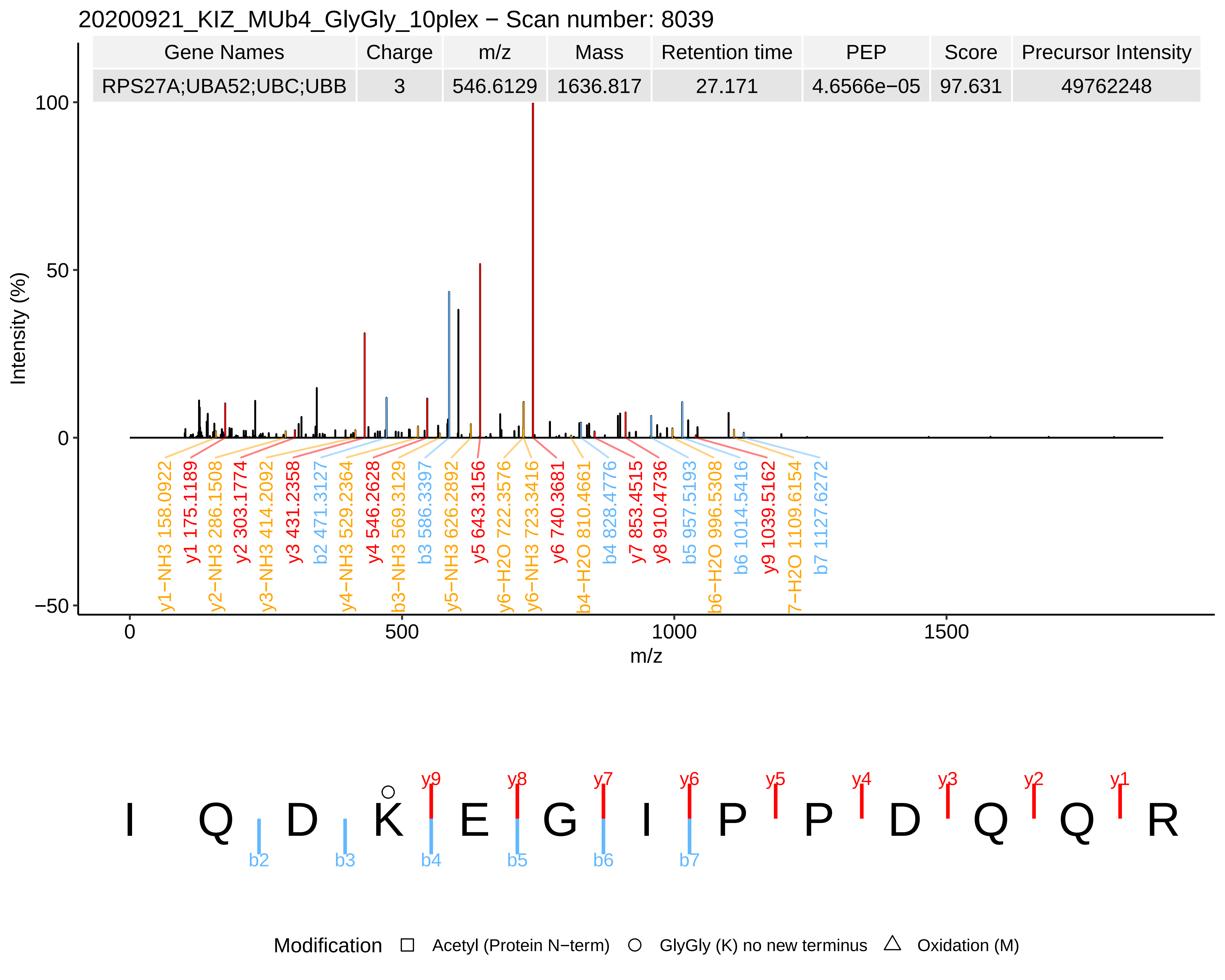
20200921_KIZ_MUb4_GlyGly_10plex − Scan number: 8039
Gene Names
Charge
m/z
Mass
Retention time
PEP
Score
Precursor Intensity
RPS27A;UBA52;UBC;UBB
3
546.6129
1636.817
27.171
4.6566e−05
97.631
49762248
Intensity (%)
100
50
0
−50
0
500
1000
1500
m/z
y1−NH3 158.0922
y1 175.1189
y2−NH3 286.1508
y2 303.1774
y3−NH3 414.2092
y3 431.2358
b2 471.3127
y4−NH3 529.2364
y4 546.2628
b3−NH3 569.3129
b3 586.3397
y5−NH3 626.2892
y5 643.3156
y6−H2O 722.3576
y6−NH3 723.3416
y6 740.3681
b4−H2O 810.4661
b4 828.4776
y7 853.4515
y8 910.4736
b5 957.5193
b6−H2O 996.5308
b6 1014.5416
y9 1039.5162
7−H2O 1109.6154
b7 1127.6272
I Q D K E G I P P D Q Q R
y9 y8 y7 y6 y5 y4 y3 y2 y1
b2 b3 b4 b5 b6 b7
Modification
Acetyl (Protein N−term)
GlyGly (K) no new terminus
Oxidation (M)

20200921_KIZ_MUb4_GlyGly_10plex − Scan number: 8180

Gene Names	Charge	m/z	Mass	Retention time	PEP	Score	Precursor Intensity
RPS27A;UBA52;UBC;UBB	3	546.6129	1636.817	27.693	1.8068e−05	101.43	36915932

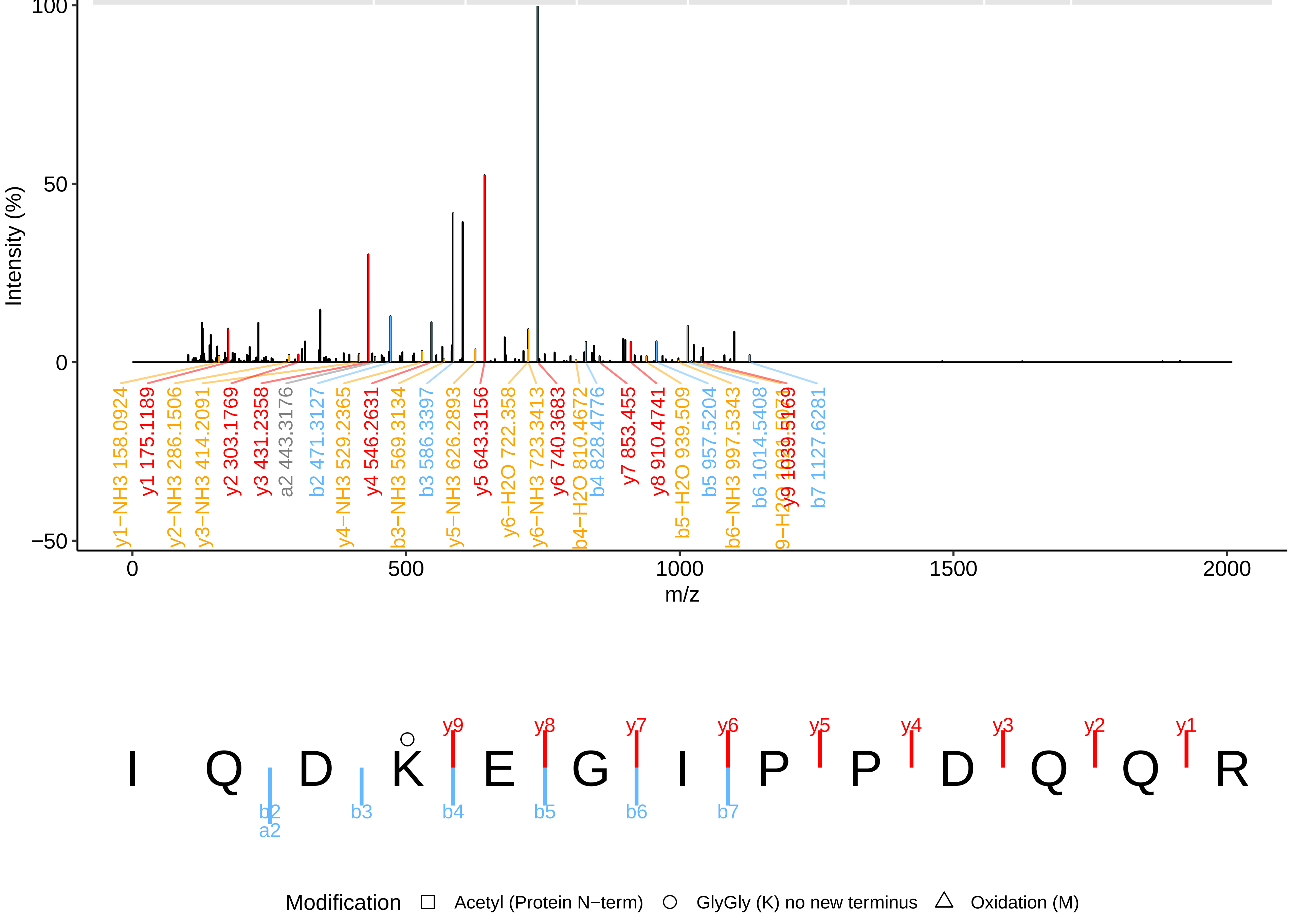

20200921_KIZ_MUb4_GlyGly_10plex − Scan number: 8323

Gene Names	Charge	m/z	Mass	Retention time	PEP	Score	Precursor Intensity
RPS27A;UBA52;UBC;UBB	3	546.6129	1636.817	28.208	4.6566e−05	97.631	32367460

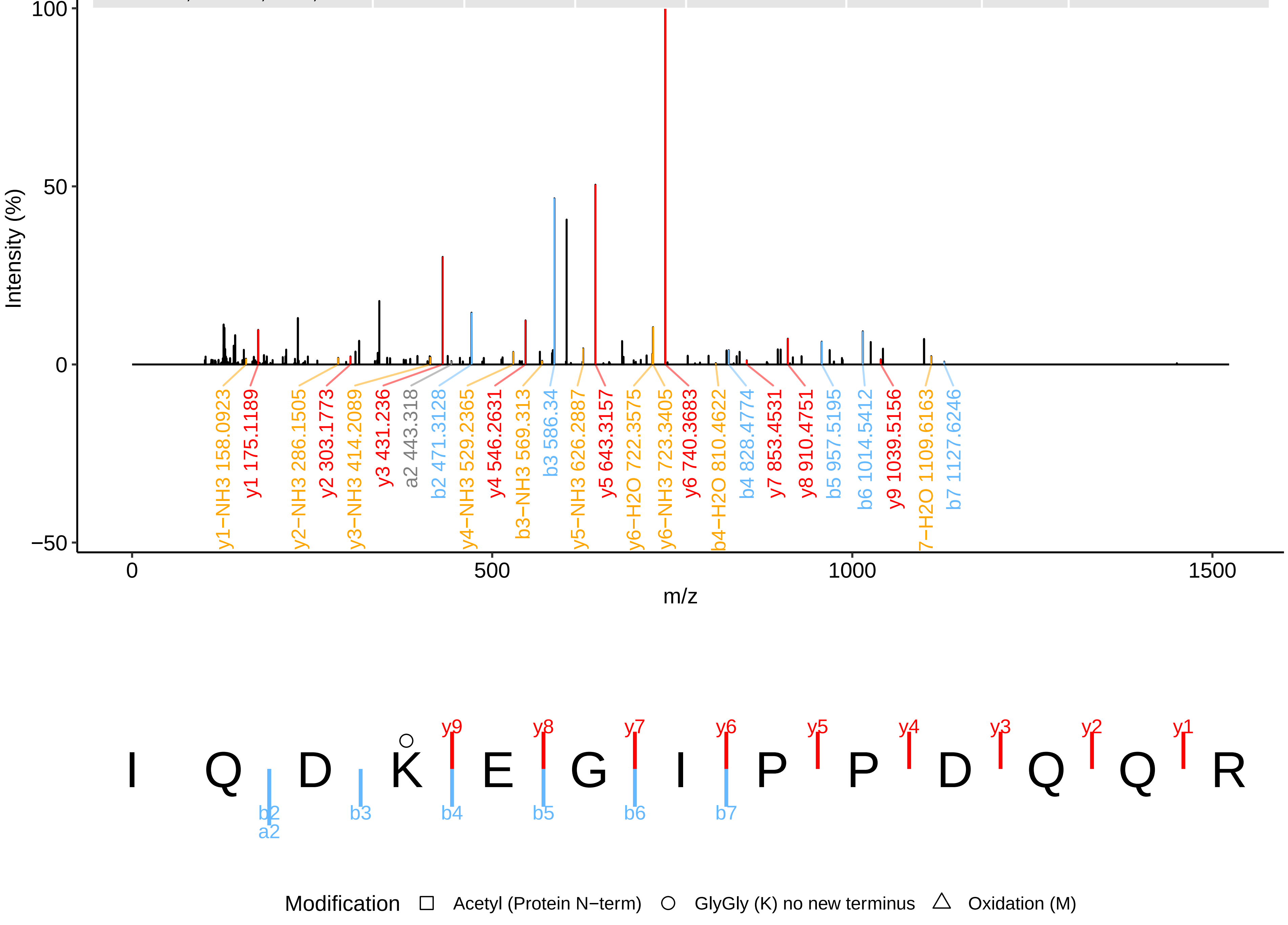

20200921_KIZ_MUb4_GlyGly_10plex − Scan number: 8601

Gene Names	Charge	m/z	Mass	Retention time	PEP	Score	Precursor Intensity
RPS27A;UBA52;UBC;UBB	3	546.6129	1636.817	29.241	4.6566e−05	97.631	18535728

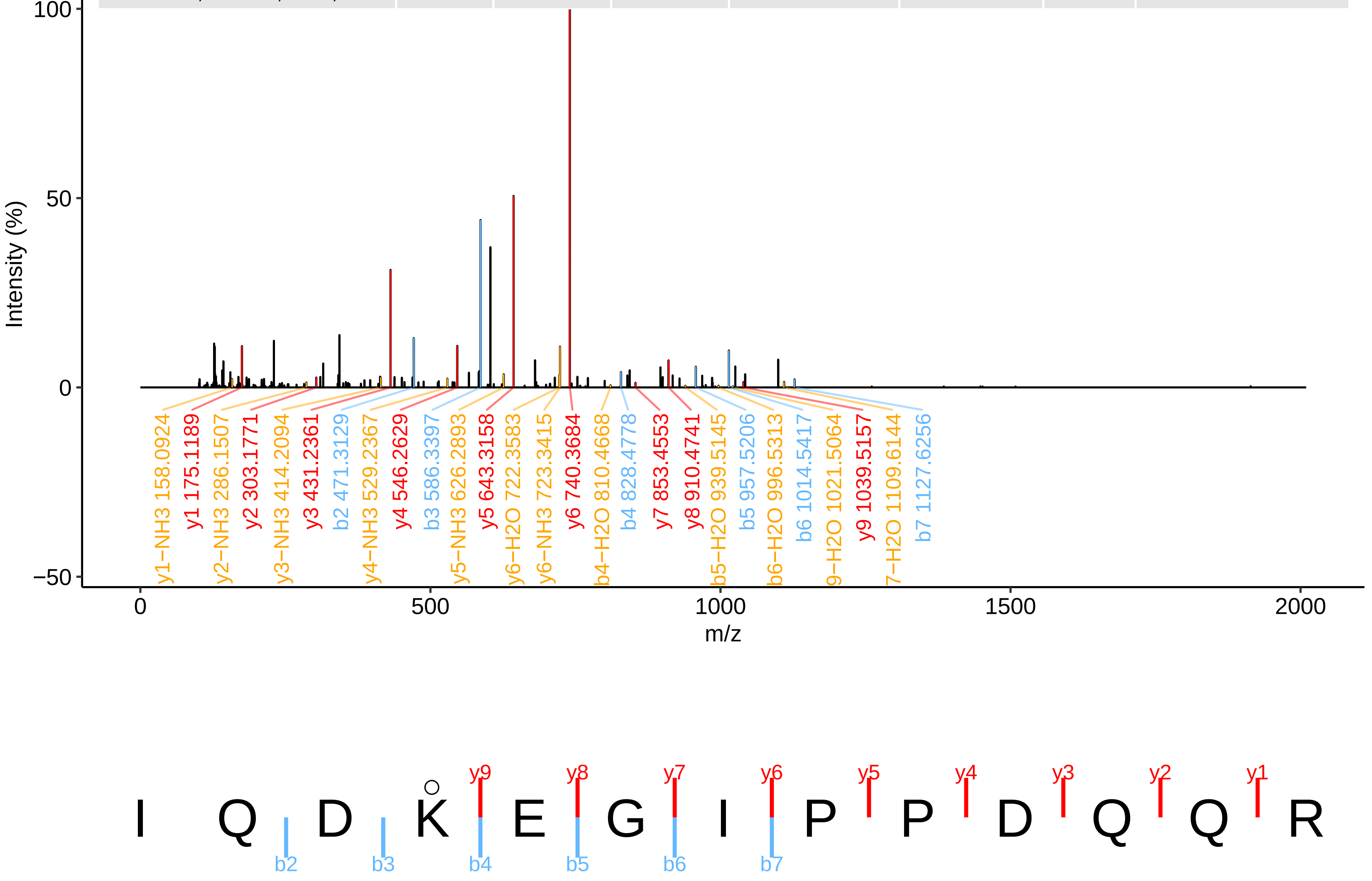

Modification □ Acetyl (Protein N−term) ○ GlyGly (K) no new terminus △ Oxidation (M)

20200921_KIZ_MUb4_GlyGly_10plex − Scan number: 8742

Gene Names	Charge	m/z	Mass	Retention time	PEP	Score	Precursor Intensity
RPS27A;UBA52;UBC;UBB	3	546.6129	1636.817	29.753	0.00037233	92.65	14195808

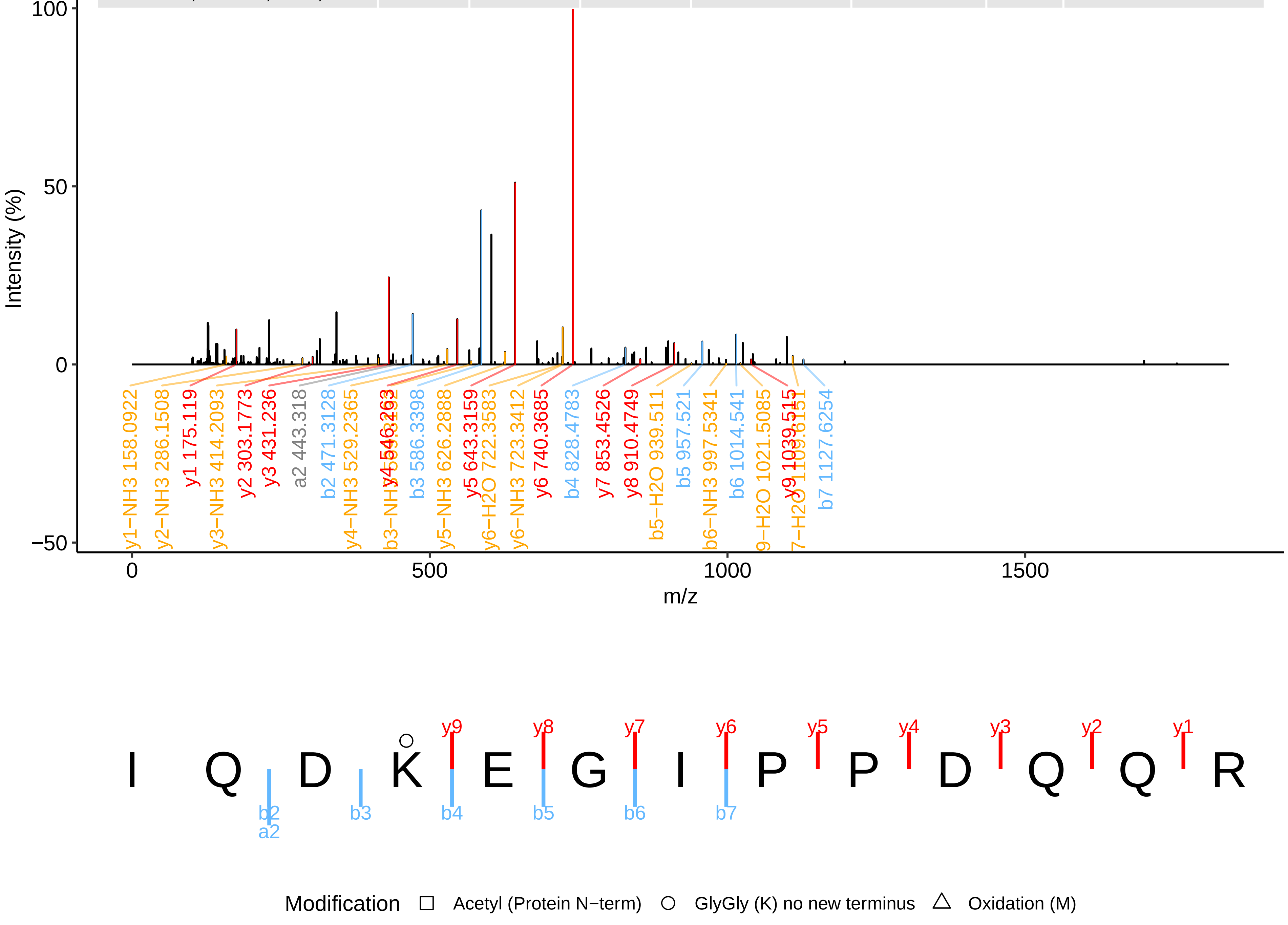

20200921_KIZ_MUb4_GlyGly_10plex − Scan number: 8878

Gene Names	Charge	m/z	Mass	Retention time	PEP	Score	Precursor Intensity
RPS27A;UBA52;UBC;UBB	3	546.6129	1636.817	30.263	0.00038494	92.457	12907870

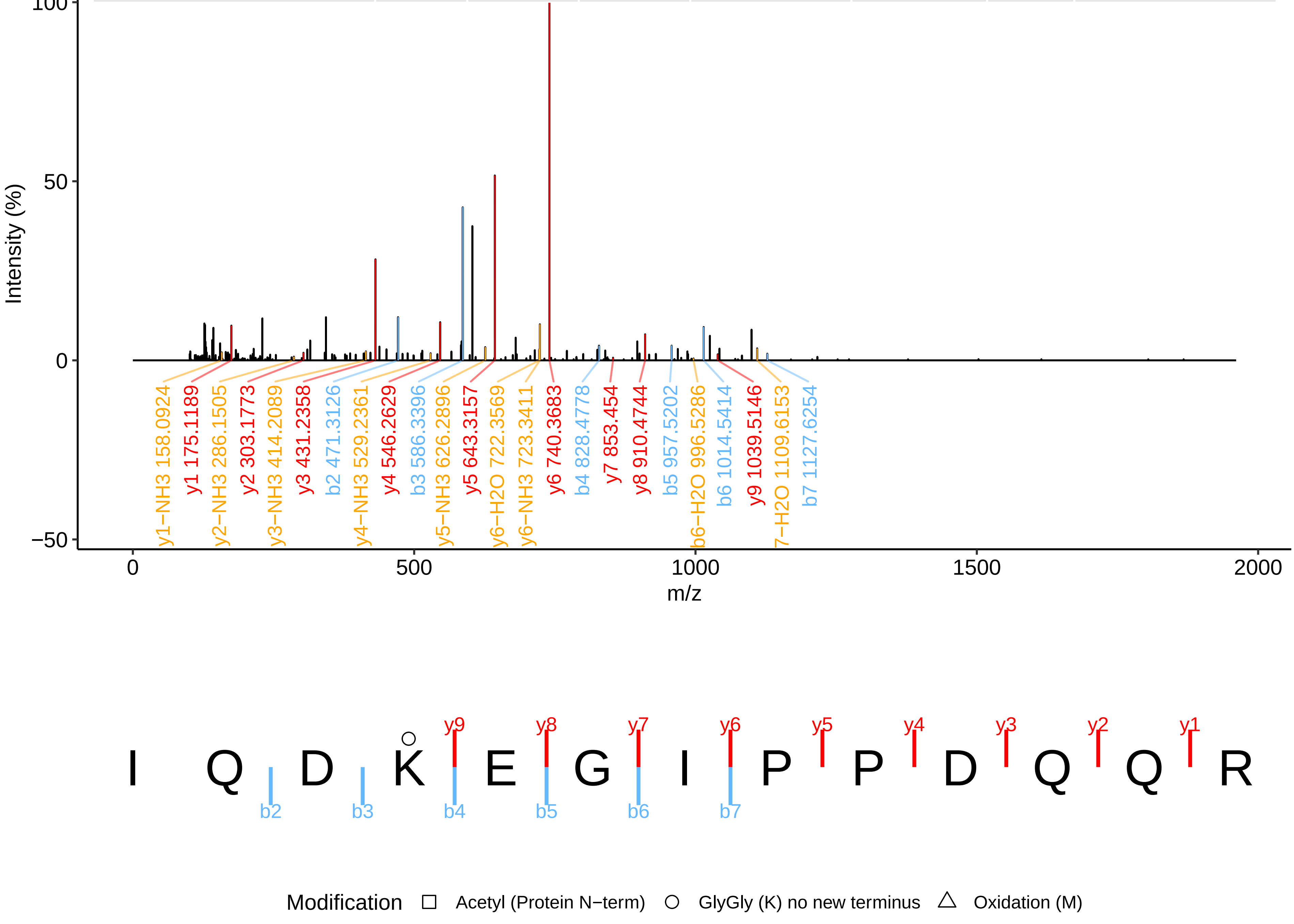

20200921_KIZ_MUb4_GlyGly_10plex − Scan number: 9073

Gene Names	Charge	m/z	Mass	Retention time	PEP	Score	Precursor Intensity
RPS27A;UBA52;UBC;UBB	3	546.6129	1636.817	31.018	0.040865	56.65	6833556

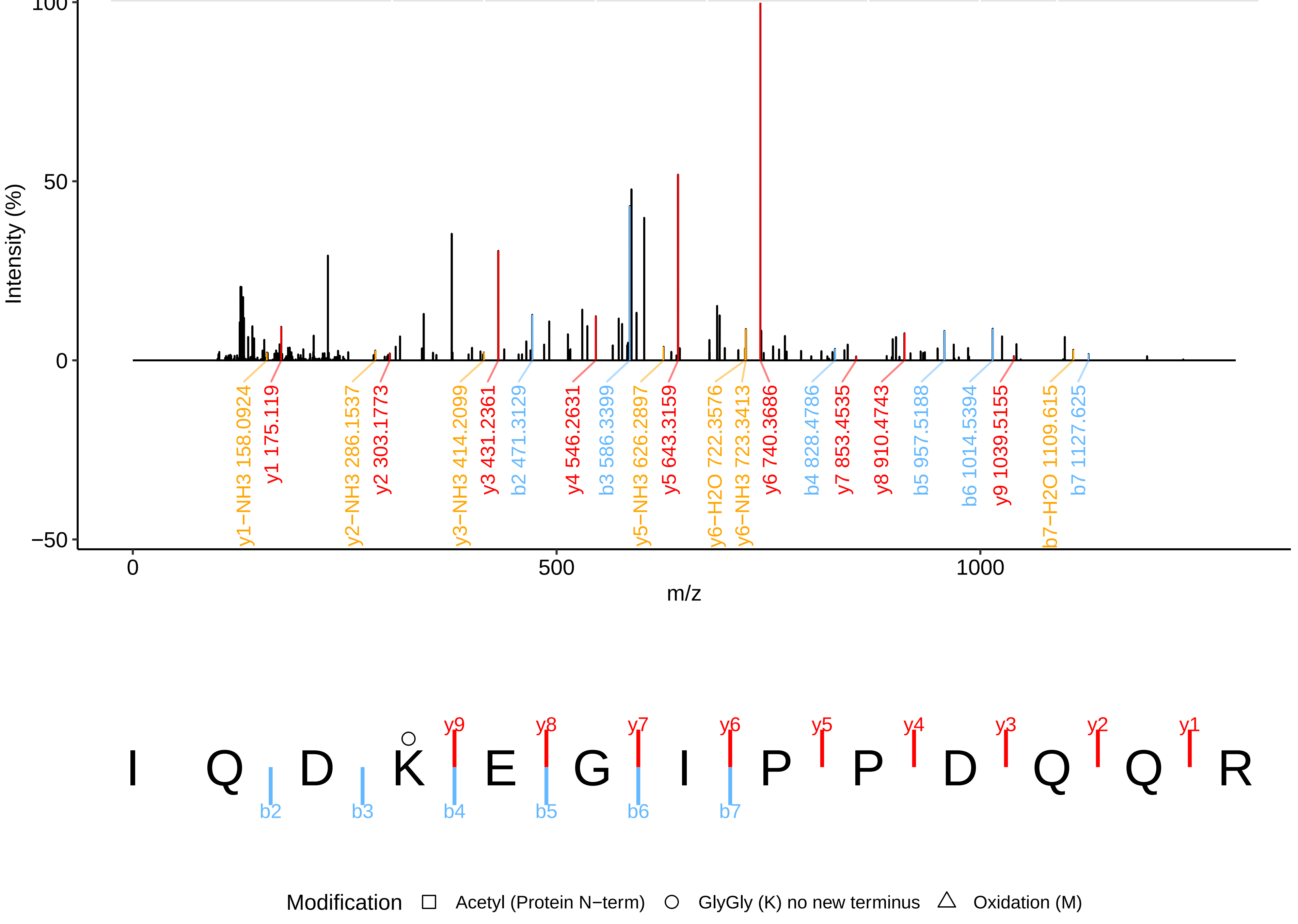

20200921_KIZ_MUb4_GlyGly_10plex − Scan number: 9220

Gene Names	Charge	m/z	Mass	Retention time	PEP	Score	Precursor Intensity
RPS27A;UBA52;UBC;UBB	3	546.6129	1636.817	31.549	0.0011222	81.185	5026621

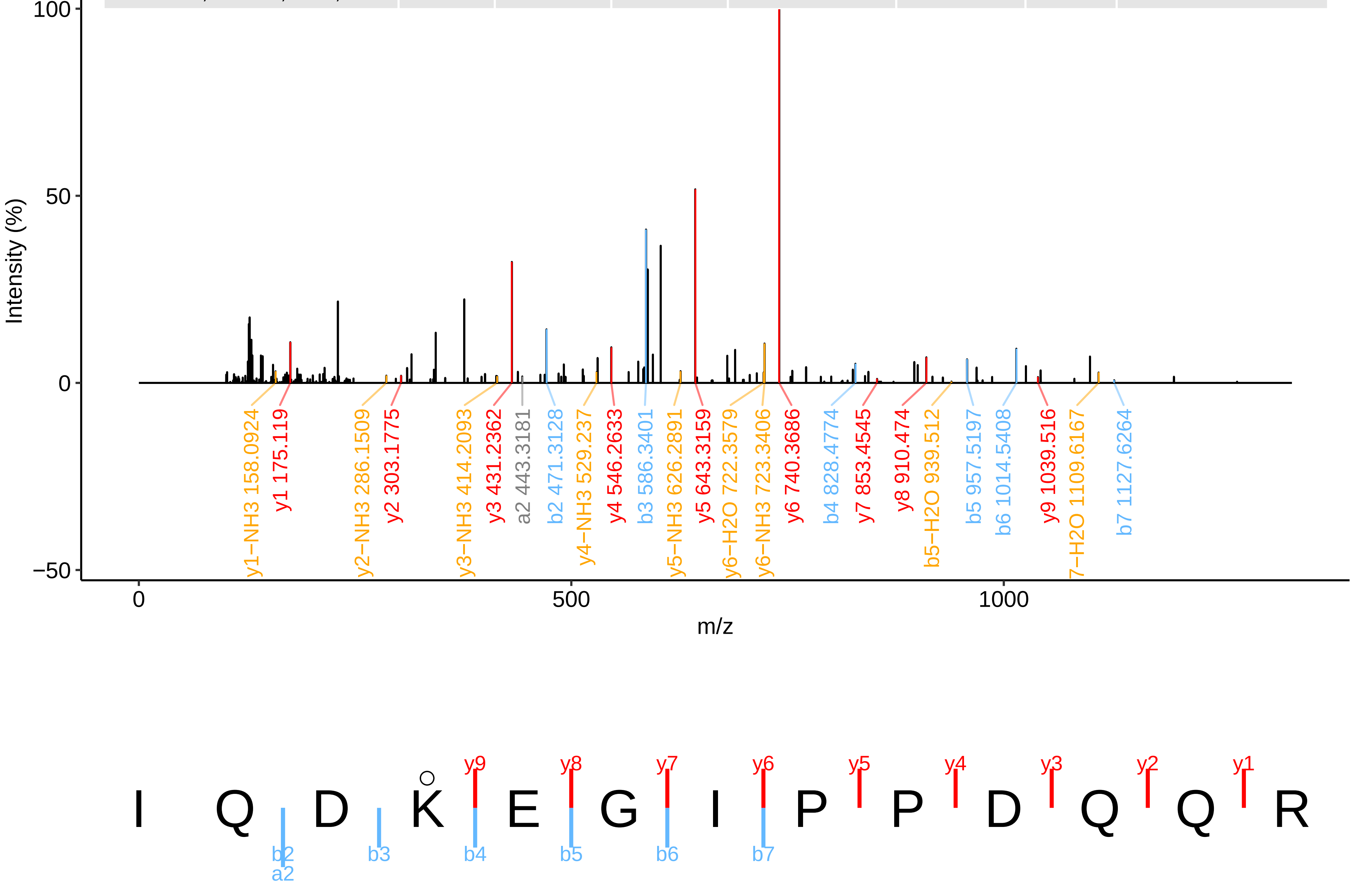

Modification □ Acetyl (Protein N−term) ○ GlyGly (K) no new terminus △ Oxidation (M)

20200921_KIZ_MUb4_GlyGly_10plex − Scan number: 9443

Gene Names	Charge	m/z	Mass	Retention time	PEP	Score	Precursor Intensity
RPS27A;UBA52;UBC;UBB	3	546.6129	1636.817	32.329	4.6566e−05	97.631	NA

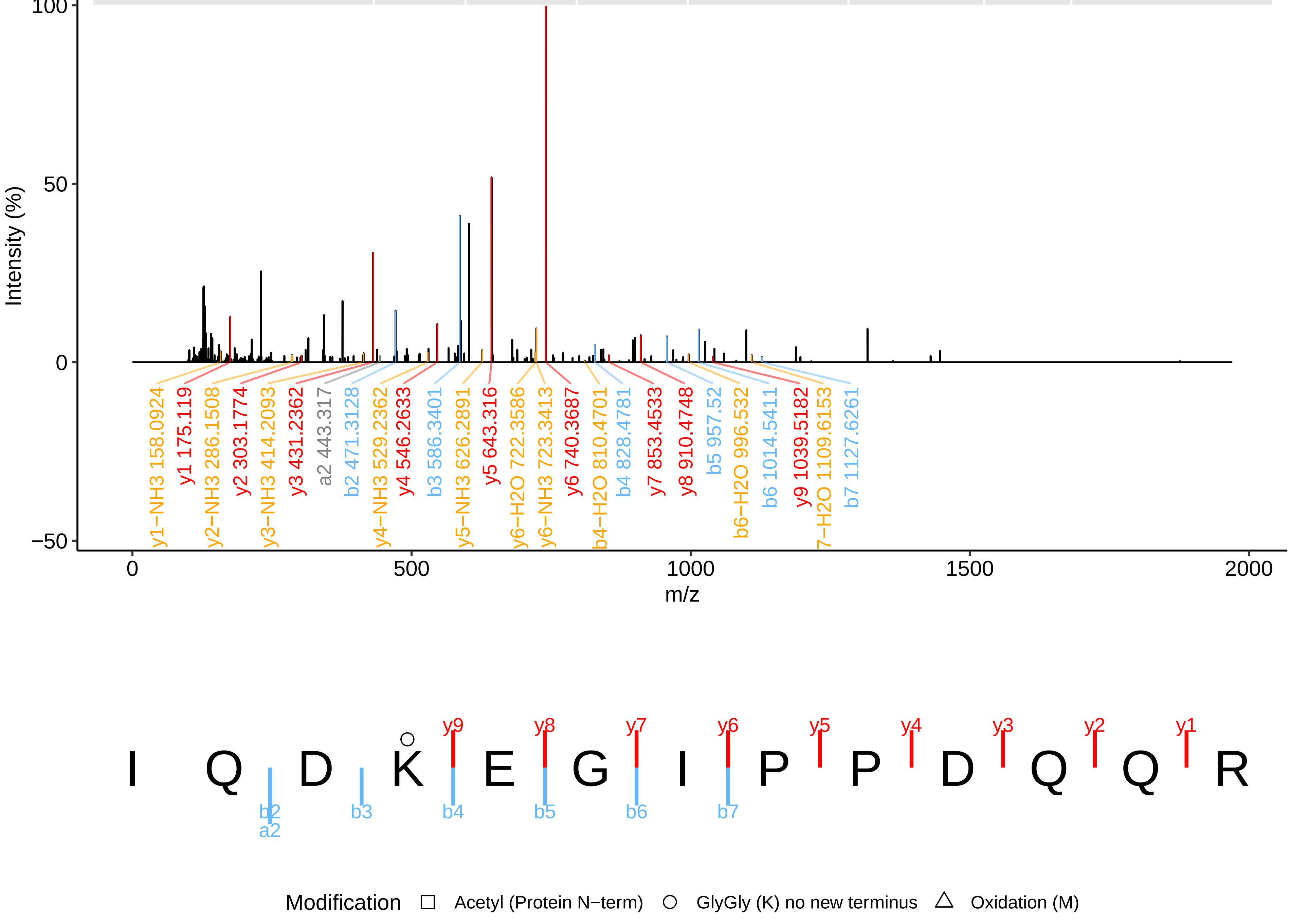

20200921_KIZ_MUb4_GlyGly_10plex_2 − Scan number: 6714

Gene Names	Charge	m/z	Mass	Retention time	PEP	Score	Precursor Intensity
RPS27A;UBA52;UBC;UBB	3	546.6129	1636.817	24.802	0.00023294	94.781	2590713

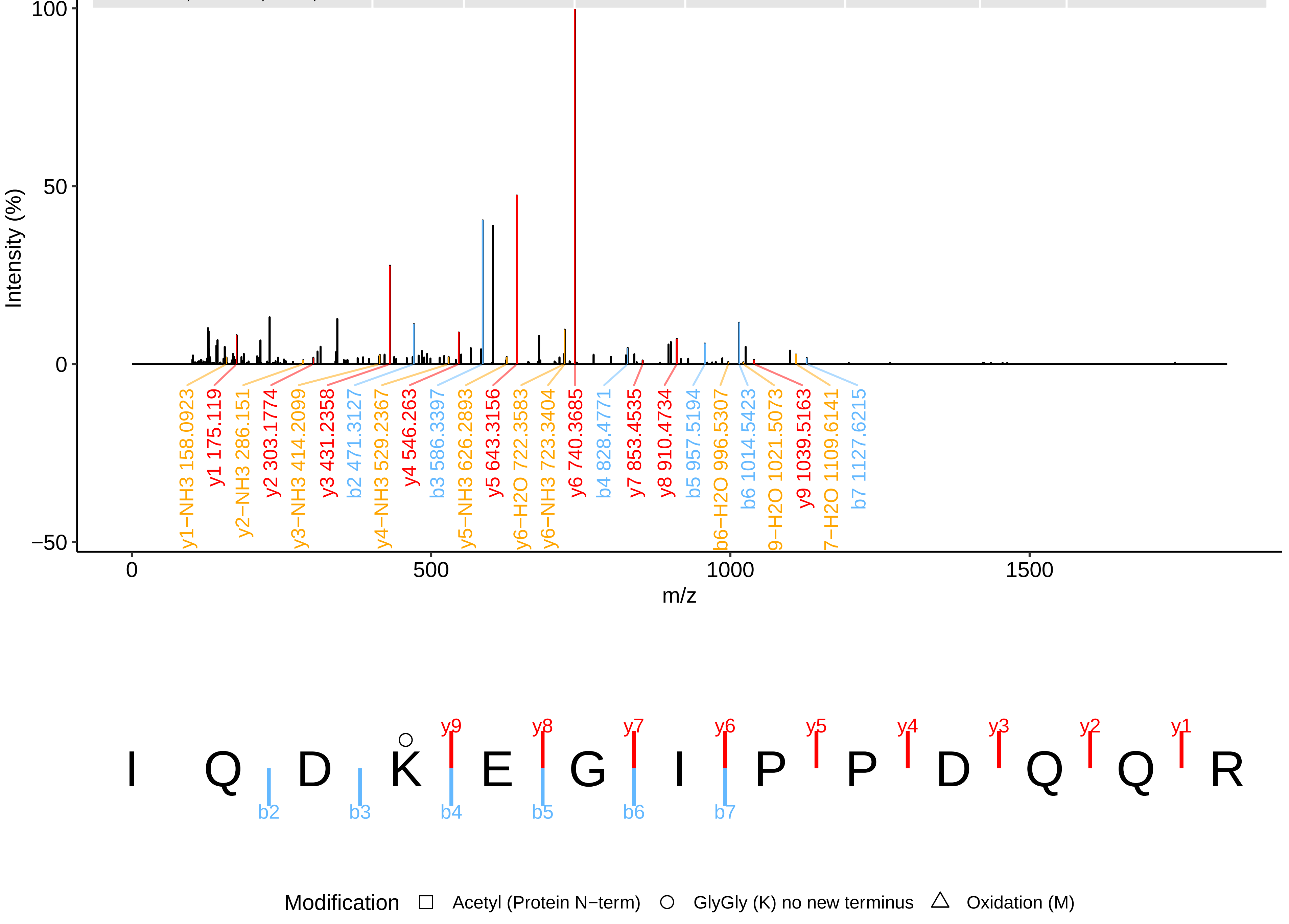

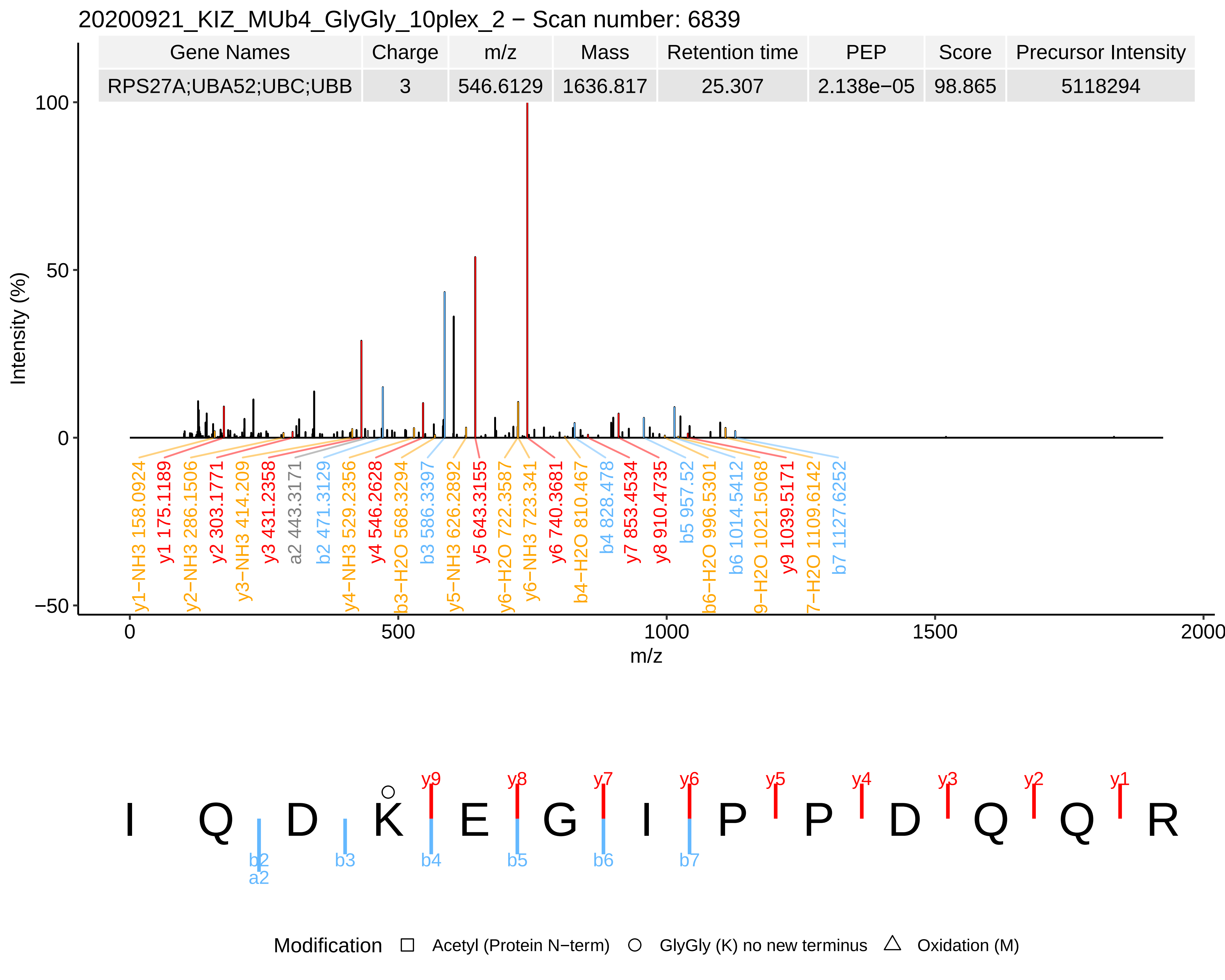
20200921_KIZ_MUb4_GlyGly_10plex_2 − Scan number: 6839
Gene Names
RPS27A;UBA52;UBC;UBB
Charge
3
m/z
546.6129
Mass
1636.817
Retention time
25.307
PEP
2.138e−05
Score
98.865
Precursor Intensity
5118294
Intensity (%)
m/z
y1−NH3 158.0924
y1 175.1189
y2−NH3 286.1506
y2 303.1771
y3−NH3 414.209
y3 431.2358
a2 443.3171
b2 471.3129
y4−NH3 529.2356
y4 546.2628
b3−H2O 568.3294
b3 586.3397
y5−NH3 626.2892
y5 643.3155
y6−H2O 722.3587
y6−NH3 723.341
y6 740.3681
b4−H2O 810.467
b4 828.478
y7 853.4534
y8 910.4735
b5 957.52
b6−H2O 996.5301
b6 1014.5412
y9−H2O 1021.5068
y9 1039.5171
y7−H2O 1109.6142
b7 1127.6252
I Q D K E G I P P D Q Q R
Modification
Acetyl (Protein N−term)
GlyGly (K) no new terminus
Oxidation (M)

20200921_KIZ_MUb4_GlyGly_10plex_2 − Scan number: 6964

Gene Names	Charge	m/z	Mass	Retention time	PEP	Score	Precursor Intensity
RPS27A;UBA52;UBC;UBB	3	546.6129	1636.817	25.813	4.6566e−05	97.631	8510416

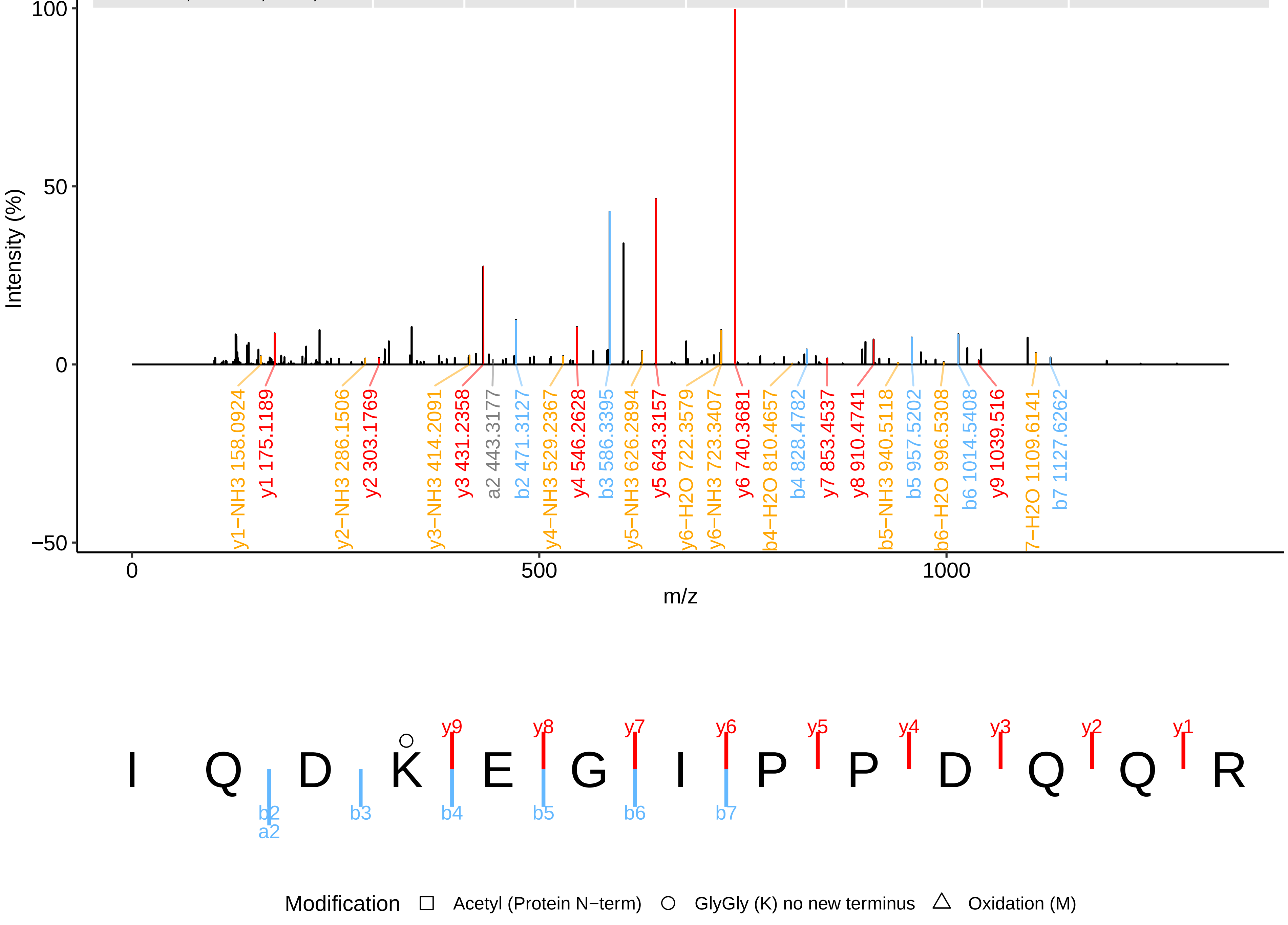

20200921_KIZ_MUb4_GlyGly_10plex_2 − Scan number: 7089

Gene Names	Charge	m/z	Mass	Retention time	PEP	Score	Precursor Intensity
RPS27A;UBA52;UBC;UBB	3	546.6129	1636.817	26.323	0.0010329	82.55	9245980

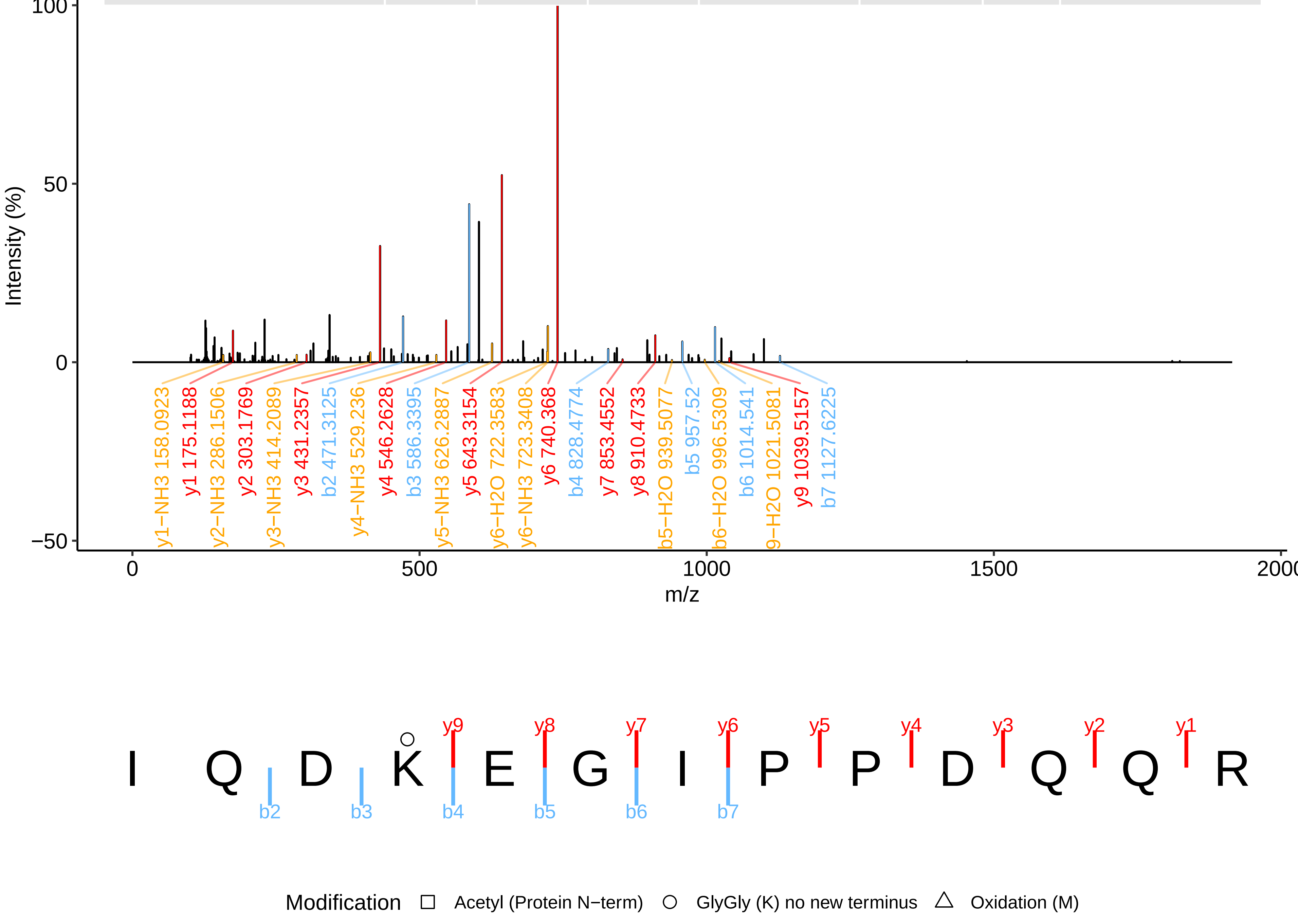

20200921_KIZ_MUb4_GlyGly_10plex_2 − Scan number: 7217

Gene Names	Charge	m/z	Mass	Retention time	PEP	Score	Precursor Intensity
RPS27A;UBA52;UBC;UBB	3	546.6129	1636.817	26.848	0.0010329	82.55	8254683

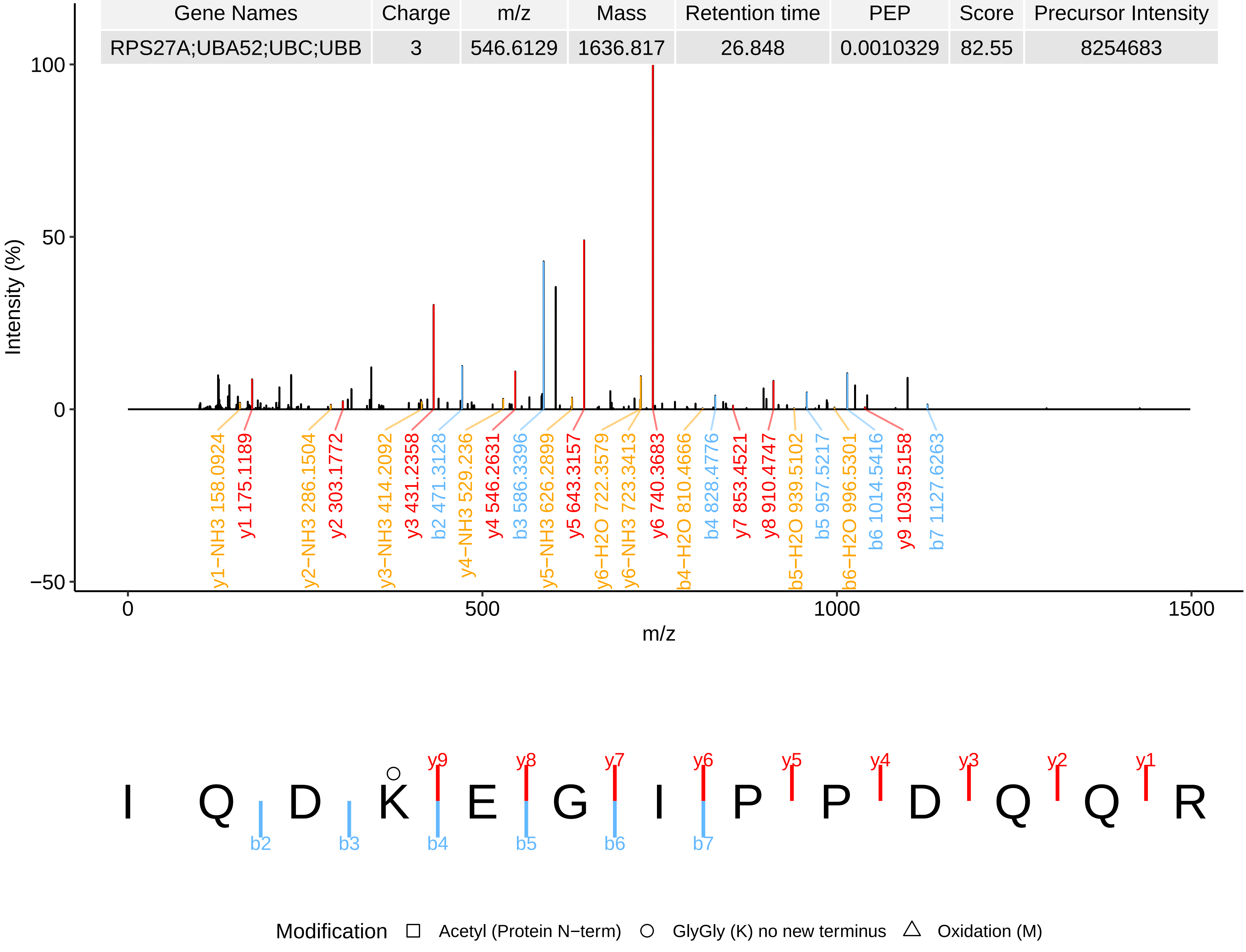

20200921_KIZ_MUb4_GlyGly_10plex_2 − Scan number: 7343

Gene Names	Charge	m/z	Mass	Retention time	PEP	Score	Precursor Intensity
RPS27A;UBA52;UBC;UBB	3	546.6129	1636.817	27.362	1.0913e−05	106.97	5550370

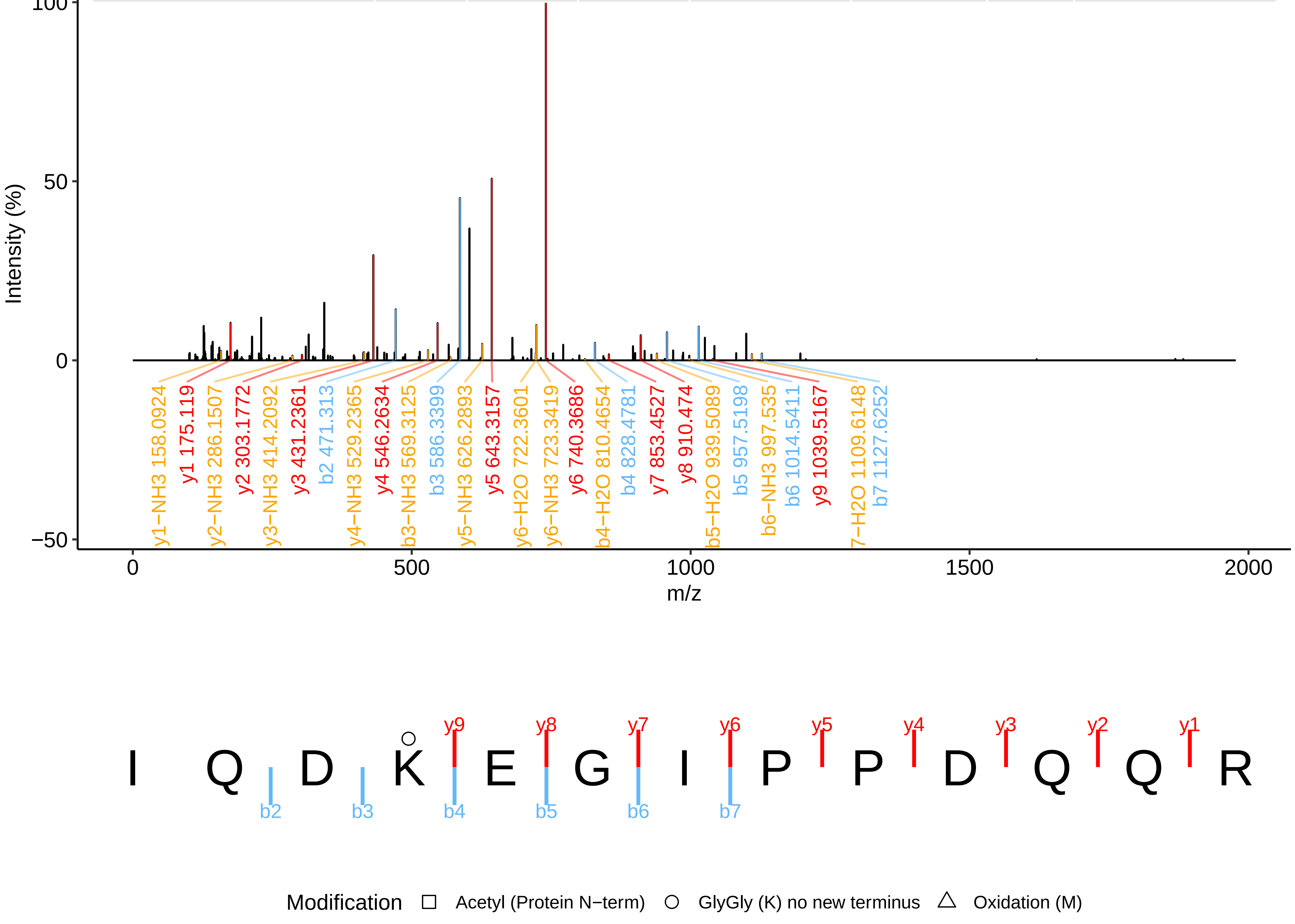

20200921_KIZ_MUb4_GlyGly_10plex_2 − Scan number: 7466

Gene Names	Charge	m/z	Mass	Retention time	PEP	Score	Precursor Intensity
RPS27A;UBA52;UBC;UBB	3	546.6129	1636.817	27.868	0.00023294	94.781	7735794

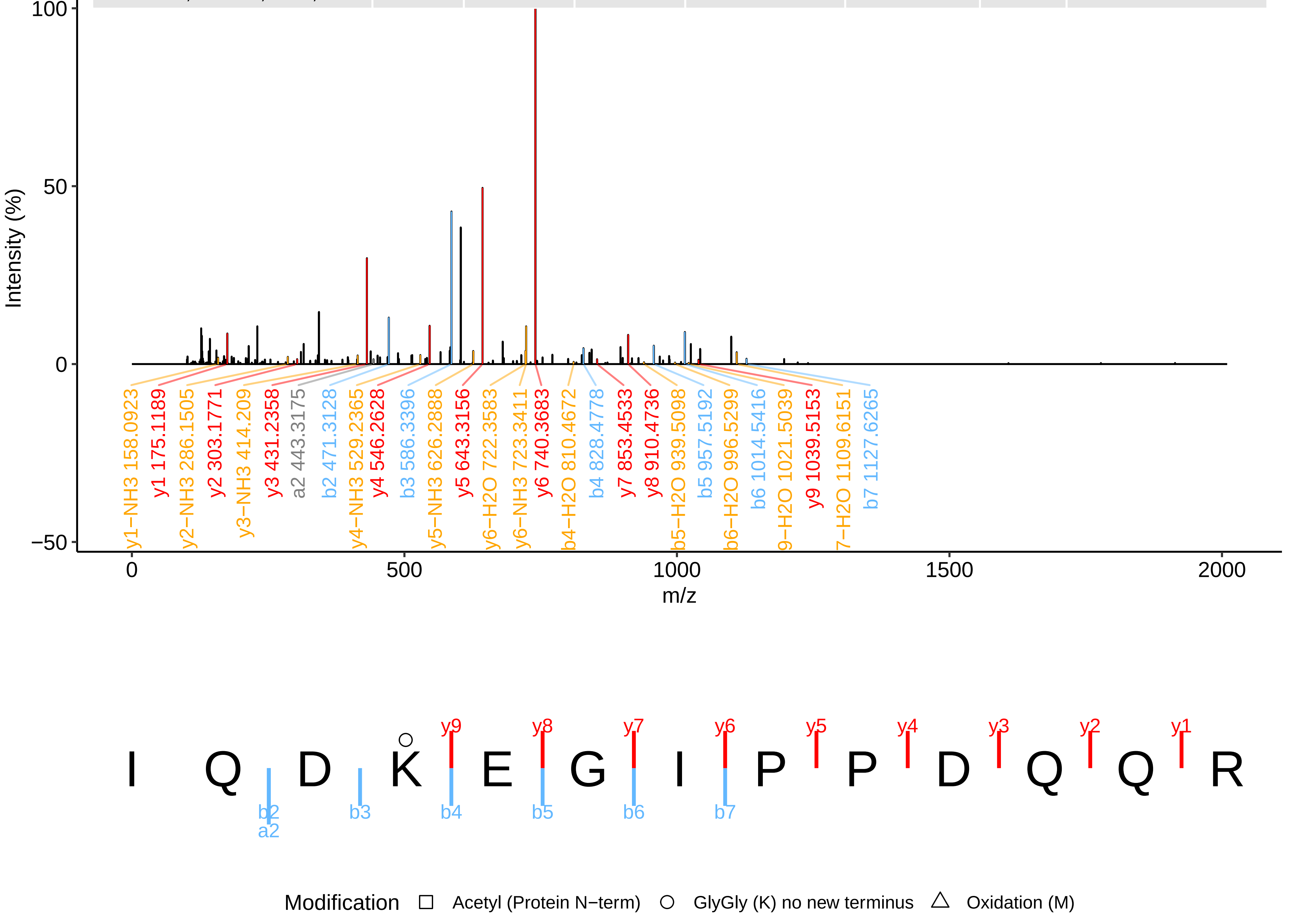

20200921_KIZ_MUb4_GlyGly_10plex_2 − Scan number: 7592

Gene Names	Charge	m/z	Mass	Retention time	PEP	Score	Precursor Intensity
RPS27A;UBA52;UBC;UBB	3	546.6129	1636.817	28.378	0.00023294	94.781	5891093

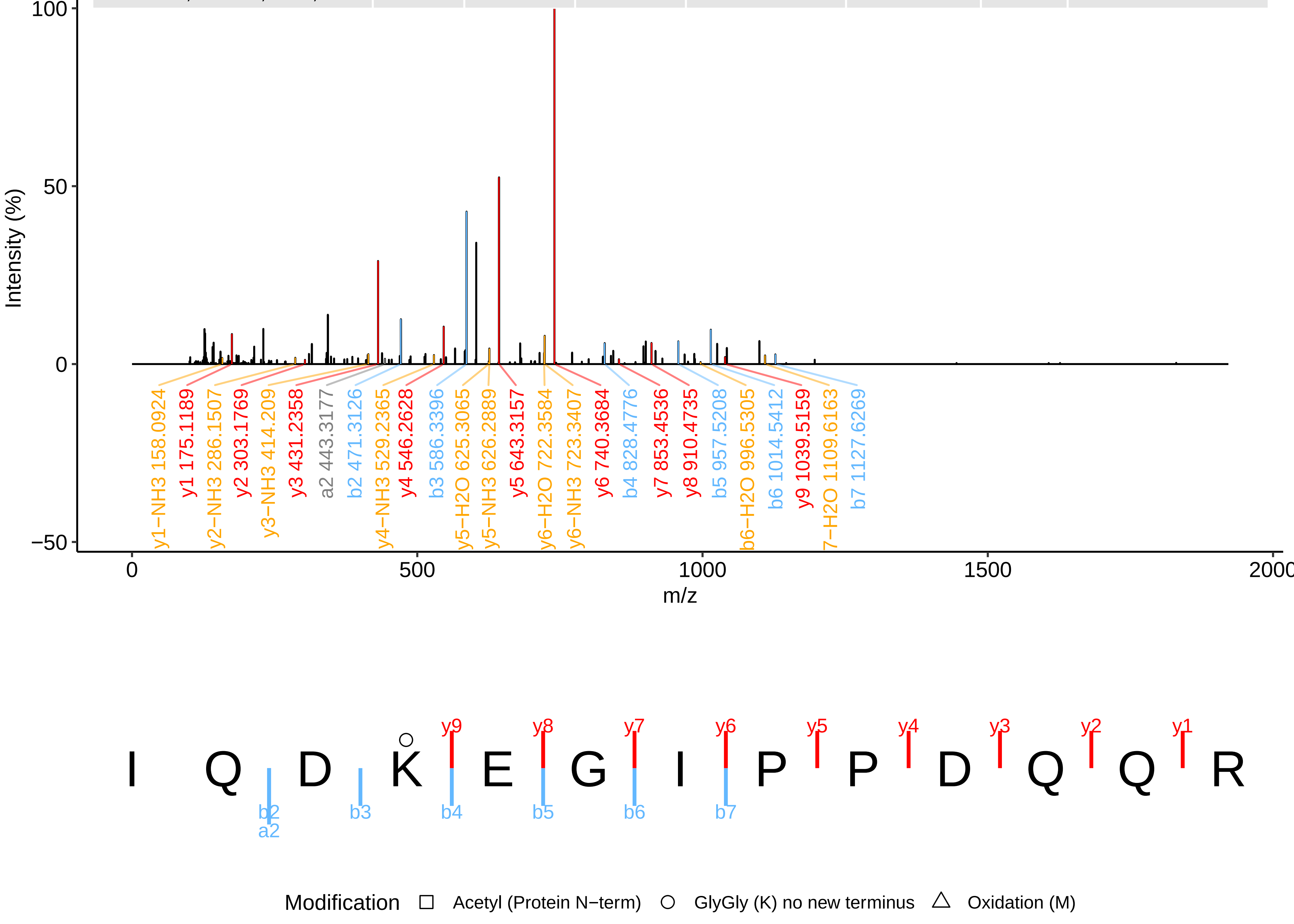

20200921_KIZ_MUb4_GlyGly_10plex_2 − Scan number: 7720

Gene Names	Charge	m/z	Mass	Retention time	PEP	Score	Precursor Intensity
RPS27A;UBA52;UBC;UBB	3	546.6129	1636.817	28.895	0.00037326	92.636	5463008

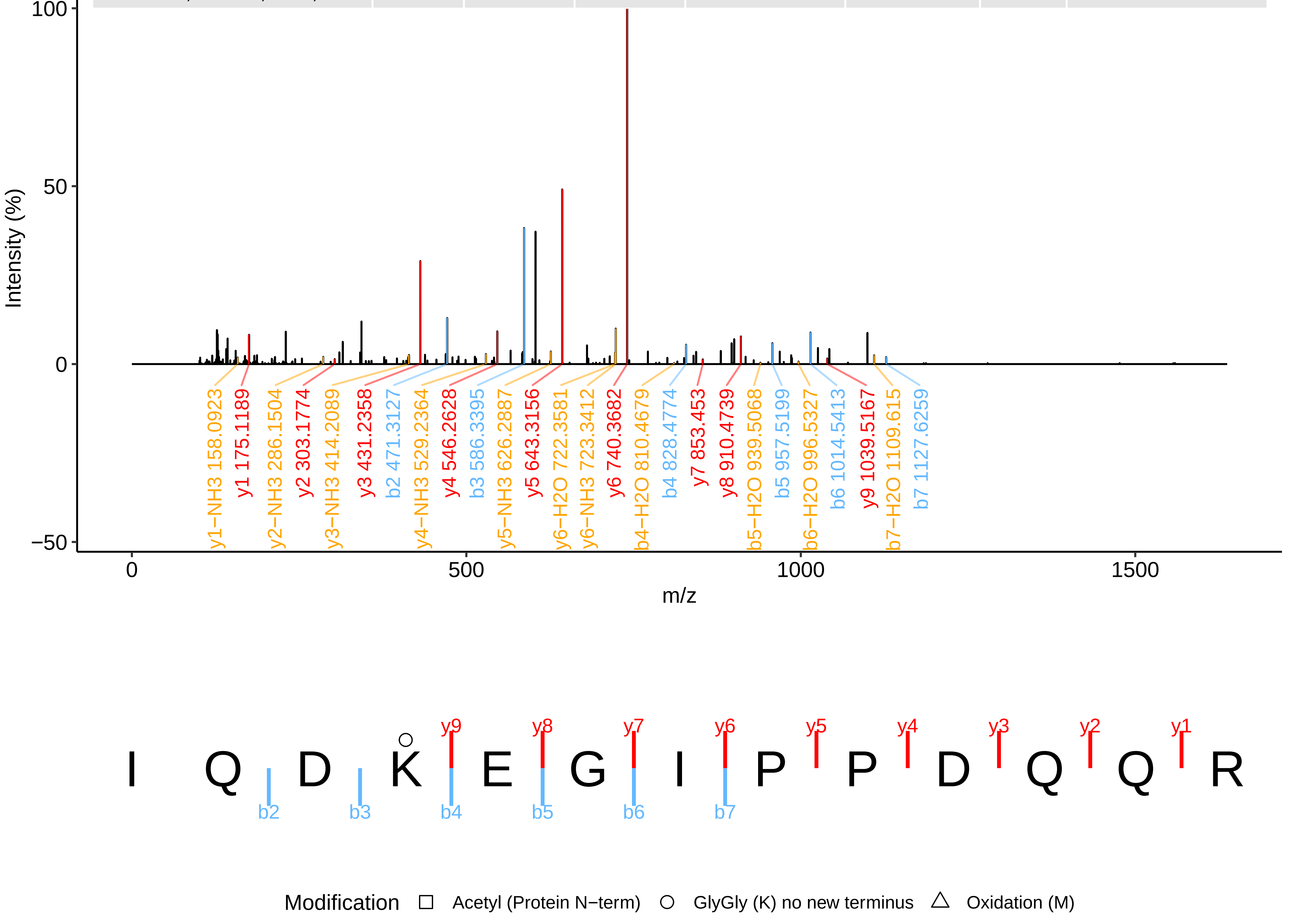

20200921_KIZ_MUb4_GlyGly_10plex_2 − Scan number: 7845

Gene Names	Charge	m/z	Mass	Retention time	PEP	Score	Precursor Intensity
RPS27A;UBA52;UBC;UBB	3	546.6129	1636.817	29.404	6.9967e−05	97.273	2803326

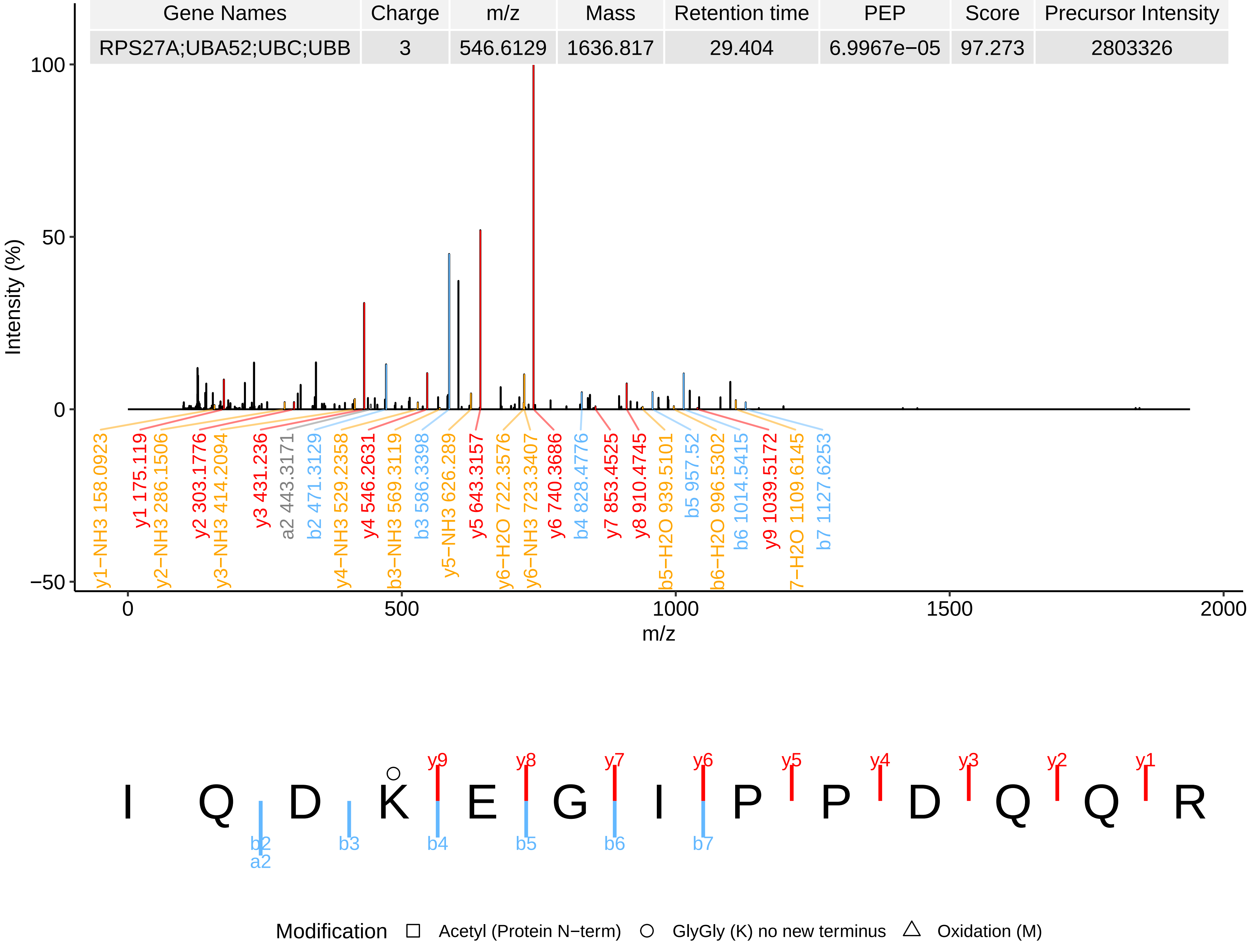

20200921_KIZ_MUb4_GlyGly_10plex_2 − Scan number: 7972

Gene Names	Charge	m/z	Mass	Retention time	PEP	Score	Precursor Intensity
RPS27A;UBA52;UBC;UBB	3	546.6129	1636.817	29.924	1.6393e−05	102.73	2853999

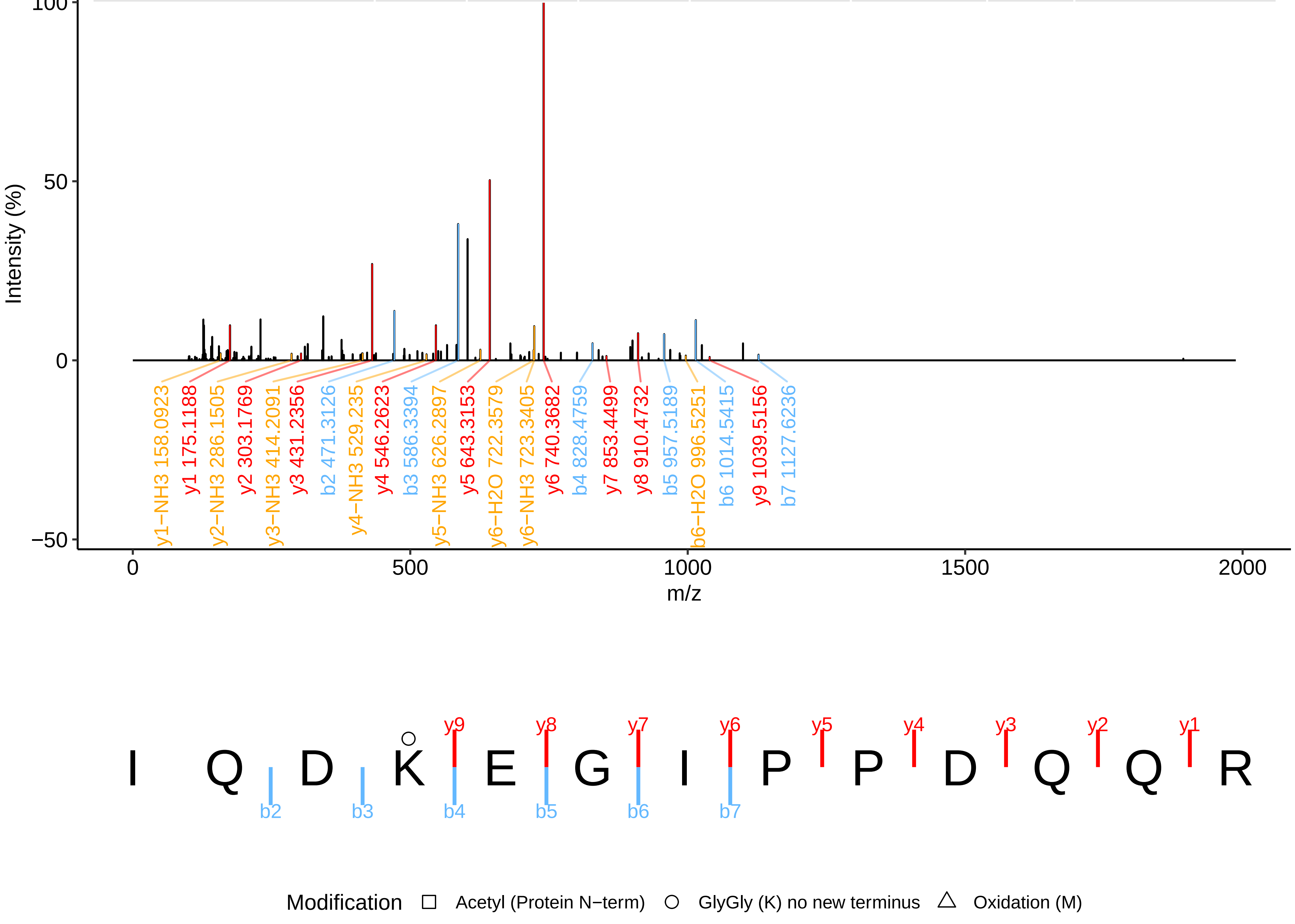

20200921_KIZ_MUb4_GlyGly_10plex_2 − Scan number: 8095

Gene Names	Charge	m/z	Mass	Retention time	PEP	Score	Precursor Intensity
RPS27A;UBA52;UBC;UBB	3	546.6129	1636.817	30.437	0.00040494	92.151	2270774

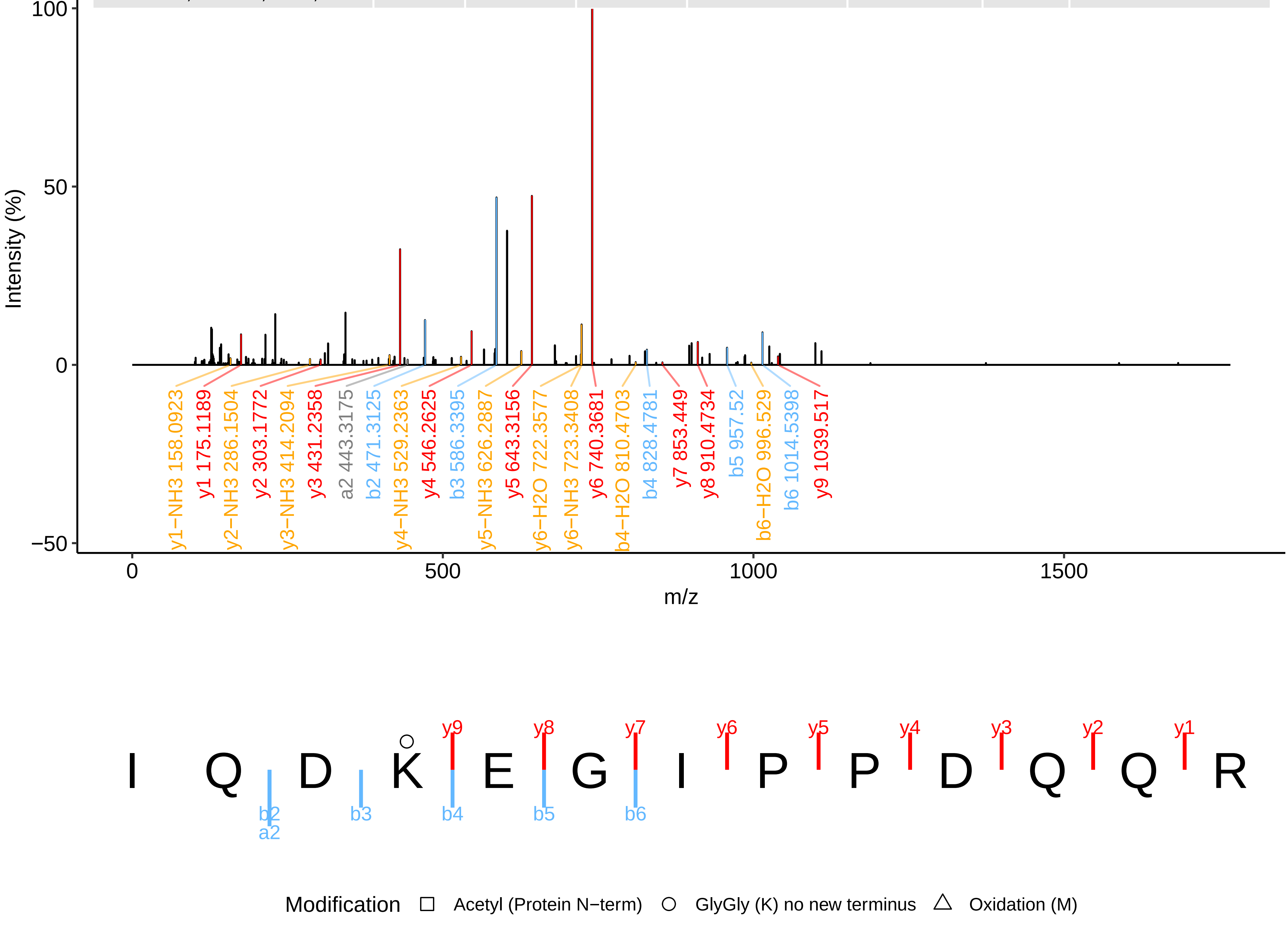

20200921_KIZ_MUb4_GlyGly_10plex_2 − Scan number: 8235

Gene Names	Charge	m/z	Mass	Retention time	PEP	Score	Precursor Intensity
RPS27A;UBA52;UBC;UBB	3	546.6129	1636.817	30.965	0.0078253	68.262	4702182

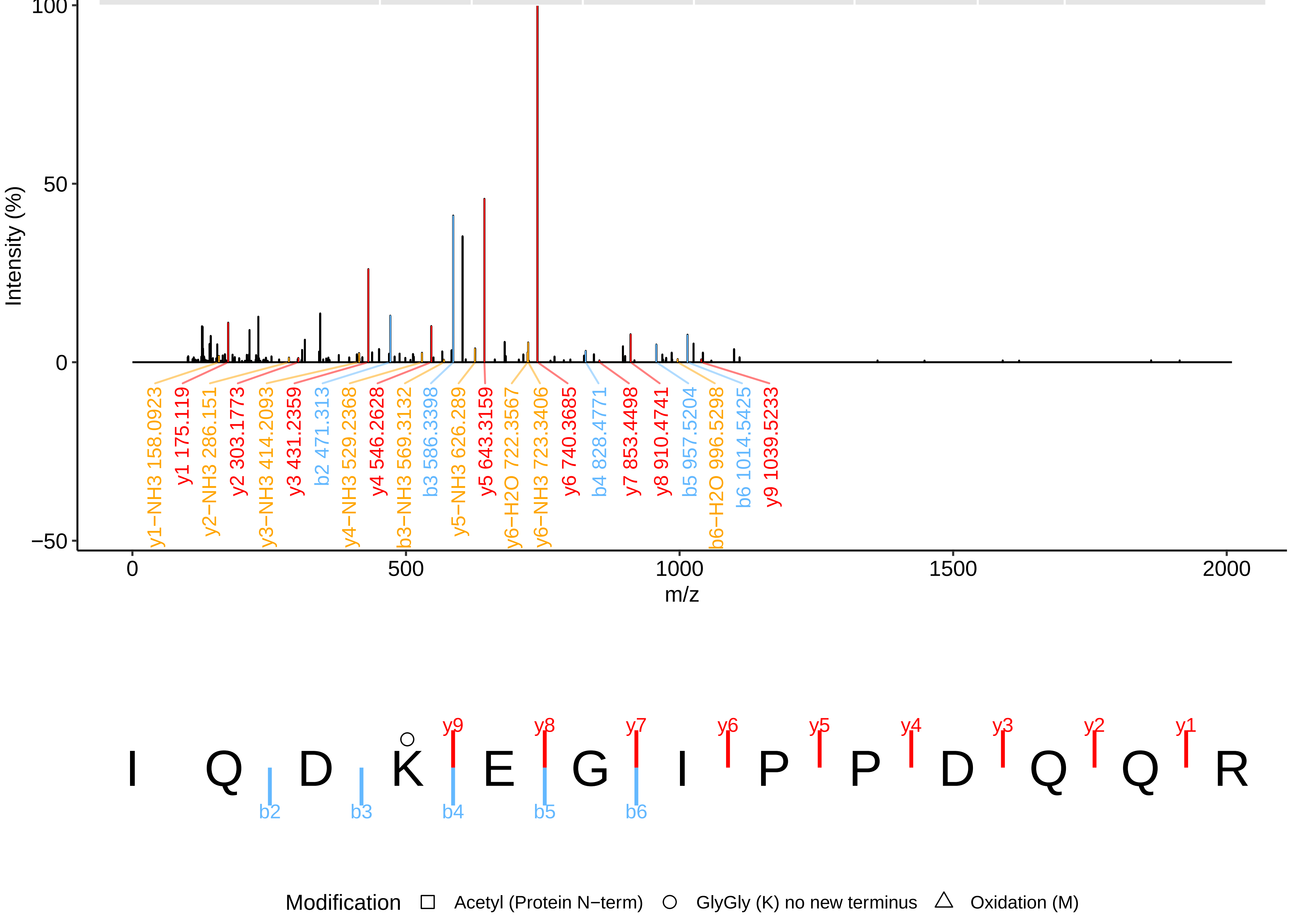

20200921_KIZ_MUb4_GlyGly_10plex_2 − Scan number: 8360

Gene Names	Charge	m/z	Mass	Retention time	PEP	Score	Precursor Intensity
RPS27A;UBA52;UBC;UBB	3	546.6129	1636.817	31.472	0.0010752	81.904	978205

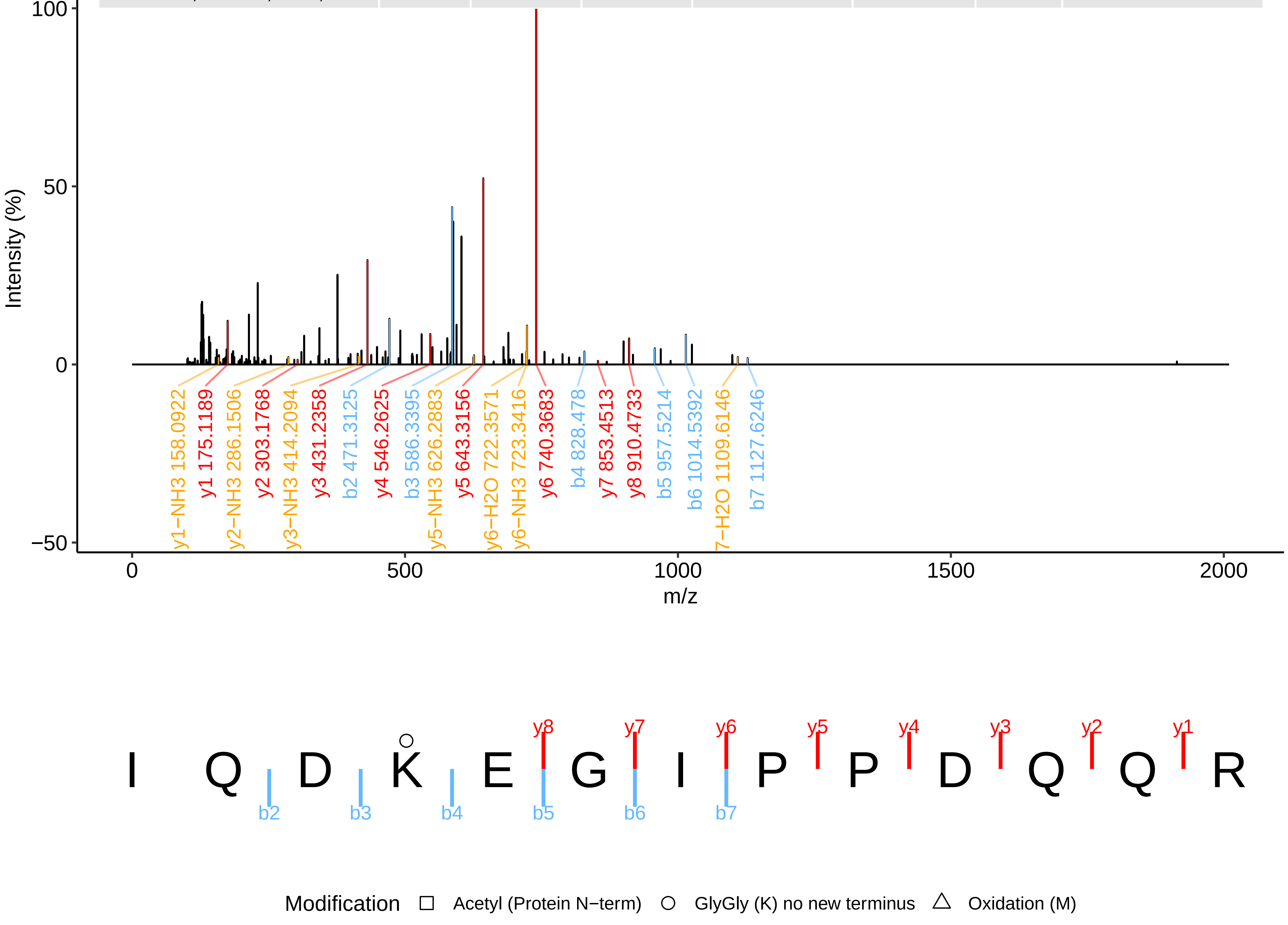

20200921_KIZ_MUb4_GlyGly_10plex_2 − Scan number: 8486

Gene Names	Charge	m/z	Mass	Retention time	PEP	Score	Precursor Intensity
RPS27A;UBA52;UBC;UBB	3	546.6129	1636.817	31.983	0.038609	57.136	856866.2

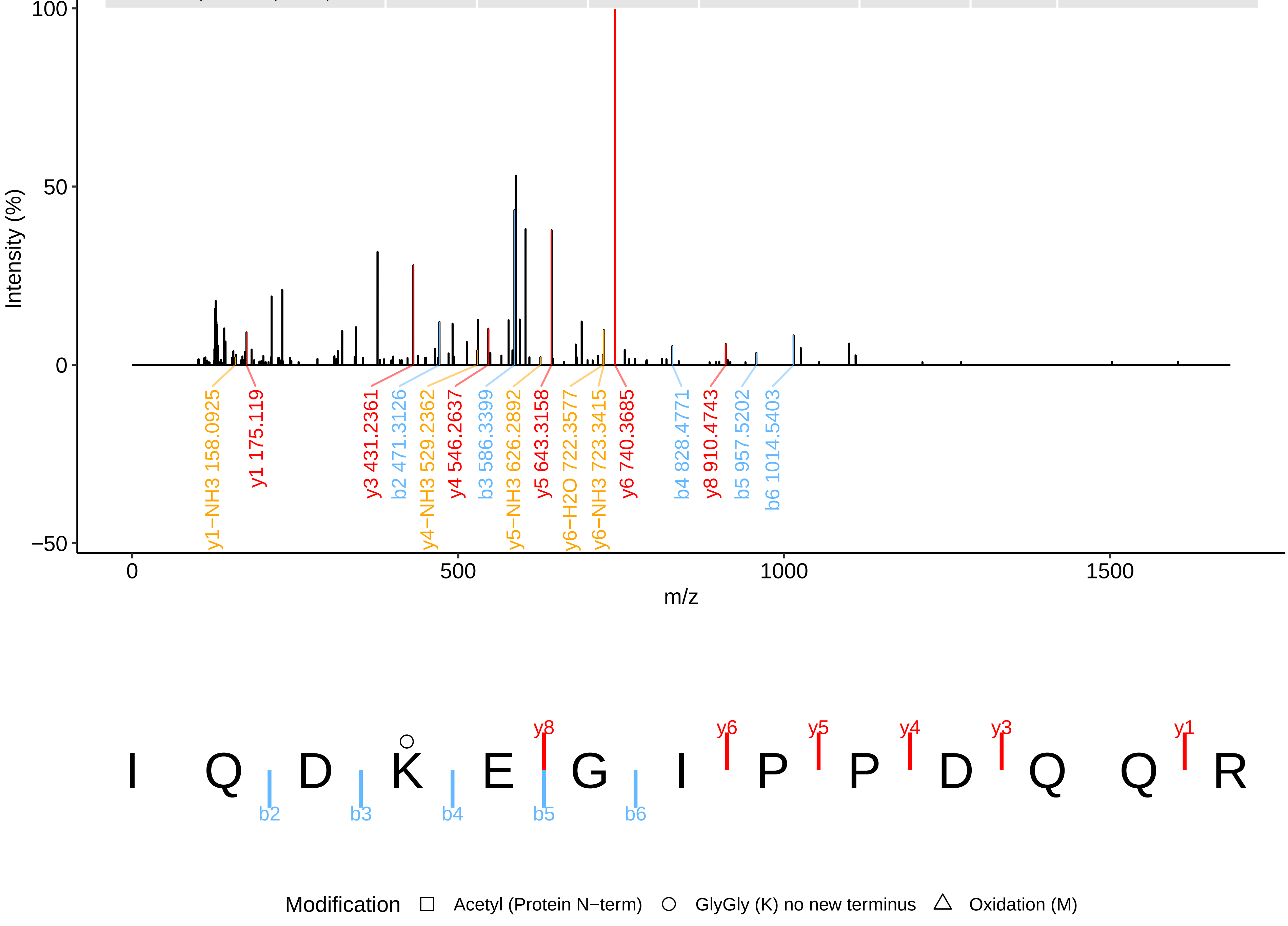

20200921_KIZ_MUb4_GlyGly_10plex_2 − Scan number: 8610

Gene Names	Charge	m/z	Mass	Retention time	PEP	Score	Precursor Intensity
RPS27A;UBA52;UBC;UBB	3	546.6129	1636.817	32.489	0.00090743	84.468	1598124

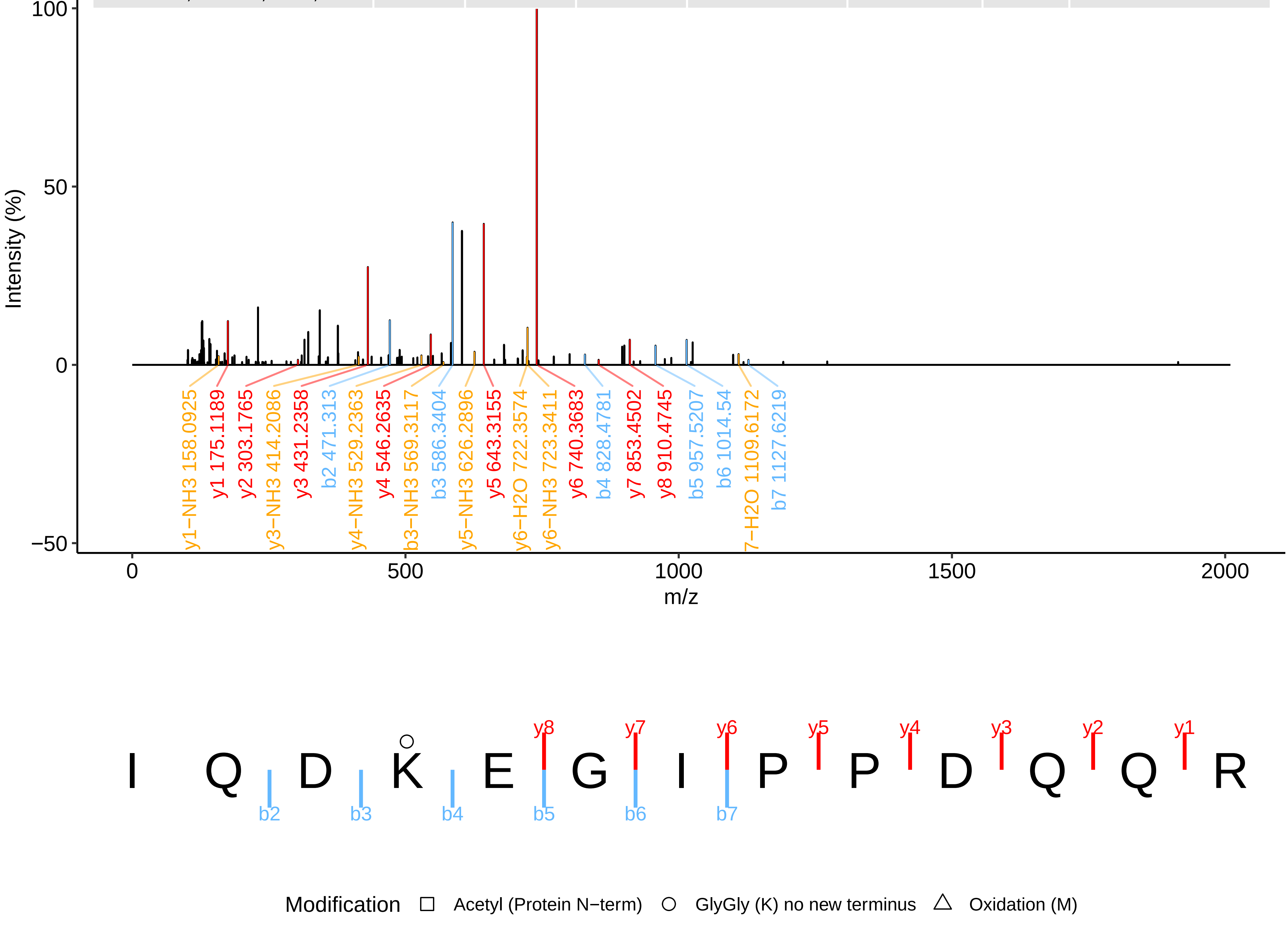

20200921_KIZ_MUb4_GlyGly_10plex_2 − Scan number: 6590

Gene Names	Charge	m/z	Mass	Retention time	PEP	Score	Precursor Intensity
RPS27A;UBA52;UBC;UBB	3	546.6129	1636.817	24.291	0.0082892	68.83	1329191

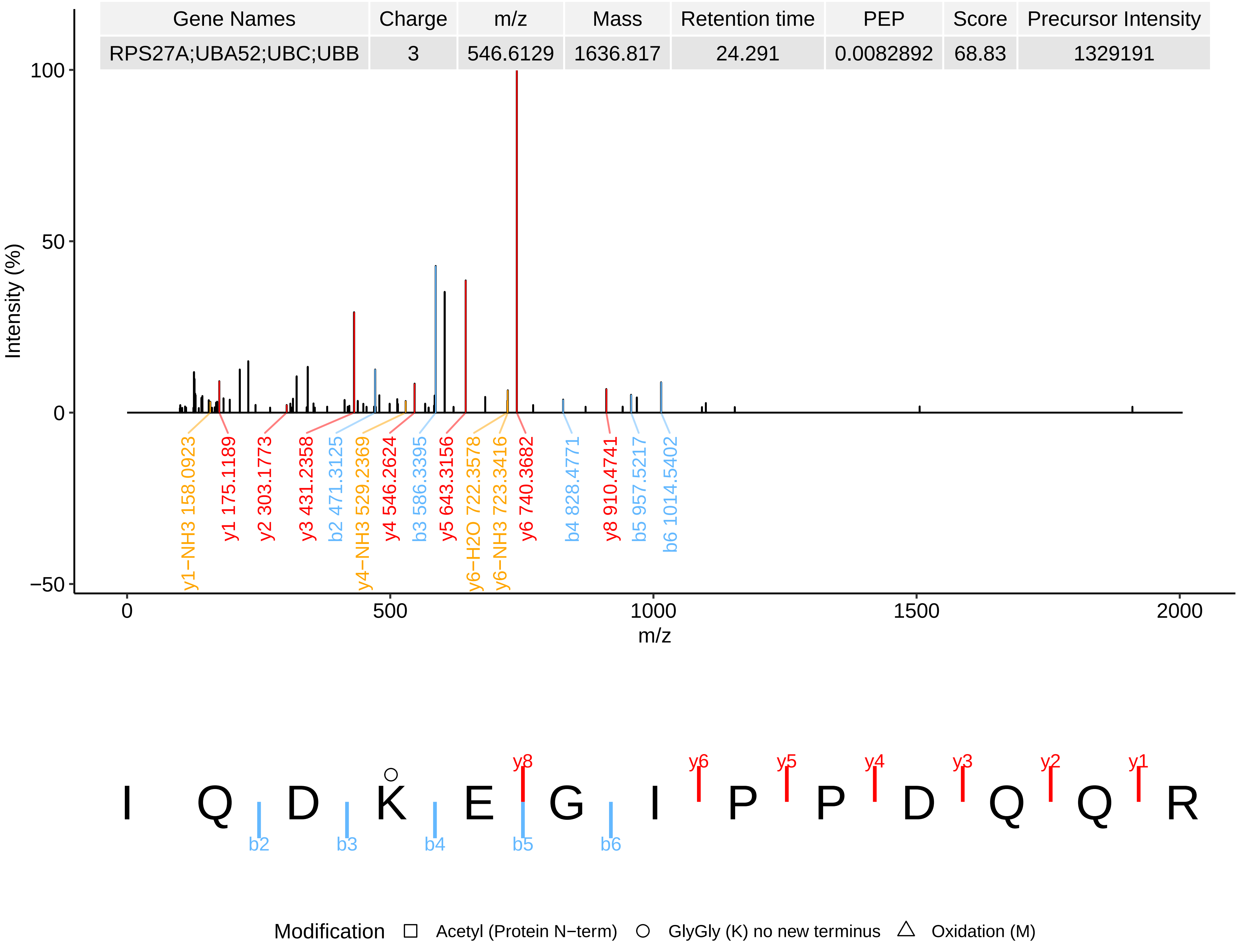

20200924_KIZ_MUb5_GlyGly_1 − Scan number: 6456

Gene Names	Charge	m/z	Mass	Retention time	PEP	Score	Precursor Intensity
RPS27A;UBA52;UBC;UBB	3	546.6129	1636.817	20.306	1.9022e−05	100.69	29034404

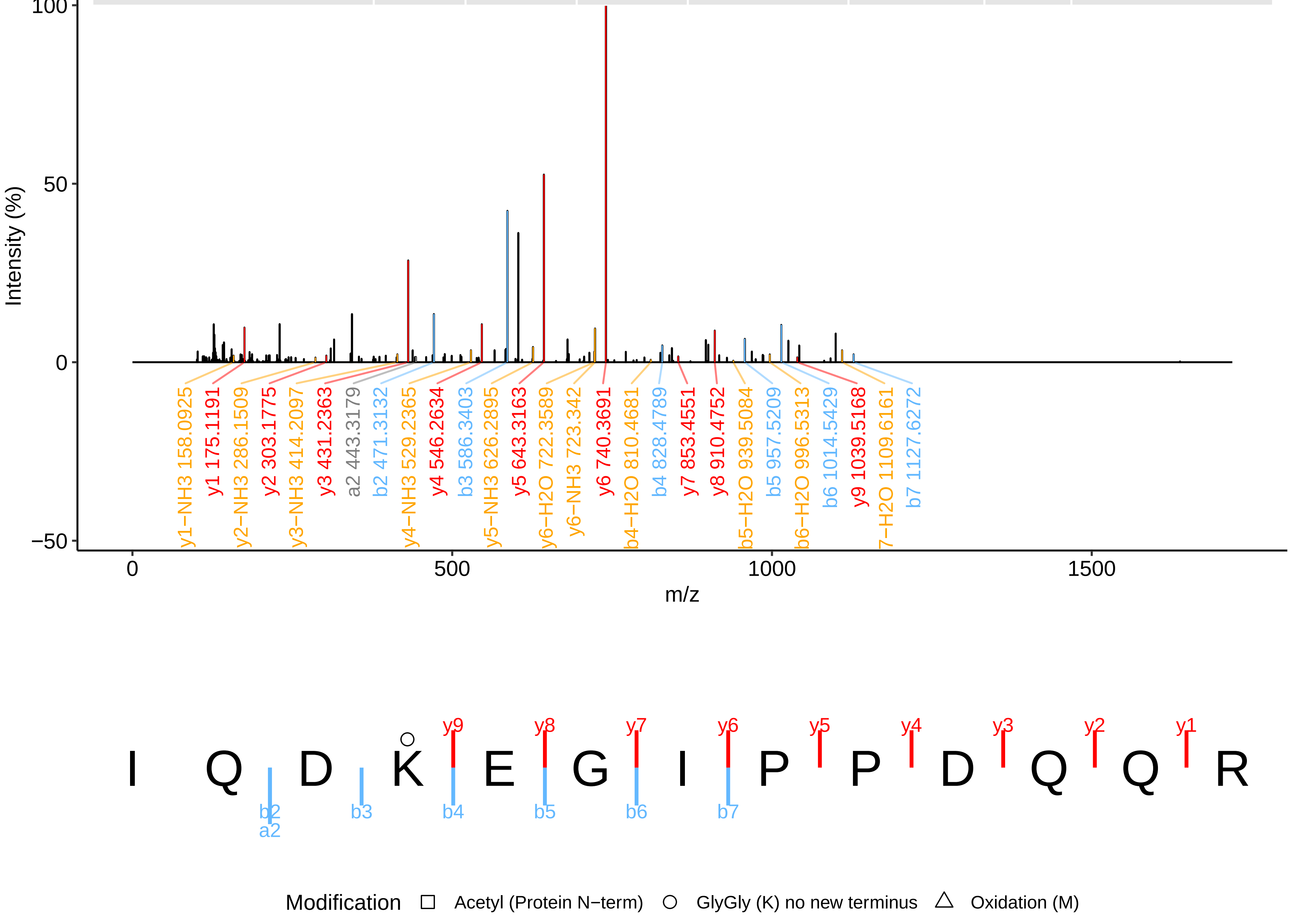

20200924_KIZ_MUb5_GlyGly_1 − Scan number: 6606

Gene Names	Charge	m/z	Mass	Retention time	PEP	Score	Precursor Intensity
RPS27A;UBA52;UBC;UBB	3	546.6129	1636.817	20.813	0.00023294	94.781	28301696

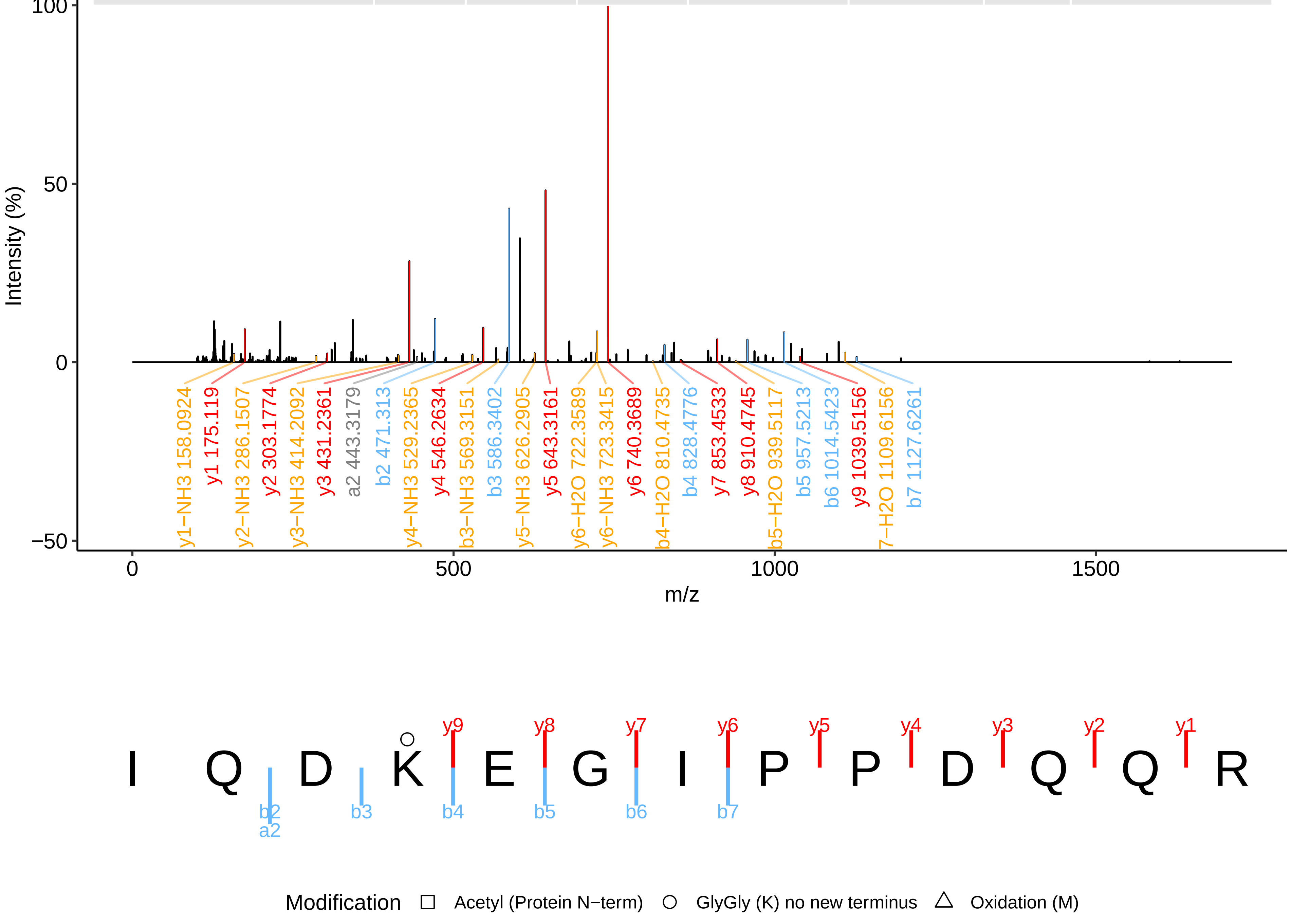

20200924_KIZ_MUb5_GlyGly_1 − Scan number: 6757

Gene Names	Charge	m/z	Mass	Retention time	PEP	Score	Precursor Intensity
RPS27A;UBA52;UBC;UBB	3	546.6129	1636.817	21.336	1.1422e−05	106.58	51178540

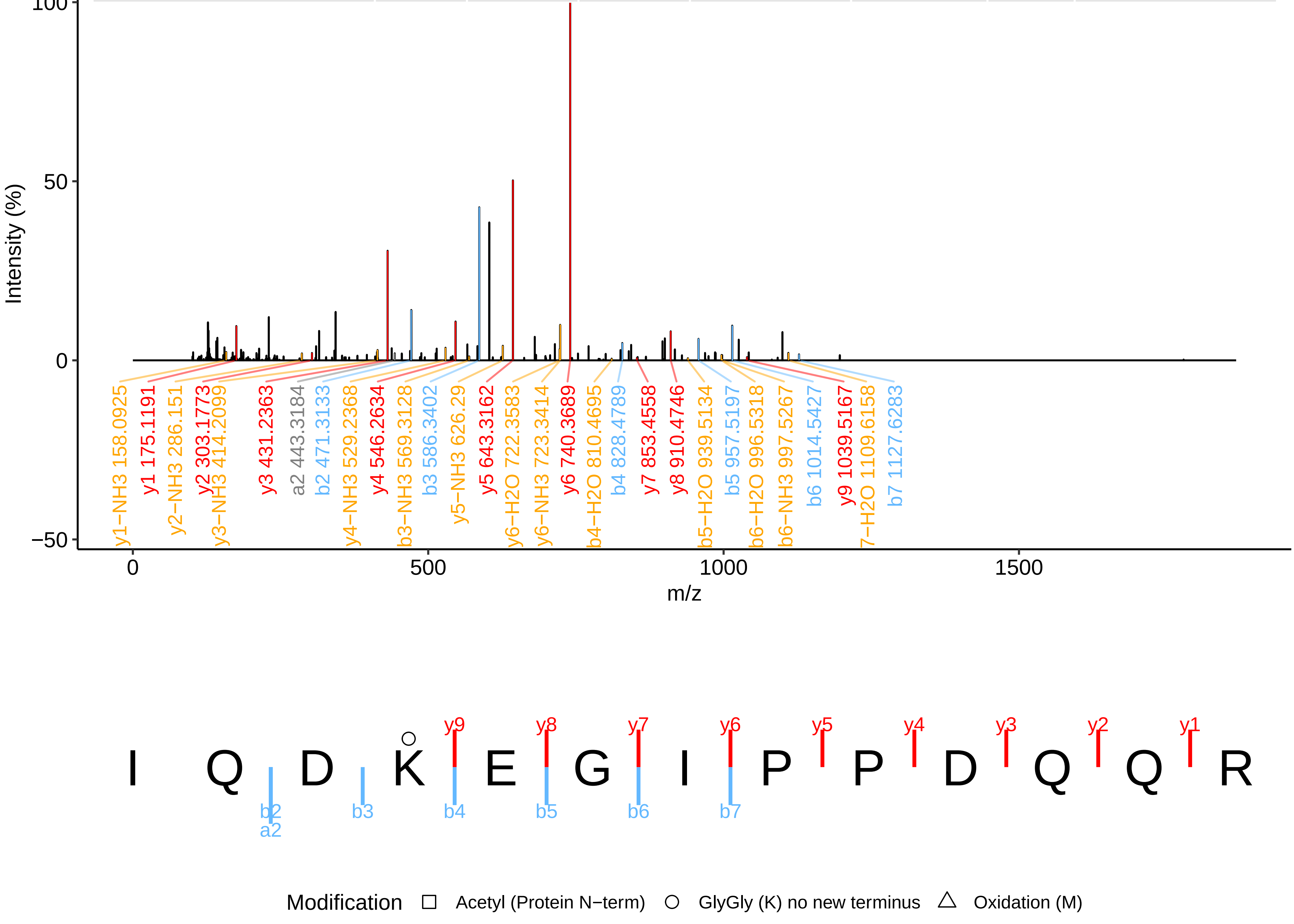

20200924_KIZ_MUb5_GlyGly_1 − Scan number: 6908

Gene Names	Charge	m/z	Mass	Retention time	PEP	Score	Precursor Intensity
RPS27A;UBA52;UBC;UBB	3	546.6129	1636.817	21.84	0.00075898	86.738	66624468

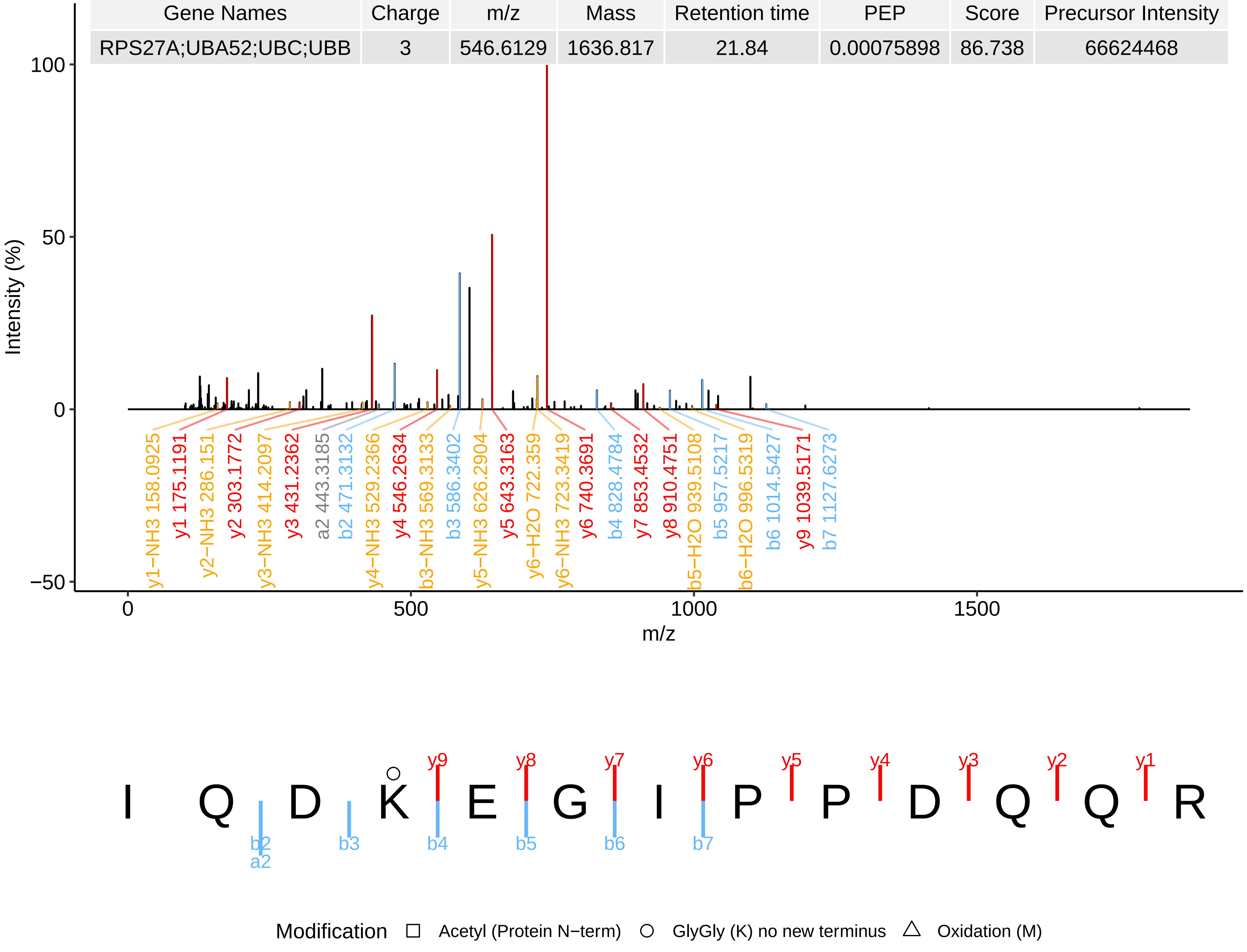

20200924_KIZ_MUb5_GlyGly_1 − Scan number: 7059

Gene Names	Charge	m/z	Mass	Retention time	PEP	Score	Precursor Intensity
RPS27A;UBA52;UBC;UBB	3	546.6129	1636.817	22.347	0.00023294	94.781	84149864

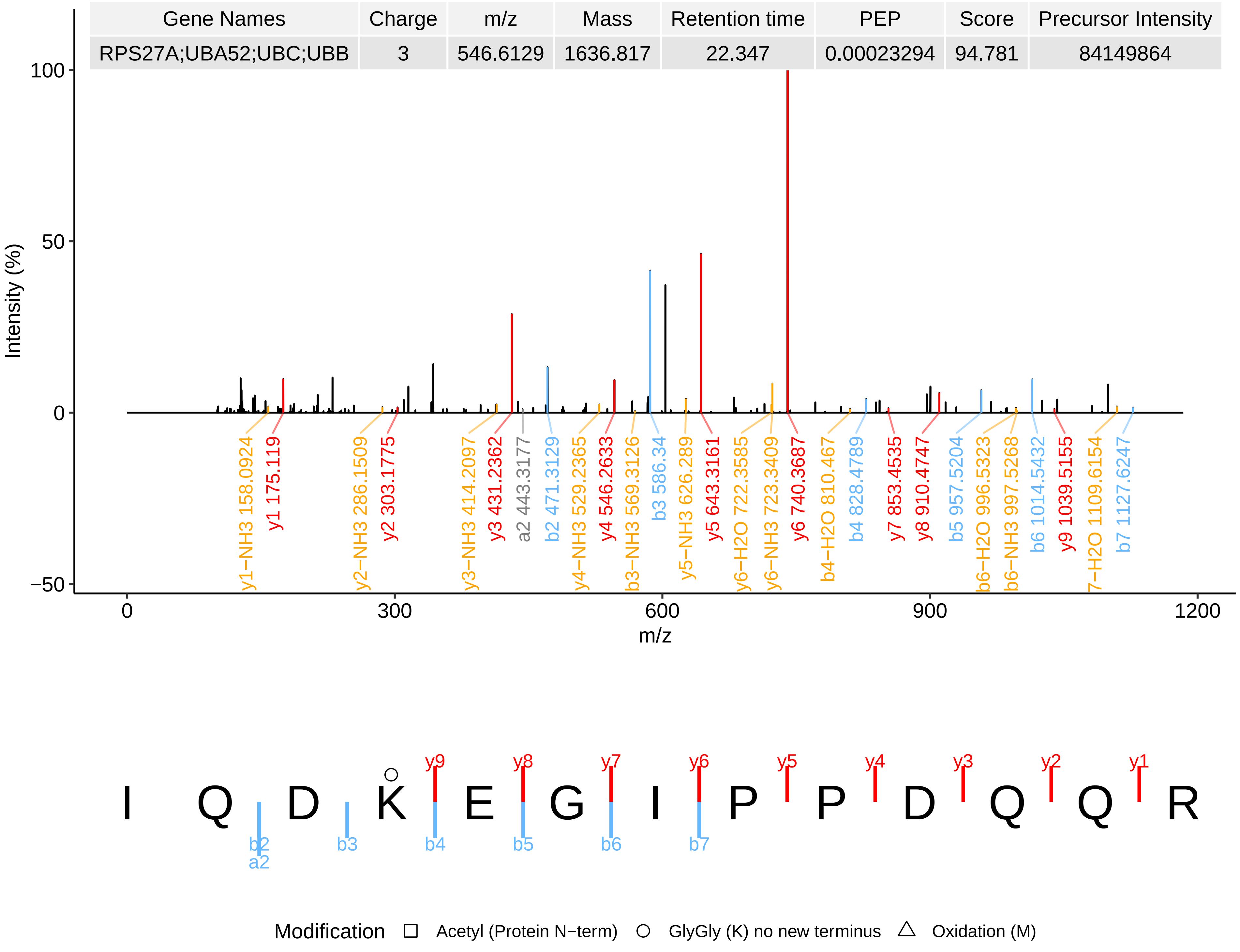

20200924_KIZ_MUb5_GlyGly_1 − Scan number: 7215

Gene Names	Charge	m/z	Mass	Retention time	PEP	Score	Precursor Intensity
RPS27A;UBA52;UBC;UBB	3	546.6129	1636.817	22.871	0.00065802	88.282	63739500

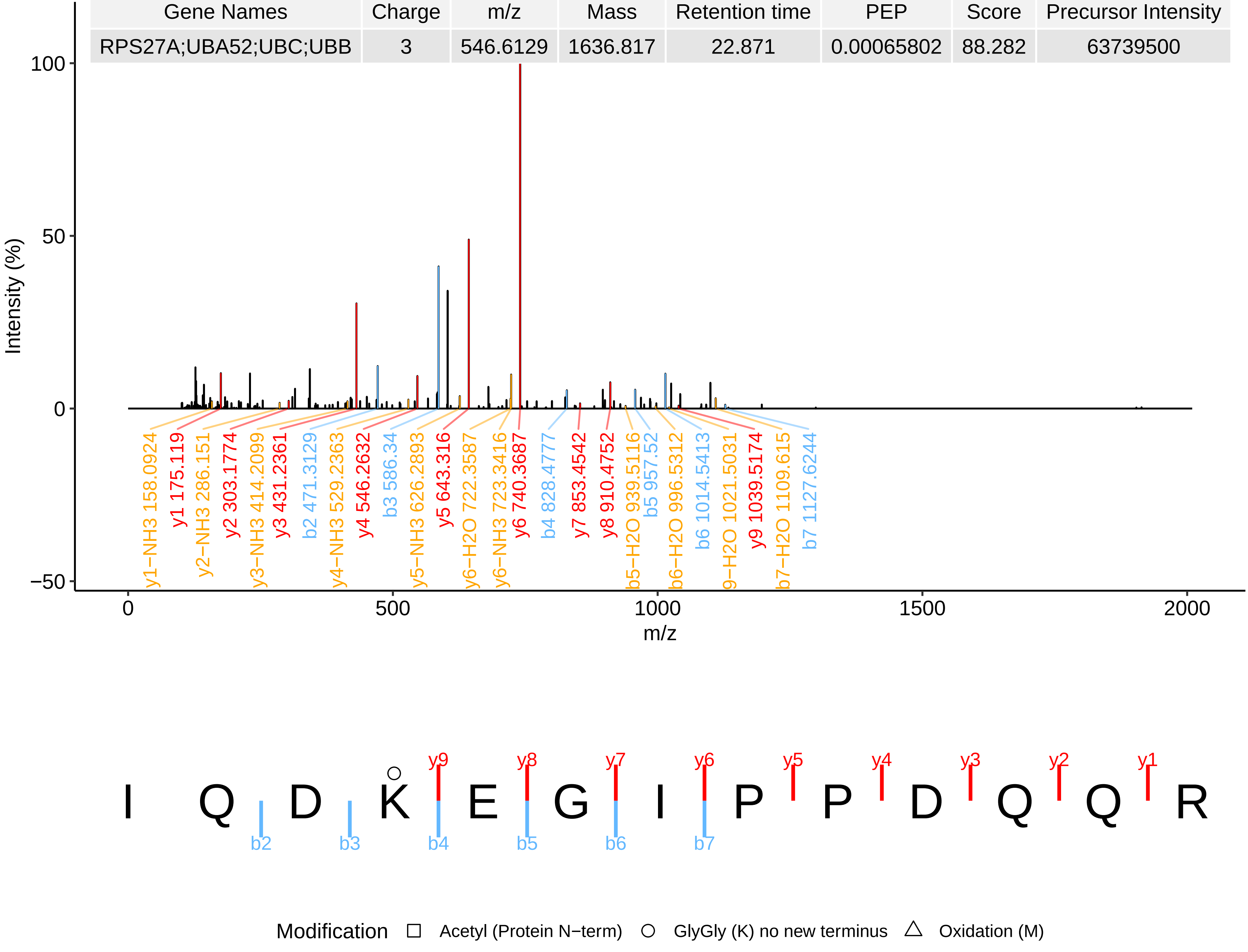

20200924_KIZ_MUb5_GlyGly_1 − Scan number: 7370

Gene Names	Charge	m/z	Mass	Retention time	PEP	Score	Precursor Intensity
RPS27A;UBA52;UBC;UBB	3	546.6129	1636.817	23.379	0.00065802	88.282	41436096

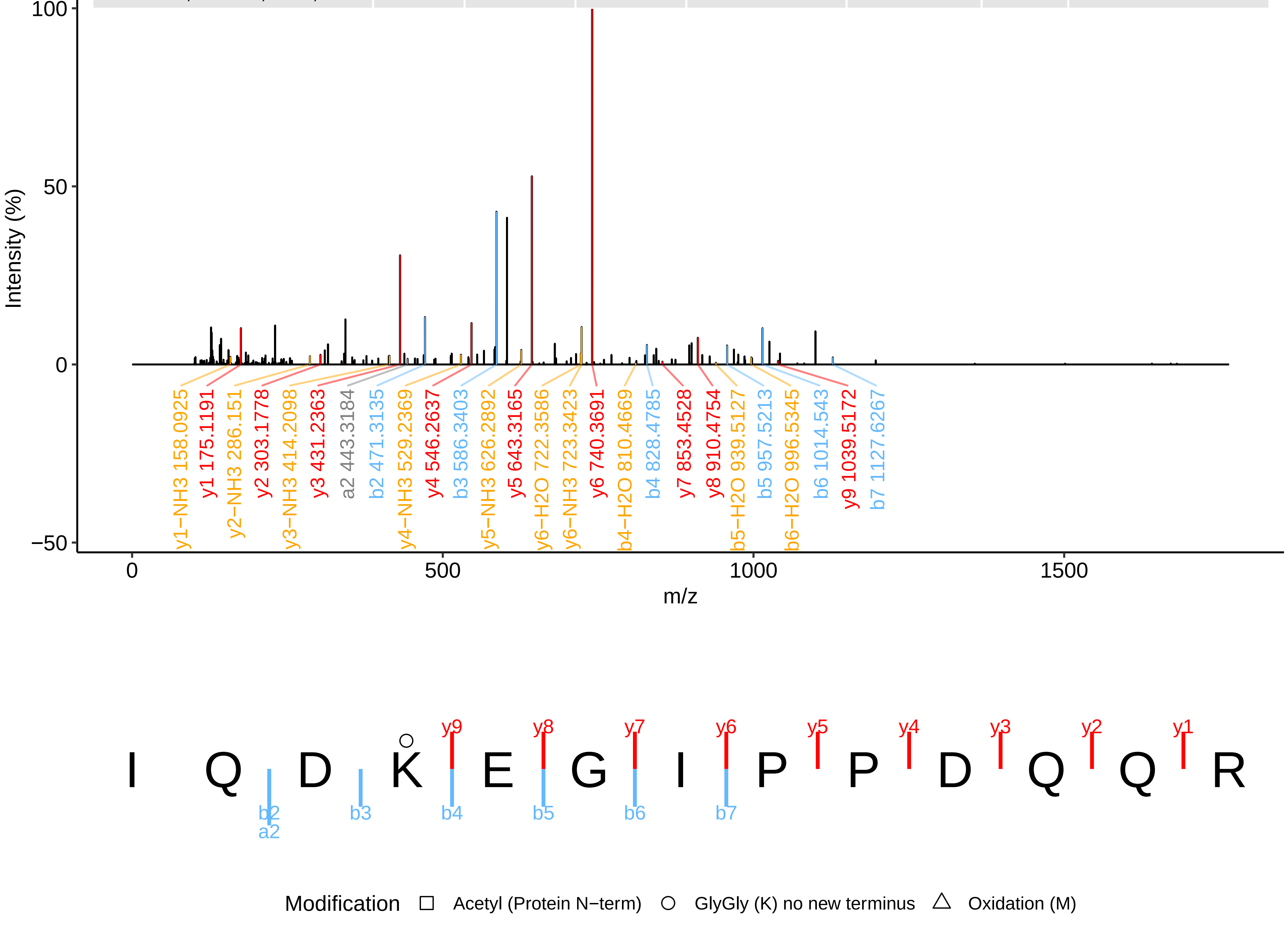

20200924_KIZ_MUb5_GlyGly_1 − Scan number: 7520

Gene Names	Charge	m/z	Mass	Retention time	PEP	Score	Precursor Intensity
RPS27A;UBA52;UBC;UBB	3	546.6129	1636.817	23.895	4.6566e−05	97.631	52641716

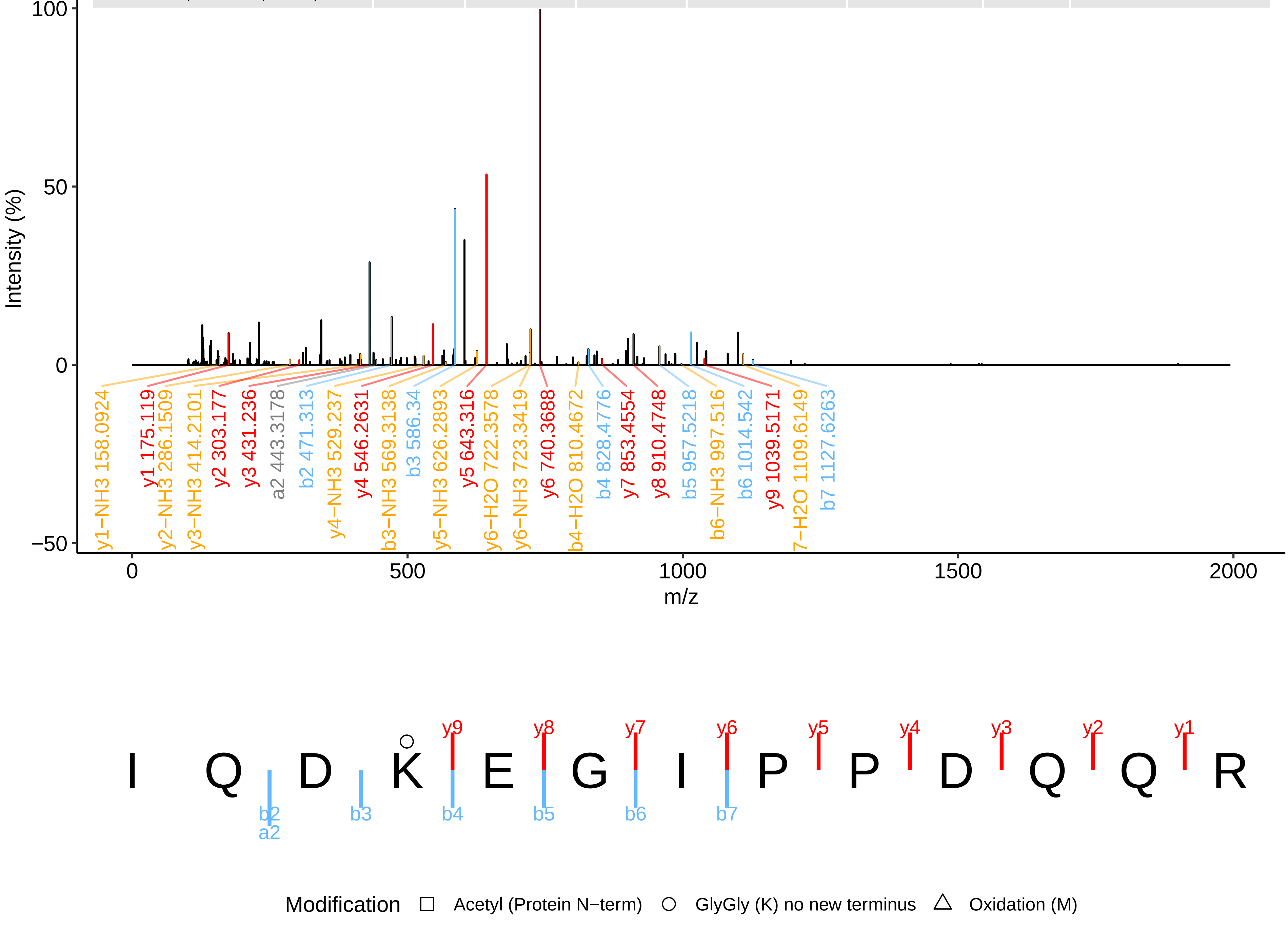

20200924_KIZ_MUb5_GlyGly_1 − Scan number: 7671

Gene Names	Charge	m/z	Mass	Retention time	PEP	Score	Precursor Intensity
RPS27A;UBA52;UBC;UBB	3	546.6129	1636.817	24.406	6.9967e−05	97.273	32138760

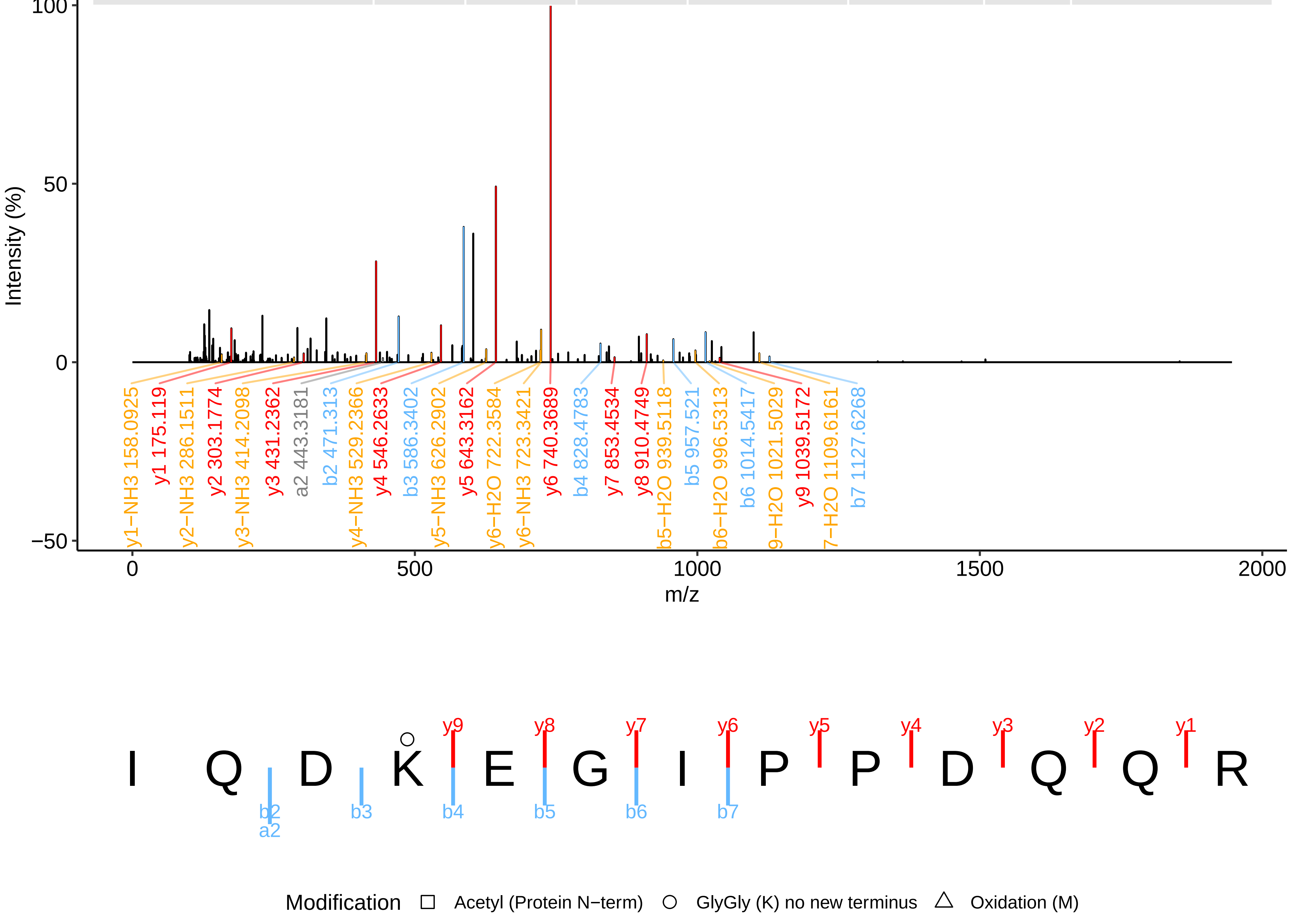

20200924_KIZ_MUb5_GlyGly_1 − Scan number: 6305

Gene Names	Charge	m/z	Mass	Retention time	PEP	Score	Precursor Intensity
RPS27A;UBA52;UBC;UBB	3	546.6129	1636.817	19.804	3.6954e−05	101.43	NA

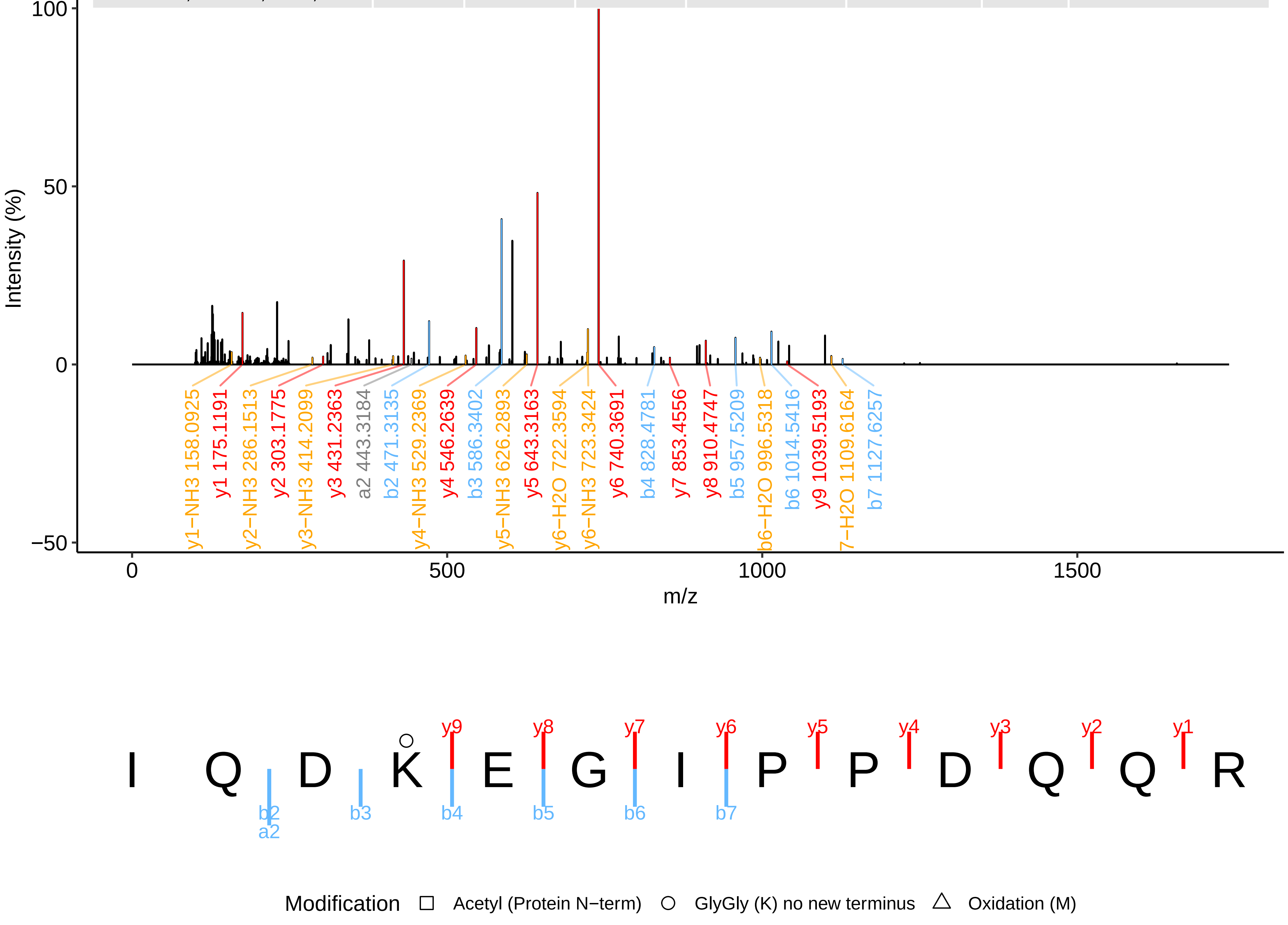

20200924_KIZ_MUb5_GlyGly_1 − Scan number: 6844

Gene Names	Charge	m/z	Mass	Retention time	PEP	Score	Precursor Intensity
RPS27A;UBA52;UBC;UBB	2	819.4157	1636.817	21.631	8.9633e−18	174.23	NA

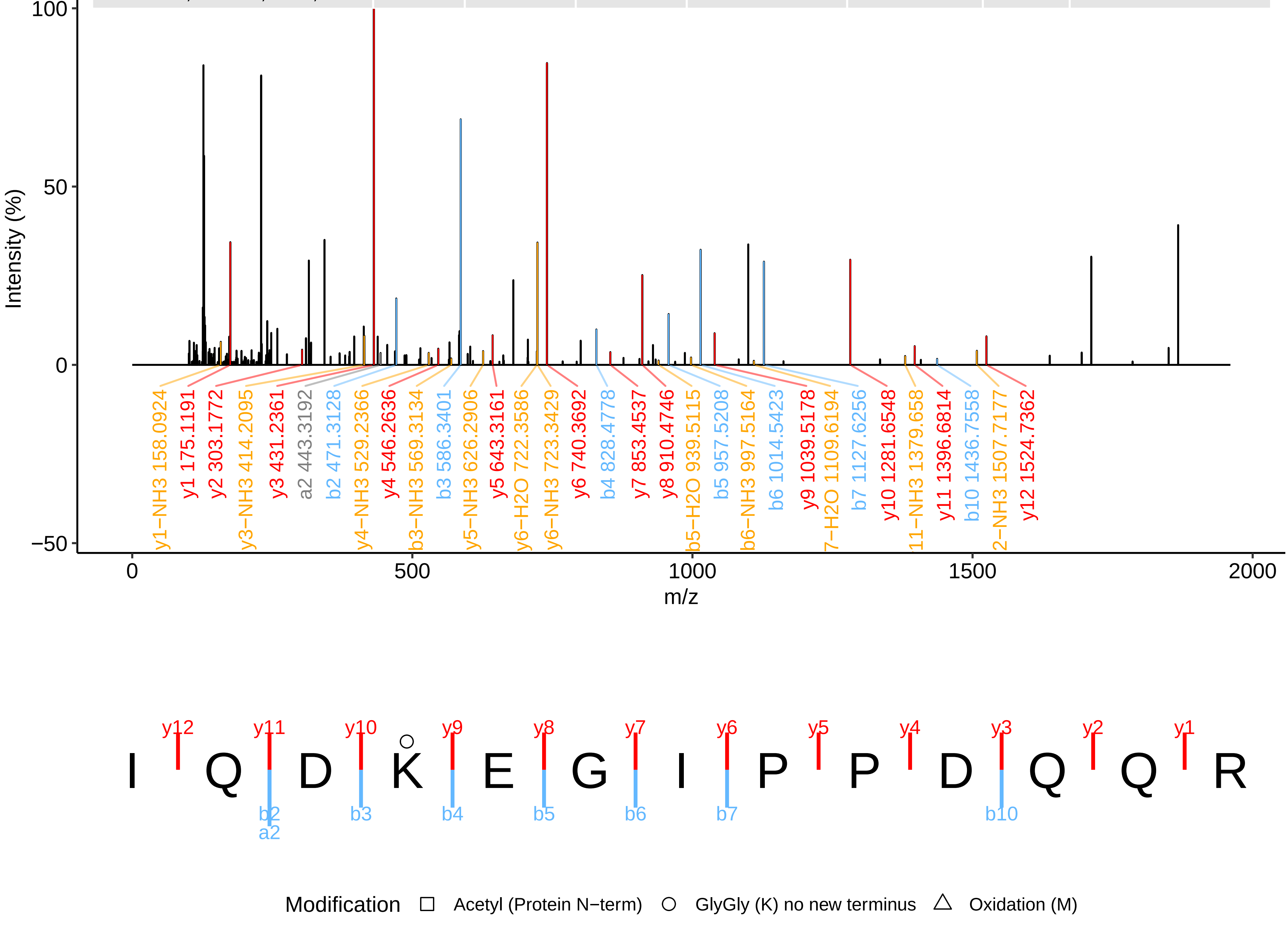

20200924_KIZ_MUb5_GlyGly_1 − Scan number: 7176

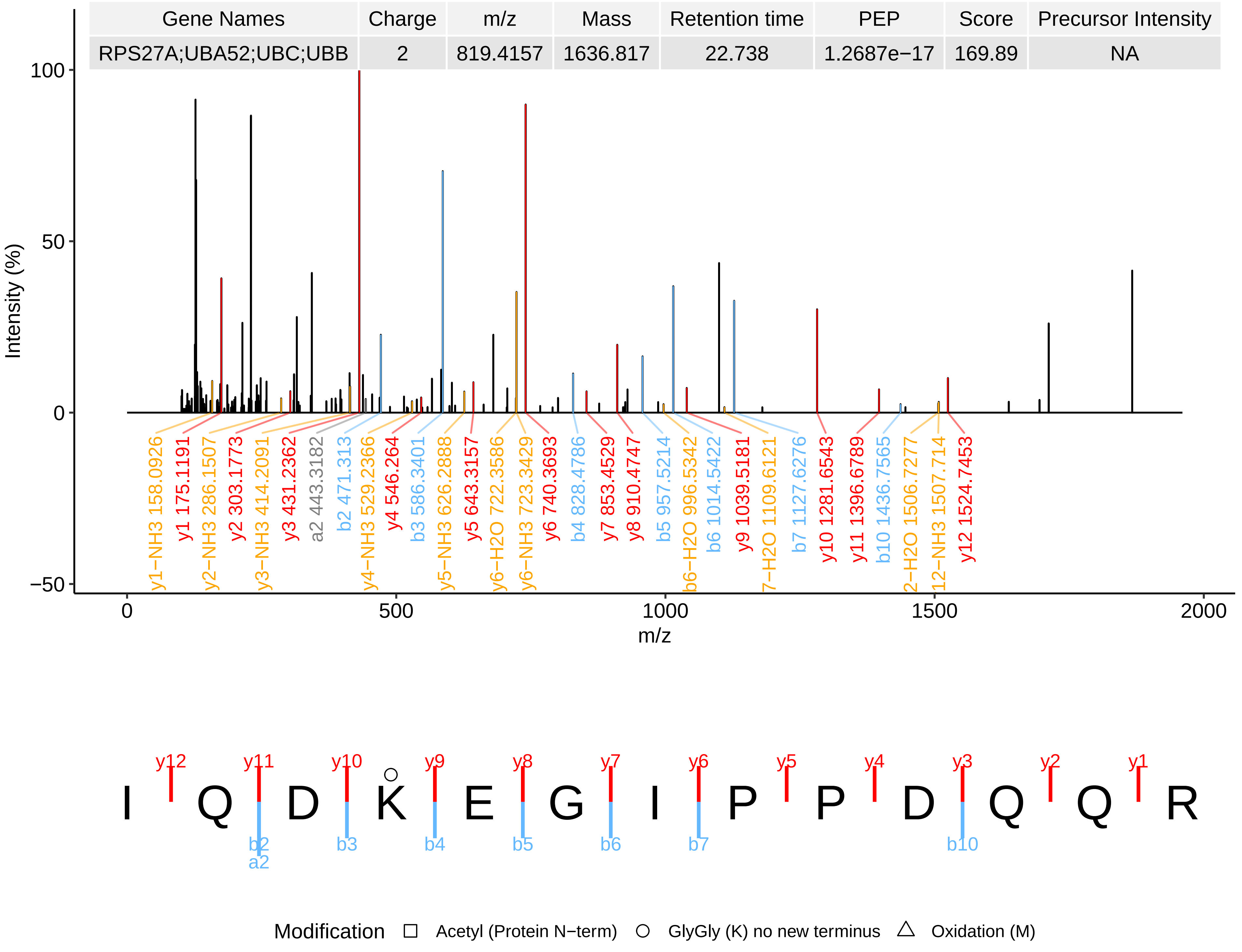

20200924_KIZ_MUb5_GlyGly_1 − Scan number: 7824

Gene Names	Charge	m/z	Mass	Retention time	PEP	Score	Precursor Intensity
RPS27A;UBA52;UBC;UBB	3	546.6129	1636.817	24.922	0.00053351	92.457	27049226

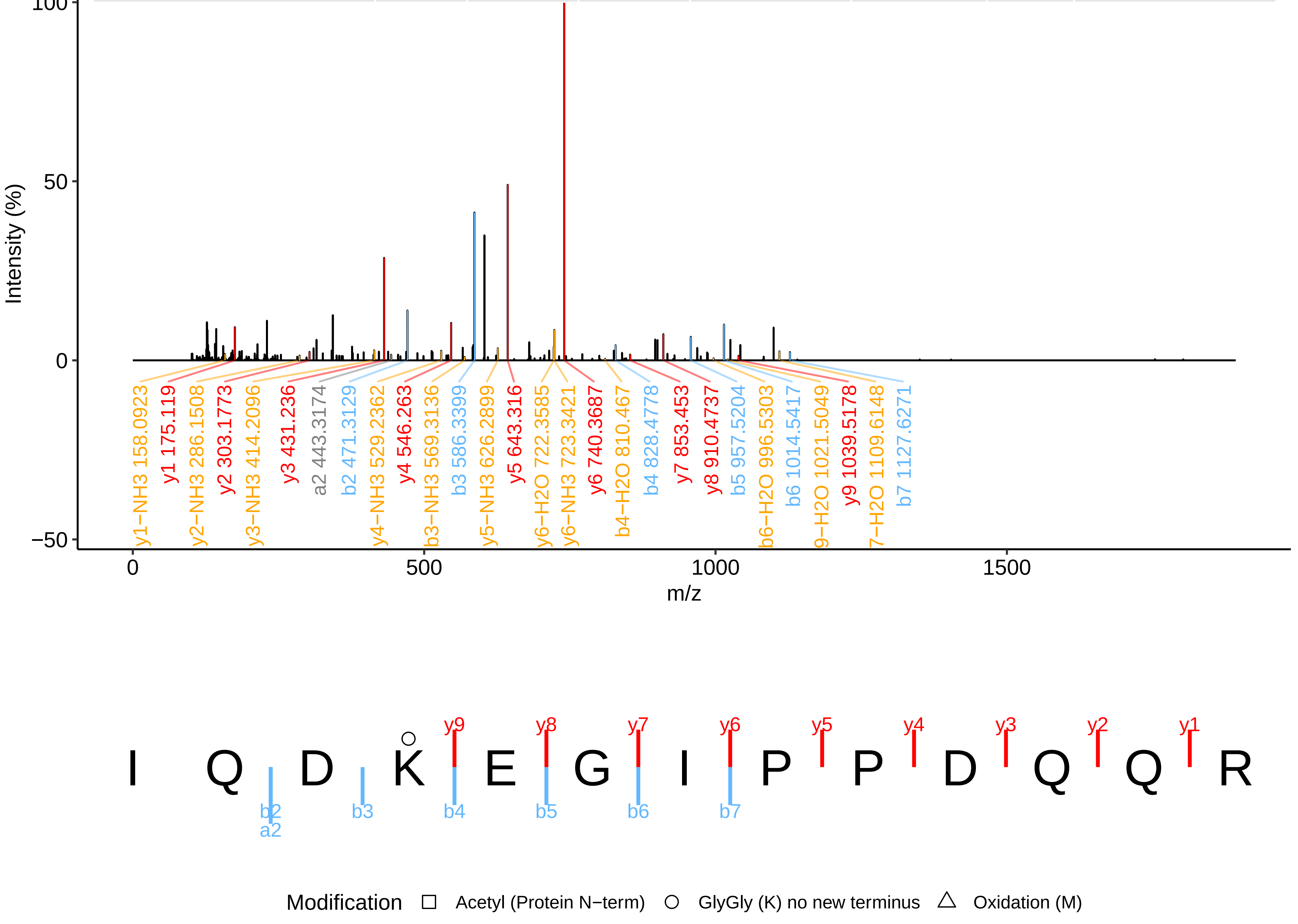

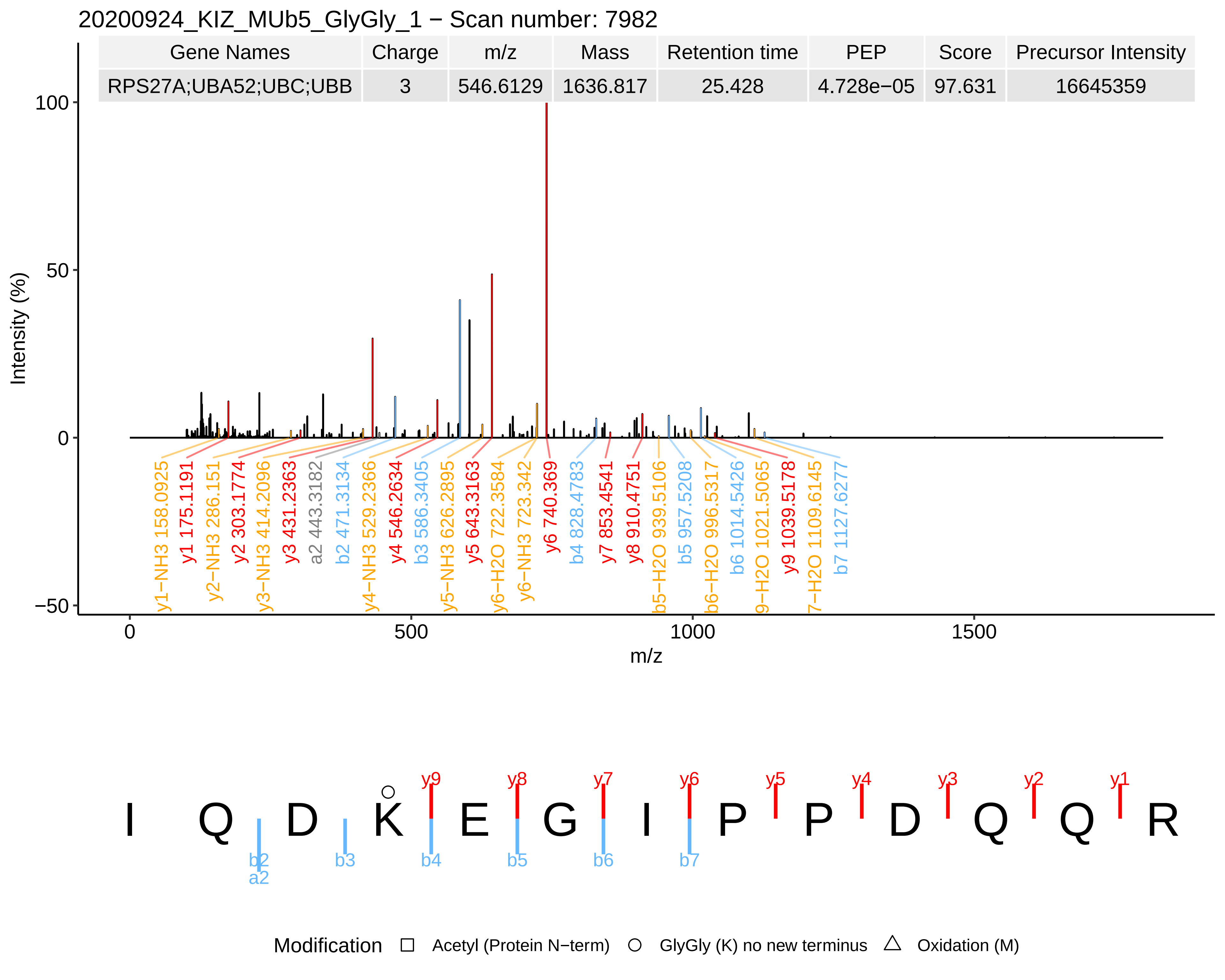

20200924_KIZ_MUb5_GlyGly_1 − Scan number: 7982
Gene Names | Charge | m/z | Mass | Retention time | PEP | Score | Precursor Intensity
RPS27A;UBA52;UBC;UBB | 3 | 546.6129 | 1636.817 | 25.428 | 4.728e−05 | 97.631 | 16645359
Intensity (%)
100
50
0
−50
0
500
1000
1500
m/z
y1−NH3 158.0925
y1 175.1191
y2−NH3 286.151
y2 303.1774
y3−NH3 414.2096
y3 431.2363
a2 443.3182
b2 471.3134
y4−NH3 529.2366
y4 546.2634
b3 586.3405
y5−NH3 626.2895
y5 643.3163
y6−H2O 722.3584
y6−NH3 723.342
y6 740.369
b4 828.4783
y7 853.4541
y8 910.4751
b5−H2O 939.5106
b5 957.5208
b6−H2O 996.5317
b6 1014.5426
9−H2O 1021.5065
y9 1039.5178
7−H2O 1109.6145
b7 1127.6277
I Q D K E G I P P D Q Q R
y9 y8 y7 y6 y5 y4 y3 y2 y1
b2 a2 b3 b4 b5 b6 b7
Modification
Acetyl (Protein N−term)
GlyGly (K) no new terminus
Oxidation (M)

20200924_KIZ_MUb5_GlyGly_2 − Scan number: 5977

Gene Names	Charge	m/z	Mass	Retention time	PEP	Score	Precursor Intensity
RPS27A;UBA52;UBC;UBB	3	546.6129	1636.817	22.244	4.6566e−05	97.631	6371993

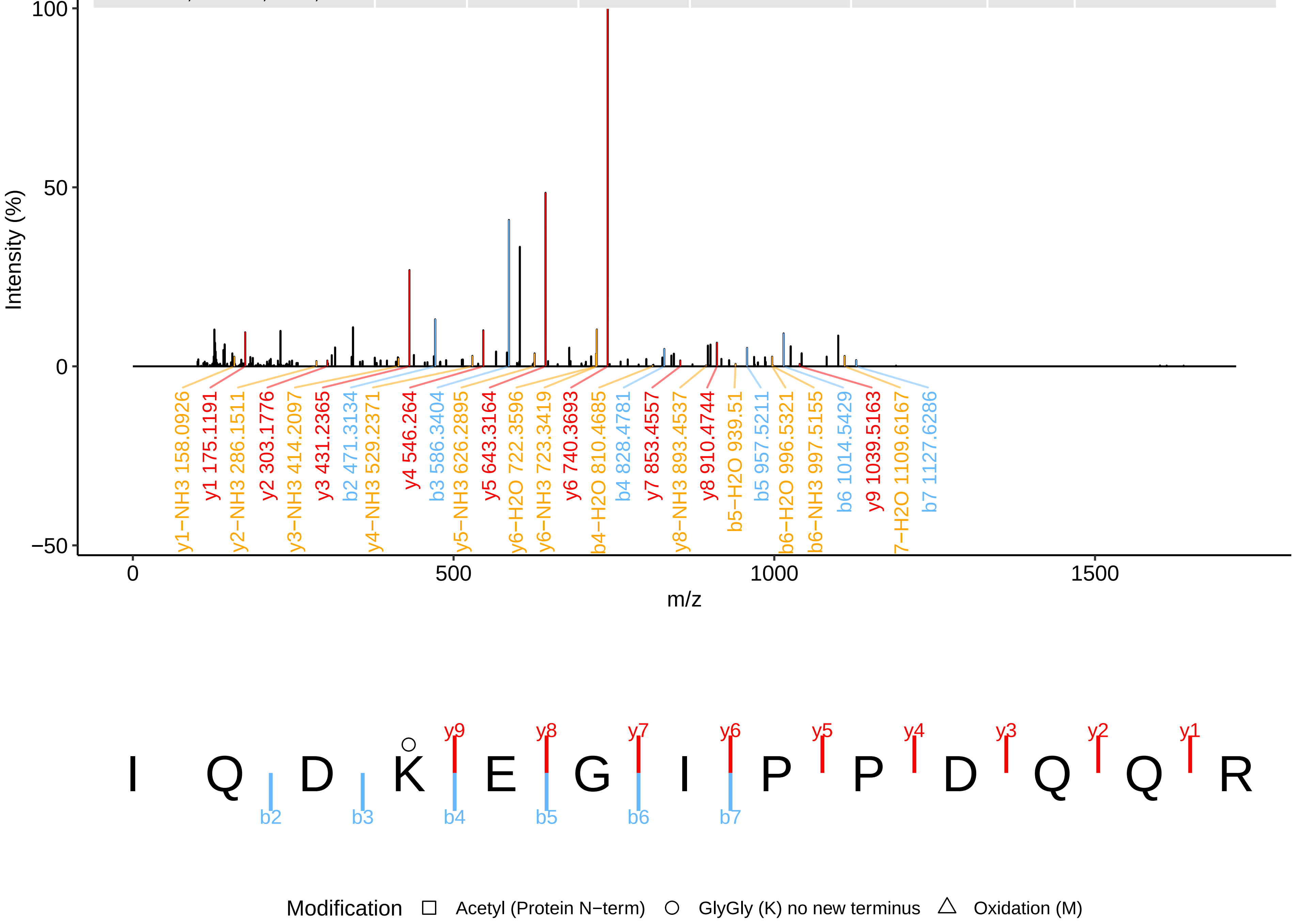

20200924_KIZ_MUb5_GlyGly_2 − Scan number: 6111

Gene Names	Charge	m/z	Mass	Retention time	PEP	Score	Precursor Intensity
RPS27A;UBA52;UBC;UBB	3	546.6129	1636.817	22.76	0.00038494	92.457	13830742

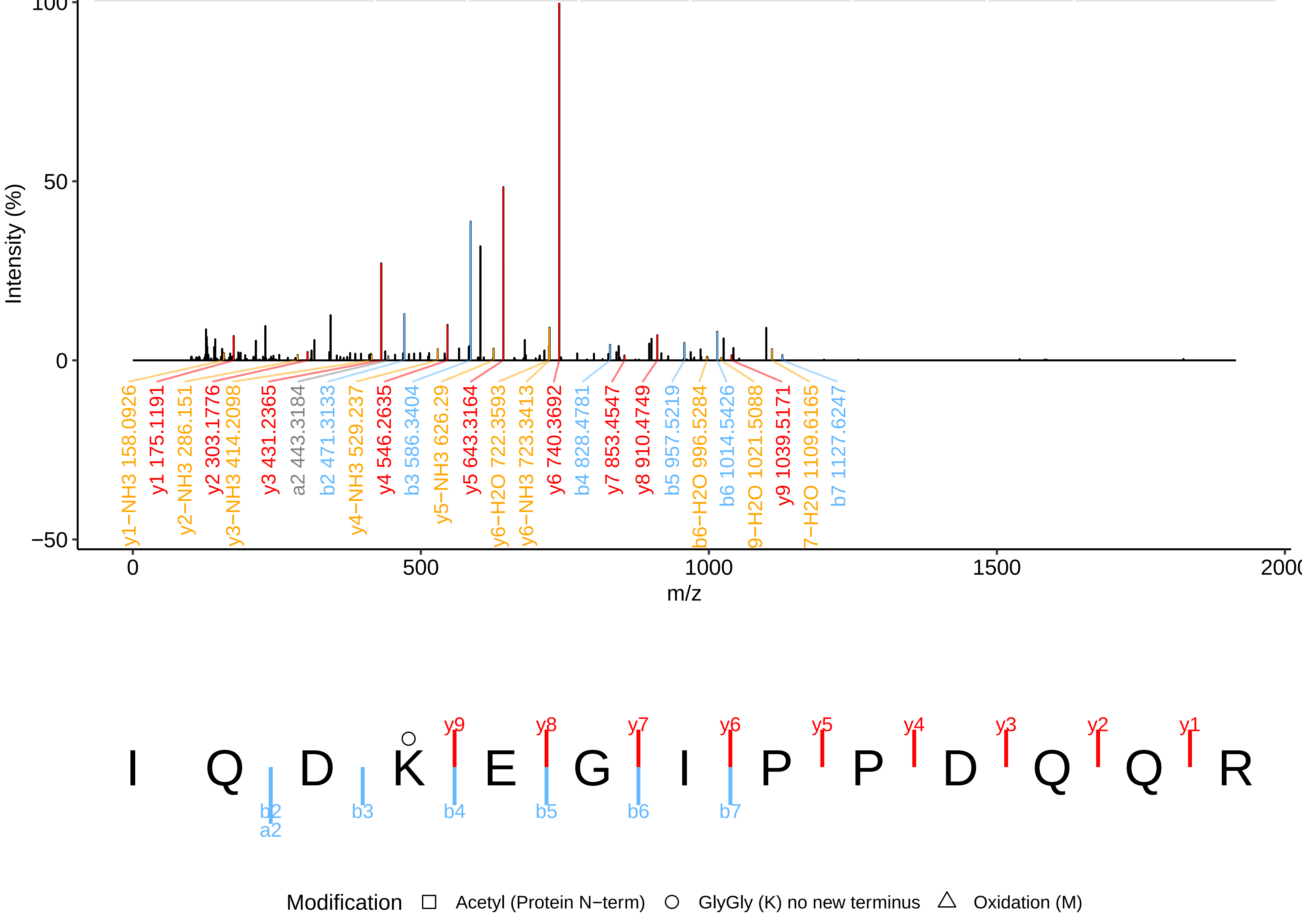

20200924_KIZ_MUb5_GlyGly_2 − Scan number: 6243

Gene Names	Charge	m/z	Mass	Retention time	PEP	Score	Precursor Intensity
RPS27A;UBA52;UBC;UBB	3	546.6129	1636.817	23.271	4.6566e−05	97.631	NA

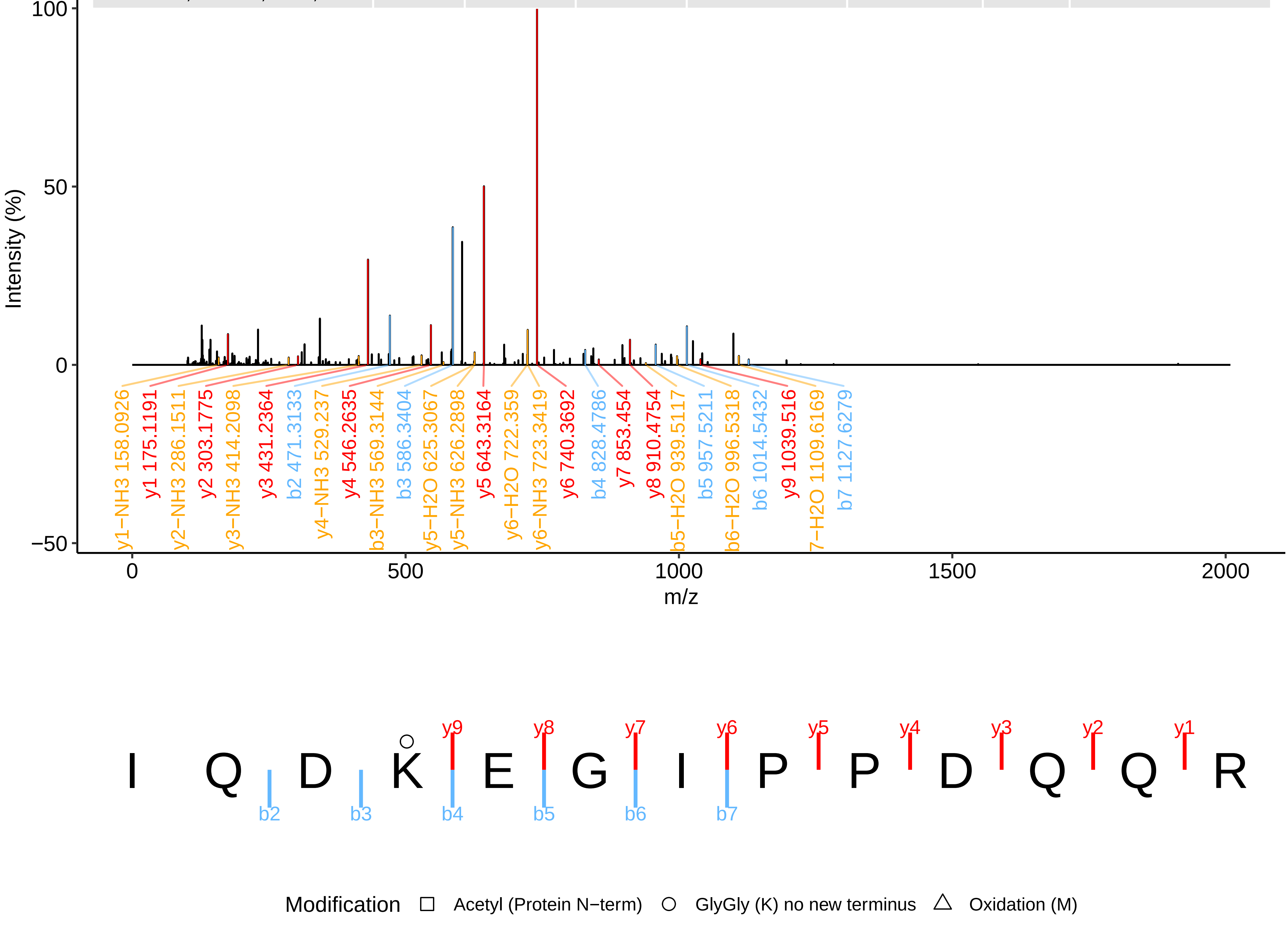

20200924_KIZ_MUb5_GlyGly_2 − Scan number: 6377

Gene Names	Charge	m/z	Mass	Retention time	PEP	Score	Precursor Intensity
RPS27A;UBA52;UBC;UBB	3	546.6129	1636.817	23.779	4.6566e−05	97.631	21384074

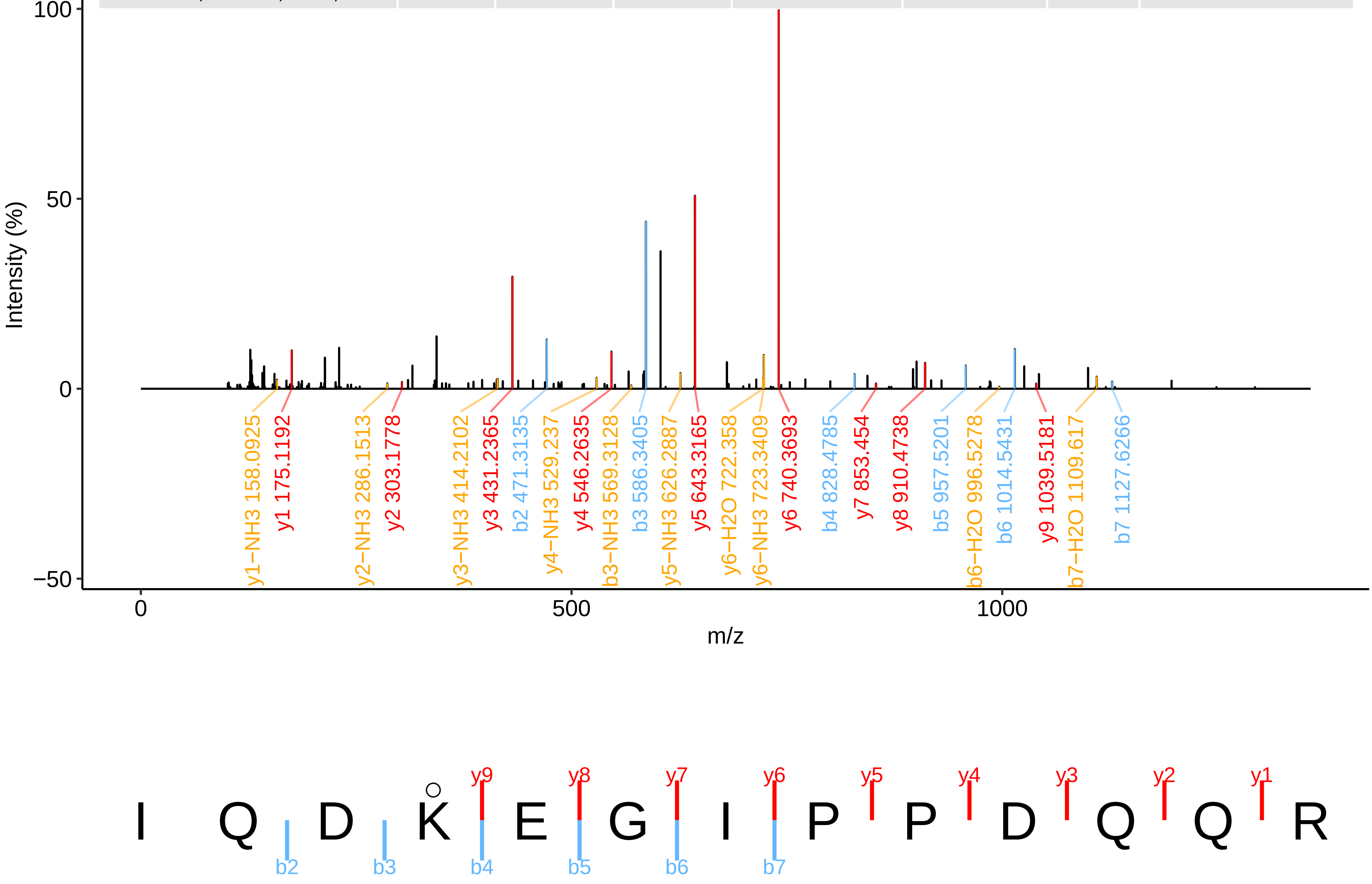

Modification □ Acetyl (Protein N−term) ○ GlyGly (K) no new terminus △ Oxidation (M)

20200924_KIZ_MUb5_GlyGly_2 − Scan number: 6512

Gene Names	Charge	m/z	Mass	Retention time	PEP	Score	Precursor Intensity
RPS27A;UBA52;UBC;UBB	3	546.6129	1636.817	24.295	1.6393e−05	102.73	15672055

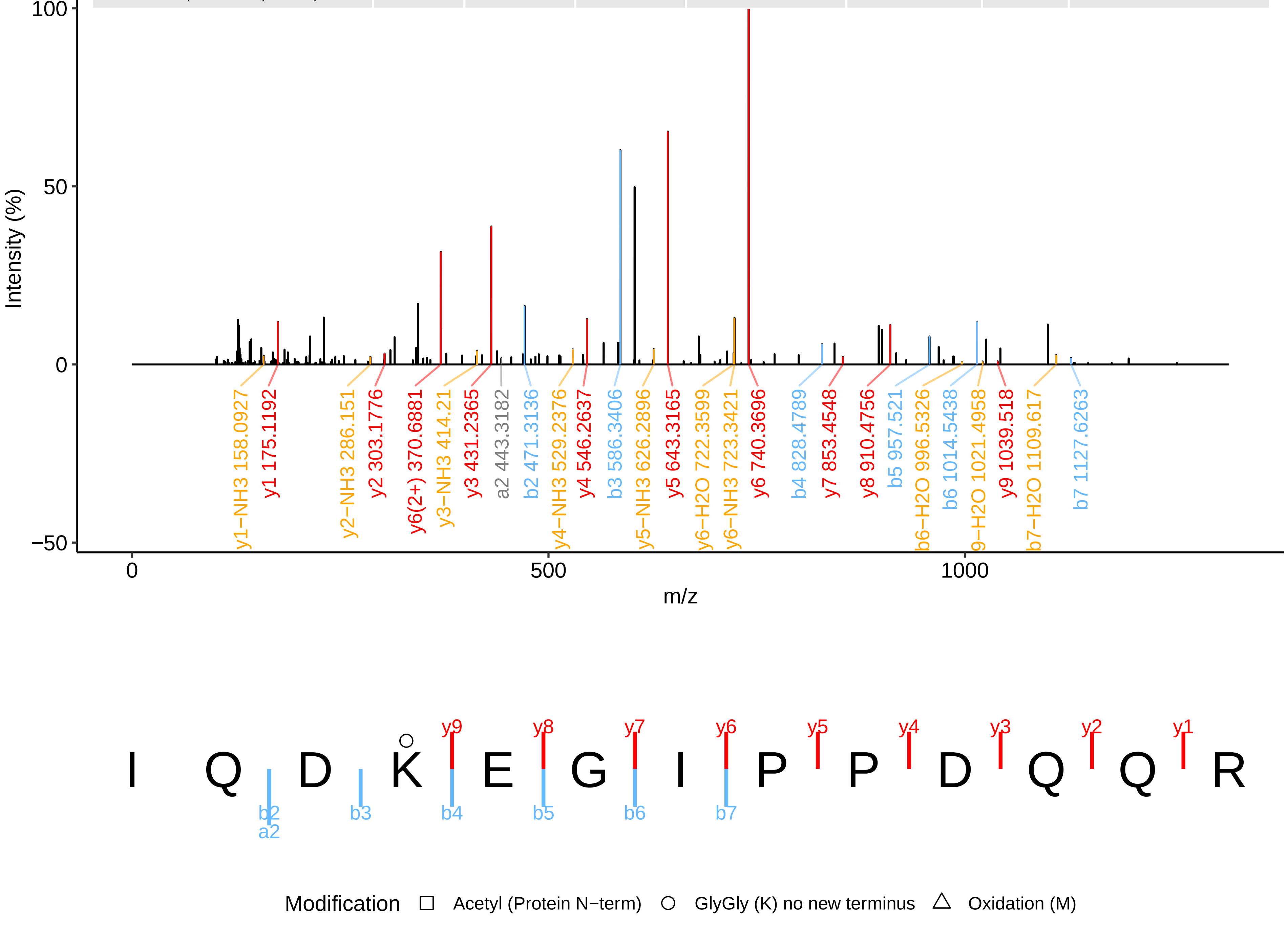

20200924_KIZ_MUb5_GlyGly_2 − Scan number: 6643

Gene Names	Charge	m/z	Mass	Retention time	PEP	Score	Precursor Intensity
RPS27A;UBA52;UBC;UBB	3	546.6129	1636.817	24.8	1.179e−05	106.29	17348922

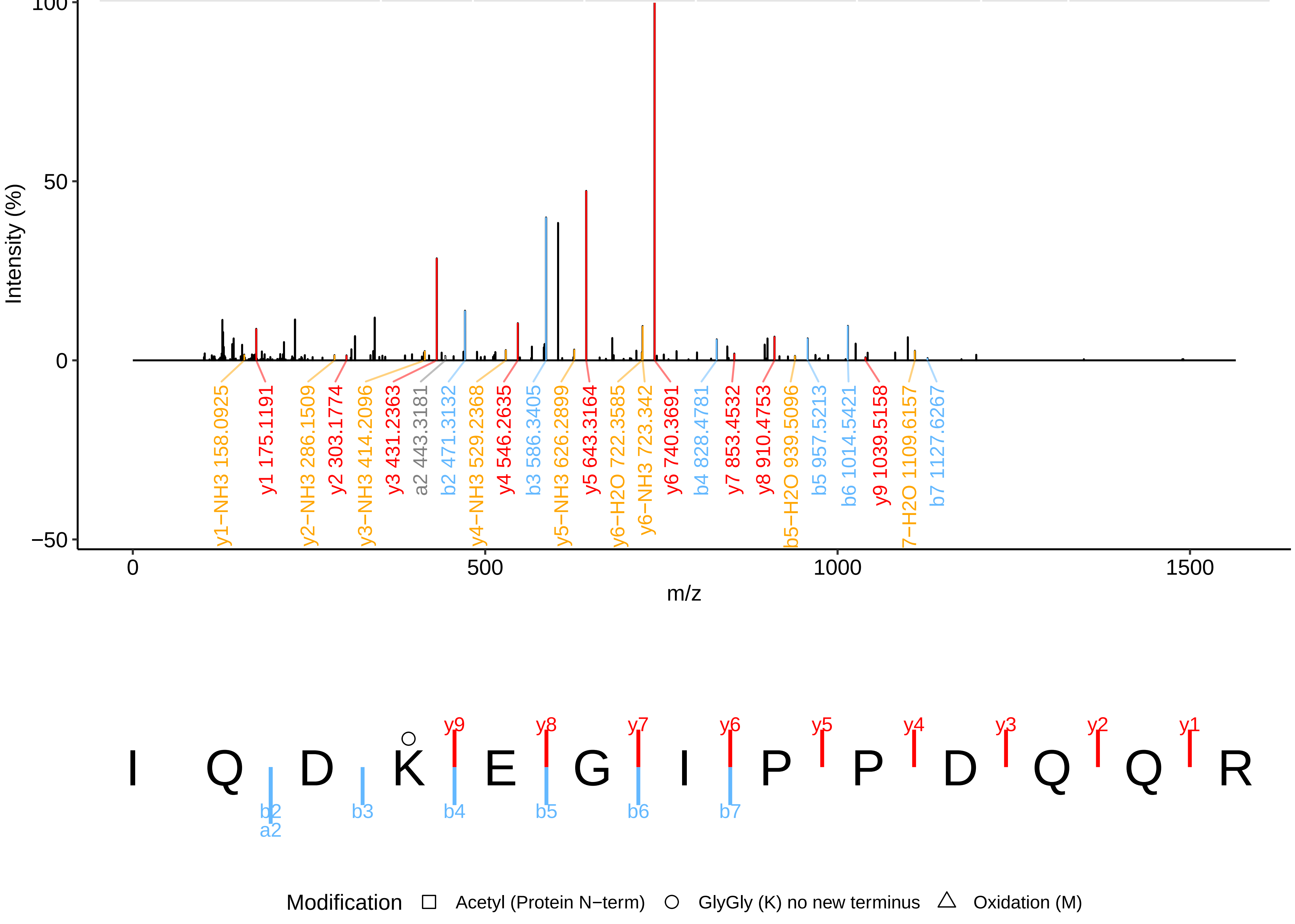

20200924_KIZ_MUb5_GlyGly_2 − Scan number: 6778

Gene Names	Charge	m/z	Mass	Retention time	PEP	Score	Precursor Intensity
RPS27A;UBA52;UBC;UBB	3	546.6129	1636.817	25.3	0.00088185	84.859	22678766

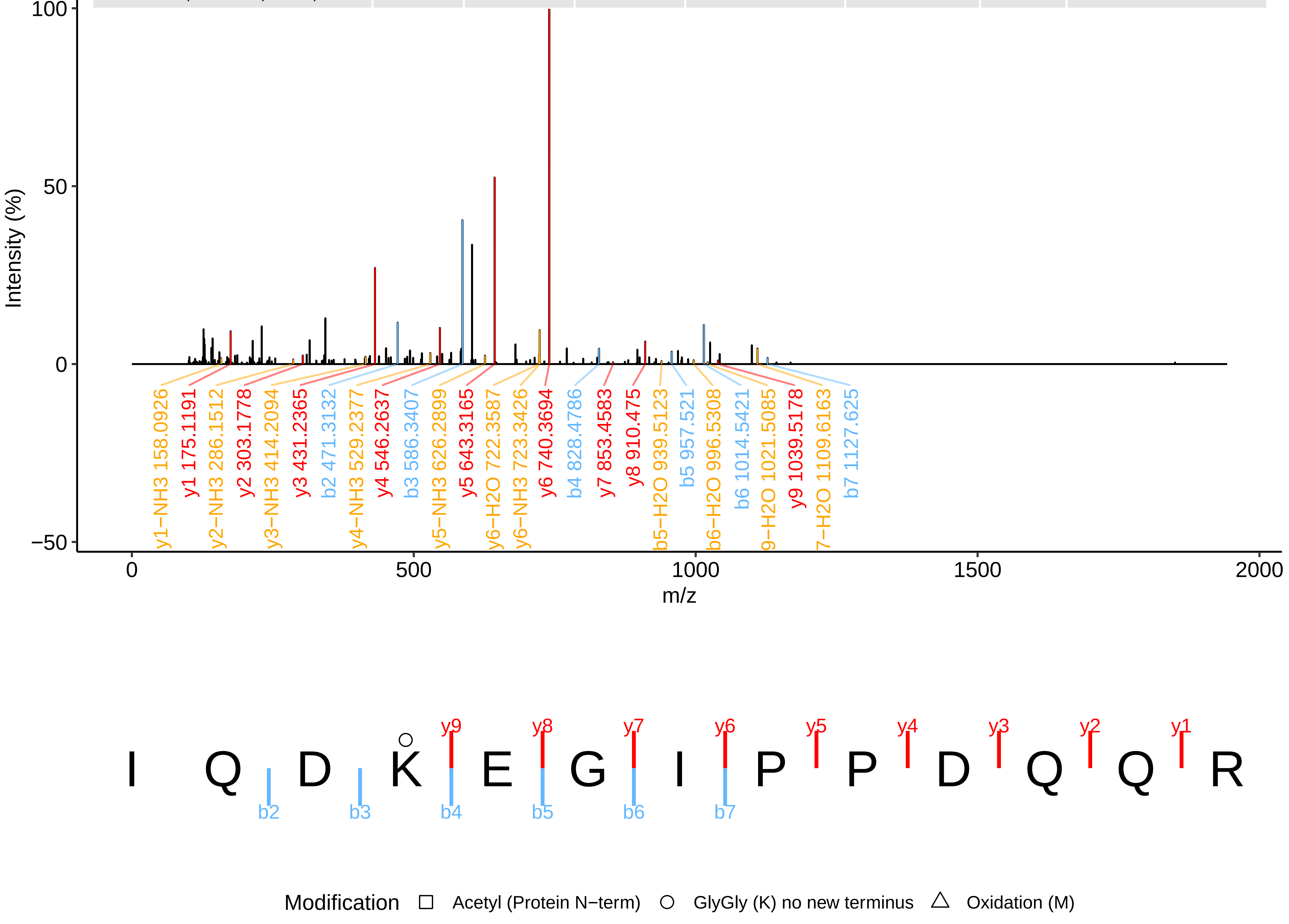

20200924_KIZ_MUb5_GlyGly_2 − Scan number: 6917

Gene Names	Charge	m/z	Mass	Retention time	PEP	Score	Precursor Intensity
RPS27A;UBA52;UBC;UBB	3	546.6129	1636.817	25.804	1.1422e−05	106.58	14508983

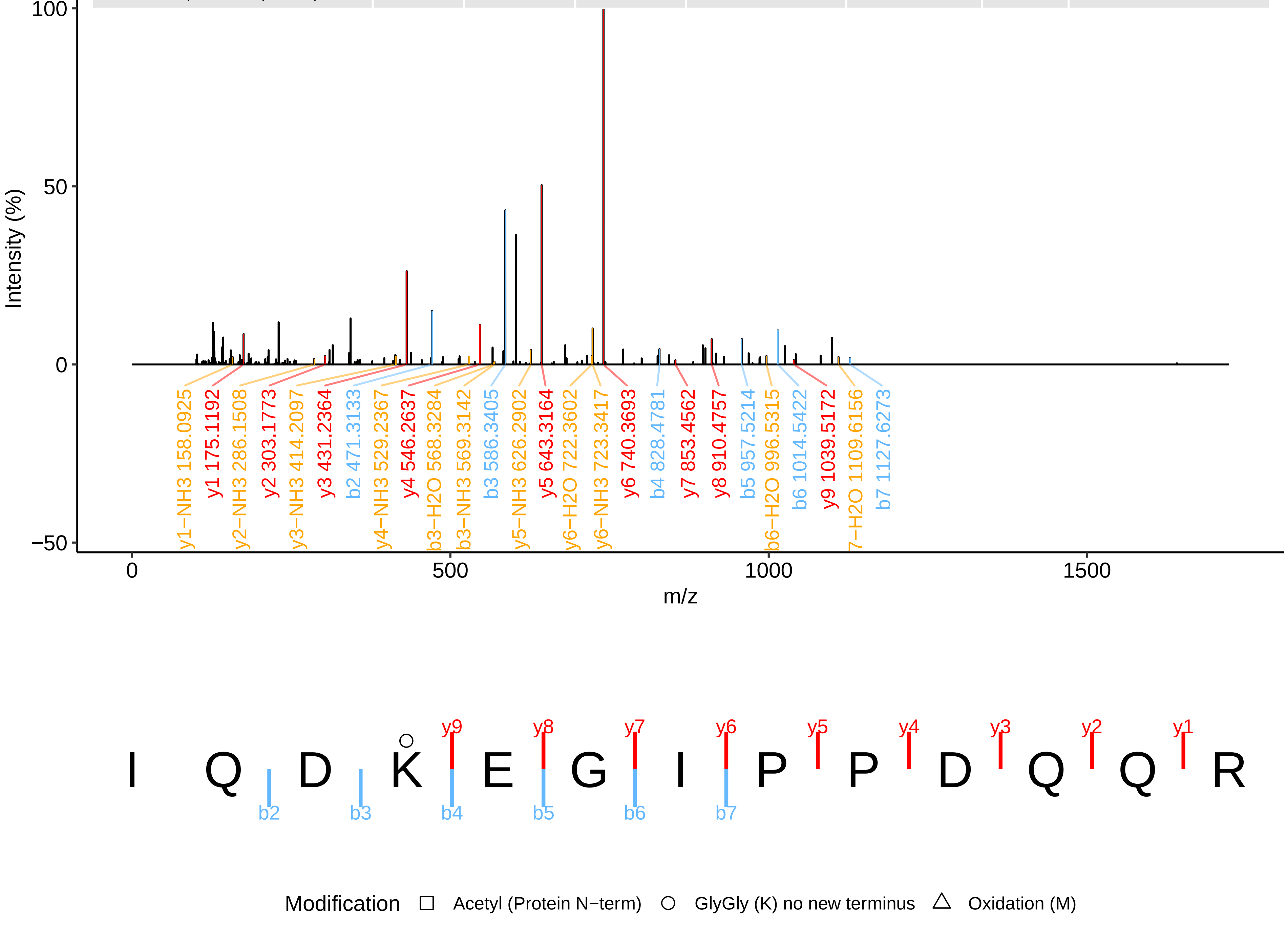

20200924_KIZ_MUb5_GlyGly_2 − Scan number: 7057

Gene Names	Charge	m/z	Mass	Retention time	PEP	Score	Precursor Intensity
RPS27A;UBA52;UBC;UBB	3	546.6129	1636.817	26.319	0.00037326	92.636	8401748

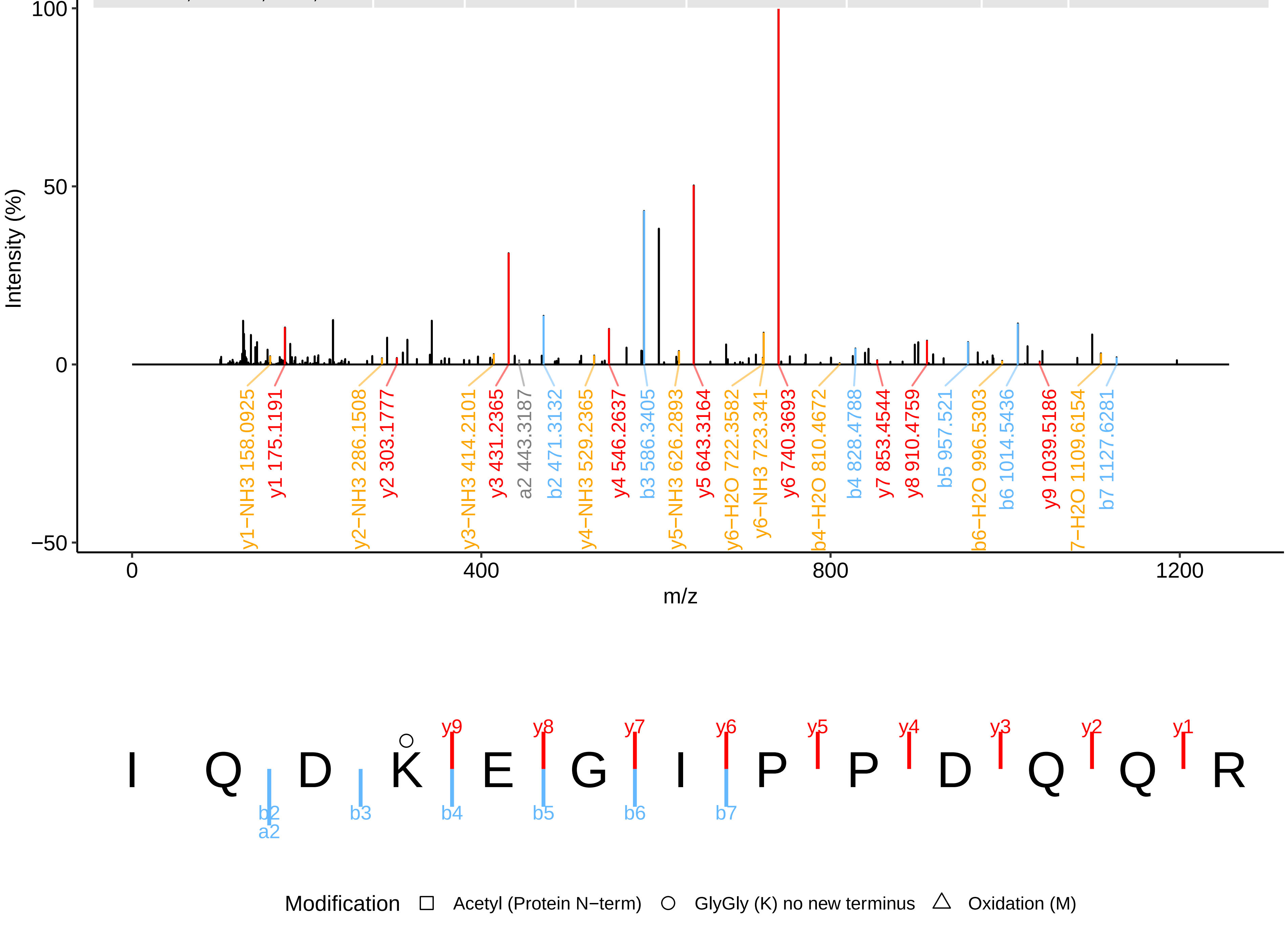

20200924_KIZ_MUb5_GlyGly_2 − Scan number: 7194

Gene Names	Charge	m/z	Mass	Retention time	PEP	Score	Precursor Intensity
RPS27A;UBA52;UBC;UBB	3	546.6129	1636.817	26.833	1.4365e−05	104.3	10217412

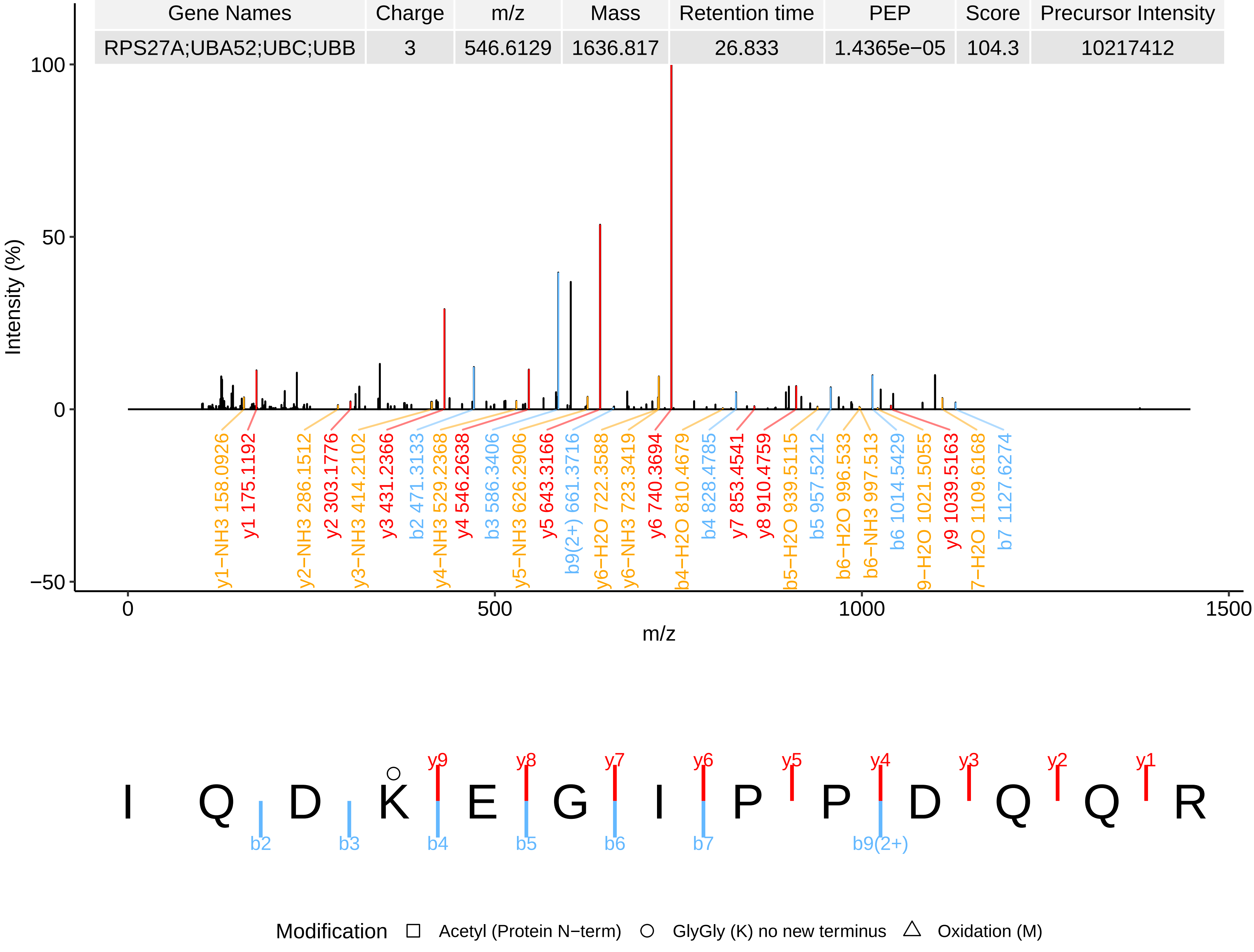

20200924_KIZ_MUb5_GlyGly_2 − Scan number: 7327

Gene Names	Charge	m/z	Mass	Retention time	PEP	Score	Precursor Intensity
RPS27A;UBA52;UBC;UBB	3	546.6129	1636.817	27.342	1.179e−05	106.29	6331214

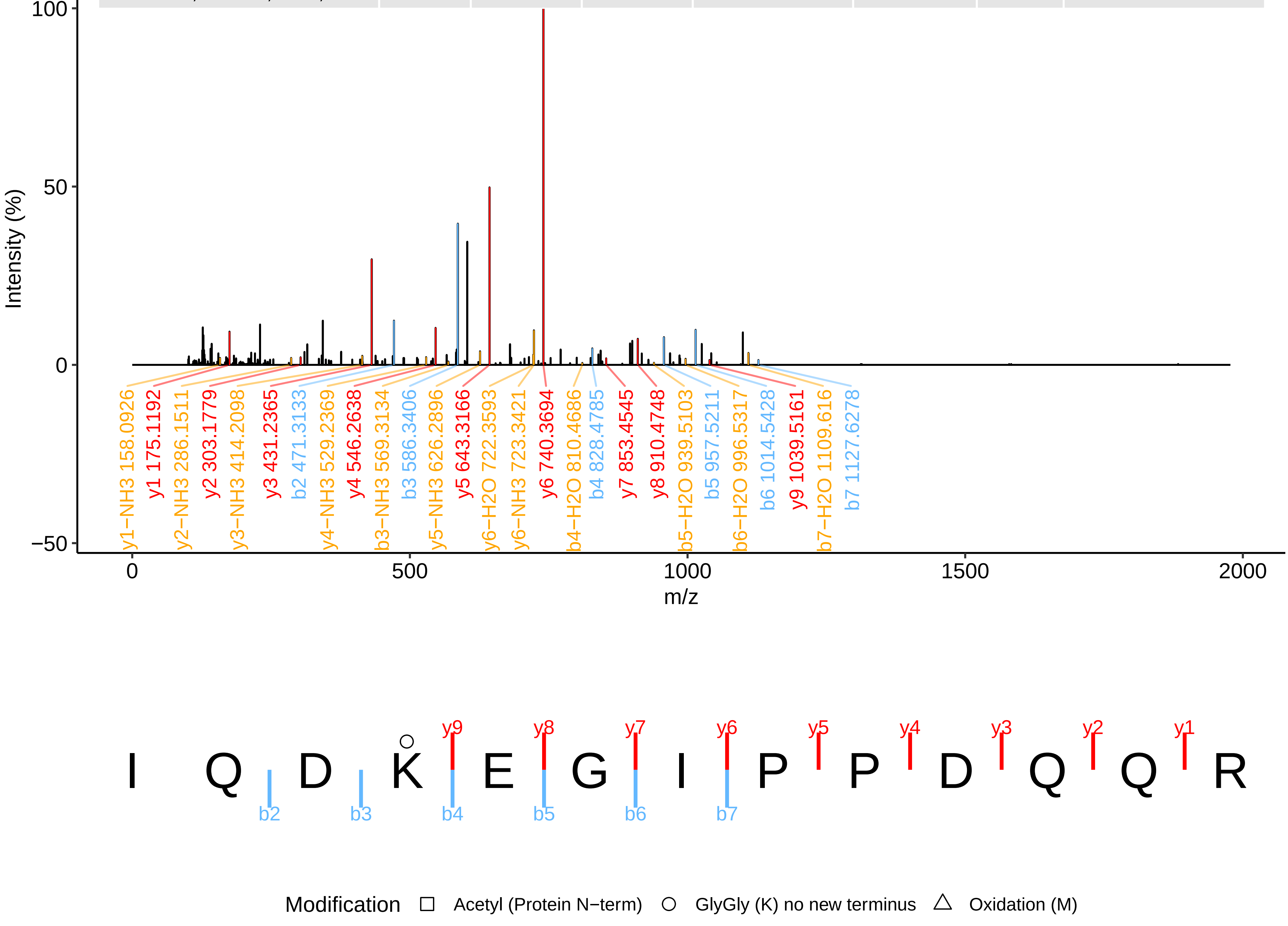

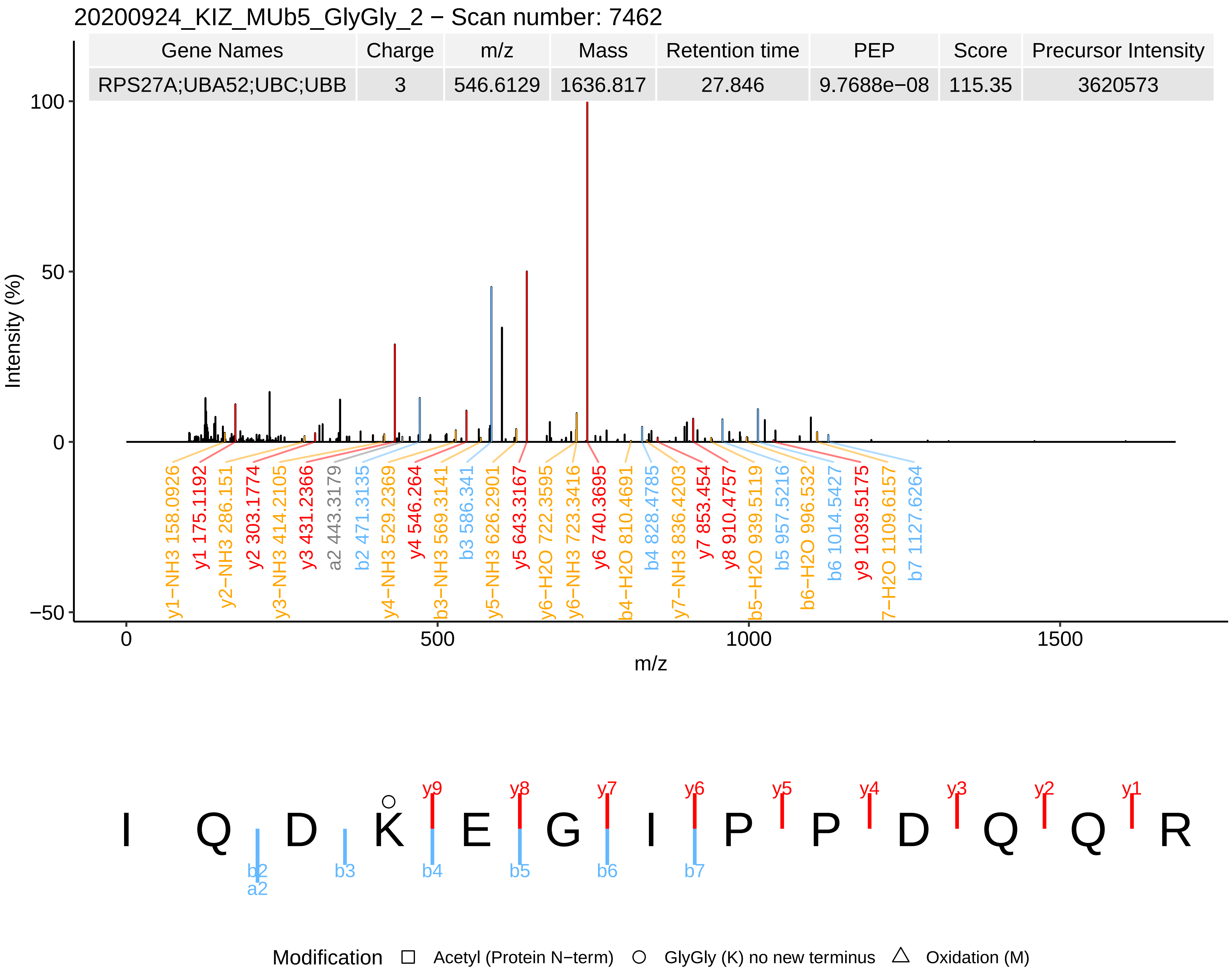

20200924_KIZ_MUb5_GlyGly_2 − Scan number: 7462

Gene Names	Charge	m/z	Mass	Retention time	PEP	Score	Precursor Intensity
RPS27A;UBA52;UBC;UBB	3	546.6129	1636.817	27.846	9.7688e−08	115.35	3620573

Intensity (%)
m/z
y1−NH3 158.0926
y1 175.1192
y2−NH3 286.151
y2 303.1774
y3−NH3 414.2105
y3 431.2366
a2 443.3179
b2 471.3135
y4−NH3 529.2369
y4 546.264
b3−NH3 569.3141
b3 586.341
y5−NH3 626.2901
y5 643.3167
y6−H2O 722.3595
y6−NH3 723.3416
y6 740.3695
b4−H2O 810.4691
b4 828.4785
y7−NH3 836.4203
y7 853.454
y8 910.4757
b5−H2O 939.5119
b5 957.5216
b6−H2O 996.532
b6 1014.5427
y9 1039.5175
y7−H2O 1109.6157
b7 1127.6264
I Q D K E G I P P D Q Q R
Modification
Acetyl (Protein N−term)
GlyGly (K) no new terminus
Oxidation (M)

20200924_KIZ_MUb5_GlyGly_2 − Scan number: 7768

Gene Names	Charge	m/z	Mass	Retention time	PEP	Score	Precursor Intensity
RPS27A;UBA52;UBC;UBB	3	546.6129	1636.817	29.019	0.0029015	77.367	4346691

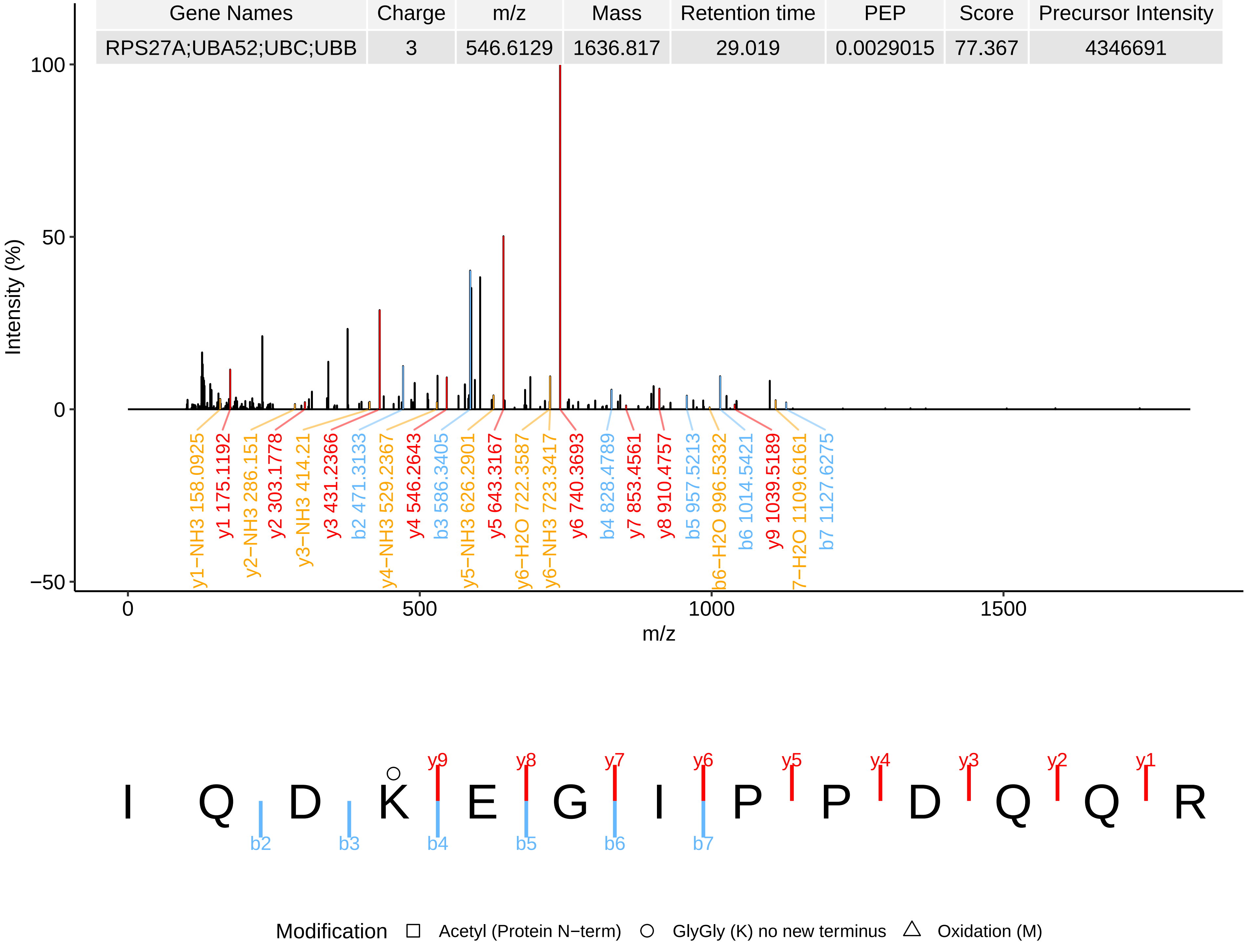

20200924_KIZ_MUb5_GlyGly_2 − Scan number: 7905

Gene Names	Charge	m/z	Mass	Retention time	PEP	Score	Precursor Intensity
RPS27A;UBA52;UBC;UBB	3	546.6129	1636.817	29.538	0.0055783	72.417	3983732

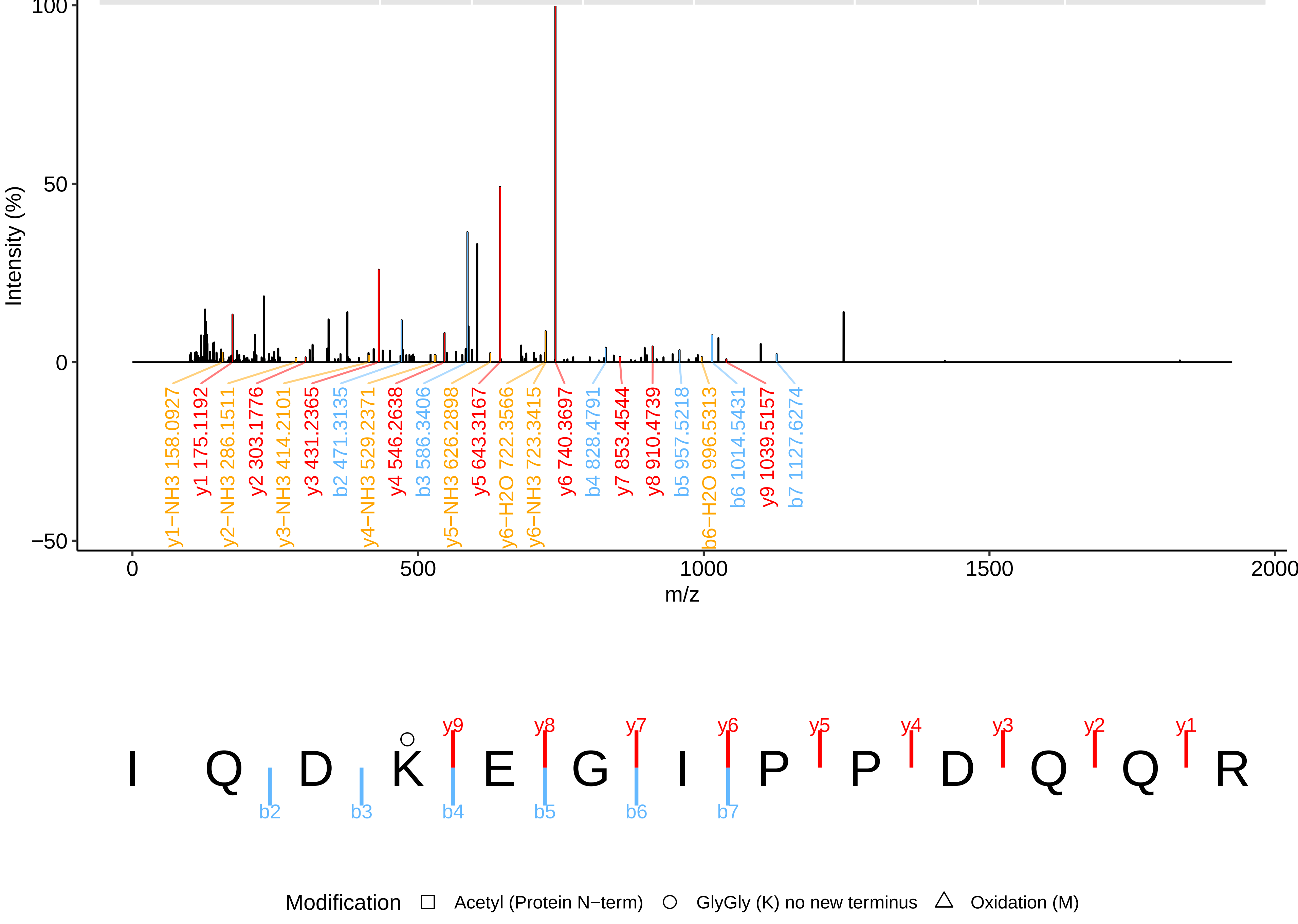

20200924_KIZ_MUb5_GlyGly_2 − Scan number: 8192

Gene Names	Charge	m/z	Mass	Retention time	PEP	Score	Precursor Intensity
RPS27A;UBA52;UBC;UBB	3	546.6129	1636.817	30.584	0.010187	63.894	5312255

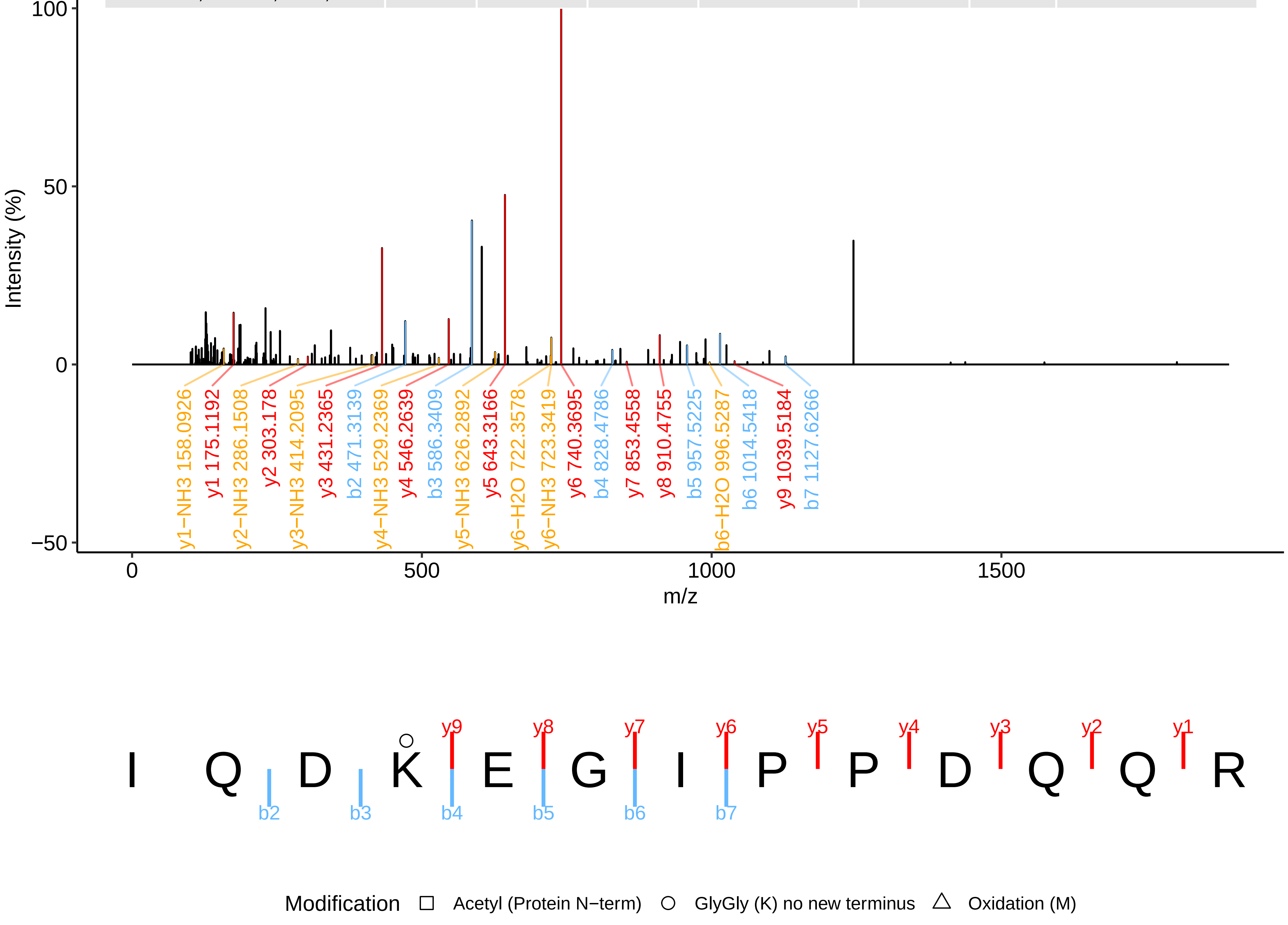

20200924_KIZ_MUb5_GlyGly_2 − Scan number: 8036

Gene Names	Charge	m/z	Mass	Retention time	PEP	Score	Precursor Intensity
RPS27A;UBA52;UBC;UBB	3	546.6129	1636.817	30.045	0.0064295	72.031	2474259

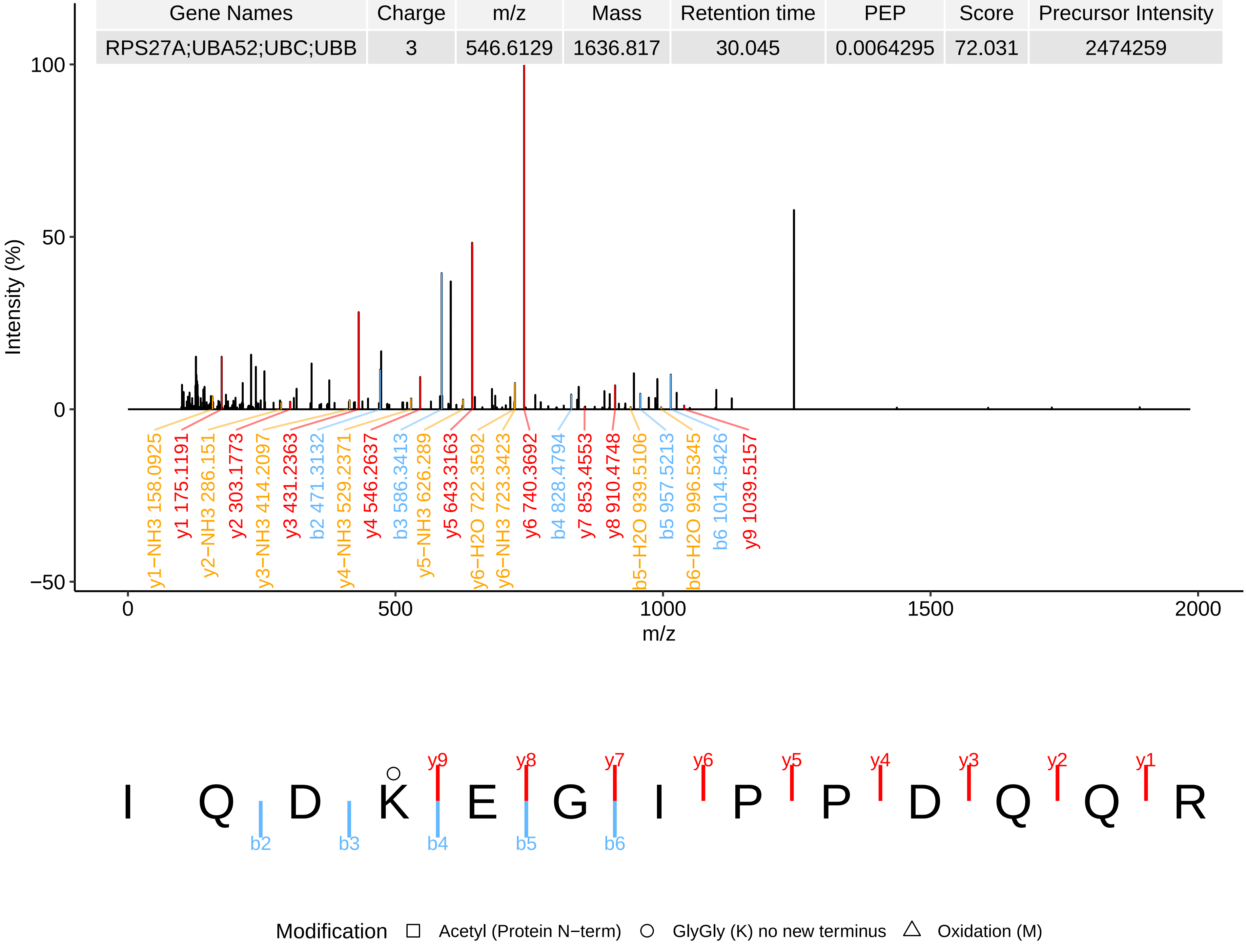

20200924_KIZ_MUb5_GlyGly_2 − Scan number: 8329

Gene Names	Charge	m/z	Mass	Retention time	PEP	Score	Precursor Intensity
RPS27A;UBA52;UBC;UBB	3	546.6129	1636.817	31.12	0.0073931	70.373	741527.1

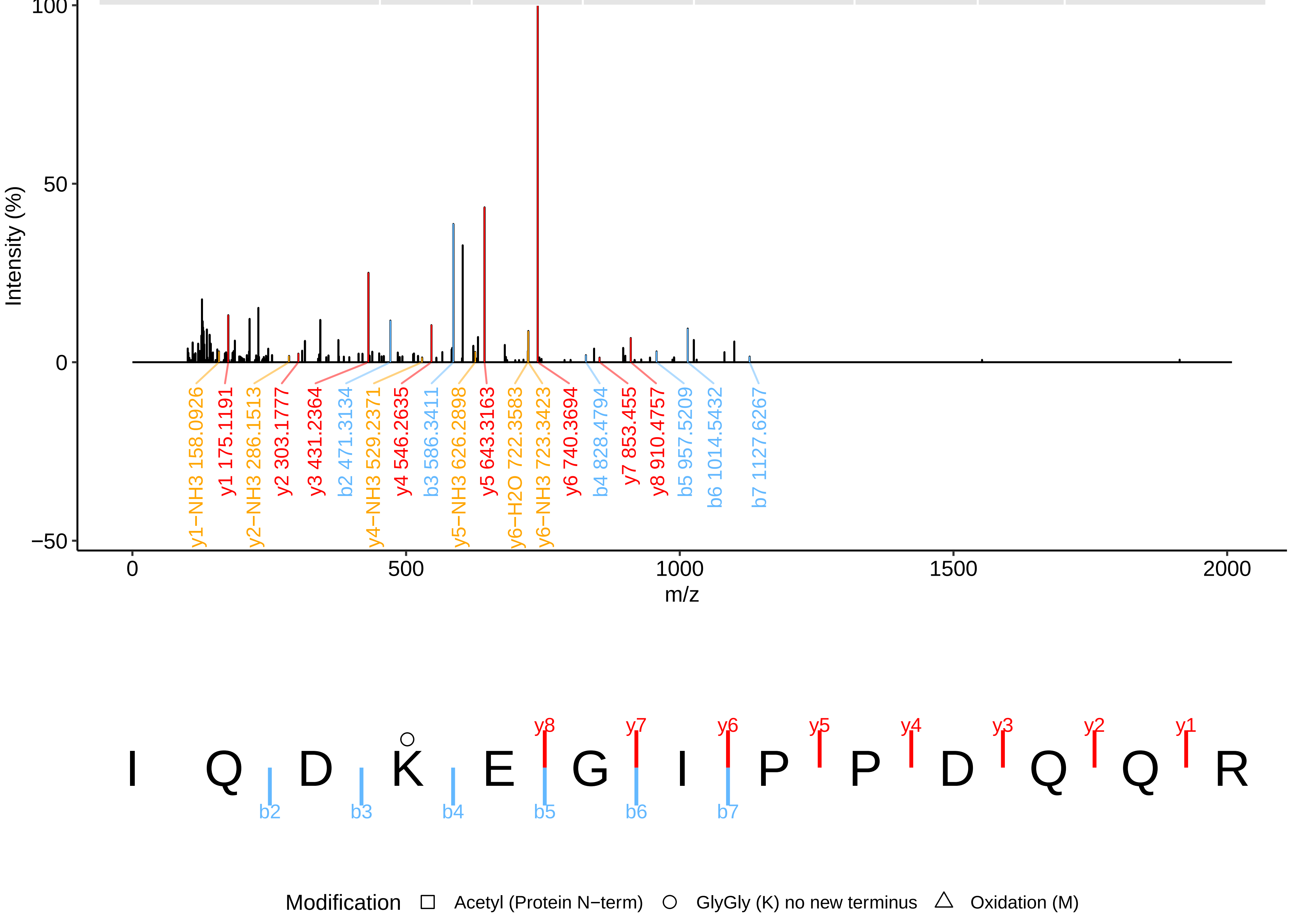

20200921_KIZ_MUb4_GlyGly_10plex − Scan number: 19679

Gene Names	Charge	m/z	Mass	Retention time	PEP	Score	Precursor Intensity
HTRA2	3	559.6613	1675.962	71.017	0.020423	59.709	1183570

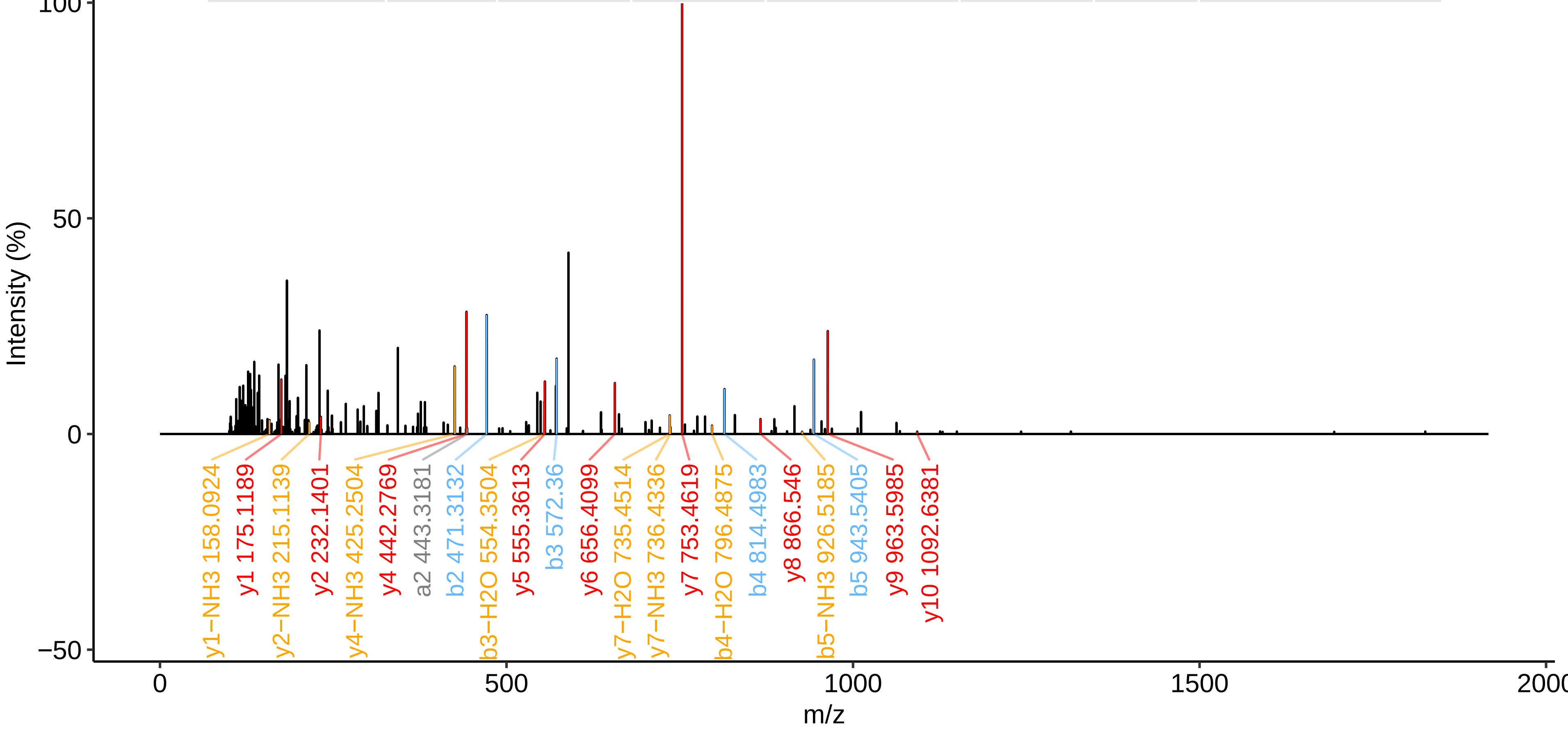

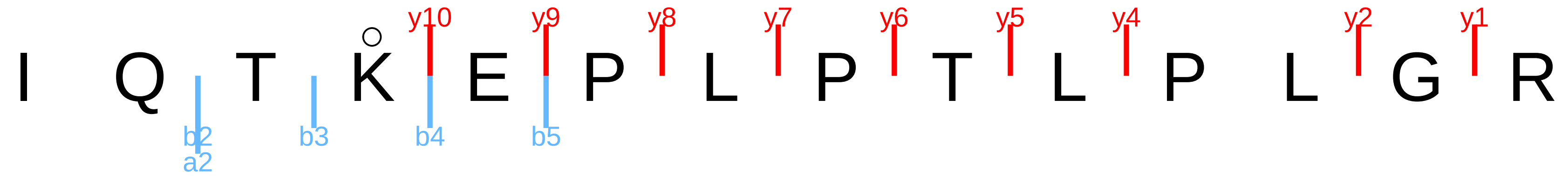

Modification □ Acetyl (Protein N−term) ○ GlyGly (K) no new terminus △ Oxidation (M)

20200921_KIZ_MUb4_GlyGly_10plex_2 − Scan number: 18154

Gene Names	Charge	m/z	Mass	Retention time	PEP	Score	Precursor Intensity
HTRA2	3	559.6613	1675.962	71.058	0.001568	78.401	688906.4

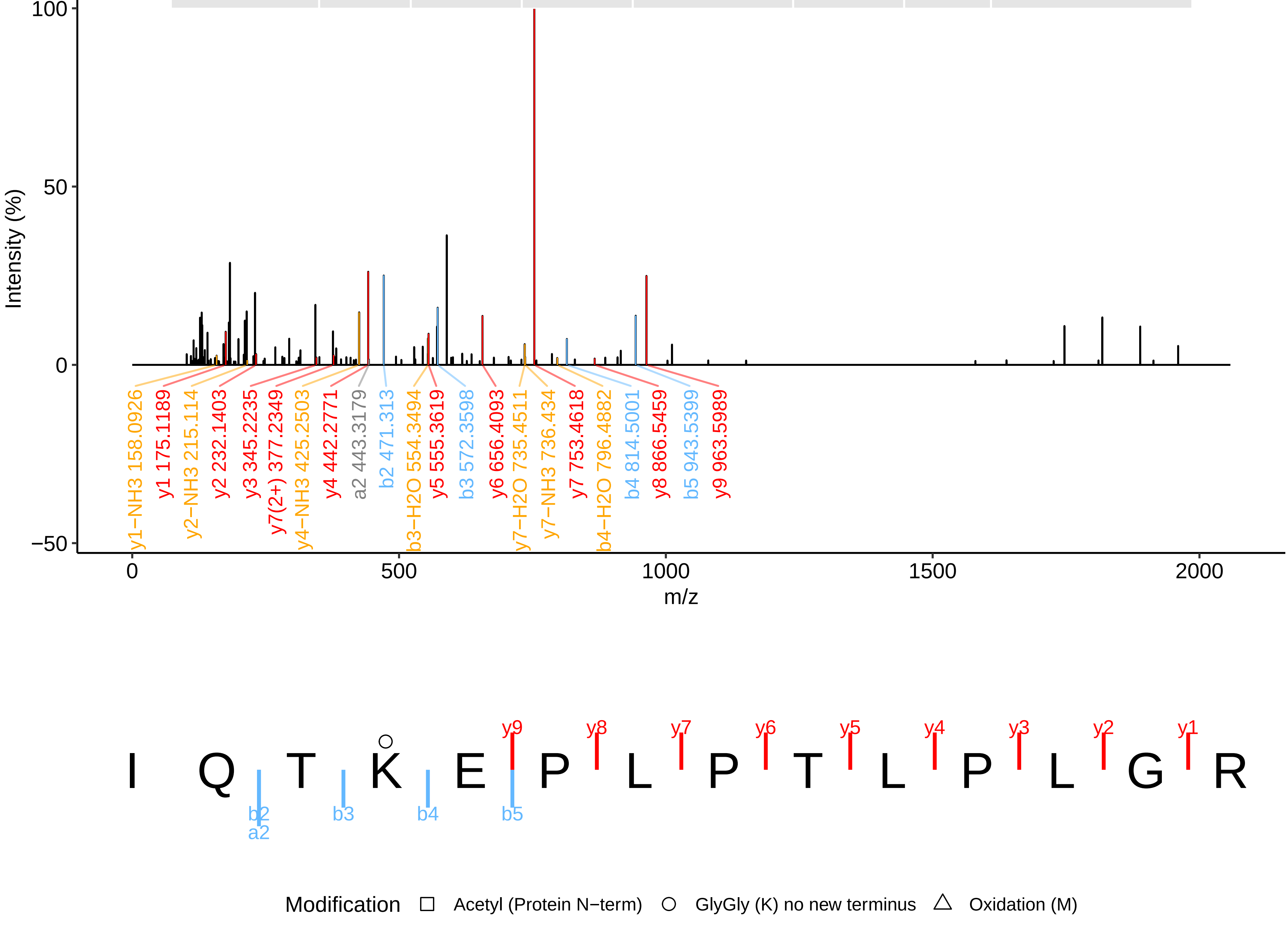

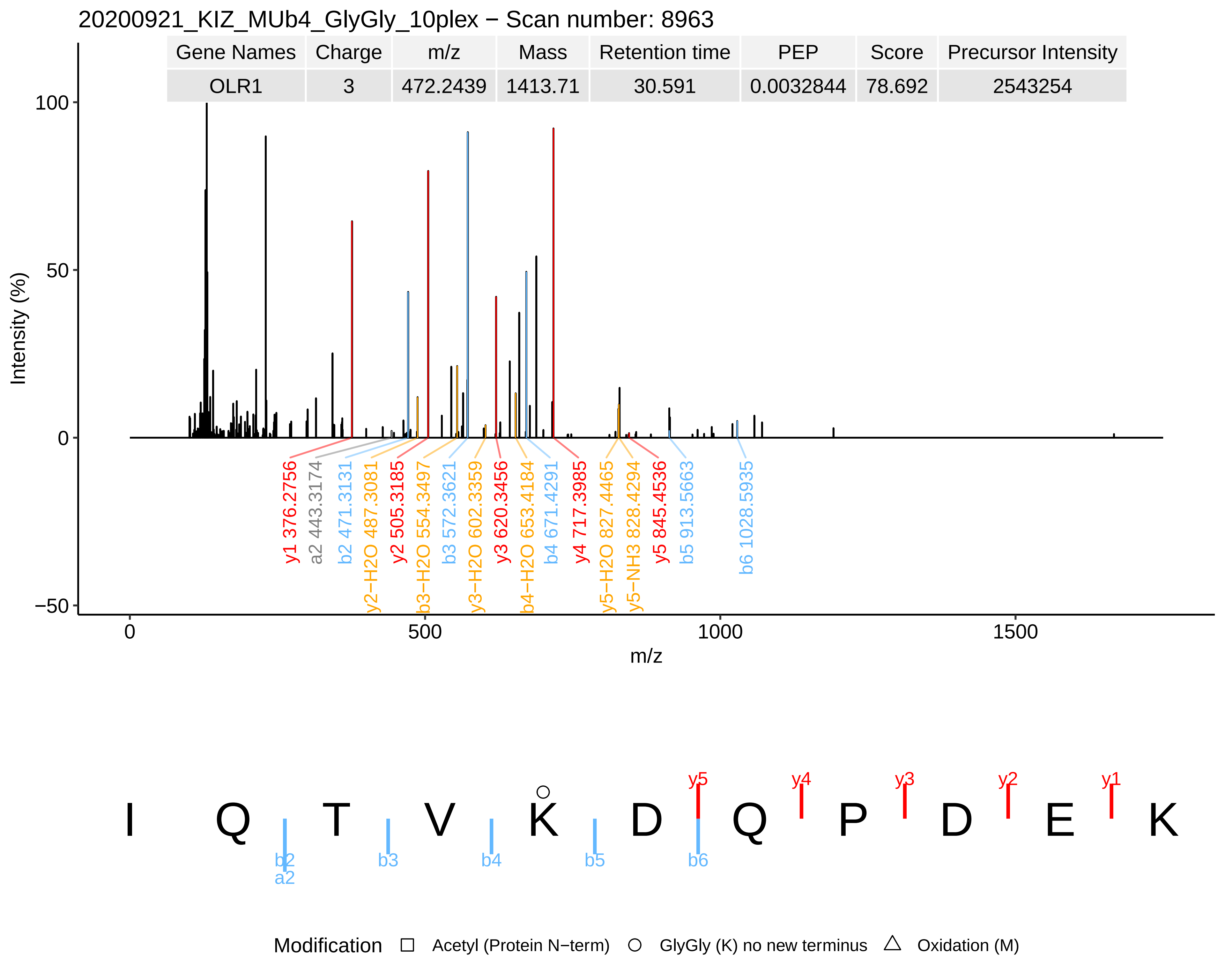
20200921_KIZ_MUb4_GlyGly_10plex − Scan number: 8963
Gene Names | Charge | m/z | Mass | Retention time | PEP | Score | Precursor Intensity
OLR1 | 3 | 472.2439 | 1413.71 | 30.591 | 0.0032844 | 78.692 | 2543254
Intensity (%)
100
50
0
−50
m/z
0
500
1000
1500
y1 376.2756
a2 443.3174
b2 471.3131
y2−H2O 487.3081
y2 505.3185
b3−H2O 554.3497
b3 572.3621
y3−H2O 602.3359
y3 620.3456
b4−H2O 653.4184
b4 671.4291
y4 717.3985
y5−H2O 827.4465
y5−NH3 828.4294
y5 845.4536
b5 913.5663
b6 1028.5935
I Q T V K D Q P D E K
y5 y4 y3 y2 y1
b2 a2 b3 b4 b5 b6
Modification
Acetyl (Protein N−term)
GlyGly (K) no new terminus
Oxidation (M)

20200921_KIZ_MUb4_GlyGly_10plex − Scan number: 9096

Gene Names	Charge	m/z	Mass	Retention time	PEP	Score	Precursor Intensity
OLR1	3	472.2439	1413.71	31.105	0.05803	55.04	5780796

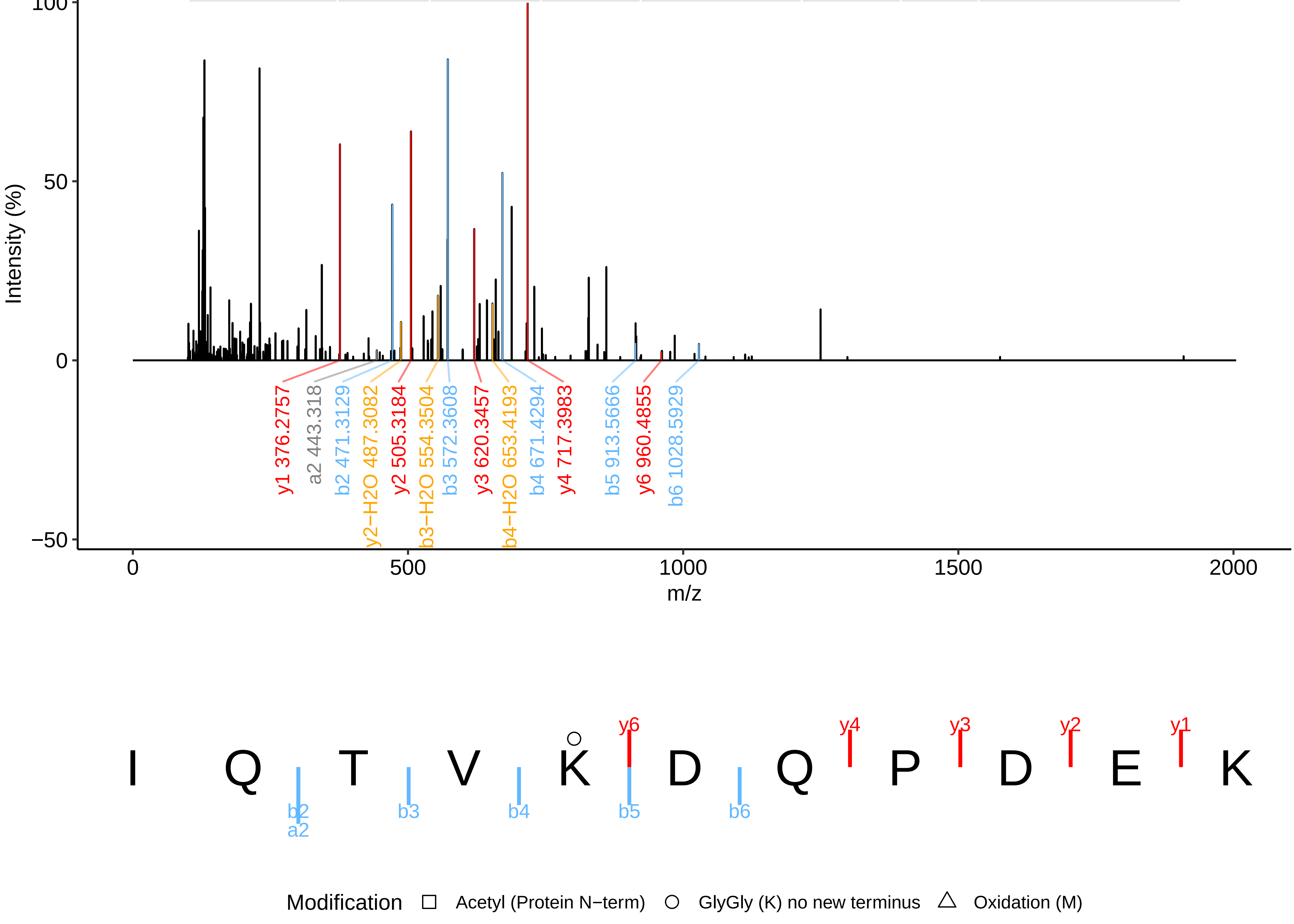

20200921_KIZ_MUb4_GlyGly_10plex_2 − Scan number: 8263

Gene Names	Charge	m/z	Mass	Retention time	PEP	Score	Precursor Intensity
OLR1	3	472.2439	1413.71	31.083	0.0065198	74.76	1218190

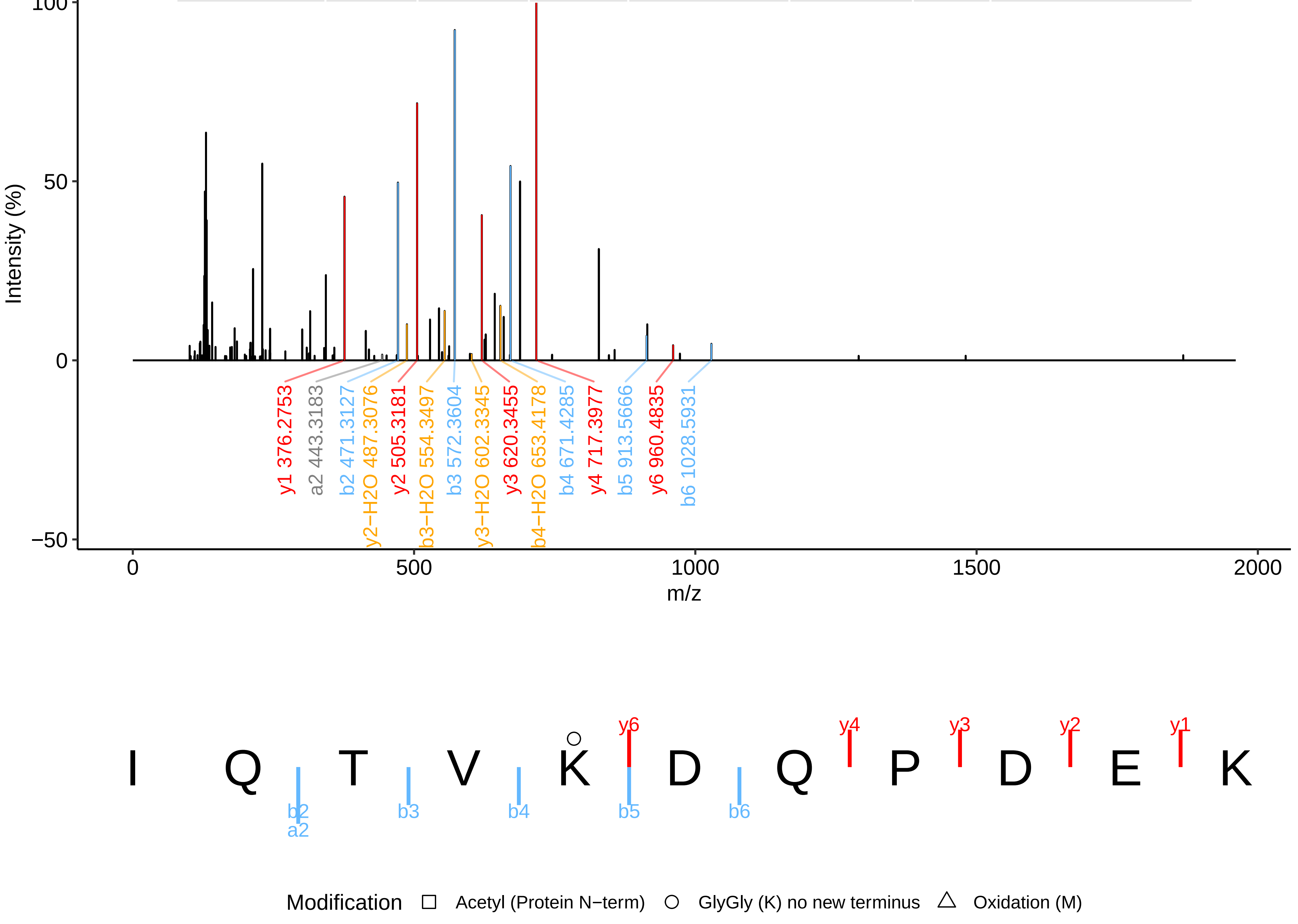

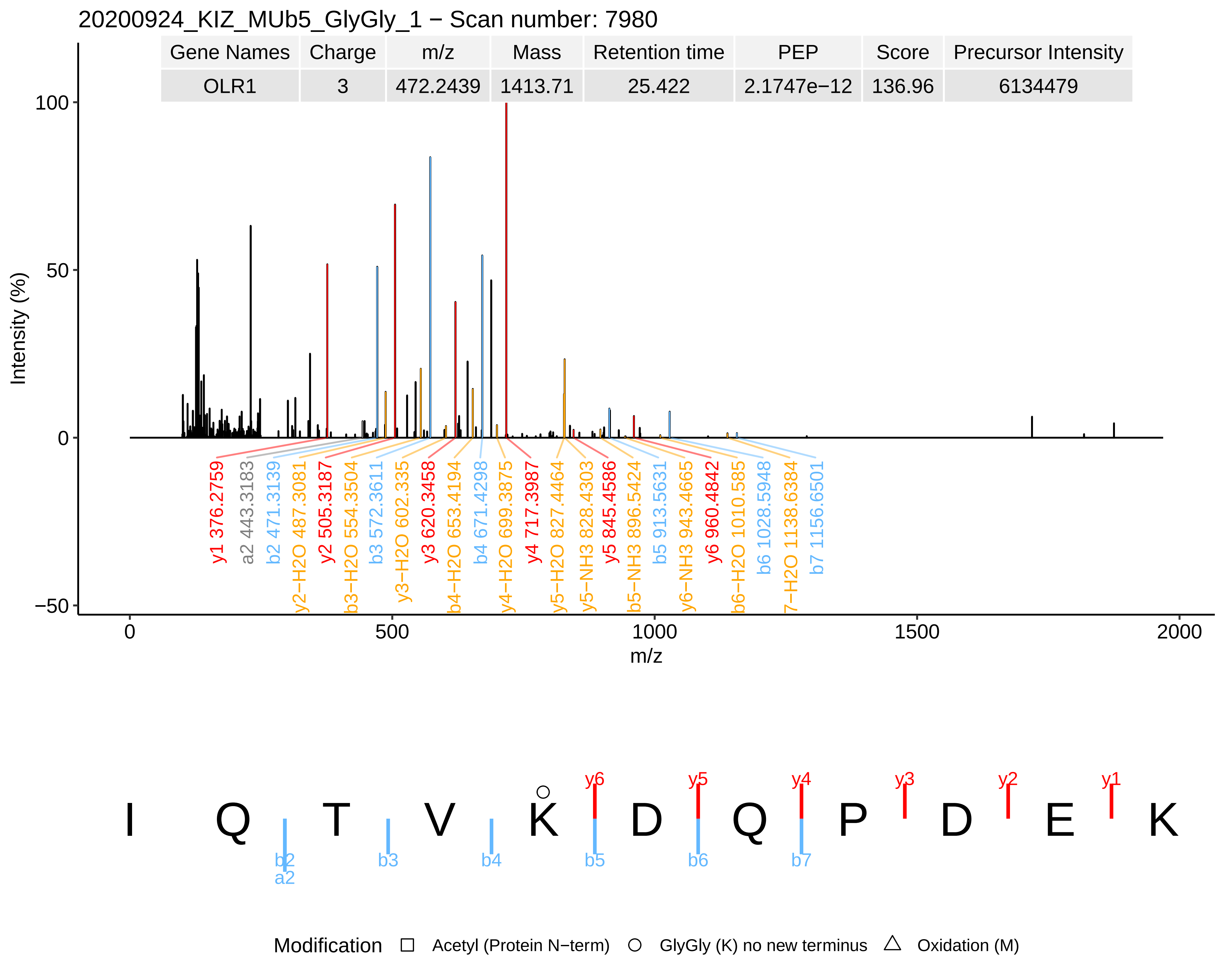

20200924_KIZ_MUb5_GlyGly_1 − Scan number: 7980
Gene Names | Charge | m/z | Mass | Retention time | PEP | Score | Precursor Intensity
OLR1 | 3 | 472.2439 | 1413.71 | 25.422 | 2.1747e−12 | 136.96 | 6134479
Intensity (%)
m/z
y1 376.2759
a2 443.3183
b2 471.3139
y2−H2O 487.3081
y2 505.3187
b3−H2O 554.3504
b3 572.3611
y3−H2O 602.335
y3 620.3458
b4−H2O 653.4194
b4 671.4298
y4−H2O 699.3875
y4 717.3987
y5−H2O 827.4464
y5−NH3 828.4303
y5 845.4586
b5−NH3 896.5424
b5 913.5631
y6−NH3 943.4665
y6 960.4842
b6−H2O 1010.585
b6 1028.5948
b7−H2O 1138.6384
b7 1156.6501
I Q T V K D Q P D E K
Modification
Acetyl (Protein N−term)
GlyGly (K) no new terminus
Oxidation (M)

20200924_KIZ_MUb5_GlyGly_1 − Scan number: 8128

Gene Names	Charge	m/z	Mass	Retention time	PEP	Score	Precursor Intensity
OLR1	3	472.2439	1413.71	25.925	0.00029578	95.401	31356768

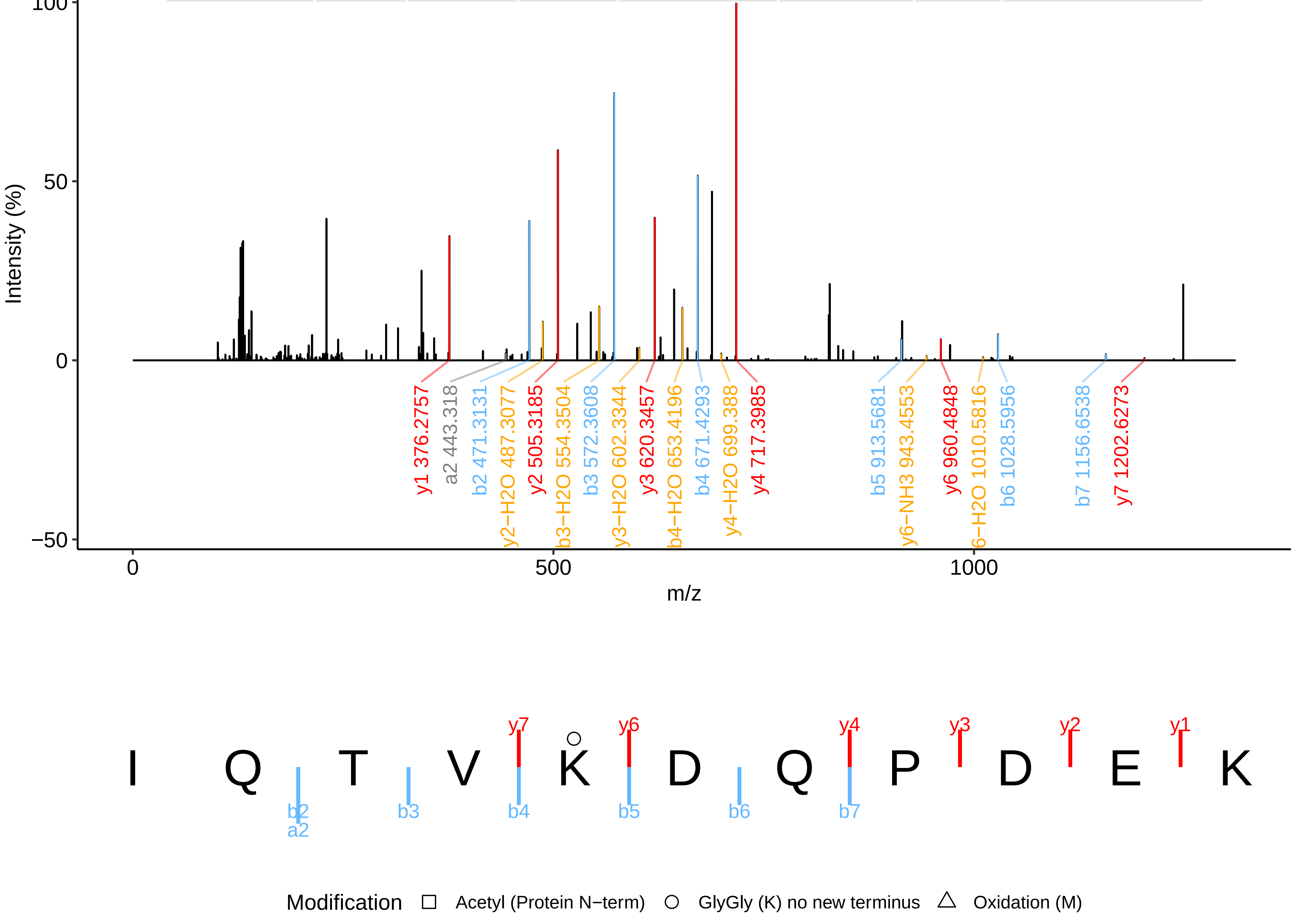

20200924_KIZ_MUb5_GlyGly_1 − Scan number: 8291

Gene Names	Charge	m/z	Mass	Retention time	PEP	Score	Precursor Intensity
OLR1	3	472.2439	1413.71	26.479	4.4525e−05	98.156	5500963

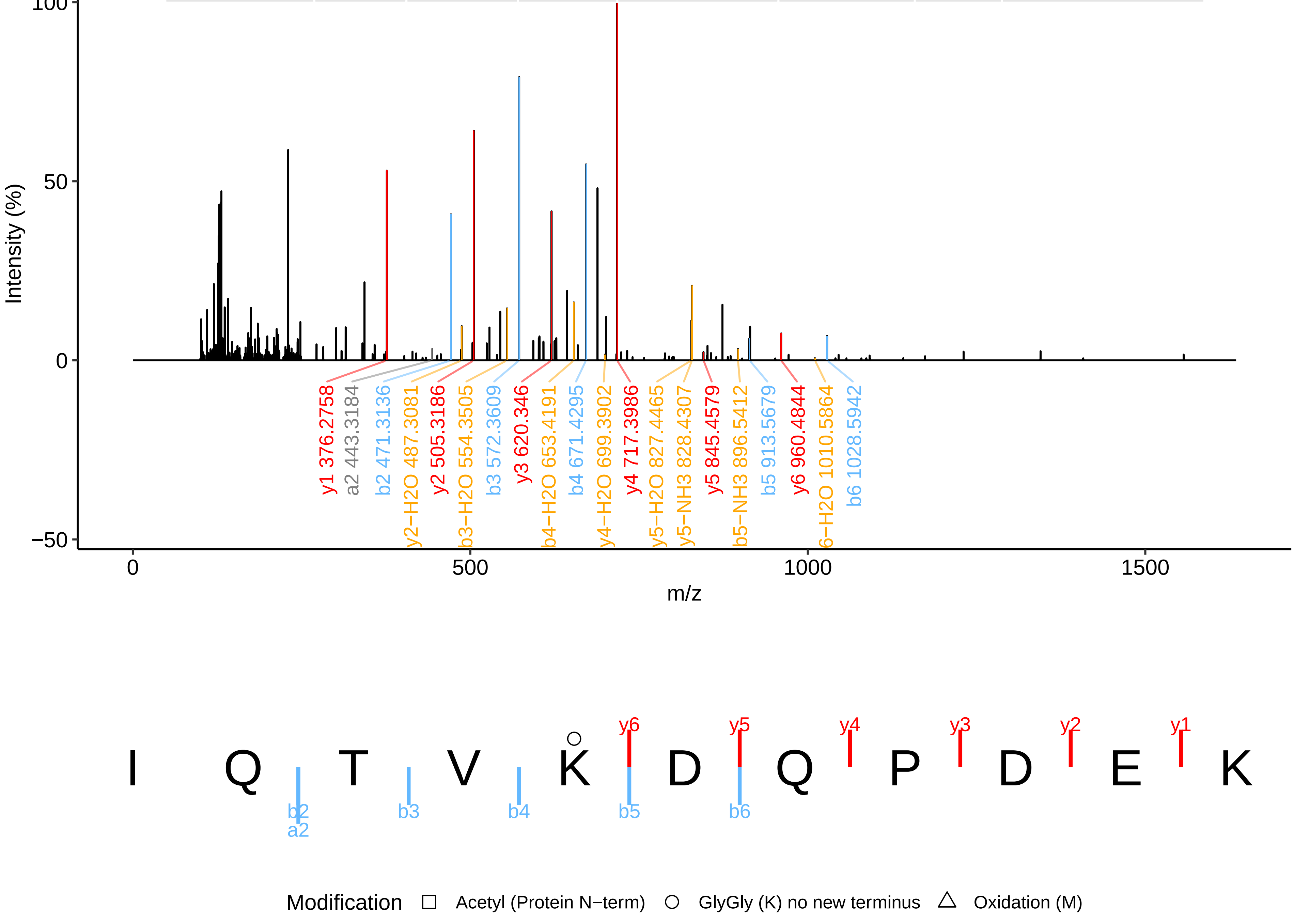

20200924_KIZ_MUb5_GlyGly_2 − Scan number: 7475

Gene Names	Charge	m/z	Mass	Retention time	PEP	Score	Precursor Intensity
OLR1	3	472.2439	1413.71	27.903	0.001107	87.001	2567731

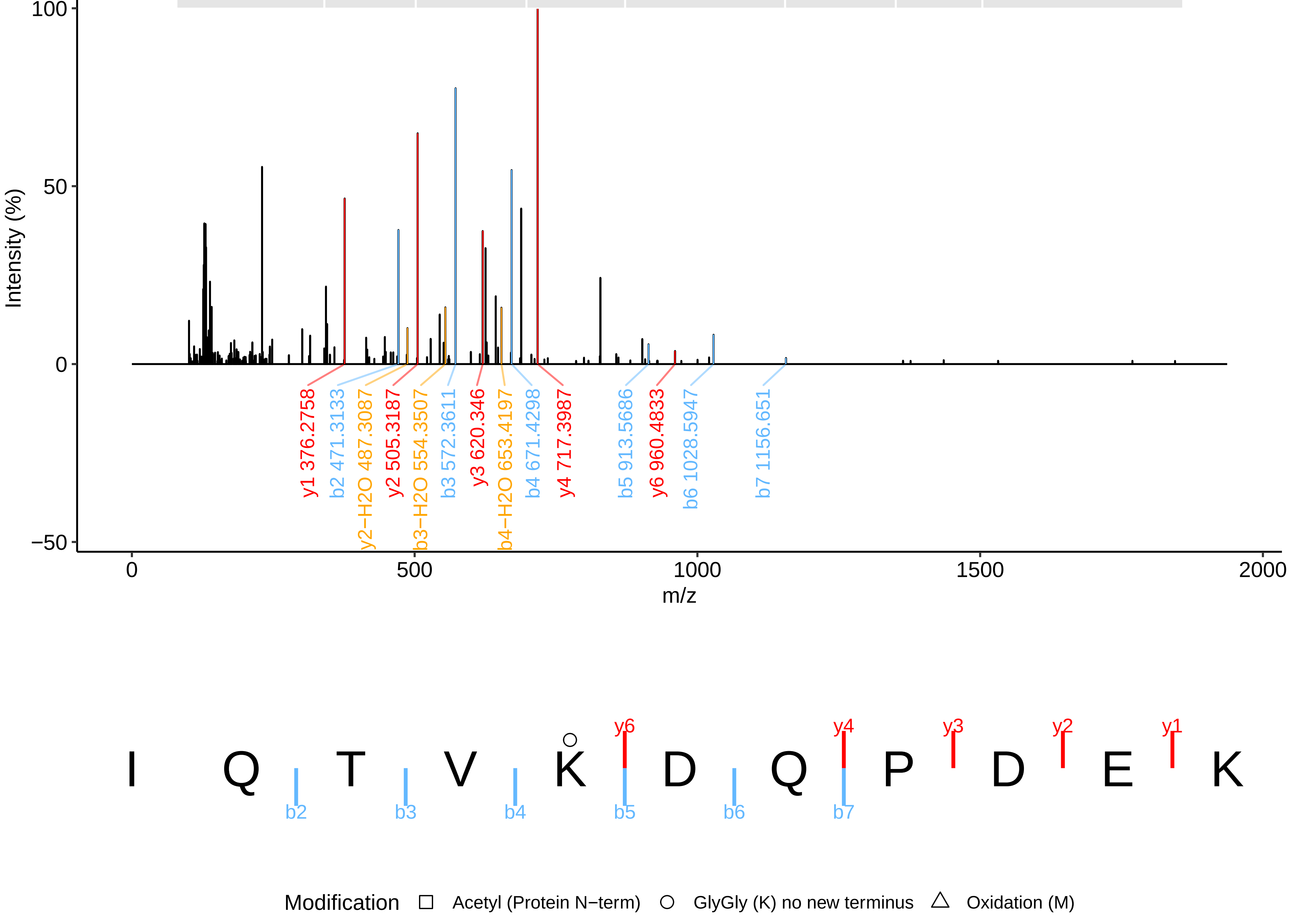

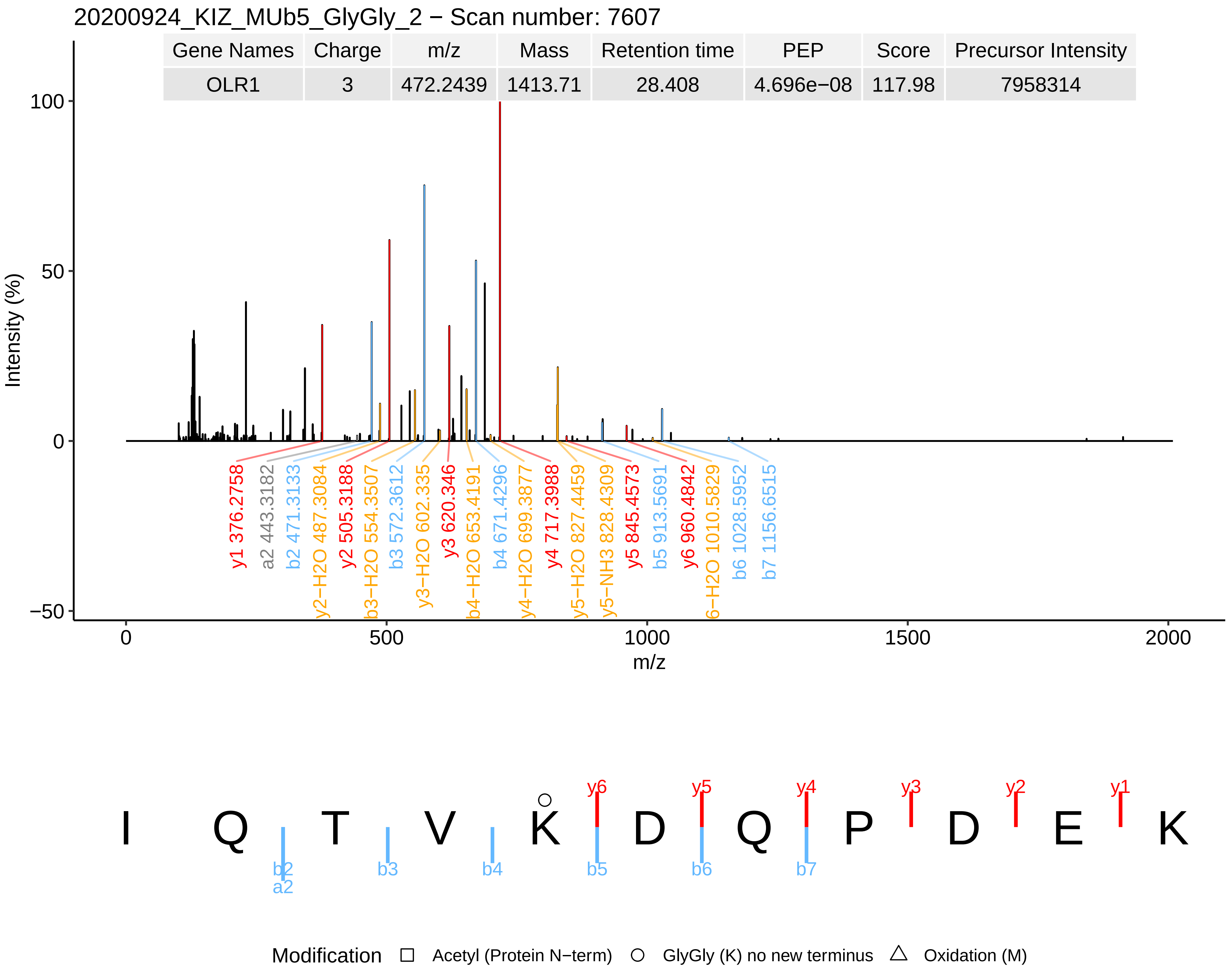

20200924_KIZ_MUb5_GlyGly_2 − Scan number: 7607
Gene Names | Charge | m/z | Mass | Retention time | PEP | Score | Precursor Intensity
OLR1 | 3 | 472.2439 | 1413.71 | 28.408 | 4.696e−08 | 117.98 | 7958314
Intensity (%)
m/z
y1 376.2758
a2 443.3182
b2 471.3133
y2−H2O 487.3084
y2 505.3188
b3−H2O 554.3507
b3 572.3612
y3−H2O 602.335
y3 620.346
b4−H2O 653.4191
b4 671.4296
y4−H2O 699.3877
y4 717.3988
y5−H2O 827.4459
y5−NH3 828.4309
y5 845.4573
b5 913.5691
y6 960.4842
y6−H2O 1010.5829
b6 1028.5952
b7 1156.6515
I Q T V K D Q P D E K
y6 y5 y4 y3 y2 y1
b2 a2 b3 b4 b5 b6 b7
Modification
Acetyl (Protein N−term)
GlyGly (K) no new terminus
Oxidation (M)

20201118_KIZ_MUb6_GlyGly_1 − Scan number: 7713

Gene Names	Charge	m/z	Mass	Retention time	PEP	Score	Precursor Intensity
OLR1	3	472.2439	1413.71	24.44	0.00010765	117.86	NA

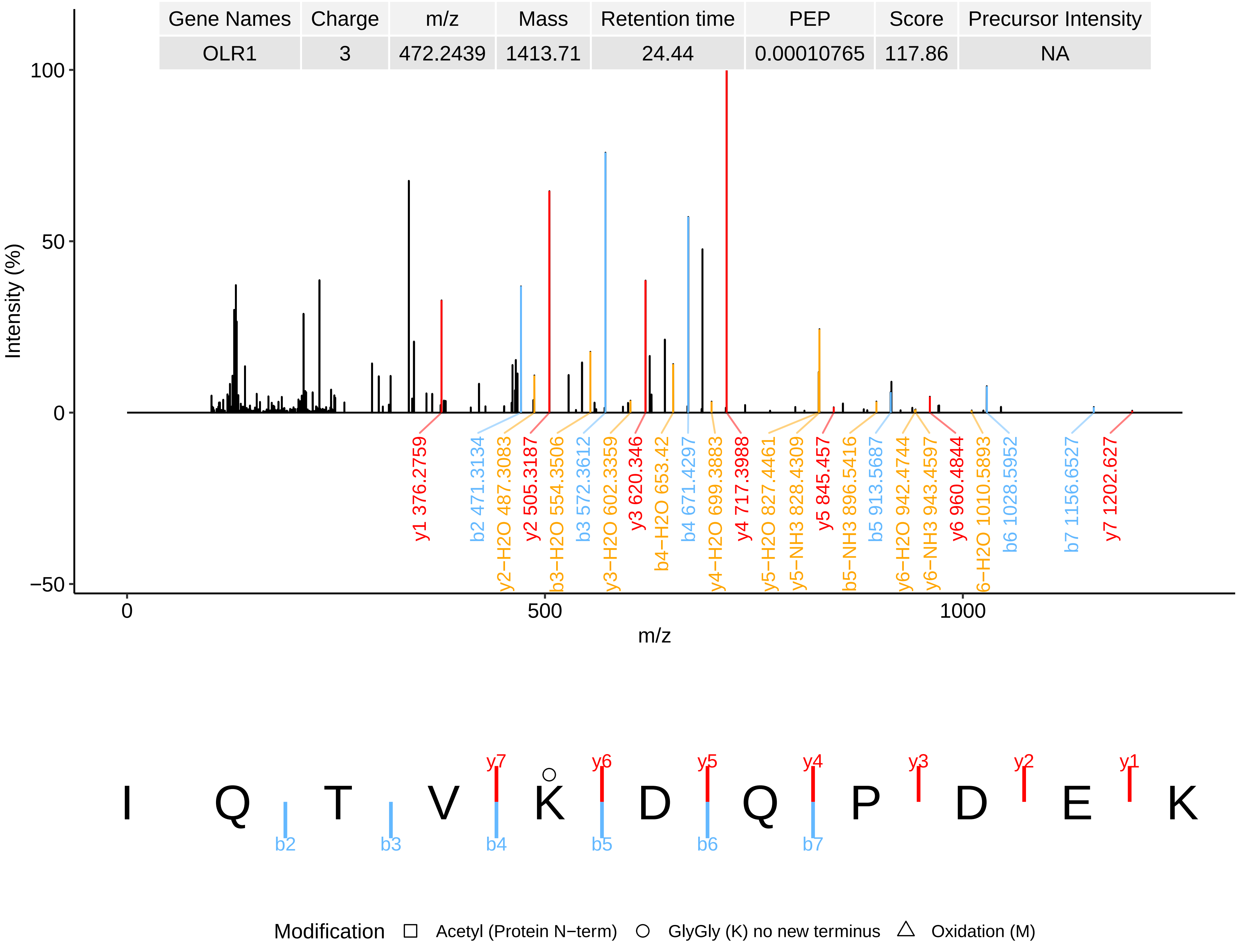

20201118_KIZ_MUb6_GlyGly_2 − Scan number: 9337

Gene Names	Charge	m/z	Mass	Retention time	PEP	Score	Precursor Intensity
OLR1	3	472.2439	1413.71	31.662	0.00062795	91.961	2799985

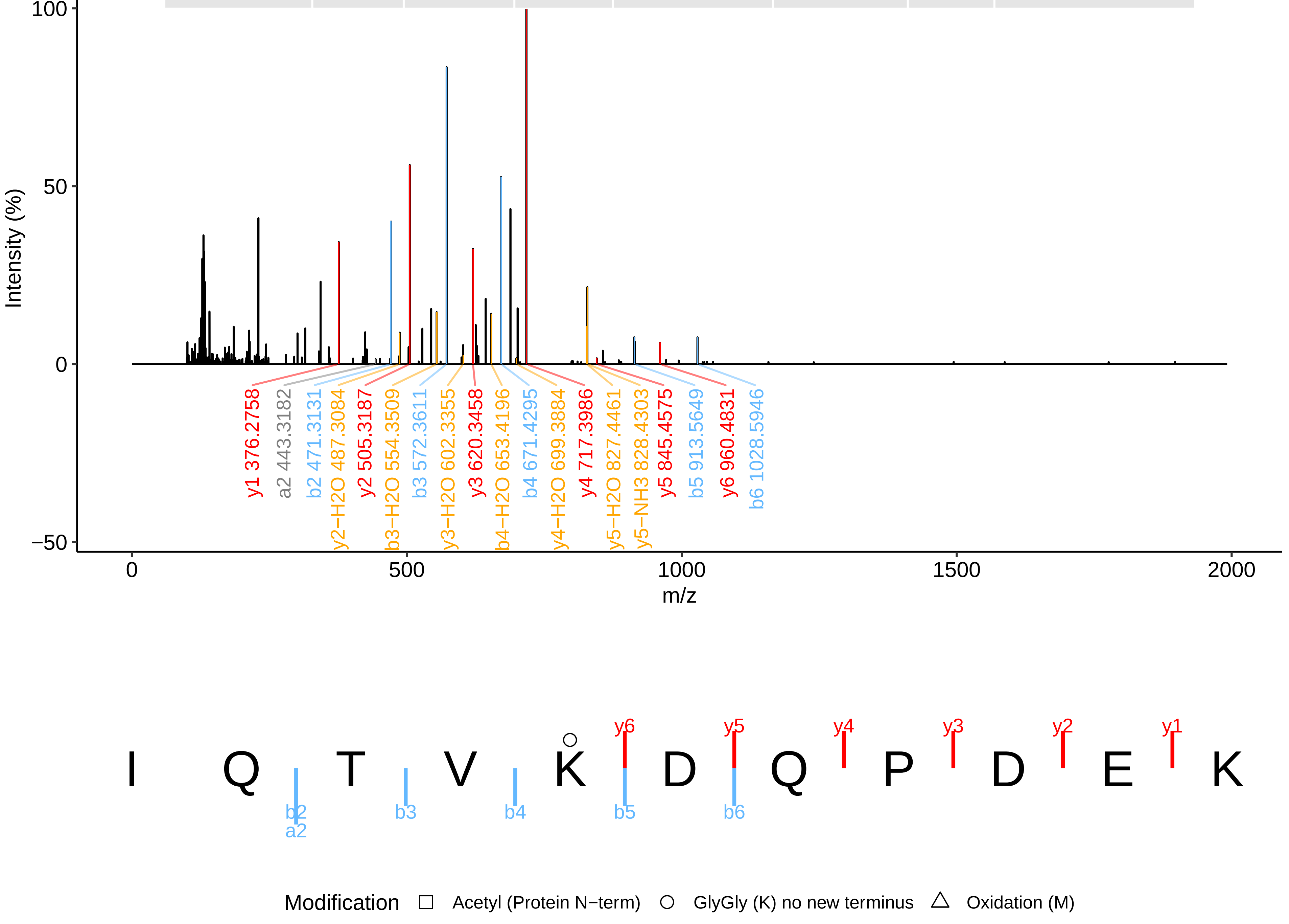

20200921_KIZ_MUb4_GlyGly_10plex − Scan number: 5240

Gene Names	Charge	m/z	Mass	Retention time	PEP	Score	Precursor Intensity
ATP2B2;ATP2B1;ATP2B3	4	404.4603	1613.812	17.127	0.03153	75.088	6109262

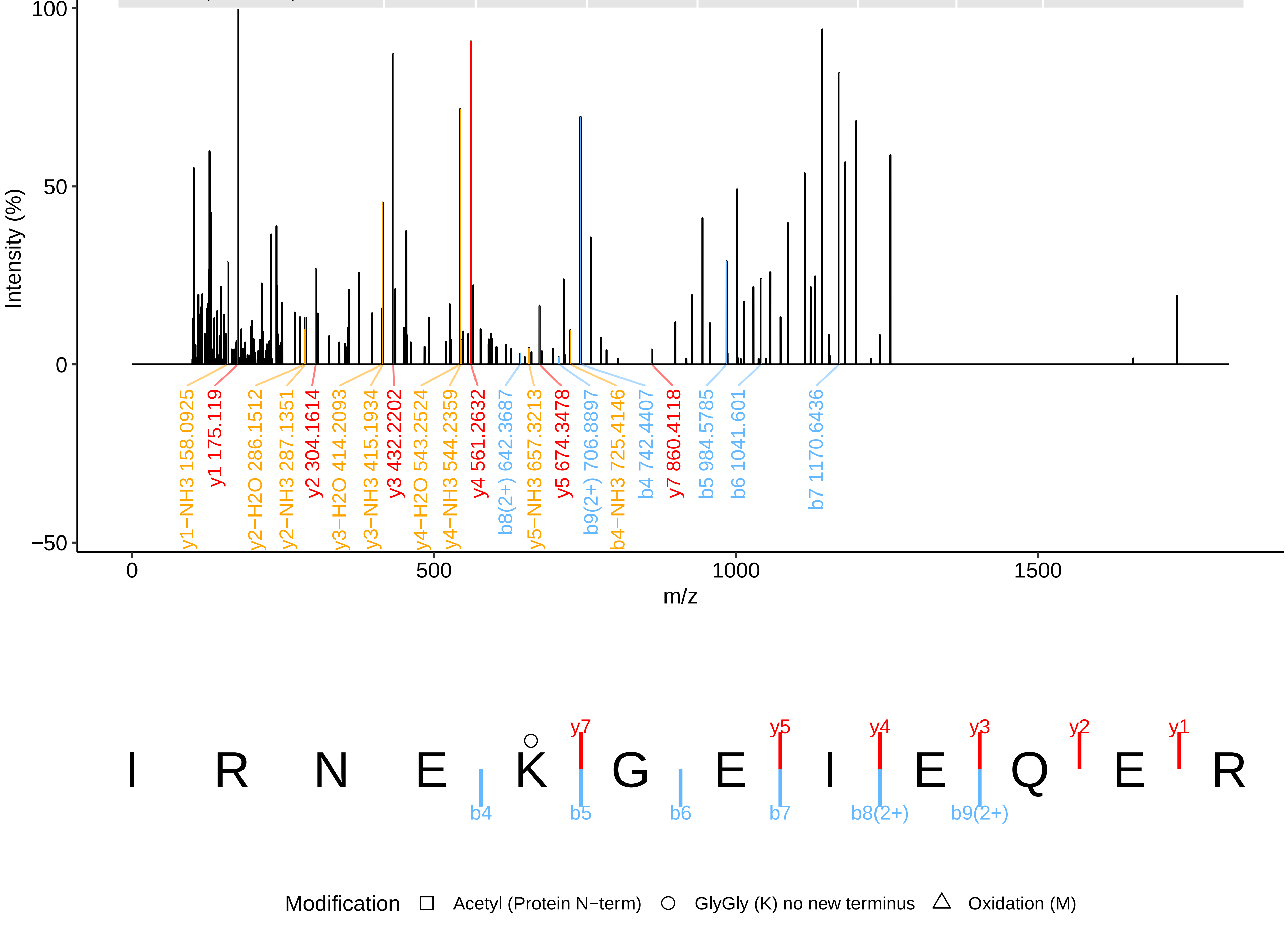

20200924_KIZ_MUb5_GlyGly_1 − Scan number: 11320

Gene Names	Charge	m/z	Mass	Retention time	PEP	Score	Precursor Intensity
ZC3H14	3	462.2488	1383.725	36.653	0.00045055	99.522	6465239

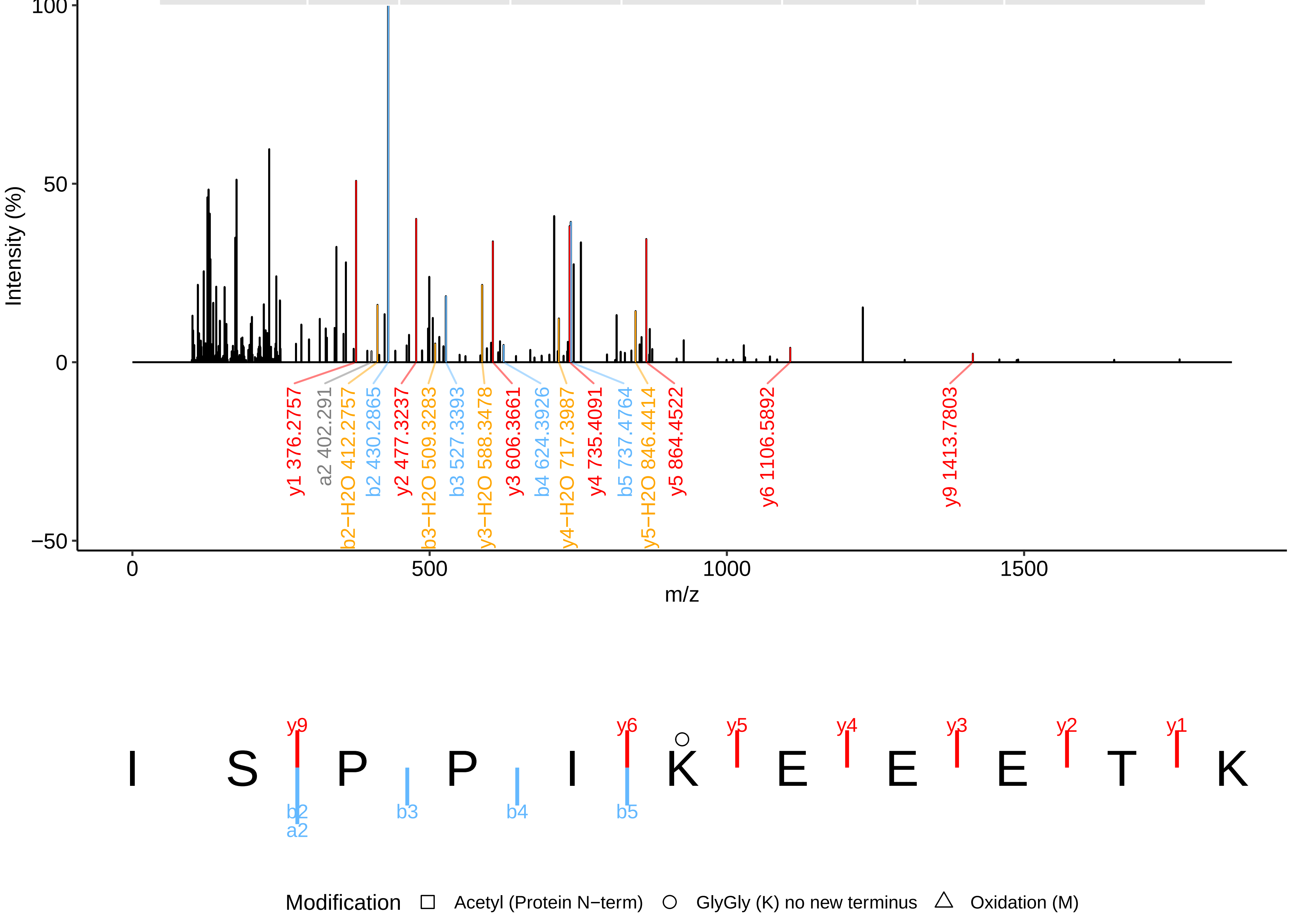

20200921_KIZ_MUb4_GlyGly_10plex_2 − Scan number: 12128

Gene Names	Charge	m/z	Mass	Retention time	PEP	Score	Precursor Intensity
SRP14	3	468.928	1403.762	46.856	0.0007644	87.184	540543.9

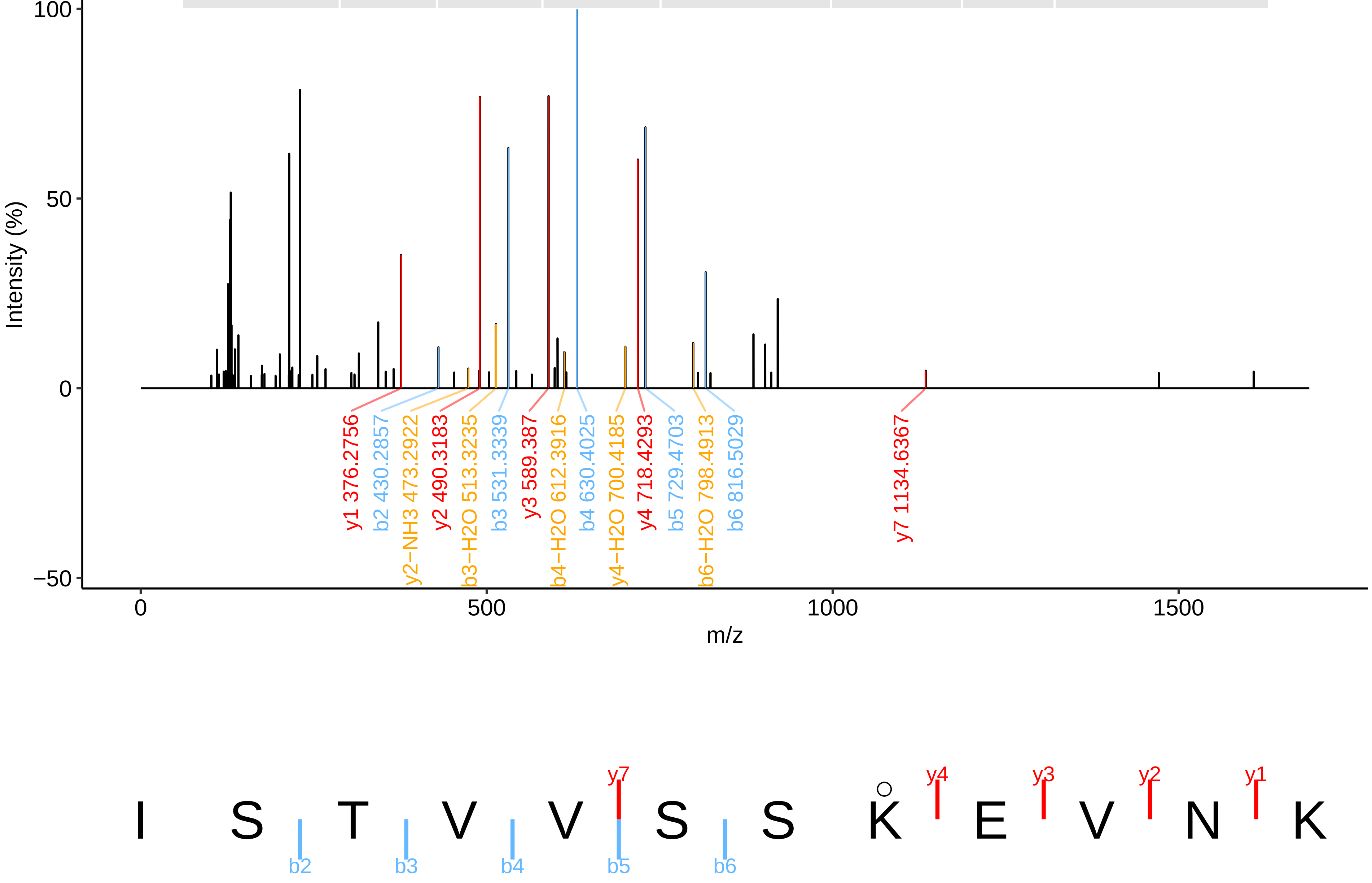

Modification □ Acetyl (Protein N−term) ○ GlyGly (K) no new terminus △ Oxidation (M)

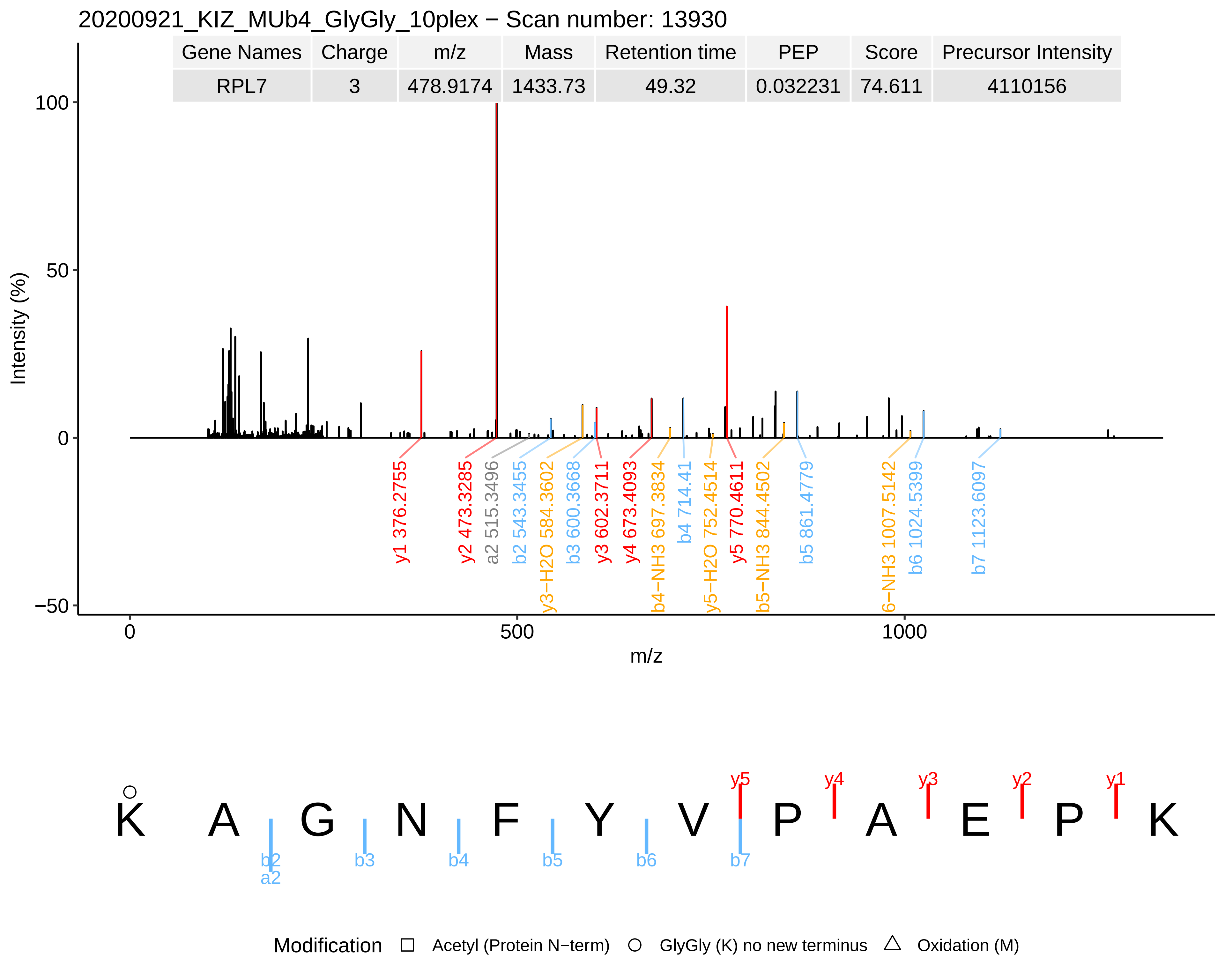

20200921_KIZ_MUb4_GlyGly_10plex − Scan number: 13930
Gene Names | Charge | m/z | Mass | Retention time | PEP | Score | Precursor Intensity
RPL7 | 3 | 478.9174 | 1433.73 | 49.32 | 0.032231 | 74.611 | 4110156
Intensity (%)
100
50
0
−50
0
500
1000
m/z
y1 376.2755
y2 473.3285
a2 515.3496
b2 543.3455
y3−H2O 584.3602
b3 600.3668
y3 602.3711
y4 673.4093
b4−NH3 697.3834
b4 714.41
y5−H2O 752.4514
y5 770.4611
b5−NH3 844.4502
b5 861.4779
6−NH3 1007.5142
b6 1024.5399
b7 1123.6097
K A G N F Y V P A E P K
y5 y4 y3 y2 y1
b2 a2 b3 b4 b5 b6 b7
Modification
Acetyl (Protein N−term)
GlyGly (K) no new terminus
Oxidation (M)

20200921_KIZ_MUb4_GlyGly_10plex − Scan number: 13663

Gene Names	Charge	m/z	Mass	Retention time	PEP	Score	Precursor Intensity
ARPC2	4	292.1616	1164.617	48.284	0.089591	45.997	5825941

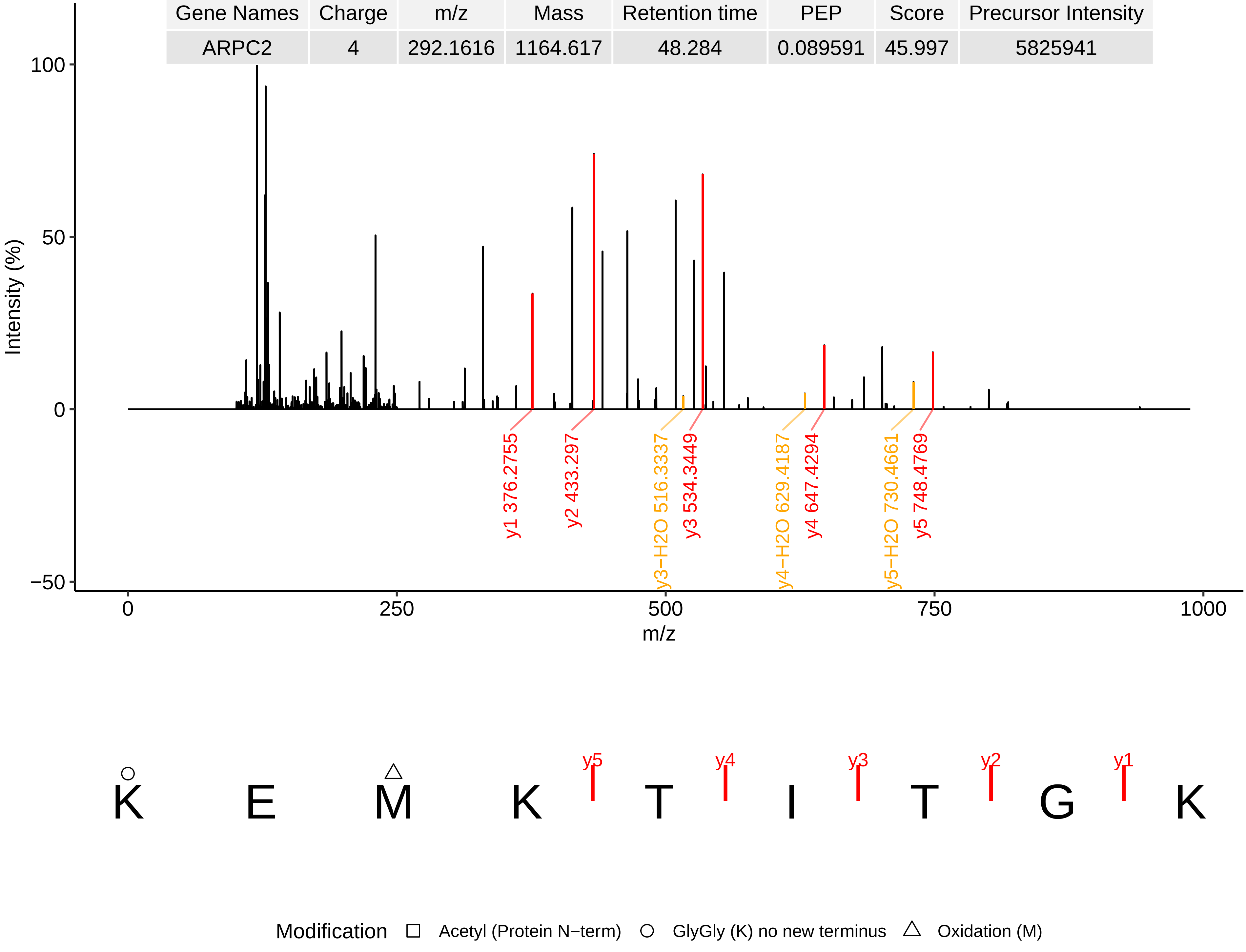

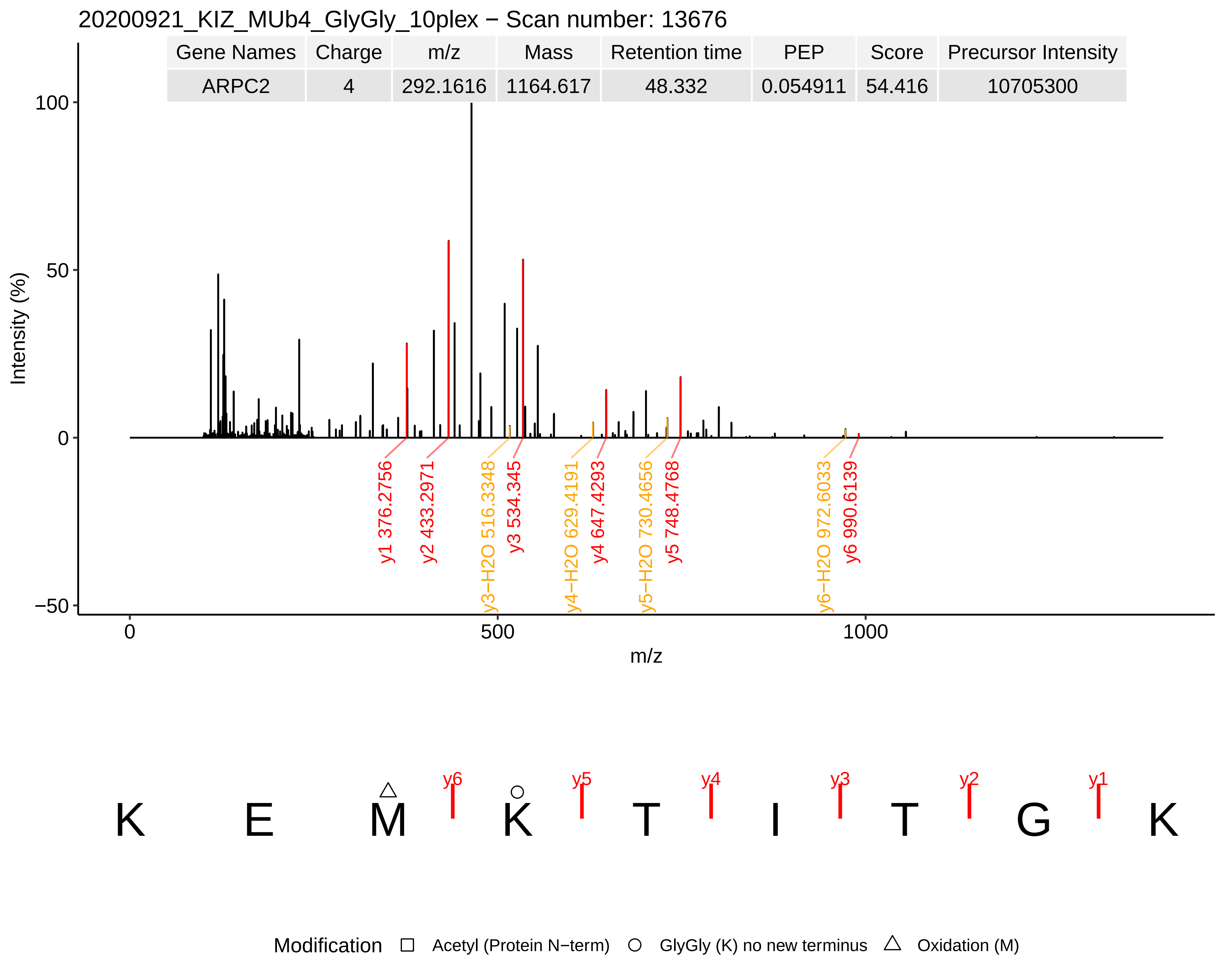
20200921_KIZ_MUb4_GlyGly_10plex − Scan number: 13676
Gene Names | Charge | m/z | Mass | Retention time | PEP | Score | Precursor Intensity
ARPC2 | 4 | 292.1616 | 1164.617 | 48.332 | 0.054911 | 54.416 | 10705300
Intensity (%)
m/z
y1 376.2756
y2 433.2971
y3−H2O 516.3348
y3 534.345
y4−H2O 629.4191
y4 647.4293
y5−H2O 730.4656
y5 748.4768
y6−H2O 972.6033
y6 990.6139
K E M K T I T G K
y6 y5 y4 y3 y2 y1
Modification
Acetyl (Protein N−term)
GlyGly (K) no new terminus
Oxidation (M)

20200921_KIZ_MUb4_GlyGly_10plex − Scan number: 12011

Gene Names	Charge	m/z	Mass	Retention time	PEP	Score	Precursor Intensity
CISD1	4	356.1712	1420.656	41.99	5.9659e−06	112.13	11731682

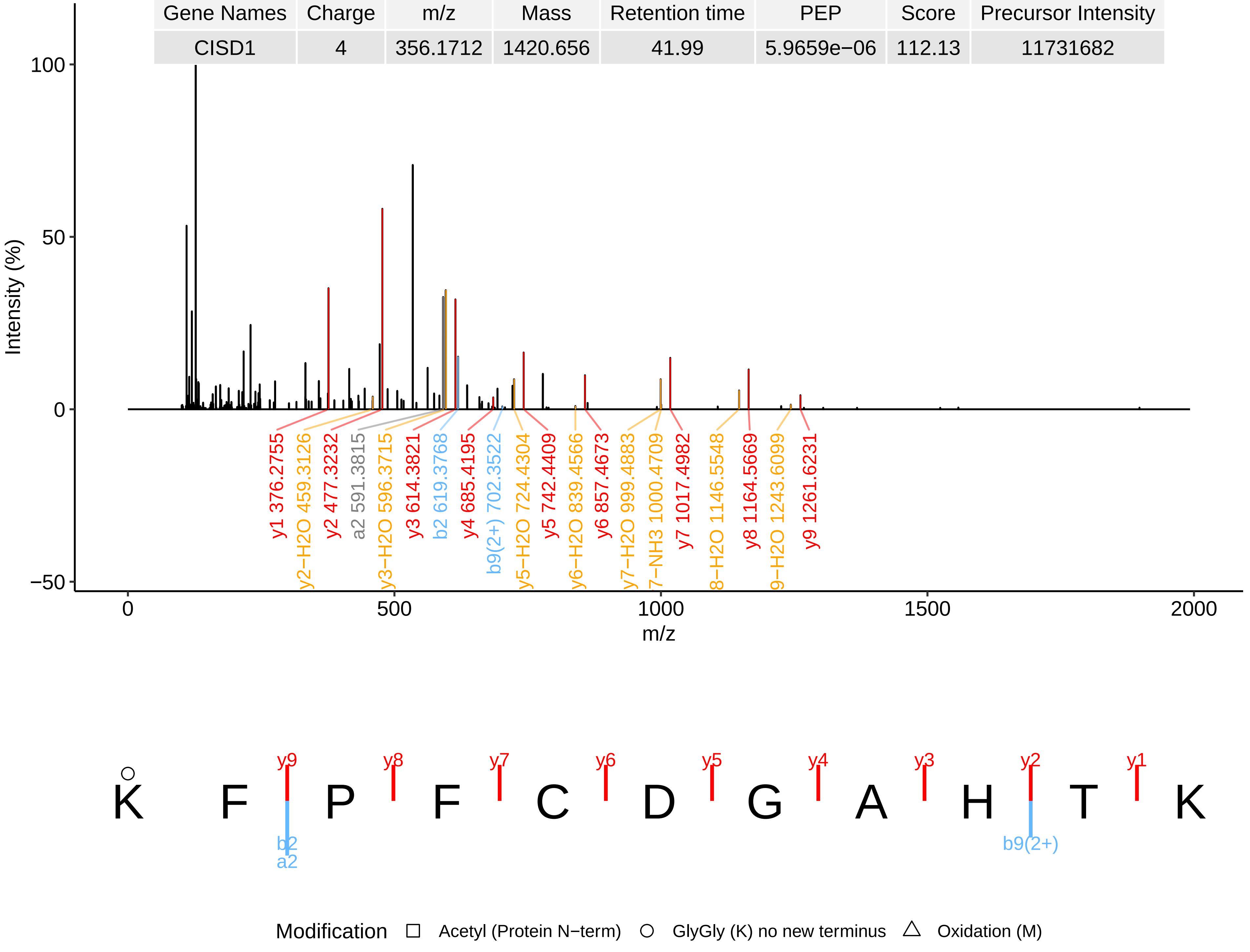

20200921_KIZ_MUb4_GlyGly_10plex_2 − Scan number: 11123

Gene Names	Charge	m/z	Mass	Retention time	PEP	Score	Precursor Intensity
CISD1	4	356.1712	1420.656	42.738	0.020927	60.49	939519.9

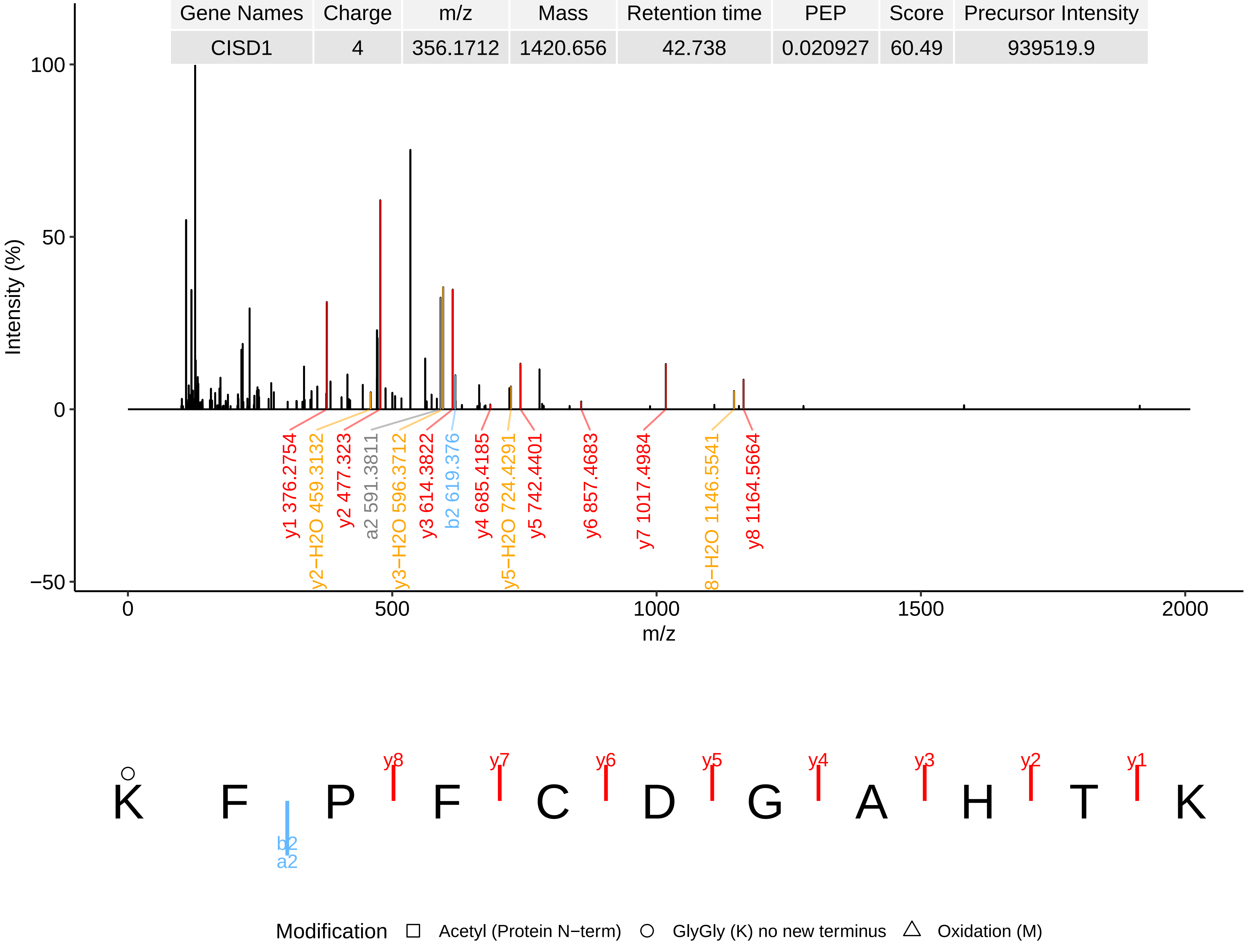

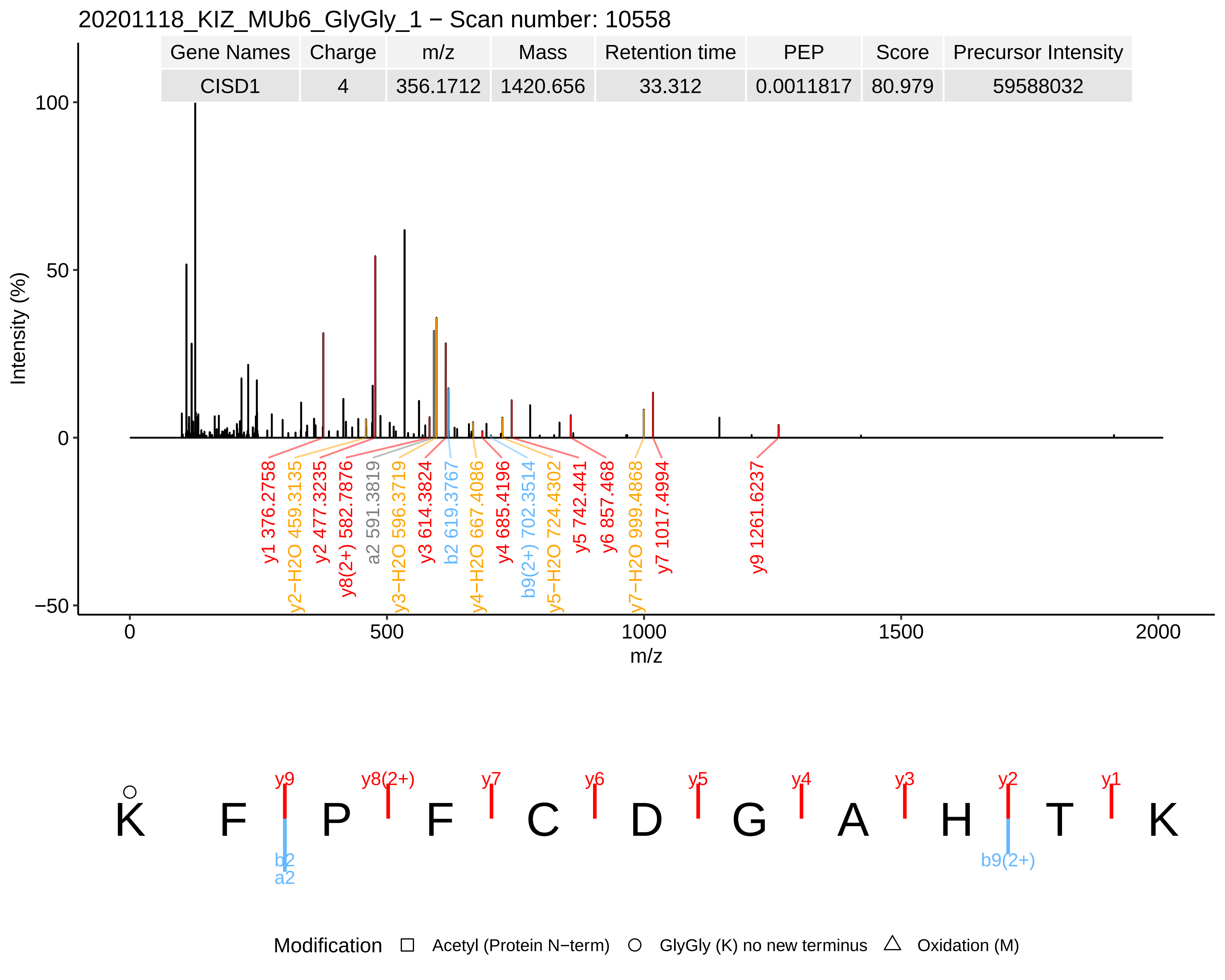

20201118_KIZ_MUb6_GlyGly_1 − Scan number: 10558
Gene Names | Charge | m/z | Mass | Retention time | PEP | Score | Precursor Intensity
CISD1 | 4 | 356.1712 | 1420.656 | 33.312 | 0.0011817 | 80.979 | 59588032
Intensity (%)
m/z
y1 376.2758
y2−H2O 459.3135
y2 477.3235
y8(2+) 582.7876
a2 591.3819
y3−H2O 596.3719
y3 614.3824
b2 619.3767
y4−H2O 667.4086
y4 685.4196
b9(2+) 702.3514
y5−H2O 724.4302
y5 742.441
y6 857.468
y7−H2O 999.4868
y7 1017.4994
y9 1261.6237
K F P F C D G A H T K
y9 y8(2+) y7 y6 y5 y4 y3 y2 y1
b2 a2 b9(2+)
Modification
Acetyl (Protein N−term)
GlyGly (K) no new terminus
Oxidation (M)

20201118_KIZ_MUb6_GlyGly_2 − Scan number: 13107

Gene Names	Charge	m/z	Mass	Retention time	PEP	Score	Precursor Intensity
CISD1	4	356.1712	1420.656	44.155	0.069102	47.154	14490059

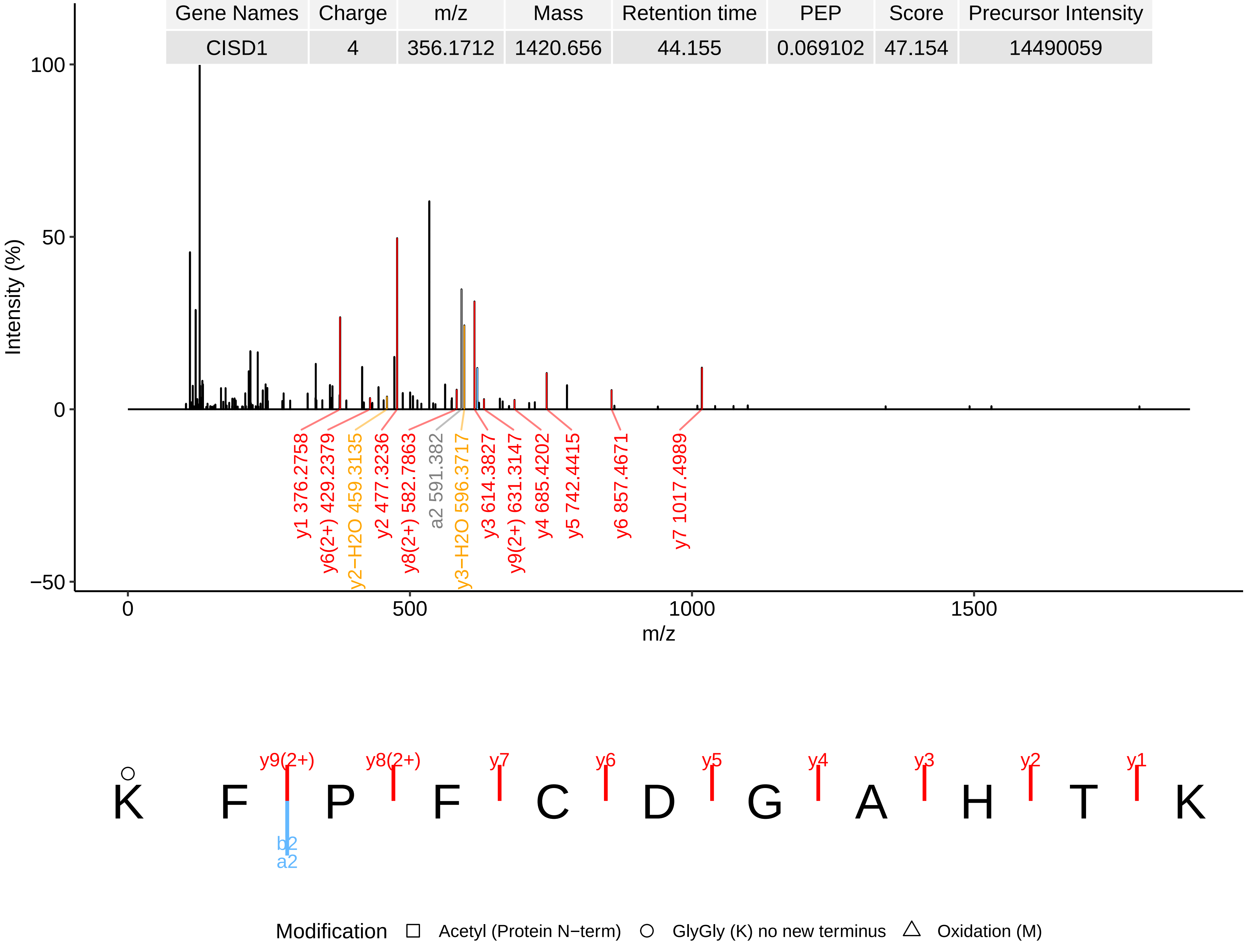

20200921_KIZ_MUb4_GlyGly_10plex − Scan number: 18842

Gene Names	Charge	m/z	Mass	Retention time	PEP	Score	Precursor Intensity
COX5B	3	476.933	1427.777	67.864	0.032854	74.46	4964905

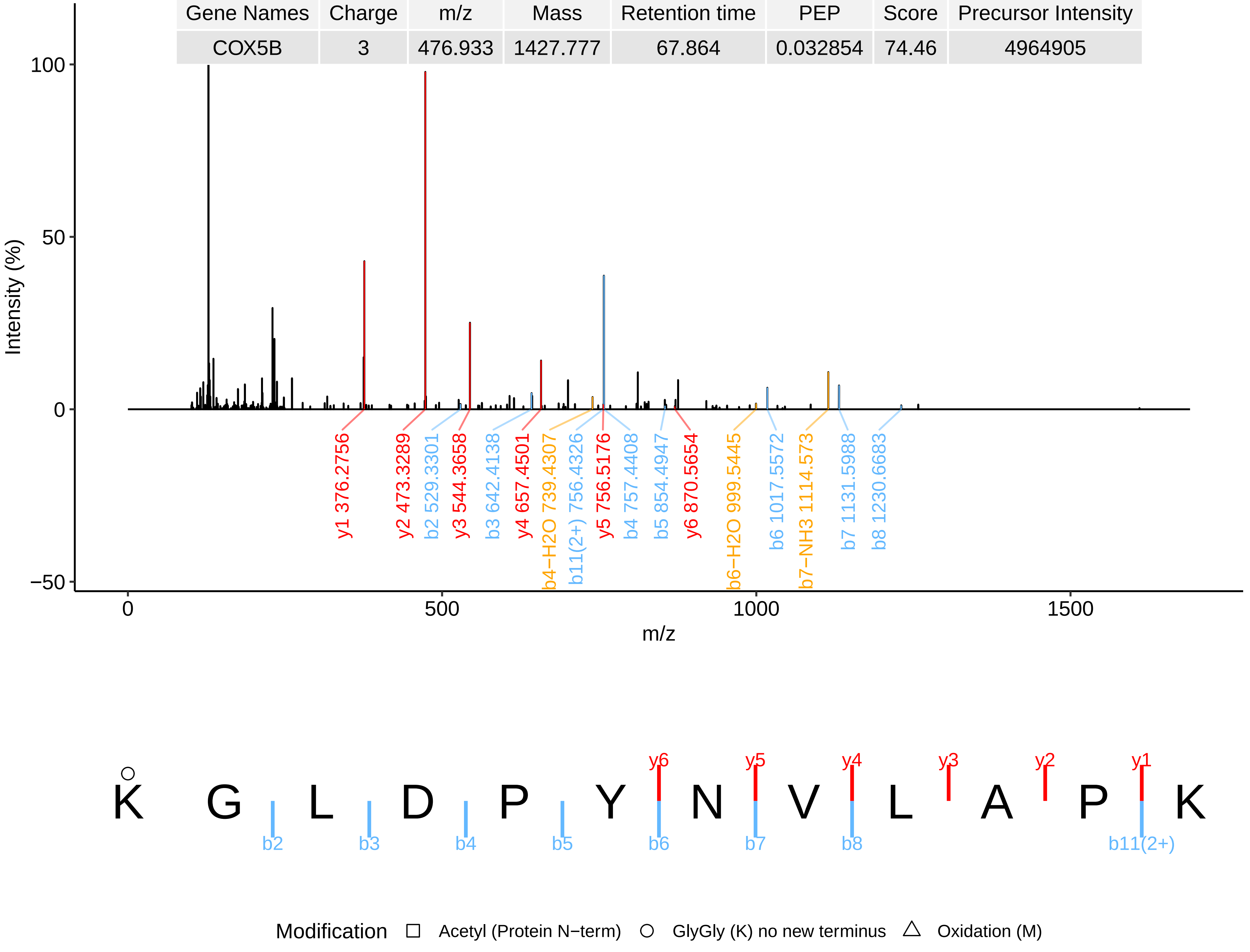

20200921_KIZ_MUb4_GlyGly_10plex − Scan number: 10317

Gene Names	Charge	m/z	Mass	Retention time	PEP	Score	Precursor Intensity
STAM;STAM2	4	350.19	1396.731	35.671	1.1388e−12	140.3	5737808

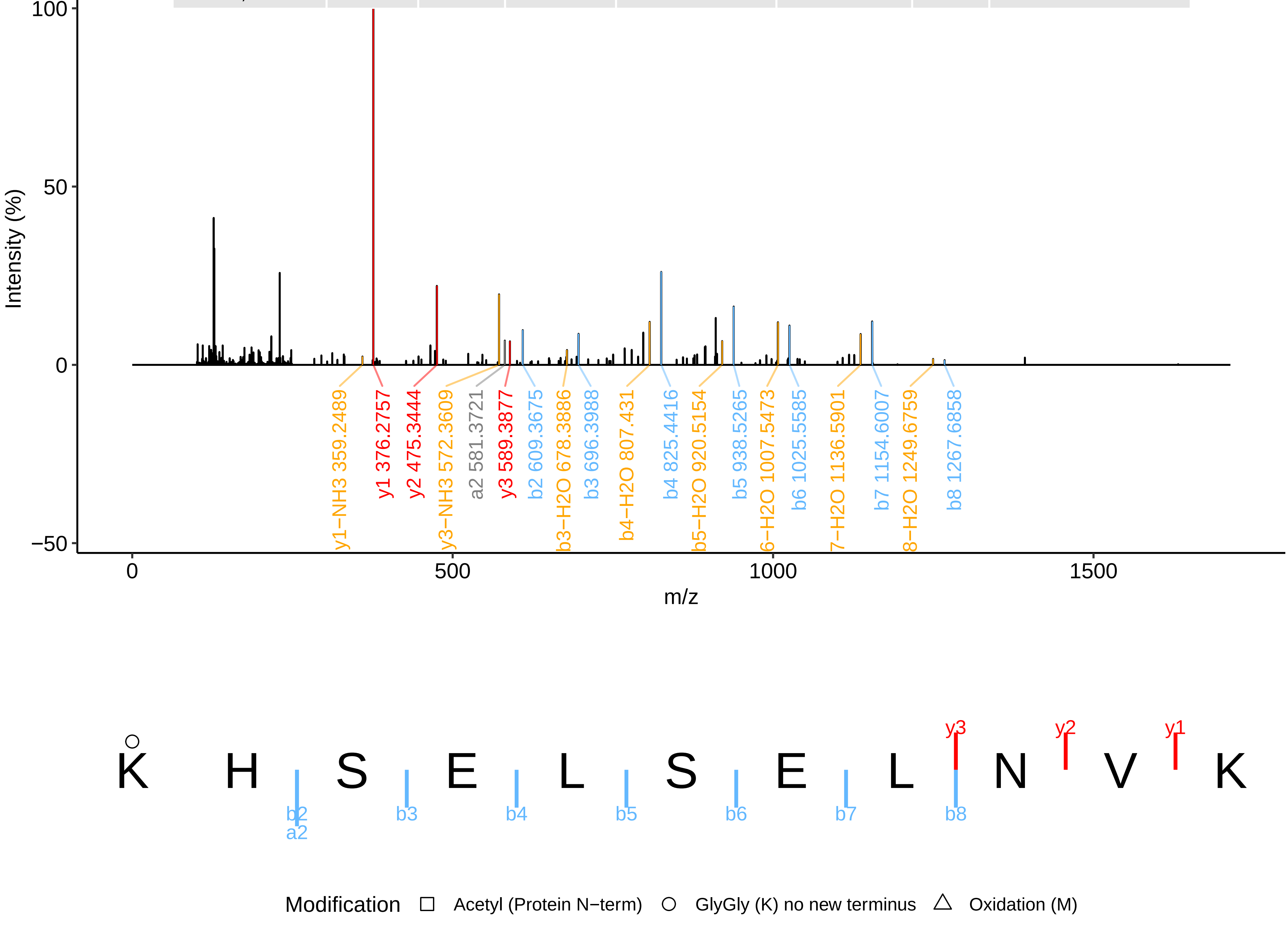

20200921_KIZ_MUb4_GlyGly_10plex_2 − Scan number: 9592

Gene Names	Charge	m/z	Mass	Retention time	PEP	Score	Precursor Intensity
STAM;STAM2	4	350.19	1396.731	36.515	0.00026004	94.616	917352.2

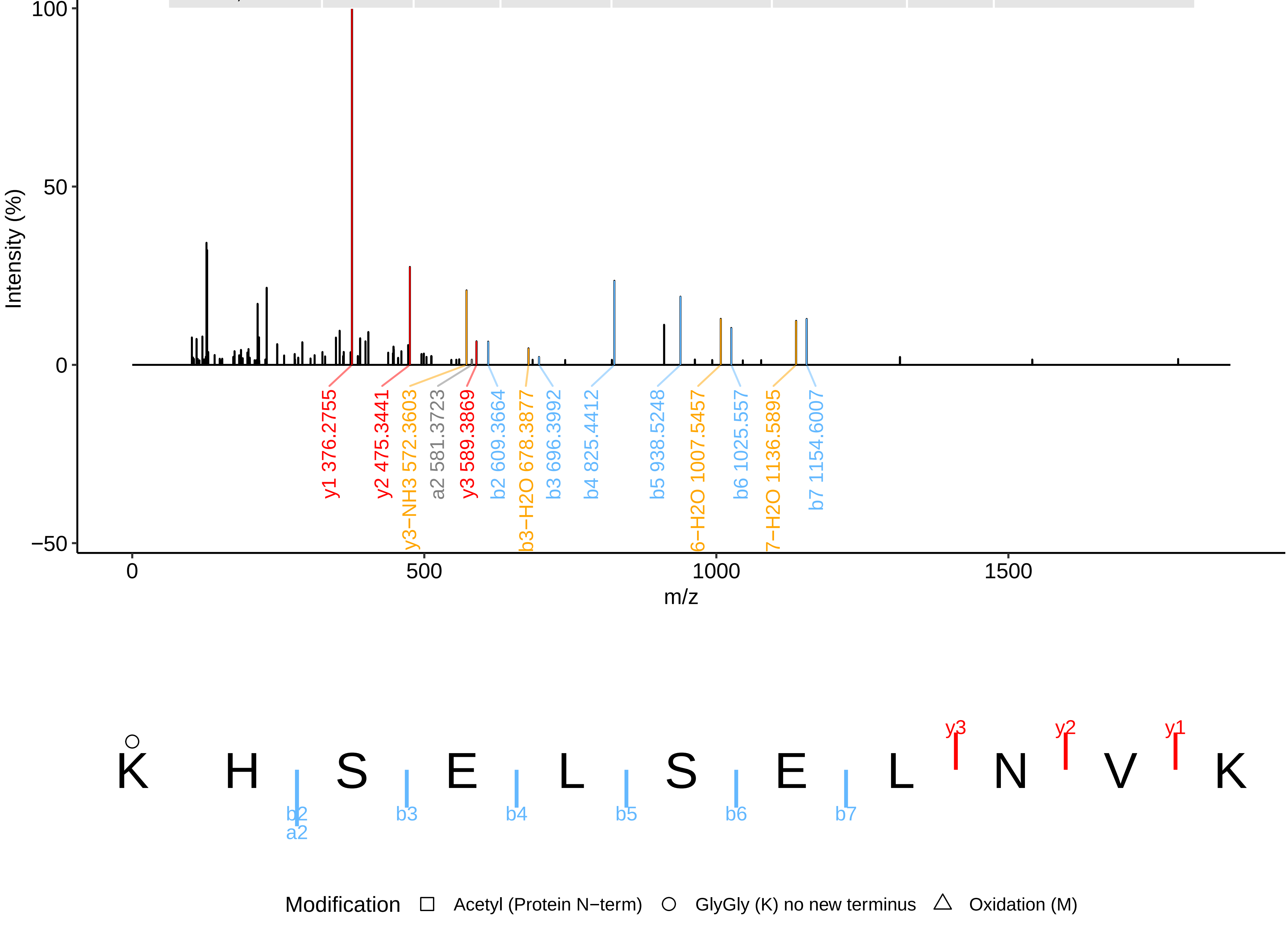

20200921_KIZ_MUb4_GlyGly_10plex_2 − Scan number: 11357

Gene Names	Charge	m/z	Mass	Retention time	PEP	Score	Precursor Intensity
RDH11;RDH12	4	348.1979	1388.762	43.696	0.092767	65.179	2793463

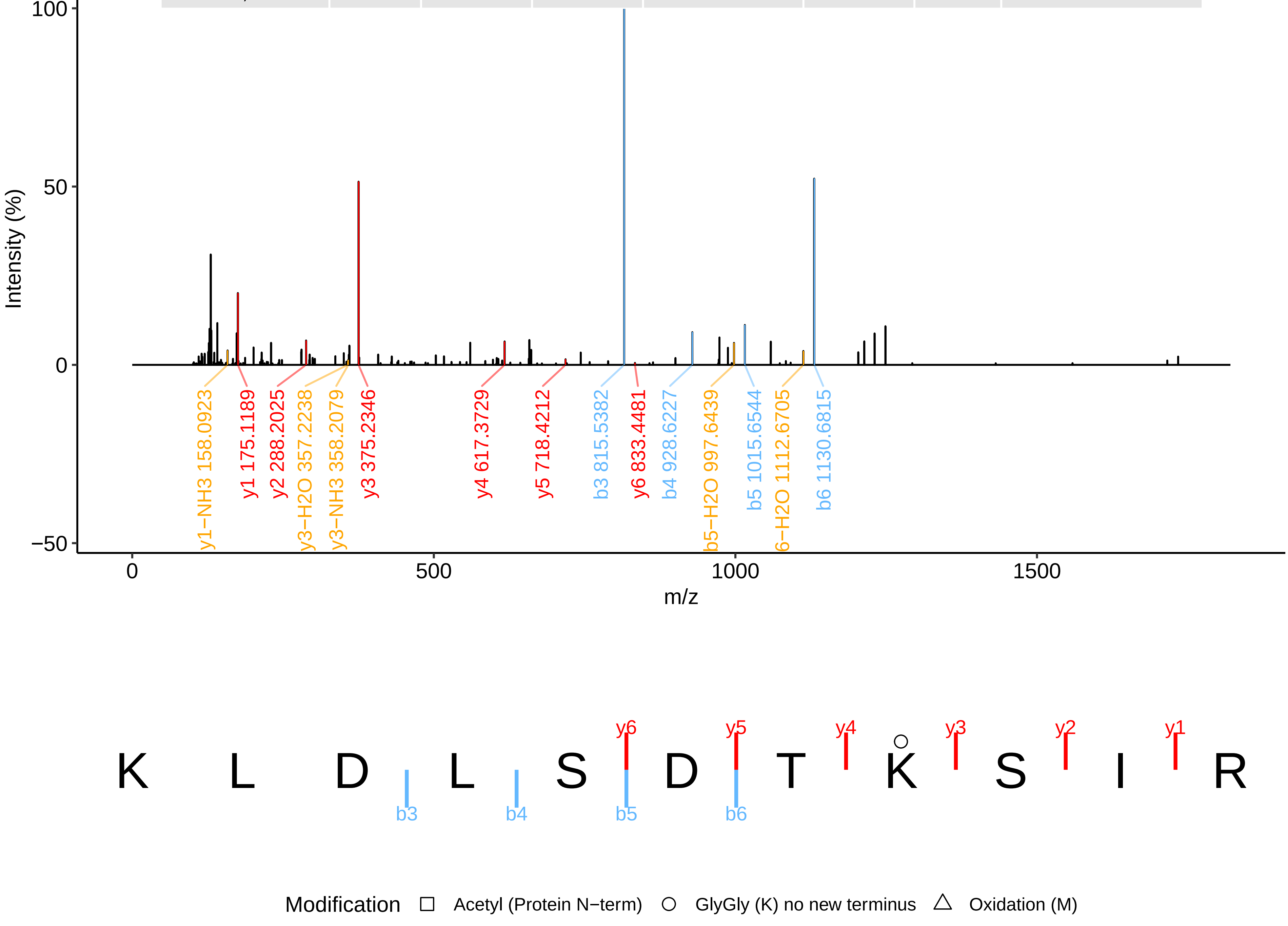

20201118_KIZ_MUb6_GlyGly_2 − Scan number: 35296

Gene Names	Charge	m/z	Mass	Retention time	PEP	Score	Precursor Intensity
MPHOSPH8	2	838.4417	1674.869	114.46	0.021209	60.563	14524934

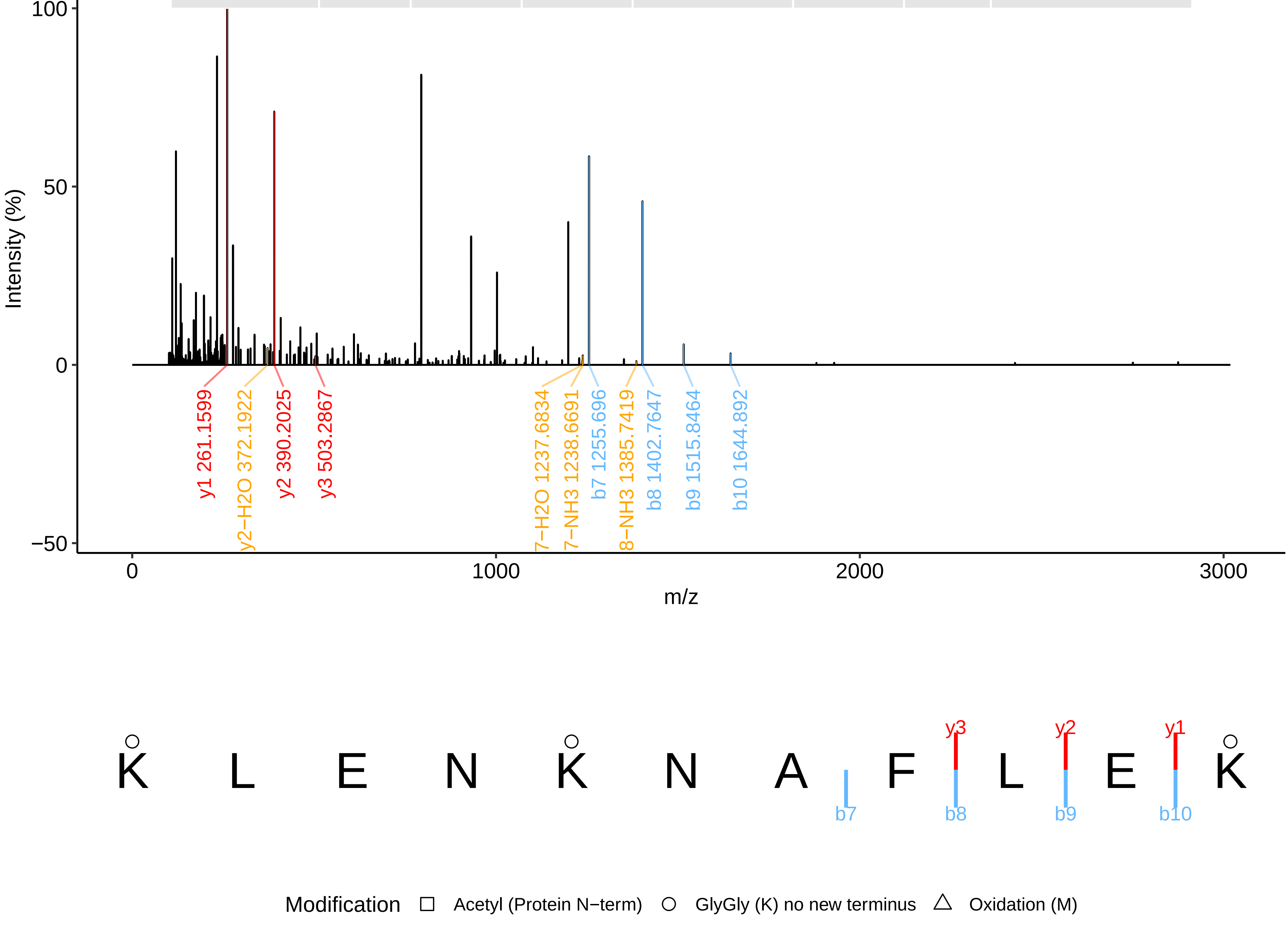

20200921_KIZ_MUb4_GlyGly_10plex − Scan number: 15988

Gene Names	Charge	m/z	Mass	Retention time	PEP	Score	Precursor Intensity
LAMTOR1	3	470.6048	1408.793	57.299	0.015065	78.763	2643449

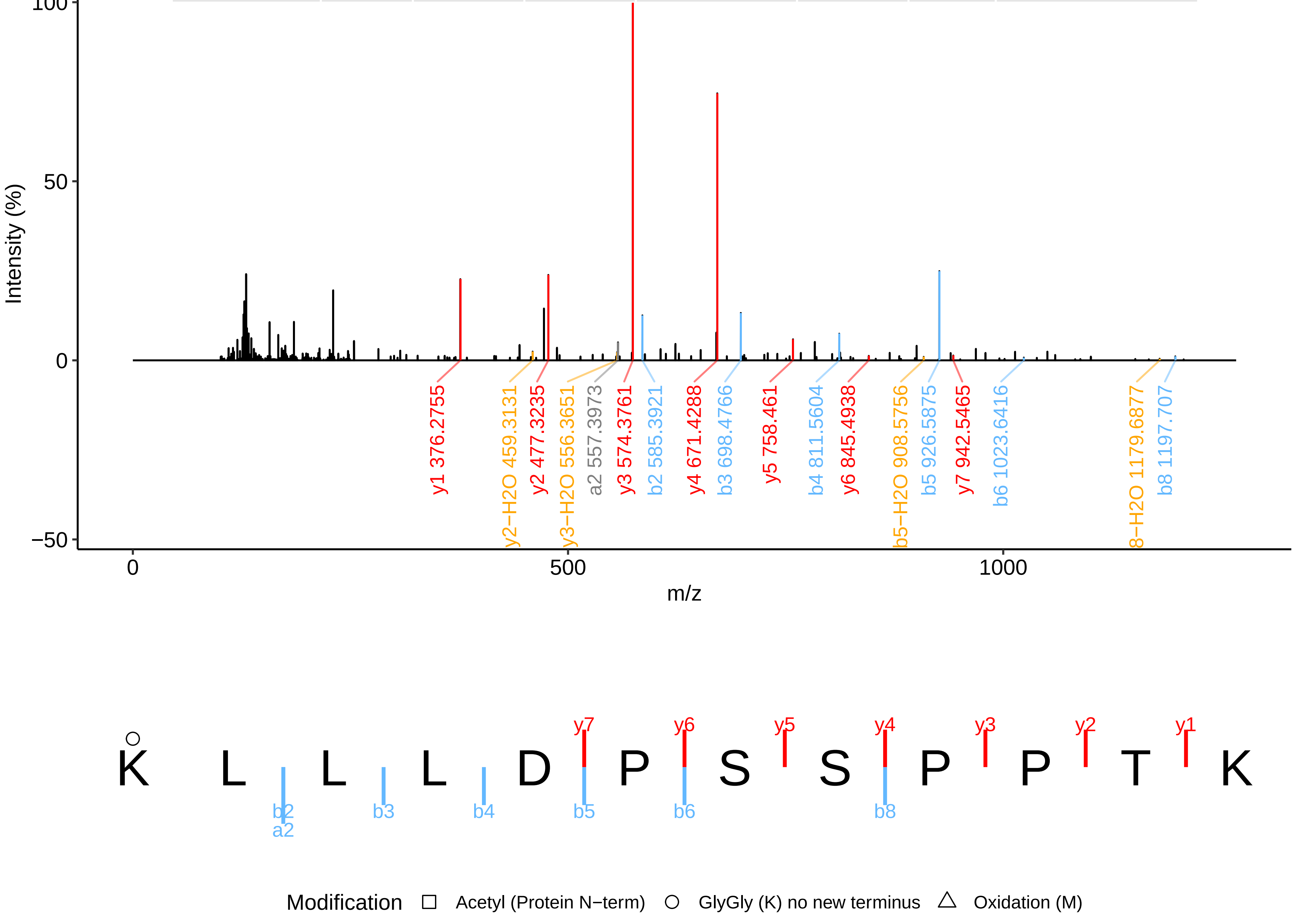

20200921_KIZ_MUb4_GlyGly_10plex − Scan number: 10000

Gene Names	Charge	m/z	Mass	Retention time	PEP	Score	Precursor Intensity
MAP4K4	3	476.5673	1426.68	34.45	0.047314	70.963	5653332

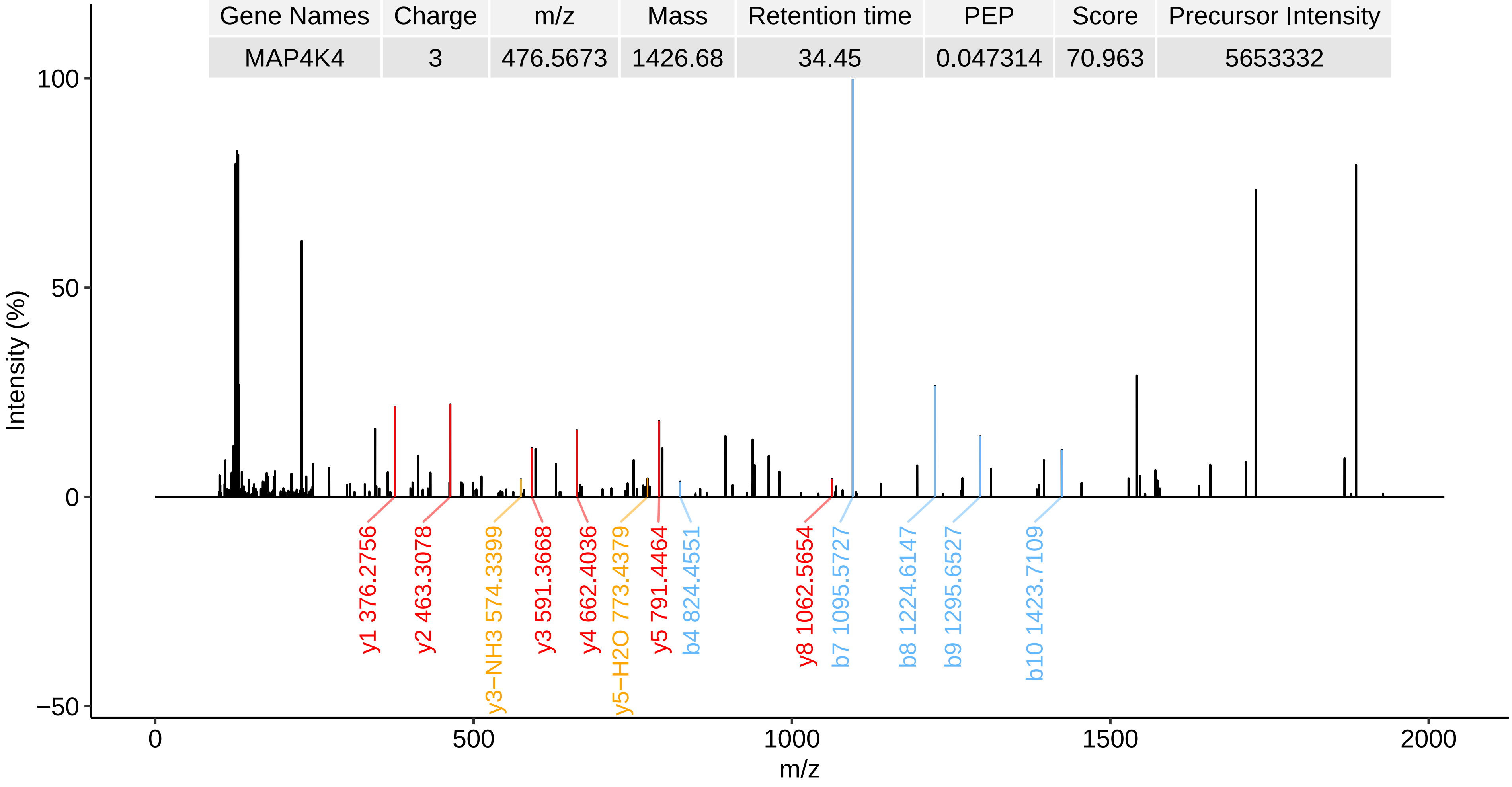

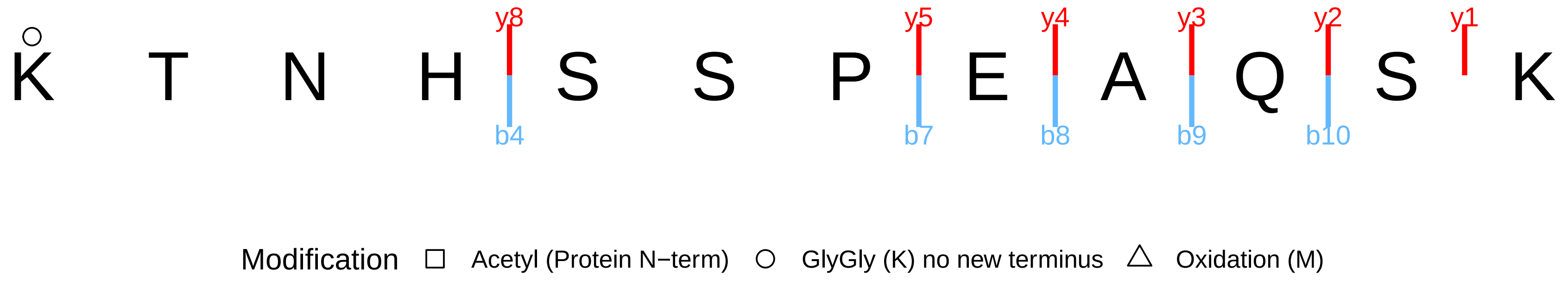

20200924_KIZ_MUb5_GlyGly_1 − Scan number: 9056

Gene Names	Charge	m/z	Mass	Retention time	PEP	Score	Precursor Intensity
MAP4K4	3	476.5673	1426.68	29.092	0.050463	66.989	3127977

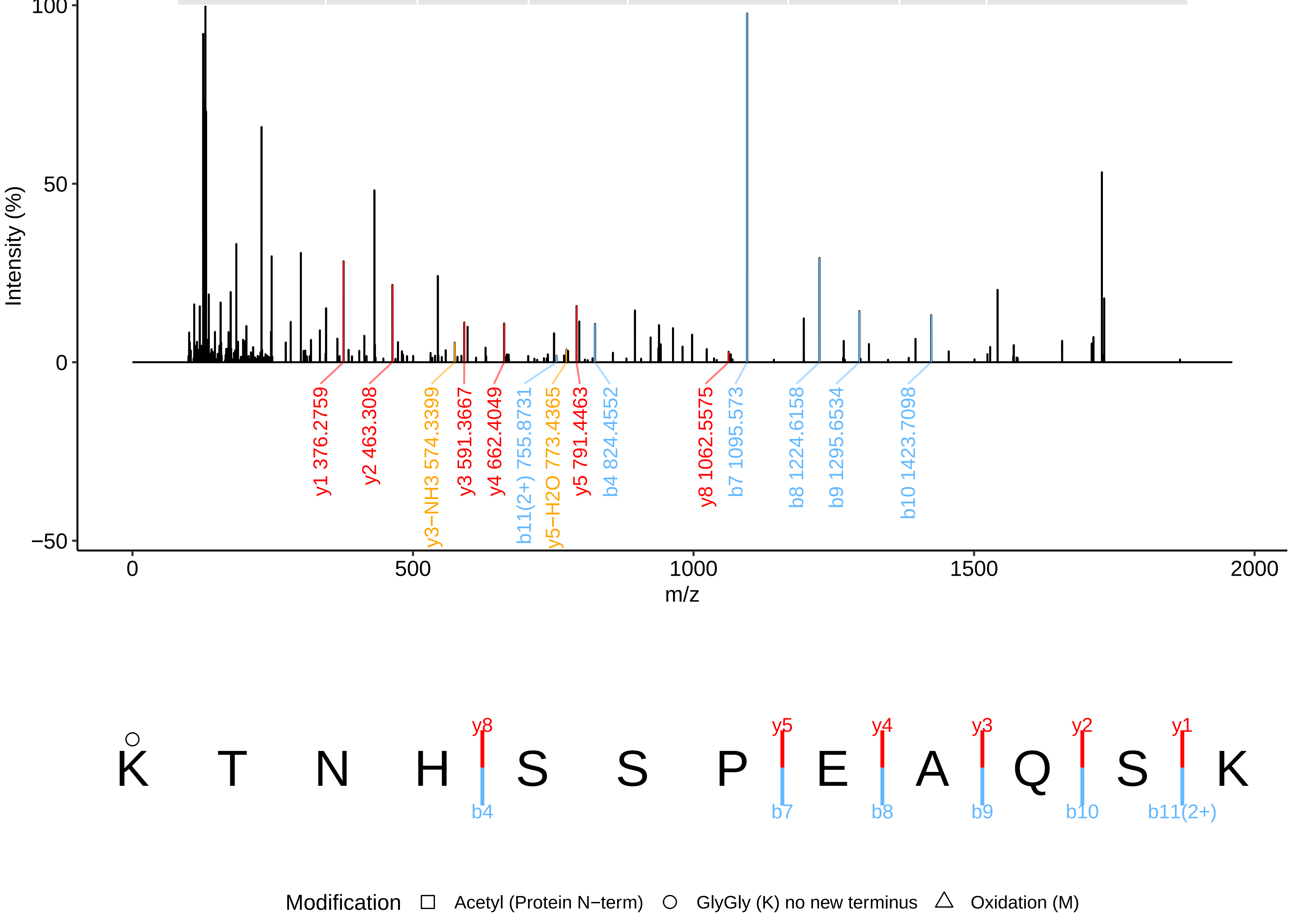

20200921_KIZ_MUb4_GlyGly_10plex − Scan number: 9164

Gene Names	Charge	m/z	Mass	Retention time	PEP	Score	Precursor Intensity
ASXH1;ASXL1	3	396.8839	1187.63	31.349	0.00036295	119.16	7287695

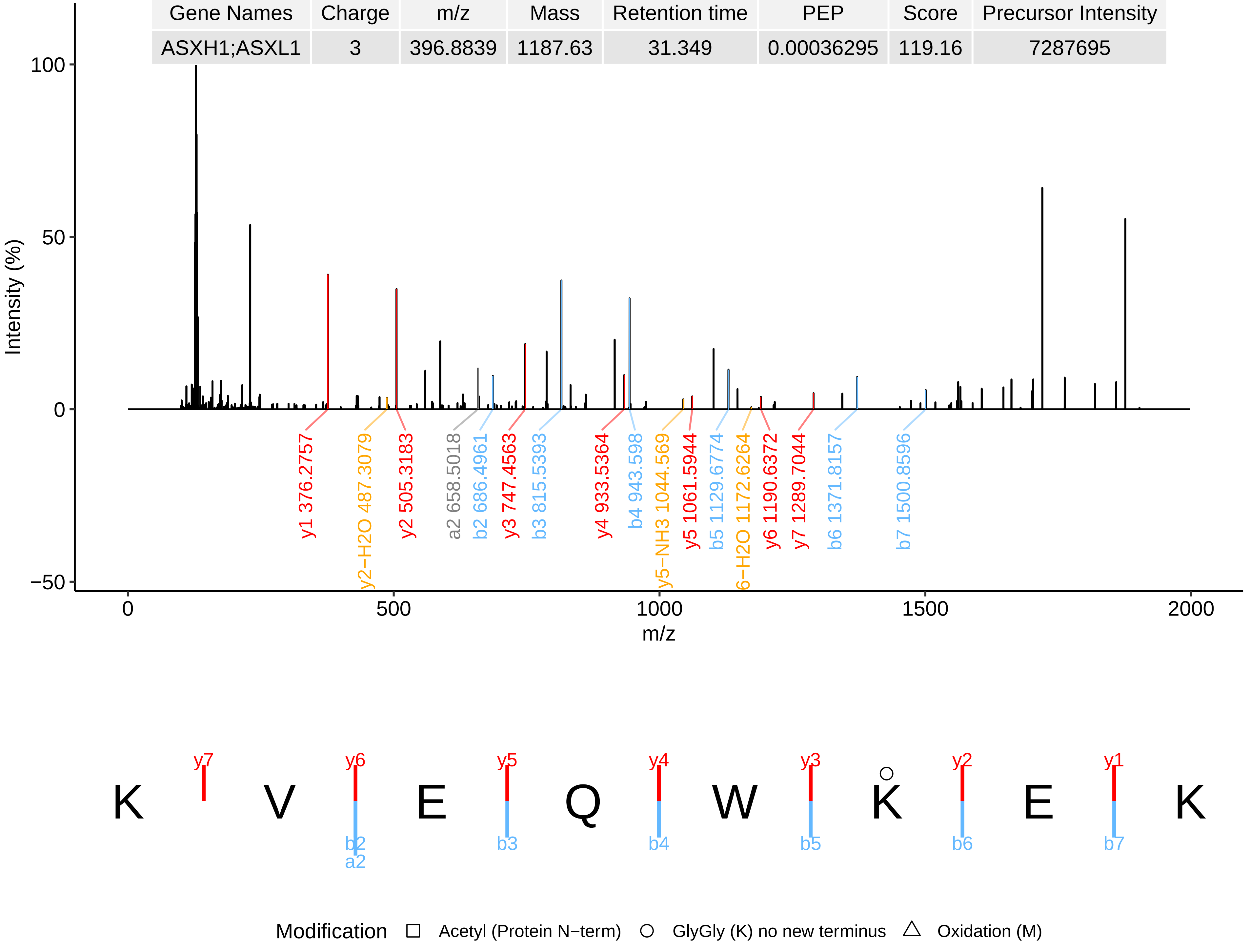

20200921_KIZ_MUb4_GlyGly_10plex_2 − Scan number: 8553

Gene Names	Charge	m/z	Mass	Retention time	PEP	Score	Precursor Intensity
ASXH1;ASXL1	3	396.8839	1187.63	32.258	0.0024923	103.55	1220729

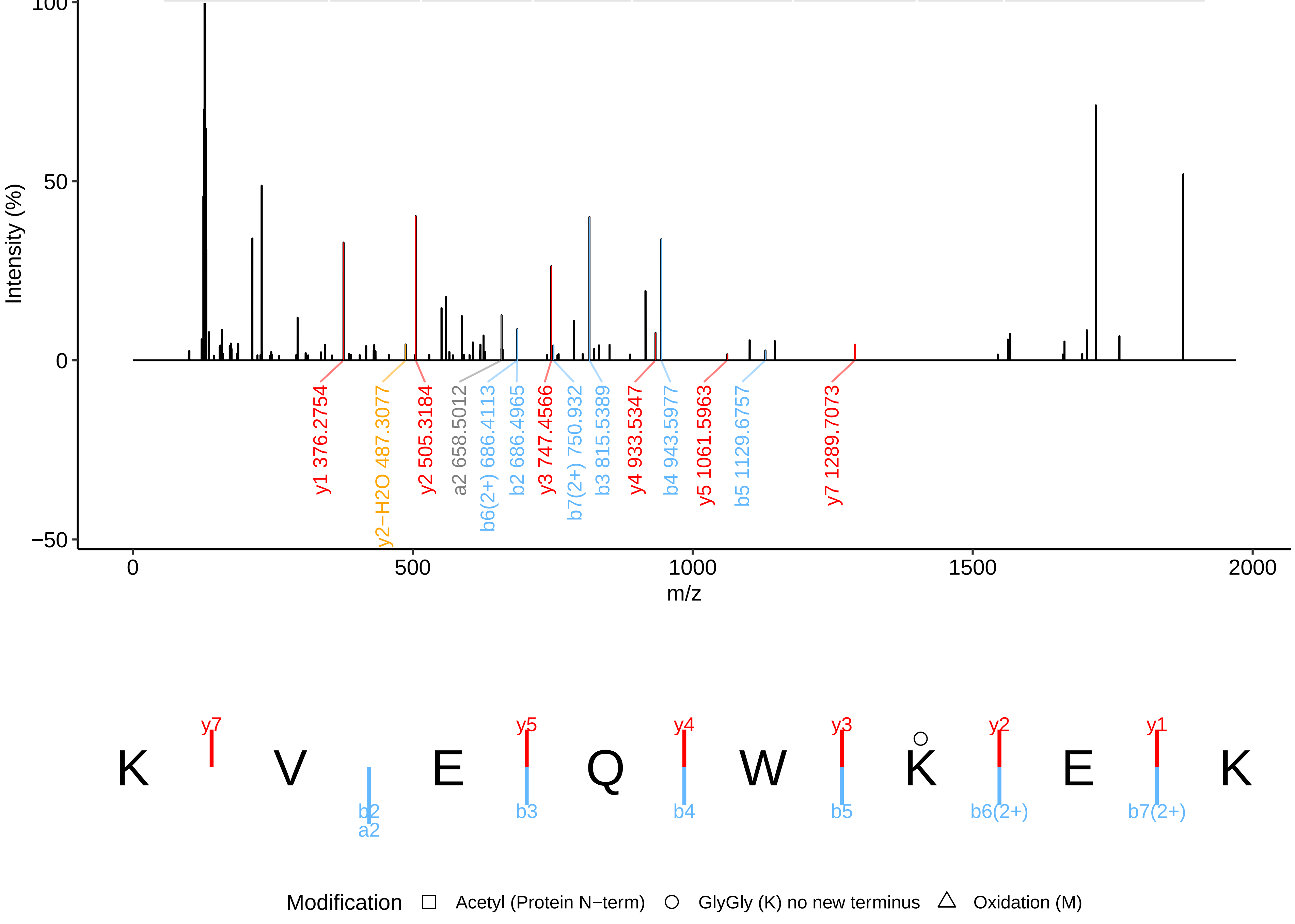

20200924_KIZ_MUb5_GlyGly_1 − Scan number: 7776

Gene Names	Charge	m/z	Mass	Retention time	PEP	Score	Precursor Intensity
ASXH1;ASXL1	3	396.8839	1187.63	24.764	4.9938e−05	147.75	NA

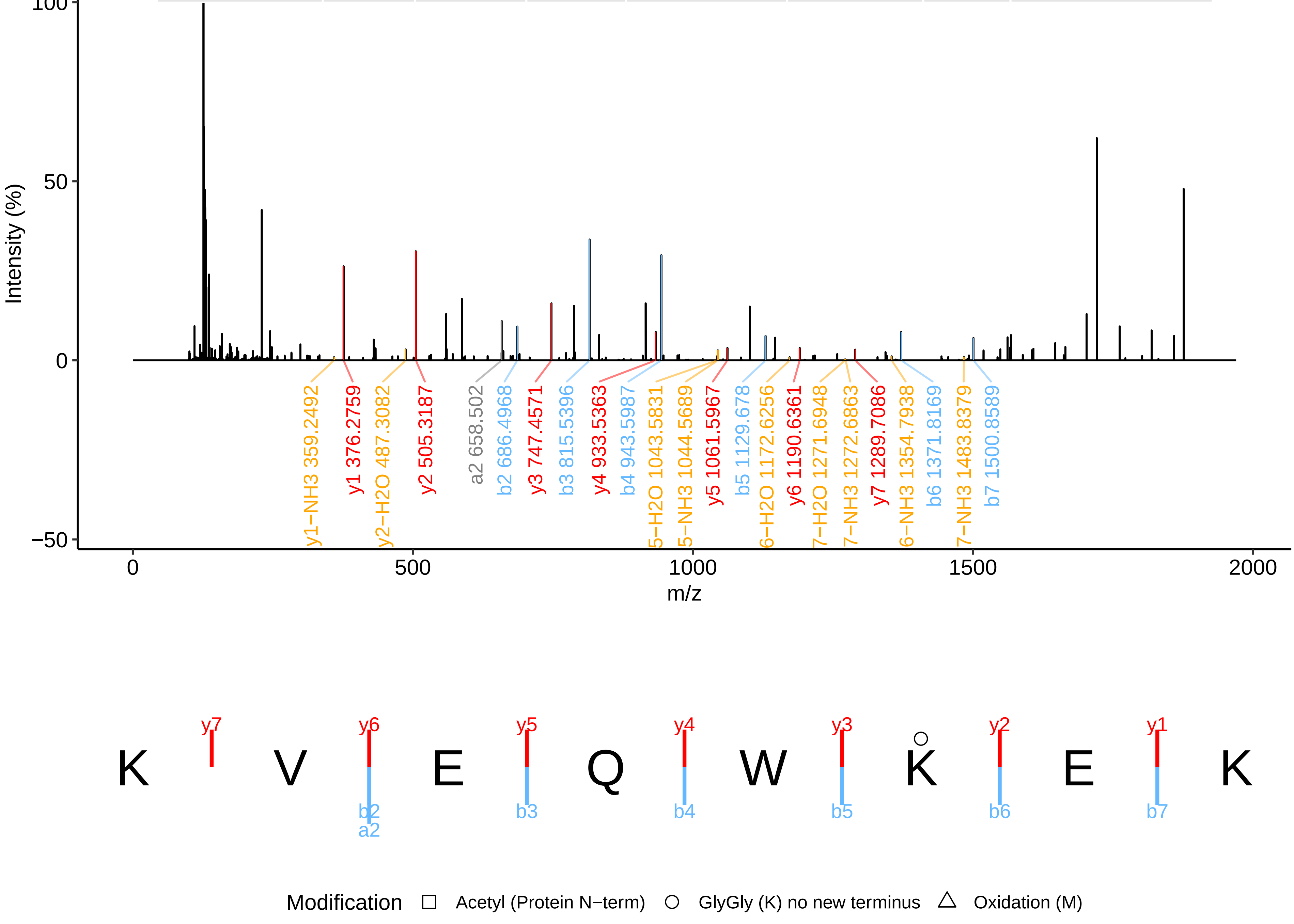

20200924_KIZ_MUb5_GlyGly_1 − Scan number: 7931

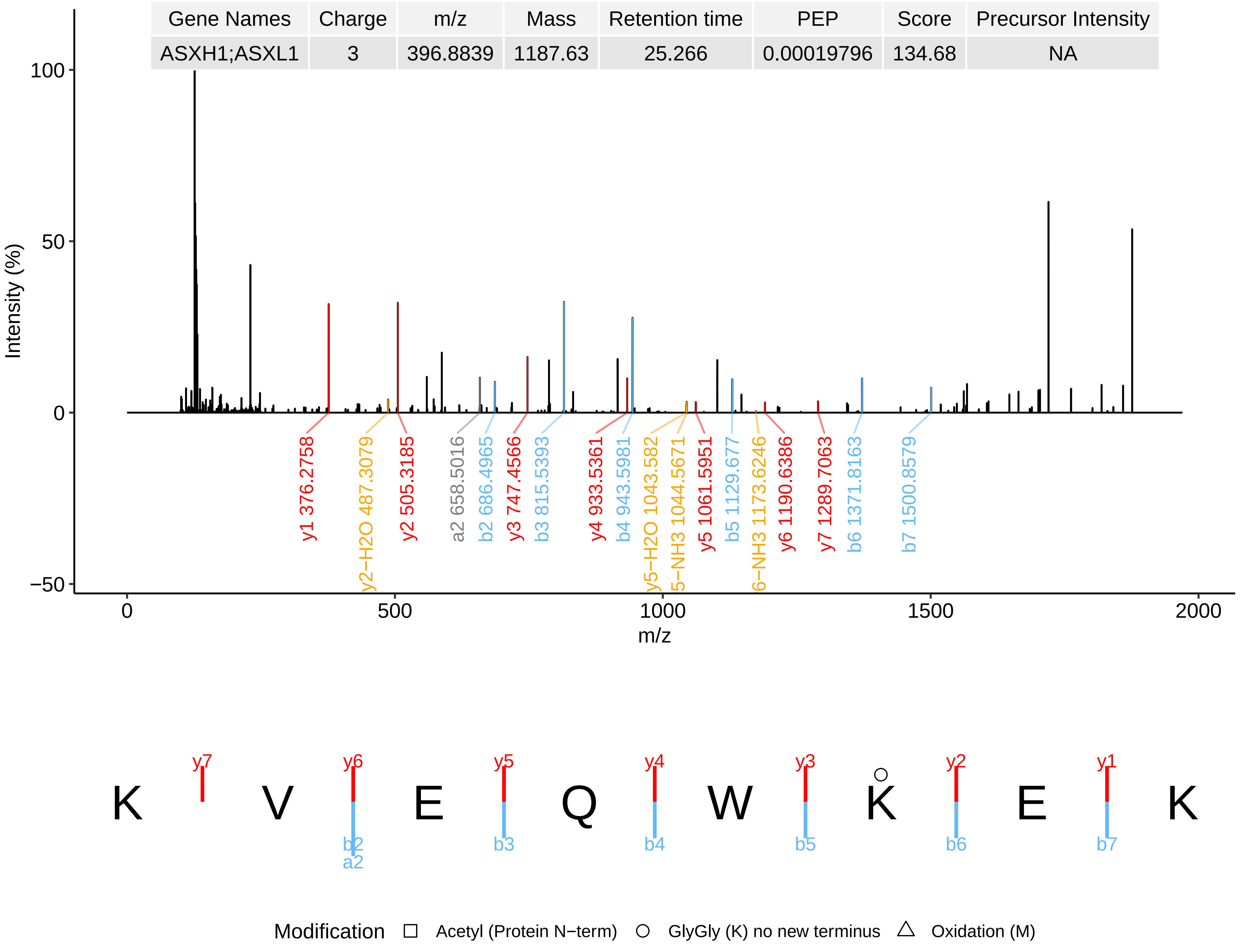

20200924_KIZ_MUb5_GlyGly_1 − Scan number: 7626

Gene Names	Charge	m/z	Mass	Retention time	PEP	Score	Precursor Intensity
ASXH1;ASXL1	3	396.8839	1187.63	24.244	0.0055989	97.456	NA

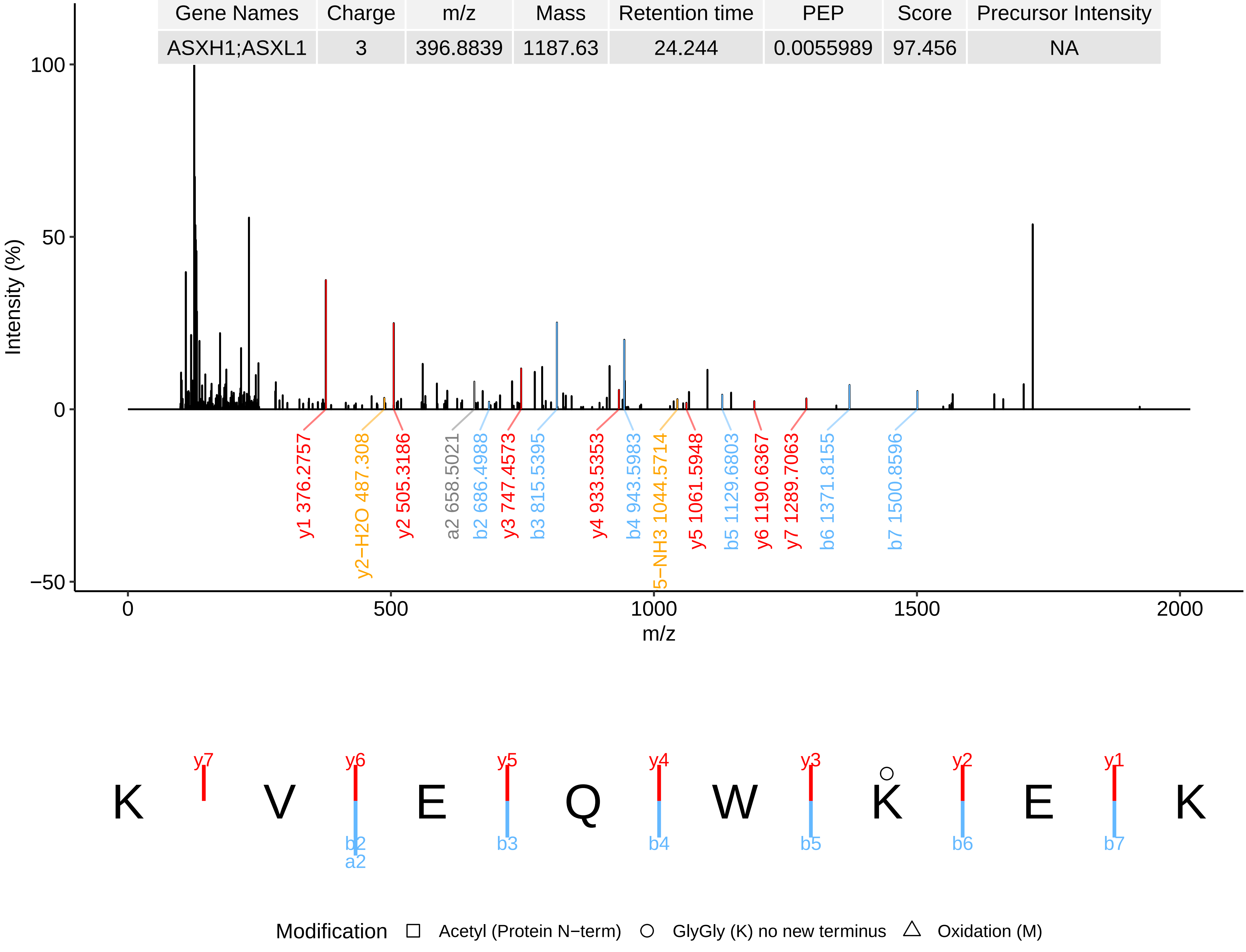

20200924_KIZ_MUb5_GlyGly_2 − Scan number: 7706

Gene Names	Charge	m/z	Mass	Retention time	PEP	Score	Precursor Intensity
ASXH1;ASXL1	3	396.8839	1187.63	28.779	0.015255	84.17	1403985

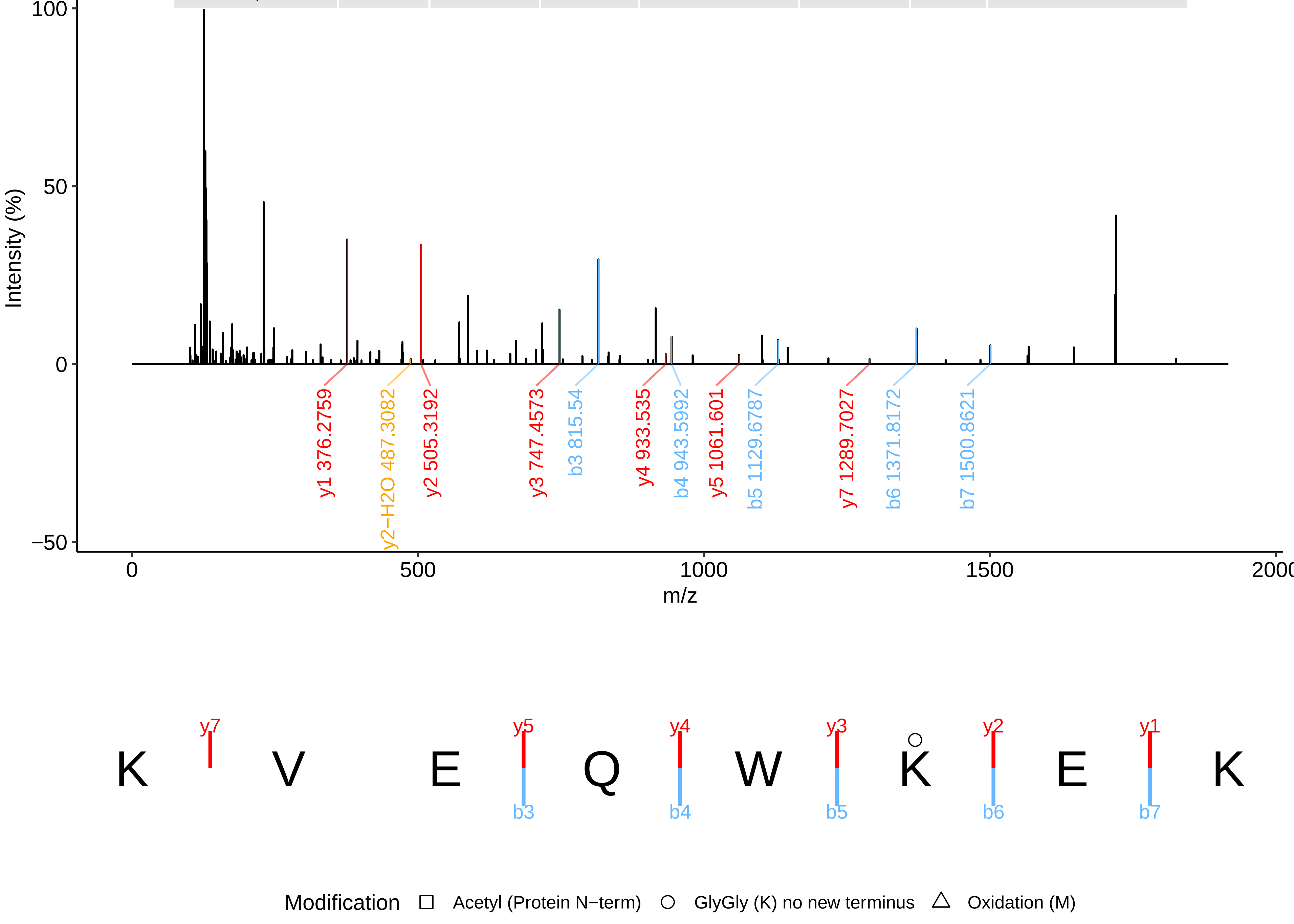

20201118_KIZ_MUb6_GlyGly_1 − Scan number: 7727

Gene Names	Charge	m/z	Mass	Retention time	PEP	Score	Precursor Intensity
ASXH1;ASXL1	4	297.9147	1187.63	24.484	0.088759	76.827	50276980

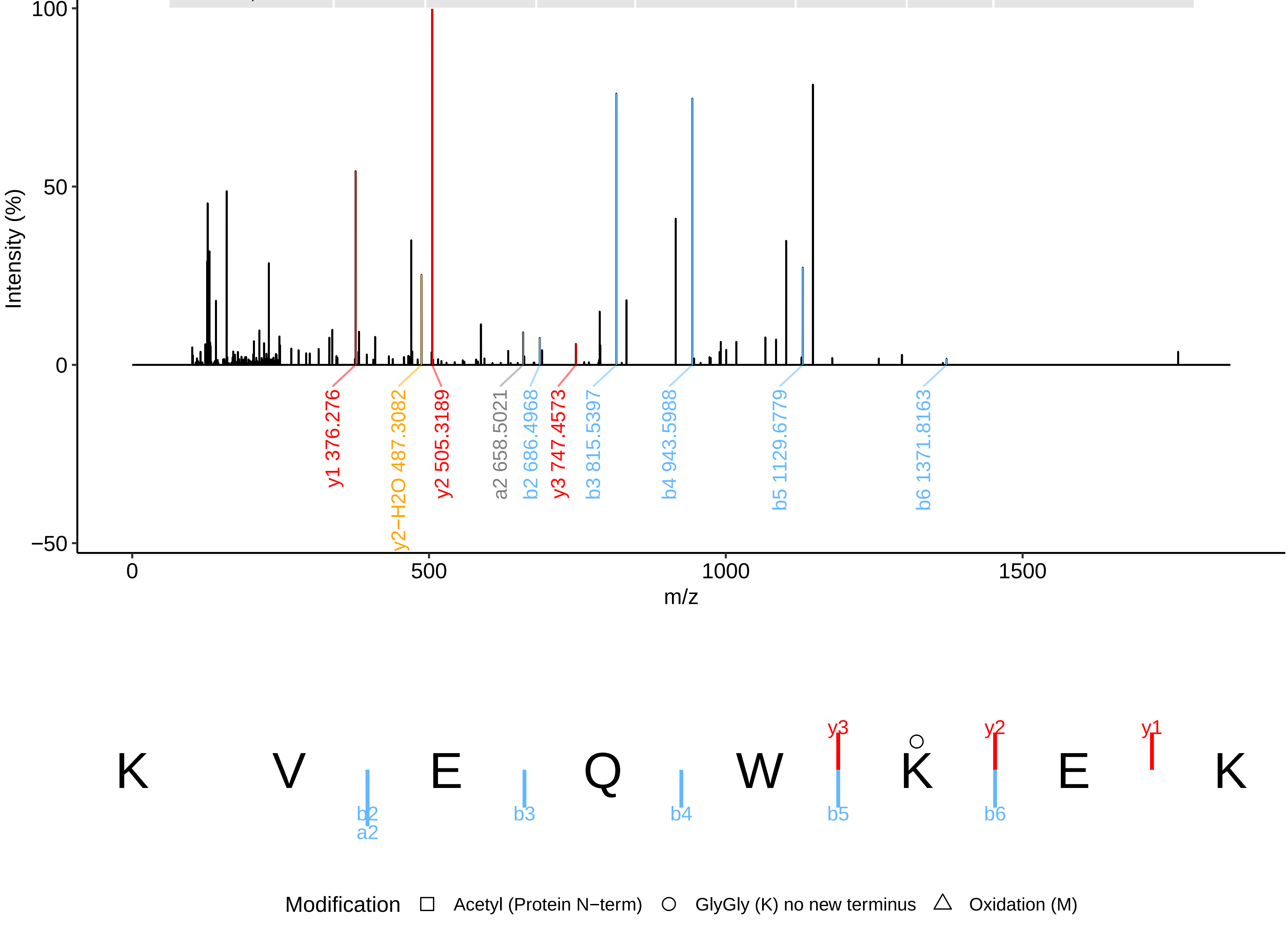

20200921_KIZ_MUb4_GlyGly_10plex_2 − Scan number: 6719

Gene Names	Charge	m/z	Mass	Retention time	PEP	Score	Precursor Intensity
IMMT	3	481.5915	1441.753	24.822	0.0096616	70.942	539152.8

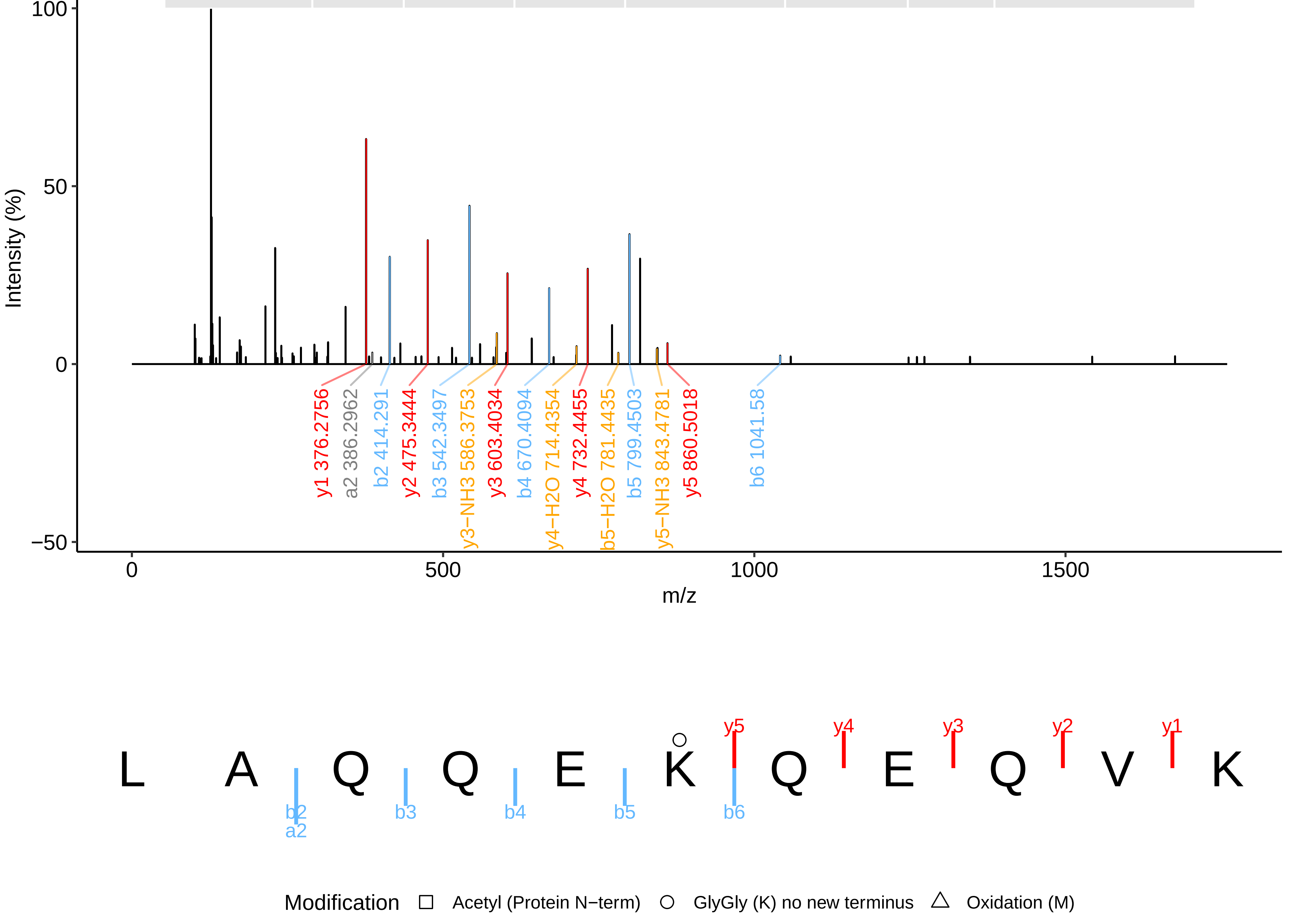

20200921_KIZ_MUb4_GlyGly_10plex − Scan number: 16087

Gene Names	Charge	m/z	Mass	Retention time	PEP	Score	Precursor Intensity
USP24	3	478.5997	1432.777	57.699	6.9182e−09	124.42	4119615

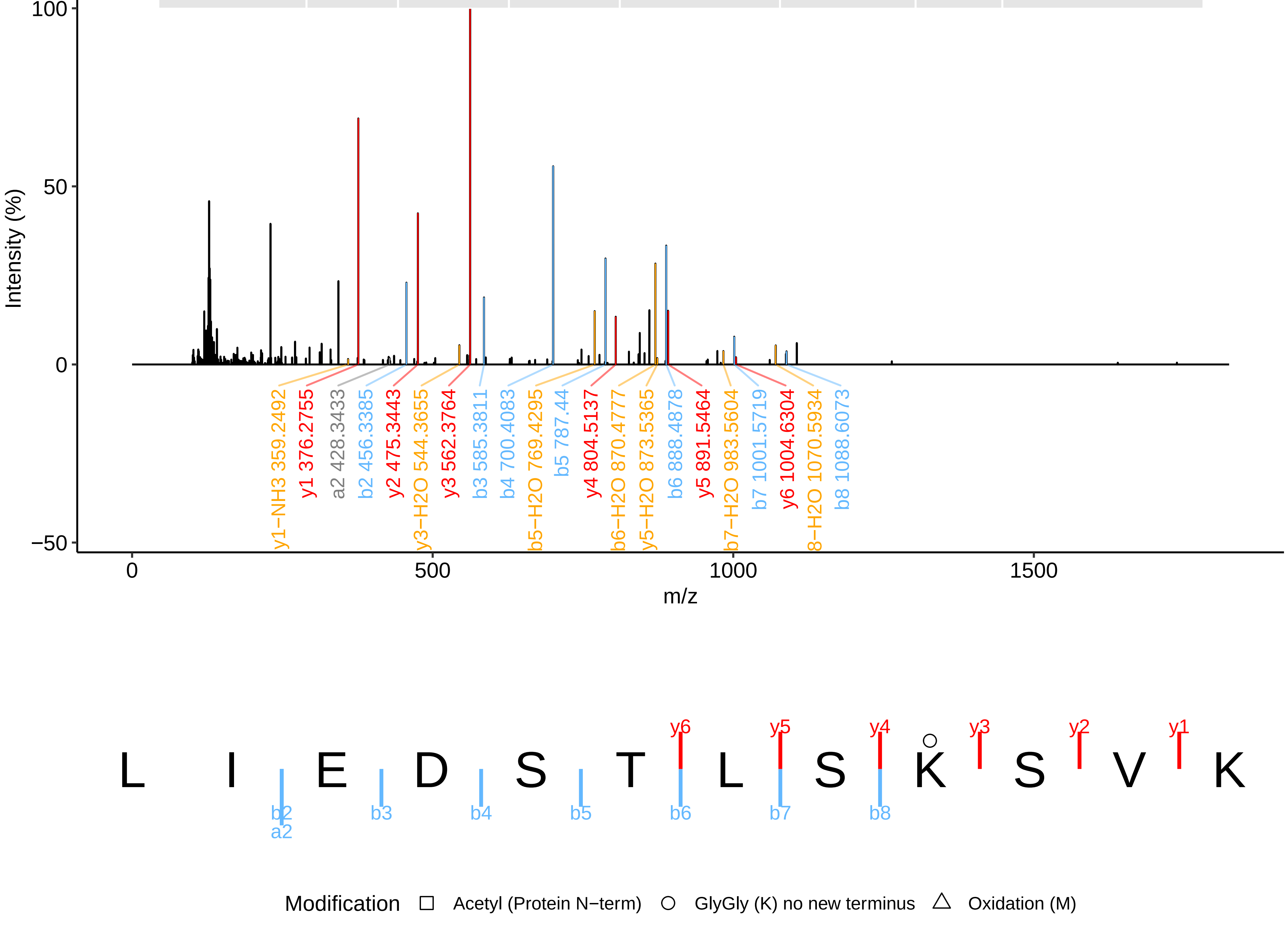

20200921_KIZ_MUb4_GlyGly_10plex_2 − Scan number: 14937

Gene Names	Charge	m/z	Mass	Retention time	PEP	Score	Precursor Intensity
USP24	3	478.5997	1432.777	58.314	3.9314e−06	112.13	1053746

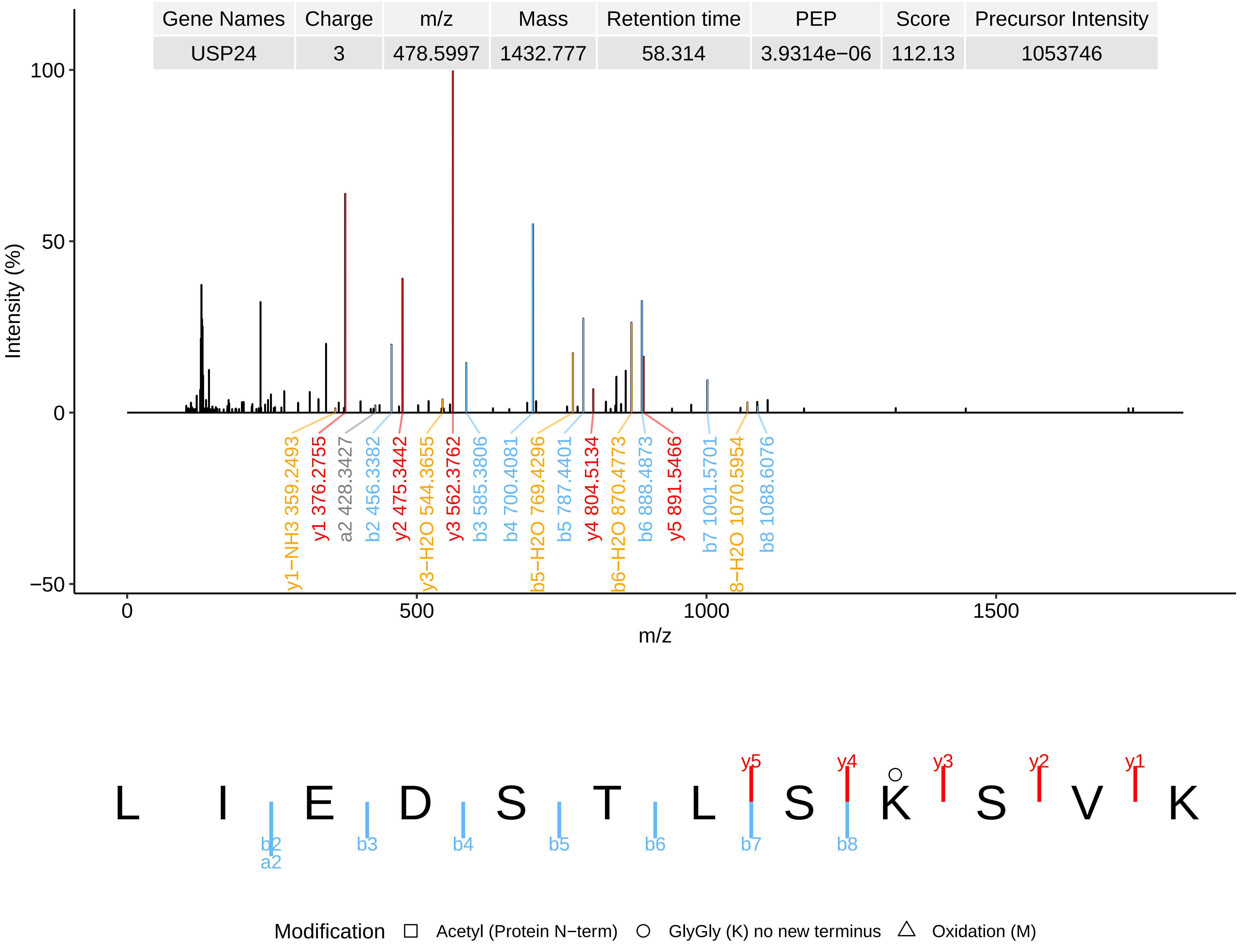

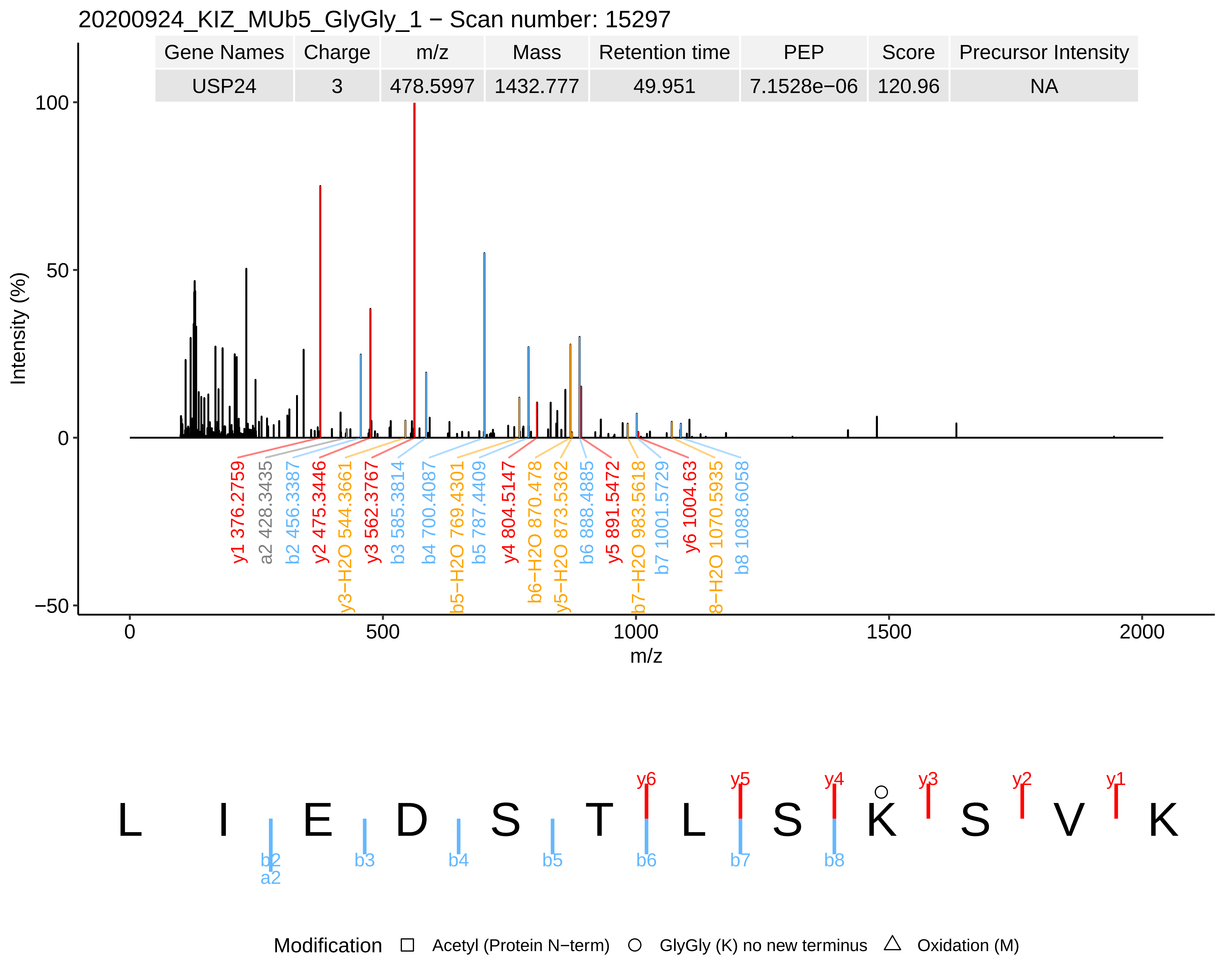

Gene Names	Charge	m/z	Mass	Retention time	PEP	Score	Precursor Intensity
USP24	3	478.5997	1432.777	49.951	7.1528e−06	120.96	NA

20200924_KIZ_MUb5_GlyGly_1 − Scan number: 21456

Gene Names	Charge	m/z	Mass	Retention time	PEP	Score	Precursor Intensity
ATXN3	3	547.6371	1639.889	69.115	0.006038	72.705	7789312

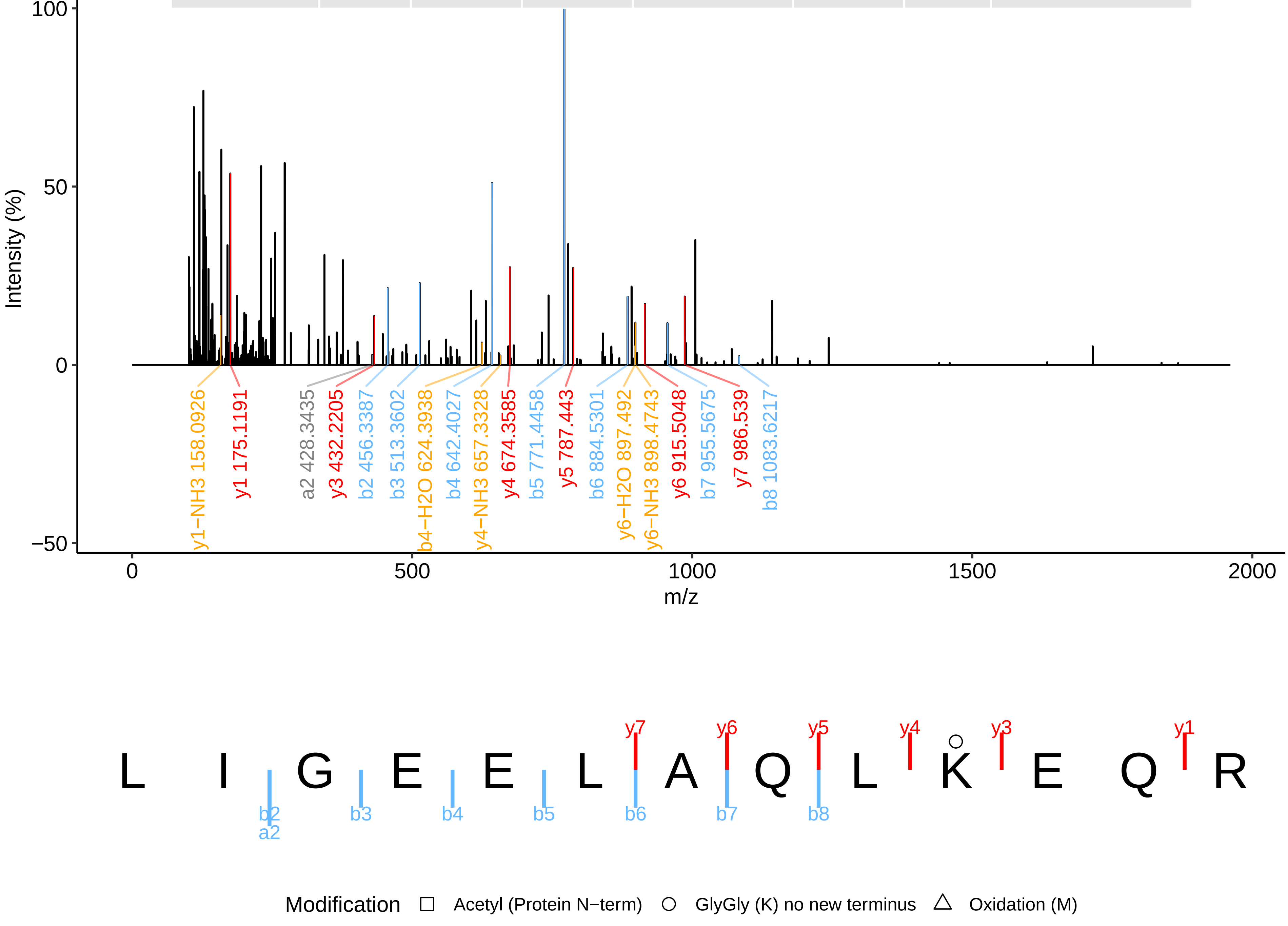

20200924_KIZ_MUb5_GlyGly_1 − Scan number: 21515

Gene Names	Charge	m/z	Mass	Retention time	PEP	Score	Precursor Intensity
ATXN3	3	547.6371	1639.889	69.303	0.063025	55.196	NA

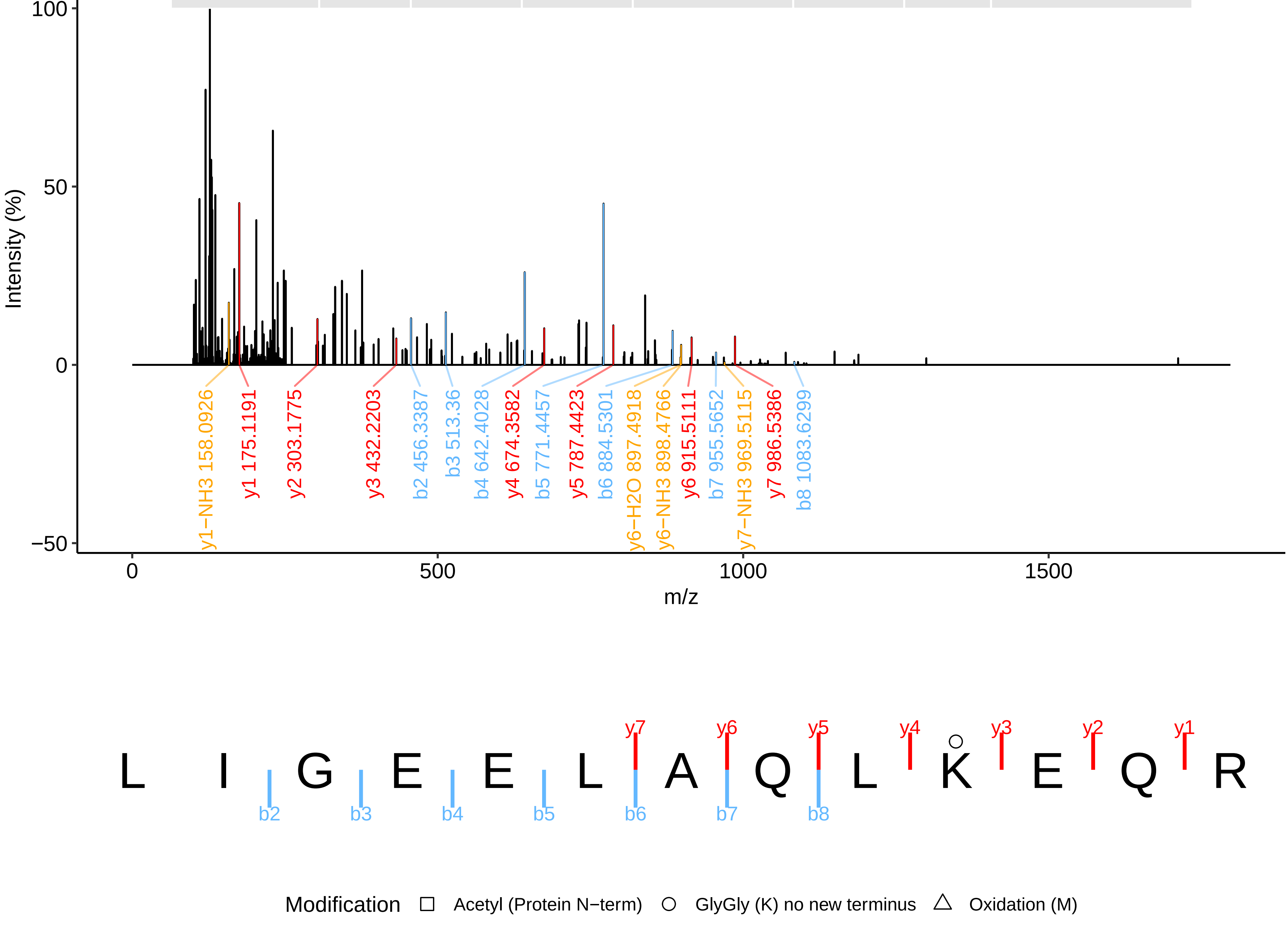

20200921_KIZ_MUb4_GlyGly_10plex_2 − Scan number: 20579

Gene Names	Charge	m/z	Mass	Retention time	PEP	Score	Precursor Intensity
EIF3A	3	548.631	1642.871	80.905	0.0001924	95.401	953743.8

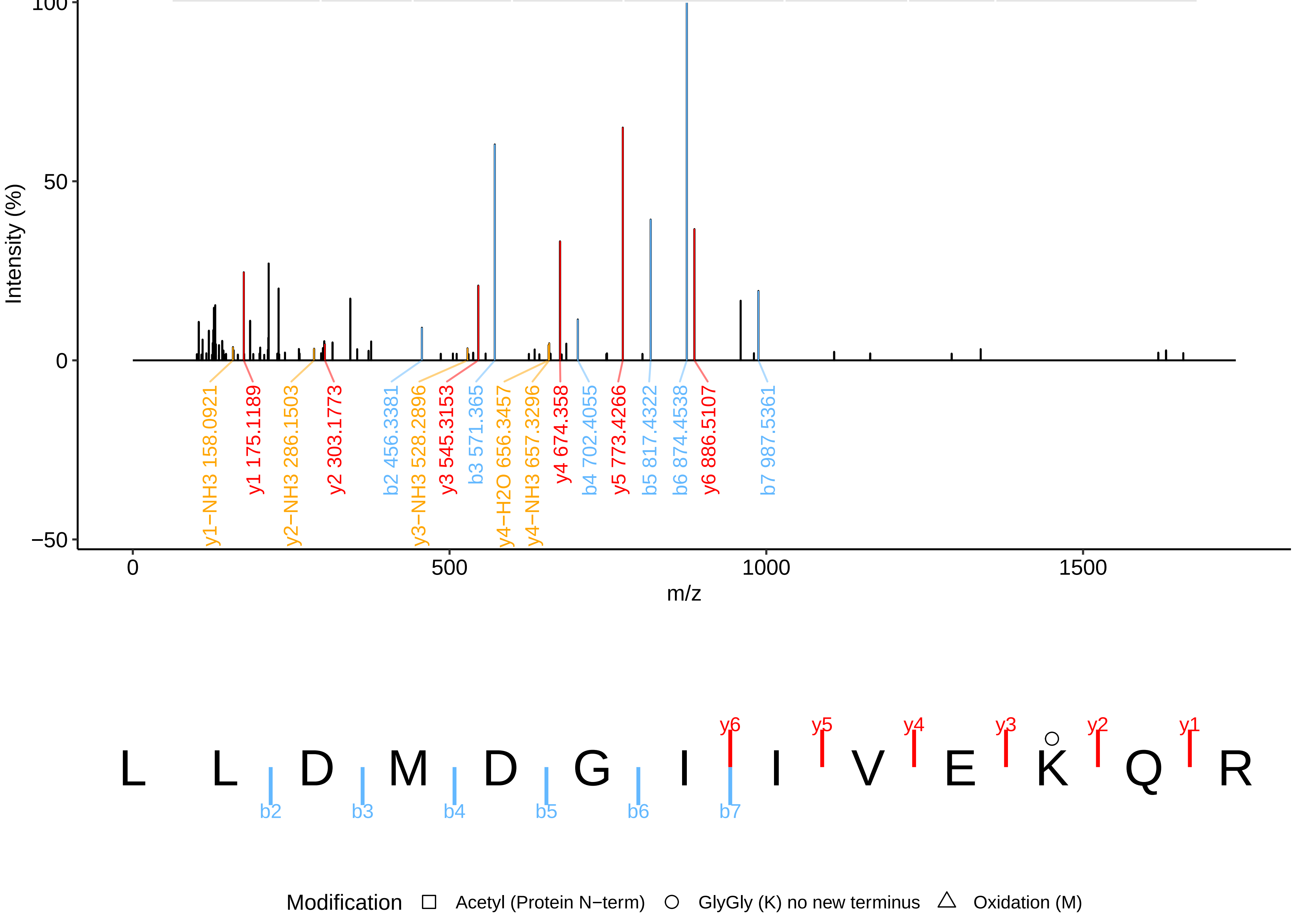

20200921_KIZ_MUb4_GlyGly_10plex_2 − Scan number: 15838

Gene Names	Charge	m/z	Mass	Retention time	PEP	Score	Precursor Intensity
EIF3A	3	553.9627	1658.866	62.011	0.035102	60.47	3344308

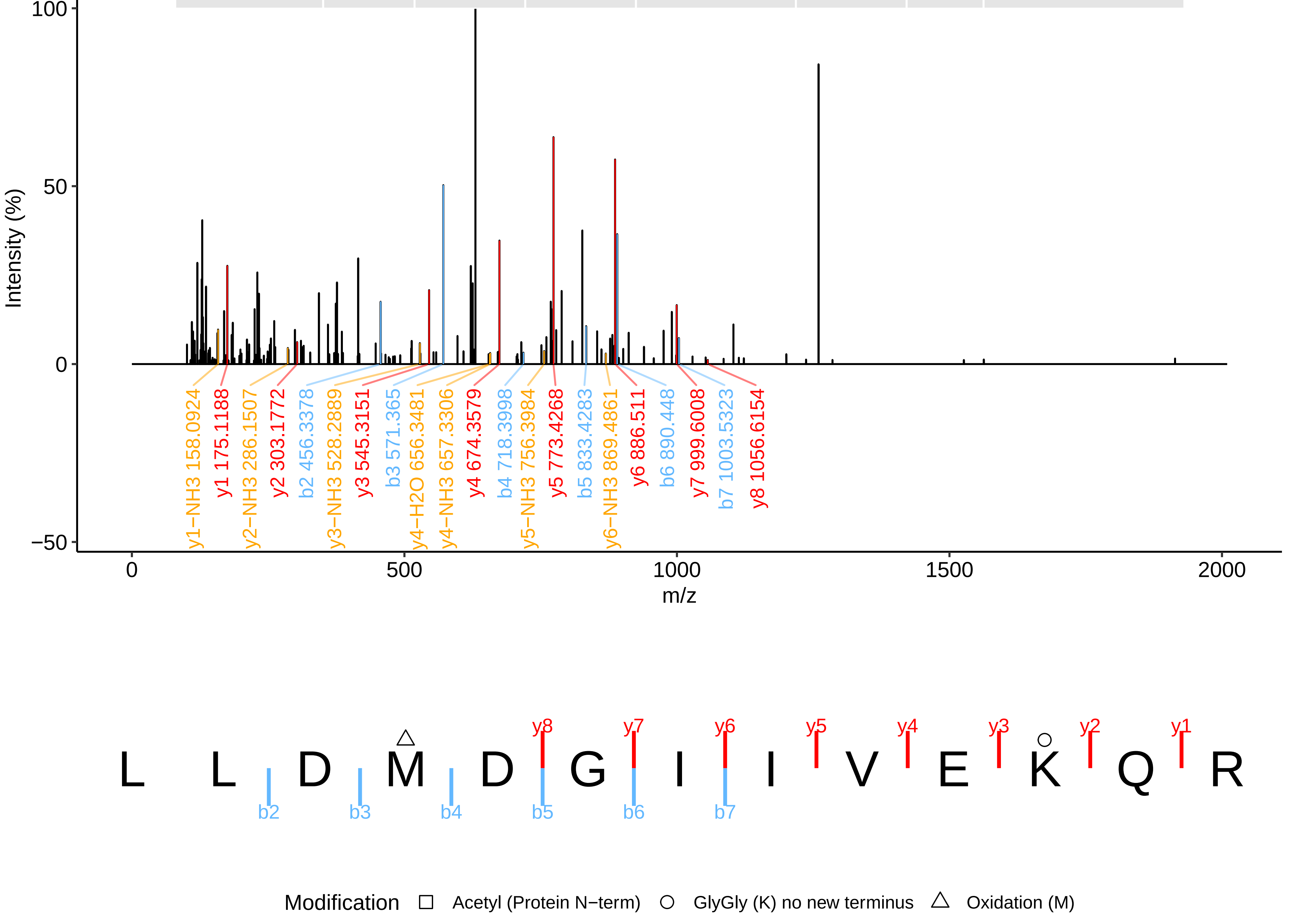

20200921_KIZ_MUb4_GlyGly_10plex_2 − Scan number: 11908

Gene Names	Charge	m/z	Mass	Retention time	PEP	Score	Precursor Intensity
ENO1	3	477.5877	1429.741	45.951	0.0052268	77.64	NA

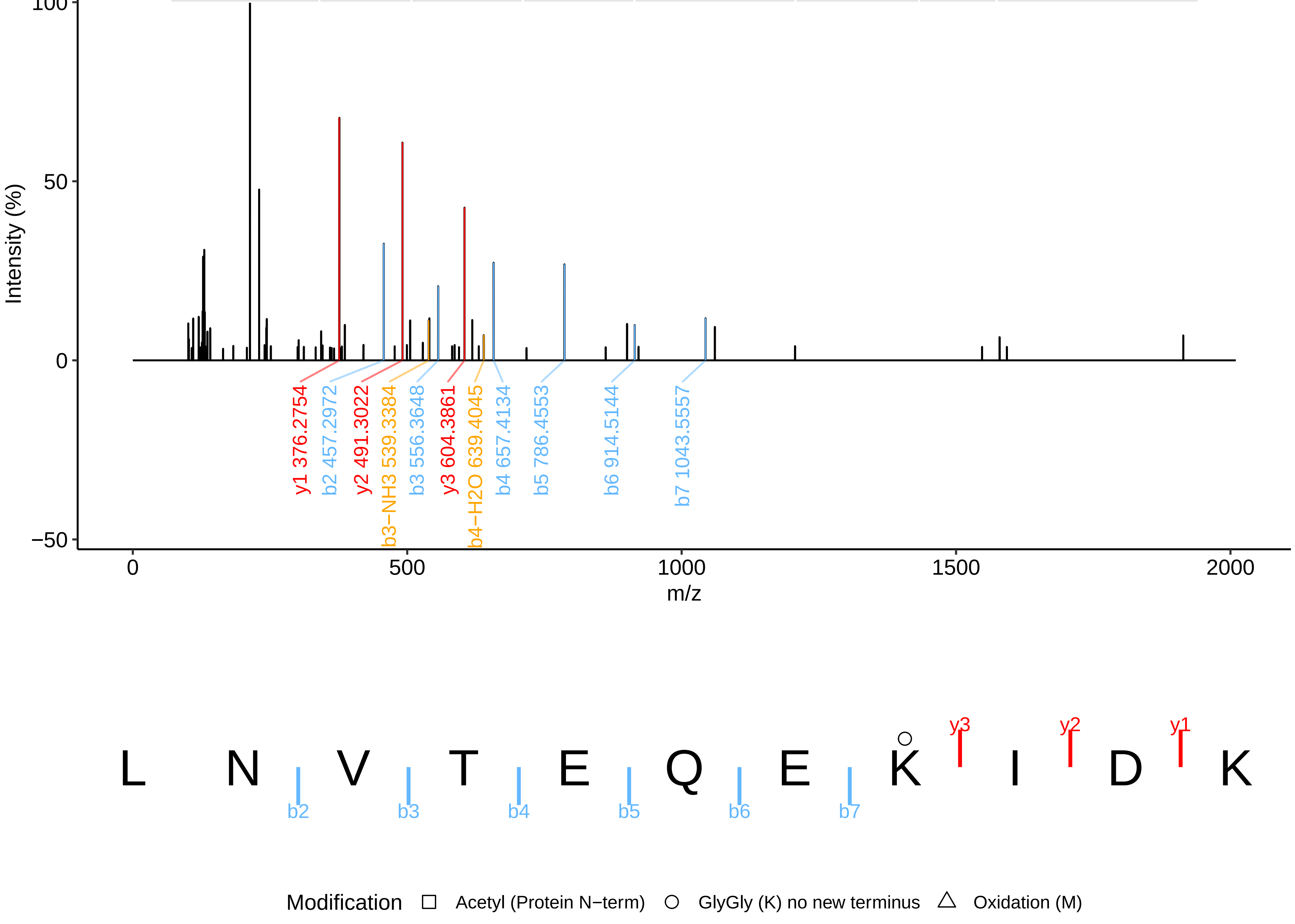

20200921_KIZ_MUb4_GlyGly_10plex − Scan number: 17641

Gene Names	Charge	m/z	Mass	Retention time	PEP	Score	Precursor Intensity
TOMM20	3	480.928	1439.762	63.366	3.4255e−07	115.14	10410754

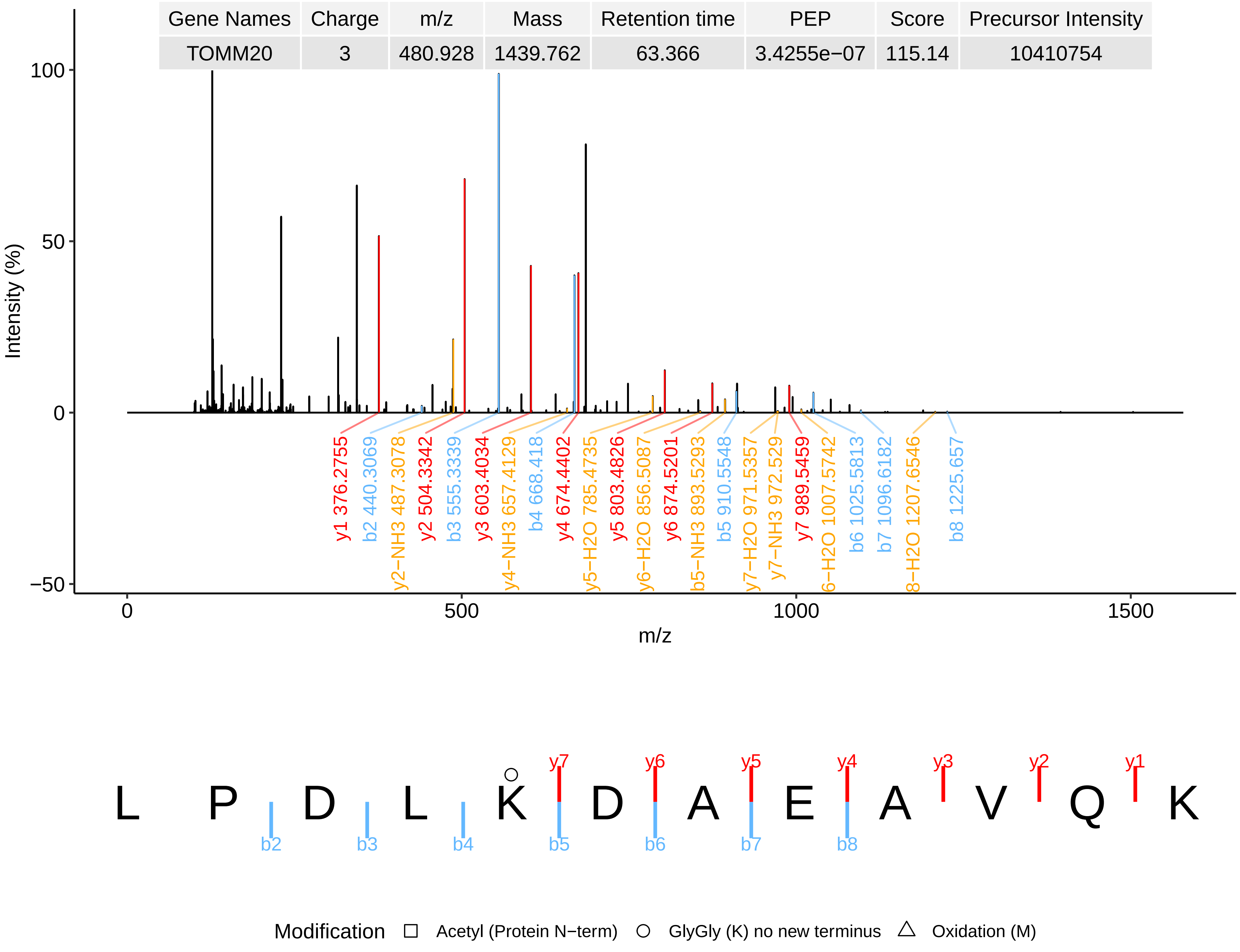

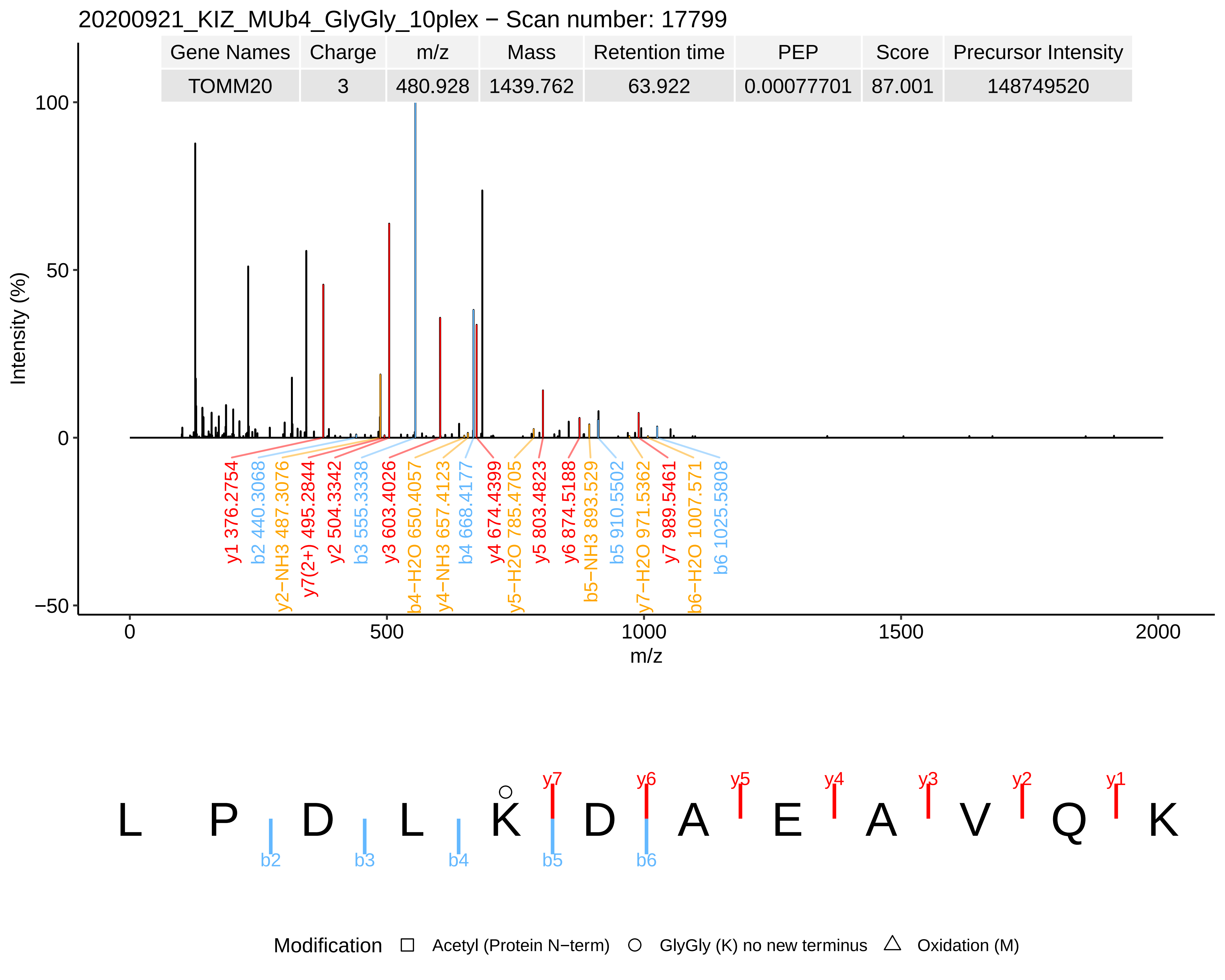

20200921_KIZ_MUb4_GlyGly_10plex − Scan number: 17799
Gene Names | Charge | m/z | Mass | Retention time | PEP | Score | Precursor Intensity
TOMM20 | 3 | 480.928 | 1439.762 | 63.922 | 0.00077701 | 87.001 | 148749520
Intensity (%)
100
50
0
−50
0
500
1000
1500
2000
m/z
y1 376.2754
b2 440.3068
y2−NH3 487.3076
y7(2+) 495.2844
y2 504.3342
b3 555.3338
y3 603.4026
b4−H2O 650.4057
y4−NH3 657.4123
b4 668.4177
y4 674.4399
y5−H2O 785.4705
y5 803.4823
y6 874.5188
b5−NH3 893.529
b5 910.5502
y7−H2O 971.5362
y7 989.5461
b6−H2O 1007.571
b6 1025.5808
L P D L K D A E A V Q K
y7 y6 y5 y4 y3 y2 y1
b2 b3 b4 b5 b6
Modification
Acetyl (Protein N−term)
GlyGly (K) no new terminus
Oxidation (M)

20200921_KIZ_MUb4_GlyGly_10plex − Scan number: 17946

Gene Names	Charge	m/z	Mass	Retention time	PEP	Score	Precursor Intensity
TOMM20	3	480.928	1439.762	64.455	0.011526	70.089	9520203

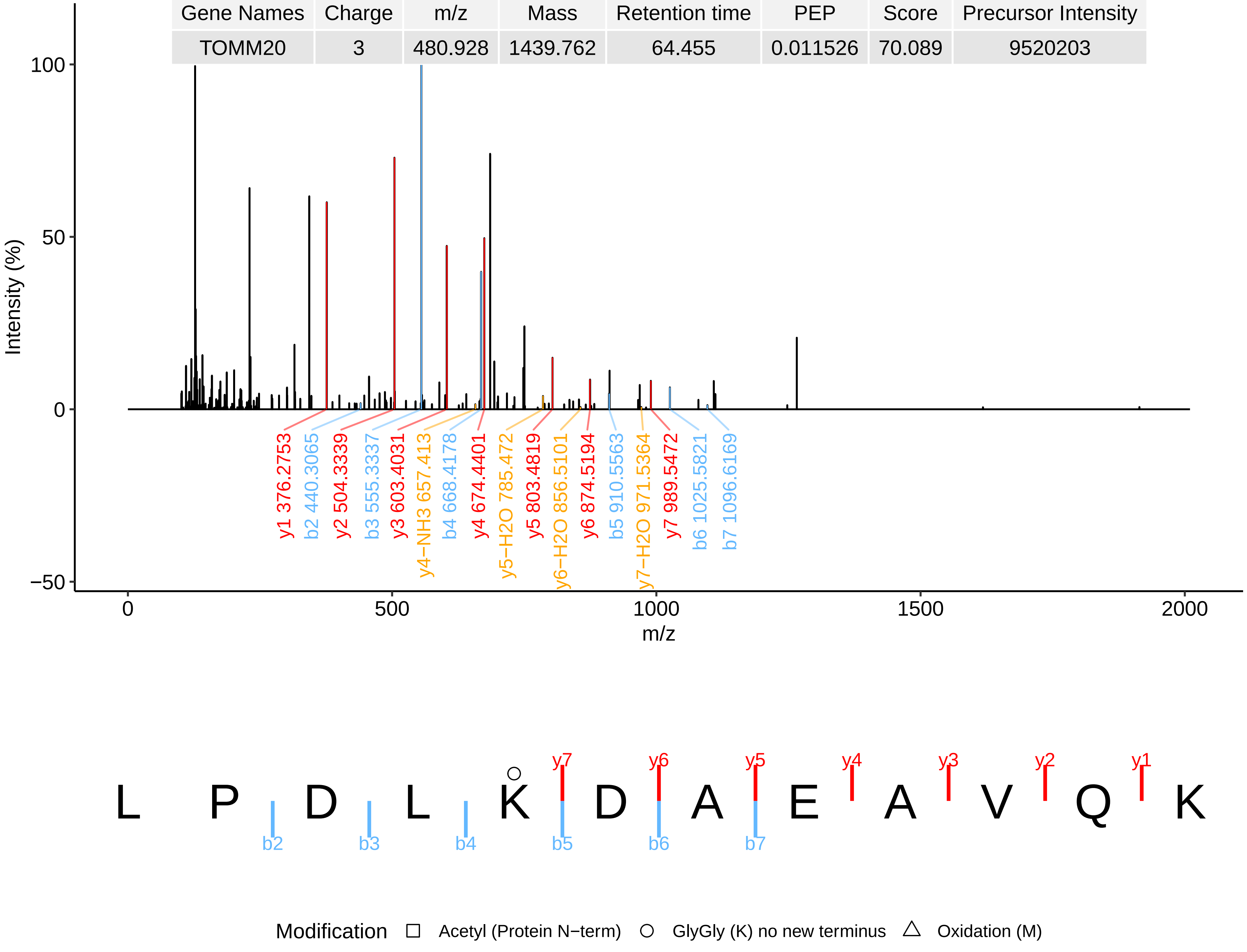

20200921_KIZ_MUb4_GlyGly_10plex_2 − Scan number: 16311

Gene Names	Charge	m/z	Mass	Retention time	PEP	Score	Precursor Intensity
TOMM20	3	480.928	1439.762	63.667	0.0067662	72.29	7758588

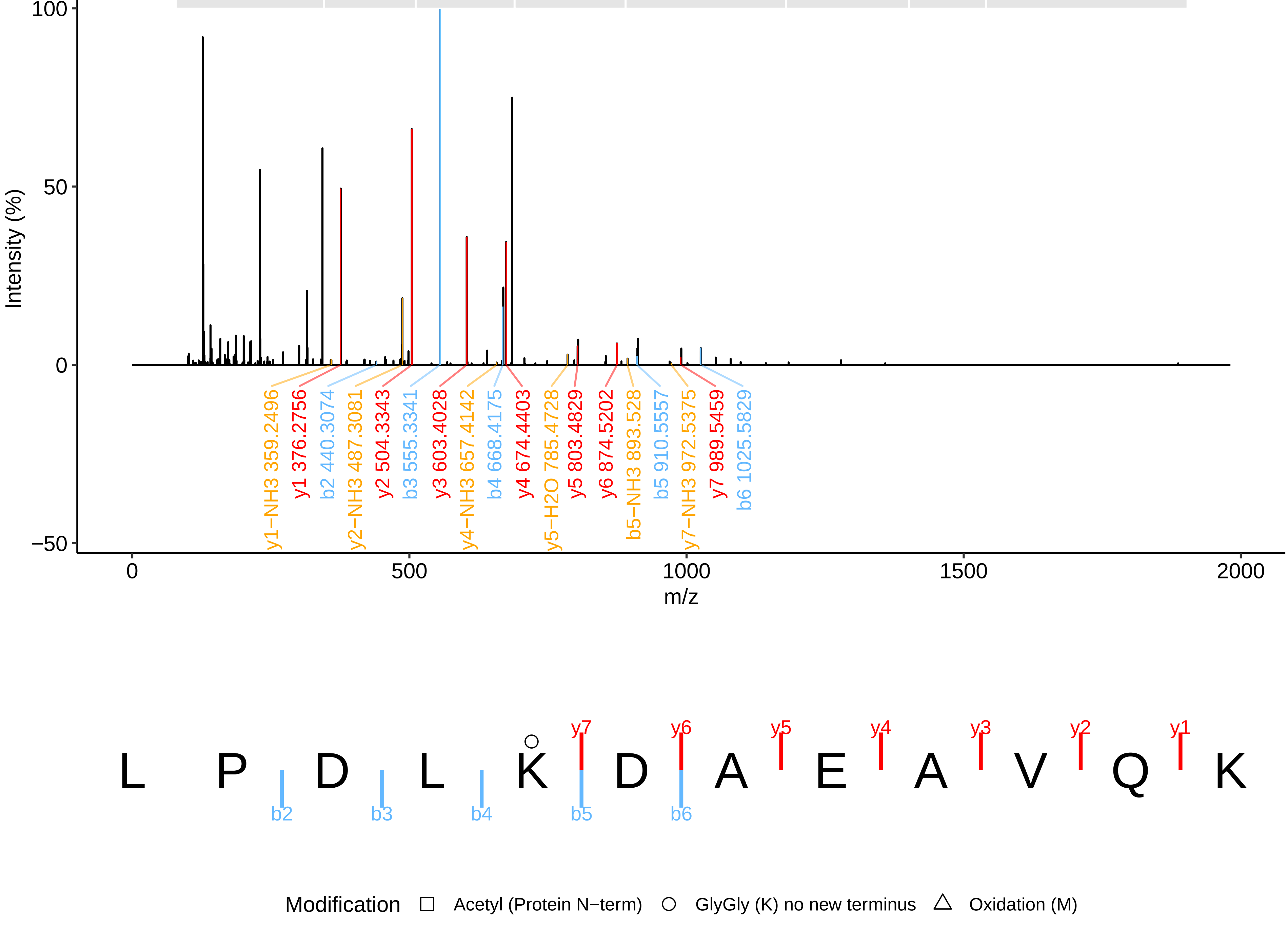

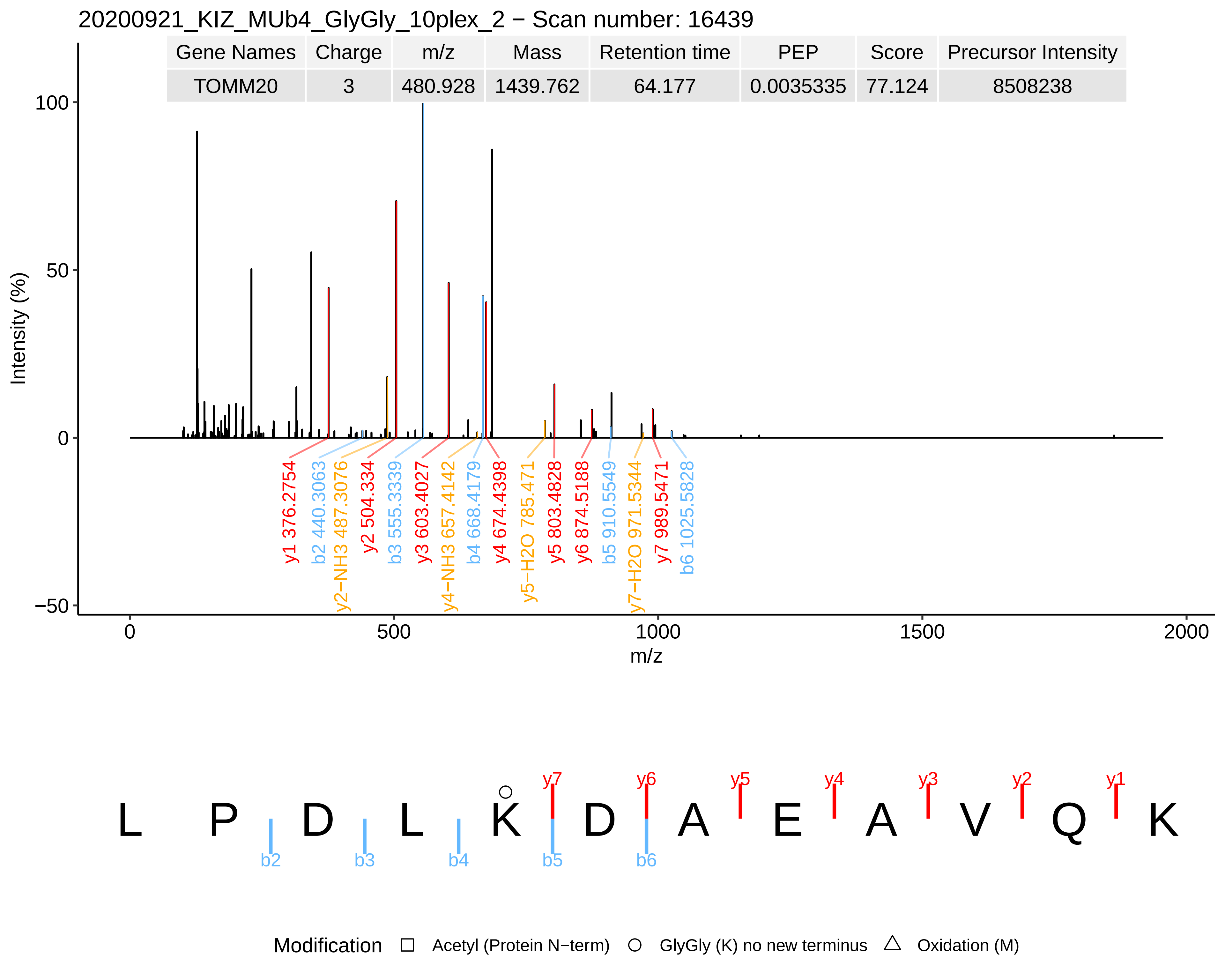
20200921_KIZ_MUb4_GlyGly_10plex_2 − Scan number: 16439
Gene Names | Charge | m/z | Mass | Retention time | PEP | Score | Precursor Intensity
TOMM20 | 3 | 480.928 | 1439.762 | 64.177 | 0.0035335 | 77.124 | 8508238
Intensity (%)
100
50
0
−50
0
500
1000
1500
2000
m/z
y1 376.2754
b2 440.3063
y2−NH3 487.3076
y2 504.334
b3 555.3339
y3 603.4027
y4−NH3 657.4142
b4 668.4179
y4 674.4398
y5−H2O 785.471
y5 803.4828
y6 874.5188
b5 910.5549
y7−H2O 971.5344
y7 989.5471
b6 1025.5828
L P D L K D A E A V Q K
y7 y6 y5 y4 y3 y2 y1
b2 b3 b4 b5 b6
Modification
Acetyl (Protein N−term)
GlyGly (K) no new terminus
Oxidation (M)

20200921_KIZ_MUb4_GlyGly_10plex_2 − Scan number: 16292

Gene Names	Charge	m/z	Mass	Retention time	PEP	Score	Precursor Intensity
TOMM20	3	480.928	1439.762	63.596	0.0013282	84.289	NA

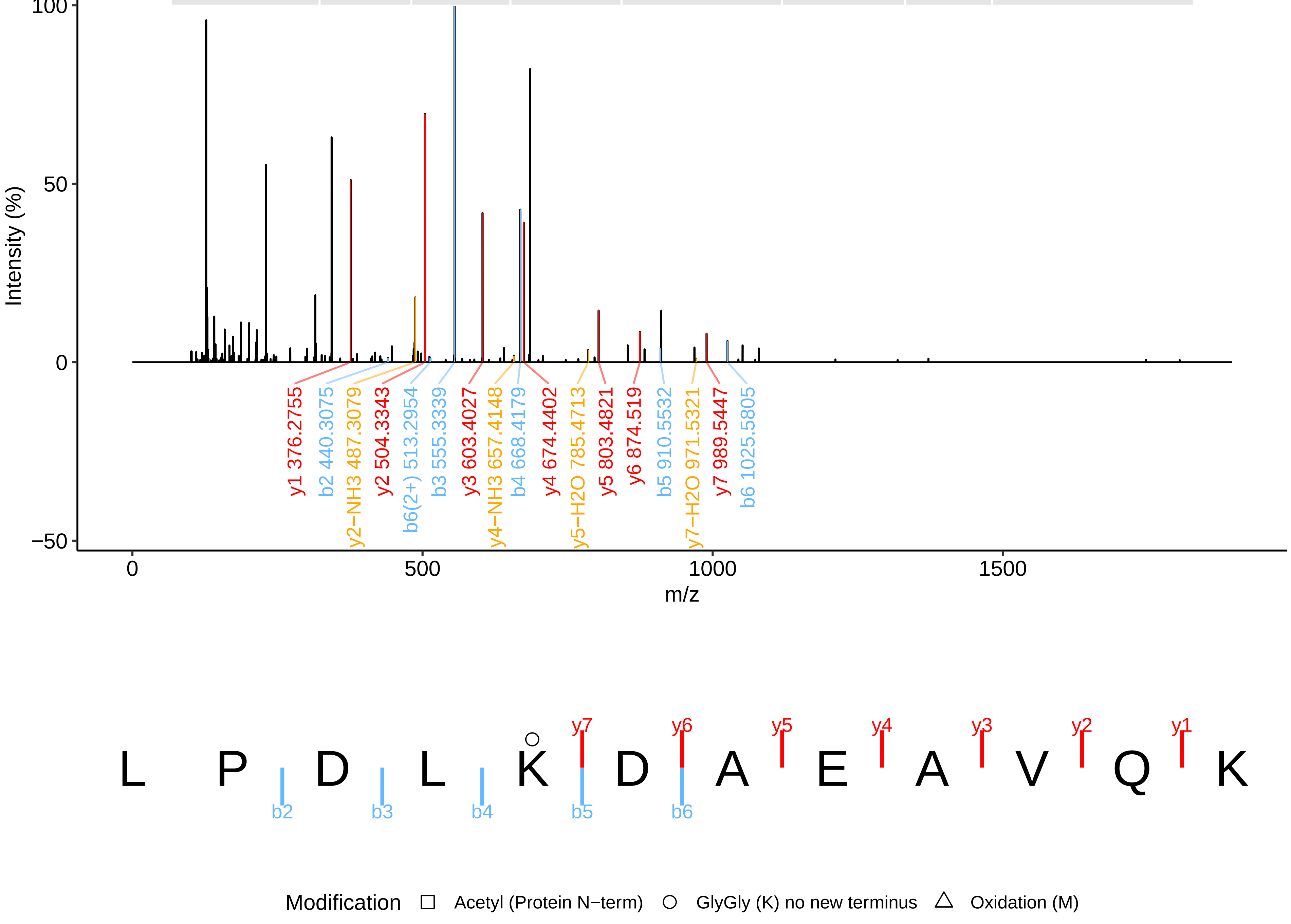

20200924_KIZ_MUb5_GlyGly_1 − Scan number: 17286

Gene Names	Charge	m/z	Mass	Retention time	PEP	Score	Precursor Intensity
TOMM20	3	480.928	1439.762	55.815	0.0082385	70.089	12880648

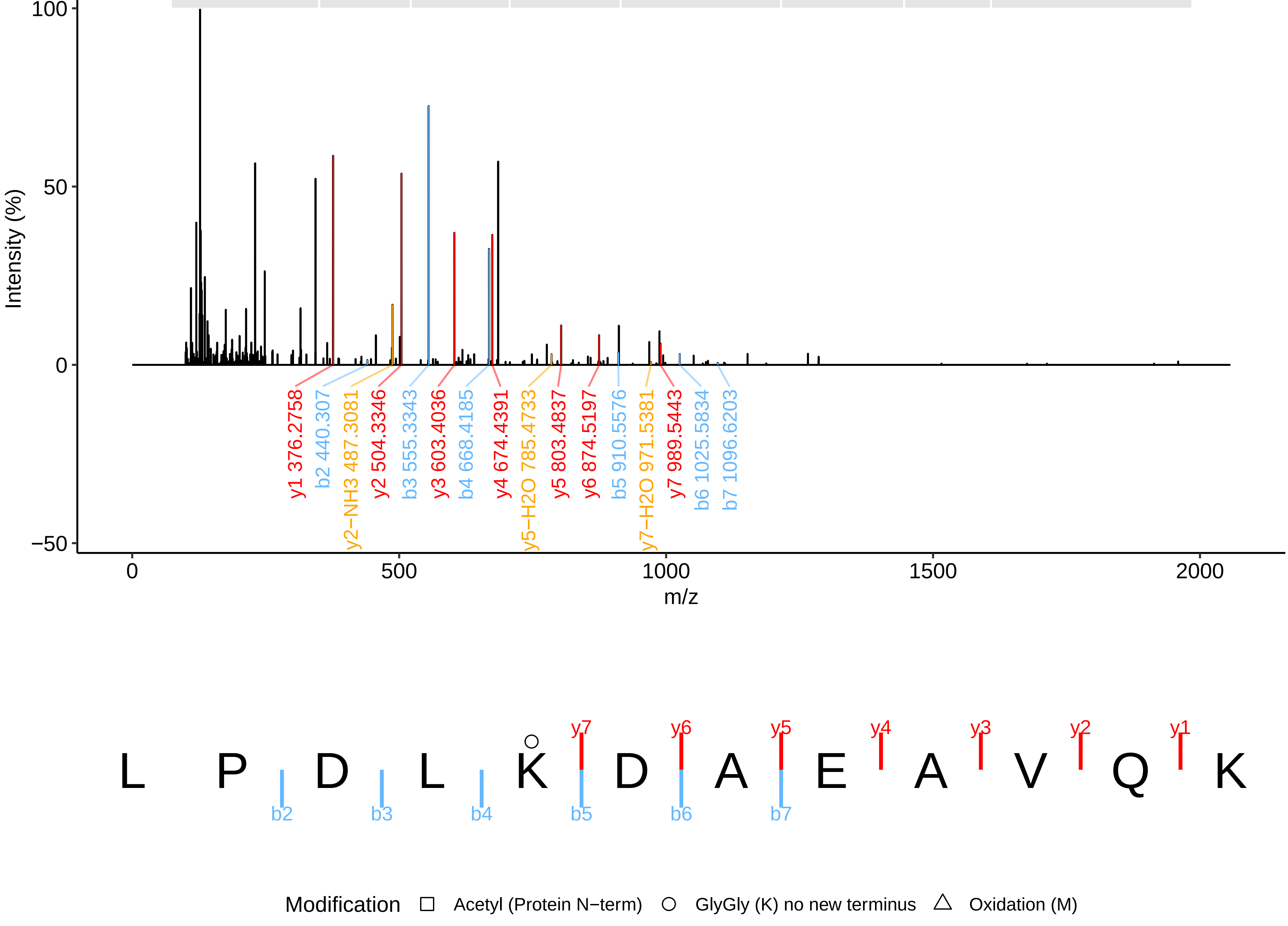

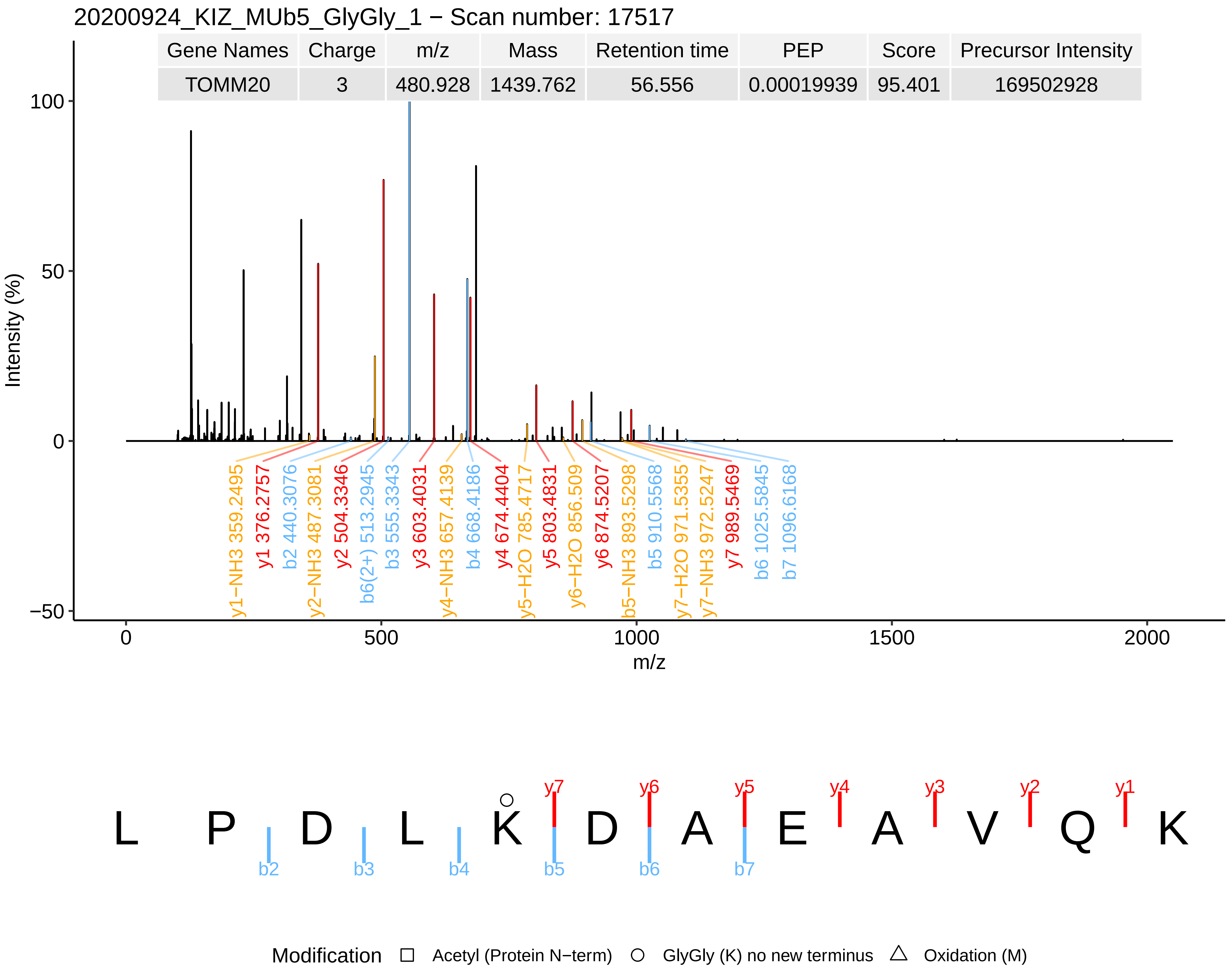

20200924_KIZ_MUb5_GlyGly_1 − Scan number: 17517
Gene Names
Charge
m/z
Mass
Retention time
PEP
Score
Precursor Intensity
TOMM20
3
480.928
1439.762
56.556
0.00019939
95.401
169502928
Intensity (%)
100
50
0
−50
0
500
1000
1500
2000
m/z
y1−NH3 359.2495
y1 376.2757
b2 440.3076
y2−NH3 487.3081
y2 504.3346
b6(2+) 513.2945
b3 555.3343
y3 603.4031
y4−NH3 657.4139
b4 668.4186
y4 674.4404
y5−H2O 785.4717
y5 803.4831
y6−H2O 856.509
y6 874.5207
b5−NH3 893.5298
b5 910.5568
y7−H2O 971.5355
y7−NH3 972.5247
y7 989.5469
b6 1025.5845
b7 1096.6168
L P D L K D A E A V Q K
y7
y6
y5
y4
y3
y2
y1
b2
b3
b4
b5
b6
b7
Modification
Acetyl (Protein N−term)
GlyGly (K) no new terminus
Oxidation (M)

20200924_KIZ_MUb5_GlyGly_1 − Scan number: 17666

Gene Names	Charge	m/z	Mass	Retention time	PEP	Score	Precursor Intensity
TOMM20	3	480.928	1439.762	57.067	1.0646e−05	106.49	146252224

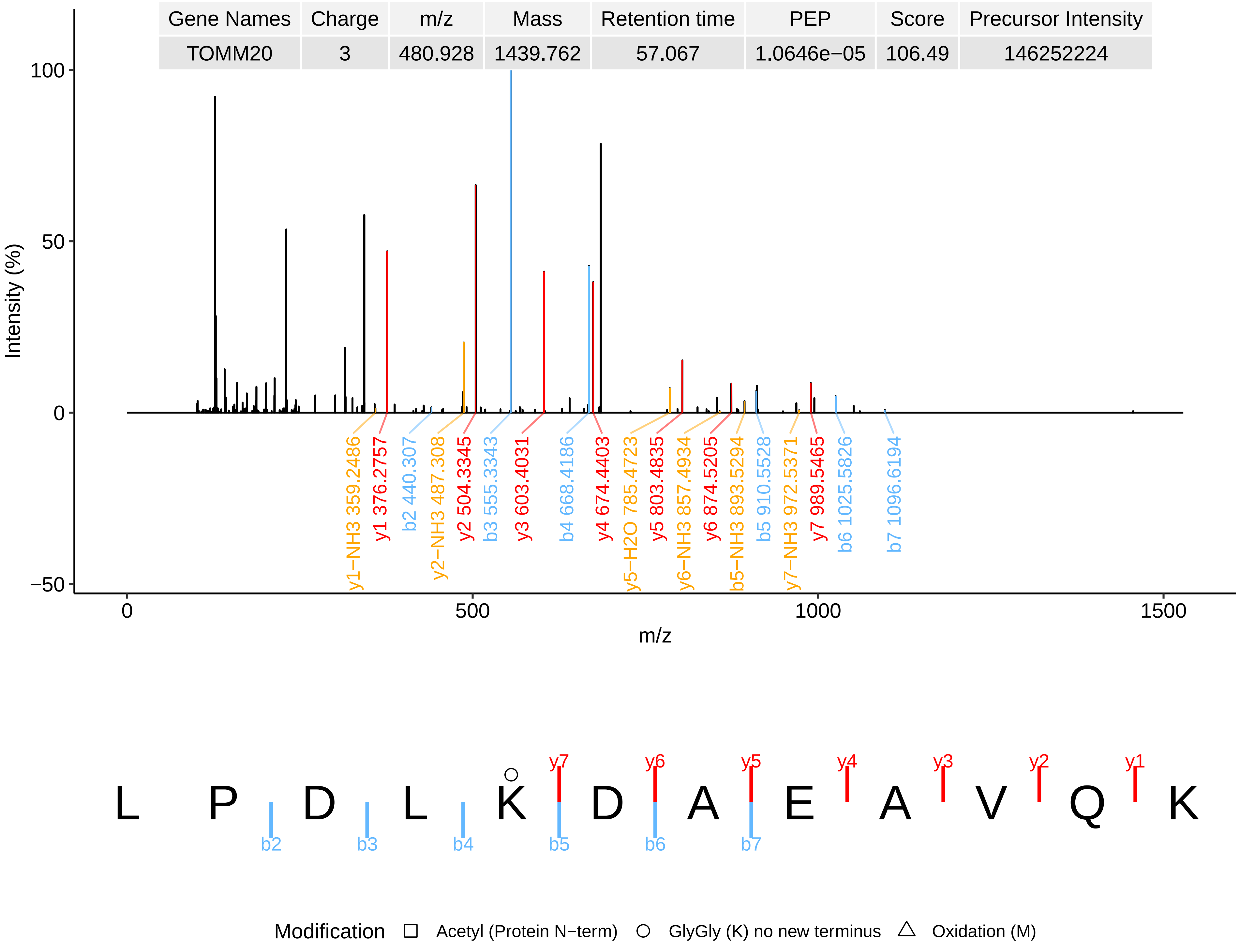

20200924_KIZ_MUb5_GlyGly_1 − Scan number: 17818

Gene Names	Charge	m/z	Mass	Retention time	PEP	Score	Precursor Intensity
TOMM20	3	480.928	1439.762	57.576	0.0014959	82.543	NA

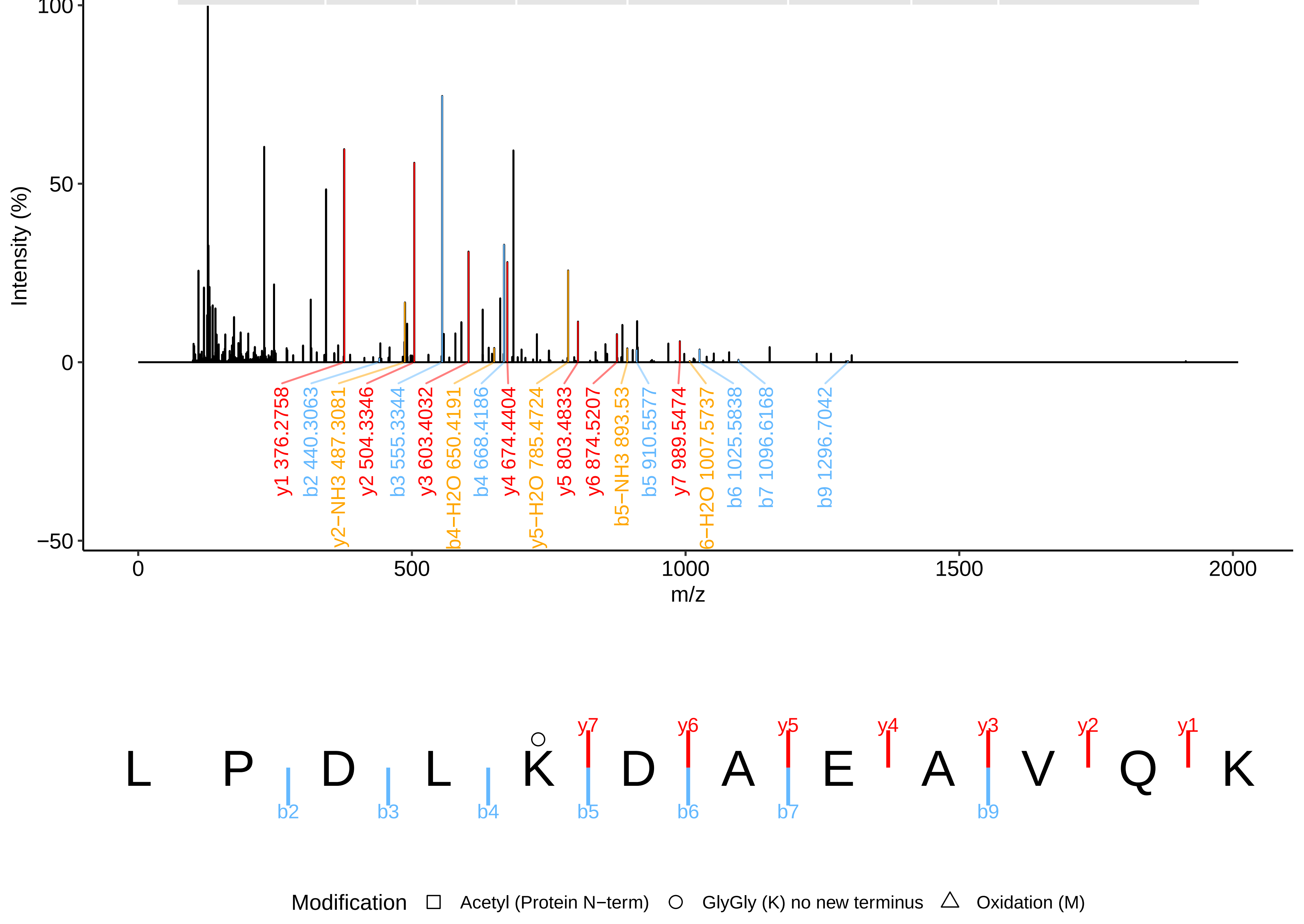

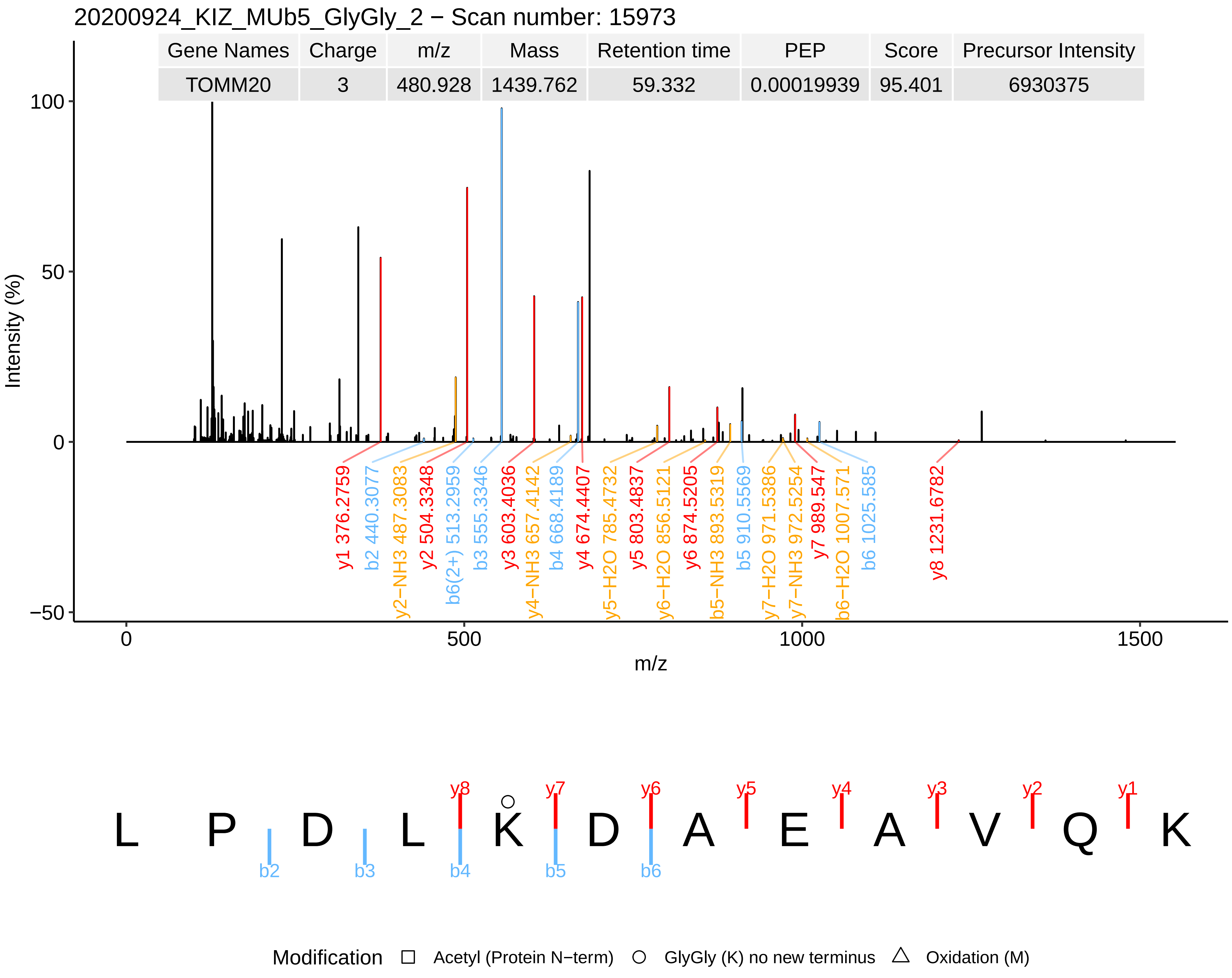
20200924_KIZ_MUb5_GlyGly_2 − Scan number: 15973
Gene Names
Charge
m/z
Mass
Retention time
PEP
Score
Precursor Intensity
TOMM20
3
480.928
1439.762
59.332
0.00019939
95.401
6930375
Intensity (%)
100
50
0
−50
0
500
1000
1500
m/z
y1 376.2759
b2 440.3077
y2−NH3 487.3083
y2 504.3348
b6(2+) 513.2959
b3 555.3346
y3 603.4036
y4−NH3 657.4142
b4 668.4189
y4 674.4407
y5−H2O 785.4732
y5 803.4837
y6−H2O 856.5121
y6 874.5205
b5−NH3 893.5319
b5 910.5569
y7−H2O 971.5386
y7−NH3 972.5254
y7 989.547
b6−H2O 1007.571
b6 1025.585
y8 1231.6782
L P D L K D A E A V Q K
y8 y7 y6 y5 y4 y3 y2 y1
b2 b3 b4 b5 b6
Modification
Acetyl (Protein N−term)
GlyGly (K) no new terminus
Oxidation (M)

20200924_KIZ_MUb5_GlyGly_2 − Scan number: 16119

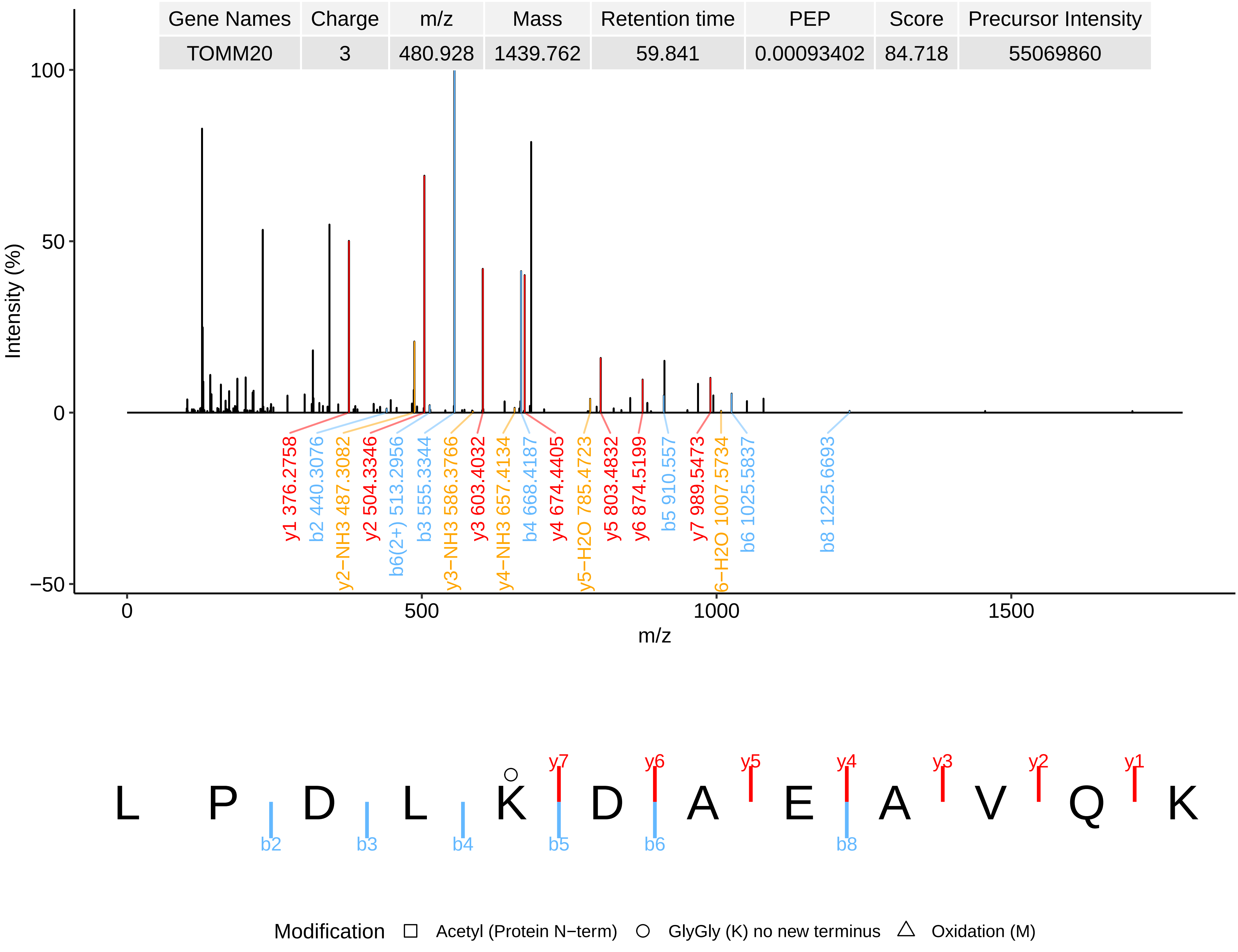

20200924_KIZ_MUb5_GlyGly_2 − Scan number: 16263

Gene Names	Charge	m/z	Mass	Retention time	PEP	Score	Precursor Intensity
TOMM20	3	480.928	1439.762	60.344	0.00019939	95.401	35633512

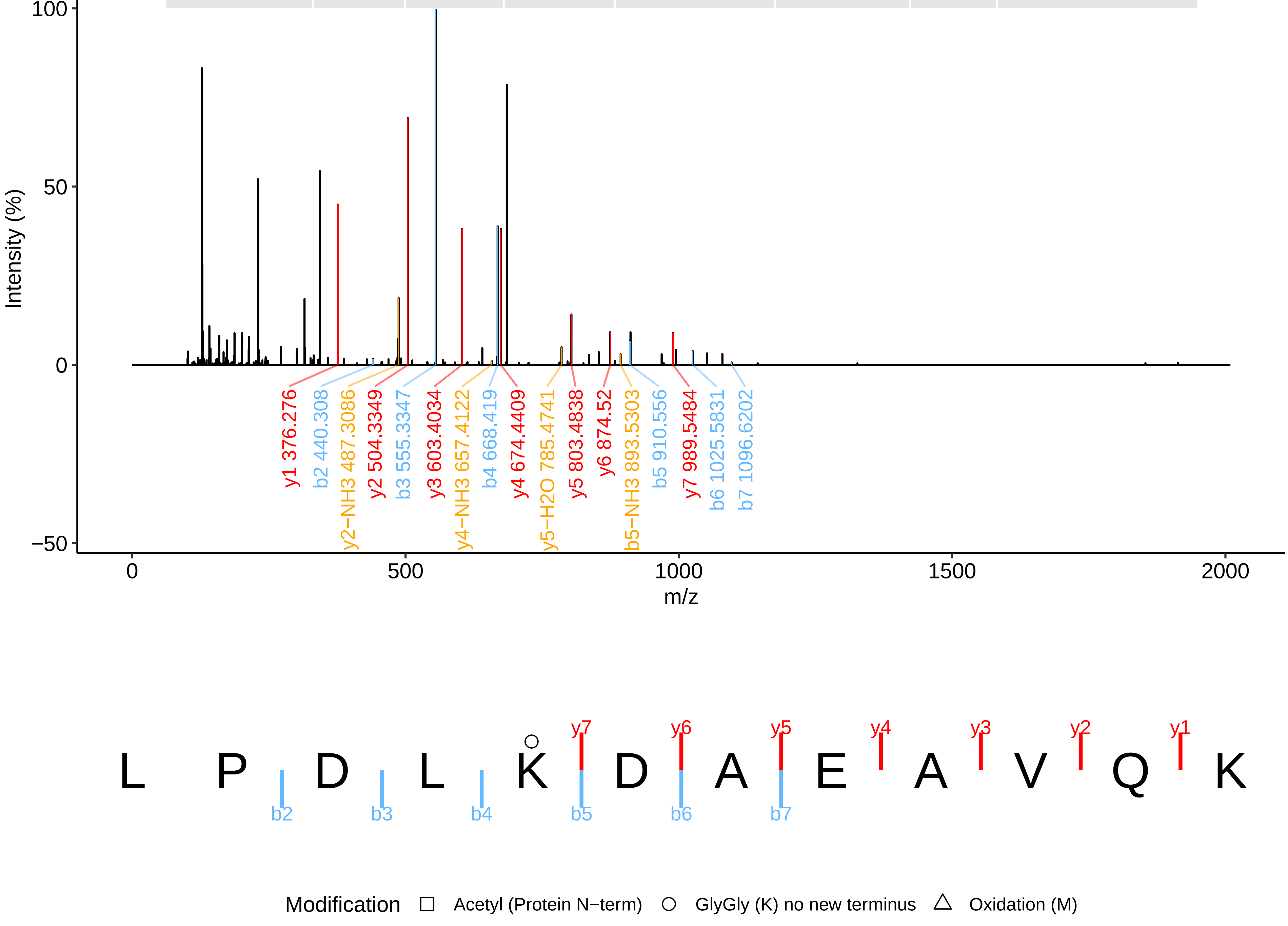

20200924_KIZ_MUb5_GlyGly_2 − Scan number: 16401

Gene Names	Charge	m/z	Mass	Retention time	PEP	Score	Precursor Intensity
TOMM20	3	480.928	1439.762	60.854	0.00026127	95.401	NA

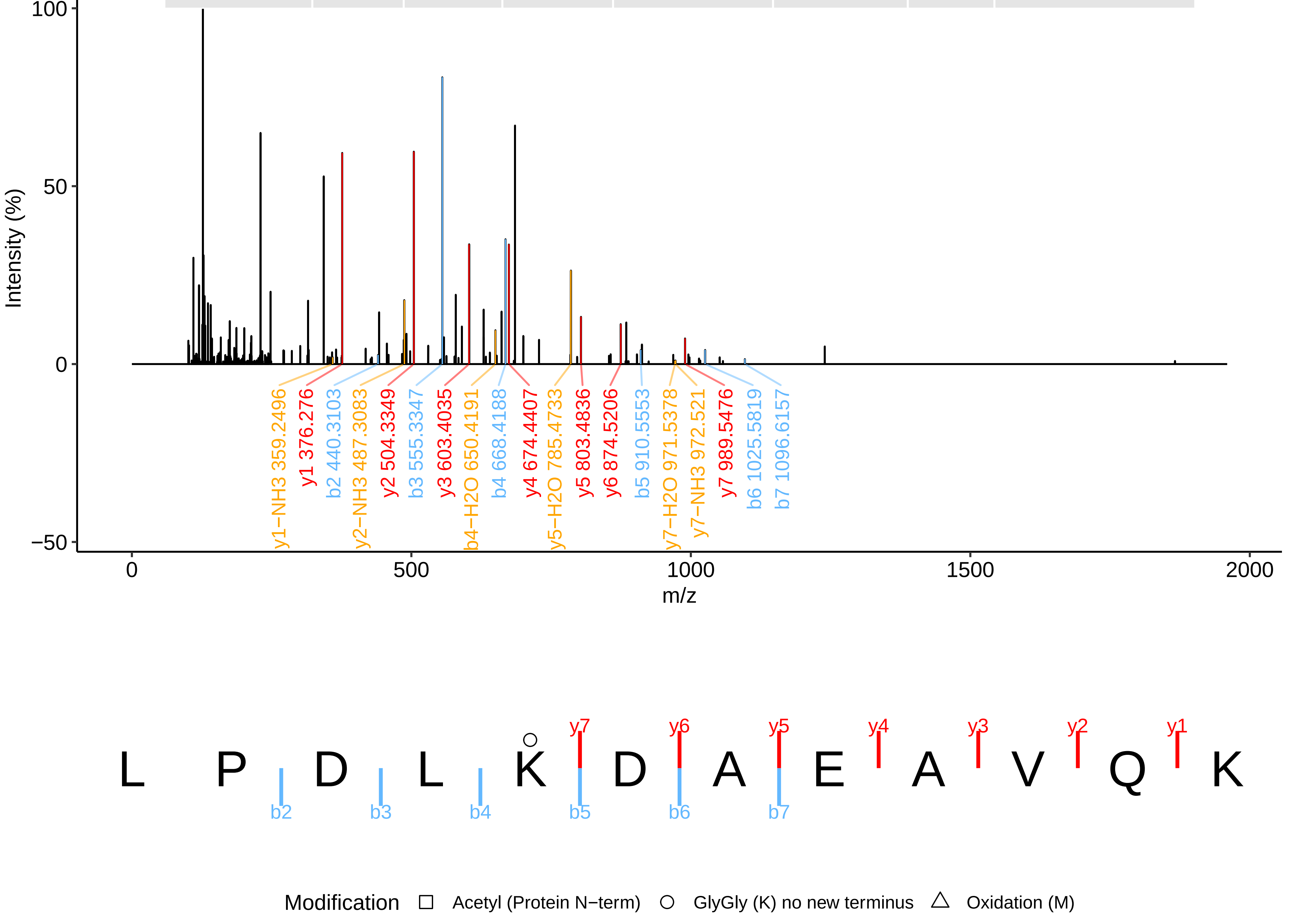

20201118_KIZ_MUb6_GlyGly_1 − Scan number: 17660

Gene Names	Charge	m/z	Mass	Retention time	PEP	Score	Precursor Intensity
TOMM20	3	480.928	1439.762	55.563	0.00043282	93.614	27633392

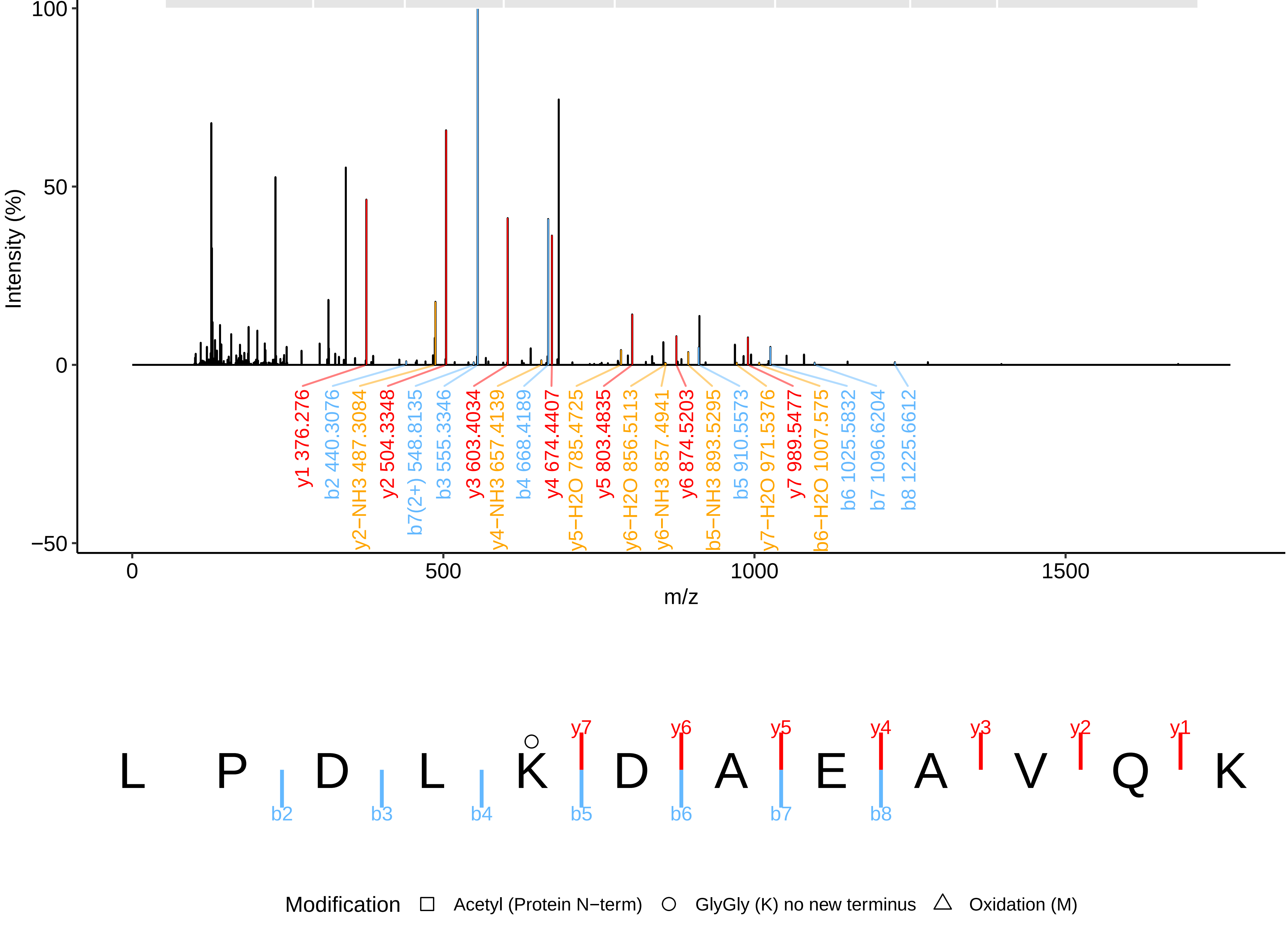

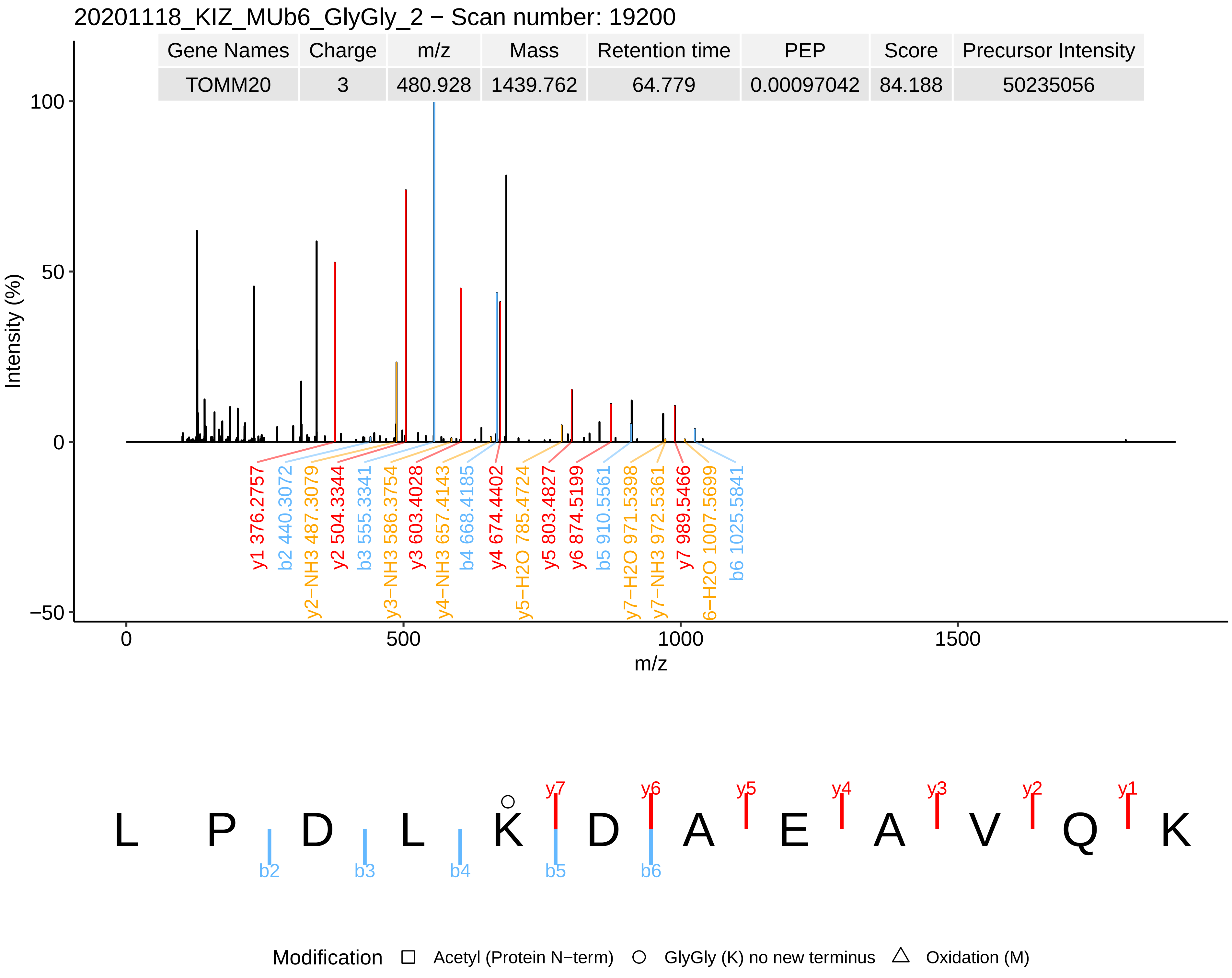

20201118_KIZ_MUb6_GlyGly_2 − Scan number: 19200
Gene Names | Charge | m/z | Mass | Retention time | PEP | Score | Precursor Intensity
TOMM20 | 3 | 480.928 | 1439.762 | 64.779 | 0.00097042 | 84.188 | 50235056
Intensity (%)
m/z
y1 376.2757
b2 440.3072
y2−NH3 487.3079
y2 504.3344
b3 555.3341
y3−NH3 586.3754
y3 603.4028
y4−NH3 657.4143
b4 668.4185
y4 674.4402
y5−H2O 785.4724
y5 803.4827
y6 874.5199
b5 910.5561
y7−H2O 971.5398
y7−NH3 972.5361
y7 989.5466
6−H2O 1007.5699
b6 1025.5841
L P D L K D A E A V Q K
y7 y6 y5 y4 y3 y2 y1
b2 b3 b4 b5 b6
Modification
Acetyl (Protein N−term)
GlyGly (K) no new terminus
Oxidation (M)

20200921_KIZ_MUb4_GlyGly_10plex − Scan number: 8472

Gene Names	Charge	m/z	Mass	Retention time	PEP	Score	Precursor Intensity
TARDBP;TDP43	3	542.6129	1624.817	28.756	1.4435e−05	104.24	6202844

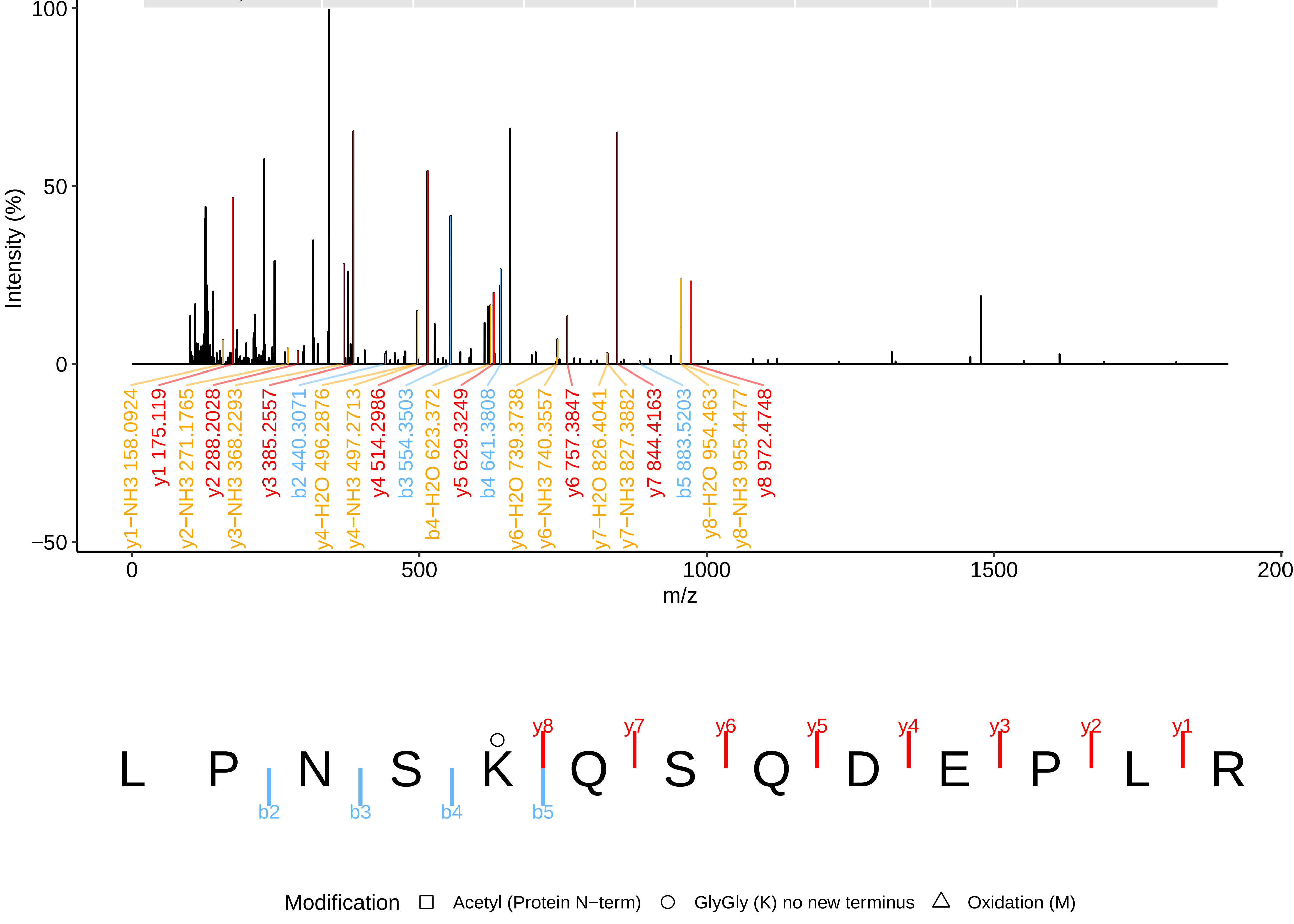

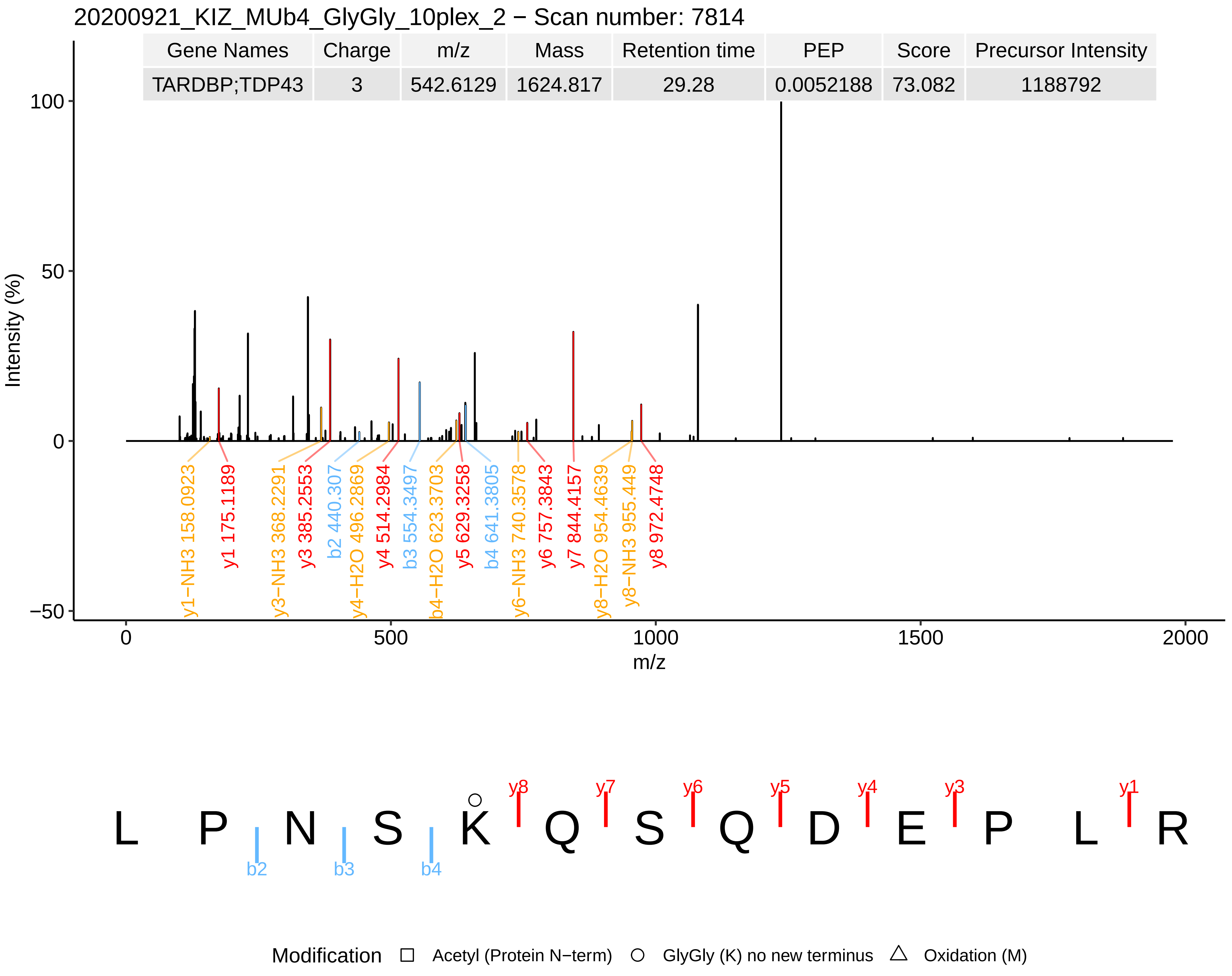

20200921_KIZ_MUb4_GlyGly_10plex_2 − Scan number: 7814
Gene Names | Charge | m/z | Mass | Retention time | PEP | Score | Precursor Intensity
TARDBP;TDP43 | 3 | 542.6129 | 1624.817 | 29.28 | 0.0052188 | 73.082 | 1188792
Intensity (%)
100
50
0
−50
0
500
1000
1500
2000
m/z
y1−NH3 158.0923
y1 175.1189
y3−NH3 368.2291
y3 385.2553
b2 440.307
y4−H2O 496.2869
y4 514.2984
b3 554.3497
b4−H2O 623.3703
y5 629.3258
b4 641.3805
y6−NH3 740.3578
y6 757.3843
y7 844.4157
y8−H2O 954.4639
y8−NH3 955.449
y8 972.4748
L P N S K Q S Q D E P L R
b2 b3 b4
y8 y7 y6 y5 y4 y3 y1
Modification
Acetyl (Protein N−term)
GlyGly (K) no new terminus
Oxidation (M)

20200924_KIZ_MUb5_GlyGly_1 − Scan number: 7458

Gene Names	Charge	m/z	Mass	Retention time	PEP	Score	Precursor Intensity
TARDBP;TDP43	3	542.6129	1624.817	23.687	2.931e−05	104.24	3438480

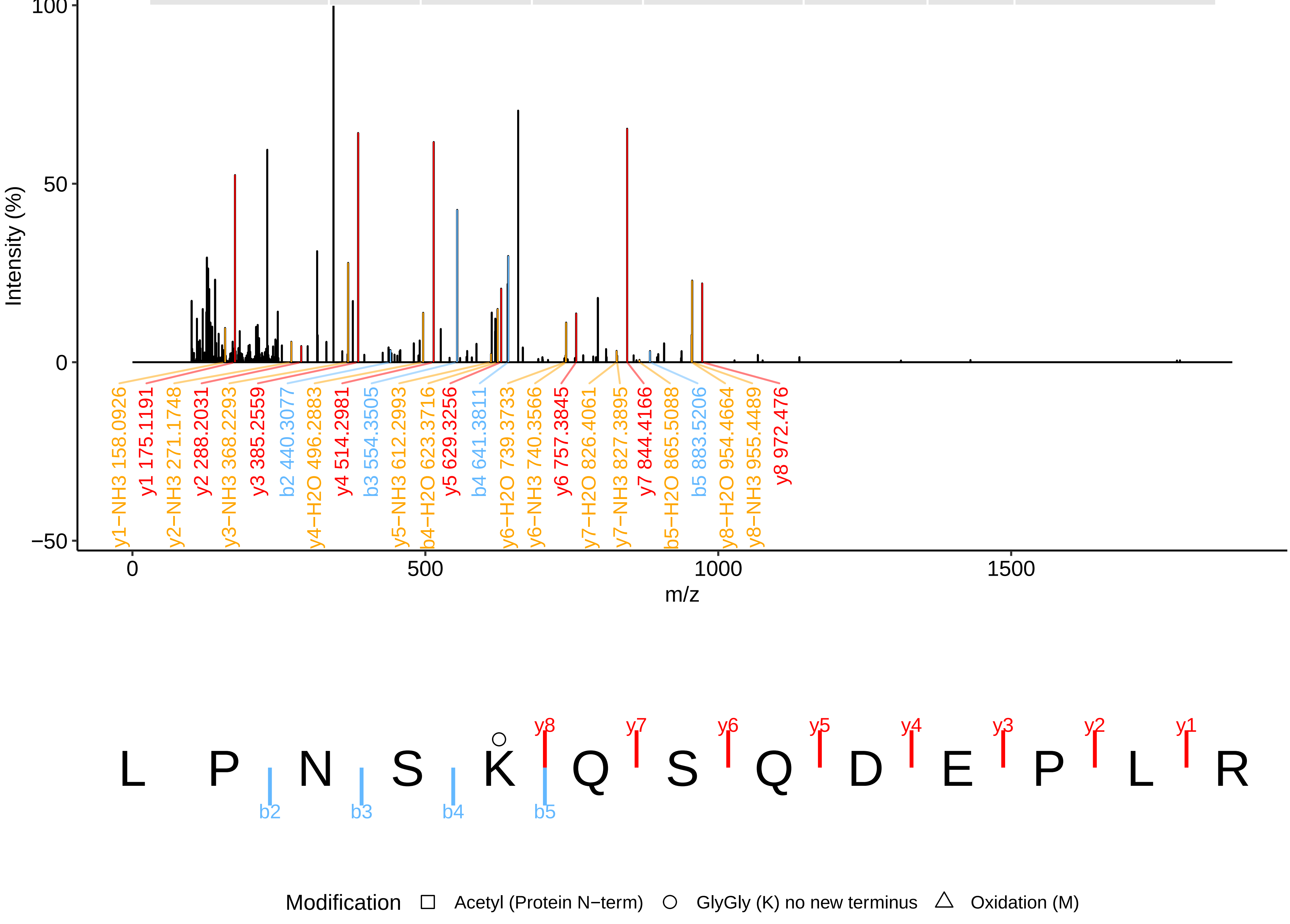

20200921_KIZ_MUb4_GlyGly_10plex − Scan number: 19109

Gene Names	Charge	m/z	Mass	Retention time	PEP	Score	Precursor Intensity
RPS27A;UBA52;UBC;UBB	3	465.927	1394.759	68.804	0.0095644	68.132	64944048

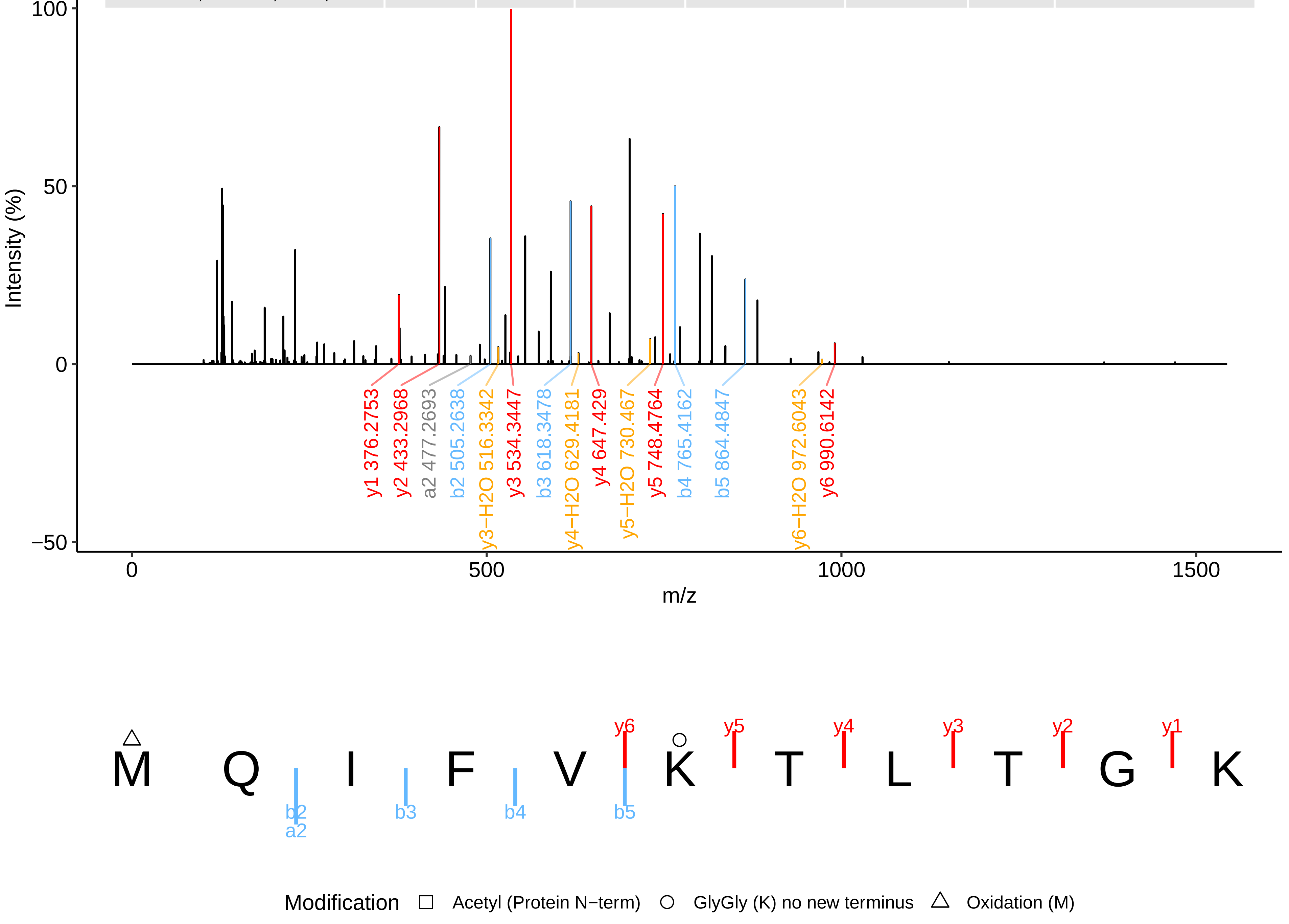

20200921_KIZ_MUb4_GlyGly_10plex − Scan number: 19240

Gene Names	Charge	m/z	Mass	Retention time	PEP	Score	Precursor Intensity
RPS27A;UBA52;UBC;UBB	3	465.927	1394.759	69.314	0.0011866	83.081	NA

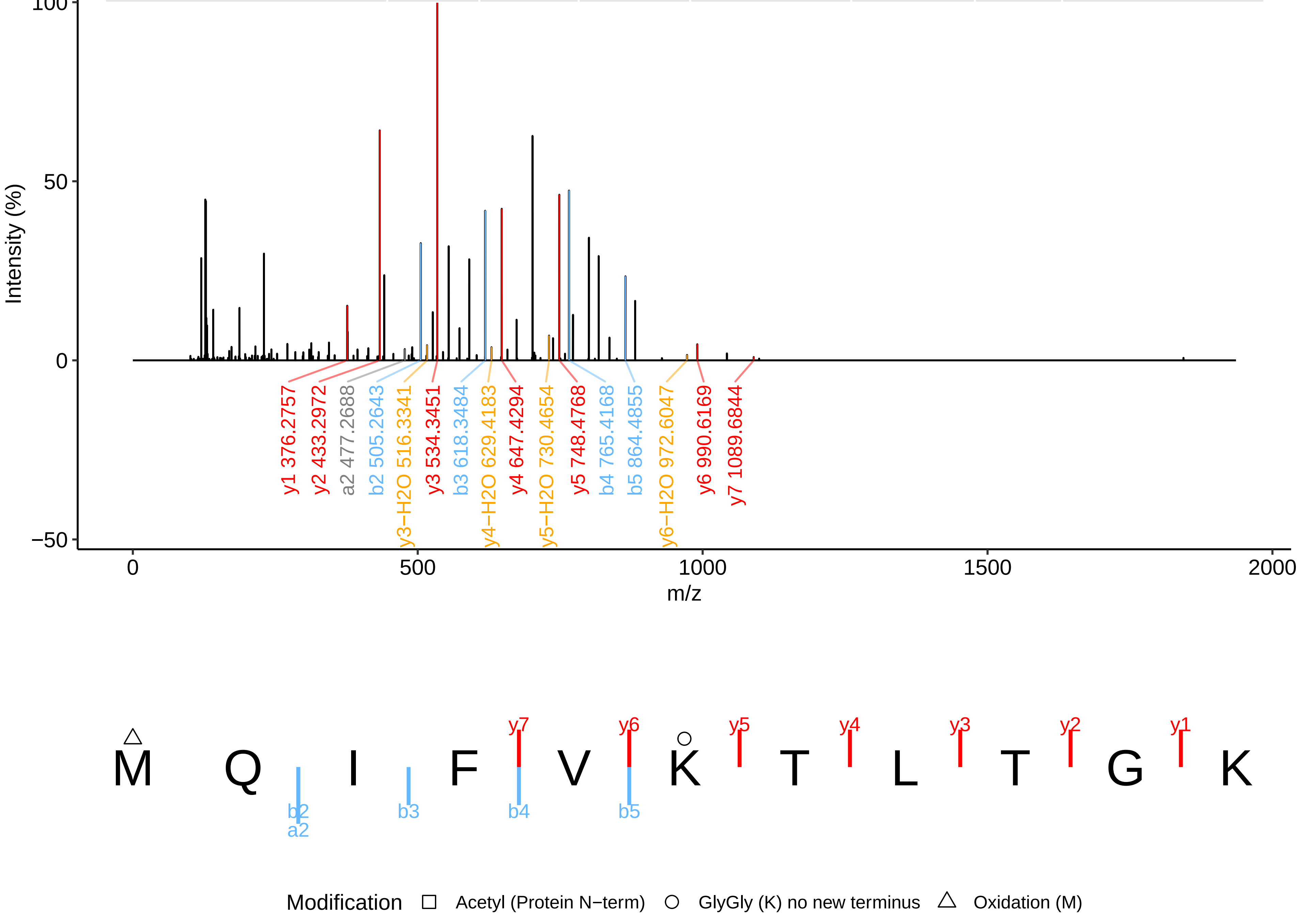

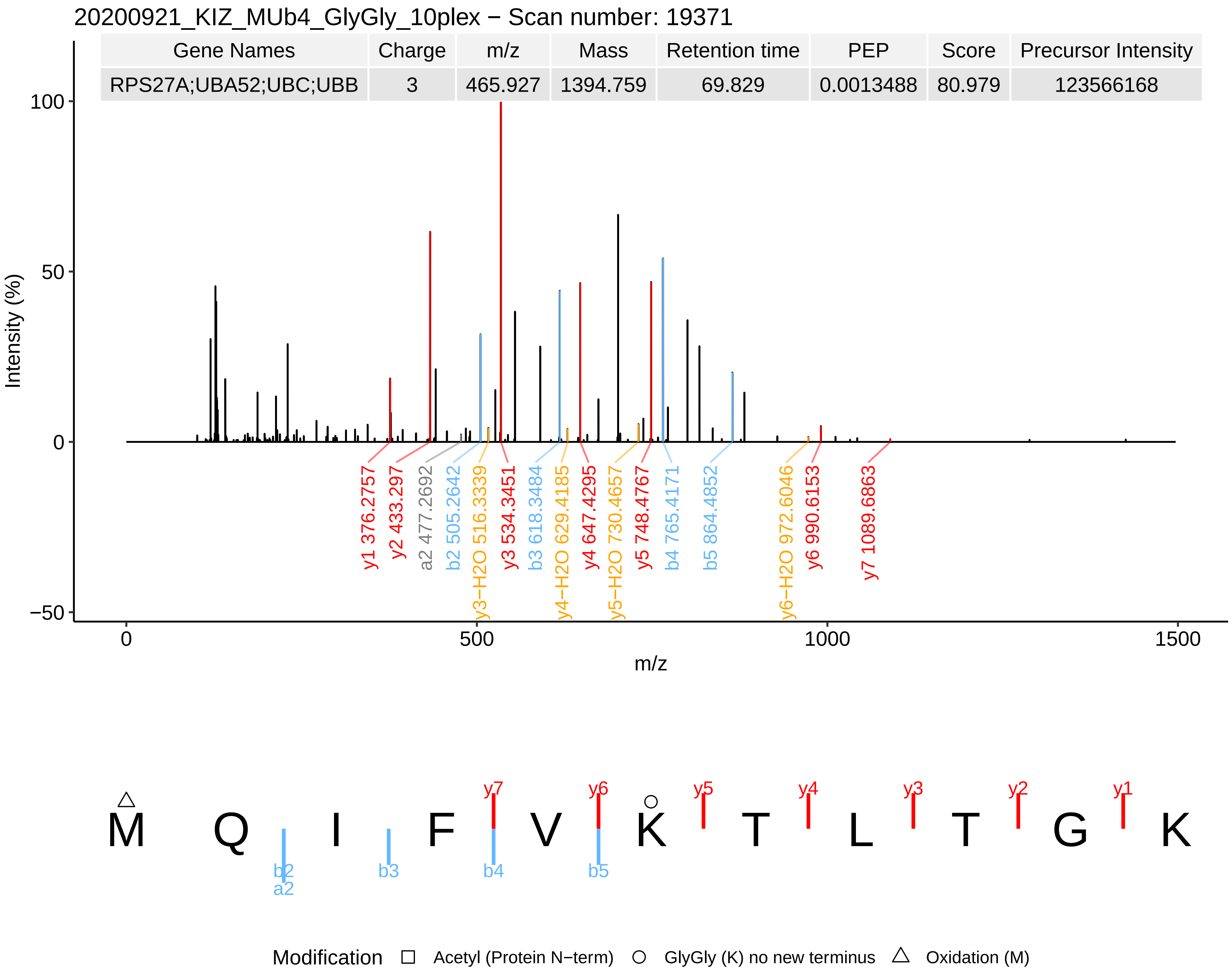

20200921_KIZ_MUb4_GlyGly_10plex − Scan number: 19371
Gene Names | Charge | m/z | Mass | Retention time | PEP | Score | Precursor Intensity
RPS27A;UBA52;UBC;UBB | 3 | 465.927 | 1394.759 | 69.829 | 0.0013488 | 80.979 | 123566168
Intensity (%)
m/z
y1 376.2757
y2 433.297
a2 477.2692
b2 505.2642
y3−H2O 516.3339
y3 534.3451
b3 618.3484
y4−H2O 629.4185
y4 647.4295
y5−H2O 730.4657
y5 748.4767
b4 765.4171
b5 864.4852
y6−H2O 972.6046
y6 990.6153
y7 1089.6863
M Q I F V K T L T G K
Modification □ Acetyl (Protein N−term) ○ GlyGly (K) no new terminus △ Oxidation (M)

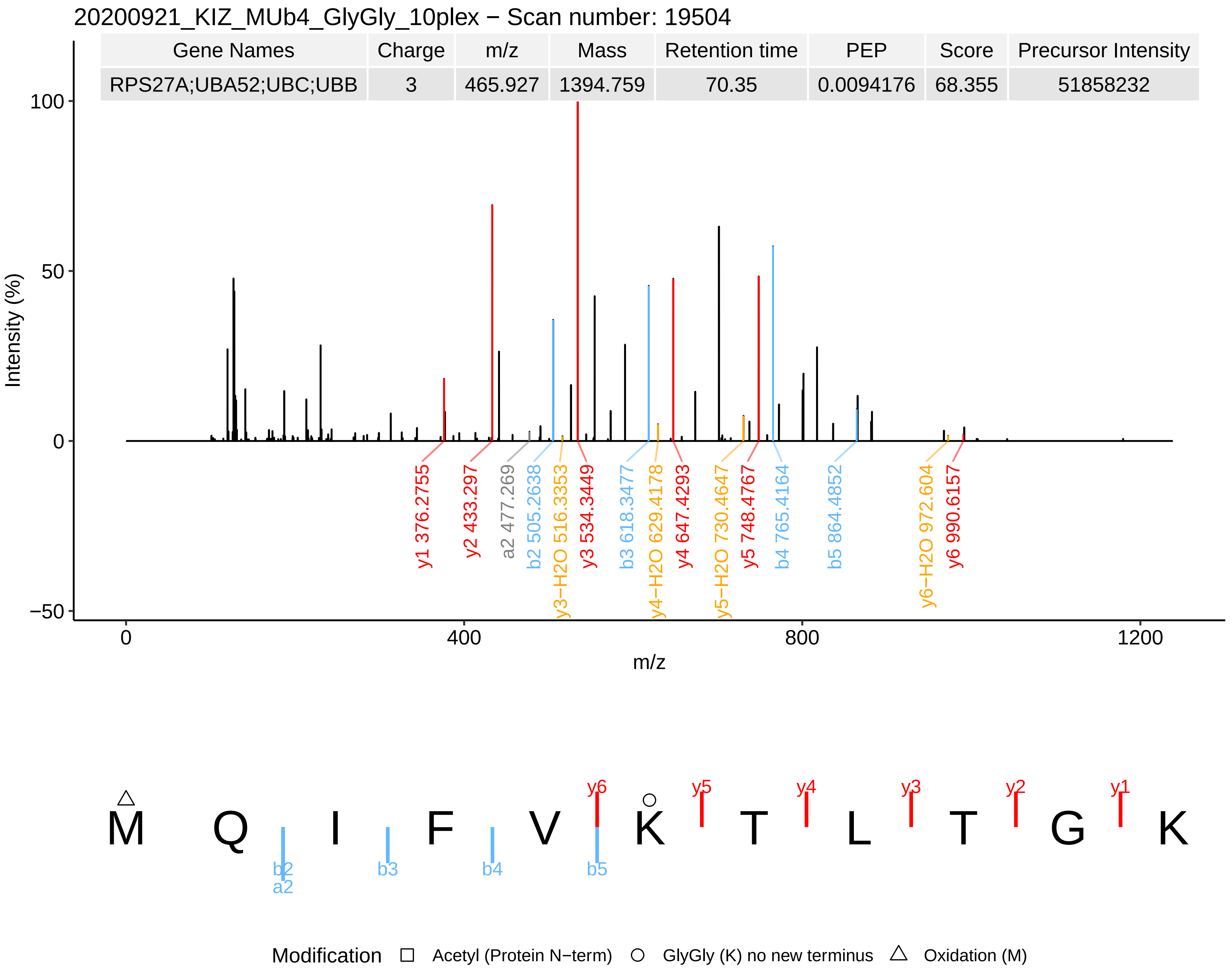
20200921_KIZ_MUb4_GlyGly_10plex − Scan number: 19504
Gene Names | Charge | m/z | Mass | Retention time | PEP | Score | Precursor Intensity
RPS27A;UBA52;UBC;UBB | 3 | 465.927 | 1394.759 | 70.35 | 0.0094176 | 68.355 | 51858232
Intensity (%)
m/z
y1 376.2755
y2 433.297
a2 477.269
b2 505.2638
y3−H2O 516.3353
y3 534.3449
b3 618.3477
y4−H2O 629.4178
y4 647.4293
y5−H2O 730.4647
y5 748.4767
b4 765.4164
b5 864.4852
y6−H2O 972.604
y6 990.6157
M Q I F V K T L T G K
y6 y5 y4 y3 y2 y1
b2 a2 b3 b4 b5
Modification
Acetyl (Protein N−term)
GlyGly (K) no new terminus
Oxidation (M)

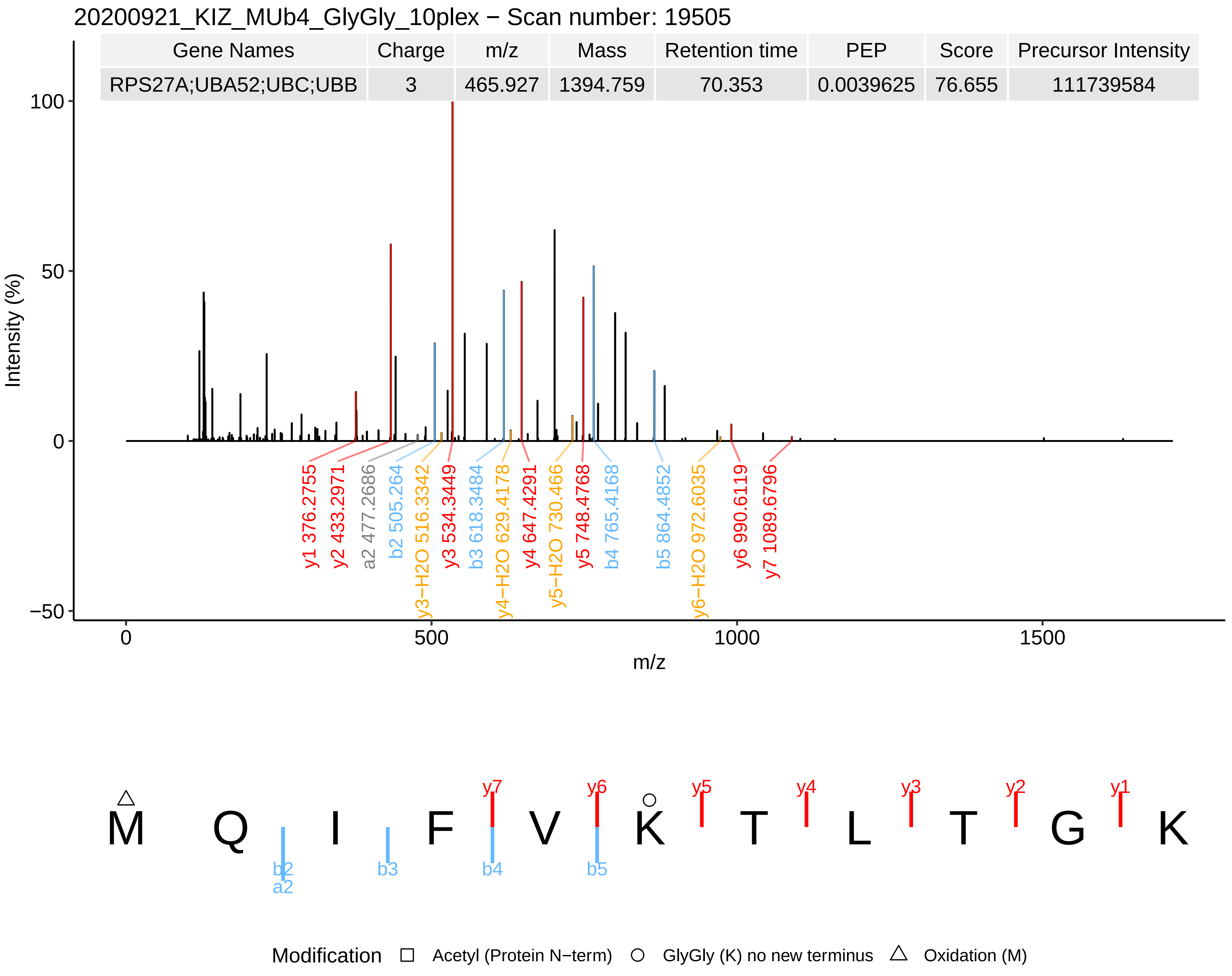
20200921_KIZ_MUb4_GlyGly_10plex − Scan number: 19505
Gene Names | Charge | m/z | Mass | Retention time | PEP | Score | Precursor Intensity
RPS27A;UBA52;UBC;UBB | 3 | 465.927 | 1394.759 | 70.353 | 0.0039625 | 76.655 | 111739584
Intensity (%)
100
50
0
−50
0
500
1000
1500
m/z
y1 376.2755
y2 433.2971
a2 477.2686
b2 505.264
y3−H2O 516.3342
y3 534.3449
b3 618.3484
y4−H2O 629.4178
y4 647.4291
y5−H2O 730.466
y5 748.4768
b4 765.4168
b5 864.4852
y6−H2O 972.6035
y6 990.6119
y7 1089.6796
M Q I F V K T L T G K
y7 y6 y5 y4 y3 y2 y1
b2 a2 b3 b4 b5
Modification
Acetyl (Protein N−term)
GlyGly (K) no new terminus
Oxidation (M)

20200921_KIZ_MUb4_GlyGly_10plex − Scan number: 19636

Gene Names	Charge	m/z	Mass	Retention time	PEP	Score	Precursor Intensity
RPS27A;UBA52;UBC;UBB	3	465.927	1394.759	70.855	0.0026496	78.653	33811944

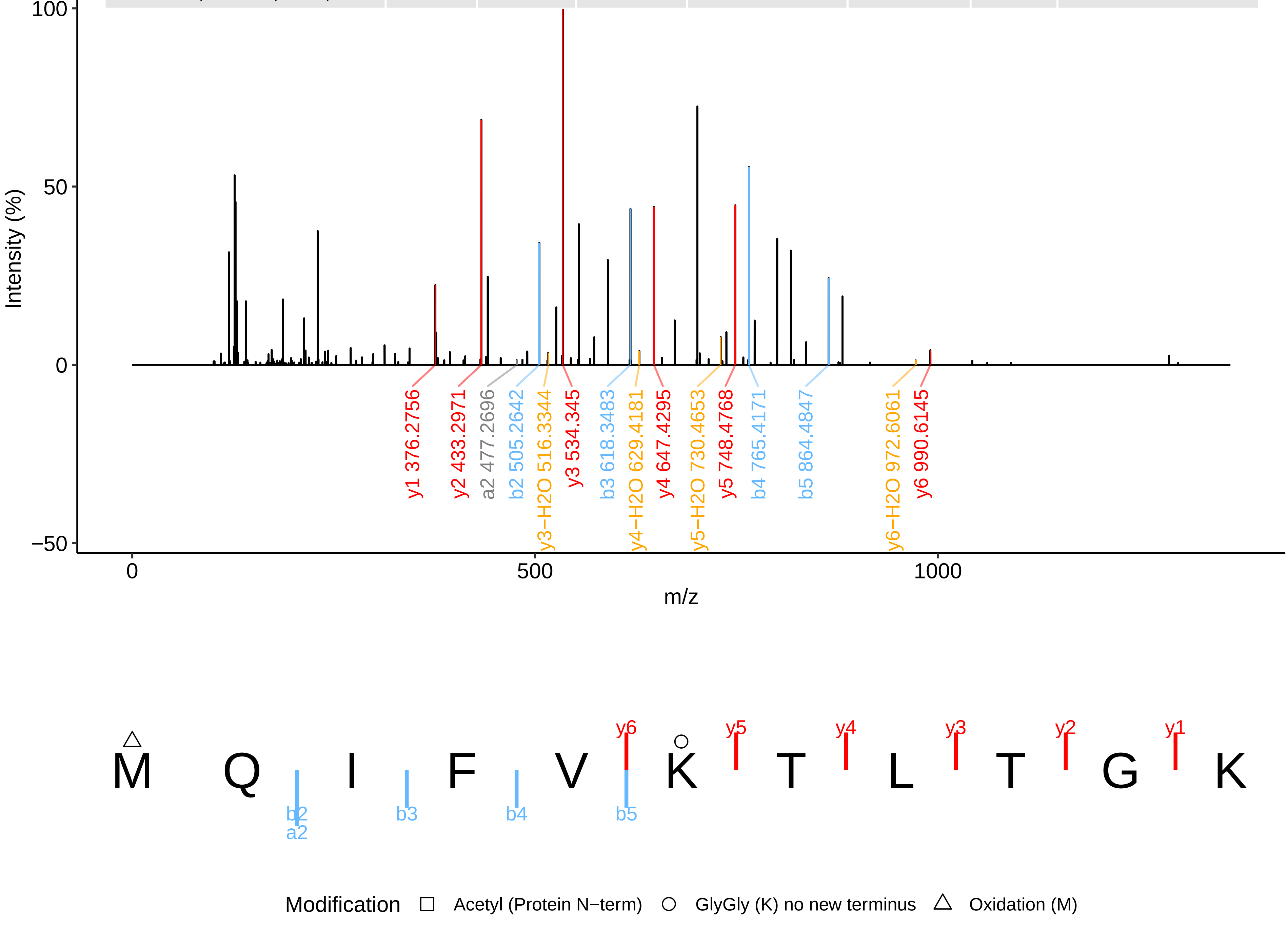

20200921_KIZ_MUb4_GlyGly_10plex − Scan number: 19773

Gene Names	Charge	m/z	Mass	Retention time	PEP	Score	Precursor Intensity
RPS27A;UBA52;UBC;UBB	3	465.927	1394.759	71.364	0.0095644	68.132	18652560

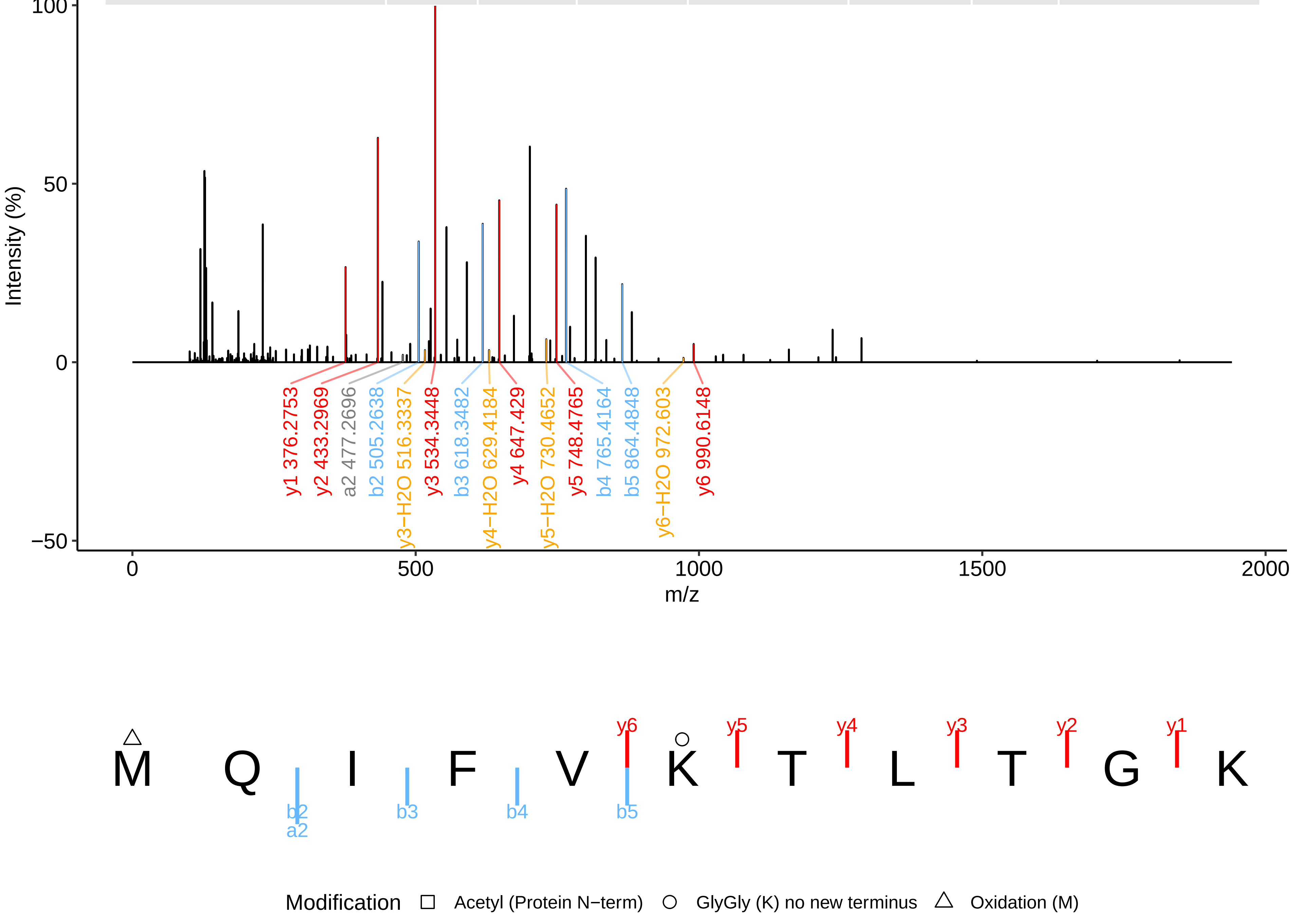

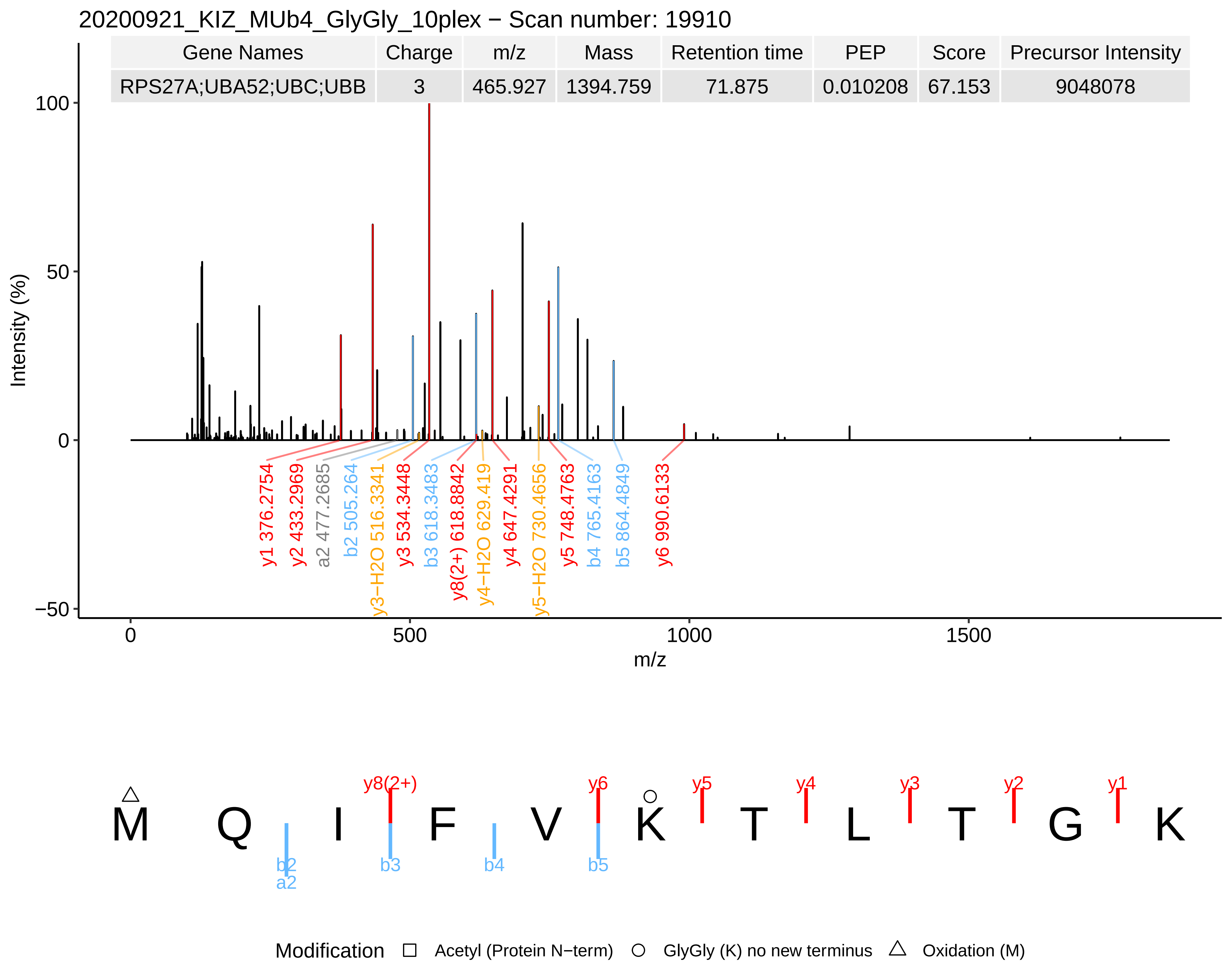
20200921_KIZ_MUb4_GlyGly_10plex − Scan number: 19910
Gene Names | Charge | m/z | Mass | Retention time | PEP | Score | Precursor Intensity
RPS27A;UBA52;UBC;UBB | 3 | 465.927 | 1394.759 | 71.875 | 0.010208 | 67.153 | 9048078
Intensity (%)
m/z
y1 376.2754
y2 433.2969
a2 477.2685
b2 505.264
y3−H2O 516.3341
y3 534.3448
b3 618.3483
y8(2+) 618.8842
y4−H2O 629.419
y4 647.4291
y5−H2O 730.4656
y5 748.4763
b4 765.4163
b5 864.4849
y6 990.6133
M Q I F V K T L T G K
y8(2+) y6 y5 y4 y3 y2 y1
b2 a2 b3 b4 b5
Modification □ Acetyl (Protein N−term) ○ GlyGly (K) no new terminus △ Oxidation (M)

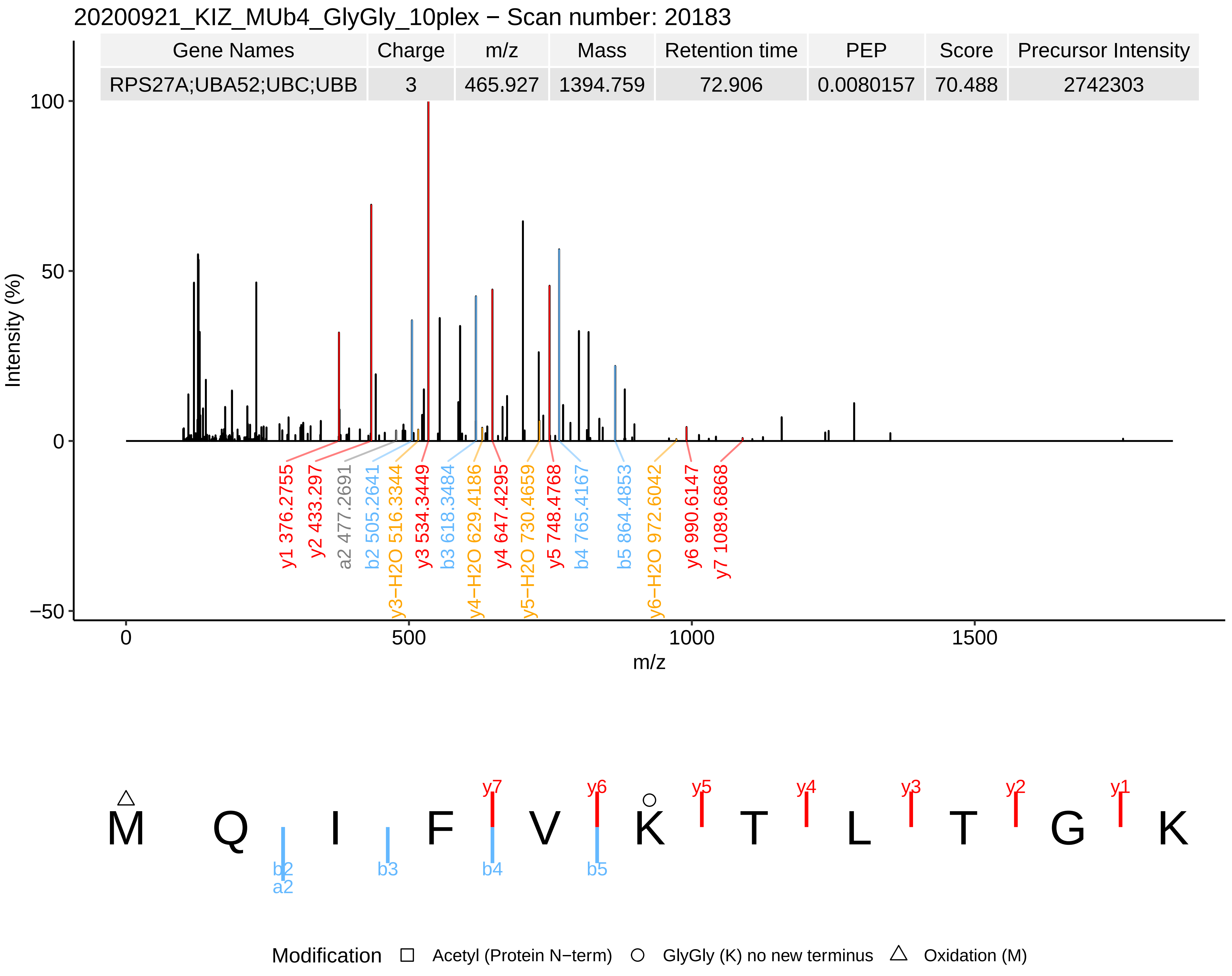
20200921_KIZ_MUb4_GlyGly_10plex − Scan number: 20183
Gene Names
RPS27A;UBA52;UBC;UBB
Charge
3
m/z
465.927
Mass
1394.759
Retention time
72.906
PEP
0.0080157
Score
70.488
Precursor Intensity
2742303
Intensity (%)
m/z
y1 376.2755
y2 433.297
a2 477.2691
b2 505.2641
y3−H2O 516.3344
y3 534.3449
b3 618.3484
y4−H2O 629.4186
y4 647.4295
y5−H2O 730.4659
y5 748.4768
b4 765.4167
b5 864.4853
y6−H2O 972.6042
y6 990.6147
y7 1089.6868
M Q I F V K T L T G K
y7 y6 y5 y4 y3 y2 y1
b2 a2 b3 b4 b5
Modification
Acetyl (Protein N−term)
GlyGly (K) no new terminus
Oxidation (M)

20200921_KIZ_MUb4_GlyGly_10plex − Scan number: 27739

Gene Names	Charge	m/z	Mass	Retention time	PEP	Score	Precursor Intensity
RPS27A;UBA52;UBC;UBB	3	465.927	1394.759	101.3	0.015978	62.408	2285104

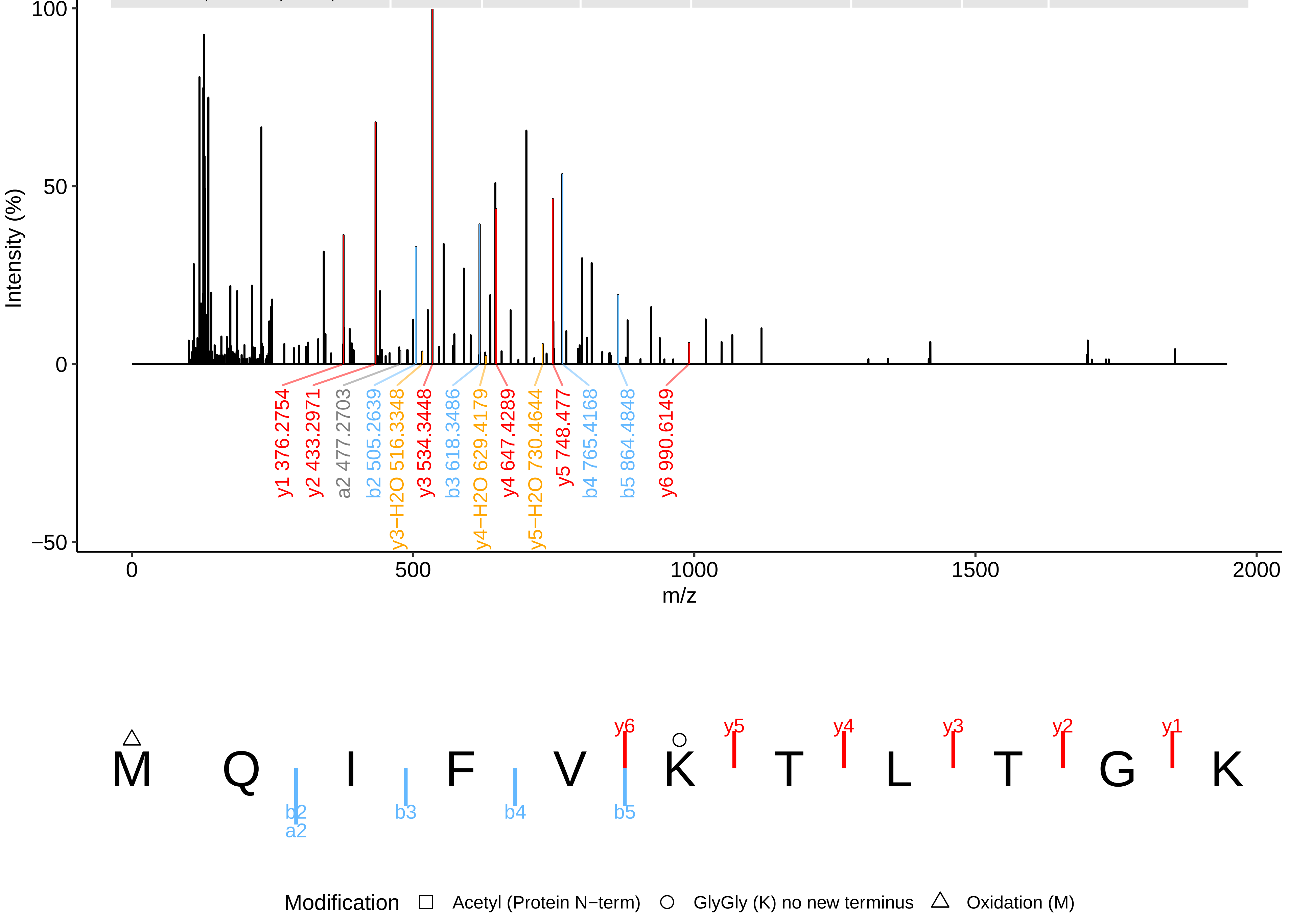

20200921_KIZ_MUb4_GlyGly_10plex − Scan number: 28008

Gene Names	Charge	m/z	Mass	Retention time	PEP	Score	Precursor Intensity
RPS27A;UBA52;UBC;UBB	3	465.927	1394.759	102.37	0.0026496	78.653	NA

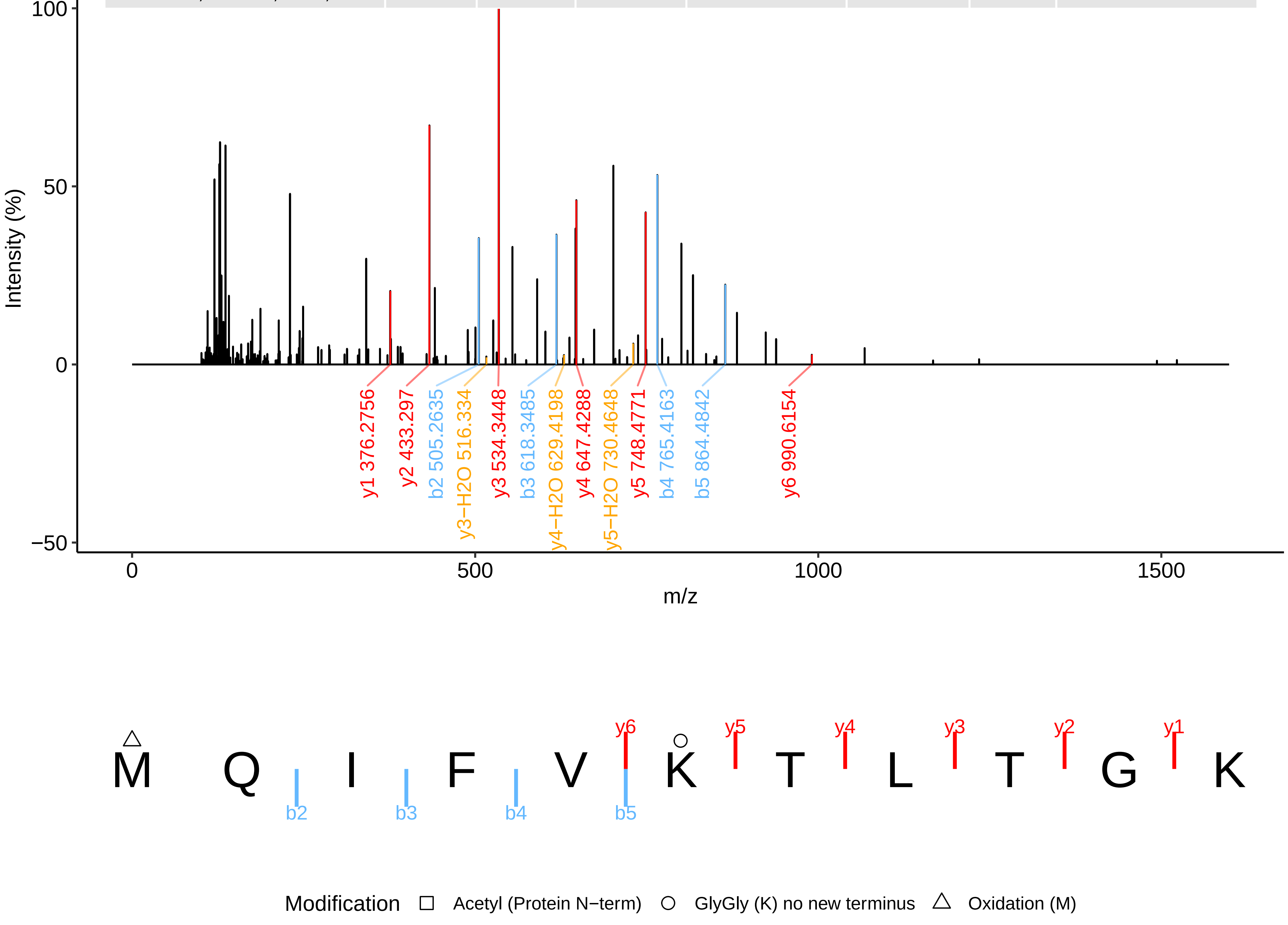

20200921_KIZ_MUb4_GlyGly_10plex − Scan number: 28139

Gene Names	Charge	m/z	Mass	Retention time	PEP	Score	Precursor Intensity
RPS27A;UBA52;UBC;UBB	3	465.927	1394.759	102.89	0.015978	62.408	NA

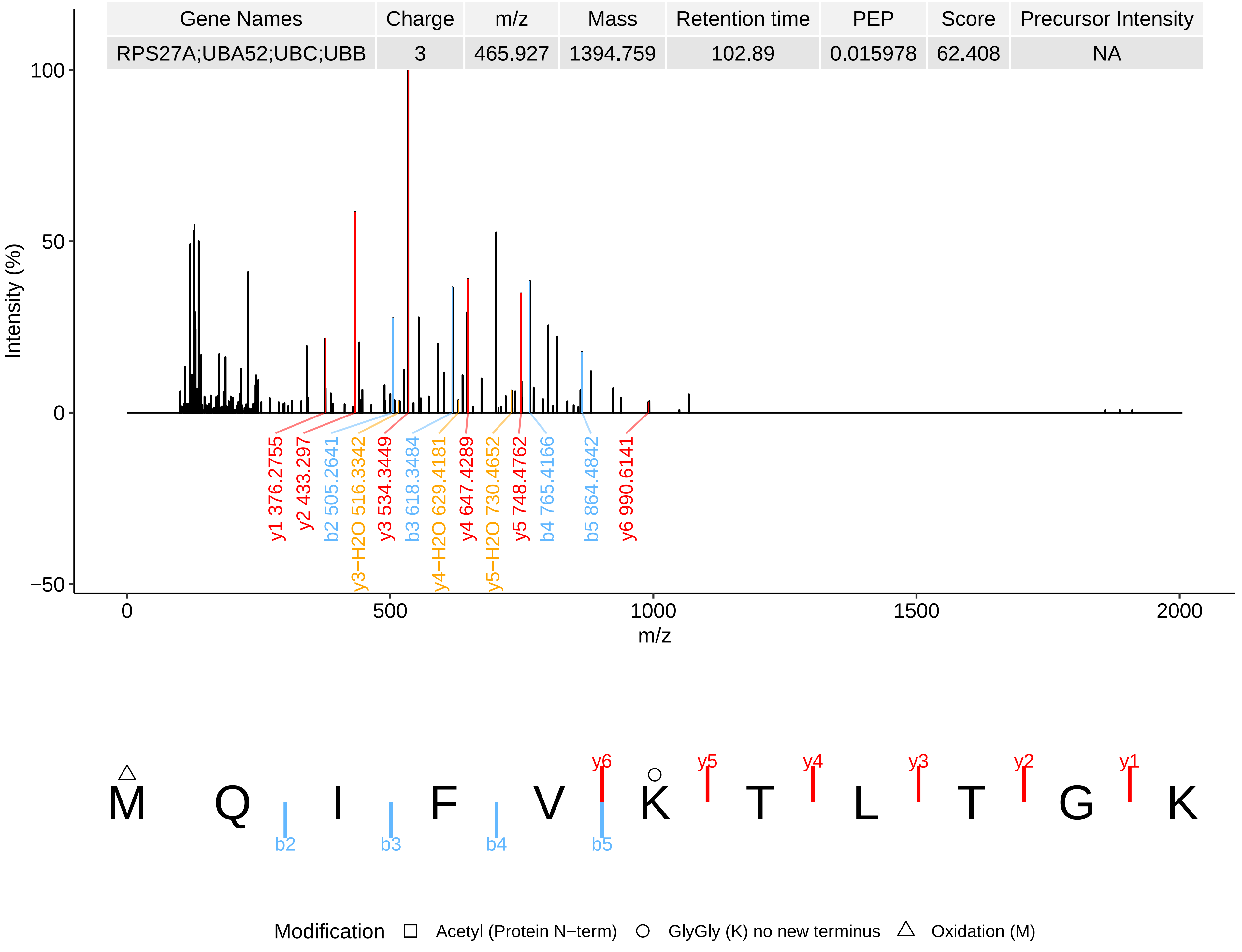

20200921_KIZ_MUb4_GlyGly_10plex − Scan number: 28271

Gene Names	Charge	m/z	Mass	Retention time	PEP	Score	Precursor Intensity
RPS27A;UBA52;UBC;UBB	3	465.927	1394.759	103.4	0.010208	67.153	4293459

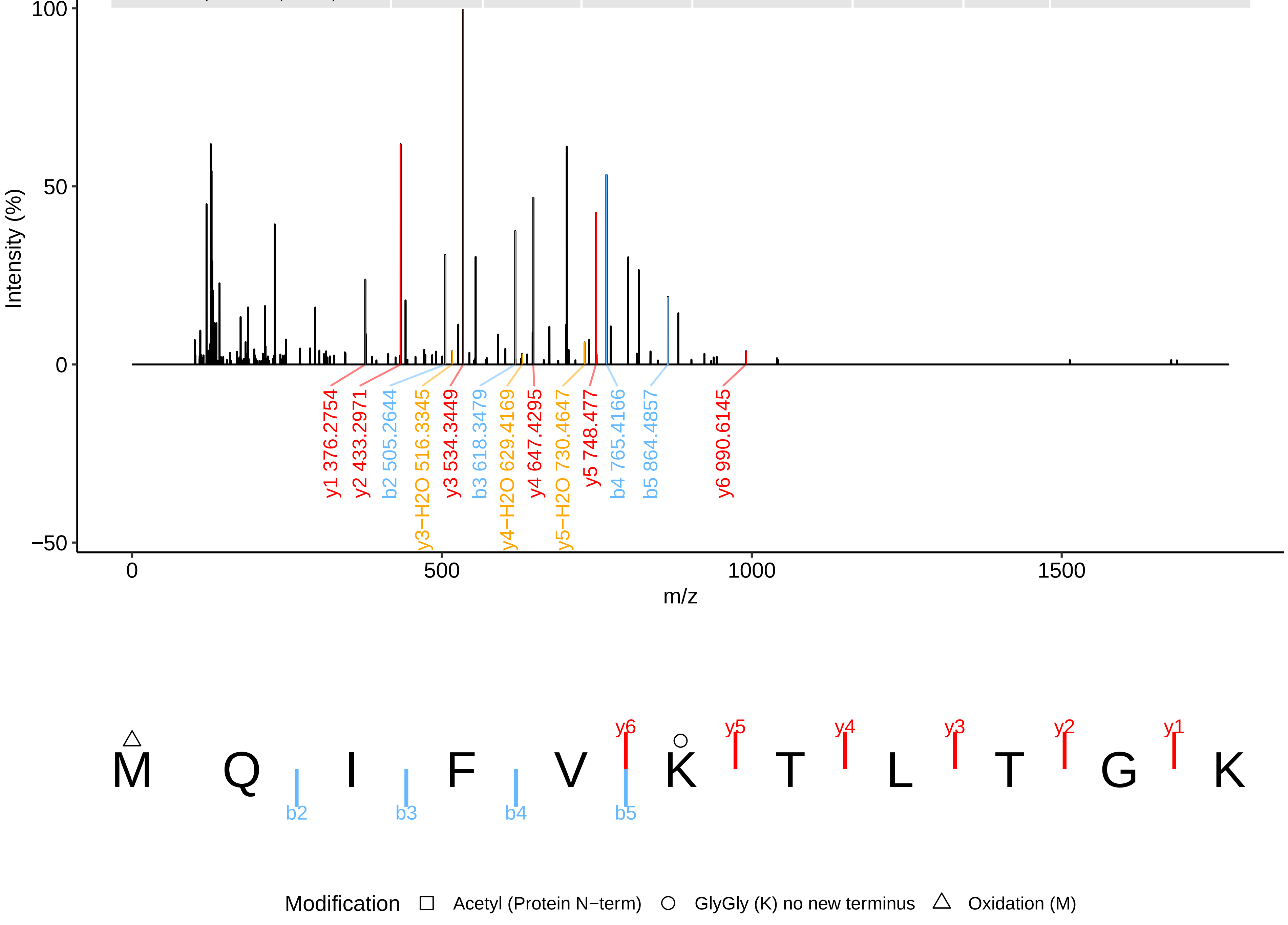

20200921_KIZ_MUb4_GlyGly_10plex − Scan number: 27876

Gene Names	Charge	m/z	Mass	Retention time	PEP	Score	Precursor Intensity
RPS27A;UBA52;UBC;UBB	3	465.927	1394.759	101.86	0.01426	72.652	1697122

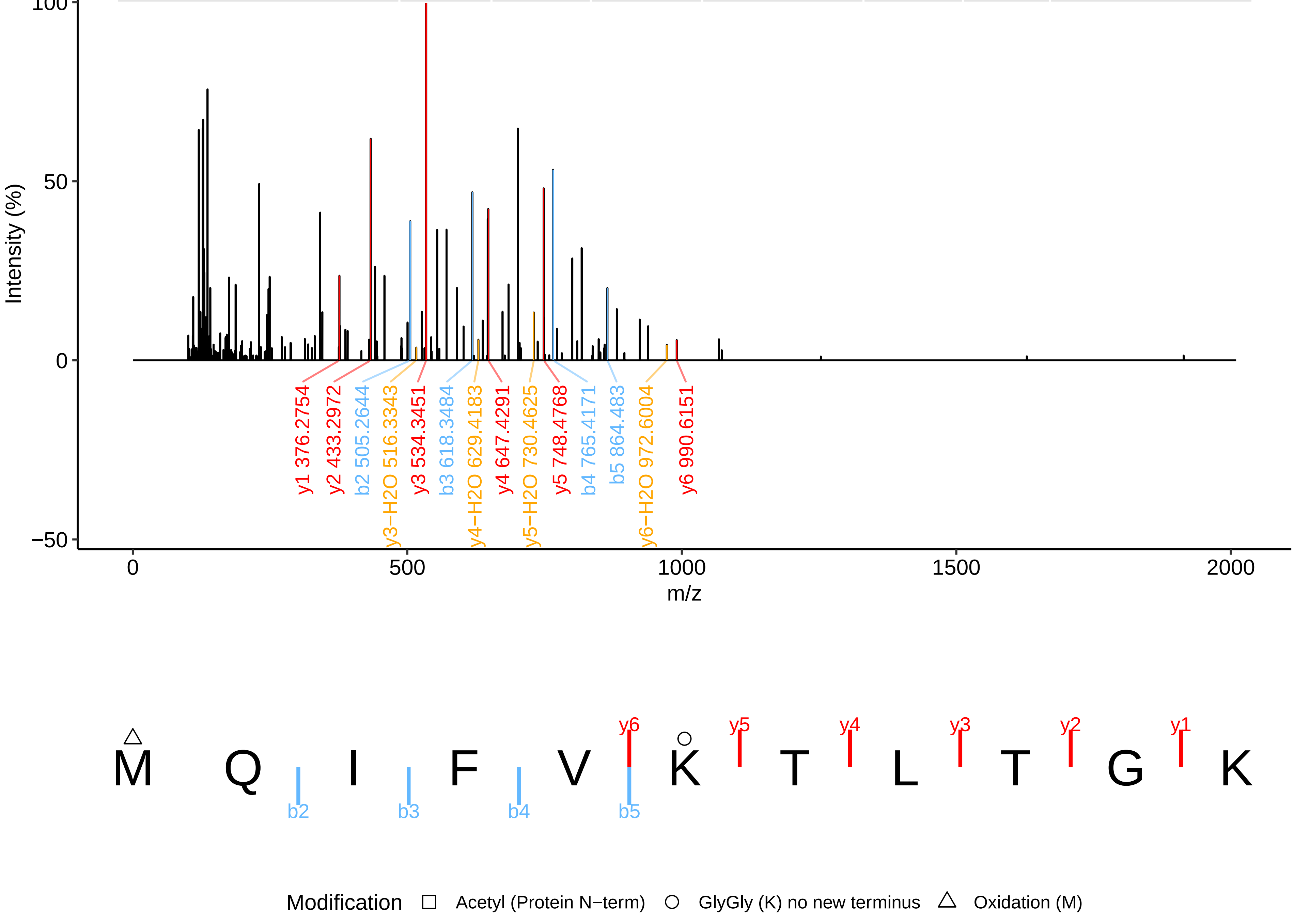

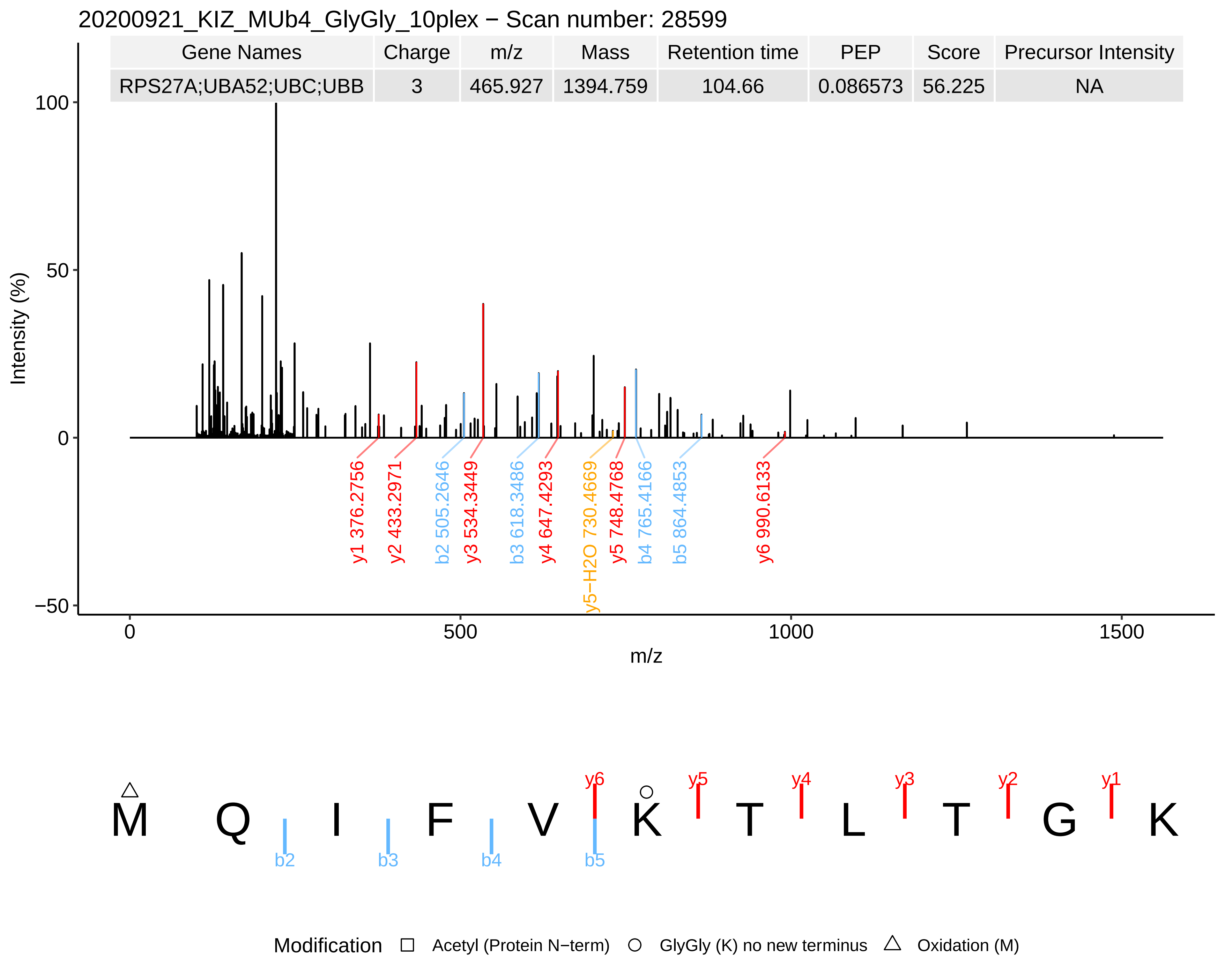
20200921_KIZ_MUb4_GlyGly_10plex − Scan number: 28599
Gene Names | Charge | m/z | Mass | Retention time | PEP | Score | Precursor Intensity
RPS27A;UBA52;UBC;UBB | 3 | 465.927 | 1394.759 | 104.66 | 0.086573 | 56.225 | NA
Intensity (%)
m/z
y1 376.2756
y2 433.2971
b2 505.2646
y3 534.3449
b3 618.3486
y4 647.4293
y5−H2O 730.4669
y5 748.4768
b4 765.4166
b5 864.4853
y6 990.6133
M Q I F V K T L T G K
Modification
Acetyl (Protein N−term)
GlyGly (K) no new terminus
Oxidation (M)

20200921_KIZ_MUb4_GlyGly_10plex_2 − Scan number: 17418

Gene Names	Charge	m/z	Mass	Retention time	PEP	Score	Precursor Intensity
RPS27A;UBA52;UBC;UBB	3	465.927	1394.759	68.122	0.0065935	72.652	813690.4

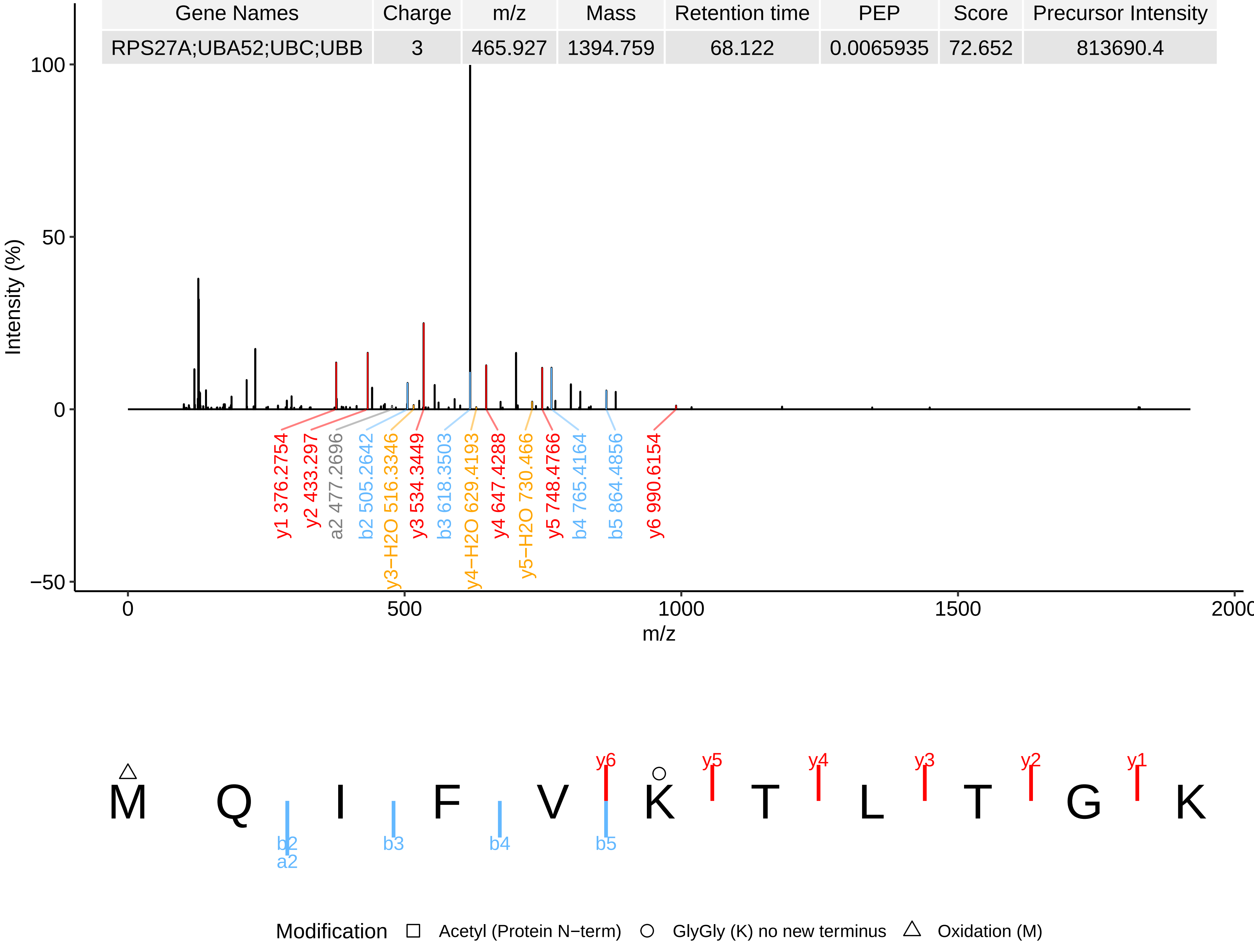

20200921_KIZ_MUb4_GlyGly_10plex_2 − Scan number: 17426

Gene Names	Charge	m/z	Mass	Retention time	PEP	Score	Precursor Intensity
RPS27A;UBA52;UBC;UBB	3	465.927	1394.759	68.15	0.07461	48.188	3131668

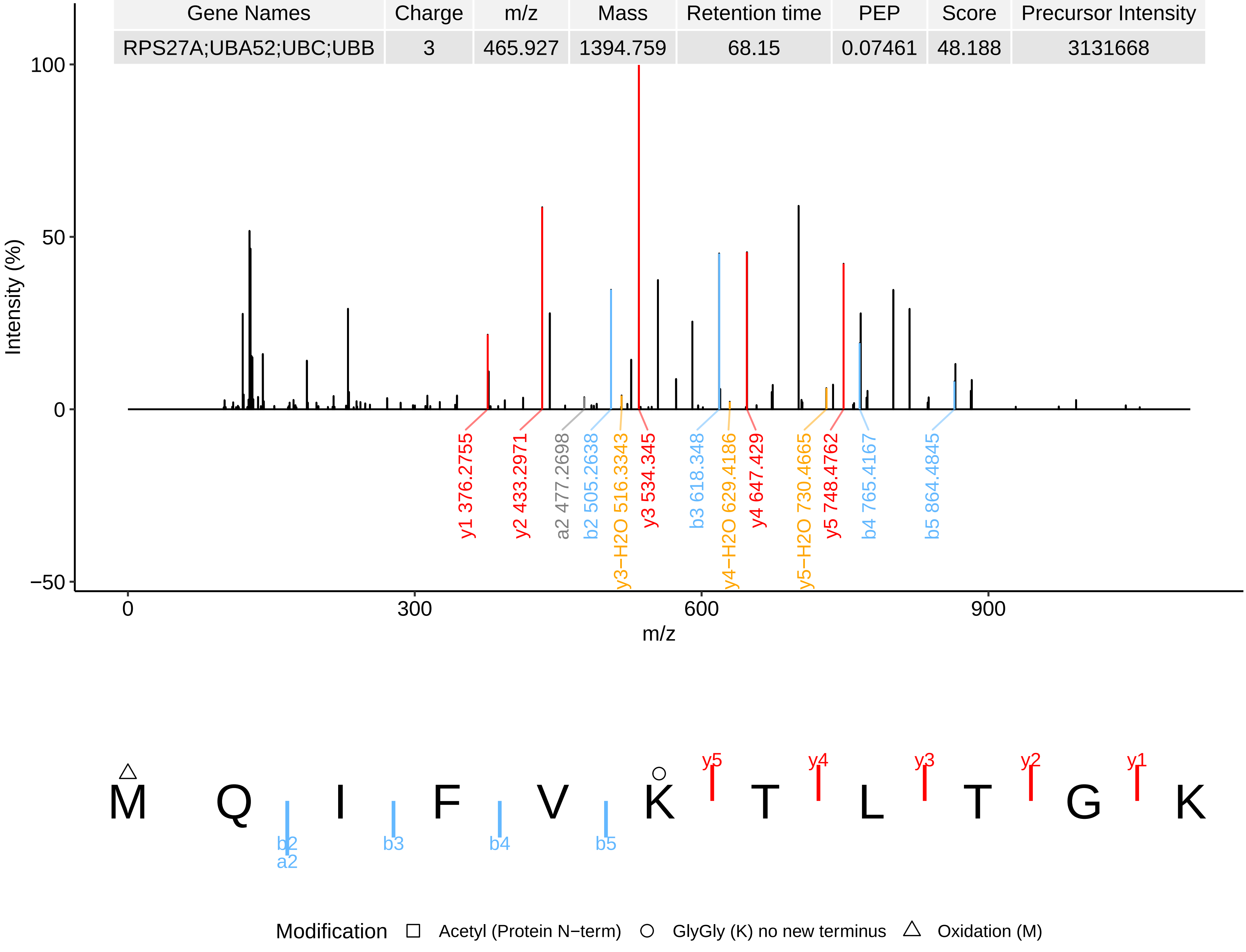

20200921_KIZ_MUb4_GlyGly_10plex_2 − Scan number: 17501

Gene Names	Charge	m/z	Mass	Retention time	PEP	Score	Precursor Intensity
RPS27A;UBA52;UBC;UBB	3	465.927	1394.759	68.445	0.0026496	78.653	19609856

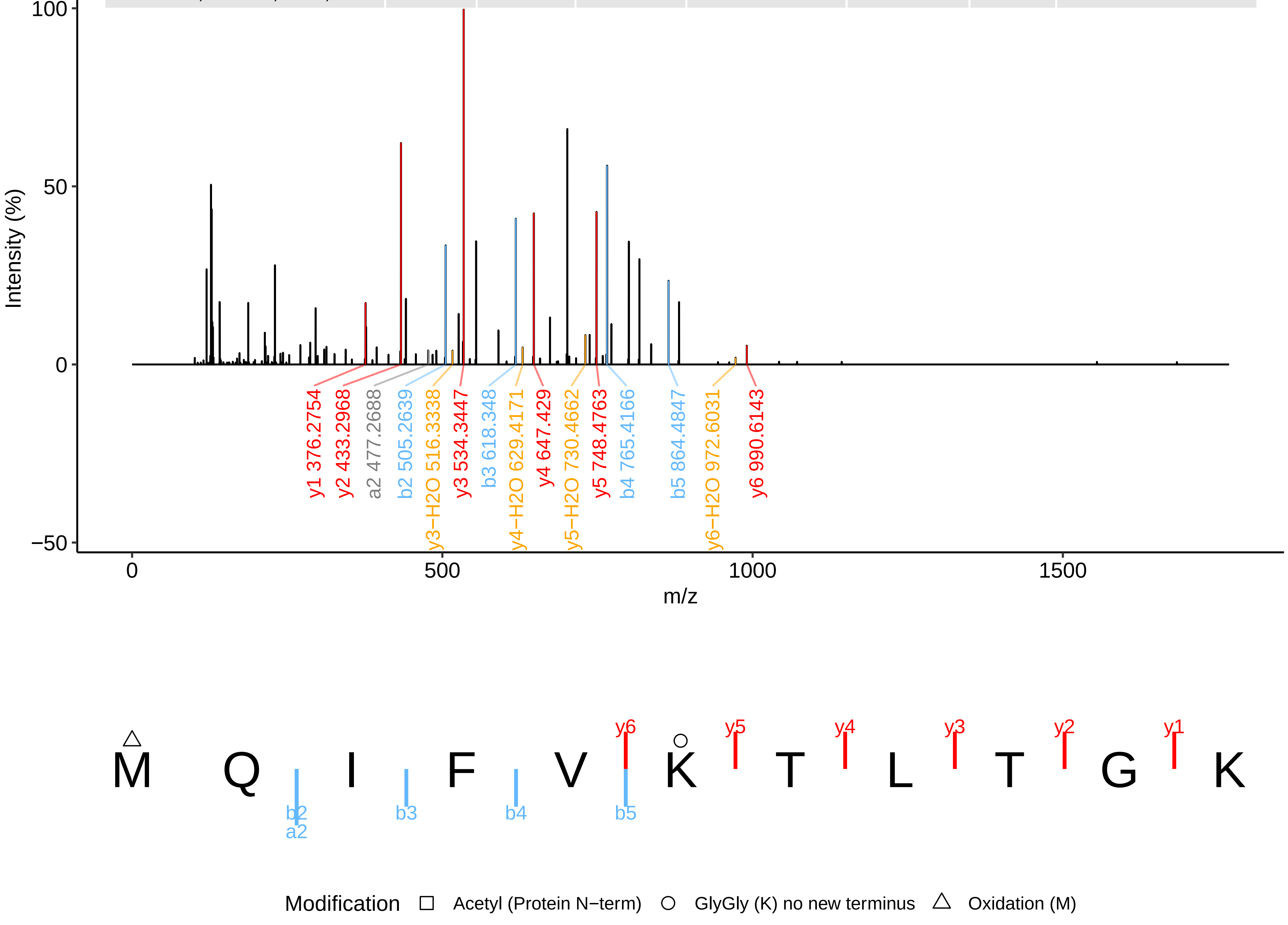

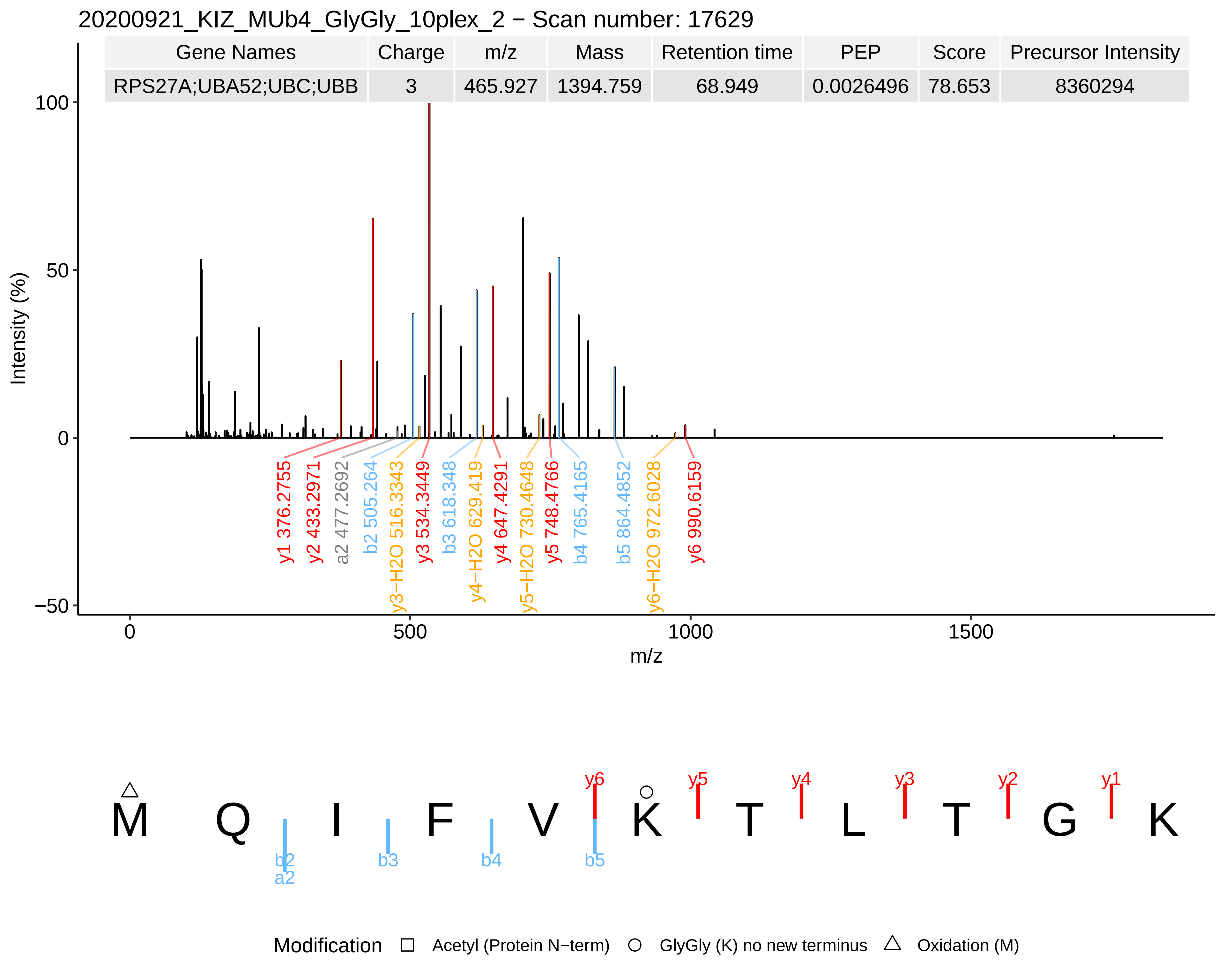

20200921_KIZ_MUb4_GlyGly_10plex_2 − Scan number: 17629

Gene Names	Charge	m/z	Mass	Retention time	PEP	Score	Precursor Intensity
RPS27A;UBA52;UBC;UBB	3	465.927	1394.759	68.949	0.0026496	78.653	8360294

Intensity (%)
100
50
0
−50
0
500
1000
1500
m/z
y1 376.2755
y2 433.2971
a2 477.2692
b2 505.264
y3−H2O 516.3343
y3 534.3449
b3 618.348
y4−H2O 629.419
y4 647.4291
y5−H2O 730.4648
y5 748.4766
b4 765.4165
b5 864.4852
y6−H2O 972.6028
y6 990.6159
M Q I F V K T L T G K
y6 y5 y4 y3 y2 y1
b2 a2 b3 b4 b5
Modification
Acetyl (Protein N−term)
GlyGly (K) no new terminus
Oxidation (M)

20200921_KIZ_MUb4_GlyGly_10plex_2 − Scan number: 17759

Gene Names	Charge	m/z	Mass	Retention time	PEP	Score	Precursor Intensity
RPS27A;UBA52;UBC;UBB	3	465.927	1394.759	69.476	0.0026496	78.653	6208767

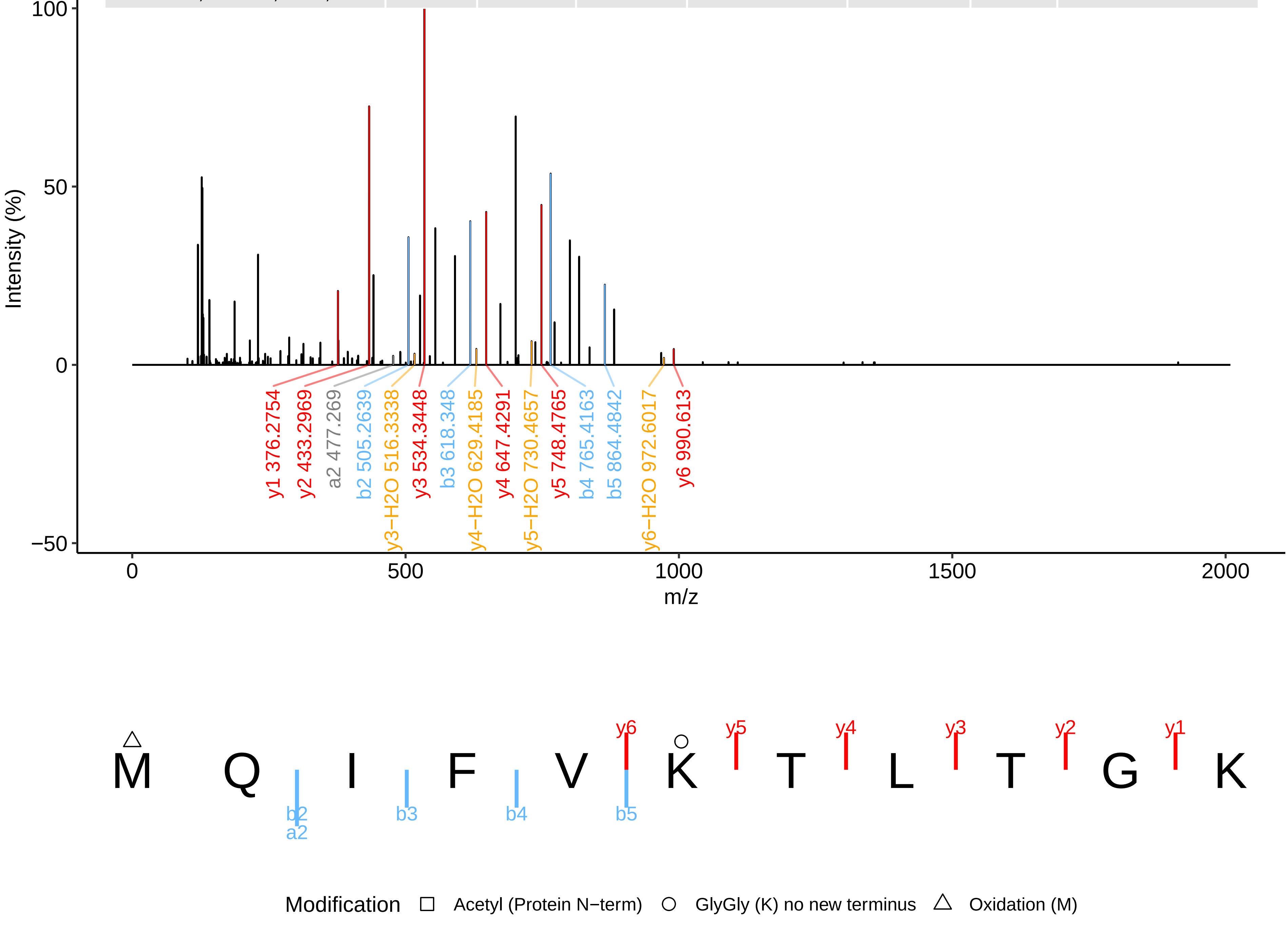

20200921_KIZ_MUb4_GlyGly_10plex_2 − Scan number: 17650

Gene Names	Charge	m/z	Mass	Retention time	PEP	Score	Precursor Intensity
RPS27A;UBA52;UBC;UBB	2	698.3869	1394.759	69.036	0.061734	61.161	1052490

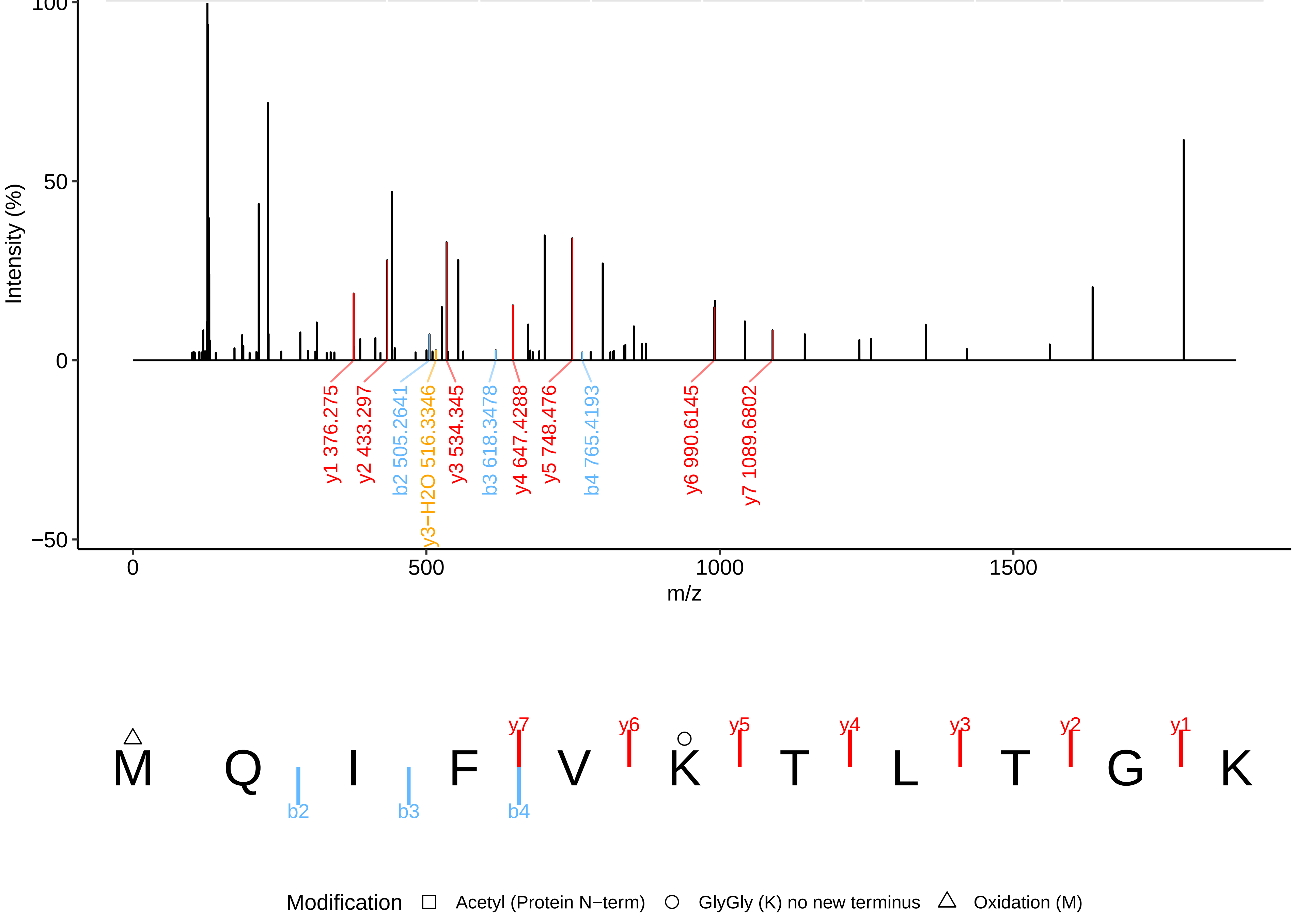

20200921_KIZ_MUb4_GlyGly_10plex_2 − Scan number: 17467

Gene Names	Charge	m/z	Mass	Retention time	PEP	Score	Precursor Intensity
RPS27A;UBA52;UBC;UBB	2	698.3869	1394.759	68.304	0.00033948	109.42	NA

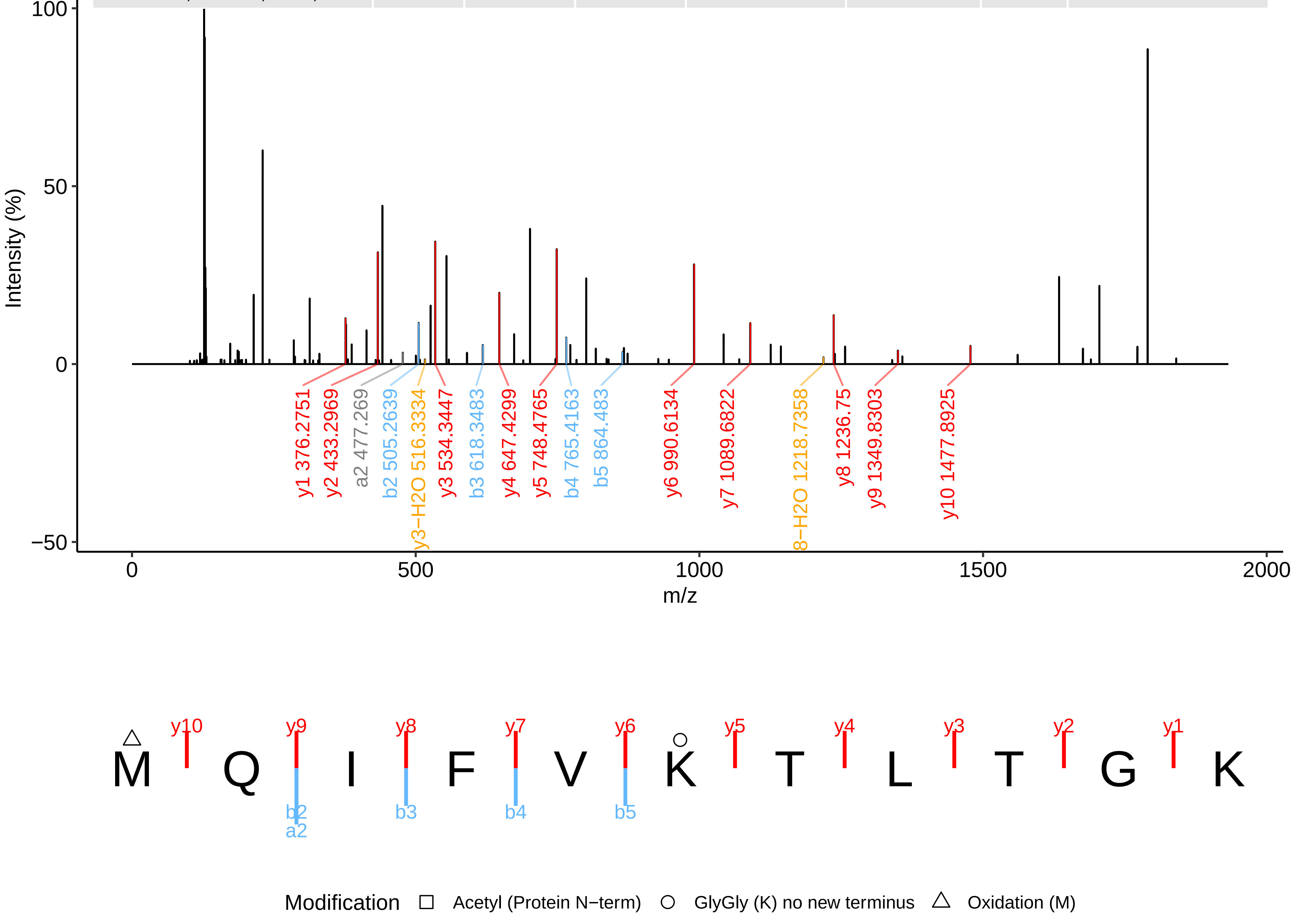

20200924_KIZ_MUb5_GlyGly_1 − Scan number: 18057

Gene Names	Charge	m/z	Mass	Retention time	PEP	Score	Precursor Intensity
RPS27A;UBA52;UBC;UBB	3	465.927	1394.759	58.373	0.010793	66.262	20346900

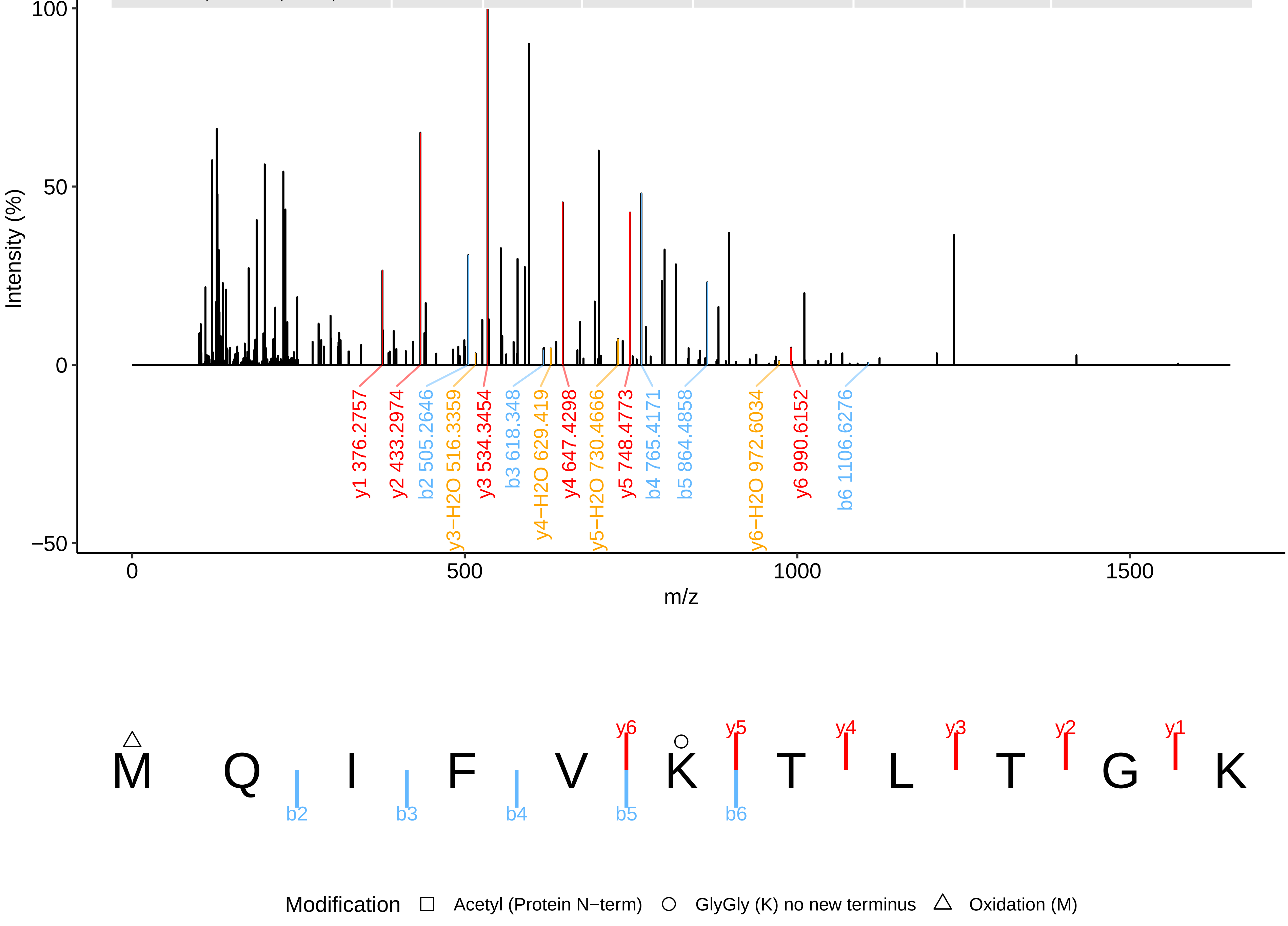

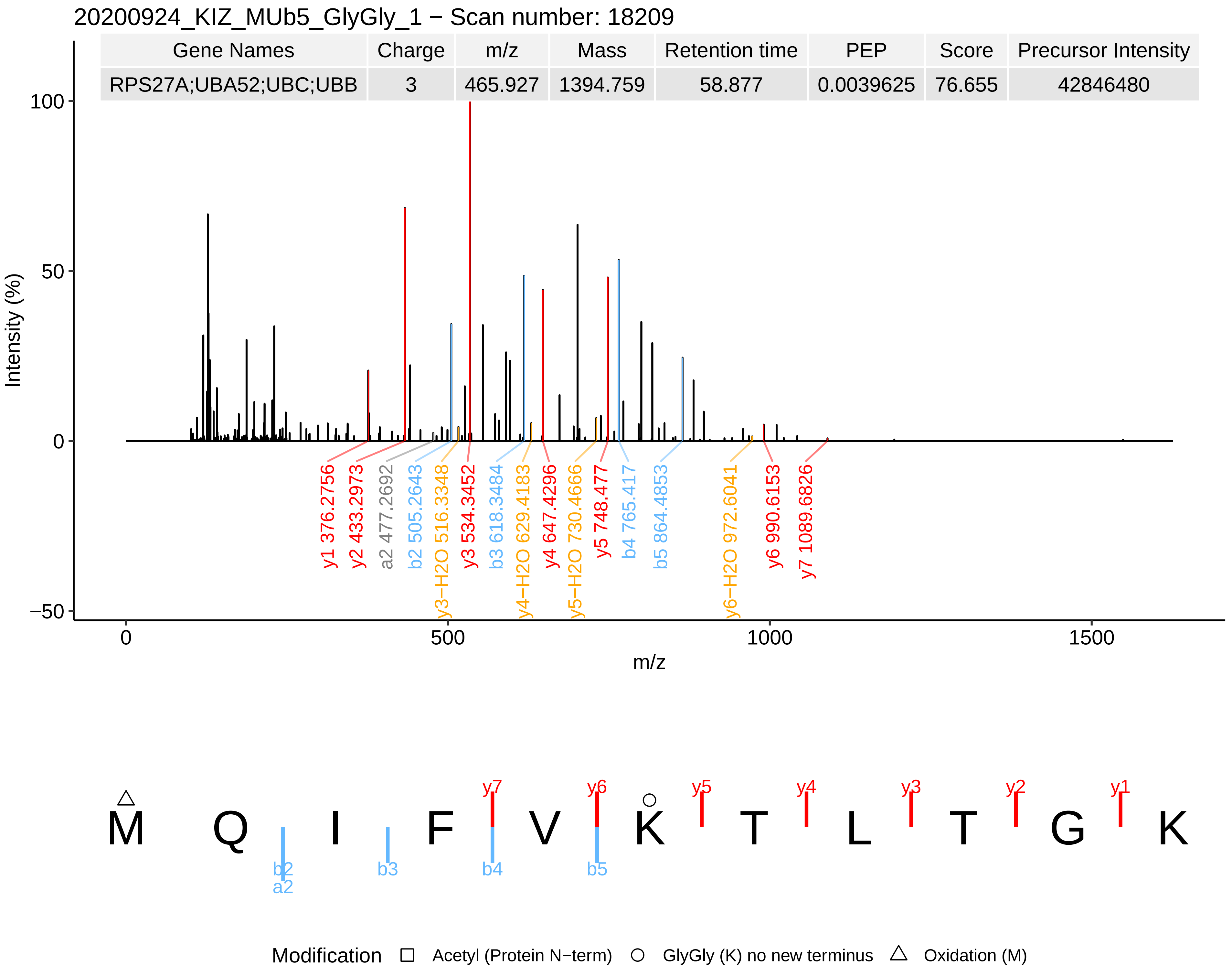
20200924_KIZ_MUb5_GlyGly_1 − Scan number: 18209
Gene Names | Charge | m/z | Mass | Retention time | PEP | Score | Precursor Intensity
RPS27A;UBA52;UBC;UBB | 3 | 465.927 | 1394.759 | 58.877 | 0.0039625 | 76.655 | 42846480
Intensity (%)
m/z
y1 376.2756
y2 433.2973
a2 477.2692
b2 505.2643
y3−H2O 516.3348
y3 534.3452
b3 618.3484
y4−H2O 629.4183
y4 647.4296
y5−H2O 730.4666
y5 748.477
b4 765.417
b5 864.4853
y6−H2O 972.6041
y6 990.6153
y7 1089.6826
M Q I F V K T L T G K
Modification
Acetyl (Protein N−term)
GlyGly (K) no new terminus
Oxidation (M)

20200924_KIZ_MUb5_GlyGly_1 − Scan number: 18367

Gene Names	Charge	m/z	Mass	Retention time	PEP	Score	Precursor Intensity
RPS27A;UBA52;UBC;UBB	3	465.927	1394.759	59.385	0.010208	67.153	39292348

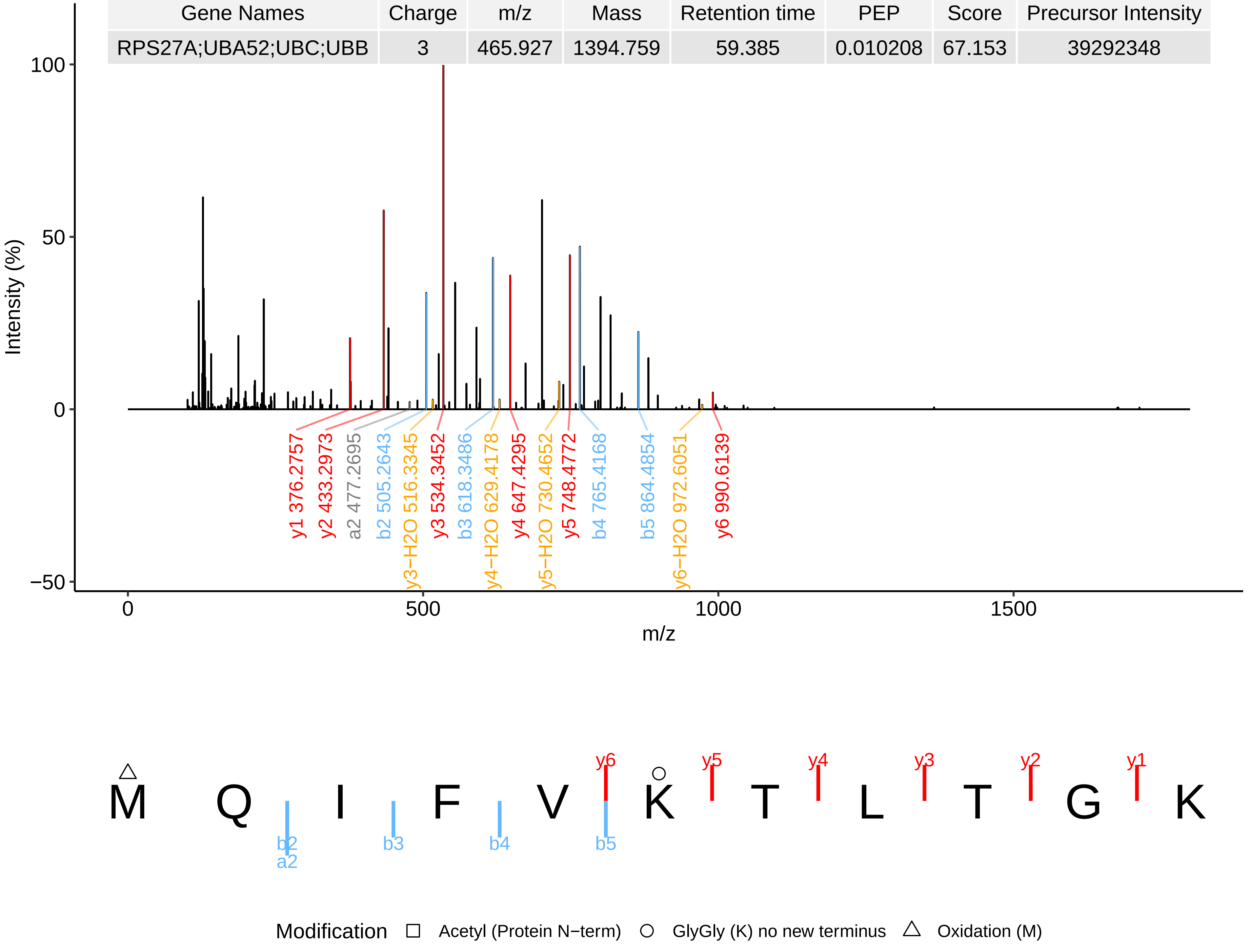

20200924_KIZ_MUb5_GlyGly_1 − Scan number: 18527

Gene Names	Charge	m/z	Mass	Retention time	PEP	Score	Precursor Intensity
RPS27A;UBA52;UBC;UBB	3	465.927	1394.759	59.888	0.0059271	73.666	59805012

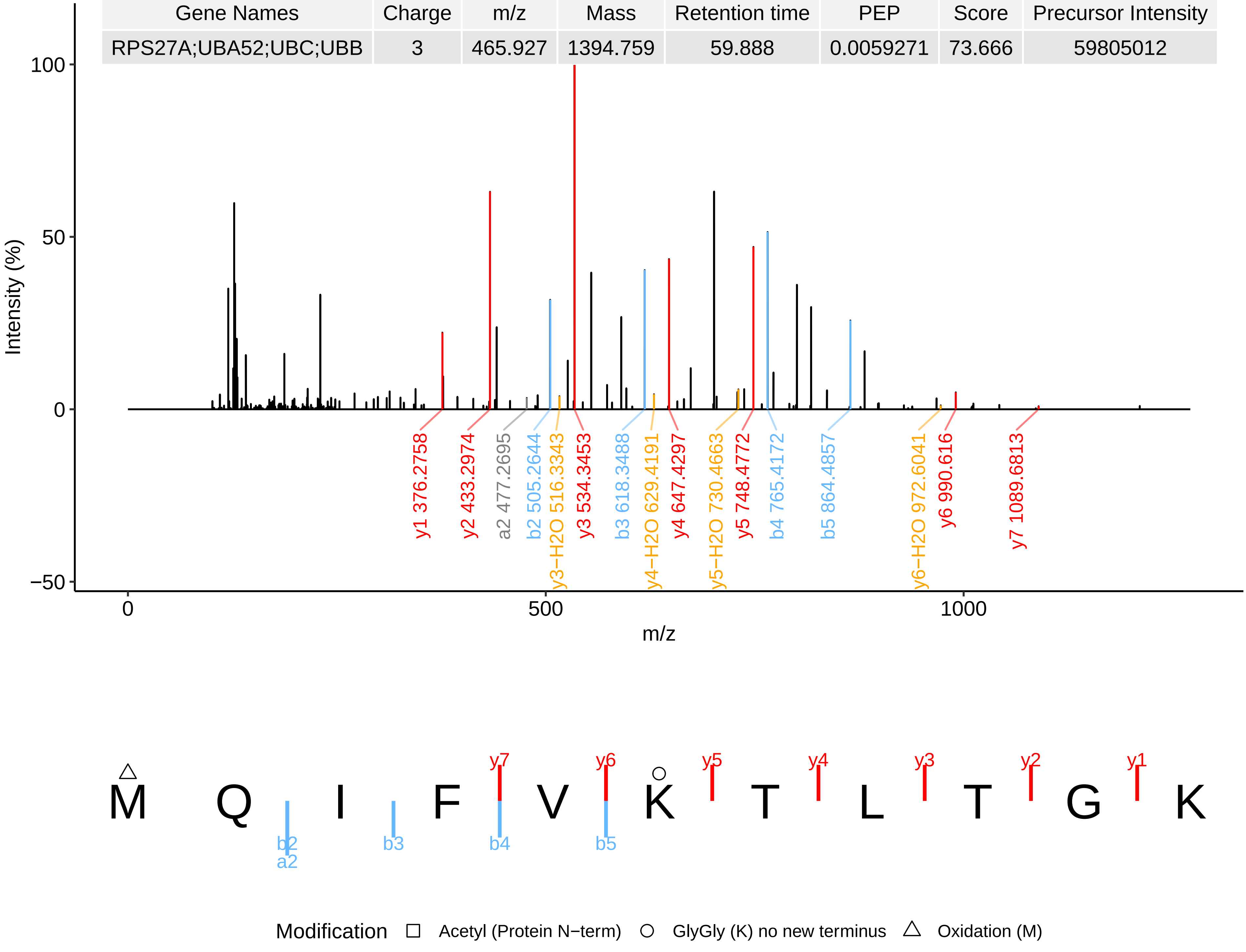

20200924_KIZ_MUb5_GlyGly_1 − Scan number: 18687

Gene Names	Charge	m/z	Mass	Retention time	PEP	Score	Precursor Intensity
RPS27A;UBA52;UBC;UBB	3	465.927	1394.759	60.393	0.0026496	78.653	44896732

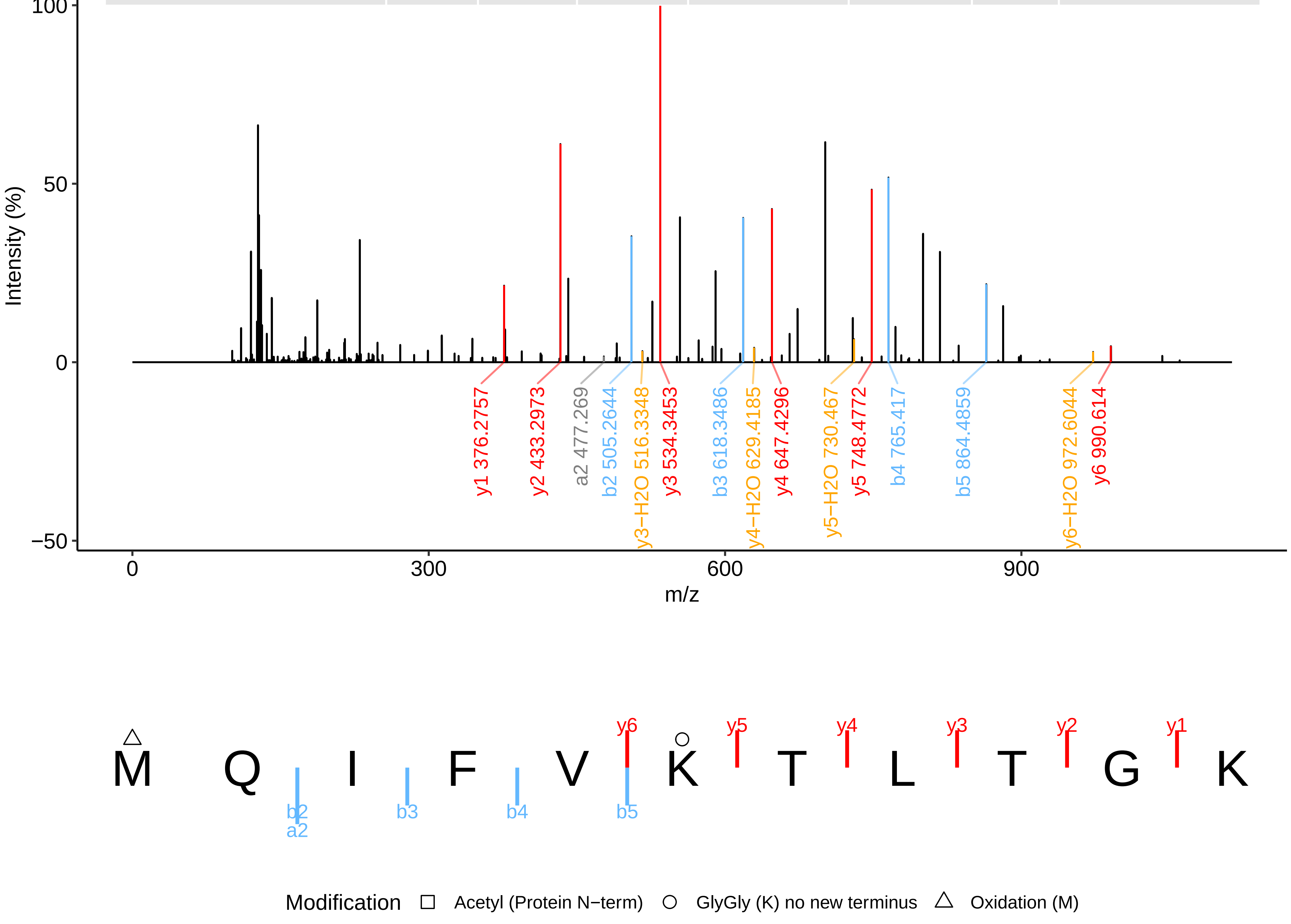

20200924_KIZ_MUb5_GlyGly_1 − Scan number: 18846

Gene Names	Charge	m/z	Mass	Retention time	PEP	Score	Precursor Intensity
RPS27A;UBA52;UBC;UBB	3	465.927	1394.759	60.899	0.13229	51.013	11981669

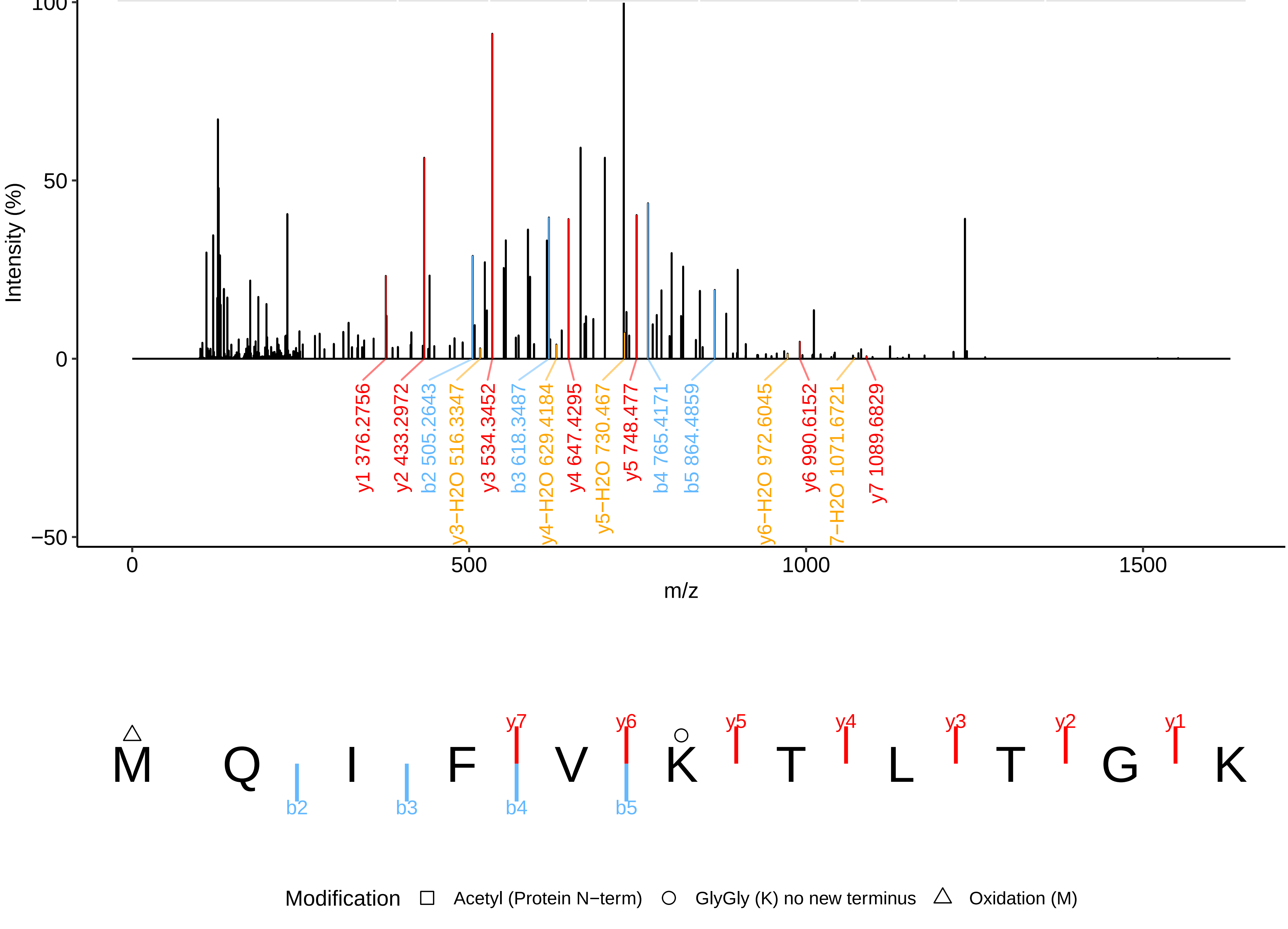

20200924_KIZ_MUb5_GlyGly_2 − Scan number: 16852

Gene Names	Charge	m/z	Mass	Retention time	PEP	Score	Precursor Intensity
RPS27A;UBA52;UBC;UBB	3	465.927	1394.759	62.531	0.0080157	70.488	4614183

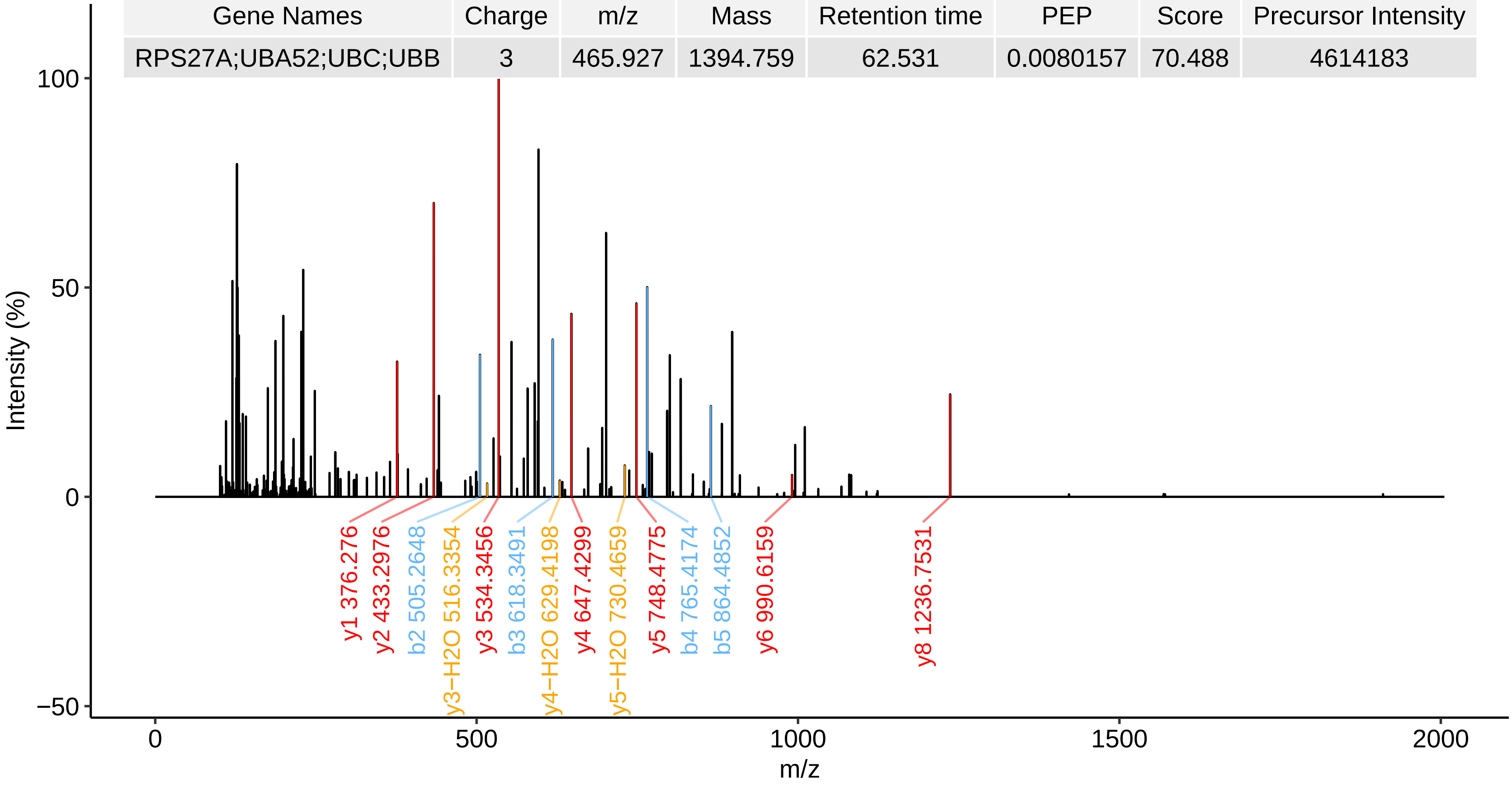

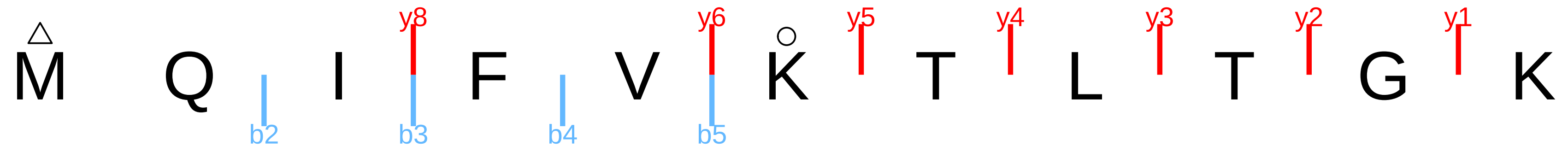

Modification □ Acetyl (Protein N−term) ○ GlyGly (K) no new terminus △ Oxidation (M)

20200924_KIZ_MUb5_GlyGly_2 − Scan number: 16990

Gene Names	Charge	m/z	Mass	Retention time	PEP	Score	Precursor Intensity
RPS27A;UBA52;UBC;UBB	3	465.927	1394.759	63.031	0.0039625	76.655	20649184

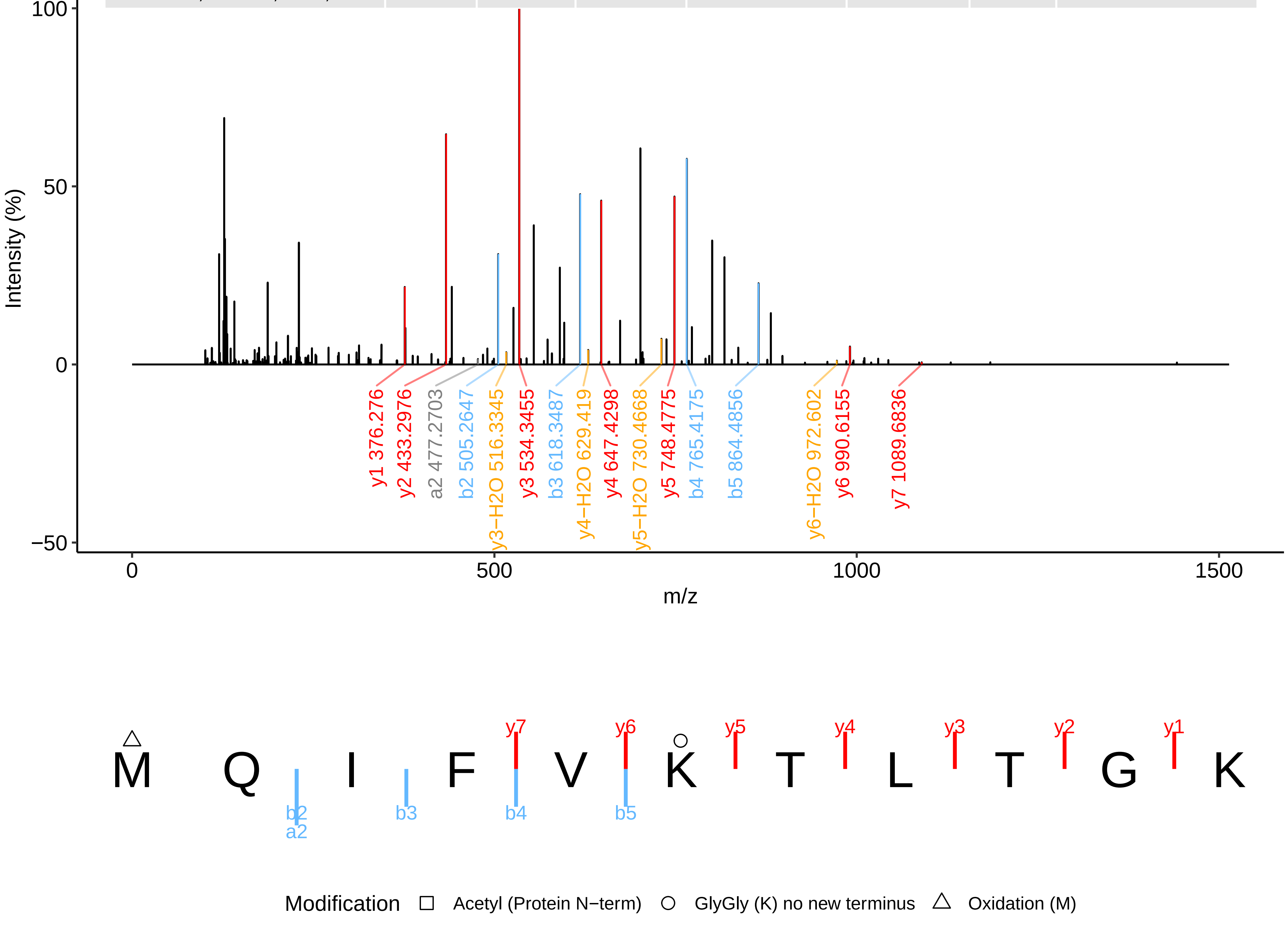

20200924_KIZ_MUb5_GlyGly_2 − Scan number: 17129

Gene Names	Charge	m/z	Mass	Retention time	PEP	Score	Precursor Intensity
RPS27A;UBA52;UBC;UBB	3	465.927	1394.759	63.538	0.00077317	88.441	34875900

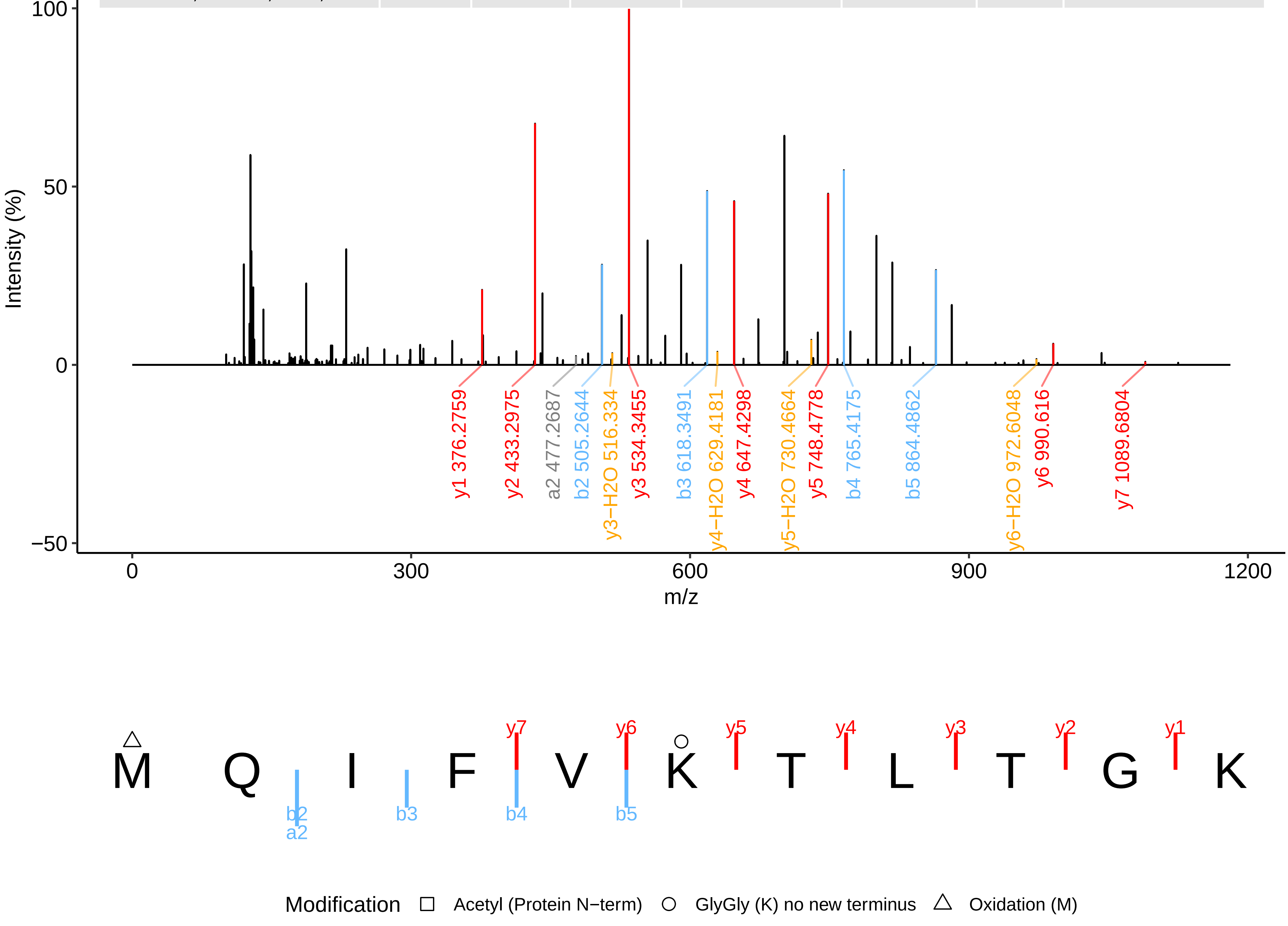

20200924_KIZ_MUb5_GlyGly_2 − Scan number: 17276

Gene Names	Charge	m/z	Mass	Retention time	PEP	Score	Precursor Intensity
RPS27A;UBA52;UBC;UBB	3	465.927	1394.759	64.043	0.0065935	72.652	48357824

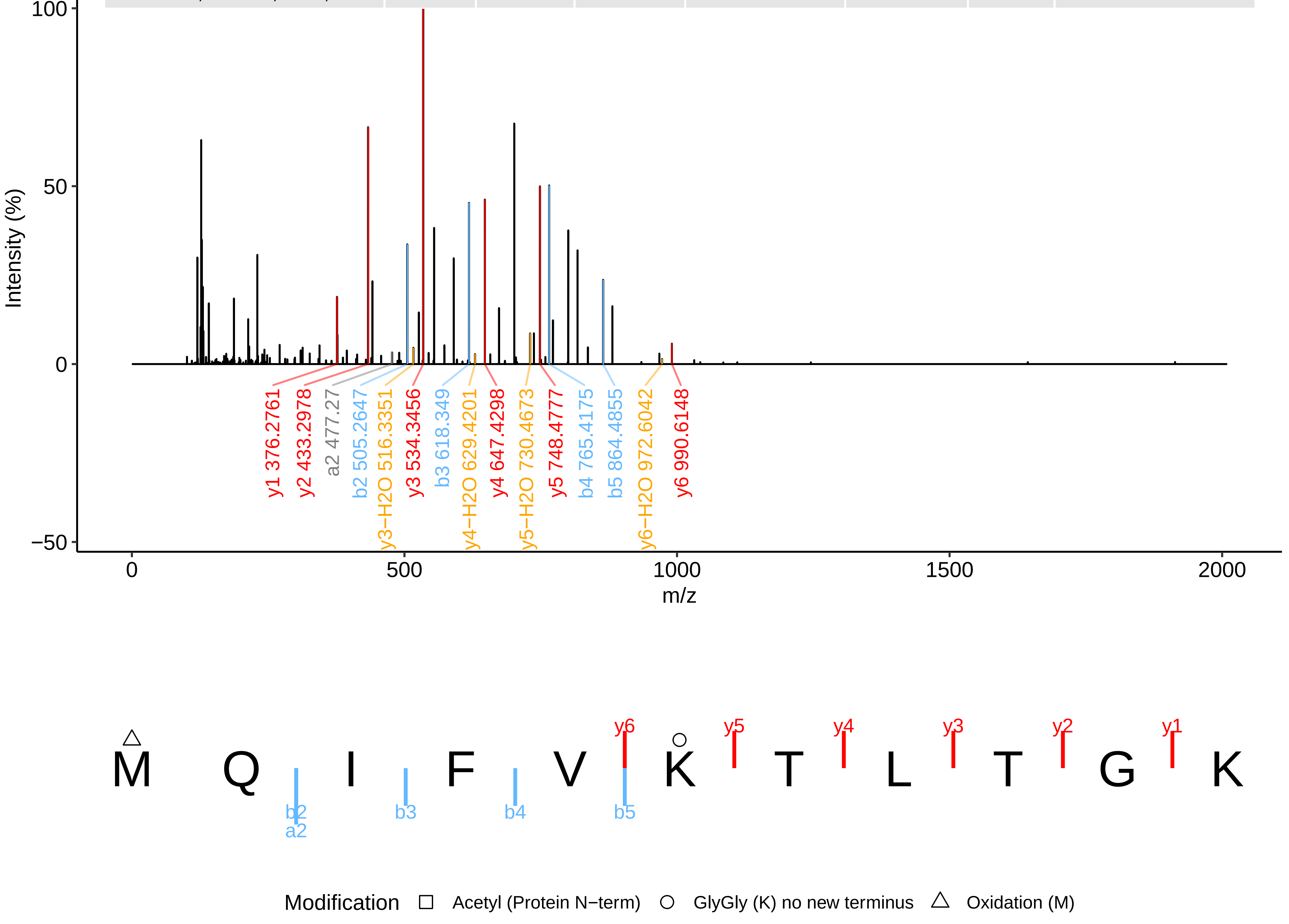

20200924_KIZ_MUb5_GlyGly_2 − Scan number: 17557

Gene Names	Charge	m/z	Mass	Retention time	PEP	Score	Precursor Intensity
RPS27A;UBA52;UBC;UBB	3	465.927	1394.759	65.072	0.0013488	80.979	8177236

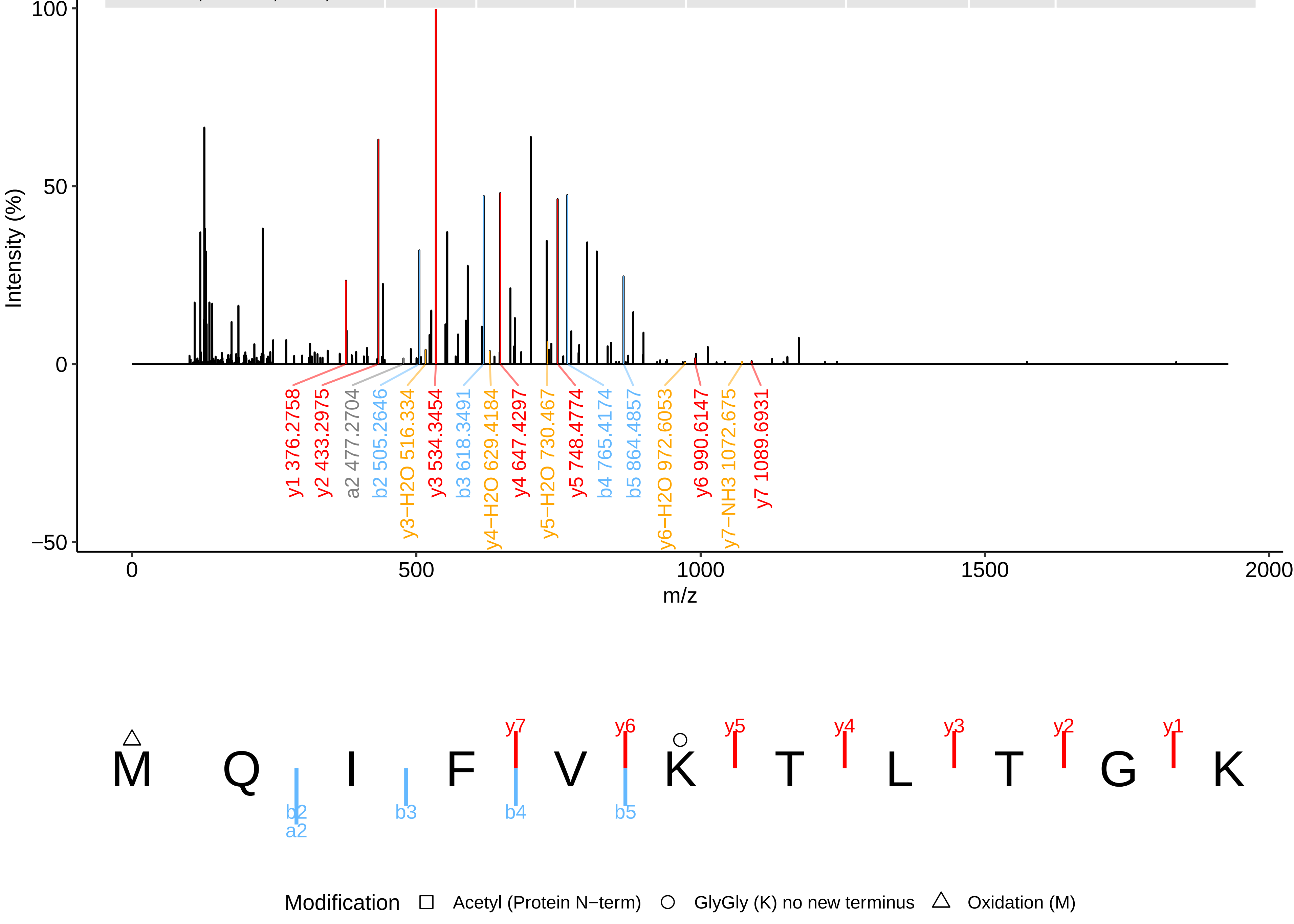

20200924_KIZ_MUb5_GlyGly_2 − Scan number: 17418

Gene Names	Charge	m/z	Mass	Retention time	PEP	Score	Precursor Intensity
RPS27A;UBA52;UBC;UBB	3	465.927	1394.759	64.556	0.0018083	88.441	29633574

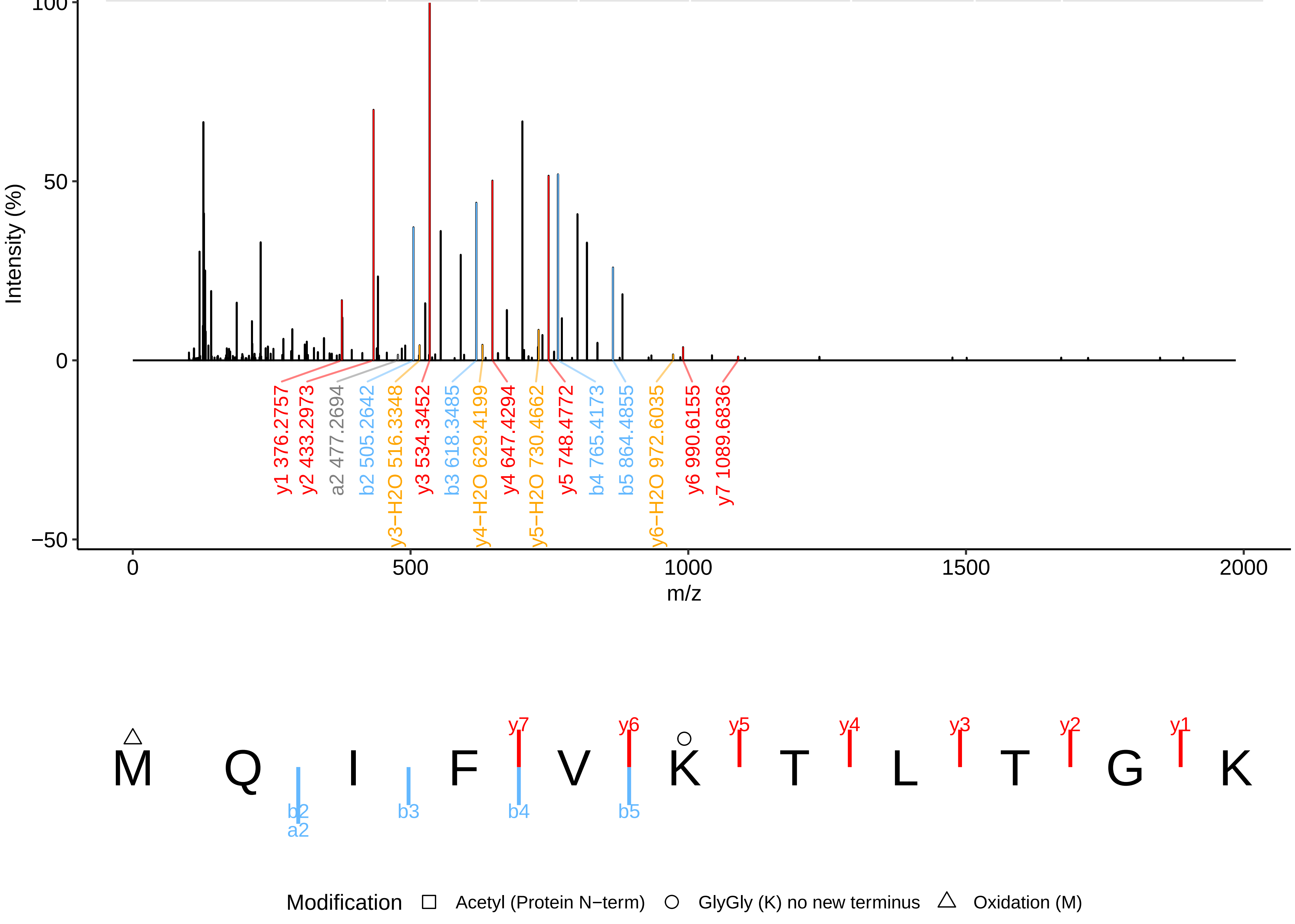

20201118_KIZ_MUb6_GlyGly_1 − Scan number: 18596

Gene Names	Charge	m/z	Mass	Retention time	PEP	Score	Precursor Intensity
RPS27A;UBA52;UBC;UBB	3	465.927	1394.759	58.45	0.0095644	68.132	46531272

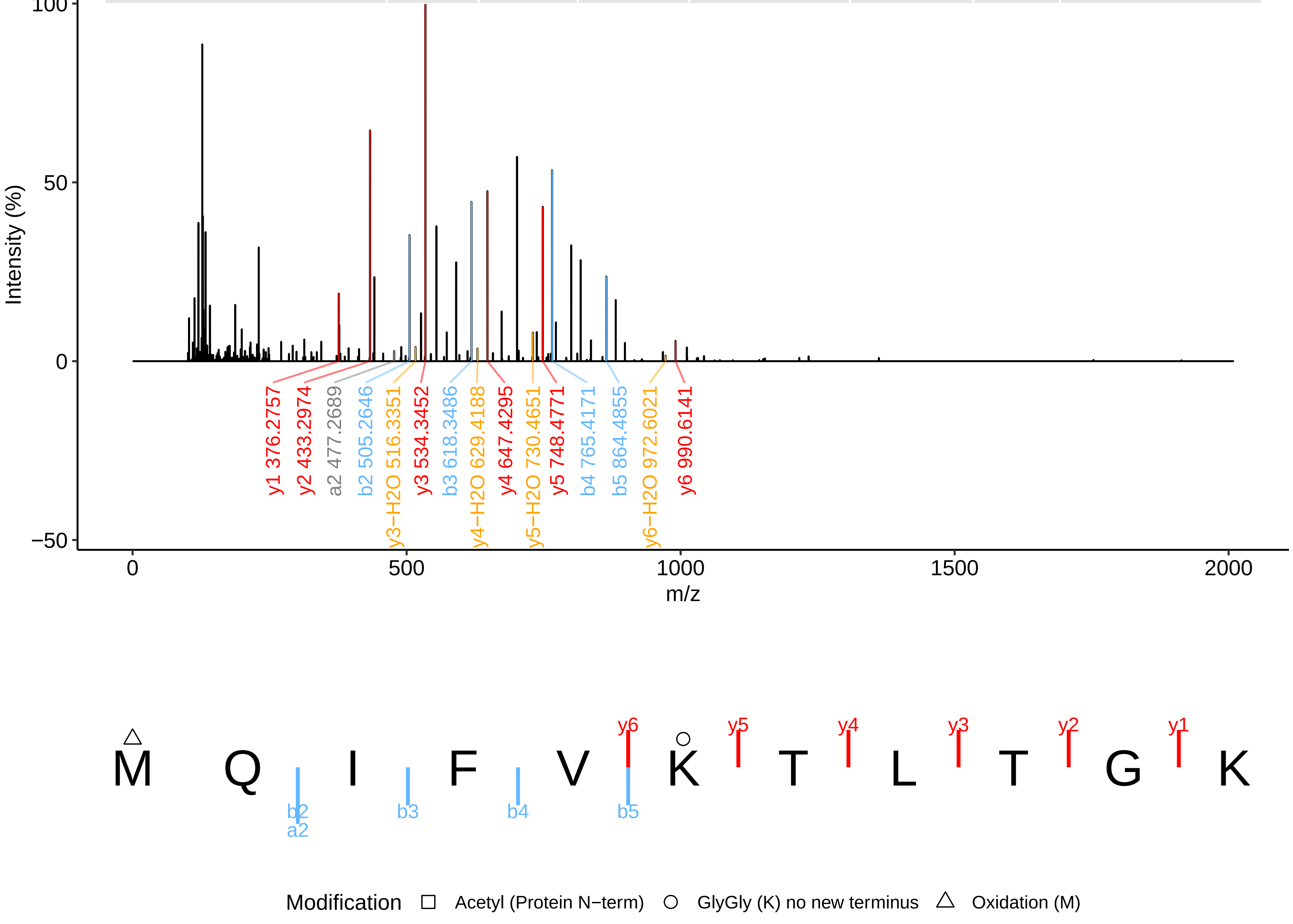

20201118_KIZ_MUb6_GlyGly_1 − Scan number: 18795

Gene Names	Charge	m/z	Mass	Retention time	PEP	Score	Precursor Intensity
RPS27A;UBA52;UBC;UBB	3	465.927	1394.759	59.037	0.0094176	68.355	66674272

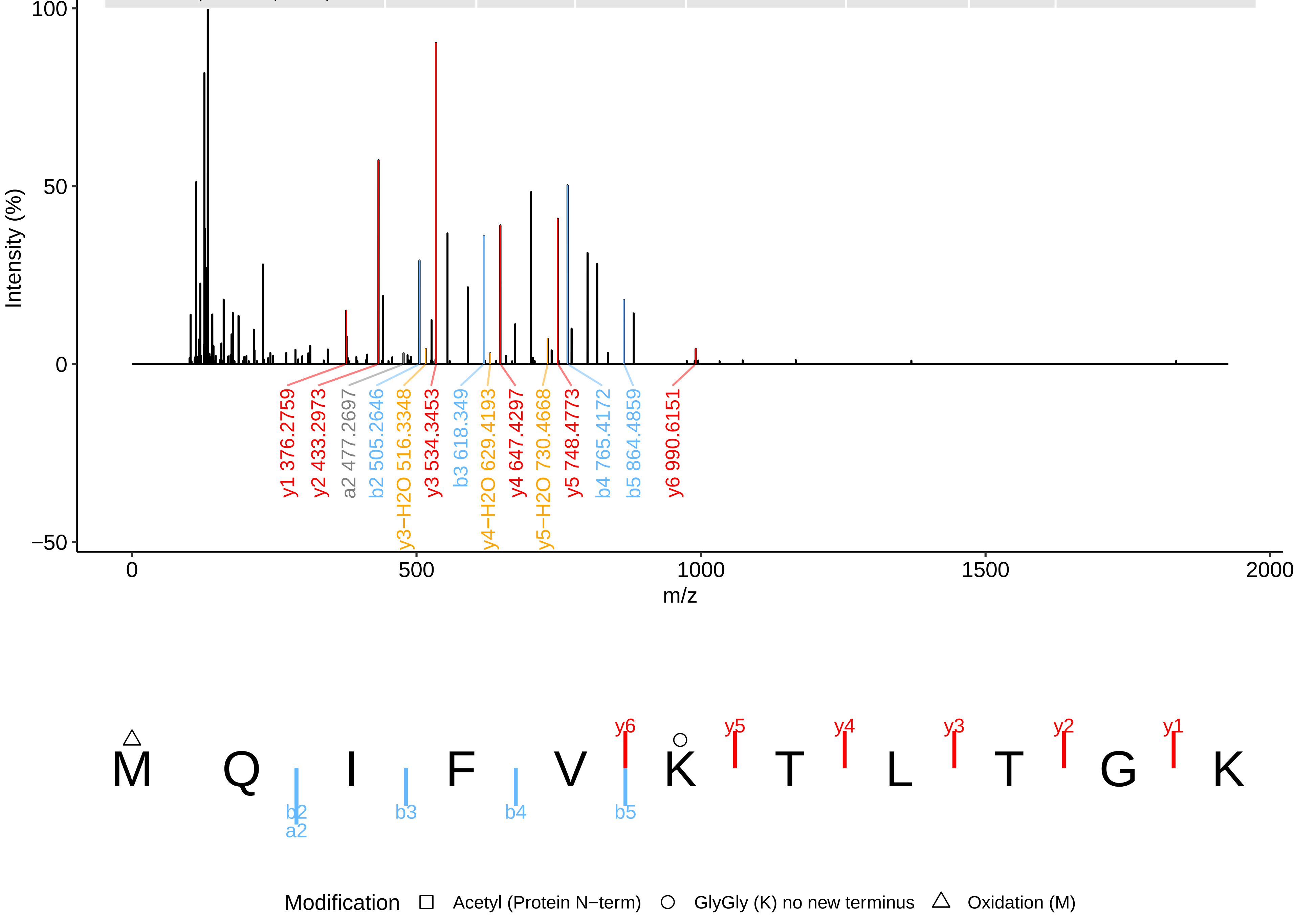

20201118_KIZ_MUb6_GlyGly_1 − Scan number: 18968

Gene Names	Charge	m/z	Mass	Retention time	PEP	Score	Precursor Intensity
RPS27A;UBA52;UBC;UBB	3	465.927	1394.759	59.541	0.0065935	72.652	194005776

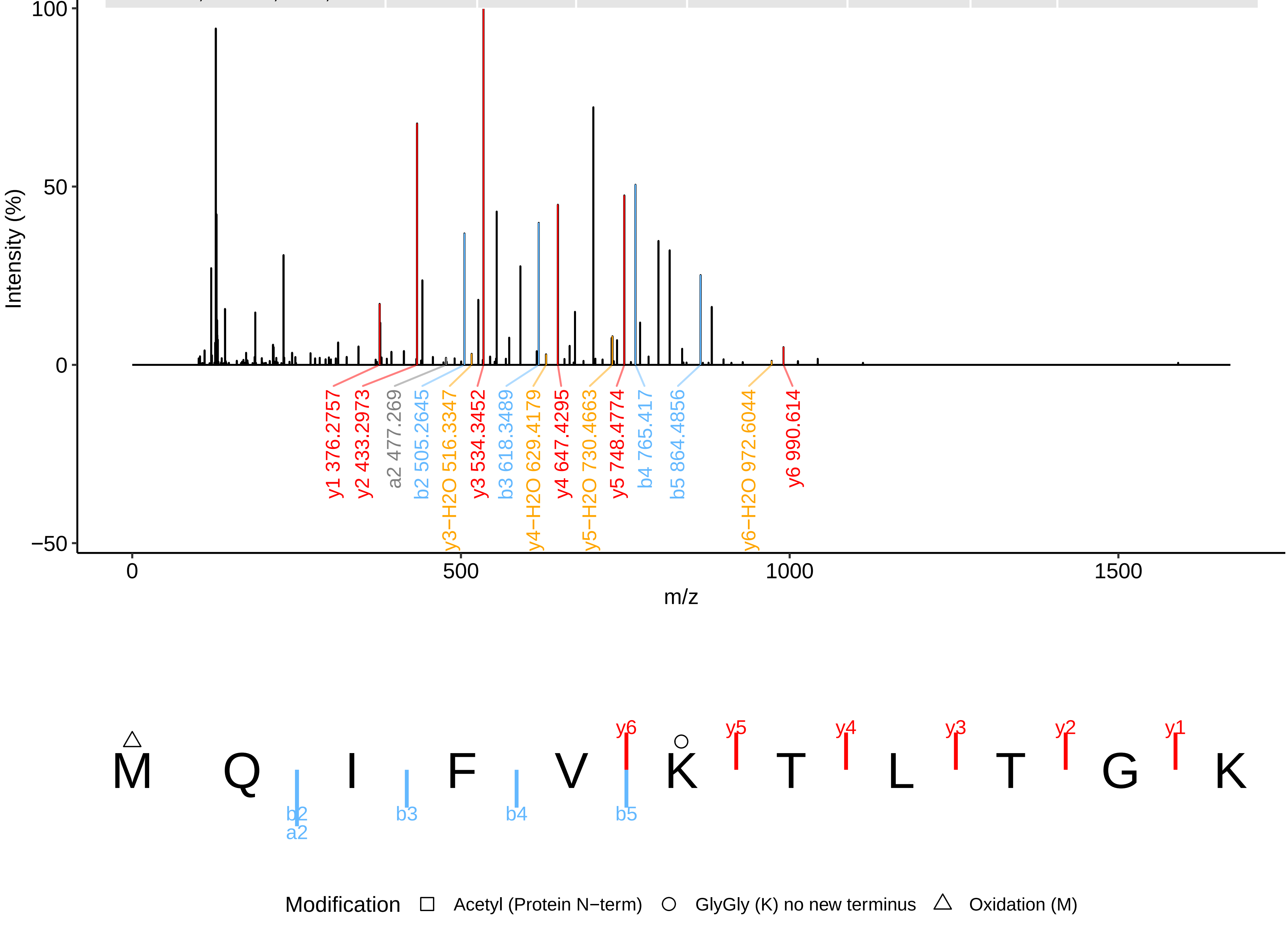

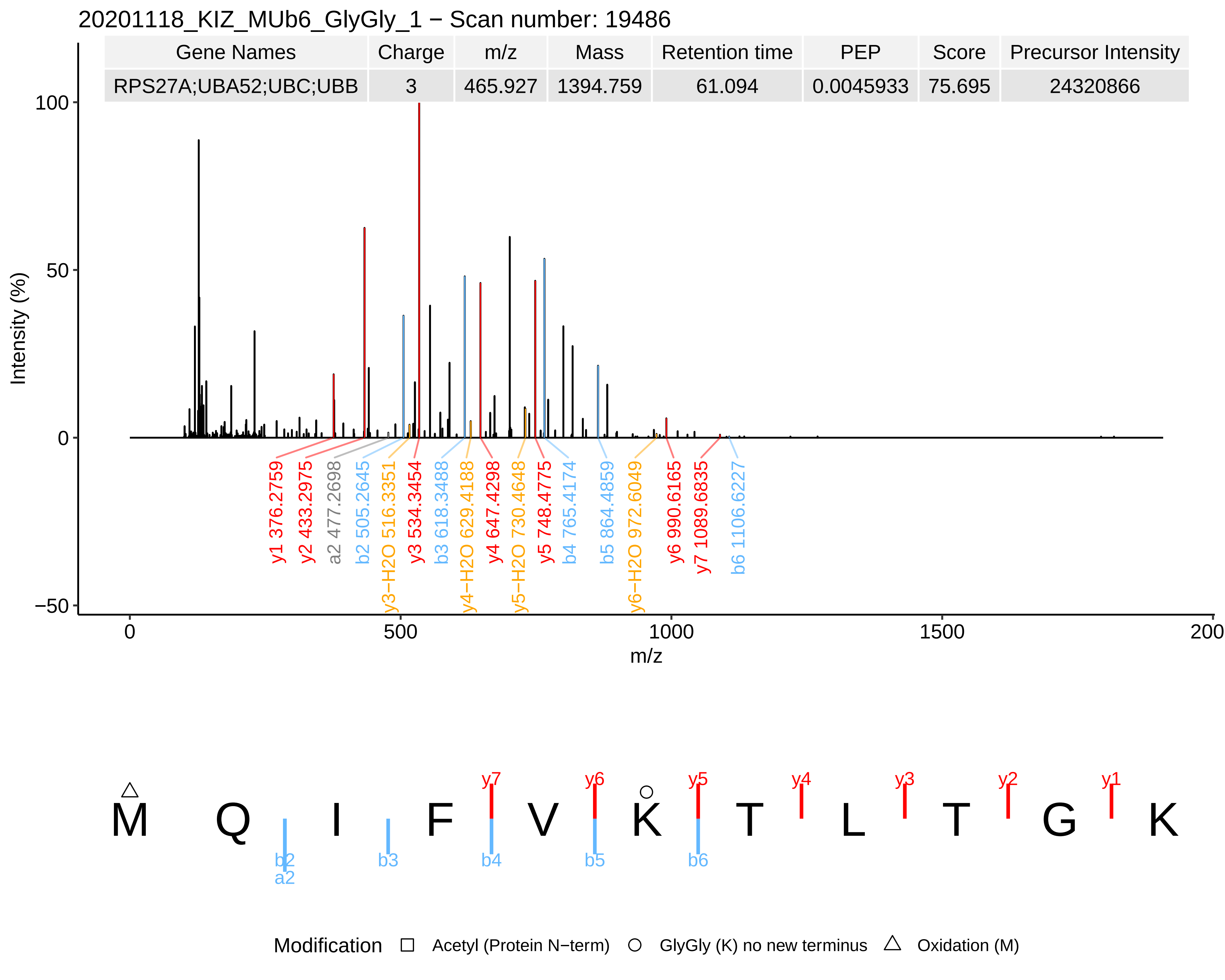

Gene Names	Charge	m/z	Mass	Retention time	PEP	Score	Precursor Intensity
RPS27A;UBA52;UBC;UBB	3	465.927	1394.759	61.094	0.0045933	75.695	24320866

20201118_KIZ_MUb6_GlyGly_1 − Scan number: 19528

Gene Names	Charge	m/z	Mass	Retention time	PEP	Score	Precursor Intensity
RPS27A;UBA52;UBC;UBB	3	465.927	1394.759	61.222	0.009697	67.93	16820064

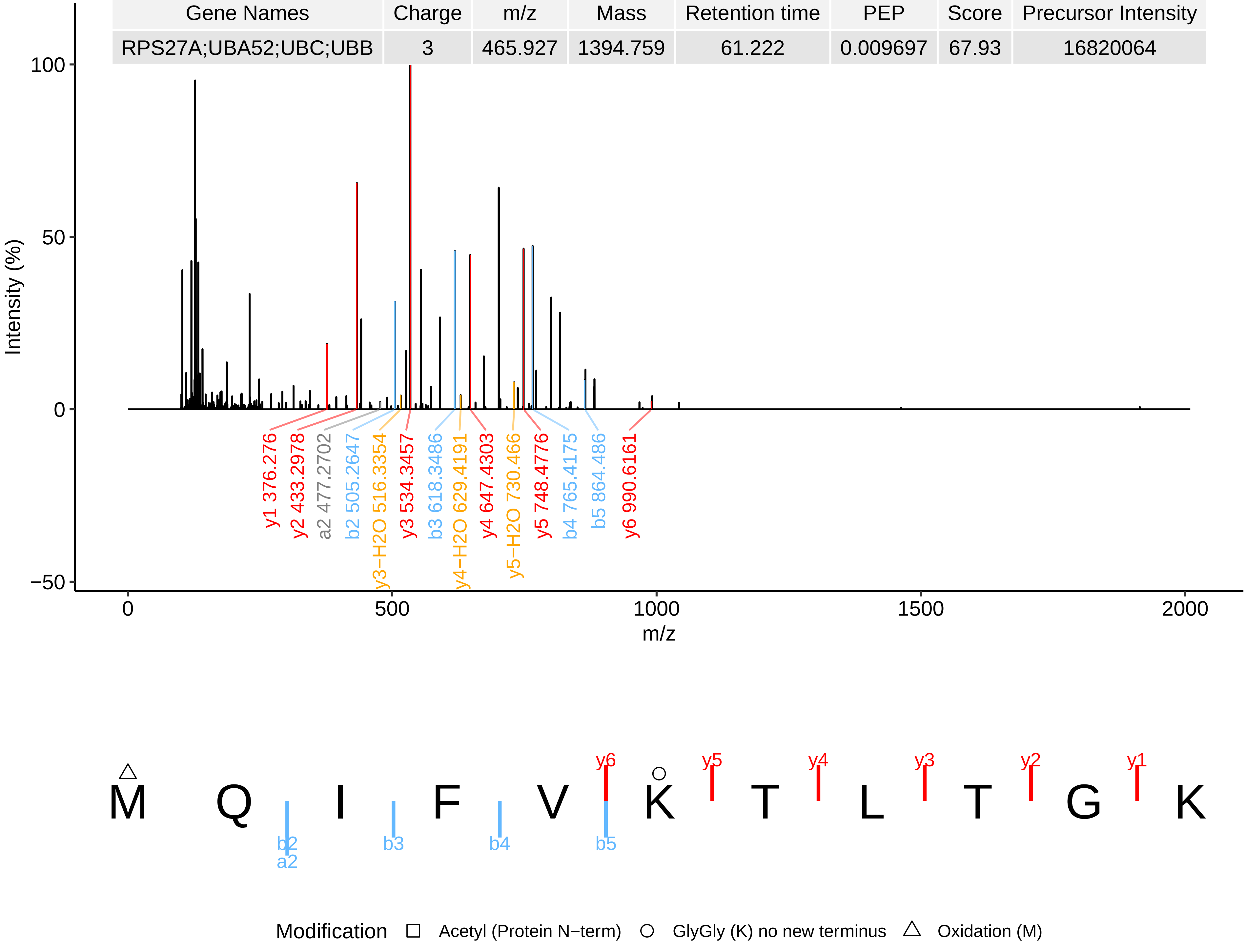

20201118_KIZ_MUb6_GlyGly_1 − Scan number: 19866

Gene Names	Charge	m/z	Mass	Retention time	PEP	Score	Precursor Intensity
RPS27A;UBA52;UBC;UBB	3	465.927	1394.759	62.239	0.0095644	68.132	37376348

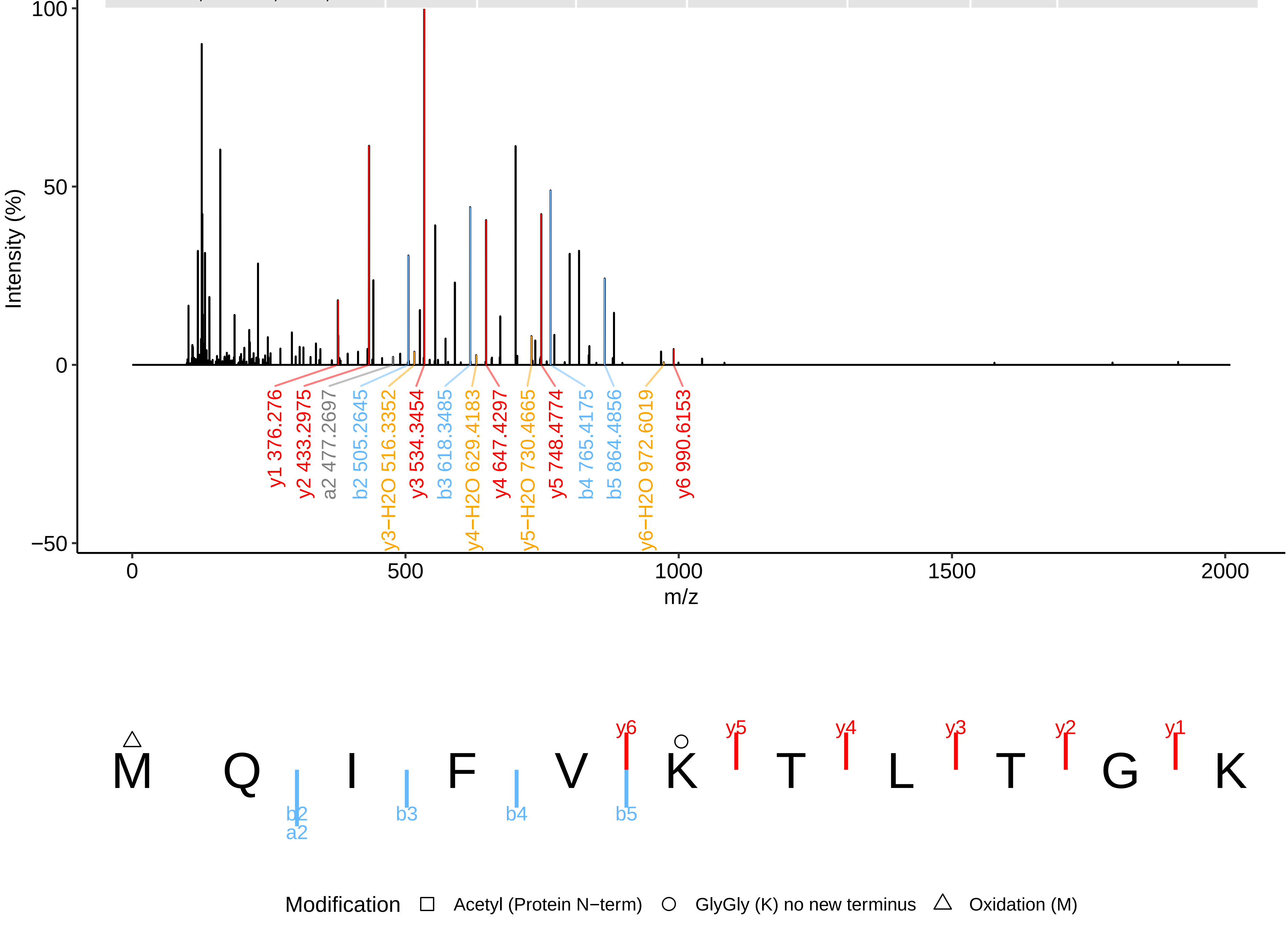

20201118_KIZ_MUb6_GlyGly_1 − Scan number: 21506

Gene Names	Charge	m/z	Mass	Retention time	PEP	Score	Precursor Intensity
RPS27A;UBA52;UBC;UBB	3	465.927	1394.759	67.093	0.0039625	76.655	15679021

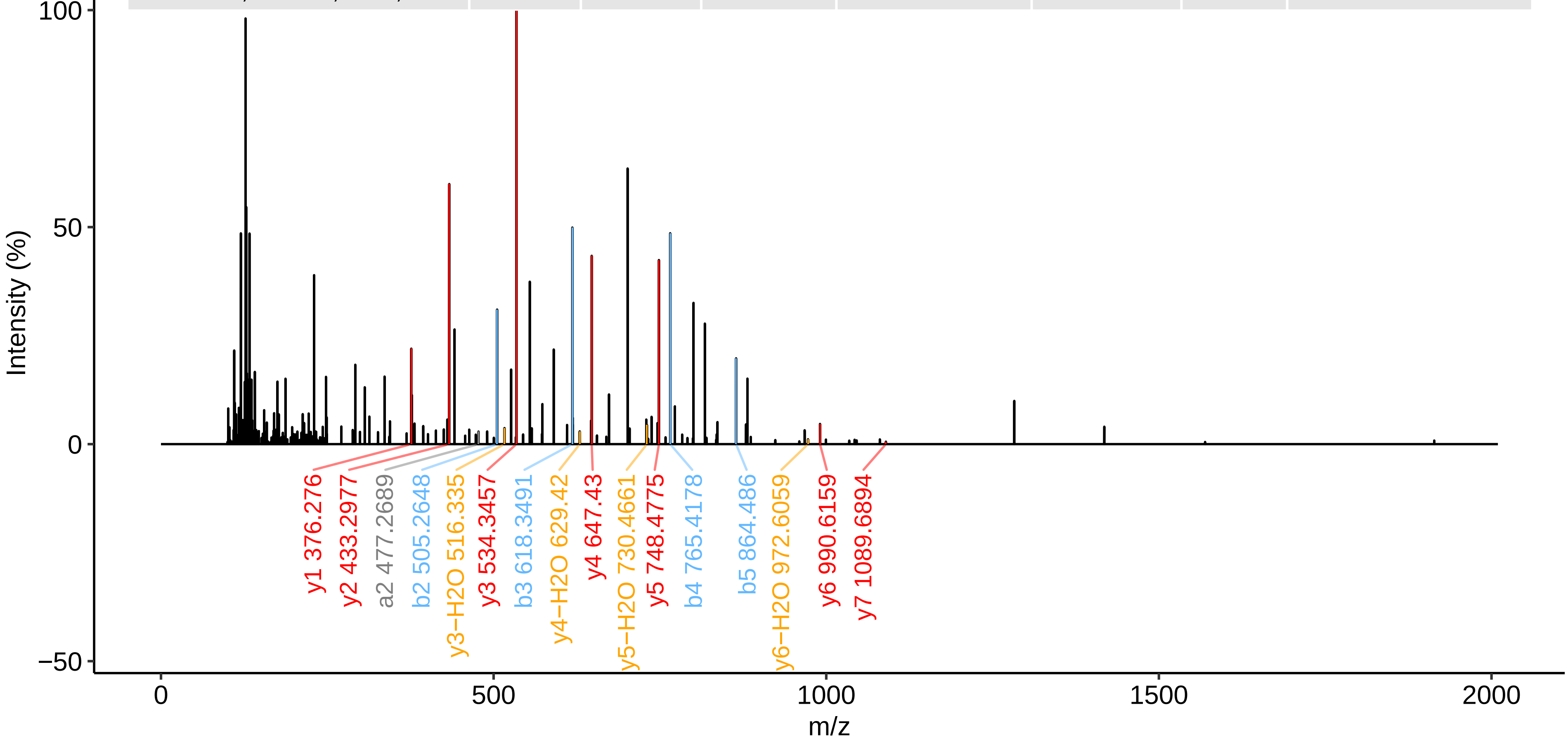

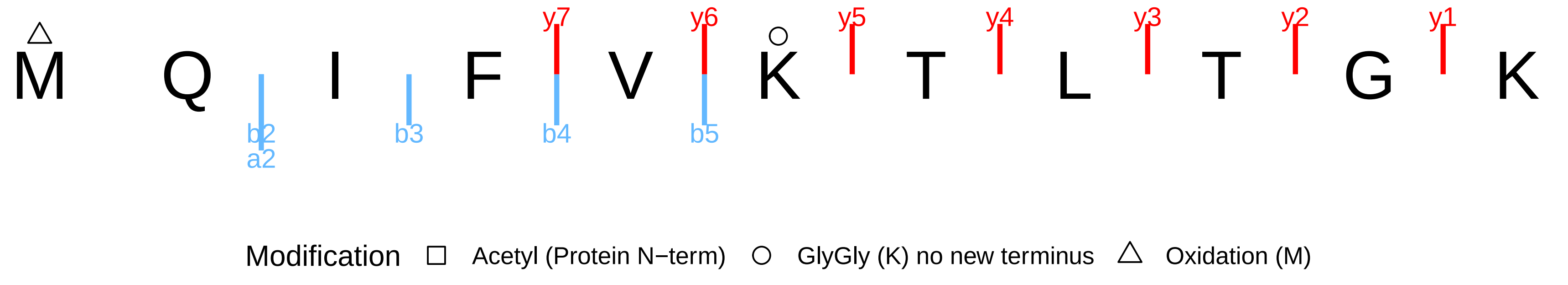

20201118_KIZ_MUb6_GlyGly_1 − Scan number: 19697

Gene Names	Charge	m/z	Mass	Retention time	PEP	Score	Precursor Intensity
RPS27A;UBA52;UBC;UBB	3	465.927	1394.759	61.73	0.019104	69.451	34534904

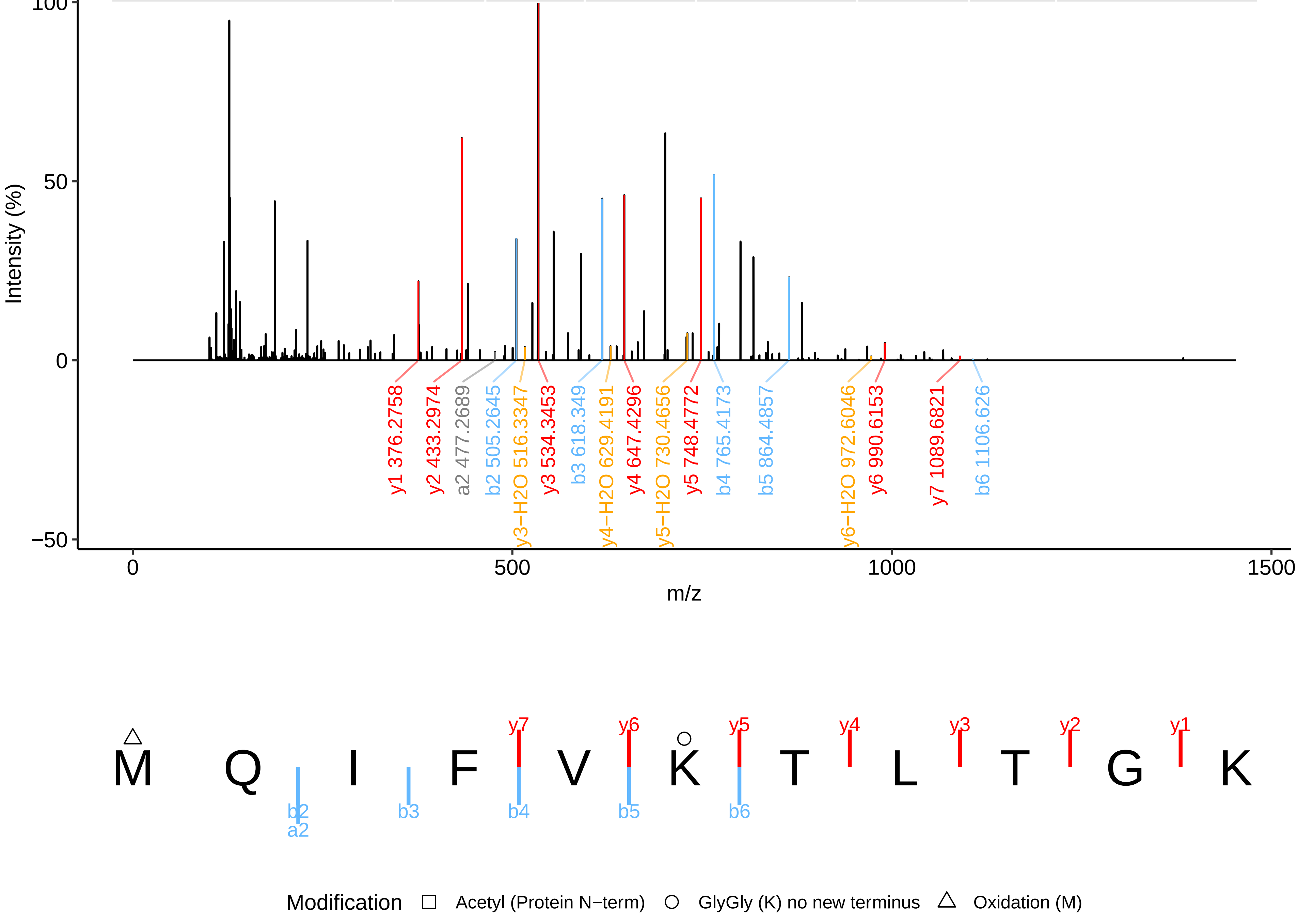

20201118_KIZ_MUb6_GlyGly_1 − Scan number: 20039

Gene Names	Charge	m/z	Mass	Retention time	PEP	Score	Precursor Intensity
RPS27A;UBA52;UBC;UBB	3	465.927	1394.759	62.751	0.0094669	75.819	23682064

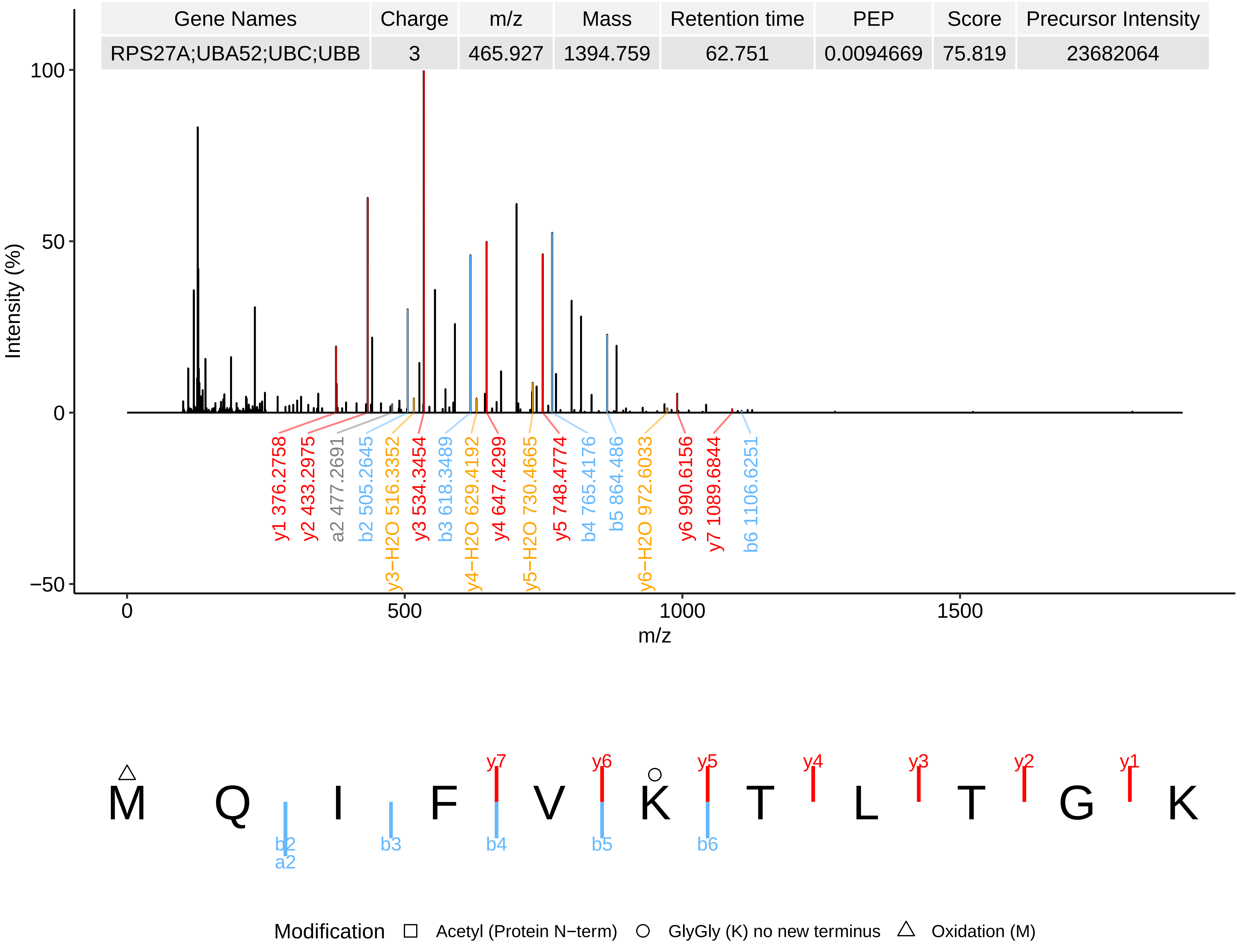

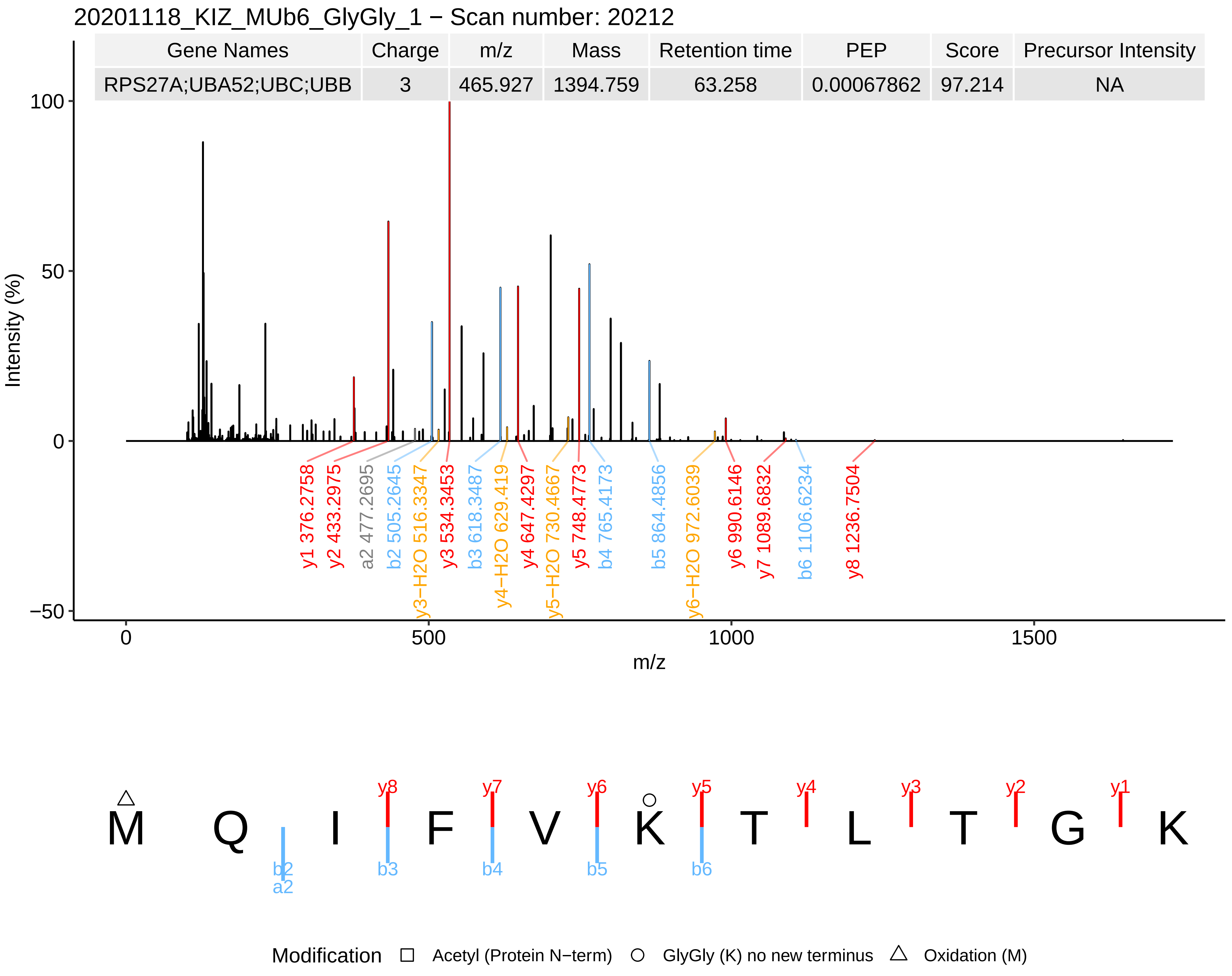

20201118_KIZ_MUb6_GlyGly_1 − Scan number: 20212
Gene Names
Charge
m/z
Mass
Retention time
PEP
Score
Precursor Intensity
RPS27A;UBA52;UBC;UBB
3
465.927
1394.759
63.258
0.00067862
97.214
NA
Intensity (%)
m/z
y1 376.2758
y2 433.2975
a2 477.2695
b2 505.2645
y3−H2O 516.3347
y3 534.3453
b3 618.3487
y4−H2O 629.419
y4 647.4297
y5−H2O 730.4667
y5 748.4773
b4 765.4173
b5 864.4856
y6−H2O 972.6039
y6 990.6146
y7 1089.6832
b6 1106.6234
y8 1236.7504
M Q I F V K T L T G K
Modification
Acetyl (Protein N−term)
GlyGly (K) no new terminus
Oxidation (M)

20201118_KIZ_MUb6_GlyGly_1 − Scan number: 20395

Gene Names	Charge	m/z	Mass	Retention time	PEP	Score	Precursor Intensity
RPS27A;UBA52;UBC;UBB	3	465.927	1394.759	63.801	0.0027692	80.979	19274410

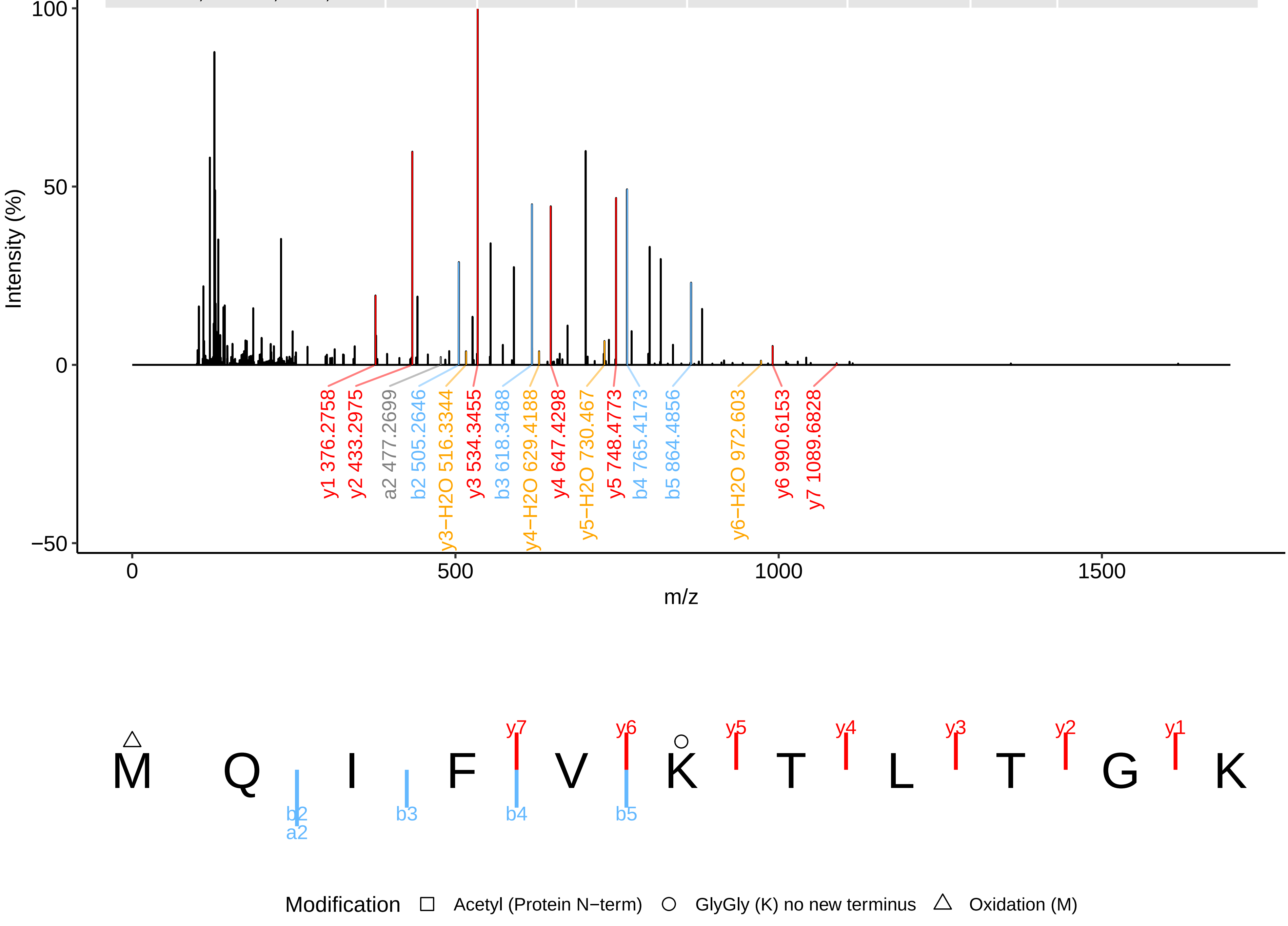

20201118_KIZ_MUb6_GlyGly_2 − Scan number: 21053

Gene Names	Charge	m/z	Mass	Retention time	PEP	Score	Precursor Intensity
RPS27A;UBA52;UBC;UBB	3	465.927	1394.759	70.932	0.0011866	83.081	11380577

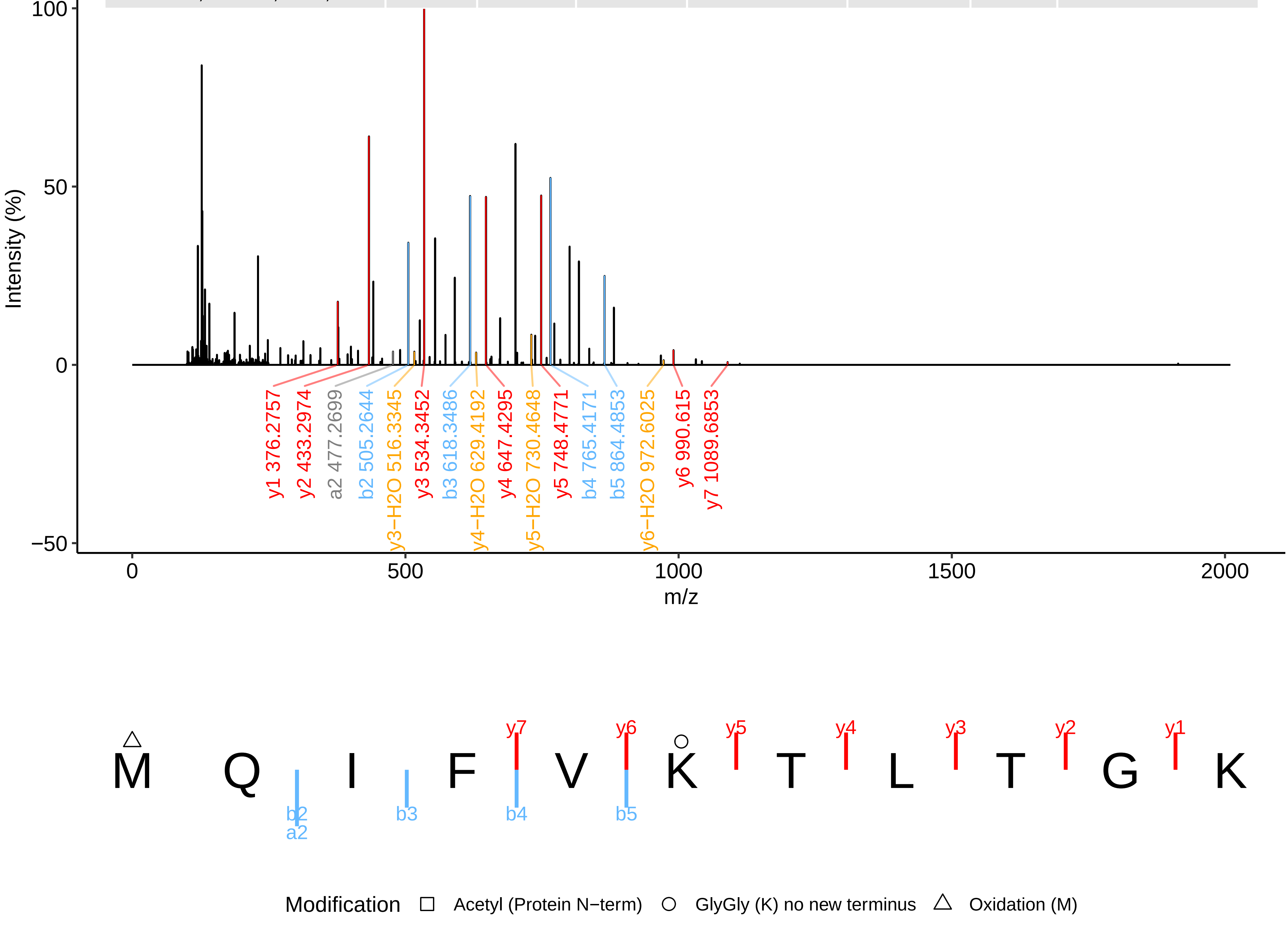

20201118_KIZ_MUb6_GlyGly_2 − Scan number: 21224

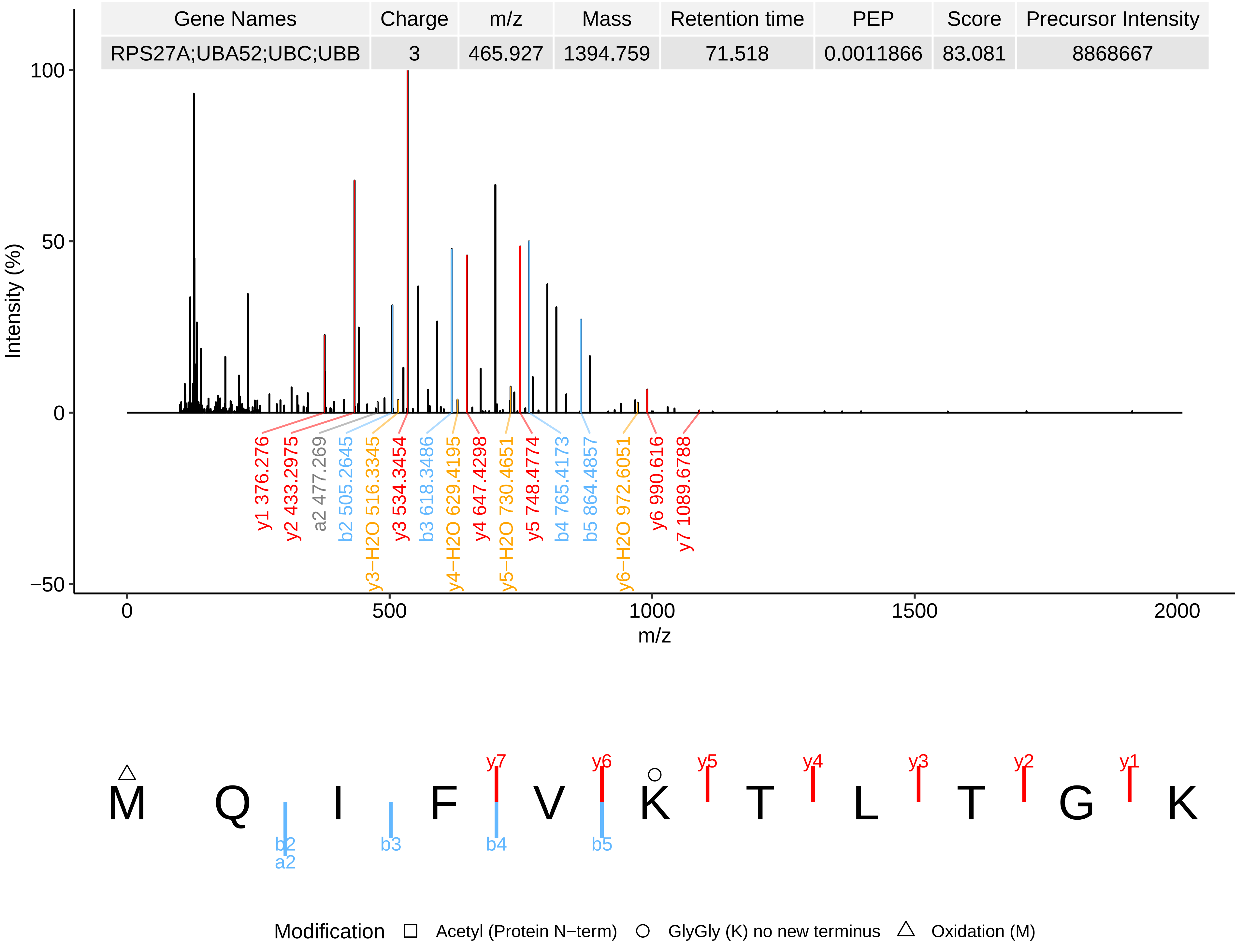

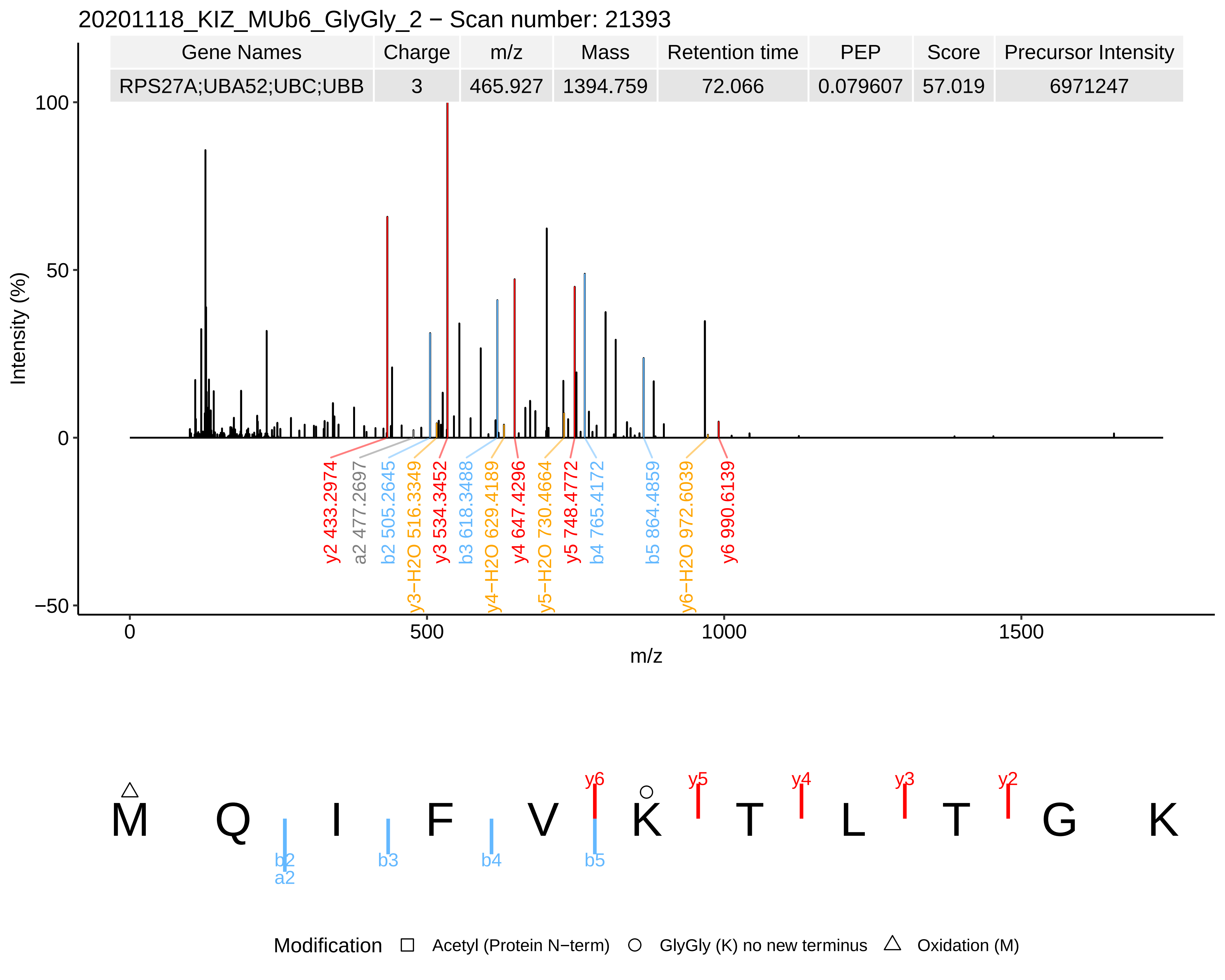
20201118_KIZ_MUb6_GlyGly_2 − Scan number: 21393
Gene Names | Charge | m/z | Mass | Retention time | PEP | Score | Precursor Intensity
RPS27A;UBA52;UBC;UBB | 3 | 465.927 | 1394.759 | 72.066 | 0.079607 | 57.019 | 6971247
Intensity (%)
m/z
y2 433.2974
a2 477.2697
b2 505.2645
y3−H2O 516.3349
y3 534.3452
b3 618.3488
y4−H2O 629.4189
y4 647.4296
y5−H2O 730.4664
y5 748.4772
b4 765.4172
b5 864.4859
y6−H2O 972.6039
y6 990.6139
M Q I F V K T L T G K
y6 y5 y4 y3 y2
b2 a2 b3 b4 b5
Modification
Acetyl (Protein N−term)
GlyGly (K) no new terminus
Oxidation (M)

20200921_KIZ_MUb4_GlyGly_10plex − Scan number: 9217

Gene Names	Charge	m/z	Mass	Retention time	PEP	Score	Precursor Intensity
ADRM1	3	469.9098	1406.707	31.539	0.094058	42.426	4868916

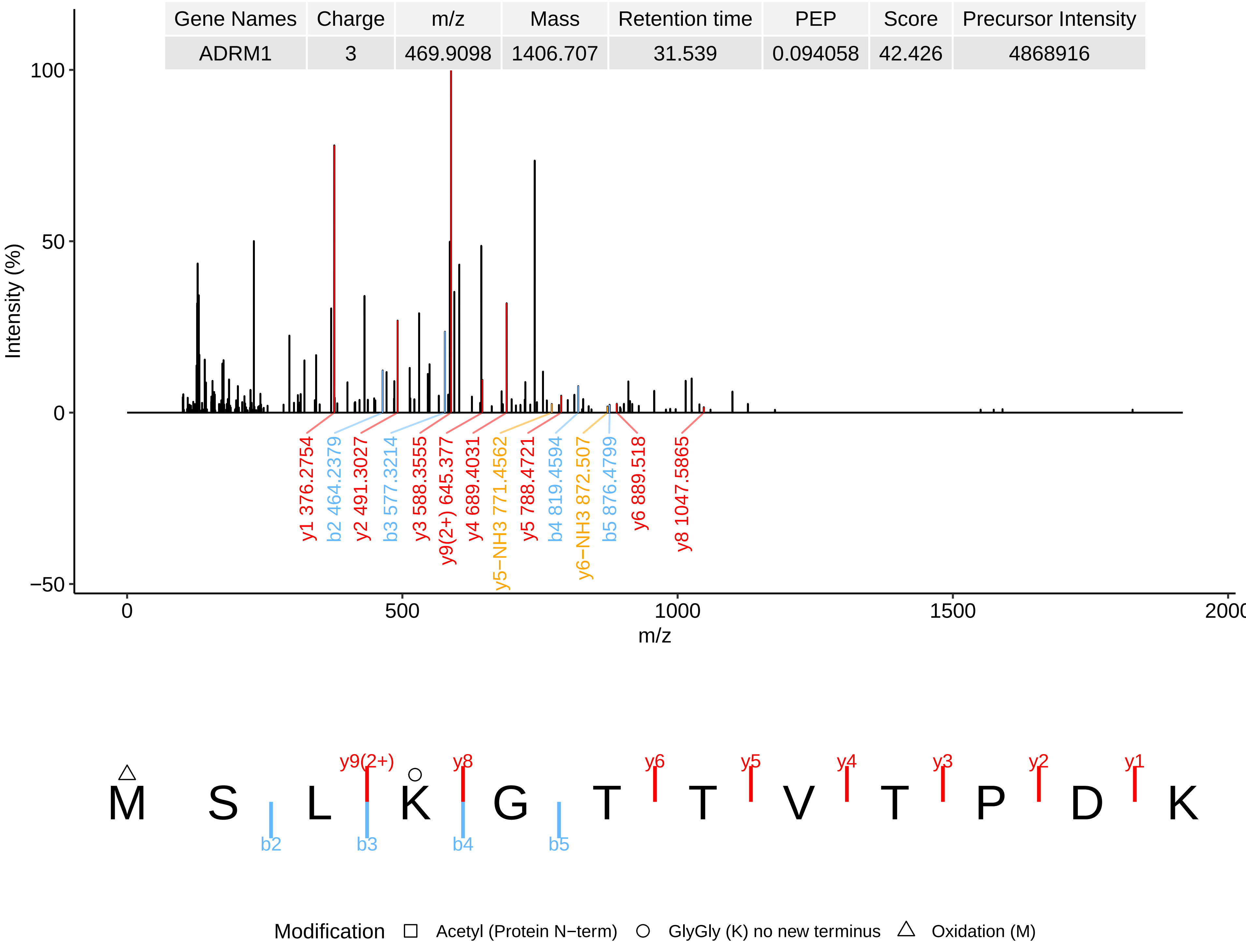

20200921_KIZ_MUb4_GlyGly_10plex_2 − Scan number: 8374

Gene Names	Charge	m/z	Mass	Retention time	PEP	Score	Precursor Intensity
ADRM1	3	469.9098	1406.707	31.529	0.082445	45.79	1767355

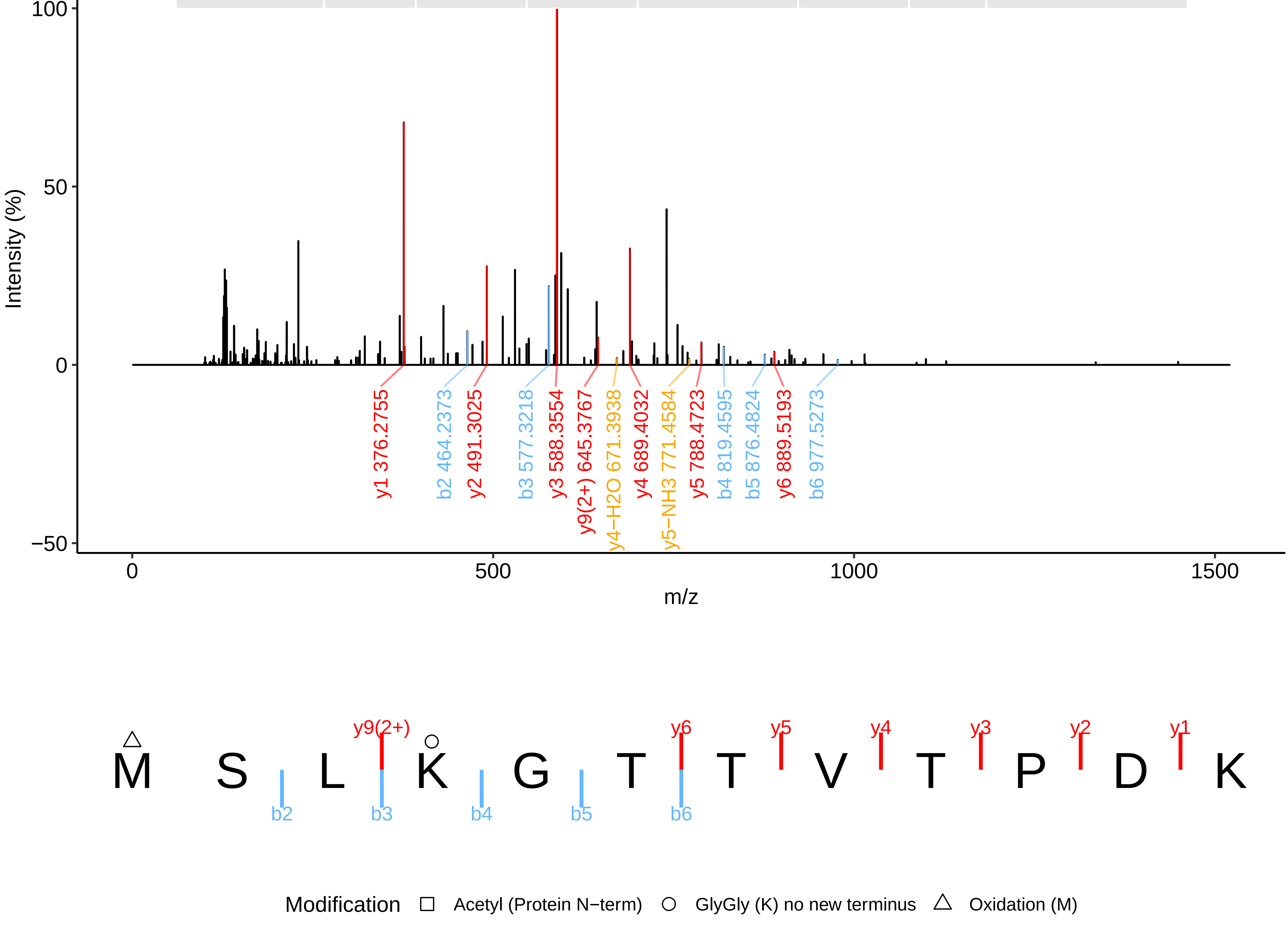

20200921_KIZ_MUb4_GlyGly_10plex_2 − Scan number: 11969

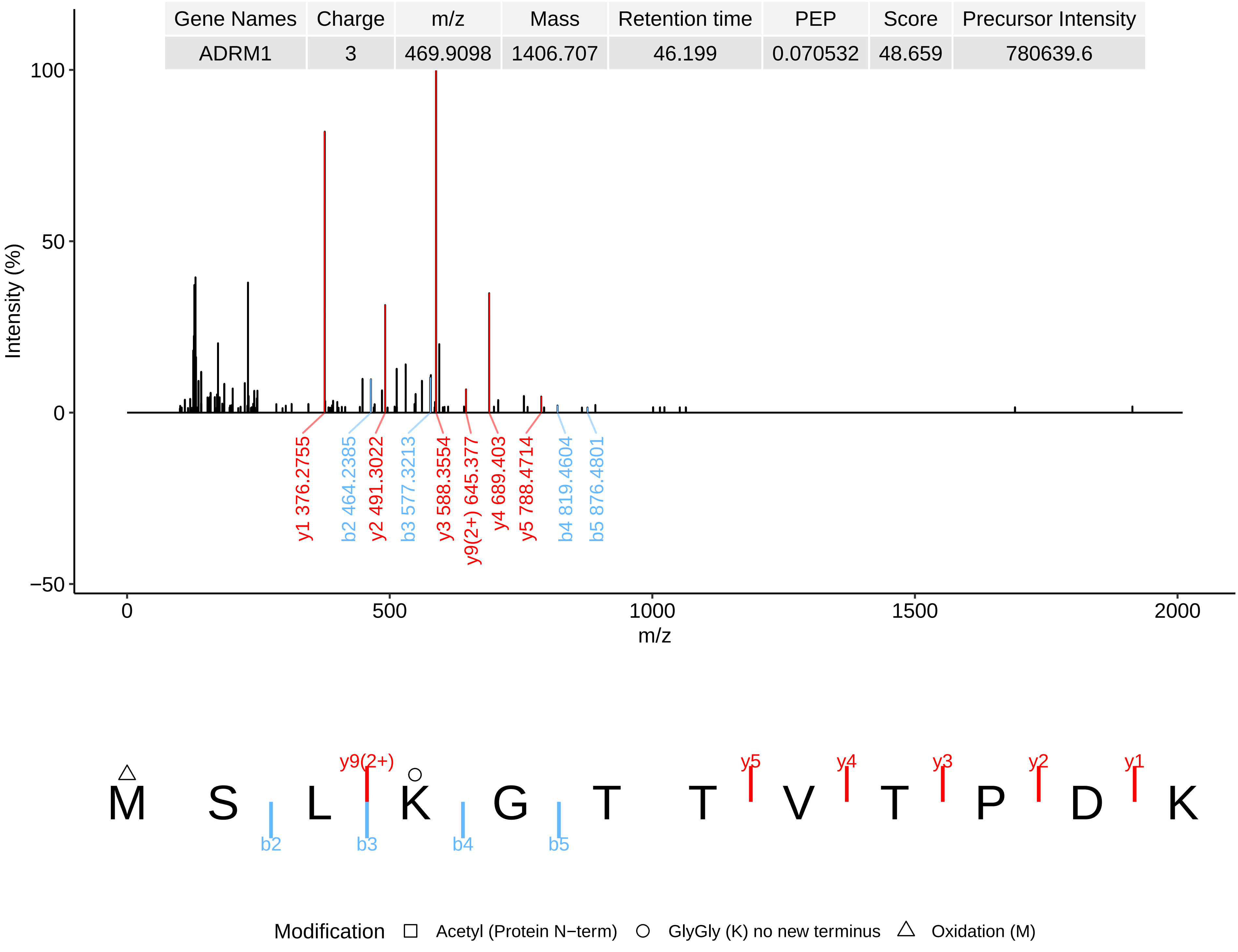

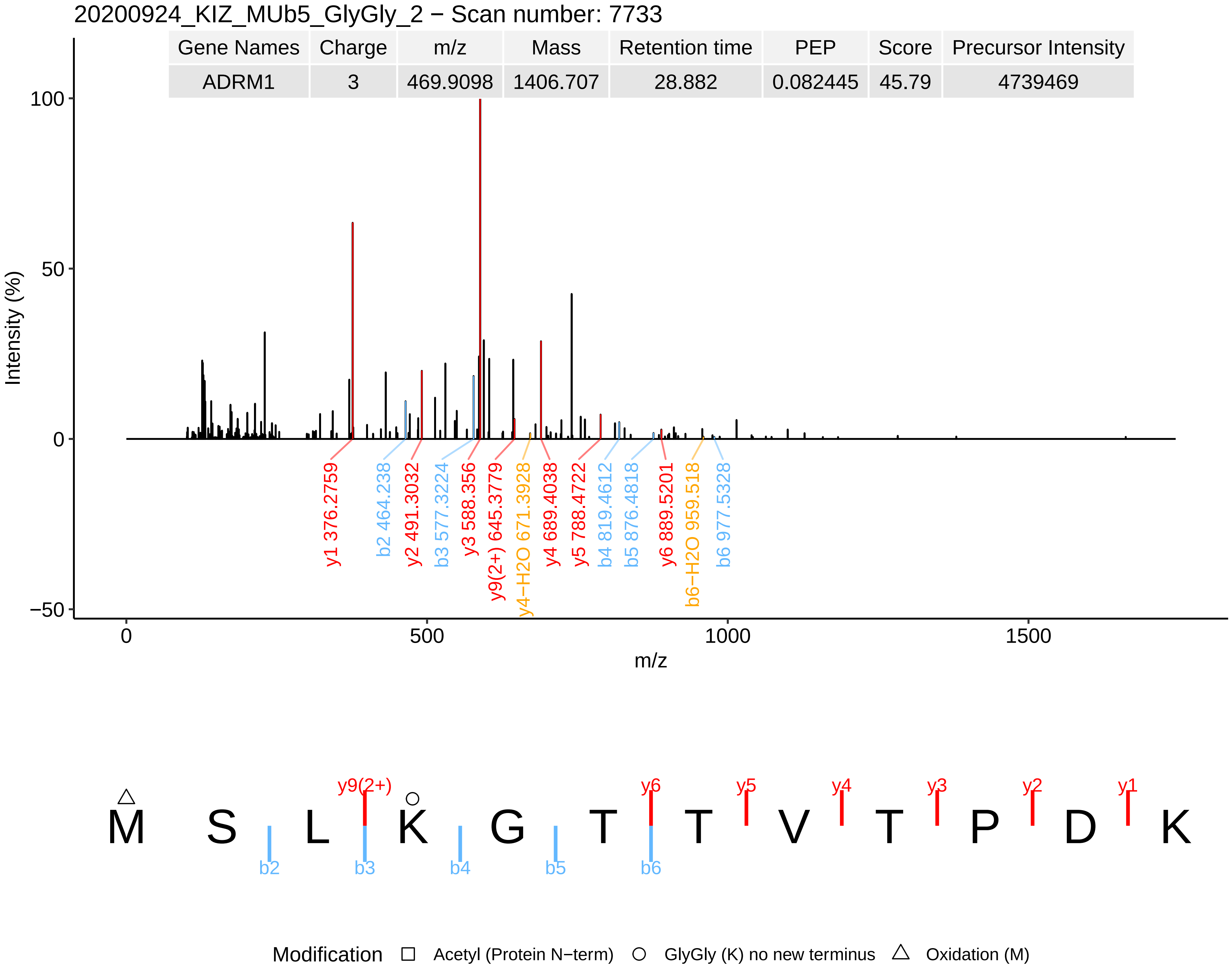
20200924_KIZ_MUb5_GlyGly_2 − Scan number: 7733
Gene Names | Charge | m/z | Mass | Retention time | PEP | Score | Precursor Intensity
ADRM1 | 3 | 469.9098 | 1406.707 | 28.882 | 0.082445 | 45.79 | 4739469
Intensity (%)
m/z
y1 376.2759
b2 464.238
y2 491.3032
b3 577.3224
y3 588.356
y9(2+) 645.3779
y4−H2O 671.3928
y4 689.4038
y5 788.4722
b4 819.4612
b5 876.4818
y6 889.5201
b6−H2O 959.518
b6 977.5328
M S L K G T T V T P D K
y9(2+) y6 y5 y4 y3 y2 y1
b2 b3 b4 b5 b6
Modification
Acetyl (Protein N−term)
GlyGly (K) no new terminus
Oxidation (M)

20201118_KIZ_MUb6_GlyGly_1 − Scan number: 12093

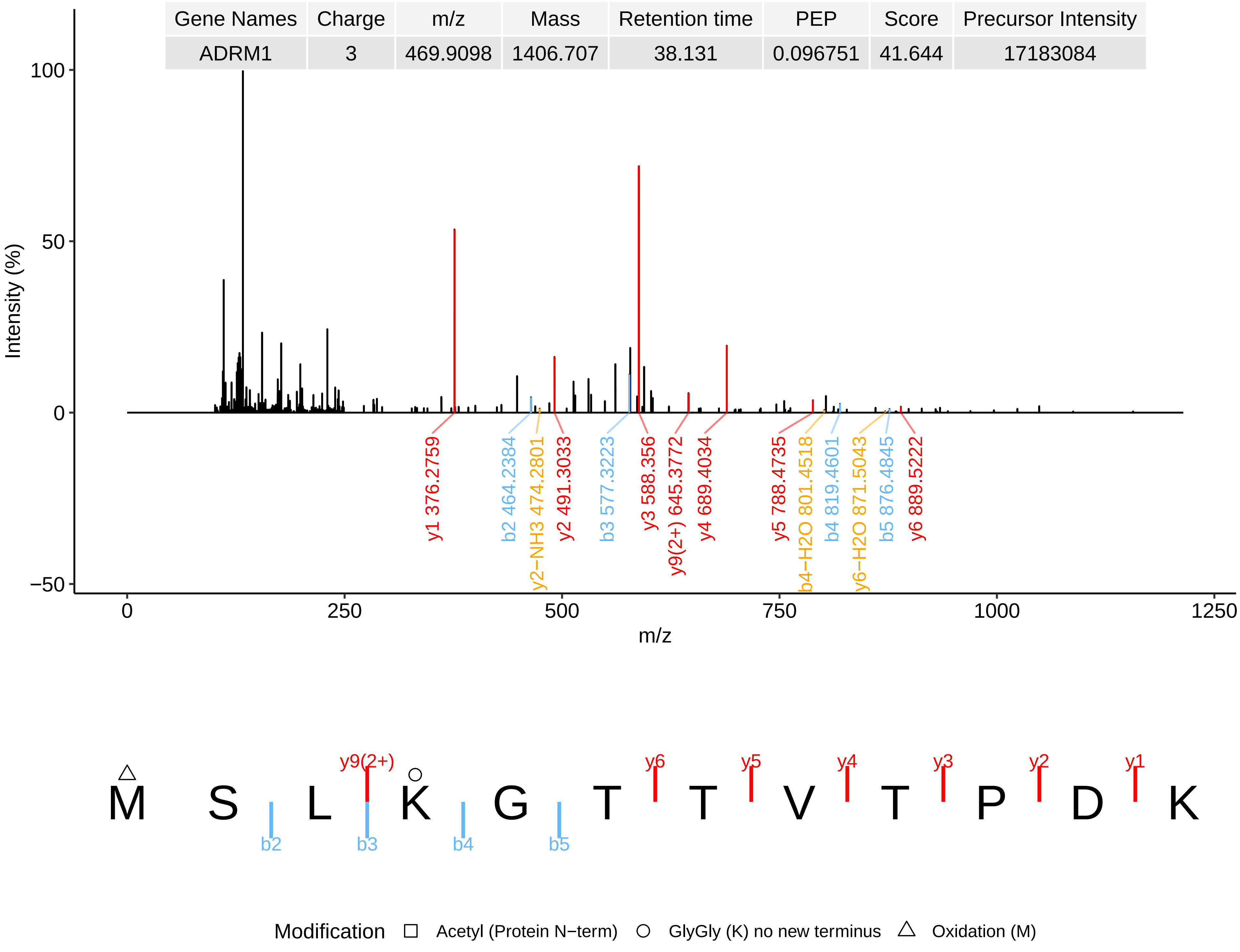

20200921_KIZ_MUb4_GlyGly_10plex − Scan number: 12992

Gene Names	Charge	m/z	Mass	Retention time	PEP	Score	Precursor Intensity
ADRM1	3	464.5781	1390.713	45.716	0.00052285	90.697	27905450

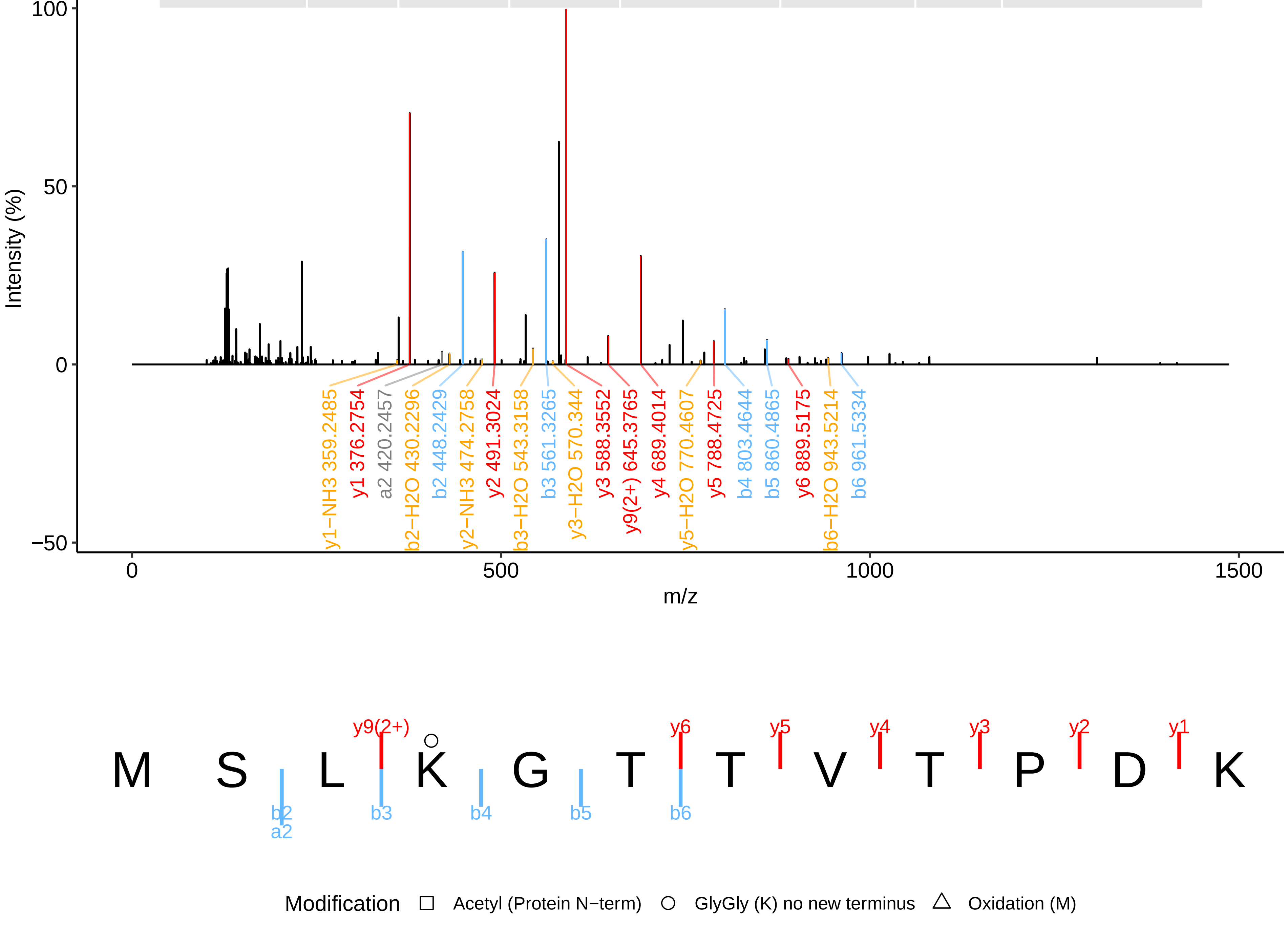

20200921_KIZ_MUb4_GlyGly_10plex − Scan number: 13005

Gene Names	Charge	m/z	Mass	Retention time	PEP	Score	Precursor Intensity
ADRM1	3	464.5781	1390.713	45.756	0.00097176	84.169	31438608

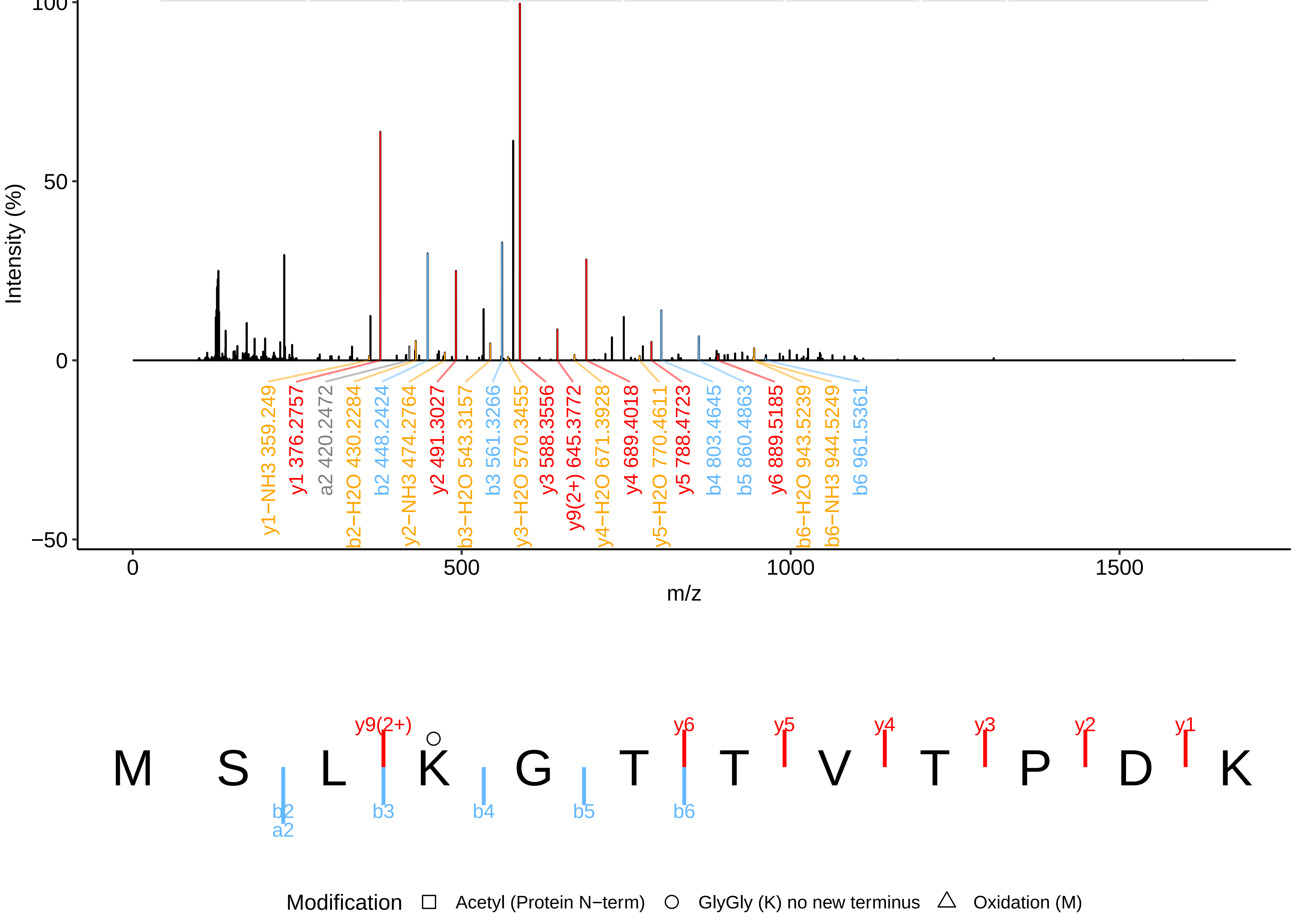

20200921_KIZ_MUb4_GlyGly_10plex_2 − Scan number: 11929

Gene Names	Charge	m/z	Mass	Retention time	PEP	Score	Precursor Intensity
ADRM1	3	464.5781	1390.713	46.037	0.088296	48.659	445904.6

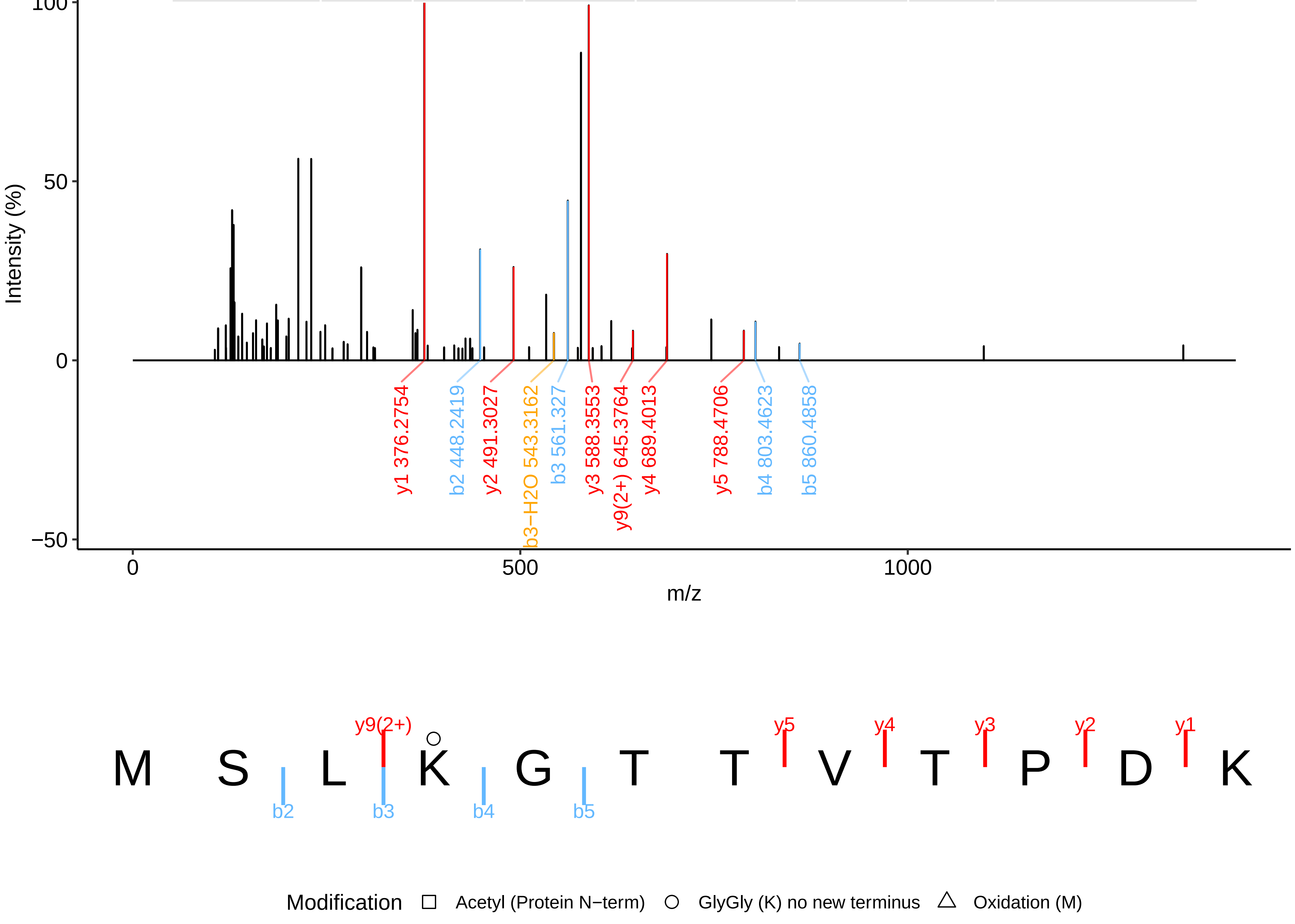

20200924_KIZ_MUb5_GlyGly_1 − Scan number: 11963

Gene Names	Charge	m/z	Mass	Retention time	PEP	Score	Precursor Intensity
ADRM1	3	464.5781	1390.713	38.859	0.00069712	88.163	8792021

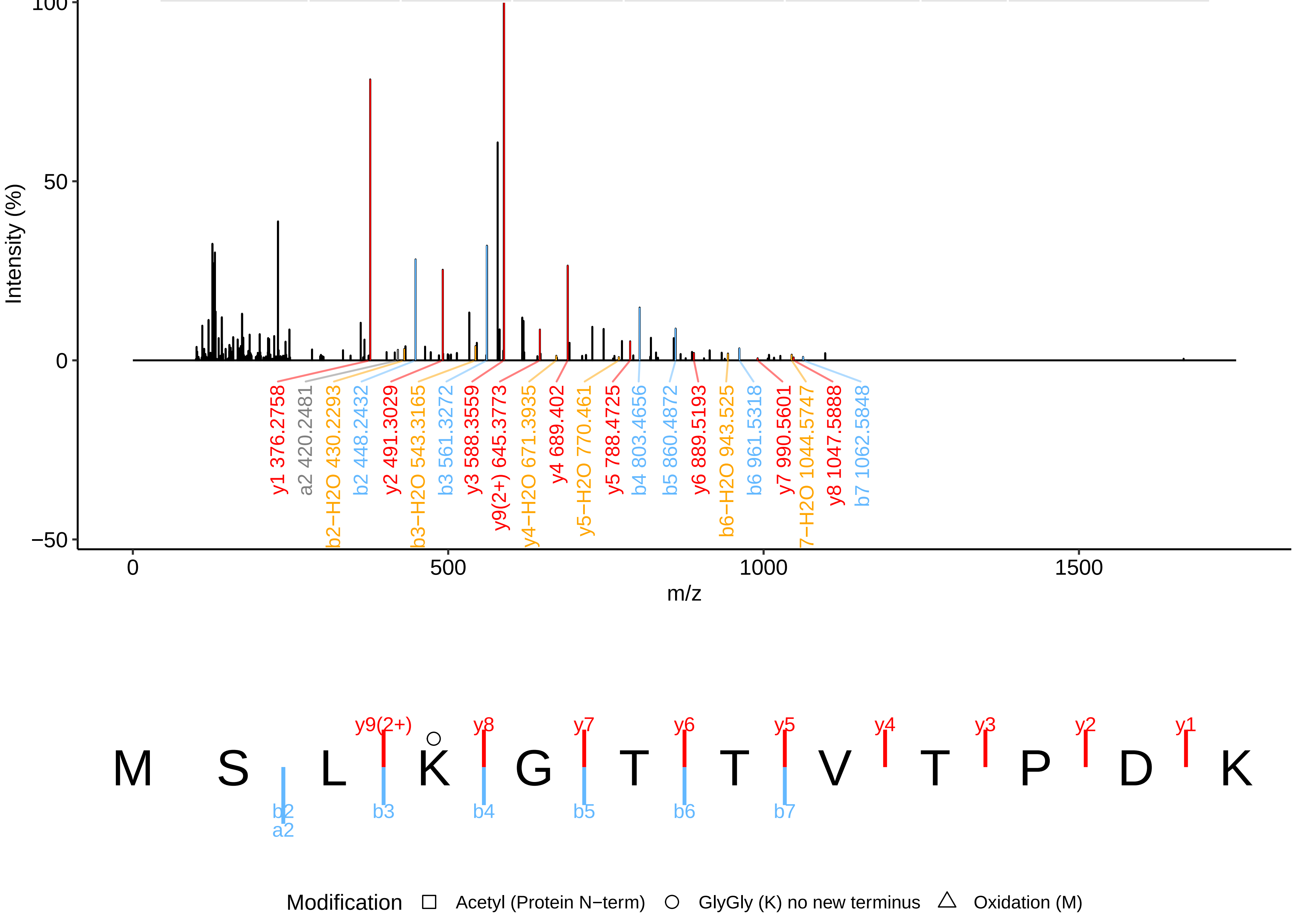

20200924_KIZ_MUb5_GlyGly_1 − Scan number: 11999

Gene Names	Charge	m/z	Mass	Retention time	PEP	Score	Precursor Intensity
ADRM1	3	464.5781	1390.713	38.976	1.7936e−05	100.38	22298978

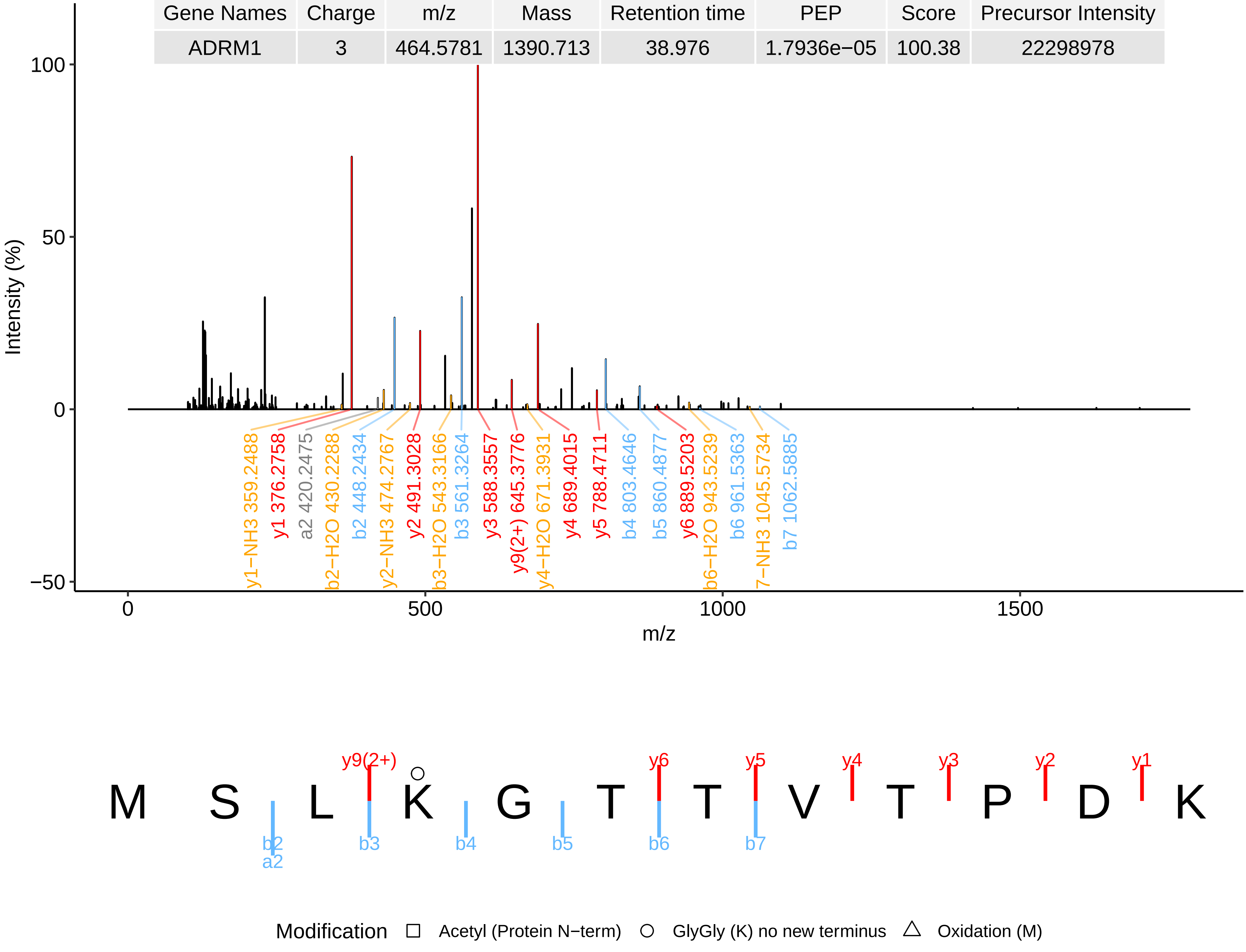

20200924_KIZ_MUb5_GlyGly_1 − Scan number: 12148

Gene Names	Charge	m/z	Mass	Retention time	PEP	Score	Precursor Intensity
ADRM1	3	464.5781	1390.713	39.479	5.1265e−06	111.12	153213808

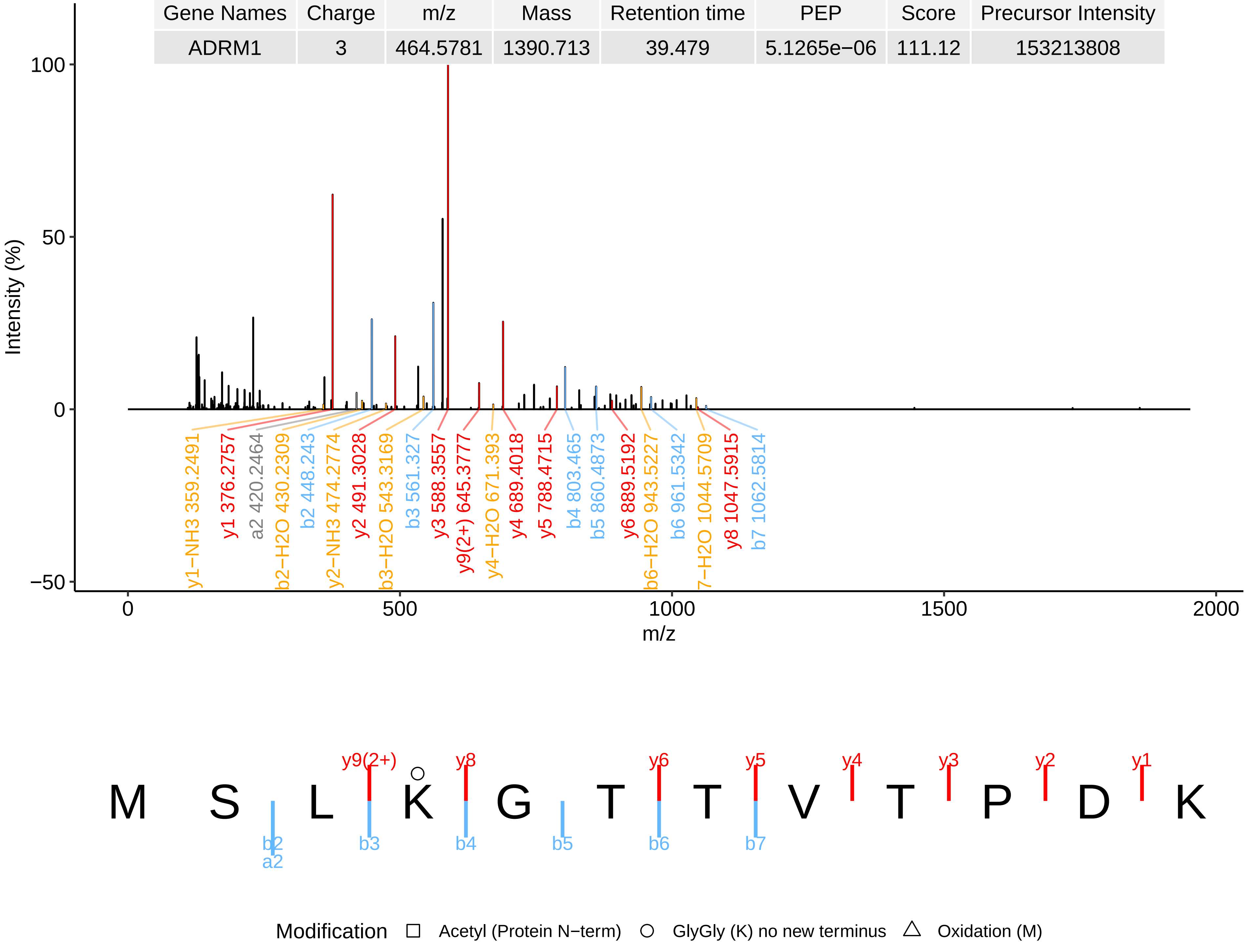

20200924_KIZ_MUb5_GlyGly_1 − Scan number: 12300

Gene Names	Charge	m/z	Mass	Retention time	PEP	Score	Precursor Intensity
ADRM1	3	464.5781	1390.713	40.003	5.759e−05	97.463	34612664

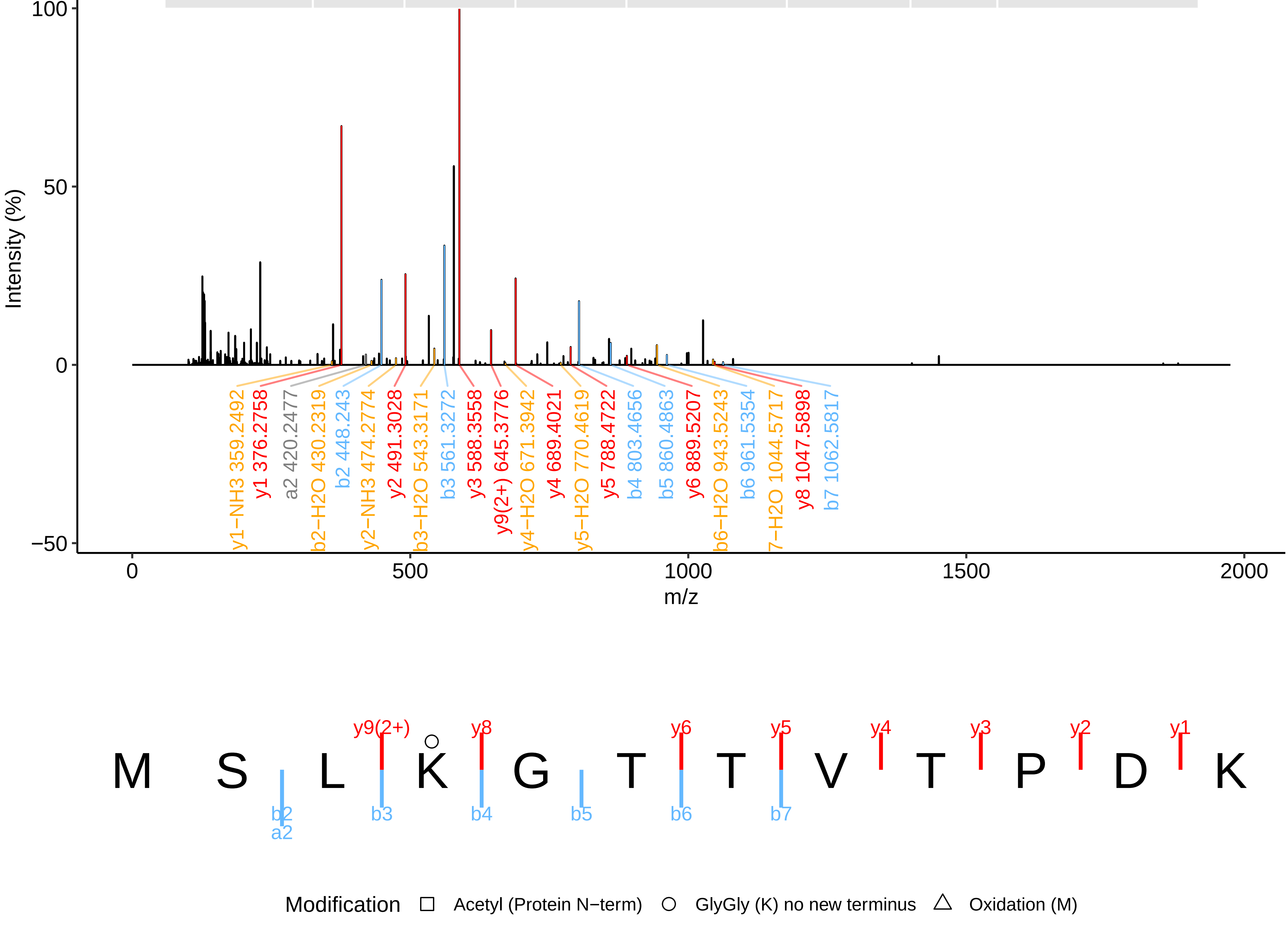

20200924_KIZ_MUb5_GlyGly_2 − Scan number: 11058

Gene Names	Charge	m/z	Mass	Retention time	PEP	Score	Precursor Intensity
ADRM1	3	464.5781	1390.713	41.301	0.0059797	73.466	4403396

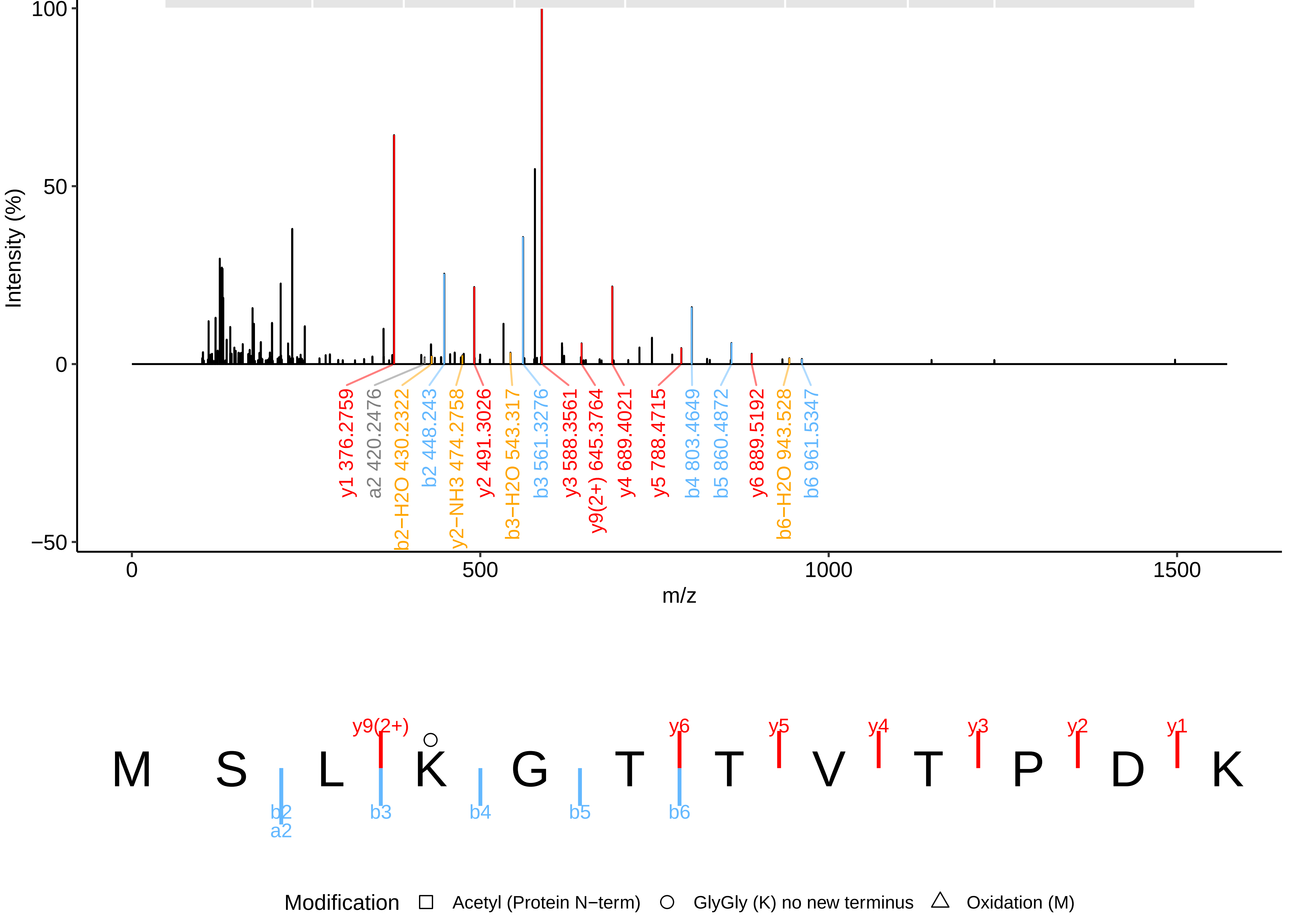

20200924_KIZ_MUb5_GlyGly_2 − Scan number: 11095

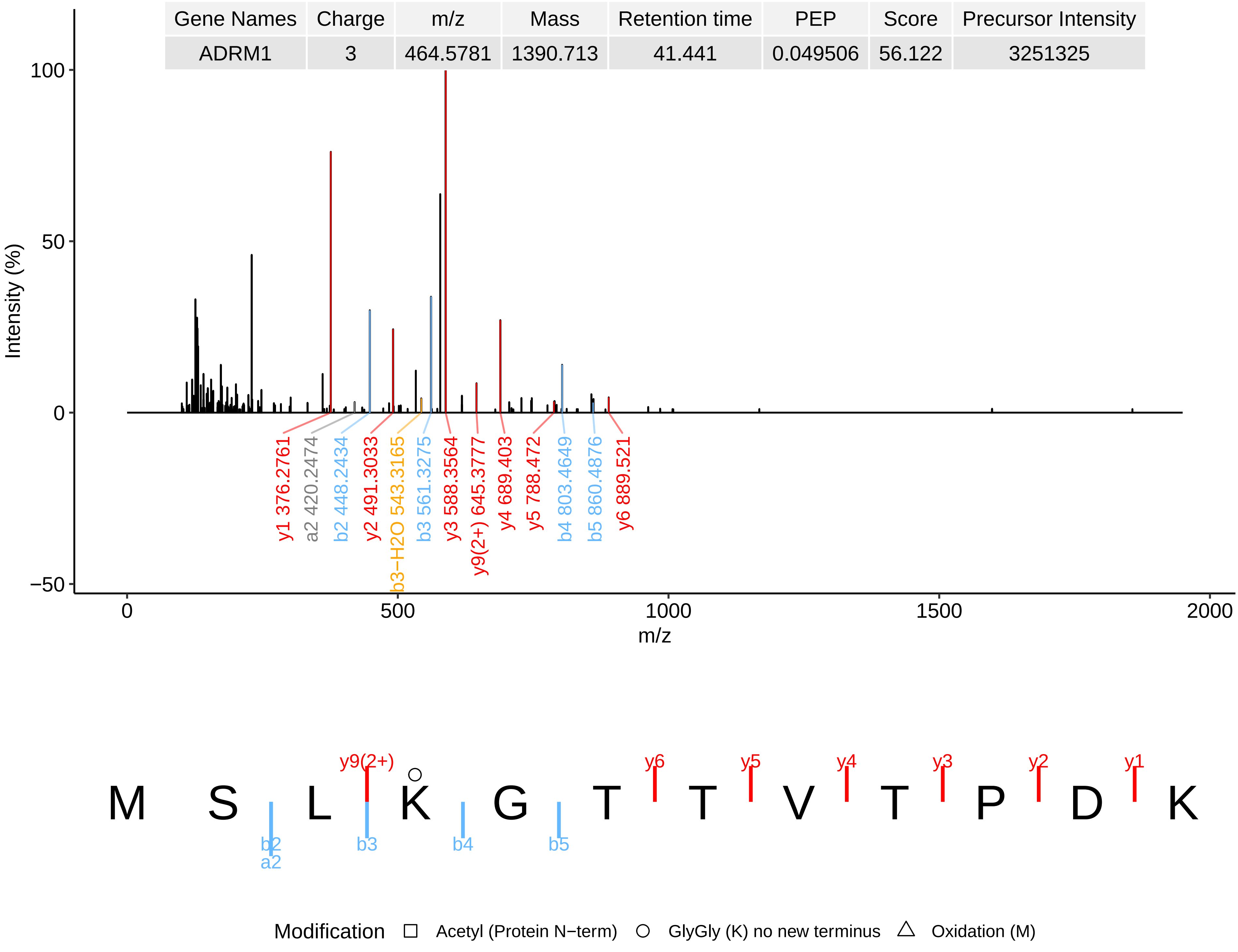

20200924_KIZ_MUb5_GlyGly_2 − Scan number: 11229

Gene Names	Charge	m/z	Mass	Retention time	PEP	Score	Precursor Intensity
ADRM1	3	464.5781	1390.713	41.944	1.4844e−05	102.97	27463864

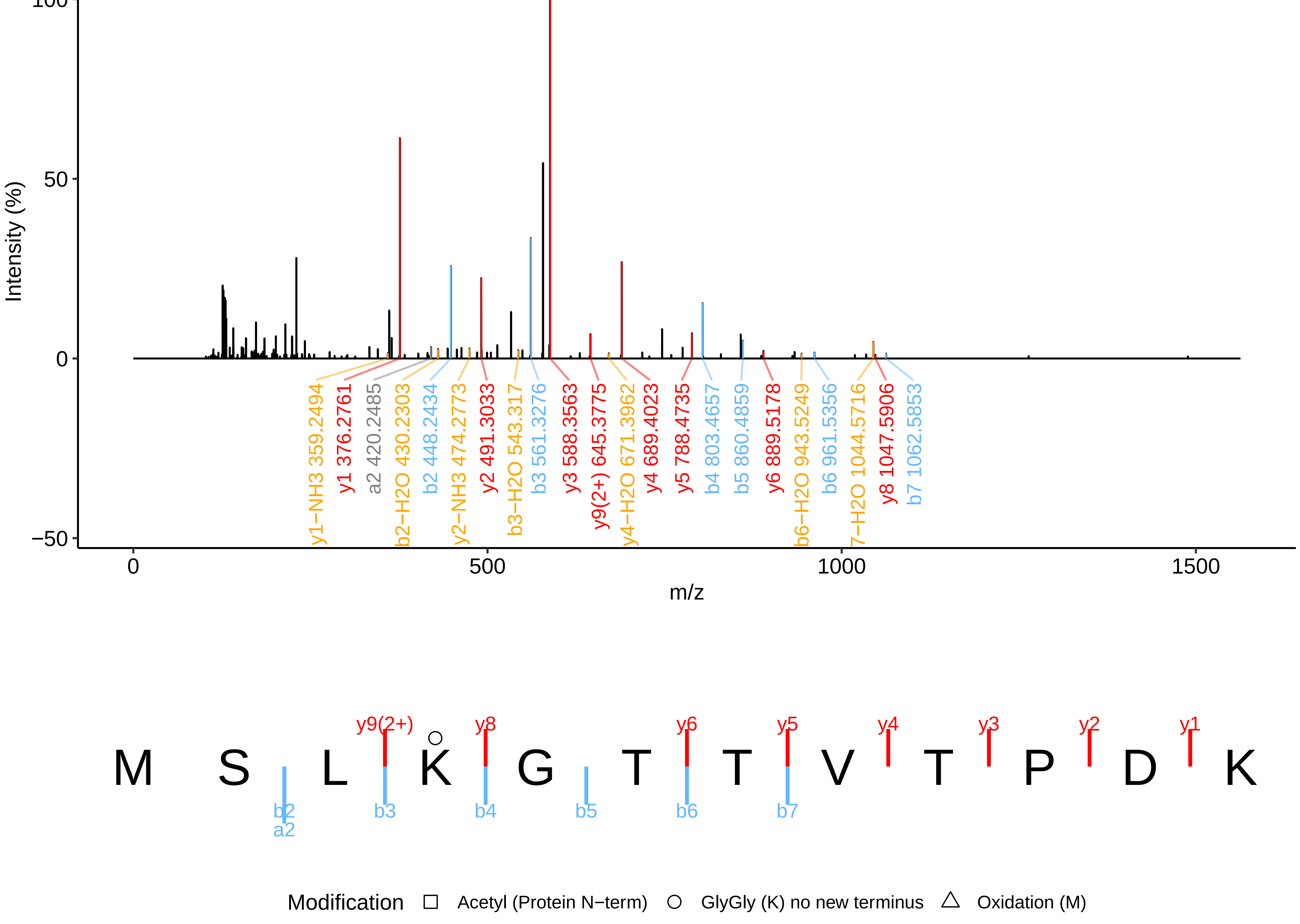

20200924_KIZ_MUb5_GlyGly_2 − Scan number: 11361

Gene Names	Charge	m/z	Mass	Retention time	PEP	Score	Precursor Intensity
ADRM1	3	464.5781	1390.713	42.456	5.759e−05	97.463	10477051

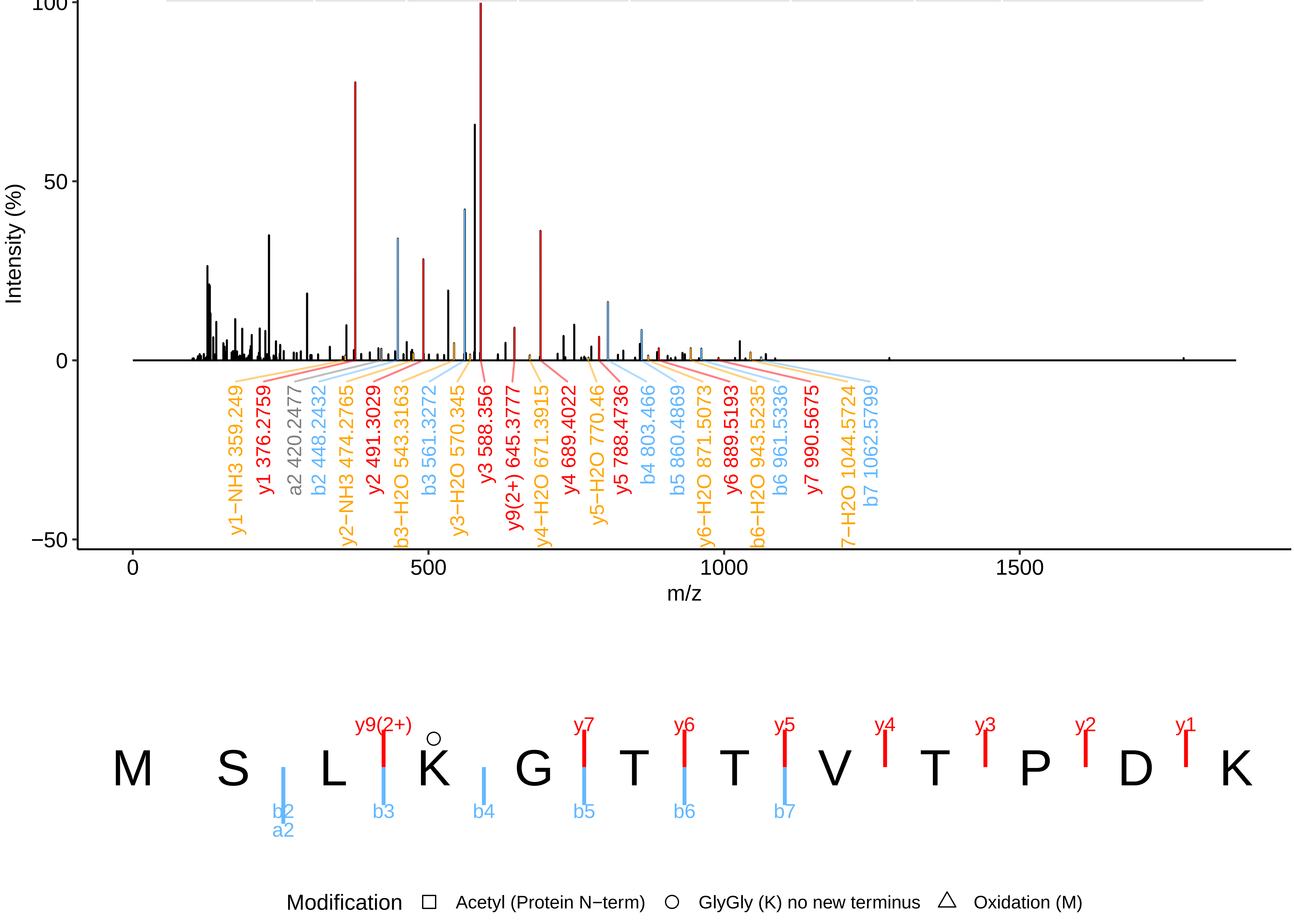

20200924_KIZ_MUb5_GlyGly_2 − Scan number: 11494

Gene Names	Charge	m/z	Mass	Retention time	PEP	Score	Precursor Intensity
ADRM1	3	464.5781	1390.713	42.969	0.0072628	71.548	2495207

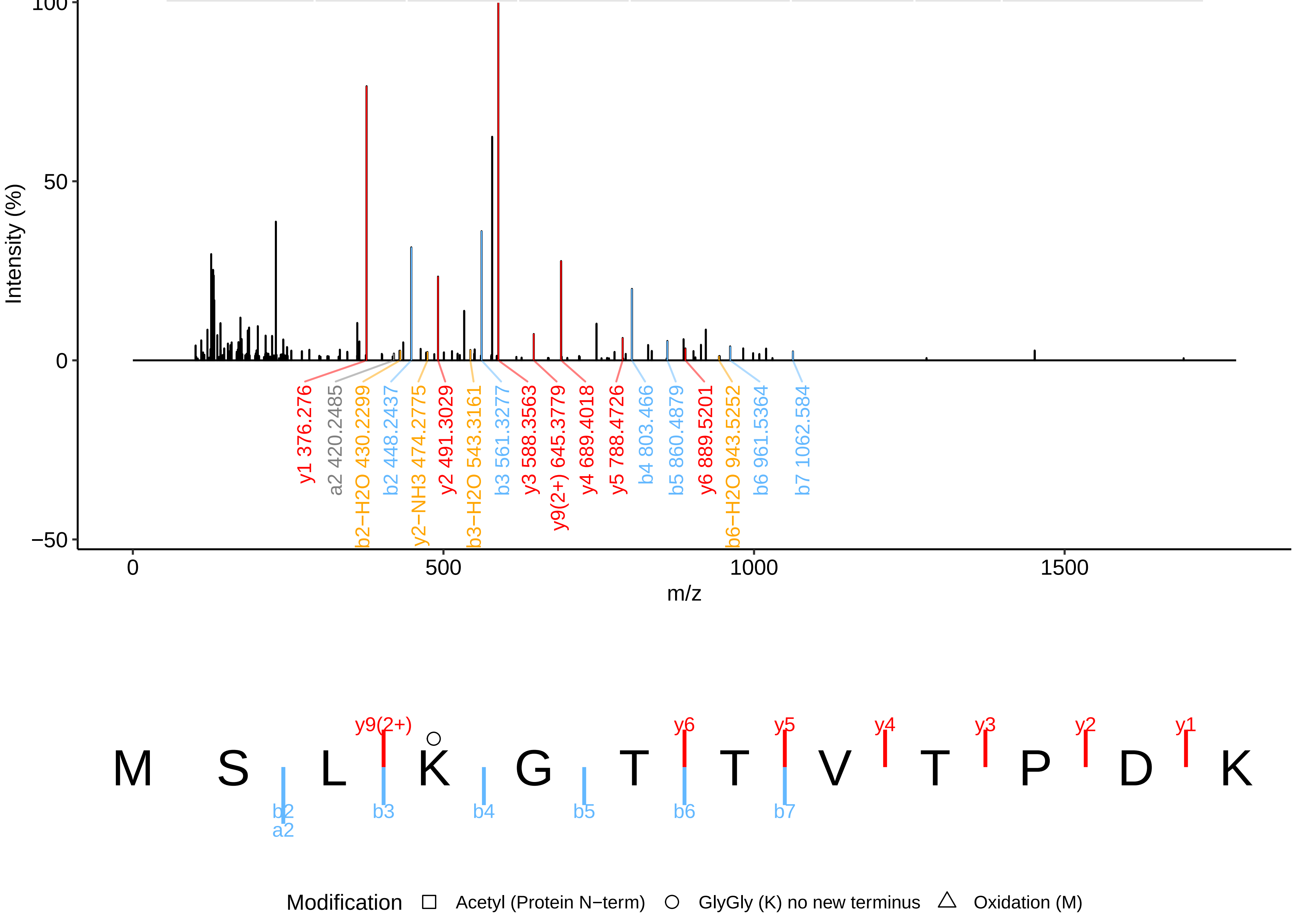

20201118_KIZ_MUb6_GlyGly_1 − Scan number: 12059

Gene Names	Charge	m/z	Mass	Retention time	PEP	Score	Precursor Intensity
ADRM1	3	464.5781	1390.713	38.025	1.857e−05	99.844	22928426

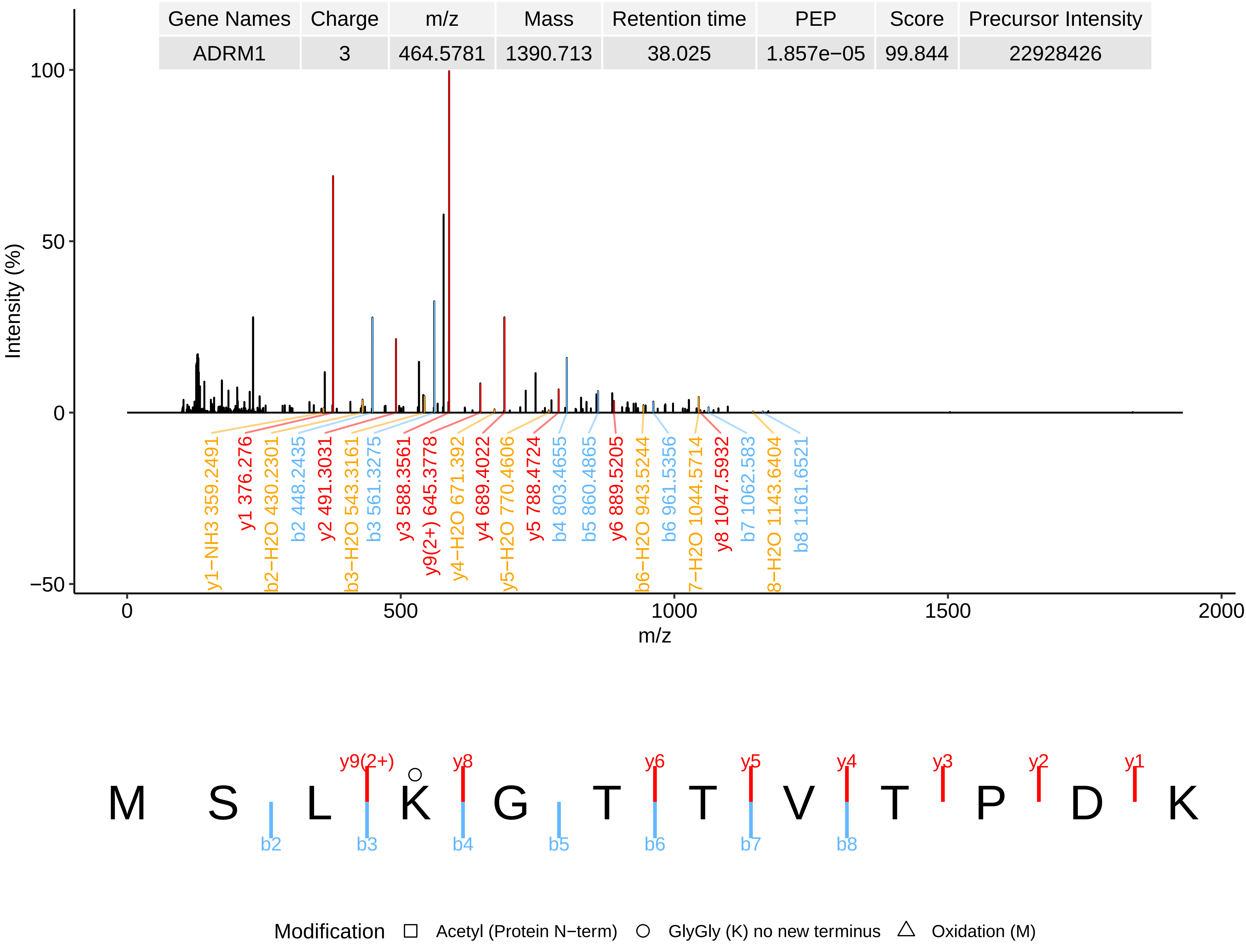

20201118_KIZ_MUb6_GlyGly_2 − Scan number: 13939

Gene Names	Charge	m/z	Mass	Retention time	PEP	Score	Precursor Intensity
ADRM1	3	464.5781	1390.713	46.944	1.4844e−05	102.97	23986316

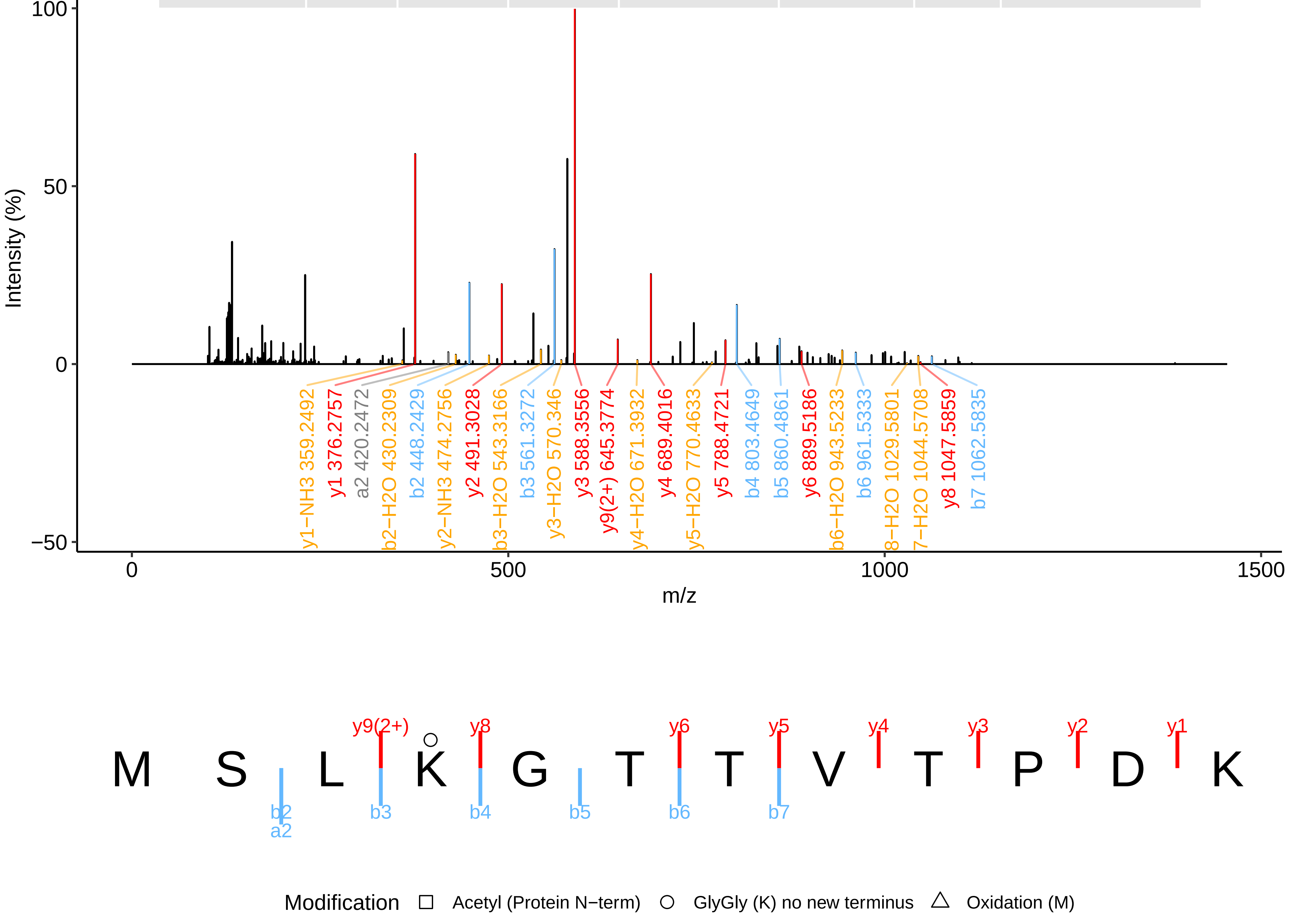

20200924_KIZ_MUb5_GlyGly_1 − Scan number: 22237

Gene Names	Charge	m/z	Mass	Retention time	PEP	Score	Precursor Intensity
C12orf60	3	480.5525	1438.636	71.556	0.09237	54.776	41473428

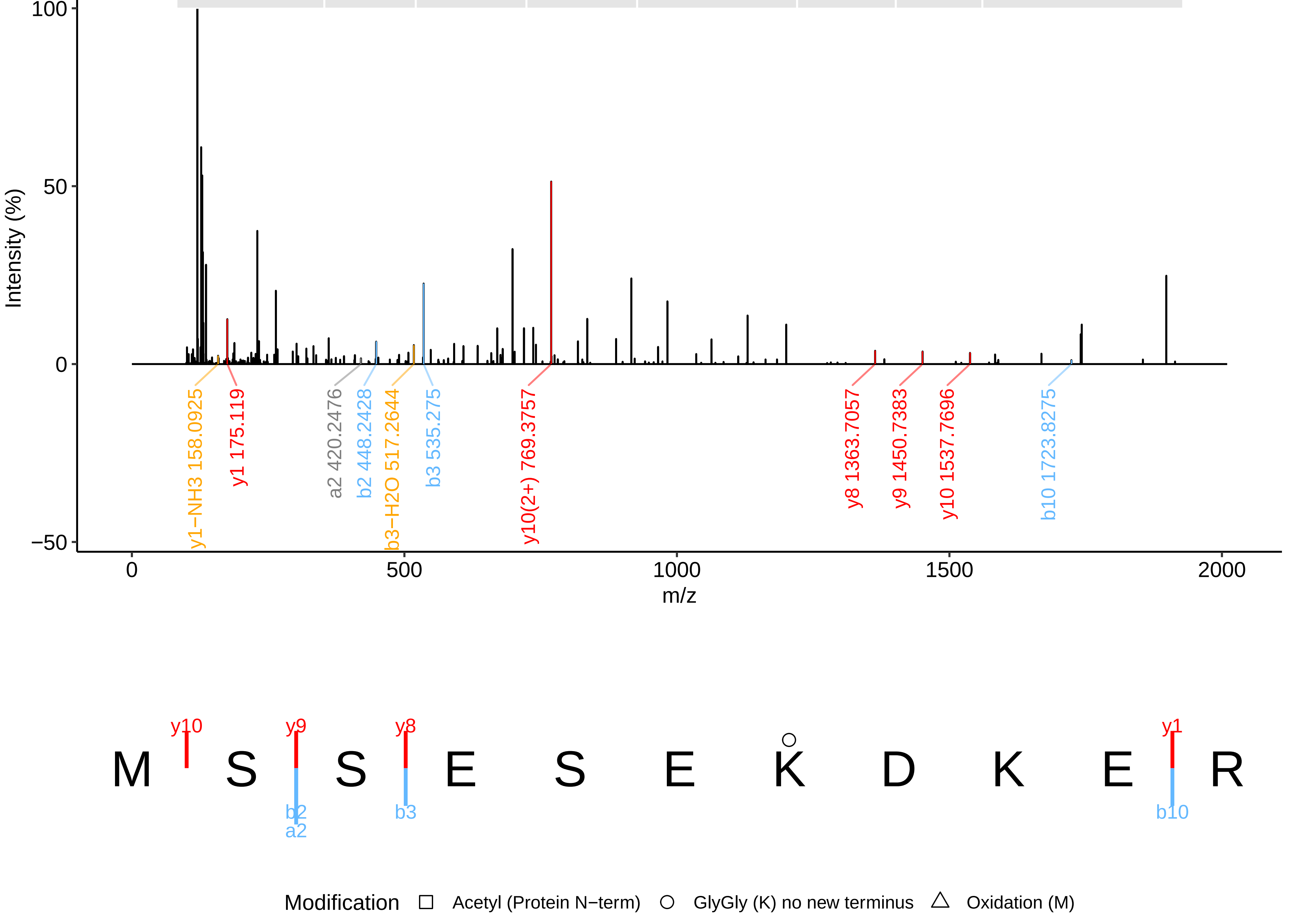

20200924_KIZ_MUb5_GlyGly_1 − Scan number: 22558

Gene Names	Charge	m/z	Mass	Retention time	PEP	Score	Precursor Intensity
C12orf60	3	480.5525	1438.636	72.573	0.081785	56.094	17818300

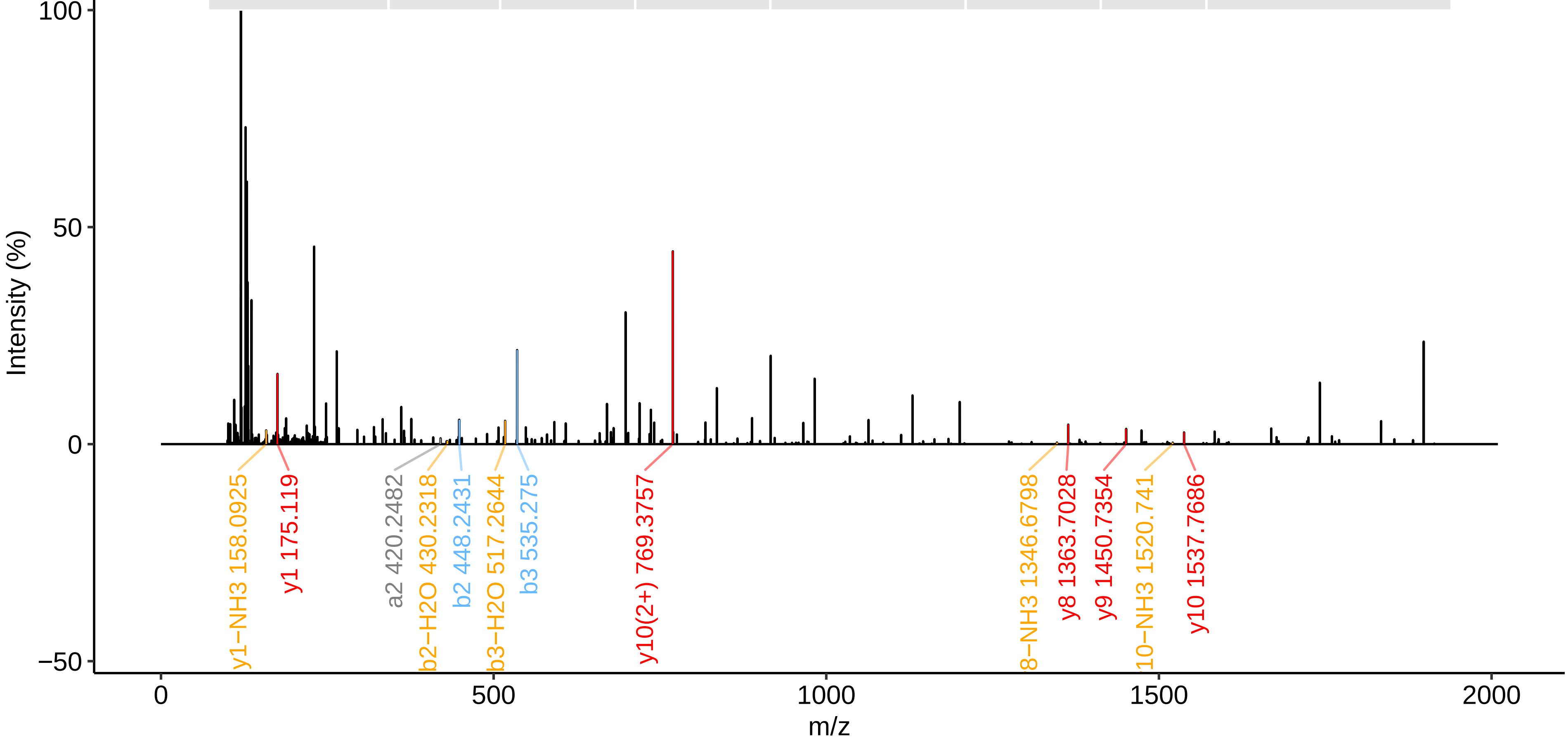

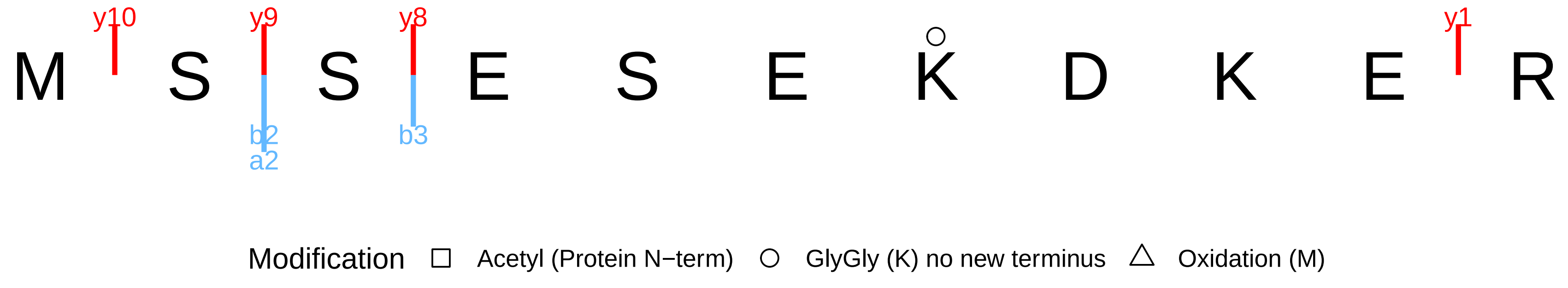

20200921_KIZ_MUb4_GlyGly_10plex − Scan number: 26368

Gene Names	Charge	m/z	Mass	Retention time	PEP	Score	Precursor Intensity
YWHAE	3	544.6458	1630.916	96.061	1.0146e−37	185.59	10508232

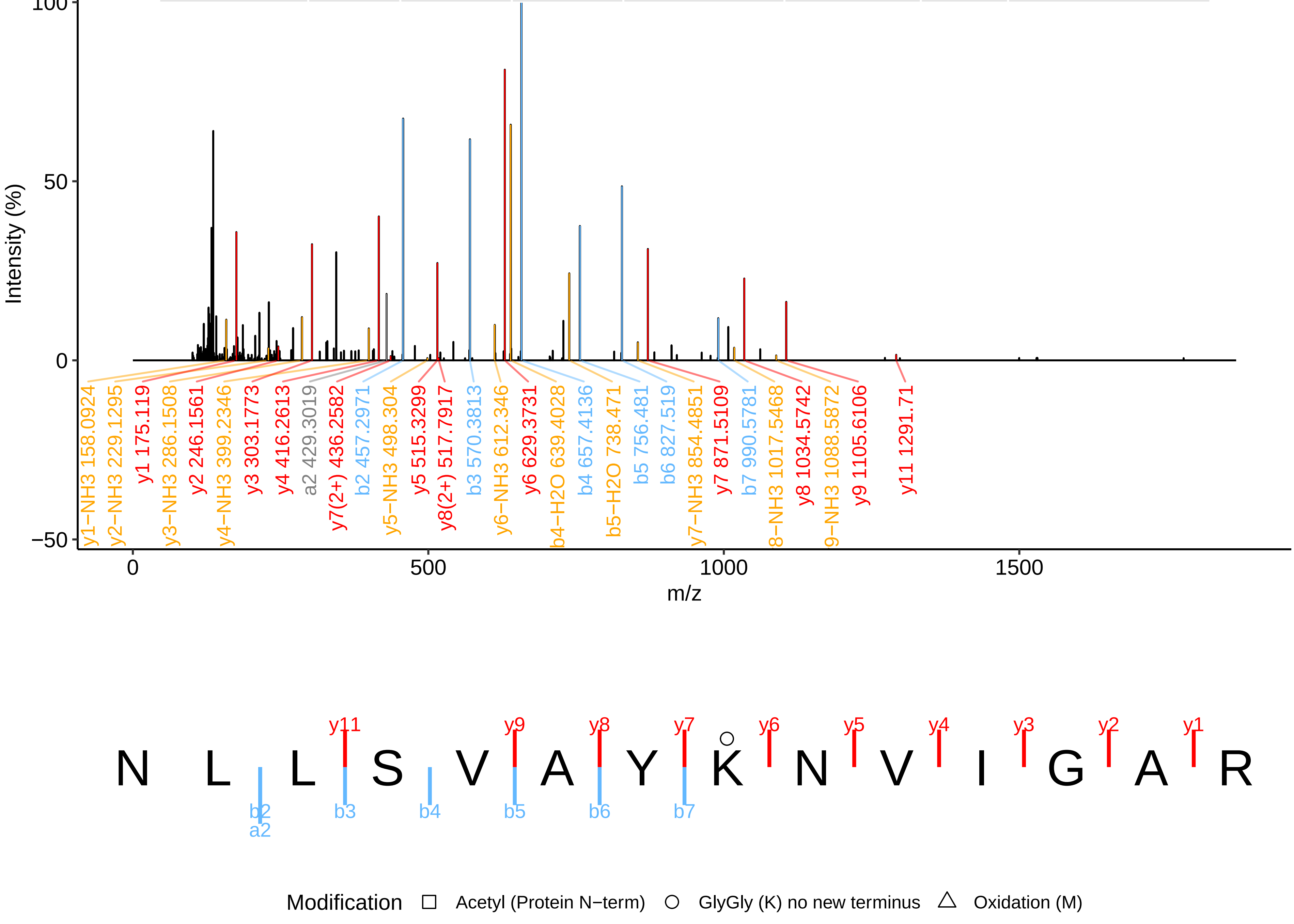

20200921_KIZ_MUb4_GlyGly_10plex_2 − Scan number: 22419

Gene Names	Charge	m/z	Mass	Retention time	PEP	Score	Precursor Intensity
YWHAE	3	544.6458	1630.916	88.373	1.3652e−19	151.78	883716.8

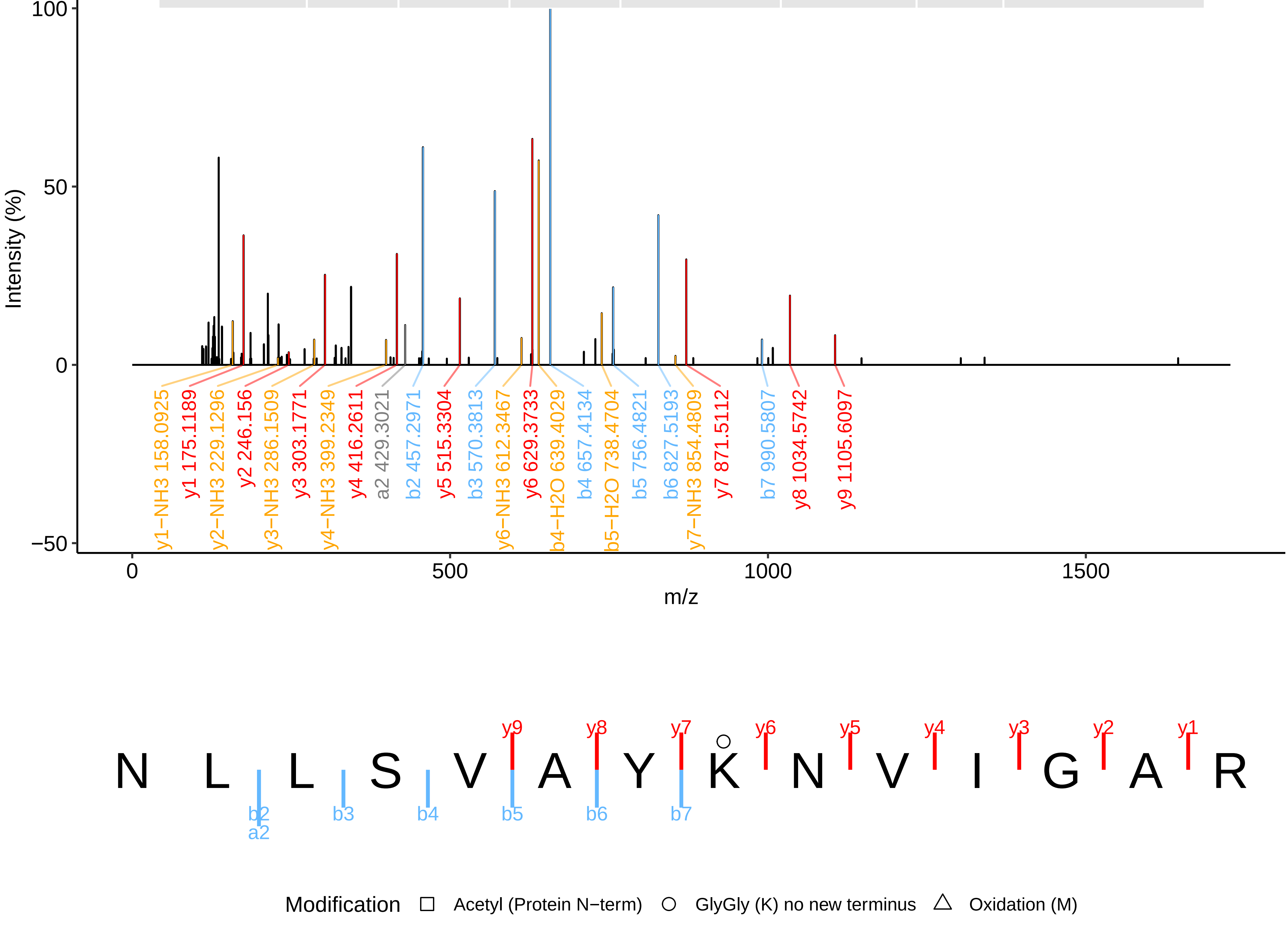

20201118_KIZ_MUb6_GlyGly_1 − Scan number: 25053

Gene Names	Charge	m/z	Mass	Retention time	PEP	Score	Precursor Intensity
YWHAE	3	544.6458	1630.916	76.905	1.1118e−13	148.68	20493218

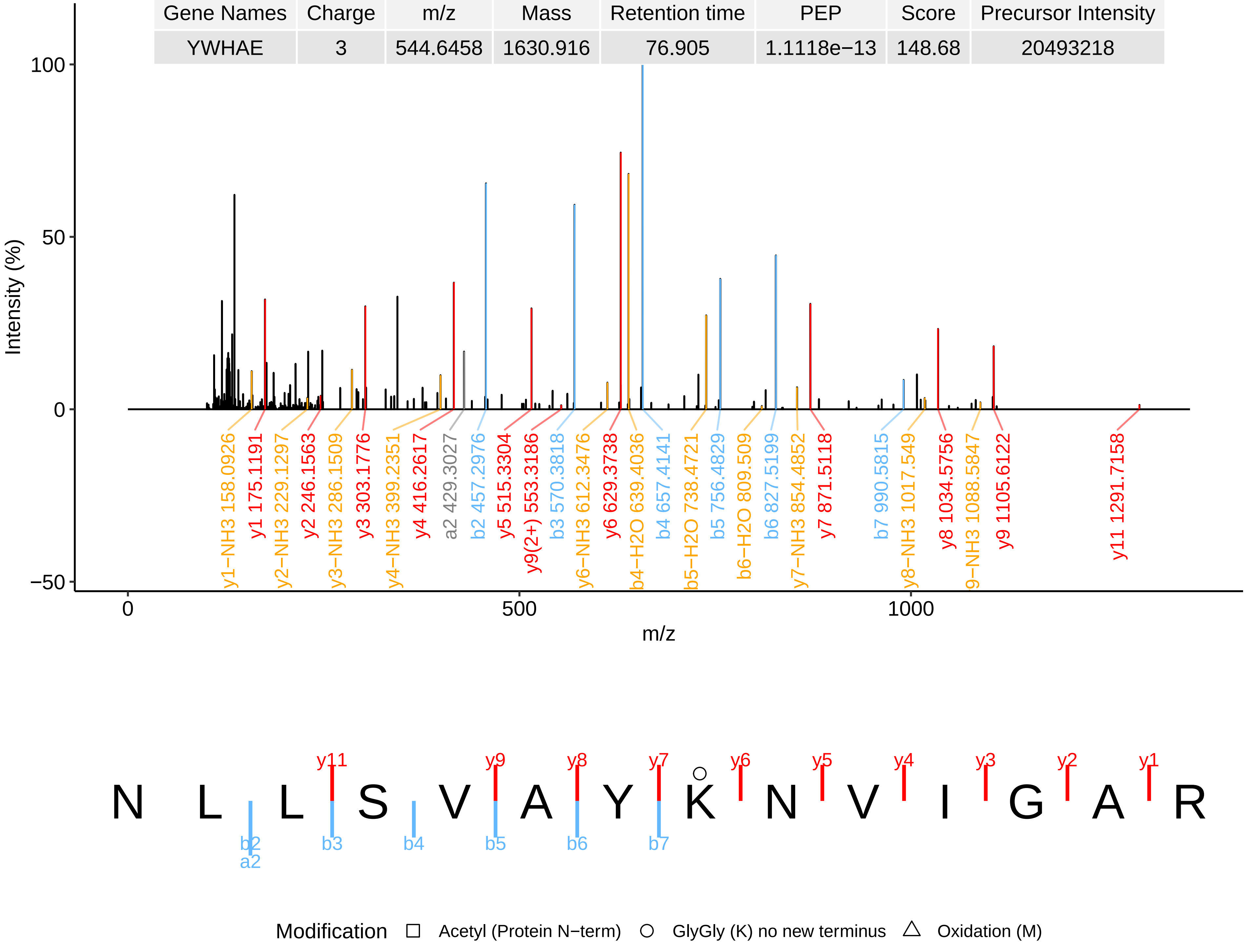

20200921_KIZ_MUb4_GlyGly_10plex − Scan number: 25245

Gene Names	Charge	m/z	Mass	Retention time	PEP	Score	Precursor Intensity
YWHAG;YWHAB;YWHAZ;YWHAH	3	539.9739	1616.9	91.823	4.5856e−20	162.32	33320746

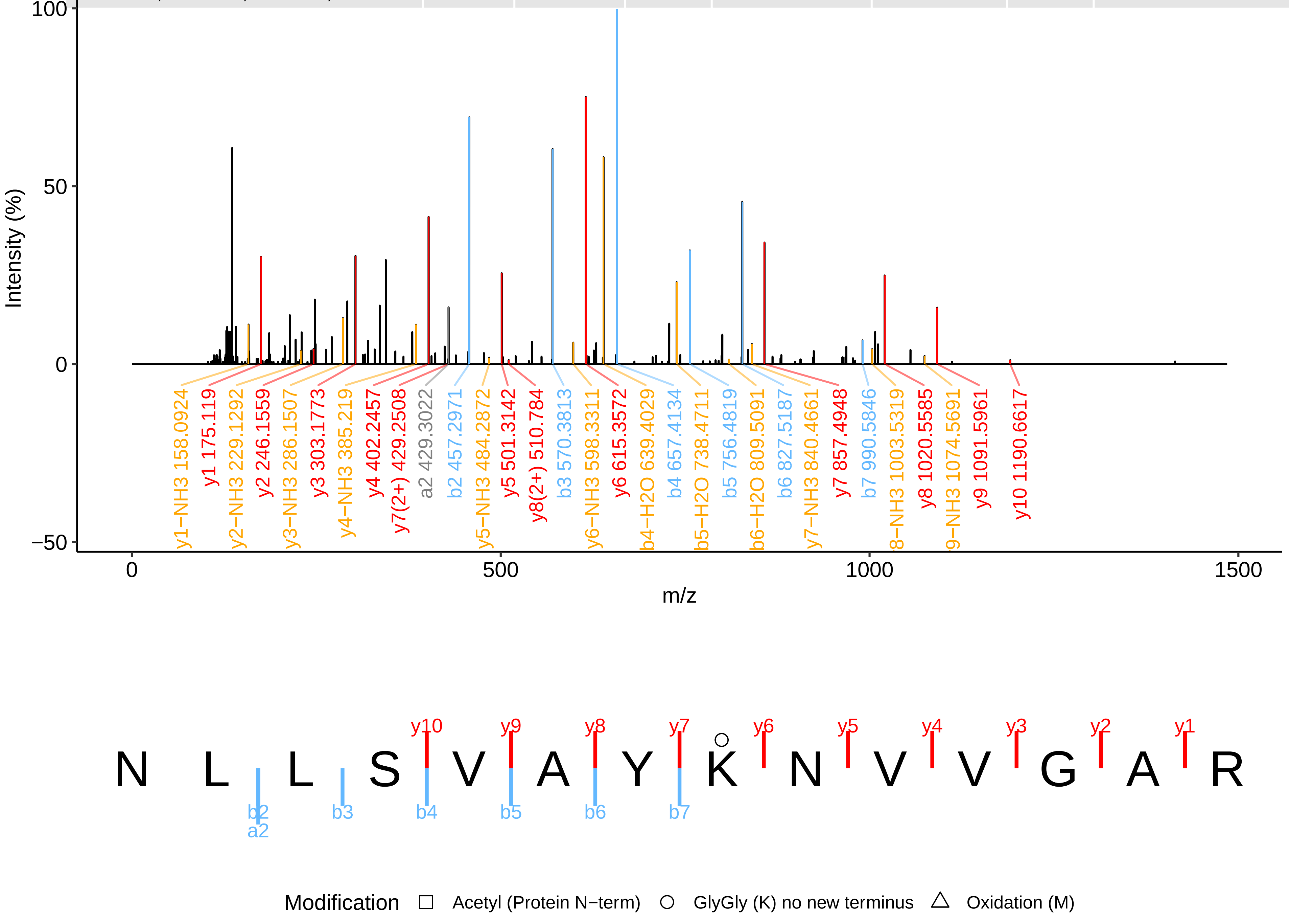

20200921_KIZ_MUb4_GlyGly_10plex_2 − Scan number: 21245

Gene Names	Charge	m/z	Mass	Retention time	PEP	Score	Precursor Intensity
YWHAG;YWHAB;YWHAZ;YWHAH	3	539.9739	1616.9	83.621	3.504e−14	148.68	1597844

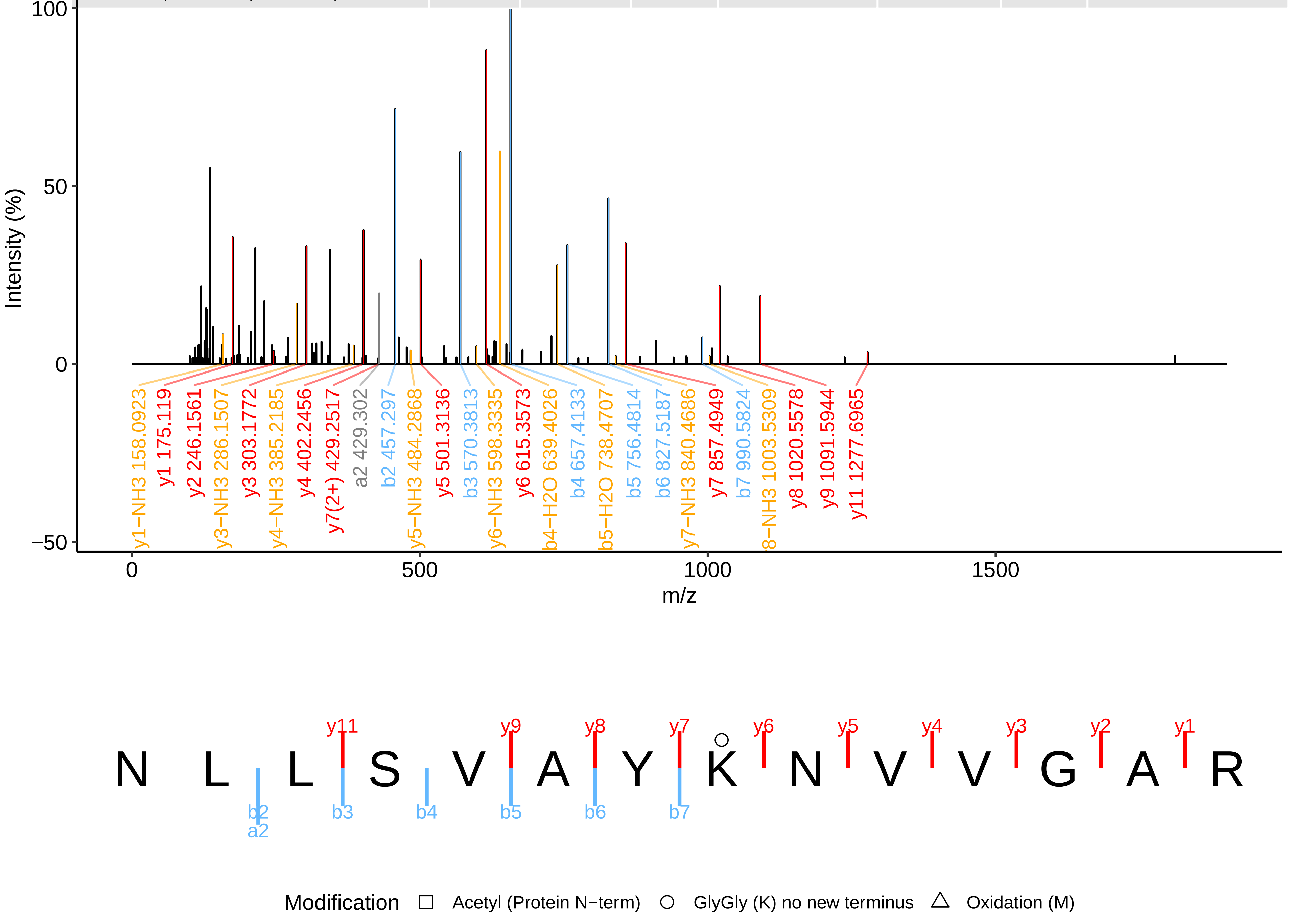

20200924_KIZ_MUb5_GlyGly_1 − Scan number: 23447

Gene Names	Charge	m/z	Mass	Retention time	PEP	Score	Precursor Intensity
YWHAG;YWHAB;YWHAZ;YWHAH	3	539.9739	1616.9	75.364	0.0027966	74.649	8631588

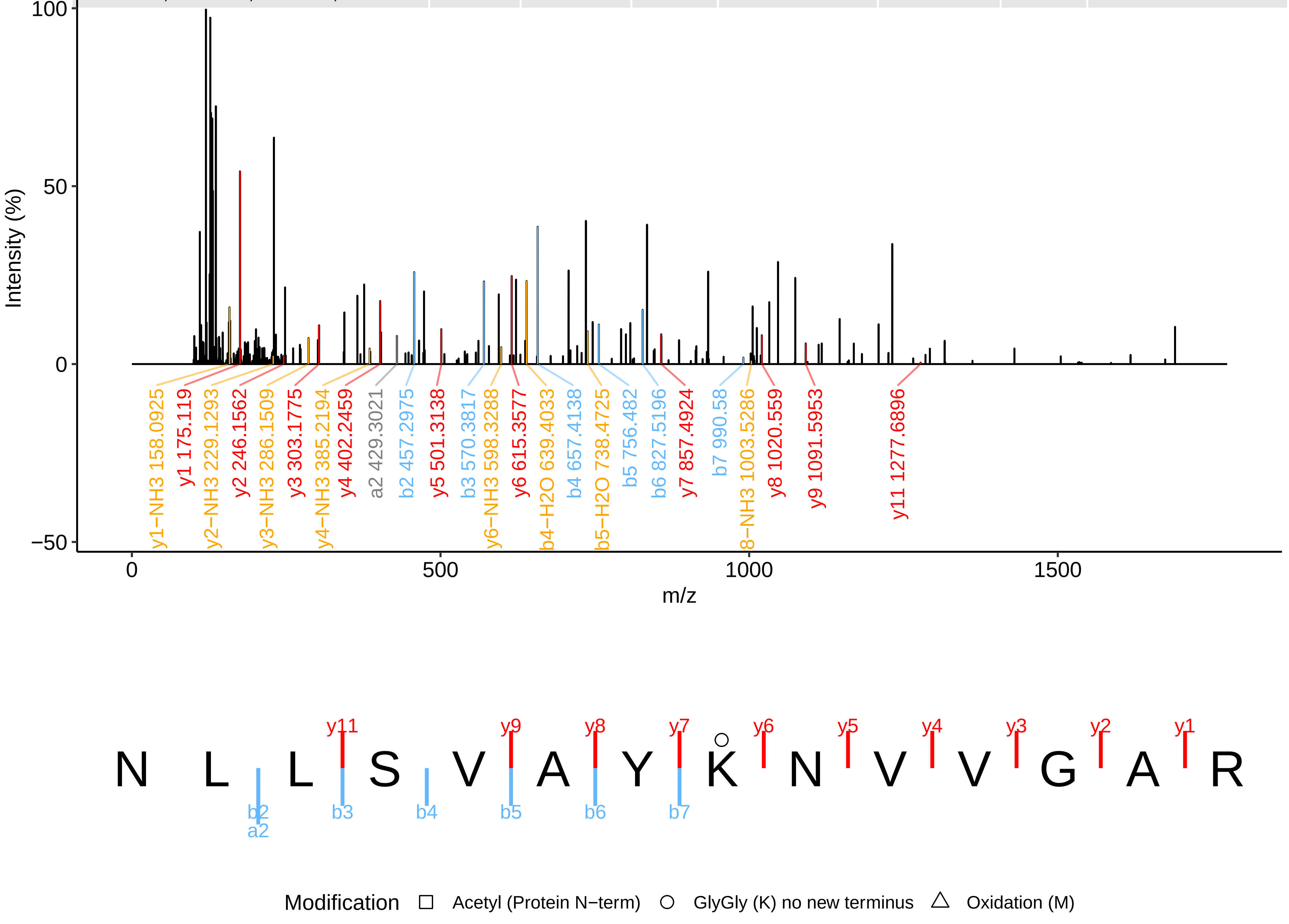

20200924_KIZ_MUb5_GlyGly_2 − Scan number: 21025

Gene Names	Charge	m/z	Mass	Retention time	PEP	Score	Precursor Intensity
YWHAG;YWHAB;YWHAZ;YWHAH	3	539.9739	1616.9	77.808	0.04761	56.286	8542795

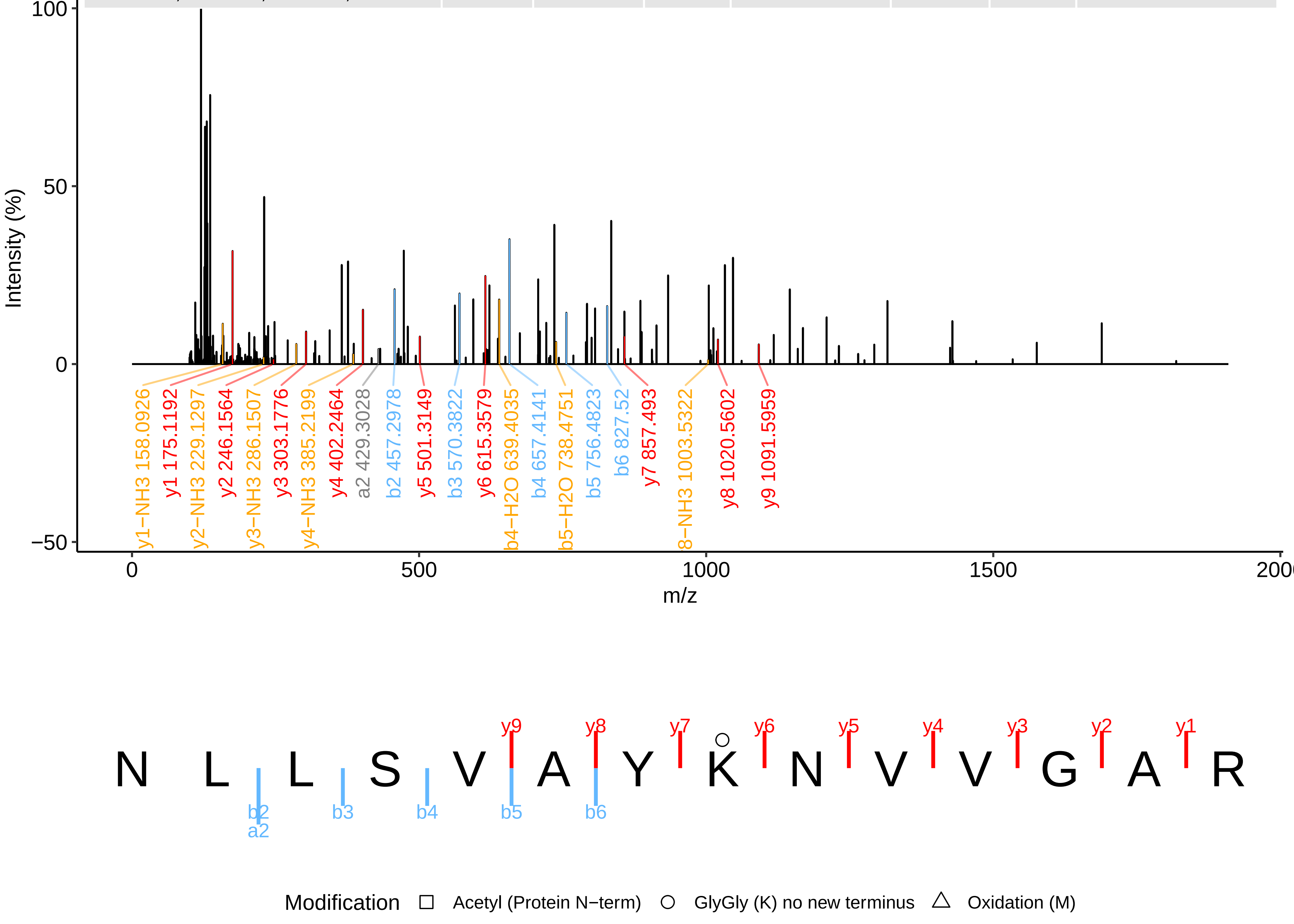

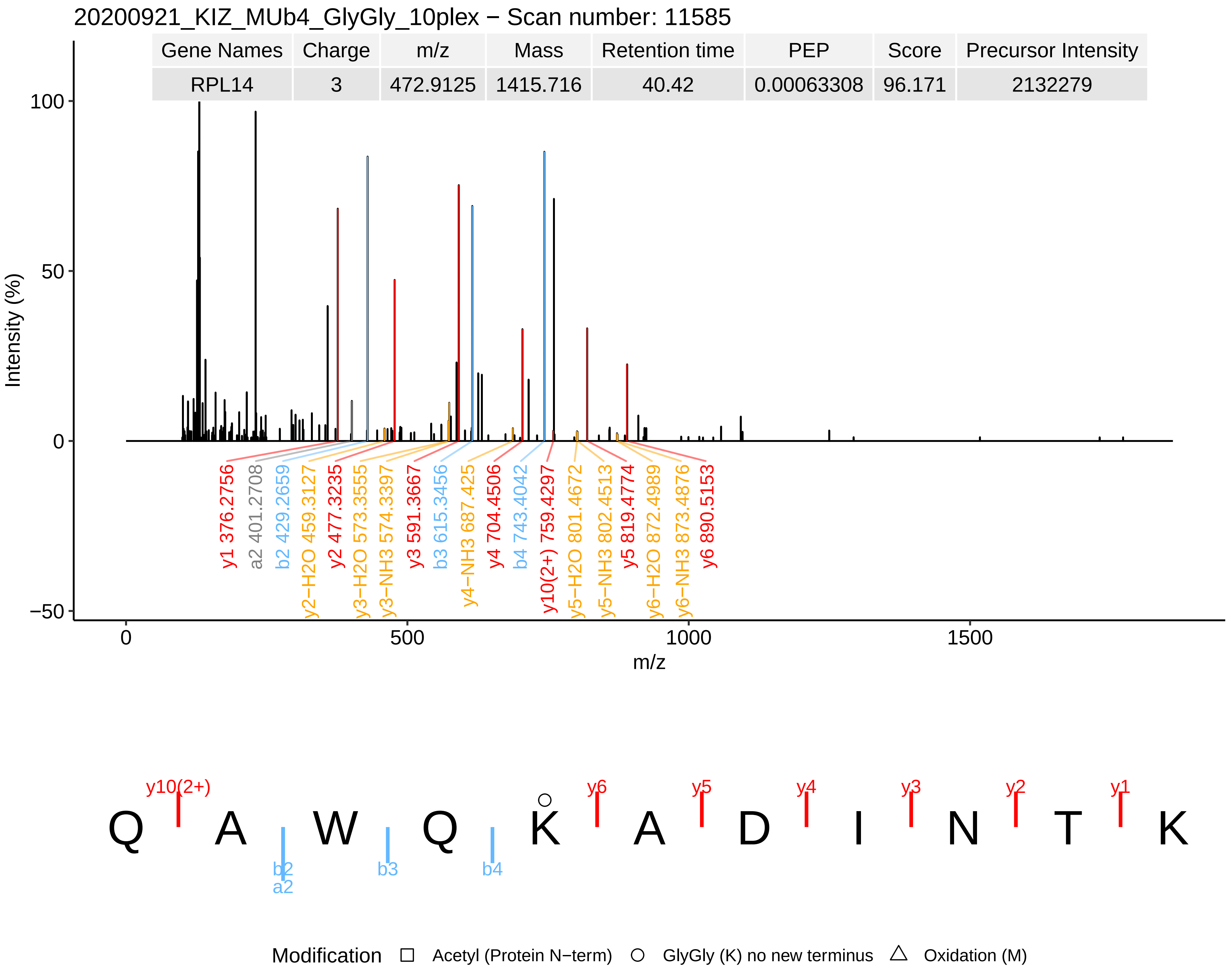
20200921_KIZ_MUb4_GlyGly_10plex − Scan number: 11585
Gene Names | Charge | m/z | Mass | Retention time | PEP | Score | Precursor Intensity
RPL14 | 3 | 472.9125 | 1415.716 | 40.42 | 0.00063308 | 96.171 | 2132279
Intensity (%)
100
50
0
−50
m/z
0
500
1000
1500
y1 376.2756
a2 401.2708
b2 429.2659
y2−H2O 459.3127
y2 477.3235
y3−H2O 573.3555
y3−NH3 574.3397
y3 591.3667
b3 615.3456
y4−NH3 687.425
y4 704.4506
b4 743.4042
y10(2+) 759.4297
y5−H2O 801.4672
y5−NH3 802.4513
y5 819.4774
y6−H2O 872.4989
y6−NH3 873.4876
y6 890.5153
Q A W Q K A D I N T K
y10(2+)
y6
y5
y4
y3
y2
y1
b2
a2
b3
b4
Modification
Acetyl (Protein N−term)
GlyGly (K) no new terminus
Oxidation (M)

20200921_KIZ_MUb4_GlyGly_10plex_2 − Scan number: 19286

Gene Names	Charge	m/z	Mass	Retention time	PEP	Score	Precursor Intensity
GNAS	3	466.9366	1397.788	75.679	0.075033	51.211	1776493

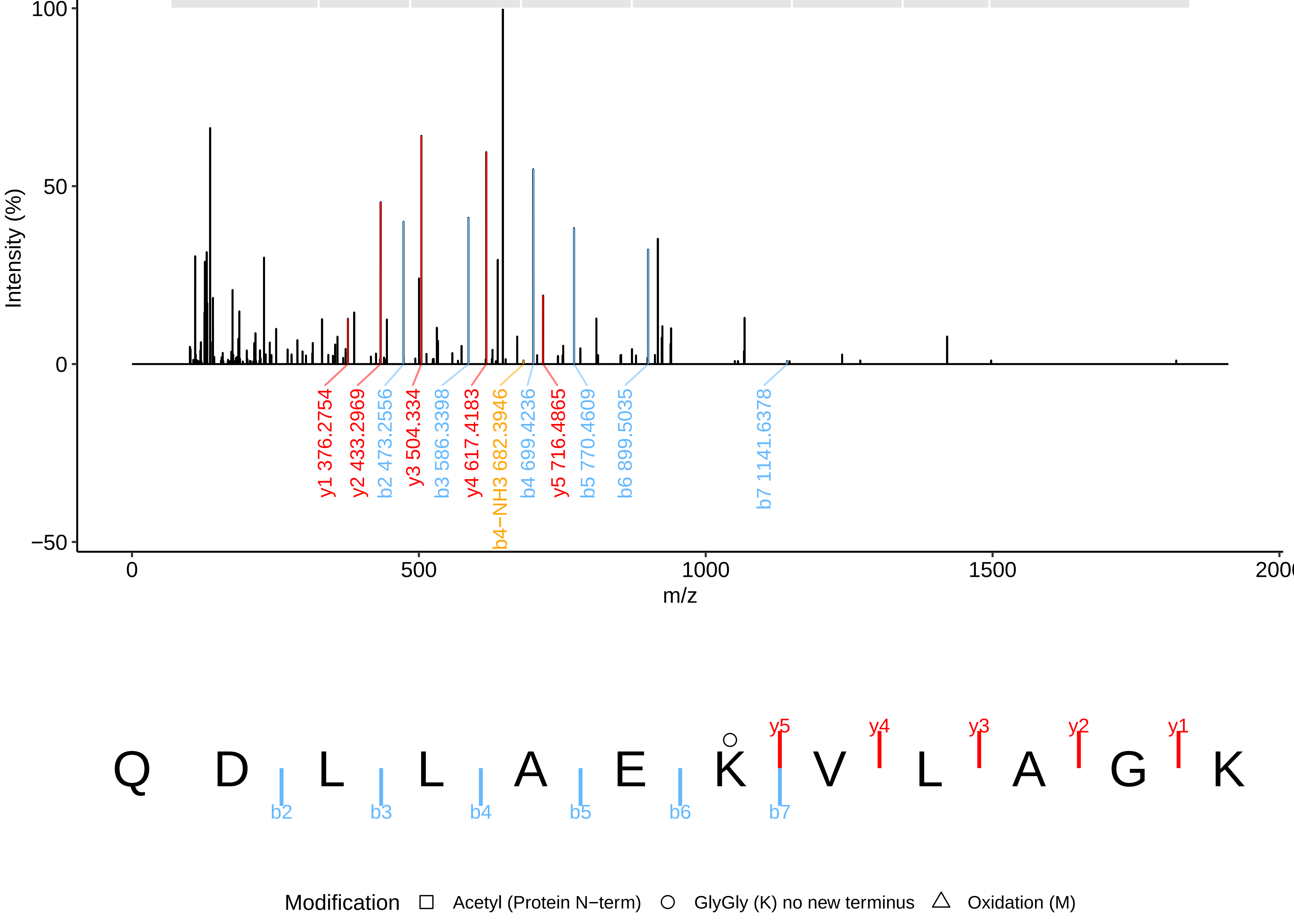

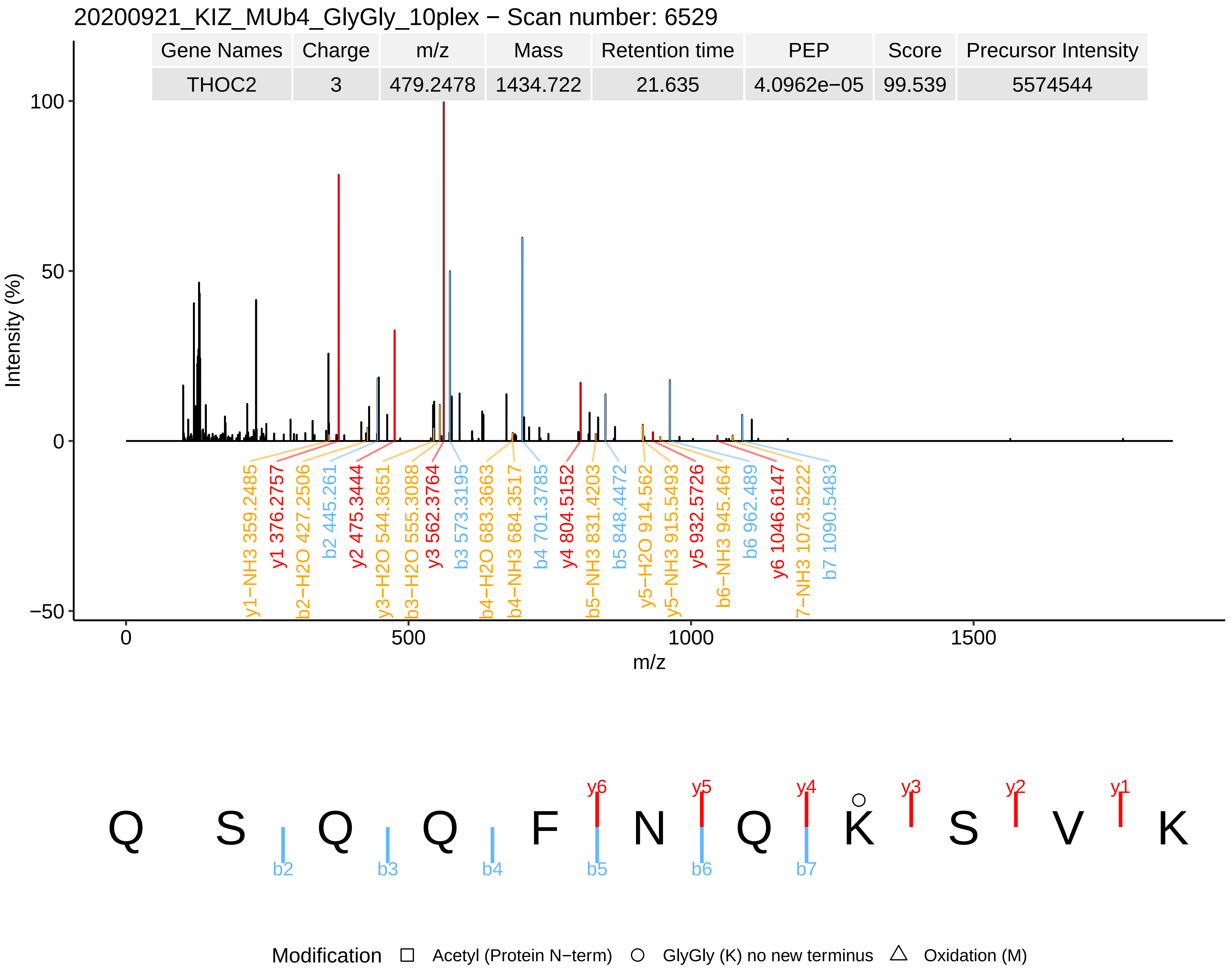

20200921_KIZ_MUb4_GlyGly_10plex − Scan number: 6529
Gene Names | Charge | m/z | Mass | Retention time | PEP | Score | Precursor Intensity
THOC2 | 3 | 479.2478 | 1434.722 | 21.635 | 4.0962e−05 | 99.539 | 5574544
Intensity (%)
m/z
y1−NH3 359.2485
y1 376.2757
b2−H2O 427.2506
b2 445.261
y2 475.3444
y3−H2O 544.3651
b3−H2O 555.3088
y3 562.3764
b3 573.3195
b4−H2O 683.3663
b4−NH3 684.3517
b4 701.3785
y4 804.5152
b5−NH3 831.4203
b5 848.4472
y5−H2O 914.562
y5−NH3 915.5493
y5 932.5726
b6−NH3 945.464
b6 962.489
y6 1046.6147
b7−NH3 1073.5222
b7 1090.5483
Q S Q Q F N Q K S V K
Modification
Acetyl (Protein N−term)
GlyGly (K) no new terminus
Oxidation (M)

20200921_KIZ_MUb4_GlyGly_10plex_2 − Scan number: 6030

Gene Names	Charge	m/z	Mass	Retention time	PEP	Score	Precursor Intensity
THOC2	3	479.2478	1434.722	21.983	0.078818	51.013	494344.8

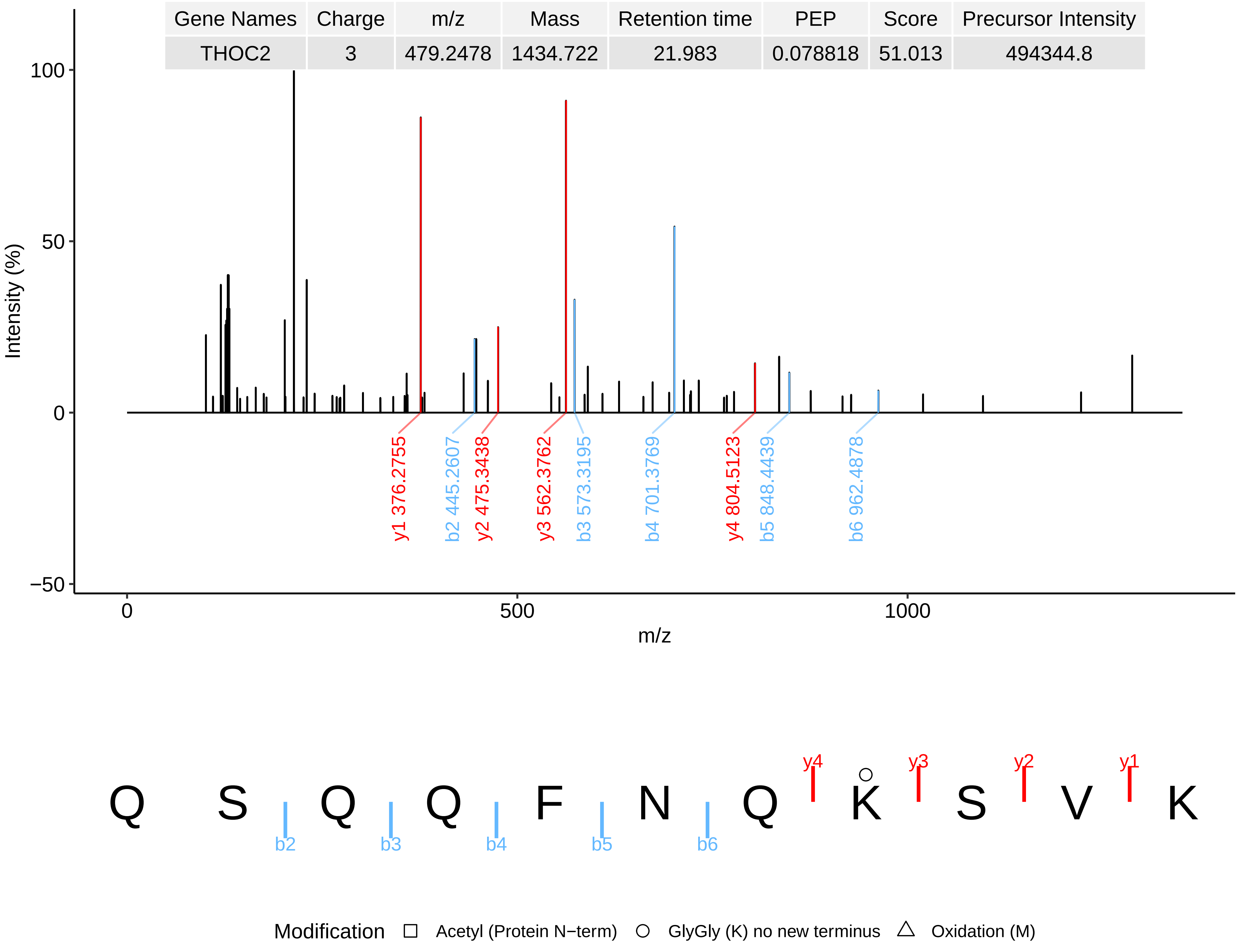

20200921_KIZ_MUb4_GlyGly_10plex_2 − Scan number: 6033

Gene Names	Charge	m/z	Mass	Retention time	PEP	Score	Precursor Intensity
THOC2	3	479.2478	1434.722	21.995	0.095856	47.712	666375.1

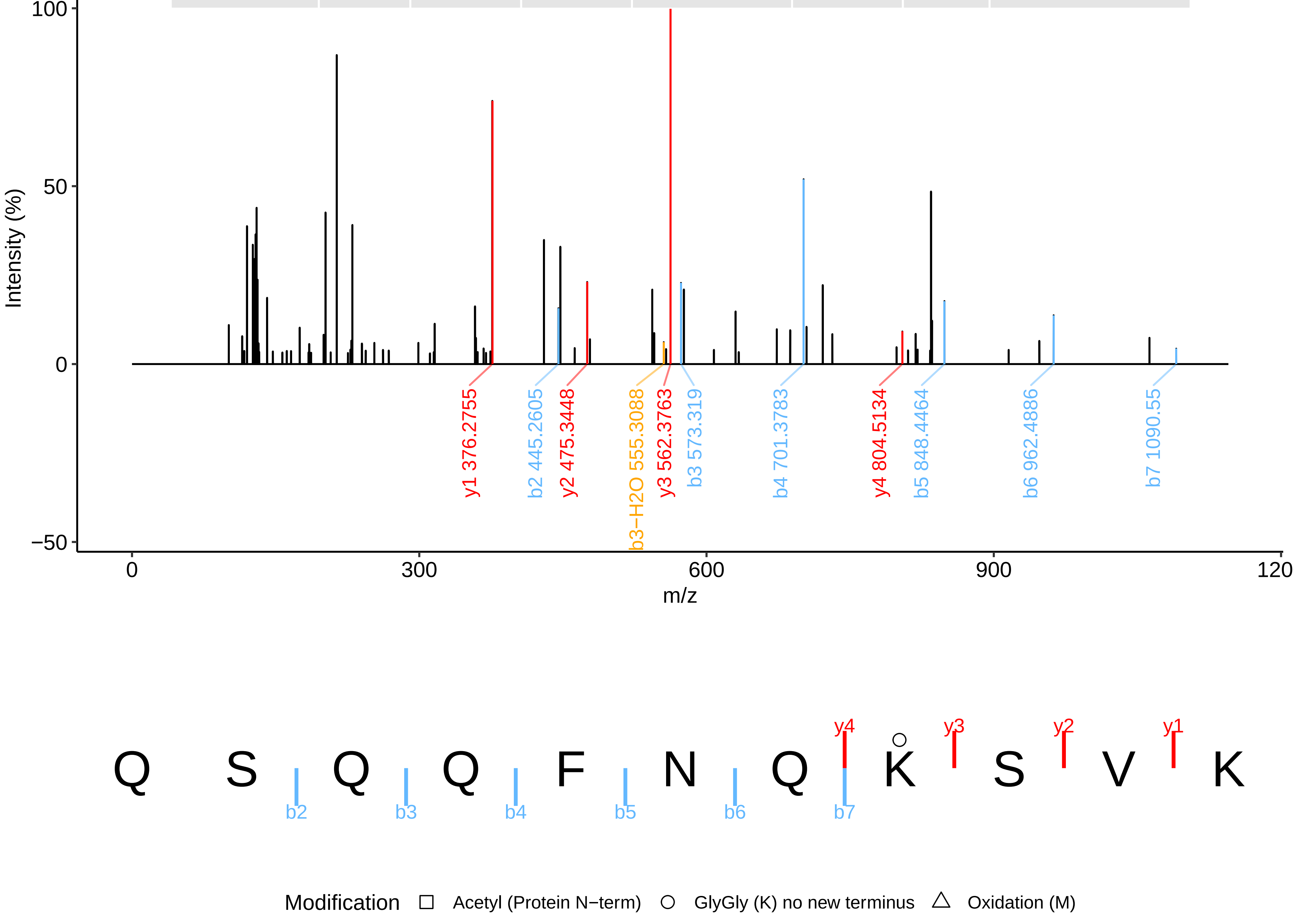

20200921_KIZ_MUb4_GlyGly_10plex − Scan number: 18539

Gene Names	Charge	m/z	Mass	Retention time	PEP	Score	Precursor Intensity
EEF1A1;EEF1A1P5	3	481.284	1440.83	66.707	0.0053513	73.887	5228446

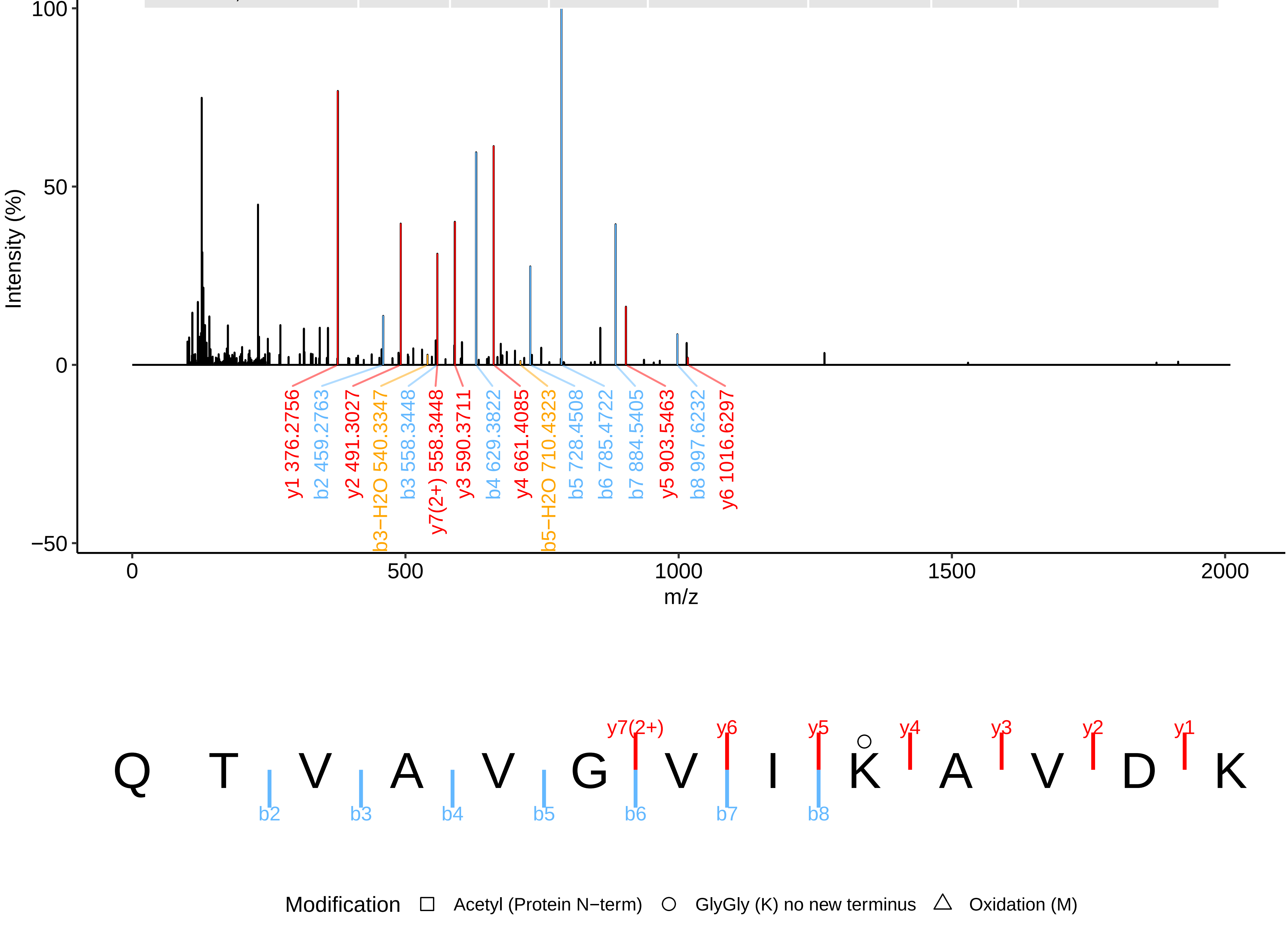

20200921_KIZ_MUb4_GlyGly_10plex_2 − Scan number: 16877

Gene Names	Charge	m/z	Mass	Retention time	PEP	Score	Precursor Intensity
EEF1A1;EEF1A1P5	3	481.284	1440.83	65.933	0.00023642	94.728	2384991

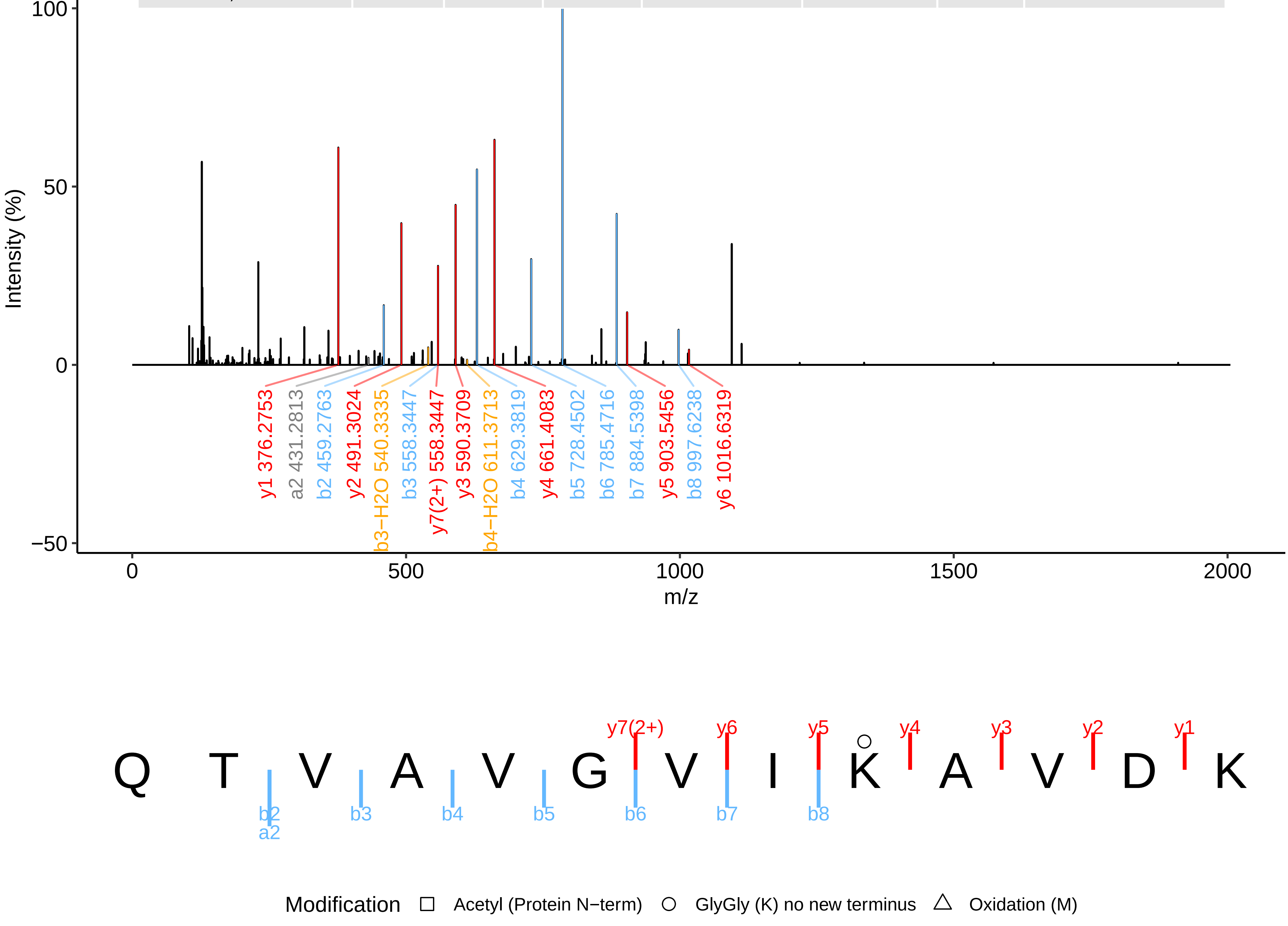

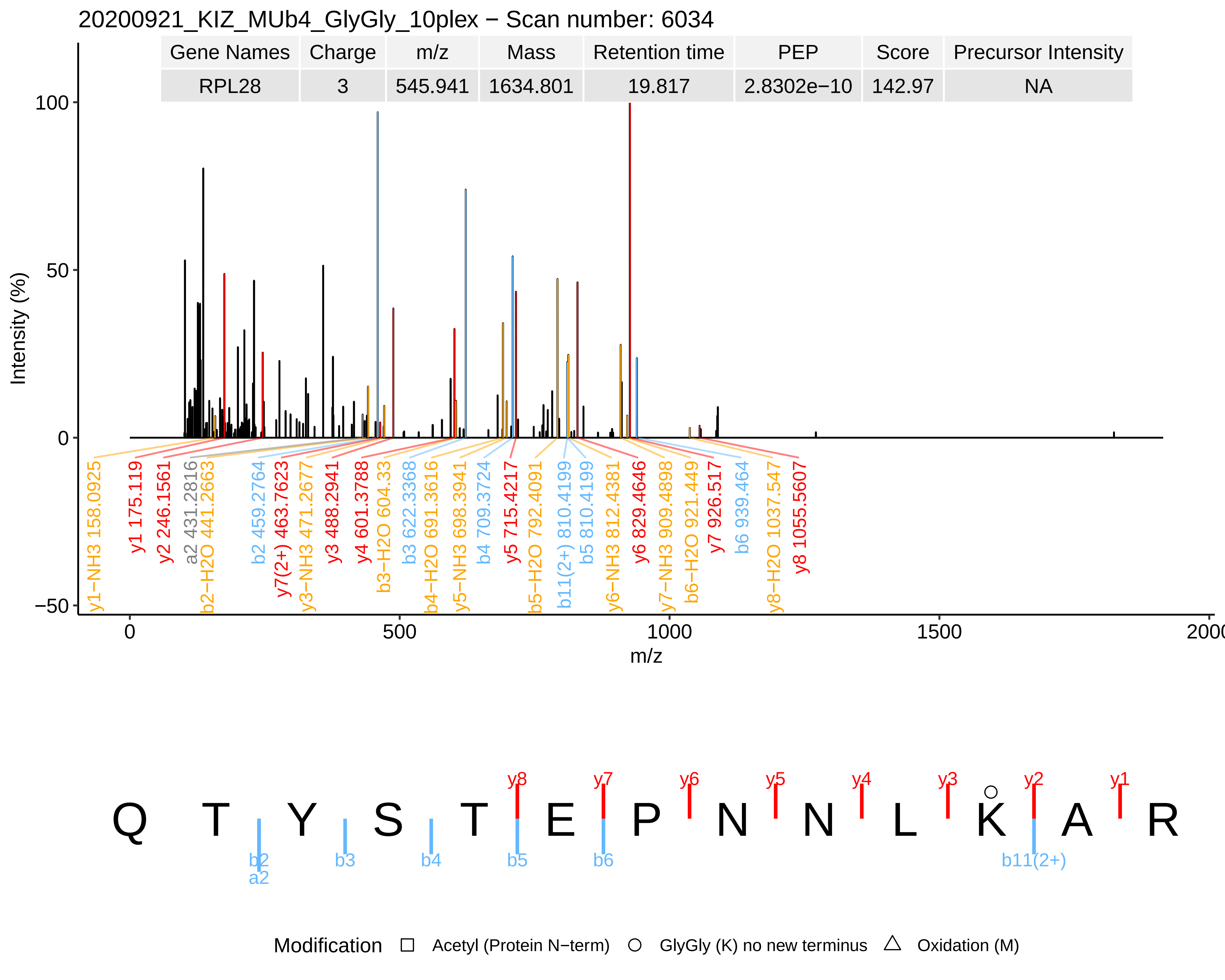
20200921_KIZ_MUb4_GlyGly_10plex − Scan number: 6034
Gene Names
Charge
m/z
Mass
Retention time
PEP
Score
Precursor Intensity
RPL28
3
545.941
1634.801
19.817
2.8302e−10
142.97
NA
Intensity (%)
100
50
0
−50
m/z
0
500
1000
1500
2000
y1−NH3 158.0925
y1 175.119
y2 246.1561
a2 431.2816
b2−H2O 441.2663
b2 459.2764
y7(2+) 463.7623
y3−NH3 471.2677
y3 488.2941
y4 601.3788
b3−H2O 604.33
b3 622.3368
b4−H2O 691.3616
y5−NH3 698.3941
b4 709.3724
y5 715.4217
b5−H2O 792.4091
b11(2+) 810.4199
b5 810.4199
y6−NH3 812.4381
y6 829.4646
y7−NH3 909.4898
b6−H2O 921.449
y7 926.517
b6 939.464
y8−H2O 1037.547
y8 1055.5607
Q T Y S T E P N N L K A R
y8 y7 y6 y5 y4 y3 y2 y1
b2 a2 b3 b4 b5 b6 b11(2+)
Modification
Acetyl (Protein N−term)
GlyGly (K) no new terminus
Oxidation (M)

20200921_KIZ_MUb4_GlyGly_10plex_2 − Scan number: 3058

Gene Names	Charge	m/z	Mass	Retention time	PEP	Score	Precursor Intensity
FAF2	4	354.6864	1414.716	10.056	0.014181	83.31	1560949

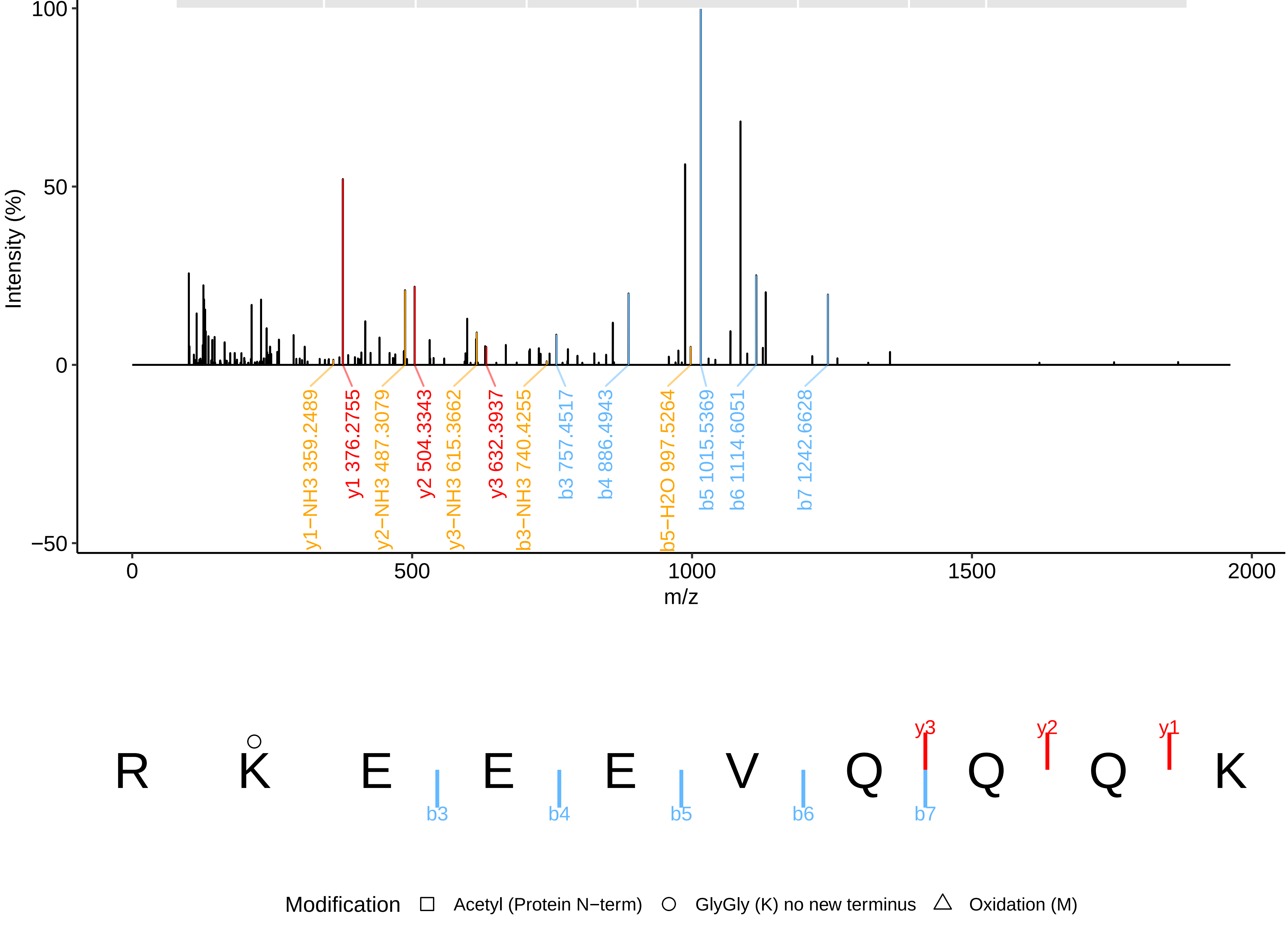

20200924_KIZ_MUb5_GlyGly_1 − Scan number: 37162

Gene Names	Charge	m/z	Mass	Retention time	PEP	Score	Precursor Intensity
NSMF	2	836.9455	1671.877	118.27	0.058616	52.79	NA

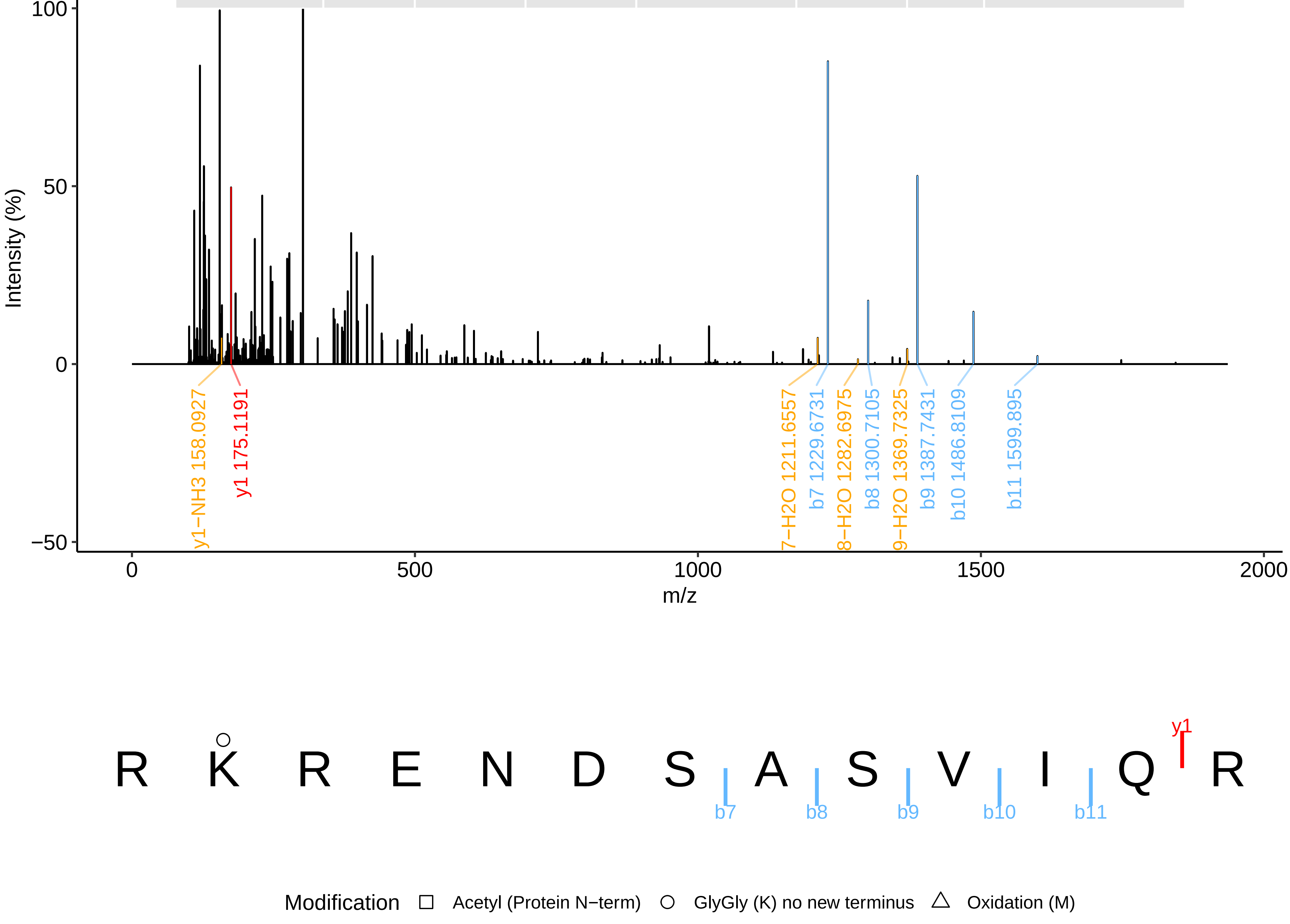

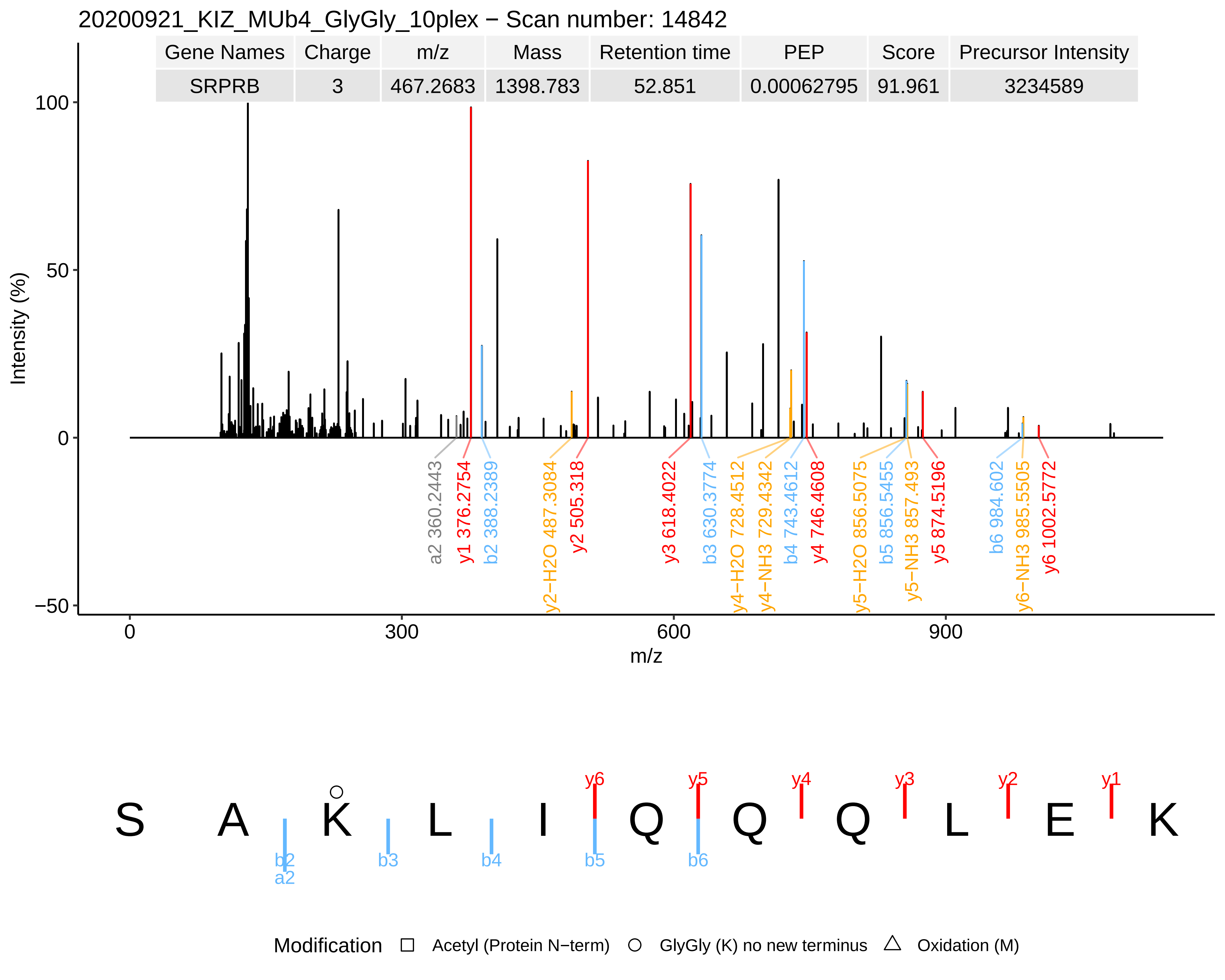
20200921_KIZ_MUb4_GlyGly_10plex − Scan number: 14842
Gene Names | Charge | m/z | Mass | Retention time | PEP | Score | Precursor Intensity
SRPRB | 3 | 467.2683 | 1398.783 | 52.851 | 0.00062795 | 91.961 | 3234589
Intensity (%)
100
50
0
−50
0
300
600
900
m/z
a2 360.2443
y1 376.2754
b2 388.2389
y2−H2O 487.3084
y2 505.318
y3 618.4022
b3 630.3774
y4−H2O 728.4512
y4−NH3 729.4342
b4 743.4612
y4 746.4608
y5−H2O 856.5075
b5 856.5455
y5−NH3 857.493
y5 874.5196
b6 984.602
y6−NH3 985.5505
y6 1002.5772
S A K L I Q Q Q L E K
y6 y5 y4 y3 y2 y1
b2 a2 b3 b4 b5 b6
Modification
Acetyl (Protein N−term)
GlyGly (K) no new terminus
Oxidation (M)

20200921_KIZ_MUb4_GlyGly_10plex − Scan number: 25134

Gene Names	Charge	m/z	Mass	Retention time	PEP	Score	Precursor Intensity
CHIC2	3	548.9495	1643.827	91.419	0.0014961	80.17	2929720

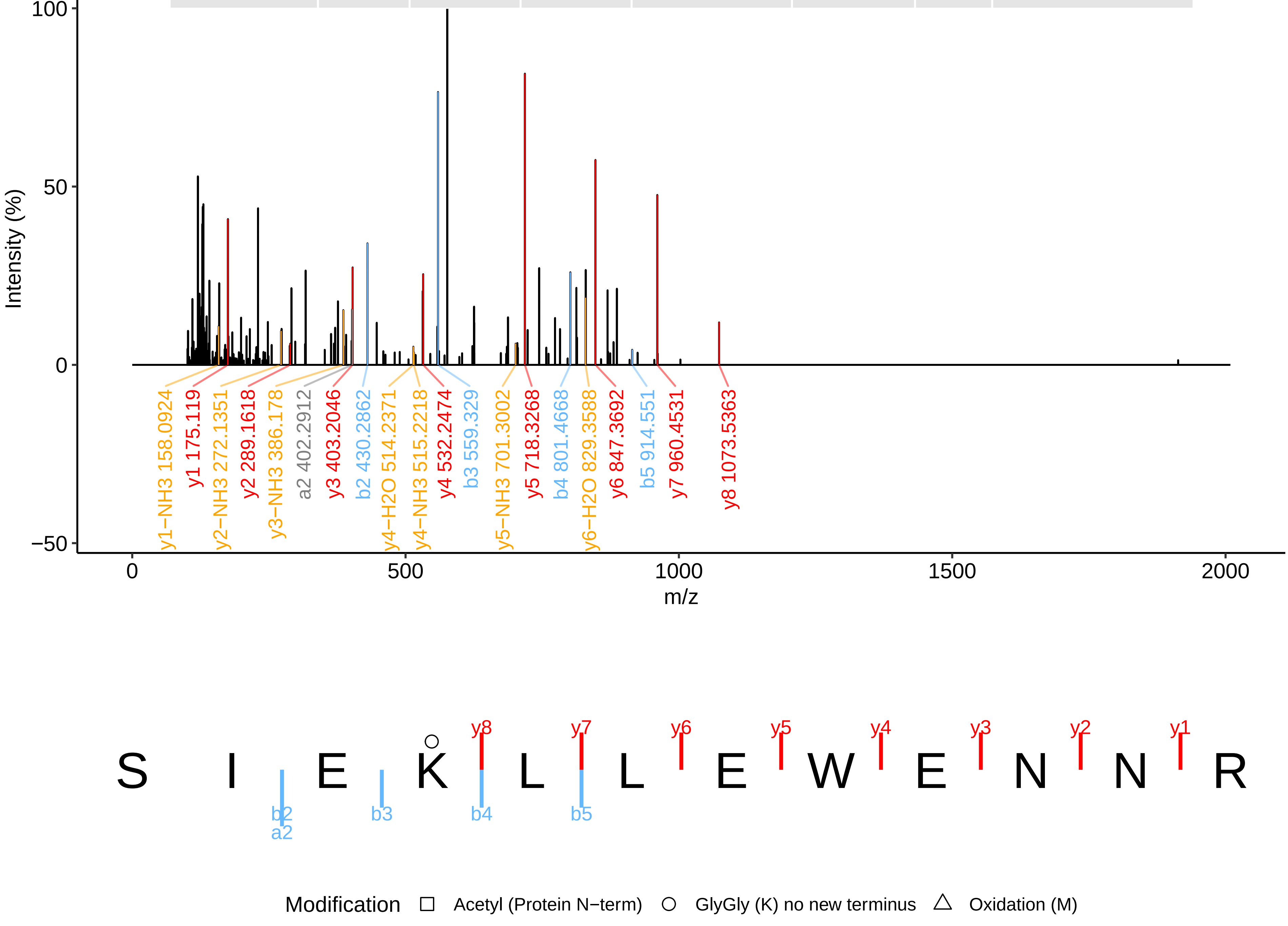

20200924_KIZ_MUb5_GlyGly_1 − Scan number: 23708

Gene Names	Charge	m/z	Mass	Retention time	PEP	Score	Precursor Intensity
CHIC2	3	548.9495	1643.827	76.187	0.054157	57.52	NA

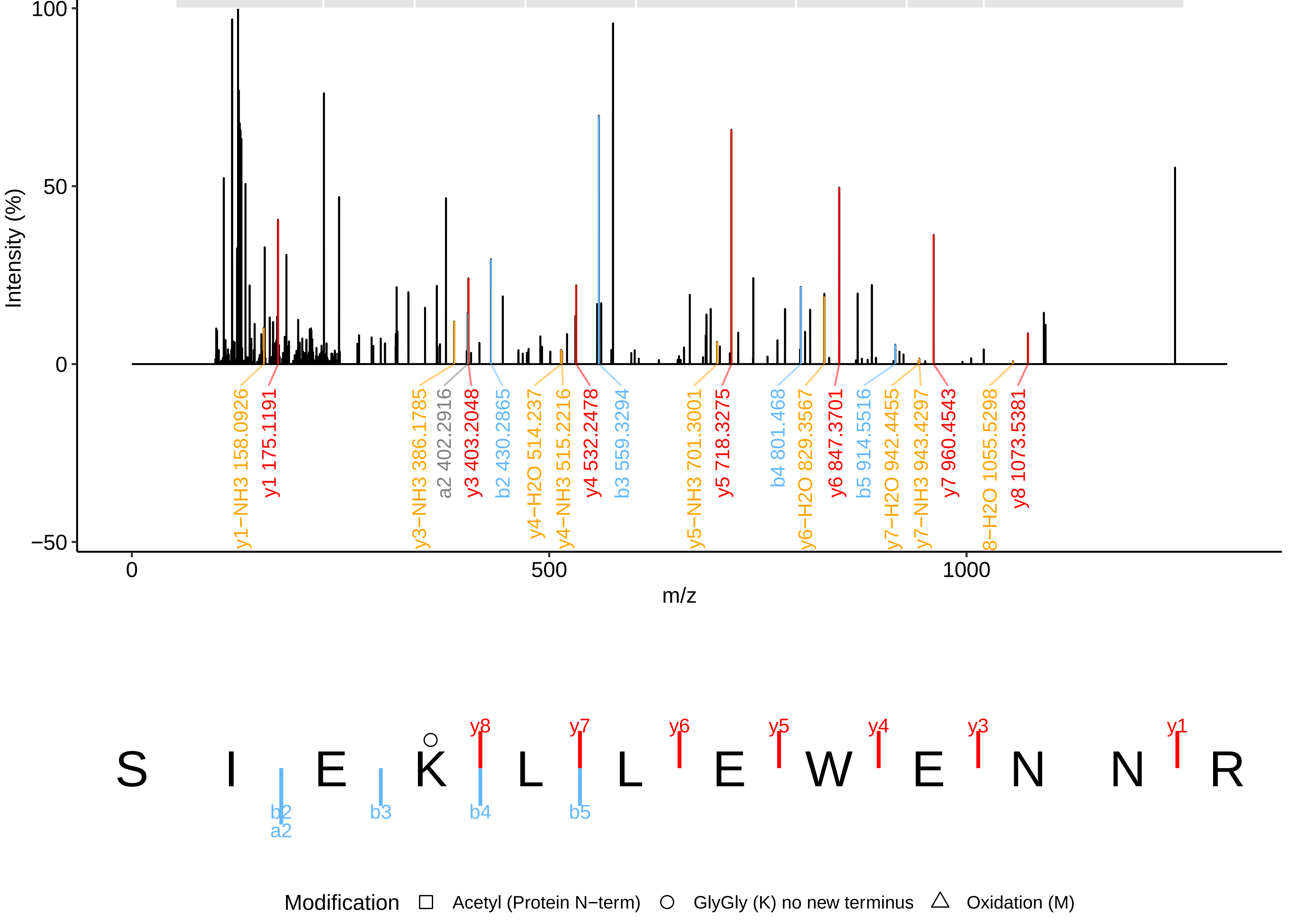

20200921_KIZ_MUb4_GlyGly_10plex − Scan number: 13876

Gene Names	Charge	m/z	Mass	Retention time	PEP	Score	Precursor Intensity
SMC3	3	470.9174	1409.73	49.1	0.001072	87.363	3939059

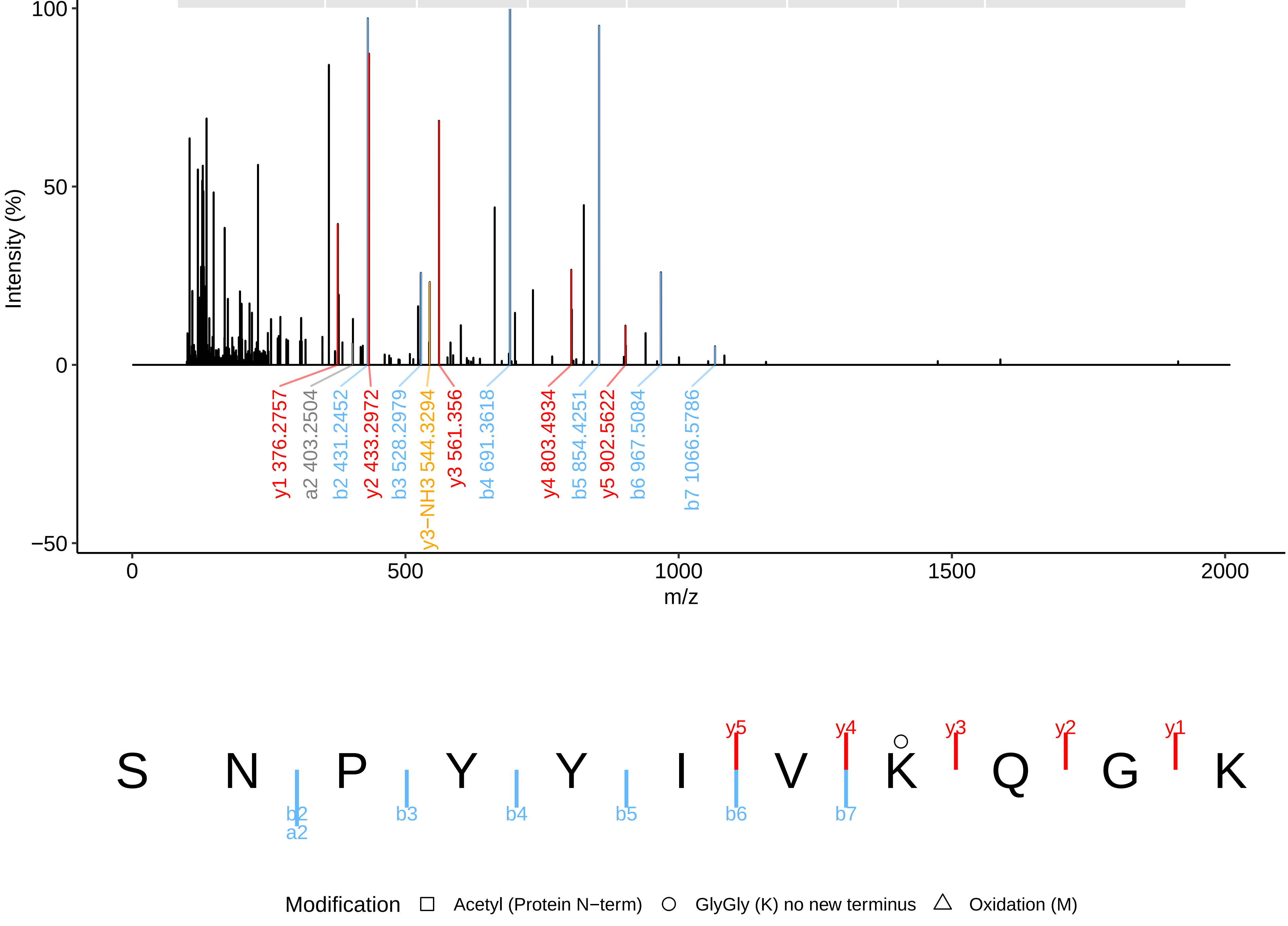

20200921_KIZ_MUb4_GlyGly_10plex − Scan number: 7794

Gene Names	Charge	m/z	Mass	Retention time	PEP	Score	Precursor Intensity
RAD18	3	463.9159	1388.726	26.286	0.0007477	86.911	NA

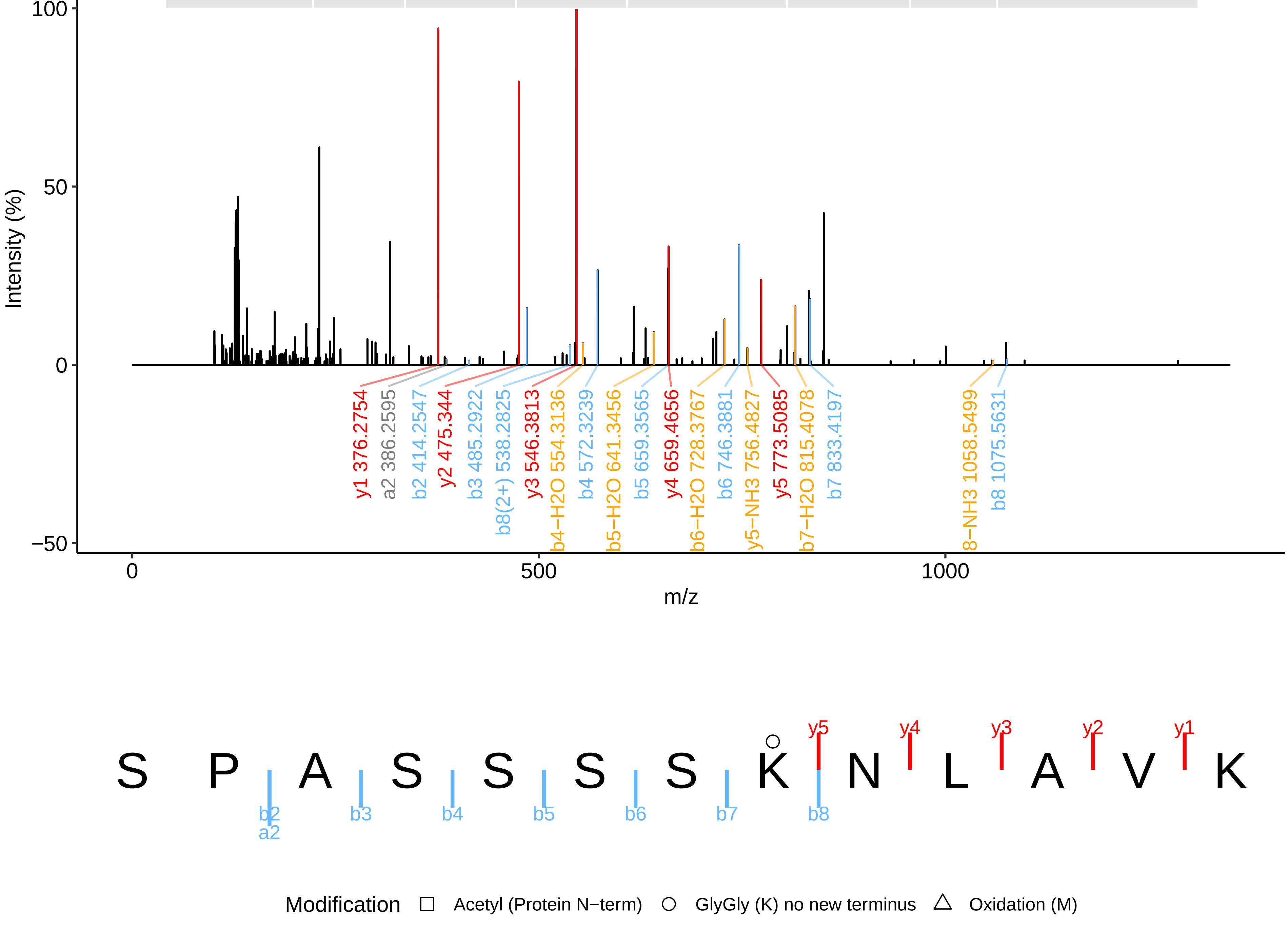

20200921_KIZ_MUb4_GlyGly_10plex_2 − Scan number: 7195

Gene Names	Charge	m/z	Mass	Retention time	PEP	Score	Precursor Intensity
RAD18	3	463.9159	1388.726	26.76	0.00090672	84.479	707925.4

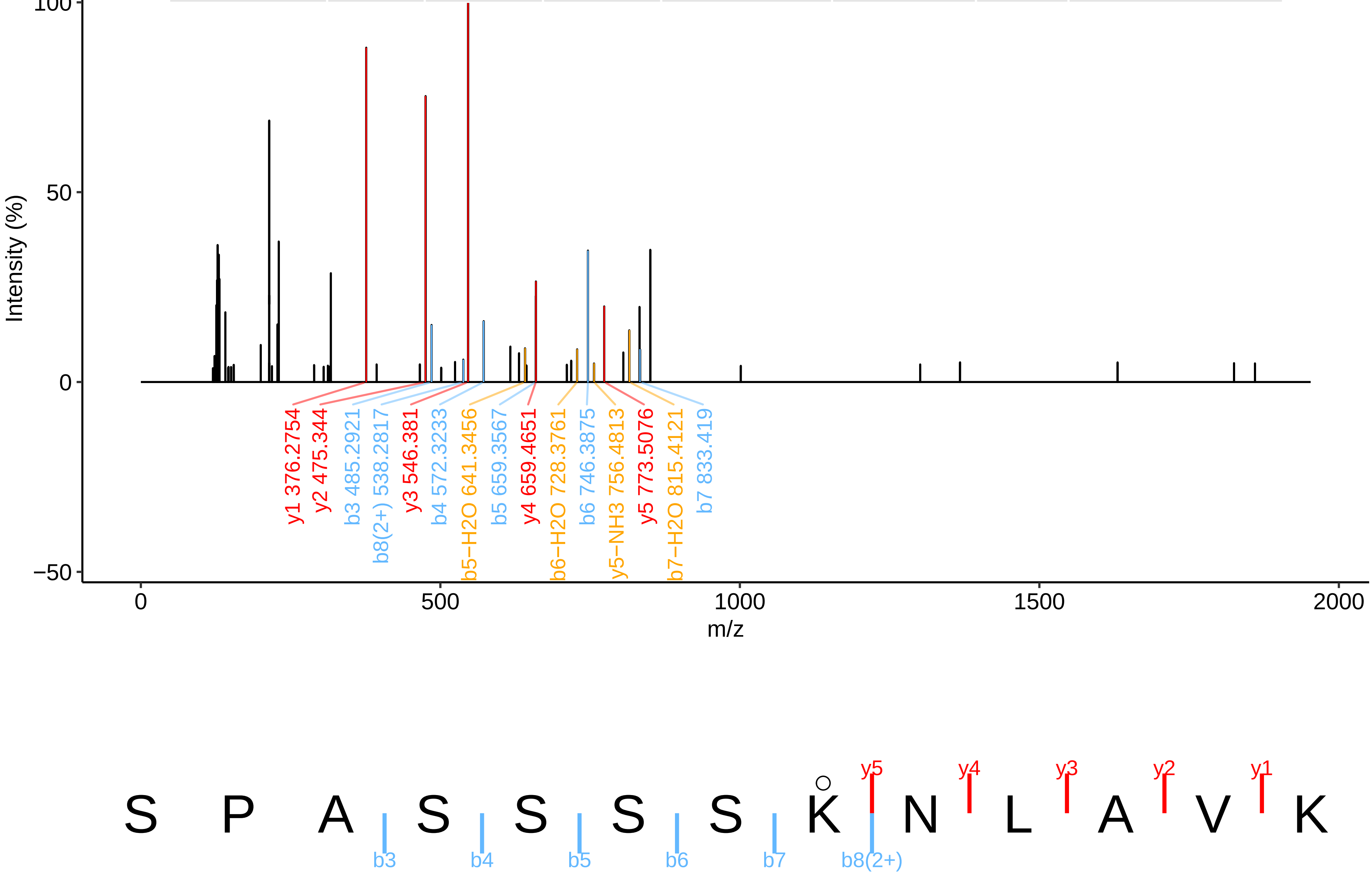

Modification □ Acetyl (Protein N−term) ○ GlyGly (K) no new terminus △ Oxidation (M)

20200924_KIZ_MUb5_GlyGly_1 − Scan number: 21914

Gene Names	Charge	m/z	Mass	Retention time	PEP	Score	Precursor Intensity
ZNF532	2	838.9172	1675.82	70.566	0.09754	40.352	NA

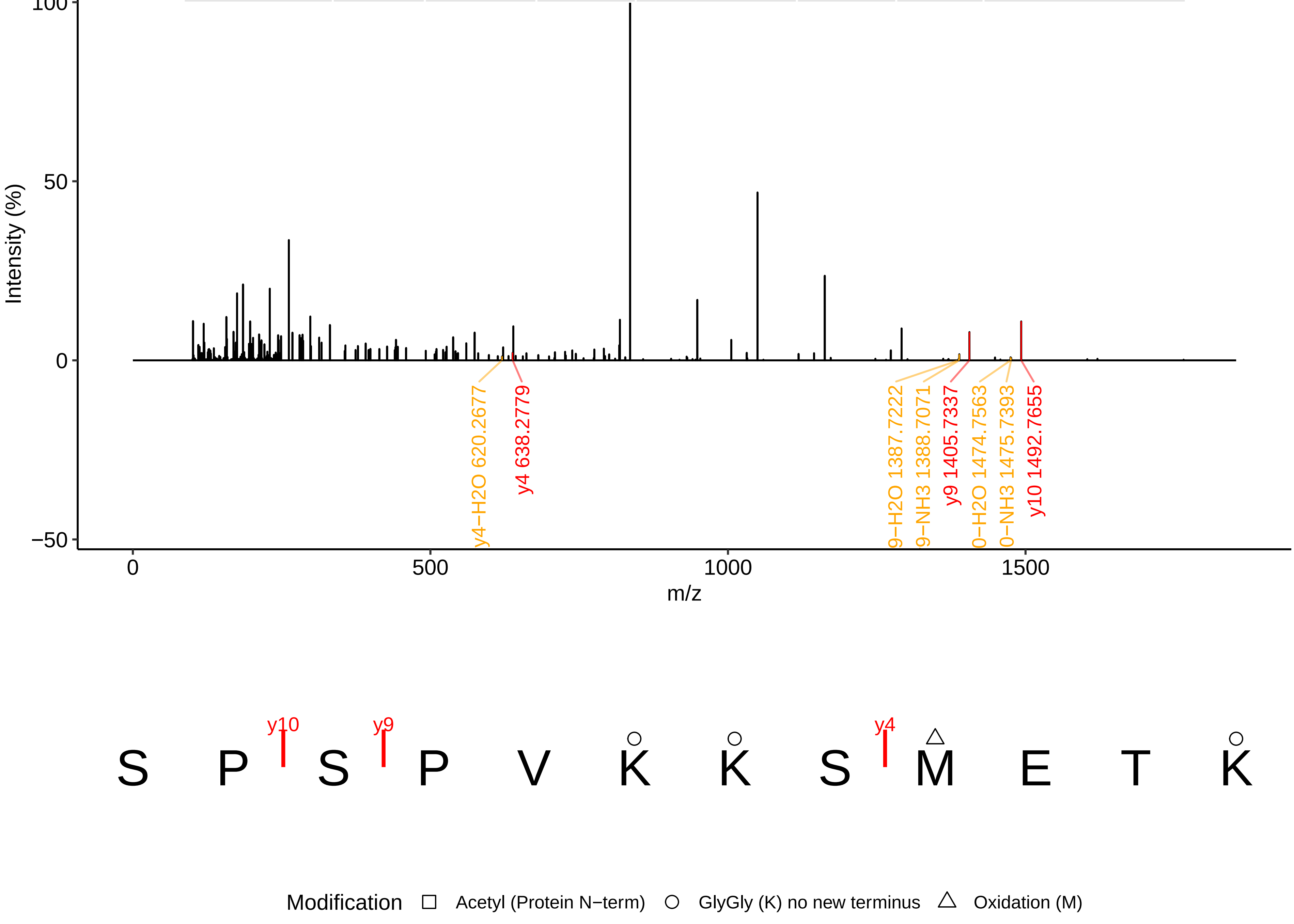

20201118_KIZ_MUb6_GlyGly_1 − Scan number: 2468

Gene Names	Charge	m/z	Mass	Retention time	PEP	Score	Precursor Intensity
GNAS	4	405.1948	1616.75	7.5662	0.059843	68.515	59333312

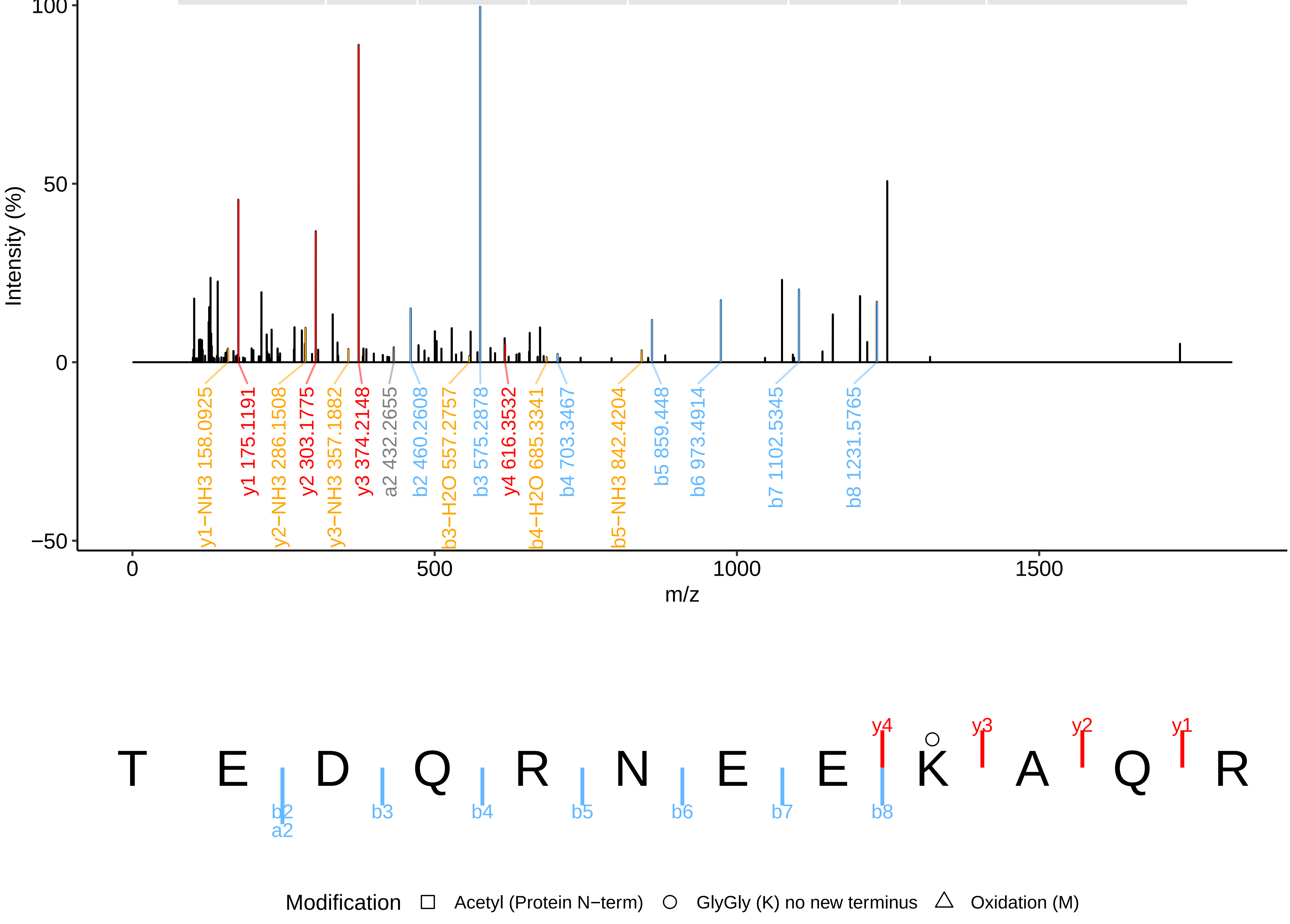

20201118_KIZ_MUb6_GlyGly_2 − Scan number: 2492

Gene Names	Charge	m/z	Mass	Retention time	PEP	Score	Precursor Intensity
GNAS	4	405.1948	1616.75	7.7542	5.0662e−06	114.76	8701303

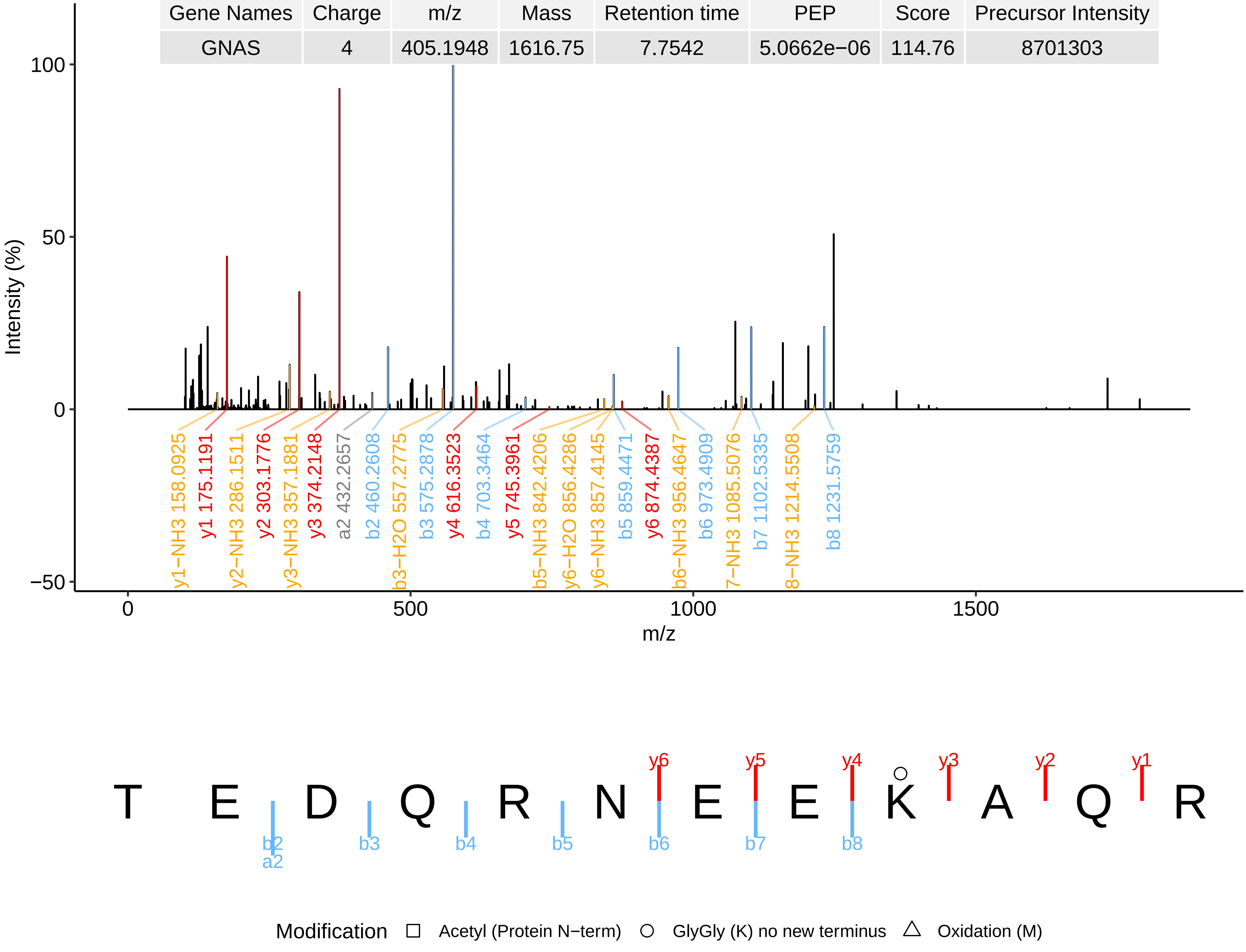

20200924_KIZ_MUb5_GlyGly_1 − Scan number: 5592

Gene Names	Charge	m/z	Mass	Retention time	PEP	Score	Precursor Intensity
BASP1	3	471.572	1411.694	17.427	0.00069111	90.793	4258860

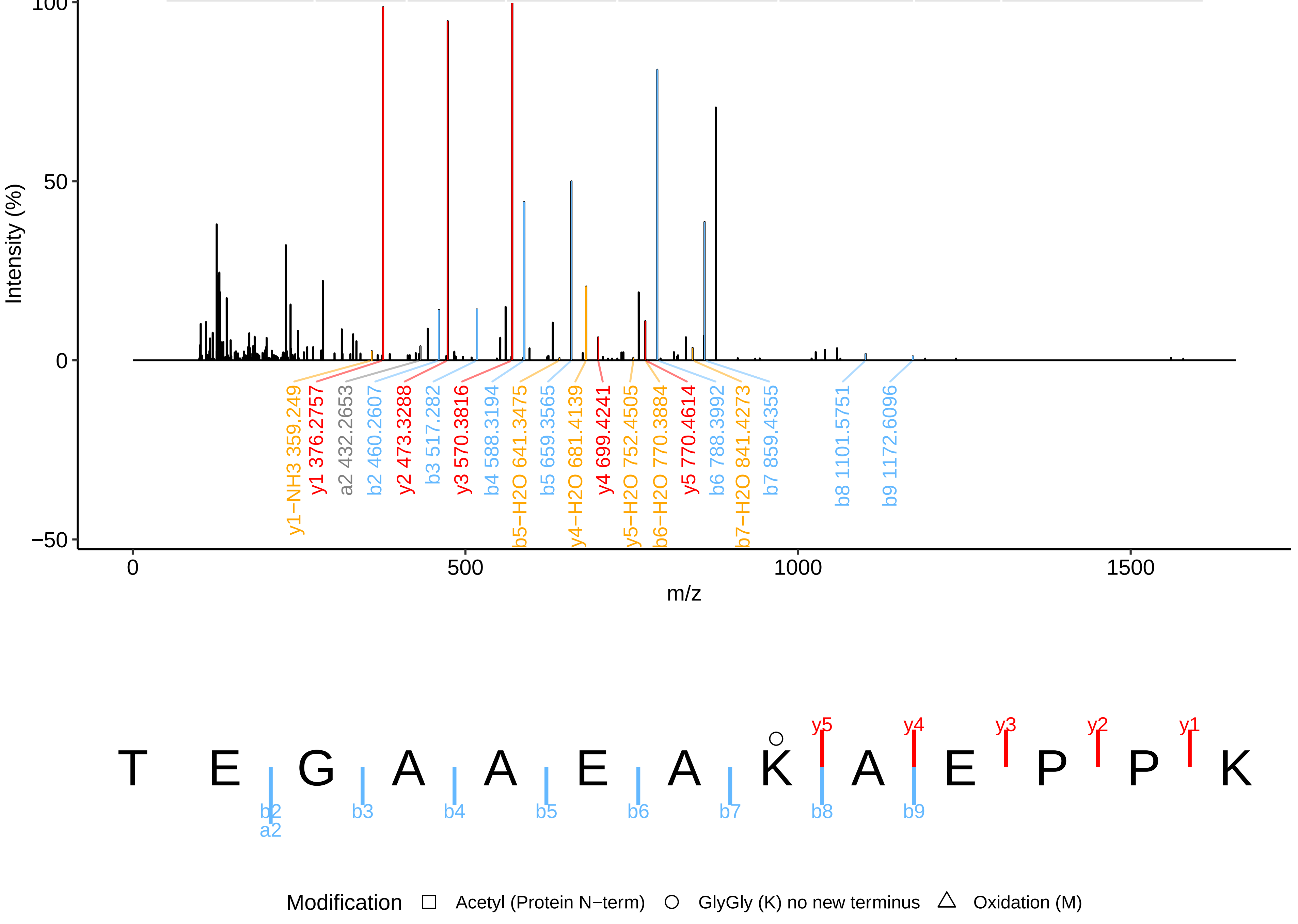

20200924_KIZ_MUb5_GlyGly_1 − Scan number: 5616

Gene Names	Charge	m/z	Mass	Retention time	PEP	Score	Precursor Intensity
BASP1	3	471.572	1411.694	17.507	0.00049569	92.856	5611551

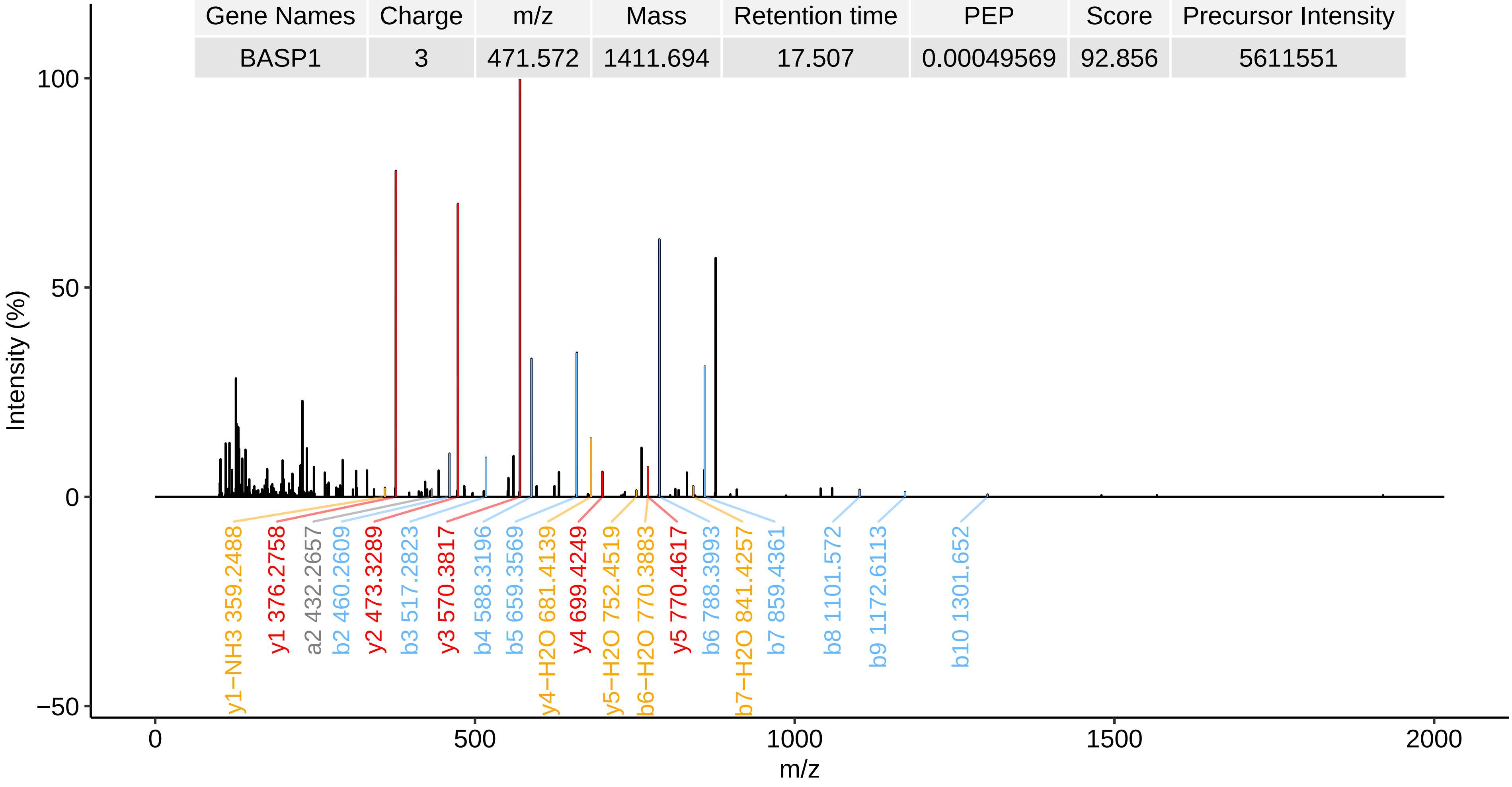

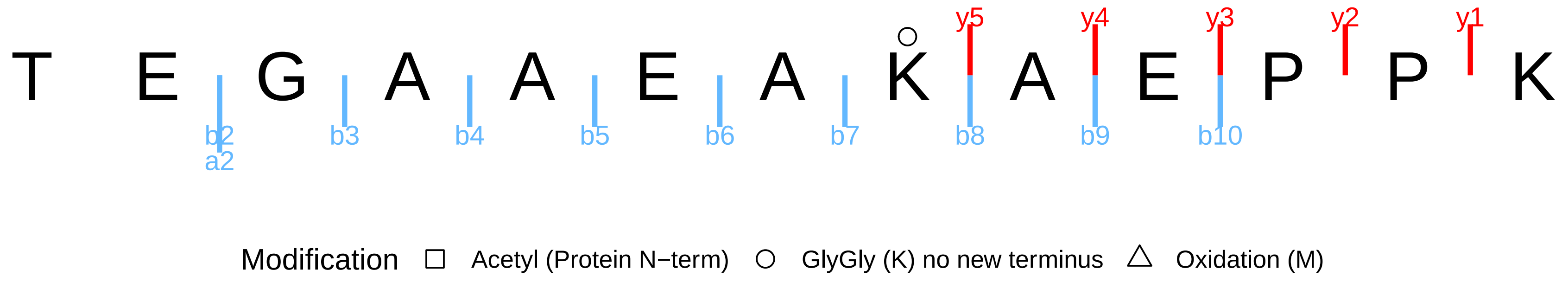

20200924_KIZ_MUb5_GlyGly_2 − Scan number: 5337

Gene Names	Charge	m/z	Mass	Retention time	PEP	Score	Precursor Intensity
BASP1	3	471.572	1411.694	19.793	0.010188	65.563	1564029

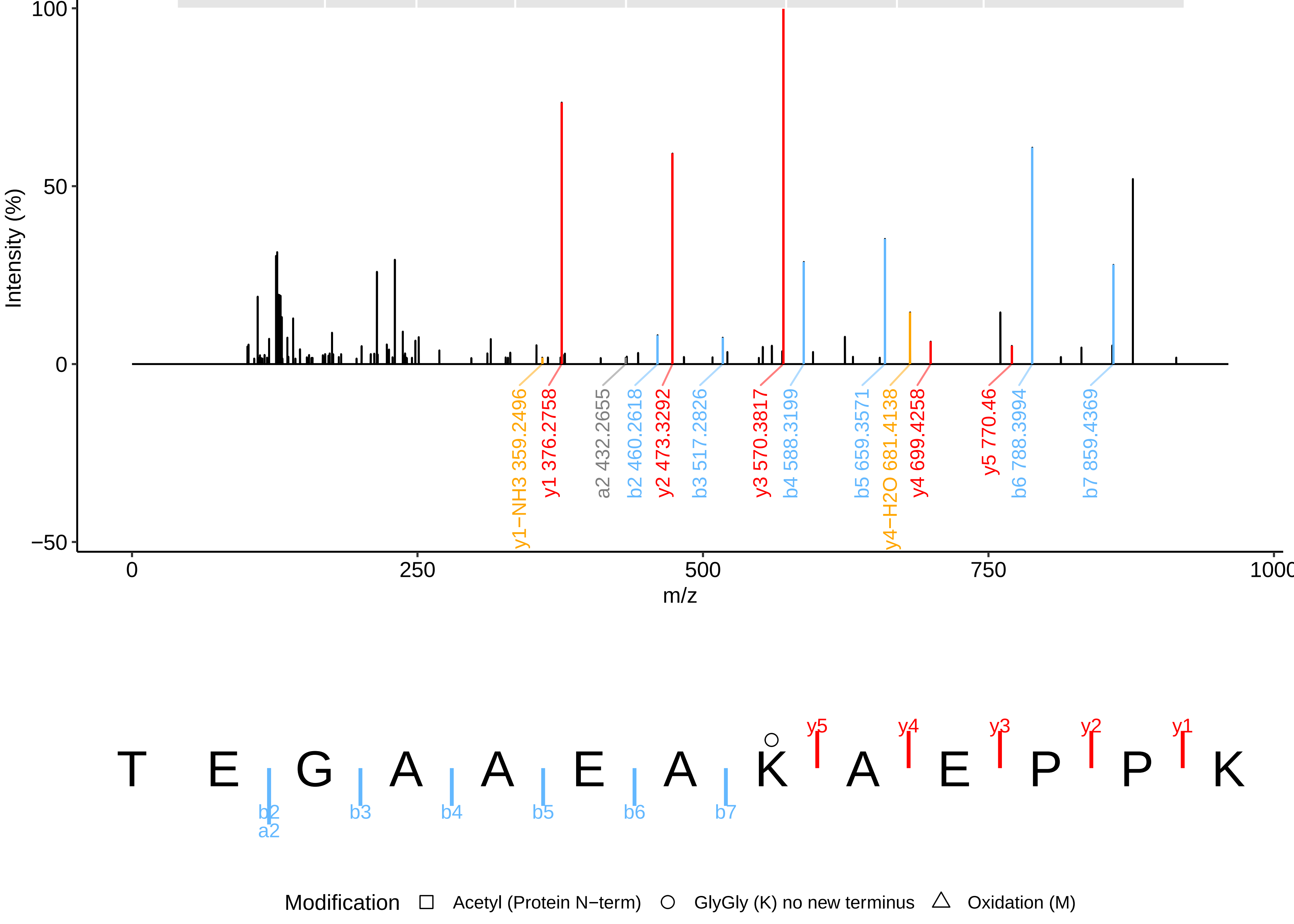

20201118_KIZ_MUb6_GlyGly_2 − Scan number: 6507

Gene Names	Charge	m/z	Mass	Retention time	PEP	Score	Precursor Intensity
BASP1	3	471.572	1411.694	22.292	0.043314	56.122	2157744

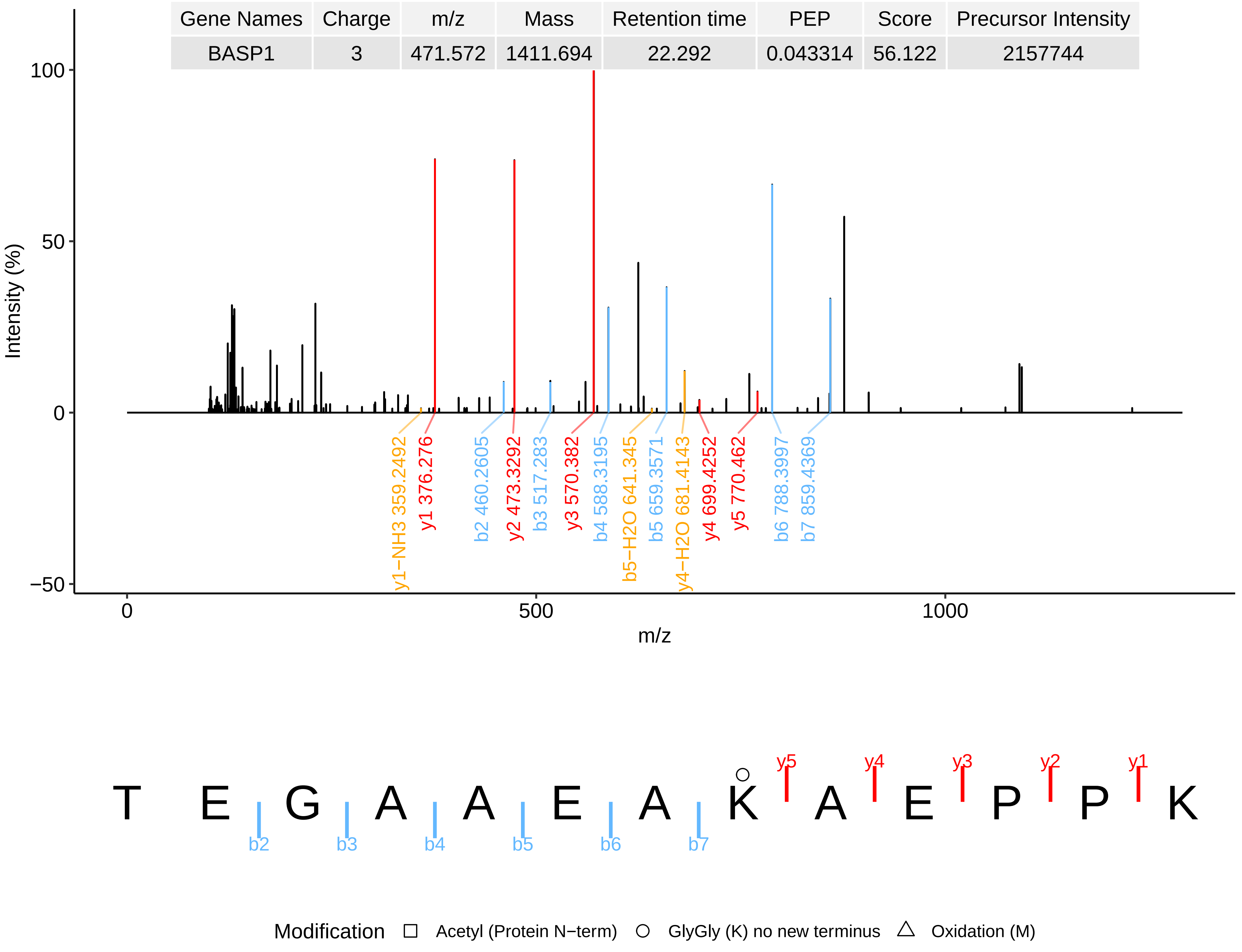

20200921_KIZ_MUb4_GlyGly_10plex − Scan number: 18418

Gene Names	Charge	m/z	Mass	Retention time	PEP	Score	Precursor Intensity
OLR1	3	474.5927	1420.756	66.232	0.0082541	72.652	3774963

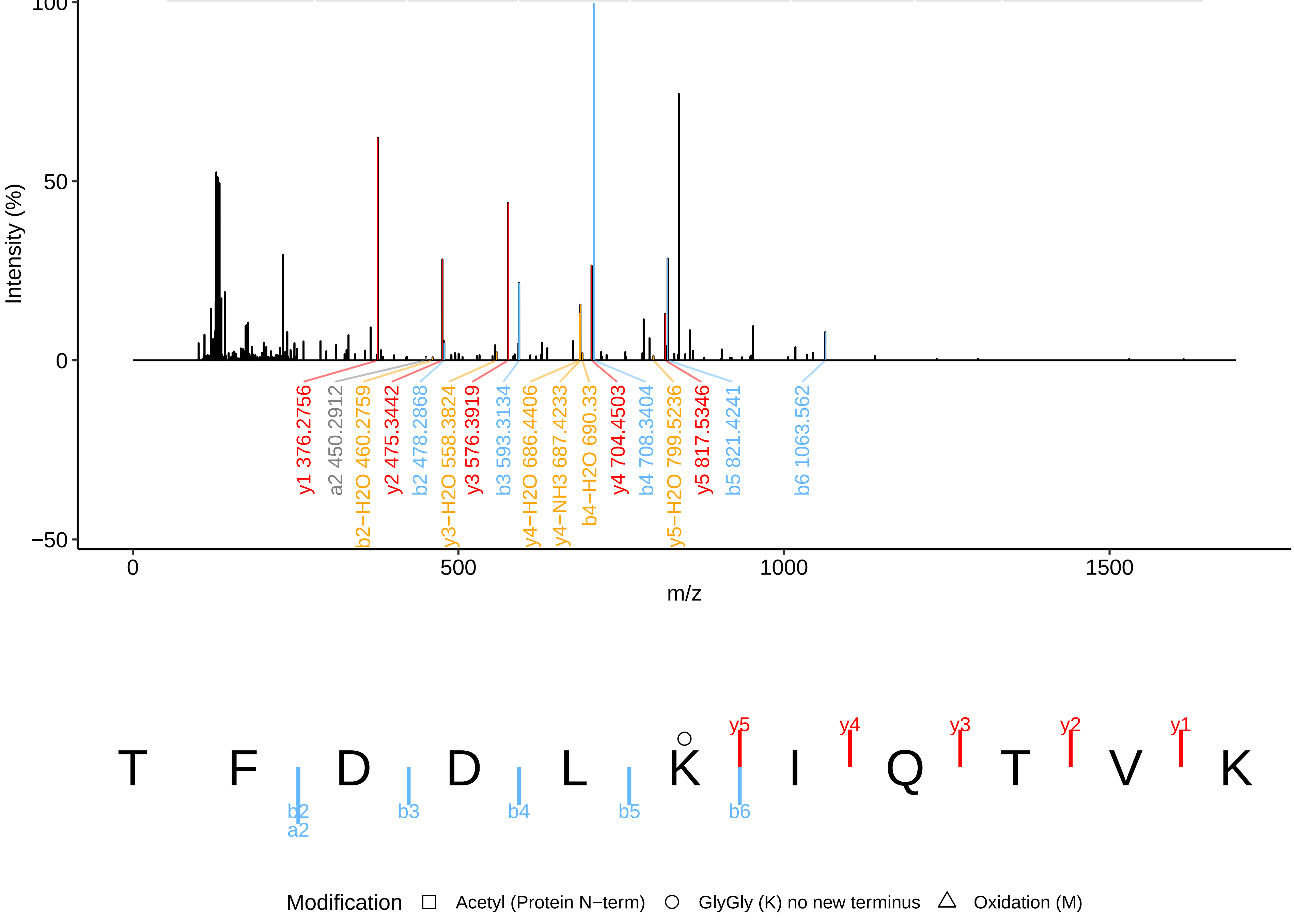

20200921_KIZ_MUb4_GlyGly_10plex_2 − Scan number: 16949

Gene Names	Charge	m/z	Mass	Retention time	PEP	Score	Precursor Intensity
OLR1	3	474.5927	1420.756	66.219	0.00081248	90.05	1147107

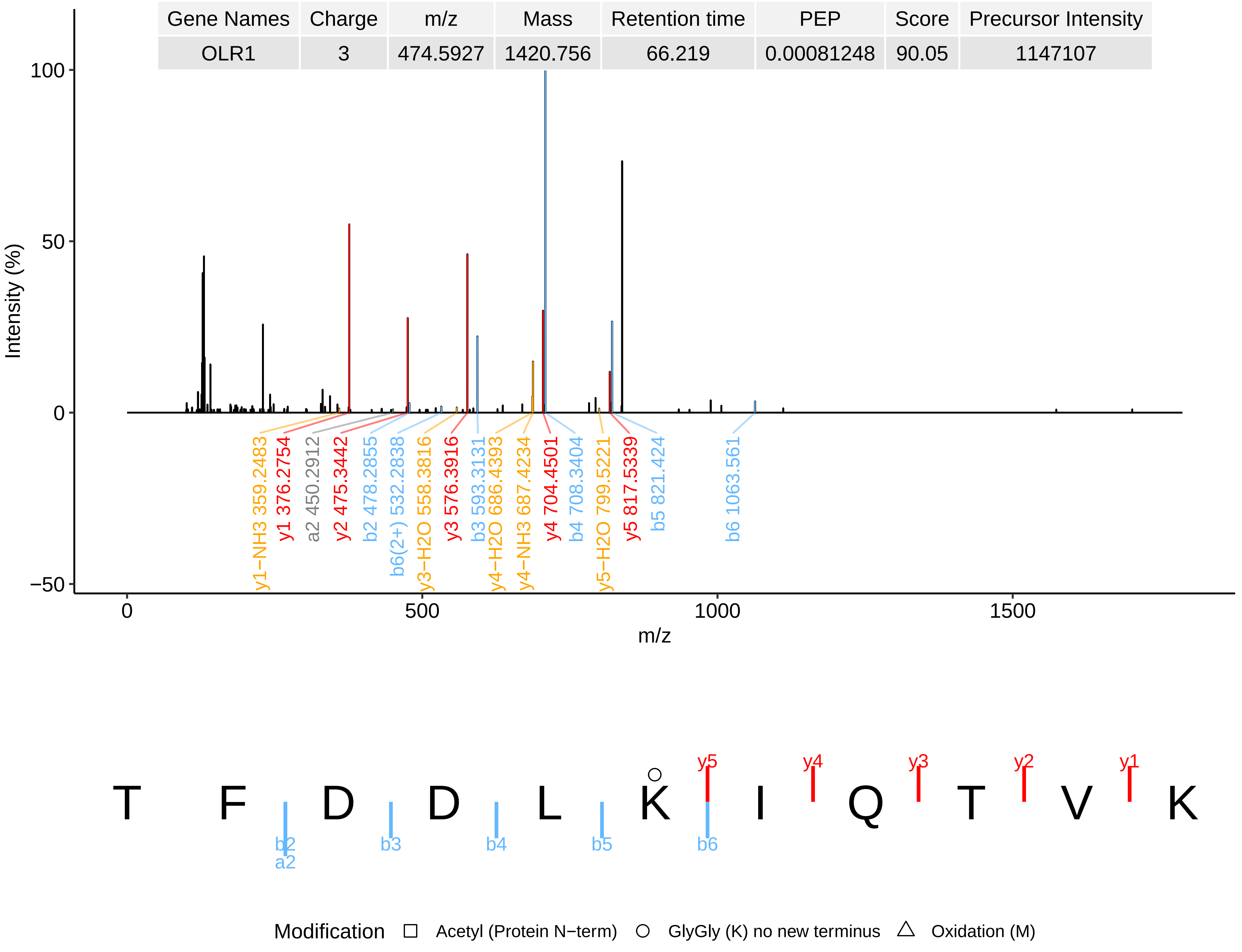

20200924_KIZ_MUb5_GlyGly_1 − Scan number: 18008

Gene Names	Charge	m/z	Mass	Retention time	PEP	Score	Precursor Intensity
OLR1	3	474.5927	1420.756	58.206	0.020002	62.408	8251064

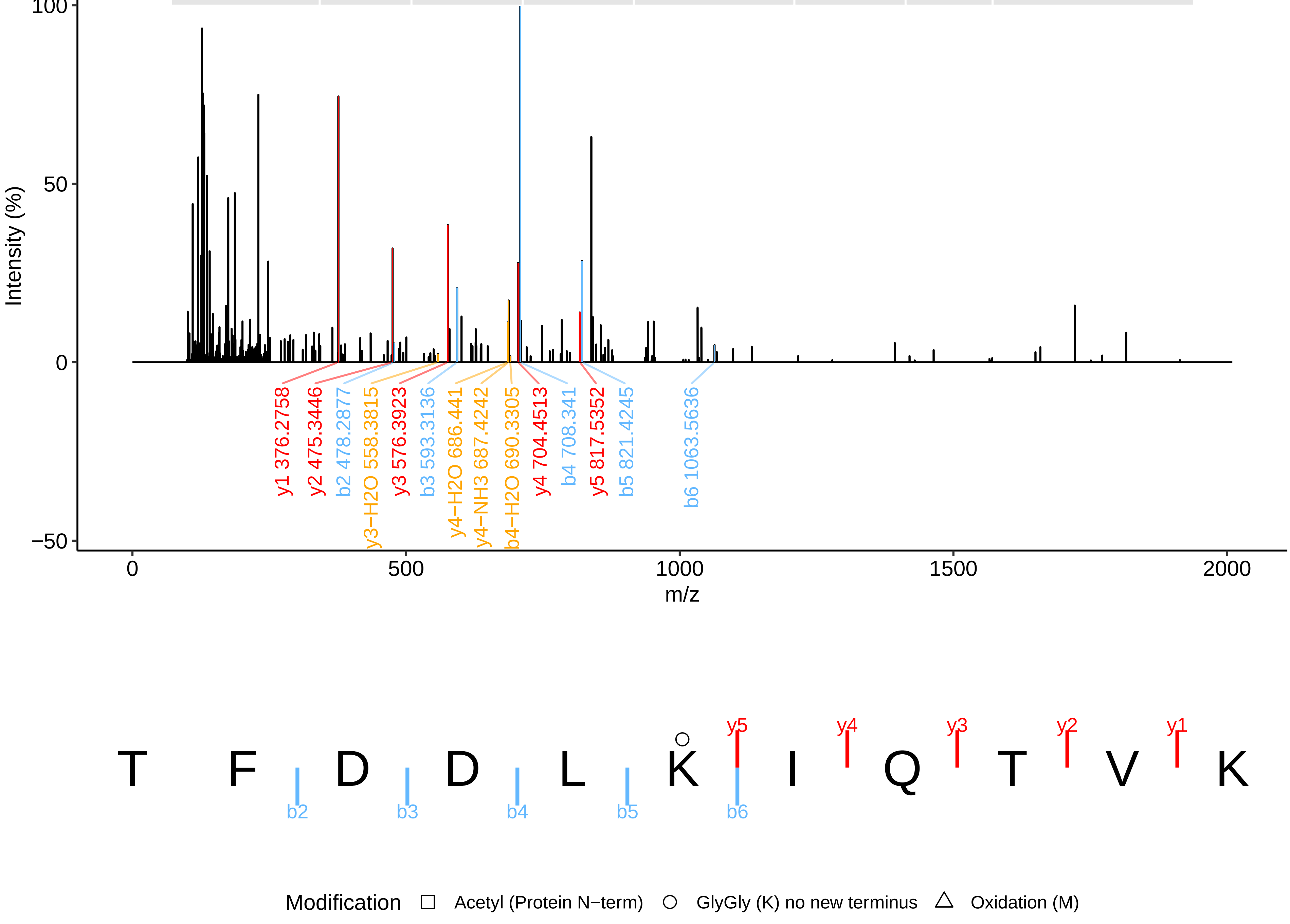

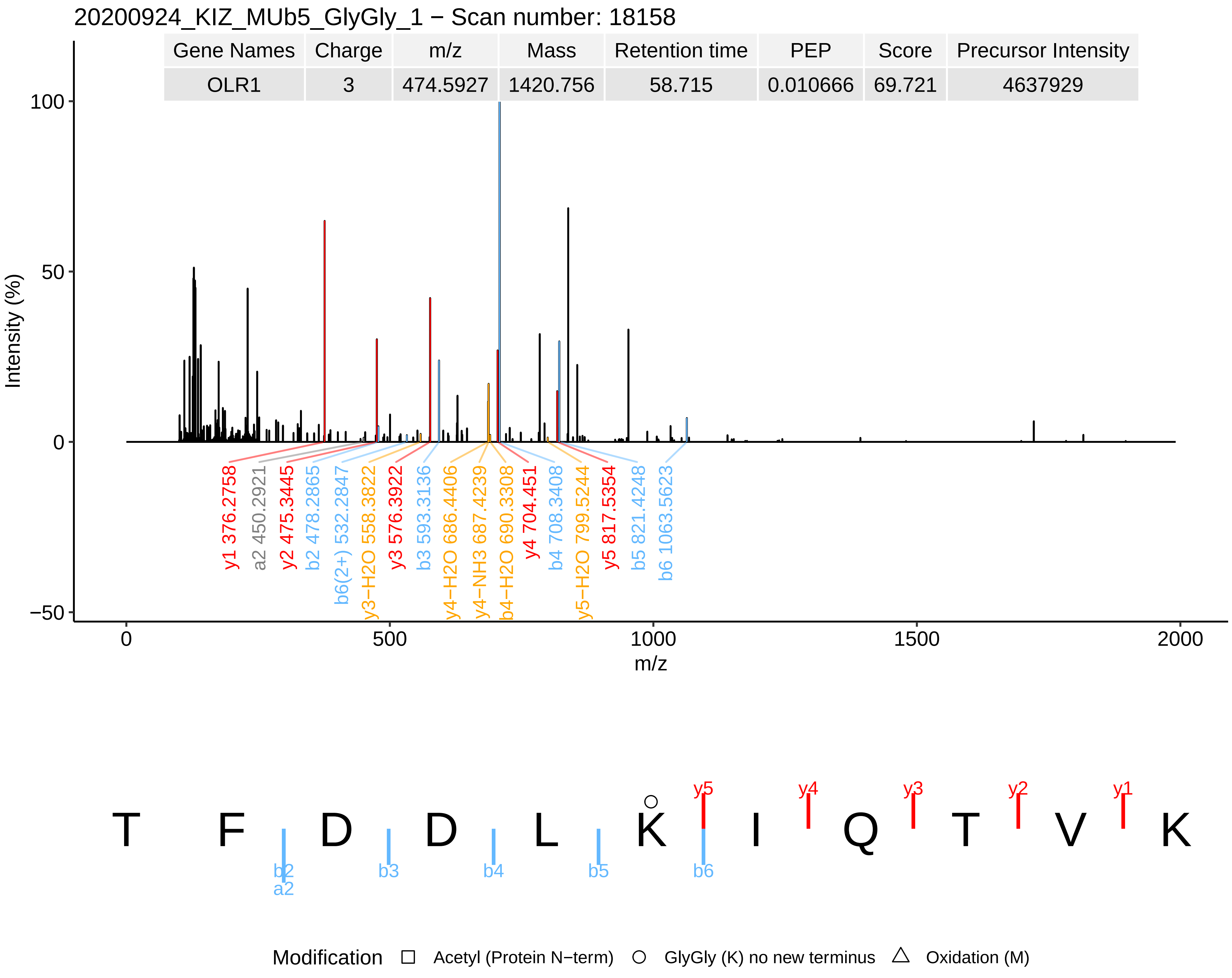

20200924_KIZ_MUb5_GlyGly_1 − Scan number: 18158
Gene Names | Charge | m/z | Mass | Retention time | PEP | Score | Precursor Intensity
OLR1 | 3 | 474.5927 | 1420.756 | 58.715 | 0.010666 | 69.721 | 4637929
Intensity (%)
m/z
y1 376.2758
a2 450.2921
y2 475.3445
b2 478.2865
b6(2+) 532.2847
y3−H2O 558.3822
y3 576.3922
b3 593.3136
y4−H2O 686.4406
y4−NH3 687.4239
b4−H2O 690.3308
y4 704.451
b4 708.3408
y5−H2O 799.5244
y5 817.5354
b5 821.4248
b6 1063.5623
T F D D L K I Q T V K
Modification
Acetyl (Protein N−term)
GlyGly (K) no new terminus
Oxidation (M)

20200924_KIZ_MUb5_GlyGly_1 − Scan number: 18167

Gene Names	Charge	m/z	Mass	Retention time	PEP	Score	Precursor Intensity
OLR1	3	474.5927	1420.756	58.742	0.0082541	72.652	5880538

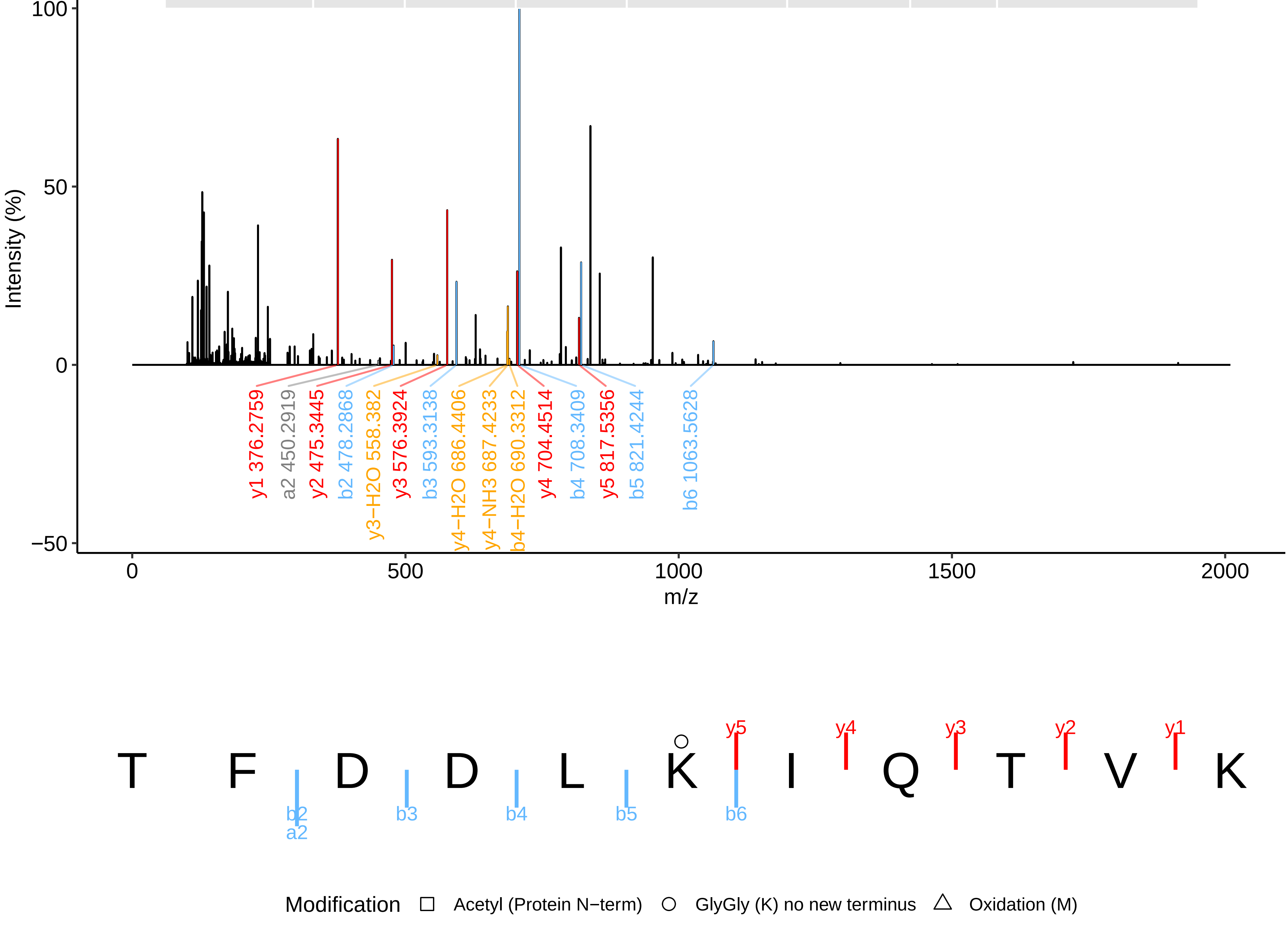

20200924_KIZ_MUb5_GlyGly_2 − Scan number: 16680

Gene Names	Charge	m/z	Mass	Retention time	PEP	Score	Precursor Intensity
OLR1	3	474.5927	1420.756	61.885	0.11246	49.632	NA

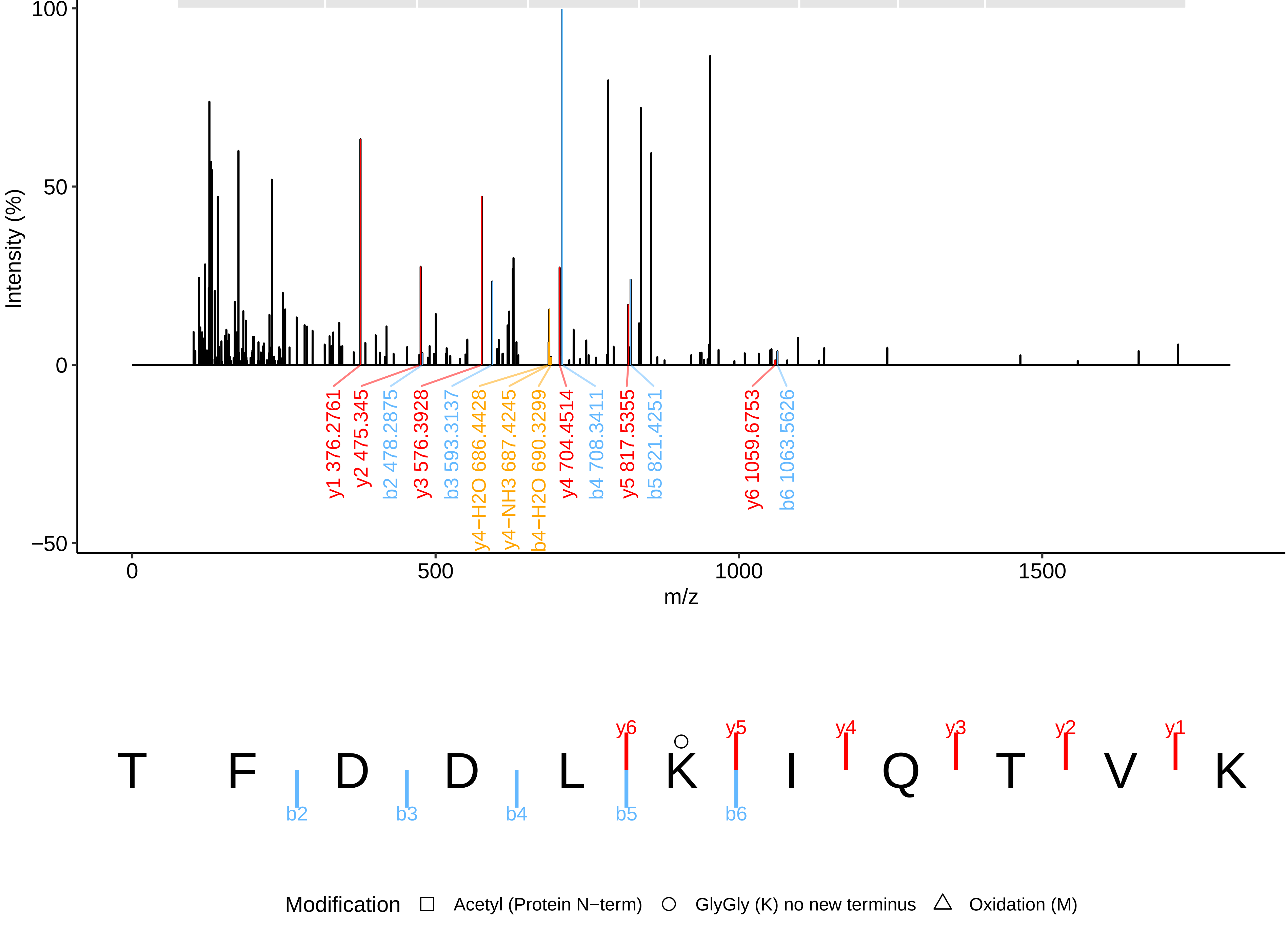

20200924_KIZ_MUb5_GlyGly_2 − Scan number: 16816

Gene Names	Charge	m/z	Mass	Retention time	PEP	Score	Precursor Intensity
OLR1	3	474.5927	1420.756	62.397	0.039896	60.255	3651398

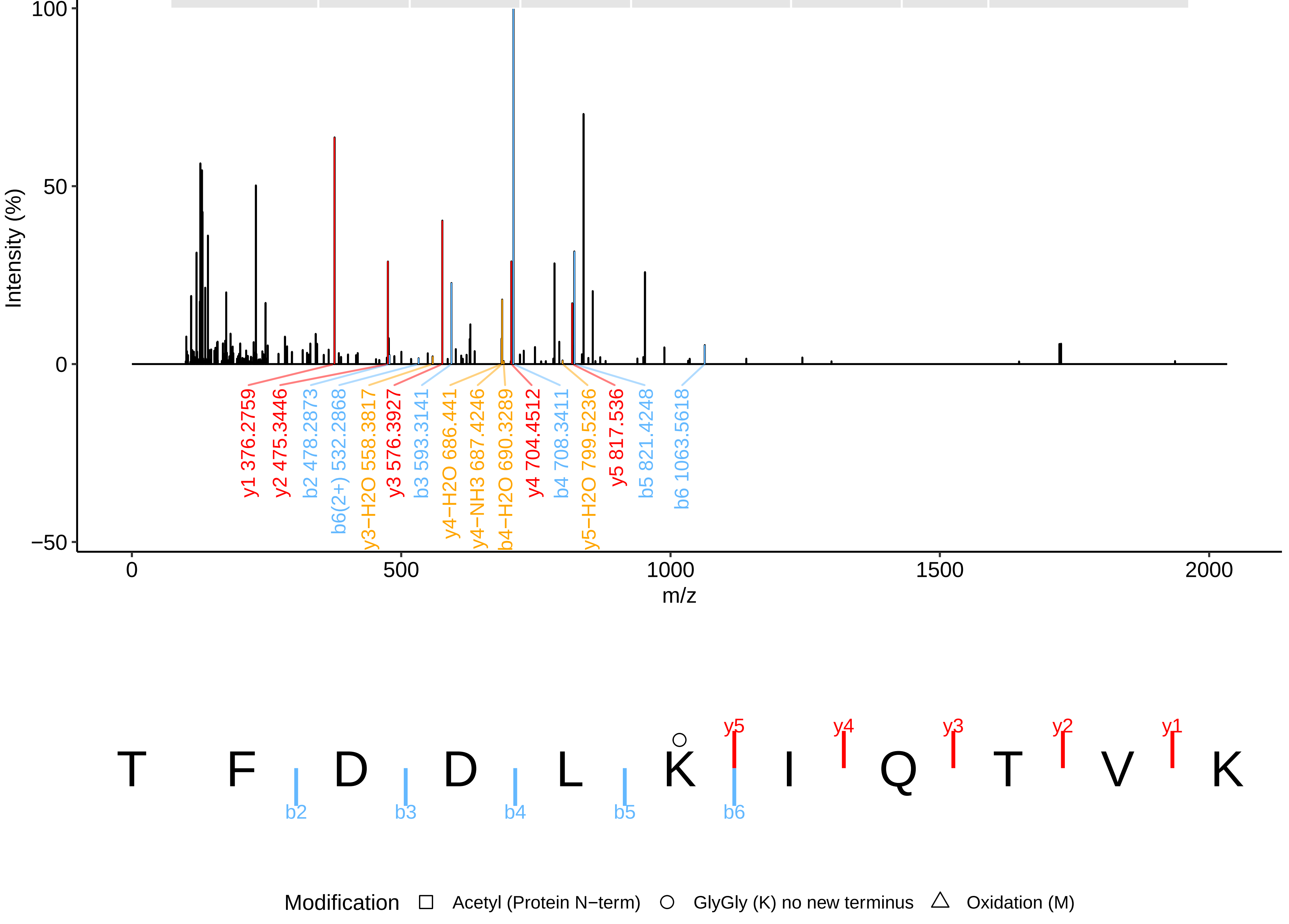

20201118_KIZ_MUb6_GlyGly_1 − Scan number: 18283

Gene Names	Charge	m/z	Mass	Retention time	PEP	Score	Precursor Intensity
OLR1	3	474.5927	1420.756	57.501	0.0082541	72.652	14705241

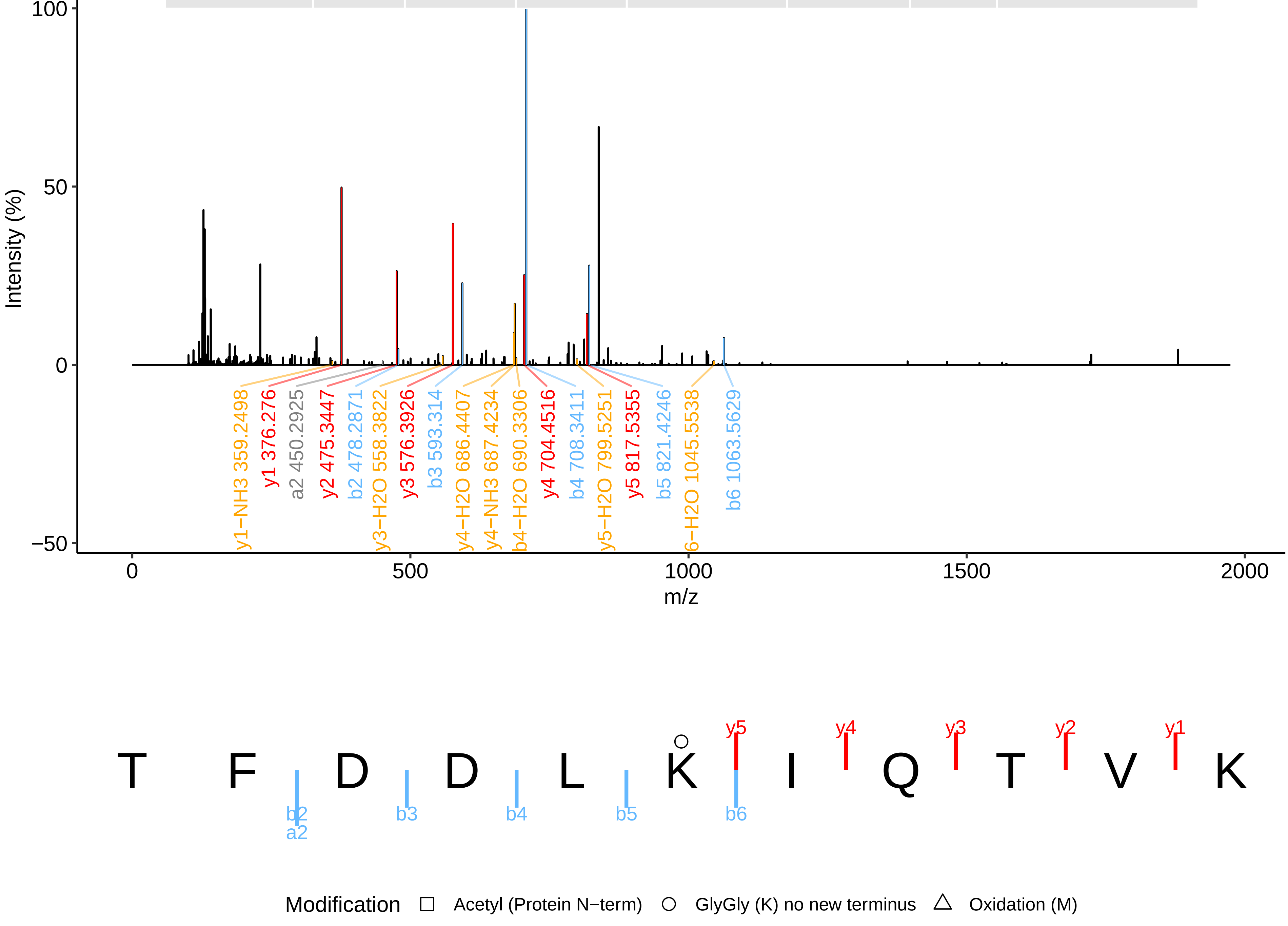

20201118_KIZ_MUb6_GlyGly_2 − Scan number: 20032

Gene Names	Charge	m/z	Mass	Retention time	PEP	Score	Precursor Intensity
OLR1	3	474.5927	1420.756	67.557	0.00081248	90.05	10162849

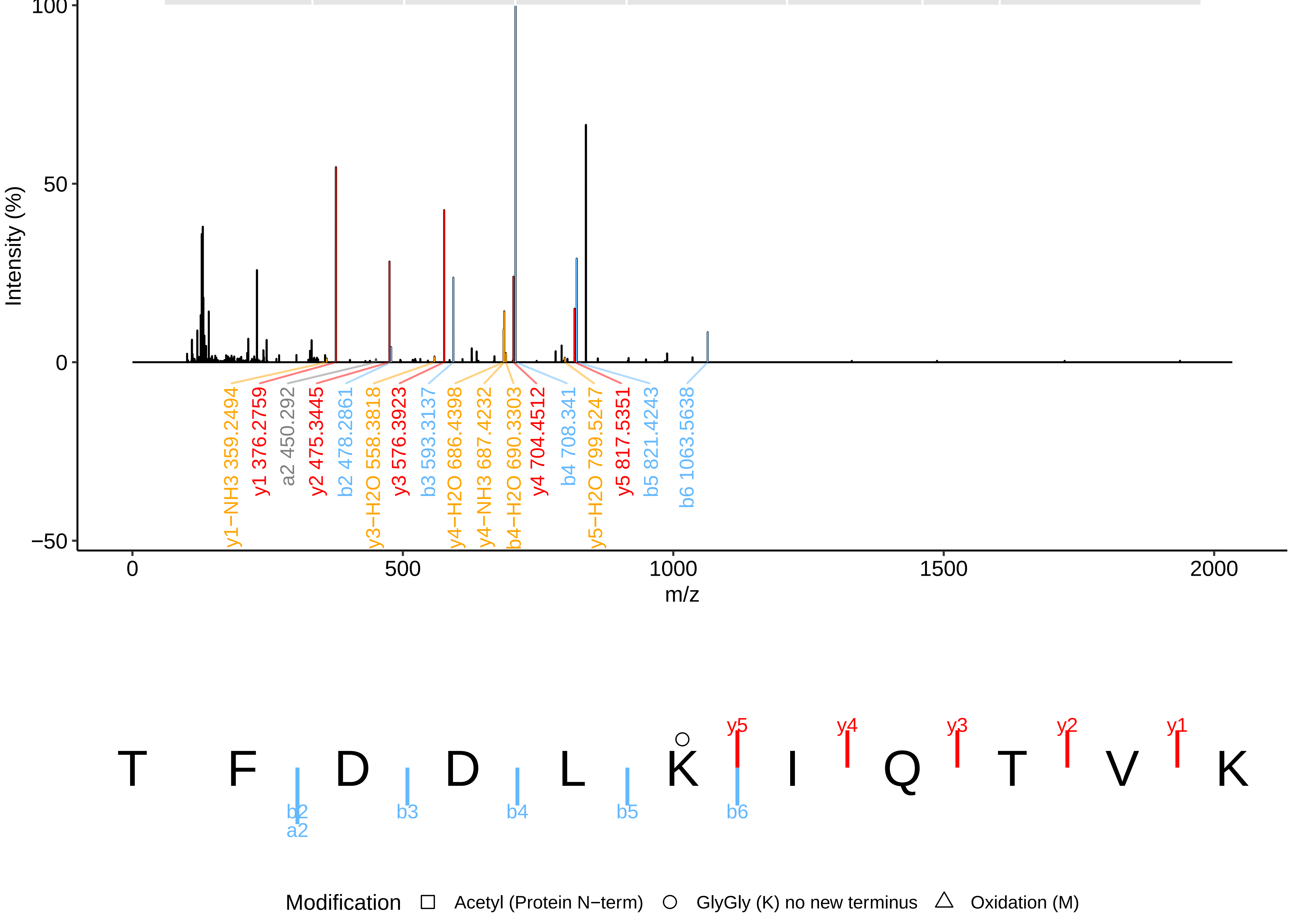

20201118_KIZ_MUb6_GlyGly_2 − Scan number: 20043

Gene Names	Charge	m/z	Mass	Retention time	PEP	Score	Precursor Intensity
OLR1	3	474.5927	1420.756	67.596	0.00058128	92.445	20346100

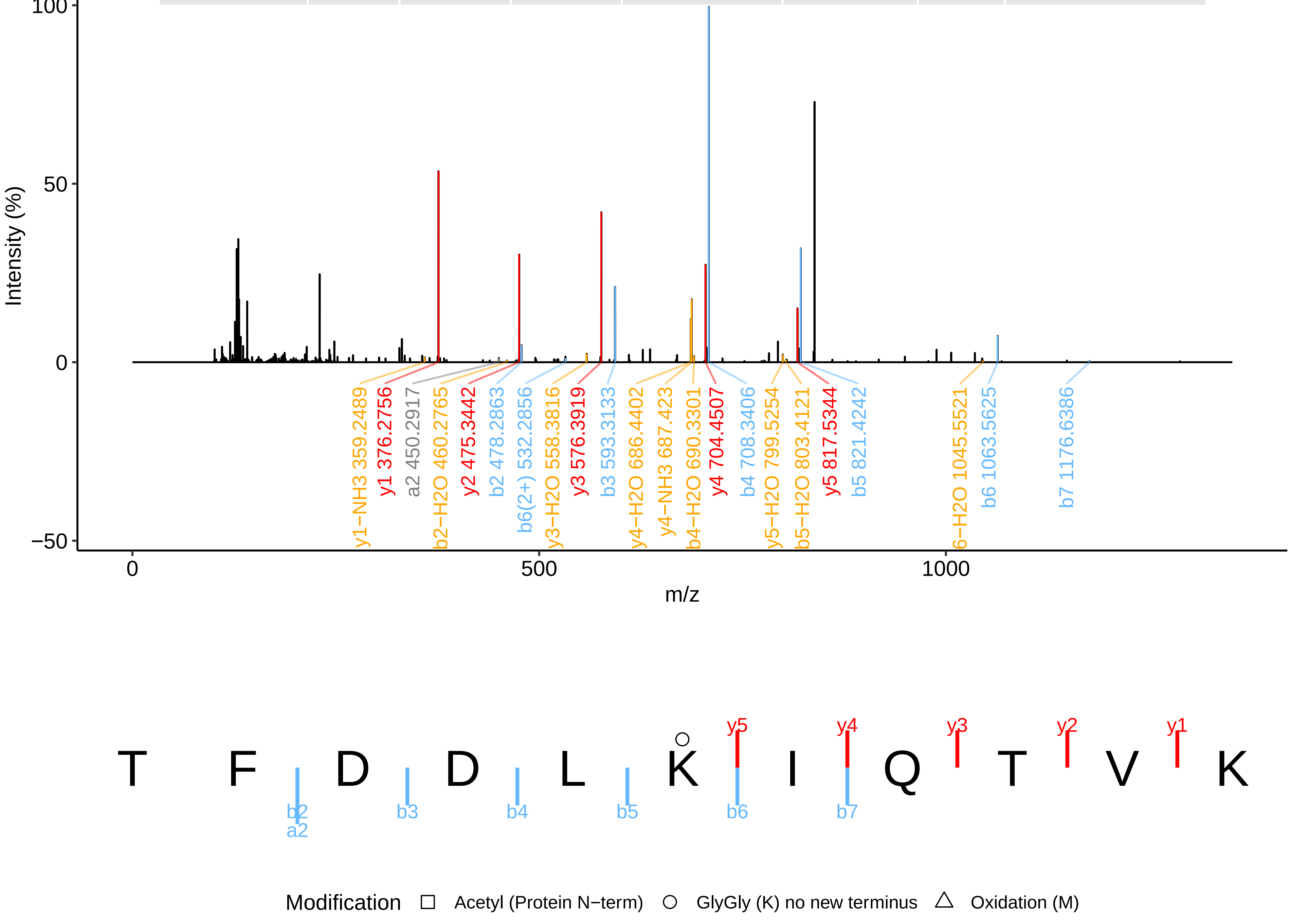

20200921_KIZ_MUb4_GlyGly_10plex_2 − Scan number: 19420

Gene Names	Charge	m/z	Mass	Retention time	PEP	Score	Precursor Intensity
MFAP3L;MFAP3	3	464.6013	1390.782	76.226	0.015682	63.624	1301004

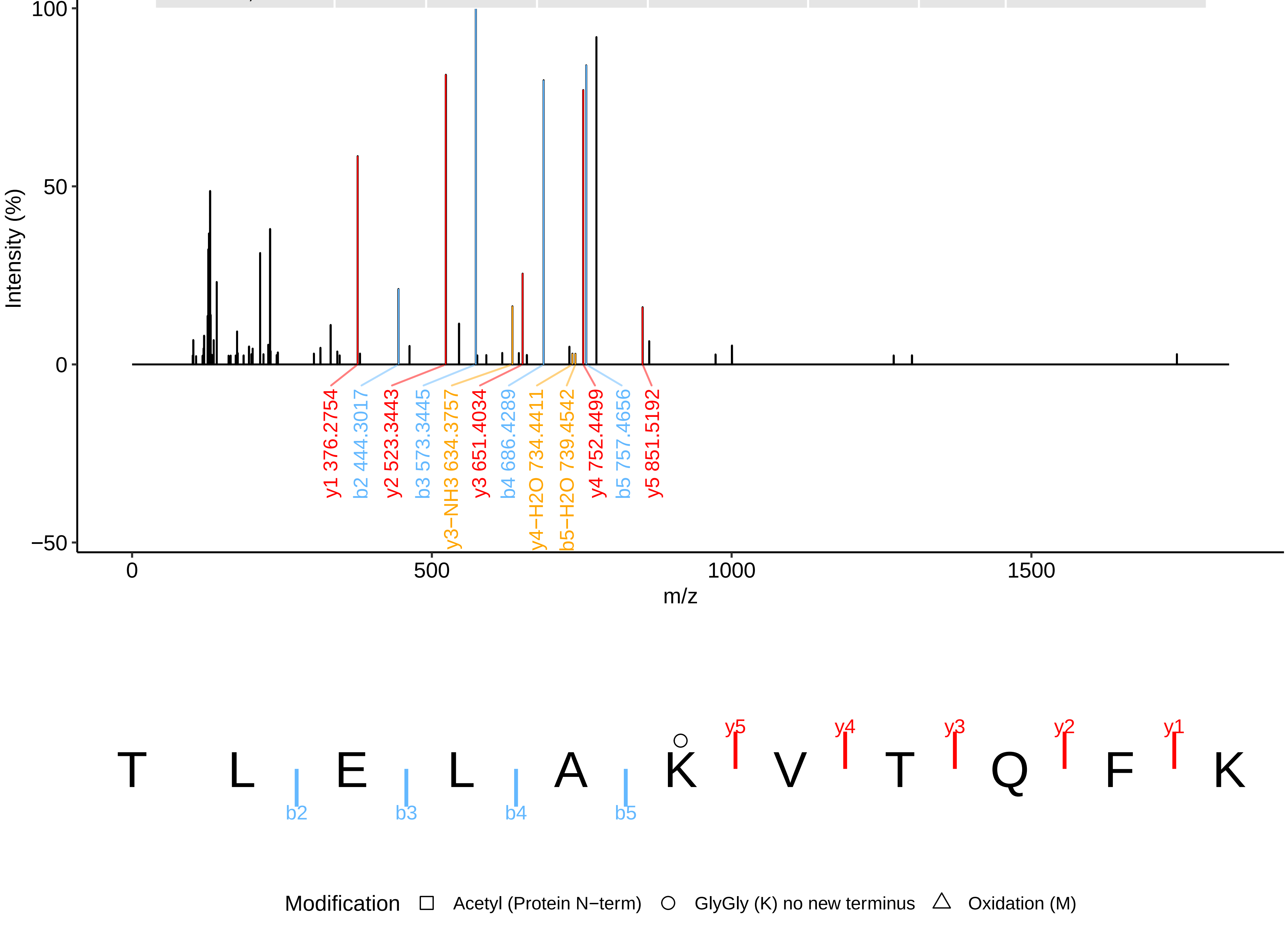

20200921_KIZ_MUb4_GlyGly_10plex − Scan number: 13082

Gene Names	Charge	m/z	Mass	Retention time	PEP	Score	Precursor Intensity
TMEM230	3	477.2718	1428.794	46.017	3.1569e−05	97.86	9375596

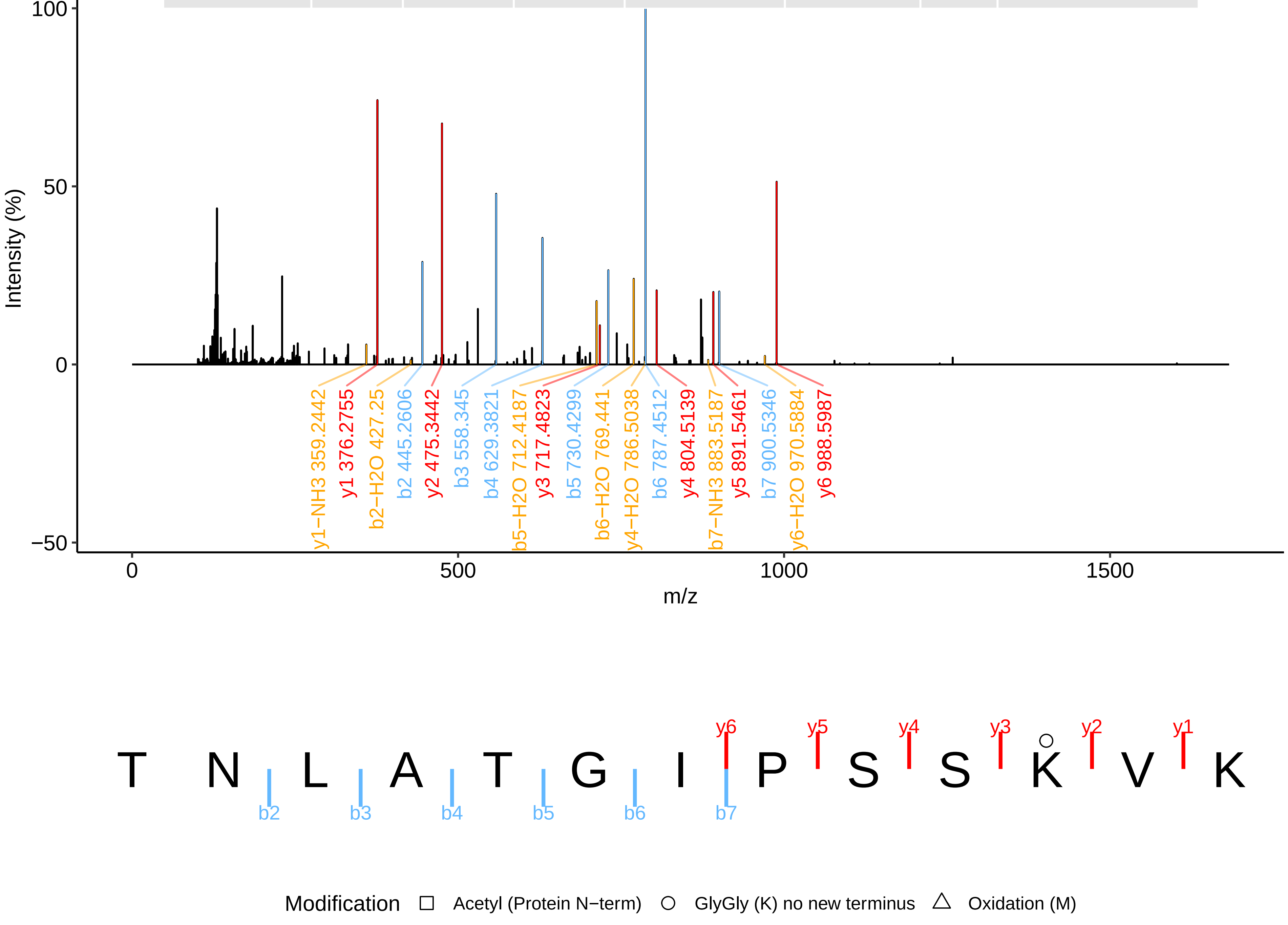

20200921_KIZ_MUb4_GlyGly_10plex_2 − Scan number: 12007

Gene Names	Charge	m/z	Mass	Retention time	PEP	Score	Precursor Intensity
TMEM230	3	477.2718	1428.794	46.361	0.0011369	80.96	987828.9

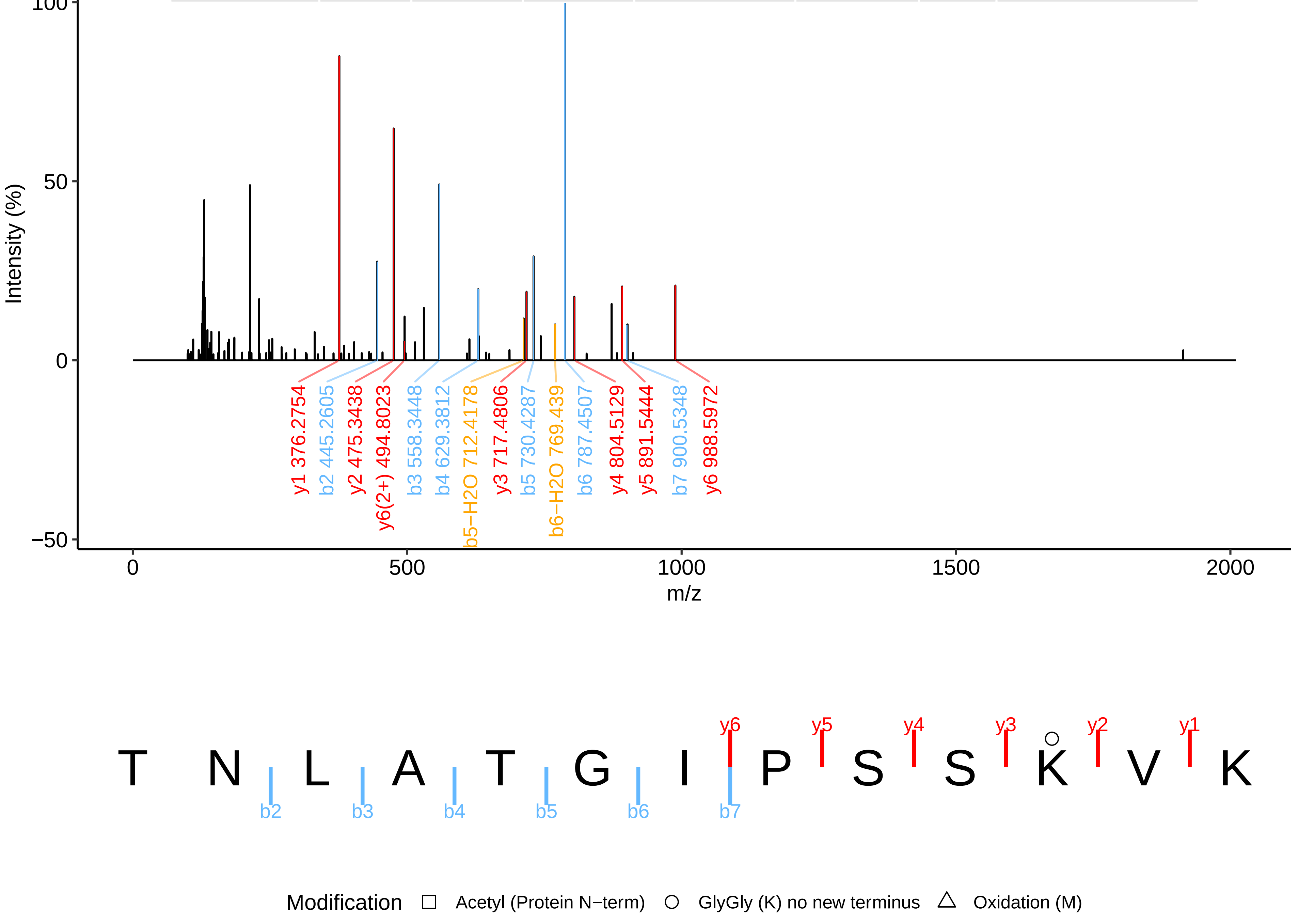

20200921_KIZ_MUb4_GlyGly_10plex_2 − Scan number: 12053

Gene Names	Charge	m/z	Mass	Retention time	PEP	Score	Precursor Intensity
TMEM230	3	477.2718	1428.794	46.548	0.069133	50.563	234549.1

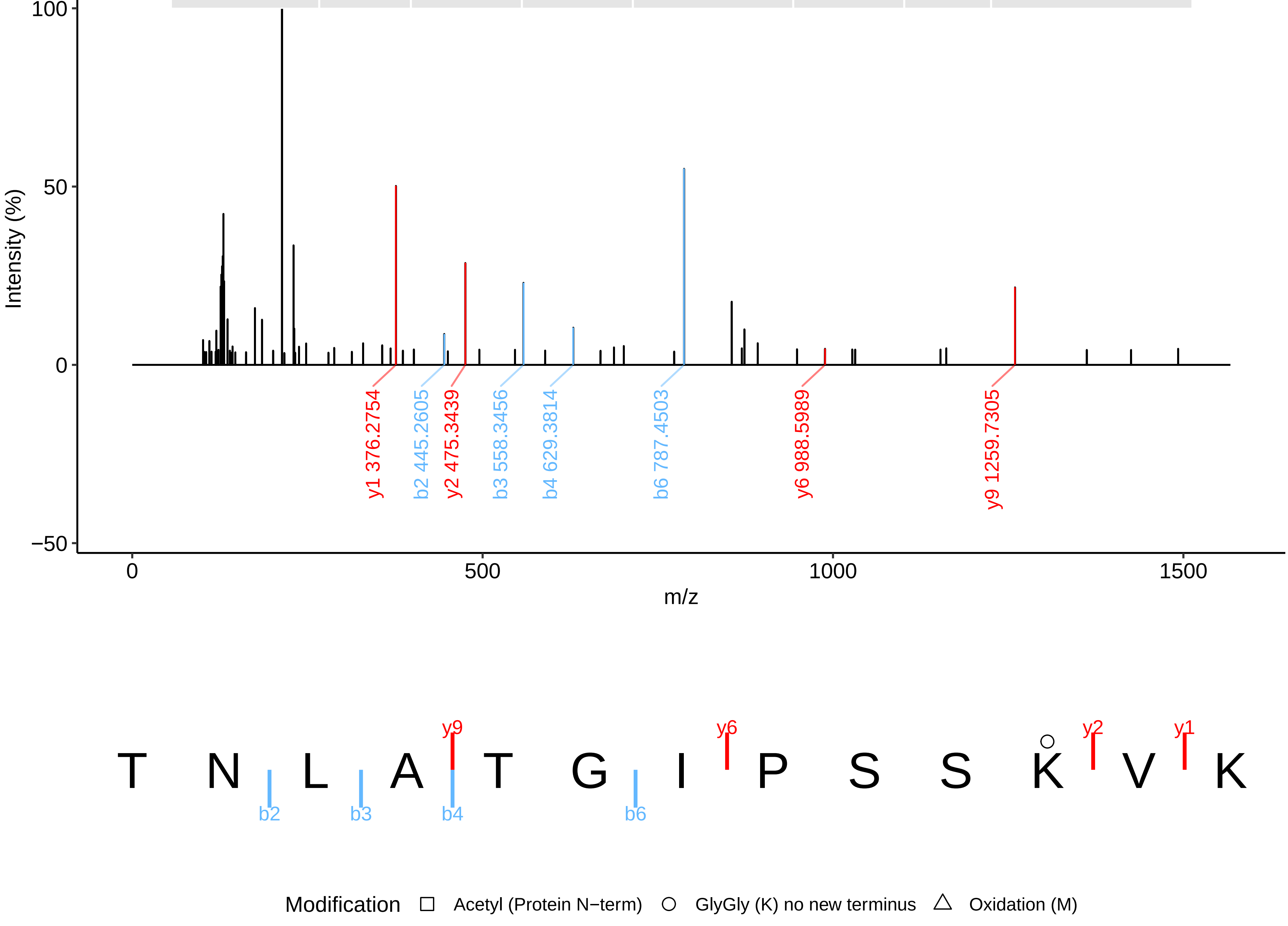

20200921_KIZ_MUb4_GlyGly_10plex − Scan number: 11514

Gene Names	Charge	m/z	Mass	Retention time	PEP	Score	Precursor Intensity
CYC1	3	539.2969	1614.869	40.147	9.9247e−05	88.427	8869658

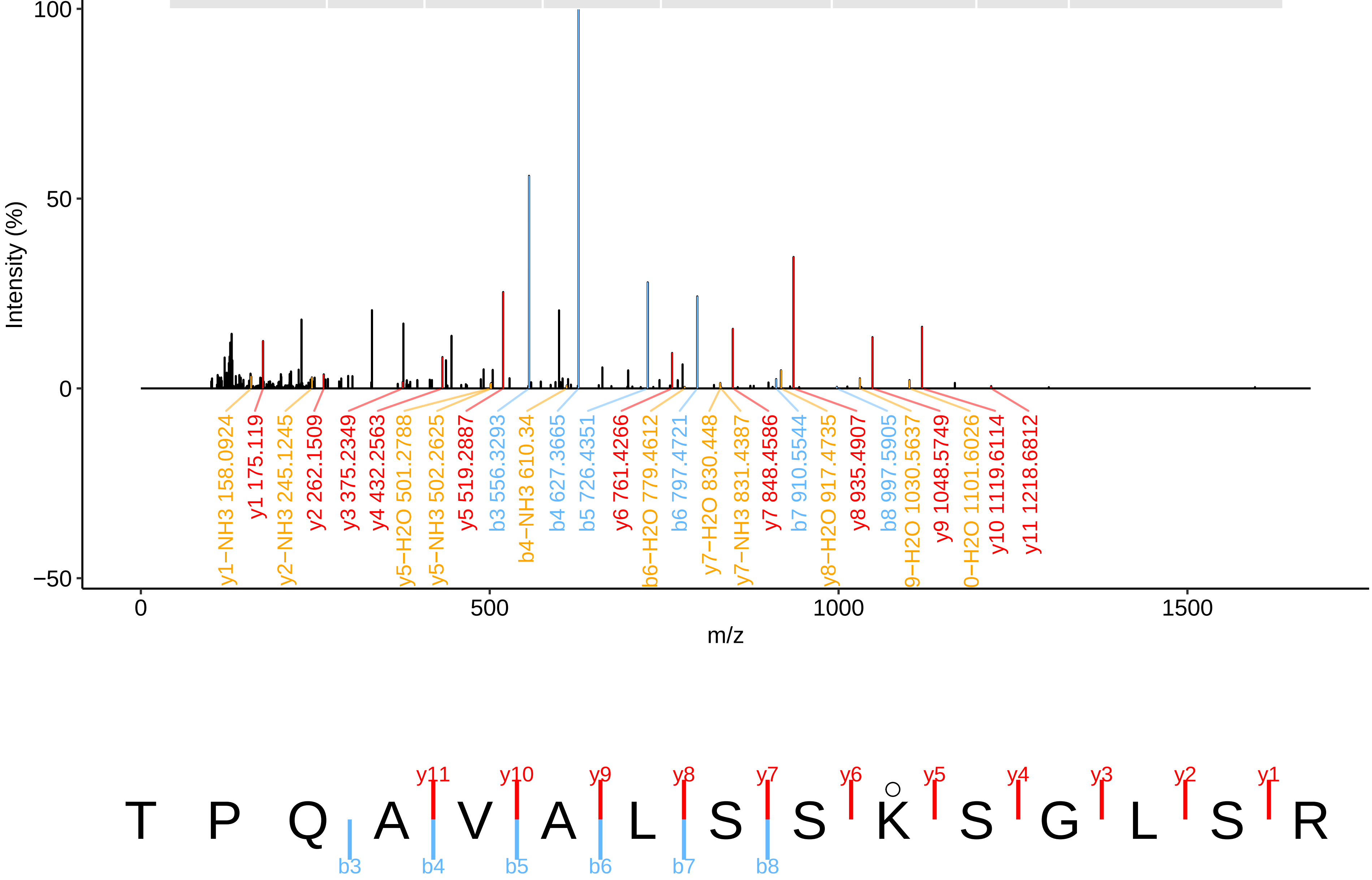

Modification □ Acetyl (Protein N−term) ○ GlyGly (K) no new terminus △ Oxidation (M)

20200921_KIZ_MUb4_GlyGly_10plex − Scan number: 14719

Gene Names	Charge	m/z	Mass	Retention time	PEP	Score	Precursor Intensity
VAT1	3	464.8918	1391.654	52.355	0.0082541	72.652	5282805

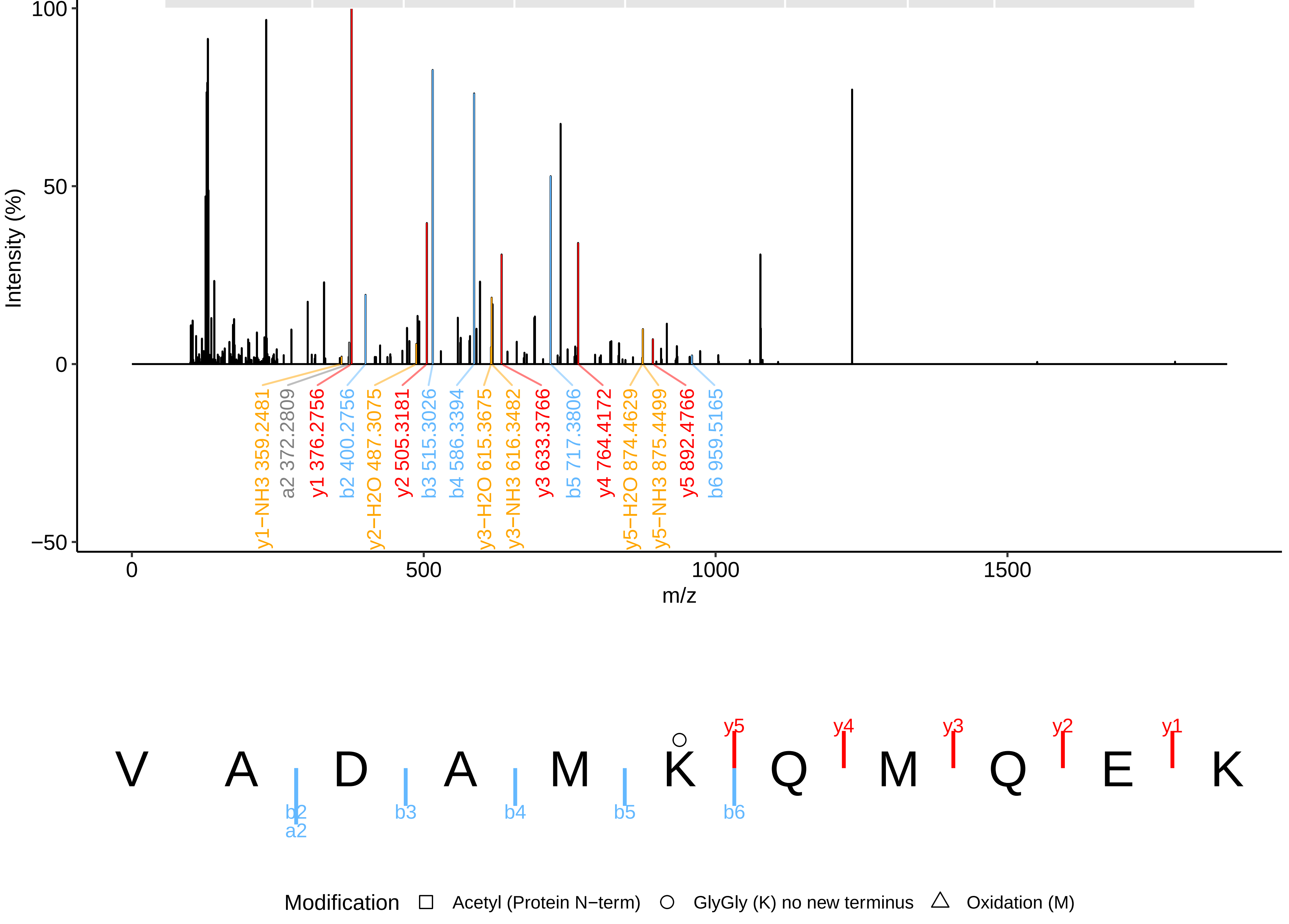

20200921_KIZ_MUb4_GlyGly_10plex_2 − Scan number: 13567

Gene Names	Charge	m/z	Mass	Retention time	PEP	Score	Precursor Intensity
VAT1	3	464.8918	1391.654	52.708	0.0084042	74.944	446448.4

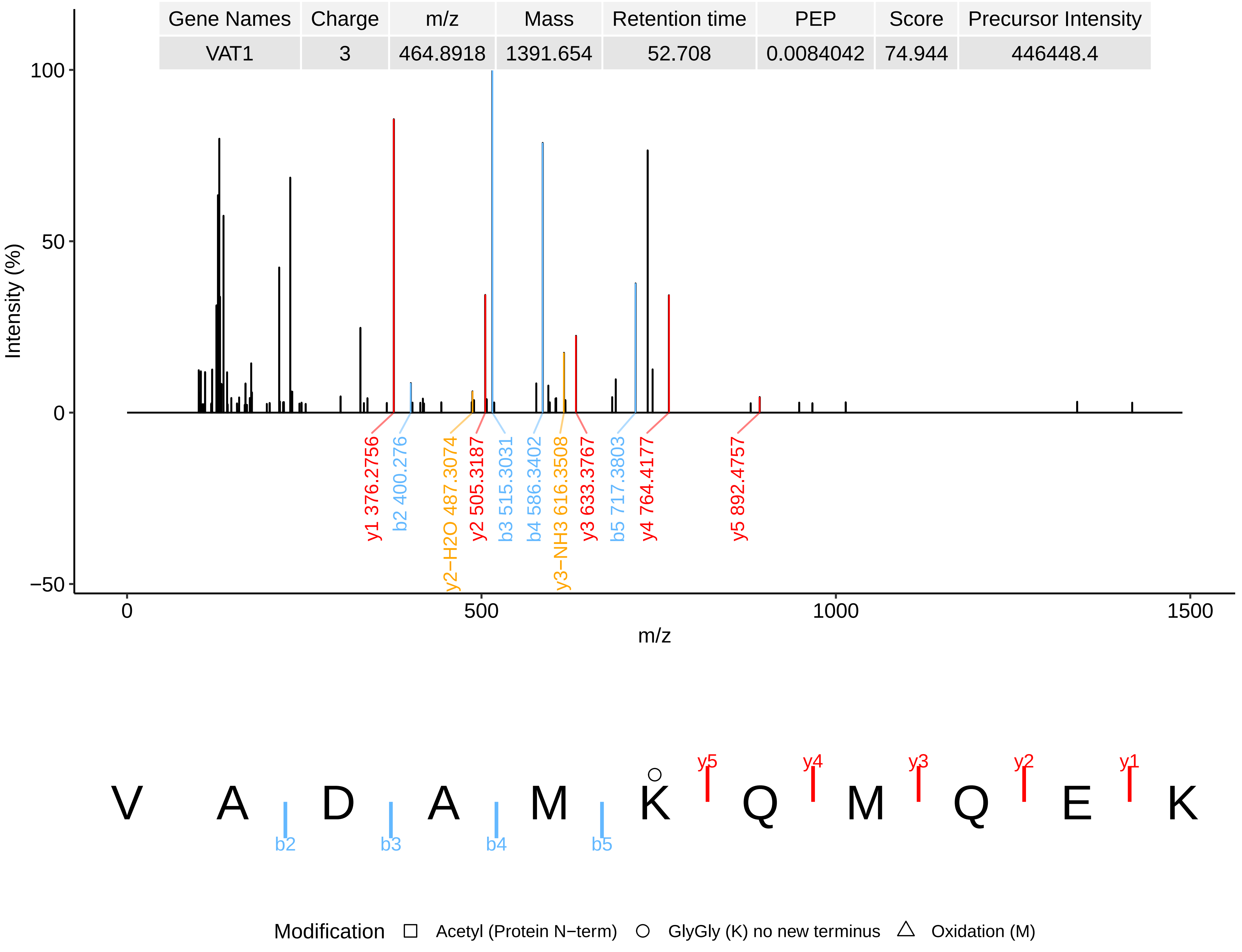

20200921_KIZ_MUb4_GlyGly_10plex − Scan number: 12762

Gene Names	Charge	m/z	Mass	Retention time	PEP	Score	Precursor Intensity
EIF3D	3	542.262	1623.764	44.857	0.018492	73.927	NA

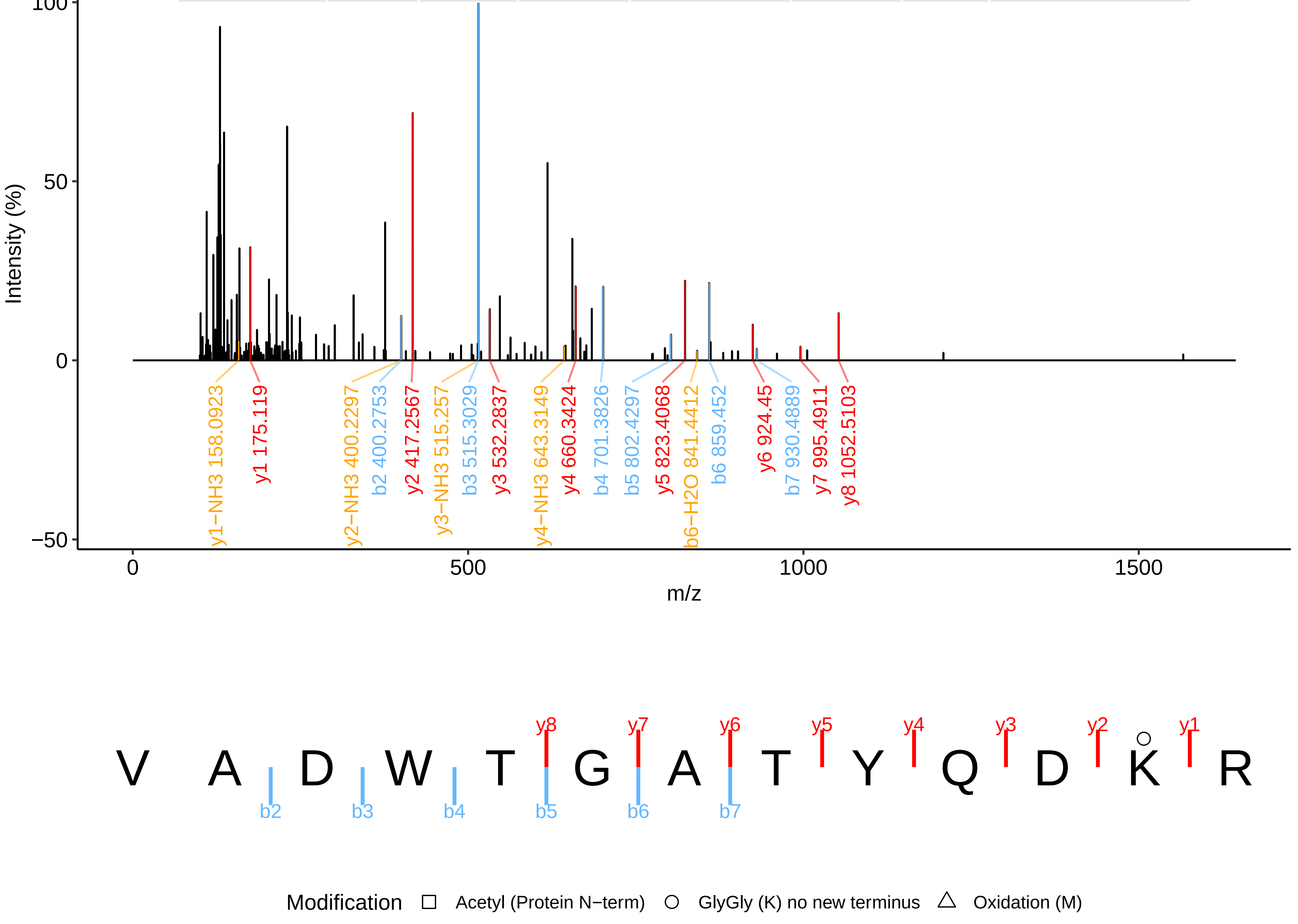

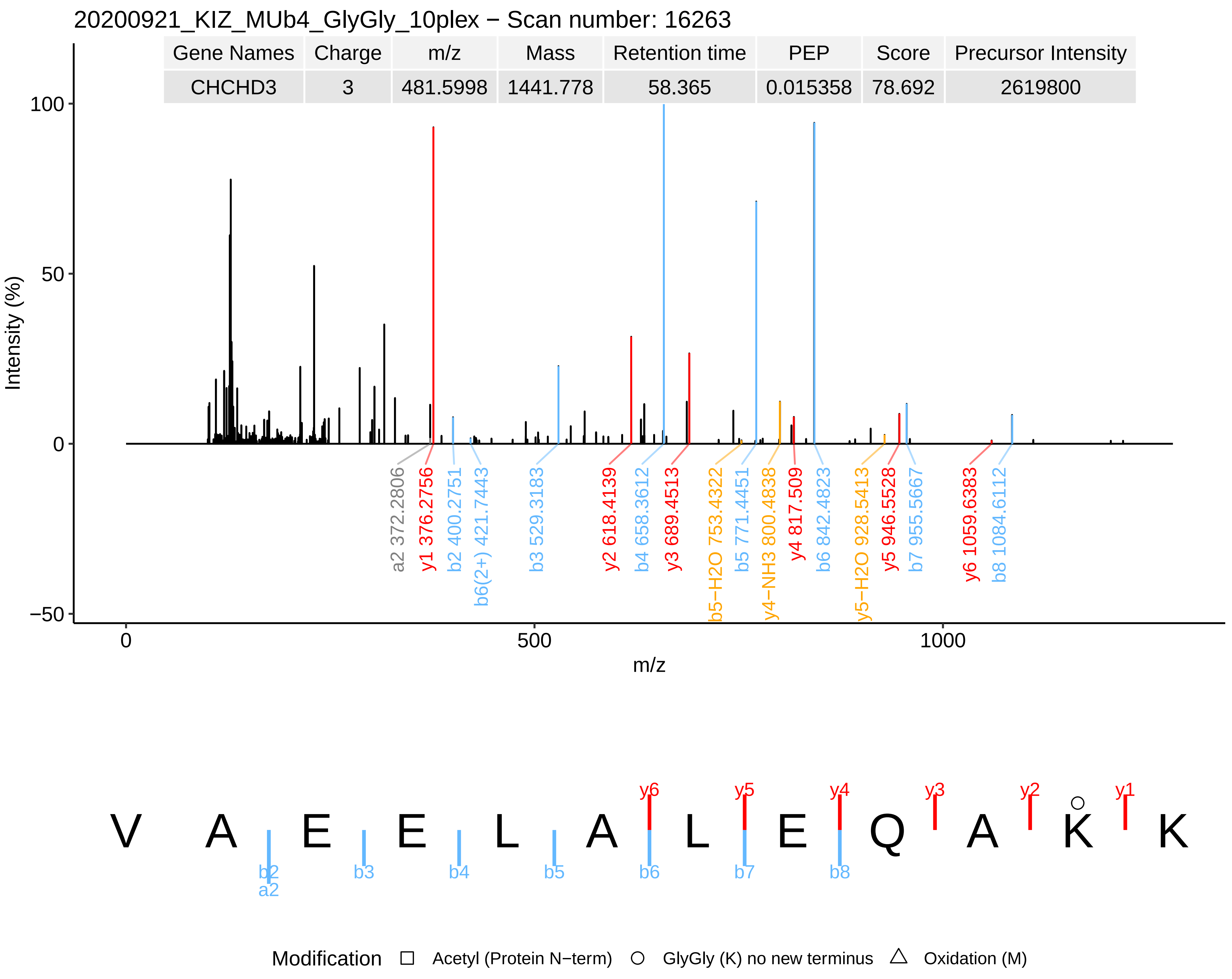

20200921_KIZ_MUb4_GlyGly_10plex − Scan number: 16263
Gene Names | Charge | m/z | Mass | Retention time | PEP | Score | Precursor Intensity
CHCHD3 | 3 | 481.5998 | 1441.778 | 58.365 | 0.015358 | 78.692 | 2619800
Intensity (%)
m/z
a2 372.2806
y1 376.2756
b2 400.2751
b6(2+) 421.7443
b3 529.3183
y2 618.4139
b4 658.3612
y3 689.4513
b5−H2O 753.4322
b5 771.4451
y4−NH3 800.4838
y4 817.509
b6 842.4823
y5−H2O 928.5413
y5 946.5528
b7 955.5667
y6 1059.6383
b8 1084.6112
V A E E L A L E Q A K K
Modification
Acetyl (Protein N−term)
GlyGly (K) no new terminus
Oxidation (M)

20200921_KIZ_MUb4_GlyGly_10plex_2 − Scan number: 15106

Gene Names	Charge	m/z	Mass	Retention time	PEP	Score	Precursor Intensity
CHCHD3	3	481.5998	1441.778	59.008	0.0073625	80.979	1189418

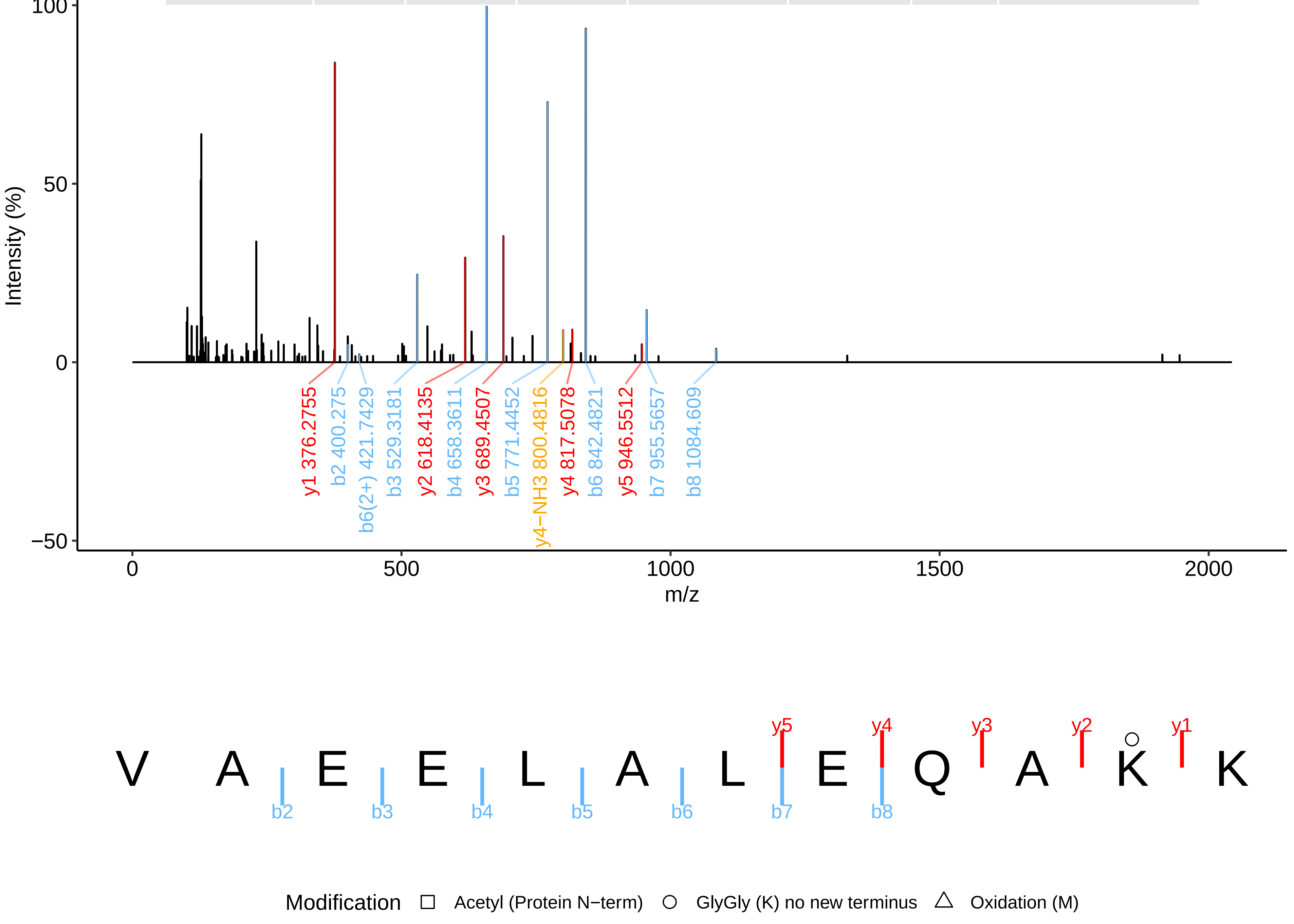

20200921_KIZ_MUb4_GlyGly_10plex_2 − Scan number: 15888

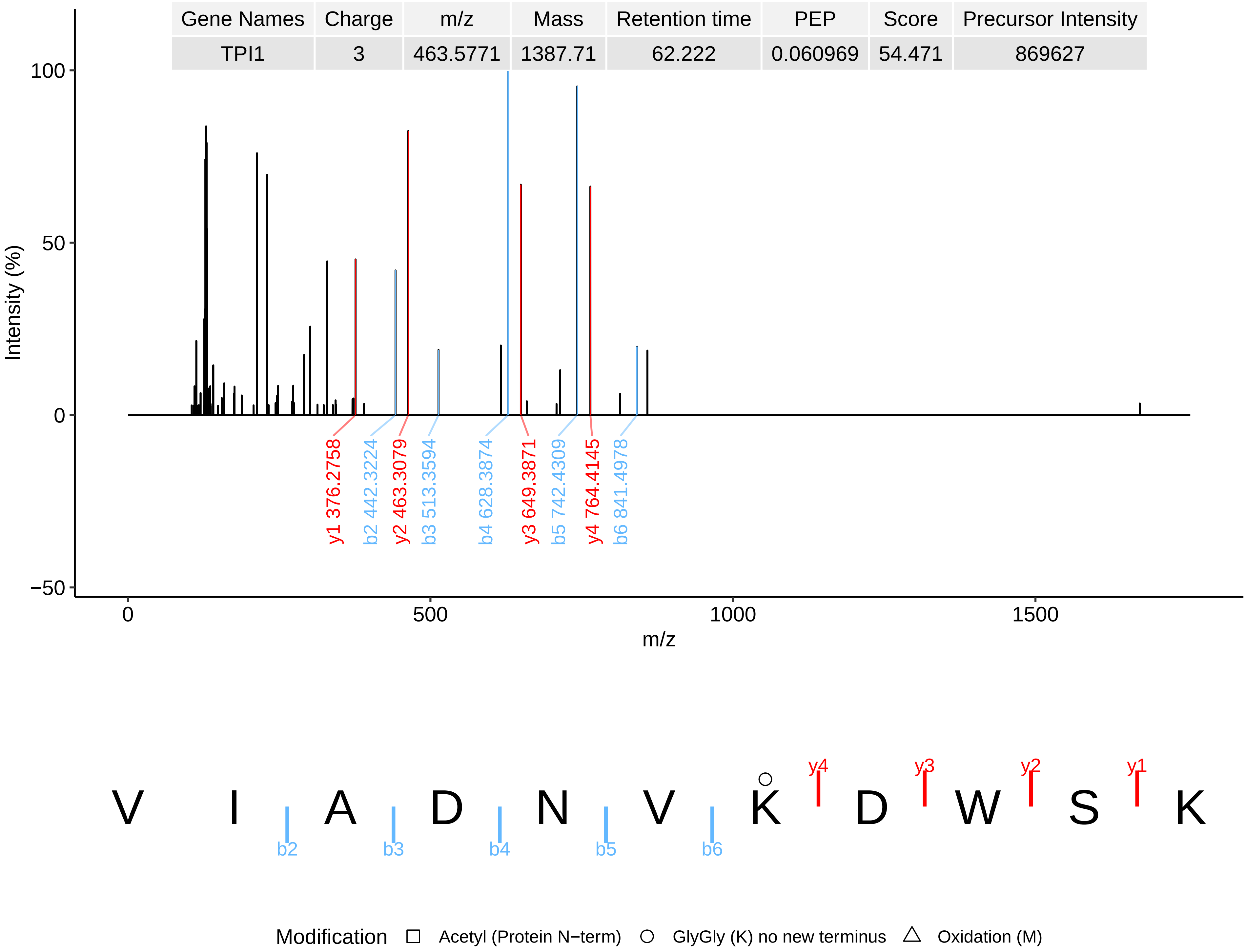

20200921_KIZ_MUb4_GlyGly_10plex_2 − Scan number: 18380

Gene Names	Charge	m/z	Mass	Retention time	PEP	Score	Precursor Intensity
RBBP7;RBBP4	3	479.2575	1434.751	71.967	0.022926	63.283	691243.4

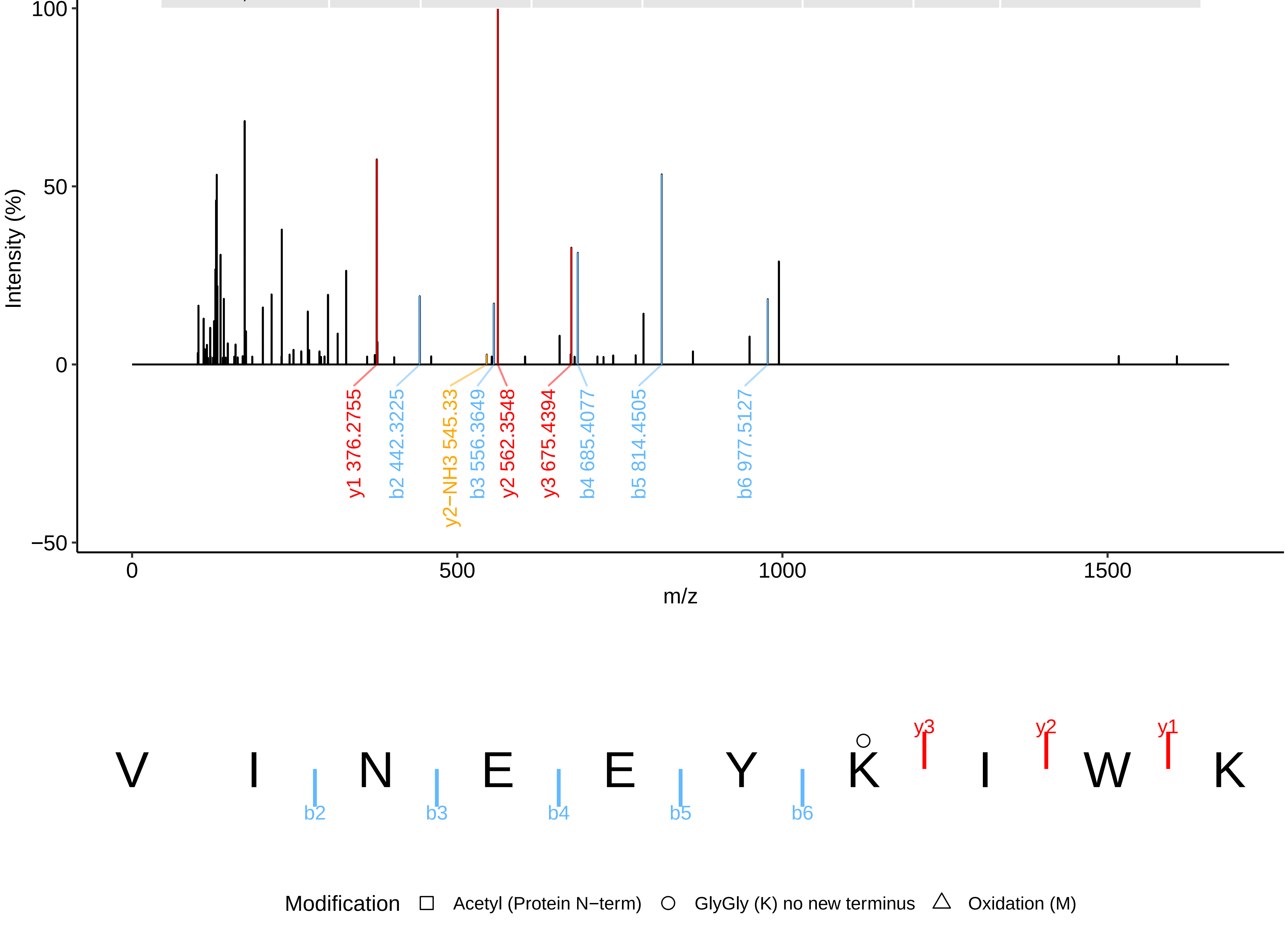

20200924_KIZ_MUb5_GlyGly_1 − Scan number: 19562

Gene Names	Charge	m/z	Mass	Retention time	PEP	Score	Precursor Intensity
RBBP7;RBBP4	3	479.2575	1434.751	63.246	0.033803	61.353	NA

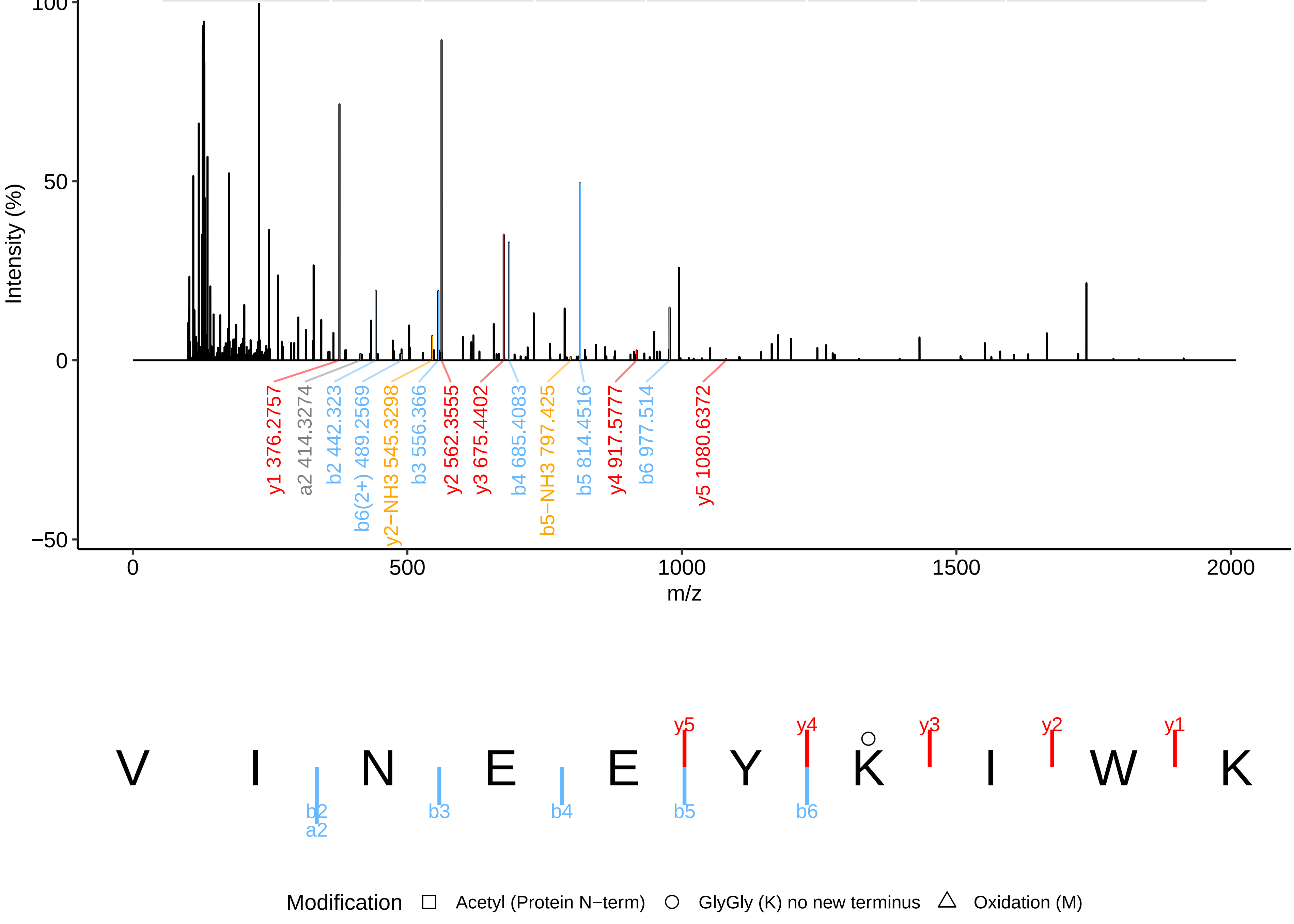

20200924_KIZ_MUb5_GlyGly_2 − Scan number: 18065

Gene Names	Charge	m/z	Mass	Retention time	PEP	Score	Precursor Intensity
RBBP7;RBBP4	3	479.2575	1434.751	66.977	0.010986	74.162	NA

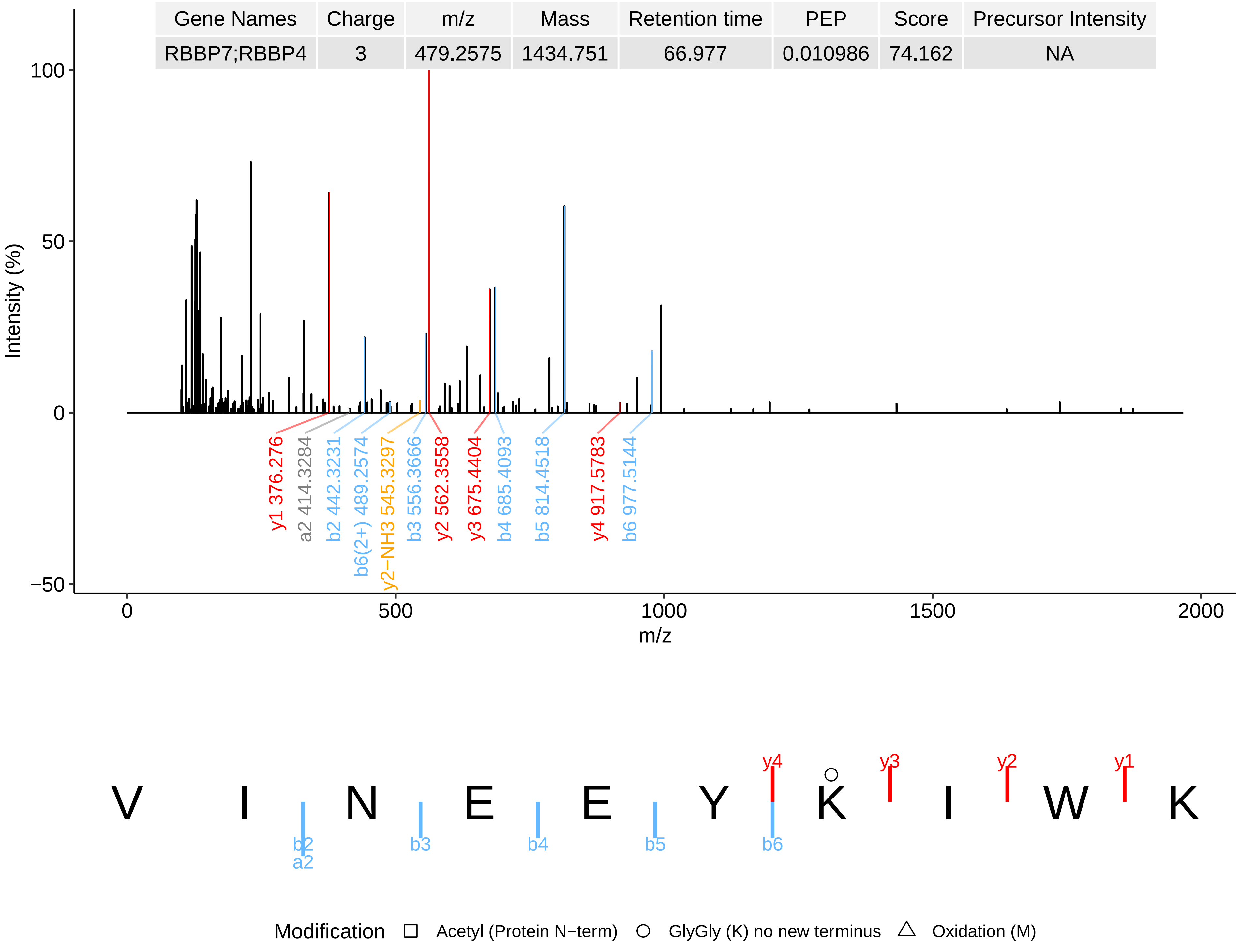

20200921_KIZ_MUb4_GlyGly_10plex_2 − Scan number: 17145

Gene Names	Charge	m/z	Mass	Retention time	PEP	Score	Precursor Intensity
SLC27A2	3	471.6025	1411.786	67.001	0.10256	45.915	1149164

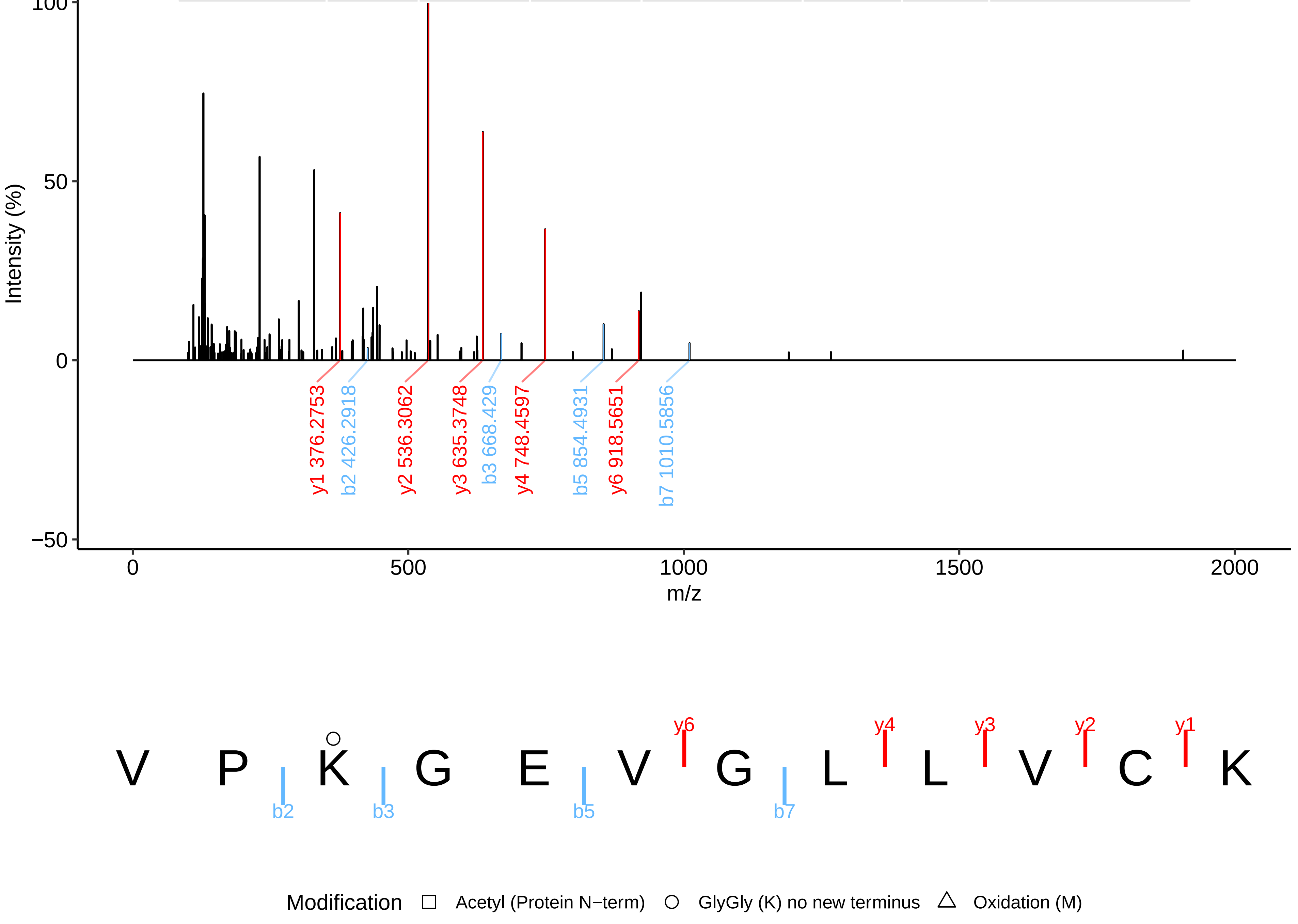

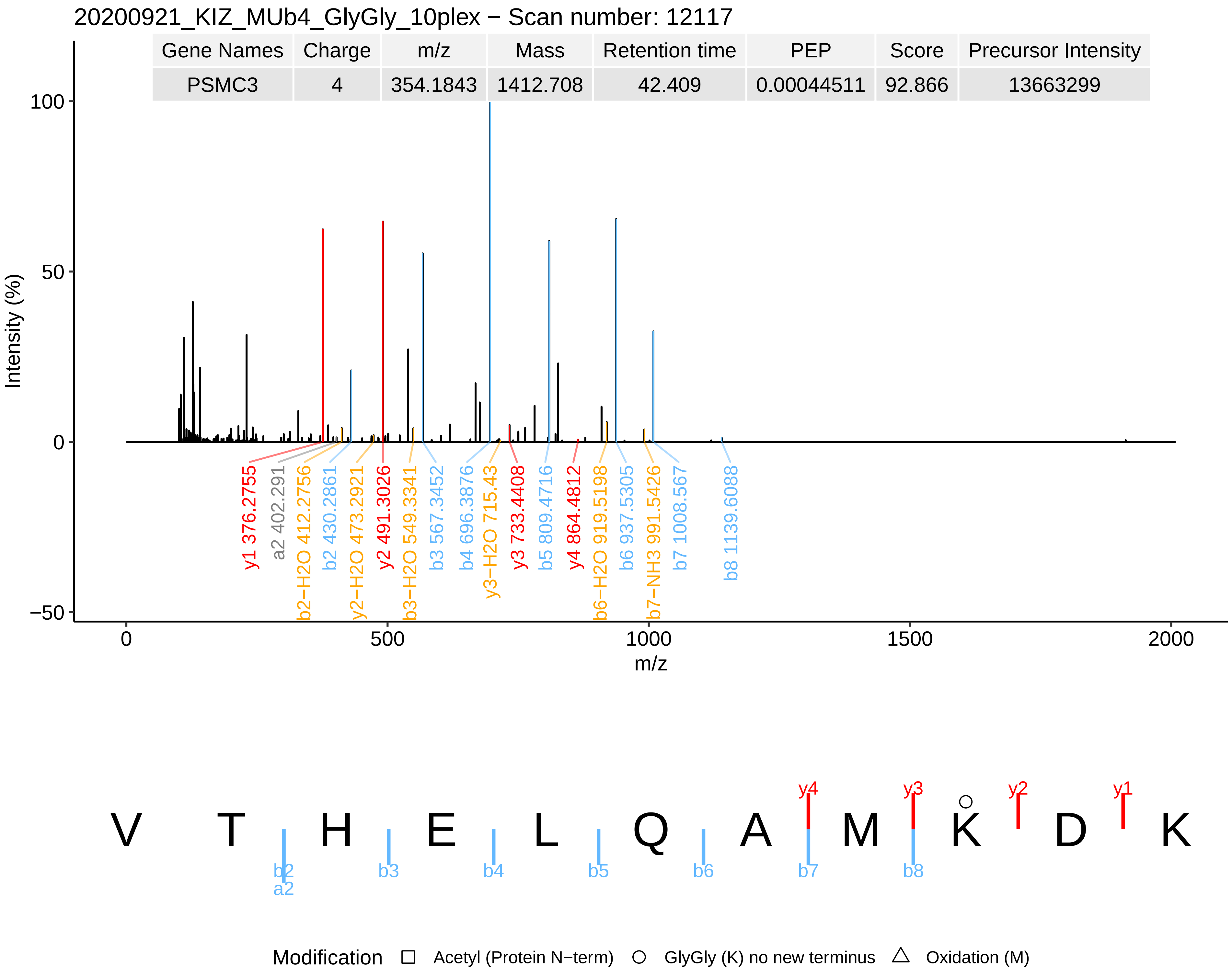

20200921_KIZ_MUb4_GlyGly_10plex − Scan number: 12117
Gene Names | Charge | m/z | Mass | Retention time | PEP | Score | Precursor Intensity
PSMC3 | 4 | 354.1843 | 1412.708 | 42.409 | 0.00044511 | 92.866 | 13663299
Intensity (%)
100
50
0
−50
0
500
1000
1500
2000
m/z
y1 376.2755
a2 402.291
b2−H2O 412.2756
b2 430.2861
y2−H2O 473.2921
y2 491.3026
b3−H2O 549.3341
b3 567.3452
b4 696.3876
y3−H2O 715.43
y3 733.4408
b5 809.4716
y4 864.4812
b6−H2O 919.5198
b6 937.5305
b7−NH3 991.5426
b7 1008.567
b8 1139.6088
V T H E L Q A M K D K
y4 y3 y2 y1
b2 a2 b3 b4 b5 b6 b7 b8
Modification
Acetyl (Protein N−term)
GlyGly (K) no new terminus
Oxidation (M)

20200921_KIZ_MUb4_GlyGly_10plex − Scan number: 12136

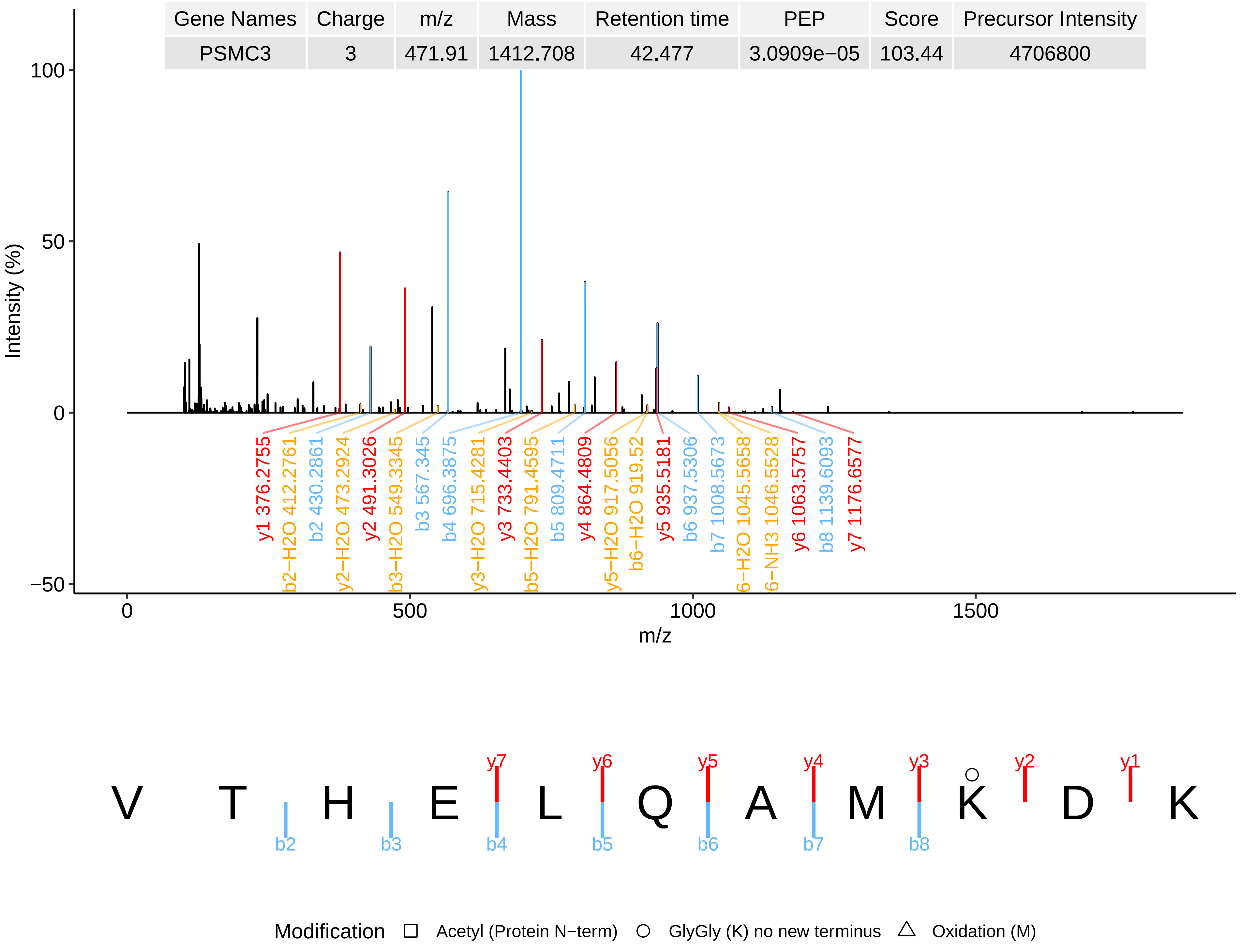

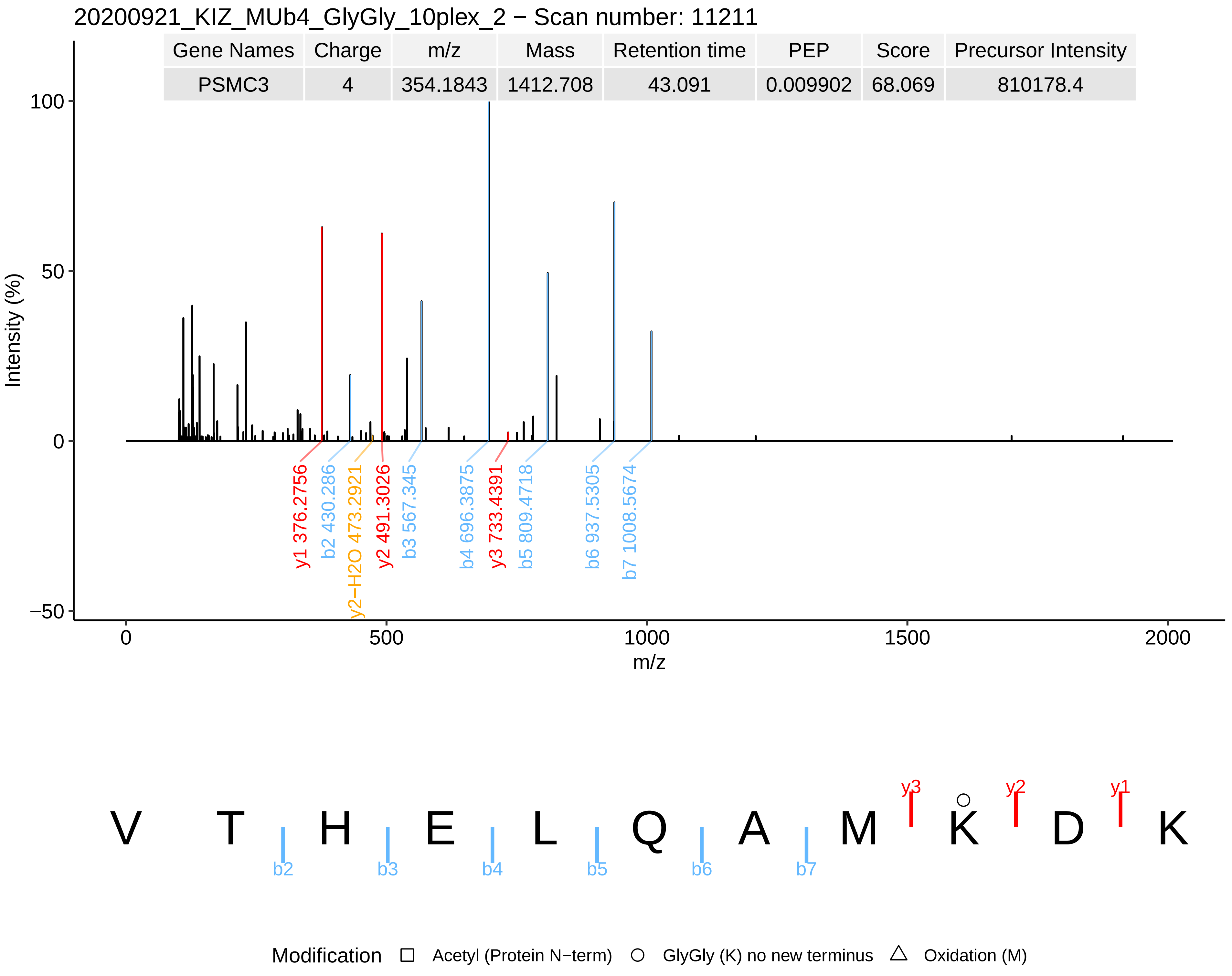

20200921_KIZ_MUb4_GlyGly_10plex_2 − Scan number: 11211
Gene Names | Charge | m/z | Mass | Retention time | PEP | Score | Precursor Intensity
PSMC3 | 4 | 354.1843 | 1412.708 | 43.091 | 0.009902 | 68.069 | 810178.4
Intensity (%)
100
50
0
−50
y1 376.2756
b2 430.286
y2−H2O 473.2921
y2 491.3026
b3 567.345
b4 696.3875
y3 733.4391
b5 809.4718
b6 937.5305
b7 1008.5674
0
500
1000
1500
2000
m/z
V T H E L Q A M K D K
b2 b3 b4 b5 b6 b7
y3 y2 y1
Modification
Acetyl (Protein N−term)
GlyGly (K) no new terminus
Oxidation (M)

20200921_KIZ_MUb4_GlyGly_10plex_2 − Scan number: 11237

Gene Names	Charge	m/z	Mass	Retention time	PEP	Score	Precursor Intensity
PSMC3	3	471.91	1412.708	43.196	0.0057501	75.695	1421128

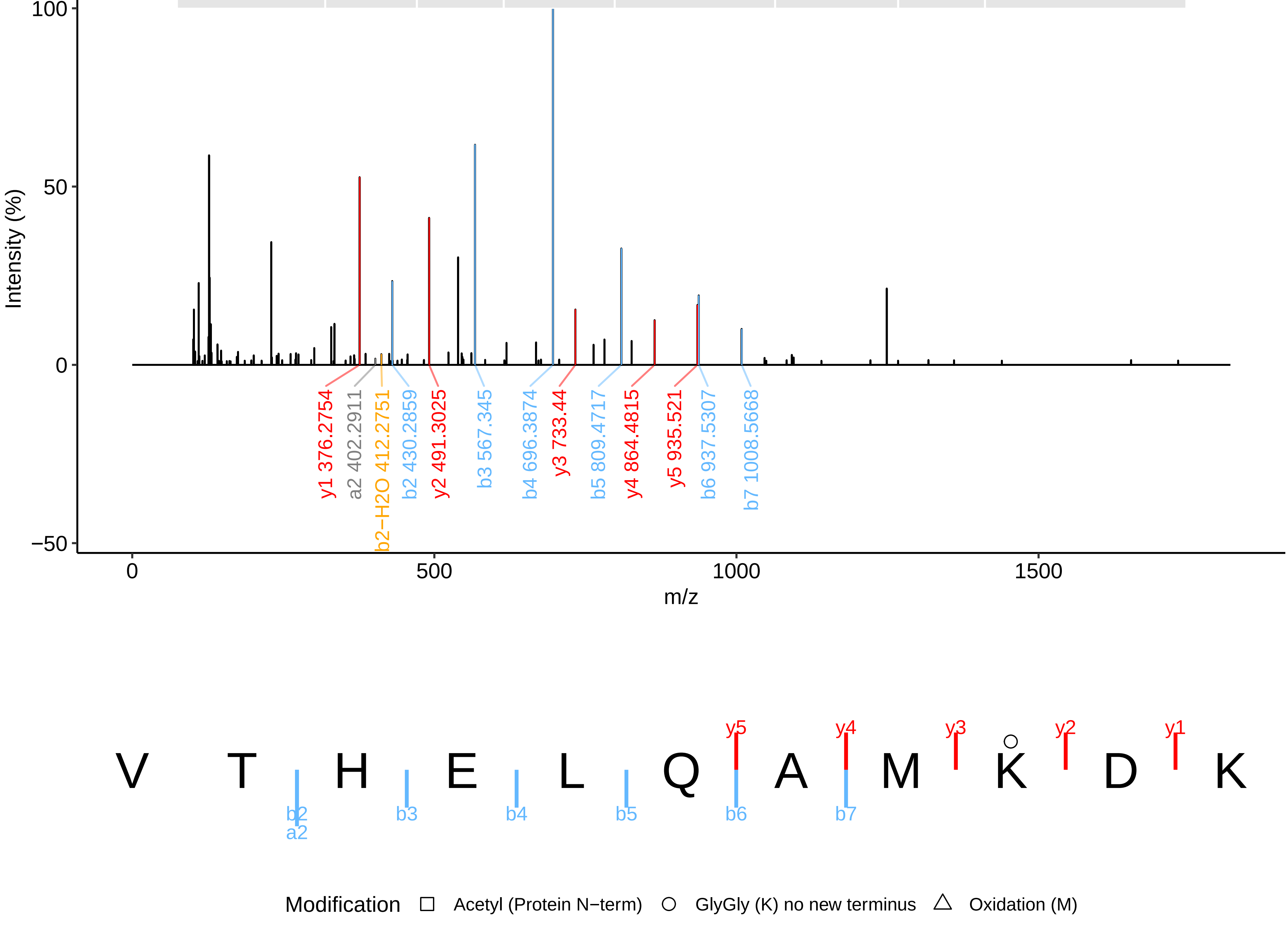

20200924_KIZ_MUb5_GlyGly_1 − Scan number: 10759

Gene Names	Charge	m/z	Mass	Retention time	PEP	Score	Precursor Intensity
PSMC3	4	354.1843	1412.708	34.779	0.010481	67.214	11118642

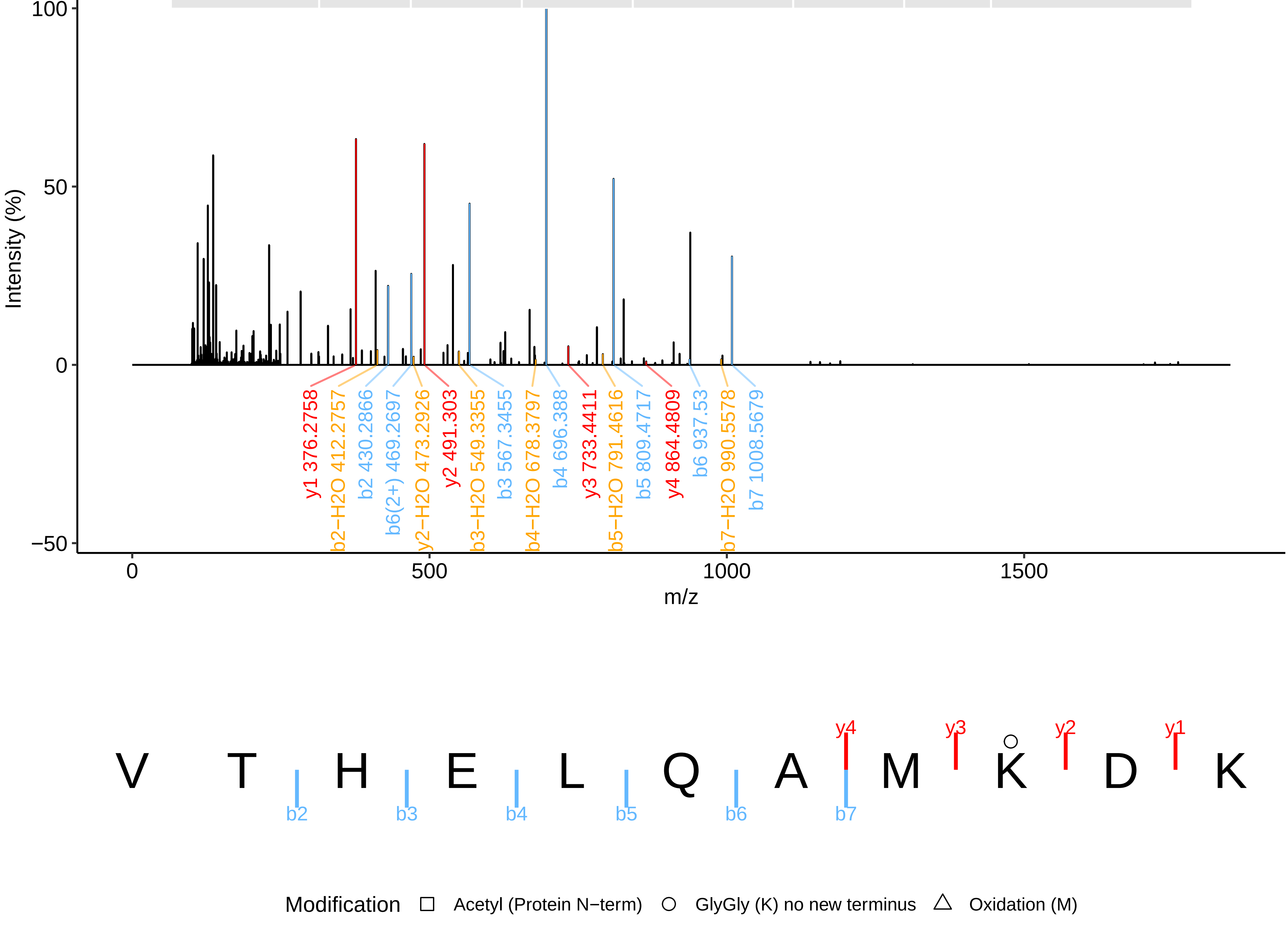

20200924_KIZ_MUb5_GlyGly_1 − Scan number: 10930

Gene Names	Charge	m/z	Mass	Retention time	PEP	Score	Precursor Intensity
PSMC3	4	354.1843	1412.708	35.379	0.010924	66.56	4659390

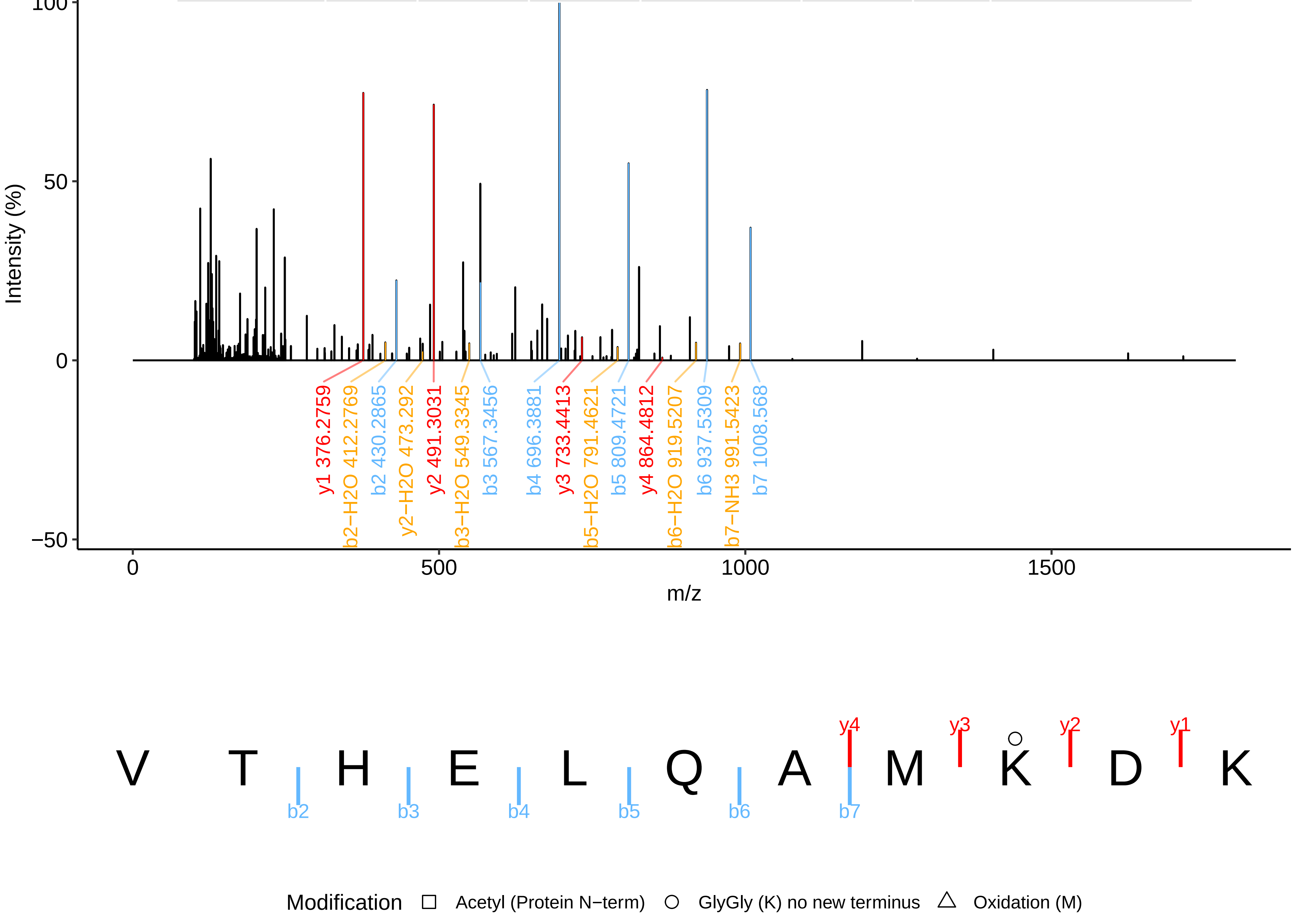

20200924_KIZ_MUb5_GlyGly_1 − Scan number: 10576

Gene Names	Charge	m/z	Mass	Retention time	PEP	Score	Precursor Intensity
PSMC3	3	471.91	1412.708	34.164	0.00011219	117.23	NA

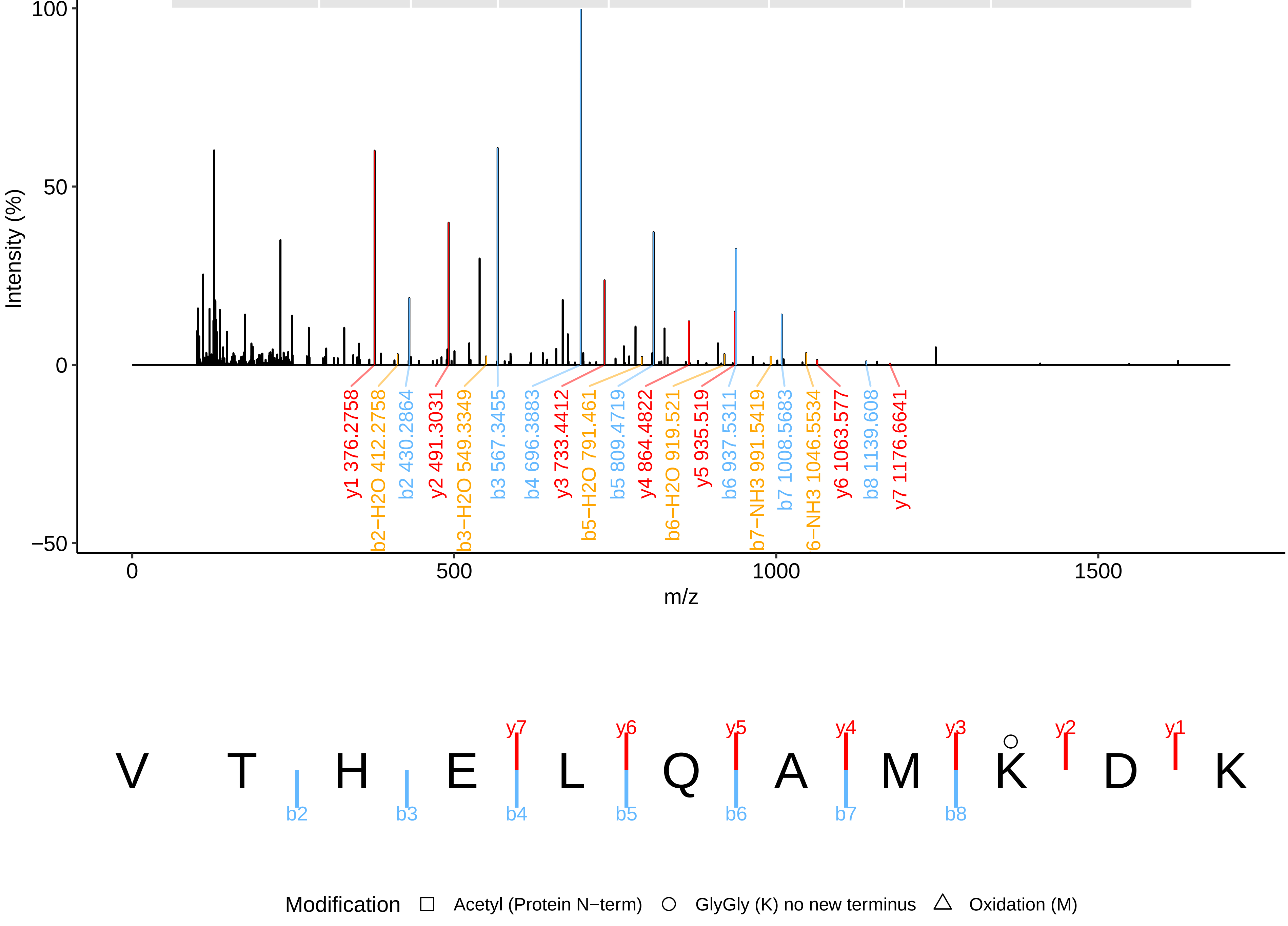

20200921_KIZ_MUb4_GlyGly_10plex_2 − Scan number: 5412

Gene Names	Charge	m/z	Mass	Retention time	PEP	Score	Precursor Intensity
HSPA4	3	478.5792	1432.716	19.443	0.11046	41.621	387709.3

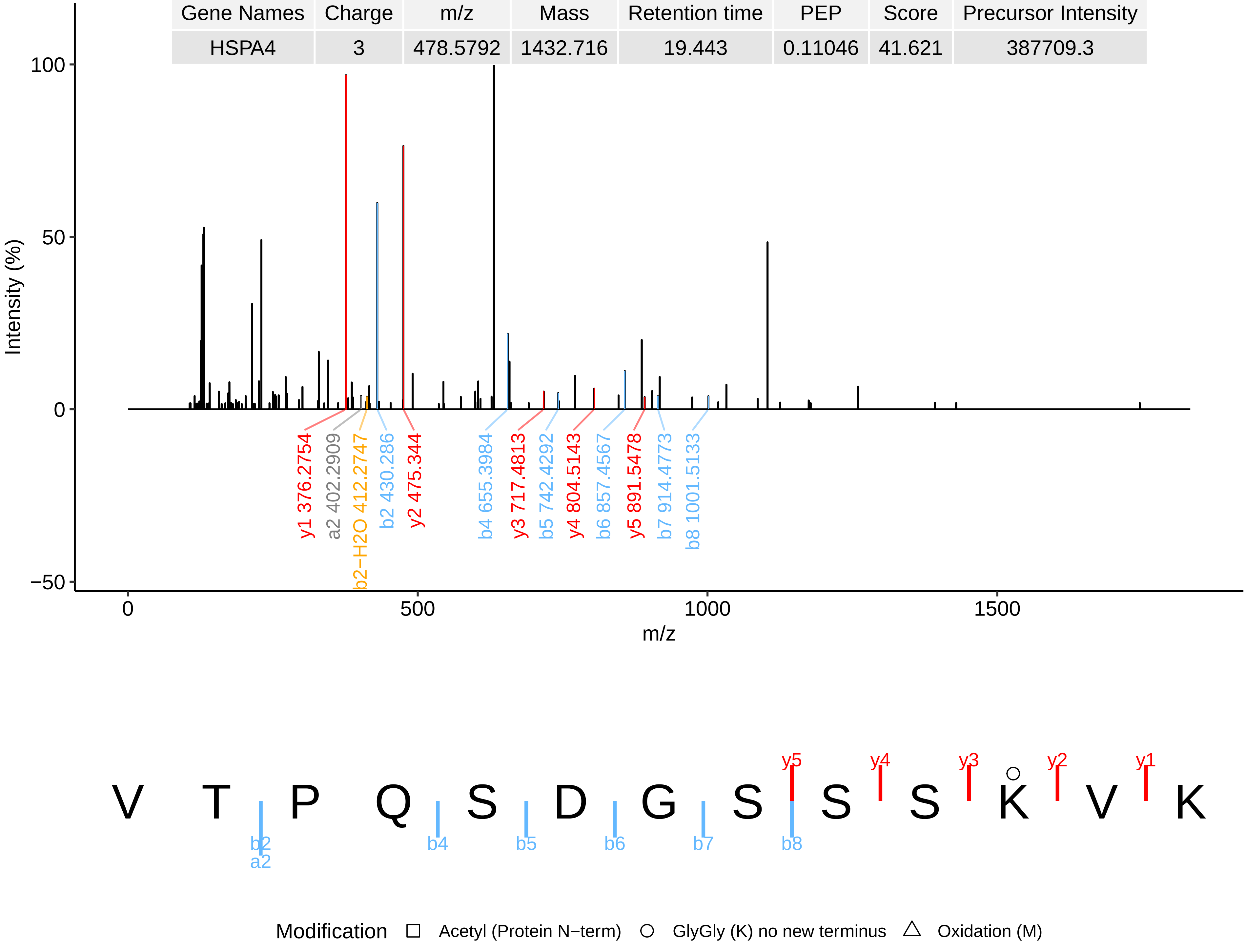

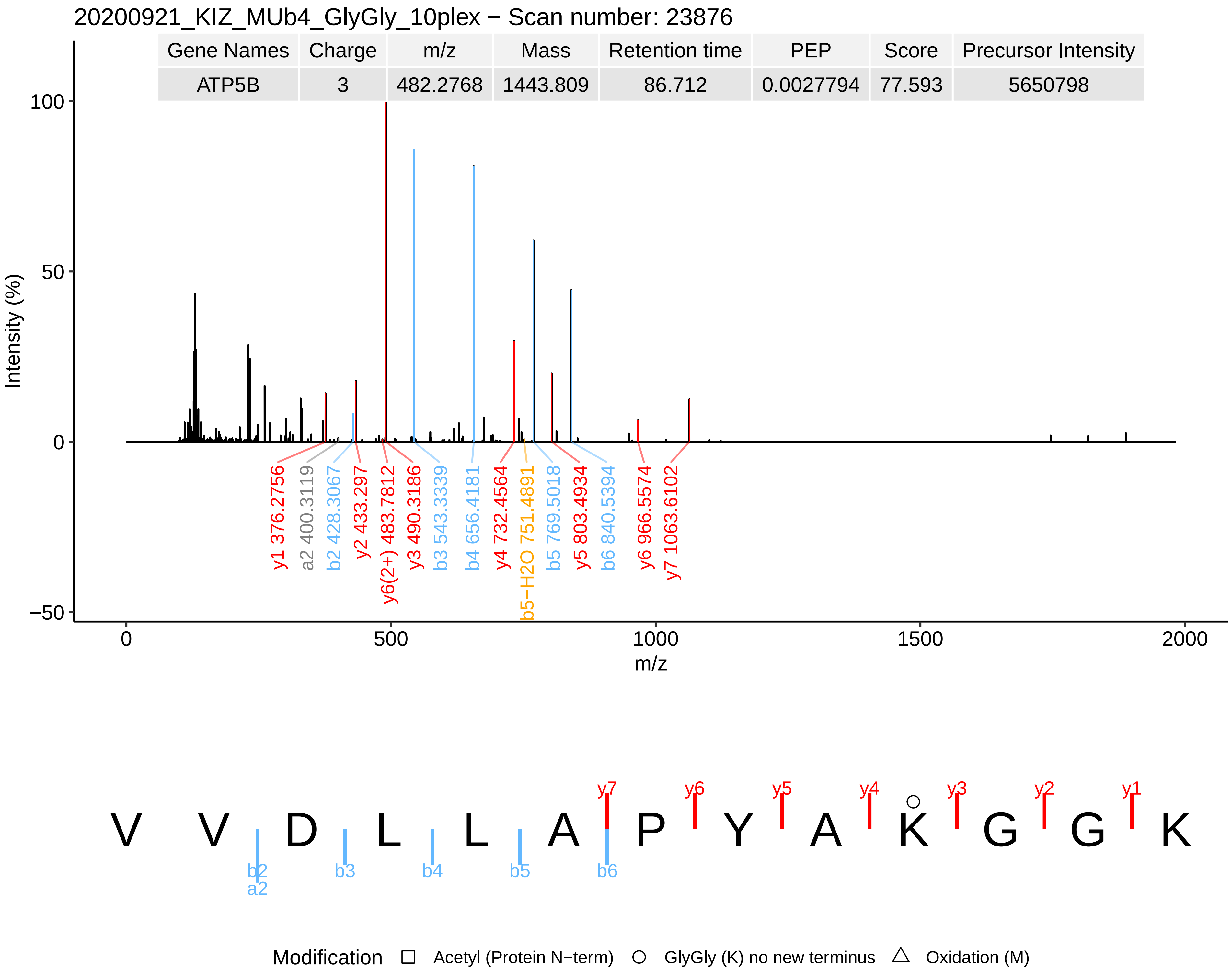

20200921_KIZ_MUb4_GlyGly_10plex − Scan number: 23876
Gene Names | Charge | m/z | Mass | Retention time | PEP | Score | Precursor Intensity
ATP5B | 3 | 482.2768 | 1443.809 | 86.712 | 0.0027794 | 77.593 | 5650798
Intensity (%)
m/z
y1 376.2756
a2 400.3119
b2 428.3067
y2 433.297
y6(2+) 483.7812
y3 490.3186
b3 543.3339
b4 656.4181
y4 732.4564
b5−H2O 751.4891
b5 769.5018
y5 803.4934
b6 840.5394
y6 966.5574
y7 1063.6102
V V D L L A P Y A K G G K
Modification
Acetyl (Protein N−term)
GlyGly (K) no new terminus
Oxidation (M)

20200921_KIZ_MUb4_GlyGly_10plex − Scan number: 24019

Gene Names	Charge	m/z	Mass	Retention time	PEP	Score	Precursor Intensity
ATP5B	3	482.2768	1443.809	87.236	0.0010502	87.001	3003457

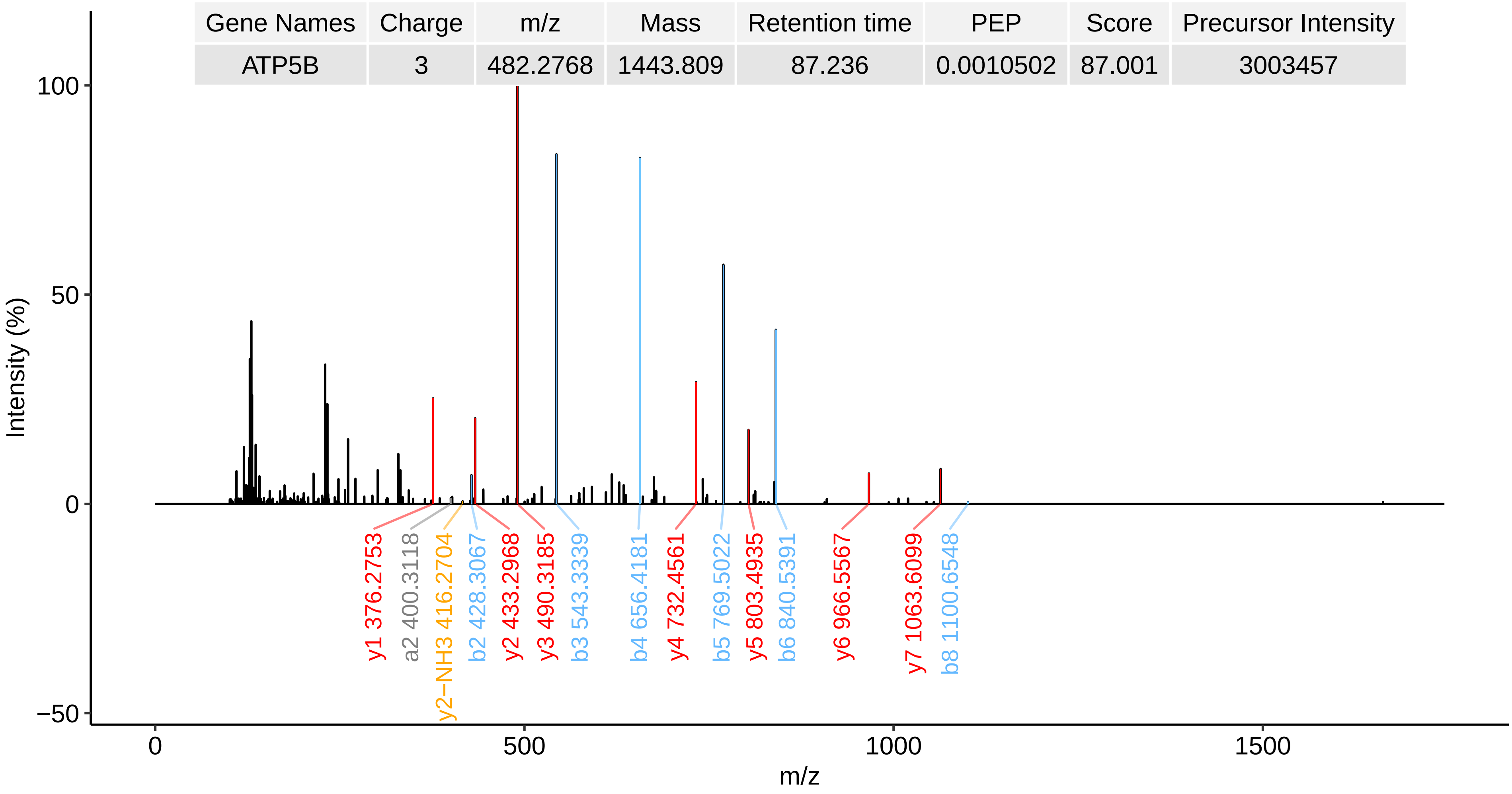

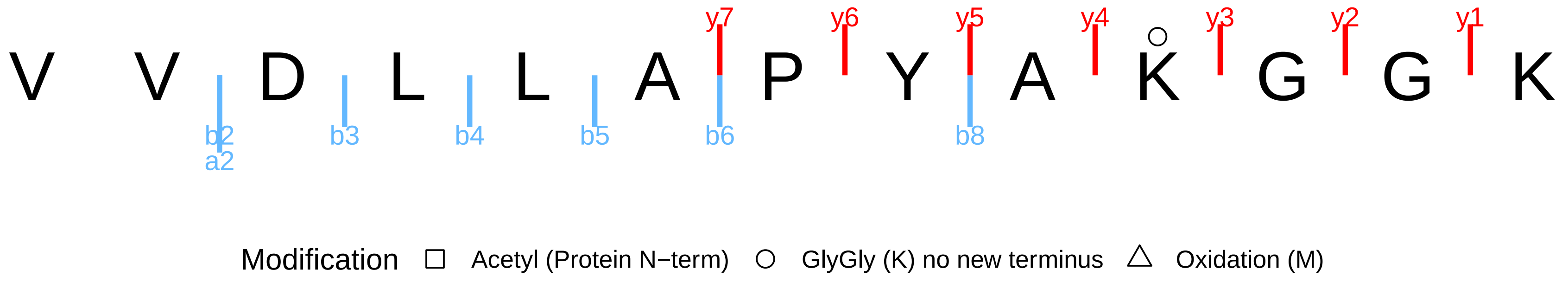

20200921_KIZ_MUb4_GlyGly_10plex_2 − Scan number: 20731

Gene Names	Charge	m/z	Mass	Retention time	PEP	Score	Precursor Intensity
ATP5B	3	482.2768	1443.809	81.528	0.0082607	67.456	1657079

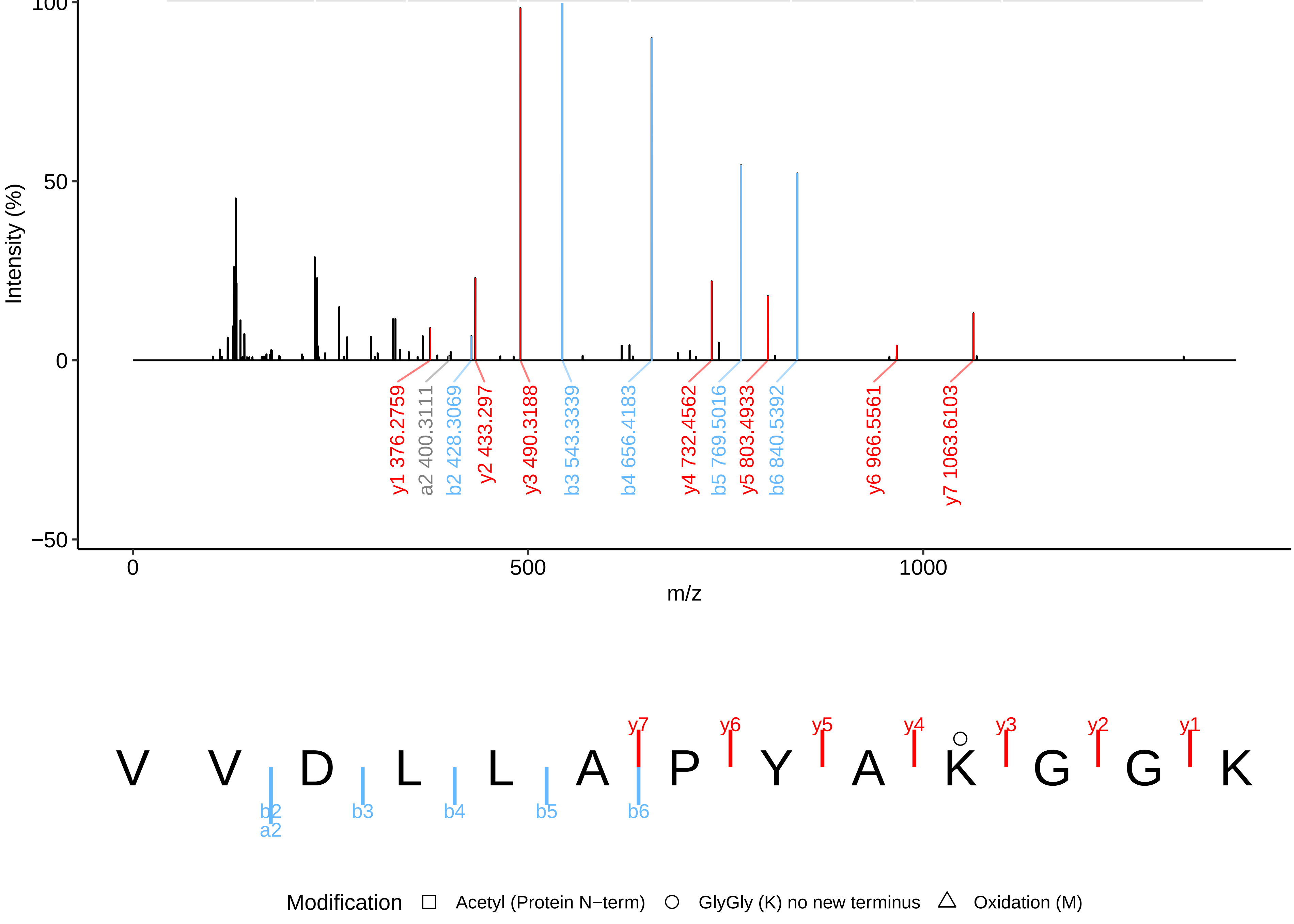

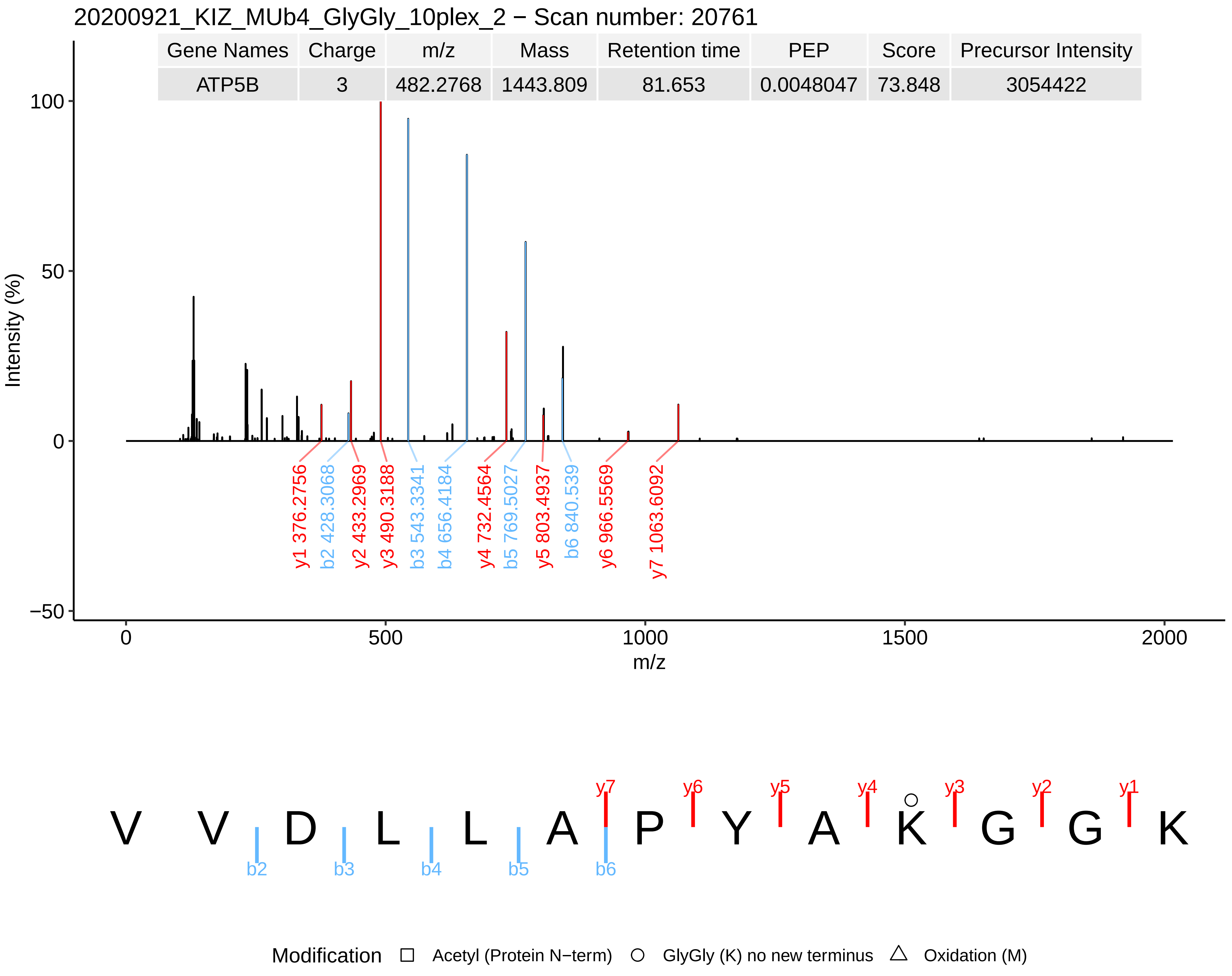
20200921_KIZ_MUb4_GlyGly_10plex_2 − Scan number: 20761
Gene Names | Charge | m/z | Mass | Retention time | PEP | Score | Precursor Intensity
ATP5B | 3 | 482.2768 | 1443.809 | 81.653 | 0.0048047 | 73.848 | 3054422
Intensity (%)
100
50
0
−50
0
500
1000
1500
2000
m/z
y1 376.2756
b2 428.3068
y2 433.2969
y3 490.3188
b3 543.3341
b4 656.4184
y4 732.4564
b5 769.5027
y5 803.4937
b6 840.539
y6 966.5569
y7 1063.6092
V V D L L A P Y A K G G K
y7 y6 y5 y4 y3 y2 y1
b2 b3 b4 b5 b6
Modification
Acetyl (Protein N−term)
GlyGly (K) no new terminus
Oxidation (M)

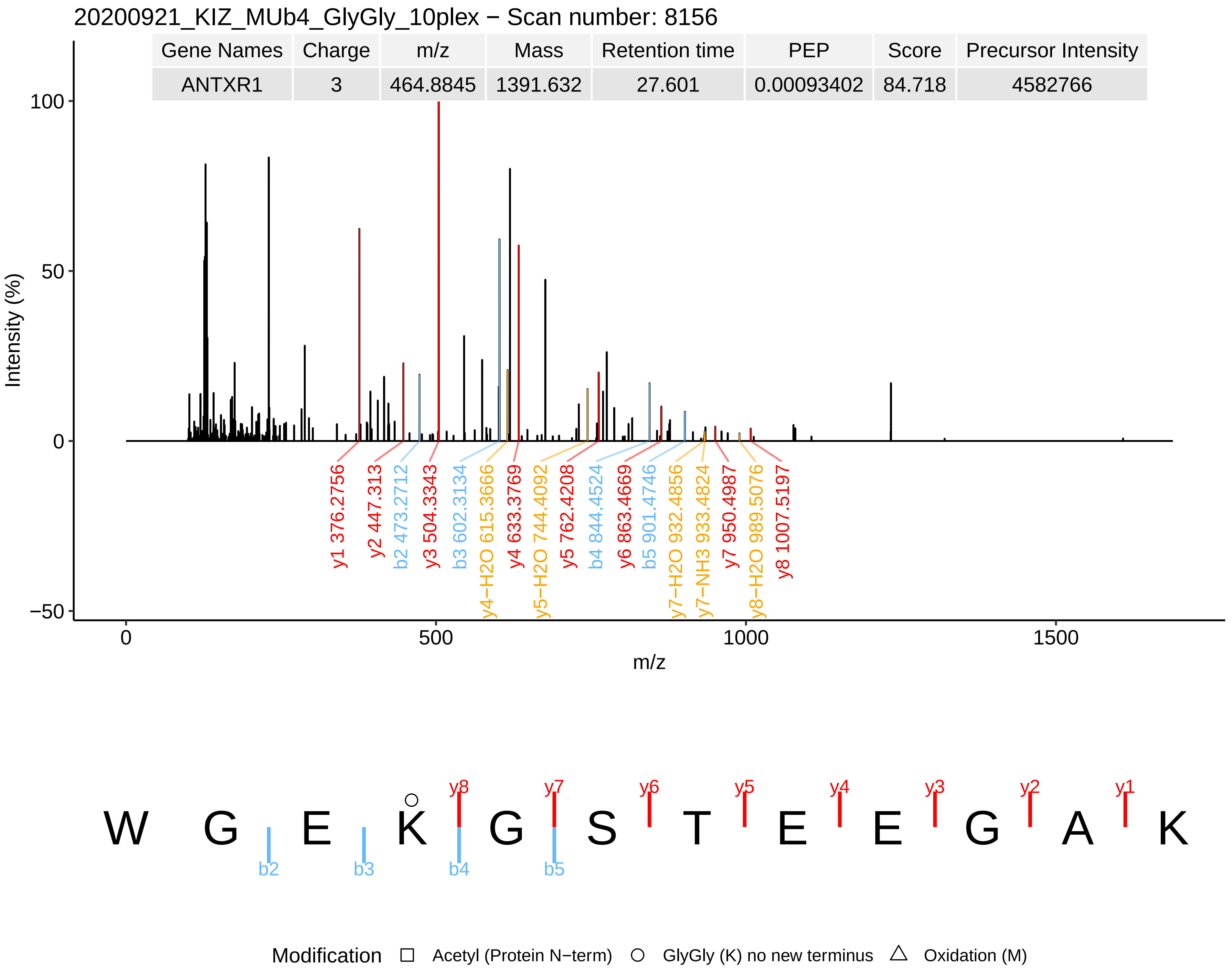
20200921_KIZ_MUb4_GlyGly_10plex − Scan number: 8156
Gene Names | Charge | m/z | Mass | Retention time | PEP | Score | Precursor Intensity
ANTXR1 | 3 | 464.8845 | 1391.632 | 27.601 | 0.00093402 | 84.718 | 4582766
Intensity (%)
100
50
0
−50
0
500
1000
1500
m/z
y1 376.2756
y2 447.313
b2 473.2712
y3 504.3343
b3 602.3134
y4−H2O 615.3666
y4 633.3769
y5−H2O 744.4092
y5 762.4208
b4 844.4524
y6 863.4669
b5 901.4746
y7−H2O 932.4856
y7−NH3 933.4824
y7 950.4987
y8−H2O 989.5076
y8 1007.5197
W G E K G S T E E G A K
y8 y7 y6 y5 y4 y3 y2 y1
b2 b3 b4 b5
Modification
Acetyl (Protein N−term)
GlyGly (K) no new terminus
Oxidation (M)

20200921_KIZ_MUb4_GlyGly_10plex_2 − Scan number: 20463

Gene Names	Charge	m/z	Mass	Retention time	PEP	Score	Precursor Intensity
NAP1L1	3	542.9595	1625.857	80.437	0.004125	88.596	1787935

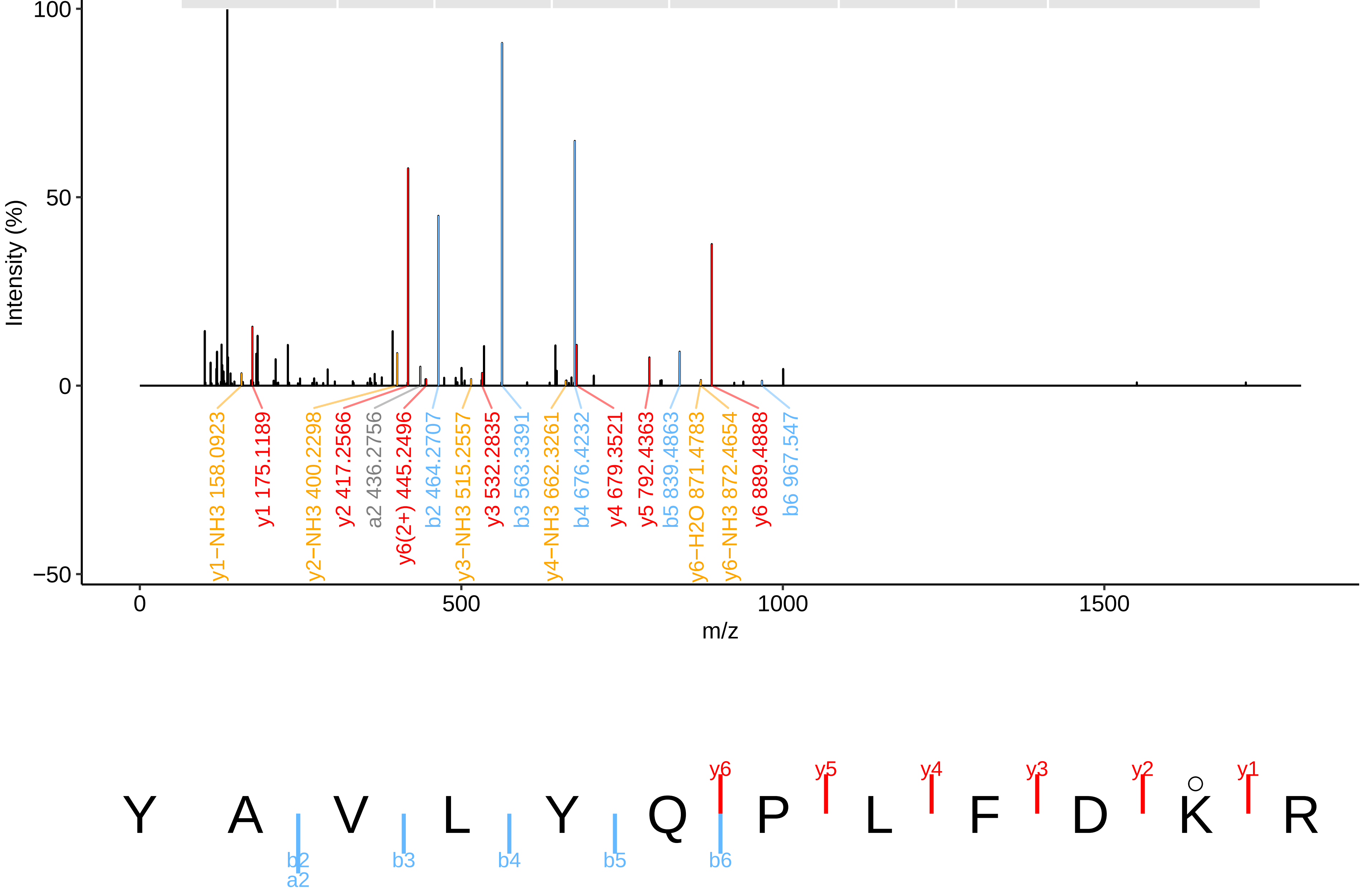

Modification □ Acetyl (Protein N−term) ○ GlyGly (K) no new terminus △ Oxidation (M)

20200924_KIZ_MUb5_GlyGly_2 − Scan number: 20232

Gene Names	Charge	m/z	Mass	Retention time	PEP	Score	Precursor Intensity
NAP1L1	3	542.9595	1625.857	74.982	6.1352e−08	120.77	4524191

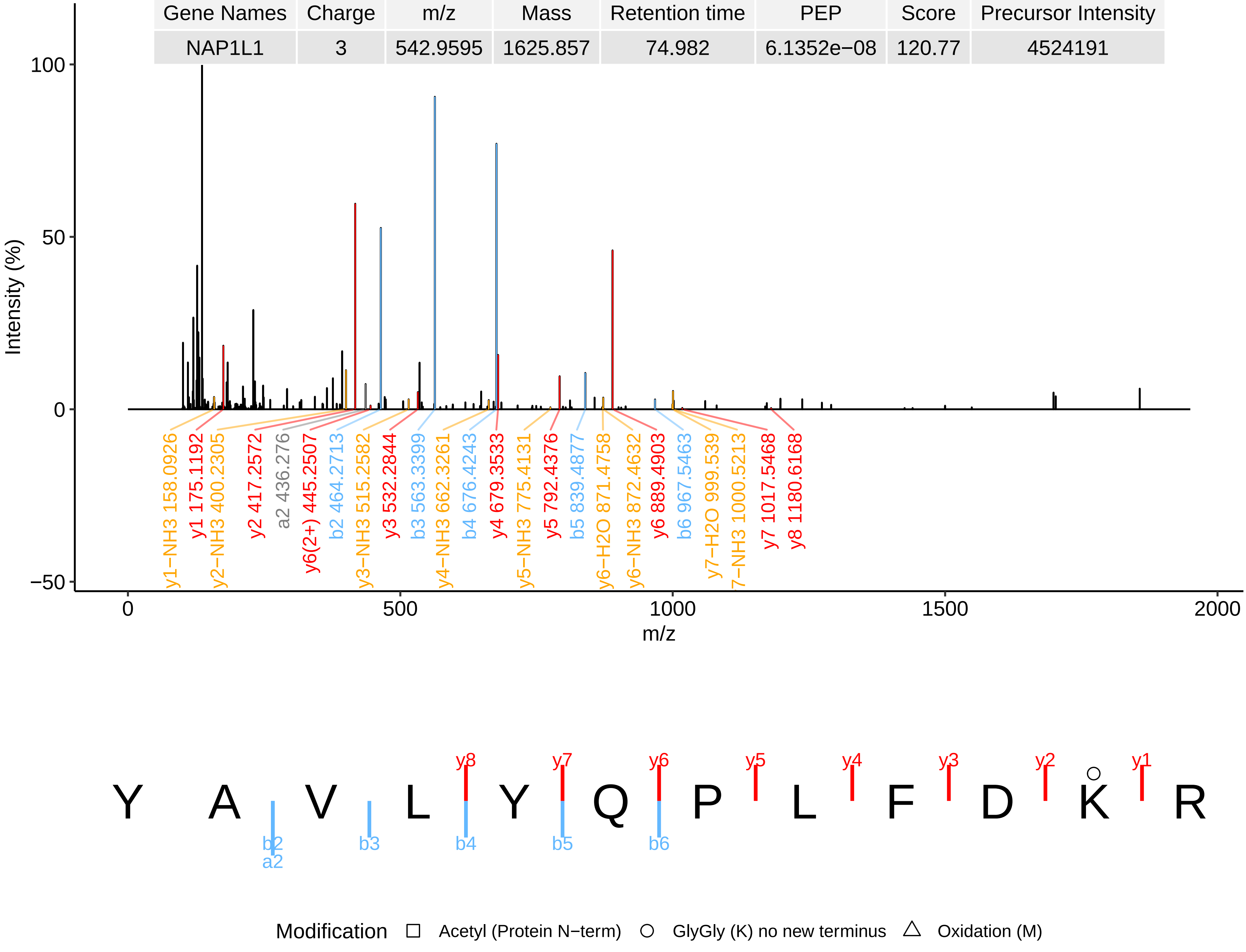

20200924_KIZ_MUb5_GlyGly_2 − Scan number: 20247

Gene Names	Charge	m/z	Mass	Retention time	PEP	Score	Precursor Intensity
NAP1L1	3	542.9595	1625.857	75.039	1.2798e−05	113.69	9428863

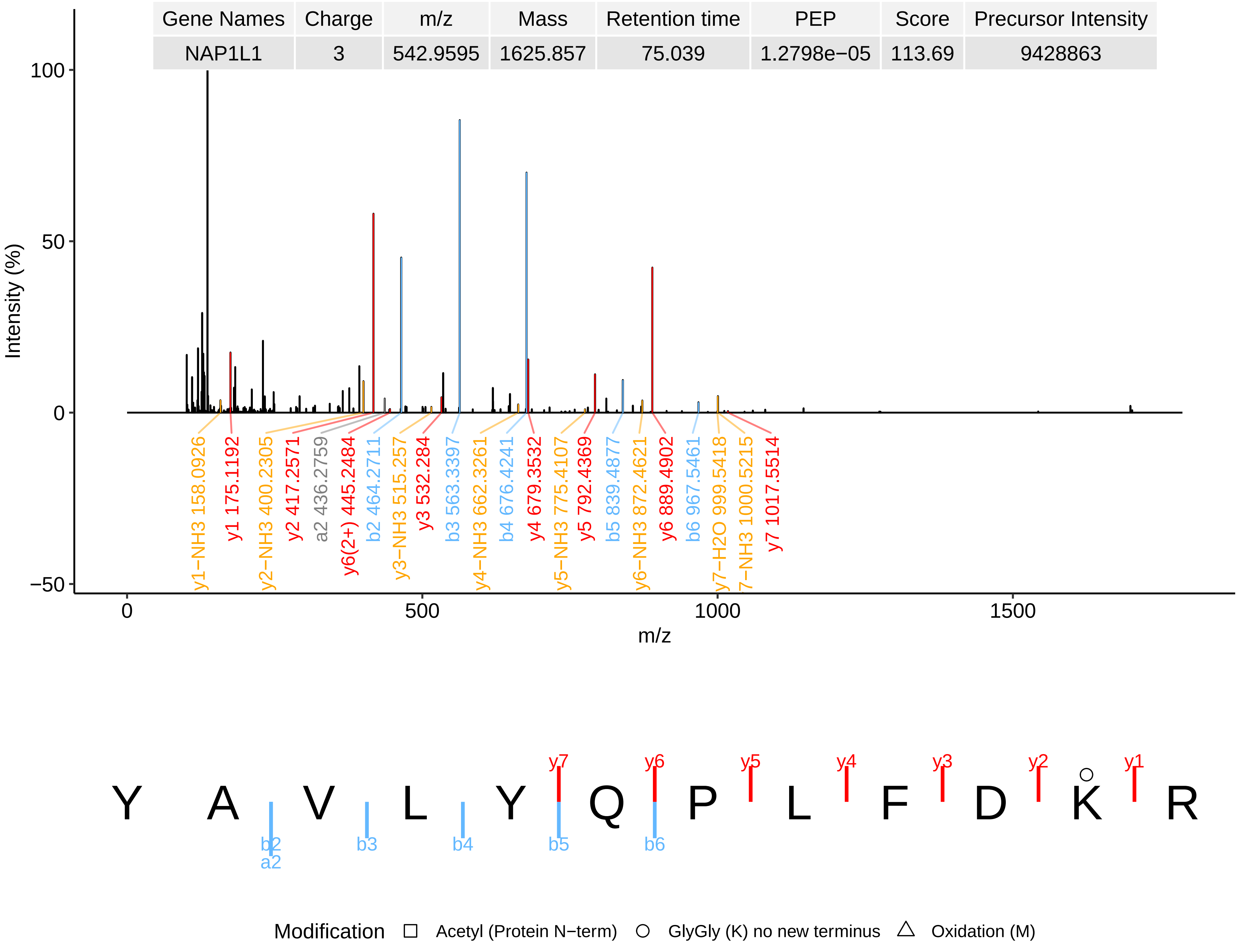

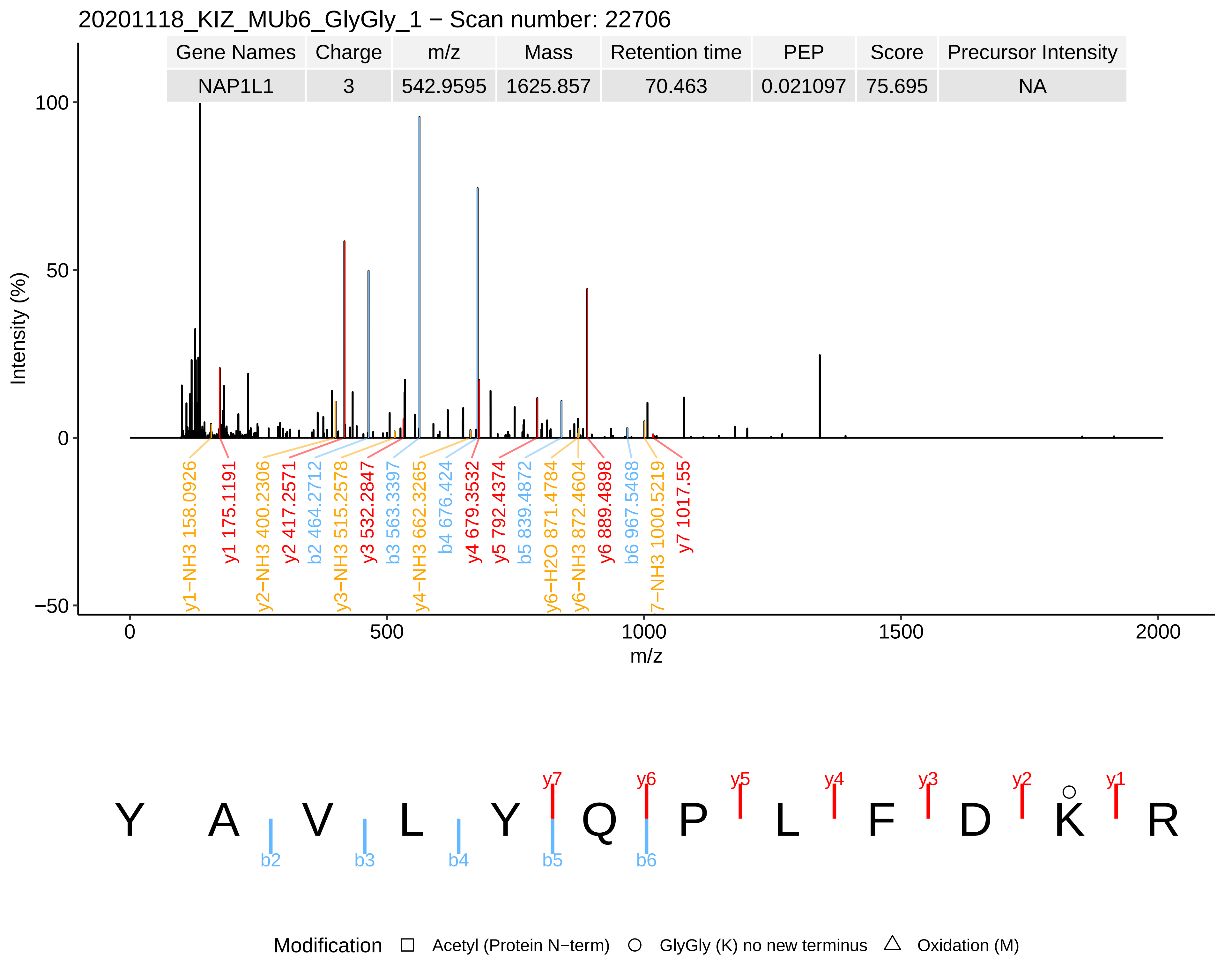
20201118_KIZ_MUb6_GlyGly_1 − Scan number: 22706
Gene Names | Charge | m/z | Mass | Retention time | PEP | Score | Precursor Intensity
NAP1L1 | 3 | 542.9595 | 1625.857 | 70.463 | 0.021097 | 75.695 | NA
Intensity (%)
100
50
0
−50
y1−NH3 158.0926
y1 175.1191
y2−NH3 400.2306
y2 417.2571
b2 464.2712
y3−NH3 515.2578
y3 532.2847
b3 563.3397
y4−NH3 662.3265
b4 676.424
y4 679.3532
y5 792.4374
b5 839.4872
y6−H2O 871.4784
y6−NH3 872.4604
y6 889.4898
b6 967.5468
7−NH3 1000.5219
y7 1017.55
0
500
1000
1500
2000
m/z
Y A V L Y Q P L F D K R
y7 y6 y5 y4 y3 y2 y1
b2 b3 b4 b5 b6
Modification
Acetyl (Protein N−term)
GlyGly (K) no new terminus
Oxidation (M)

20201118_KIZ_MUb6_GlyGly_2 − Scan number: 24391

Gene Names	Charge	m/z	Mass	Retention time	PEP	Score	Precursor Intensity
NAP1L1	3	542.9595	1625.857	81.684	0.00066931	96.113	5836391

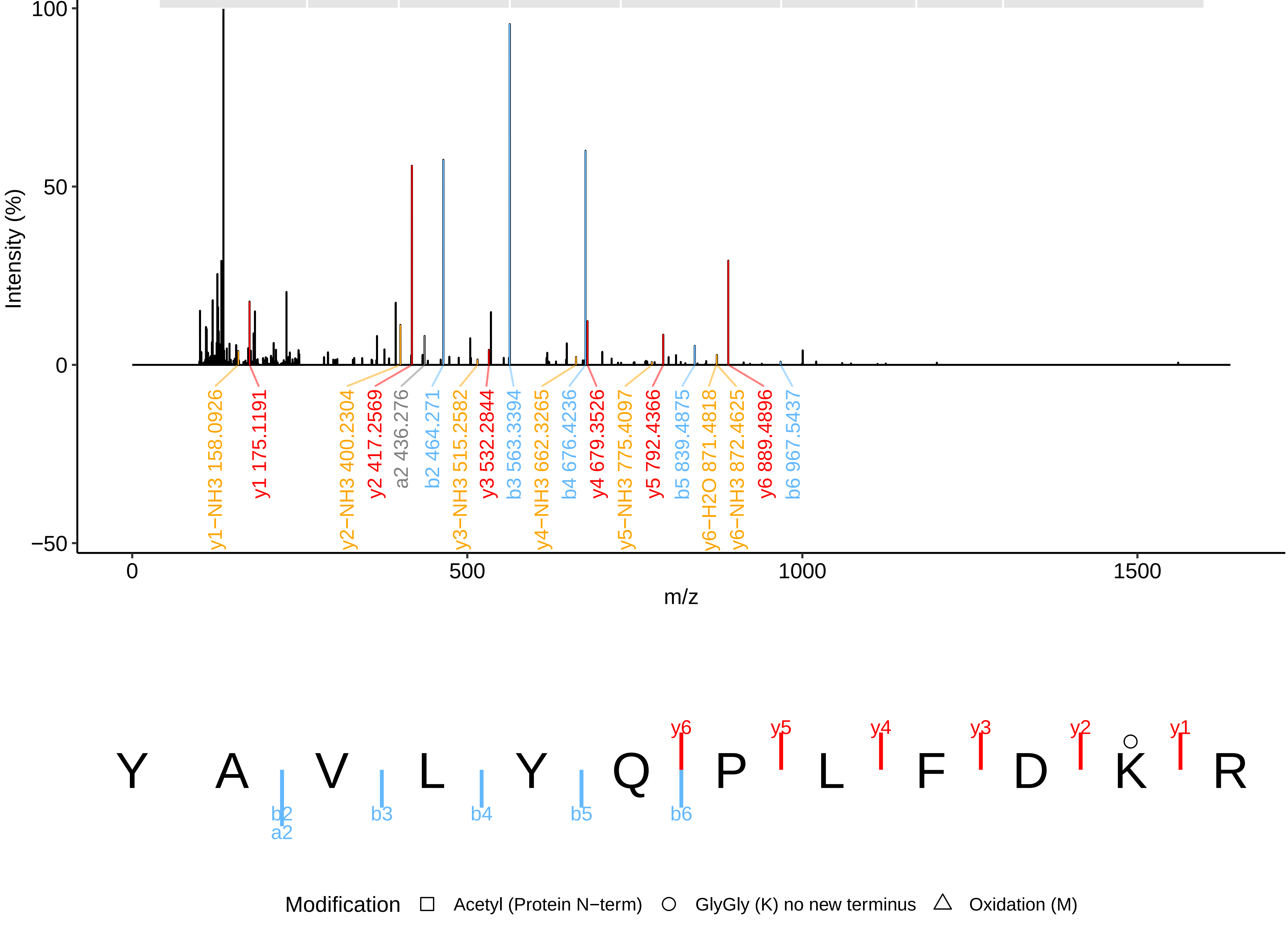

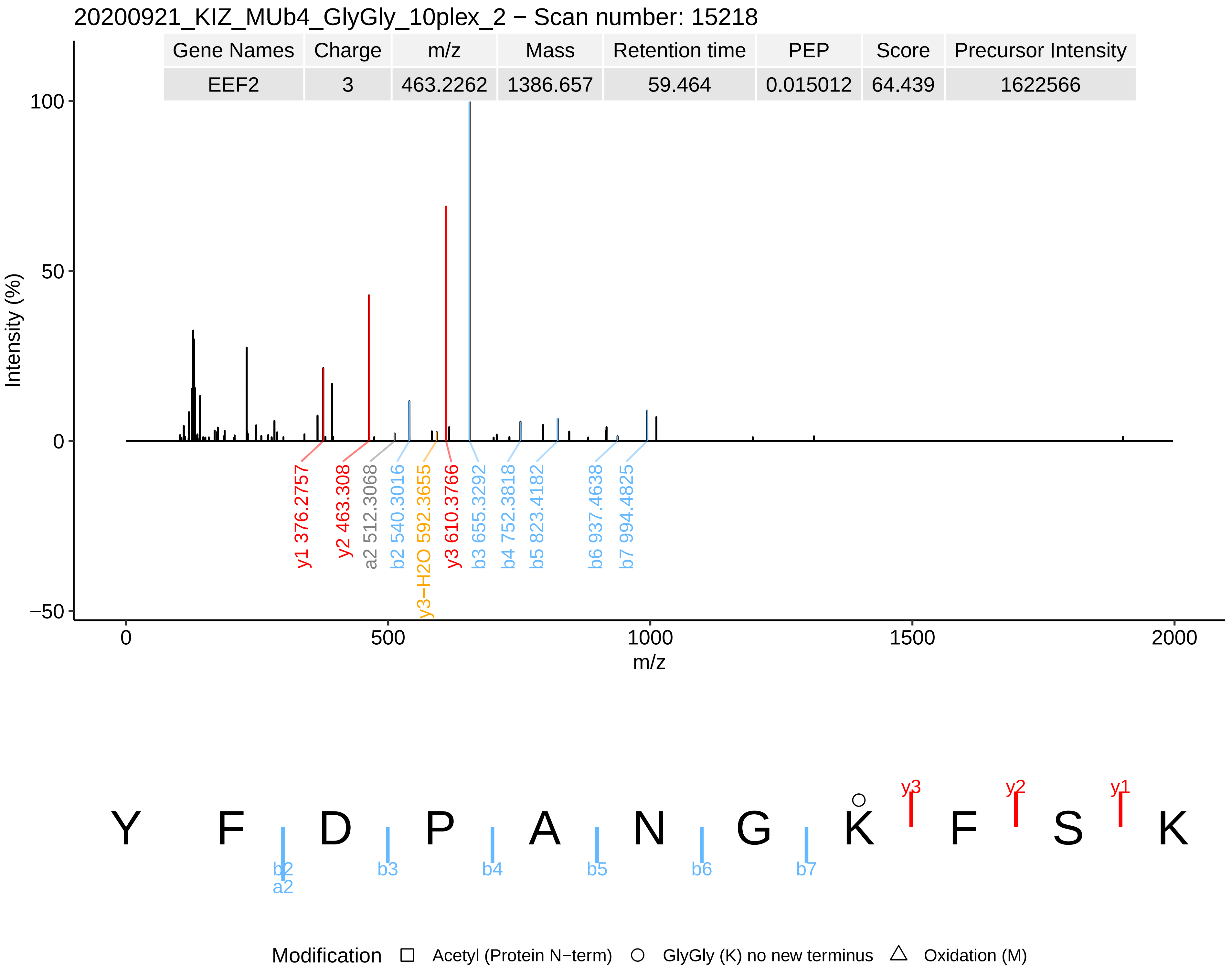
20200921_KIZ_MUb4_GlyGly_10plex_2 − Scan number: 15218
Gene Names | Charge | m/z | Mass | Retention time | PEP | Score | Precursor Intensity
EEF2 | 3 | 463.2262 | 1386.657 | 59.464 | 0.015012 | 64.439 | 1622566
Intensity (%)
100
50
0
−50
0
500
1000
1500
2000
m/z
y1 376.2757
y2 463.308
a2 512.3068
b2 540.3016
y3−H2O 592.3655
y3 610.3766
b3 655.3292
b4 752.3818
b5 823.4182
b6 937.4638
b7 994.4825
Y F D P A N G K F S K
y3 y2 y1
b2 a2 b3 b4 b5 b6 b7
Modification
Acetyl (Protein N−term)
GlyGly (K) no new terminus
Oxidation (M)

20200924_KIZ_MUb5_GlyGly_2 − Scan number: 14751

Gene Names	Charge	m/z	Mass	Retention time	PEP	Score	Precursor Intensity
EEF2	3	463.2262	1386.657	55.118	0.0083836	74.962	NA

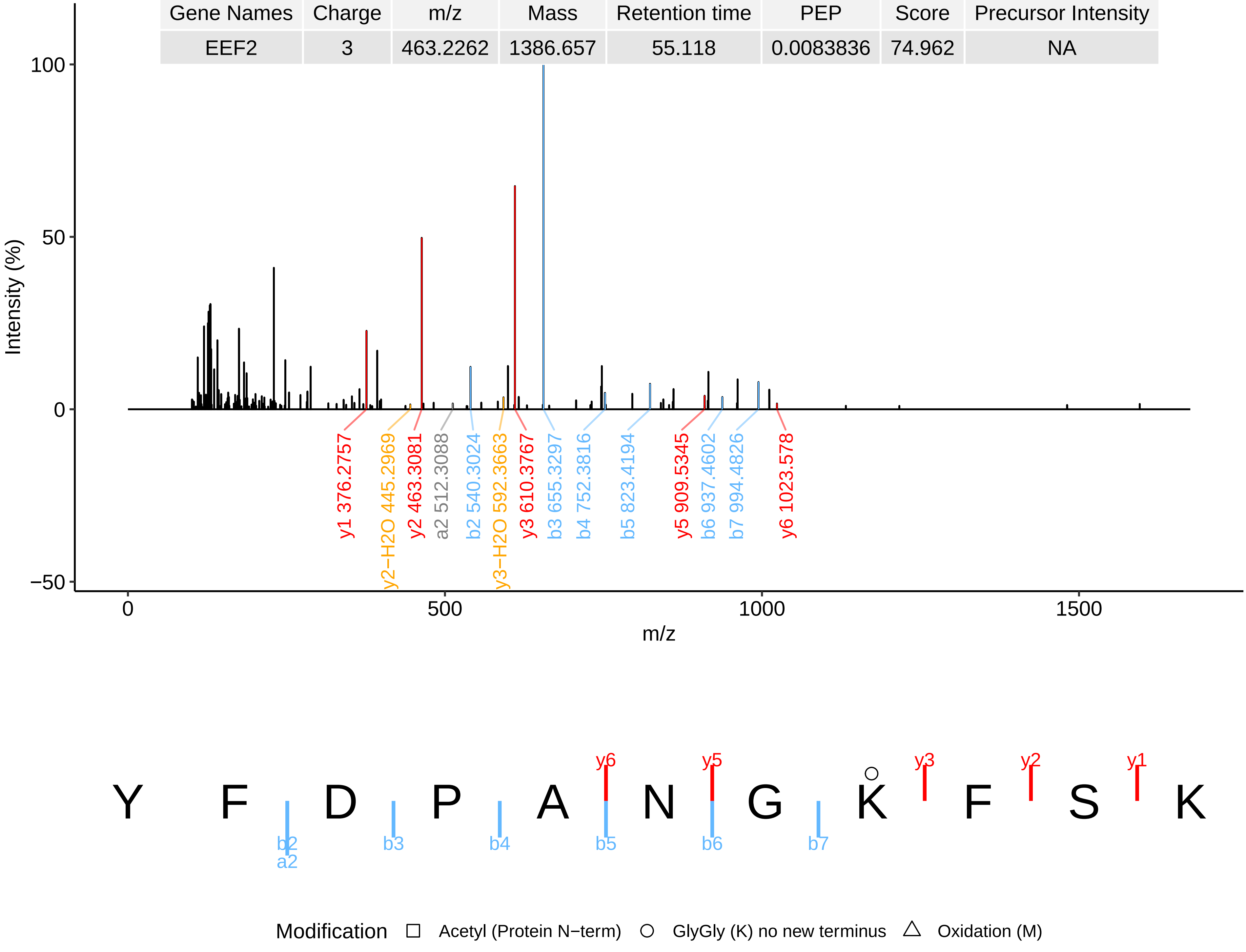

20200921_KIZ_MUb4_GlyGly_10plex − Scan number: 14376

Gene Names	Charge	m/z	Mass	Retention time	PEP	Score	Precursor Intensity
YWHAZ	3	465.2368	1392.688	51.081	0.0037063	87.001	1978363

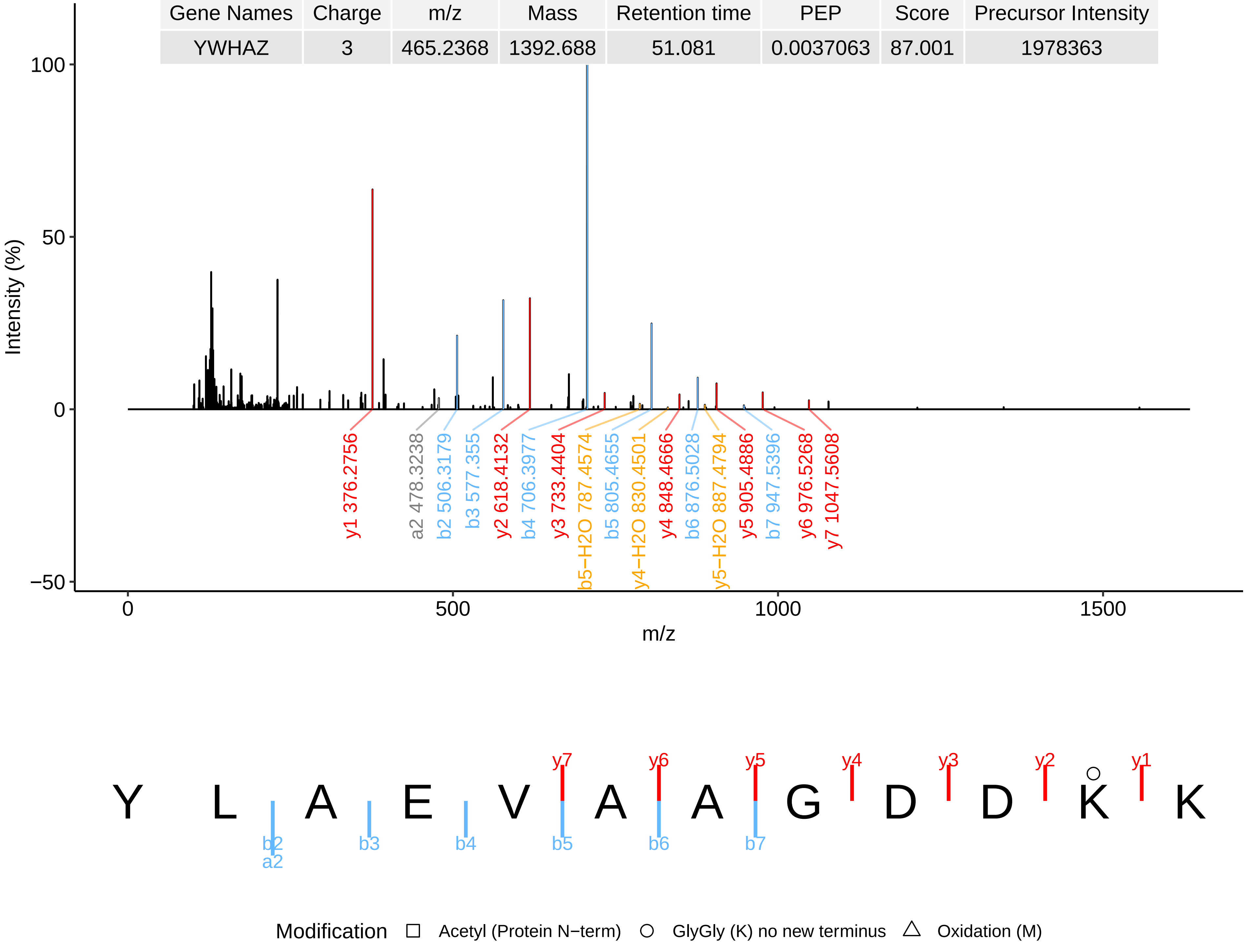

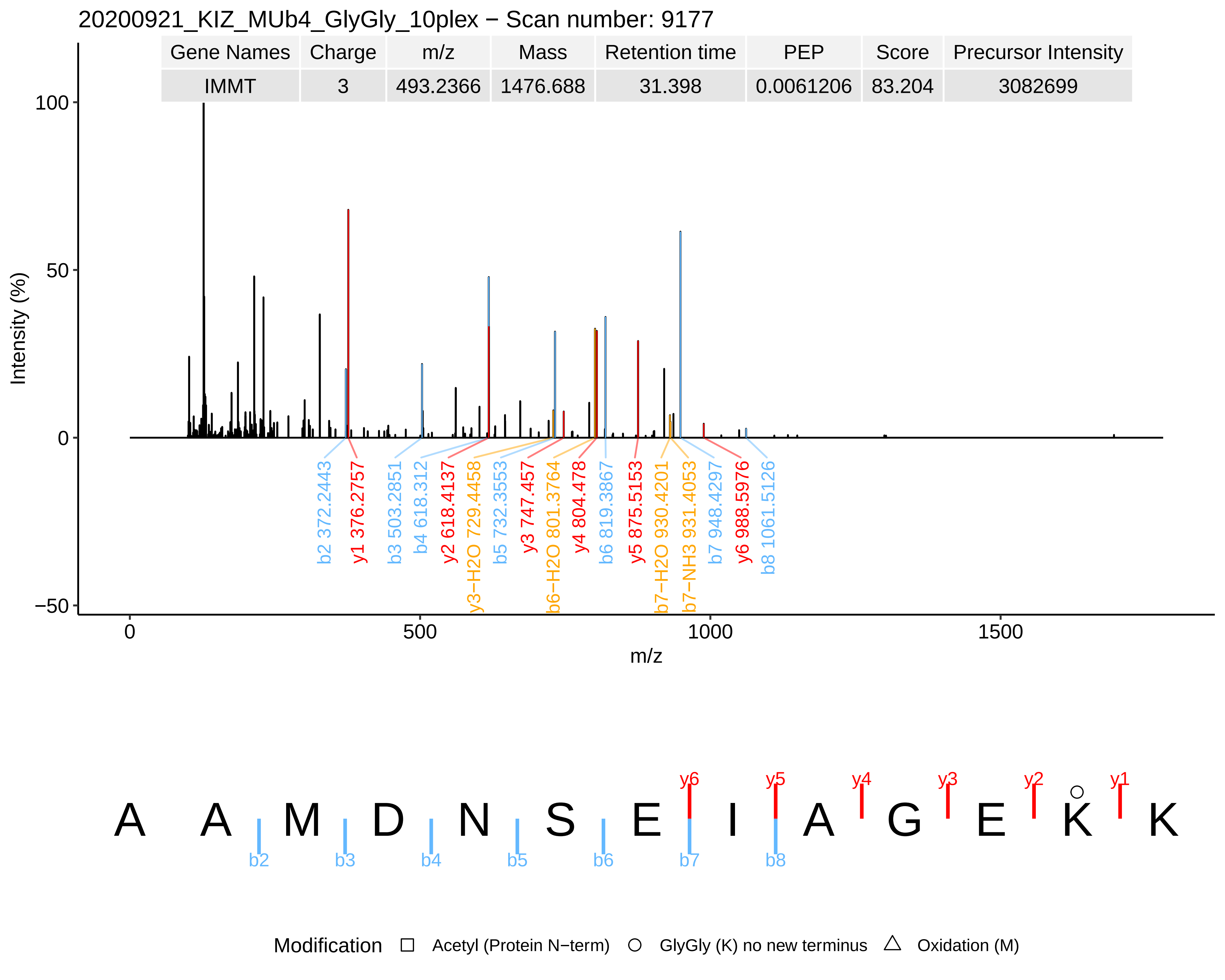

20200921_KIZ_MUb4_GlyGly_10plex − Scan number: 9177
Gene Names | Charge | m/z | Mass | Retention time | PEP | Score | Precursor Intensity
IMMT | 3 | 493.2366 | 1476.688 | 31.398 | 0.0061206 | 83.204 | 3082699
Intensity (%)
m/z
b2 372.2443
y1 376.2757
b3 503.2851
b4 618.312
y2 618.4137
y3−H2O 729.4458
b5 732.3553
y3 747.457
b6−H2O 801.3764
y4 804.478
b6 819.3867
y5 875.5153
b7−H2O 930.4201
b7−NH3 931.4053
b7 948.4297
y6 988.5976
b8 1061.5126
A A M D N S E I A G E K K
Modification
Acetyl (Protein N−term)
GlyGly (K) no new terminus
Oxidation (M)

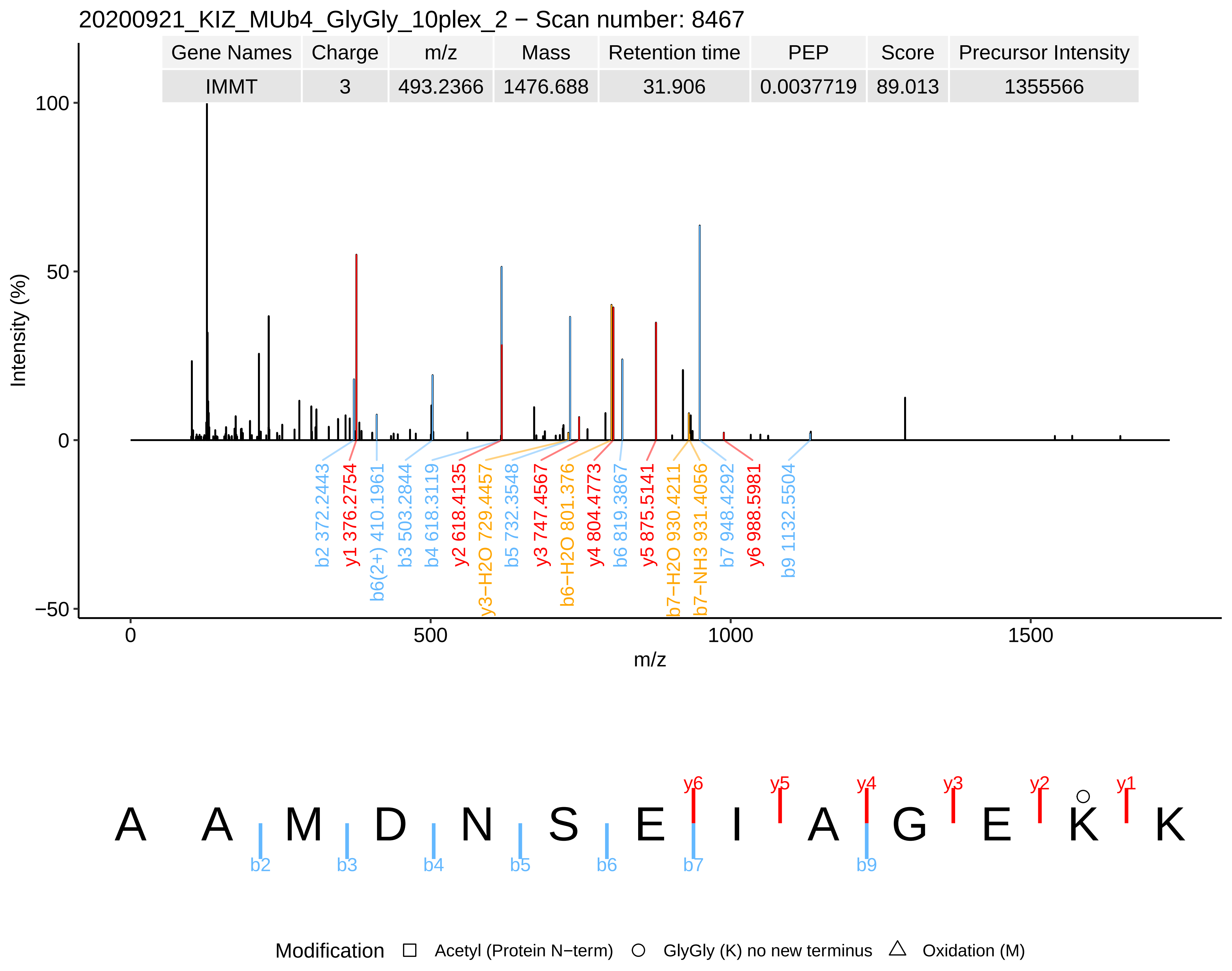
20200921_KIZ_MUb4_GlyGly_10plex_2 − Scan number: 8467
Gene Names	Charge	m/z	Mass	Retention time	PEP	Score	Precursor Intensity
IMMT	3	493.2366	1476.688	31.906	0.0037719	89.013	1355566
Intensity (%)
m/z
b2 372.2443
y1 376.2754
b6(2+) 410.1961
b3 503.2844
b4 618.3119
y2 618.4135
y3−H2O 729.4457
b5 732.3548
y3 747.4567
b6−H2O 801.376
y4 804.4773
b6 819.3867
y5 875.5141
b7−H2O 930.4211
b7−NH3 931.4056
b7 948.4292
y6 988.5981
b9 1132.5504
A A M D N S E I A G E K K
y6 y5 y4 y3 y2 y1
b2 b3 b4 b5 b6 b7 b9
Modification
Acetyl (Protein N−term)
GlyGly (K) no new terminus
Oxidation (M)

20200921_KIZ_MUb4_GlyGly_10plex − Scan number: 9024

Gene Names	Charge	m/z	Mass	Retention time	PEP	Score	Precursor Intensity
PHB2	3	572.9711	1715.891	30.832	3.1448e−06	104.55	5325910

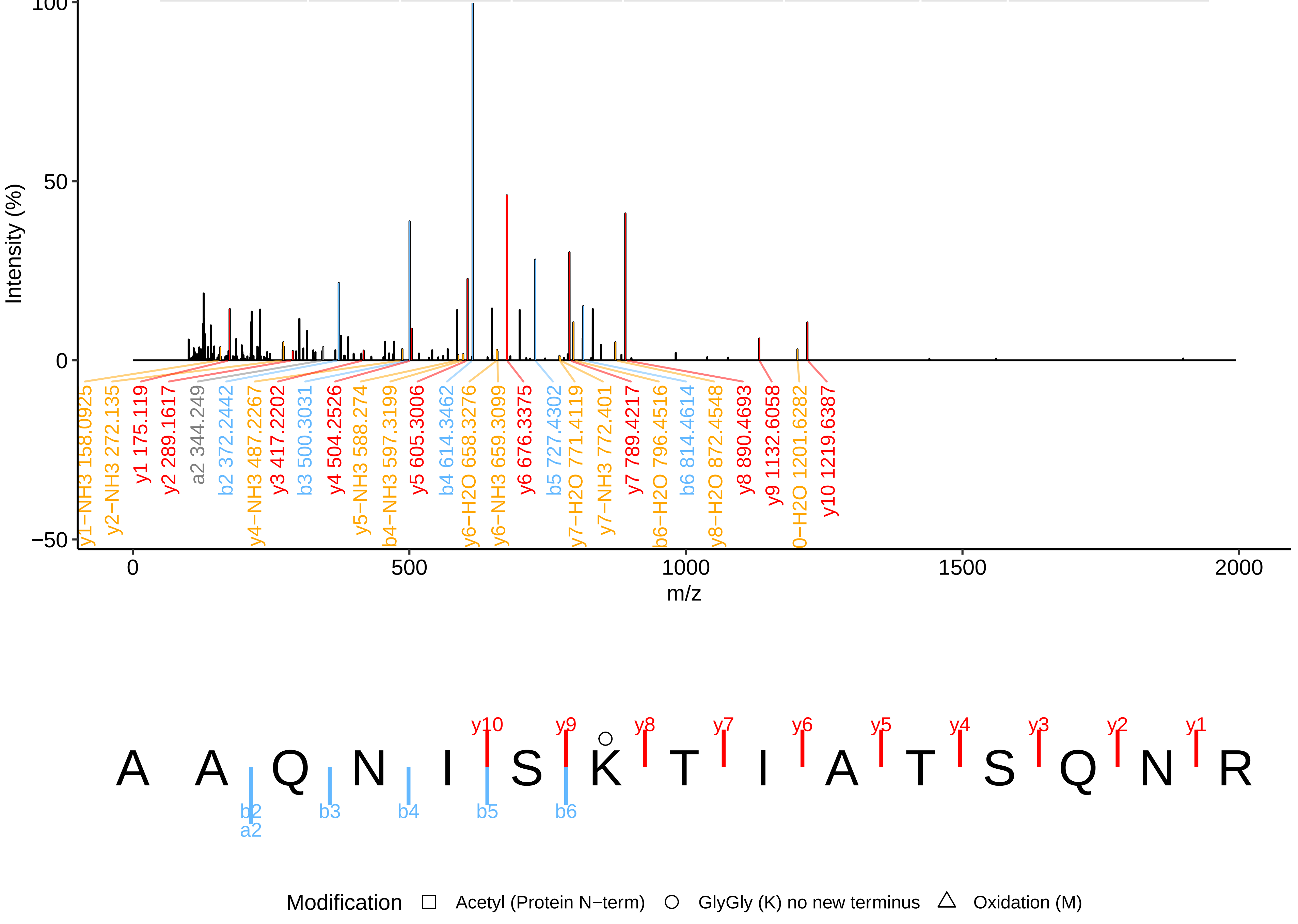

20200921_KIZ_MUb4_GlyGly_10plex − Scan number: 9031

Gene Names	Charge	m/z	Mass	Retention time	PEP	Score	Precursor Intensity
PHB2	3	572.9711	1715.891	30.862	0.00092324	77.367	5064324

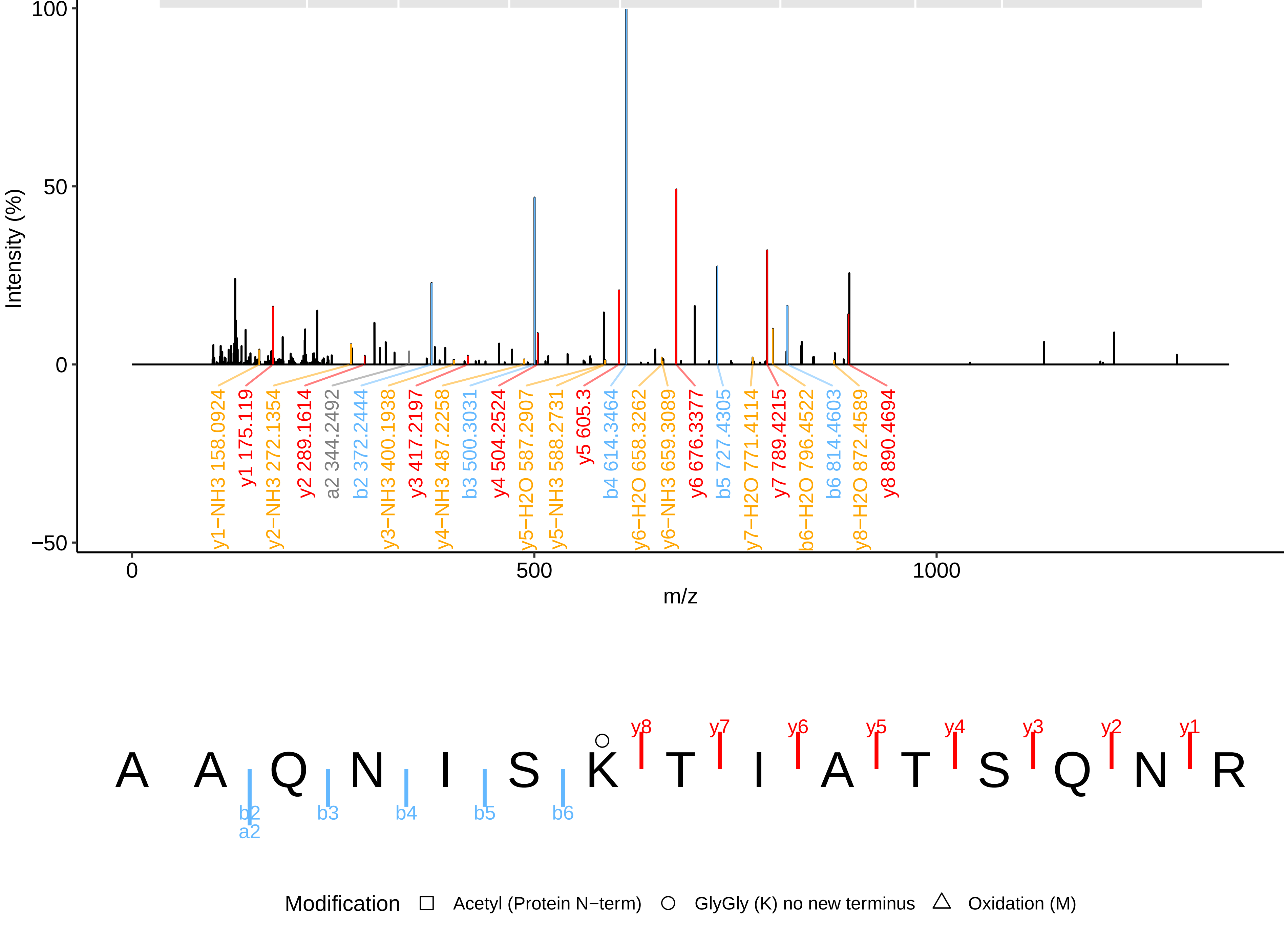

20200921_KIZ_MUb4_GlyGly_10plex_2 − Scan number: 8356

Gene Names	Charge	m/z	Mass	Retention time	PEP	Score	Precursor Intensity
PHB2	3	572.9711	1715.891	31.456	4.4265e−06	100.11	1515170

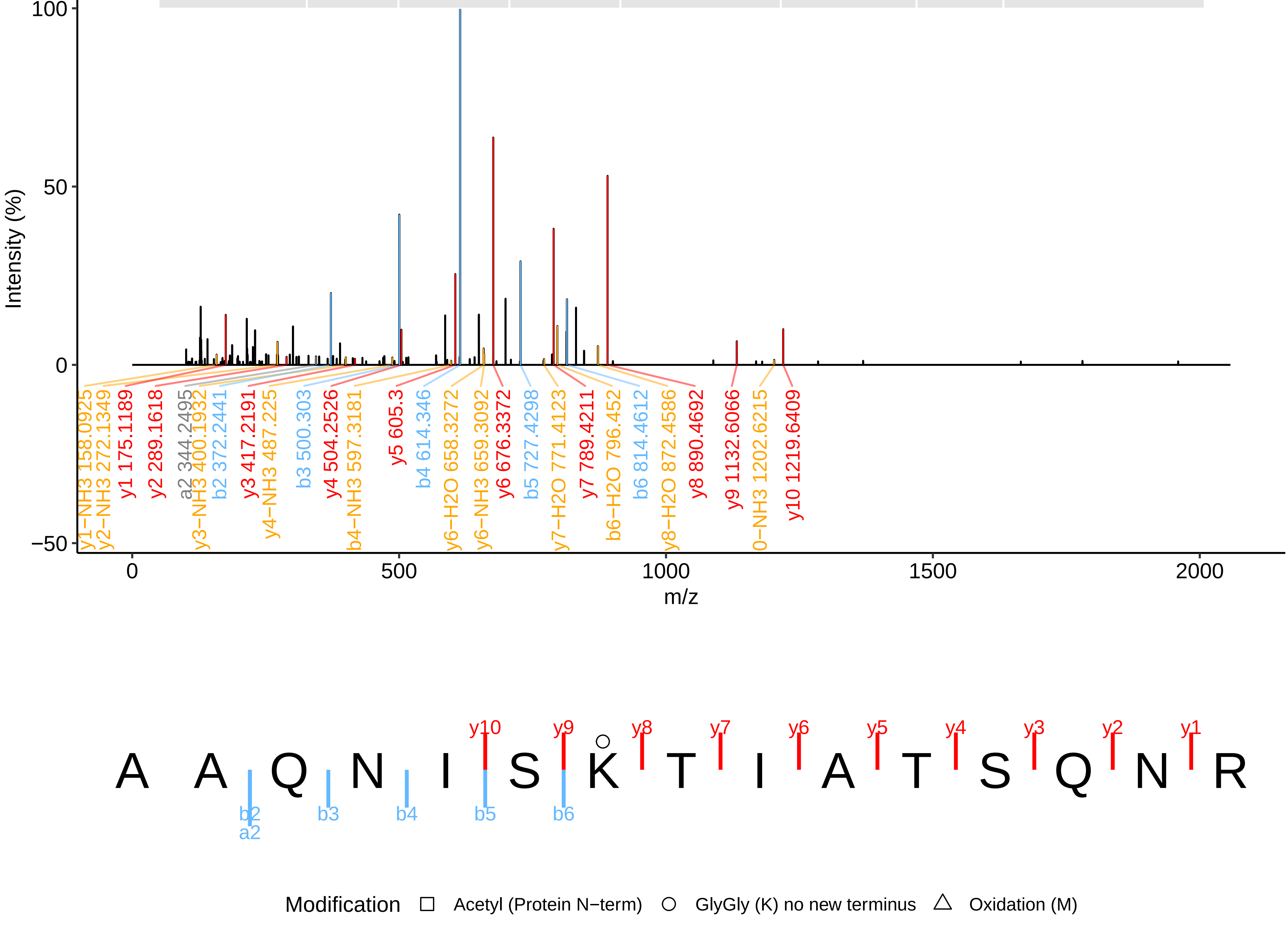

20200924_KIZ_MUb5_GlyGly_1 − Scan number: 8008

Gene Names	Charge	m/z	Mass	Retention time	PEP	Score	Precursor Intensity
PHB2	3	572.9711	1715.891	25.516	3.1537e−05	96.707	4846917

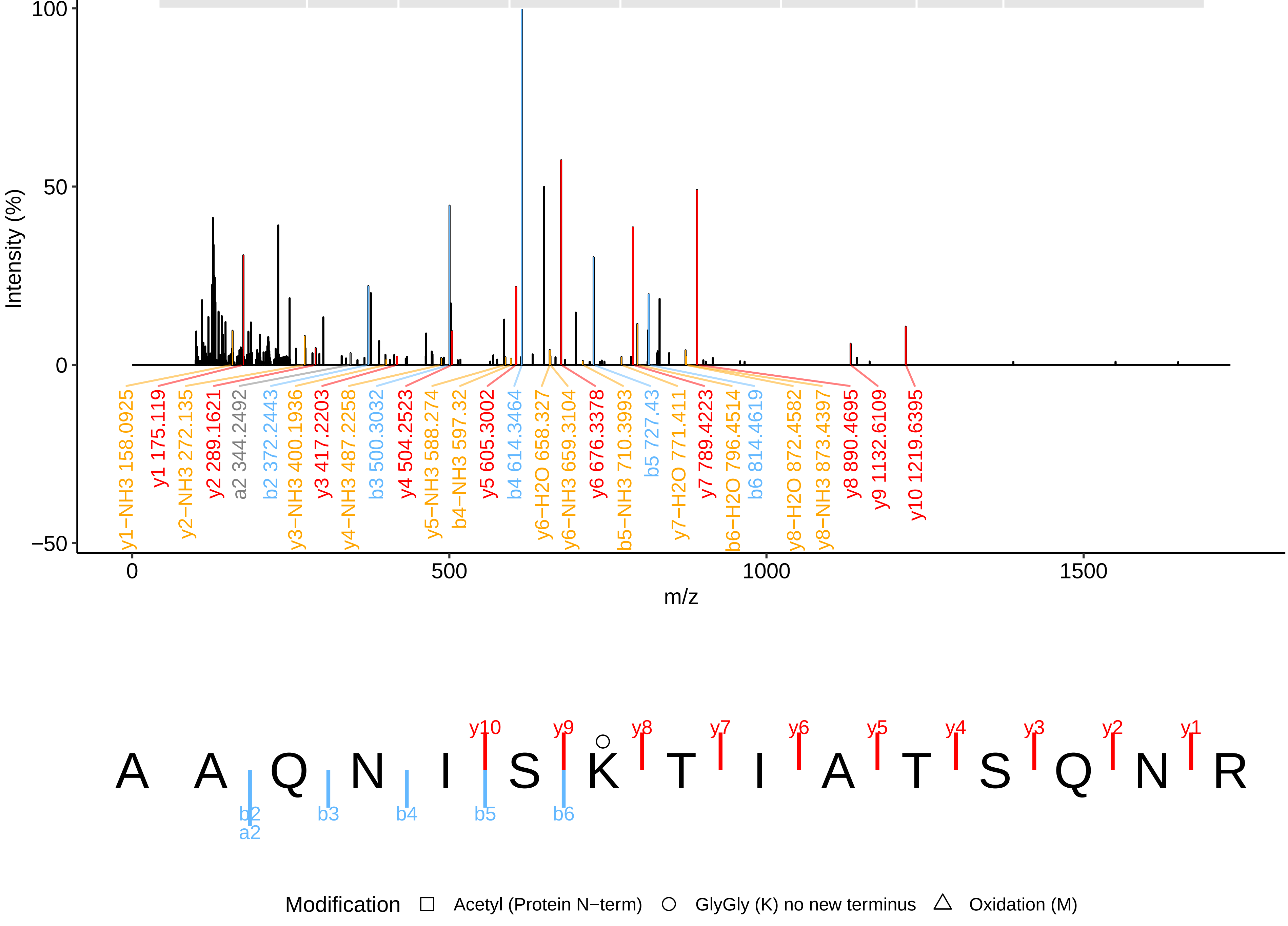

20200921_KIZ_MUb4_GlyGly_10plex − Scan number: 11978

Gene Names	Charge	m/z	Mass	Retention time	PEP	Score	Precursor Intensity
RPS2	3	488.566	1462.676	41.864	0.0073146	71.555	13951697

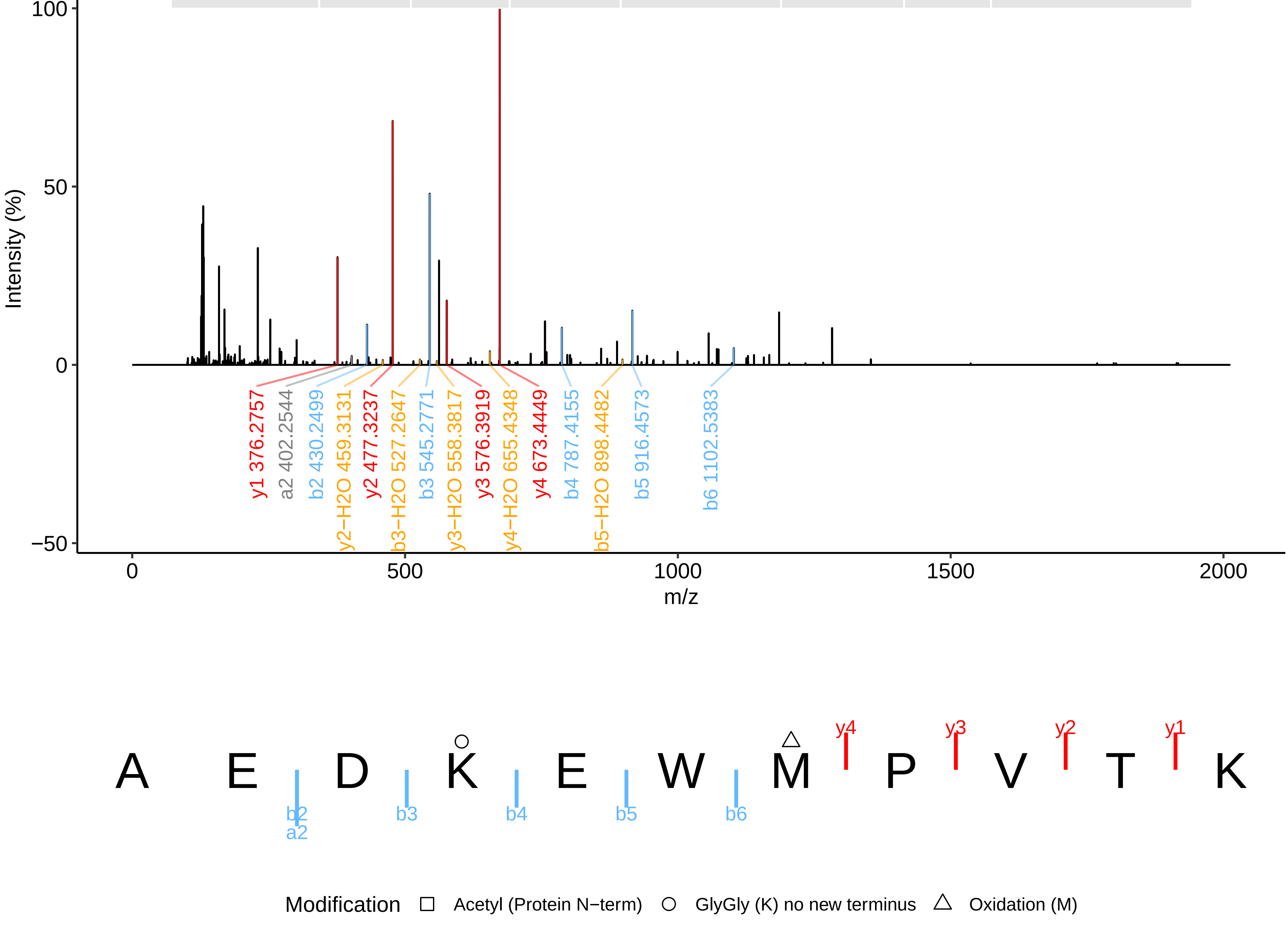

20200921_KIZ_MUb4_GlyGly_10plex_2 − Scan number: 11030

Gene Names	Charge	m/z	Mass	Retention time	PEP	Score	Precursor Intensity
RPS2	3	488.566	1462.676	42.351	0.11023	53.528	3247425

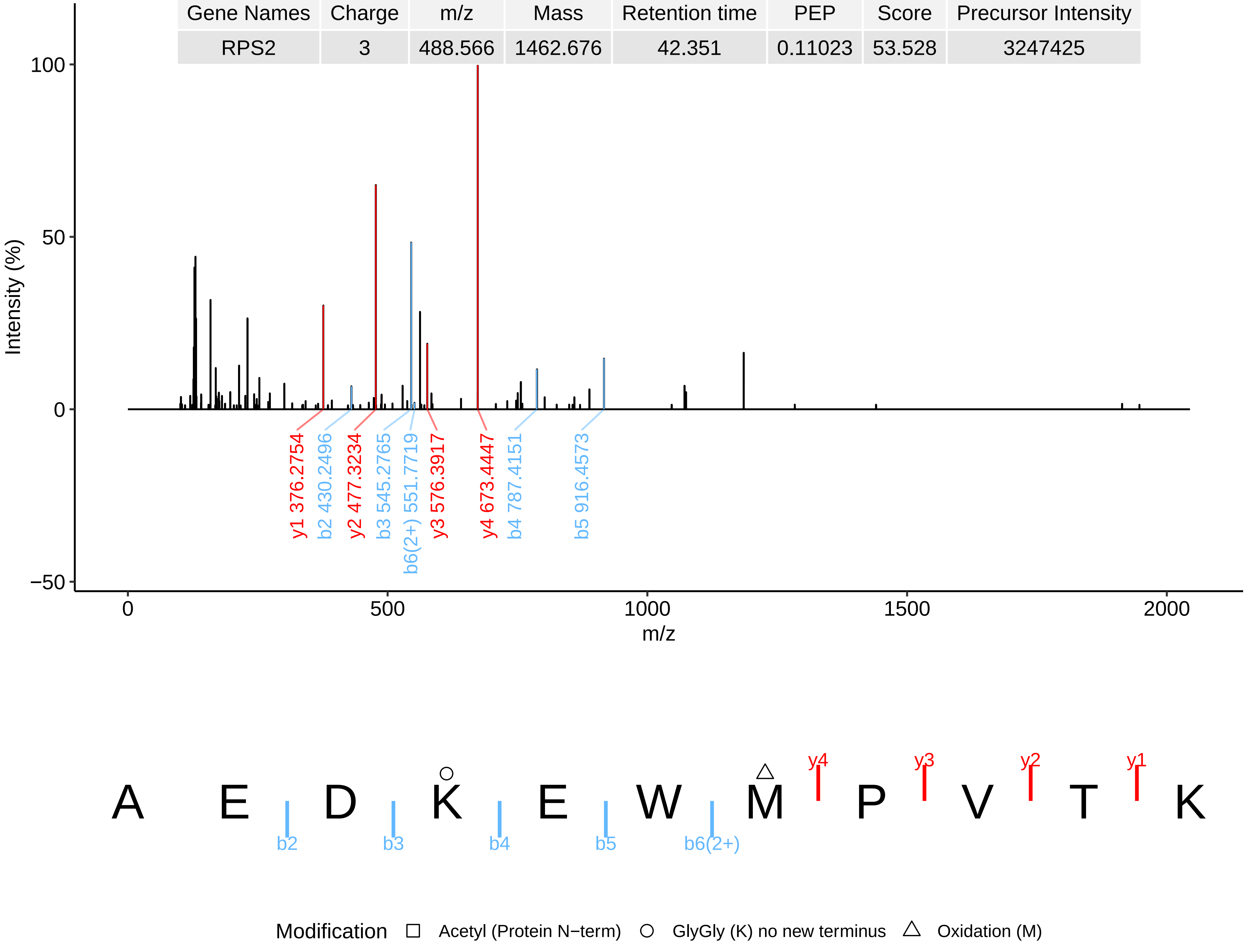

20200921_KIZ_MUb4_GlyGly_10plex − Scan number: 4398

Gene Names	Charge	m/z	Mass	Retention time	PEP	Score	Precursor Intensity
PGAM1;PGAM4	4	364.1903	1452.732	14.333	1.2124e−13	138.42	29454556

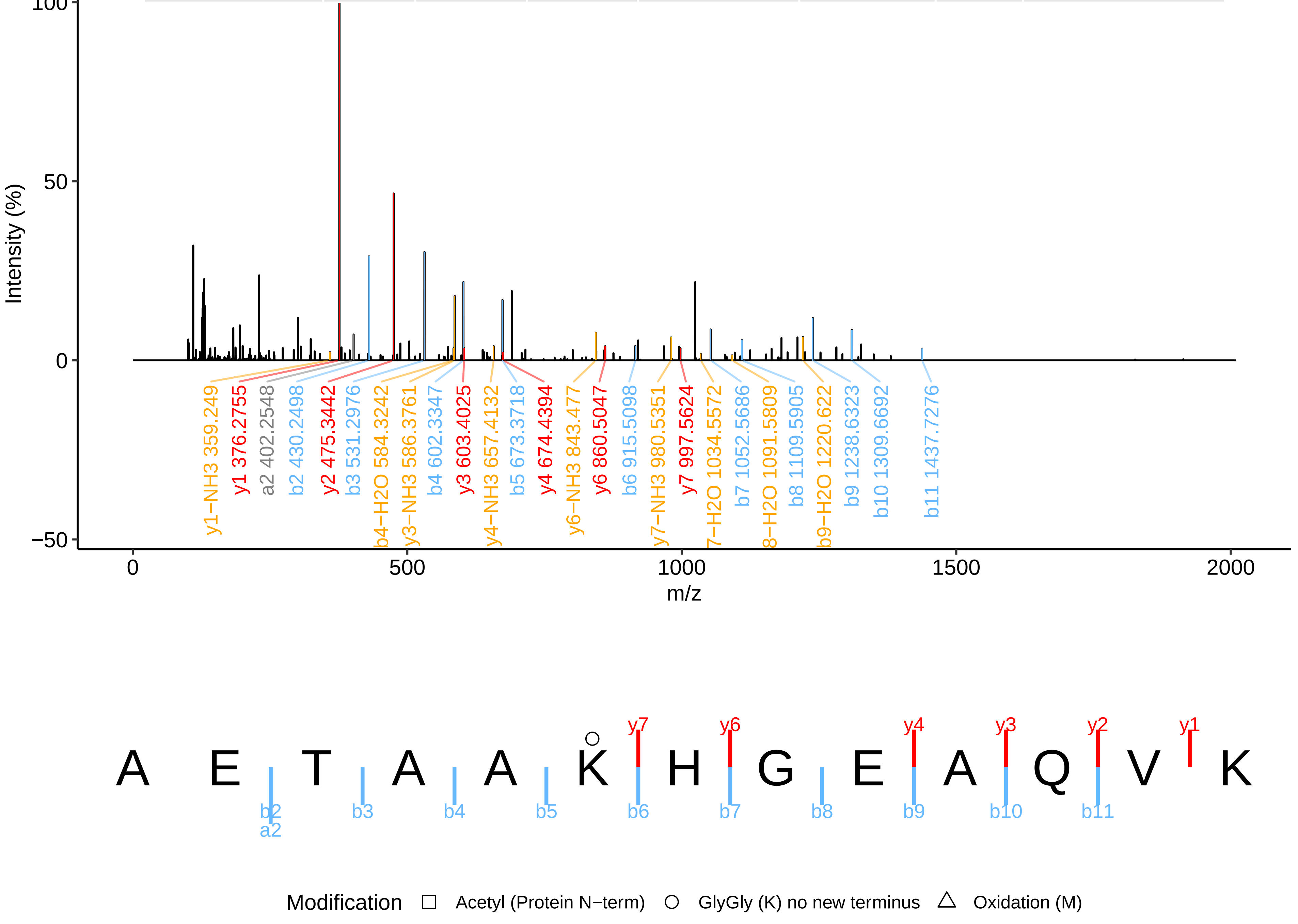

20200921_KIZ_MUb4_GlyGly_10plex_2 − Scan number: 4113

Gene Names	Charge	m/z	Mass	Retention time	PEP	Score	Precursor Intensity
PGAM1;PGAM4	3	485.2513	1452.732	14.246	2.1193e−05	99.011	560768.3

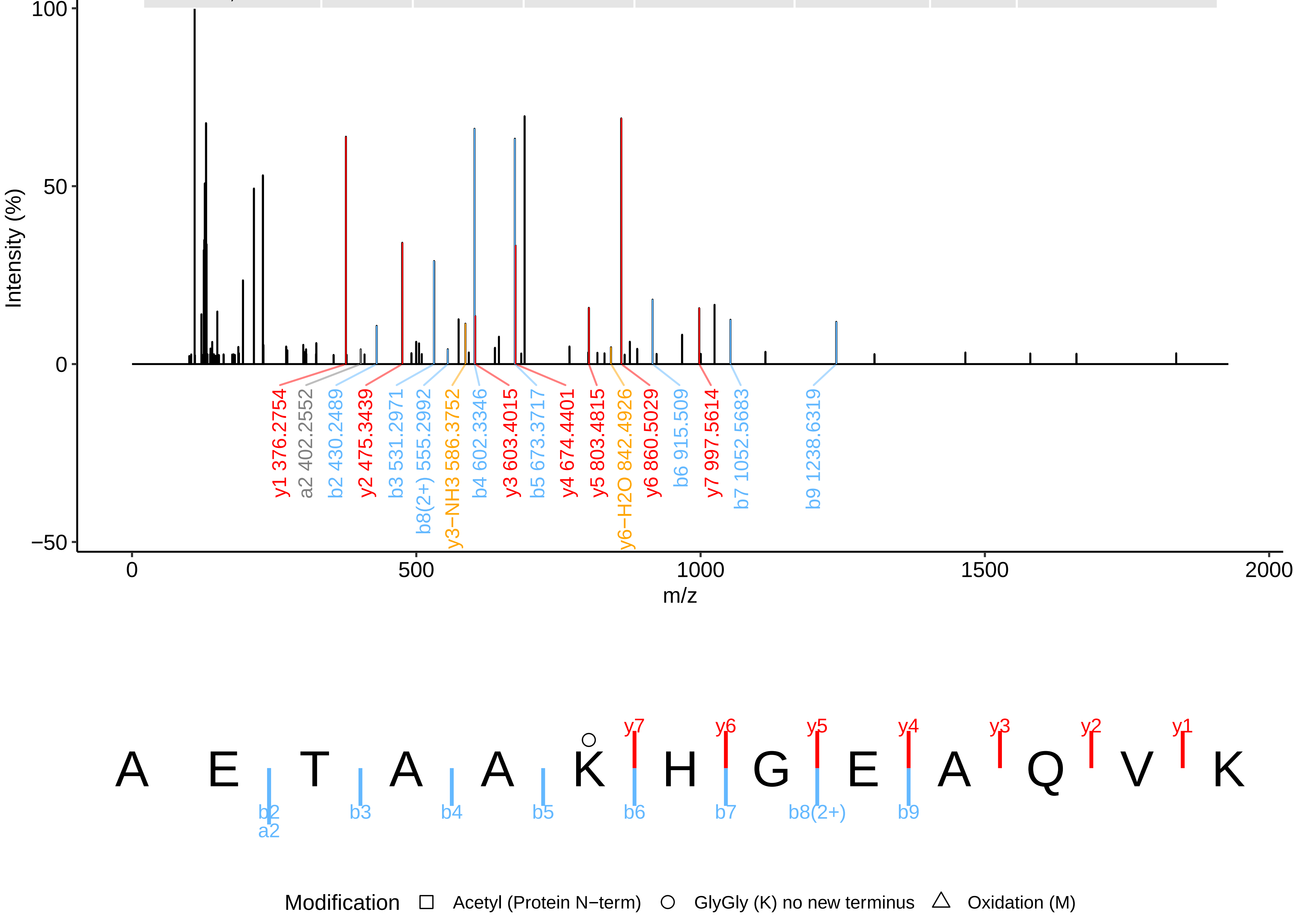

20200924_KIZ_MUb5_GlyGly_1 − Scan number: 3643

Gene Names	Charge	m/z	Mass	Retention time	PEP	Score	Precursor Intensity
PGAM1;PGAM4	3	485.2513	1452.732	11.139	5.2949e−10	136.99	9410210

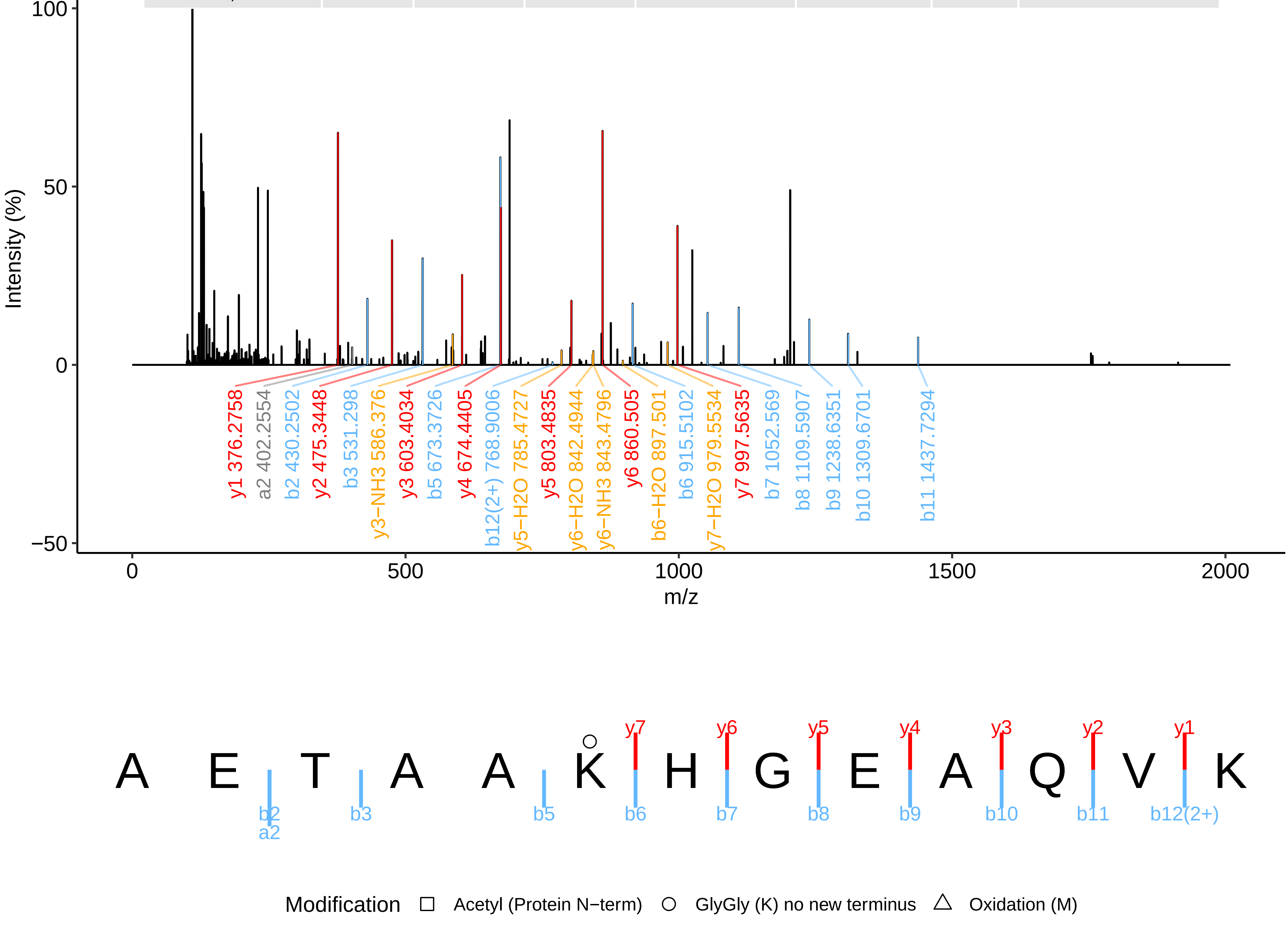

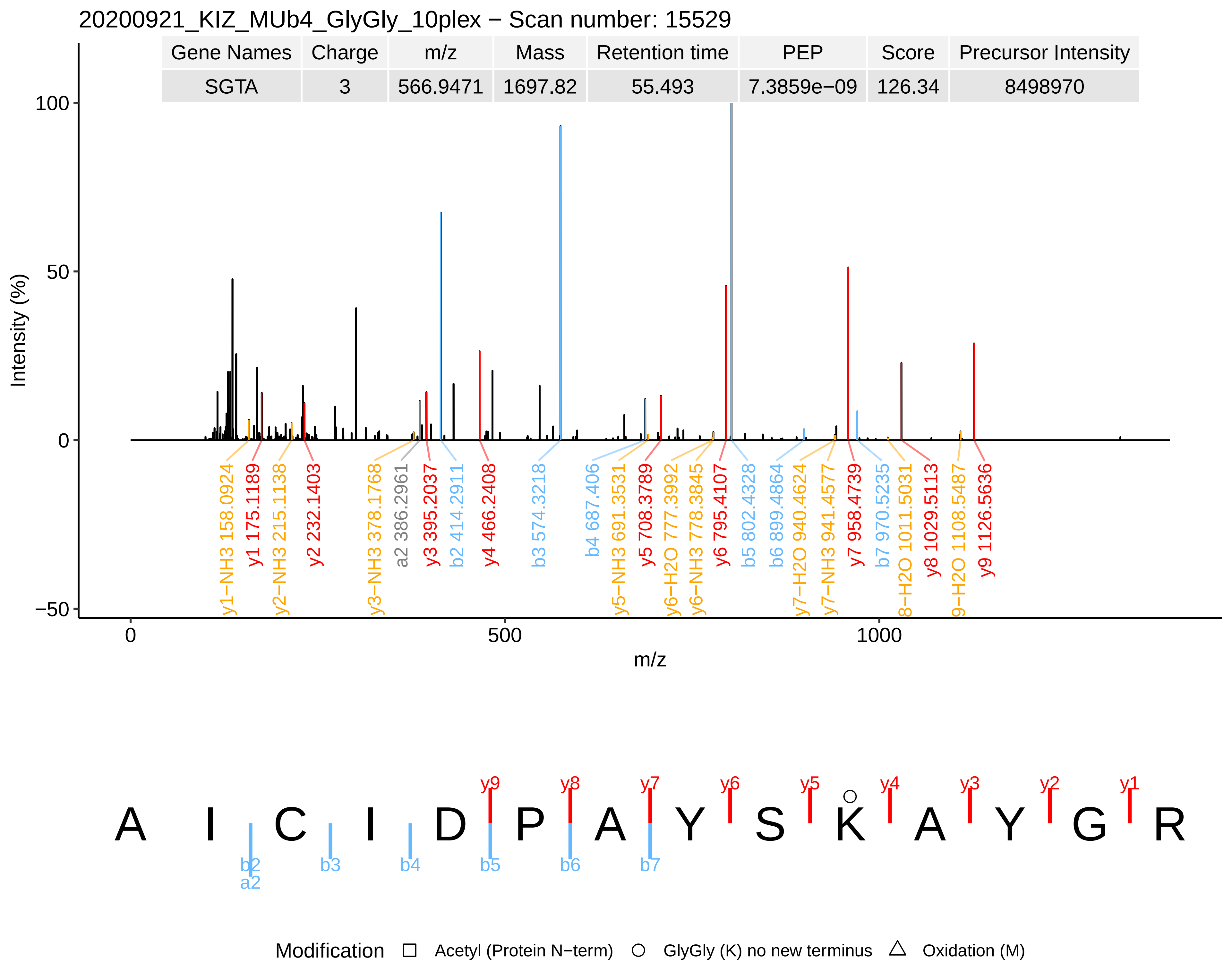

20200921_KIZ_MUb4_GlyGly_10plex − Scan number: 15529
Gene Names | Charge | m/z | Mass | Retention time | PEP | Score | Precursor Intensity
SGTA | 3 | 566.9471 | 1697.82 | 55.493 | 7.3859e−09 | 126.34 | 8498970
Intensity (%)
m/z
y1−NH3 158.0924
y1 175.1189
y2−NH3 215.1138
y2 232.1403
y3−NH3 378.1768
a2 386.2961
y3 395.2037
b2 414.2911
y4 466.2408
b3 574.3218
b4 687.406
y5−NH3 691.3531
y5 708.3789
y6−H2O 777.3992
y6−NH3 778.3845
y6 795.4107
b5 802.4328
b6 899.4864
y7−H2O 940.4624
y7−NH3 941.4577
y7 958.4739
b7 970.5235
y8−H2O 1011.5031
y8 1029.5113
y9−H2O 1108.5487
y9 1126.5636
A I C I D P A Y S K A Y G R
Modification
Acetyl (Protein N−term)
GlyGly (K) no new terminus
Oxidation (M)

20200921_KIZ_MUb4_GlyGly_10plex_2 − Scan number: 14361

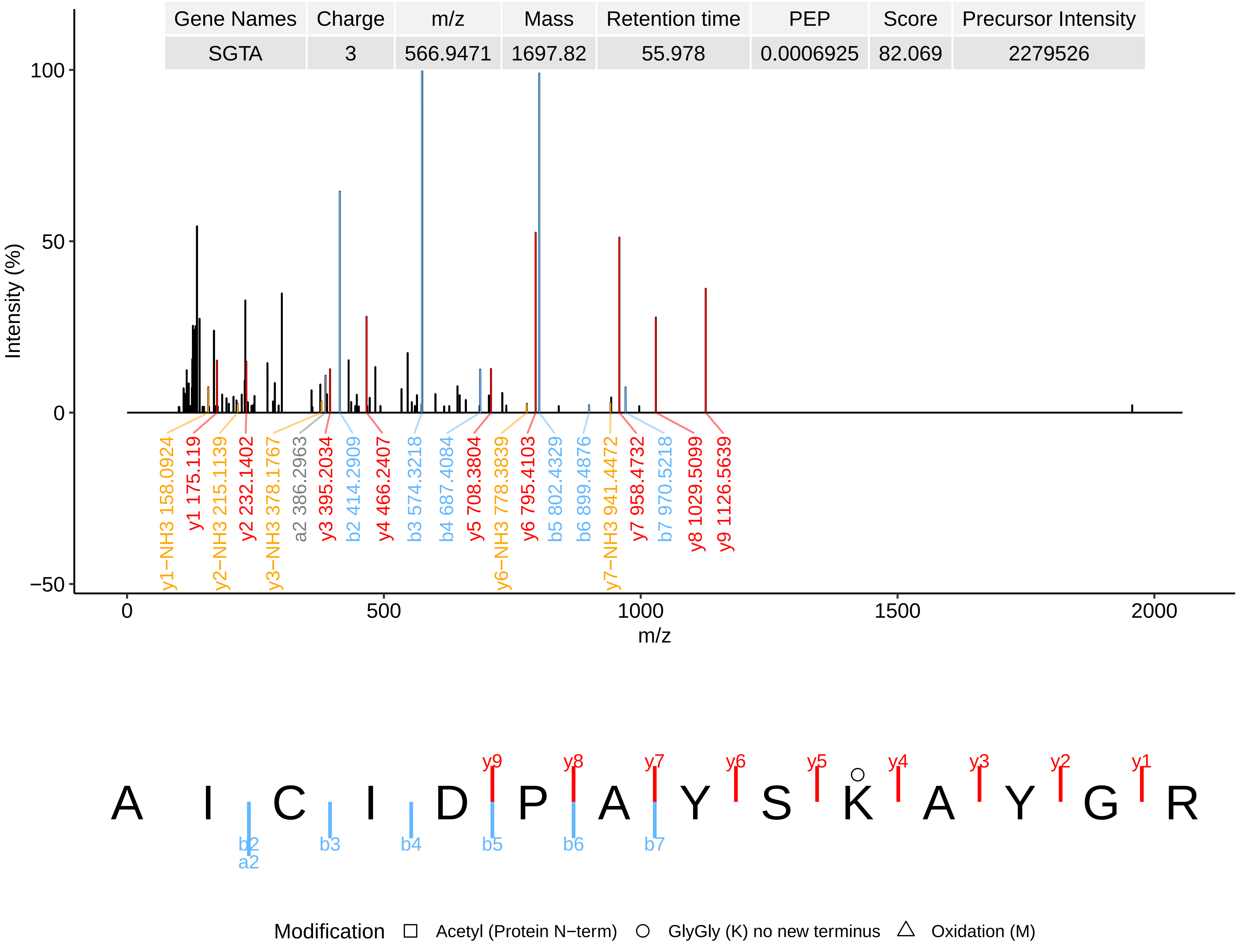

20200924_KIZ_MUb5_GlyGly_1 − Scan number: 14735

Gene Names	Charge	m/z	Mass	Retention time	PEP	Score	Precursor Intensity
SGTA	3	566.9471	1697.82	48.135	8.0116e−20	158.34	23306354

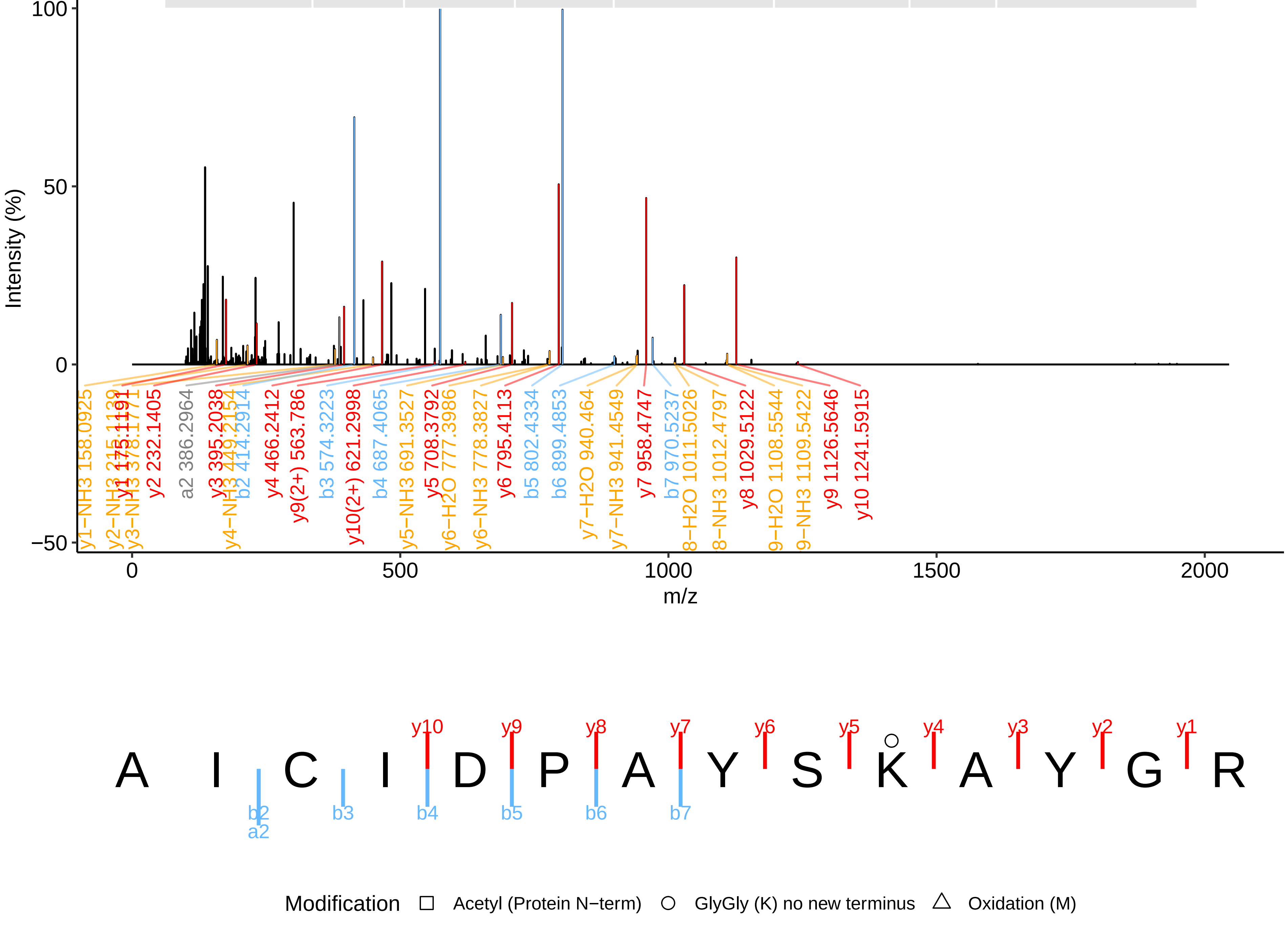

20200924_KIZ_MUb5_GlyGly_2 − Scan number: 13862

Gene Names	Charge	m/z	Mass	Retention time	PEP	Score	Precursor Intensity
SGTA	3	566.9471	1697.82	51.844	2.7168e−10	132.59	3876827

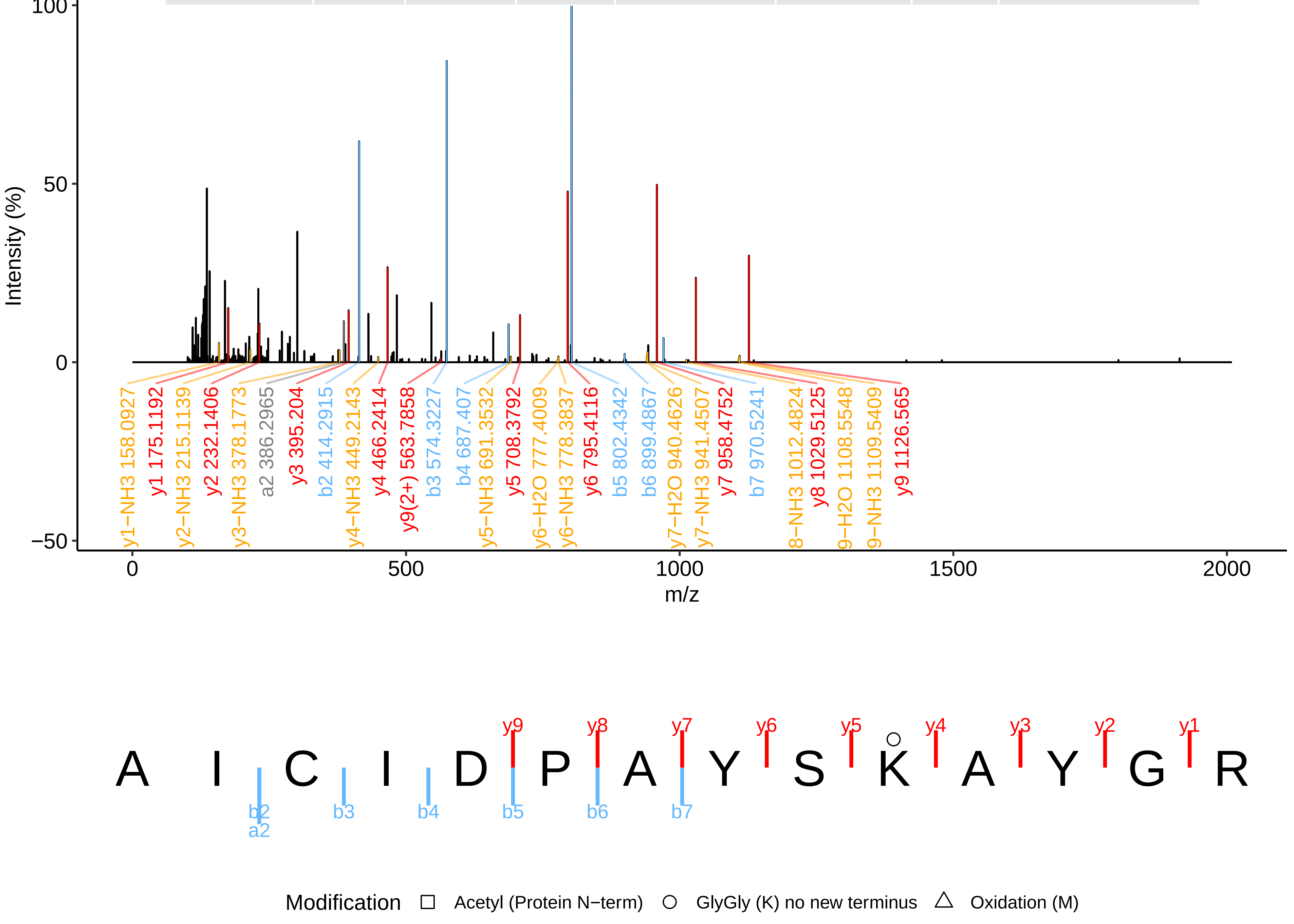

20201118_KIZ_MUb6_GlyGly_2 − Scan number: 16628

Gene Names	Charge	m/z	Mass	Retention time	PEP	Score	Precursor Intensity
SGTA	3	566.9471	1697.82	56.196	5.1422e−09	128.31	4838561

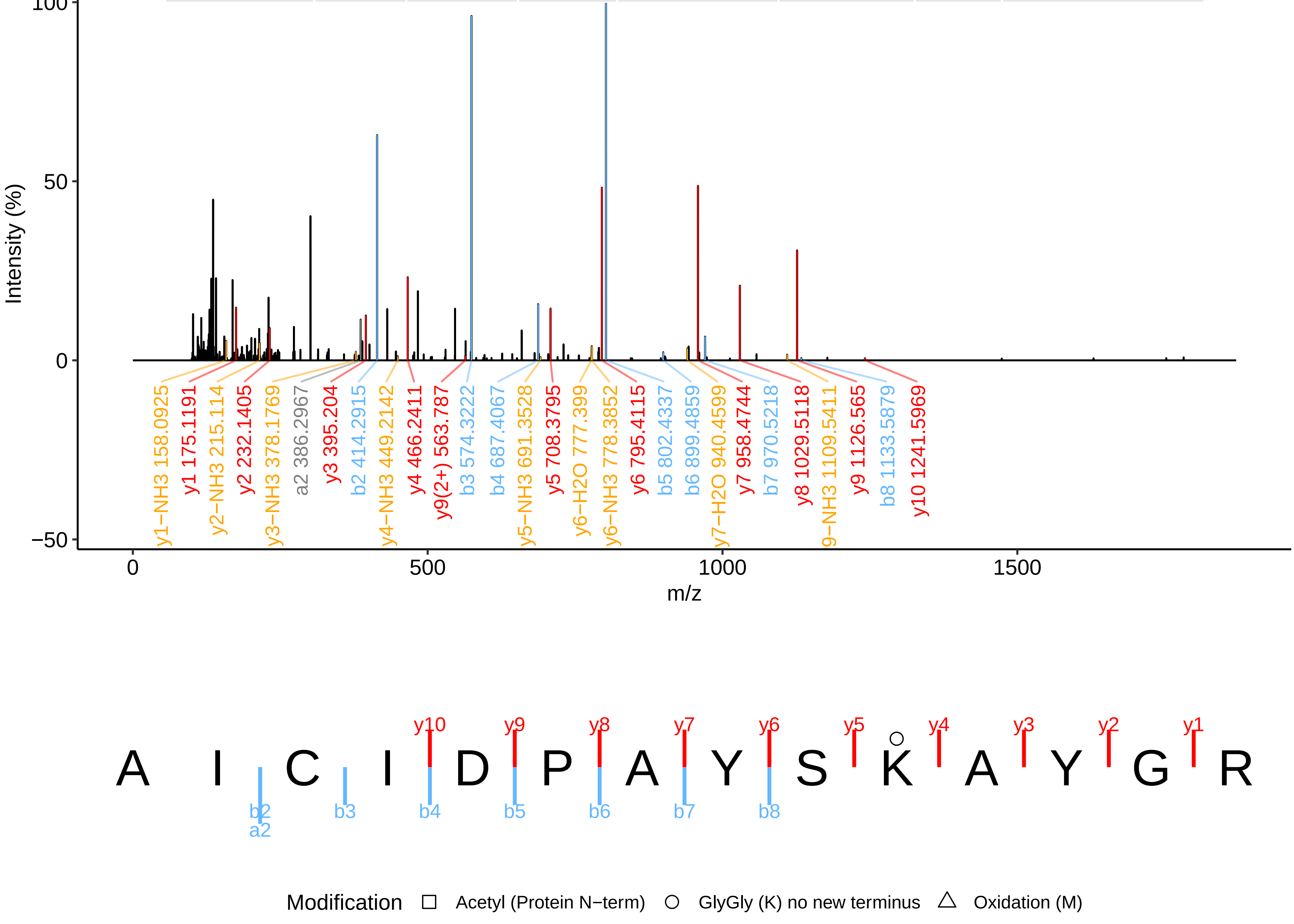

20200921_KIZ_MUb4_GlyGly_10plex − Scan number: 10626

Gene Names	Charge	m/z	Mass	Retention time	PEP	Score	Precurso
ST1H1E;HIST1H1D;HIST1H1A;HIST1H1T;HIST1H1C	3	564.9482	1691.823	36.829	2.0016e−09	126.1	413

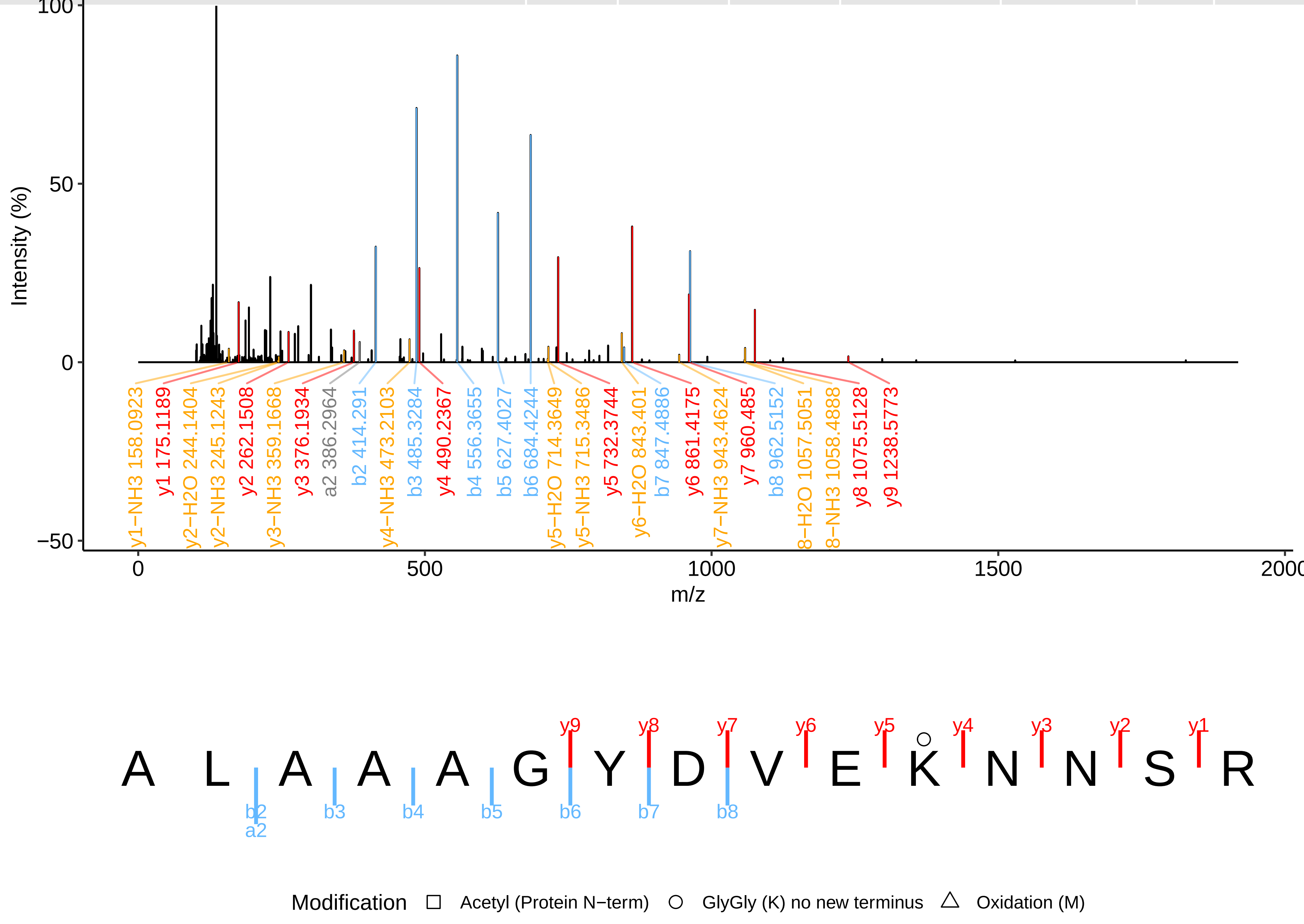

20200924_KIZ_MUb5_GlyGly_1 − Scan number: 9598

Gene Names	Charge	m/z	Mass	Retention time	PEP	Score	Precurs
ST1H1E;HIST1H1D;HIST1H1A;HIST1H1T;HIST1H1C	3	564.9482	1691.823	30.888	9.8735e−23	174.39	

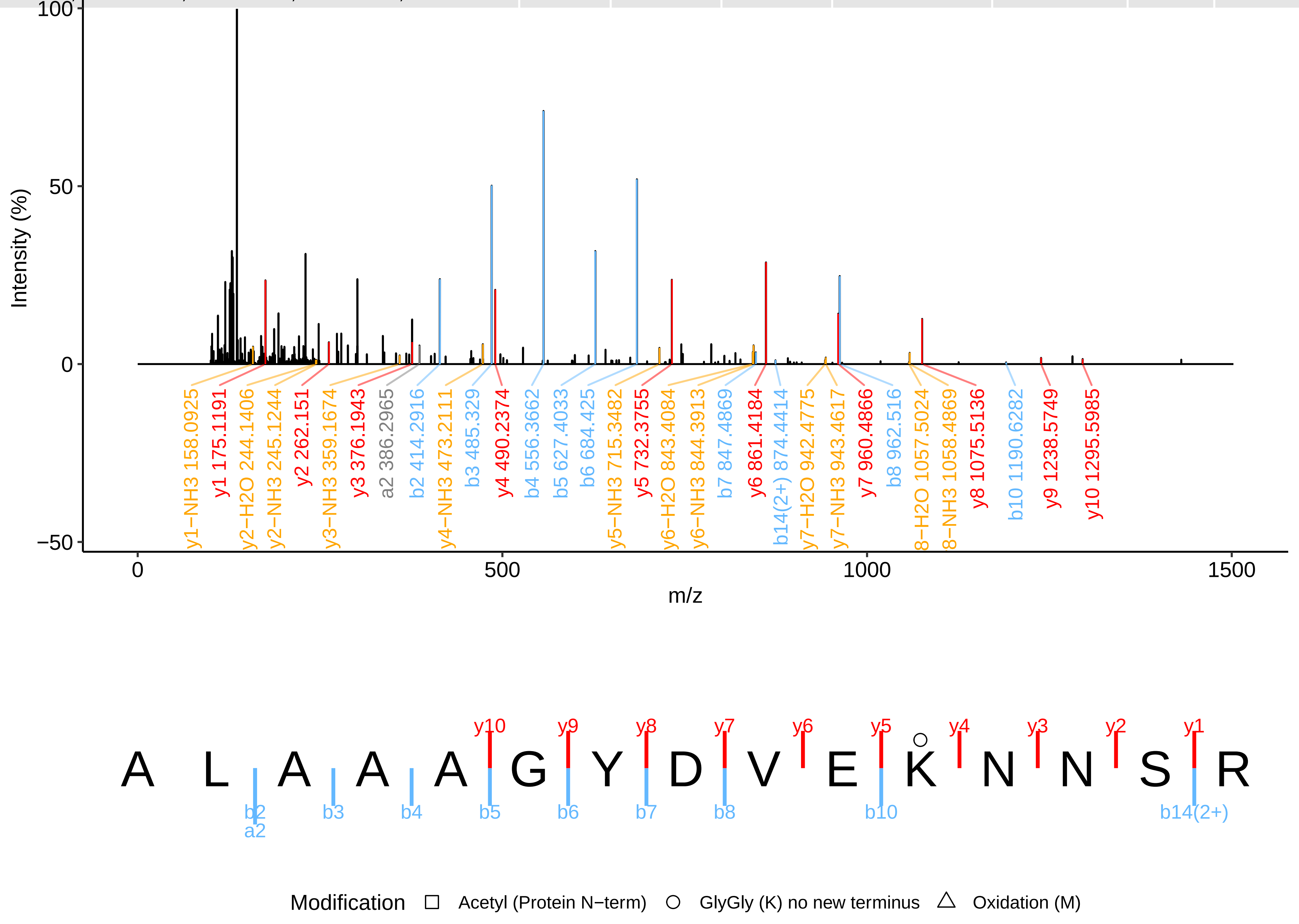

20200921_KIZ_MUb4_GlyGly_10plex_2 − Scan number: 20894

Gene Names	Charge	m/z	Mass	Retention time	PEP	Score	Precursor Intensity
POLD1	3	573.9895	1718.947	82.198	0.046372	56.482	985307.4

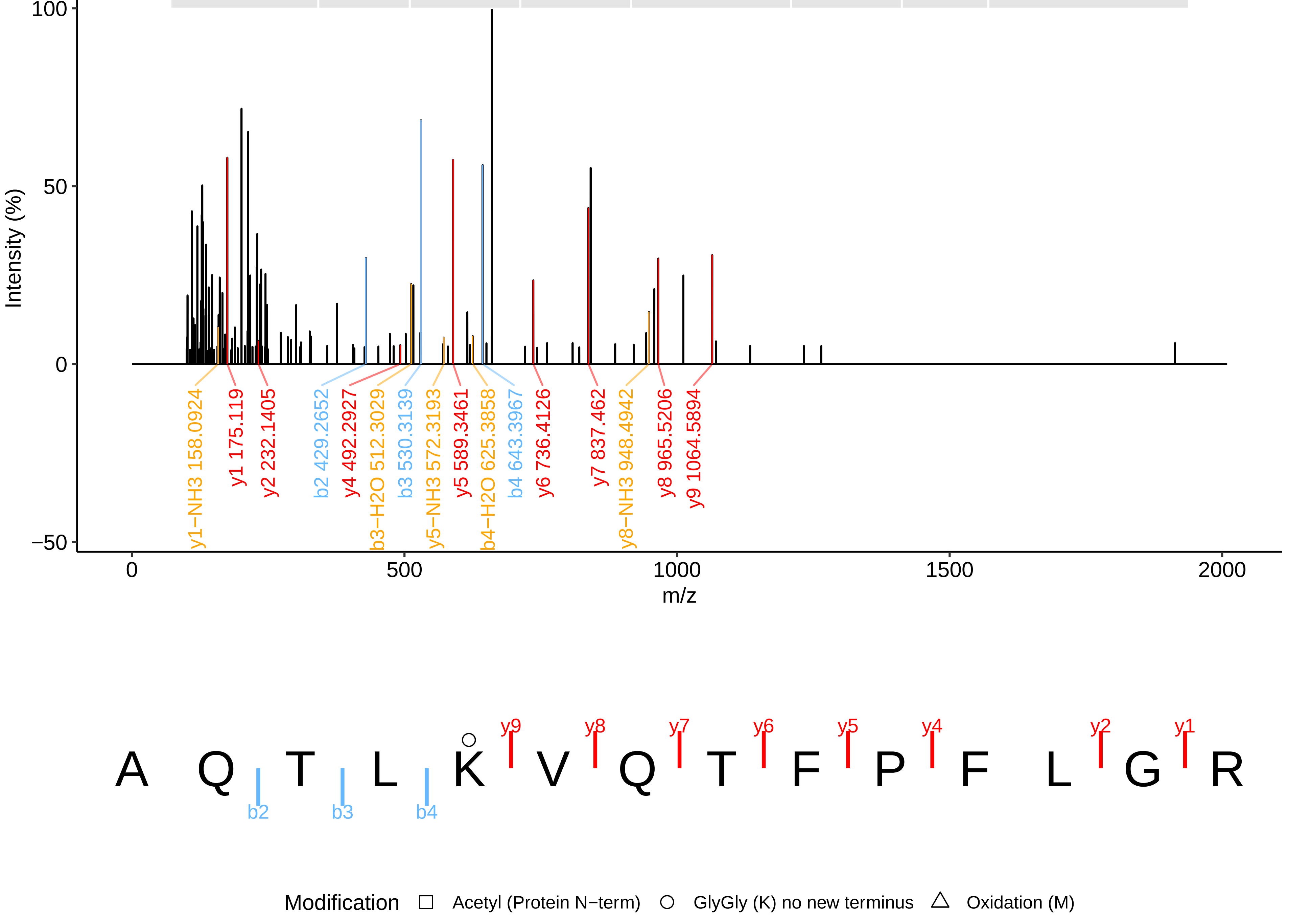

20200921_KIZ_MUb4_GlyGly_10plex − Scan number: 10674

Gene Names	Charge	m/z	Mass	Retention time	PEP	Score	Precursor Intensity
PSMA6	4	379.1941	1512.747	37.01	0.062229	70.942	3604326

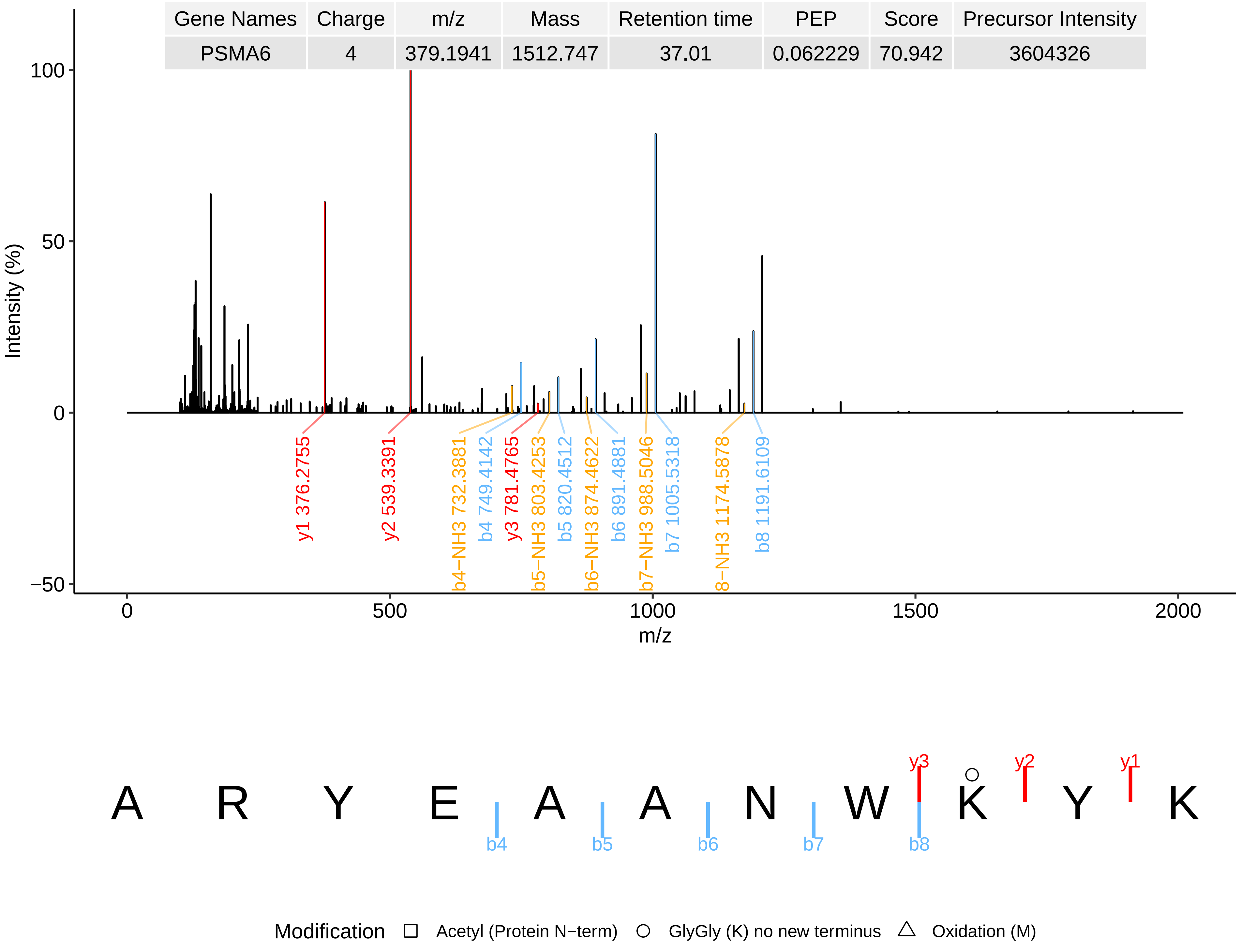

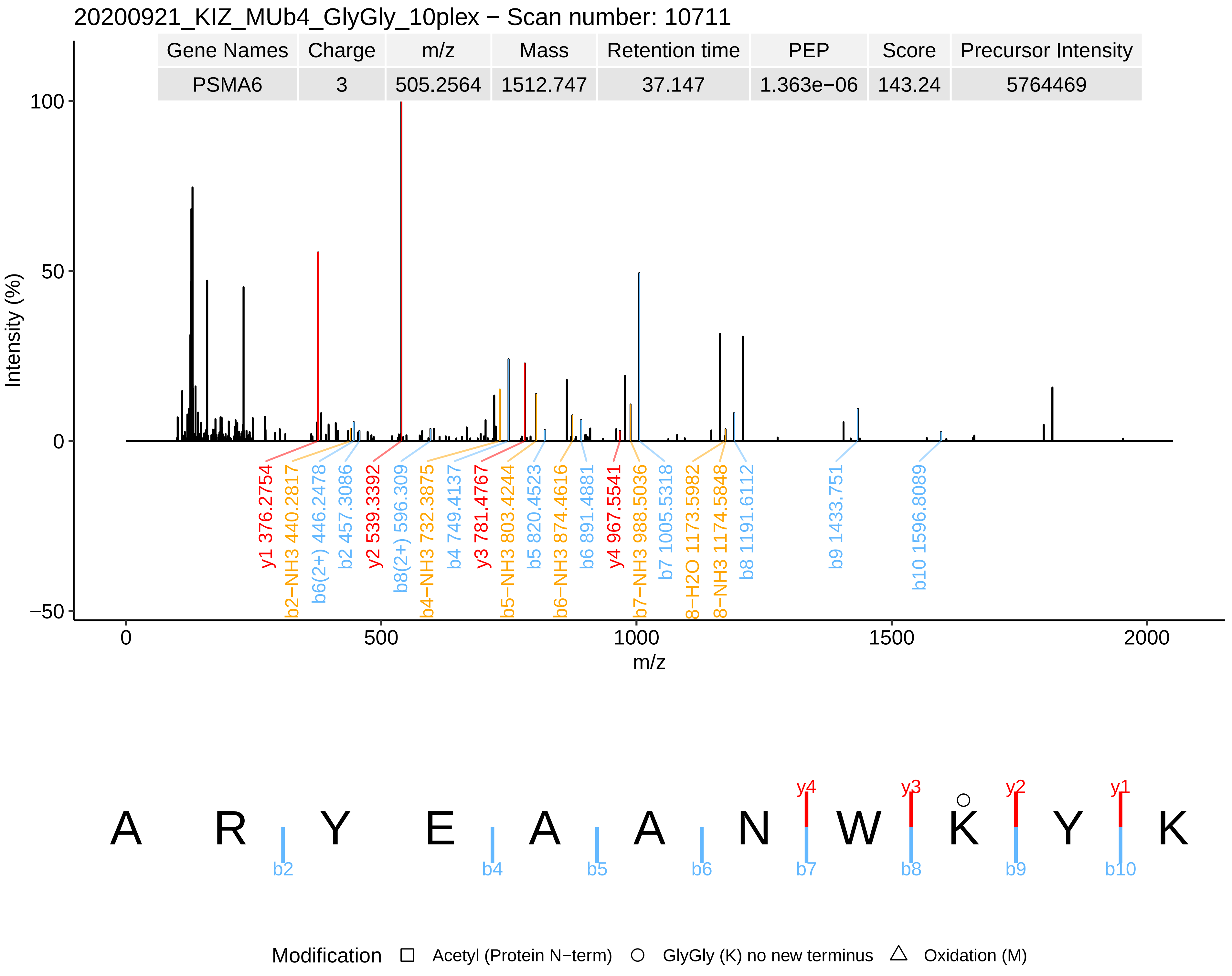

20200921_KIZ_MUb4_GlyGly_10plex − Scan number: 10711
Gene Names | Charge | m/z | Mass | Retention time | PEP | Score | Precursor Intensity
PSMA6 | 3 | 505.2564 | 1512.747 | 37.147 | 1.363e−06 | 143.24 | 5764469
Intensity (%)
m/z
y1 376.2754
b2−NH3 440.2817
b6(2+) 446.2478
b2 457.3086
y2 539.3392
b8(2+) 596.309
b4−NH3 732.3875
b4 749.4137
y3 781.4767
b5−NH3 803.4244
b5 820.4523
b6−NH3 874.4616
b6 891.4881
y4 967.5541
b7−NH3 988.5036
b7 1005.5318
8−H2O 1173.5982
8−NH3 1174.5848
b8 1191.6112
b9 1433.751
b10 1596.8089
A R Y E A A N W K Y K
Modification
Acetyl (Protein N−term)
GlyGly (K) no new terminus
Oxidation (M)

20200921_KIZ_MUb4_GlyGly_10plex_2 − Scan number: 9948

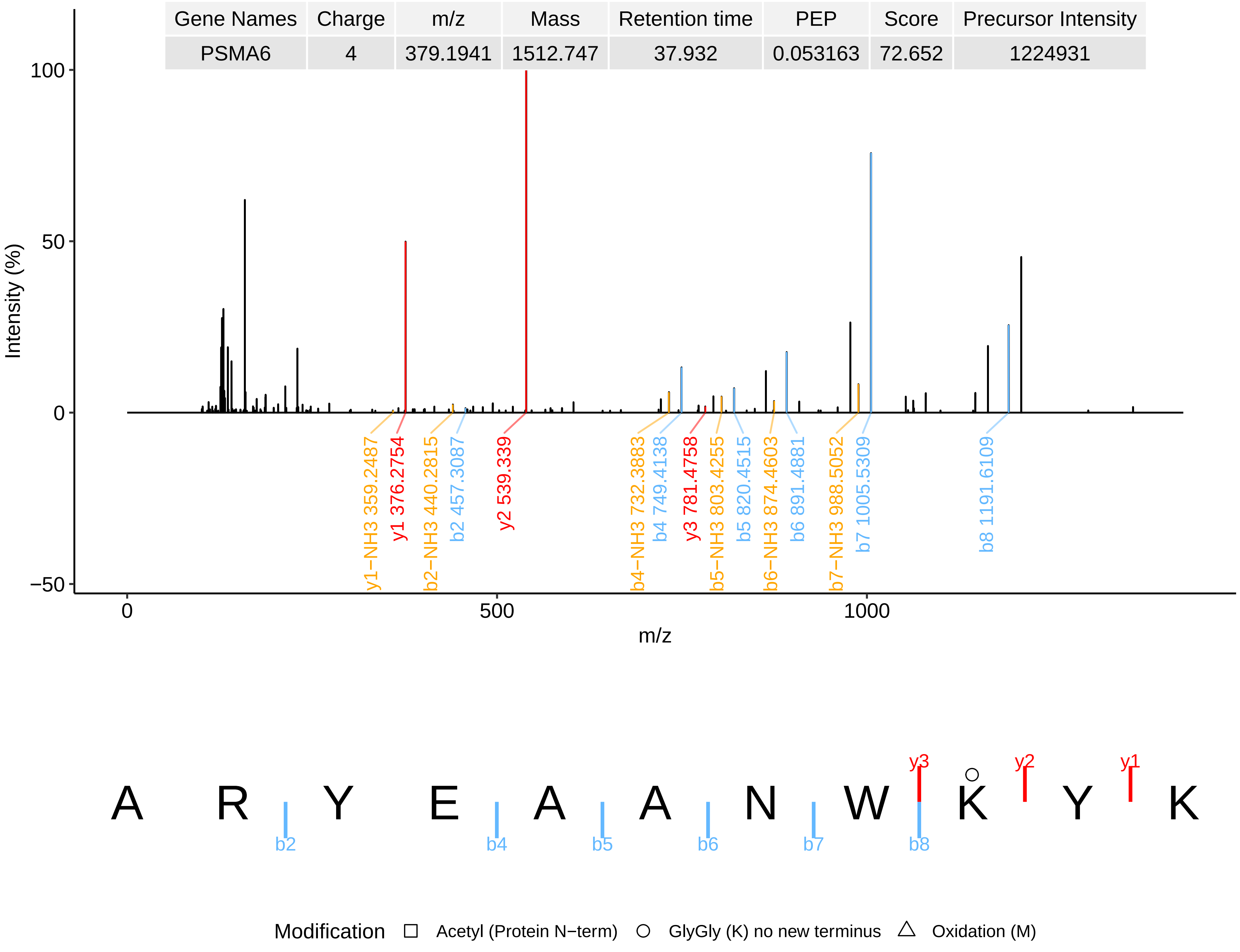

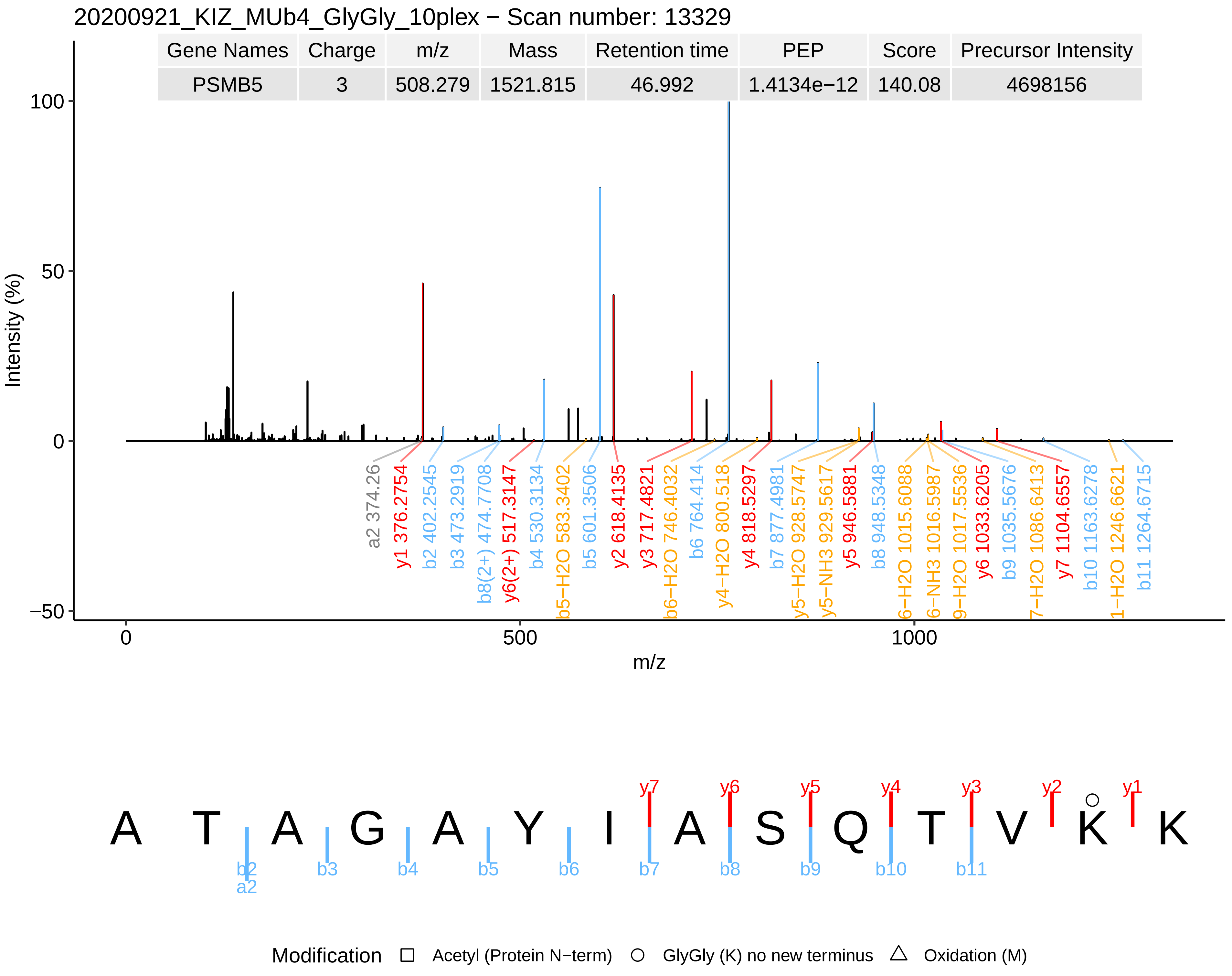
20200921_KIZ_MUb4_GlyGly_10plex − Scan number: 13329
Gene Names
Charge
m/z
Mass
Retention time
PEP
Score
Precursor Intensity
PSMB5
3
508.279
1521.815
46.992
1.4134e−12
140.08
4698156
Intensity (%)
m/z
a2 374.26
y1 376.2754
b2 402.2545
b3 473.2919
b8(2+) 474.7708
y6(2+) 517.3147
b4 530.3134
b5−H2O 583.3402
b5 601.3506
y2 618.4135
y3 717.4821
b6−H2O 746.4032
b6 764.414
y4−H2O 800.518
y4 818.5297
b7 877.4981
y5−H2O 928.5747
y5−NH3 929.5617
y5 946.5881
b8 948.5348
6−H2O 1015.6088
6−NH3 1016.5987
9−H2O 1017.5536
y6 1033.6205
b9 1035.5676
7−H2O 1086.6413
y7 1104.6557
b10 1163.6278
1−H2O 1246.6621
b11 1264.6715
A T A G A Y I A S Q T V K K
Modification
Acetyl (Protein N−term)
GlyGly (K) no new terminus
Oxidation (M)

20200921_KIZ_MUb4_GlyGly_10plex − Scan number: 13408

Gene Names	Charge	m/z	Mass	Retention time	PEP	Score	Precursor Intensity
PSMB5	3	508.279	1521.815	47.295	0.0029502	87.18	4397123

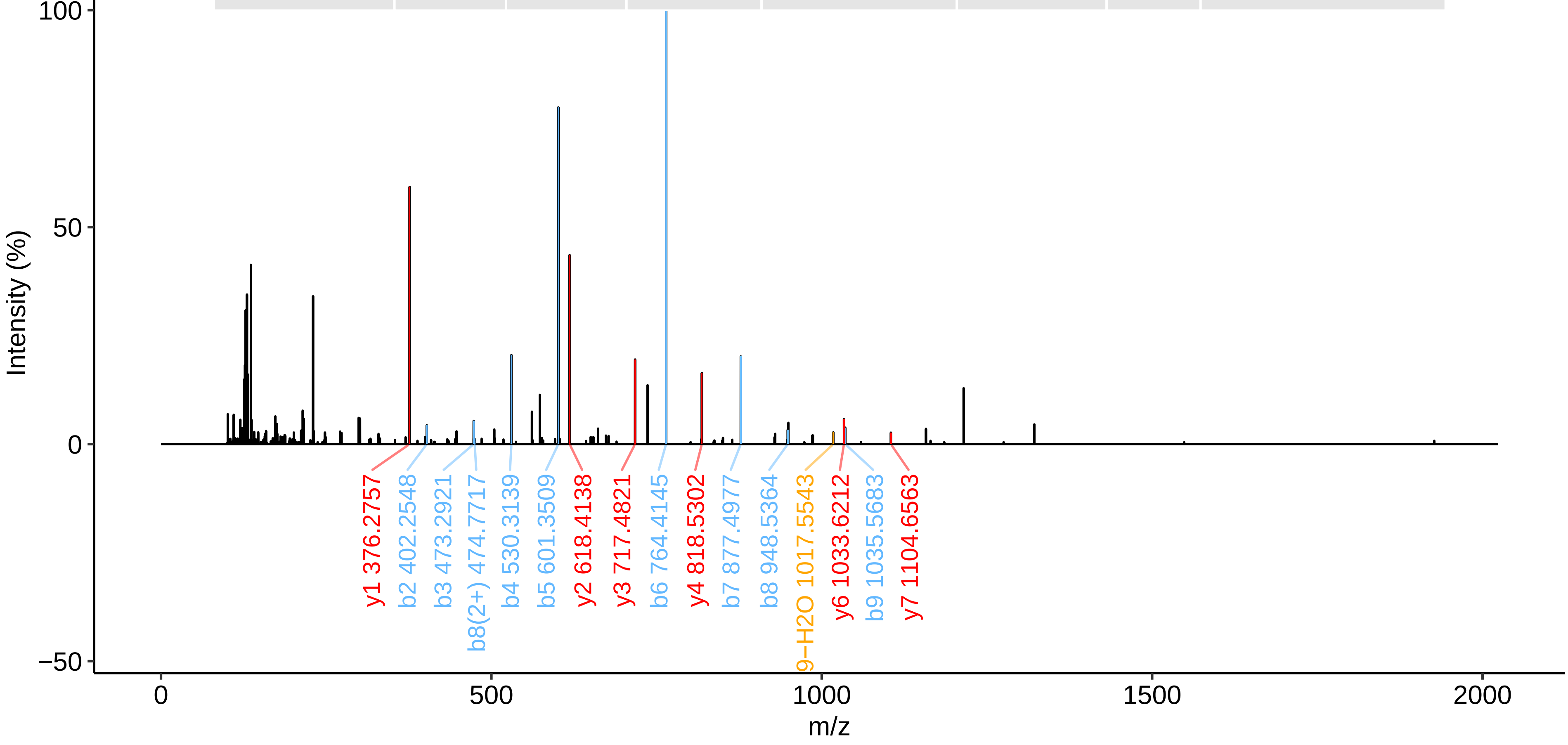

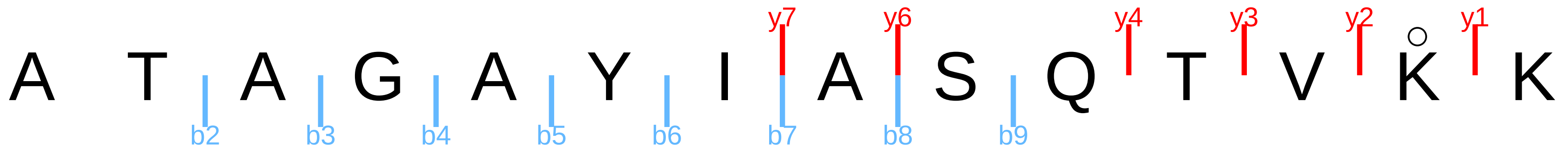

Modification □ Acetyl (Protein N−term) ○ GlyGly (K) no new terminus △ Oxidation (M)

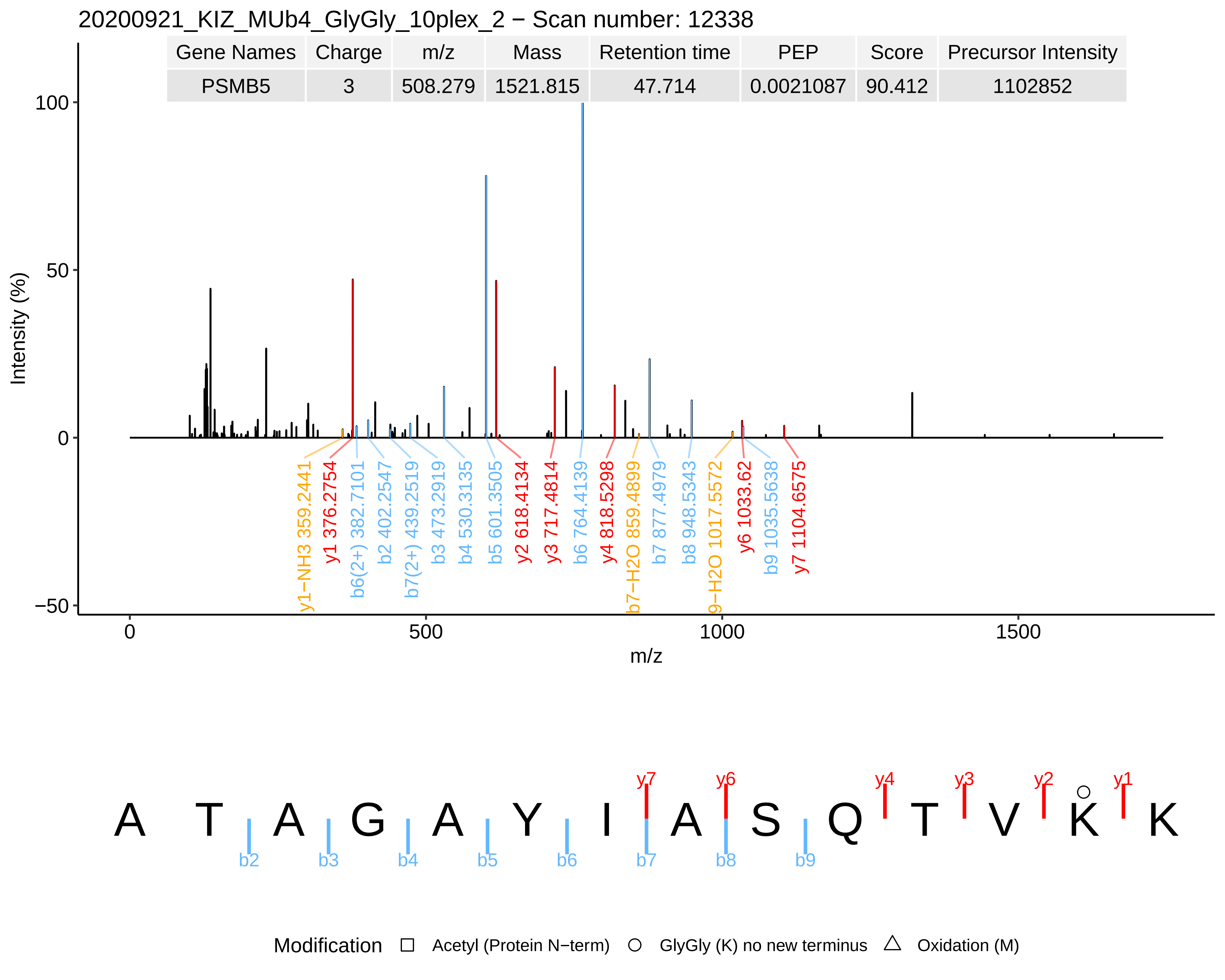
20200921_KIZ_MUb4_GlyGly_10plex_2 − Scan number: 12338
Gene Names | Charge | m/z | Mass | Retention time | PEP | Score | Precursor Intensity
PSMB5 | 3 | 508.279 | 1521.815 | 47.714 | 0.0021087 | 90.412 | 1102852
Intensity (%)
100
50
0
−50
0
500
1000
1500
m/z
y1−NH3 359.2441
y1 376.2754
b6(2+) 382.7101
b2 402.2547
b7(2+) 439.2519
b3 473.2919
b4 530.3135
b5 601.3505
y2 618.4134
y3 717.4814
b6 764.4139
y4 818.5298
b7−H2O 859.4899
b7 877.4979
b8 948.5343
9−H2O 1017.5572
y6 1033.62
b9 1035.5638
y7 1104.6575
A T A G A Y I A S Q T V K K
y7 y6 y4 y3 y2 y1
b2 b3 b4 b5 b6 b7 b8 b9
Modification
Acetyl (Protein N−term)
GlyGly (K) no new terminus
Oxidation (M)

20200921_KIZ_MUb4_GlyGly_10plex − Scan number: 10936

Gene Names	Charge	m/z	Mass	Retention time	PEP	Score	Precursor Intensity
ABCG2	3	491.5912	1471.752	38.018	1.1224e−05	106.01	8383400

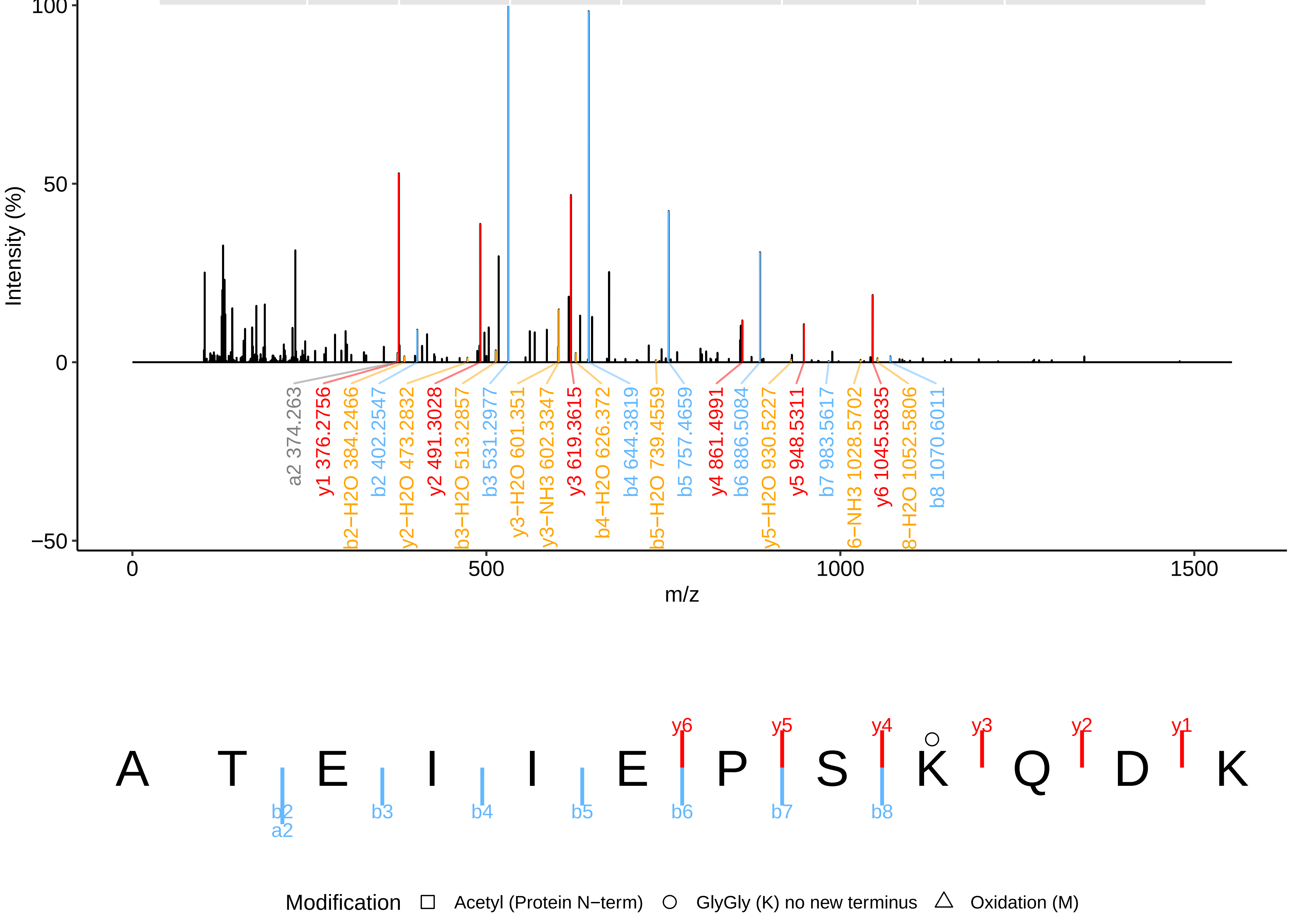

20200921_KIZ_MUb4_GlyGly_10plex_2 − Scan number: 10079

Gene Names	Charge	m/z	Mass	Retention time	PEP	Score	Precursor Intensity
ABCG2	3	491.5912	1471.752	38.466	0.00077701	87.001	1910293

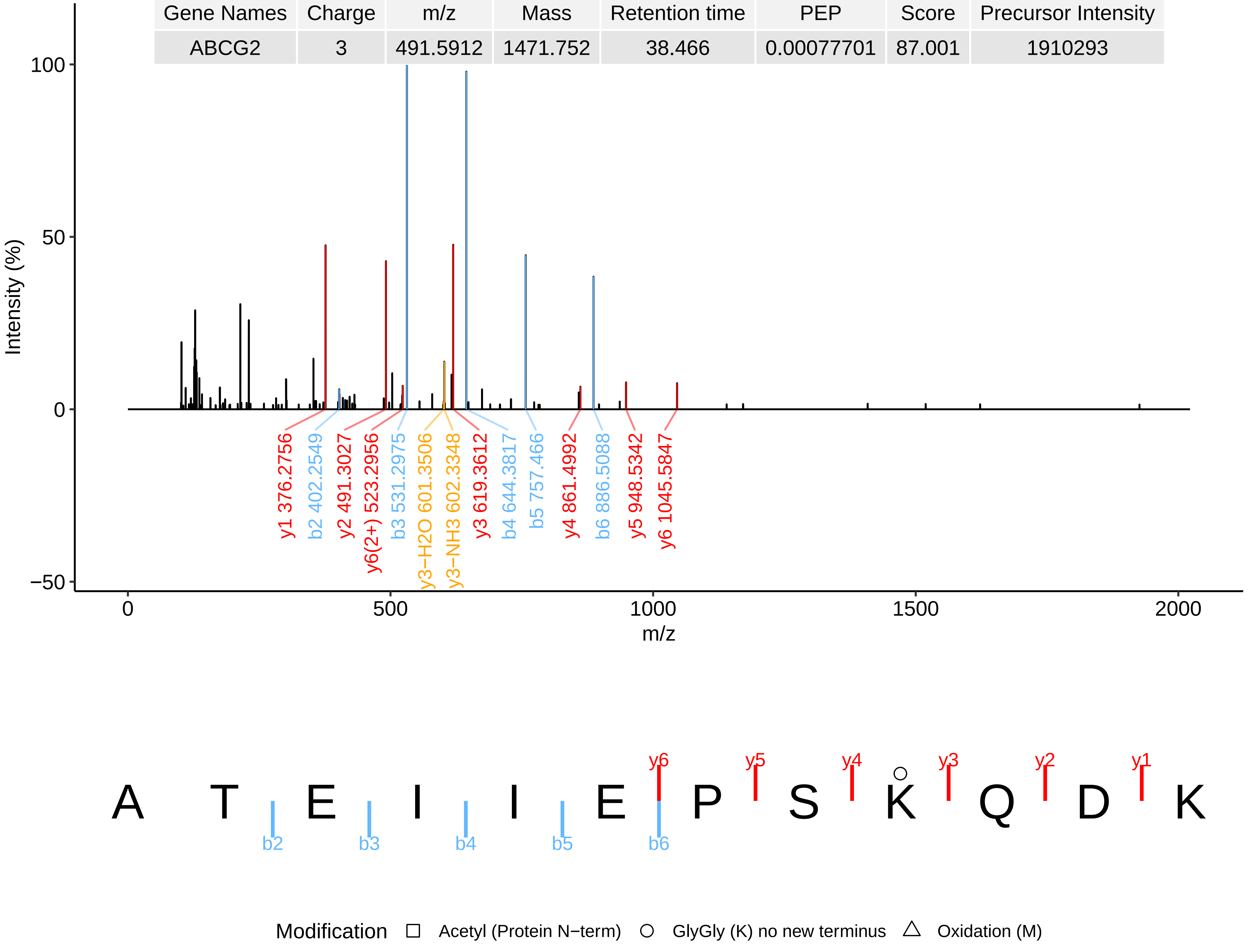

20200924_KIZ_MUb5_GlyGly_2 − Scan number: 9179

Gene Names	Charge	m/z	Mass	Retention time	PEP	Score	Precursor Intensity
ABCG2	3	491.5912	1471.752	34.307	0.0013282	84.289	NA

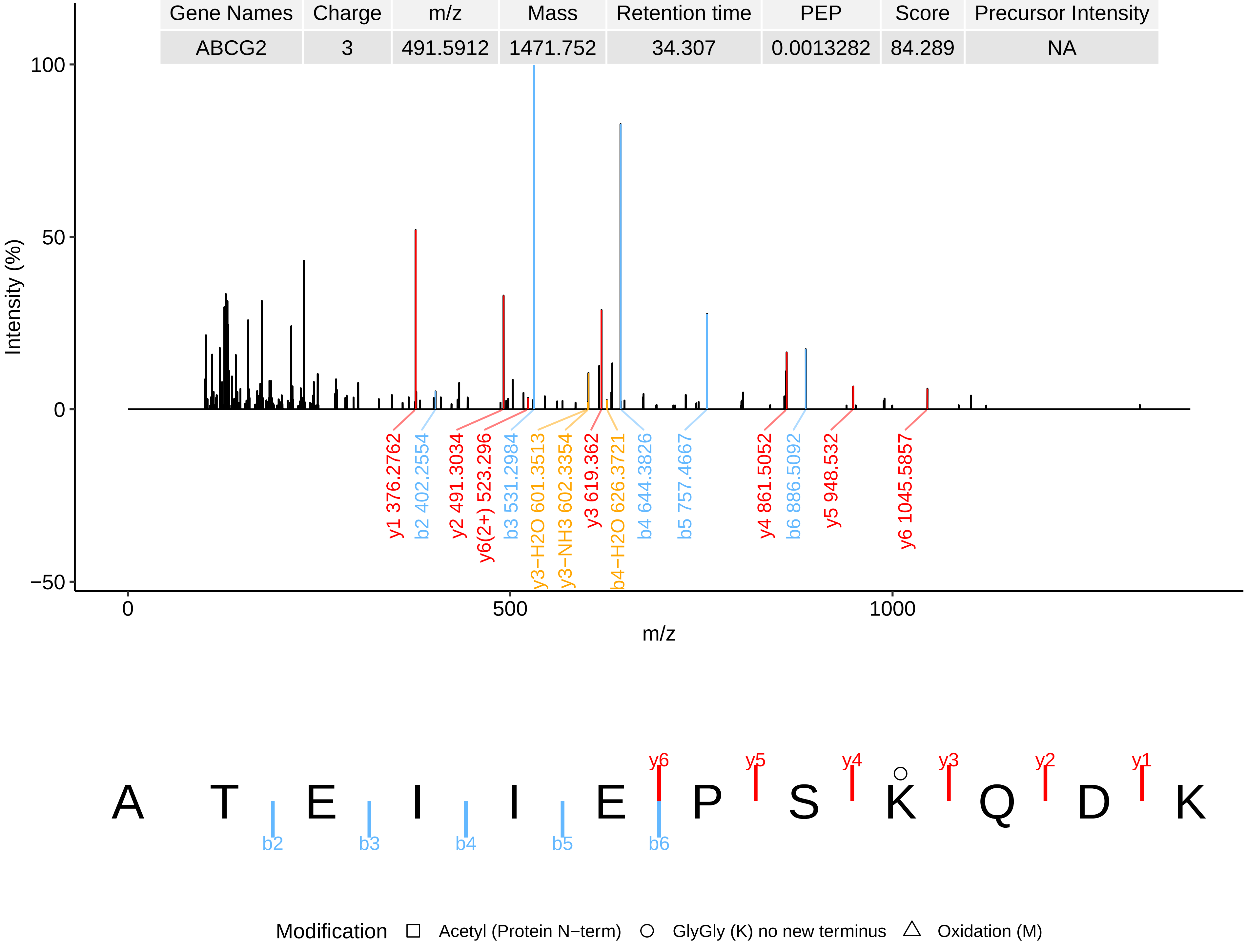

20200921_KIZ_MUb4_GlyGly_10plex_2 − Scan number: 18020

Gene Names	Charge	m/z	Mass	Retention time	PEP	Score	Precursor Intensity
RPSA	3	576.9967	1727.968	70.524	0.023865	58.159	1062822

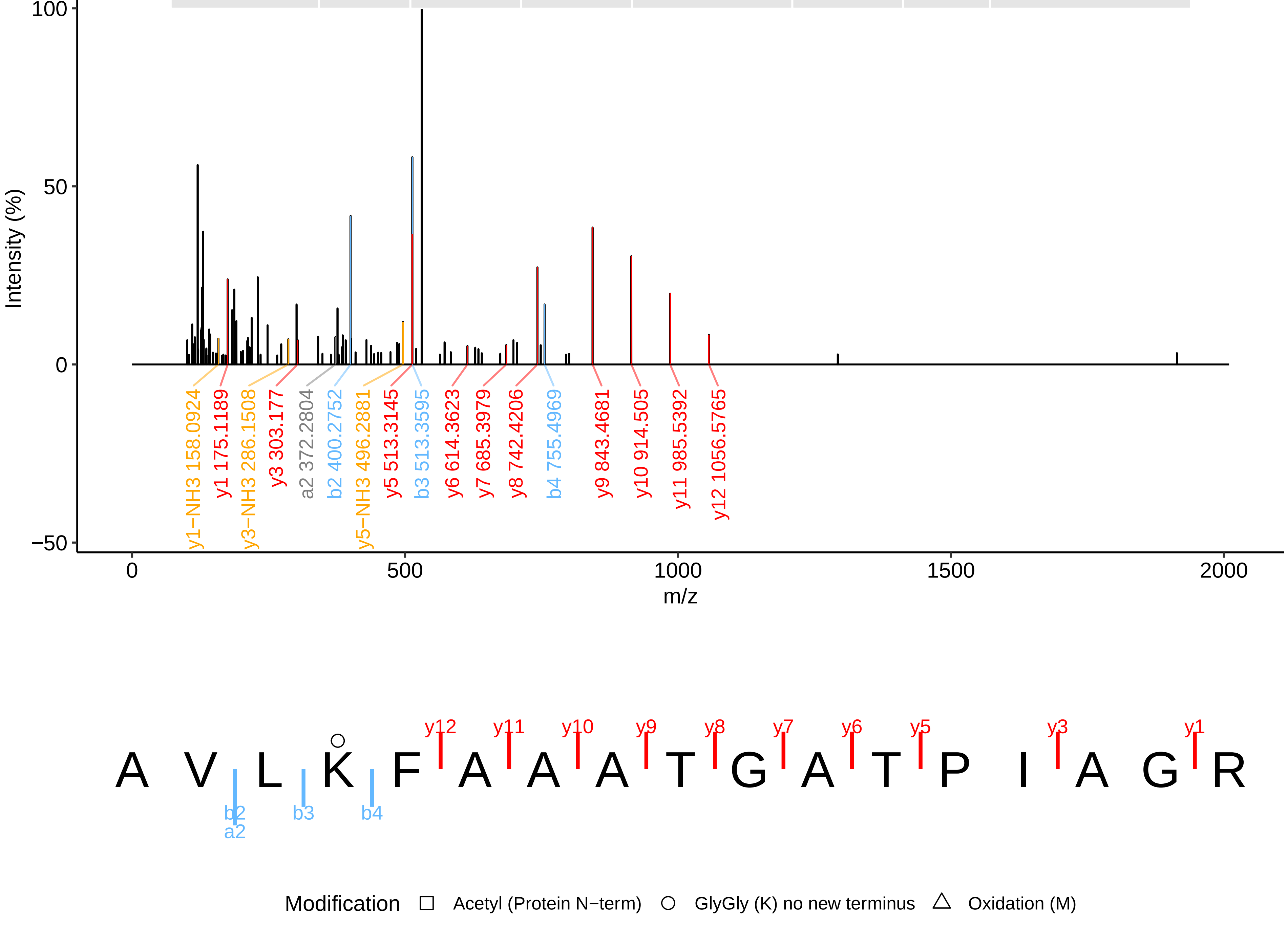

20200921_KIZ_MUb4_GlyGly_10plex − Scan number: 26166

Gene Names	Charge	m/z	Mass	Retention time	PEP	Score	Precursor Intensity
SRSF1	3	568.286	1701.836	95.278	0.00013735	96.64	NA

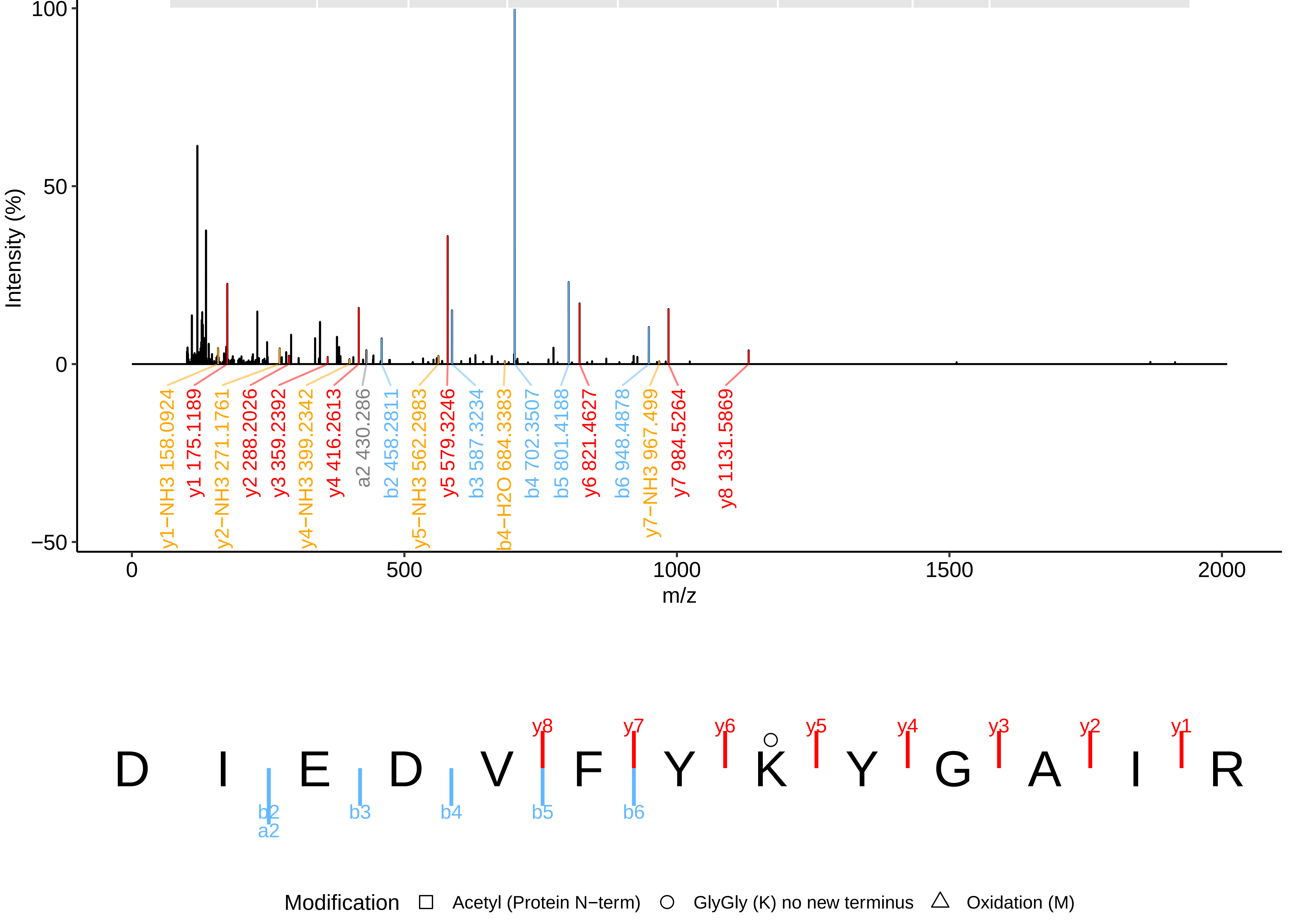

20200921_KIZ_MUb4_GlyGly_10plex − Scan number: 14867

Gene Names	Charge	m/z	Mass	Retention time	PEP	Score	Precursor Intensity
BAG1	4	426.223	1700.863	52.951	1.2581e−12	134.56	2050727

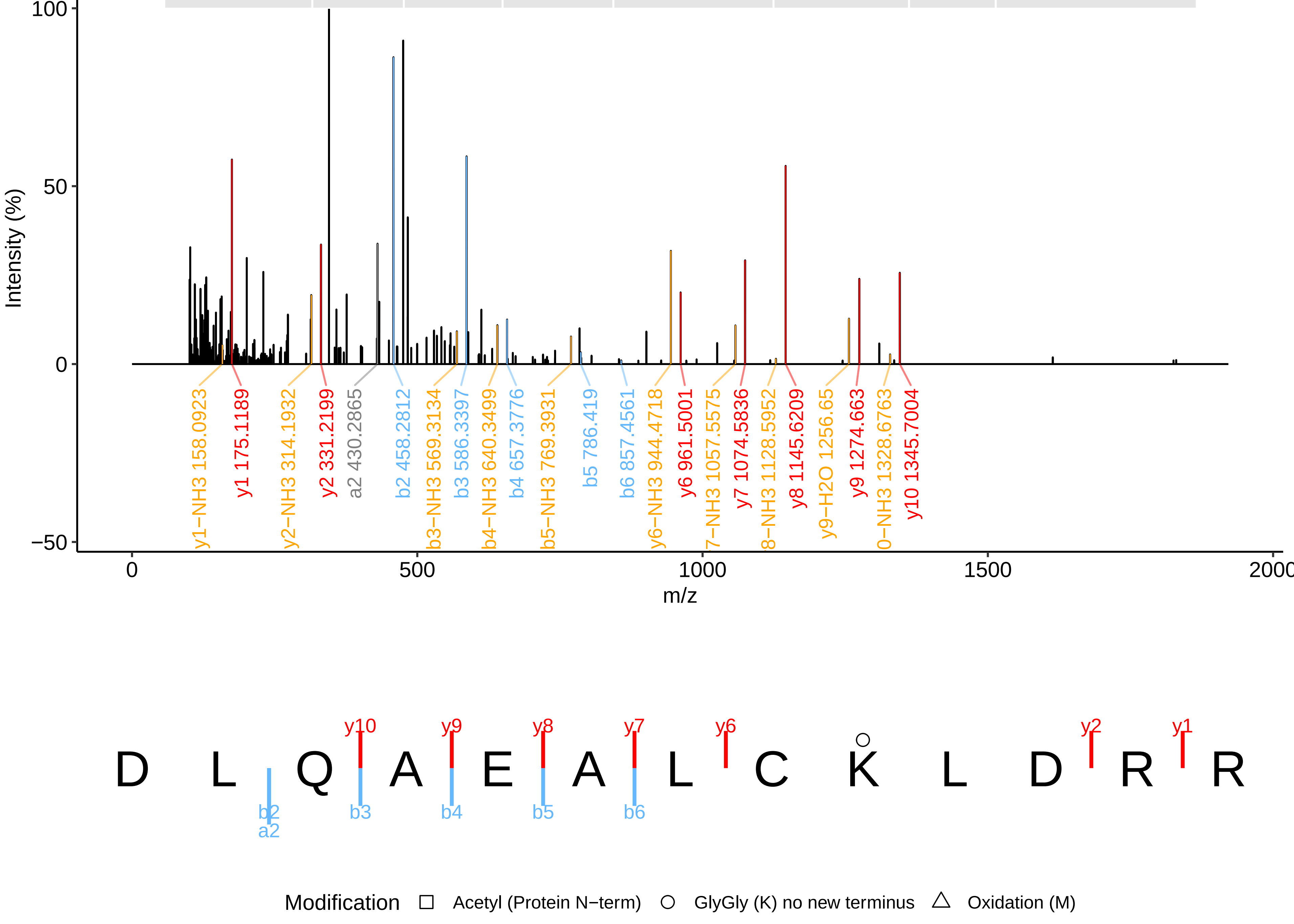

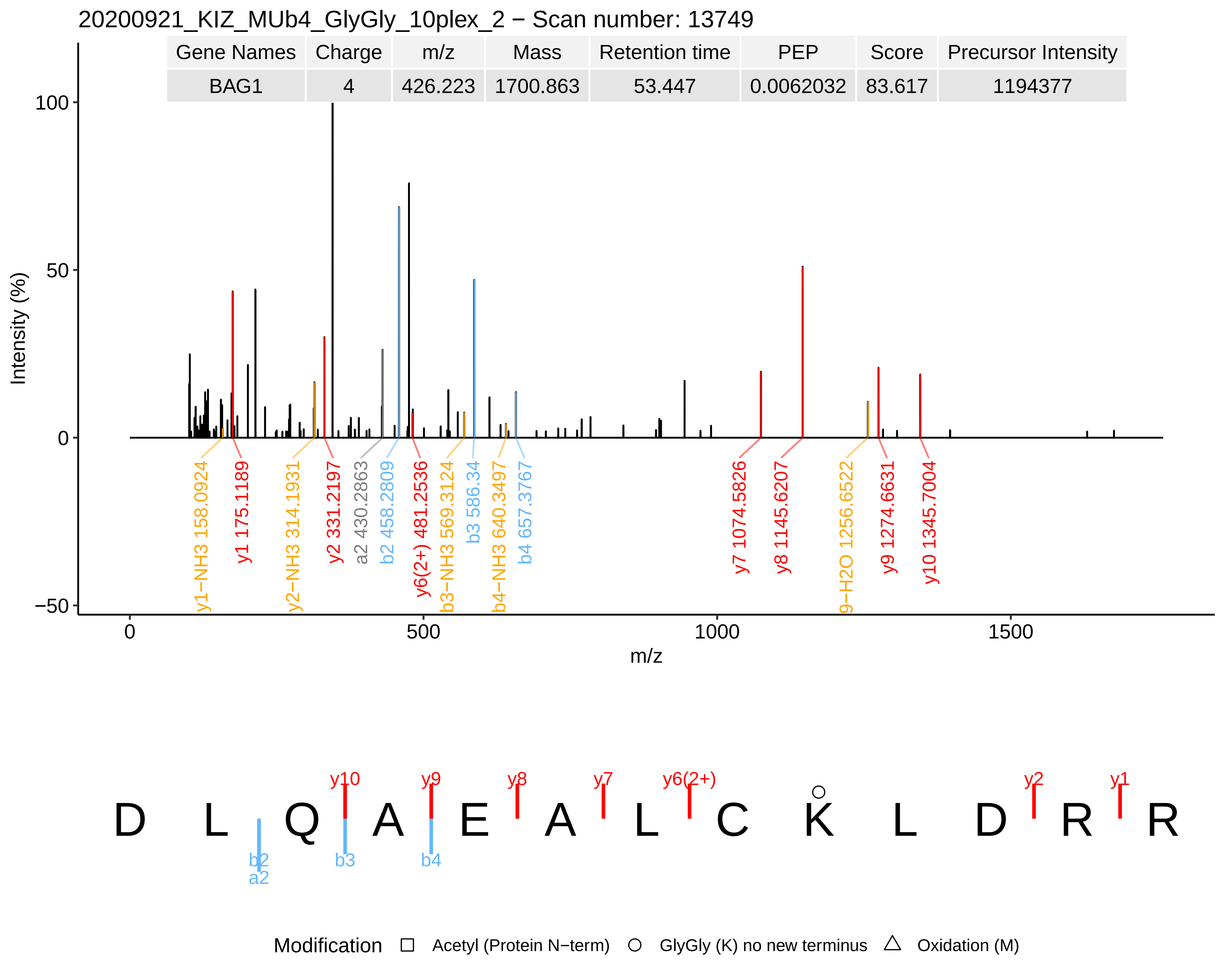

20200921_KIZ_MUb4_GlyGly_10plex_2 − Scan number: 13749
Gene Names | Charge | m/z | Mass | Retention time | PEP | Score | Precursor Intensity
BAG1 | 4 | 426.223 | 1700.863 | 53.447 | 0.0062032 | 83.617 | 1194377
Intensity (%)
m/z
y1−NH3 158.0924
y1 175.1189
y2−NH3 314.1931
y2 331.2197
a2 430.2863
b2 458.2809
y6(2+) 481.2536
b3−NH3 569.3124
b3 586.34
b4−NH3 640.3497
b4 657.3767
y7 1074.5826
y8 1145.6207
y9−H2O 1256.6522
y9 1274.6631
y10 1345.7004
D L Q A E A L C K L D R R
Modification
Acetyl (Protein N−term)
GlyGly (K) no new terminus
Oxidation (M)

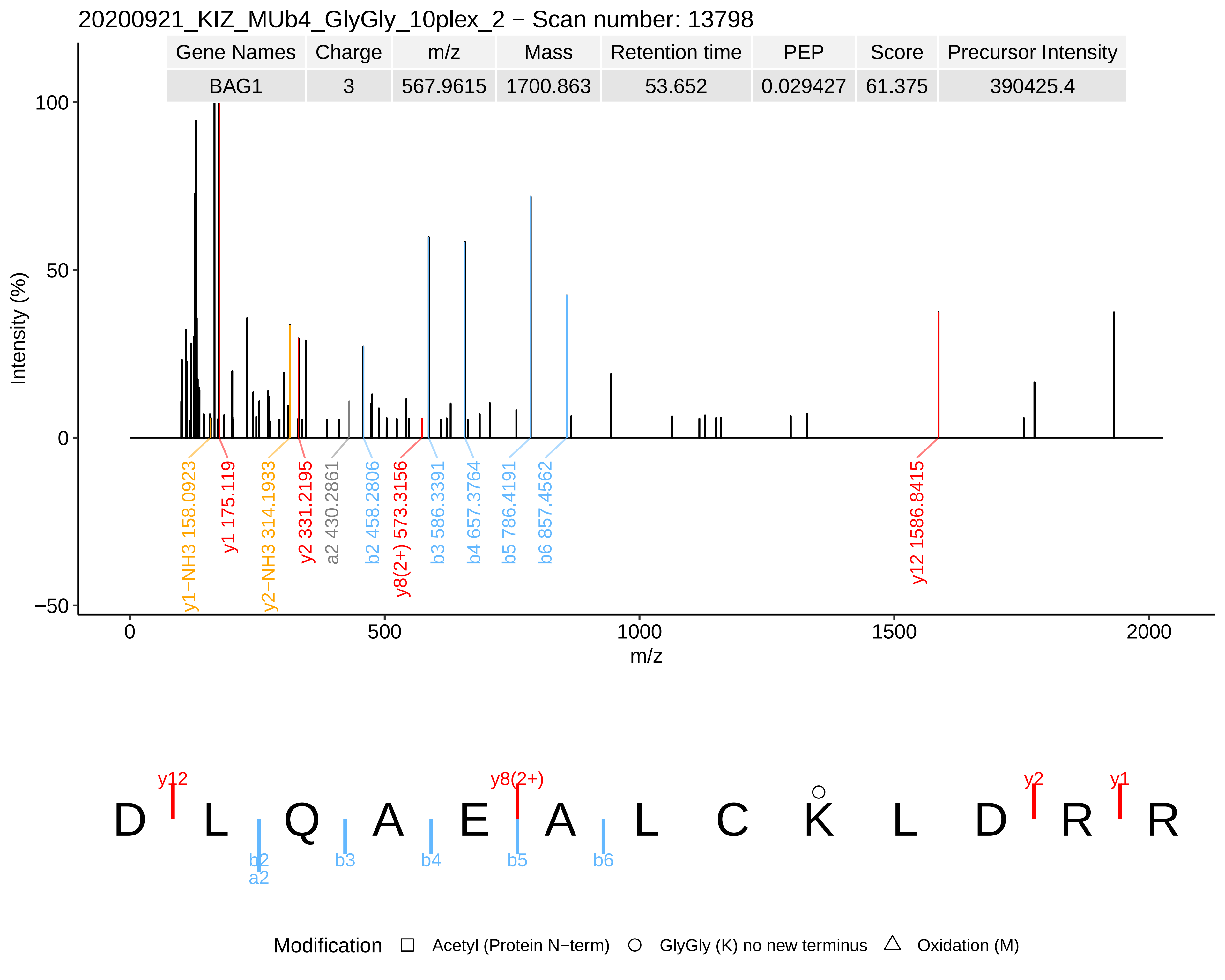
20200921_KIZ_MUb4_GlyGly_10plex_2 − Scan number: 13798
Gene Names | Charge | m/z | Mass | Retention time | PEP | Score | Precursor Intensity
BAG1 | 3 | 567.9615 | 1700.863 | 53.652 | 0.029427 | 61.375 | 390425.4
Intensity (%)
100
50
0
−50
0
500
1000
1500
2000
m/z
y1−NH3 158.0923
y1 175.119
y2−NH3 314.1933
y2 331.2195
a2 430.2861
b2 458.2806
y8(2+) 573.3156
b3 586.3391
b4 657.3764
b5 786.4191
b6 857.4562
y12 1586.8415
D L Q A E A L C K L D R R
y12
y8(2+)
y2
y1
b2
a2
b3
b4
b5
b6
Modification
Acetyl (Protein N−term)
GlyGly (K) no new terminus
Oxidation (M)

20200924_KIZ_MUb5_GlyGly_1 − Scan number: 13918

Gene Names	Charge	m/z	Mass	Retention time	PEP	Score	Precursor Intensity
BAG1	4	426.223	1700.863	45.422	2.2823e−08	128.54	7787088

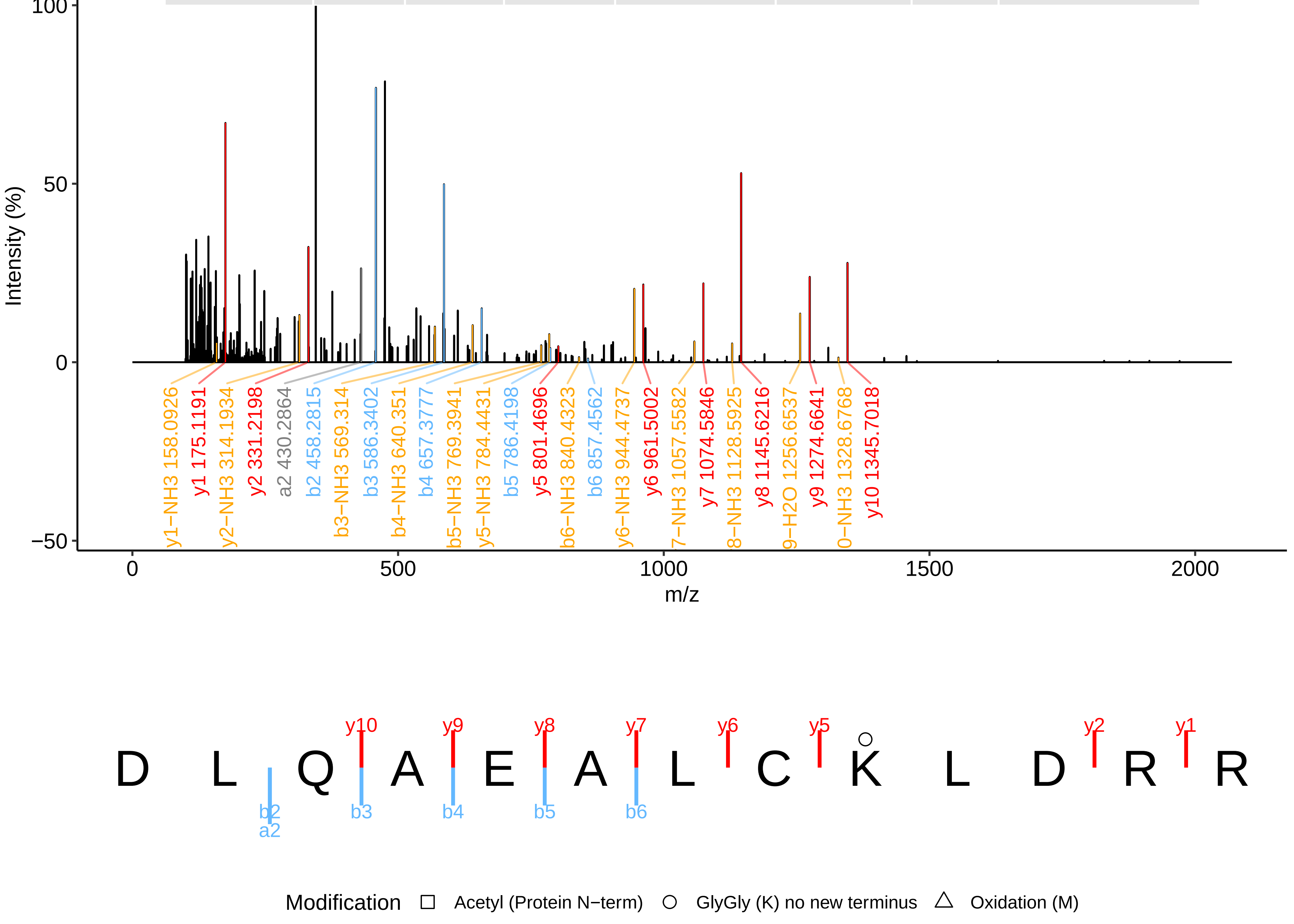

20201118_KIZ_MUb6_GlyGly_1 − Scan number: 14071

Gene Names	Charge	m/z	Mass	Retention time	PEP	Score	Precursor Intensity
BAG1	4	426.223	1700.863	44.479	4.3831e−19	151.78	26271832

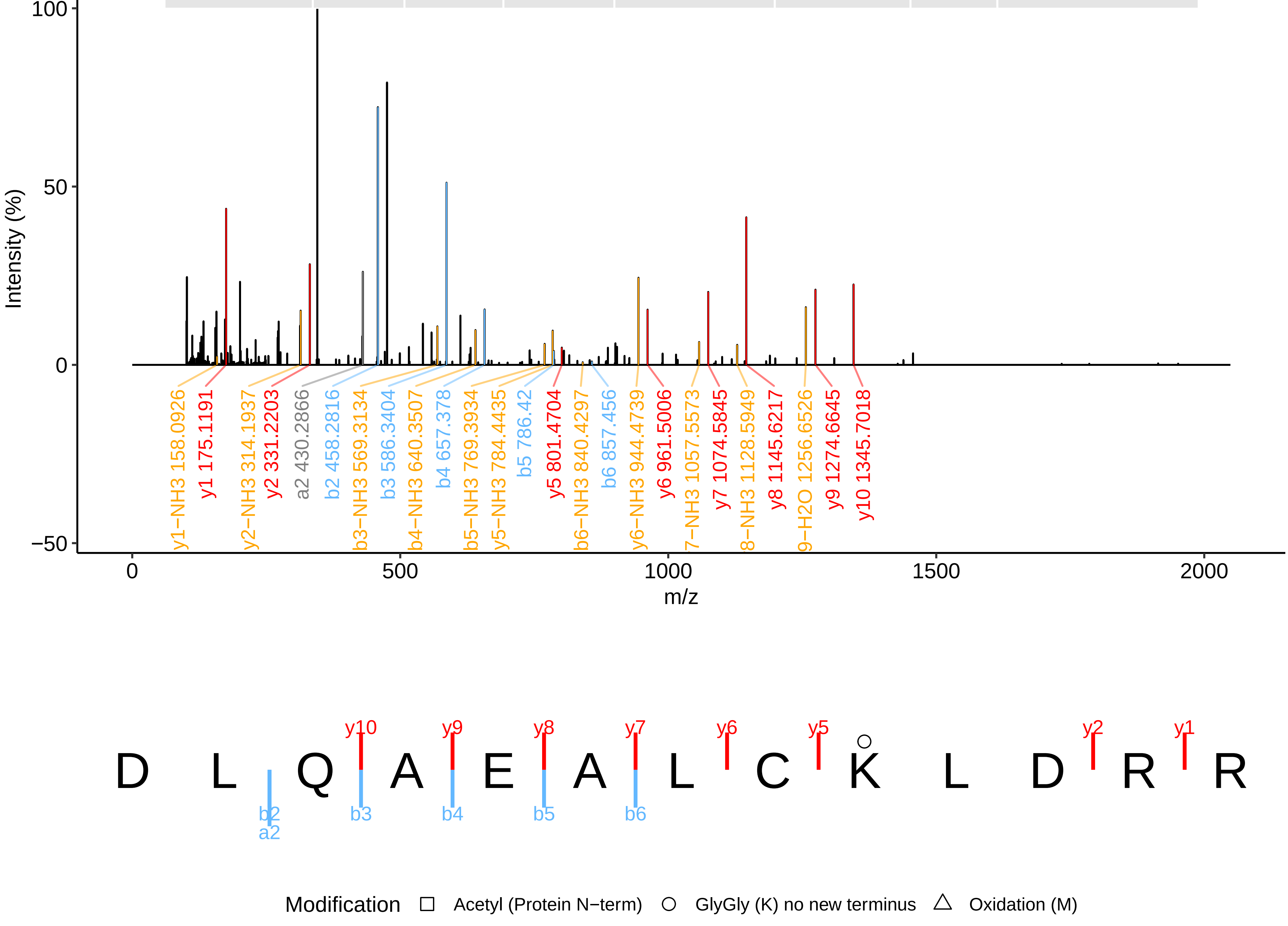

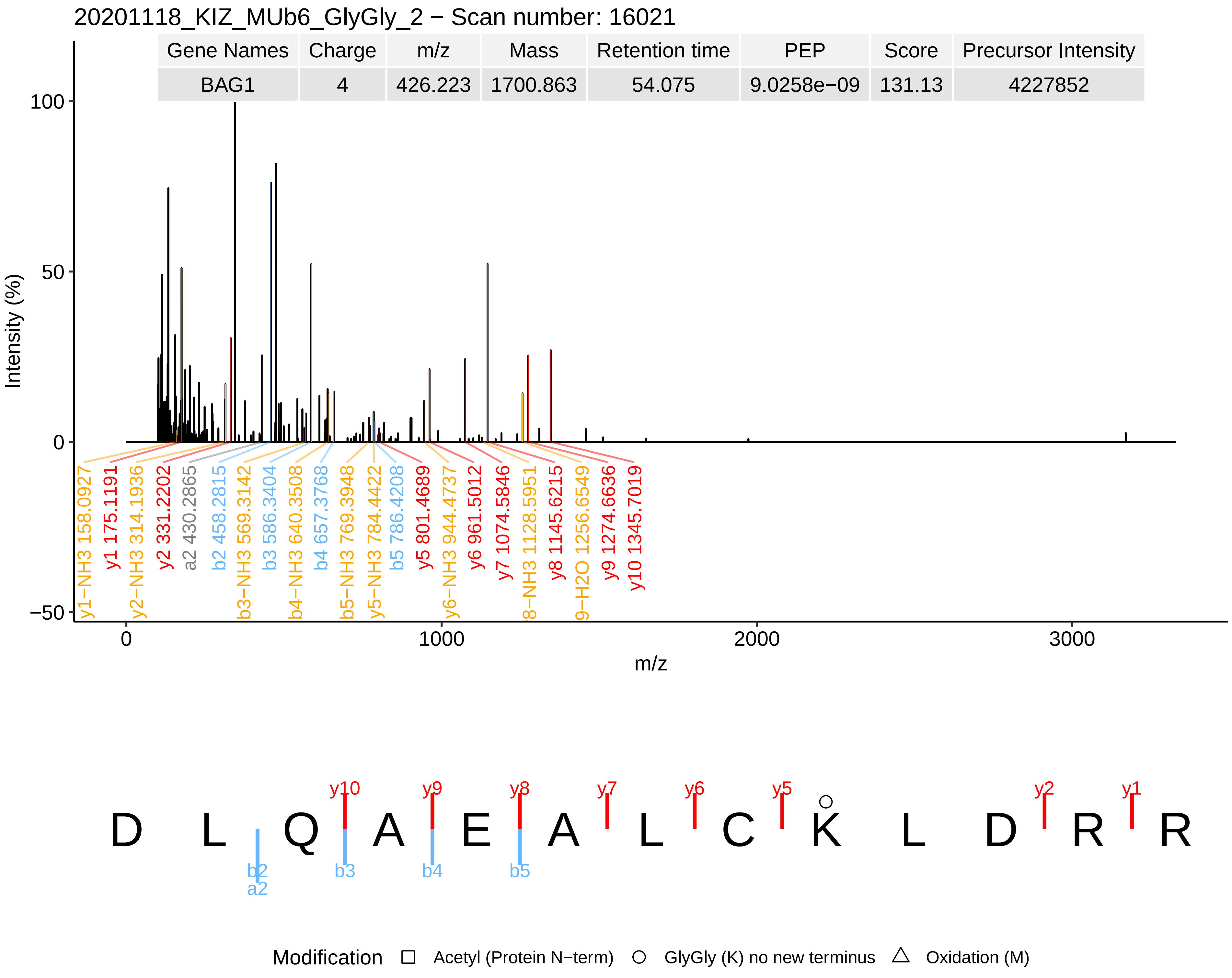

20201118_KIZ_MUb6_GlyGly_2 − Scan number: 16021
Gene Names | Charge | m/z | Mass | Retention time | PEP | Score | Precursor Intensity
BAG1 | 4 | 426.223 | 1700.863 | 54.075 | 9.0258e−09 | 131.13 | 4227852
Intensity (%)
m/z
y1−NH3 158.0927
y1 175.1191
y2−NH3 314.1936
y2 331.2202
a2 430.2865
b2 458.2815
b3−NH3 569.3142
b3 586.3404
b4−NH3 640.3508
b4 657.3768
b5−NH3 769.3948
y5−NH3 784.4422
b5 786.4208
y5 801.4689
y6−NH3 944.4737
y6 961.5012
y7 1074.5846
8−NH3 1128.5951
y8 1145.6215
9−H2O 1256.6549
y9 1274.6636
y10 1345.7019
D L Q A E A L C K L D R R
y10 y9 y8 y7 y6 y5 y2 y1
b2 a2 b3 b4 b5
Modification
Acetyl (Protein N−term)
GlyGly (K) no new terminus
Oxidation (M)

20201118_KIZ_MUb6_GlyGly_2 − Scan number: 16062

Gene Names	Charge	m/z	Mass	Retention time	PEP	Score	Precursor Intensity
BAG1	3	567.9615	1700.863	54.22	6.3925e−05	100.22	5131038

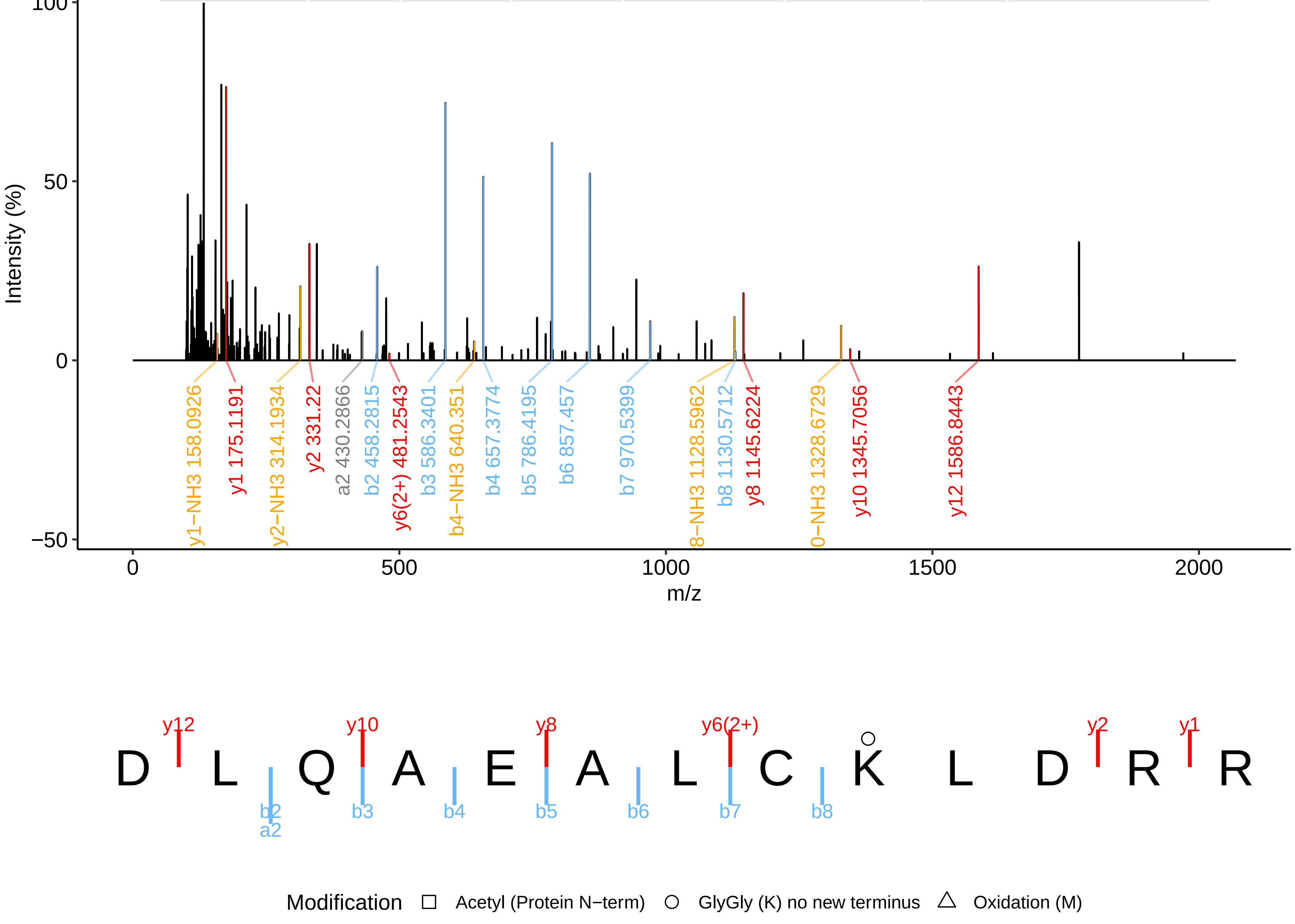

20200921_KIZ_MUb4_GlyGly_10plex_2 − Scan number: 17598

Gene Names	Charge	m/z	Mass	Retention time	PEP	Score	Precursor Intensity
ABCC2	3	566.2812	1695.822	68.828	1.2841e−07	124.07	758989.1

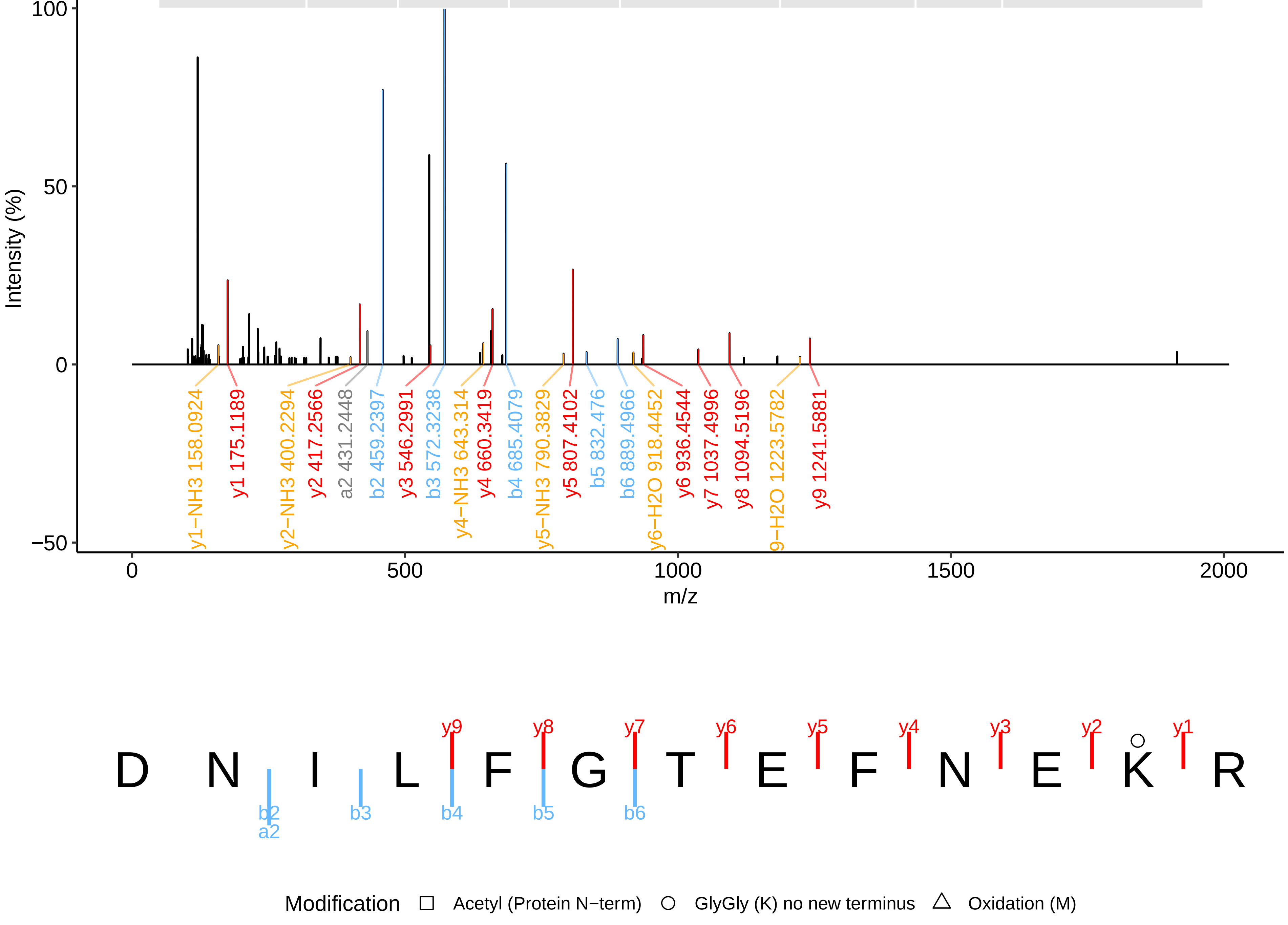

20200921_KIZ_MUb4_GlyGly_10plex_2 − Scan number: 13487

Gene Names	Charge	m/z	Mass	Retention time	PEP	Score	Precursor Intensity
DNAJC13	3	499.2452	1494.714	52.375	0.084325	49.423	435958.9

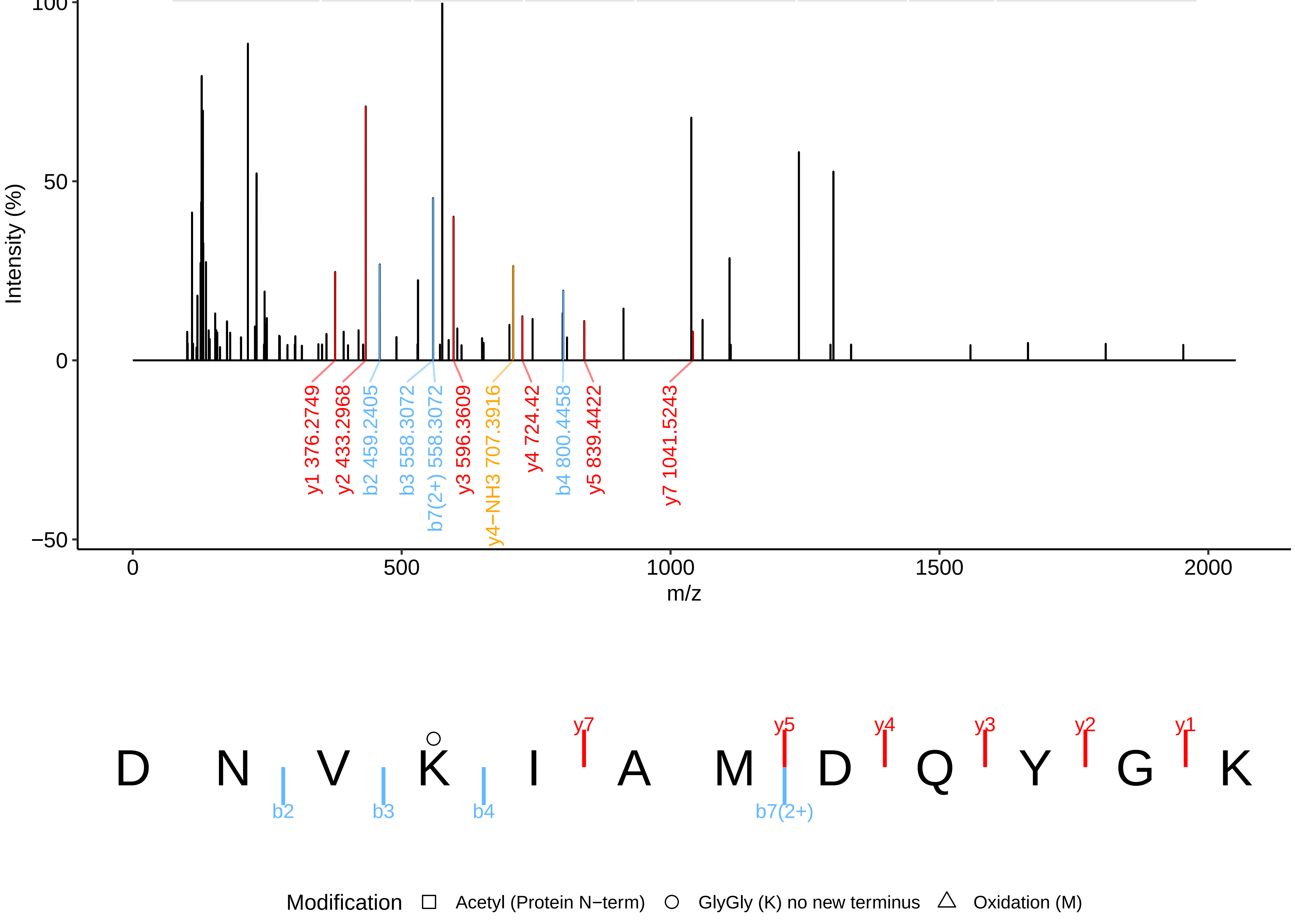

20200921_KIZ_MUb4_GlyGly_10plex_2 − Scan number: 21427

Gene Names	Charge	m/z	Mass	Retention time	PEP	Score	Precursor Intensity
PLXNB2	3	494.5927	1480.756	84.369	0.01684	62.408	733255.1

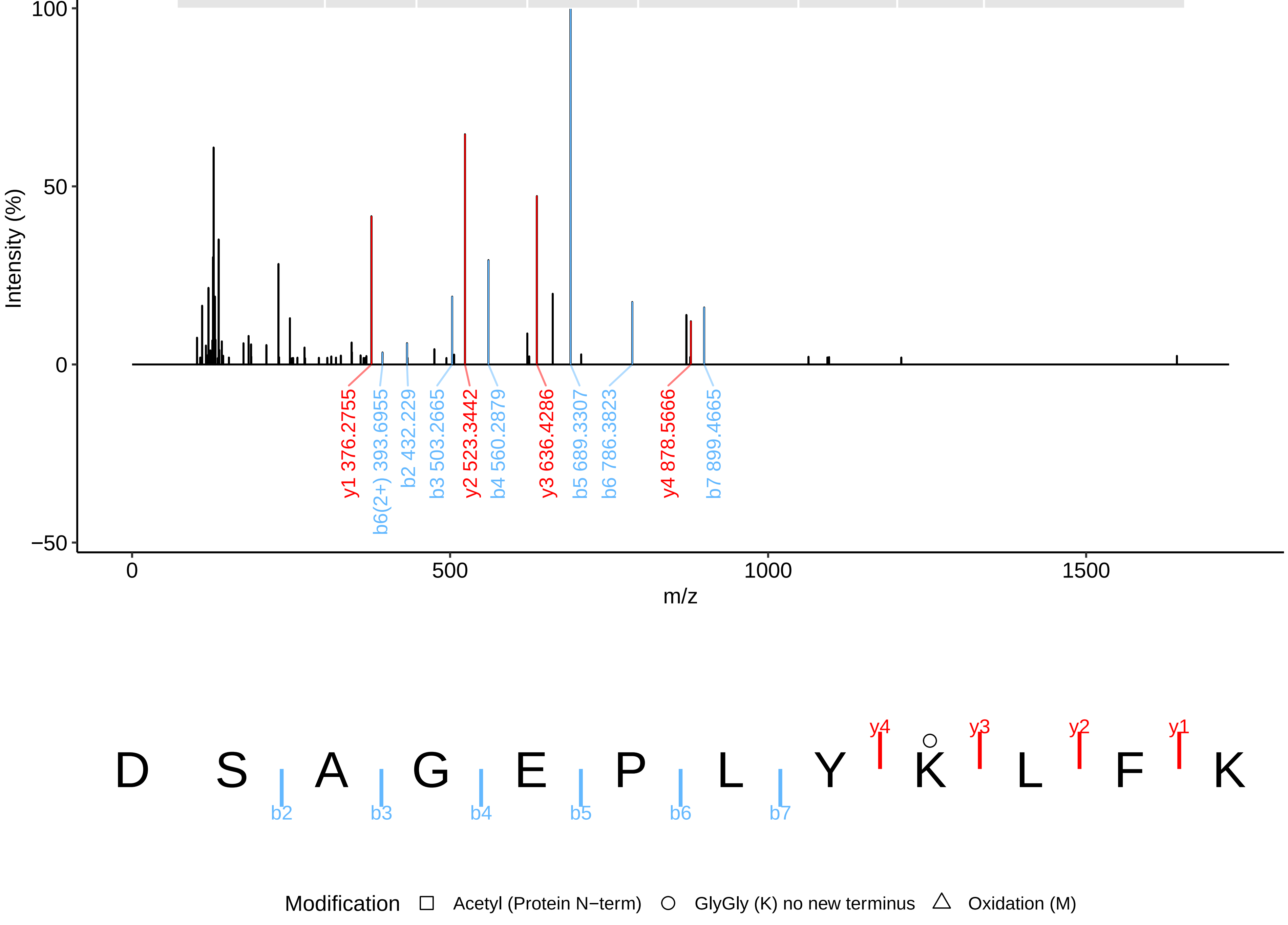

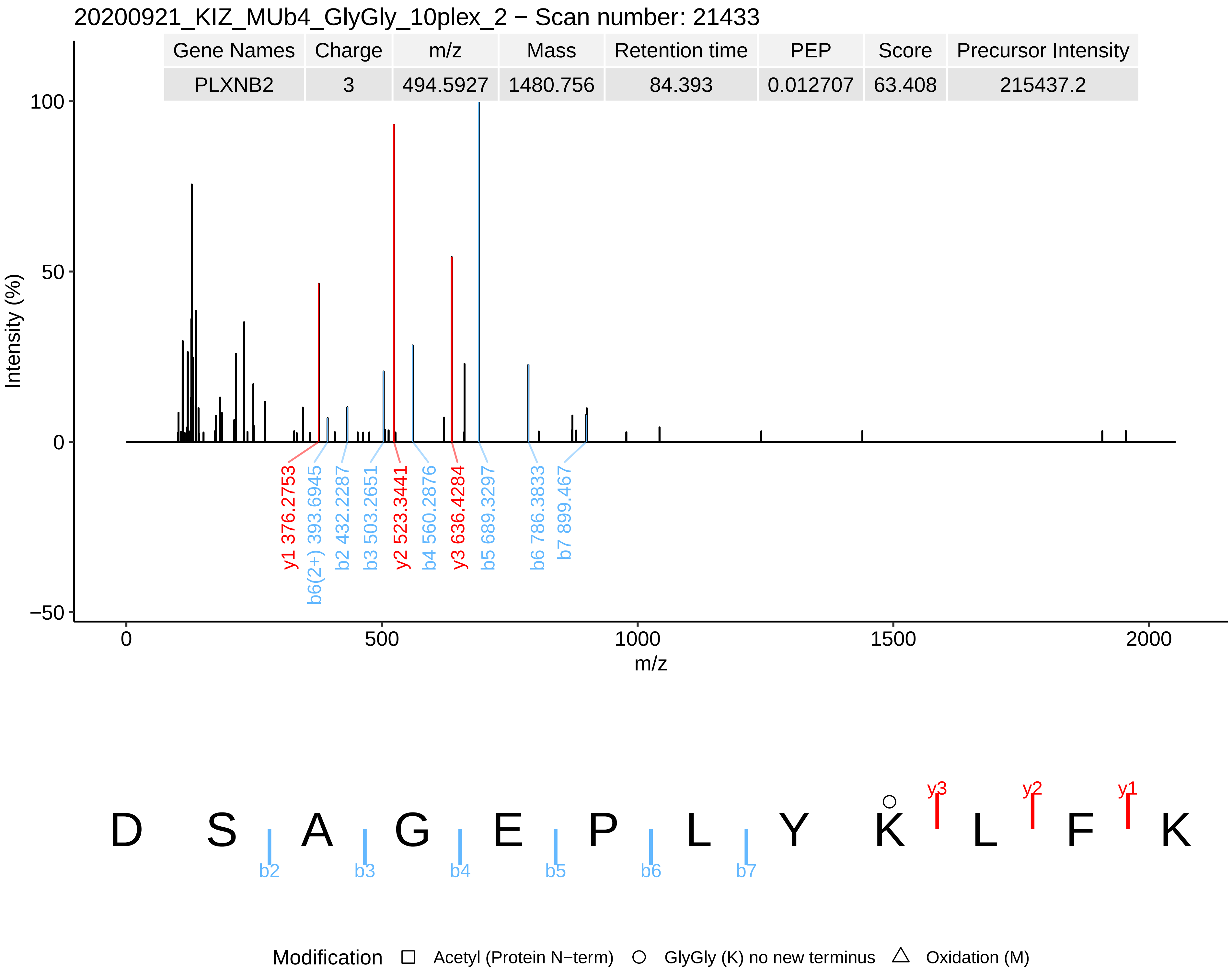

20200921_KIZ_MUb4_GlyGly_10plex_2 − Scan number: 21433

Gene Names	Charge	m/z	Mass	Retention time	PEP	Score	Precursor Intensity
PLXNB2	3	494.5927	1480.756	84.393	0.012707	63.408	215437.2

Intensity (%)
100
50
0
−50
y1 376.2753
b6(2+) 393.6945
b2 432.2287
b3 503.2651
y2 523.3441
b4 560.2876
y3 636.4284
b5 689.3297
b6 786.3833
b7 899.467
0
500
1000
1500
2000
m/z
D S A G E P L Y K L F K
b2 b3 b4 b5 b6 b7
y3 y2 y1
Modification
Acetyl (Protein N−term)
GlyGly (K) no new terminus
Oxidation (M)

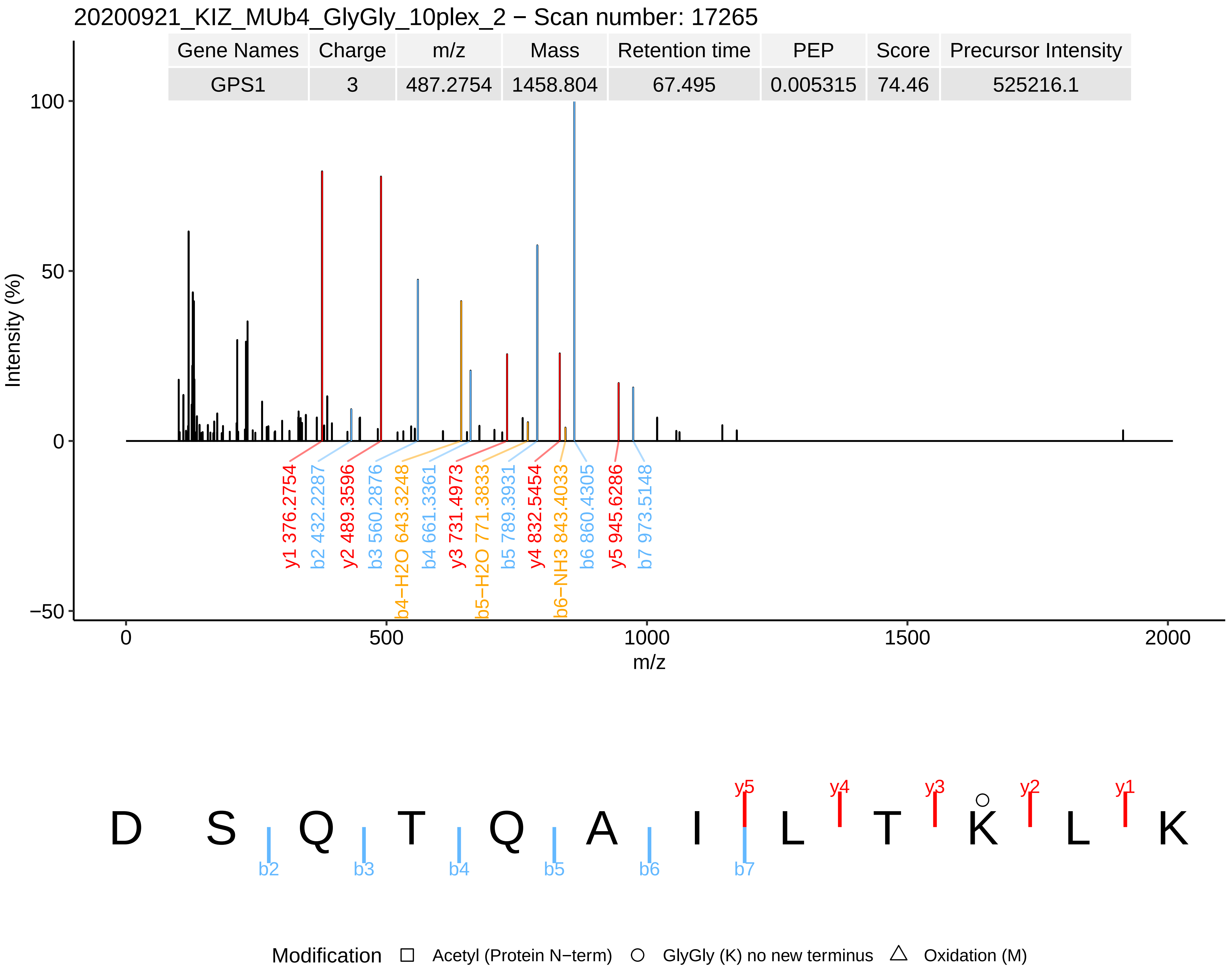
20200921_KIZ_MUb4_GlyGly_10plex_2 − Scan number: 17265
Gene Names | Charge | m/z | Mass | Retention time | PEP | Score | Precursor Intensity
GPS1 | 3 | 487.2754 | 1458.804 | 67.495 | 0.005315 | 74.46 | 525216.1
Intensity (%)
m/z
y1 376.2754
b2 432.2287
y2 489.3596
b3 560.2876
b4−H2O 643.3248
b4 661.3361
y3 731.4973
b5−H2O 771.3833
b5 789.3931
y4 832.5454
b6−NH3 843.4033
b6 860.4305
y5 945.6286
b7 973.5148
D S Q T Q A I L T K L K
Modification □ Acetyl (Protein N−term) ○ GlyGly (K) no new terminus △ Oxidation (M)

20200921_KIZ_MUb4_GlyGly_10plex − Scan number: 7017

Gene Names	Charge	m/z	Mass	Retention time	PEP	Score	Precursor Intensity
FAF2	3	577.2832	1728.828	23.426	3.9607e−28	174.72	9502200

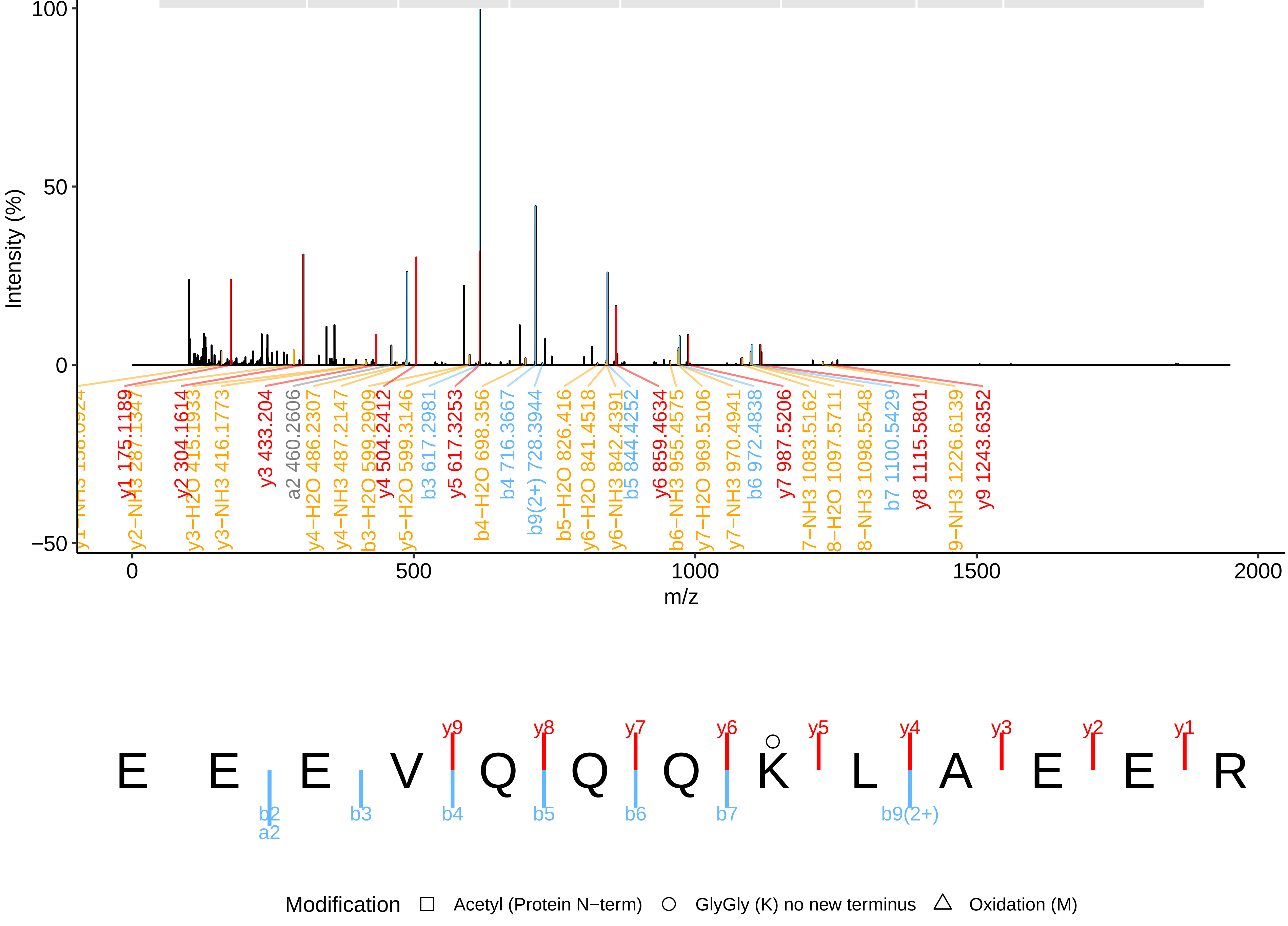

20200921_KIZ_MUb4_GlyGly_10plex − Scan number: 7034

Gene Names	Charge	m/z	Mass	Retention time	PEP	Score	Precursor Intensity
FAF2	3	577.2832	1728.828	23.491	2.8592e−09	129.37	13458240

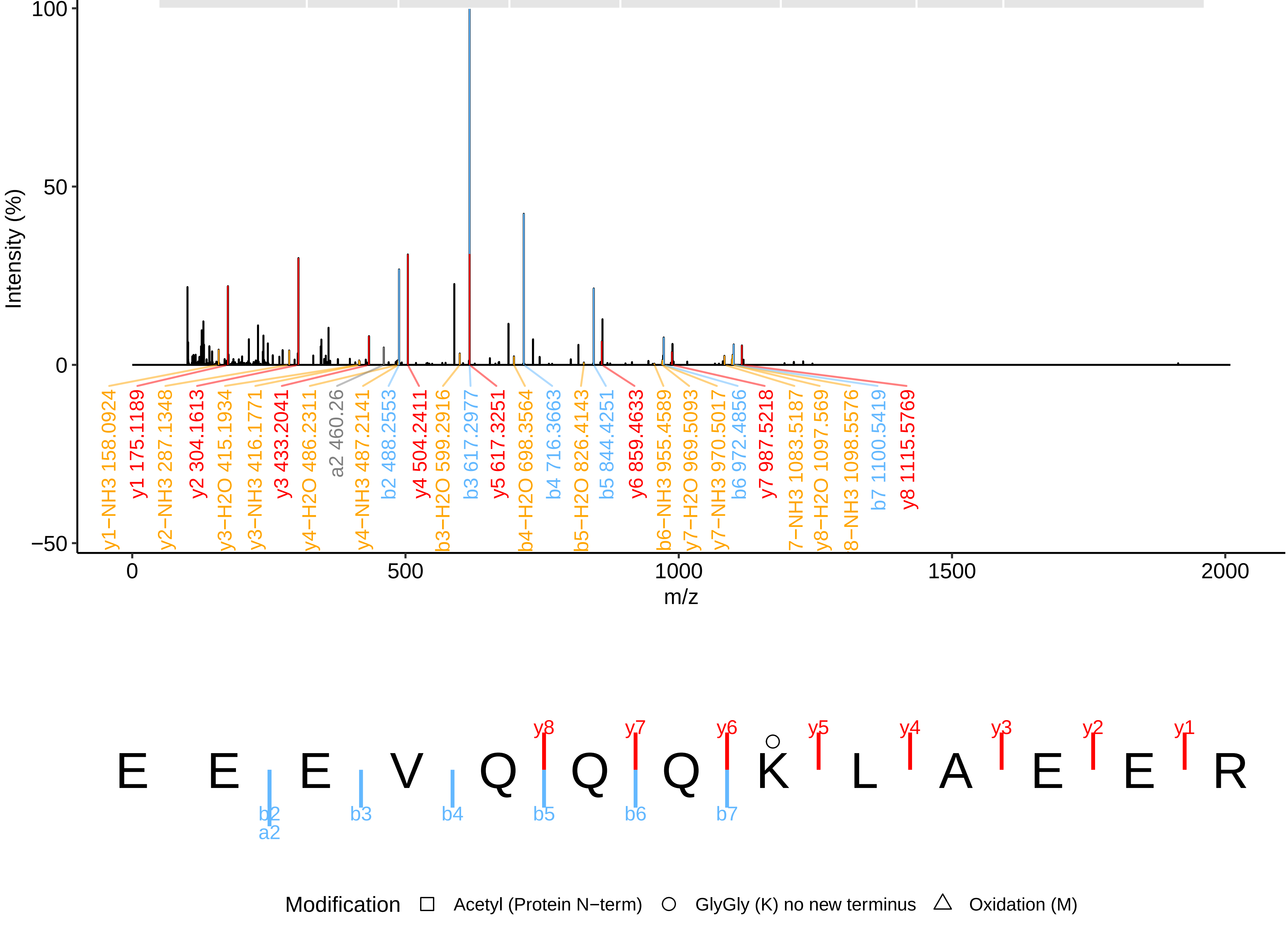

20200921_KIZ_MUb4_GlyGly_10plex_2 − Scan number: 6469

Gene Names	Charge	m/z	Mass	Retention time	PEP	Score	Precursor Intensity
FAF2	3	577.2832	1728.828	23.787	1.3457e−05	105	1017742

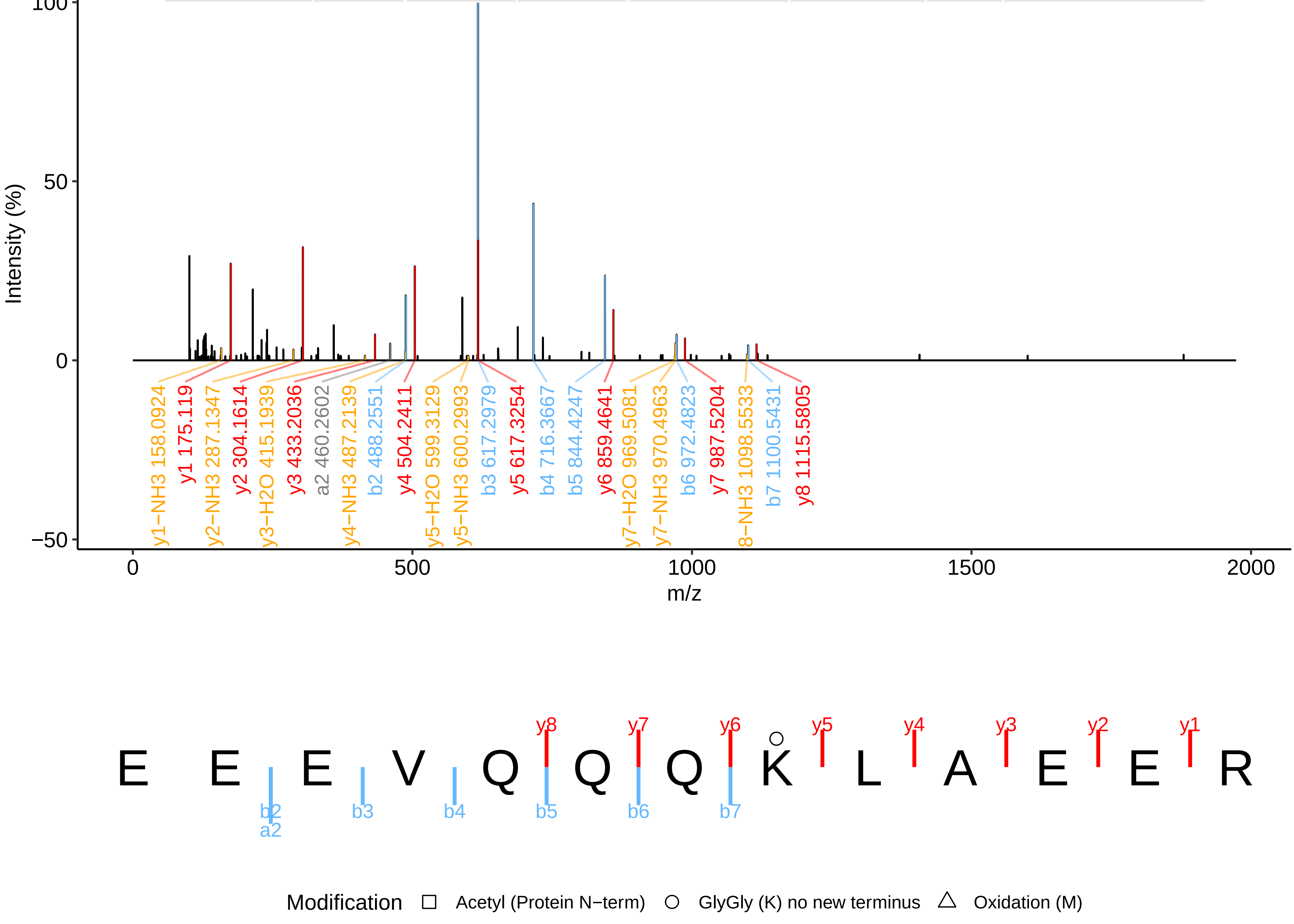

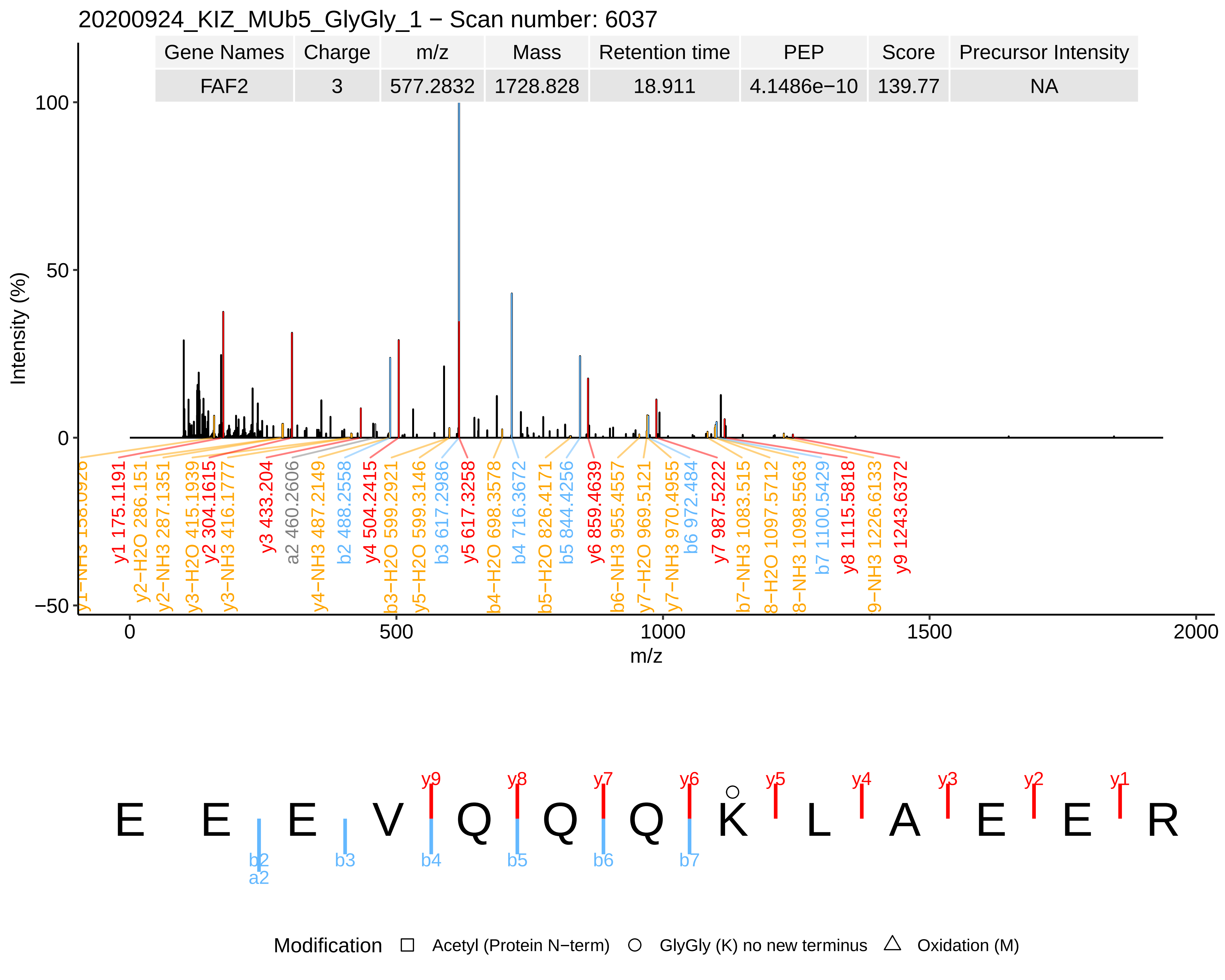
20200924_KIZ_MUb5_GlyGly_1 − Scan number: 6037
Gene Names | Charge | m/z | Mass | Retention time | PEP | Score | Precursor Intensity
FAF2 | 3 | 577.2832 | 1728.828 | 18.911 | 4.1486e−10 | 139.77 | NA
Intensity (%)
m/z
y1 175.1191
y2−H2O 286.151
y2−NH3 287.1351
y3−H2O 415.1939
y2 304.1615
y3−NH3 416.1777
y3 433.204
a2 460.2606
y4−NH3 487.2149
b2 488.2558
y4 504.2415
b3−H2O 599.2921
y5−H2O 599.3146
b3 617.2986
y5 617.3258
b4−H2O 698.3578
b4 716.3672
b5−H2O 826.4171
b5 844.4256
y6 859.4639
b6−NH3 955.4557
y7−H2O 969.5121
y7−NH3 970.4955
b6 972.484
y7 987.5222
b7−NH3 1083.515
y8−H2O 1097.5712
y8−NH3 1098.5563
b7 1100.5429
y8 1115.5818
y9−NH3 1226.6133
y9 1243.6372
E E E V Q Q Q K L A E E R
y9 y8 y7 y6 y5 y4 y3 y2 y1
b2 a2 b3 b4 b5 b6 b7
Modification
Acetyl (Protein N−term)
GlyGly (K) no new terminus
Oxidation (M)

20200924_KIZ_MUb5_GlyGly_2 − Scan number: 5786

Gene Names	Charge	m/z	Mass	Retention time	PEP	Score	Precursor Intensity
FAF2	3	577.2832	1728.828	21.525	2.448e−08	126.95	1019334

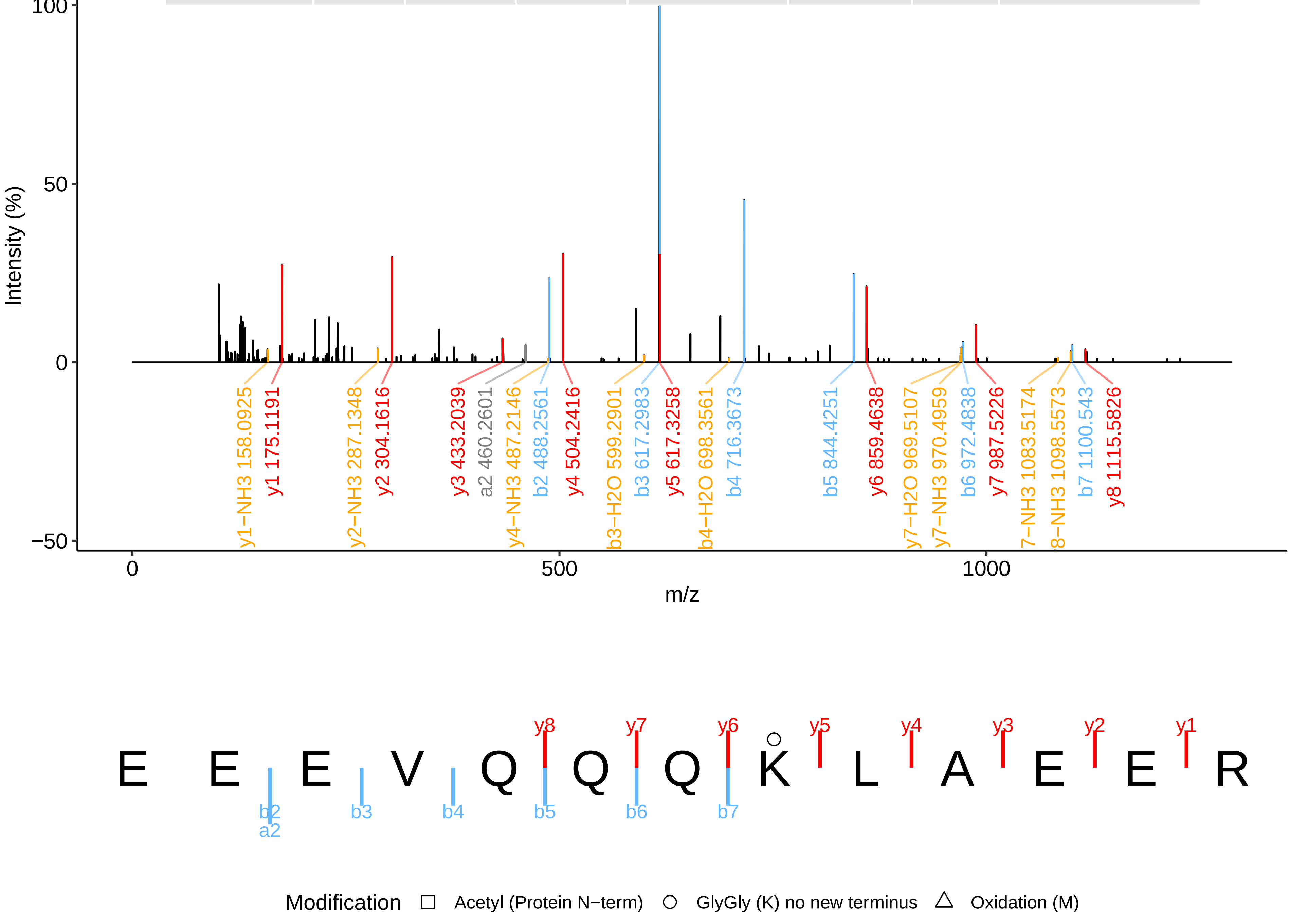

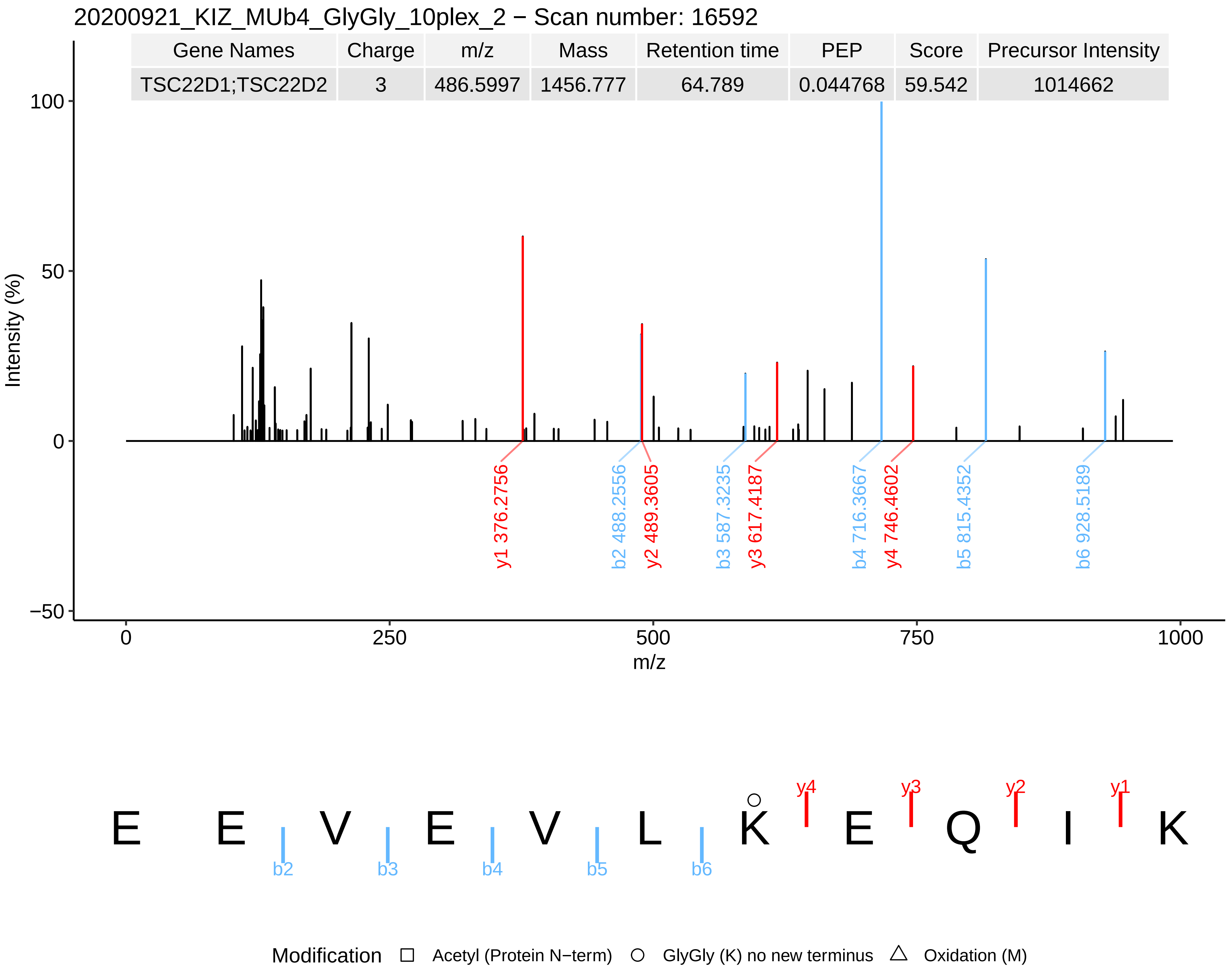

20200921_KIZ_MUb4_GlyGly_10plex_2 − Scan number: 16592
Gene Names | Charge | m/z | Mass | Retention time | PEP | Score | Precursor Intensity
TSC22D1;TSC22D2 | 3 | 486.5997 | 1456.777 | 64.789 | 0.044768 | 59.542 | 1014662
Intensity (%)
m/z
y1 376.2756
b2 488.2556
y2 489.3605
b3 587.3235
y3 617.4187
b4 716.3667
y4 746.4602
b5 815.4352
b6 928.5189
E E V E V L K E Q I K
Modification
Acetyl (Protein N−term)
GlyGly (K) no new terminus
Oxidation (M)

20200921_KIZ_MUb4_GlyGly_10plex − Scan number: 19349

Gene Names	Charge	m/z	Mass	Retention time	PEP	Score	Precursor Inte
ACTB;ACTG2;ACTG1;ACTA1;ACTC1;ACTA2	3	506.285	1515.833	69.75	1.3632e−05	104.87	2644595

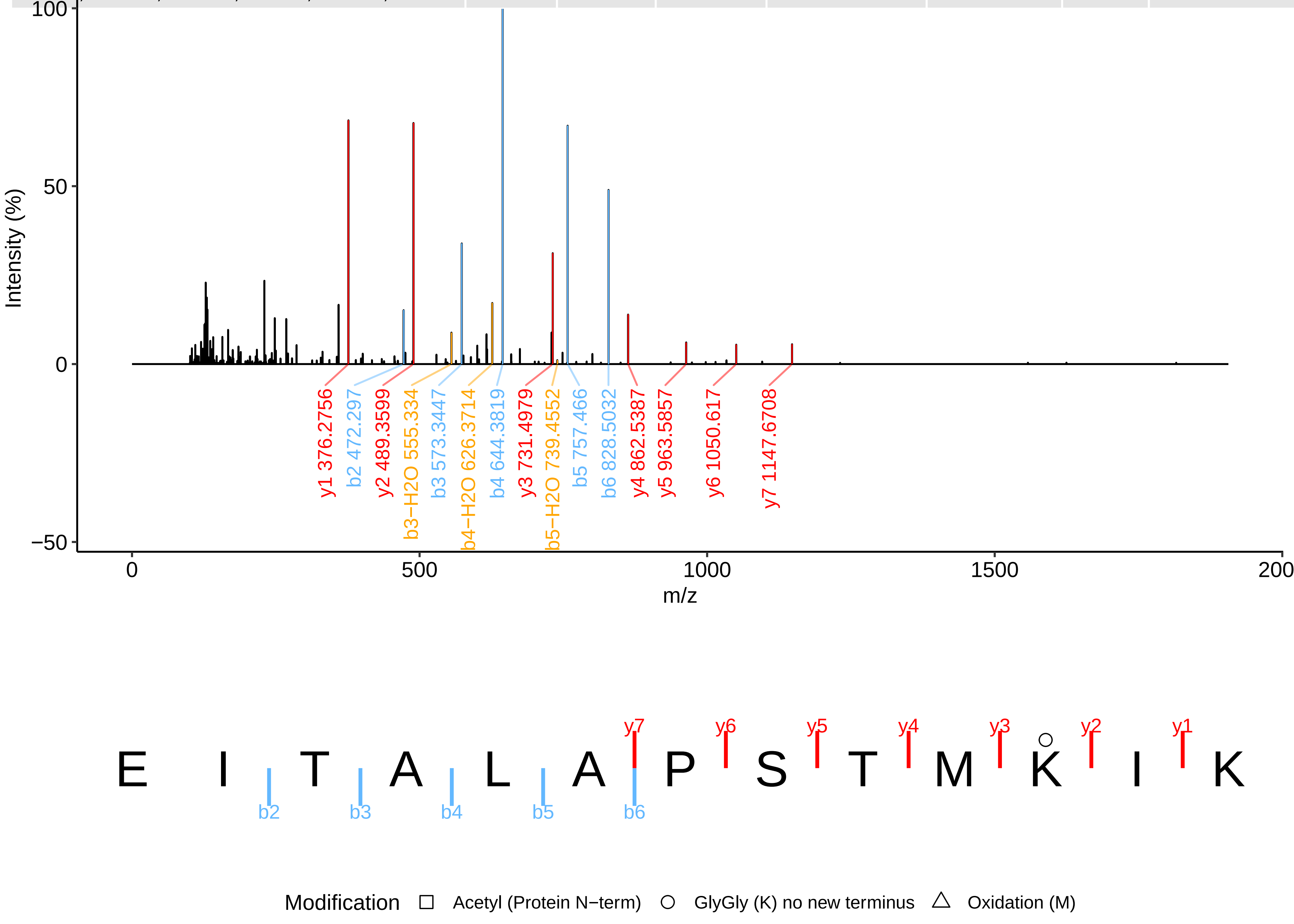

20200921_KIZ_MUb4_GlyGly_10plex − Scan number: 19484

Gene Names	Charge	m/z	Mass	Retention time	PEP	Score	Precursor Inte
ACTB;ACTG2;ACTG1;ACTA1;ACTC1;ACTA2	3	506.285	1515.833	70.277	1.5071e−05	103.75	4455279

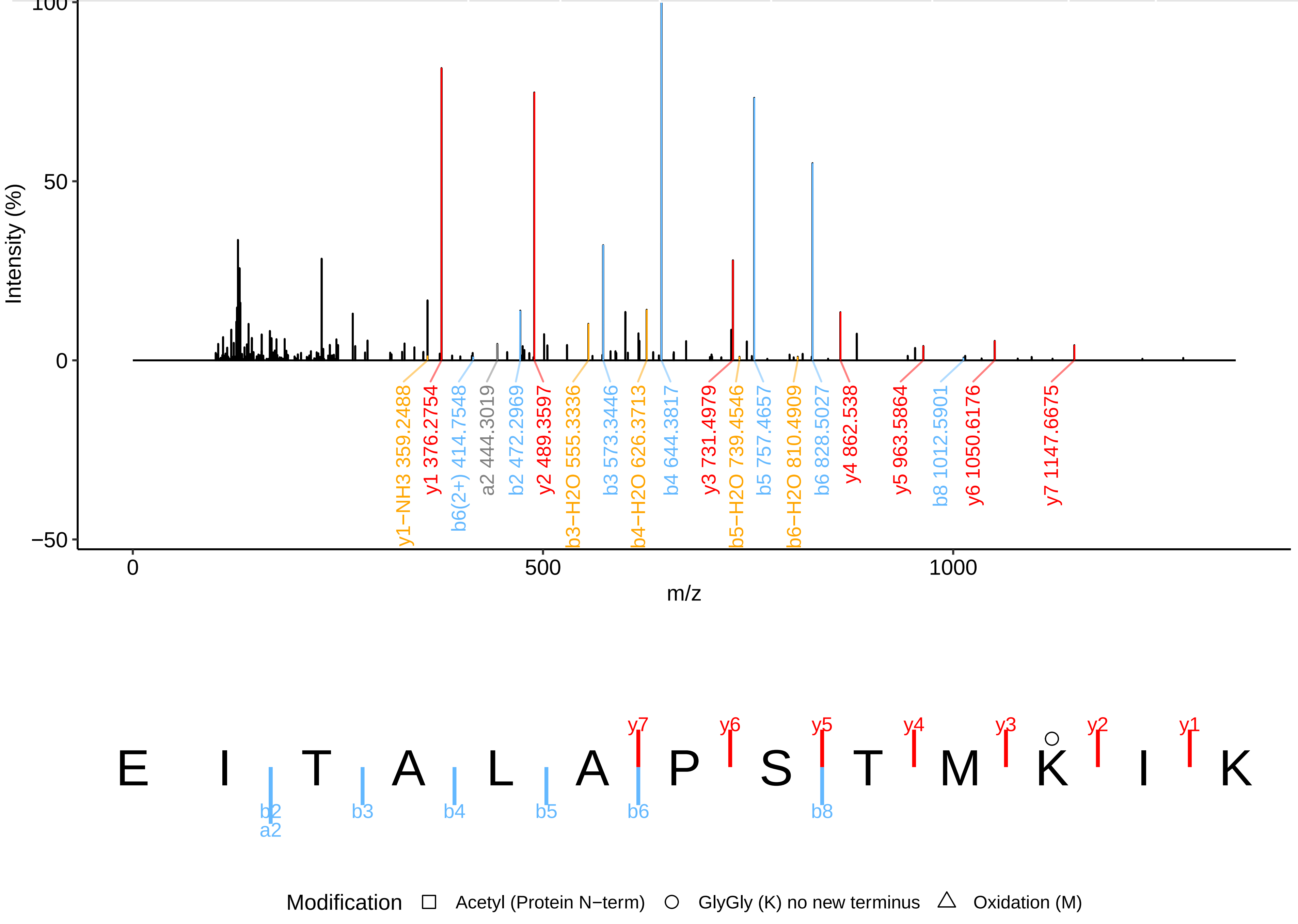

20200921_KIZ_MUb4_GlyGly_10plex_2 − Scan number: 17810

Gene Names	Charge	m/z	Mass	Retention time	PEP	Score	Precursor Inter
ACTB;ACTG2;ACTG1;ACTA1;ACTC1;ACTA2	3	506.285	1515.833	69.678	0.0011061	81.431	1127147

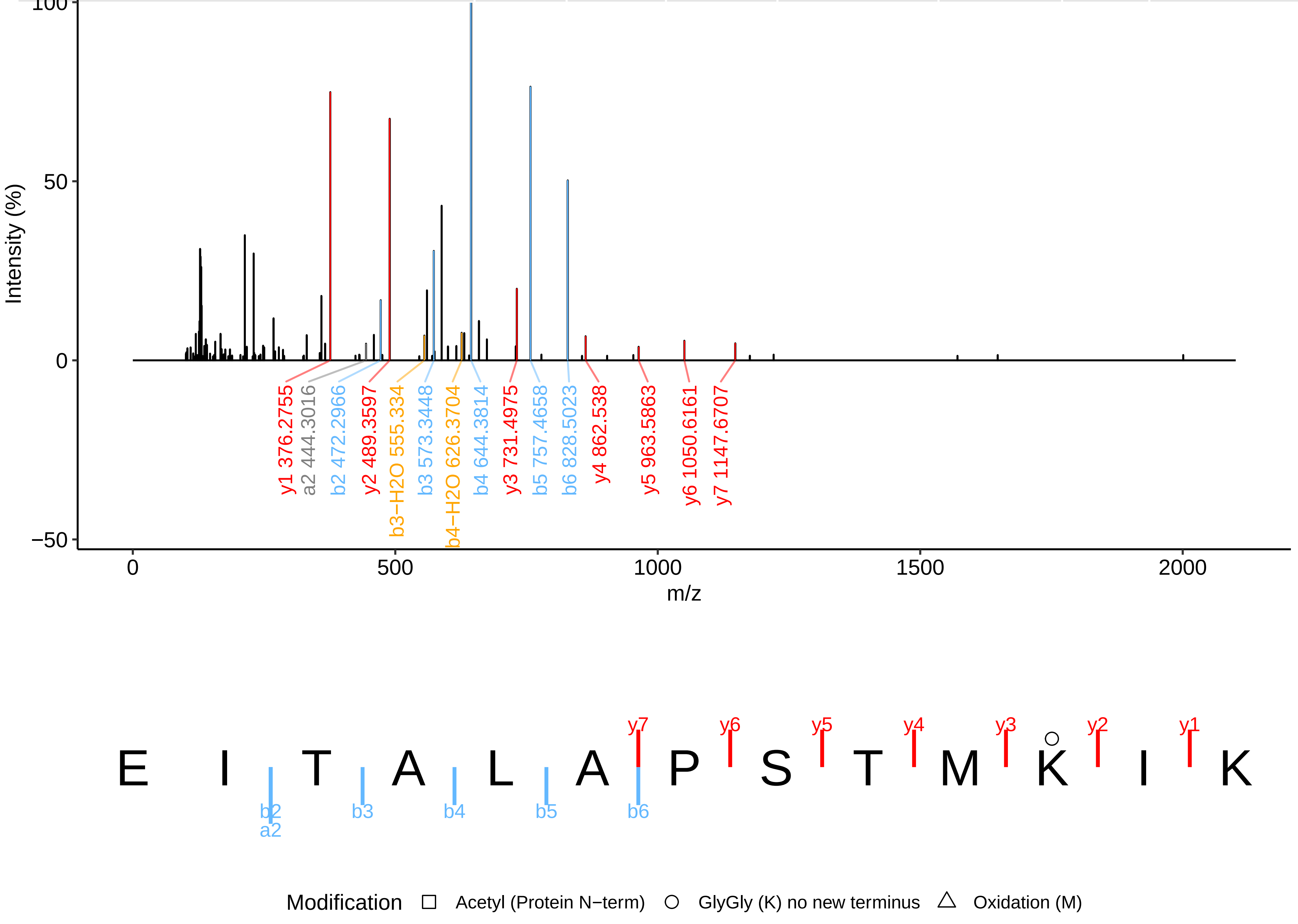

20200924_KIZ_MUb5_GlyGly_1 − Scan number: 18821

Gene Names	Charge	m/z	Mass	Retention time	PEP	Score	Precursor Inte
ACTB;ACTG2;ACTG1;ACTA1;ACTC1;ACTA2	3	506.285	1515.833	60.823	1.6531e−05	102.62	5589222

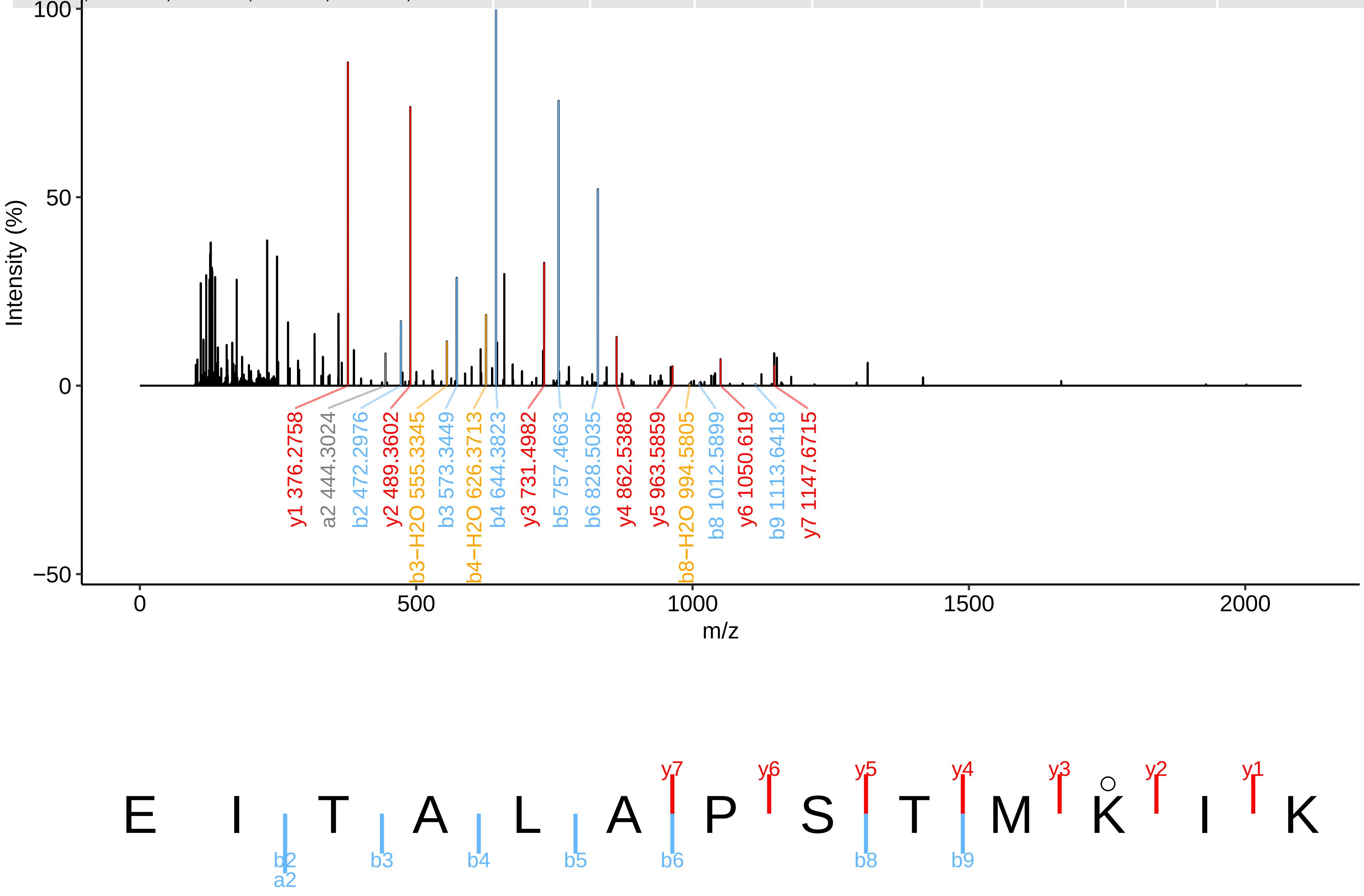

Modification □ Acetyl (Protein N−term) ○ GlyGly (K) no new terminus △ Oxidation (M)

20200924_KIZ_MUb5_GlyGly_1 − Scan number: 18981

Gene Names	Charge	m/z	Mass	Retention time	PEP	Score	Precursor Inter
ACTB;ACTG2;ACTG1;ACTA1;ACTC1;ACTA2	3	506.285	1515.833	61.329	3.6508e−05	101.6	13976964

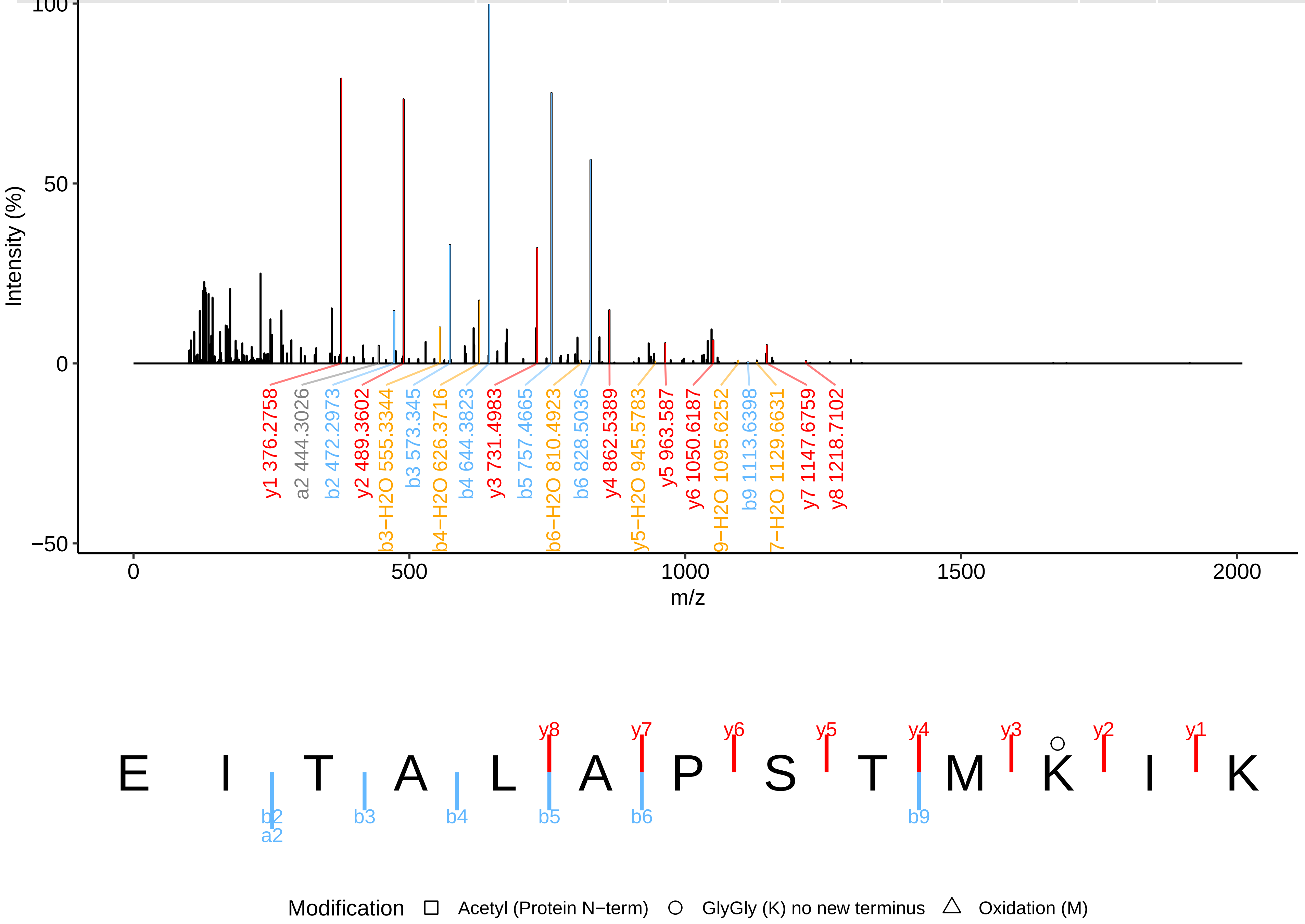

20200924_KIZ_MUb5_GlyGly_2 − Scan number: 17518

Gene Names	Charge	m/z	Mass	Retention time	PEP	Score	Precursor Inte
ACTB;ACTG2;ACTG1;ACTA1;ACTC1;ACTA2	3	506.285	1515.833	64.929	1.2897e−08	117.23	3477600

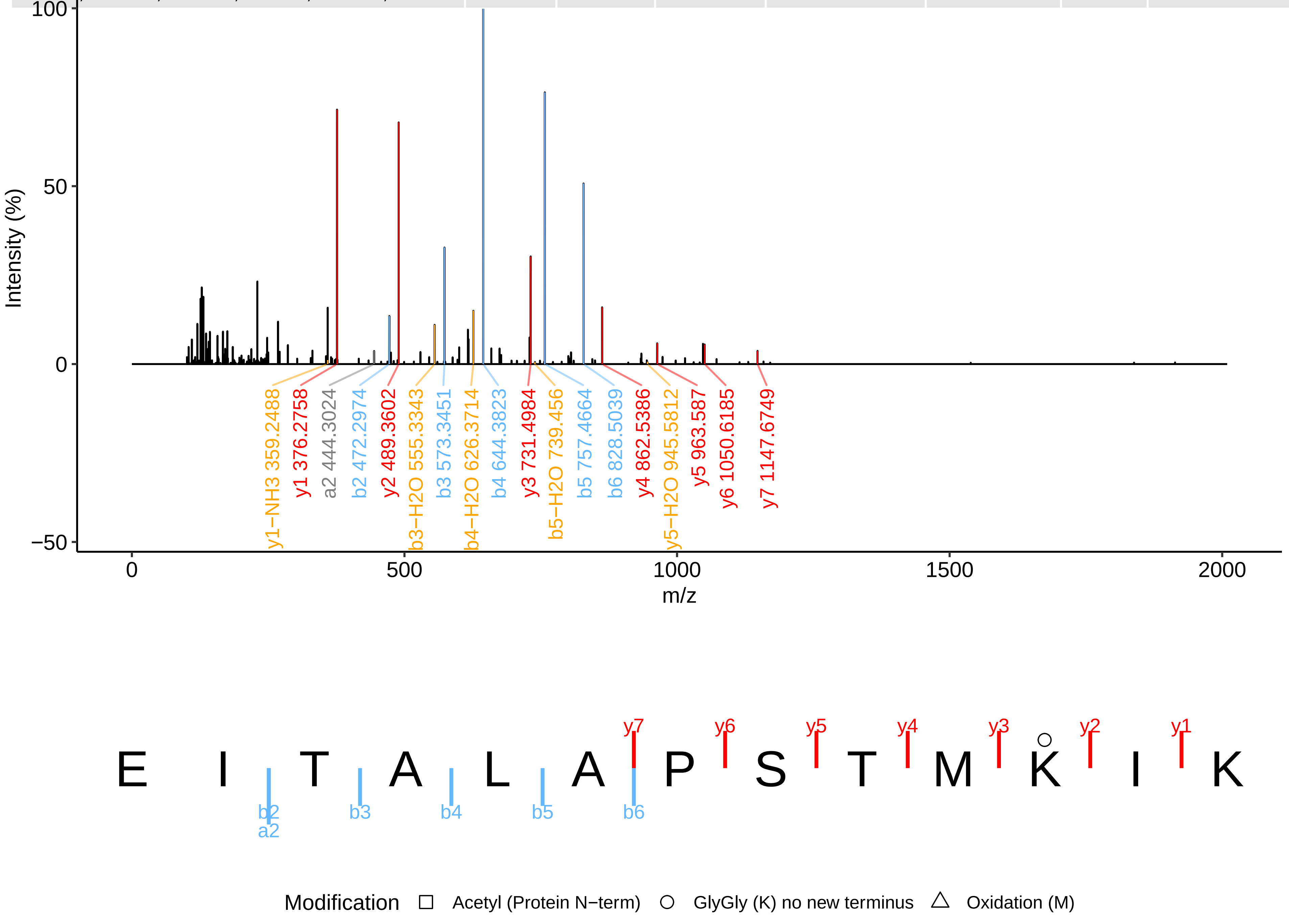

20200924_KIZ_MUb5_GlyGly_2 − Scan number: 17380

Gene Names	Charge	m/z	Mass	Retention time	PEP	Score	Precursor Inter
ACTB;ACTG2;ACTG1;ACTA1;ACTC1;ACTA2	3	506.285	1515.833	64.425	0.0011597	85.845	3133523

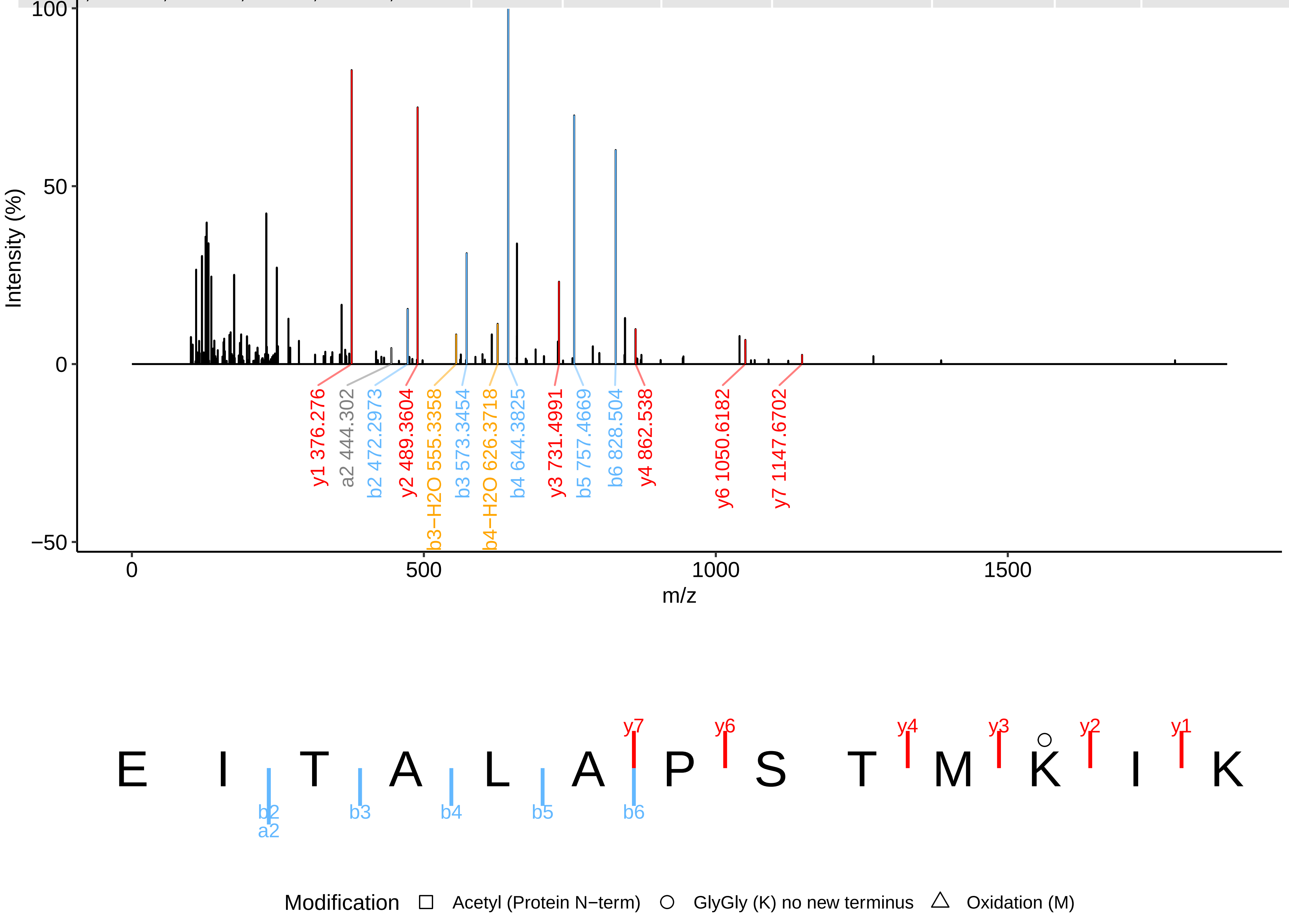

20200921_KIZ_MUb4_GlyGly_10plex_2 − Scan number: 17207

Gene Names	Charge	m/z	Mass	Retention time	PEP	Score	Precursor Intensity
ABHD17A	3	573.9686	1718.884	67.258	1.2267e−14	149.69	2528998

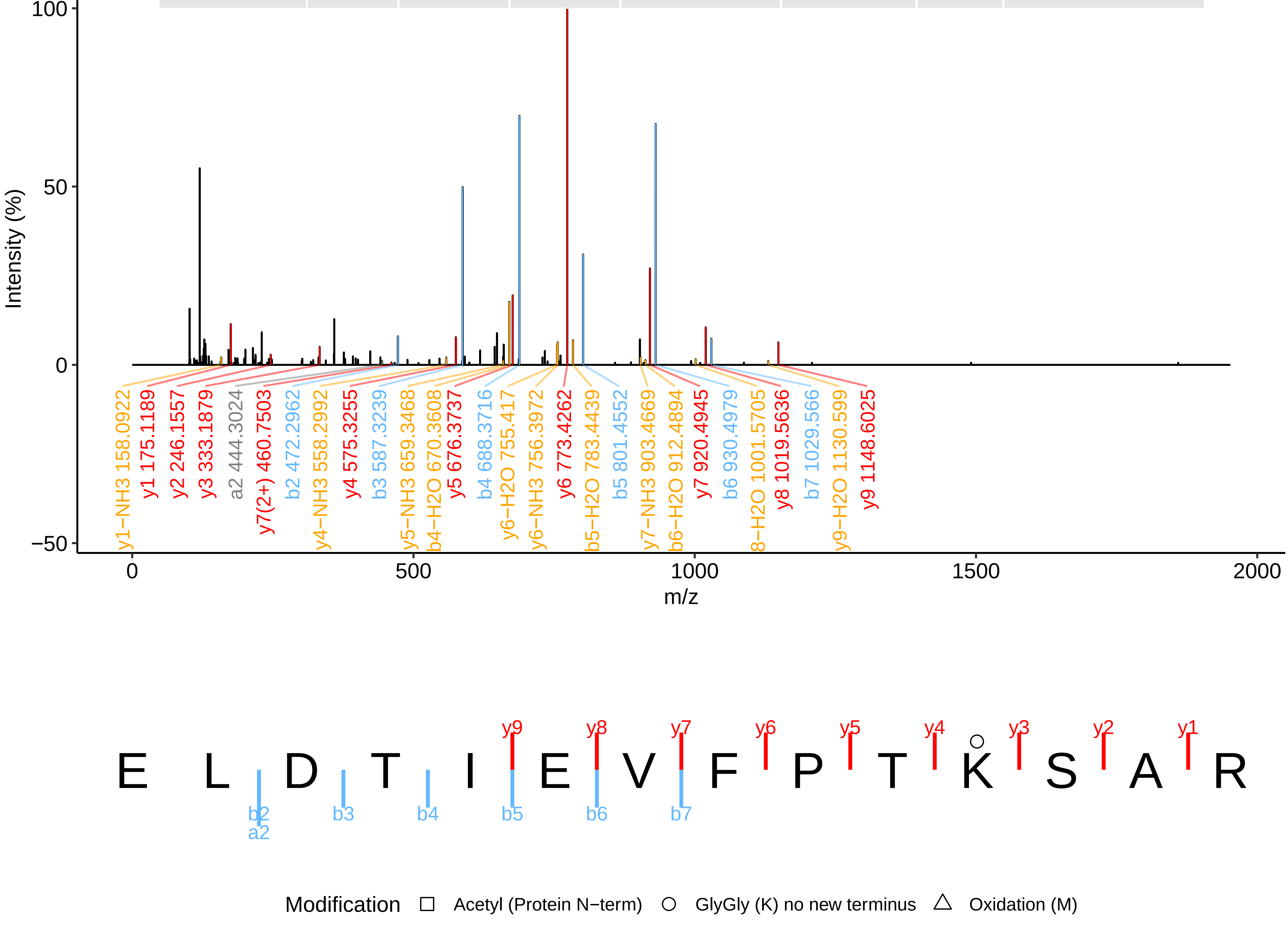

20200921_KIZ_MUb4_GlyGly_10plex − Scan number: 16081

Gene Names	Charge	m/z	Mass	Retention time	PEP	Score	Precursor Intensity
TPR	3	563.2918	1686.854	57.673	5.3892e−13	146.37	2864525

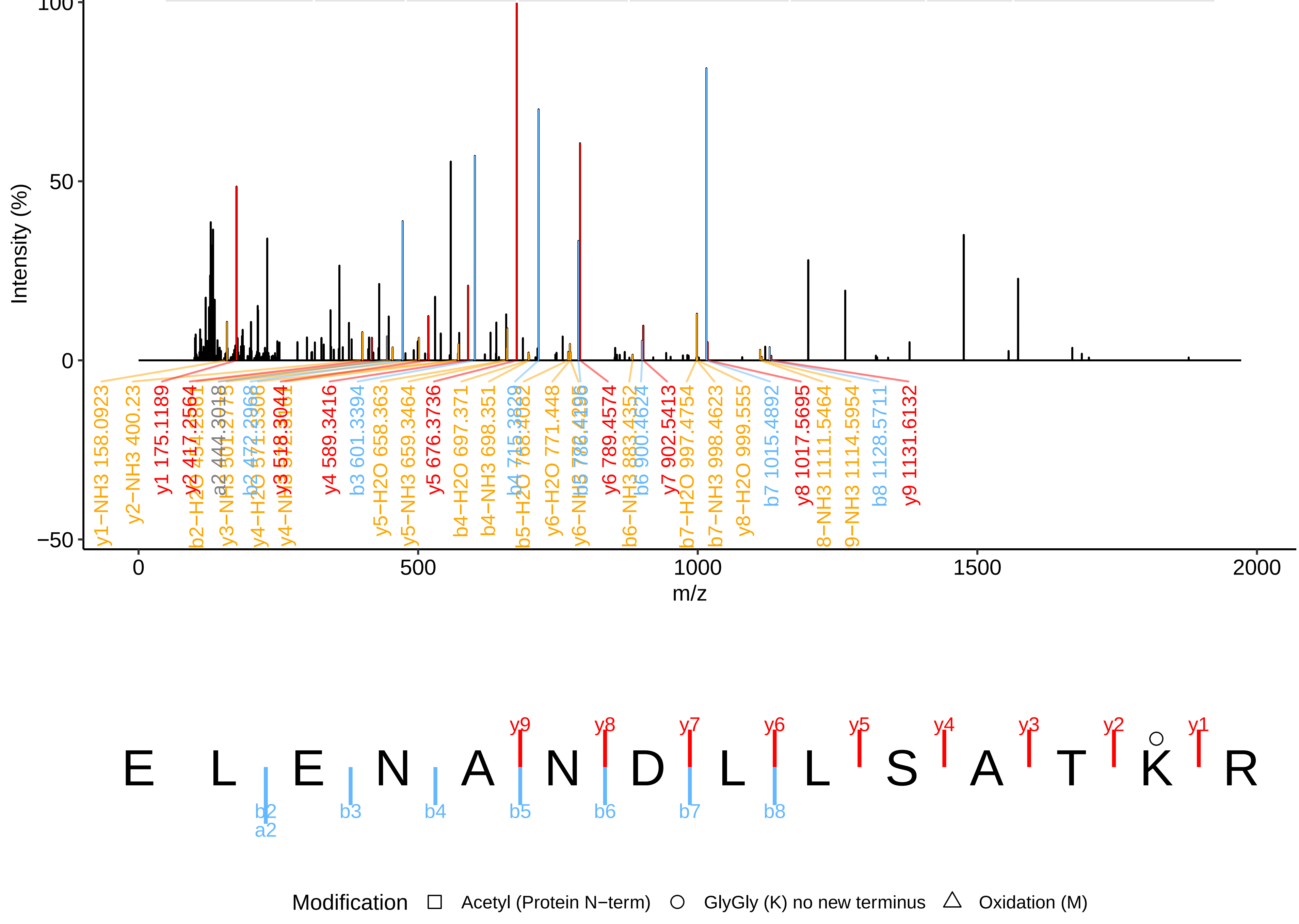

20200921_KIZ_MUb4_GlyGly_10plex − Scan number: 19393

Gene Names	Charge	m/z	Mass	Retention time	PEP	Score	Precursor Intensity
S100A6	3	491.6155	1471.825	69.916	0.0015898	81.565	2687536

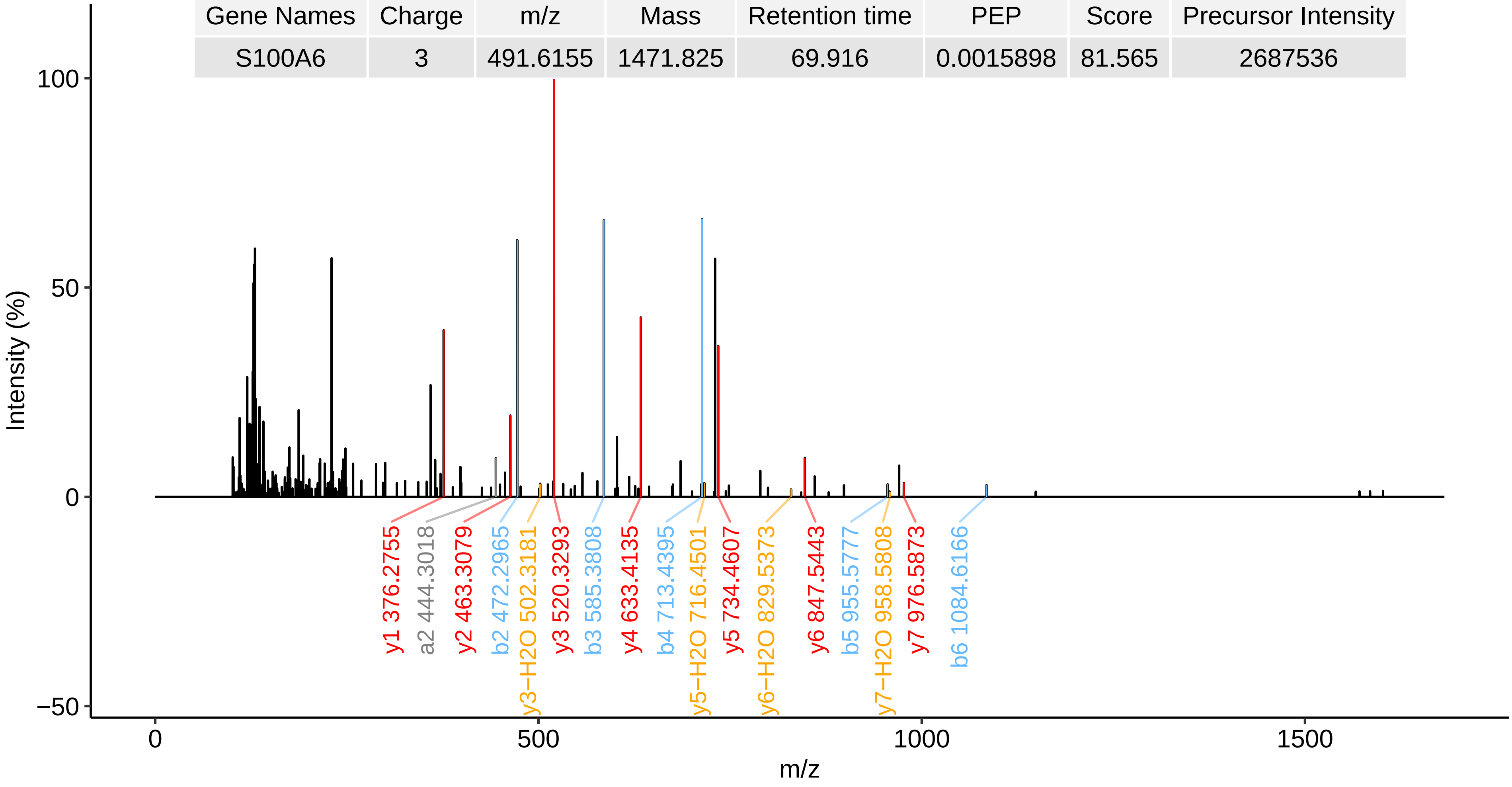

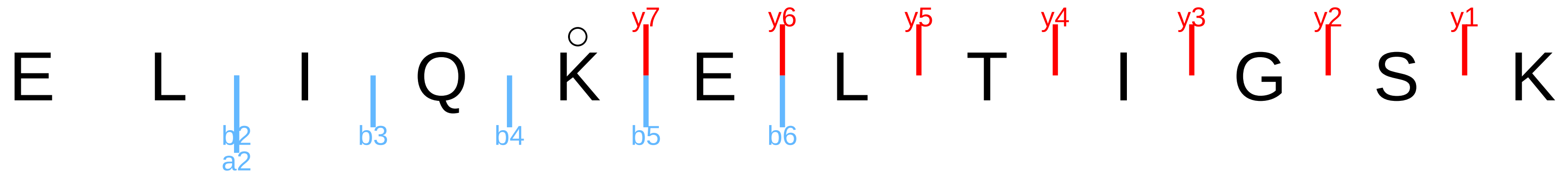

Modification □ Acetyl (Protein N−term) ○ GlyGly (K) no new terminus △ Oxidation (M)

20200921_KIZ_MUb4_GlyGly_10plex_2 − Scan number: 17630

Gene Names	Charge	m/z	Mass	Retention time	PEP	Score	Precursor Intensity
S100A6	3	491.6155	1471.825	68.953	0.0011557	81.494	1179966

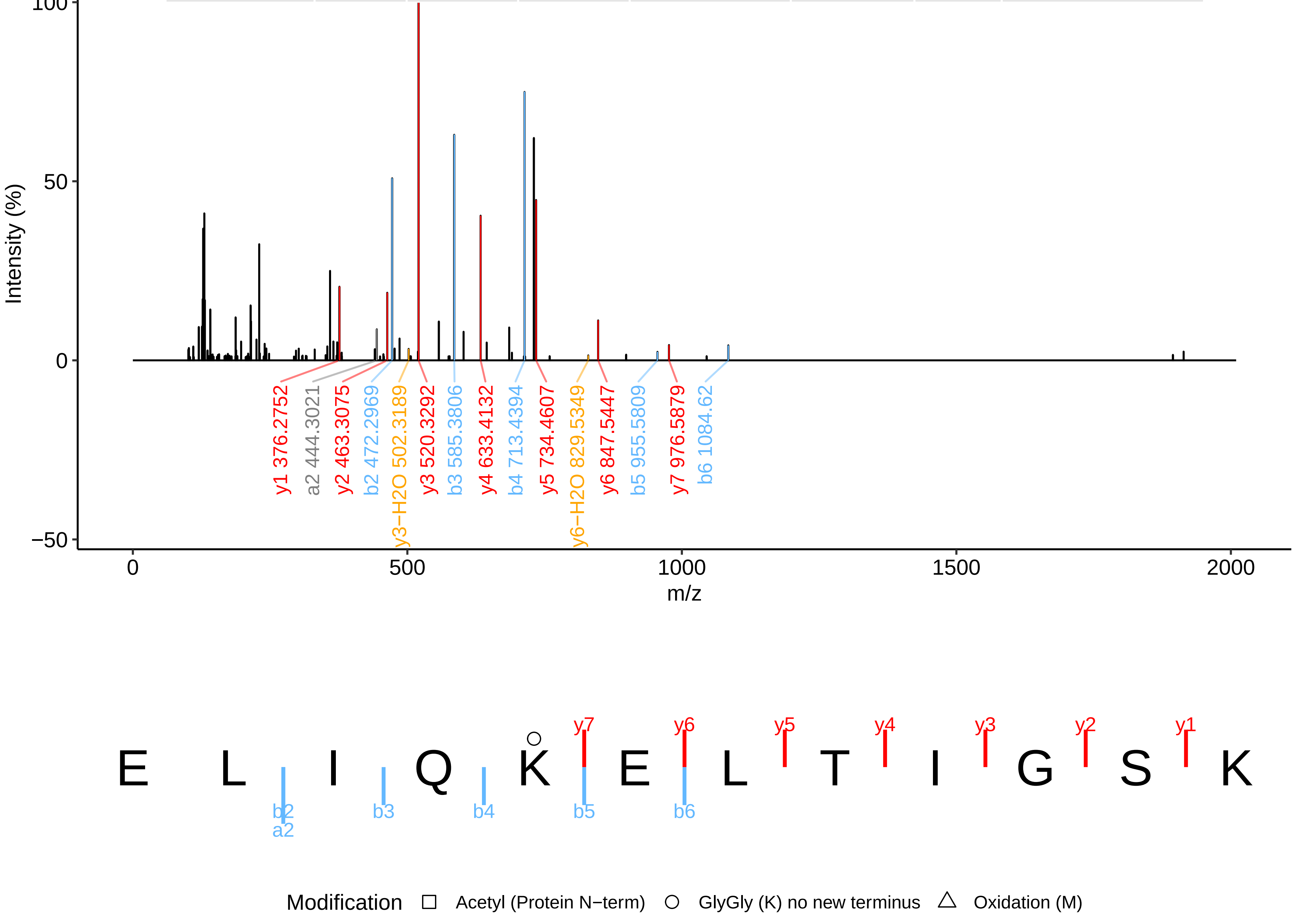

20200921_KIZ_MUb4_GlyGly_10plex − Scan number: 11800

Gene Names	Charge	m/z	Mass	Retention time	PEP	Score	Precursor Intensity
DNAJA1	3	488.9158	1463.726	41.224	0.00019827	95.417	3636426

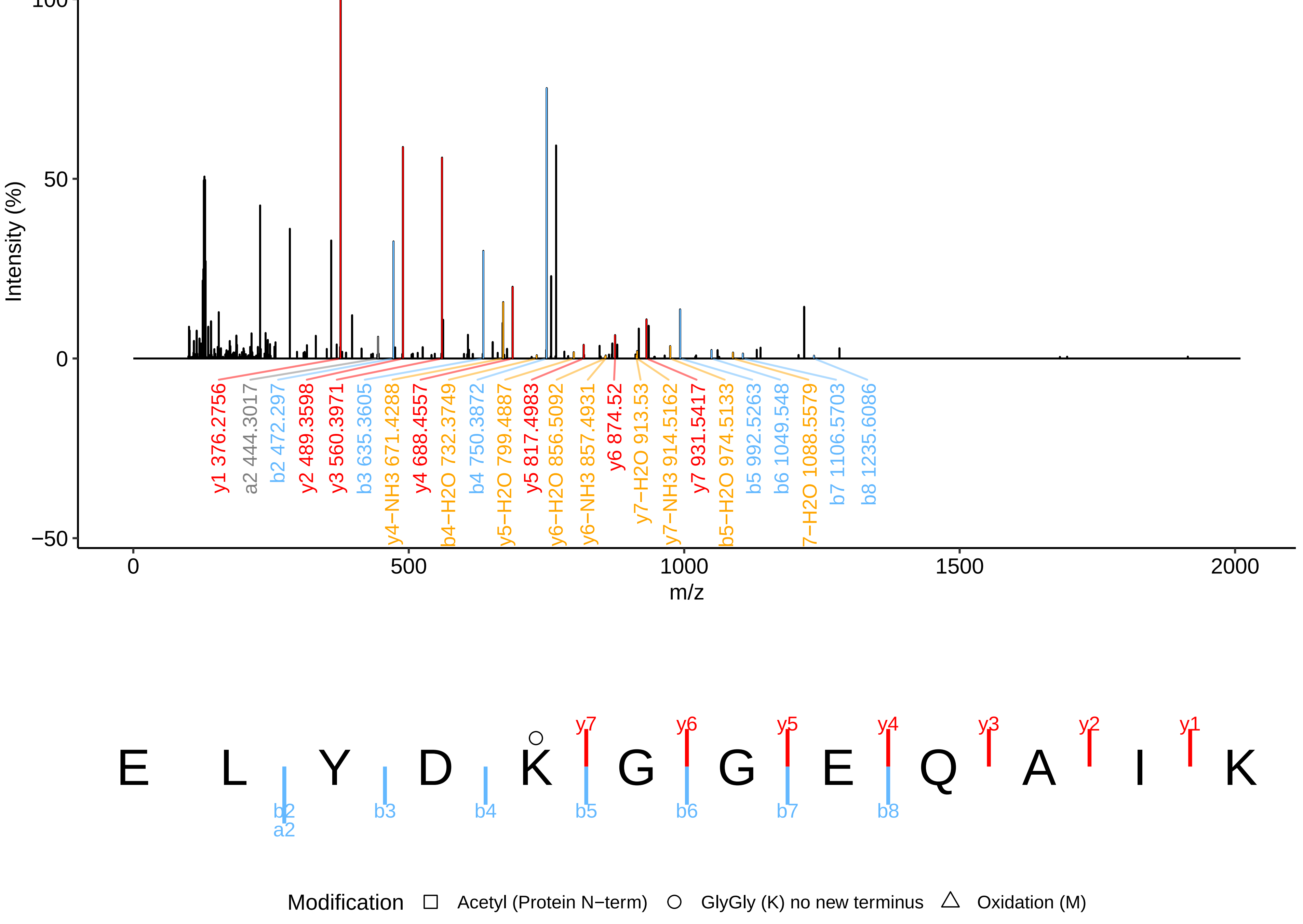

20200921_KIZ_MUb4_GlyGly_10plex_2 − Scan number: 10838

Gene Names	Charge	m/z	Mass	Retention time	PEP	Score	Precursor Intensity
DNAJA1	3	488.9158	1463.726	41.562	0.09904	46.592	403815.8

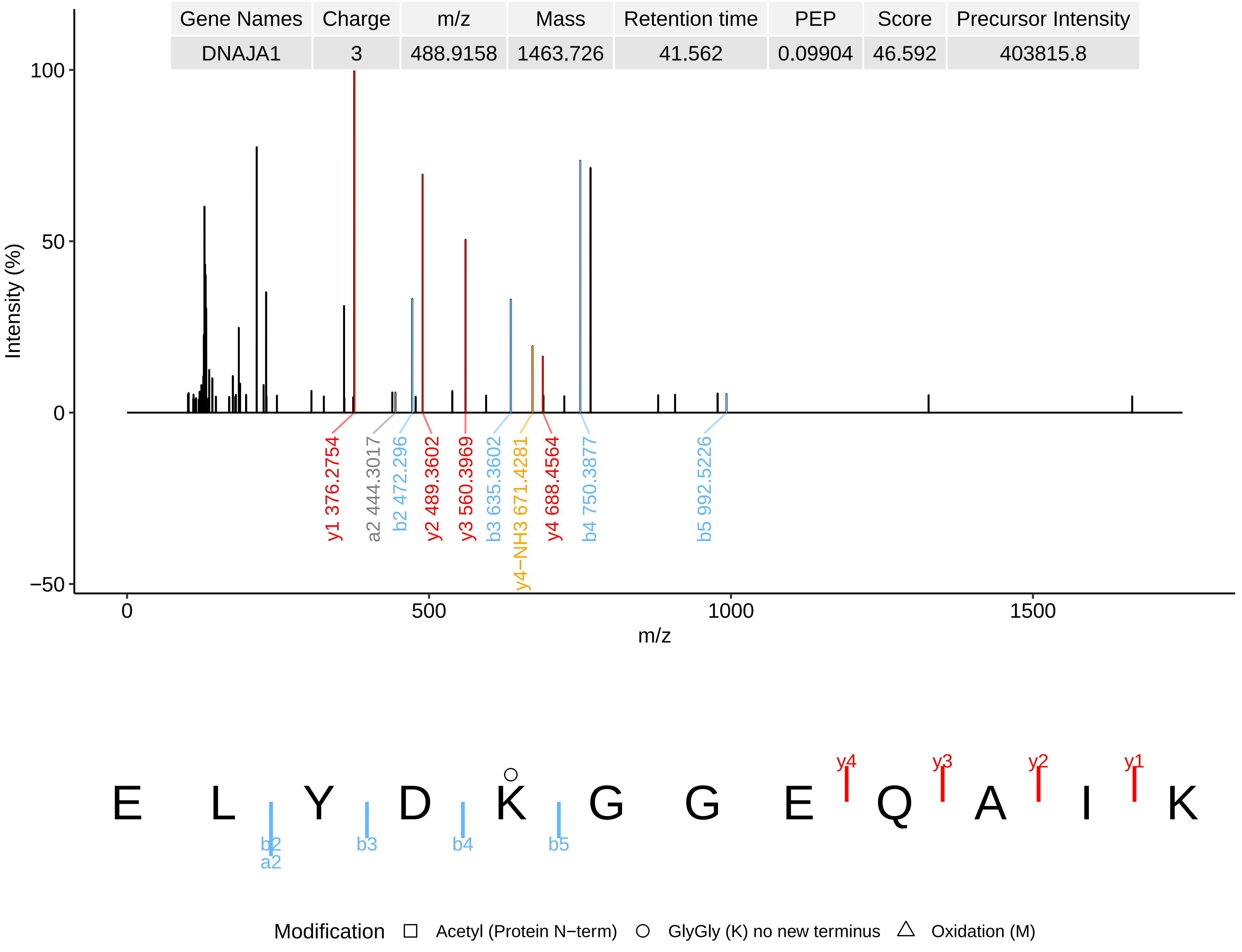

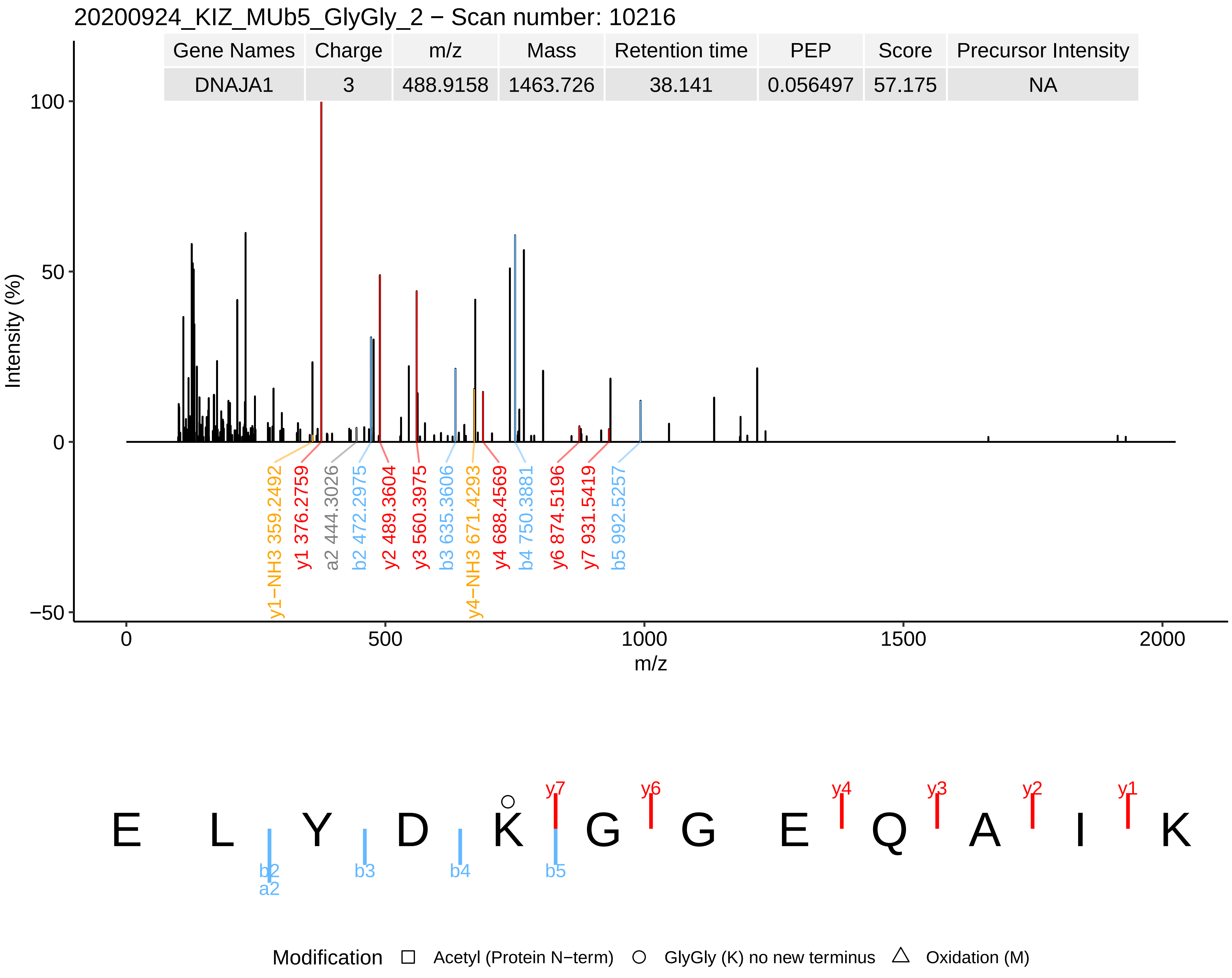

20200924_KIZ_MUb5_GlyGly_2 − Scan number: 10216
Gene Names | Charge | m/z | Mass | Retention time | PEP | Score | Precursor Intensity
DNAJA1 | 3 | 488.9158 | 1463.726 | 38.141 | 0.056497 | 57.175 | NA
Intensity (%)
100
50
0
−50
0
500
1000
1500
2000
m/z
y1−NH3 359.2492
y1 376.2759
a2 444.3026
b2 472.2975
y2 489.3604
y3 560.3975
b3 635.3606
y4−NH3 671.4293
y4 688.4569
b4 750.3881
y6 874.5196
y7 931.5419
b5 992.5257
E L Y D K G G E Q A I K
y7 y6 y4 y3 y2 y1
b2 a2 b3 b4 b5
Modification
Acetyl (Protein N−term)
GlyGly (K) no new terminus
Oxidation (M)

20200921_KIZ_MUb4_GlyGly_10plex − Scan number: 9059

Gene Names	Charge	m/z	Mass	Retention time	PEP	Score	Precursor Intensity
CCT8	3	498.2424	1491.705	30.968	1.0133e−08	120.57	13030404

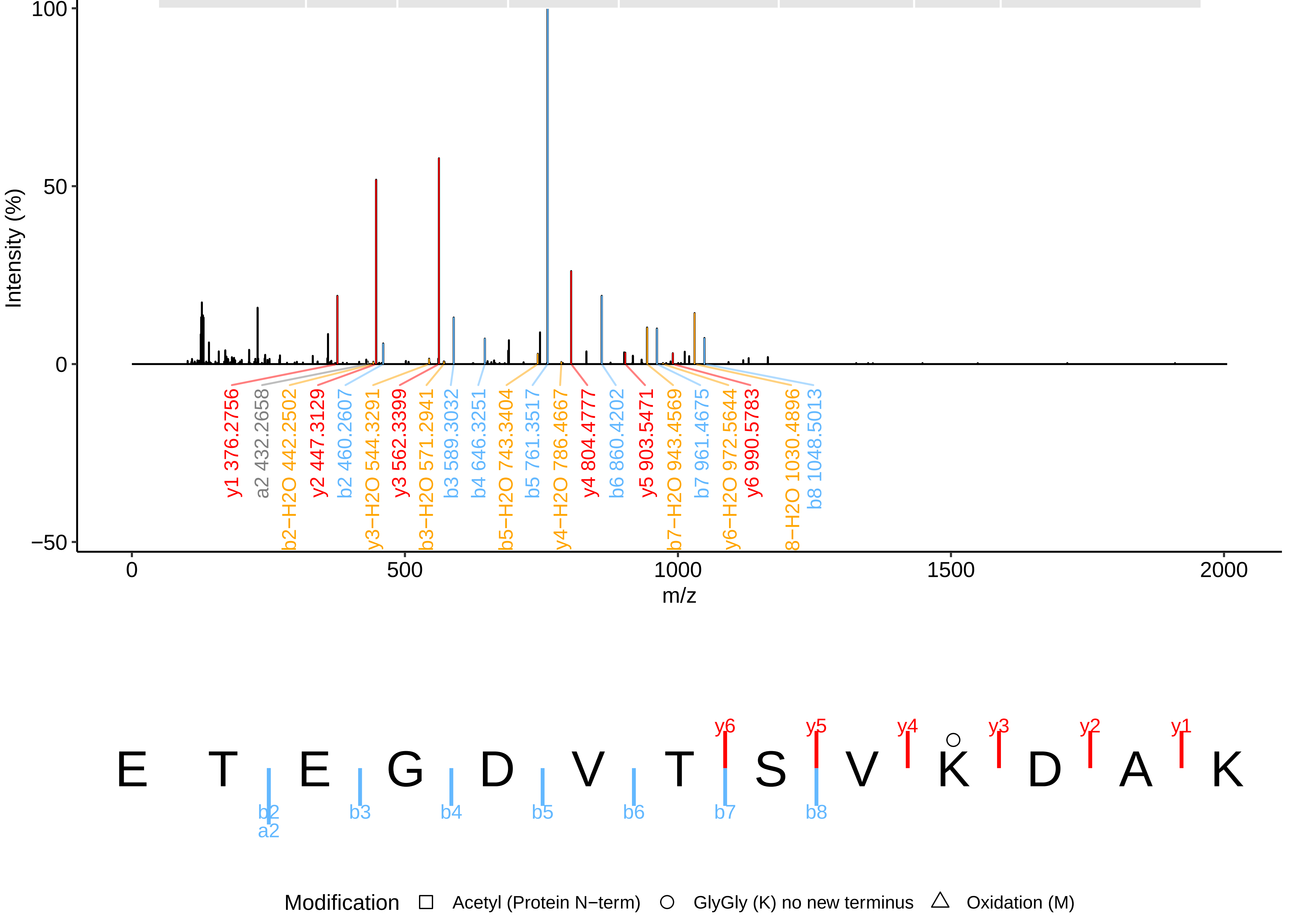

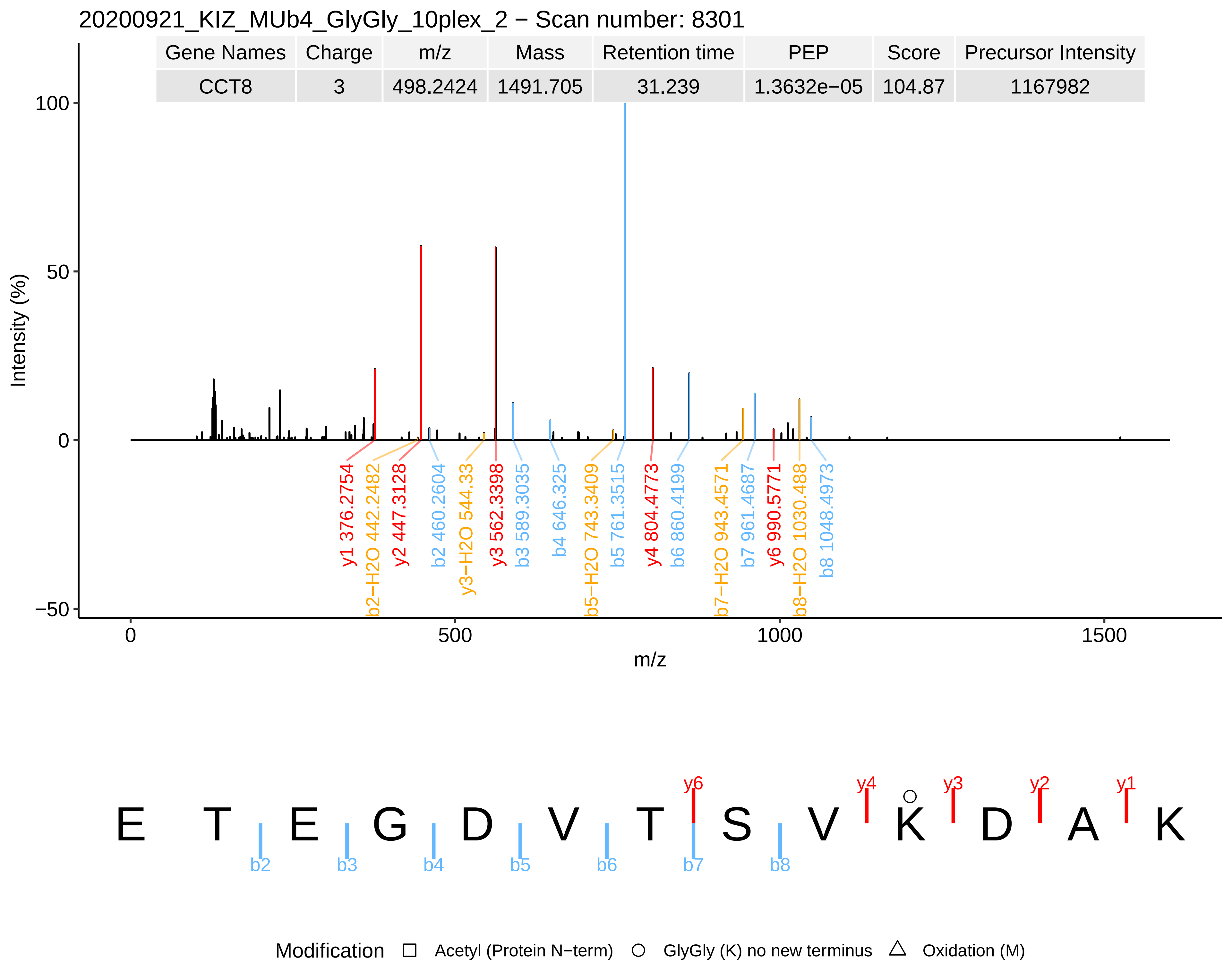

20200921_KIZ_MUb4_GlyGly_10plex_2 − Scan number: 8301
Gene Names | Charge | m/z | Mass | Retention time | PEP | Score | Precursor Intensity
CCT8 | 3 | 498.2424 | 1491.705 | 31.239 | 1.3632e−05 | 104.87 | 1167982
Intensity (%)
m/z
y1 376.2754
b2−H2O 442.2482
y2 447.3128
b2 460.2604
y3−H2O 544.33
y3 562.3398
b3 589.3035
b4 646.325
b5−H2O 743.3409
b5 761.3515
y4 804.4773
b6 860.4199
b7−H2O 943.4571
b7 961.4687
y6 990.5771
b8−H2O 1030.488
b8 1048.4973
E T E G D V T S V K D A K
y6 y4 y3 y2 y1
b2 b3 b4 b5 b6 b7 b8
Modification
Acetyl (Protein N−term)
GlyGly (K) no new terminus
Oxidation (M)

20200924_KIZ_MUb5_GlyGly_1 − Scan number: 8150

Gene Names	Charge	m/z	Mass	Retention time	PEP	Score	Precursor Intensity
CCT8	3	498.2424	1491.705	25.989	4.6871e−06	111.79	5848786

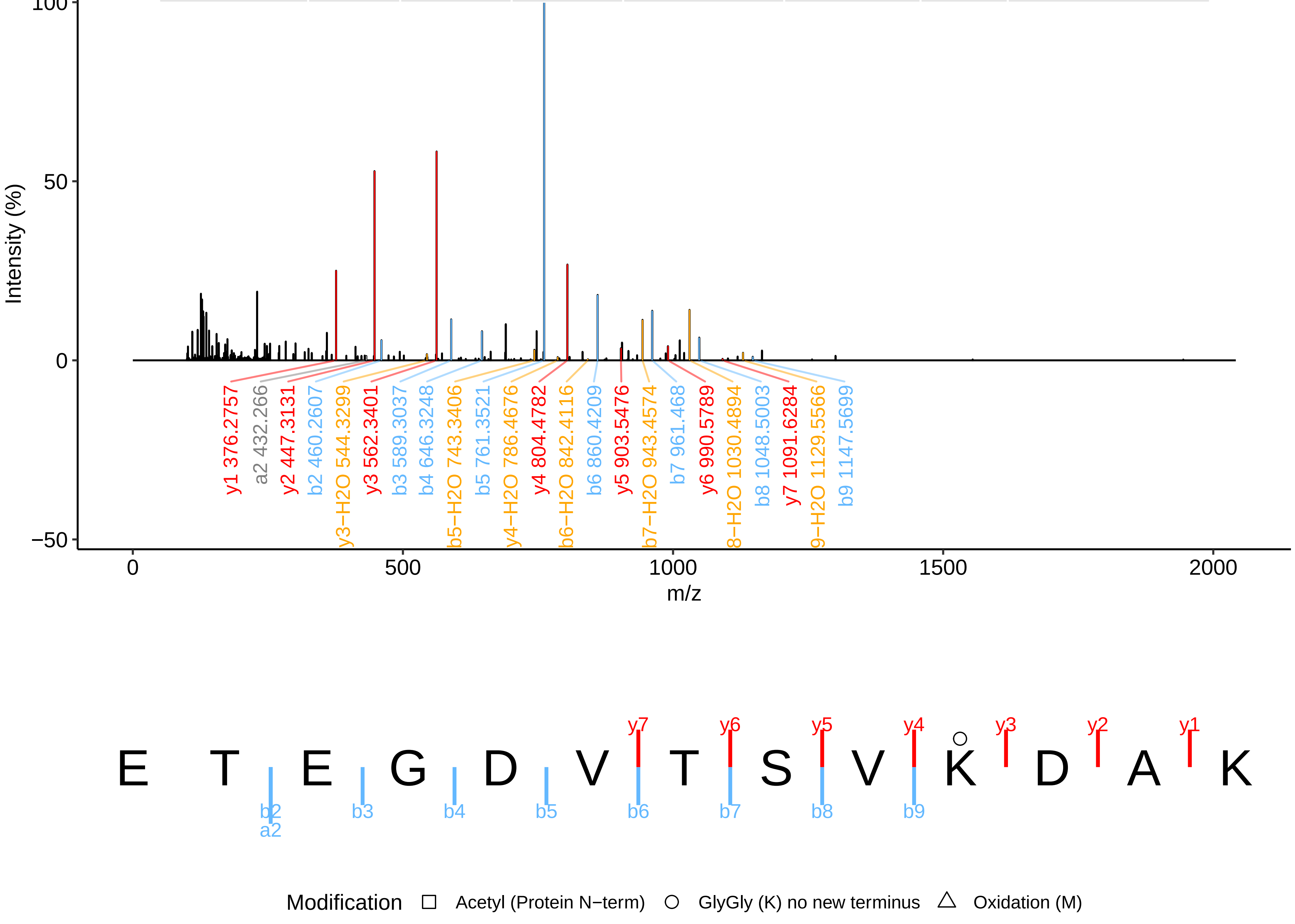

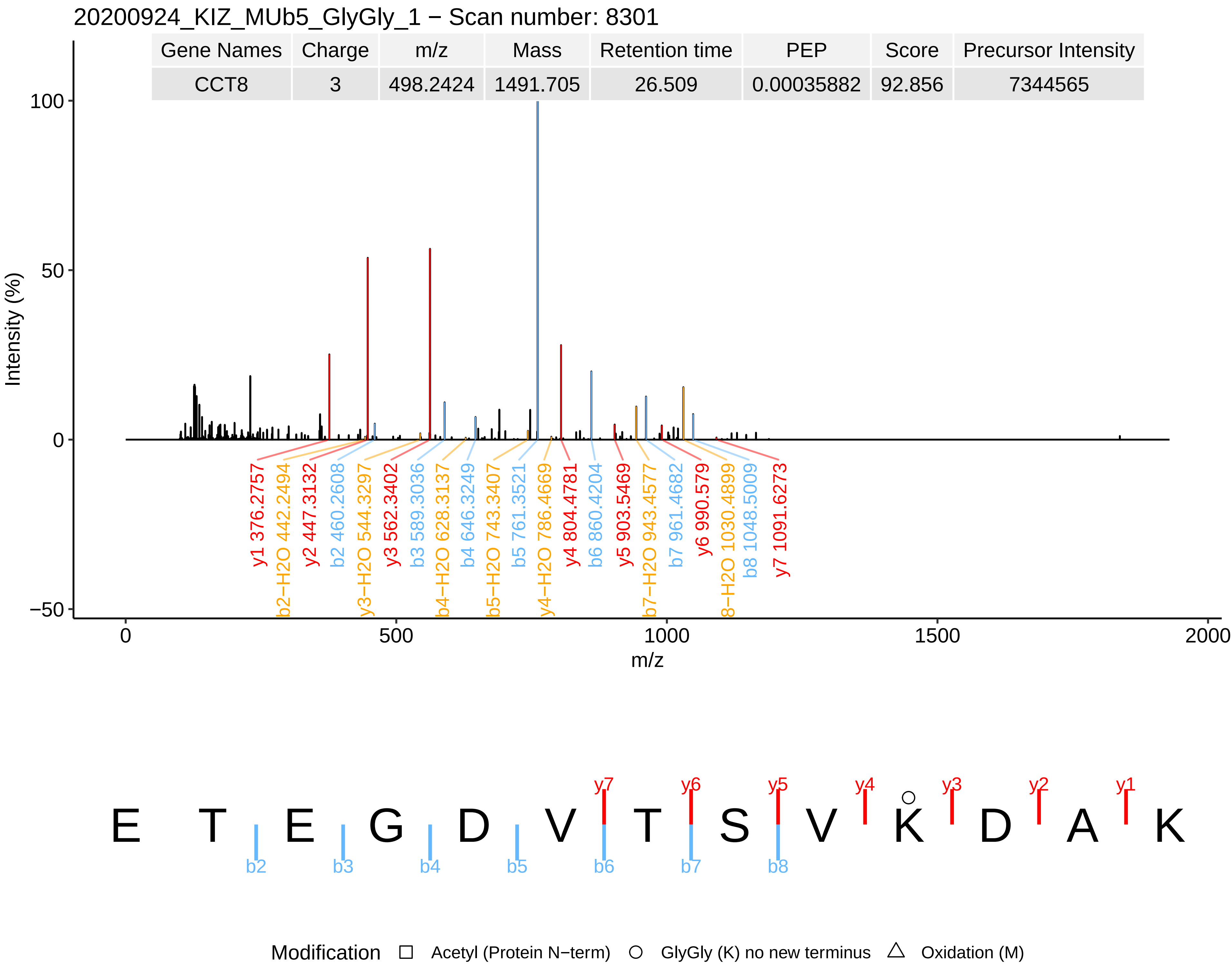
20200924_KIZ_MUb5_GlyGly_1 − Scan number: 8301
Gene Names | Charge | m/z | Mass | Retention time | PEP | Score | Precursor Intensity
CCT8 | 3 | 498.2424 | 1491.705 | 26.509 | 0.00035882 | 92.856 | 7344565
Intensity (%)
100
50
0
−50
0
500
1000
1500
2000
m/z
y1 376.2757
b2−H2O 442.2494
y2 447.3132
b2 460.2608
y3−H2O 544.3297
y3 562.3402
b3 589.3036
b4−H2O 628.3137
b4 646.3249
b5−H2O 743.3407
b5 761.3521
y4−H2O 786.4669
y4 804.4781
b6 860.4204
y5 903.5469
b7−H2O 943.4577
b7 961.4682
y6 990.579
b8−H2O 1030.4899
b8 1048.5009
y7 1091.6273
E T E G D V T S V K D A K
y7 y6 y5 y4 y3 y2 y1
b2 b3 b4 b5 b6 b7 b8
Modification
Acetyl (Protein N−term)
GlyGly (K) no new terminus
Oxidation (M)

20201118_KIZ_MUb6_GlyGly_2 − Scan number: 9150

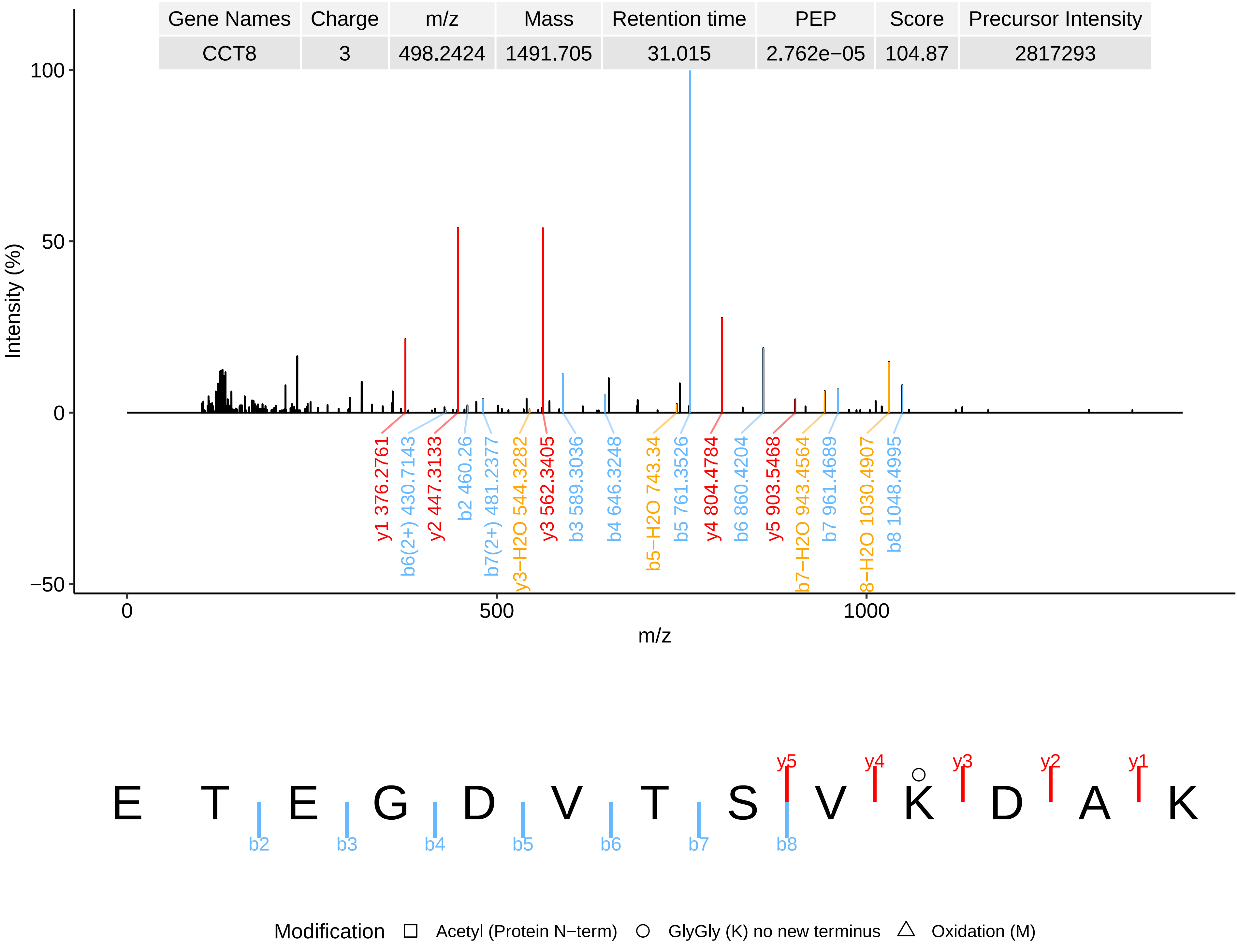

20200921_KIZ_MUb4_GlyGly_10plex − Scan number: 6169

Gene Names	Charge	m/z	Mass	Retention time	PEP	Score	Precursor Intensity
MET	4	378.4376	1509.721	20.318	2.2041e−05	97.911	4667703

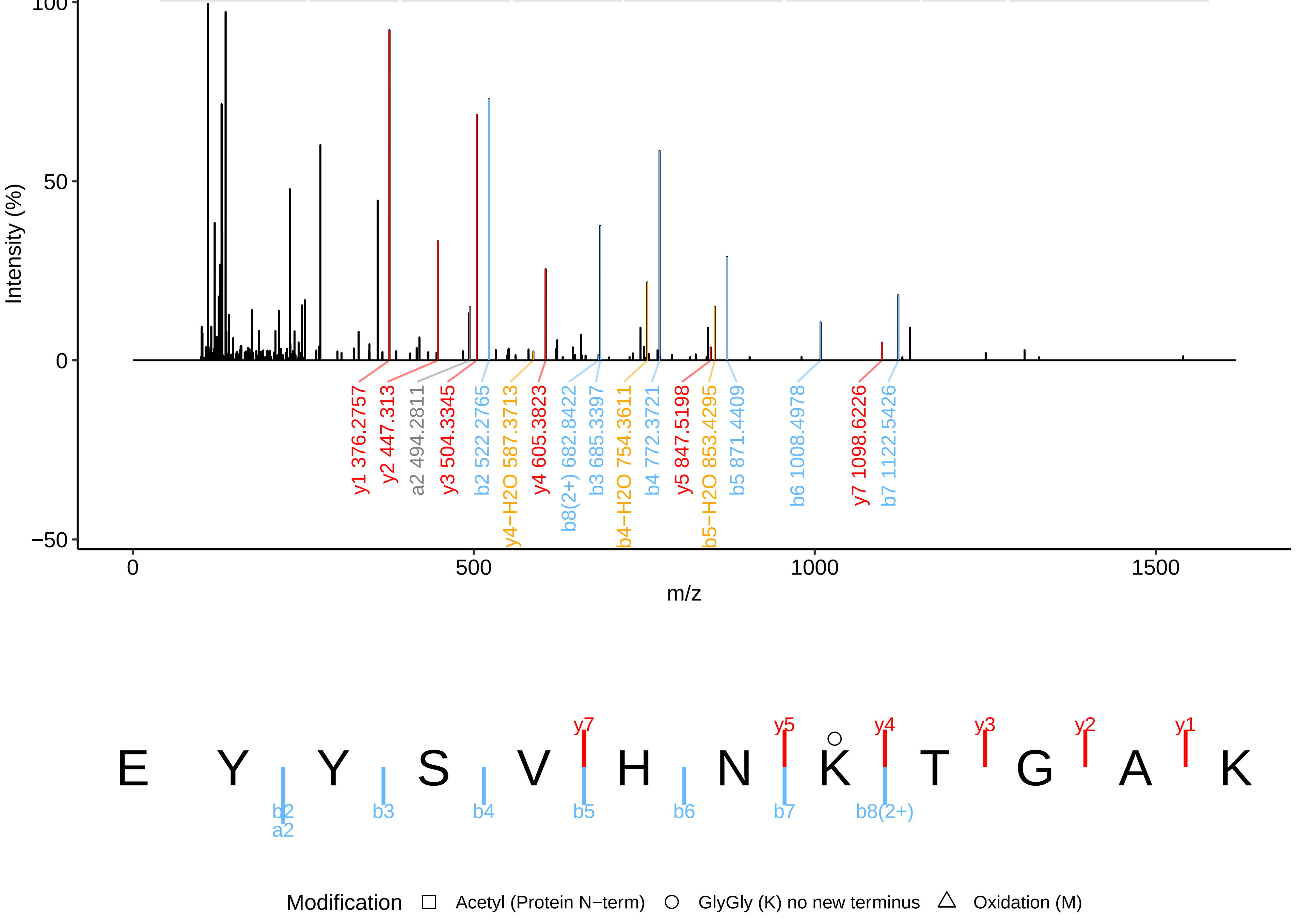

20200921_KIZ_MUb4_GlyGly_10plex − Scan number: 29516

Gene Names	Charge	m/z	Mass	Retention time	PEP	Score	Precursor Intensity
APEX2	3	583.0016	1745.983	108.24	5.2547e−05	99.011	NA

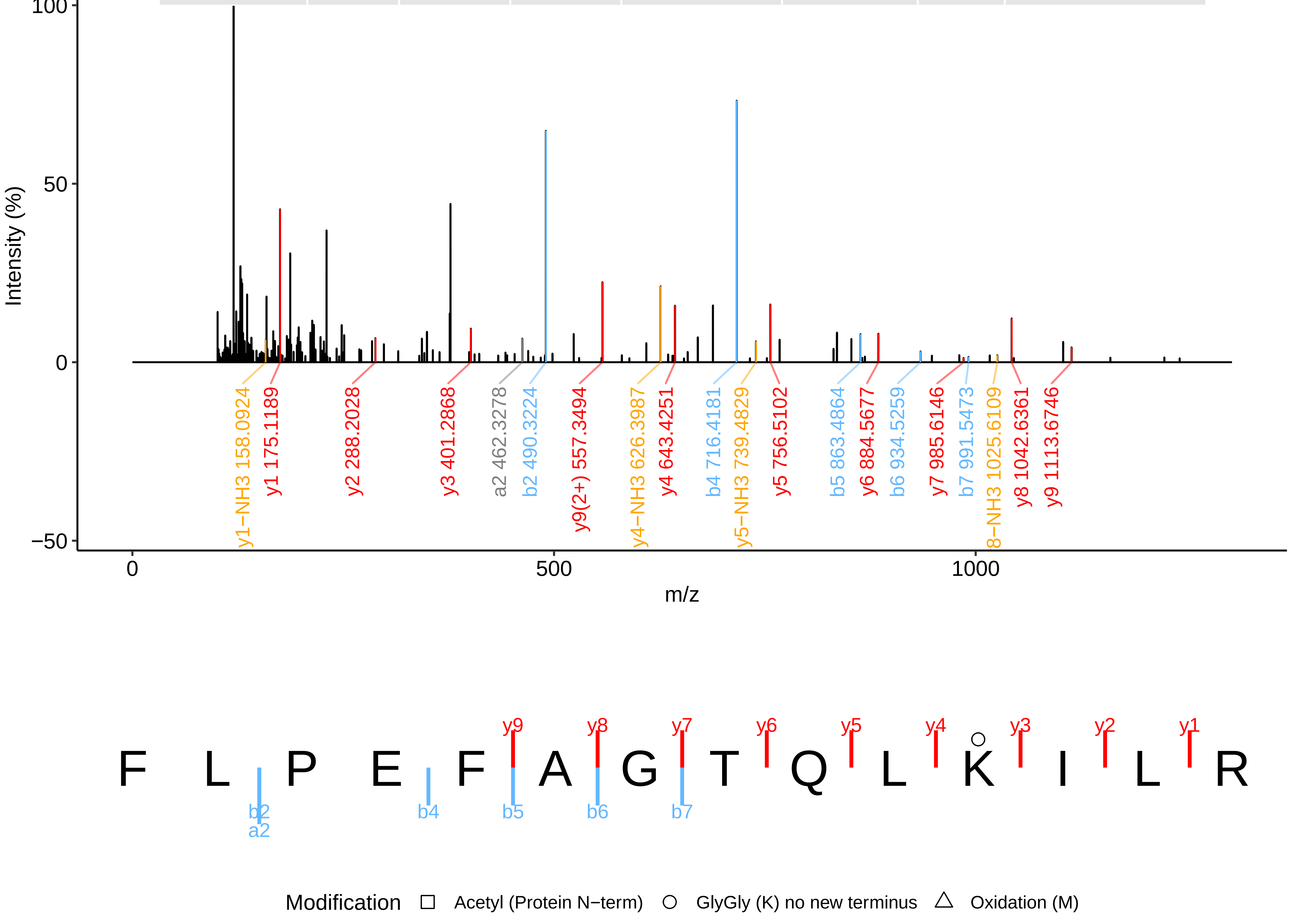

20200921_KIZ_MUb4_GlyGly_10plex − Scan number: 14312

Gene Names	Charge	m/z	Mass	Retention time	PEP	Score	Precursor Intensity
WRNIP1	3	584.3076	1749.901	50.816	4.8604e−20	156.24	4007056

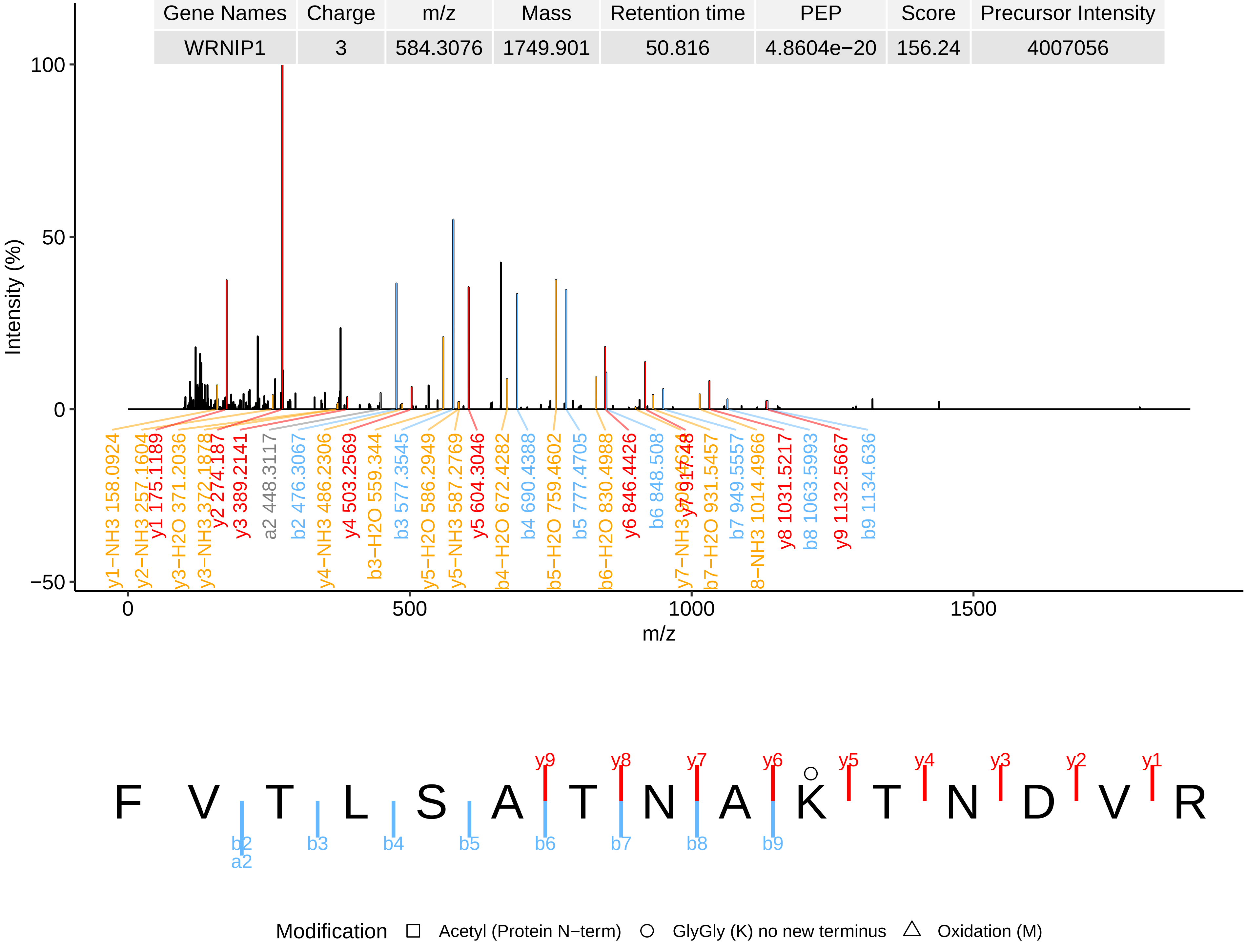

20200921_KIZ_MUb4_GlyGly_10plex_2 − Scan number: 13188

Gene Names	Charge	m/z	Mass	Retention time	PEP	Score	Precursor Intensity
WRNIP1	3	584.3076	1749.901	51.154	0.0024888	70.622	327443.6

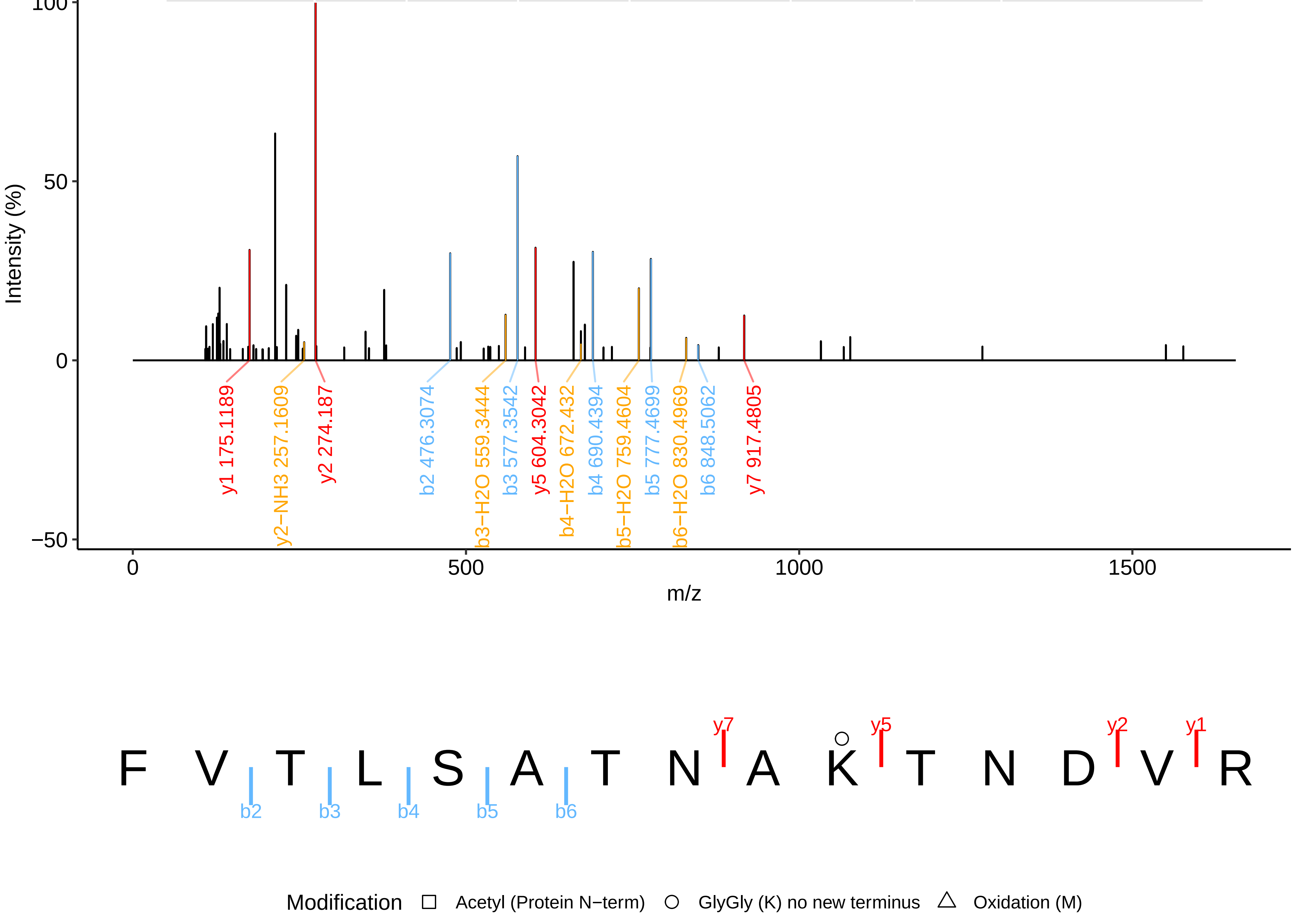

20200921_KIZ_MUb4_GlyGly_10plex_2 − Scan number: 13217

Gene Names	Charge	m/z	Mass	Retention time	PEP	Score	Precursor Intensity
WRNIP1	3	584.3076	1749.901	51.273	2.4474e−28	179.46	978318.2

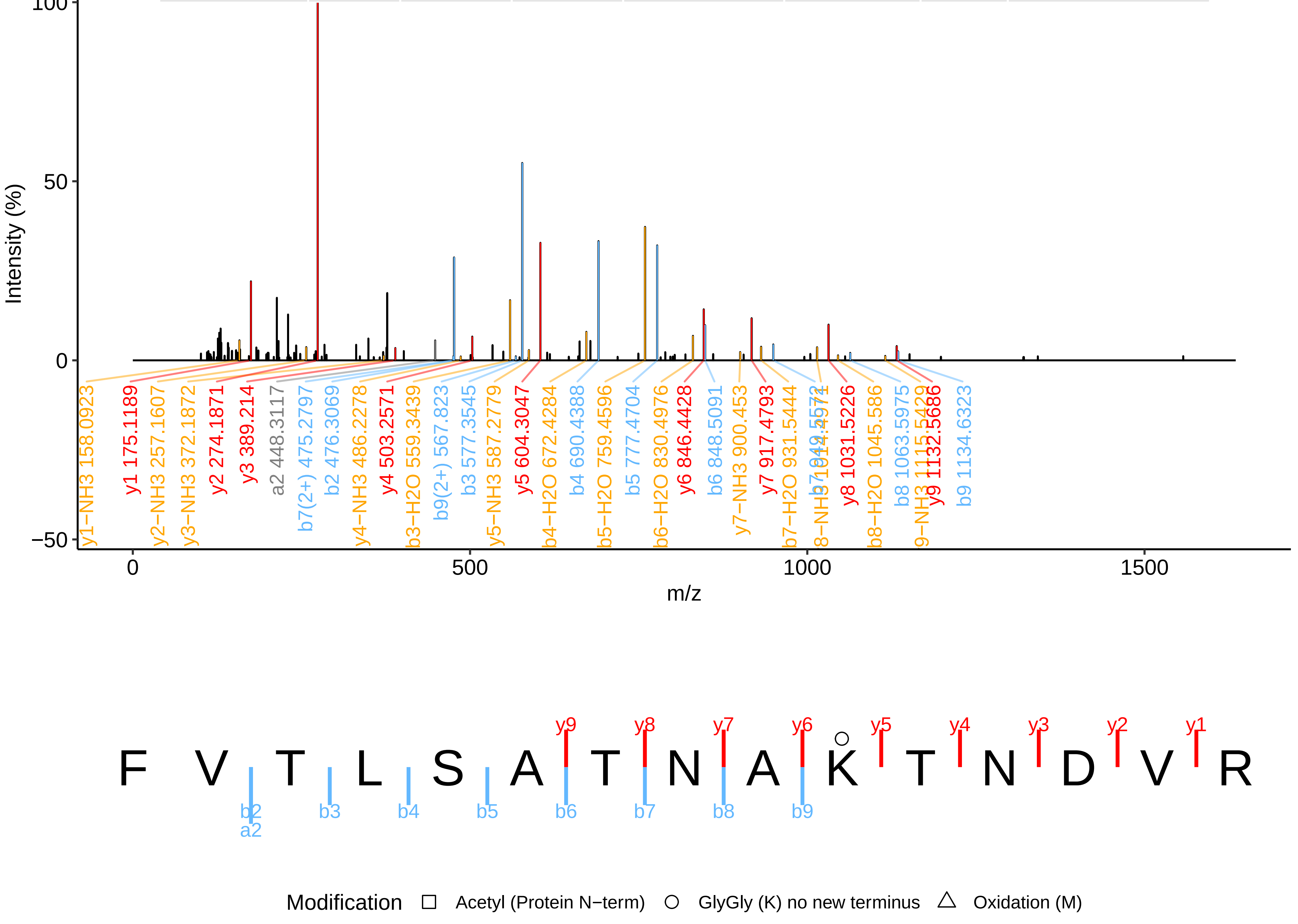

20200924_KIZ_MUb5_GlyGly_1 − Scan number: 13278

Gene Names	Charge	m/z	Mass	Retention time	PEP	Score	Precursor Intensity
WRNIP1	3	584.3076	1749.901	43.27	1.08e−38	200.9	12132073

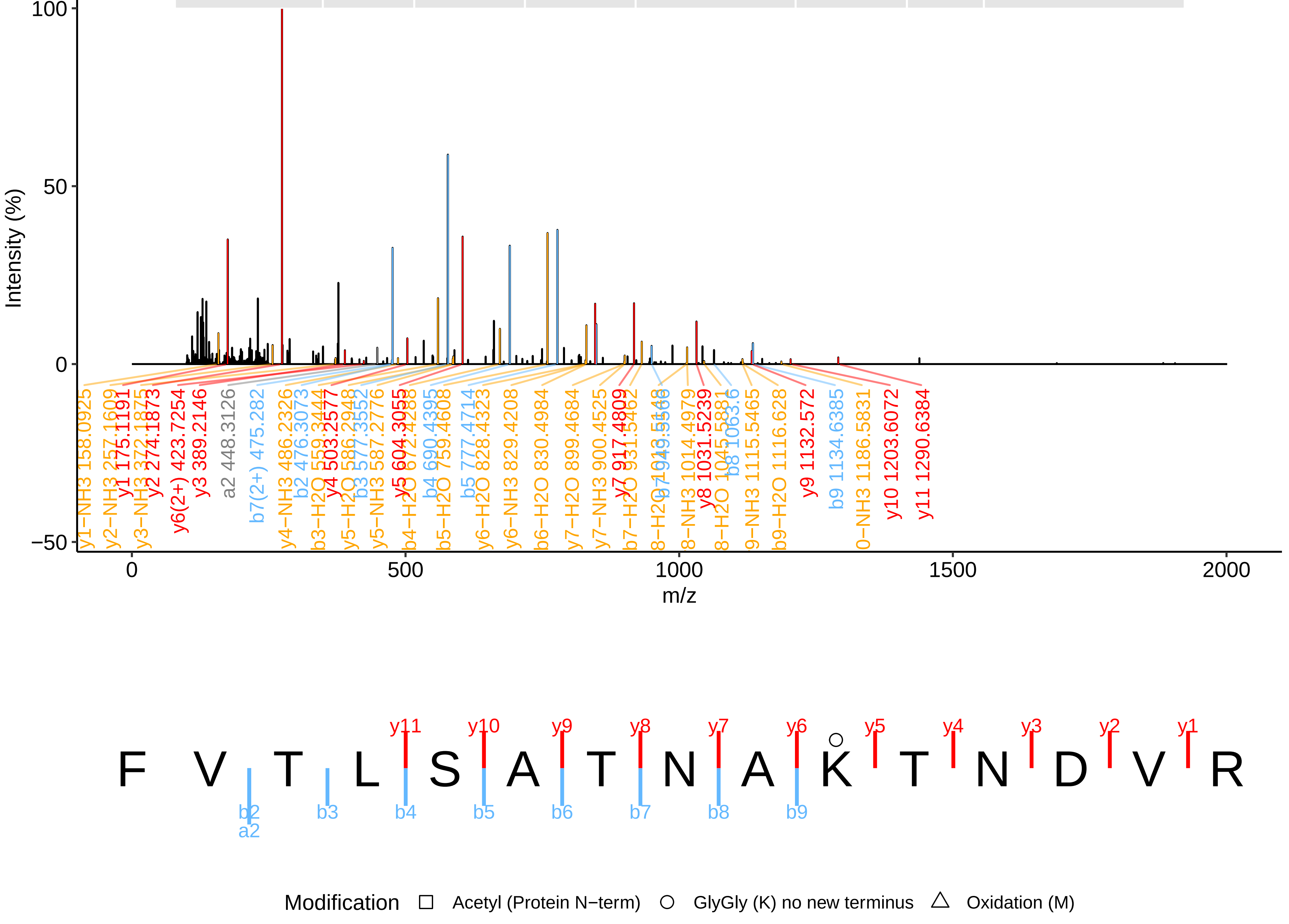

20200924_KIZ_MUb5_GlyGly_2 − Scan number: 12368

Gene Names	Charge	m/z	Mass	Retention time	PEP	Score	Precursor Intensity
WRNIP1	3	584.3076	1749.901	46.225	0.0020943	72.321	2270005

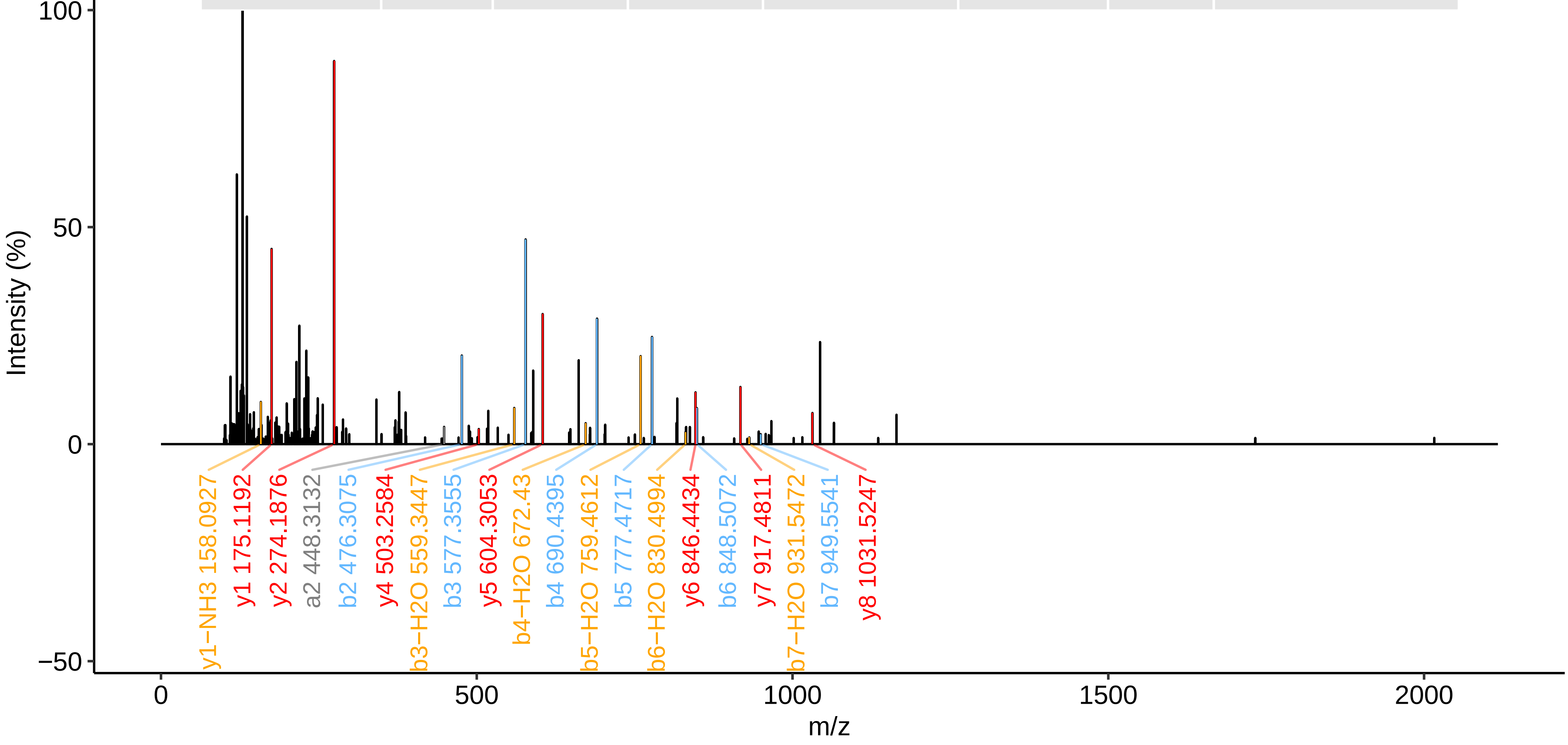

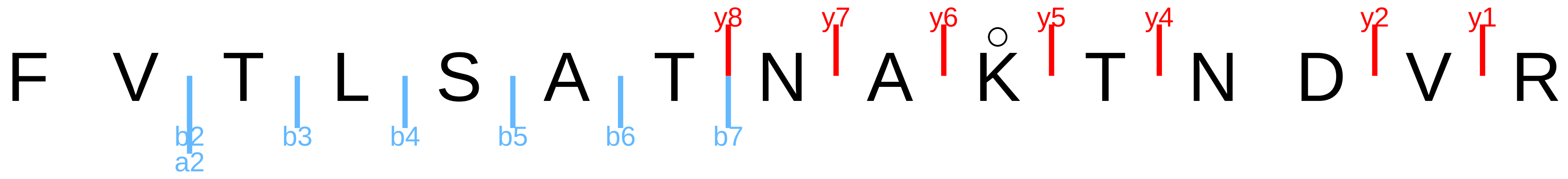

Modification □ Acetyl (Protein N−term) ○ GlyGly (K) no new terminus △ Oxidation (M)

20200921_KIZ_MUb4_GlyGly_10plex_2 − Scan number: 10595

Gene Names	Charge	m/z	Mass	Retention time	PEP	Score	Precursor Intensity
RPL23	3	487.2573	1458.75	40.566	0.0018936	77.527	1068484

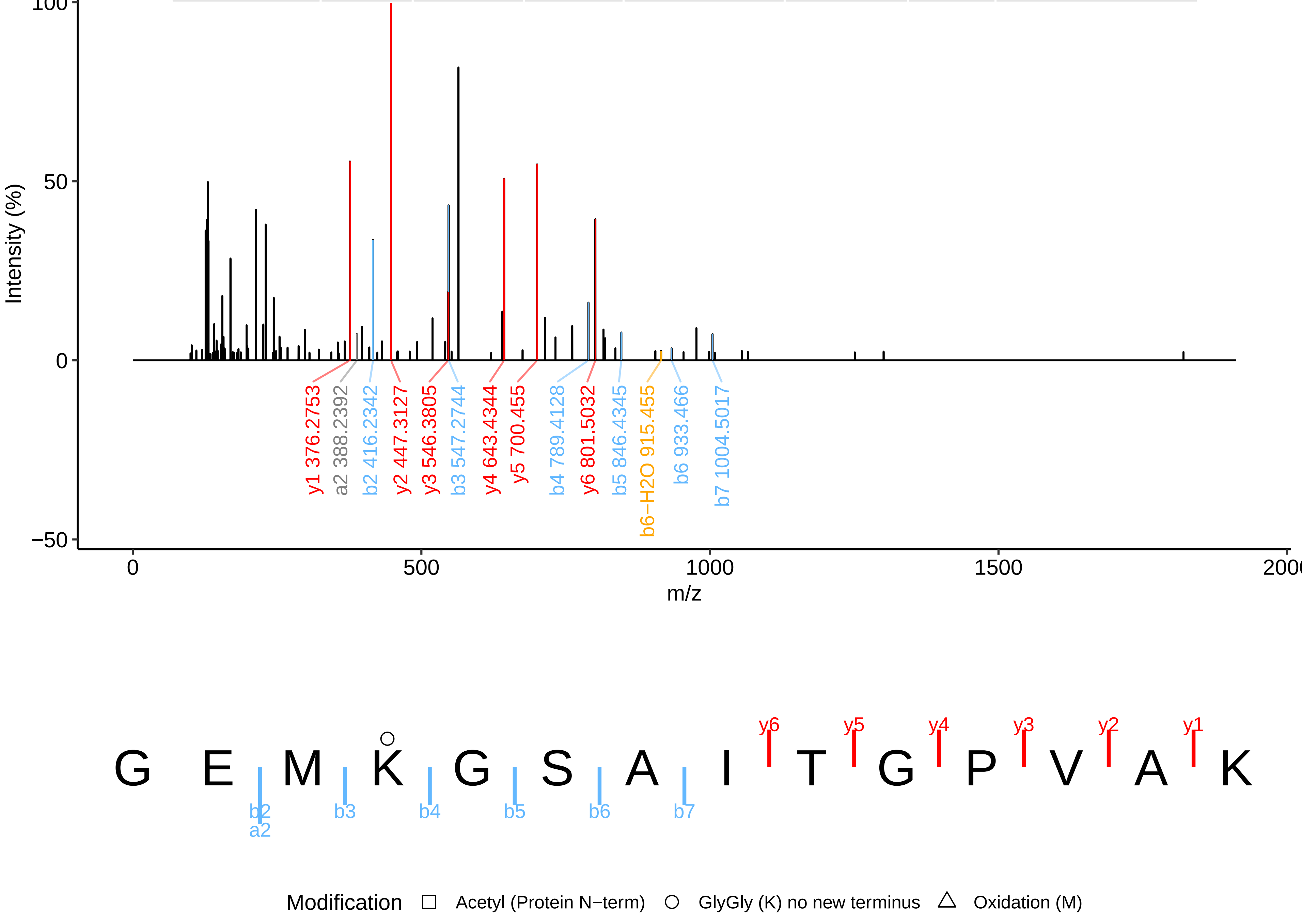

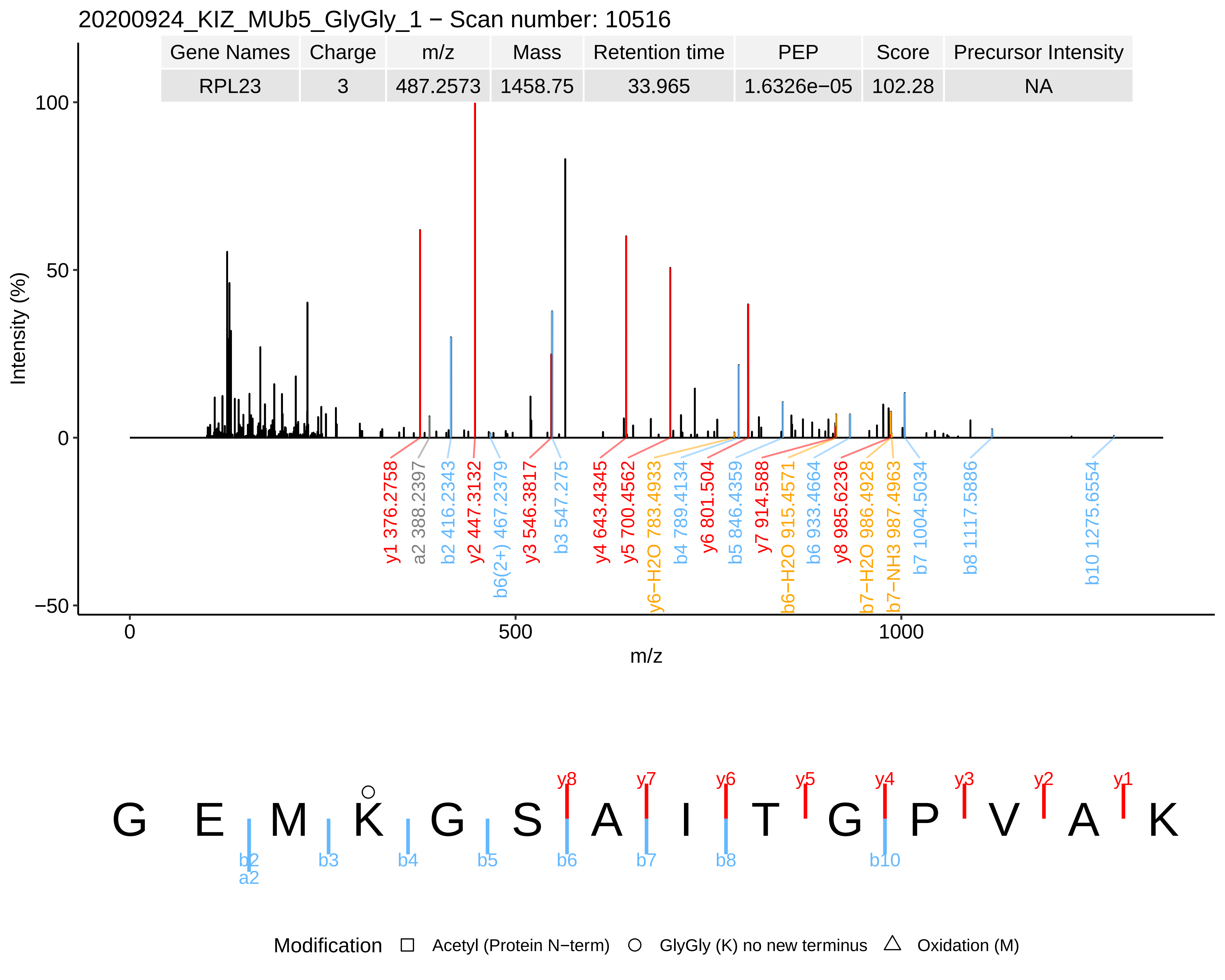
20200924_KIZ_MUb5_GlyGly_1 − Scan number: 10516
Gene Names | Charge | m/z | Mass | Retention time | PEP | Score | Precursor Intensity
RPL23 | 3 | 487.2573 | 1458.75 | 33.965 | 1.6326e−05 | 102.28 | NA
Intensity (%)
100
50
0
−50
0
500
1000
m/z
y1 376.2758
a2 388.2397
b2 416.2343
y2 447.3132
b6(2+) 467.2379
y3 546.3817
b3 547.275
y4 643.4345
y5 700.4562
y6−H2O 783.4933
b4 789.4134
y6 801.504
b5 846.4359
y7 914.588
b6−H2O 915.4571
b6 933.4664
y8 985.6236
b7−H2O 986.4928
b7−NH3 987.4963
b7 1004.5034
b8 1117.5886
b10 1275.6554
G E M K G S A I T G P V A K
y8 y7 y6 y5 y4 y3 y2 y1
b2 a2 b3 b4 b5 b6 b7 b8 b10
Modification
Acetyl (Protein N−term)
GlyGly (K) no new terminus
Oxidation (M)

20200924_KIZ_MUb5_GlyGly_2 − Scan number: 9780

Gene Names	Charge	m/z	Mass	Retention time	PEP	Score	Precursor Intensity
RPL23	3	487.2573	1458.75	36.551	0.00028465	93.494	2101792

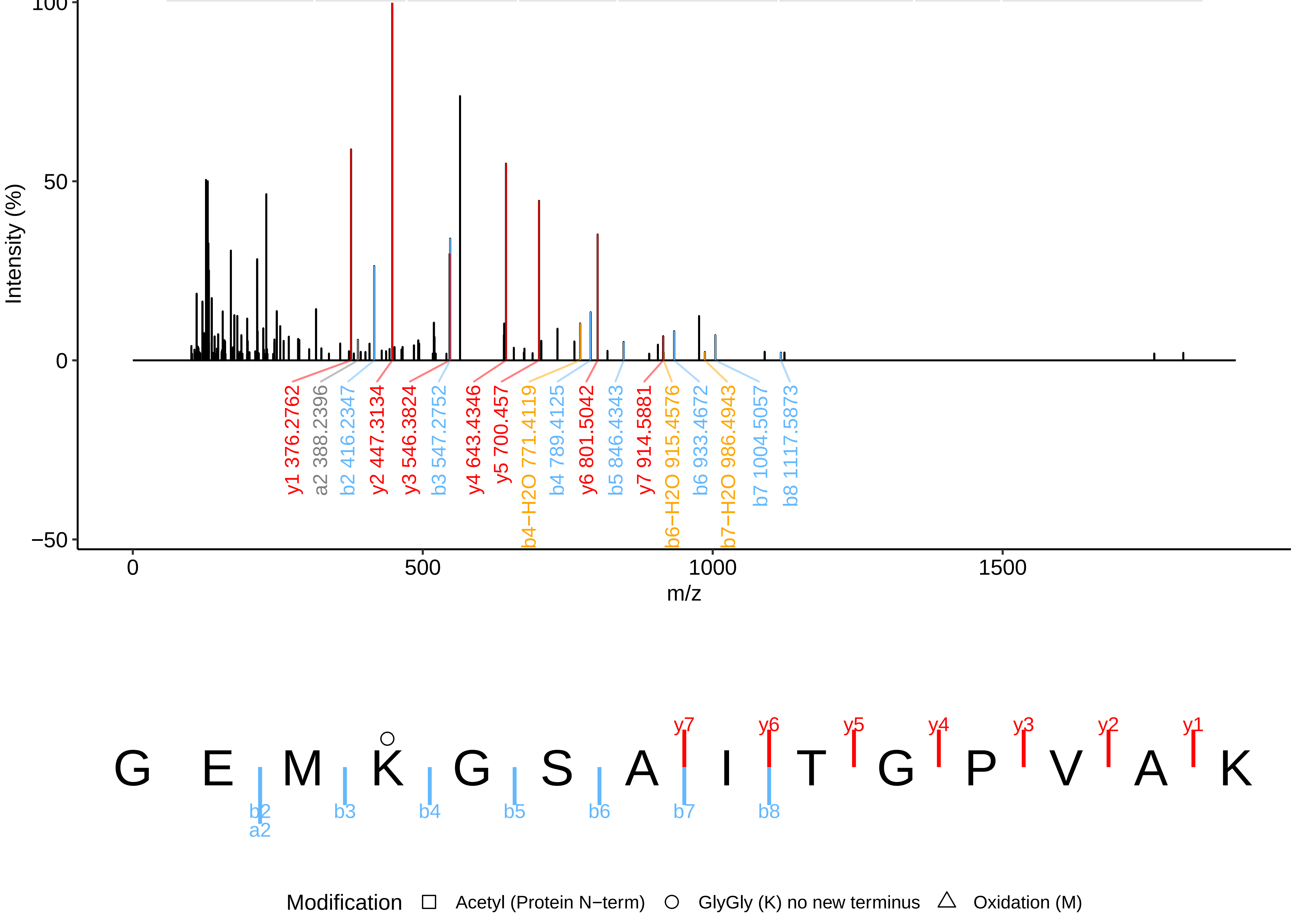

20200921_KIZ_MUb4_GlyGly_10plex − Scan number: 13290

Gene Names	Charge	m/z	Mass	Retention time	PEP	Score	Precursor Intensity
CCT7	3	501.6106	1501.81	46.84	1.9383e−08	115.82	9289749

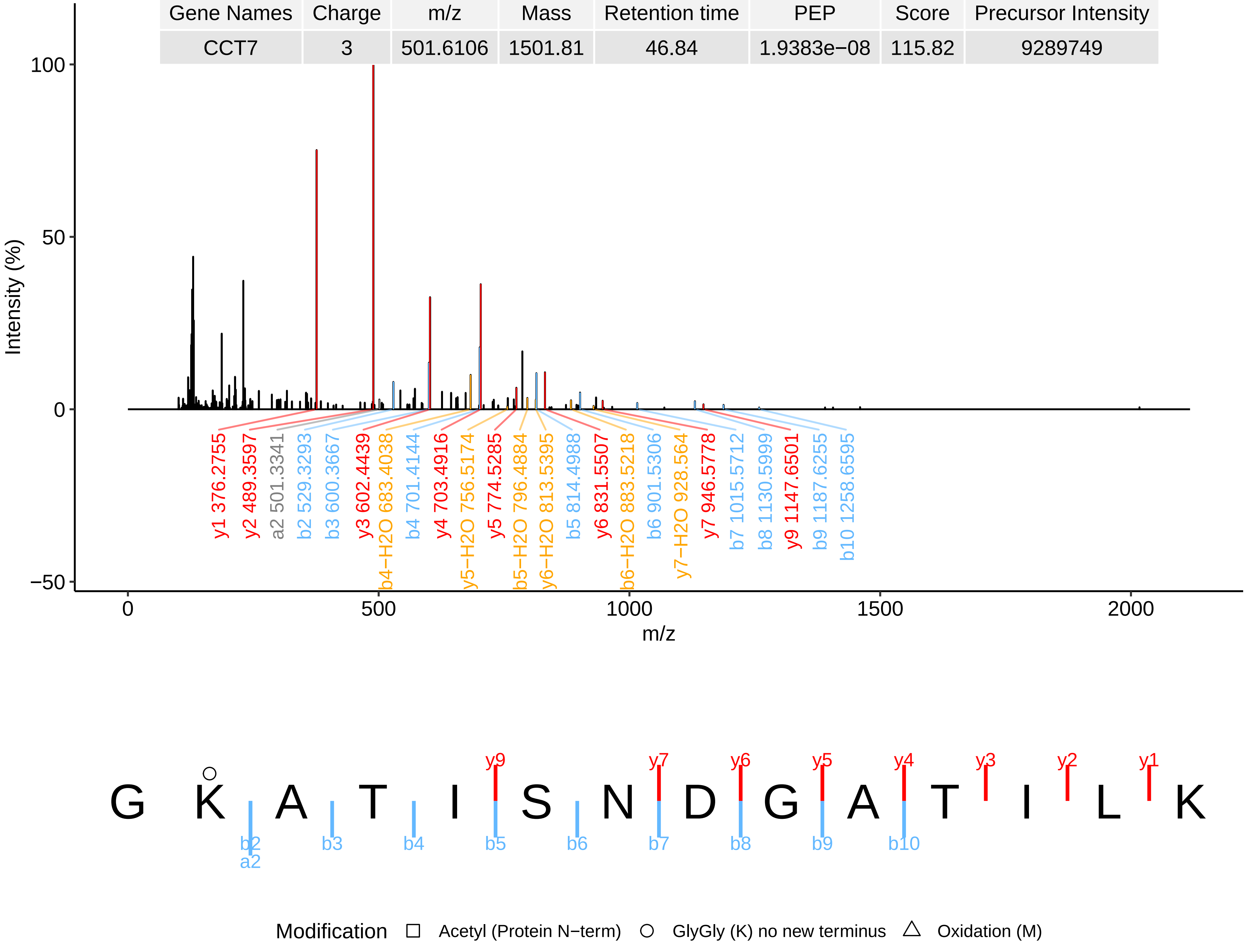

20200921_KIZ_MUb4_GlyGly_10plex_2 − Scan number: 12204

Gene Names	Charge	m/z	Mass	Retention time	PEP	Score	Precursor Intensity
LYN	3	488.2389	1461.695	47.167	0.023448	60.401	735756.1

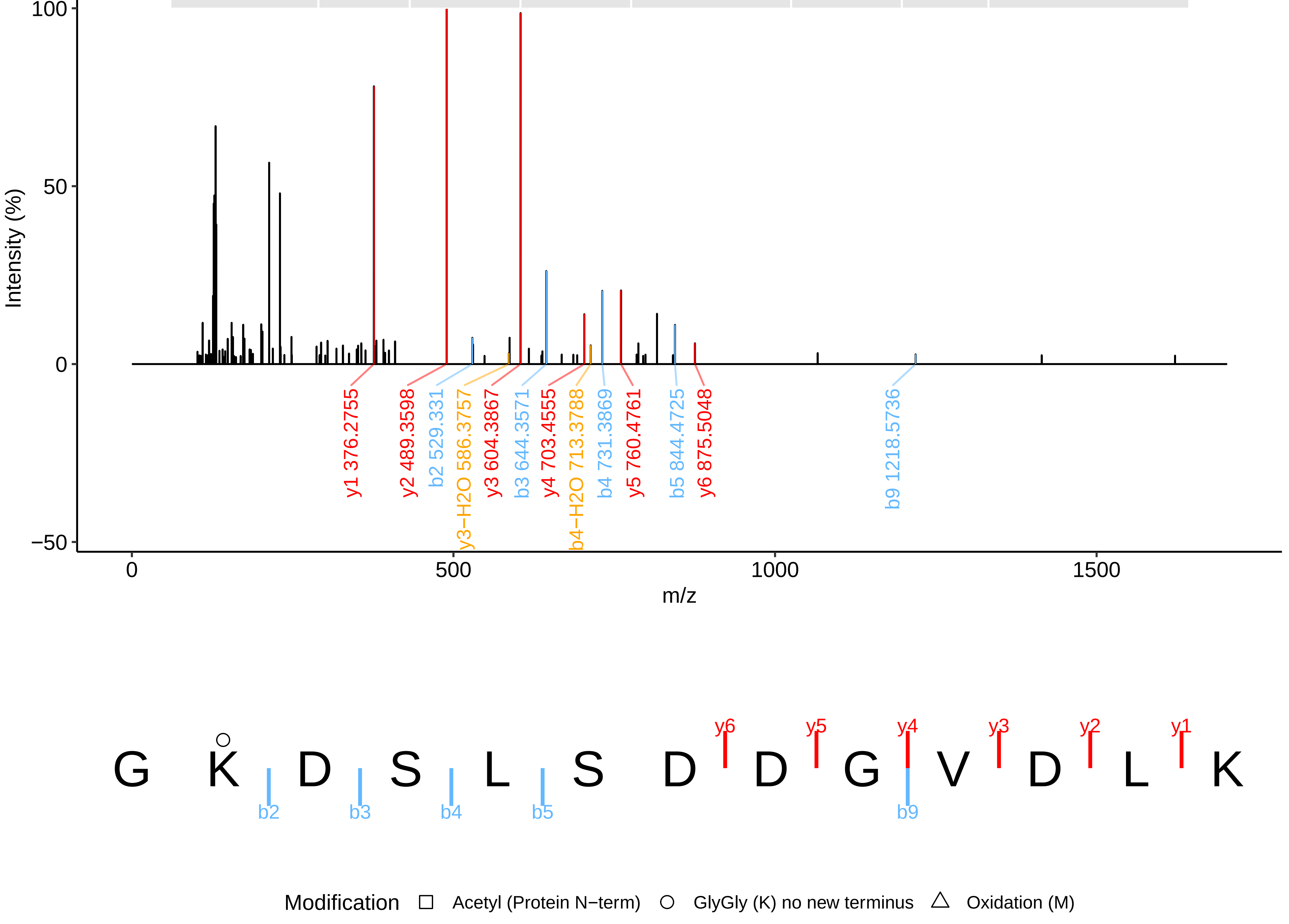

20200924_KIZ_MUb5_GlyGly_1 − Scan number: 12341

Gene Names	Charge	m/z	Mass	Retention time	PEP	Score	Precursor Intensity
LYN	3	488.2389	1461.695	40.144	0.00031814	93.478	8781688

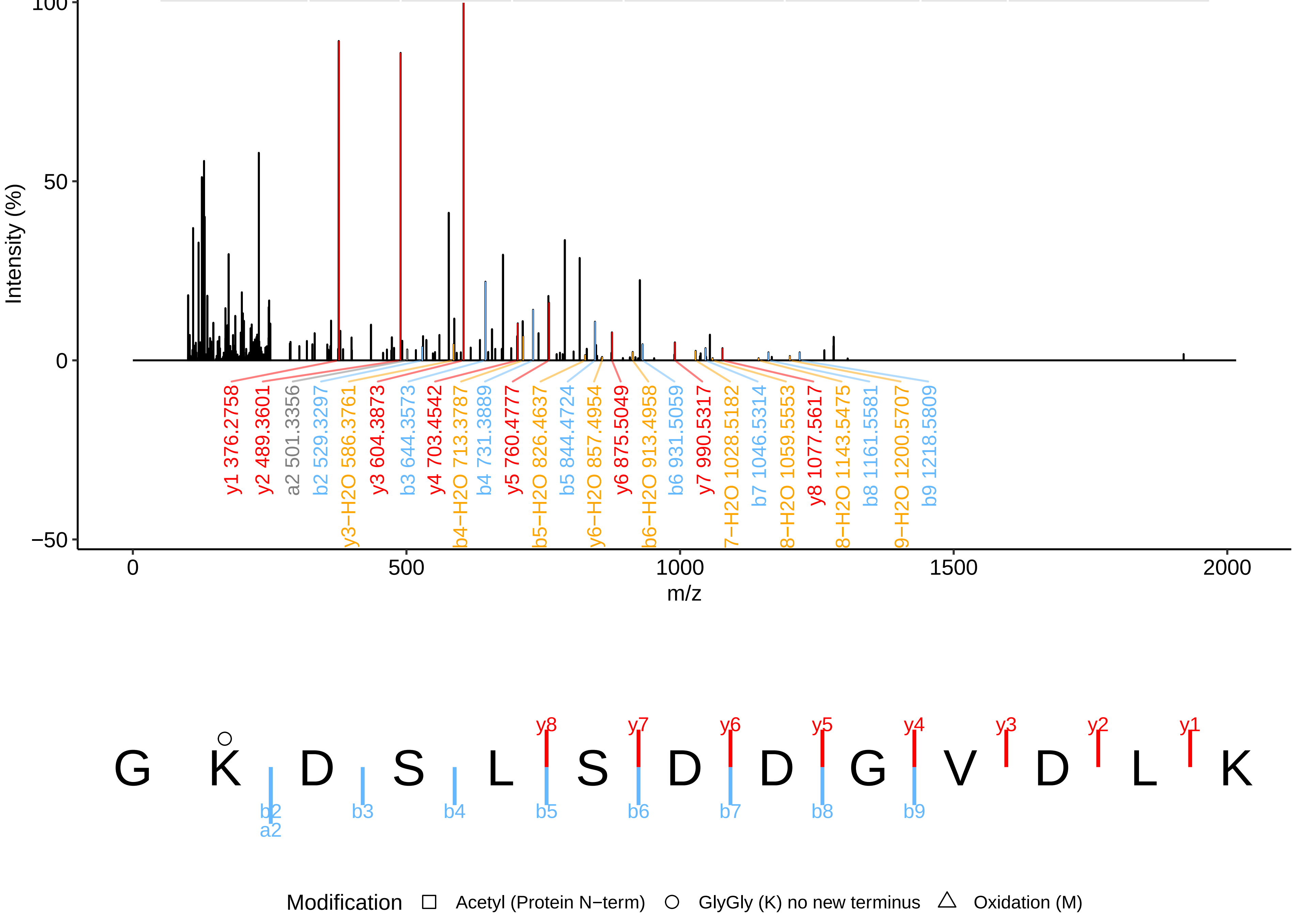

20200924_KIZ_MUb5_GlyGly_2 − Scan number: 11537

Gene Names	Charge	m/z	Mass	Retention time	PEP	Score	Precursor Intensity
LYN	3	488.2389	1461.695	43.138	0.0042605	75.764	1610556

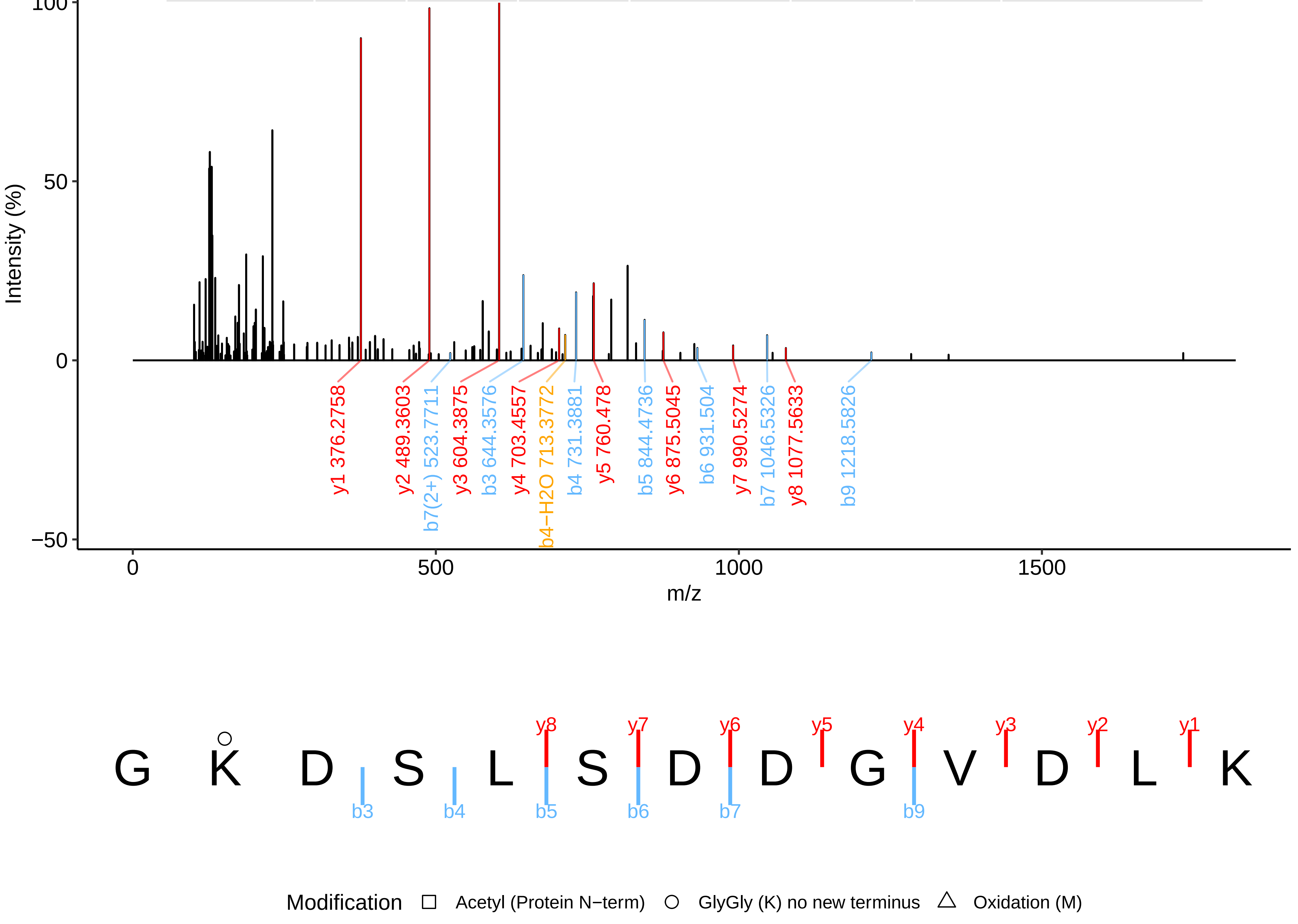

20200921_KIZ_MUb4_GlyGly_10plex − Scan number: 13744

Gene Names	Charge	m/z	Mass	Retention time	PEP	Score	Precursor Intensity
SPG20	3	488.2719	1461.794	48.593	1.0353e−05	107.09	3993815

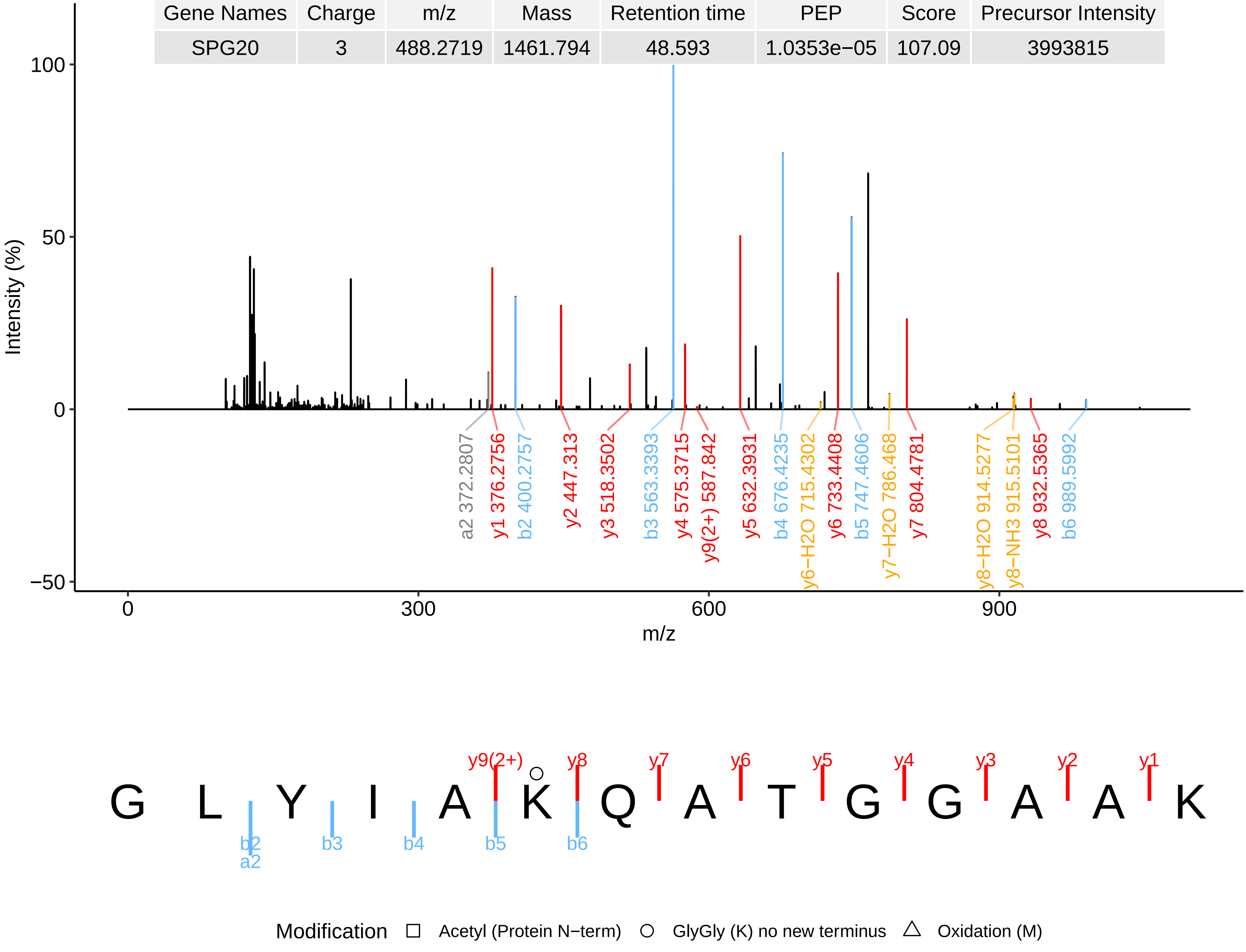

20200921_KIZ_MUb4_GlyGly_10plex_2 − Scan number: 12678

Gene Names	Charge	m/z	Mass	Retention time	PEP	Score	Precursor Intensity
SPG20	3	488.2719	1461.794	49.103	0.0015968	78.324	949396.2

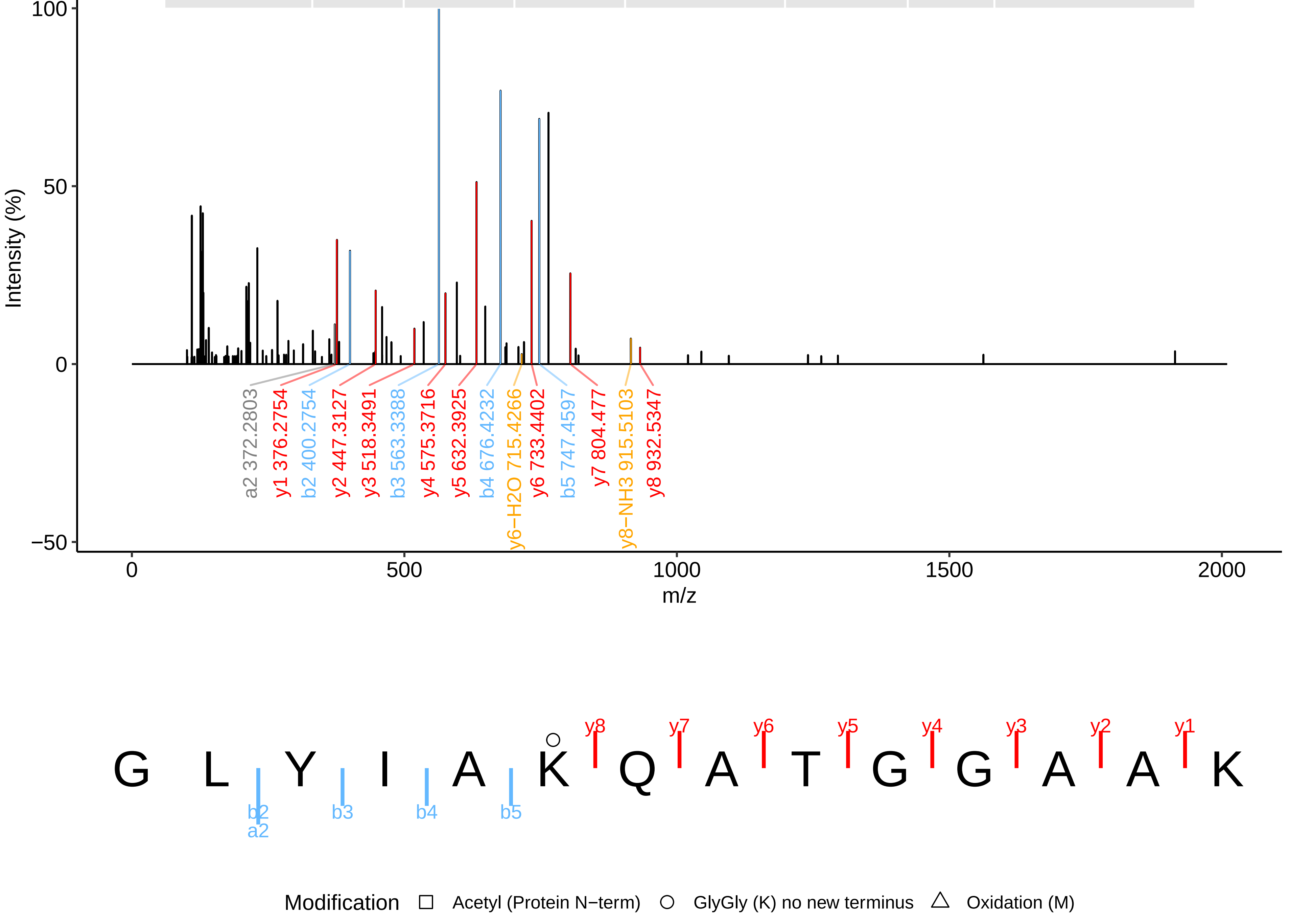

20200921_KIZ_MUb4_GlyGly_10plex − Scan number: 16051

Gene Names	Charge	m/z	Mass	Retention time	PEP	Score	Precursor Intensity
GPRC5C	3	493.5998	1477.778	57.555	8.2877e−10	131.82	1854648

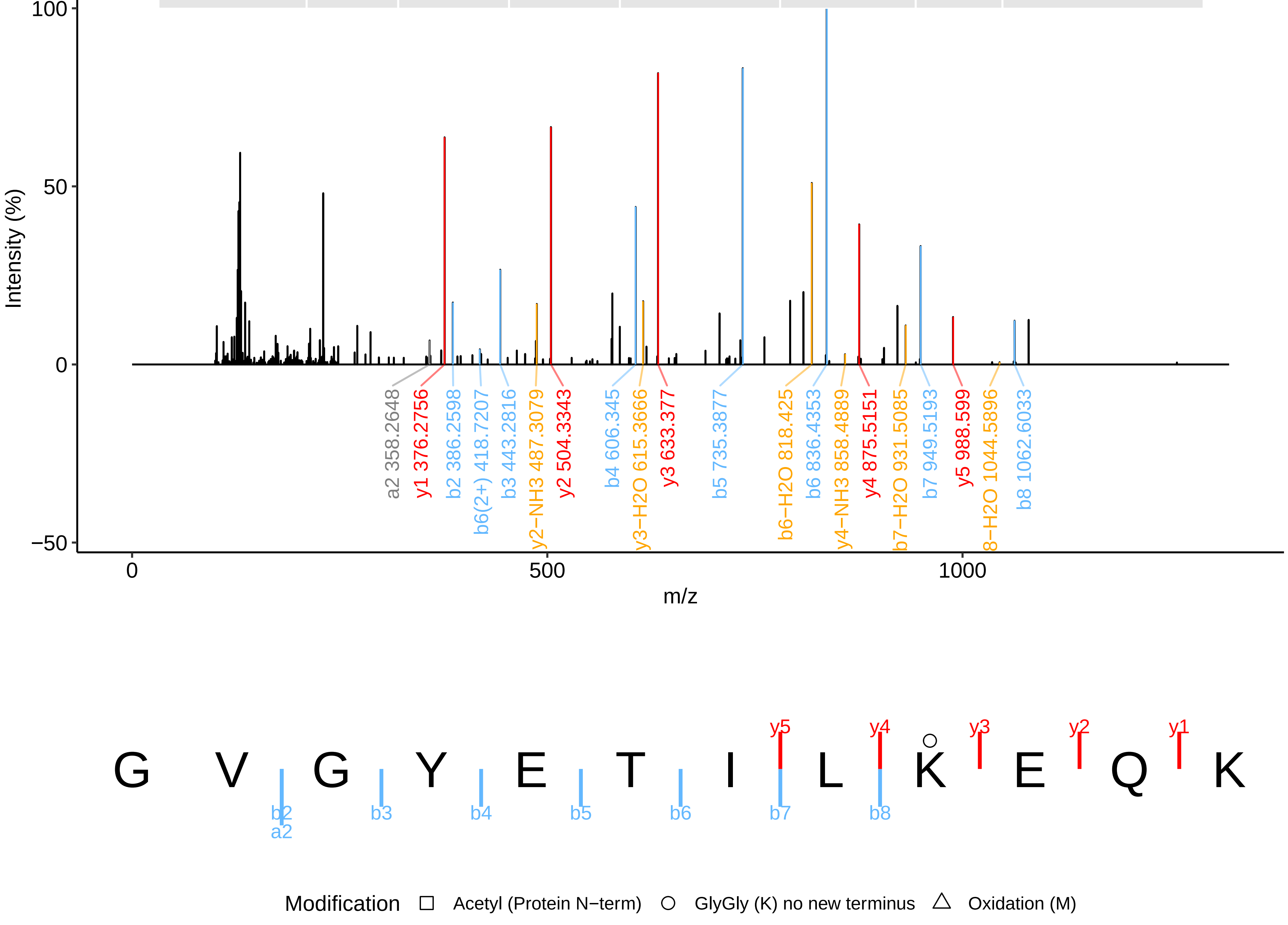

20200921_KIZ_MUb4_GlyGly_10plex_2 − Scan number: 14910

Gene Names	Charge	m/z	Mass	Retention time	PEP	Score	Precursor Intensity
GPRC5C	3	493.5998	1477.778	58.204	4.4474e−09	127.42	1392841

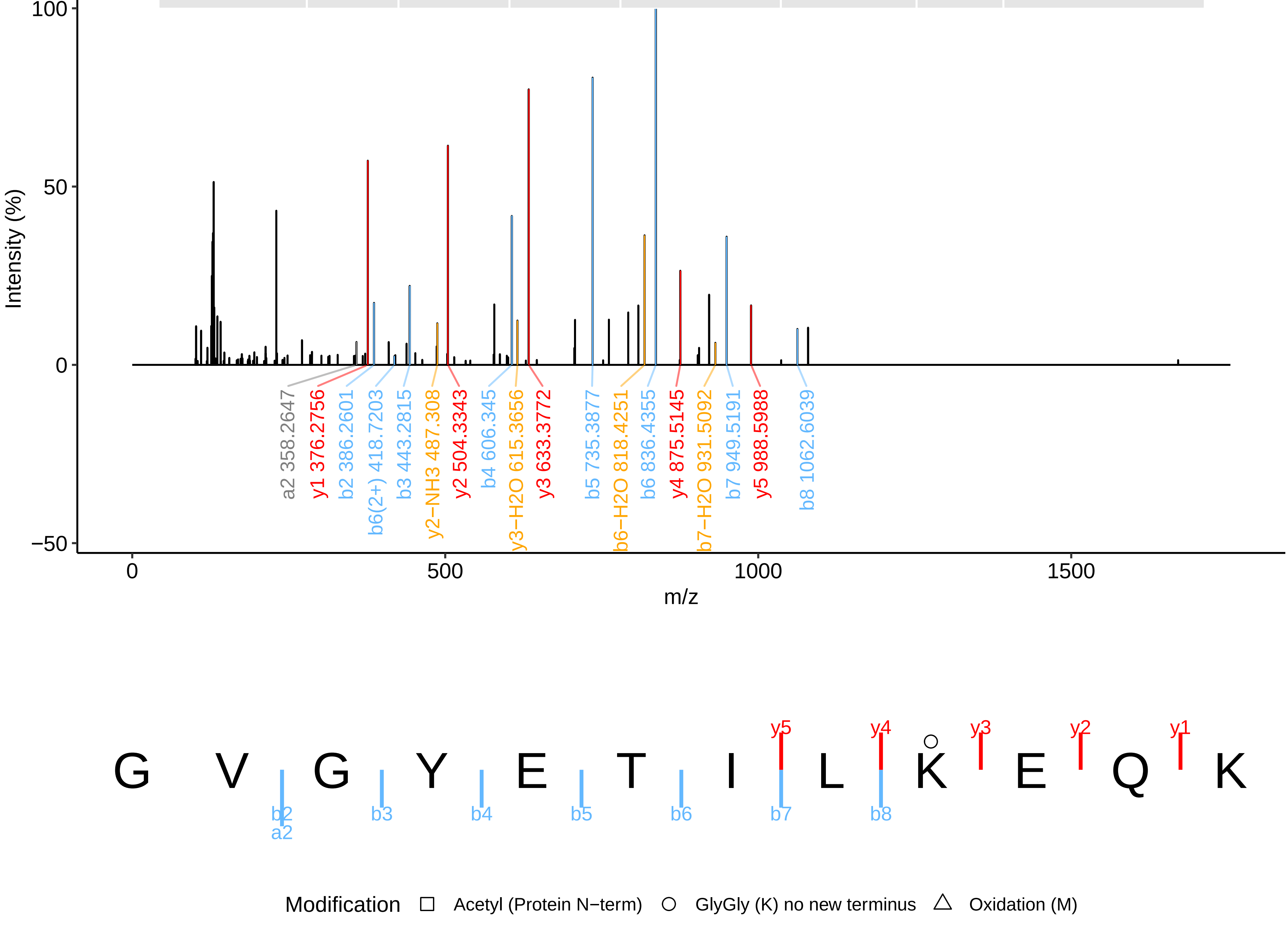

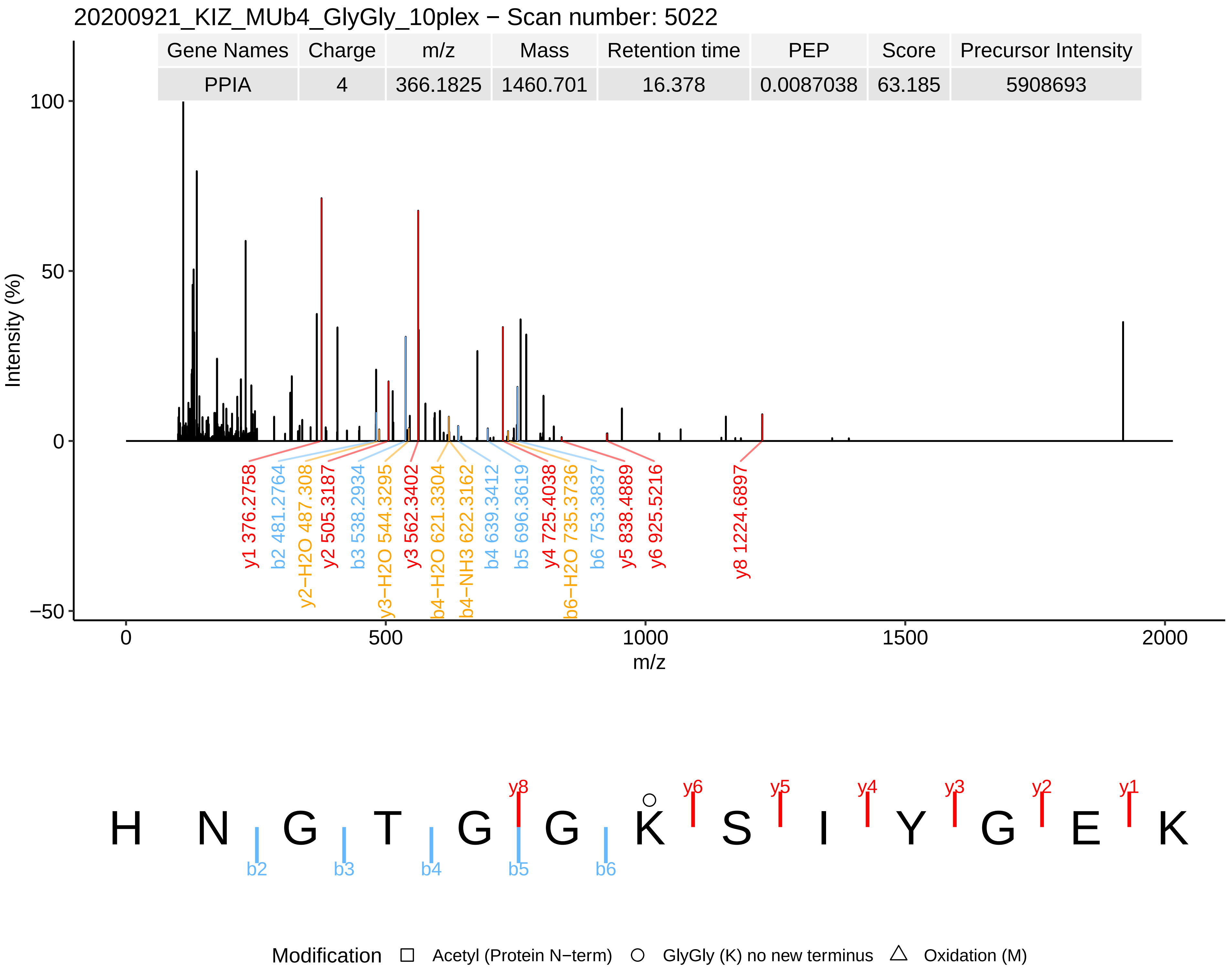
20200921_KIZ_MUb4_GlyGly_10plex − Scan number: 5022
Gene Names | Charge | m/z | Mass | Retention time | PEP | Score | Precursor Intensity
PPIA | 4 | 366.1825 | 1460.701 | 16.378 | 0.0087038 | 63.185 | 5908693
Intensity (%)
100
50
0
−50
0
500
1000
1500
2000
m/z
y1 376.2758
b2 481.2764
y2−H2O 487.308
y2 505.3187
b3 538.2934
y3−H2O 544.3295
y3 562.3402
b4−H2O 621.3304
b4−NH3 622.3162
b4 639.3412
b5 696.3619
y4 725.4038
b6−H2O 735.3736
b6 753.3837
y5 838.4889
y6 925.5216
y8 1224.6897
H N G T G G K S I Y G E K
y8 y6 y5 y4 y3 y2 y1
b2 b3 b4 b5 b6
Modification
Acetyl (Protein N−term)
GlyGly (K) no new terminus
Oxidation (M)

20200921_KIZ_MUb4_GlyGly_10plex − Scan number: 26526

Gene Names	Charge	m/z	Mass	Retention time	PEP	Score	Precursor Intensity
PTBP1;PTBP3	3	493.612	1477.814	96.659	0.011793	69.815	3109875

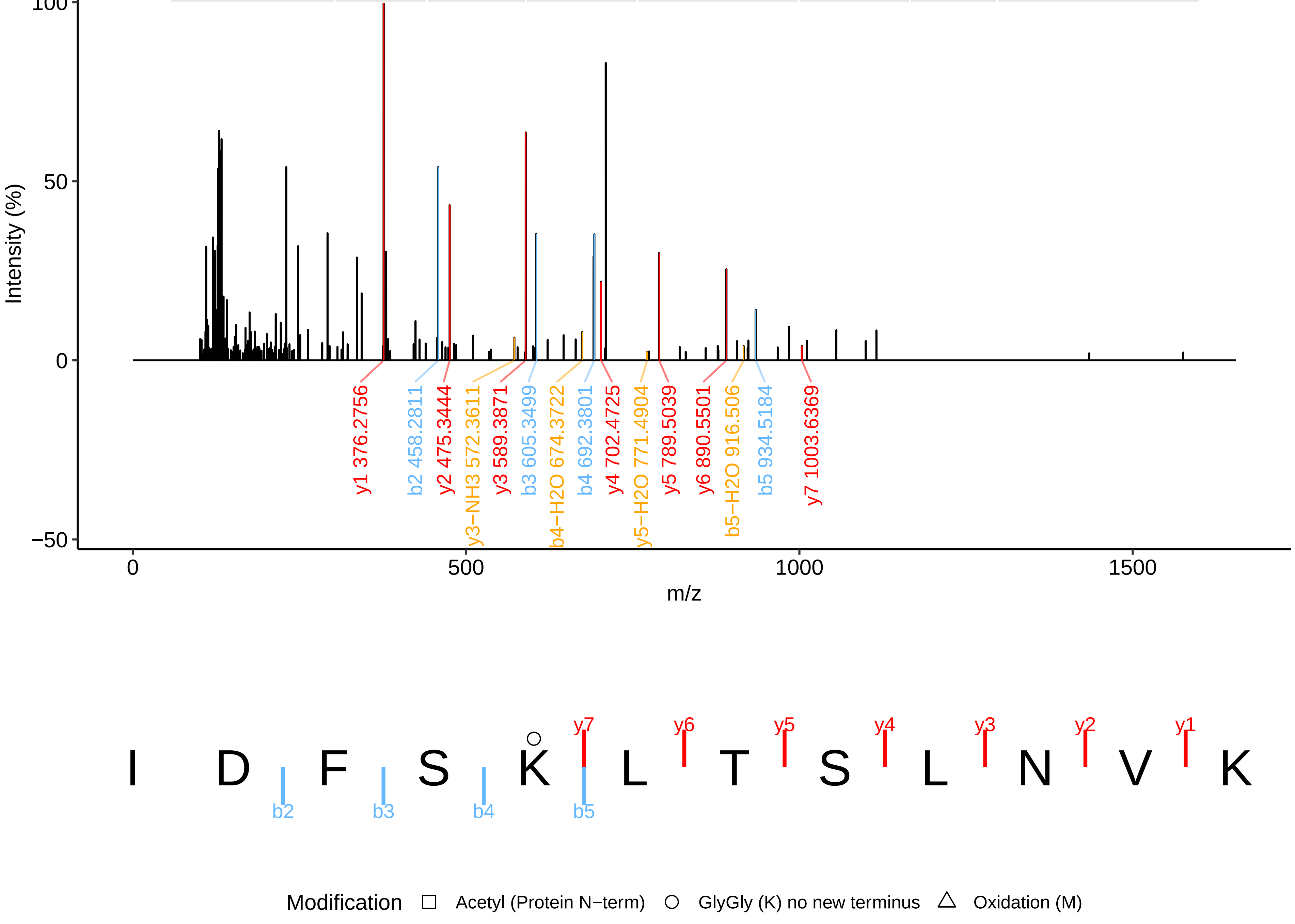

20200921_KIZ_MUb4_GlyGly_10plex_2 − Scan number: 24969

Gene Names	Charge	m/z	Mass	Retention time	PEP	Score	Precursor Intensity
POLD1	3	581.6719	1741.994	98.708	0.00012052	95.651	464565.5

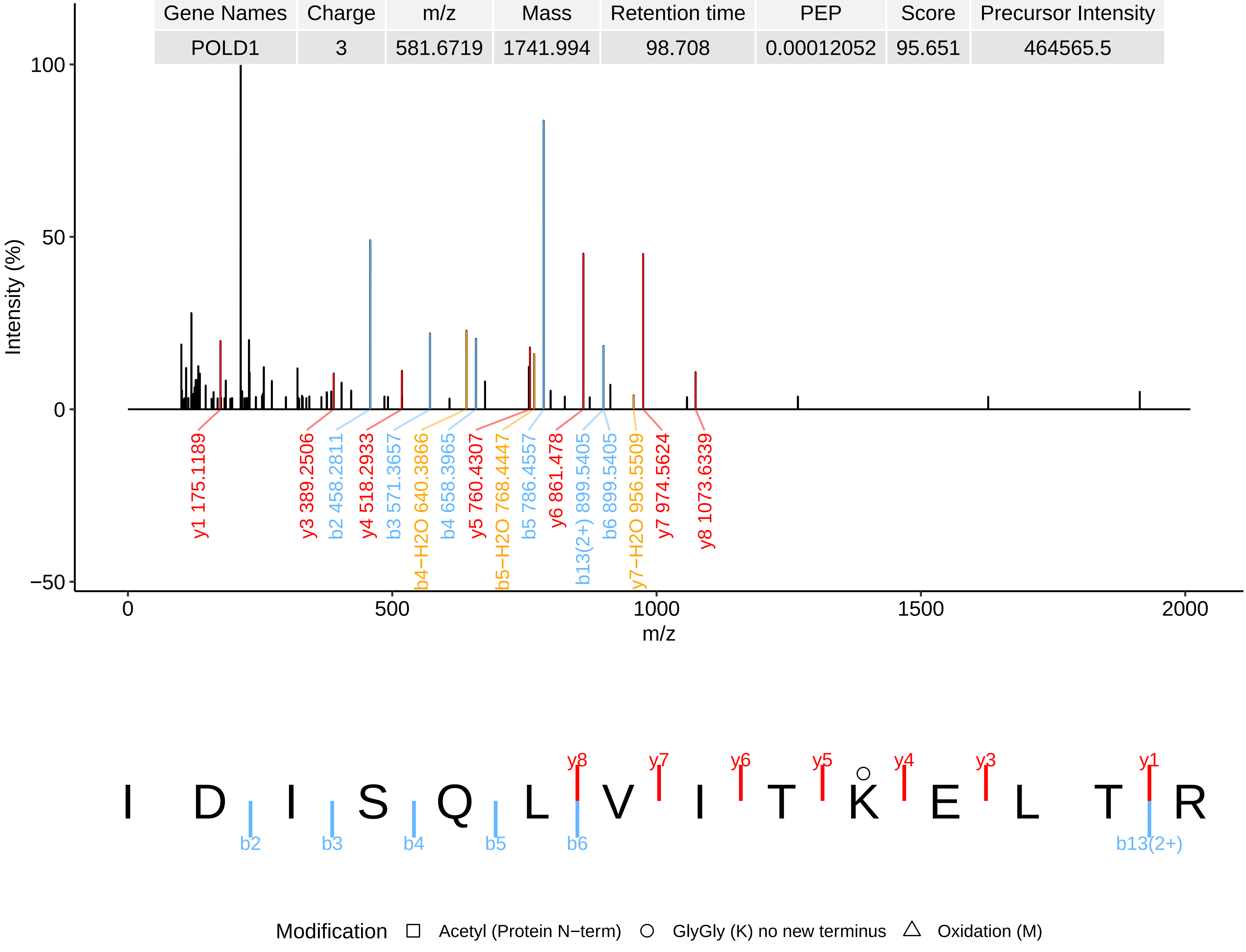

20200921_KIZ_MUb4_GlyGly_10plex − Scan number: 14883

Gene Names	Charge	m/z	Mass	Retention time	PEP	Score	Precursor Intensity
SSBP1	3	577.5983	1729.773	53.009	2.8802e−09	129.34	6283413

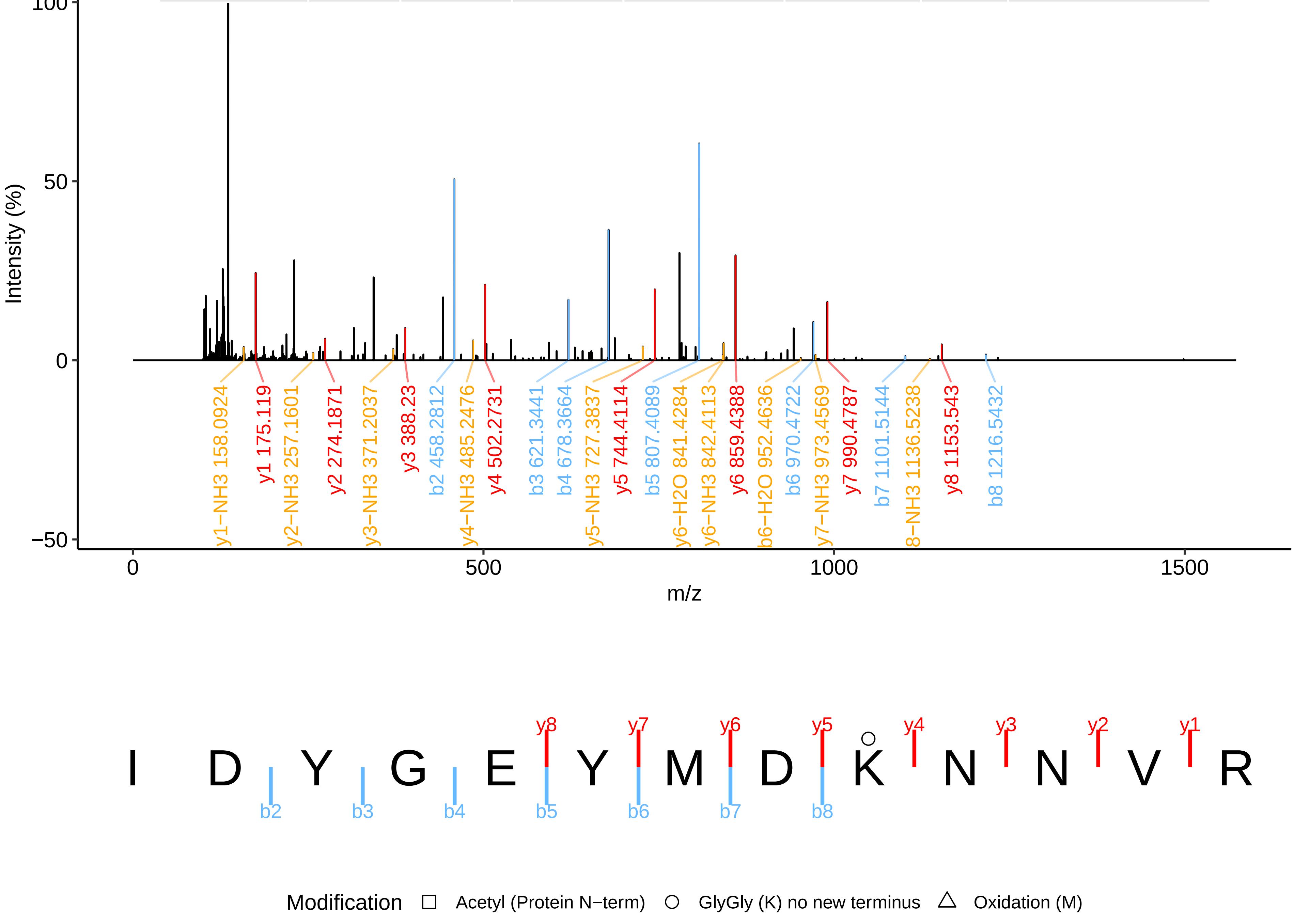

20200921_KIZ_MUb4_GlyGly_10plex_2 − Scan number: 13708

Gene Names	Charge	m/z	Mass	Retention time	PEP	Score	Precursor Intensity
SSBP1	3	577.5983	1729.773	53.277	8.6844e−09	122.33	954356

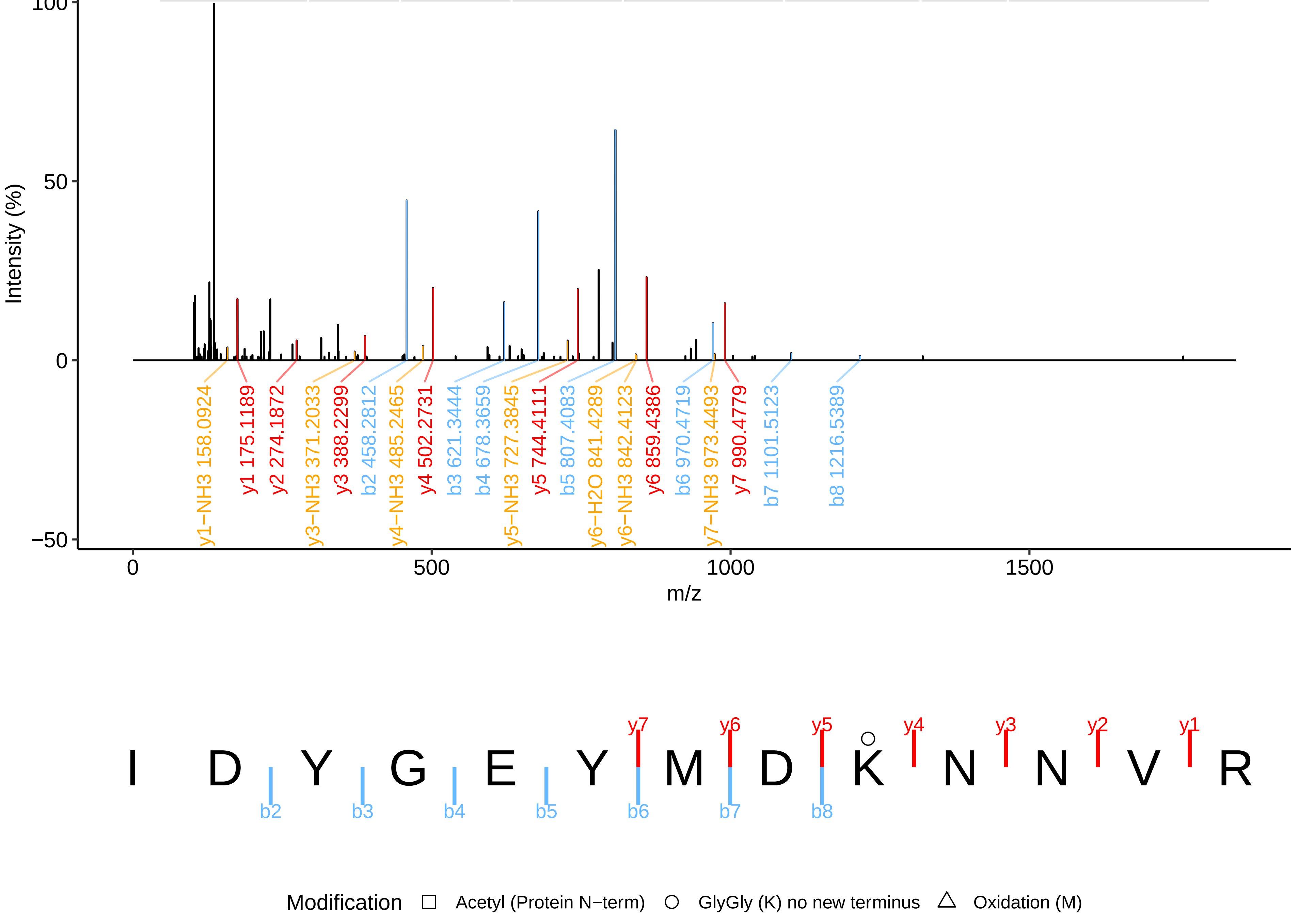

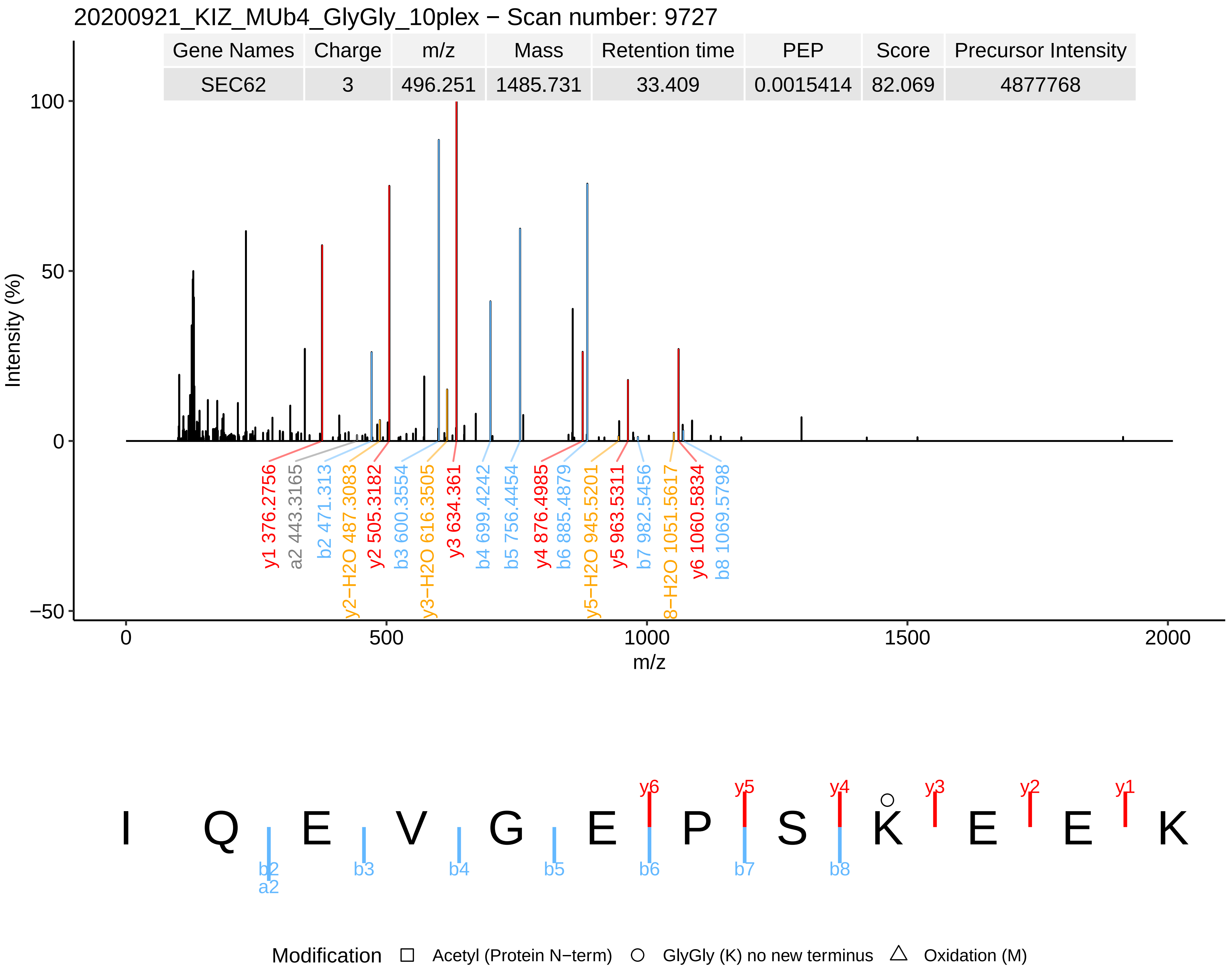
20200921_KIZ_MUb4_GlyGly_10plex − Scan number: 9727
Gene Names
Charge
m/z
Mass
Retention time
PEP
Score
Precursor Intensity
SEC62
3
496.251
1485.731
33.409
0.0015414
82.069
4877768
Intensity (%)
m/z
y1 376.2756
a2 443.3165
b2 471.313
y2−H2O 487.3083
y2 505.3182
b3 600.3554
y3−H2O 616.3505
y3 634.361
b4 699.4242
b5 756.4454
y4 876.4985
b6 885.4879
y5−H2O 945.5201
y5 963.5311
b7 982.5456
8−H2O 1051.5617
y6 1060.5834
b8 1069.5798
I Q E V G E P S K E E K
y6 y5 y4 y3 y2 y1
b2 a2 b3 b4 b5 b6 b7 b8
Modification
Acetyl (Protein N−term)
GlyGly (K) no new terminus
Oxidation (M)

20200921_KIZ_MUb4_GlyGly_10plex_2 − Scan number: 8948

Gene Names	Charge	m/z	Mass	Retention time	PEP	Score	Precursor Intensity
SEC62	3	496.251	1485.731	33.878	0.018229	62.14	1132912

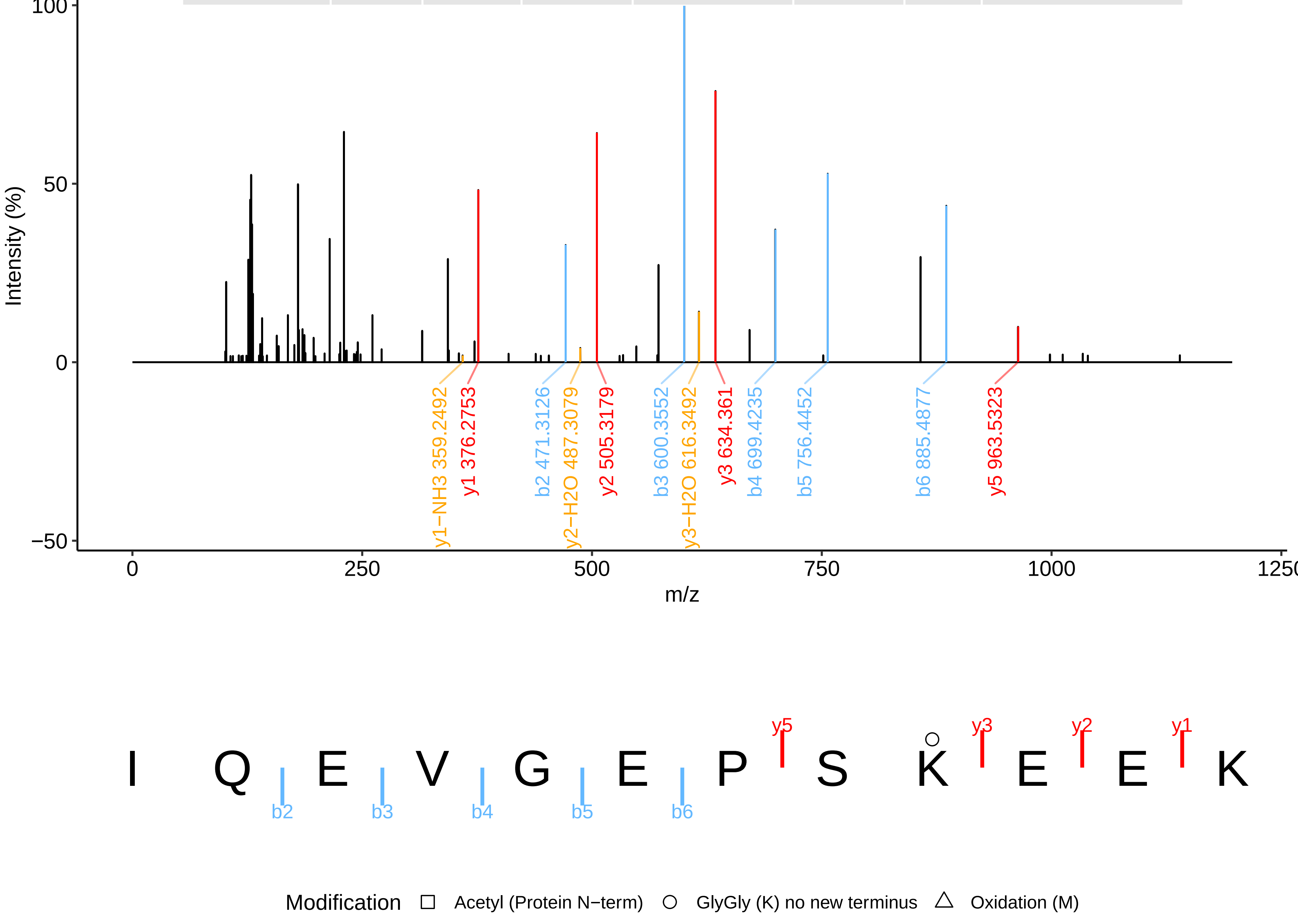

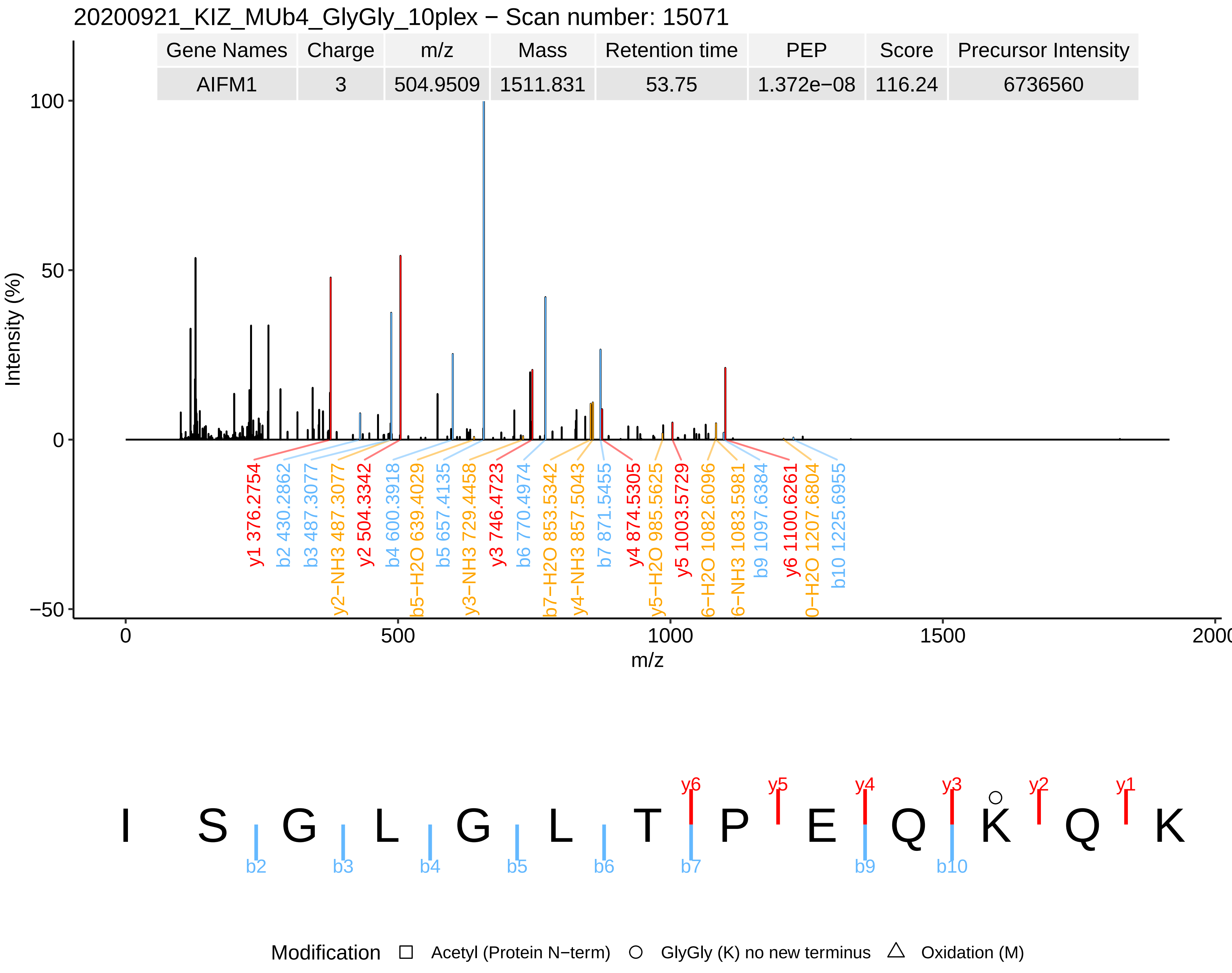
20200921_KIZ_MUb4_GlyGly_10plex − Scan number: 15071
Gene Names | Charge | m/z | Mass | Retention time | PEP | Score | Precursor Intensity
AIFM1 | 3 | 504.9509 | 1511.831 | 53.75 | 1.372e−08 | 116.24 | 6736560
Intensity (%)
m/z
y1 376.2754
b2 430.2862
b3 487.3077
y2−NH3 487.3077
y2 504.3342
b4 600.3918
b5−H2O 639.4029
b5 657.4135
y3−NH3 729.4458
y3 746.4723
b6 770.4974
b7−H2O 853.5342
y4−NH3 857.5043
b7 871.5455
y4 874.5305
y5−H2O 985.5625
y5 1003.5729
6−H2O 1082.6096
6−NH3 1083.5981
b9 1097.6384
y6 1100.6261
0−H2O 1207.6804
b10 1225.6955
I S G L G L T P E Q K Q K
y6 y5 y4 y3 y2 y1
b2 b3 b4 b5 b6 b7 b9 b10
Modification
Acetyl (Protein N−term)
GlyGly (K) no new terminus
Oxidation (M)

20200921_KIZ_MUb4_GlyGly_10plex_2 − Scan number: 13916

Gene Names	Charge	m/z	Mass	Retention time	PEP	Score	Precursor Intensity
AIFM1	3	504.9509	1511.831	54.135	1.2897e−08	117.23	908492.2

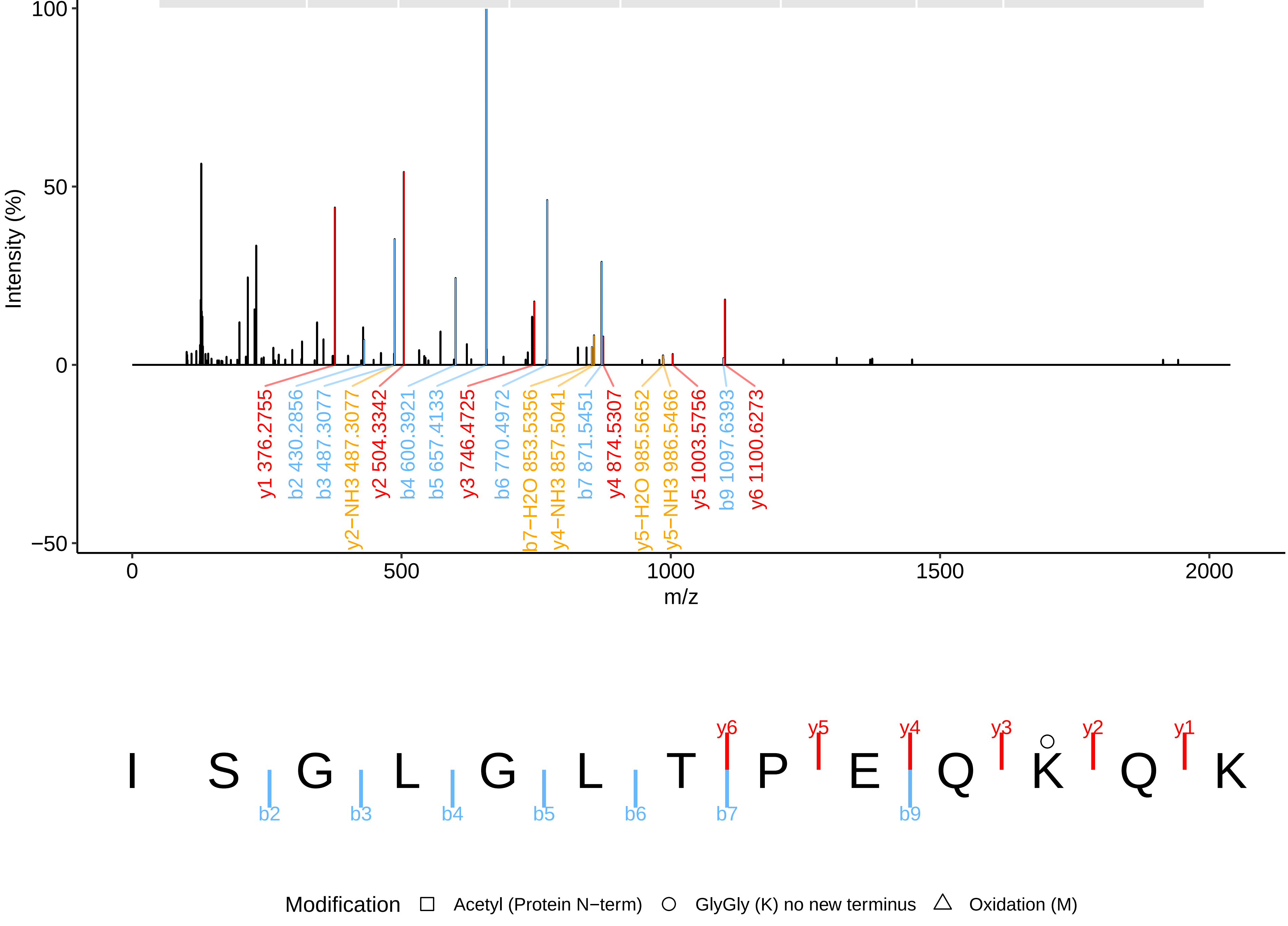

20200921_KIZ_MUb4_GlyGly_10plex − Scan number: 11764

Gene Names	Charge	m/z	Mass	Retention time	PEP	Score	Precursor Intensity
RPS10;RPS10P5	3	561.941	1682.801	41.083	6.6167e−13	135.72	6671244

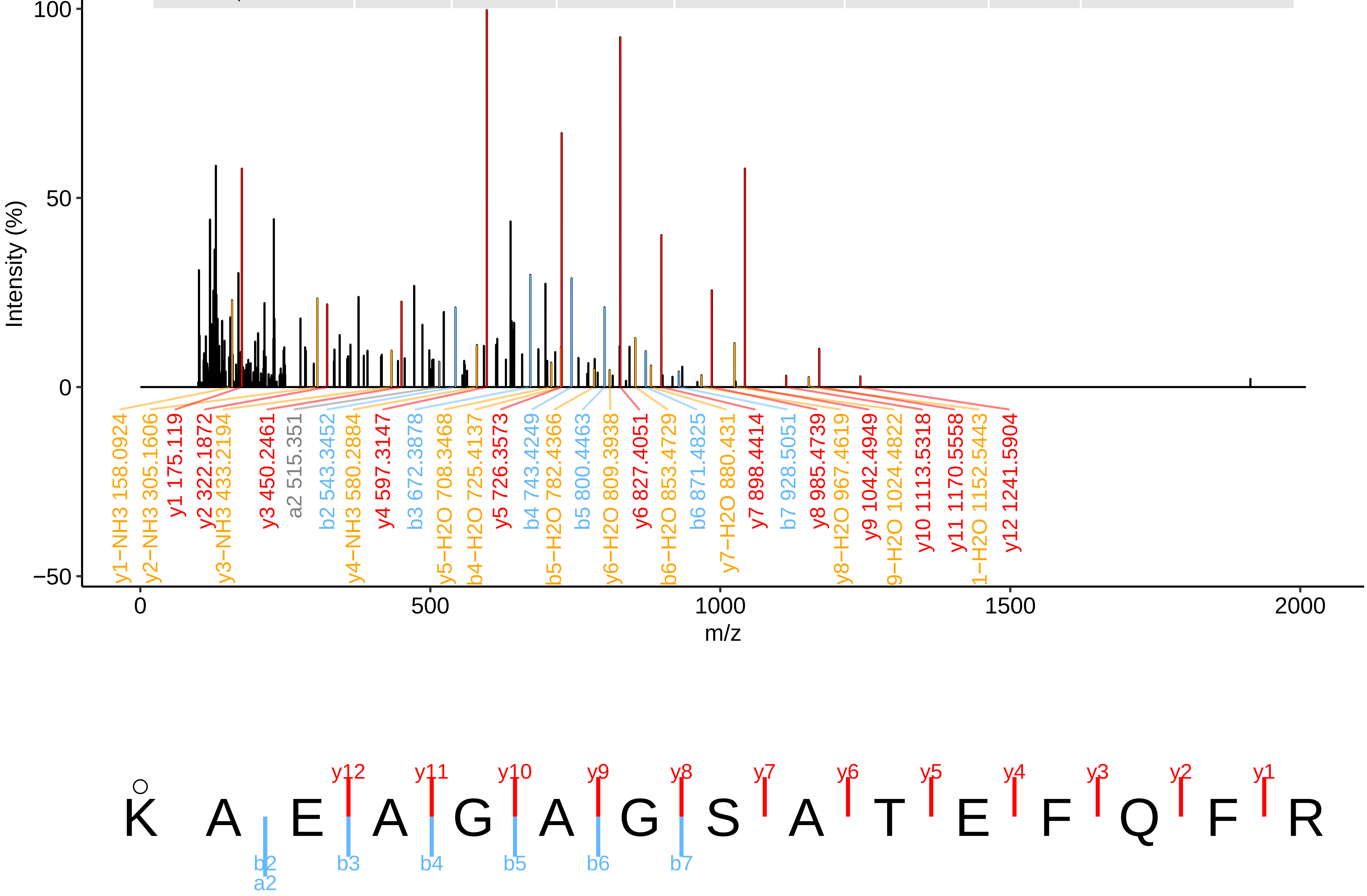

Modification □ Acetyl (Protein N−term) ○ GlyGly (K) no new terminus △ Oxidation (M)

20200921_KIZ_MUb4_GlyGly_10plex_2 − Scan number: 12652

Gene Names	Charge	m/z	Mass	Retention time	PEP	Score	Precursor Intensity
MARC1	4	370.2057	1476.794	48.993	0.0071298	87.001	1316911

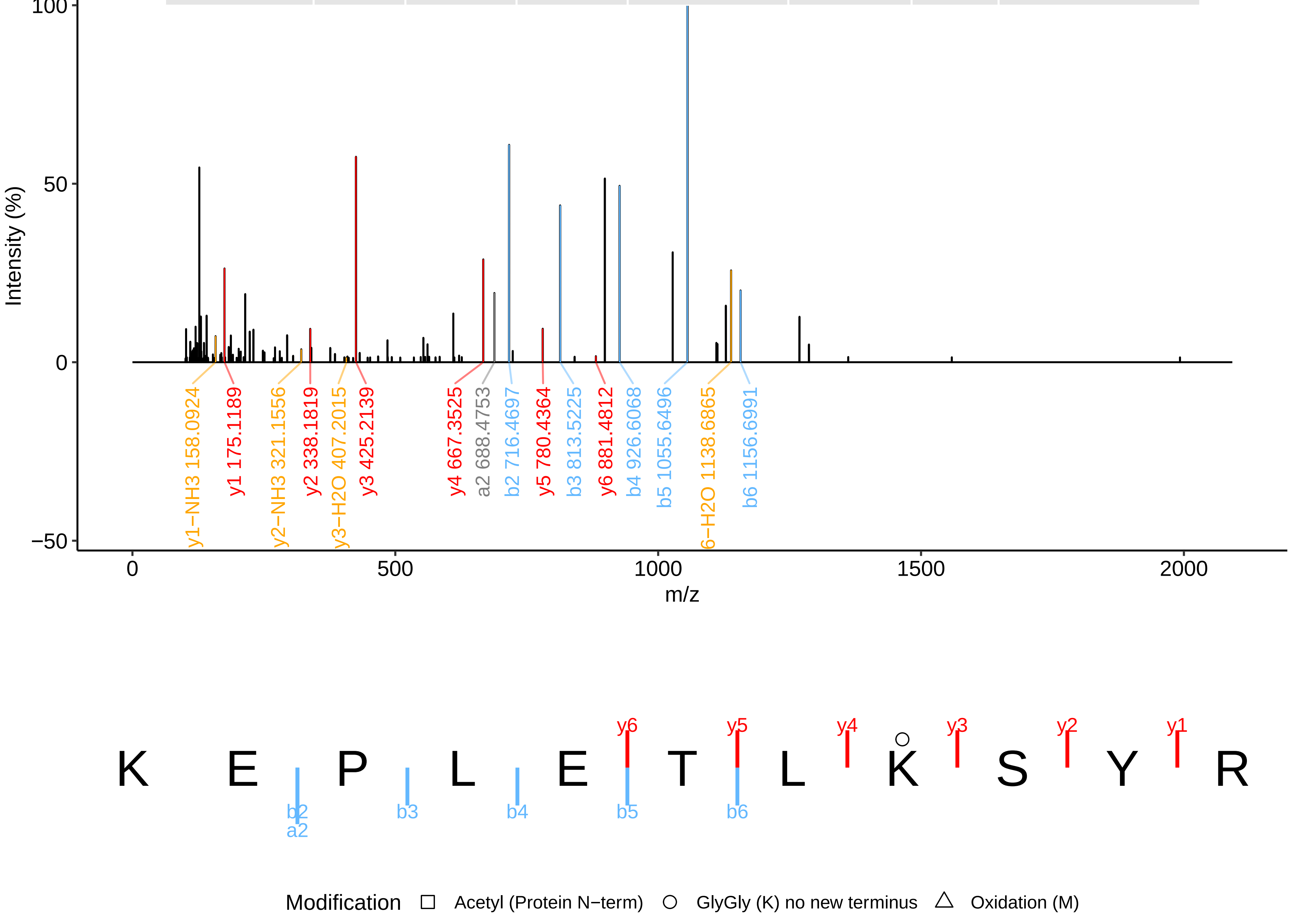

20200924_KIZ_MUb5_GlyGly_1 − Scan number: 29564

Gene Names	Charge	m/z	Mass	Retention time	PEP	Score	Precursor Intensity
	3	504.2781	1509.813	94.327	0.079607	56.359	26656828

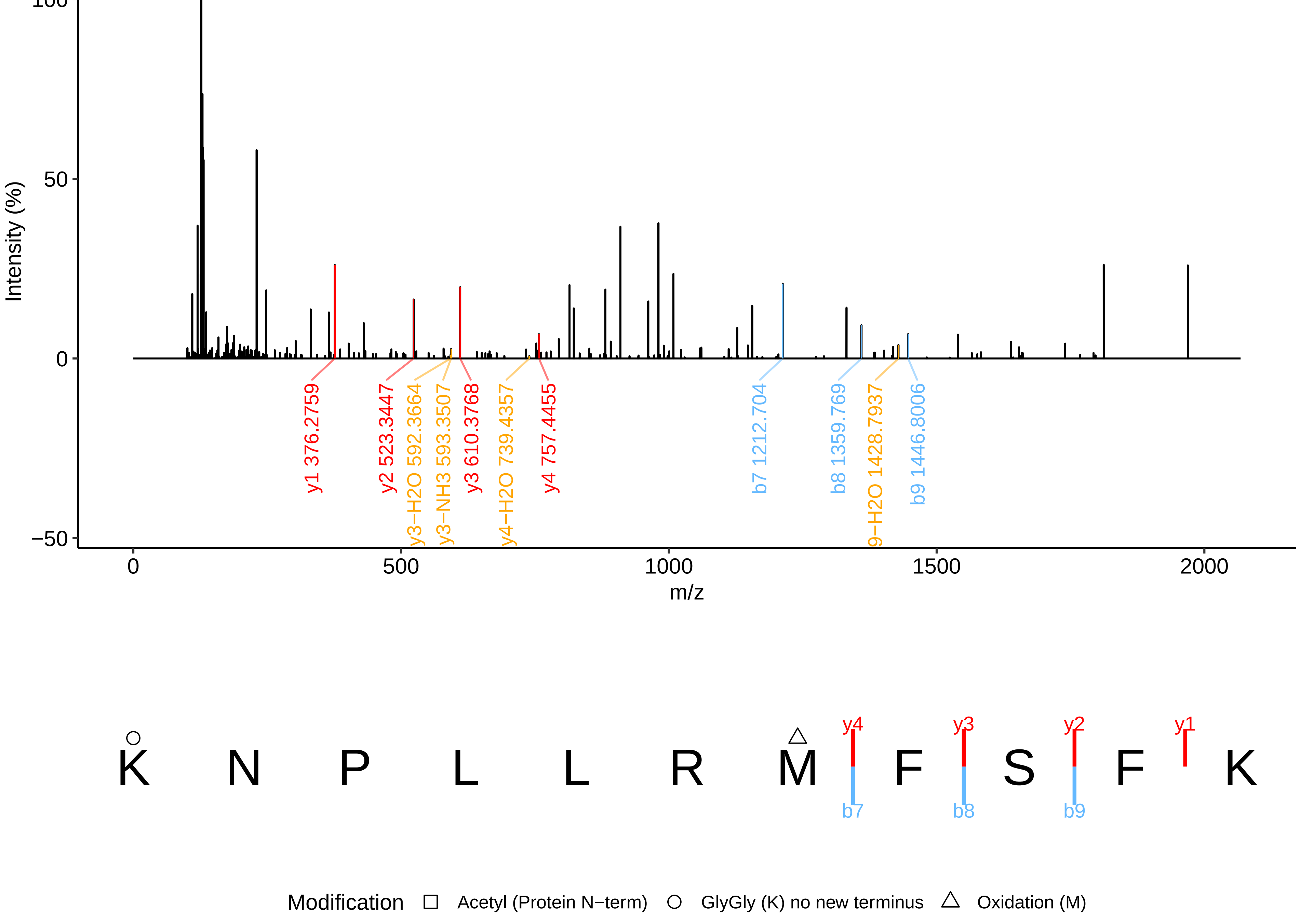

20200924_KIZ_MUb5_GlyGly_1 − Scan number: 29042

Gene Names	Charge	m/z	Mass	Retention time	PEP	Score	Precursor Intensity
	3	504.2781	1509.813	92.719	0.11252	45.077	15253500

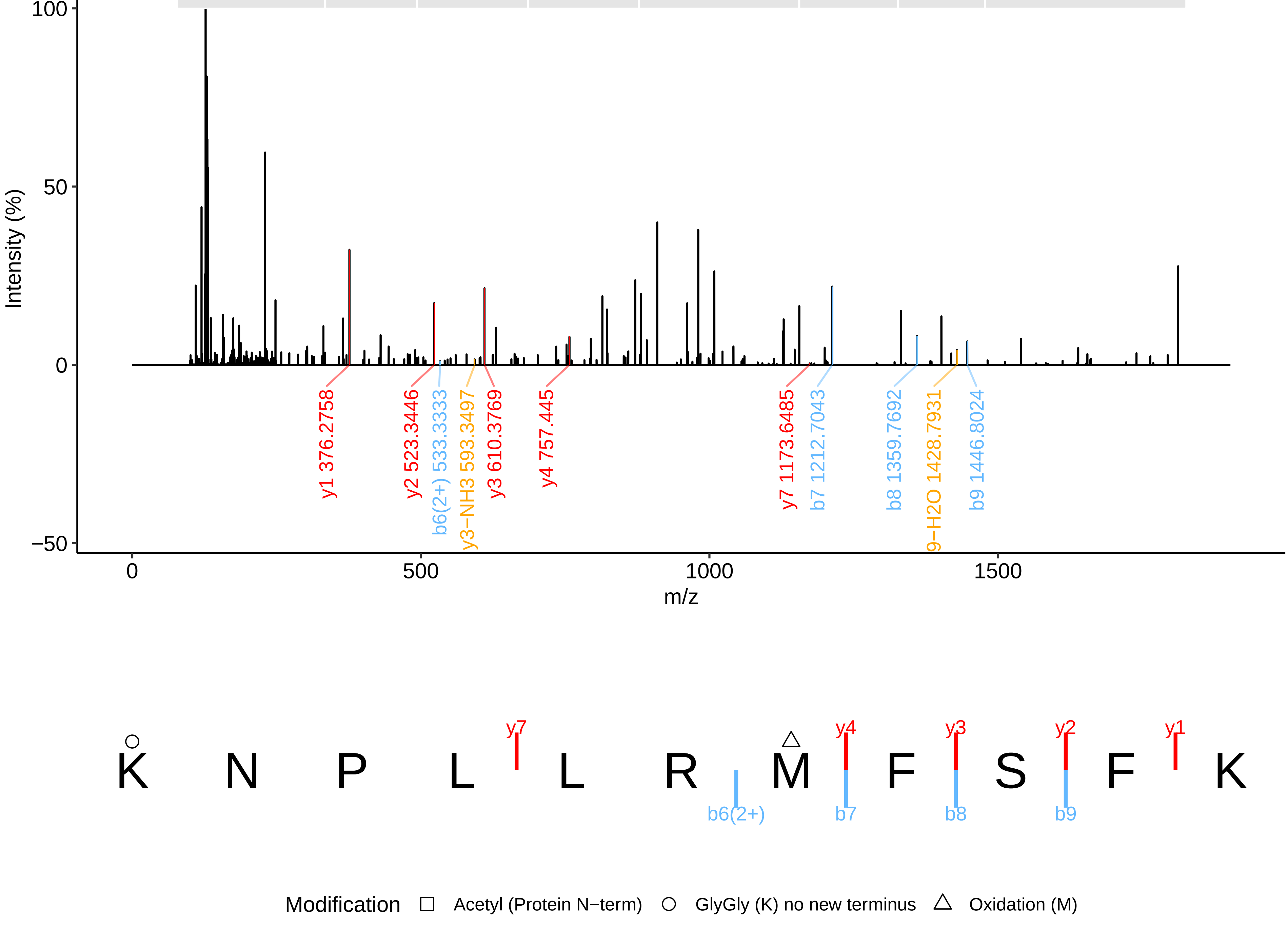

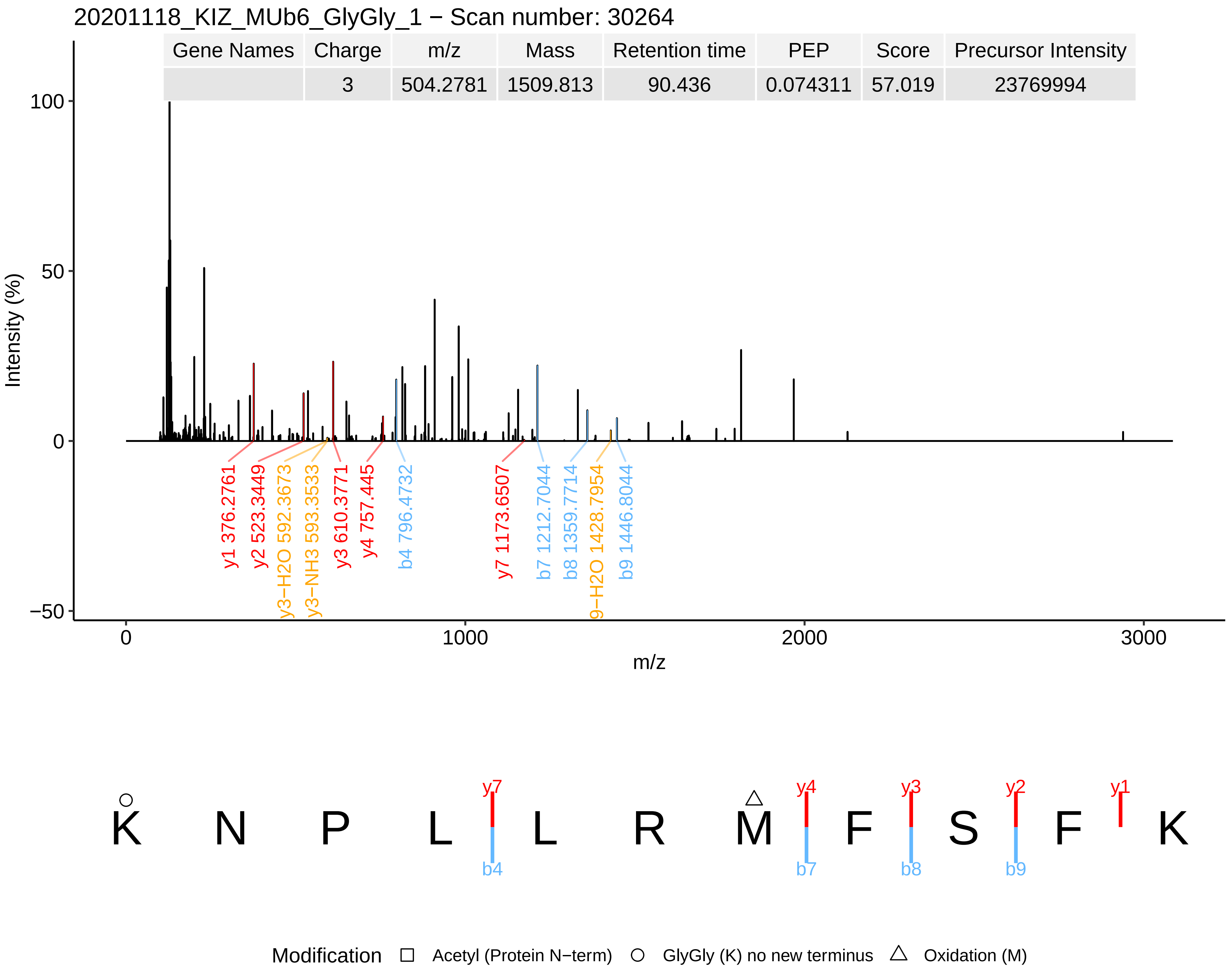
20201118_KIZ_MUb6_GlyGly_1 − Scan number: 30264
Gene Names
Charge
m/z
Mass
Retention time
PEP
Score
Precursor Intensity
3
504.2781
1509.813
90.436
0.074311
57.019
23769994
Intensity (%)
m/z
y1 376.2761
y2 523.3449
y3−H2O 592.3673
y3−NH3 593.3533
y3 610.3771
y4 757.445
b4 796.4732
y7 1173.6507
b7 1212.7044
b8 1359.7714
b9 1446.8044
K N P L L R M F S F K
Modification
Acetyl (Protein N−term)
GlyGly (K) no new terminus
Oxidation (M)

20200924_KIZ_MUb5_GlyGly_1 − Scan number: 29174

Gene Names	Charge	m/z	Mass	Retention time	PEP	Score	Precursor Intensity
HES4	2	844.4547	1686.895	93.112	0.066844	47.198	NA

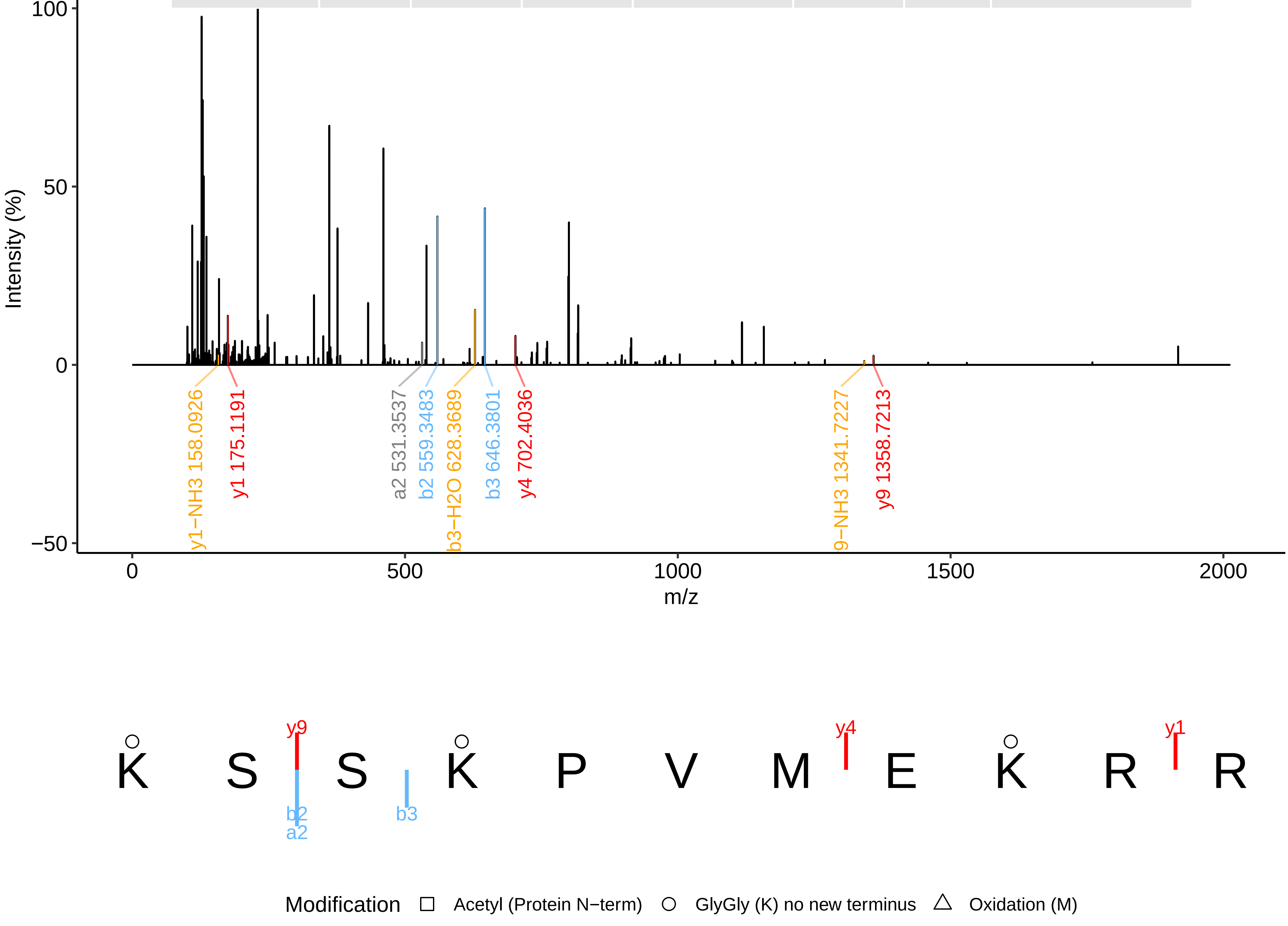

20200921_KIZ_MUb4_GlyGly_10plex − Scan number: 25327

Gene Names	Charge	m/z	Mass	Retention time	PEP	Score	Precursor Intensity
PDCD6IP	3	579.3212	1734.942	92.119	1.6894e−08	128.76	2638437

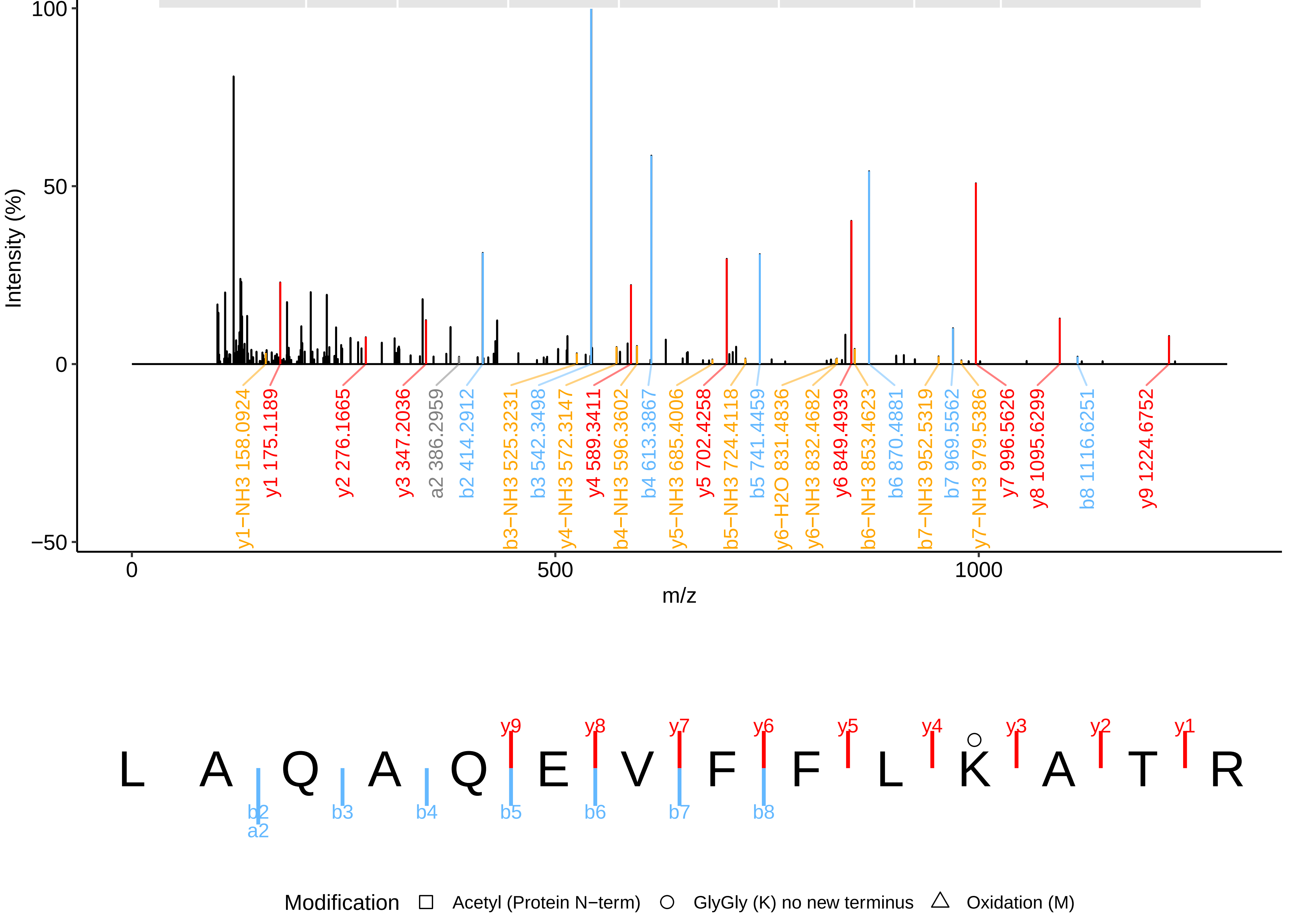

20200921_KIZ_MUb4_GlyGly_10plex_2 − Scan number: 21708

Gene Names	Charge	m/z	Mass	Retention time	PEP	Score	Precursor Intensity
PDCD6IP	3	579.3212	1734.942	85.497	0.0035704	73.022	700375.8

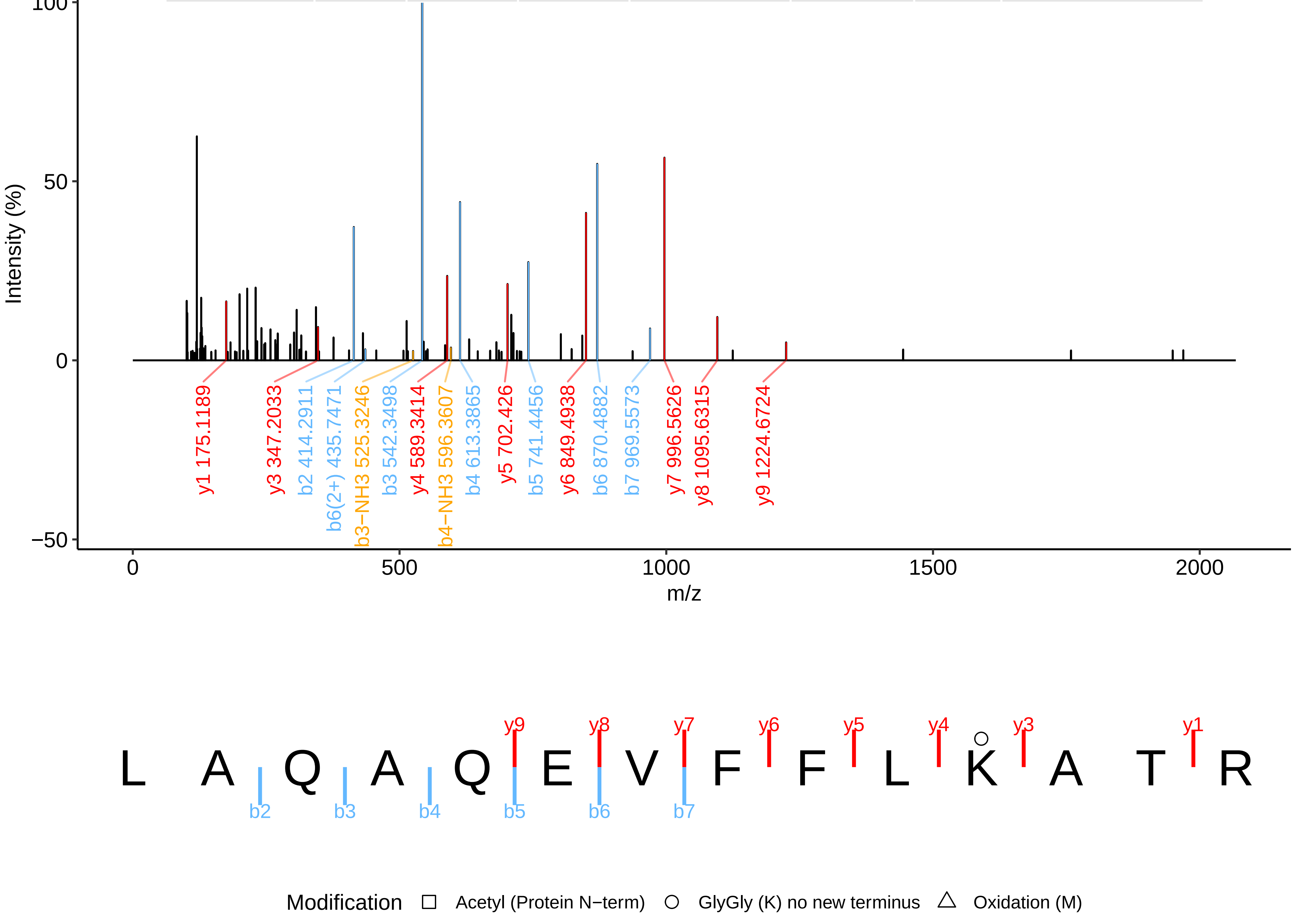

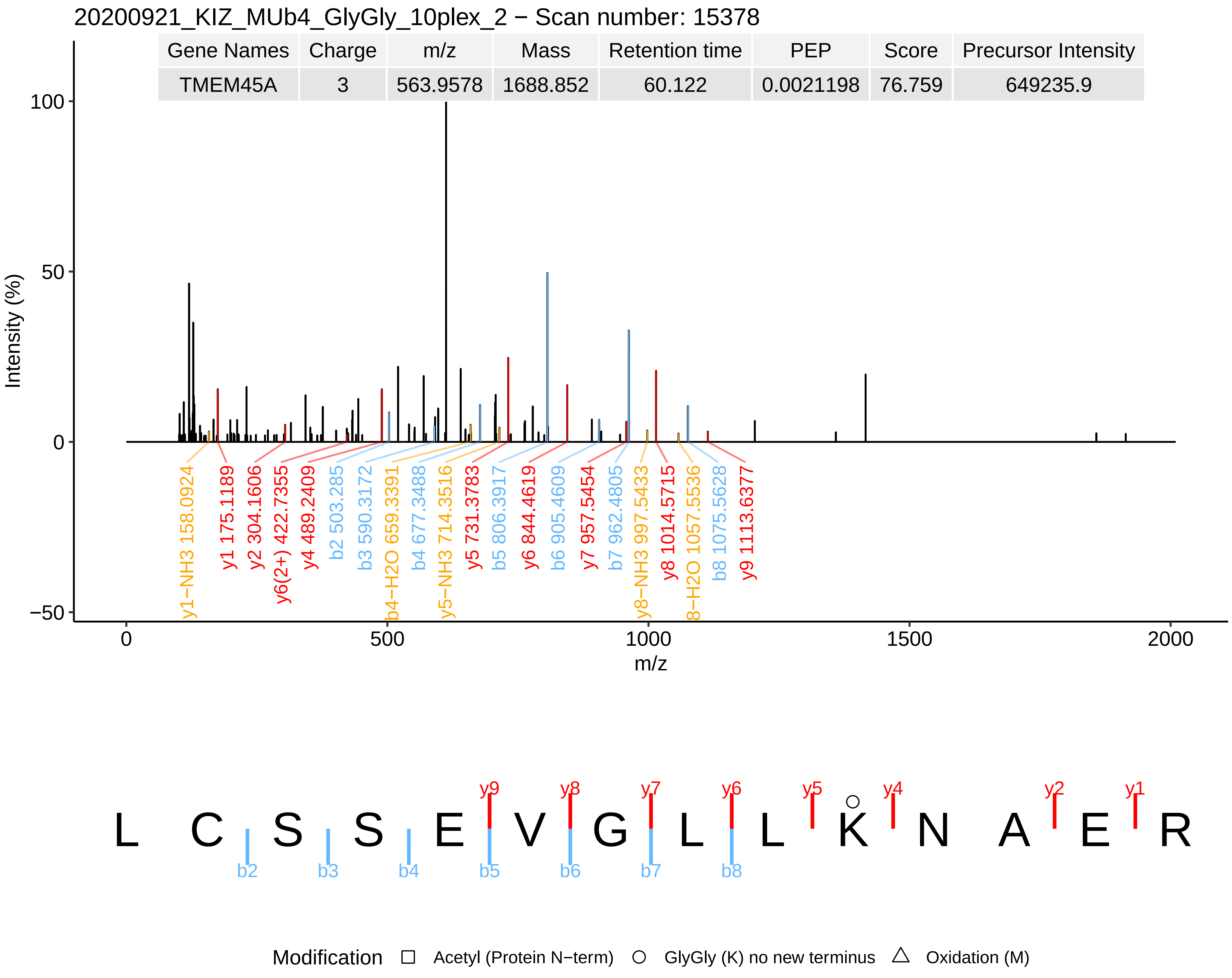
20200921_KIZ_MUb4_GlyGly_10plex_2 − Scan number: 15378
Gene Names | Charge | m/z | Mass | Retention time | PEP | Score | Precursor Intensity
TMEM45A | 3 | 563.9578 | 1688.852 | 60.122 | 0.0021198 | 76.759 | 649235.9
Intensity (%)
100
50
0
−50
0
500
1000
1500
2000
m/z
y1−NH3 158.0924
y1 175.1189
y2 304.1606
y6(2+) 422.7355
y4 489.2409
b2 503.285
b3 590.3172
b4−H2O 659.3391
b4 677.3488
y5−NH3 714.3516
y5 731.3783
b5 806.3917
y6 844.4619
b6 905.4609
y7 957.5454
b7 962.4805
y8−NH3 997.5433
y8 1014.5715
b8−H2O 1057.5536
b8 1075.5628
y9 1113.6377
L C S S E V G L L K N A E R
y9 y8 y7 y6 y5 y4 y2 y1
b2 b3 b4 b5 b6 b7 b8
Modification
Acetyl (Protein N−term)
GlyGly (K) no new terminus
Oxidation (M)

20200921_KIZ_MUb4_GlyGly_10plex_2 − Scan number: 15048

Gene Names	Charge	m/z	Mass	Retention time	PEP	Score	Precursor Inter
TUBA1C;TUBA1B;TUBA4A;TUBAL3;TUBA8	4	378.4394	1509.729	58.767	0.053748	54.259	1575465

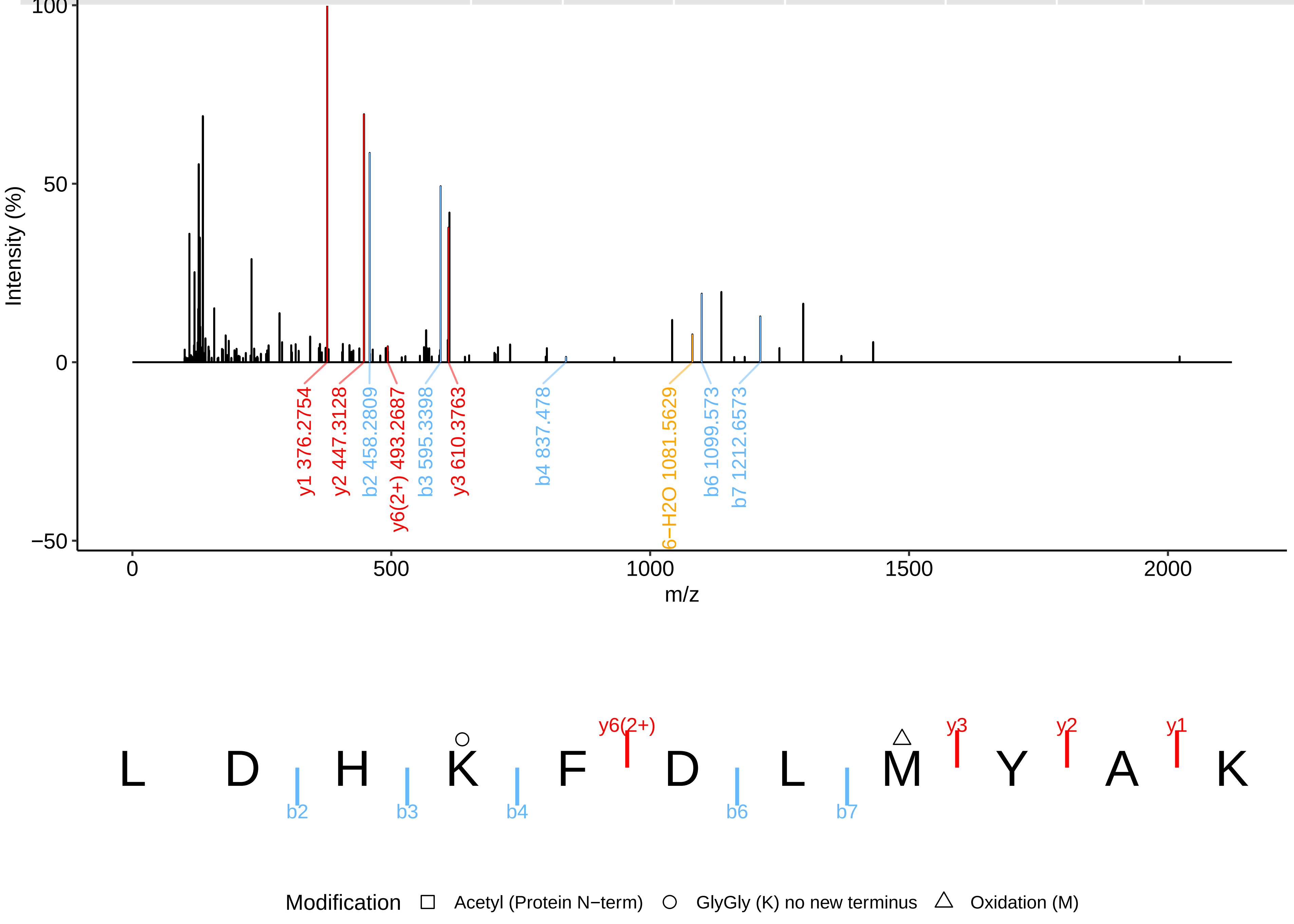

20200921_KIZ_MUb4_GlyGly_10plex_2 − Scan number: 15909

Gene Names	Charge	m/z	Mass	Retention time	PEP	Score	Precursor Intensity
BCORL1;BCOR	3	561.6564	1681.947	62.307	0.0013122	79.089	579991.4

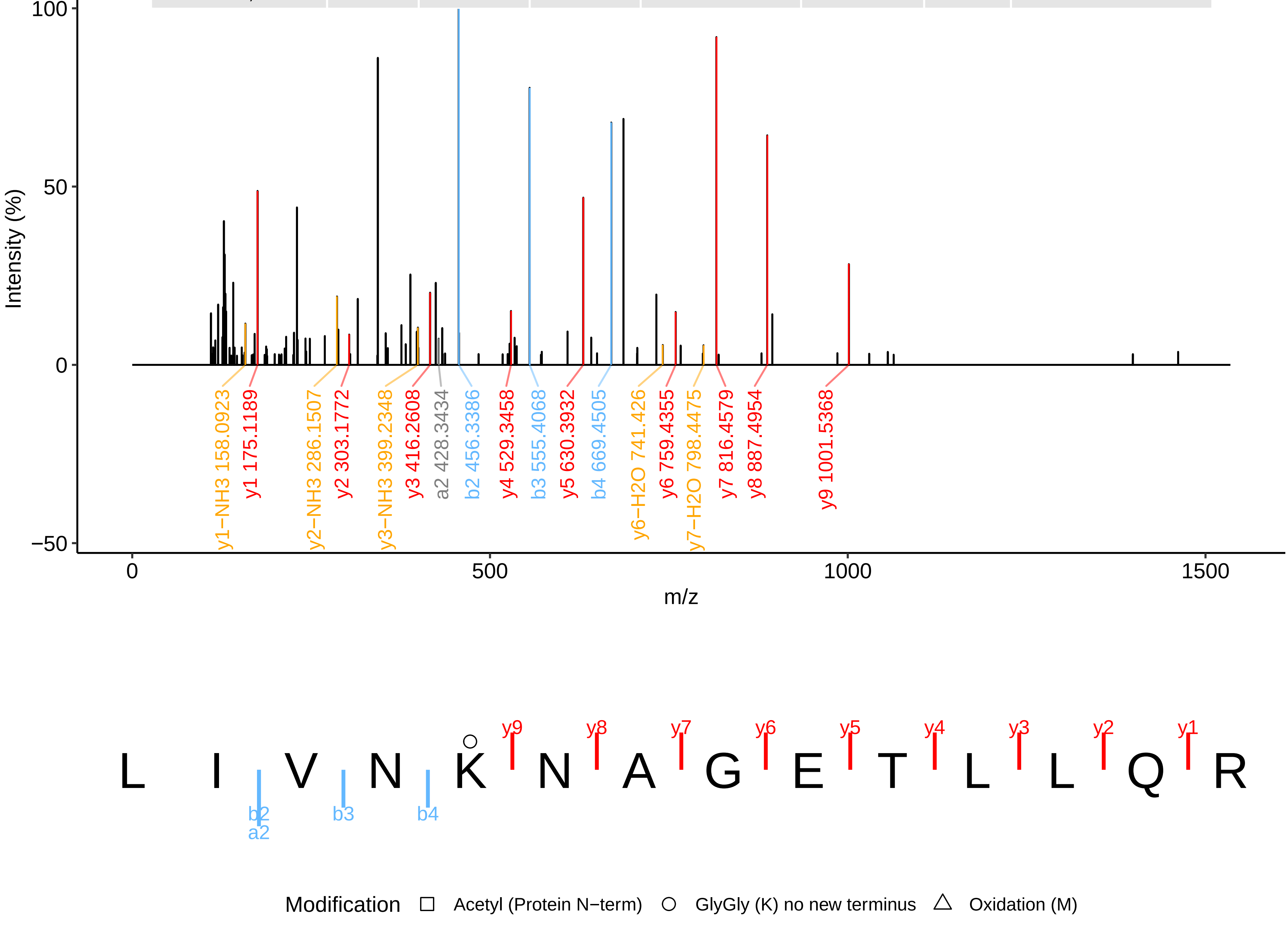

20200921_KIZ_MUb4_GlyGly_10plex_2 − Scan number: 16688

Gene Names	Charge	m/z	Mass	Retention time	PEP	Score	Precursor Intensity
SLC25A24	3	578.9722	1733.895	65.182	5.6411e−14	147.73	4945149

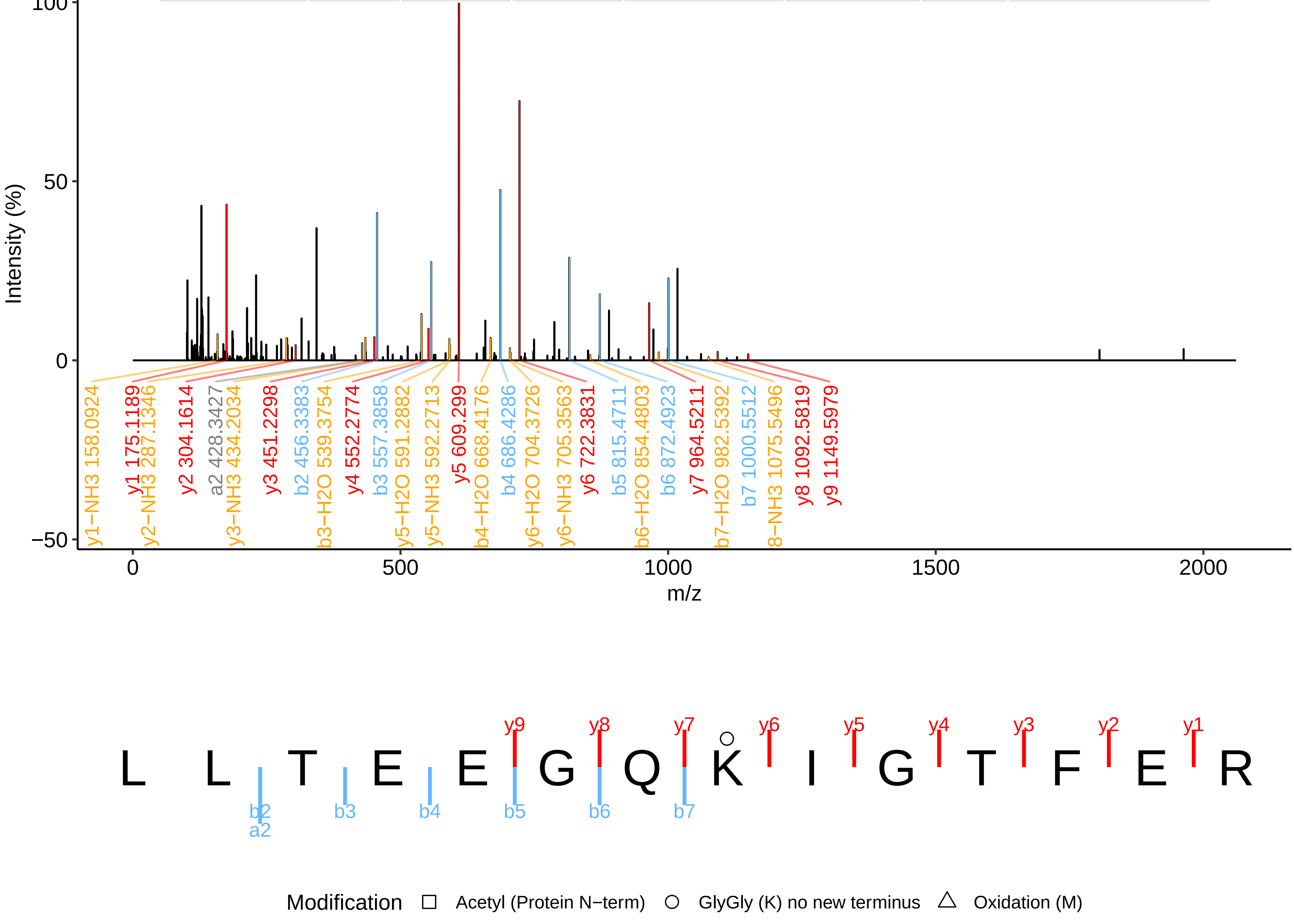

20200924_KIZ_MUb5_GlyGly_1 − Scan number: 17797

Gene Names	Charge	m/z	Mass	Retention time	PEP	Score	Precursor Intensity
SLC25A24	3	578.9722	1733.895	57.505	4.9331e−05	99.991	NA

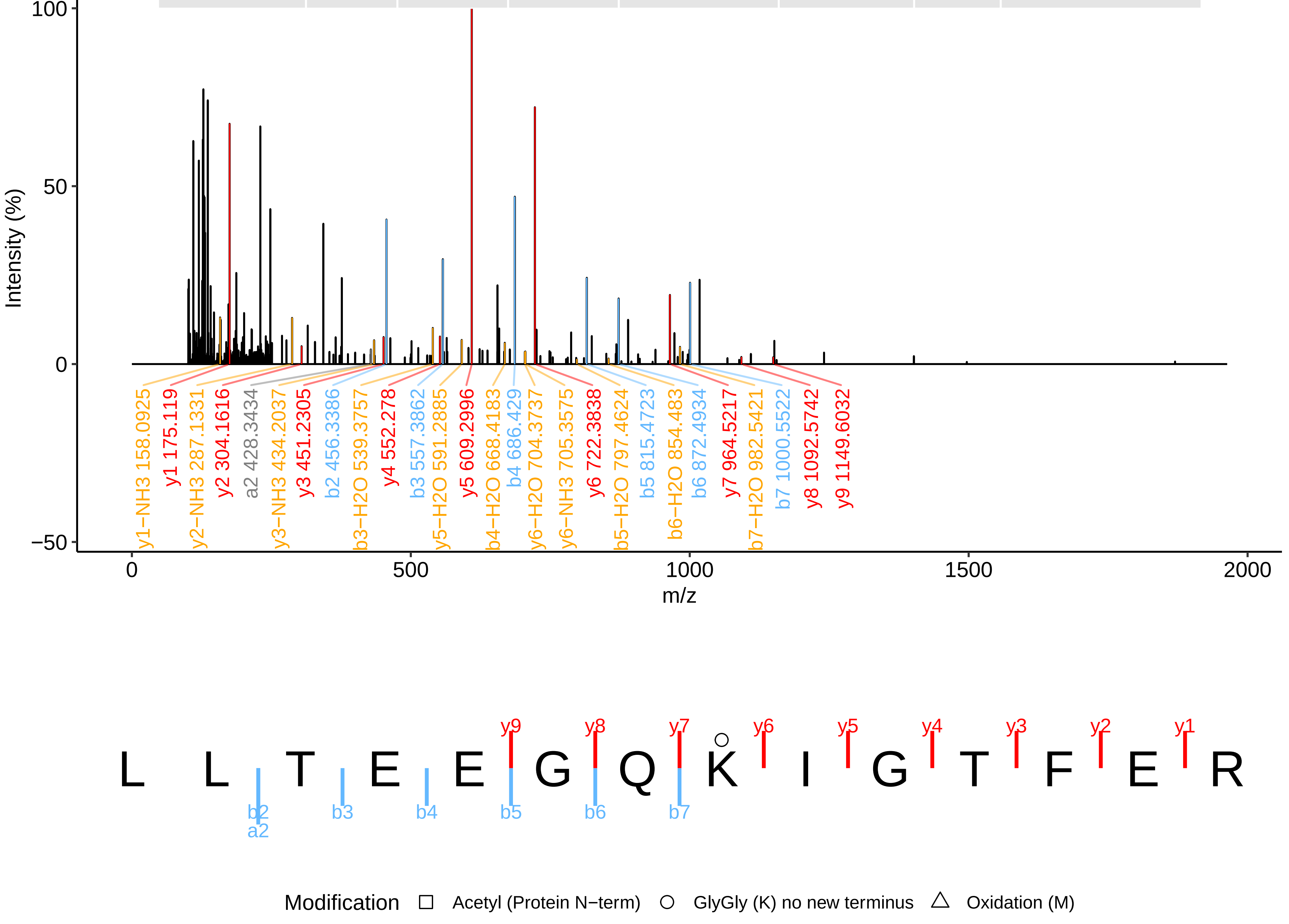

20200921_KIZ_MUb4_GlyGly_10plex − Scan number: 19805

Gene Names	Charge	m/z	Mass	Retention time	PEP	Score	Precursor Intensity
DAG1	3	507.6155	1519.825	71.484	7.1539e−07	115.33	8760658

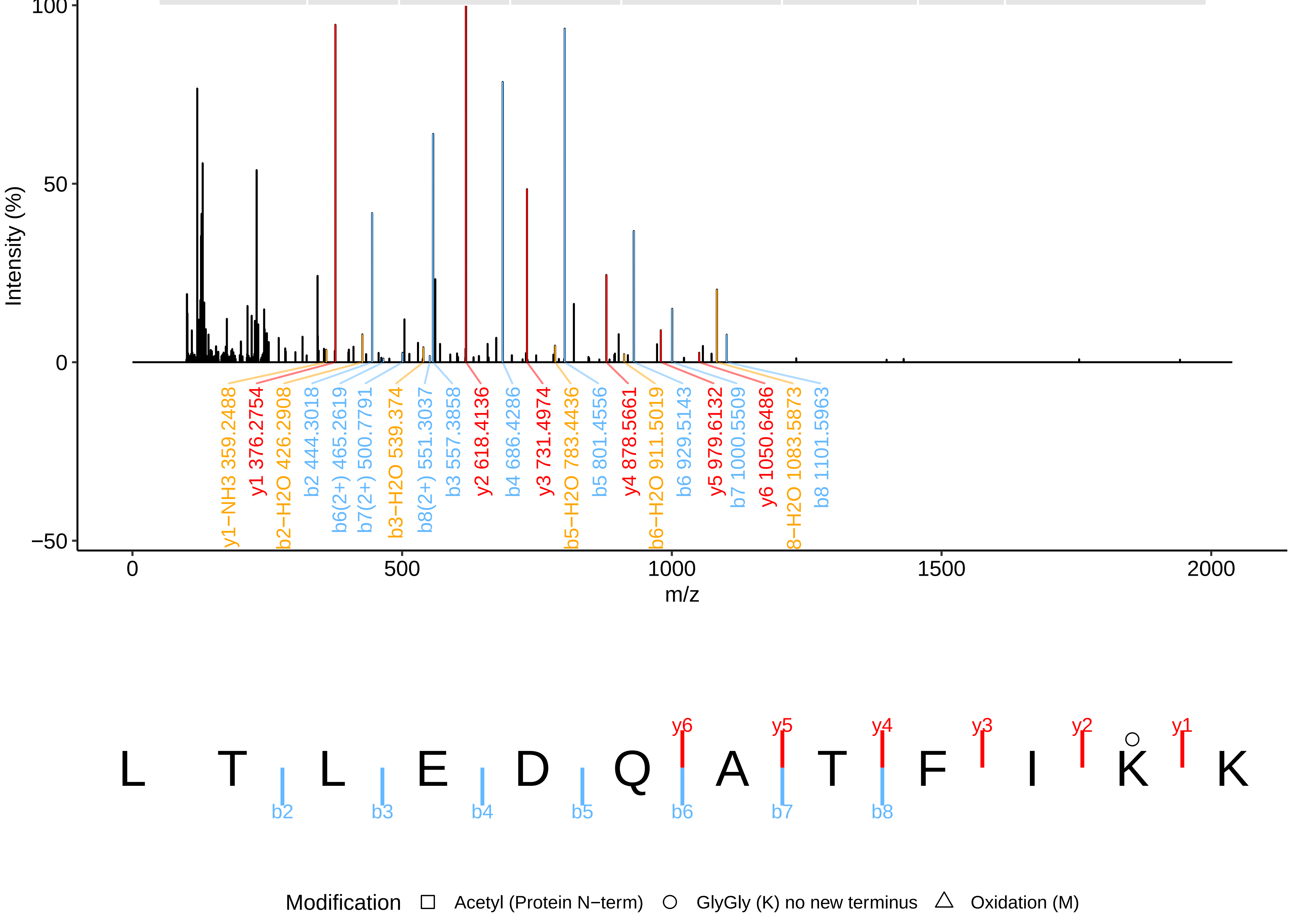

20200921_KIZ_MUb4_GlyGly_10plex_2 − Scan number: 18185

Gene Names	Charge	m/z	Mass	Retention time	PEP	Score	Precursor Intensity
DAG1	3	507.6155	1519.825	71.185	2.0248e−08	128.85	2616457

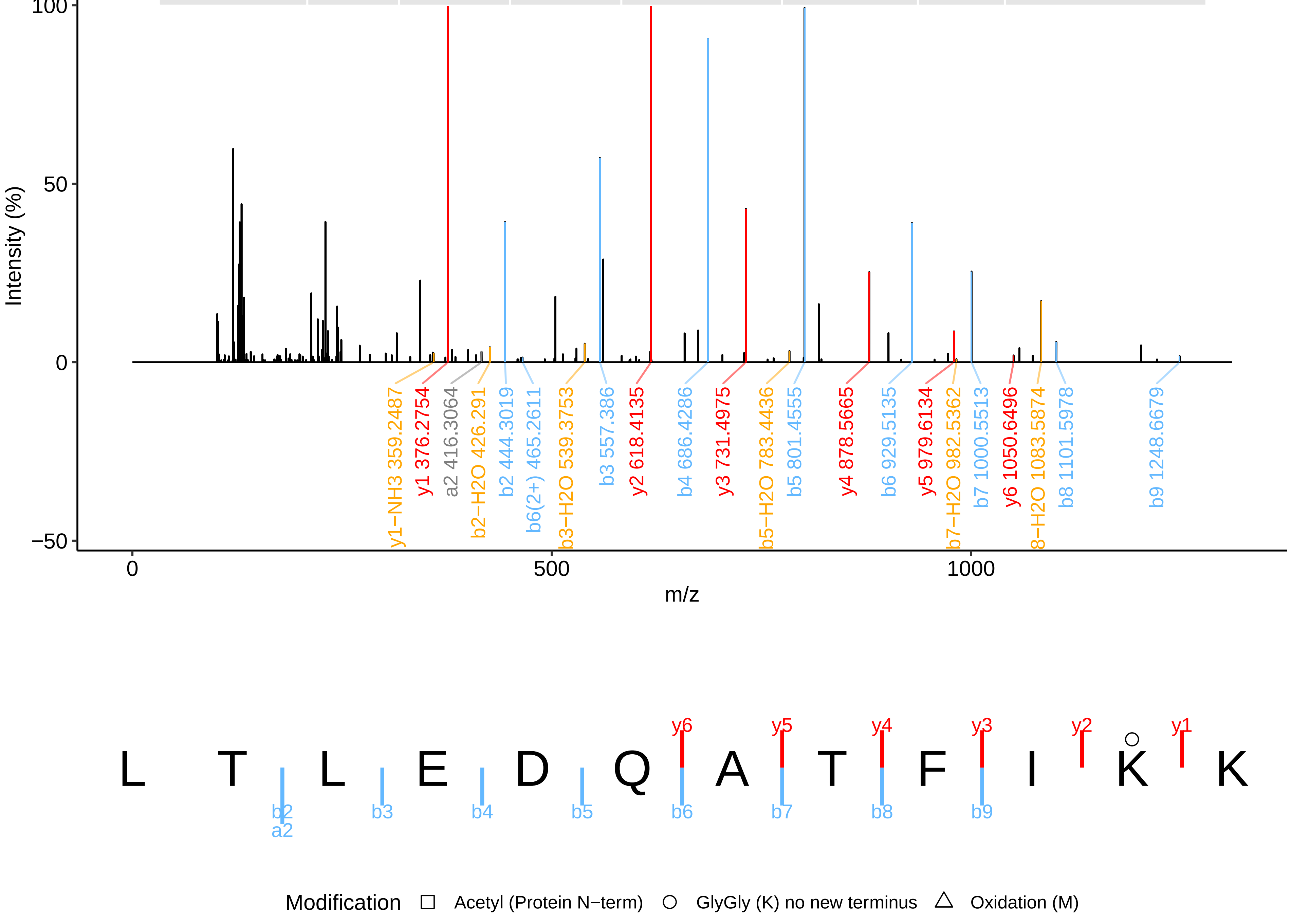

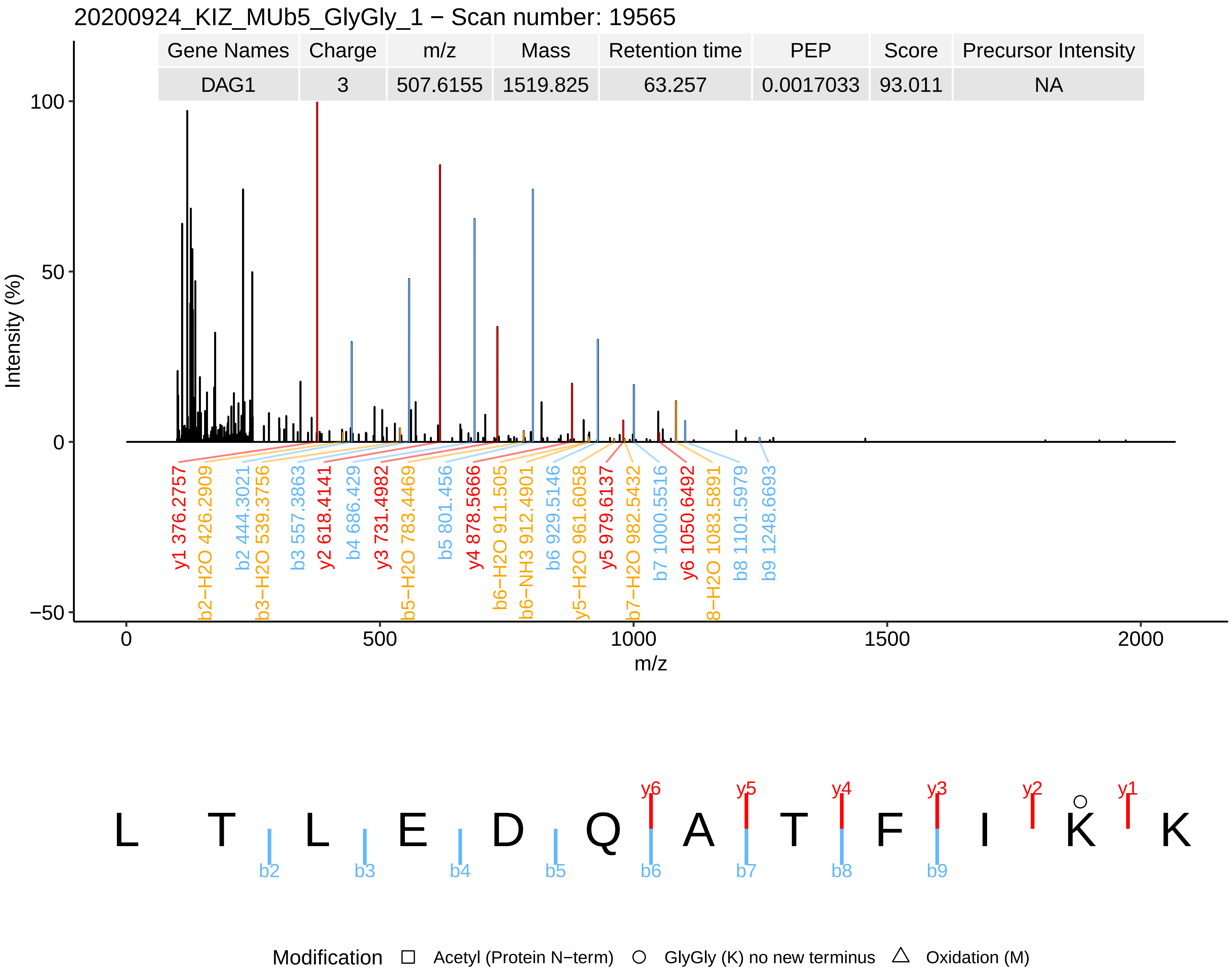

20200924_KIZ_MUb5_GlyGly_1 − Scan number: 19565
Gene Names | Charge | m/z | Mass | Retention time | PEP | Score | Precursor Intensity
DAG1 | 3 | 507.6155 | 1519.825 | 63.257 | 0.0017033 | 93.011 | NA
Intensity (%)
100
50
0
−50
0
500
1000
1500
2000
m/z
y1 376.2757
b2−H2O 426.2909
b2 444.3021
b3−H2O 539.3756
b3 557.3863
y2 618.4141
b4 686.429
y3 731.4982
b5−H2O 783.4469
b5 801.456
y4 878.5666
b6−H2O 911.505
b6−NH3 912.4901
b6 929.5146
y5−H2O 961.6058
y5 979.6137
b7−H2O 982.5432
b7 1000.5516
y6 1050.6492
b8−H2O 1083.5891
b8 1101.5979
b9 1248.6693
L T L E D Q A T F I K K
y6 y5 y4 y3 y2 y1
b2 b3 b4 b5 b6 b7 b8 b9
Modification
Acetyl (Protein N−term)
GlyGly (K) no new terminus
Oxidation (M)

20201118_KIZ_MUb6_GlyGly_1 − Scan number: 20000

Gene Names	Charge	m/z	Mass	Retention time	PEP	Score	Precursor Intensity
DAG1	3	507.6155	1519.825	62.633	8.0413e−06	128.85	14237698

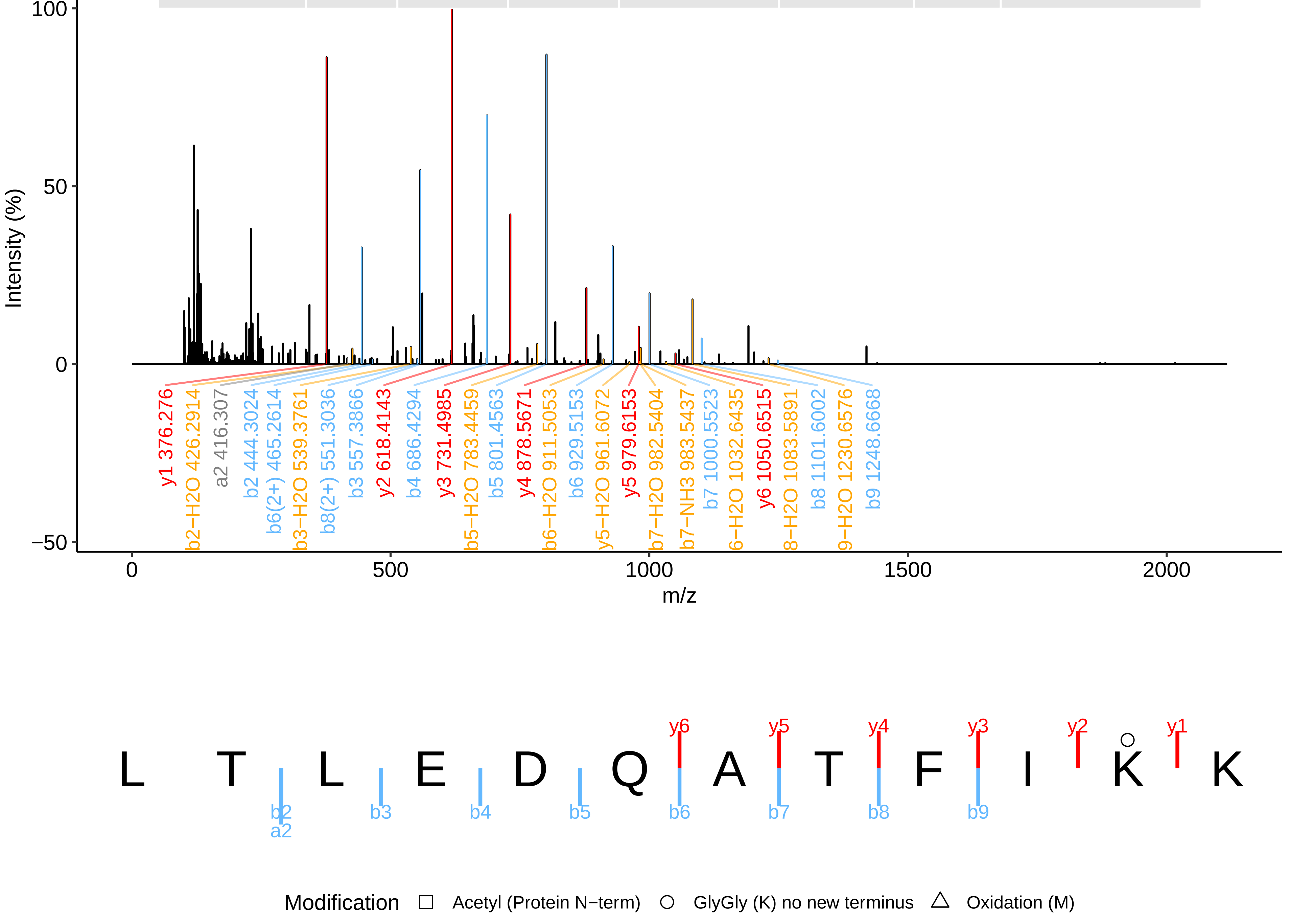

20201118_KIZ_MUb6_GlyGly_2 − Scan number: 21800

Gene Names	Charge	m/z	Mass	Retention time	PEP	Score	Precursor Intensity
DAG1	3	507.6155	1519.825	73.371	7.6509e−08	117.79	9706250

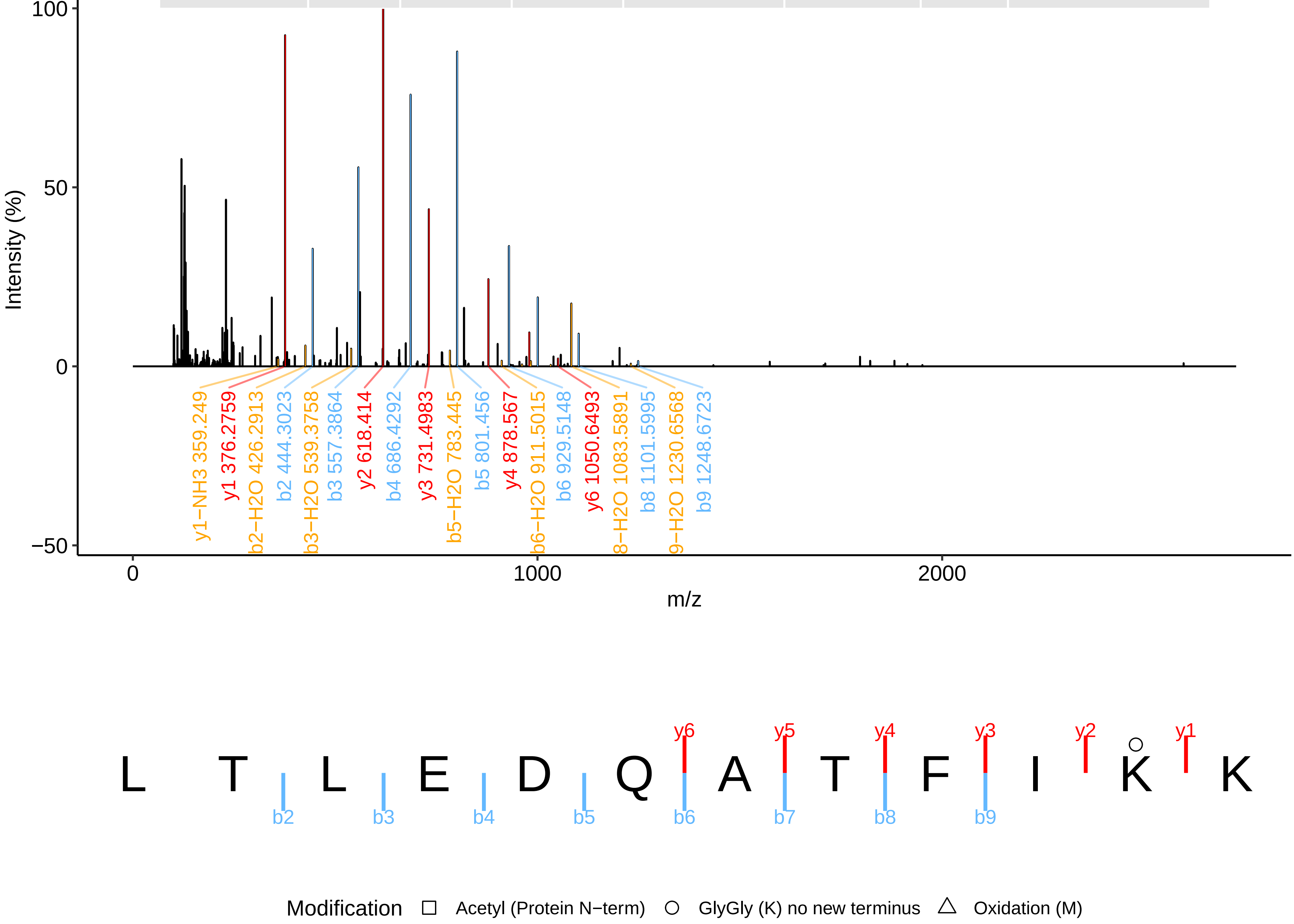

20201118_KIZ_MUb6_GlyGly_2 − Scan number: 21806

Gene Names	Charge	m/z	Mass	Retention time	PEP	Score	Precursor Intensity
DAG1	3	507.6155	1519.825	73.395	3.9393e−05	110.08	17260384

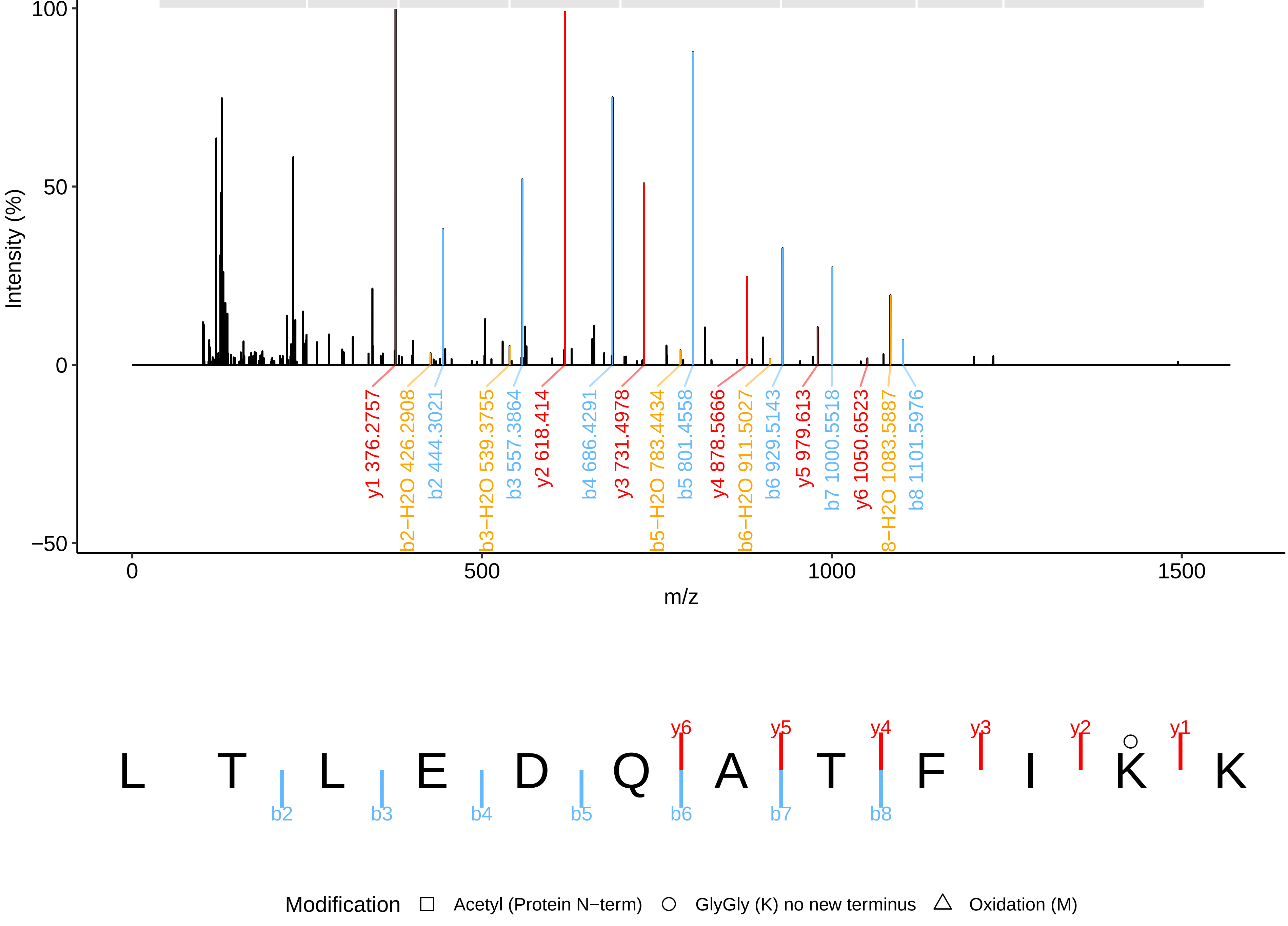

20200921_KIZ_MUb4_GlyGly_10plex − Scan number: 14870

Gene Names	Charge	m/z	Mass	Retention time	PEP	Score	Precursor Intensity
PTBP1;PTBP3	3	508.2668	1521.779	52.962	0.0010835	82.543	4425695

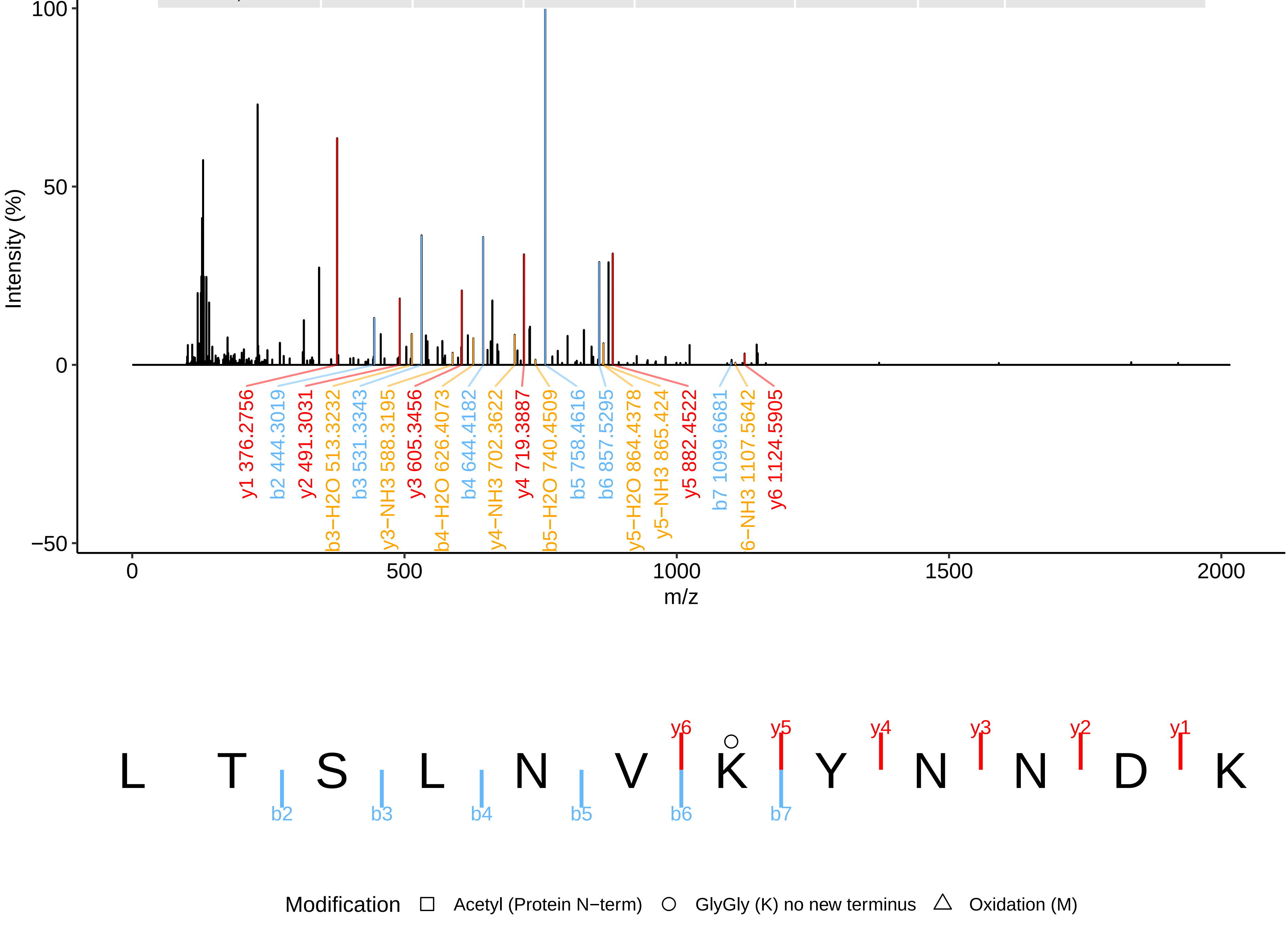

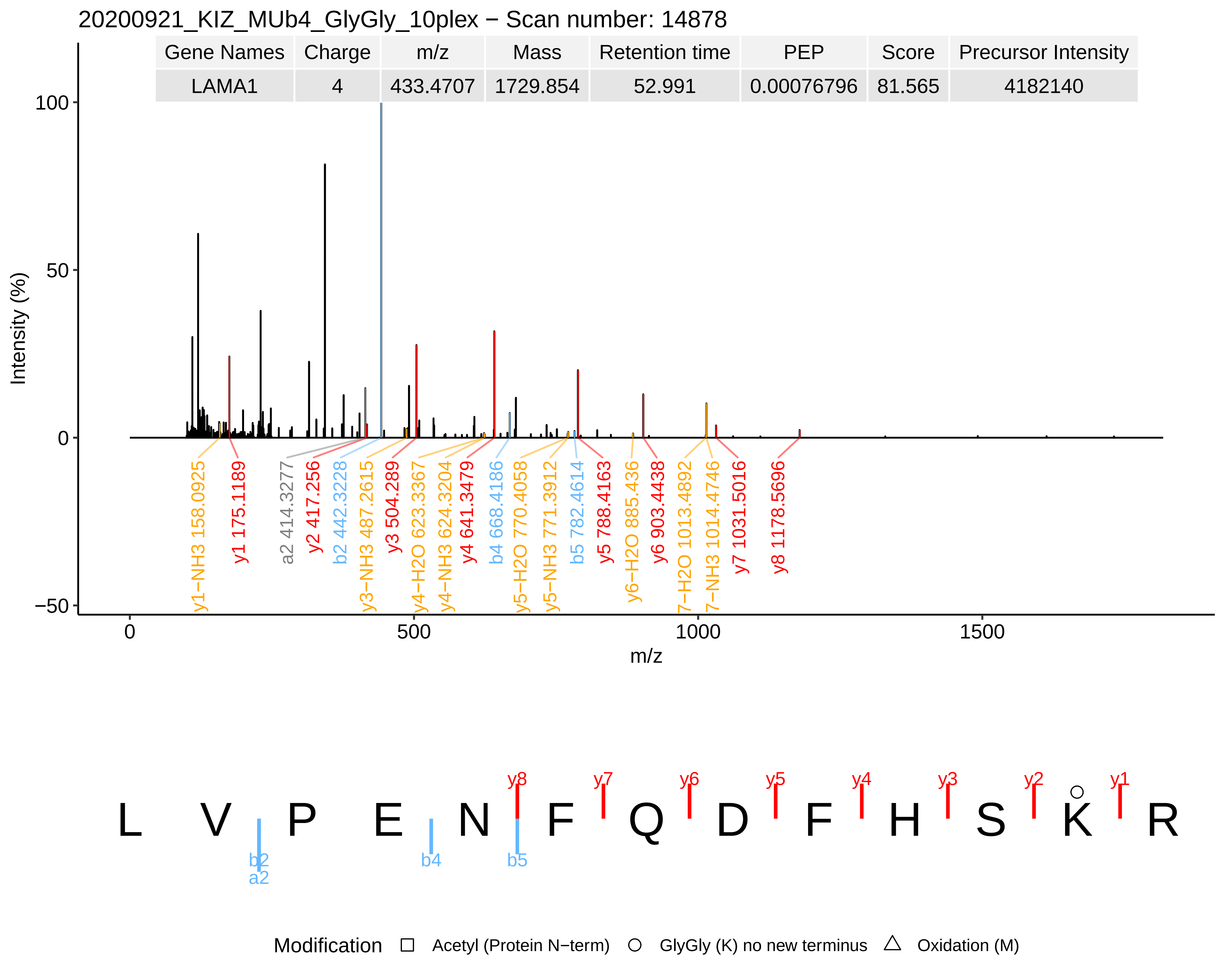
20200921_KIZ_MUb4_GlyGly_10plex − Scan number: 14878
Gene Names | Charge | m/z | Mass | Retention time | PEP | Score | Precursor Intensity
LAMA1 | 4 | 433.4707 | 1729.854 | 52.991 | 0.00076796 | 81.565 | 4182140
Intensity (%)
100
50
0
−50
m/z
0
500
1000
1500
y1−NH3 158.0925
y1 175.1189
a2 414.3277
y2 417.256
b2 442.3228
y3−NH3 487.2615
y3 504.289
y4−H2O 623.3367
y4−NH3 624.3204
y4 641.3479
b4 668.4186
y5−H2O 770.4058
y5−NH3 771.3912
b5 782.4614
y5 788.4163
y6−H2O 885.436
y6 903.4438
7−H2O 1013.4892
7−NH3 1014.4746
y7 1031.5016
y8 1178.5696
L V P E N F Q D F H S K R
y8 y7 y6 y5 y4 y3 y2 y1
b2 a2 b4 b5
Modification
Acetyl (Protein N−term)
GlyGly (K) no new terminus
Oxidation (M)

20201118_KIZ_MUb6_GlyGly_1 − Scan number: 15993

Gene Names	Charge	m/z	Mass	Retention time	PEP	Score	Precursor Intensity
	4	434.4865	1733.917	50.559	0.086149	41.092	15195506

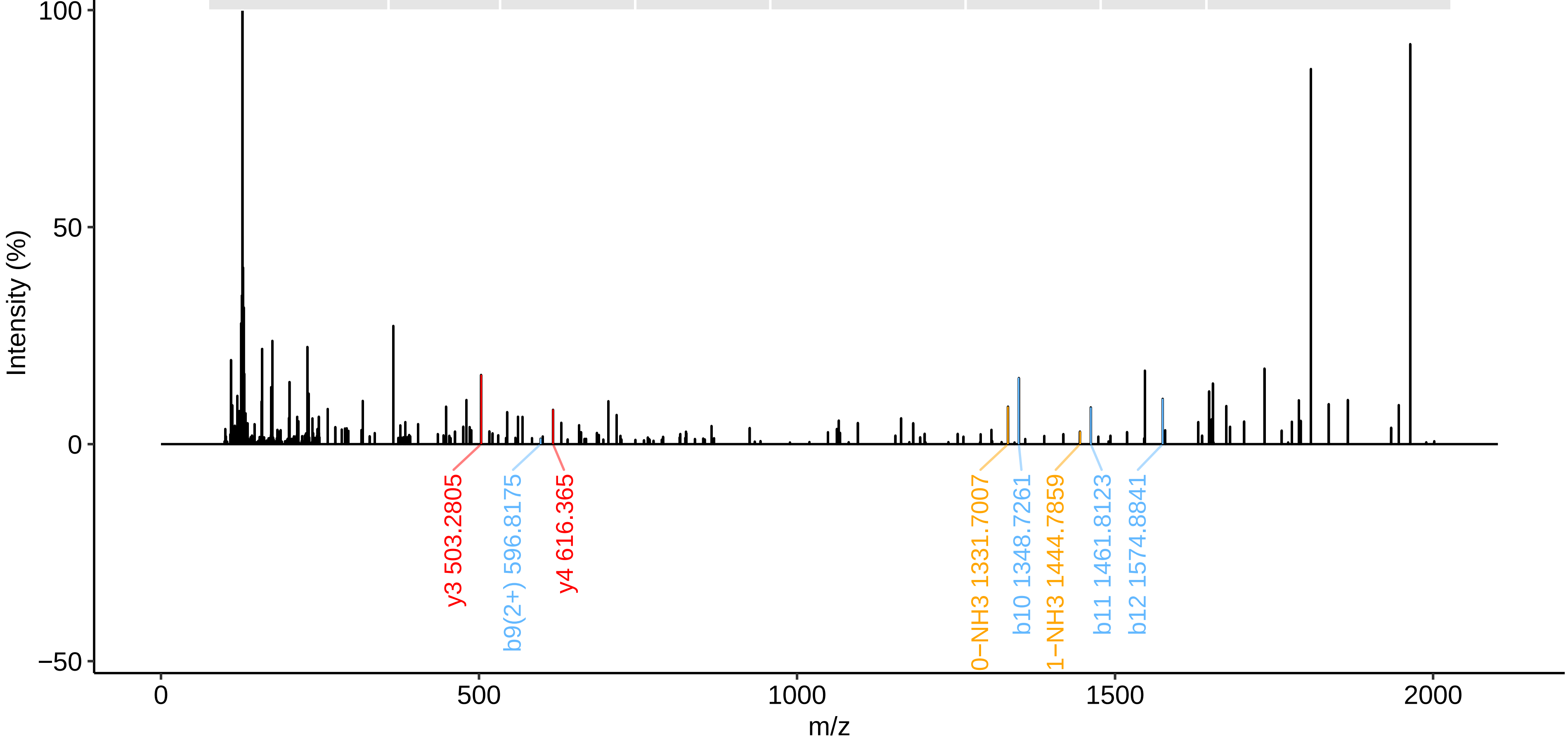

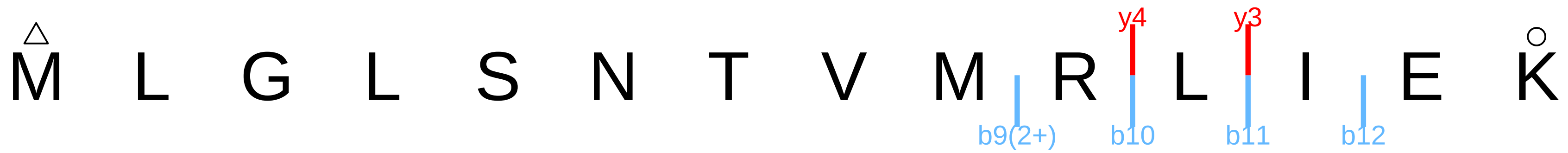

Modification □ Acetyl (Protein N−term) ○ GlyGly (K) no new terminus △ Oxidation (M)

20200921_KIZ_MUb4_GlyGly_10plex_2 − Scan number: 6524

Gene Names	Charge	m/z	Mass	Retention time	PEP	Score	Precursor Intensity
LANCL1	3	574.9226	1721.746	24.022	0.0014098	83.204	2406332

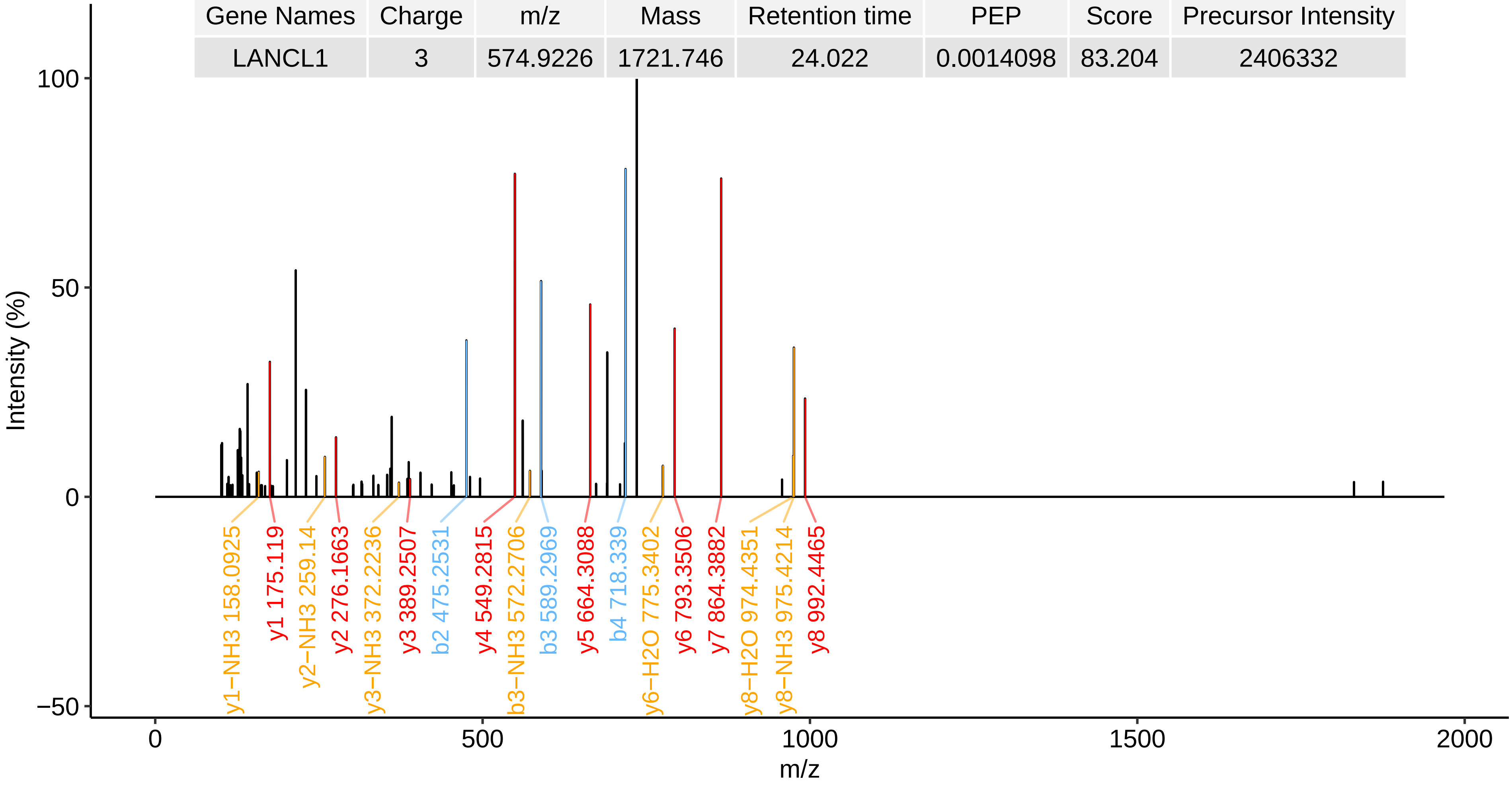

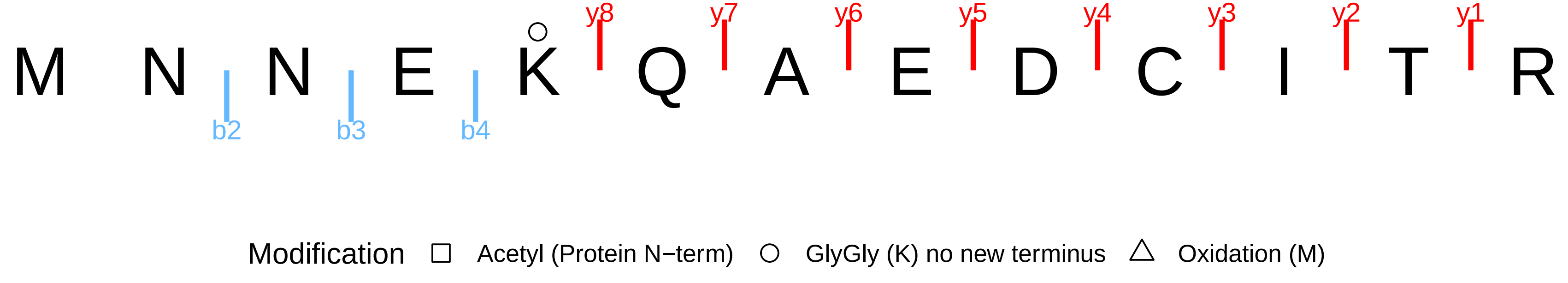

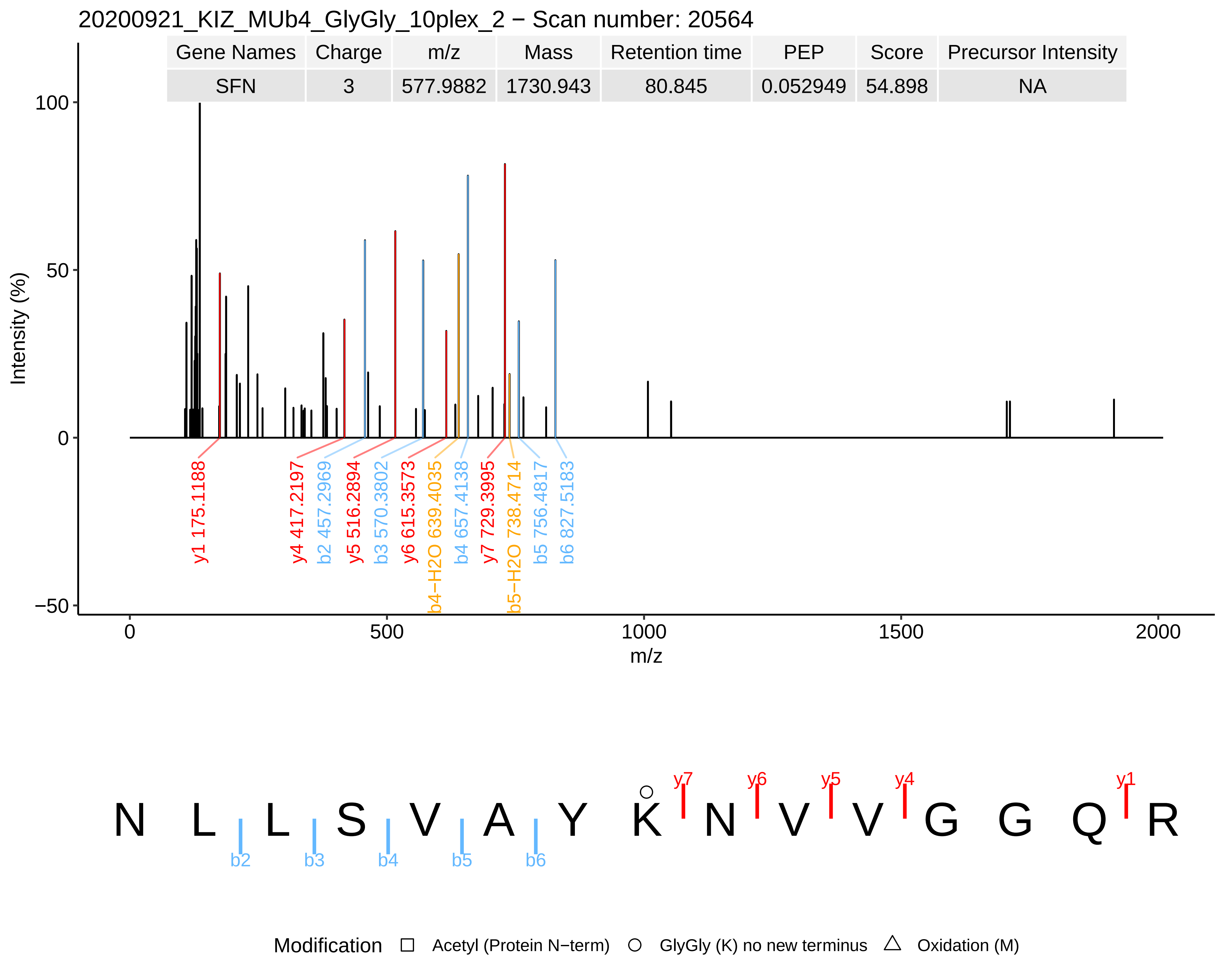
20200921_KIZ_MUb4_GlyGly_10plex_2 − Scan number: 20564
Gene Names | Charge | m/z | Mass | Retention time | PEP | Score | Precursor Intensity
SFN | 3 | 577.9882 | 1730.943 | 80.845 | 0.052949 | 54.898 | NA
Intensity (%)
m/z
y1 175.1188
y4 417.2197
b2 457.2969
y5 516.2894
b3 570.3802
y6 615.3573
b4−H2O 639.4035
b4 657.4138
y7 729.3995
b5−H2O 738.4714
b5 756.4817
b6 827.5183
N L L S V A Y K N V V G G Q R
y7 y6 y5 y4 y1
b2 b3 b4 b5 b6
Modification
Acetyl (Protein N−term)
GlyGly (K) no new terminus
Oxidation (M)

20200921_KIZ_MUb4_GlyGly_10plex − Scan number: 16711

Gene Names	Charge	m/z	Mass	Retention time	PEP	Score	Precursor Intensity
HSPA8	3	581.3234	1740.948	60.114	6.3679e−10	118.2	14882212

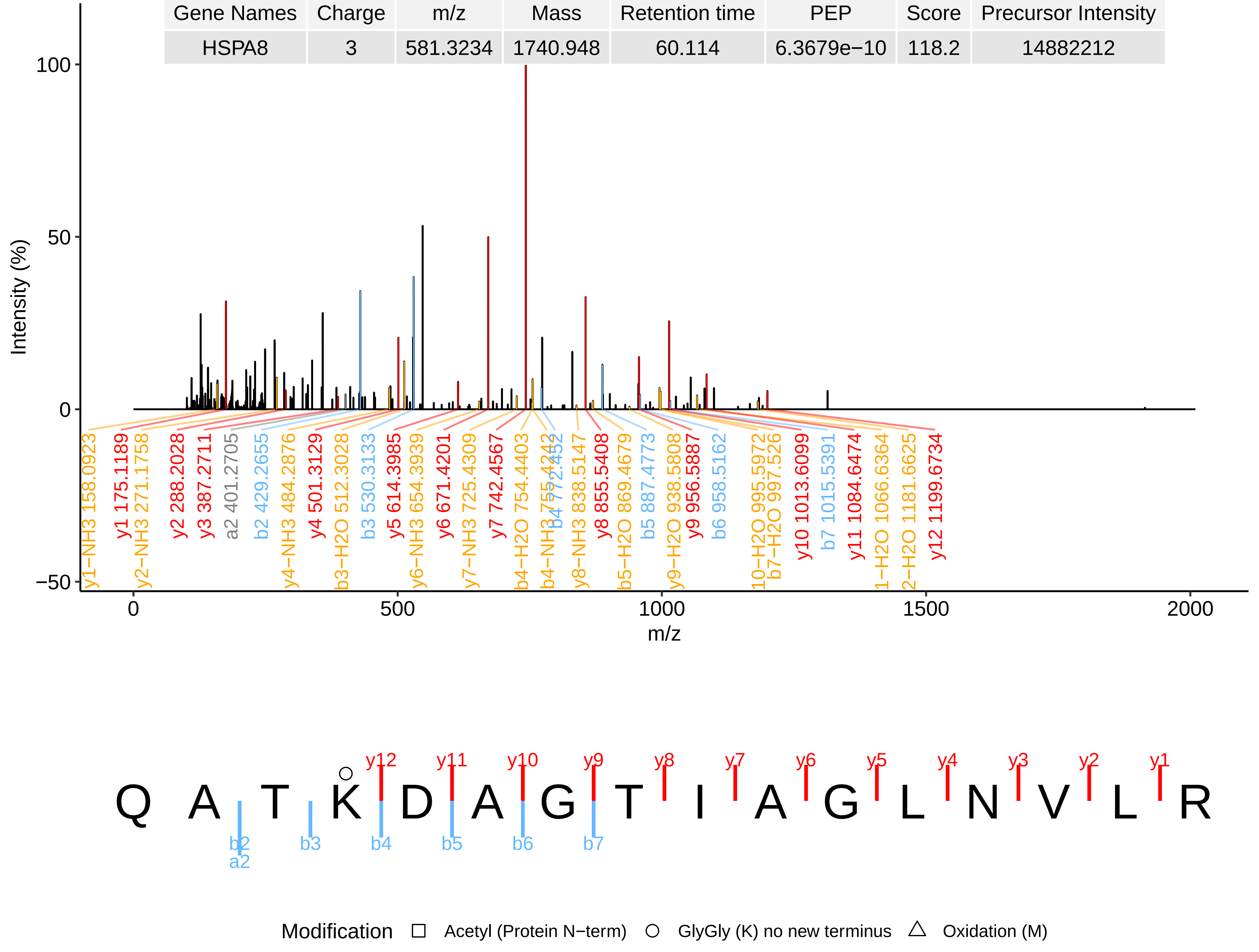

20200921_KIZ_MUb4_GlyGly_10plex − Scan number: 16750

Gene Names	Charge	m/z	Mass	Retention time	PEP	Score	Precursor Intensity
HSPA8	2	871.4814	1740.948	60.275	5.9784e−14	140.91	5273262

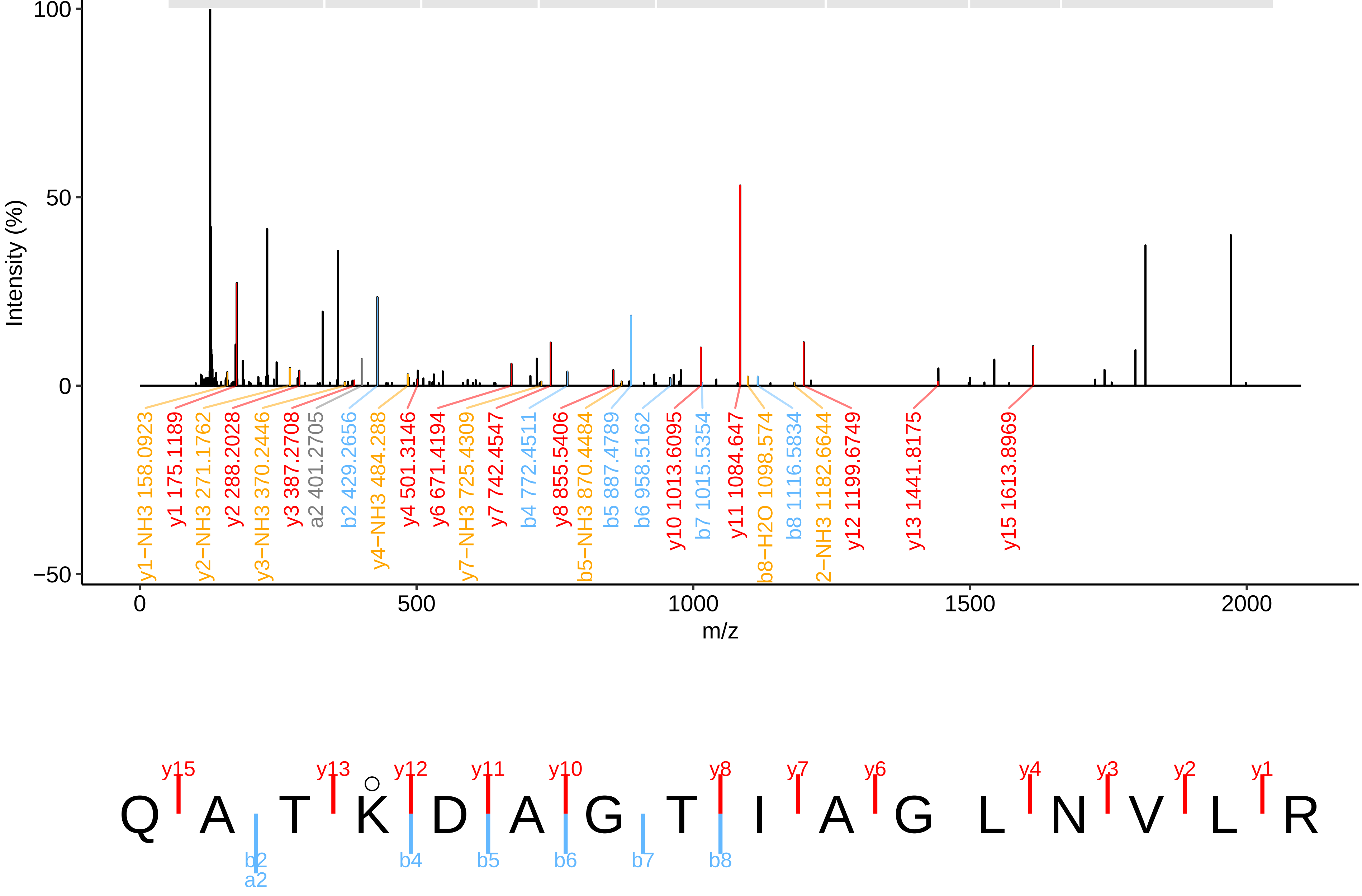

Modification □ Acetyl (Protein N−term) ○ GlyGly (K) no new terminus △ Oxidation (M)

20200921_KIZ_MUb4_GlyGly_10plex_2 − Scan number: 15509

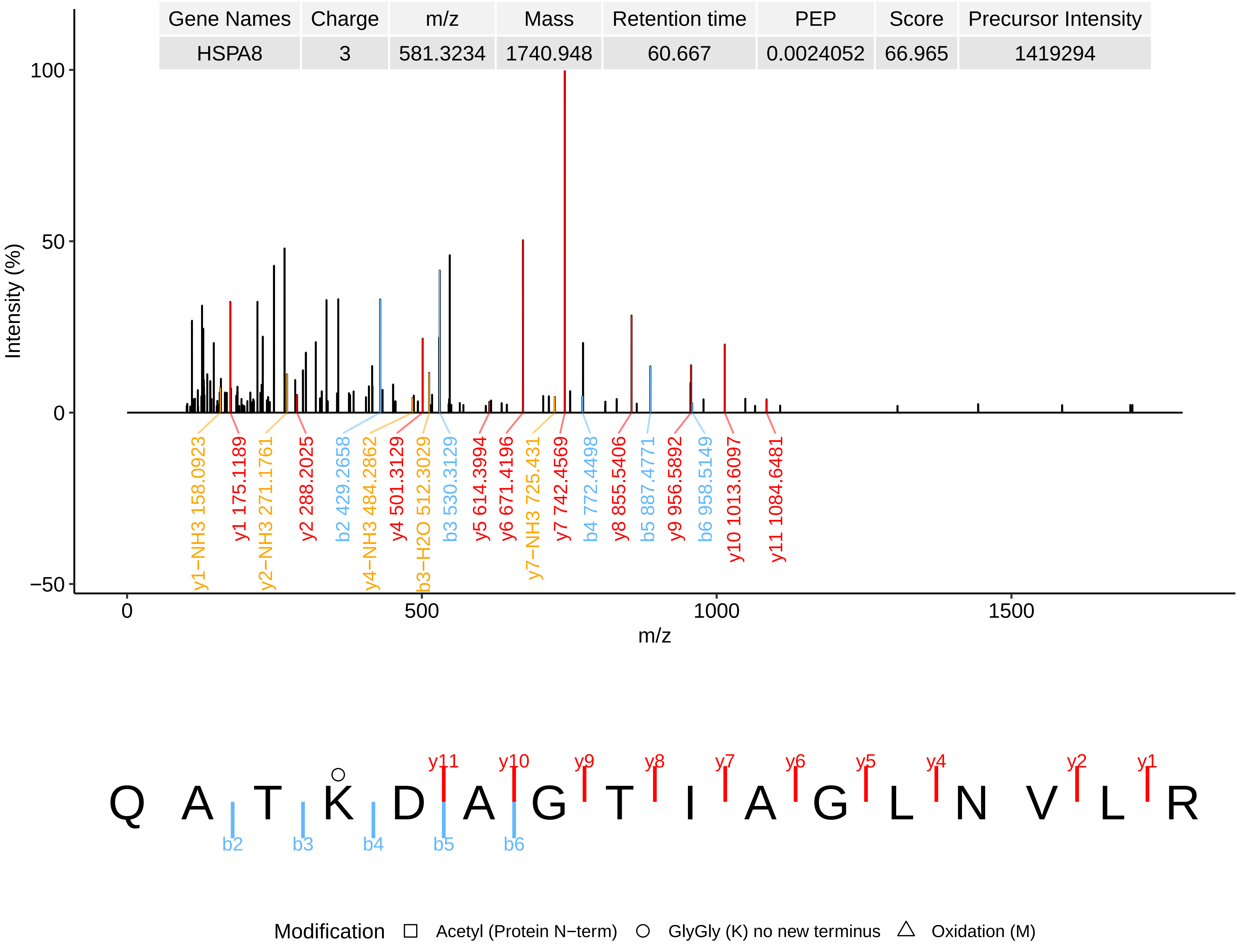

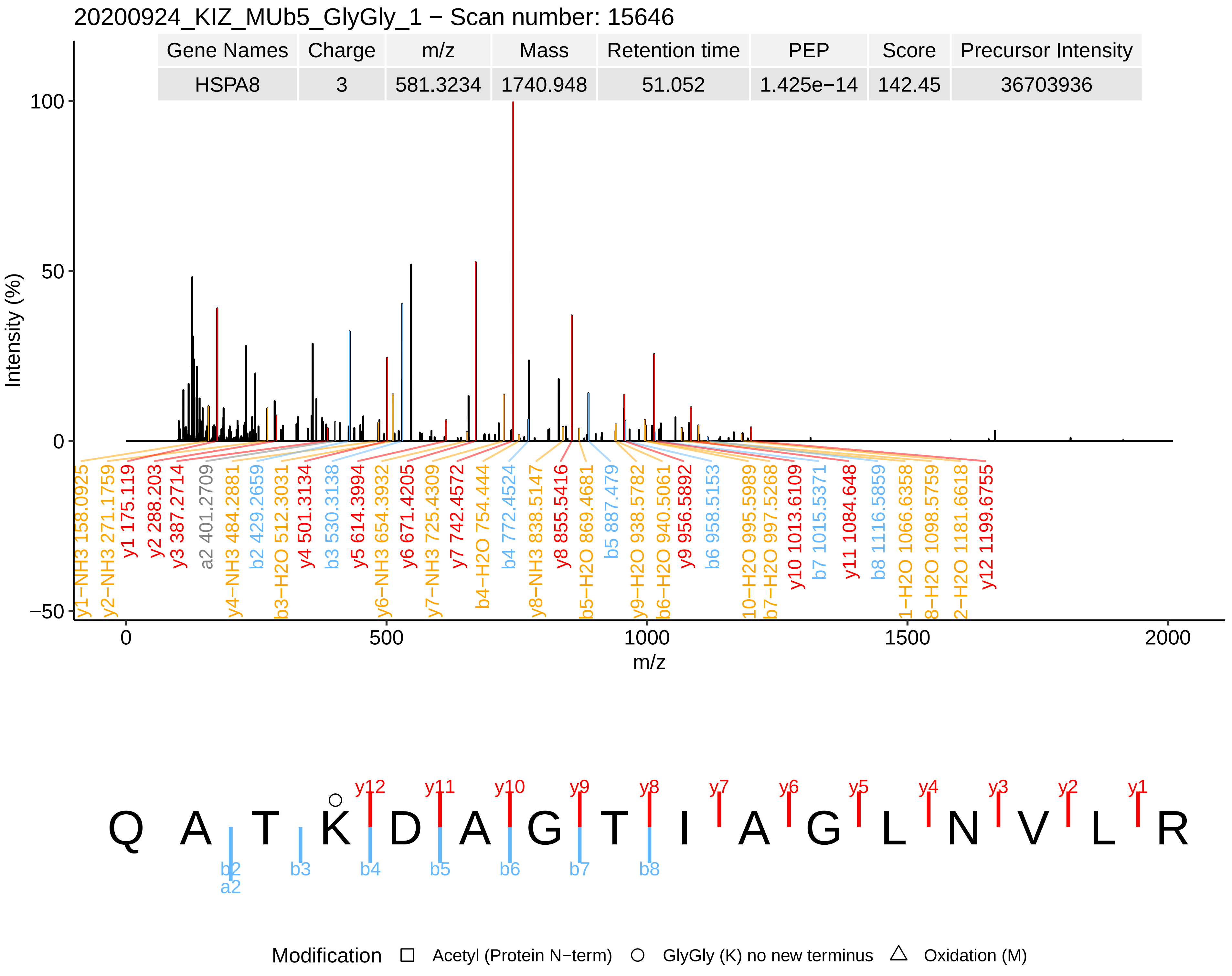
20200924_KIZ_MUb5_GlyGly_1 − Scan number: 15646
Gene Names
Charge
m/z
Mass
Retention time
PEP
Score
Precursor Intensity
HSPA8
3
581.3234
1740.948
51.052
1.425e−14
142.45
36703936
Intensity (%)
m/z
y1−NH3 158.0925
y2−NH3 271.1759
y1 175.119
y2 288.203
y3 387.2714
a2 401.2709
y4−NH3 484.2881
b2 429.2659
b3−H2O 512.3031
y4 501.3134
b3 530.3138
y5 614.3994
y6−NH3 654.3932
y6 671.4205
y7−NH3 725.4309
y7 742.4572
b4−H2O 754.444
b4 772.4524
y8−NH3 838.5147
y8 855.5416
b5−H2O 869.4681
b5 887.479
y9−H2O 938.5782
b6−H2O 940.5061
y9 956.5892
b6 958.5153
10−H2O 995.5989
b7−H2O 997.5268
y10 1013.6109
b7 1015.5371
y11 1084.648
b8 1116.5859
1−H2O 1066.6358
8−H2O 1098.5759
2−H2O 1181.6618
y12 1199.6755
Q A T K D A G T I A G L N V L R
y12 y11 y10 y9 y8 y7 y6 y5 y4 y3 y2 y1
b2 a2 b3 b4 b5 b6 b7 b8
Modification
Acetyl (Protein N−term)
GlyGly (K) no new terminus
Oxidation (M)

20200924_KIZ_MUb5_GlyGly_2 − Scan number: 14801

Gene Names	Charge	m/z	Mass	Retention time	PEP	Score	Precursor Intensity
HSPA8	3	581.3234	1740.948	55.297	2.3841e−07	105.79	2783077

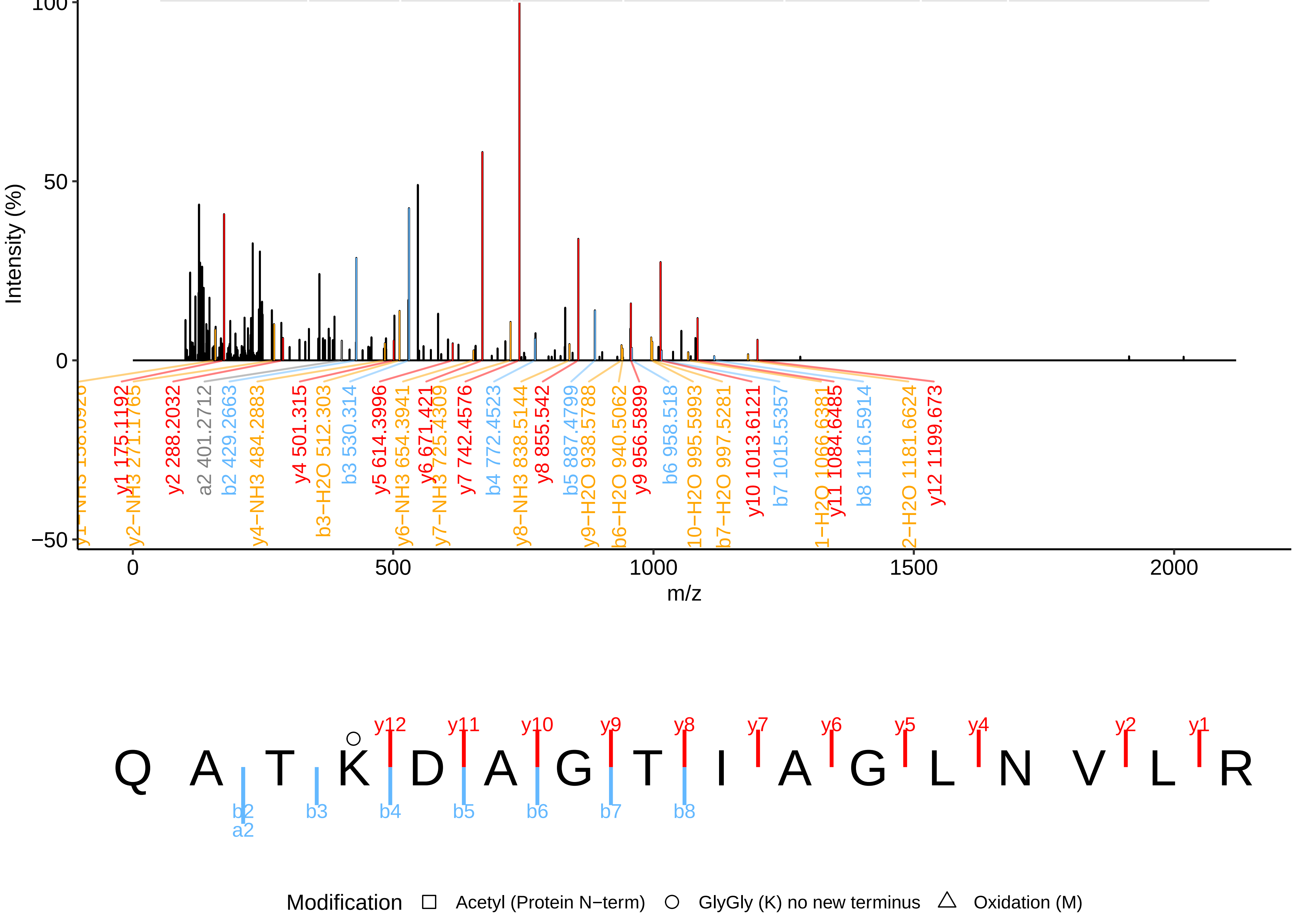

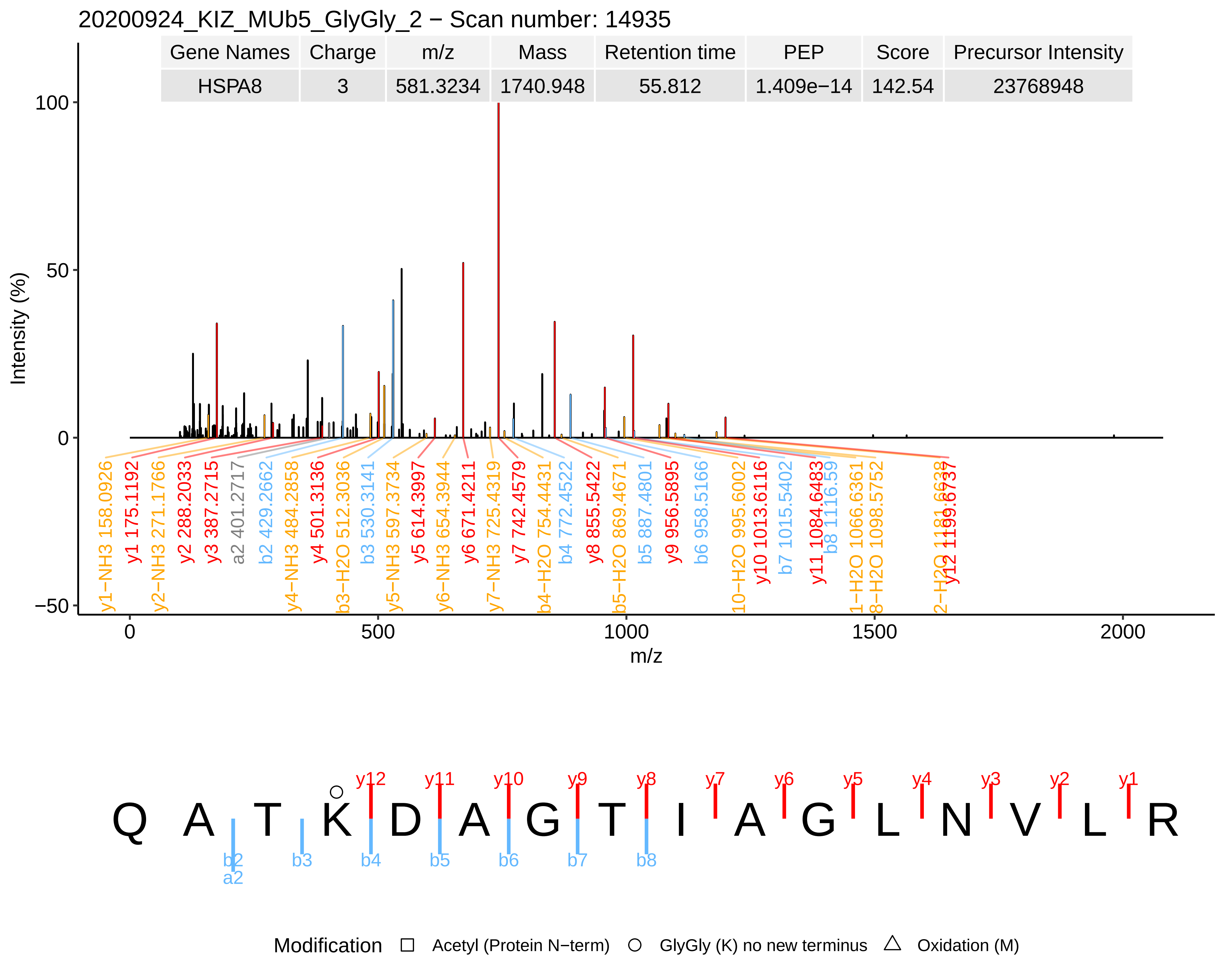
20200924_KIZ_MUb5_GlyGly_2 − Scan number: 14935
Gene Names | Charge | m/z | Mass | Retention time | PEP | Score | Precursor Intensity
HSPA8 | 3 | 581.3234 | 1740.948 | 55.812 | 1.409e−14 | 142.54 | 23768948
Intensity (%)
m/z
y1−NH3 158.0926
y1 175.1192
y2−NH3 271.1766
y2 288.2033
y3 387.2715
a2 401.2717
b2 429.2662
y4−NH3 484.2858
y4 501.3136
b3−H2O 512.3036
b3 530.3141
y5−NH3 597.3734
y5 614.3997
y6−NH3 654.3944
y6 671.4211
y7−NH3 725.4319
y7 742.4579
b4−H2O 754.4431
b4 772.4522
y8 855.5422
b5−H2O 869.4671
b5 887.4801
y9 956.5895
b6 958.5166
y10 1013.6116
b7 1015.5402
y11 1084.6483
b8 1116.59
y12 1199.6737
Q A T K D A G T I A G L N V L R
y12 y11 y10 y9 y8 y7 y6 y5 y4 y3 y2 y1
b2 a2 b3 b4 b5 b6 b7 b8
Modification
Acetyl (Protein N−term)
GlyGly (K) no new terminus
Oxidation (M)

20200924_KIZ_MUb5_GlyGly_2 − Scan number: 15073

Gene Names	Charge	m/z	Mass	Retention time	PEP	Score	Precursor Intensity
HSPA8	3	581.3234	1740.948	56.333	7.8299e−11	131.03	4909085

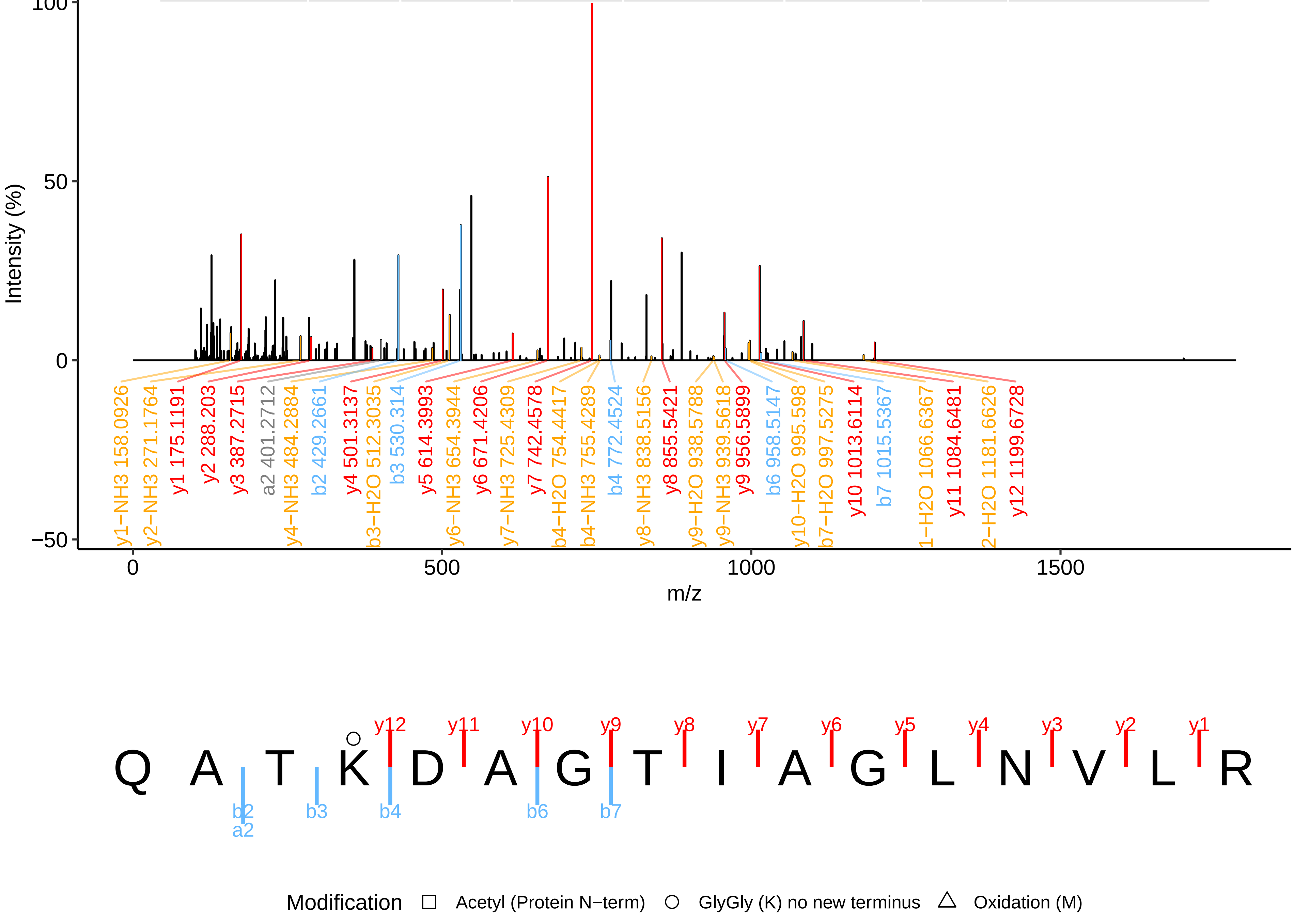

20201118_KIZ_MUb6_GlyGly_1 − Scan number: 15918

Gene Names	Charge	m/z	Mass	Retention time	PEP	Score	Precursor Intensity
HSPA8	3	581.3234	1740.948	50.339	1.5959e−15	149.37	18737242

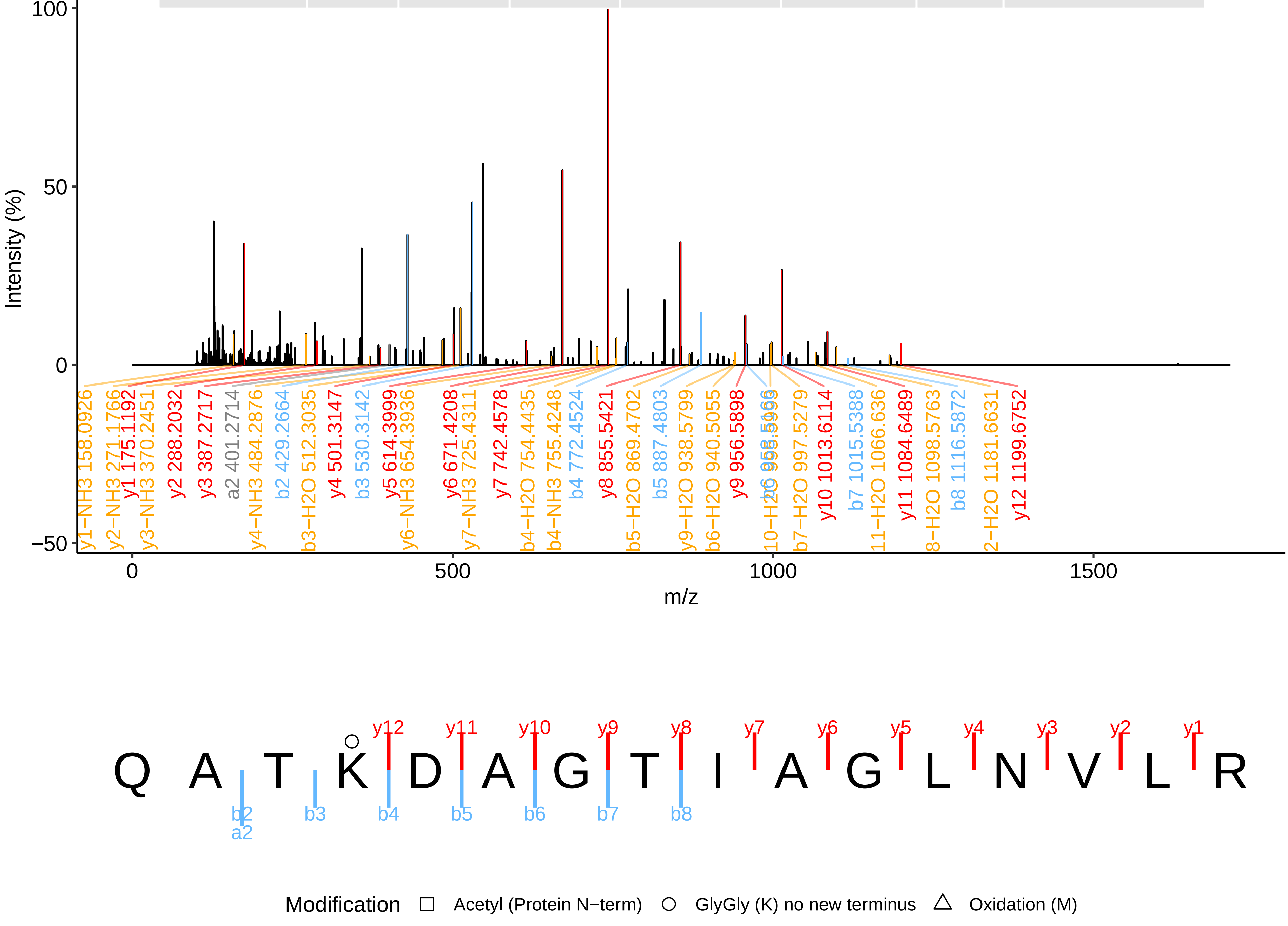

20201118_KIZ_MUb6_GlyGly_2 − Scan number: 18130

Gene Names	Charge	m/z	Mass	Retention time	PEP	Score	Precursor Intensity
HSPA8	3	581.3234	1740.948	61.322	1.9718e−07	107.46	17060384

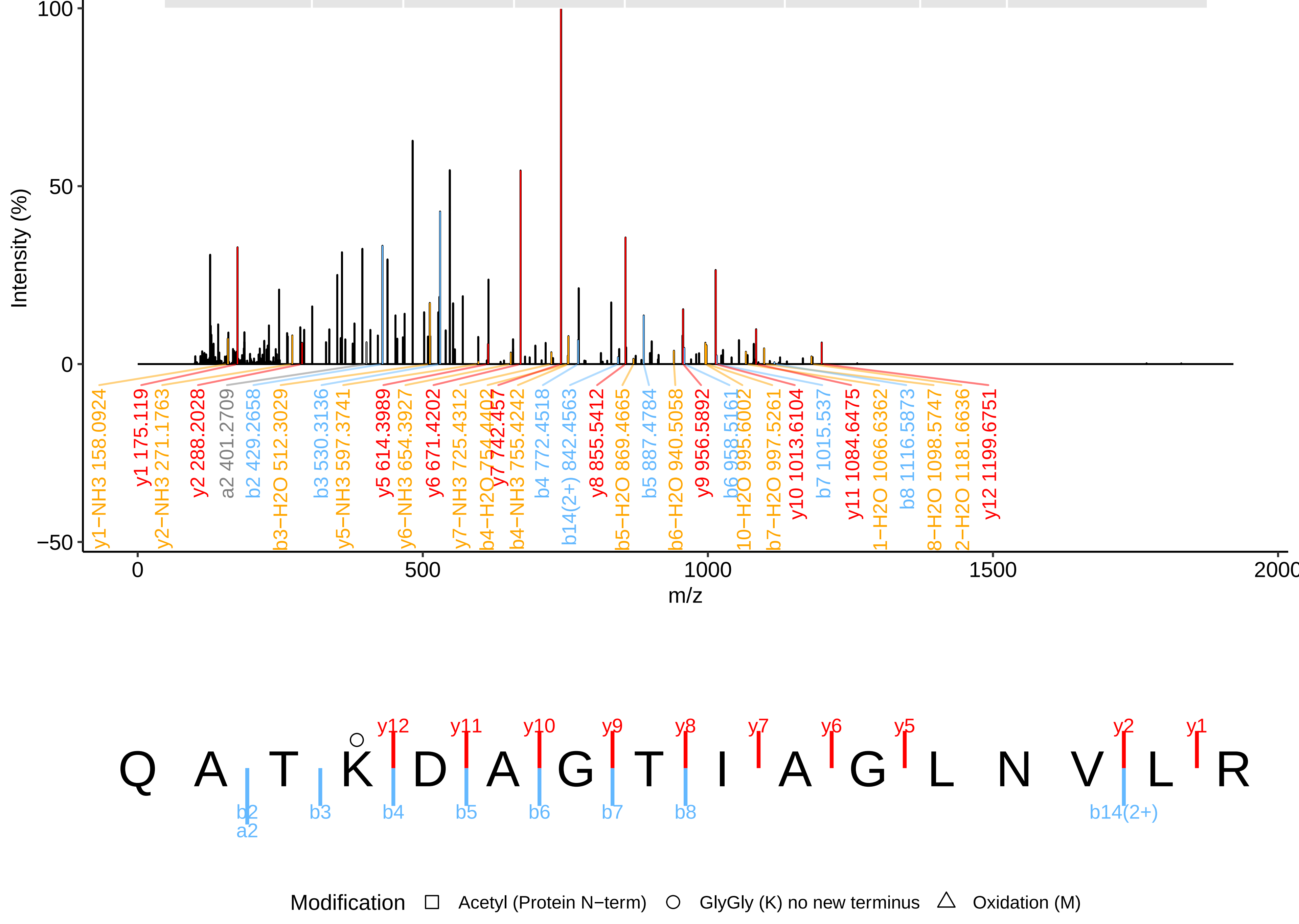

20200921_KIZ_MUb4_GlyGly_10plex − Scan number: 19602

Gene Names	Charge	m/z	Mass	Retention time	PEP	Score	Precursor Intensity
HSPA1B;HSPA1A;HSPA1L	3	580.6636	1738.969	70.733	7.4506e−06	91.07	5775968

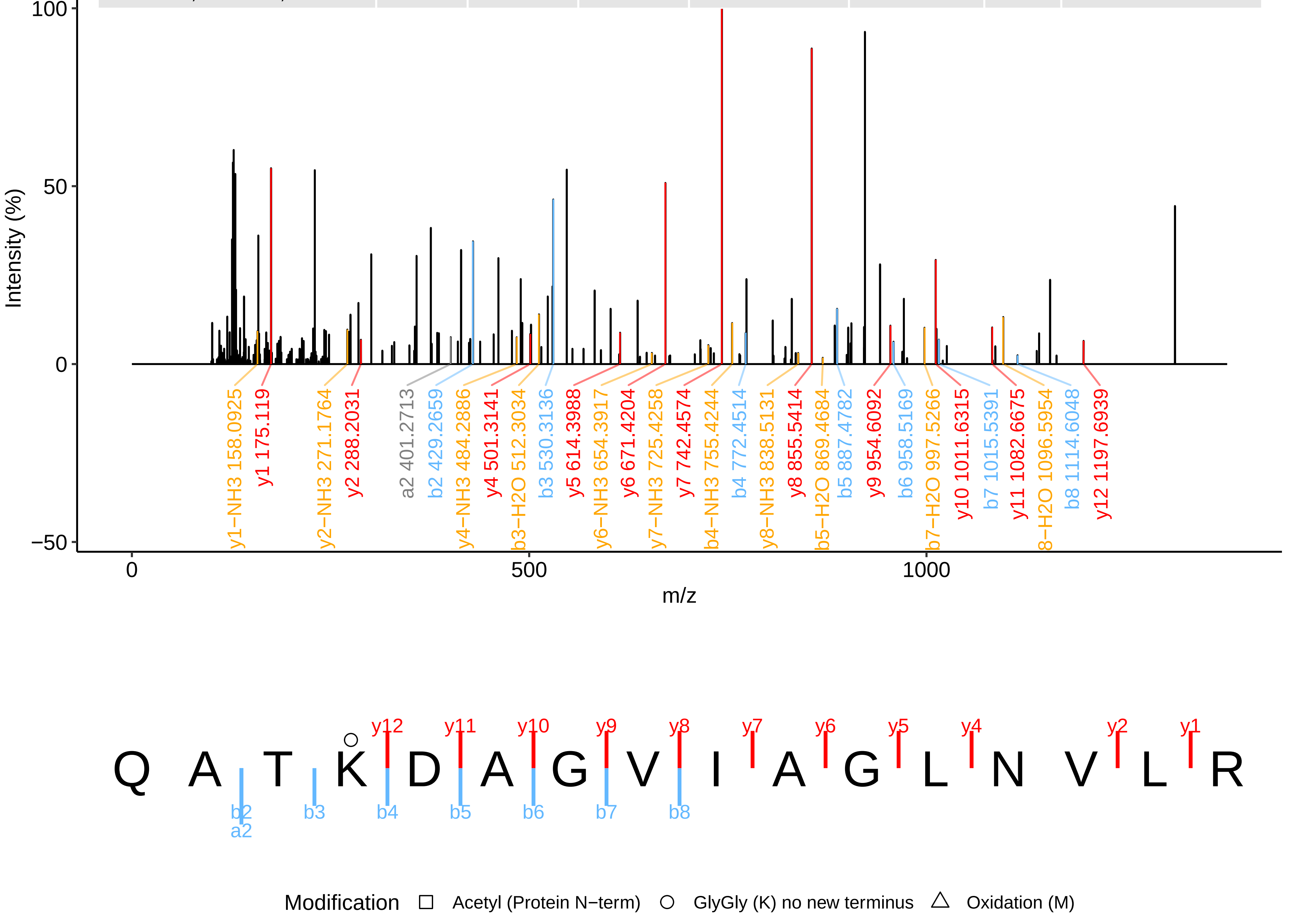

20200921_KIZ_MUb4_GlyGly_10plex − Scan number: 19740

Gene Names	Charge	m/z	Mass	Retention time	PEP	Score	Precursor Intensity
HSPA1B;HSPA1A;HSPA1L	3	580.6636	1738.969	71.247	2.6326e−14	135.86	21156808

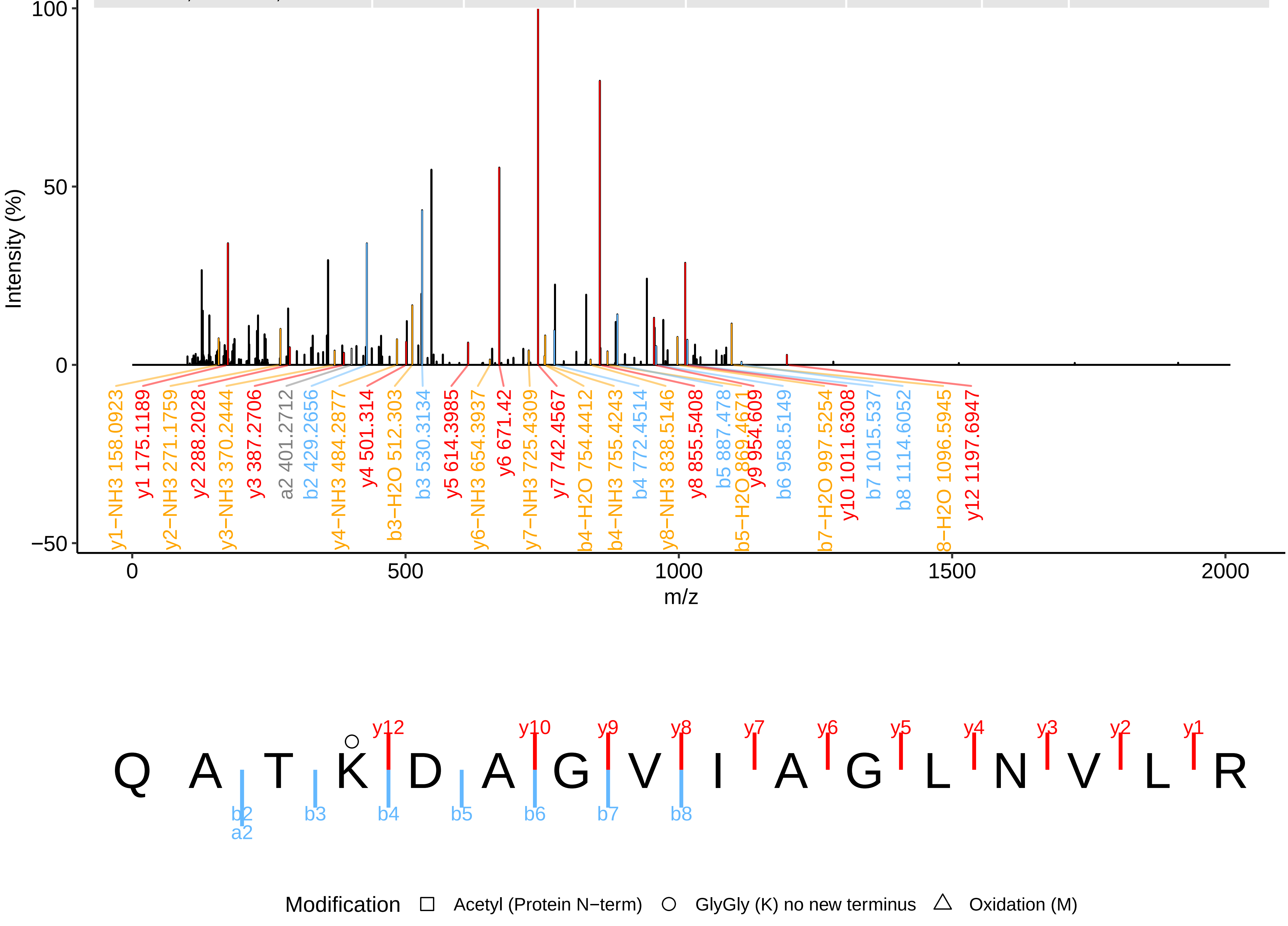

20200921_KIZ_MUb4_GlyGly_10plex − Scan number: 19880

Gene Names	Charge	m/z	Mass	Retention time	PEP	Score	Precursor Intensity
HSPA1B;HSPA1A;HSPA1L	3	580.6636	1738.969	71.763	3.9293e−10	123.8	10698288

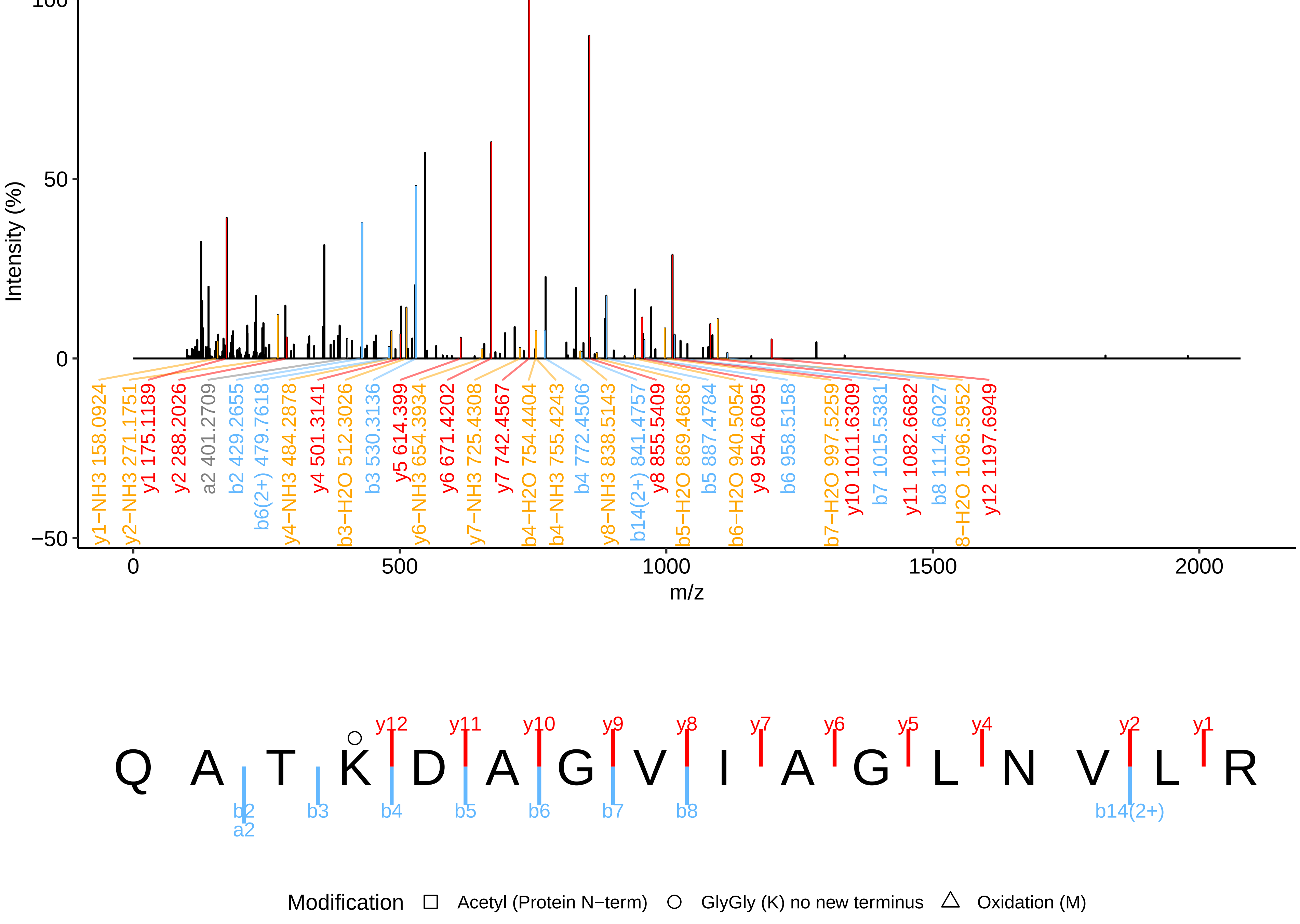

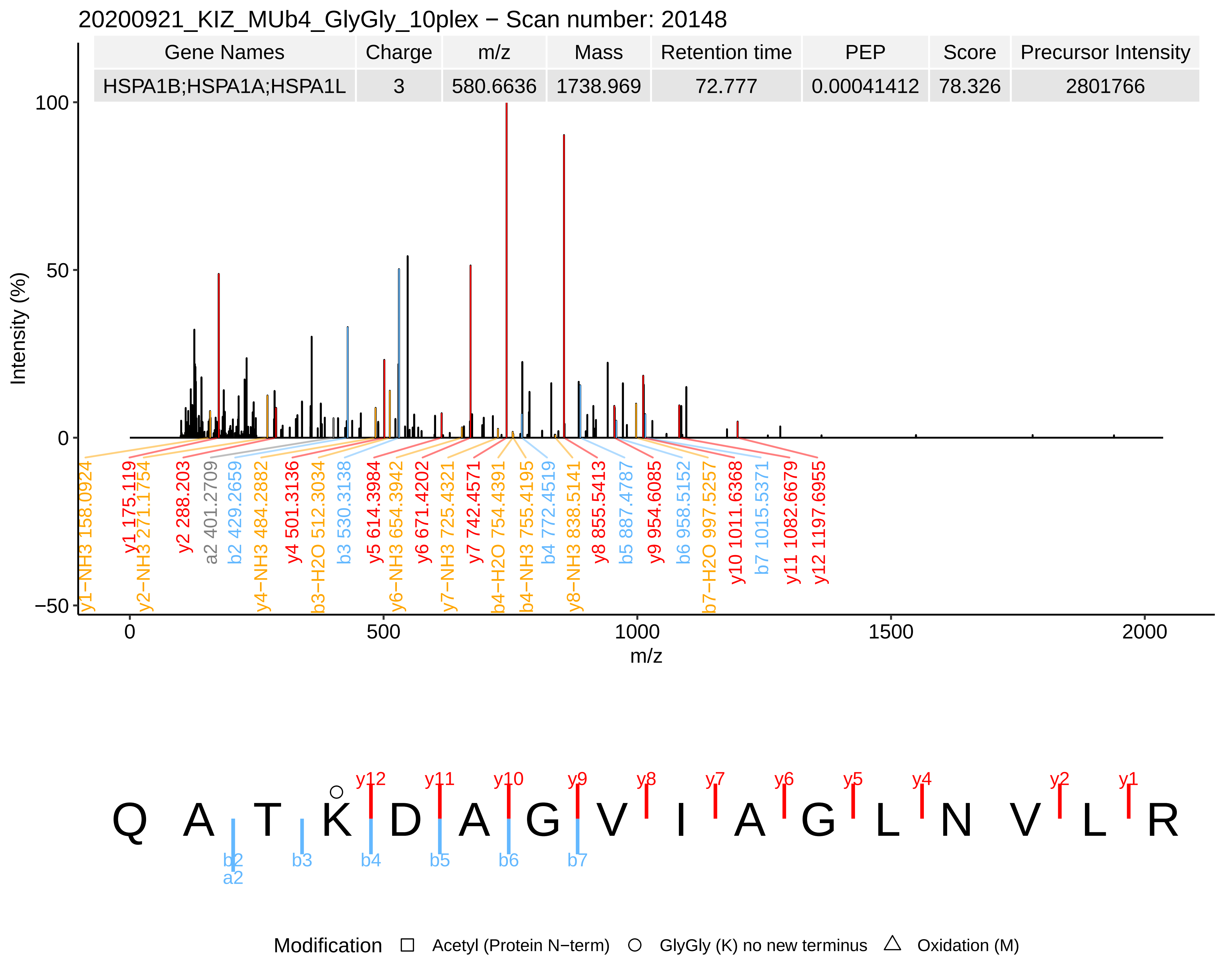

20200921_KIZ_MUb4_GlyGly_10plex − Scan number: 20148
Gene Names | Charge | m/z | Mass | Retention time | PEP | Score | Precursor Intensity
HSPA1B;HSPA1A;HSPA1L | 3 | 580.6636 | 1738.969 | 72.777 | 0.00041412 | 78.326 | 2801766
Intensity (%)
100
50
0
−50
0
500
1000
1500
2000
m/z
y1−NH3 158.0924
y1 175.119
y2−NH3 271.1754
y2 288.203
a2 401.2709
b2 429.2659
y4−NH3 484.2882
y4 501.3136
b3−H2O 512.3034
b3 530.3138
y5 614.3984
y6−NH3 654.3942
y6 671.4202
y7−NH3 725.4321
y7 742.4571
b4−H2O 754.4391
b4−NH3 755.4195
b4 772.4519
y8−NH3 838.5141
y8 855.5413
b5 887.4787
y9 954.6085
b6 958.5152
b7−H2O 997.5257
y10 1011.6368
b7 1015.5371
y11 1082.6679
y12 1197.6955
Q A T K D A G V I A G L N V L R
y12 y11 y10 y9 y8 y7 y6 y5 y4 y2 y1
b2 a2 b3 b4 b5 b6 b7
Modification
Acetyl (Protein N−term)
GlyGly (K) no new terminus
Oxidation (M)

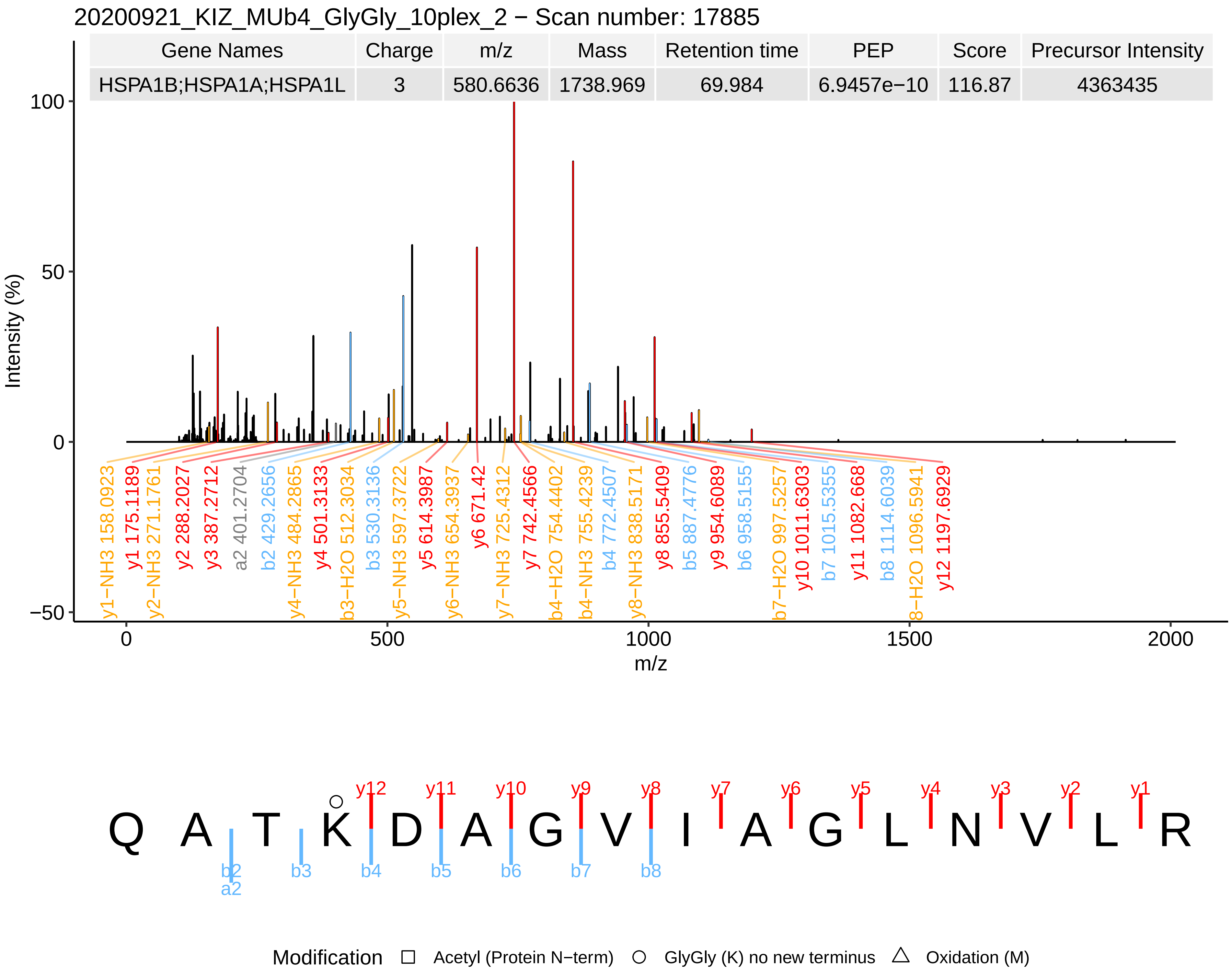
20200921_KIZ_MUb4_GlyGly_10plex_2 − Scan number: 17885
Gene Names | Charge | m/z | Mass | Retention time | PEP | Score | Precursor Intensity
HSPA1B;HSPA1A;HSPA1L | 3 | 580.6636 | 1738.969 | 69.984 | 6.9457e−10 | 116.87 | 4363435
Intensity (%)
m/z
y1−NH3 158.0923
y1 175.1189
y2−NH3 271.1761
y2 288.2027
y3 387.2712
a2 401.2704
b2 429.2656
y4−NH3 484.2865
y4 501.3133
b3−H2O 512.3034
b3 530.3136
y5−NH3 597.3722
y5 614.3987
y6−NH3 654.3937
y6 671.42
y7−NH3 725.4312
y7 742.4566
b4−H2O 754.4402
b4−NH3 755.4239
b4 772.4507
y8−NH3 838.5171
y8 855.5409
b5 887.4776
y9 954.6089
b6 958.5155
b7−H2O 997.5257
y10 1011.6303
b7 1015.5355
y11 1082.668
b8 1114.6039
8−H2O 1096.5941
y12 1197.6929
Q A T K D A G V I A G L N V L R
Modification
Acetyl (Protein N−term)
GlyGly (K) no new terminus
Oxidation (M)

20200924_KIZ_MUb5_GlyGly_1 − Scan number: 18733

Gene Names	Charge	m/z	Mass	Retention time	PEP	Score	Precursor Intensity
HSPA1B;HSPA1A;HSPA1L	3	580.6636	1738.969	60.537	5.2479e−10	120.77	15052180

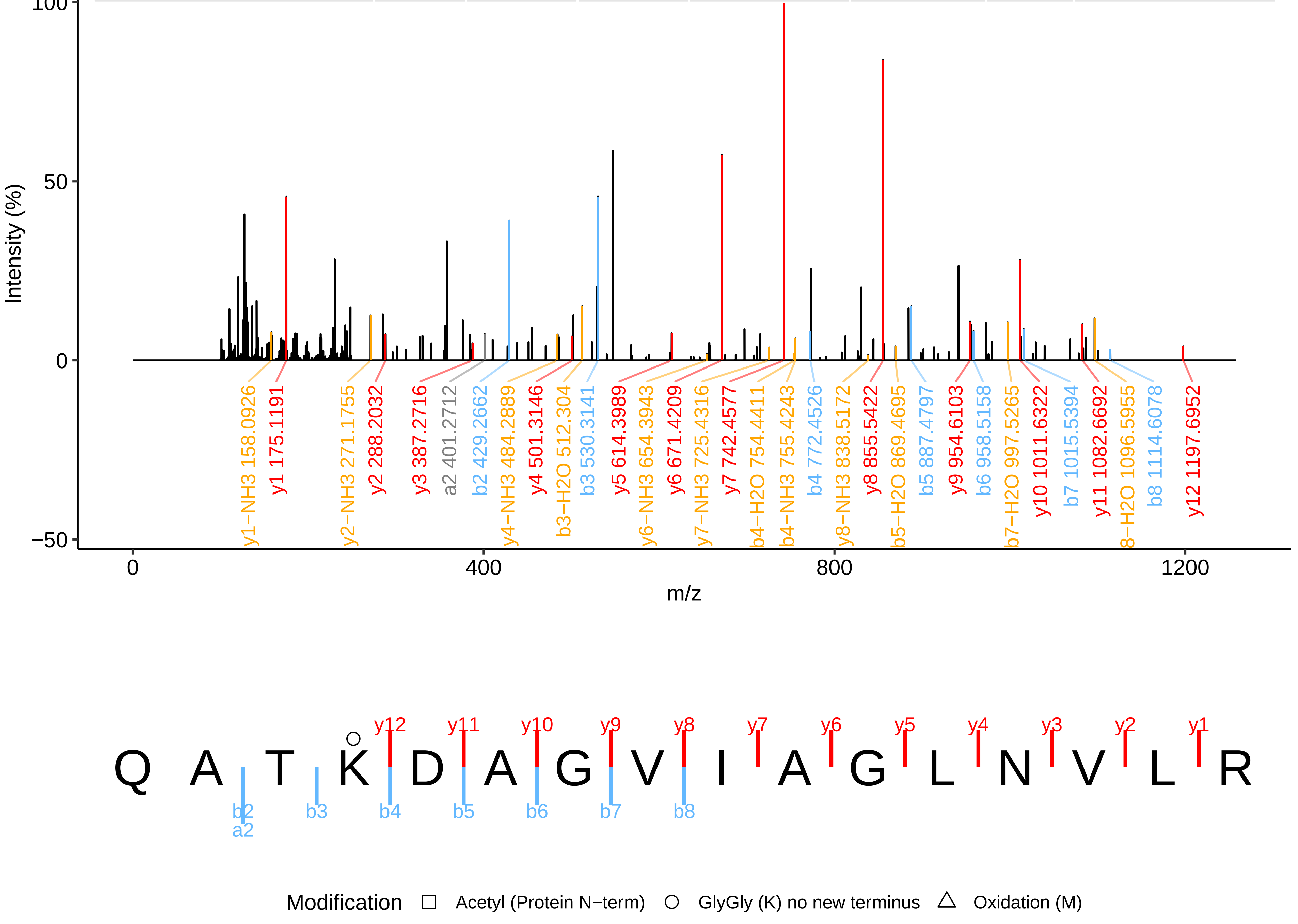

20200924_KIZ_MUb5_GlyGly_1 − Scan number: 18889

Gene Names	Charge	m/z	Mass	Retention time	PEP	Score	Precursor Intensity
HSPA1B;HSPA1A;HSPA1L	3	580.6636	1738.969	61.037	1.6758e−07	108.66	39652540

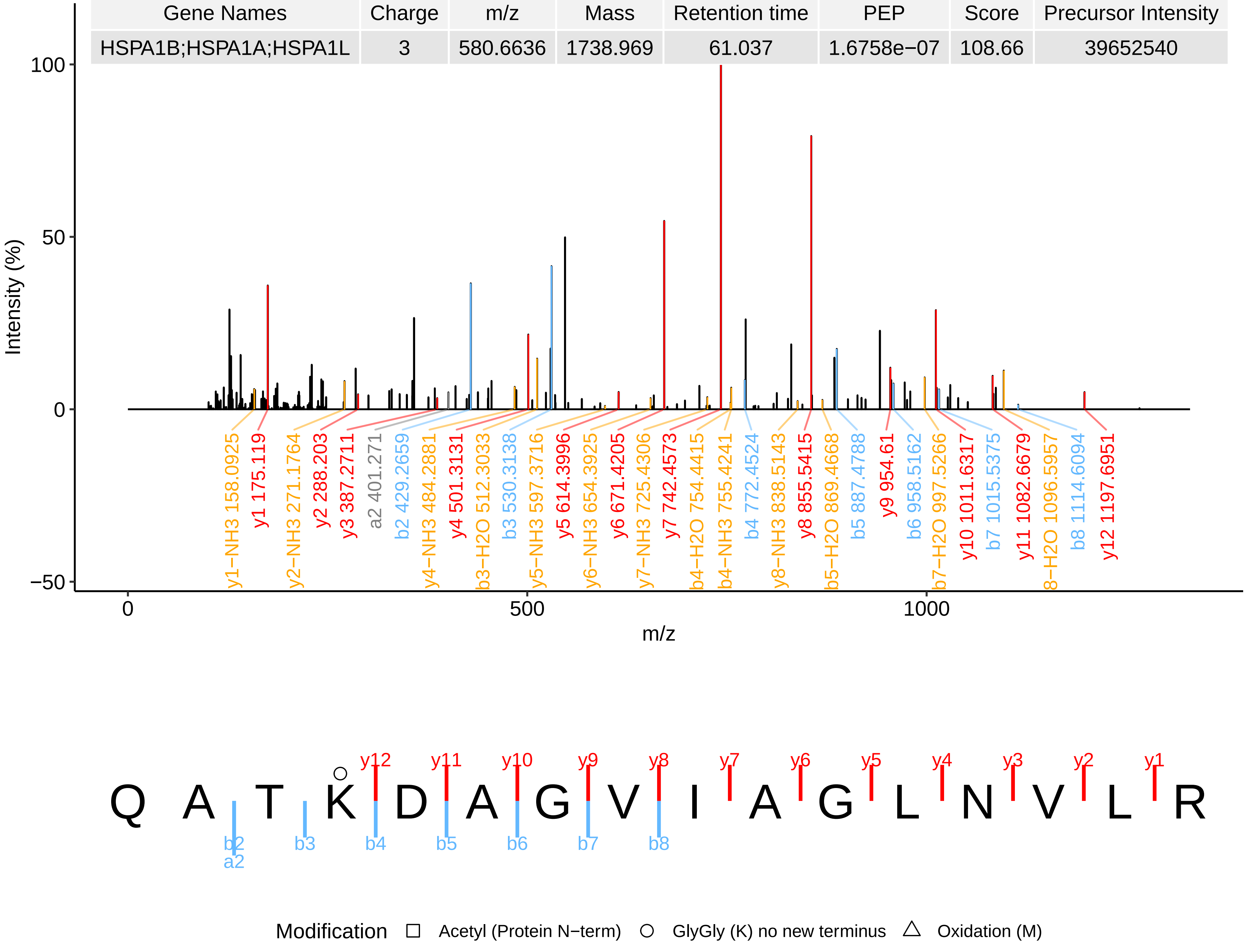

20200924_KIZ_MUb5_GlyGly_1 − Scan number: 19042

Gene Names	Charge	m/z	Mass	Retention time	PEP	Score	Precursor Intensity
HSPA1B;HSPA1A;HSPA1L	3	580.6636	1738.969	61.544	3.0697e−14	133.47	34109232

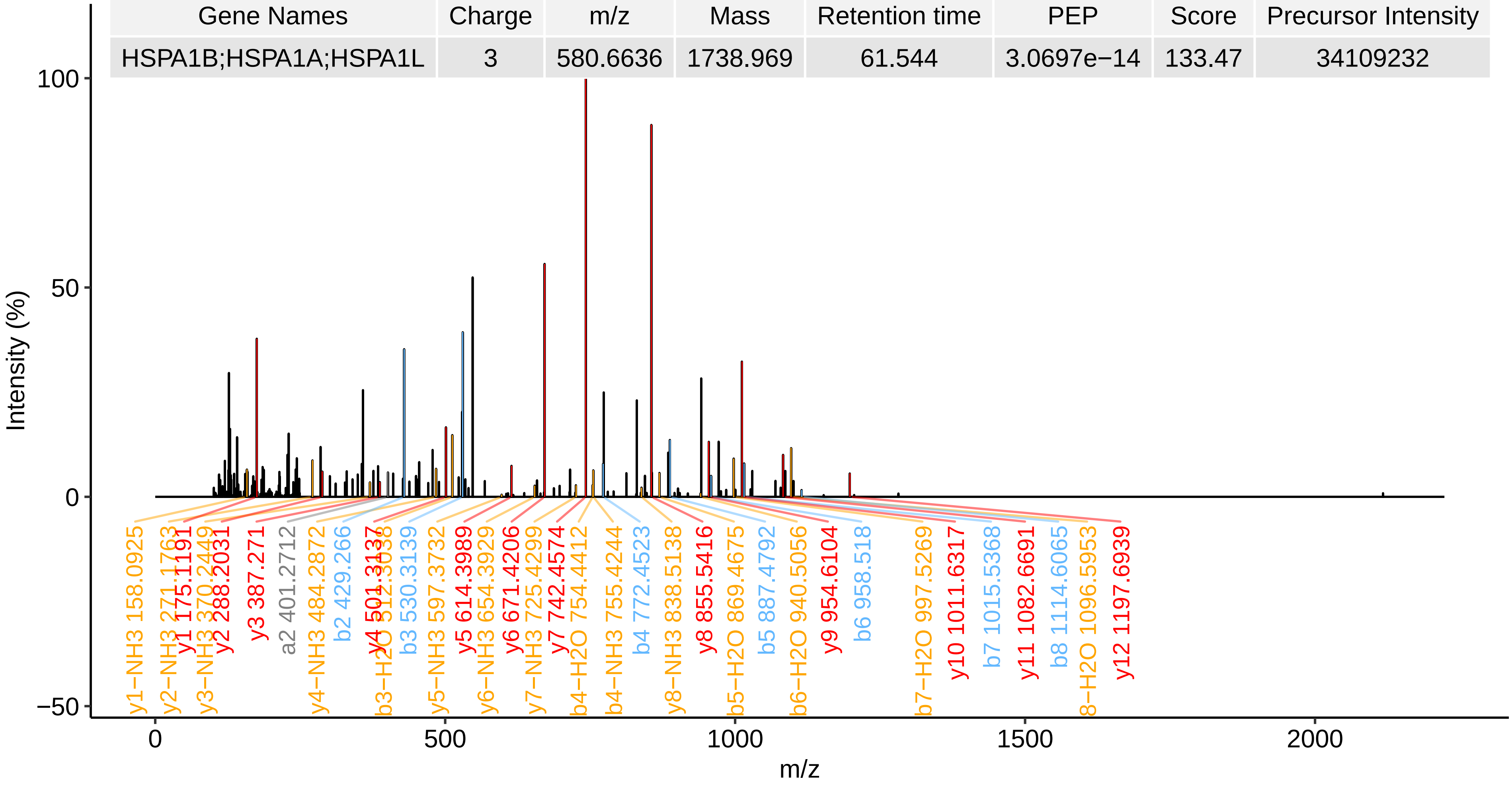

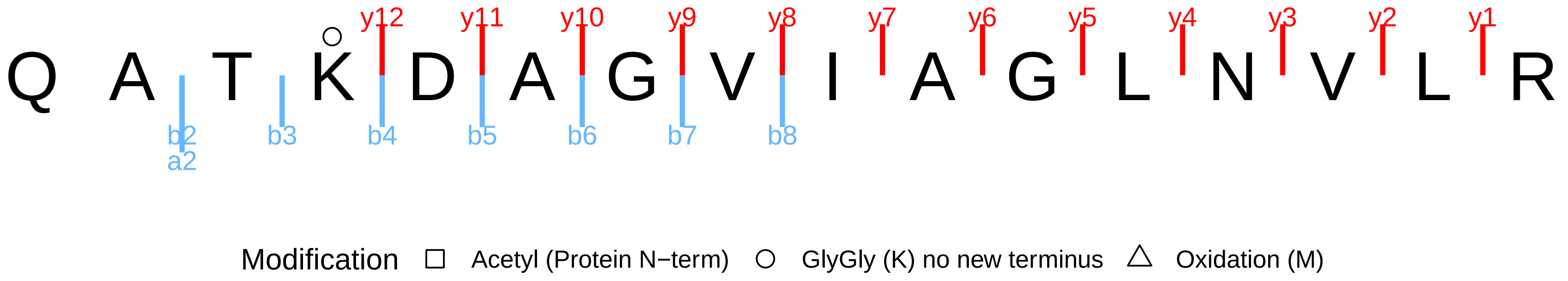

20200924_KIZ_MUb5_GlyGly_2 − Scan number: 17390

Gene Names	Charge	m/z	Mass	Retention time	PEP	Score	Precursor Intensity
HSPA1B;HSPA1A;HSPA1L	3	580.6636	1738.969	64.459	2.183e−07	106.6	5988054

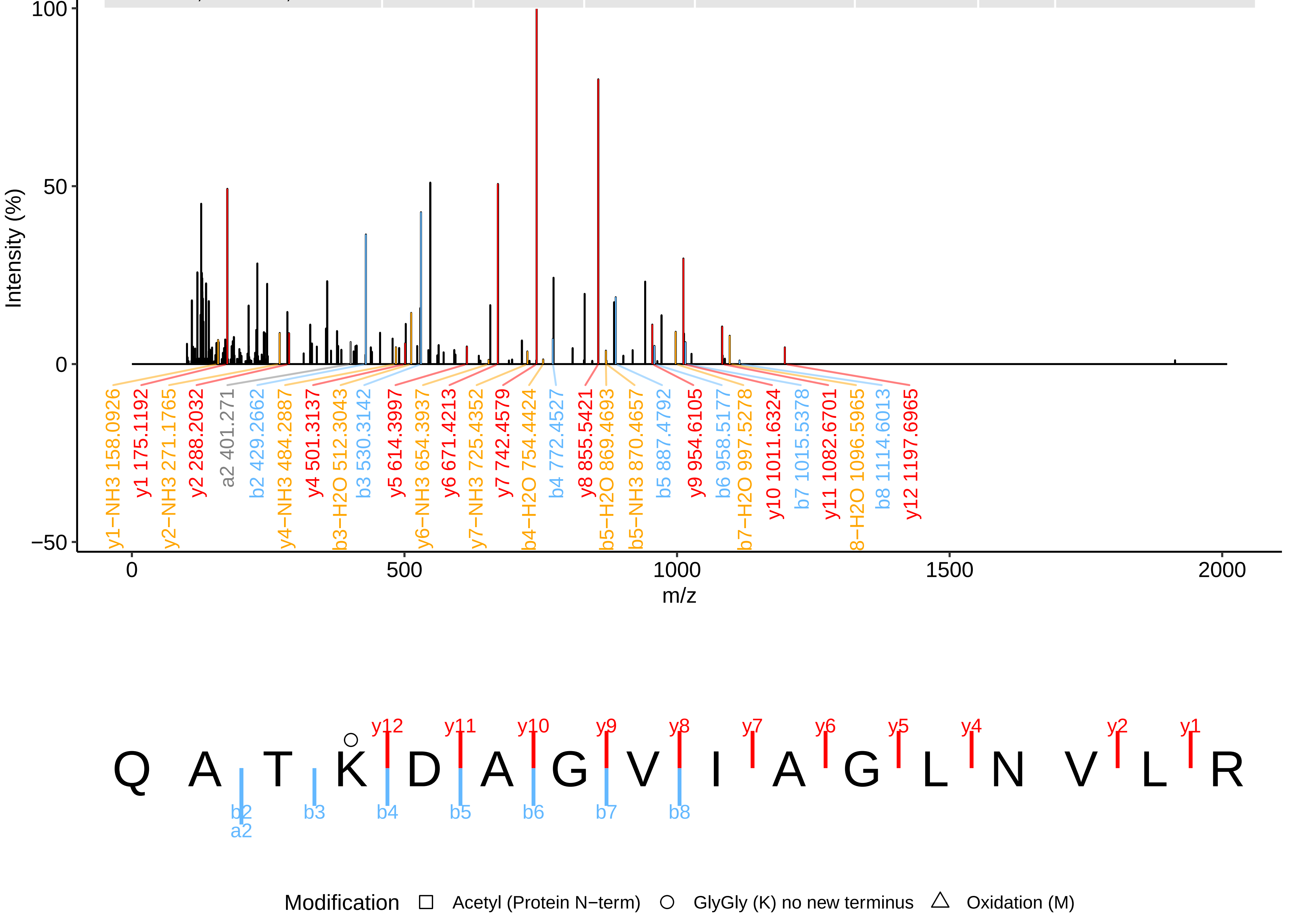

20200924_KIZ_MUb5_GlyGly_2 − Scan number: 17556

Gene Names	Charge	m/z	Mass	Retention time	PEP	Score	Precursor Intensity
HSPA1B;HSPA1A;HSPA1L	3	580.6636	1738.969	65.069	1.7932e−10	128.71	14271865

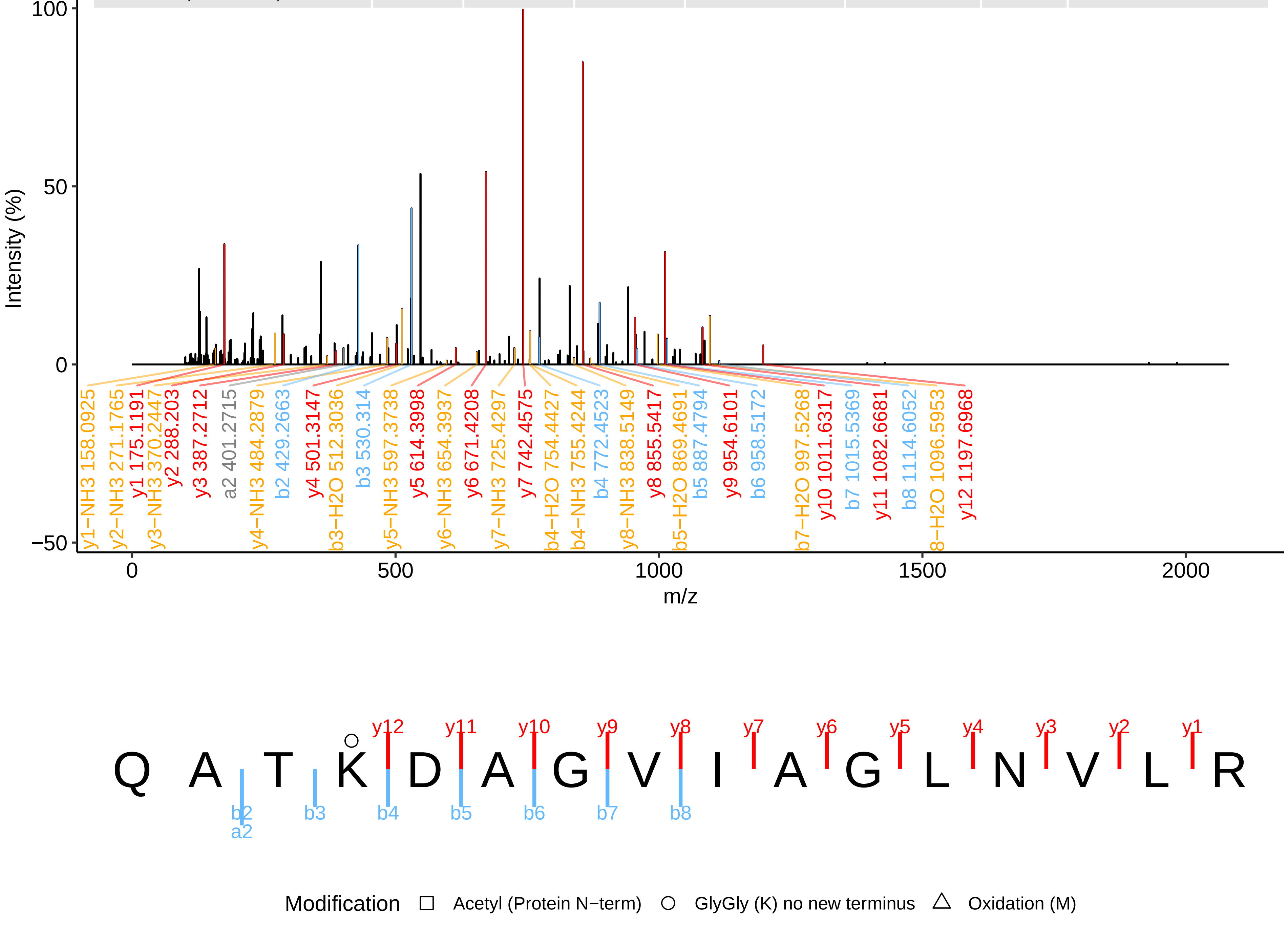

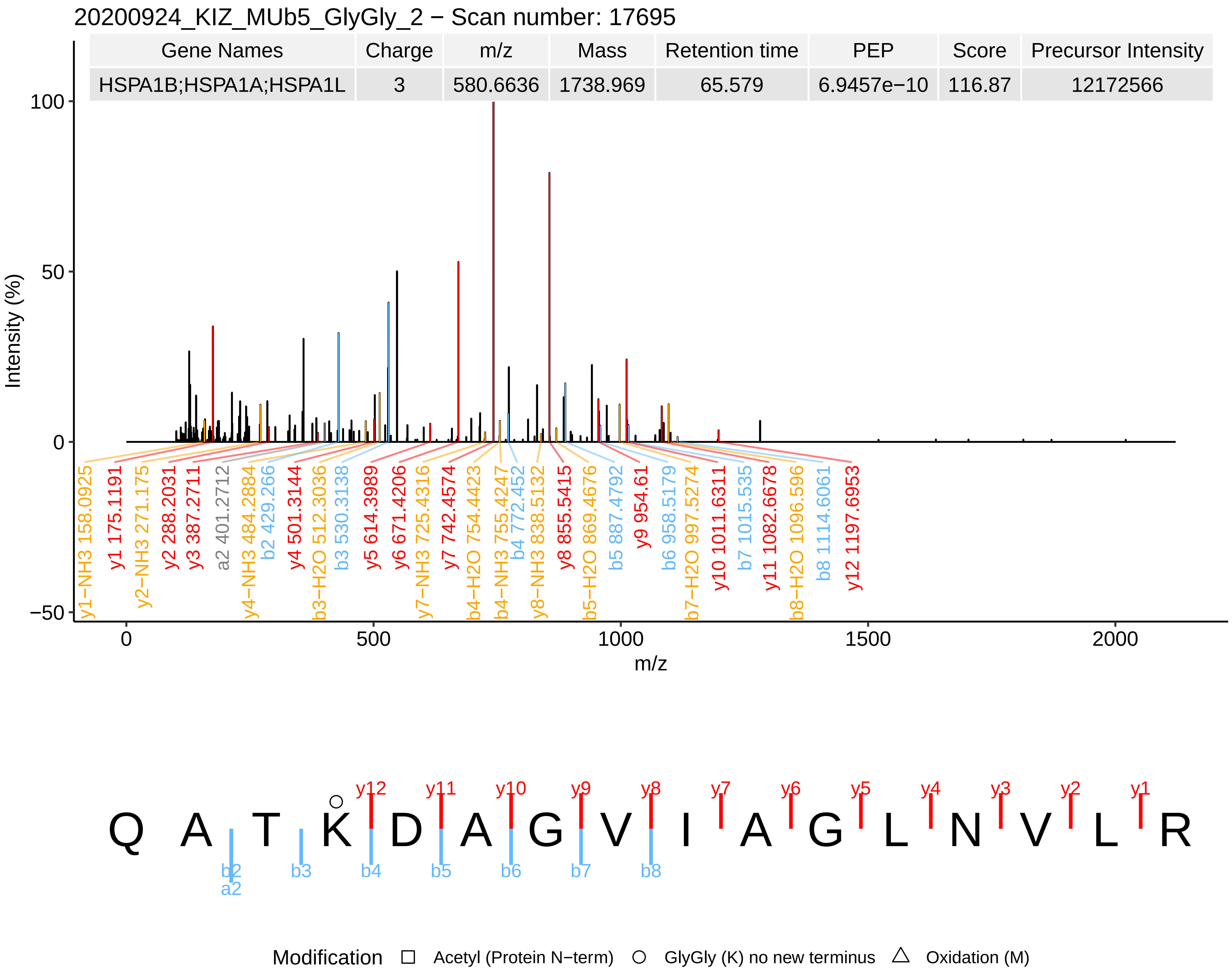
20200924_KIZ_MUb5_GlyGly_2 − Scan number: 17695
Gene Names
Charge
m/z
Mass
Retention time
PEP
Score
Precursor Intensity
HSPA1B;HSPA1A;HSPA1L
3
580.6636
1738.969
65.579
6.9457e−10
116.87
12172566
Intensity (%)
m/z
y1−NH3 158.0925
y1 175.1191
y2−NH3 271.175
y2 288.2031
y3 387.2711
a2 401.2712
y4−NH3 484.2884
b2 429.266
y4 501.3144
b3−H2O 512.3036
b3 530.3138
y5 614.3989
y6 671.4206
y7−NH3 725.4316
y7 742.4574
b4−H2O 754.4423
b4−NH3 755.4247
b4 772.452
y8−NH3 838.5132
y8 855.5415
b5−H2O 869.4676
b5 887.4792
y9 954.61
b6 958.5179
b7−H2O 997.5274
y10 1011.6311
b7 1015.535
y11 1082.6678
b8−H2O 1096.596
b8 1114.6061
y12 1197.6953
Q A T K D A G V I A G L N V L R
y12 y11 y10 y9 y8 y7 y6 y5 y4 y3 y2 y1
b2 a2 b3 b4 b5 b6 b7 b8
Modification
Acetyl (Protein N−term)
GlyGly (K) no new terminus
Oxidation (M)

20201118_KIZ_MUb6_GlyGly_1 − Scan number: 19153

Gene Names	Charge	m/z	Mass	Retention time	PEP	Score	Precursor Intensity
HSPA1B;HSPA1A;HSPA1L	3	580.6636	1738.969	60.108	1.8812e−07	107.82	33300010

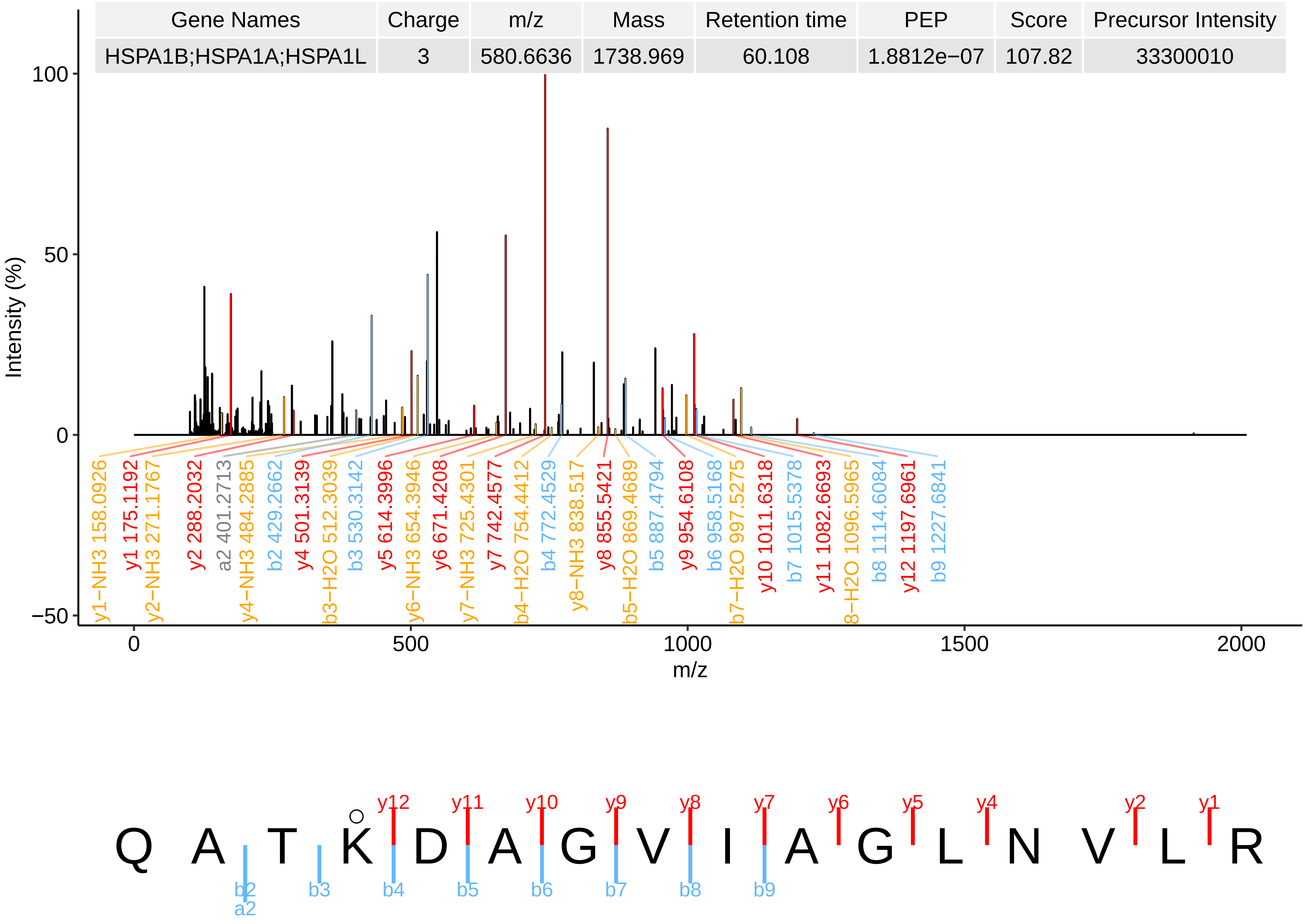

Modification ☐ Acetyl (Protein N−term) ○ GlyGly (K) no new terminus △ Oxidation (M)

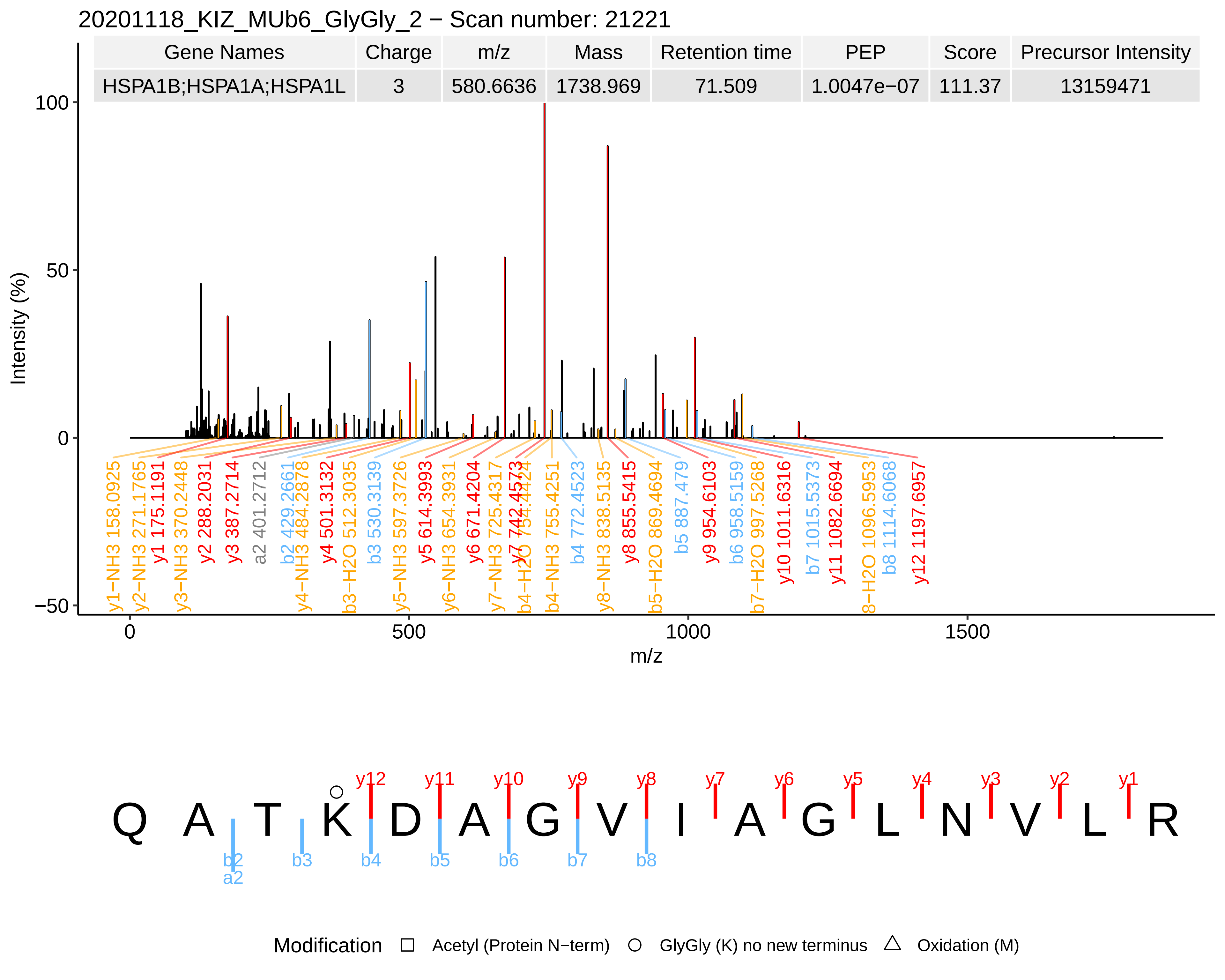
20201118_KIZ_MUb6_GlyGly_2 − Scan number: 21221
Gene Names | Charge | m/z | Mass | Retention time | PEP | Score | Precursor Intensity
HSPA1B;HSPA1A;HSPA1L | 3 | 580.6636 | 1738.969 | 71.509 | 1.0047e−07 | 111.37 | 13159471
Intensity (%)
m/z
y1−NH3 158.0925
y2−NH3 271.1765
y1 175.1191
y3−NH3 370.2448
y2 288.2031
y3 387.2714
a2 401.2712
b2 429.2661
y4−NH3 484.2878
y4 501.3132
b3−H2O 512.3035
b3 530.3139
y5−NH3 597.3726
y5 614.3993
y6−NH3 654.3931
y6 671.4204
y7−NH3 725.4317
y7 742.4573
b4−H2O 754.4424
b4−NH3 755.4251
b4 772.4523
y8−NH3 838.5135
y8 855.5415
b5−H2O 869.4694
b5 887.479
y9 954.6103
b6 958.5159
b7−H2O 997.5268
y10 1011.6316
b7 1015.5373
y11 1082.6694
b8−H2O 1096.5953
b8 1114.6068
y12 1197.6957
Q A T K D A G V I A G L N V L R
y12 y11 y10 y9 y8 y7 y6 y5 y4 y3 y2 y1
b2 a2 b3 b4 b5 b6 b7 b8
Modification
Acetyl (Protein N−term)
GlyGly (K) no new terminus
Oxidation (M)

20200921_KIZ_MUb4_GlyGly_10plex − Scan number: 13596

Gene Names	Charge	m/z	Mass	Retention time	PEP	Score	Precursor Intensity
HUWE1	3	506.9264	1517.757	48.018	0.011213	70.412	6008571

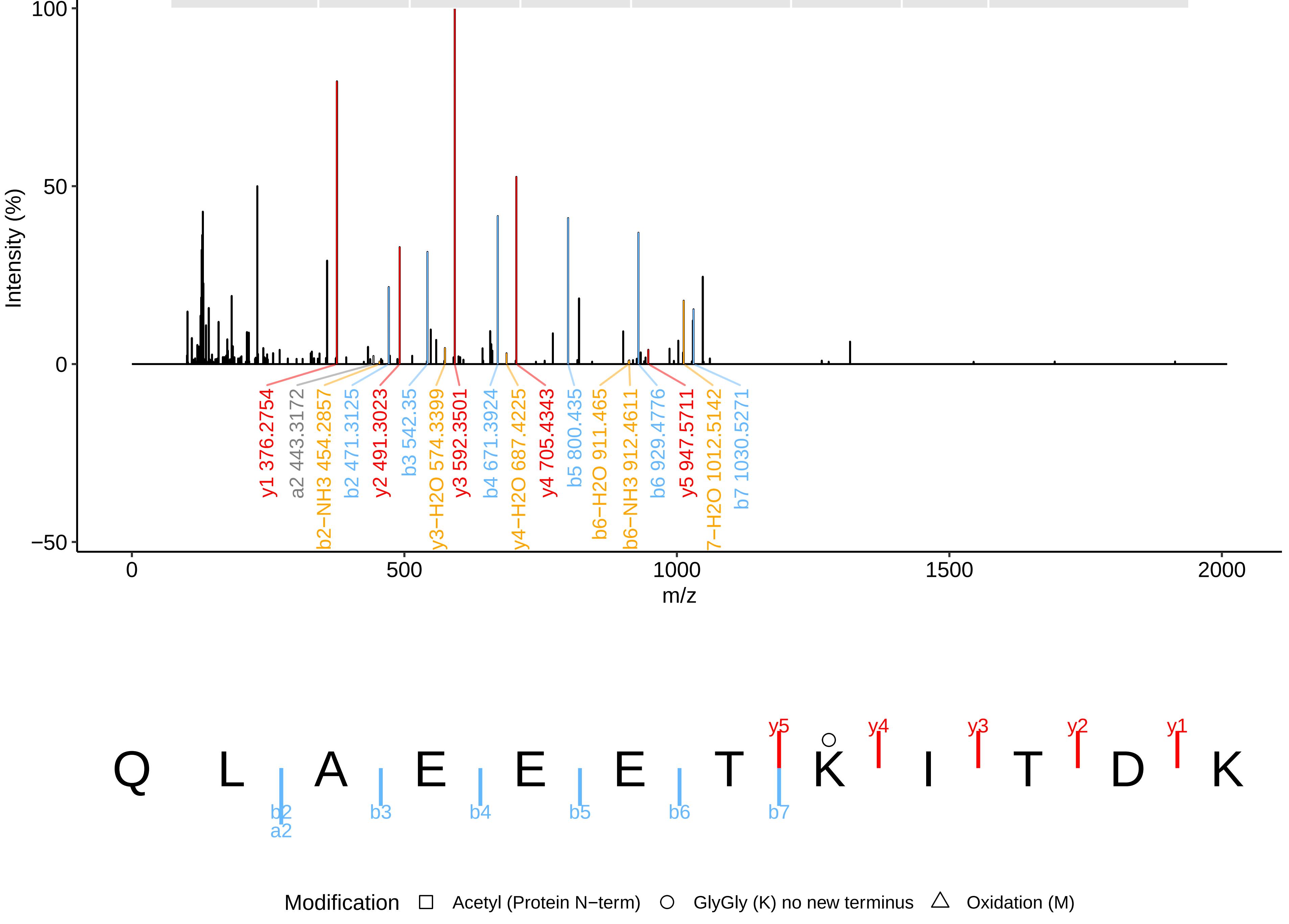

20200924_KIZ_MUb5_GlyGly_1 − Scan number: 12655

Gene Names	Charge	m/z	Mass	Retention time	PEP	Score	Precursor Intensity
HUWE1	3	506.9264	1517.757	41.206	0.063408	53.448	5939500

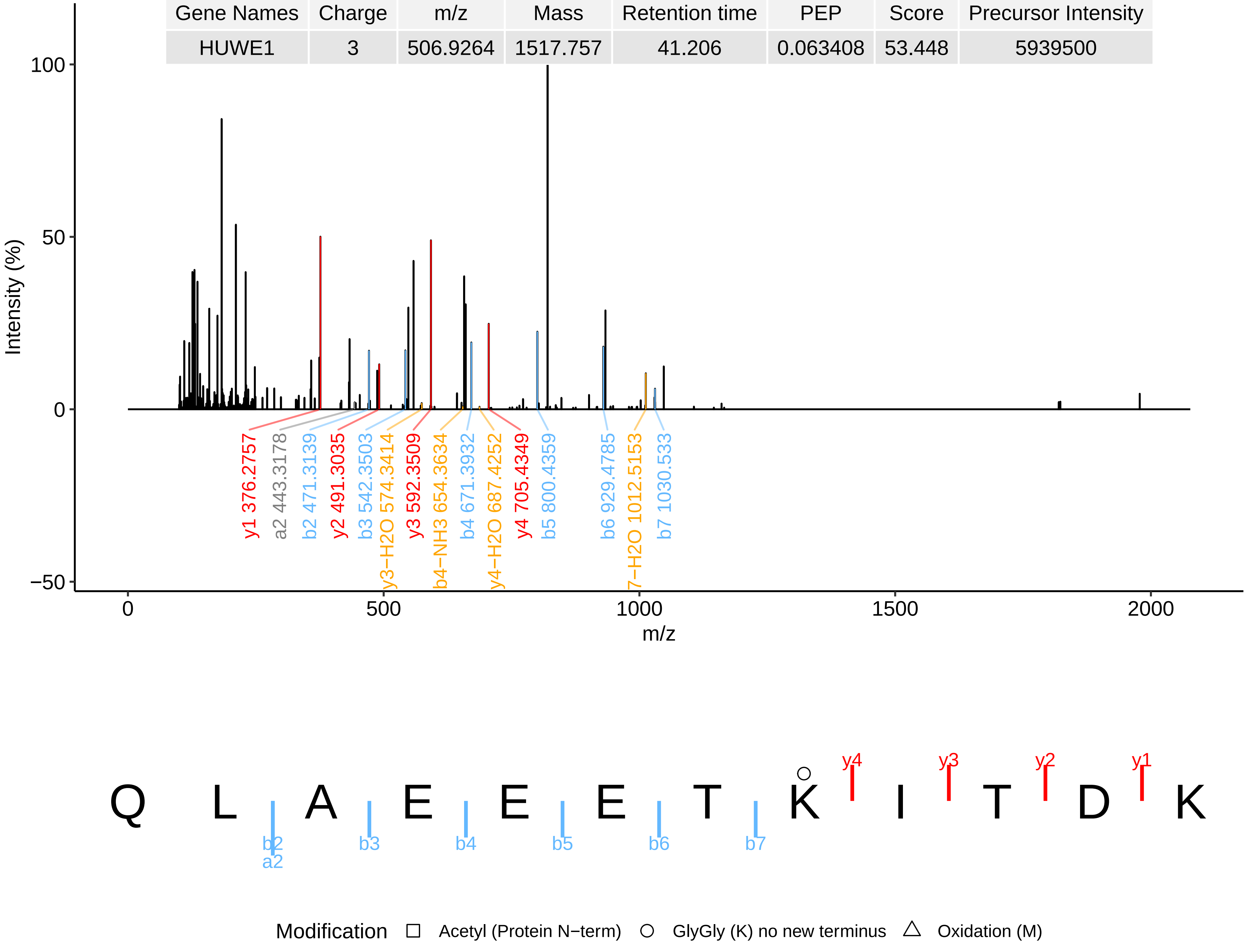

20200921_KIZ_MUb4_GlyGly_10plex − Scan number: 13121

Gene Names	Charge	m/z	Mass	Retention time	PEP	Score	Precursor Intensity
BIRC6	3	499.623	1495.847	46.168	1.1278e−05	111.06	5851949

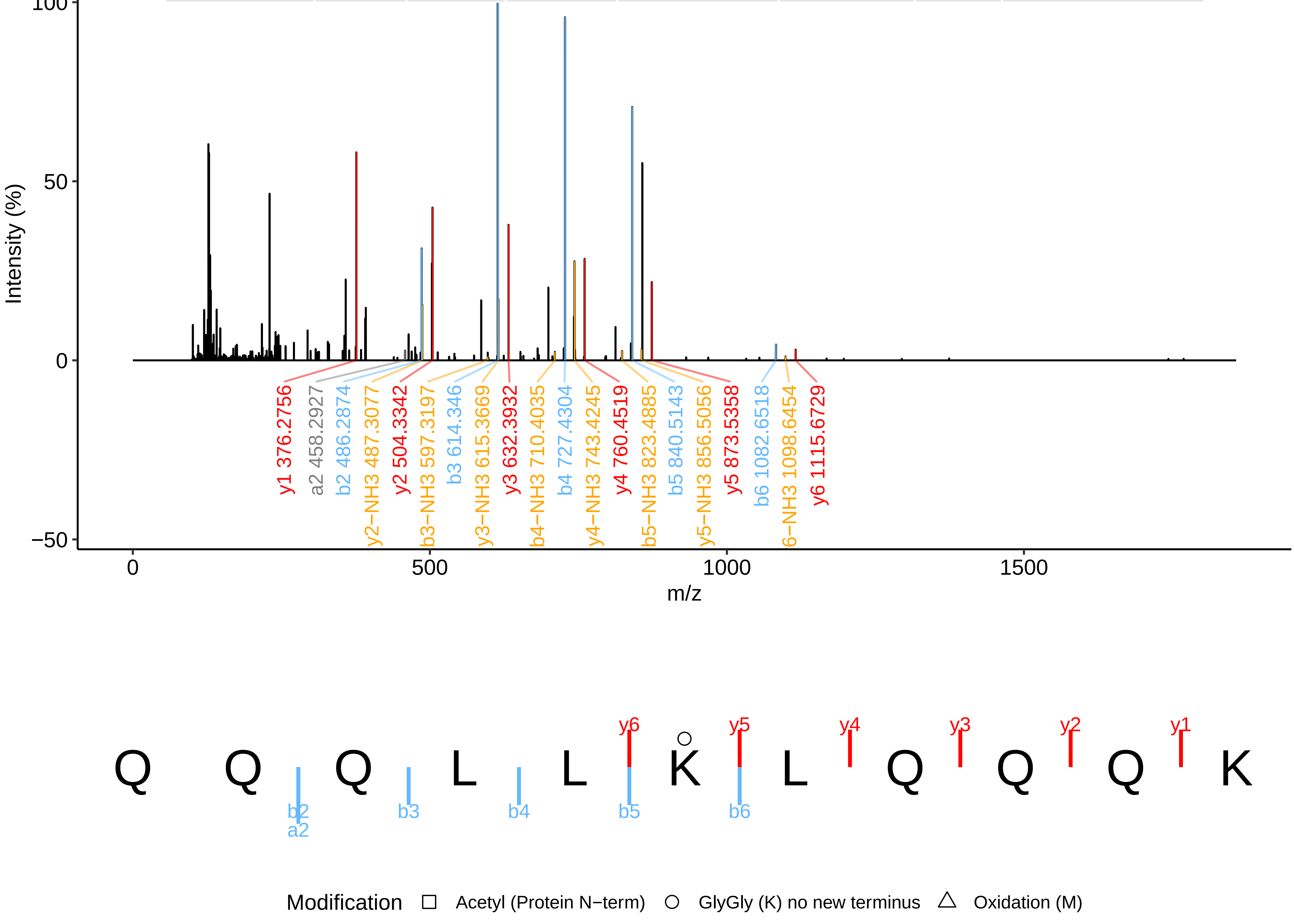

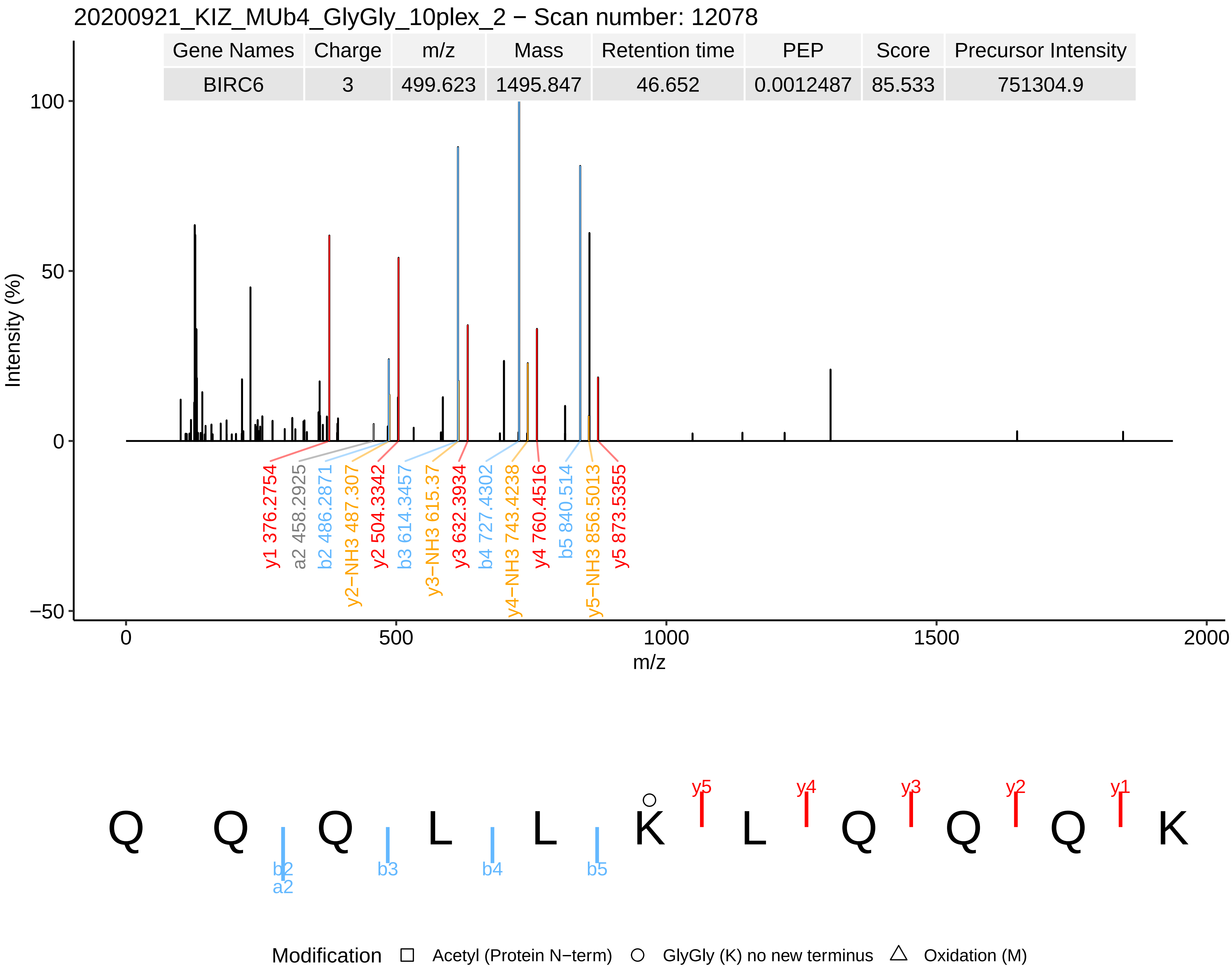
20200921_KIZ_MUb4_GlyGly_10plex_2 − Scan number: 12078
Gene Names
Charge
m/z
Mass
Retention time
PEP
Score
Precursor Intensity
BIRC6
3
499.623
1495.847
46.652
0.0012487
85.533
751304.9
Intensity (%)
m/z
y1 376.2754
a2 458.2925
b2 486.2871
y2−NH3 487.307
y2 504.3342
b3 614.3457
y3−NH3 615.37
y3 632.3934
b4 727.4302
y4−NH3 743.4238
y4 760.4516
b5 840.514
y5−NH3 856.5013
y5 873.5355
Q Q Q L L K L Q Q Q K
y5 y4 y3 y2 y1
b2 a2 b3 b4 b5
Modification
Acetyl (Protein N−term)
GlyGly (K) no new terminus
Oxidation (M)

20200921_KIZ_MUb4_GlyGly_10plex − Scan number: 9823

Gene Names	Charge	m/z	Mass	Retention time	PEP	Score	Precursor Intensity
STEAP4	3	489.5734	1465.698	33.779	0.00093402	84.718	3351025

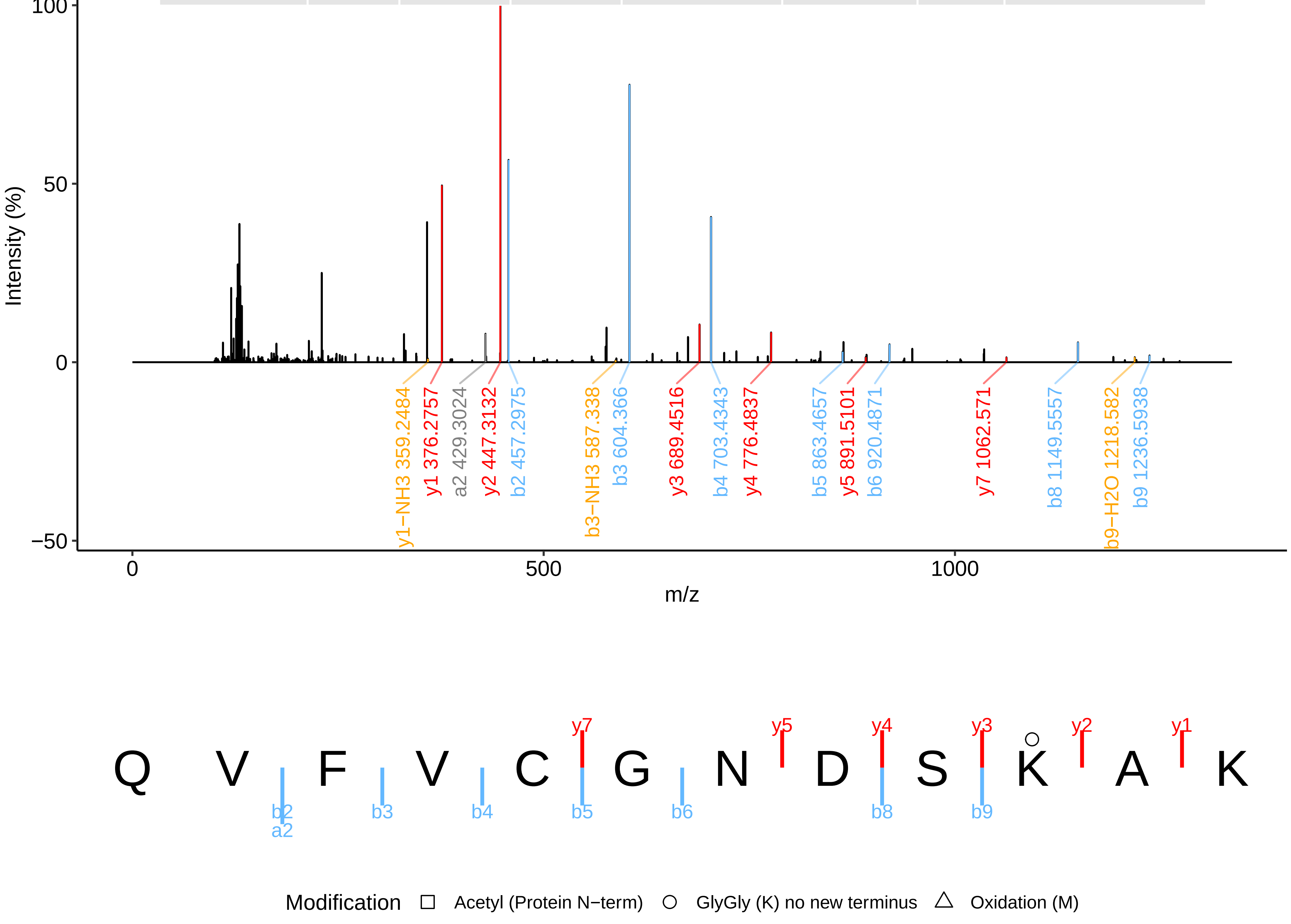

20200921_KIZ_MUb4_GlyGly_10plex − Scan number: 9813

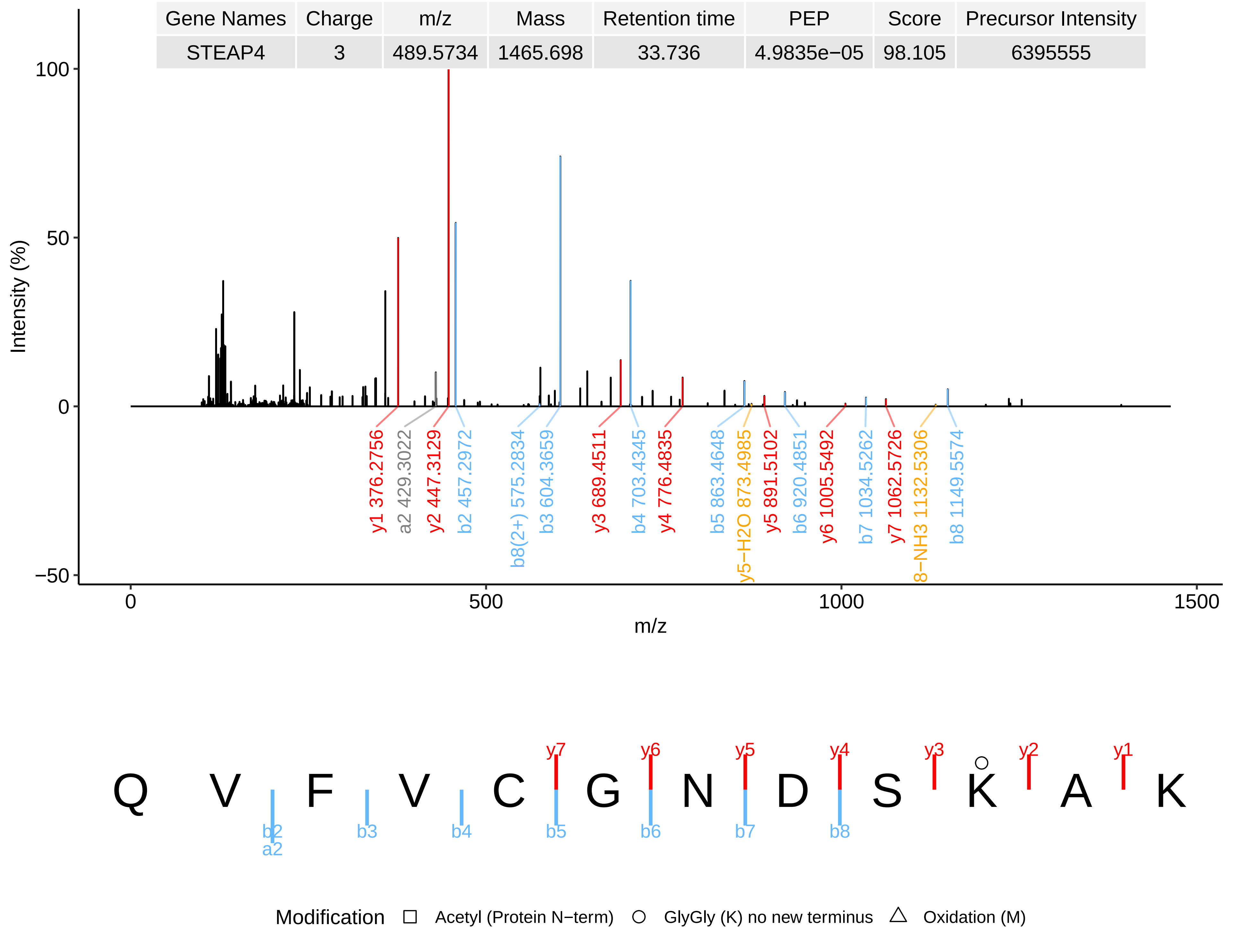

Gene Names	Charge	m/z	Mass	Retention time	PEP	Score	Precursor Intensity
STEAP4	3	489.5734	1465.698	33.736	4.9835e−05	98.105	6395555

20200921_KIZ_MUb4_GlyGly_10plex_2 − Scan number: 9036

Gene Names	Charge	m/z	Mass	Retention time	PEP	Score	Precursor Intensity
STEAP4	3	489.5734	1465.698	34.234	7.4711e−05	97.214	1093896

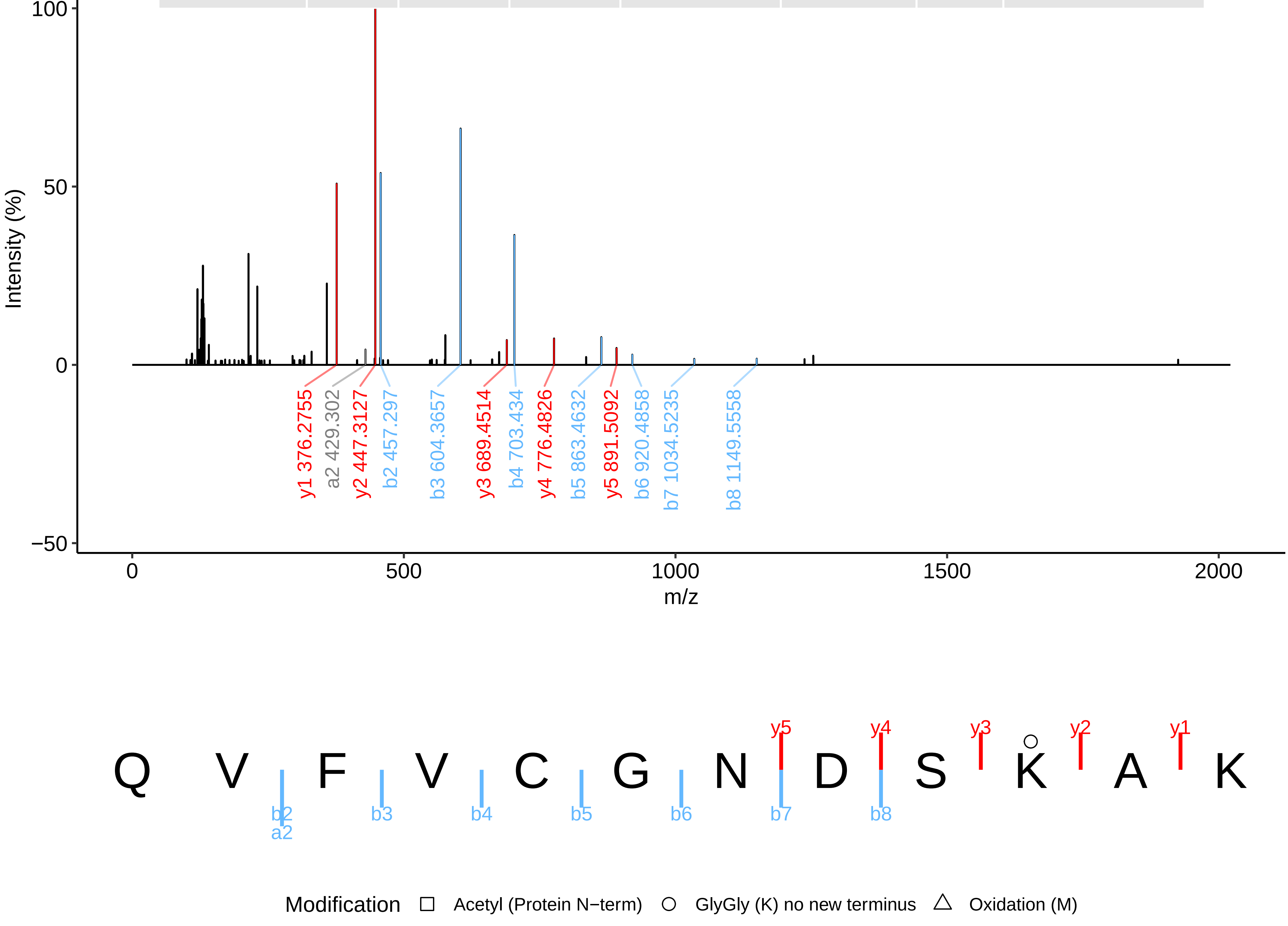

20200924_KIZ_MUb5_GlyGly_1 − Scan number: 8843

Gene Names	Charge	m/z	Mass	Retention time	PEP	Score	Precursor Intensity
STEAP4	3	489.5734	1465.698	28.353	0.0008565	85.845	5897615

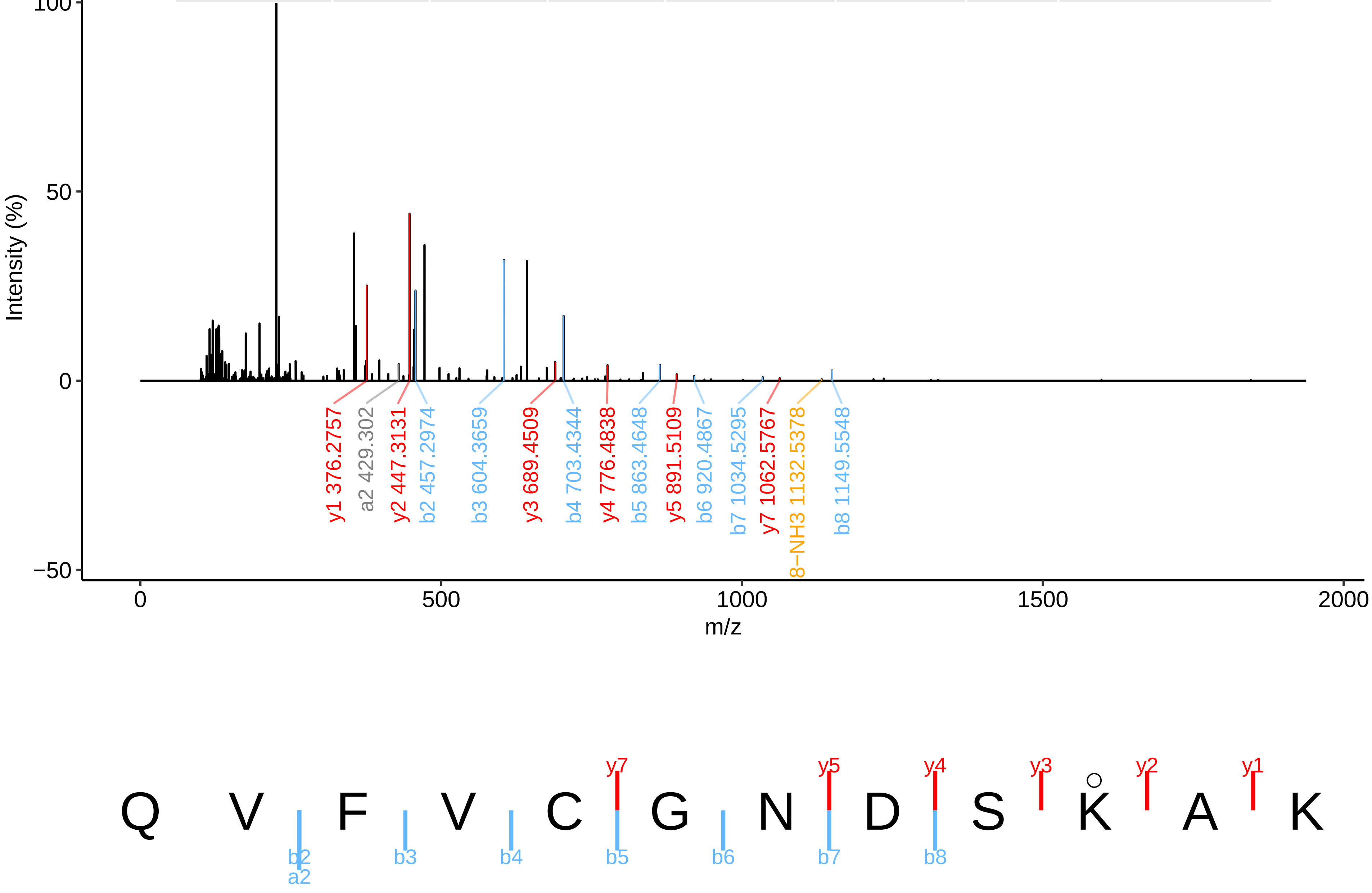

Modification □ Acetyl (Protein N−term) ○ GlyGly (K) no new terminus △ Oxidation (M)

20200924_KIZ_MUb5_GlyGly_1 − Scan number: 8691

Gene Names	Charge	m/z	Mass	Retention time	PEP	Score	Precursor Intensity
STEAP4	3	489.5734	1465.698	27.825	3.3516e−05	105.17	NA

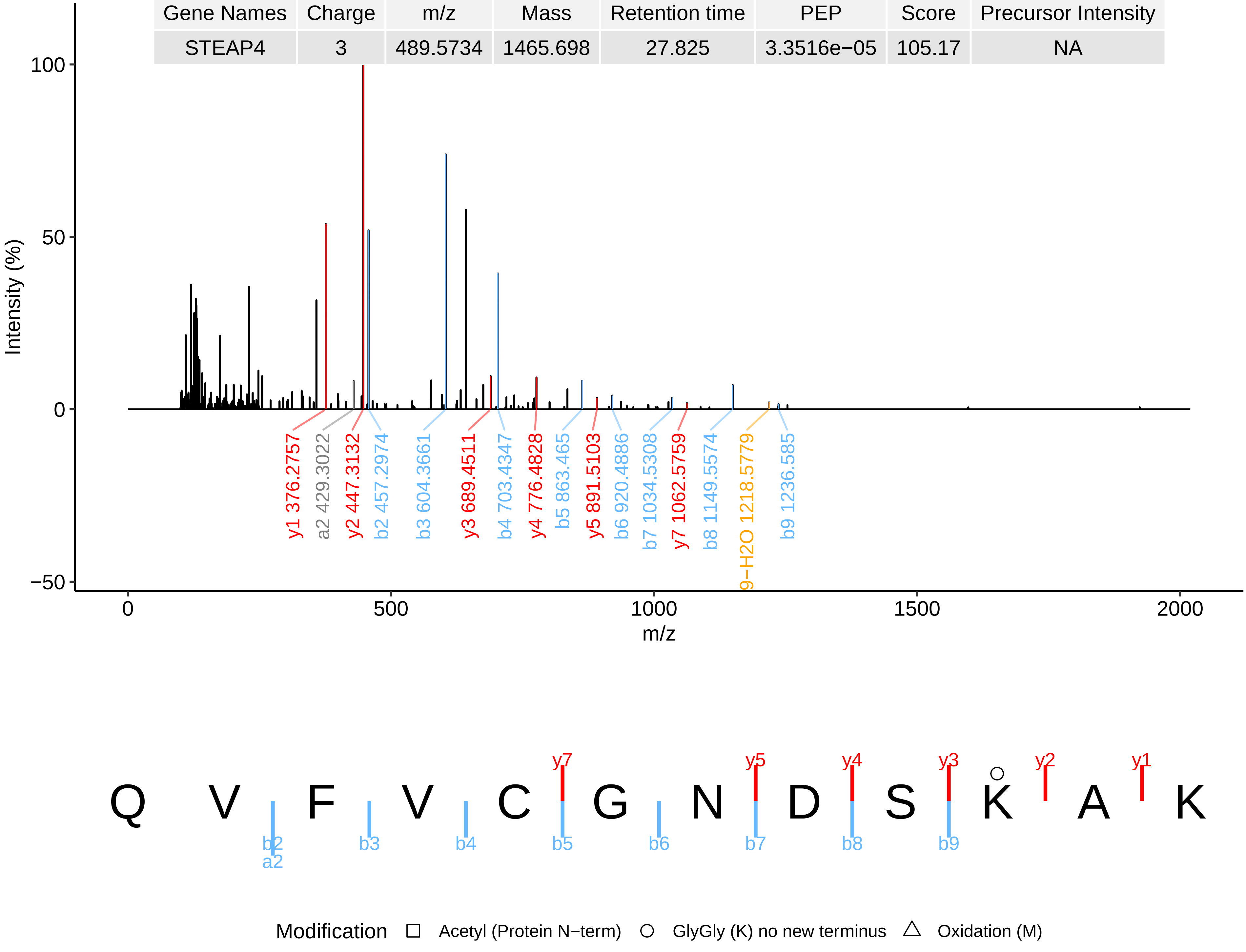

20200924_KIZ_MUb5_GlyGly_2 − Scan number: 8273

Gene Names	Charge	m/z	Mass	Retention time	PEP	Score	Precursor Intensity
STEAP4	3	489.5734	1465.698	30.897	0.040278	59.57	NA

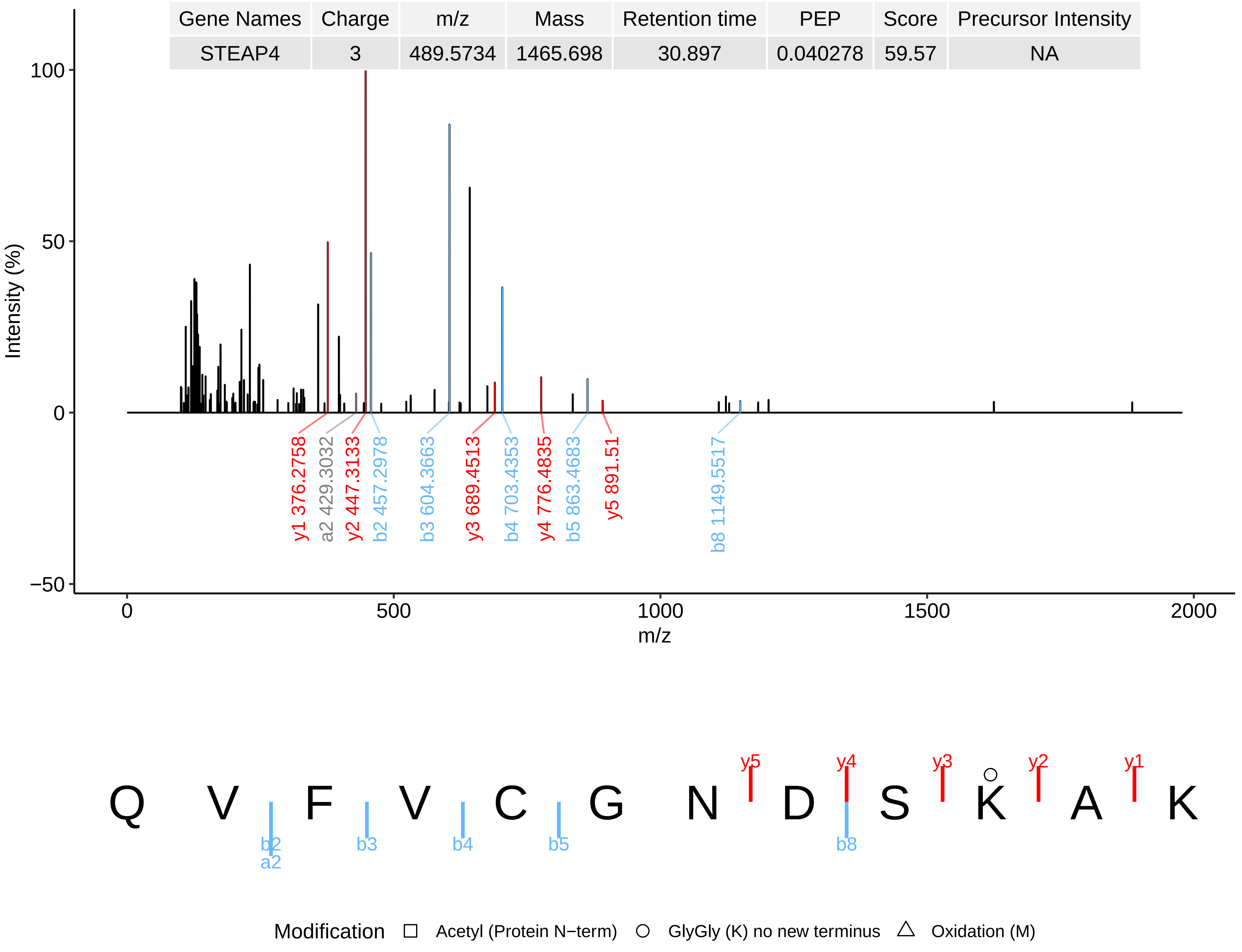

20200921_KIZ_MUb4_GlyGly_10plex_2 − Scan number: 6885

Gene Names	Charge	m/z	Mass	Retention time	PEP	Score	Precursor Intensity
DNAJB12	3	581.5958	1741.766	25.494	0.0009171	82.102	630956.2

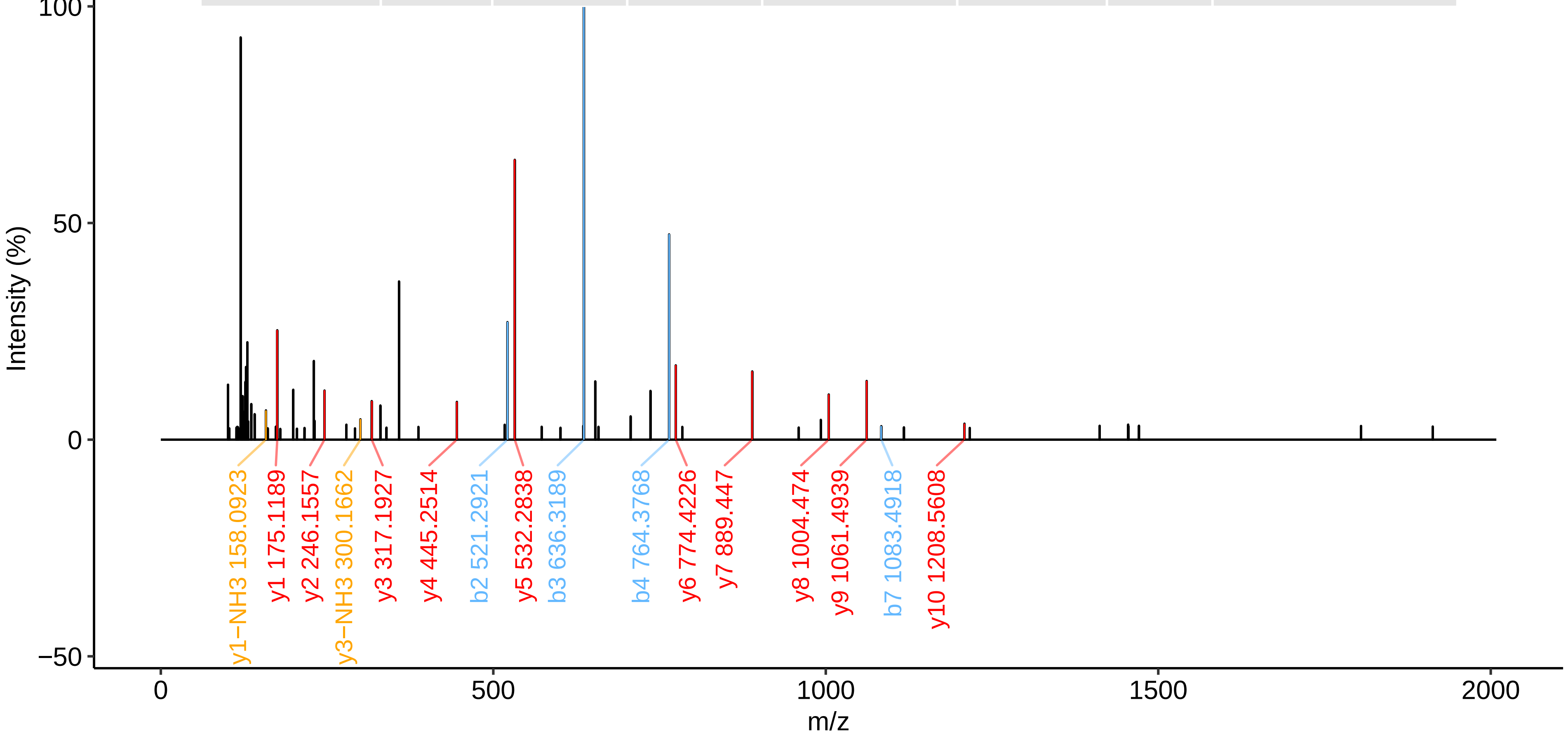

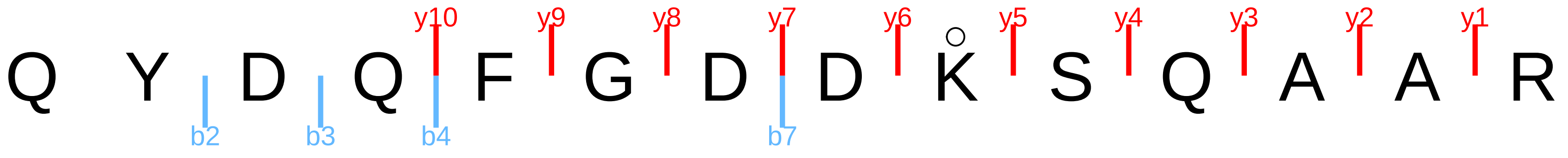

Modification □ Acetyl (Protein N−term) ○ GlyGly (K) no new terminus △ Oxidation (M)

20200924_KIZ_MUb5_GlyGly_1 − Scan number: 6568

Gene Names	Charge	m/z	Mass	Retention time	PEP	Score	Precursor Intensity
DNAJB12	3	581.5958	1741.766	20.678	1.157e−13	145.1	6244330

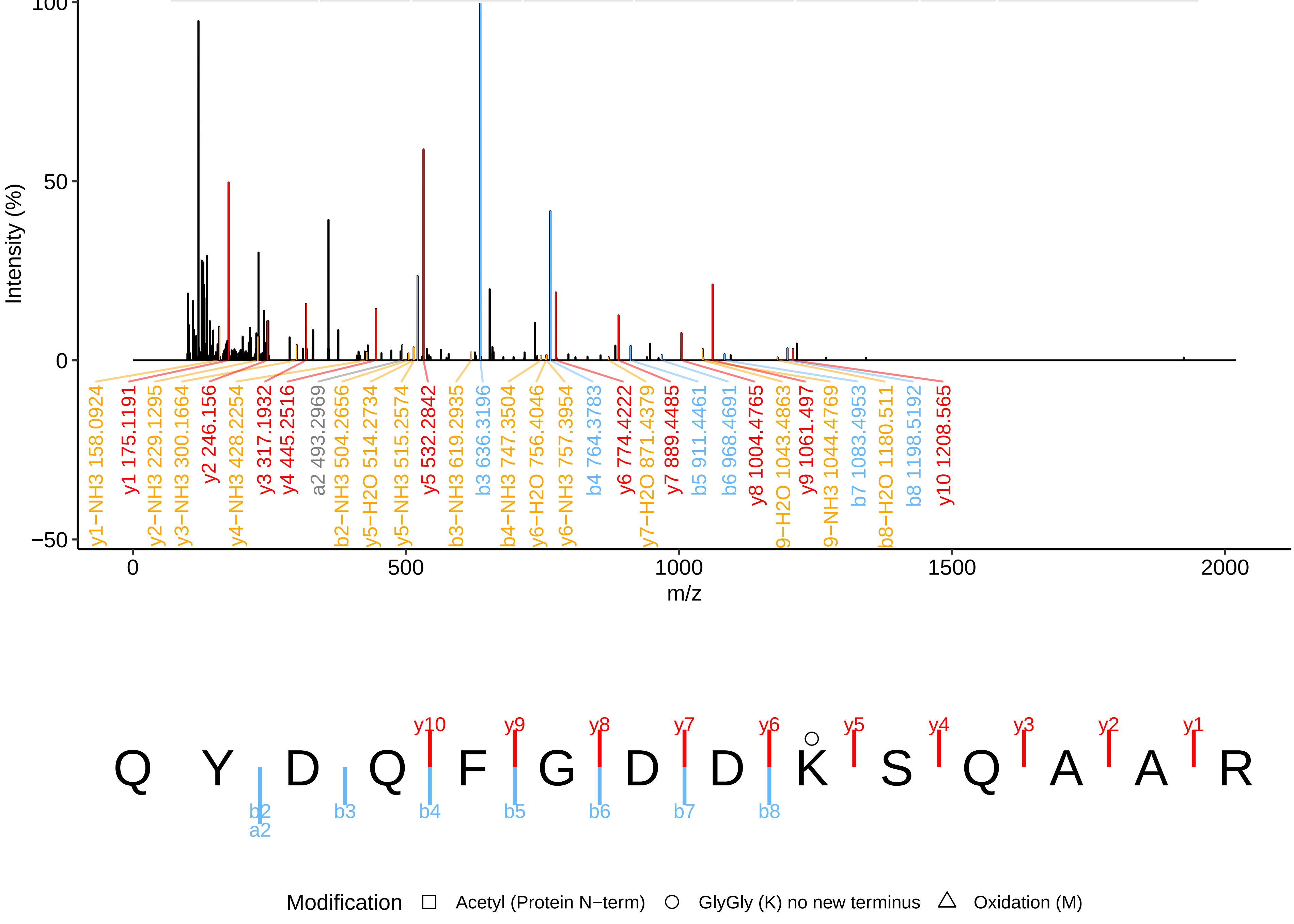

20200924_KIZ_MUb5_GlyGly_1 − Scan number: 35052

Gene Names	Charge	m/z	Mass	Retention time	PEP	Score	Precursor Intensity
DNAJC21	2	856.4579	1710.901	111.56	0.073741	47.823	12564230

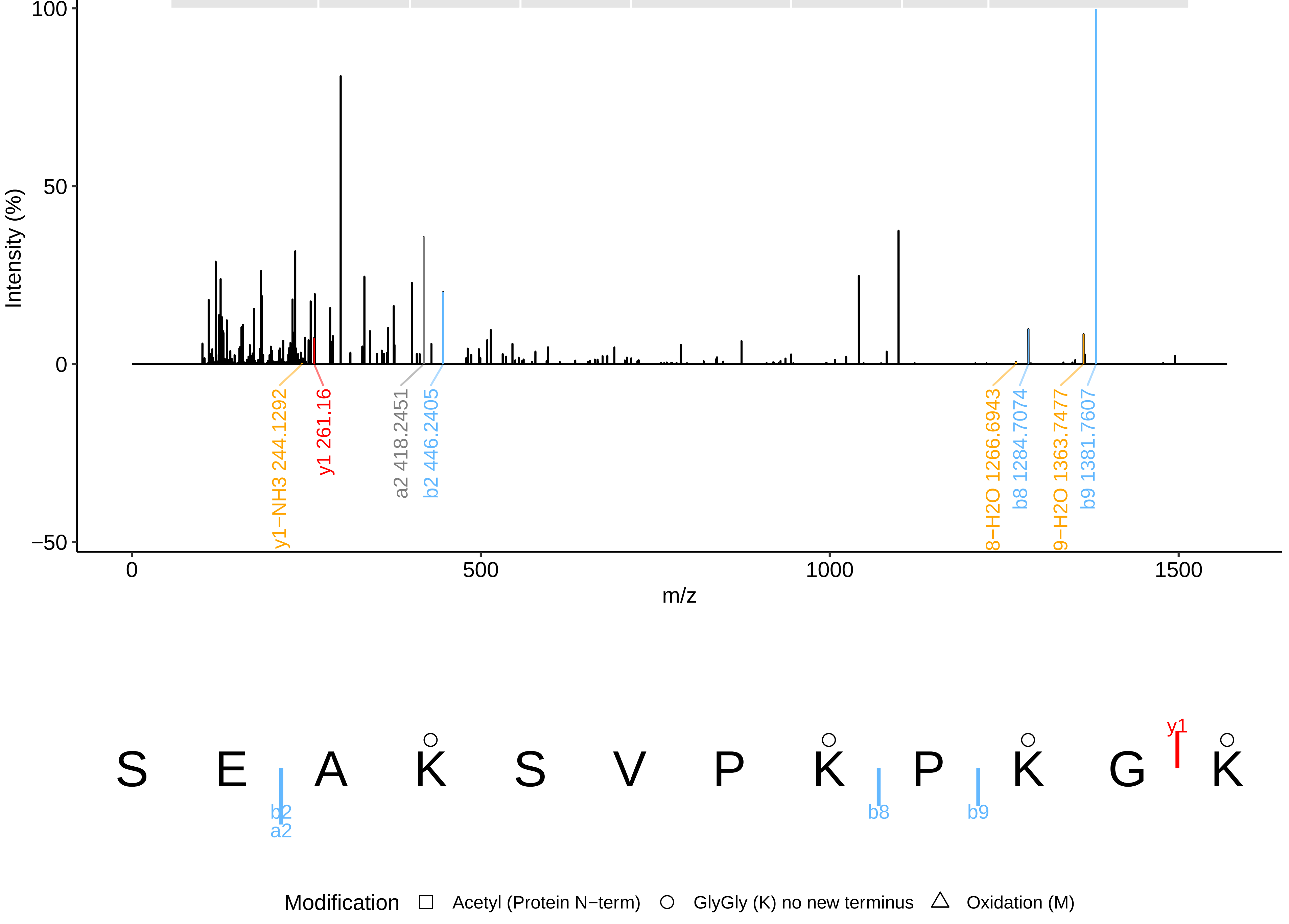

20200924_KIZ_MUb5_GlyGly_2 − Scan number: 30888

Gene Names	Charge	m/z	Mass	Retention time	PEP	Score	Precursor Intensity
DNAJC21	2	856.4579	1710.901	114.64	0.081355	45.997	5259900

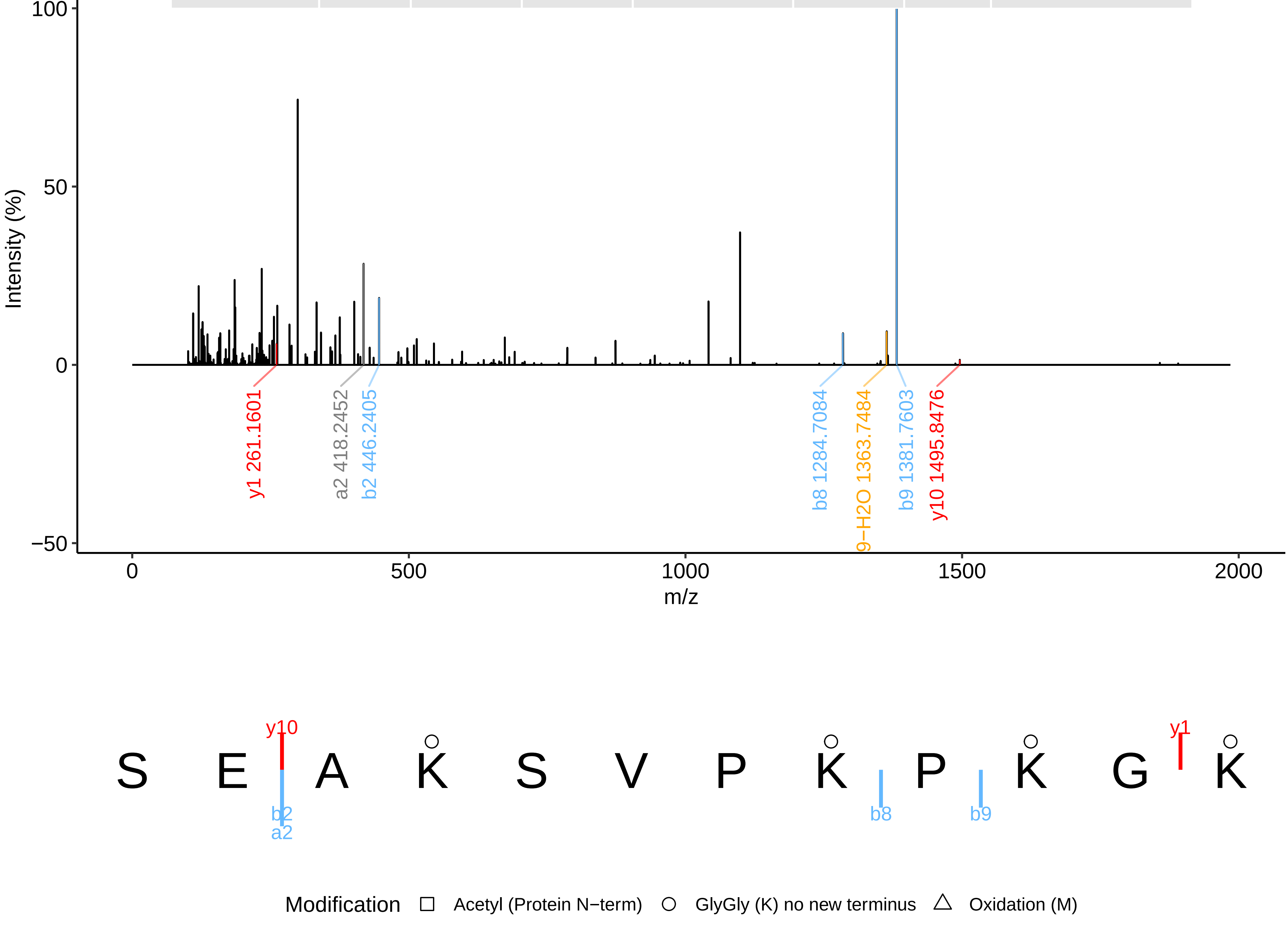

20200921_KIZ_MUb4_GlyGly_10plex − Scan number: 27086

Gene Names	Charge	m/z	Mass	Retention time	PEP	Score	Precursor Intensity
PLEC	3	576.9879	1727.942	98.799	4.1512e−06	112.08	2184517

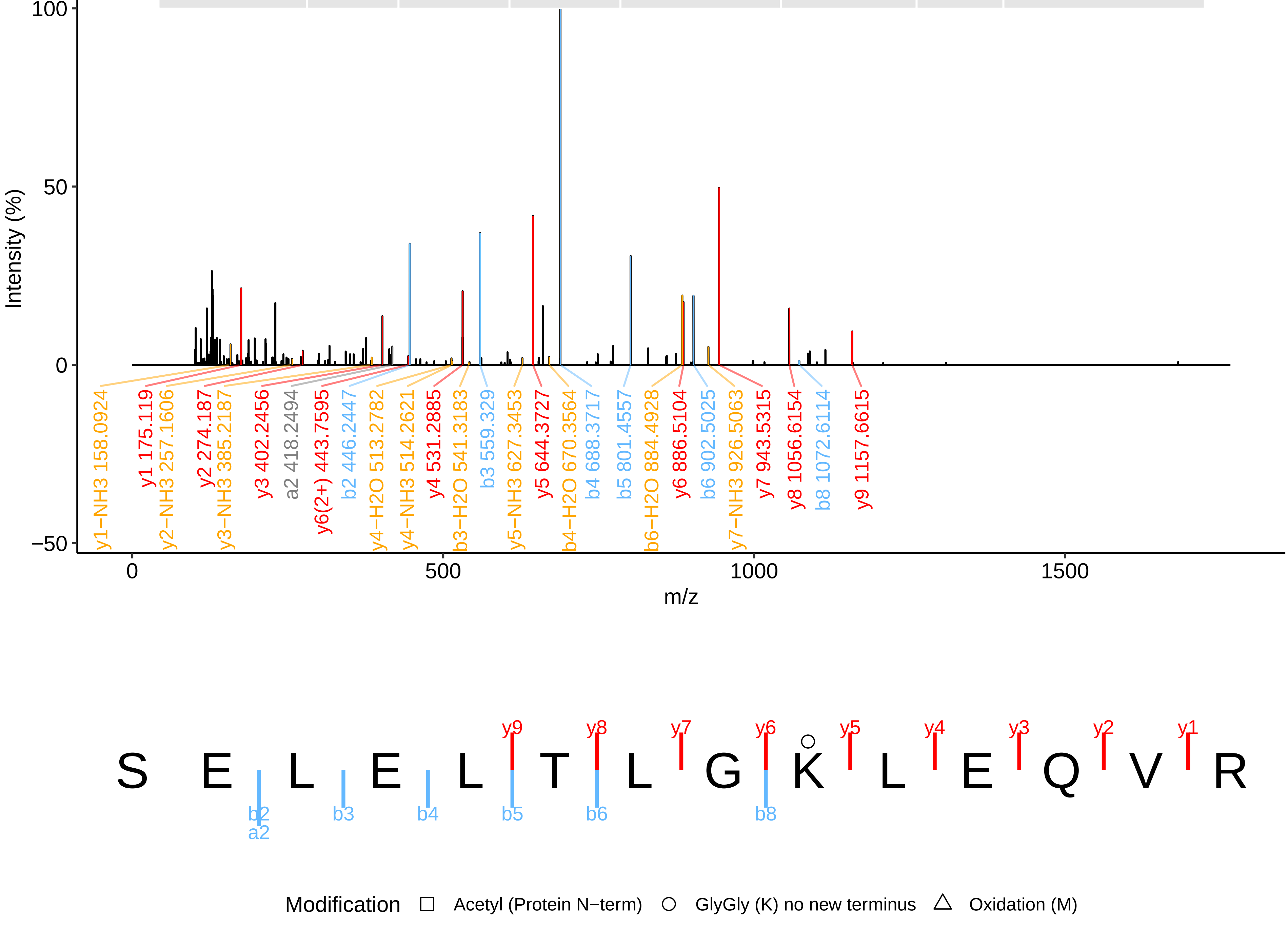

20200921_KIZ_MUb4_GlyGly_10plex − Scan number: 27249

Gene Names	Charge	m/z	Mass	Retention time	PEP	Score	Precursor Intensity
PLEC	3	576.9879	1727.942	99.421	3.247e−13	135.81	7507498

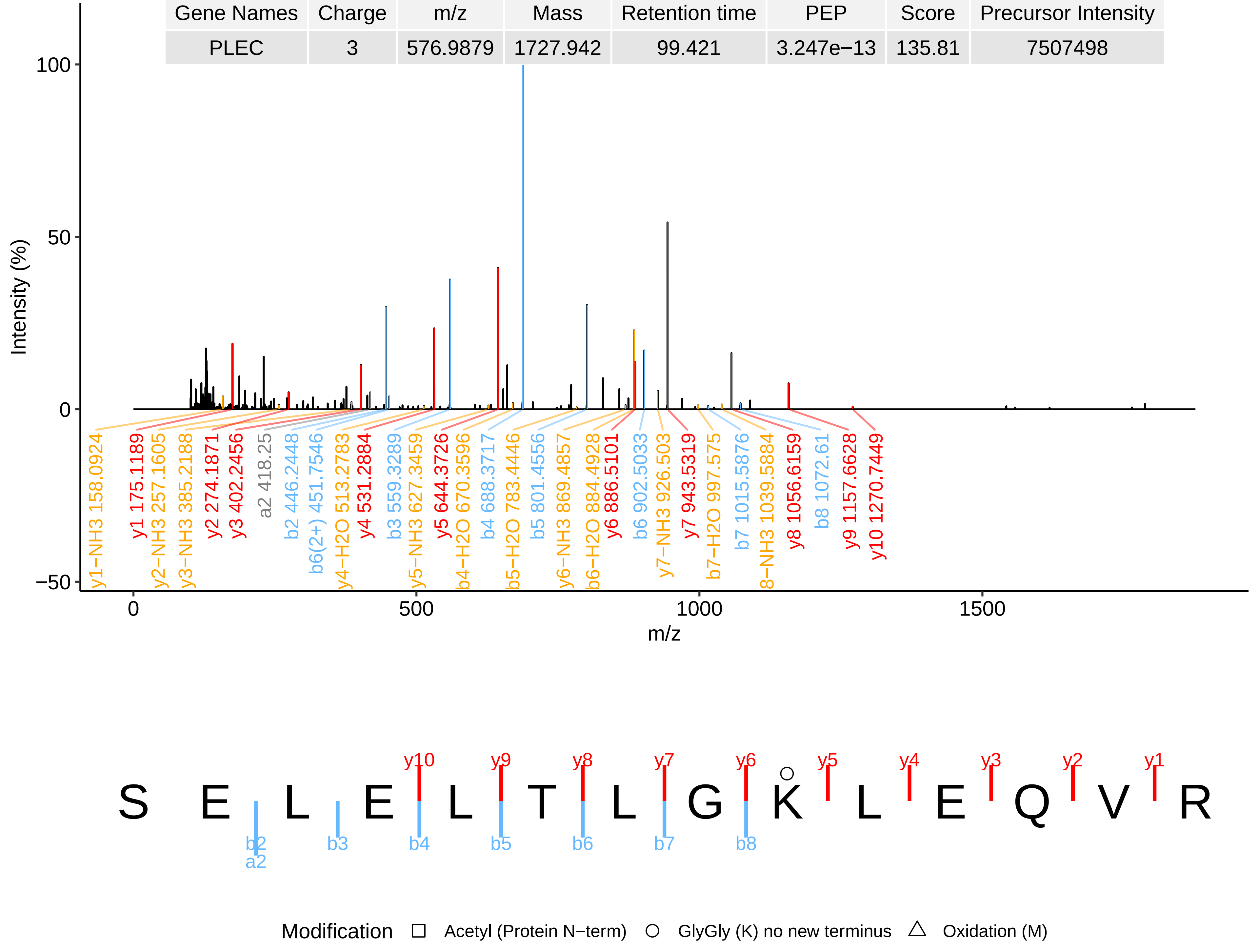

20200921_KIZ_MUb4_GlyGly_10plex_2 − Scan number: 23254

Gene Names	Charge	m/z	Mass	Retention time	PEP	Score	Precursor Intensity
PLEC	3	576.9879	1727.942	91.766	2.1217e−05	98.337	787077.8

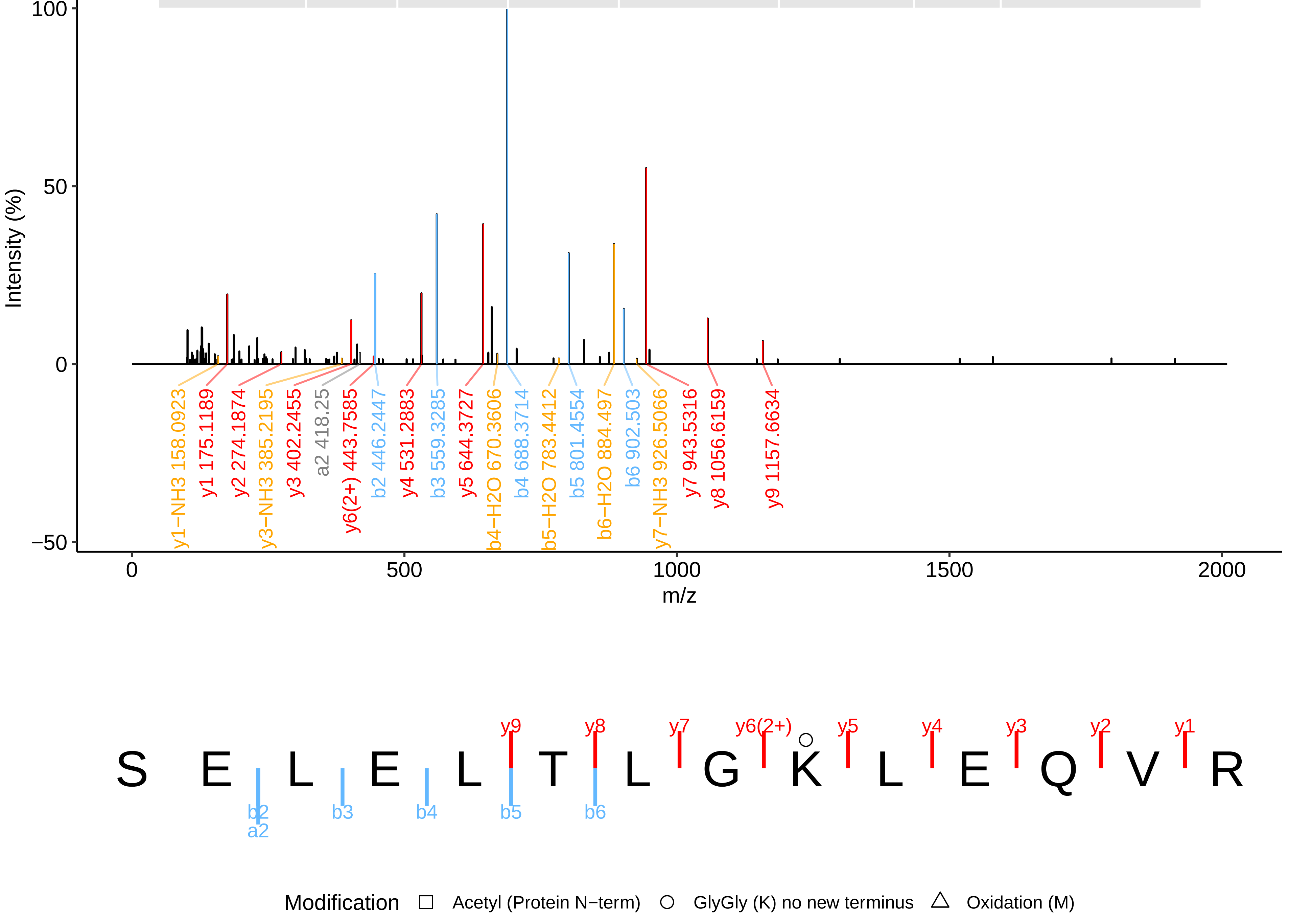

20200921_KIZ_MUb4_GlyGly_10plex − Scan number: 14173

Gene Names	Charge	m/z	Mass	Retention time	PEP	Score	Precursor Intensity
GMPS	3	501.2705	1500.79	50.268	0.0013023	93.51	5489838

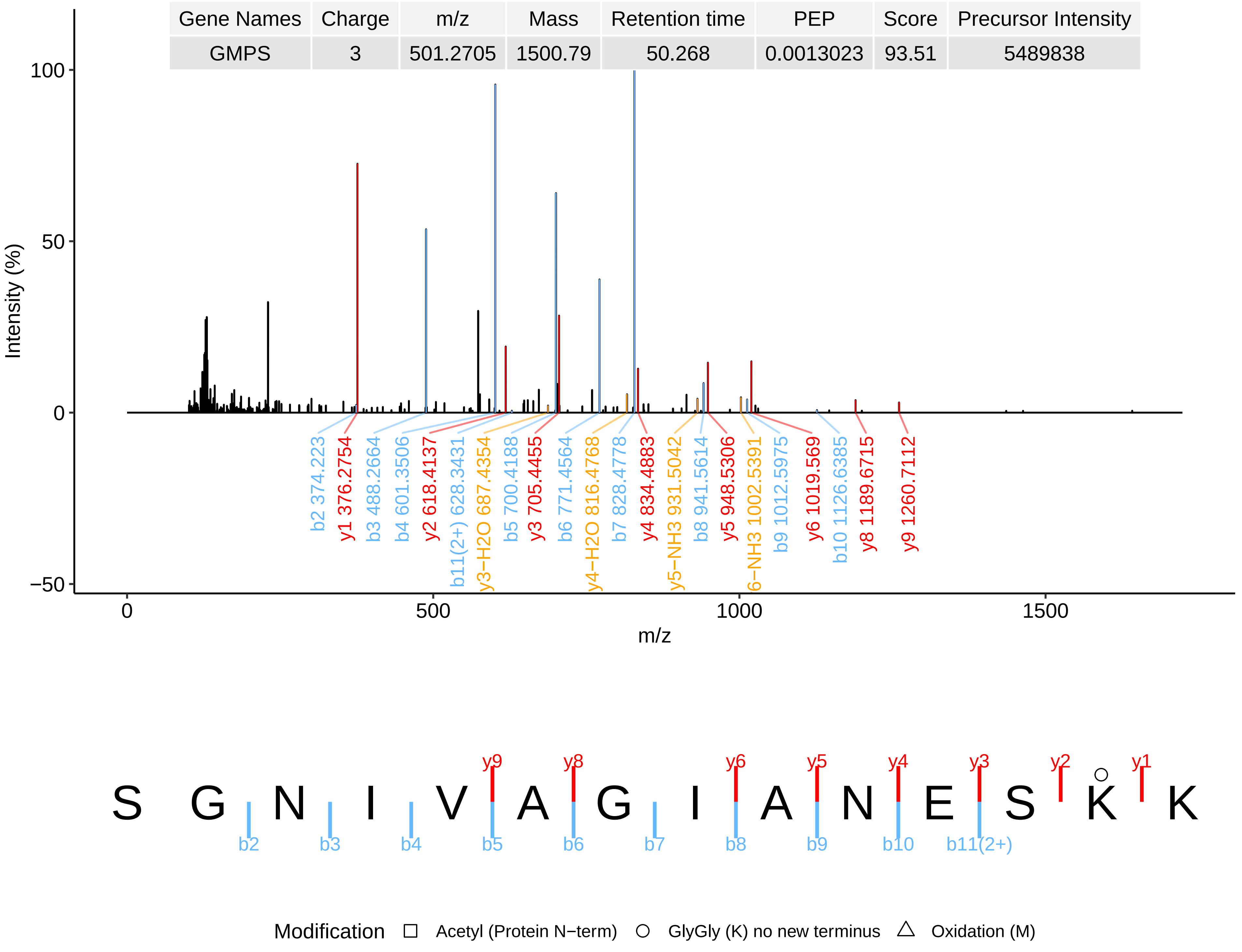

20200921_KIZ_MUb4_GlyGly_10plex_2 − Scan number: 5169

Gene Names	Charge	m/z	Mass	Retention time	PEP	Score	Precursor Intensity
CBX3	3	500.2216	1497.643	18.46	0.051146	56.951	229580.1

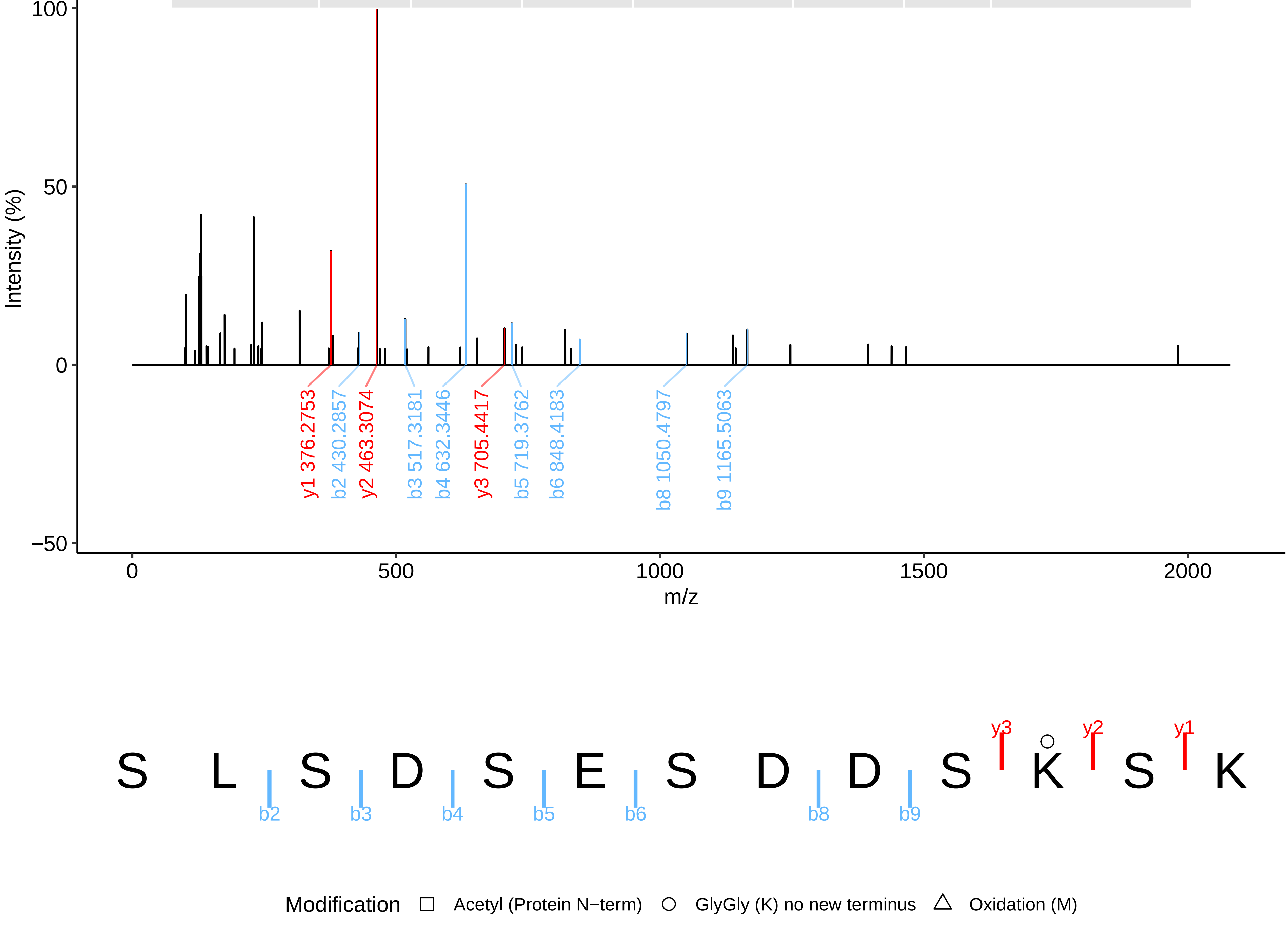

20200921_KIZ_MUb4_GlyGly_10plex − Scan number: 10543

Gene Names	Charge	m/z	Mass	Retention time	PEP	Score	Precursor Intensity
HNRNPA1	3	581.613	1741.817	36.521	6.6846e−15	146.59	6321635

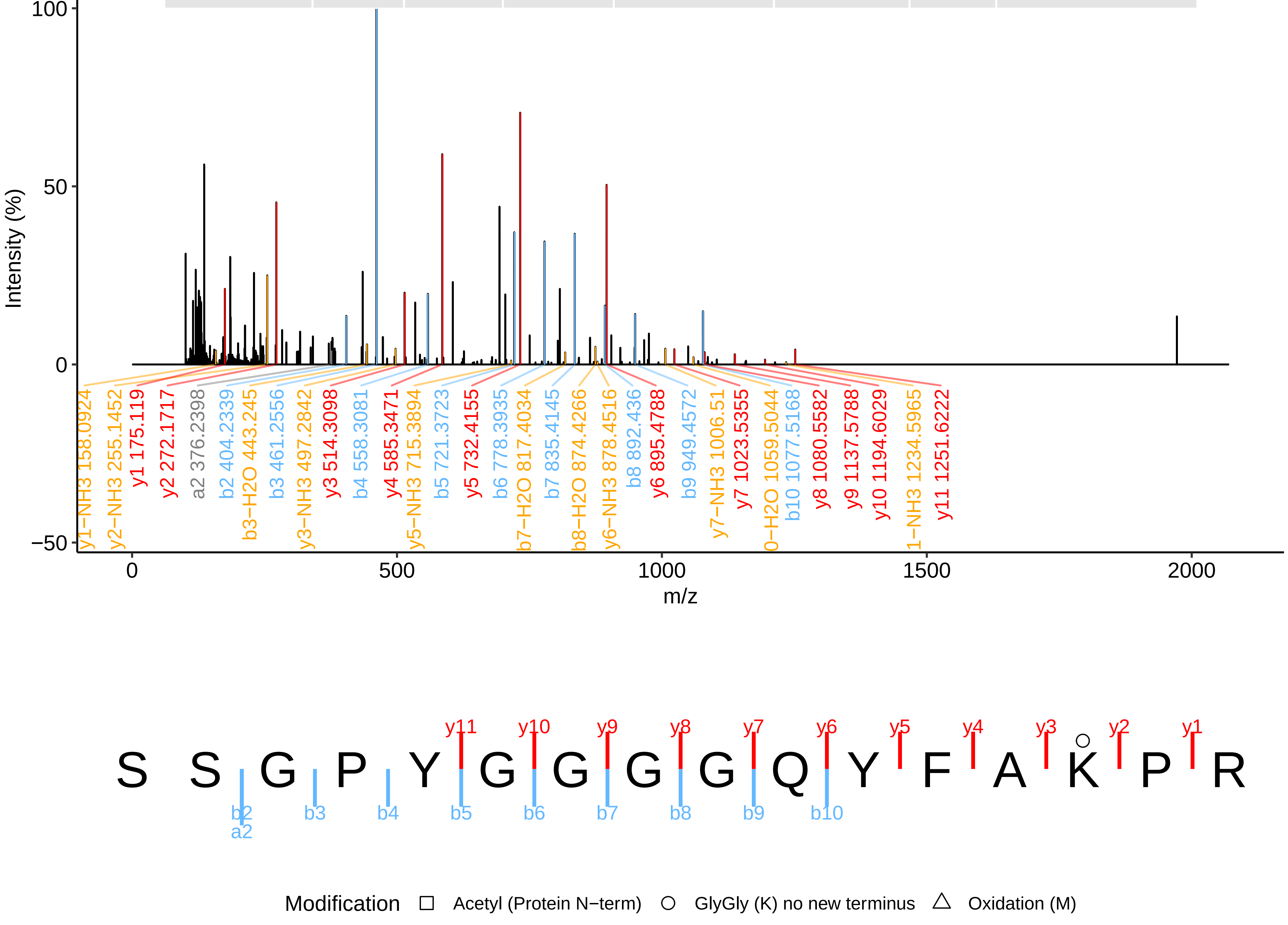

20200921_KIZ_MUb4_GlyGly_10plex_2 − Scan number: 9741

Gene Names	Charge	m/z	Mass	Retention time	PEP	Score	Precursor Intensity
HNRNPA1	3	581.613	1741.817	37.127	1.8176e−14	140.31	568617.2

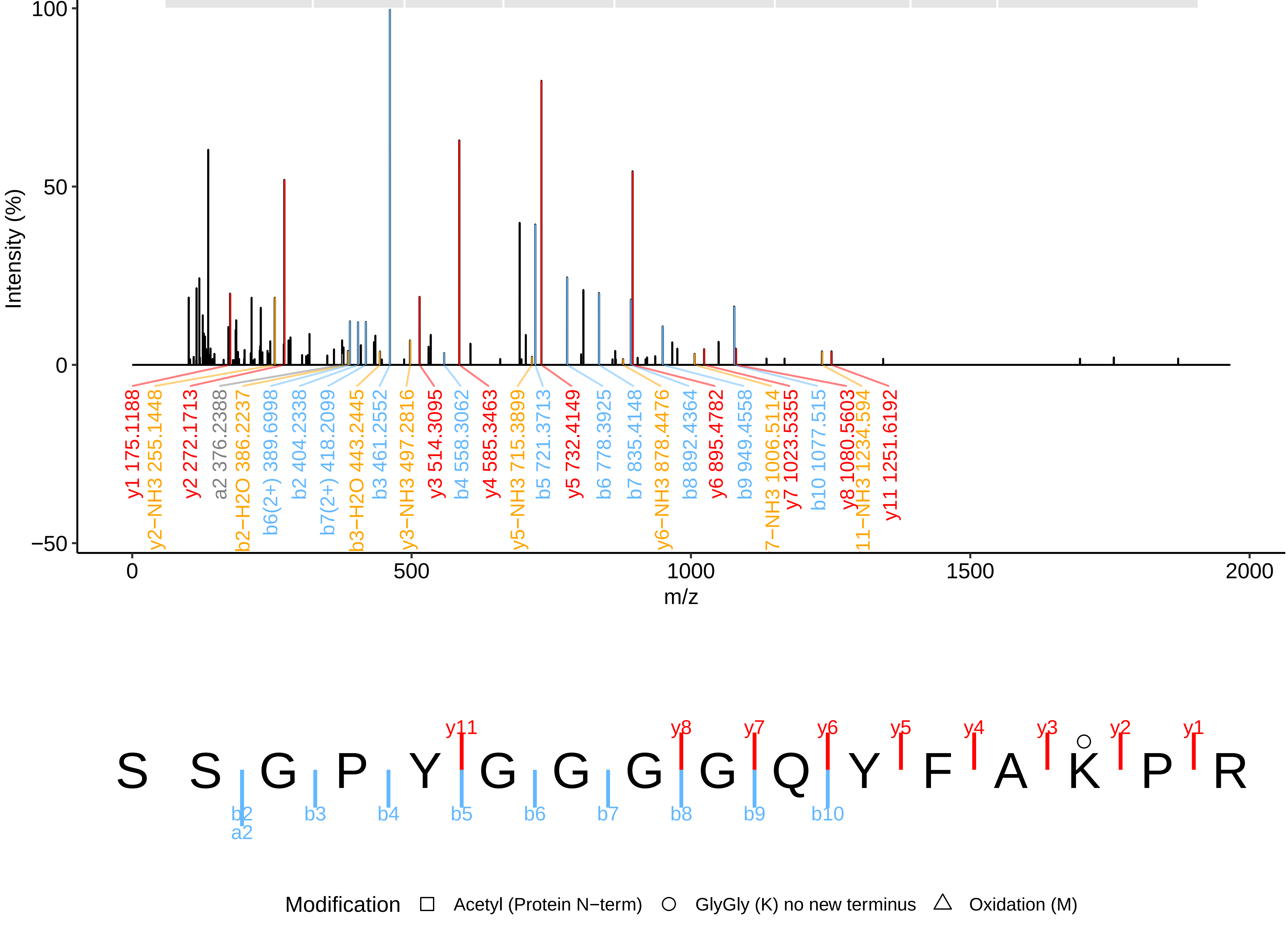

20200921_KIZ_MUb4_GlyGly_10plex − Scan number: 17352

Gene Names	Charge	m/z	Mass	Retention time	PEP	Score	Precursor Intensity
CAPZB	3	505.5998	1513.778	62.405	0.057448	57.034	17738708

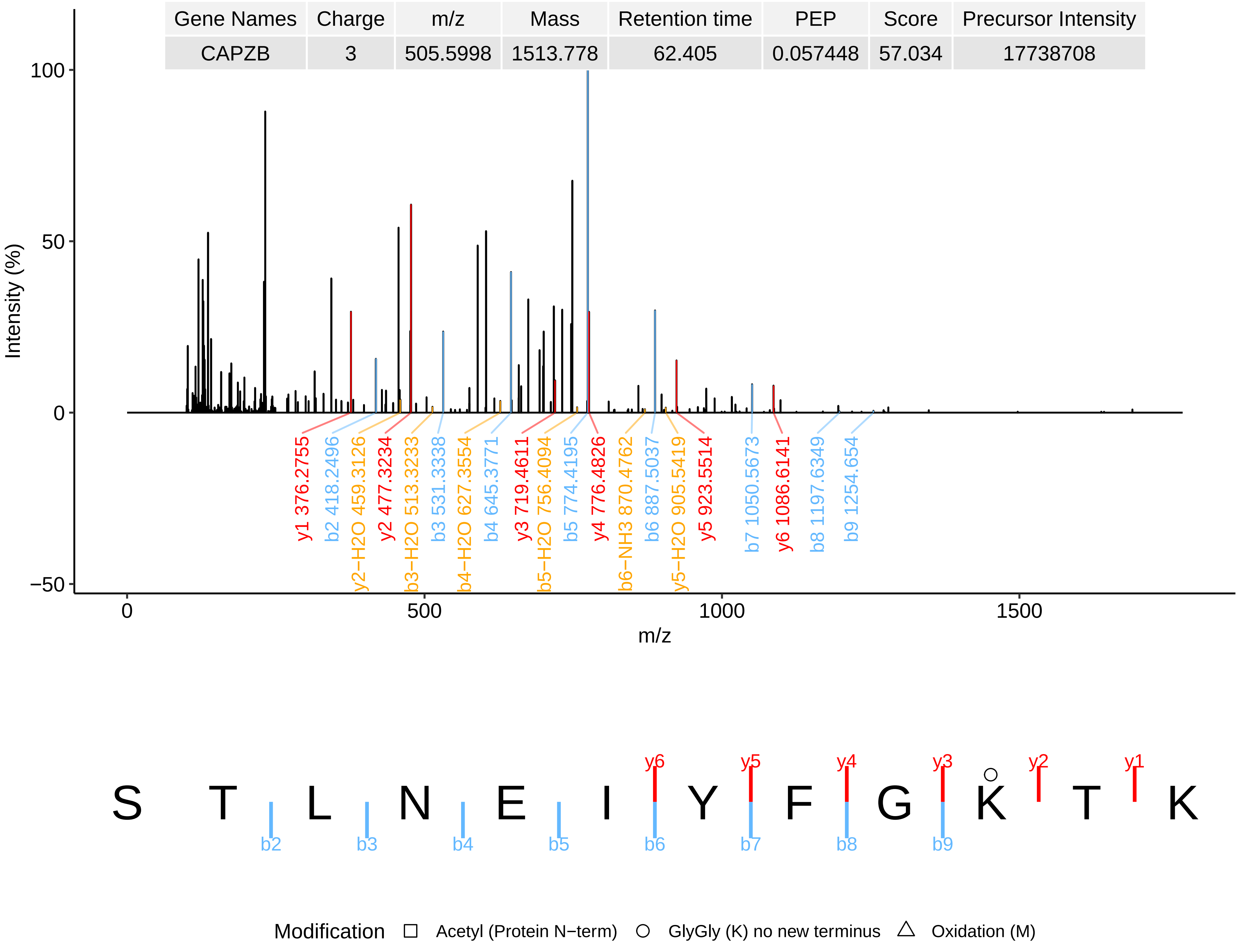

20201118_KIZ_MUb6_GlyGly_1 − Scan number: 16844

Gene Names	Charge	m/z	Mass	Retention time	PEP	Score	Precursor Intensity
CAPZB	3	505.5998	1513.778	53.064	0.006051	73.36	24043058

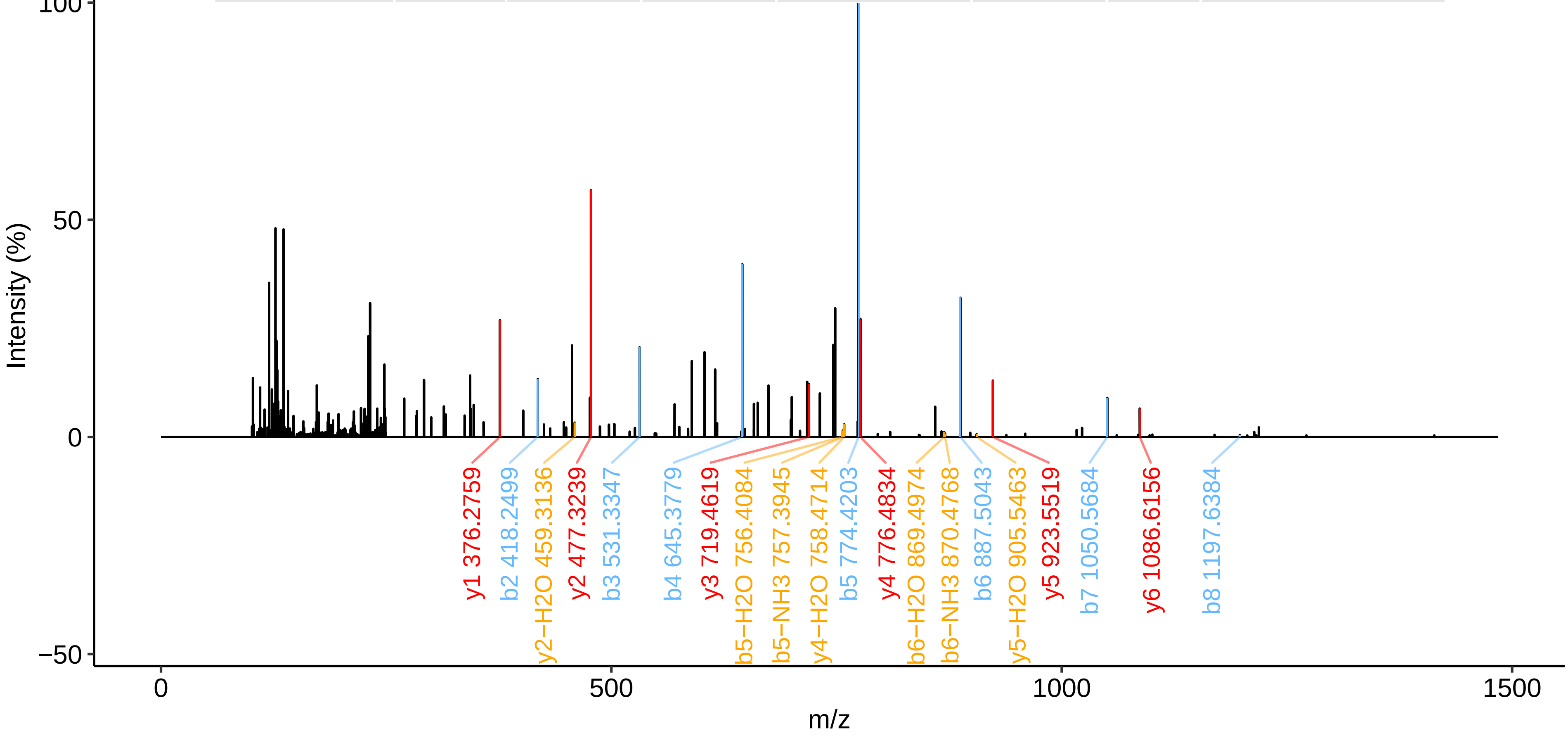

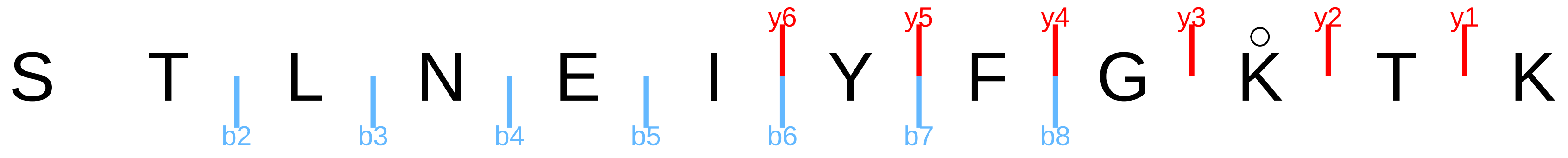

Modification □ Acetyl (Protein N−term) ○ GlyGly (K) no new terminus △ Oxidation (M)

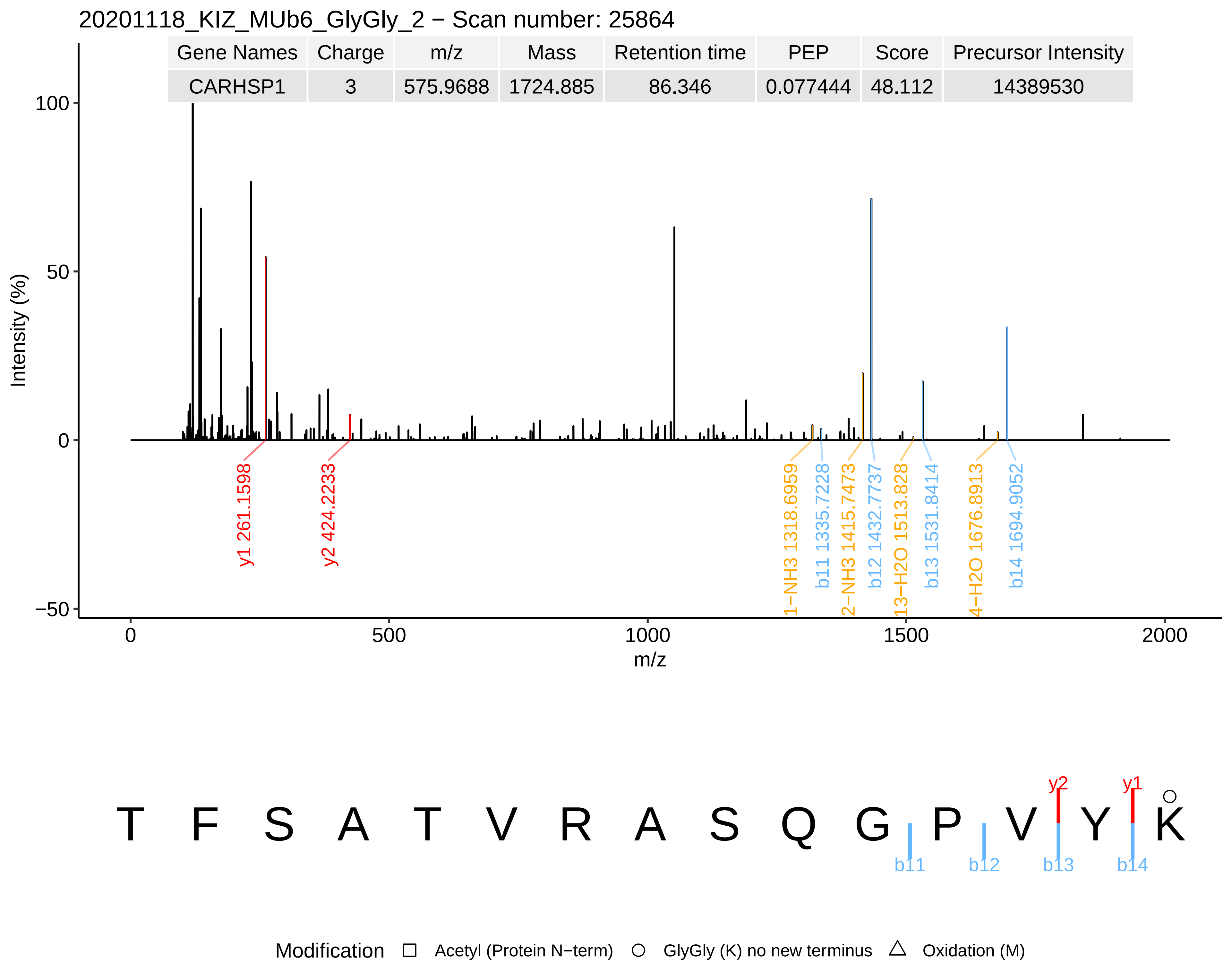
20201118_KIZ_MUb6_GlyGly_2 − Scan number: 25864
Gene Names | Charge | m/z | Mass | Retention time | PEP | Score | Precursor Intensity
CARHSP1 | 3 | 575.9688 | 1724.885 | 86.346 | 0.077444 | 48.112 | 14389530
Intensity (%)
m/z
y1 261.1598
y2 424.2233
1−NH3 1318.6959
b11 1335.7228
2−NH3 1415.7473
b12 1432.7737
13−H2O 1513.828
b13 1531.8414
4−H2O 1676.8913
b14 1694.9052
T F S A T V R A S Q G P V Y K
y2
y1
b11
b12
b13
b14
Modification
Acetyl (Protein N−term)
GlyGly (K) no new terminus
Oxidation (M)

20200921_KIZ_MUb4_GlyGly_10plex_2 − Scan number: 13696

Gene Names	Charge	m/z	Mass	Retention time	PEP	Score	Precursor Intensity
SLC7A1	3	486.9255	1457.755	53.229	0.0079715	70.488	941306.1

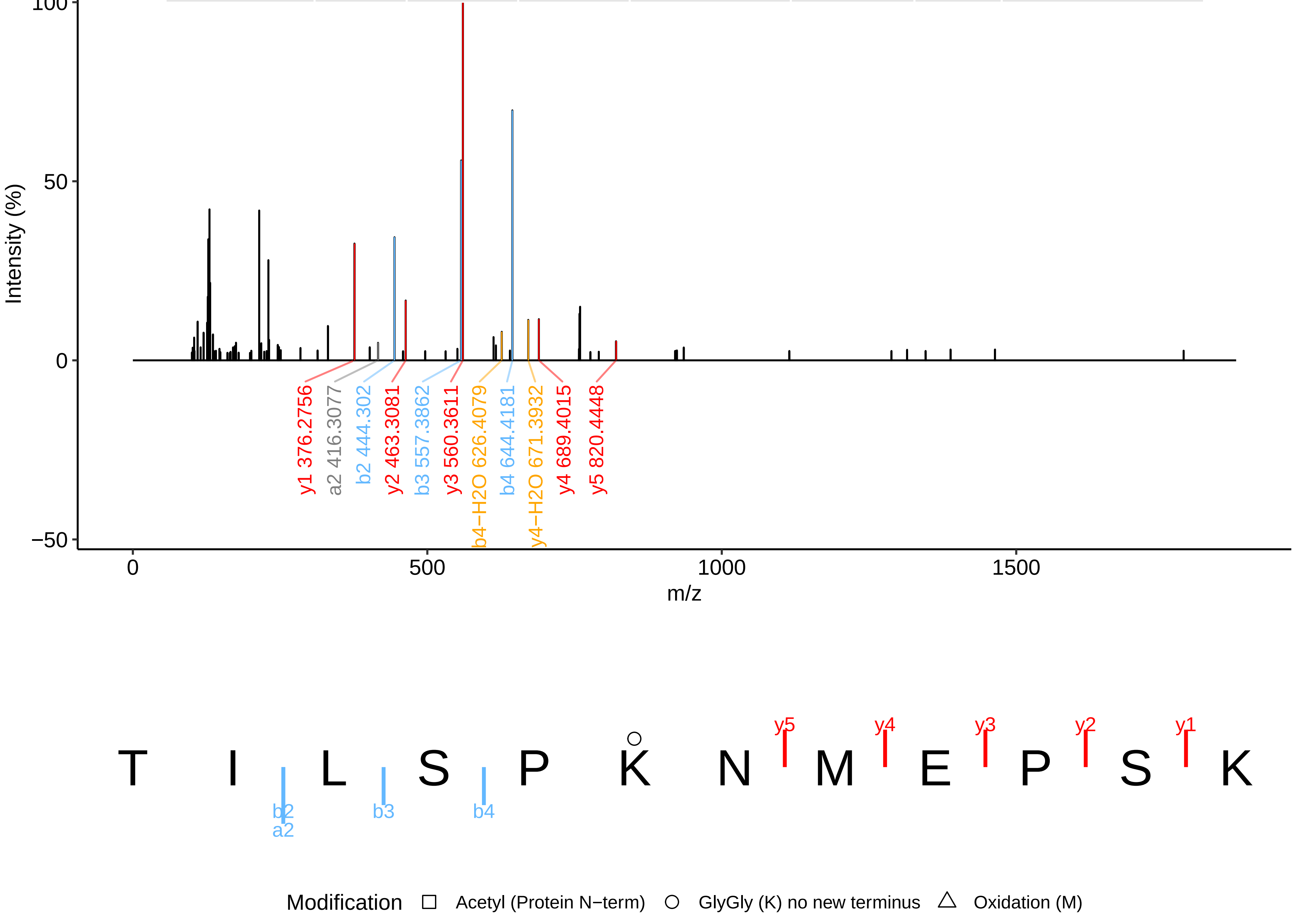

20200921_KIZ_MUb4_GlyGly_10plex_2 − Scan number: 13707

Gene Names	Charge	m/z	Mass	Retention time	PEP	Score	Precursor Intensity
SLC7A1	3	486.9255	1457.755	53.272	0.0011264	81.92	1095723

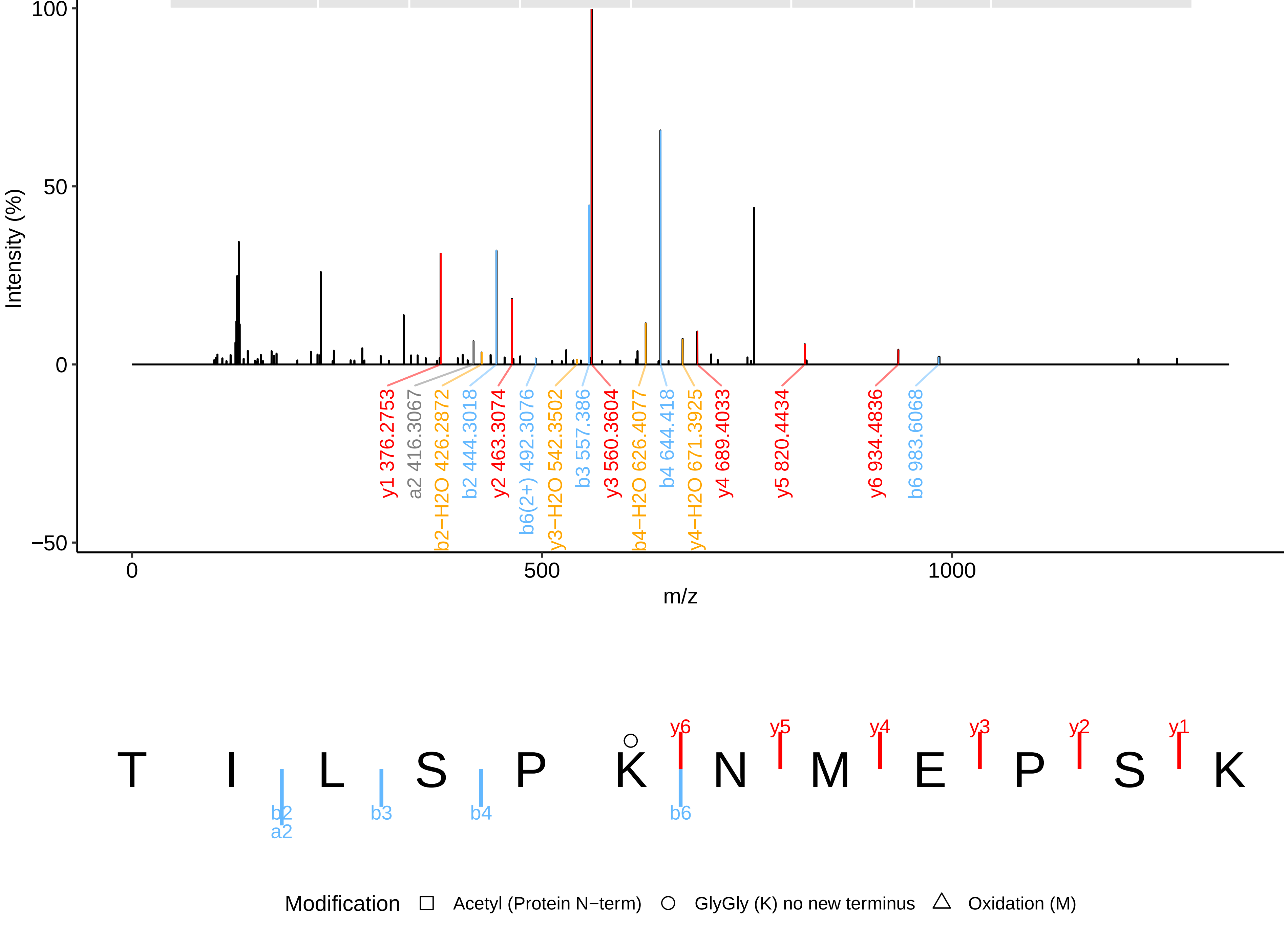

20200924_KIZ_MUb5_GlyGly_1 − Scan number: 13865

Gene Names	Charge	m/z	Mass	Retention time	PEP	Score	Precursor Intensity
SLC7A1	3	486.9255	1457.755	45.233	0.005315	74.46	7079631

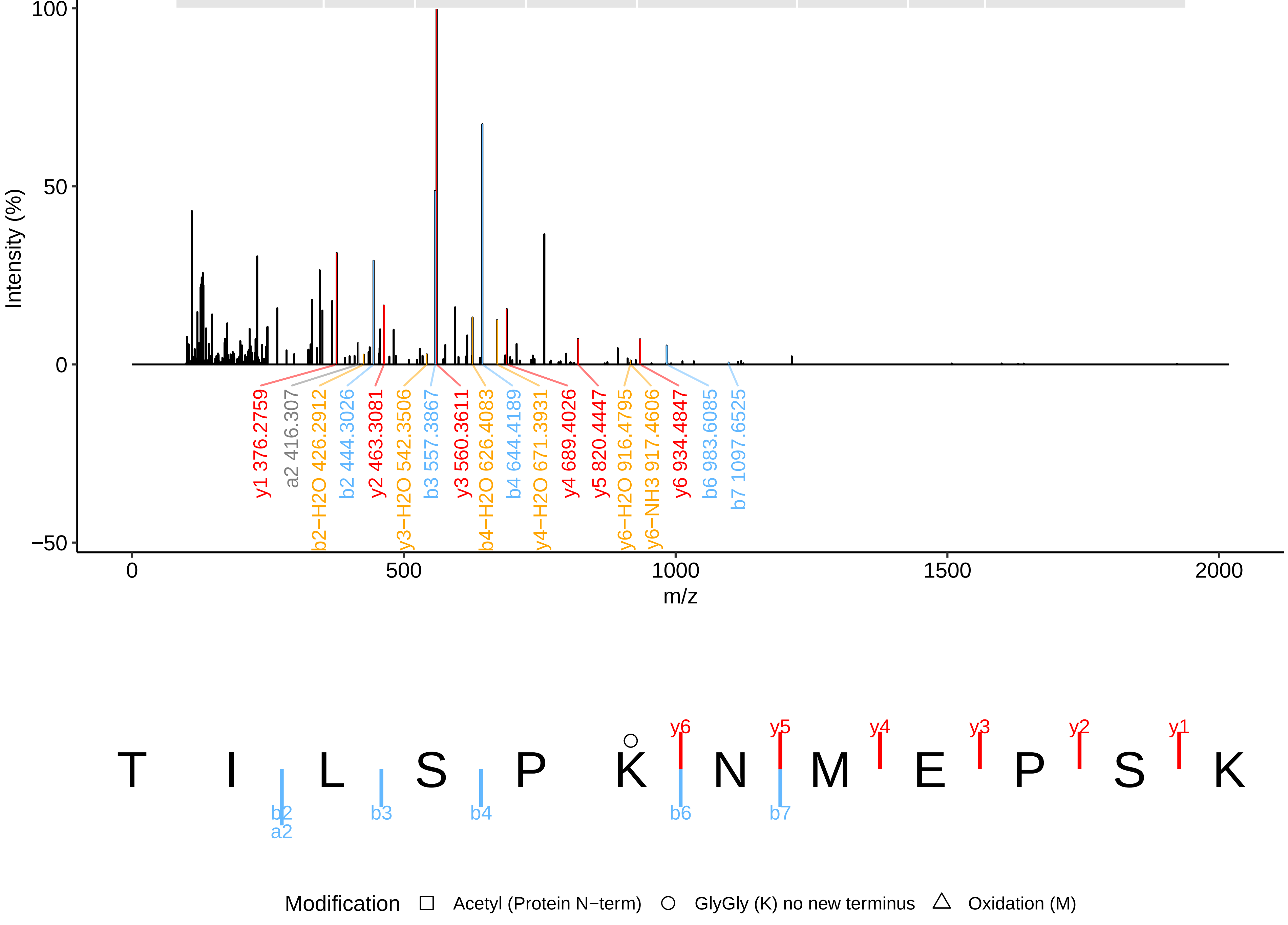

20200924_KIZ_MUb5_GlyGly_1 − Scan number: 14033

Gene Names	Charge	m/z	Mass	Retention time	PEP	Score	Precursor Intensity
SLC7A1	3	486.9255	1457.755	45.8	0.00059155	91.961	6486686

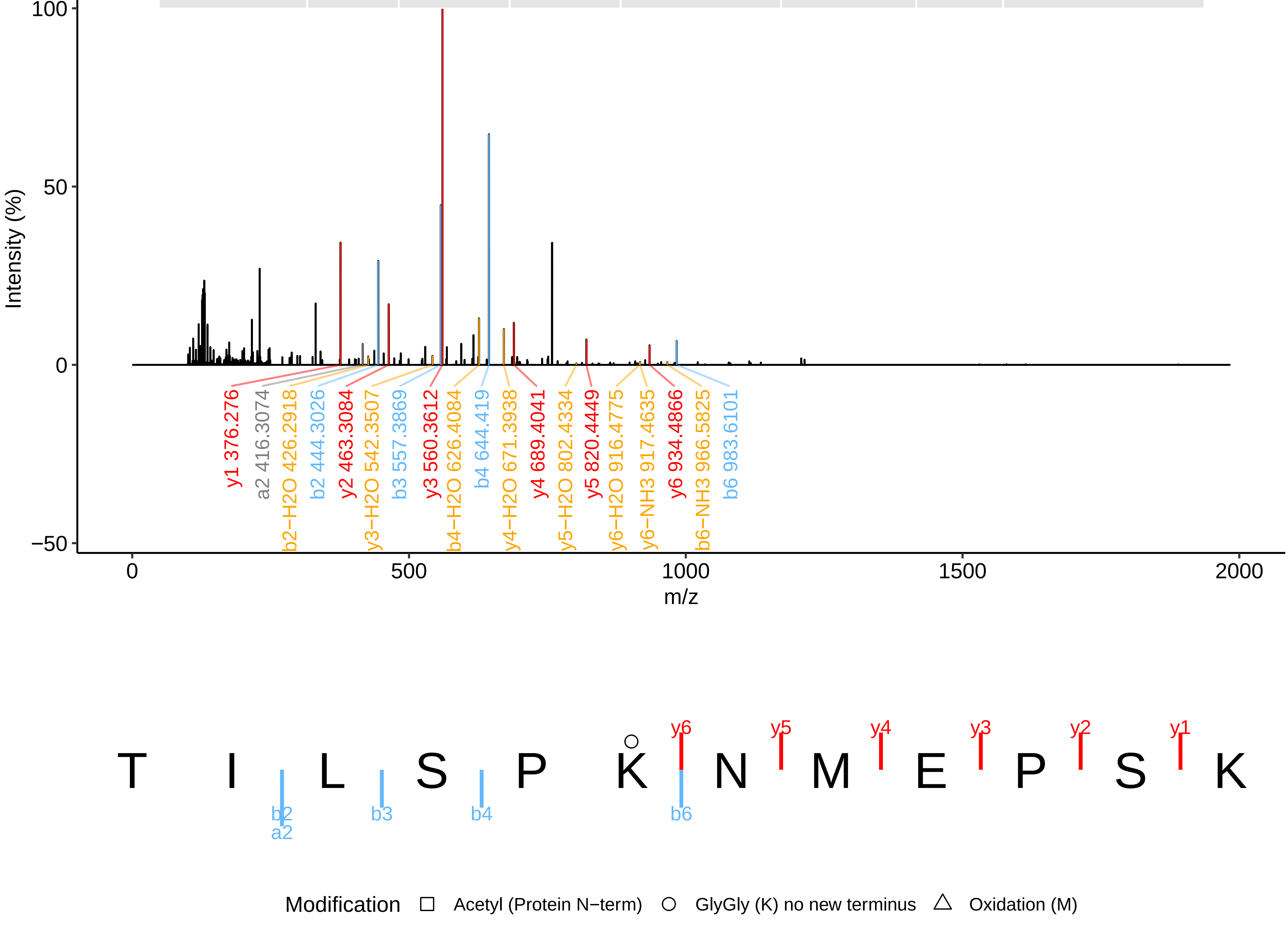

20200924_KIZ_MUb5_GlyGly_2 − Scan number: 12991

Gene Names	Charge	m/z	Mass	Retention time	PEP	Score	Precursor Intensity
SLC7A1	3	486.9255	1457.755	48.579	0.00059155	91.961	NA

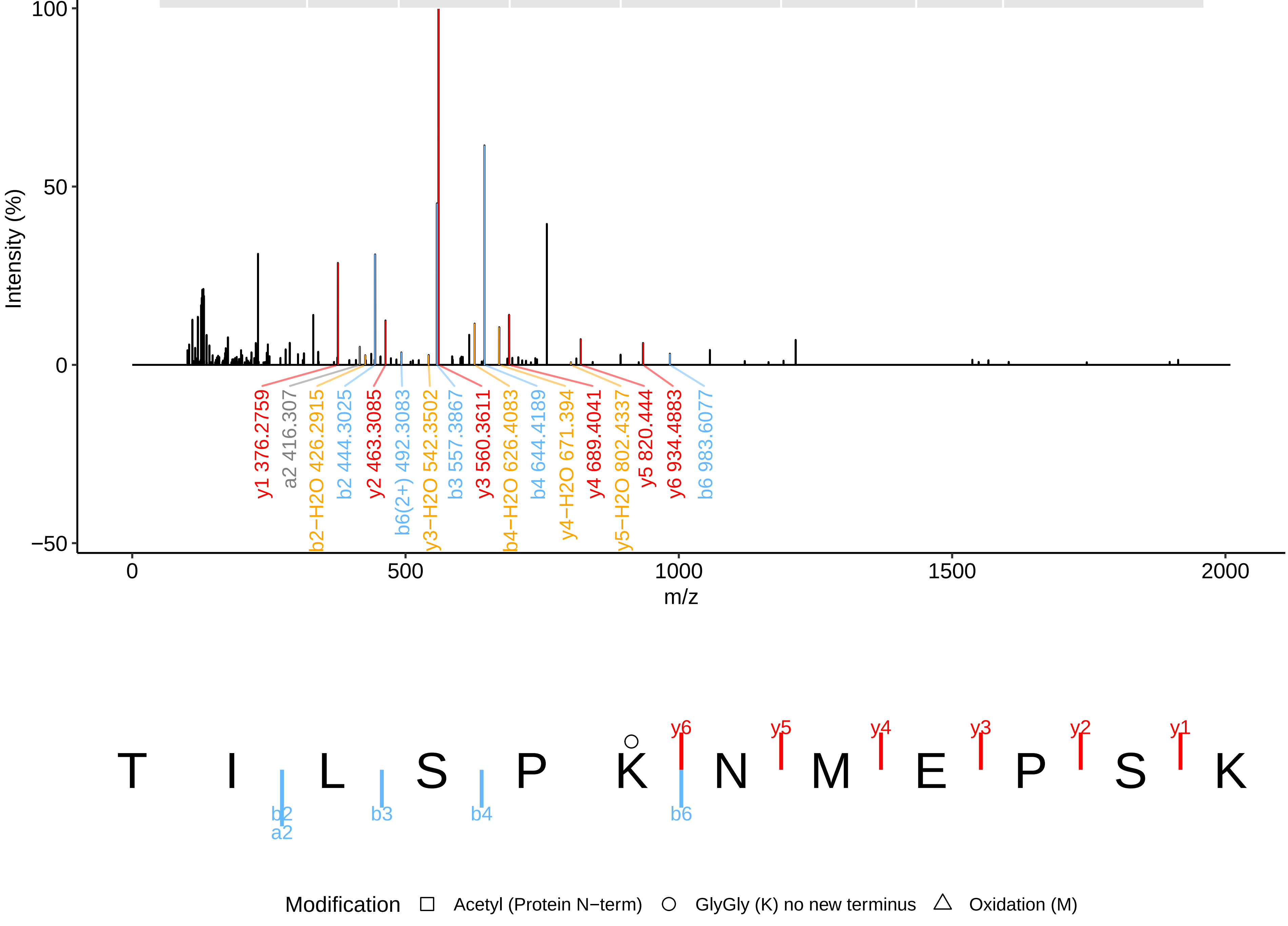

20200924_KIZ_MUb5_GlyGly_2 − Scan number: 13123

Gene Names	Charge	m/z	Mass	Retention time	PEP	Score	Precursor Intensity
SLC7A1	3	486.9255	1457.755	49.091	0.046343	58.674	2190853

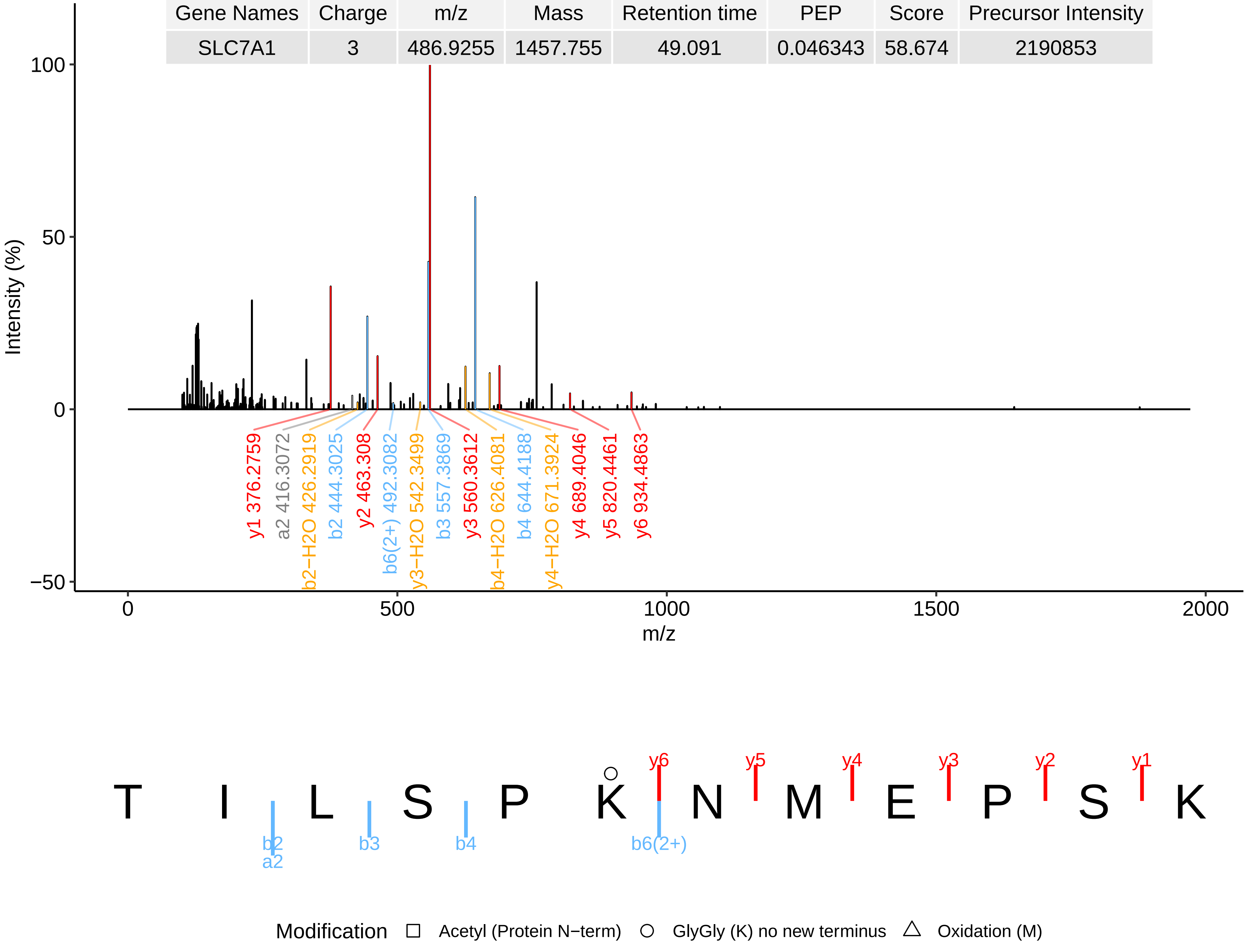

20201118_KIZ_MUb6_GlyGly_2 − Scan number: 16078

Gene Names	Charge	m/z	Mass	Retention time	PEP	Score	Precursor Intensity
SLC7A1	3	486.9255	1457.755	54.28	0.0067662	72.29	6812157

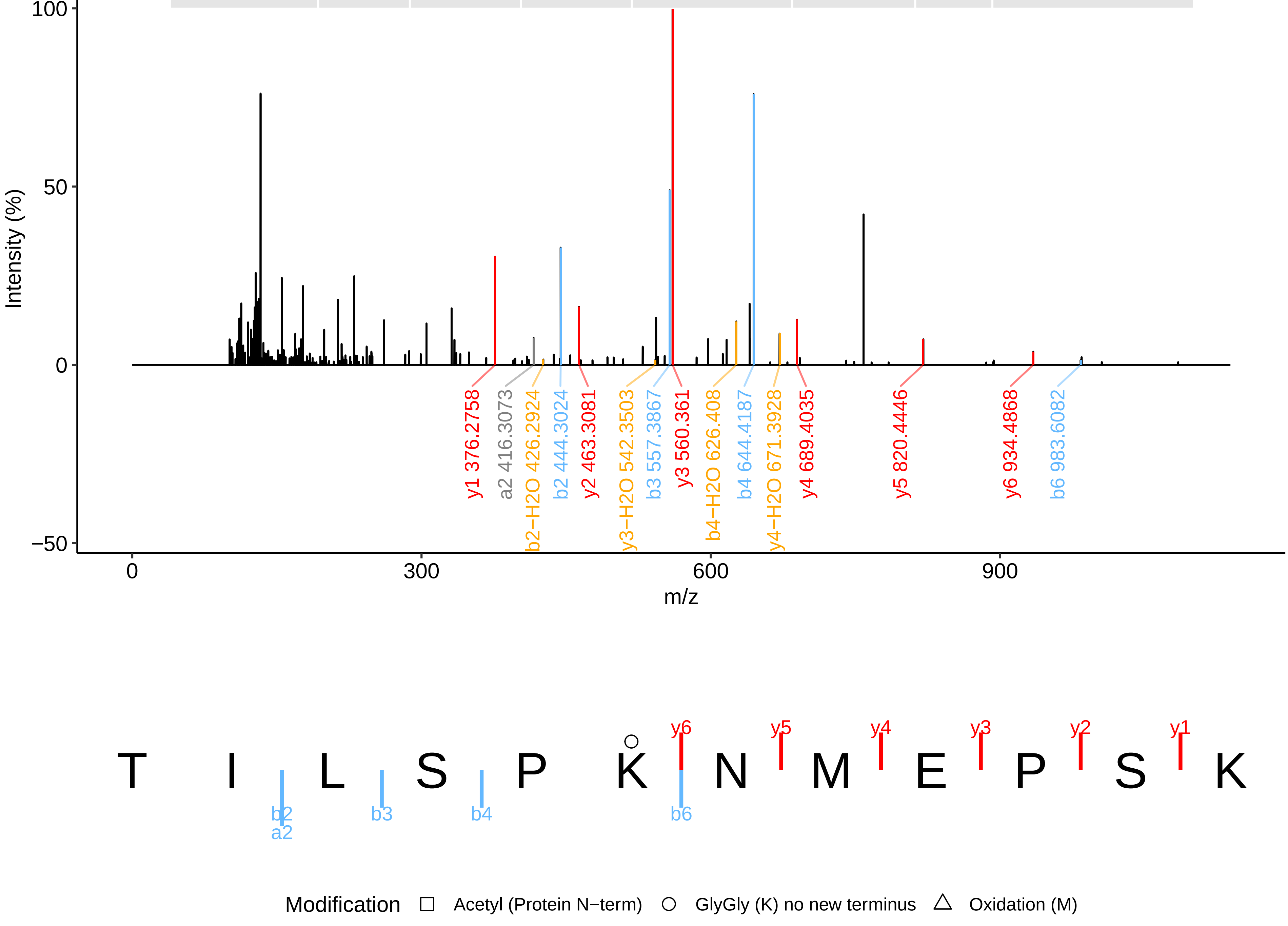

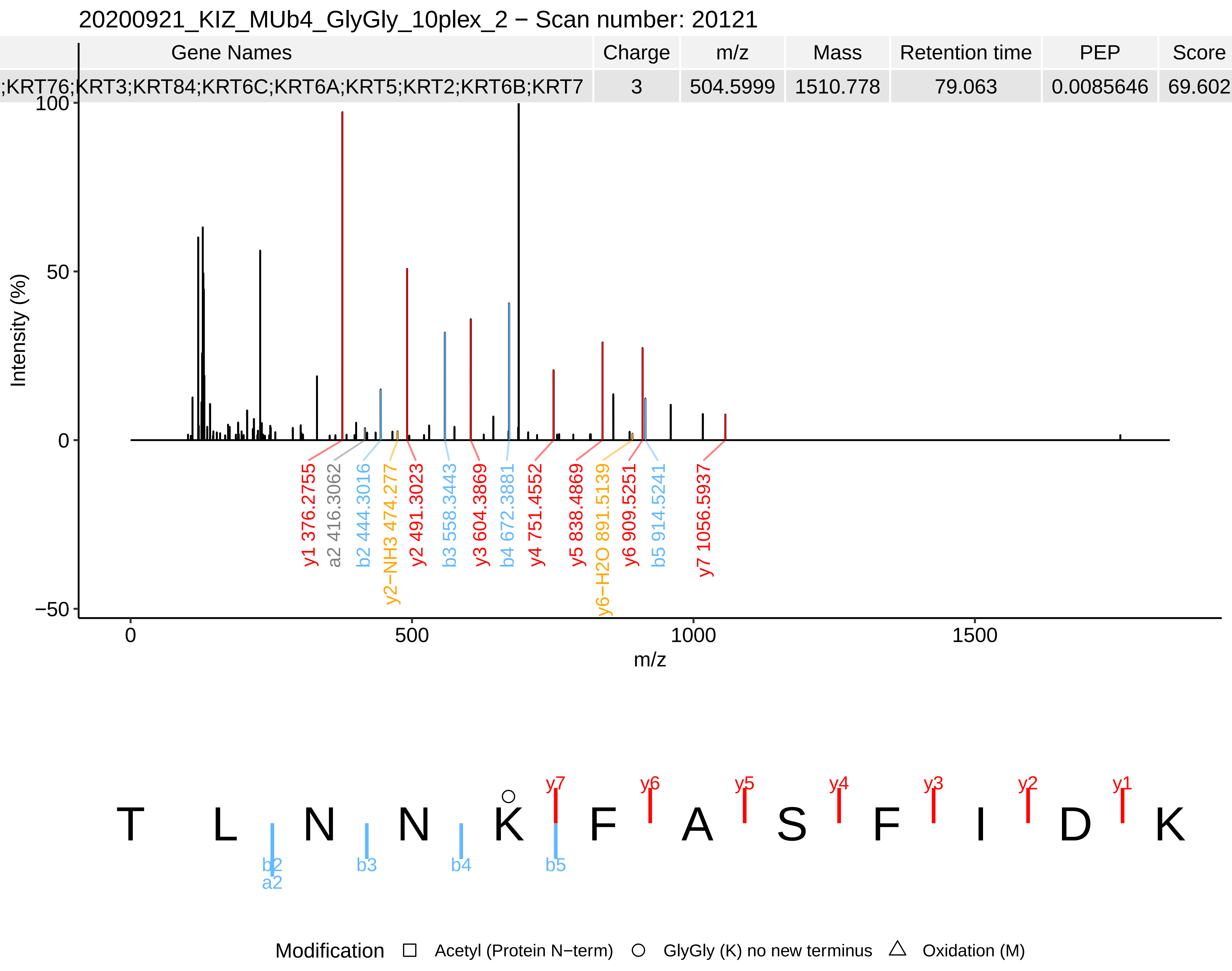
20200921_KIZ_MUb4_GlyGly_10plex_2 − Scan number: 20121
Gene Names | Charge | m/z | Mass | Retention time | PEP | Score
;KRT76;KRT3;KRT84;KRT6C;KRT6A;KRT5;KRT2;KRT6B;KRT7 | 3 | 504.5999 | 1510.778 | 79.063 | 0.0085646 | 69.602
Intensity (%)
m/z
y1 376.2755
a2 416.3062
b2 444.3016
y2−NH3 474.277
y2 491.3023
b3 558.3443
y3 604.3869
b4 672.3881
y4 751.4552
y5 838.4869
y6−H2O 891.5139
y6 909.5251
b5 914.5241
y7 1056.5937
T L N N K F A S F I D K
y7 y6 y5 y4 y3 y2 y1
b2 a2 b3 b4 b5
Modification
Acetyl (Protein N−term)
GlyGly (K) no new terminus
Oxidation (M)

20200921_KIZ_MUb4_GlyGly_10plex_2 − Scan number: 20133

Gene Names	Charge	m/z	Mass	Retention time	PEP	Score
5;KRT76;KRT3;KRT84;KRT6C;KRT6A;KRT5;KRT2;KRT6B;KRT7	3	504.5999	1510.778	79.112	0.071583	51.875

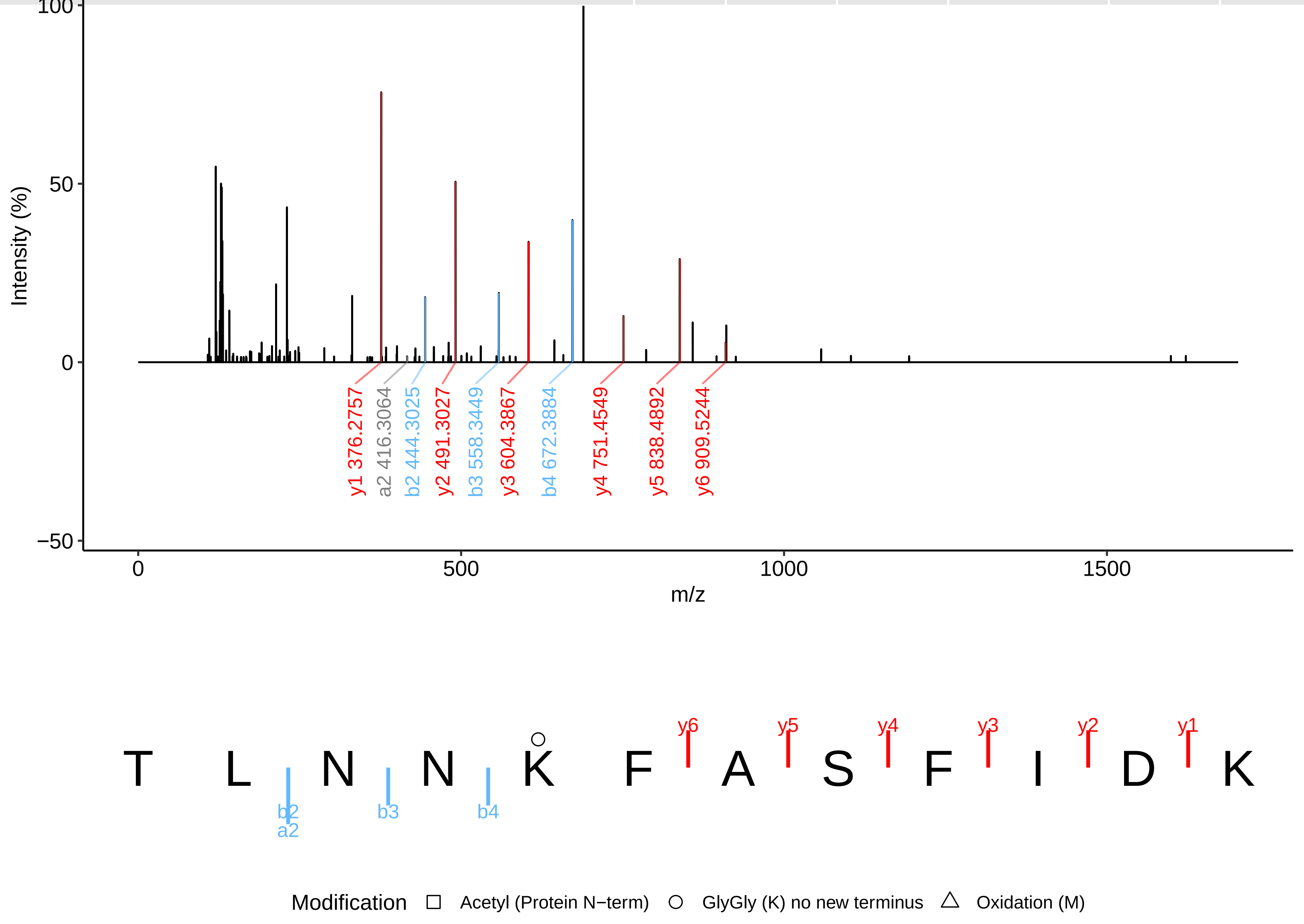

20200921_KIZ_MUb4_GlyGly_10plex − Scan number: 11645

Gene Names	Charge	m/z	Mass	Retention time	PEP	Score	Precursor Intensity
VIM;DES	3	567.9516	1700.833	40.636	1.6661e−05	102.52	NA

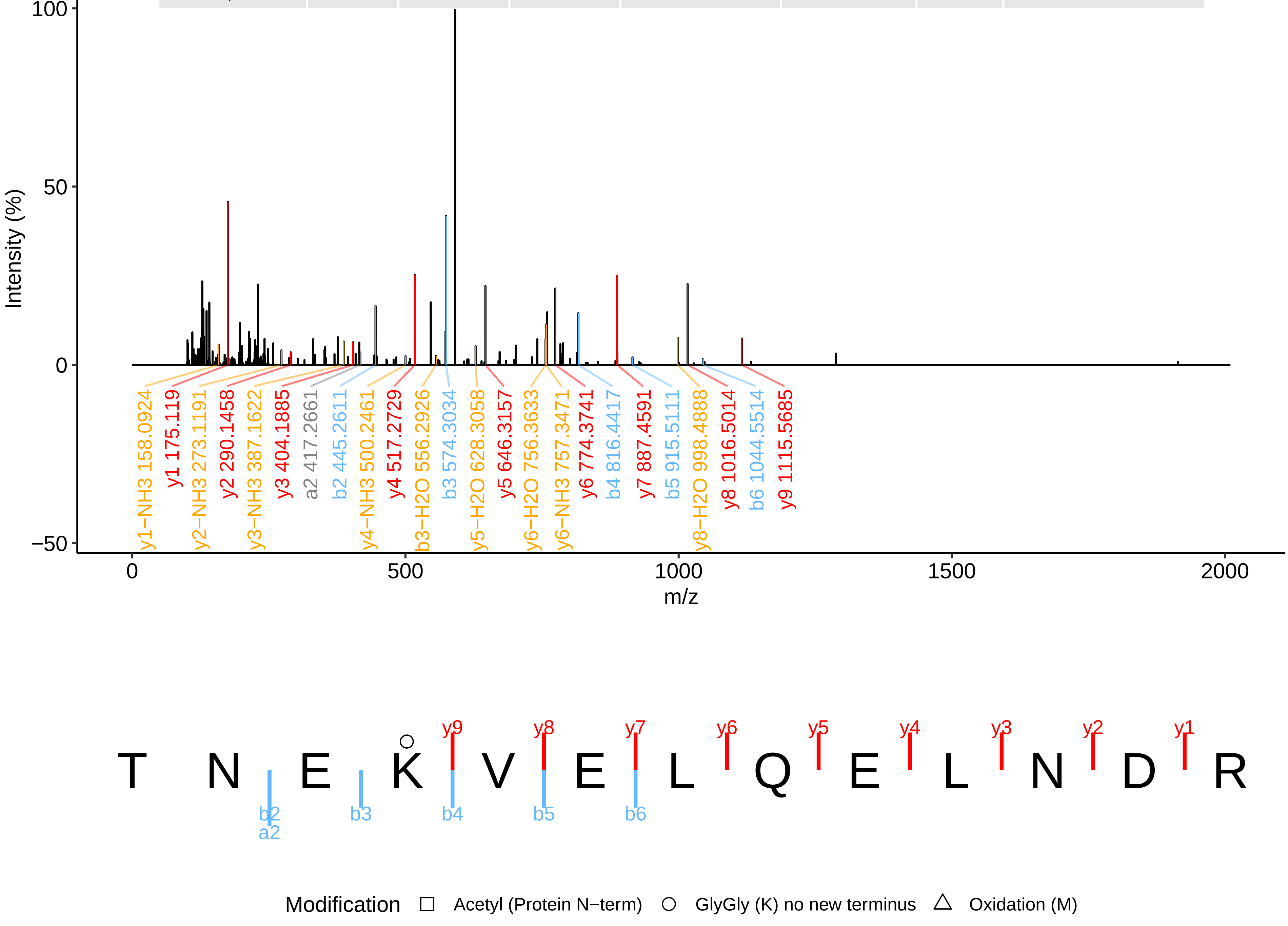

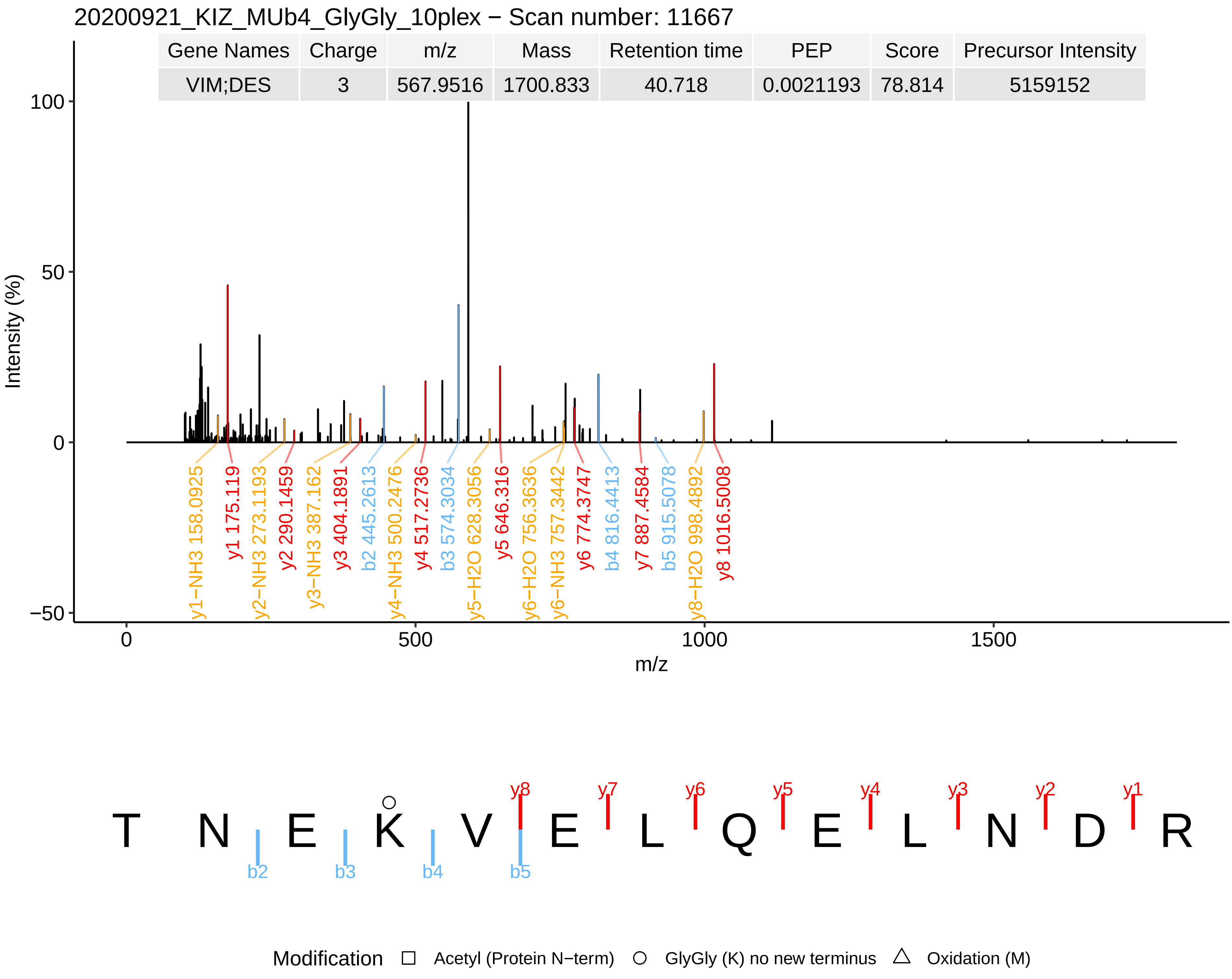

20200921_KIZ_MUb4_GlyGly_10plex − Scan number: 11667
Gene Names | Charge | m/z | Mass | Retention time | PEP | Score | Precursor Intensity
VIM;DES | 3 | 567.9516 | 1700.833 | 40.718 | 0.0021193 | 78.814 | 5159152
Intensity (%)
100
50
0
−50
0
500
1000
1500
m/z
y1−NH3 158.0925
y1 175.119
y2−NH3 273.1193
y2 290.1459
y3−NH3 387.162
y3 404.1891
b2 445.2613
y4−NH3 500.2476
y4 517.2736
b3 574.3034
y5−H2O 628.3056
y5 646.316
y6−H2O 756.3636
y6−NH3 757.3442
y6 774.3747
b4 816.4413
y7 887.4584
b5 915.5078
y8−H2O 998.4892
y8 1016.5008
T N E K V E L Q E L N D R
y8 y7 y6 y5 y4 y3 y2 y1
b2 b3 b4 b5
Modification
Acetyl (Protein N−term)
GlyGly (K) no new terminus
Oxidation (M)

20200921_KIZ_MUb4_GlyGly_10plex_2 − Scan number: 10701

Gene Names	Charge	m/z	Mass	Retention time	PEP	Score	Precursor Intensity
VIM;DES	3	567.9516	1700.833	40.996	9.3656e−06	108.17	738264.4

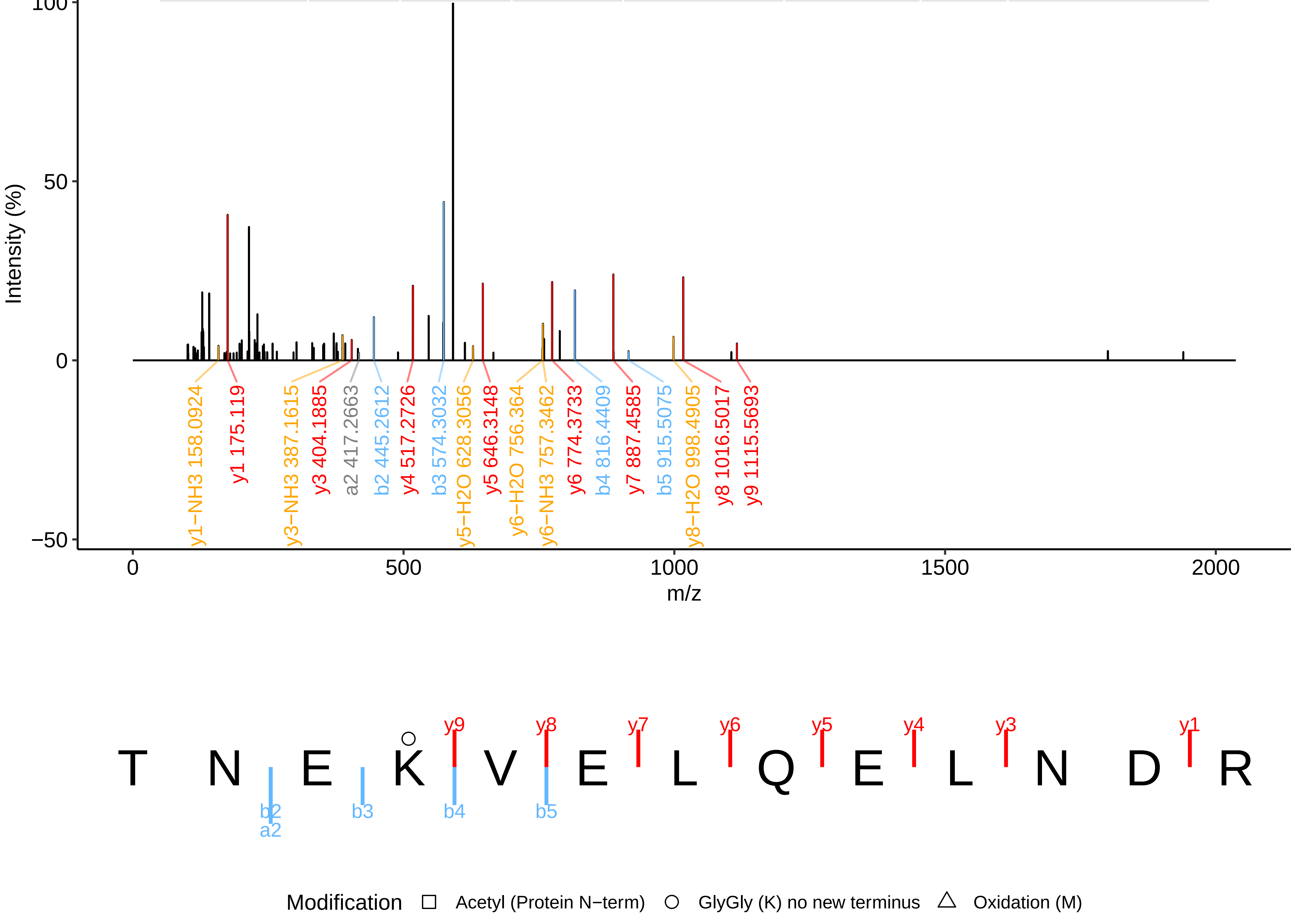

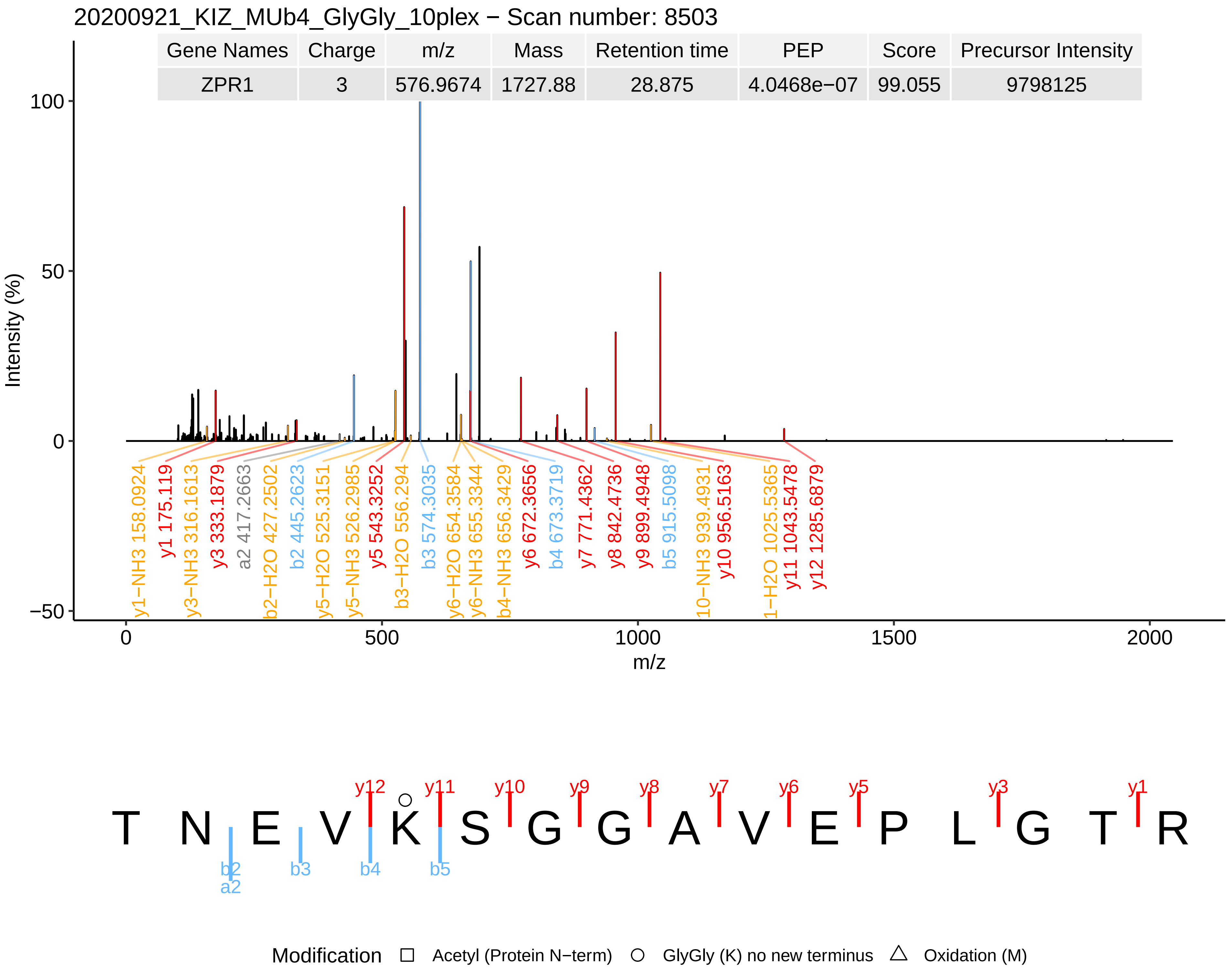
20200921_KIZ_MUb4_GlyGly_10plex − Scan number: 8503
Gene Names | Charge | m/z | Mass | Retention time | PEP | Score | Precursor Intensity
ZPR1 | 3 | 576.9674 | 1727.88 | 28.875 | 4.0468e−07 | 99.055 | 9798125
Intensity (%)
100
50
0
−50
y1−NH3 158.0924
y1 175.119
y3−NH3 316.1613
y3 333.1879
a2 417.2663
b2−H2O 427.2502
b2 445.2623
y5−H2O 525.3151
y5−NH3 526.2985
y5 543.3252
b3−H2O 556.294
b3 574.3035
y6−H2O 654.3584
y6−NH3 655.3344
b4−NH3 656.3429
y6 672.3656
b4 673.3719
y7 771.4362
y8 842.4736
y9 899.4948
b5 915.5098
10−NH3 939.4931
y10 956.5163
1−H2O 1025.5365
y11 1043.5478
y12 1285.6879
0
500
1000
1500
2000
m/z
T N E V K S G G A V E P L G T R
y12 y11 y10 y9 y8 y7 y6 y5 y3 y1
b2 a2 b3 b4 b5
Modification
Acetyl (Protein N−term)
GlyGly (K) no new terminus
Oxidation (M)

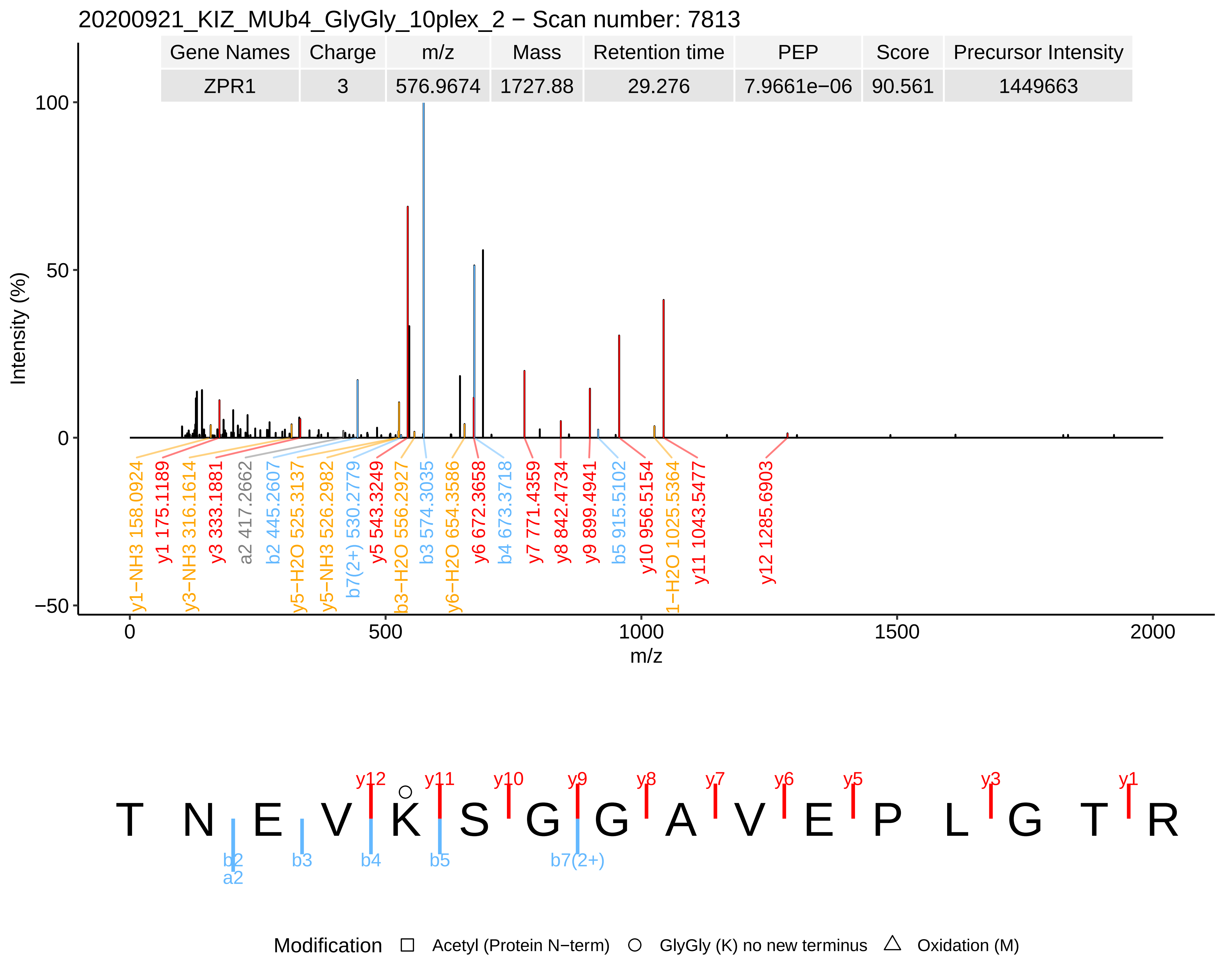
20200921_KIZ_MUb4_GlyGly_10plex_2 − Scan number: 7813
Gene Names | Charge | m/z | Mass | Retention time | PEP | Score | Precursor Intensity
ZPR1 | 3 | 576.9674 | 1727.88 | 29.276 | 7.9661e−06 | 90.561 | 1449663
Intensity (%)
100
50
0
−50
0
500
1000
1500
2000
m/z
y1−NH3 158.0924
y1 175.1189
y3−NH3 316.1614
y3 333.1881
a2 417.2662
b2 445.2607
y5−H2O 525.3137
y5−NH3 526.2982
b7(2+) 530.2779
y5 543.3249
b3−H2O 556.2927
b3 574.3035
y6−H2O 654.3586
y6 672.3658
b4 673.3718
y7 771.4359
y8 842.4734
y9 899.4941
b5 915.5102
y10 956.5154
1−H2O 1025.5364
y11 1043.5477
y12 1285.6903
y12 y11 y10 y9 y8 y7 y6 y5 y3 y1
T N E V K S G G A V E P L G T R
b2 a2 b3 b4 b5 b7(2+)
Modification
Acetyl (Protein N−term)
GlyGly (K) no new terminus
Oxidation (M)

20201118_KIZ_MUb6_GlyGly_1 − Scan number: 7204

Gene Names	Charge	m/z	Mass	Retention time	PEP	Score	Precursor Intensity
ZPR1	3	576.9674	1727.88	22.828	0.00016089	82.349	NA

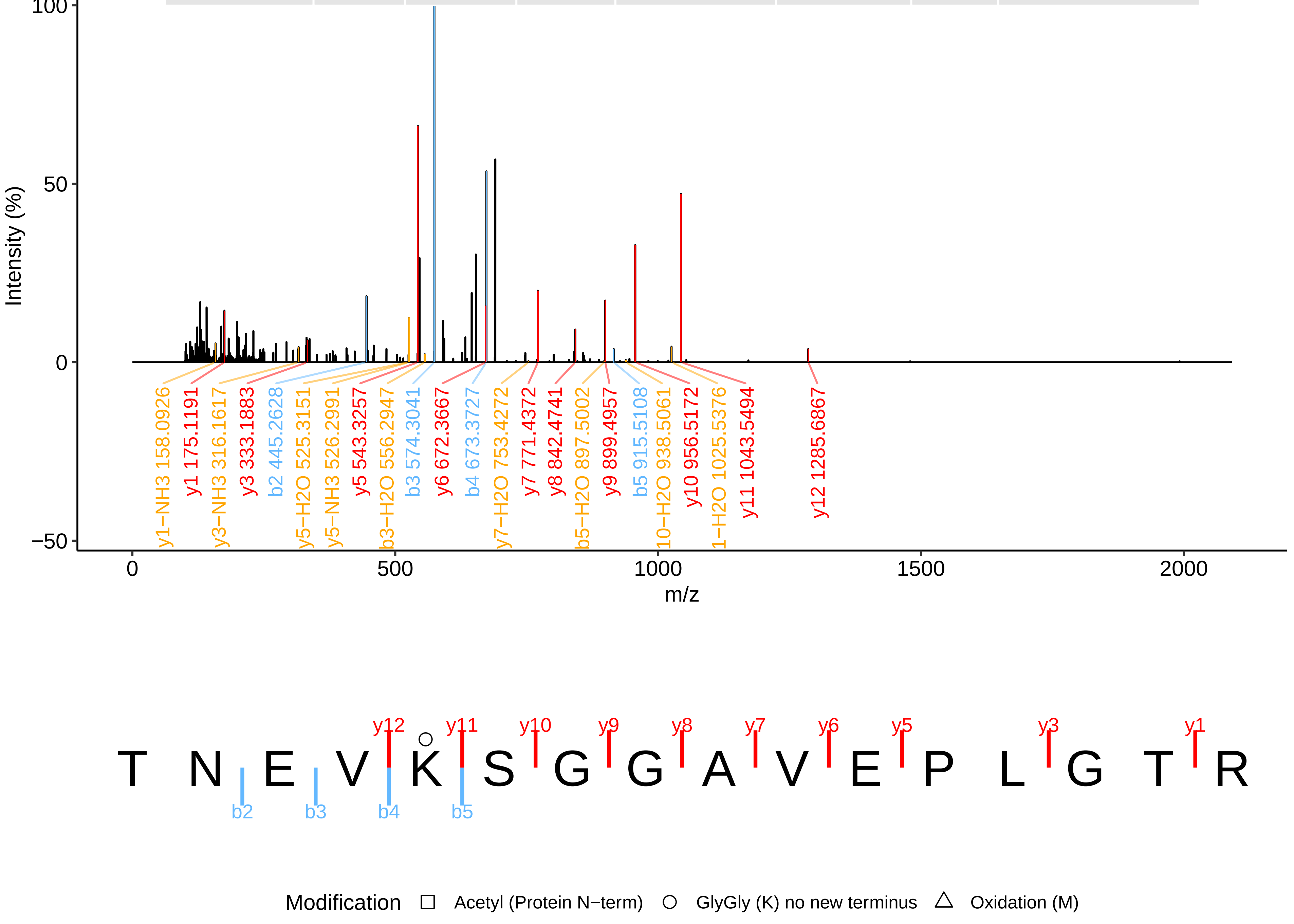

20200921_KIZ_MUb4_GlyGly_10plex − Scan number: 7405

Gene Names	Charge	m/z	Mass	Retention time	PEP	Score	Precursor Intensity
NRAS	4	364.4454	1453.753	24.847	0.00072357	85.522	11408756

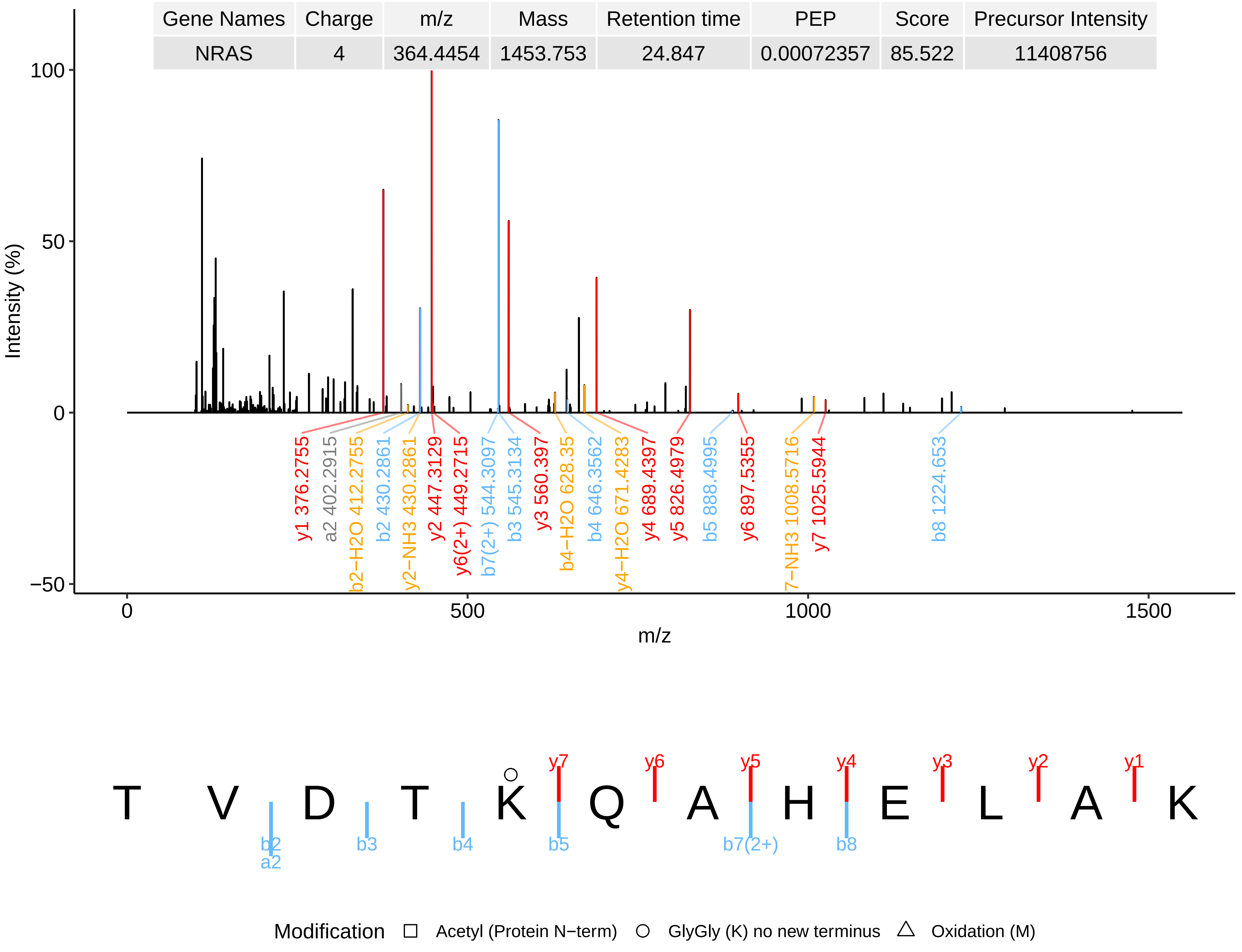

20200921_KIZ_MUb4_GlyGly_10plex_2 − Scan number: 6830

Gene Names	Charge	m/z	Mass	Retention time	PEP	Score	Precursor Intensity
NRAS	4	364.4454	1453.753	25.271	0.046854	54.7	1737576

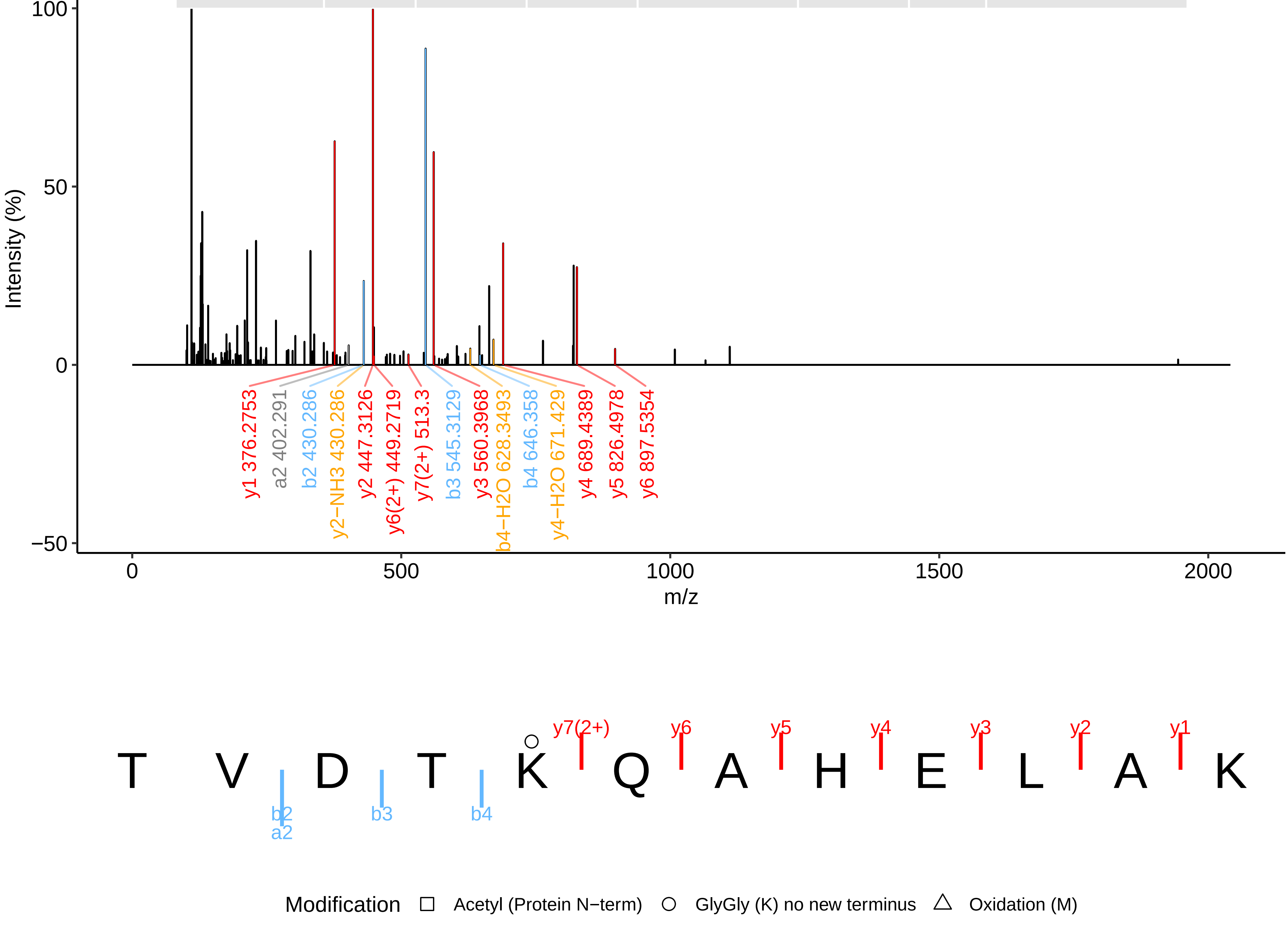

20201118_KIZ_MUb6_GlyGly_1 − Scan number: 6059

Gene Names	Charge	m/z	Mass	Retention time	PEP	Score	Precursor Intensity
NRAS	4	364.4454	1453.753	19.167	0.012641	62.694	25928808

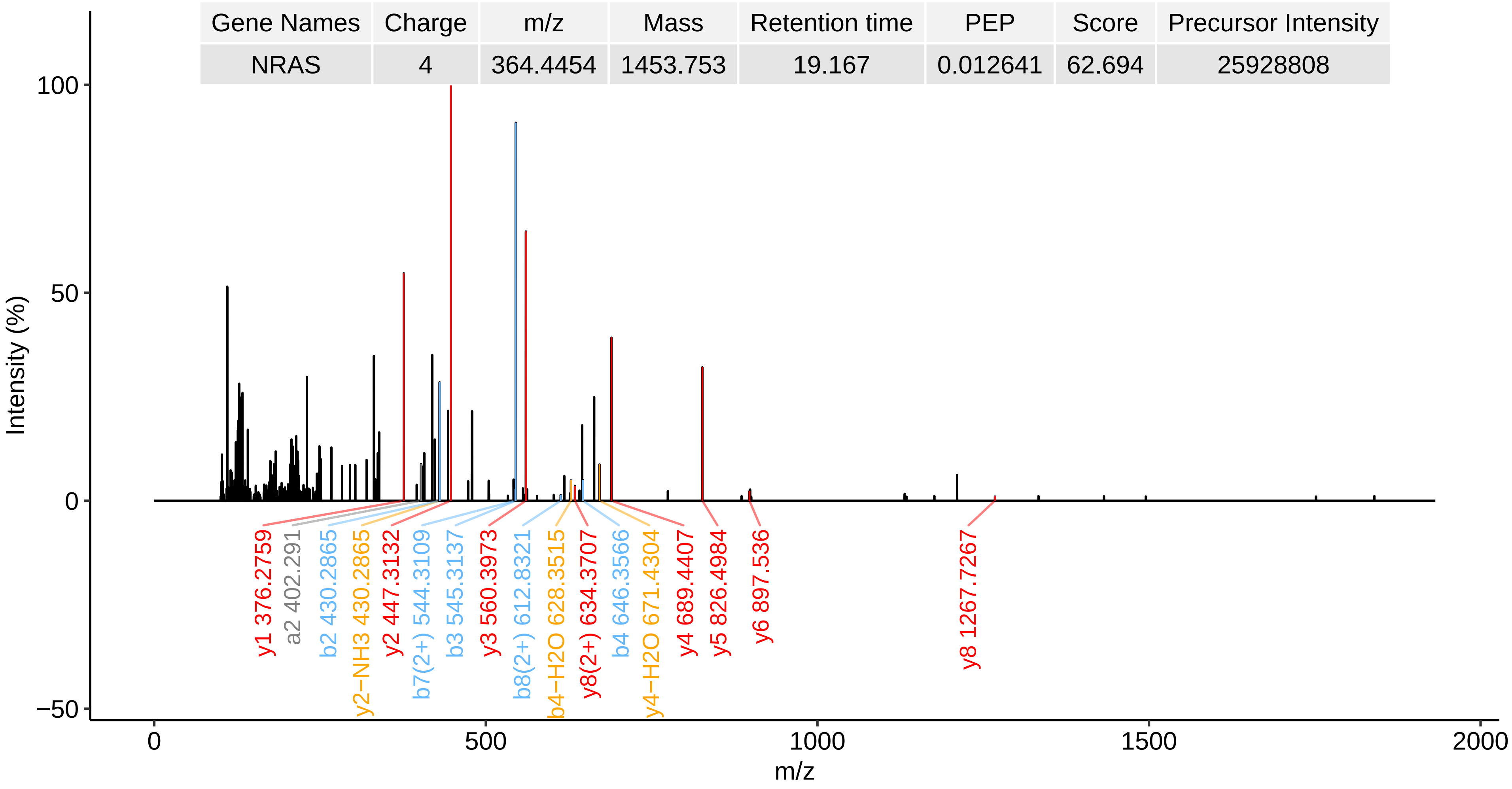

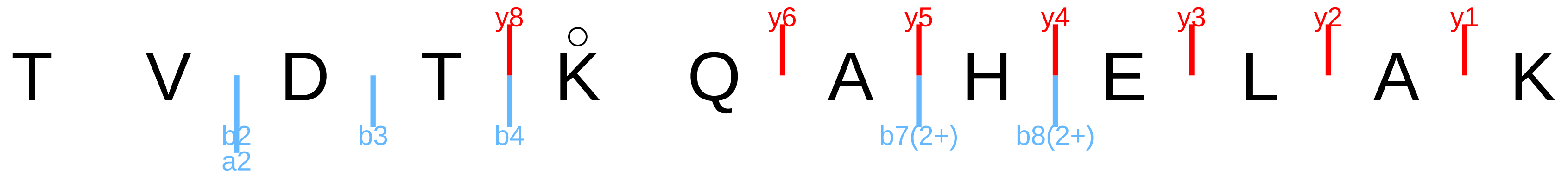

Modification □ Acetyl (Protein N−term) ○ GlyGly (K) no new terminus △ Oxidation (M)

20200921_KIZ_MUb4_GlyGly_10plex_2 − Scan number: 12774

Gene Names	Charge	m/z	Mass	Retention time	PEP	Score	Precursor Intensity
EEF2	3	489.2718	1464.794	49.498	0.0074098	69.03	1208743

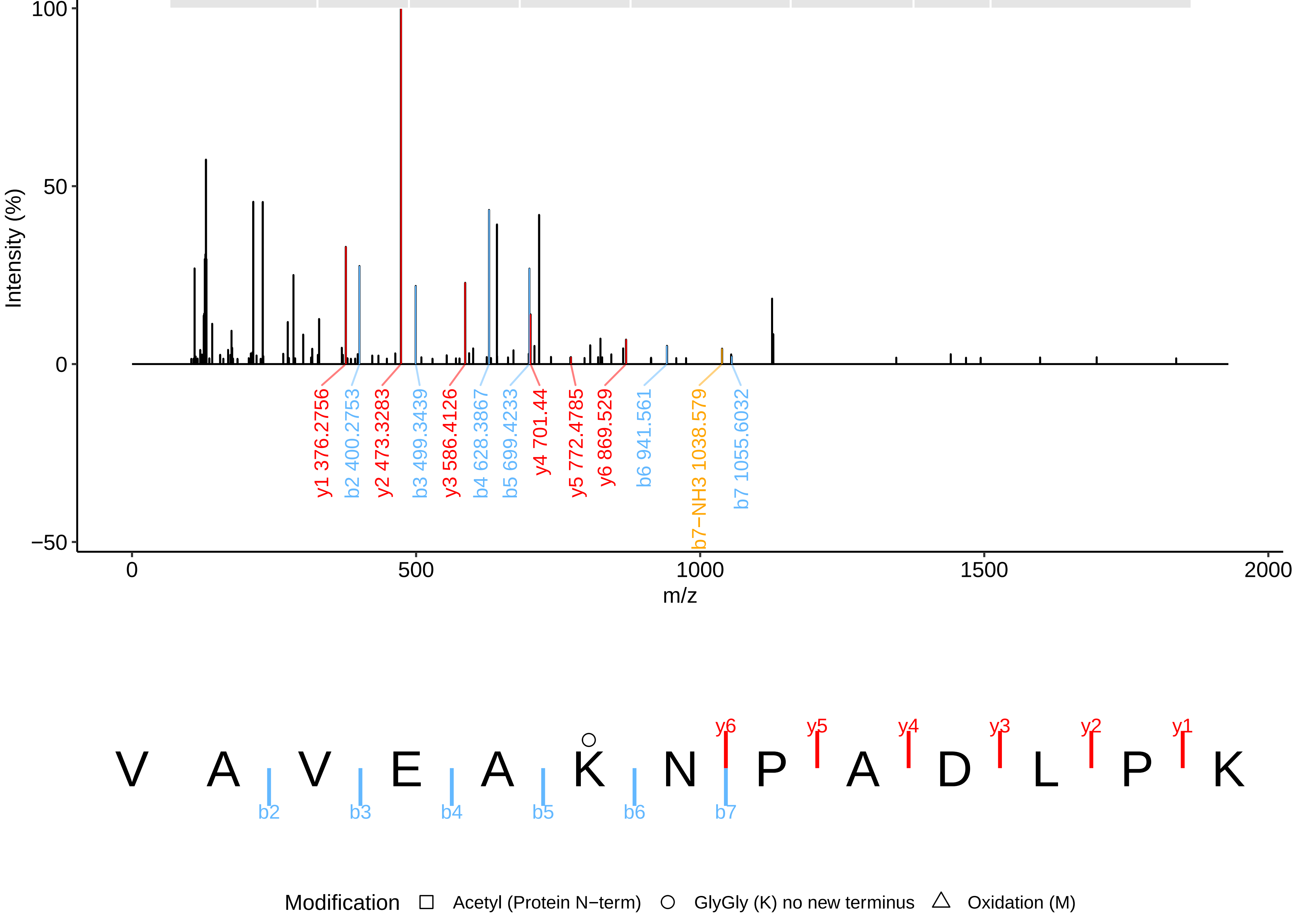

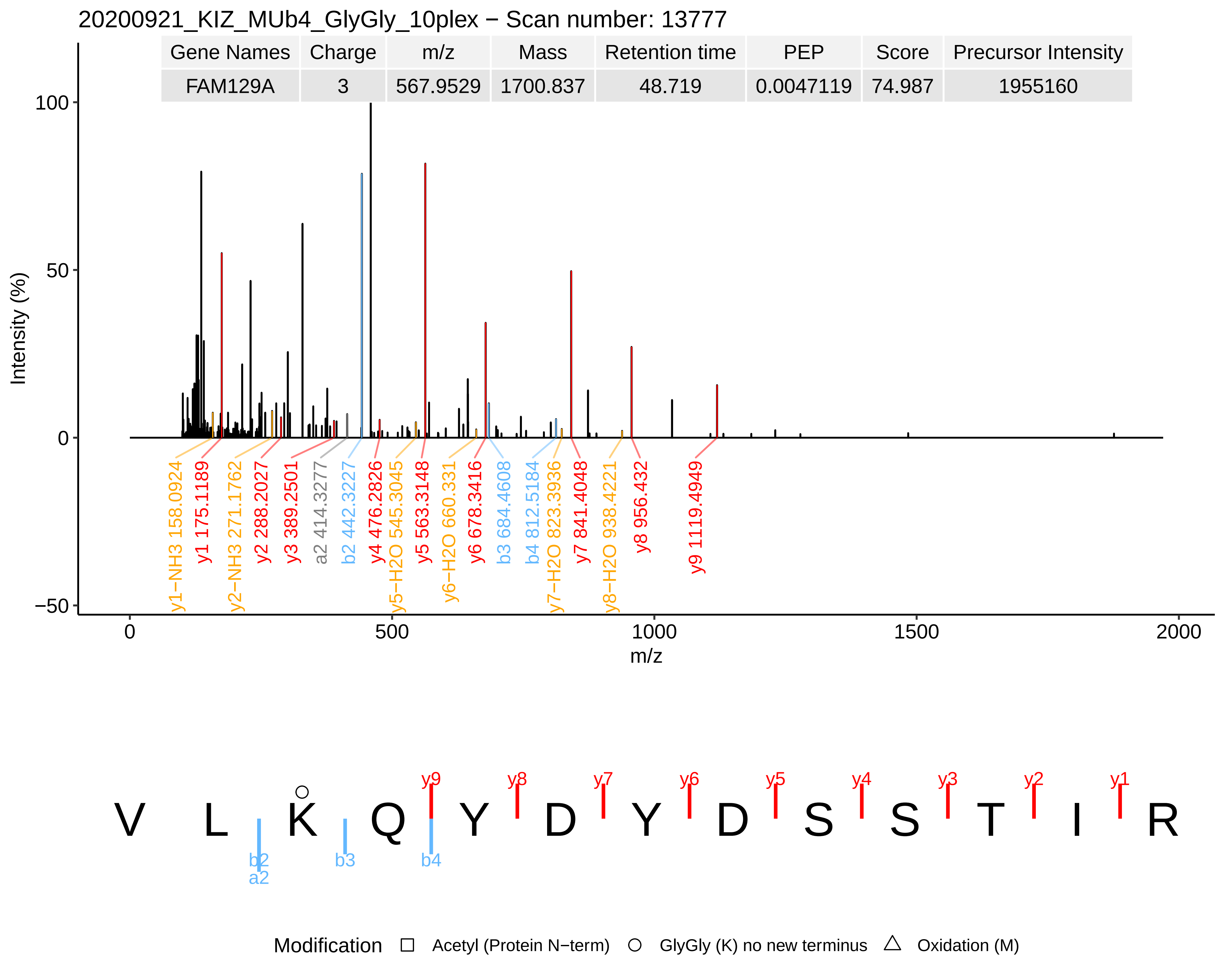
20200921_KIZ_MUb4_GlyGly_10plex − Scan number: 13777
Gene Names
Charge
m/z
Mass
Retention time
PEP
Score
Precursor Intensity
FAM129A
3
567.9529
1700.837
48.719
0.0047119
74.987
1955160
Intensity (%)
100
50
0
−50
0
500
1000
1500
2000
m/z
y1−NH3 158.0924
y1 175.1189
y2−NH3 271.1762
y2 288.2027
y3 389.2501
a2 414.3277
b2 442.3227
y4 476.2826
y5−H2O 545.3045
y5 563.3148
y6−H2O 660.331
y6 678.3416
b3 684.4608
b4 812.5184
y7−H2O 823.3936
y7 841.4048
y8−H2O 938.4221
y8 956.432
y9 1119.4949
V L K Q Y D Y D S S T I R
y9 y8 y7 y6 y5 y4 y3 y2 y1
b2 a2 b3 b4
Modification
Acetyl (Protein N−term)
GlyGly (K) no new terminus
Oxidation (M)

20200921_KIZ_MUb4_GlyGly_10plex − Scan number: 11936

Gene Names	Charge	m/z	Mass	Retention time	PEP	Score	Precursor Intensity
KCTD10	3	486.9315	1457.773	41.705	9.899e−20	162.26	7364884

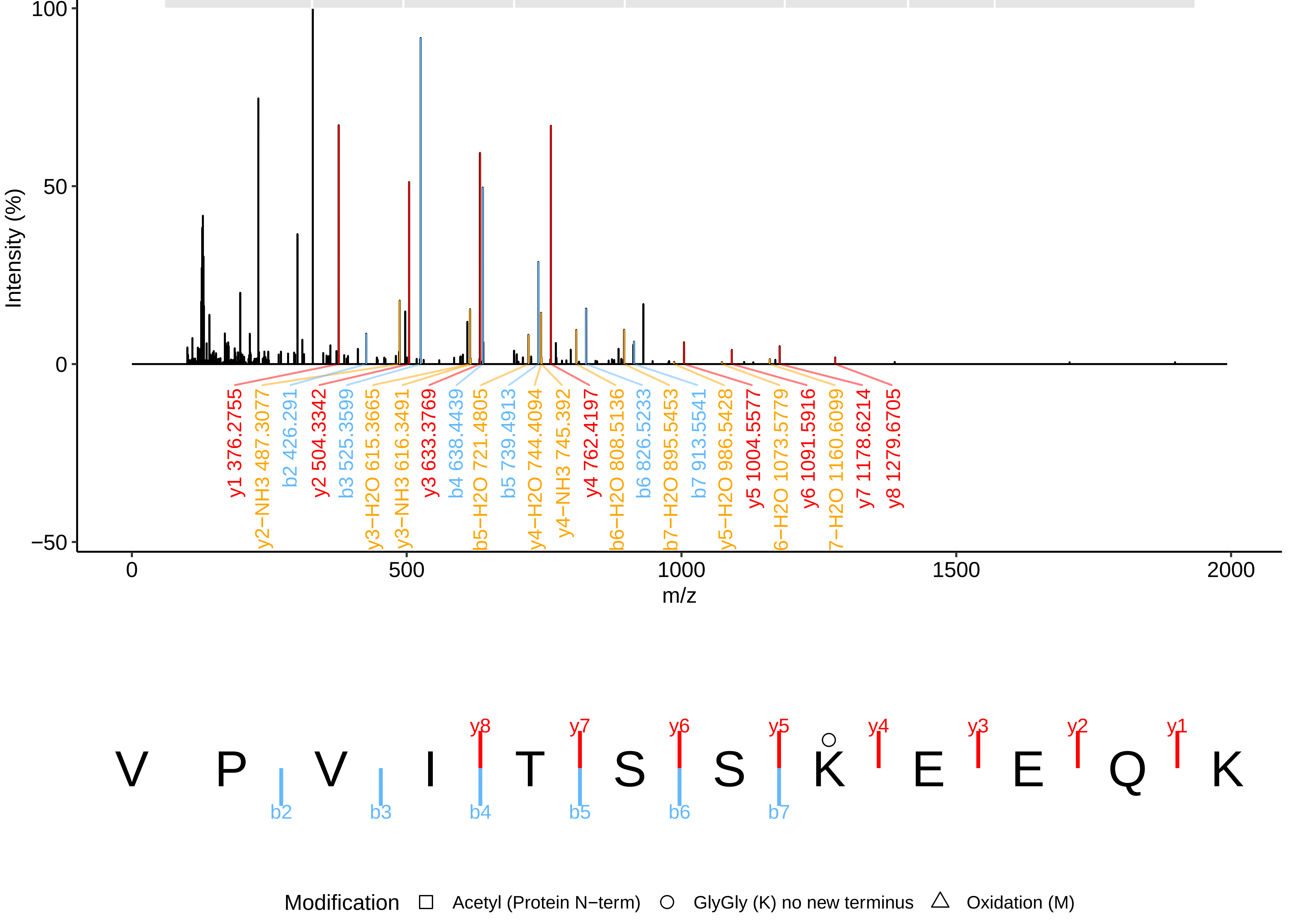

20200921_KIZ_MUb4_GlyGly_10plex_2 − Scan number: 10983

Gene Names	Charge	m/z	Mass	Retention time	PEP	Score	Precursor Intensity
KCTD10	3	486.9315	1457.773	42.167	0.074807	54.471	685366.5

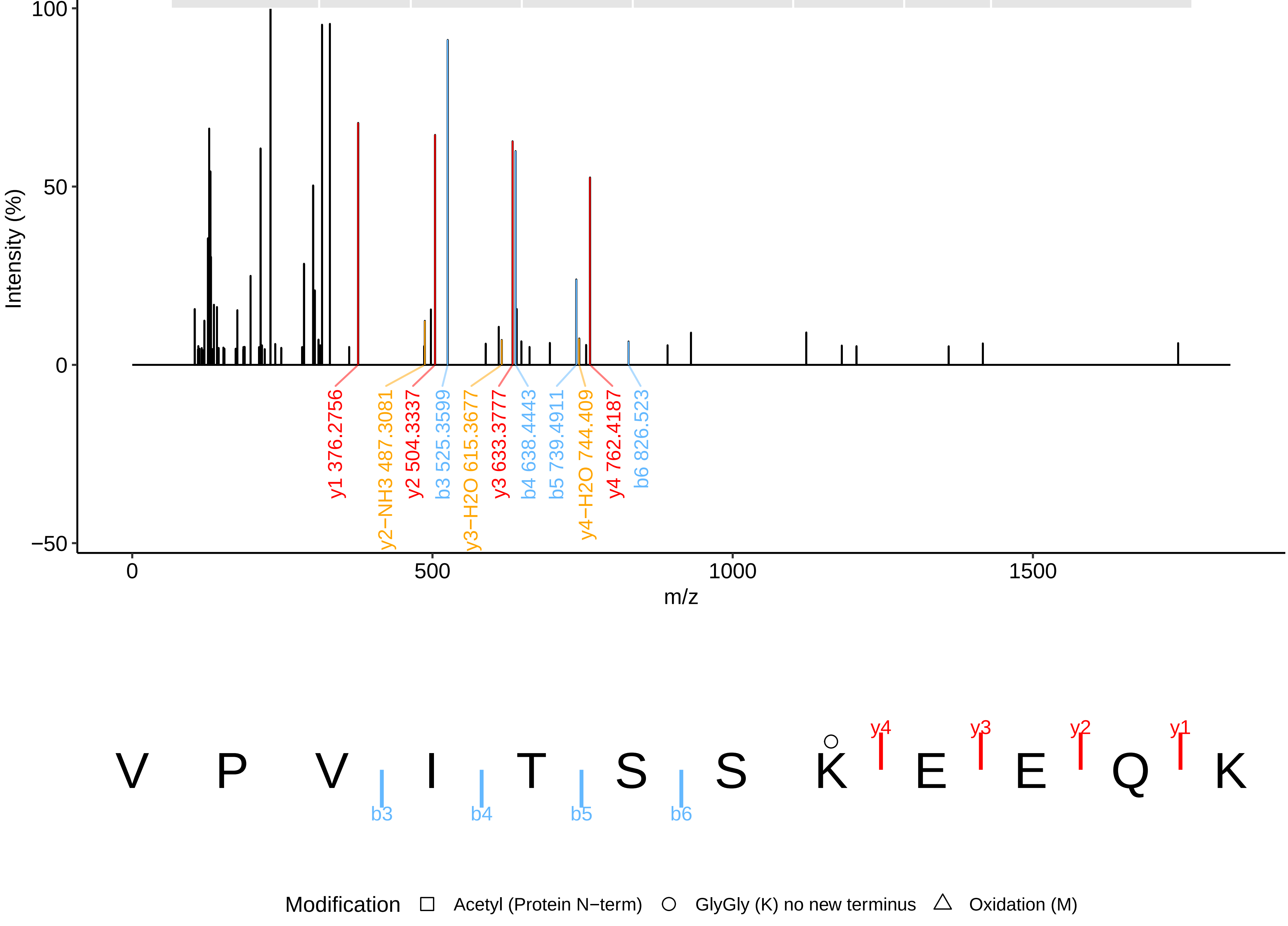

20200921_KIZ_MUb4_GlyGly_10plex_2 − Scan number: 18378

Gene Names	Charge	m/z	Mass	Retention time	PEP	Score	Precursor Intensity
AUP1	3	581.3234	1740.948	71.959	2.5739e−06	106.52	867357.8

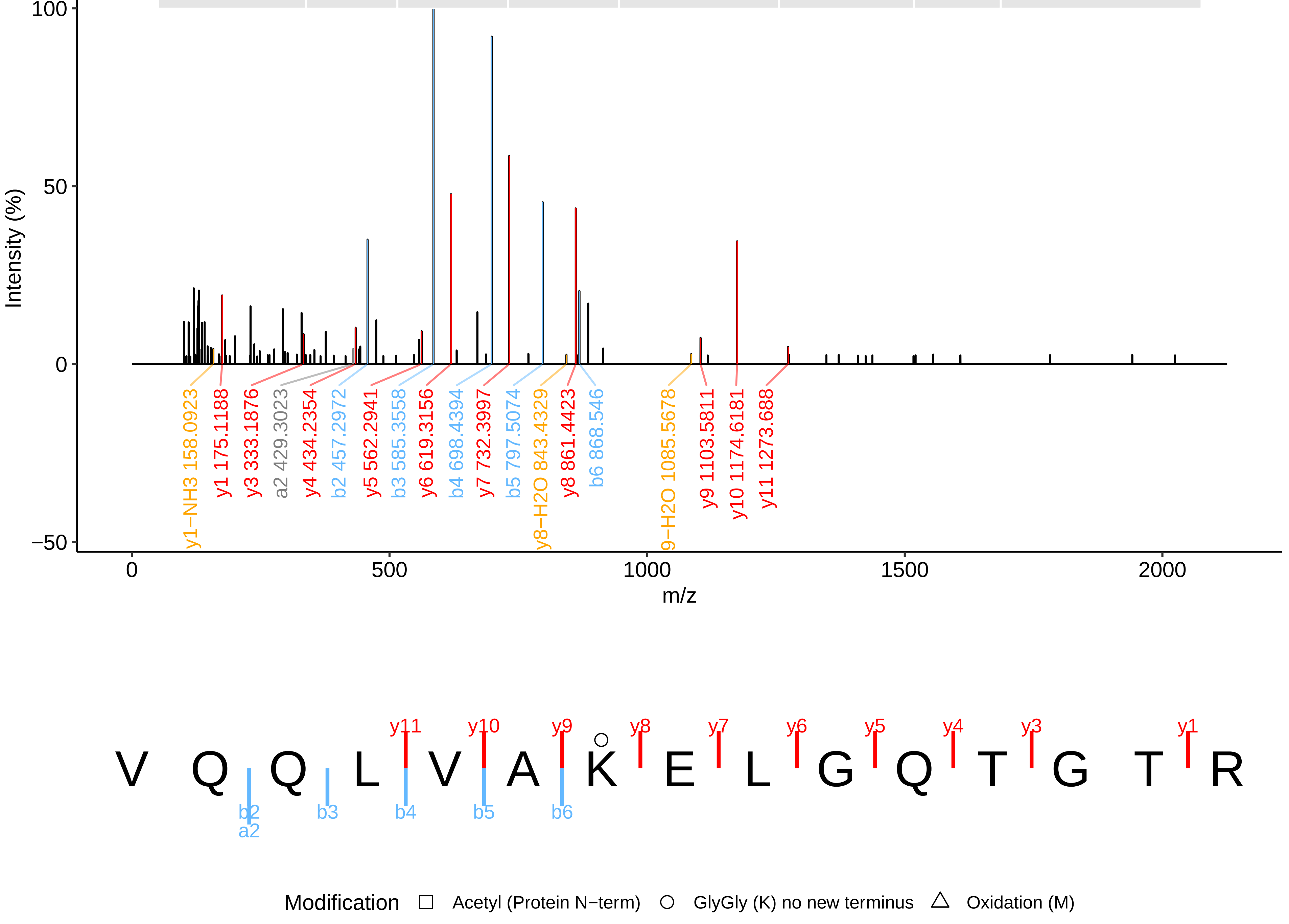

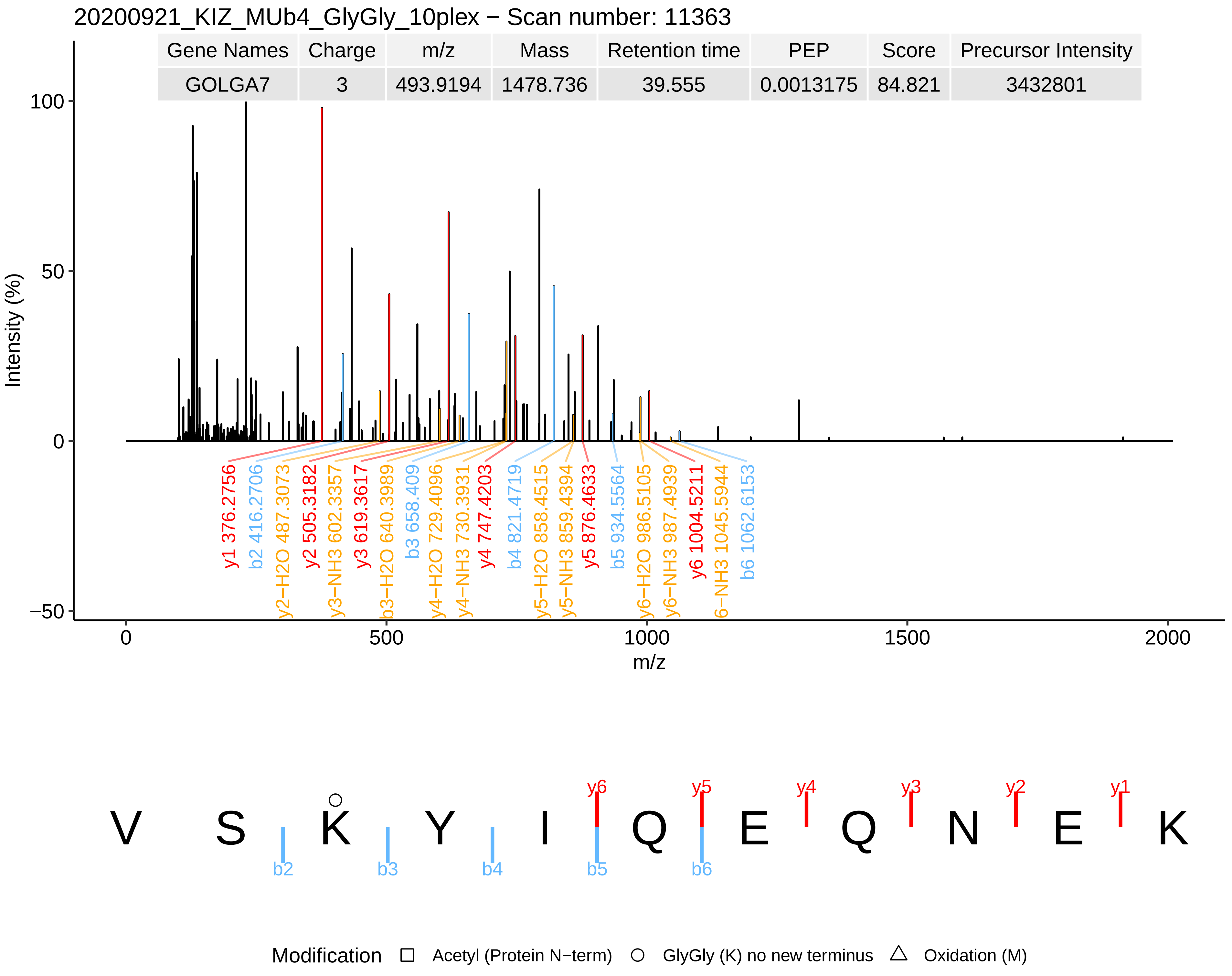
20200921_KIZ_MUb4_GlyGly_10plex − Scan number: 11363
Gene Names | Charge | m/z | Mass | Retention time | PEP | Score | Precursor Intensity
GOLGA7 | 3 | 493.9194 | 1478.736 | 39.555 | 0.0013175 | 84.821 | 3432801
Intensity (%)
100
50
0
−50
0
500
1000
1500
2000
m/z
y1 376.2756
b2 416.2706
y2−H2O 487.3073
y2 505.3182
y3−NH3 602.3357
y3 619.3617
b3−H2O 640.3989
b3 658.409
y4−H2O 729.4096
y4−NH3 730.3931
y4 747.4203
b4 821.4719
y5−H2O 858.4515
y5−NH3 859.4394
y5 876.4633
b5 934.5564
y6−H2O 986.5105
y6−NH3 987.4939
y6 1004.5211
6−NH3 1045.5944
b6 1062.6153
V S K Y I Q E Q N E K
y6 y5 y4 y3 y2 y1
b2 b3 b4 b5 b6
Modification
Acetyl (Protein N−term)
GlyGly (K) no new terminus
Oxidation (M)

20200921_KIZ_MUb4_GlyGly_10plex_2 − Scan number: 10477

Gene Names	Charge	m/z	Mass	Retention time	PEP	Score	Precursor Intensity
GOLGA7	3	493.9194	1478.736	40.086	0.0016261	81.625	1272123

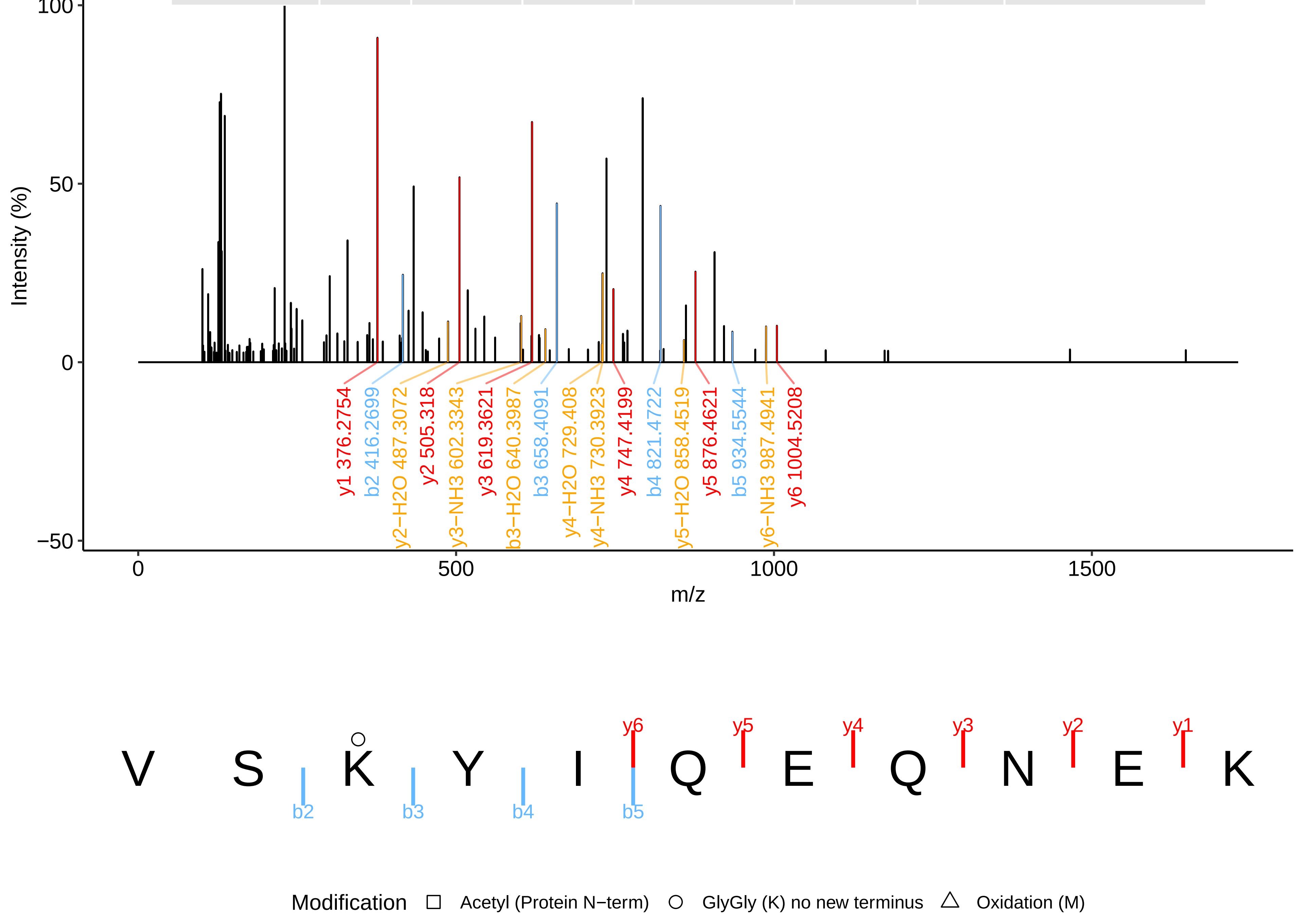

20200921_KIZ_MUb4_GlyGly_10plex_2 − Scan number: 10482

Gene Names	Charge	m/z	Mass	Retention time	PEP	Score	Precursor Intensity
GOLGA7	3	493.9194	1478.736	40.107	0.00069052	91.313	2230592

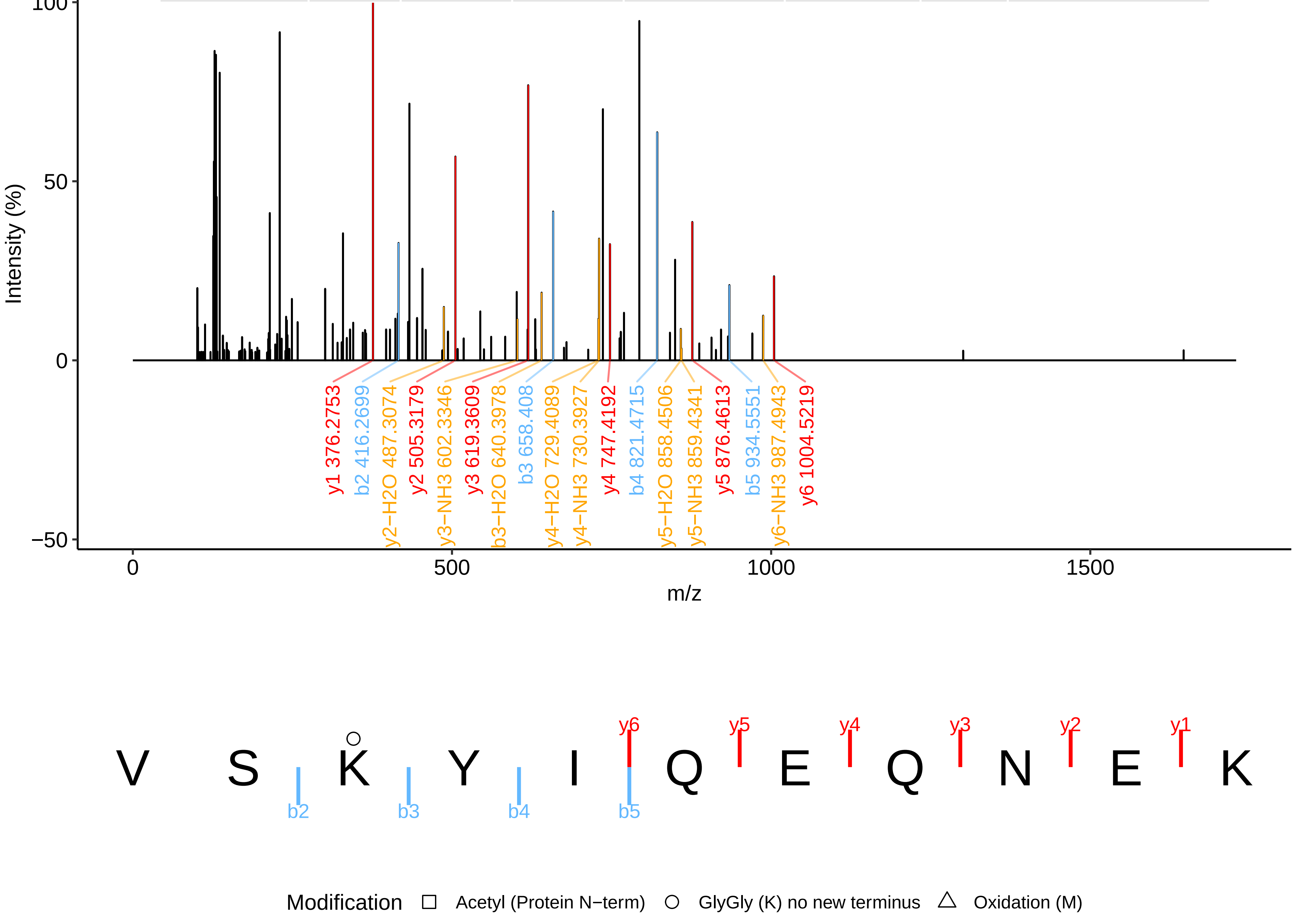

20200924_KIZ_MUb5_GlyGly_1 − Scan number: 10137

Gene Names	Charge	m/z	Mass	Retention time	PEP	Score	Precursor Intensity
GOLGA7	3	493.9194	1478.736	32.677	0.015981	68.515	8756697

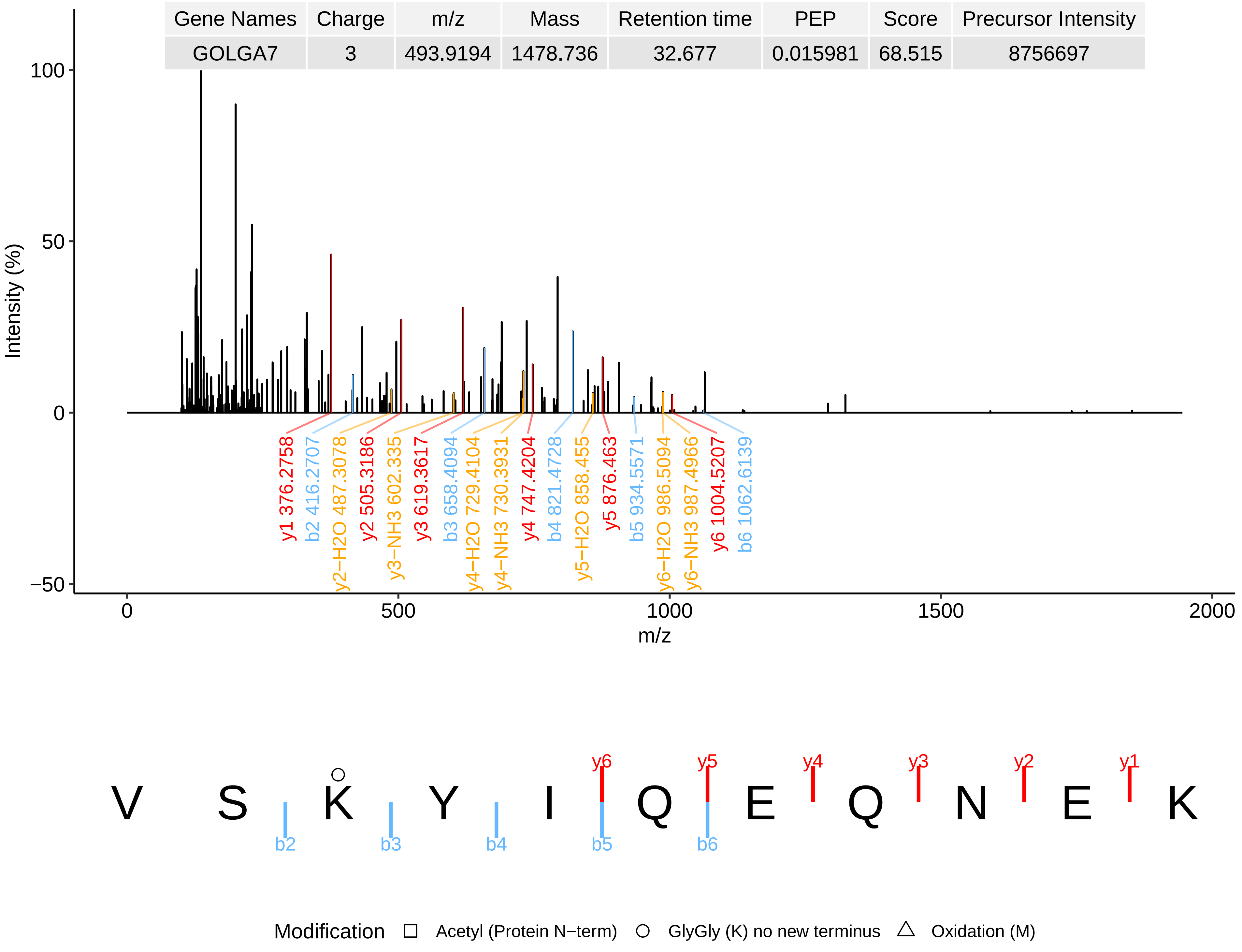

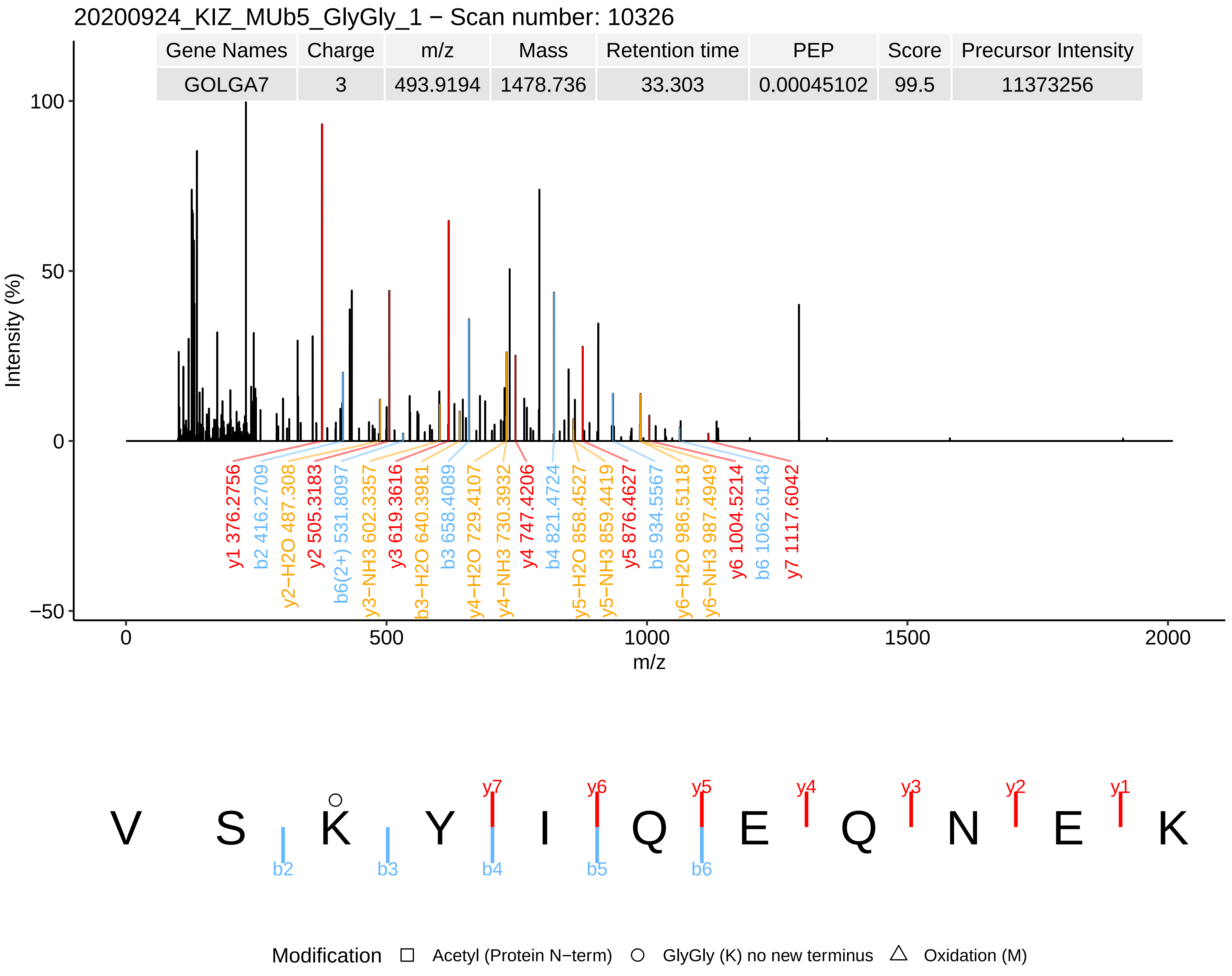

20200924_KIZ_MUb5_GlyGly_1 − Scan number: 10326
Gene Names | Charge | m/z | Mass | Retention time | PEP | Score | Precursor Intensity
GOLGA7 | 3 | 493.9194 | 1478.736 | 33.303 | 0.00045102 | 99.5 | 11373256
Intensity (%)
m/z
y1 376.2756
b2 416.2709
y2−H2O 487.308
y2 505.3183
b6(2+) 531.8097
y3−NH3 602.3357
y3 619.3616
b3−H2O 640.3981
b3 658.4089
y4−H2O 729.4107
y4−NH3 730.3932
y4 747.4206
b4 821.4724
y5−H2O 858.4527
y5−NH3 859.4419
y5 876.4627
b5 934.5567
y6−H2O 986.5118
y6−NH3 987.4949
y6 1004.5214
b6 1062.6148
y7 1117.6042
V S K Y I Q E Q N E K
y7 y6 y5 y4 y3 y2 y1
b2 b3 b4 b5 b6
Modification
Acetyl (Protein N−term)
GlyGly (K) no new terminus
Oxidation (M)

20201118_KIZ_MUb6_GlyGly_2 − Scan number: 12185

Gene Names	Charge	m/z	Mass	Retention time	PEP	Score	Precursor Intensity
GOLGA7	3	493.9194	1478.736	40.999	0.00023086	110.08	5485716

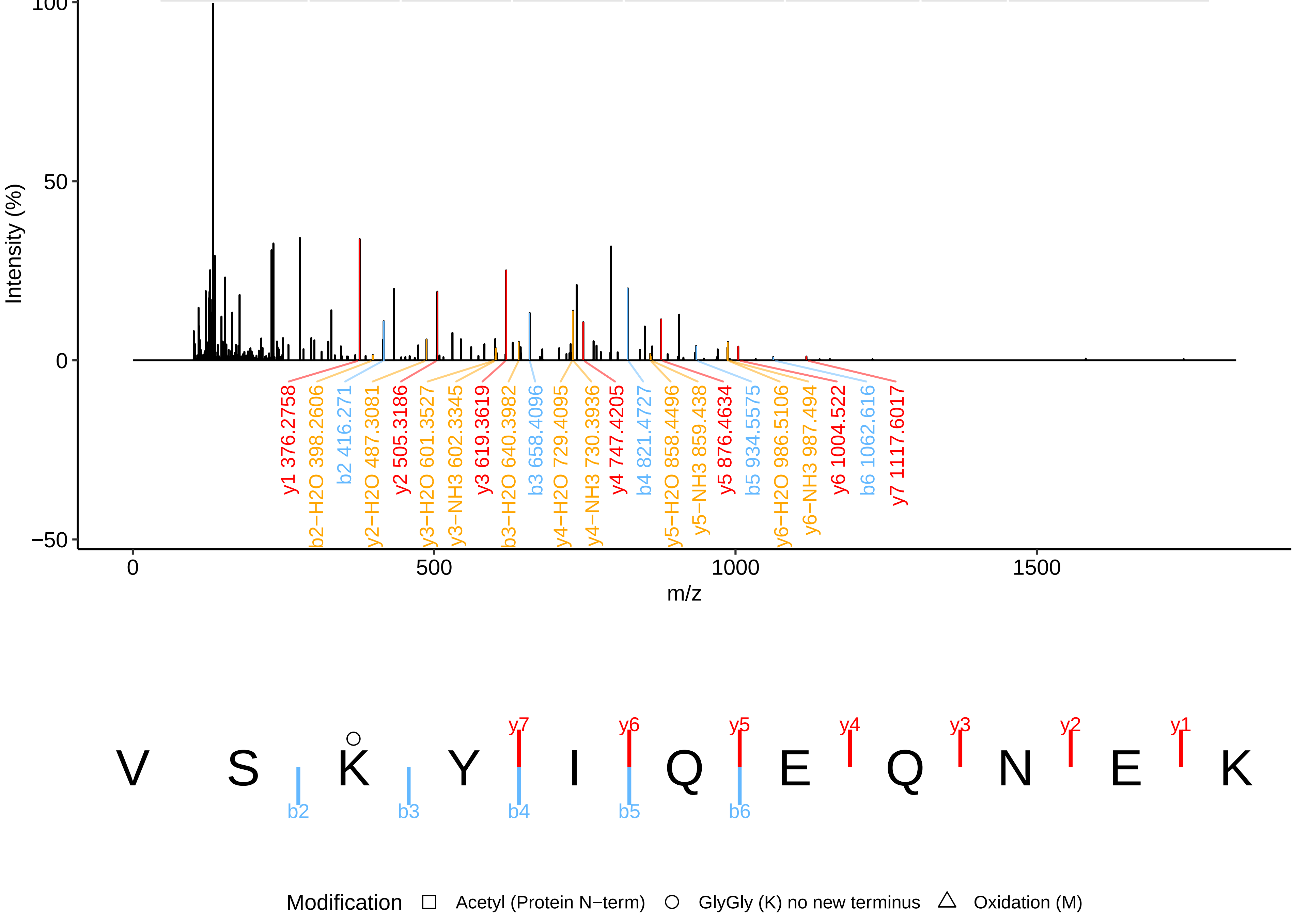

20200921_KIZ_MUb4_GlyGly_10plex − Scan number: 14995

Gene Names	Charge	m/z	Mass	Retention time	PEP	Score	Precursor Intensity
ITM2B	3	507.6071	1519.8	53.446	9.5926e−06	107.99	4869120

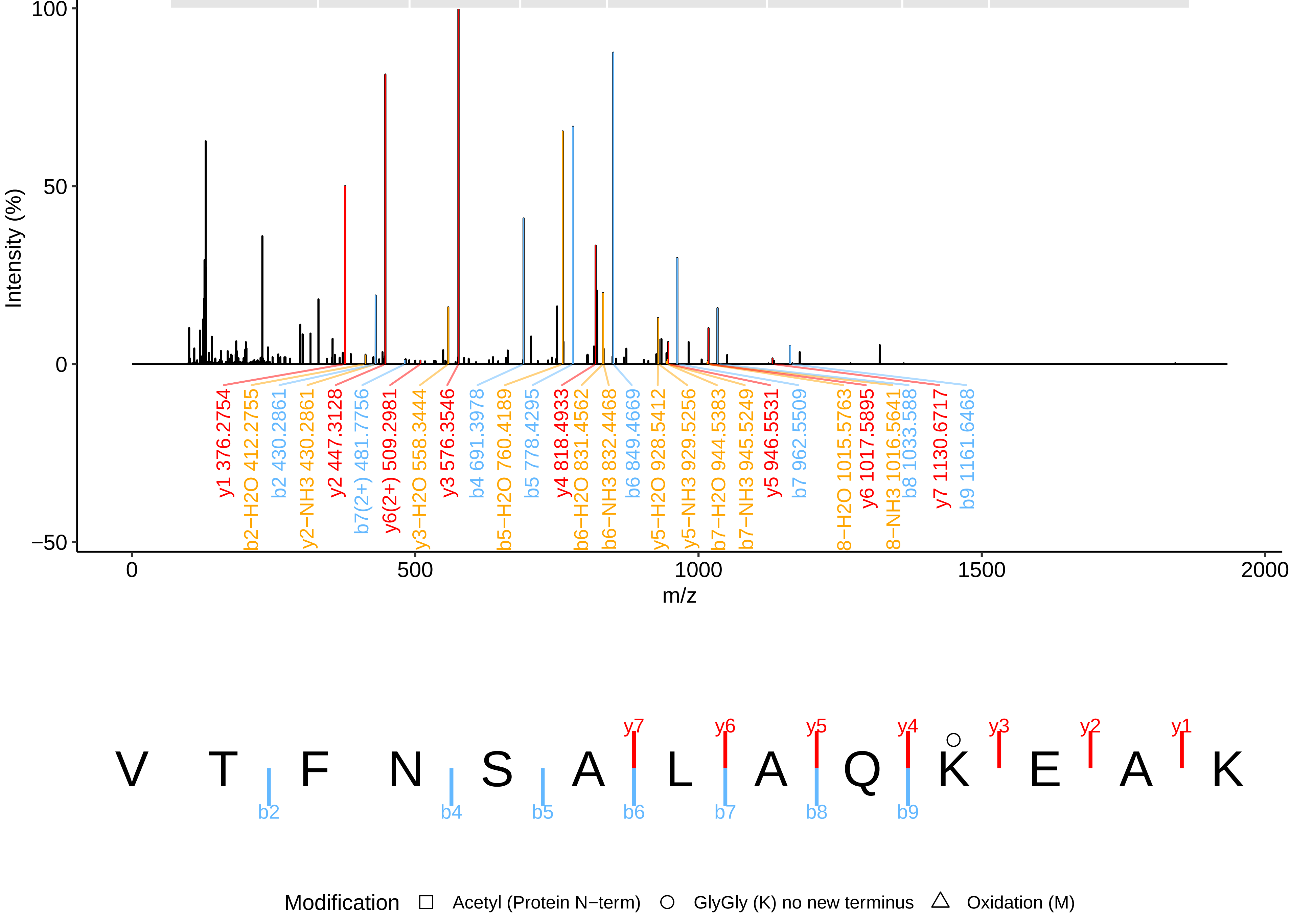

20200921_KIZ_MUb4_GlyGly_10plex_2 − Scan number: 13873

Gene Names	Charge	m/z	Mass	Retention time	PEP	Score	Precursor Intensity
ITM2B	3	507.6071	1519.8	53.963	9.3656e−06	108.17	1647934

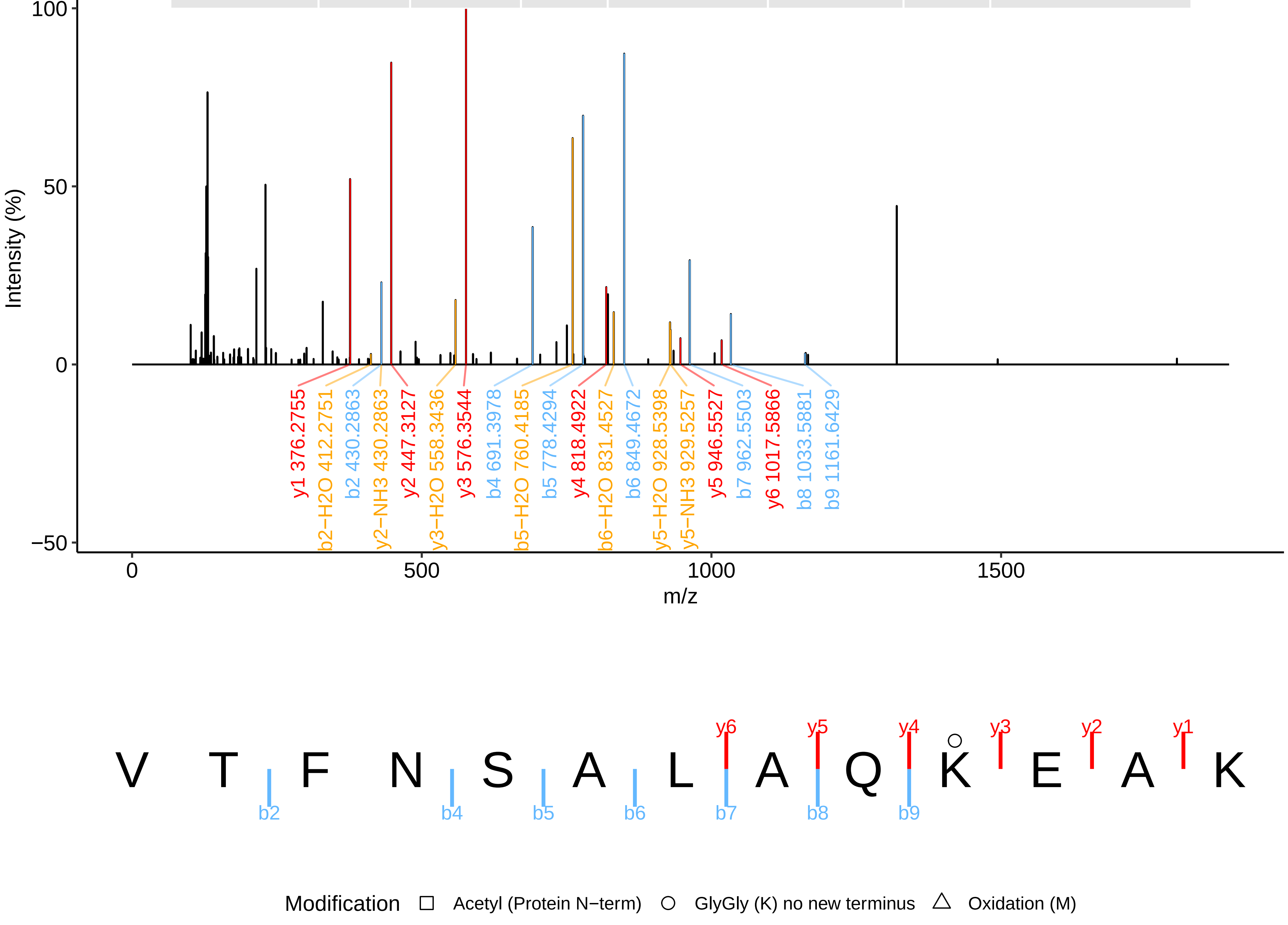

20200924_KIZ_MUb5_GlyGly_1 − Scan number: 13939

Gene Names	Charge	m/z	Mass	Retention time	PEP	Score	Precursor Intensity
ITM2B	3	507.6071	1519.8	45.493	1.3422e−05	105.03	11932559

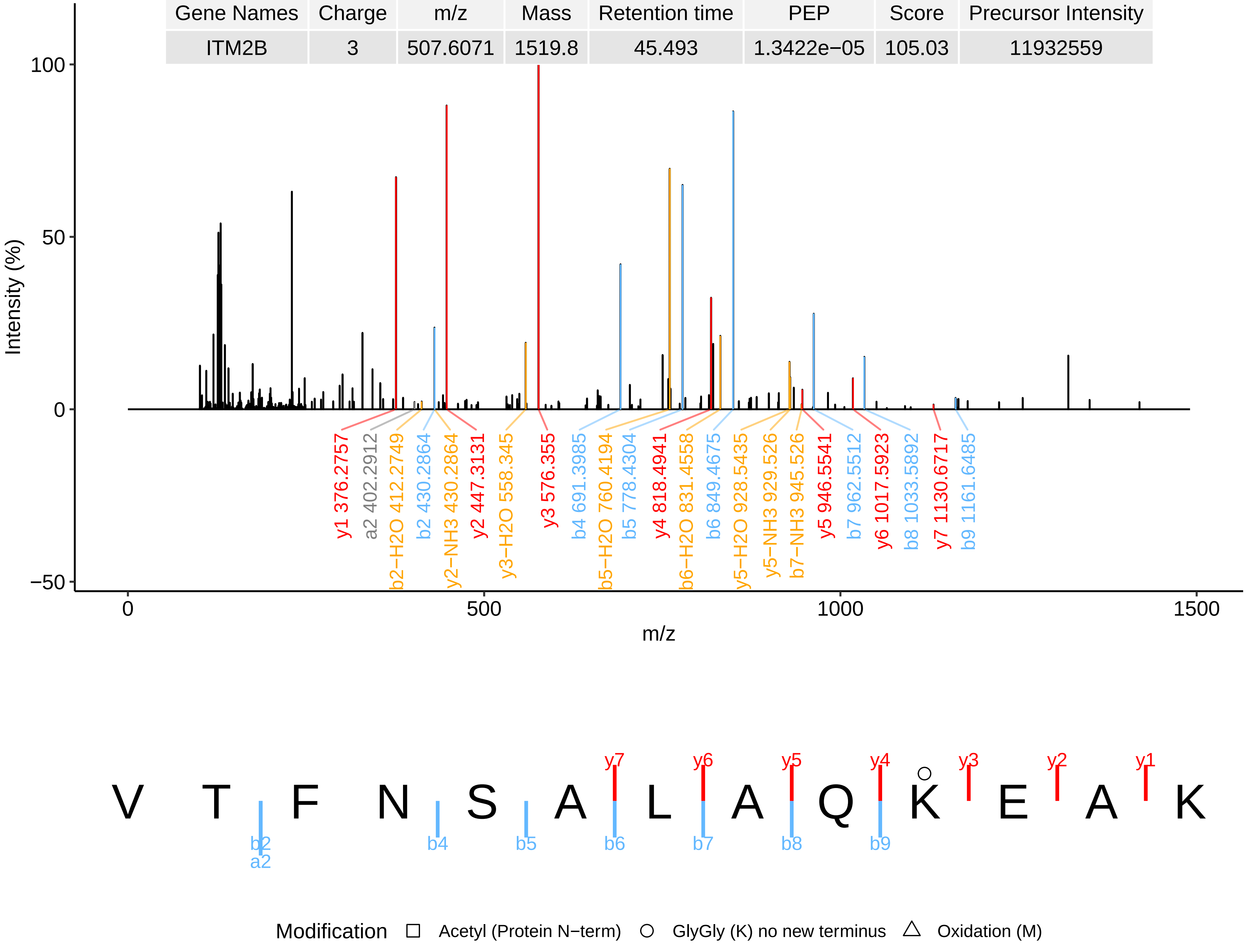

20200924_KIZ_MUb5_GlyGly_1 − Scan number: 14092

Gene Names	Charge	m/z	Mass	Retention time	PEP	Score	Precursor Intensity
ITM2B	3	507.6071	1519.8	46.002	3.9199e−07	115.12	42821048

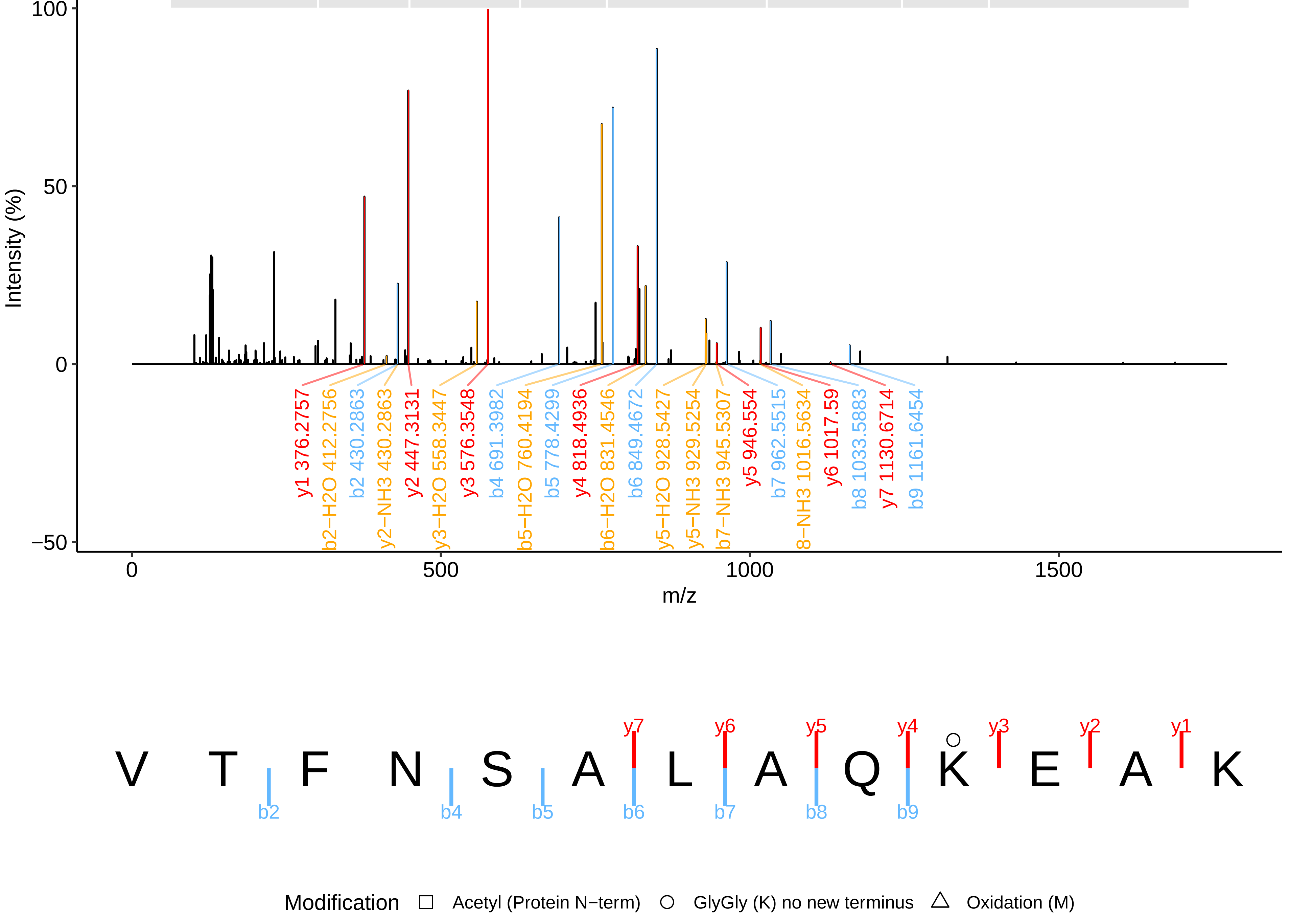

20200924_KIZ_MUb5_GlyGly_1 − Scan number: 14247

Gene Names	Charge	m/z	Mass	Retention time	PEP	Score	Precursor Intensity
ITM2B	3	507.6071	1519.8	46.506	0.00078078	86.405	26419084

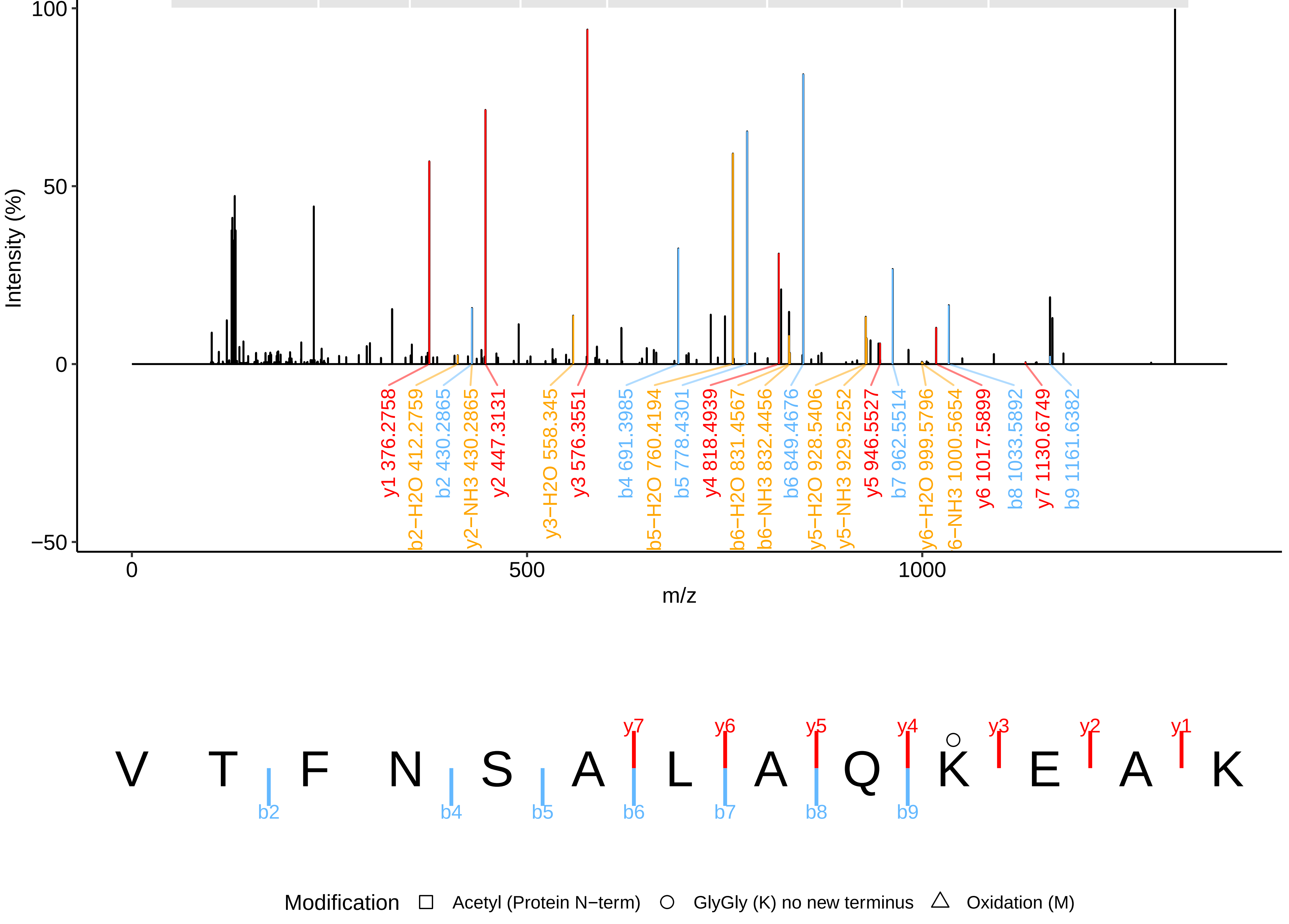

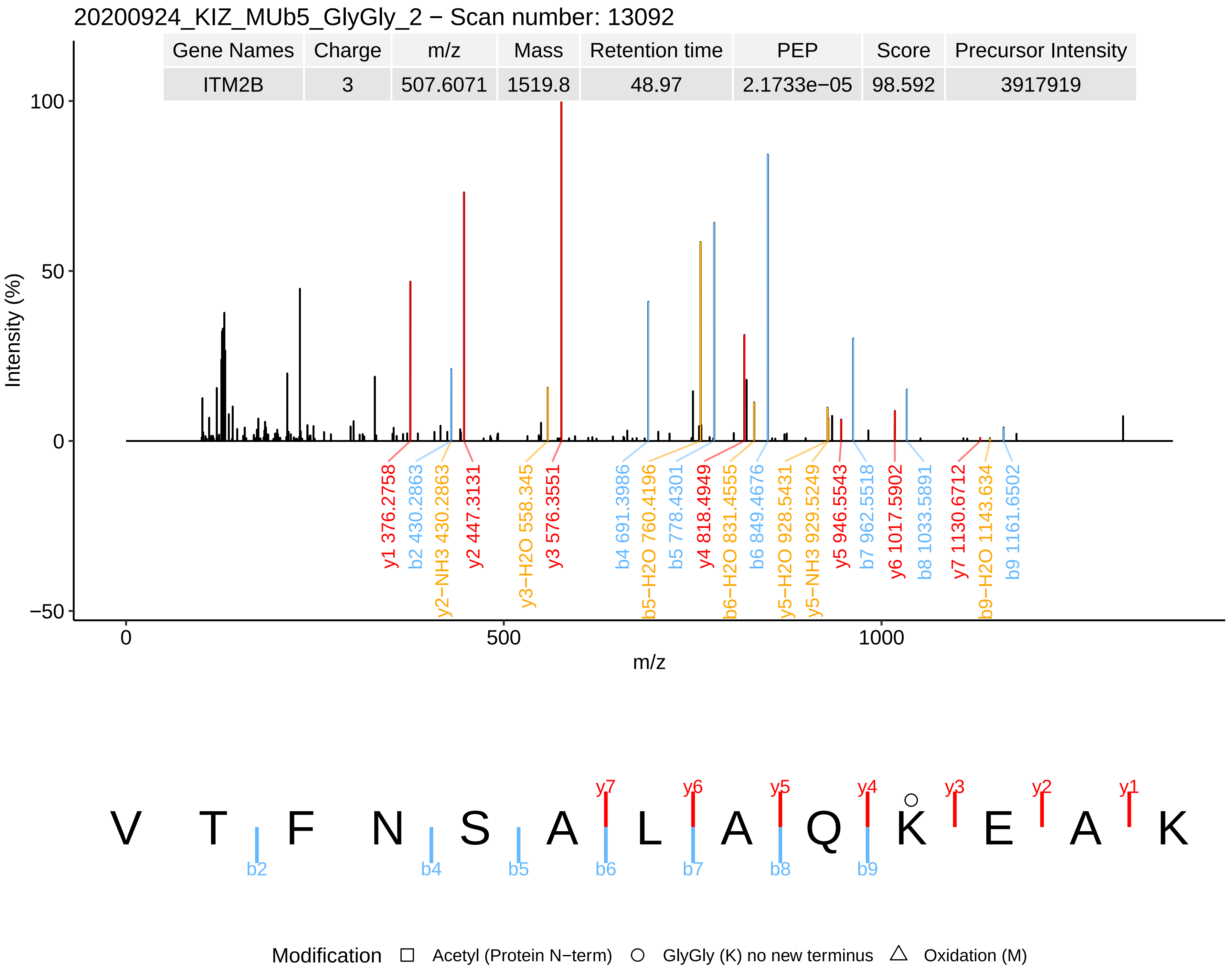
20200924_KIZ_MUb5_GlyGly_2 − Scan number: 13092
Gene Names | Charge | m/z | Mass | Retention time | PEP | Score | Precursor Intensity
ITM2B | 3 | 507.6071 | 1519.8 | 48.97 | 2.1733e−05 | 98.592 | 3917919
Intensity (%)
m/z
y1 376.2758
b2 430.2863
y2−NH3 430.2863
y2 447.3131
y3−H2O 558.345
y3 576.3551
b4 691.3986
b5−H2O 760.4196
b5 778.4301
y4 818.4949
b6−H2O 831.4555
b6 849.4676
y5−H2O 928.5431
y5−NH3 929.5249
y5 946.5543
b7 962.5518
y6 1017.5902
b8 1033.5891
y7 1130.6712
b9−H2O 1143.634
b9 1161.6502
V T F N S A L A Q K E A K
y7 y6 y5 y4 y3 y2 y1
b2 b4 b5 b6 b7 b8 b9
Modification
Acetyl (Protein N−term)
GlyGly (K) no new terminus
Oxidation (M)

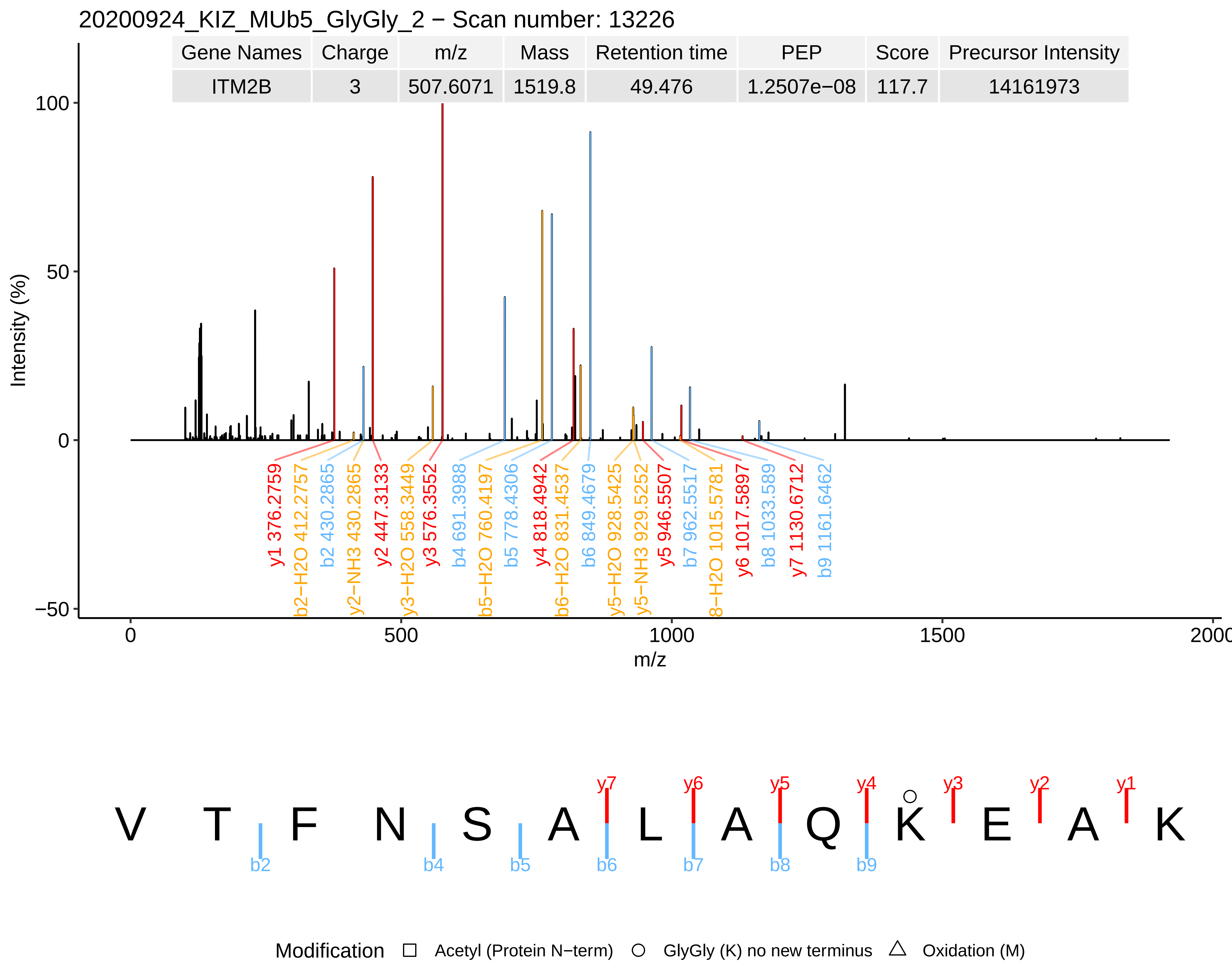
20200924_KIZ_MUb5_GlyGly_2 − Scan number: 13226
Gene Names
Charge
m/z
Mass
Retention time
PEP
Score
Precursor Intensity
ITM2B
3
507.6071
1519.8
49.476
1.2507e−08
117.7
14161973
Intensity (%)
m/z
y1 376.2759
b2−H2O 412.2757
b2 430.2865
y2−NH3 430.2865
y2 447.3133
y3−H2O 558.3449
y3 576.3552
b4 691.3988
b5−H2O 760.4197
b5 778.4306
y4 818.4942
b6−H2O 831.4537
b6 849.4679
y5−H2O 928.5425
y5−NH3 929.5252
y5 946.5507
b7 962.5517
8−H2O 1015.5781
y6 1017.5897
b8 1033.589
y7 1130.6712
b9 1161.6462
V T F N S A L A Q K E A K
Modification
Acetyl (Protein N−term)
GlyGly (K) no new terminus
Oxidation (M)

20200924_KIZ_MUb5_GlyGly_2 − Scan number: 13360

Gene Names	Charge	m/z	Mass	Retention time	PEP	Score	Precursor Intensity
ITM2B	3	507.6071	1519.8	49.983	0.00017733	95.631	9963410

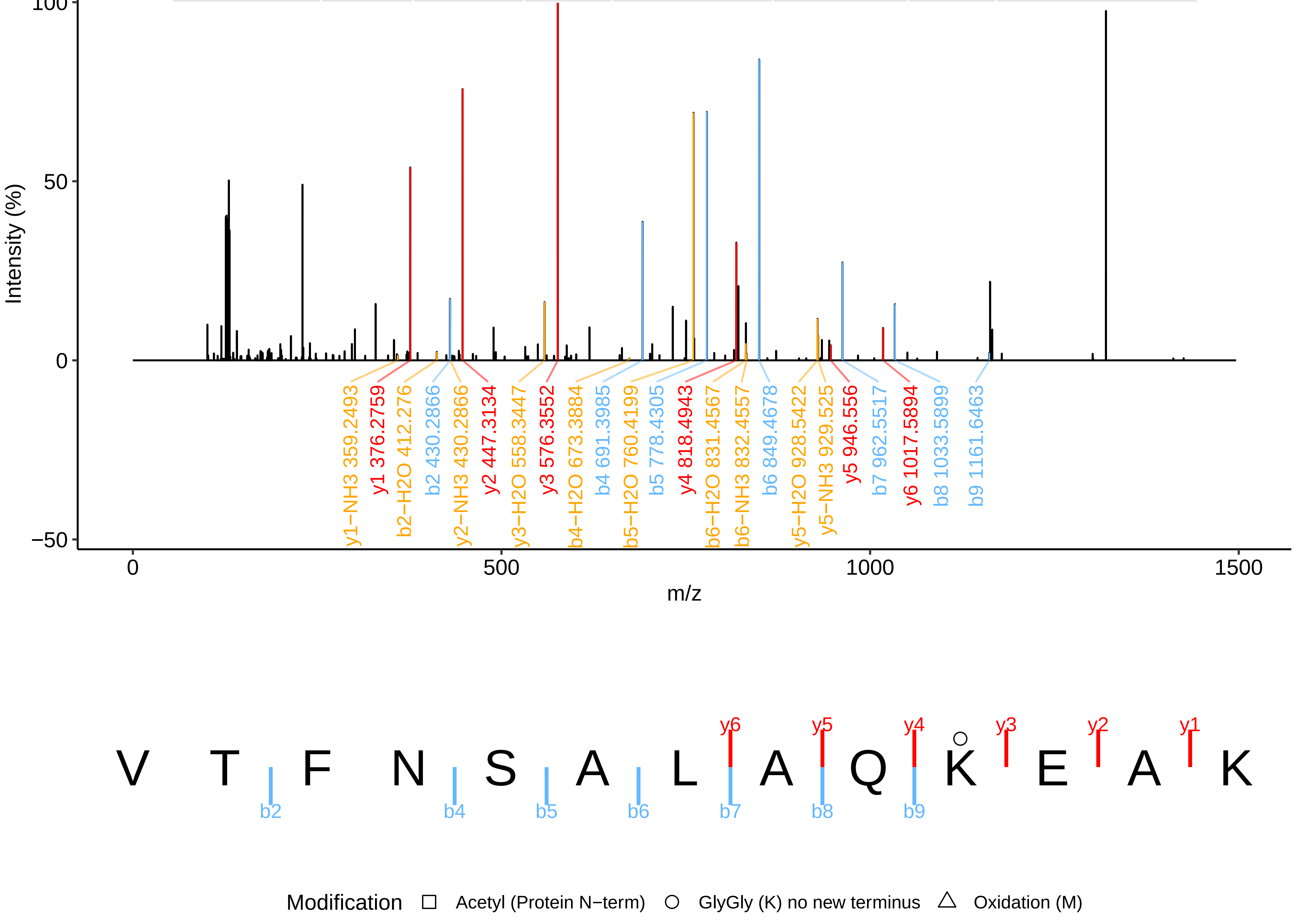

20201118_KIZ_MUb6_GlyGly_1 − Scan number: 14306

Gene Names	Charge	m/z	Mass	Retention time	PEP	Score	Precursor Intensity
ITM2B	3	507.6071	1519.8	45.251	1.1102e−06	114.56	20828474

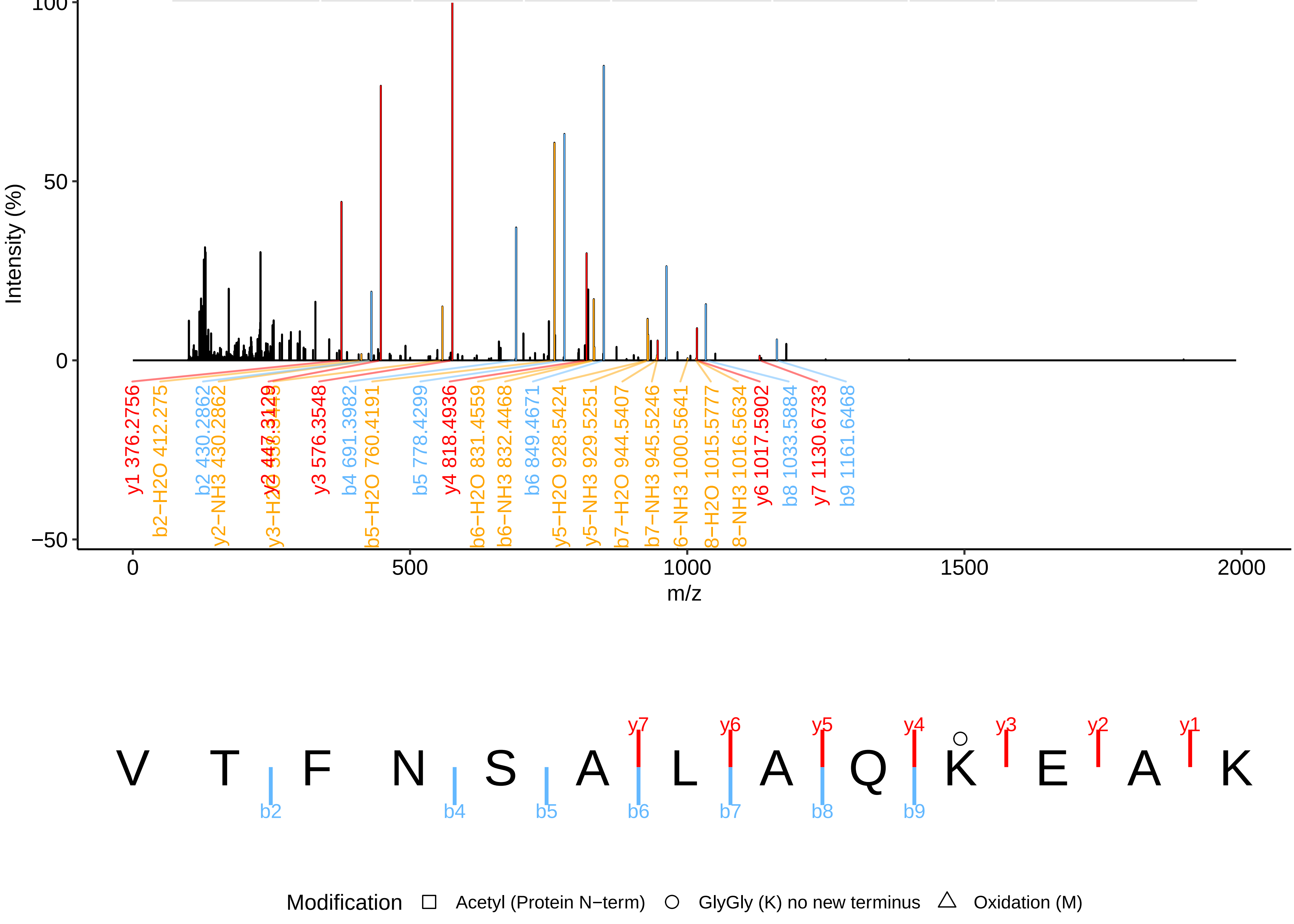

20201118_KIZ_MUb6_GlyGly_2 − Scan number: 16304

Gene Names	Charge	m/z	Mass	Retention time	PEP	Score	Precursor Intensity
ITM2B	3	507.6071	1519.8	55.074	3.9199e−07	115.12	16598062

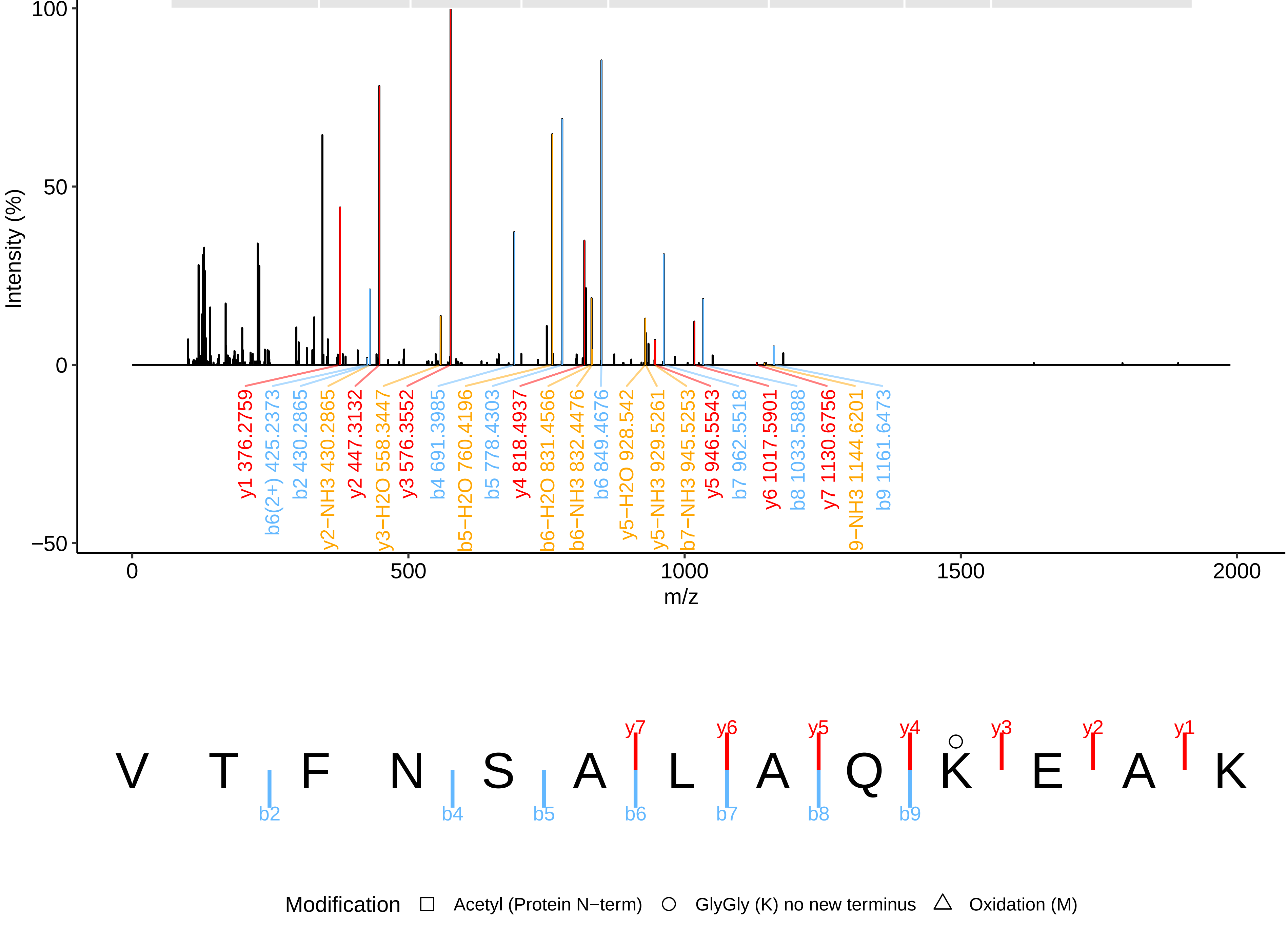

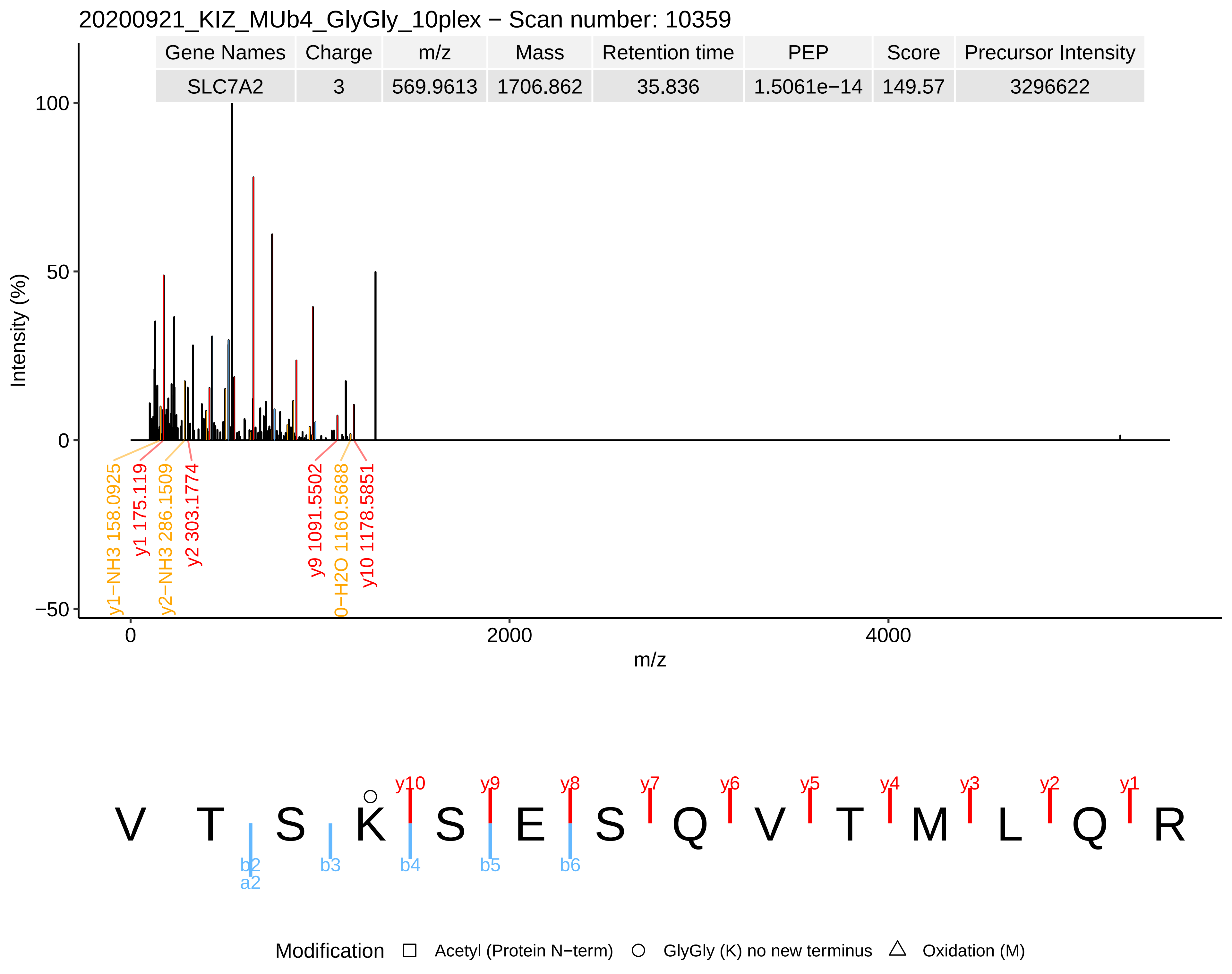
20200921_KIZ_MUb4_GlyGly_10plex − Scan number: 10359
Gene Names | Charge | m/z | Mass | Retention time | PEP | Score | Precursor Intensity
SLC7A2 | 3 | 569.9613 | 1706.862 | 35.836 | 1.5061e−14 | 149.57 | 3296622
Intensity (%)
100
50
0
−50
0
2000
4000
m/z
y1−NH3 158.0925
y1 175.119
y2−NH3 286.1509
y2 303.1774
y9 1091.5502
0−H2O 1160.5688
y10 1178.5851
V T S K S E S Q V T M L Q R
y10 y9 y8 y7 y6 y5 y4 y3 y2 y1
b2 a2 b3 b4 b5 b6
Modification
Acetyl (Protein N−term)
GlyGly (K) no new terminus
Oxidation (M)

20200921_KIZ_MUb4_GlyGly_10plex_2 − Scan number: 9538

Gene Names	Charge	m/z	Mass	Retention time	PEP	Score	Precursor Intensity
SLC7A2	3	569.9613	1706.862	36.289	0.00048717	86.944	814037.6

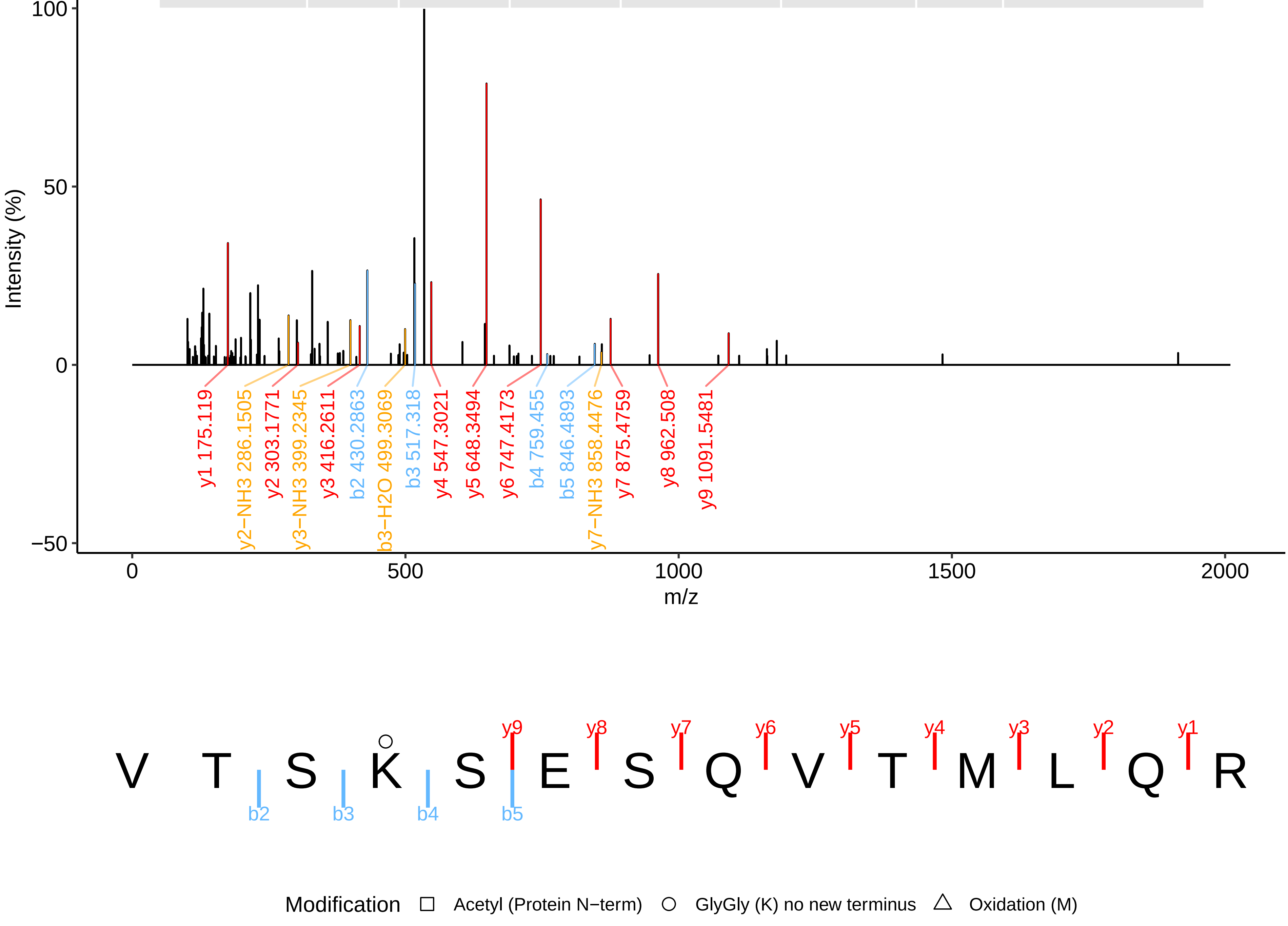

20200921_KIZ_MUb4_GlyGly_10plex_2 − Scan number: 9542

Gene Names	Charge	m/z	Mass	Retention time	PEP	Score	Precursor Intensity
SLC7A2	3	569.9613	1706.862	36.304	0.0147	61.212	486880.6

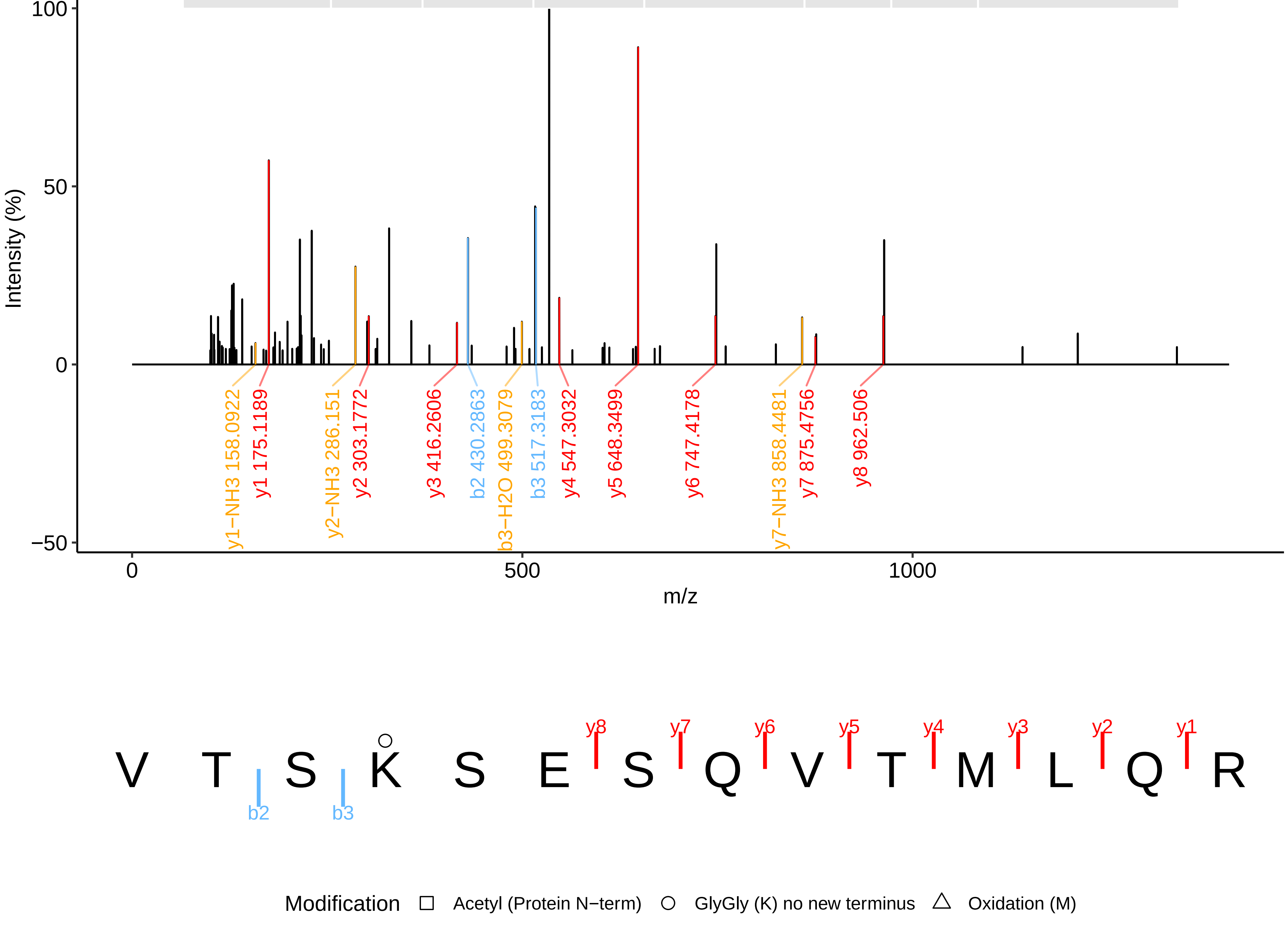

20200921_KIZ_MUb4_GlyGly_10plex − Scan number: 6362

Gene Names	Charge	m/z	Mass	Retention time	PEP	Score	Precursor Intensity
MIB1	4	424.9592	1695.808	21.02	0.0016143	72.898	4042587

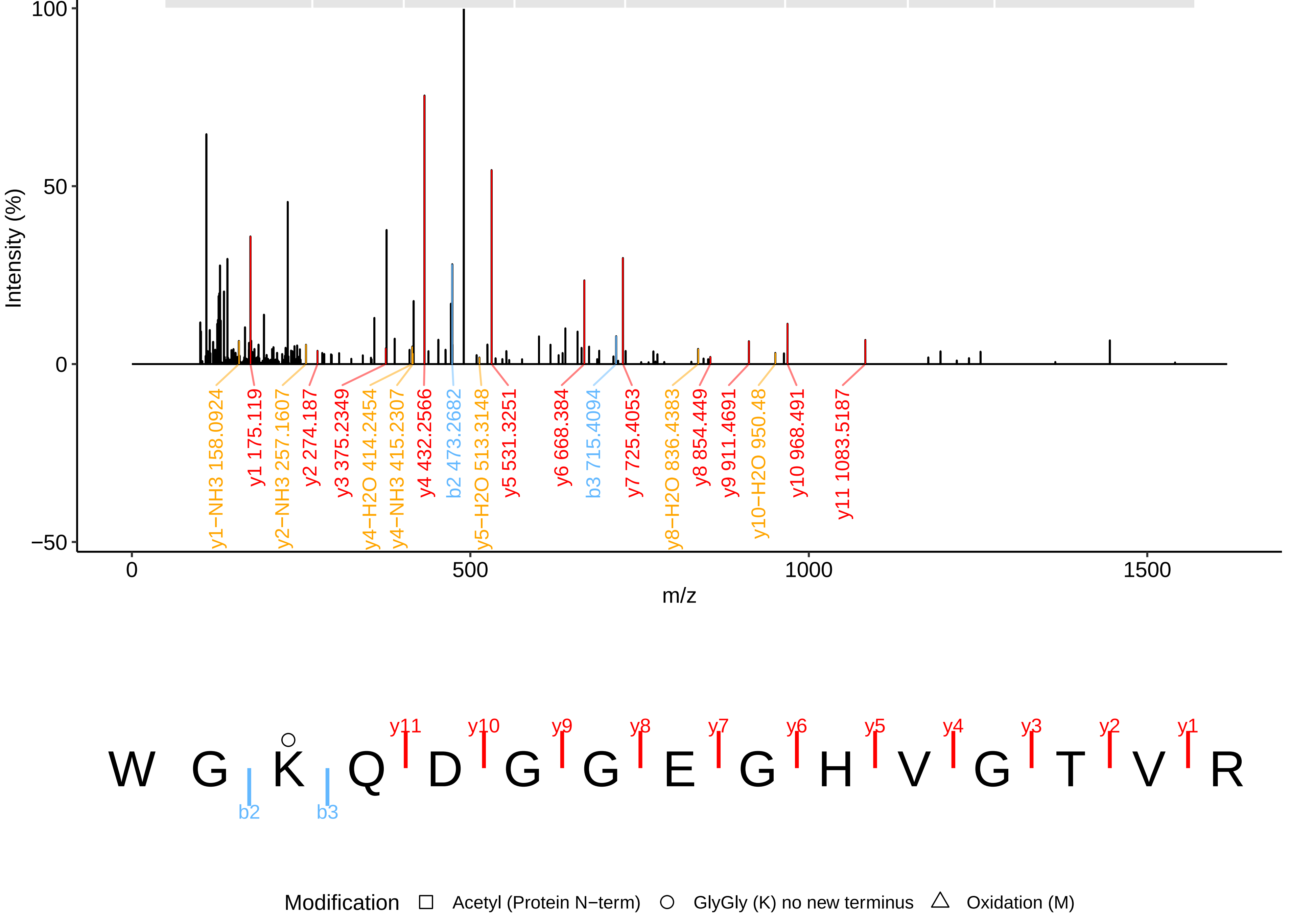

20200921_KIZ_MUb4_GlyGly_10plex − Scan number: 12746

Gene Names	Charge	m/z	Mass	Retention time	PEP	Score	Precursor I
HSP90AA1;HSP90AA2P;HSP90AB1;HSP90AB2P	3	498.9193	1493.736	44.797	1.8258e−05	108.35	55557

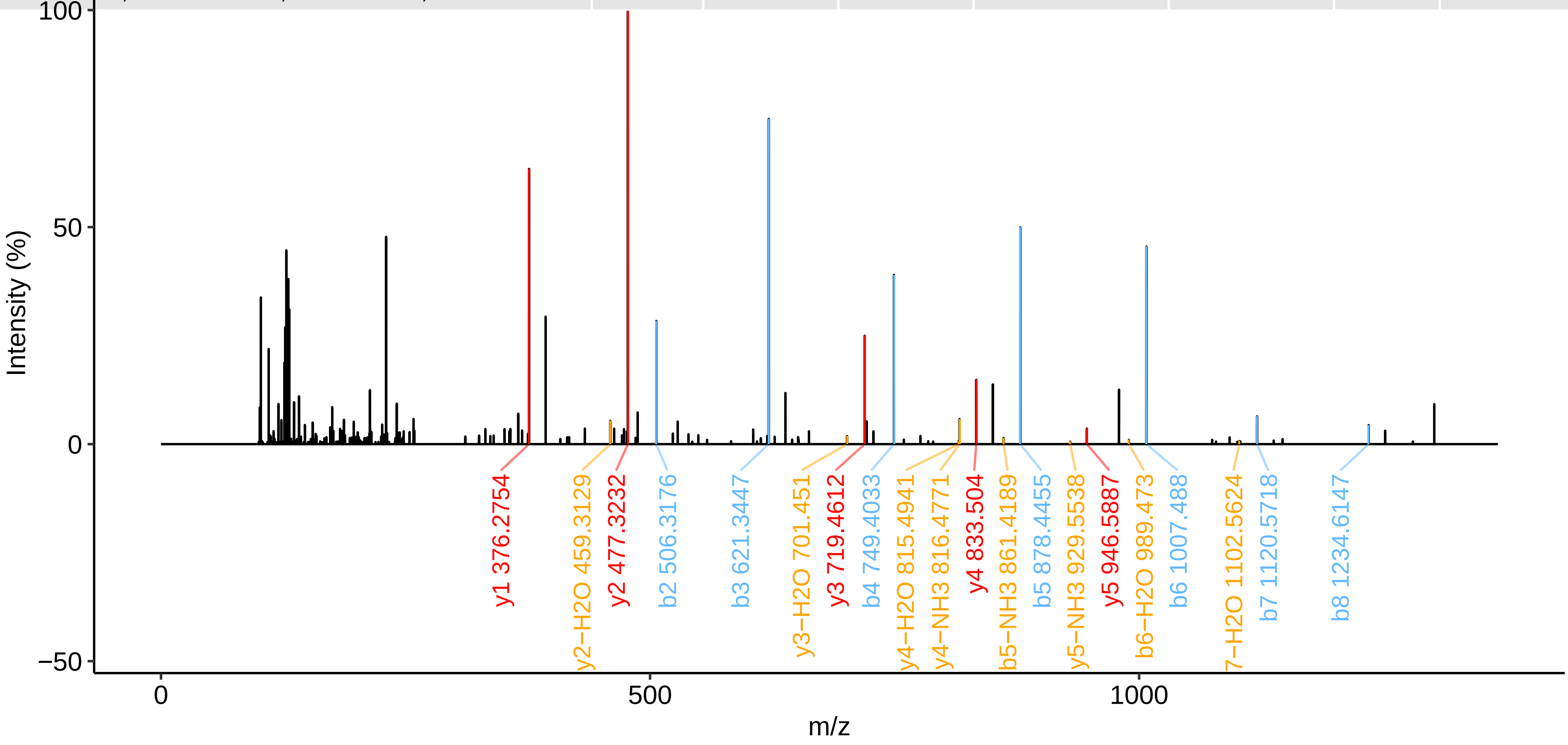

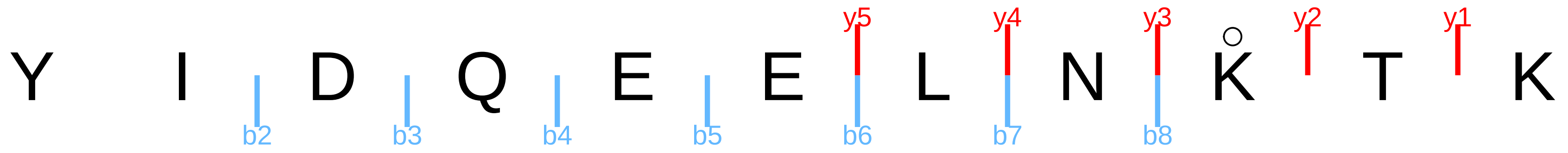

Modification □ Acetyl (Protein N−term) ○ GlyGly (K) no new terminus △ Oxidation (M)

20200921_KIZ_MUb4_GlyGly_10plex_2 − Scan number: 11615

Gene Names	Charge	m/z	Mass	Retention time	PEP	Score	Precursor In
HSP90AA1;HSP90AA2P;HSP90AB1;HSP90AB2P	3	498.9193	1493.736	44.729	0.067423	56.225	456127

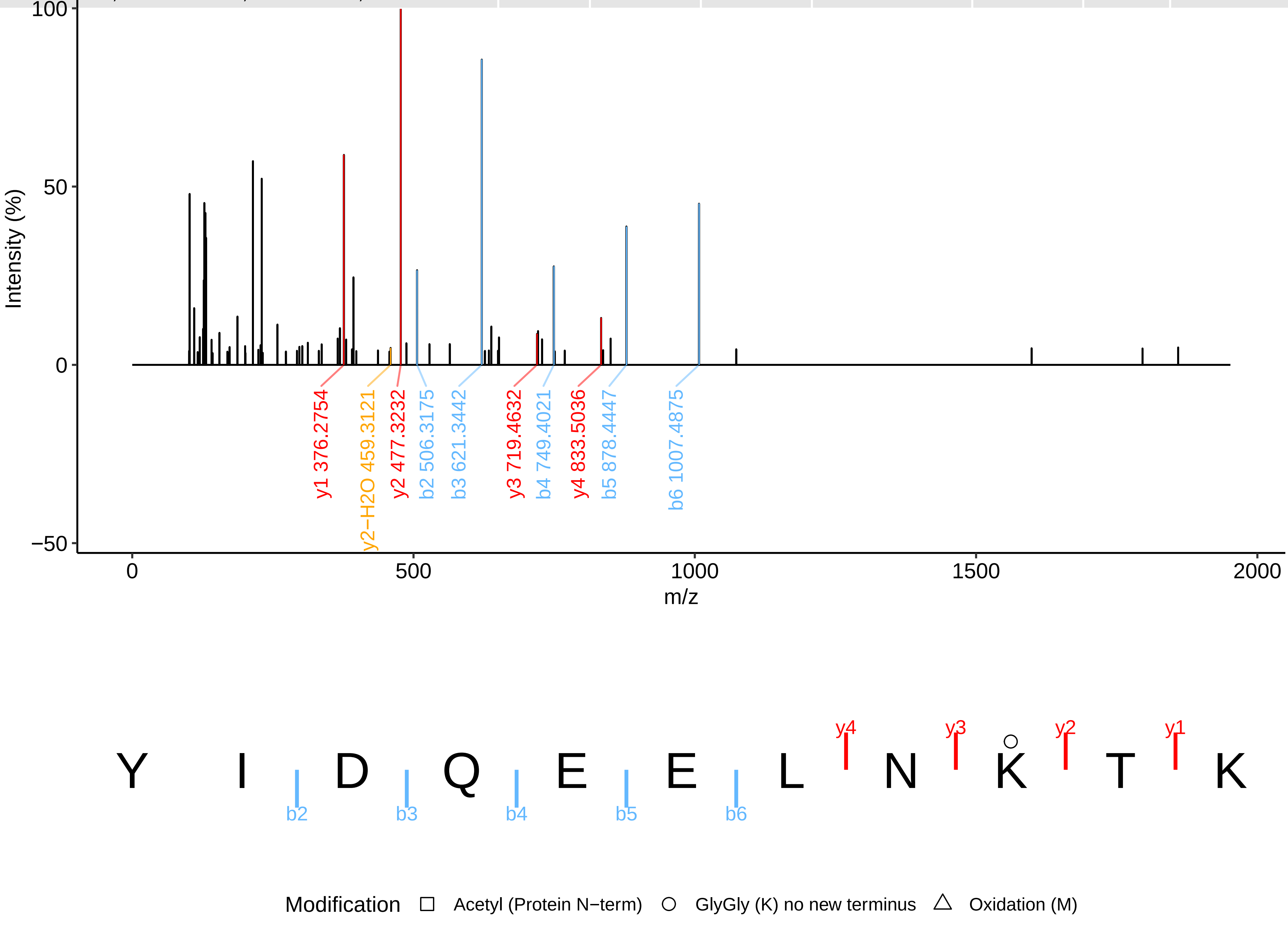

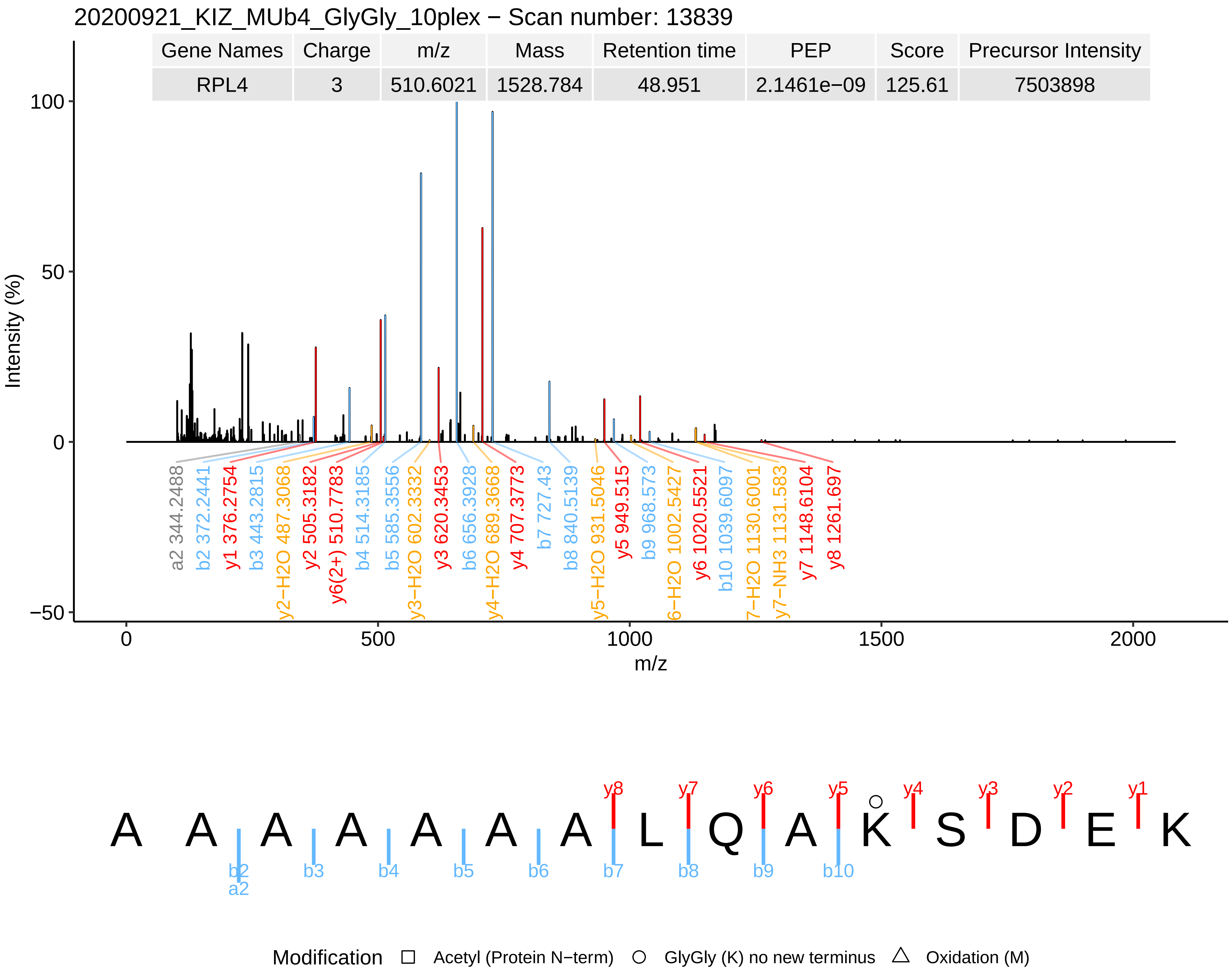
20200921_KIZ_MUb4_GlyGly_10plex − Scan number: 13839
Gene Names | Charge | m/z | Mass | Retention time | PEP | Score | Precursor Intensity
RPL4 | 3 | 510.6021 | 1528.784 | 48.951 | 2.1461e−09 | 125.61 | 7503898
Intensity (%)
100
50
0
−50
0
500
1000
1500
2000
m/z
a2 344.2488
b2 372.2441
y1 376.2754
b3 443.2815
y2−H2O 487.3068
y2 505.3182
y6(2+) 510.7783
b4 514.3185
b5 585.3556
y3−H2O 602.3332
y3 620.3453
b6 656.3928
y4−H2O 689.3668
y4 707.3773
b7 727.43
b8 840.5139
y5−H2O 931.5046
y5 949.515
b9 968.573
y6−H2O 1002.5427
y6 1020.5521
b10 1039.6097
y7−H2O 1130.6001
y7−NH3 1131.583
y7 1148.6104
y8 1261.697
A A A A A A A L Q A K S D E K
y8 y7 y6 y5 y4 y3 y2 y1
b2 a2 b3 b4 b5 b6 b7 b8 b9 b10
Modification
Acetyl (Protein N−term)
GlyGly (K) no new terminus
Oxidation (M)

20200921_KIZ_MUb4_GlyGly_10plex_2 − Scan number: 12747

Gene Names	Charge	m/z	Mass	Retention time	PEP	Score	Precursor Intensity
RPL4	3	510.6021	1528.784	49.382	4.3015e−09	118.37	852376.6

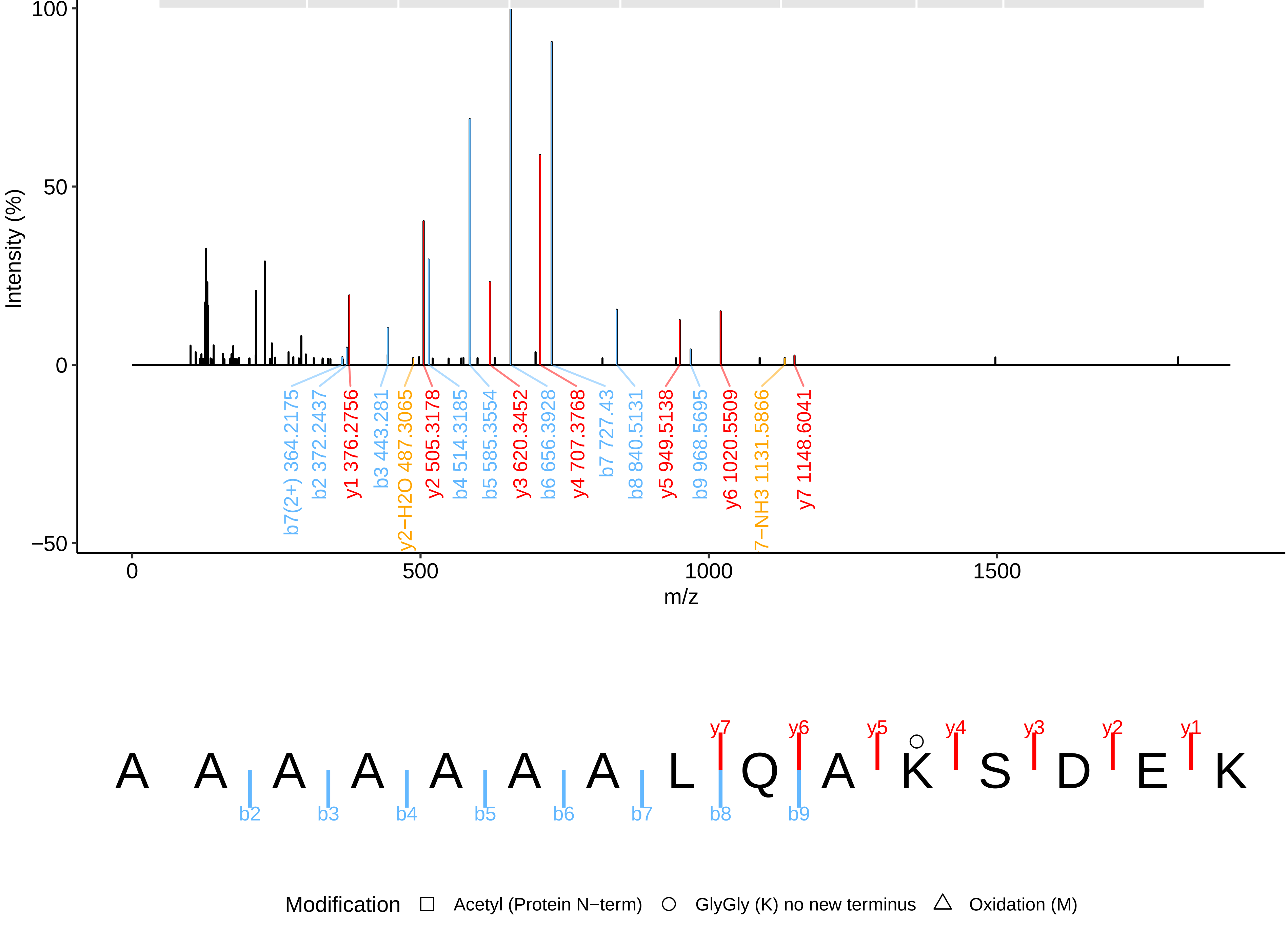

20200921_KIZ_MUb4_GlyGly_10plex_2 − Scan number: 18924

Gene Names	Charge	m/z	Mass	Retention time	PEP	Score	Precursor Intensity
ATP11A	3	514.9544	1541.841	74.201	0.0007167	81.494	442365.4

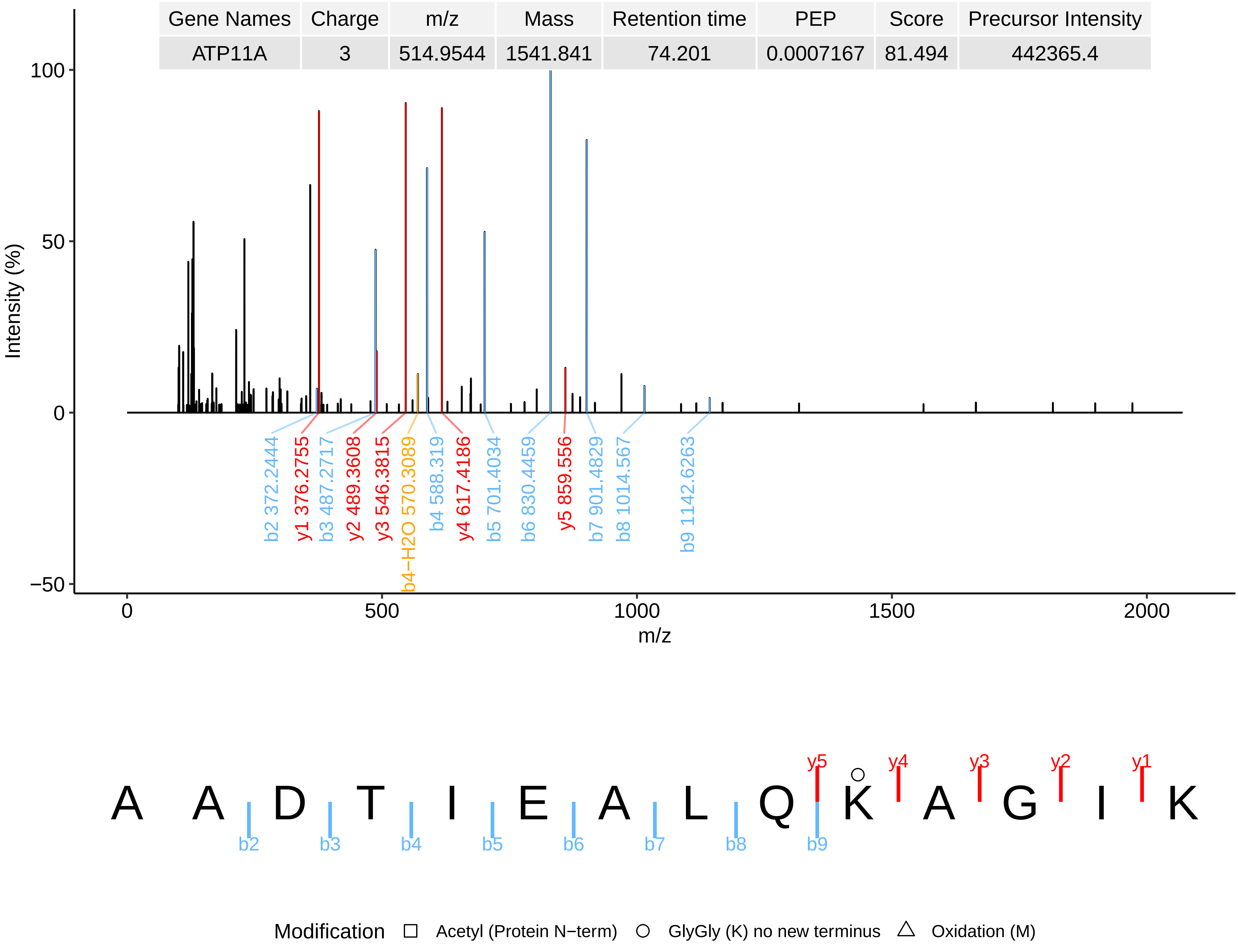

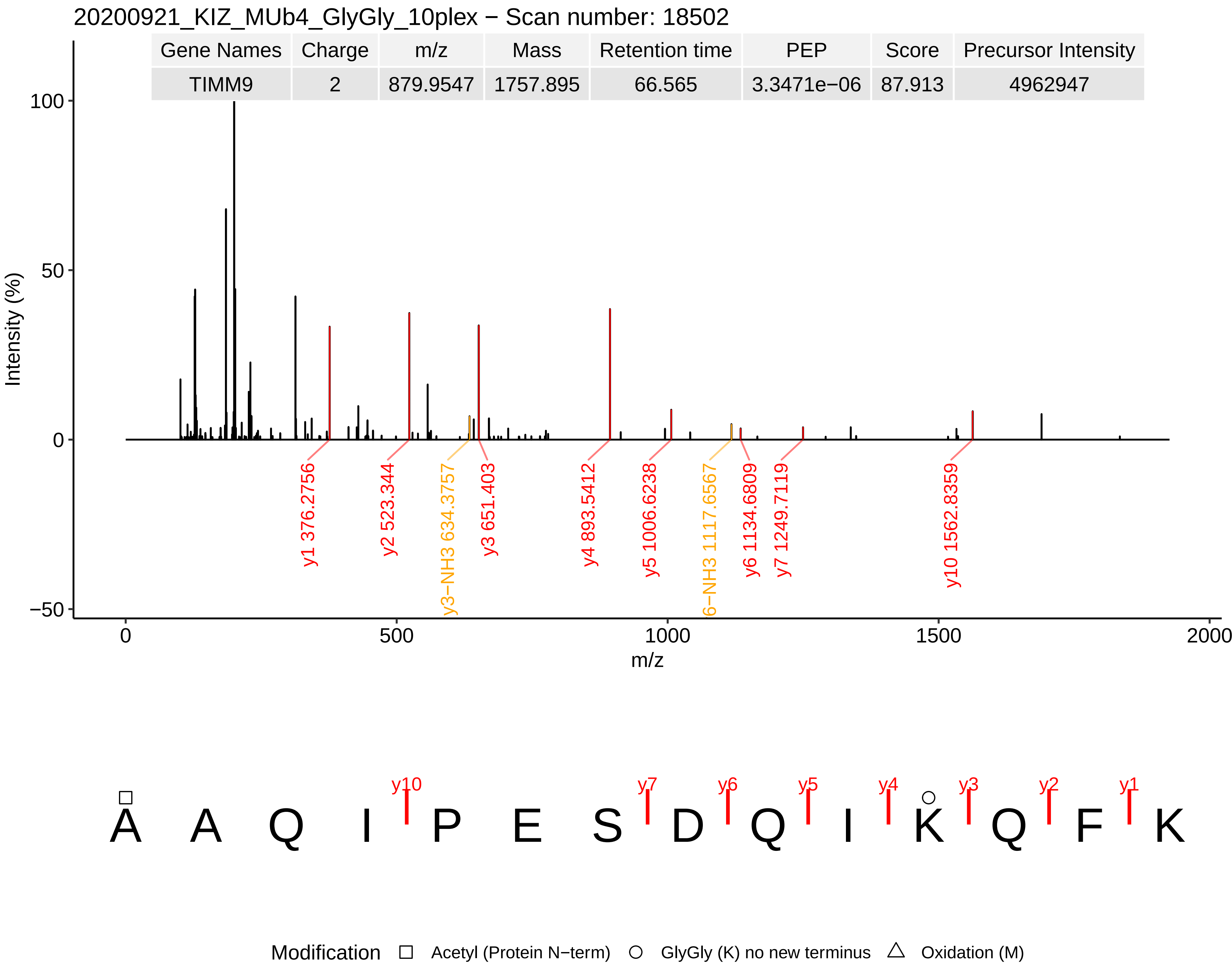
20200921_KIZ_MUb4_GlyGly_10plex − Scan number: 18502
Gene Names | Charge | m/z | Mass | Retention time | PEP | Score | Precursor Intensity
TIMM9 | 2 | 879.9547 | 1757.895 | 66.565 | 3.3471e−06 | 87.913 | 4962947
Intensity (%)
m/z
y1 376.2756
y2 523.344
y3−NH3 634.3757
y3 651.403
y4 893.5412
y5 1006.6238
y6−NH3 1117.6567
y6 1134.6809
y7 1249.7119
y10 1562.8359
A A Q I P E S D Q I K Q F K
Modification
Acetyl (Protein N−term)
GlyGly (K) no new terminus
Oxidation (M)

20200921_KIZ_MUb4_GlyGly_10plex_2 − Scan number: 16970

Gene Names	Charge	m/z	Mass	Retention time	PEP	Score	Precursor Intensity
TIMM9	2	879.9547	1757.895	66.307	0.011162	47.261	NA

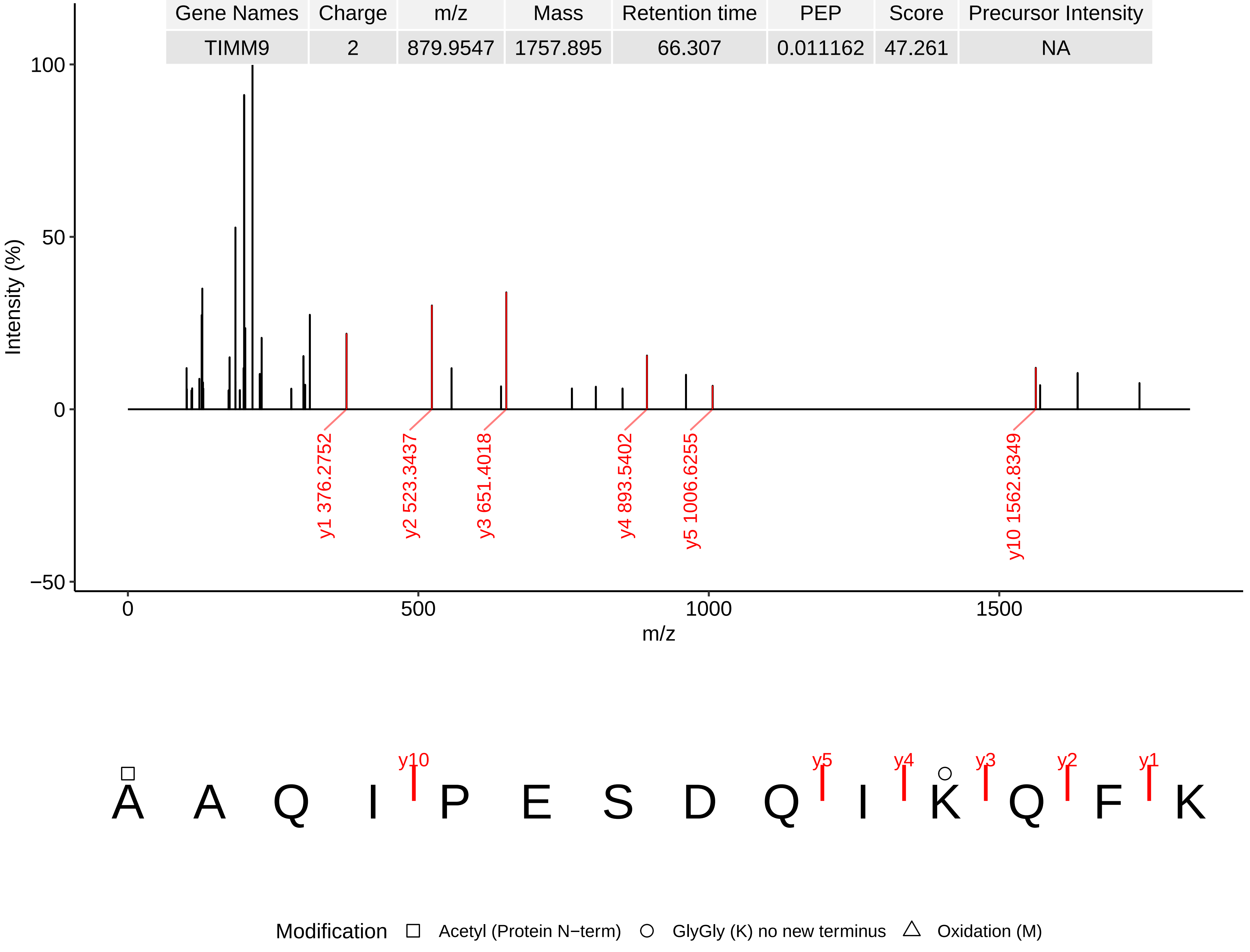

20200921_KIZ_MUb4_GlyGly_10plex − Scan number: 27682

Gene Names	Charge	m/z	Mass	Retention time	PEP	Score	Precursor Intensity
TOMM70A	3	521.6168	1561.829	101.09	0.00011133	96.64	3667541

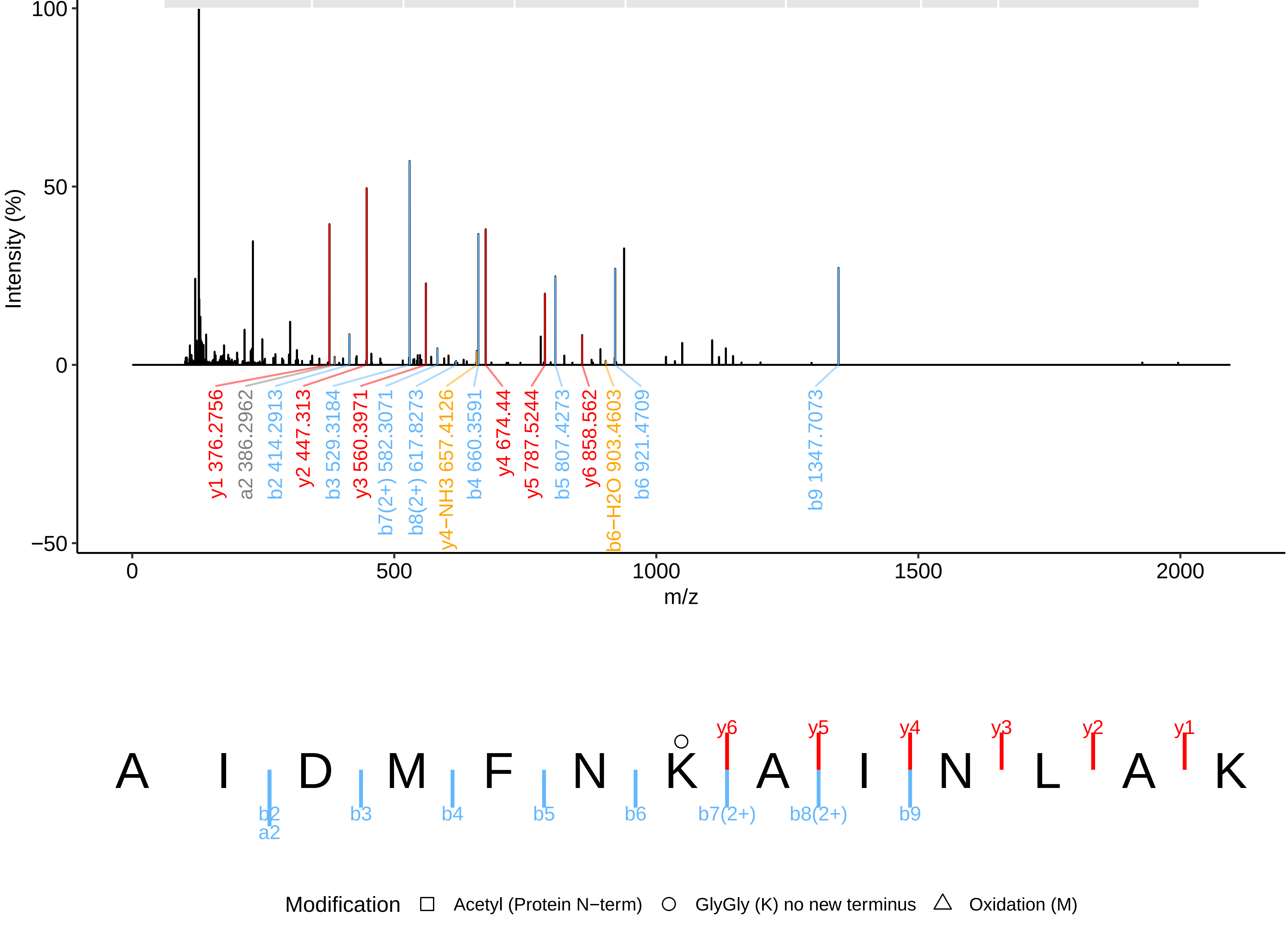

20201118_KIZ_MUb6_GlyGly_2 − Scan number: 24321

Gene Names	Charge	m/z	Mass	Retention time	PEP	Score	Precursor Intensity
PABPC1;PABPC4	3	522.6046	1564.792	81.444	0.00055881	92.19	19370202

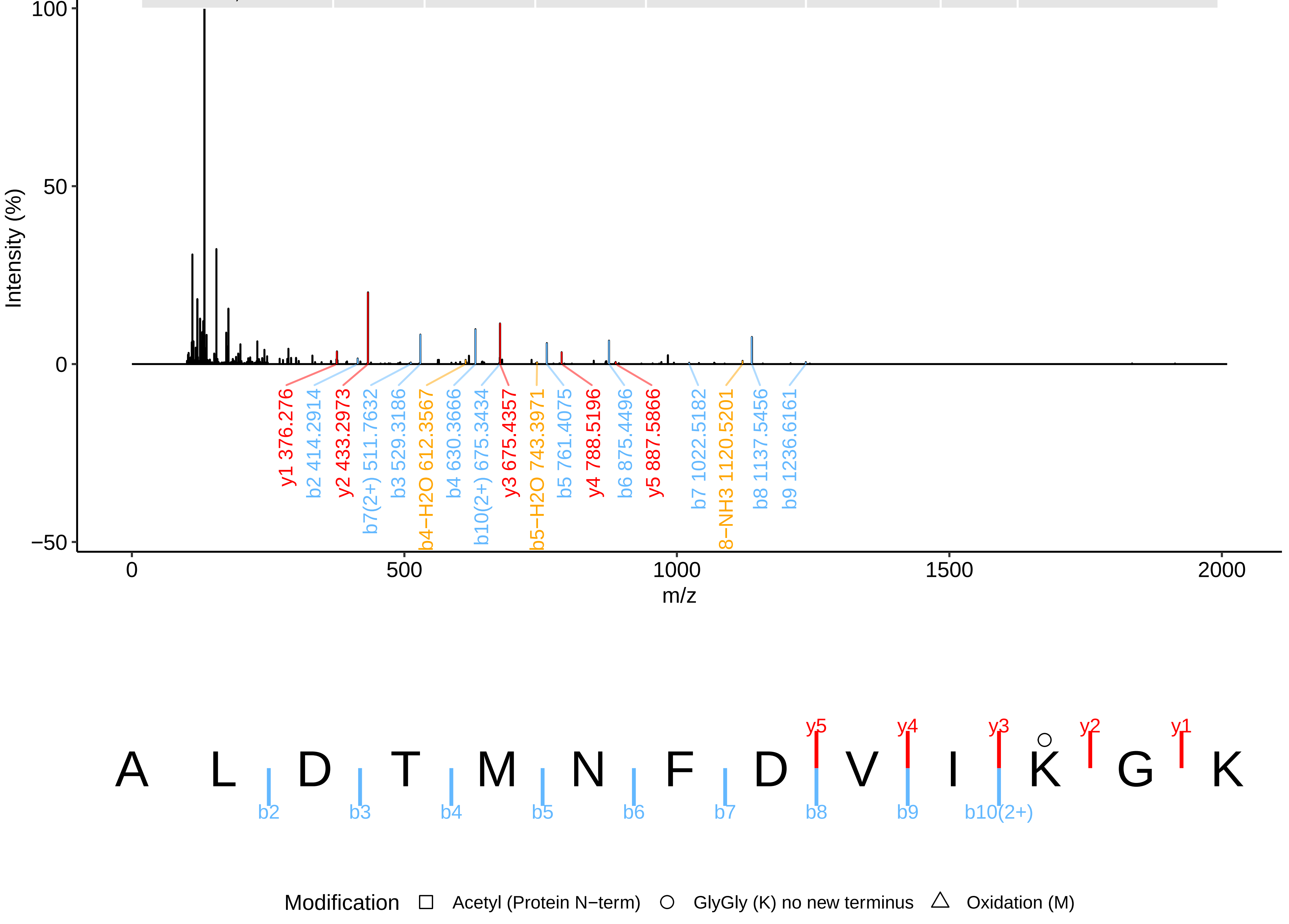

20200921_KIZ_MUb4_GlyGly_10plex − Scan number: 15641

Gene Names	Charge	m/z	Mass	Retention time	PEP	Score	Precursor Intensity
LYPLA1	3	589.9831	1766.928	55.929	0.0012044	76.156	2841007

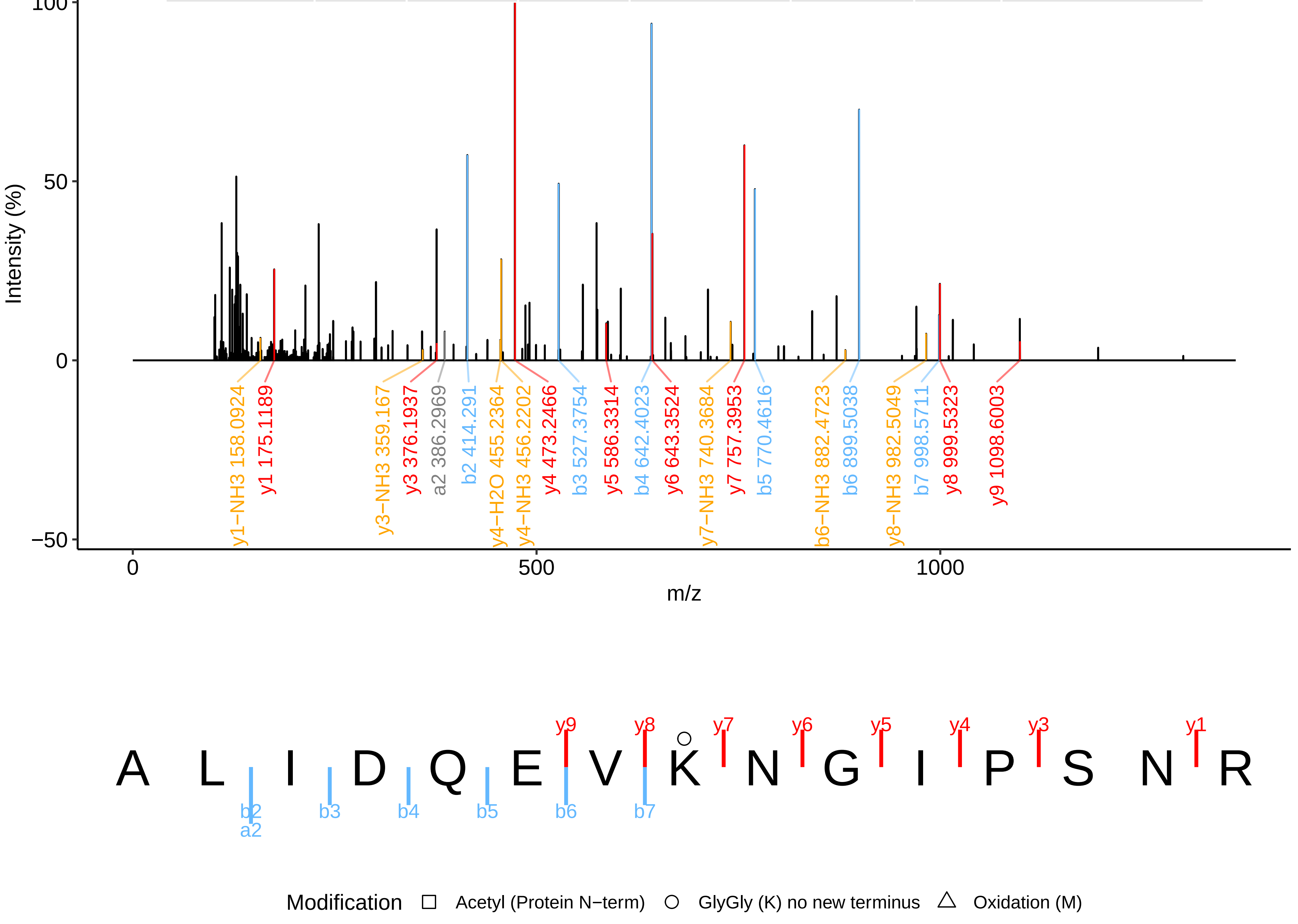

20200924_KIZ_MUb5_GlyGly_2 − Scan number: 13980

Gene Names	Charge	m/z	Mass	Retention time	PEP	Score	Precursor Intensity
LYPLA1	3	589.9831	1766.928	52.277	0.039525	57.288	839216.2

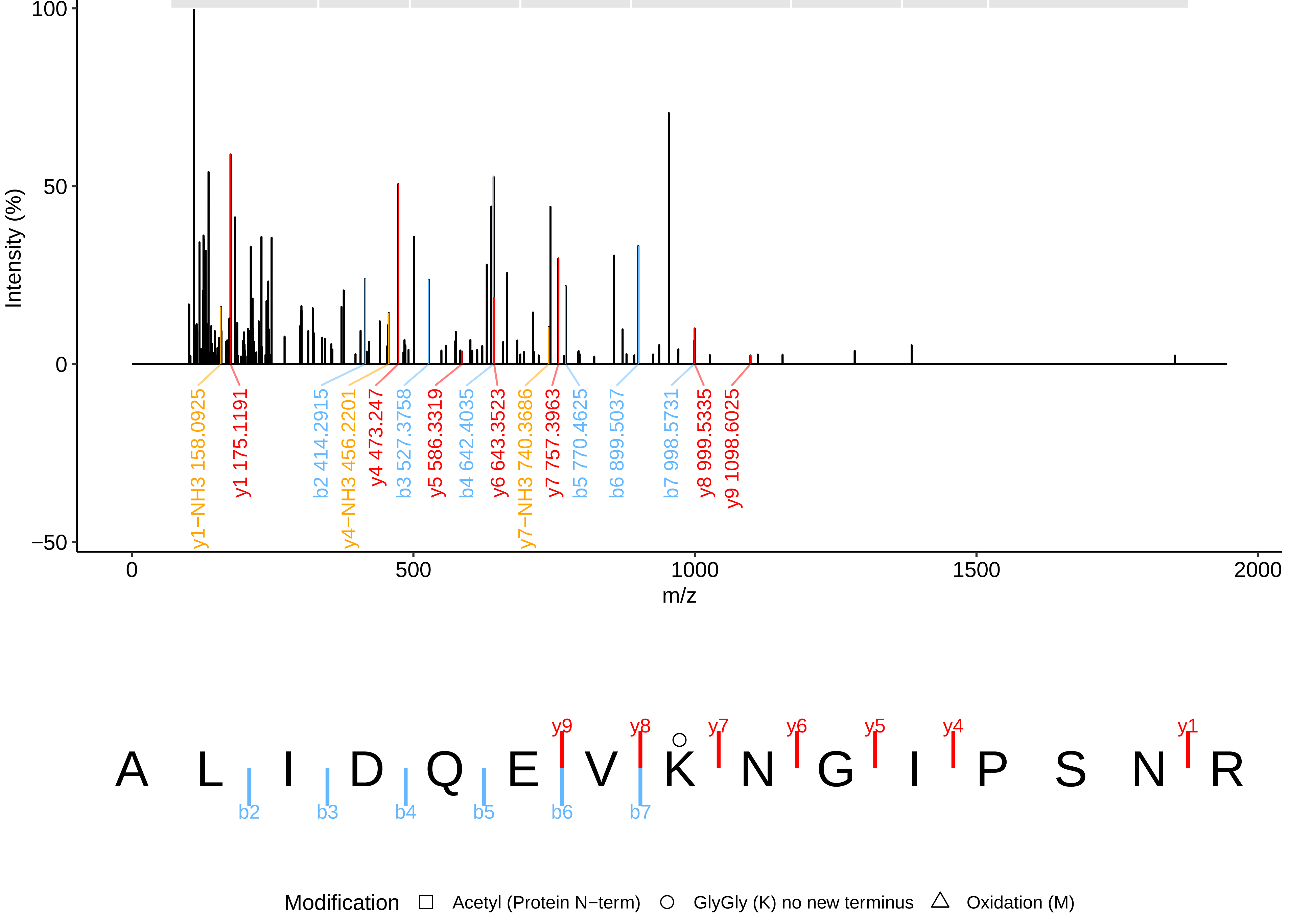

20200921_KIZ_MUb4_GlyGly_10plex − Scan number: 18355

Gene Names	Charge	m/z	Mass	Retention time	PEP	Score	Precursor Intensity
VCP	2	902.9498	1803.885	65.981	1.9664e−10	87.618	6683901

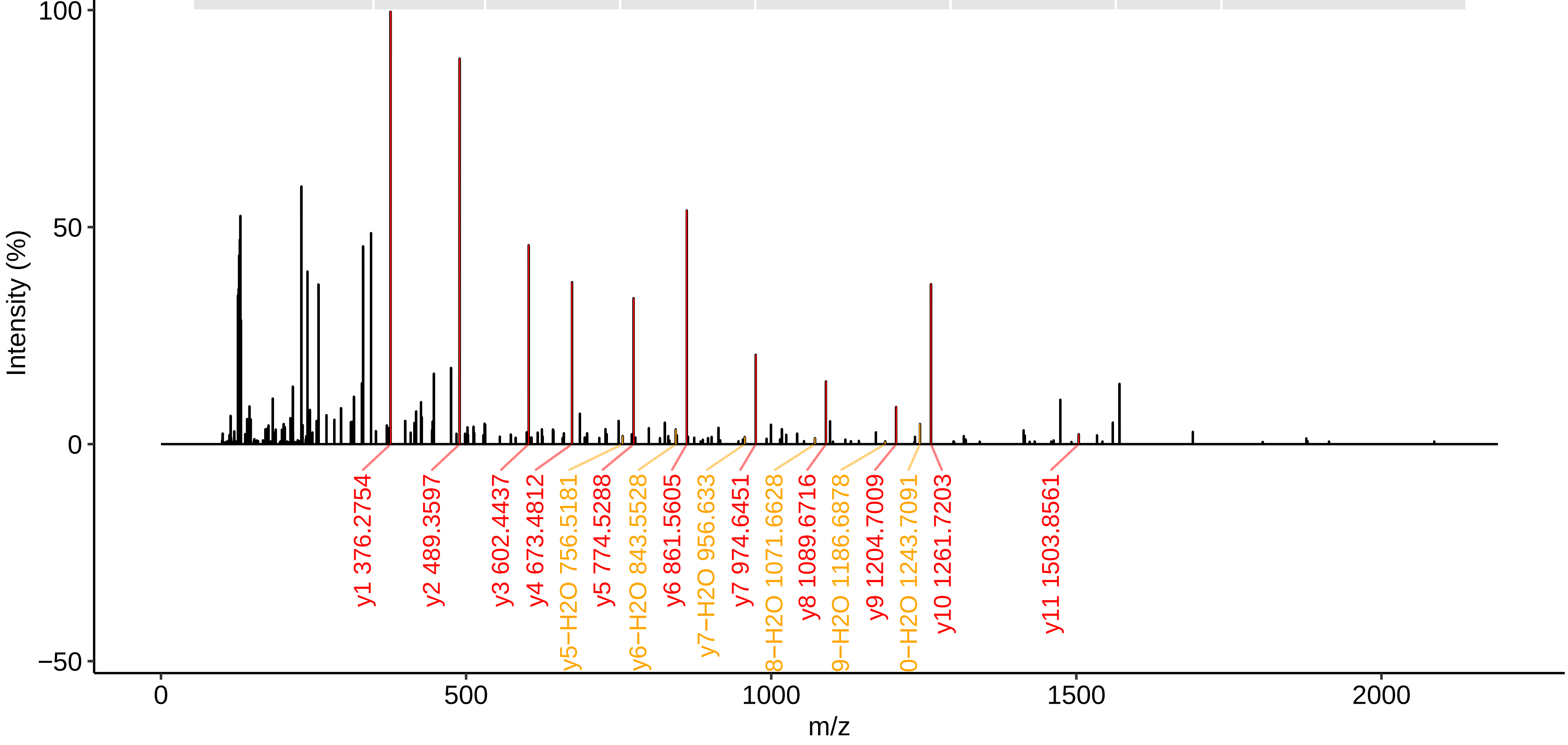

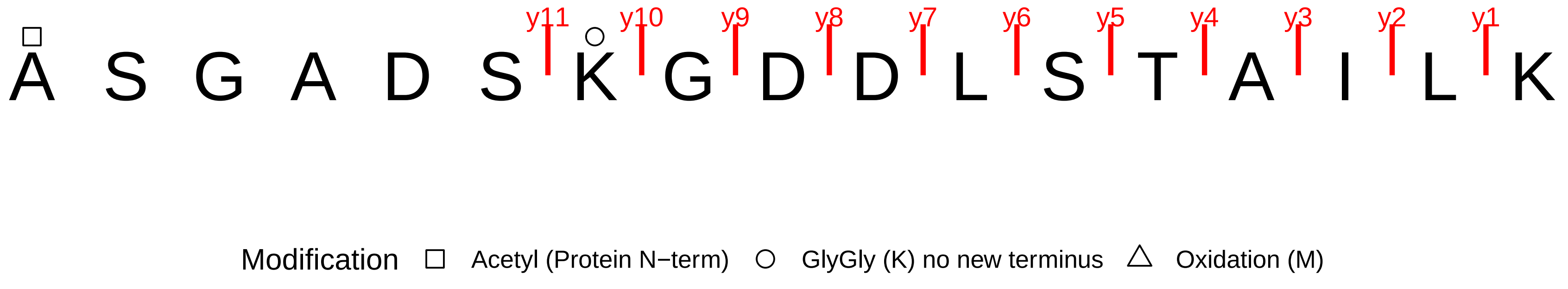

20200921_KIZ_MUb4_GlyGly_10plex − Scan number: 18362

Gene Names	Charge	m/z	Mass	Retention time	PEP	Score	Precursor Intensity
VCP	2	902.9498	1803.885	66.007	3.6648e−10	78.692	NA

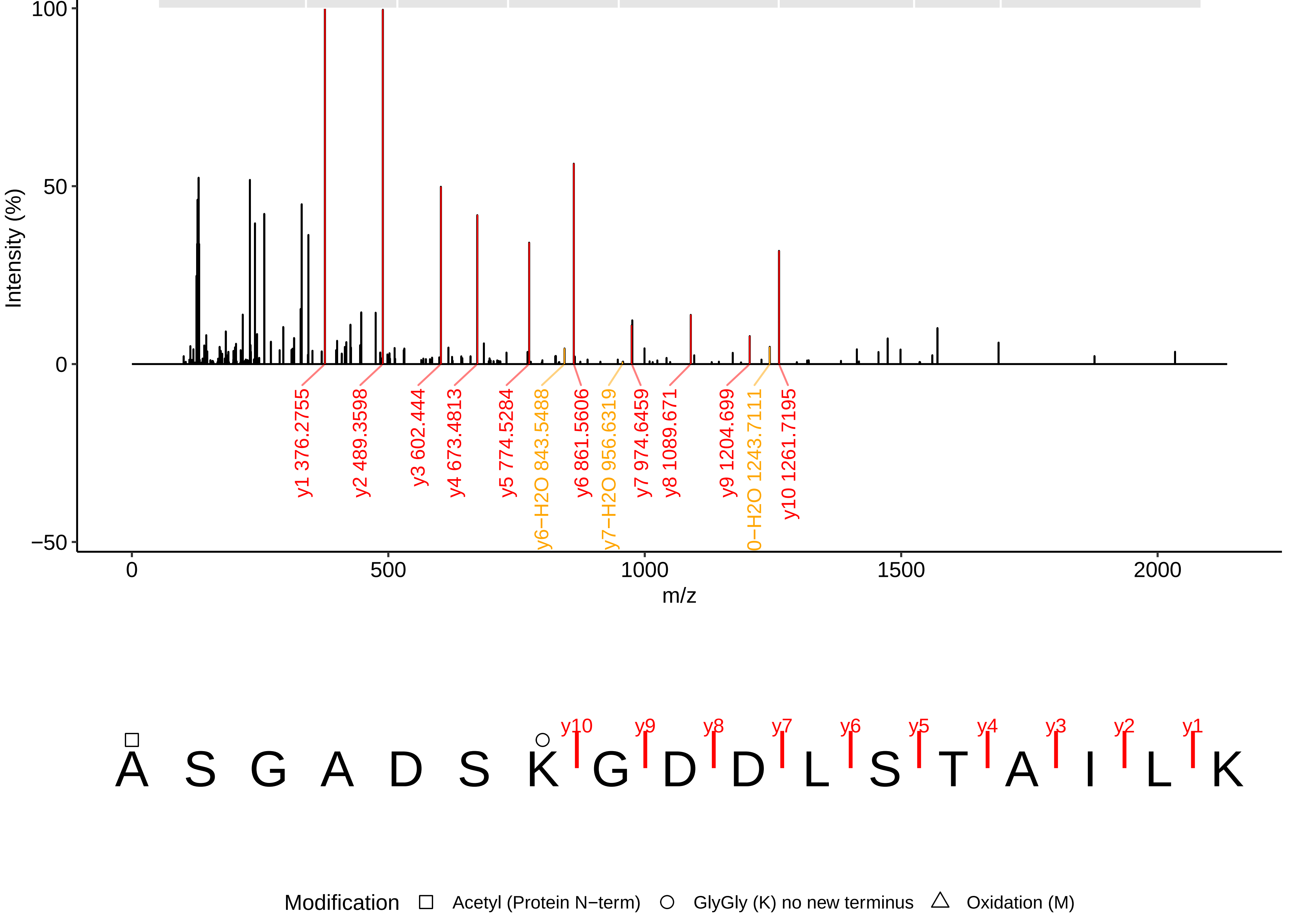

20200921_KIZ_MUb4_GlyGly_10plex − Scan number: 18377

Gene Names	Charge	m/z	Mass	Retention time	PEP	Score	Precursor Intensity
VCP	2	902.9498	1803.885	66.068	2.6048e−13	89.283	15418717

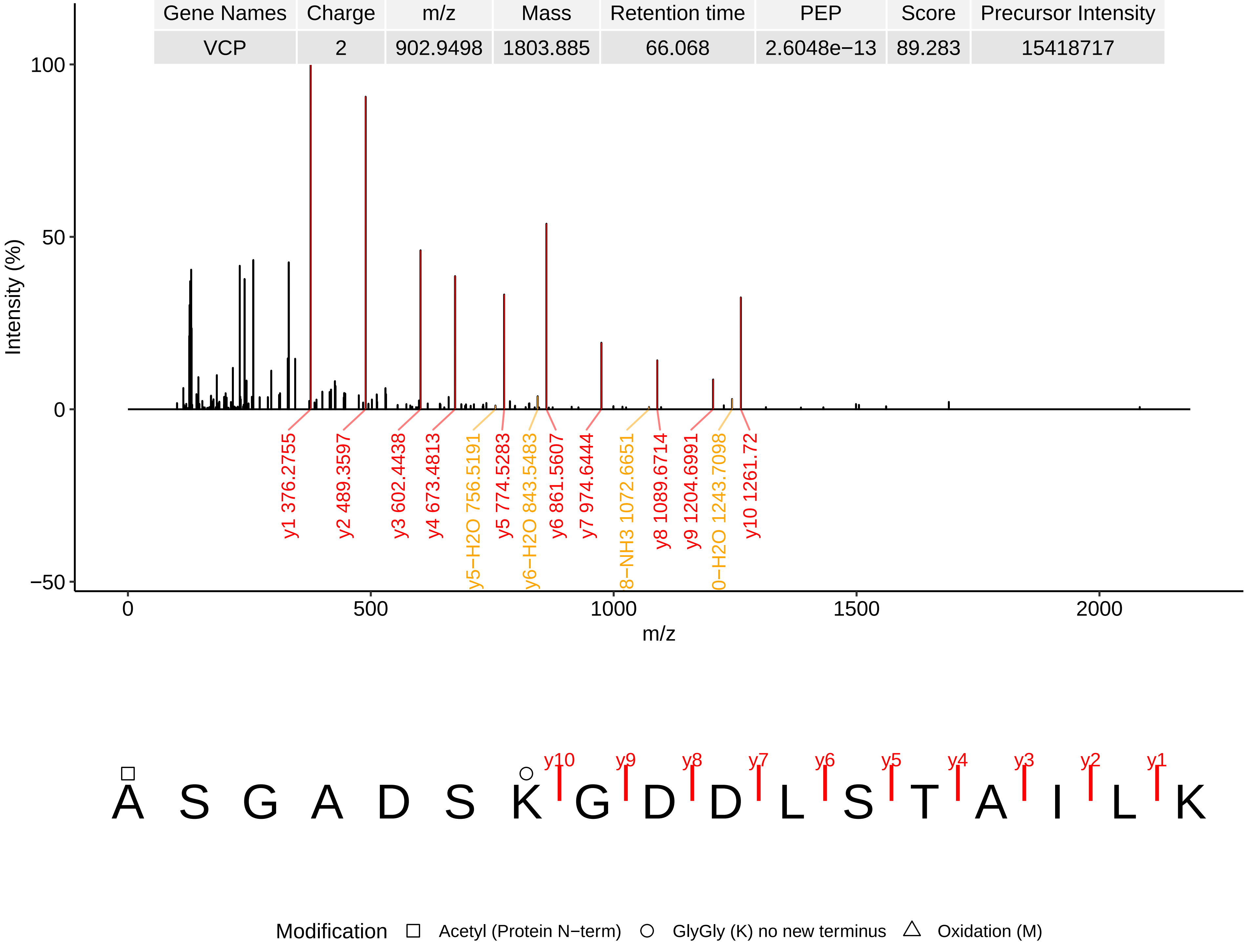

20200921_KIZ_MUb4_GlyGly_10plex − Scan number: 18519

Gene Names	Charge	m/z	Mass	Retention time	PEP	Score	Precursor Intensity
VCP	2	902.9498	1803.885	66.635	2.9544e−05	89.283	NA

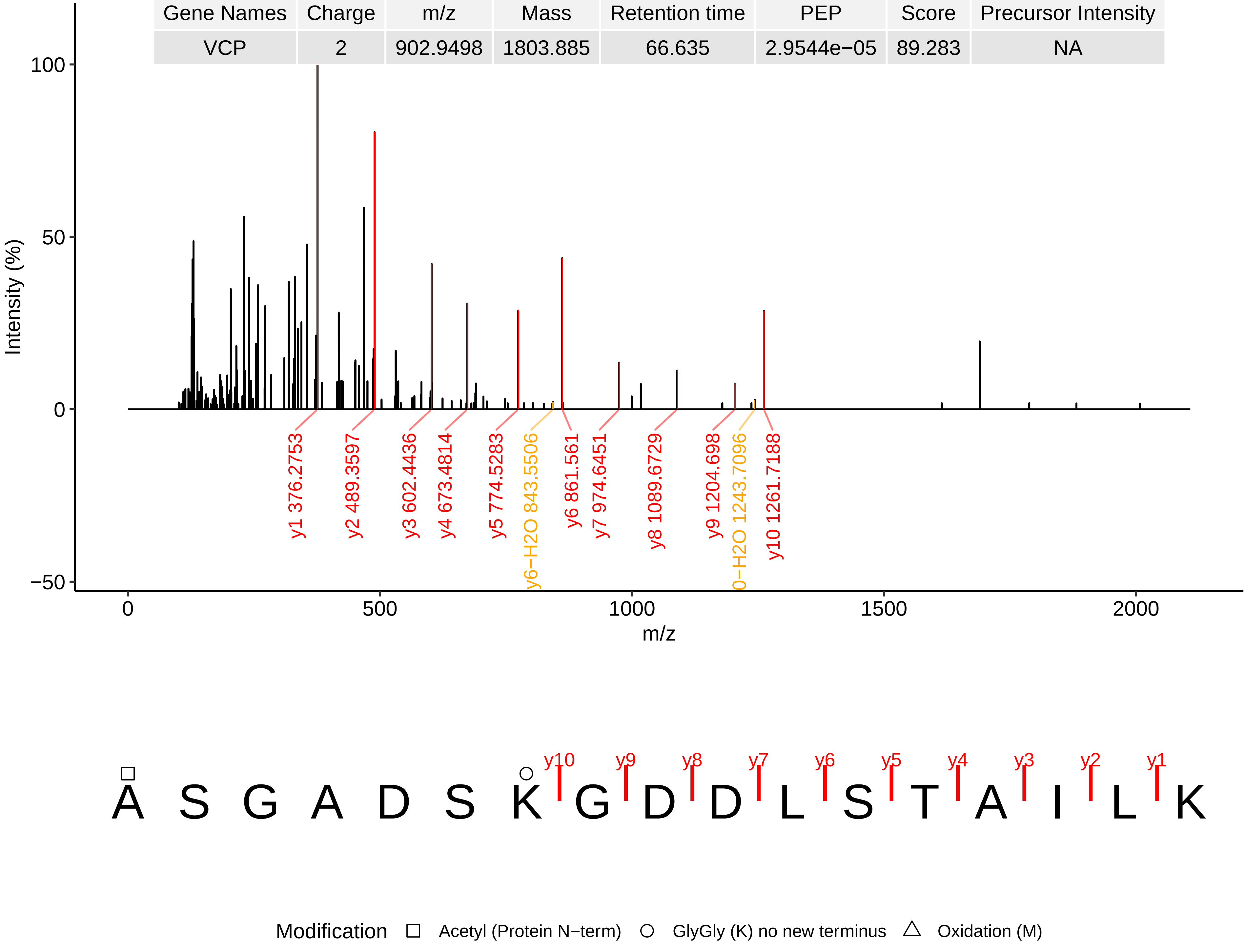

20200924_KIZ_MUb5_GlyGly_1 − Scan number: 18498

Gene Names	Charge	m/z	Mass	Retention time	PEP	Score	Precursor Intensity
VCP	2	902.9498	1803.885	59.798	1.2644e−39	138.75	13112171

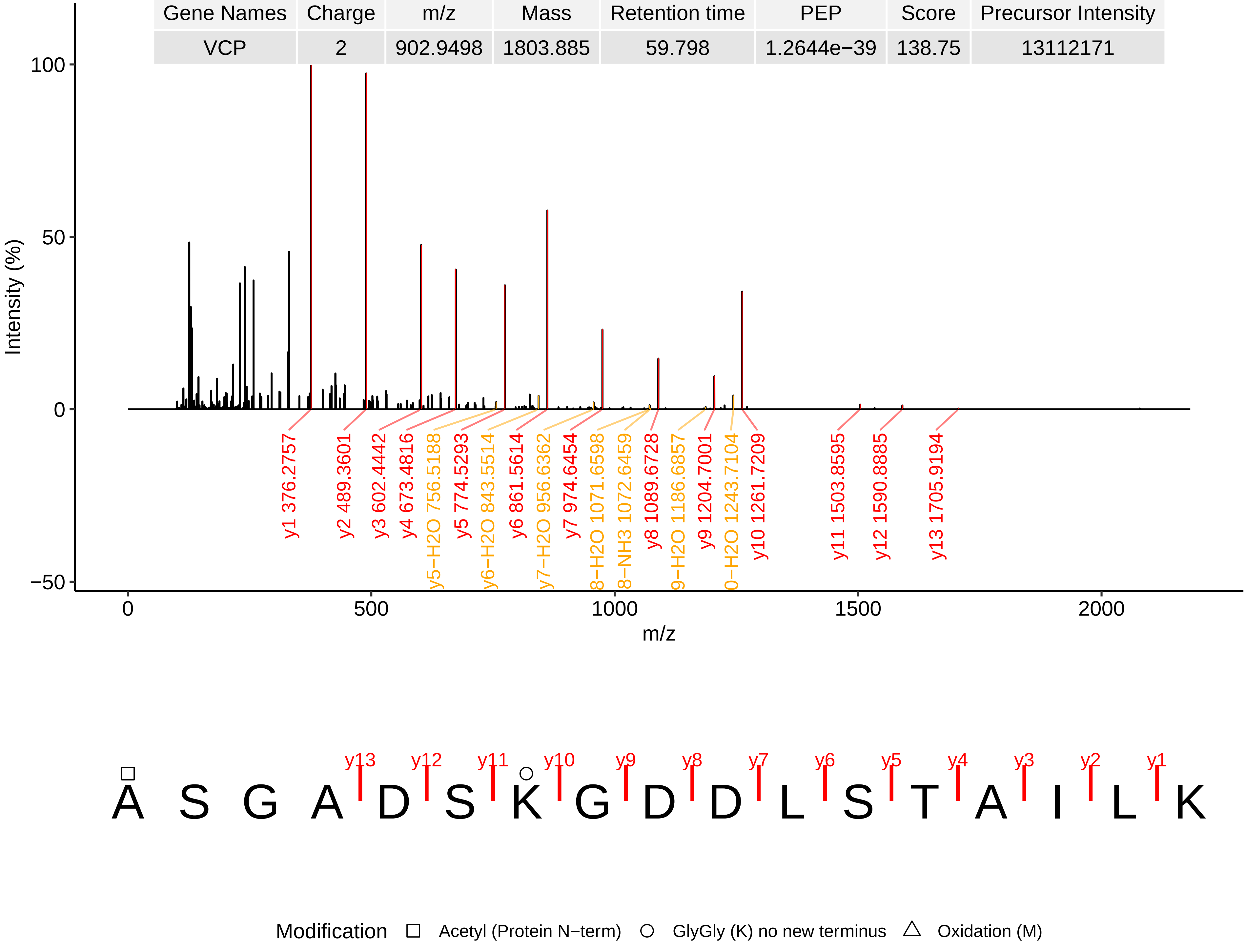

20200924_KIZ_MUb5_GlyGly_1 − Scan number: 18657

Gene Names	Charge	m/z	Mass	Retention time	PEP	Score	Precursor Intensity
VCP	2	902.9498	1803.885	60.299	1.9198e−31	129.84	8804622

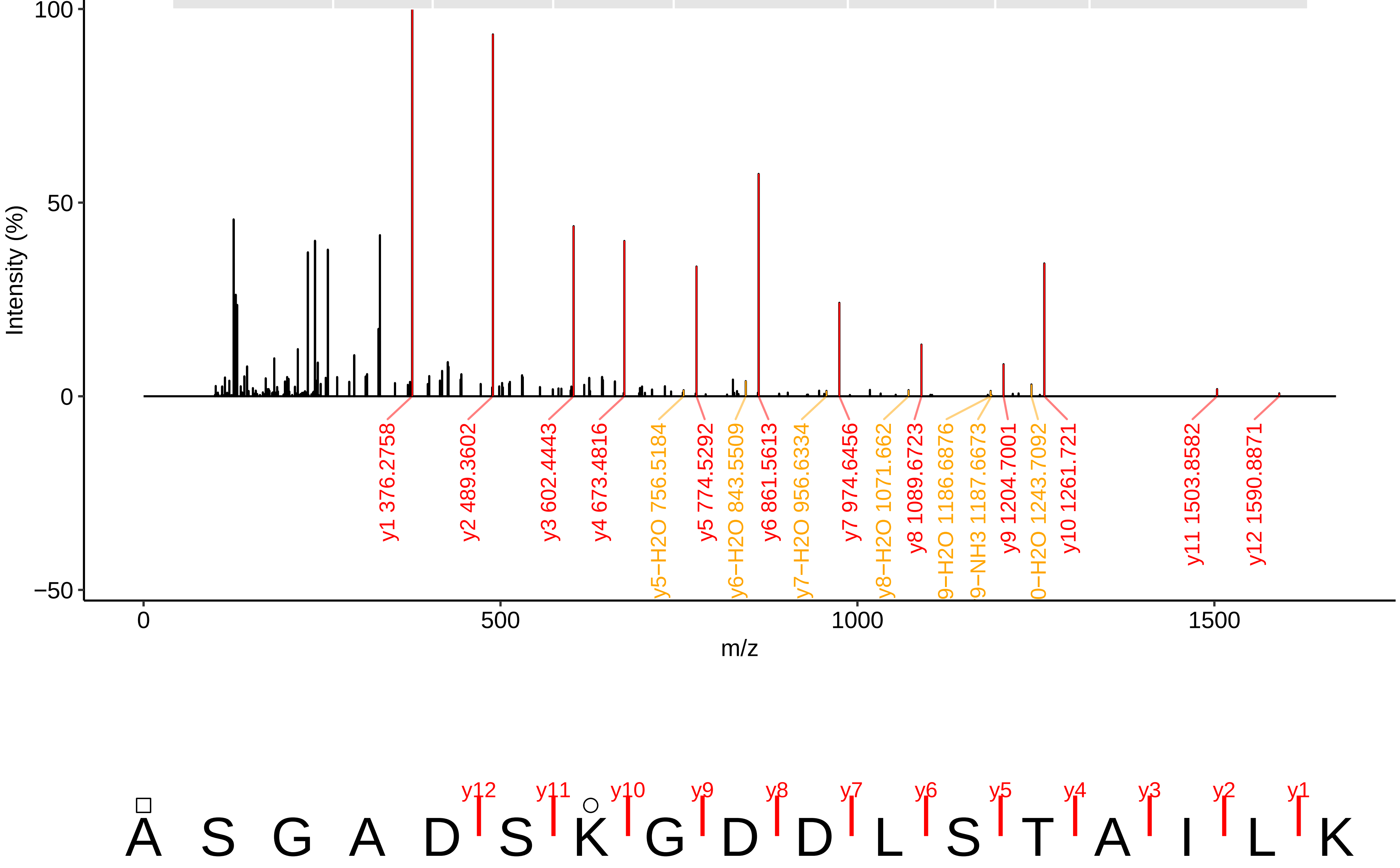

Modification □ Acetyl (Protein N−term) ○ GlyGly (K) no new terminus △ Oxidation (M)

20200924_KIZ_MUb5_GlyGly_2 − Scan number: 16855

Gene Names	Charge	m/z	Mass	Retention time	PEP	Score	Precursor Intensity
VCP	2	902.9498	1803.885	62.541	2.6048e−13	78.692	3303543

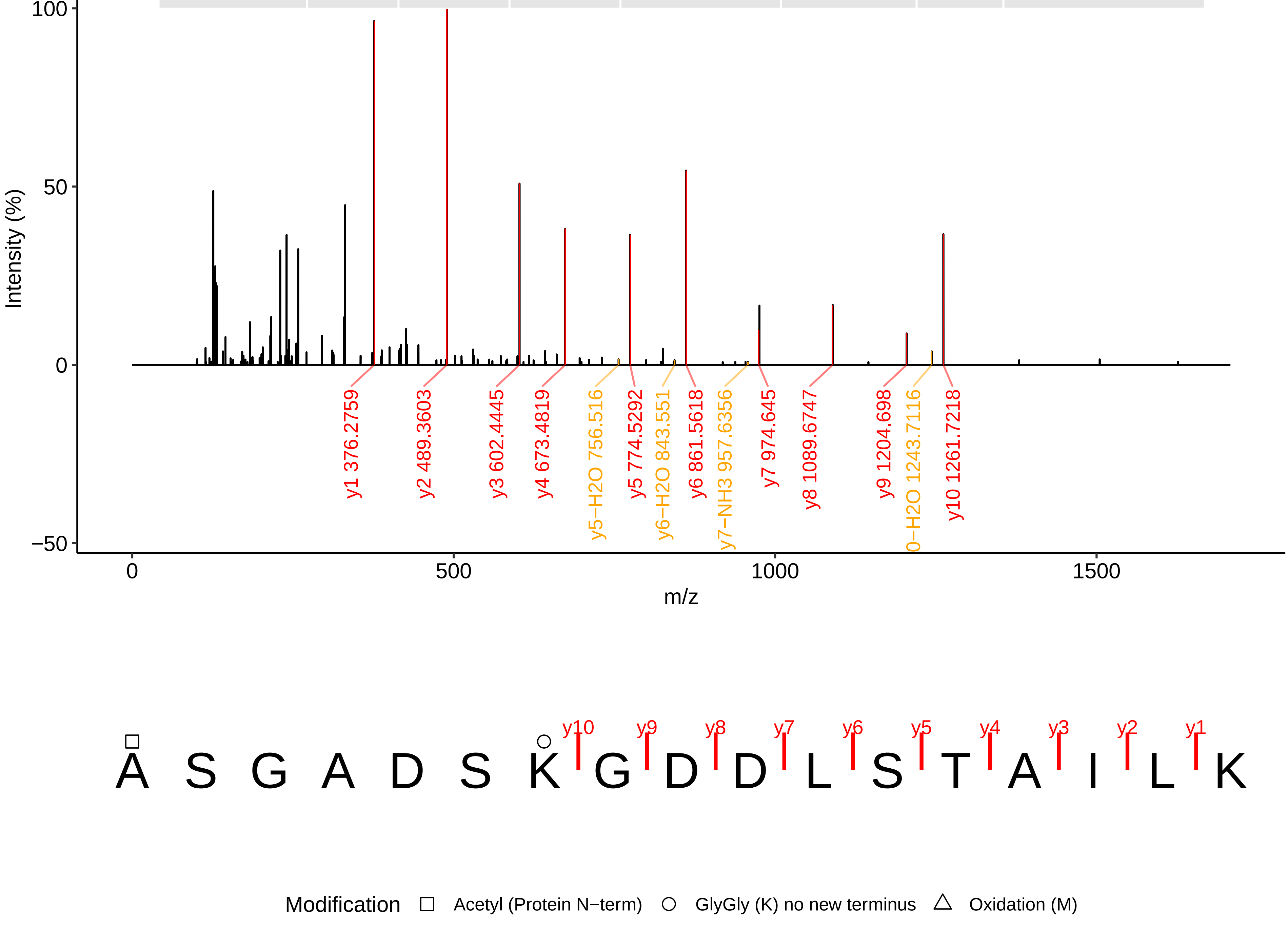

20201118_KIZ_MUb6_GlyGly_1 − Scan number: 18455

Gene Names	Charge	m/z	Mass	Retention time	PEP	Score	Precursor Intensity
VCP	2	902.9498	1803.885	58.034	4.6975e−29	129.84	NA

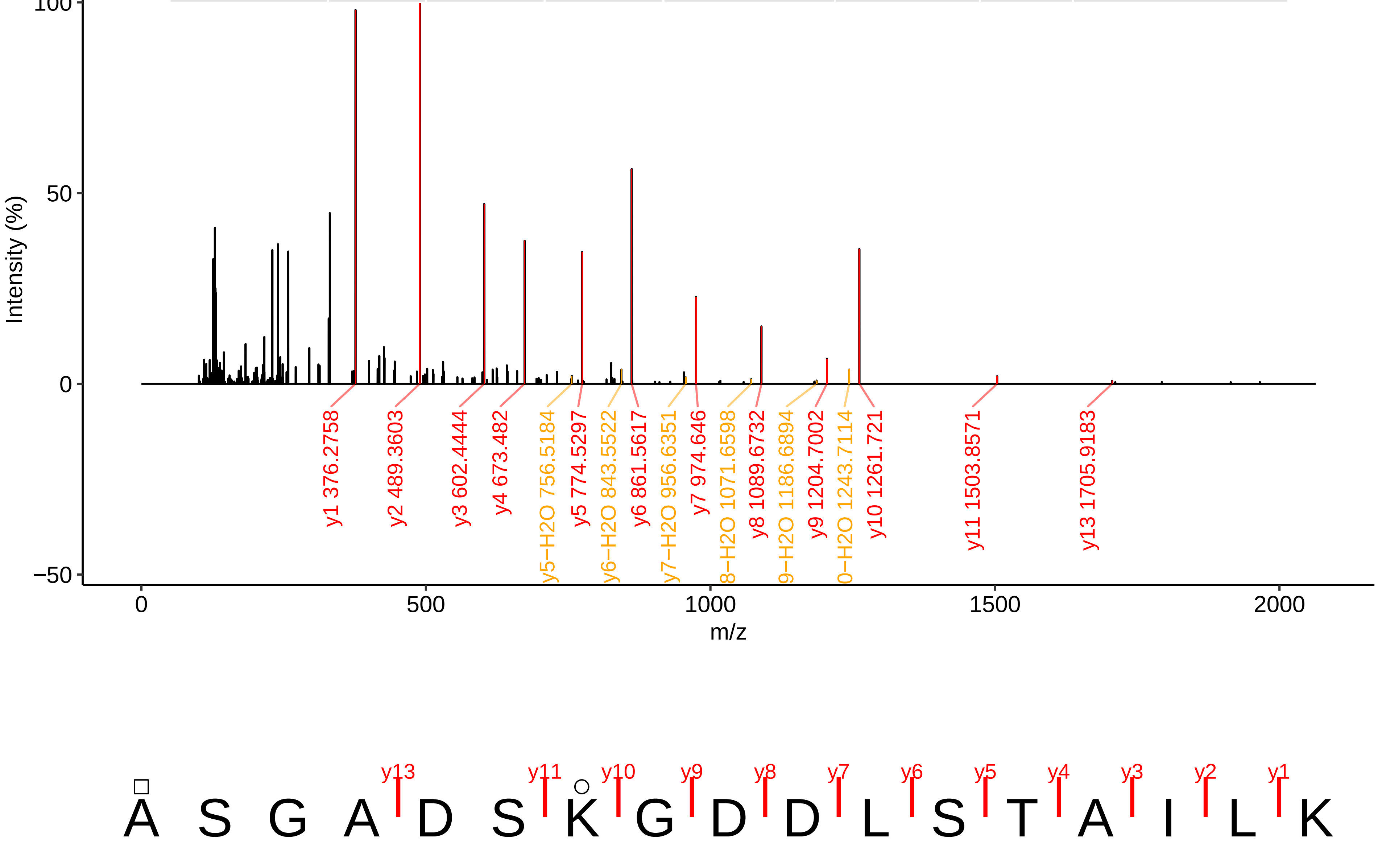

Modification □ Acetyl (Protein N−term) ○ GlyGly (K) no new terminus △ Oxidation (M)

20201118_KIZ_MUb6_GlyGly_2 − Scan number: 19465

Gene Names	Charge	m/z	Mass	Retention time	PEP	Score	Precursor Intensity
VCP	3	602.3023	1803.885	65.683	0.00055968	44.196	13332836

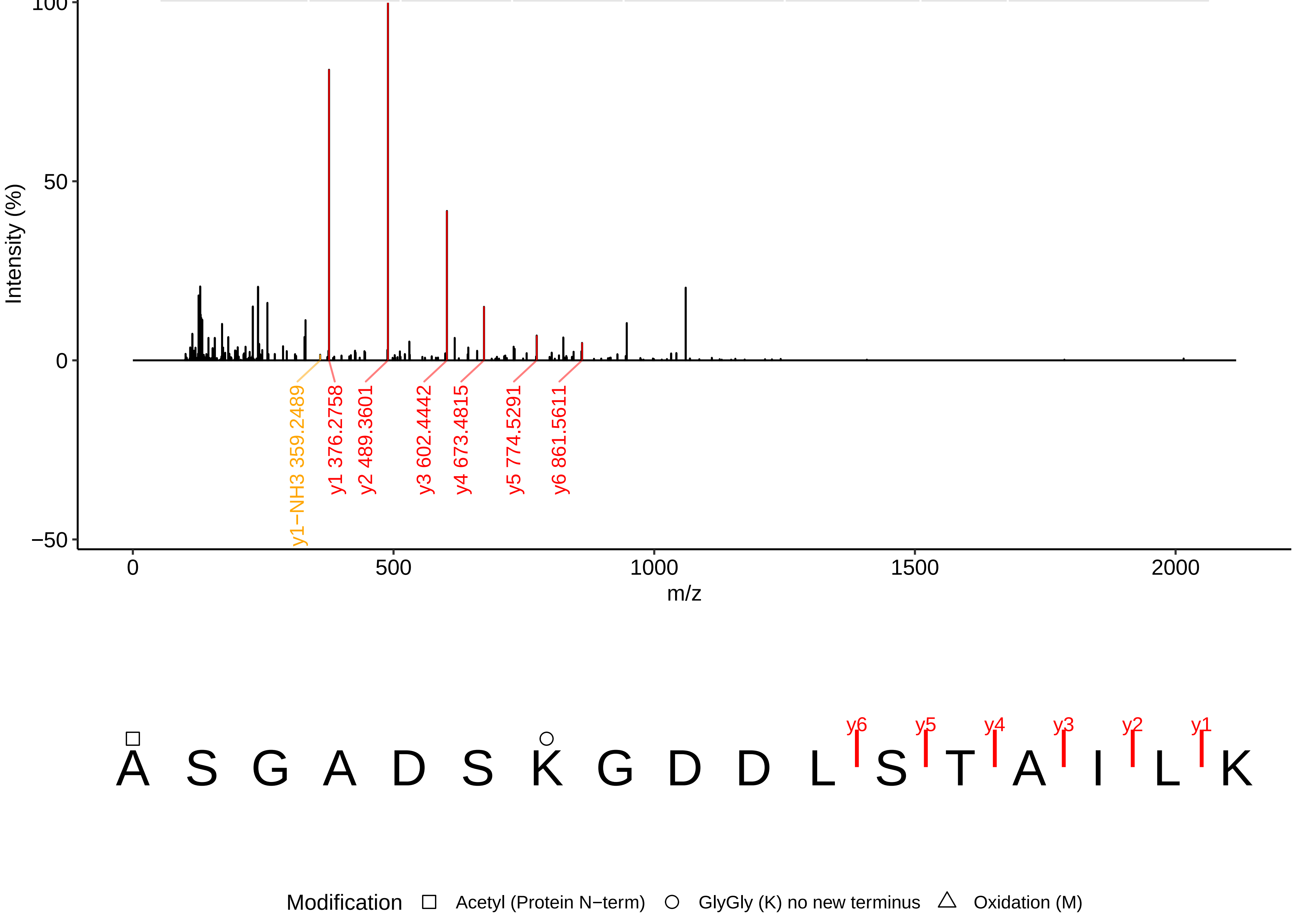

20201118_KIZ_MUb6_GlyGly_2 − Scan number: 19470

Gene Names	Charge	m/z	Mass	Retention time	PEP	Score	Precursor Intensity
VCP	2	902.9498	1803.885	65.703	1.7031e−17	106.49	5466346

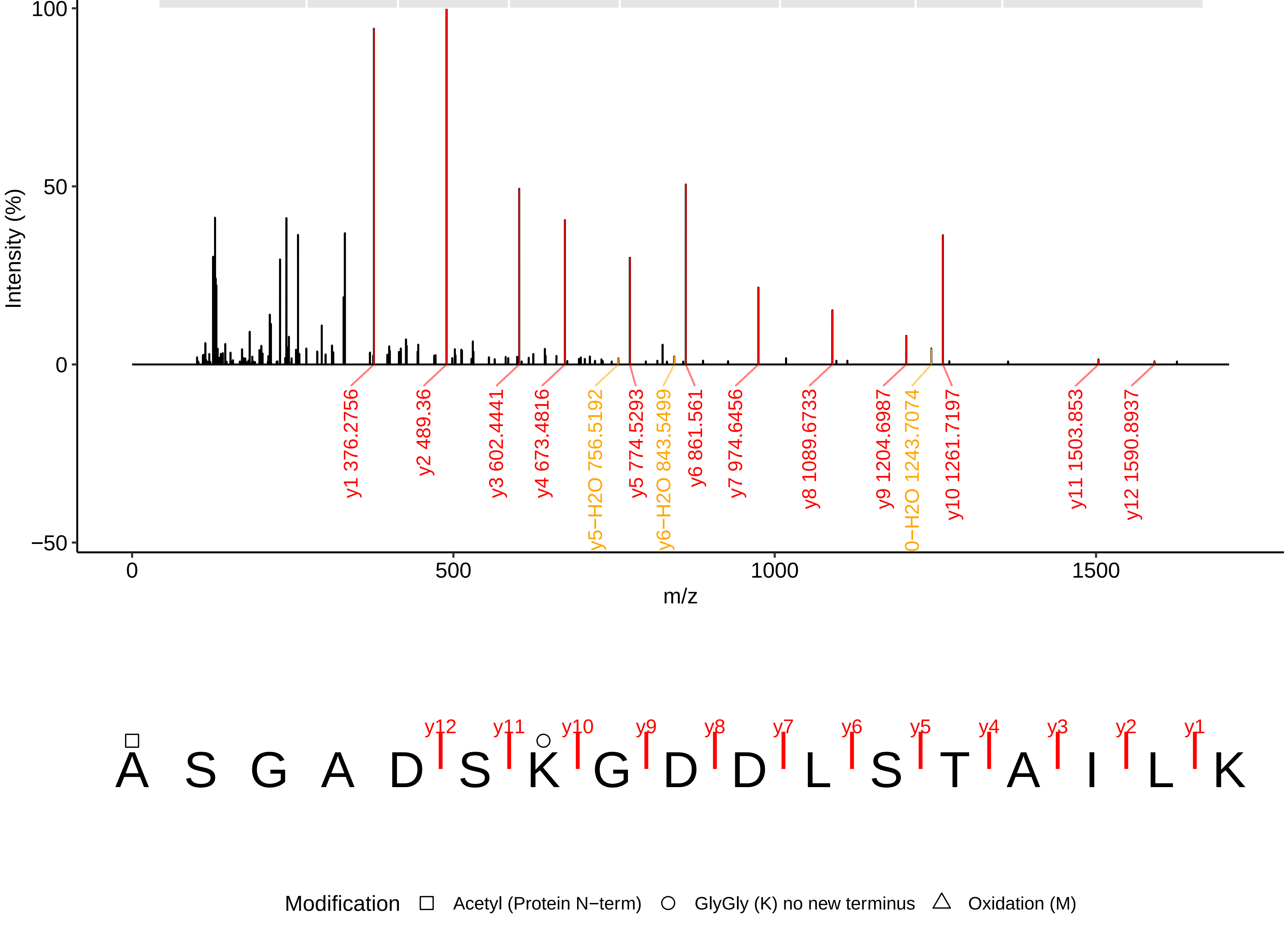

20200921_KIZ_MUb4_GlyGly_10plex − Scan number: 13444

Gene Names	Charge	m/z	Mass	Retention time	PEP	Score	Precursor Intensity
NCL	3	520.2827	1557.826	47.435	2.2362e−05	98.105	3357166

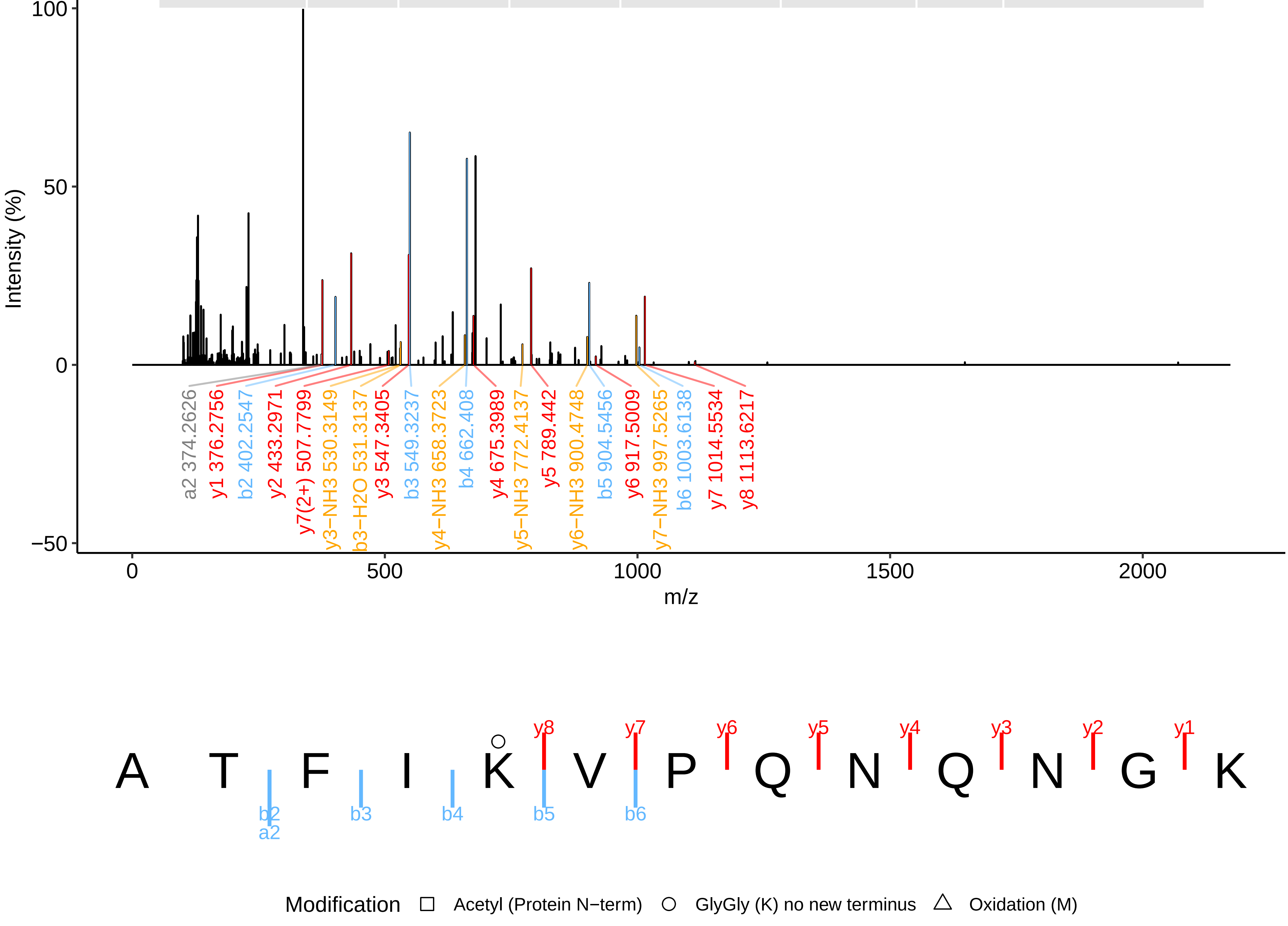

20200921_KIZ_MUb4_GlyGly_10plex − Scan number: 11542

Gene Names	Charge	m/z	Mass	Retention time	PEP	Score	Precursor Intensity
CALM2;CALM3;CALM1	3	608.6316	1822.873	40.253	0.075982	44.312	12913977

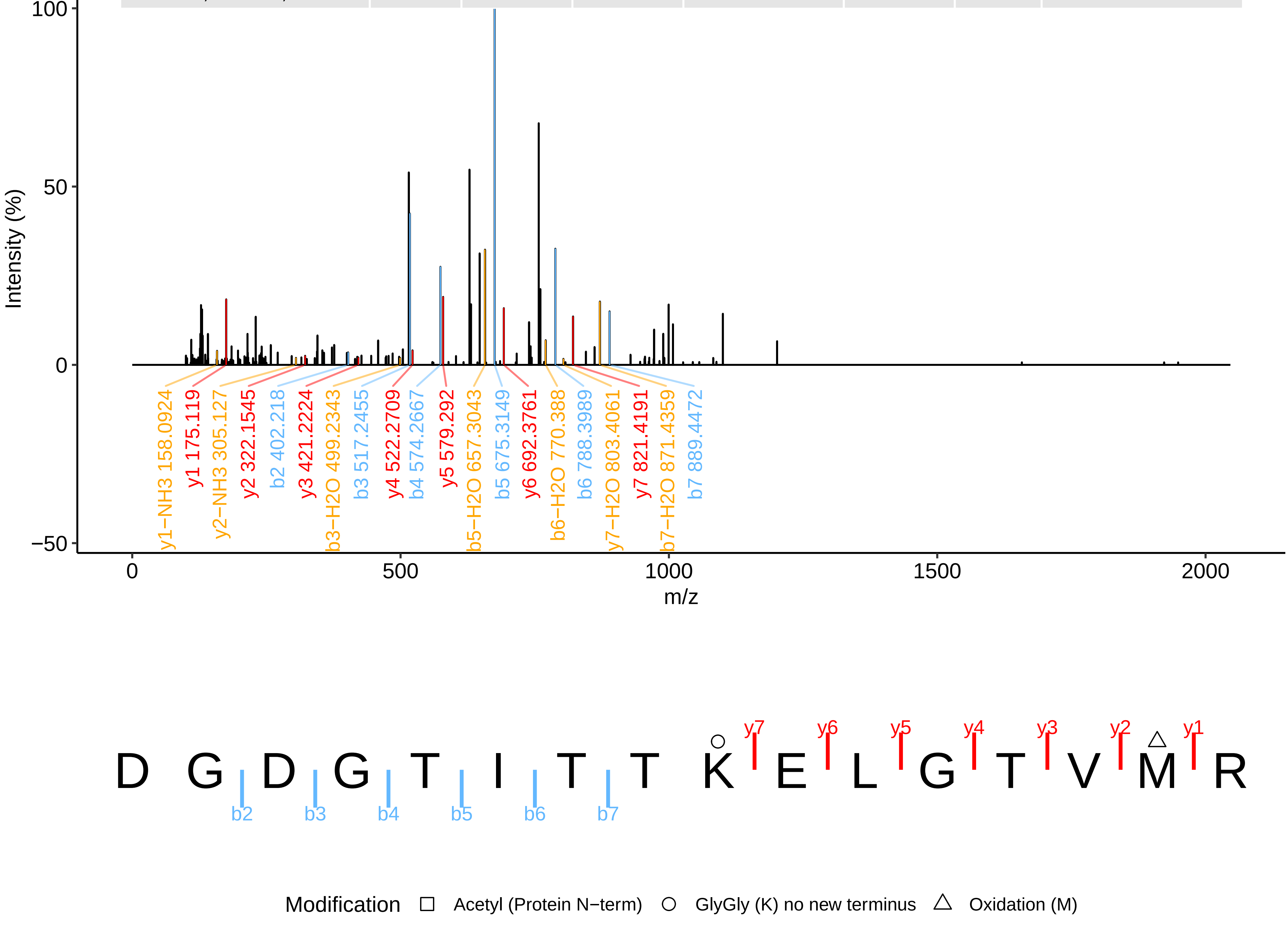

20200921_KIZ_MUb4_GlyGly_10plex_2 − Scan number: 10567

Gene Names	Charge	m/z	Mass	Retention time	PEP	Score	Precursor Intensity
CALM2;CALM3;CALM1	3	608.6316	1822.873	40.452	0.037404	53.981	1205390

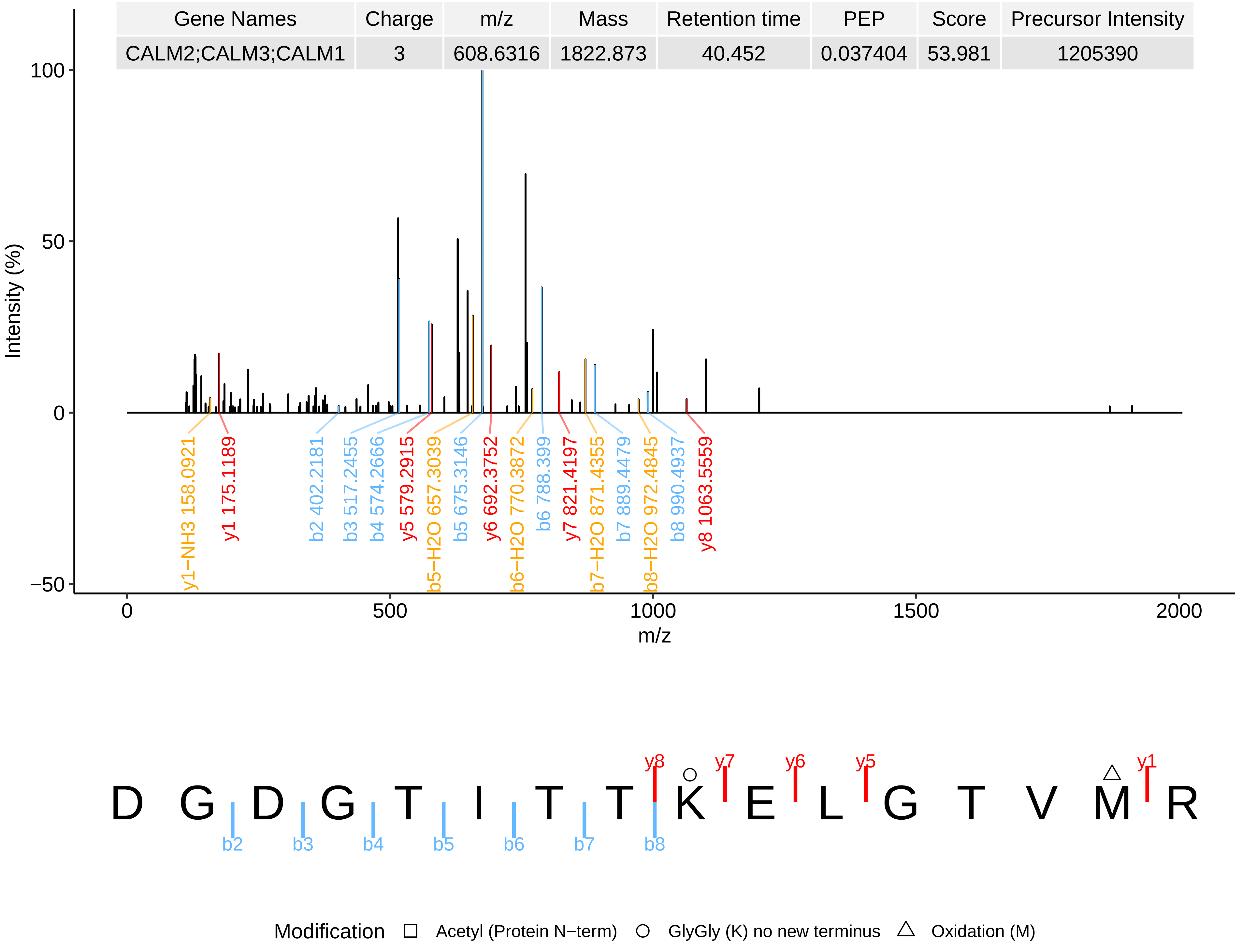

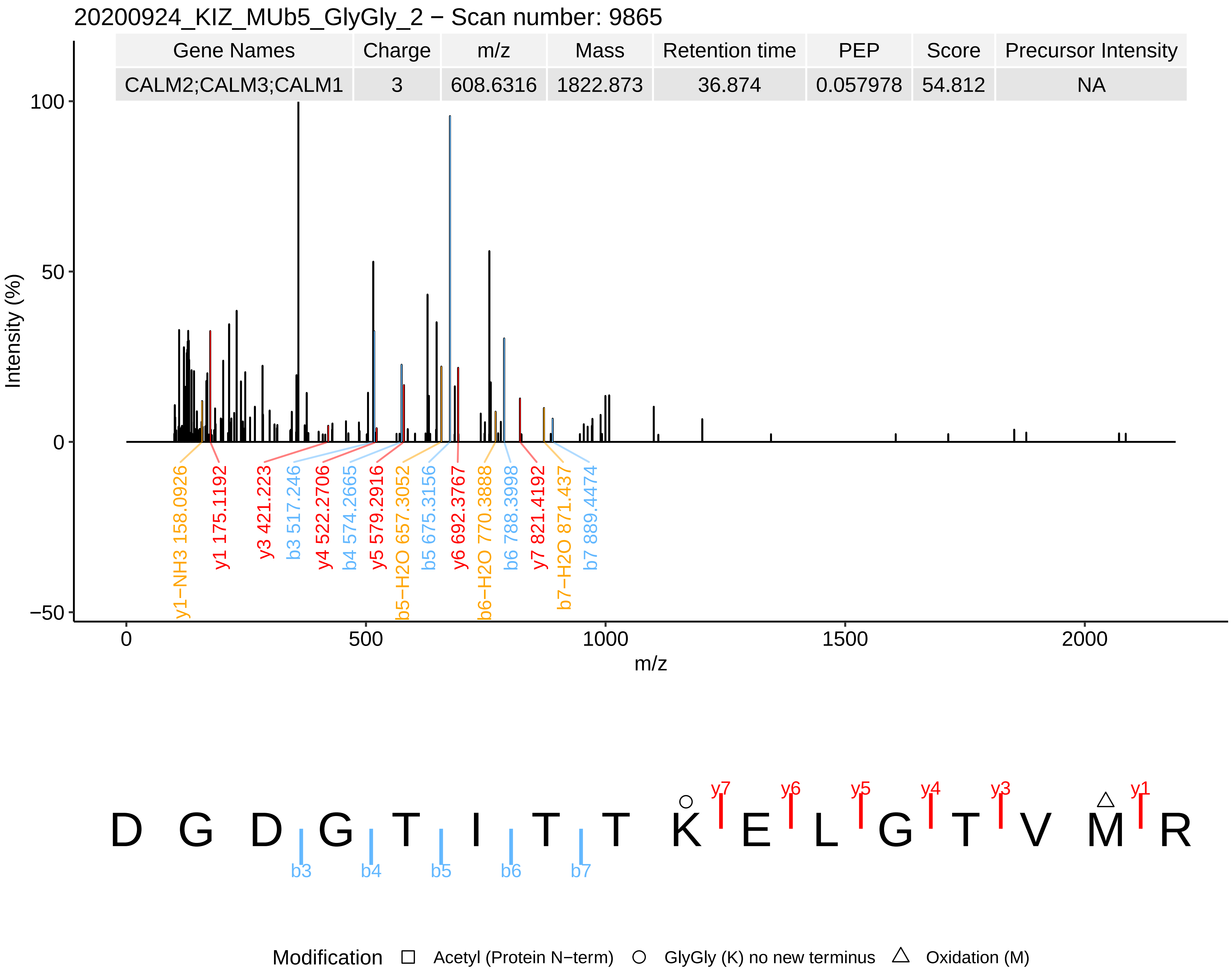
20200924_KIZ_MUb5_GlyGly_2 − Scan number: 9865
Gene Names | Charge | m/z | Mass | Retention time | PEP | Score | Precursor Intensity
CALM2;CALM3;CALM1 | 3 | 608.6316 | 1822.873 | 36.874 | 0.057978 | 54.812 | NA
Intensity (%)
100
50
0
−50
0
500
1000
1500
2000
m/z
y1−NH3 158.0926
y1 175.1192
y3 421.223
b3 517.246
y4 522.2706
b4 574.2665
y5 579.2916
b5−H2O 657.3052
b5 675.3156
y6 692.3767
b6−H2O 770.3888
b6 788.3998
y7 821.4192
b7−H2O 871.437
b7 889.4474
D G D G T I T T K E L G T V M R
y7 y6 y5 y4 y3 y1
b3 b4 b5 b6 b7
Modification
Acetyl (Protein N−term)
GlyGly (K) no new terminus
Oxidation (M)

20200921_KIZ_MUb4_GlyGly_10plex − Scan number: 15823

Gene Names	Charge	m/z	Mass	Retention time	PEP	Score	Precursor Intensity
CALM2;CALM3;CALM1	3	603.3	1806.878	56.65	5.0652e−06	93.424	6510309

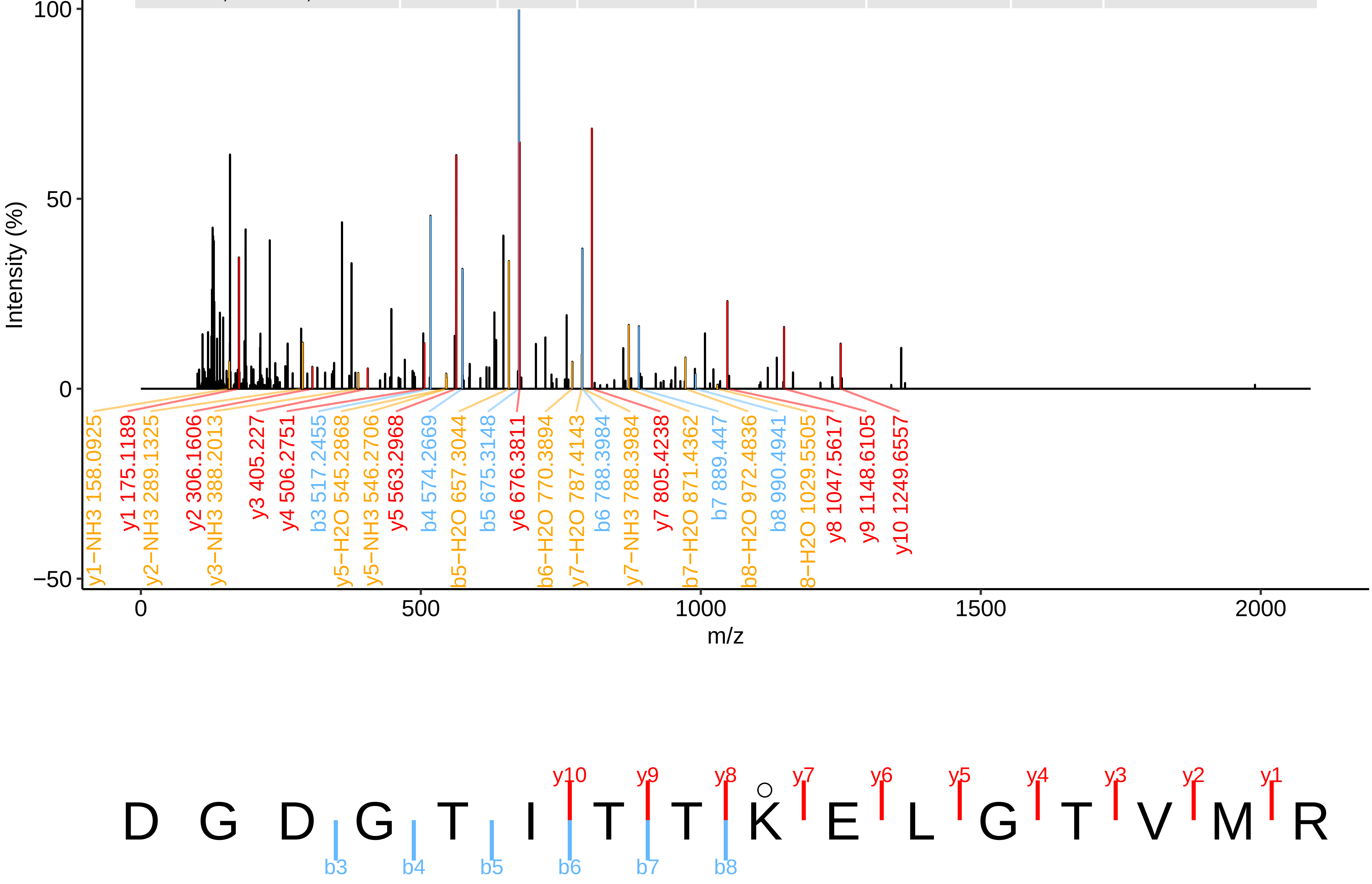

Modification □ Acetyl (Protein N−term) ○ GlyGly (K) no new terminus △ Oxidation (M)

20200921_KIZ_MUb4_GlyGly_10plex_2 − Scan number: 14506

Gene Names	Charge	m/z	Mass	Retention time	PEP	Score	Precursor Intensity
CALM2;CALM3;CALM1	3	603.3	1806.878	56.567	1.8438e−07	107.98	2398020

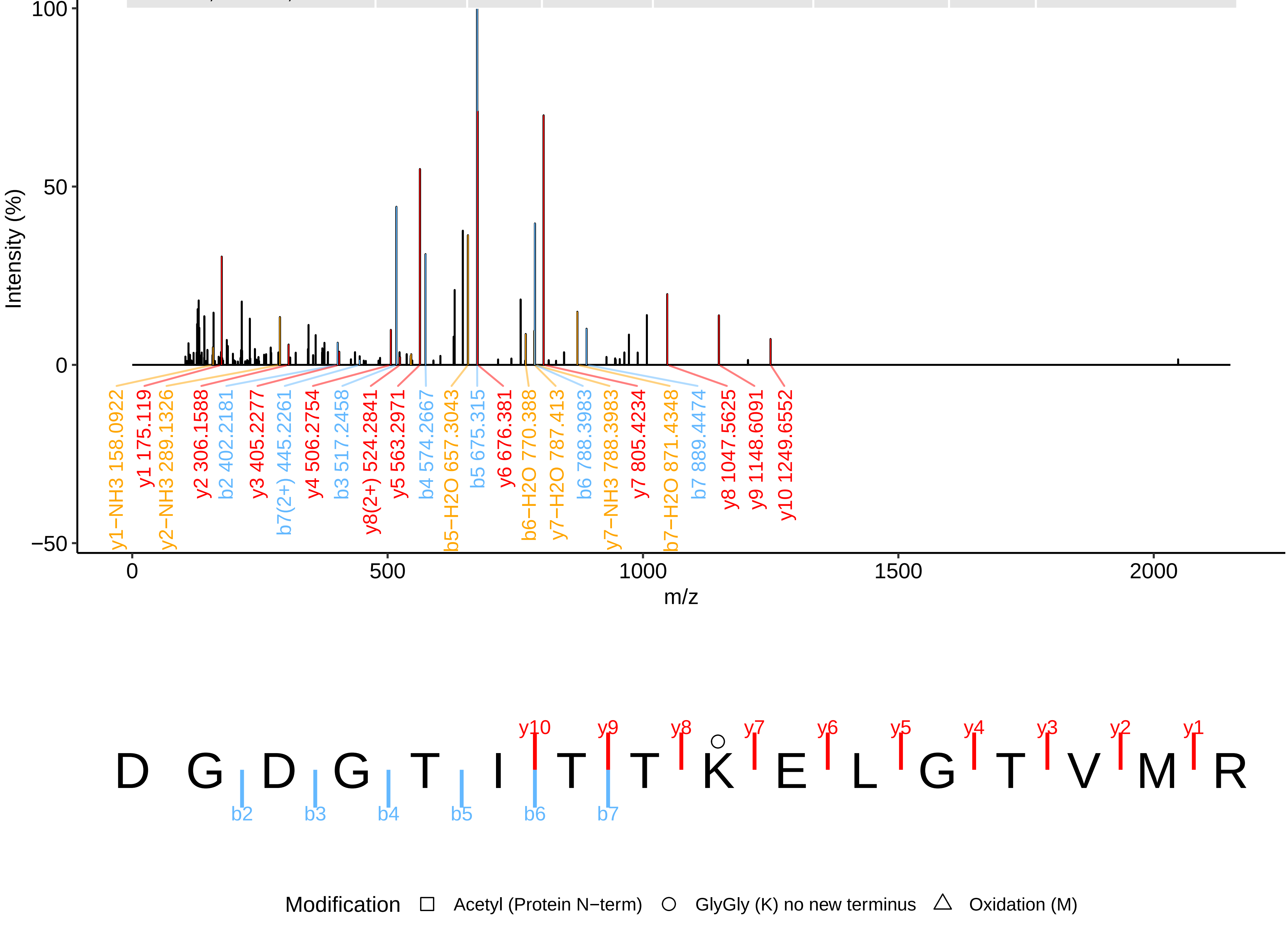

20200924_KIZ_MUb5_GlyGly_1 − Scan number: 14945

Gene Names	Charge	m/z	Mass	Retention time	PEP	Score	Precursor Intensity
CALM2;CALM3;CALM1	3	603.3	1806.878	48.82	6.3641e−10	118.21	61142932

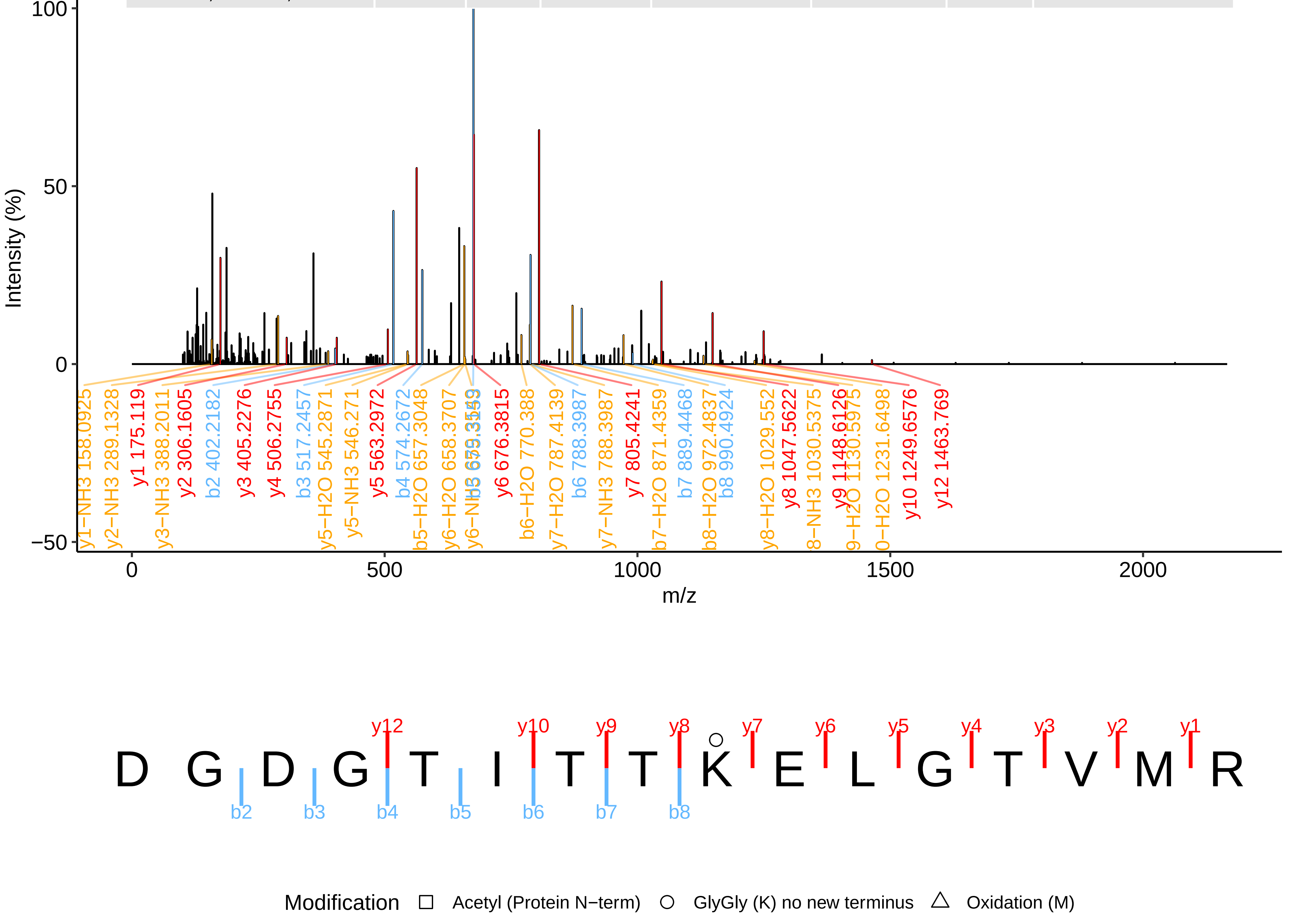

20200924_KIZ_MUb5_GlyGly_2 − Scan number: 13958

Gene Names	Charge	m/z	Mass	Retention time	PEP	Score	Precursor Intensity
CALM2;CALM3;CALM1	3	603.3	1806.878	52.201	6.8759e−10	117.03	10146637

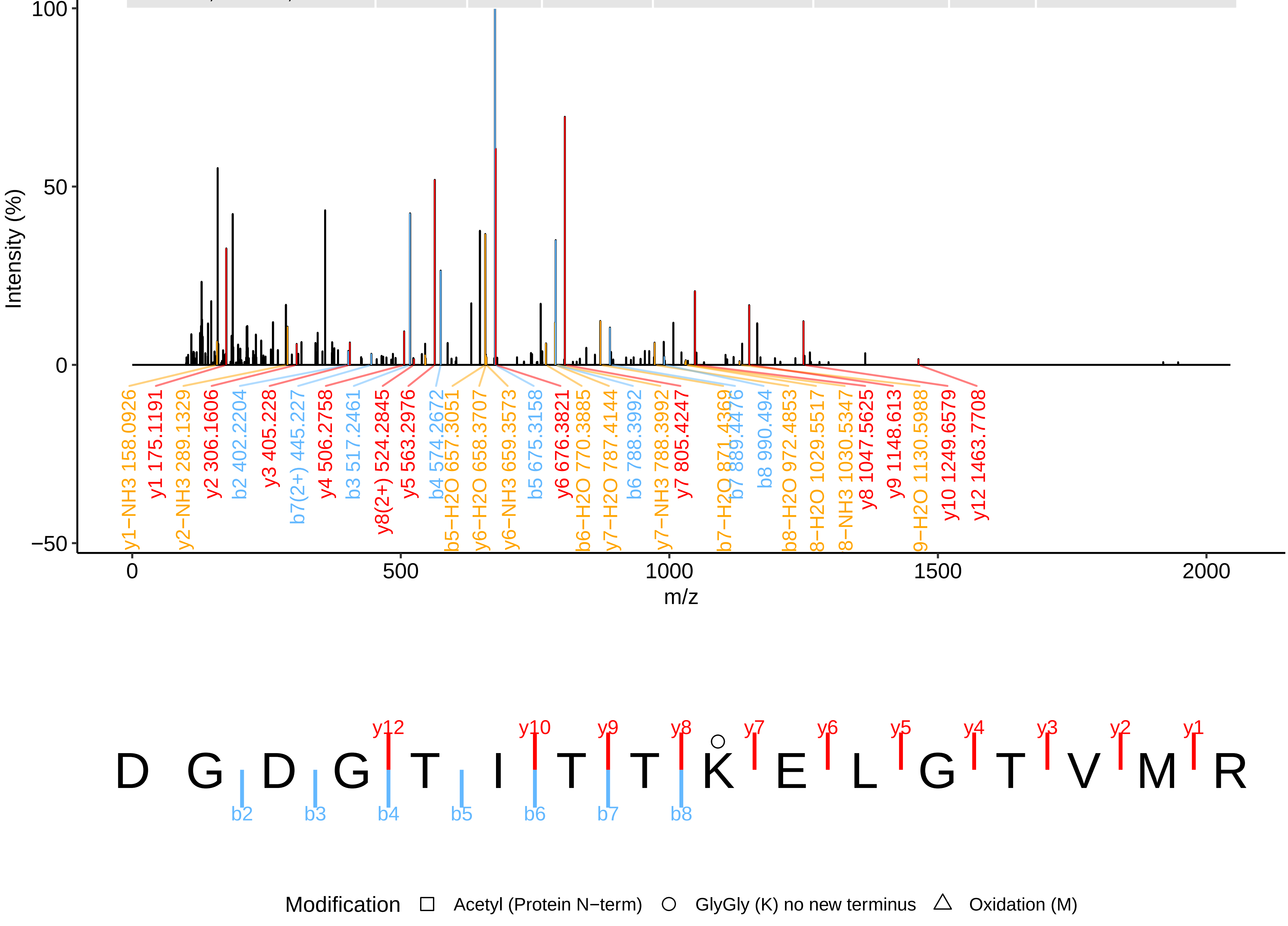

20200924_KIZ_MUb5_GlyGly_2 − Scan number: 14111

Gene Names	Charge	m/z	Mass	Retention time	PEP	Score	Precursor Intensity
CALM2;CALM3;CALM1	3	603.3	1806.878	52.756	6.3098e−10	118.33	7075544

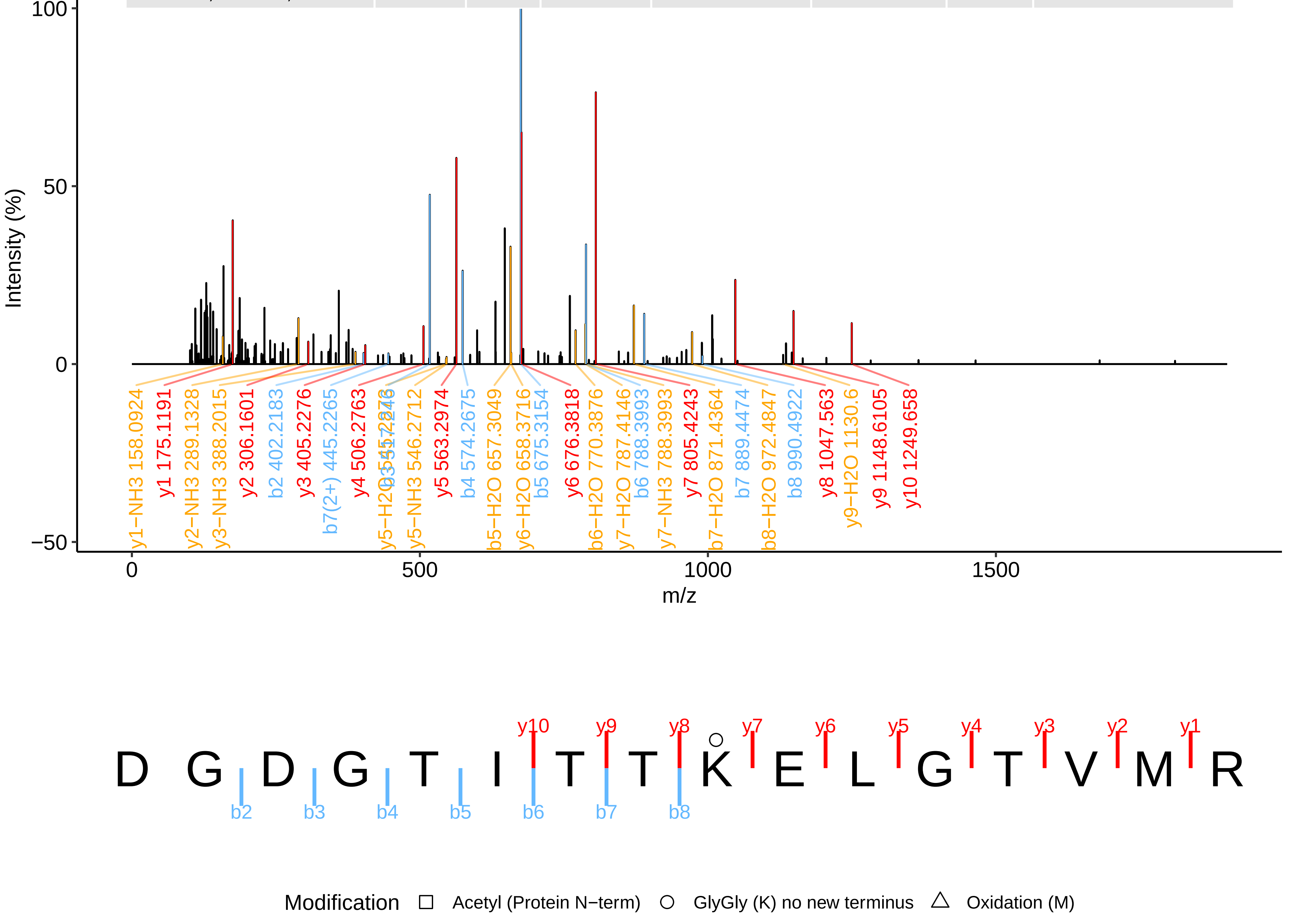

20201118_KIZ_MUb6_GlyGly_2 − Scan number: 16768

Gene Names	Charge	m/z	Mass	Retention time	PEP	Score	Precursor Intensity
CALM2;CALM3;CALM1	3	603.3	1806.878	56.667	2.8375e−20	150.34	9263697

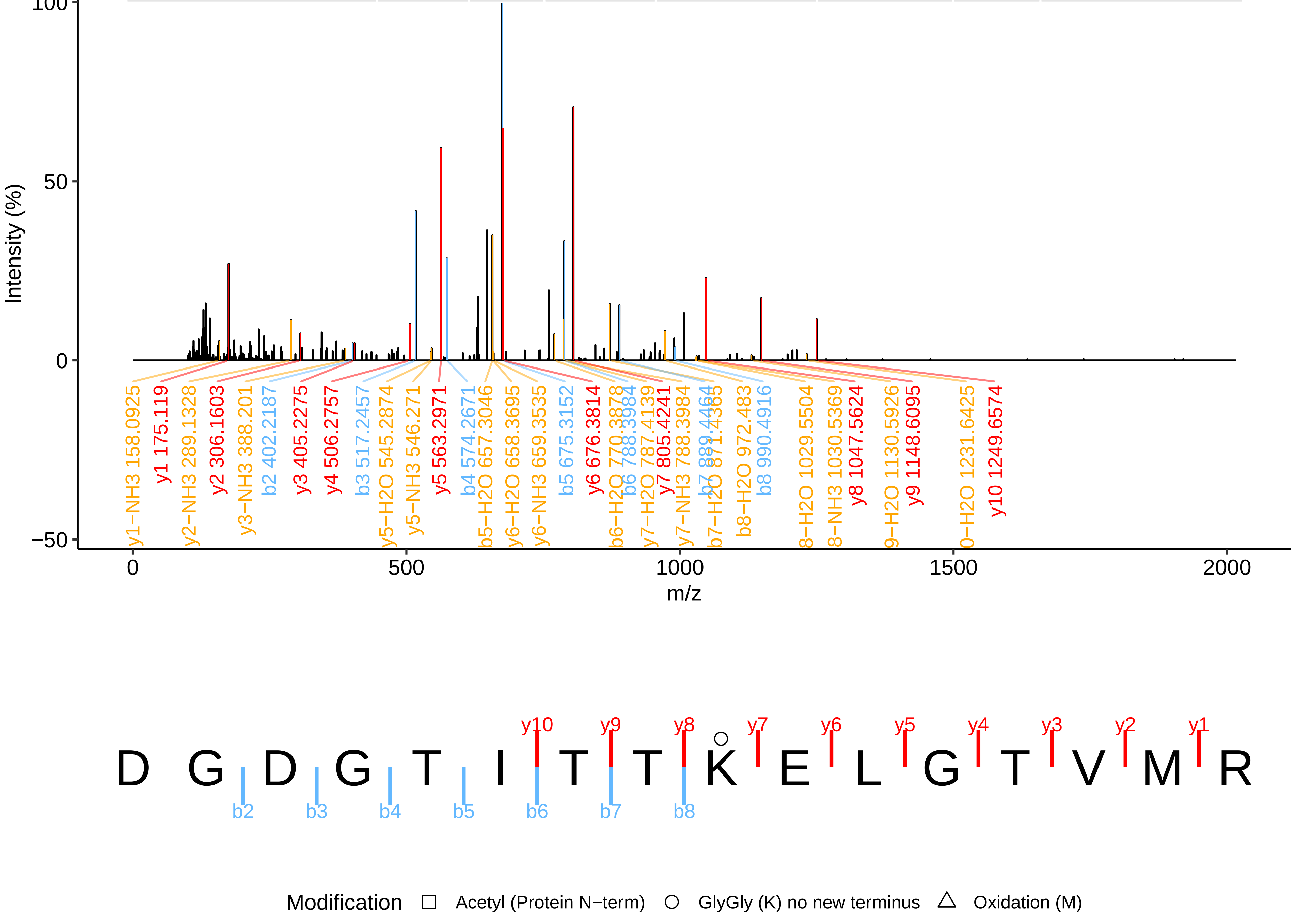

20201118_KIZ_MUb6_GlyGly_2 − Scan number: 16745

Gene Names	Charge	m/z	Mass	Retention time	PEP	Score	Precursor Intensity
CALM2;CALM3;CALM1	3	603.3	1806.878	56.584	7.2518e−14	141	5099326

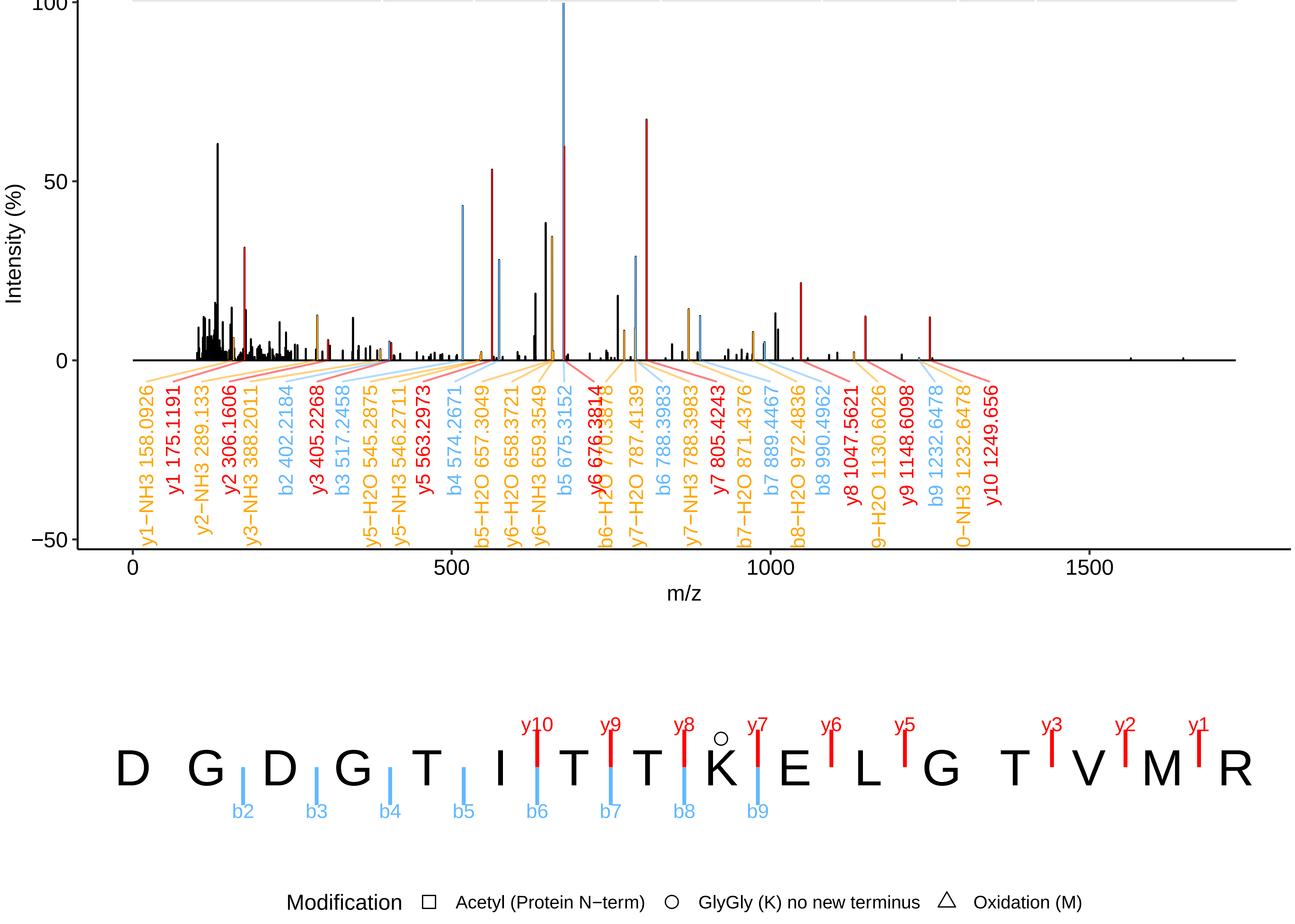

20200921_KIZ_MUb4_GlyGly_10plex − Scan number: 15593

Gene Names	Charge	m/z	Mass	Retention time	PEP	Score	Precursor Intensity
KRT8	3	529.9457	1586.815	55.737	0.00038095	89.466	1786113

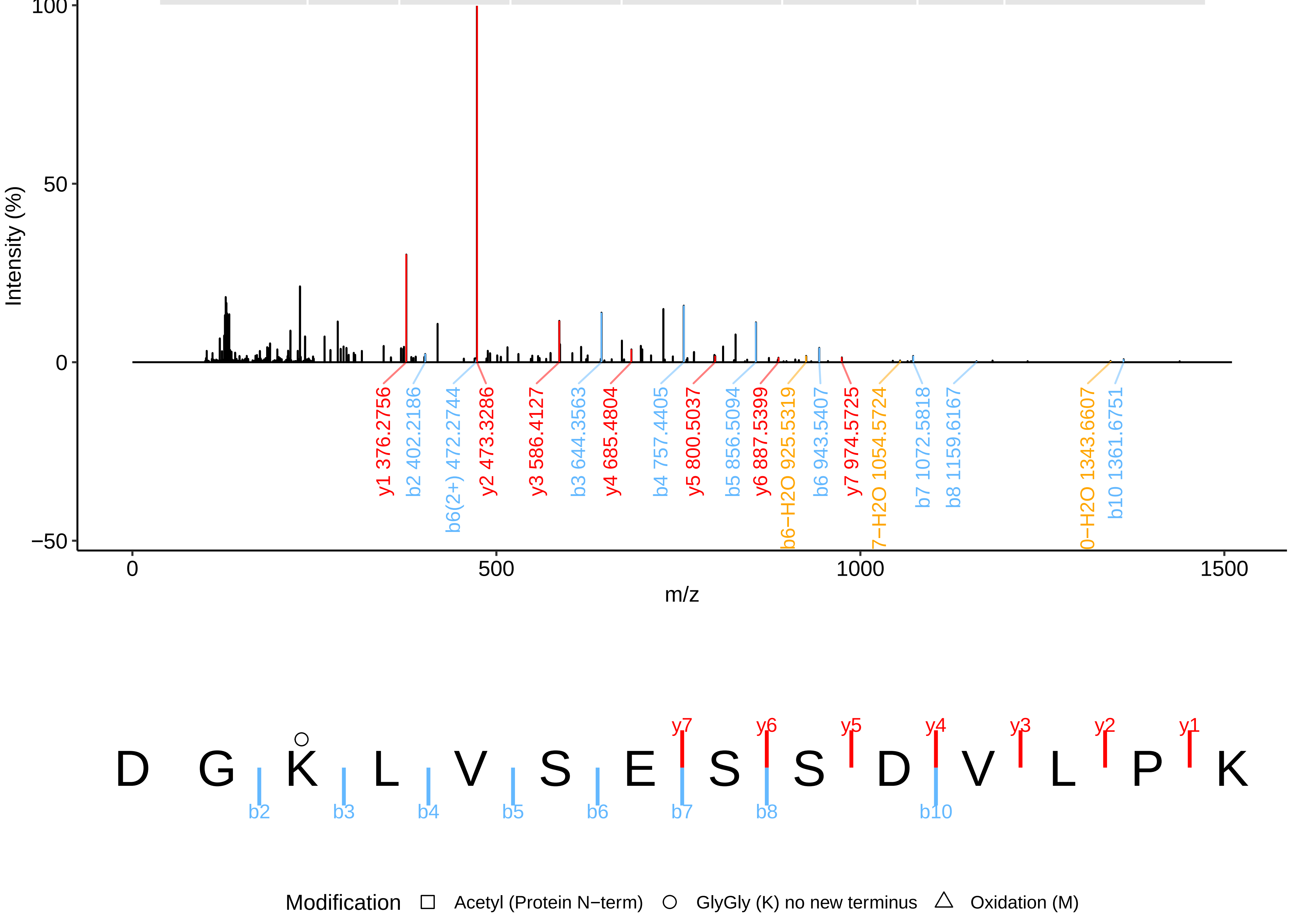

20200921_KIZ_MUb4_GlyGly_10plex_2 − Scan number: 14413

Gene Names	Charge	m/z	Mass	Retention time	PEP	Score	Precursor Intensity
KRT8	3	529.9457	1586.815	56.194	0.035148	55.841	1119750

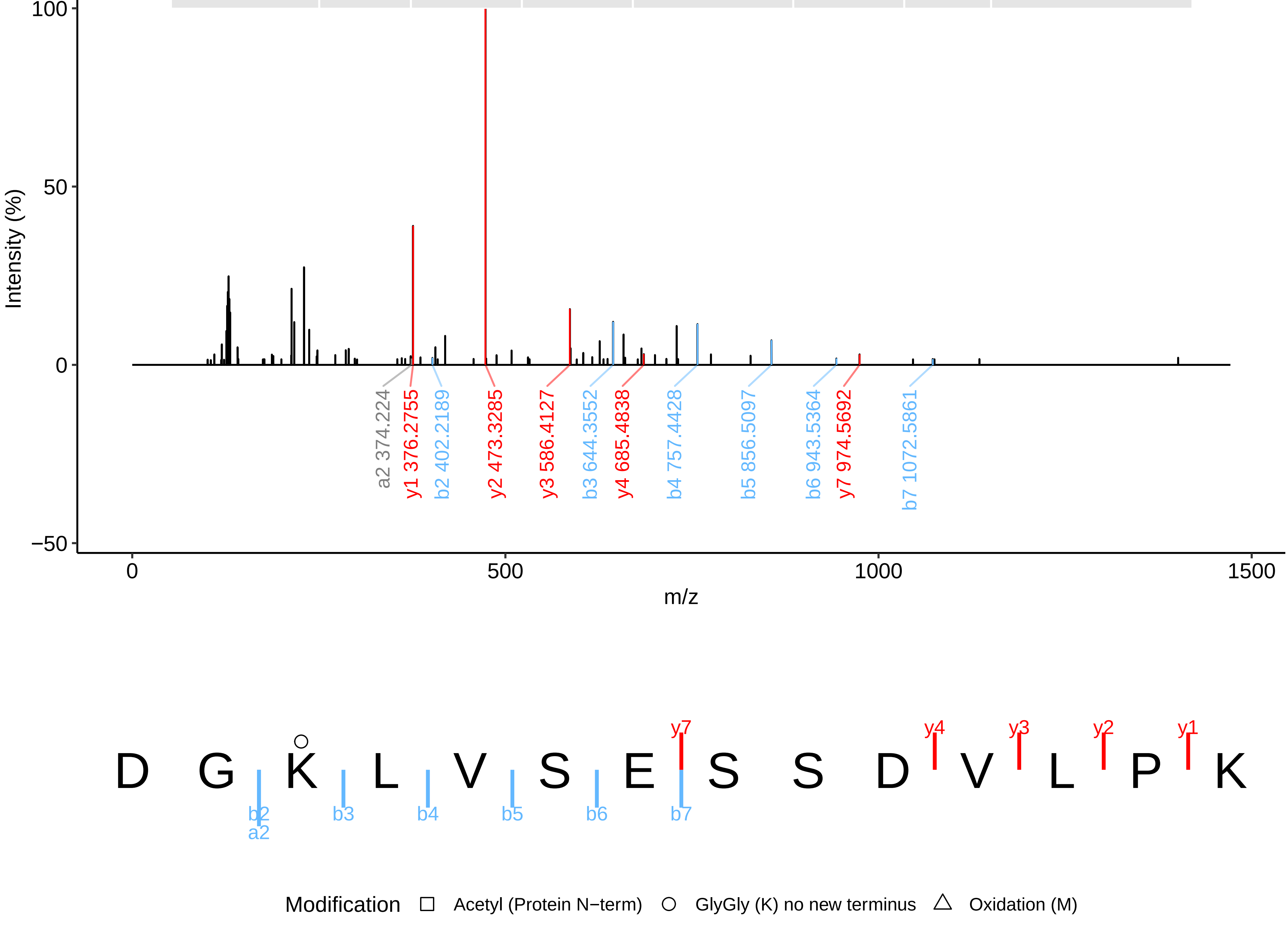

20200921_KIZ_MUb4_GlyGly_10plex − Scan number: 28335

Gene Names	Charge	m/z	Mass	Retention time	PEP	Score	Precursor Intensity
VDAC2	3	519.2804	1554.82	103.66	0.0030004	77.185	2941830

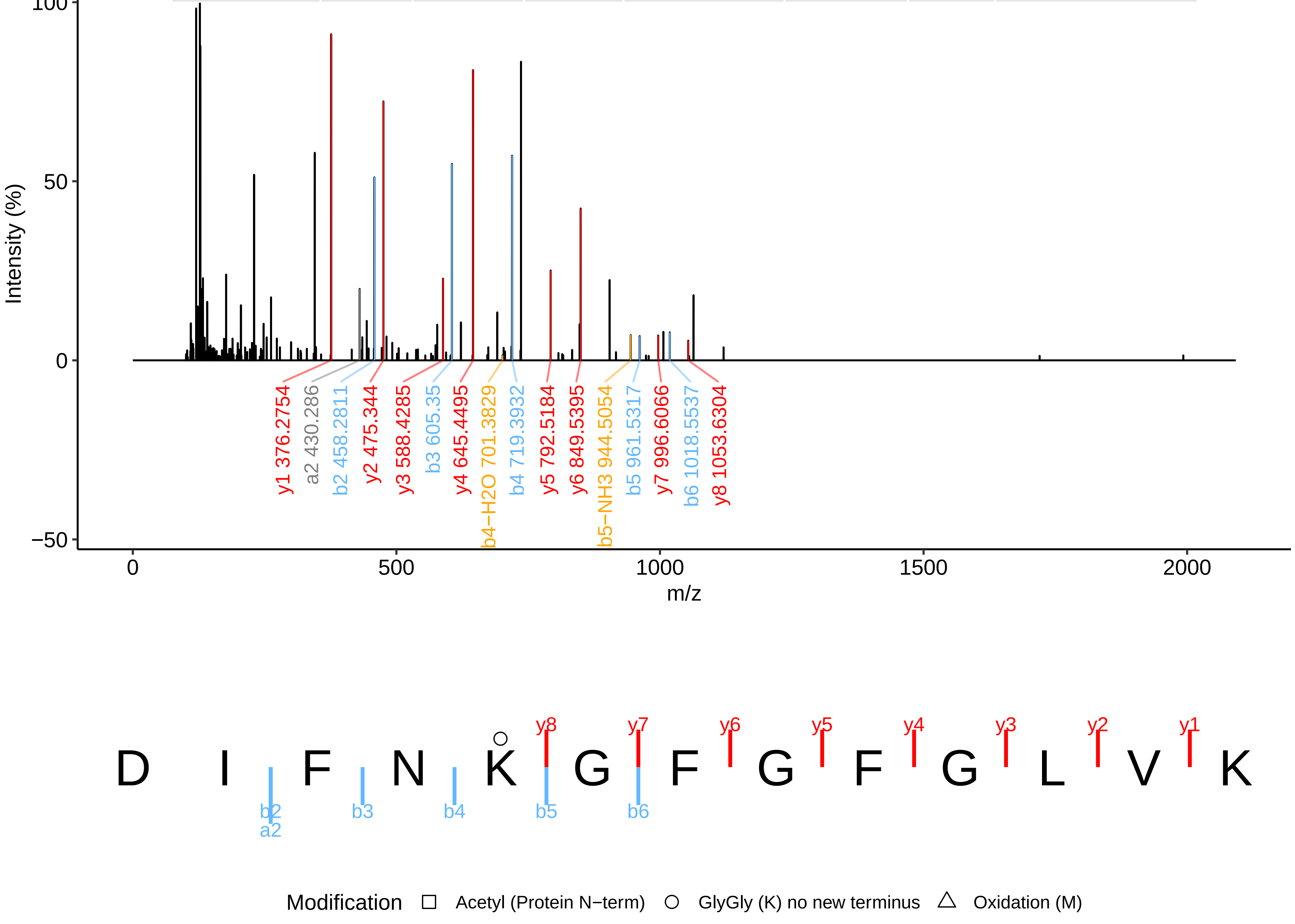

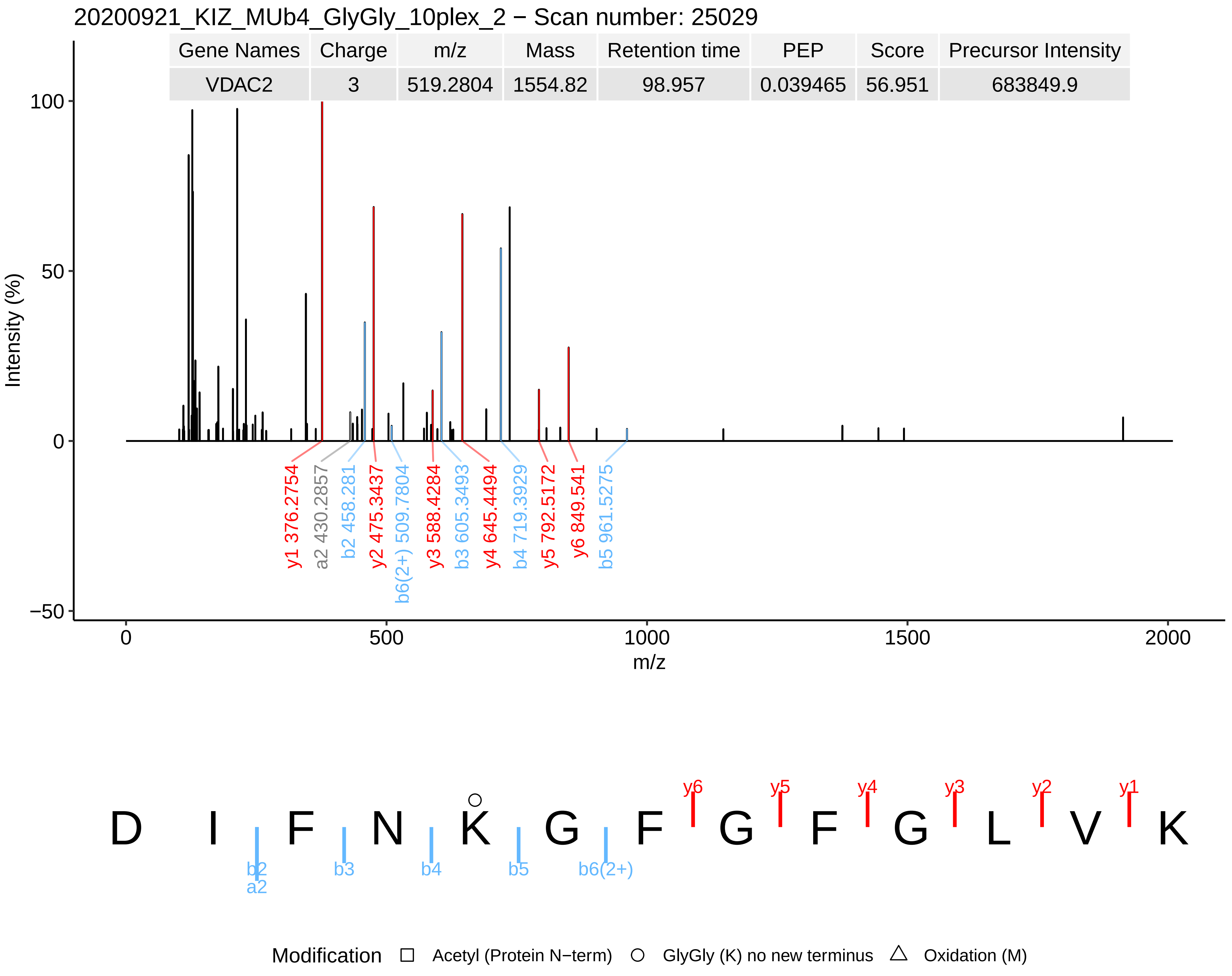

20200921_KIZ_MUb4_GlyGly_10plex_2 − Scan number: 25029
Gene Names | Charge | m/z | Mass | Retention time | PEP | Score | Precursor Intensity
VDAC2 | 3 | 519.2804 | 1554.82 | 98.957 | 0.039465 | 56.951 | 683849.9
Intensity (%)
m/z
y1 376.2754
a2 430.2857
b2 458.281
y2 475.3437
b6(2+) 509.7804
y3 588.4284
b3 605.3493
y4 645.4494
b4 719.3929
y5 792.5172
y6 849.541
b5 961.5275
D I F N K G F G F G L V K
y6 y5 y4 y3 y2 y1
b2 a2 b3 b4 b5 b6(2+)
Modification Acetyl (Protein N−term) GlyGly (K) no new terminus Oxidation (M)

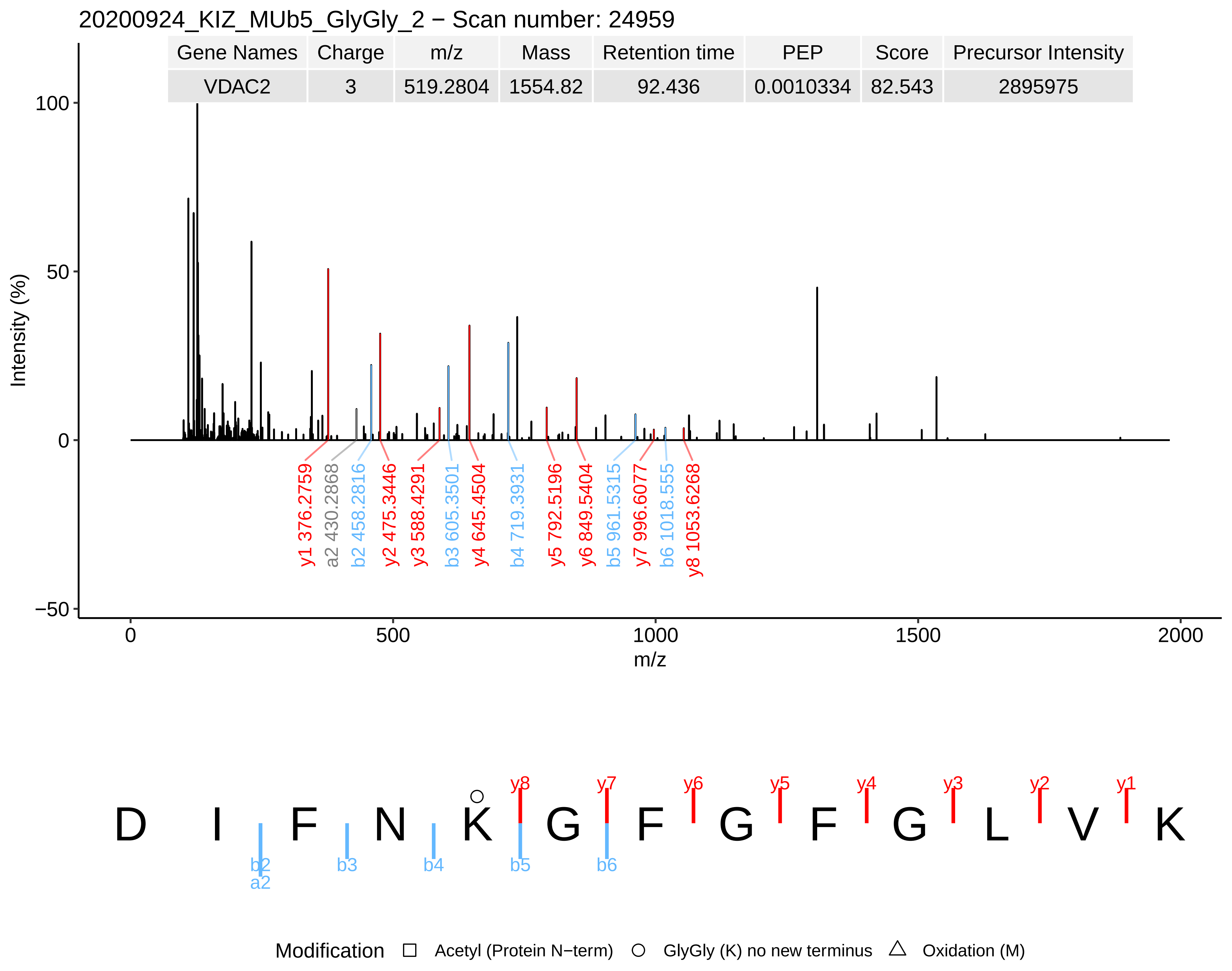
20200924_KIZ_MUb5_GlyGly_2 − Scan number: 24959
Gene Names
Charge
m/z
Mass
Retention time
PEP
Score
Precursor Intensity
VDAC2
3
519.2804
1554.82
92.436
0.0010334
82.543
2895975
Intensity (%)
100
50
0
−50
0
500
1000
1500
2000
m/z
y1 376.2759
a2 430.2868
b2 458.2816
y2 475.3446
y3 588.4291
b3 605.3501
y4 645.4504
b4 719.3931
y5 792.5196
y6 849.5404
b5 961.5315
y7 996.6077
b6 1018.555
y8 1053.6268
D I F N K G F G F G L V K
y8
y7
y6
y5
y4
y3
y2
y1
b2
a2
b3
b4
b5
b6
Modification
Acetyl (Protein N−term)
GlyGly (K) no new terminus
Oxidation (M)

20200921_KIZ_MUb4_GlyGly_10plex_2 − Scan number: 12270

Gene Names	Charge	m/z	Mass	Retention time	PEP	Score	Precursor Intensity
ITFG3	4	452.48	1805.891	47.434	1.5309e−08	116.51	1583437

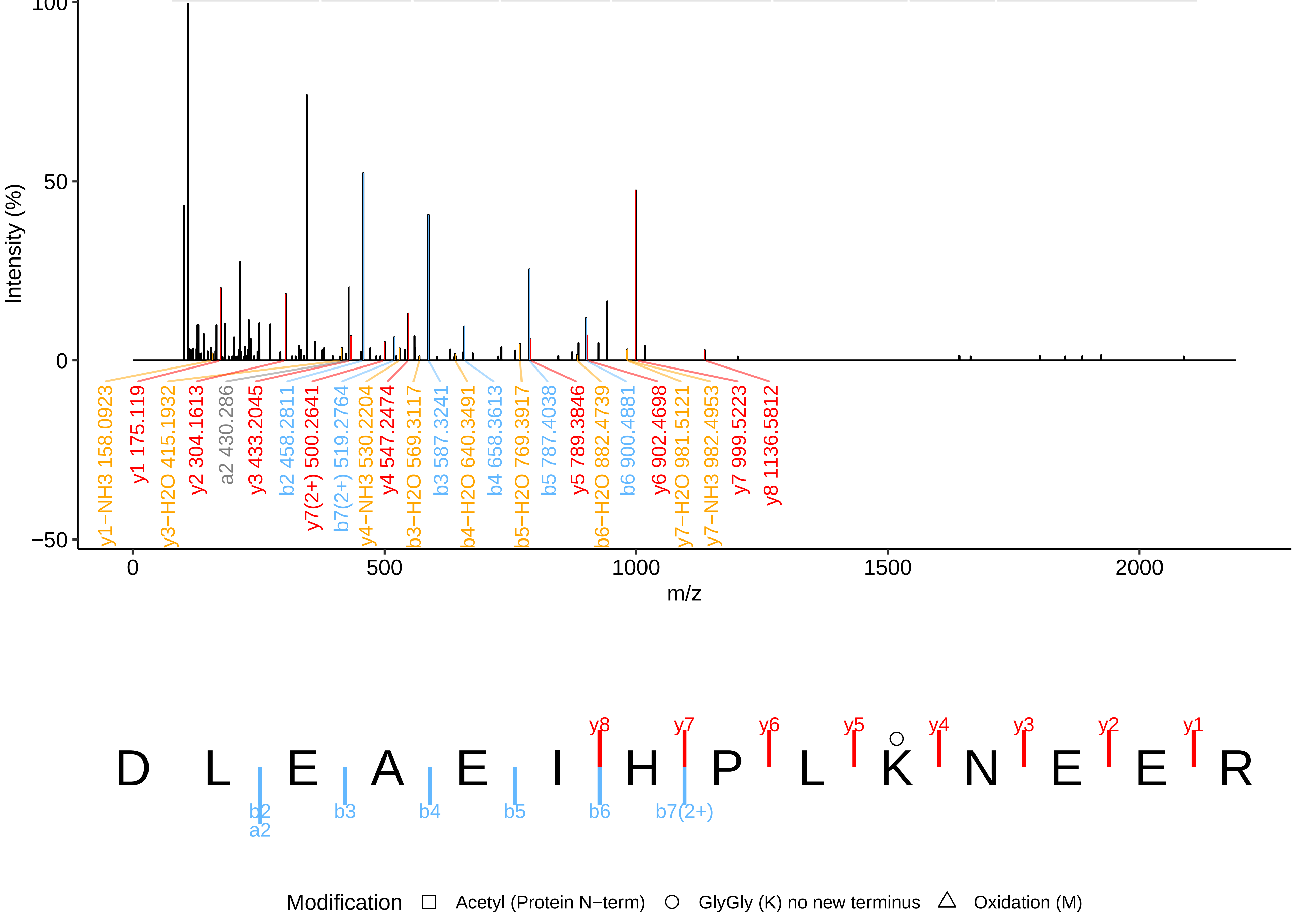

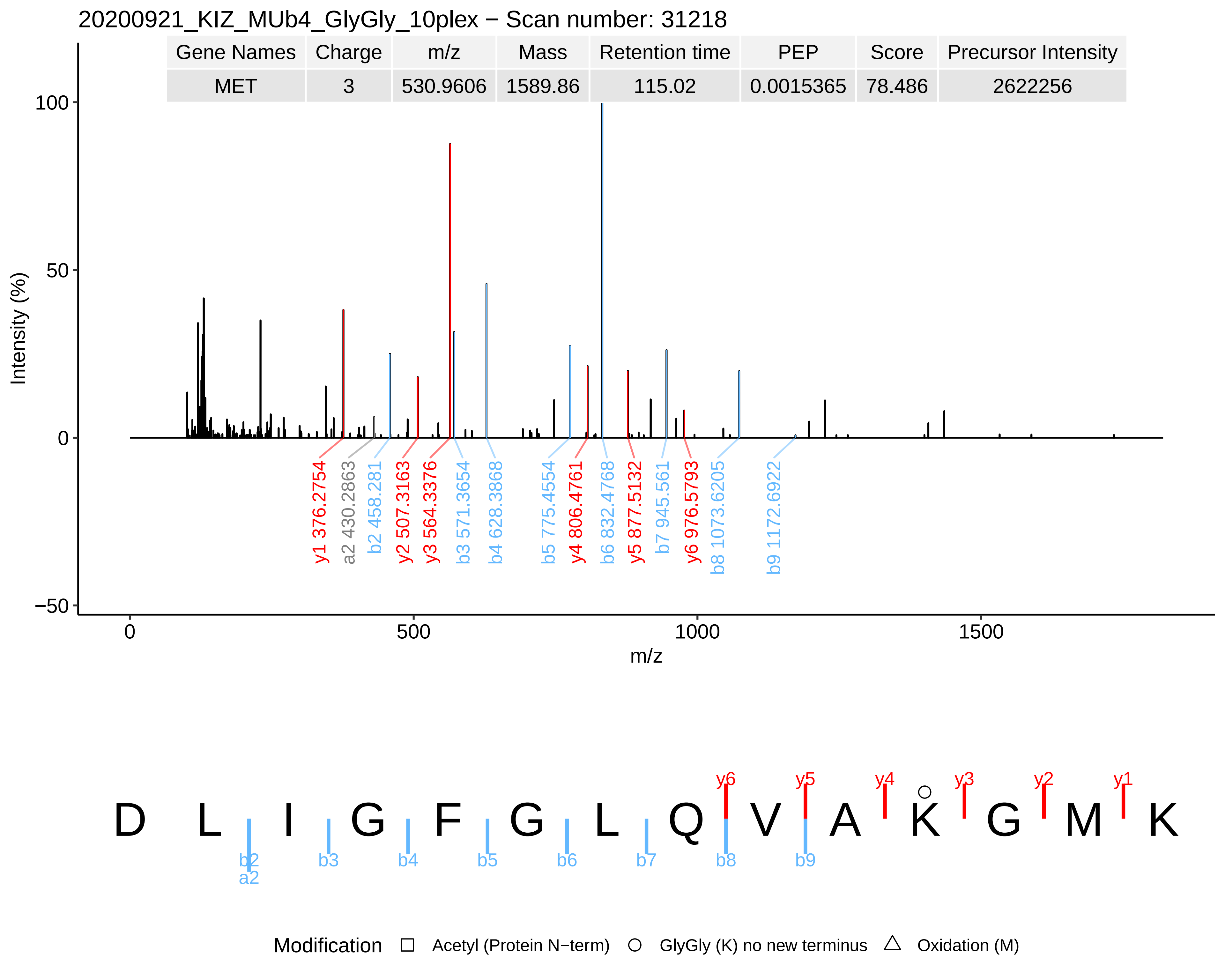
20200921_KIZ_MUb4_GlyGly_10plex − Scan number: 31218
Gene Names | Charge | m/z | Mass | Retention time | PEP | Score | Precursor Intensity
MET | 3 | 530.9606 | 1589.86 | 115.02 | 0.0015365 | 78.486 | 2622256
Intensity (%)
100
50
0
−50
0
500
1000
1500
m/z
y1 376.2754
a2 430.2863
b2 458.281
y2 507.3163
y3 564.3376
b3 571.3654
b4 628.3868
b5 775.4554
y4 806.4761
b6 832.4768
y5 877.5132
b7 945.561
y6 976.5793
b8 1073.6205
b9 1172.6922
D L I G F G L Q V A K G M K
y6 y5 y4 y3 y2 y1
b2 a2 b3 b4 b5 b6 b7 b8 b9
Modification
Acetyl (Protein N−term)
GlyGly (K) no new terminus
Oxidation (M)

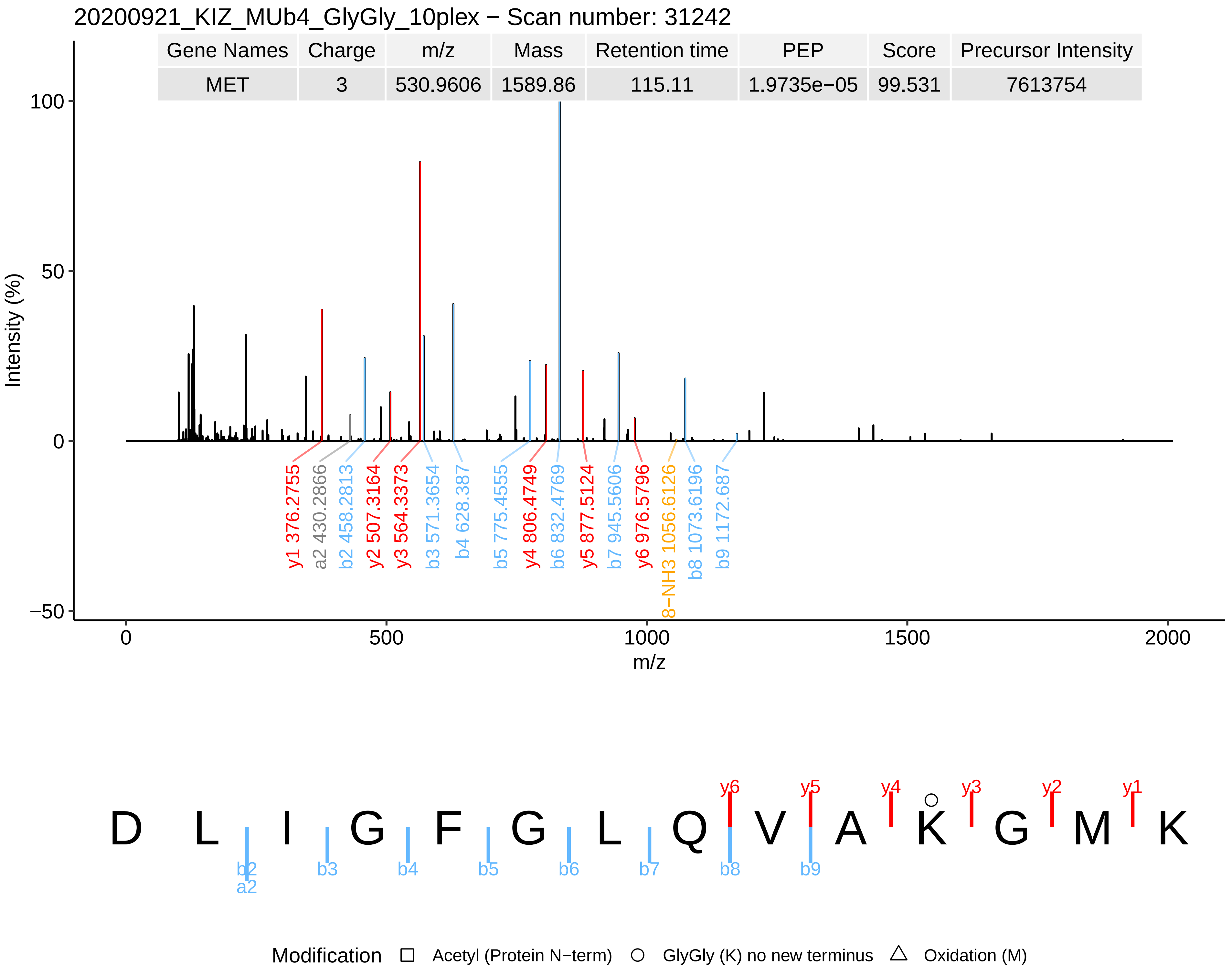

20200921_KIZ_MUb4_GlyGly_10plex − Scan number: 31242
Gene Names | Charge | m/z | Mass | Retention time | PEP | Score | Precursor Intensity
MET | 3 | 530.9606 | 1589.86 | 115.11 | 1.9735e−05 | 99.531 | 7613754
Intensity (%)
m/z
y1 376.2755
a2 430.2866
b2 458.2813
y2 507.3164
y3 564.3373
b3 571.3654
b4 628.387
b5 775.4555
y4 806.4749
b6 832.4769
y5 877.5124
b7 945.5606
y6 976.5796
8−NH3 1056.6126
b8 1073.6196
b9 1172.687
D L I G F G L Q V A K G M K
Modification
Acetyl (Protein N−term)
GlyGly (K) no new terminus
Oxidation (M)

20200921_KIZ_MUb4_GlyGly_10plex_2 − Scan number: 18006

Gene Names	Charge	m/z	Mass	Retention time	PEP	Score	Precursor Intensity
CDC2;CDK1	3	509.2789	1524.815	70.466	0.071583	51.875	1718776

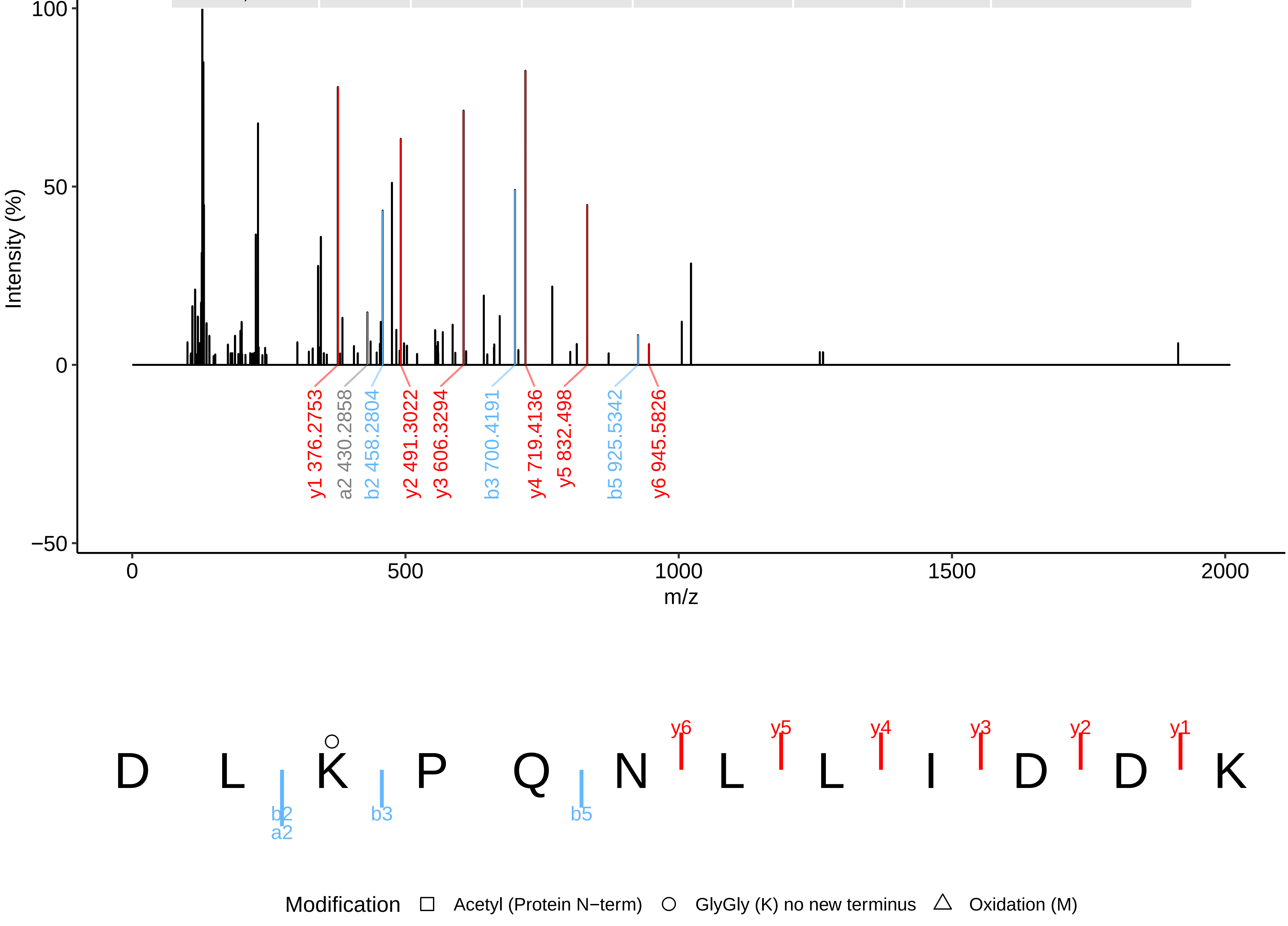

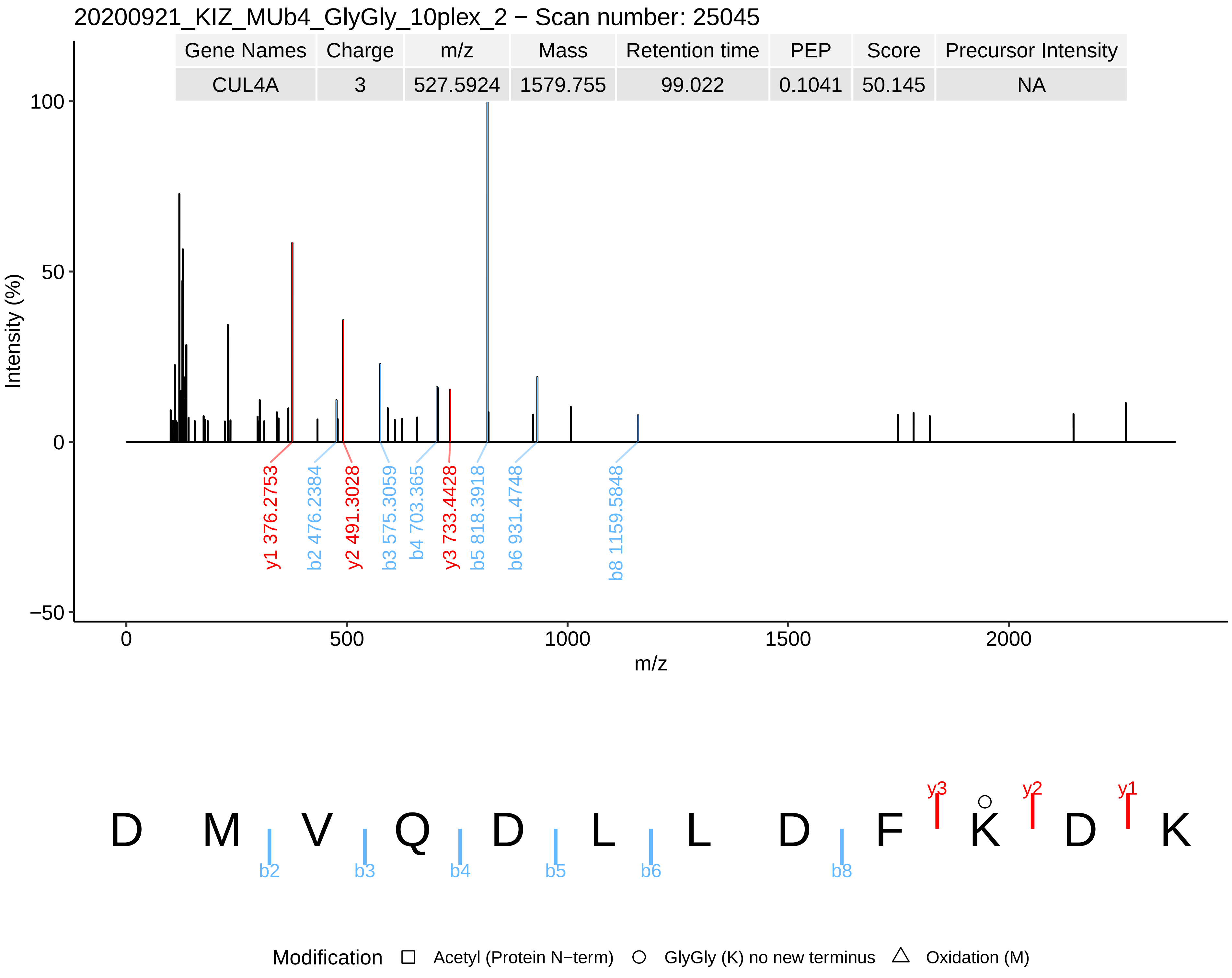
20200921_KIZ_MUb4_GlyGly_10plex_2 − Scan number: 25045
Gene Names
Charge
m/z
Mass
Retention time
PEP
Score
Precursor Intensity
CUL4A
3
527.5924
1579.755
99.022
0.1041
50.145
NA
Intensity (%)
m/z
y1 376.2753
b2 476.2384
y2 491.3028
b3 575.3059
b4 703.365
y3 733.4428
b5 818.3918
b6 931.4748
b8 1159.5848
D M V Q D L L D F K D K
Modification
Acetyl (Protein N−term)
GlyGly (K) no new terminus
Oxidation (M)

20201118_KIZ_MUb6_GlyGly_1 − Scan number: 3158

Gene Names	Charge	m/z	Mass	Retention time	PEP	Score	Precursor Intensity
OLR1	4	340.6679	1358.643	9.7341	3.6663e−05	113.22	12004840

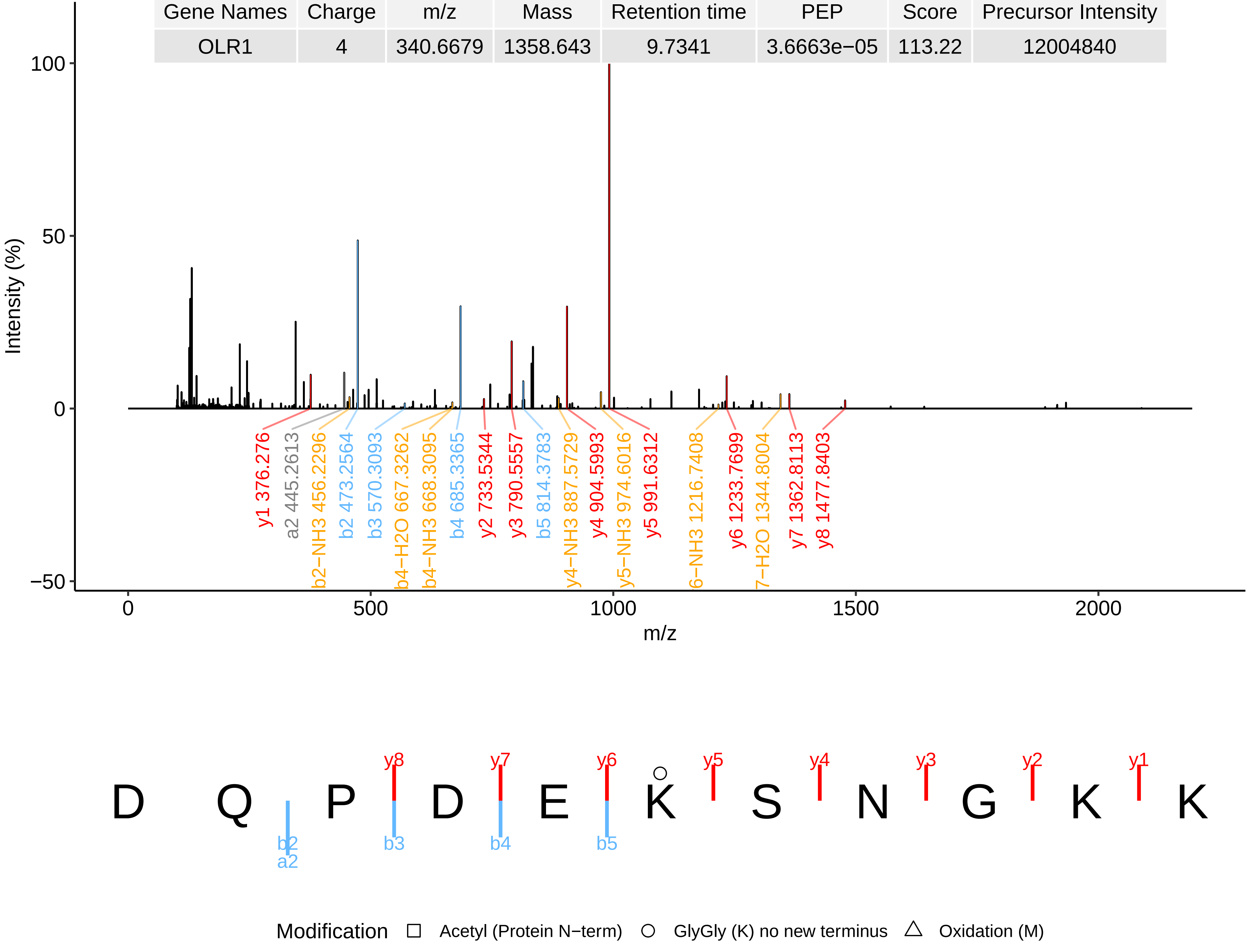

20201118_KIZ_MUb6_GlyGly_2 − Scan number: 3556

Gene Names	Charge	m/z	Mass	Retention time	PEP	Score	Precursor Intensity
OLR1	4	340.6679	1358.643	11.809	0.008195	85.288	3391890

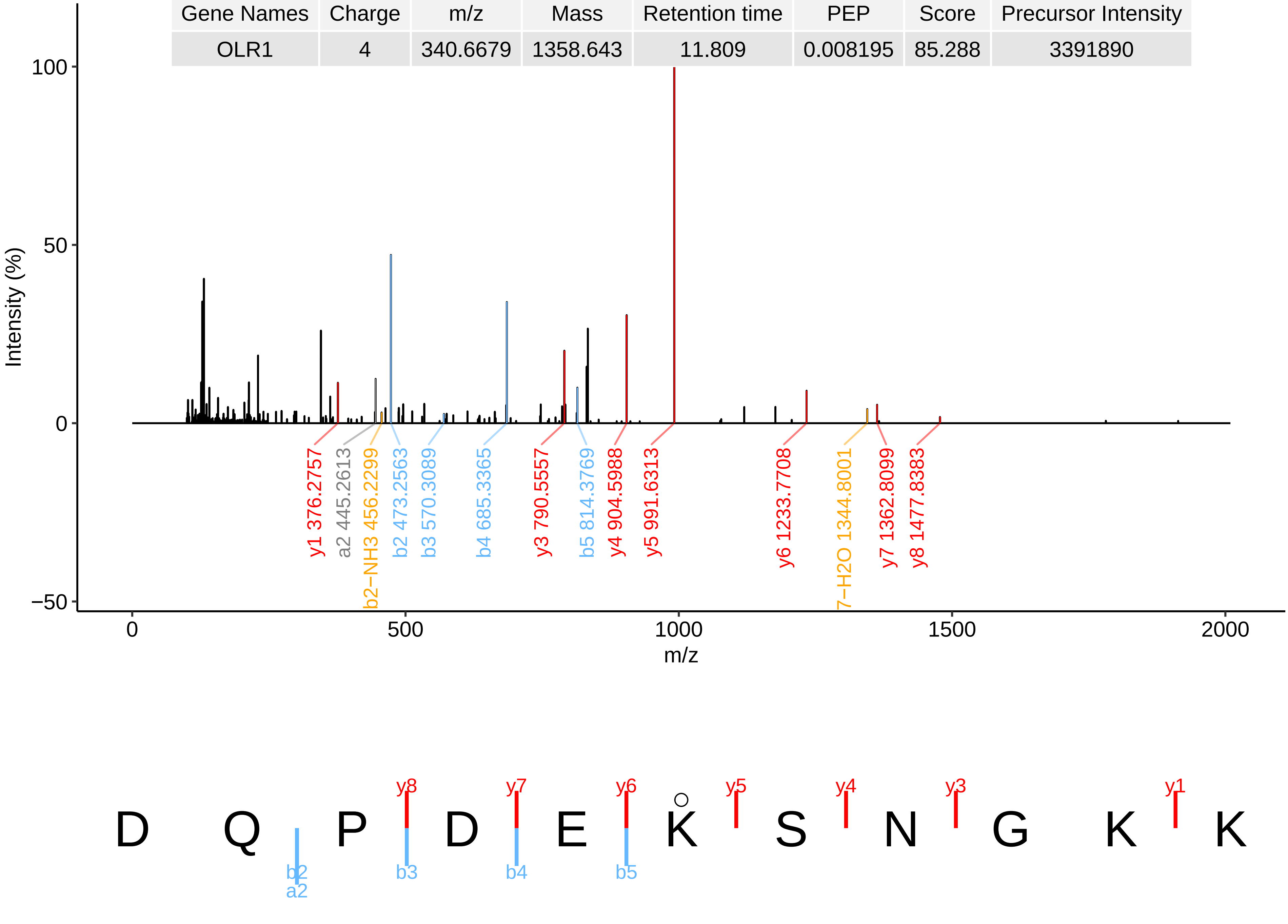

Modification □ Acetyl (Protein N−term) ○ GlyGly (K) no new terminus △ Oxidation (M)

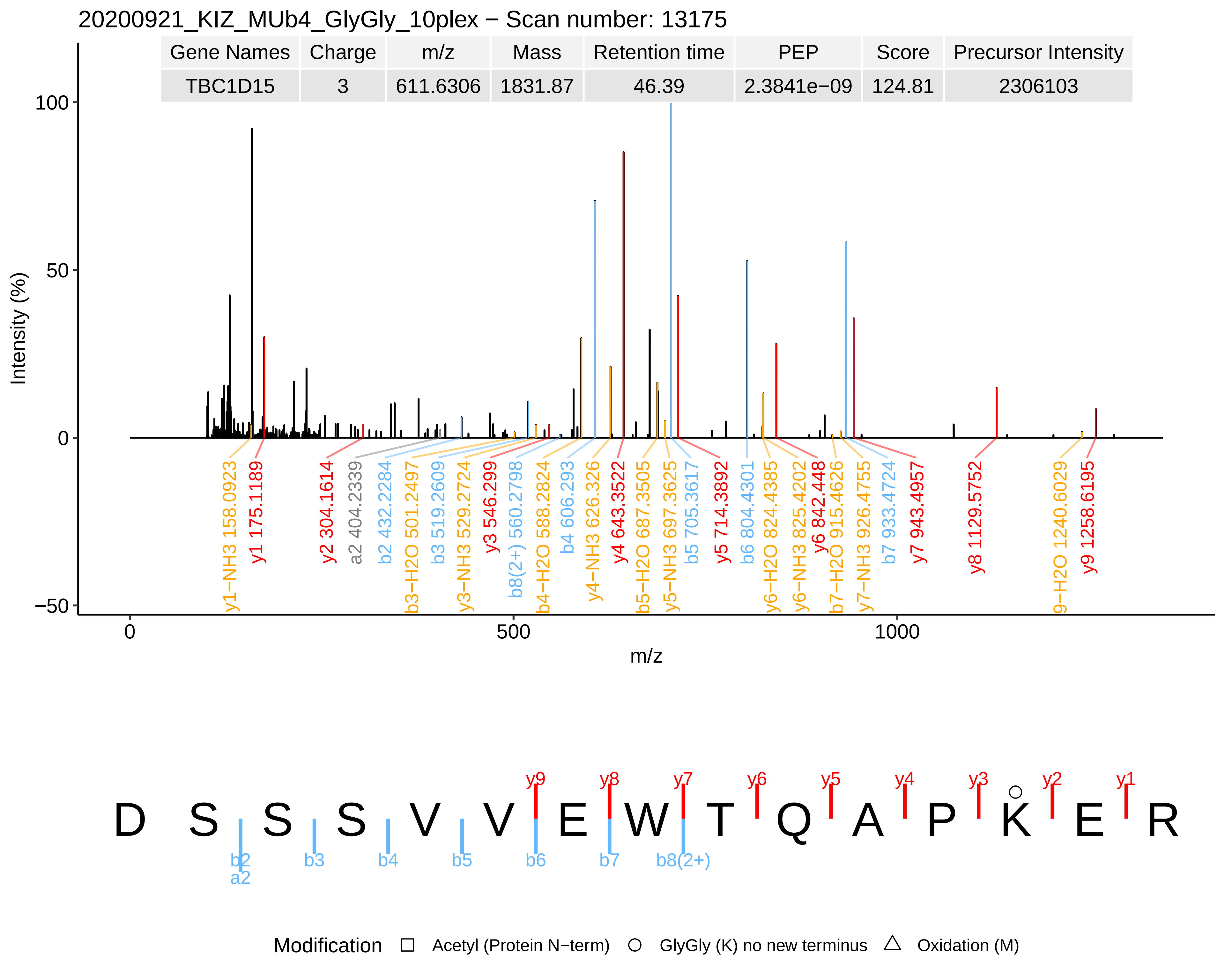
20200921_KIZ_MUb4_GlyGly_10plex − Scan number: 13175
Gene Names | Charge | m/z | Mass | Retention time | PEP | Score | Precursor Intensity
TBC1D15 | 3 | 611.6306 | 1831.87 | 46.39 | 2.3841e−09 | 124.81 | 2306103
Intensity (%)
m/z
y1−NH3 158.0923
y1 175.1189
y2 304.1614
a2 404.2339
b2 432.2284
b3−H2O 501.2497
b3 519.2609
y3−NH3 529.2724
y3 546.299
b8(2+) 560.2798
b4−H2O 588.2824
b4 606.293
y4−NH3 626.326
y4 643.3522
b5−H2O 687.3505
y5−NH3 697.3625
b5 705.3617
y5 714.3892
b6 804.4301
y6−H2O 824.4385
y6−NH3 825.4202
y6 842.448
b7−H2O 915.4626
y7−NH3 926.4755
b7 933.4724
y7 943.4957
y8 1129.5752
y9−H2O 1240.6029
y9 1258.6195
D S S S V V E W T Q A P K E R
Modification □ Acetyl (Protein N−term) ○ GlyGly (K) no new terminus △ Oxidation (M)

20200921_KIZ_MUb4_GlyGly_10plex_2 − Scan number: 12099

Gene Names	Charge	m/z	Mass	Retention time	PEP	Score	Precursor Intensity
TBC1D15	3	611.6306	1831.87	46.739	5.4834e−05	92.939	814308.9

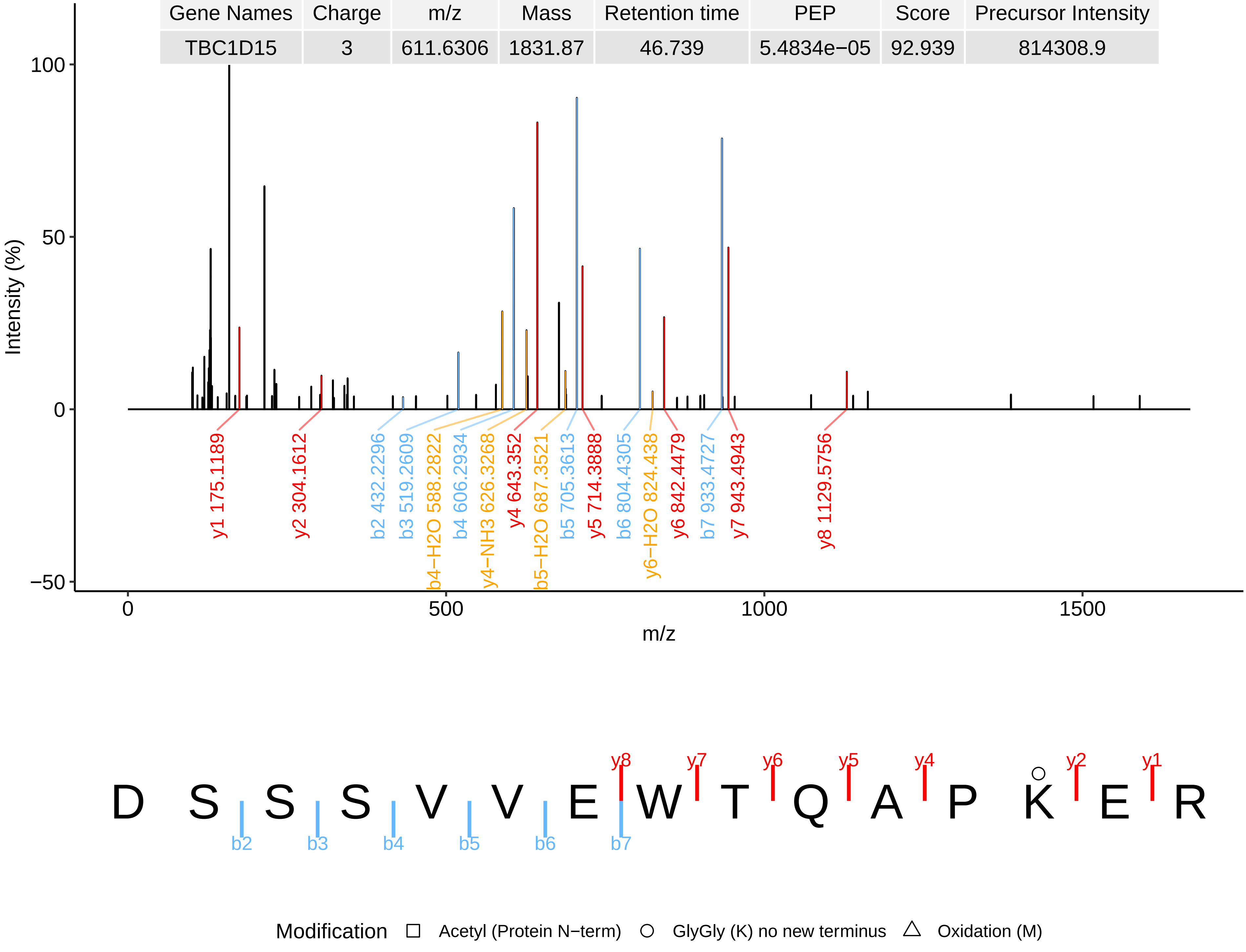

20200921_KIZ_MUb4_GlyGly_10plex_2 − Scan number: 12111

Gene Names	Charge	m/z	Mass	Retention time	PEP	Score	Precursor Intensity
TBC1D15	3	611.6306	1831.87	46.787	0.089683	45.614	357683.6

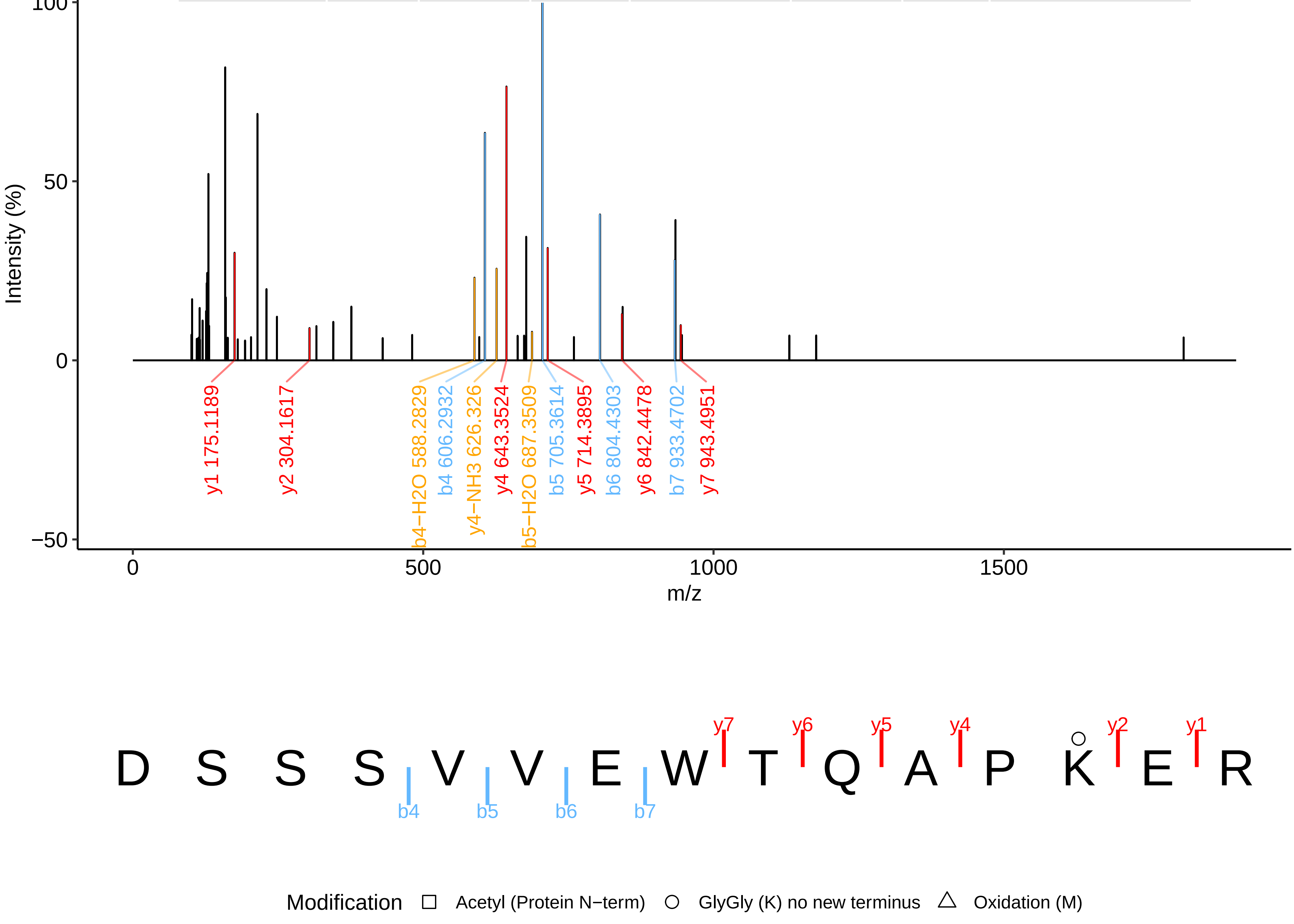

20200921_KIZ_MUb4_GlyGly_10plex − Scan number: 8876

Gene Names	Charge	m/z	Mass	Retention time	PEP	Score	Precursor Intensity
RPS20	4	441.4825	1761.901	30.259	0.024996	57.796	8986950

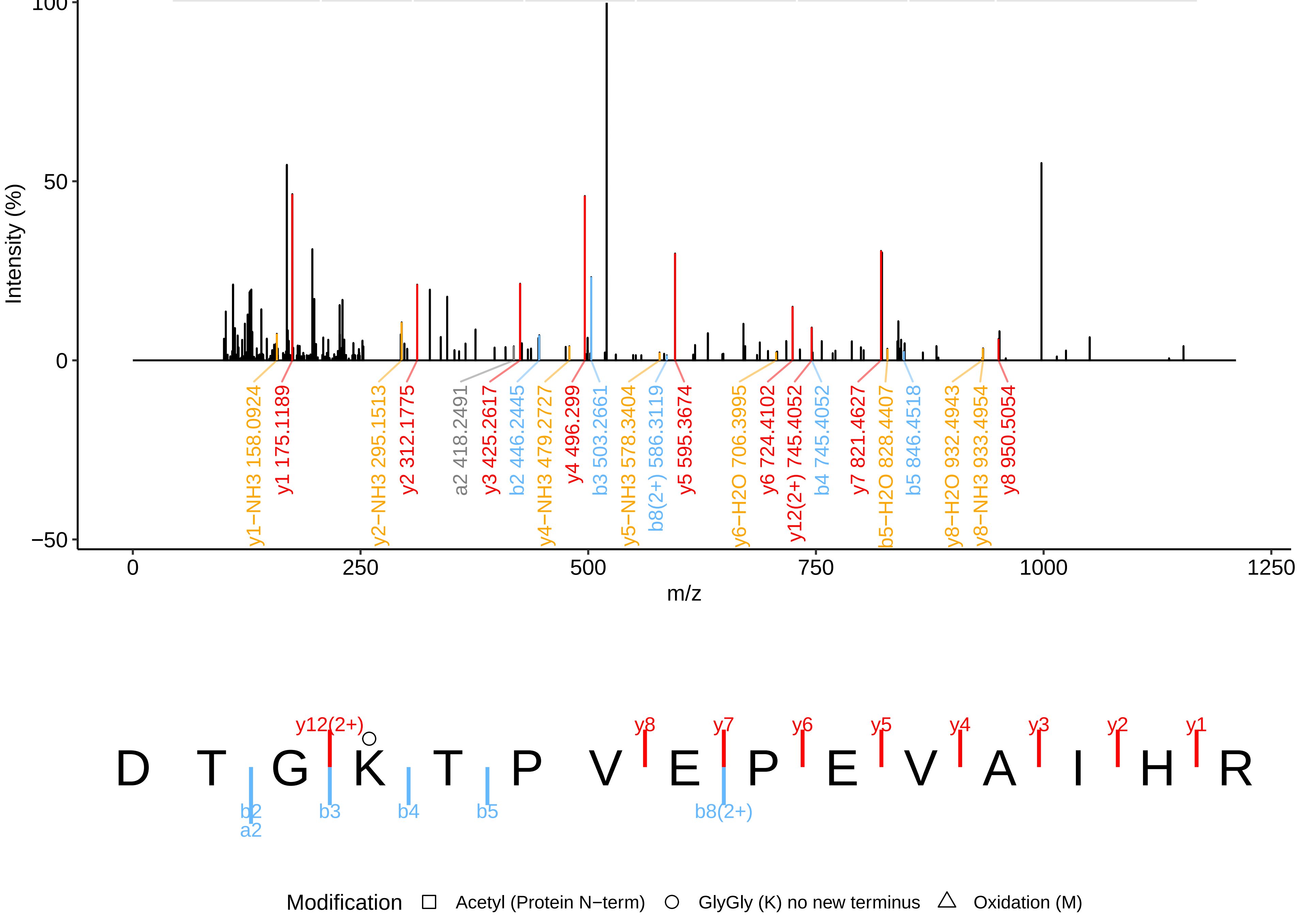

20200921_KIZ_MUb4_GlyGly_10plex − Scan number: 8938

Gene Names	Charge	m/z	Mass	Retention time	PEP	Score	Precursor Intensity
RPS20	3	588.3076	1761.901	30.492	0.0021203	72.209	7367209

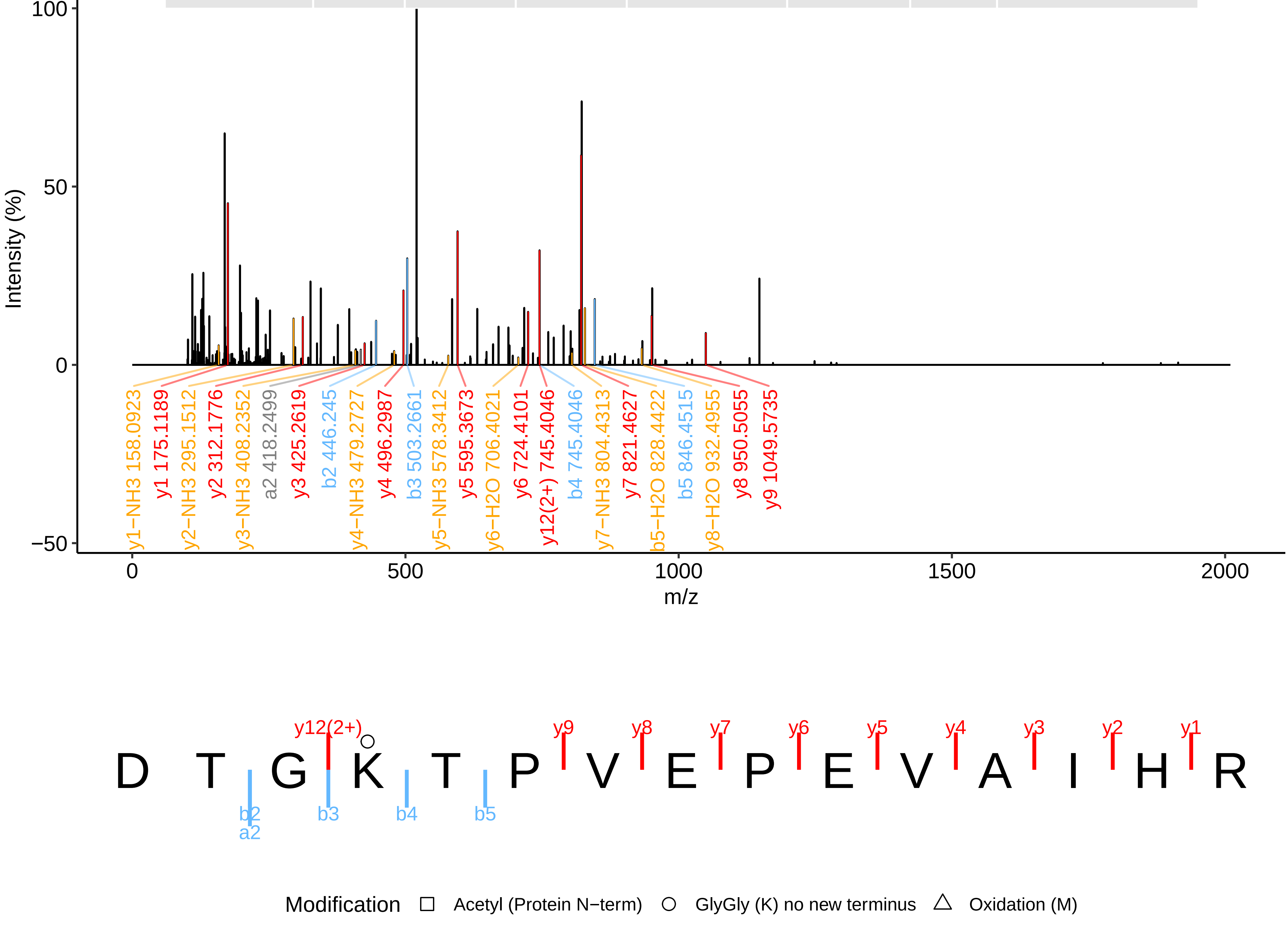

20200921_KIZ_MUb4_GlyGly_10plex_2 − Scan number: 8200

Gene Names	Charge	m/z	Mass	Retention time	PEP	Score	Precursor Intensity
RPS20	4	441.4825	1761.901	30.842	1.7696e−06	107.99	5305092

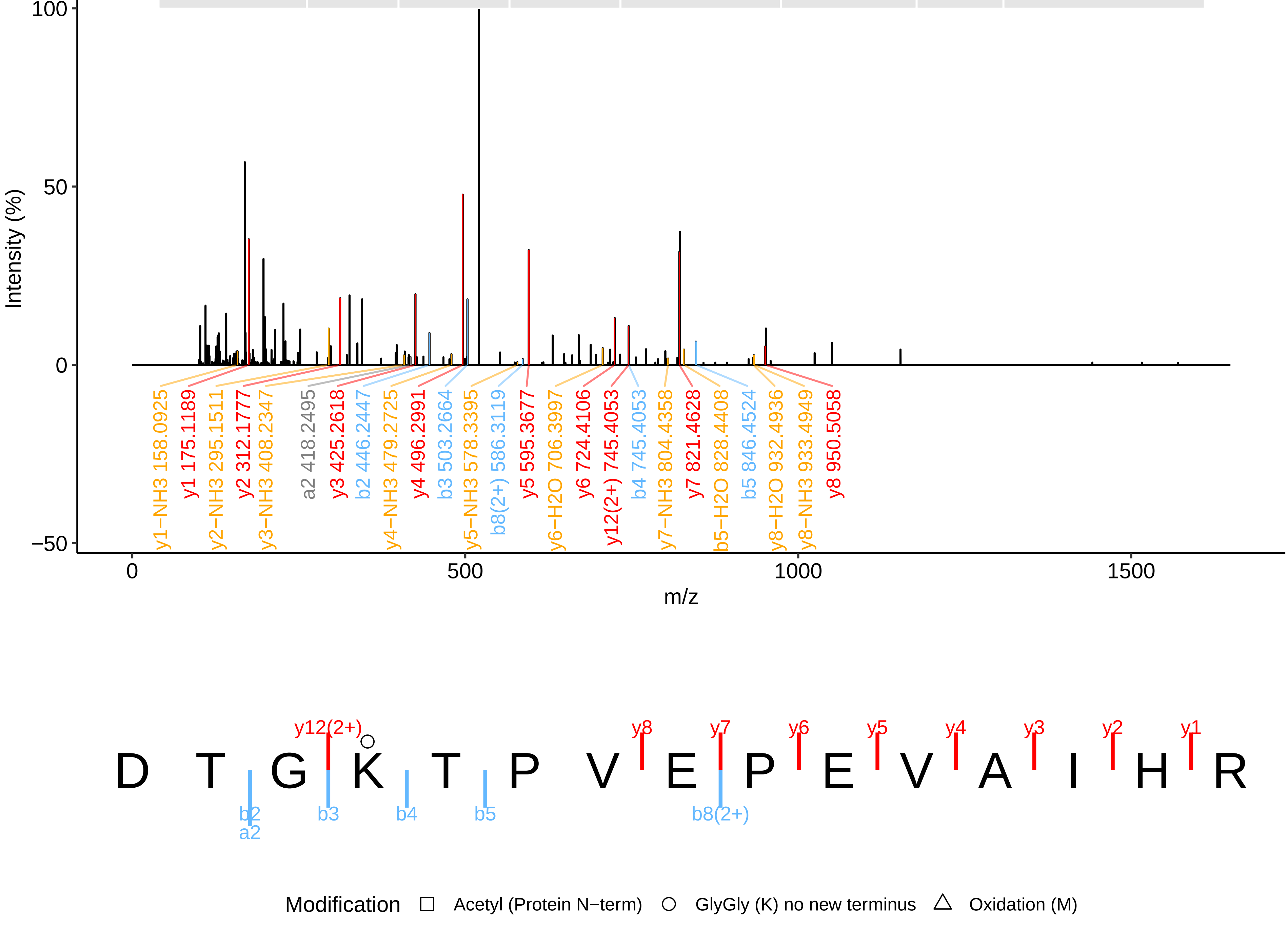

20200921_KIZ_MUb4_GlyGly_10plex_2 − Scan number: 8233

Gene Names	Charge	m/z	Mass	Retention time	PEP	Score	Precursor Intensity
RPS20	3	588.3076	1761.901	30.956	3.8804e−06	109.83	2942412

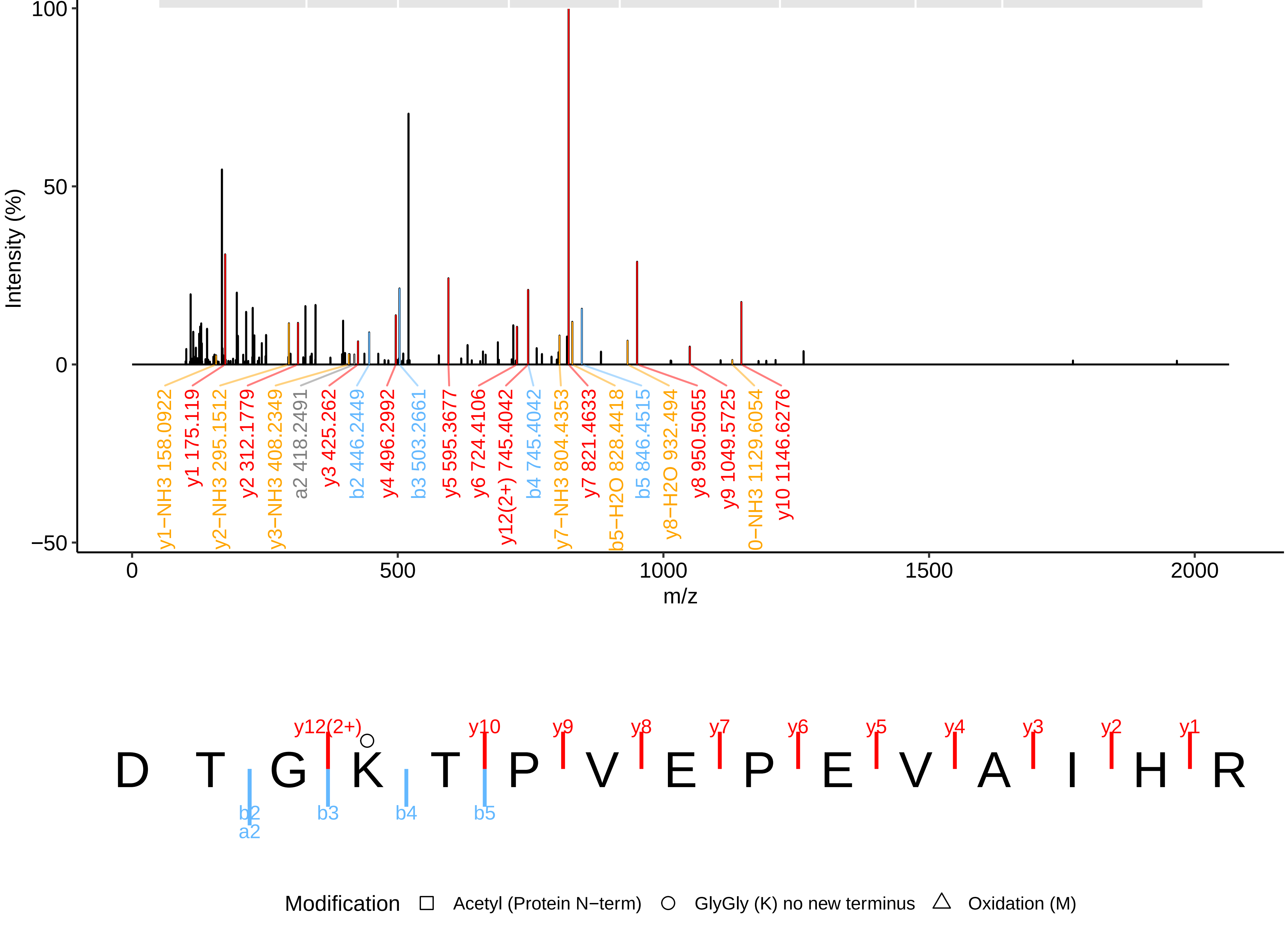

20200924_KIZ_MUb5_GlyGly_1 − Scan number: 7852

Gene Names	Charge	m/z	Mass	Retention time	PEP	Score	Precursor Intensity
RPS20	4	441.4825	1761.901	25.014	0.0009532	76.358	17461188

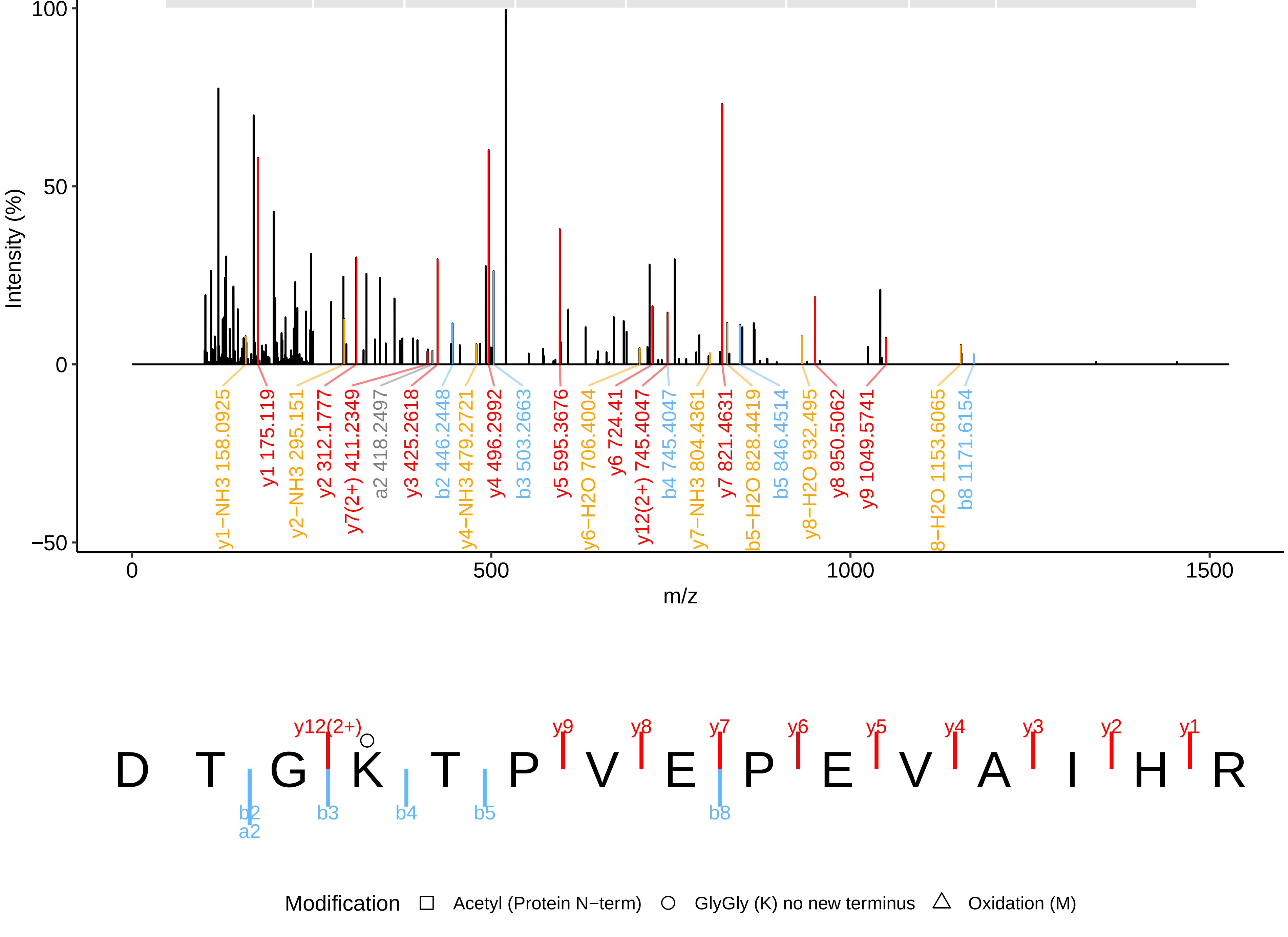

20200924_KIZ_MUb5_GlyGly_1 − Scan number: 8015

Gene Names	Charge	m/z	Mass	Retention time	PEP	Score	Precursor Intensity
RPS20	4	441.4825	1761.901	25.534	4.0604e−07	113.72	22727912

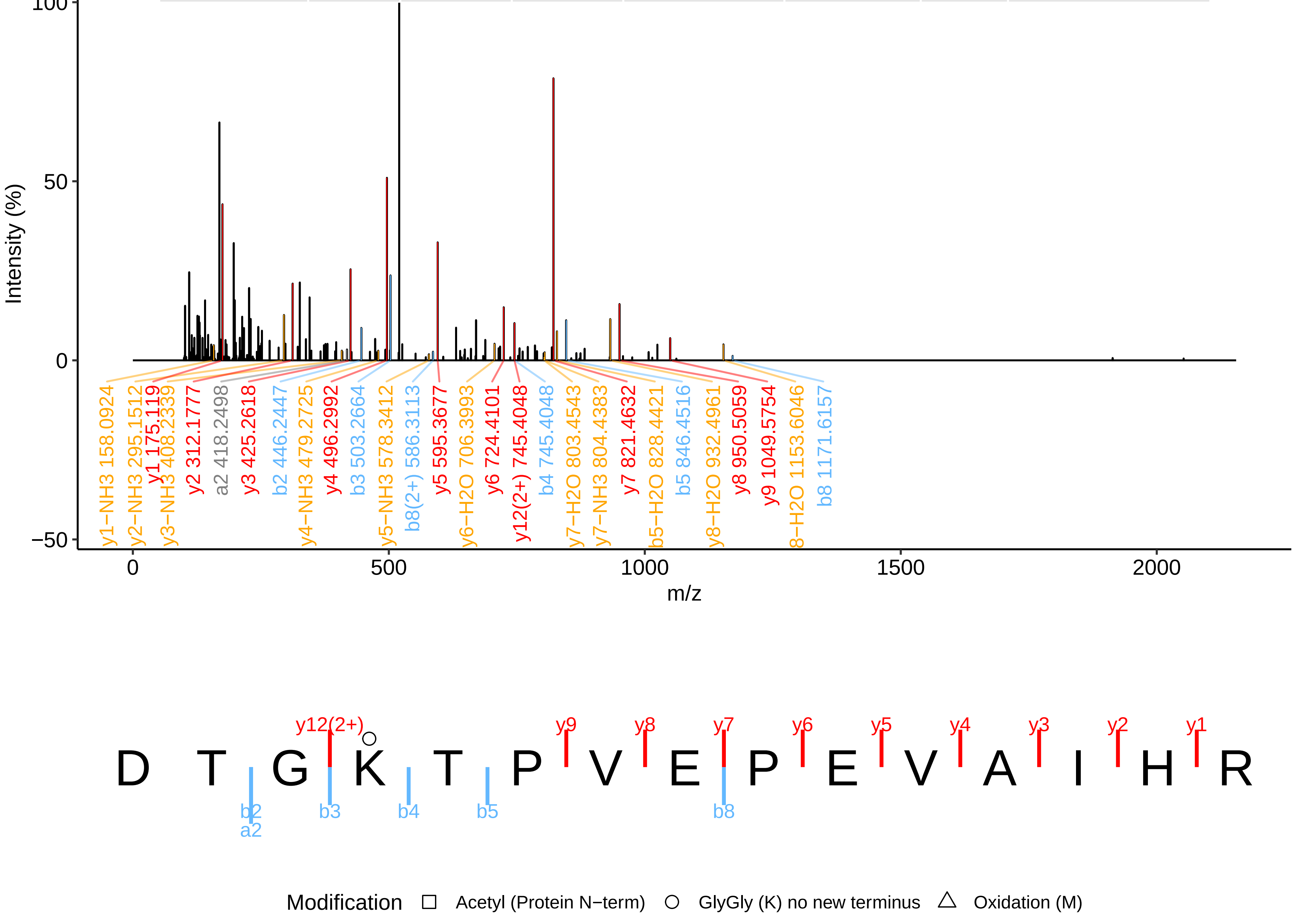

20200924_KIZ_MUb5_GlyGly_1 − Scan number: 8239

Gene Names	Charge	m/z	Mass	Retention time	PEP	Score	Precursor Intensity
RPS20	4	441.4825	1761.901	26.297	1.3308e−06	109.84	7794666

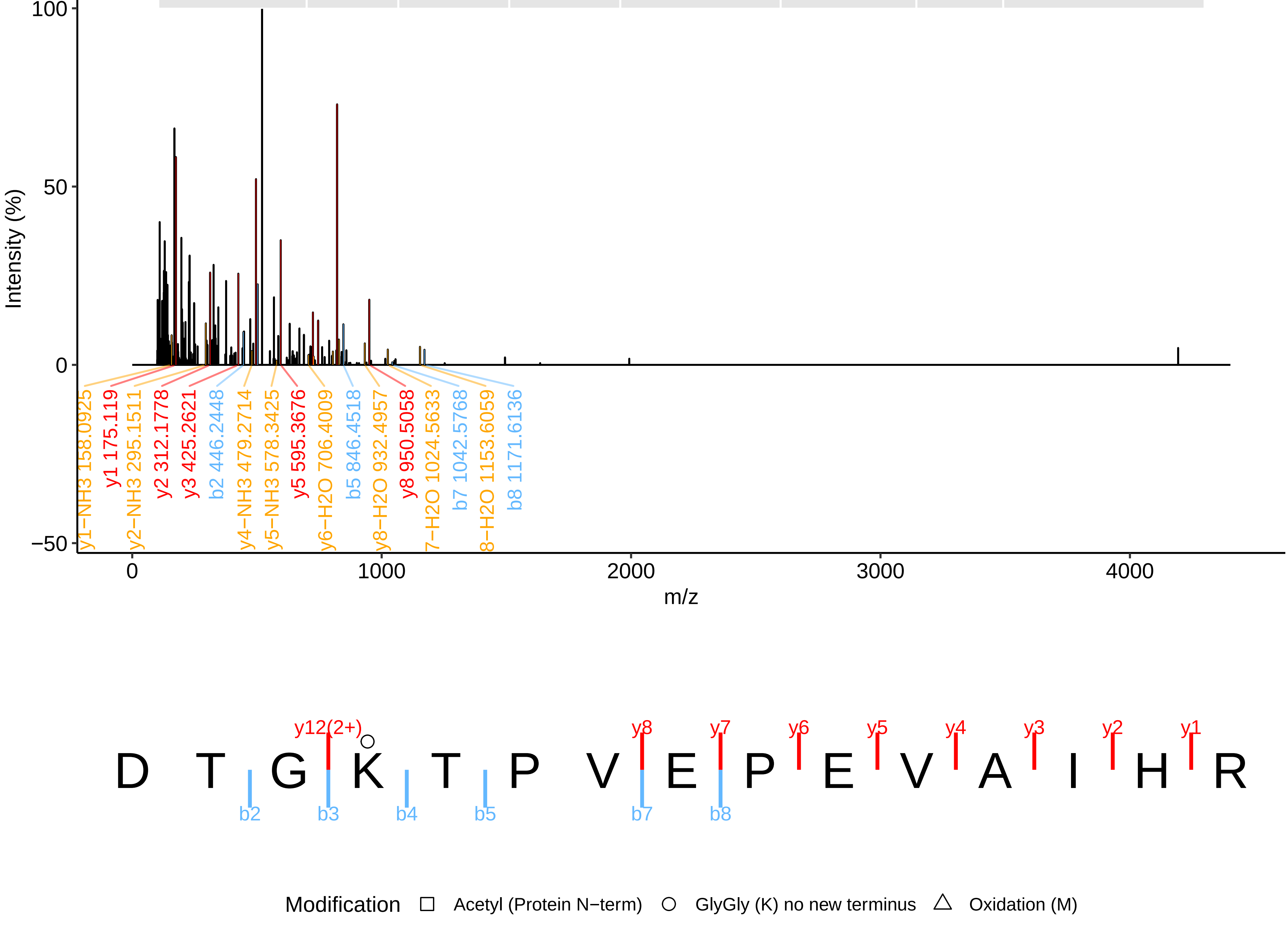

20200924_KIZ_MUb5_GlyGly_1 − Scan number: 7908

Gene Names	Charge	m/z	Mass	Retention time	PEP	Score	Precursor Intensity
RPS20	3	588.3076	1761.901	25.195	9.2456e−06	102.65	6969190

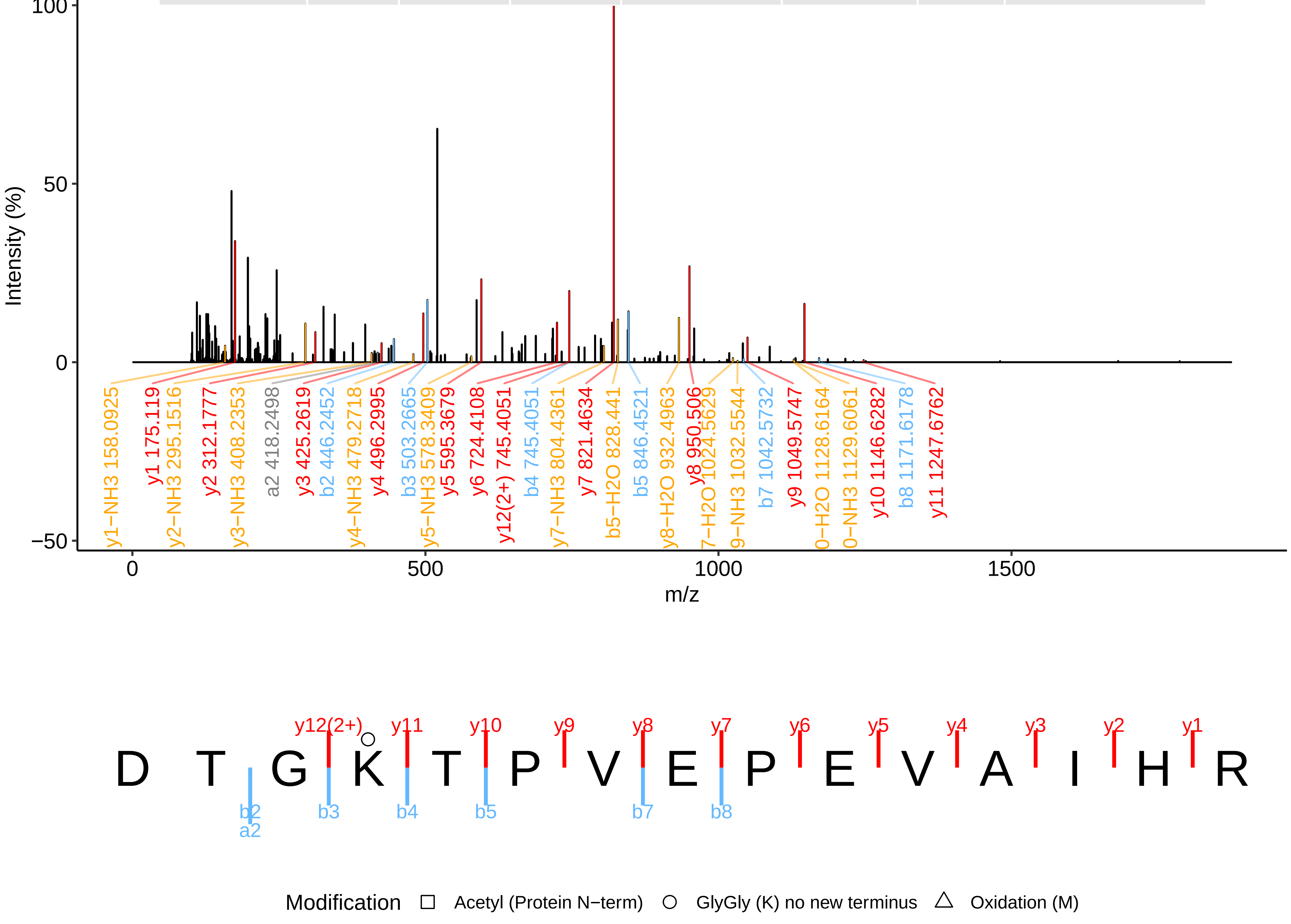

20200924_KIZ_MUb5_GlyGly_1 − Scan number: 8069

Gene Names	Charge	m/z	Mass	Retention time	PEP	Score	Precursor Intensity
RPS20	3	588.3076	1761.901	25.711	6.1958e−09	127.36	NA

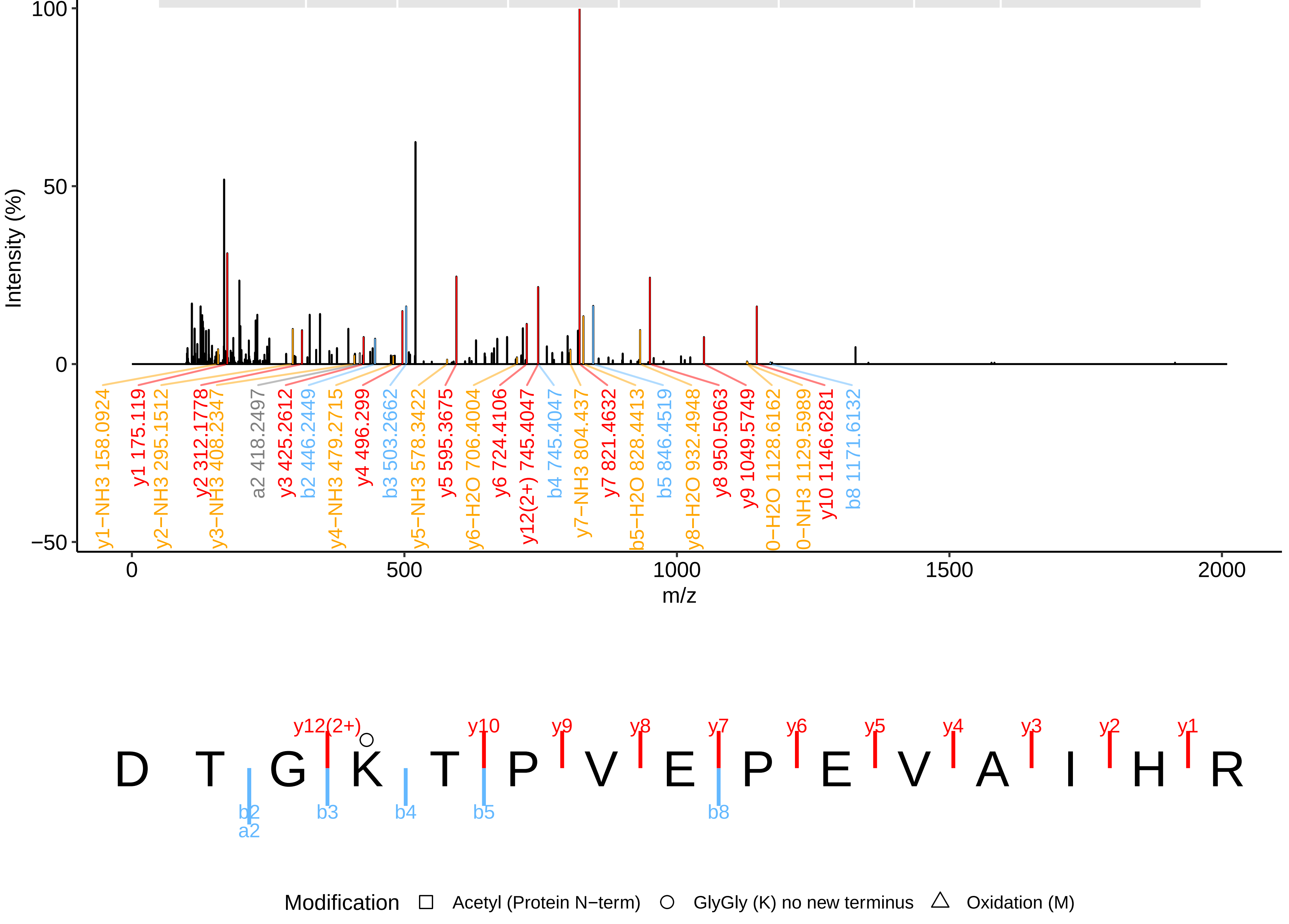

20200924_KIZ_MUb5_GlyGly_2 − Scan number: 7307

Gene Names	Charge	m/z	Mass	Retention time	PEP	Score	Precursor Intensity
RPS20	4	441.4825	1761.901	27.268	6.0251e−05	91.076	5250978

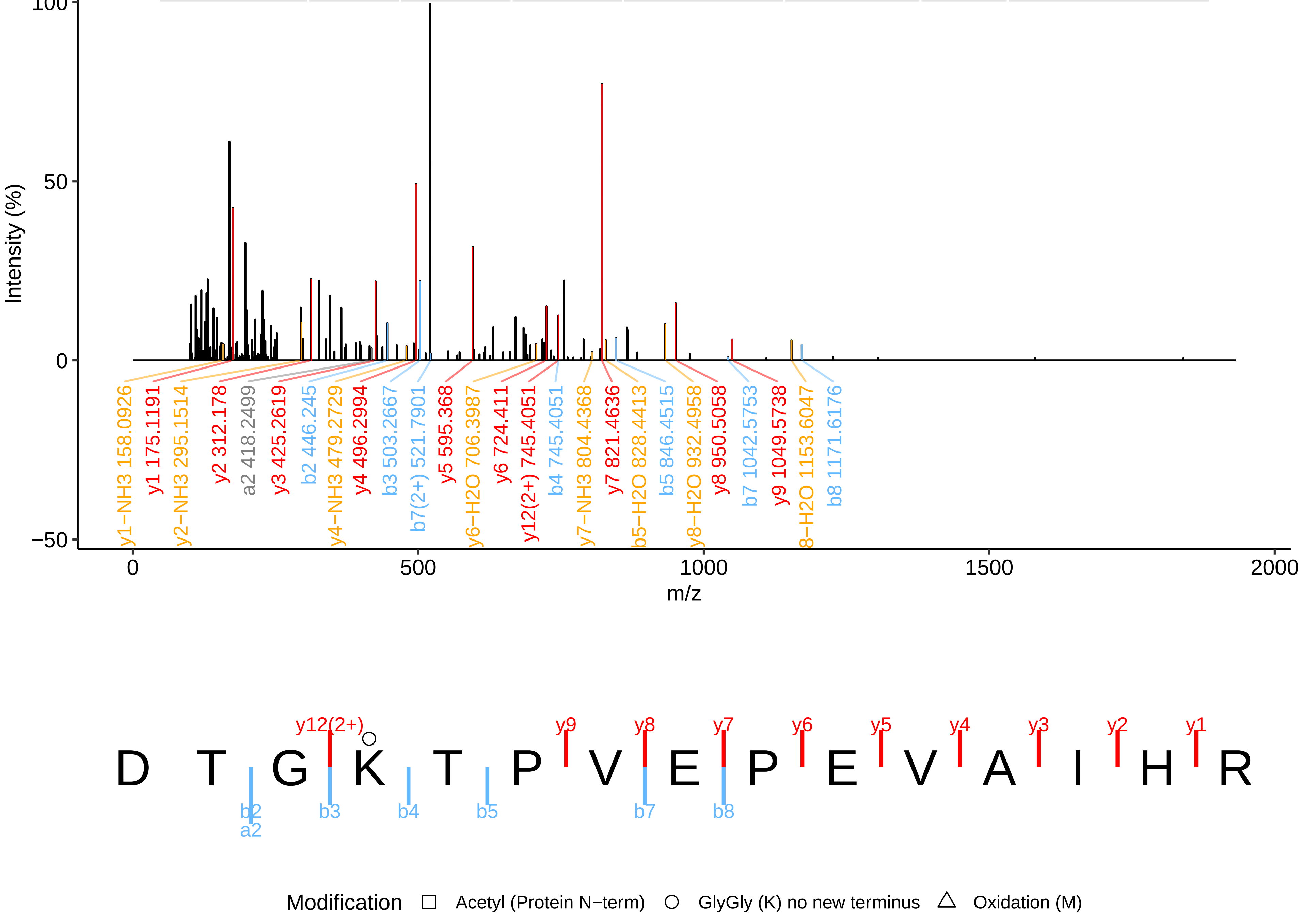

20200924_KIZ_MUb5_GlyGly_2 − Scan number: 7444

Gene Names	Charge	m/z	Mass	Retention time	PEP	Score	Precursor Intensity
RPS20	4	441.4825	1761.901	27.781	3.6005e−09	118.13	6683939

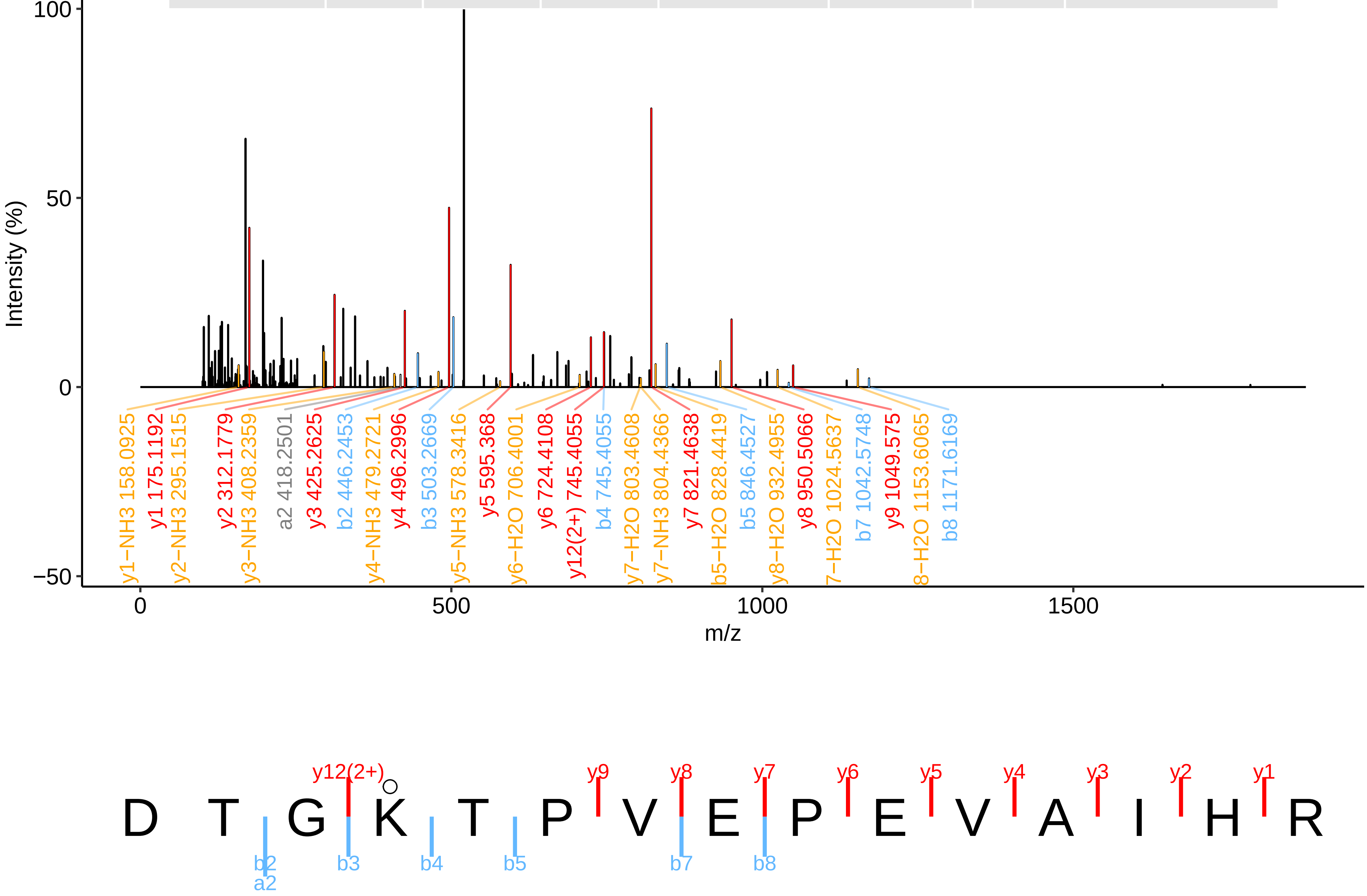

Modification □ Acetyl (Protein N−term) ○ GlyGly (K) no new terminus △ Oxidation (M)

20200924_KIZ_MUb5_GlyGly_2 − Scan number: 7575

Gene Names	Charge	m/z	Mass	Retention time	PEP	Score	Precursor Intensity
RPS20	4	441.4825	1761.901	28.287	3.6005e−09	118.13	6052833

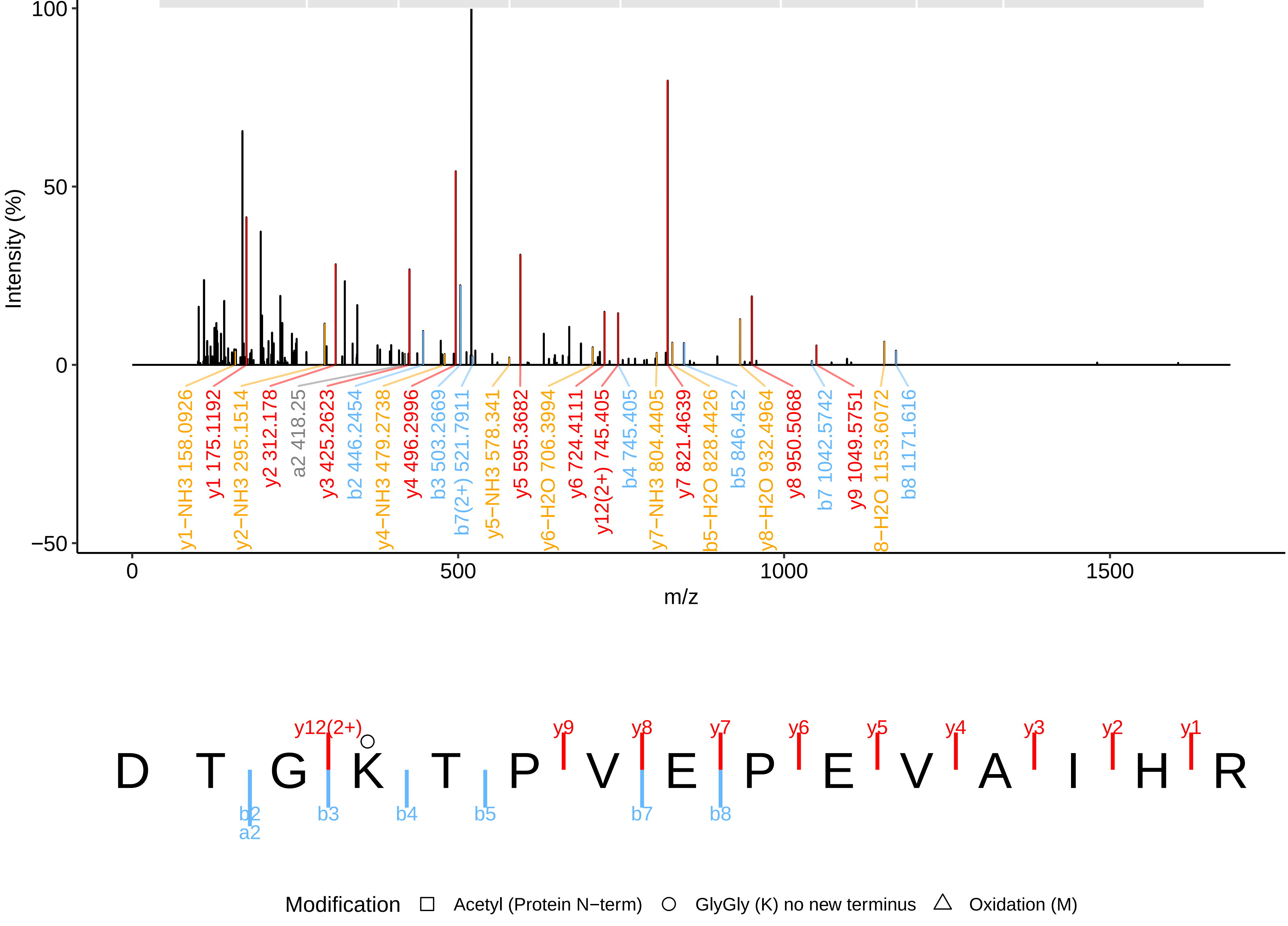

20200924_KIZ_MUb5_GlyGly_2 − Scan number: 7427

Gene Names	Charge	m/z	Mass	Retention time	PEP	Score	Precursor Intensity
RPS20	3	588.3076	1761.901	27.724	0.0046701	71.279	2467978

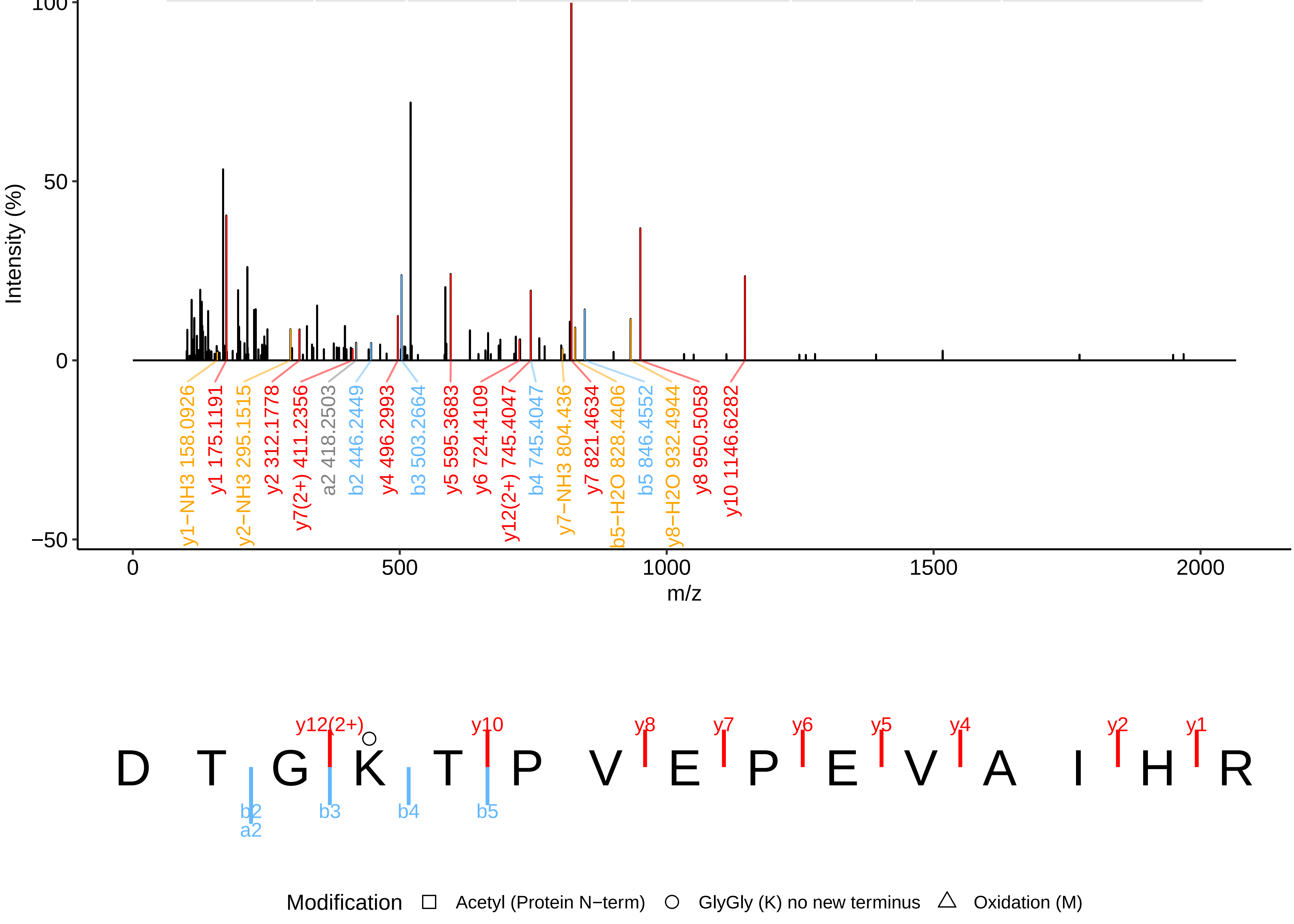

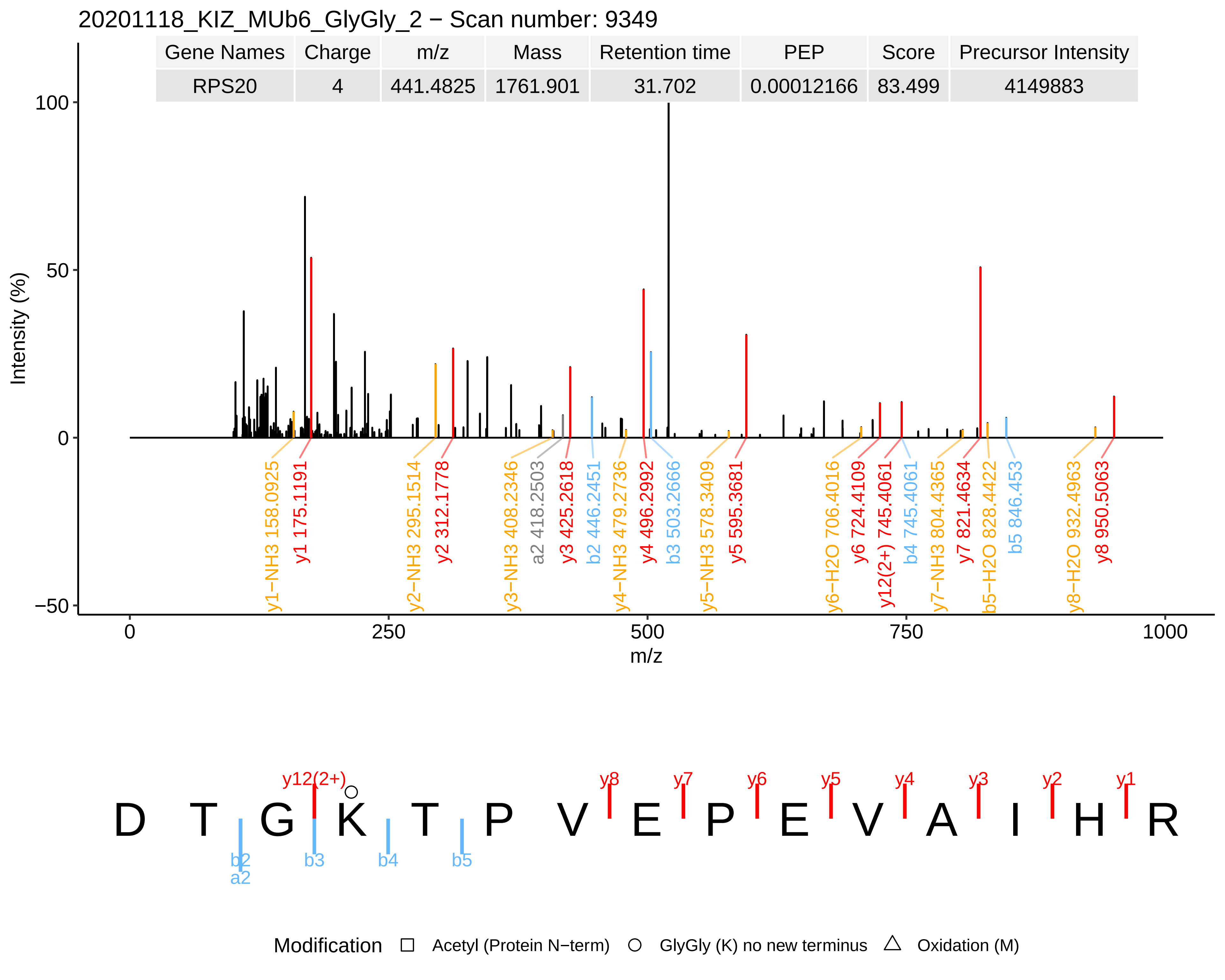

20201118_KIZ_MUb6_GlyGly_2 − Scan number: 9349
Gene Names | Charge | m/z | Mass | Retention time | PEP | Score | Precursor Intensity
RPS20 | 4 | 441.4825 | 1761.901 | 31.702 | 0.00012166 | 83.499 | 4149883
Intensity (%)
m/z
y1−NH3 158.0925
y1 175.1191
y2−NH3 295.1514
y2 312.1778
y3−NH3 408.2346
a2 418.2503
y3 425.2618
b2 446.2451
y4−NH3 479.2736
y4 496.2992
b3 503.2666
y5−NH3 578.3409
y5 595.3681
y6−H2O 706.4016
y6 724.4109
y12(2+) 745.4061
b4 745.4061
y7−NH3 804.4365
y7 821.4634
b5−H2O 828.4422
b5 846.453
y8−H2O 932.4963
y8 950.5063
D T G K T P V E P E V A I H R
Modification □ Acetyl (Protein N−term) ○ GlyGly (K) no new terminus △ Oxidation (M)

20201118_KIZ_MUb6_GlyGly_2 − Scan number: 9357

Gene Names	Charge	m/z	Mass	Retention time	PEP	Score	Precursor Intensity
RPS20	4	441.4825	1761.901	31.731	1.3315e−06	109.83	8668710

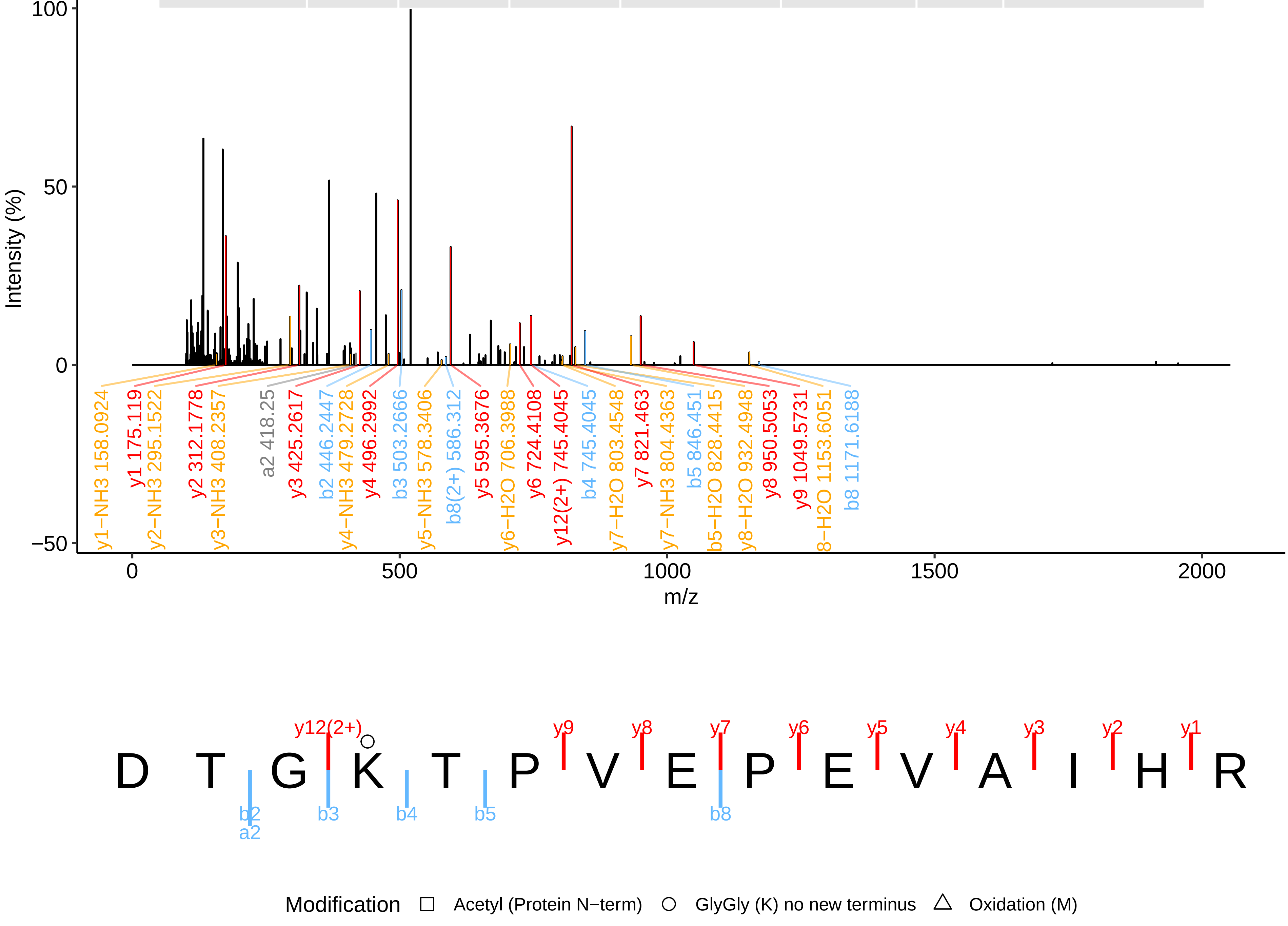

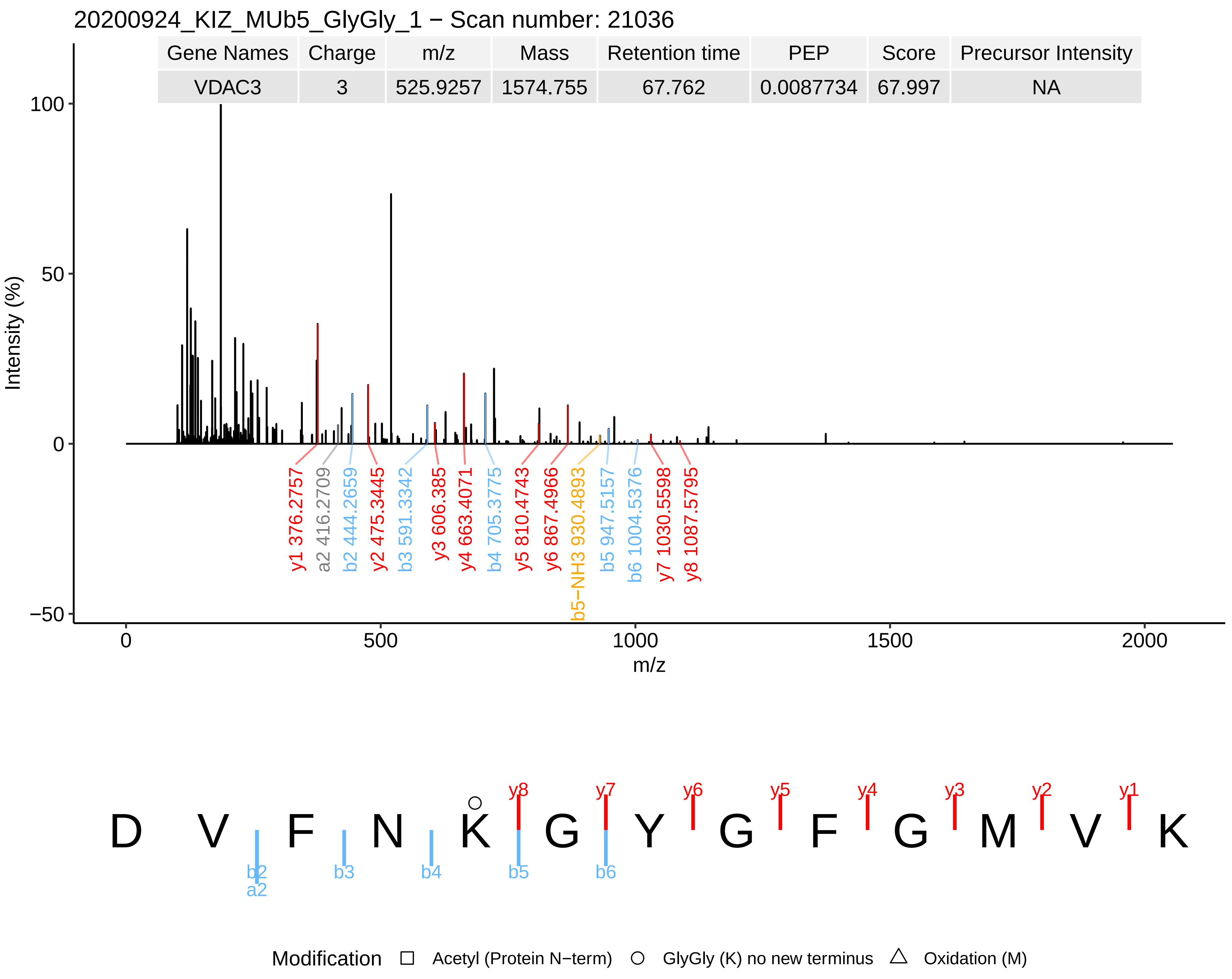

20200924_KIZ_MUb5_GlyGly_1 − Scan number: 21036

Gene Names	Charge	m/z	Mass	Retention time	PEP	Score	Precursor Intensity
VDAC3	3	525.9257	1574.755	67.762	0.0087734	67.997	NA

Intensity (%)
m/z
y1 376.2757
a2 416.2709
b2 444.2659
y2 475.3445
b3 591.3342
y3 606.385
y4 663.4071
b4 705.3775
y5 810.4743
y6 867.4966
b5−NH3 930.4893
b5 947.5157
b6 1004.5376
y7 1030.5598
y8 1087.5795
D V F N K G Y G F G M V K
Modification
Acetyl (Protein N−term)
GlyGly (K) no new terminus
Oxidation (M)

20200921_KIZ_MUb4_GlyGly_10plex − Scan number: 26329

Gene Names	Charge	m/z	Mass	Retention time	PEP	Score	Precursor Intensity
VDAC1	3	520.2803	1557.819	95.909	0.00096309	83.617	2678902

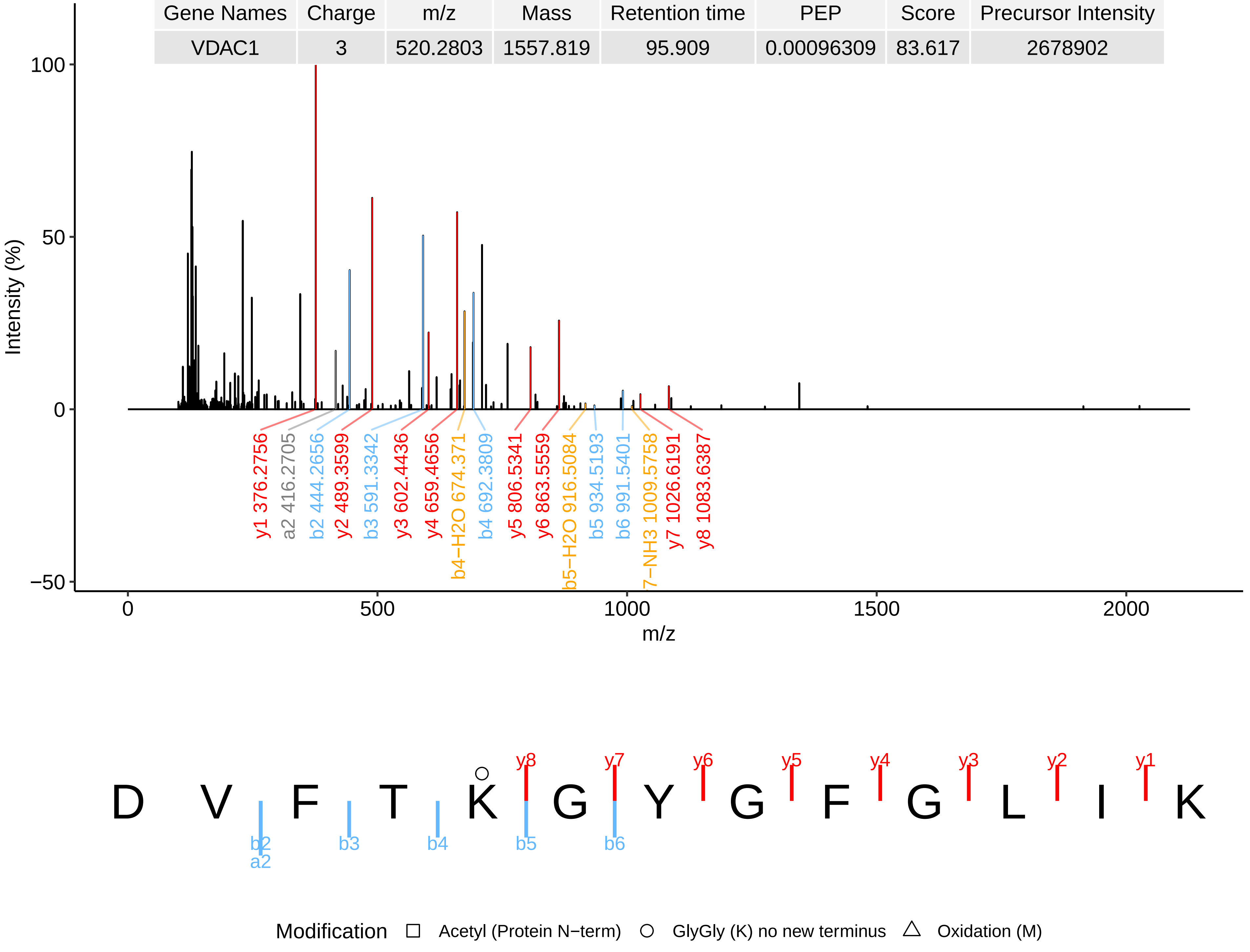

20200921_KIZ_MUb4_GlyGly_10plex − Scan number: 26451

Gene Names	Charge	m/z	Mass	Retention time	PEP	Score	Precursor Intensity
VDAC1	3	520.2803	1557.819	96.382	0.0082607	67.456	6293919

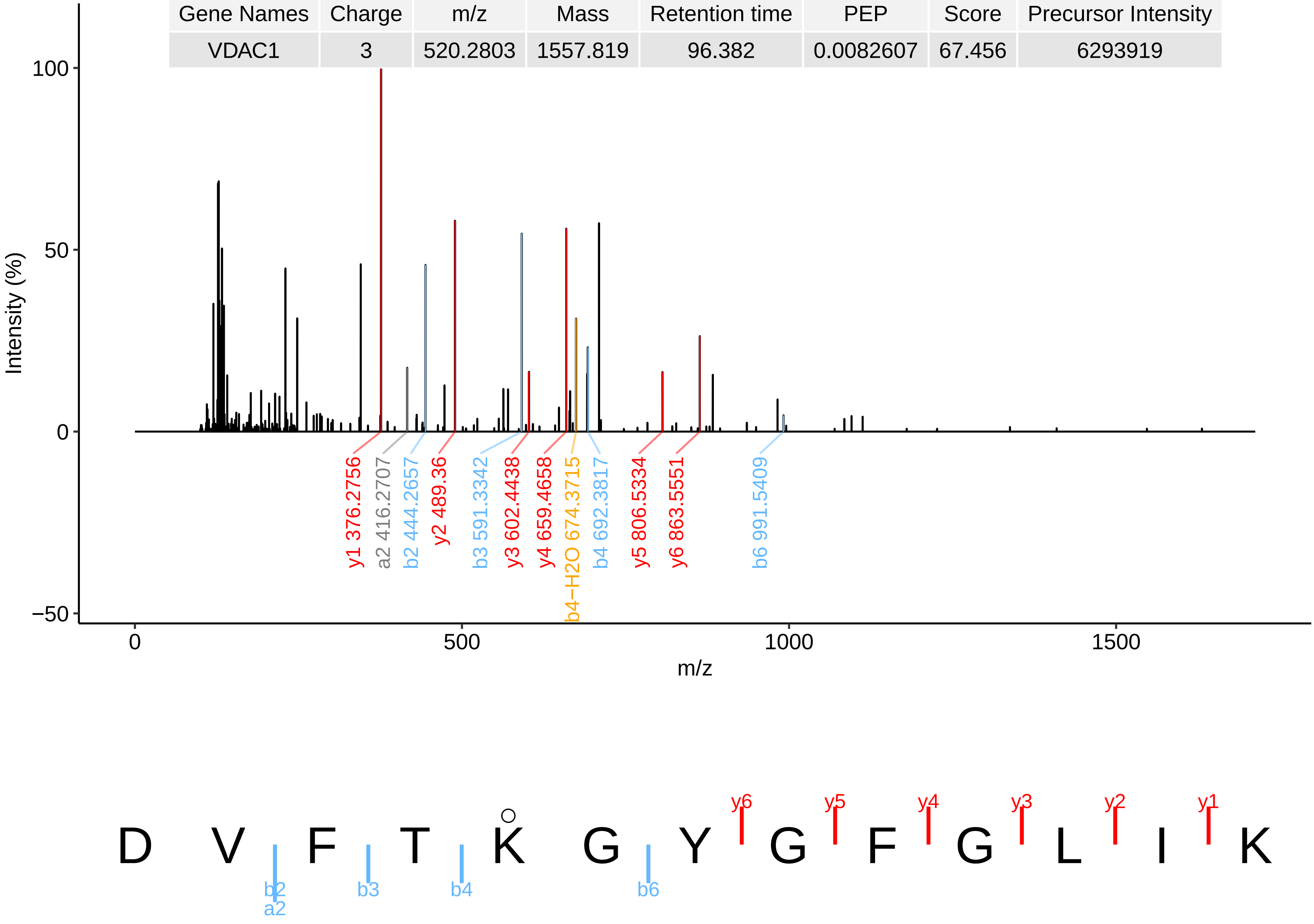

Modification □ Acetyl (Protein N−term) ○ GlyGly (K) no new terminus △ Oxidation (M)

20201118_KIZ_MUb6_GlyGly_1 − Scan number: 25918

Gene Names	Charge	m/z	Mass	Retention time	PEP	Score	Precursor Intensity
VDAC1	3	520.2803	1557.819	79.148	0.041963	56.414	46546236

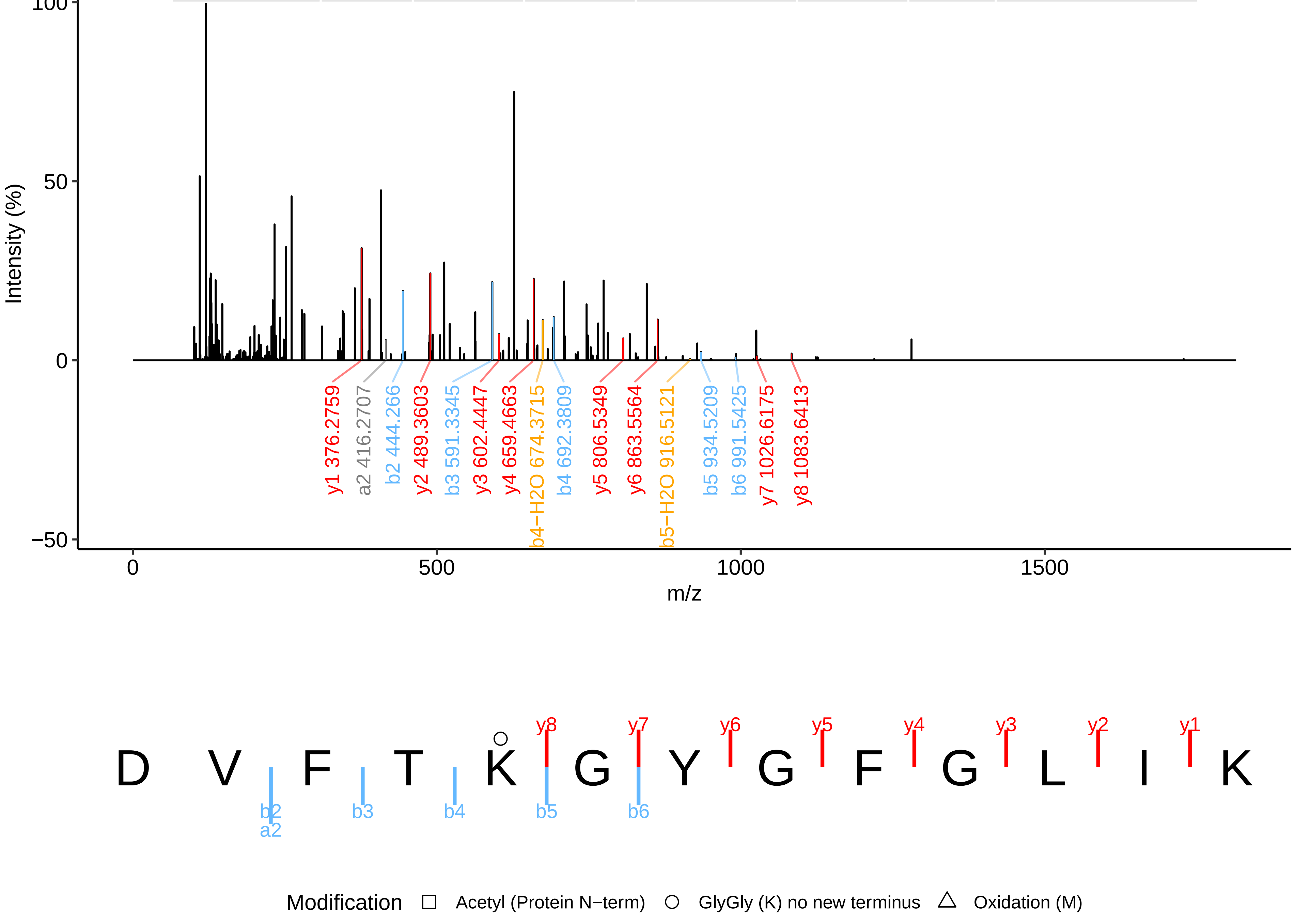

20200921_KIZ_MUb4_GlyGly_10plex_2 − Scan number: 13463

Gene Names	Charge	m/z	Mass	Retention time	PEP	Score	Precursor Intensity
EIF1AX;EIF1AY	3	512.5949	1534.763	52.278	0.0069008	72.089	1144205

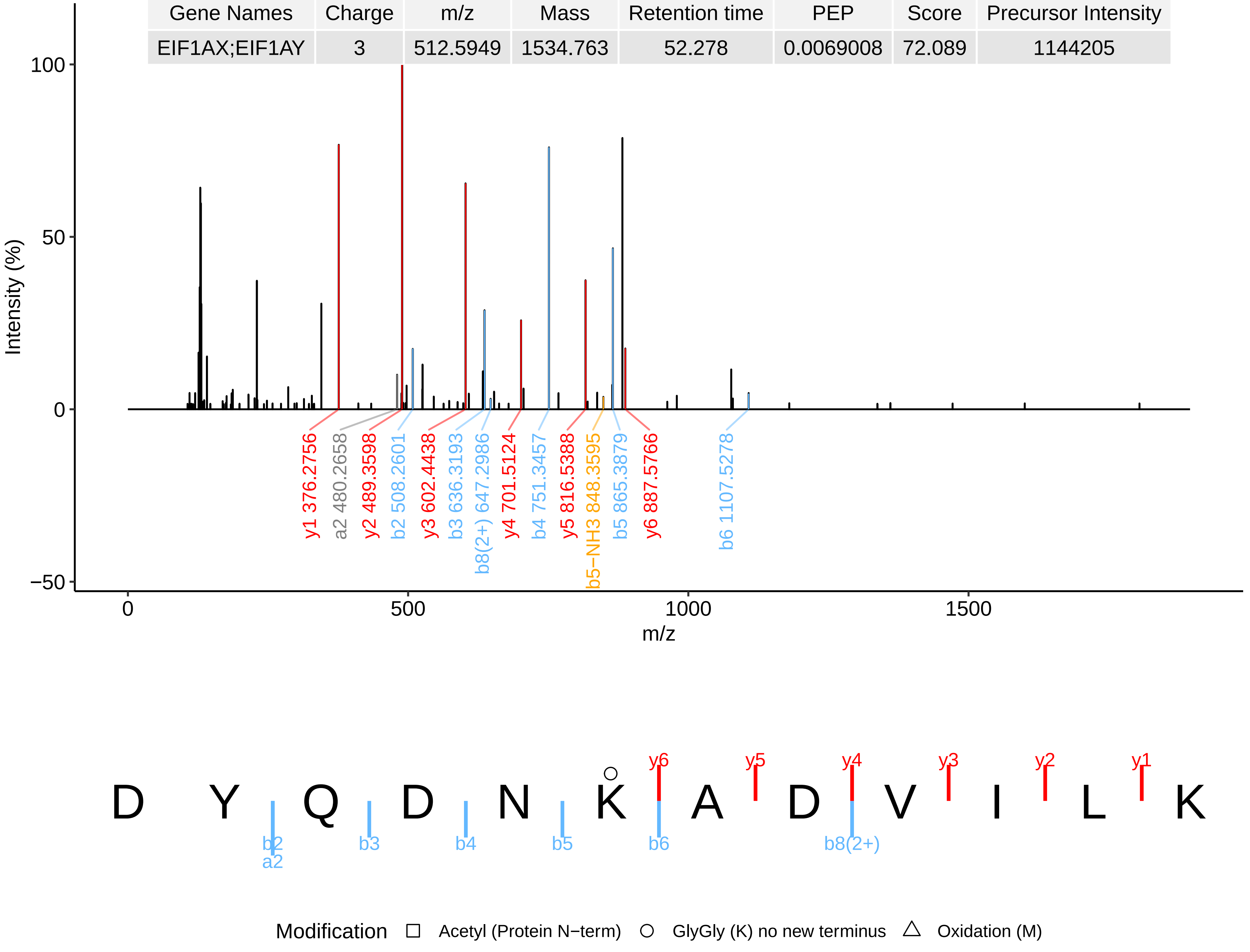

20200924_KIZ_MUb5_GlyGly_1 − Scan number: 13770

Gene Names	Charge	m/z	Mass	Retention time	PEP	Score	Precursor Intensity
EIF1AX;EIF1AY	3	512.5949	1534.763	44.916	0.00060149	91.858	11178678

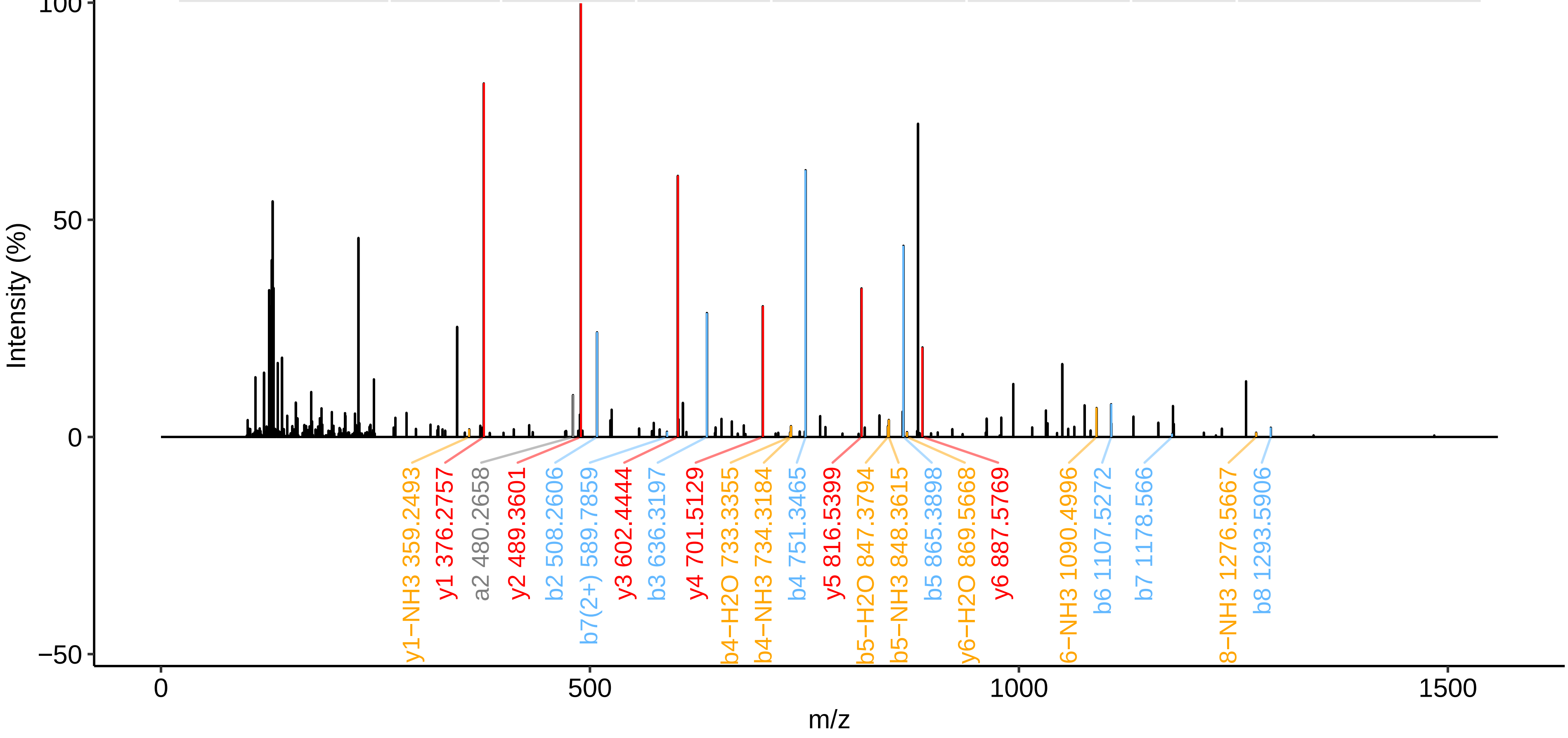

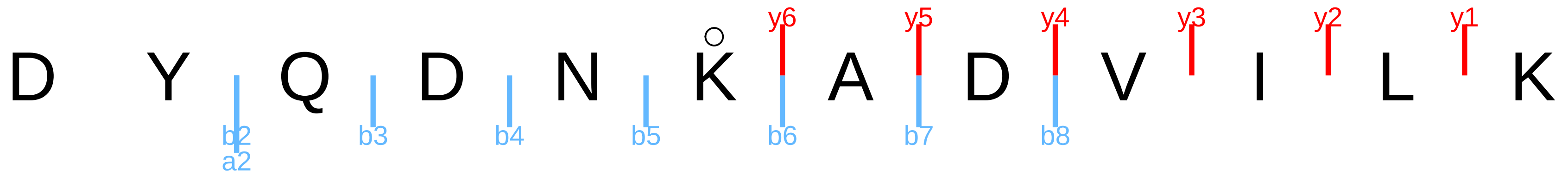

Modification ☐ Acetyl (Protein N−term) ○ GlyGly (K) no new terminus △ Oxidation (M)

20200924_KIZ_MUb5_GlyGly_2 − Scan number: 12870

Gene Names	Charge	m/z	Mass	Retention time	PEP	Score	Precursor Intensity
EIF1AX;EIF1AY	3	512.5949	1534.763	48.133	0.0042342	77.593	2382440

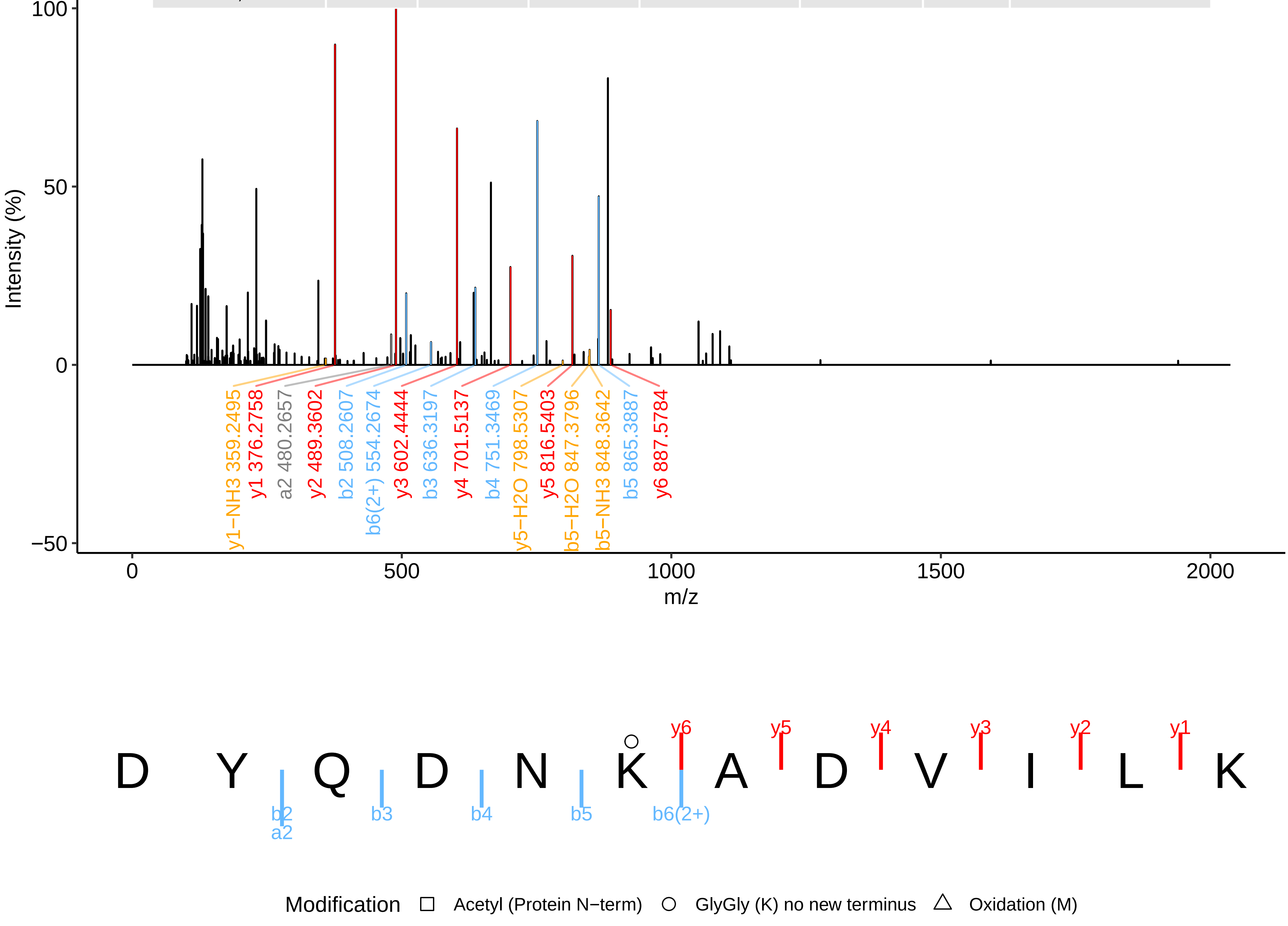

20200921_KIZ_MUb4_GlyGly_10plex_2 − Scan number: 7043

Gene Names	Charge	m/z	Mass	Retention time	PEP	Score	Precursor Intensity
TDP2	3	520.2459	1557.716	26.145	0.0043955	84.718	655958.1

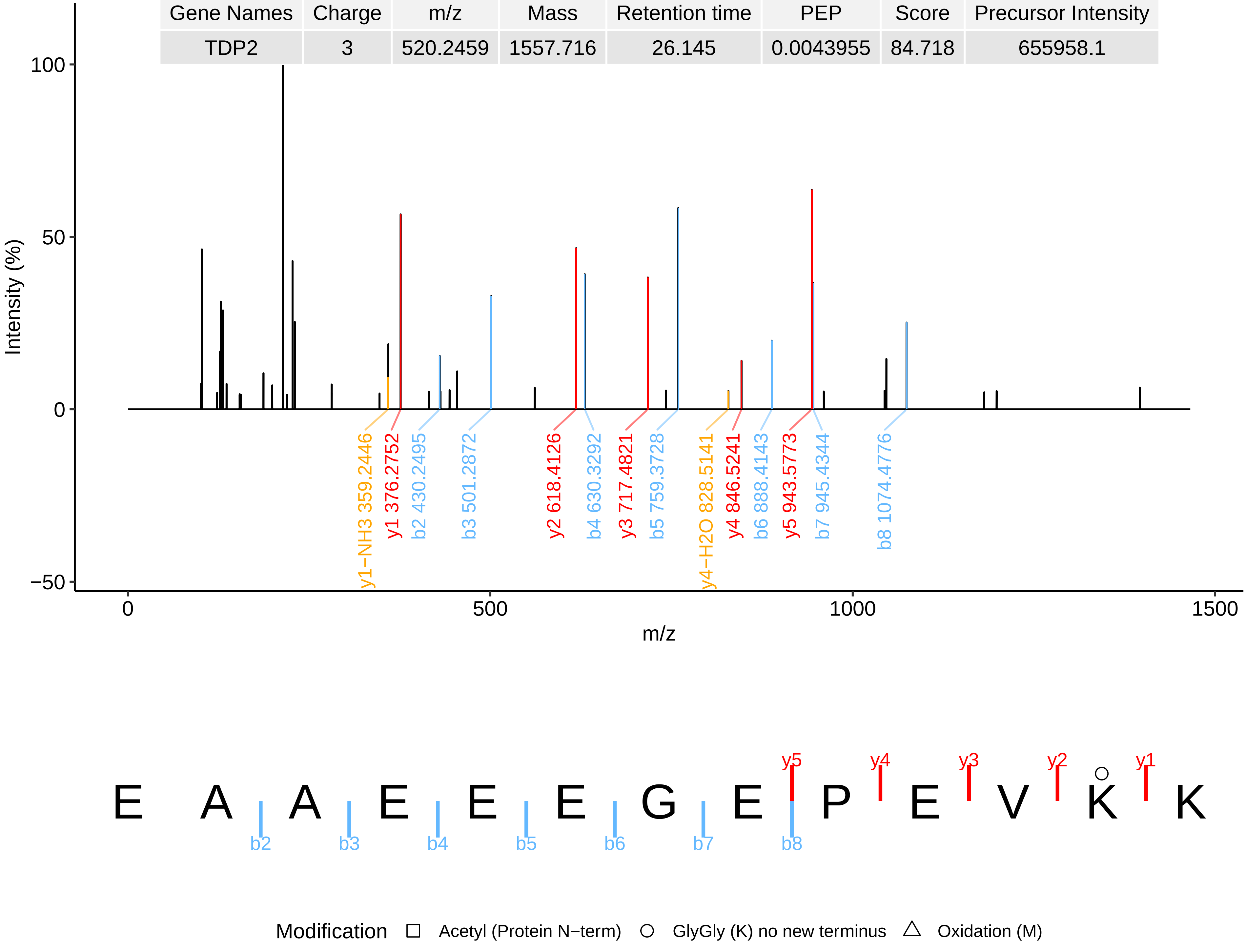

20200921_KIZ_MUb4_GlyGly_10plex − Scan number: 22092

Gene Names	Charge	m/z	Mass	Retention time	PEP	Score	Precursor Intensity
ABCB6	3	531.2875	1590.841	80.113	0.0014219	83.314	NA

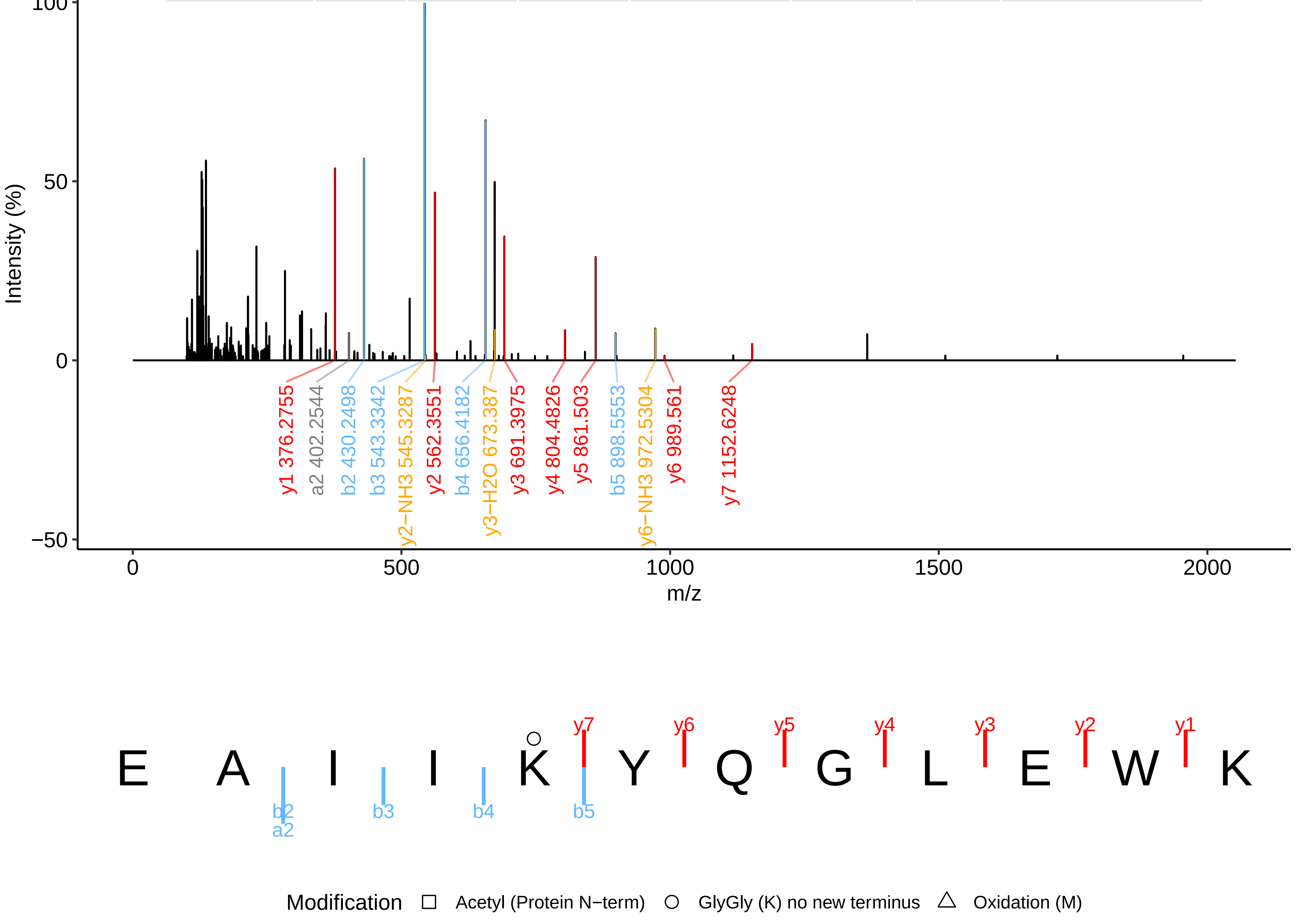

20200921_KIZ_MUb4_GlyGly_10plex − Scan number: 22622

Gene Names	Charge	m/z	Mass	Retention time	PEP	Score	Precursor Intensity
ABCB6	3	531.2875	1590.841	82.082	0.0011788	85.845	2737720

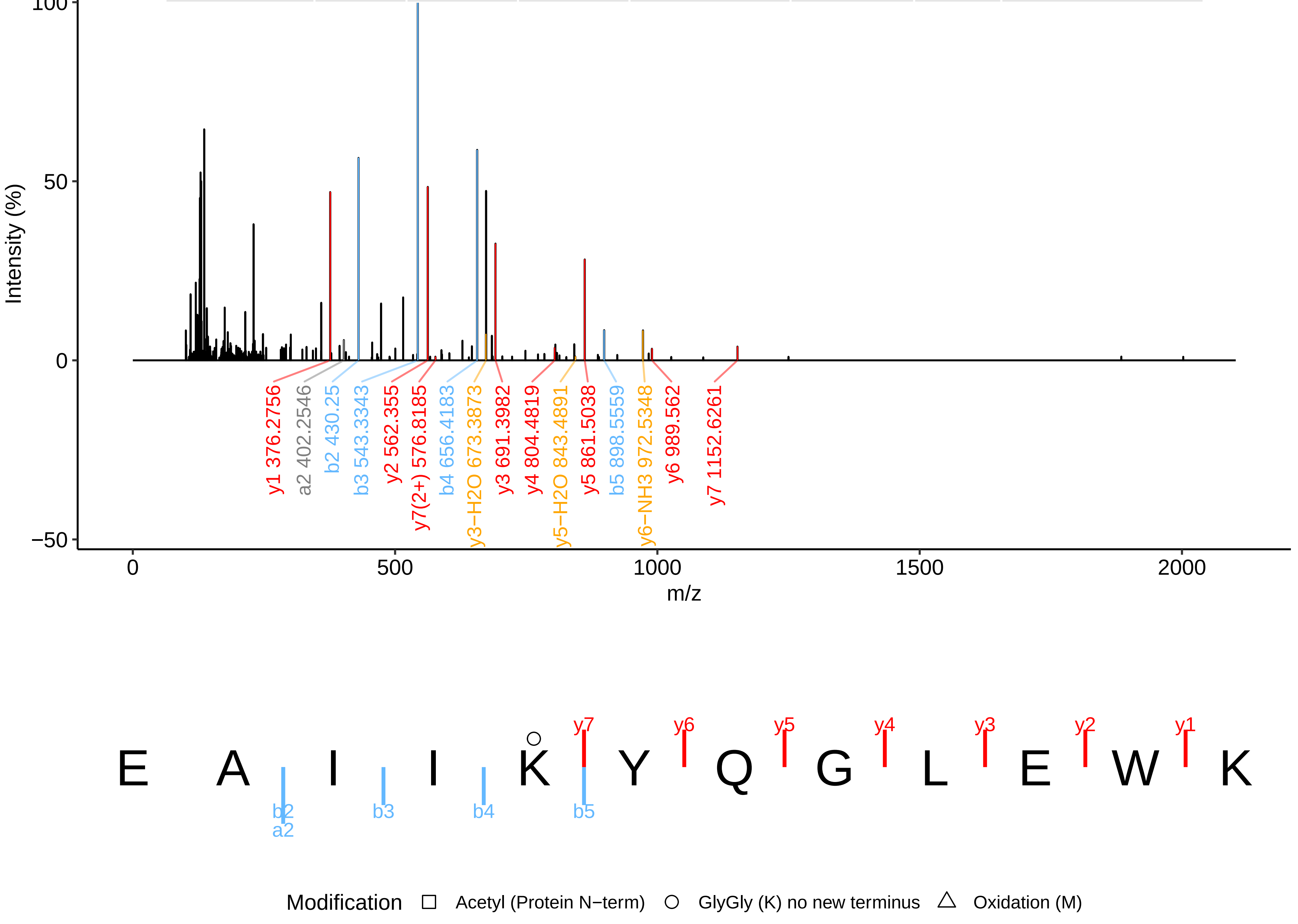

20200921_KIZ_MUb4_GlyGly_10plex_2 − Scan number: 19618

Gene Names	Charge	m/z	Mass	Retention time	PEP	Score	Precursor Intensity
ABCB6	3	531.2875	1590.841	77.022	0.00052859	90.614	2213278

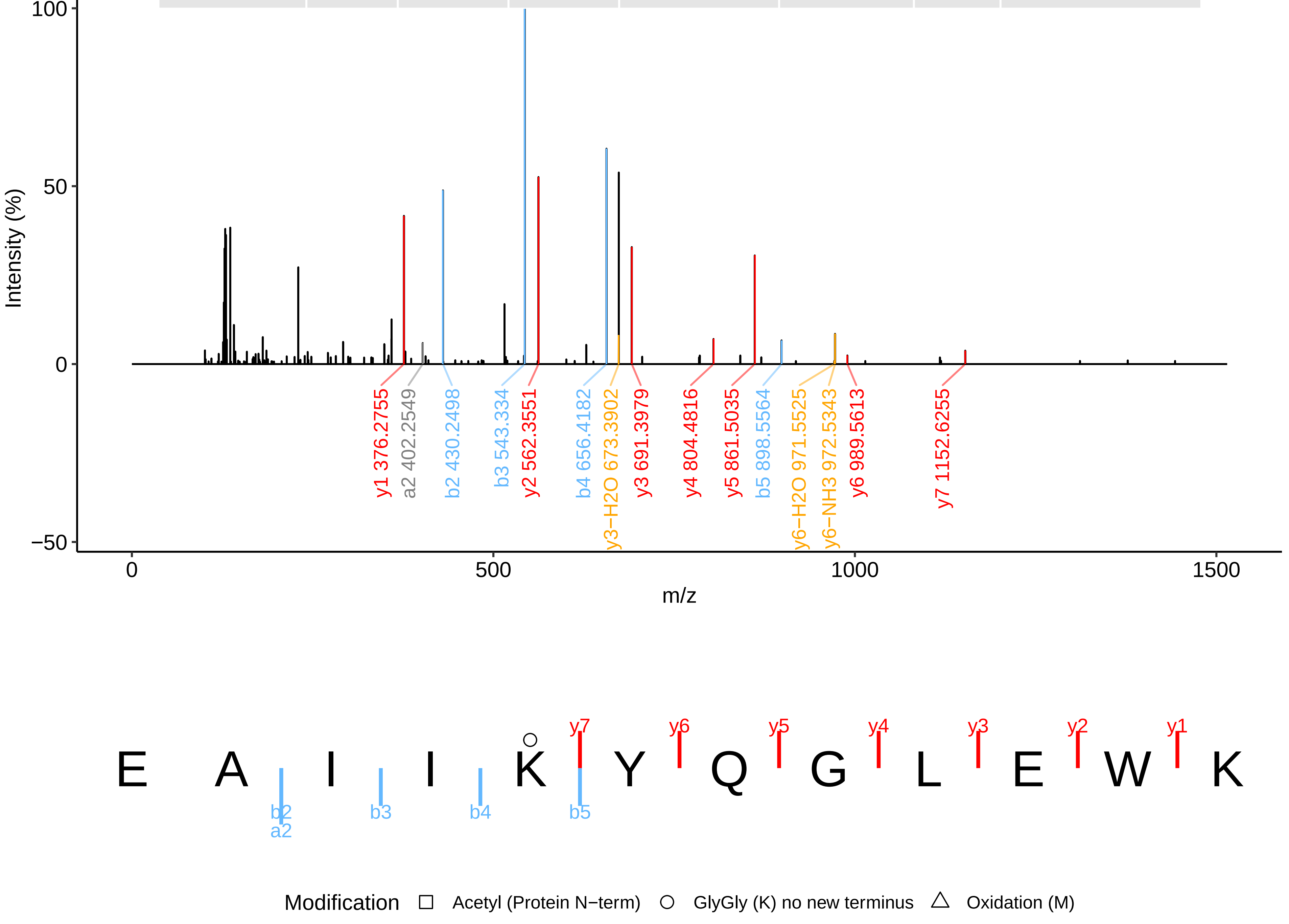

20200924_KIZ_MUb5_GlyGly_1 − Scan number: 21511

Gene Names	Charge	m/z	Mass	Retention time	PEP	Score	Precursor Intensity
ABCB6	3	531.2875	1590.841	69.289	0.0003774	94.191	NA

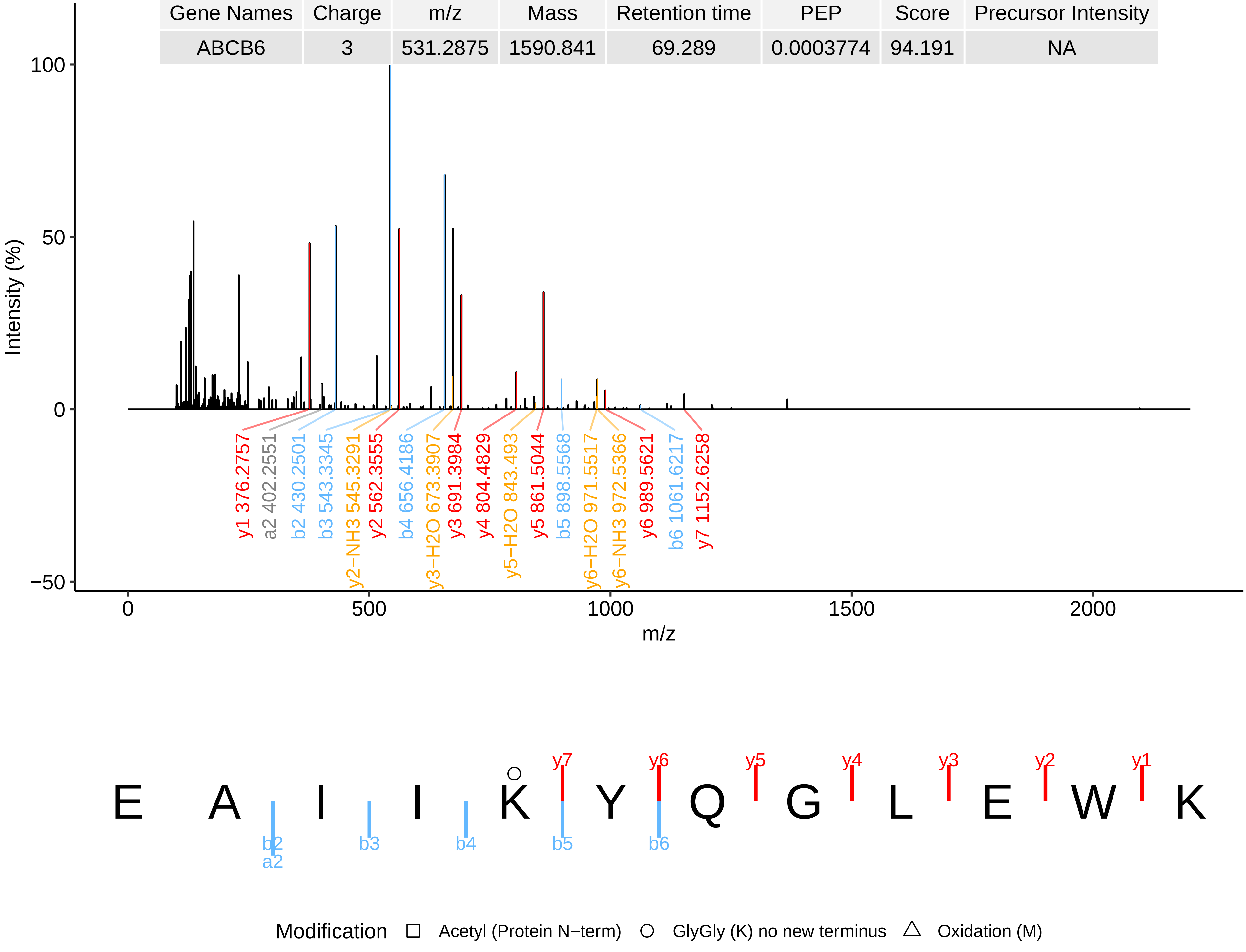

20200924_KIZ_MUb5_GlyGly_2 − Scan number: 19440

Gene Names	Charge	m/z	Mass	Retention time	PEP	Score	Precursor Intensity
ABCB6	3	531.2875	1590.841	72.062	0.0016738	80.69	4636828

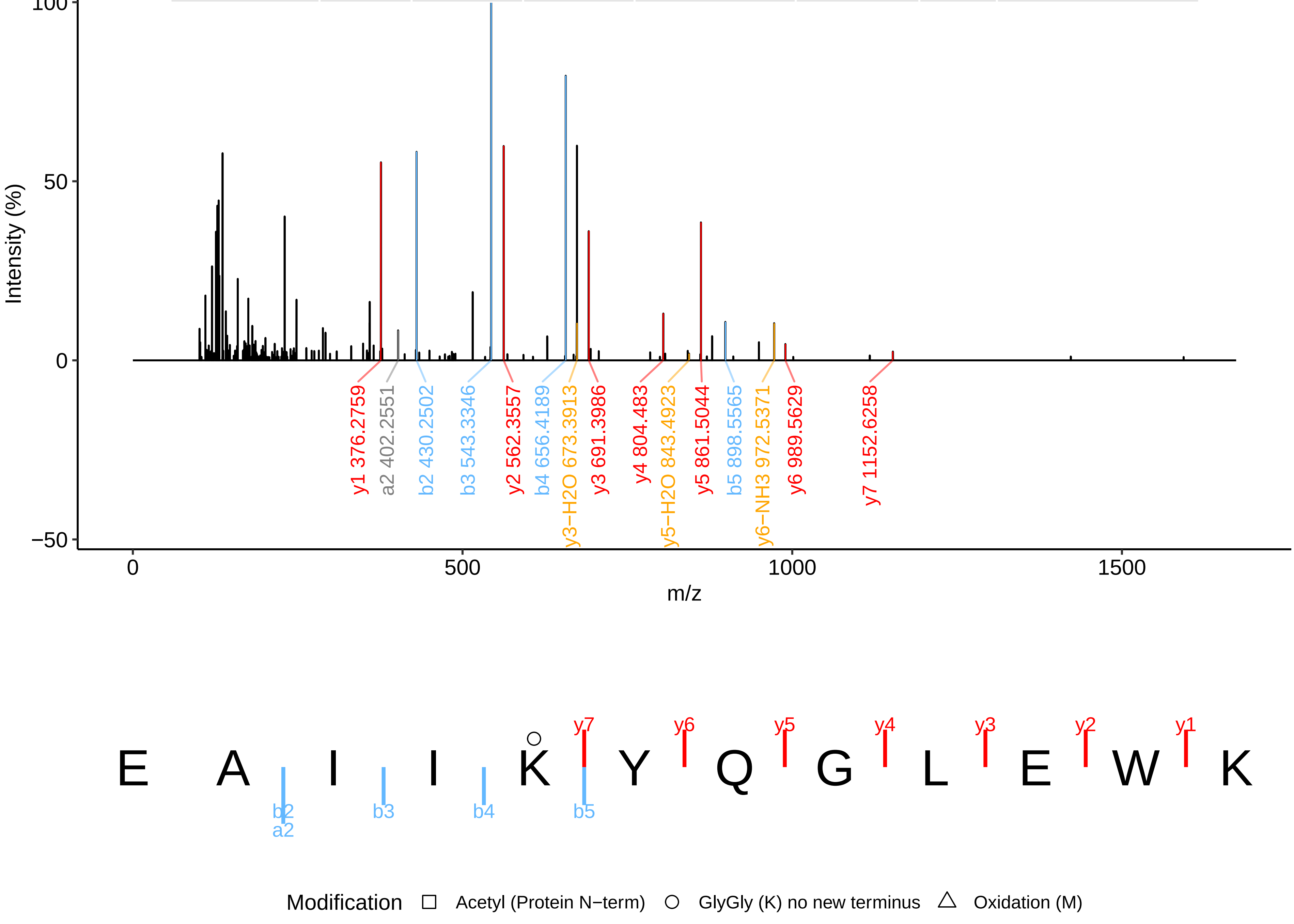

20200924_KIZ_MUb5_GlyGly_2 − Scan number: 19575

Gene Names	Charge	m/z	Mass	Retention time	PEP	Score	Precursor Intensity
ABCB6	3	531.2875	1590.841	72.567	0.00096994	88.021	NA

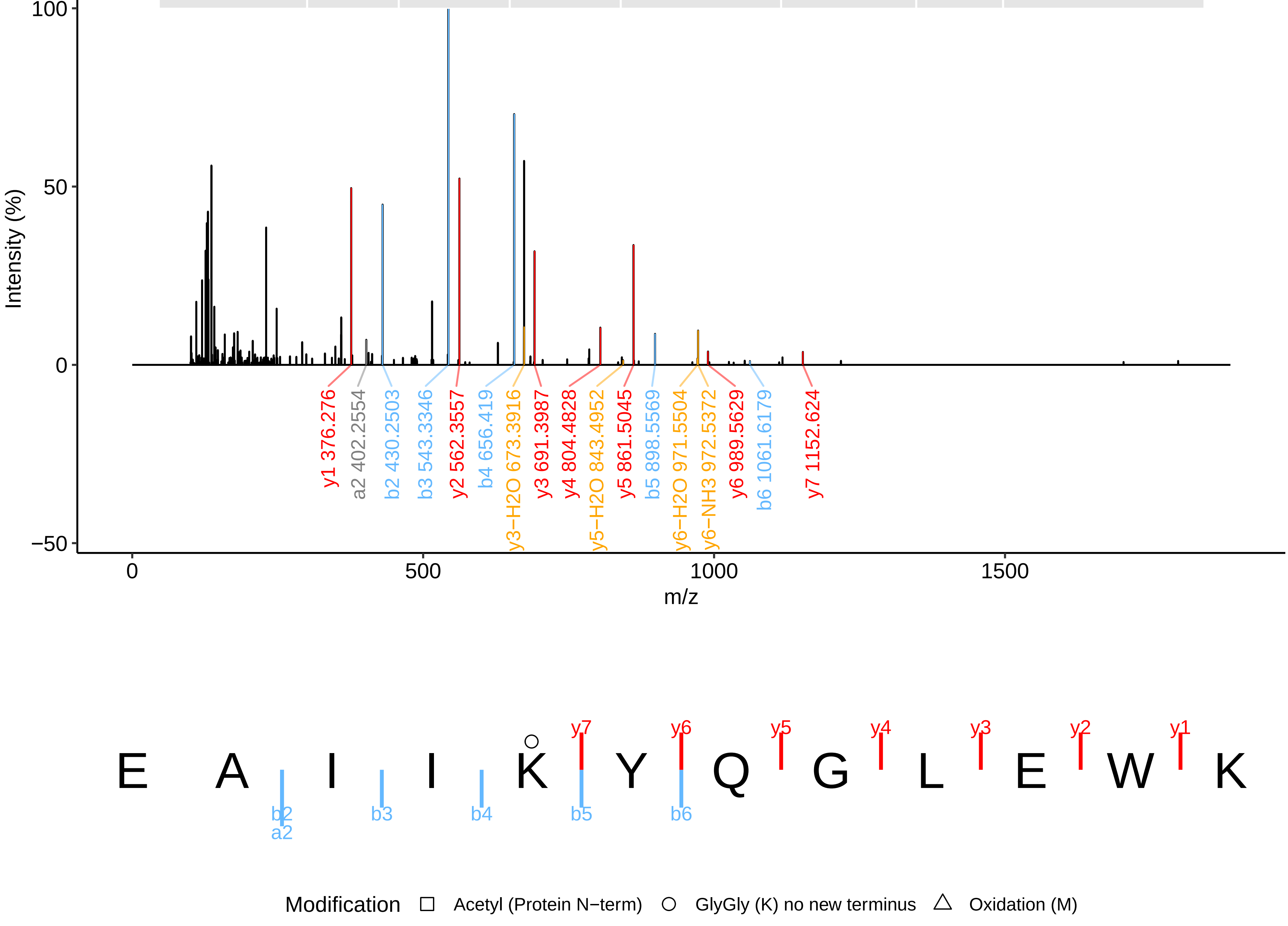

20201118_KIZ_MUb6_GlyGly_1 − Scan number: 21786

Gene Names	Charge	m/z	Mass	Retention time	PEP	Score	Precursor Intensity
ABCB6	3	531.2875	1590.841	67.904	0.00011416	96.64	21266362

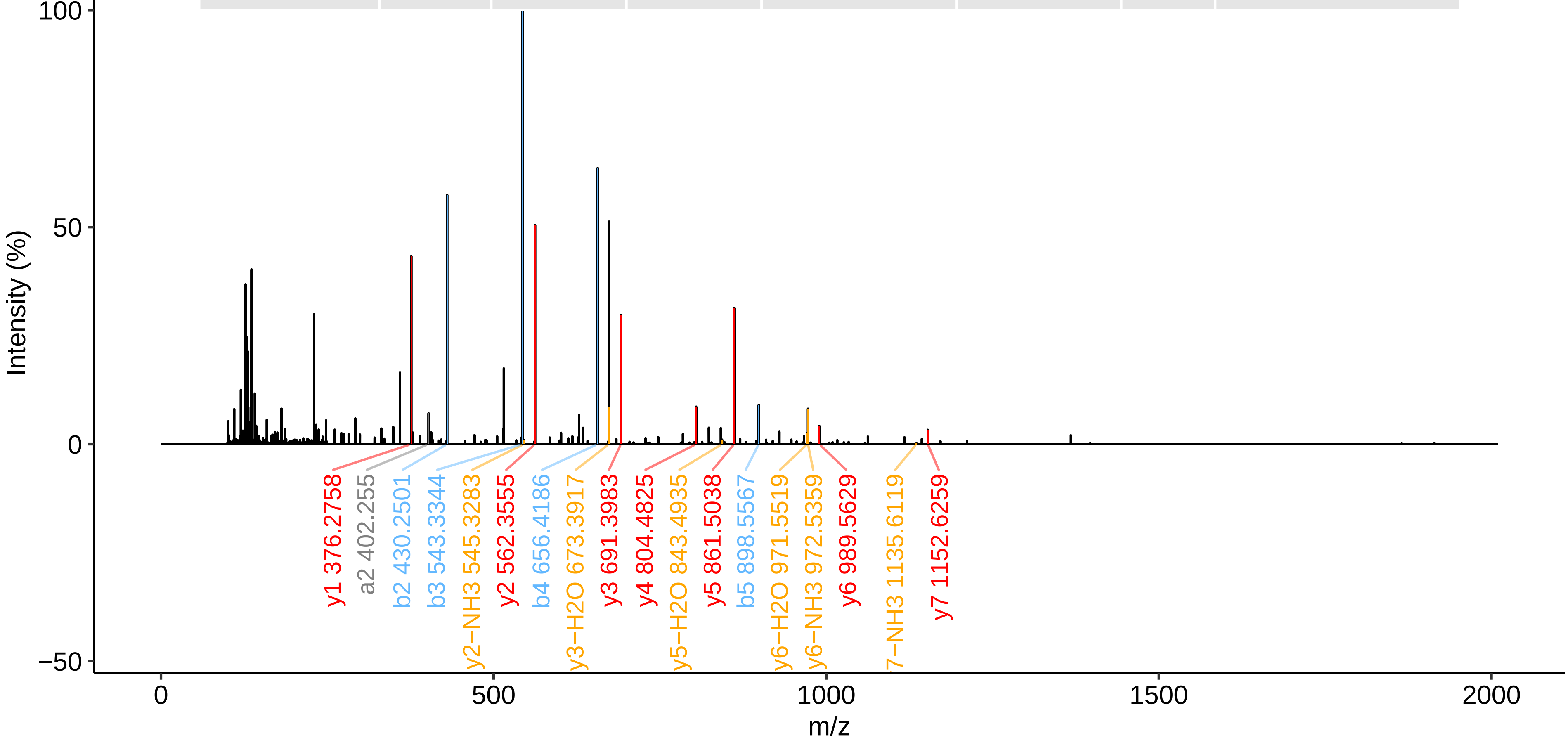

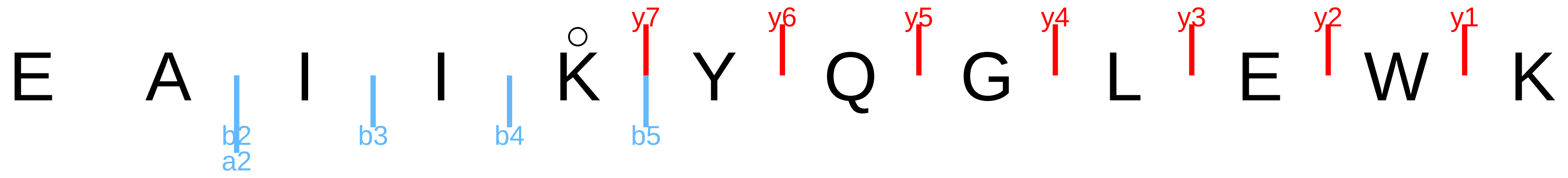

Modification □ Acetyl (Protein N−term) ○ GlyGly (K) no new terminus △ Oxidation (M)

20201118_KIZ_MUb6_GlyGly_2 − Scan number: 23556

Gene Names	Charge	m/z	Mass	Retention time	PEP	Score	Precursor Intensity
ABCB6	3	531.2875	1590.841	78.989	0.00052859	90.614	13257812

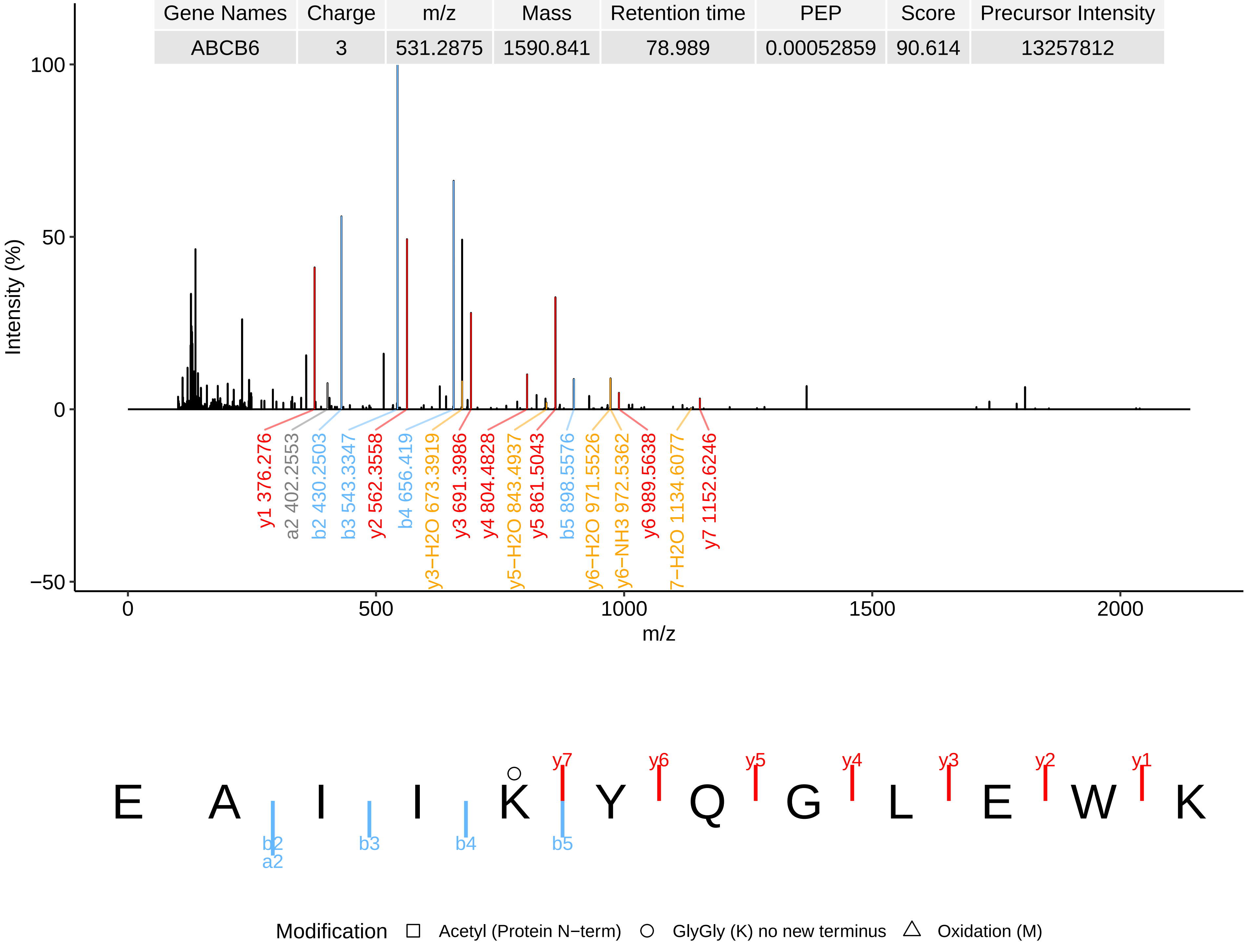

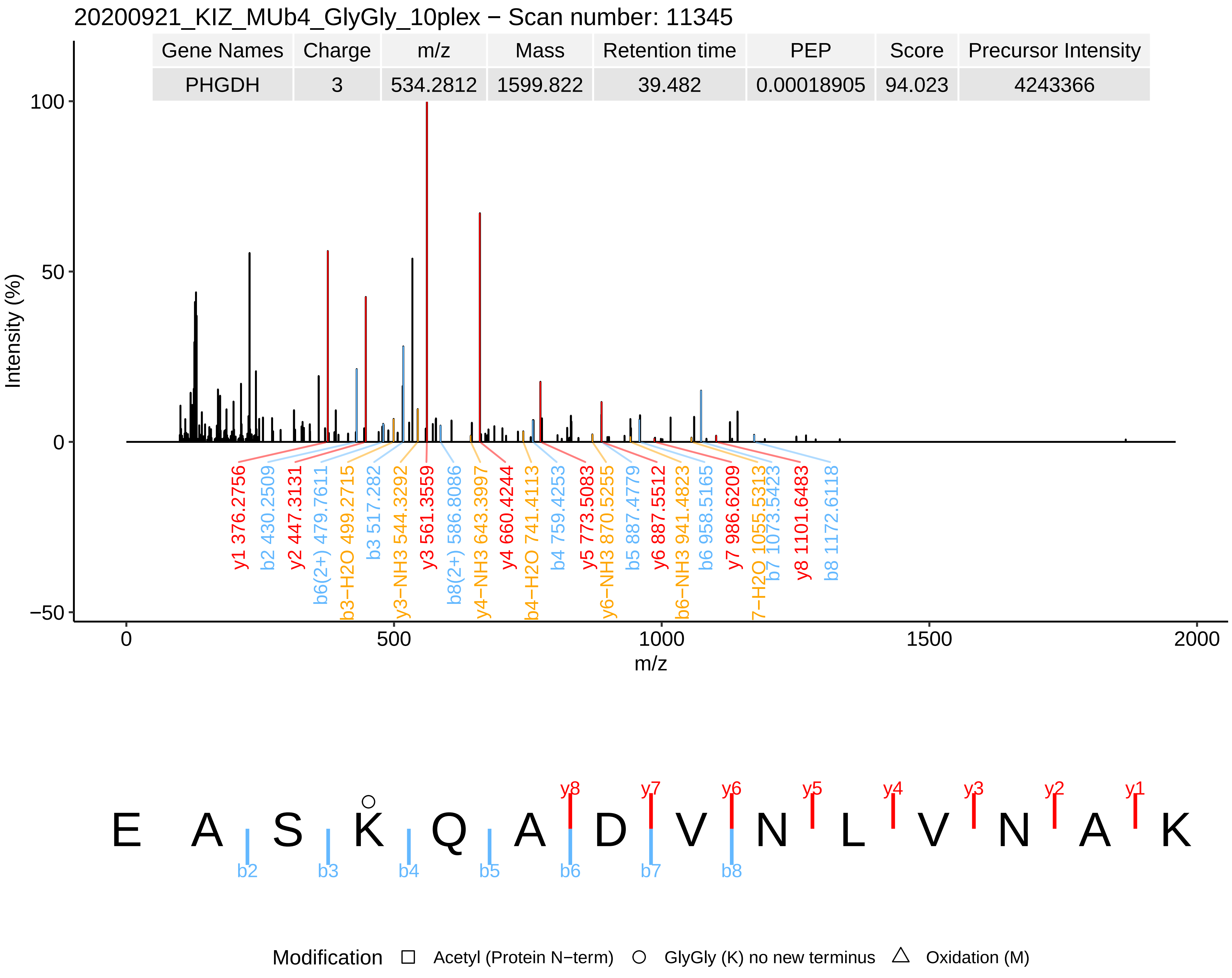
20200921_KIZ_MUb4_GlyGly_10plex − Scan number: 11345
Gene Names | Charge | m/z | Mass | Retention time | PEP | Score | Precursor Intensity
PHGDH | 3 | 534.2812 | 1599.822 | 39.482 | 0.00018905 | 94.023 | 4243366
Intensity (%)
m/z
y1 376.2756
b2 430.2509
y2 447.3131
b6(2+) 479.7611
b3−H2O 499.2715
b3 517.282
y3−NH3 544.3292
y3 561.3559
b8(2+) 586.8086
y4−NH3 643.3997
y4 660.4244
b4−H2O 741.4113
b4 759.4253
y5 773.5083
y6−NH3 870.5255
b5 887.4779
y6 887.5512
b6−NH3 941.4823
b6 958.5165
y7 986.6209
y7−H2O 1055.5313
b7 1073.5423
y8 1101.6483
b8 1172.6118
E A S K Q A D V N L V N A K
y8 y7 y6 y5 y4 y3 y2 y1
b2 b3 b4 b5 b6 b7 b8
Modification
Acetyl (Protein N−term)
GlyGly (K) no new terminus
Oxidation (M)

20200921_KIZ_MUb4_GlyGly_10plex_2 − Scan number: 10436

Gene Names	Charge	m/z	Mass	Retention time	PEP	Score	Precursor Intensity
PHGDH	3	534.2812	1599.822	39.927	0.088698	43.03	424210.8

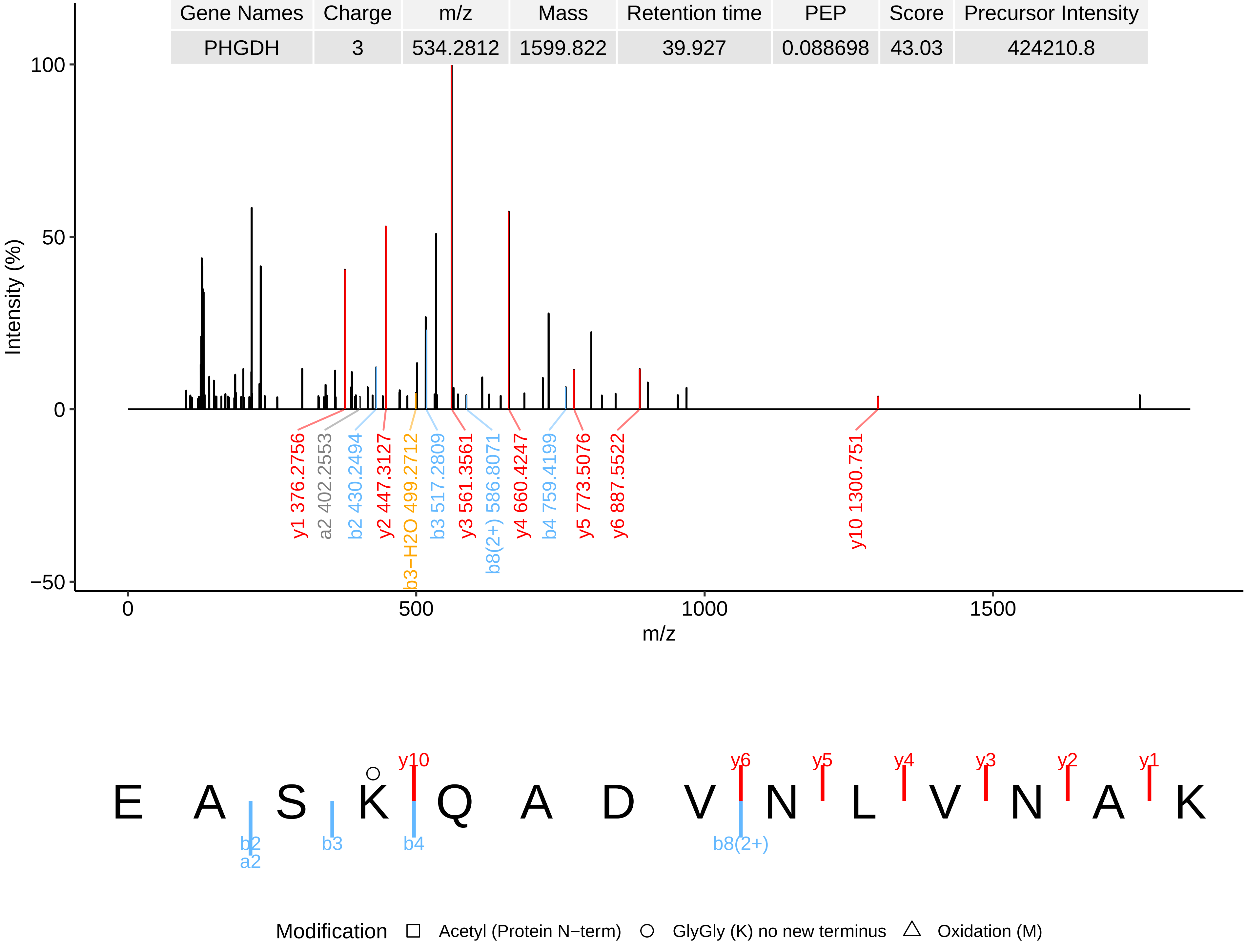

20200921_KIZ_MUb4_GlyGly_10plex_2 − Scan number: 11694

Gene Names	Charge	m/z	Mass	Retention time	PEP	Score	Precursor Intensity
YBX1;YBX3	4	382.9584	1527.804	45.063	0.085078	41.54	449011.7

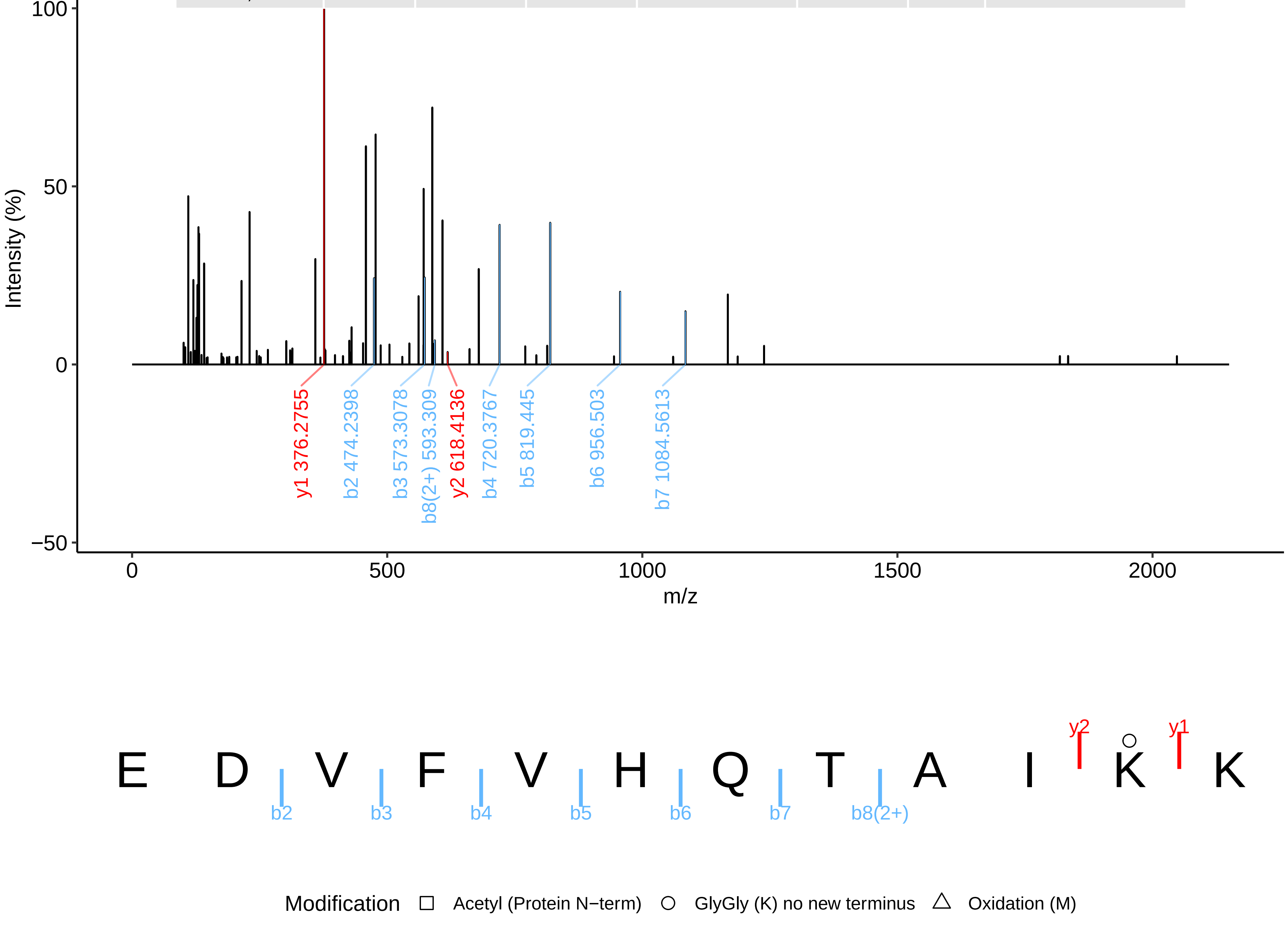

20200921_KIZ_MUb4_GlyGly_10plex − Scan number: 9568

Gene Names	Charge	m/z	Mass	Retention time	PEP	Score	Precursor Intensity
BAG6;BAT3	4	442.2352	1764.912	32.794	0.061781	48.608	1082831

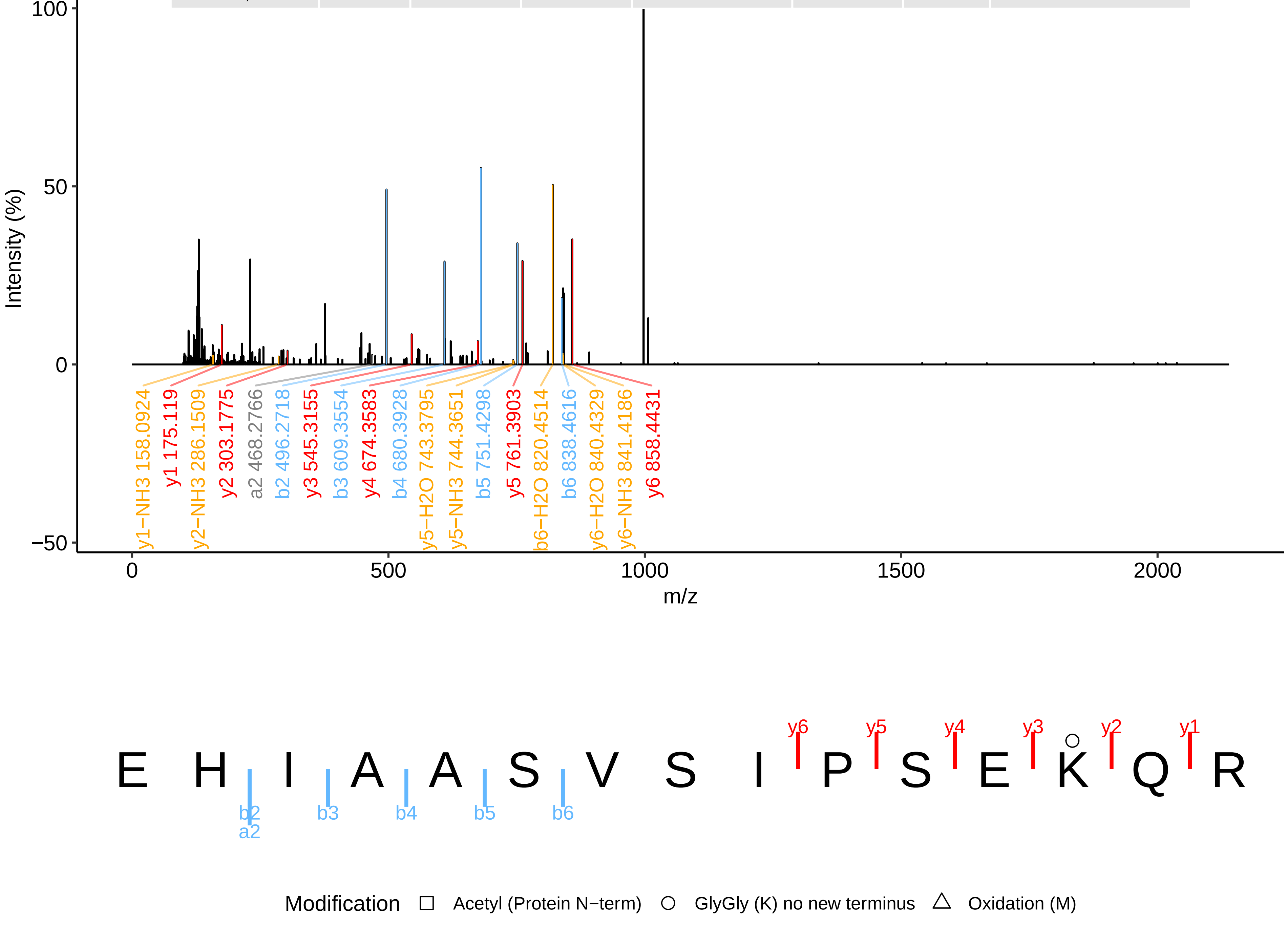

20200921_KIZ_MUb4_GlyGly_10plex_2 − Scan number: 8818

Gene Names	Charge	m/z	Mass	Retention time	PEP	Score	Precursor Intensity
BAG6;BAT3	4	442.2352	1764.912	33.35	0.0070152	62.287	789559.5

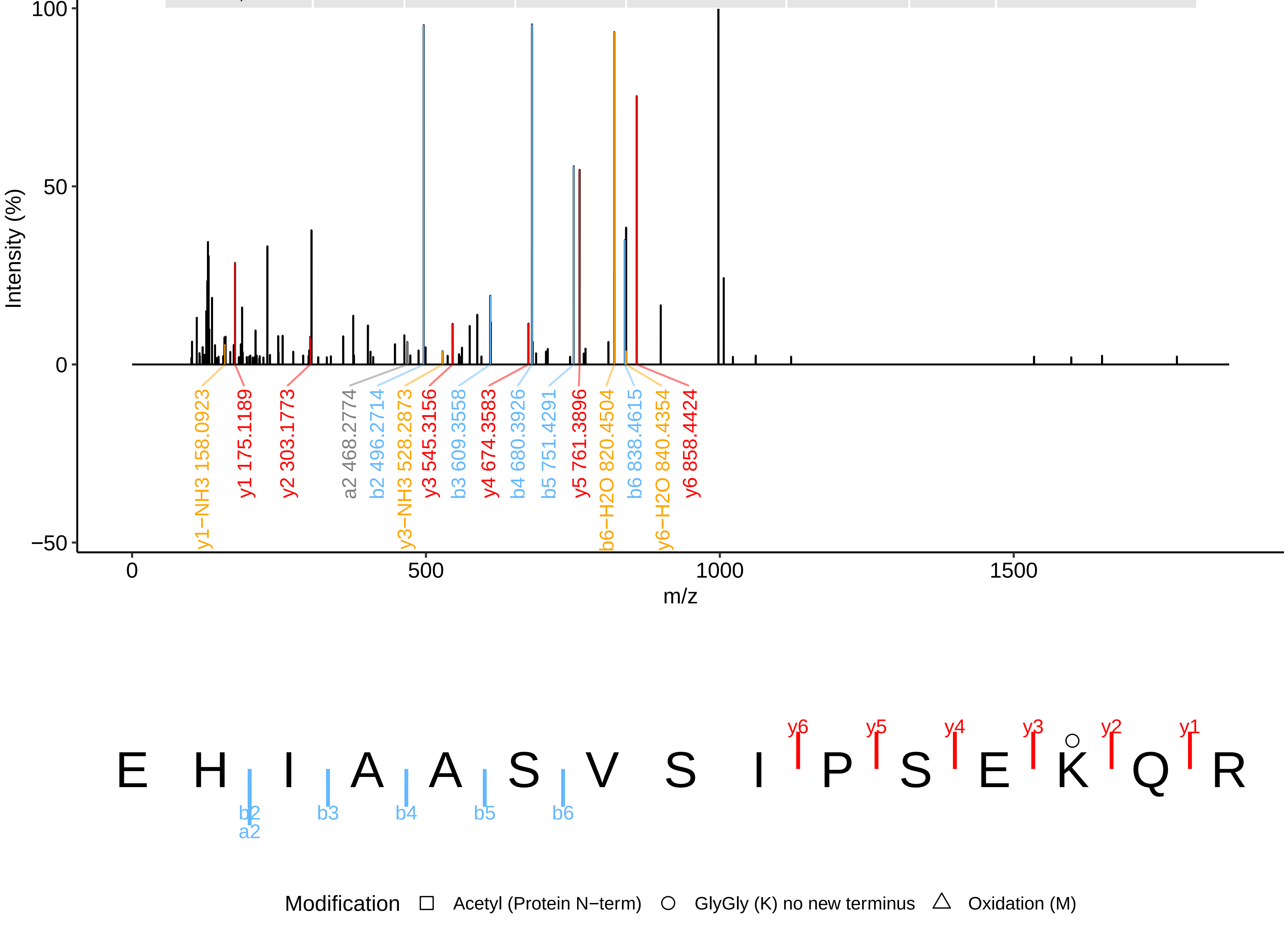

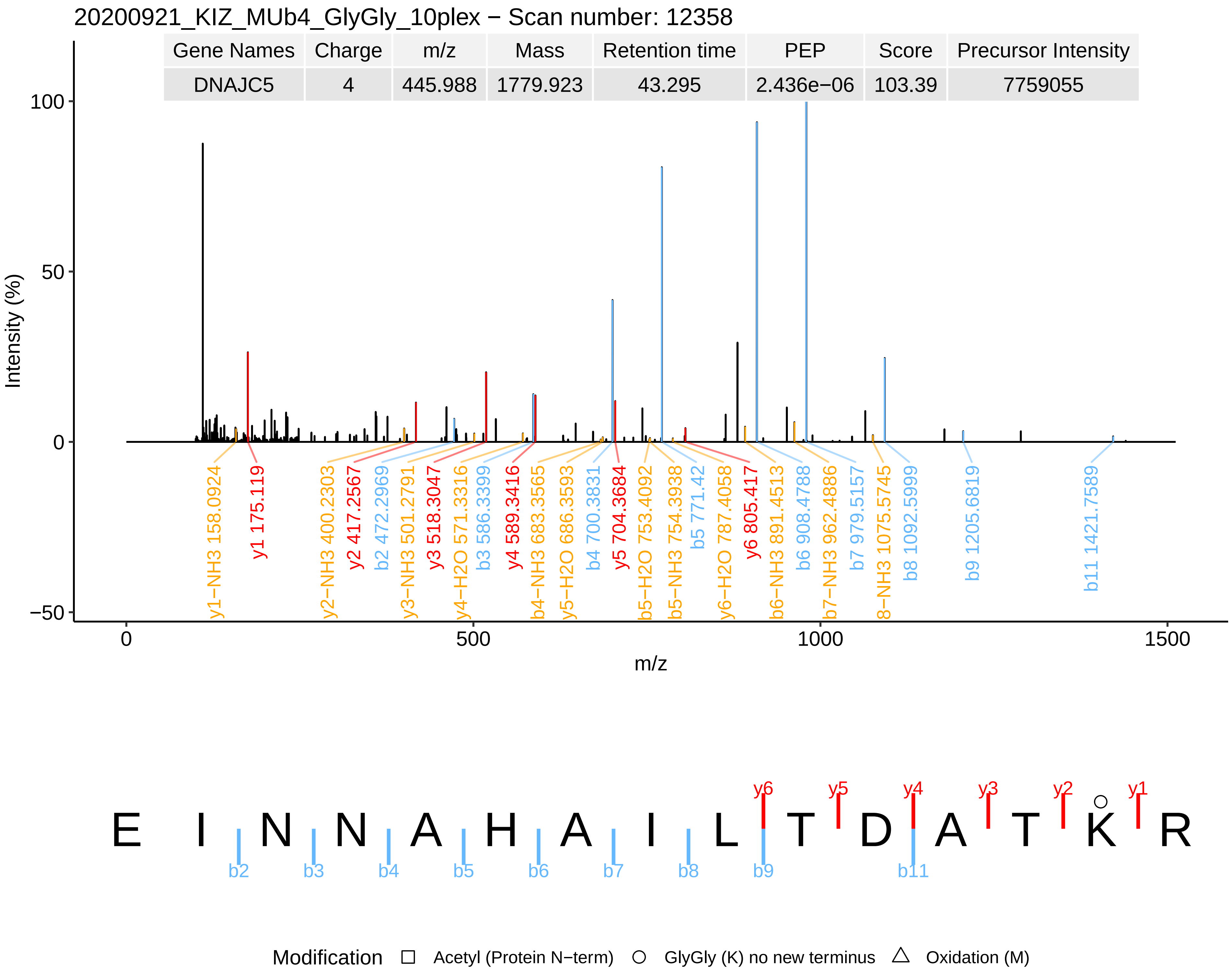
20200921_KIZ_MUb4_GlyGly_10plex − Scan number: 12358
Gene Names | Charge | m/z | Mass | Retention time | PEP | Score | Precursor Intensity
DNAJC5 | 4 | 445.988 | 1779.923 | 43.295 | 2.436e−06 | 103.39 | 7759055
Intensity (%)
m/z
y1−NH3 158.0924
y1 175.119
y2−NH3 400.2303
y2 417.2567
b2 472.2969
y3−NH3 501.2791
y3 518.3047
y4−H2O 571.3316
b3 586.3399
y4 589.3416
b4−NH3 683.3565
y5−H2O 686.3593
b4 700.3831
y5 704.3684
b5−H2O 753.4092
b5−NH3 754.3938
b5 771.42
y6−H2O 787.4058
y6 805.417
b6−NH3 891.4513
b6 908.4788
b7−NH3 962.4886
b7 979.5157
8−NH3 1075.5745
b8 1092.5999
b9 1205.6819
b11 1421.7589
E I N N A H A I L T D A T K R
Modification
Acetyl (Protein N−term)
GlyGly (K) no new terminus
Oxidation (M)

20200921_KIZ_MUb4_GlyGly_10plex − Scan number: 15841

Gene Names	Charge	m/z	Mass	Retention time	PEP	Score	Precursor Inte
ACTB;ACTG2;ACTG1;ACTA1;ACTC1;ACTA2	3	511.6166	1531.828	56.723	0.00085803	81.92	2035497

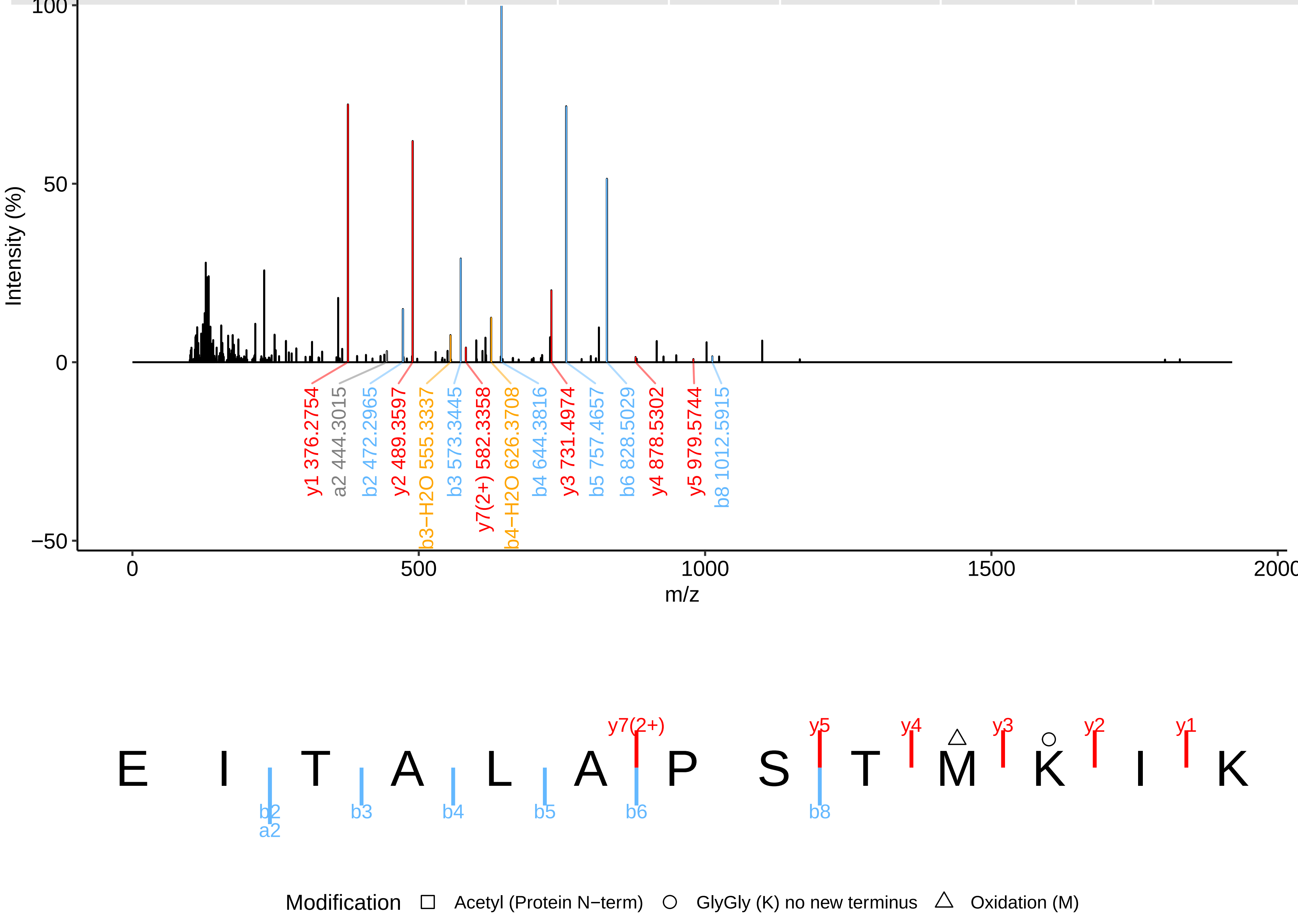

20200921_KIZ_MUb4_GlyGly_10plex_2 − Scan number: 14689

Gene Names	Charge	m/z	Mass	Retention time	PEP	Score	Precursor Inten
ACTB;ACTG2;ACTG1;ACTA1;ACTC1;ACTA2	3	511.6166	1531.828	57.306	0.021813	59.57	899630.5

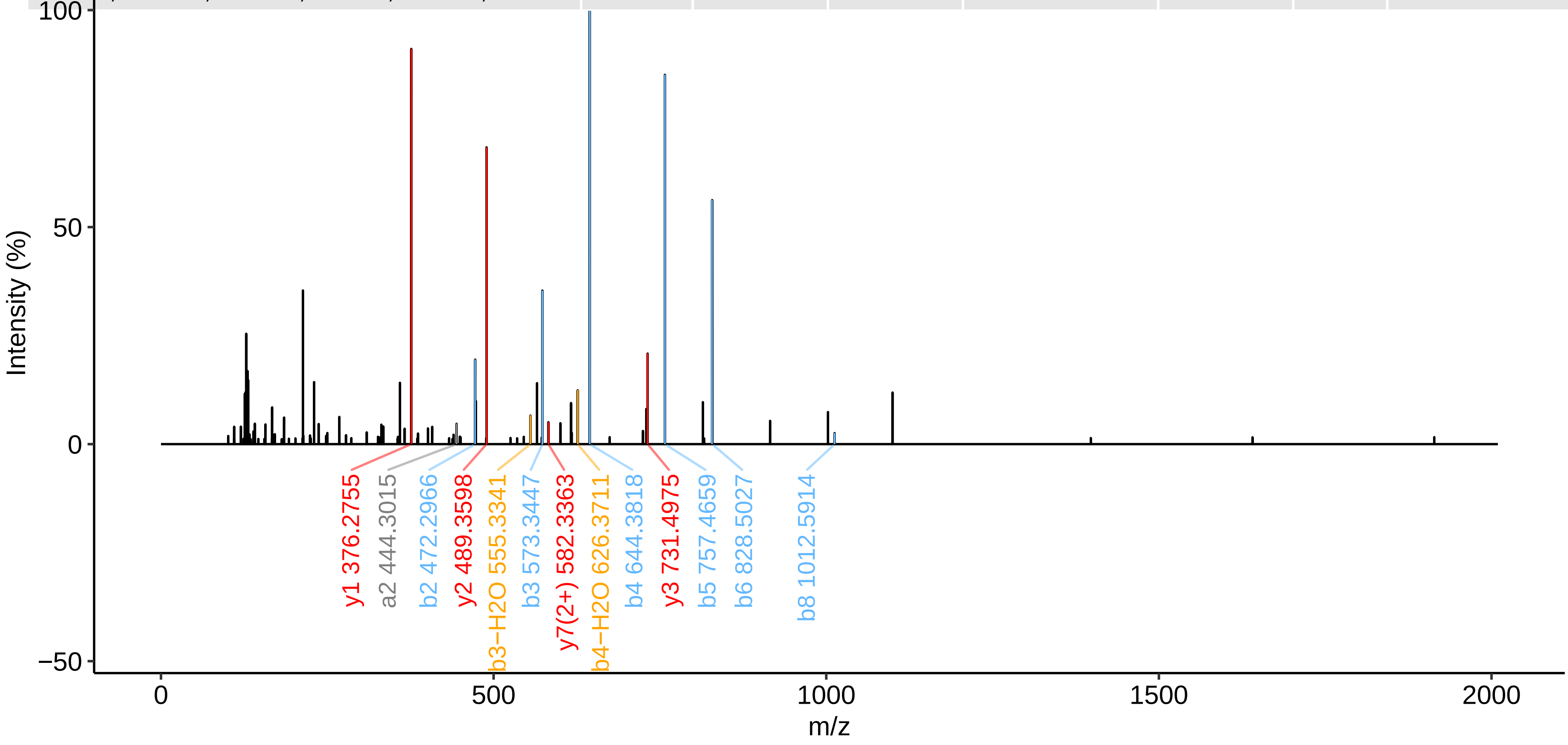

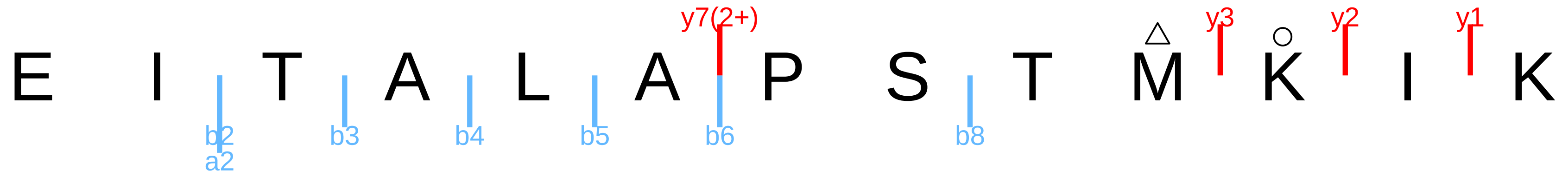

Modification □ Acetyl (Protein N−term) ○ GlyGly (K) no new terminus △ Oxidation (M)

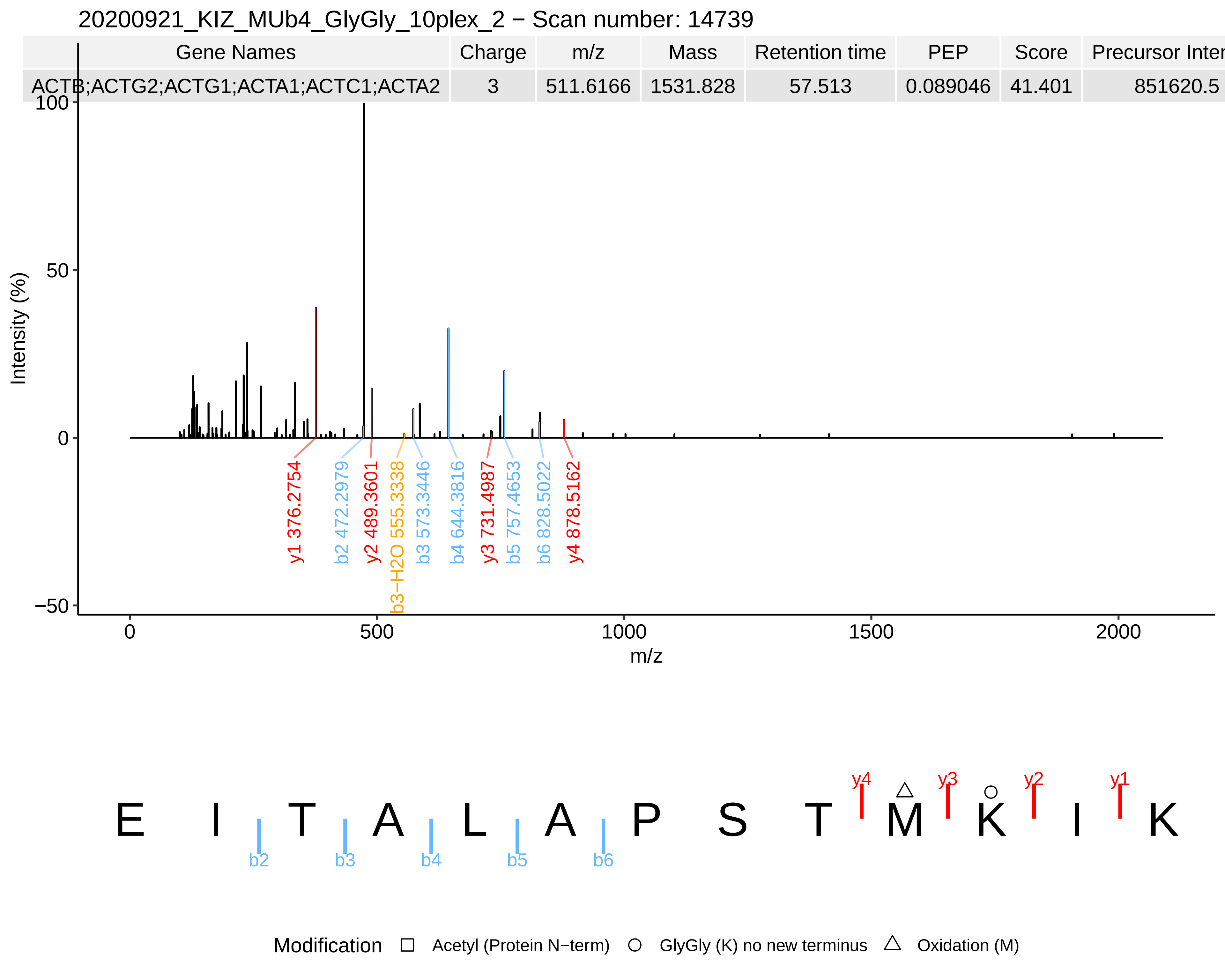
20200921_KIZ_MUb4_GlyGly_10plex_2 − Scan number: 14739
Gene Names | Charge | m/z | Mass | Retention time | PEP | Score | Precursor Inter
ACTB;ACTG2;ACTG1;ACTA1;ACTC1;ACTA2 | 3 | 511.6166 | 1531.828 | 57.513 | 0.089046 | 41.401 | 851620.5
Intensity (%)
100
50
0
−50
y1 376.2754
b2 472.2979
y2 489.3601
b3−H2O 555.3338
b3 573.3446
b4 644.3816
y3 731.4987
b5 757.4653
b6 828.5022
y4 878.5162
0
500
1000
1500
2000
m/z
E I T A L A P S T M K I K
y4 y3 y2 y1
b2 b3 b4 b5 b6
Modification
Acetyl (Protein N−term)
GlyGly (K) no new terminus
Oxidation (M)

20200924_KIZ_MUb5_GlyGly_2 − Scan number: 14187

Gene Names	Charge	m/z	Mass	Retention time	PEP	Score	Precursor Inten
ACTB;ACTG2;ACTG1;ACTA1;ACTC1;ACTA2	3	511.6166	1531.828	53.034	0.11727	51.013	2889090

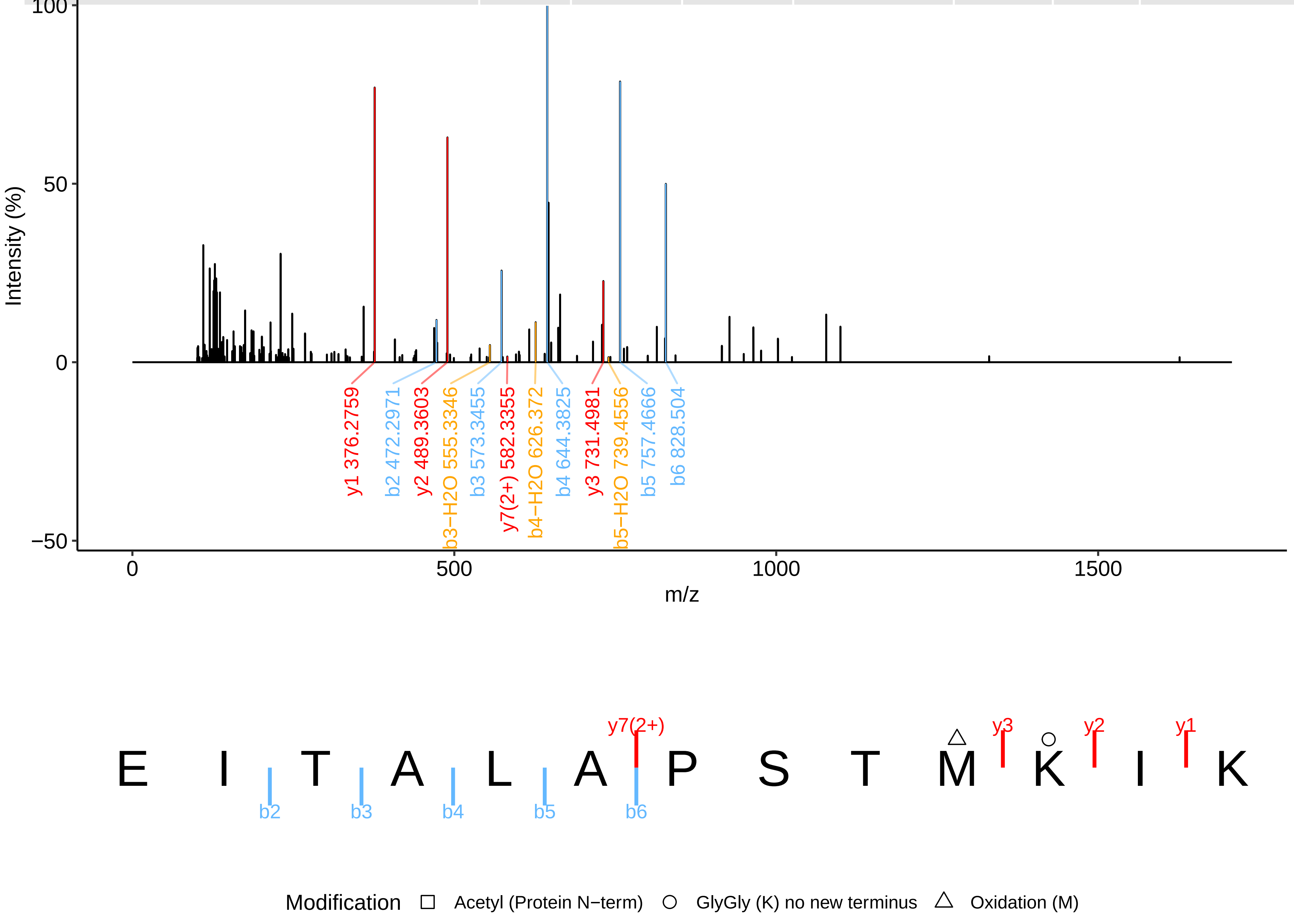

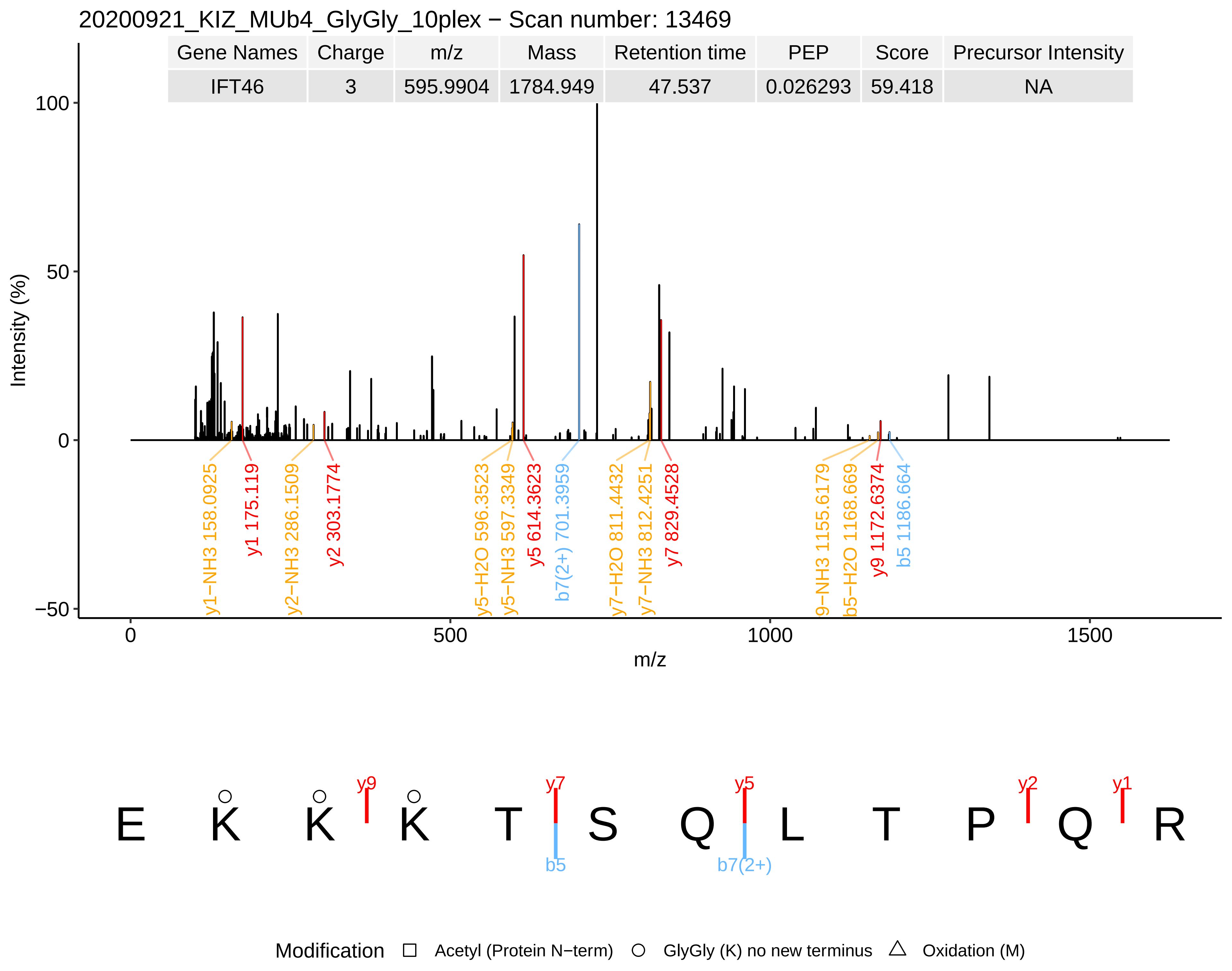
20200921_KIZ_MUb4_GlyGly_10plex − Scan number: 13469
Gene Names
Charge
m/z
Mass
Retention time
PEP
Score
Precursor Intensity
IFT46
3
595.9904
1784.949
47.537
0.026293
59.418
NA
Intensity (%)
100
50
0
−50
0
500
1000
1500
m/z
y1−NH3 158.0925
y1 175.119
y2−NH3 286.1509
y2 303.1774
y5−H2O 596.3523
y5−NH3 597.3349
y5 614.3623
b7(2+) 701.3959
y7−H2O 811.4432
y7−NH3 812.4251
y7 829.4528
9−NH3 1155.6179
b5−H2O 1168.669
y9 1172.6374
b5 1186.664
E
K
K
K
T
S
Q
L
T
P
Q
R
y9
y7
y5
y2
y1
b5
b7(2+)
Modification
Acetyl (Protein N−term)
GlyGly (K) no new terminus
Oxidation (M)

20200921_KIZ_MUb4_GlyGly_10plex − Scan number: 18329

Gene Names	Charge	m/z	Mass	Retention time	PEP	Score	Precursor Intensity
S100A6	3	586.6513	1756.932	65.887	3.93e−09	119.62	24073708

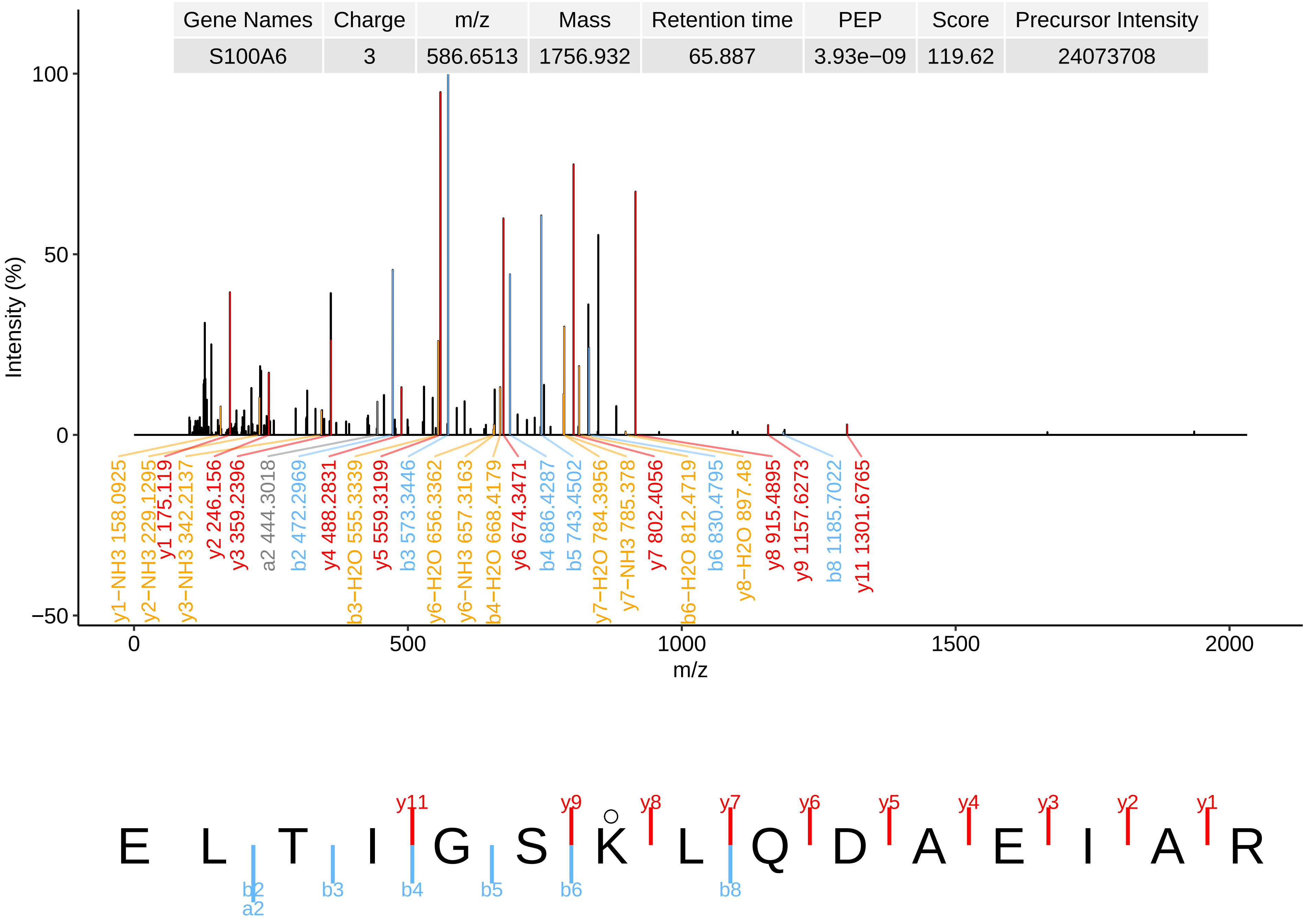

Modification □ Acetyl (Protein N−term) ○ GlyGly (K) no new terminus △ Oxidation (M)

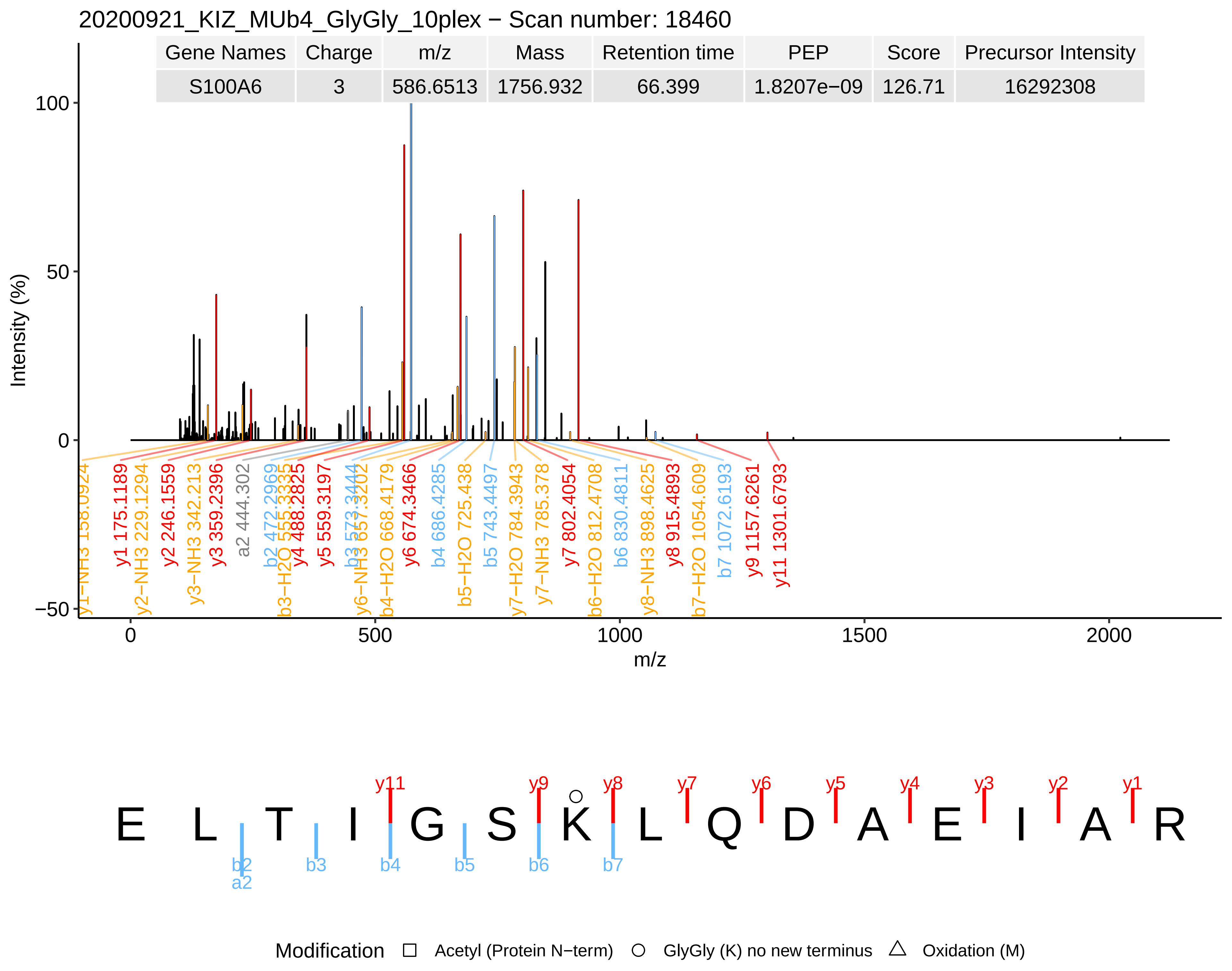
20200921_KIZ_MUb4_GlyGly_10plex − Scan number: 18460
Gene Names | Charge | m/z | Mass | Retention time | PEP | Score | Precursor Intensity
S100A6 | 3 | 586.6513 | 1756.932 | 66.399 | 1.8207e−09 | 126.71 | 16292308
Intensity (%)
m/z
y1−NH3 158.0924
y1 175.1189
y2−NH3 229.1294
y2 246.1559
y3−NH3 342.213
y3 359.2396
a2 444.302
b2 472.2969
b3−H2O 555.3335
y4 488.2825
y5 559.3197
b3 573.3444
y6−NH3 657.3202
b4−H2O 668.4179
y6 674.3466
b4 686.4285
b5−H2O 725.438
b5 743.4497
y7−H2O 784.3943
y7−NH3 785.378
y7 802.4054
b6−H2O 812.4708
b6 830.4811
y8−NH3 898.4625
y8 915.4893
b7−H2O 1054.609
b7 1072.6193
y9 1157.6261
y11 1301.6793
E L T I G S K L Q D A E I A R
Modification
Acetyl (Protein N−term)
GlyGly (K) no new terminus
Oxidation (M)

20200921_KIZ_MUb4_GlyGly_10plex − Scan number: 18727

Gene Names	Charge	m/z	Mass	Retention time	PEP	Score	Precursor Intensity
S100A6	3	586.6513	1756.932	67.414	1.5212e−09	127.71	11400032

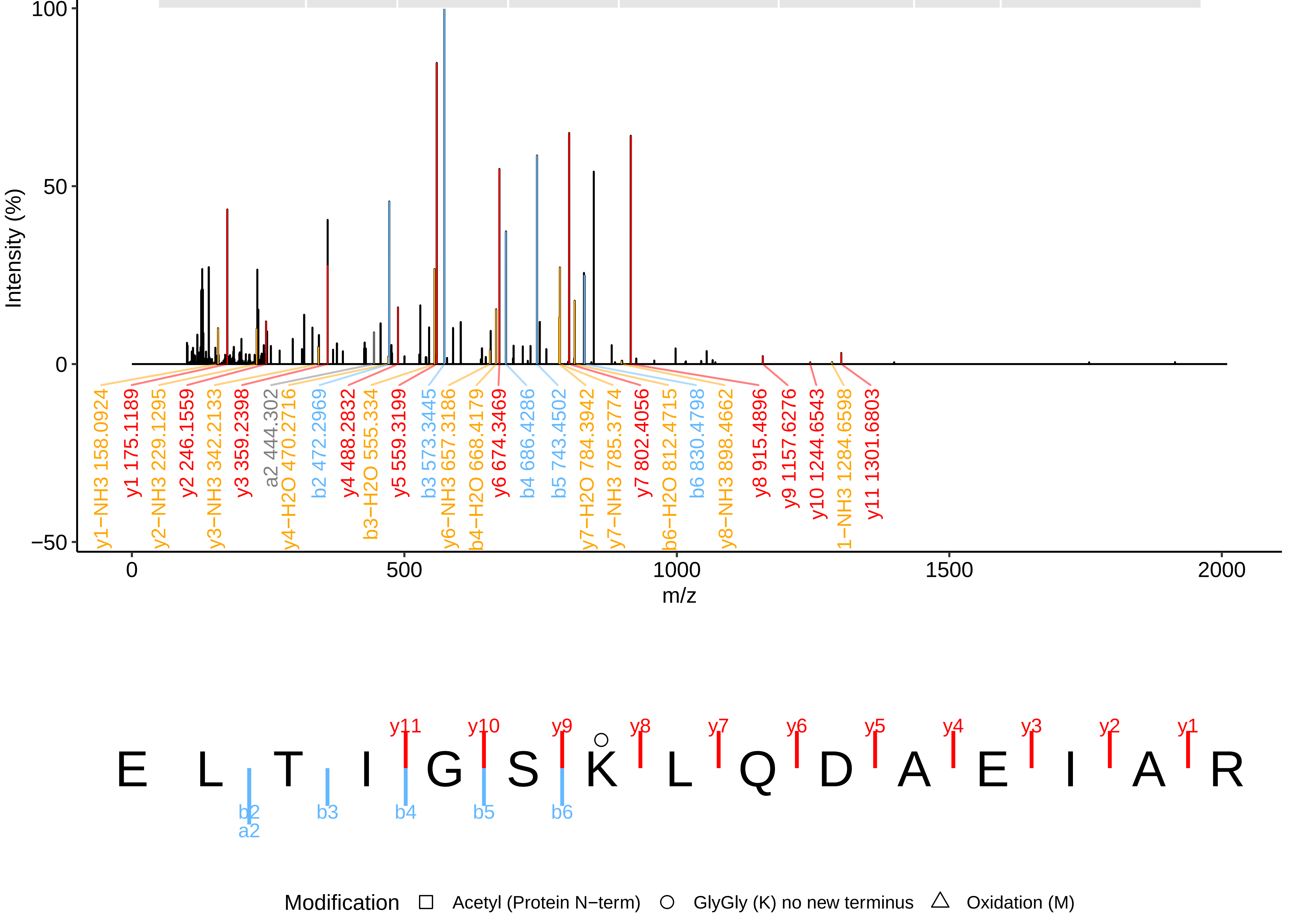

20200921_KIZ_MUb4_GlyGly_10plex − Scan number: 18857

Gene Names	Charge	m/z	Mass	Retention time	PEP	Score	Precursor Intensity
S100A6	3	586.6513	1756.932	67.922	6.5608e−05	91.845	1887578

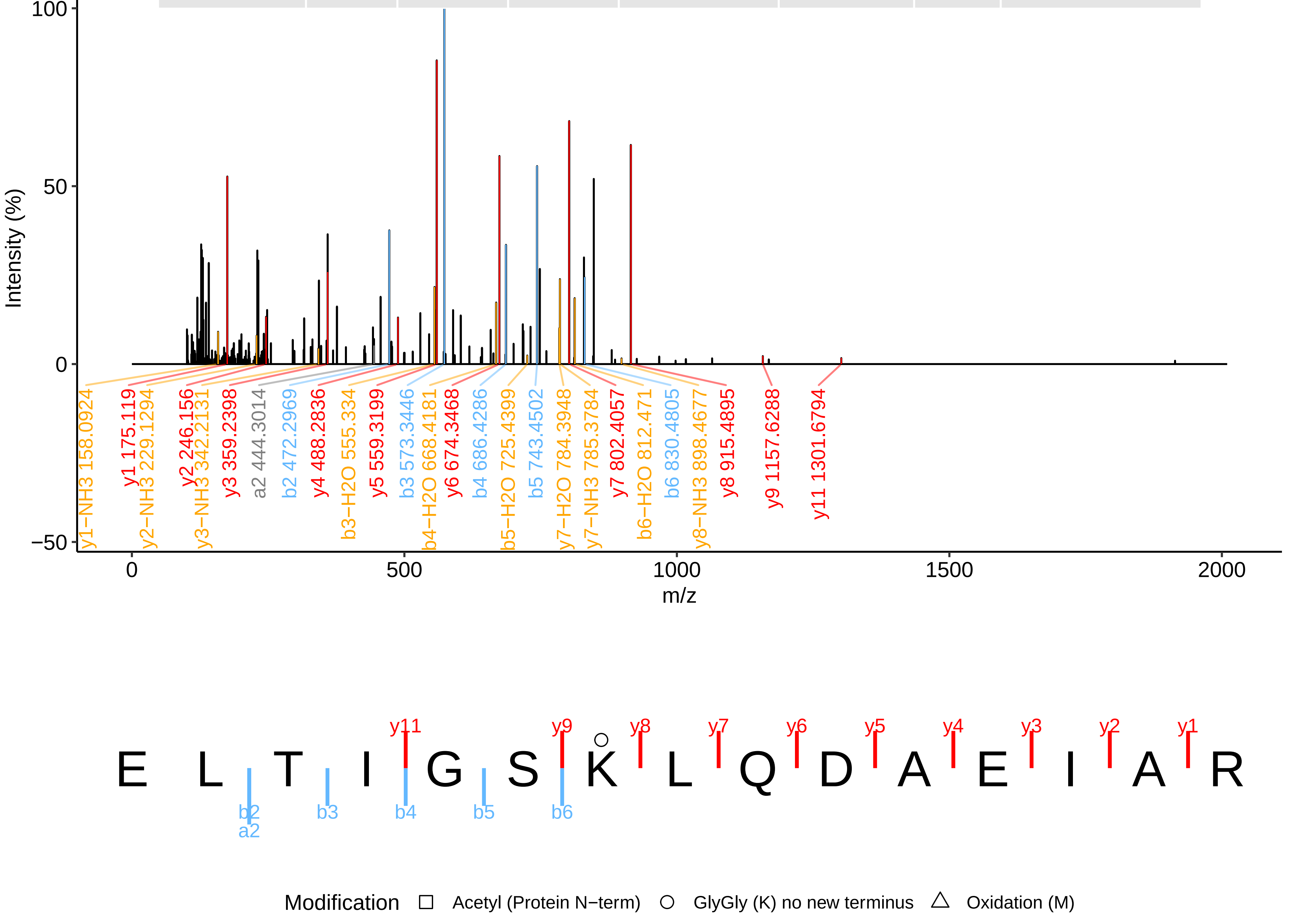

20200921_KIZ_MUb4_GlyGly_10plex − Scan number: 18197

Gene Names	Charge	m/z	Mass	Retention time	PEP	Score	Precursor Intensity
S100A6	3	586.6513	1756.932	65.384	0.00030088	83.856	5319636

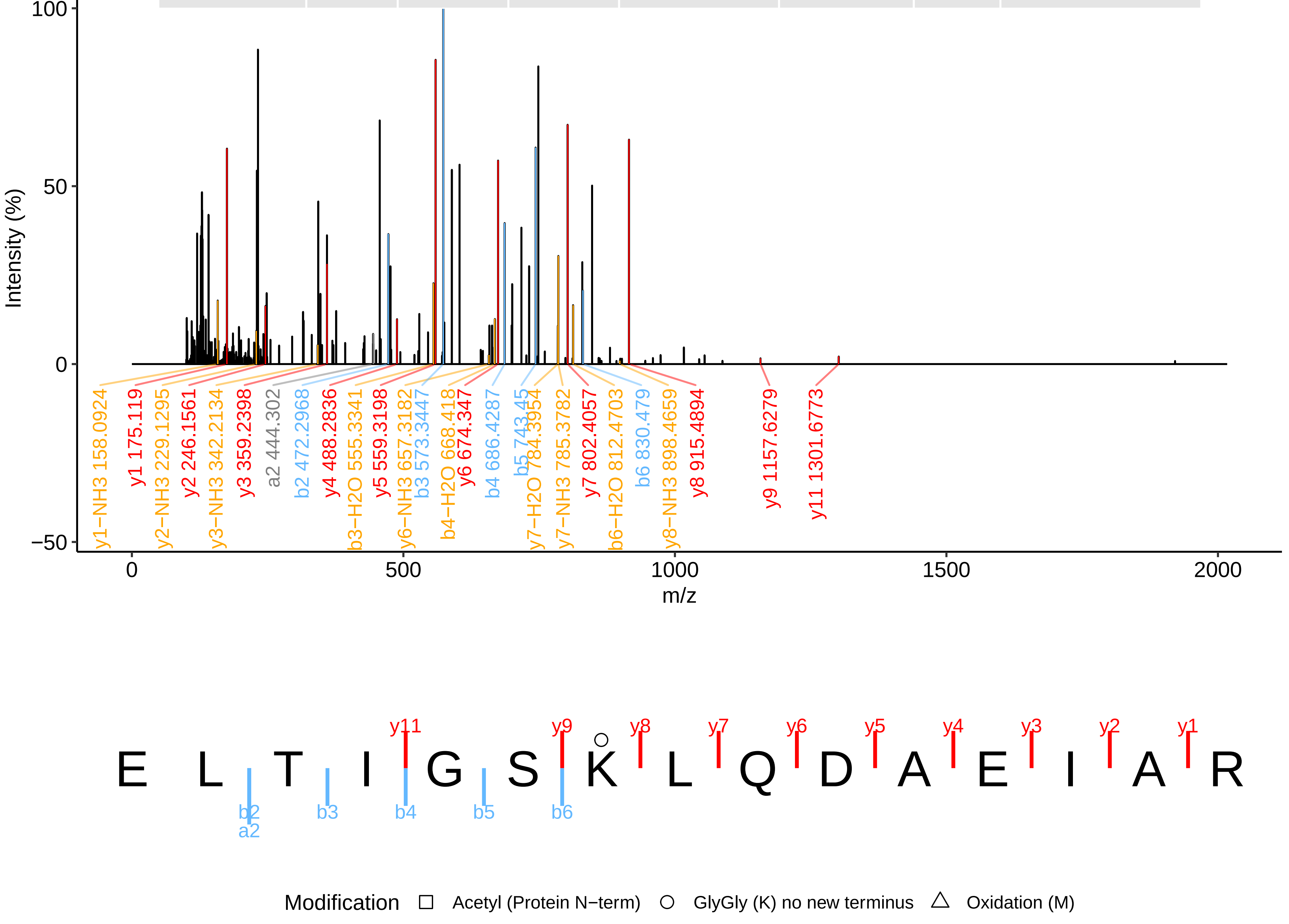

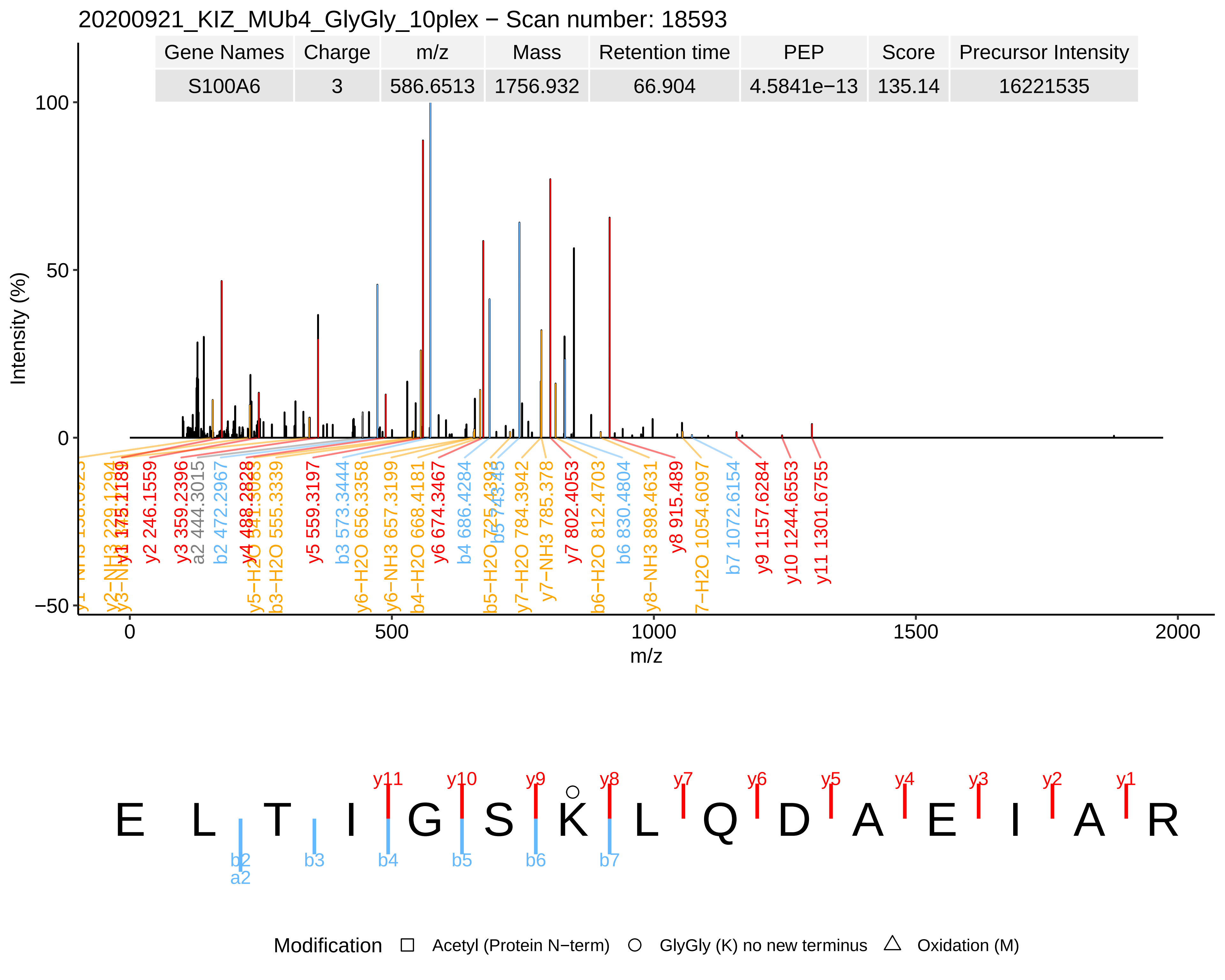
20200921_KIZ_MUb4_GlyGly_10plex − Scan number: 18593
Gene Names | Charge | m/z | Mass | Retention time | PEP | Score | Precursor Intensity
S100A6 | 3 | 586.6513 | 1756.932 | 66.904 | 4.5841e−13 | 135.14 | 16221535
Intensity (%)
m/z
y1 175.1189
y2 246.1559
y3 359.2396
a2 444.3015
b2 472.2967
y4 488.2828
b3−H2O 555.3339
y5 559.3197
b3 573.3444
y6−H2O 656.3358
y6−NH3 657.3199
b4−H2O 668.4181
y6 674.3467
b4 686.4284
y7−H2O 784.3942
y7−NH3 785.378
y7 802.4053
b6−H2O 812.4703
b6 830.4804
y8−NH3 898.4631
y8 915.489
b7 1072.6154
y9 1157.6284
y10 1244.6553
y11 1301.6755
E L T I G S K L Q D A E I A R
y11 y10 y9 y8 y7 y6 y5 y4 y3 y2 y1
b2 b3 b4 b5 b6 b7
a2
Modification
Acetyl (Protein N−term)
GlyGly (K) no new terminus
Oxidation (M)

20200921_KIZ_MUb4_GlyGly_10plex_2 − Scan number: 16624

Gene Names	Charge	m/z	Mass	Retention time	PEP	Score	Precursor Intensity
S100A6	3	586.6513	1756.932	64.922	2.5585e−06	106.58	3685345

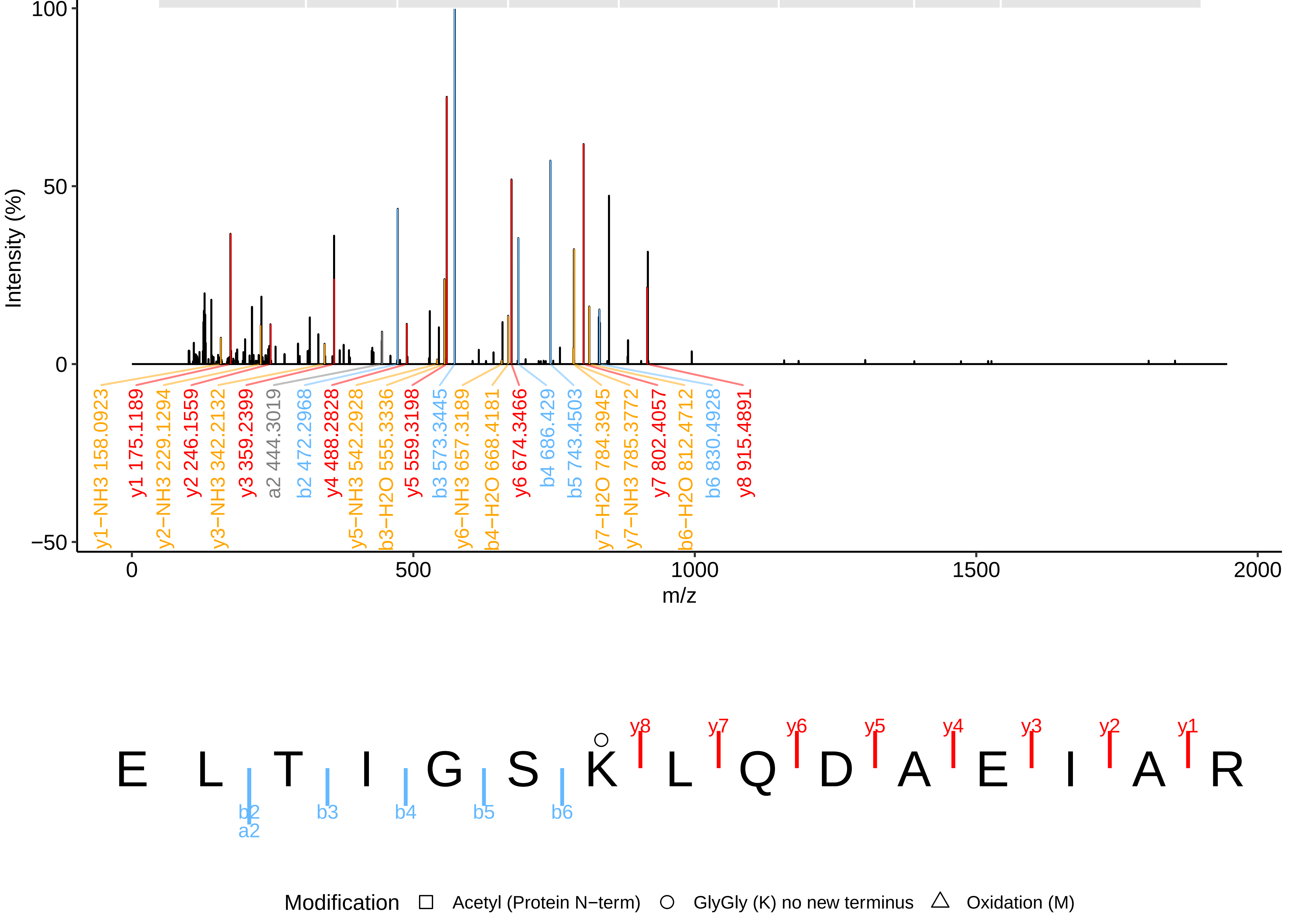

20200921_KIZ_MUb4_GlyGly_10plex_2 − Scan number: 16754

Gene Names	Charge	m/z	Mass	Retention time	PEP	Score	Precursor Intensity
S100A6	3	586.6513	1756.932	65.44	0.00017524	80.706	992739.1

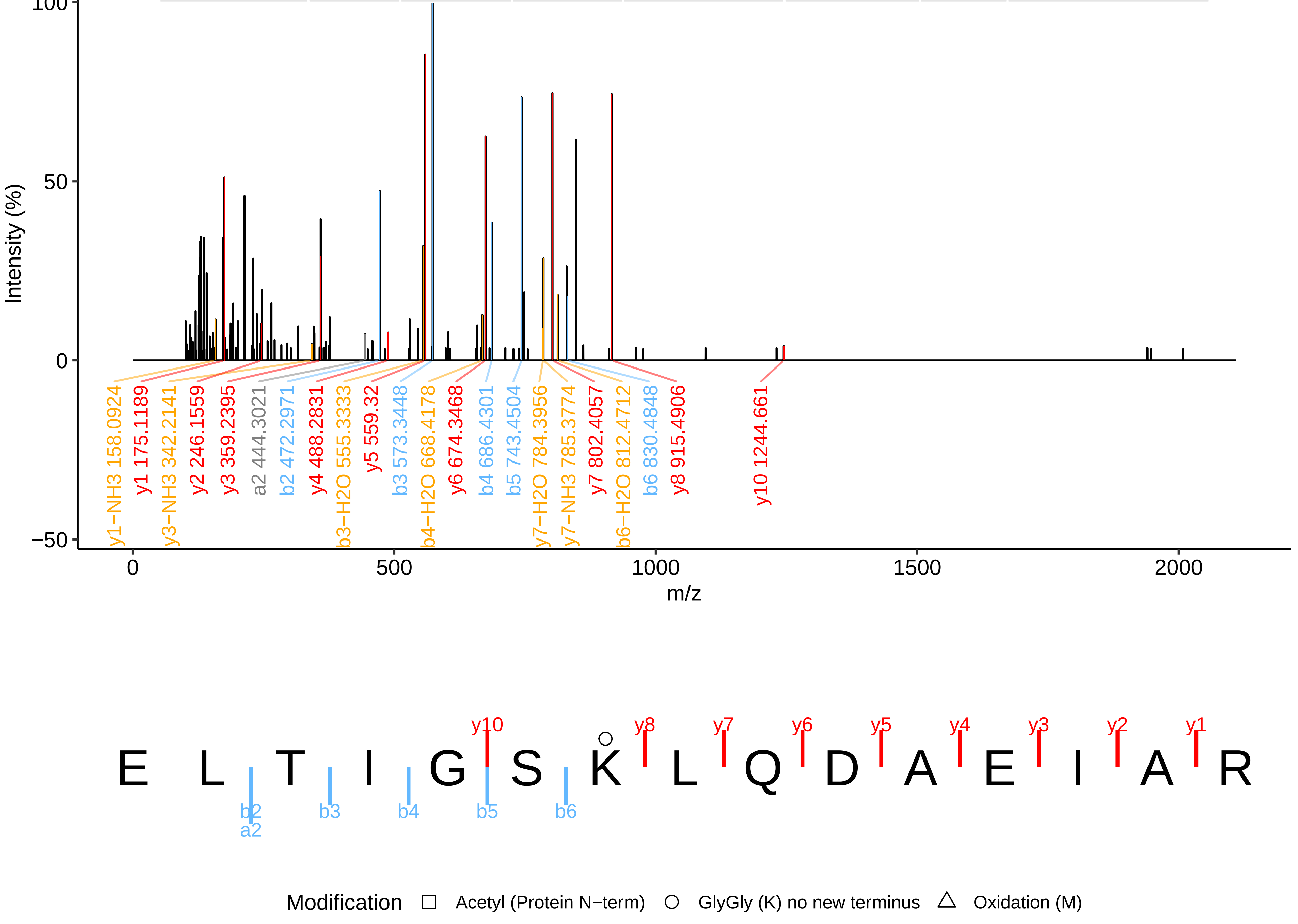

20200924_KIZ_MUb5_GlyGly_1 − Scan number: 17679

Gene Names	Charge	m/z	Mass	Retention time	PEP	Score	Precursor Intensity
S100A6	3	586.6513	1756.932	57.111	9.0683e−14	137.93	7169743

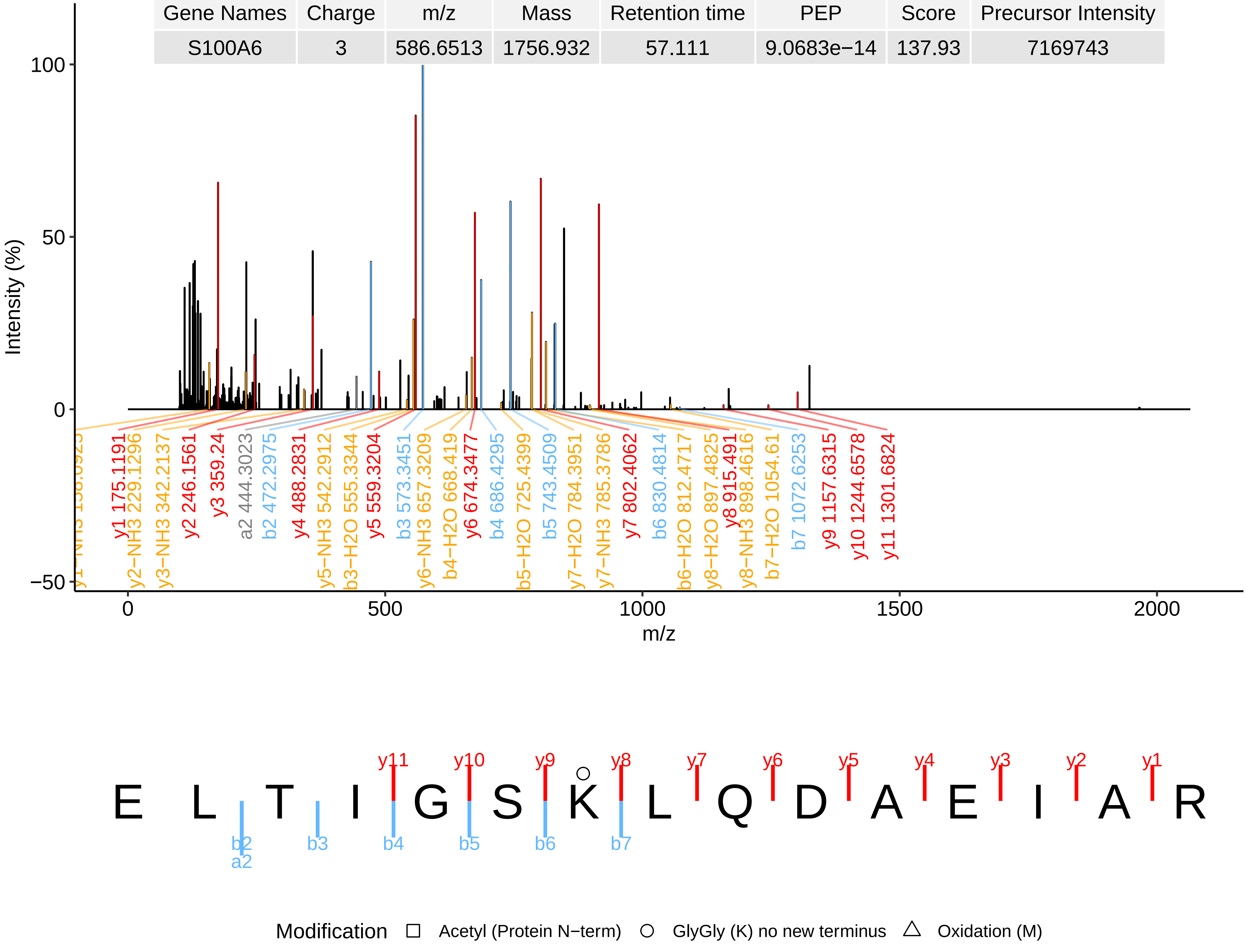

20200924_KIZ_MUb5_GlyGly_1 − Scan number: 17832

Gene Names	Charge	m/z	Mass	Retention time	PEP	Score	Precursor Intensity
S100A6	3	586.6513	1756.932	57.621	5.5384e−20	154.65	19693186

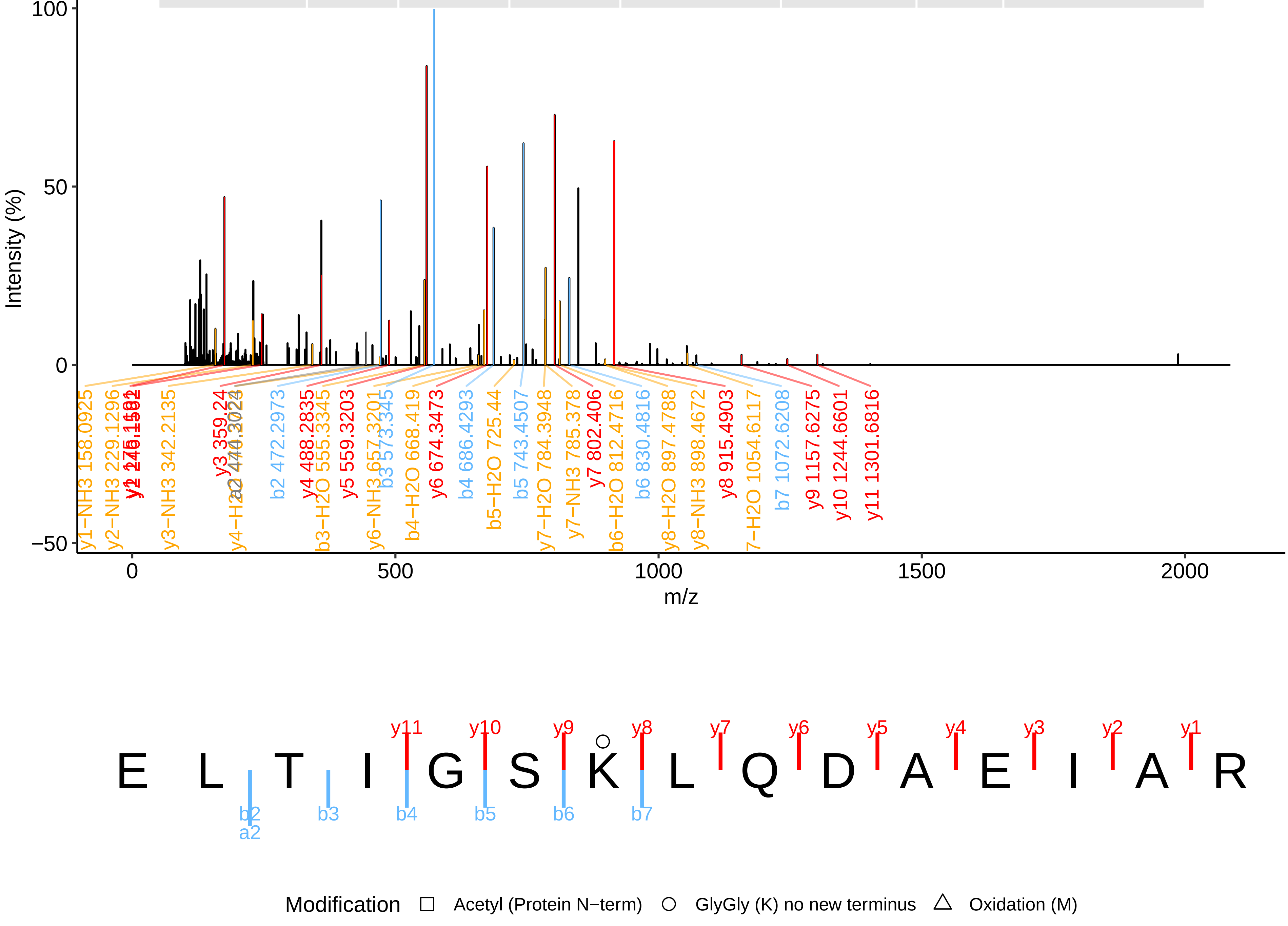

20200924_KIZ_MUb5_GlyGly_2 − Scan number: 16438

Gene Names	Charge	m/z	Mass	Retention time	PEP	Score	Precursor Intensity
S100A6	3	586.6513	1756.932	60.99	8.5585e−21	165.64	5045731

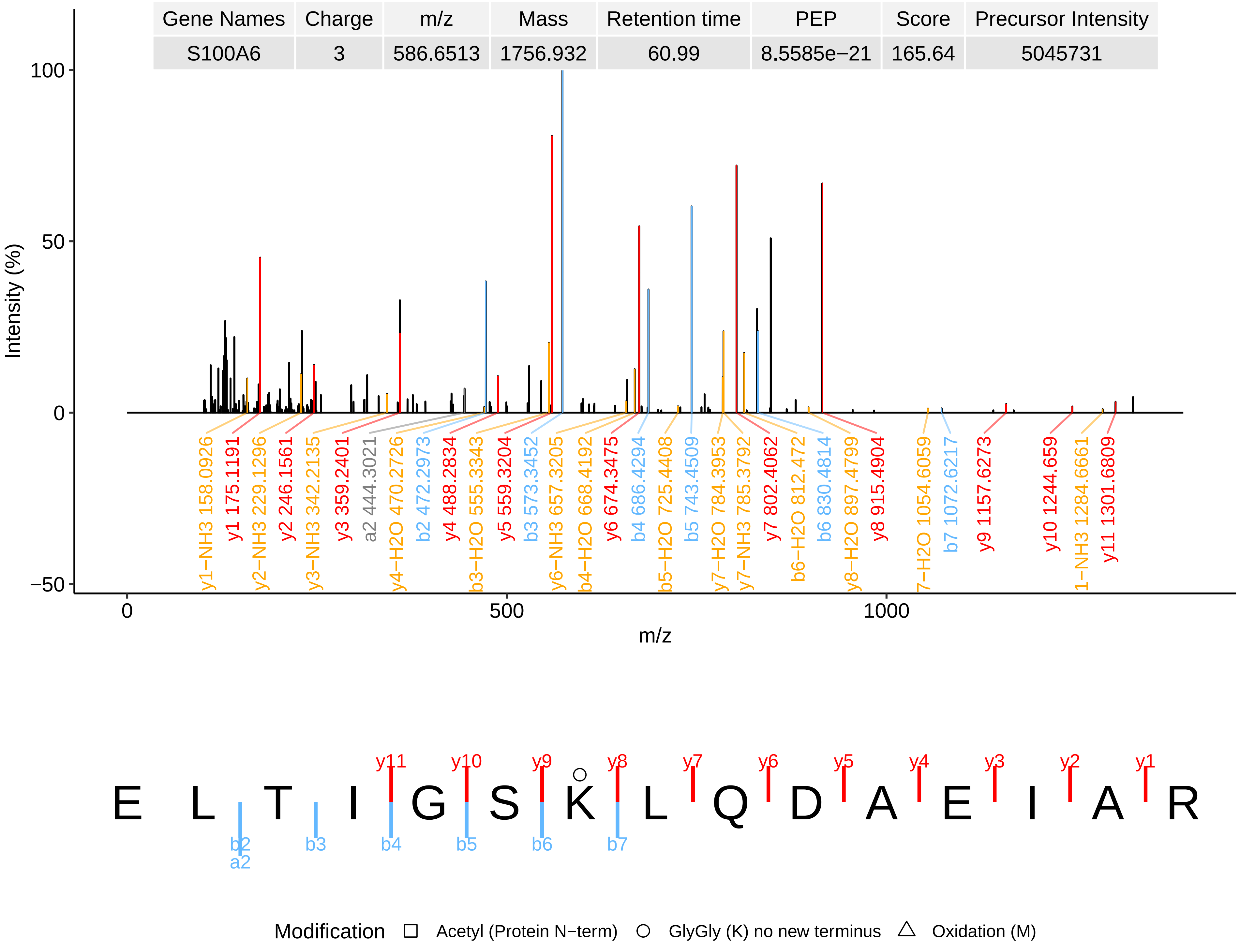

20201118_KIZ_MUb6_GlyGly_1 − Scan number: 17948

Gene Names	Charge	m/z	Mass	Retention time	PEP	Score	Precursor Intensity
S100A6	3	586.6513	1756.932	56.449	5.5384e−20	154.65	22739332

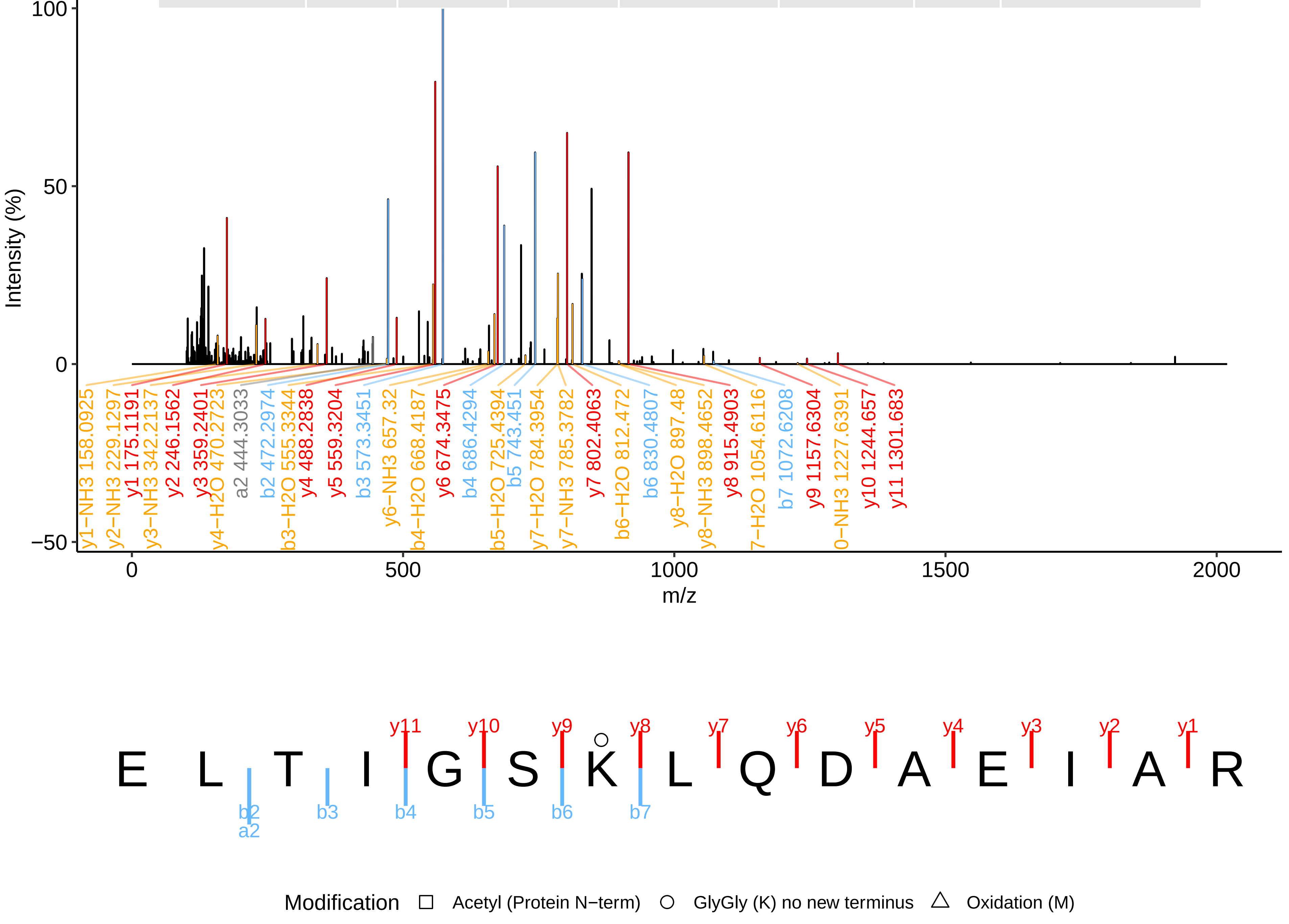

20201118_KIZ_MUb6_GlyGly_2 − Scan number: 19482

Gene Names	Charge	m/z	Mass	Retention time	PEP	Score	Precursor Intensity
S100A6	3	586.6513	1756.932	65.74	2.1291e−14	147.35	7105916

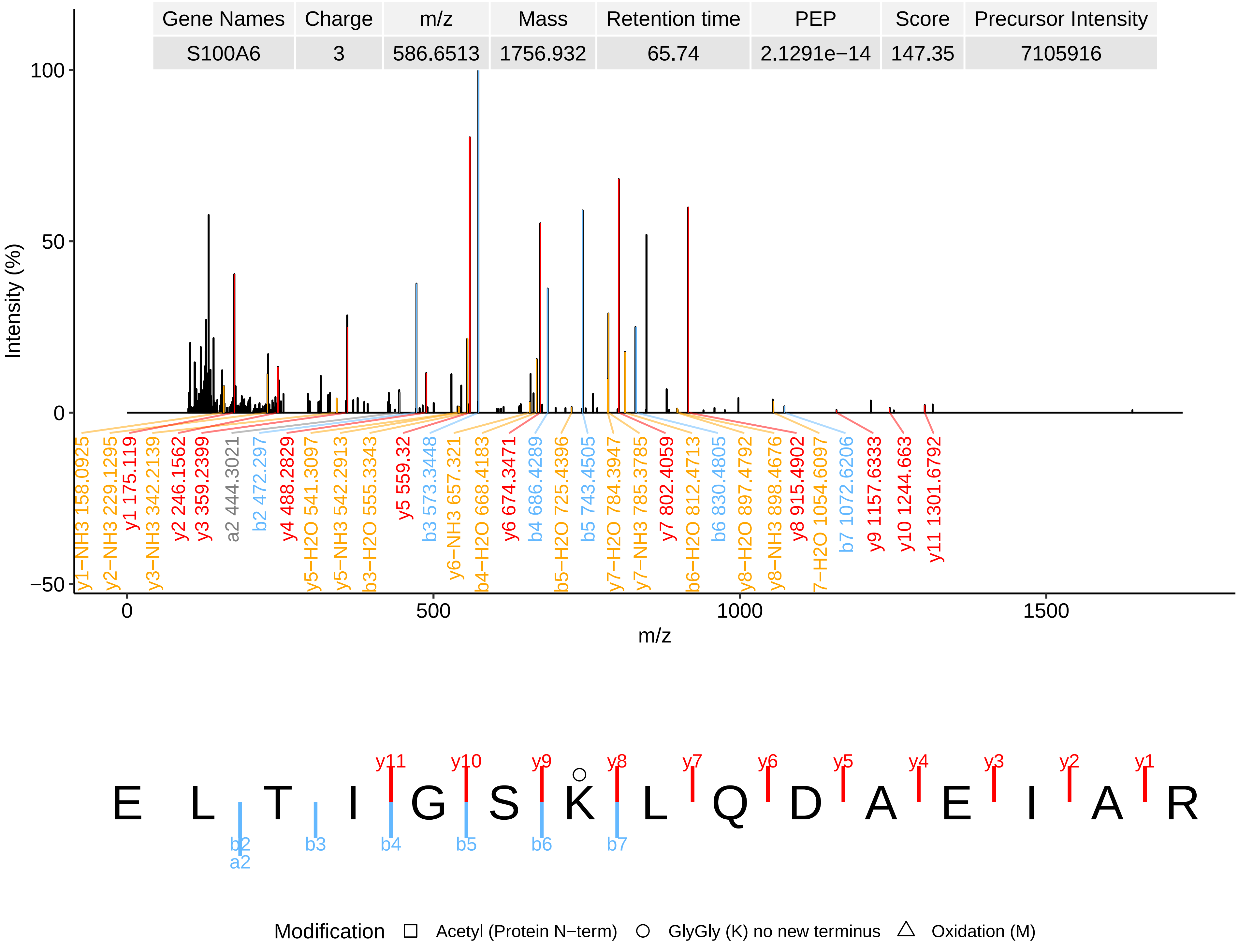

20200921_KIZ_MUb4_GlyGly_10plex − Scan number: 12320

Gene Names	Charge	m/z	Mass	Retention time	PEP	Score	Precursor Intensity
ALDOA	3	512.5949	1534.763	43.15	2.6032e−06	113.24	10559561

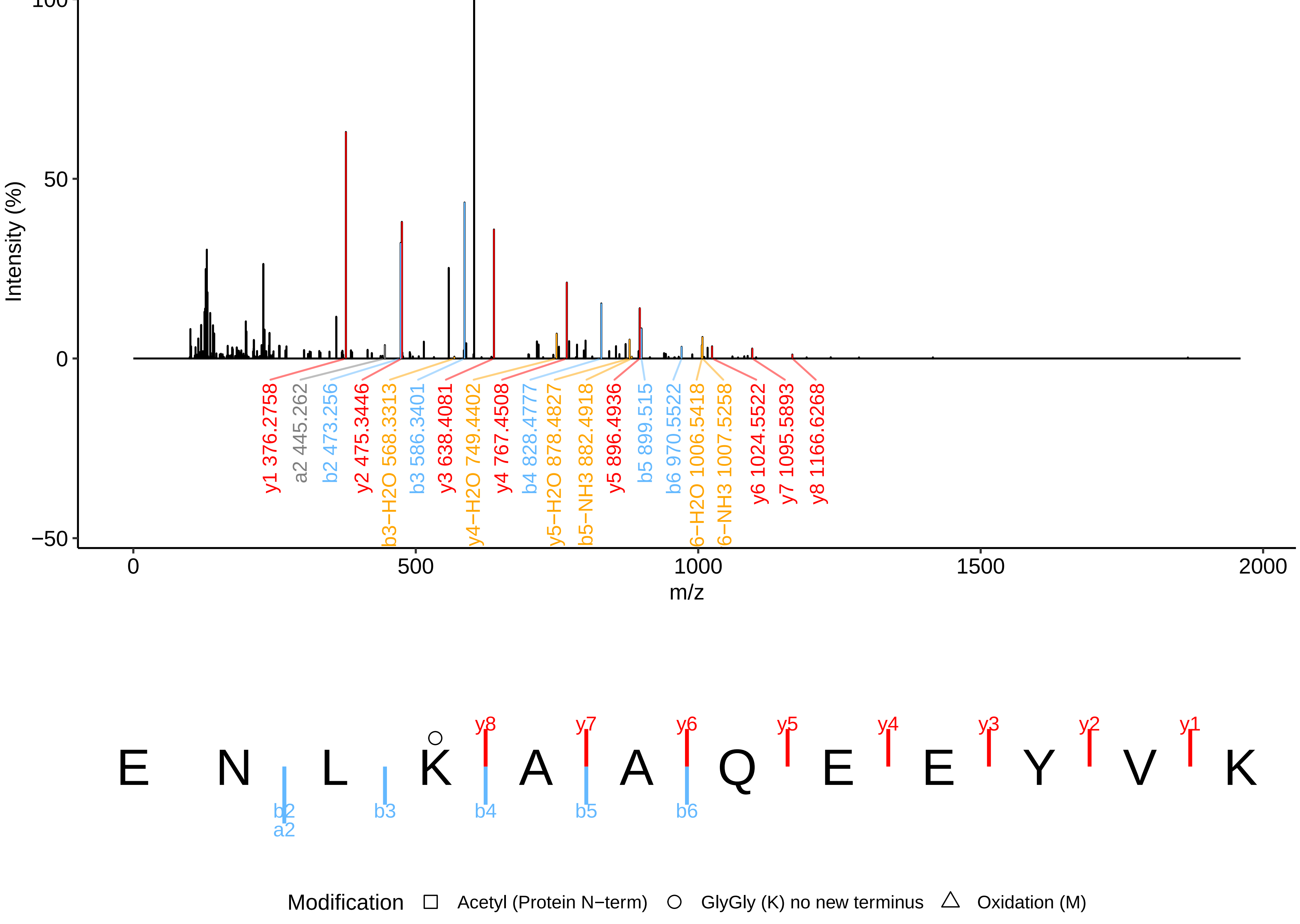

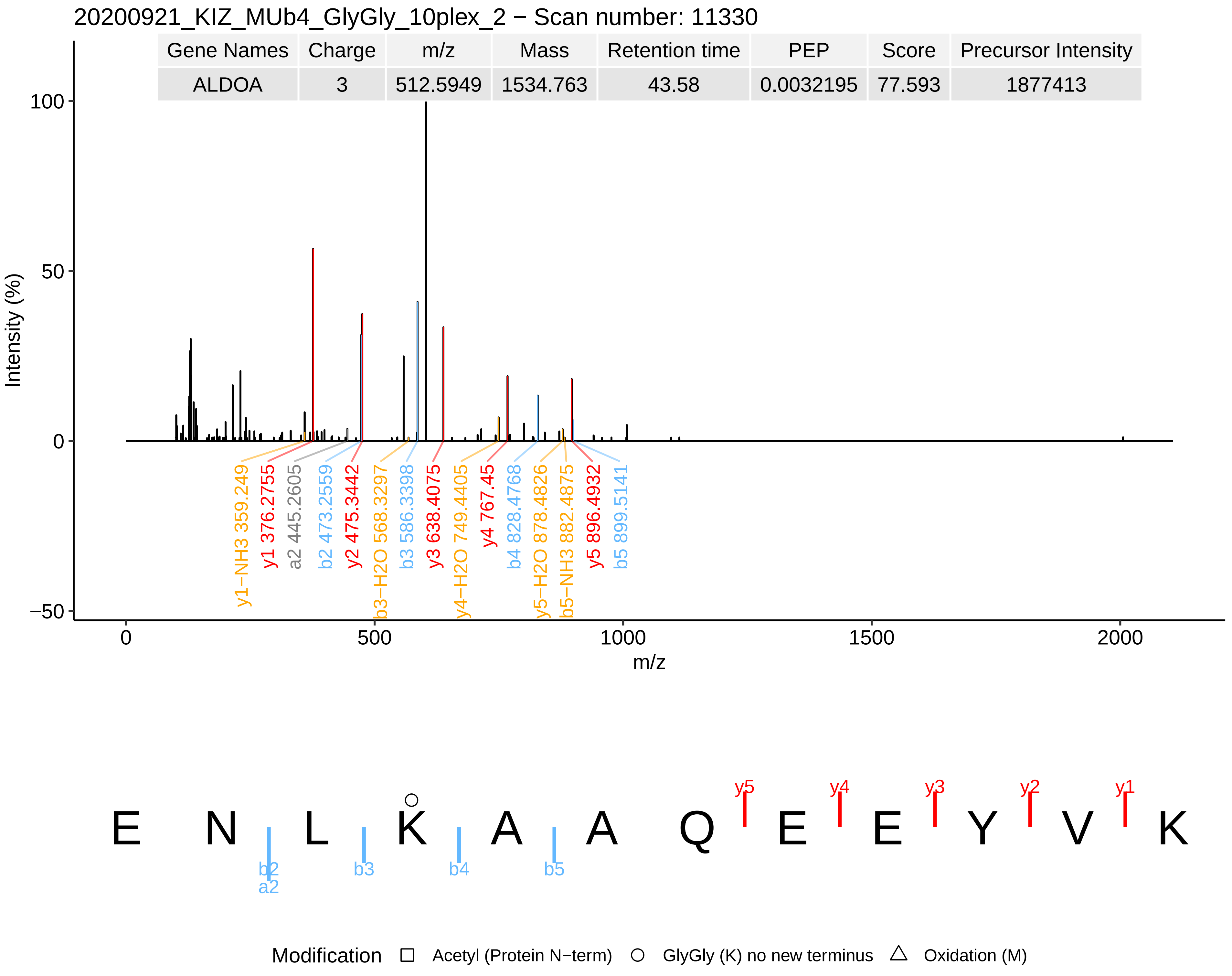
20200921_KIZ_MUb4_GlyGly_10plex_2 − Scan number: 11330
Gene Names | Charge | m/z | Mass | Retention time | PEP | Score | Precursor Intensity
ALDOA | 3 | 512.5949 | 1534.763 | 43.58 | 0.0032195 | 77.593 | 1877413
Intensity (%)
100
50
0
−50
0
500
1000
1500
2000
m/z
y1−NH3 359.249
y1 376.2755
a2 445.2605
b2 473.2559
y2 475.3442
b3−H2O 568.3297
b3 586.3398
y3 638.4075
y4−H2O 749.4405
y4 767.45
b4 828.4768
y5−H2O 878.4826
b5−NH3 882.4875
y5 896.4932
b5 899.5141
E N L K A A Q E E Y V K
y5 y4 y3 y2 y1
b2 a2 b3 b4 b5
Modification
Acetyl (Protein N−term)
GlyGly (K) no new terminus
Oxidation (M)

20200921_KIZ_MUb4_GlyGly_10plex_2 − Scan number: 11337

Gene Names	Charge	m/z	Mass	Retention time	PEP	Score	Precursor Intensity
ALDOA	3	512.5949	1534.763	43.611	8.5647e−09	122.42	3460151

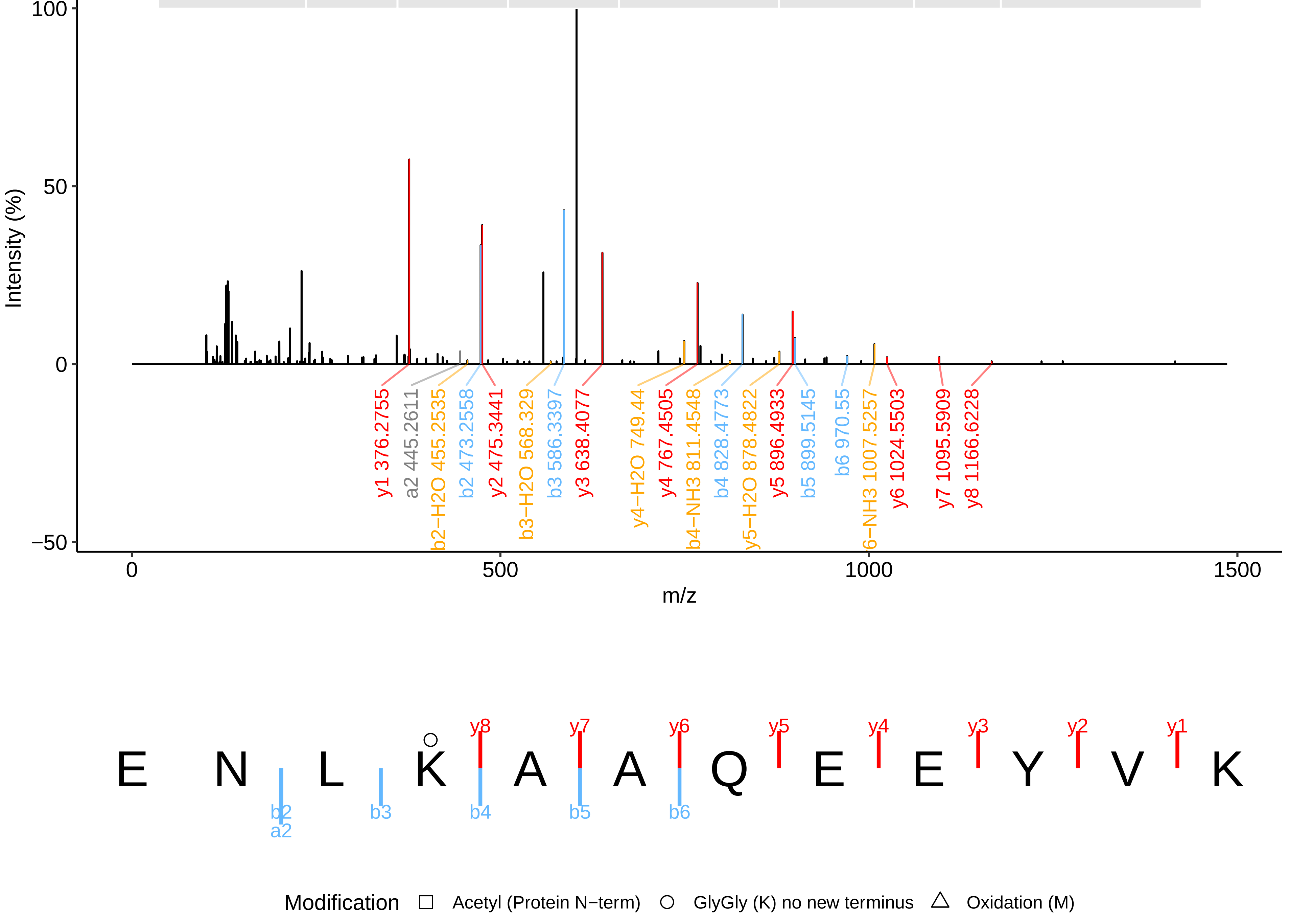

20200924_KIZ_MUb5_GlyGly_1 − Scan number: 11407

Gene Names	Charge	m/z	Mass	Retention time	PEP	Score	Precursor Intensity
ALDOA	3	512.5949	1534.763	36.951	3.8824e−05	102.87	6921349

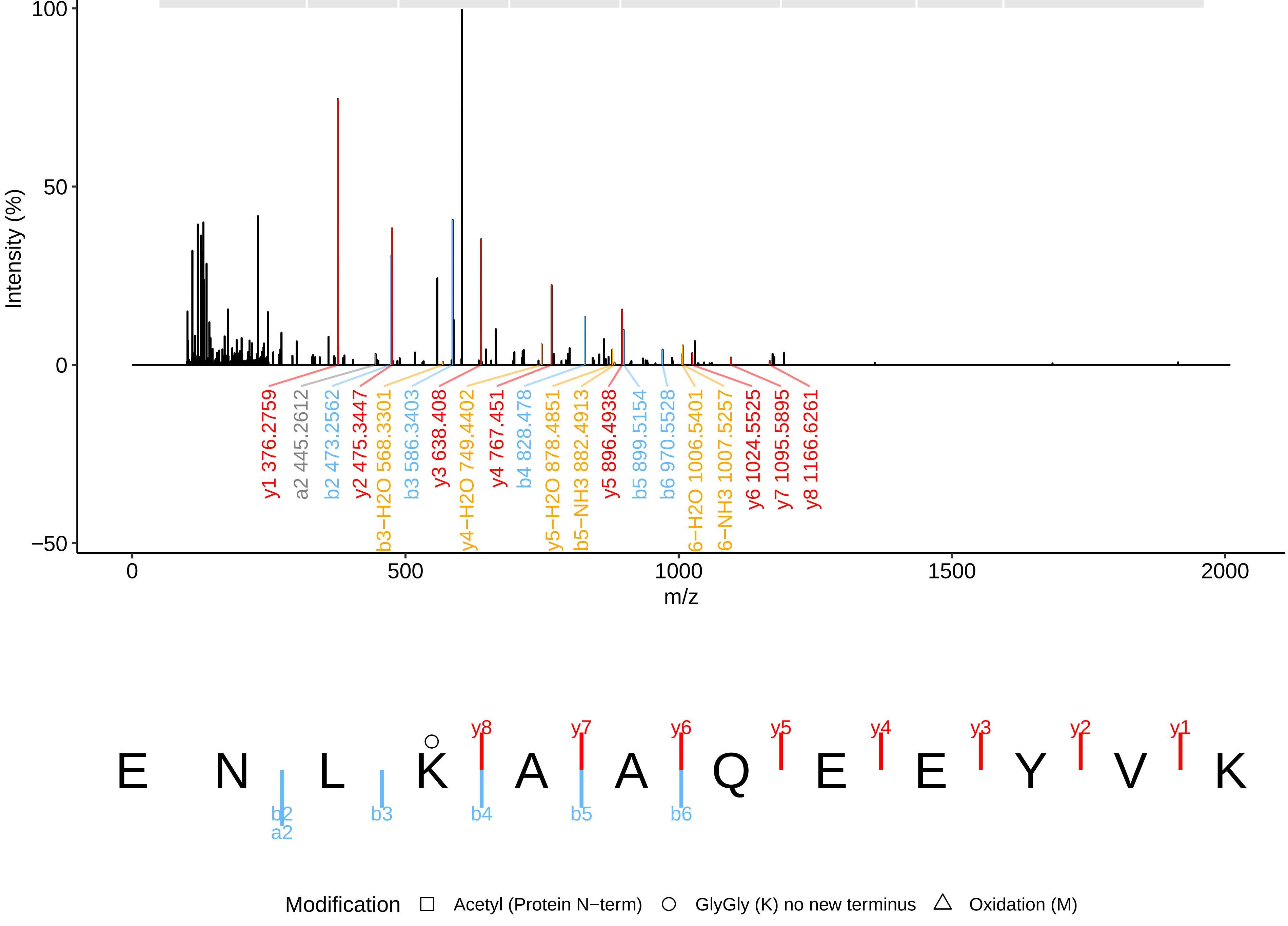

20200921_KIZ_MUb4_GlyGly_10plex − Scan number: 4561

Gene Names	Charge	m/z	Mass	Retention time	PEP	Score	Precursor Intensity
MCL1	4	401.7019	1602.778	14.868	0.00010333	102.5	6953594

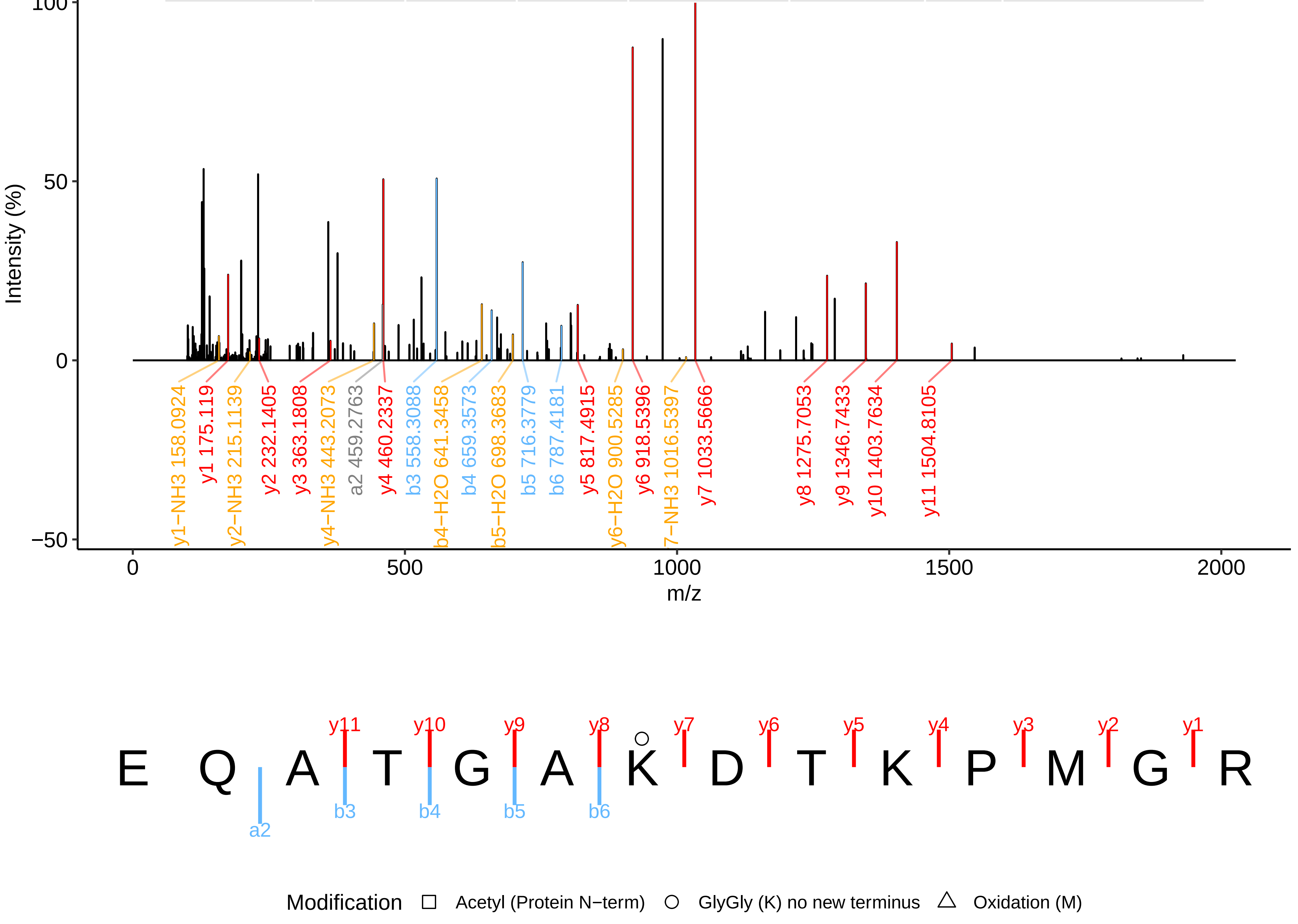

20200921_KIZ_MUb4_GlyGly_10plex − Scan number: 10843

Gene Names	Charge	m/z	Mass	Retention time	PEP	Score	Precursor Intensity
GPRC5C	3	513.5804	1537.719	37.666	0.0024497	78.744	22652472

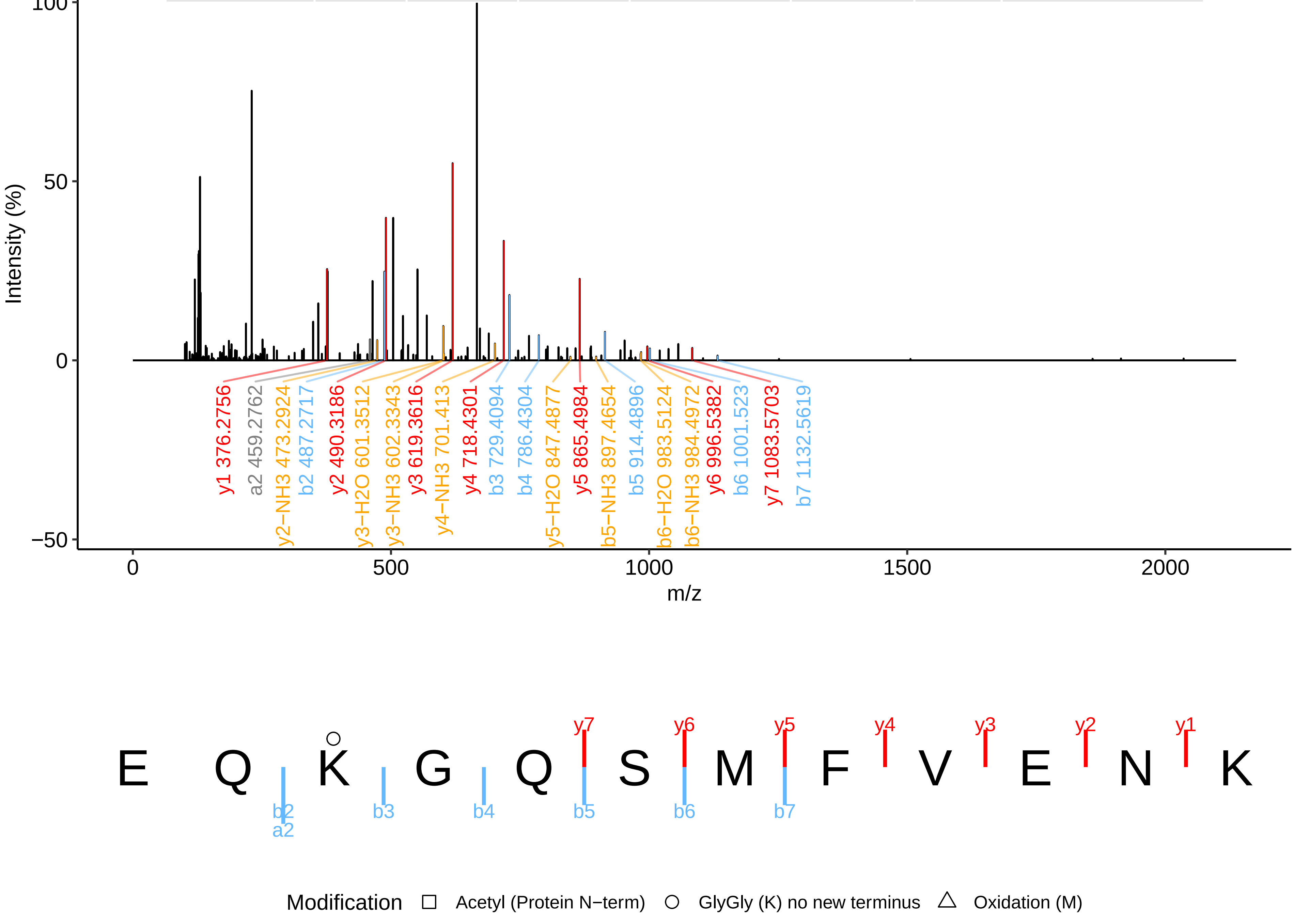

20200921_KIZ_MUb4_GlyGly_10plex_2 − Scan number: 10008

Gene Names	Charge	m/z	Mass	Retention time	PEP	Score	Precursor Intensity
GPRC5C	3	513.5804	1537.719	38.171	0.00028736	94.122	992178.4

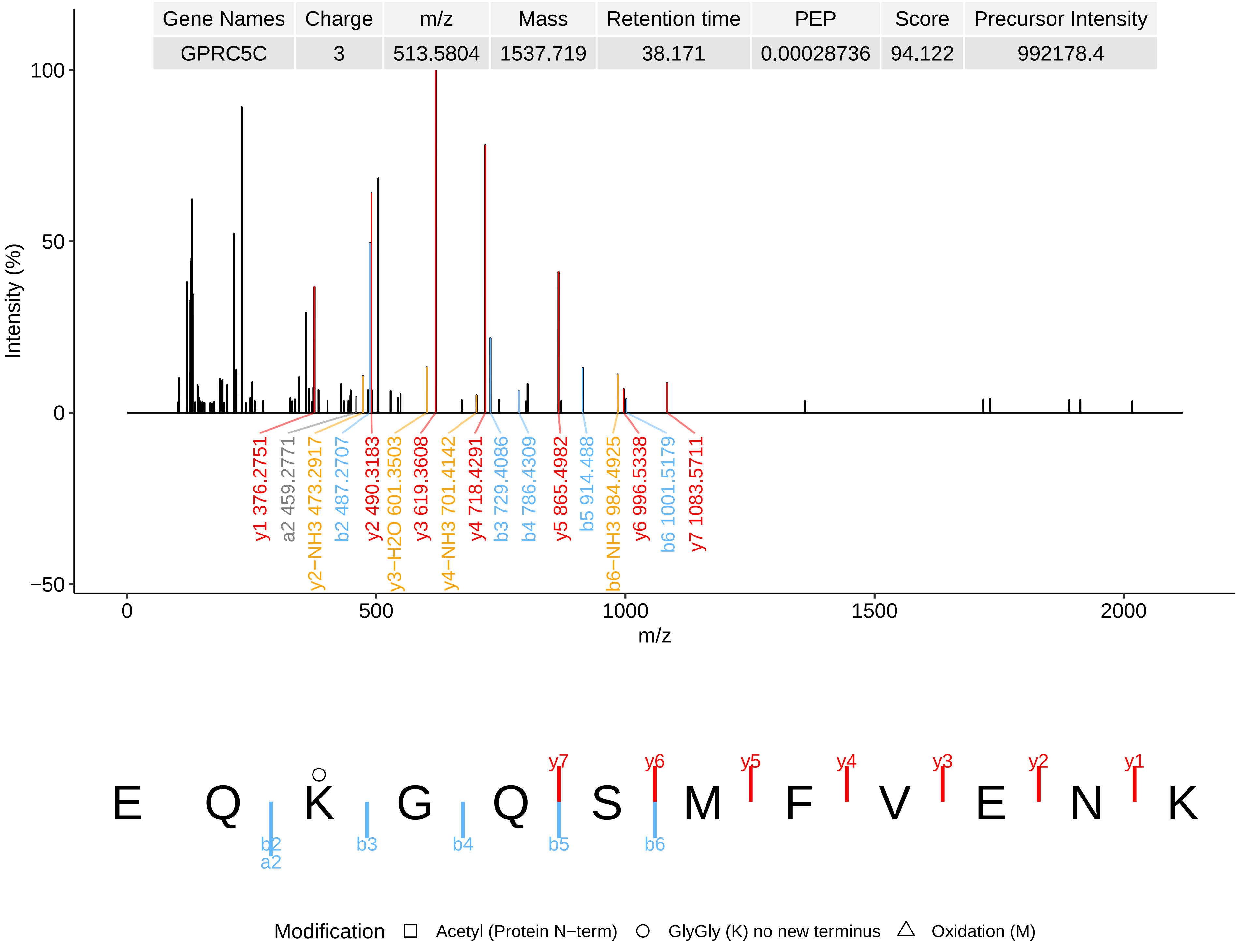

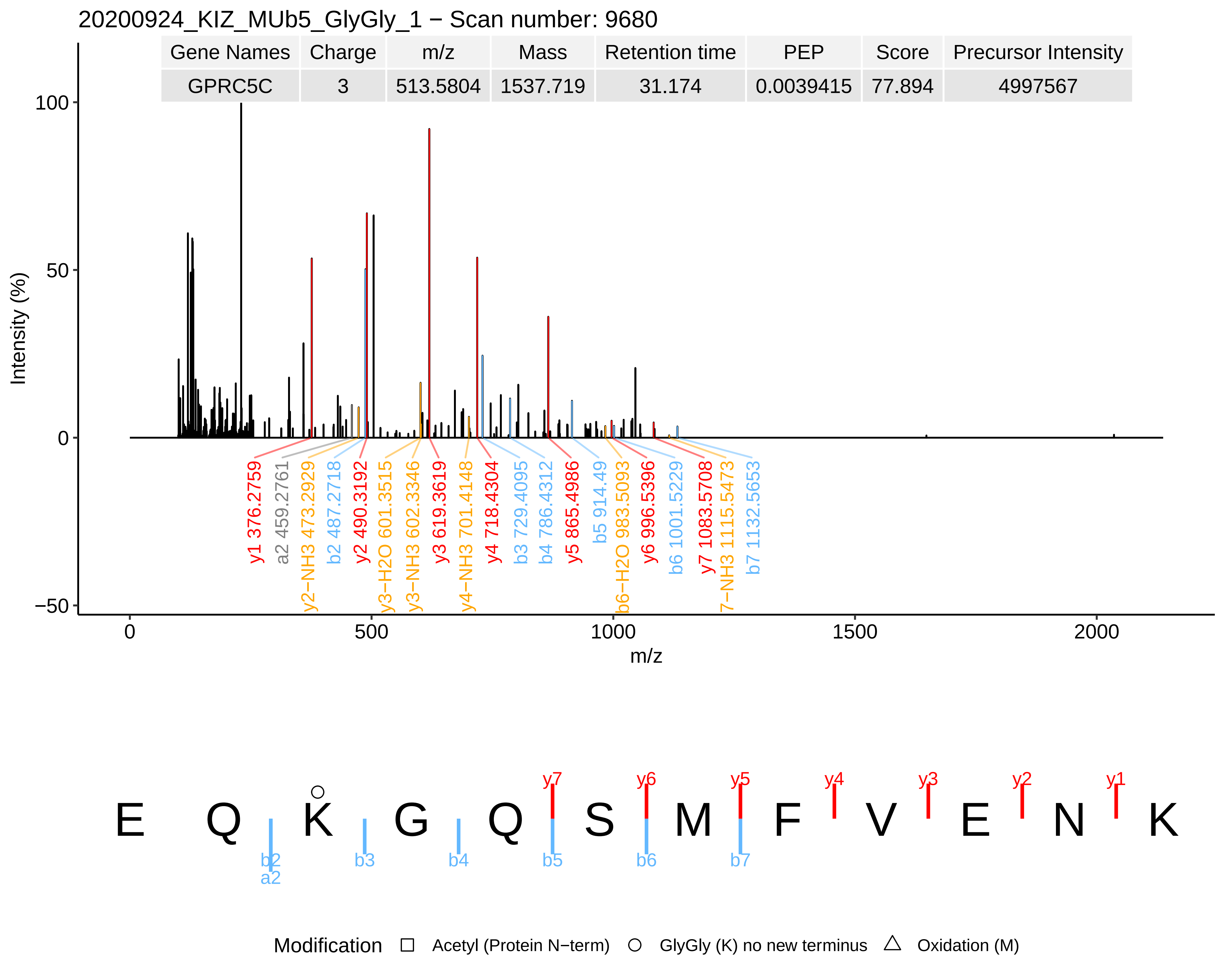

20200924_KIZ_MUb5_GlyGly_1 − Scan number: 9680
Gene Names | Charge | m/z | Mass | Retention time | PEP | Score | Precursor Intensity
GPRC5C | 3 | 513.5804 | 1537.719 | 31.174 | 0.0039415 | 77.894 | 4997567
Intensity (%)
m/z
y1 376.2759
a2 459.2761
y2−NH3 473.2929
b2 487.2718
y2 490.3192
y3−H2O 601.3515
y3−NH3 602.3346
y3 619.3619
y4−NH3 701.4148
y4 718.4304
b3 729.4095
b4 786.4312
y5 865.4986
b5 914.49
b6−H2O 983.5093
y6 996.5396
b6 1001.5229
y7 1083.5708
7−NH3 1115.5473
b7 1132.5653
E Q K G Q S M F V E N K
Modification
Acetyl (Protein N−term)
GlyGly (K) no new terminus
Oxidation (M)

20200924_KIZ_MUb5_GlyGly_1 − Scan number: 9681

Gene Names	Charge	m/z	Mass	Retention time	PEP	Score	Precursor Intensity
GPRC5C	3	513.5804	1537.719	31.178	0.00055947	92.295	4997567

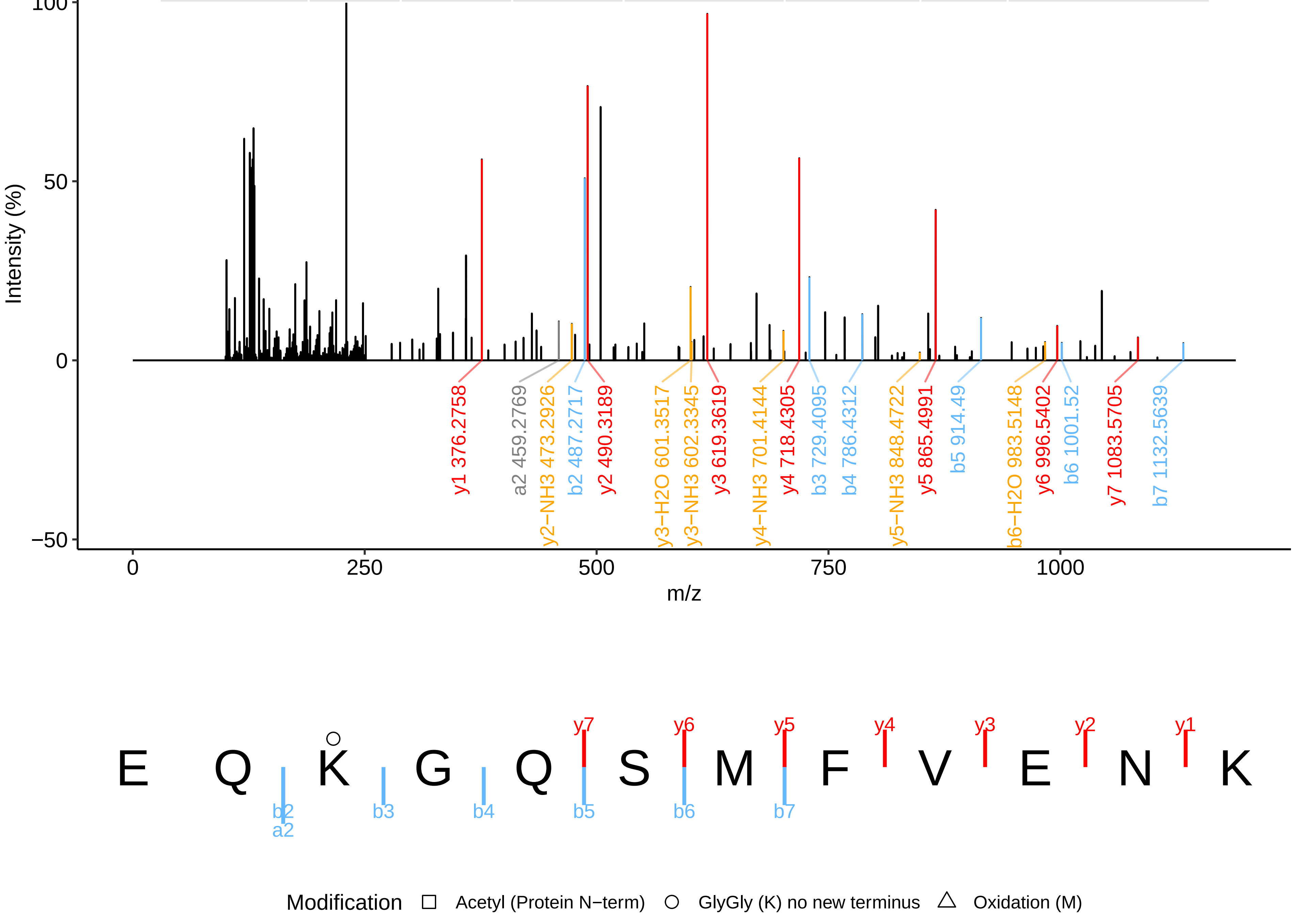

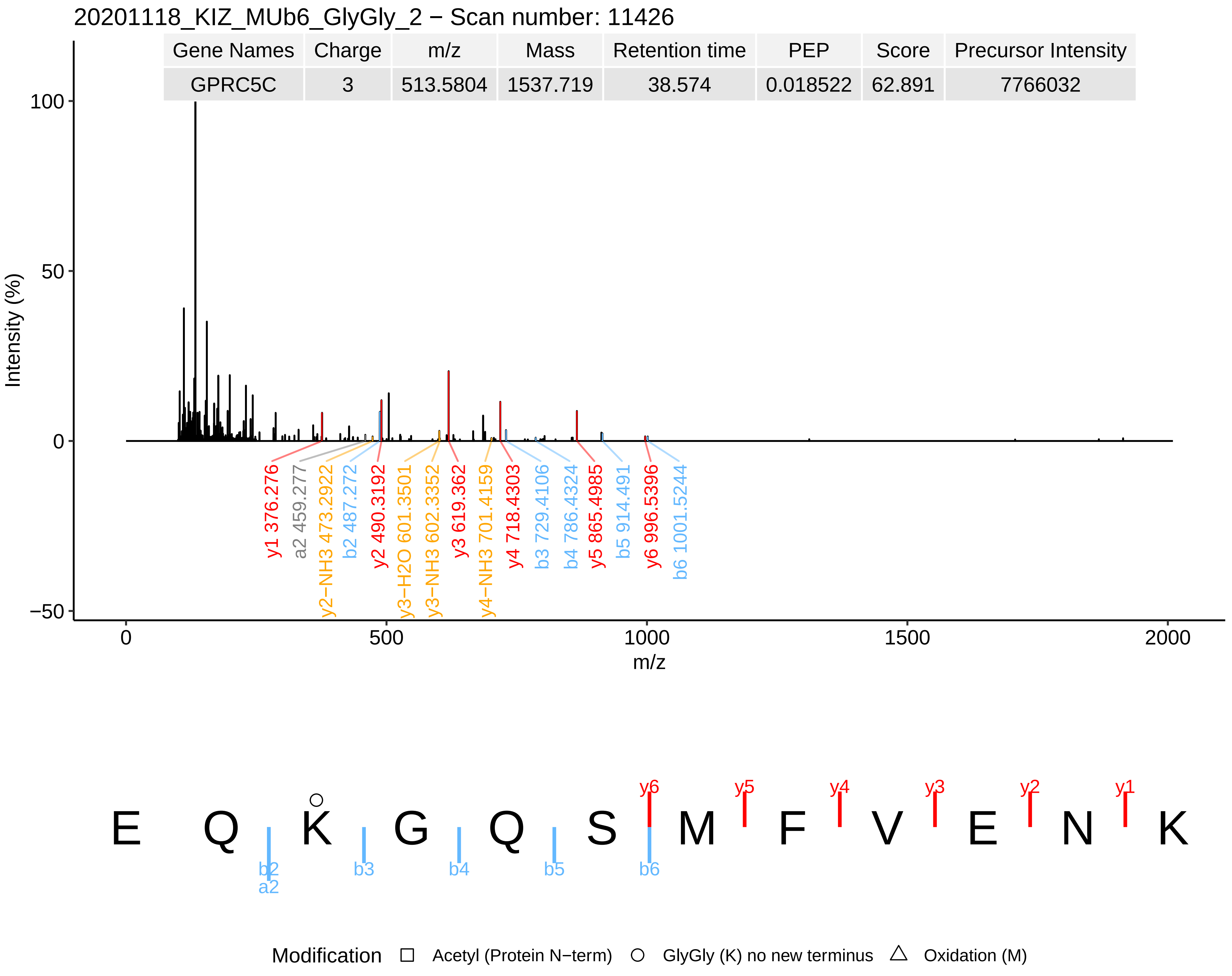

20201118_KIZ_MUb6_GlyGly_2 − Scan number: 11426
Gene Names | Charge | m/z | Mass | Retention time | PEP | Score | Precursor Intensity
GPRC5C | 3 | 513.5804 | 1537.719 | 38.574 | 0.018522 | 62.891 | 7766032
Intensity (%)
m/z
y1 376.276
a2 459.277
y2−NH3 473.2922
b2 487.272
y2 490.3192
y3−H2O 601.3501
y3−NH3 602.3352
y3 619.362
y4−NH3 701.4159
y4 718.4303
b3 729.4106
b4 786.4324
y5 865.4985
b5 914.491
y6 996.5396
b6 1001.5244
E Q K G Q S M F V E N K
Modification
Acetyl (Protein N−term)
GlyGly (K) no new terminus
Oxidation (M)

20200921_KIZ_MUb4_GlyGly_10plex − Scan number: 13905

Gene Names	Charge	m/z	Mass	Retention time	PEP	Score	Precursor Intensity
HSPA9	3	602.3001	1803.879	49.219	2.1461e−09	125.61	2612474

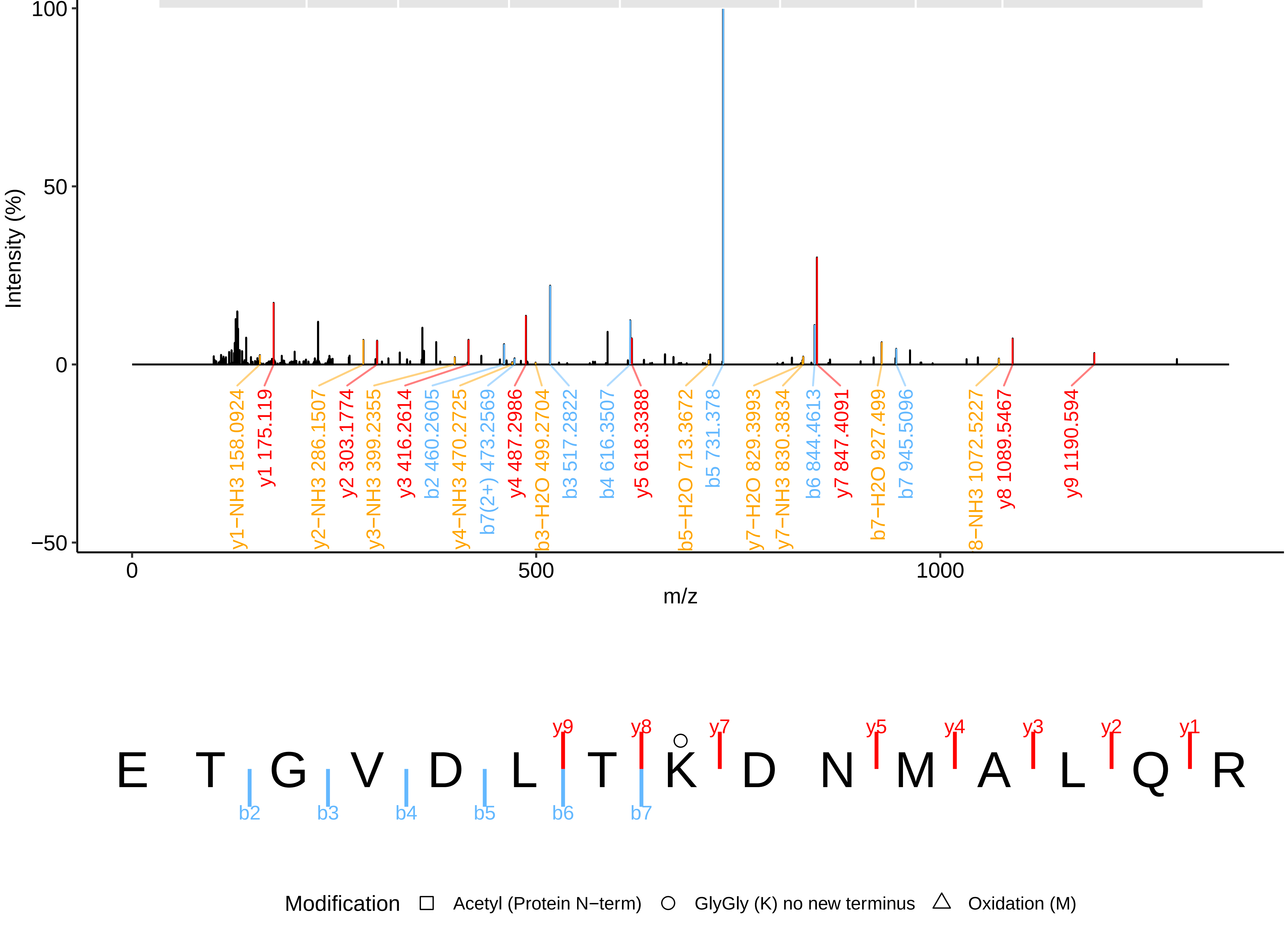

20200921_KIZ_MUb4_GlyGly_10plex − Scan number: 13766

Gene Names	Charge	m/z	Mass	Retention time	PEP	Score	Precursor Intensity
SLC16A3	4	393.2031	1568.784	48.675	0.00018157	95.094	9068446

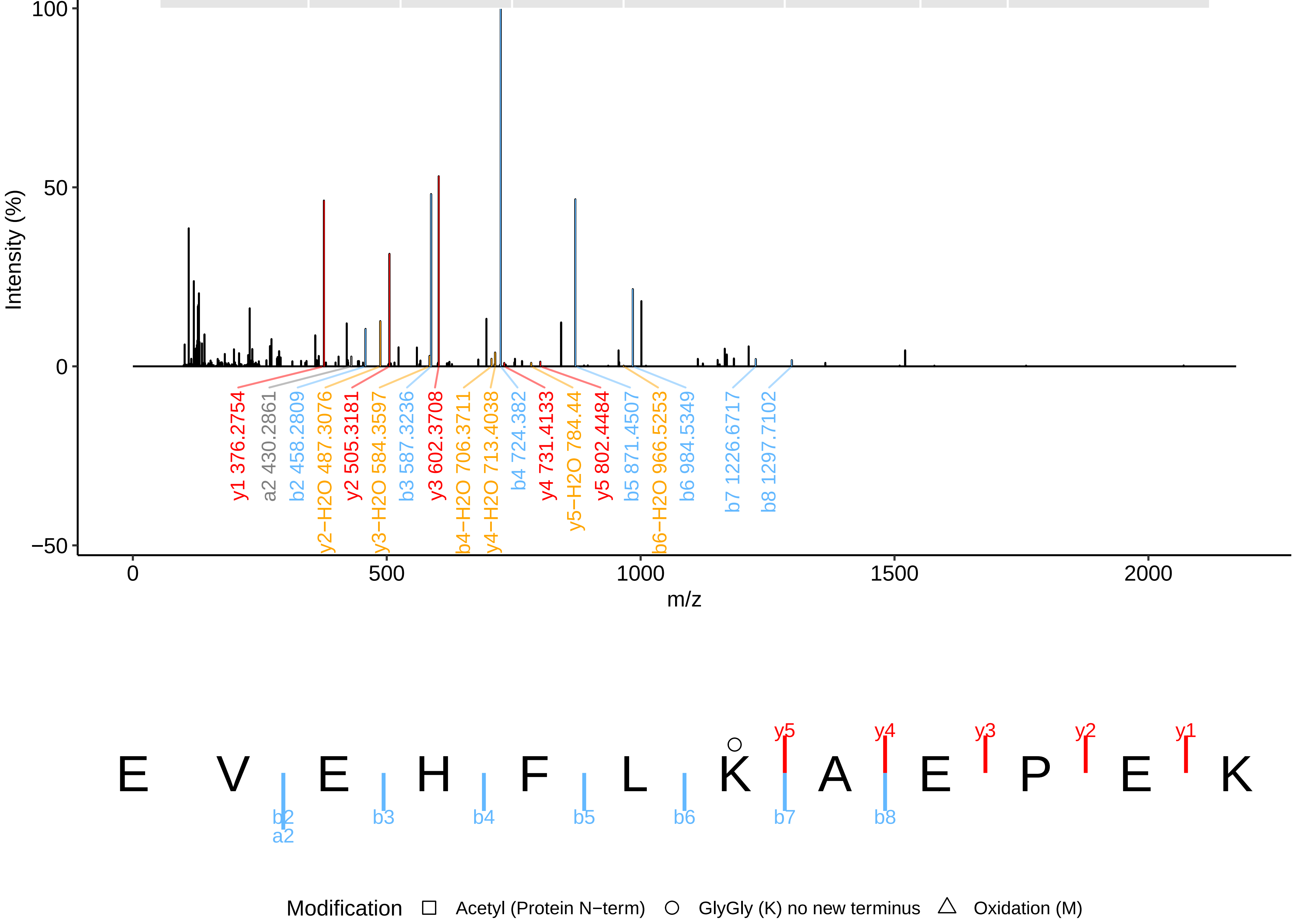

20200921_KIZ_MUb4_GlyGly_10plex_2 − Scan number: 12694

Gene Names	Charge	m/z	Mass	Retention time	PEP	Score	Precursor Intensity
SLC16A3	4	393.2031	1568.784	49.166	0.0042935	74.611	1229476

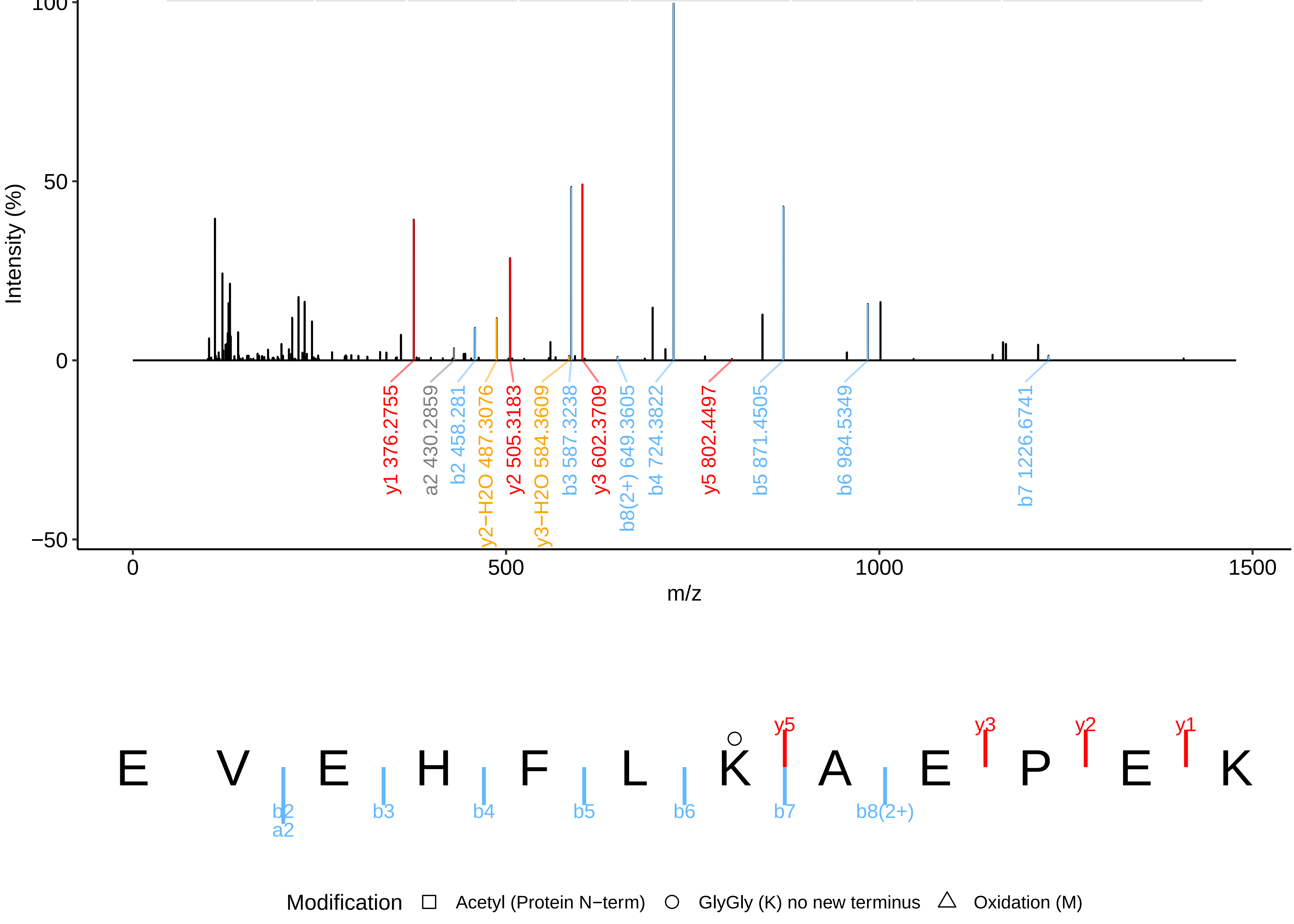

20200921_KIZ_MUb4_GlyGly_10plex_2 − Scan number: 12702

Gene Names	Charge	m/z	Mass	Retention time	PEP	Score	Precursor Intensity
SLC16A3	3	523.9351	1568.784	49.201	0.0051145	74.76	989436.1

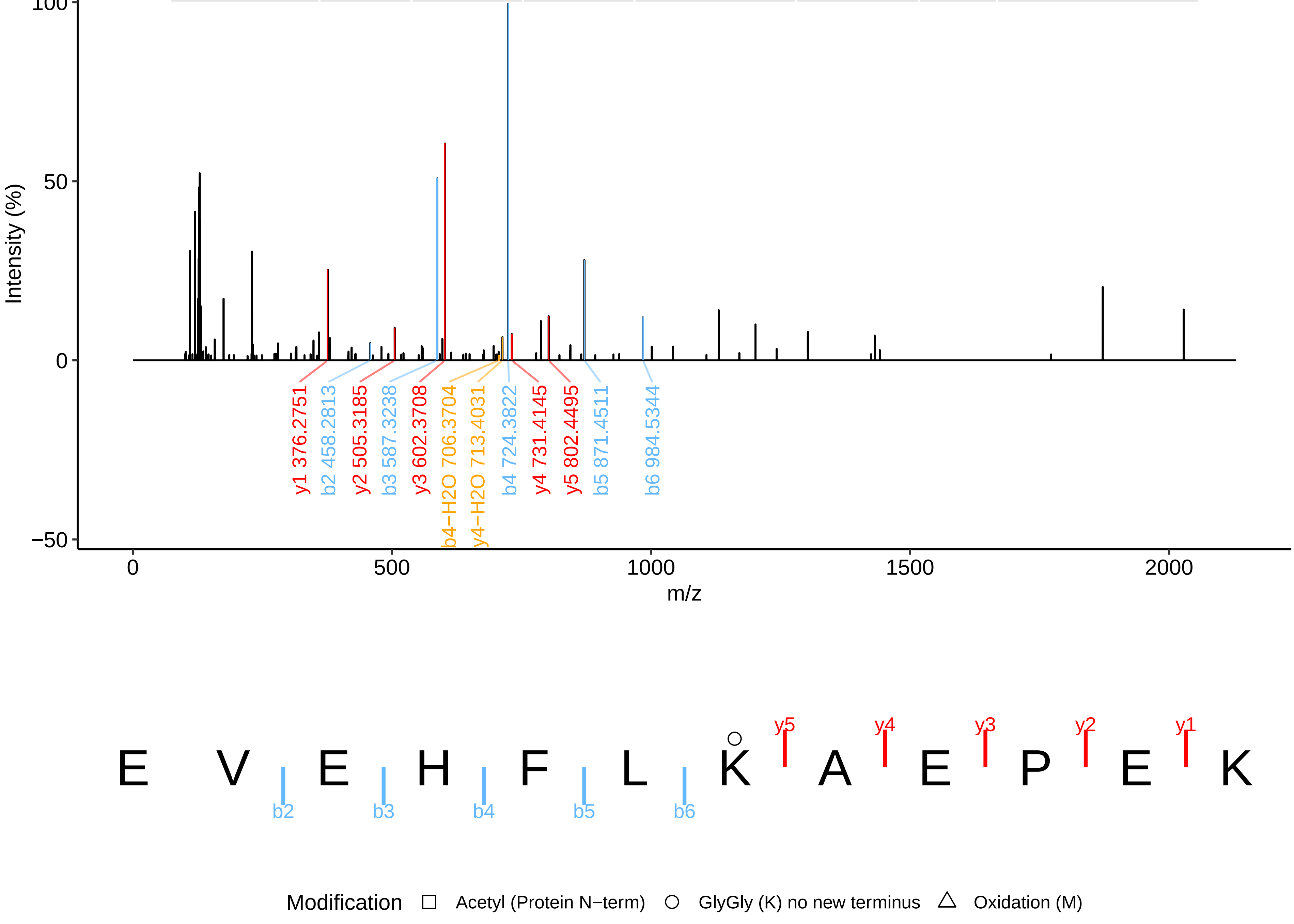

20200924_KIZ_MUb5_GlyGly_1 − Scan number: 12591

Gene Names	Charge	m/z	Mass	Retention time	PEP	Score	Precursor Intensity
SLC16A3	3	523.9351	1568.784	40.995	0.0023891	79.492	3166712

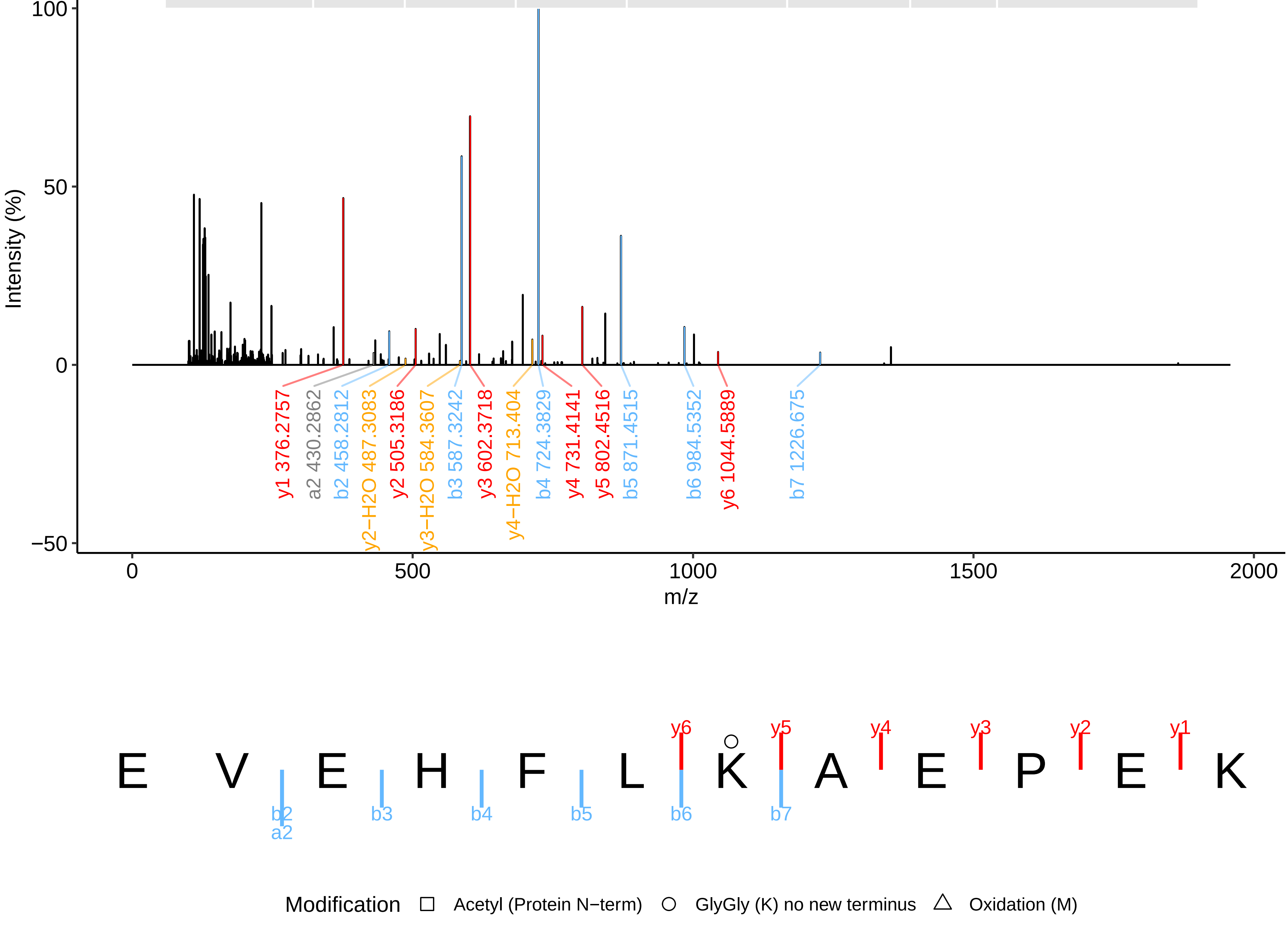

20201118_KIZ_MUb6_GlyGly_2 − Scan number: 15038

Gene Names	Charge	m/z	Mass	Retention time	PEP	Score	Precursor Intensity
SLC16A3	4	393.2031	1568.784	50.752	0.006908	69.864	15806197

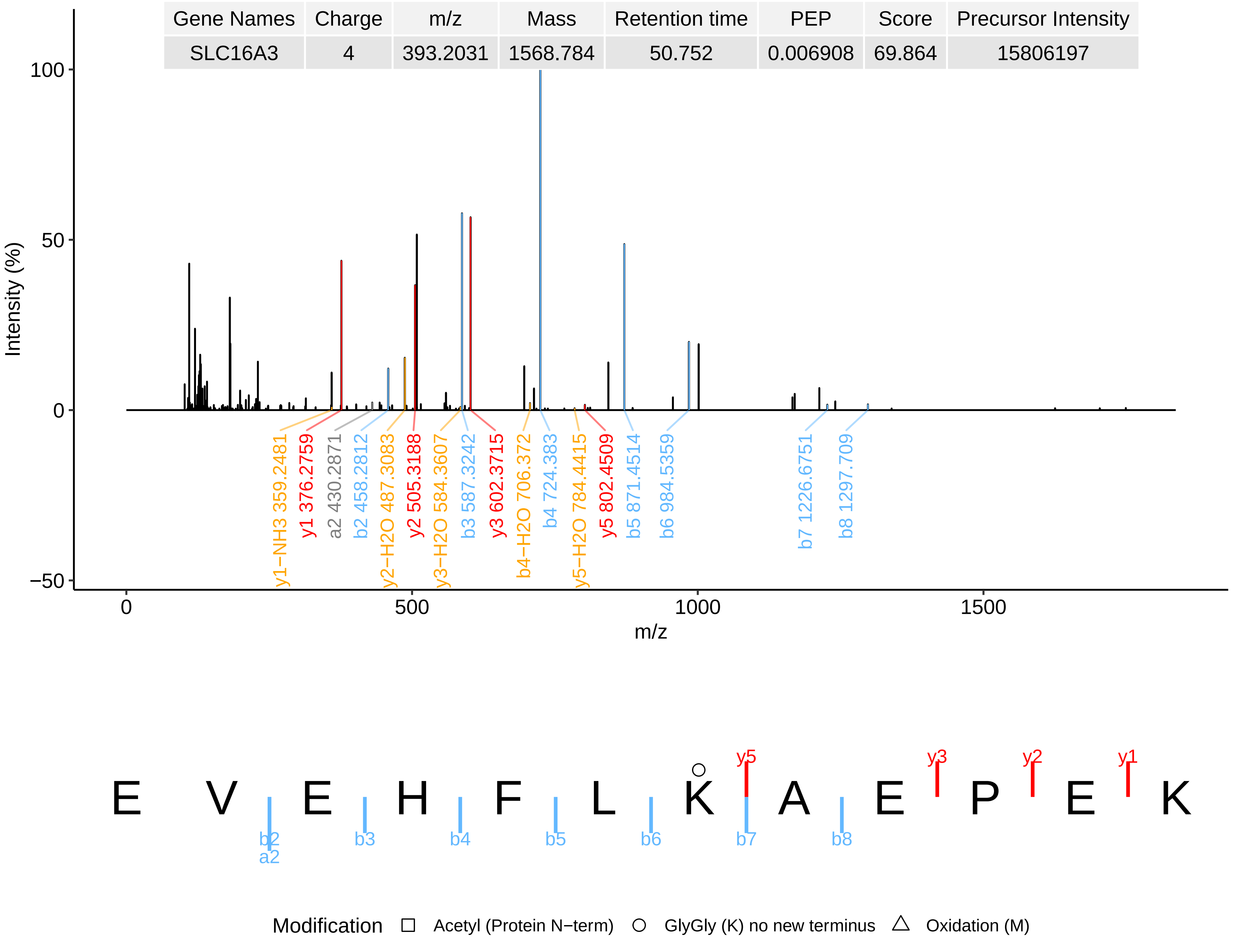

20200921_KIZ_MUb4_GlyGly_10plex_2 − Scan number: 17050

Gene Names	Charge	m/z	Mass	Retention time	PEP	Score	Precursor Intensity
SMARCE1	3	605.2954	1812.864	66.623	7.302e−08	122.46	1187091

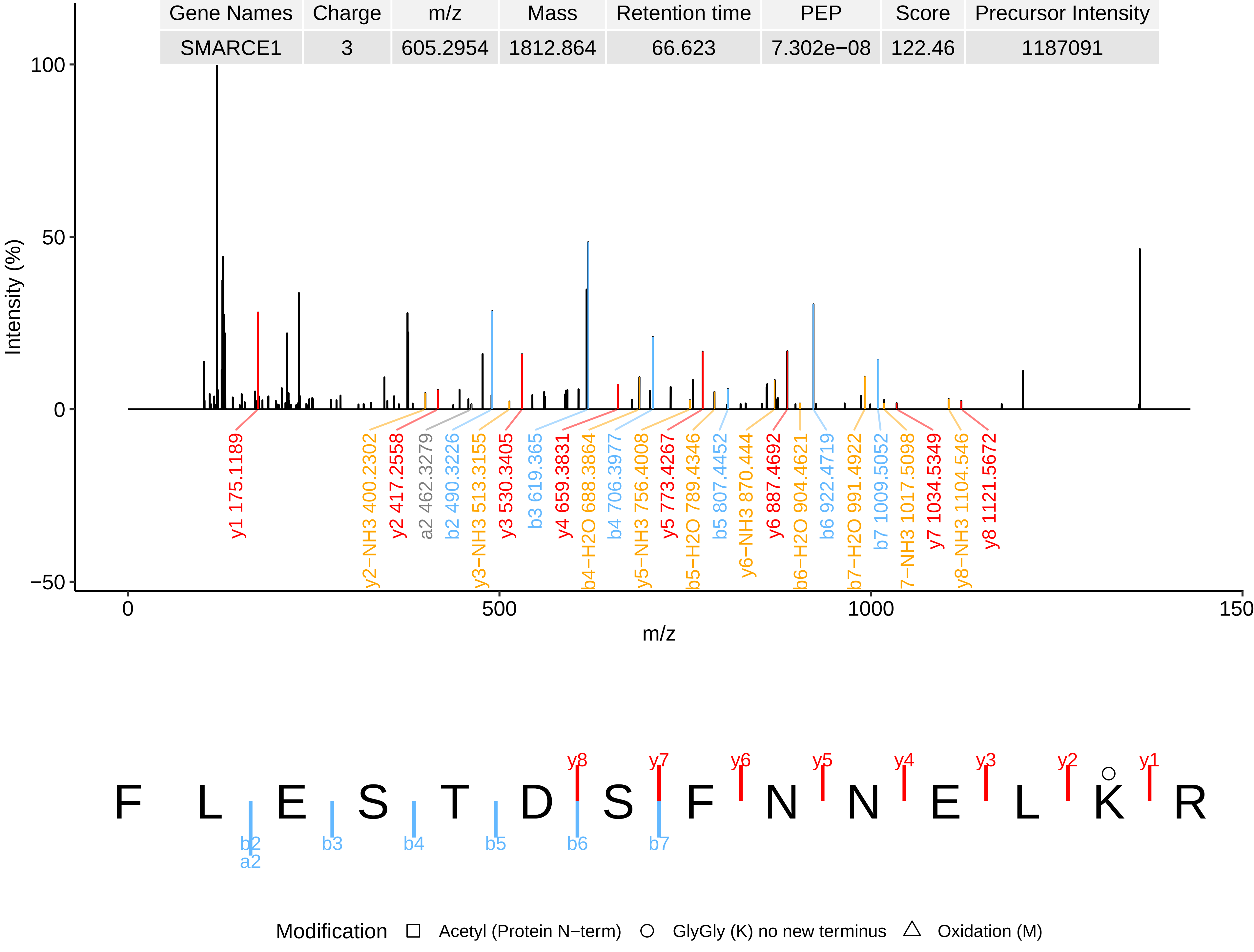

20200924_KIZ_MUb5_GlyGly_1 − Scan number: 18116

Gene Names	Charge	m/z	Mass	Retention time	PEP	Score	Precursor Intensity
SMARCE1	3	605.2954	1812.864	58.581	1.6733e−21	175.48	6005239

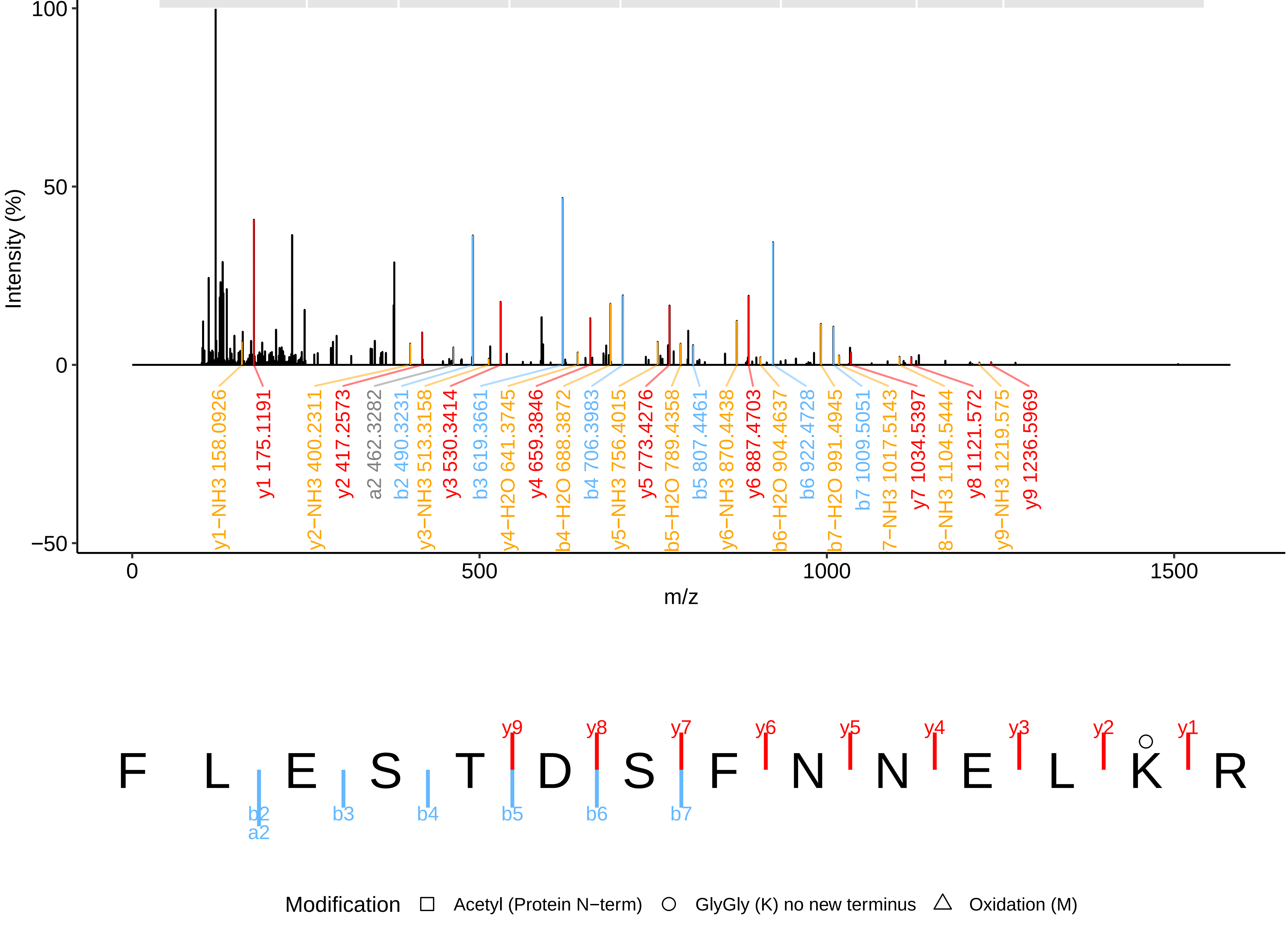

20200924_KIZ_MUb5_GlyGly_2 − Scan number: 16936

Gene Names	Charge	m/z	Mass	Retention time	PEP	Score	Precursor Intensity
SMARCE1	3	605.2954	1812.864	62.849	2.1189e−07	119.21	NA

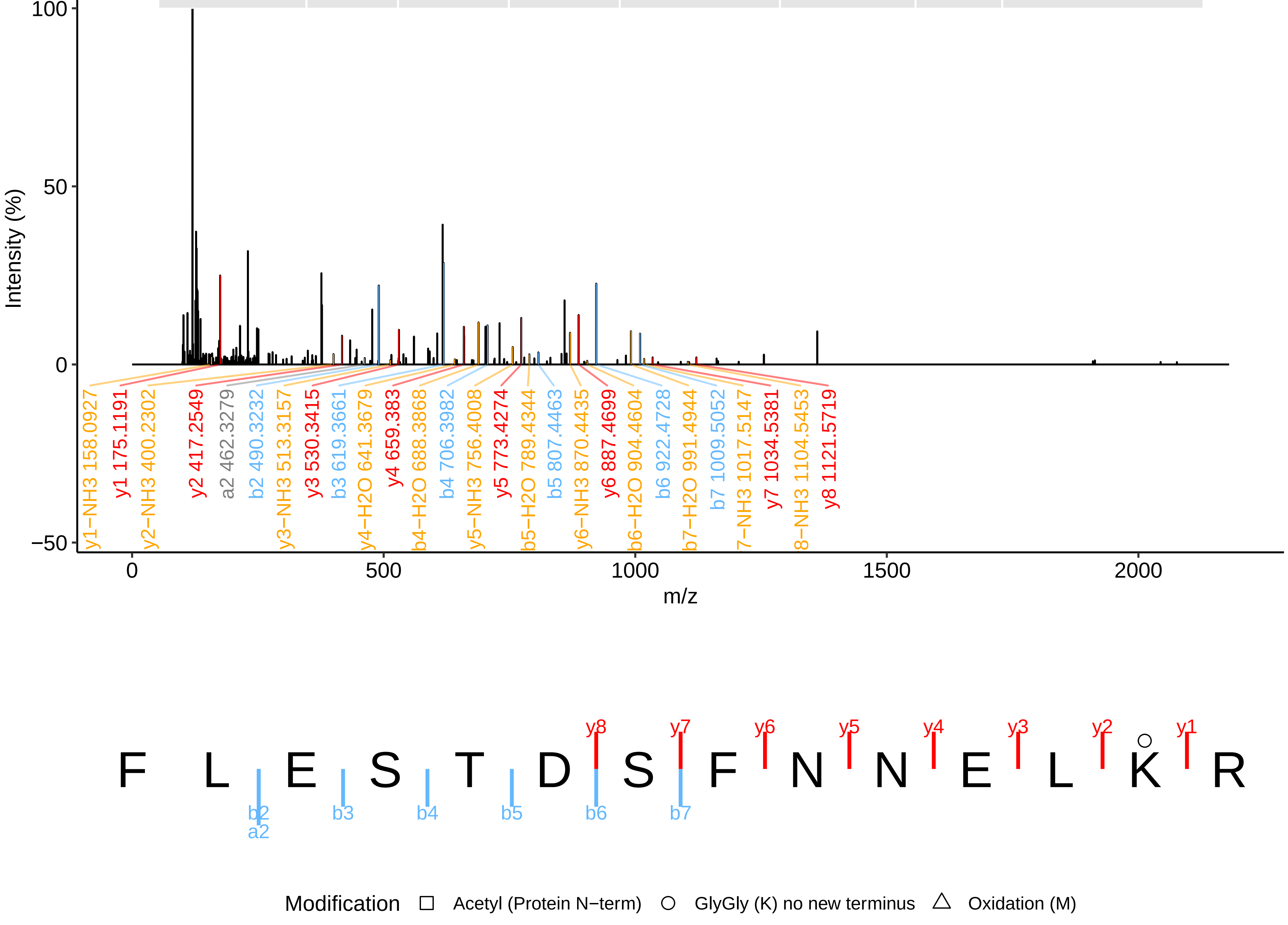

20201118_KIZ_MUb6_GlyGly_1 − Scan number: 18080

Gene Names	Charge	m/z	Mass	Retention time	PEP	Score	Precursor Intensity
SMARCE1	3	605.2954	1812.864	56.863	4.5982e−37	190.1	13668738

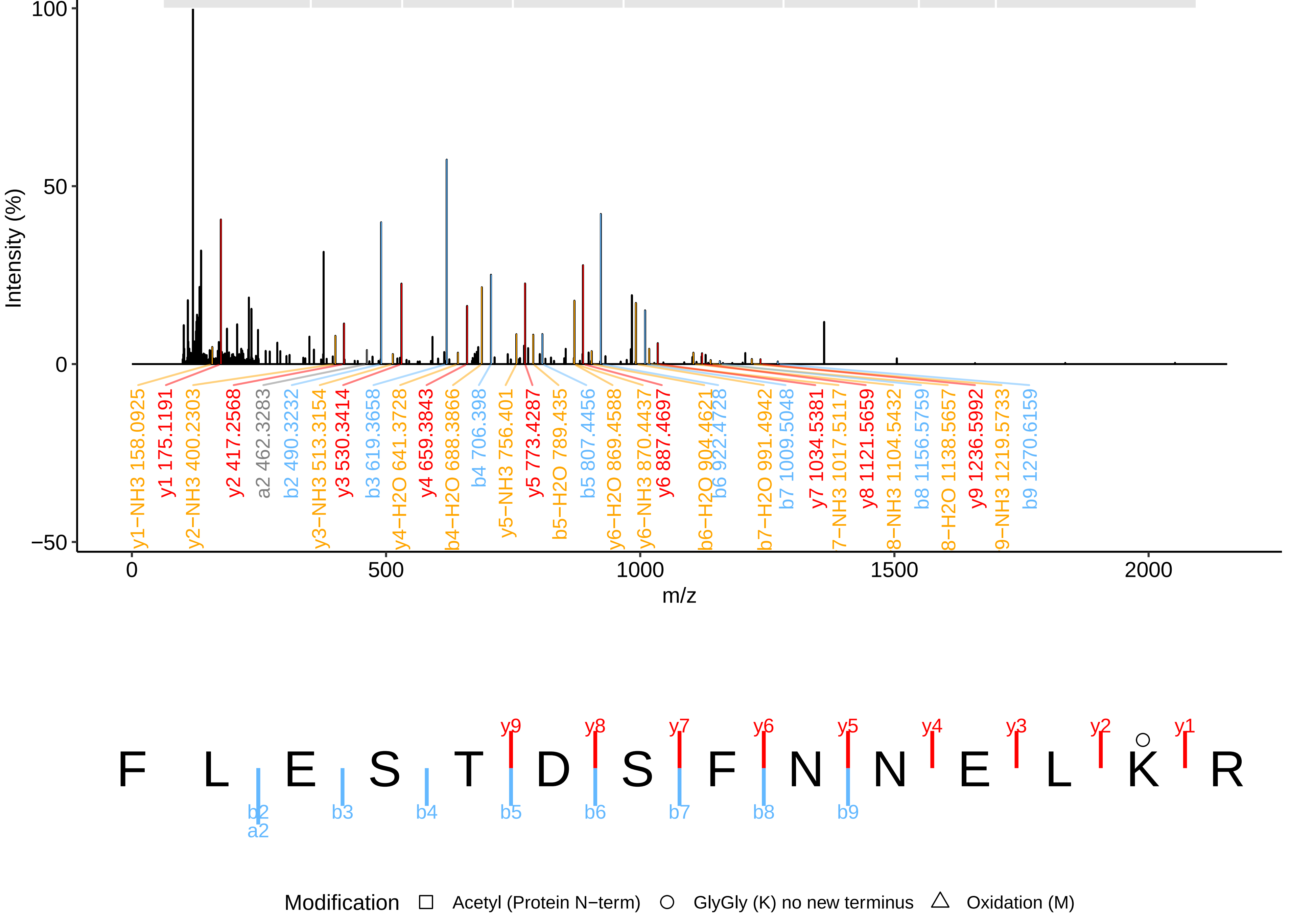

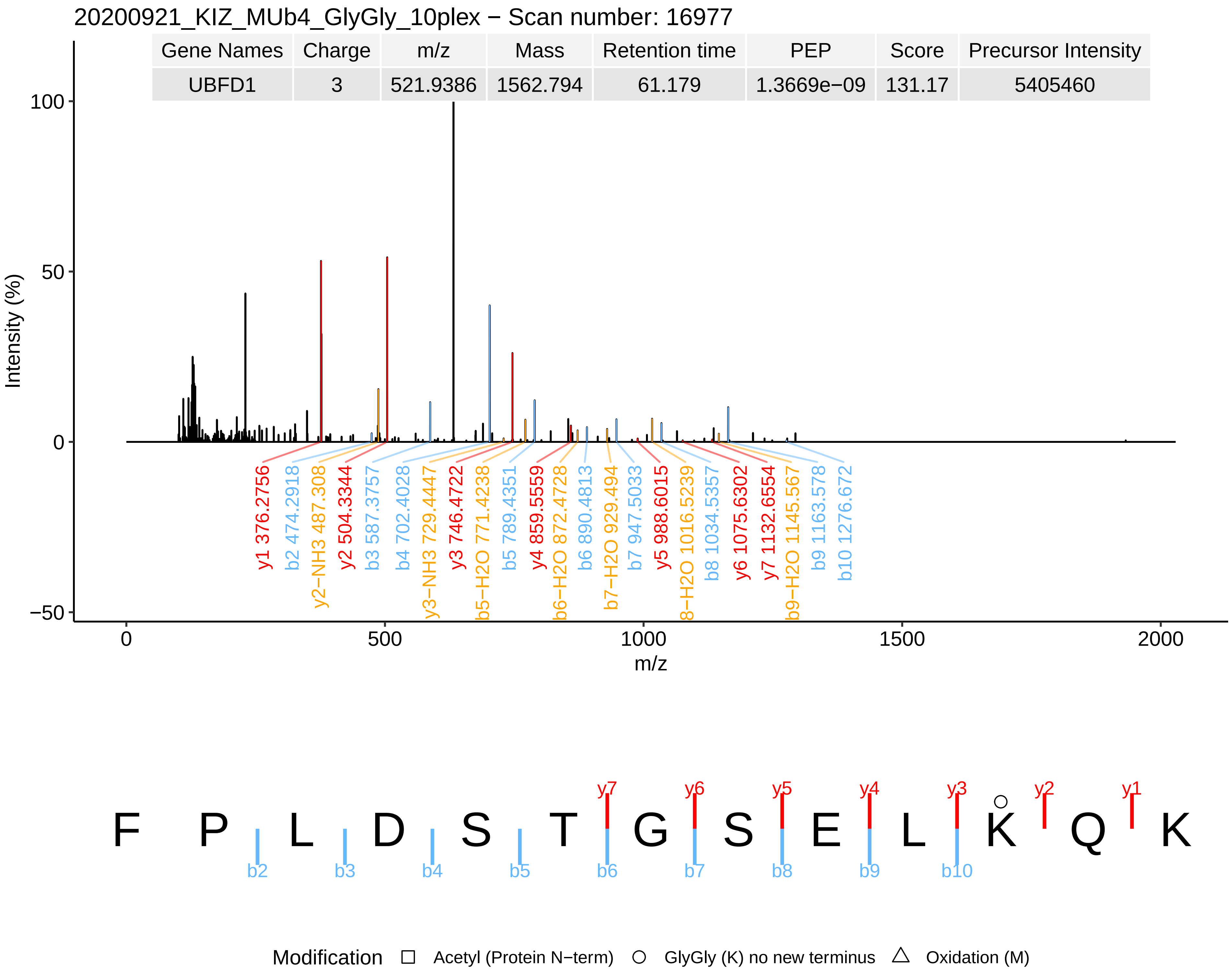
20200921_KIZ_MUb4_GlyGly_10plex − Scan number: 16977
Gene Names | Charge | m/z | Mass | Retention time | PEP | Score | Precursor Intensity
UBFD1 | 3 | 521.9386 | 1562.794 | 61.179 | 1.3669e−09 | 131.17 | 5405460
Intensity (%)
100
50
0
−50
0
500
1000
1500
2000
m/z
y1 376.2756
b2 474.2918
y2−NH3 487.308
y2 504.3344
b3 587.3757
b4 702.4028
y3−NH3 729.4447
y3 746.4722
b5−H2O 771.4238
b5 789.4351
y4 859.5559
b6−H2O 872.4728
b6 890.4813
b7−H2O 929.494
b7 947.5033
y5 988.6015
8−H2O 1016.5239
b8 1034.5357
y6 1075.6302
y7 1132.6554
b9−H2O 1145.567
b9 1163.578
b10 1276.672
F P L D S T G S E L K Q K
y7 y6 y5 y4 y3 y2 y1
b2 b3 b4 b5 b6 b7 b8 b9 b10
Modification
Acetyl (Protein N−term)
GlyGly (K) no new terminus
Oxidation (M)

20200921_KIZ_MUb4_GlyGly_10plex − Scan number: 19997

Gene Names	Charge	m/z	Mass	Retention time	PEP	Score	Precursor Intensity
UFD1L	3	599.6611	1795.961	72.201	0.07107	49.223	NA

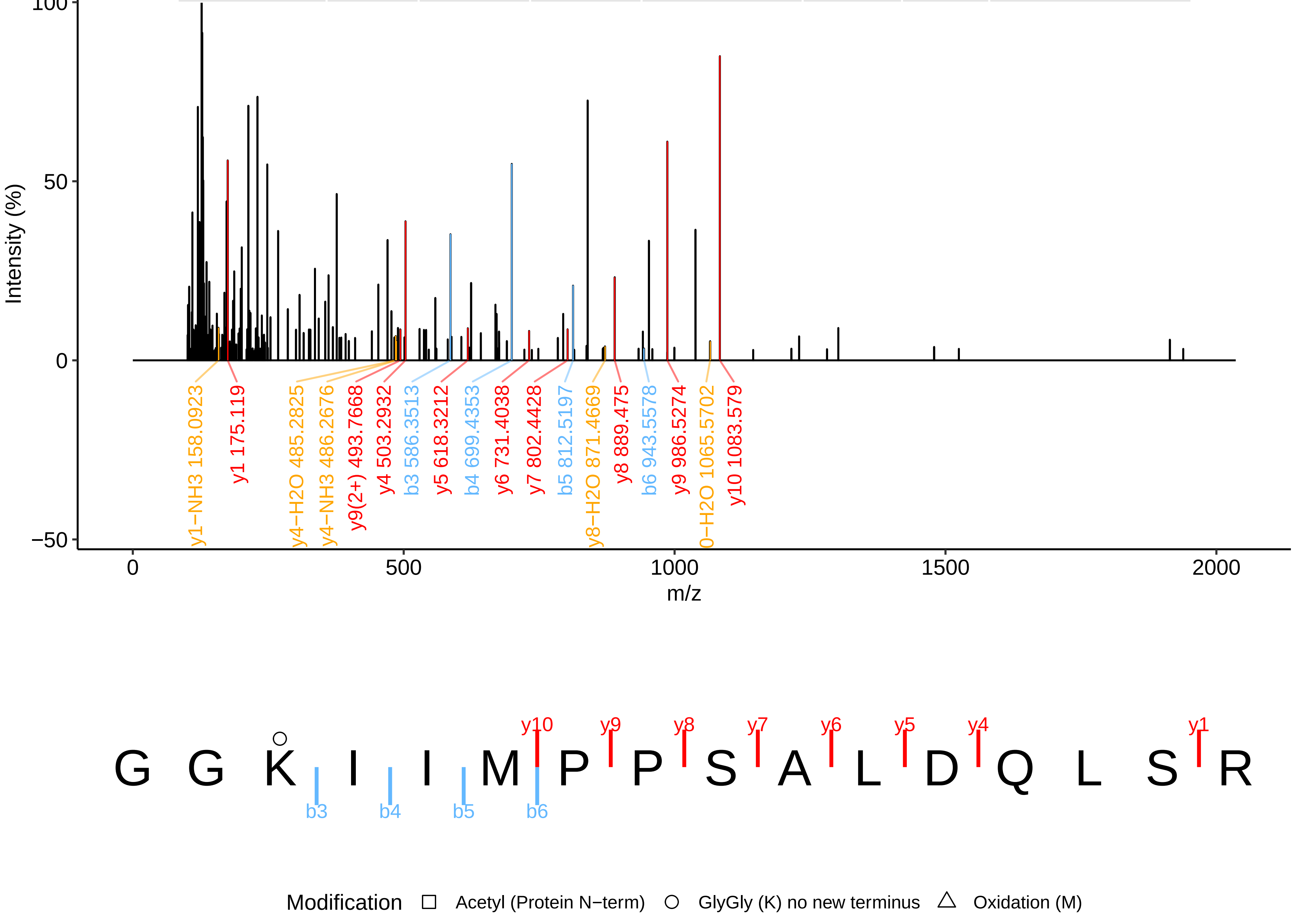

20200921_KIZ_MUb4_GlyGly_10plex − Scan number: 6808

Gene Names	Charge	m/z	Mass	Retention time	PEP	Score	Precursor Intensity
STX4	4	399.7089	1594.806	22.657	1.7451e−05	99.011	2408610

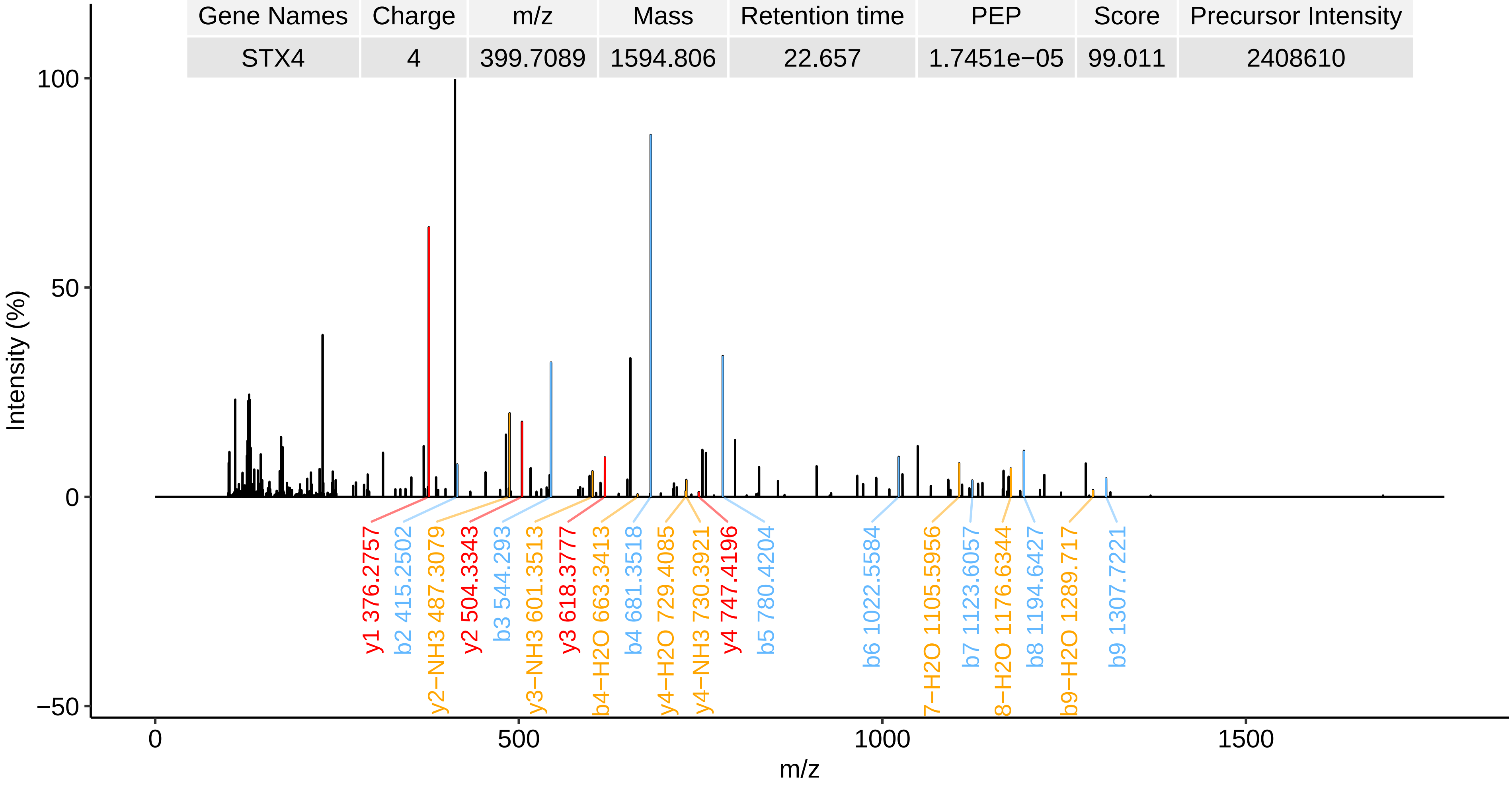

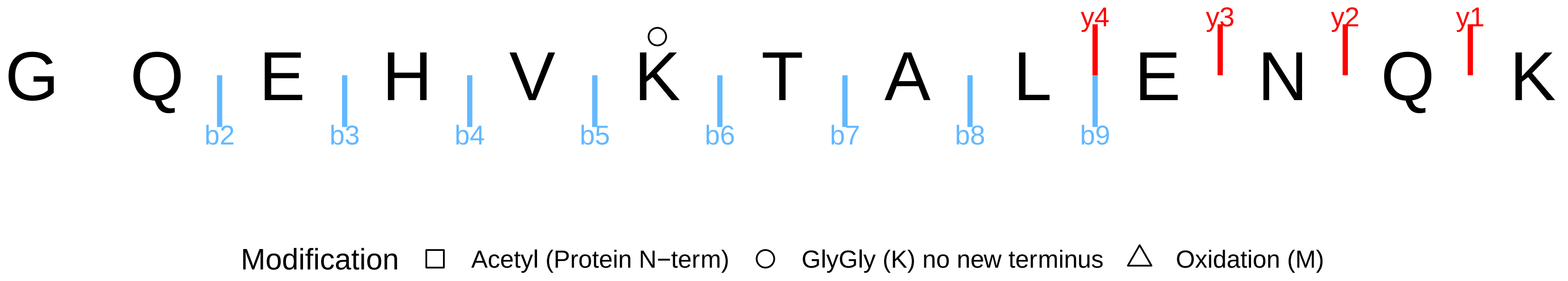

20200921_KIZ_MUb4_GlyGly_10plex_2 − Scan number: 22016

Gene Names	Charge	m/z	Mass	Retention time	PEP	Score	Precursor Intensity
RAD23B	3	528.9542	1583.841	86.749	2.8204e−05	97.911	1575418

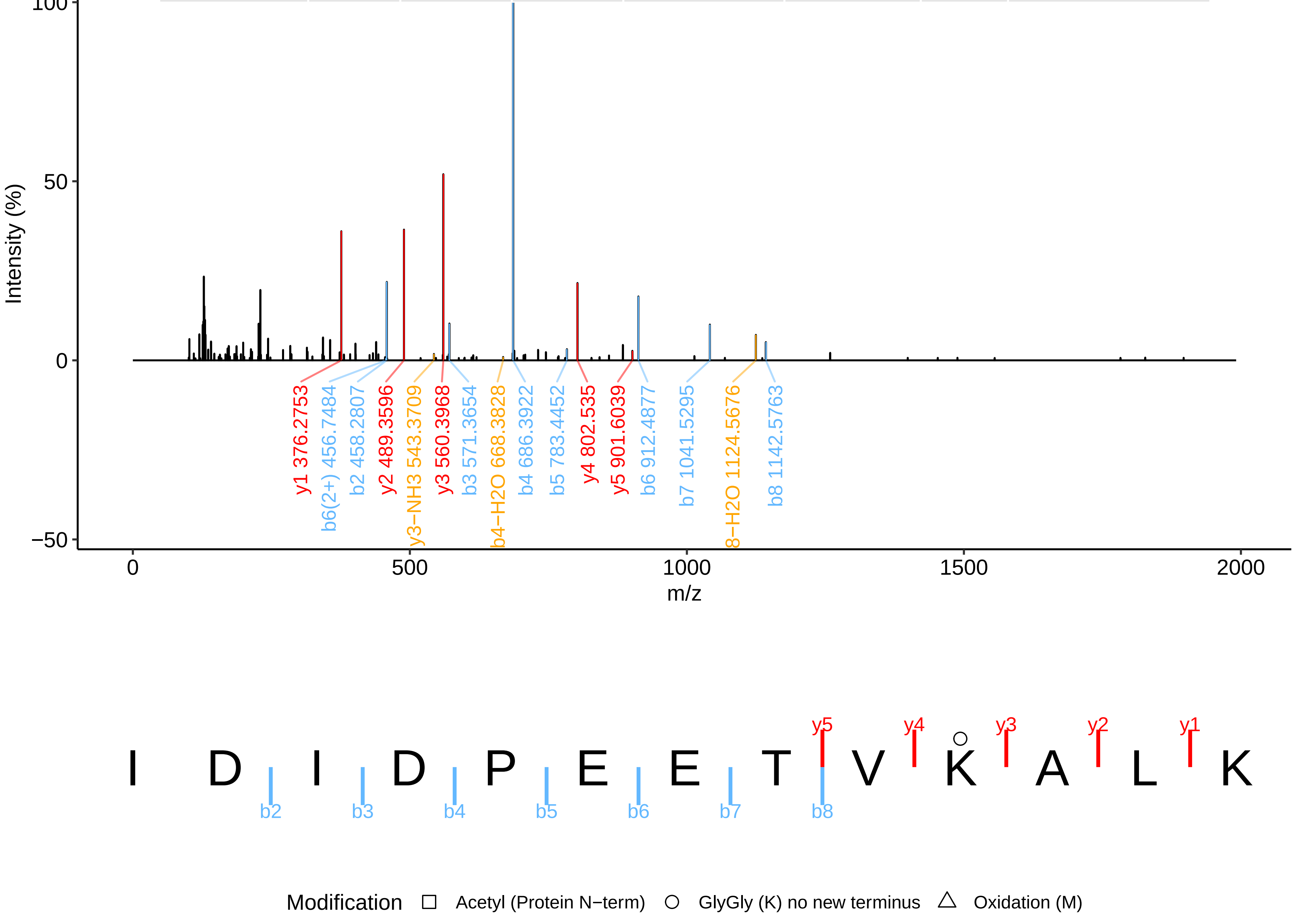

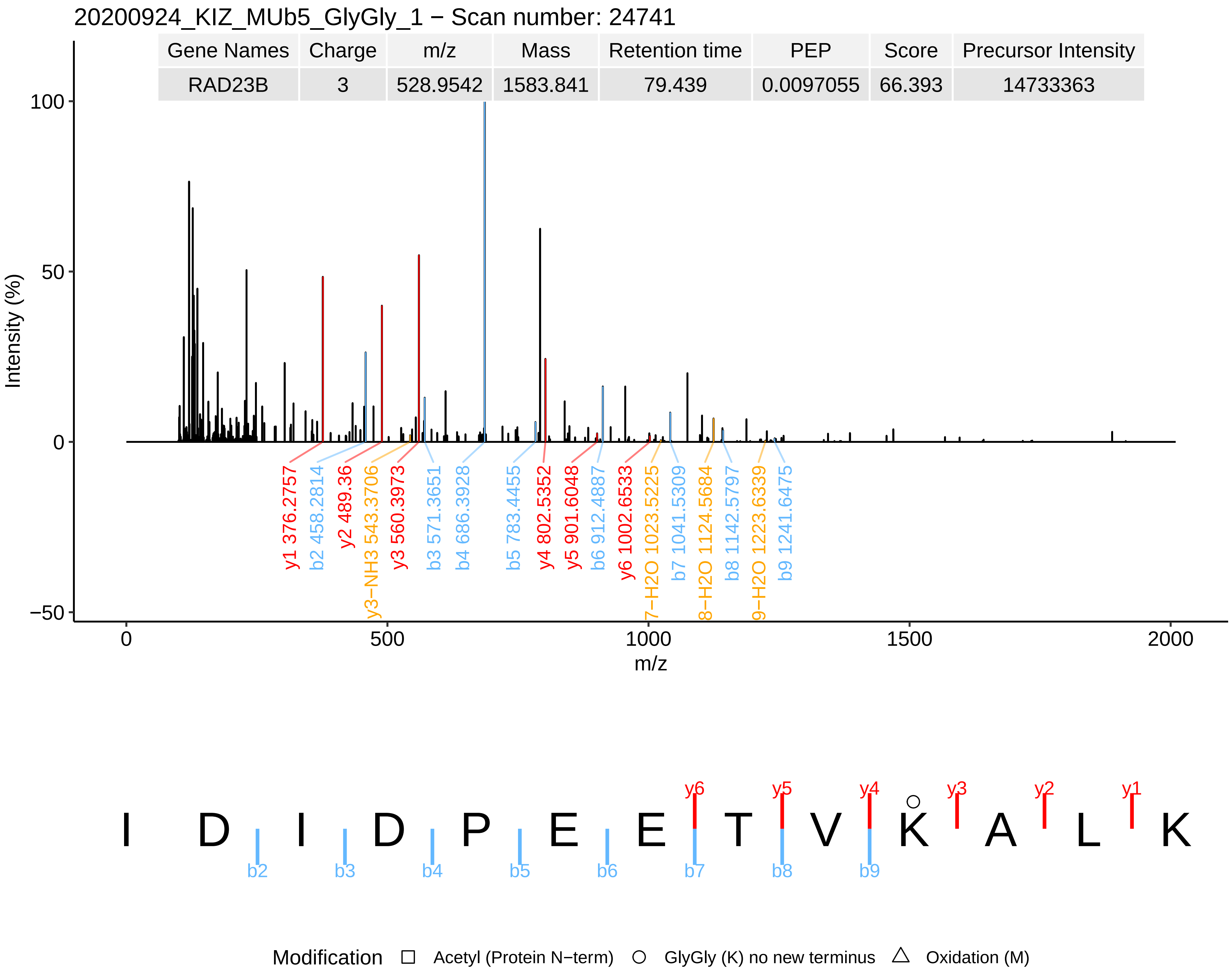

20200924_KIZ_MUb5_GlyGly_1 − Scan number: 24741
Gene Names | Charge | m/z | Mass | Retention time | PEP | Score | Precursor Intensity
RAD23B | 3 | 528.9542 | 1583.841 | 79.439 | 0.0097055 | 66.393 | 14733363
Intensity (%)
100
50
0
−50
0
500
1000
1500
2000
m/z
y1 376.2757
b2 458.2814
y2 489.36
y3−NH3 543.3706
y3 560.3973
b3 571.3651
b4 686.3928
b5 783.4455
y4 802.5352
y5 901.6048
b6 912.4887
y6 1002.6533
7−H2O 1023.5225
b7 1041.5309
8−H2O 1124.5684
b8 1142.5797
9−H2O 1223.6339
b9 1241.6475
I D I D P E E T V K A L K
y6 y5 y4 y3 y2 y1
b2 b3 b4 b5 b6 b7 b8 b9
Modification
Acetyl (Protein N−term)
GlyGly (K) no new terminus
Oxidation (M)

20200921_KIZ_MUb4_GlyGly_10plex − Scan number: 19290

Gene Names	Charge	m/z	Mass	Retention time	PEP	Score	Precursor Intensity
SLC3A2	3	534.2774	1599.81	69.512	1.0663e−06	111.74	13674314

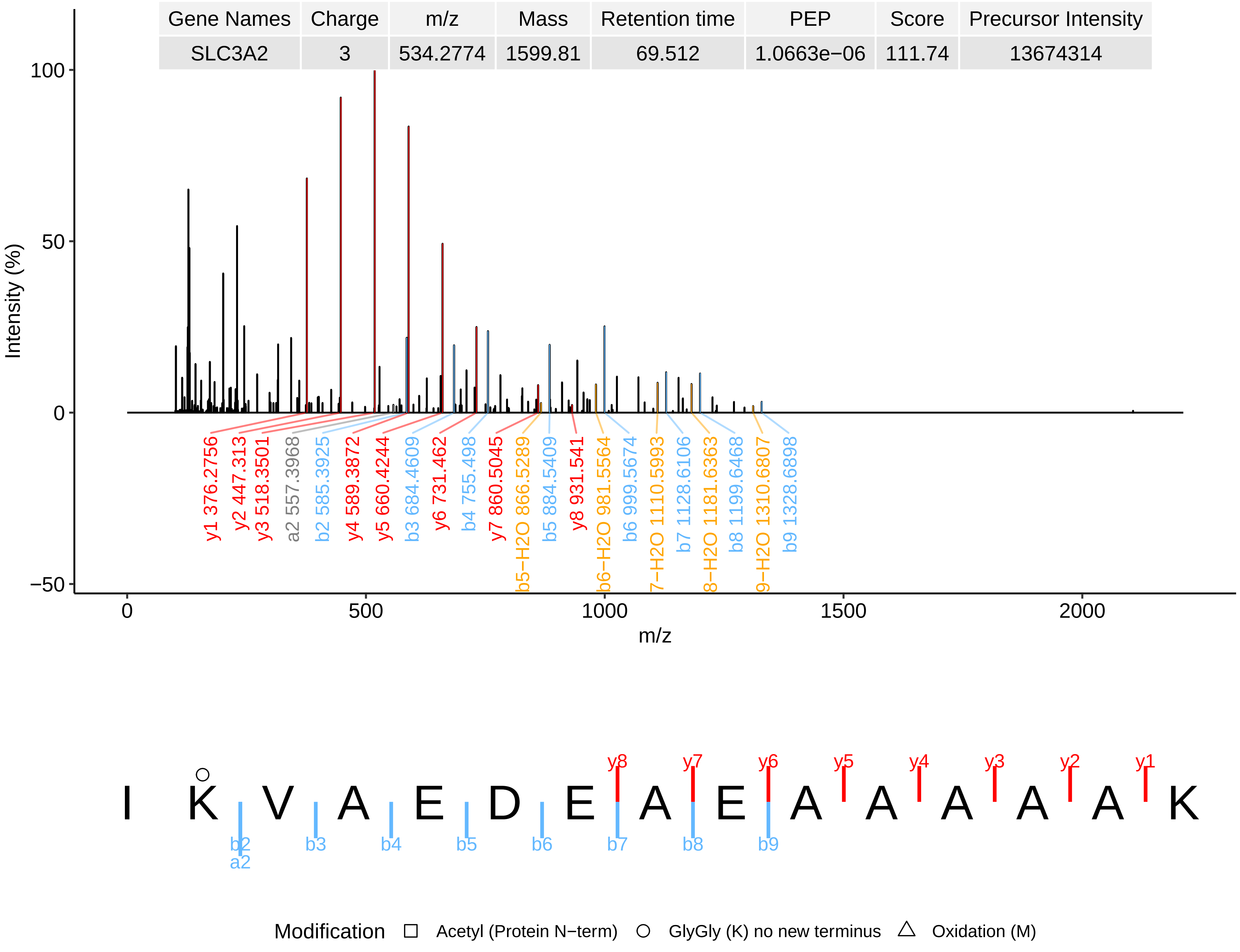

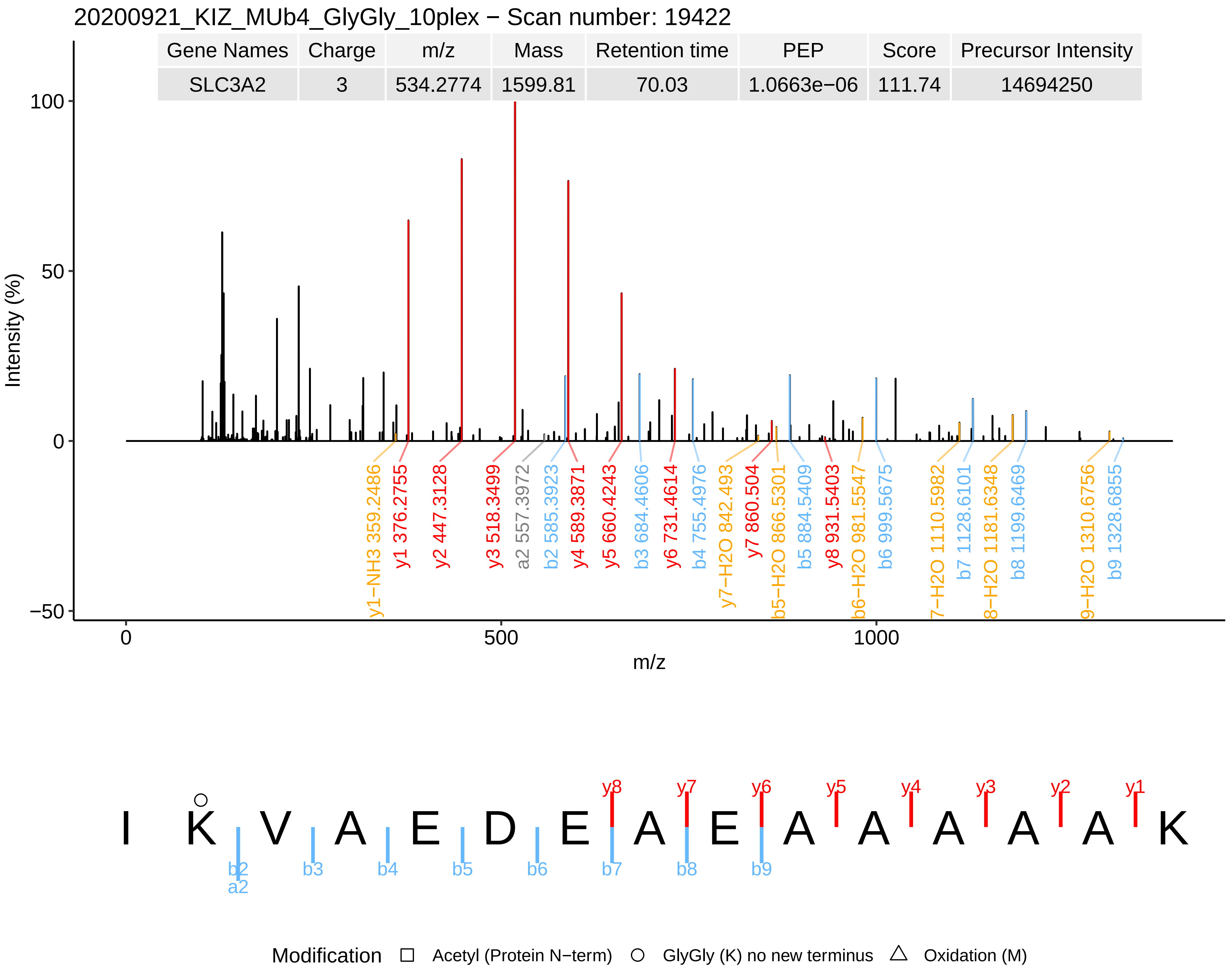

20200921_KIZ_MUb4_GlyGly_10plex − Scan number: 19422
Gene Names
Charge
m/z
Mass
Retention time
PEP
Score
Precursor Intensity
SLC3A2
3
534.2774
1599.81
70.03
1.0663e−06
111.74
14694250
Intensity (%)
100
50
0
−50
0
500
1000
m/z
y1−NH3 359.2486
y1 376.2755
y2 447.3128
y3 518.3499
a2 557.3972
b2 585.3923
y4 589.3871
y5 660.4243
b3 684.4606
y6 731.4614
b4 755.4976
y7−H2O 842.493
y7 860.504
b5−H2O 866.5301
b5 884.5409
y8 931.5403
b6−H2O 981.5547
b6 999.5675
7−H2O 1110.5982
b7 1128.6101
8−H2O 1181.6348
b8 1199.6469
9−H2O 1310.6756
b9 1328.6855
I K V A E D E A E A A A A A K
y8 y7 y6 y5 y4 y3 y2 y1
b2 b3 b4 b5 b6 b7 b8 b9
a2
Modification
Acetyl (Protein N−term)
GlyGly (K) no new terminus
Oxidation (M)

20200921_KIZ_MUb4_GlyGly_10plex − Scan number: 19555

Gene Names	Charge	m/z	Mass	Retention time	PEP	Score	Precursor Intensity
SLC3A2	3	534.2774	1599.81	70.548	7.1884e−05	91.207	4416138

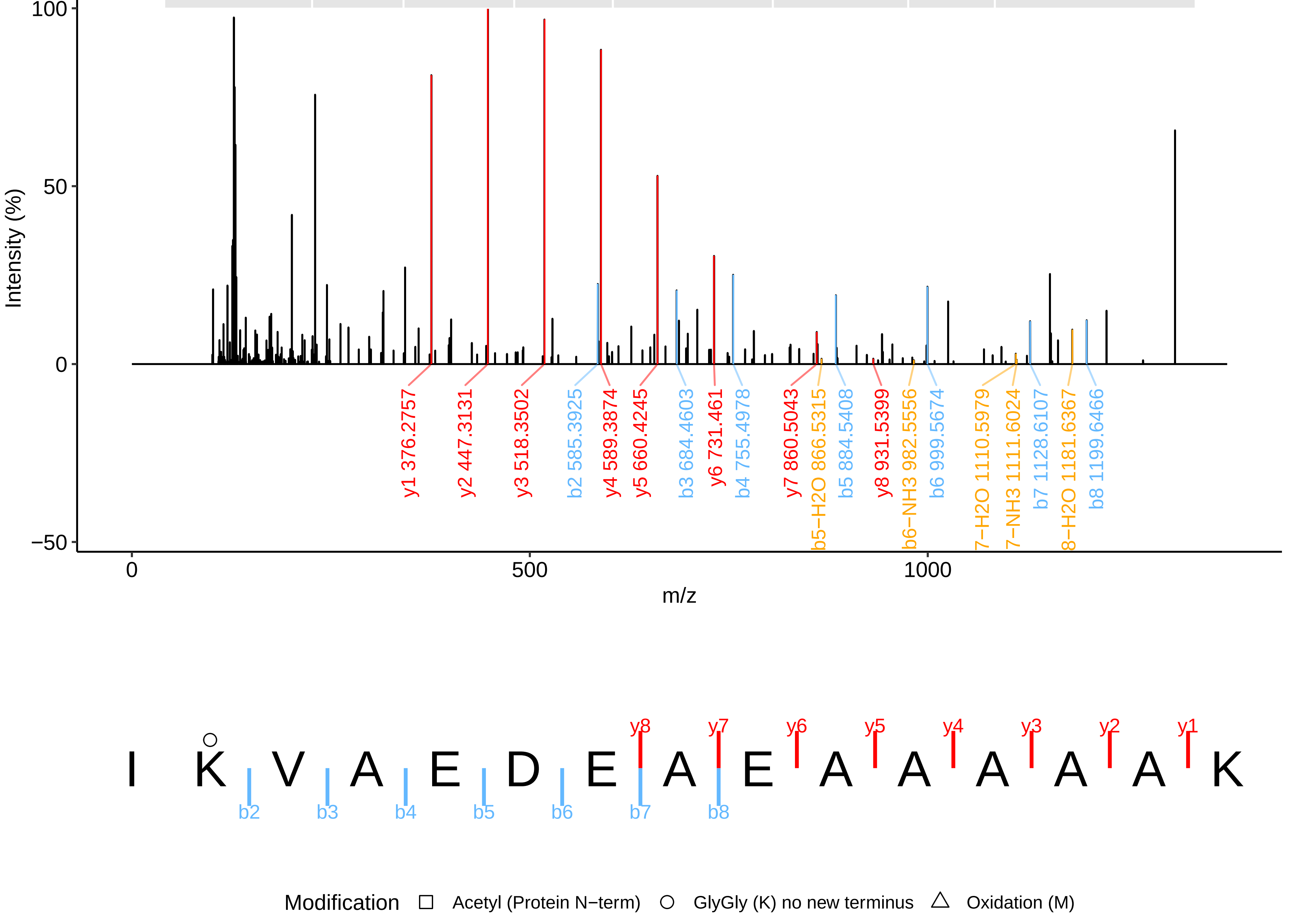

20200921_KIZ_MUb4_GlyGly_10plex − Scan number: 19018

Gene Names	Charge	m/z	Mass	Retention time	PEP	Score	Precursor Intensity
SLC3A2	3	534.2774	1599.81	68.492	0.00014681	91.207	8091663

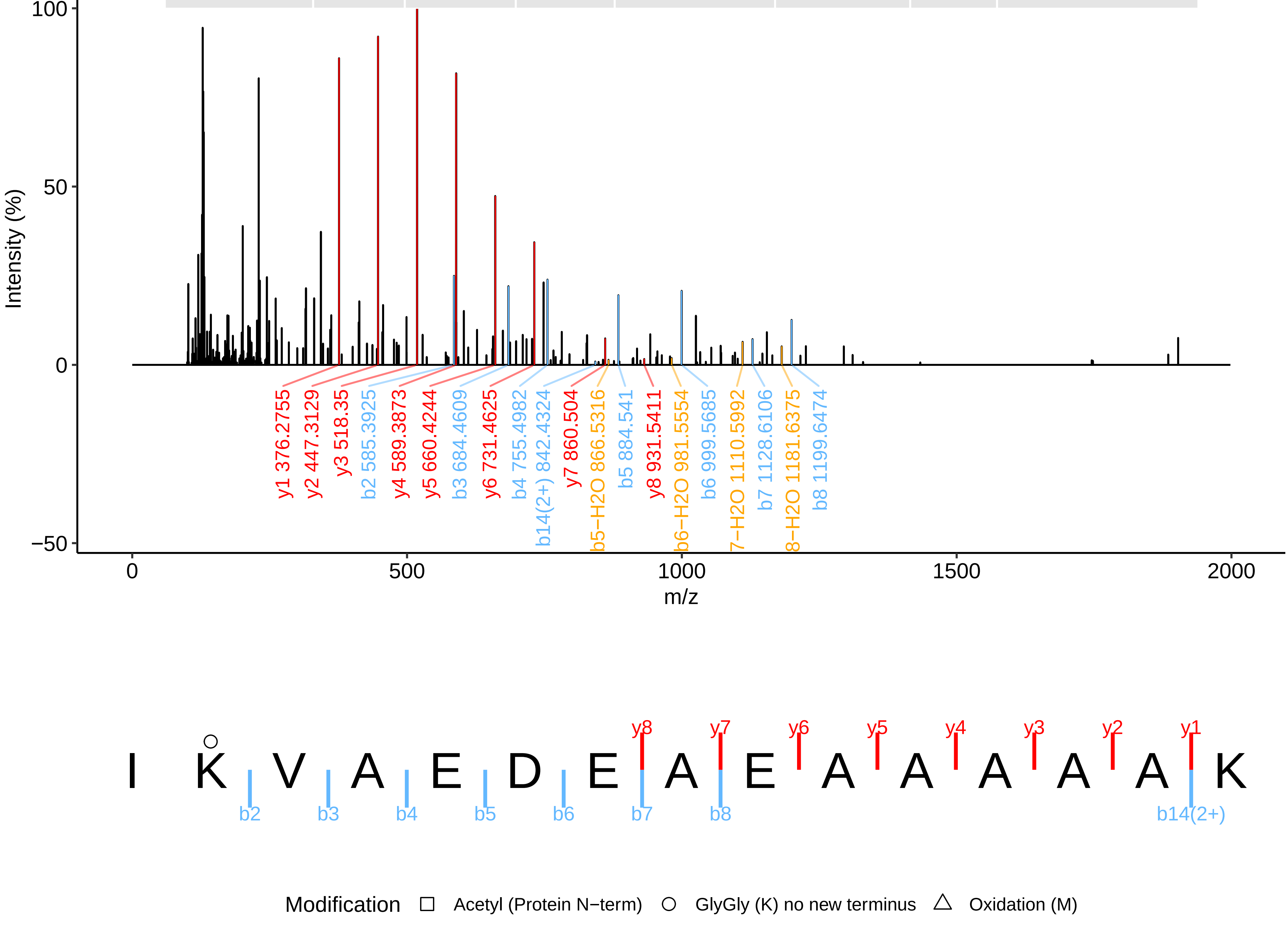

20200921_KIZ_MUb4_GlyGly_10plex_2 − Scan number: 16654

Gene Names	Charge	m/z	Mass	Retention time	PEP	Score	Precursor Intensity
SLC3A2	3	534.2774	1599.81	65.043	1.0663e−06	111.74	2765648

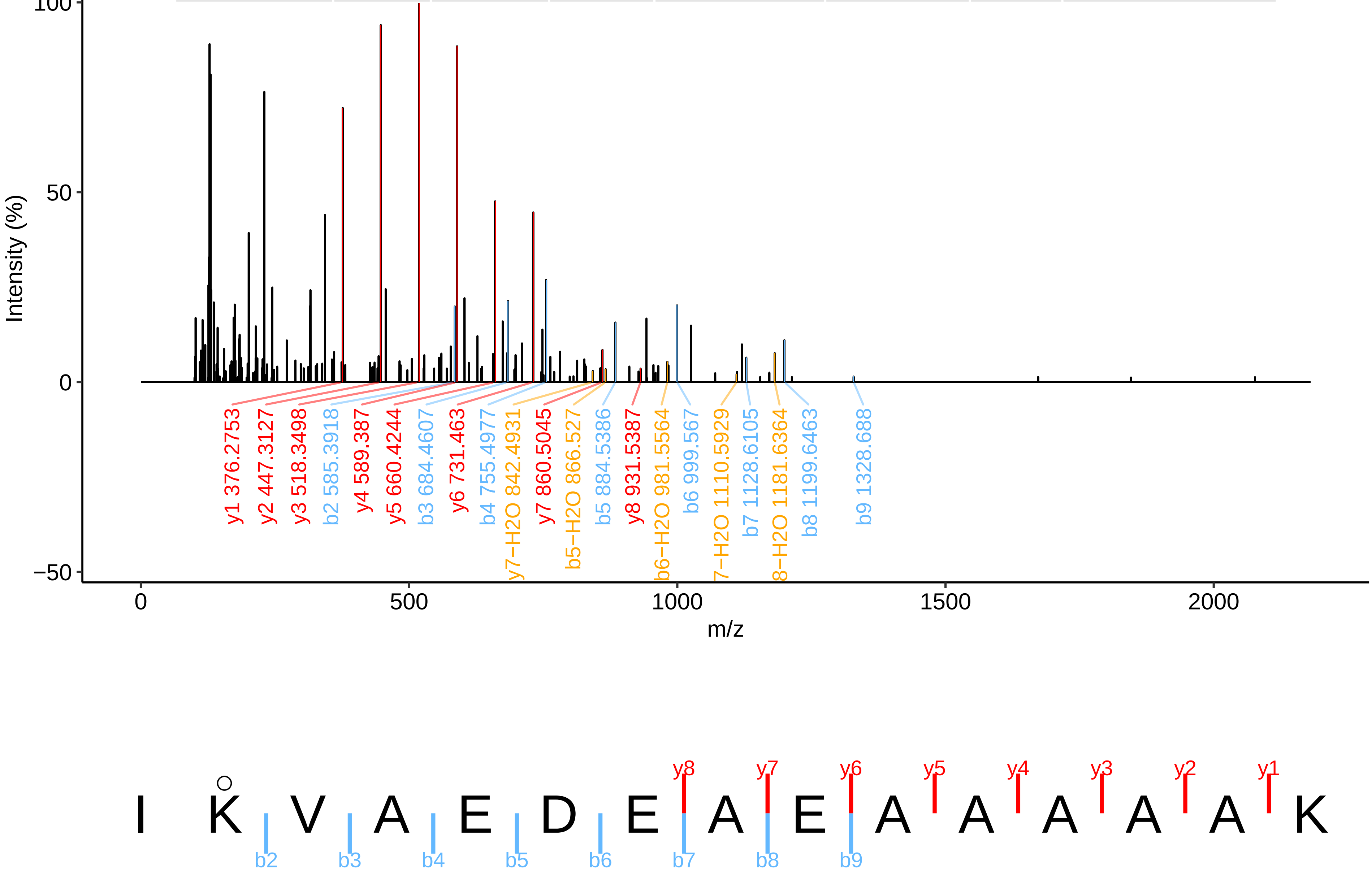

Modification □ Acetyl (Protein N−term) ○ GlyGly (K) no new terminus △ Oxidation (M)

20200924_KIZ_MUb5_GlyGly_1 − Scan number: 17725

Gene Names	Charge	m/z	Mass	Retention time	PEP	Score	Precursor Intensity
SLC3A2	3	534.2774	1599.81	57.26	7.0206e−06	97.797	19002718

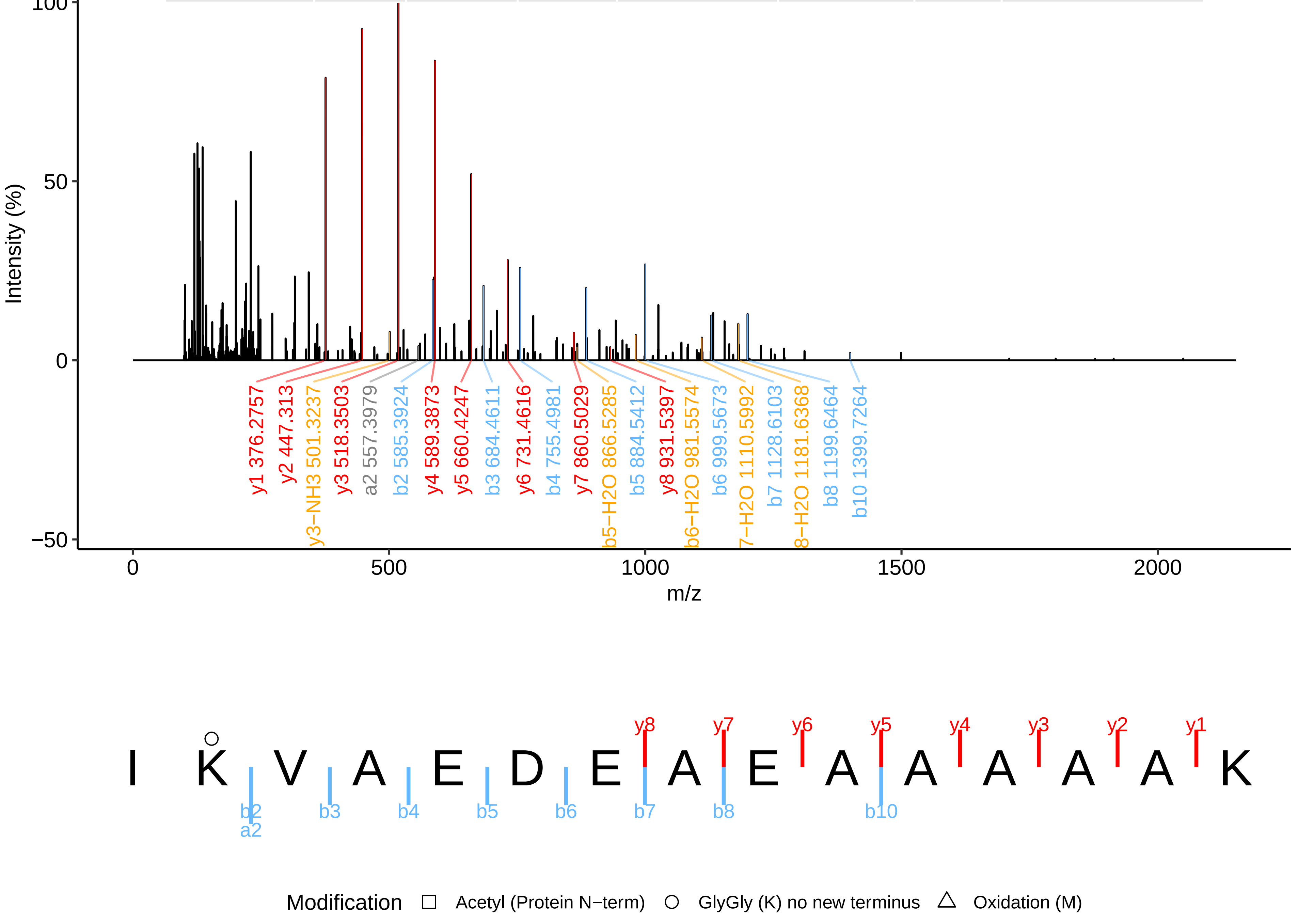

20200924_KIZ_MUb5_GlyGly_1 − Scan number: 17877

Gene Names	Charge	m/z	Mass	Retention time	PEP	Score	Precursor Intensity
SLC3A2	3	534.2774	1599.81	57.767	1.8864e−09	126.48	12989452

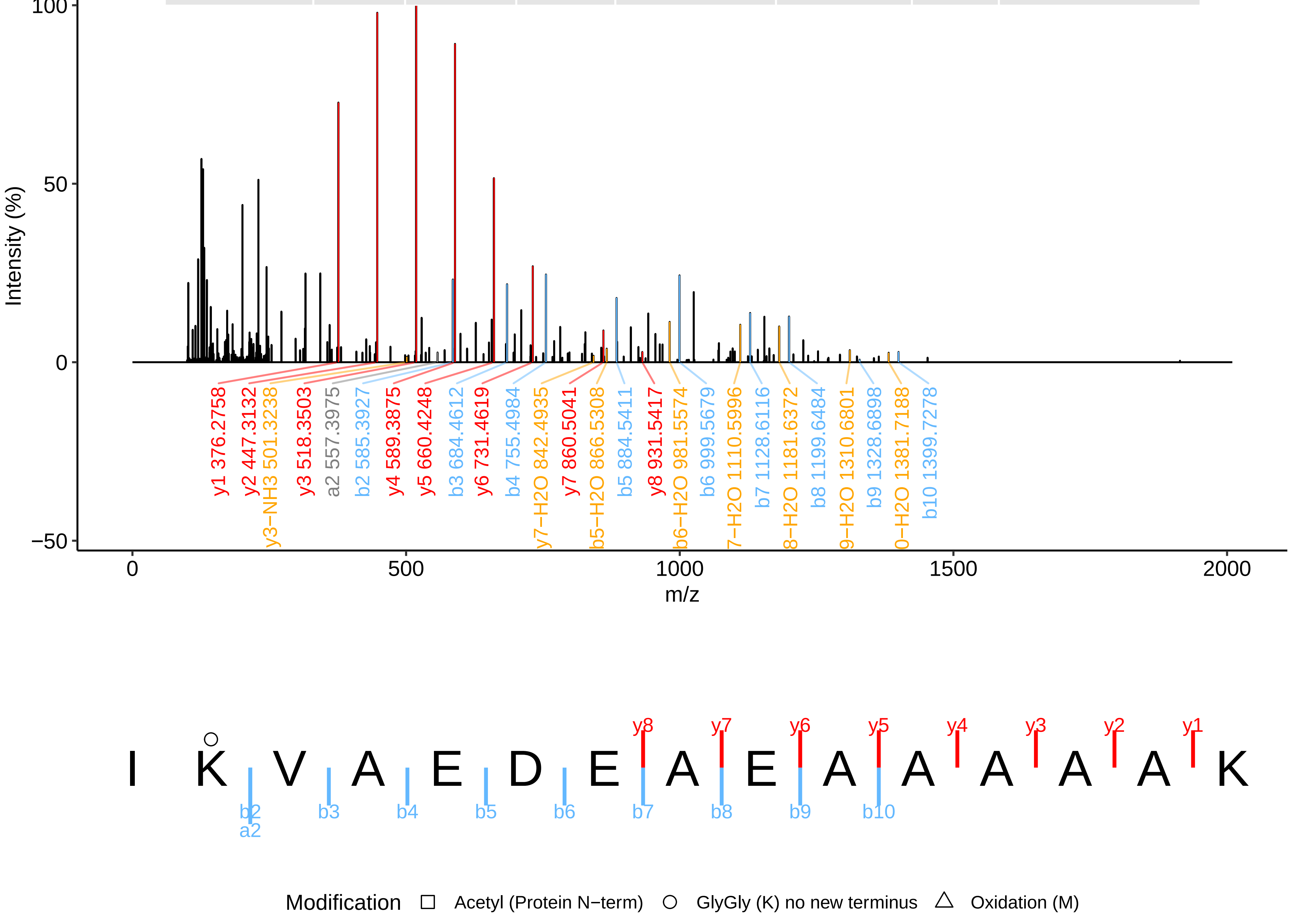

20200924_KIZ_MUb5_GlyGly_2 − Scan number: 16369

Gene Names	Charge	m/z	Mass	Retention time	PEP	Score	Precursor Intensity
SLC3A2	3	534.2774	1599.81	60.737	0.00011248	87.083	3404046

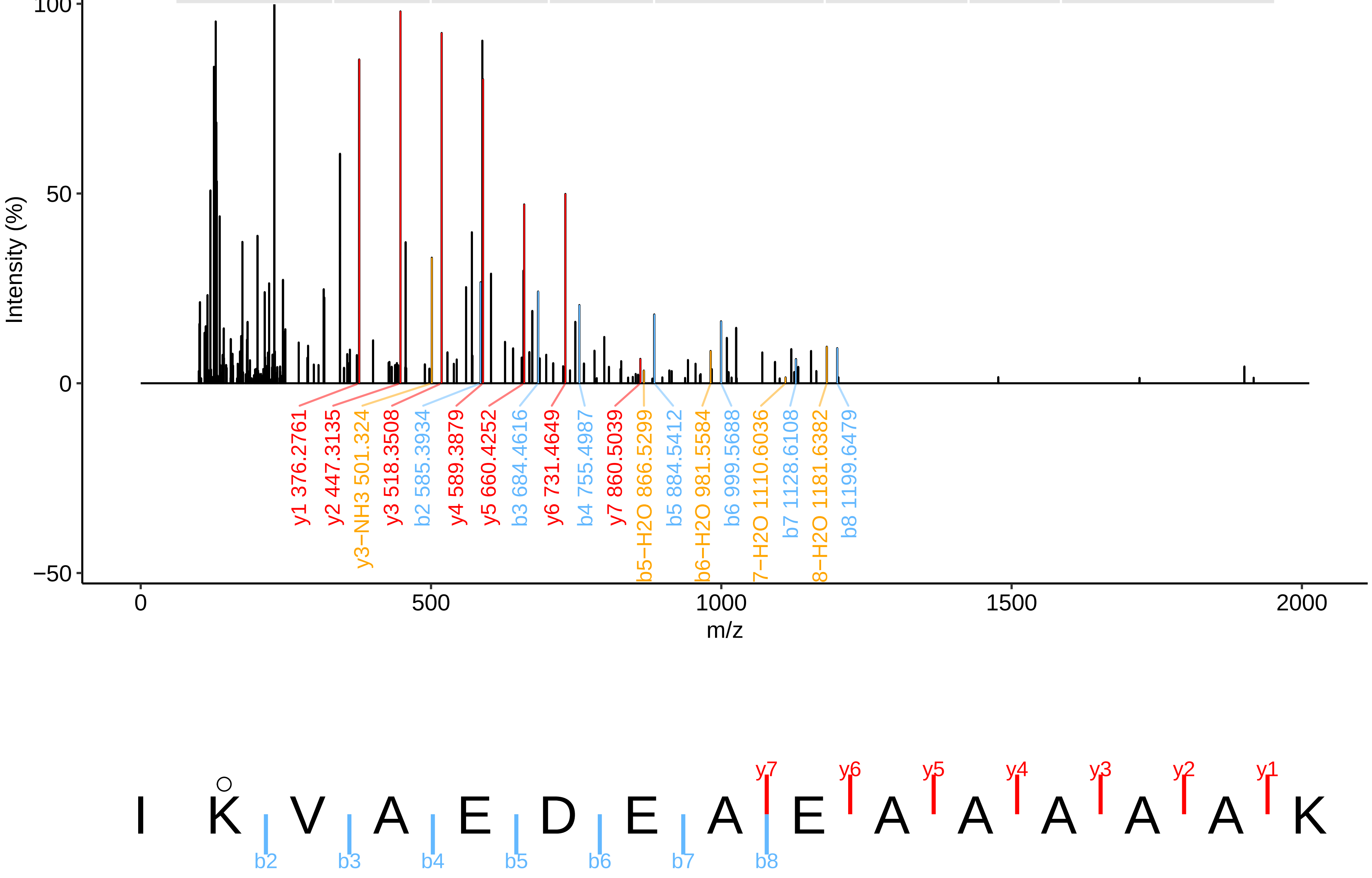

Modification □ Acetyl (Protein N−term) ○ GlyGly (K) no new terminus △ Oxidation (M)

20200924_KIZ_MUb5_GlyGly_2 − Scan number: 16383

Gene Names	Charge	m/z	Mass	Retention time	PEP	Score	Precursor Intensity
SLC3A2	3	534.2774	1599.81	60.79	0.00014586	83.692	2801568

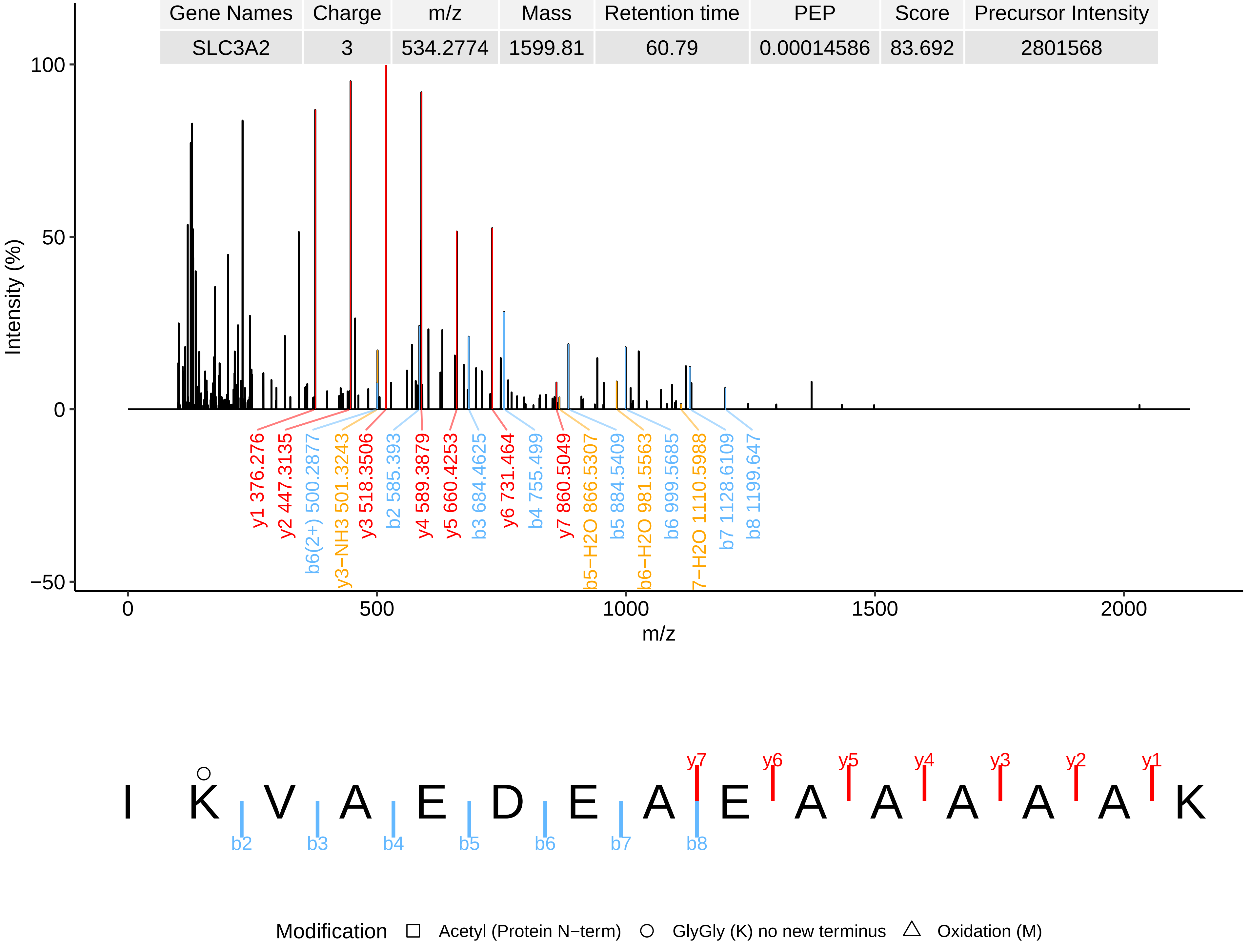

20200924_KIZ_MUb5_GlyGly_2 − Scan number: 16663

Gene Names	Charge	m/z	Mass	Retention time	PEP	Score	Precursor Intensity
SLC3A2	3	534.2774	1599.81	61.815	4.3286e−06	100.45	6888567

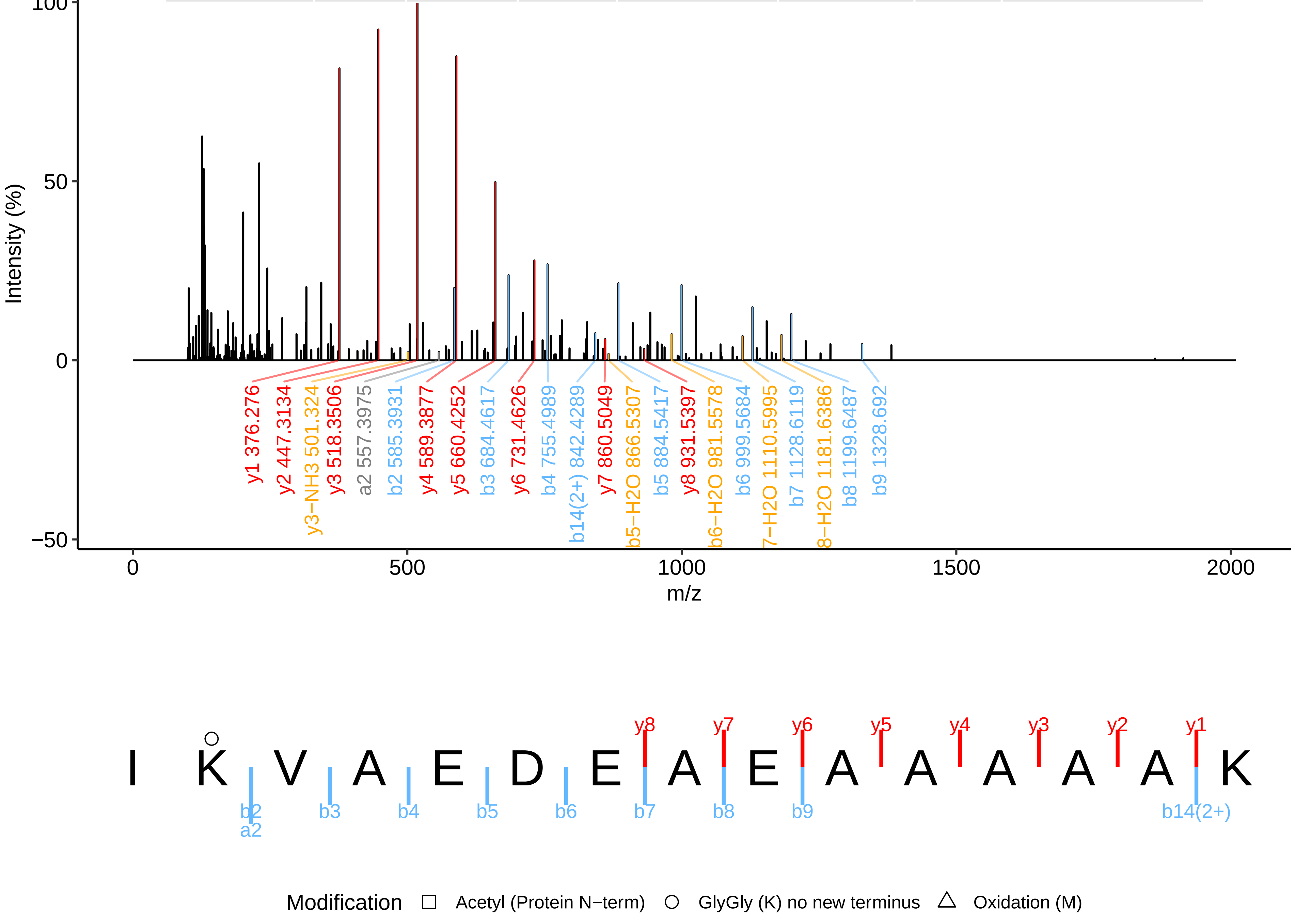

20201118_KIZ_MUb6_GlyGly_1 − Scan number: 17853

Gene Names	Charge	m/z	Mass	Retention time	PEP	Score	Precursor Intensity
SLC3A2	3	534.2774	1599.81	56.155	4.3727e−09	118.13	19920126

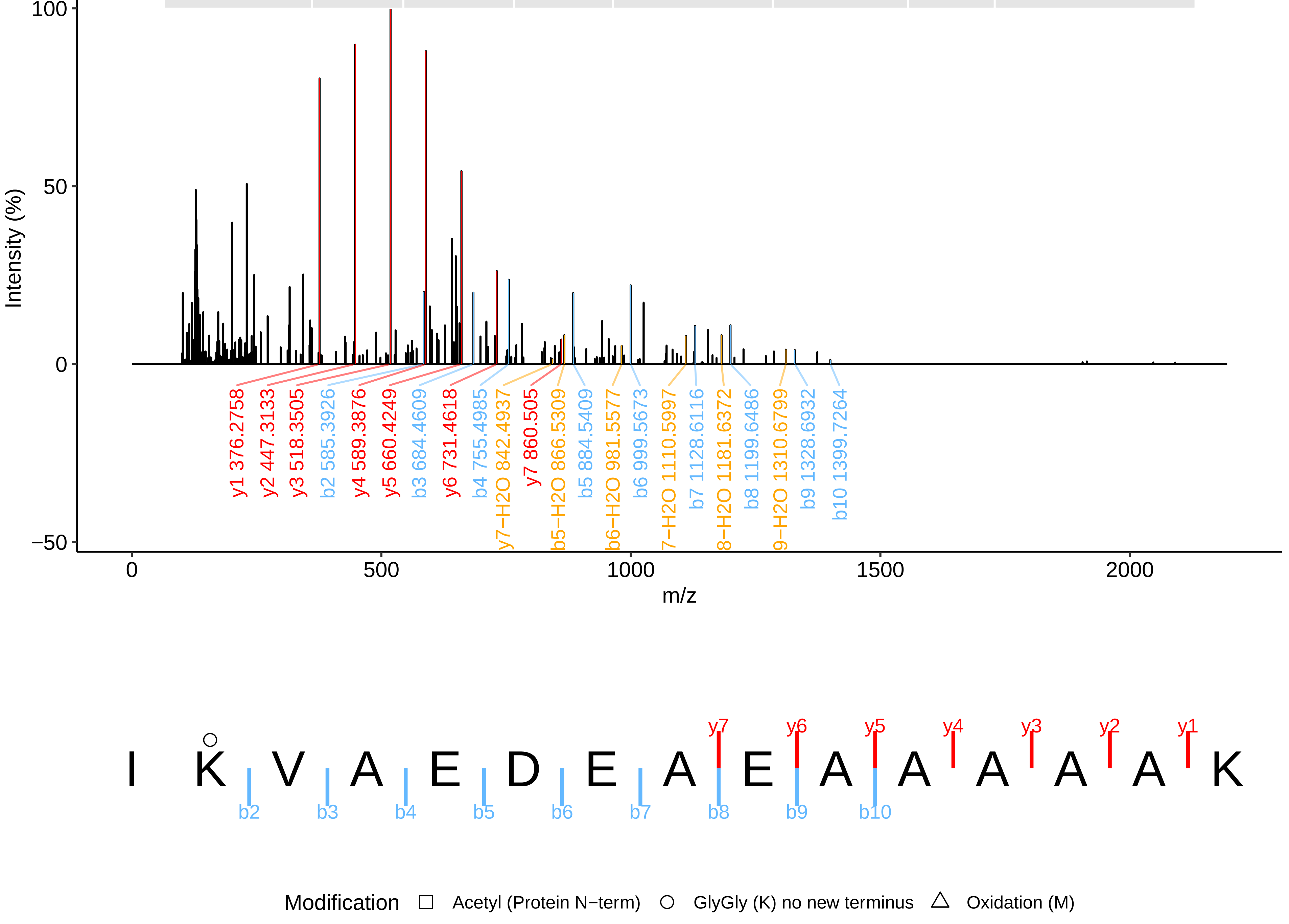

20201118_KIZ_MUb6_GlyGly_1 − Scan number: 17870

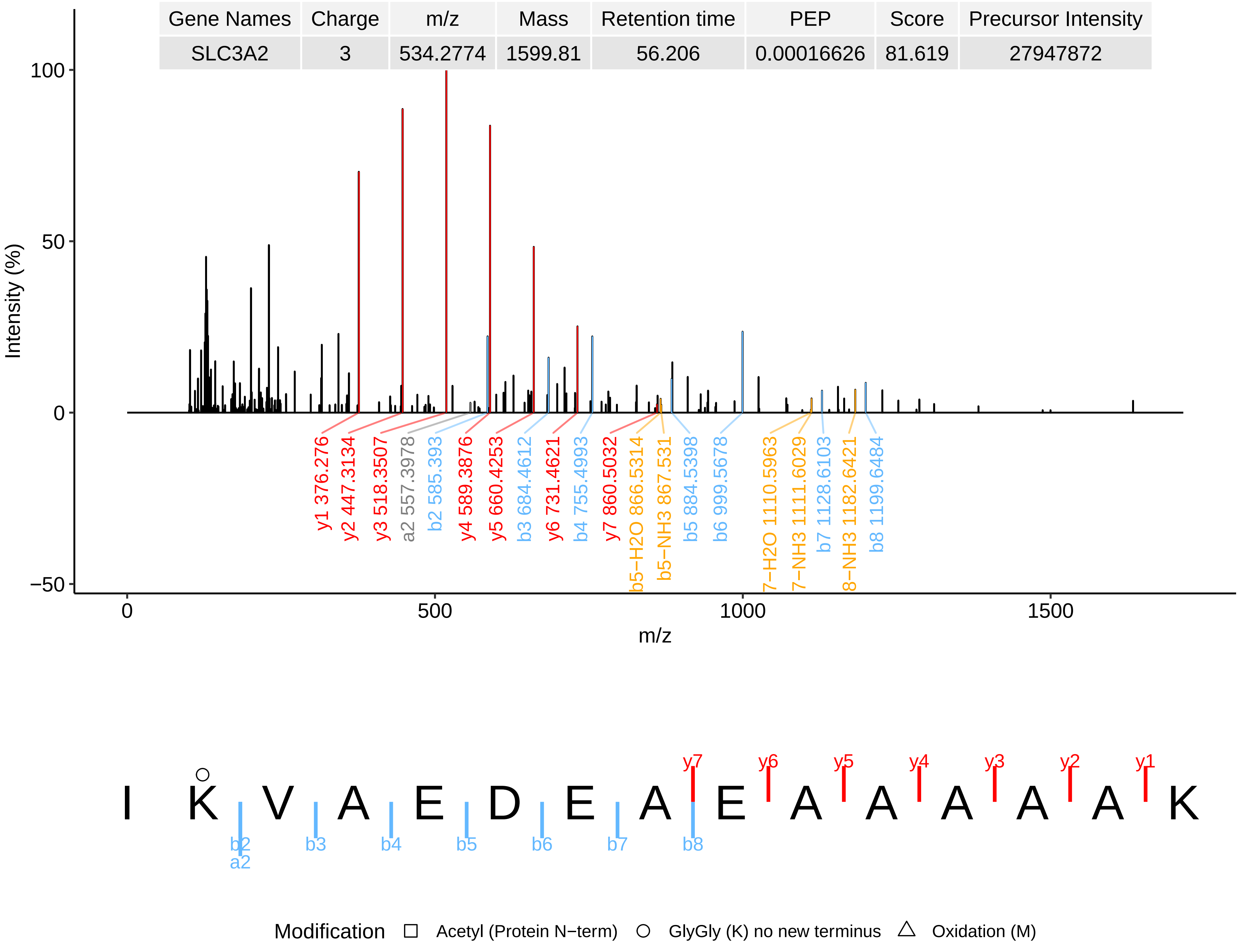

20201118_KIZ_MUb6_GlyGly_2 − Scan number: 19435

Gene Names	Charge	m/z	Mass	Retention time	PEP	Score	Precursor Intensity
SLC3A2	3	534.2774	1599.81	65.582	2.2605e−06	107.61	10897579

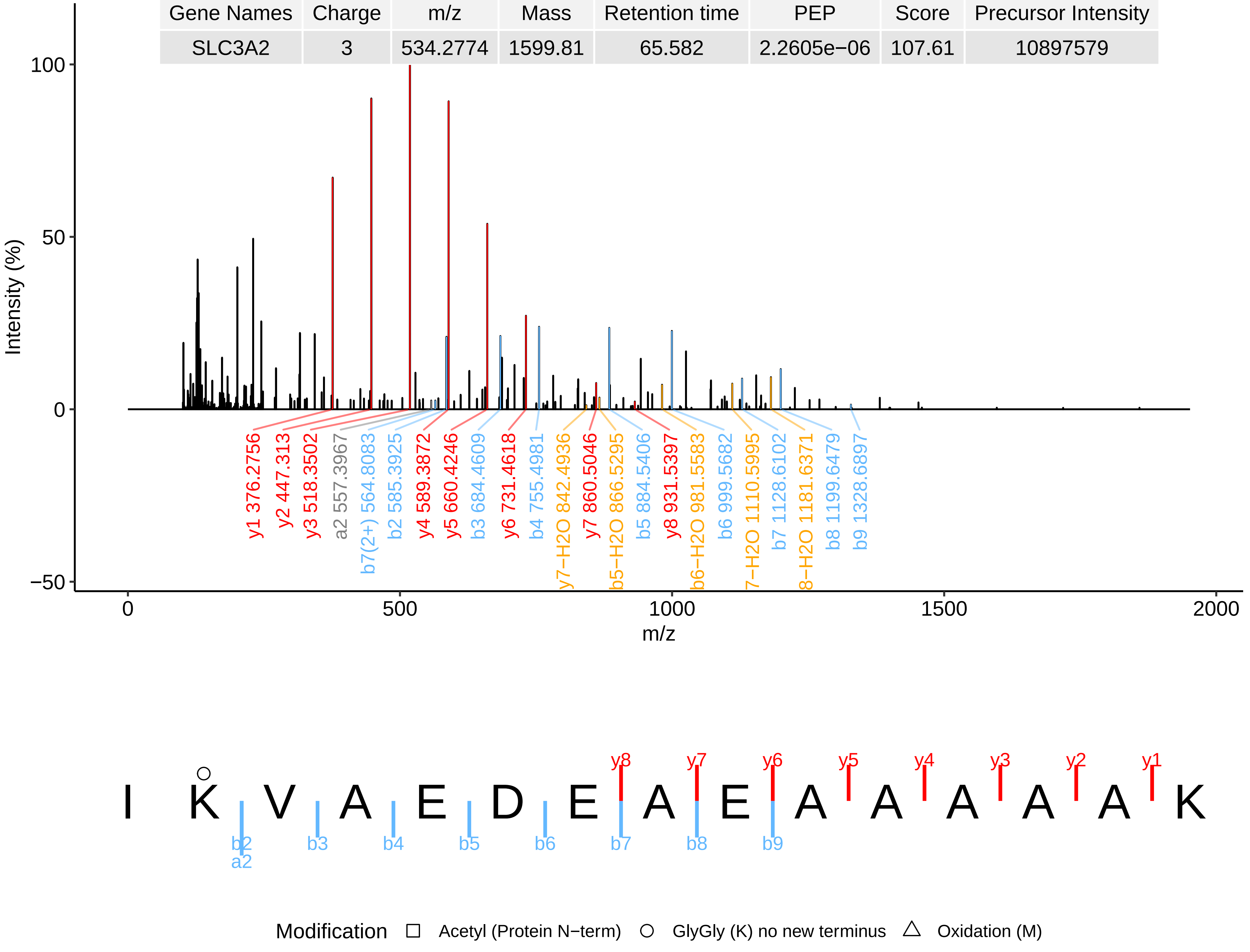

20200921_KIZ_MUb4_GlyGly_10plex_2 − Scan number: 16738

Gene Names	Charge	m/z	Mass	Retention time	PEP	Score	Precursor Intensity
RAD23B	3	512.935	1535.783	65.376	0.00028256	94.191	1015524

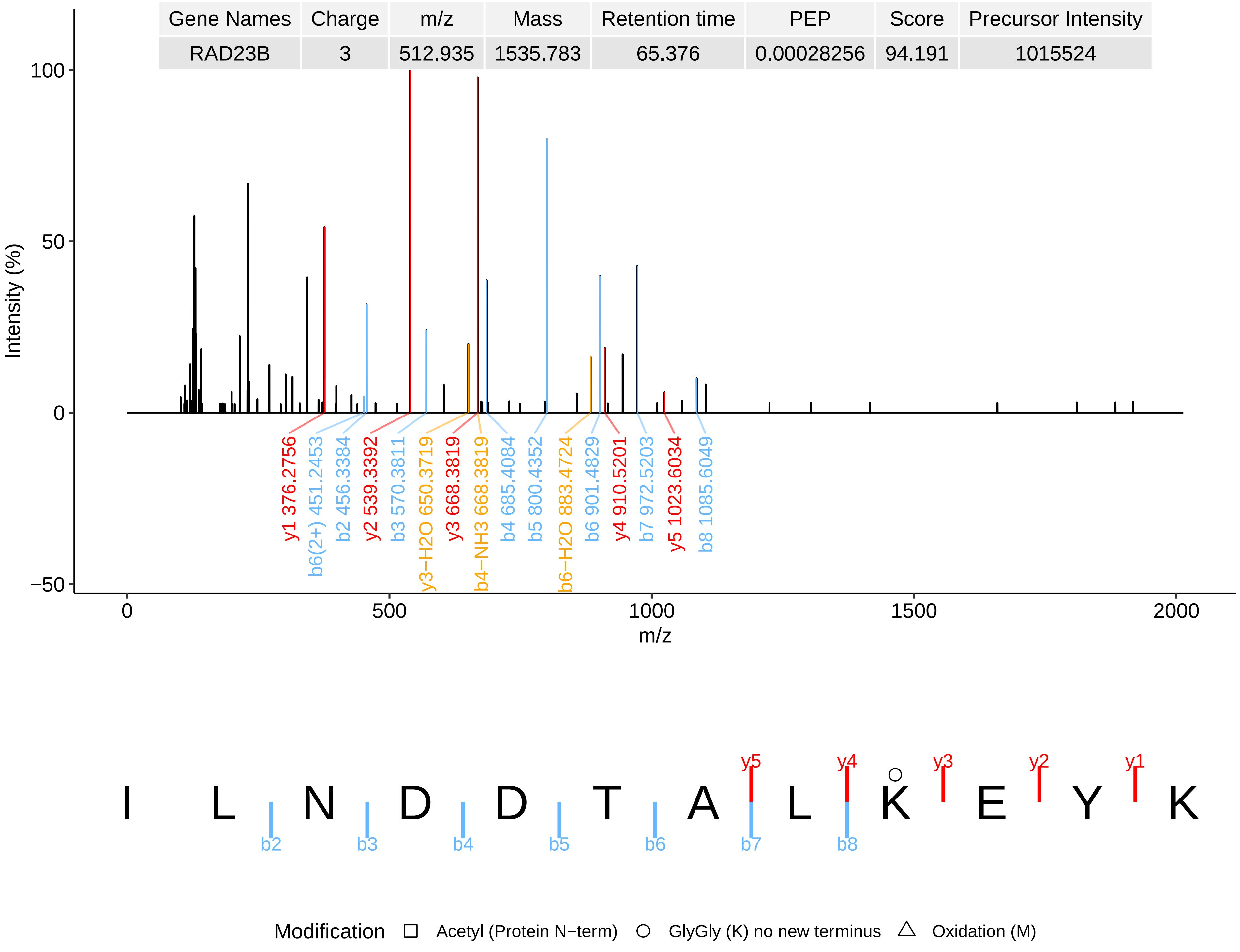

20200921_KIZ_MUb4_GlyGly_10plex − Scan number: 19226

Gene Names	Charge	m/z	Mass	Retention time	PEP	Score	Precursor Intensity
ITM2C	3	515.6192	1543.836	69.264	0.00021068	93.51	7085483

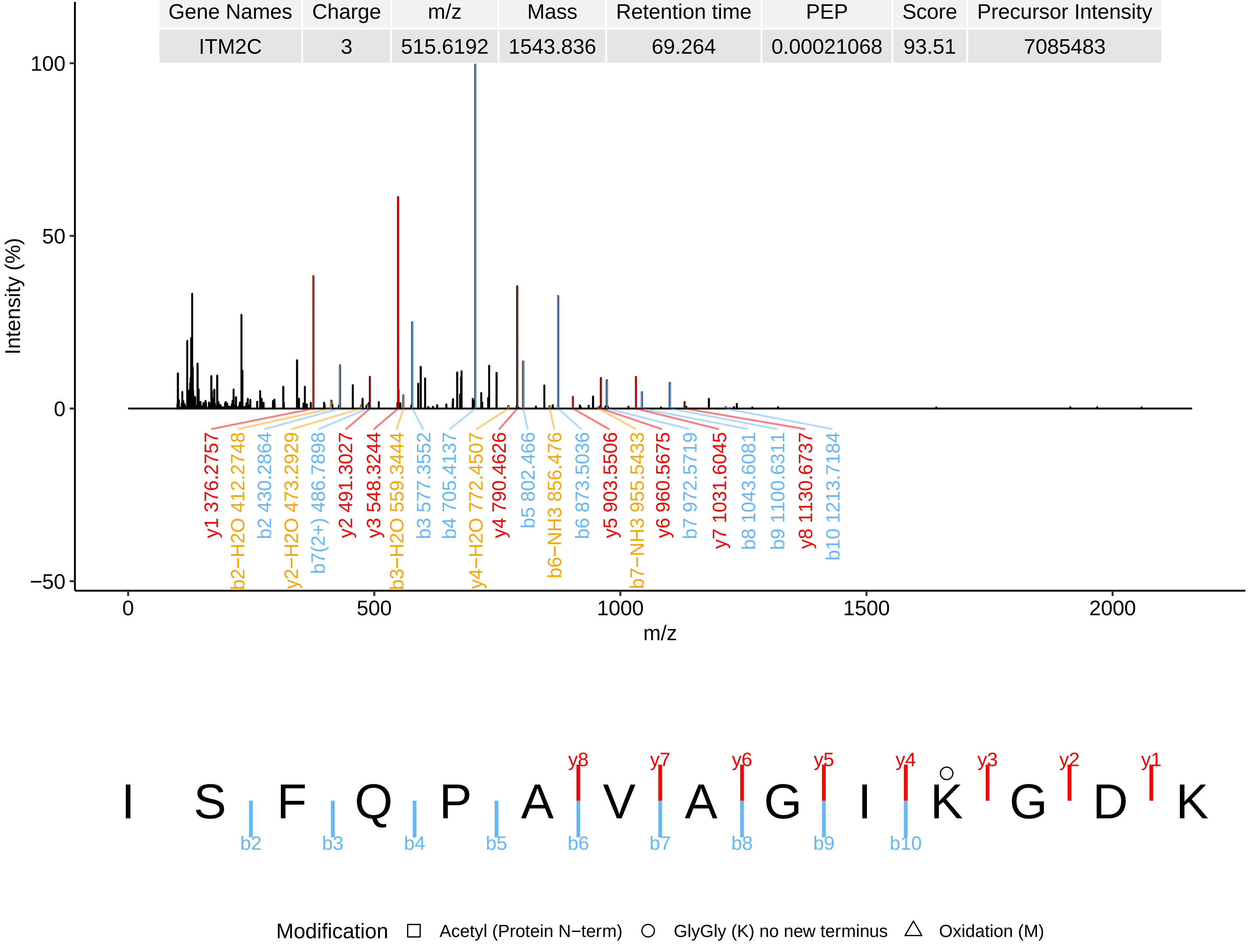

20200921_KIZ_MUb4_GlyGly_10plex − Scan number: 5106

Gene Names	Charge	m/z	Mass	Retention time	PEP	Score	Precursor Intensity
BASP1	3	511.246	1530.716	16.667	0.00012238	99.481	2884265

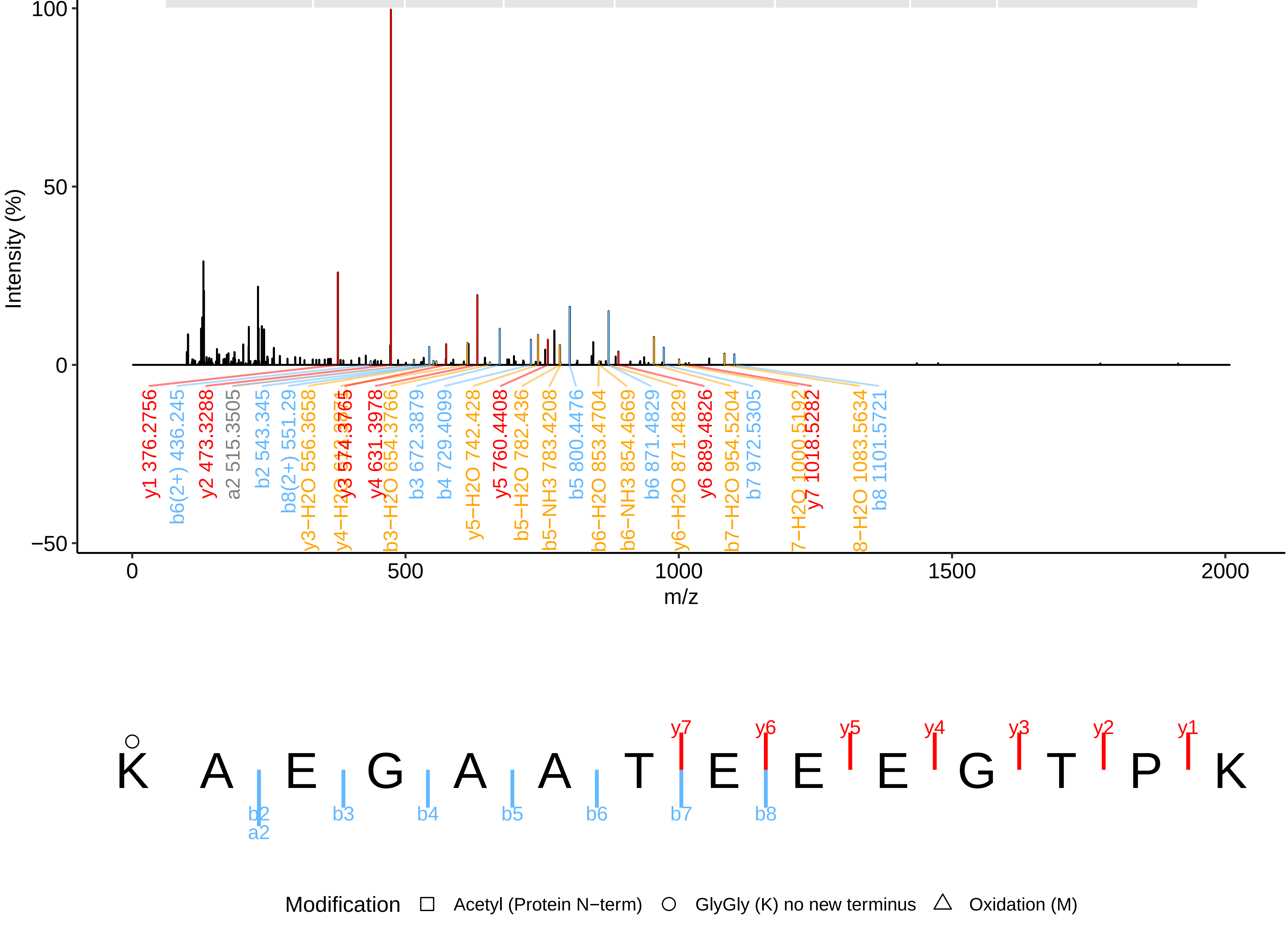

20200921_KIZ_MUb4_GlyGly_10plex_2 − Scan number: 23739

Gene Names	Charge	m/z	Mass	Retention time	PEP	Score	Precursor Intensity
RAD23A	3	596.3402	1785.999	93.723	2.8339e−05	95.631	974631.2

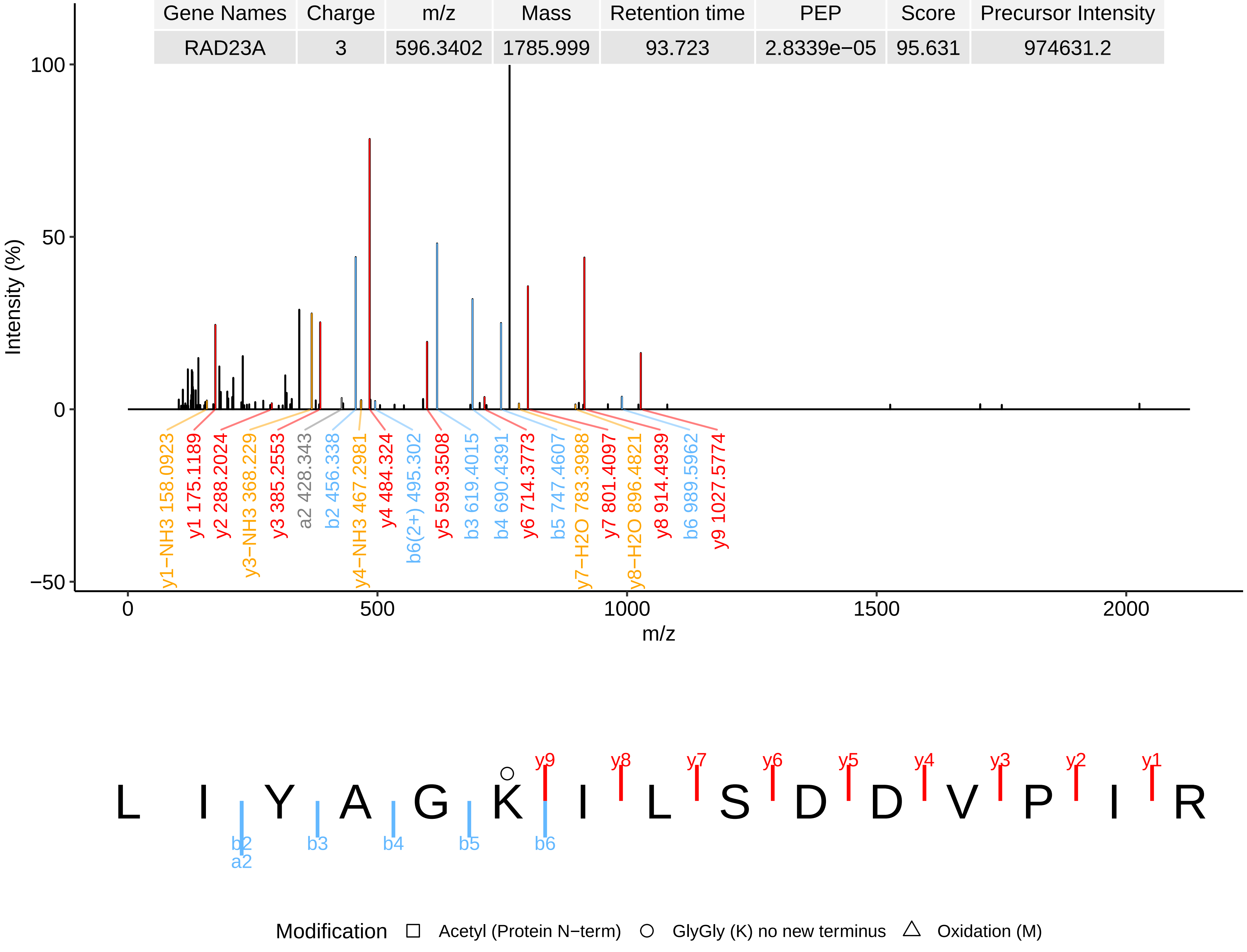

20200924_KIZ_MUb5_GlyGly_2 − Scan number: 23601

Gene Names	Charge	m/z	Mass	Retention time	PEP	Score	Precursor Intensity
RAD23A	3	596.3402	1785.999	87.412	0.091251	45.33	3290772

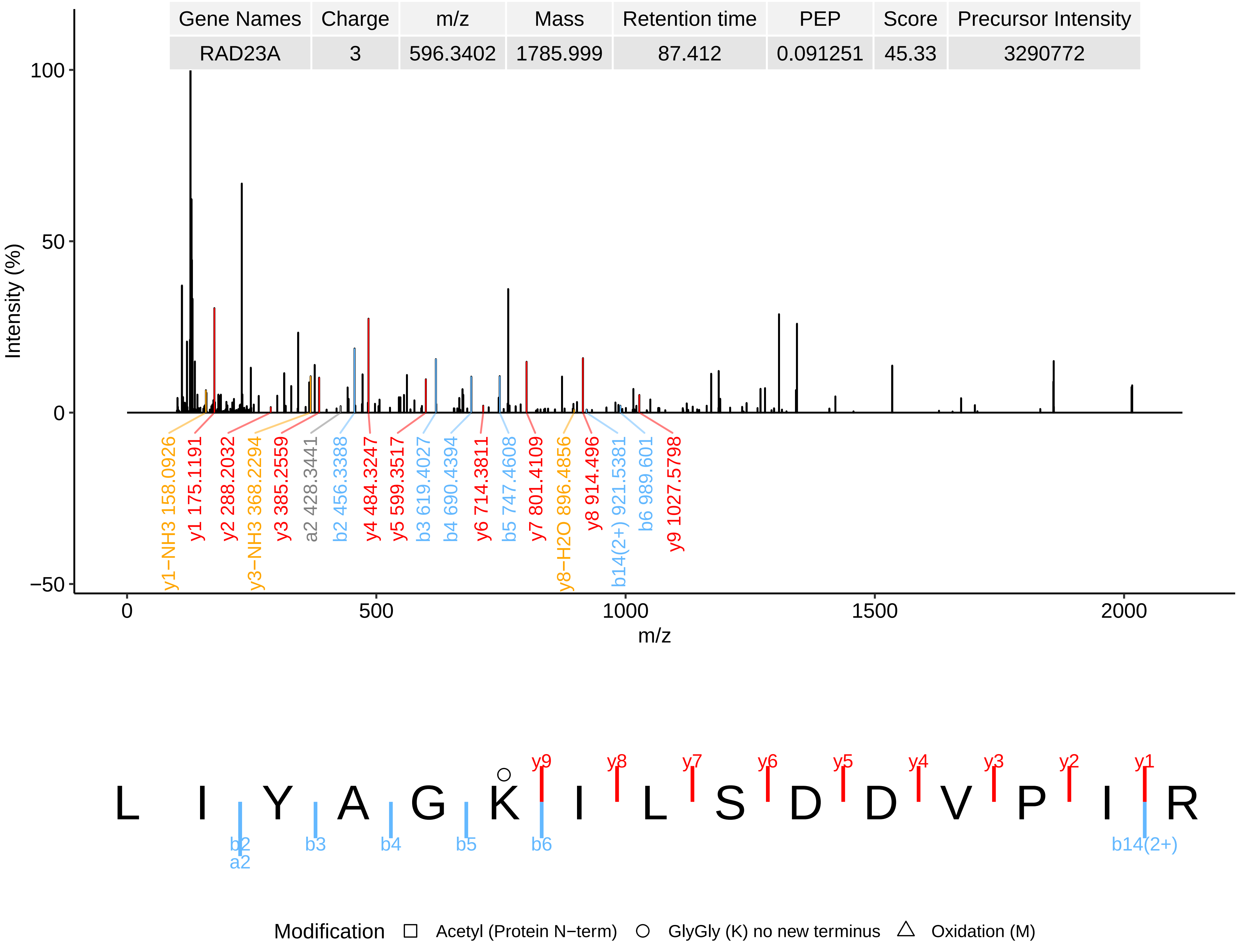

20200921_KIZ_MUb4_GlyGly_10plex − Scan number: 13195

Gene Names	Charge	m/z	Mass	Retention time	PEP	Score	Precursor Intensity
NEDD8;NEDD8−MDP1	3	513.9206	1538.74	46.469	4.0818e−09	127.87	3564058

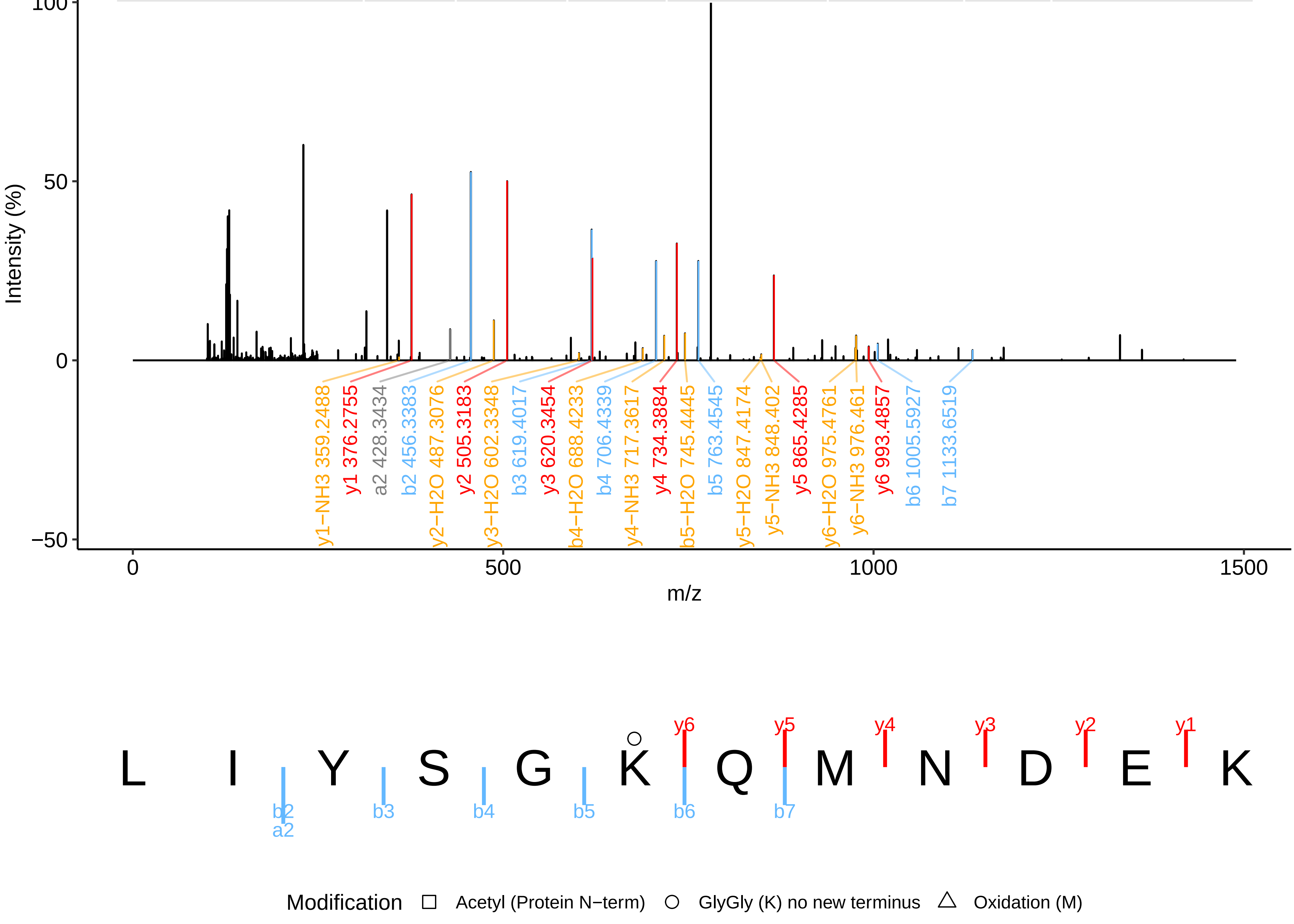

20200921_KIZ_MUb4_GlyGly_10plex_2 − Scan number: 12163

Gene Names	Charge	m/z	Mass	Retention time	PEP	Score	Precursor Intensity
NEDD8;NEDD8−MDP1	3	513.9206	1538.74	46.998	0.0092775	68.536	1589801

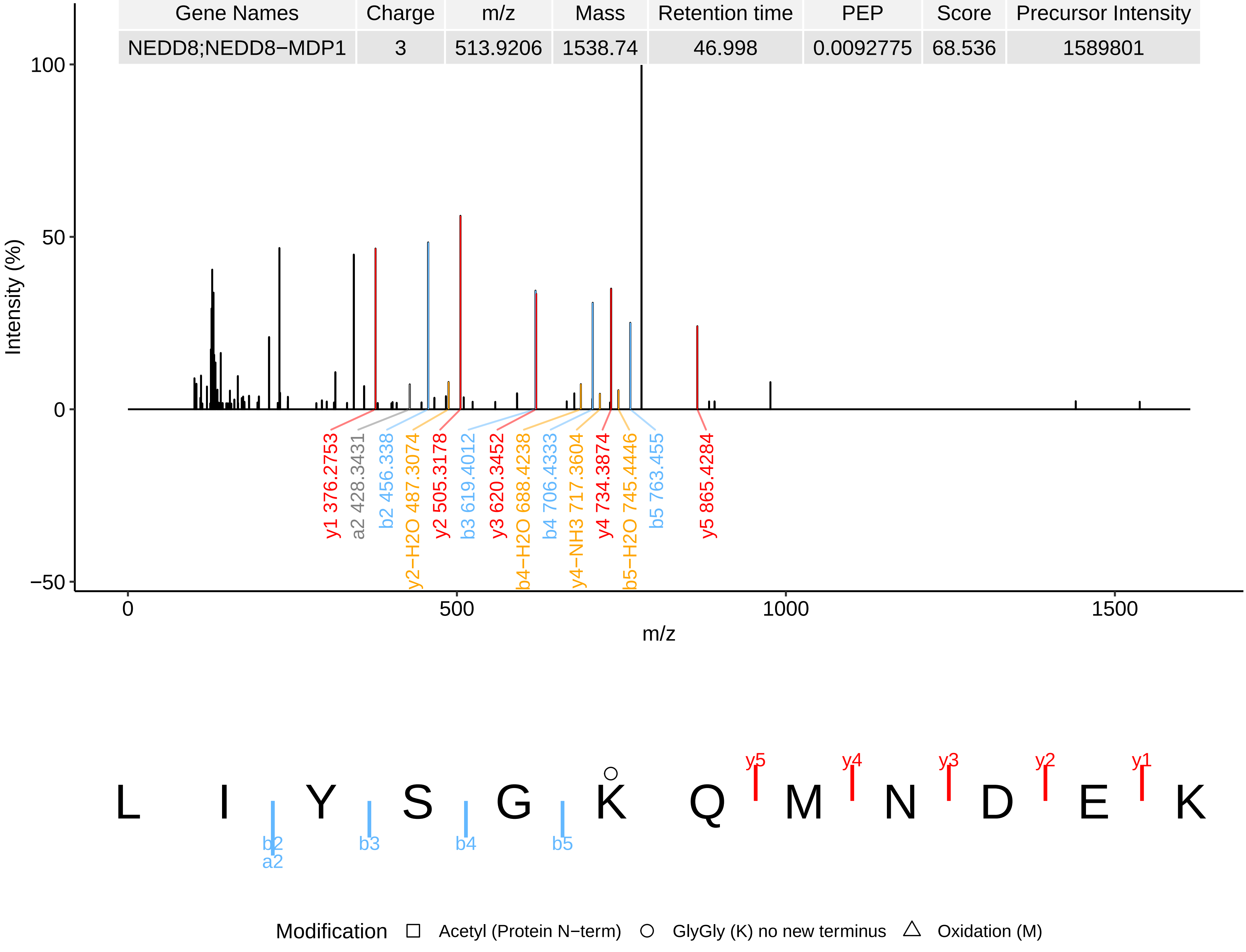

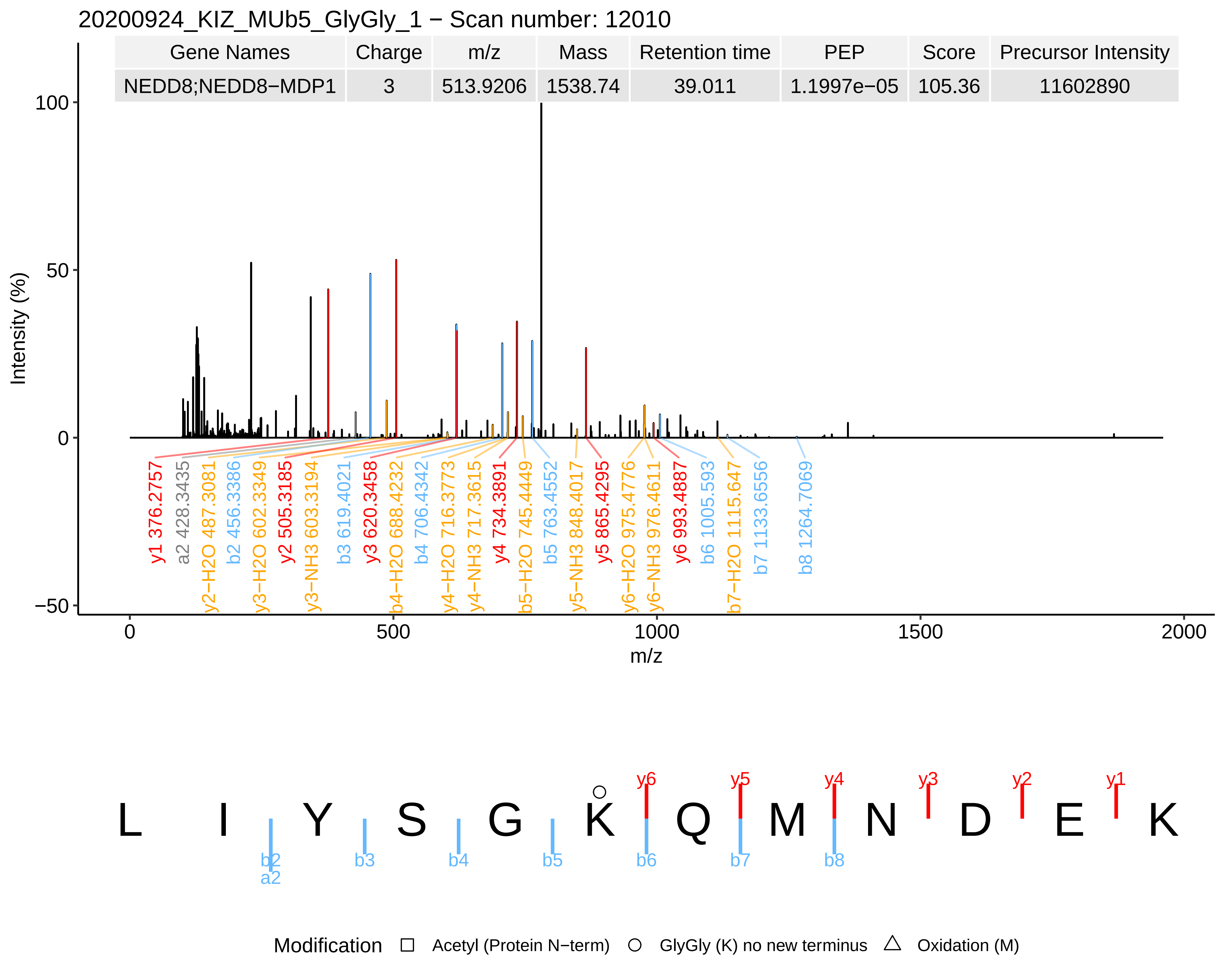
20200924_KIZ_MUb5_GlyGly_1 − Scan number: 12010
Gene Names | Charge | m/z | Mass | Retention time | PEP | Score | Precursor Intensity
NEDD8;NEDD8−MDP1 | 3 | 513.9206 | 1538.74 | 39.011 | 1.1997e−05 | 105.36 | 11602890
Intensity (%)
m/z
y1 376.2757
a2 428.3435
y2−H2O 487.3081
b2 456.3386
y3−H2O 602.3349
y2 505.3185
y3−NH3 603.3194
b3 619.4021
y3 620.3458
b4−H2O 688.4232
b4 706.4342
y4−H2O 716.3773
y4−NH3 717.3615
y4 734.3891
b5−H2O 745.4449
b5 763.4552
y5−NH3 848.4017
y5 865.4295
y6−H2O 975.4776
y6−NH3 976.4611
y6 993.4887
b6 1005.593
b7−H2O 1115.647
b7 1133.6556
b8 1264.7069
L I Y S G K Q M N D E K
y6 y5 y4 y3 y2 y1
b2 a2 b3 b4 b5 b6 b7 b8
Modification
Acetyl (Protein N−term)
GlyGly (K) no new terminus
Oxidation (M)

20200924_KIZ_MUb5_GlyGly_1 − Scan number: 12161

Gene Names	Charge	m/z	Mass	Retention time	PEP	Score	Precursor Intensity
NEDD8;NEDD8−MDP1	3	513.9206	1538.74	39.519	5.46e−09	126.19	54615332

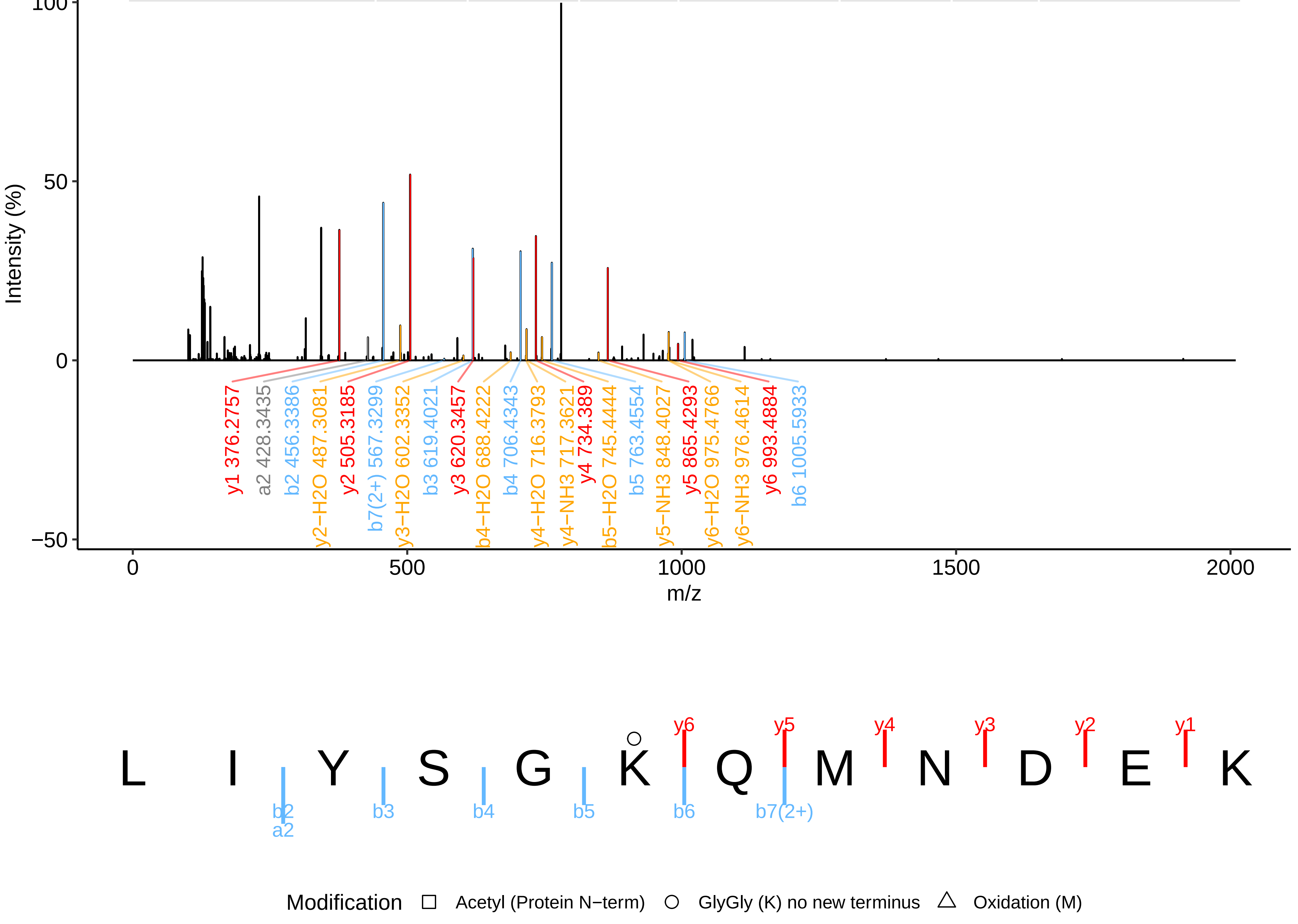

20200924_KIZ_MUb5_GlyGly_1 − Scan number: 12308

Gene Names	Charge	m/z	Mass	Retention time	PEP	Score	Precursor Intensity
NEDD8;NEDD8−MDP1	3	513.9206	1538.74	40.027	2.5e−13	138.99	16462134

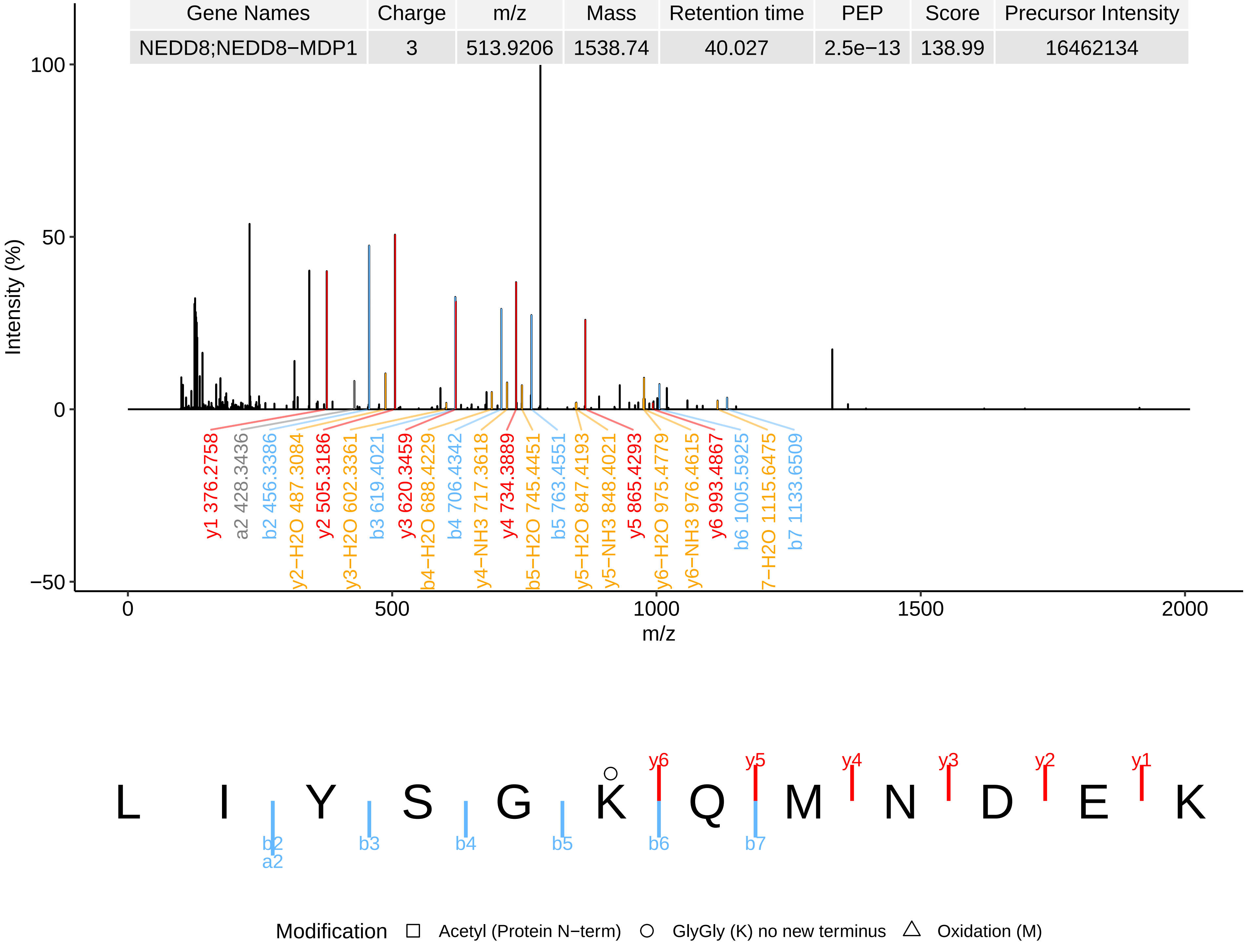

20200924_KIZ_MUb5_GlyGly_2 − Scan number: 11262

Gene Names	Charge	m/z	Mass	Retention time	PEP	Score	Precursor Intensity
NEDD8;NEDD8−MDP1	3	513.9206	1538.74	42.073	4.0818e−09	127.87	4303840

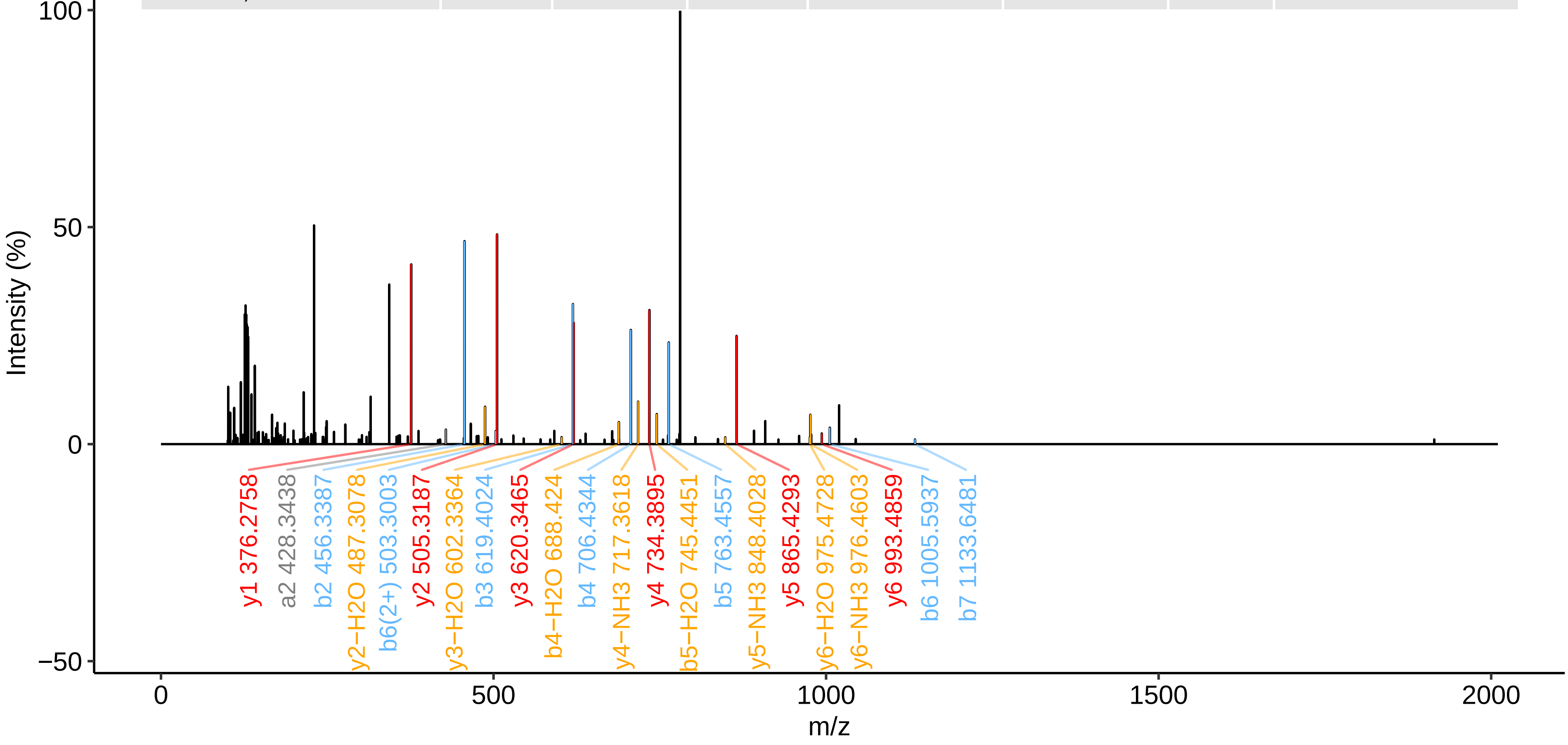

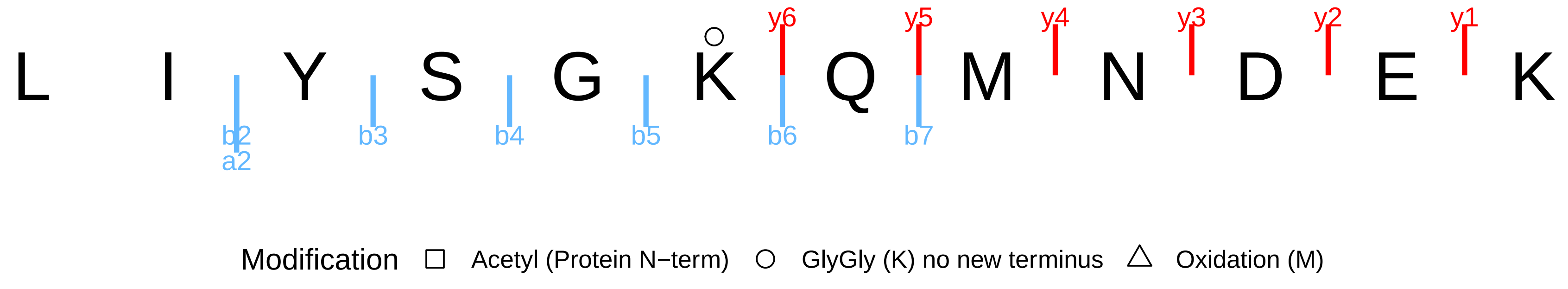

20200924_KIZ_MUb5_GlyGly_2 − Scan number: 11456

Gene Names	Charge	m/z	Mass	Retention time	PEP	Score	Precursor Intensity
NEDD8;NEDD8−MDP1	3	513.9206	1538.74	42.827	4.0818e−09	127.87	8801591

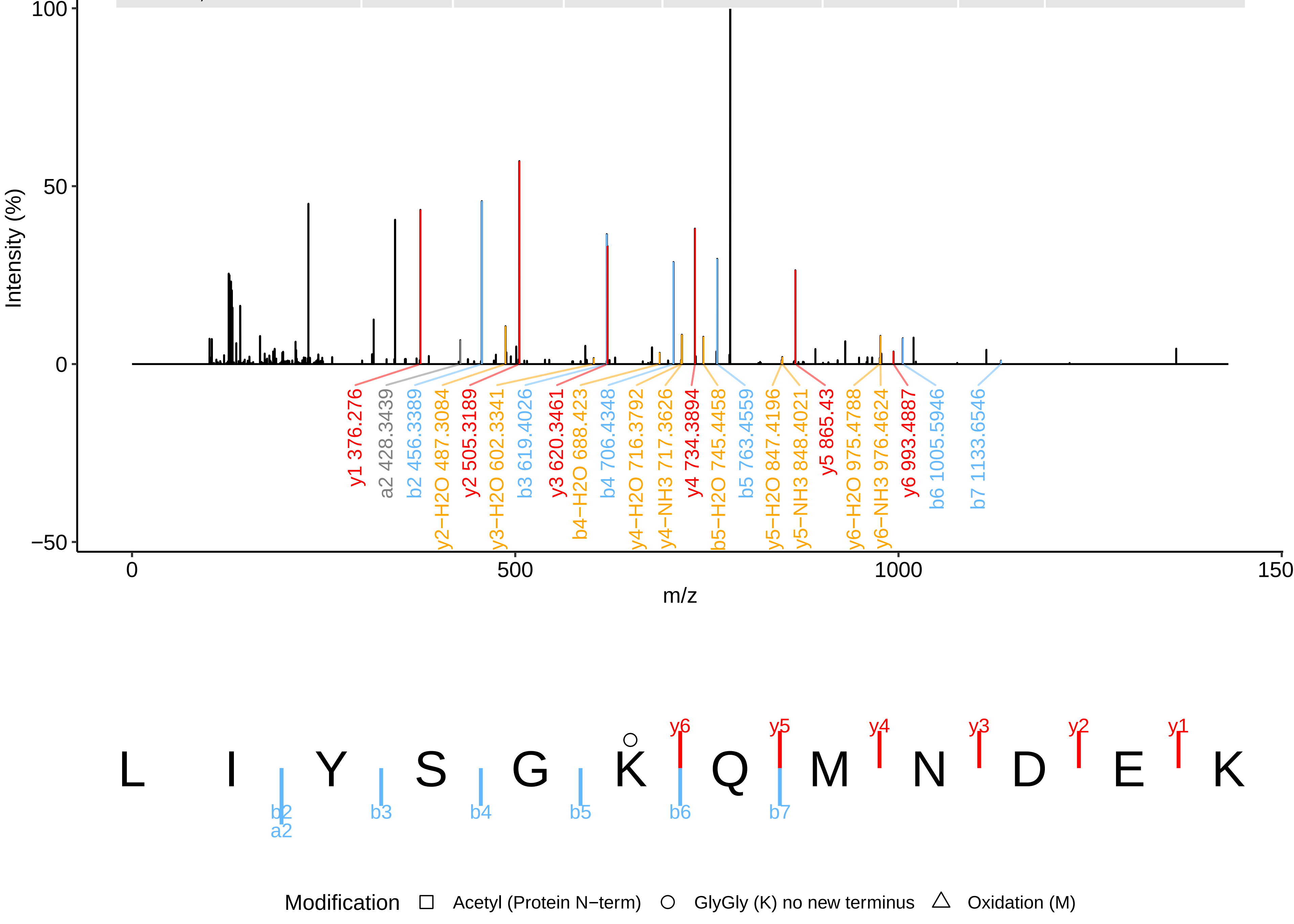

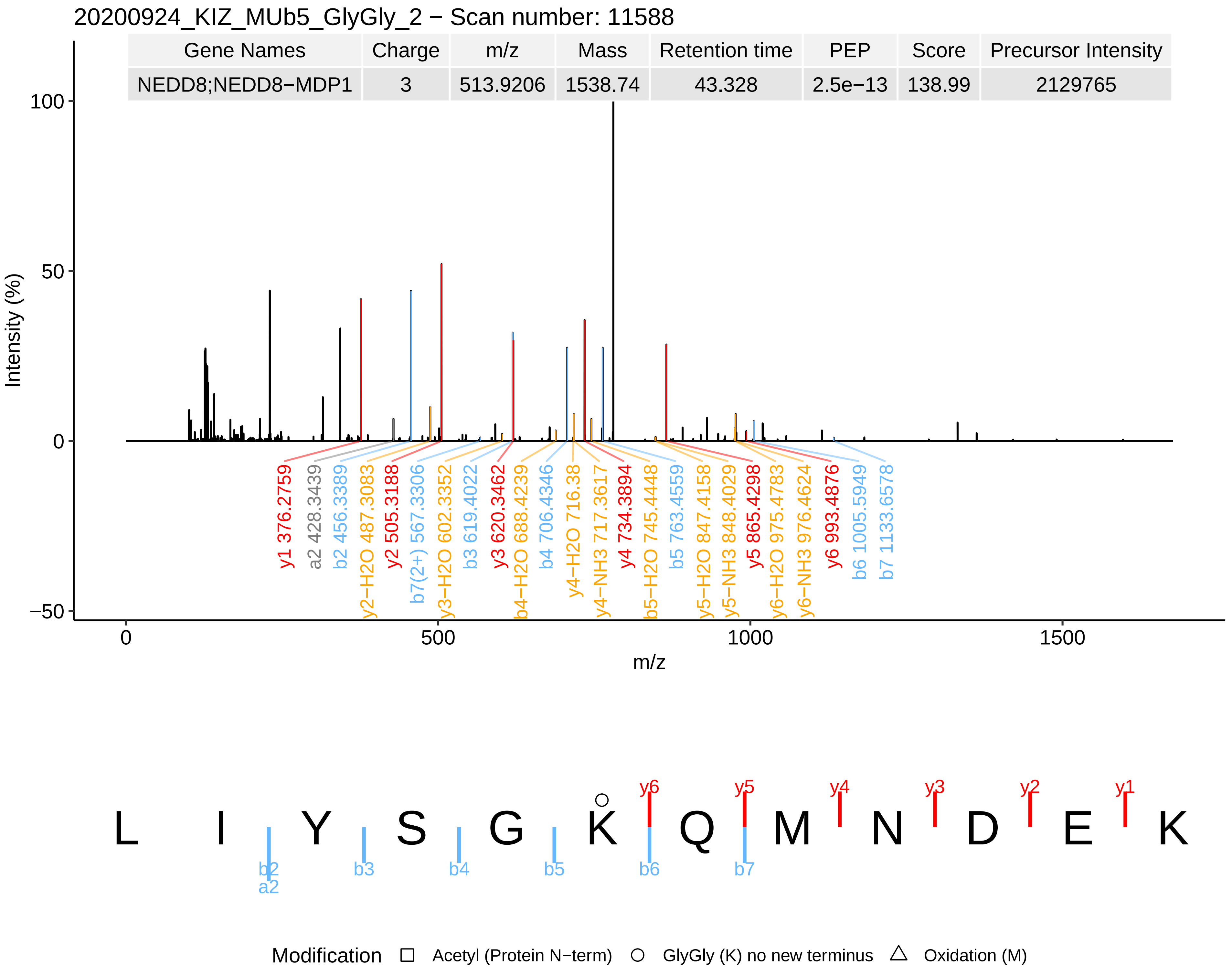
20200924_KIZ_MUb5_GlyGly_2 − Scan number: 11588
Gene Names | Charge | m/z | Mass | Retention time | PEP | Score | Precursor Intensity
NEDD8;NEDD8−MDP1 | 3 | 513.9206 | 1538.74 | 43.328 | 2.5e−13 | 138.99 | 2129765
Intensity (%)
m/z
y1 376.2759
a2 428.3439
b2 456.3389
y2−H2O 487.3083
y2 505.3188
b7(2+) 567.3306
y3−H2O 602.3352
b3 619.4022
y3 620.3462
b4−H2O 688.4239
b4 706.4346
y4−H2O 716.38
y4−NH3 717.3617
y4 734.3894
b5−H2O 745.4448
b5 763.4559
y5−H2O 847.4158
y5−NH3 848.4029
y5 865.4298
y6−H2O 975.4783
y6−NH3 976.4624
y6 993.4876
b6 1005.5949
b7 1133.6578
L I Y S G K Q M N D E K
Modification
Acetyl (Protein N−term)
GlyGly (K) no new terminus
Oxidation (M)

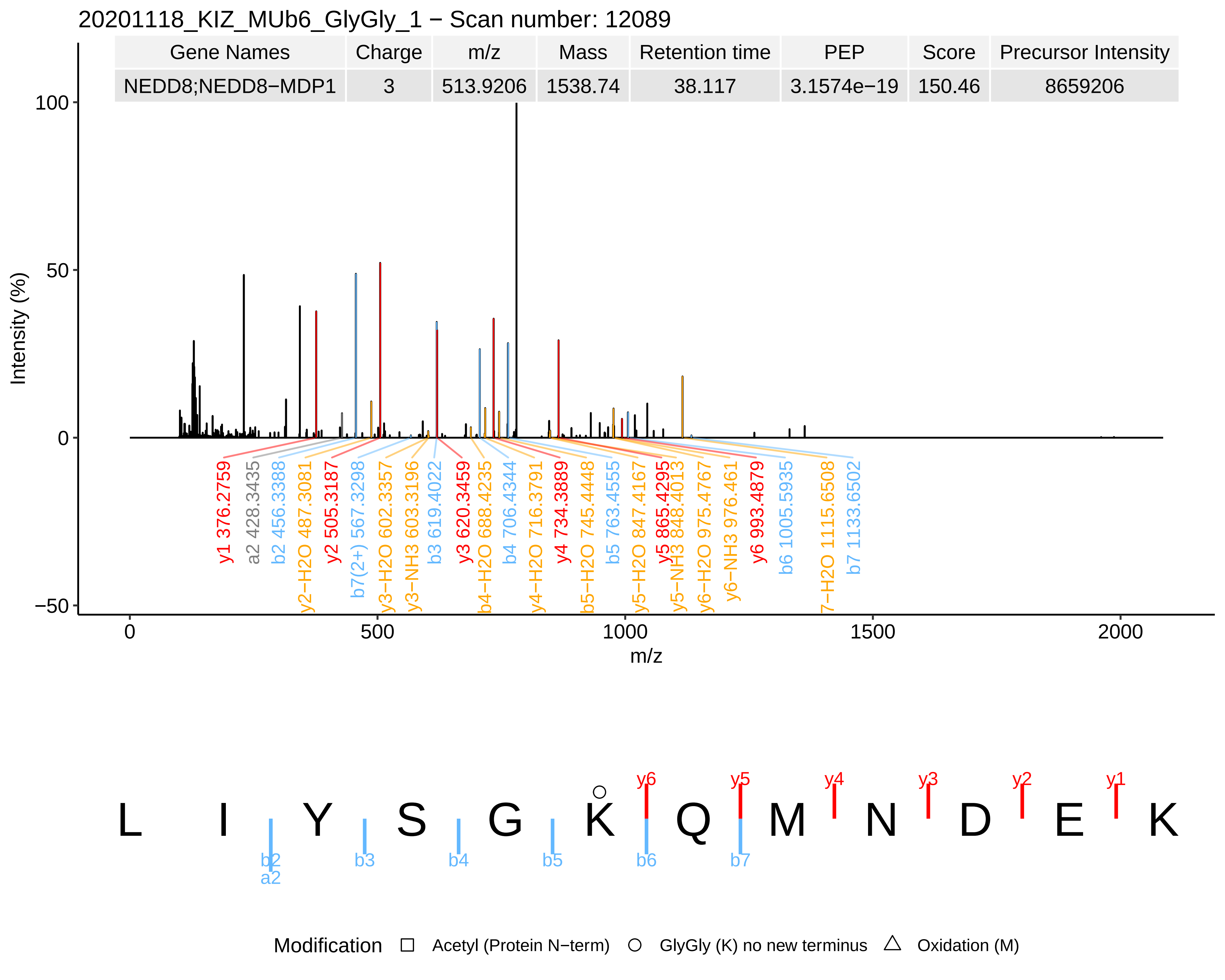
20201118_KIZ_MUb6_GlyGly_1 − Scan number: 12089
Gene Names | Charge | m/z | Mass | Retention time | PEP | Score | Precursor Intensity
NEDD8;NEDD8−MDP1 | 3 | 513.9206 | 1538.74 | 38.117 | 3.1574e−19 | 150.46 | 8659206
Intensity (%)
m/z
y1 376.2759
a2 428.3435
b2 456.3388
y2−H2O 487.3081
y2 505.3187
b7(2+) 567.3298
y3−H2O 602.3357
y3−NH3 603.3196
b3 619.4022
y3 620.3459
b4−H2O 688.4235
b4 706.4344
y4−H2O 716.3791
y4 734.3889
b5−H2O 745.4448
b5 763.4555
y5−H2O 847.4167
y5 865.4295
y5−NH3 848.4013
y6−H2O 975.4767
y6−NH3 976.461
y6 993.4879
b6 1005.5935
b7−H2O 1115.6508
b7 1133.6502
L I Y S G K Q M N D E K
Modification
Acetyl (Protein N−term)
GlyGly (K) no new terminus
Oxidation (M)

20201118_KIZ_MUb6_GlyGly_1 − Scan number: 12112

Gene Names	Charge	m/z	Mass	Retention time	PEP	Score	Precursor Intensity
NEDD8;NEDD8−MDP1	3	513.9206	1538.74	38.188	7.1933e−06	109.39	14944004

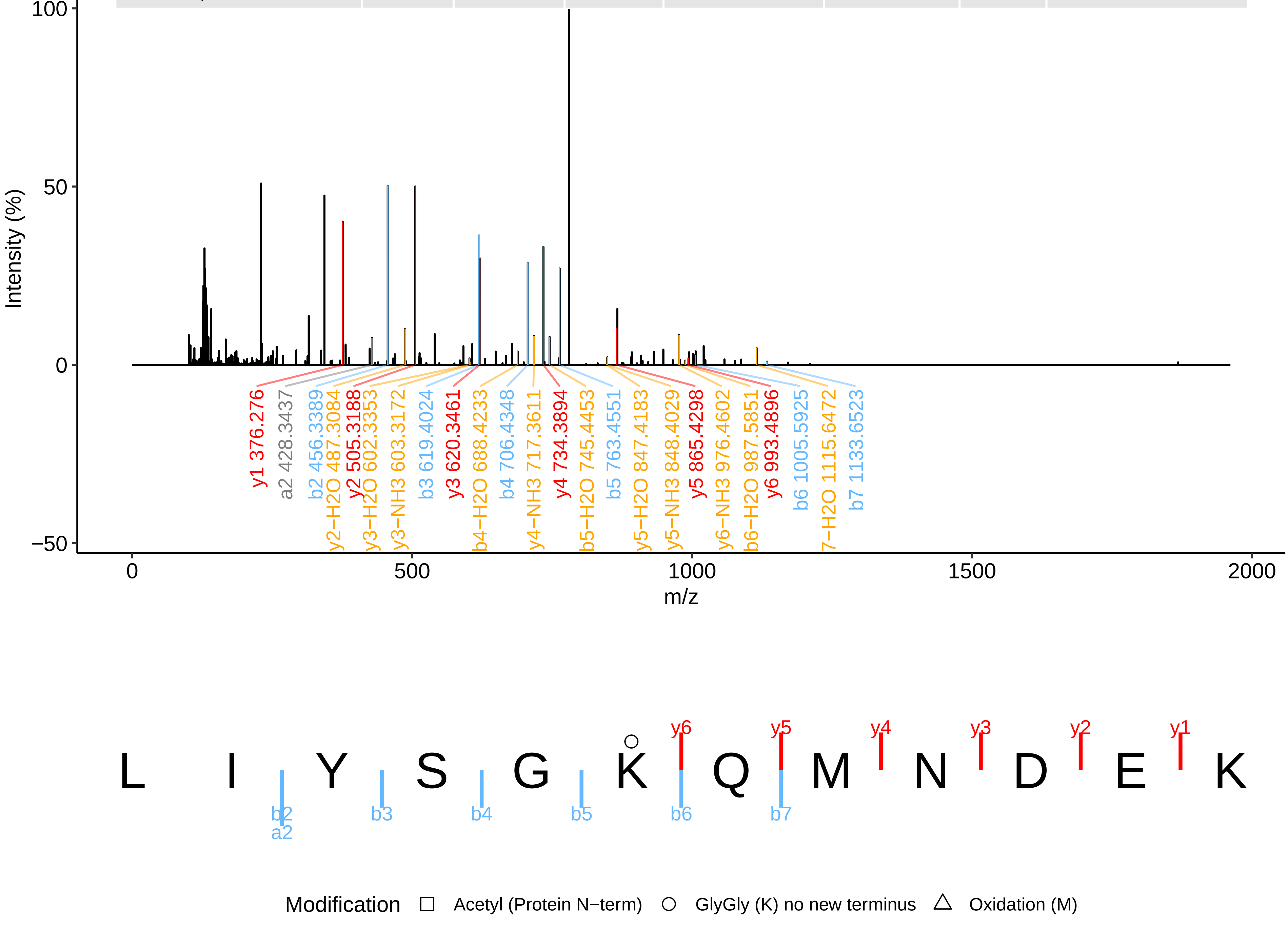

20200921_KIZ_MUb4_GlyGly_10plex − Scan number: 10529

Gene Names	Charge	m/z	Mass	Retention time	PEP	Score	Precursor Intensity
NEDD8;NEDD8−MDP1	3	519.2522	1554.735	36.467	0.00058894	93.345	NA

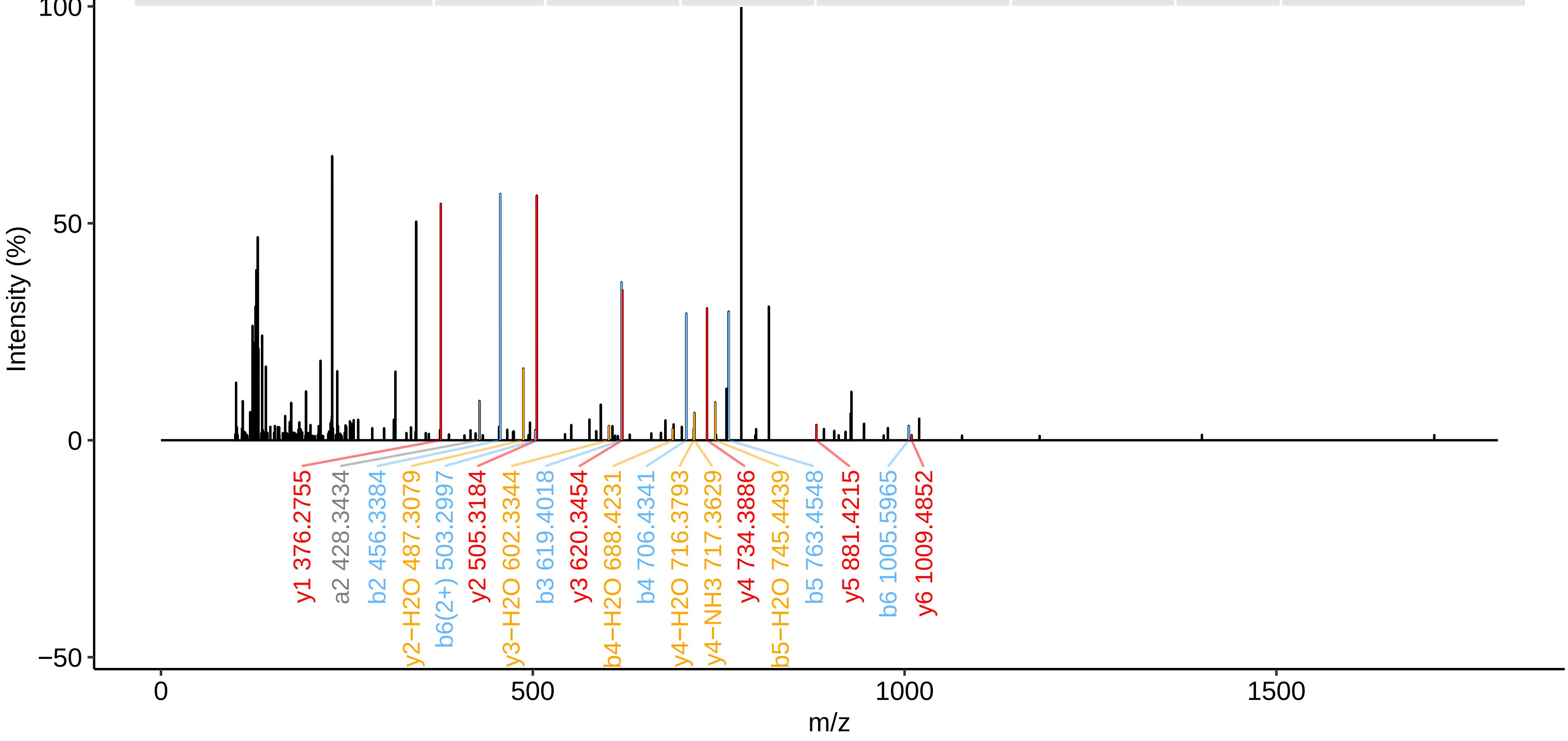

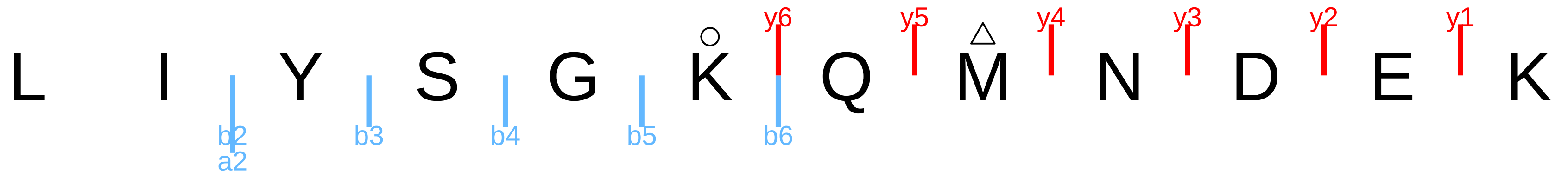

Modification □ Acetyl (Protein N−term) ○ GlyGly (K) no new terminus △ Oxidation (M)

20200921_KIZ_MUb4_GlyGly_10plex_2 − Scan number: 9743

Gene Names	Charge	m/z	Mass	Retention time	PEP	Score	Precursor Intensity
NEDD8;NEDD8−MDP1	3	519.2522	1554.735	37.136	0.0074111	68.536	1684086

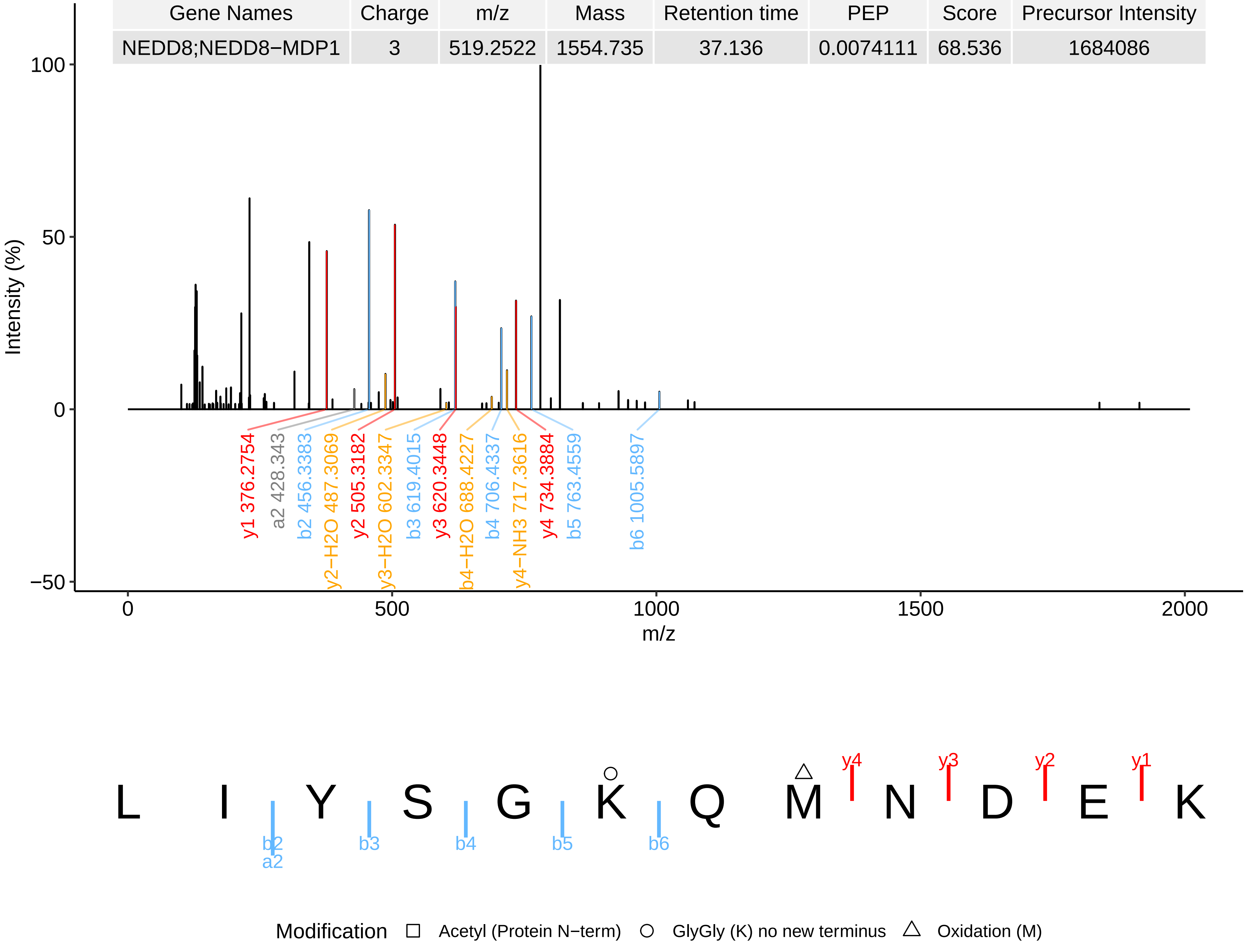

20200924_KIZ_MUb5_GlyGly_1 − Scan number: 9479

Gene Names	Charge	m/z	Mass	Retention time	PEP	Score	Precursor Intensity
NEDD8;NEDD8−MDP1	3	519.2522	1554.735	30.475	0.00033995	92.112	5931857

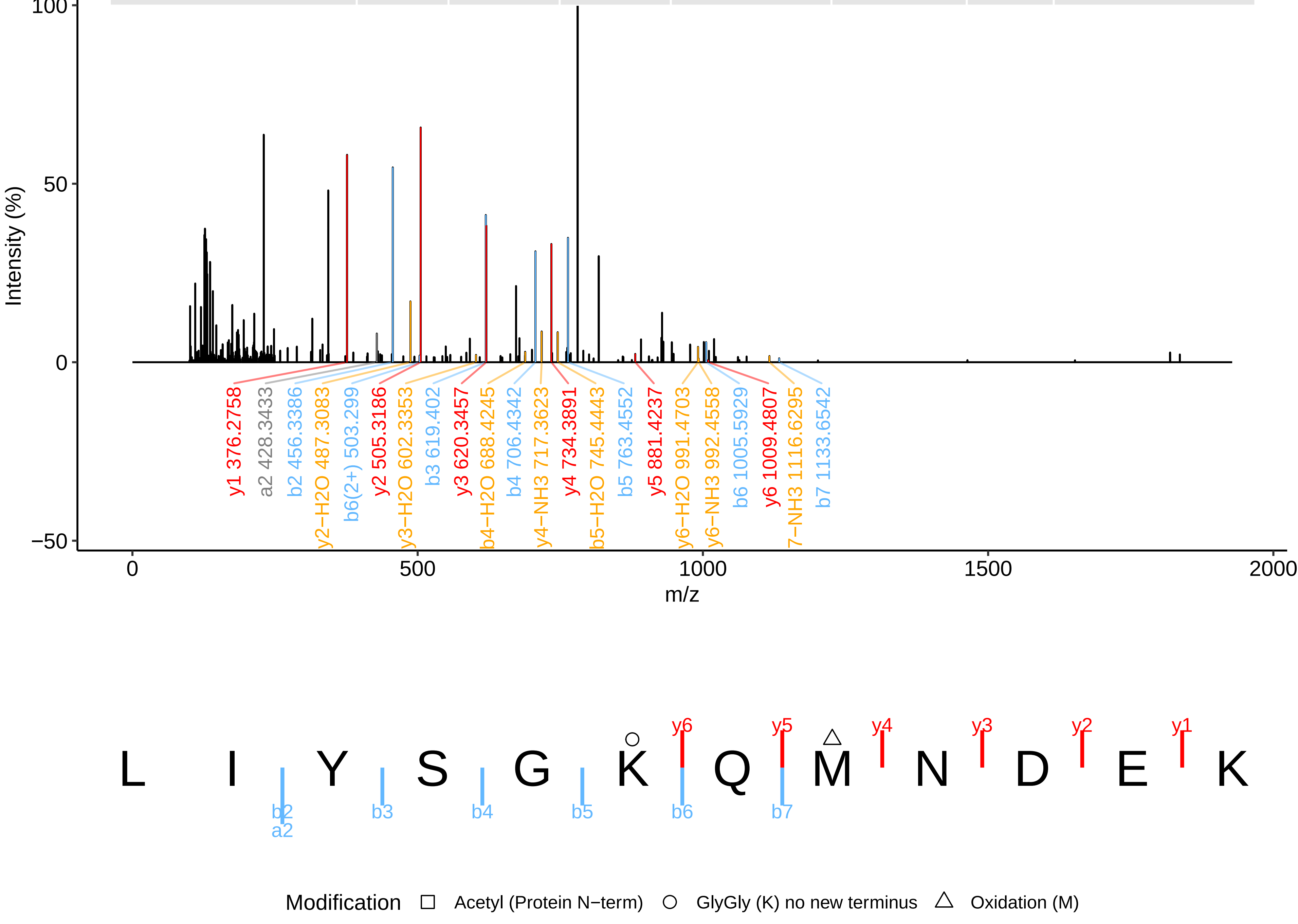

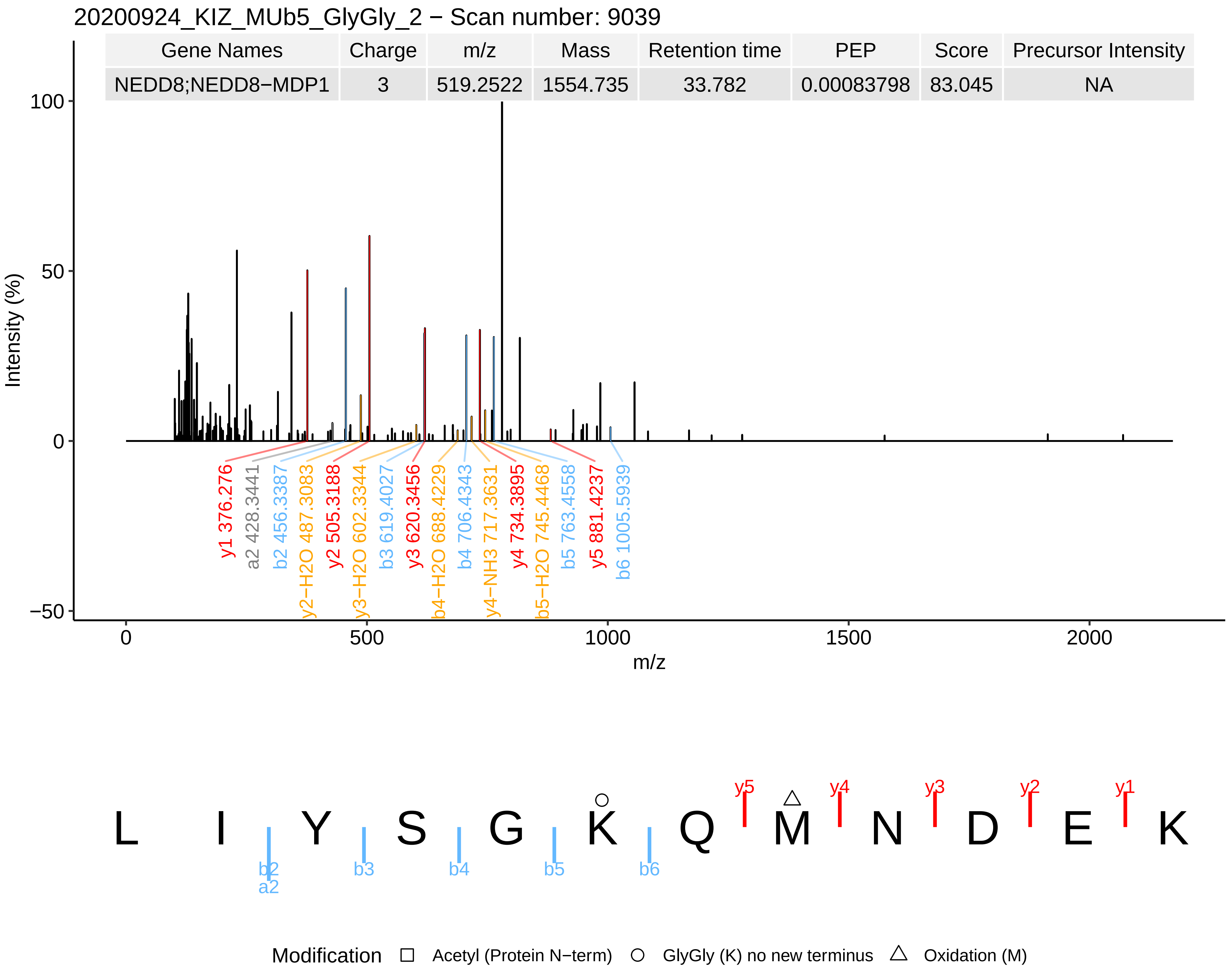

20200924_KIZ_MUb5_GlyGly_2 − Scan number: 9039
Gene Names | Charge | m/z | Mass | Retention time | PEP | Score | Precursor Intensity
NEDD8;NEDD8−MDP1 | 3 | 519.2522 | 1554.735 | 33.782 | 0.00083798 | 83.045 | NA
Intensity (%)
m/z
y1 376.276
a2 428.3441
b2 456.3387
y2−H2O 487.3083
y2 505.3188
y3−H2O 602.3344
b3 619.4027
y3 620.3456
b4−H2O 688.4229
b4 706.4343
y4−NH3 717.3631
y4 734.3895
b5−H2O 745.4468
b5 763.4558
y5 881.4237
b6 1005.5939
L I Y S G K Q M N D E K
y5 y4 y3 y2 y1
b2 a2 b3 b4 b5 b6
Modification □ Acetyl (Protein N−term) ○ GlyGly (K) no new terminus △ Oxidation (M)

20200924_KIZ_MUb5_GlyGly_1 − Scan number: 22454

Gene Names	Charge	m/z	Mass	Retention time	PEP	Score	Precursor Intensity
CPS1	3	609.6454	1825.915	72.261	0.14843	42.568	NA

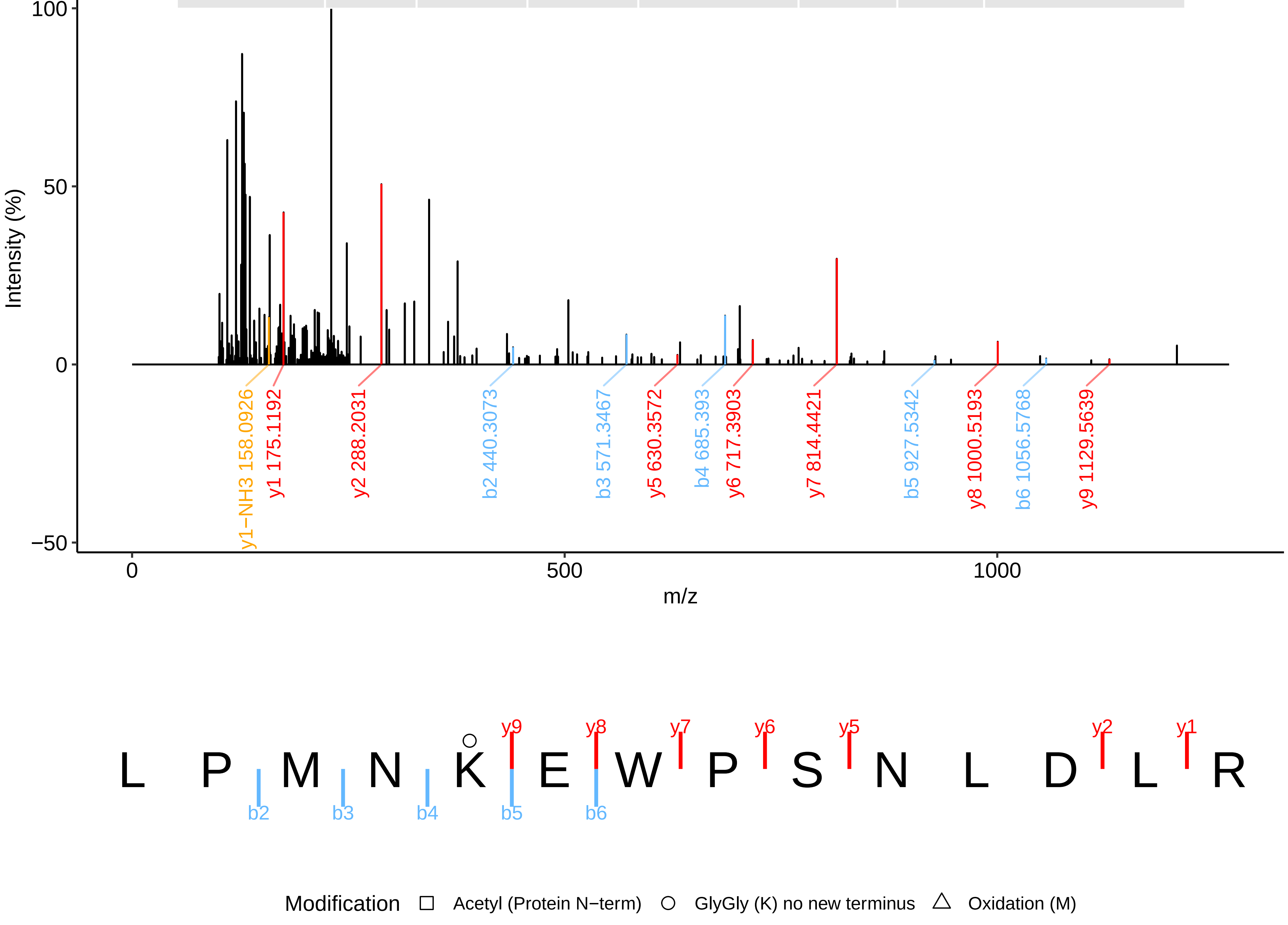

20200921_KIZ_MUb4_GlyGly_10plex_2 − Scan number: 12382

Gene Names	Charge	m/z	Mass	Retention time	PEP	Score	Precursor Intensity
USP14	3	595.6549	1783.943	47.893	0.0054535	67.964	743323.2

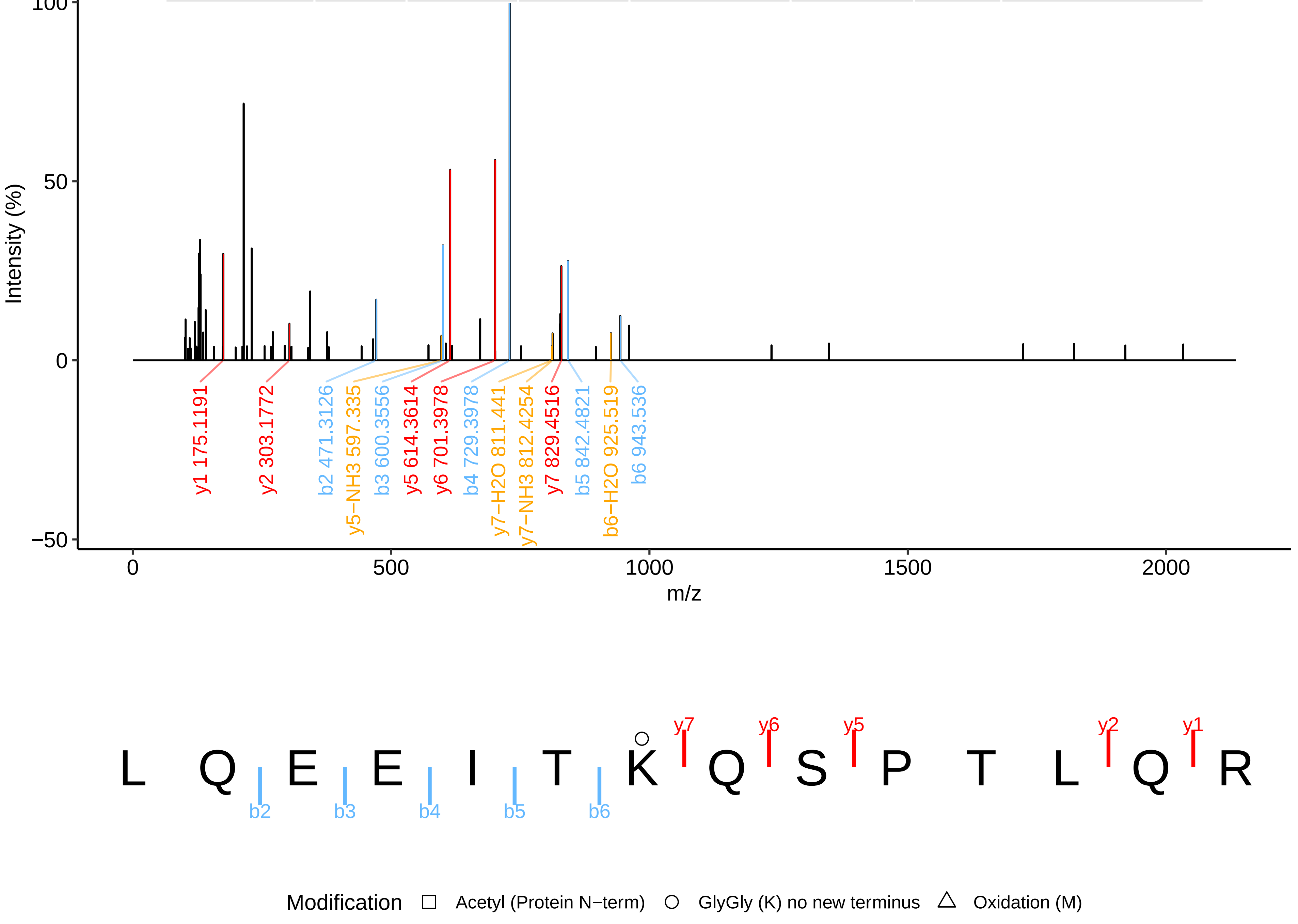

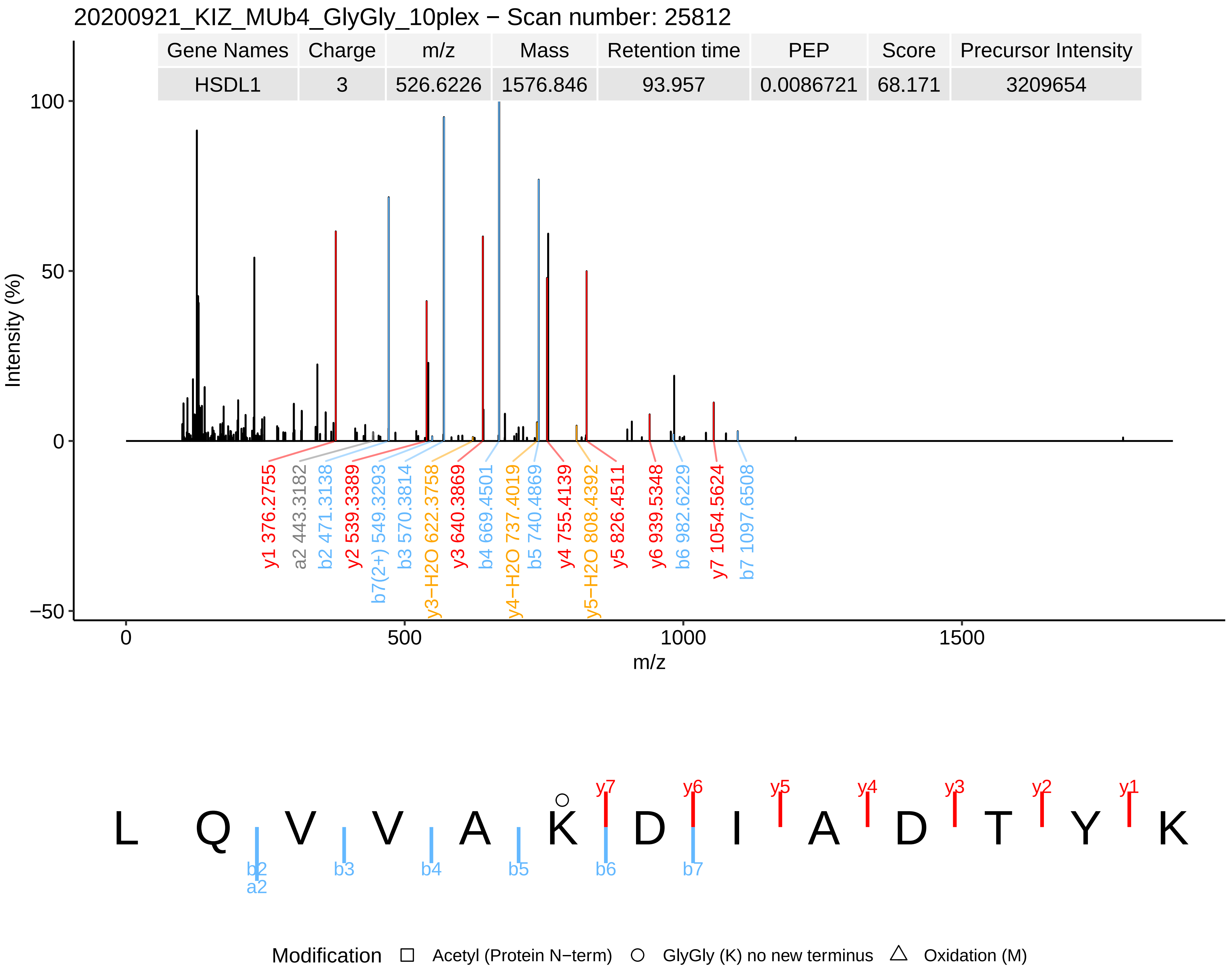
20200921_KIZ_MUb4_GlyGly_10plex − Scan number: 25812
Gene Names | Charge | m/z | Mass | Retention time | PEP | Score | Precursor Intensity
HSDL1 | 3 | 526.6226 | 1576.846 | 93.957 | 0.0086721 | 68.171 | 3209654
Intensity (%)
100
50
0
−50
0
500
1000
1500
m/z
y1 376.2755
a2 443.3182
b2 471.3138
y2 539.3389
b7(2+) 549.3293
b3 570.3814
y3−H2O 622.3758
y3 640.3869
b4 669.4501
y4−H2O 737.4019
b5 740.4869
y4 755.4139
y5−H2O 808.4392
y5 826.4511
y6 939.5348
b6 982.6229
y7 1054.5624
b7 1097.6508
L Q V V A K D I A D T Y K
y7 y6 y5 y4 y3 y2 y1
b2 a2 b3 b4 b5 b6 b7
Modification
Acetyl (Protein N−term)
GlyGly (K) no new terminus
Oxidation (M)

20200921_KIZ_MUb4_GlyGly_10plex_2 − Scan number: 21720

Gene Names	Charge	m/z	Mass	Retention time	PEP	Score	Precursor Intensity
HSDL1	3	526.6226	1576.846	85.544	0.10776	42.209	1037067

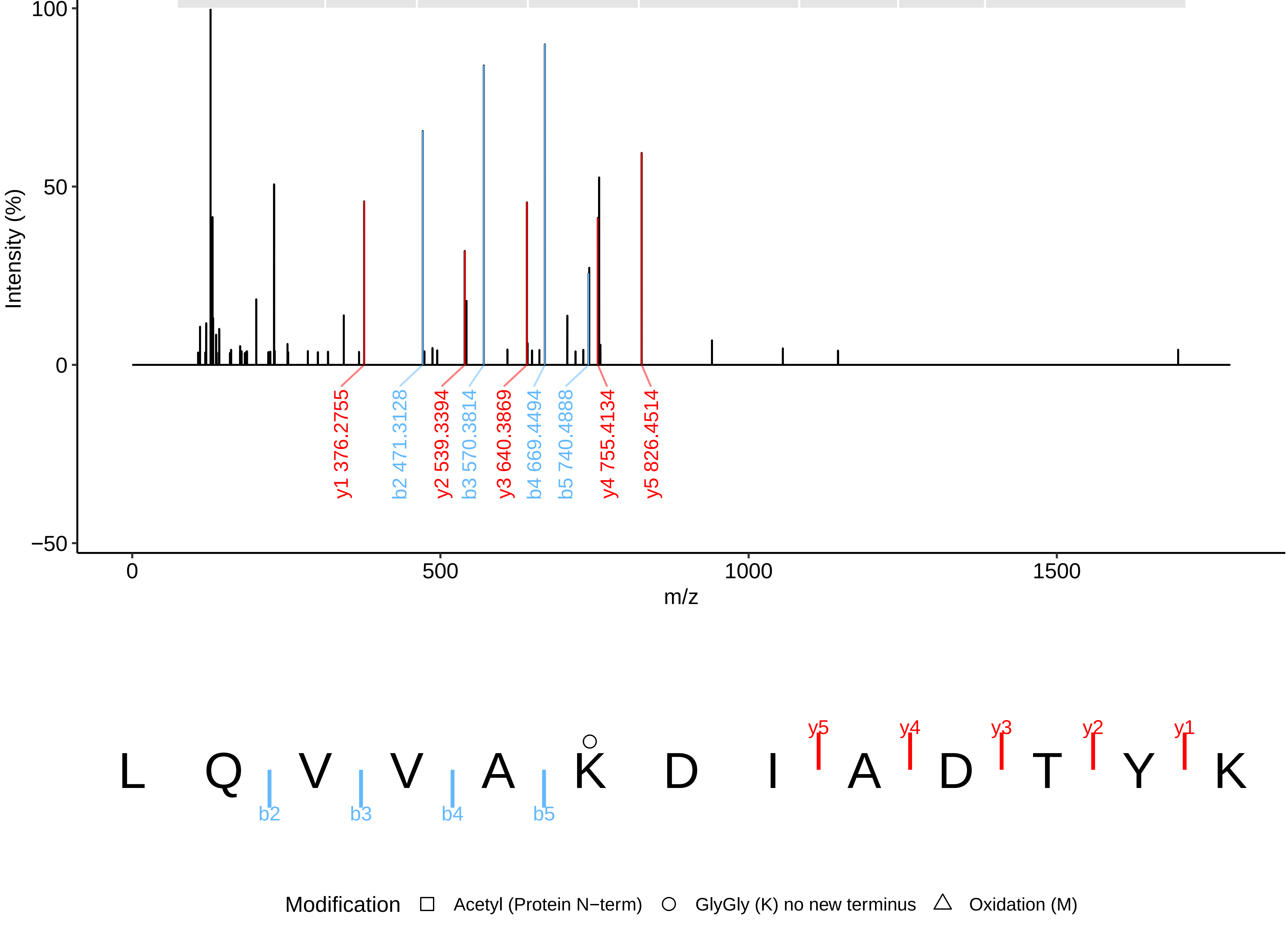

20201118_KIZ_MUb6_GlyGly_1 − Scan number: 24276

Gene Names	Charge	m/z	Mass	Retention time	PEP	Score	Precursor Intensity
HSDL1	3	526.6226	1576.846	74.783	0.00065236	91.202	26875432

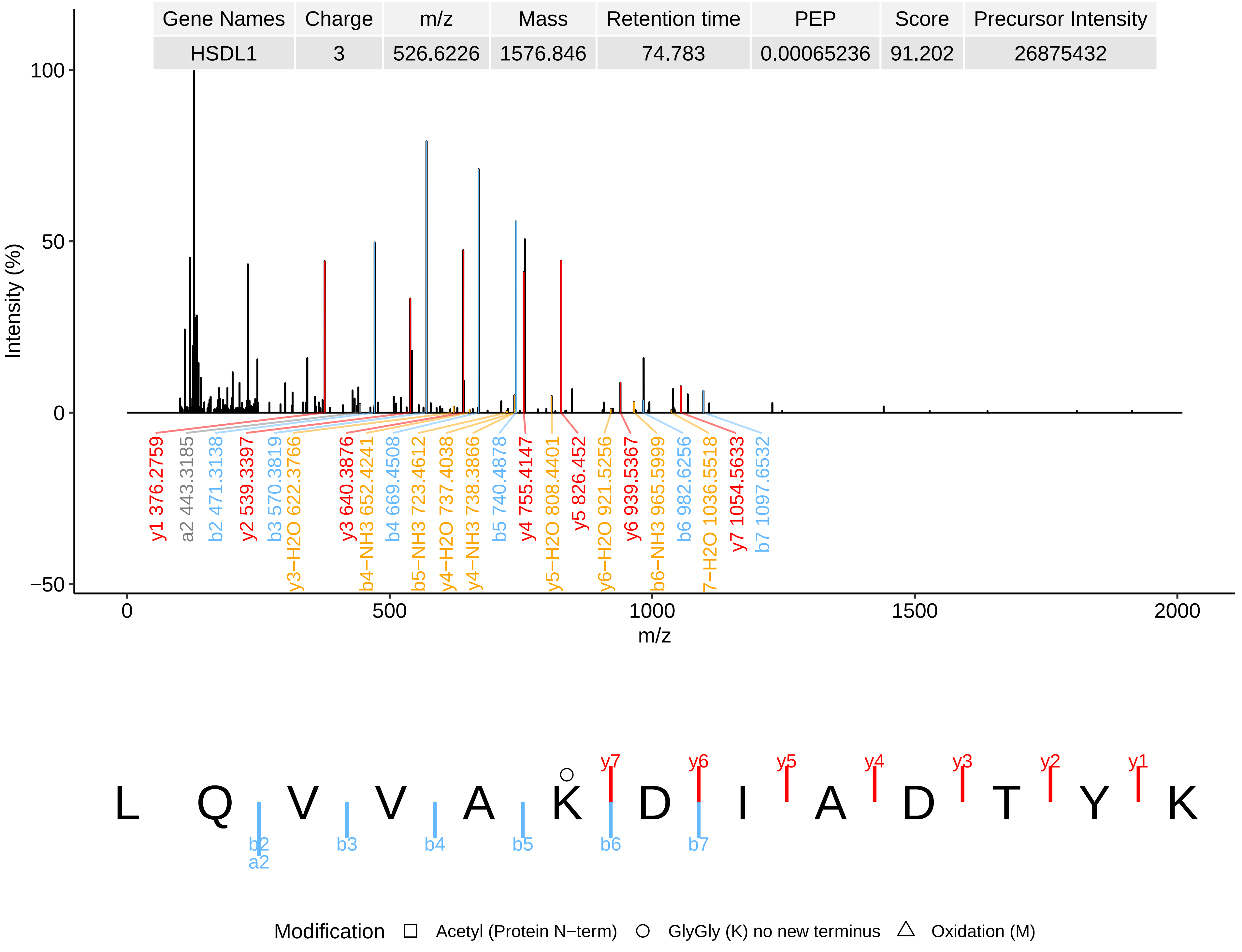

20200921_KIZ_MUb4_GlyGly_10plex − Scan number: 15537

Gene Names	Charge	m/z	Mass	Retention time	PEP	Score	Precursor Intensity
UQCRFS1;UQCRFS1P1	3	527.2643	1578.771	55.524	0.037795	55.261	4913345

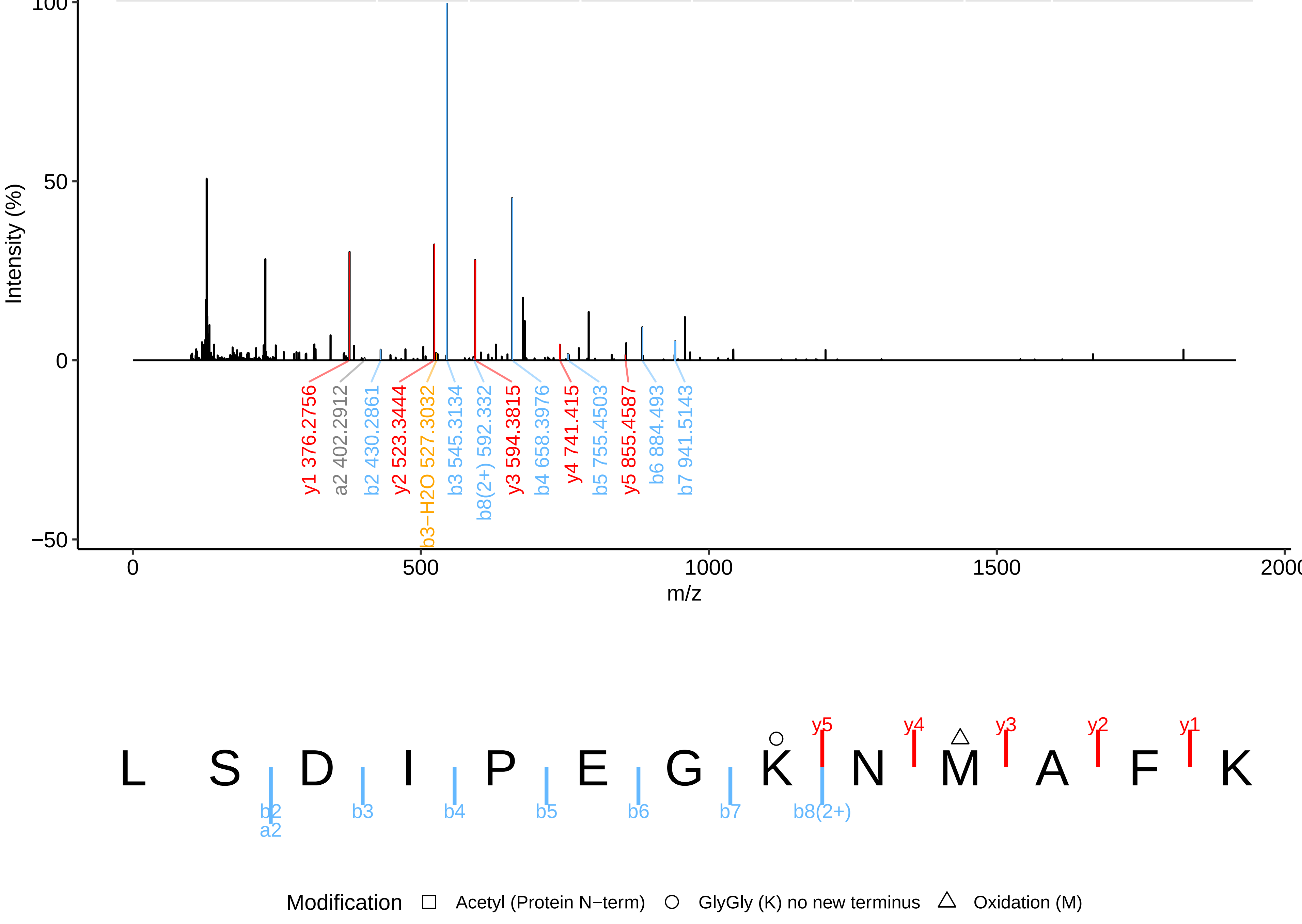

20200921_KIZ_MUb4_GlyGly_10plex_2 − Scan number: 14399

Gene Names	Charge	m/z	Mass	Retention time	PEP	Score	Precursor Intensity
UQCRFS1;UQCRFS1P1	3	527.2643	1578.771	56.136	0.0080394	64.121	470973

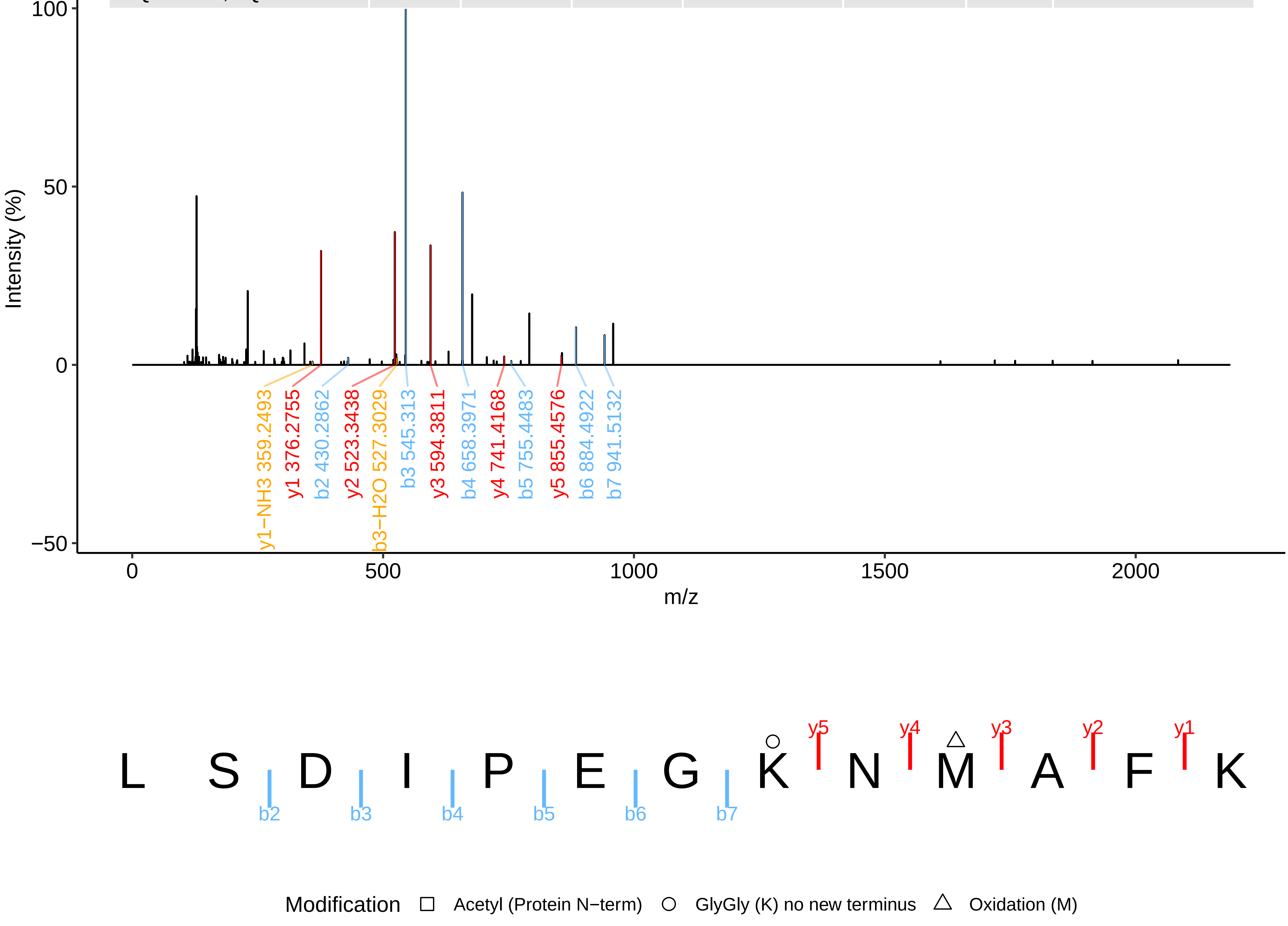

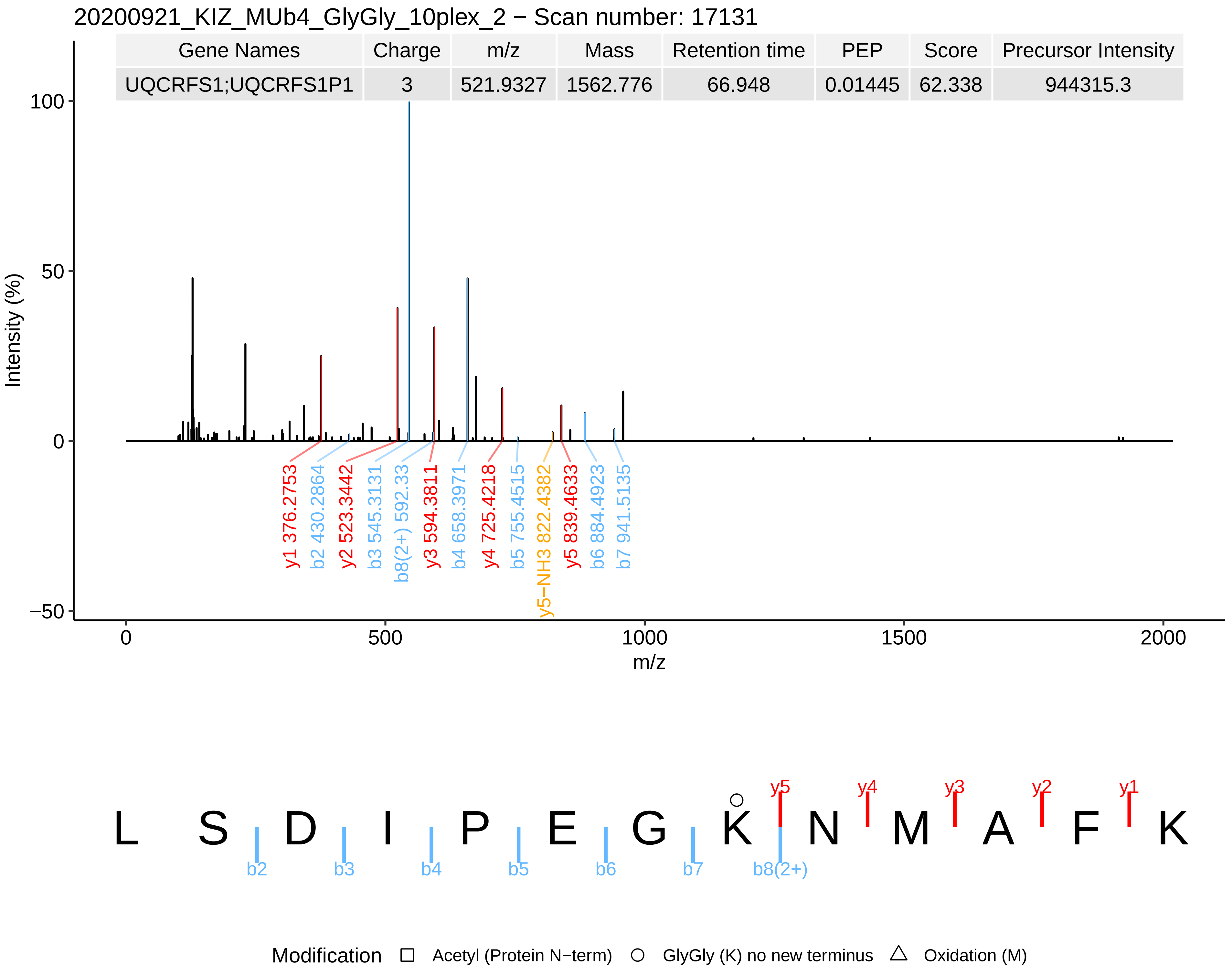

20200921_KIZ_MUb4_GlyGly_10plex_2 − Scan number: 17131
Gene Names | Charge | m/z | Mass | Retention time | PEP | Score | Precursor Intensity
UQCRFS1;UQCRFS1P1 | 3 | 521.9327 | 1562.776 | 66.948 | 0.01445 | 62.338 | 944315.3
Intensity (%)
m/z
y1 376.2753
b2 430.2864
y2 523.3442
b3 545.3131
b8(2+) 592.33
y3 594.3811
b4 658.3971
y4 725.4218
b5 755.4515
y5−NH3 822.4382
y5 839.4633
b6 884.4923
b7 941.5135
L S D I P E G K N M A F K
b2 b3 b4 b5 b6 b7 b8(2+)
y5 y4 y3 y2 y1
Modification
Acetyl (Protein N−term)
GlyGly (K) no new terminus
Oxidation (M)

20201118_KIZ_MUb6_GlyGly_2 − Scan number: 20306

Gene Names	Charge	m/z	Mass	Retention time	PEP	Score	Precursor Intensity
UQCRFS1;UQCRFS1P1	3	521.9327	1562.776	68.443	0.047314	55.261	25078892

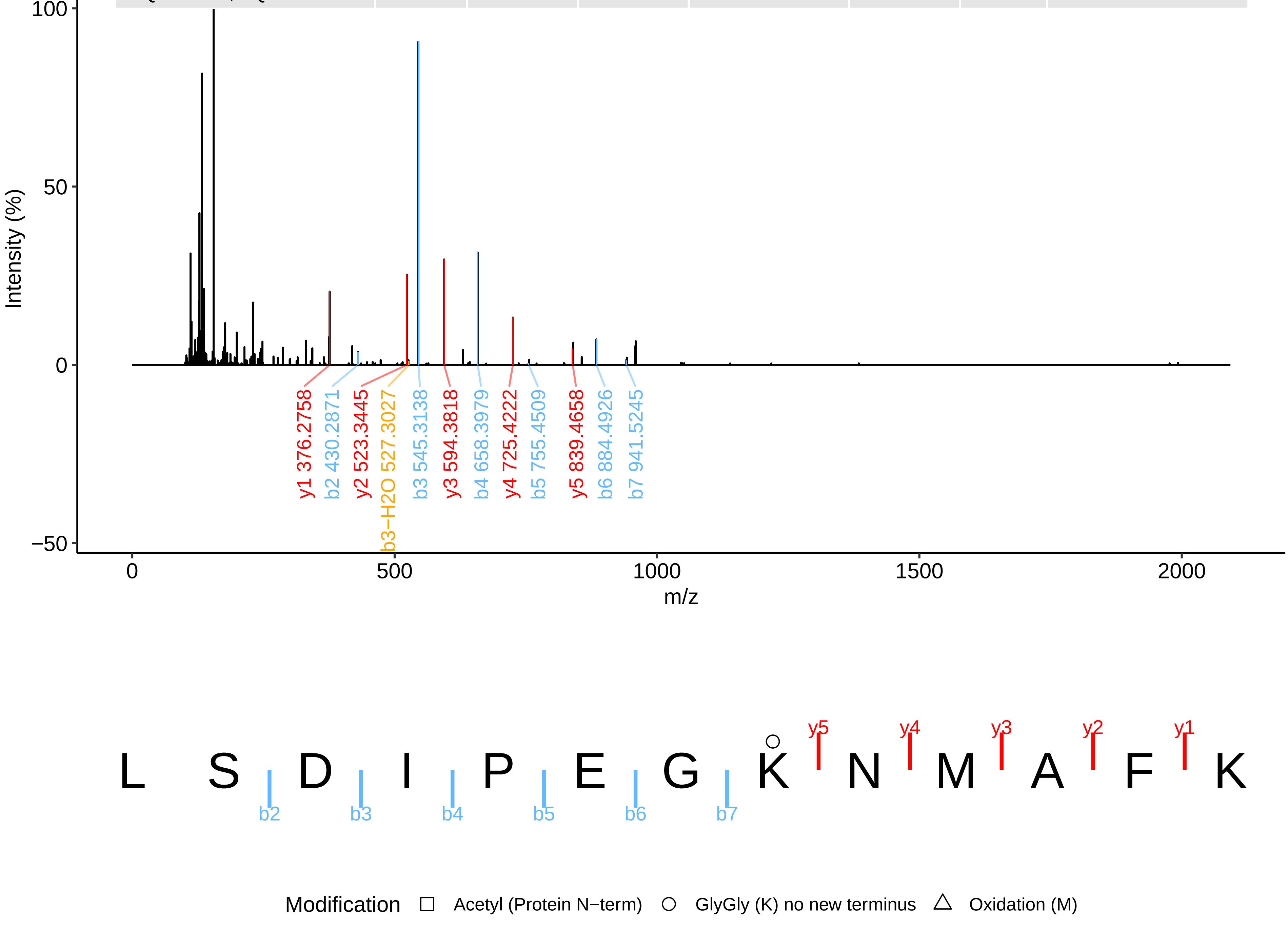

20200921_KIZ_MUb4_GlyGly_10plex_2 − Scan number: 14979

Gene Names	Charge	m/z	Mass	Retention time	PEP	Score	Precursor Intensity
UQCRC2	3	514.6105	1540.81	58.487	0.0040877	70.622	672730.9

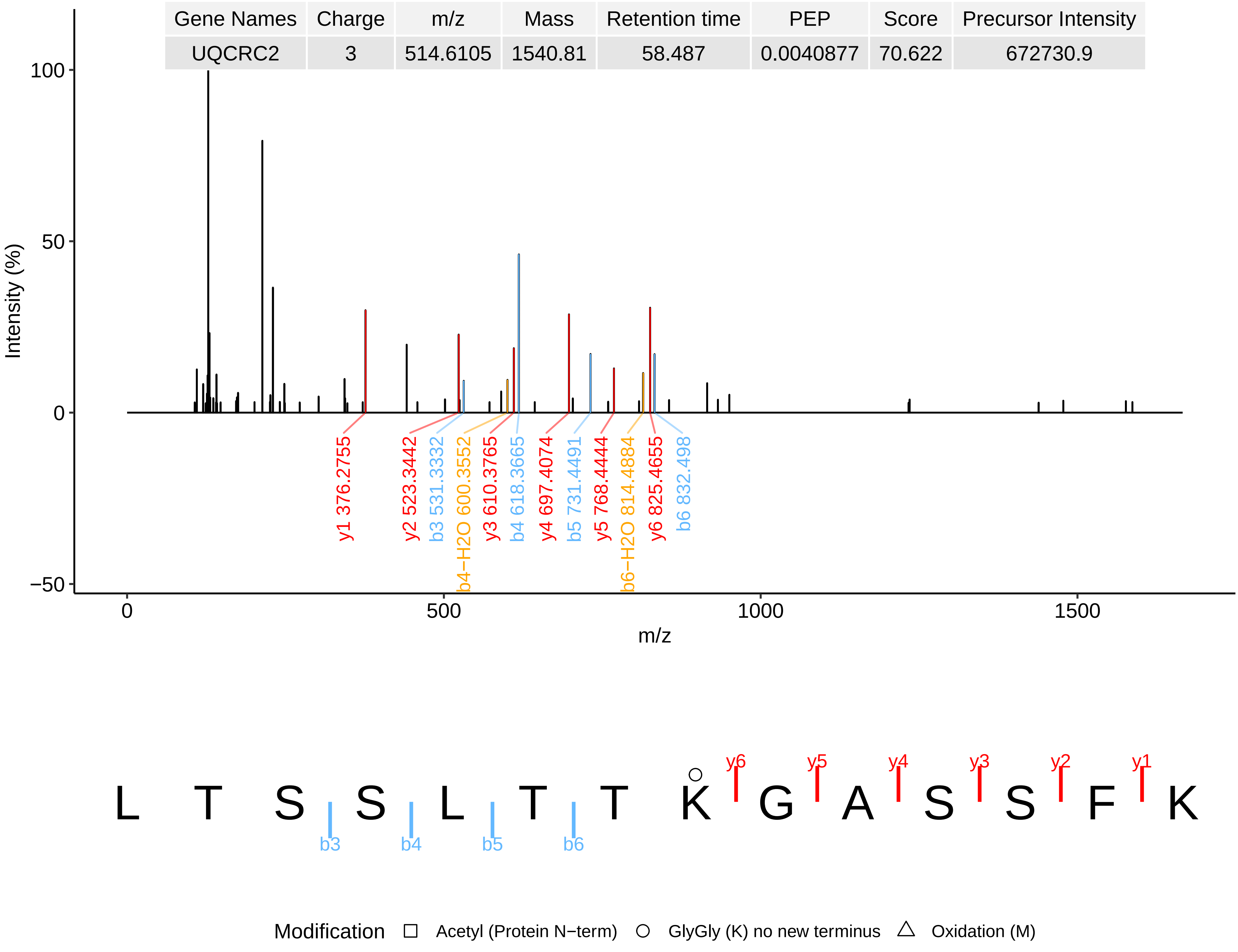

20200921_KIZ_MUb4_GlyGly_10plex_2 − Scan number: 18599

Gene Names	Charge	m/z	Mass	Retention time	PEP	Score	Precursor Intensity
PSMA1	3	585.6756	1754.005	72.871	0.00014586	83.692	825764.6

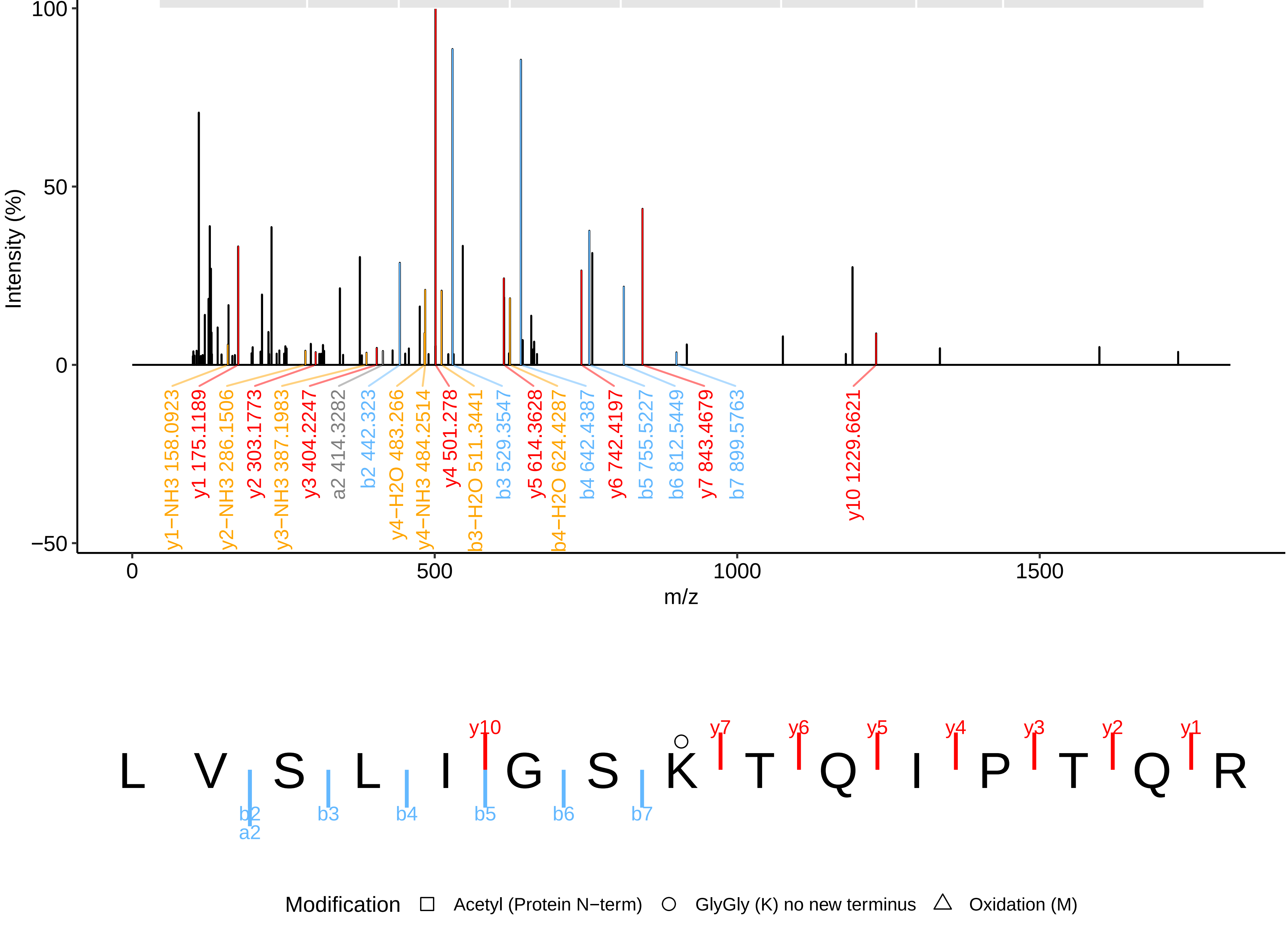

20200921_KIZ_MUb4_GlyGly_10plex_2 − Scan number: 18608

Gene Names	Charge	m/z	Mass	Retention time	PEP	Score	Precursor Intensity
PSMA1	3	585.6756	1754.005	72.911	3.0306e−09	122.64	1530032

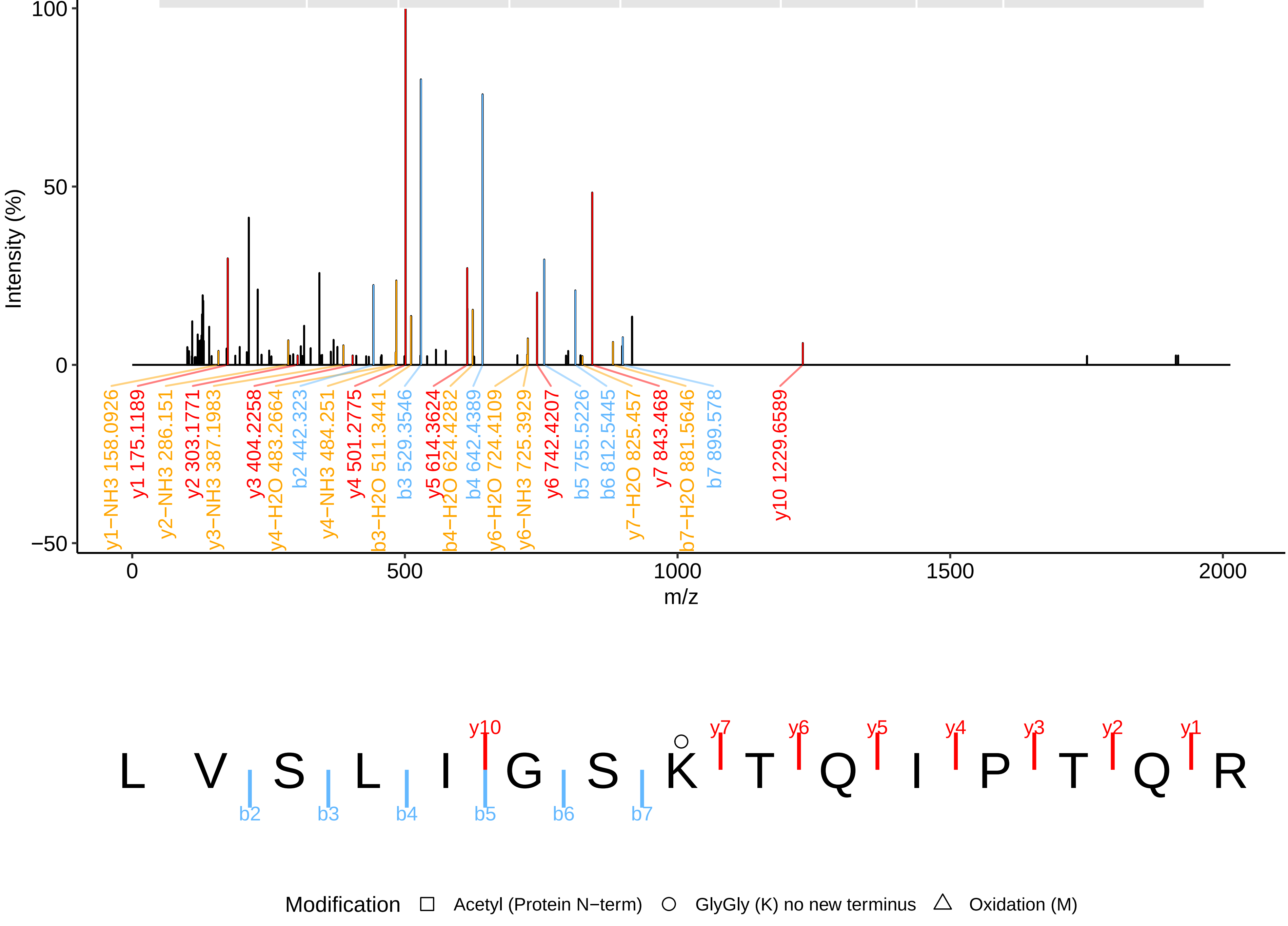

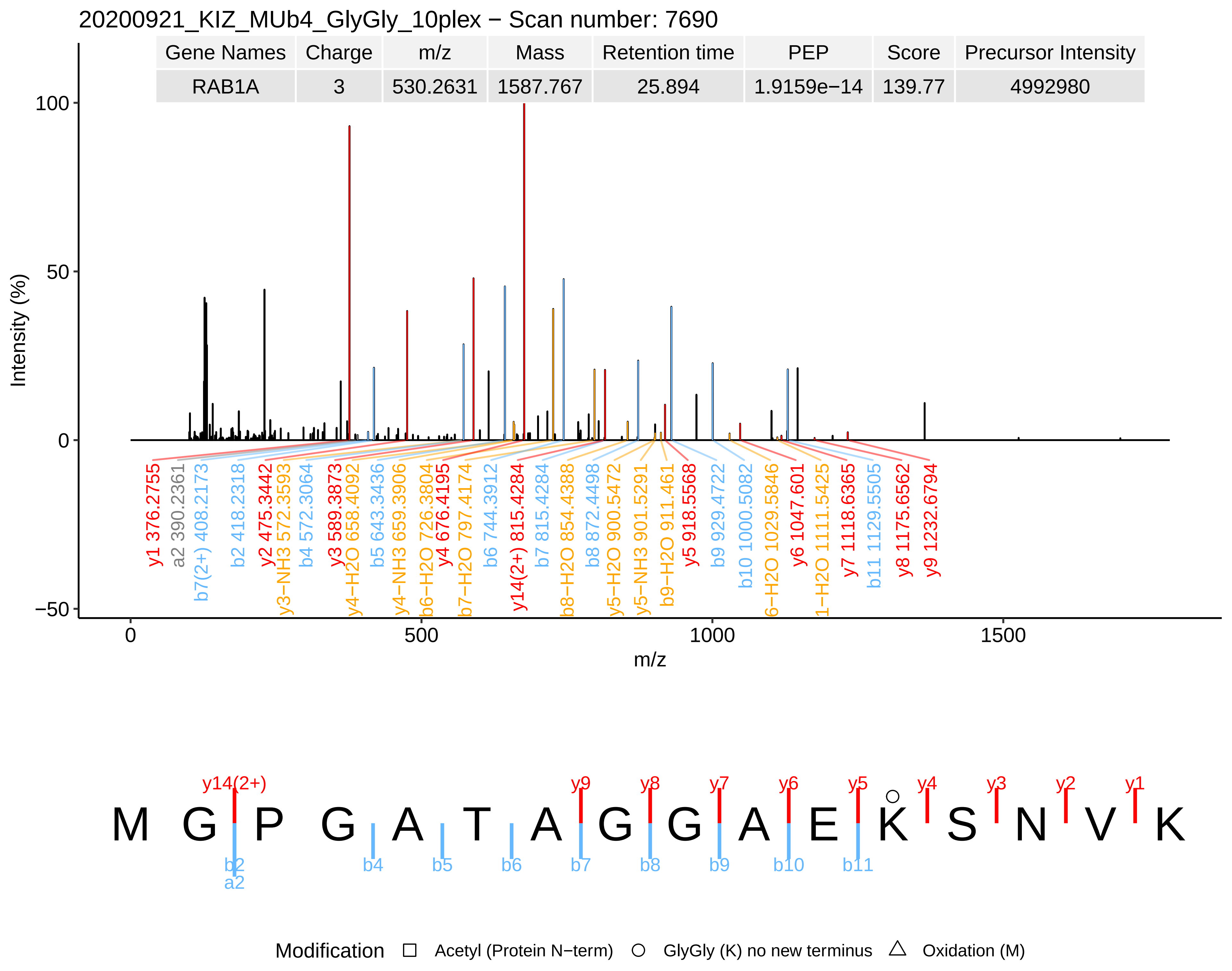
20200921_KIZ_MUb4_GlyGly_10plex − Scan number: 7690
Gene Names | Charge | m/z | Mass | Retention time | PEP | Score | Precursor Intensity
RAB1A | 3 | 530.2631 | 1587.767 | 25.894 | 1.9159e−14 | 139.77 | 4992980
Intensity (%)
m/z
y1 376.2755
a2 390.2361
b7(2+) 408.2173
b2 418.2318
y2 475.3442
y3−NH3 572.3593
b4 572.3064
y3 589.3873
y4−H2O 658.4092
b5 643.3436
y4−NH3 659.3906
b6−H2O 726.3804
y4 676.4195
b7−H2O 797.4174
b6 744.3912
y14(2+) 815.4284
b7 815.4284
b8−H2O 854.4388
b8 872.4498
y5−H2O 900.5472
y5−NH3 901.5291
b9−H2O 911.461
y5 918.5568
b9 929.4722
b10 1000.5082
6−H2O 1029.5846
y6 1047.601
1−H2O 1111.5425
y7 1118.6365
b11 1129.5505
y8 1175.6562
y9 1232.6794
M G P G A T A G G A E K S N V K
Modification
Acetyl (Protein N−term)
GlyGly (K) no new terminus
Oxidation (M)

20200921_KIZ_MUb4_GlyGly_10plex − Scan number: 7734

Gene Names	Charge	m/z	Mass	Retention time	PEP	Score	Precursor Intensity
RAB1A	3	530.2631	1587.767	26.067	1.3848e−07	109.83	NA

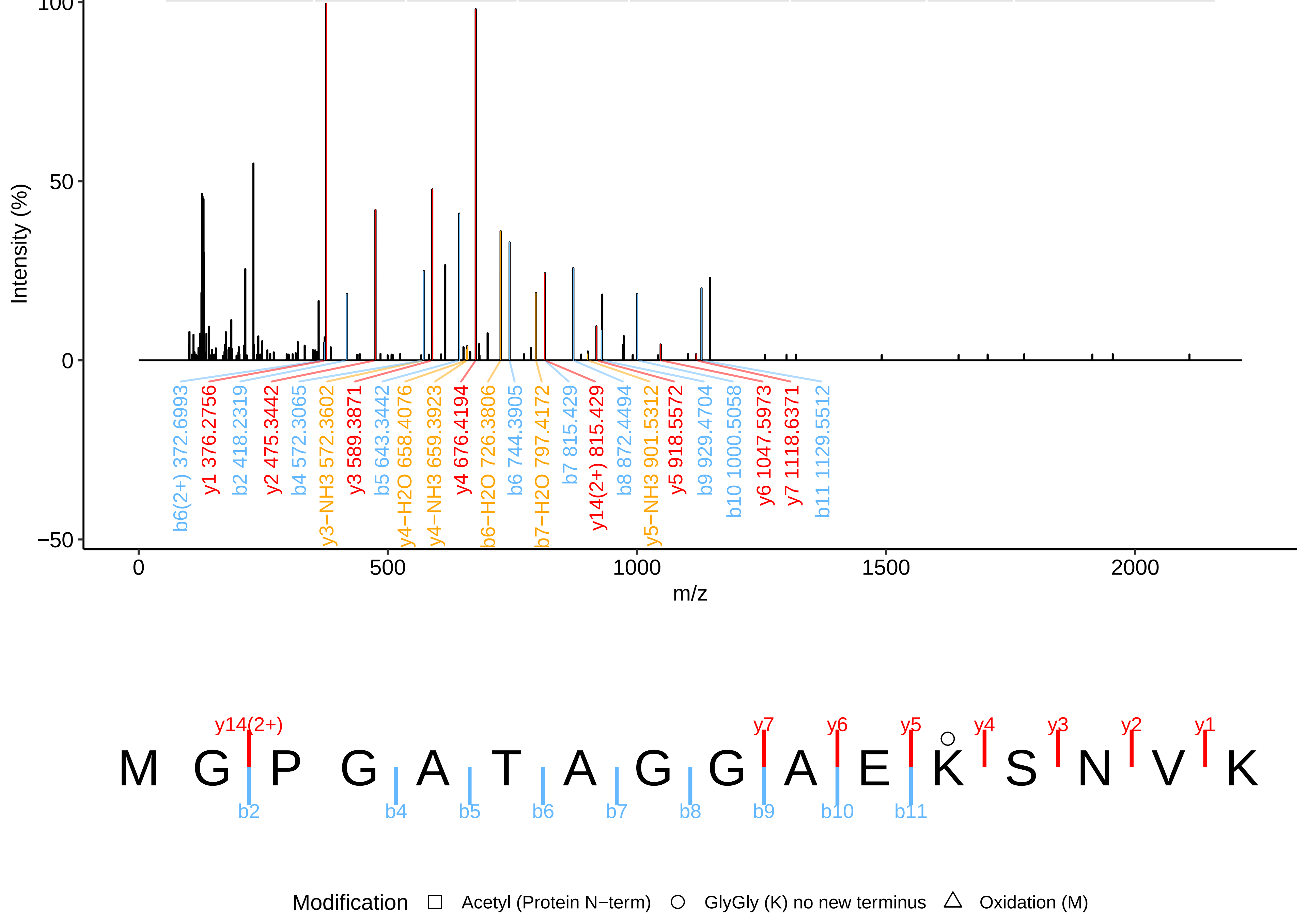

20200921_KIZ_MUb4_GlyGly_10plex_2 − Scan number: 7071

Gene Names	Charge	m/z	Mass	Retention time	PEP	Score	Precursor Intensity
RAB1A	3	530.2631	1587.767	26.254	4.2703e−10	123.02	918351.6

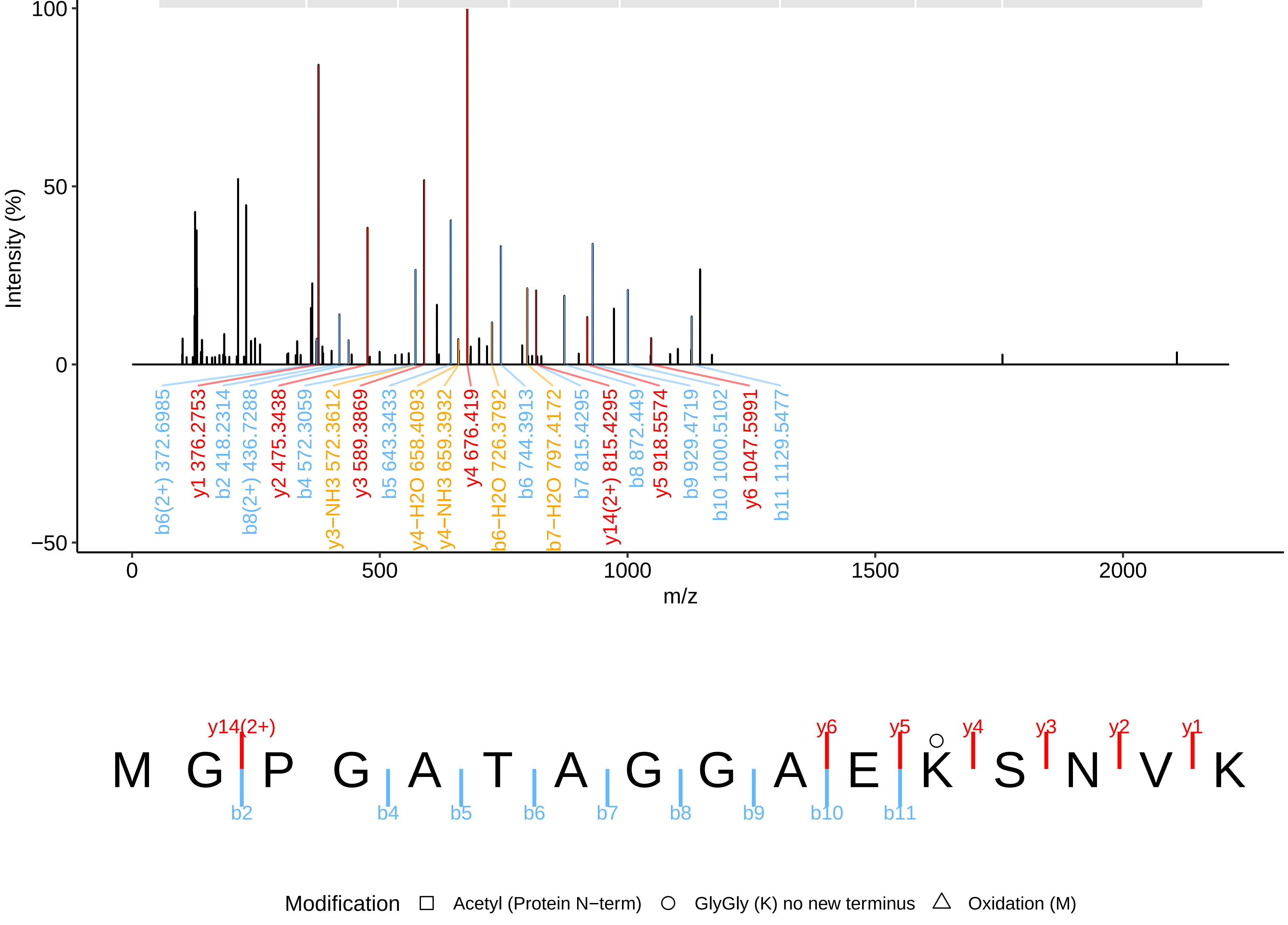

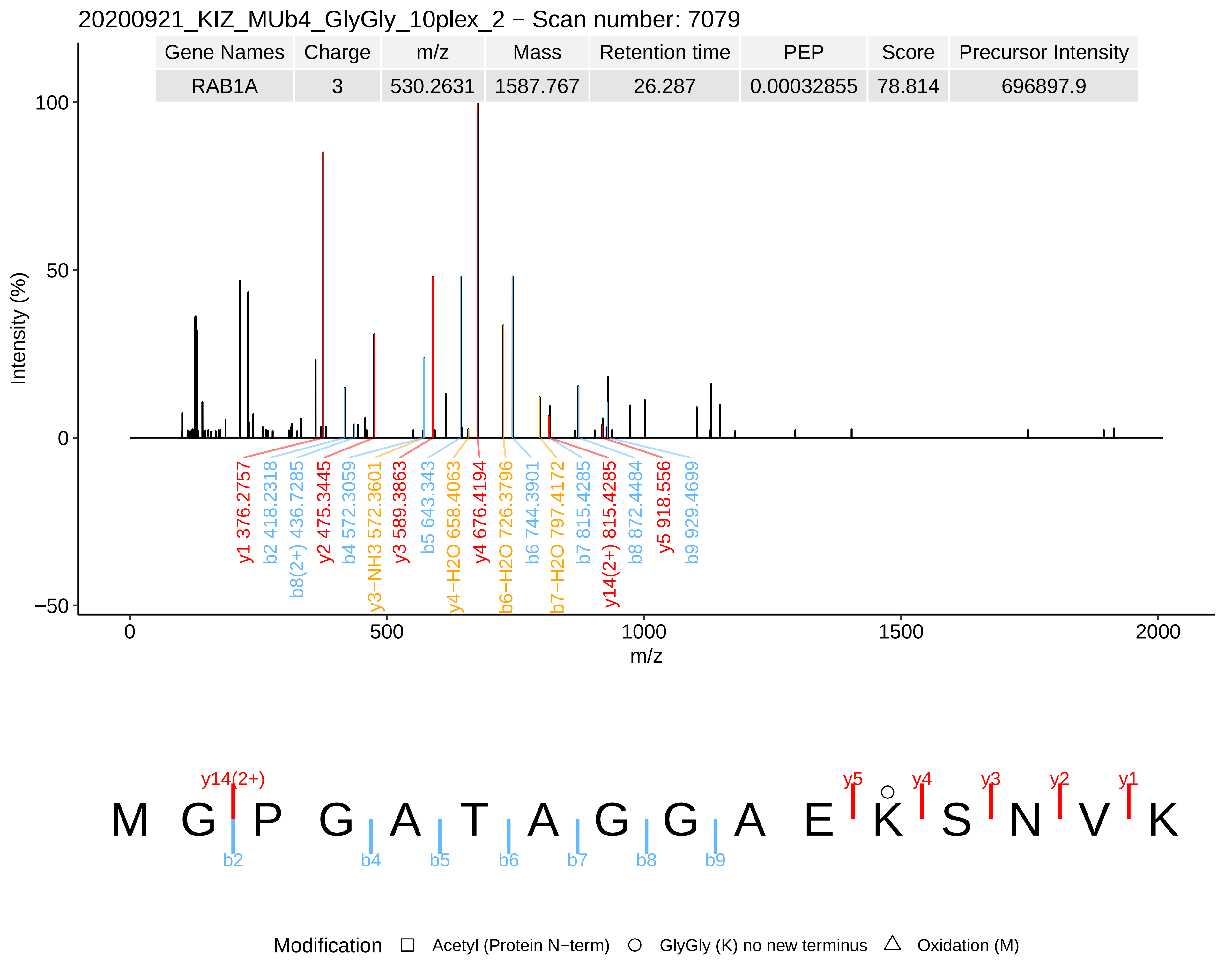

20200921_KIZ_MUb4_GlyGly_10plex_2 − Scan number: 7079
Gene Names | Charge | m/z | Mass | Retention time | PEP | Score | Precursor Intensity
RAB1A | 3 | 530.2631 | 1587.767 | 26.287 | 0.00032855 | 78.814 | 696897.9
Intensity (%)
100
50
0
−50
0
500
1000
1500
2000
m/z
y1 376.2757
b2 418.2318
b8(2+) 436.7285
y2 475.3445
b4 572.3059
y3−NH3 572.3601
y3 589.3863
b5 643.343
y4−H2O 658.4063
y4 676.4194
b6−H2O 726.3796
b6 744.3901
b7−H2O 797.4172
b7 815.4285
y14(2+) 815.4285
b8 872.4484
y5 918.556
b9 929.4699
y14(2+)
y5
y4
y3
y2
y1
M G P G A T A G G A E K S N V K
b2
b4
b5
b6
b7
b8
b9
Modification
Acetyl (Protein N−term)
GlyGly (K) no new terminus
Oxidation (M)

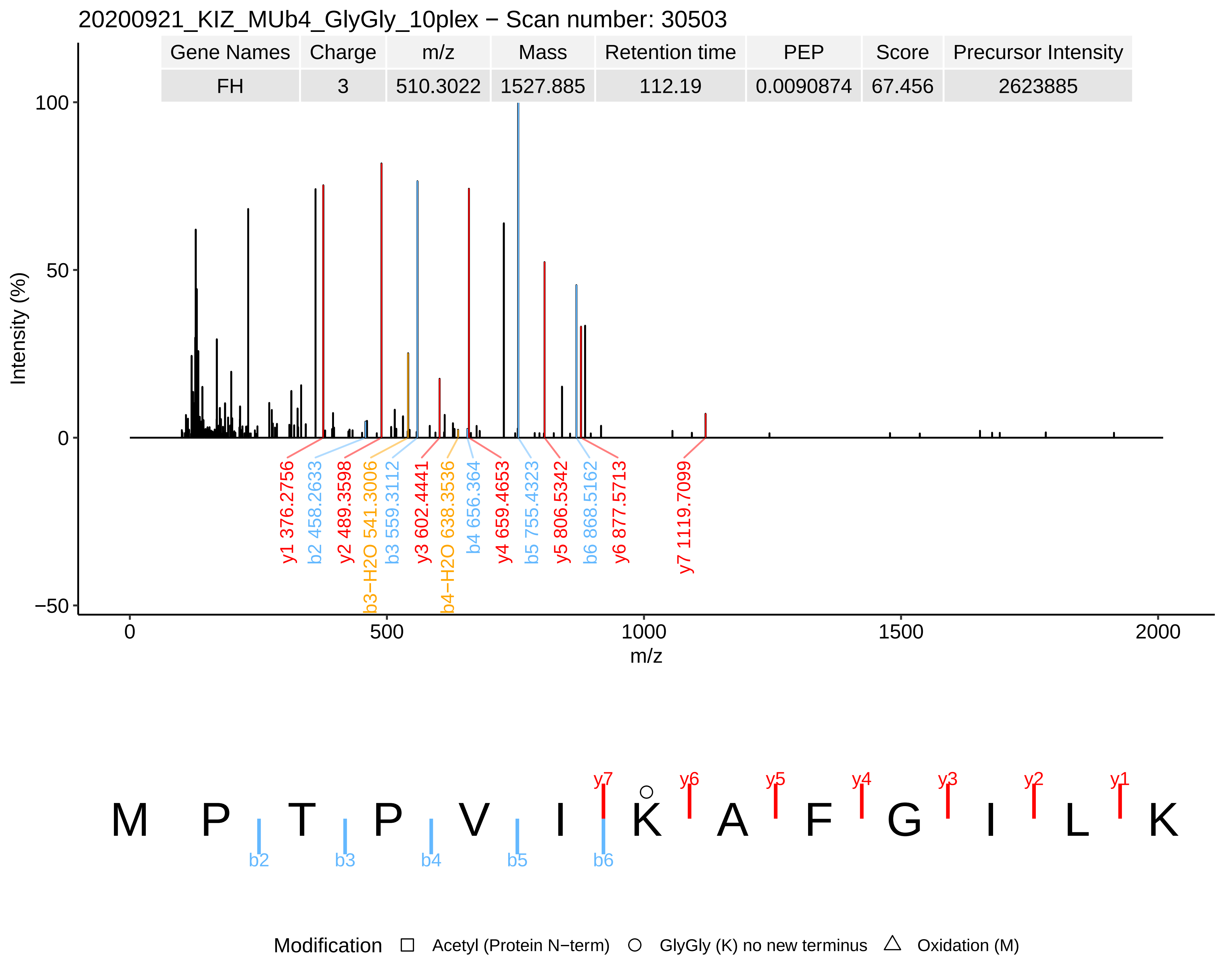
20200921_KIZ_MUb4_GlyGly_10plex − Scan number: 30503
Gene Names | Charge | m/z | Mass | Retention time | PEP | Score | Precursor Intensity
FH | 3 | 510.3022 | 1527.885 | 112.19 | 0.0090874 | 67.456 | 2623885
Intensity (%)
100
50
0
−50
0
500
1000
1500
2000
m/z
y1 376.2756
b2 458.2633
y2 489.3598
b3−H2O 541.3006
b3 559.3112
y3 602.4441
b4−H2O 638.3536
b4 656.364
y4 659.4653
b5 755.4323
y5 806.5342
b6 868.5162
y6 877.5713
y7 1119.7099
M P T P V I K A F G I L K
y7 y6 y5 y4 y3 y2 y1
b2 b3 b4 b5 b6
Modification
Acetyl (Protein N−term)
GlyGly (K) no new terminus
Oxidation (M)

20200921_KIZ_MUb4_GlyGly_10plex_2 − Scan number: 7361

Gene Names	Charge	m/z	Mass	Retention time	PEP	Score	Precursor Intensity
NME1−NME2;NME2;NME2P1	4	400.4588	1597.806	27.439	0.0015592	77.527	462216.2

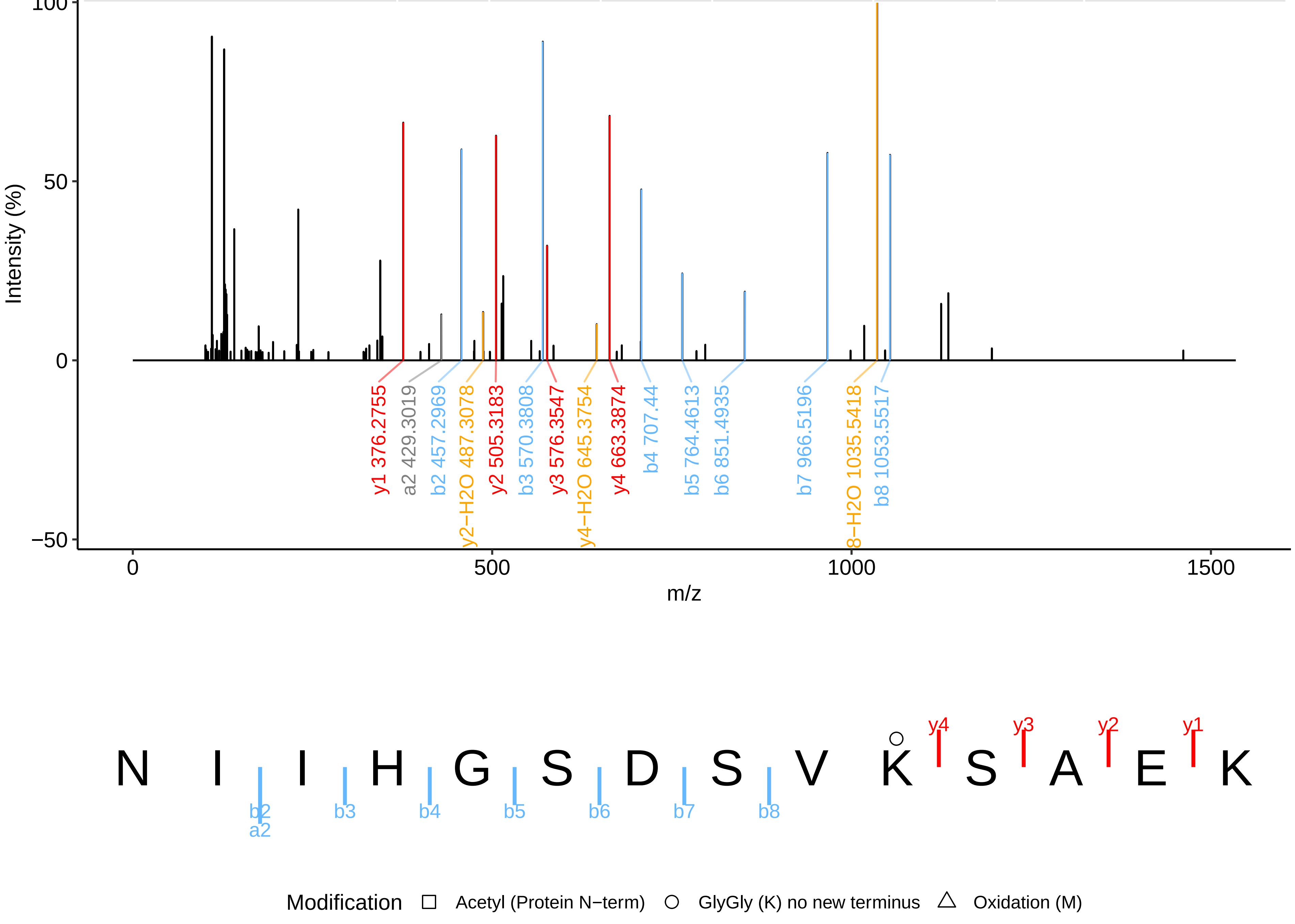

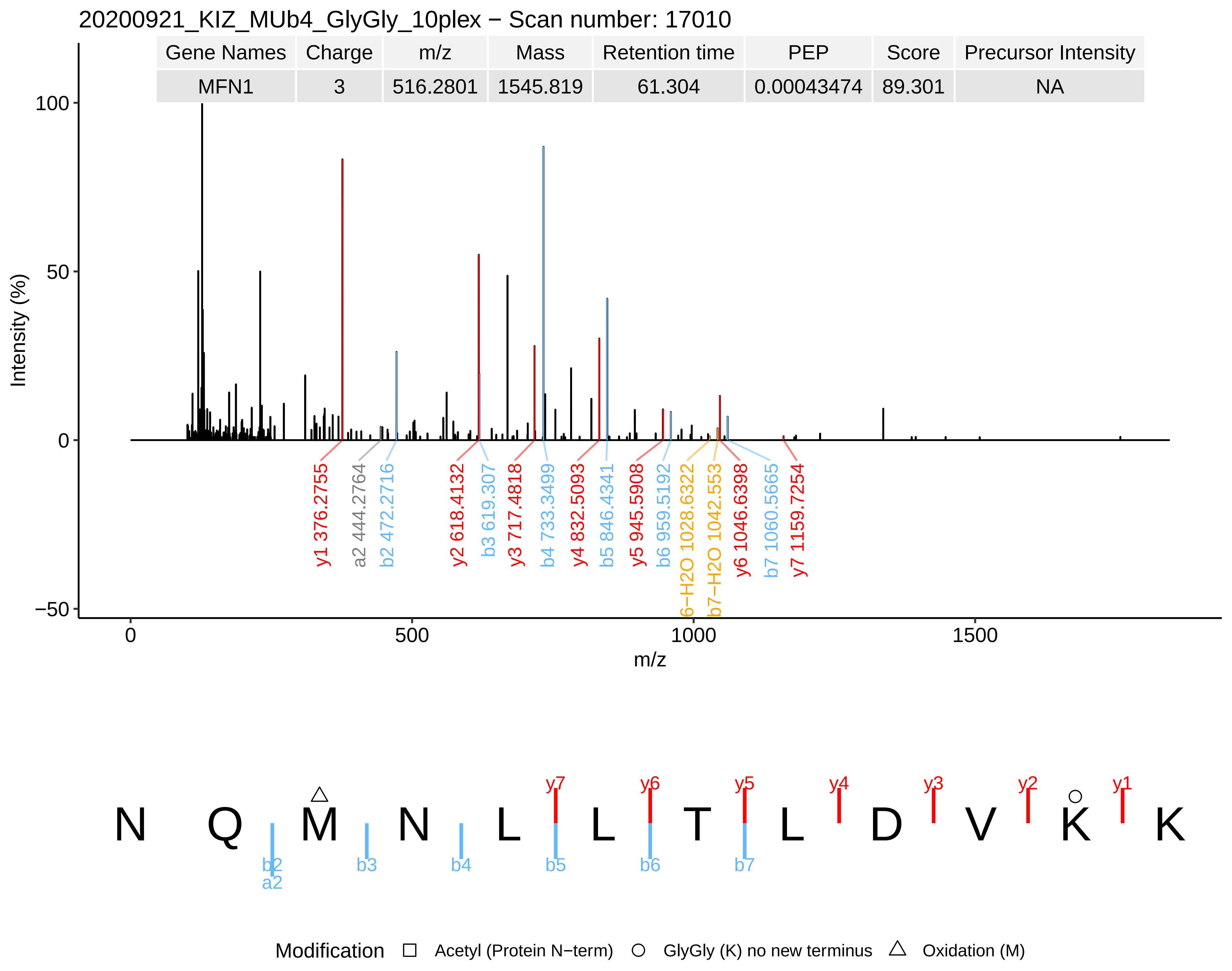
20200921_KIZ_MUb4_GlyGly_10plex − Scan number: 17010
Gene Names | Charge | m/z | Mass | Retention time | PEP | Score | Precursor Intensity
MFN1 | 3 | 516.2801 | 1545.819 | 61.304 | 0.00043474 | 89.301 | NA
Intensity (%)
m/z
y1 376.2755
a2 444.2764
b2 472.2716
y2 618.4132
b3 619.307
y3 717.4818
b4 733.3499
y4 832.5093
b5 846.4341
y5 945.5908
b6 959.5192
b6−H2O 1028.6322
b7−H2O 1042.553
y6 1046.6398
b7 1060.5665
y7 1159.7254
N Q M N L L T L D V K K
y7 y6 y5 y4 y3 y2 y1
b2 a2 b3 b4 b5 b6 b7
Modification
Acetyl (Protein N−term)
GlyGly (K) no new terminus
Oxidation (M)

20200921_KIZ_MUb4_GlyGly_10plex_2 − Scan number: 15882

Gene Names	Charge	m/z	Mass	Retention time	PEP	Score	Precursor Intensity
MFN1	3	516.2801	1545.819	62.198	6.6046e−06	107.09	2184578

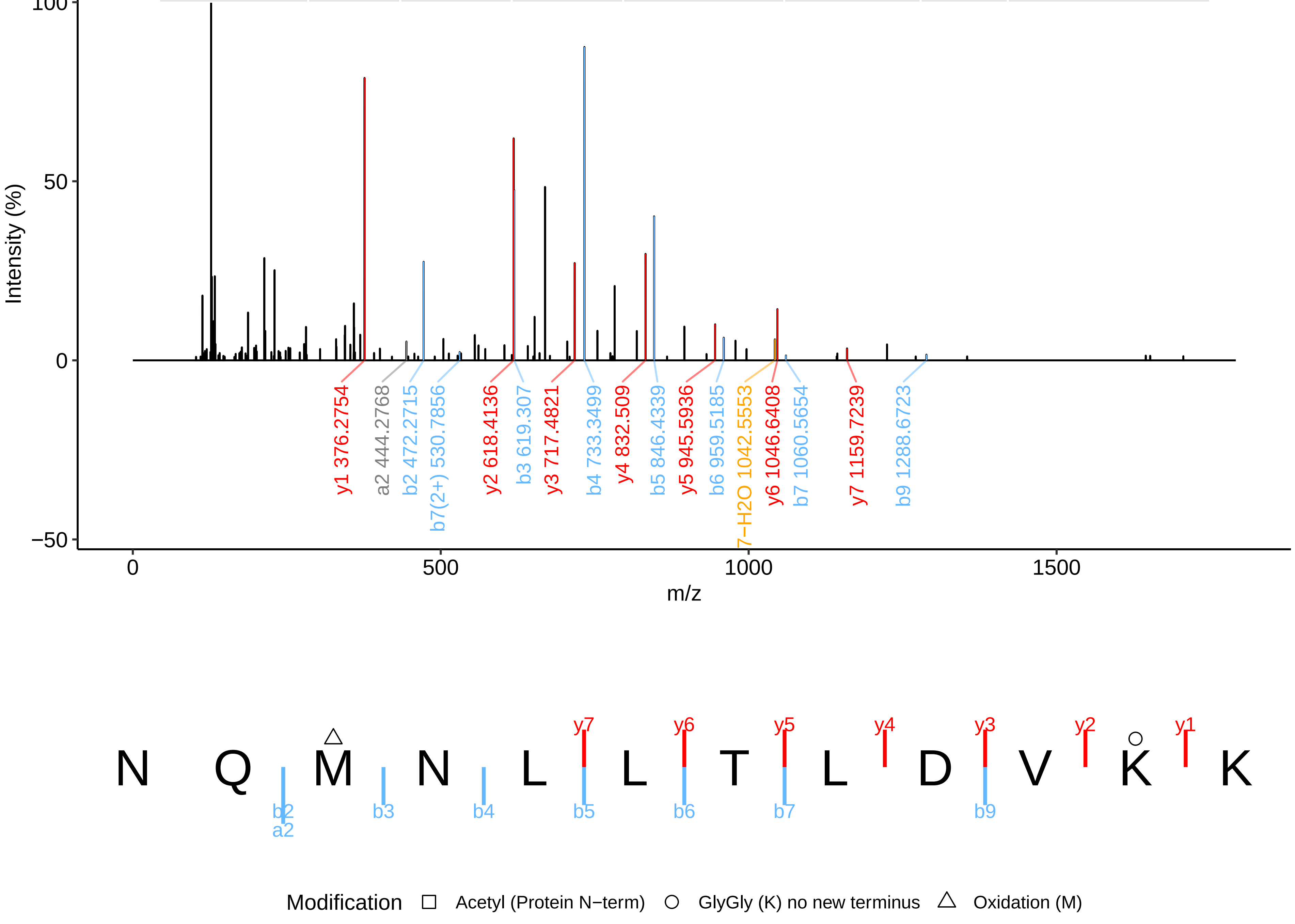

20200921_KIZ_MUb4_GlyGly_10plex − Scan number: 20827

Gene Names	Charge	m/z	Mass	Retention time	PEP	Score	Precursor Intensity
MFN1	3	510.9485	1529.824	75.334	3.5356e−05	116.01	3655991

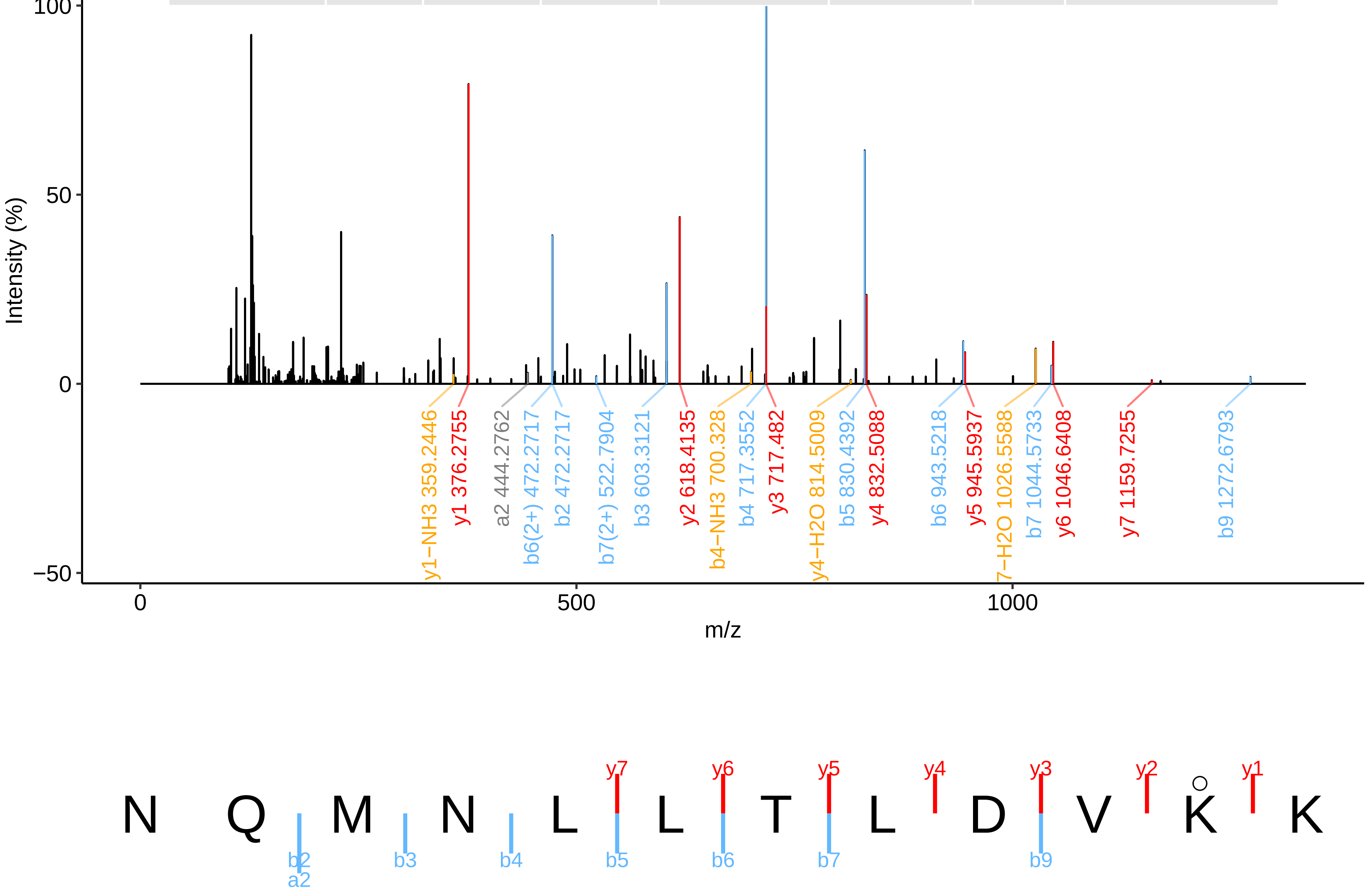

Modification □ Acetyl (Protein N−term) ○ GlyGly (K) no new terminus △ Oxidation (M)

20200921_KIZ_MUb4_GlyGly_10plex_2 − Scan number: 19035

Gene Names	Charge	m/z	Mass	Retention time	PEP	Score	Precursor Intensity
MFN1	3	510.9485	1529.824	74.655	9.0658e−05	108.37	1298458

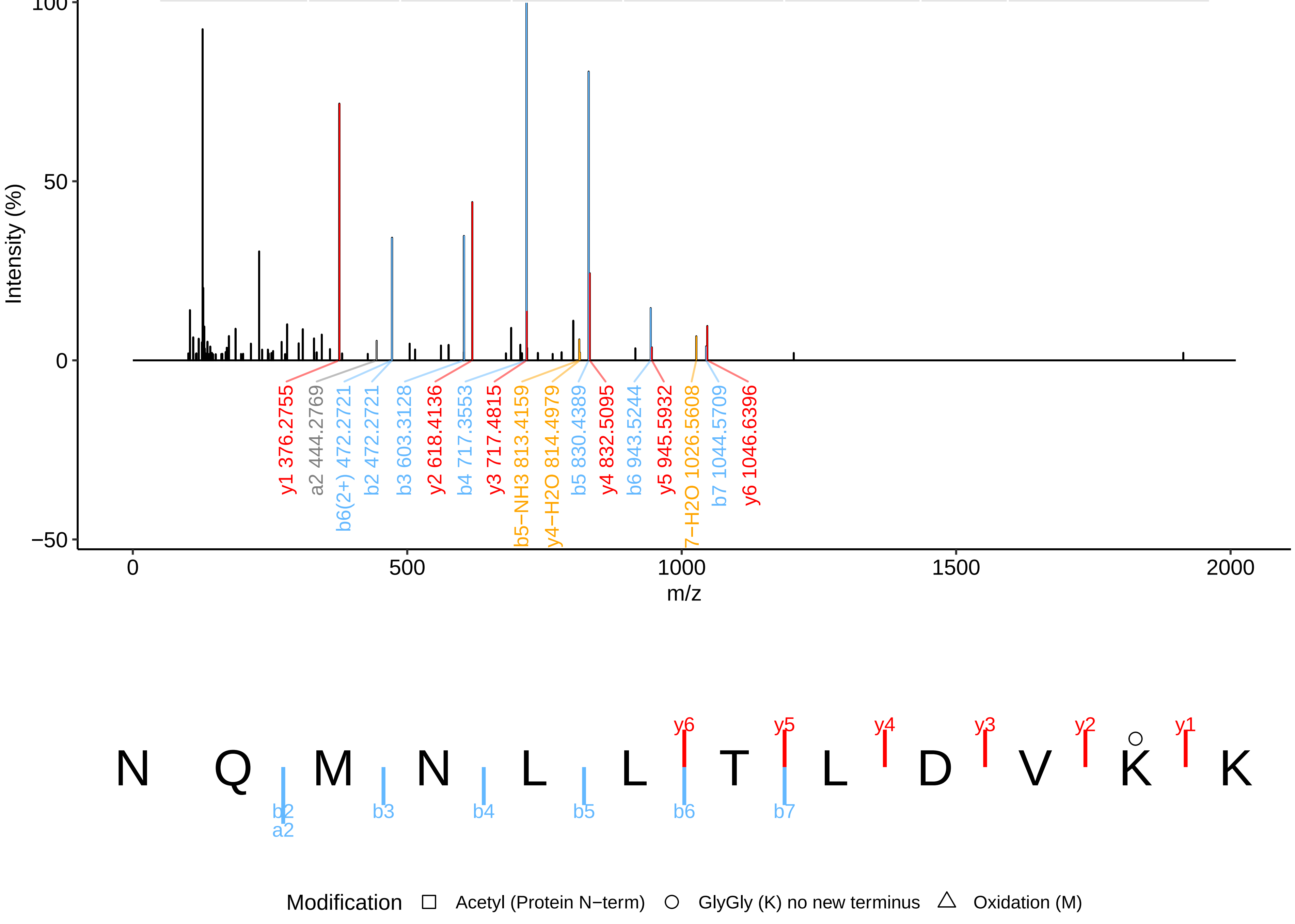

20201118_KIZ_MUb6_GlyGly_2 − Scan number: 22870

Gene Names	Charge	m/z	Mass	Retention time	PEP	Score	Precursor Intensity
MFN1	3	510.9485	1529.824	76.837	1.5778e−12	138.75	15219826

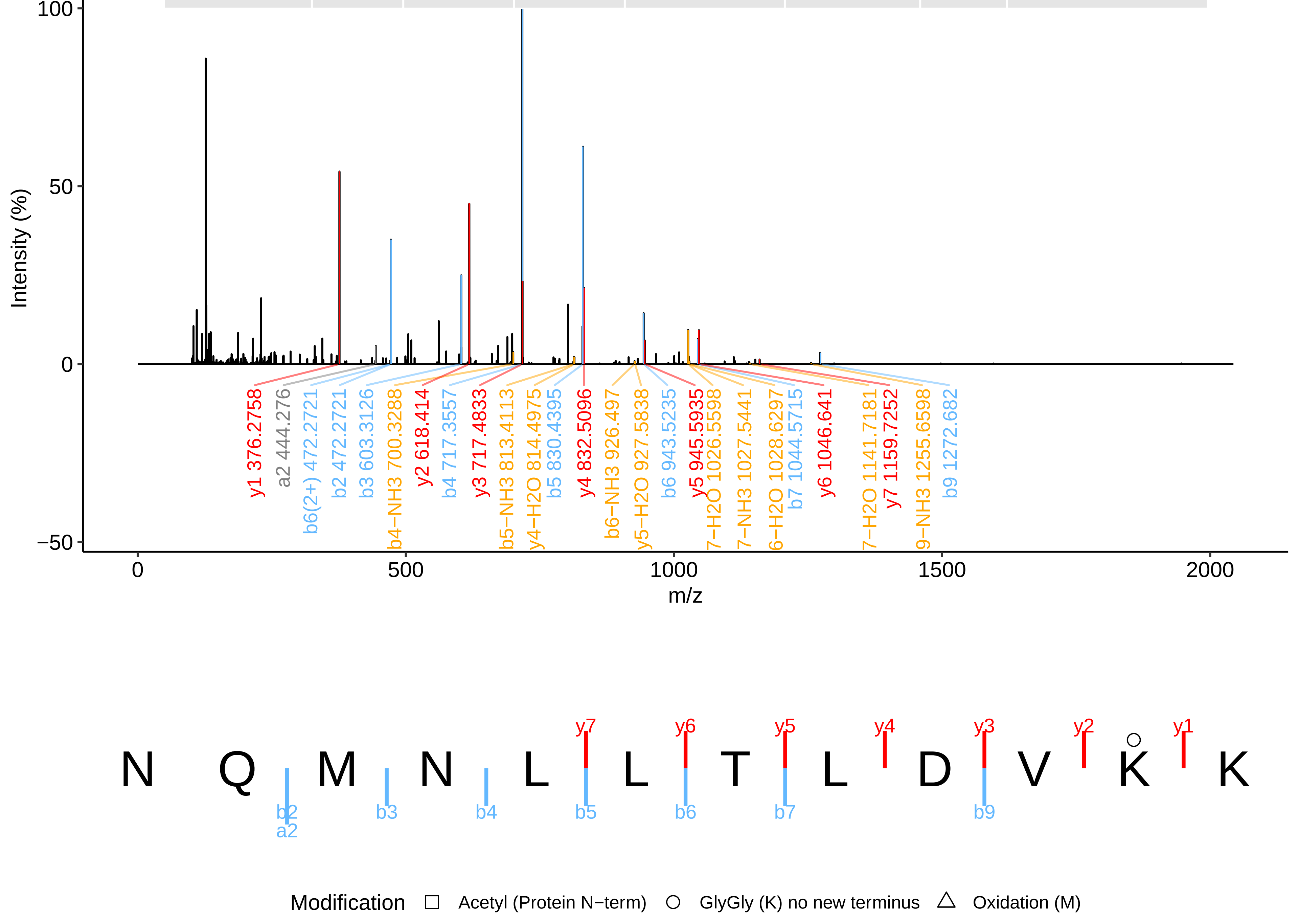

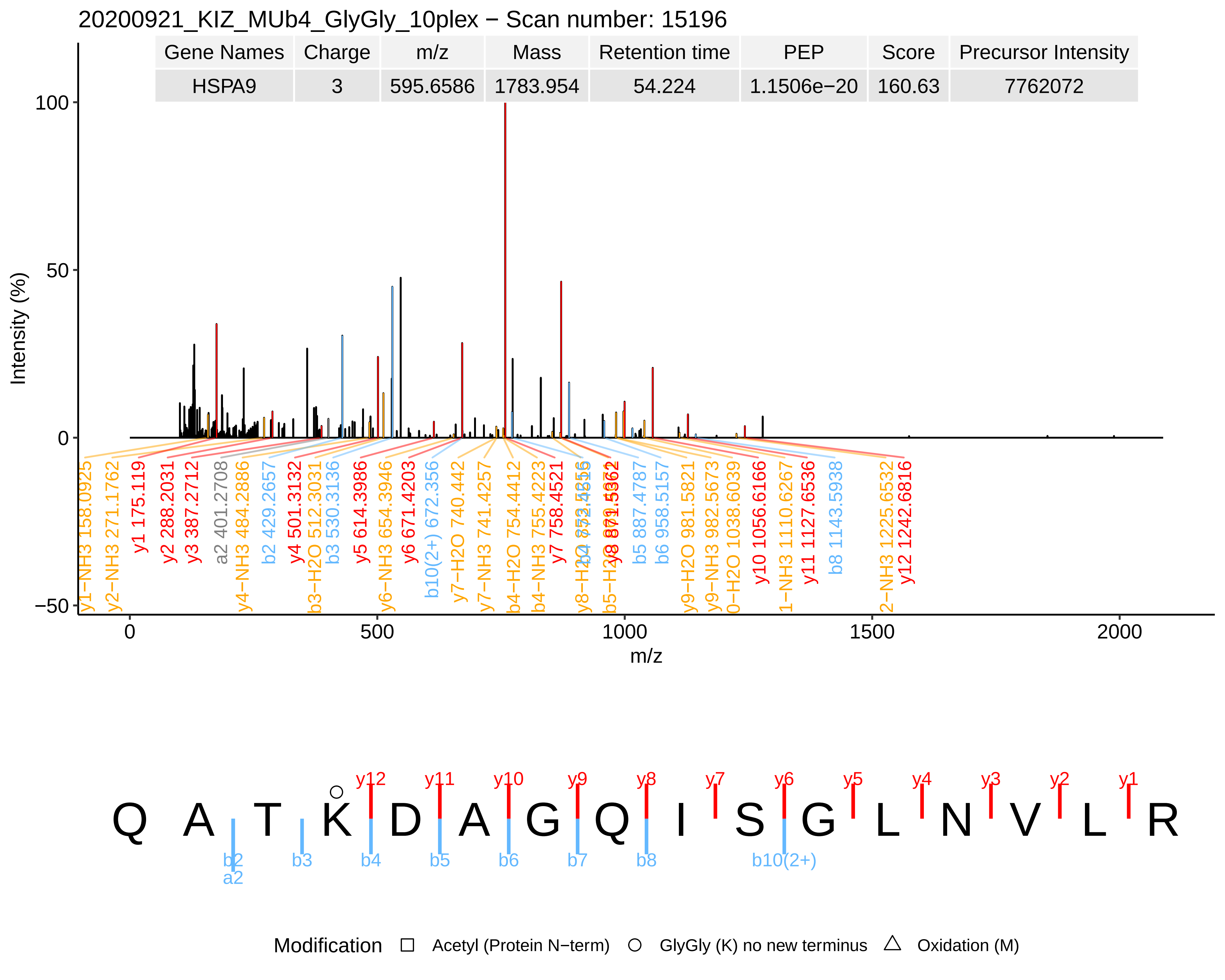

20200921_KIZ_MUb4_GlyGly_10plex − Scan number: 15196
Gene Names | Charge | m/z | Mass | Retention time | PEP | Score | Precursor Intensity
HSPA9 | 3 | 595.6586 | 1783.954 | 54.224 | 1.1506e−20 | 160.63 | 7762072
Intensity (%)
m/z
y1−NH3 158.0925
y2−NH3 271.1762
y1 175.119
y2 288.2031
y3 387.2712
a2 401.2708
y4−NH3 484.2886
b2 429.2657
y4 501.3132
b3−H2O 512.3031
b3 530.3136
y5 614.3986
y6−NH3 654.3946
y6 671.4203
b10(2+) 672.356
y7−H2O 740.442
y7−NH3 741.4257
b4−H2O 754.4412
b4−NH3 755.4223
y7 758.4521
b5 887.4787
b6 958.5157
y9−H2O 981.5821
y9−NH3 982.5673
y10 1056.6166
y11 1127.6536
b8 1143.5938
y12 1242.6816
Q A T K D A G Q I S G L N V L R
y12 y11 y10 y9 y8 y7 y6 y5 y4 y3 y2 y1
b2 a2 b3 b4 b5 b6 b7 b8 b10(2+)
Modification
Acetyl (Protein N−term)
GlyGly (K) no new terminus
Oxidation (M)

20200921_KIZ_MUb4_GlyGly_10plex_2 − Scan number: 14016

Gene Names	Charge	m/z	Mass	Retention time	PEP	Score	Precursor Intensity
HSPA9	3	595.6586	1783.954	54.552	0.0022929	67.605	761174

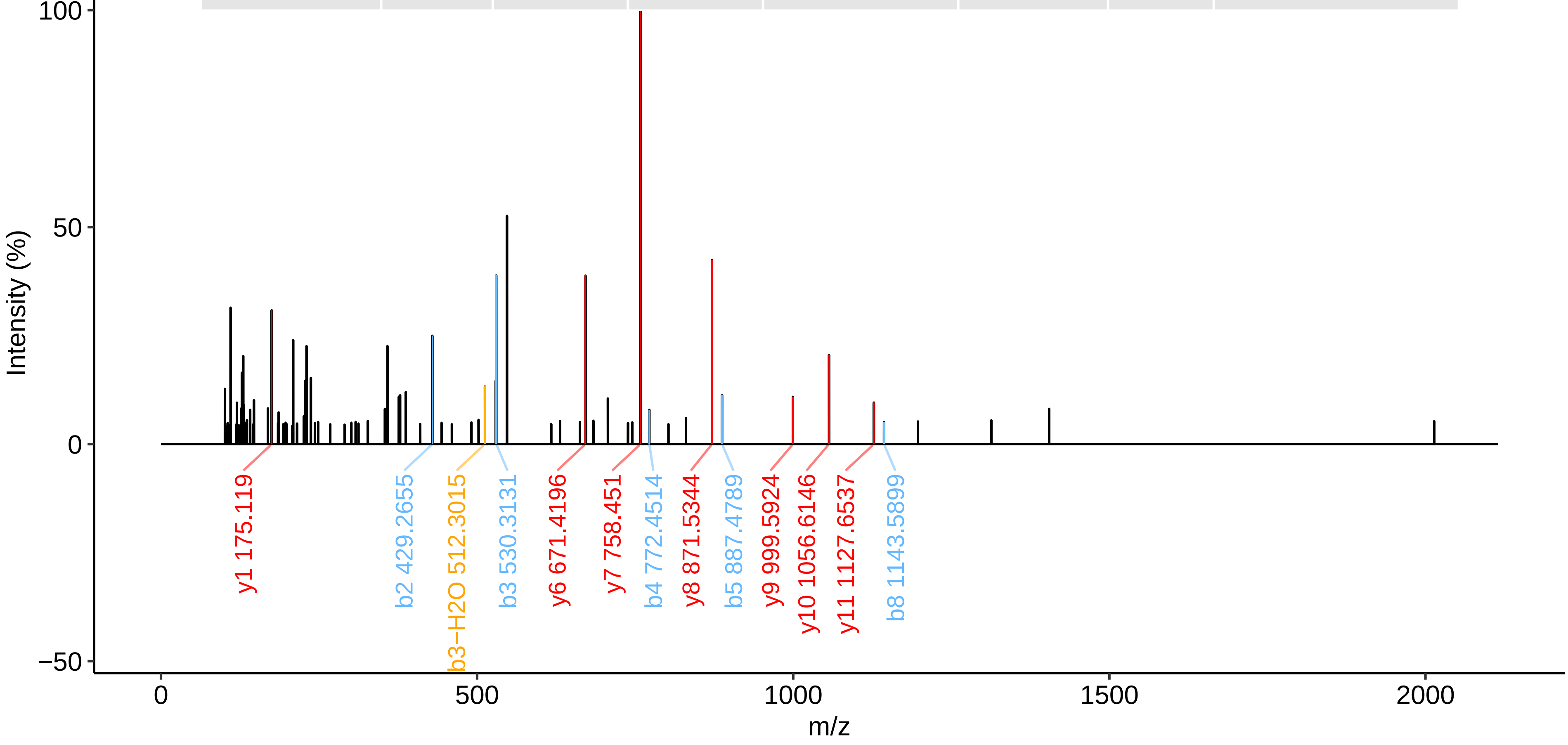

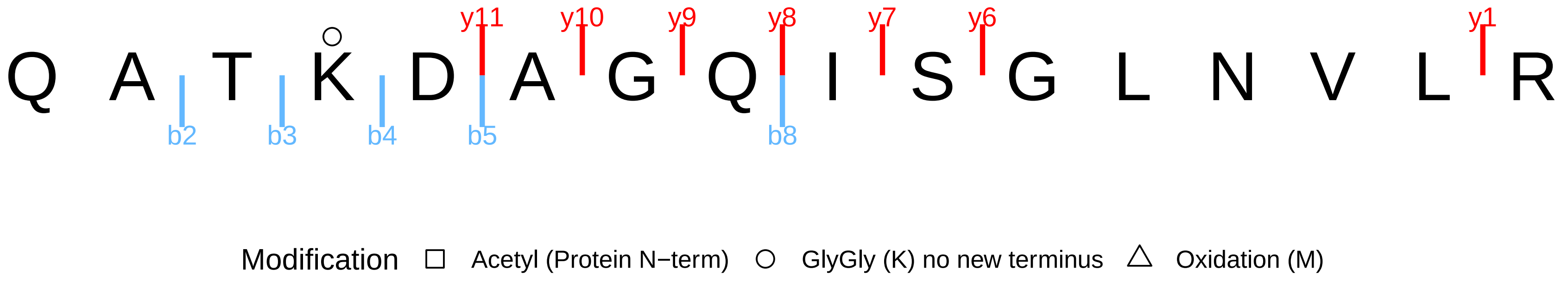

20200921_KIZ_MUb4_GlyGly_10plex_2 − Scan number: 18528

Gene Names	Charge	m/z	Mass	Retention time	PEP	Score	Precursor Intensity
ARL8B	3	591.649	1771.925	72.571	0.00069111	82.102	1018833

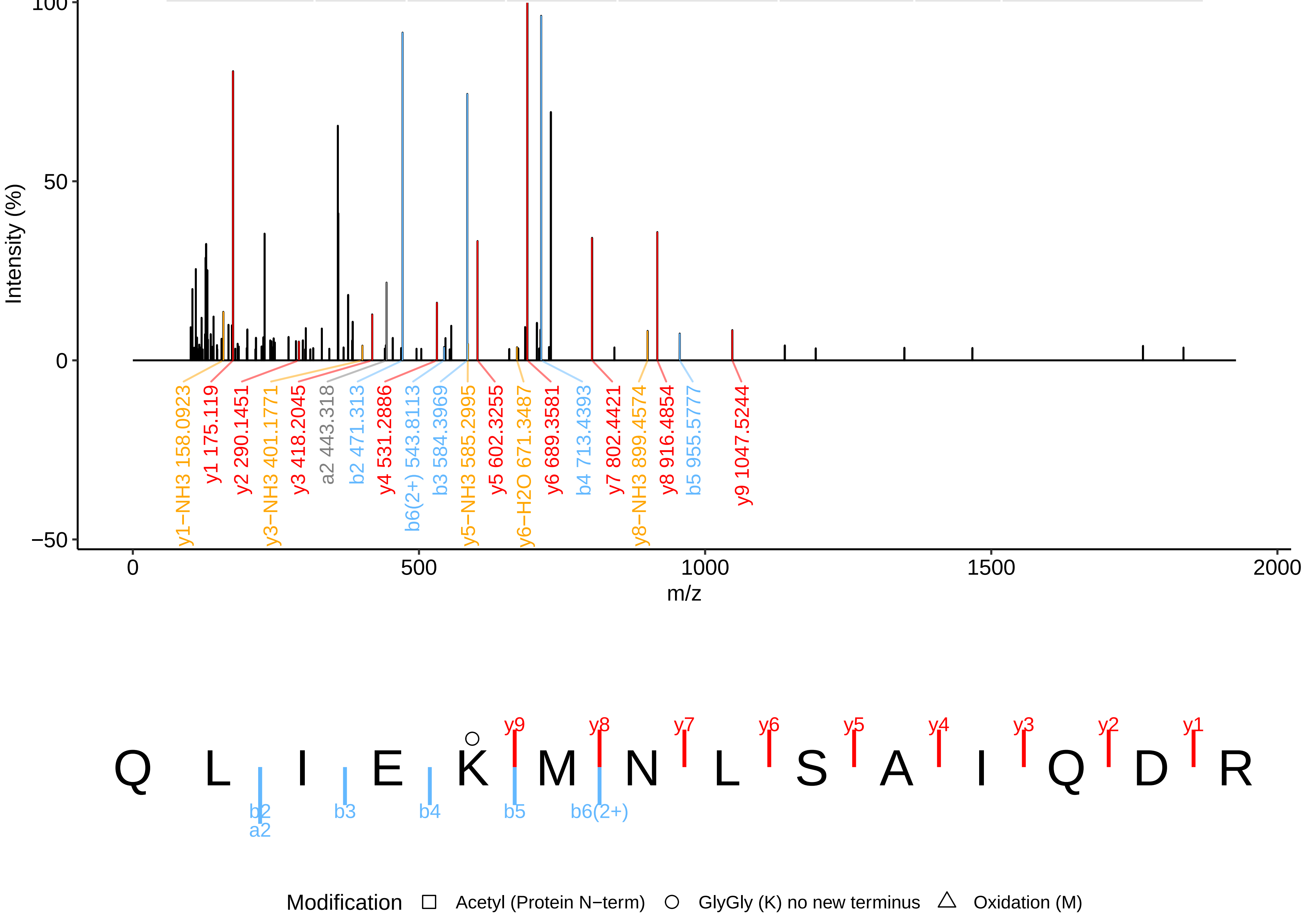

20200921_KIZ_MUb4_GlyGly_10plex − Scan number: 6295

Gene Names	Charge	m/z	Mass	Retention time	PEP	Score	Precursor Intensity
NEDD8;NEDD8−MDP1	3	515.228	1542.662	20.774	0.057182	51.875	4885392

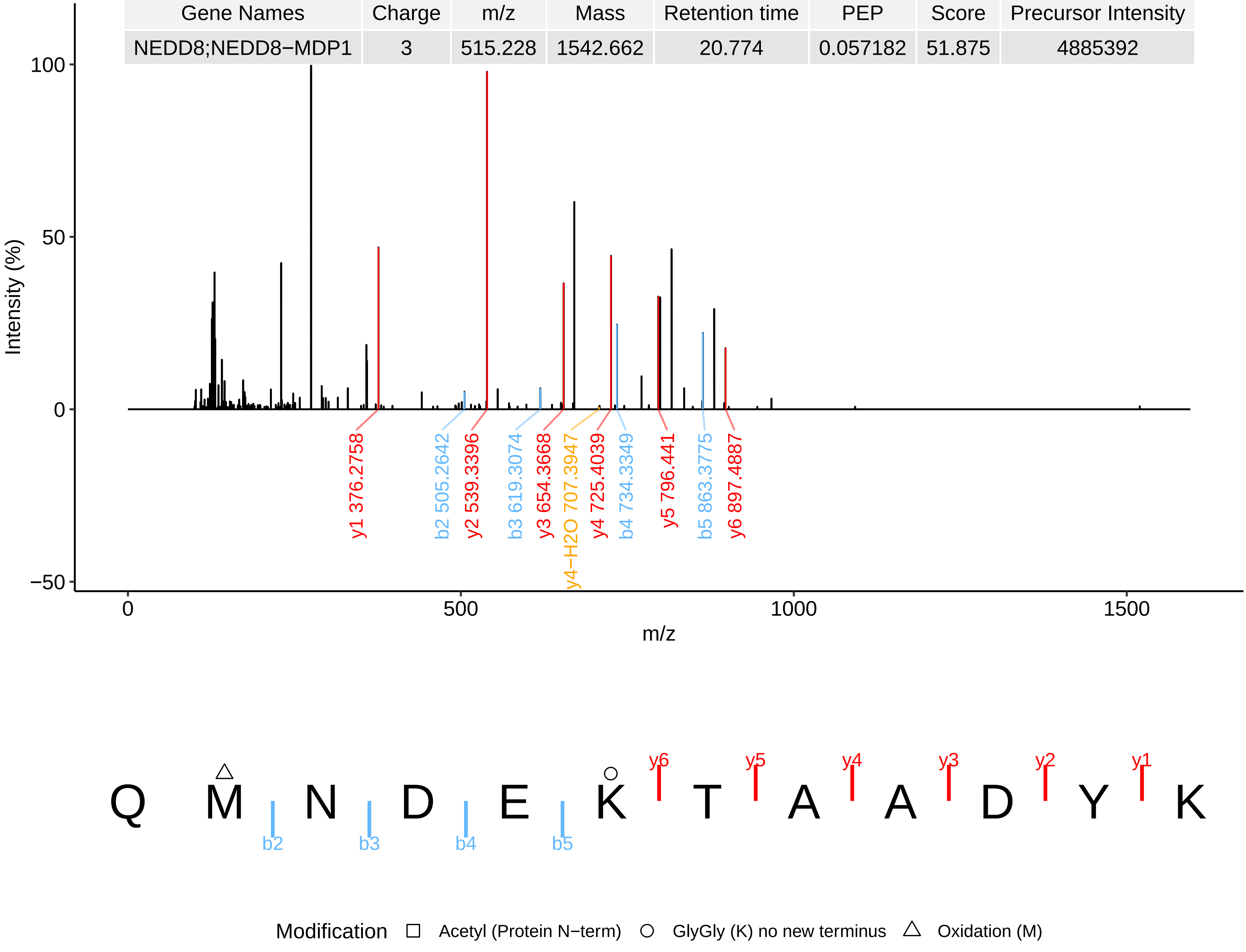

20200921_KIZ_MUb4_GlyGly_10plex − Scan number: 6335

Gene Names	Charge	m/z	Mass	Retention time	PEP	Score	Precursor Intensity
NEDD8;NEDD8−MDP1	3	515.228	1542.662	20.919	0.073623	47.915	8907840

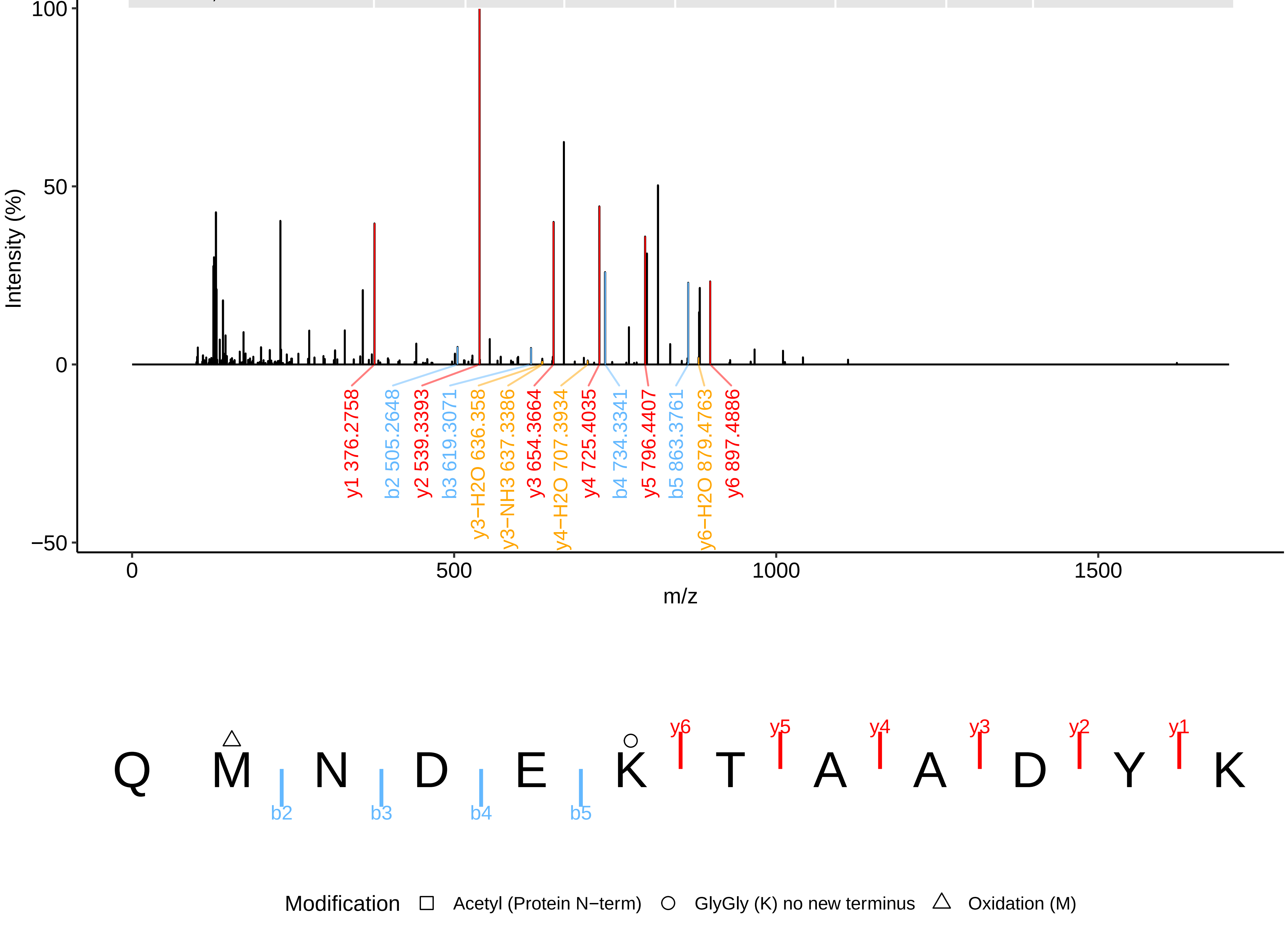

20200921_KIZ_MUb4_GlyGly_10plex − Scan number: 8495

Gene Names	Charge	m/z	Mass	Retention time	PEP	Score	Precursor Intensity
NEDD8;NEDD8−MDP1	3	509.8963	1526.667	28.844	0.0019495	79.492	4877799

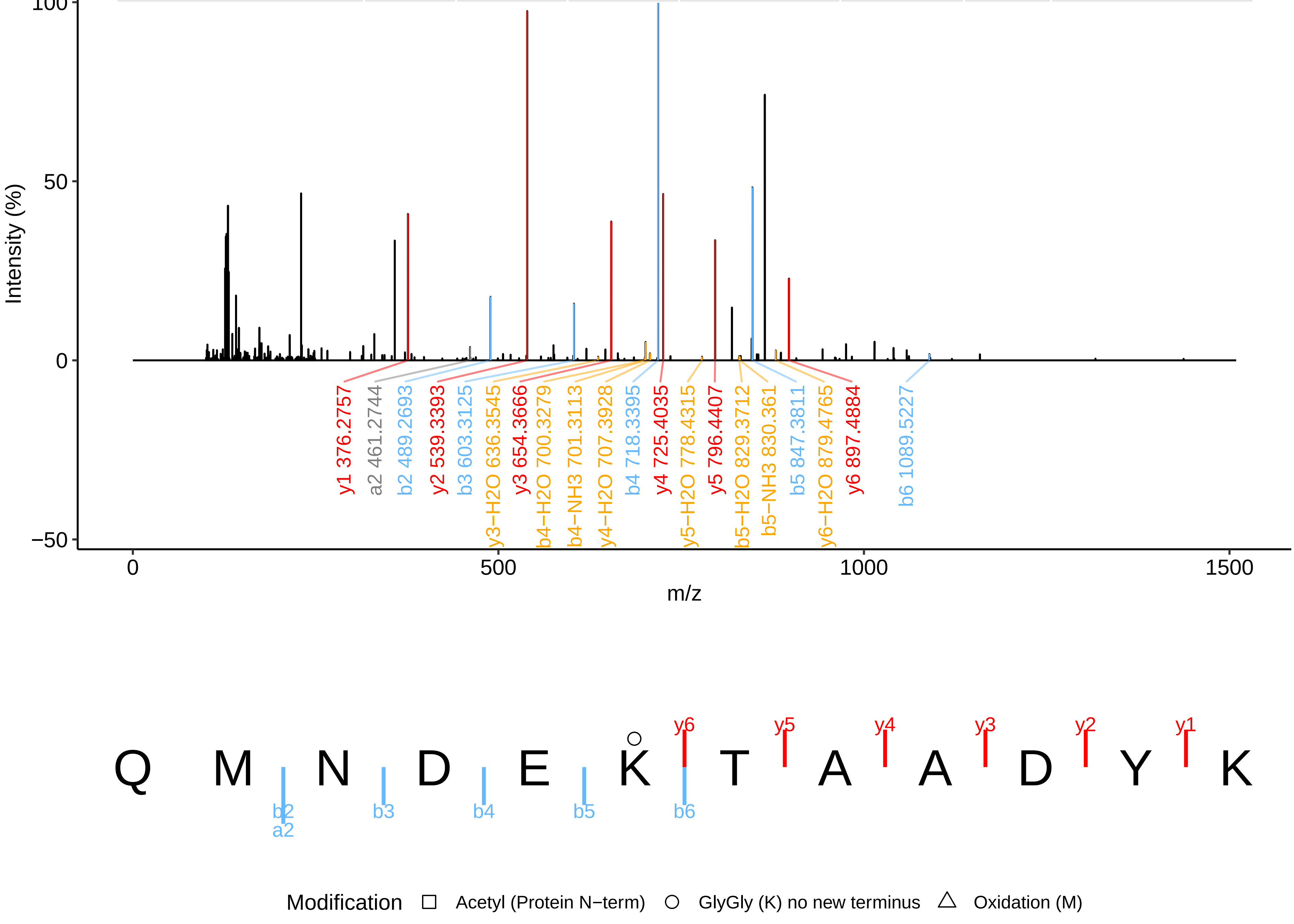

20200921_KIZ_MUb4_GlyGly_10plex_2 − Scan number: 7827

Gene Names	Charge	m/z	Mass	Retention time	PEP	Score	Precursor Intensity
NEDD8;NEDD8−MDP1	3	509.8963	1526.667	29.332	0.005315	74.46	2692929

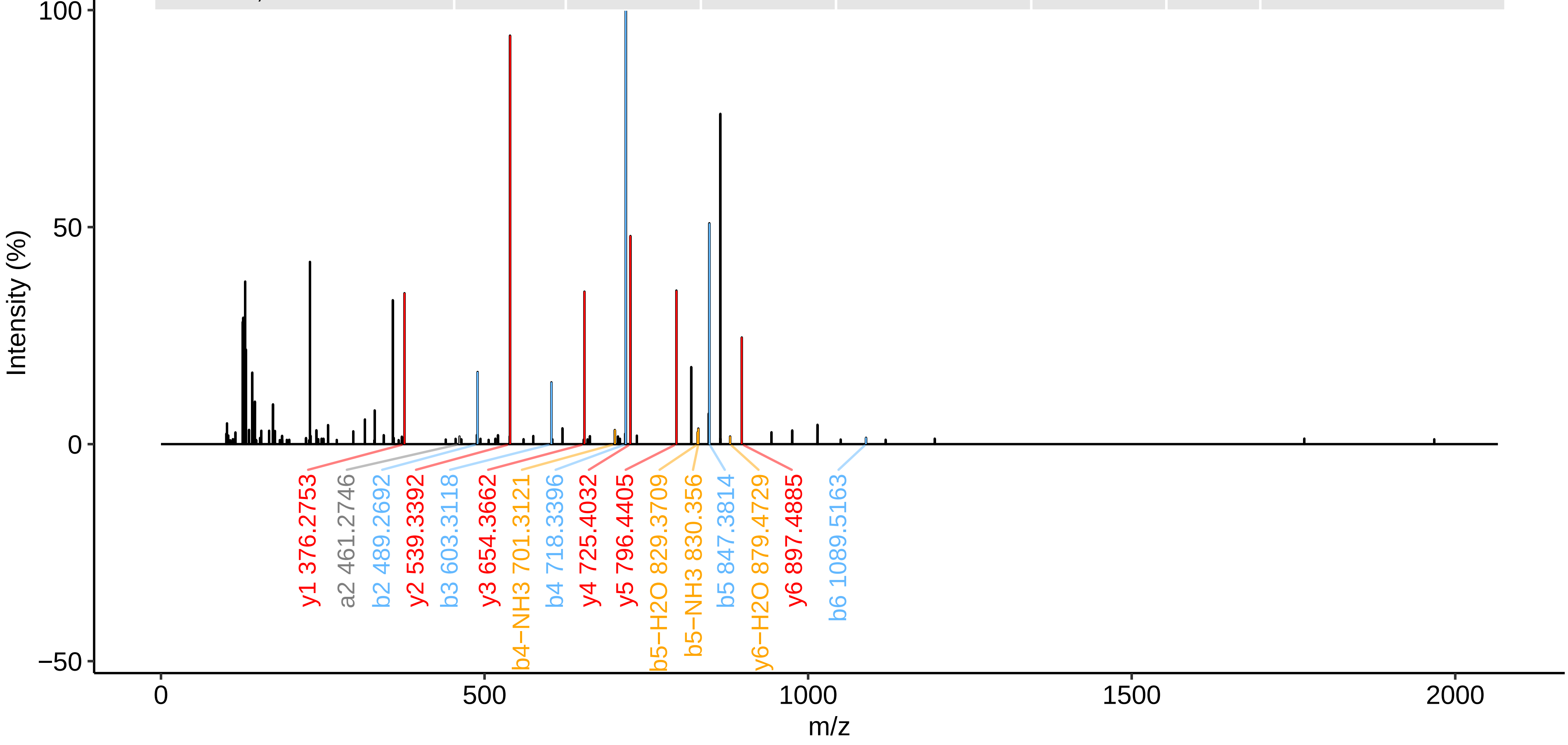

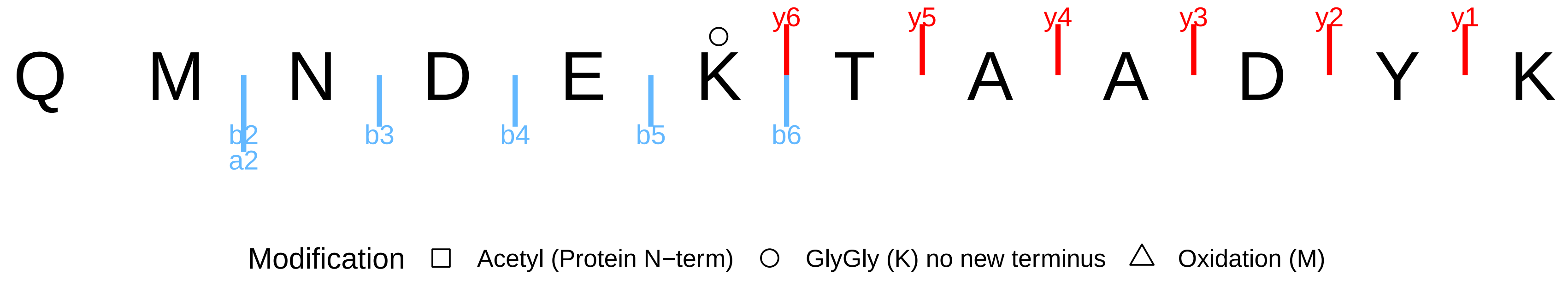

20200924_KIZ_MUb5_GlyGly_1 − Scan number: 7457

Gene Names	Charge	m/z	Mass	Retention time	PEP	Score	Precursor Intensity
NEDD8;NEDD8−MDP1	3	509.8963	1526.667	23.683	0.013287	63.091	7418749

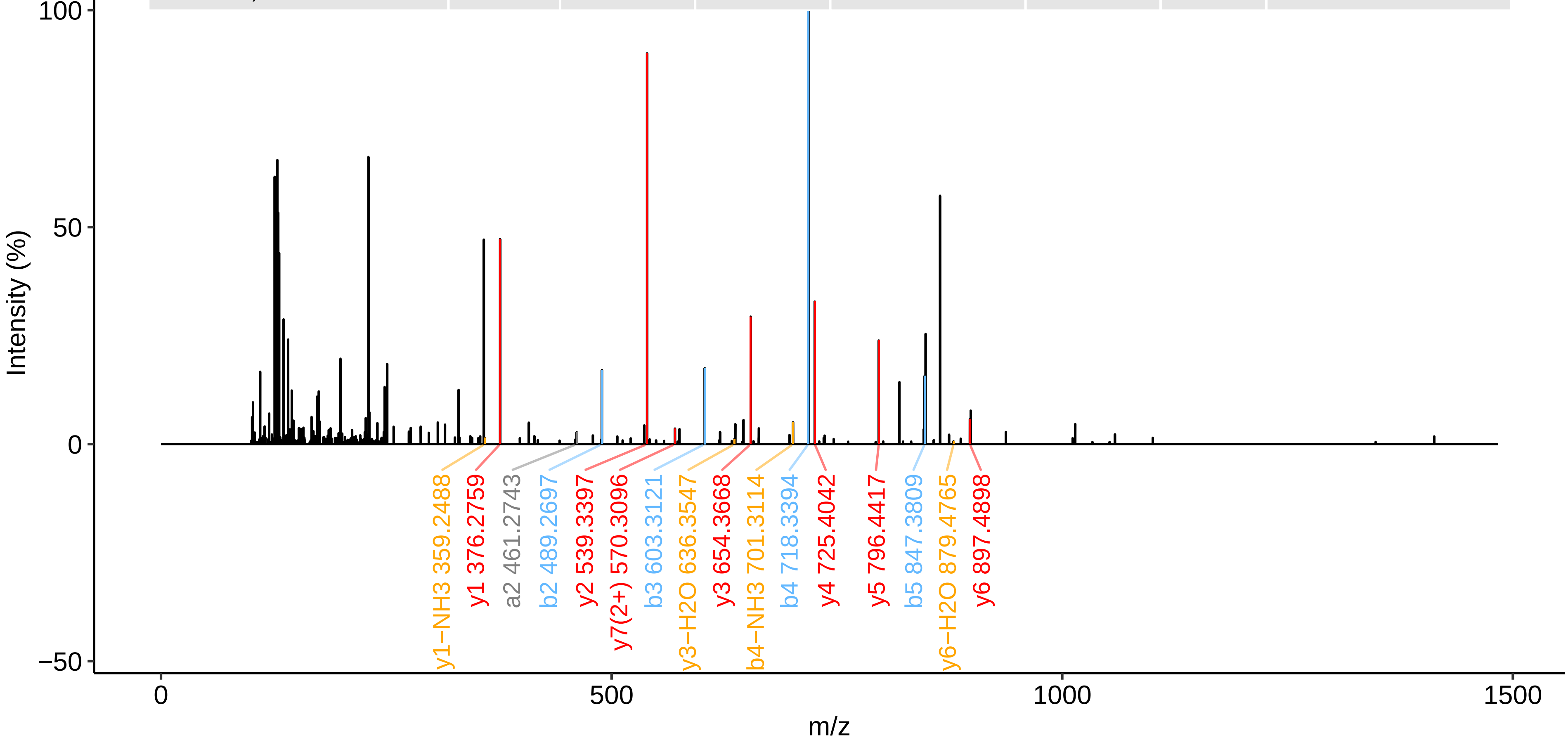

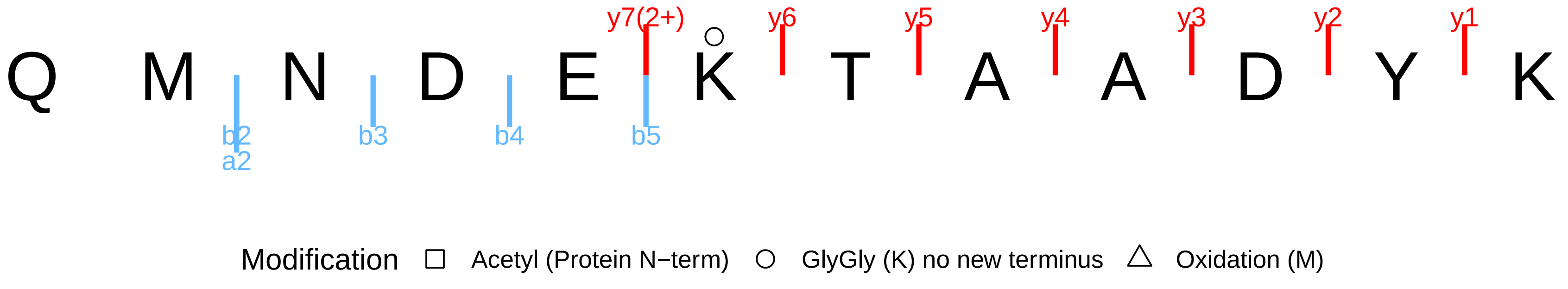

20200924_KIZ_MUb5_GlyGly_1 − Scan number: 7468

Gene Names	Charge	m/z	Mass	Retention time	PEP	Score	Precursor Intensity
NEDD8;NEDD8−MDP1	3	509.8963	1526.667	23.716	0.0013967	80.318	10398170

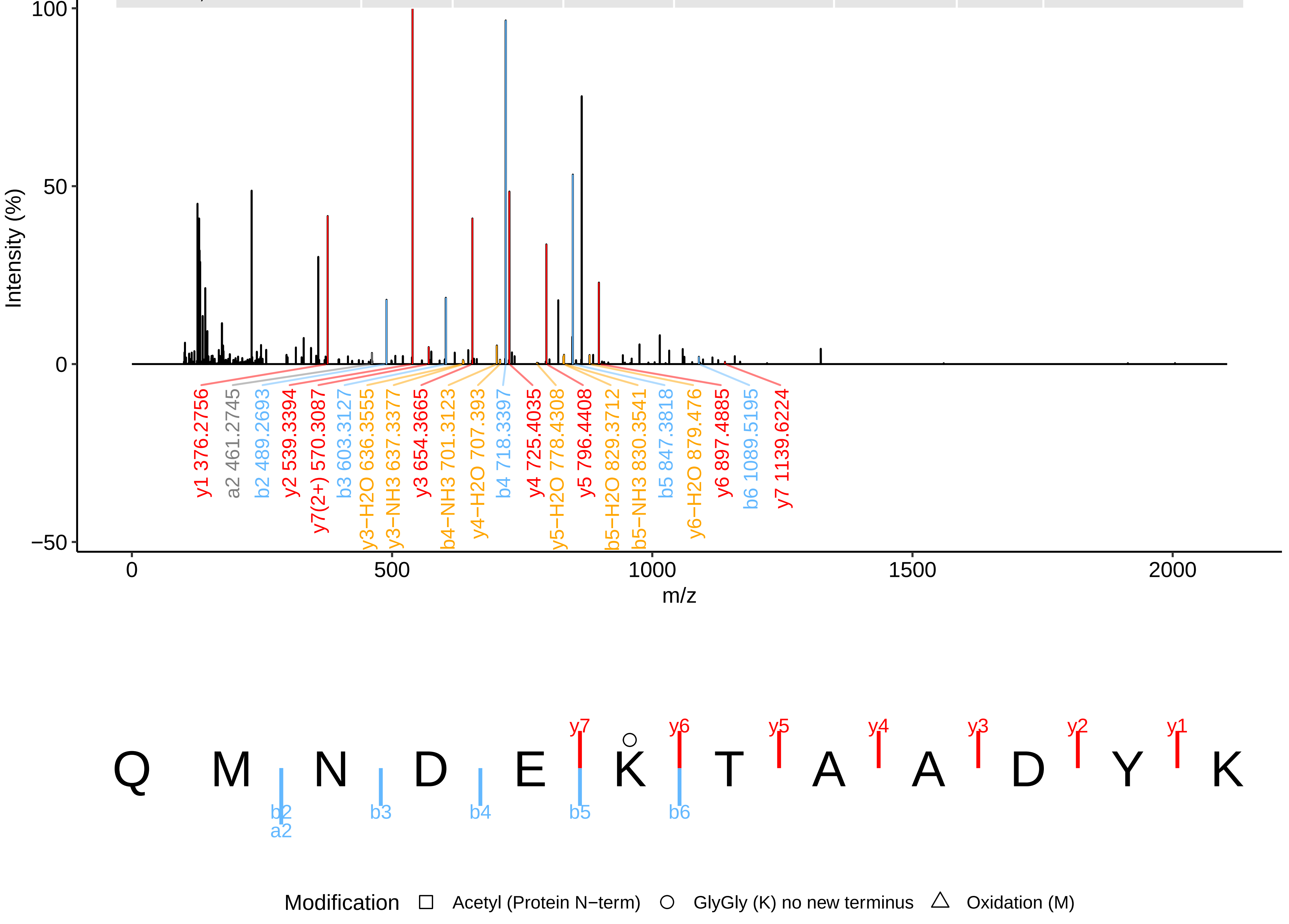

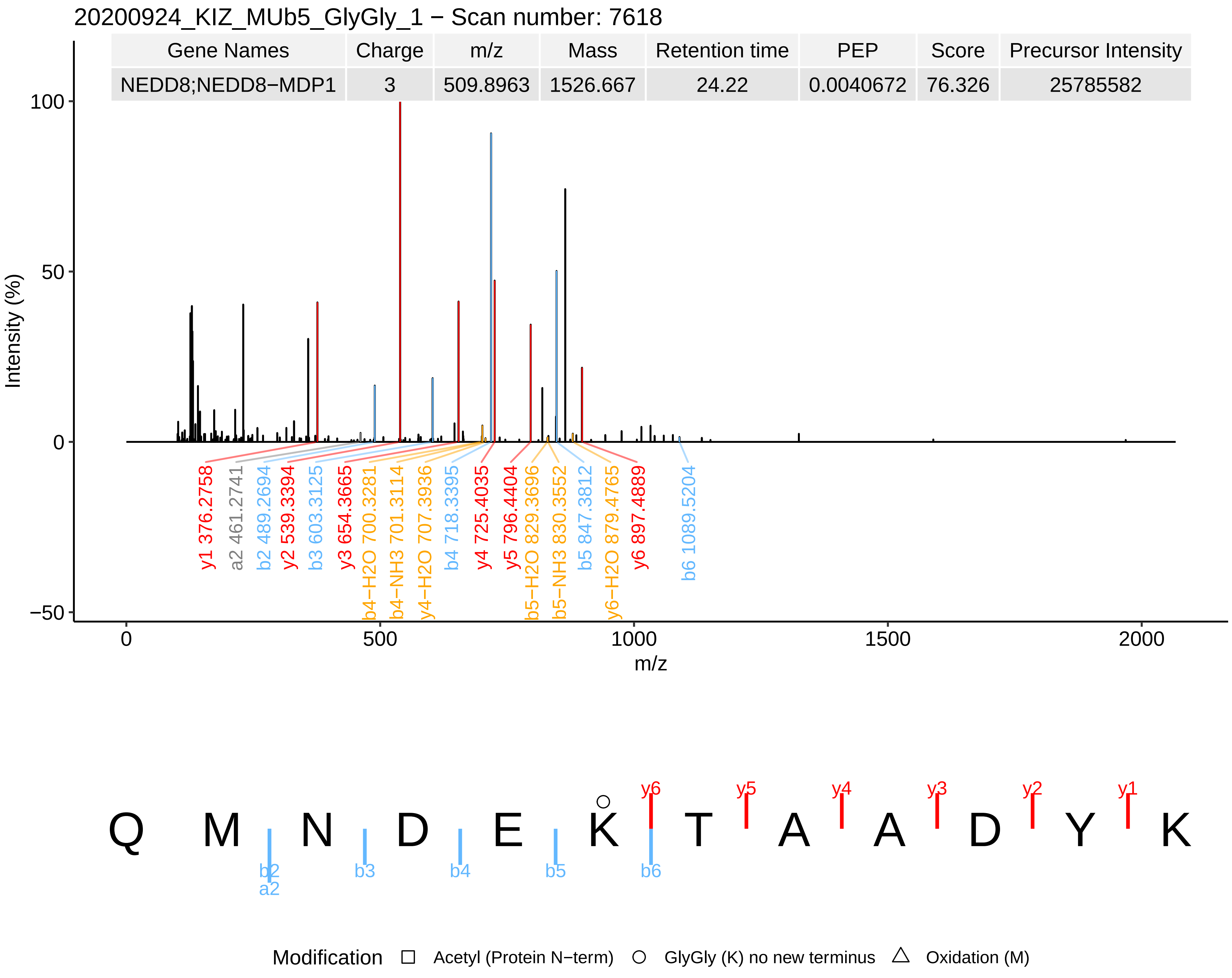

20200924_KIZ_MUb5_GlyGly_1 − Scan number: 7618
Gene Names | Charge | m/z | Mass | Retention time | PEP | Score | Precursor Intensity
NEDD8;NEDD8−MDP1 | 3 | 509.8963 | 1526.667 | 24.22 | 0.0040672 | 76.326 | 25785582
Intensity (%)
m/z
y1 376.2758
a2 461.2741
b2 489.2694
y2 539.3394
b3 603.3125
y3 654.3665
b4−H2O 700.3281
b4−NH3 701.3114
y4−H2O 707.3936
b4 718.3395
y4 725.4035
y5 796.4404
b5−H2O 829.3696
b5−NH3 830.3552
b5 847.3812
y6−H2O 879.4765
y6 897.4889
b6 1089.5204
Q M N D E K T A A D Y K
y6 y5 y4 y3 y2 y1
b2 a2 b3 b4 b5 b6
Modification
Acetyl (Protein N−term)
GlyGly (K) no new terminus
Oxidation (M)

20200924_KIZ_MUb5_GlyGly_2 − Scan number: 7059

Gene Names	Charge	m/z	Mass	Retention time	PEP	Score	Precursor Intensity
NEDD8;NEDD8−MDP1	3	509.8963	1526.667	26.328	0.010641	66.498	1984392

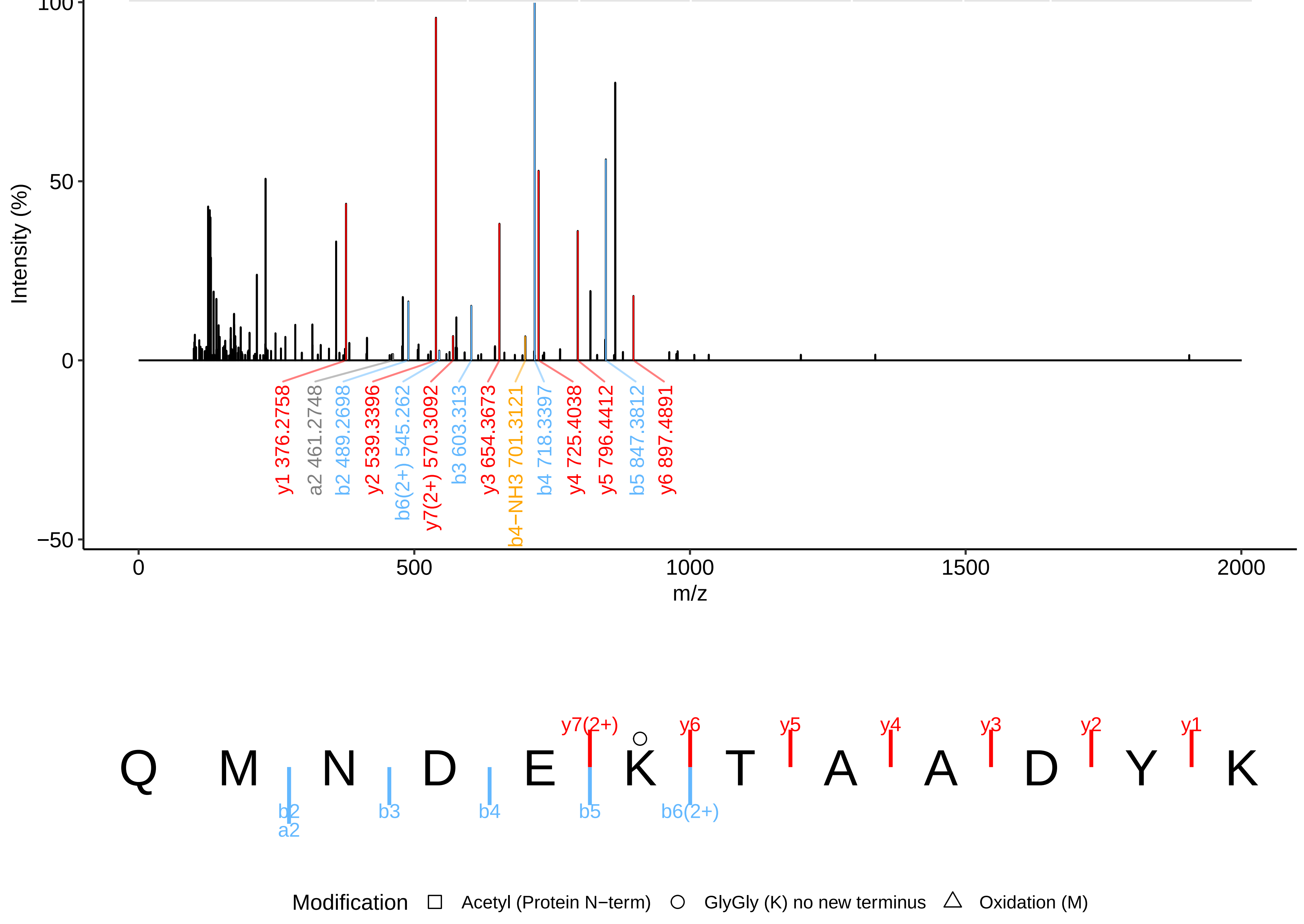

20200924_KIZ_MUb5_GlyGly_2 − Scan number: 7122

Gene Names	Charge	m/z	Mass	Retention time	PEP	Score	Precursor Intensity
NEDD8;NEDD8−MDP1	3	509.8963	1526.667	26.552	0.045197	56.951	2965268

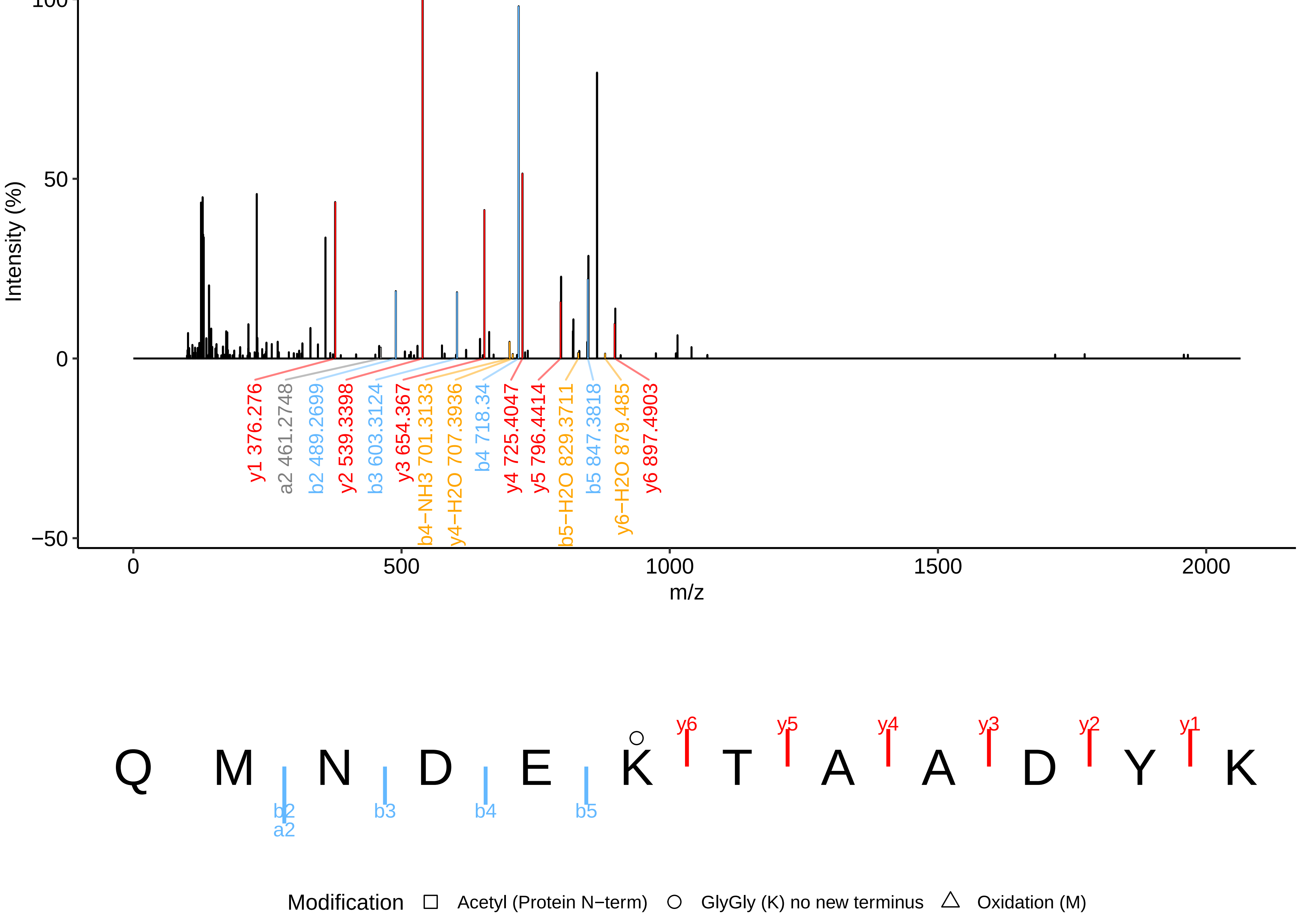

20200924_KIZ_MUb5_GlyGly_2 − Scan number: 7256

Gene Names	Charge	m/z	Mass	Retention time	PEP	Score	Precursor Intensity
NEDD8;NEDD8−MDP1	3	509.8963	1526.667	27.066	0.00093402	84.718	5432722

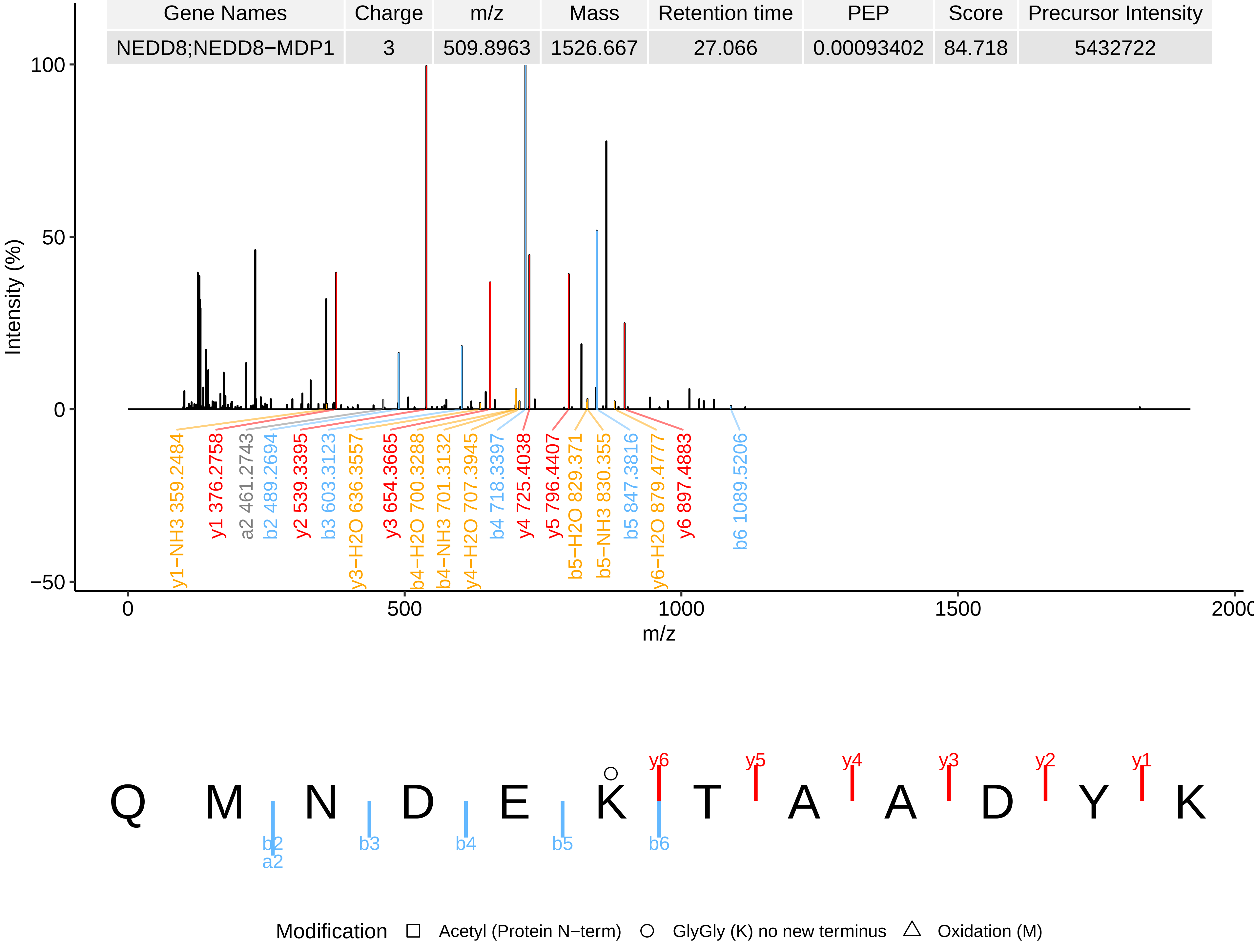

20201118_KIZ_MUb6_GlyGly_1 − Scan number: 7180

Gene Names	Charge	m/z	Mass	Retention time	PEP	Score	Precursor Intensity
NEDD8;NEDD8−MDP1	3	509.8963	1526.667	22.752	0.0031211	77.74	7202751

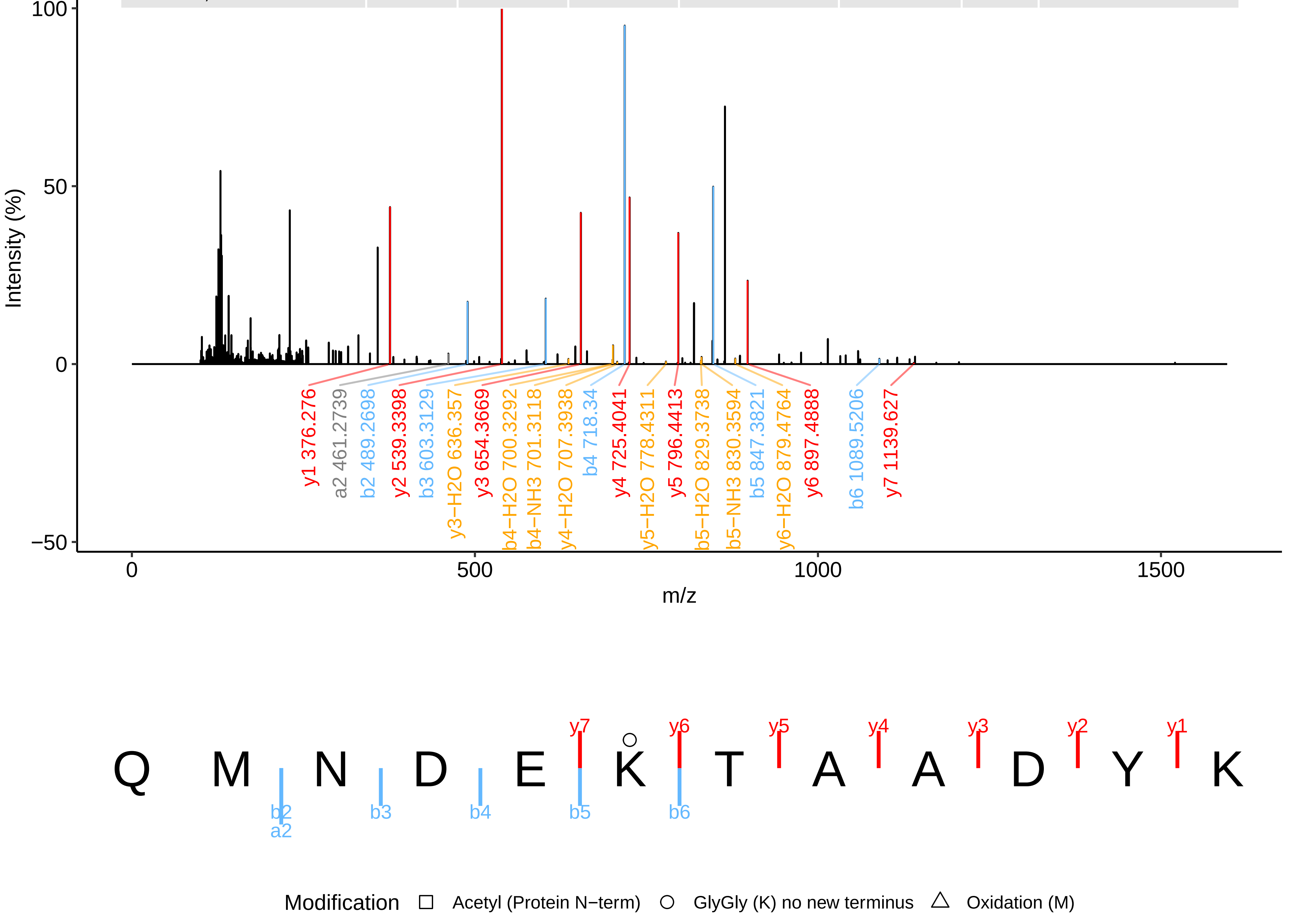

20201118_KIZ_MUb6_GlyGly_2 − Scan number: 8623

Gene Names	Charge	m/z	Mass	Retention time	PEP	Score	Precursor Intensity
NEDD8;NEDD8−MDP1	3	509.8963	1526.667	29.272	0.00065125	88.83	NA

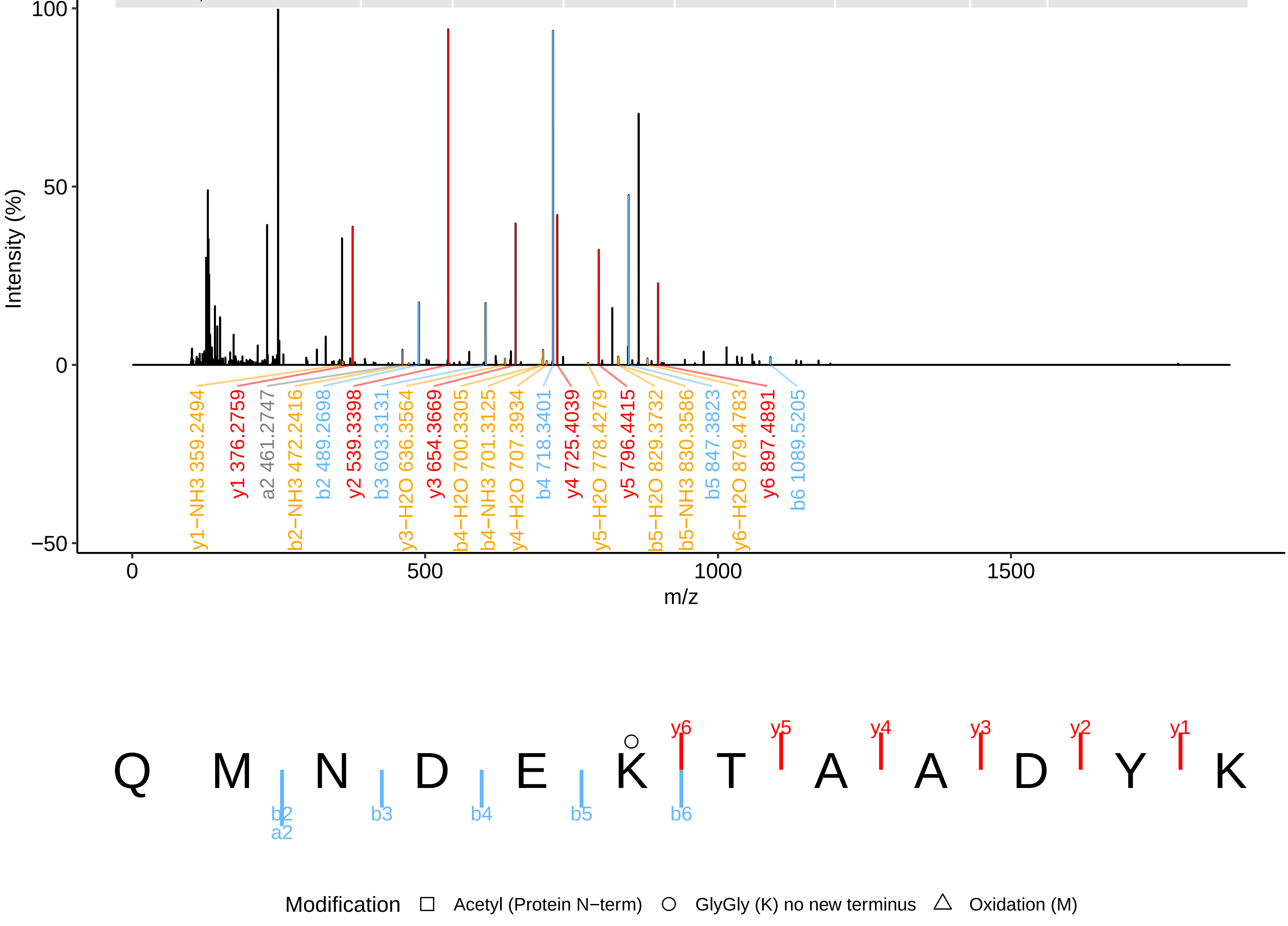

20201118_KIZ_MUb6_GlyGly_2 − Scan number: 8632

Gene Names	Charge	m/z	Mass	Retention time	PEP	Score	Precursor Intensity
NEDD8;NEDD8−MDP1	3	509.8963	1526.667	29.302	0.0011665	85.973	NA

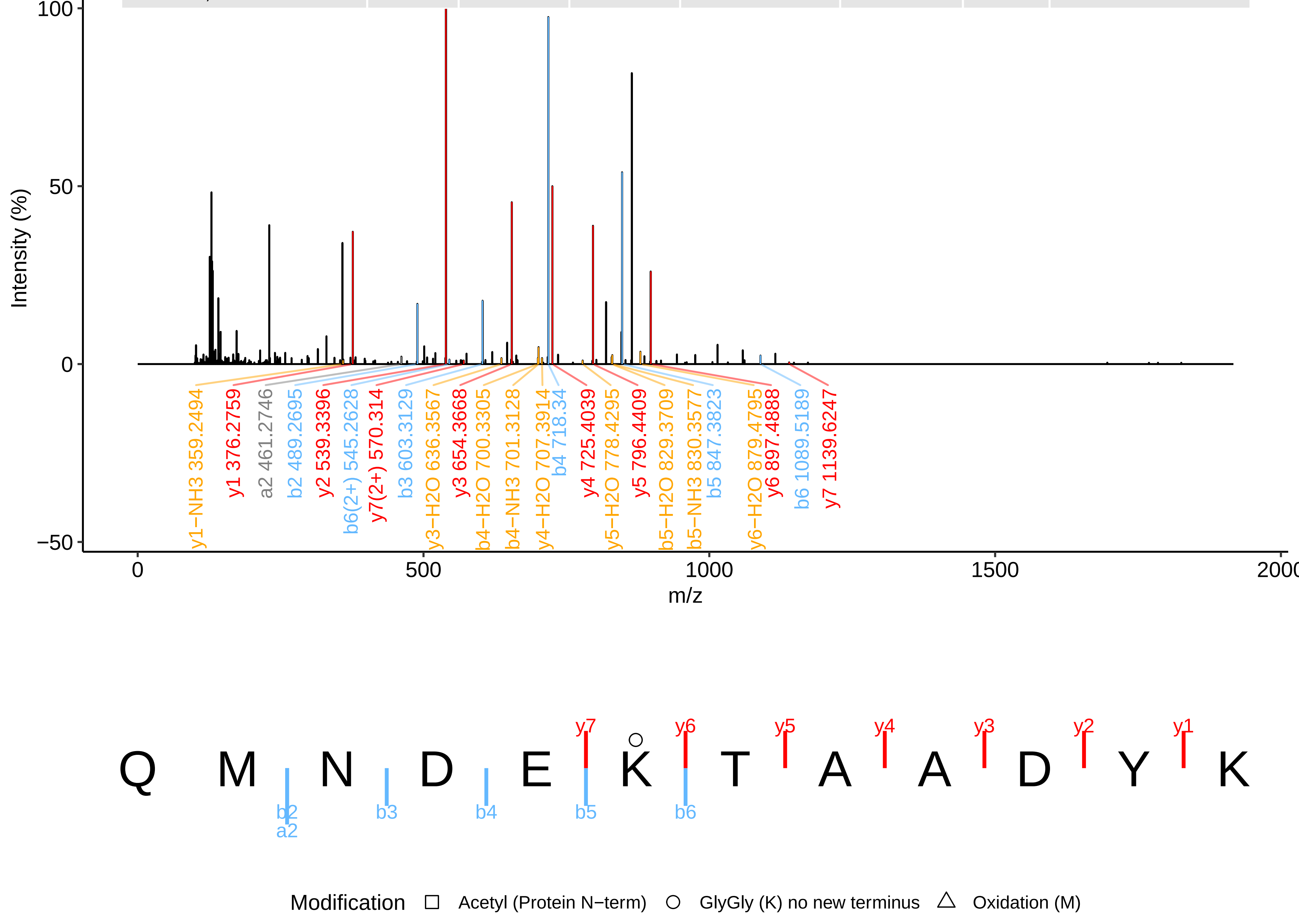

20200921_KIZ_MUb4_GlyGly_10plex − Scan number: 14663

Gene Names	Charge	m/z	Mass	Retention time	PEP	Score	Precursor Intensity
NDUFA8	3	532.2559	1593.746	52.157	1.8161e−05	100.19	22390560

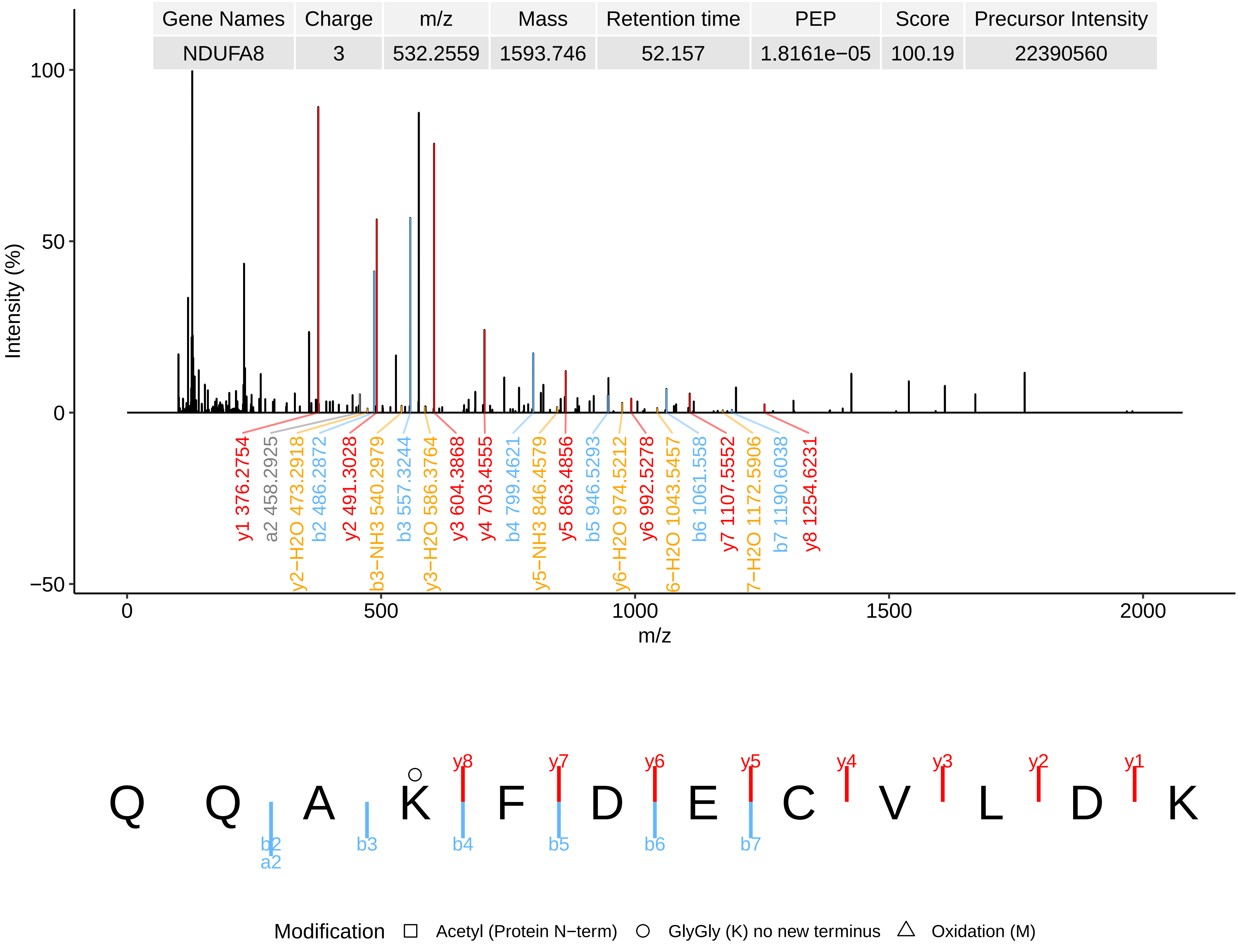

20200921_KIZ_MUb4_GlyGly_10plex − Scan number: 14669

Gene Names	Charge	m/z	Mass	Retention time	PEP	Score	Precursor Intensity
NDUFA8	3	532.2559	1593.746	52.175	0.0059797	73.466	27465456

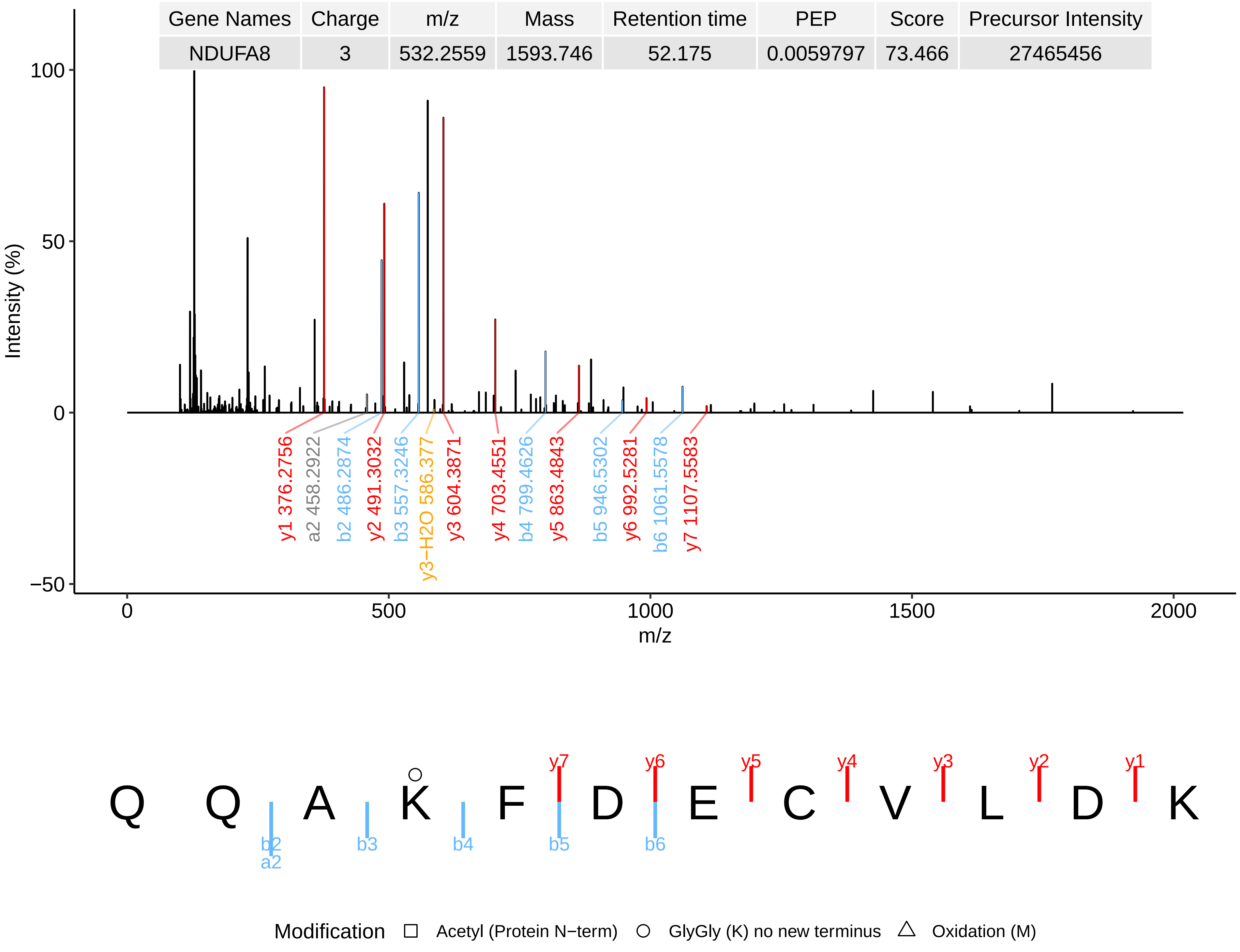

20200921_KIZ_MUb4_GlyGly_10plex − Scan number: 14801

Gene Names	Charge	m/z	Mass	Retention time	PEP	Score	Precursor Intensity
NDUFA8	3	532.2559	1593.746	52.687	1.3567e−05	104.04	17410610

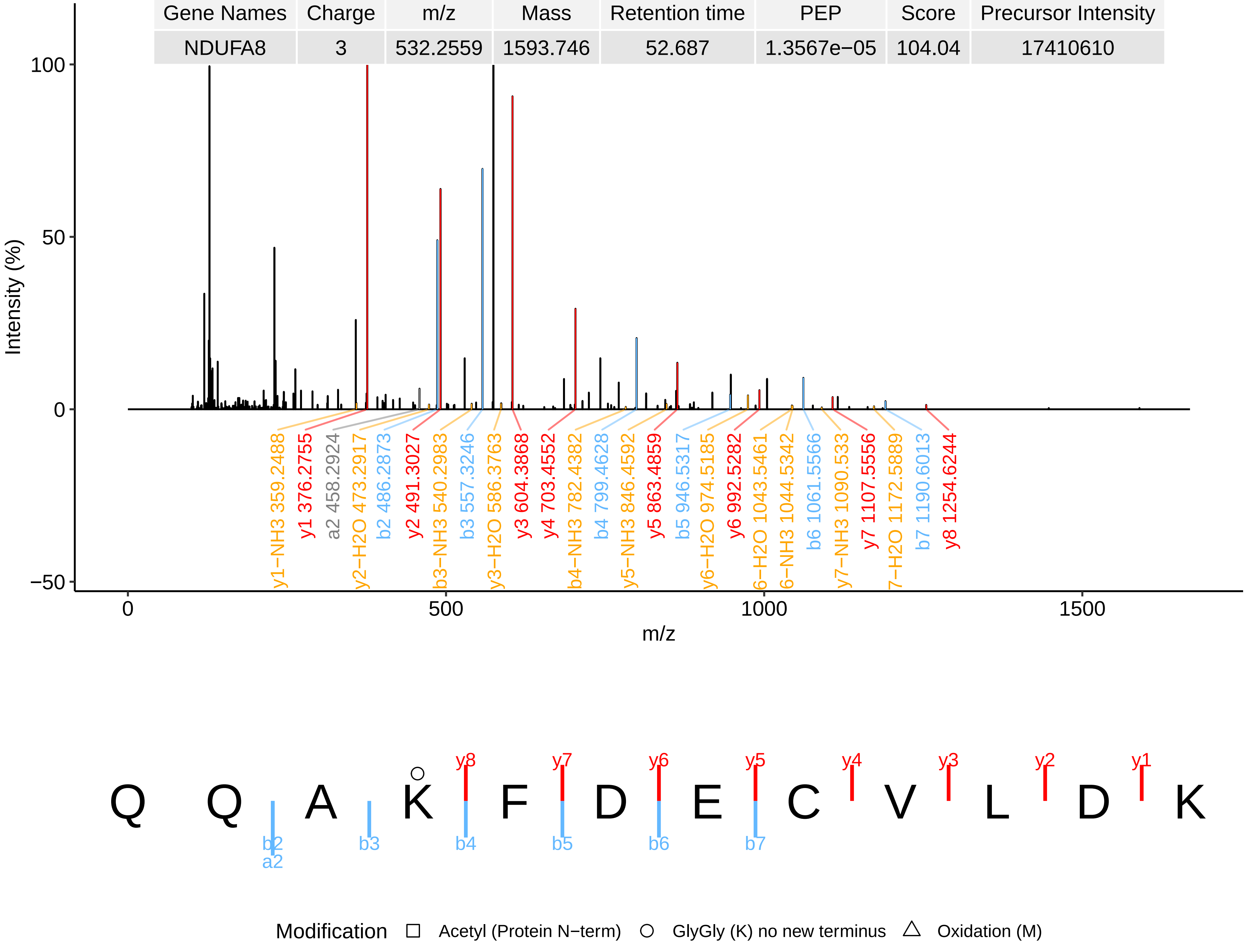

20200921_KIZ_MUb4_GlyGly_10plex − Scan number: 14731

Gene Names	Charge	m/z	Mass	Retention time	PEP	Score	Precursor Intensity
NDUFA8	2	797.8801	1593.746	52.405	3.4465e−19	203.11	NA

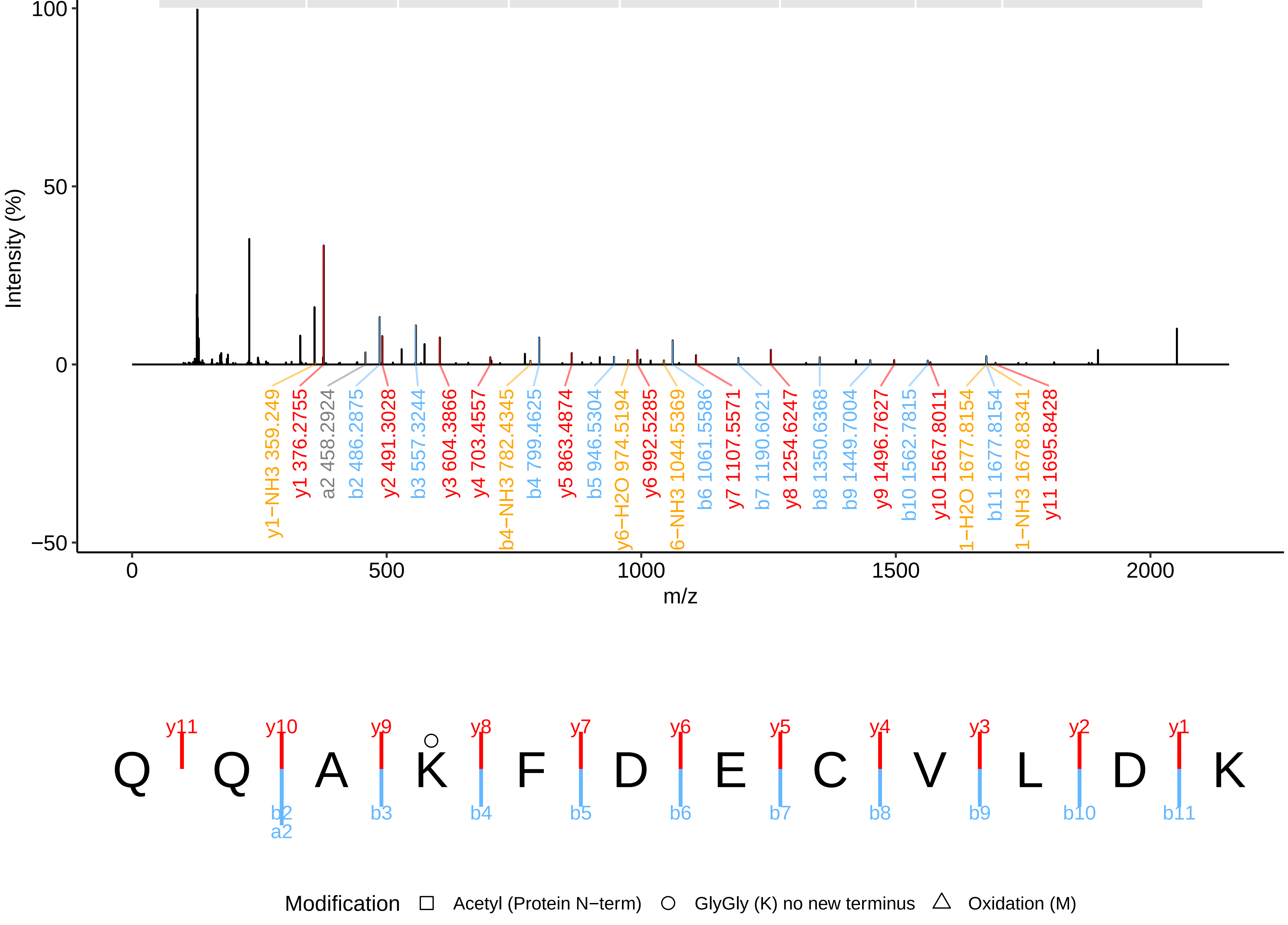

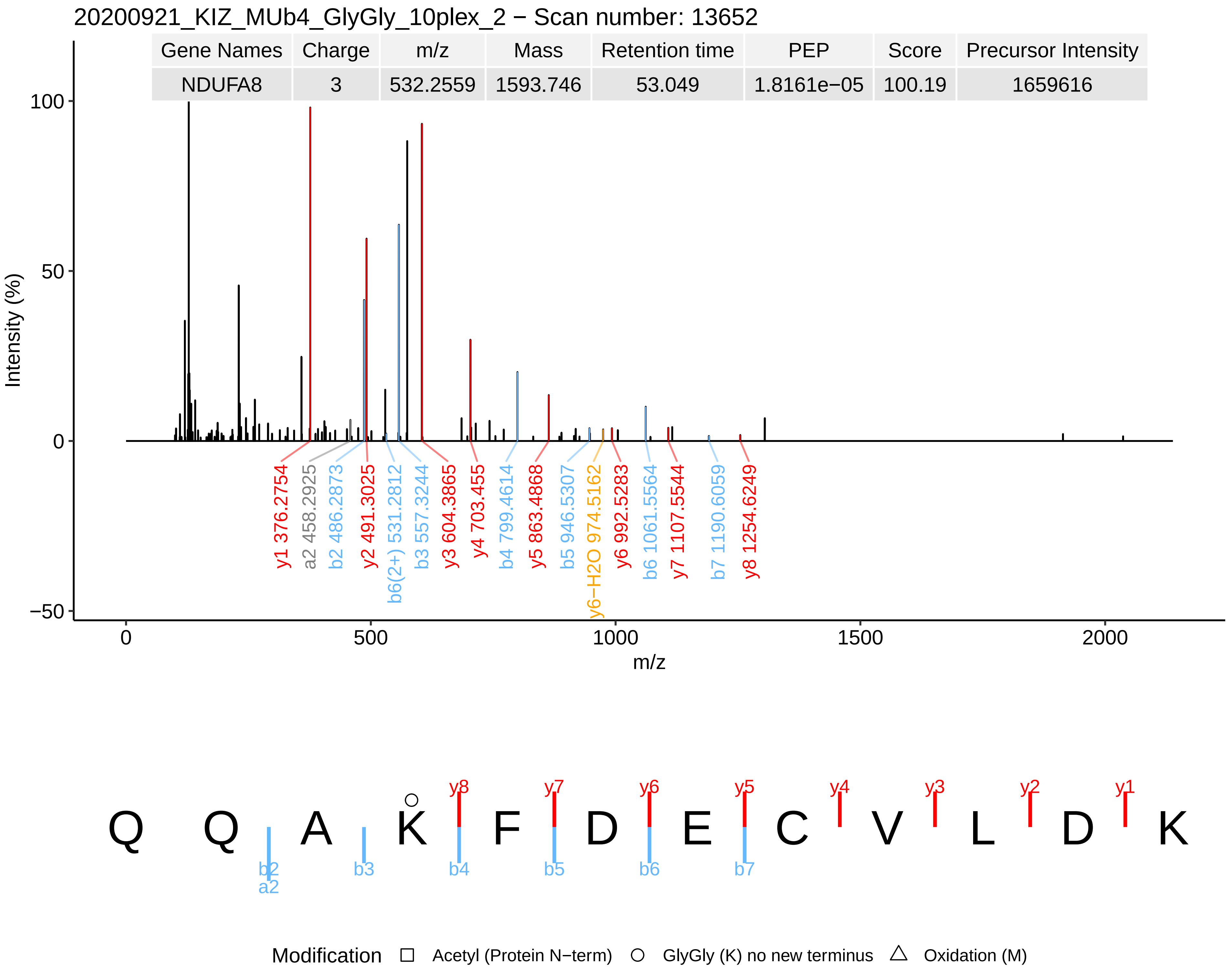

20200921_KIZ_MUb4_GlyGly_10plex_2 − Scan number: 13652
Gene Names | Charge | m/z | Mass | Retention time | PEP | Score | Precursor Intensity
NDUFA8 | 3 | 532.2559 | 1593.746 | 53.049 | 1.8161e−05 | 100.19 | 1659616
Intensity (%)
m/z
y1 376.2754
a2 458.2925
b2 486.2873
y2 491.3025
b6(2+) 531.2812
b3 557.3244
y3 604.3865
y4 703.455
b4 799.4614
y5 863.4868
b5 946.5307
y6−H2O 974.5162
y6 992.5283
b6 1061.5564
y7 1107.5544
b7 1190.6059
y8 1254.6249
Q Q A K F D E C V L D K
Modification
Acetyl (Protein N−term)
GlyGly (K) no new terminus
Oxidation (M)

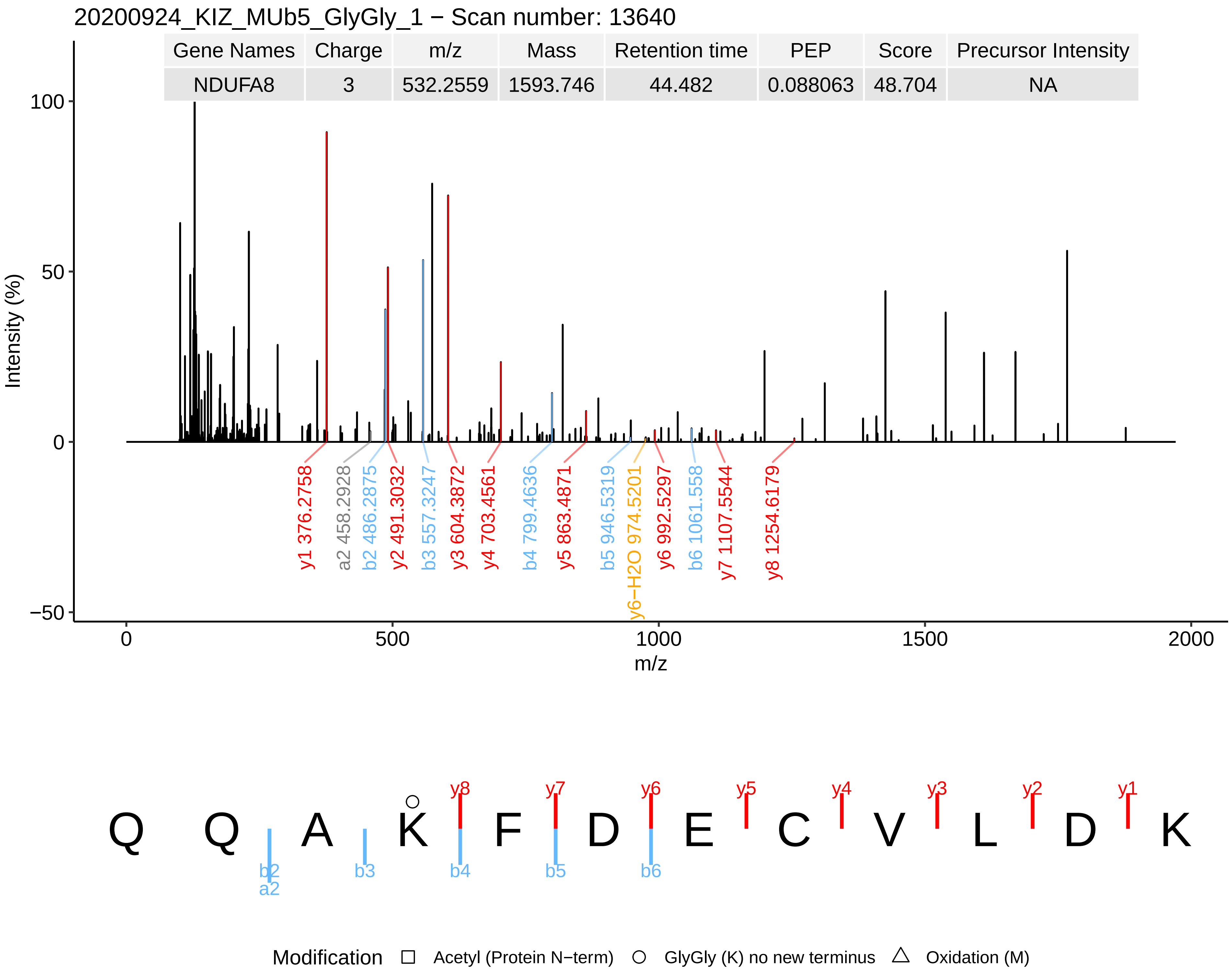
20200924_KIZ_MUb5_GlyGly_1 − Scan number: 13640
Gene Names | Charge | m/z | Mass | Retention time | PEP | Score | Precursor Intensity
NDUFA8 | 3 | 532.2559 | 1593.746 | 44.482 | 0.088063 | 48.704 | NA
Intensity (%)
100
50
0
−50
0
500
1000
1500
2000
m/z
y1 376.2758
a2 458.2928
b2 486.2875
y2 491.3032
b3 557.3247
y3 604.3872
y4 703.4561
b4 799.4636
y5 863.4871
b5 946.5319
y6−H2O 974.5201
y6 992.5297
b6 1061.558
y7 1107.5544
y8 1254.6179
Q Q A K F D E C V L D K
y8 y7 y6 y5 y4 y3 y2 y1
b2 a2 b3 b4 b5 b6
Modification
Acetyl (Protein N−term)
GlyGly (K) no new terminus
Oxidation (M)

20200924_KIZ_MUb5_GlyGly_1 − Scan number: 13648

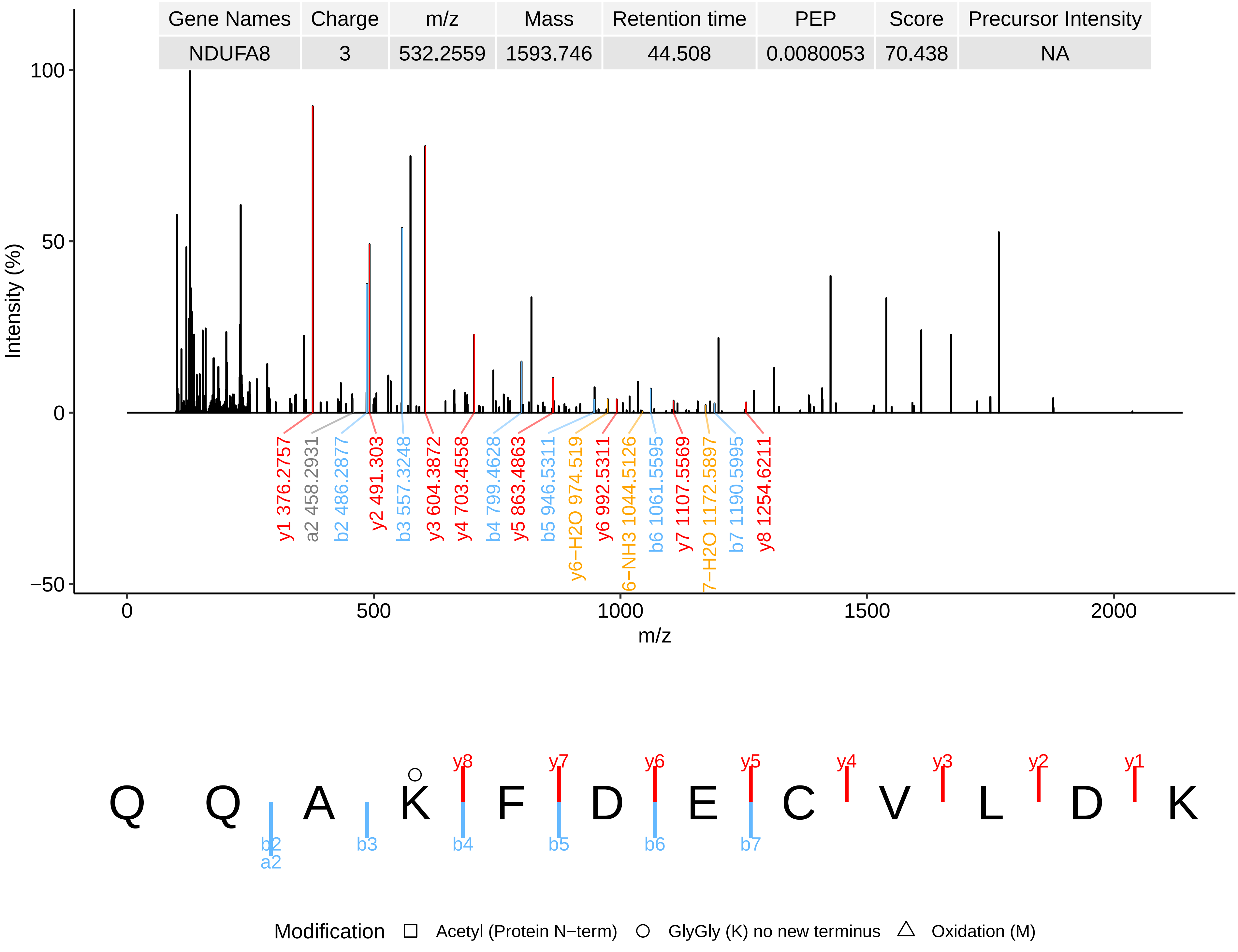

20200924_KIZ_MUb5_GlyGly_1 − Scan number: 13800

Gene Names	Charge	m/z	Mass	Retention time	PEP	Score	Precursor Intensity
NDUFA8	3	532.2559	1593.746	45.01	1.3919e−08	115.92	156637088

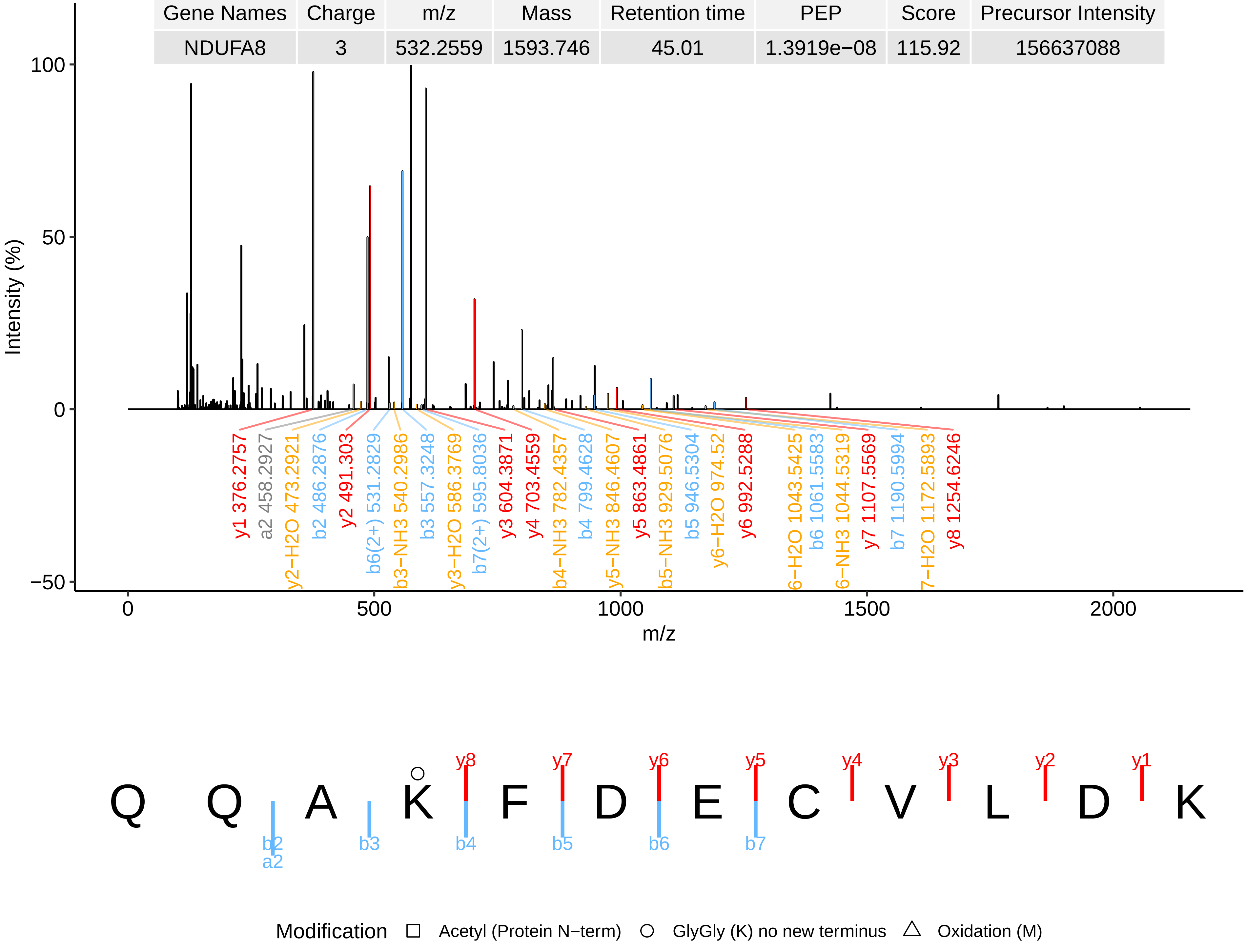

20200924_KIZ_MUb5_GlyGly_1 − Scan number: 13953

Gene Names	Charge	m/z	Mass	Retention time	PEP	Score	Precursor Intensity
NDUFA8	3	532.2559	1593.746	45.534	1.3919e−08	115.92	21693160

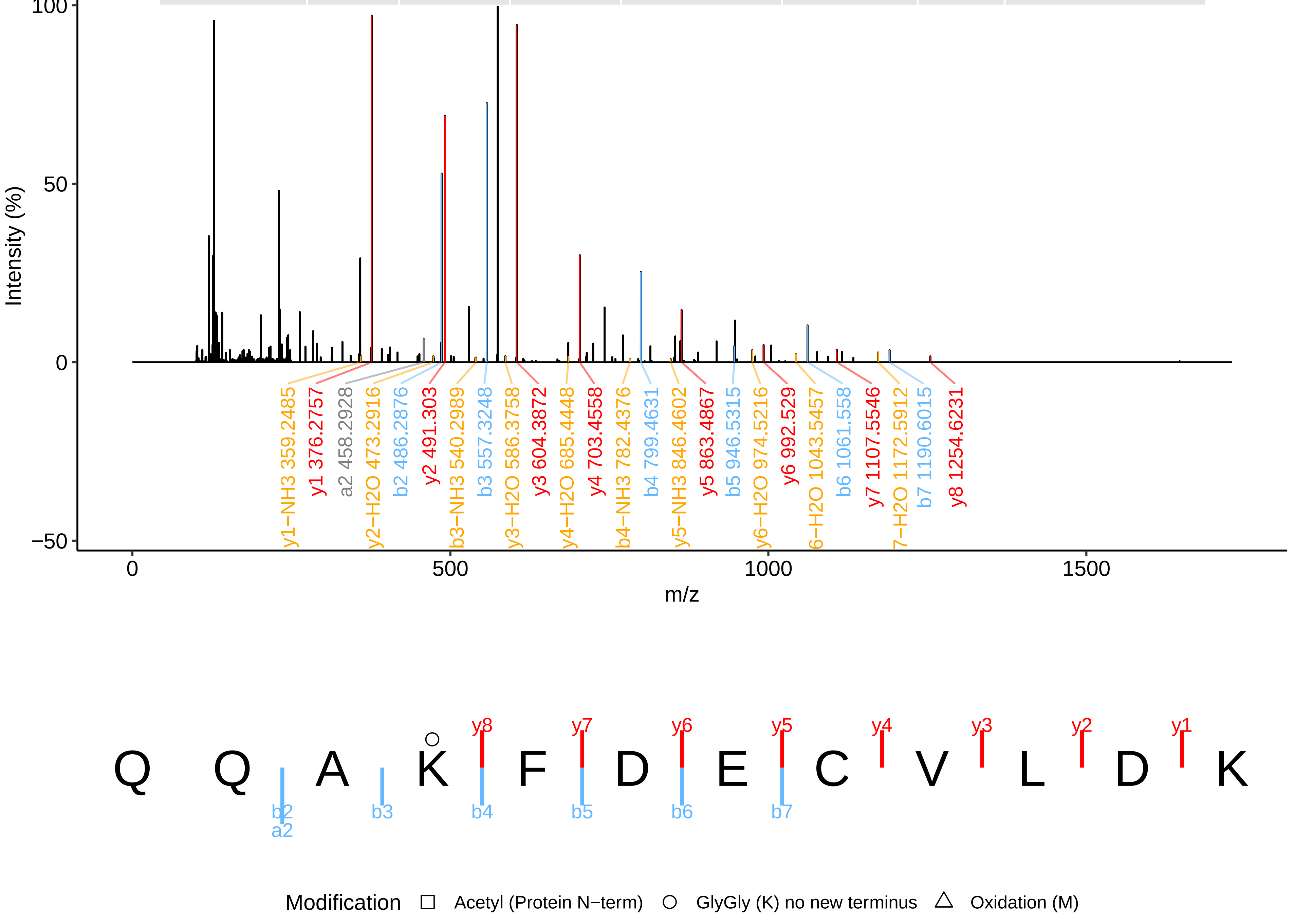

20200924_KIZ_MUb5_GlyGly_2 − Scan number: 12788

Gene Names	Charge	m/z	Mass	Retention time	PEP	Score	Precursor Intensity
NDUFA8	3	532.2559	1593.746	47.82	0.072357	51.726	3444075

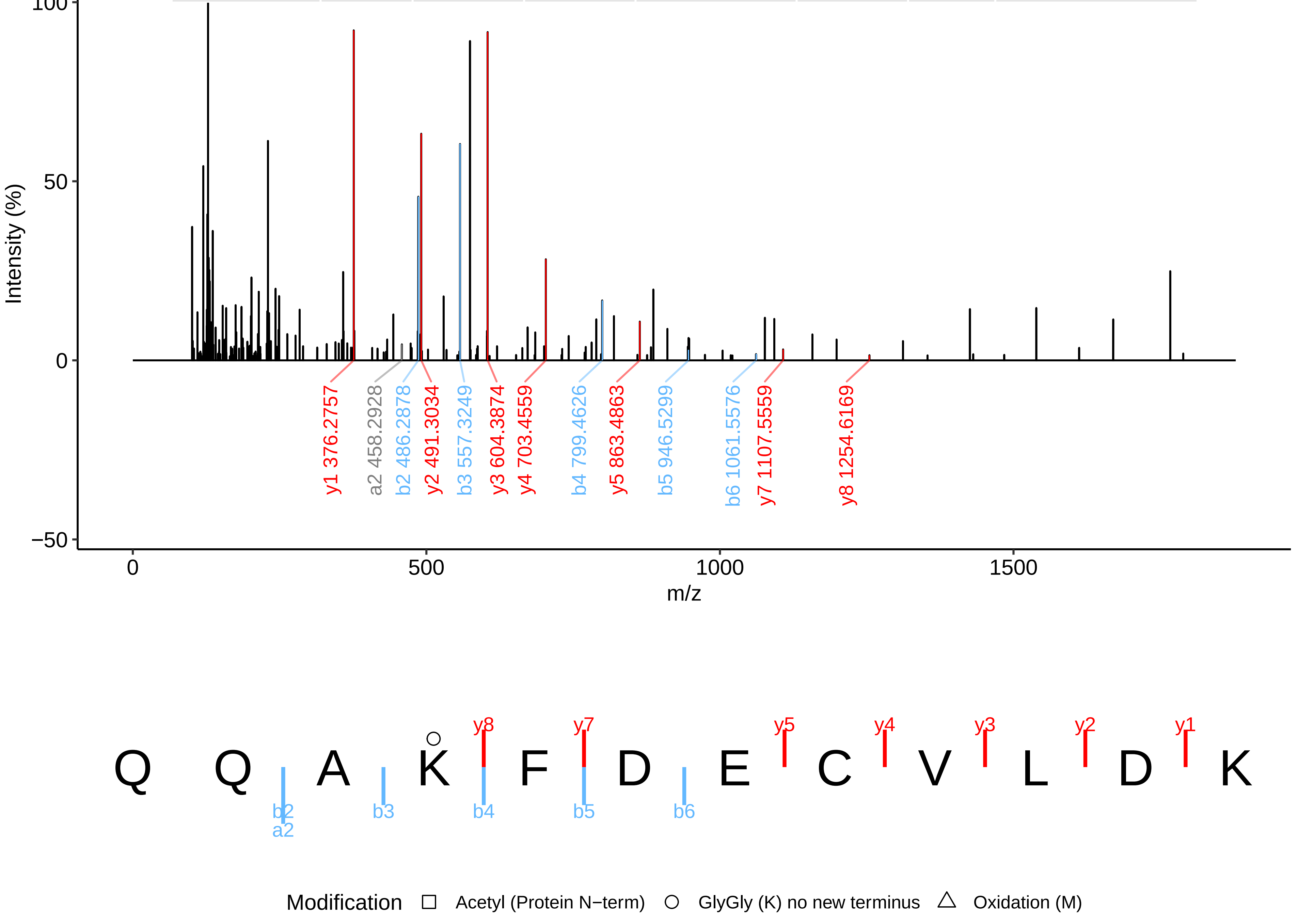

20200924_KIZ_MUb5_GlyGly_2 − Scan number: 12937

Gene Names	Charge	m/z	Mass	Retention time	PEP	Score	Precursor Intensity
NDUFA8	3	532.2559	1593.746	48.382	5.759e−05	97.463	22383868

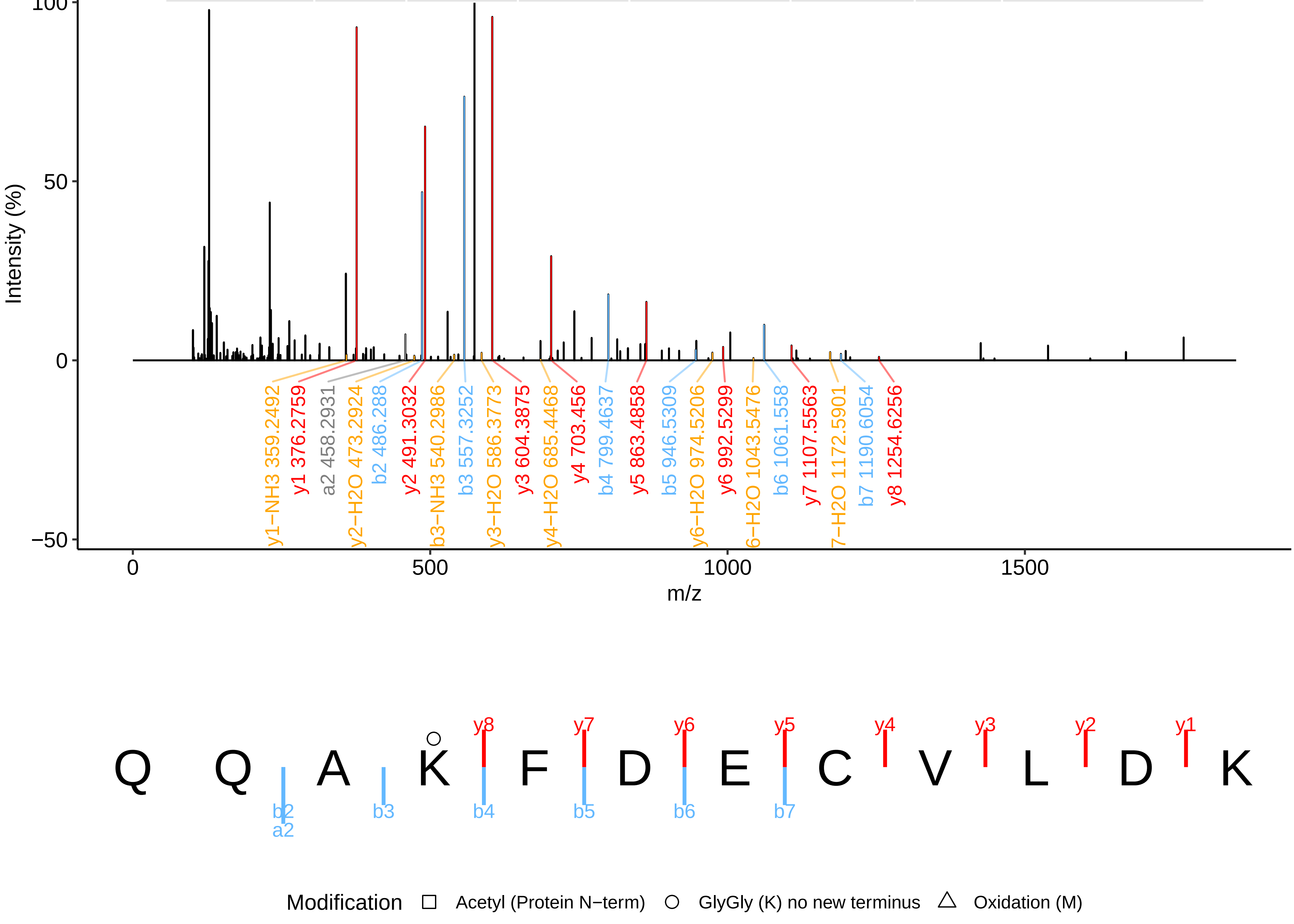

20200924_KIZ_MUb5_GlyGly_2 − Scan number: 13074

Gene Names	Charge	m/z	Mass	Retention time	PEP	Score	Precursor Intensity
NDUFA8	3	532.2559	1593.746	48.9	5.1265e−06	111.12	20852990

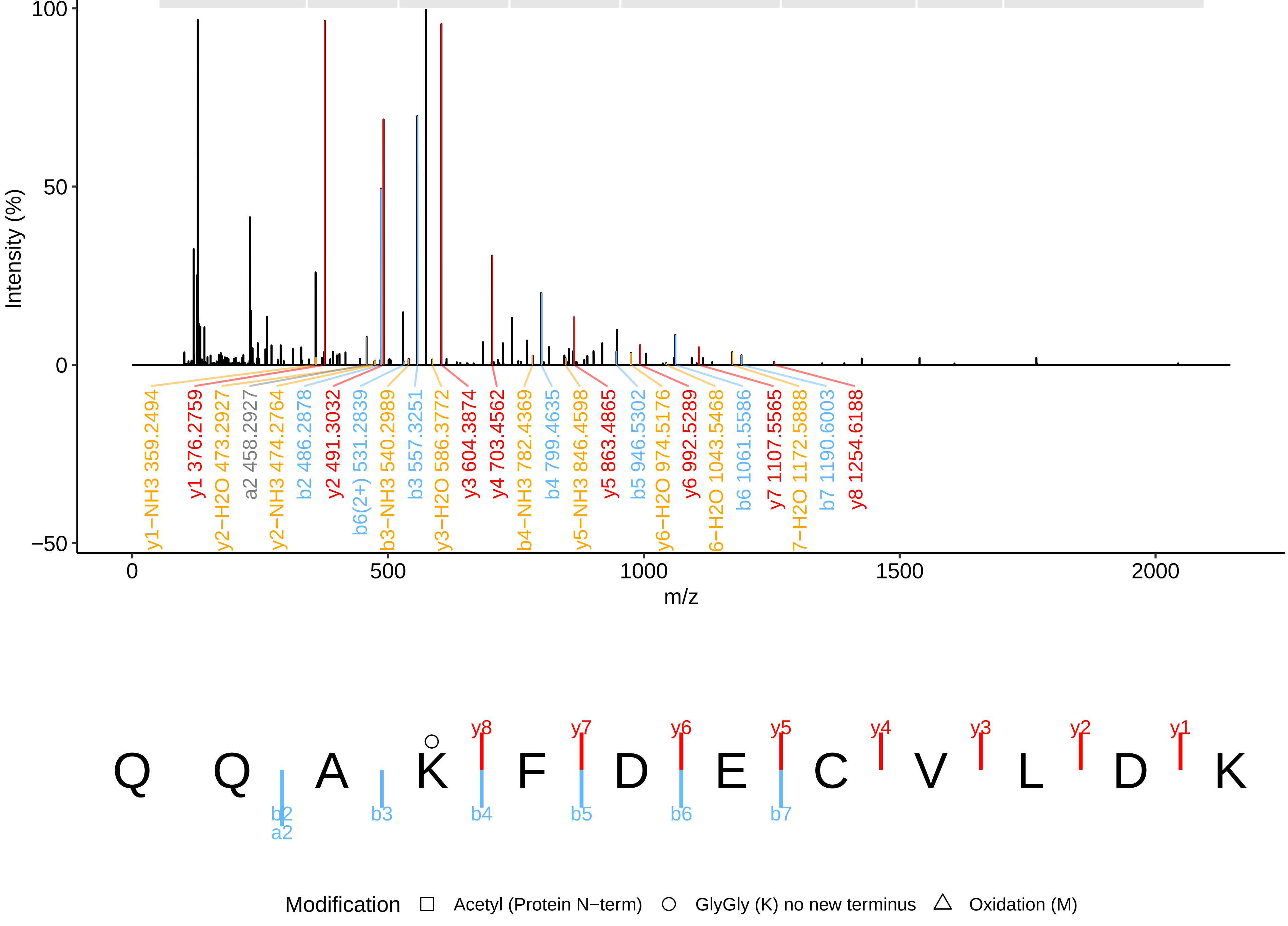

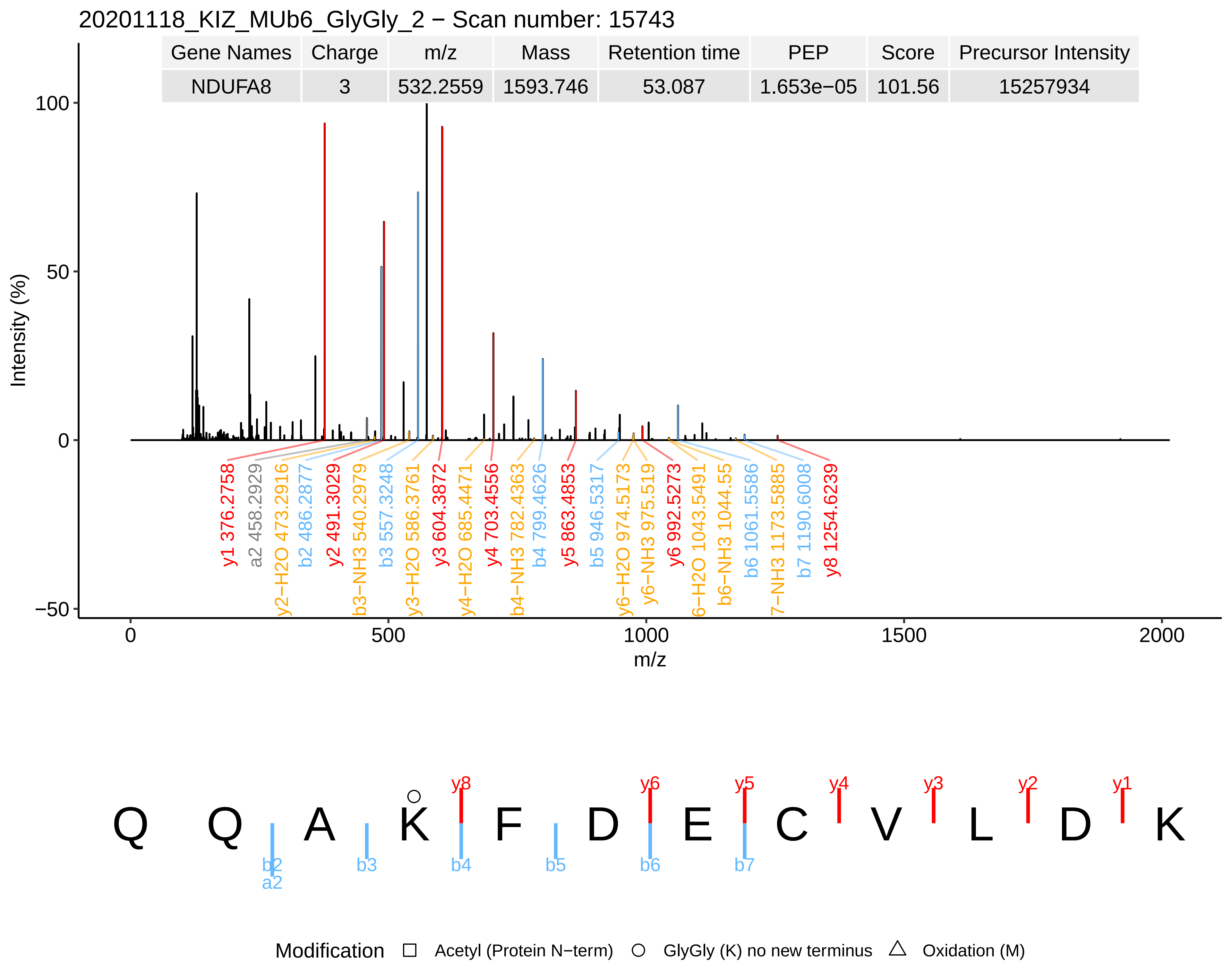
20201118_KIZ_MUb6_GlyGly_2 − Scan number: 15743
Gene Names | Charge | m/z | Mass | Retention time | PEP | Score | Precursor Intensity
NDUFA8 | 3 | 532.2559 | 1593.746 | 53.087 | 1.653e−05 | 101.56 | 15257934
Intensity (%)
m/z
y1 376.2758
a2 458.2929
y2−H2O 473.2916
b2 486.2877
y2 491.3029
b3−NH3 540.2979
b3 557.3248
y3−H2O 586.3761
y3 604.3872
y4−H2O 685.4471
y4 703.4556
b4−NH3 782.4363
b4 799.4626
y5 863.4853
b5 946.5317
y6−H2O 974.5173
y6−NH3 975.519
y6 992.5273
6−H2O 1043.5491
b6−NH3 1044.55
b6 1061.5586
7−NH3 1173.5885
b7 1190.6008
y8 1254.6239
Q Q A K F D E C V L D K
y8 y6 y5 y4 y3 y2 y1
b2 a2 b3 b4 b5 b6 b7
Modification
Acetyl (Protein N−term)
GlyGly (K) no new terminus
Oxidation (M)

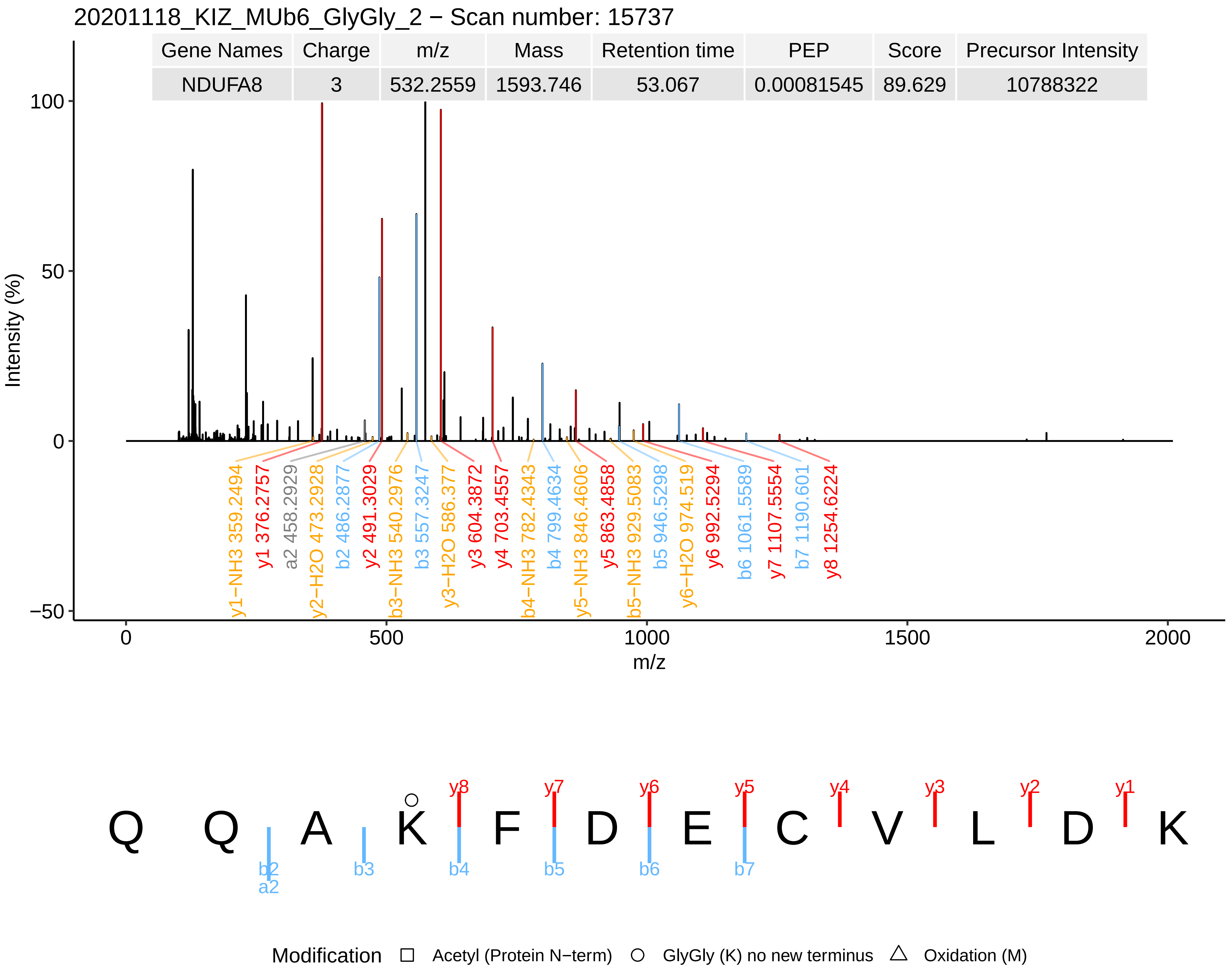
20201118_KIZ_MUb6_GlyGly_2 − Scan number: 15737
Gene Names | Charge | m/z | Mass | Retention time | PEP | Score | Precursor Intensity
NDUFA8 | 3 | 532.2559 | 1593.746 | 53.067 | 0.00081545 | 89.629 | 10788322
Intensity (%)
100
50
0
−50
0
500
1000
1500
2000
m/z
y1−NH3 359.2494
y1 376.2757
a2 458.2929
y2−H2O 473.2928
b2 486.2877
y2 491.3029
b3−NH3 540.2976
b3 557.3247
y3−H2O 586.377
y3 604.3872
y4 703.4557
b4−NH3 782.4343
b4 799.4634
y5−NH3 846.4606
y5 863.4858
b5−NH3 929.5083
b5 946.5298
y6−H2O 974.519
y6 992.5294
b6 1061.5589
y7 1107.5554
b7 1190.601
y8 1254.6224
Q Q A K F D E C V L D K
y8 y7 y6 y5 y4 y3 y2 y1
b2 a2 b3 b4 b5 b6 b7
Modification
Acetyl (Protein N−term)
GlyGly (K) no new terminus
Oxidation (M)

20200921_KIZ_MUb4_GlyGly_10plex − Scan number: 9940

Gene Names	Charge	m/z	Mass	Retention time	PEP	Score	Precursor Intensity
SCAMP2	4	457.9841	1827.908	34.225	1.4447e−12	140.27	6553393

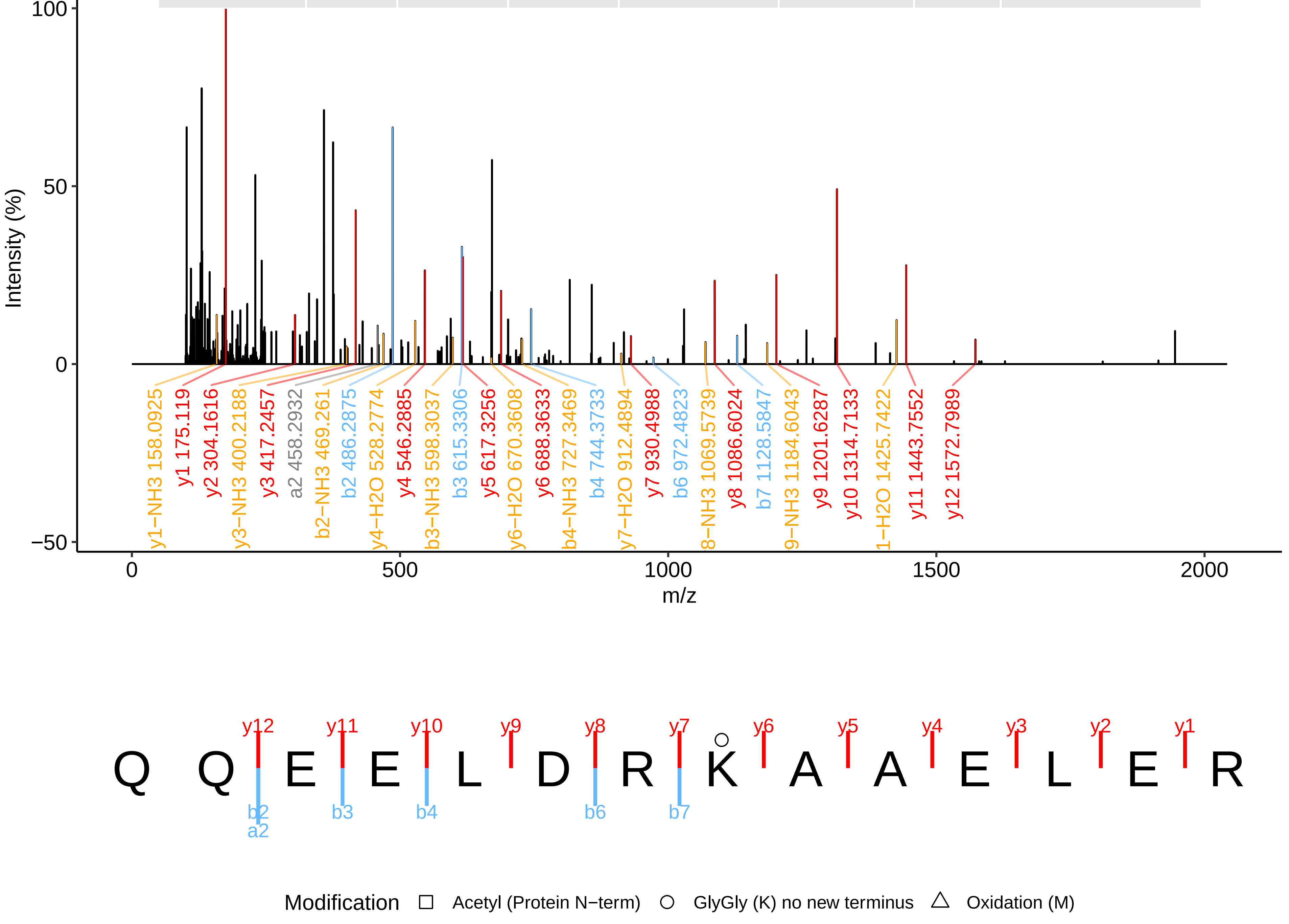

20200921_KIZ_MUb4_GlyGly_10plex − Scan number: 9974

Gene Names	Charge	m/z	Mass	Retention time	PEP	Score	Precursor Intensity
SCAMP2	3	610.3098	1827.908	34.358	0.00021676	96.163	2026808

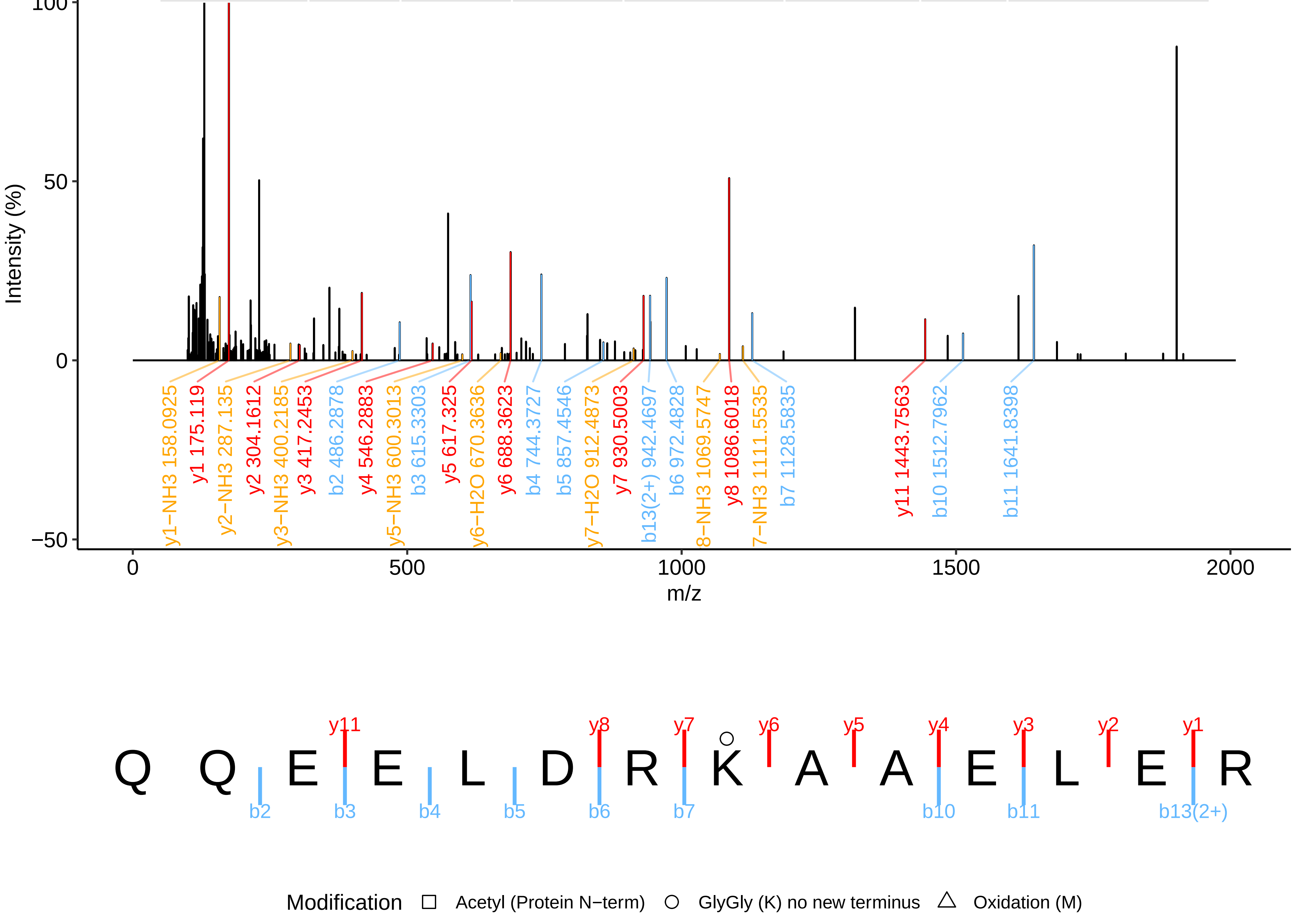

20200921_KIZ_MUb4_GlyGly_10plex_2 − Scan number: 9164

Gene Names	Charge	m/z	Mass	Retention time	PEP	Score	Precursor Intensity
SCAMP2	4	457.9841	1827.908	34.762	5.4761e−05	108.58	1025448

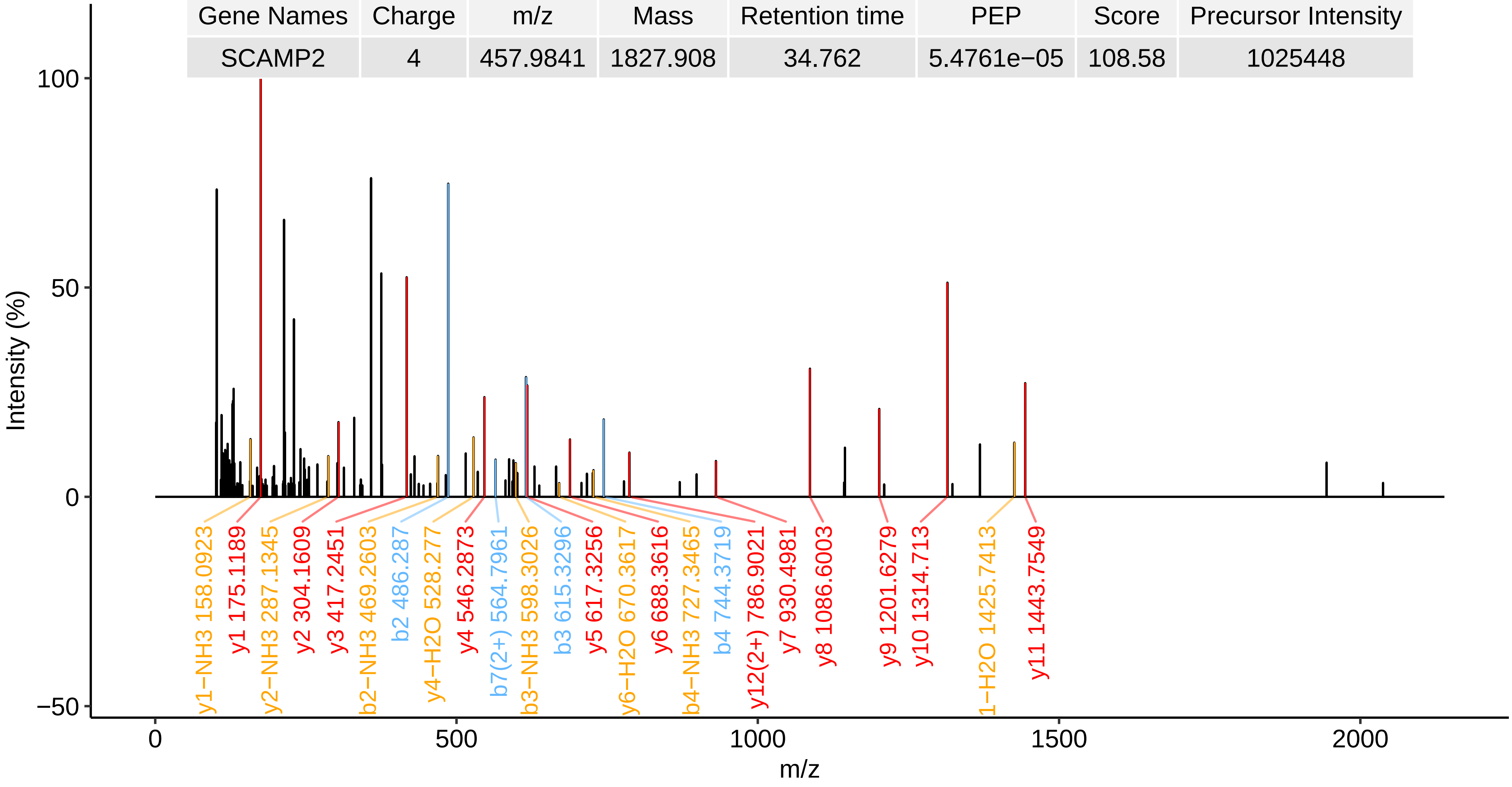

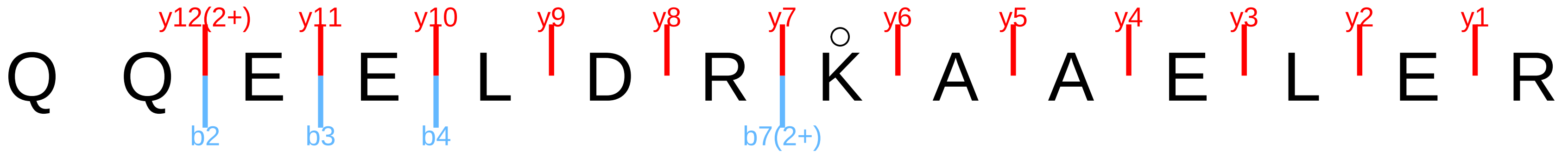

Modification □ Acetyl (Protein N−term) ○ GlyGly (K) no new terminus △ Oxidation (M)

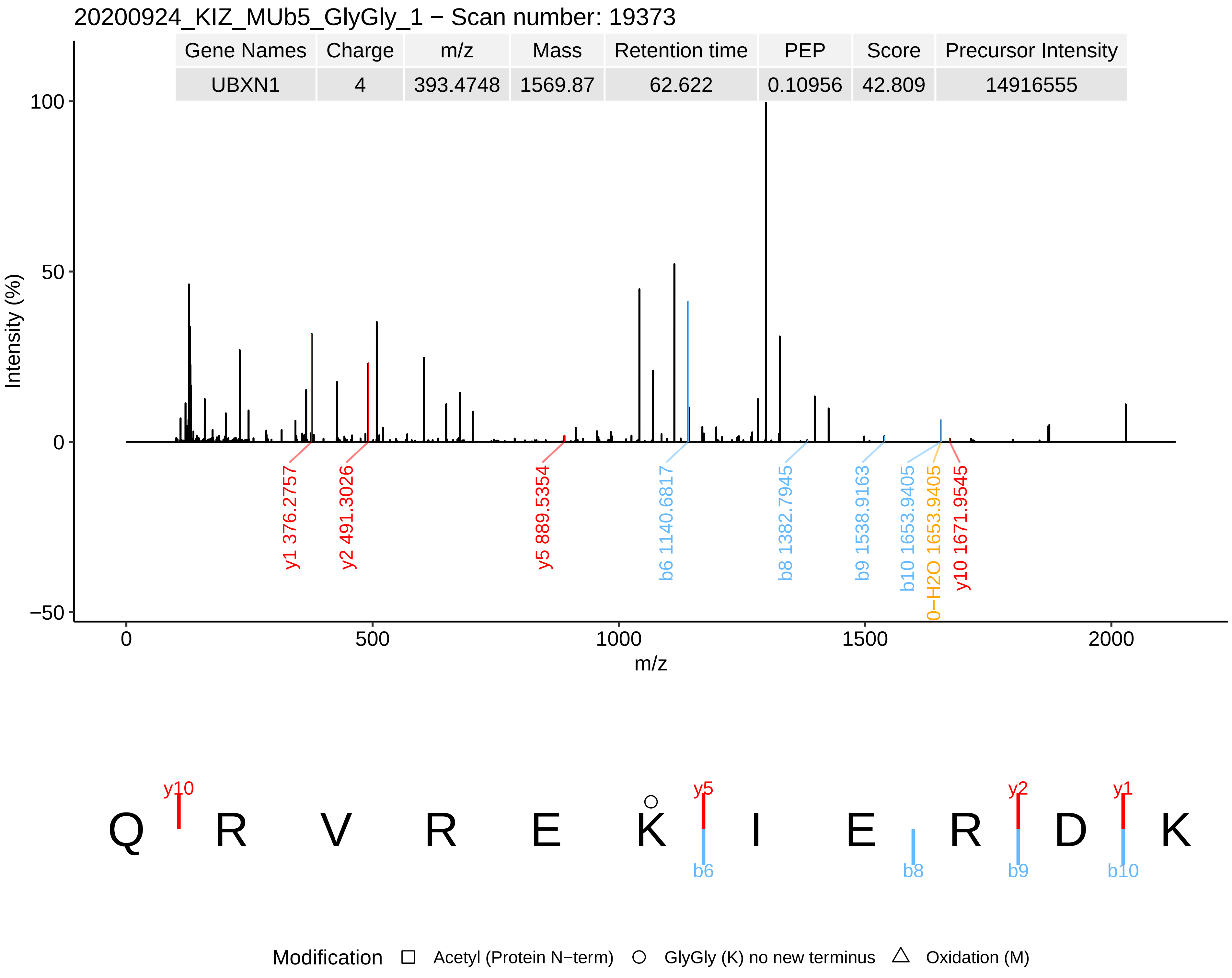
20200924_KIZ_MUb5_GlyGly_1 − Scan number: 19373
Gene Names | Charge | m/z | Mass | Retention time | PEP | Score | Precursor Intensity
UBXN1 | 4 | 393.4748 | 1569.87 | 62.622 | 0.10956 | 42.809 | 14916555
Intensity (%)
m/z
y1 376.2757
y2 491.3026
y5 889.5354
b6 1140.6817
b8 1382.7945
b9 1538.9163
b10 1653.9405
0−H2O 1653.9405
y10 1671.9545
Q R V R E K I E R D K
y10 y5 y2 y1
b6 b8 b9 b10
Modification
Acetyl (Protein N−term)
GlyGly (K) no new terminus
Oxidation (M)

20200924_KIZ_MUb5_GlyGly_1 − Scan number: 19709

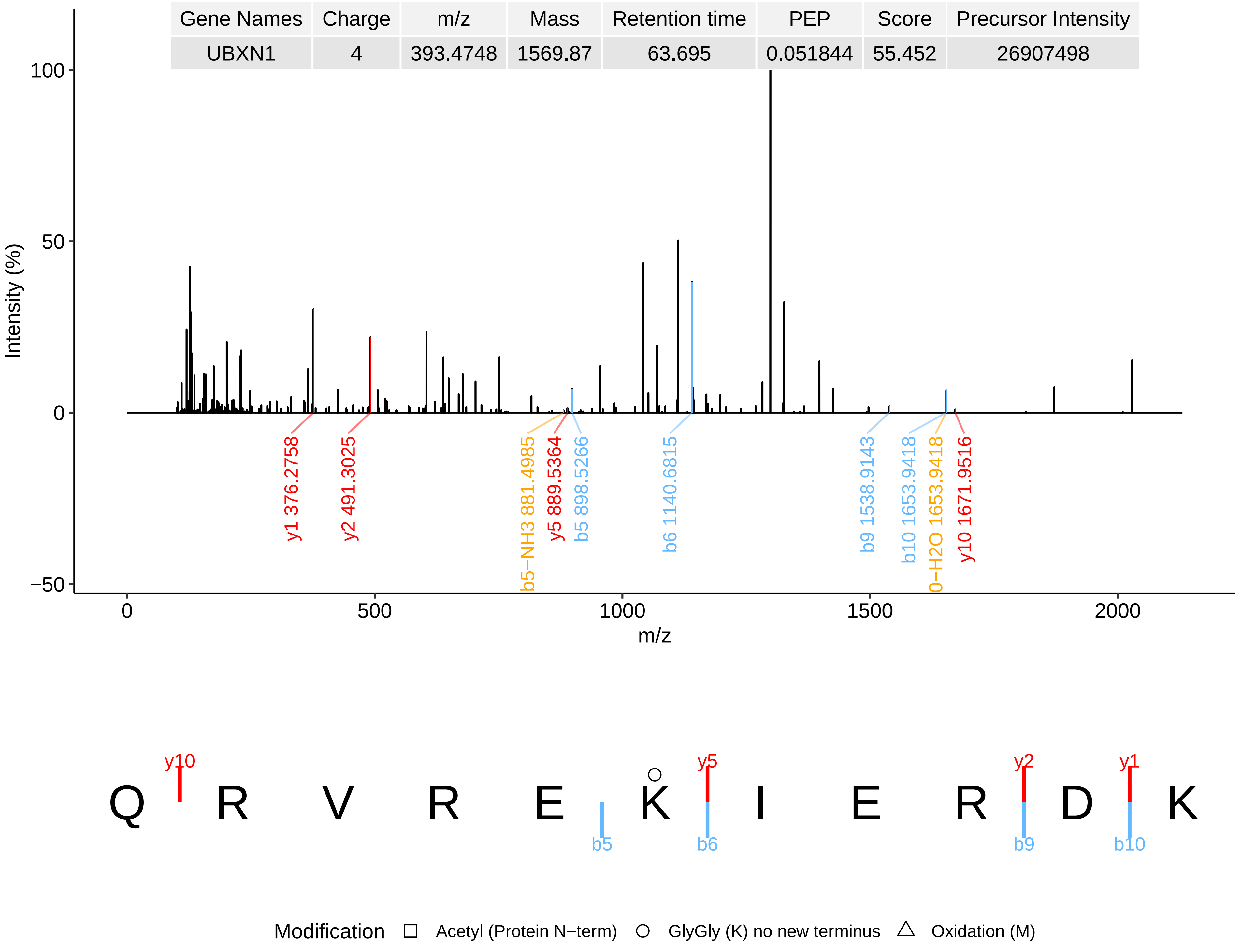

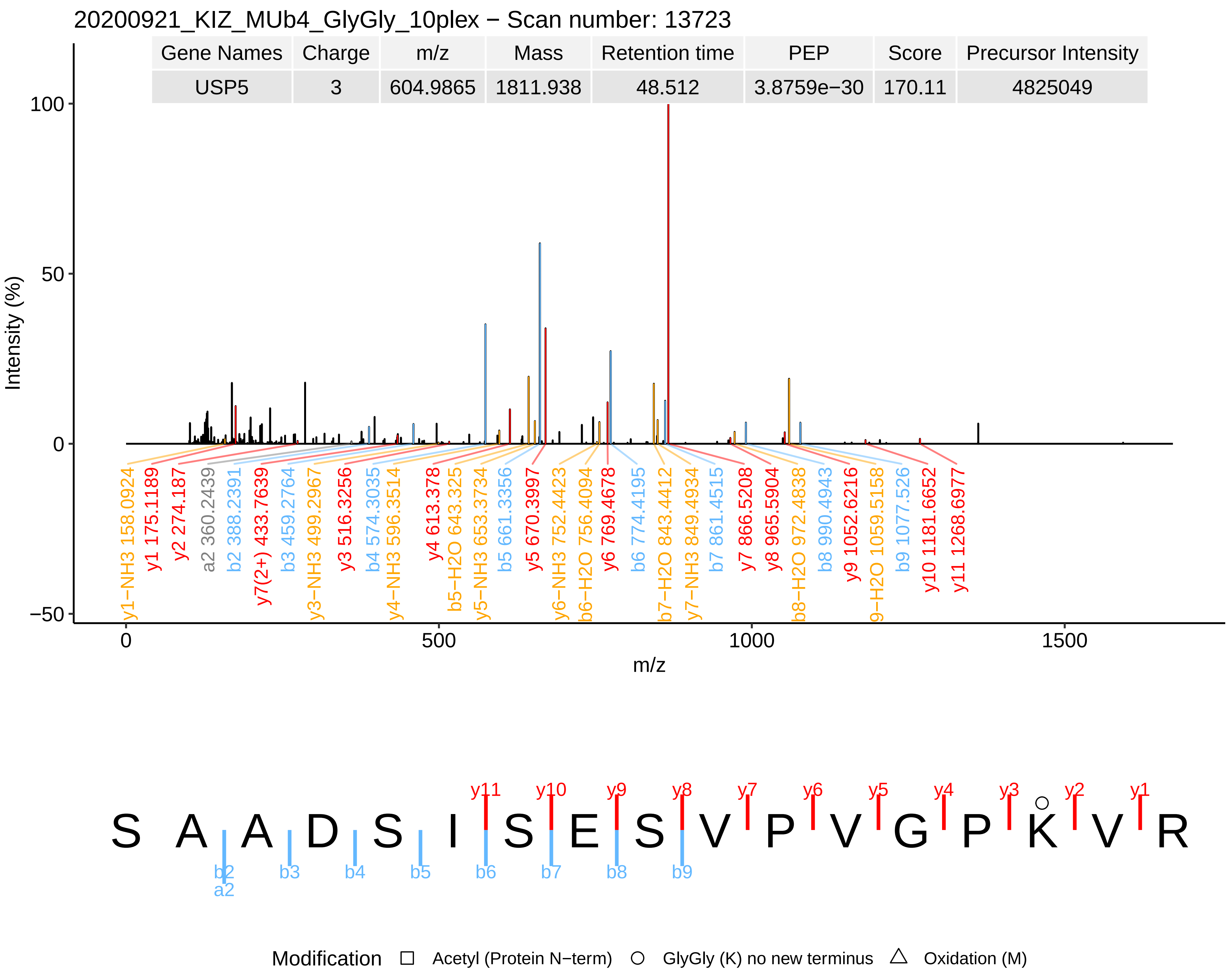
20200921_KIZ_MUb4_GlyGly_10plex − Scan number: 13723
Gene Names | Charge | m/z | Mass | Retention time | PEP | Score | Precursor Intensity
USP5 | 3 | 604.9865 | 1811.938 | 48.512 | 3.8759e−30 | 170.11 | 4825049
Intensity (%)
m/z
S A A D S I S E S V P V G P K V R
Modification
Acetyl (Protein N−term)
GlyGly (K) no new terminus
Oxidation (M)

20200921_KIZ_MUb4_GlyGly_10plex_2 − Scan number: 12575

Gene Names	Charge	m/z	Mass	Retention time	PEP	Score	Precursor Intensity
USP5	3	604.9865	1811.938	48.678	8.5473e−17	136.33	NA

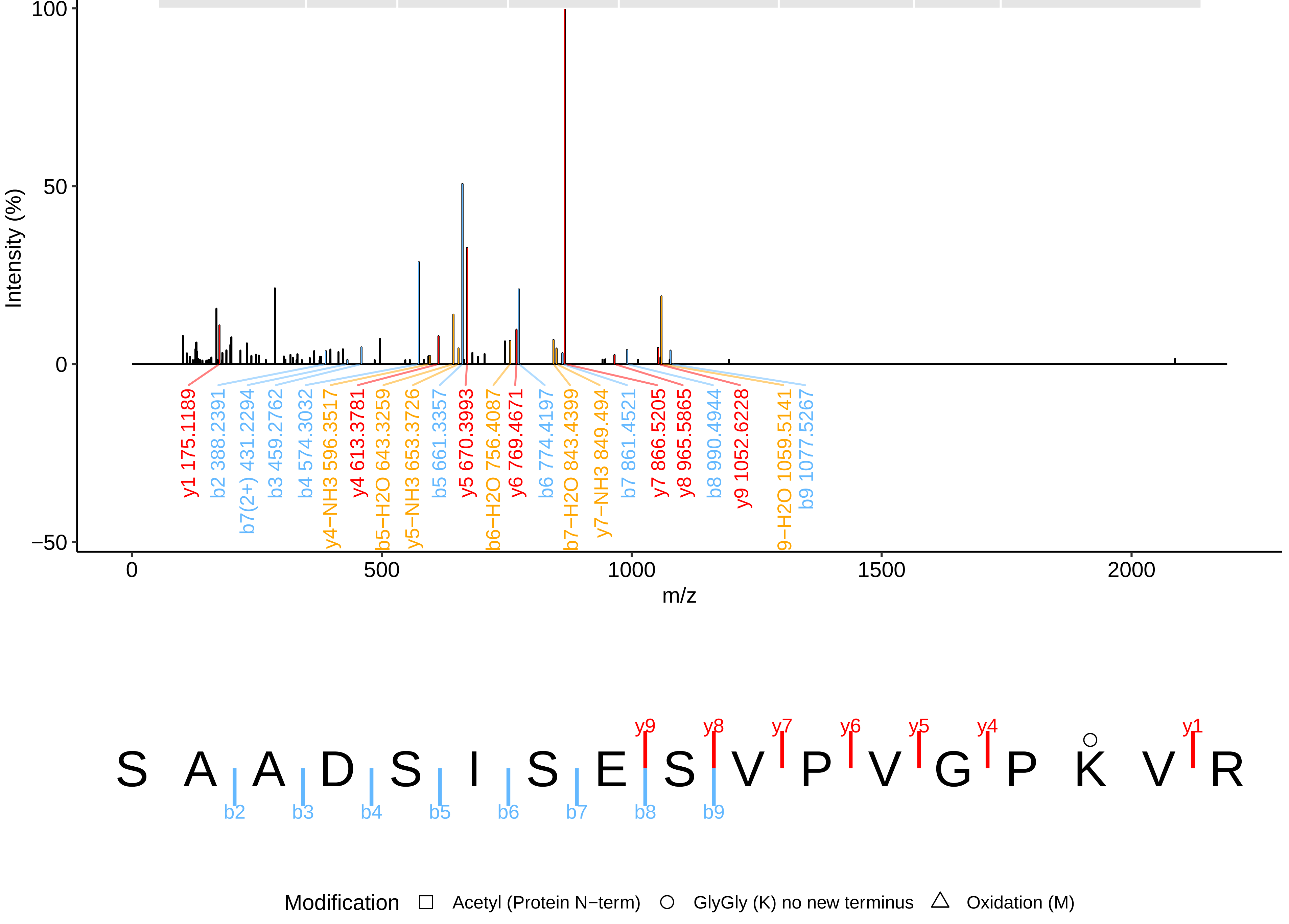

20200924_KIZ_MUb5_GlyGly_1 − Scan number: 12978

Gene Names	Charge	m/z	Mass	Retention time	PEP	Score	Precursor Intensity
USP5	3	604.9865	1811.938	42.31	0.000485	77.42	8257964

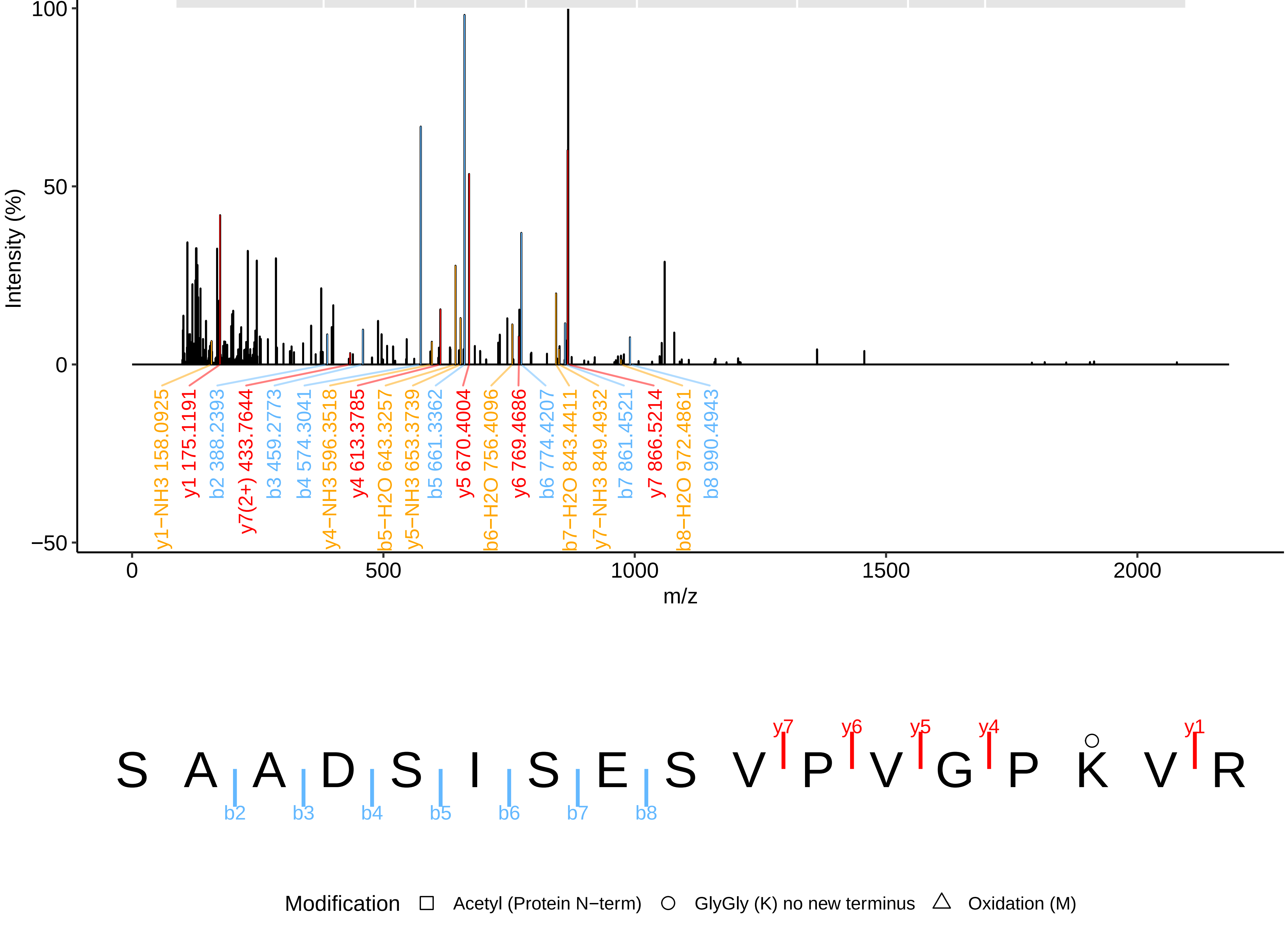

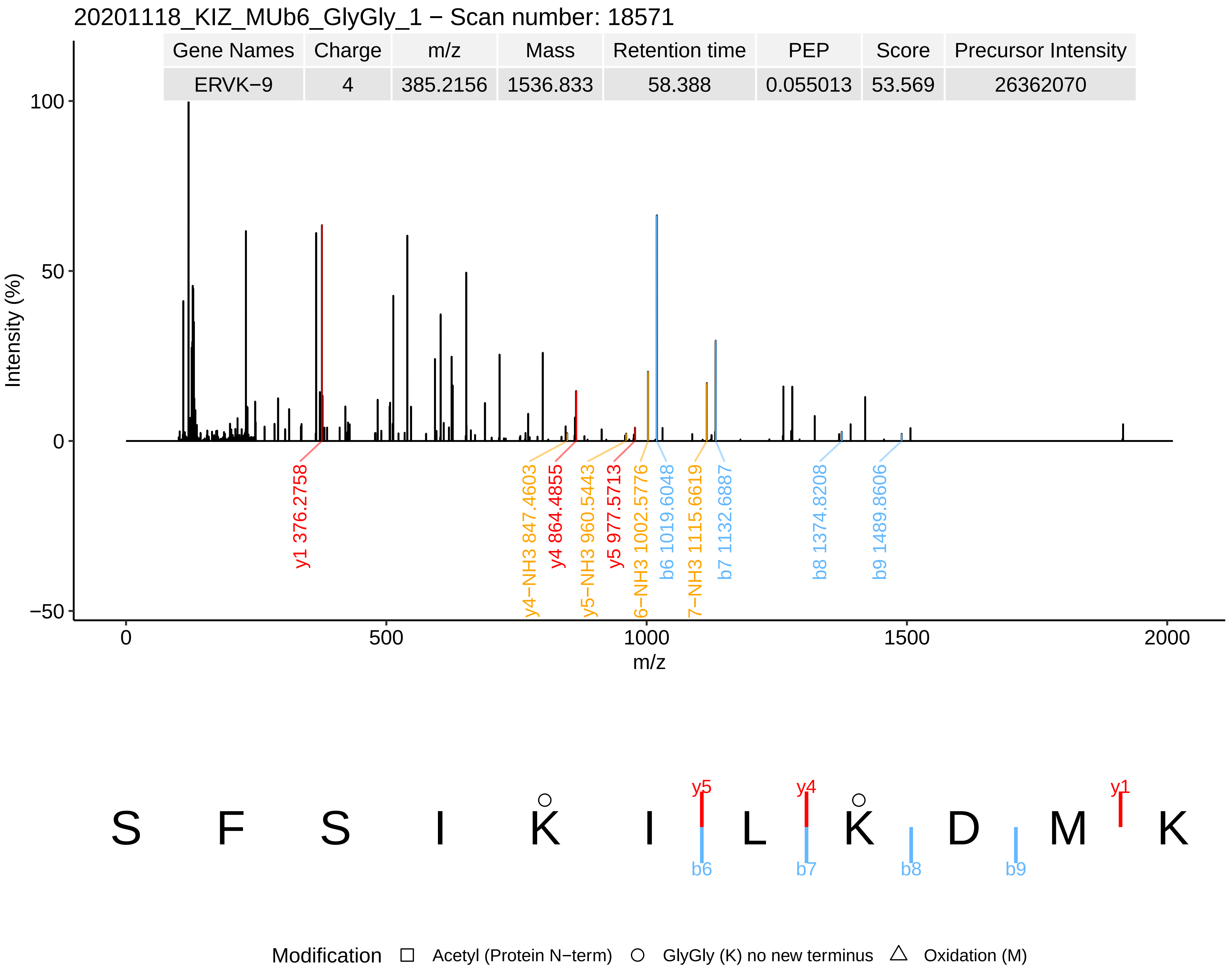
20201118_KIZ_MUb6_GlyGly_1 − Scan number: 18571
Gene Names | Charge | m/z | Mass | Retention time | PEP | Score | Precursor Intensity
ERVK−9 | 4 | 385.2156 | 1536.833 | 58.388 | 0.055013 | 53.569 | 26362070
Intensity (%)
100
50
0
−50
0
500
1000
1500
2000
m/z
y1 376.2758
y4−NH3 847.4603
y4 864.4855
y5−NH3 960.5443
y5 977.5713
b6−NH3 1002.5776
b6 1019.6048
b7−NH3 1115.6619
b7 1132.6887
b8 1374.8208
b9 1489.8606
S F S I K I L K D M K
y5
y4
y1
b6
b7
b8
b9
Modification
Acetyl (Protein N−term)
GlyGly (K) no new terminus
Oxidation (M)

20200921_KIZ_MUb4_GlyGly_10plex − Scan number: 9873

Gene Names	Charge	m/z	Mass	Retention time	PEP	Score	Precursor Intensity
ERH	3	663.3727	1987.096	33.965	1.9634e−14	87.566	3935575

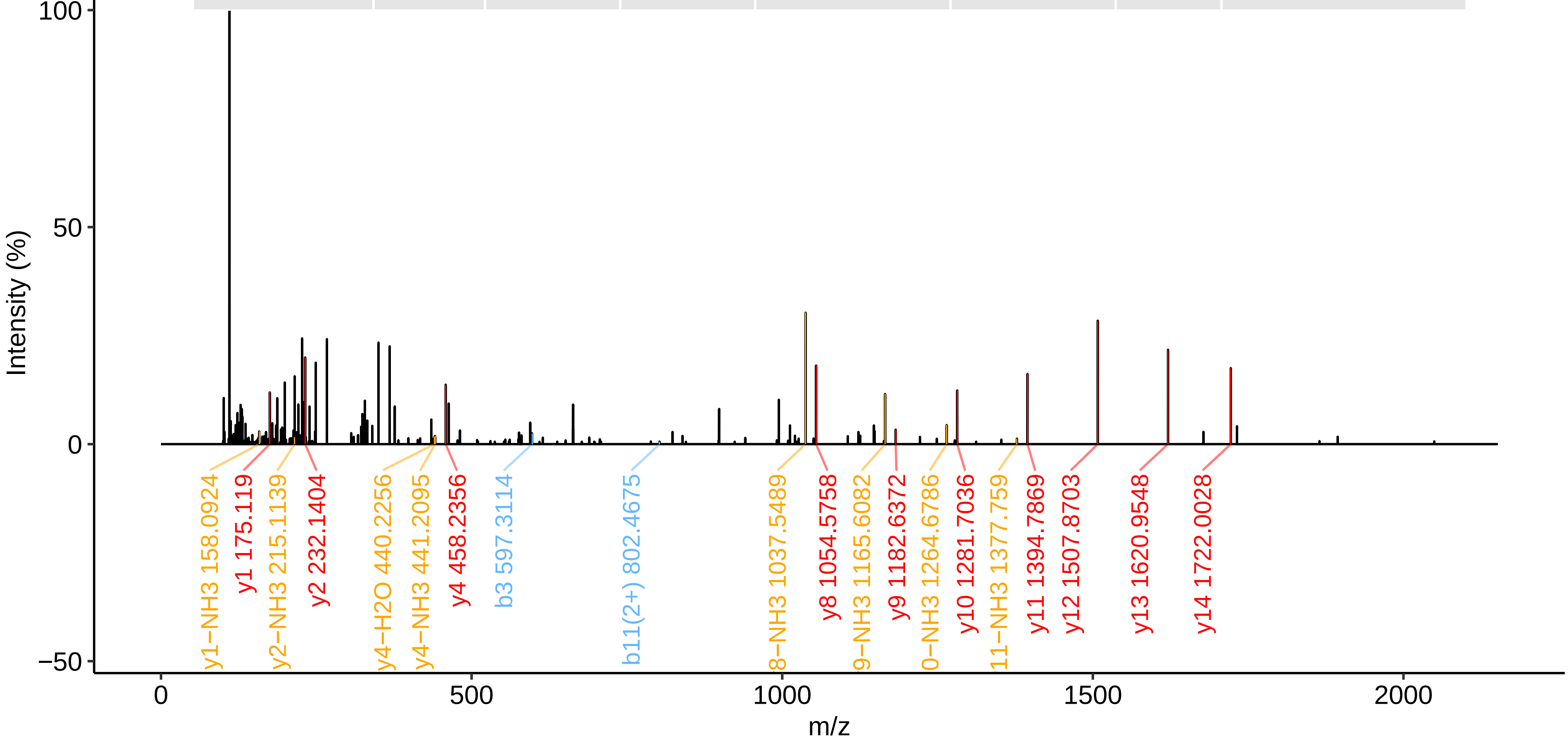

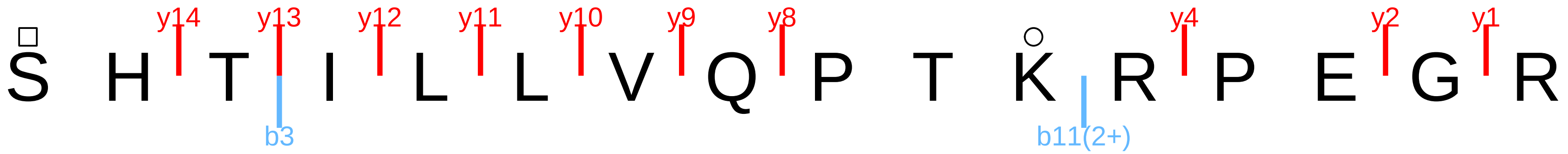

Modification □ Acetyl (Protein N−term) ○ GlyGly (K) no new terminus △ Oxidation (M)

20200921_KIZ_MUb4_GlyGly_10plex − Scan number: 9885

Gene Names	Charge	m/z	Mass	Retention time	PEP	Score	Precursor Intensity
ERH	4	497.7813	1987.096	34.009	1.8209e−07	45.14	6918246

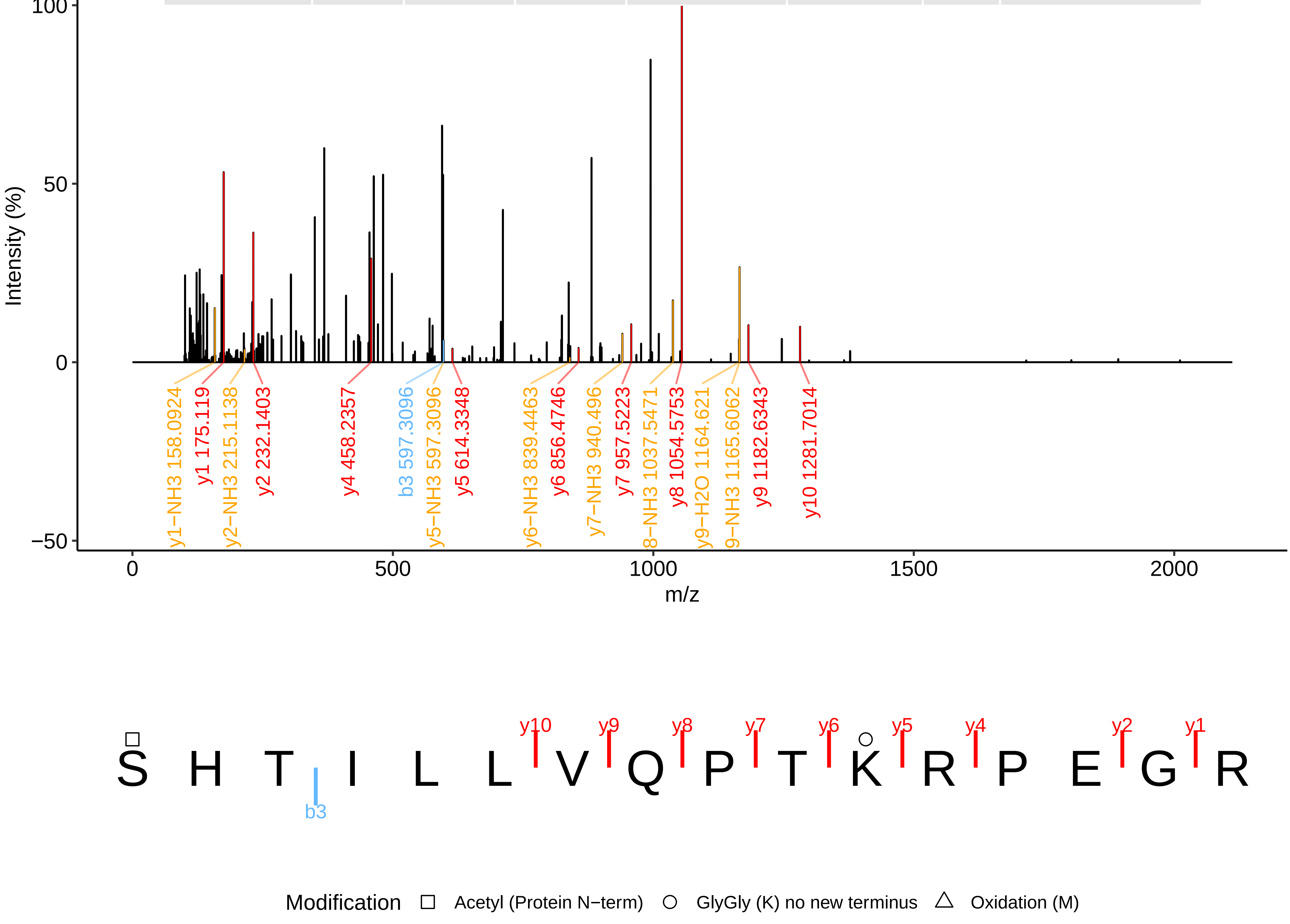

20200921_KIZ_MUb4_GlyGly_10plex_2 − Scan number: 9149

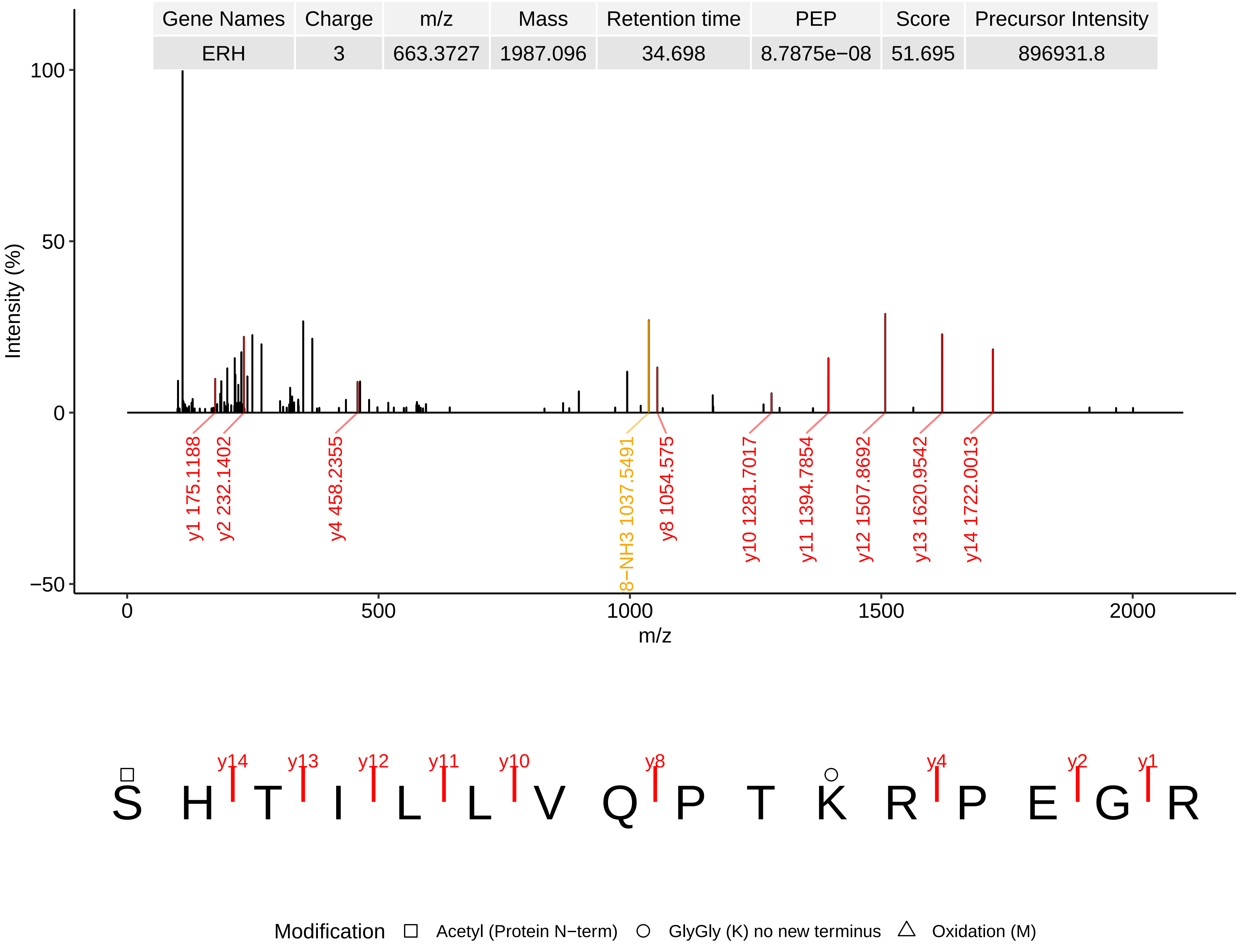

20200921_KIZ_MUb4_GlyGly_10plex_2 − Scan number: 9154

Gene Names	Charge	m/z	Mass	Retention time	PEP	Score	Precursor Intensity
ERH	4	497.7813	1987.096	34.72	3.1542e−10	48.823	783085.6

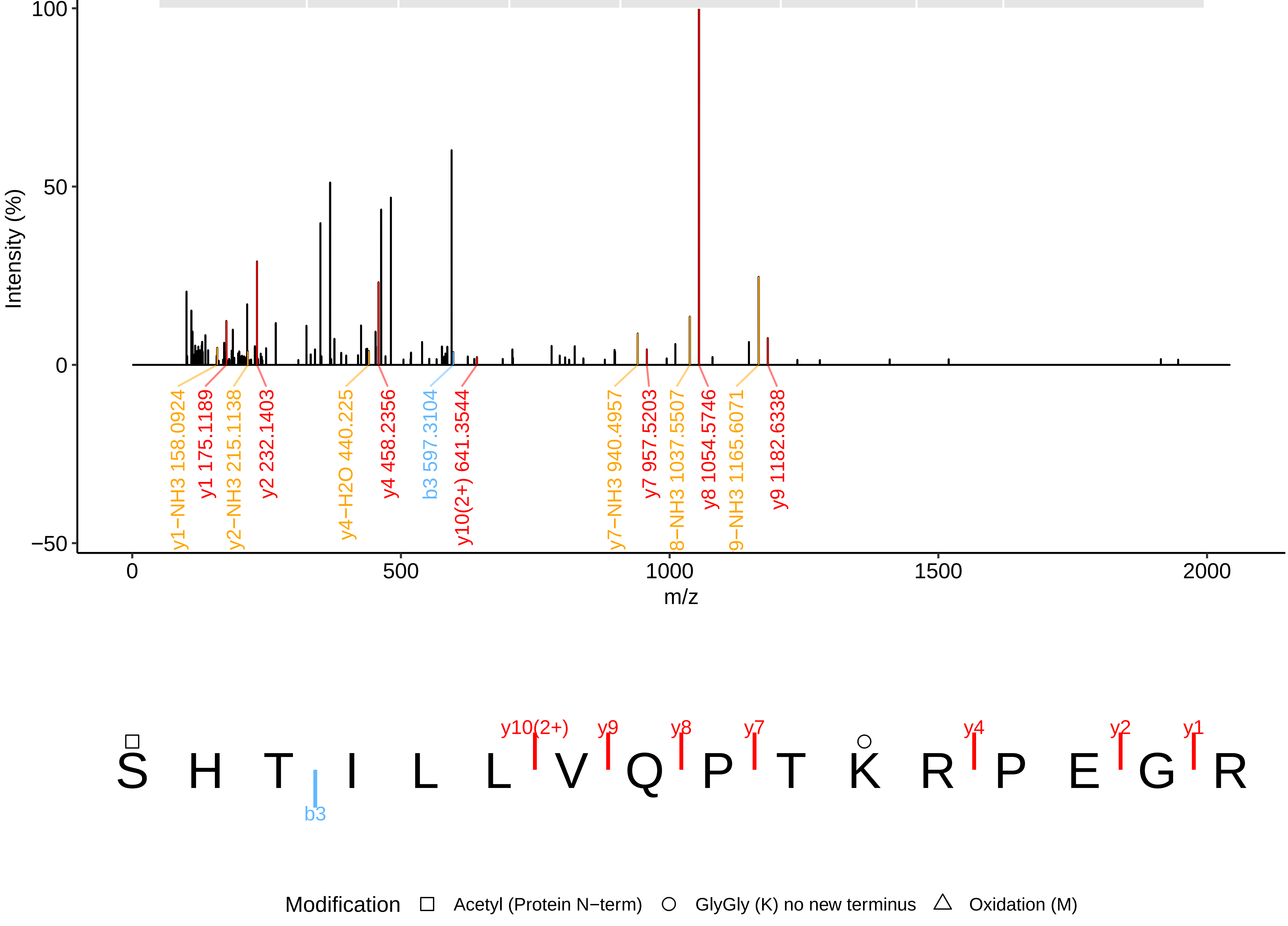

20200924_KIZ_MUb5_GlyGly_1 − Scan number: 8703

Gene Names	Charge	m/z	Mass	Retention time	PEP	Score	Precursor Intensity
ERH	4	497.7813	1987.096	27.865	2.3945e−11	68.944	12303808

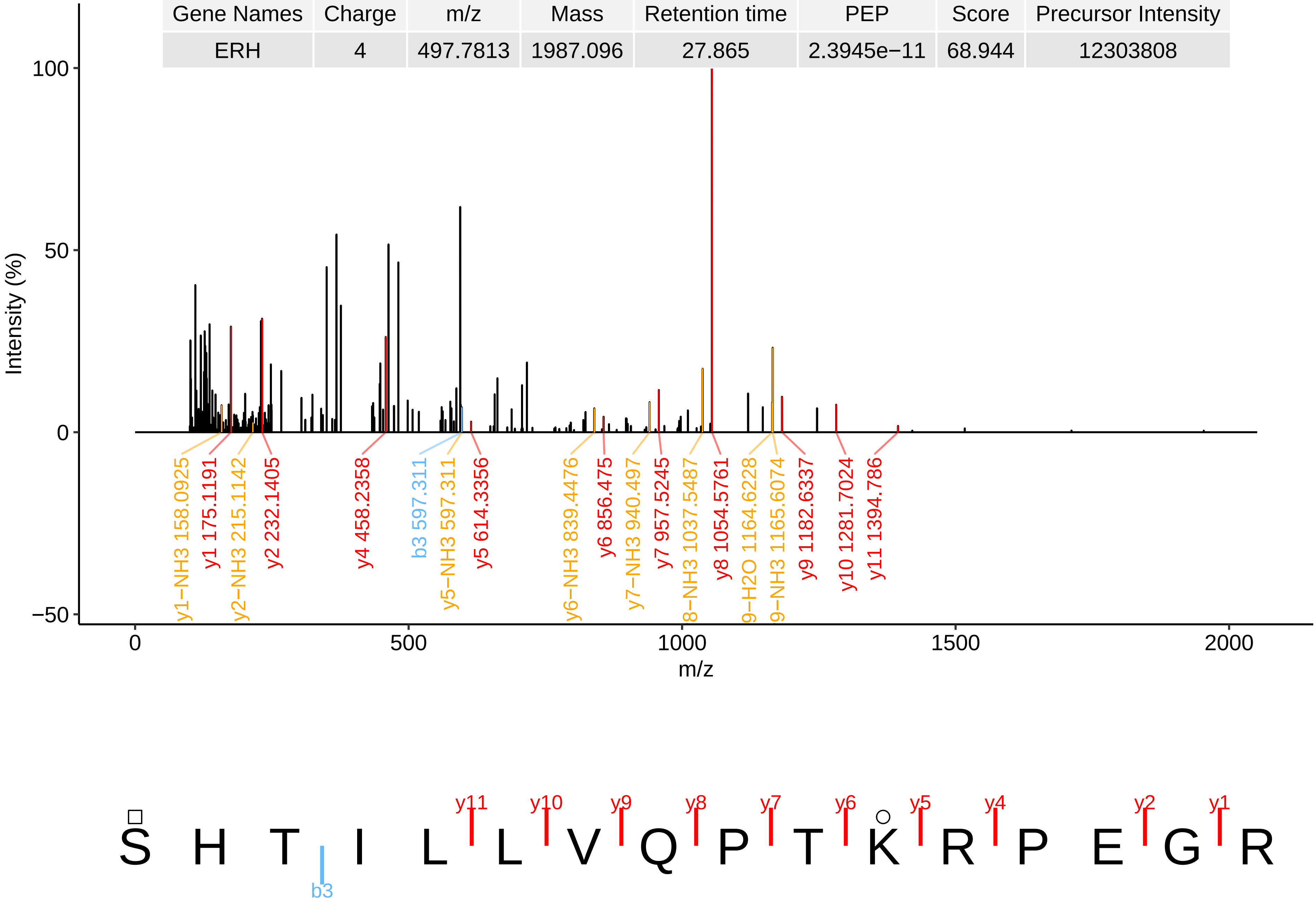

Modification □ Acetyl (Protein N−term) ○ GlyGly (K) no new terminus △ Oxidation (M)

20200924_KIZ_MUb5_GlyGly_1 − Scan number: 8746

Gene Names	Charge	m/z	Mass	Retention time	PEP	Score	Precursor Intensity
ERH	3	663.3727	1987.096	28.014	0.00030554	52.887	NA

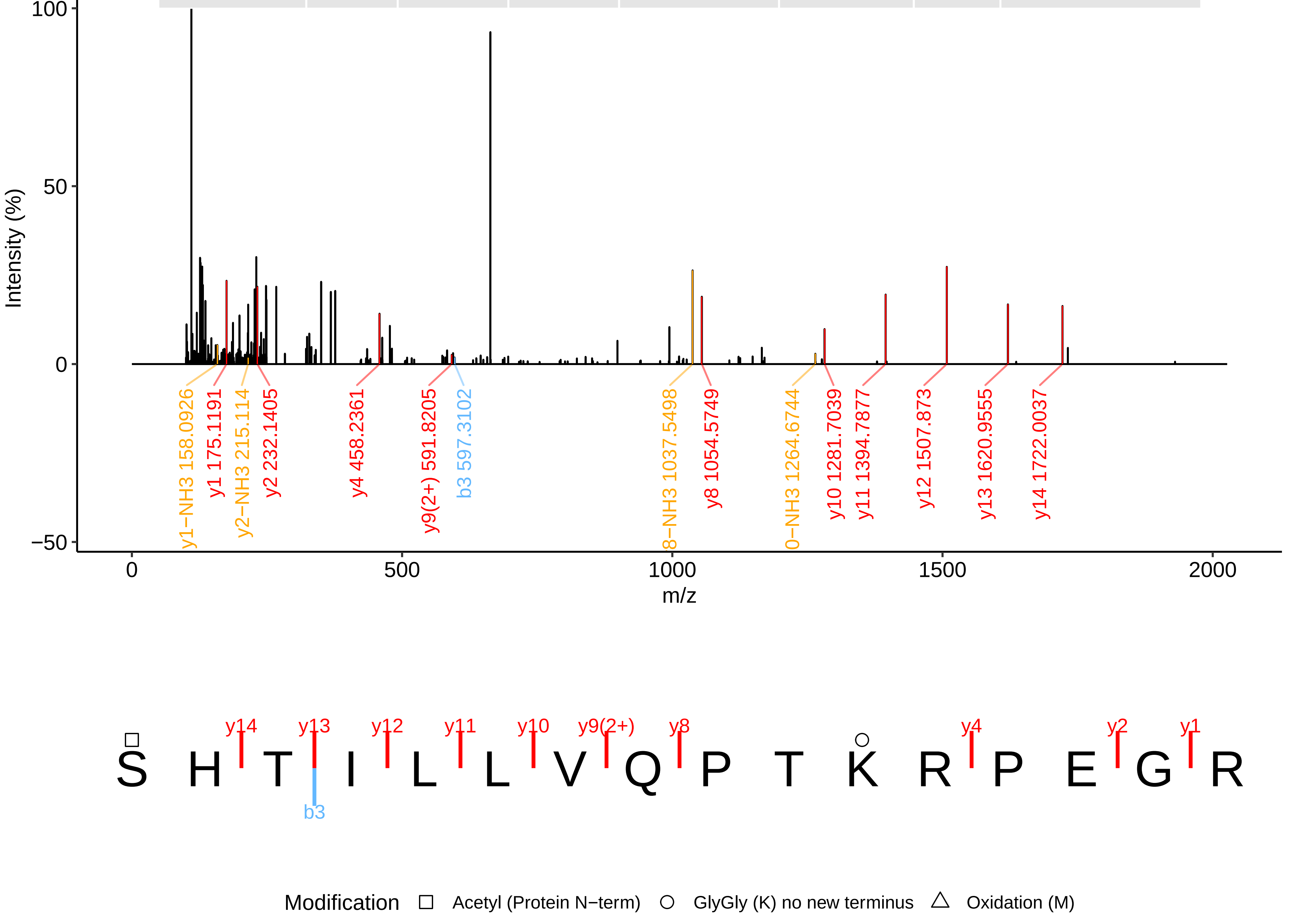

20200924_KIZ_MUb5_GlyGly_1 − Scan number: 8913

Gene Names	Charge	m/z	Mass	Retention time	PEP	Score	Precursor Intensity
ERH	3	663.3727	1987.096	28.593	8.9923e−14	89.541	2435414

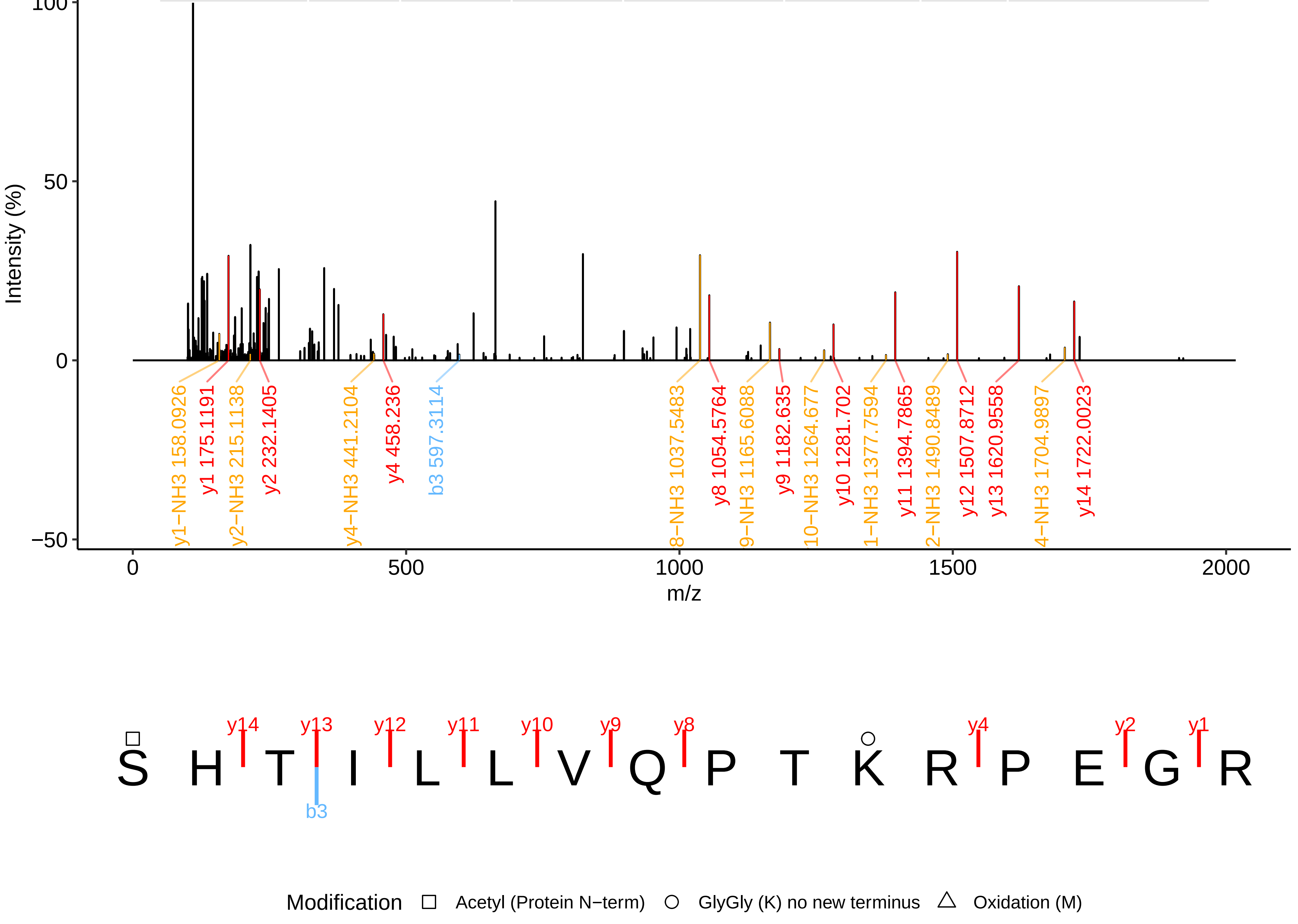

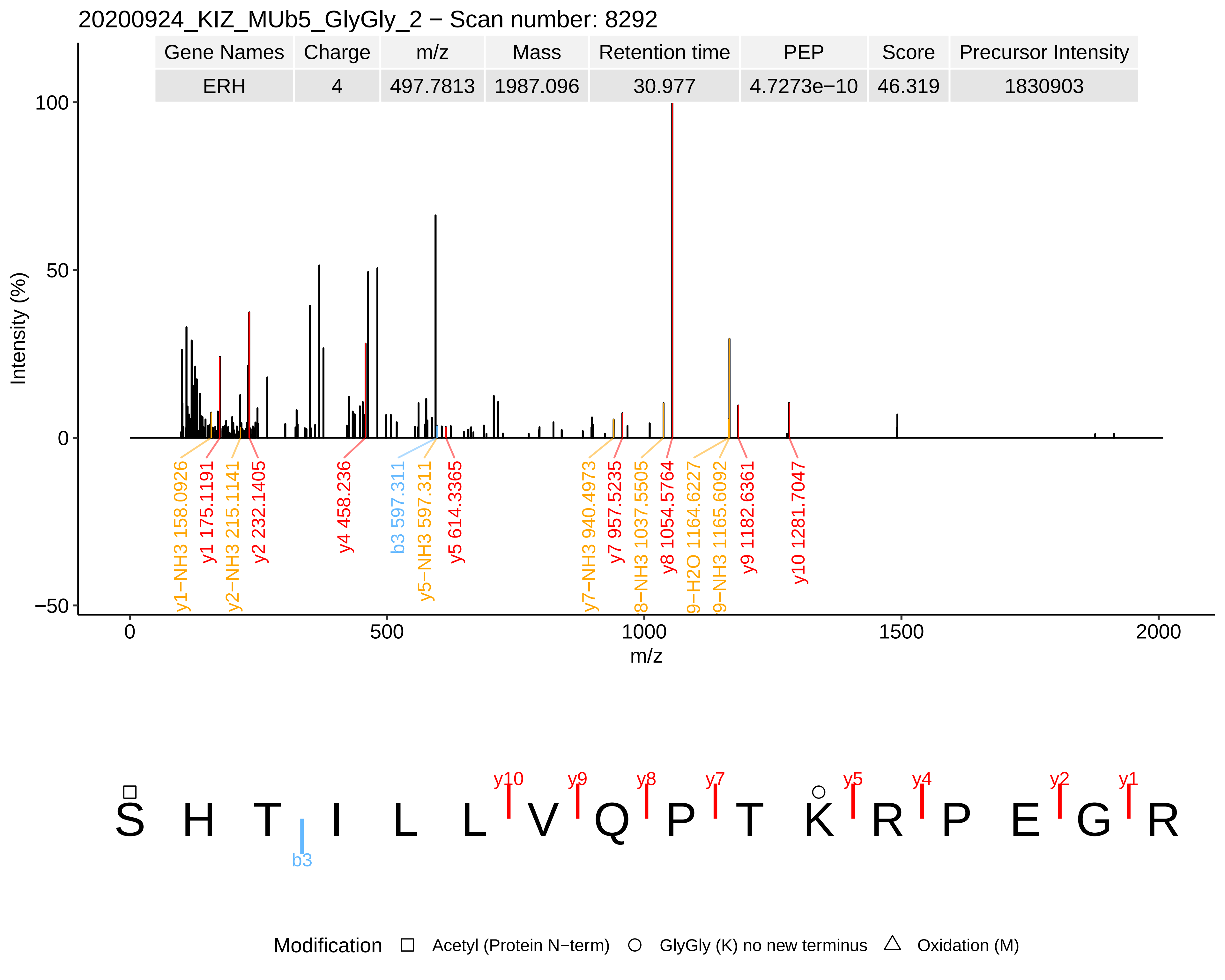

20200924_KIZ_MUb5_GlyGly_2 − Scan number: 8292
Gene Names | Charge | m/z | Mass | Retention time | PEP | Score | Precursor Intensity
ERH | 4 | 497.7813 | 1987.096 | 30.977 | 4.7273e−10 | 46.319 | 1830903
Intensity (%)
100
50
0
−50
0
500
1000
1500
2000
m/z
y1−NH3 158.0926
y1 175.1191
y2−NH3 215.1141
y2 232.1405
y4 458.236
b3 597.311
y5−NH3 597.311
y5 614.3365
y7−NH3 940.4973
y7 957.5235
8−NH3 1037.5505
y8 1054.5764
9−H2O 1164.6227
9−NH3 1165.6092
y9 1182.6361
y10 1281.7047
S H T I L L V Q P T K R P E G R
y10 y9 y8 y7 y5 y4 y2 y1
b3
Modification
Acetyl (Protein N−term)
GlyGly (K) no new terminus
Oxidation (M)

20200924_KIZ_MUb5_GlyGly_2 − Scan number: 8020

Gene Names	Charge	m/z	Mass	Retention time	PEP	Score	Precursor Intensity
ERH	3	663.3727	1987.096	29.987	0.038338	45.014	NA

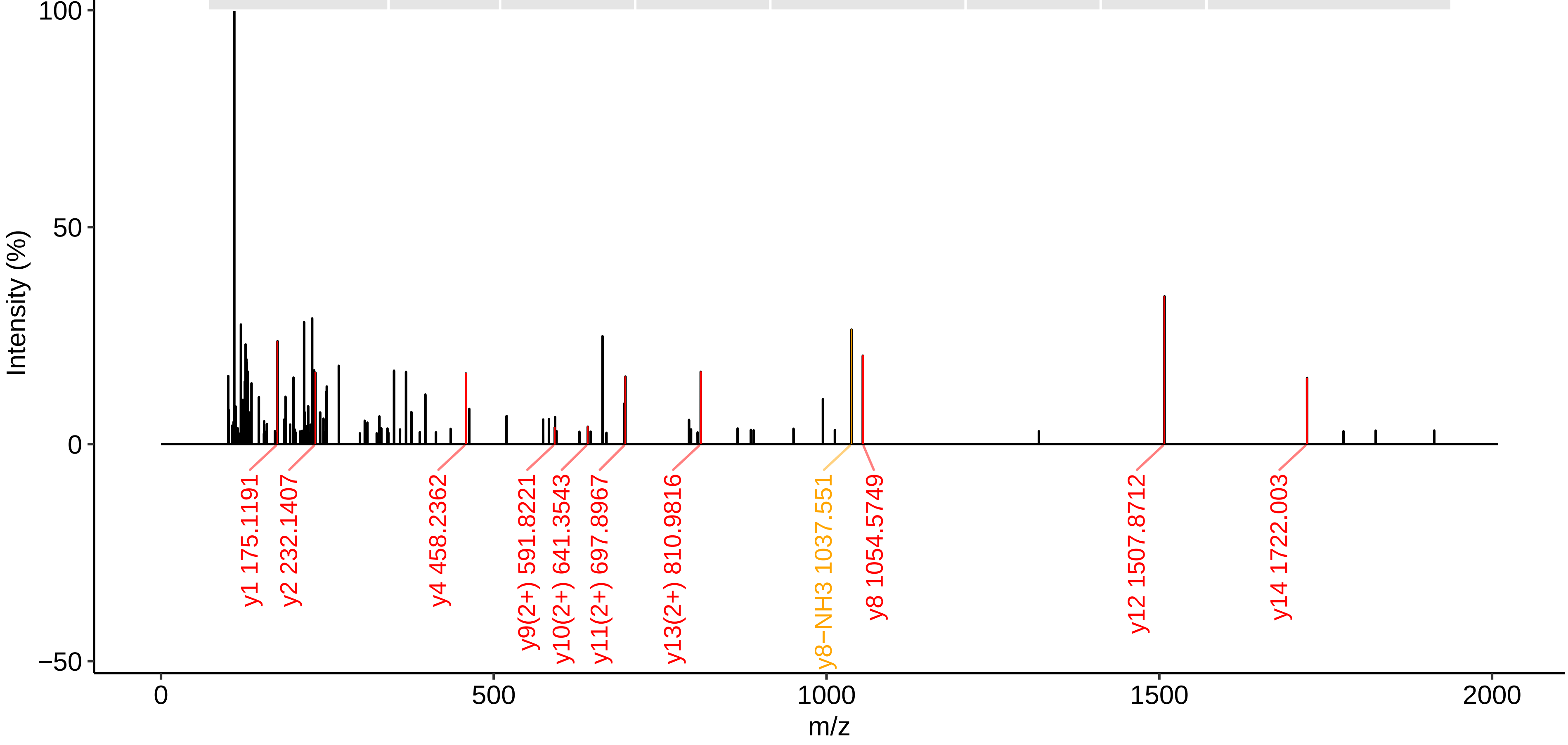

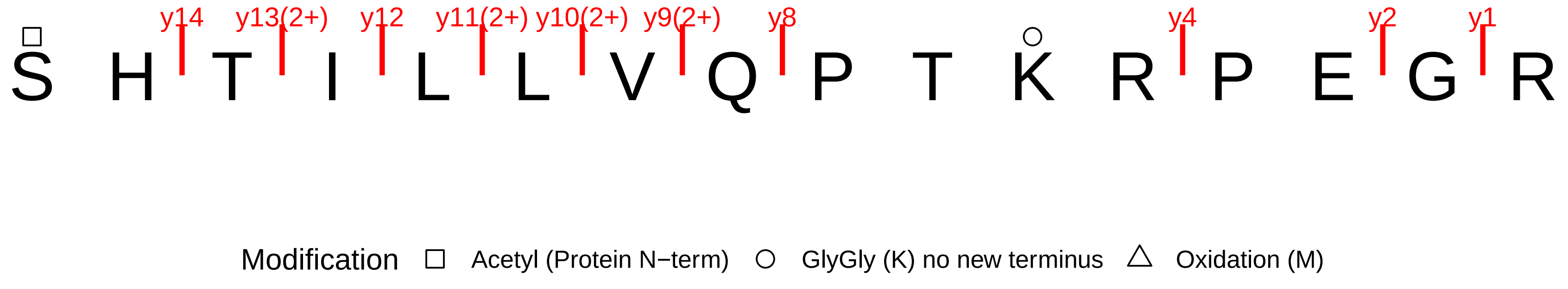

20200921_KIZ_MUb4_GlyGly_10plex_2 − Scan number: 17857

Gene Names	Charge	m/z	Mass	Retention time	PEP	Score	Precursor Intensity
VDAC2	3	598.3233	1791.948	69.87	0.0022553	65.716	1027839

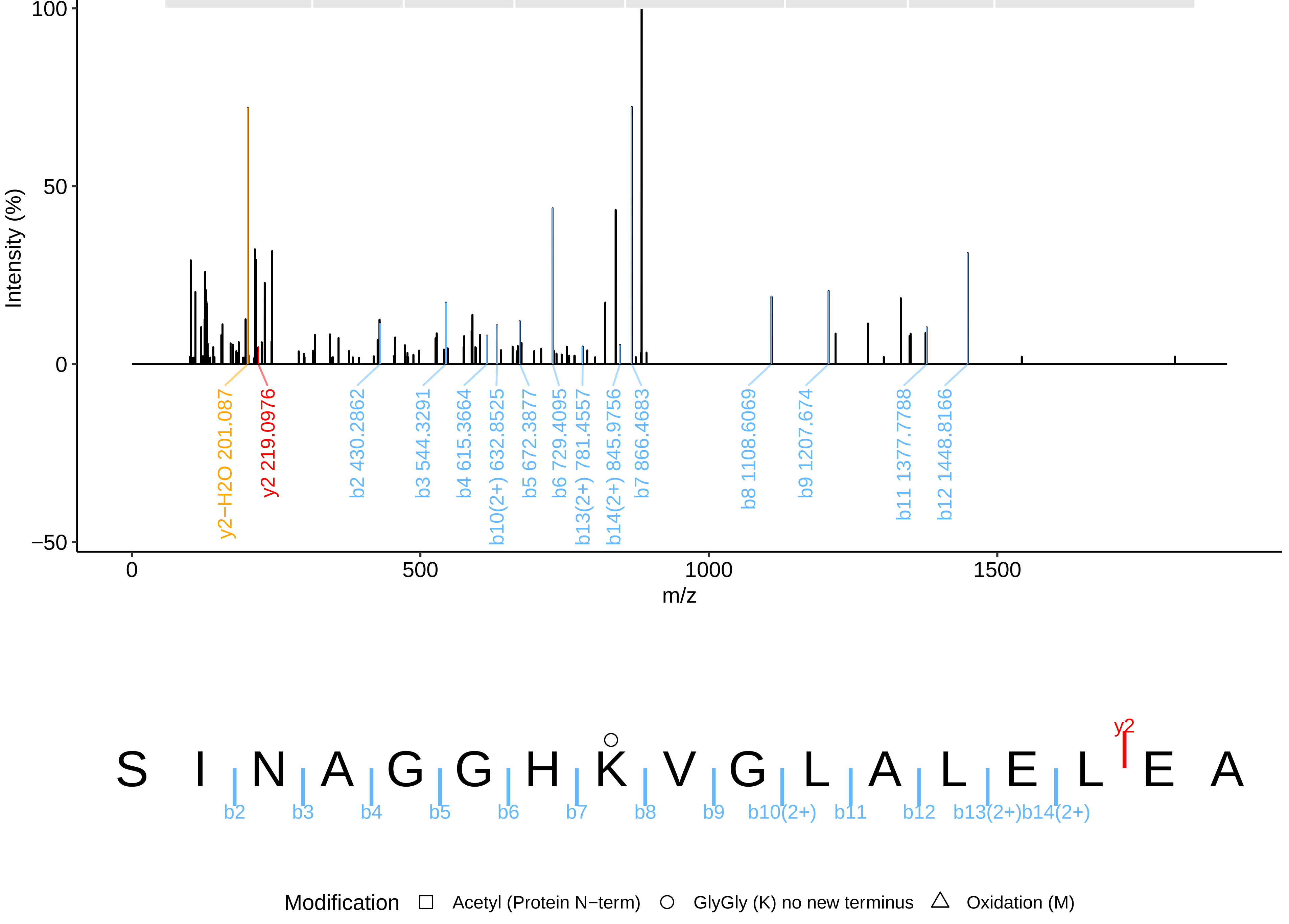

20201118_KIZ_MUb6_GlyGly_2 − Scan number: 21206

Gene Names	Charge	m/z	Mass	Retention time	PEP	Score	Precursor Intensity
VDAC2	3	598.3233	1791.948	71.465	9.9854e−10	98.035	12119332

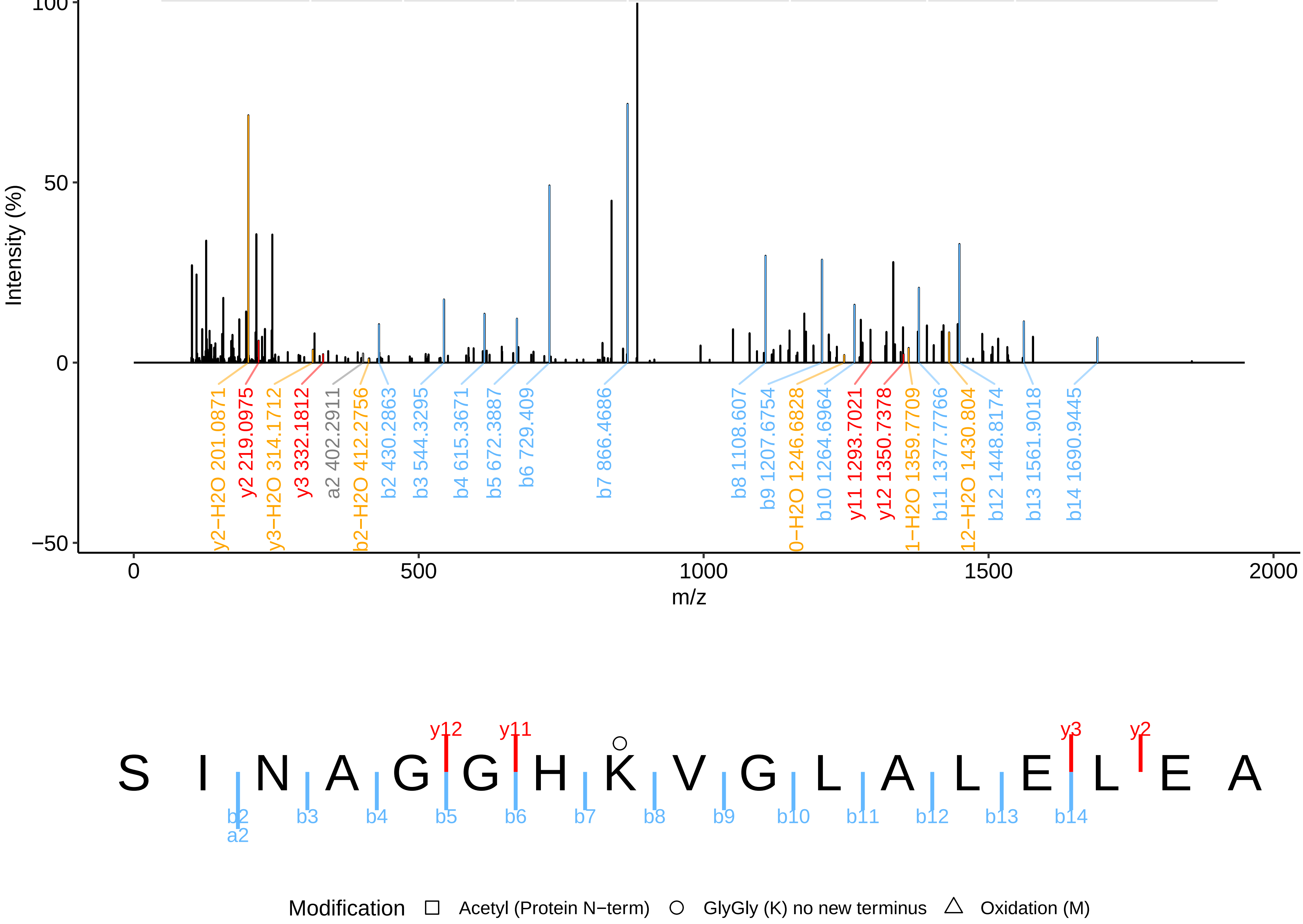

20200921_KIZ_MUb4_GlyGly_10plex − Scan number: 15706

Gene Names	Charge	m/z	Mass	Retention time	PEP	Score	Precursor Intensity
VDAC3	3	528.6107	1582.81	56.18	0.00035882	92.856	22305496

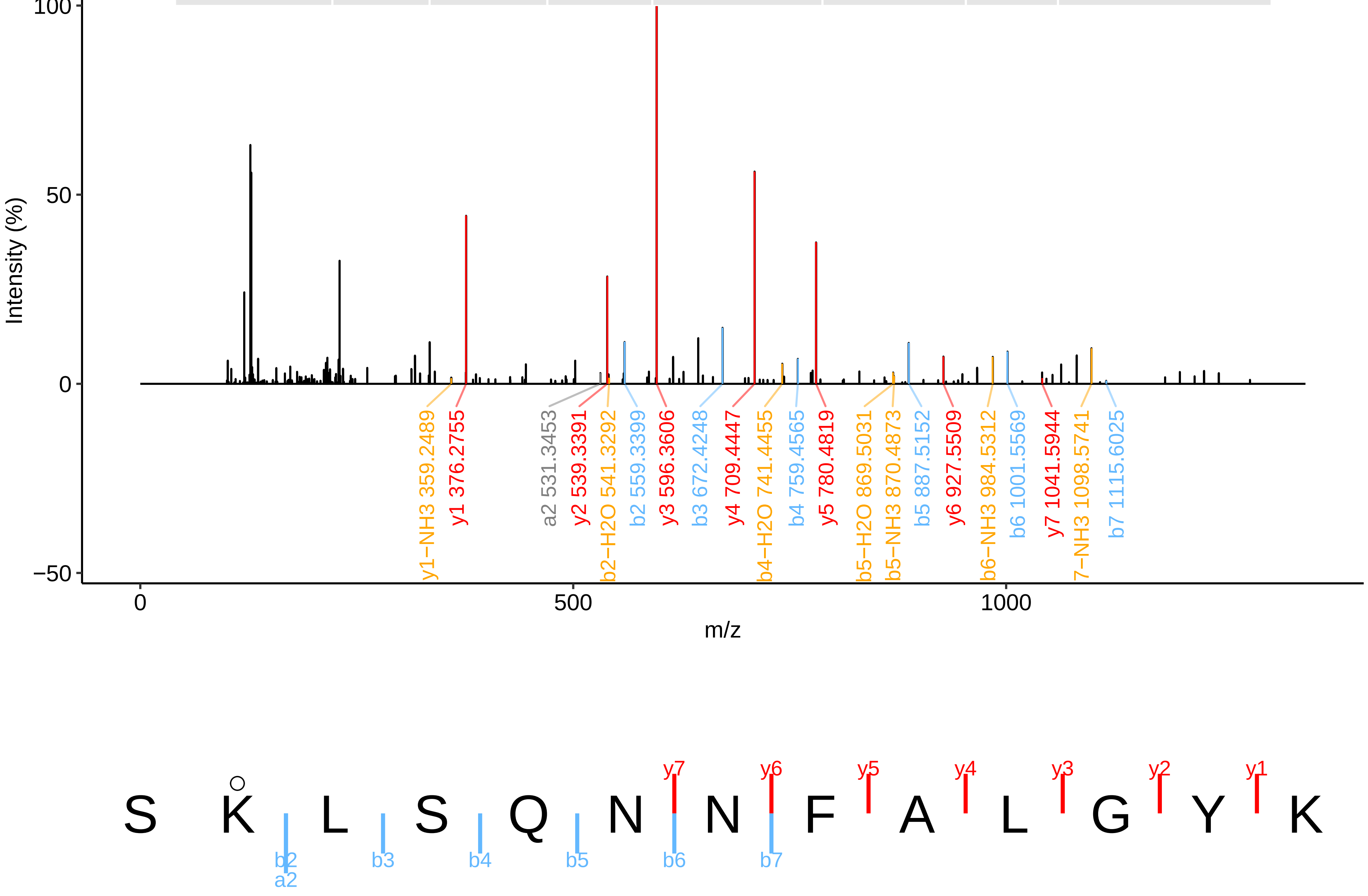

Modification □ Acetyl (Protein N−term) ○ GlyGly (K) no new terminus △ Oxidation (M)

20200921_KIZ_MUb4_GlyGly_10plex_2 − Scan number: 14581

Gene Names	Charge	m/z	Mass	Retention time	PEP	Score	Precursor Intensity
VDAC3	3	528.6107	1582.81	56.866	0.0011956	80.522	2446577

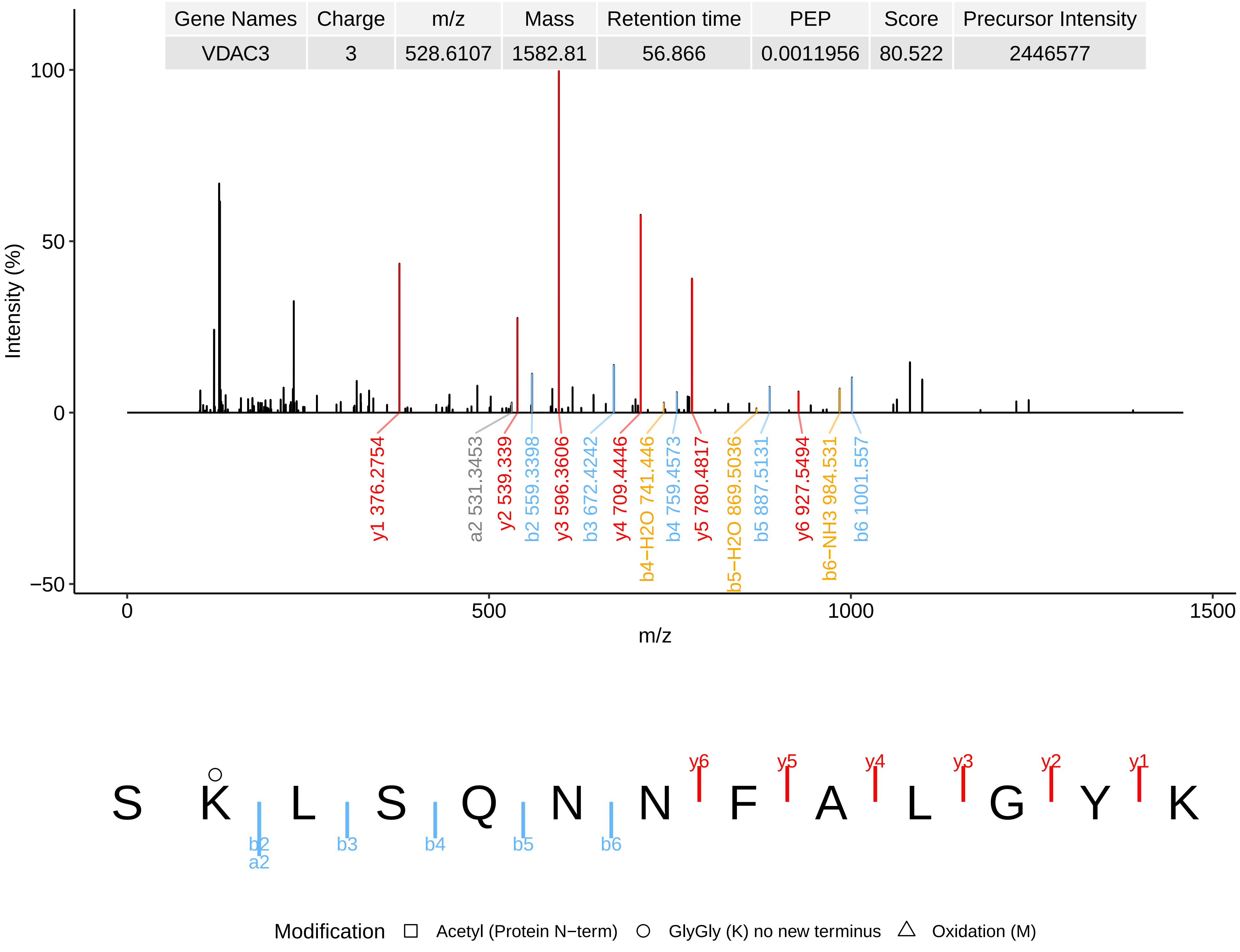

20200924_KIZ_MUb5_GlyGly_1 − Scan number: 14798

Gene Names	Charge	m/z	Mass	Retention time	PEP	Score	Precursor Intensity
VDAC3	3	528.6107	1582.81	48.336	0.00027606	94.122	44094856

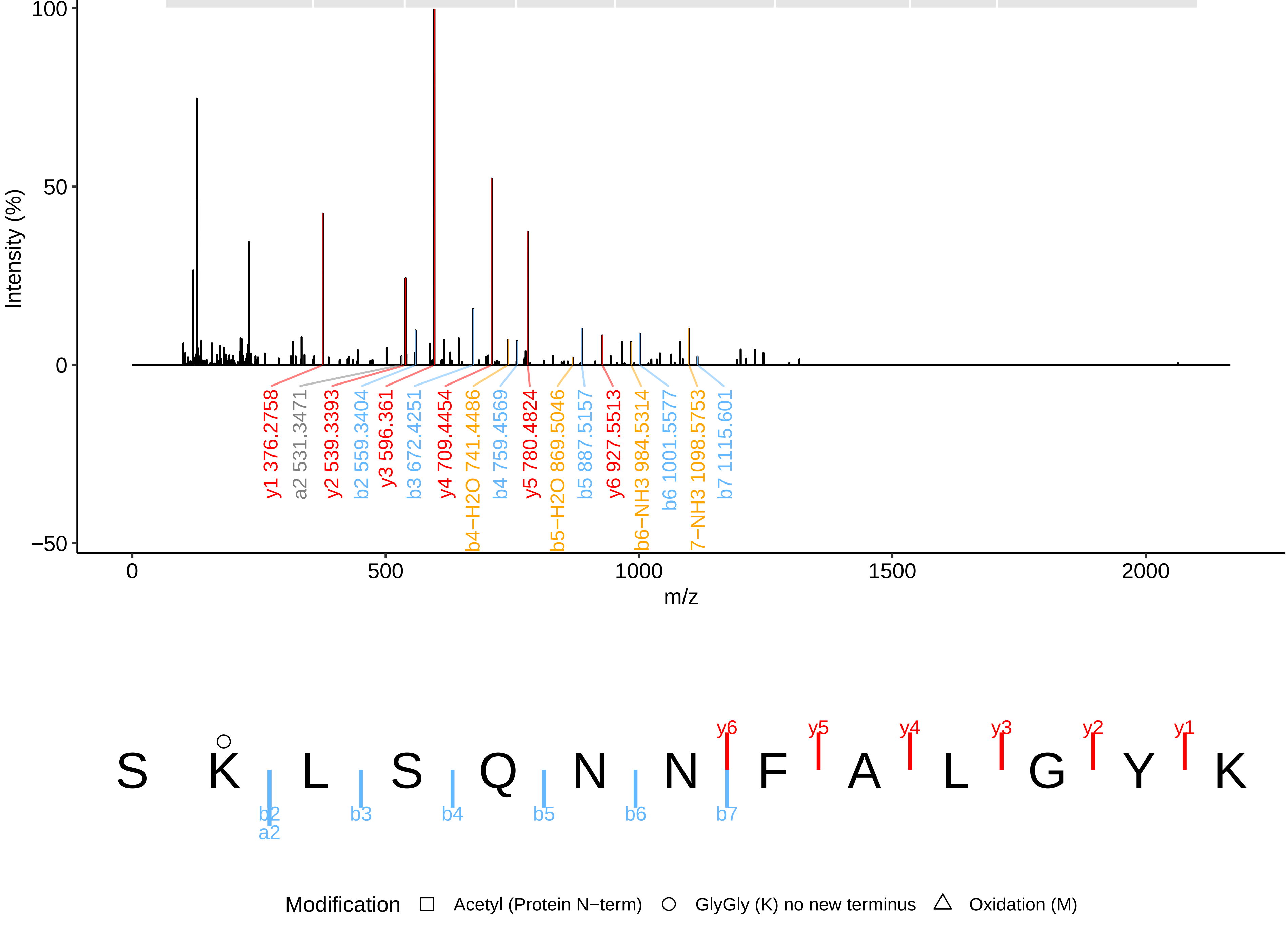

20200924_KIZ_MUb5_GlyGly_1 − Scan number: 14951

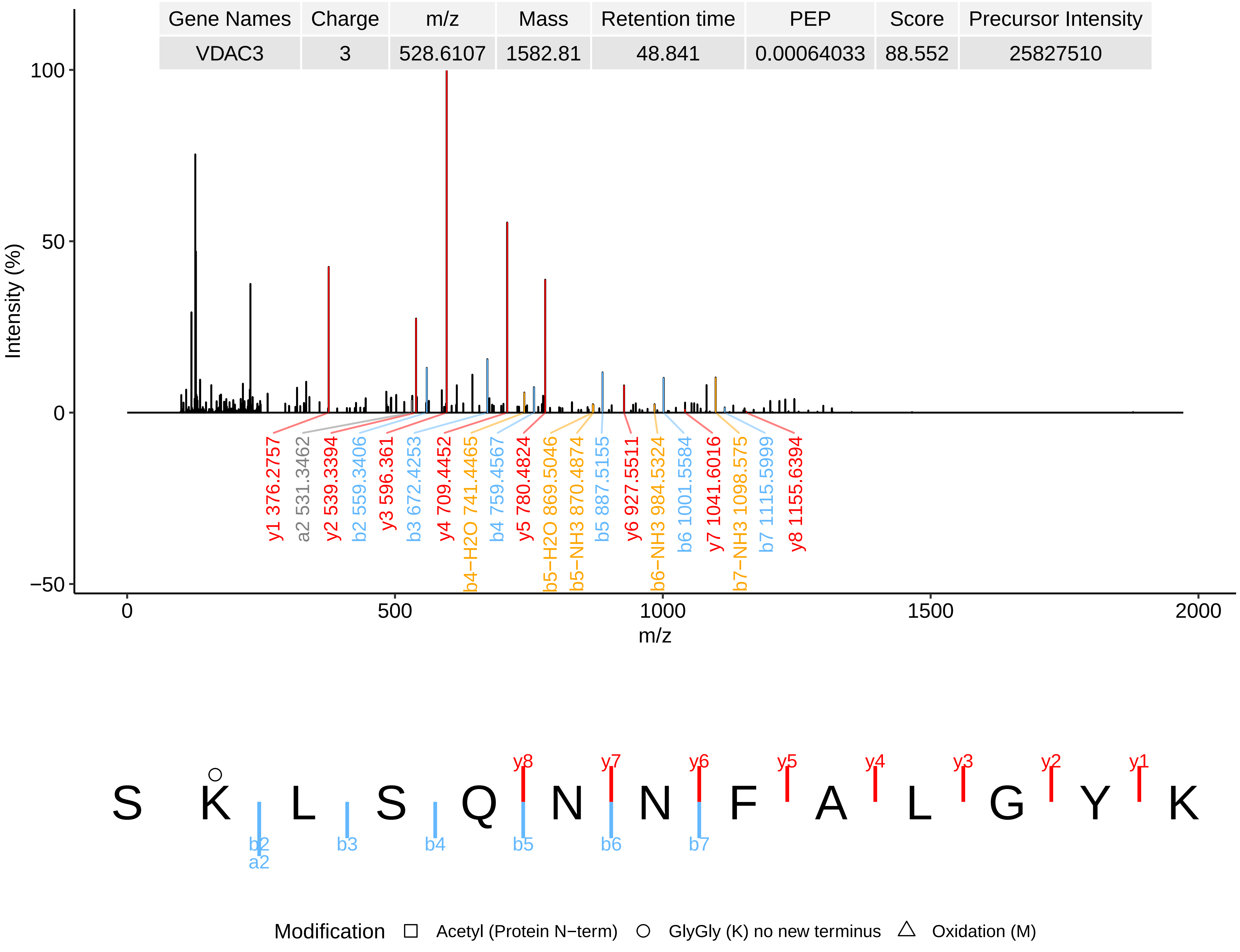

20200924_KIZ_MUb5_GlyGly_2 − Scan number: 13783

Gene Names	Charge	m/z	Mass	Retention time	PEP	Score	Precursor Intensity
VDAC3	3	528.6107	1582.81	51.548	0.0026072	77.912	3887139

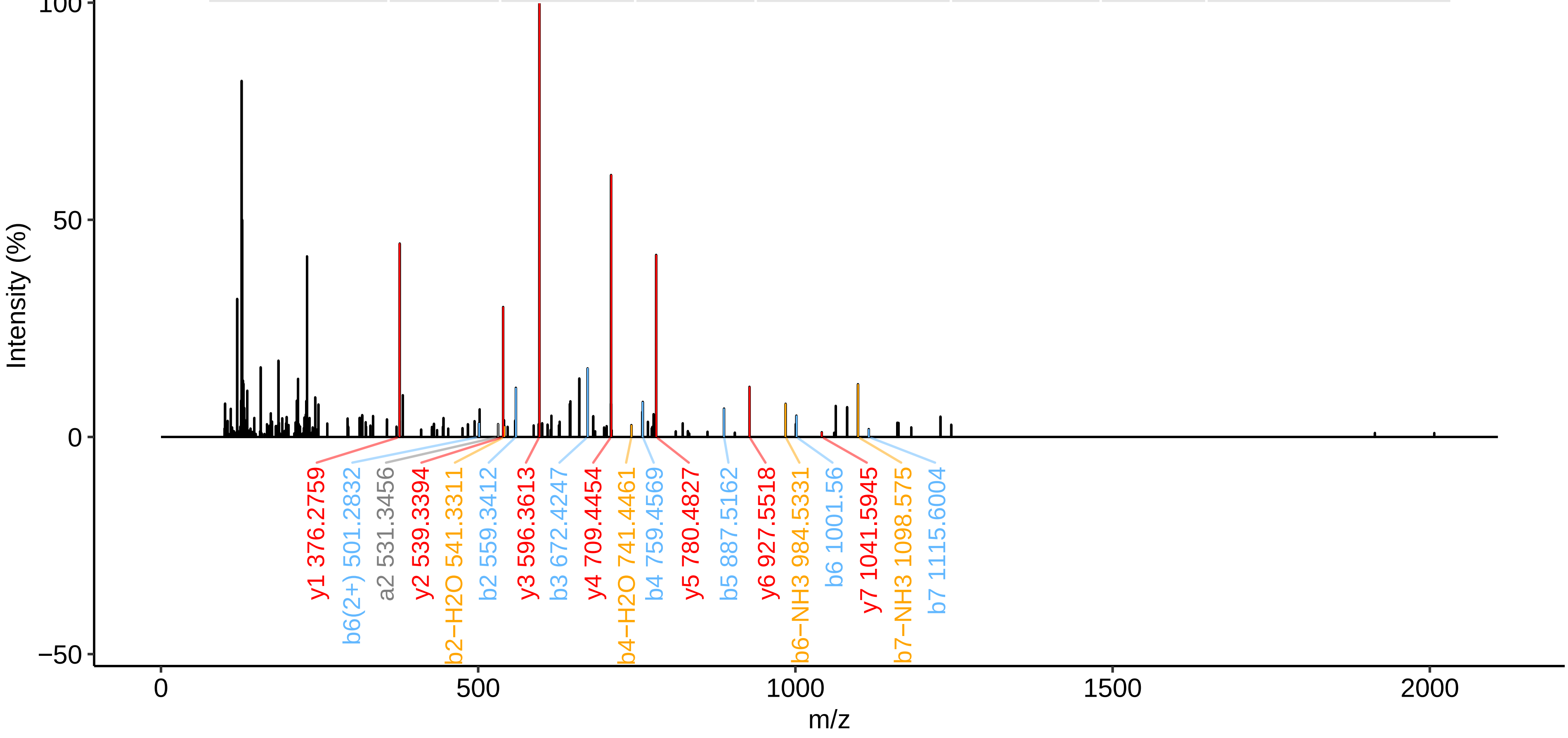

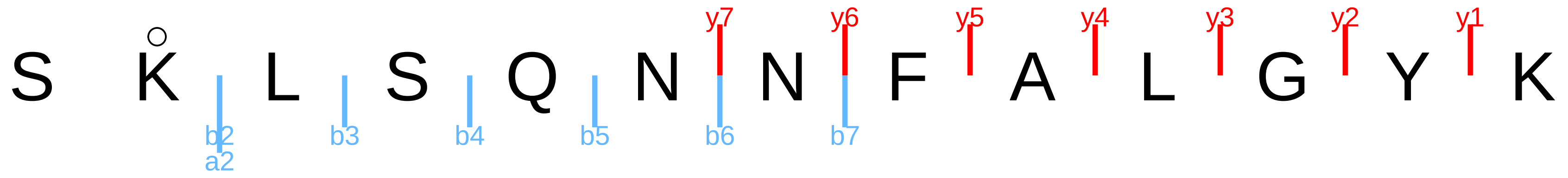

Modification □ Acetyl (Protein N−term) ○ GlyGly (K) no new terminus △ Oxidation (M)

20200924_KIZ_MUb5_GlyGly_2 − Scan number: 13839

Gene Names	Charge	m/z	Mass	Retention time	PEP	Score	Precursor Intensity
VDAC3	3	528.6107	1582.81	51.755	0.0079484	68.034	4625265

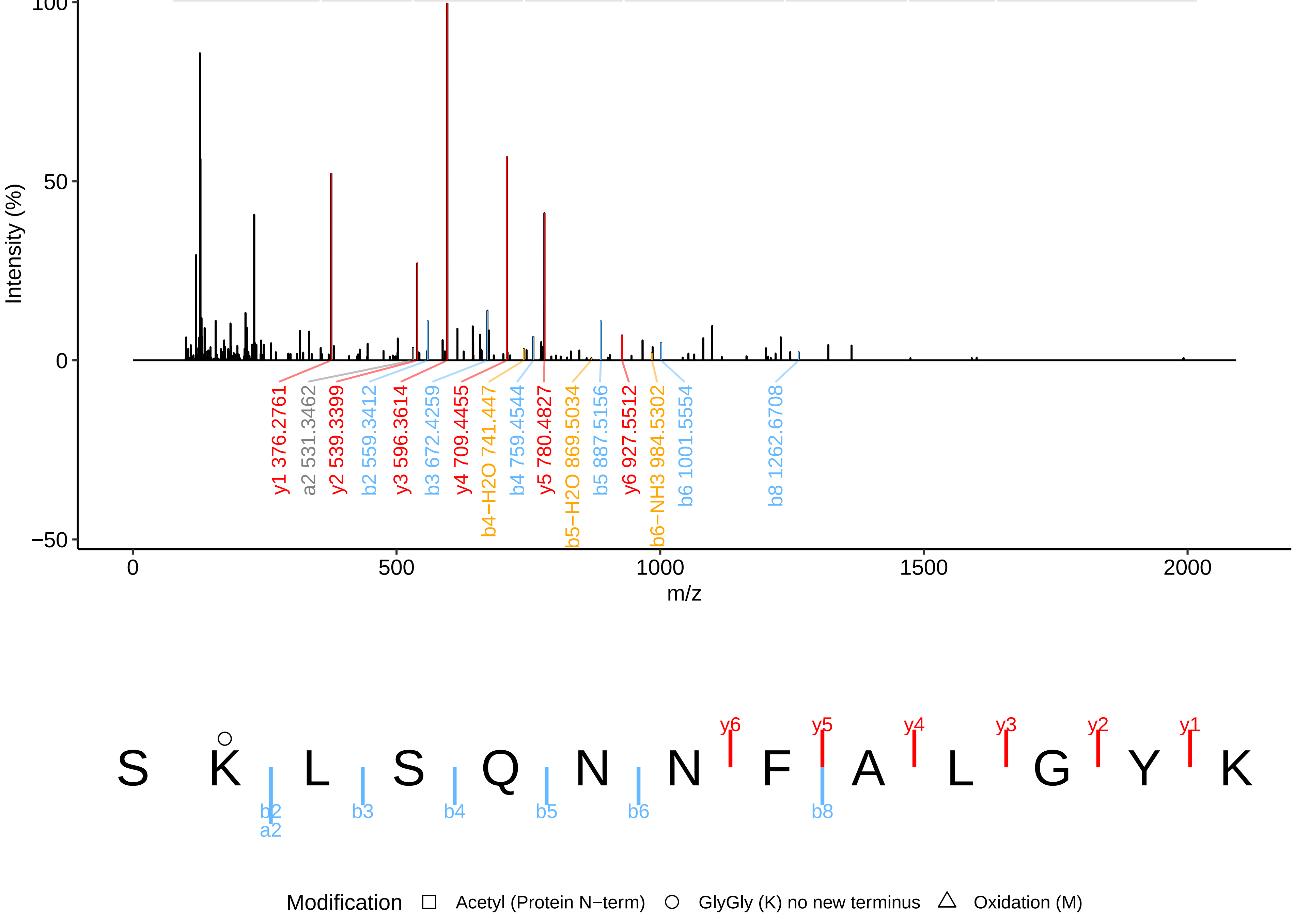

20200924_KIZ_MUb5_GlyGly_2 − Scan number: 13982

Gene Names	Charge	m/z	Mass	Retention time	PEP	Score	Precursor Intensity
VDAC3	3	528.6107	1582.81	52.283	0.00054833	89.959	13226593

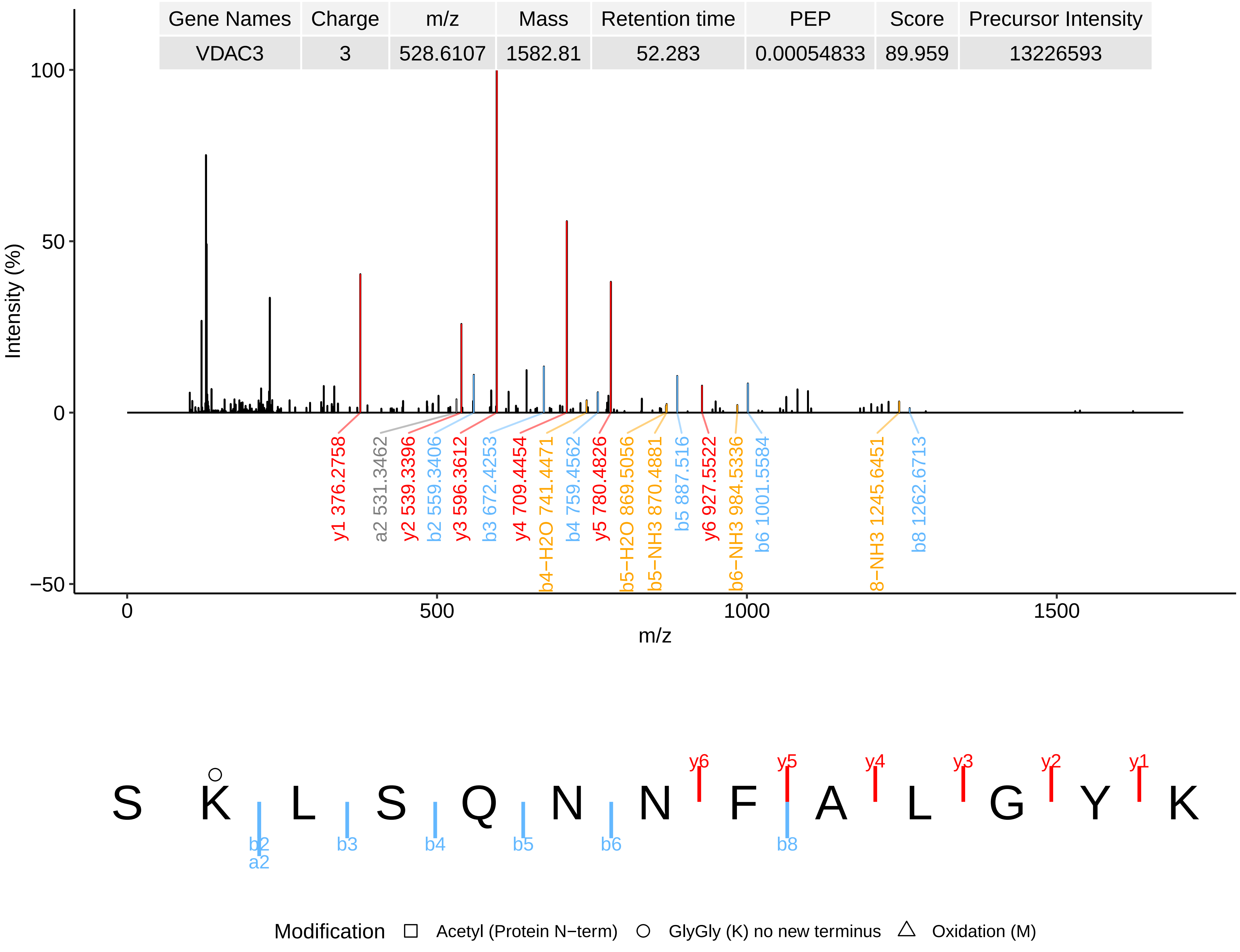

20200924_KIZ_MUb5_GlyGly_2 − Scan number: 14122

Gene Names	Charge	m/z	Mass	Retention time	PEP	Score	Precursor Intensity
VDAC3	3	528.6107	1582.81	52.796	0.00080127	89.629	4272902

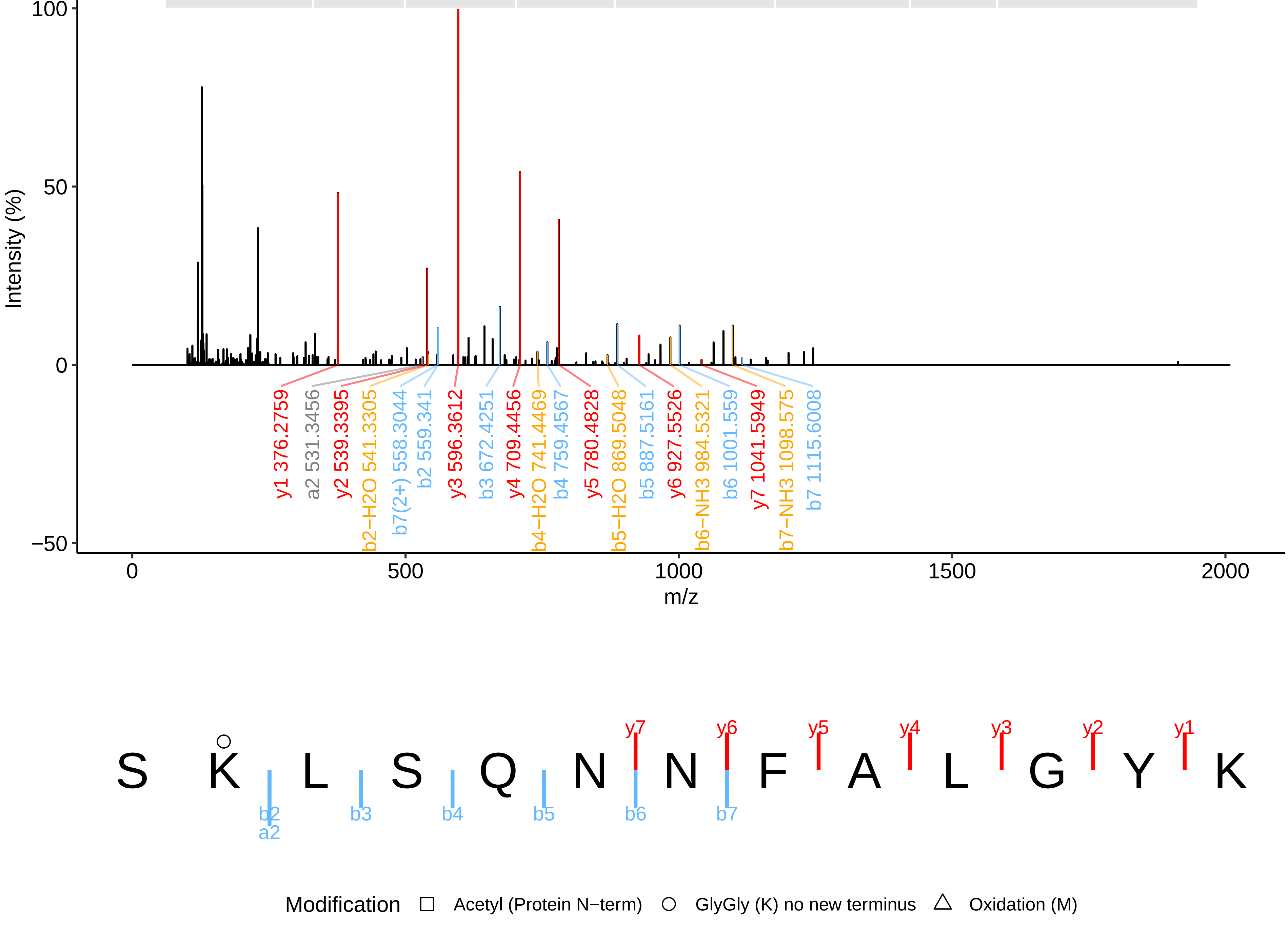

20201118_KIZ_MUb6_GlyGly_1 − Scan number: 14910

Gene Names	Charge	m/z	Mass	Retention time	PEP	Score	Precursor Intensity
VDAC3	3	528.6107	1582.81	47.171	0.0030004	77.185	25005864

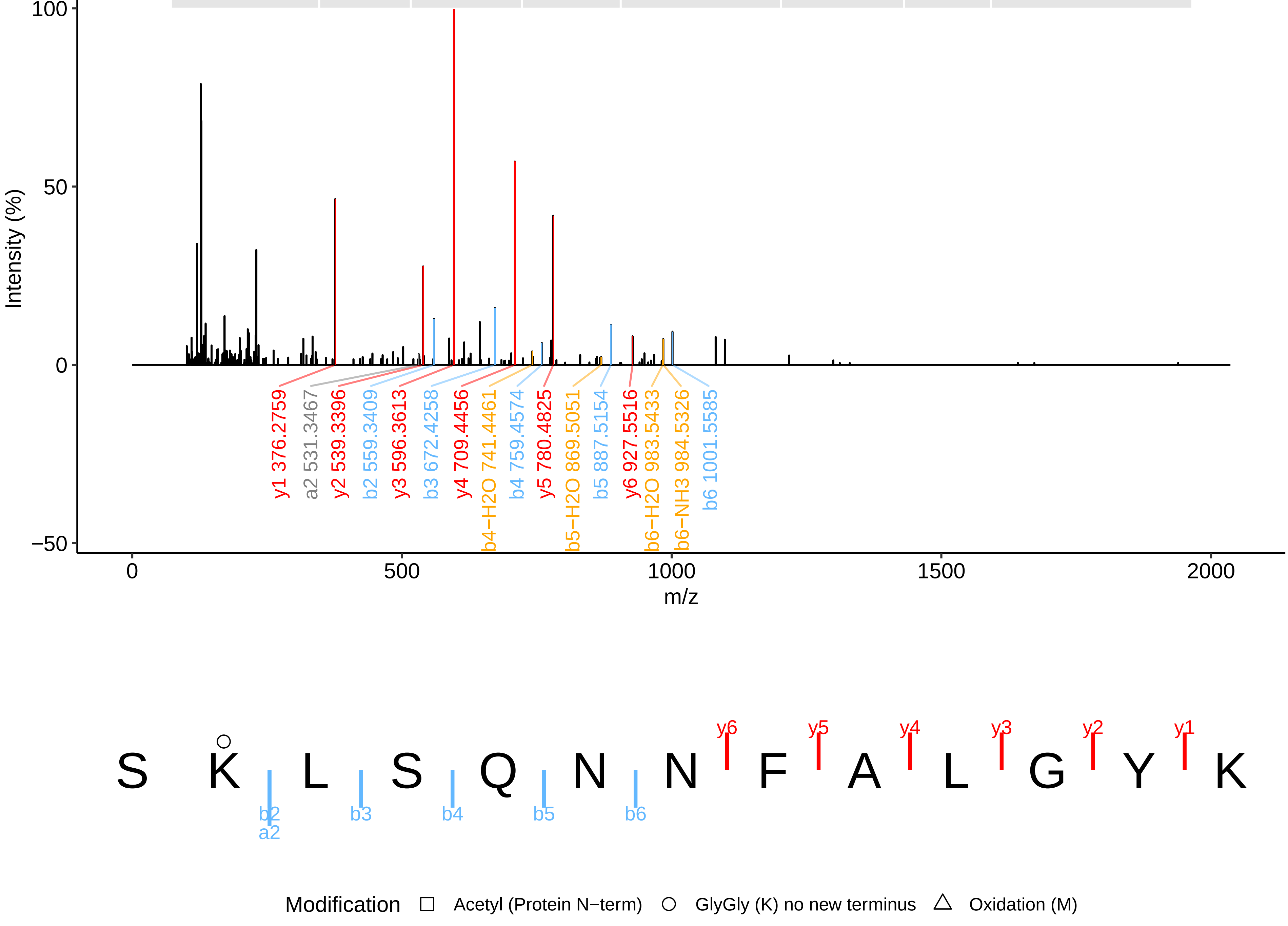

20201118_KIZ_MUb6_GlyGly_2 − Scan number: 17094

Gene Names	Charge	m/z	Mass	Retention time	PEP	Score	Precursor Intensity
VDAC3	3	528.6107	1582.81	57.784	1.4093e−05	109.84	20416242

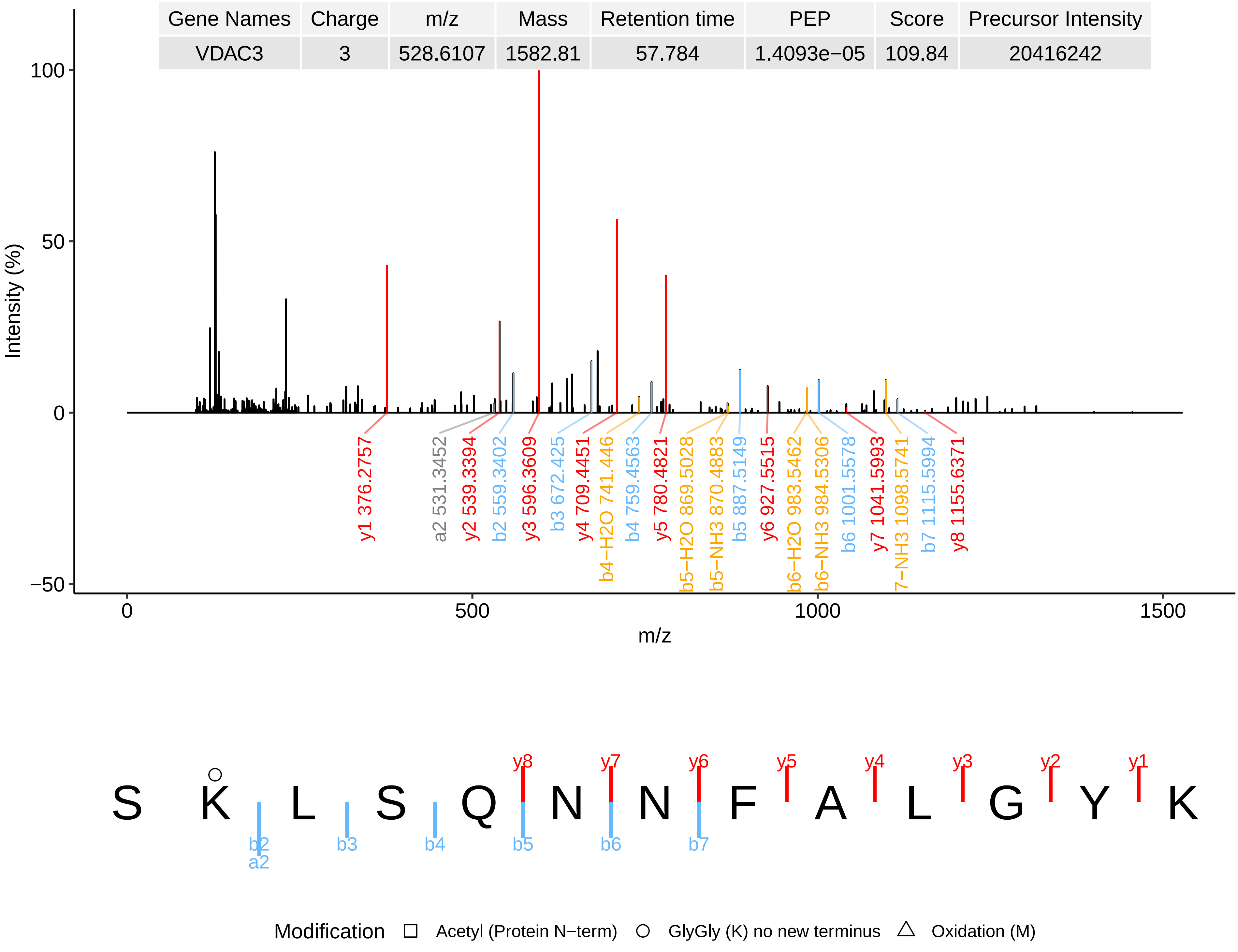

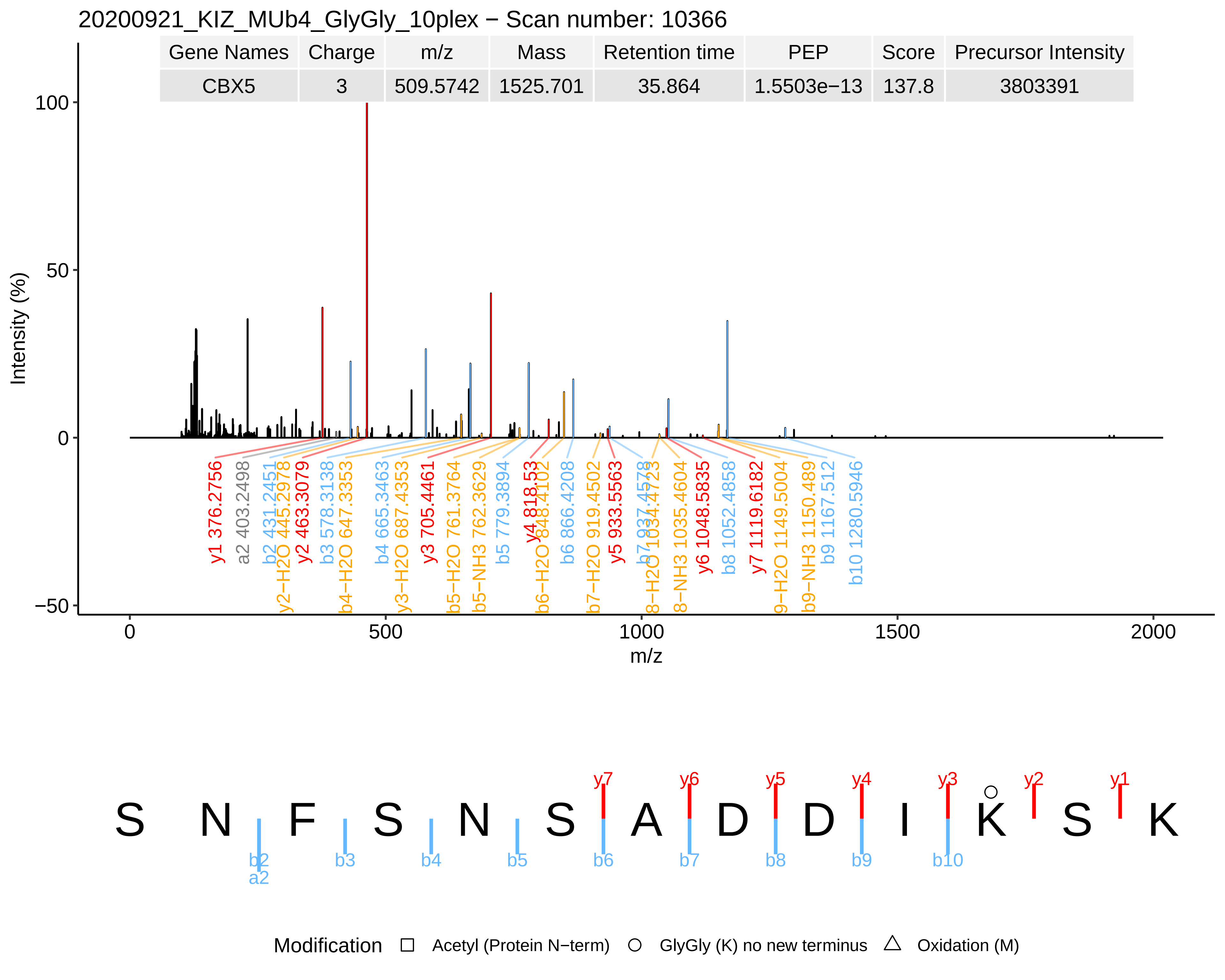

20200921_KIZ_MUb4_GlyGly_10plex − Scan number: 10366
Gene Names | Charge | m/z | Mass | Retention time | PEP | Score | Precursor Intensity
CBX5 | 3 | 509.5742 | 1525.701 | 35.864 | 1.5503e−13 | 137.8 | 3803391
Intensity (%)
100
50
0
−50
0
500
1000
1500
2000
m/z
y1 376.2756
a2 403.2498
b2 431.2451
y2−H2O 445.2978
y2 463.3079
b3 578.3138
b4−H2O 647.3353
b4 665.3463
y3−H2O 687.4353
y3 705.4461
b5−H2O 761.3764
b5−NH3 762.3629
b5 779.3894
y4 818.53
b6−H2O 848.4102
b6 866.4208
b7−H2O 919.4502
y5 933.5563
b7 937.4578
8−H2O 1034.4723
8−NH3 1035.4604
y6 1048.5835
b8 1052.4858
y7 1119.6182
9−H2O 1149.5004
b9−NH3 1150.489
b9 1167.512
b10 1280.5946
S N F S N S A D D I K S K
y7 y6 y5 y4 y3 y2 y1
b2 a2 b3 b4 b5 b6 b7 b8 b9 b10
Modification
Acetyl (Protein N−term)
GlyGly (K) no new terminus
Oxidation (M)

20200921_KIZ_MUb4_GlyGly_10plex_2 − Scan number: 9539

Gene Names	Charge	m/z	Mass	Retention time	PEP	Score	Precursor Intensity
CBX5	3	509.5742	1525.701	36.293	0.00031847	94.728	643283.4

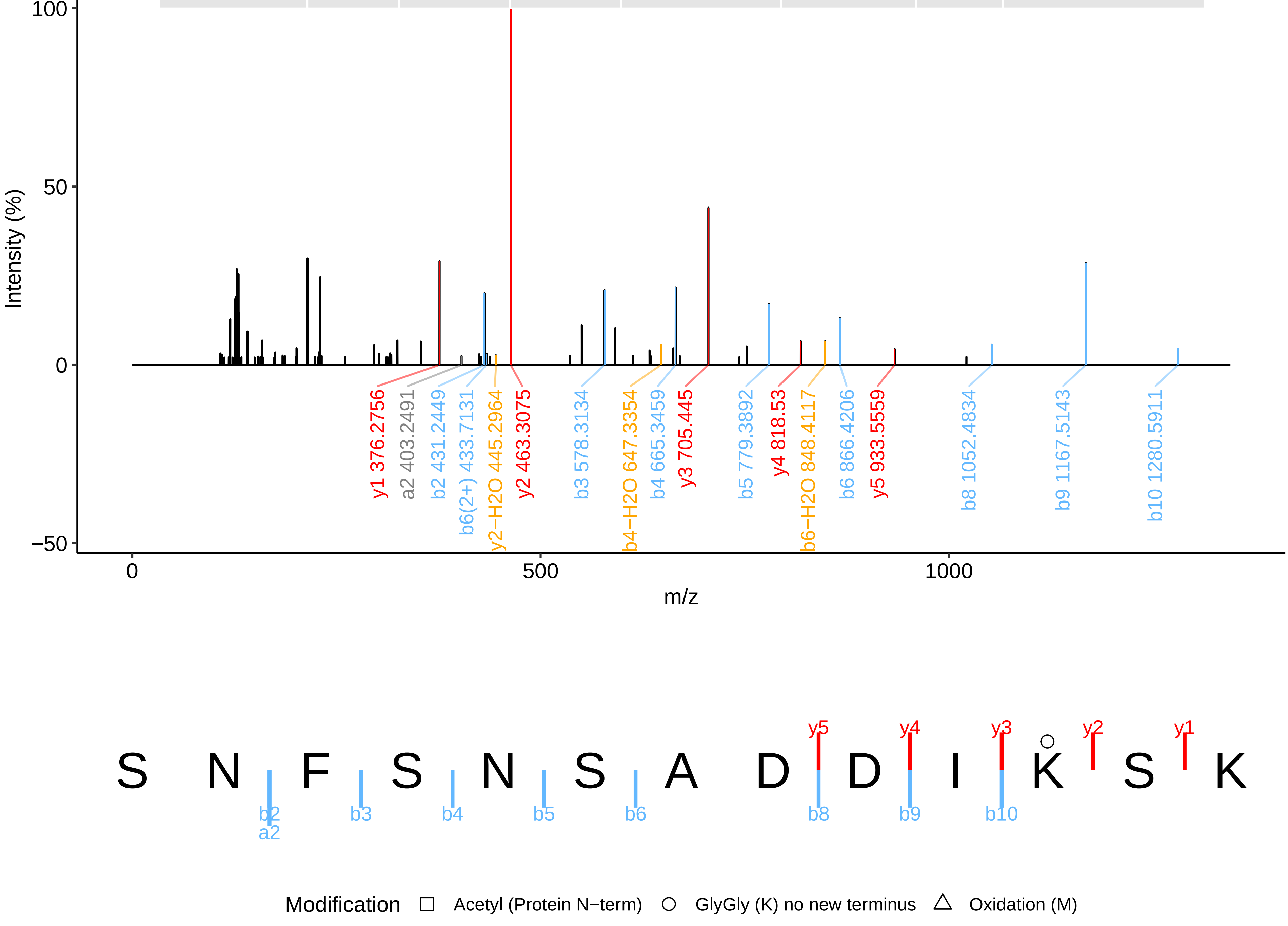

20200924_KIZ_MUb5_GlyGly_1 − Scan number: 34265

Gene Names	Charge	m/z	Mass	Retention time	PEP	Score	Precursor Intensity
MIPOL1	3	531.6313	1591.872	109.02	0.040463	72.62	31970496

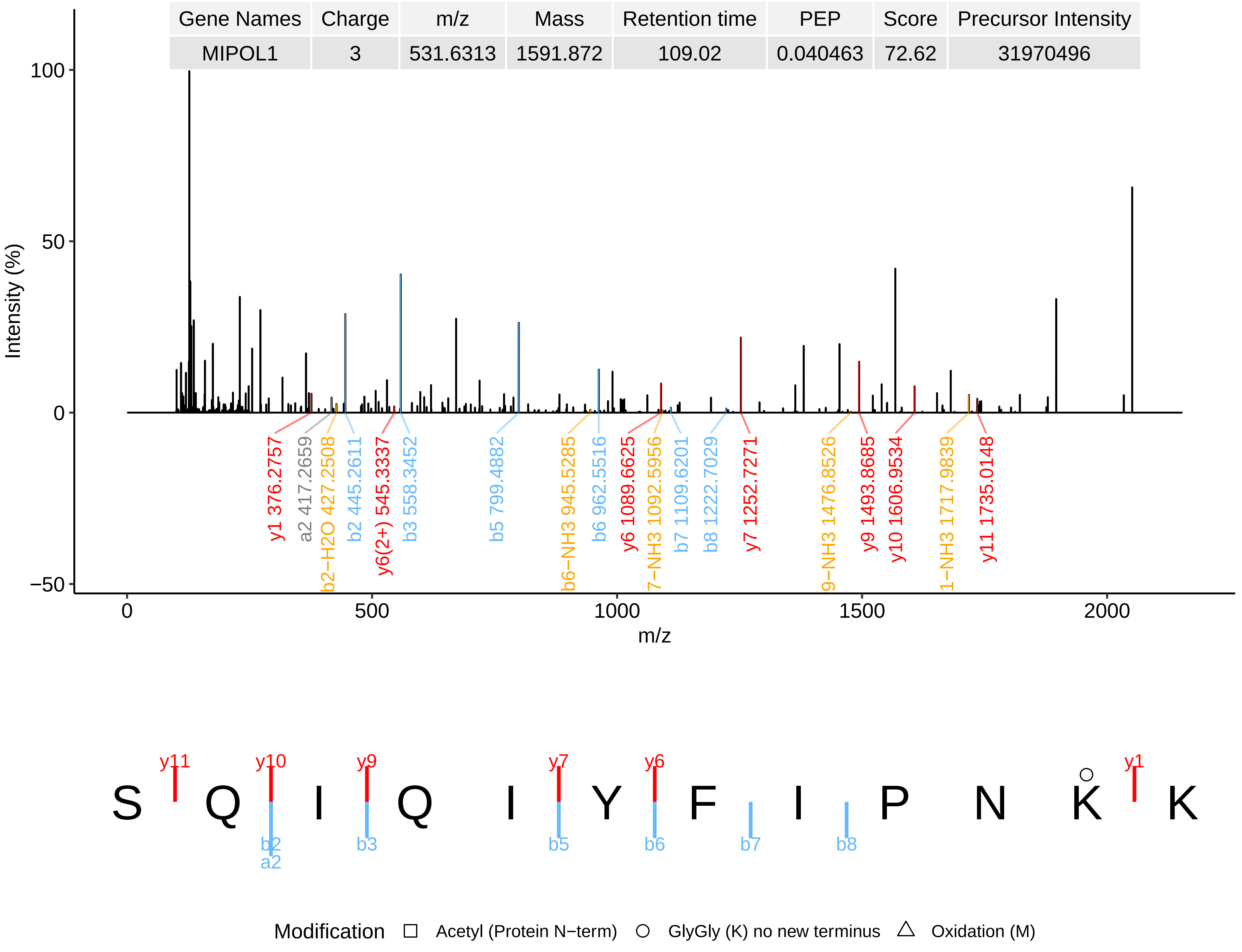

20200924_KIZ_MUb5_GlyGly_1 − Scan number: 34523

Gene Names	Charge	m/z	Mass	Retention time	PEP	Score	Precursor Intensity
MIPOL1	3	531.6313	1591.872	109.83	0.10359	61.11	32027464

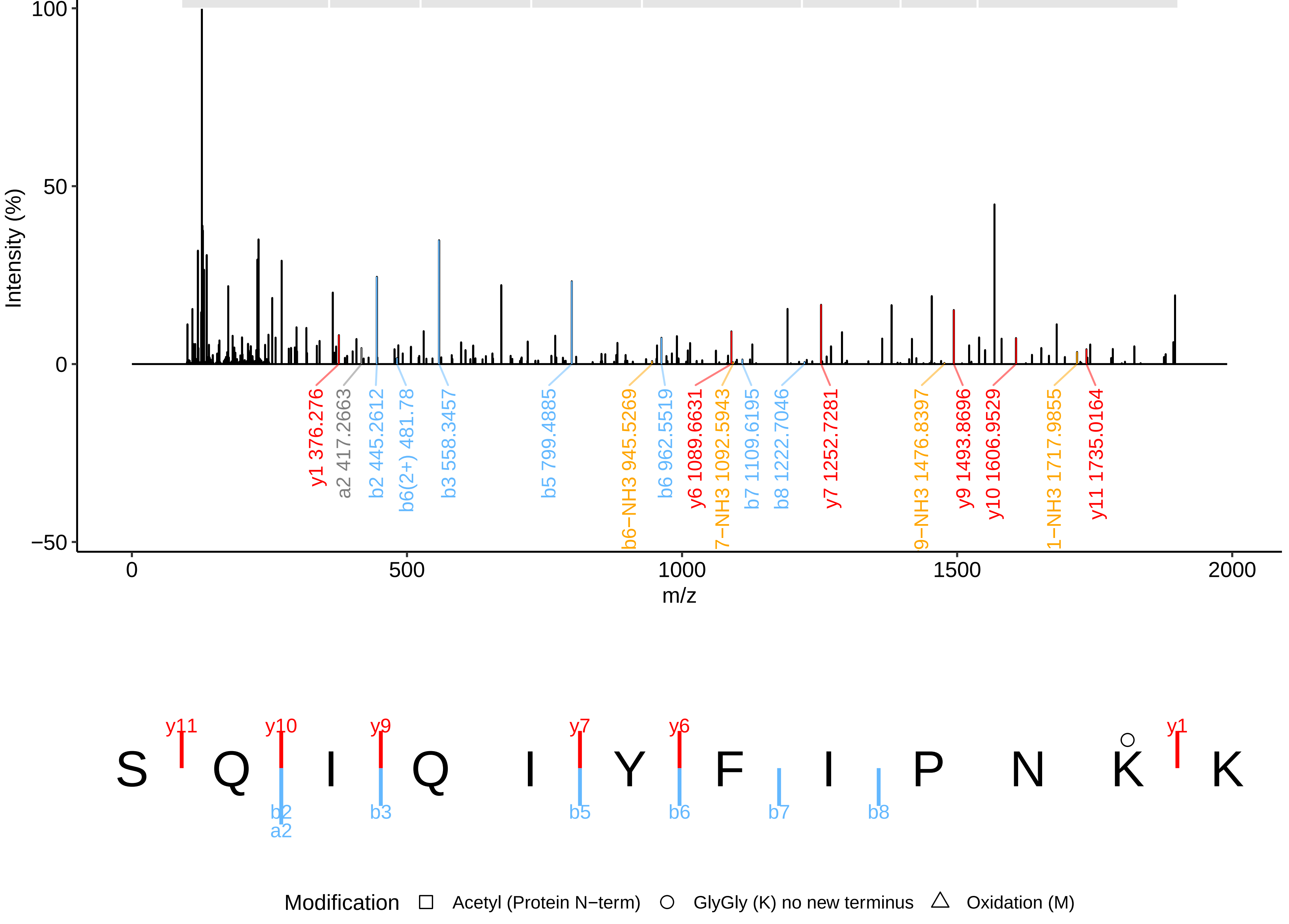

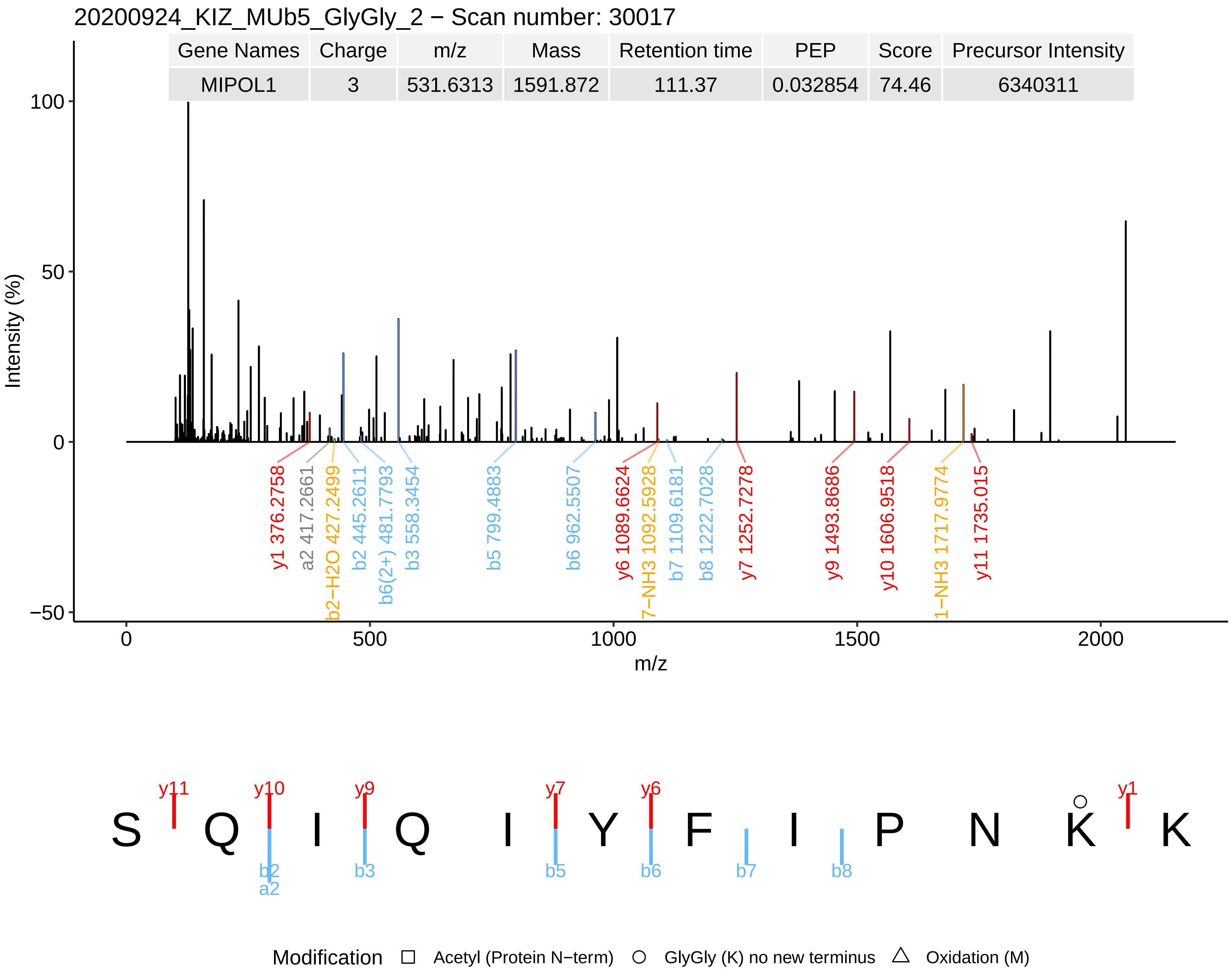
20200924_KIZ_MUb5_GlyGly_2 − Scan number: 30017
Gene Names | Charge | m/z | Mass | Retention time | PEP | Score | Precursor Intensity
MIPOL1 | 3 | 531.6313 | 1591.872 | 111.37 | 0.032854 | 74.46 | 6340311
Intensity (%)
100
50
0
−50
y1 376.2758
a2 417.2661
b2−H2O 427.2499
b2 445.2611
b6(2+) 481.7793
b3 558.3454
b5 799.4883
b6 962.5507
y6 1089.6624
7−NH3 1092.5928
b7 1109.6181
b8 1222.7028
y7 1252.7278
y9 1493.8686
y10 1606.9518
1−NH3 1717.9774
y11 1735.015
0
500
1000
1500
2000
m/z
y11
y10
y9
y7
y6
y1
S Q I Q I Y F I P N K K
b2
a2
b3
b5
b6
b7
b8
Modification
Acetyl (Protein N−term)
GlyGly (K) no new terminus
Oxidation (M)

20200924_KIZ_MUb5_GlyGly_2 − Scan number: 30182

Gene Names	Charge	m/z	Mass	Retention time	PEP	Score	Precursor Intensity
MIPOL1	3	531.6313	1591.872	111.97	0.019901	77.593	8661886

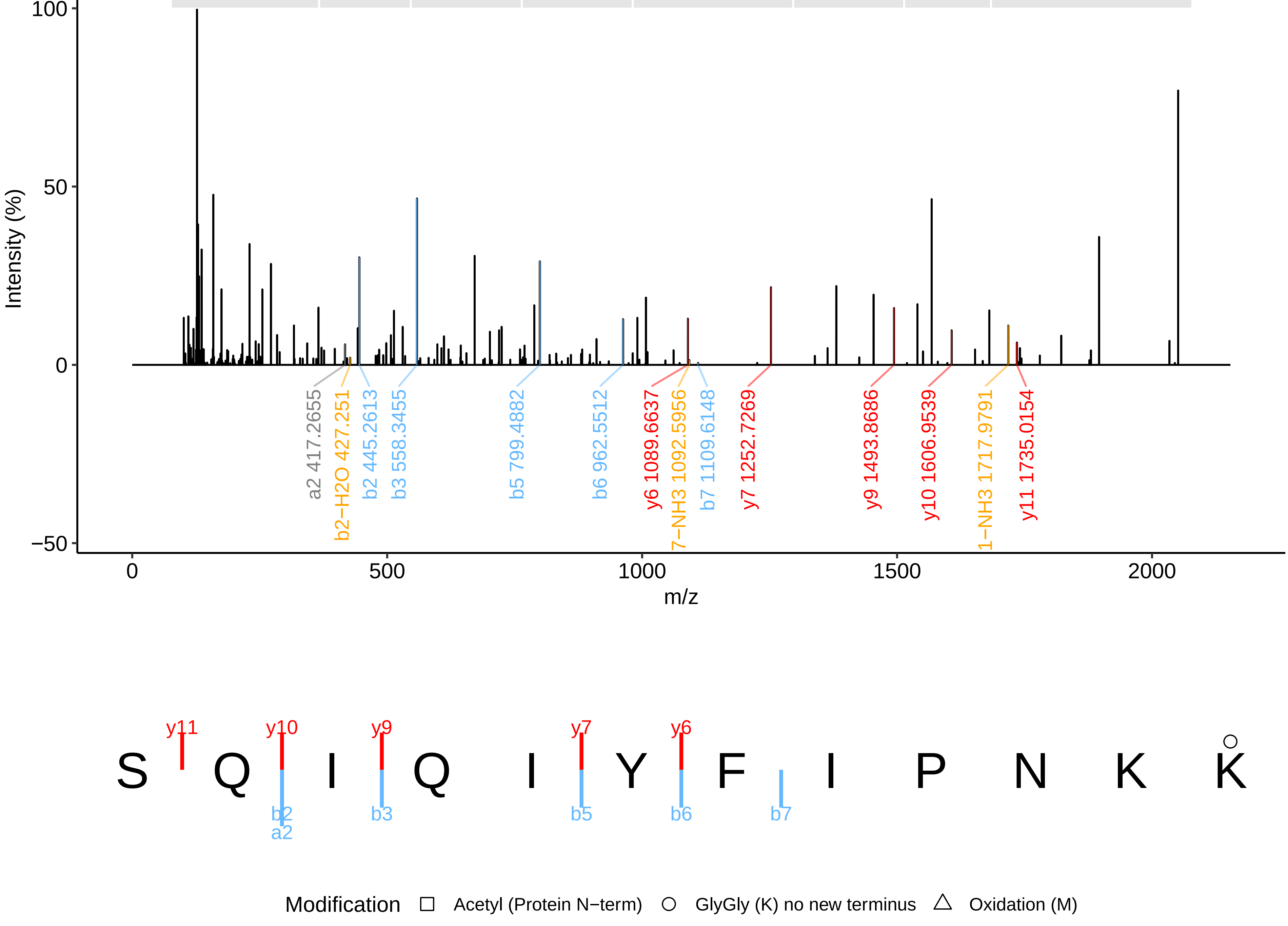

20200924_KIZ_MUb5_GlyGly_2 − Scan number: 30536

Gene Names	Charge	m/z	Mass	Retention time	PEP	Score	Precursor Intensity
MIPOL1	3	531.6313	1591.872	113.3	0.061551	67.519	8584003

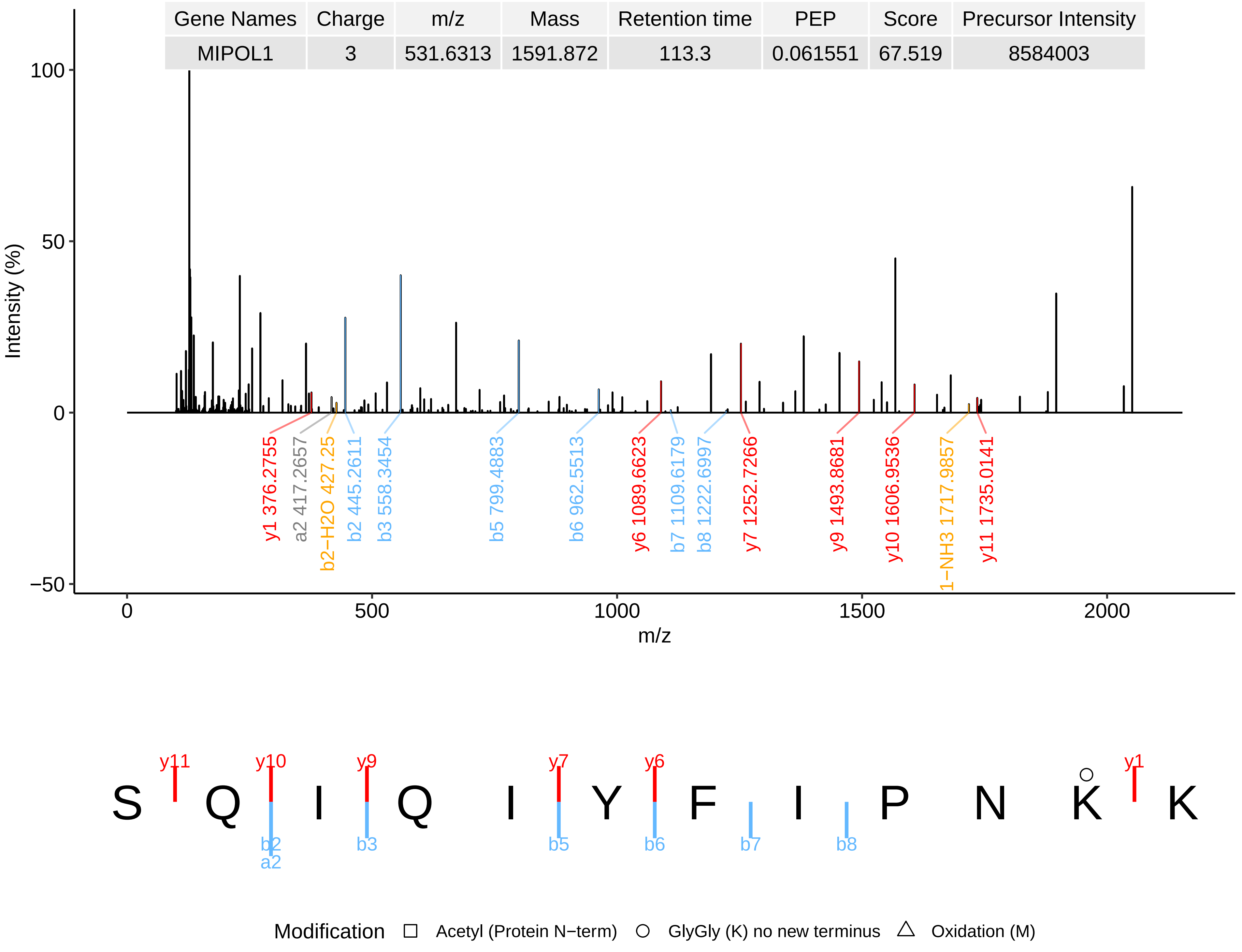

20200924_KIZ_MUb5_GlyGly_2 − Scan number: 30876

Gene Names	Charge	m/z	Mass	Retention time	PEP	Score	Precursor Intensity
MIPOL1	3	531.6313	1591.872	114.6	0.065775	66.498	10563066

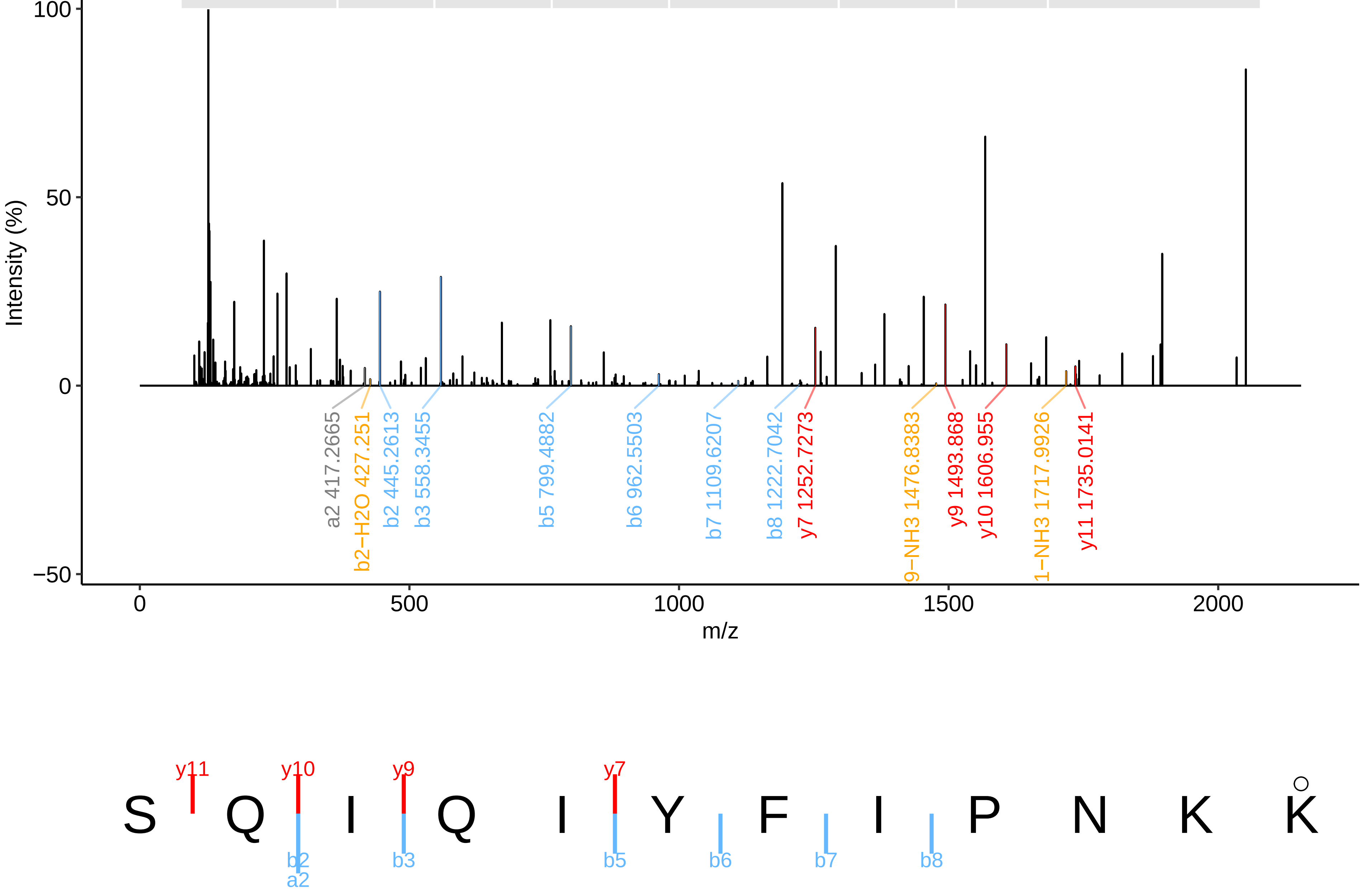

Modification □ Acetyl (Protein N−term) ○ GlyGly (K) no new terminus △ Oxidation (M)

20201118_KIZ_MUb6_GlyGly_1 − Scan number: 36677

Gene Names	Charge	m/z	Mass	Retention time	PEP	Score	Precursor Intensity
MIPOL1	3	531.6313	1591.872	106.4	0.06181	67.456	116091952

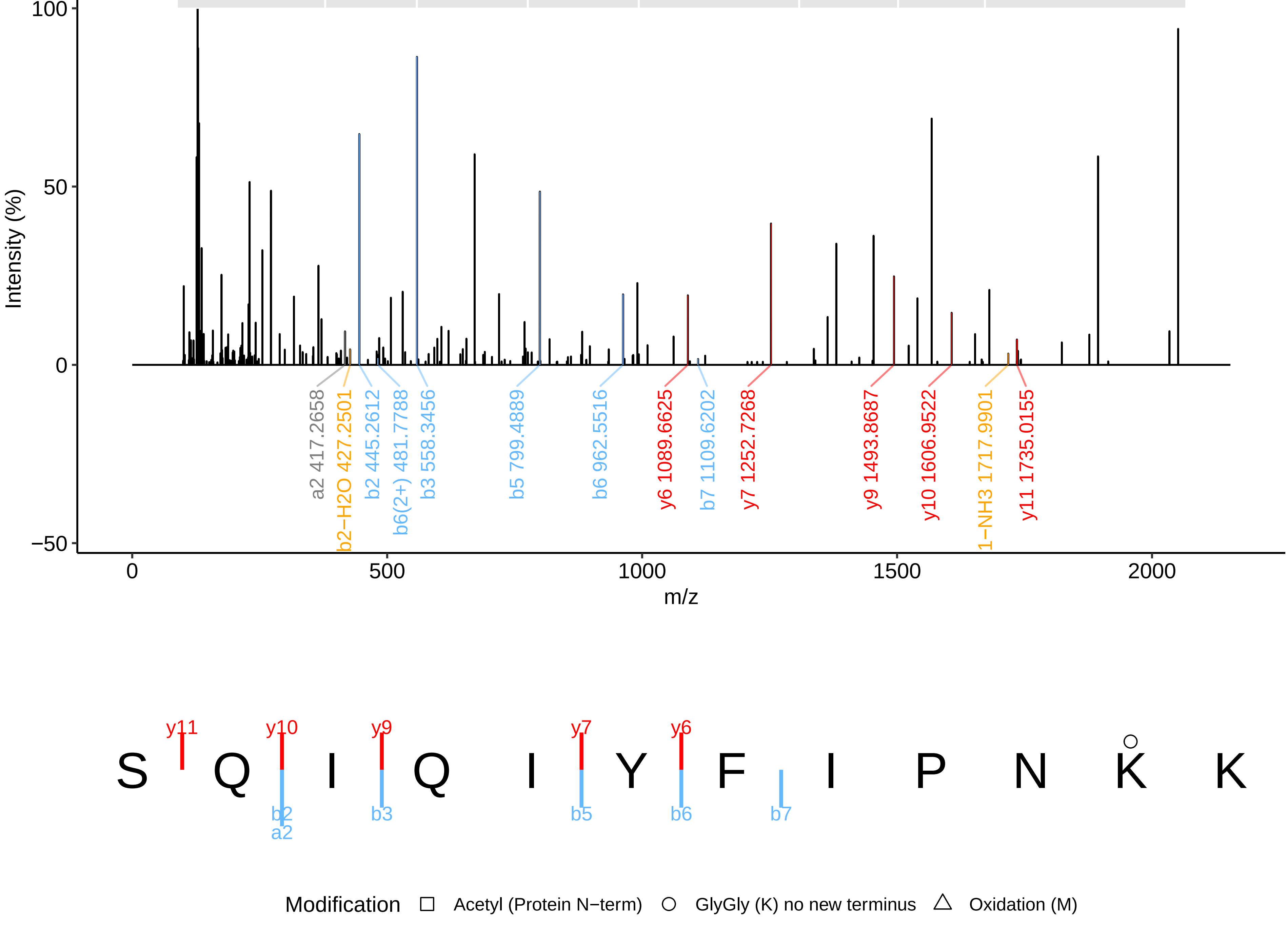

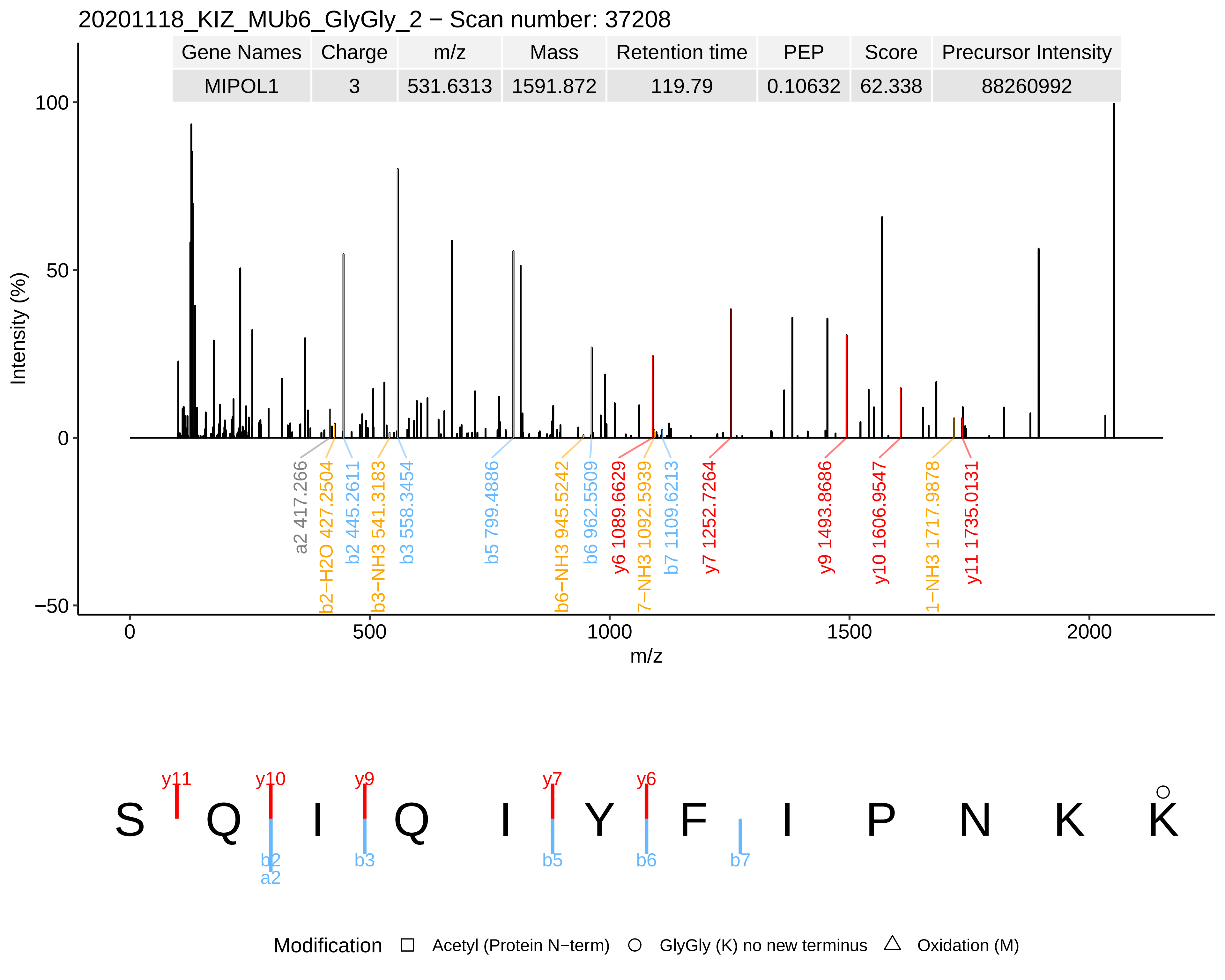
20201118_KIZ_MUb6_GlyGly_2 − Scan number: 37208
Gene Names | Charge | m/z | Mass | Retention time | PEP | Score | Precursor Intensity
MIPOL1 | 3 | 531.6313 | 1591.872 | 119.79 | 0.10632 | 62.338 | 88260992
Intensity (%)
100
50
0
−50
0
500
1000
1500
2000
m/z
a2 417.266
b2−H2O 427.2504
b2 445.2611
b3−NH3 541.3183
b3 558.3454
b5 799.4886
b6−NH3 945.5242
b6 962.5509
y6 1089.6629
7−NH3 1092.5939
b7 1109.6213
y7 1252.7264
y9 1493.8686
y10 1606.9547
1−NH3 1717.9878
y11 1735.0131
S Q I Q I Y F I P N K K
y11
y10
y9
y7
y6
b2
a2
b3
b5
b6
b7
Modification
Acetyl (Protein N−term)
GlyGly (K) no new terminus
Oxidation (M)

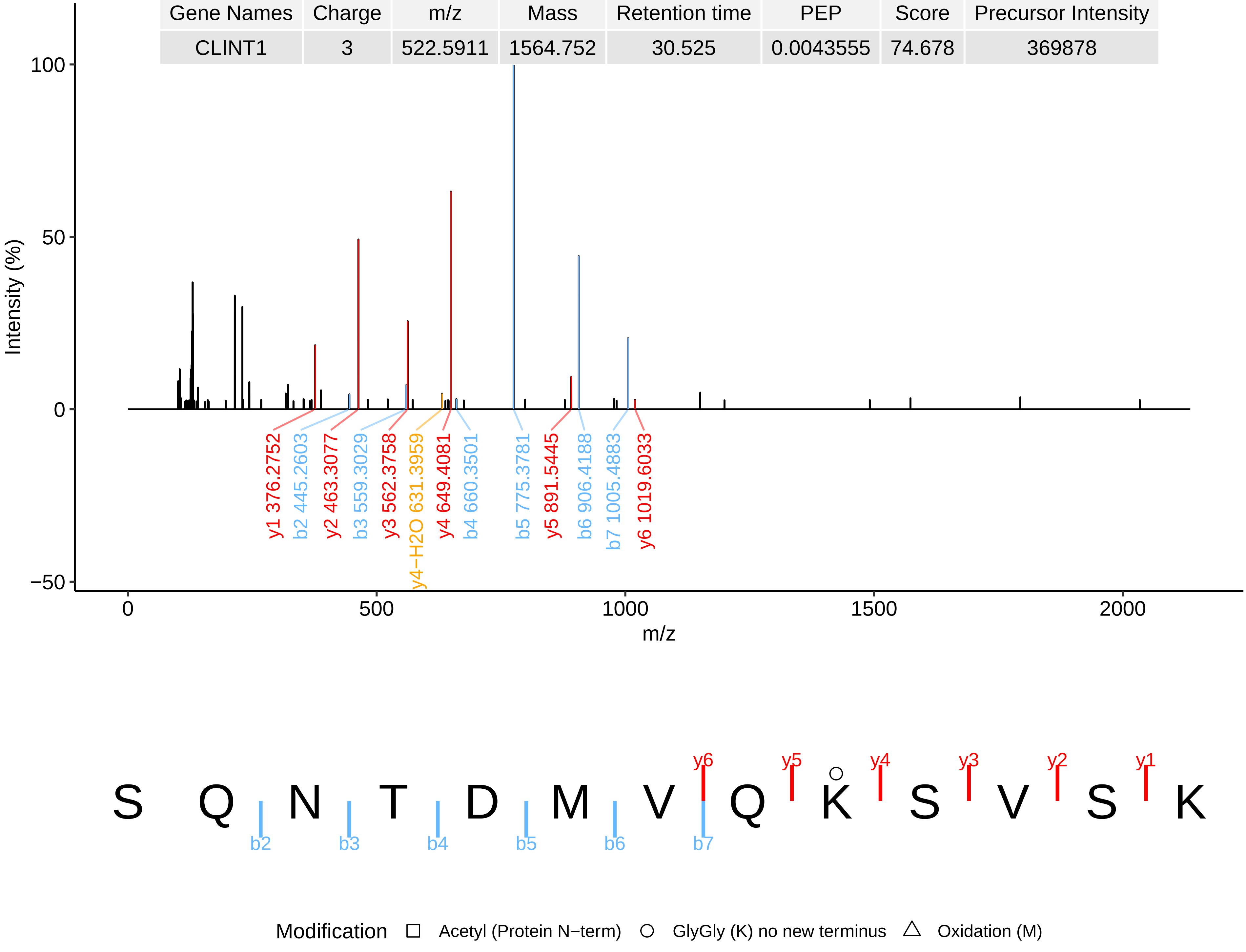

Gene Names	Charge	m/z	Mass	Retention time	PEP	Score	Precursor Intensity
CLINT1	3	522.5911	1564.752	30.525	0.0043555	74.678	369878

20200921_KIZ_MUb4_GlyGly_10plex_2 − Scan number: 17746

Gene Names	Charge	m/z	Mass	Retention time	PEP	Score	Precursor Intensity
PHB2	3	592.6636	1774.969	69.428	0.007098	66.246	NA

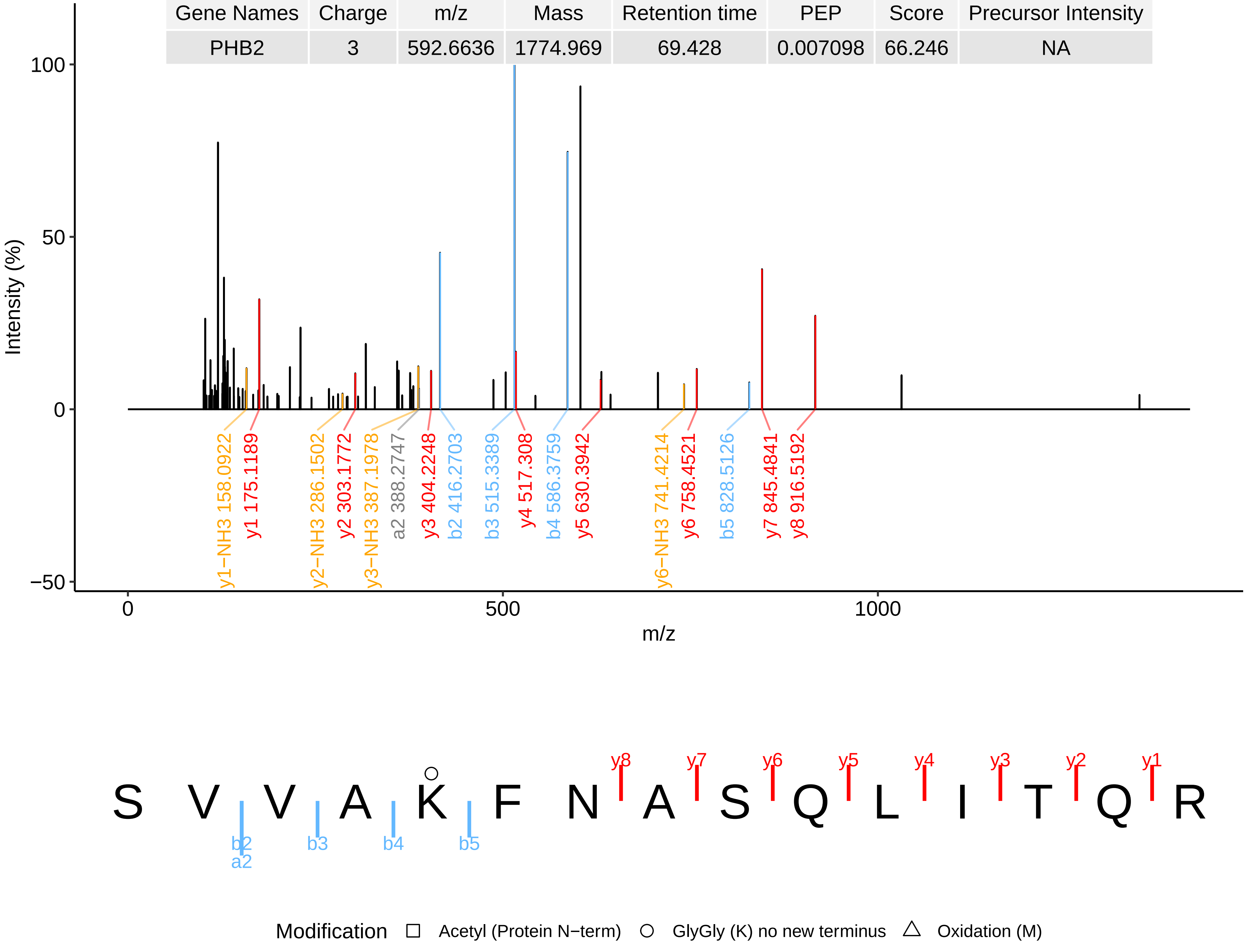

20201118_KIZ_MUb6_GlyGly_1 − Scan number: 25973

Gene Names	Charge	m/z	Mass	Retention time	PEP	Score	Precursor Intensity
PIP4K2A	3	519.6267	1555.858	79.28	0.078813	47.261	47758956

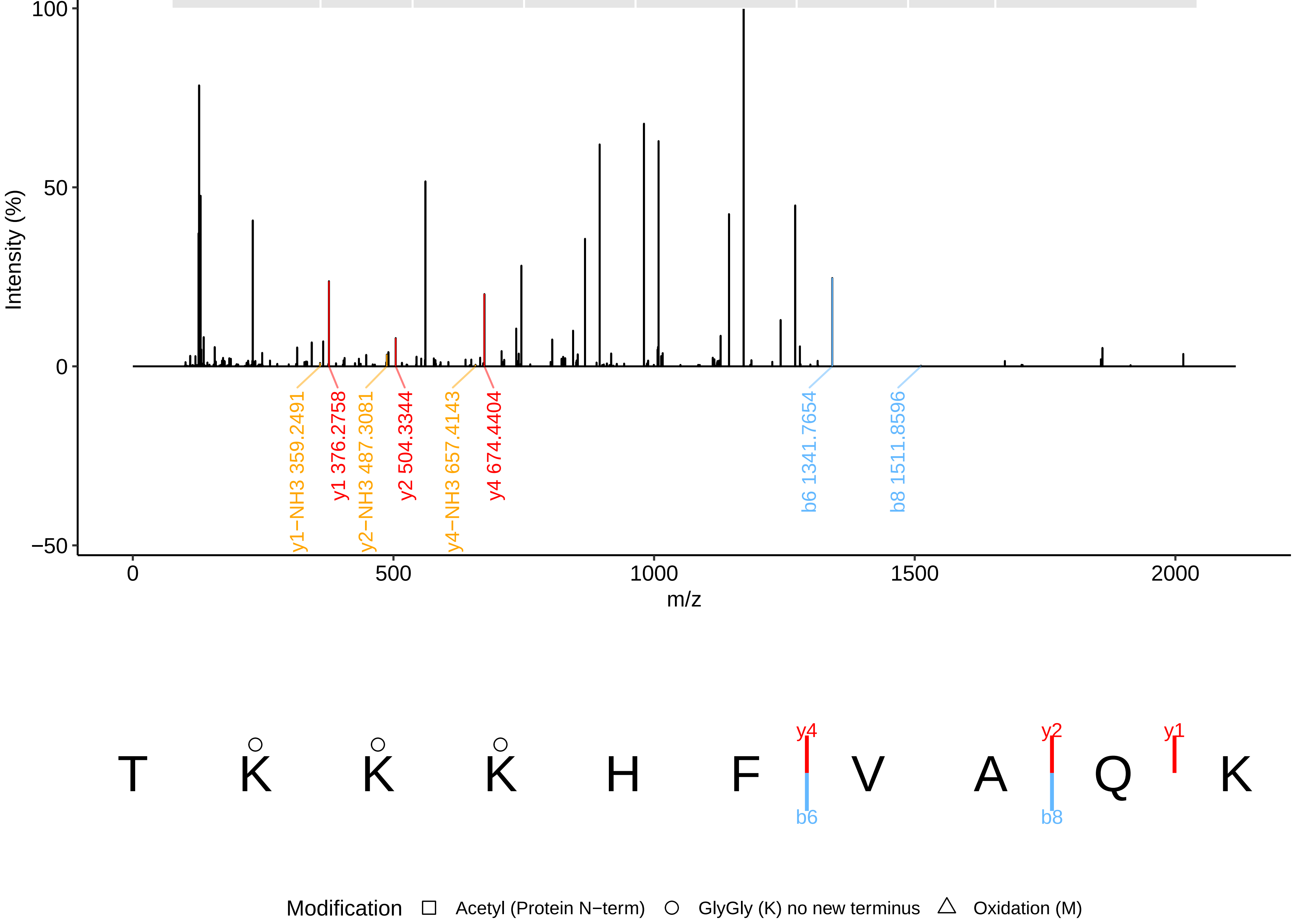

20200921_KIZ_MUb4_GlyGly_10plex − Scan number: 15156

Gene Names	Charge	m/z	Mass	Retention time	PEP	Score	Precursor Intensity
GYS1;GYS2	3	602.3149	1803.923	54.071	0.0012906	78.285	NA

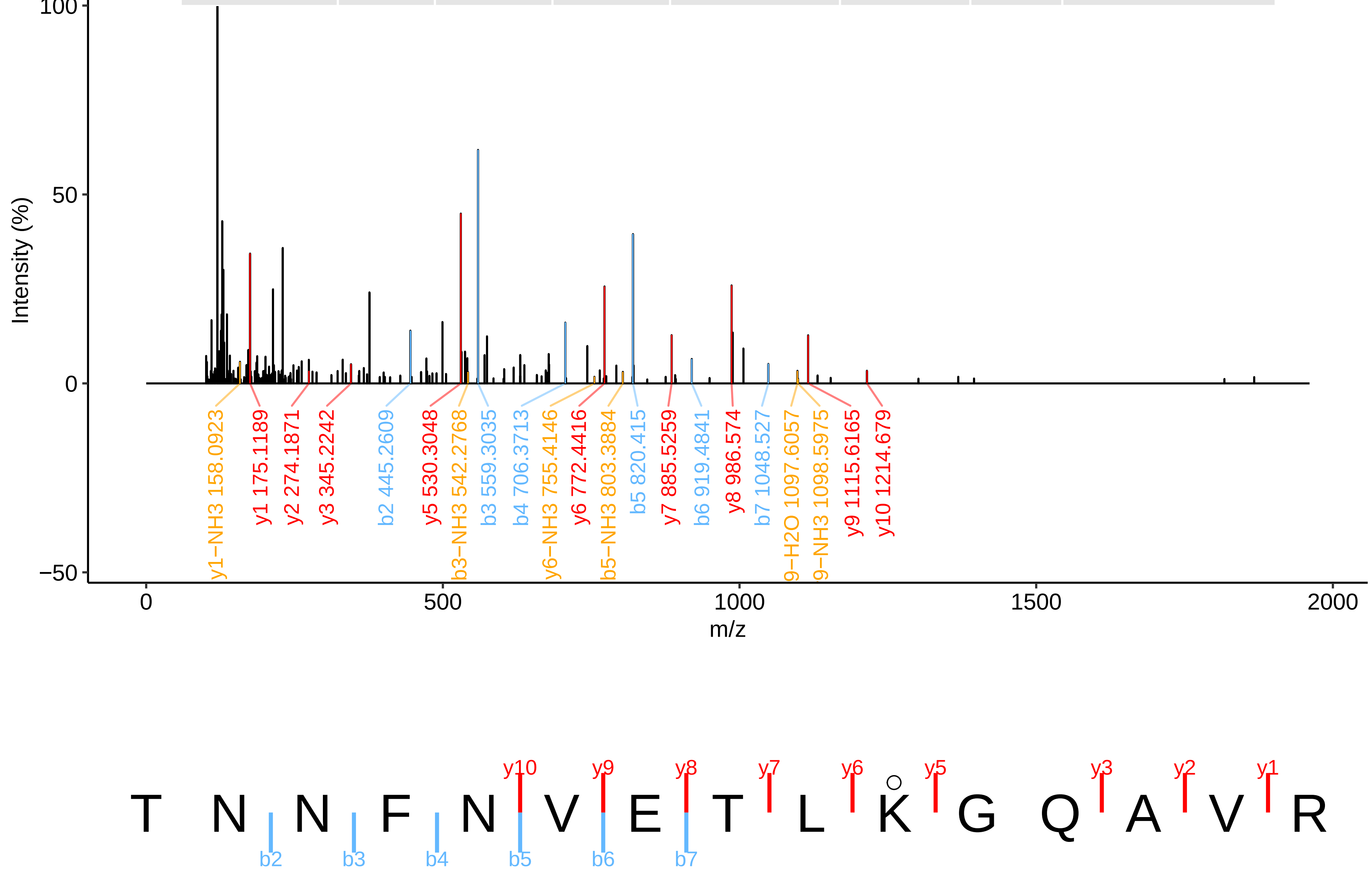

Modification □ Acetyl (Protein N−term) ○ GlyGly (K) no new terminus △ Oxidation (M)

20200921_KIZ_MUb4_GlyGly_10plex − Scan number: 15520

Gene Names	Charge	m/z	Mass	Retention time	PEP	Score	Precursor Intensity
PDCD6IP	3	529.2897	1584.847	55.458	0.00021135	93.494	2535785

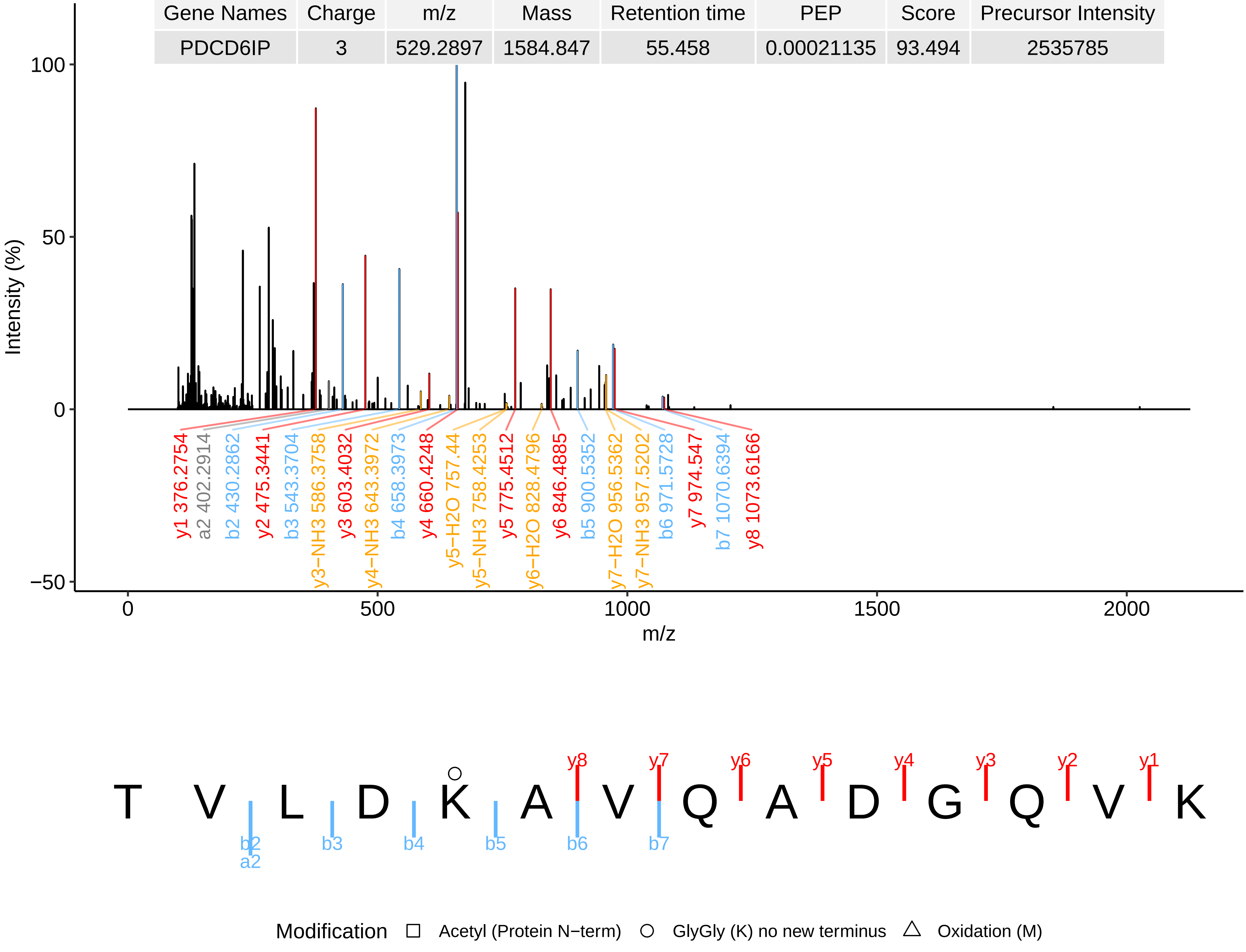

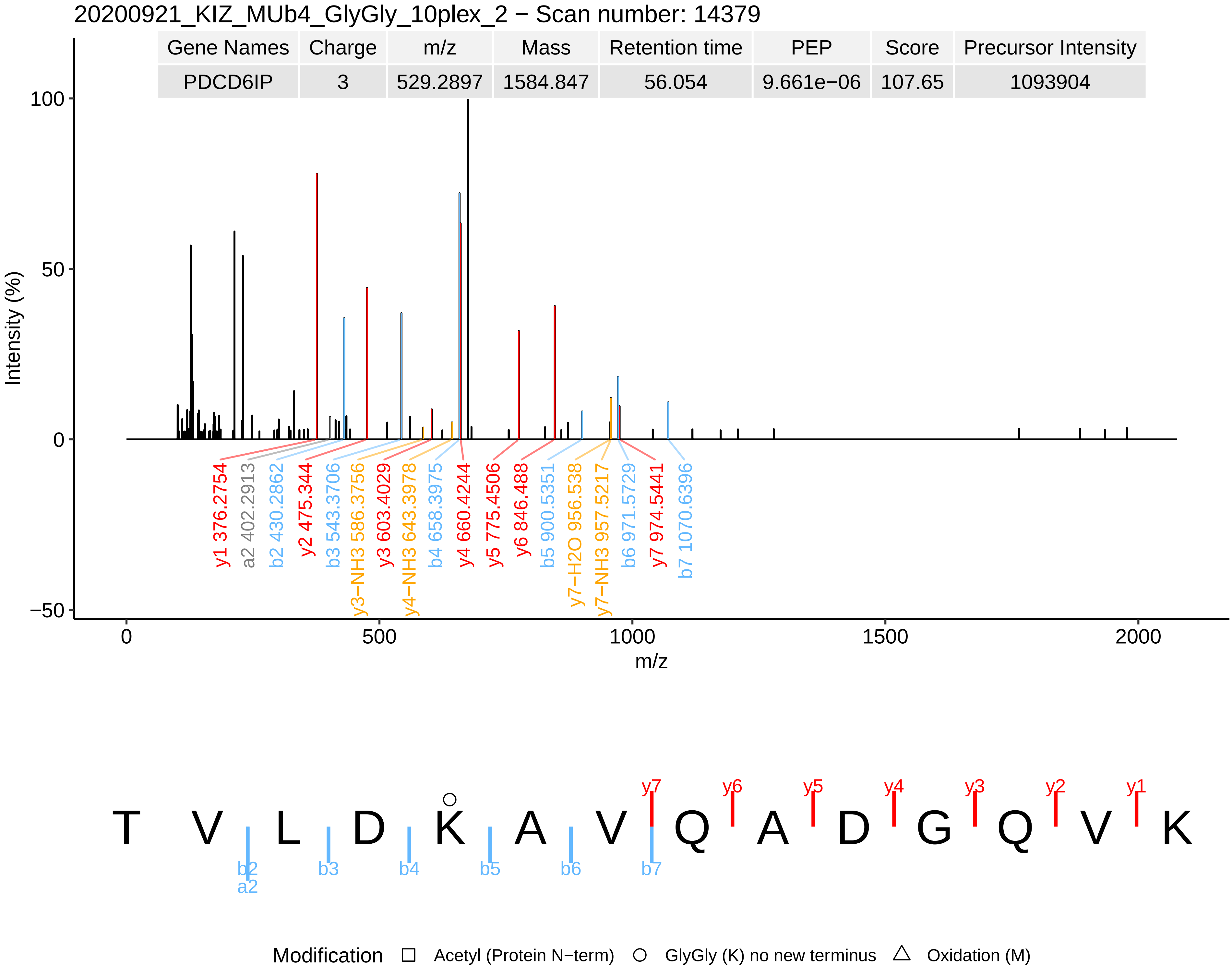

20200921_KIZ_MUb4_GlyGly_10plex_2 − Scan number: 14379
Gene Names | Charge | m/z | Mass | Retention time | PEP | Score | Precursor Intensity
PDCD6IP | 3 | 529.2897 | 1584.847 | 56.054 | 9.661e−06 | 107.65 | 1093904
Intensity (%)
m/z
y1 376.2754
a2 402.2913
b2 430.2862
y2 475.344
b3 543.3706
y3−NH3 586.3756
y3 603.4029
y4−NH3 643.3978
b4 658.3975
y4 660.4244
y5 775.4506
y6 846.488
b5 900.5351
y7−H2O 956.538
y7−NH3 957.5217
b6 971.5729
y7 974.5441
b7 1070.6396
T V L D K A V Q A D G Q V K
y7 y6 y5 y4 y3 y2 y1
b2 a2 b3 b4 b5 b6 b7
Modification
Acetyl (Protein N−term)
GlyGly (K) no new terminus
Oxidation (M)

20200921_KIZ_MUb4_GlyGly_10plex_2 − Scan number: 13204

Gene Names	Charge	m/z	Mass	Retention time	PEP	Score	Precursor Intensity
SMC4	3	516.9509	1547.831	51.221	0.081428	47.915	760963.2

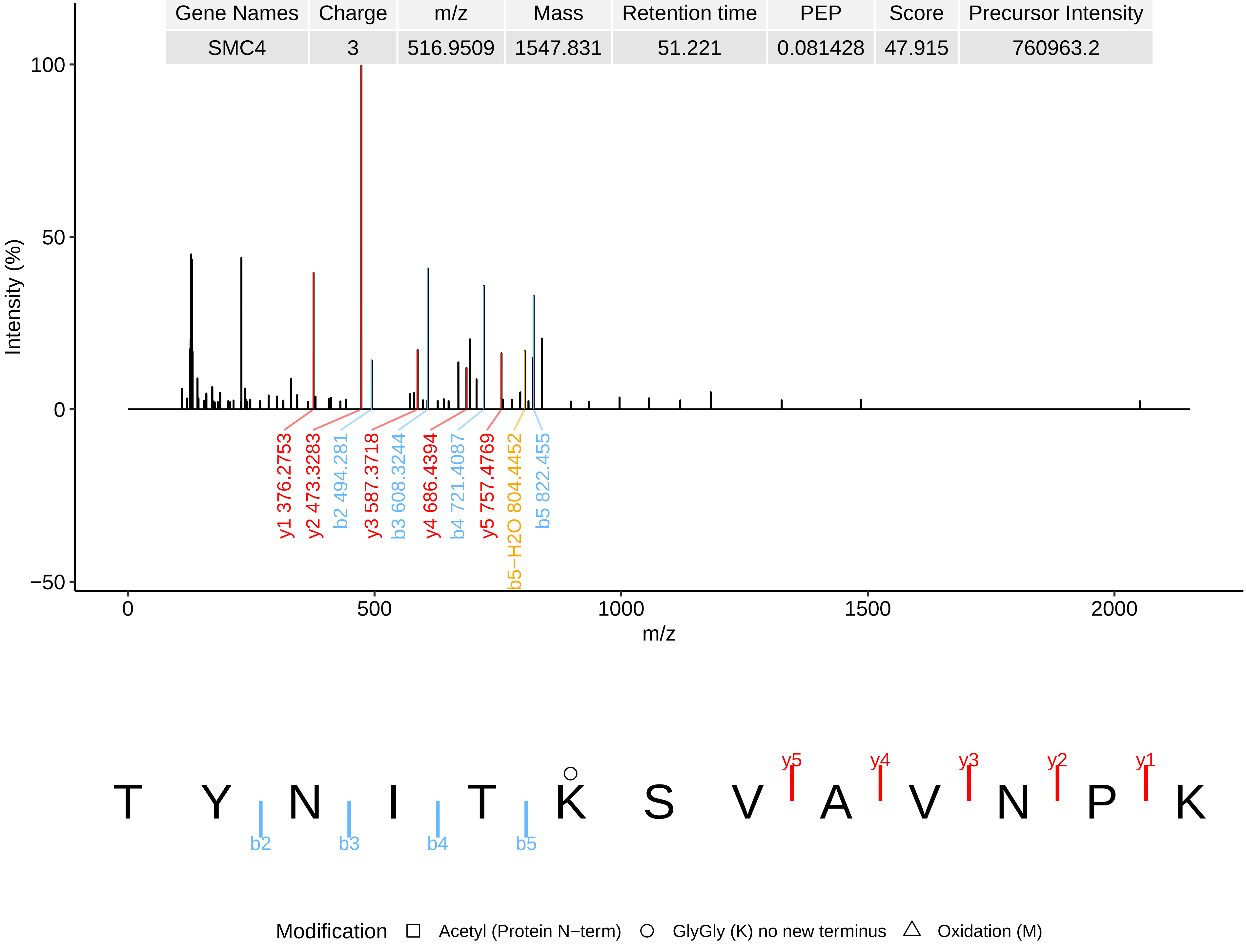

20200921_KIZ_MUb4_GlyGly_10plex − Scan number: 15007

Gene Names	Charge	m/z	Mass	Retention time	PEP	Score	Precursor Intensity
AHCY	3	529.2897	1584.847	53.494	0.00012052	95.651	2063382

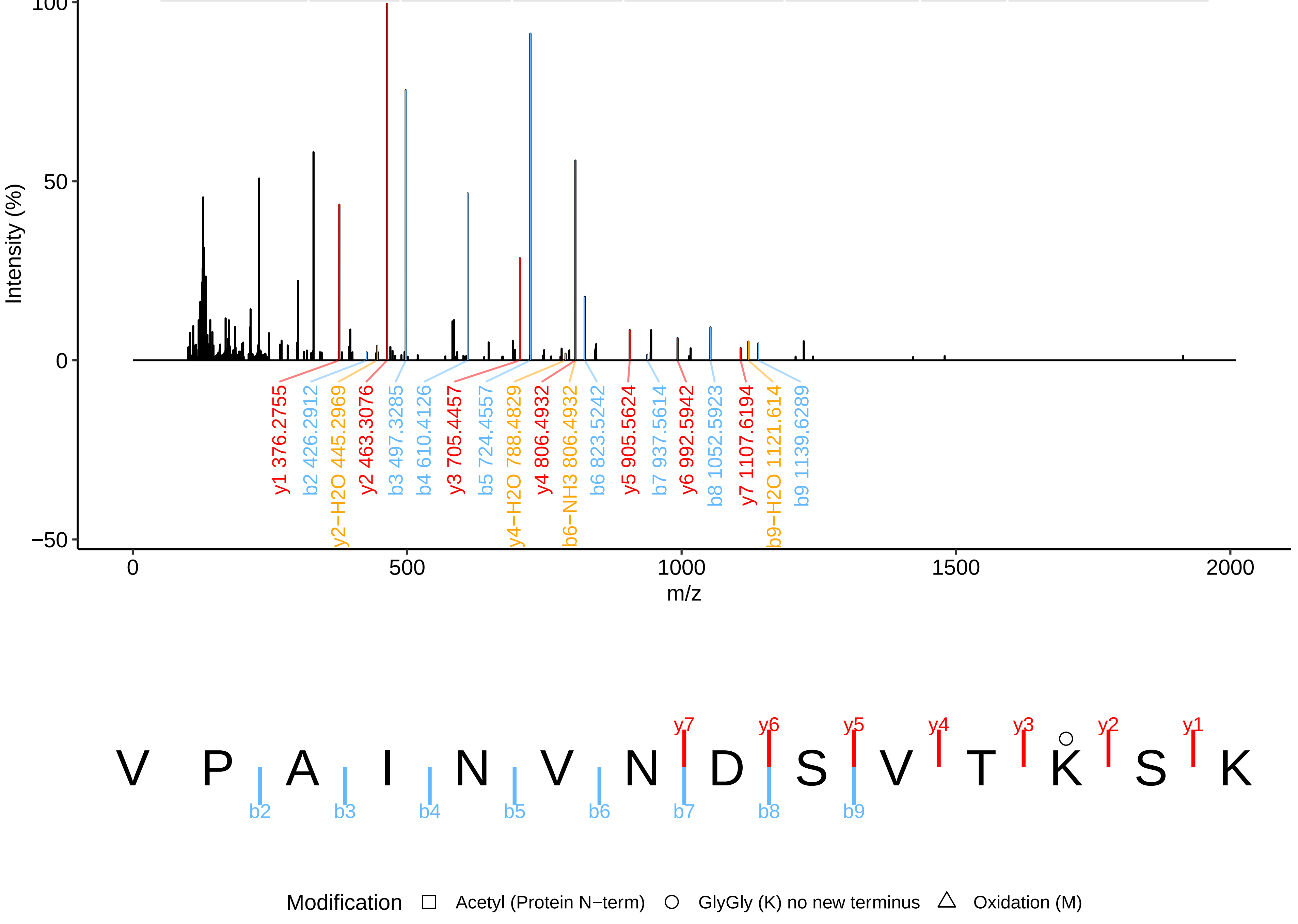

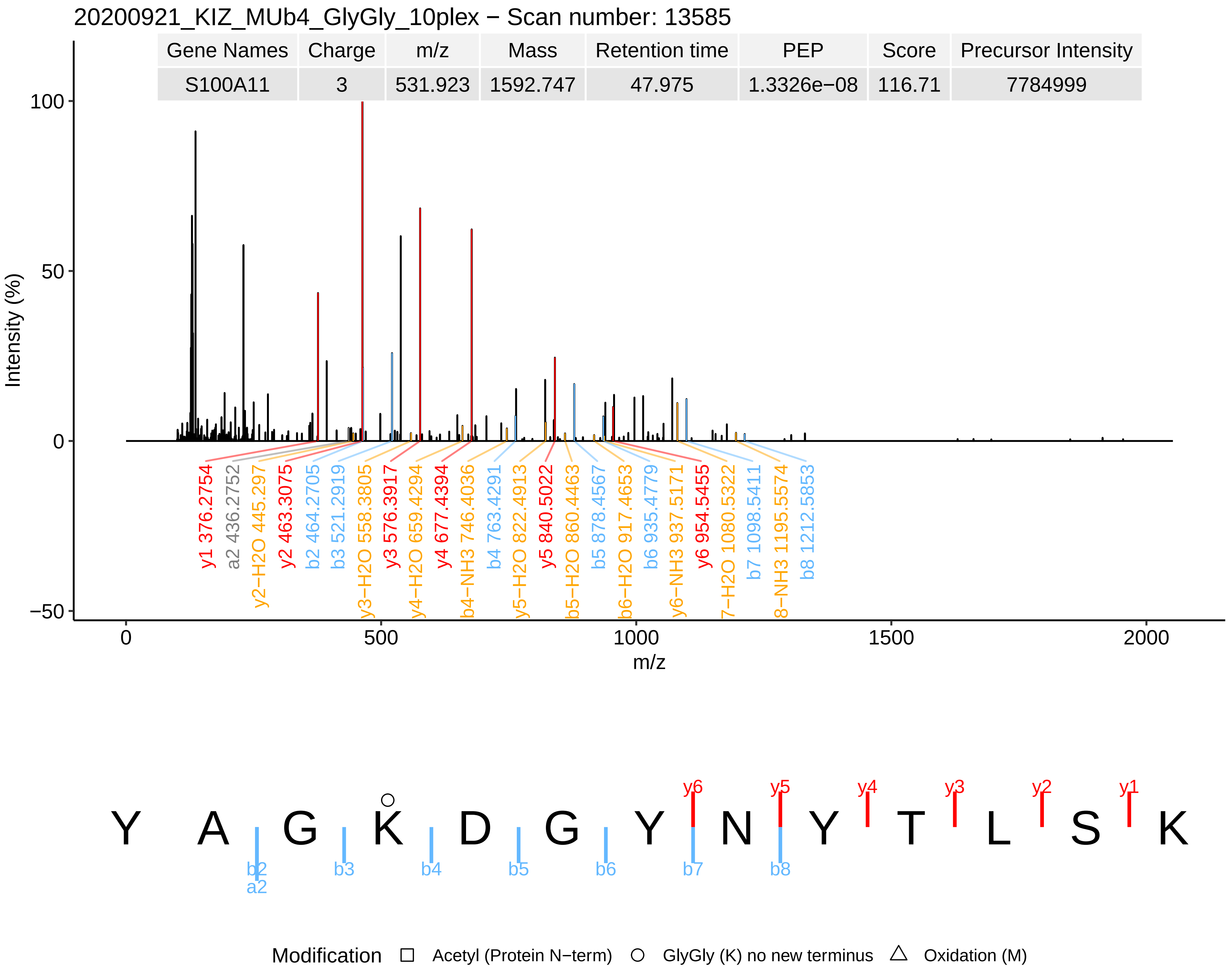

20200921_KIZ_MUb4_GlyGly_10plex − Scan number: 13585
Gene Names | Charge | m/z | Mass | Retention time | PEP | Score | Precursor Intensity
S100A11 | 3 | 531.923 | 1592.747 | 47.975 | 1.3326e−08 | 116.71 | 7784999
Intensity (%)
m/z
y1 376.2754
a2 436.2752
y2−H2O 445.297
y2 463.3075
b2 464.2705
b3 521.2919
y3−H2O 558.3805
y3 576.3917
y4−H2O 659.4294
y4 677.4394
b4−NH3 746.4036
b4 763.4291
y5−H2O 822.4913
y5 840.5022
b5−H2O 860.4463
b5 878.4567
b6−H2O 917.4653
b6 935.4779
y6−NH3 937.5171
y6 954.5455
b7−H2O 1080.5322
b7 1098.5411
b8−NH3 1195.5574
b8 1212.5853
Y A G K D G Y N Y T L S K
Modification
Acetyl (Protein N−term)
GlyGly (K) no new terminus
Oxidation (M)

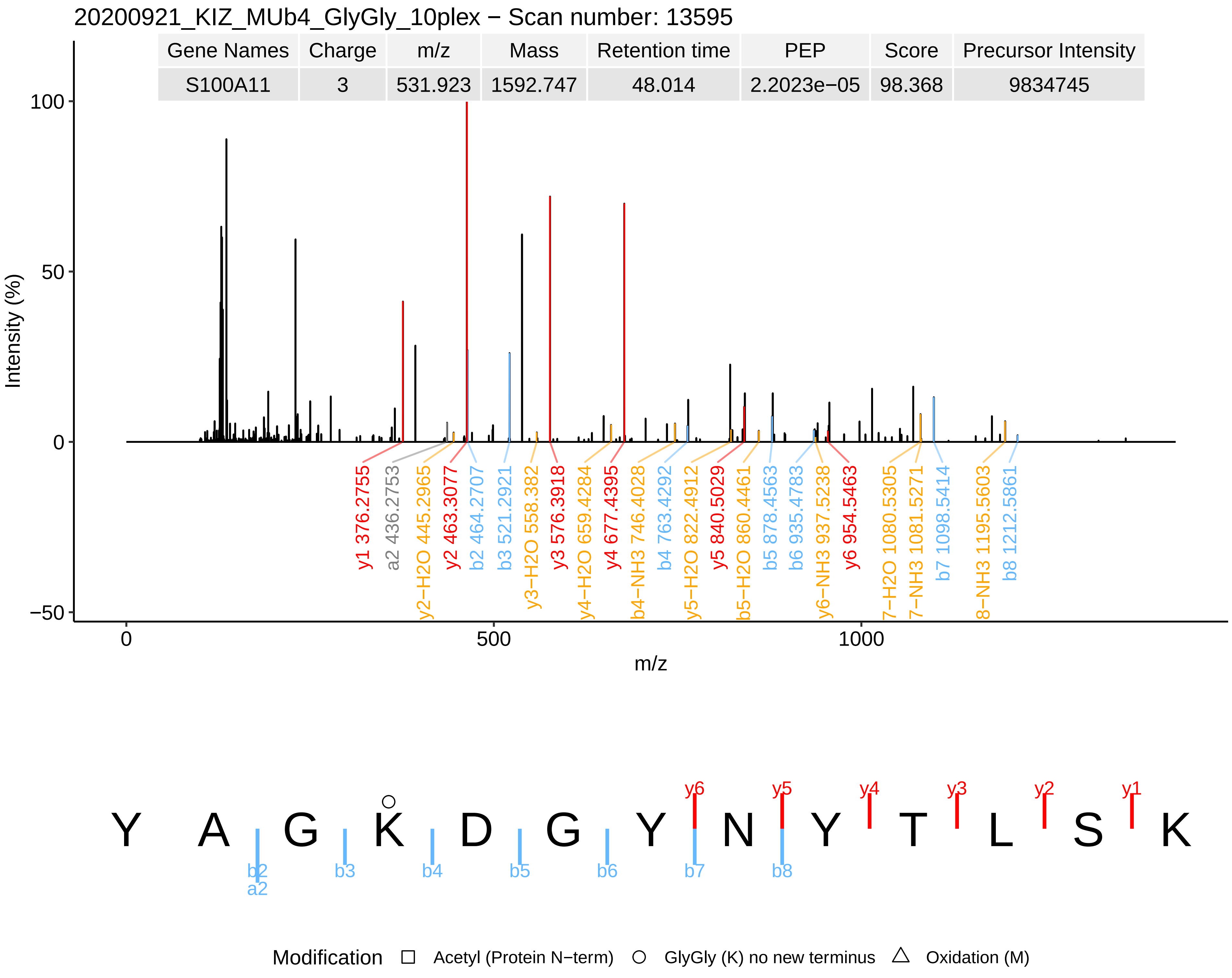
20200921_KIZ_MUb4_GlyGly_10plex − Scan number: 13595
Gene Names | Charge | m/z | Mass | Retention time | PEP | Score | Precursor Intensity
S100A11 | 3 | 531.923 | 1592.747 | 48.014 | 2.2023e−05 | 98.368 | 9834745
Intensity (%)
100
50
0
−50
0
500
1000
m/z
y1 376.2755
a2 436.2753
y2−H2O 445.2965
y2 463.3077
b2 464.2707
b3 521.2921
y3−H2O 558.382
y3 576.3918
y4−H2O 659.4284
y4 677.4395
b4−NH3 746.4028
b4 763.4292
y5−H2O 822.4912
y5 840.5029
b5−H2O 860.4461
b5 878.4563
b6 935.4783
y6−NH3 937.5238
y6 954.5463
7−H2O 1080.5305
7−NH3 1081.5271
b7 1098.5414
8−NH3 1195.5603
b8 1212.5861
Y A G K D G Y N Y T L S K
y6 y5 y4 y3 y2 y1
b2 a2 b3 b4 b5 b6 b7 b8
Modification
Acetyl (Protein N−term)
GlyGly (K) no new terminus
Oxidation (M)

20200921_KIZ_MUb4_GlyGly_10plex_2 − Scan number: 12503

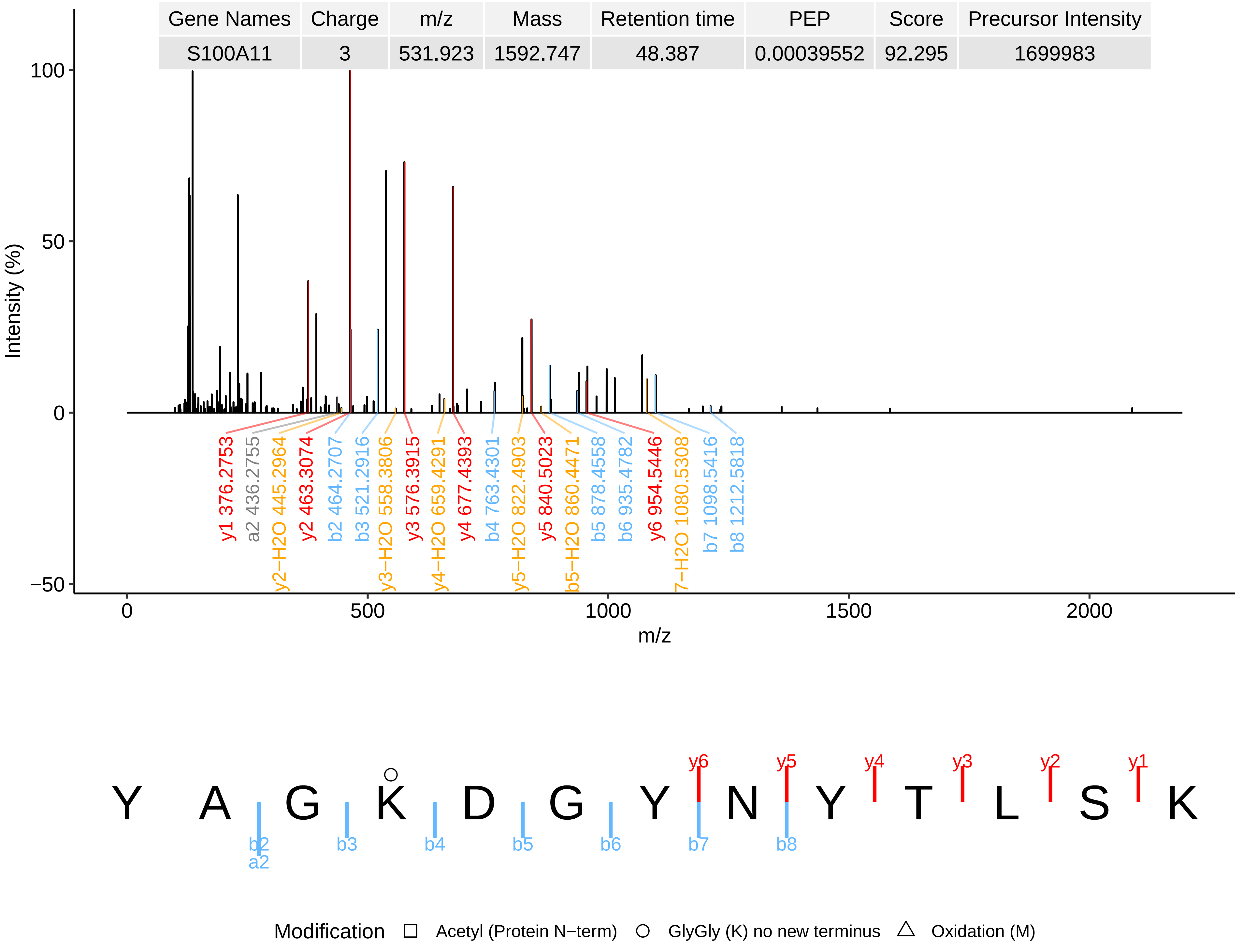

20200921_KIZ_MUb4_GlyGly_10plex_2 − Scan number: 16802

Gene Names	Charge	m/z	Mass	Retention time	PEP	Score	Precursor Intensity
PPP2R1A	3	534.9326	1601.776	65.633	0.025865	59.418	1654849

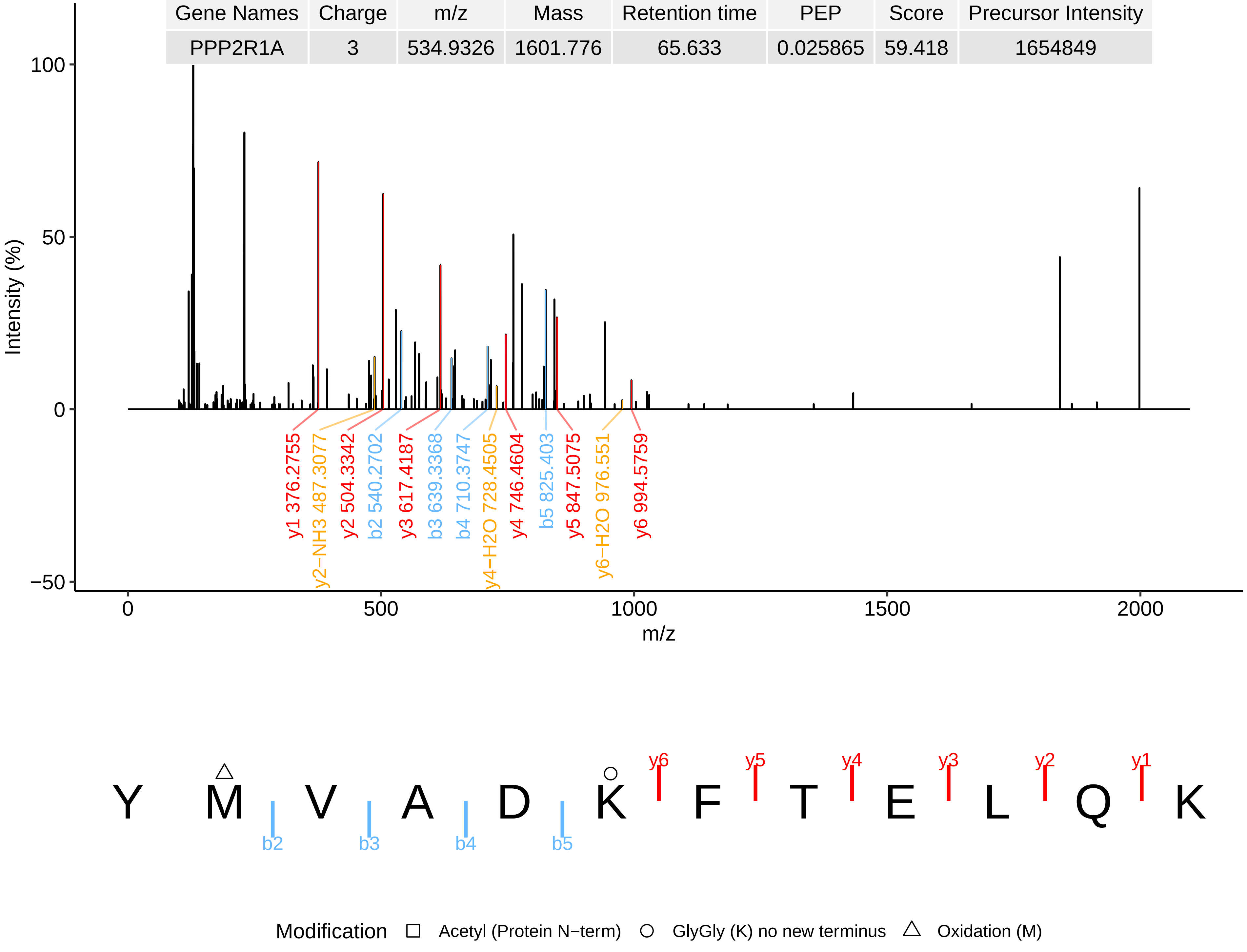

20200921_KIZ_MUb4_GlyGly_10plex_2 − Scan number: 16824

Gene Names	Charge	m/z	Mass	Retention time	PEP	Score	Precursor Intensity
PPP2R1A	3	534.9326	1601.776	65.721	0.0039778	74.962	2870139

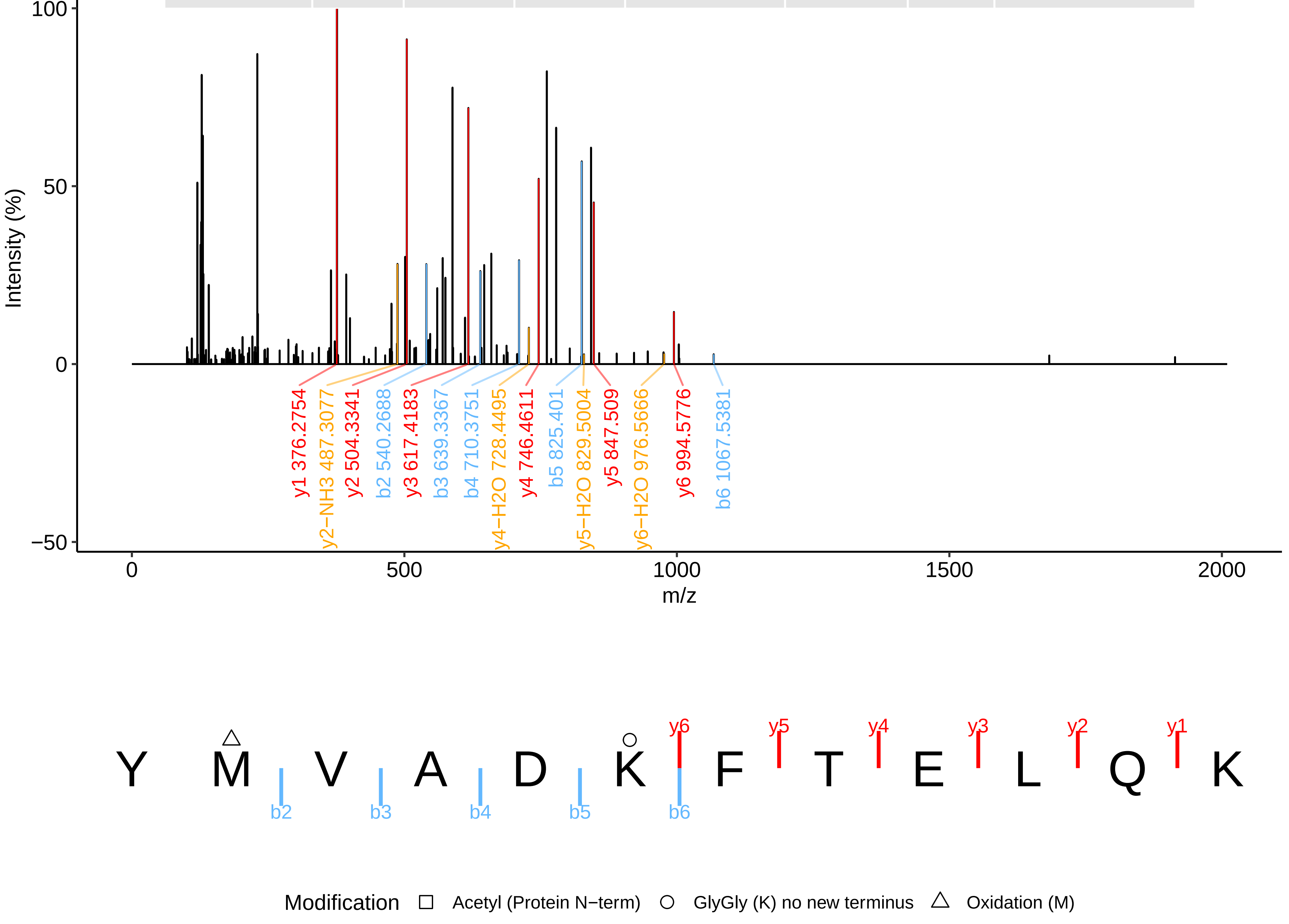

20200924_KIZ_MUb5_GlyGly_2 − Scan number: 16731

Gene Names	Charge	m/z	Mass	Retention time	PEP	Score	Precursor Intensity
PPP2R1A	3	534.9326	1601.776	62.076	0.11773	51.979	3408404

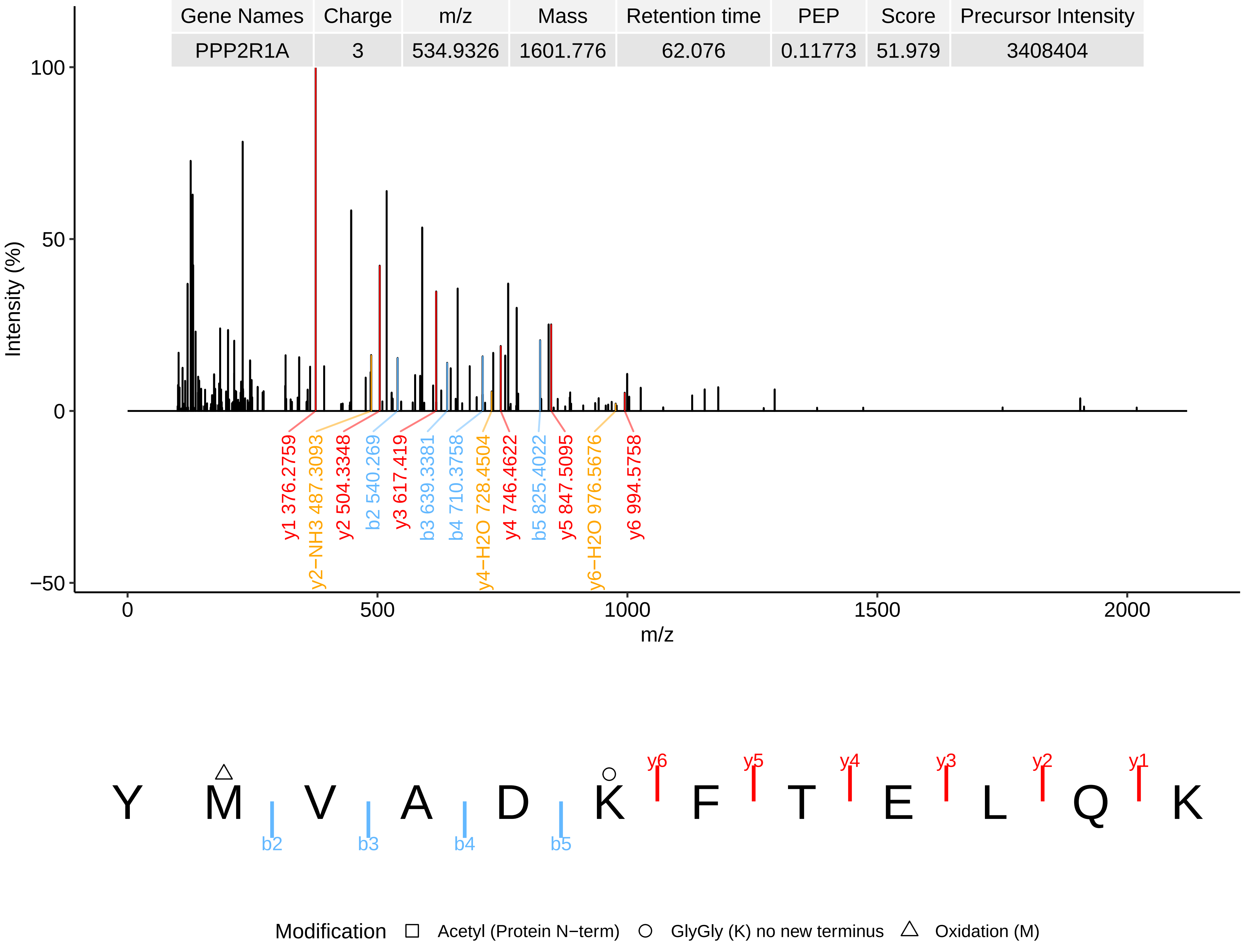

20200921_KIZ_MUb4_GlyGly_10plex_2 − Scan number: 18665

Gene Names	Charge	m/z	Mass	Retention time	PEP	Score	Precursor Intensity
PPP2R1A	3	529.601	1585.781	73.15	0.012018	64.439	1172450

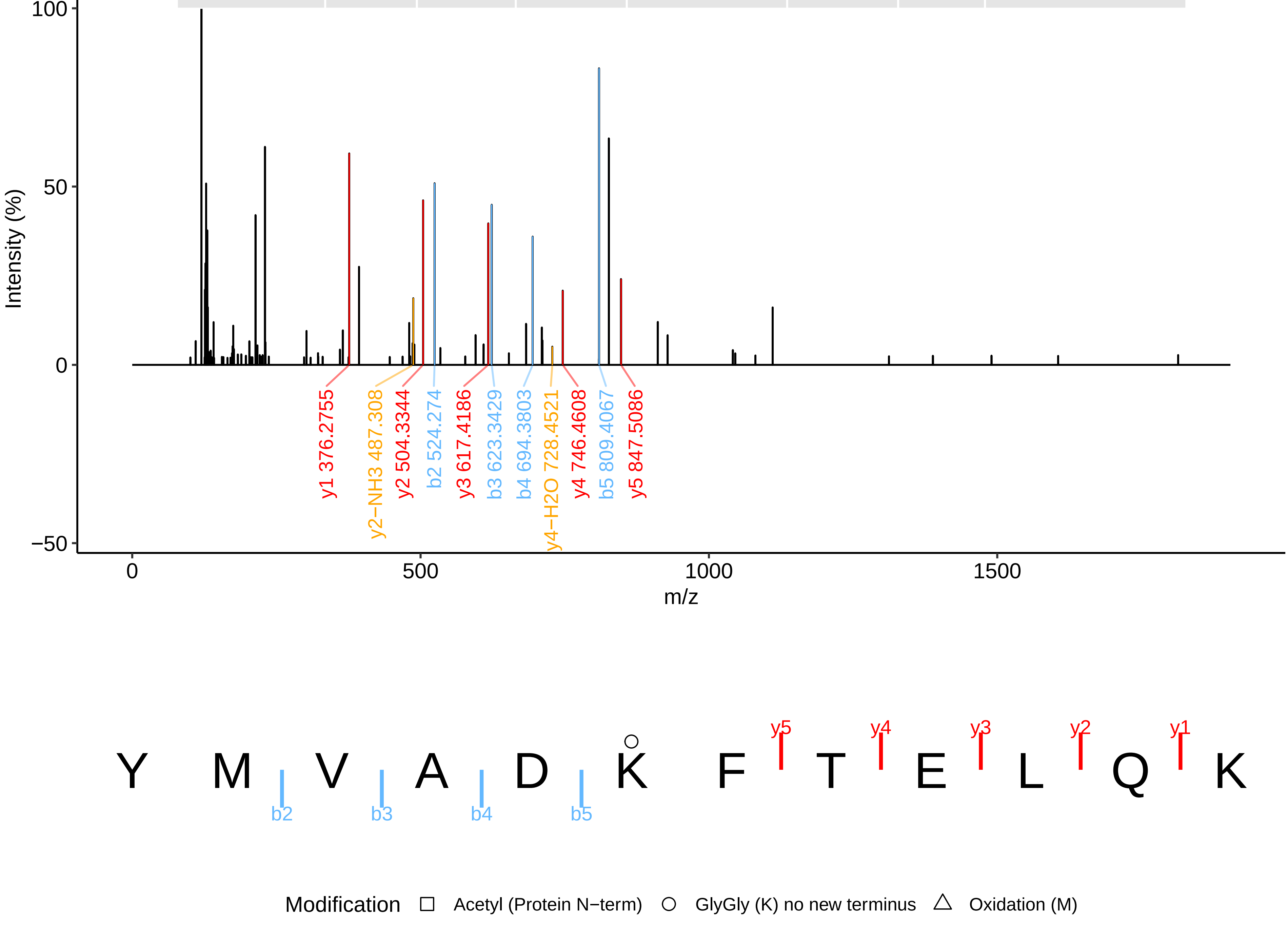

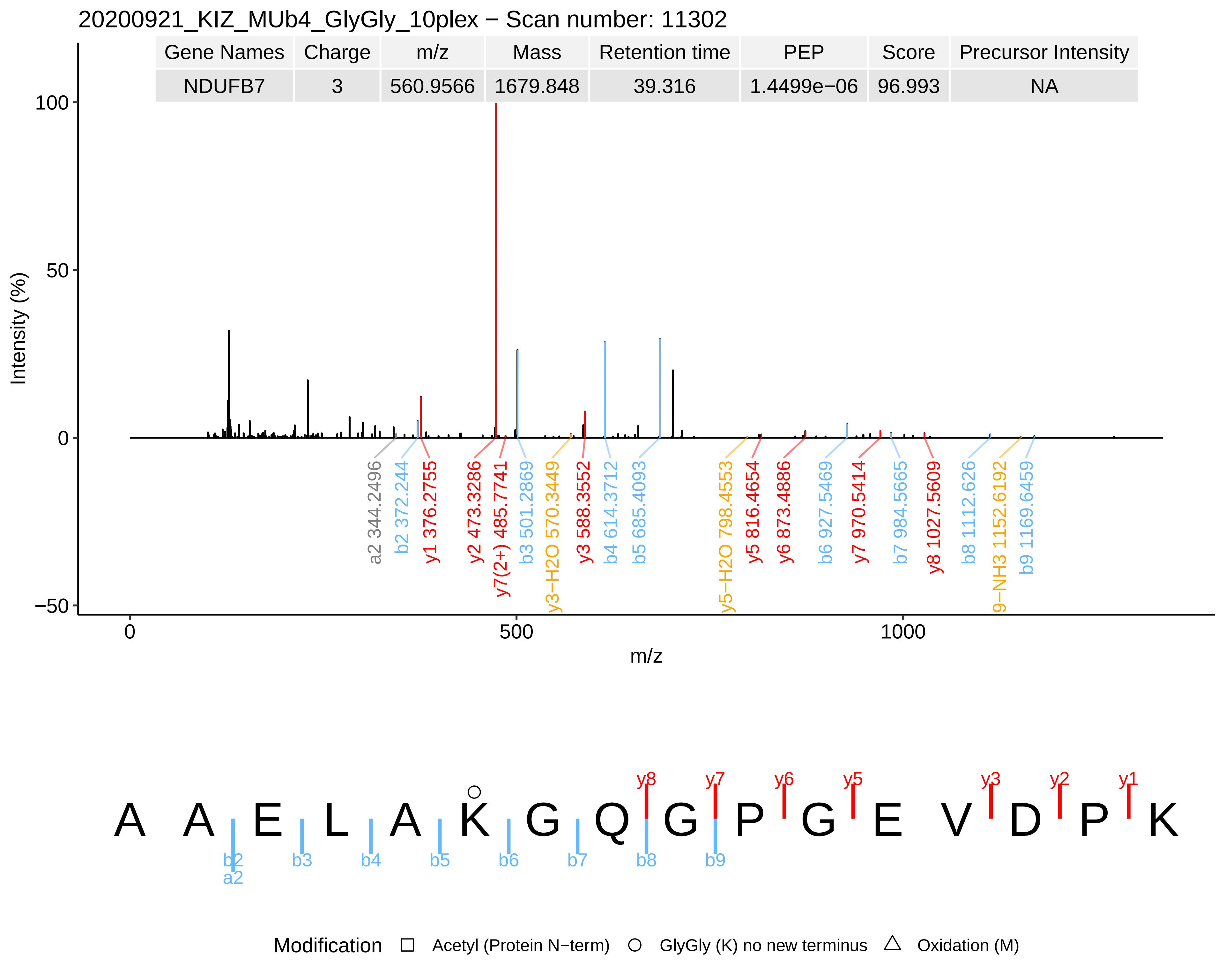
20200921_KIZ_MUb4_GlyGly_10plex − Scan number: 11302
Gene Names
Charge
m/z
Mass
Retention time
PEP
Score
Precursor Intensity
NDUFB7
3
560.9566
1679.848
39.316
1.4499e−06
96.993
NA
Intensity (%)
m/z
a2 344.2496
b2 372.244
y1 376.2755
y2 473.3286
y7(2+) 485.7741
b3 501.2869
y3−H2O 570.3449
y3 588.3552
b4 614.3712
b5 685.4093
y5−H2O 798.4553
y5 816.4654
y6 873.4886
b6 927.5469
y7 970.5414
b7 984.5665
y8 1027.5609
b8 1112.626
9−NH3 1152.6192
b9 1169.6459
A A E L A K G Q G P G E V D P K
Modification
Acetyl (Protein N−term)
GlyGly (K) no new terminus
Oxidation (M)

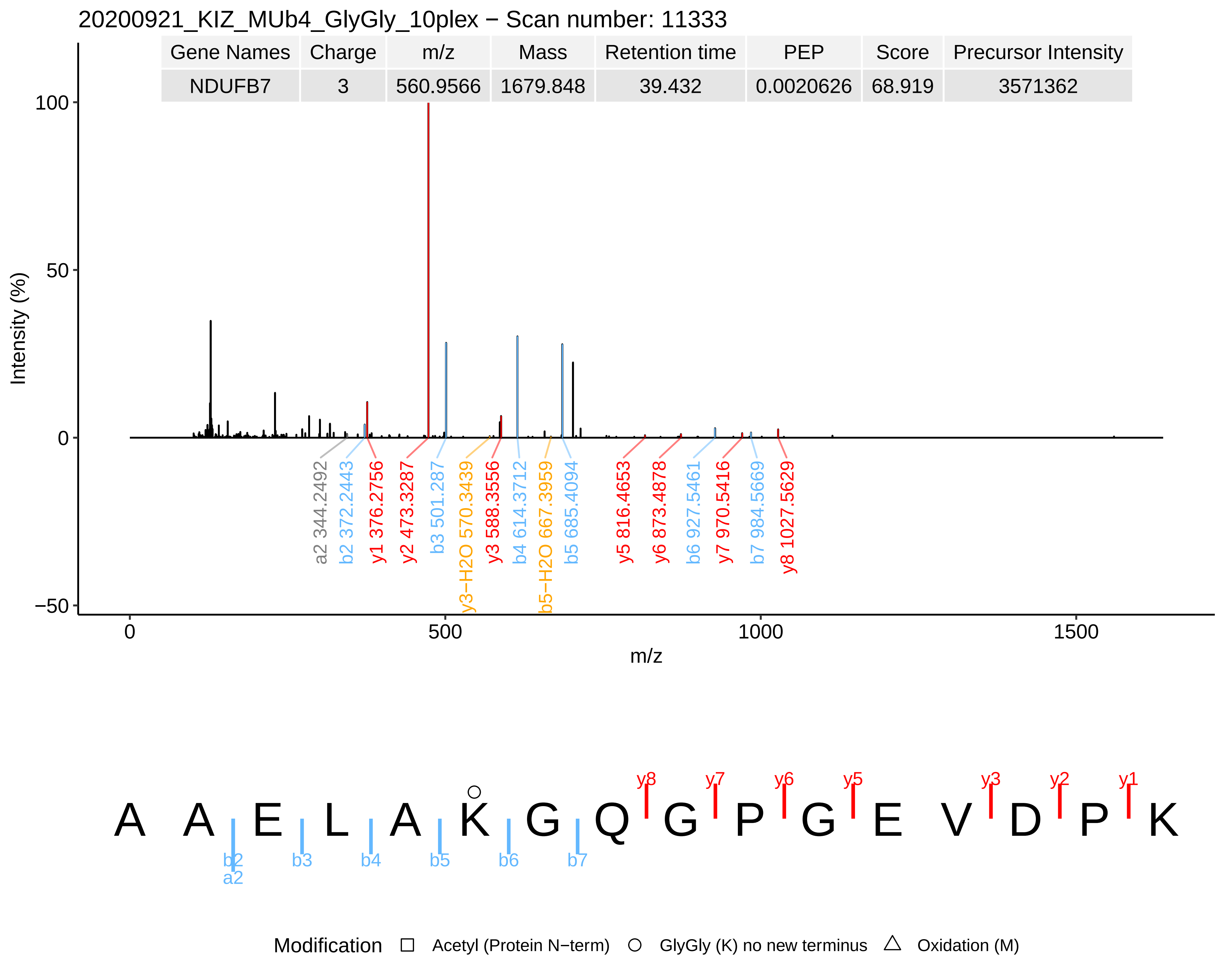

20200921_KIZ_MUb4_GlyGly_10plex − Scan number: 11333
Gene Names | Charge | m/z | Mass | Retention time | PEP | Score | Precursor Intensity
NDUFB7 | 3 | 560.9566 | 1679.848 | 39.432 | 0.0020626 | 68.919 | 3571362
Intensity (%)
m/z
a2 344.2492
b2 372.2443
y1 376.2756
y2 473.3287
b3 501.287
y3−H2O 570.3439
y3 588.3556
b4 614.3712
b5−H2O 667.3959
b5 685.4094
y5 816.4653
y6 873.4878
b6 927.5461
y7 970.5416
b7 984.5669
y8 1027.5629
A A E L A K G Q G P G E V D P K
Modification
Acetyl (Protein N−term)
GlyGly (K) no new terminus
Oxidation (M)

20200921_KIZ_MUb4_GlyGly_10plex_2 − Scan number: 10388

Gene Names	Charge	m/z	Mass	Retention time	PEP	Score	Precursor Intensity
NDUFB7	3	560.9566	1679.848	39.732	0.0032804	63.128	695257

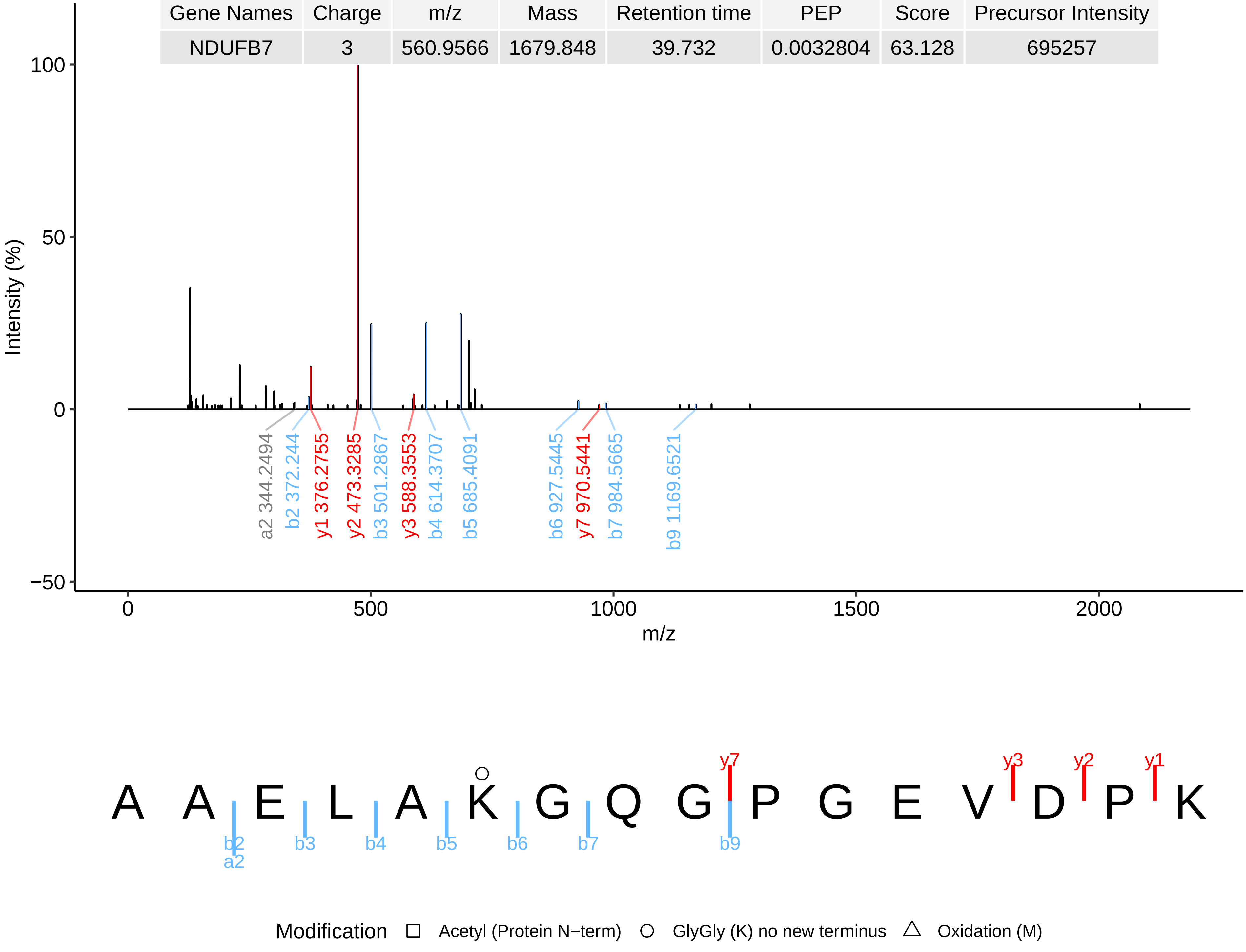

20200921_KIZ_MUb4_GlyGly_10plex − Scan number: 11976

Gene Names	Charge	m/z	Mass	Retention time	PEP	Score	Precursor Intensity
CAV1	3	630.634	1888.88	41.857	0.0012361	56.648	3898942

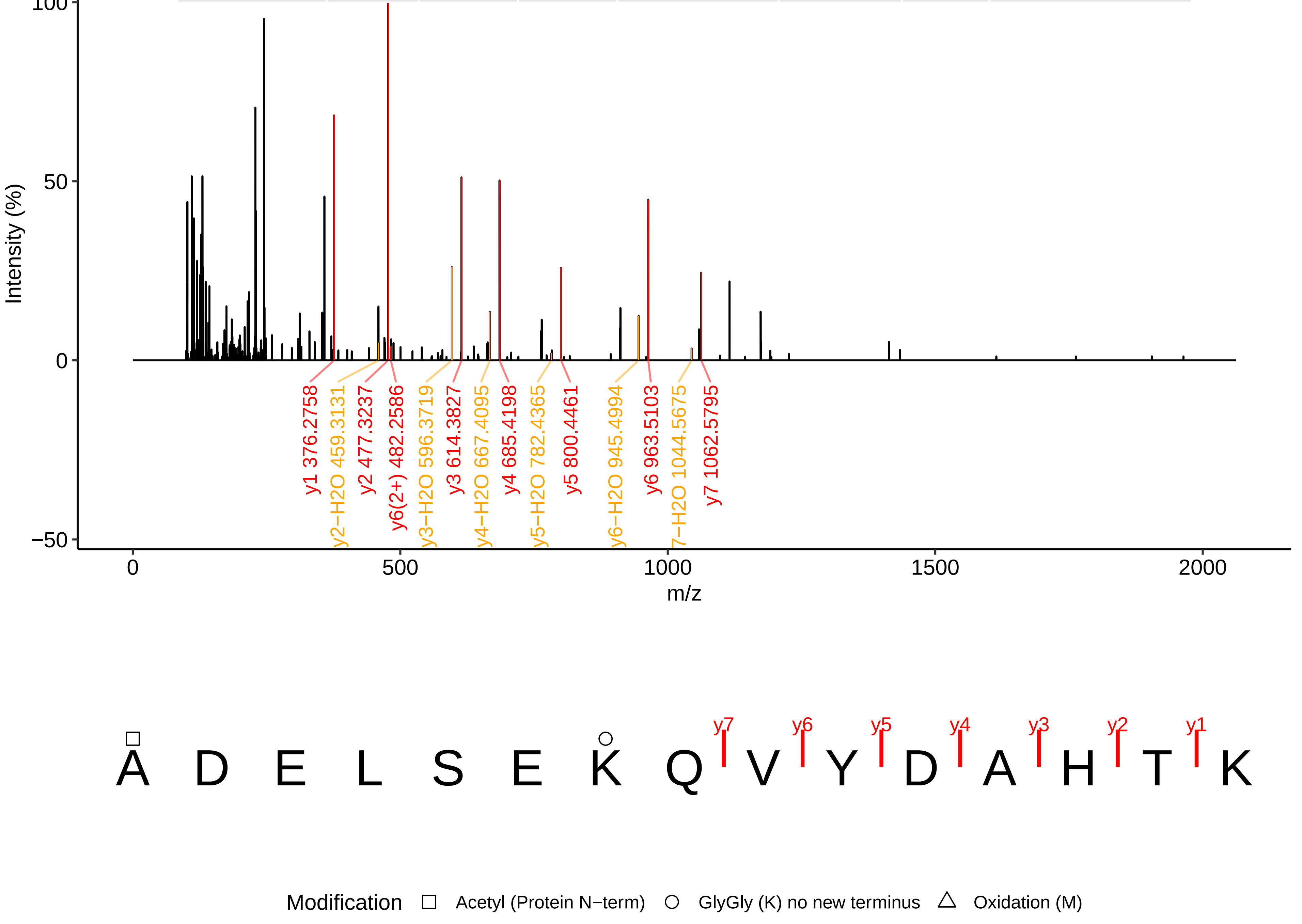

20200921_KIZ_MUb4_GlyGly_10plex − Scan number: 12116

Gene Names	Charge	m/z	Mass	Retention time	PEP	Score	Precursor Intensity
CAV1	3	630.634	1888.88	42.406	1.3155e−09	106.13	3721345

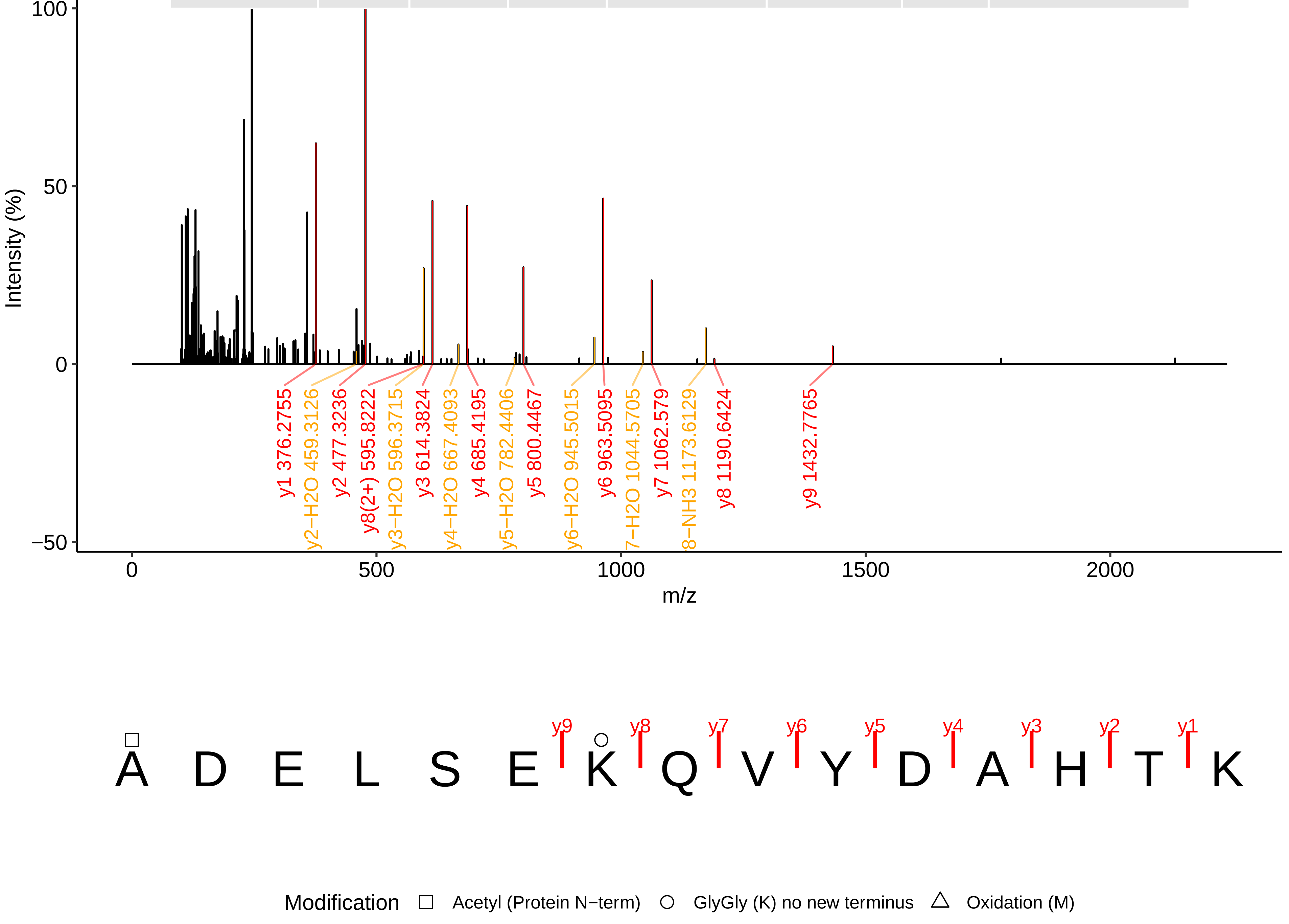

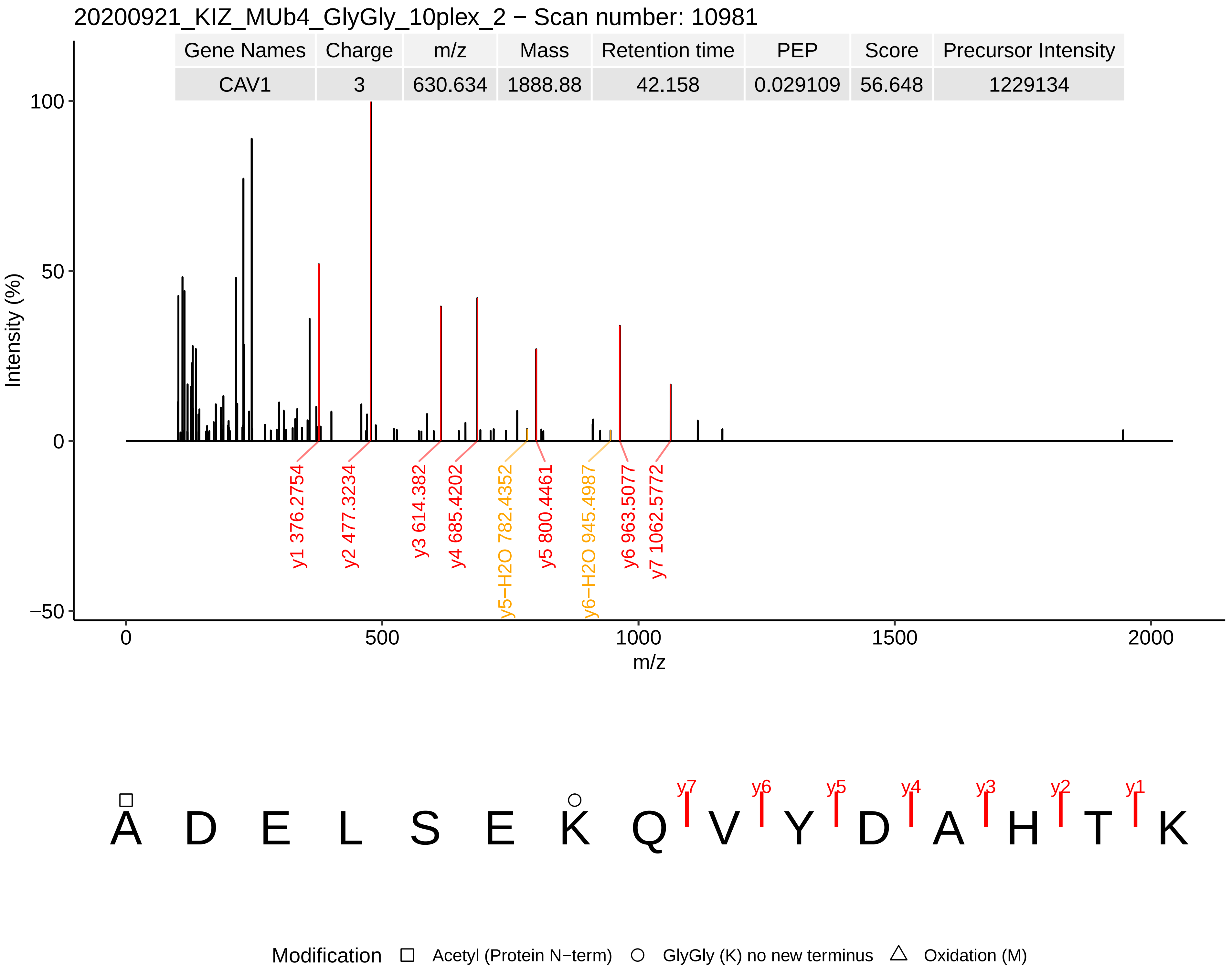
20200921_KIZ_MUb4_GlyGly_10plex_2 − Scan number: 10981
Gene Names | Charge | m/z | Mass | Retention time | PEP | Score | Precursor Intensity
CAV1 | 3 | 630.634 | 1888.88 | 42.158 | 0.029109 | 56.648 | 1229134
Intensity (%)
100
50
0
−50
y1 376.2754
y2 477.3234
y3 614.382
y4 685.4202
y5−H2O 782.4352
y5 800.4461
y6−H2O 945.4987
y6 963.5077
y7 1062.5772
0
500
1000
1500
2000
m/z
A D E L S E K Q V Y D A H T K
y7 y6 y5 y4 y3 y2 y1
Modification
Acetyl (Protein N−term)
GlyGly (K) no new terminus
Oxidation (M)

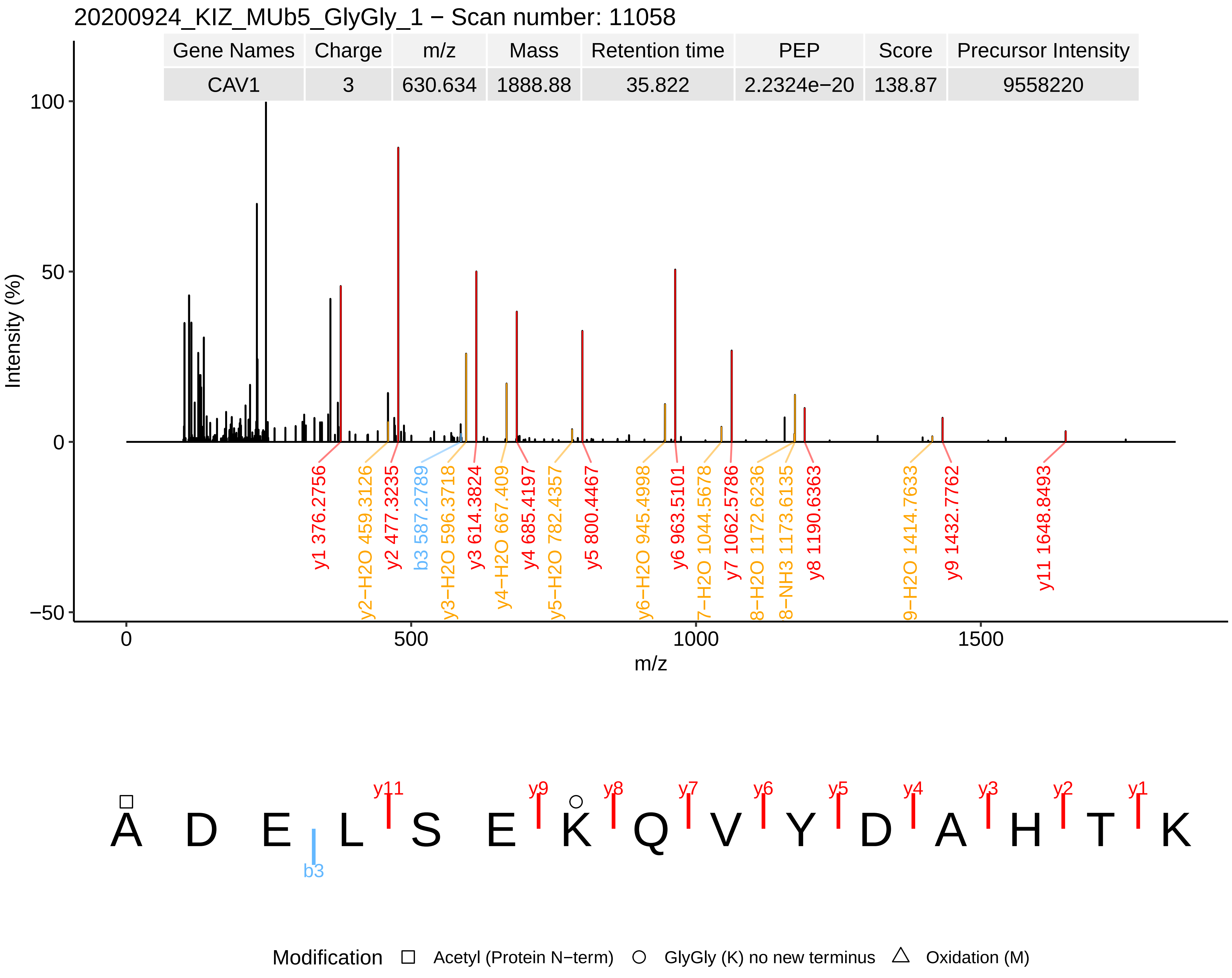
20200924_KIZ_MUb5_GlyGly_1 − Scan number: 11058
Gene Names | Charge | m/z | Mass | Retention time | PEP | Score | Precursor Intensity
CAV1 | 3 | 630.634 | 1888.88 | 35.822 | 2.2324e−20 | 138.87 | 9558220
Intensity (%)
100
50
0
−50
0
500
1000
1500
m/z
y1 376.2756
y2−H2O 459.3126
y2 477.3235
b3 587.2789
y3−H2O 596.3718
y3 614.3824
y4−H2O 667.409
y4 685.4197
y5−H2O 782.4357
y5 800.4467
y6−H2O 945.4998
y6 963.5101
7−H2O 1044.5678
y7 1062.5786
8−H2O 1172.6236
8−NH3 1173.6135
y8 1190.6363
9−H2O 1414.7633
y9 1432.7762
y11 1648.8493
A D E L S E K Q V Y D A H T K
y11 y9 y8 y7 y6 y5 y4 y3 y2 y1
b3
Modification
Acetyl (Protein N−term)
GlyGly (K) no new terminus
Oxidation (M)

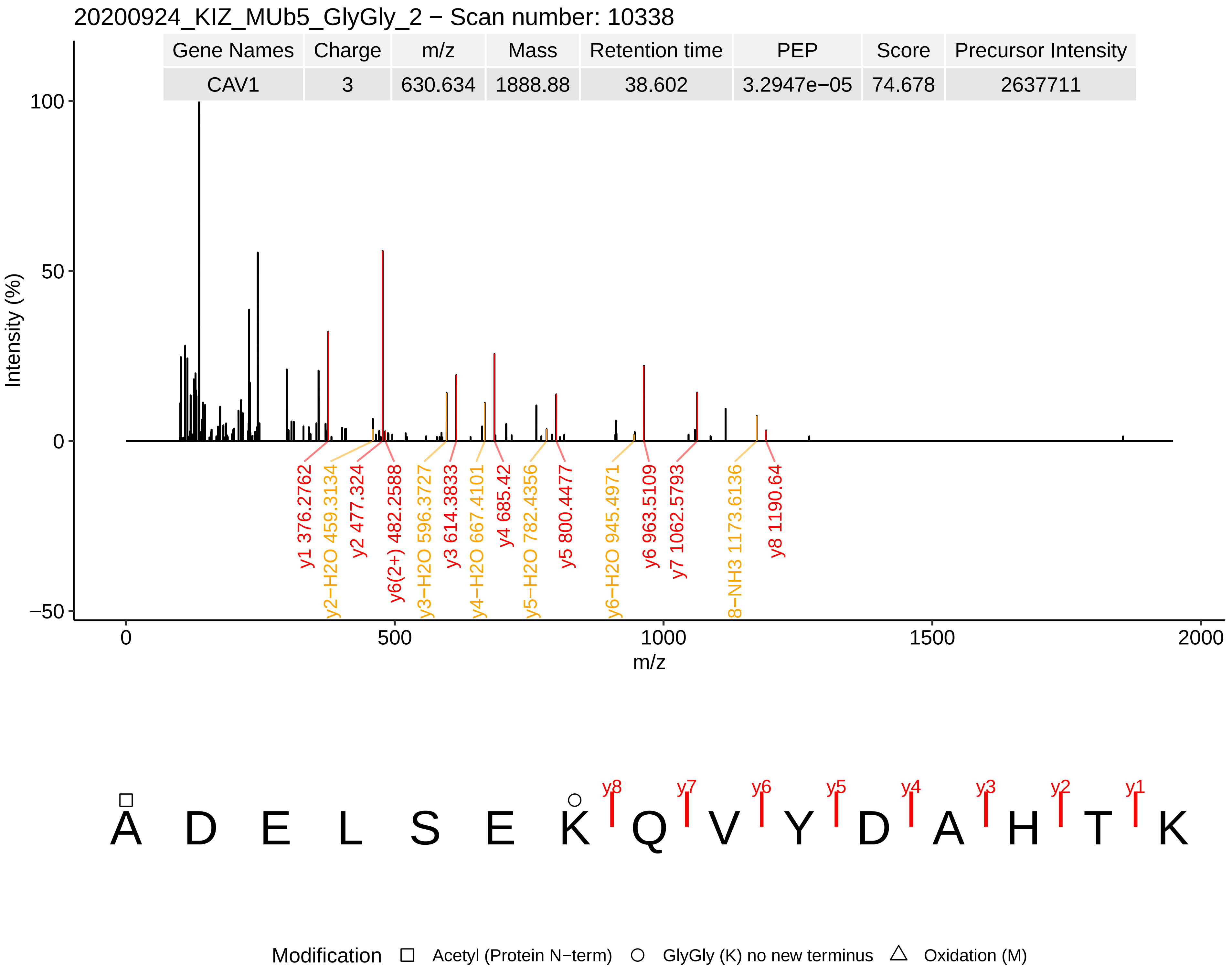
20200924_KIZ_MUb5_GlyGly_2 − Scan number: 10338
Gene Names
Charge
m/z
Mass
Retention time
PEP
Score
Precursor Intensity
CAV1
3
630.634
1888.88
38.602
3.2947e−05
74.678
2637711
Intensity (%)
m/z
y1 376.2762
y2−H2O 459.3134
y2 477.324
y6(2+) 482.2588
y3−H2O 596.3727
y3 614.3833
y4−H2O 667.4101
y4 685.42
y5−H2O 782.4356
y5 800.4477
y6−H2O 945.4971
y6 963.5109
y7 1062.5793
8−NH3 1173.6136
y8 1190.64
A D E L S E K Q V Y D A H T K
y8 y7 y6 y5 y4 y3 y2 y1
Modification
Acetyl (Protein N−term)
GlyGly (K) no new terminus
Oxidation (M)

20200924_KIZ_MUb5_GlyGly_2 − Scan number: 10417

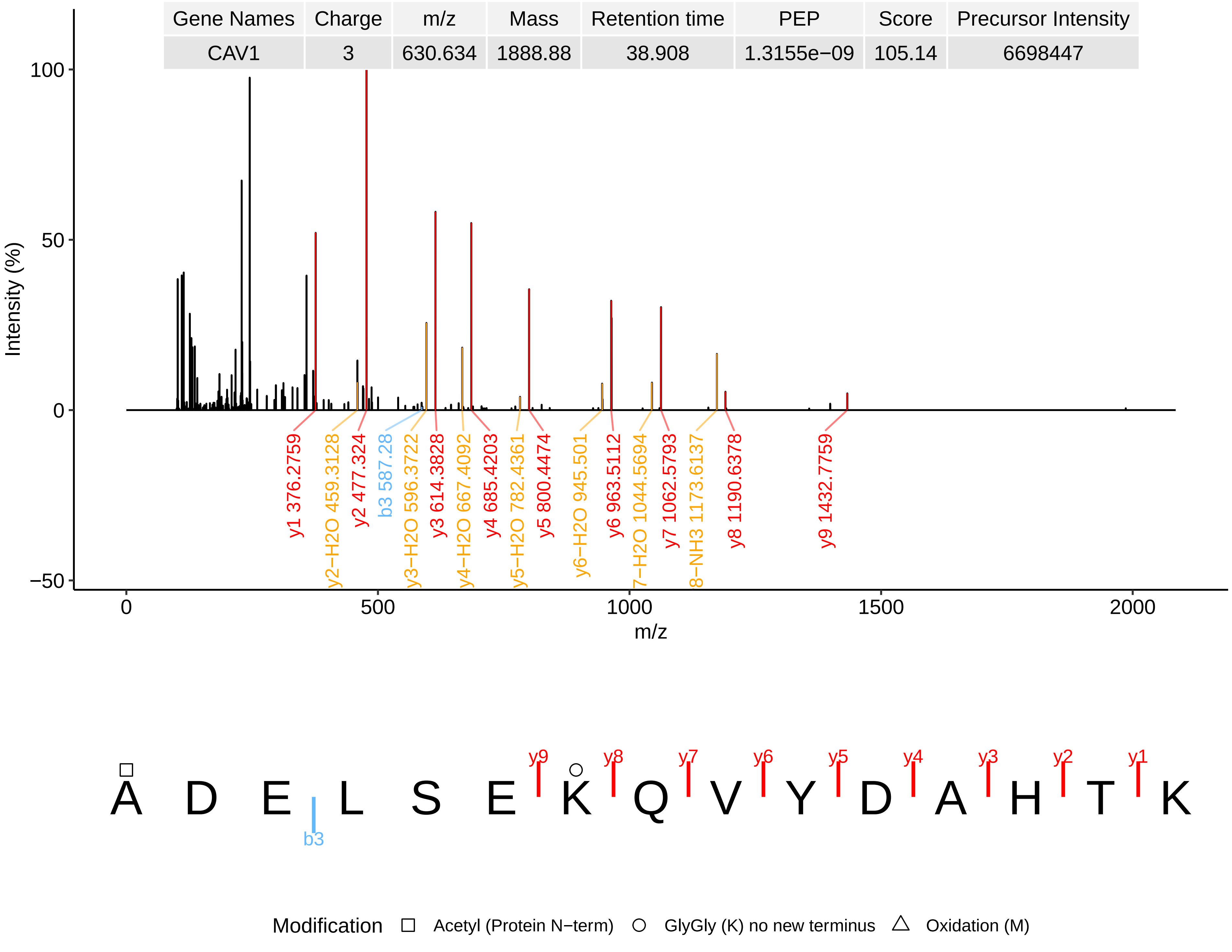

20201118_KIZ_MUb6_GlyGly_2 − Scan number: 12394

Gene Names	Charge	m/z	Mass	Retention time	PEP	Score	Precursor Intensity
CAV1	3	630.634	1888.88	41.712	1.4293e−09	101.32	5022119

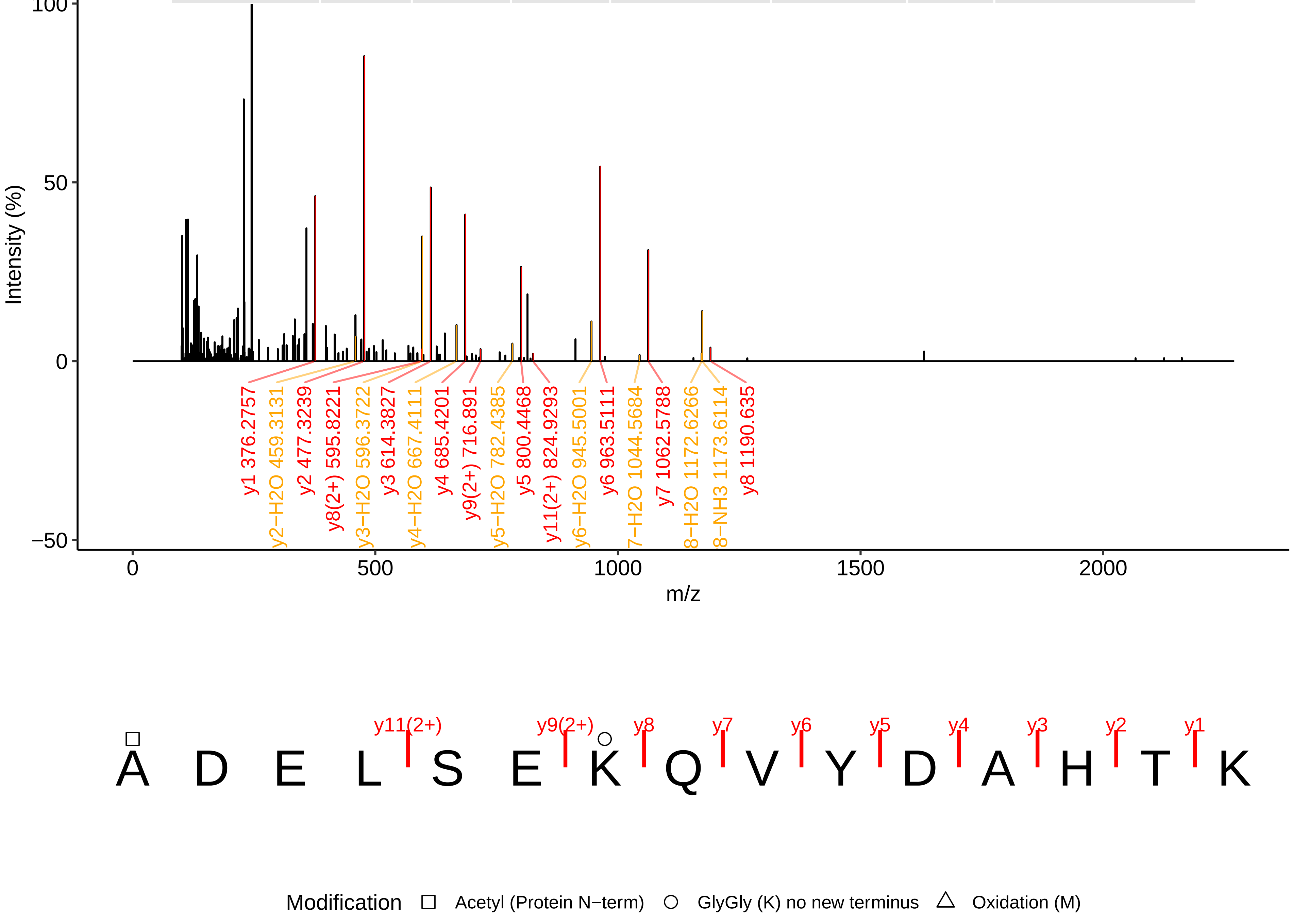

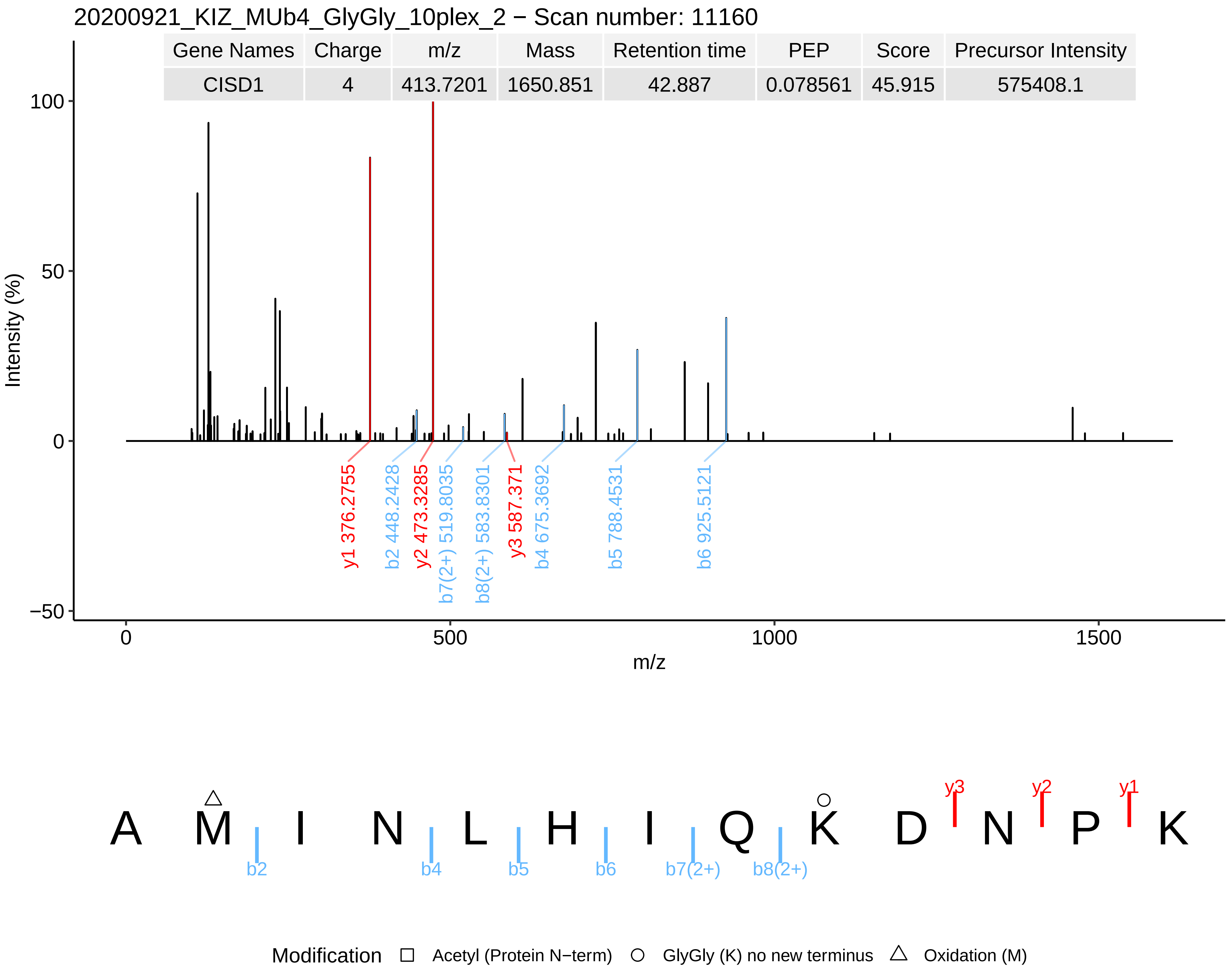
20200921_KIZ_MUb4_GlyGly_10plex_2 − Scan number: 11160
Gene Names | Charge | m/z | Mass | Retention time | PEP | Score | Precursor Intensity
CISD1 | 4 | 413.7201 | 1650.851 | 42.887 | 0.078561 | 45.915 | 575408.1
Intensity (%)
m/z
y1 376.2755
b2 448.2428
y2 473.3285
b7(2+) 519.8035
b8(2+) 583.8301
y3 587.371
b4 675.3692
b5 788.4531
b6 925.5121
A M I N L H I Q K D N P K
b2 b4 b5 b6 b7(2+) b8(2+)
y3 y2 y1
Modification
Acetyl (Protein N−term)
GlyGly (K) no new terminus
Oxidation (M)

20200921_KIZ_MUb4_GlyGly_10plex_2 − Scan number: 19929

Gene Names	Charge	m/z	Mass	Retention time	PEP	Score	Precursor Intensity
HK1	3	538.6334	1612.878	78.281	9.4401e−06	97.551	450165.8

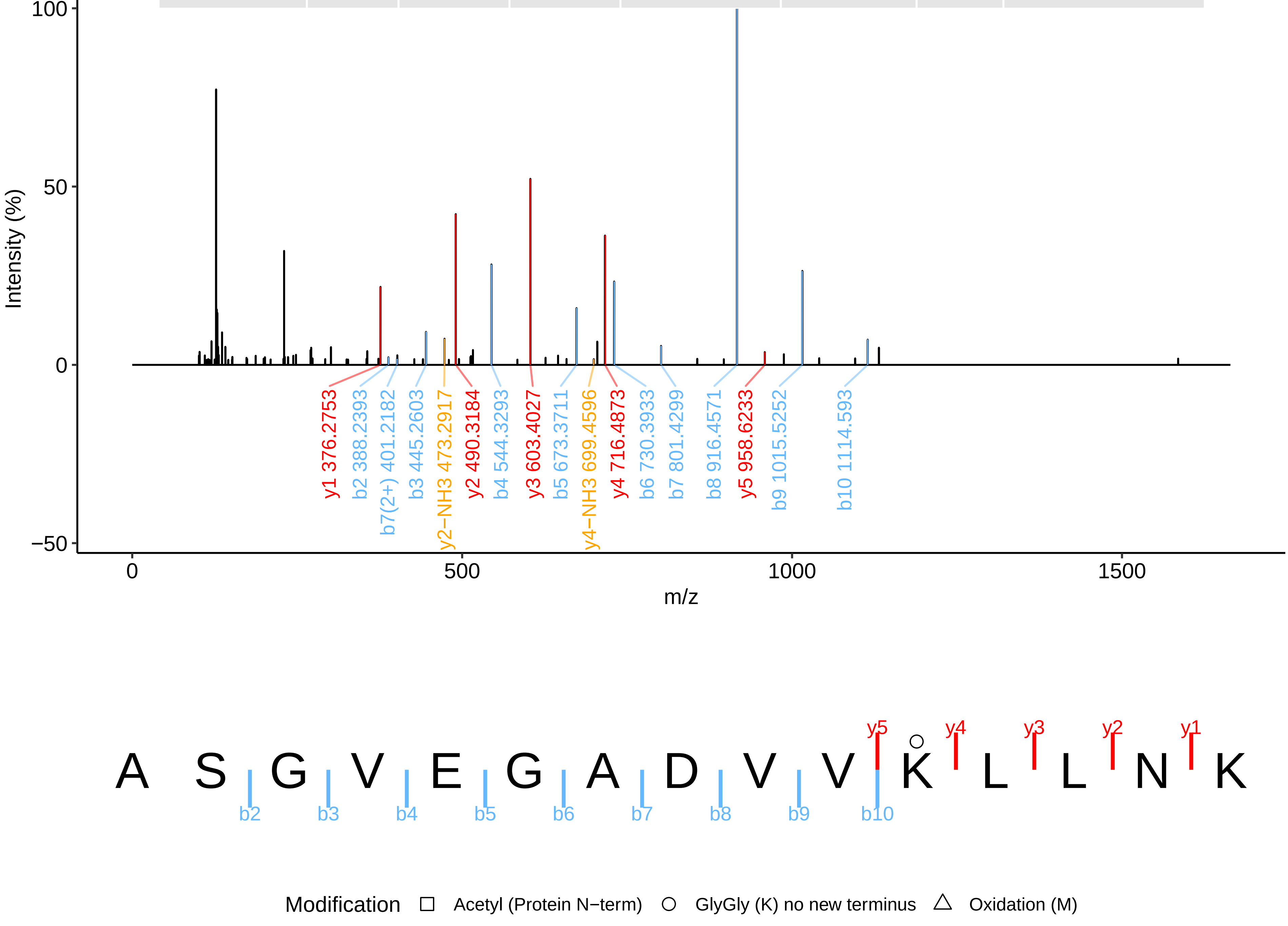

20200921_KIZ_MUb4_GlyGly_10plex − Scan number: 12930

Gene Names	Charge	m/z	Mass	Retention time	PEP	Score	Precursor Intensity
STAM	3	551.6371	1651.889	45.504	2.8773e−07	103.79	6094828

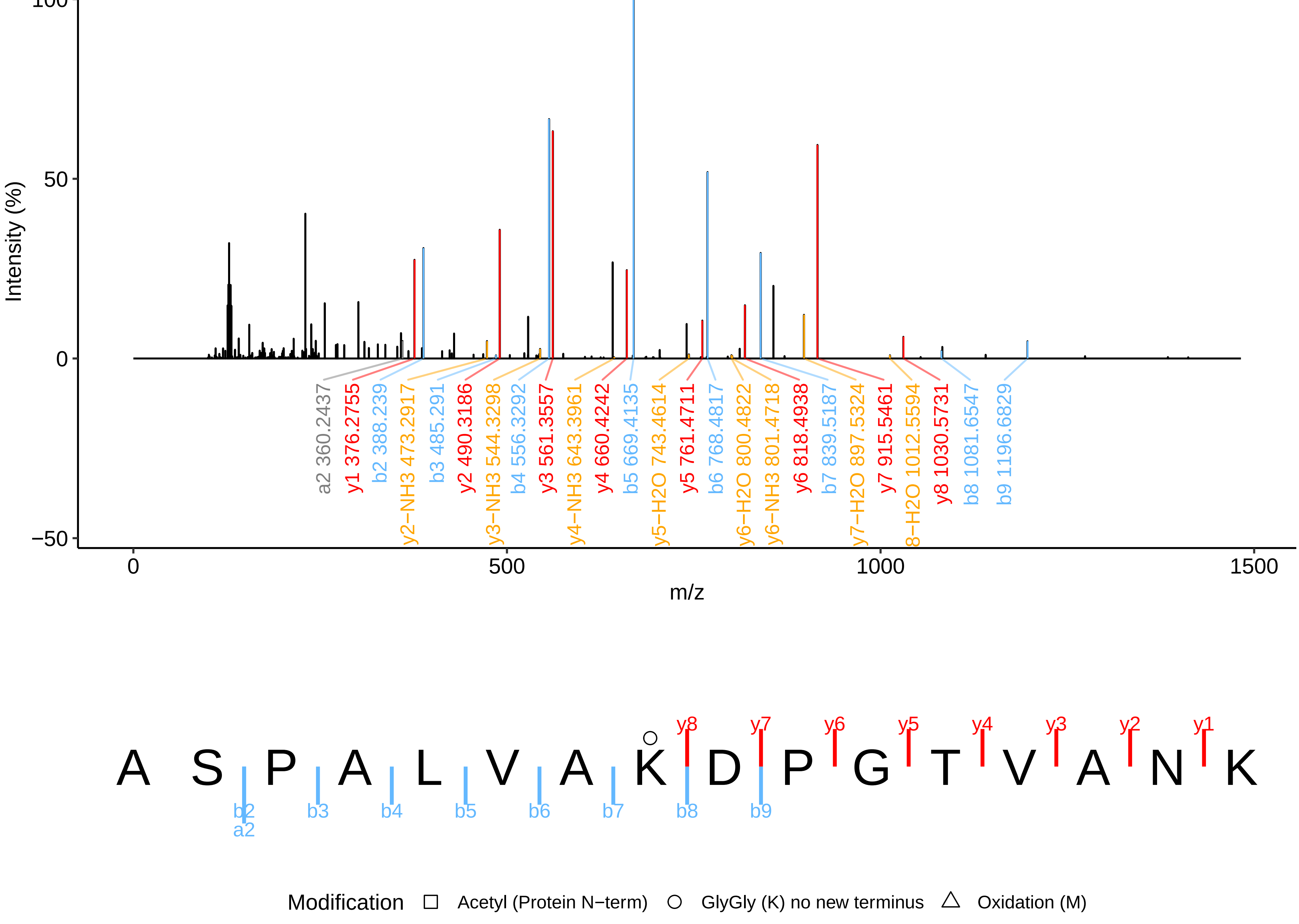

20200921_KIZ_MUb4_GlyGly_10plex_2 − Scan number: 11862

Gene Names	Charge	m/z	Mass	Retention time	PEP	Score	Precursor Intensity
STAM	3	551.6371	1651.889	45.76	8.9995e−06	89.541	390709

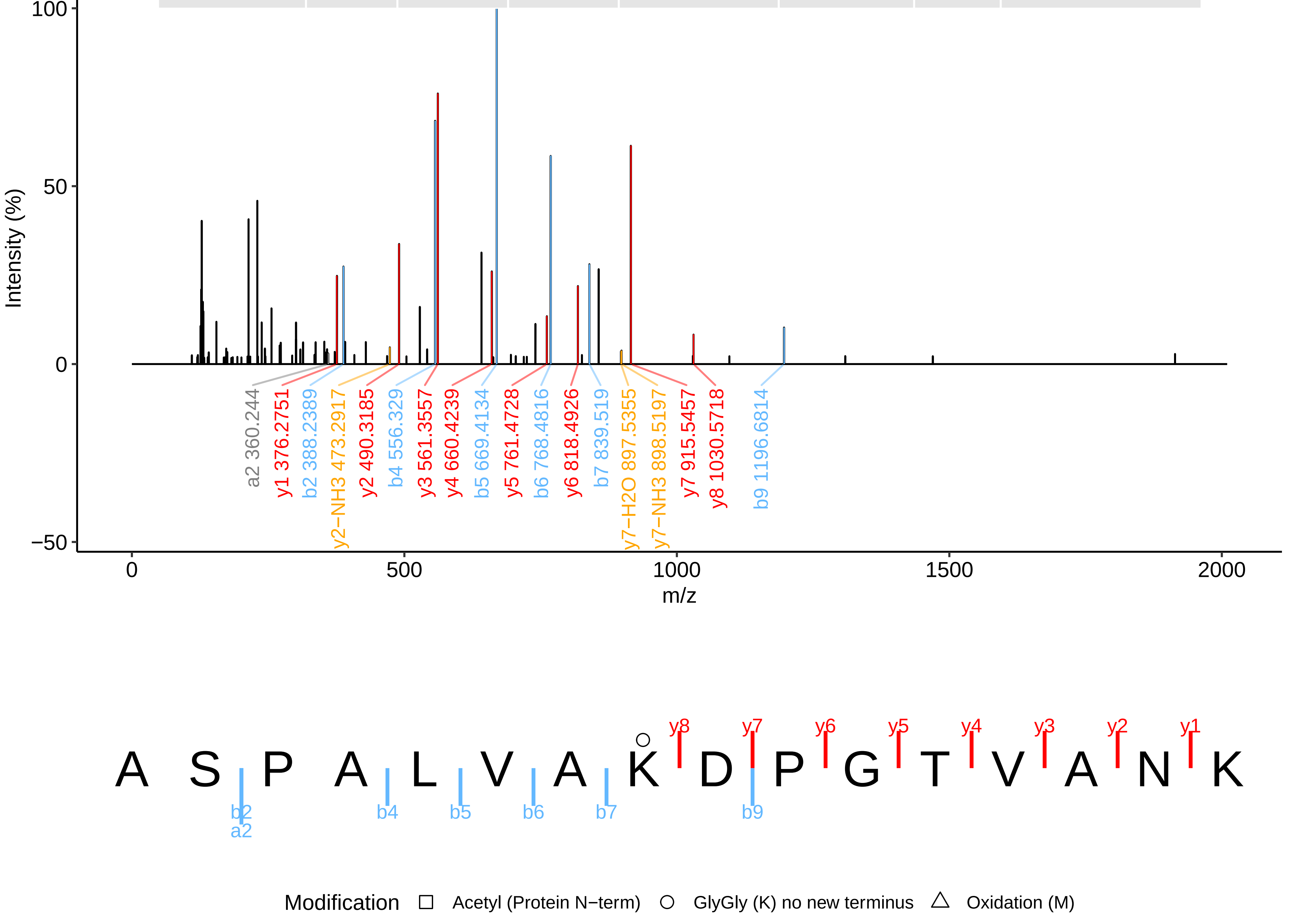

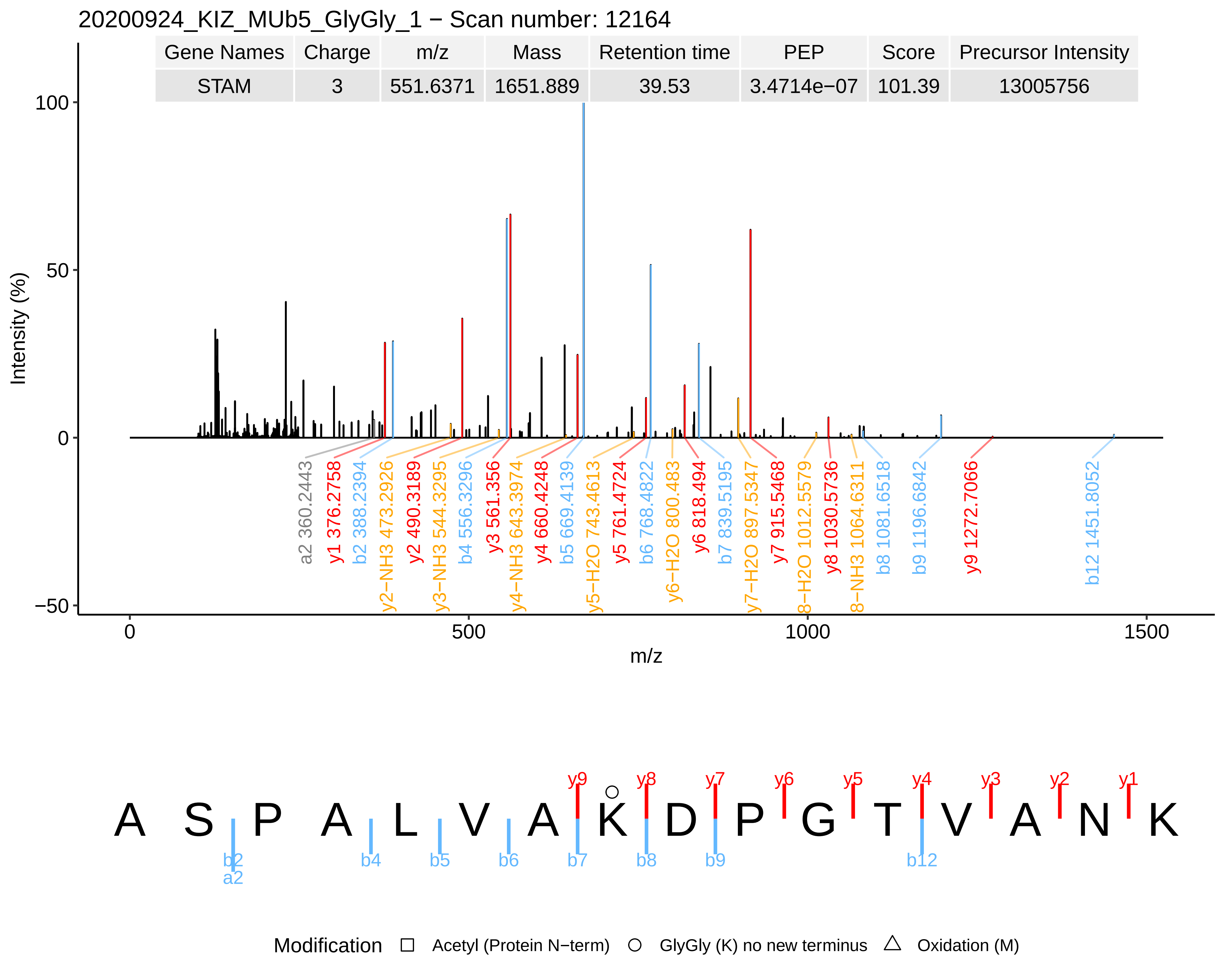
20200924_KIZ_MUb5_GlyGly_1 − Scan number: 12164
Gene Names | Charge | m/z | Mass | Retention time | PEP | Score | Precursor Intensity
STAM | 3 | 551.6371 | 1651.889 | 39.53 | 3.4714e−07 | 101.39 | 13005756
Intensity (%)
m/z
a2 360.2443
y1 376.2758
b2 388.2394
y2−NH3 473.2926
y2 490.3189
y3−NH3 544.3295
b4 556.3296
y3 561.356
y4−NH3 643.3974
y4 660.4248
b5 669.4139
y5−H2O 743.4613
y5 761.4724
b6 768.4822
y6−H2O 800.483
y6 818.494
b7 839.5195
y7−H2O 897.5347
y7 915.5468
y8−H2O 1012.5579
y8 1030.5736
y8−NH3 1064.6311
b8 1081.6518
b9 1196.6842
y9 1272.7066
b12 1451.8052
A S P A L V A K D P G T V A N K
Modification
Acetyl (Protein N−term)
GlyGly (K) no new terminus
Oxidation (M)

20200924_KIZ_MUb5_GlyGly_1 − Scan number: 12328

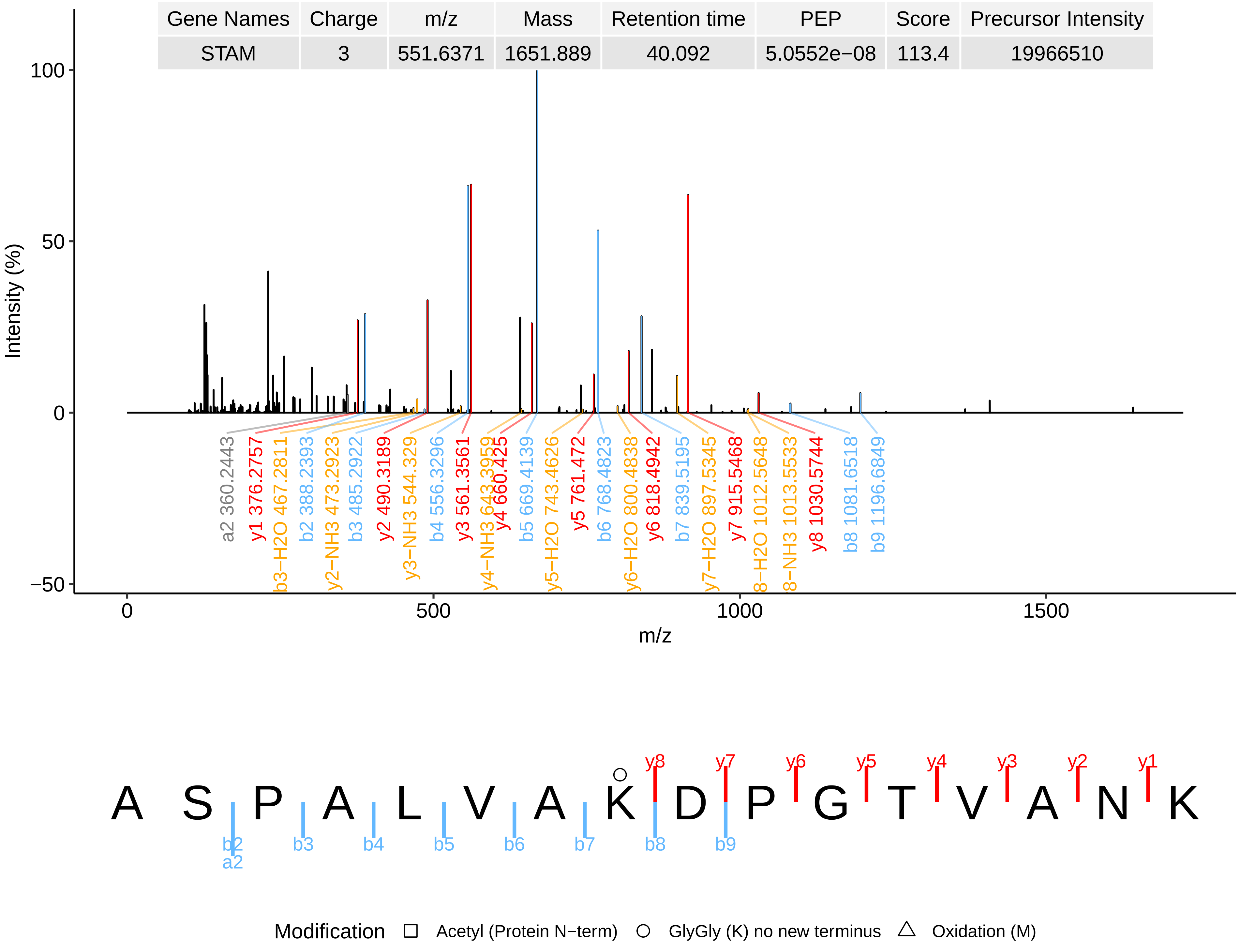

20200924_KIZ_MUb5_GlyGly_2 − Scan number: 11172

Gene Names	Charge	m/z	Mass	Retention time	PEP	Score	Precursor Intensity
STAM	3	551.6371	1651.889	41.723	1.1048e−05	87.519	2329252

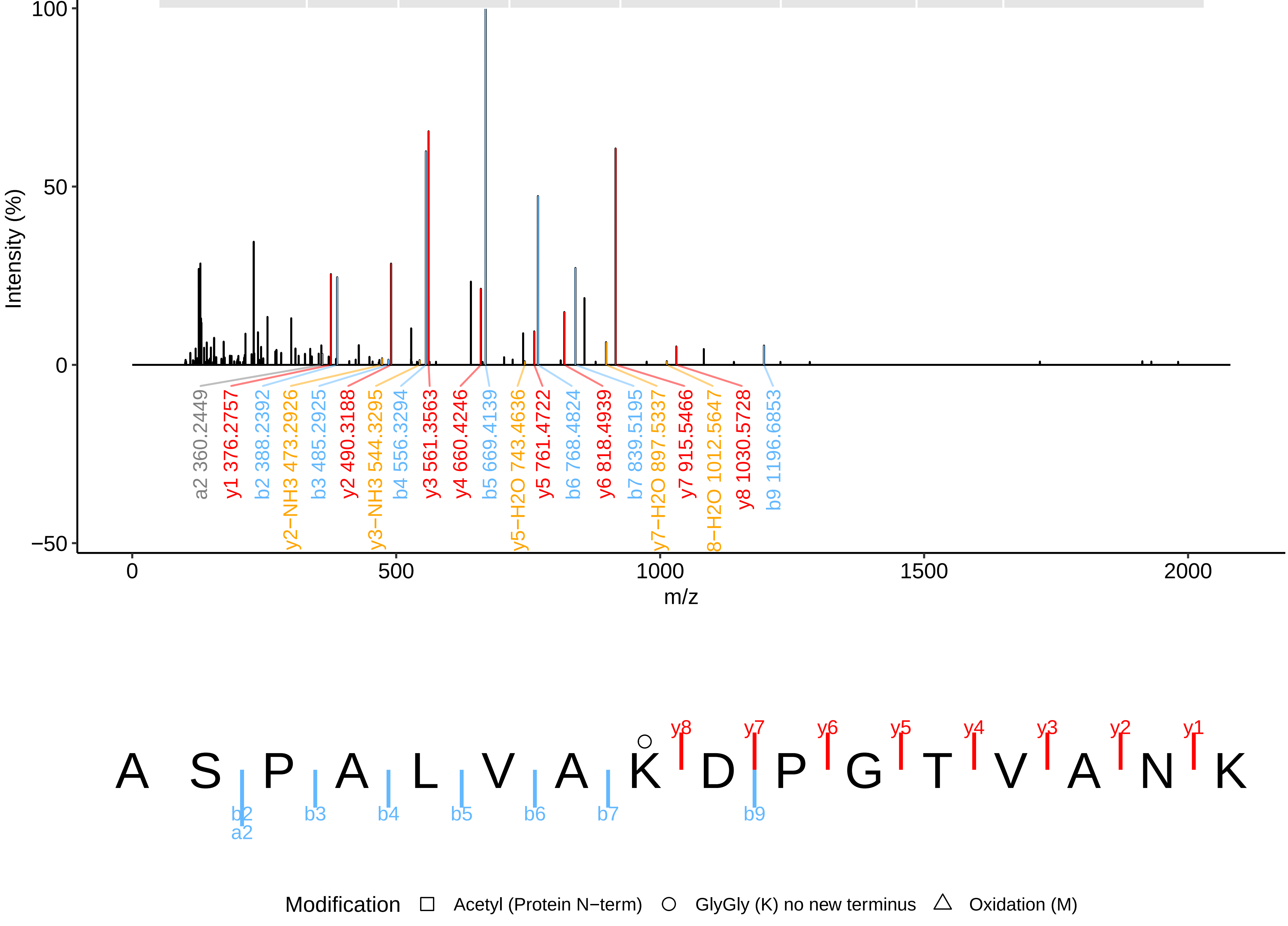

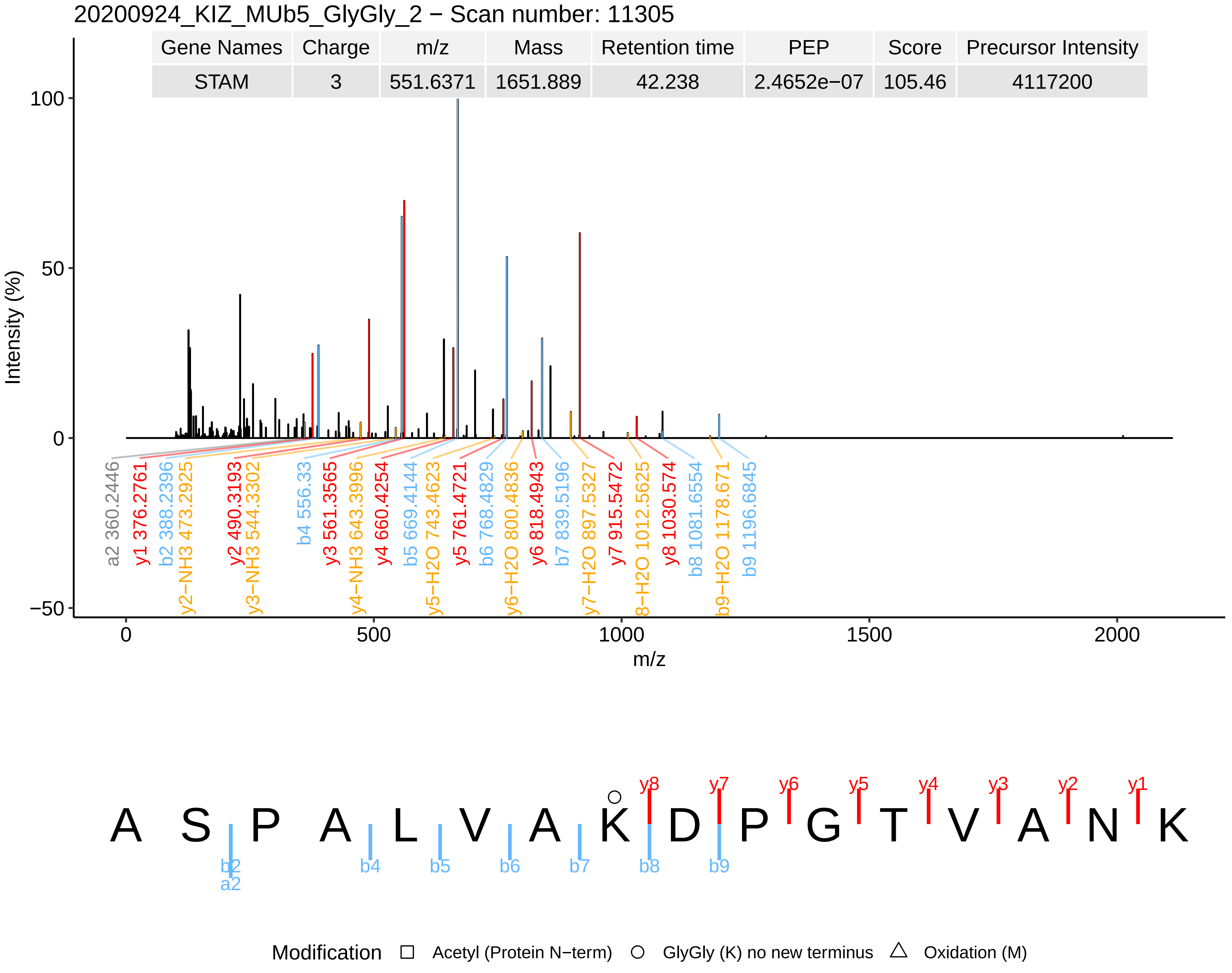

20200924_KIZ_MUb5_GlyGly_2 − Scan number: 11305
Gene Names | Charge | m/z | Mass | Retention time | PEP | Score | Precursor Intensity
STAM | 3 | 551.6371 | 1651.889 | 42.238 | 2.4652e−07 | 105.46 | 4117200
Intensity (%)
m/z
a2 360.2446
y1 376.2761
b2 388.2396
y2−NH3 473.2925
y2 490.3193
y3−NH3 544.3302
b4 556.33
y3 561.3565
y4−NH3 643.3996
y4 660.4254
b5 669.4144
y5−H2O 743.4623
y5 761.4721
b6 768.4829
y6−H2O 800.4836
y6 818.4943
b7 839.5196
y7−H2O 897.5327
y7 915.5472
8−H2O 1012.5625
y8 1030.574
b8 1081.6554
b9−H2O 1178.671
b9 1196.6845
A S P A L V A K D P G T V A N K
y8 y7 y6 y5 y4 y3 y2 y1
b2 a2 b4 b5 b6 b7 b8 b9
Modification □ Acetyl (Protein N−term) ○ GlyGly (K) no new terminus △ Oxidation (M)

20201118_KIZ_MUb6_GlyGly_1 − Scan number: 12238

Gene Names	Charge	m/z	Mass	Retention time	PEP	Score	Precursor Intensity
STAM	3	551.6371	1651.889	38.611	0.0001634	82.109	NA

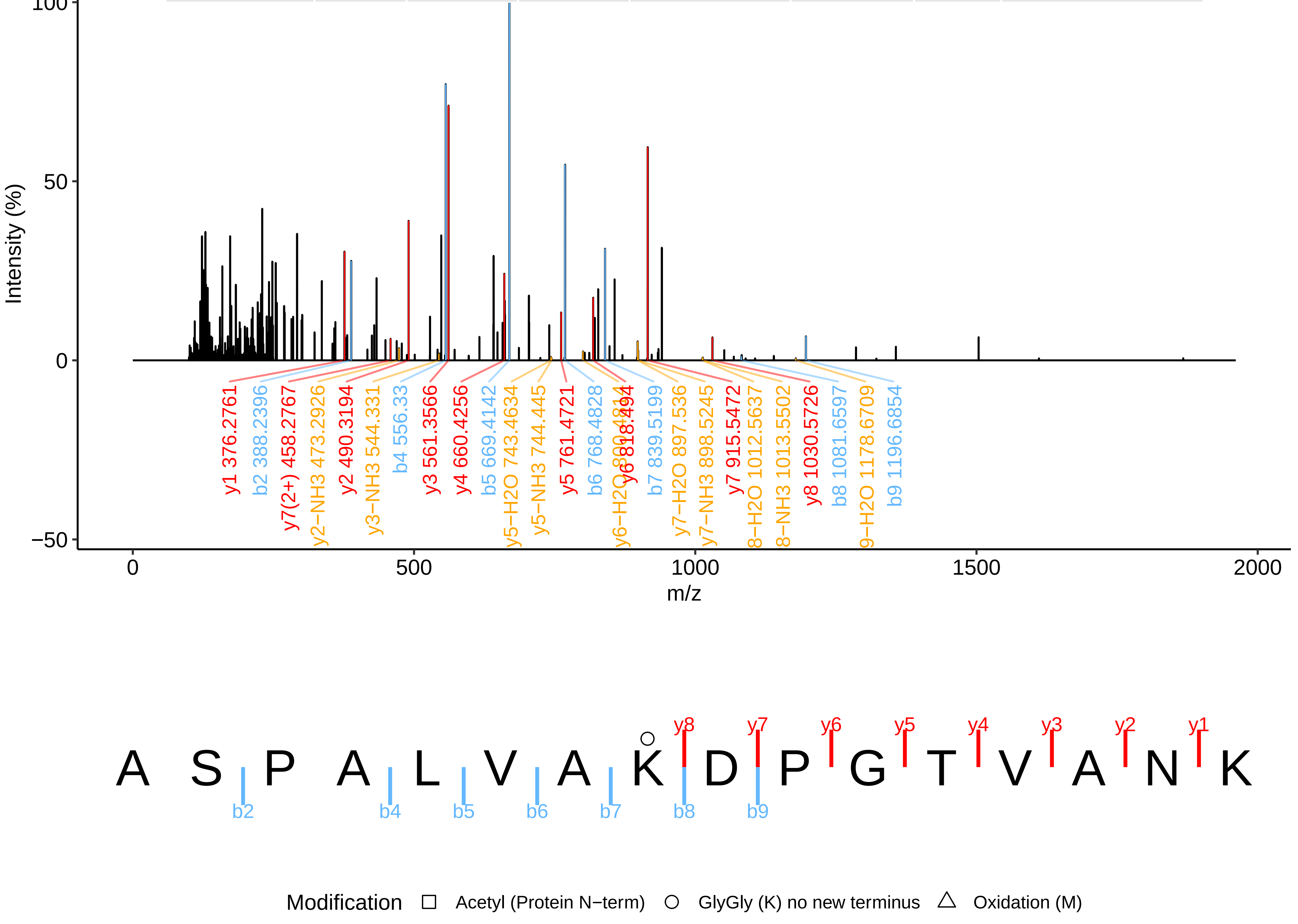

20200921_KIZ_MUb4_GlyGly_10plex − Scan number: 19383

Gene Names	Charge	m/z	Mass	Retention time	PEP	Score	Precursor Intensity
ATP1A1	3	619.011	1854.011	69.877	0.00074364	86.288	NA

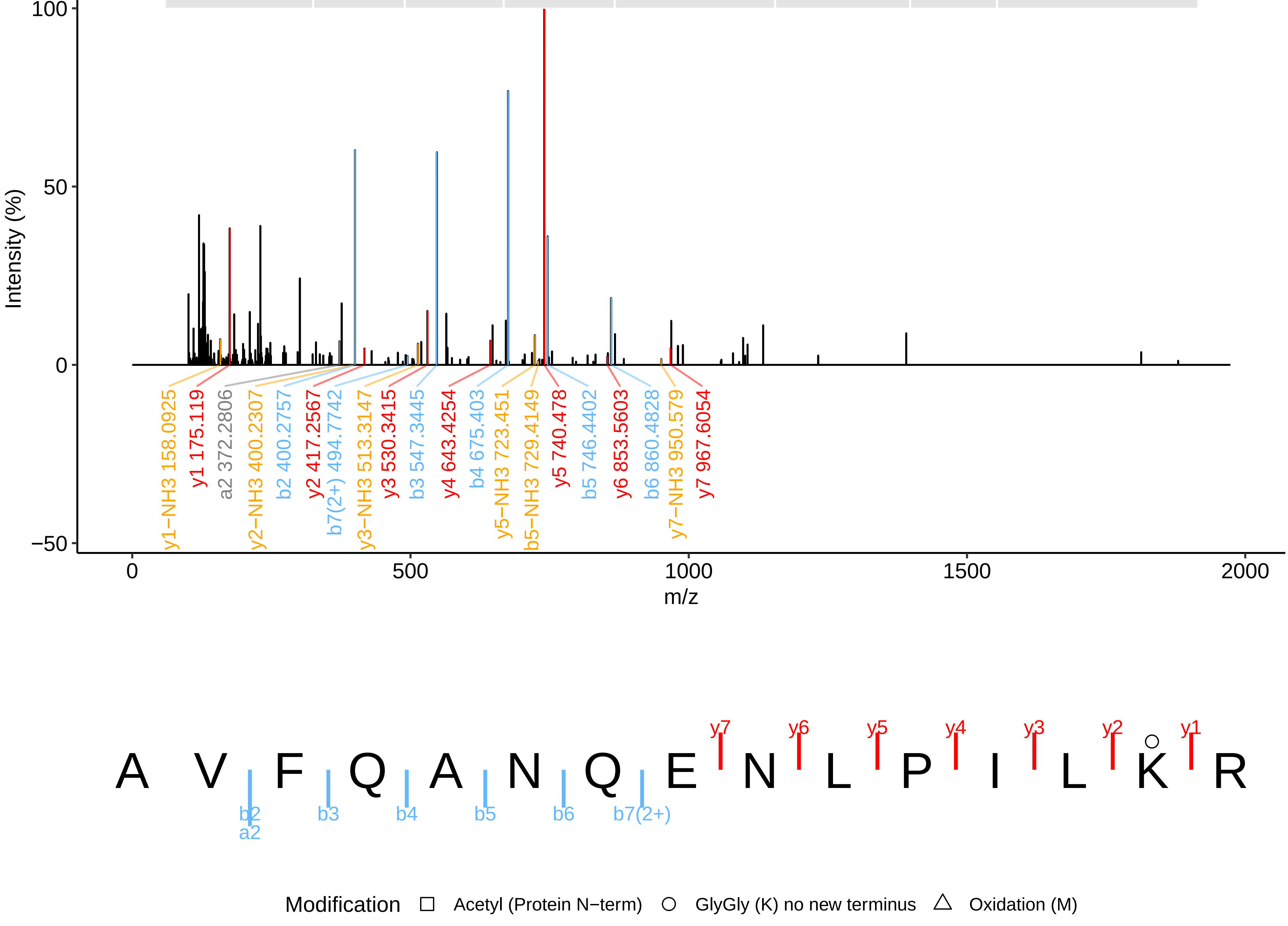

20200921_KIZ_MUb4_GlyGly_10plex − Scan number: 15580

Gene Names	Charge	m/z	Mass	Retention time	PEP	Score	Precursor Intensity
MYSM1	3	612.6235	1834.849	55.689	1.0337e−05	111.88	1483900

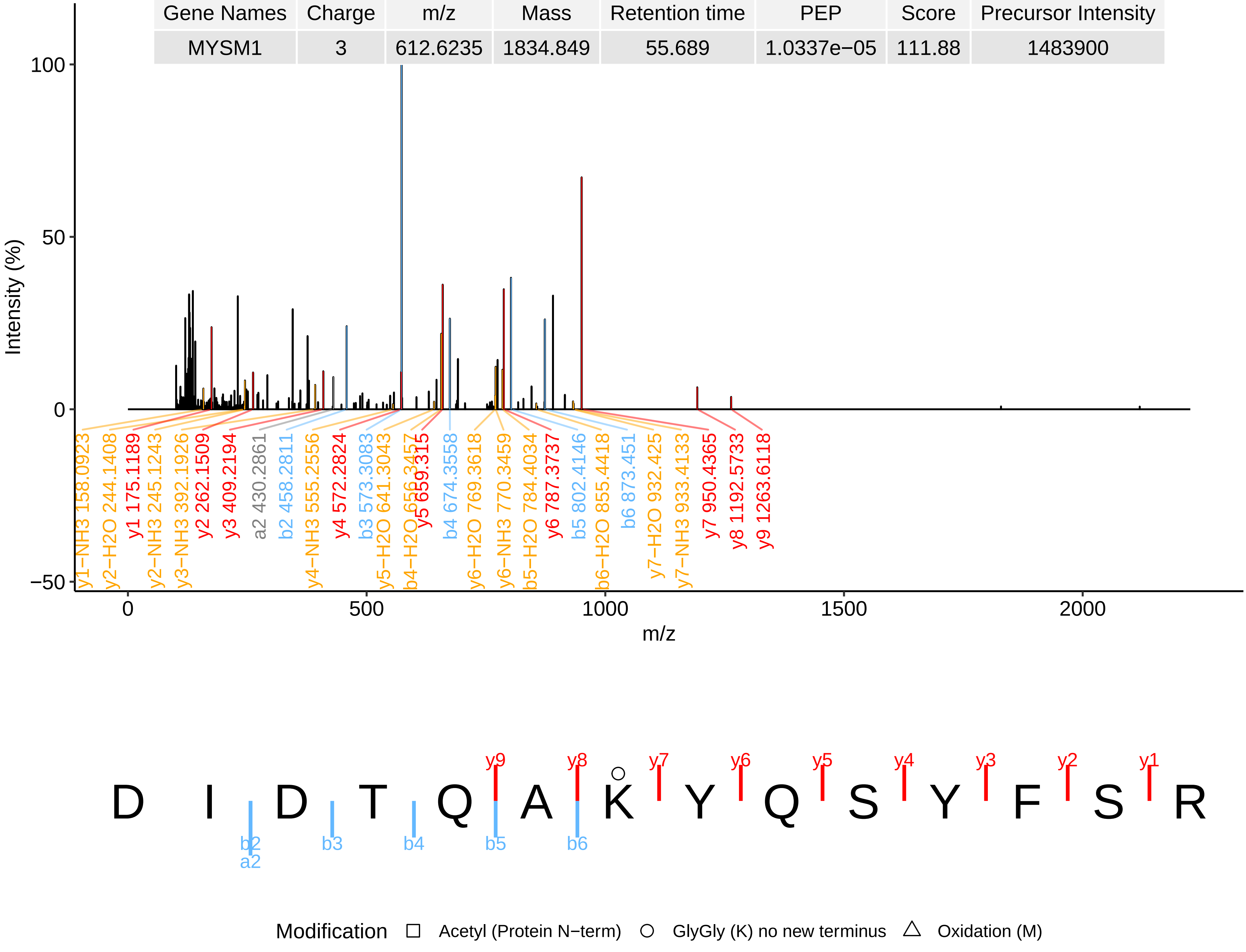

20200921_KIZ_MUb4_GlyGly_10plex_2 − Scan number: 14429

Gene Names	Charge	m/z	Mass	Retention time	PEP	Score	Precursor Intensity
MYSM1	3	612.6235	1834.849	56.259	1.0498e−05	106.97	1292137

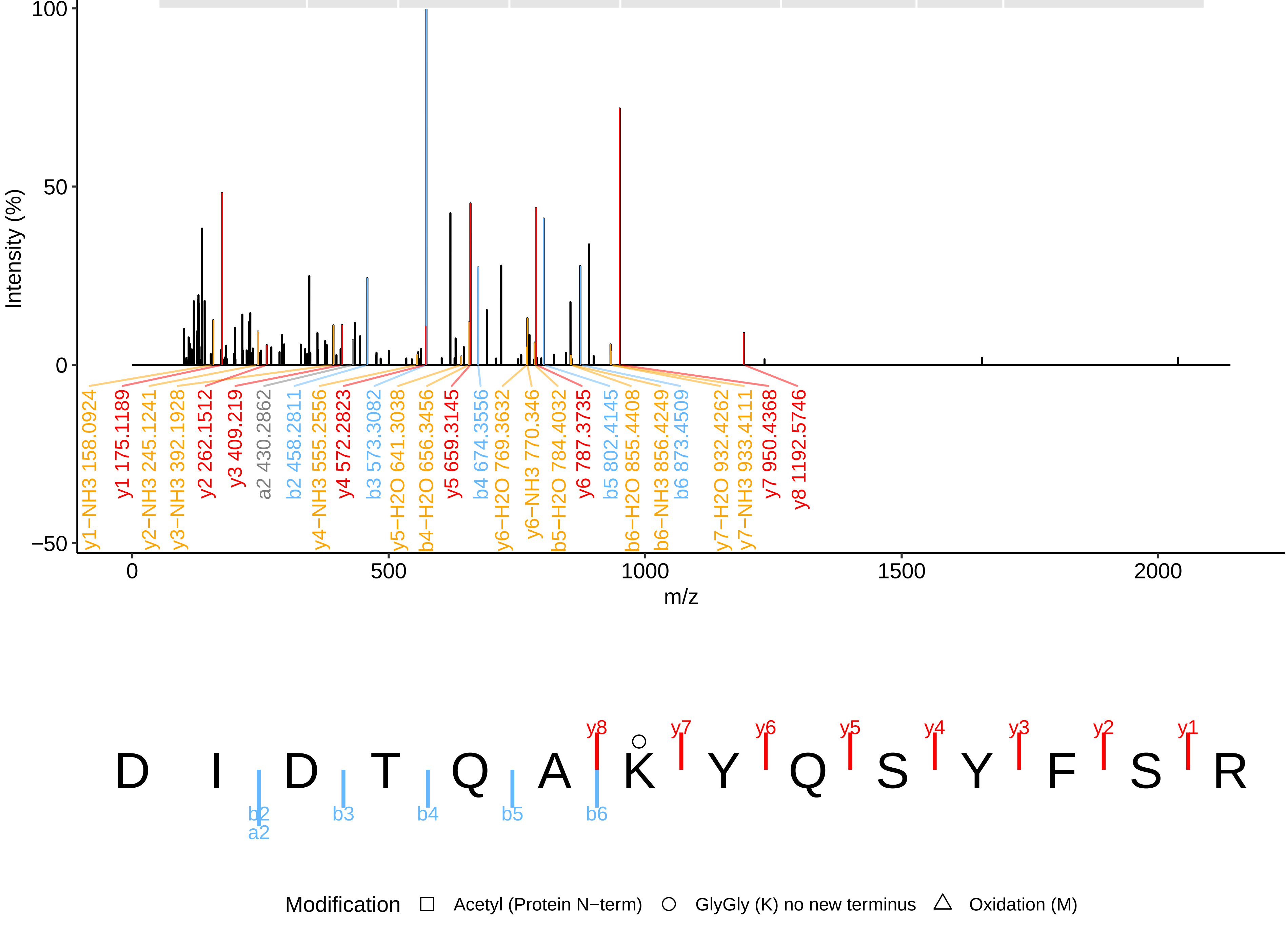

20201118_KIZ_MUb6_GlyGly_1 − Scan number: 25911

Gene Names	Charge	m/z	Mass	Retention time	PEP	Score	Precursor Intensity
GNGT1	4	365.9499	1459.771	79.132	0.04718	73.781	36744172

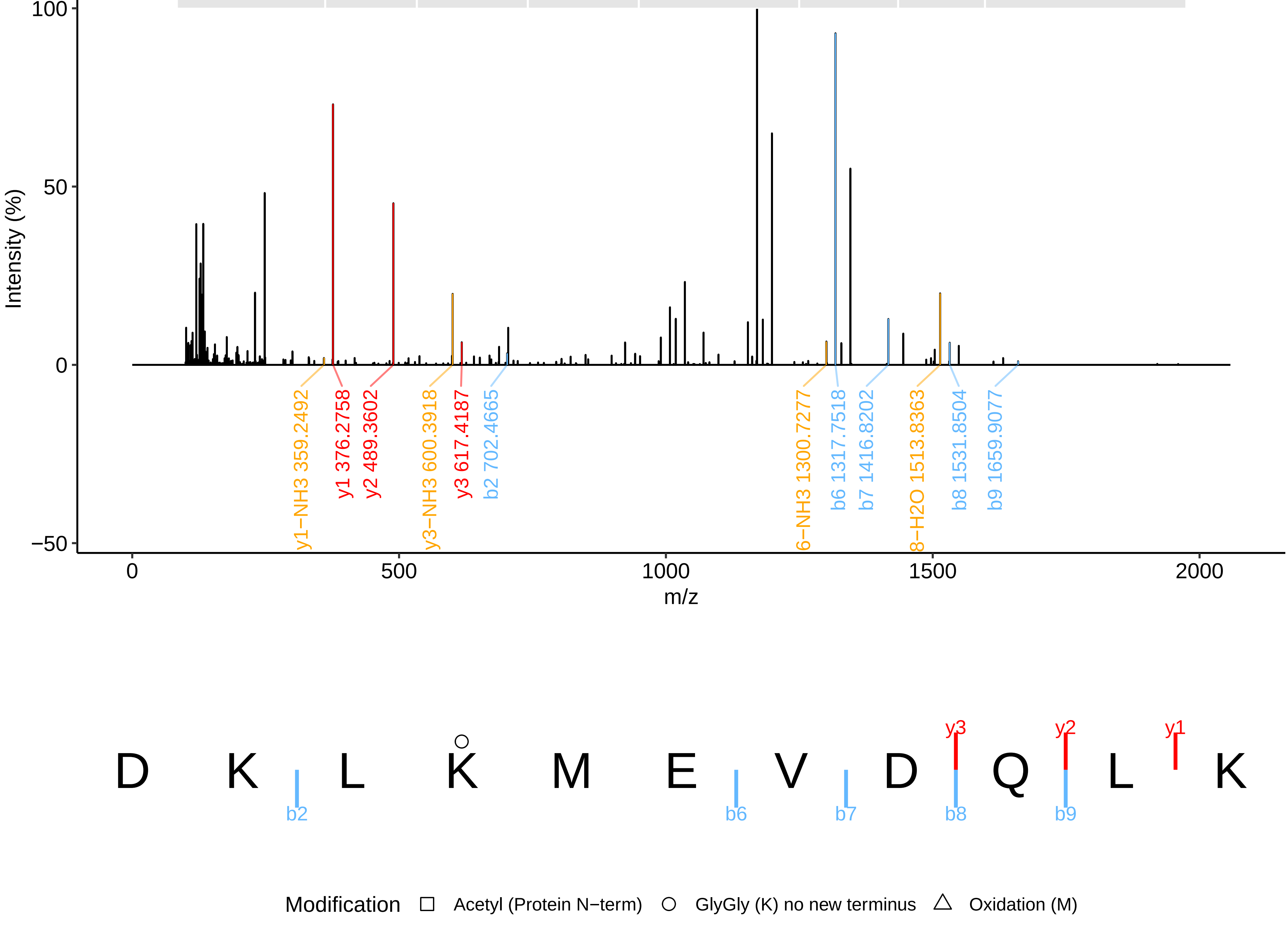

20201118_KIZ_MUb6_GlyGly_1 − Scan number: 27196

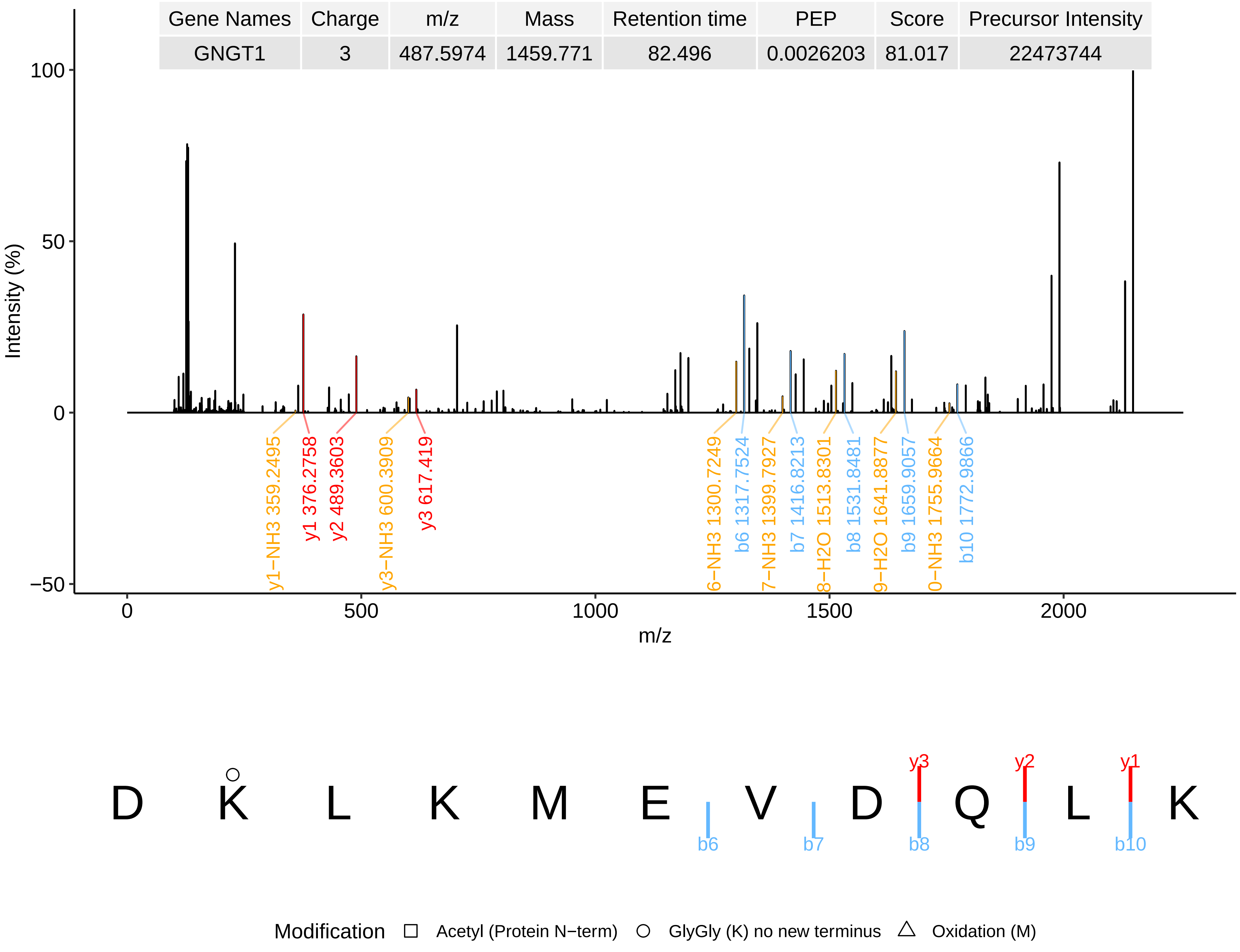

20201118_KIZ_MUb6_GlyGly_1 − Scan number: 27207

Gene Names	Charge	m/z	Mass	Retention time	PEP	Score	Precursor Intensity
GNGT1	4	365.9499	1459.771	82.523	0.091065	65.5	248320272

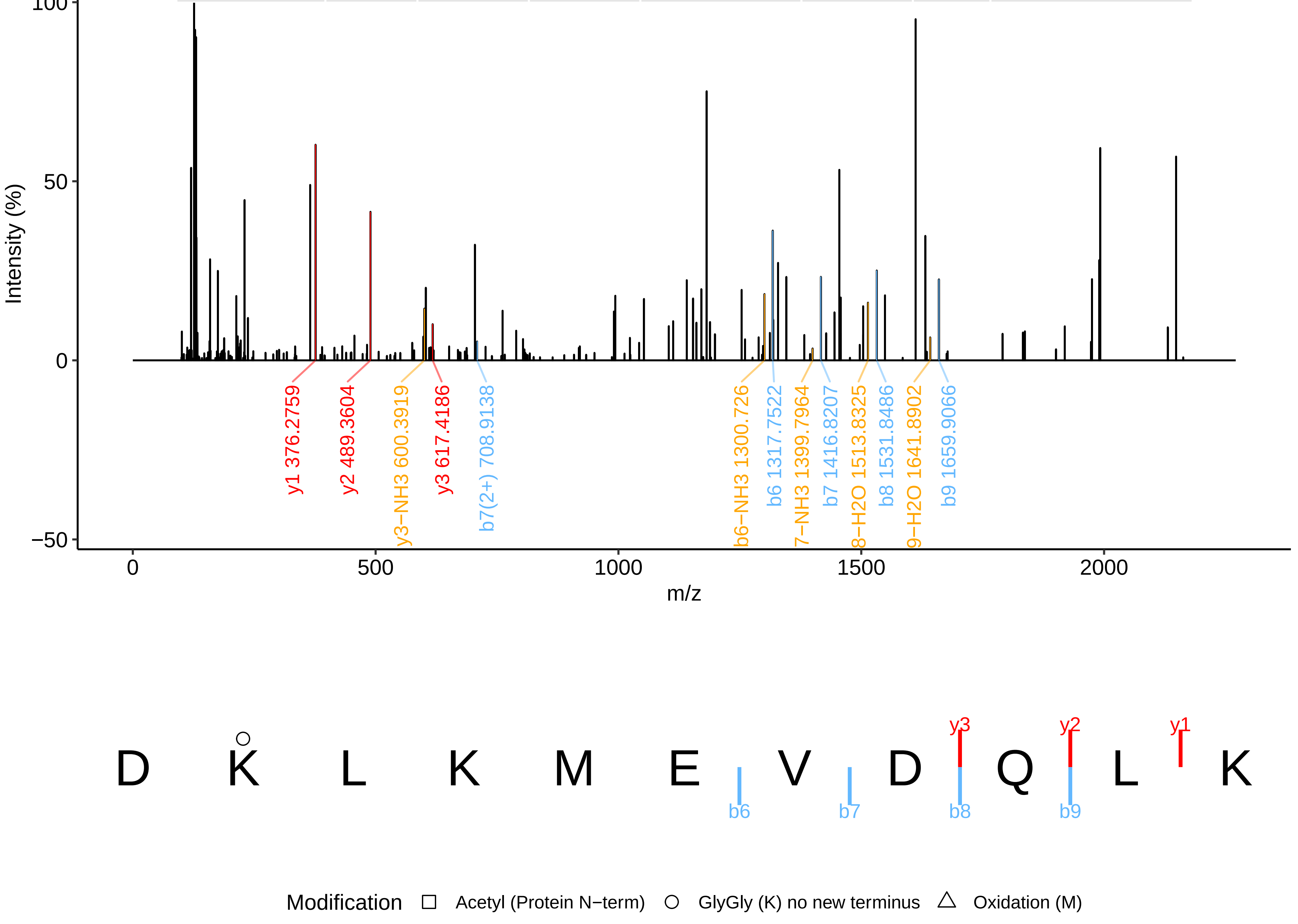

20201118_KIZ_MUb6_GlyGly_2 − Scan number: 31226

Gene Names	Charge	m/z	Mass	Retention time	PEP	Score	Precursor Intensity
GNGT1	4	365.9499	1459.771	102.77	0.010852	81.017	12643985

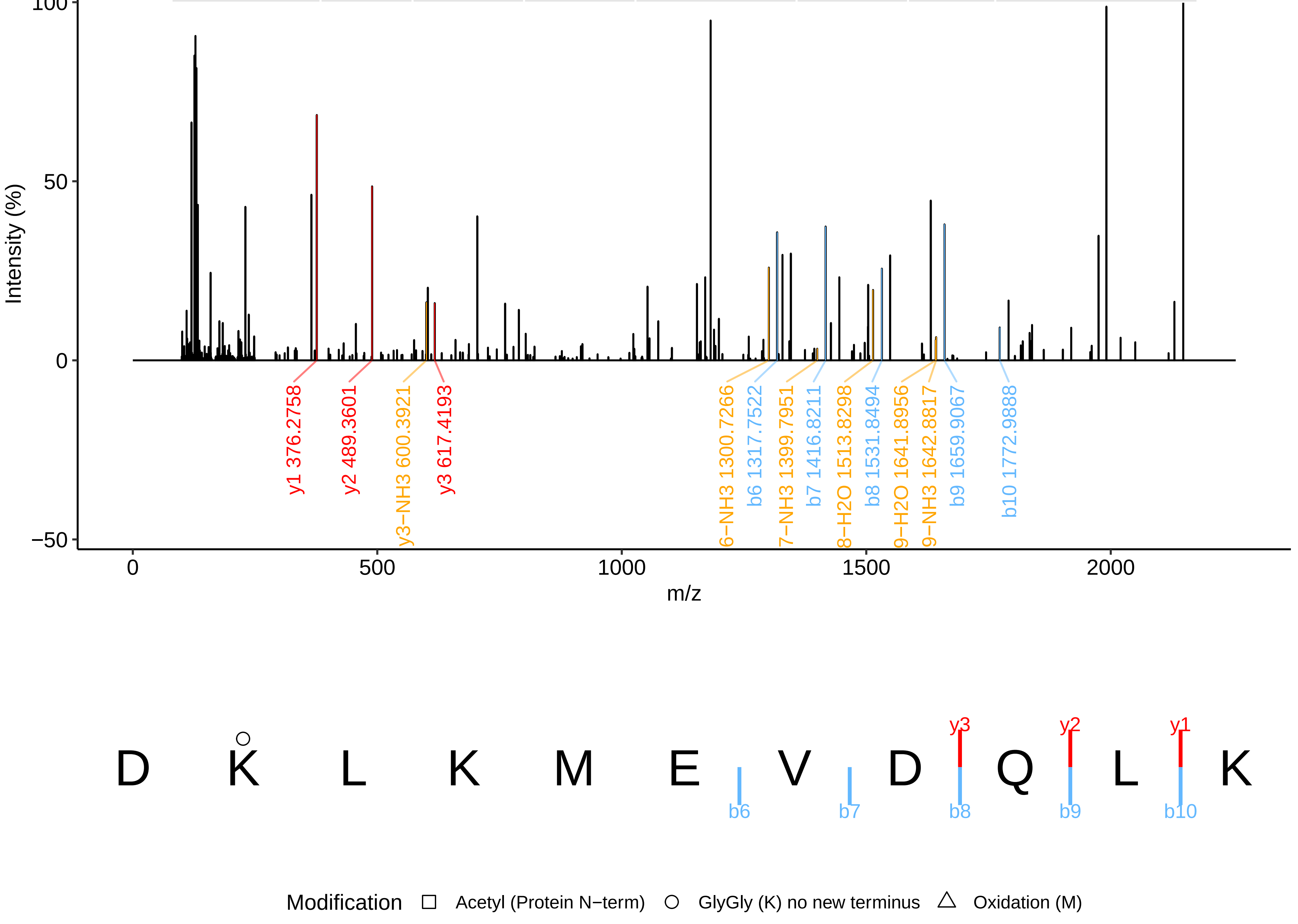

20200921_KIZ_MUb4_GlyGly_10plex_2 − Scan number: 19210

Gene Names	Charge	m/z	Mass	Retention time	PEP	Score	Precursor Intensity
ARL8A	3	561.9648	1682.873	75.373	0.00041945	88.552	1245368

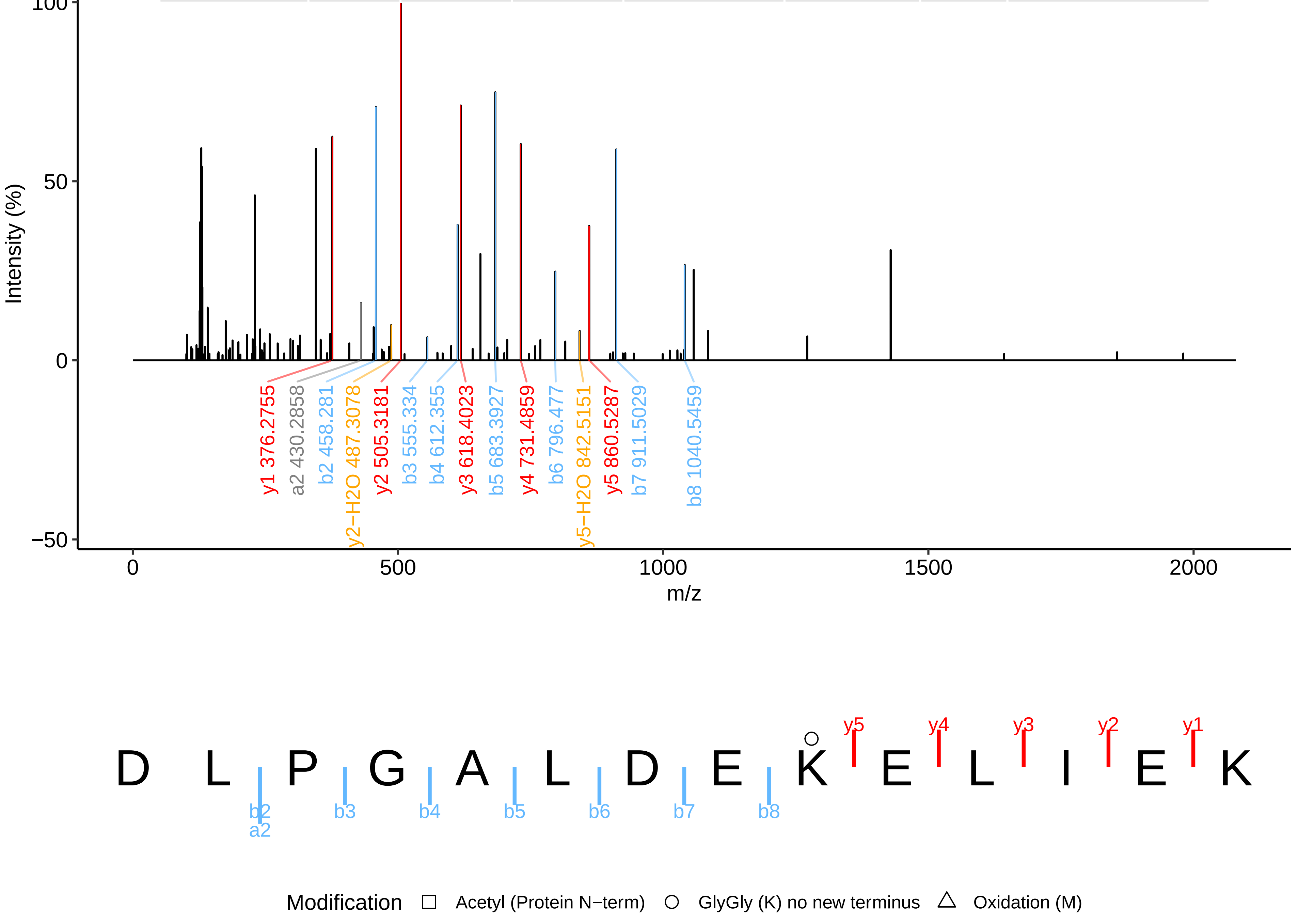

20200924_KIZ_MUb5_GlyGly_1 − Scan number: 21247

Gene Names	Charge	m/z	Mass	Retention time	PEP	Score	Precursor Intensity
ARL8A	3	561.9648	1682.873	68.437	0.00022592	94.551	NA

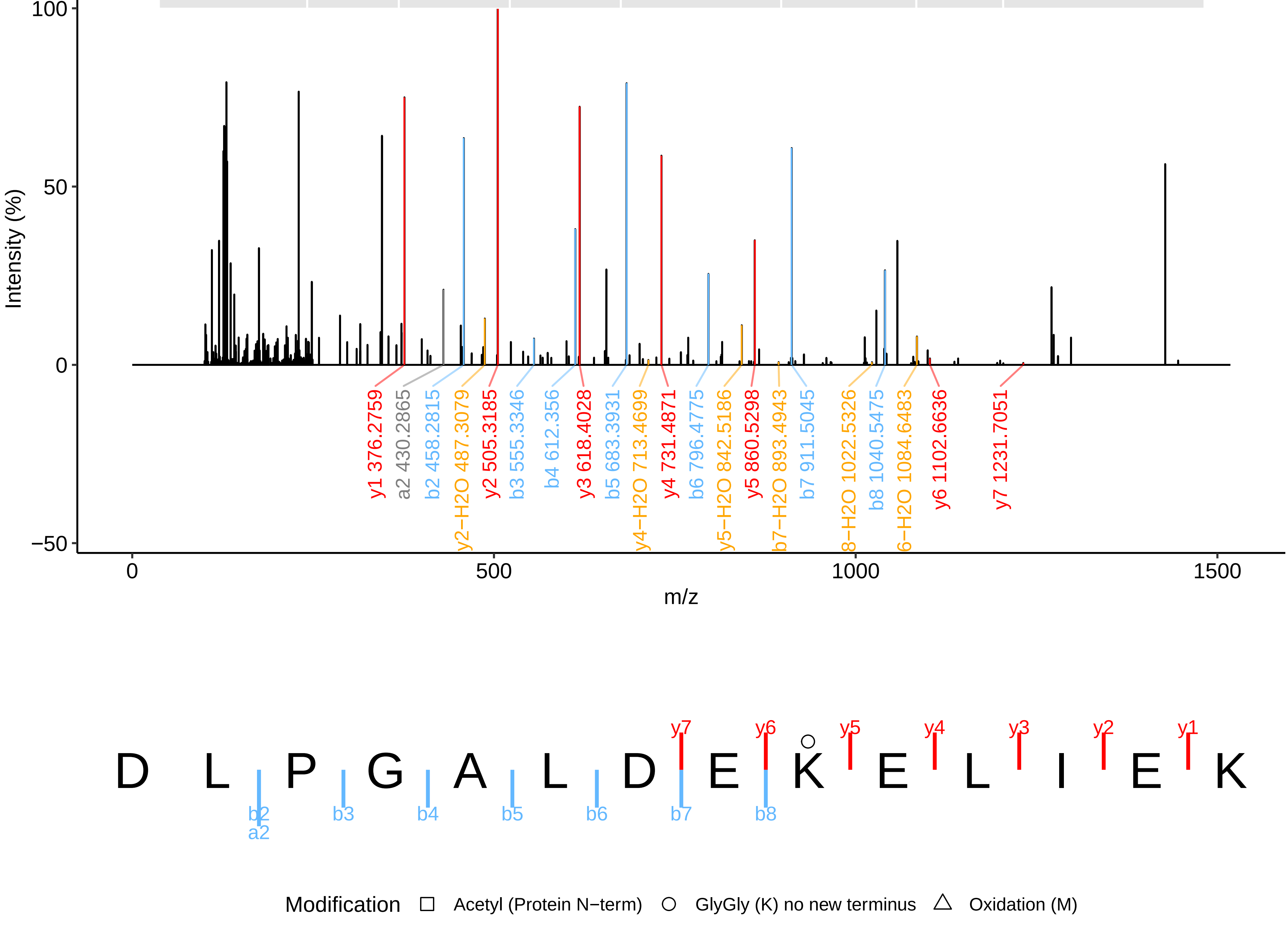

20200921_KIZ_MUb4_GlyGly_10plex − Scan number: 13299

Gene Names	Charge	m/z	Mass	Retention time	PEP	Score	Precursor Intensity
LYN	3	620.3095	1857.907	46.874	1.0383e−19	151.07	4624562

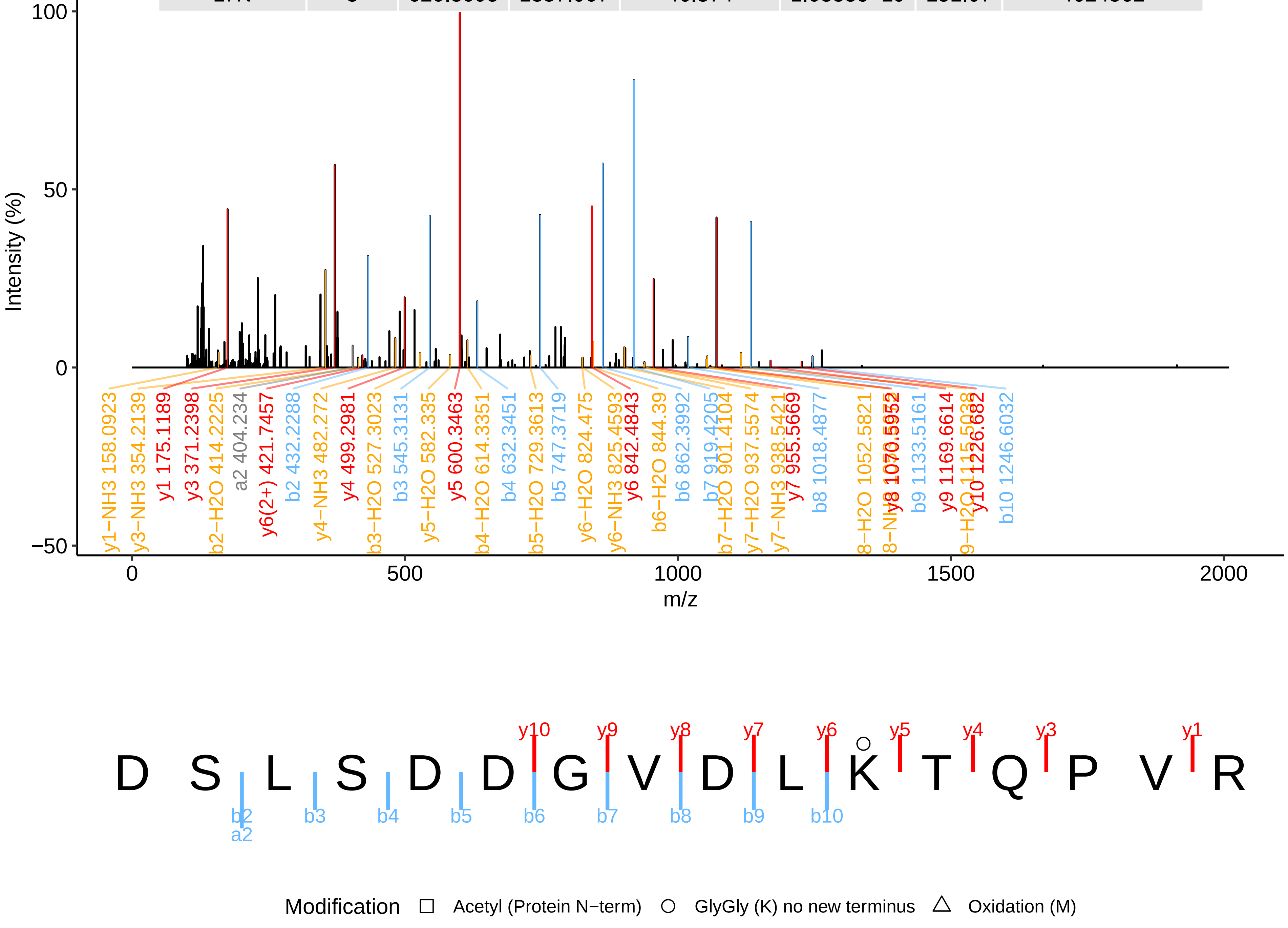

20200921_KIZ_MUb4_GlyGly_10plex_2 − Scan number: 12178

Gene Names	Charge	m/z	Mass	Retention time	PEP	Score	Precursor Intensity
LYN	3	620.3095	1857.907	47.063	9.1424e−08	111.74	994210.5

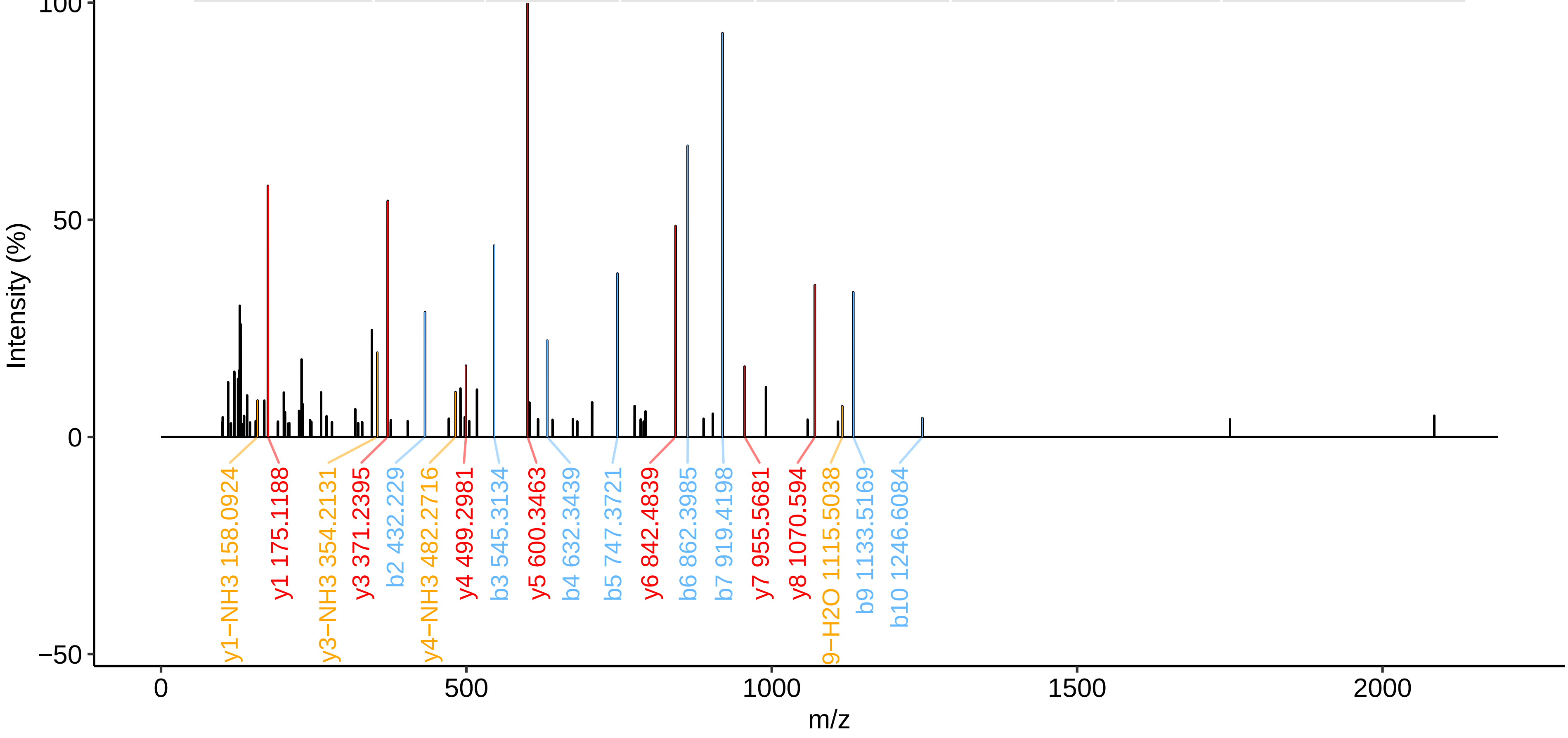

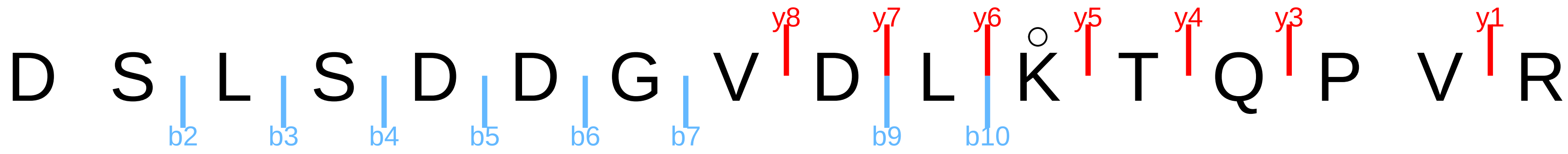

Modification □ Acetyl (Protein N−term) ○ GlyGly (K) no new terminus △ Oxidation (M)

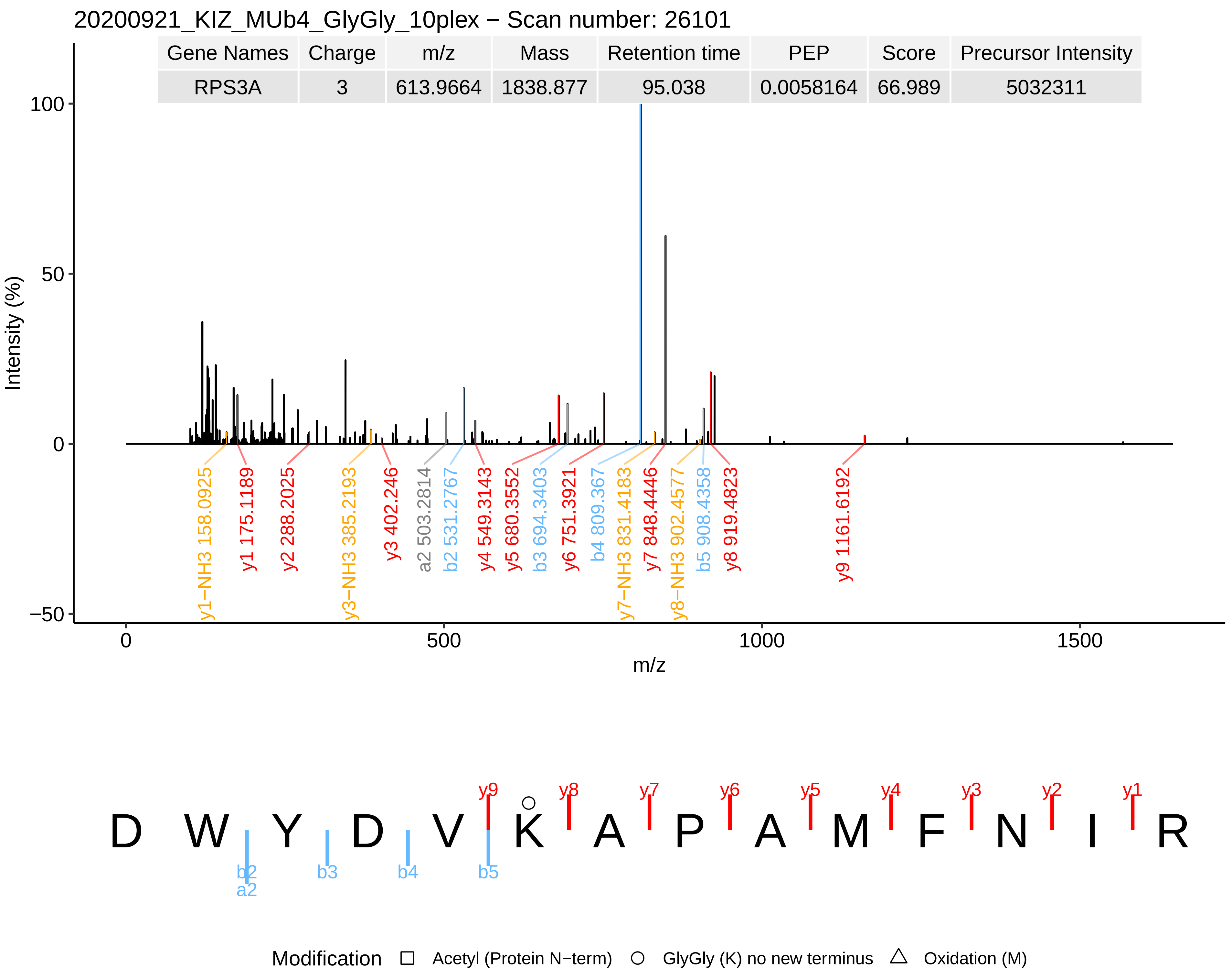
20200921_KIZ_MUb4_GlyGly_10plex − Scan number: 26101
Gene Names | Charge | m/z | Mass | Retention time | PEP | Score | Precursor Intensity
RPS3A | 3 | 613.9664 | 1838.877 | 95.038 | 0.0058164 | 66.989 | 5032311
Intensity (%)
m/z
y1−NH3 158.0925
y1 175.1189
y2 288.2025
y3−NH3 385.2193
y3 402.246
a2 503.2814
b2 531.2767
y4 549.3143
y5 680.3552
b3 694.3403
y6 751.3921
b4 809.367
y7−NH3 831.4183
y7 848.4446
y8−NH3 902.4577
b5 908.4358
y8 919.4823
y9 1161.6192
D W Y D V K A P A M F N I R
Modification
Acetyl (Protein N−term)
GlyGly (K) no new terminus
Oxidation (M)

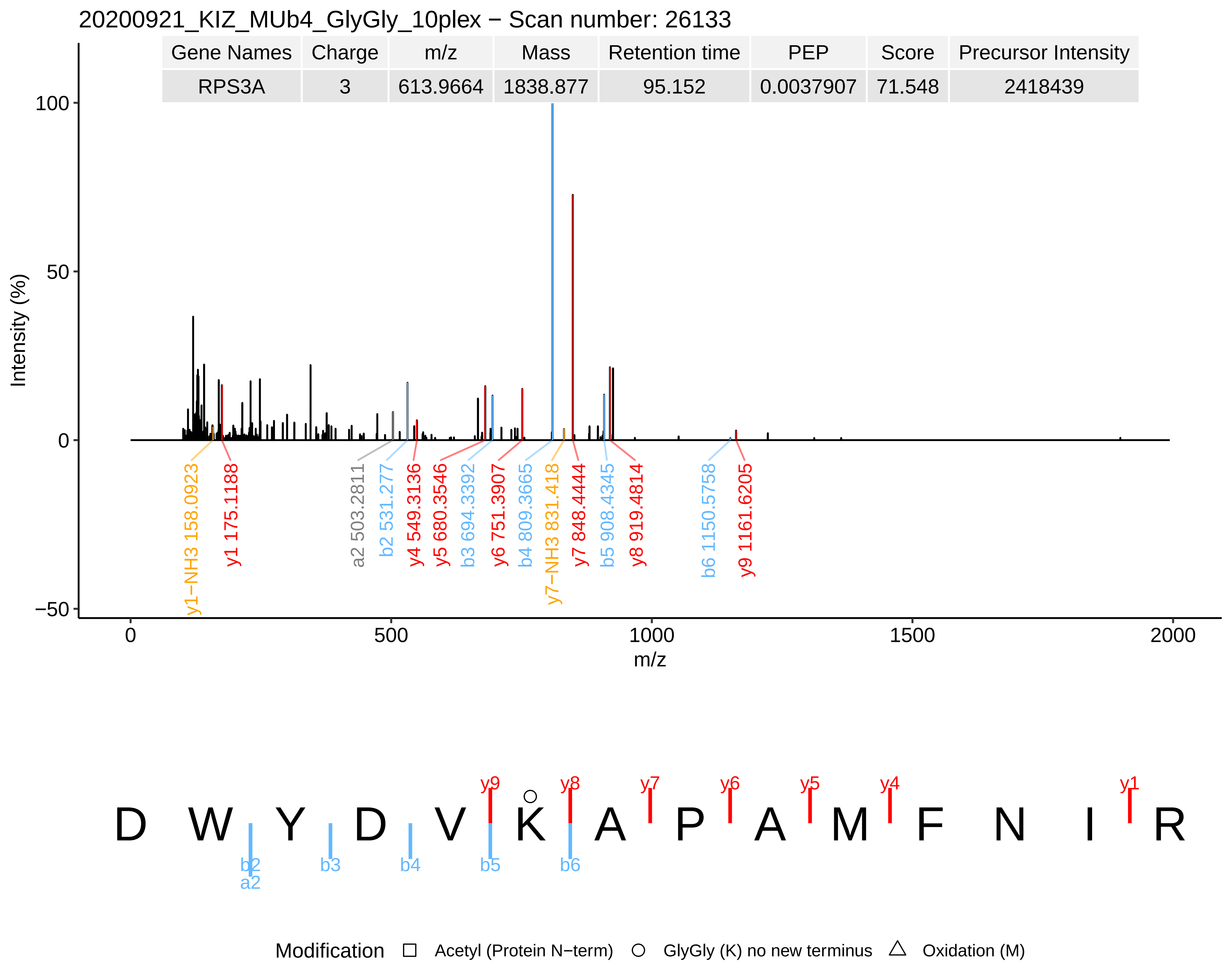
20200921_KIZ_MUb4_GlyGly_10plex − Scan number: 26133
Gene Names | Charge | m/z | Mass | Retention time | PEP | Score | Precursor Intensity
RPS3A | 3 | 613.9664 | 1838.877 | 95.152 | 0.0037907 | 71.548 | 2418439
Intensity (%)
m/z
y1−NH3 158.0923
y1 175.1188
a2 503.2811
b2 531.277
y4 549.3136
y5 680.3546
b3 694.3392
y6 751.3907
b4 809.3665
y7−NH3 831.418
y7 848.4444
b5 908.4345
y8 919.4814
b6 1150.5758
y9 1161.6205
D W Y D V K A P A M F N I R
Modification
Acetyl (Protein N−term)
GlyGly (K) no new terminus
Oxidation (M)

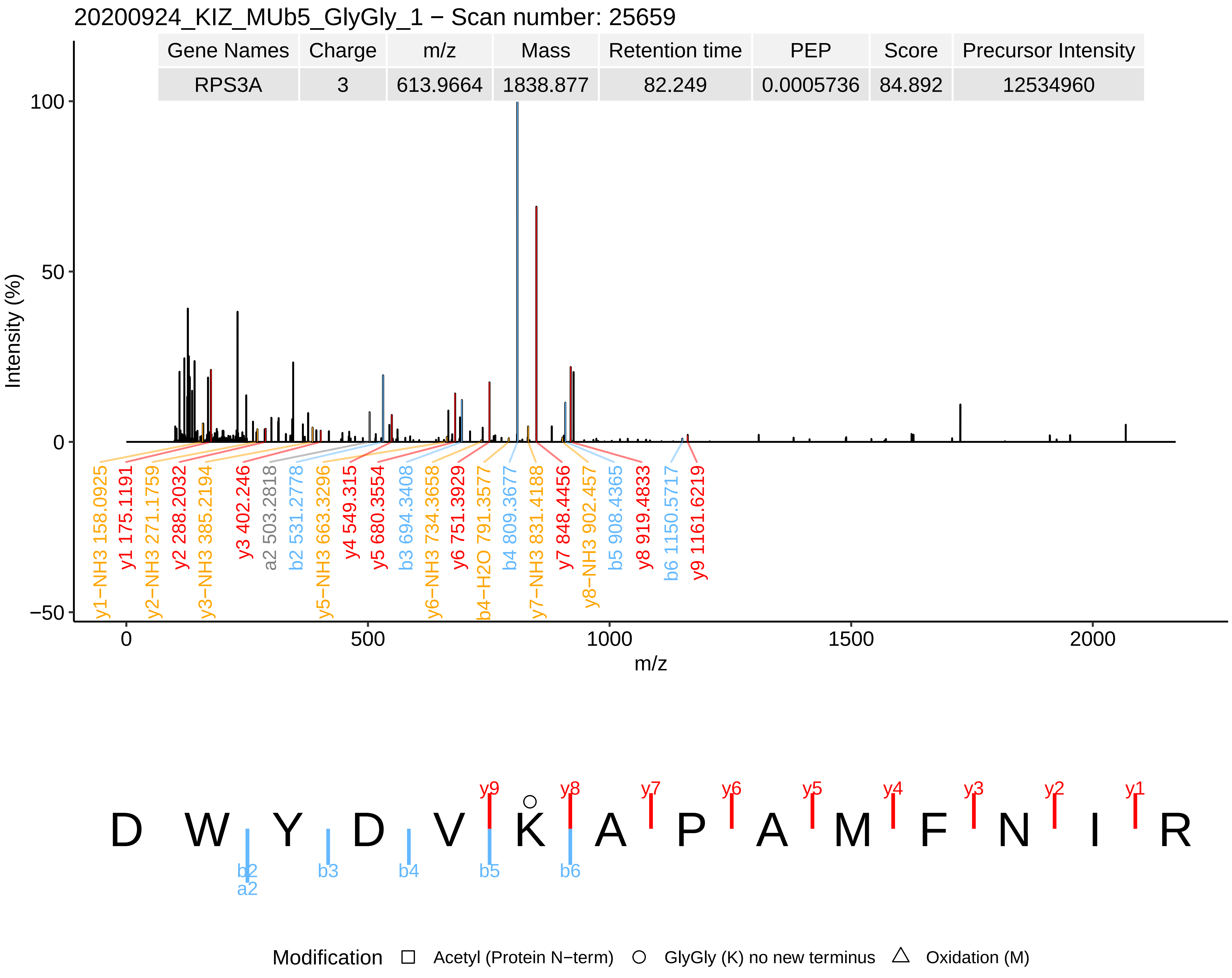
20200924_KIZ_MUb5_GlyGly_1 − Scan number: 25659
Gene Names | Charge | m/z | Mass | Retention time | PEP | Score | Precursor Intensity
RPS3A | 3 | 613.9664 | 1838.877 | 82.249 | 0.0005736 | 84.892 | 12534960
Intensity (%)
m/z
y1−NH3 158.0925
y1 175.1191
y2−NH3 271.1759
y2 288.2032
y3−NH3 385.2194
y3 402.246
a2 503.2818
b2 531.2778
y5−NH3 663.3296
y4 549.315
y5 680.3554
b3 694.3408
y6−NH3 734.3658
y6 751.3929
b4−H2O 791.3577
b4 809.3677
y7−NH3 831.4188
y7 848.4456
y8−NH3 902.457
b5 908.4365
y8 919.4833
b6 1150.5717
y9 1161.6219
D W Y D V K A P A M F N I R
Modification
Acetyl (Protein N−term)
GlyGly (K) no new terminus
Oxidation (M)

20200924_KIZ_MUb5_GlyGly_2 − Scan number: 23061

Gene Names	Charge	m/z	Mass	Retention time	PEP	Score	Precursor Intensity
RPS3A	3	613.9664	1838.877	85.395	0.0048012	69.716	4291438

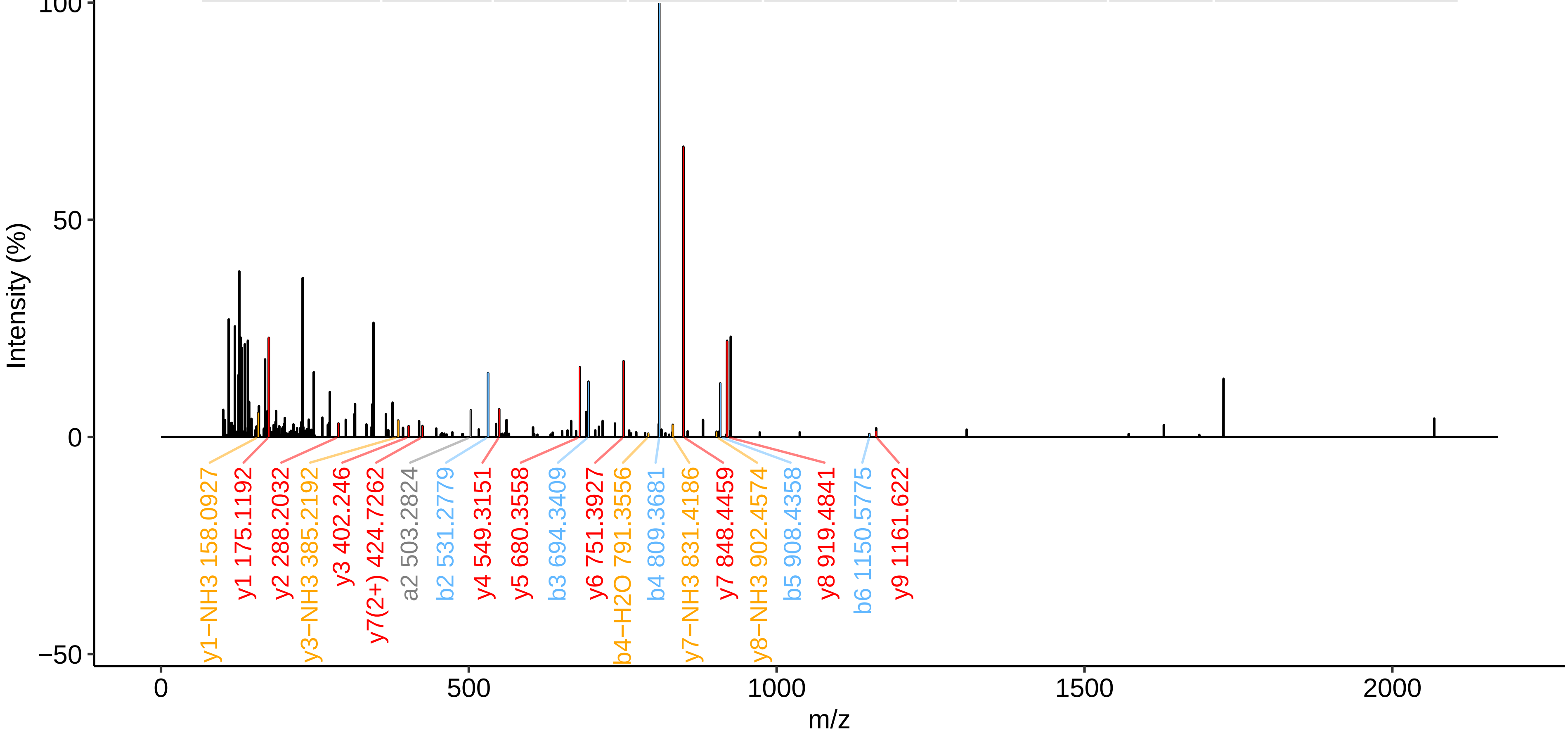

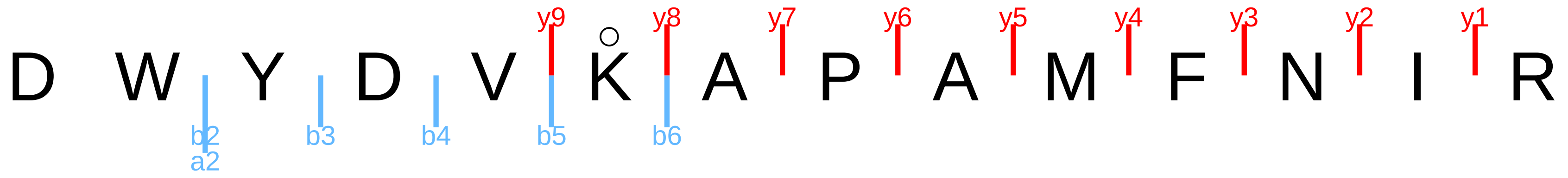

Modification □ Acetyl (Protein N−term) ○ GlyGly (K) no new terminus △ Oxidation (M)

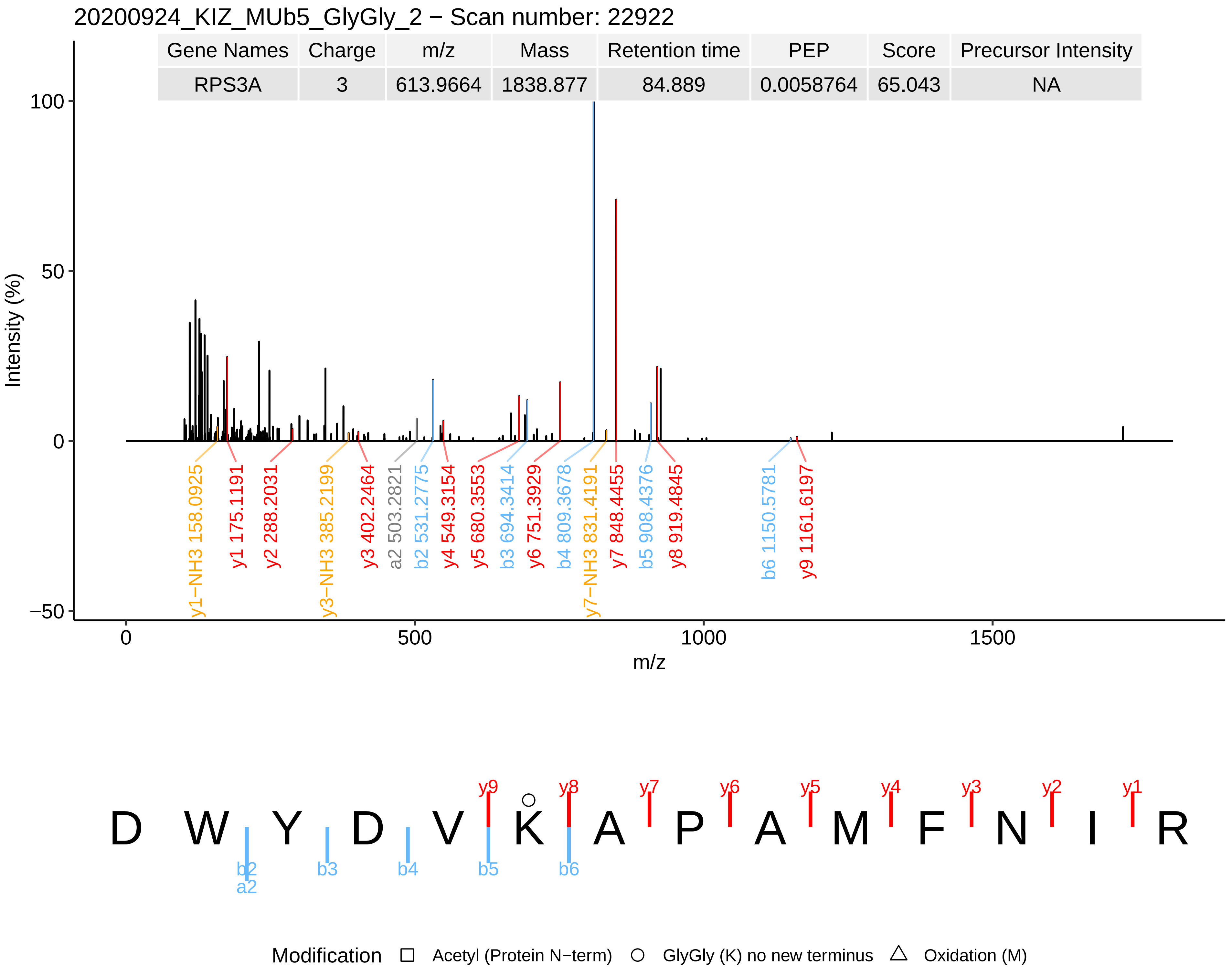
20200924_KIZ_MUb5_GlyGly_2 − Scan number: 22922
Gene Names | Charge | m/z | Mass | Retention time | PEP | Score | Precursor Intensity
RPS3A | 3 | 613.9664 | 1838.877 | 84.889 | 0.0058764 | 65.043 | NA
Intensity (%)
m/z
y1−NH3 158.0925
y1 175.1191
y2 288.2031
y3−NH3 385.2199
y3 402.2464
a2 503.2821
b2 531.2775
y4 549.3154
y5 680.3553
b3 694.3414
y6 751.3929
b4 809.3678
y7−NH3 831.4191
y7 848.4455
b5 908.4376
y8 919.4845
b6 1150.5781
y9 1161.6197
D W Y D V K A P A M F N I R
Modification
Acetyl (Protein N−term)
GlyGly (K) no new terminus
Oxidation (M)

20200921_KIZ_MUb4_GlyGly_10plex − Scan number: 9820

Gene Names	Charge	m/z	Mass	Retention time	PEP	Score	Precursor Intensity
HSPA8	4	422.7035	1686.785	33.766	0.00057267	88.187	6690208

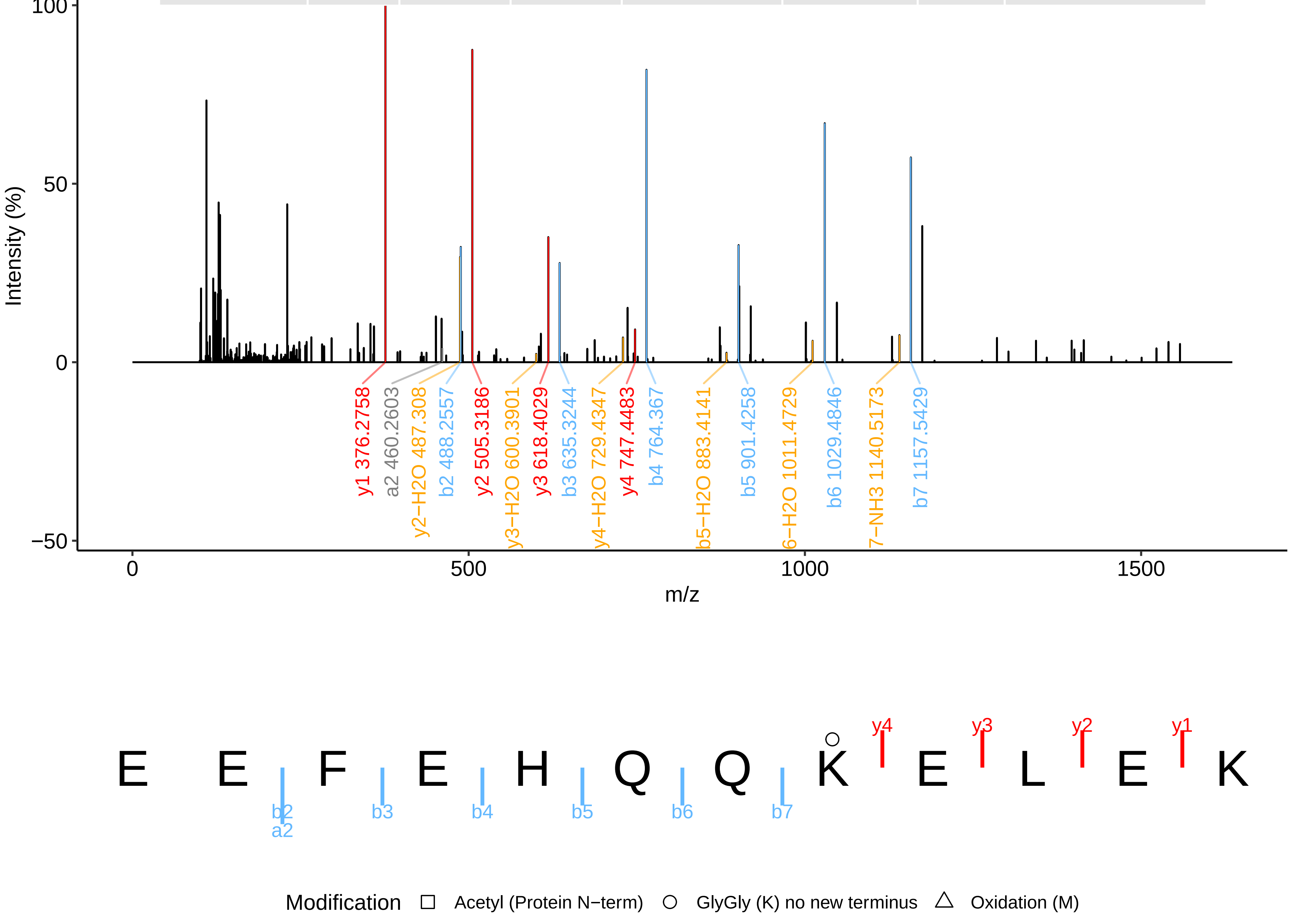

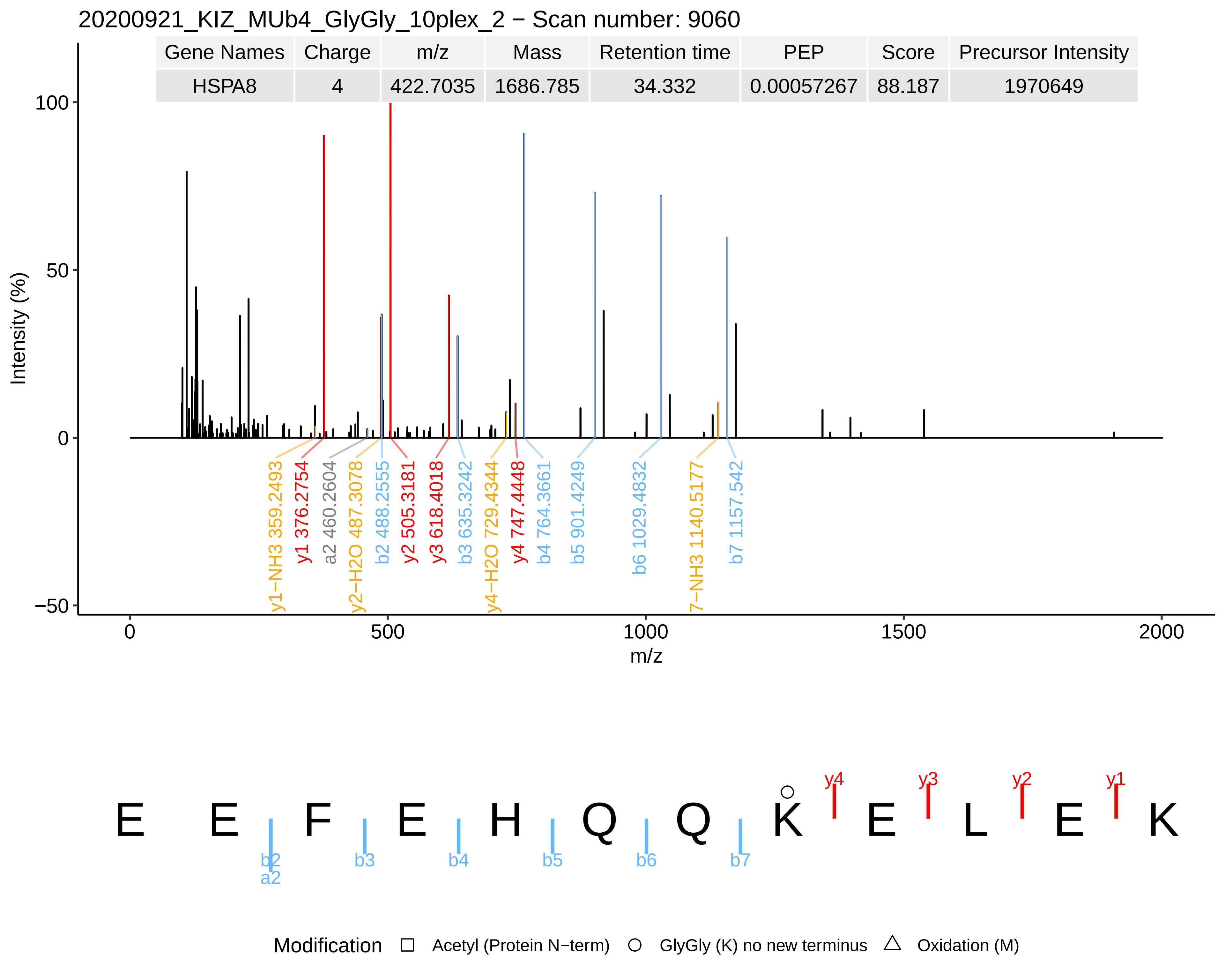
20200921_KIZ_MUb4_GlyGly_10plex_2 − Scan number: 9060
Gene Names | Charge | m/z | Mass | Retention time | PEP | Score | Precursor Intensity
HSPA8 | 4 | 422.7035 | 1686.785 | 34.332 | 0.00057267 | 88.187 | 1970649
Intensity (%)
m/z
y1−NH3 359.2493
y1 376.2754
a2 460.2604
y2−H2O 487.3078
b2 488.2555
y2 505.3181
y3 618.4018
b3 635.3242
y4−H2O 729.4344
y4 747.4448
b4 764.3661
b5 901.4249
b6 1029.4832
7−NH3 1140.5177
b7 1157.542
E E F E H Q Q K E L E K
b2 a2 b3 b4 b5 b6 b7
y4 y3 y2 y1
Modification
Acetyl (Protein N−term)
GlyGly (K) no new terminus
Oxidation (M)

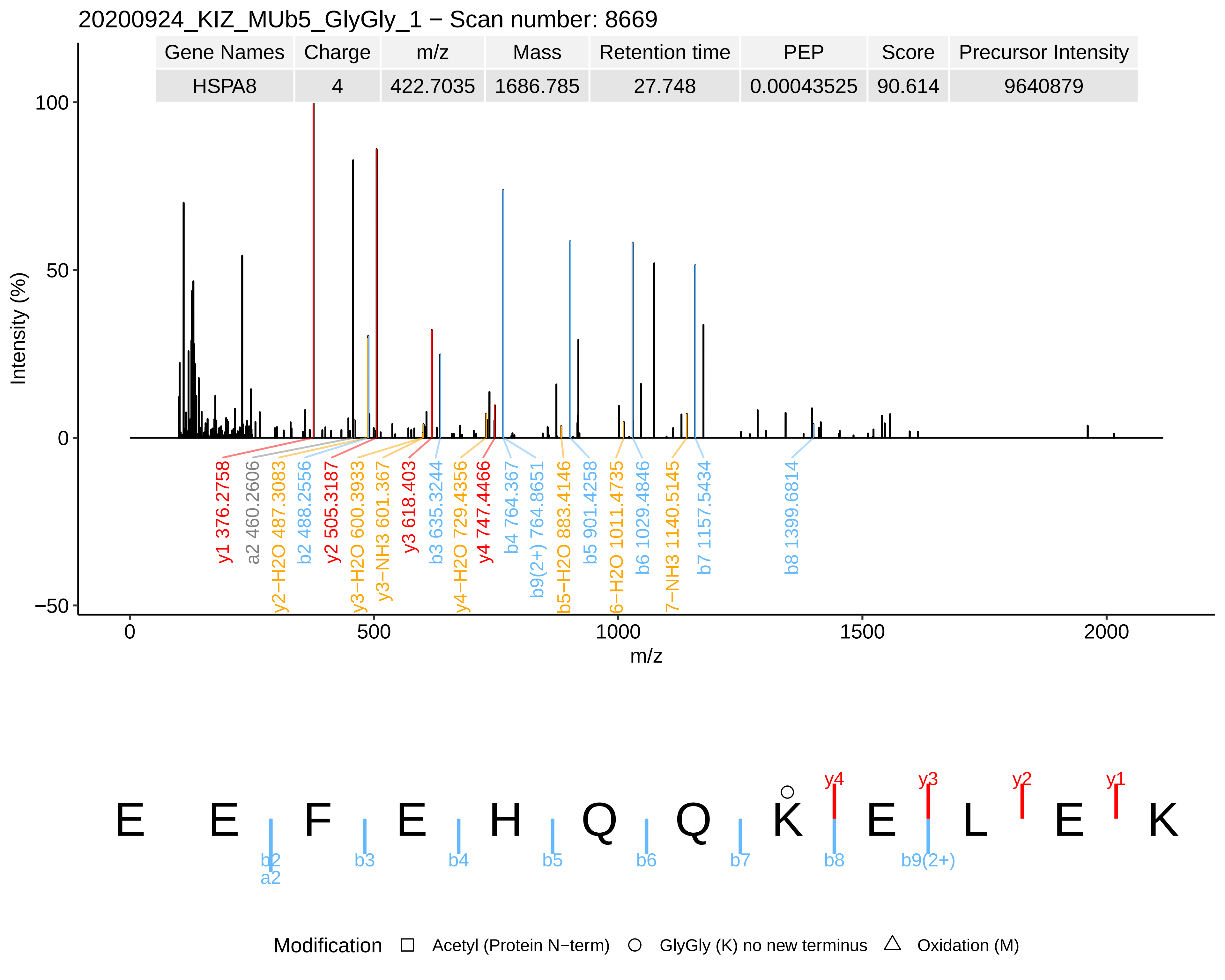
20200924_KIZ_MUb5_GlyGly_1 − Scan number: 8669
Gene Names | Charge | m/z | Mass | Retention time | PEP | Score | Precursor Intensity
HSPA8 | 4 | 422.7035 | 1686.785 | 27.748 | 0.00043525 | 90.614 | 9640879
Intensity (%)
m/z
y1 376.2758
a2 460.2606
y2−H2O 487.3083
b2 488.2556
y2 505.3187
y3−H2O 600.3933
y3−NH3 601.367
y3 618.403
b3 635.3244
y4−H2O 729.4356
y4 747.4466
b4 764.367
b9(2+) 764.8651
b5−H2O 883.4146
b5 901.4258
b6−H2O 1011.4735
b6 1029.4846
b7−NH3 1140.5145
b7 1157.5434
b8 1399.6814
E E F E H Q Q K E L E K
y4 y3 y2 y1
b2 a2 b3 b4 b5 b6 b7 b8 b9(2+)
Modification
Acetyl (Protein N−term)
GlyGly (K) no new terminus
Oxidation (M)

20200924_KIZ_MUb5_GlyGly_1 − Scan number: 8697

Gene Names	Charge	m/z	Mass	Retention time	PEP	Score	Precursor Intensity
HSPA8	4	422.7035	1686.785	27.847	1.6947e−05	98.156	12374917

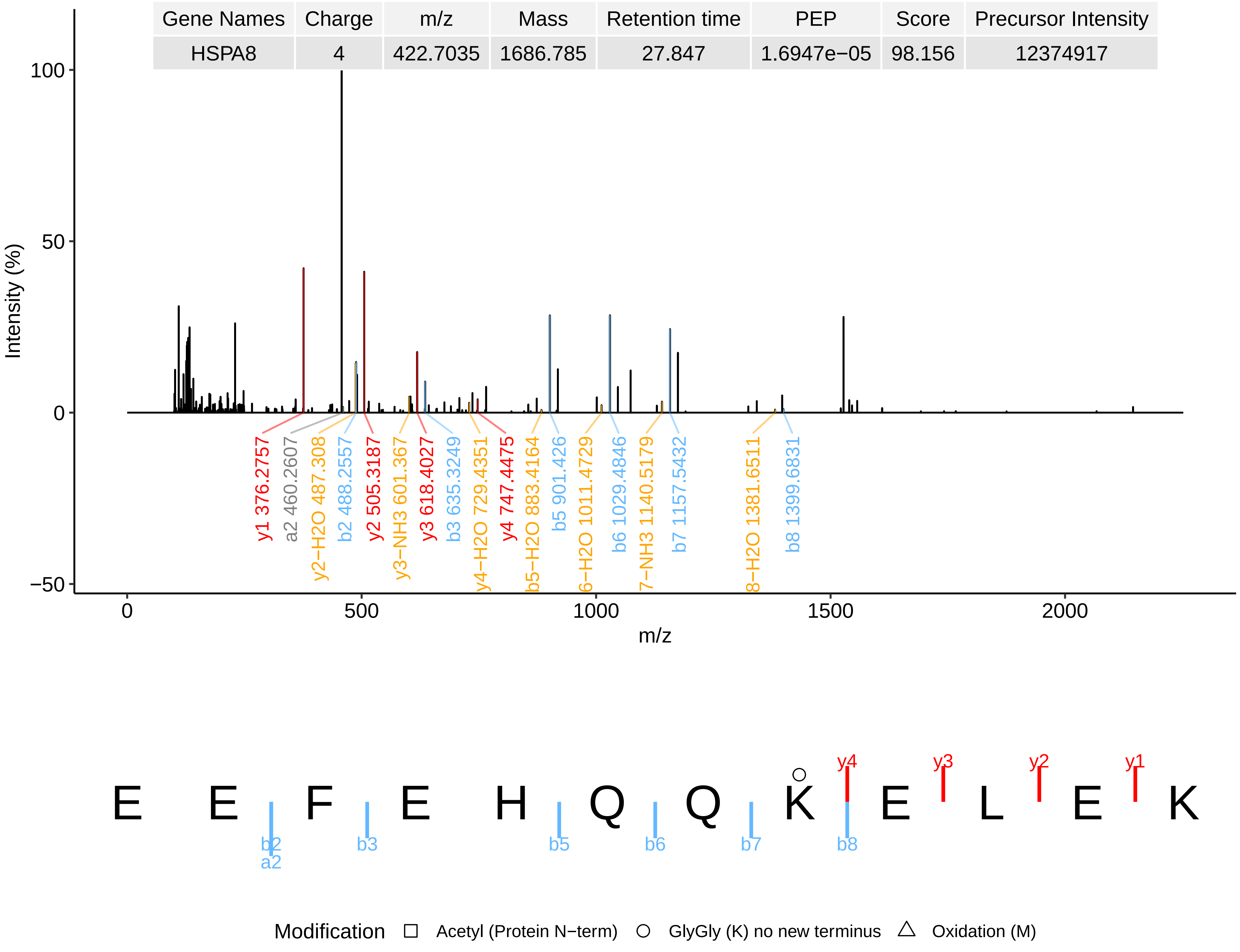

20200921_KIZ_MUb4_GlyGly_10plex − Scan number: 5336

Gene Names	Charge	m/z	Mass	Retention time	PEP	Score	Precursor Intensity
IGF2R	4	411.2102	1640.812	17.458	0.00019326	92.939	7392904

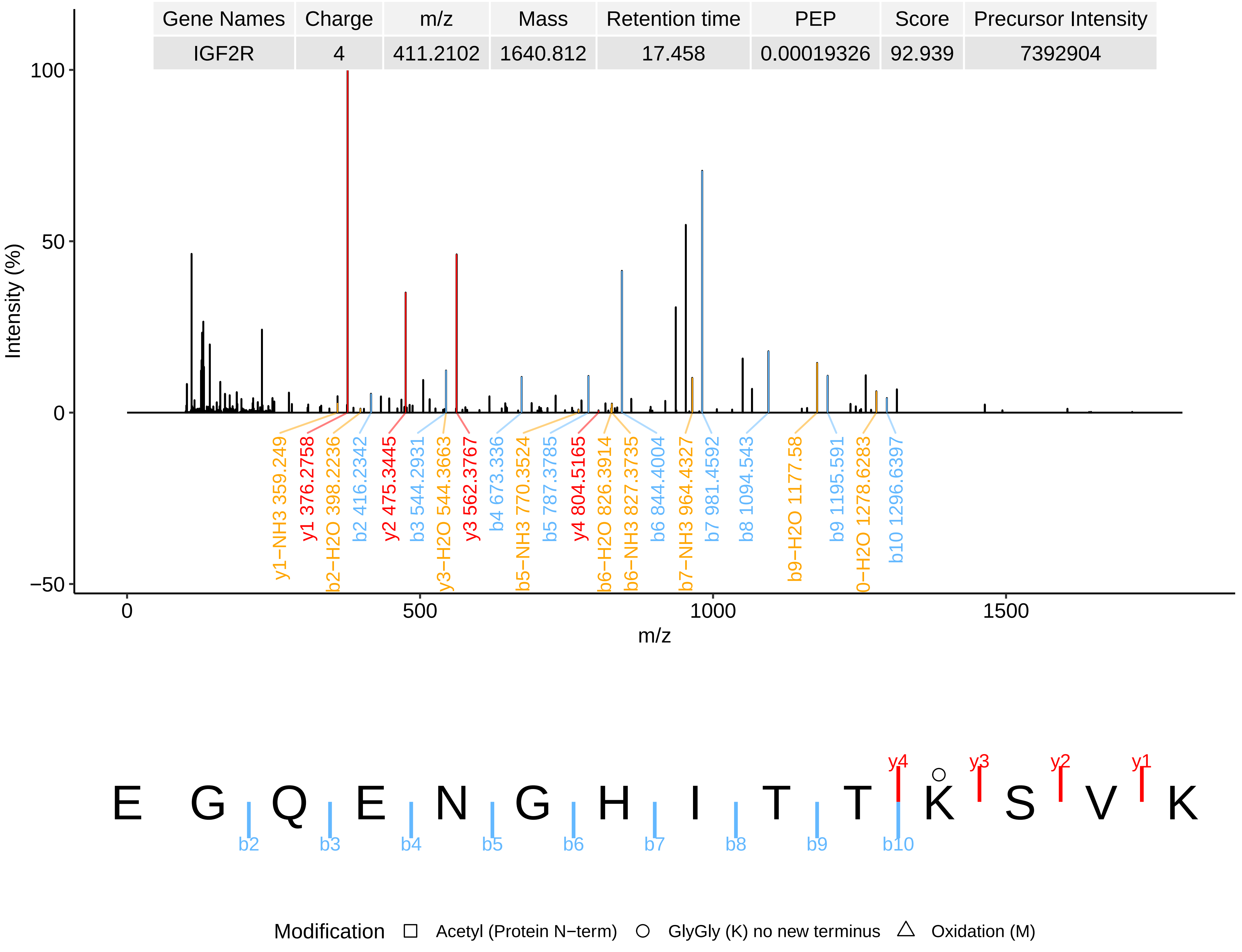

20200921_KIZ_MUb4_GlyGly_10plex_2 − Scan number: 11622

Gene Names	Charge	m/z	Mass	Retention time	PEP	Score	Precursor Intensity
HERC4	3	619.3255	1854.955	44.758	0.0013133	77.746	413278.1

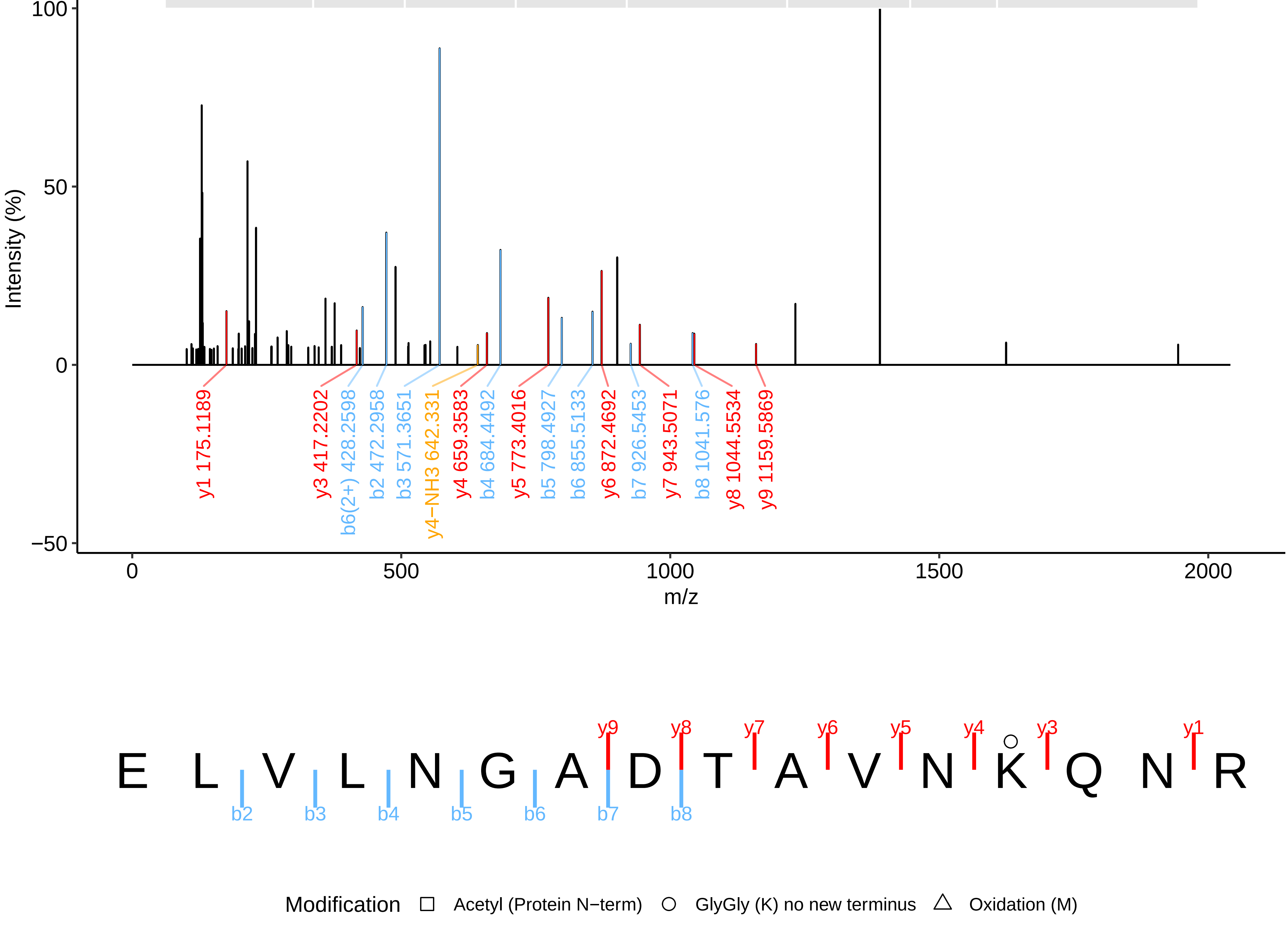

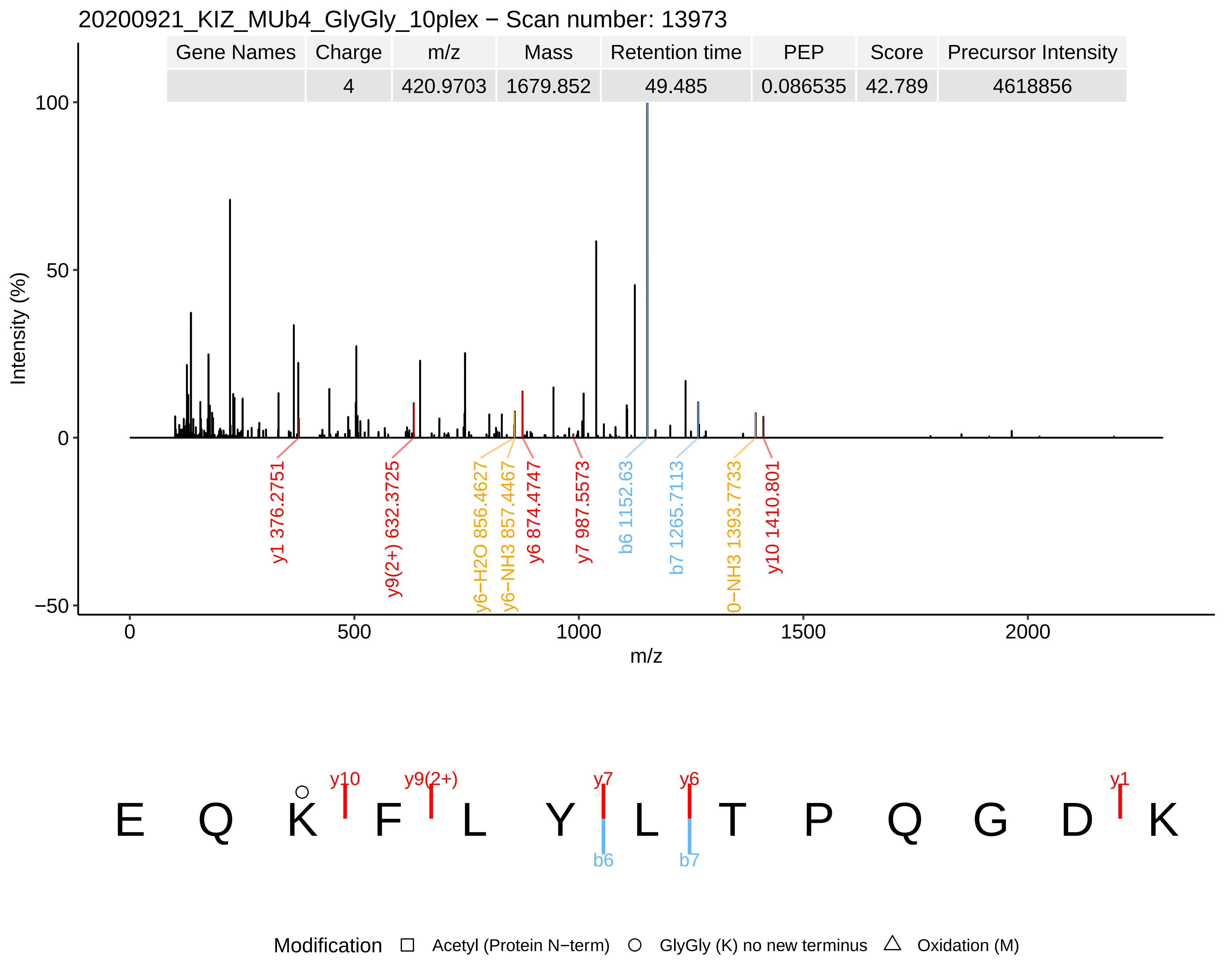

20200921_KIZ_MUb4_GlyGly_10plex − Scan number: 13973
Gene Names | Charge | m/z | Mass | Retention time | PEP | Score | Precursor Intensity
4 | 420.9703 | 1679.852 | 49.485 | 0.086535 | 42.789 | 4618856
Intensity (%)
m/z
y1 376.2751
y9(2+) 632.3725
y6−H2O 856.4627
y6−NH3 857.4467
y6 874.4747
y7 987.5573
b6 1152.63
b7 1265.7113
0−NH3 1393.7733
y10 1410.801
E Q K F L Y L T P Q G D K
y10
y9(2+)
y7
y6
y1
b6
b7
Modification
Acetyl (Protein N−term)
GlyGly (K) no new terminus
Oxidation (M)

20200921_KIZ_MUb4_GlyGly_10plex − Scan number: 19550

Gene Names	Charge	m/z	Mass	Retention time	PEP	Score	Precursor Intensity
ATAD3B;ATAD3A	3	636.3217	1905.943	70.528	1.4682e−22	169.4	4383117

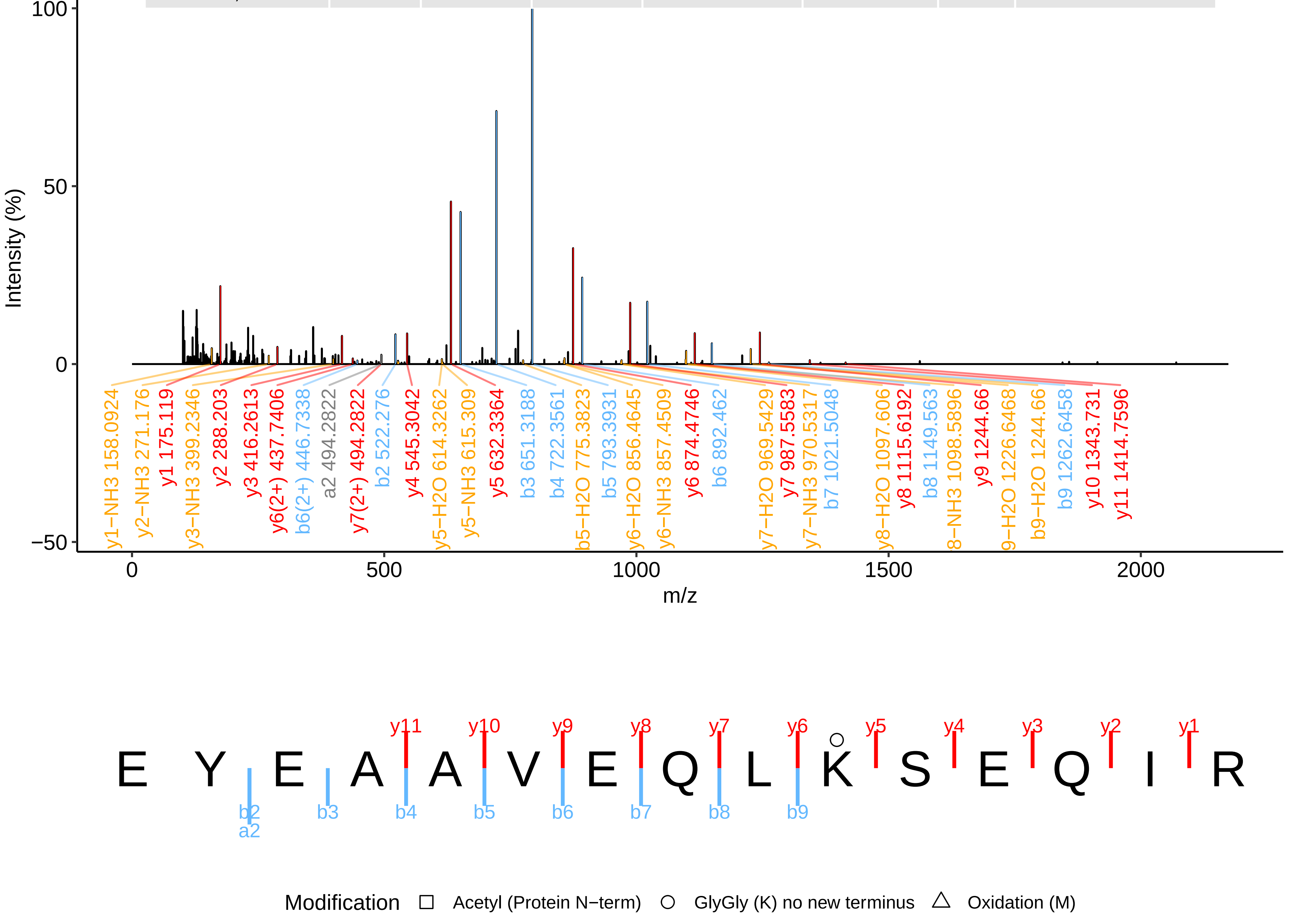

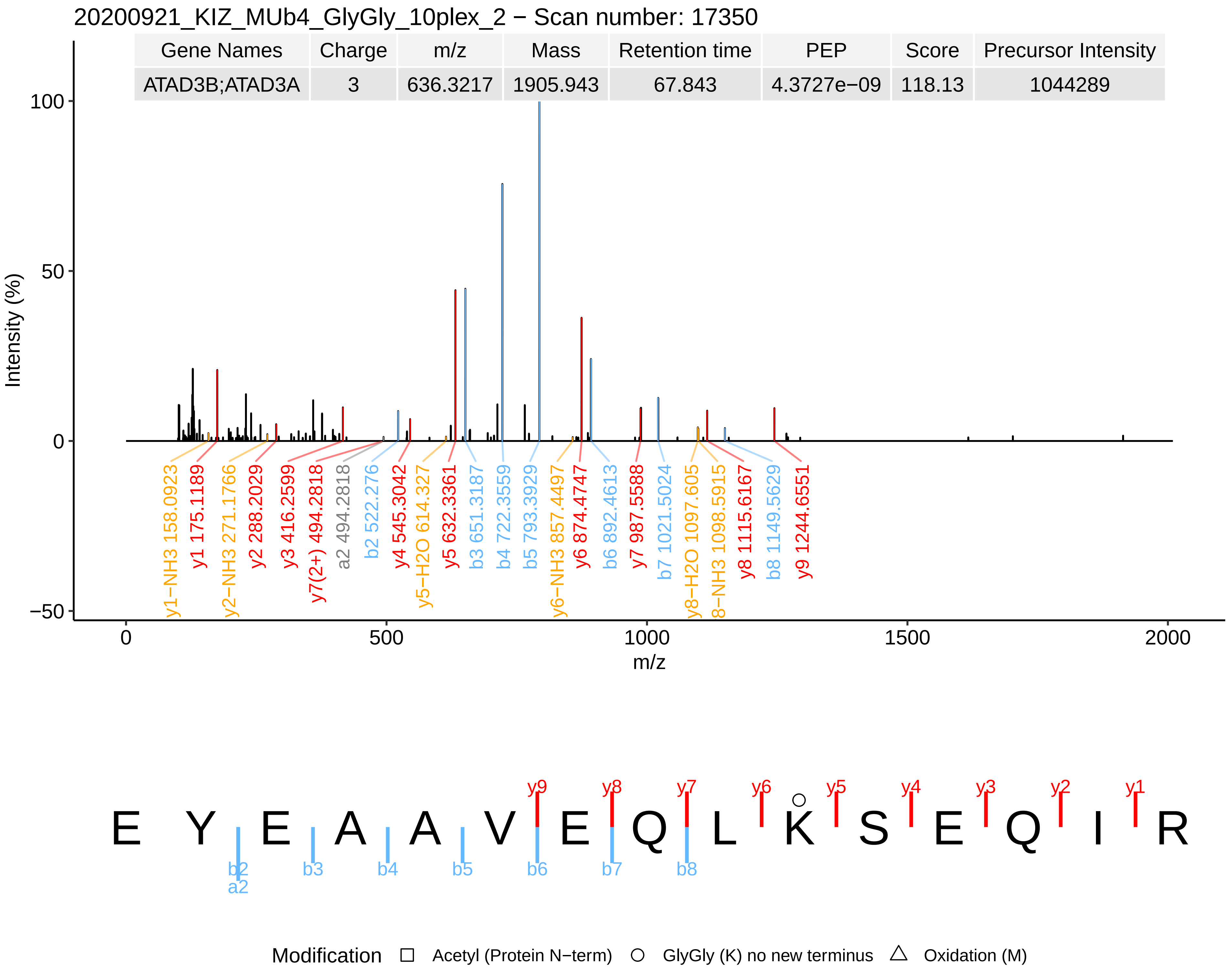
20200921_KIZ_MUb4_GlyGly_10plex_2 − Scan number: 17350
Gene Names | Charge | m/z | Mass | Retention time | PEP | Score | Precursor Intensity
ATAD3B;ATAD3A | 3 | 636.3217 | 1905.943 | 67.843 | 4.3727e−09 | 118.13 | 1044289
Intensity (%)
100
50
0
−50
0
500
1000
1500
2000
m/z
y1−NH3 158.0923
y1 175.1189
y2−NH3 271.1766
y2 288.2029
y3 416.2599
y7(2+) 494.2818
a2 494.2818
b2 522.276
y4 545.3042
y5−H2O 614.327
y5 632.3361
b3 651.3187
b4 722.3559
b5 793.3929
y6−NH3 857.4497
y6 874.4747
b6 892.4613
y7 987.5588
b7 1021.5024
y8−H2O 1097.605
8−NH3 1098.5915
y8 1115.6167
b8 1149.5629
y9 1244.6551
E Y E A A V E Q L K S E Q I R
y9 y8 y7 y6 y5 y4 y3 y2 y1
b2 a2 b3 b4 b5 b6 b7 b8
Modification
Acetyl (Protein N−term)
GlyGly (K) no new terminus
Oxidation (M)

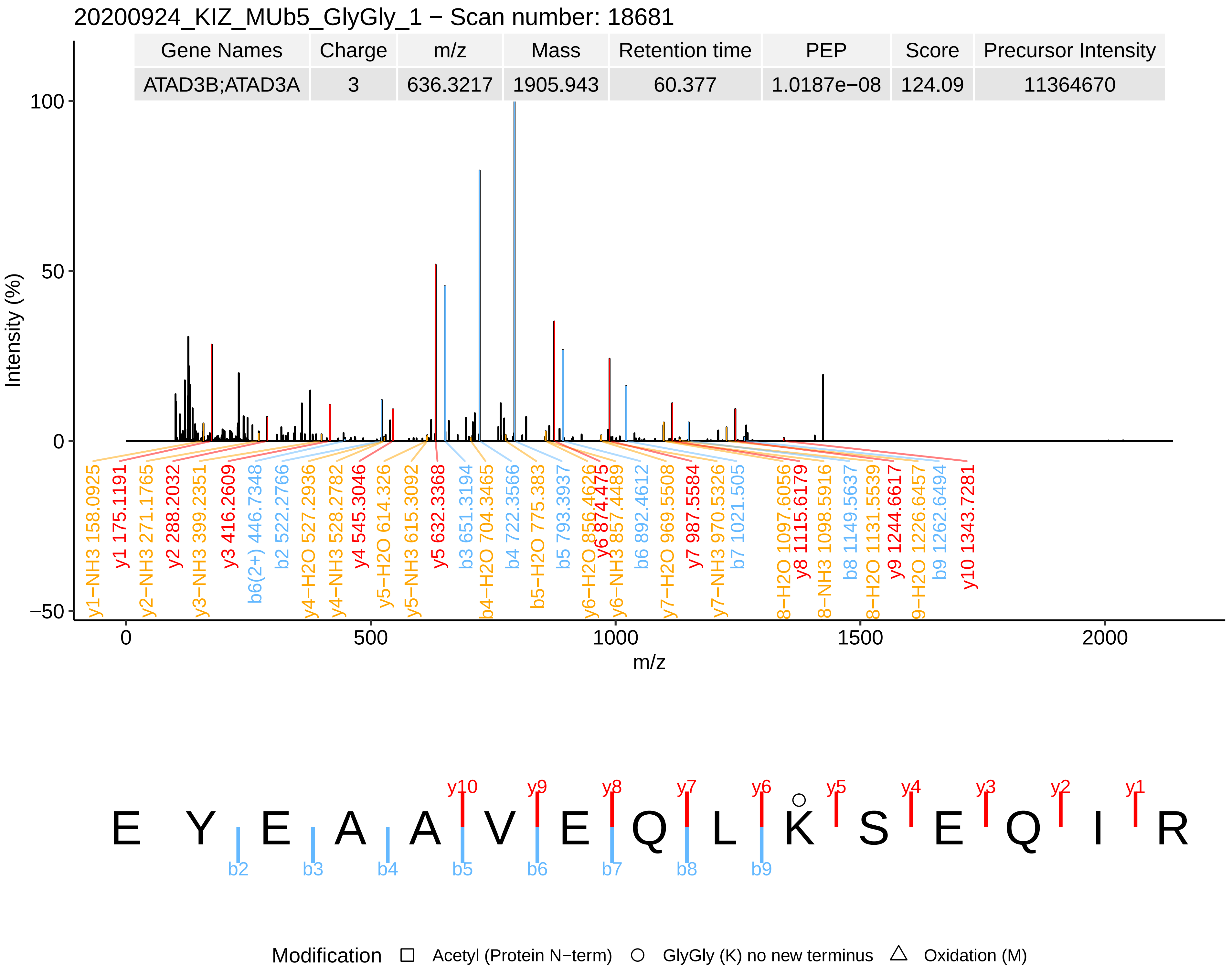
20200924_KIZ_MUb5_GlyGly_1 − Scan number: 18681
Gene Names
Charge
m/z
Mass
Retention time
PEP
Score
Precursor Intensity
ATAD3B;ATAD3A
3
636.3217
1905.943
60.377
1.0187e−08
124.09
11364670
Intensity (%)
100
50
0
−50
m/z
0
500
1000
1500
2000
y1−NH3 158.0925
y1 175.1191
y2−NH3 271.1765
y2 288.2032
y3−NH3 399.2351
y3 416.2609
b6(2+) 446.7348
b2 522.2766
y4−H2O 527.2936
y4−NH3 528.2782
y4 545.3046
y5−H2O 614.326
y5−NH3 615.3092
y5 632.3368
b3 651.3194
b4−H2O 704.3465
b4 722.3566
b5−H2O 775.383
b5 793.3937
y6−H2O 856.4626
y6 874.475
y6−NH3 857.4489
b6 892.4612
y7−H2O 969.5508
y7 987.5584
y7−NH3 970.5326
b7 1021.505
8−H2O 1097.6056
y8 1115.6179
8−NH3 1098.5916
b8 1149.5637
8−H2O 1131.5539
y9 1244.6617
9−H2O 1226.6457
b9 1262.6494
y10 1343.7281
E Y E A A V E Q L K S E Q I R
y10 y9 y8 y7 y6 y5 y4 y3 y2 y1
b2 b3 b4 b5 b6 b7 b8 b9
Modification
Acetyl (Protein N−term)
GlyGly (K) no new terminus
Oxidation (M)

20200924_KIZ_MUb5_GlyGly_2 − Scan number: 17346

Gene Names	Charge	m/z	Mass	Retention time	PEP	Score	Precursor Intensity
ATAD3B;ATAD3A	3	636.3217	1905.943	64.294	2.5802e−10	131.96	6289695

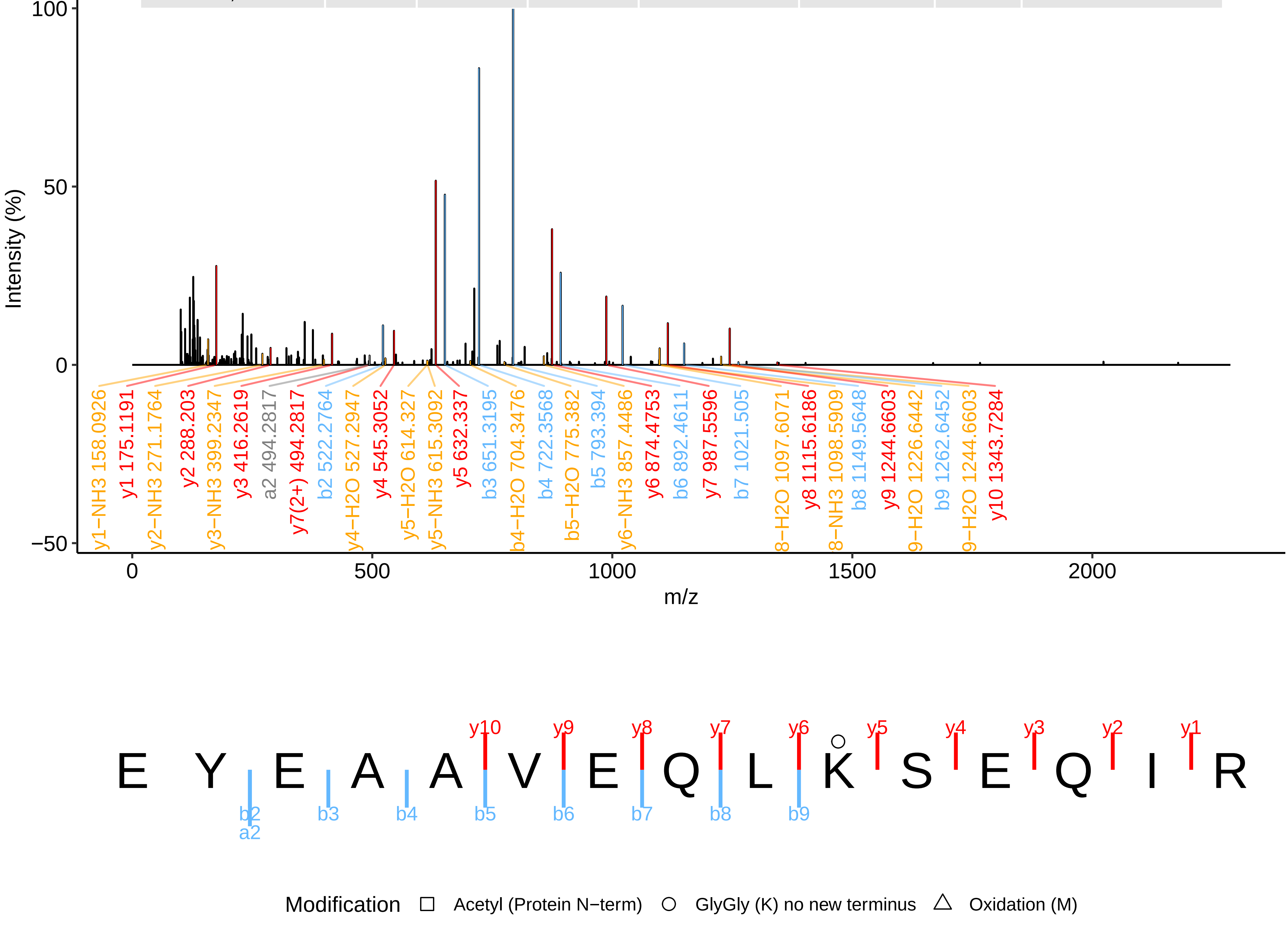

20200921_KIZ_MUb4_GlyGly_10plex − Scan number: 22428

Gene Names	Charge	m/z	Mass	Retention time	PEP	Score	Precursor Intensity
LTA4H	3	535.5912	1603.752	81.34	0.0087387	72.958	3458411

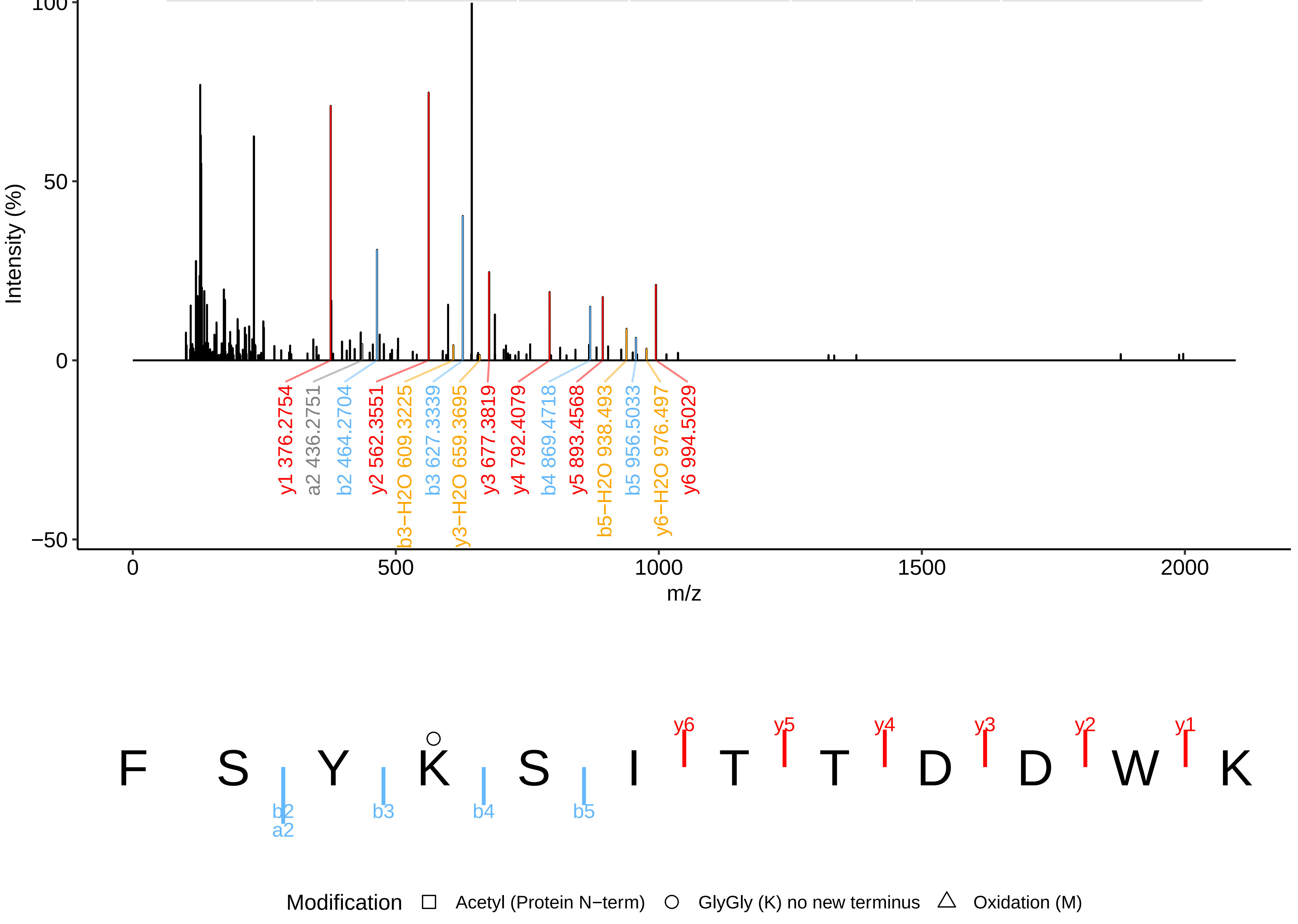

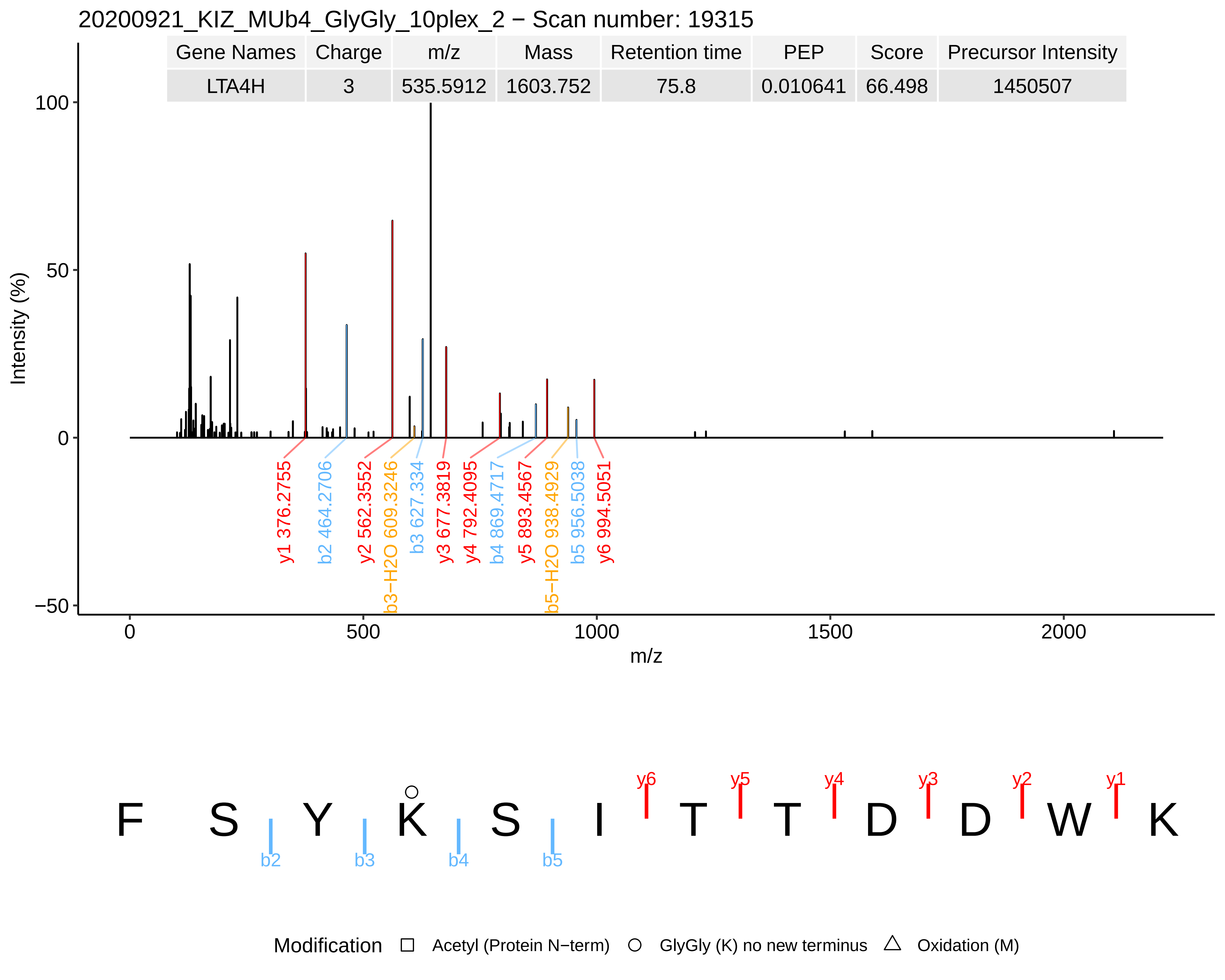
20200921_KIZ_MUb4_GlyGly_10plex_2 − Scan number: 19315
Gene Names | Charge | m/z | Mass | Retention time | PEP | Score | Precursor Intensity
LTA4H | 3 | 535.5912 | 1603.752 | 75.8 | 0.010641 | 66.498 | 1450507
Intensity (%)
m/z
y1 376.2755
b2 464.2706
y2 562.3552
b3−H2O 609.3246
b3 627.334
y3 677.3819
y4 792.4095
b4 869.4717
y5 893.4567
b5−H2O 938.4929
b5 956.5038
y6 994.5051
F S Y K S I T T D D W K
y6 y5 y4 y3 y2 y1
b2 b3 b4 b5
Modification
Acetyl (Protein N−term)
GlyGly (K) no new terminus
Oxidation (M)

20200921_KIZ_MUb4_GlyGly_10plex − Scan number: 19700

Gene Names	Charge	m/z	Mass	Retention time	PEP	Score	Precursor Intensity
HTATIP2	3	561.9615	1682.863	71.097	0.00040006	89.013	3206090

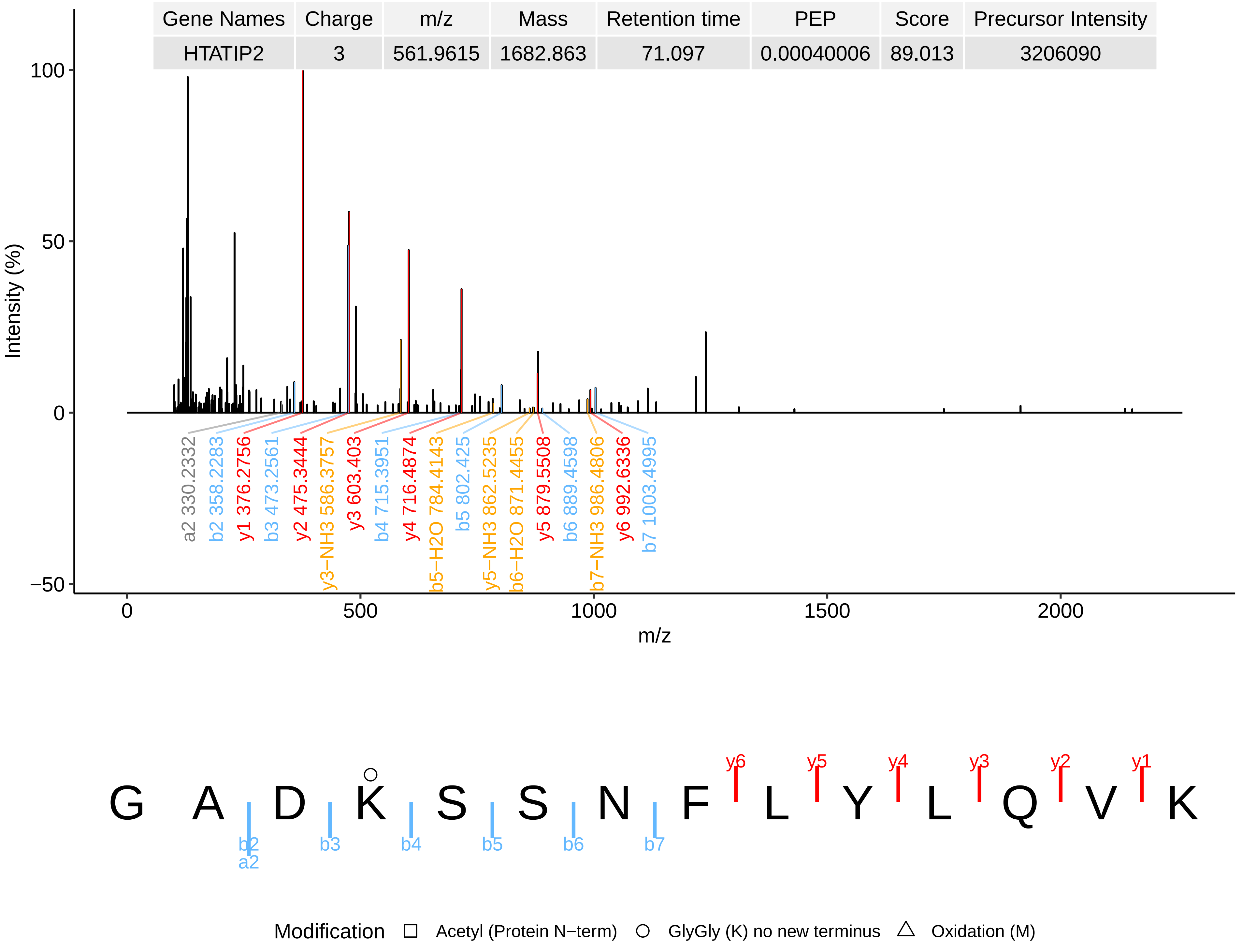

20200921_KIZ_MUb4_GlyGly_10plex_2 − Scan number: 18052

Gene Names	Charge	m/z	Mass	Retention time	PEP	Score	Precursor Intensity
HTATIP2	3	561.9615	1682.863	70.651	0.050247	51.875	594155.5

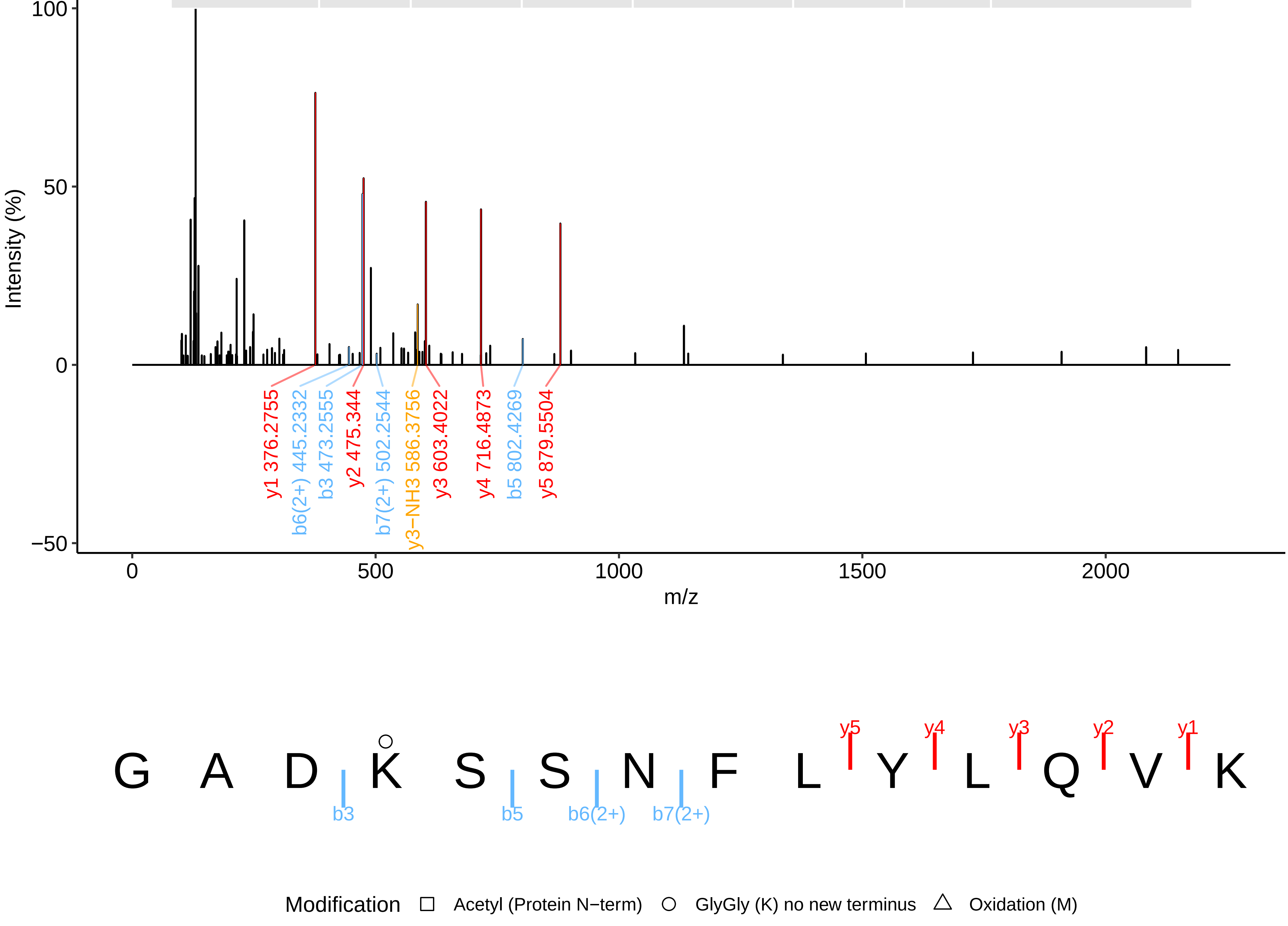

20200921_KIZ_MUb4_GlyGly_10plex_2 − Scan number: 18057

Gene Names	Charge	m/z	Mass	Retention time	PEP	Score	Precursor Intensity
HTATIP2	3	561.9615	1682.863	70.67	2.0976e−05	98.531	883025

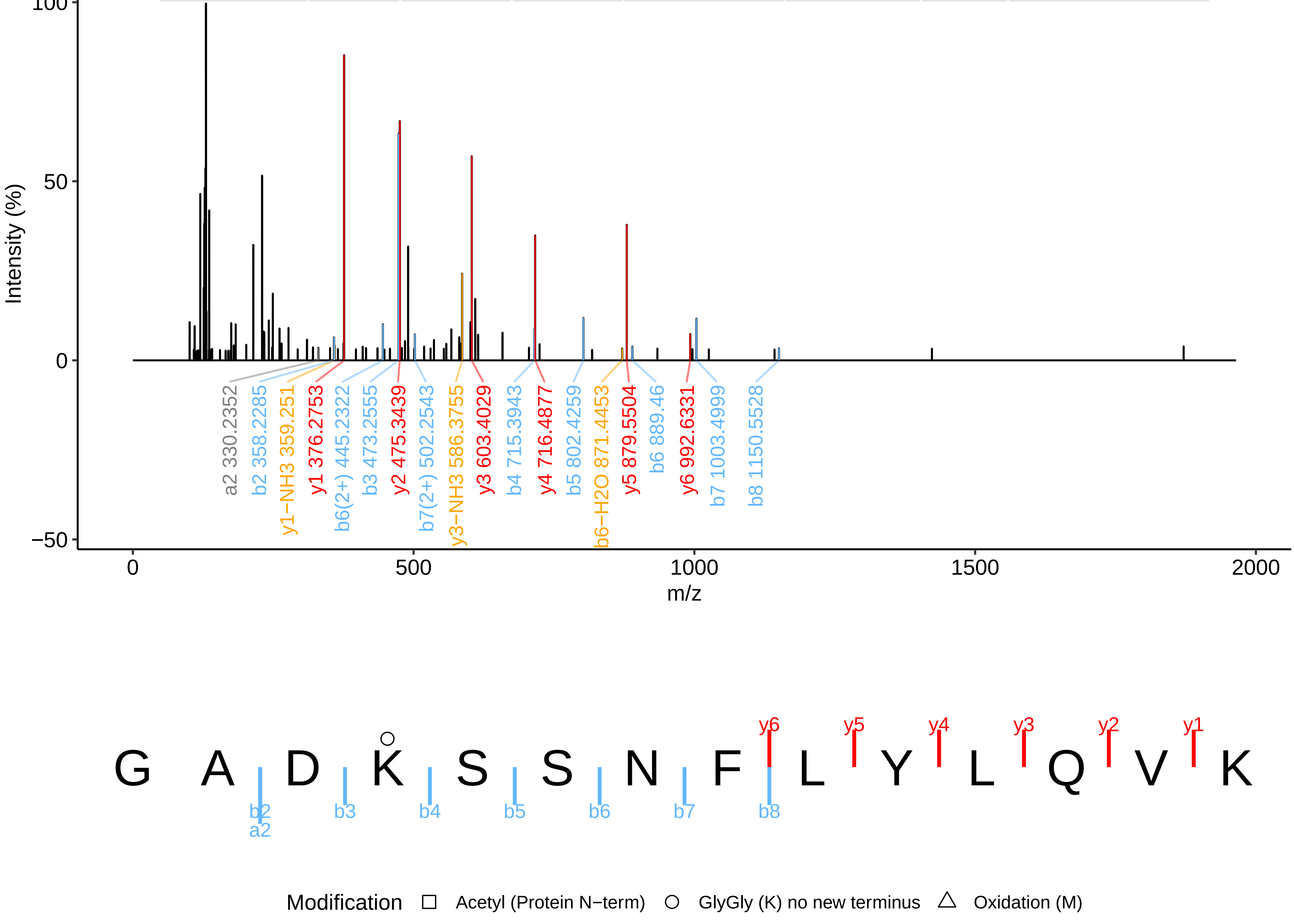

20200921_KIZ_MUb4_GlyGly_10plex − Scan number: 21357

Gene Names	Charge	m/z	Mass	Retention time	PEP	Score	Precursor Intensity
PTP4A1;PTP4A2	3	542.2946	1623.862	77.332	0.0016688	80.469	NA

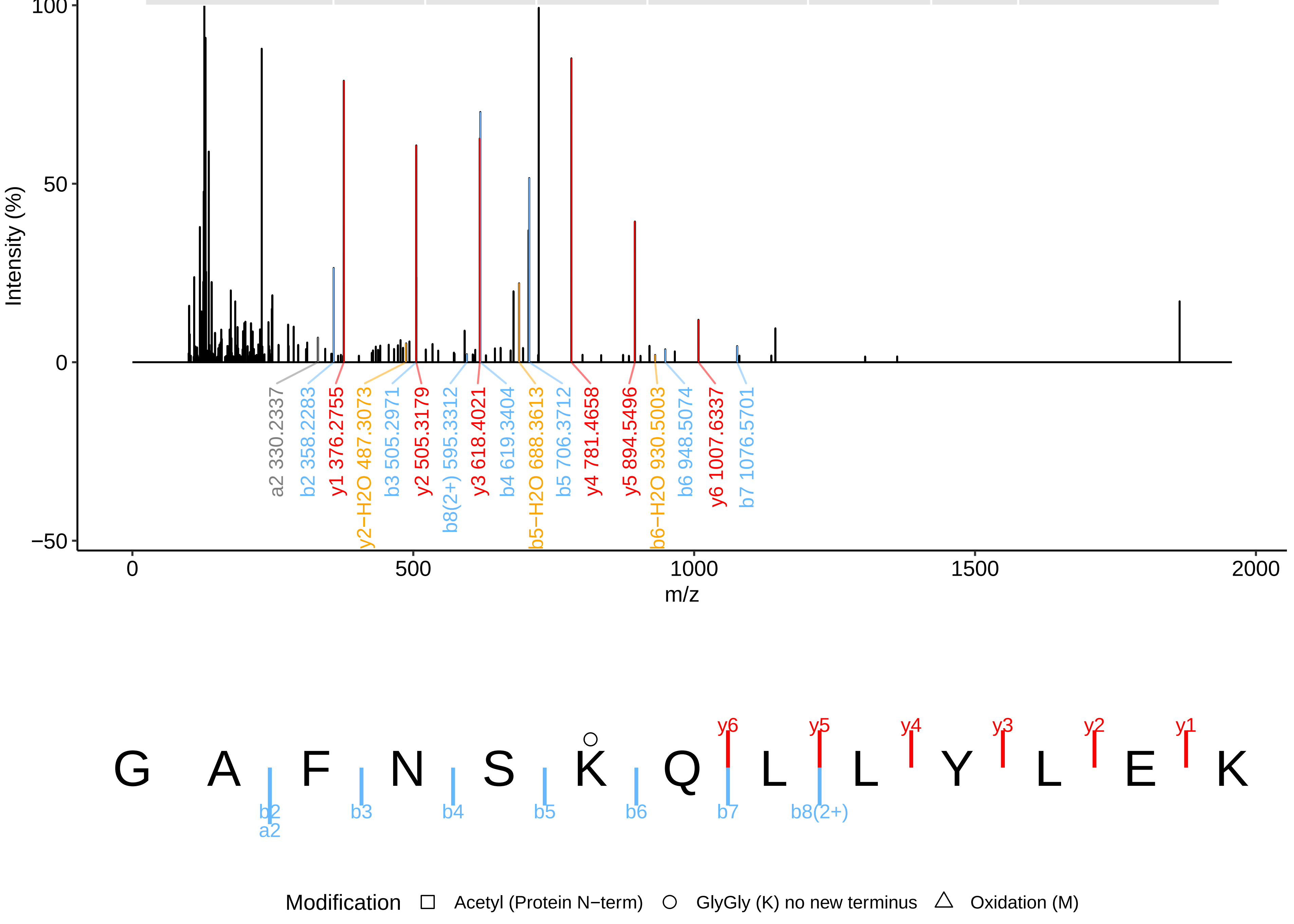

20200921_KIZ_MUb4_GlyGly_10plex_2 − Scan number: 19367

Gene Names	Charge	m/z	Mass	Retention time	PEP	Score	Precursor Intensity
PTP4A1;PTP4A2	3	542.2946	1623.862	76.01	0.00014936	96.059	1502258

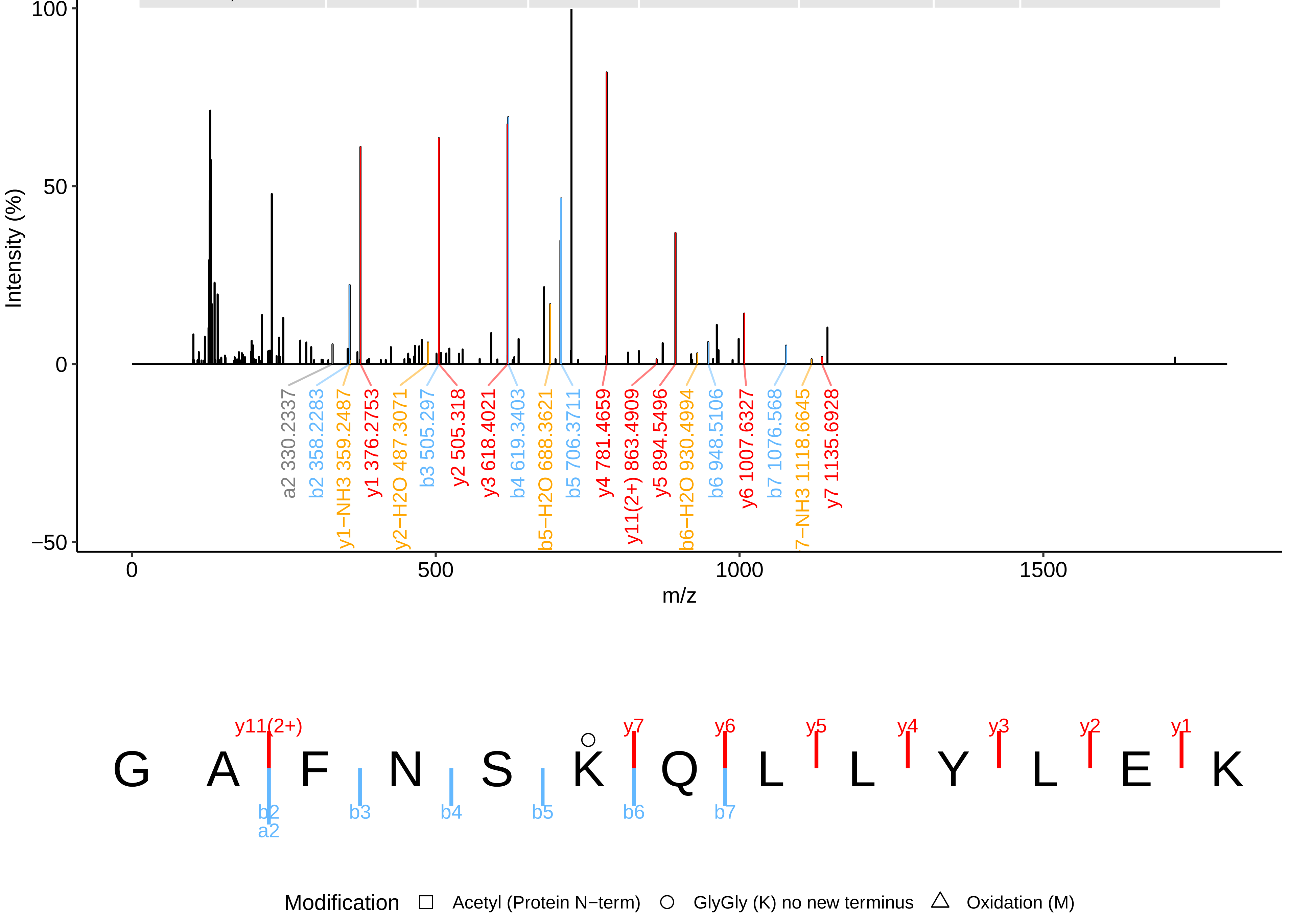

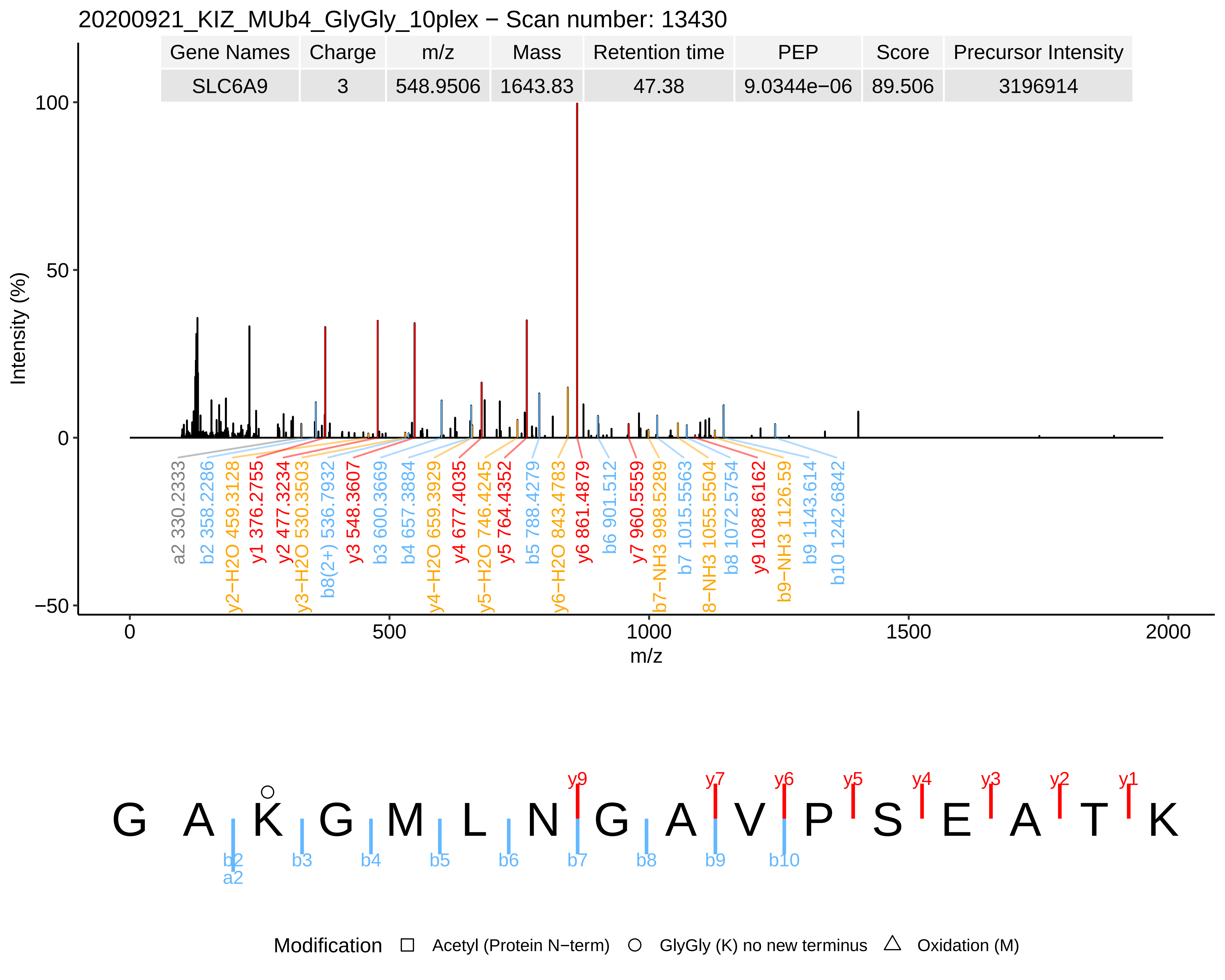

20200921_KIZ_MUb4_GlyGly_10plex − Scan number: 13430
Gene Names | Charge | m/z | Mass | Retention time | PEP | Score | Precursor Intensity
SLC6A9 | 3 | 548.9506 | 1643.83 | 47.38 | 9.0344e−06 | 89.506 | 3196914
Intensity (%)
m/z
a2 330.2333
b2 358.2286
y2−H2O 459.3128
y1 376.2755
y2 477.3234
y3−H2O 530.3503
b8(2+) 536.7932
y3 548.3607
b3 600.3669
b4 657.3884
y4−H2O 659.3929
y4 677.4035
y5−H2O 746.4245
y5 764.4352
b5 788.4279
y6−H2O 843.4783
y6 861.4879
b6 901.512
y7 960.5559
b7−NH3 998.5289
b7 1015.5563
8−NH3 1055.5504
b8 1072.5754
y9 1088.6162
b9−NH3 1126.59
b9 1143.614
b10 1242.6842
G A K G M L N G A V P S E A T K
Modification
Acetyl (Protein N−term)
GlyGly (K) no new terminus
Oxidation (M)

20200921_KIZ_MUb4_GlyGly_10plex_2 − Scan number: 19885

Gene Names	Charge	m/z	Mass	Retention time	PEP	Score	Precursor Intensity
HSPA4;HSPA4L;HSPH1	3	635.666	1903.976	78.107	0.0034462	73.156	NA

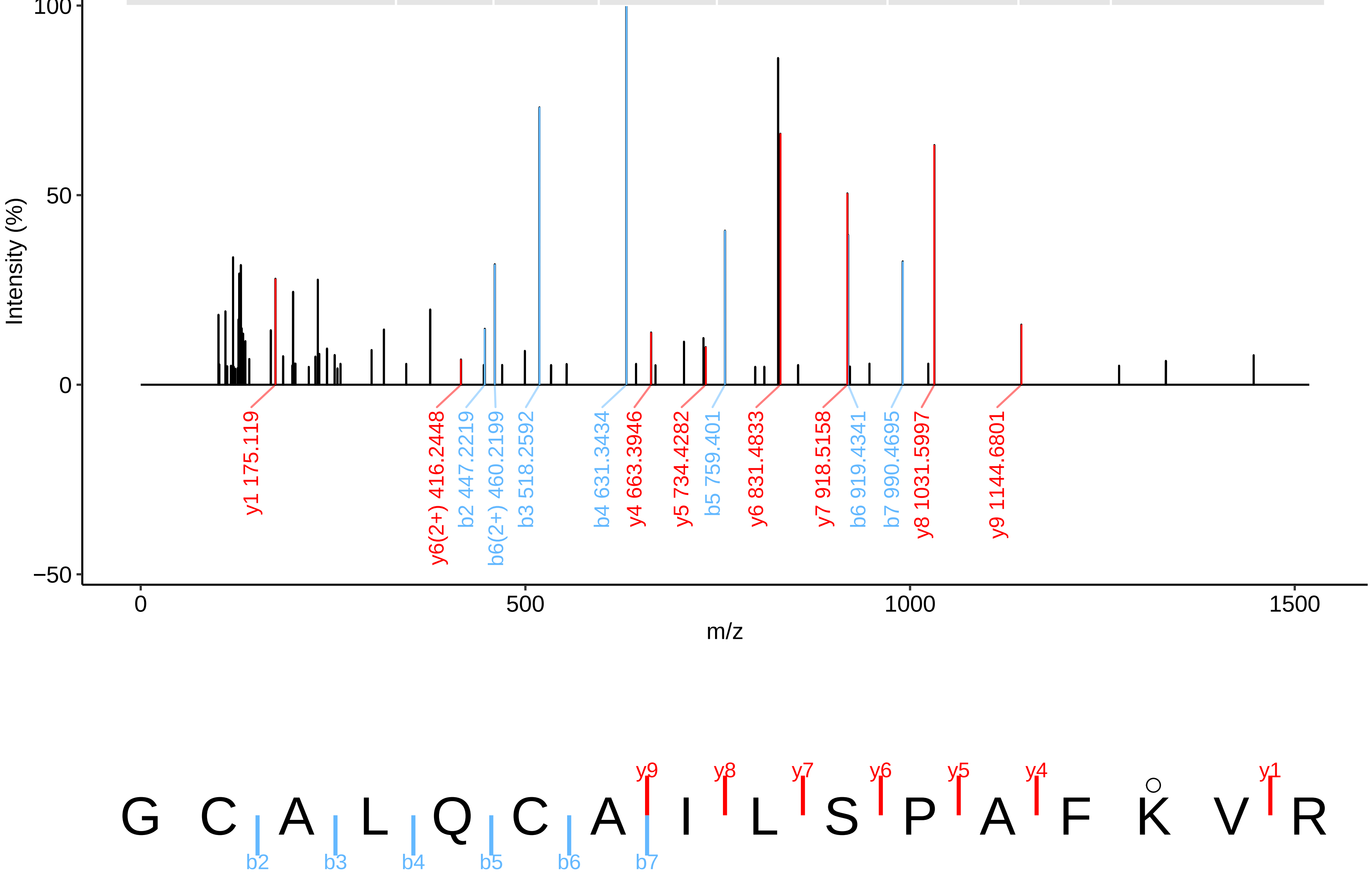

Modification □ Acetyl (Protein N−term) ○ GlyGly (K) no new terminus △ Oxidation (M)

20200921_KIZ_MUb4_GlyGly_10plex_2 − Scan number: 23493

Gene Names	Charge	m/z	Mass	Retention time	PEP	Score	Precursor Intensity
EIF4A1	3	539.2754	1614.804	92.729	0.015245	66.262	197627.2

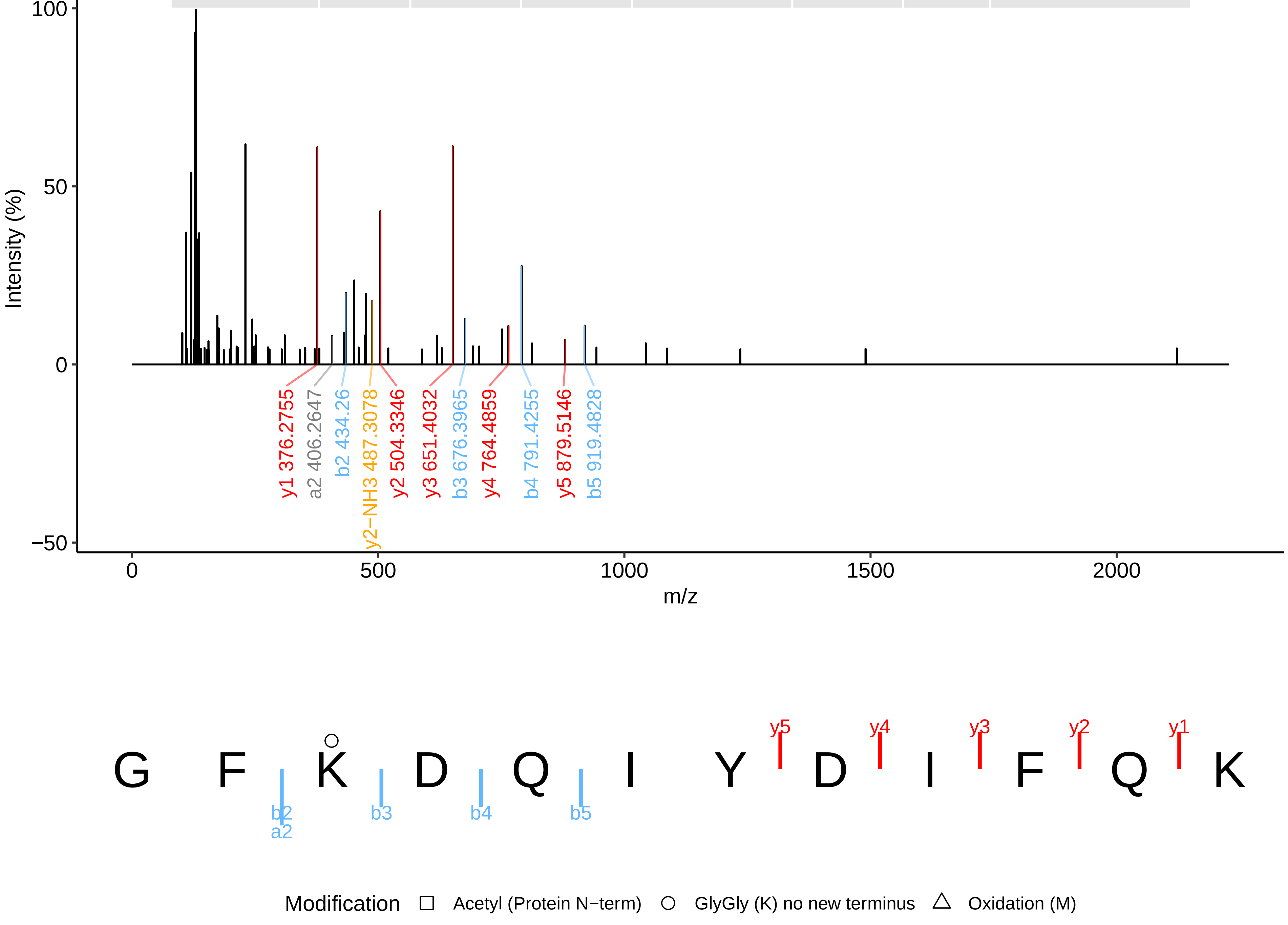

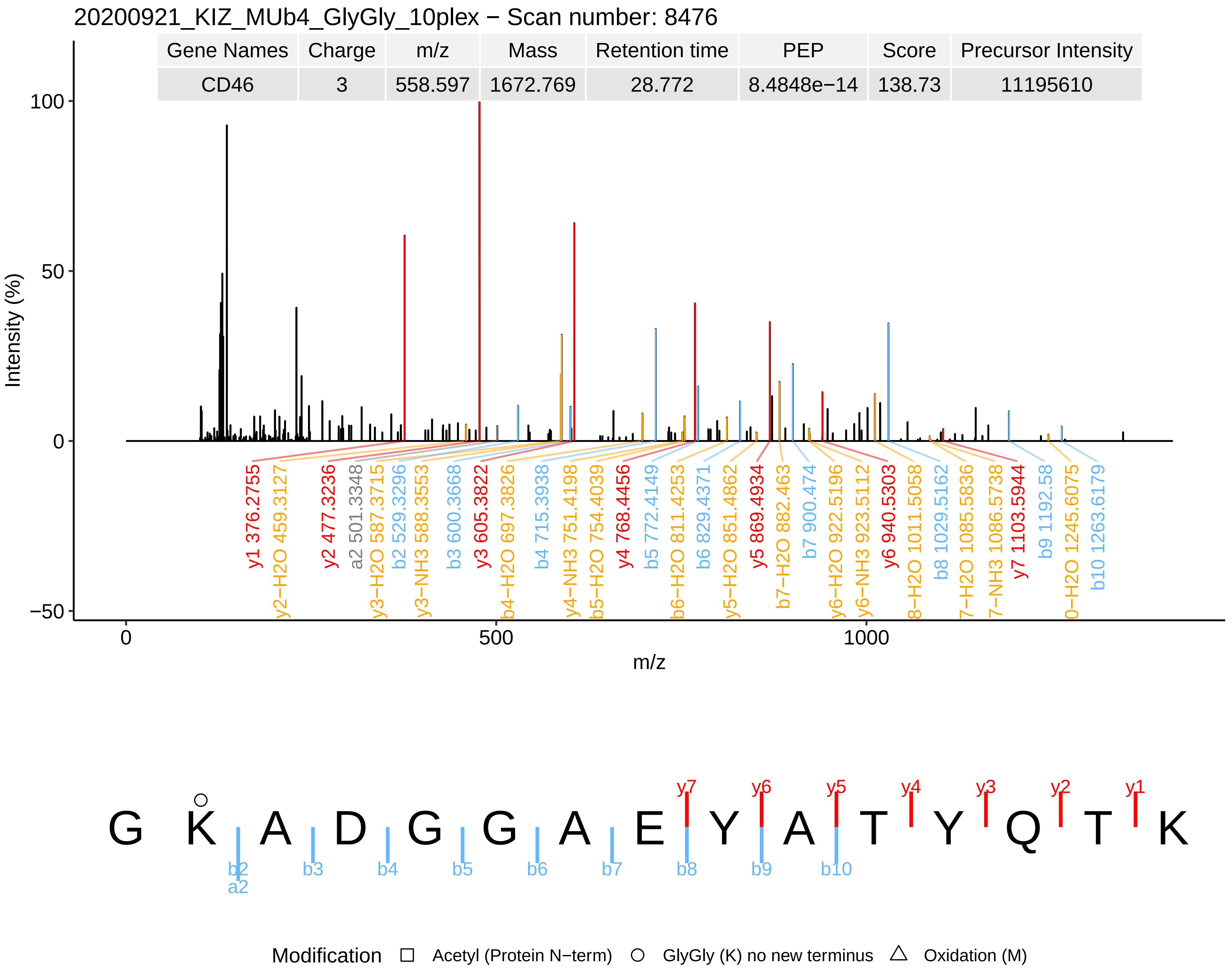

20200921_KIZ_MUb4_GlyGly_10plex − Scan number: 8476
Gene Names | Charge | m/z | Mass | Retention time | PEP | Score | Precursor Intensity
CD46 | 3 | 558.597 | 1672.769 | 28.772 | 8.4848e−14 | 138.73 | 11195610
Intensity (%)
100
50
0
−50
0
500
1000
m/z
y1 376.2755
y2−H2O 459.3127
y2 477.3236
a2 501.3348
y3−H2O 587.3715
b2 529.3296
y3−NH3 588.3553
b3 600.3668
y3 605.3822
b4−H2O 697.3826
b4 715.3938
y4−NH3 751.4198
b5−H2O 754.4039
y4 768.4456
b5 772.4149
b6−H2O 811.4253
b6 829.4371
y5−H2O 851.4862
y5 869.4934
b7−H2O 882.463
b7 900.474
y6−H2O 922.5196
y6−NH3 923.5112
y6 940.5303
8−H2O 1011.5058
b8 1029.5162
7−H2O 1085.5836
7−NH3 1086.5738
y7 1103.5944
b9 1192.58
0−H2O 1245.6075
b10 1263.6179
G K A D G G A E Y A T Y Q T K
y7 y6 y5 y4 y3 y2 y1
b2 a2 b3 b4 b5 b6 b7 b8 b9 b10
Modification
Acetyl (Protein N−term)
GlyGly (K) no new terminus
Oxidation (M)

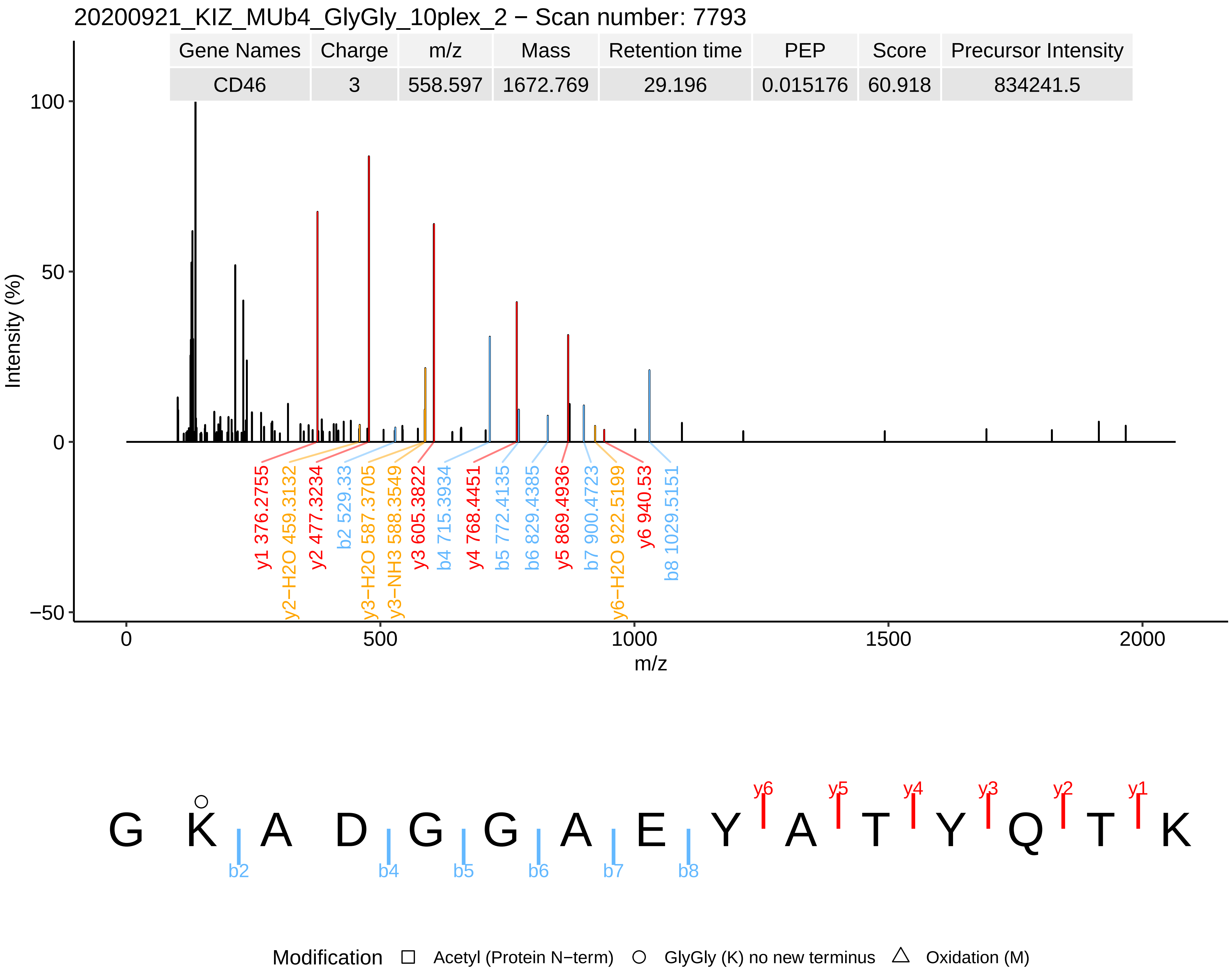
20200921_KIZ_MUb4_GlyGly_10plex_2 − Scan number: 7793
Gene Names | Charge | m/z | Mass | Retention time | PEP | Score | Precursor Intensity
CD46 | 3 | 558.597 | 1672.769 | 29.196 | 0.015176 | 60.918 | 834241.5
Intensity (%)
100
50
0
−50
0
500
1000
1500
2000
m/z
y1 376.2755
y2−H2O 459.3132
y2 477.3234
b2 529.33
y3−H2O 587.3705
y3−NH3 588.3549
y3 605.3822
b4 715.3934
y4 768.4451
b5 772.4135
b6 829.4385
y5 869.4936
b7 900.4723
y6−H2O 922.5199
y6 940.53
b8 1029.5151
G K A D G G A E Y A T Y Q T K
y6 y5 y4 y3 y2 y1
b2 b4 b5 b6 b7 b8
Modification
Acetyl (Protein N−term)
GlyGly (K) no new terminus
Oxidation (M)

20200924_KIZ_MUb5_GlyGly_1 − Scan number: 7499

Gene Names	Charge	m/z	Mass	Retention time	PEP	Score	Precursor Intensity
CD46	3	558.597	1672.769	23.823	8.4848e−14	138.73	7642855

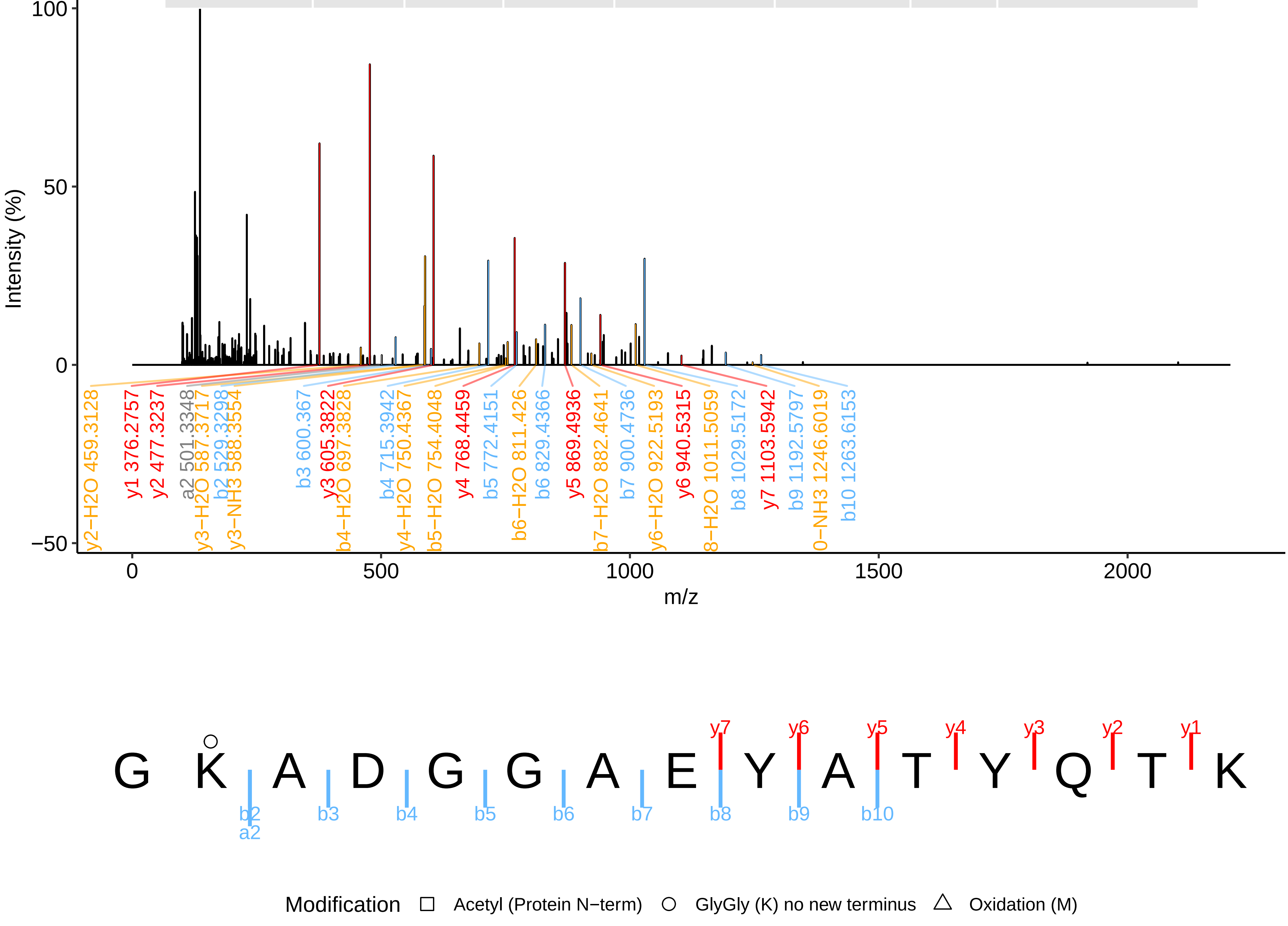

20200924_KIZ_MUb5_GlyGly_1 − Scan number: 7502

Gene Names	Charge	m/z	Mass	Retention time	PEP	Score	Precursor Intensity
CD46	3	558.597	1672.769	23.832	1.469e−06	110.35	3674575

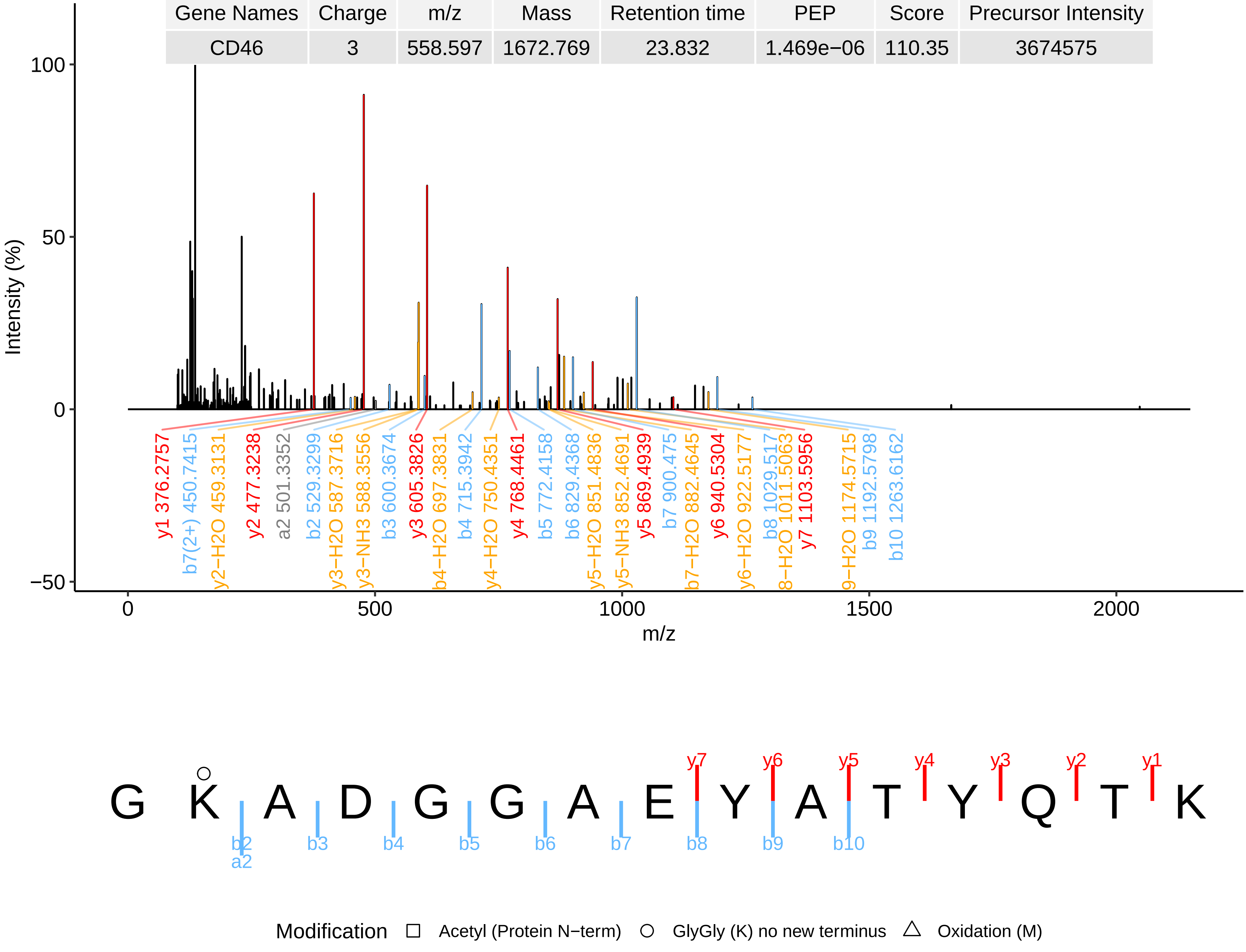

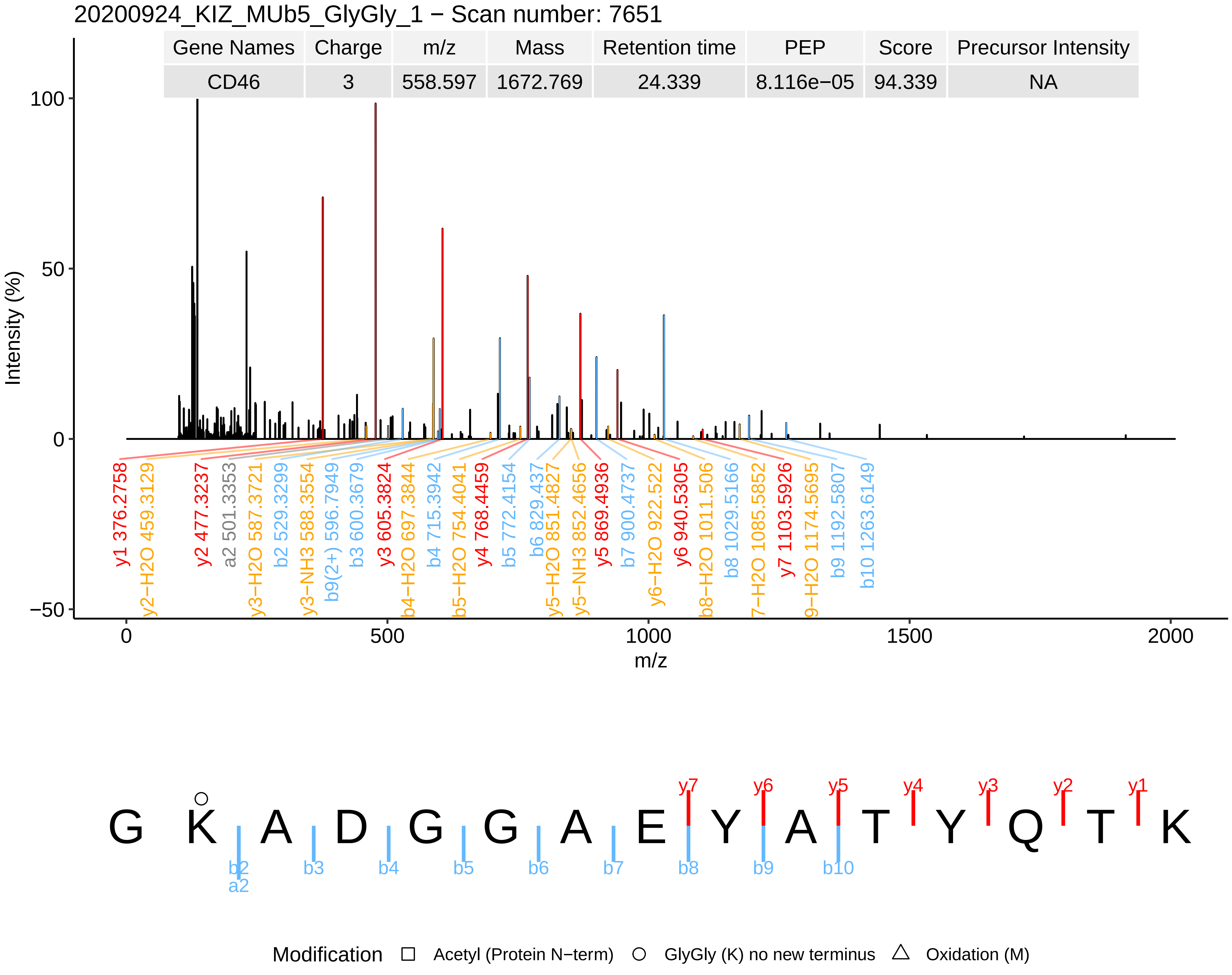
20200924_KIZ_MUb5_GlyGly_1 − Scan number: 7651
Gene Names | Charge | m/z | Mass | Retention time | PEP | Score | Precursor Intensity
CD46 | 3 | 558.597 | 1672.769 | 24.339 | 8.116e−05 | 94.339 | NA
Intensity (%)
m/z
y1 376.2758
y2−H2O 459.3129
y2 477.3237
a2 501.3353
y3−H2O 587.3721
b2 529.3299
y3−NH3 588.3554
b9(2+) 596.7949
b3 600.3679
y3 605.3824
b4−H2O 697.3844
b4 715.3942
b5−H2O 754.4041
y4 768.4459
b5 772.4154
b6 829.437
y5−H2O 851.4827
y5−NH3 852.4656
y5 869.4936
b7 900.4737
y6−H2O 922.522
y6 940.5305
b8−H2O 1011.506
b8 1029.5166
y7−H2O 1085.5852
y7 1103.5926
b9−H2O 1174.5695
b9 1192.5807
b10 1263.6149
G K A D G G A E Y A T Y Q T K
Modification □ Acetyl (Protein N−term) ○ GlyGly (K) no new terminus △ Oxidation (M)

20200921_KIZ_MUb4_GlyGly_10plex − Scan number: 28293

Gene Names	Charge	m/z	Mass	Retention time	PEP	Score	Precursor Intensity
TOMM70A	3	552.9771	1655.909	103.5	4.0472e−05	97.551	2101414

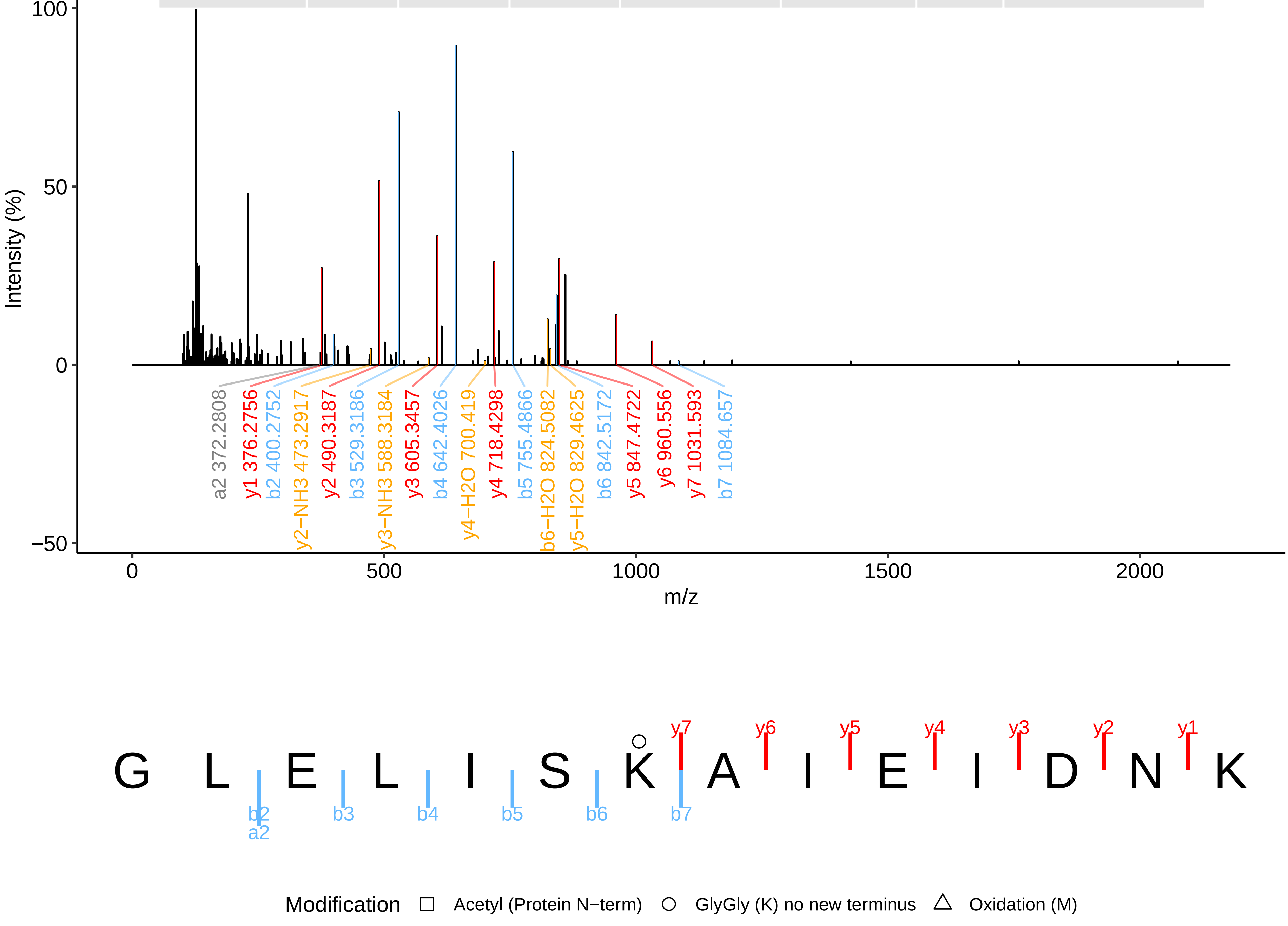

20200921_KIZ_MUb4_GlyGly_10plex − Scan number: 11950

Gene Names	Charge	m/z	Mass	Retention time	PEP	Score	Precurso
ST1H1E;HIST1H1D;HIST1H1A;HIST1H1B;HIST1H1C	3	551.6249	1651.853	41.761	6.951e−10	116.86	433

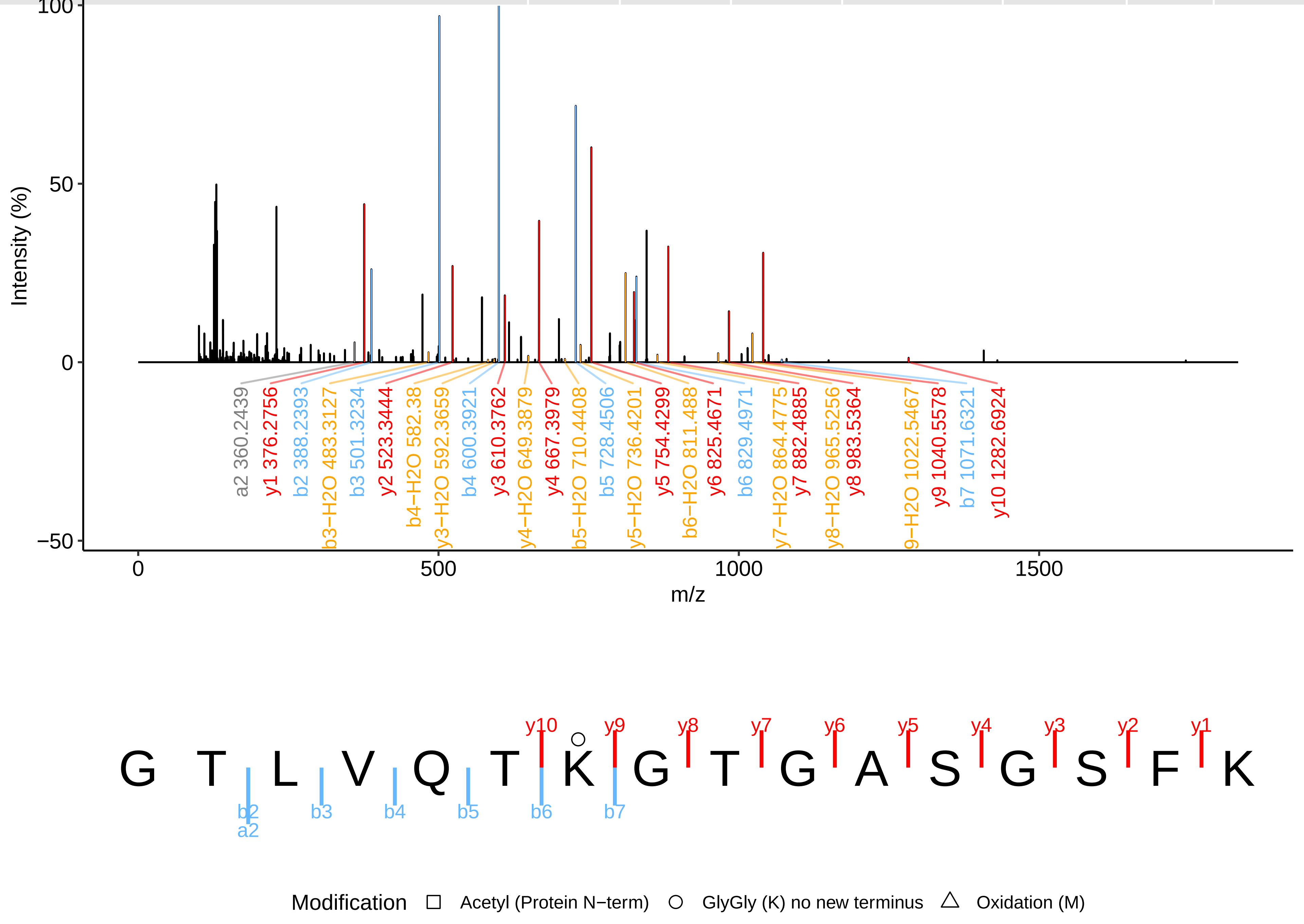

20200921_KIZ_MUb4_GlyGly_10plex − Scan number: 11961

Gene Names	Charge	m/z	Mass	Retention time	PEP	Score	Precurs
ST1H1E;HIST1H1D;HIST1H1A;HIST1H1B;HIST1H1C	3	551.6249	1651.853	41.803	1.4647e−05	83.966	58

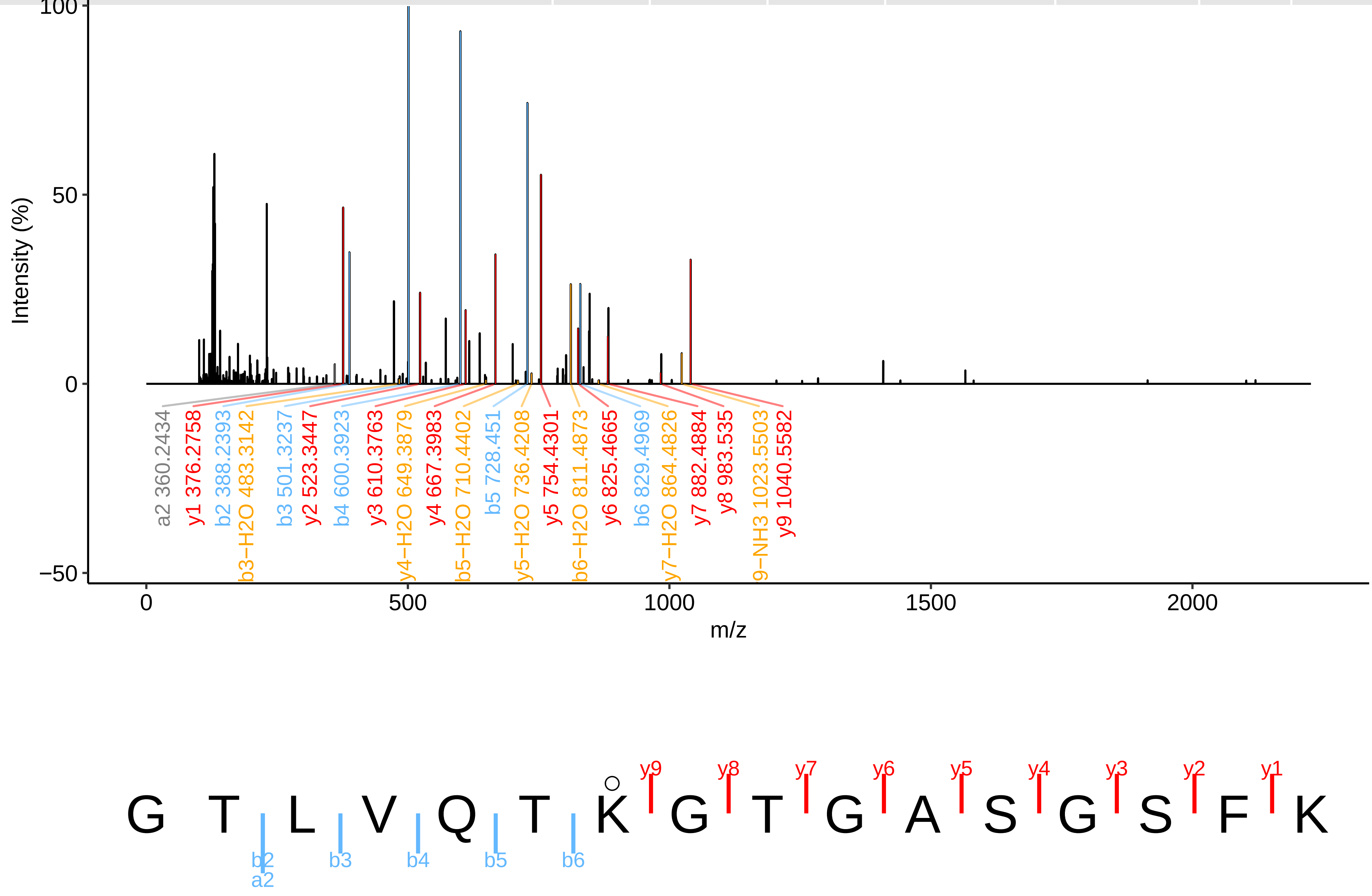

Modification □ Acetyl (Protein N−term) ○ GlyGly (K) no new terminus △ Oxidation (M)

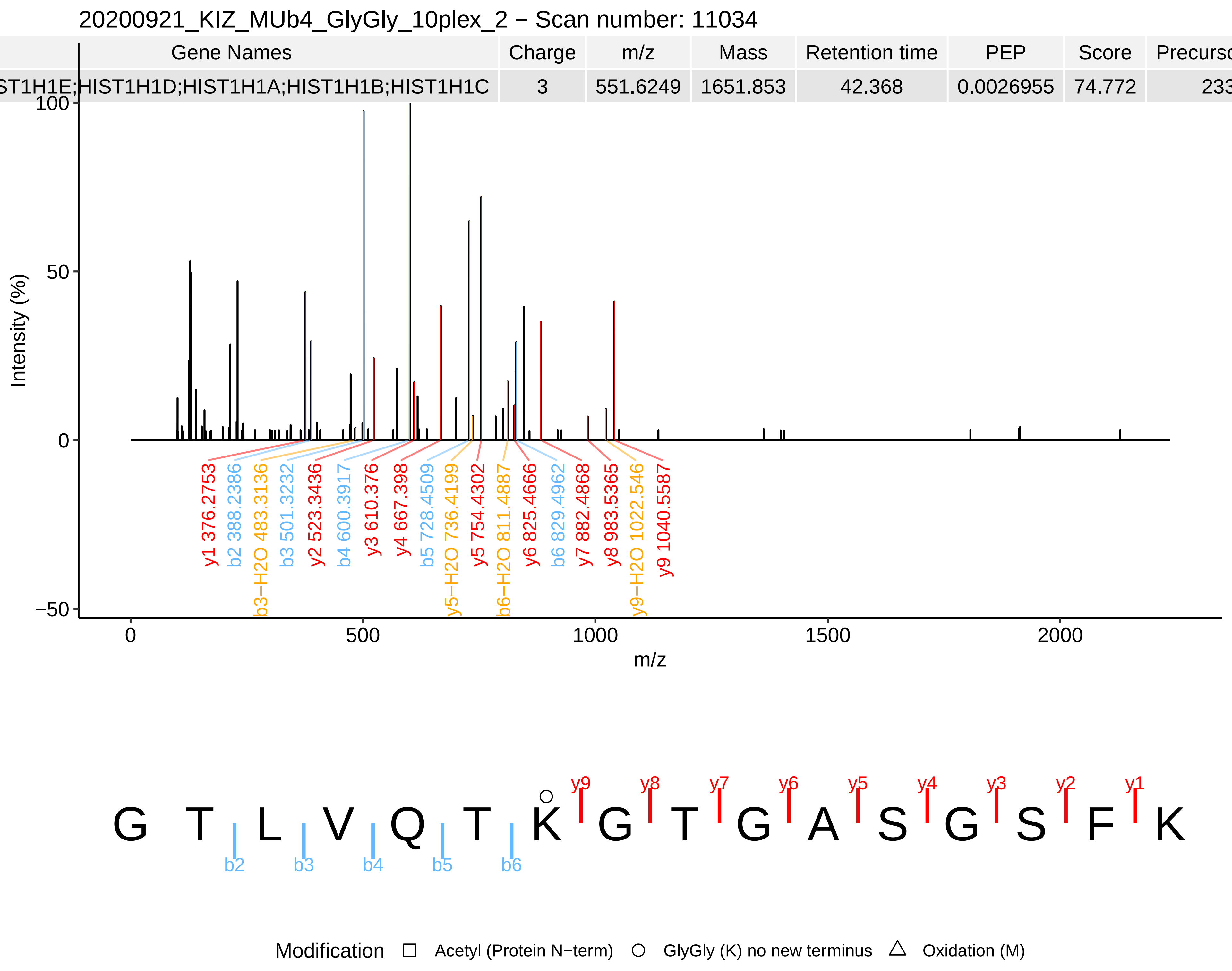

20200921_KIZ_MUb4_GlyGly_10plex_2 − Scan number: 11034
Gene Names
Charge
m/z
Mass
Retention time
PEP
Score
Precurso
ST1H1E;HIST1H1D;HIST1H1A;HIST1H1B;HIST1H1C
3
551.6249
1651.853
42.368
0.0026955
74.772
233
Intensity (%)
m/z
y1 376.2753
b2 388.2386
b3−H2O 483.3136
b3 501.3232
y2 523.3436
b4 600.3917
y3 610.376
y4 667.398
b5 728.4509
y5−H2O 736.4199
y5 754.4302
b6−H2O 811.4887
y6 825.4666
b6 829.4962
y7 882.4868
y8 983.5365
y9−H2O 1022.546
y9 1040.5587
G T L V Q T K G T G A S G S F K
Modification
Acetyl (Protein N−term)
GlyGly (K) no new terminus
Oxidation (M)

20200921_KIZ_MUb4_GlyGly_10plex − Scan number: 16184

Gene Names	Charge	m/z	Mass	Retention time	PEP	Score	Precursor Intensity
FAF1	3	550.2774	1647.81	58.058	0.002389	76.196	13007405

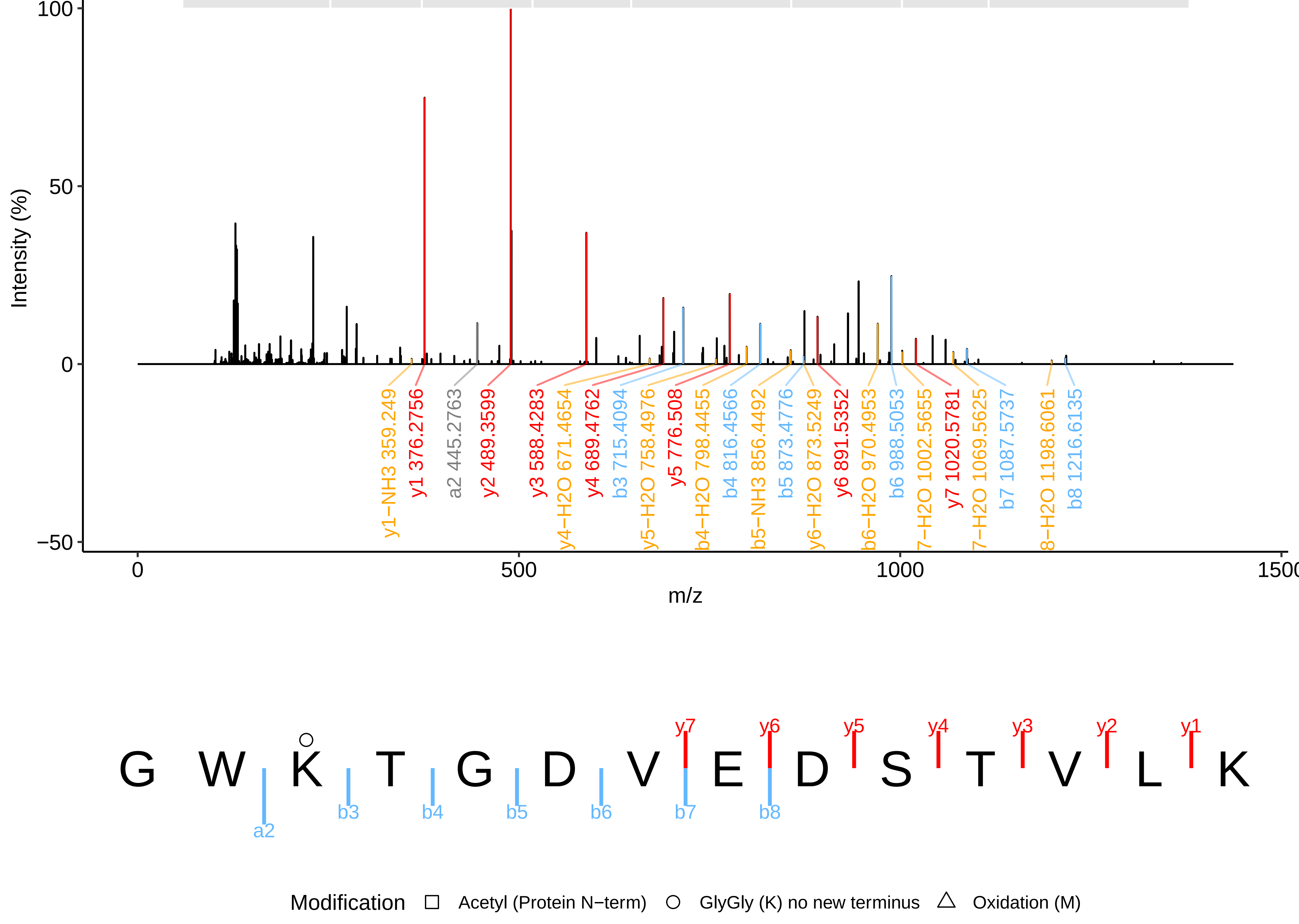

20200921_KIZ_MUb4_GlyGly_10plex − Scan number: 16189

Gene Names	Charge	m/z	Mass	Retention time	PEP	Score	Precursor Intensity
FAF1	3	550.2774	1647.81	58.074	2.0465e−05	98.943	11514367

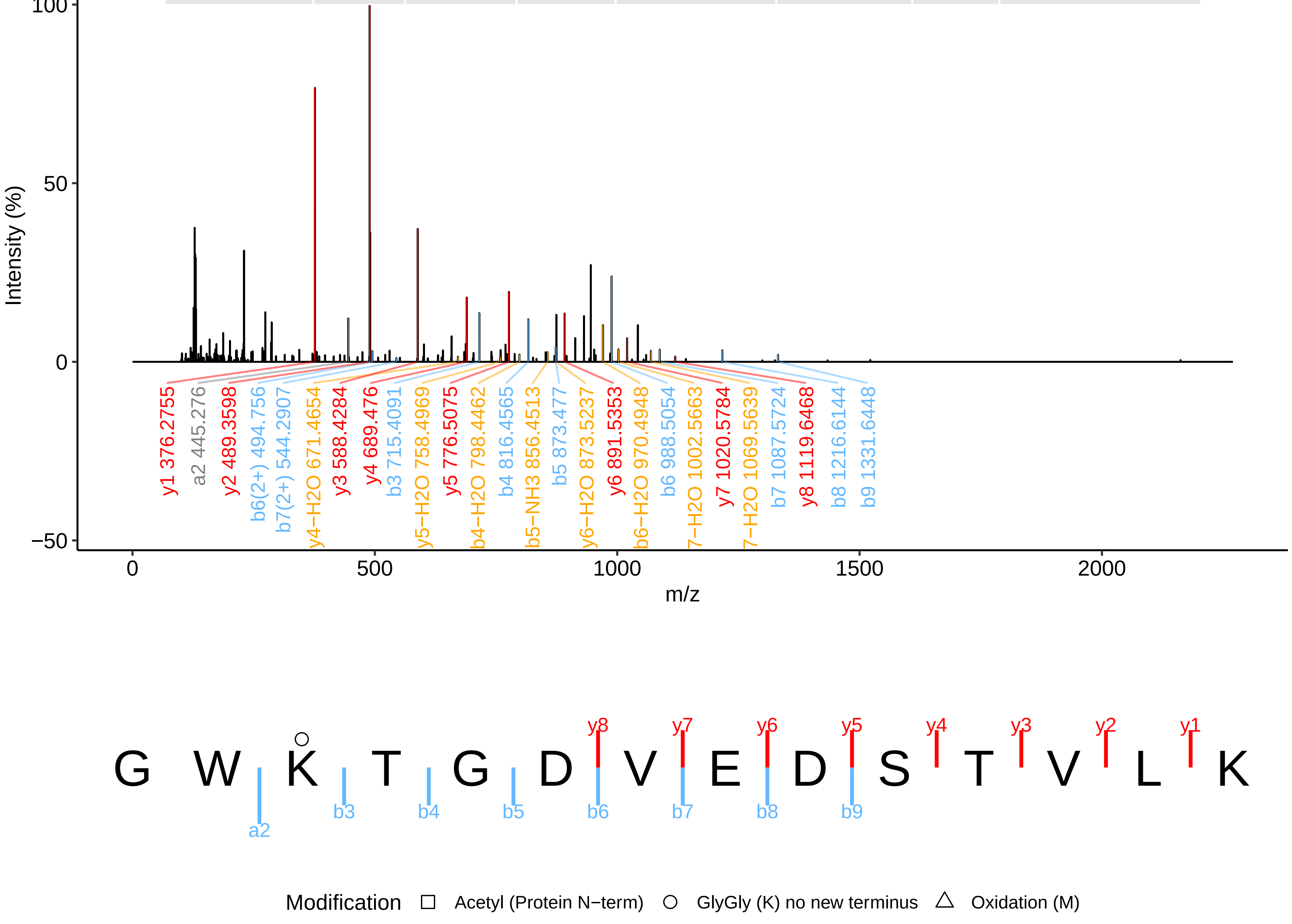

20200921_KIZ_MUb4_GlyGly_10plex_2 − Scan number: 14986

Gene Names	Charge	m/z	Mass	Retention time	PEP	Score	Precursor Intensity
FAF1	3	550.2774	1647.81	58.518	0.0006925	82.069	955821.9

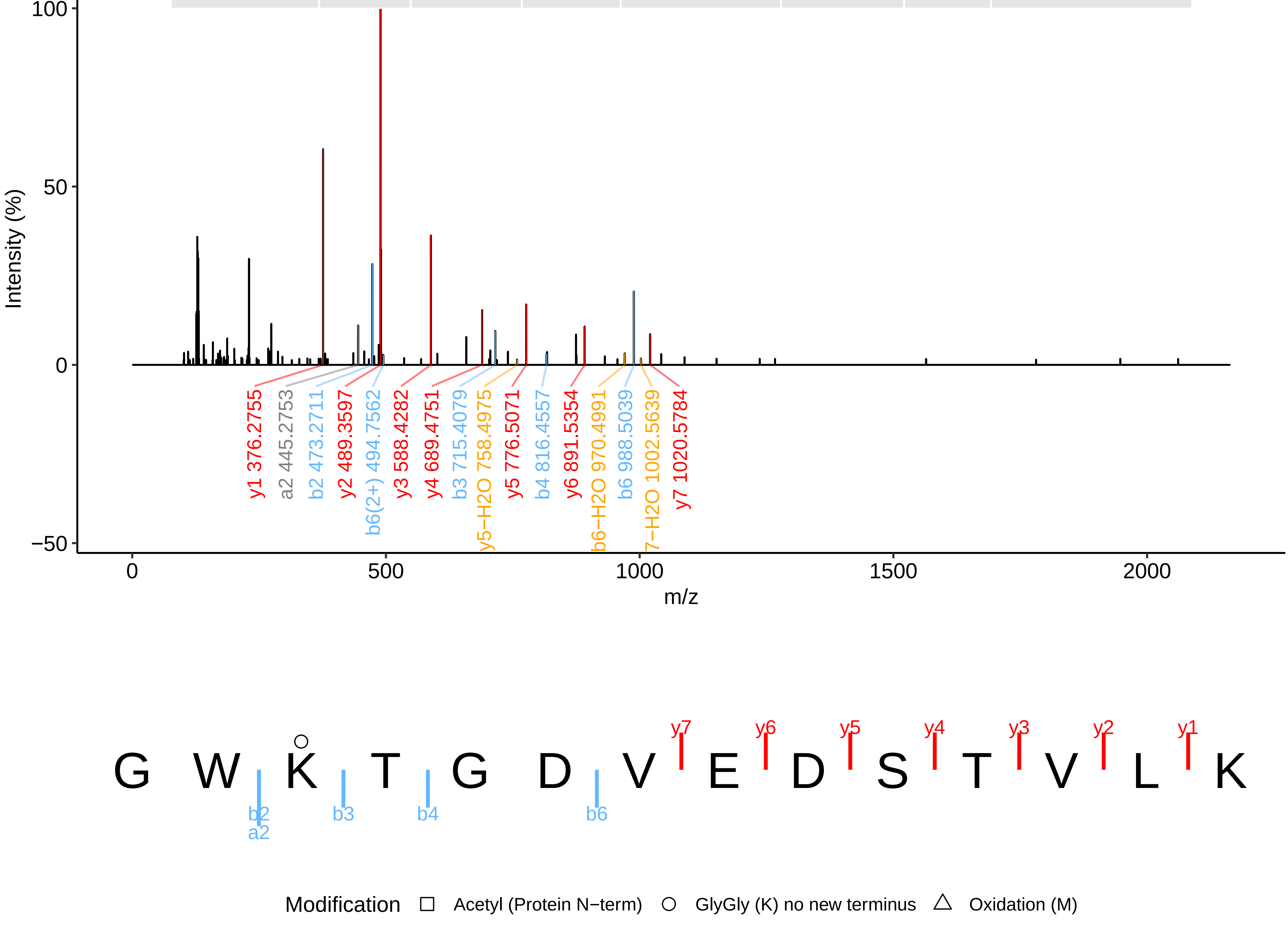

20200924_KIZ_MUb5_GlyGly_1 − Scan number: 15463

Gene Names	Charge	m/z	Mass	Retention time	PEP	Score	Precursor Intensity
FAF1	3	550.2774	1647.81	50.489	1.7014e−05	101.72	9161564

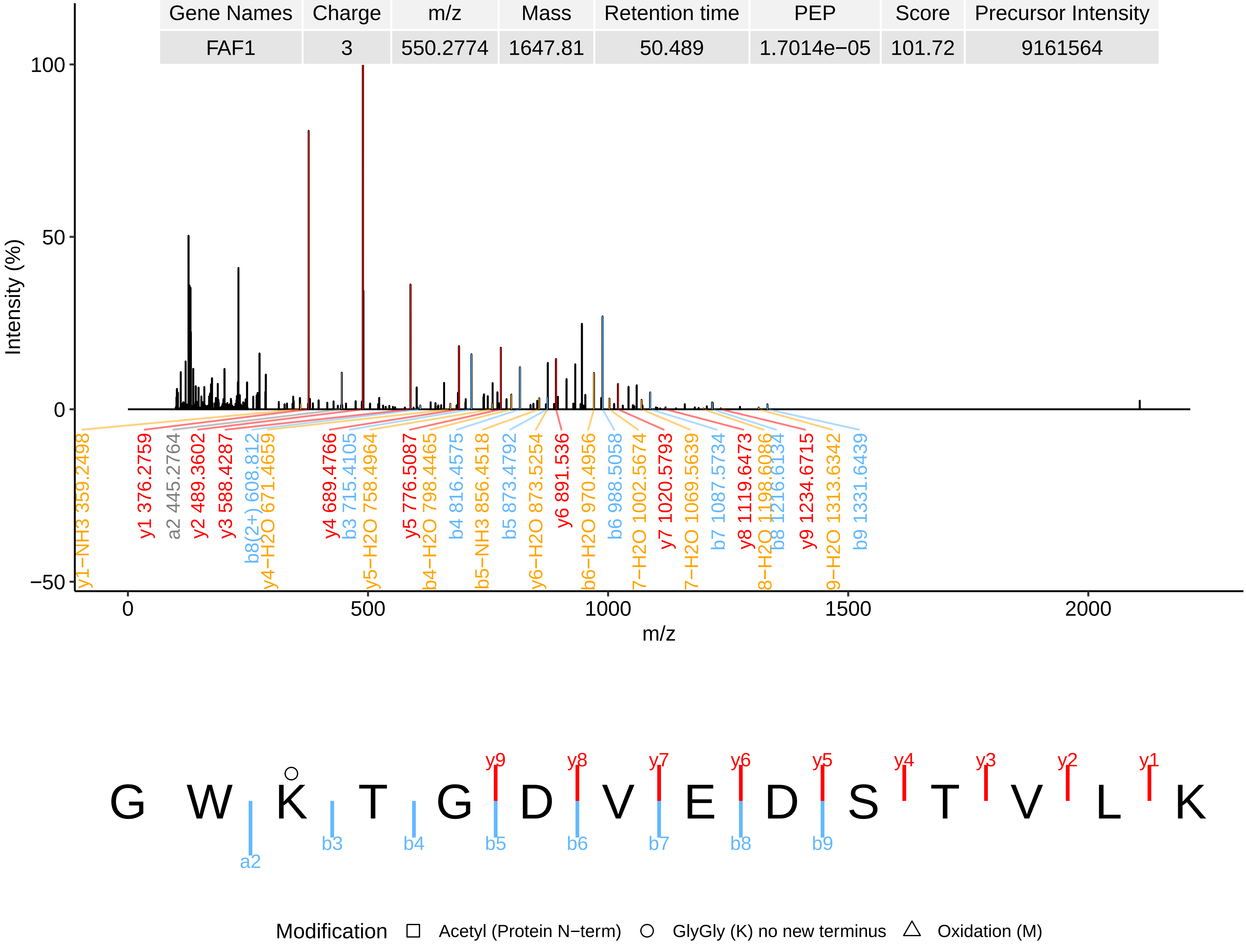

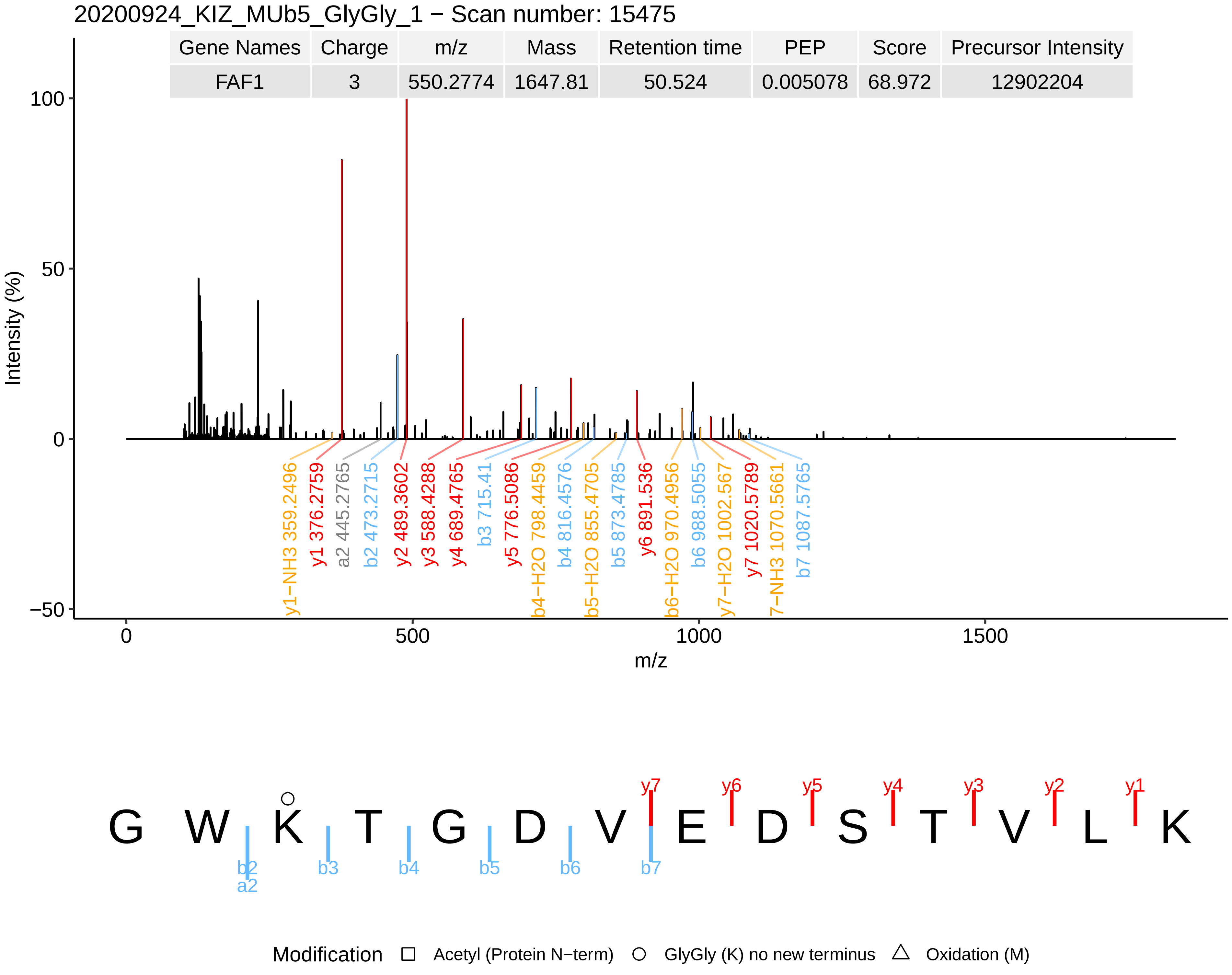
20200924_KIZ_MUb5_GlyGly_1 − Scan number: 15475
Gene Names | Charge | m/z | Mass | Retention time | PEP | Score | Precursor Intensity
FAF1 | 3 | 550.2774 | 1647.81 | 50.524 | 0.005078 | 68.972 | 12902204
Intensity (%)
100
50
0
−50
0
500
1000
1500
m/z
y1−NH3 359.2496
y1 376.2759
a2 445.2765
b2 473.2715
y2 489.3602
y3 588.4288
y4 689.4765
b3 715.41
y5 776.5086
b4−H2O 798.4459
b4 816.4576
b5−H2O 855.4705
b5 873.4785
y6 891.536
b6−H2O 970.4956
b6 988.5055
y7−H2O 1002.567
y7 1020.5789
y7−NH3 1070.5661
b7 1087.5765
G W K T G D V E D S T V L K
y7 y6 y5 y4 y3 y2 y1
b2 a2 b3 b4 b5 b6 b7
Modification
Acetyl (Protein N−term)
GlyGly (K) no new terminus
Oxidation (M)

20200921_KIZ_MUb4_GlyGly_10plex − Scan number: 22396

Gene Names	Charge	m/z	Mass	Retention time	PEP	Score	Precursor Intensity
TOMM5	3	561.9589	1682.855	81.22	0.00069111	82.102	3998982

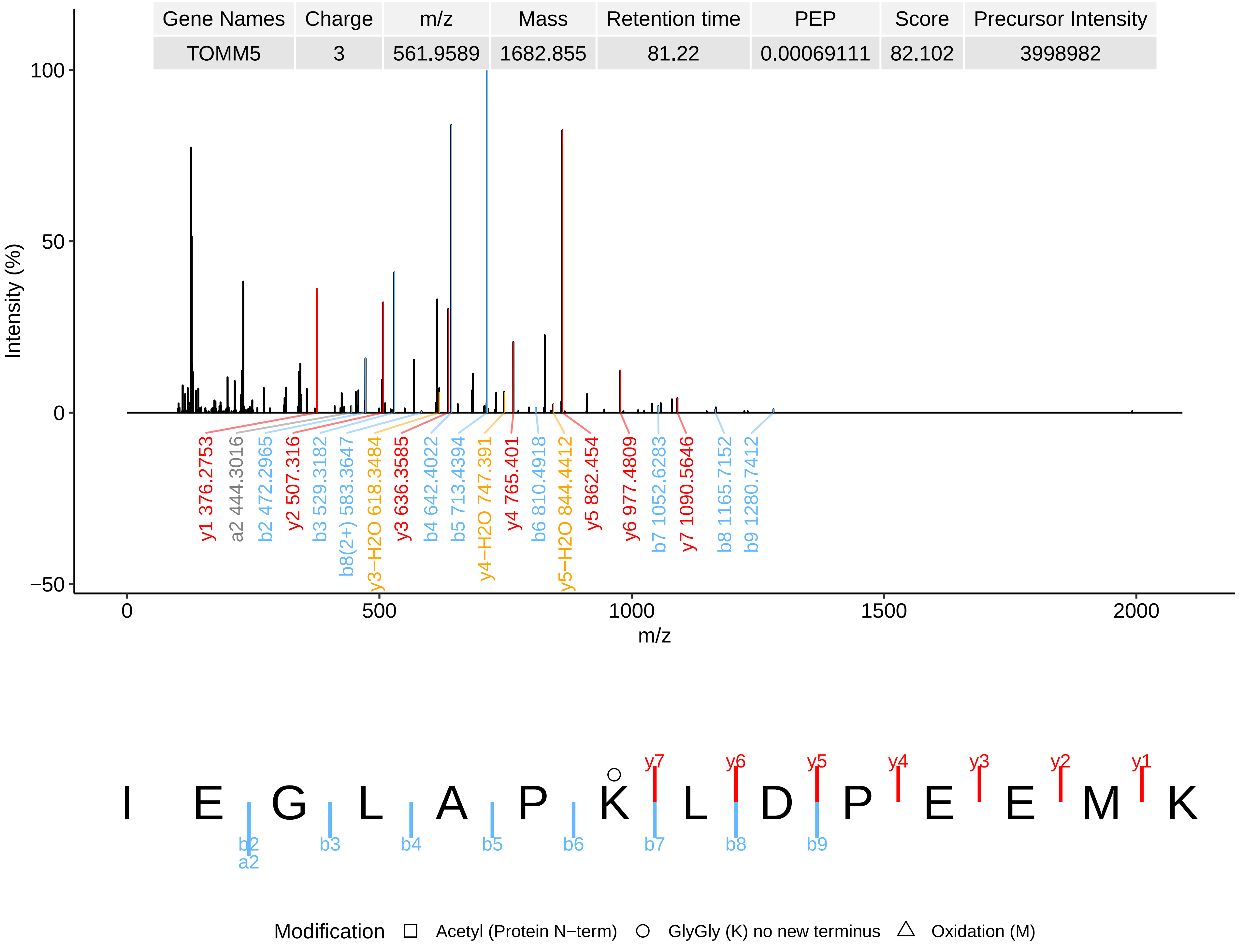

20200921_KIZ_MUb4_GlyGly_10plex − Scan number: 22567

Gene Names	Charge	m/z	Mass	Retention time	PEP	Score	Precursor Intensity
TOMM5	3	561.9589	1682.855	81.873	0.00063224	83.499	2370482

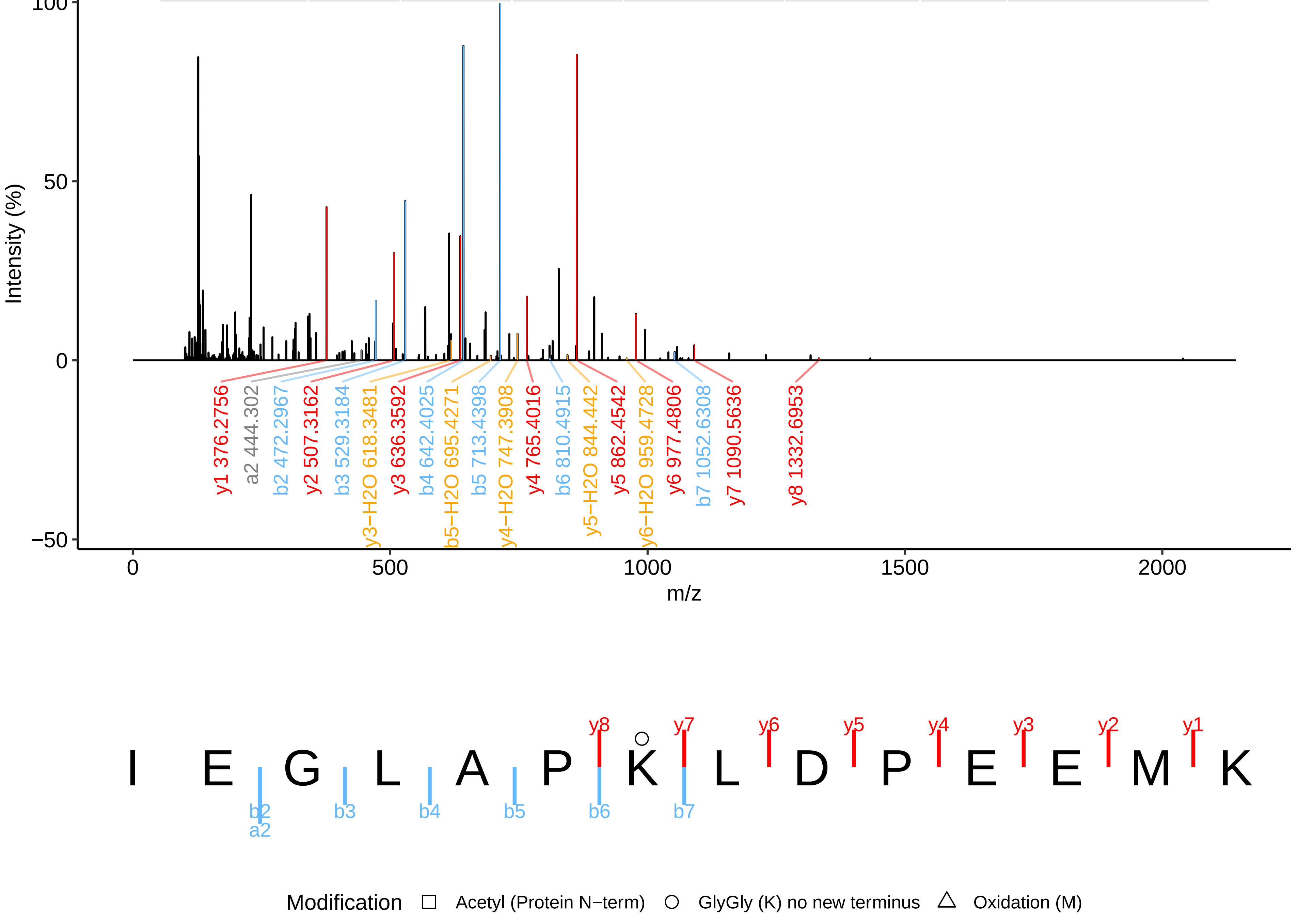

20200921_KIZ_MUb4_GlyGly_10plex_2 − Scan number: 19974

Gene Names	Charge	m/z	Mass	Retention time	PEP	Score	Precursor Intensity
TOMM5	3	561.9589	1682.855	78.457	0.027675	57.804	621974.4

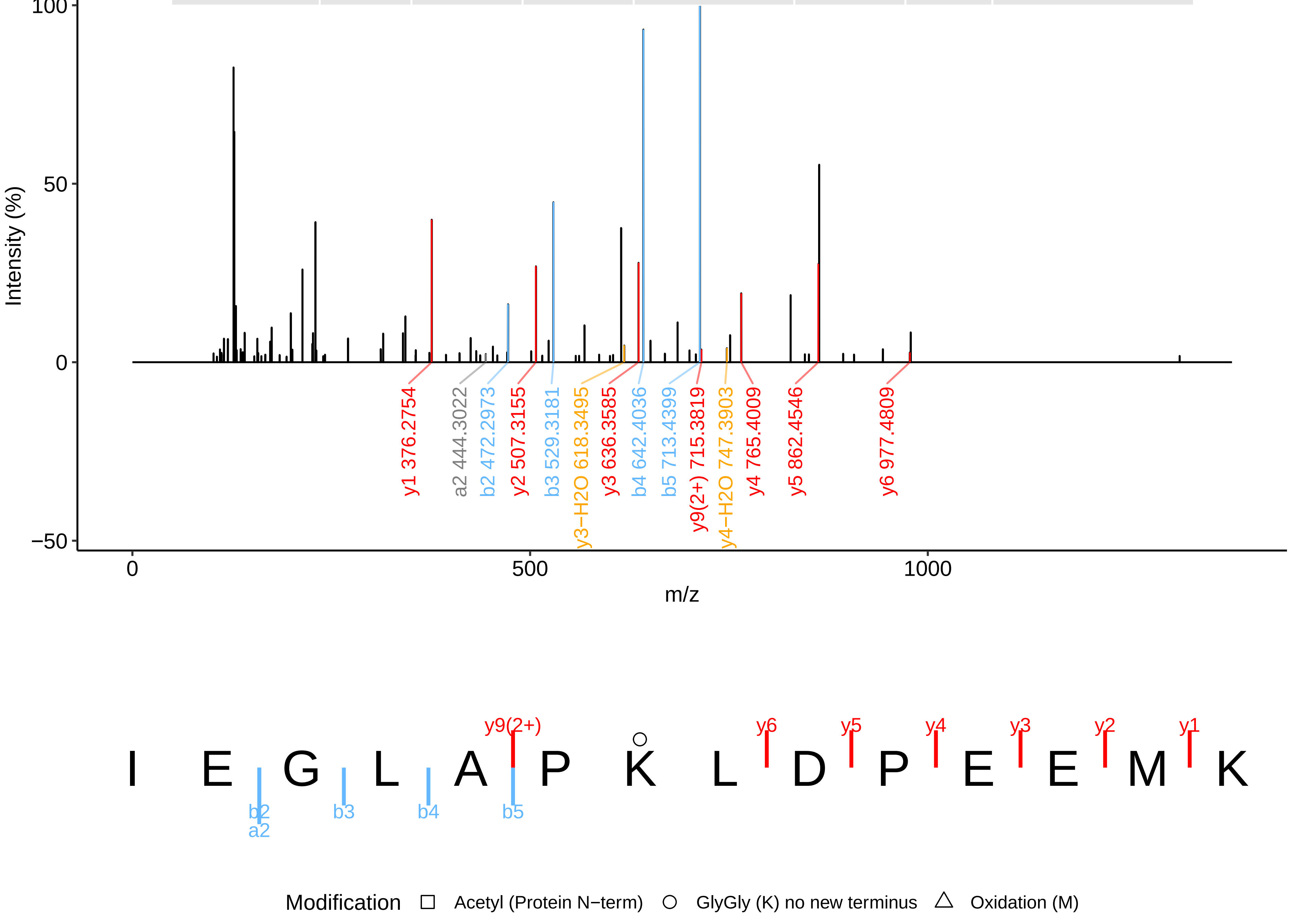

20200924_KIZ_MUb5_GlyGly_1 − Scan number: 22071

Gene Names	Charge	m/z	Mass	Retention time	PEP	Score	Precursor Intensity
TOMM5	3	561.9589	1682.855	71.057	0.00028465	93.494	28457444

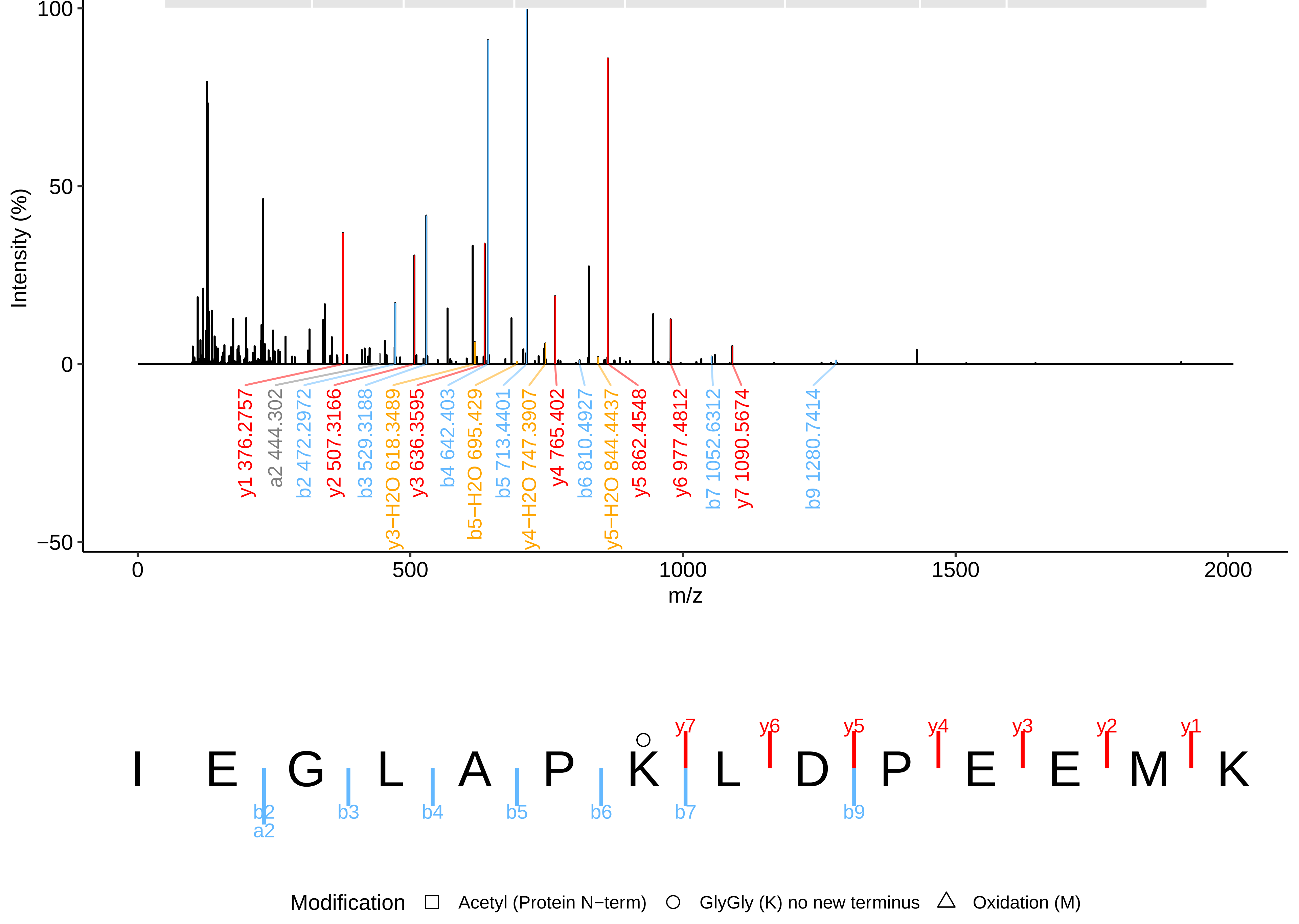

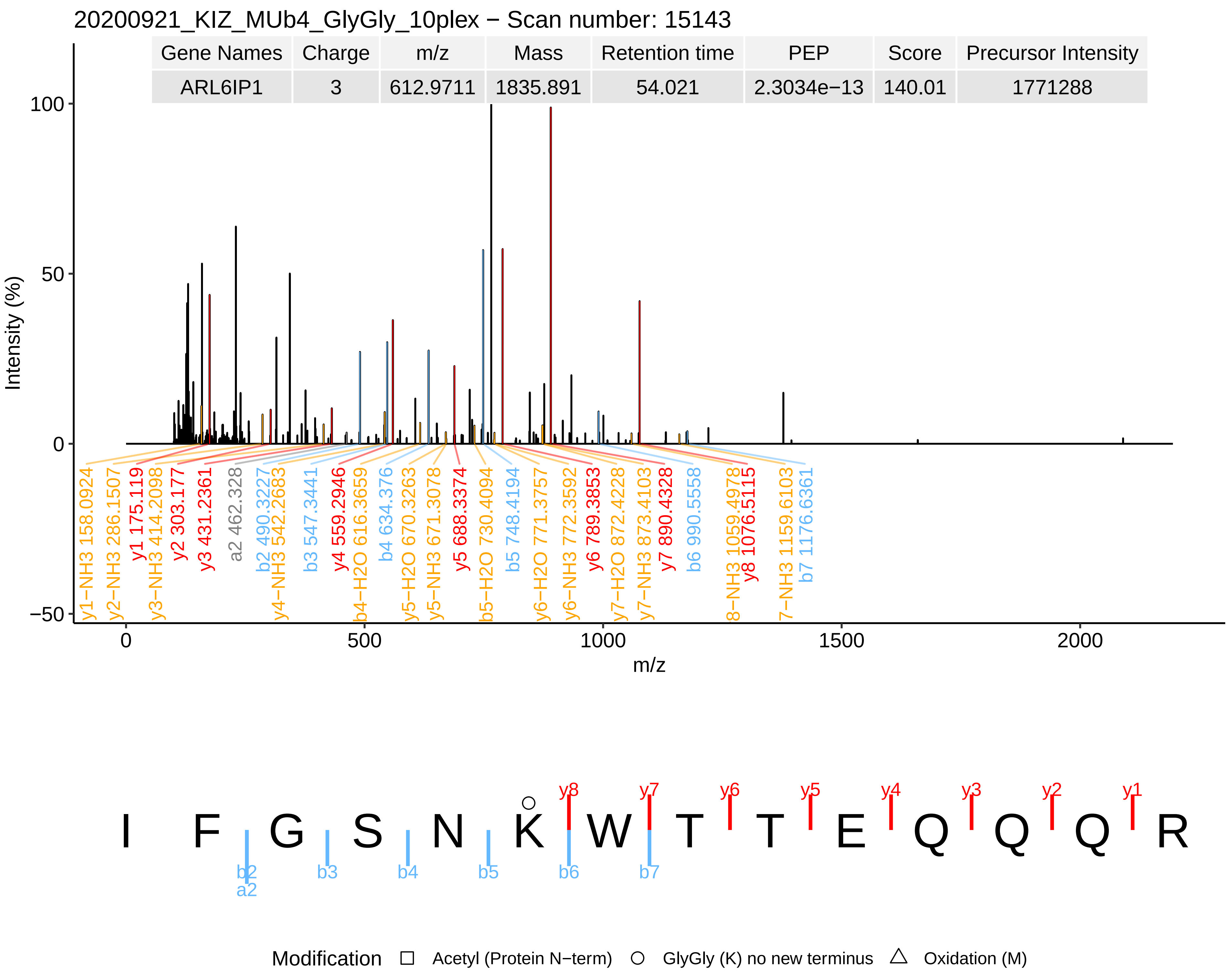
20200921_KIZ_MUb4_GlyGly_10plex − Scan number: 15143
Gene Names | Charge | m/z | Mass | Retention time | PEP | Score | Precursor Intensity
ARL6IP1 | 3 | 612.9711 | 1835.891 | 54.021 | 2.3034e−13 | 140.01 | 1771288
Intensity (%)
m/z
y1−NH3 158.0924
y2−NH3 286.1507
y1 175.119
y3−NH3 414.2098
y2 303.177
y3 431.2361
a2 462.328
b2 490.3227
y4−NH3 542.2683
b3 547.3441
y4 559.2946
b4−H2O 616.3659
b4 634.376
y5−H2O 670.3263
y5−NH3 671.3078
y5 688.3374
b5−H2O 730.4094
b5 748.4194
y6−H2O 771.3757
y6−NH3 772.3592
y6 789.3853
y7−H2O 872.4228
y7−NH3 873.4103
y7 890.4328
b6 990.5558
y8 1076.5115
b7 1176.6361
I F G S N K W T T E Q Q Q R
Modification
Acetyl (Protein N−term)
GlyGly (K) no new terminus
Oxidation (M)

20200921_KIZ_MUb4_GlyGly_10plex_2 − Scan number: 14036

Gene Names	Charge	m/z	Mass	Retention time	PEP	Score	Precursor Intensity
ARL6IP1	3	612.9711	1835.891	54.636	0.0011716	79.466	563223.5

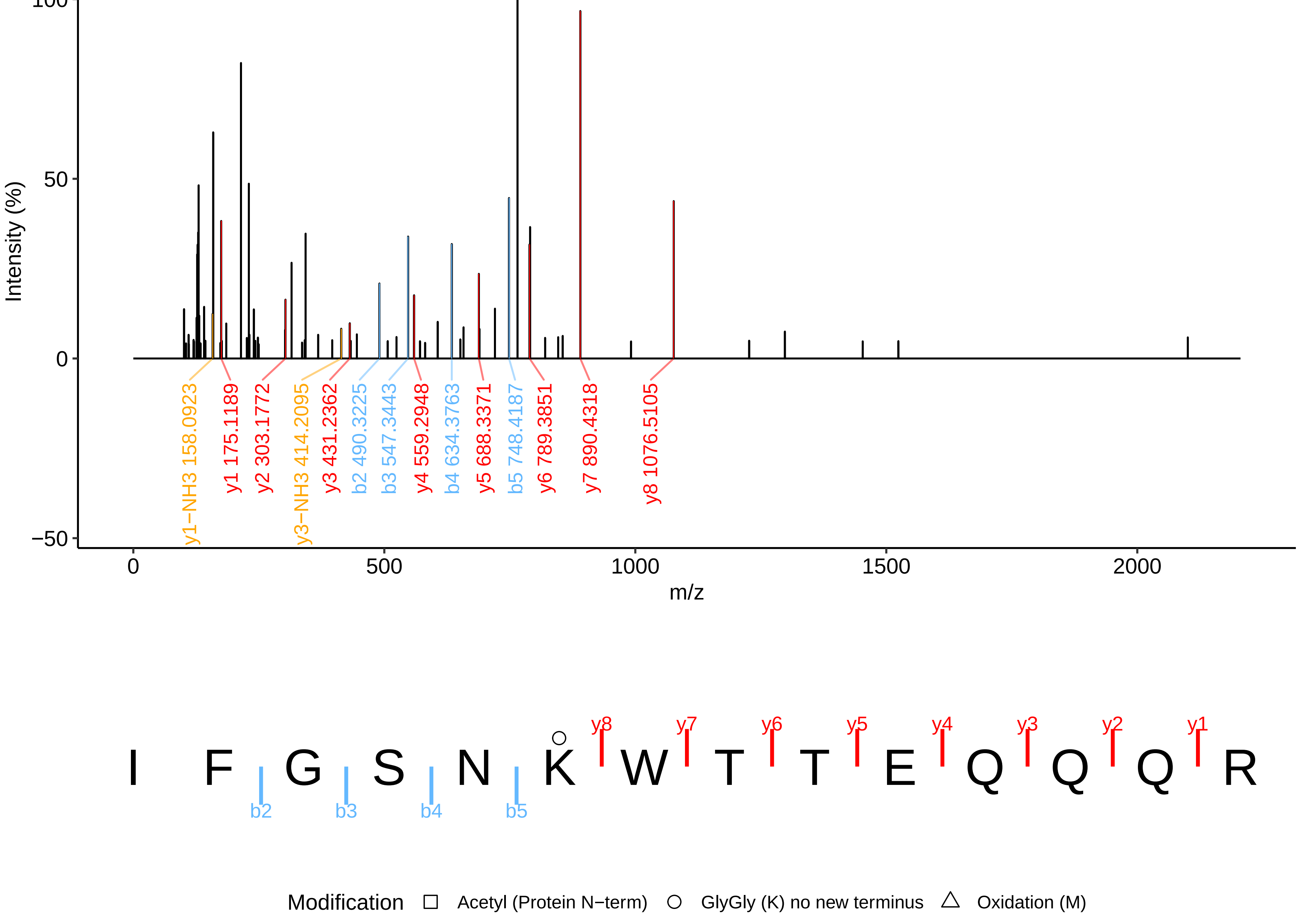

20200921_KIZ_MUb4_GlyGly_10plex_2 − Scan number: 14087

Gene Names	Charge	m/z	Mass	Retention time	PEP	Score	Precursor Intensity
ARL6IP1	3	612.9711	1835.891	54.85	0.032376	56.569	808890.3

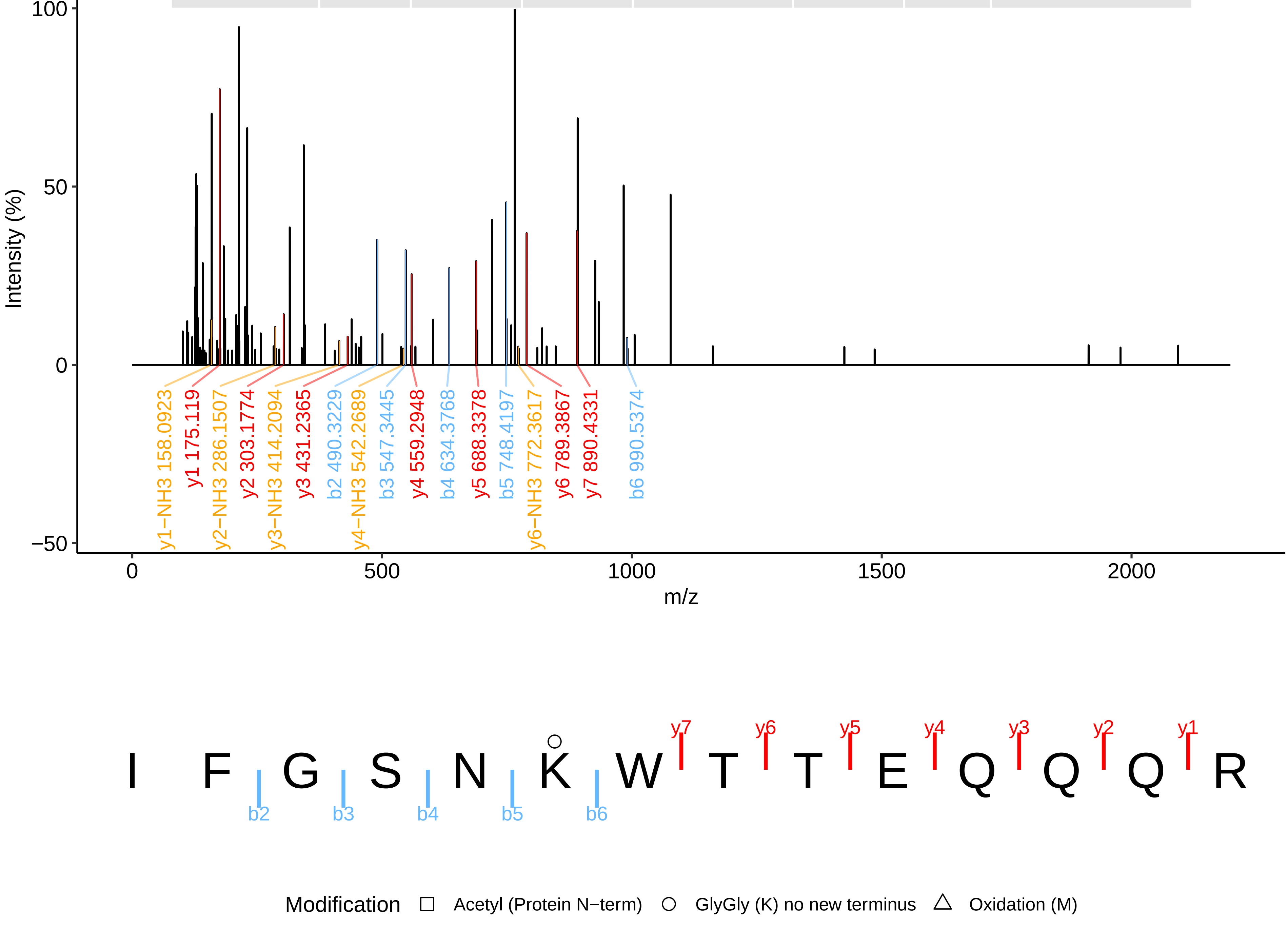

20200924_KIZ_MUb5_GlyGly_2 − Scan number: 13304

Gene Names	Charge	m/z	Mass	Retention time	PEP	Score	Precursor Intensity
ARL6IP1	3	612.9711	1835.891	49.776	0.00043586	88.163	3028925

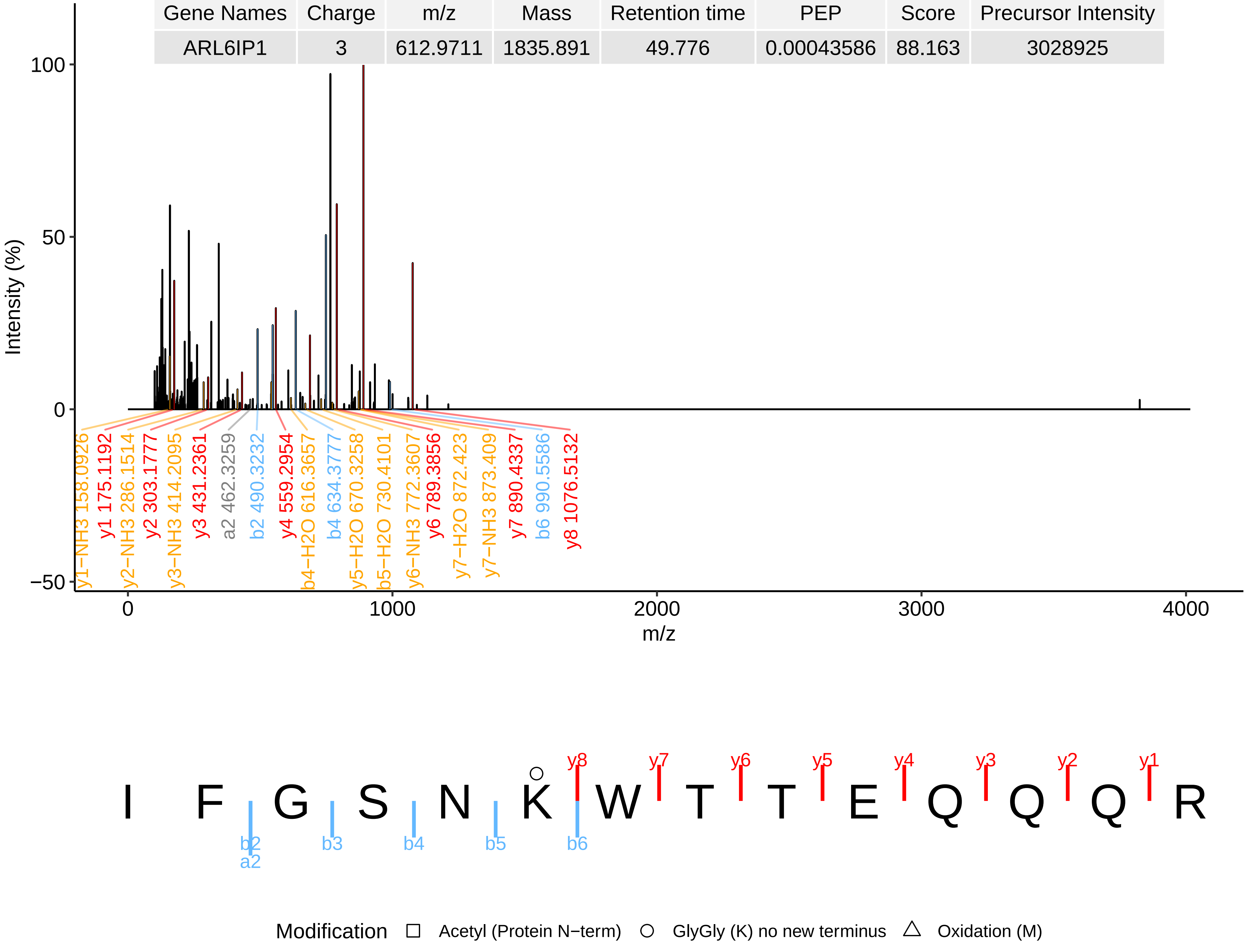

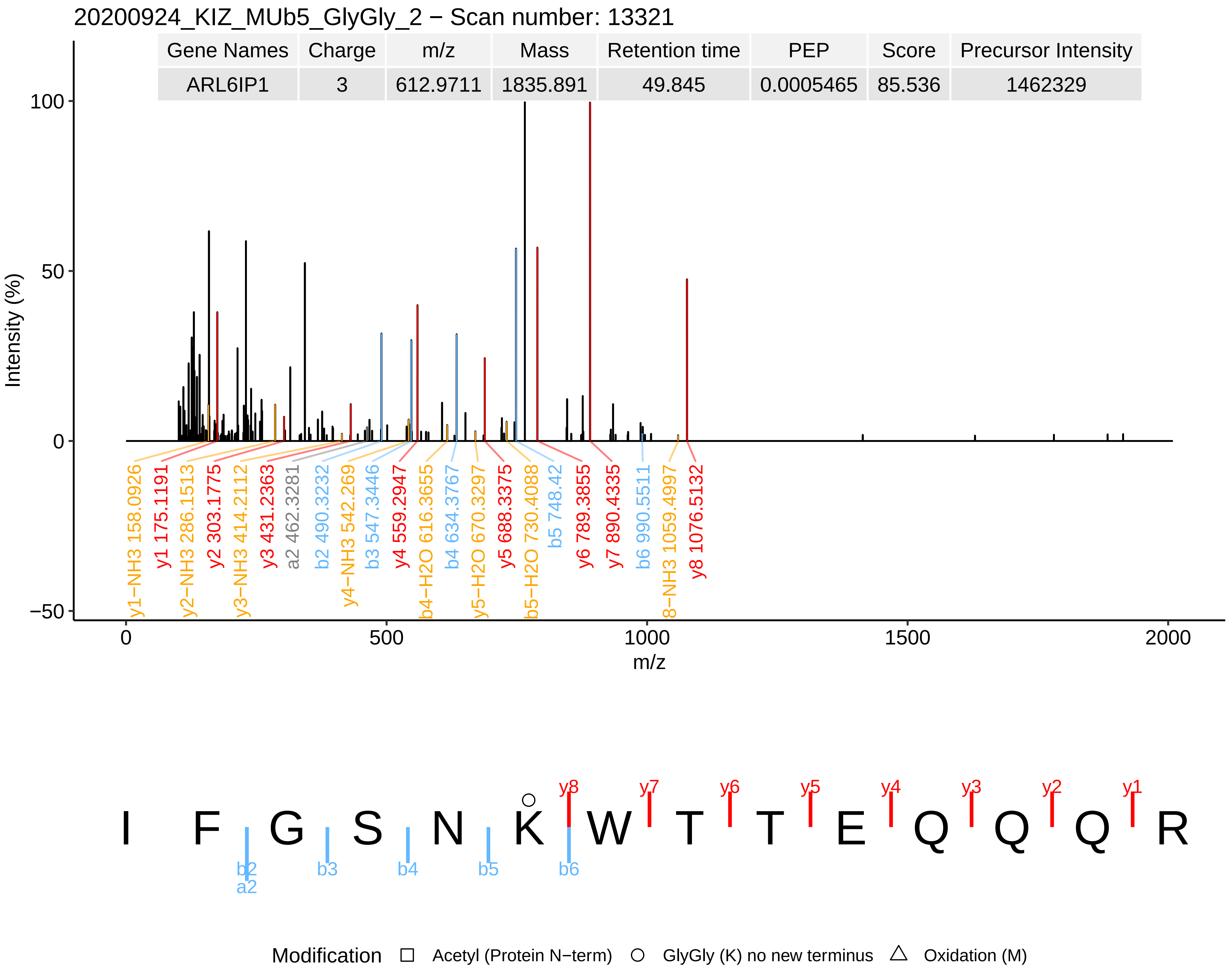

20200924_KIZ_MUb5_GlyGly_2 − Scan number: 13321
Gene Names | Charge | m/z | Mass | Retention time | PEP | Score | Precursor Intensity
ARL6IP1 | 3 | 612.9711 | 1835.891 | 49.845 | 0.0005465 | 85.536 | 1462329
Intensity (%)
m/z
y1−NH3 158.0926
y1 175.1191
y2−NH3 286.1513
y2 303.1775
y3−NH3 414.2112
y3 431.2363
a2 462.3281
b2 490.3232
y4−NH3 542.269
b3 547.3446
y4 559.2947
b4−H2O 616.3655
b4 634.3767
y5−H2O 670.3297
y5 688.3375
b5−H2O 730.4088
b5 748.42
y6 789.3855
y7 890.4335
b6 990.5511
8−NH3 1059.4997
y8 1076.5132
I F G S N K W T T E Q Q Q R
y8 y7 y6 y5 y4 y3 y2 y1
b2 a2 b3 b4 b5 b6
Modification
Acetyl (Protein N−term)
GlyGly (K) no new terminus
Oxidation (M)

20201118_KIZ_MUb6_GlyGly_2 − Scan number: 16225

Gene Names	Charge	m/z	Mass	Retention time	PEP	Score	Precursor Intensity
ARL6IP1	3	612.9711	1835.891	54.802	6.949e−07	114.87	5351021

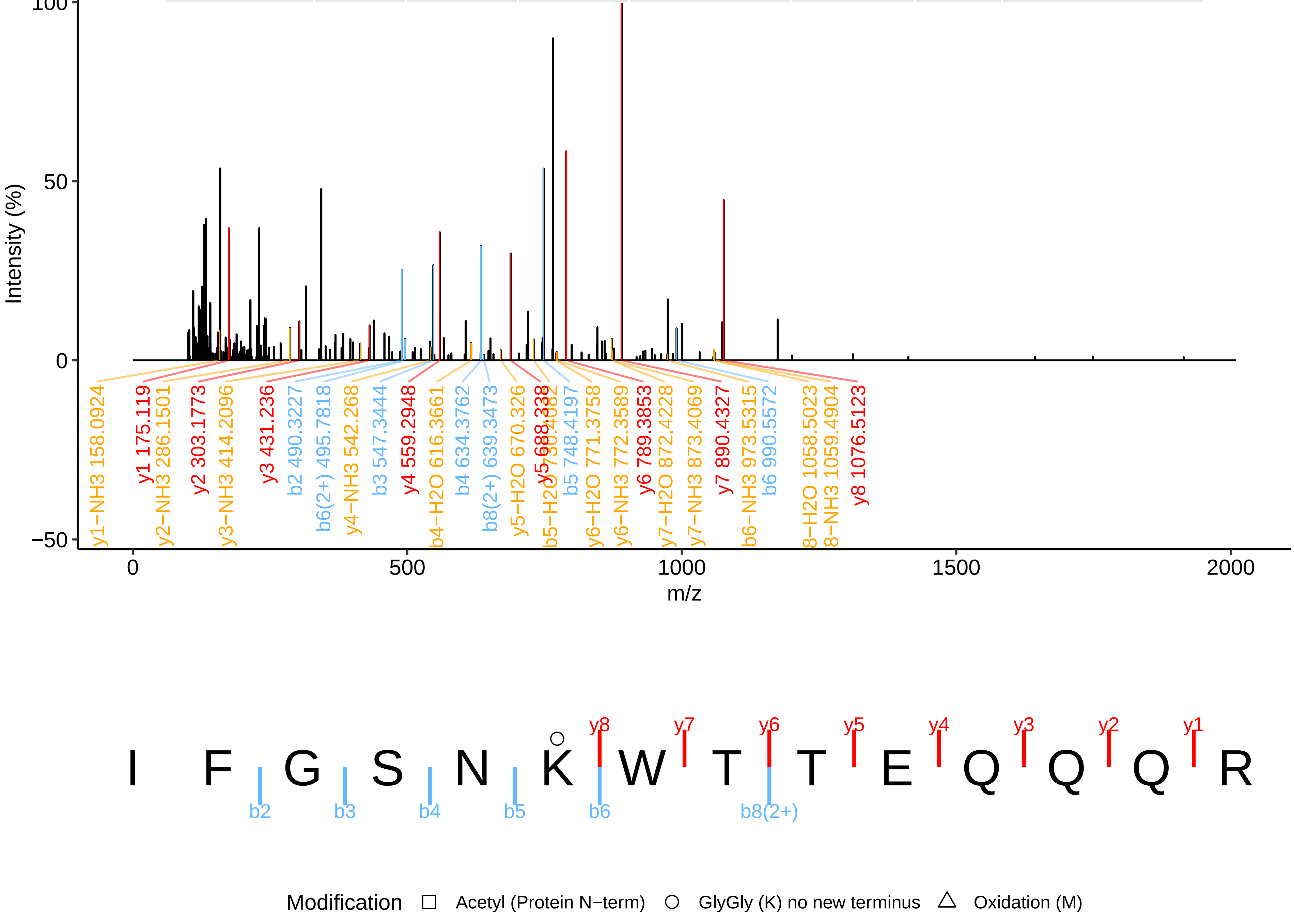

20200921_KIZ_MUb4_GlyGly_10plex − Scan number: 14871

Gene Names	Charge	m/z	Mass	Retention time	PEP	Score	Precursor Intensity
TARS	3	621.6705	1861.99	52.966	4.7185e−05	93.716	3817110

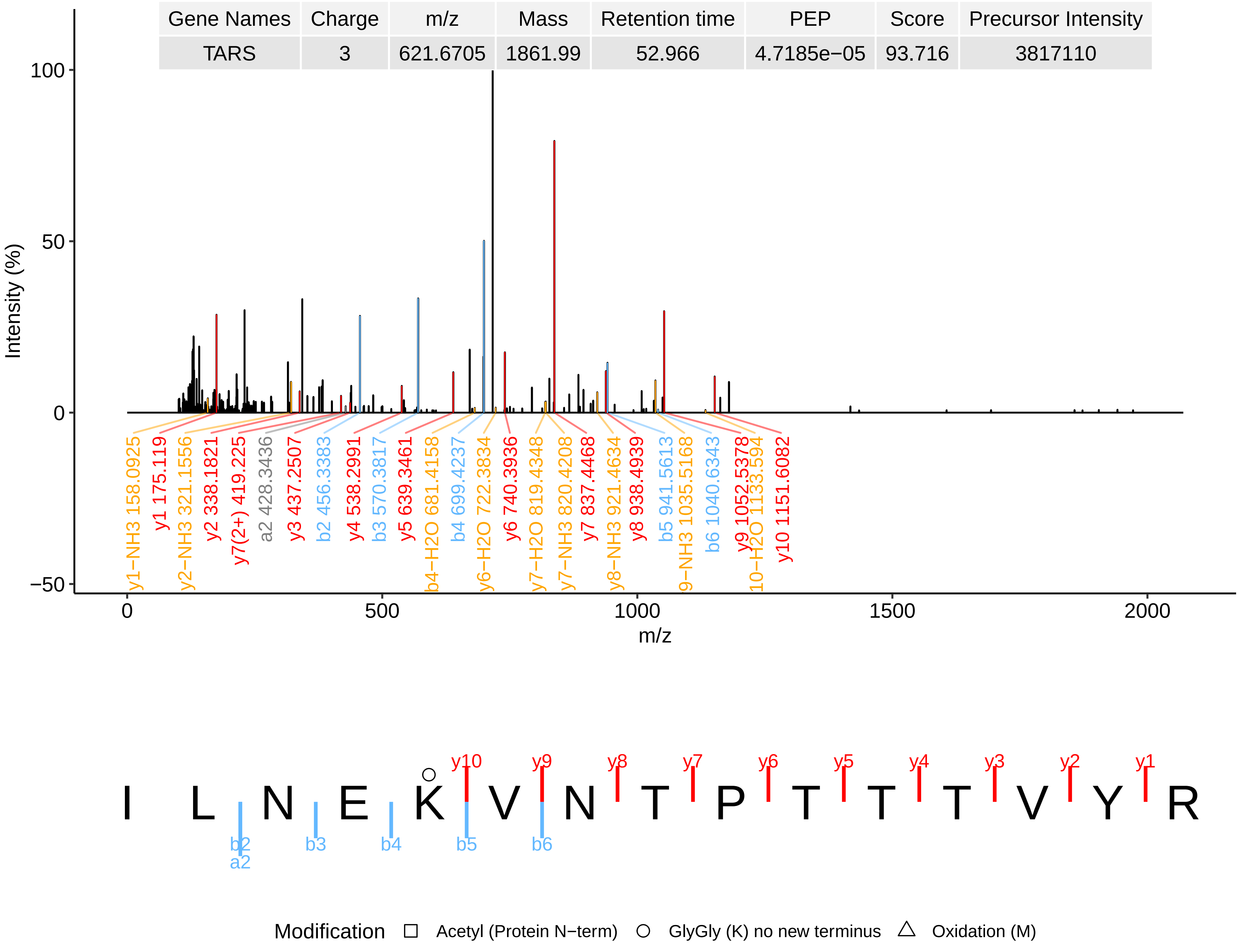

20200921_KIZ_MUb4_GlyGly_10plex_2 − Scan number: 13725

Gene Names	Charge	m/z	Mass	Retention time	PEP	Score	Precursor Intensity
TARS	3	621.6705	1861.99	53.35	0.00016613	81.632	1097105

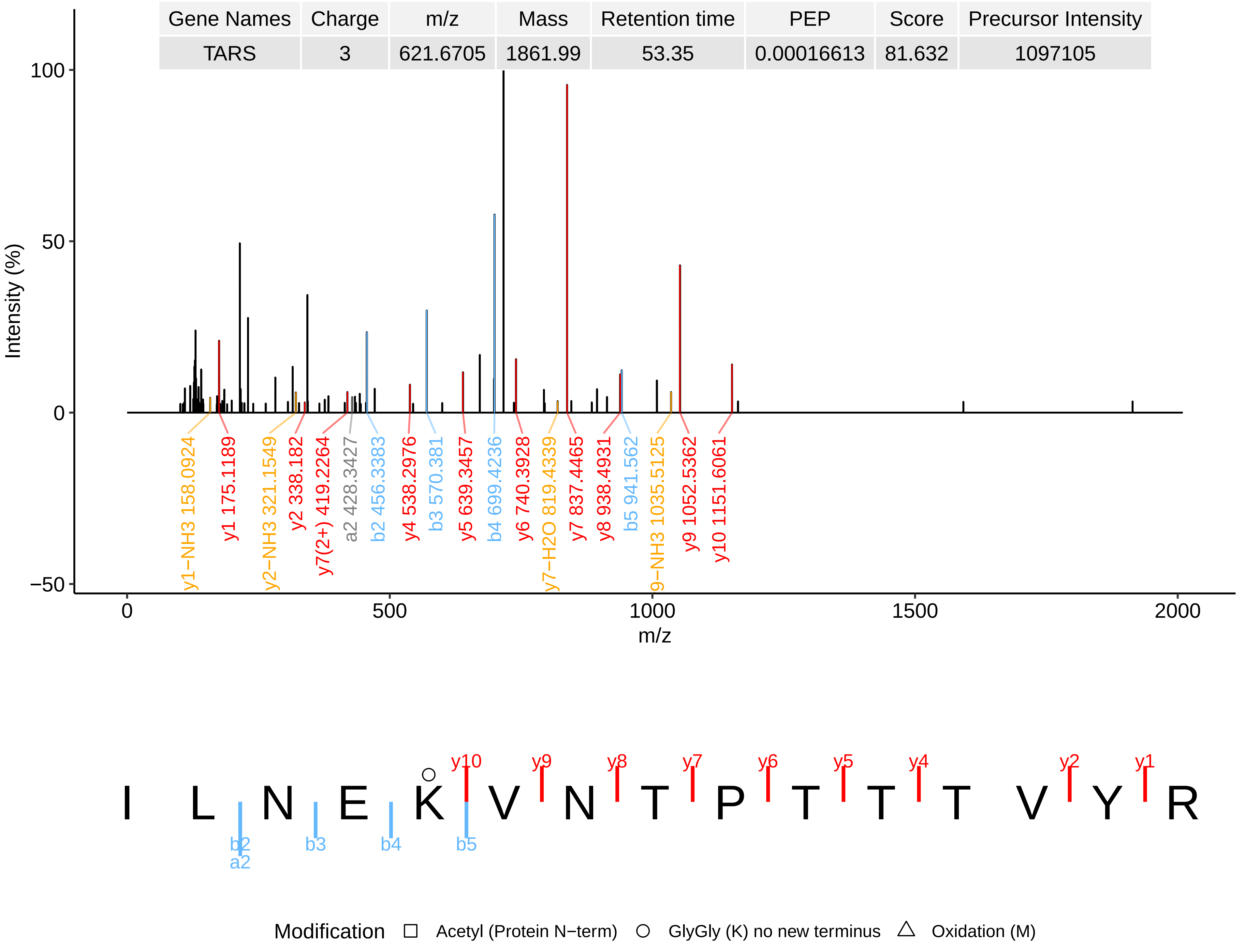

20200921_KIZ_MUb4_GlyGly_10plex_2 − Scan number: 12682

Gene Names	Charge	m/z	Mass	Retention time	PEP	Score	Precursor Intensity
MAP2K3	3	544.2898	1629.848	49.118	0.0087734	67.997	194035.9

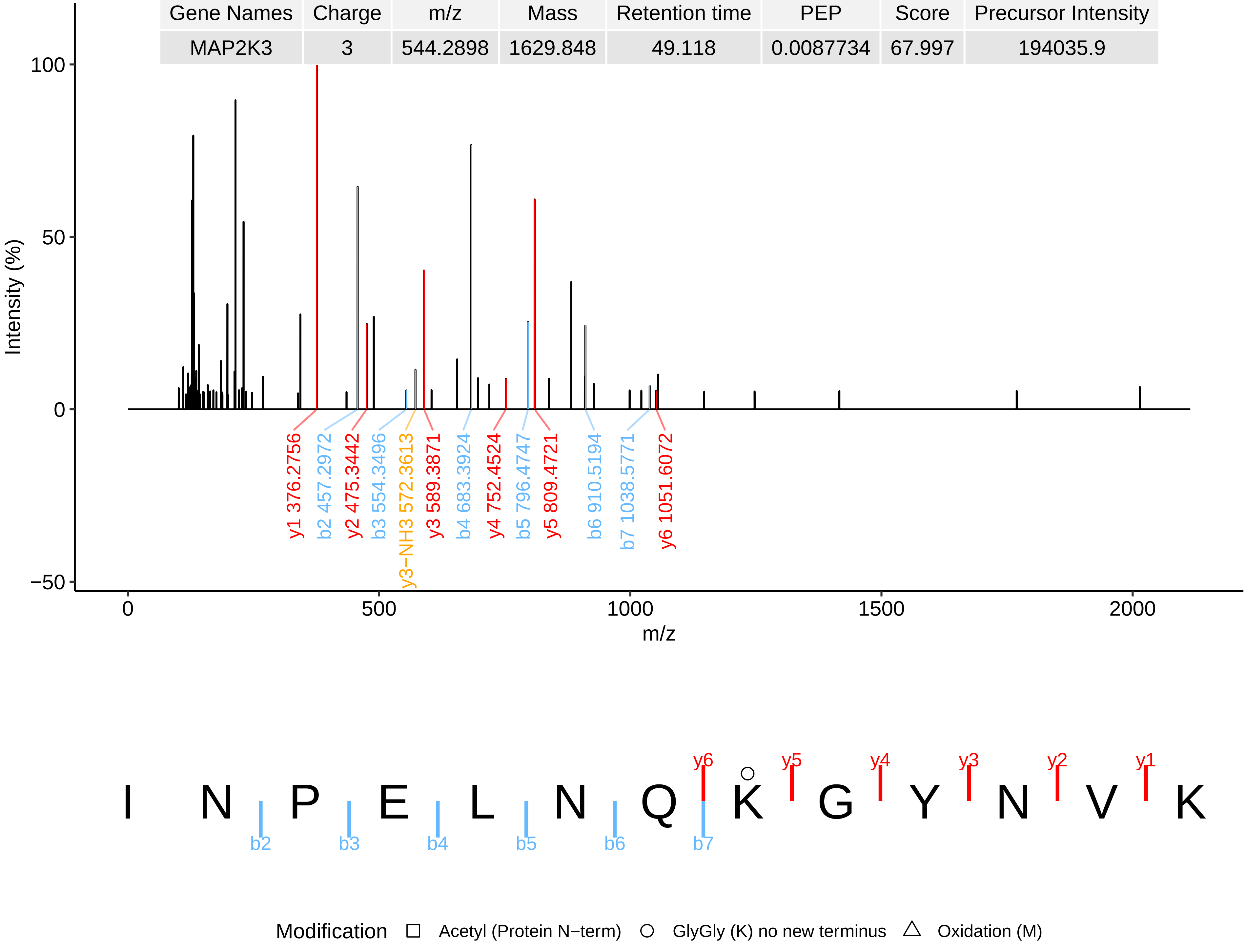

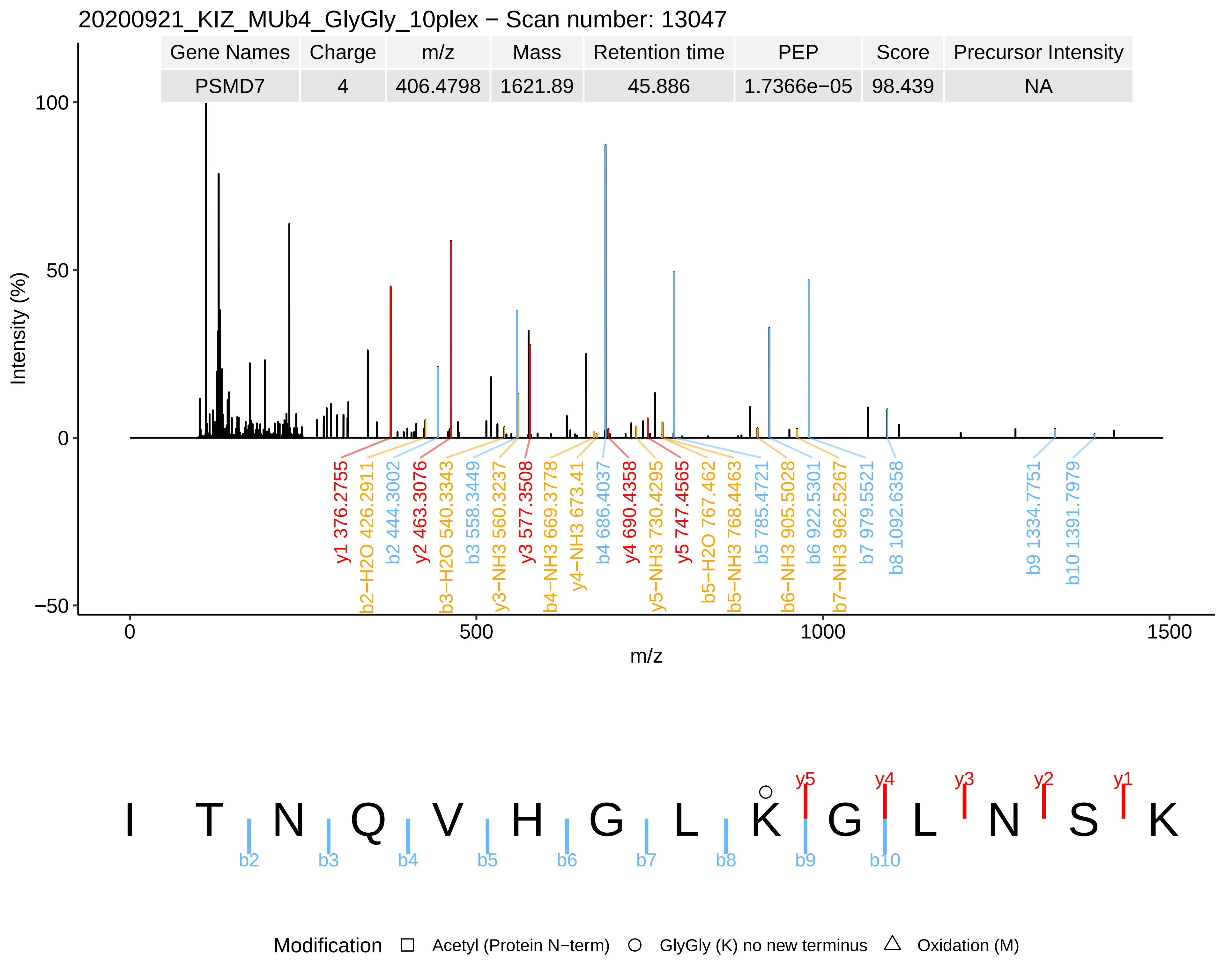

20200921_KIZ_MUb4_GlyGly_10plex − Scan number: 13047
Gene Names | Charge | m/z | Mass | Retention time | PEP | Score | Precursor Intensity
PSMD7 | 4 | 406.4798 | 1621.89 | 45.886 | 1.7366e−05 | 98.439 | NA
Intensity (%)
m/z
y1 376.2755
b2−H2O 426.2911
b2 444.3002
y2 463.3076
b3−H2O 540.3343
b3 558.3449
y3−NH3 560.3237
y3 577.3508
b4−NH3 669.3778
y4−NH3 673.41
b4 686.4037
y4 690.4358
y5−NH3 730.4295
y5 747.4565
b5−H2O 767.462
b5−NH3 768.4463
b5 785.4721
b6−NH3 905.5028
b6 922.5301
b7−NH3 962.5267
b7 979.5521
b8 1092.6358
b9 1334.7751
b10 1391.7979
I T N Q V H G L K G L N S K
Modification
Acetyl (Protein N−term)
GlyGly (K) no new terminus
Oxidation (M)

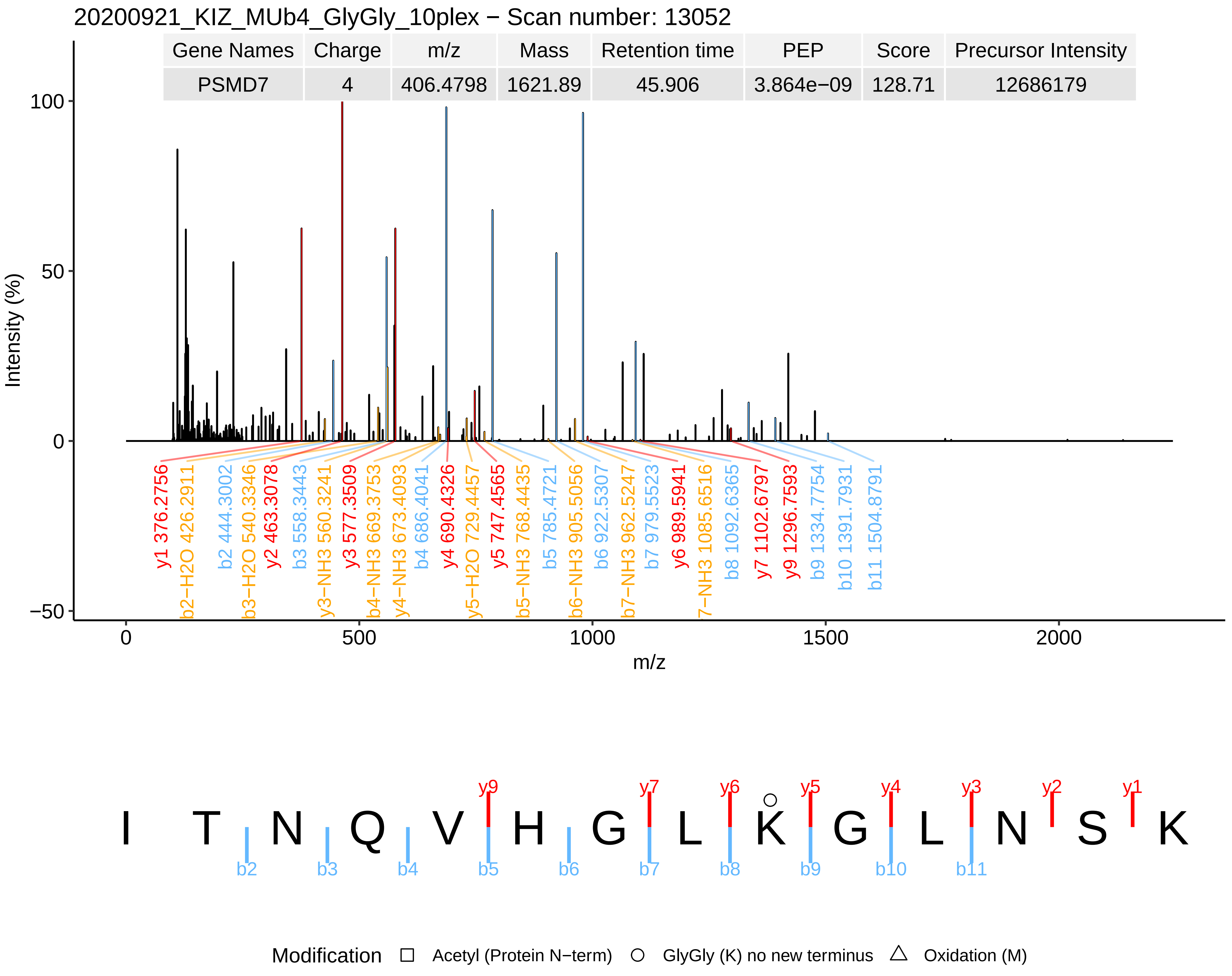
20200921_KIZ_MUb4_GlyGly_10plex − Scan number: 13052
Gene Names | Charge | m/z | Mass | Retention time | PEP | Score | Precursor Intensity
PSMD7 | 4 | 406.4798 | 1621.89 | 45.906 | 3.864e−09 | 128.71 | 12686179
Intensity (%)
100
50
0
−50
m/z
0
500
1000
1500
2000
y1 376.2756
b2−H2O 426.2911
b2 444.3002
b3−H2O 540.3346
y2 463.3078
b3 558.3443
y3−NH3 560.3241
y3 577.3509
b4−NH3 669.3753
y4−NH3 673.4093
b4 686.4041
y4 690.4326
y5−H2O 729.4457
y5 747.4565
b5−NH3 768.4435
b5 785.4721
b6−NH3 905.5056
b6 922.5307
b7−NH3 962.5247
b7 979.5523
y6 989.5941
7−NH3 1085.6516
b8 1092.6365
y7 1102.6797
y9 1296.7593
b9 1334.7754
b10 1391.7931
b11 1504.8791
I T N Q V H G L K G L N S K
y9 y7 y6 y5 y4 y3 y2 y1
b2 b3 b4 b5 b6 b7 b8 b9 b10 b11
Modification
Acetyl (Protein N−term)
GlyGly (K) no new terminus
Oxidation (M)

20200921_KIZ_MUb4_GlyGly_10plex_2 − Scan number: 12033

Gene Names	Charge	m/z	Mass	Retention time	PEP	Score	Precursor Intensity
PSMD7	4	406.4798	1621.89	46.465	0.0026678	73.91	989605.1

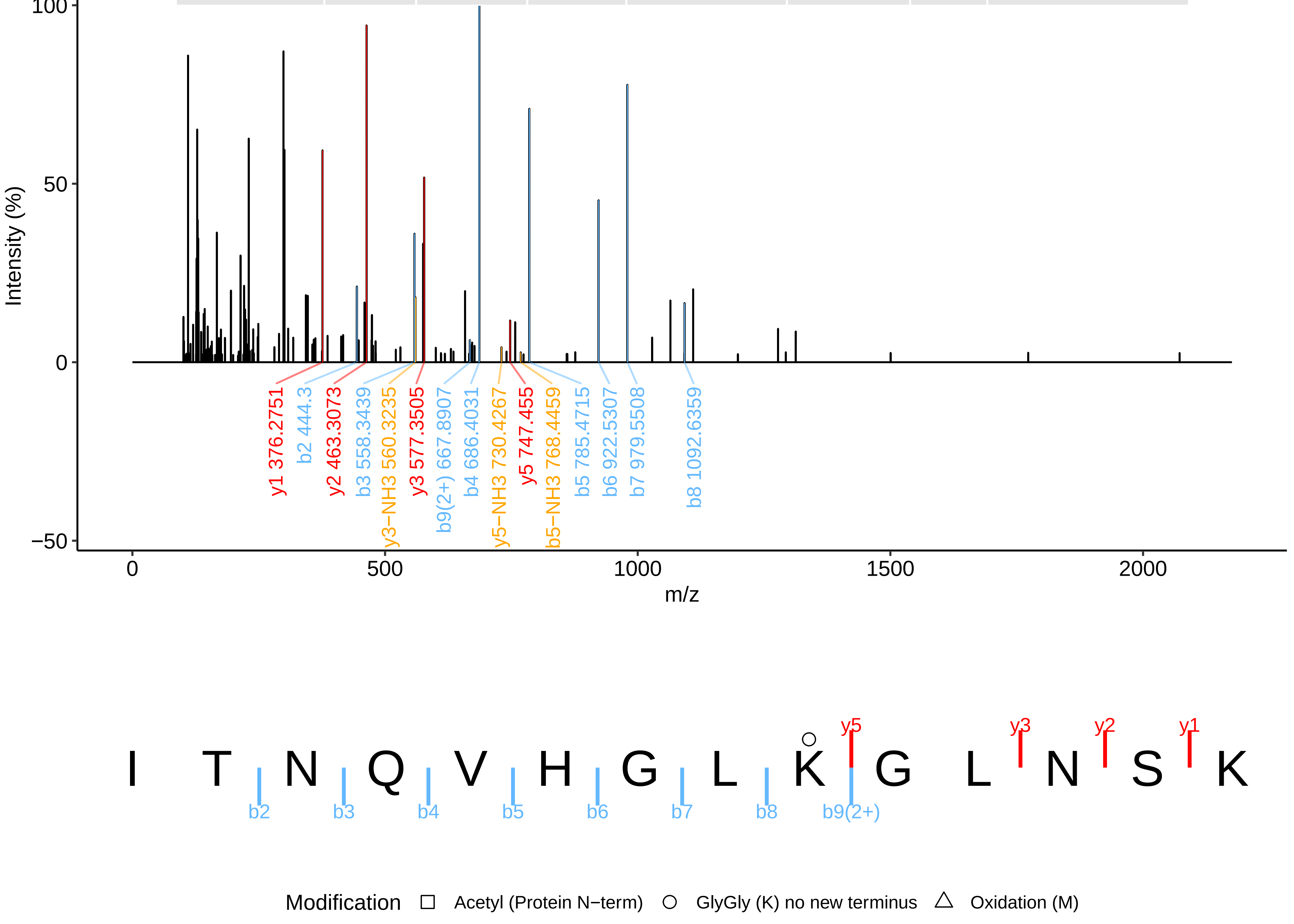

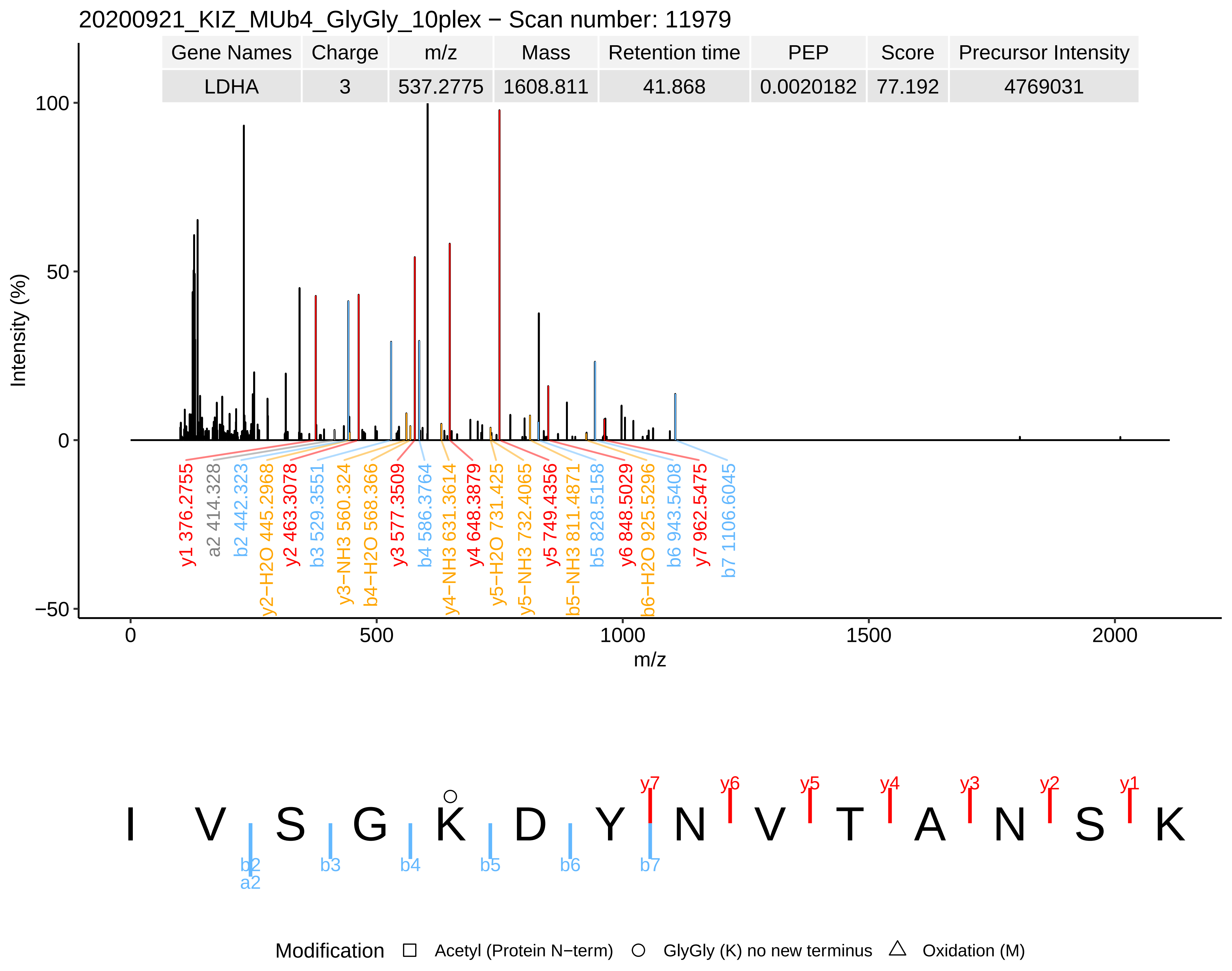
20200921_KIZ_MUb4_GlyGly_10plex − Scan number: 11979
Gene Names | Charge | m/z | Mass | Retention time | PEP | Score | Precursor Intensity
LDHA | 3 | 537.2775 | 1608.811 | 41.868 | 0.0020182 | 77.192 | 4769031
Intensity (%)
100
50
0
−50
0
500
1000
1500
2000
m/z
y1 376.2755
a2 414.328
b2 442.323
y2−H2O 445.2968
y2 463.3078
b3 529.3551
y3−NH3 560.324
b4−H2O 568.366
y3 577.3509
b4 586.3764
y4−NH3 631.3614
y4 648.3879
y5−H2O 731.425
y5−NH3 732.4065
y5 749.4356
b5−NH3 811.4871
b5 828.5158
y6 848.5029
b6−H2O 925.5296
b6 943.5408
y7 962.5475
b7 1106.6045
I V S G K D Y N V T A N S K
y7 y6 y5 y4 y3 y2 y1
b2 a2 b3 b4 b5 b6 b7
Modification
Acetyl (Protein N−term)
GlyGly (K) no new terminus
Oxidation (M)

20200924_KIZ_MUb5_GlyGly_1 − Scan number: 14206

Gene Names	Charge	m/z	Mass	Retention time	PEP	Score	Precursor Intensity
SGTA	3	550.6054	1648.794	46.369	0.00015173	98.942	NA

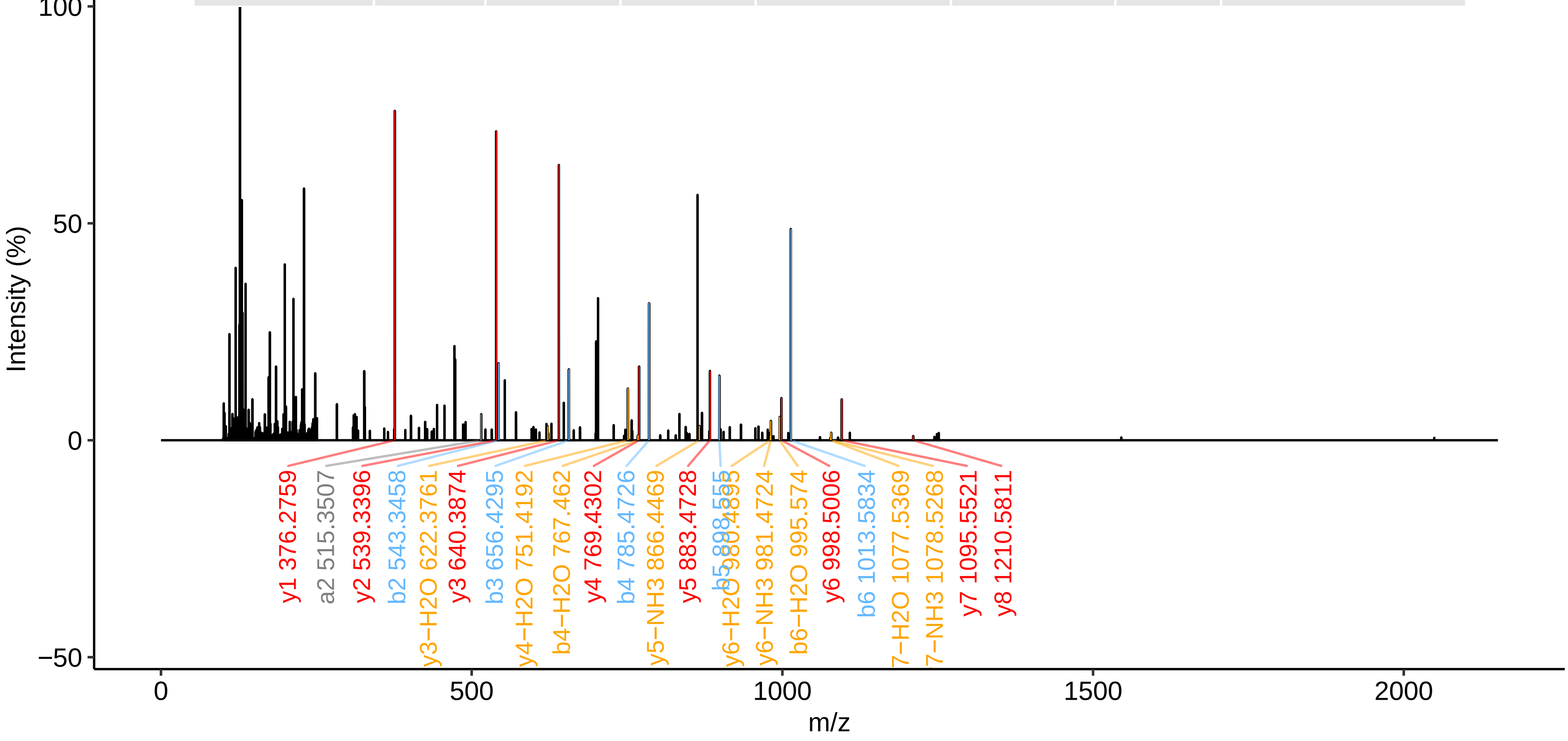

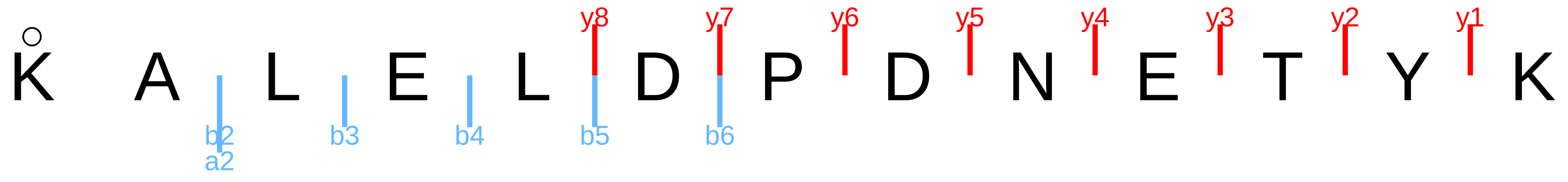

Modification □ Acetyl (Protein N−term) ○ GlyGly (K) no new terminus △ Oxidation (M)

20201118_KIZ_MUb6_GlyGly_2 − Scan number: 34875

Gene Names	Charge	m/z	Mass	Retention time	PEP	Score	Precursor Intensity
GTF3C6	4	477.5149	1906.031	113.25	0.083488	40.725	40083572

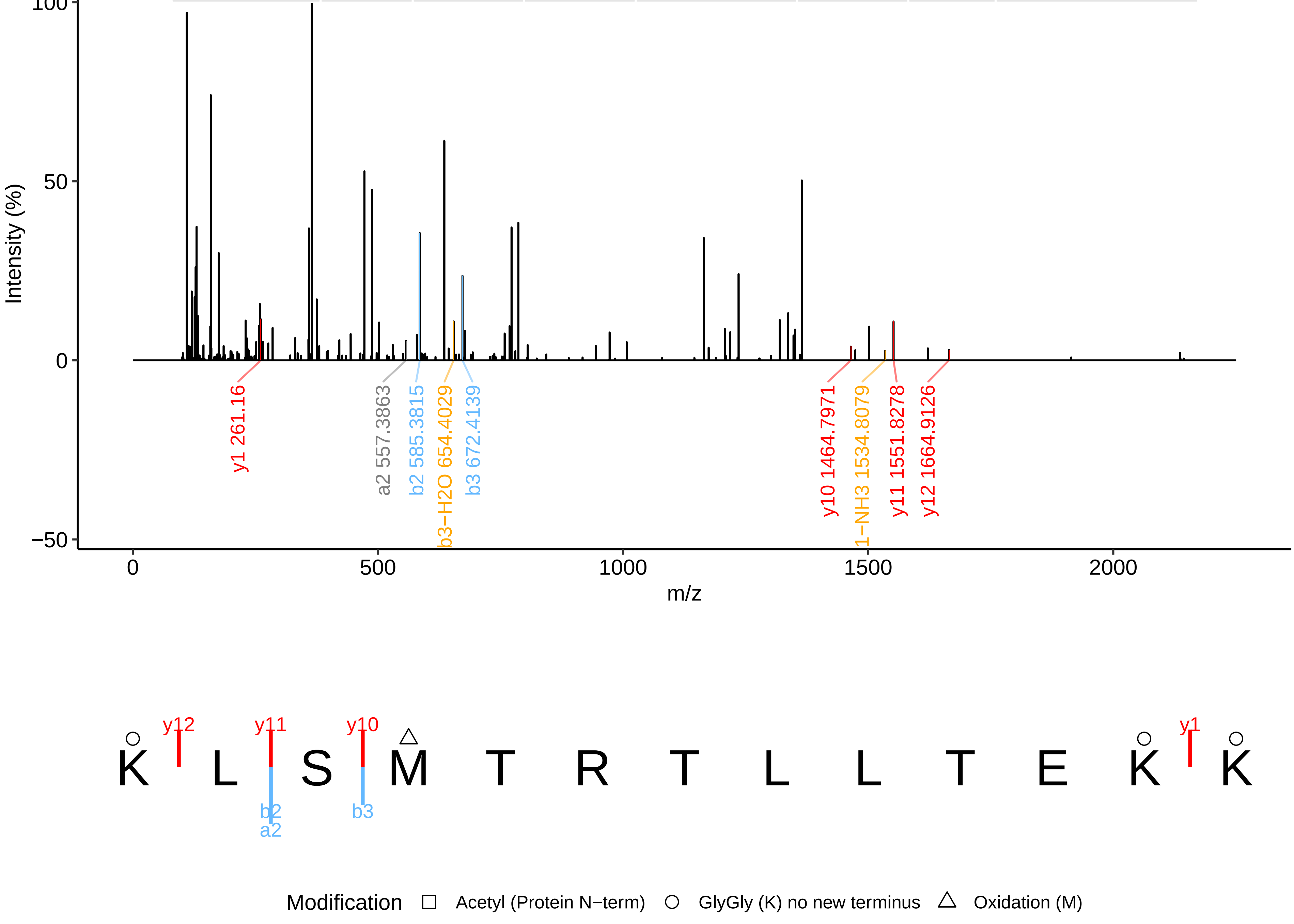

20200921_KIZ_MUb4_GlyGly_10plex − Scan number: 10693

Gene Names	Charge	m/z	Mass	Retention time	PEP	Score	Precursor Intensity
CHCHD3	4	403.4626	1609.821	37.08	0.059153	68.676	7101280

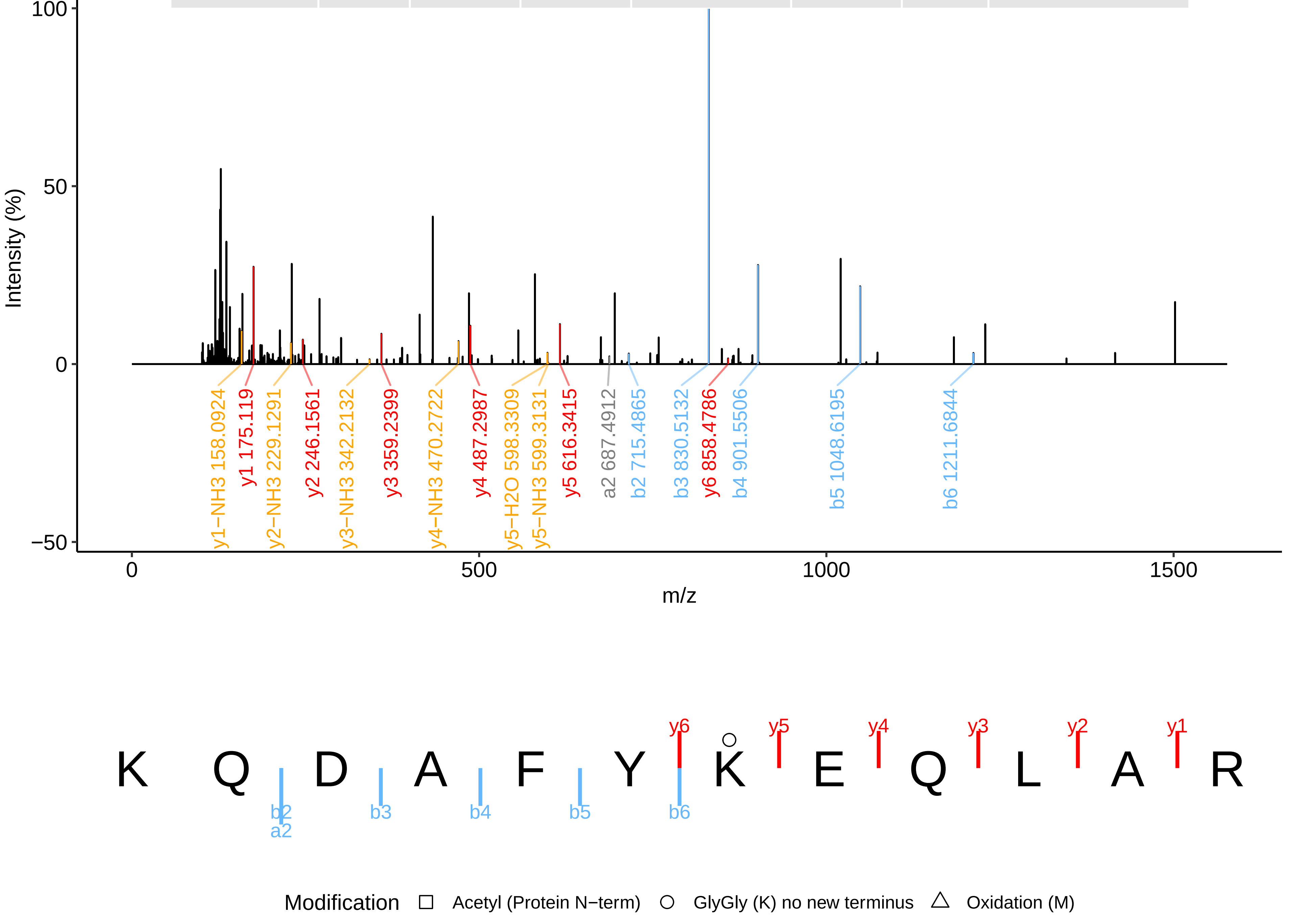

20200921_KIZ_MUb4_GlyGly_10plex_2 − Scan number: 25768

Gene Names	Charge	m/z	Mass	Retention time	PEP	Score	Precursor Intensity
VCP	3	638.6825	1913.026	101.97	7.862e−05	94.461	1353451

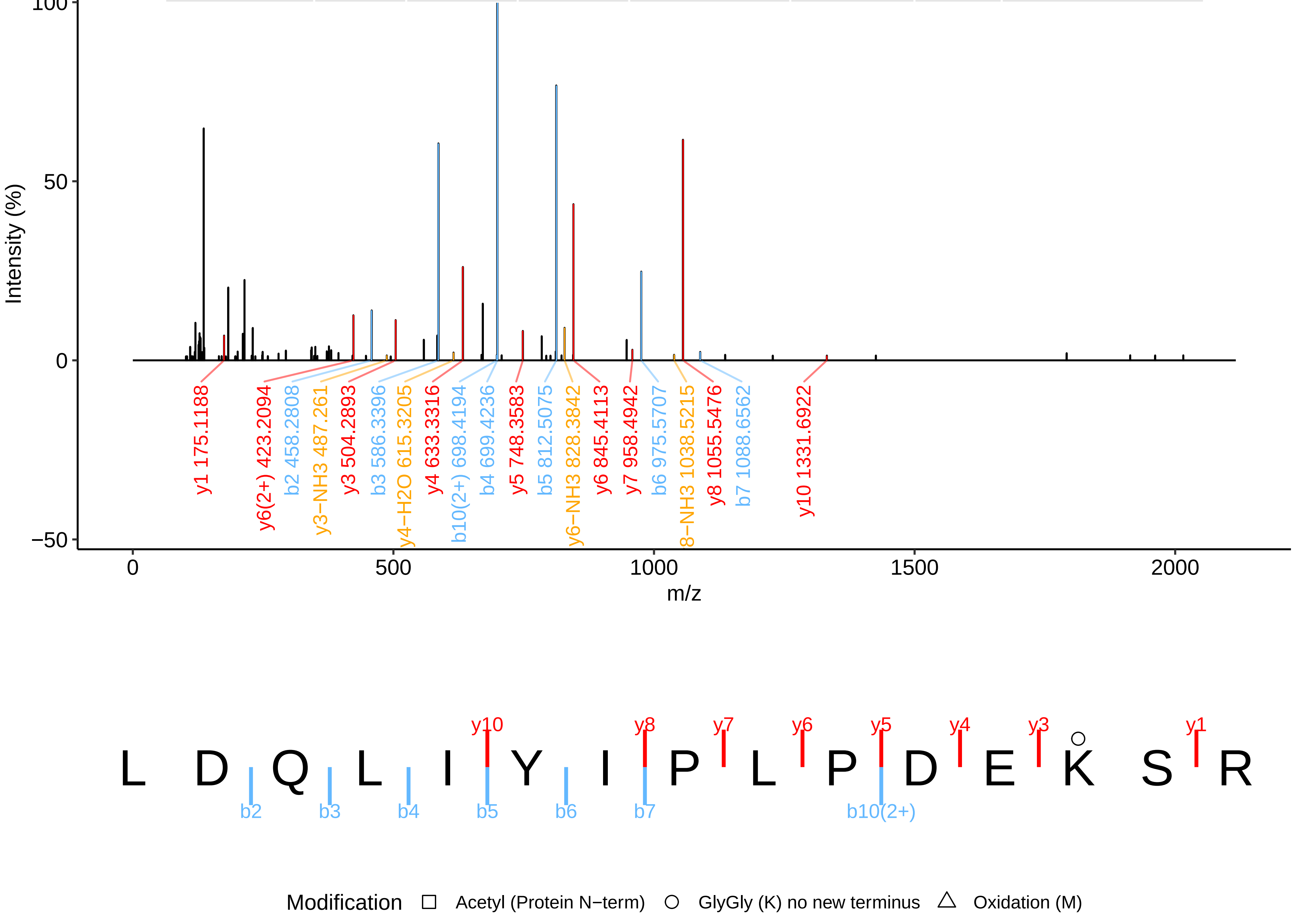

20200921_KIZ_MUb4_GlyGly_10plex − Scan number: 12758

Gene Names	Charge	m/z	Mass	Retention time	PEP	Score	Precursor Intensity
NSFL1C	3	630.9779	1889.912	44.843	5.6703e−23	166.64	11371562

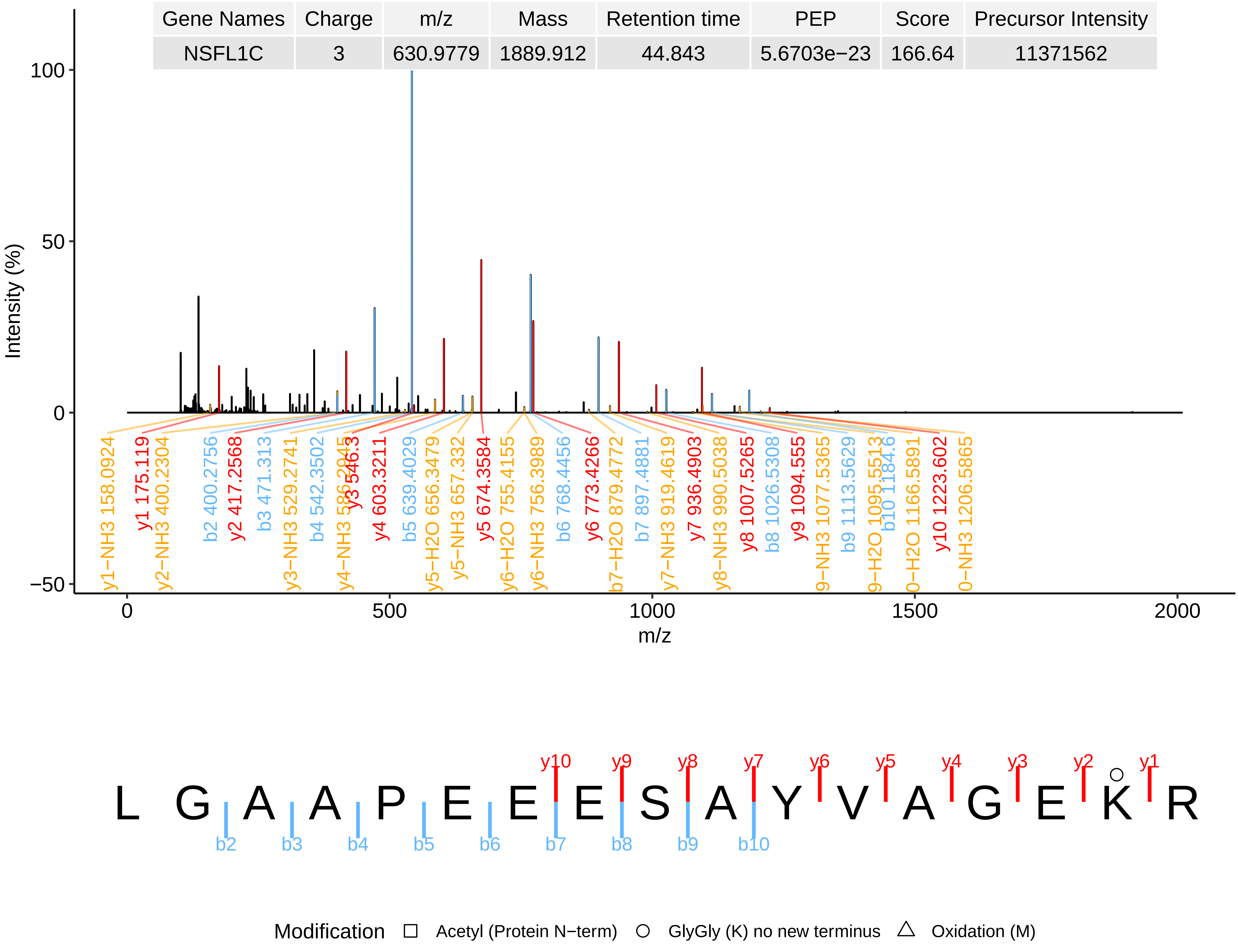

20200921_KIZ_MUb4_GlyGly_10plex_2 − Scan number: 11644

Gene Names	Charge	m/z	Mass	Retention time	PEP	Score	Precursor Intensity
NSFL1C	3	630.9779	1889.912	44.851	0.012175	67.605	523693

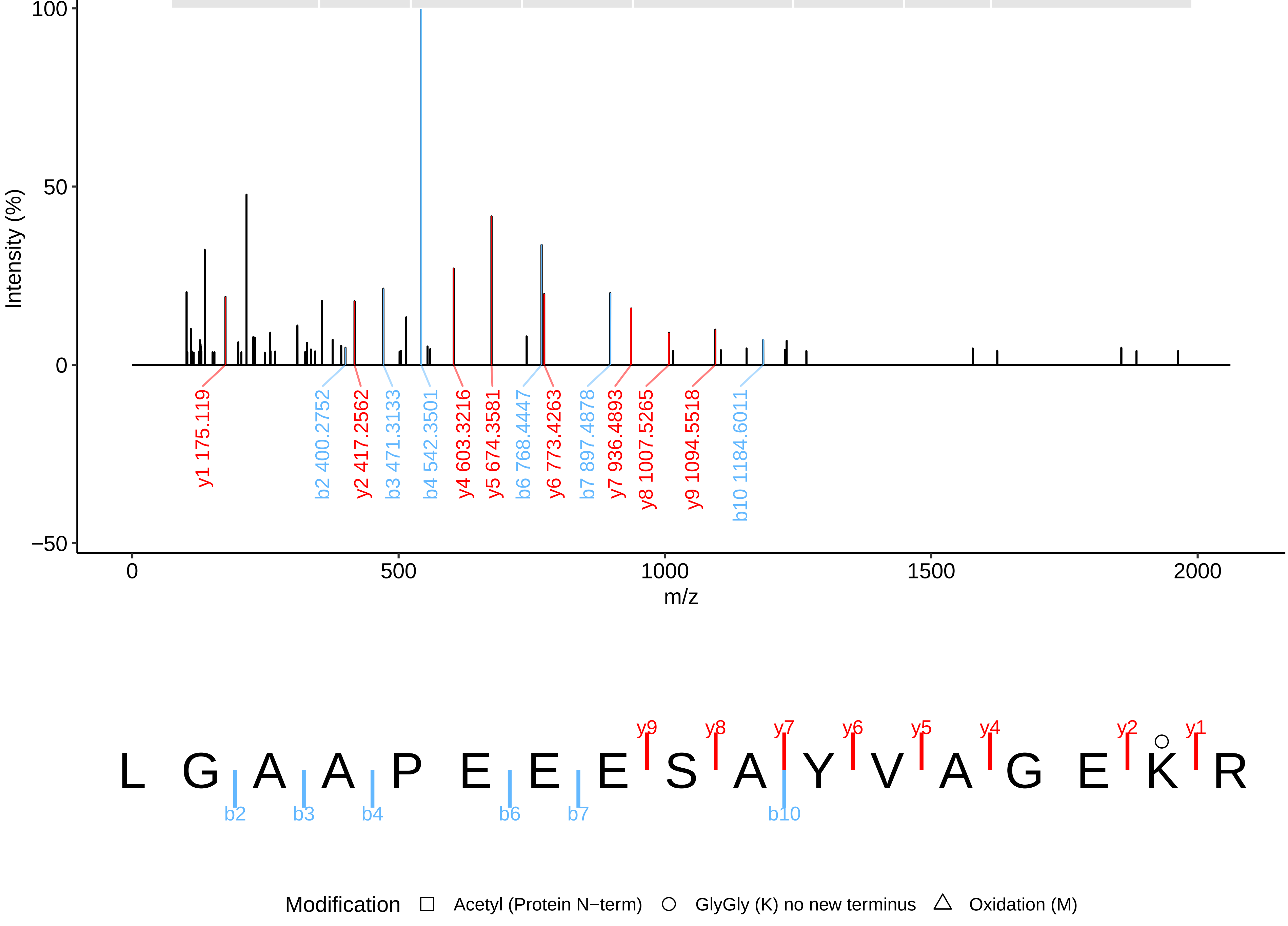

20200924_KIZ_MUb5_GlyGly_1 − Scan number: 11812

Gene Names	Charge	m/z	Mass	Retention time	PEP	Score	Precursor Intensity
NSFL1C	3	630.9779	1889.912	38.336	1.6639e−29	174.68	7590783

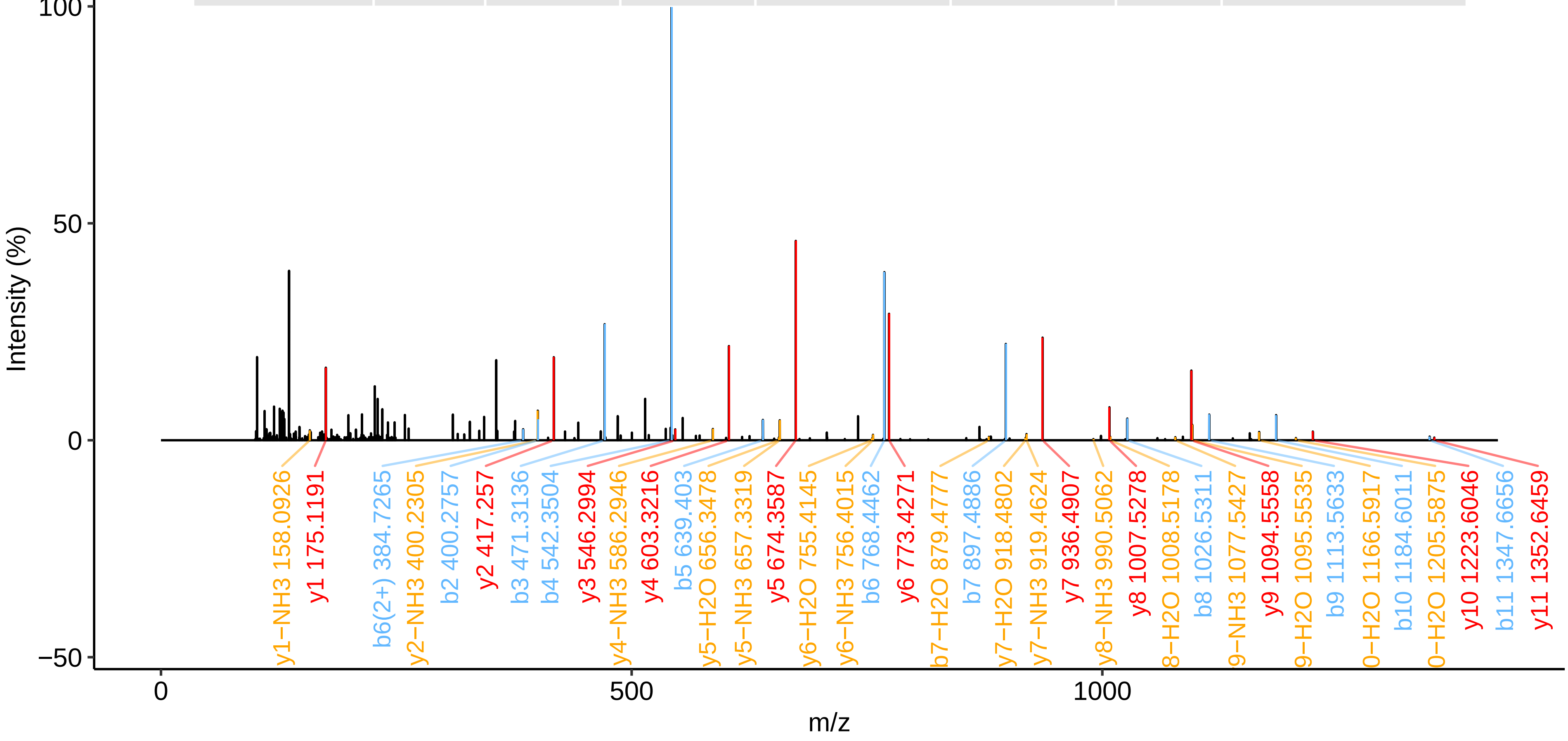

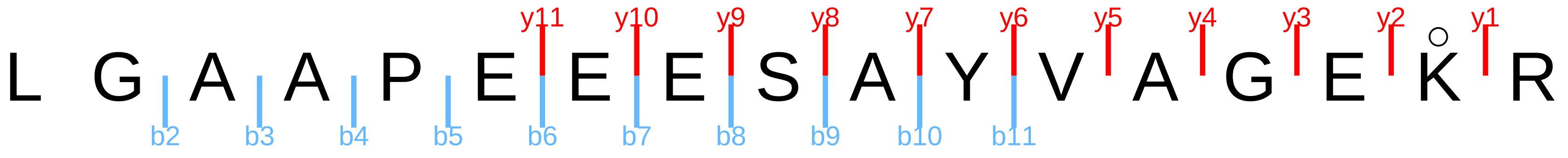

Modification □ Acetyl (Protein N−term) ○ GlyGly (K) no new terminus △ Oxidation (M)

20200924_KIZ_MUb5_GlyGly_2 − Scan number: 11037

Gene Names	Charge	m/z	Mass	Retention time	PEP	Score	Precursor Intensity
NSFL1C	3	630.9779	1889.912	41.222	2.5611e−11	121.79	2058419

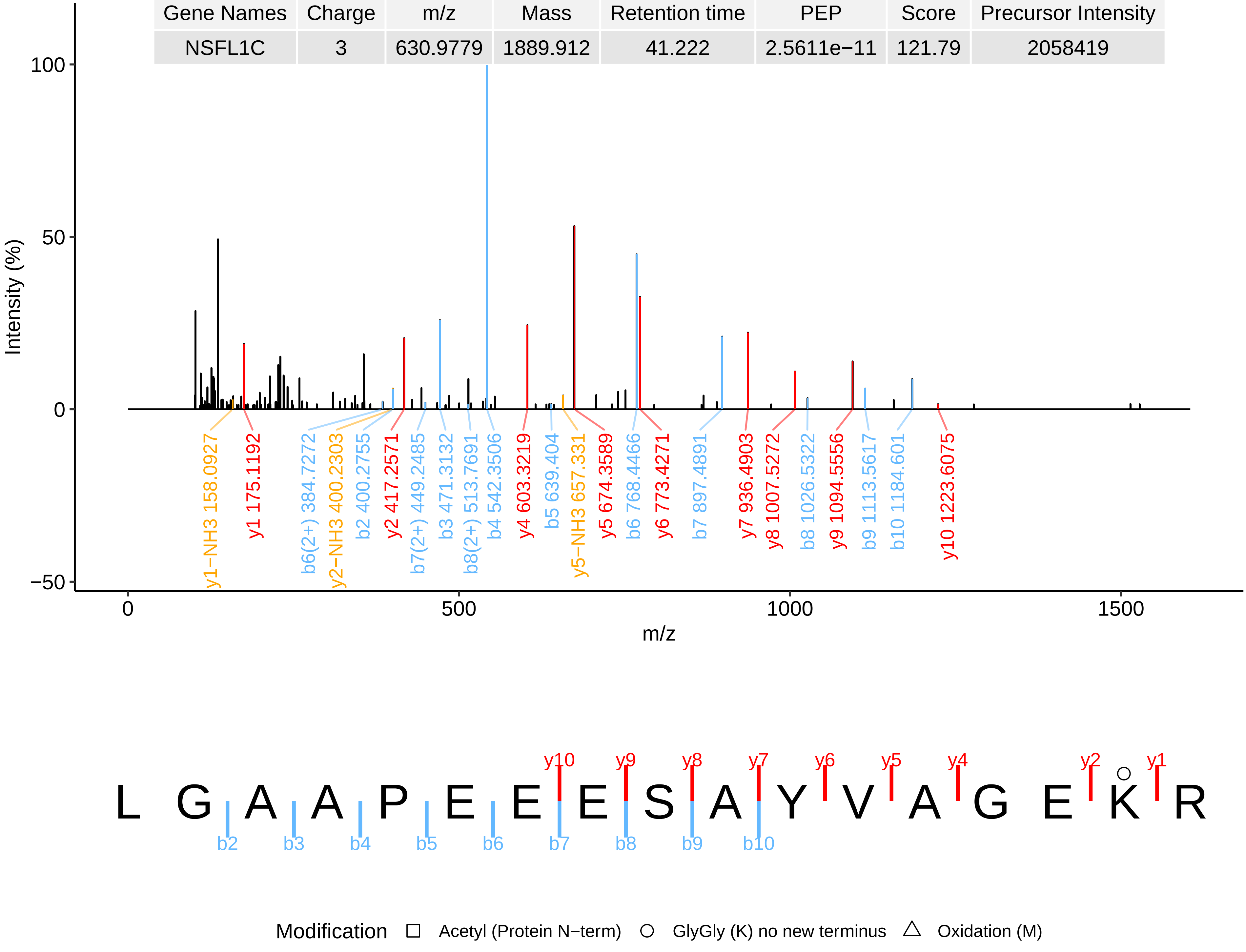

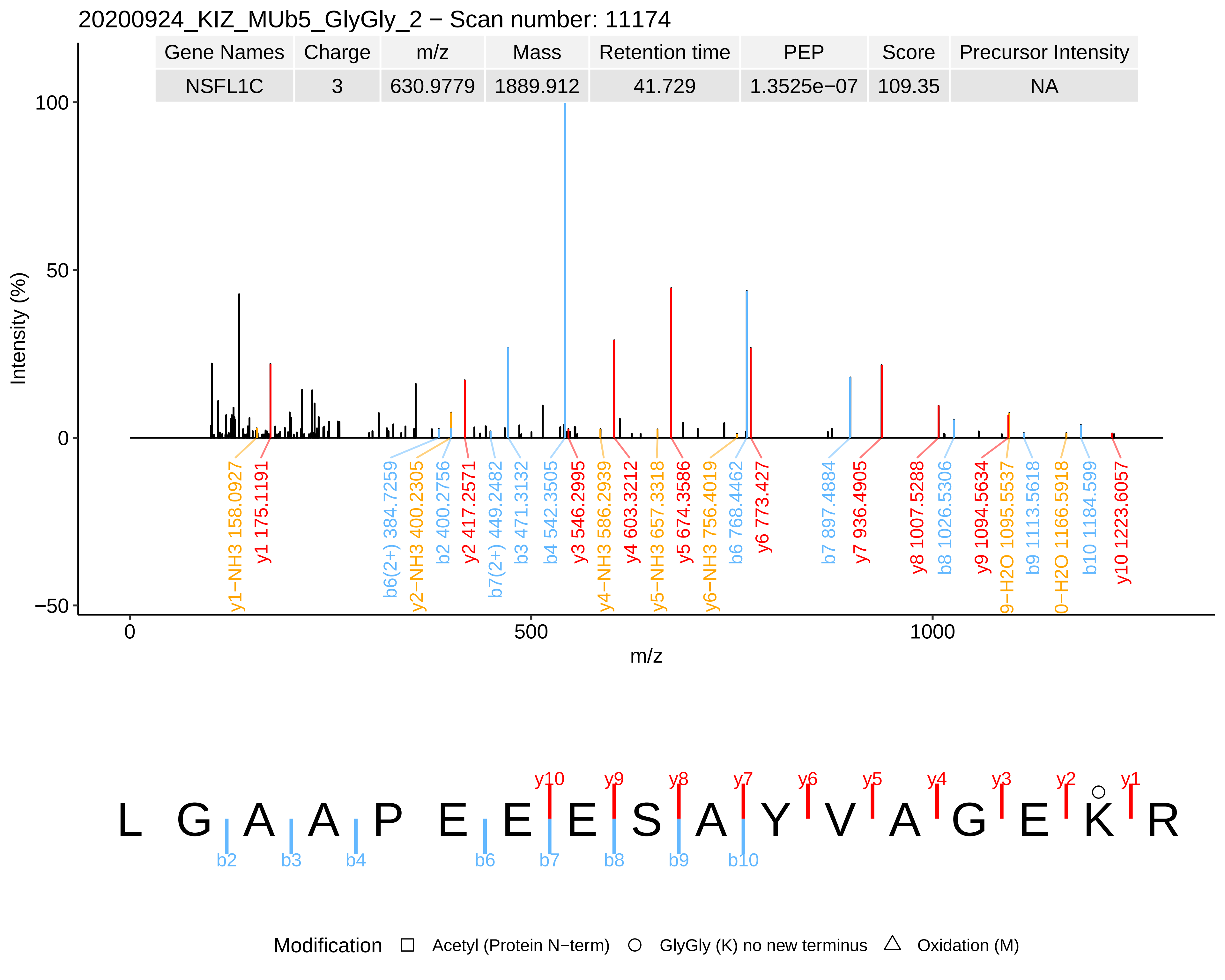

20200924_KIZ_MUb5_GlyGly_2 − Scan number: 11174
Gene Names | Charge | m/z | Mass | Retention time | PEP | Score | Precursor Intensity
NSFL1C | 3 | 630.9779 | 1889.912 | 41.729 | 1.3525e−07 | 109.35 | NA
Intensity (%)
m/z
y1−NH3 158.0927
y1 175.1191
b6(2+) 384.7259
y2−NH3 400.2305
b2 400.2756
y2 417.2571
b7(2+) 449.2482
b3 471.3132
b4 542.3505
y3 546.2995
y4−NH3 586.2939
y4 603.3212
y5−NH3 657.3318
y5 674.3586
y6−NH3 756.4019
b6 768.4462
y6 773.427
b7 897.4884
y7 936.4905
y8 1007.5288
b8 1026.5306
y9 1094.5634
9−H2O 1095.5537
b9 1113.5618
0−H2O 1166.5918
b10 1184.599
y10 1223.6057
L G A A P E E E S A Y V A G E K R
Modification
Acetyl (Protein N−term)
GlyGly (K) no new terminus
Oxidation (M)

20201118_KIZ_MUb6_GlyGly_2 − Scan number: 13309

Gene Names	Charge	m/z	Mass	Retention time	PEP	Score	Precursor Intensity
NSFL1C	3	630.9779	1889.912	44.89	1.3525e−07	109.35	2707992

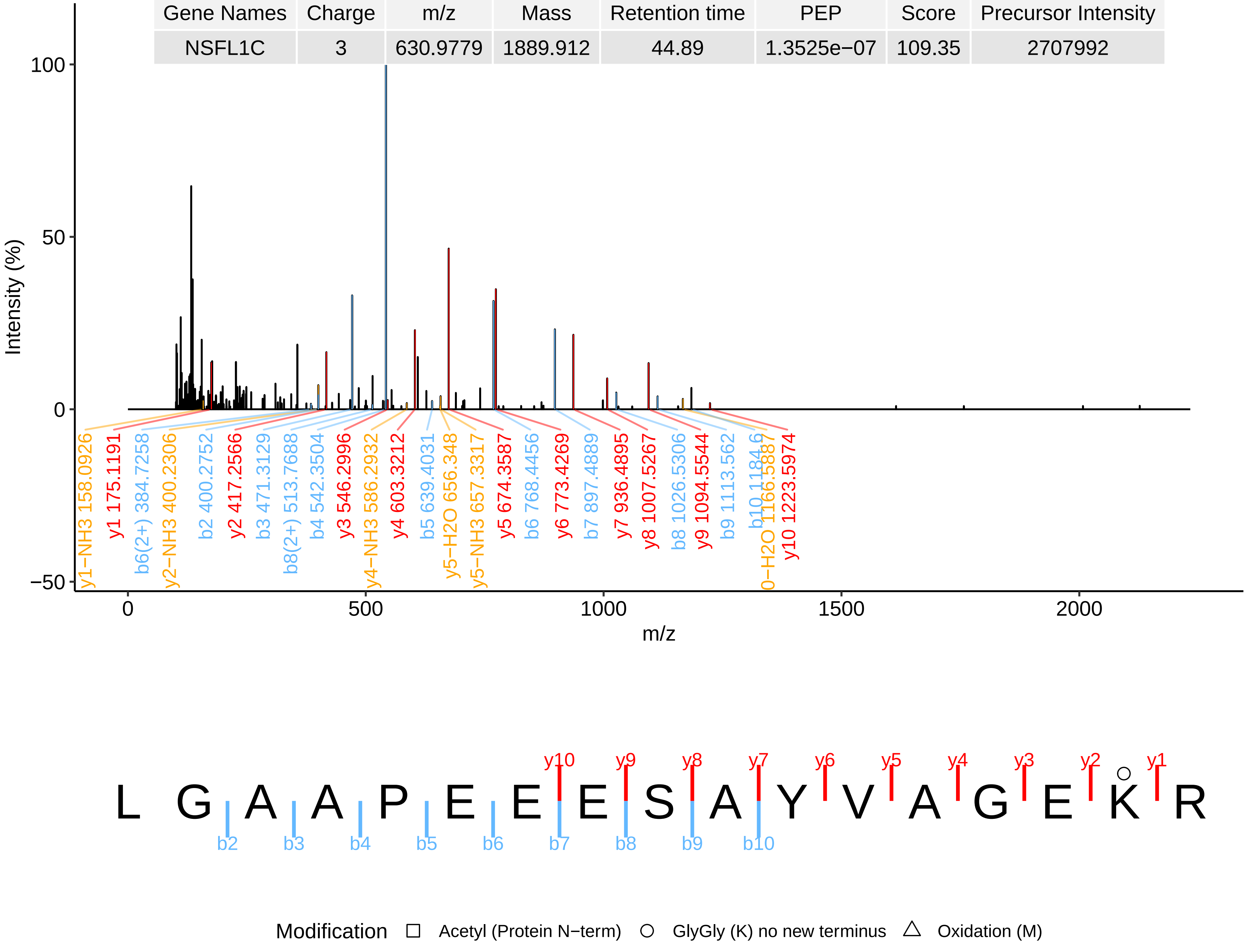

20200921_KIZ_MUb4_GlyGly_10plex − Scan number: 16073

Gene Names	Charge	m/z	Mass	Retention time	PEP	Score	Precursor Intensity
PRKDC	3	546.9493	1637.826	57.643	7.4572e−08	118.24	3593160

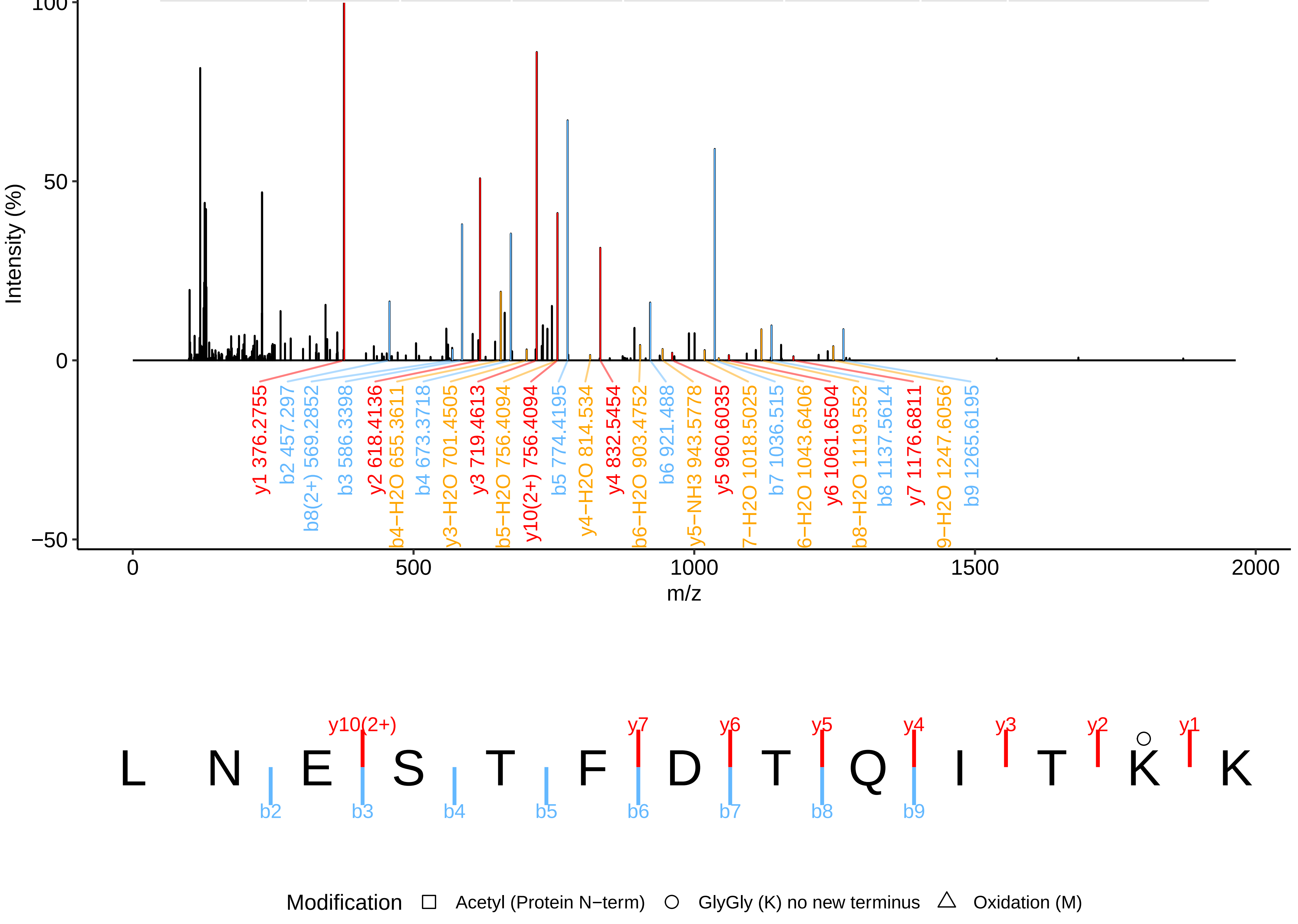

20200921_KIZ_MUb4_GlyGly_10plex_2 − Scan number: 14921

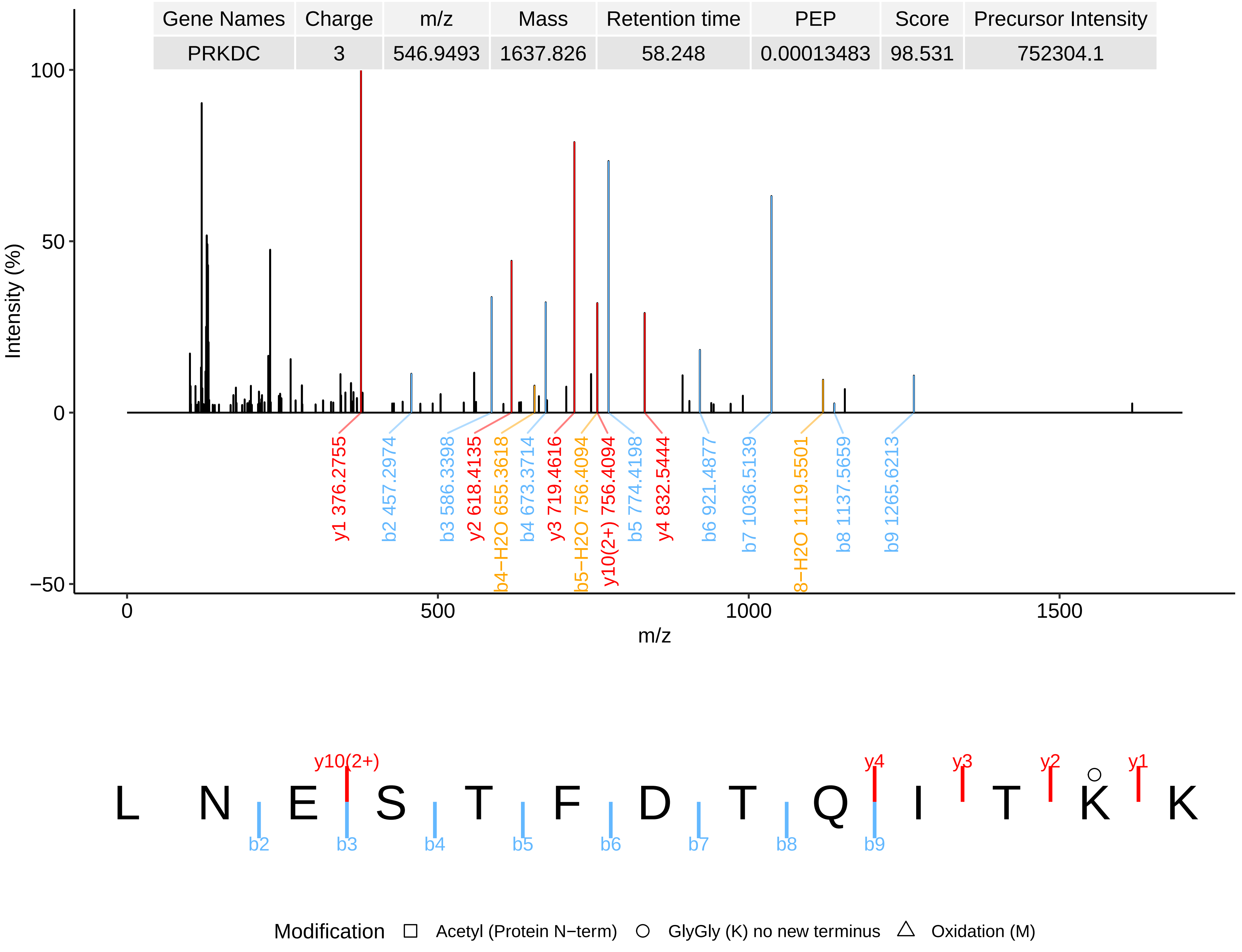

Gene Names	Charge	m/z	Mass	Retention time	PEP	Score	Precursor Intensity
PRKDC	3	546.9493	1637.826	58.248	0.00013483	98.531	752304.1

20200921_KIZ_MUb4_GlyGly_10plex_2 − Scan number: 14945

Gene Names	Charge	m/z	Mass	Retention time	PEP	Score	Precursor Intensity
PRKDC	3	546.9493	1637.826	58.346	5.7893e−05	108.17	2140811

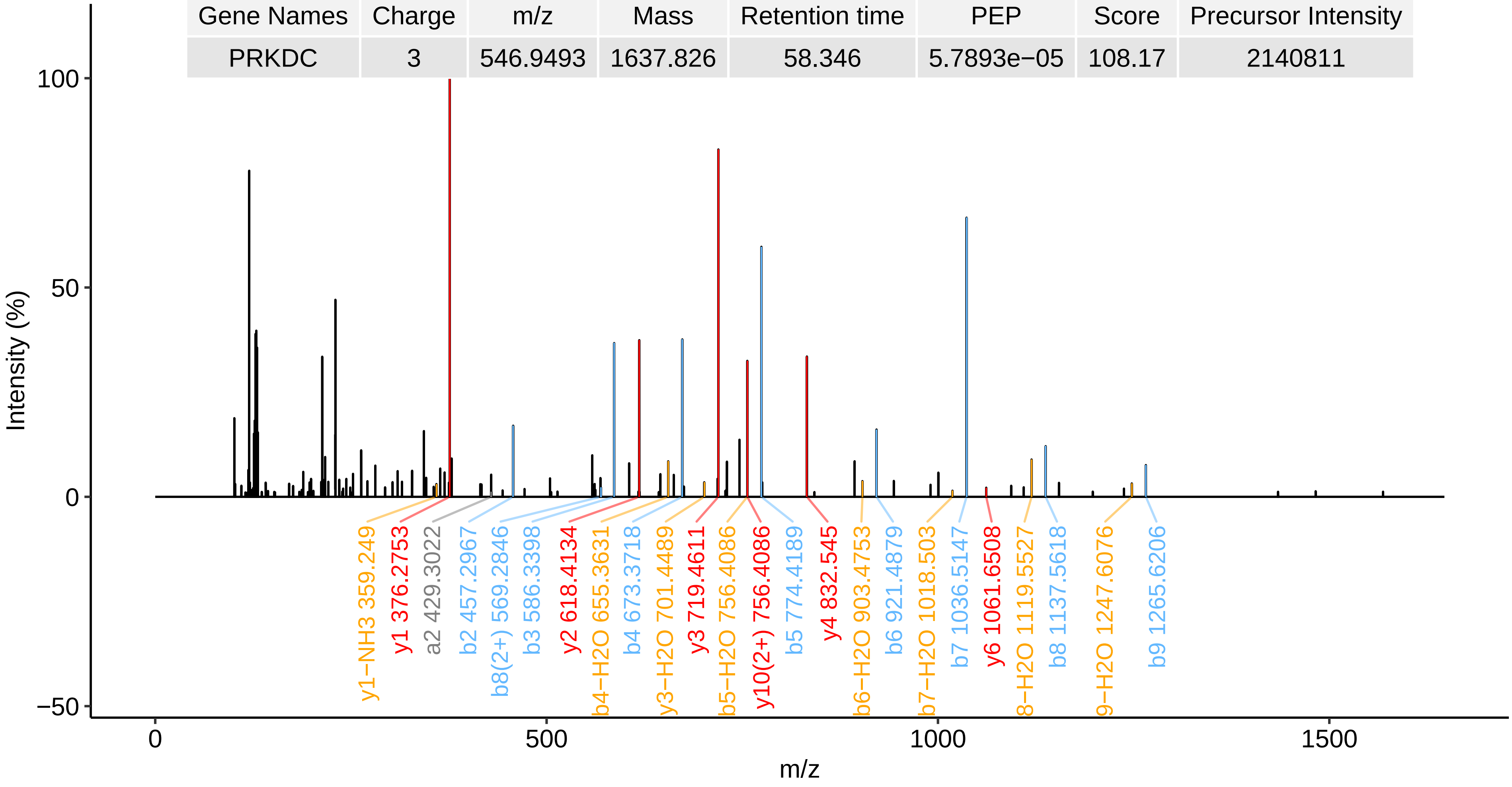

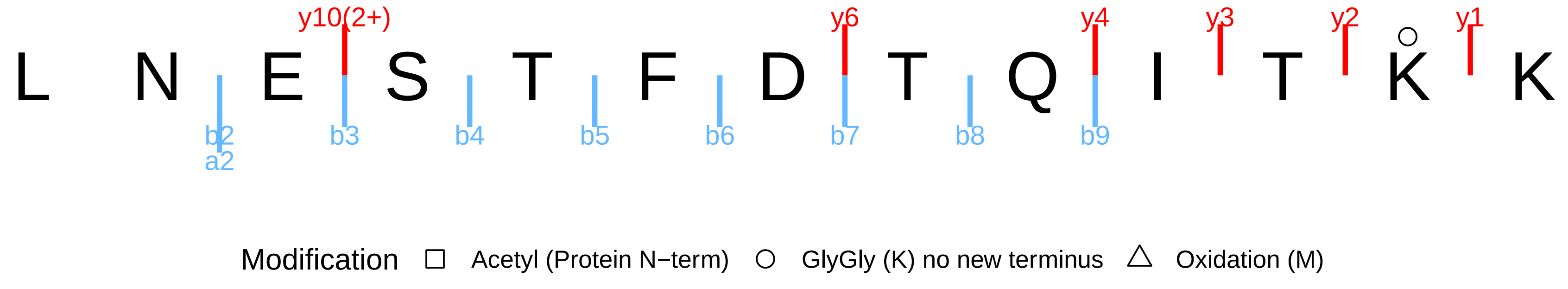

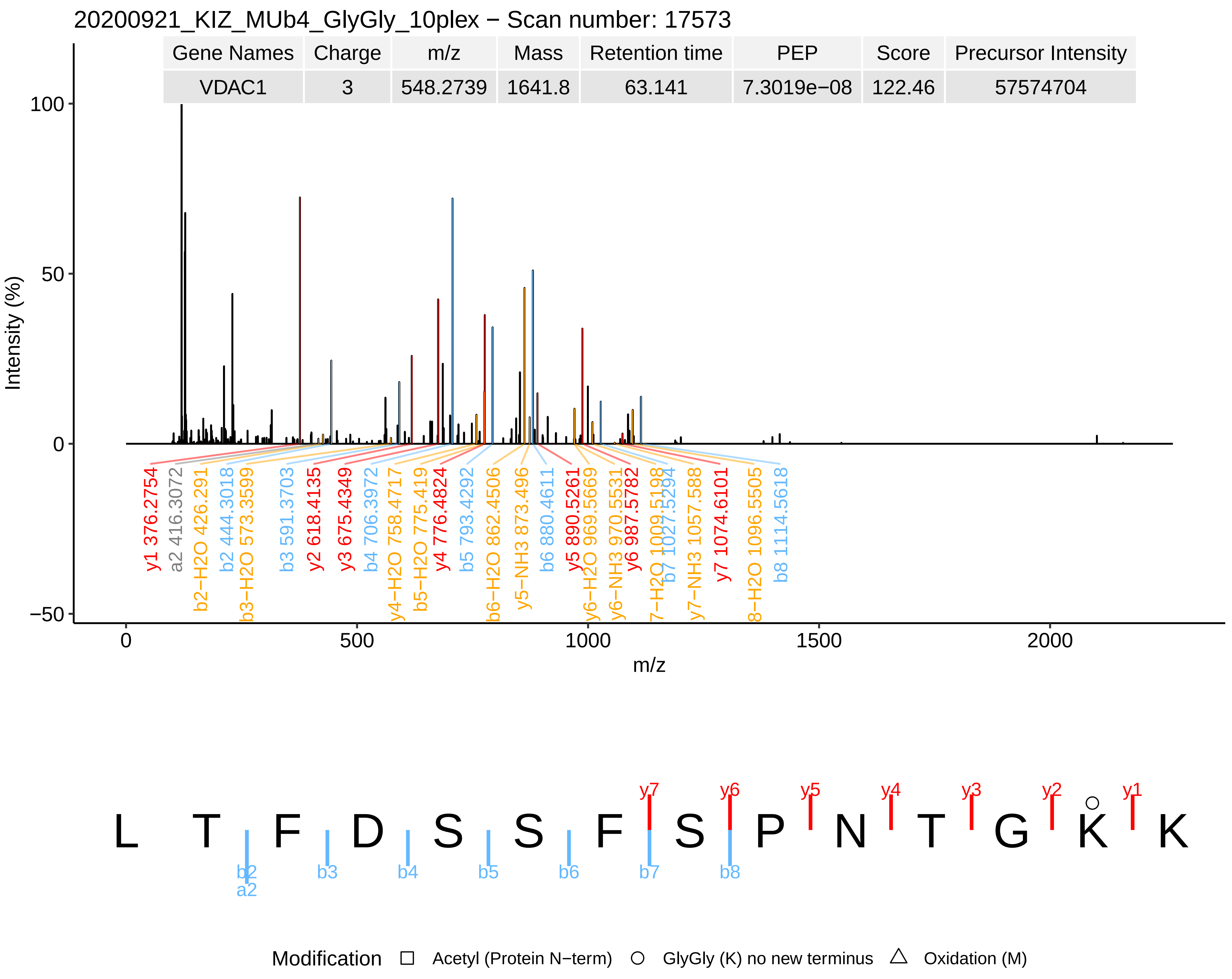
20200921_KIZ_MUb4_GlyGly_10plex − Scan number: 17573
Gene Names	Charge	m/z	Mass	Retention time	PEP	Score	Precursor Intensity
VDAC1	3	548.2739	1641.8	63.141	7.3019e−08	122.46	57574704
Intensity (%)
m/z
y1 376.2754
a2 416.3072
b2−H2O 426.291
b2 444.3018
b3−H2O 573.3599
b3 591.3703
y2 618.4135
y3 675.4349
b4 706.3972
y4−H2O 758.4717
b5−H2O 775.419
y4 776.4824
b5 793.4292
b6−H2O 862.4506
y5−NH3 873.496
b6 880.4611
y5 890.5261
y6−H2O 969.5669
y6−NH3 970.5531
y6 987.5782
b7 1027.5294
y7−NH3 1057.588
y7 1074.6101
b8 1114.5618
L T F D S S F S P N T G K K
Modification
Acetyl (Protein N−term)
GlyGly (K) no new terminus
Oxidation (M)

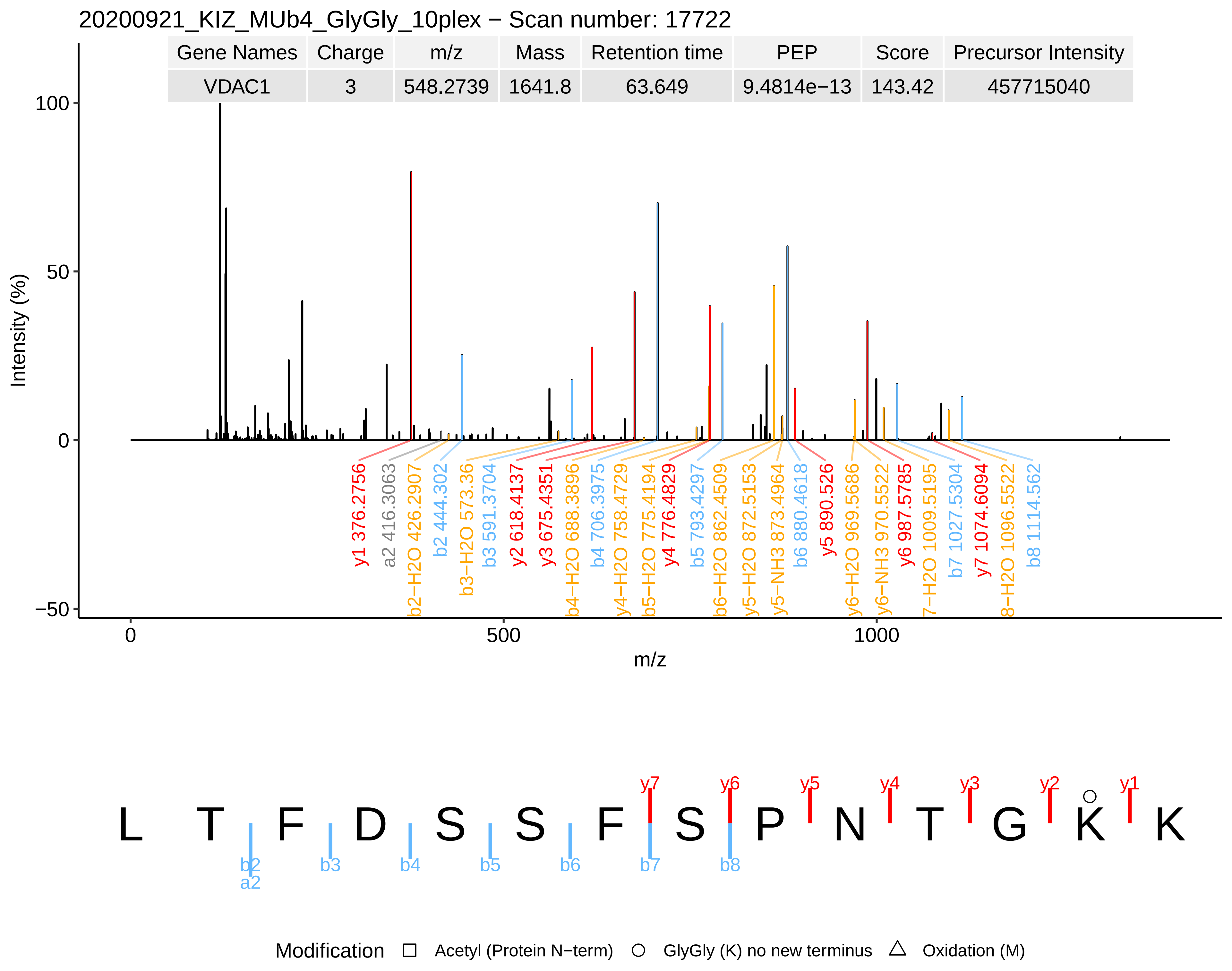
20200921_KIZ_MUb4_GlyGly_10plex − Scan number: 17722
Gene Names | Charge | m/z | Mass | Retention time | PEP | Score | Precursor Intensity
VDAC1 | 3 | 548.2739 | 1641.8 | 63.649 | 9.4814e−13 | 143.42 | 457715040
Intensity (%)
100
50
0
−50
0
500
1000
m/z
y1 376.2756
a2 416.3063
b2−H2O 426.2907
b2 444.302
b3−H2O 573.36
b3 591.3704
y2 618.4137
y3 675.4351
b4−H2O 688.3896
b4 706.3975
y4−H2O 758.4729
b5−H2O 775.4194
y4 776.4829
b5 793.4297
b6−H2O 862.4509
y5−H2O 872.5153
y5−NH3 873.4964
b6 880.4618
y5 890.526
y6−H2O 969.5686
y6−NH3 970.5522
y6 987.5785
7−H2O 1009.5195
b7 1027.5304
y7 1074.6094
8−H2O 1096.5522
b8 1114.562
L T F D S S F S P N T G K K
y7 y6 y5 y4 y3 y2 y1
b2 b3 b4 b5 b6 b7 b8
a2
Modification
Acetyl (Protein N−term)
GlyGly (K) no new terminus
Oxidation (M)

20200921_KIZ_MUb4_GlyGly_10plex − Scan number: 17865

Gene Names	Charge	m/z	Mass	Retention time	PEP	Score	Precursor Intensity
VDAC1	3	548.2739	1641.8	64.16	9.1273e−19	150.49	50578632

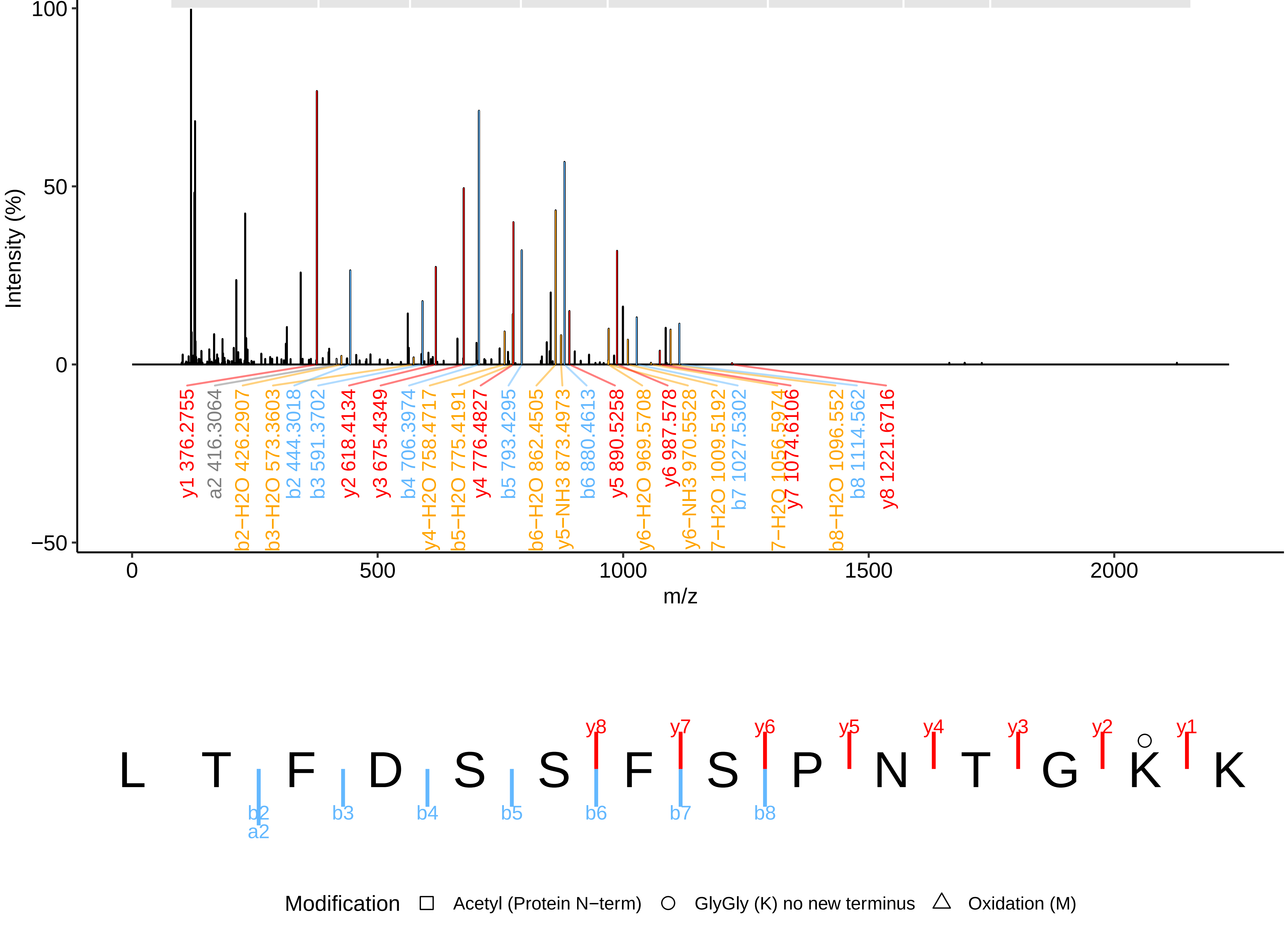

20200921_KIZ_MUb4_GlyGly_10plex − Scan number: 17742

Gene Names	Charge	m/z	Mass	Retention time	PEP	Score	Precursor Intensity
VDAC1	4	411.4572	1641.8	63.715	3.1579e−06	111.79	3376154

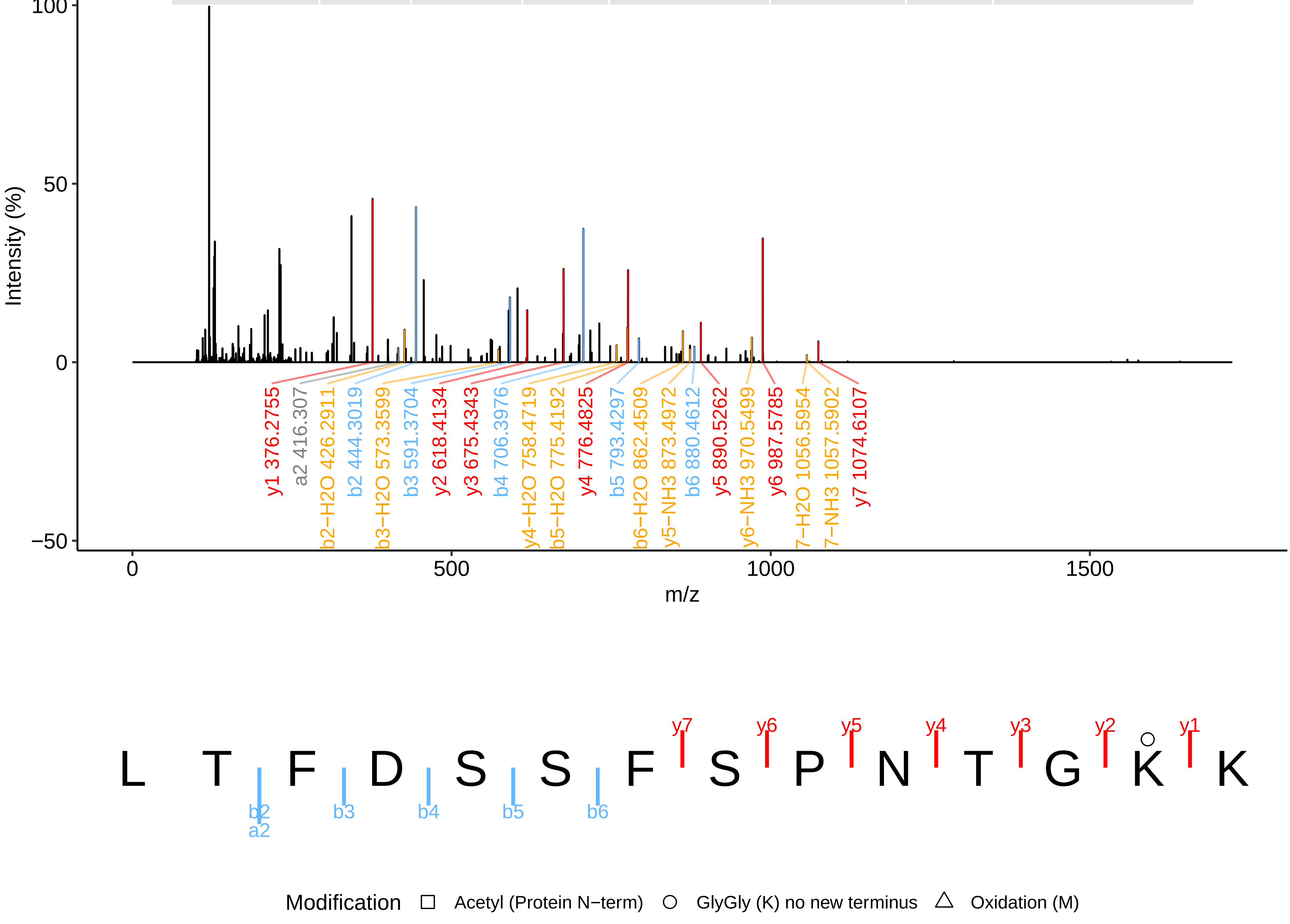

20200921_KIZ_MUb4_GlyGly_10plex − Scan number: 18005

Gene Names	Charge	m/z	Mass	Retention time	PEP	Score	Precursor Intensity
VDAC1	3	548.2739	1641.8	64.666	1.4552e−12	139.78	17401000

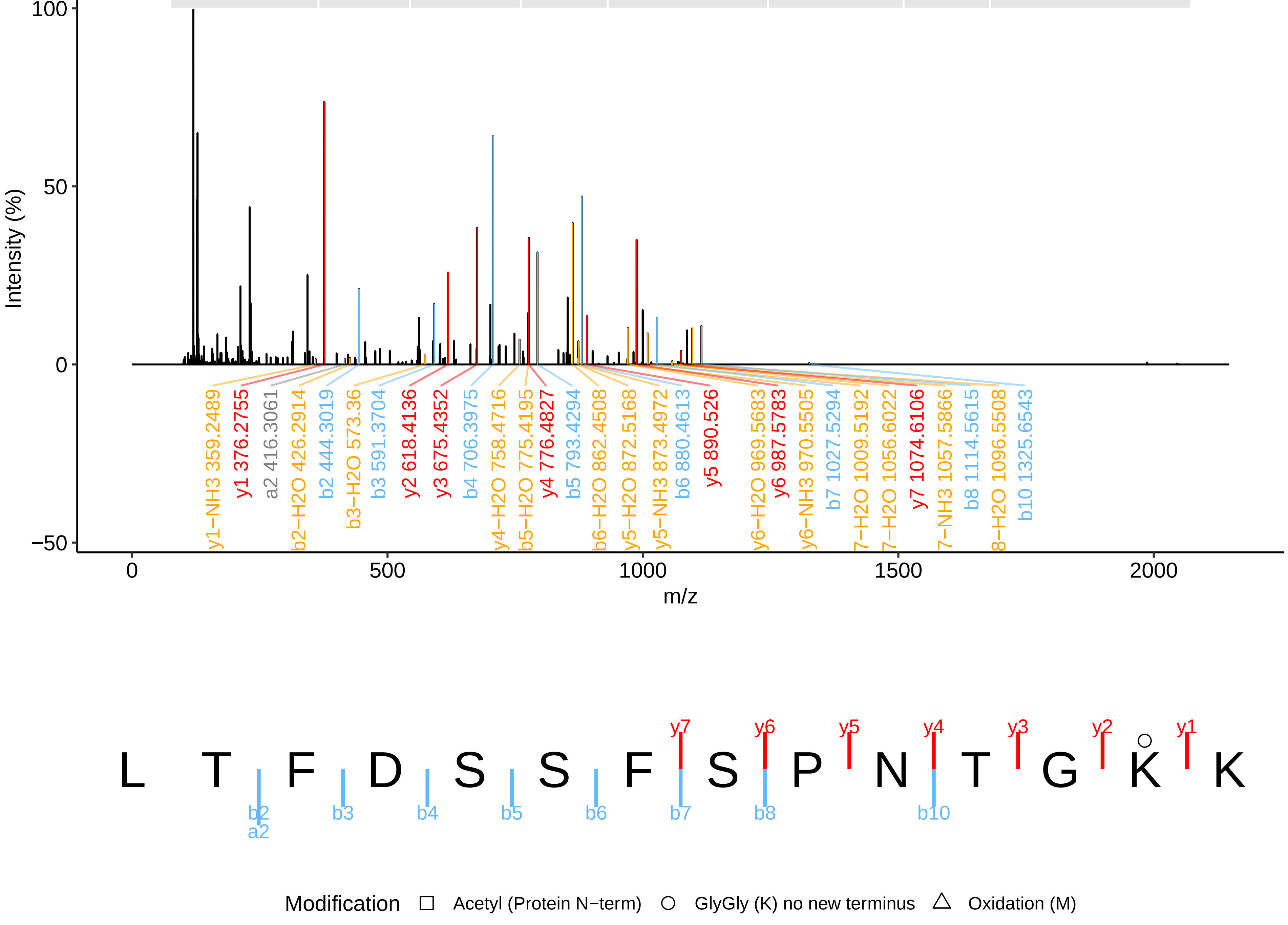

20200921_KIZ_MUb4_GlyGly_10plex − Scan number: 18143

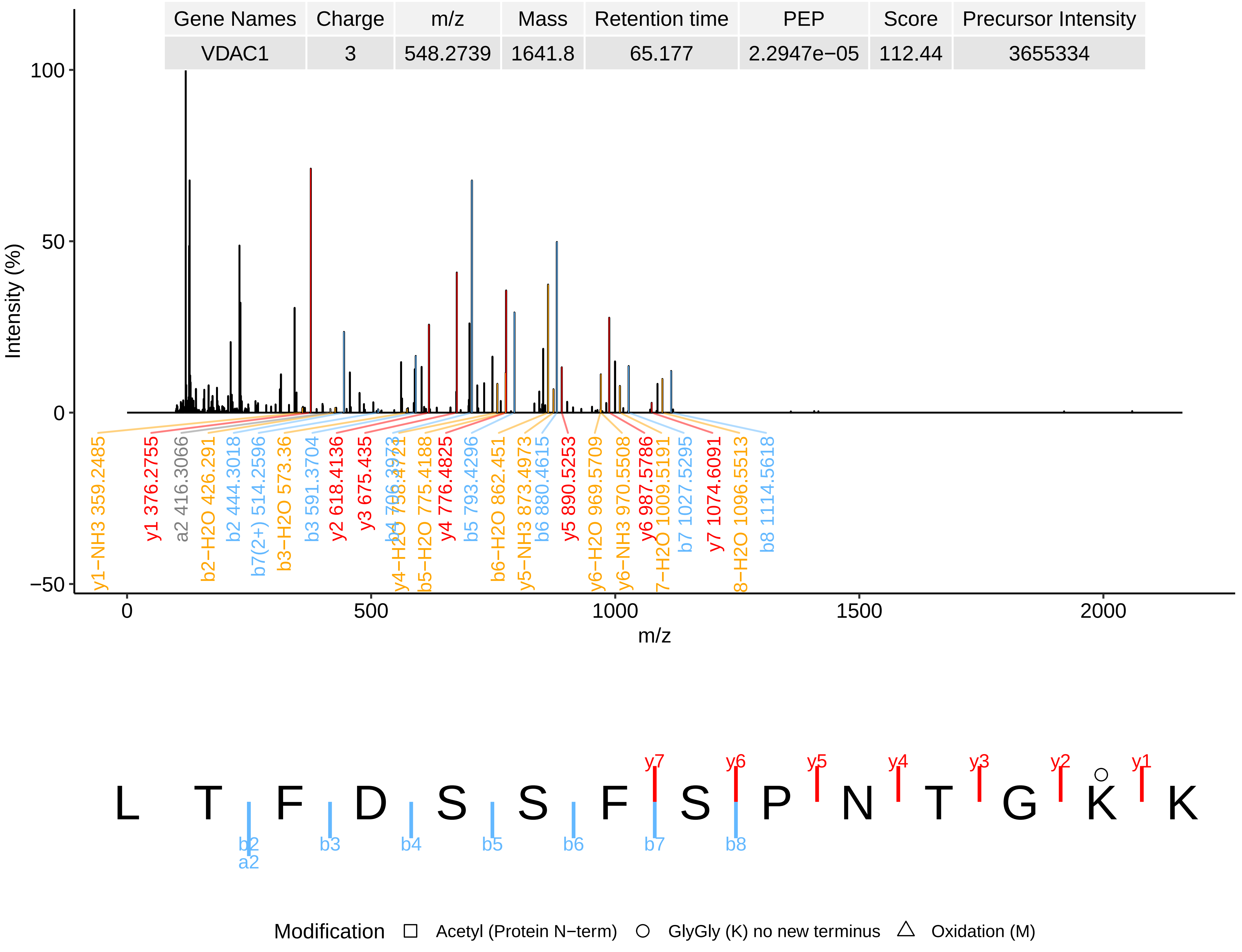

20200921_KIZ_MUb4_GlyGly_10plex − Scan number: 18278

Gene Names	Charge	m/z	Mass	Retention time	PEP	Score	Precursor Intensity
VDAC1	3	548.2739	1641.8	65.687	1.3304e−05	113.69	8488634

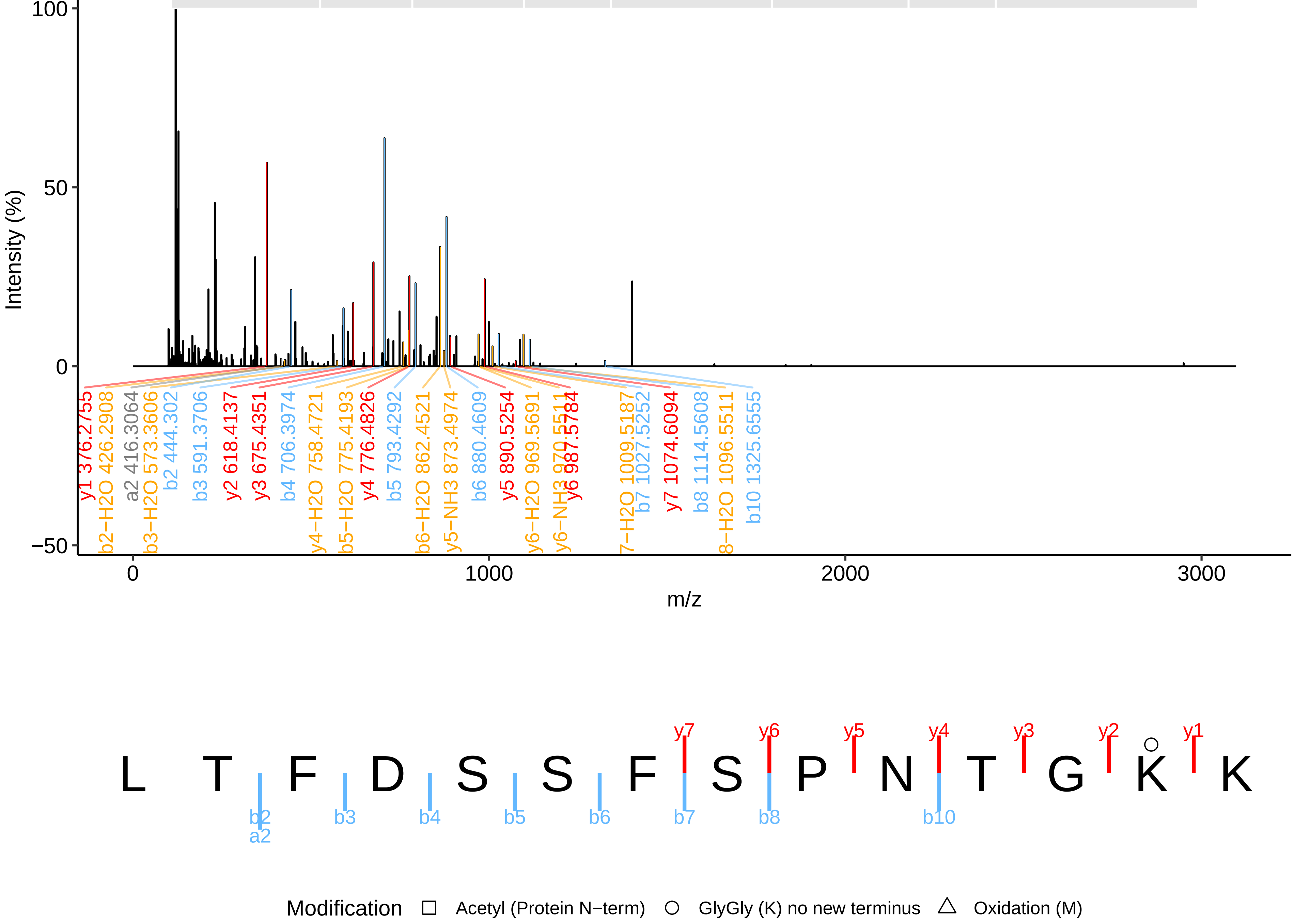

20200921_KIZ_MUb4_GlyGly_10plex − Scan number: 18408

Gene Names	Charge	m/z	Mass	Retention time	PEP	Score	Precursor Intensity
VDAC1	3	548.2739	1641.8	66.191	7.5015e−05	105.65	1669761

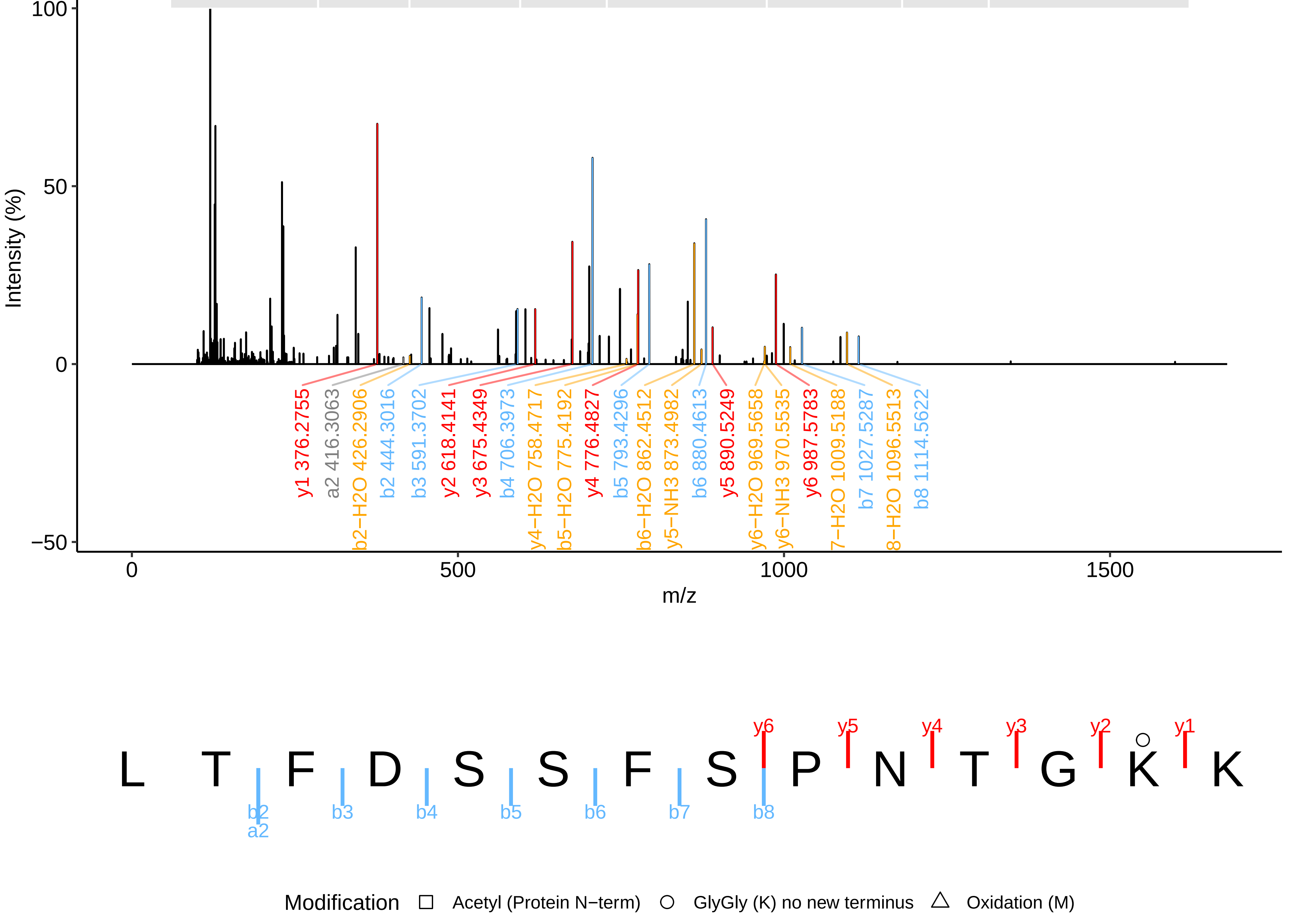

20200921_KIZ_MUb4_GlyGly_10plex − Scan number: 18544

Gene Names	Charge	m/z	Mass	Retention time	PEP	Score	Precursor Intensity
VDAC1	3	548.2739	1641.8	66.726	0.00018316	98.942	4121873

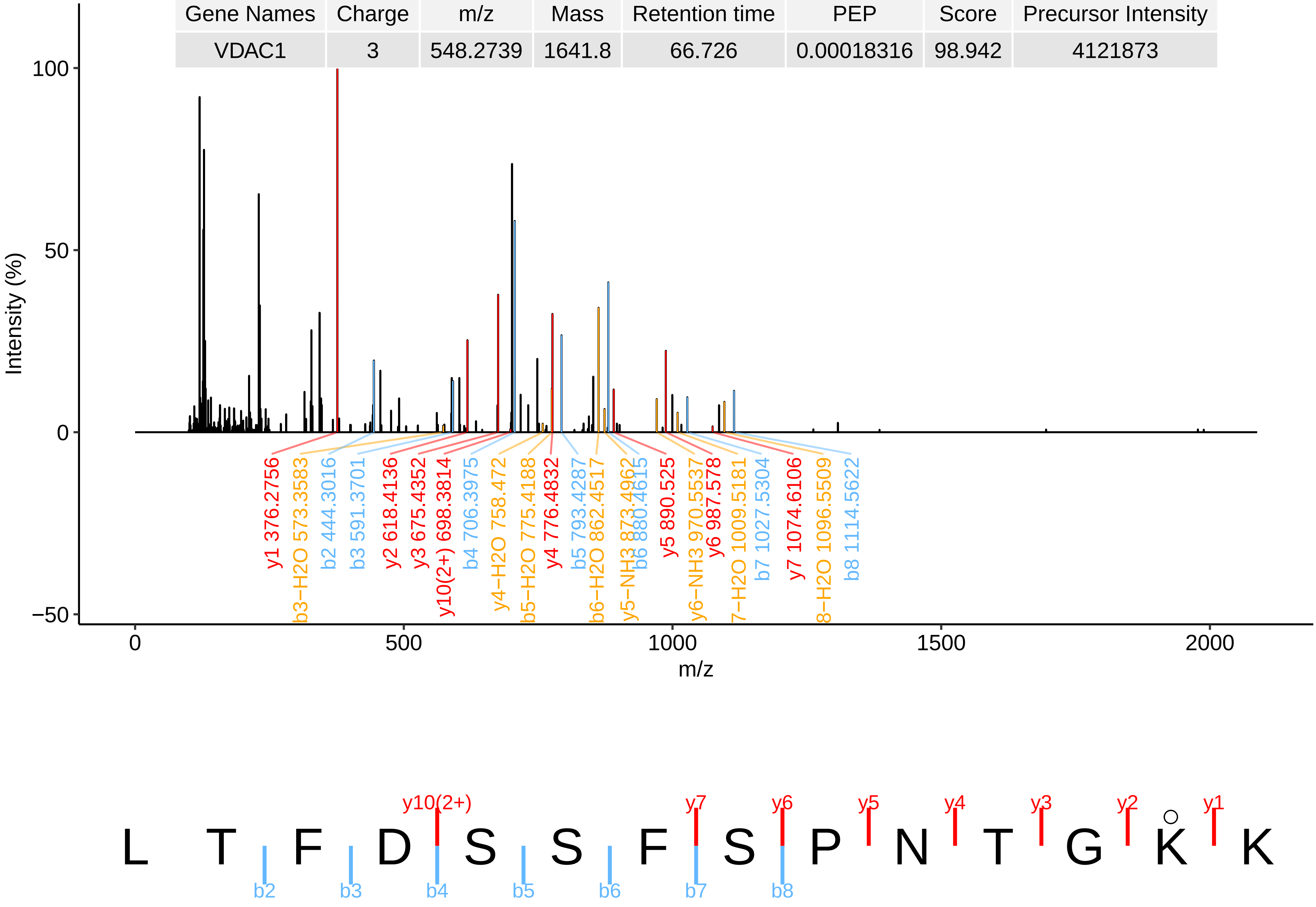

Modification □ Acetyl (Protein N−term) ○ GlyGly (K) no new terminus △ Oxidation (M)

20200921_KIZ_MUb4_GlyGly_10plex − Scan number: 18735

Gene Names	Charge	m/z	Mass	Retention time	PEP	Score	Precursor Intensity
VDAC1	3	548.2739	1641.8	67.446	0.0012189	92.295	NA

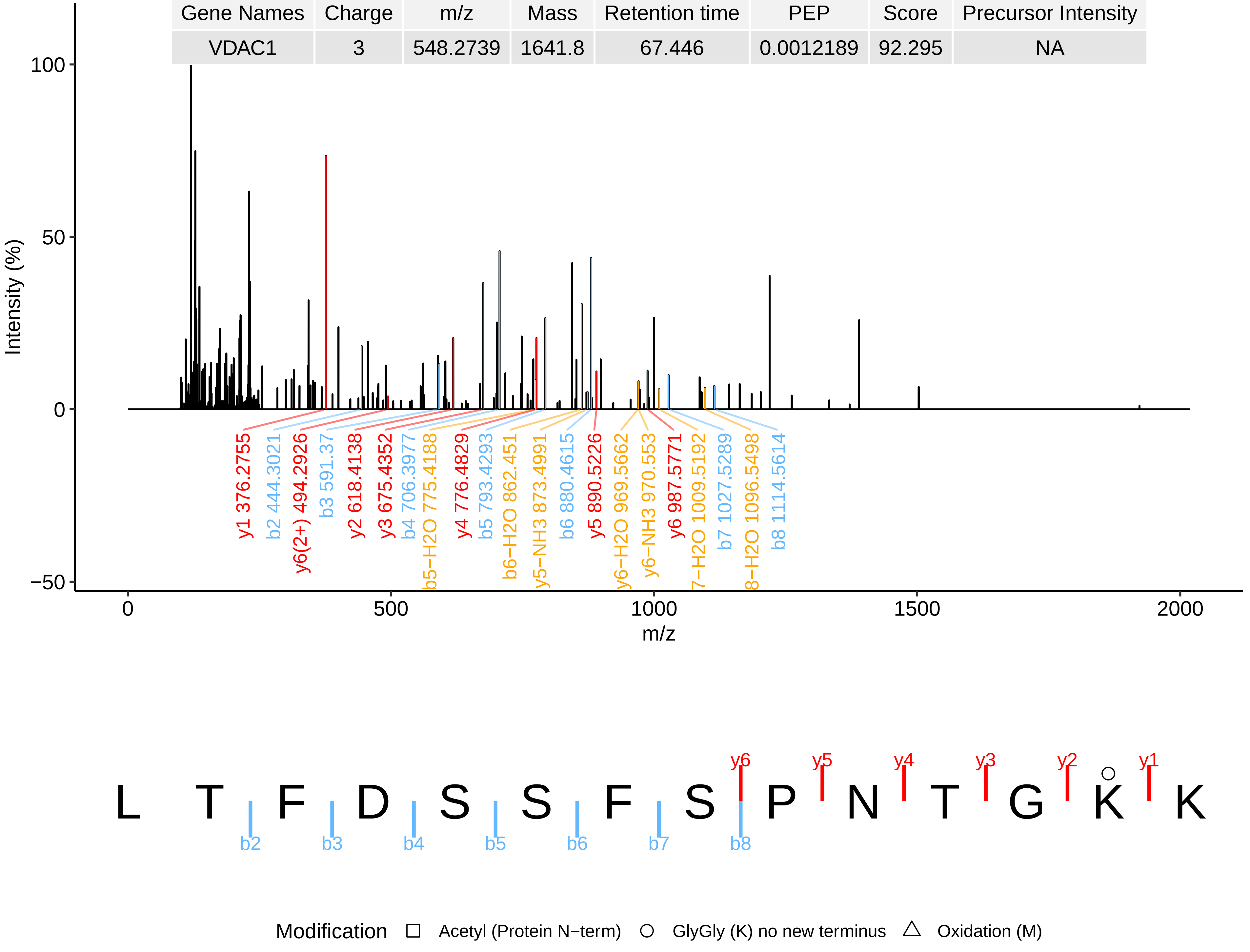

20200921_KIZ_MUb4_GlyGly_10plex_2 − Scan number: 16330

Gene Names	Charge	m/z	Mass	Retention time	PEP	Score	Precursor Intensity
VDAC1	3	548.2739	1641.8	63.749	1.9207e−08	130.1	5331756

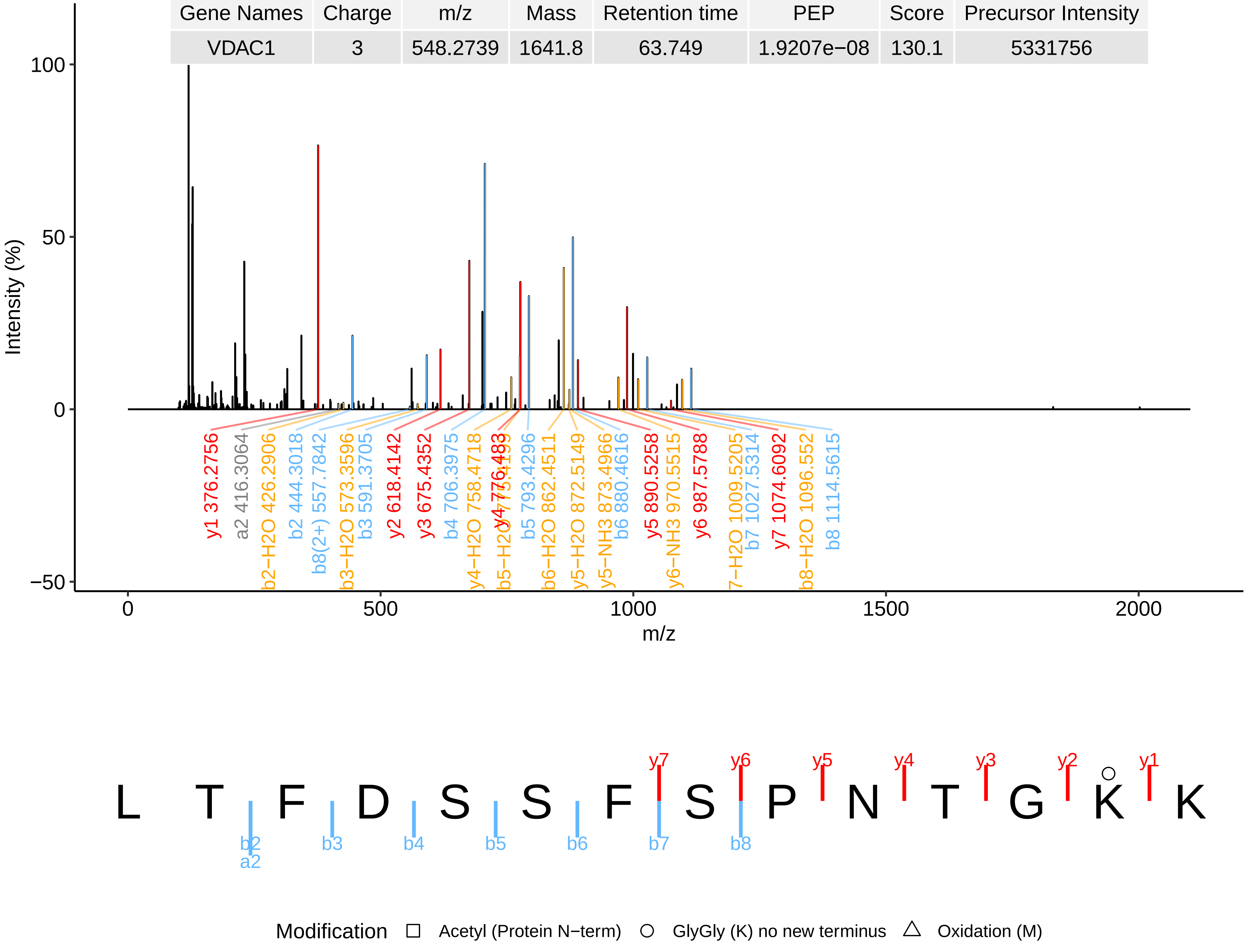

20200921_KIZ_MUb4_GlyGly_10plex_2 − Scan number: 16355

Gene Names	Charge	m/z	Mass	Retention time	PEP	Score	Precursor Intensity
VDAC1	3	548.2739	1641.8	63.848	4.8017e−13	146.79	5022428

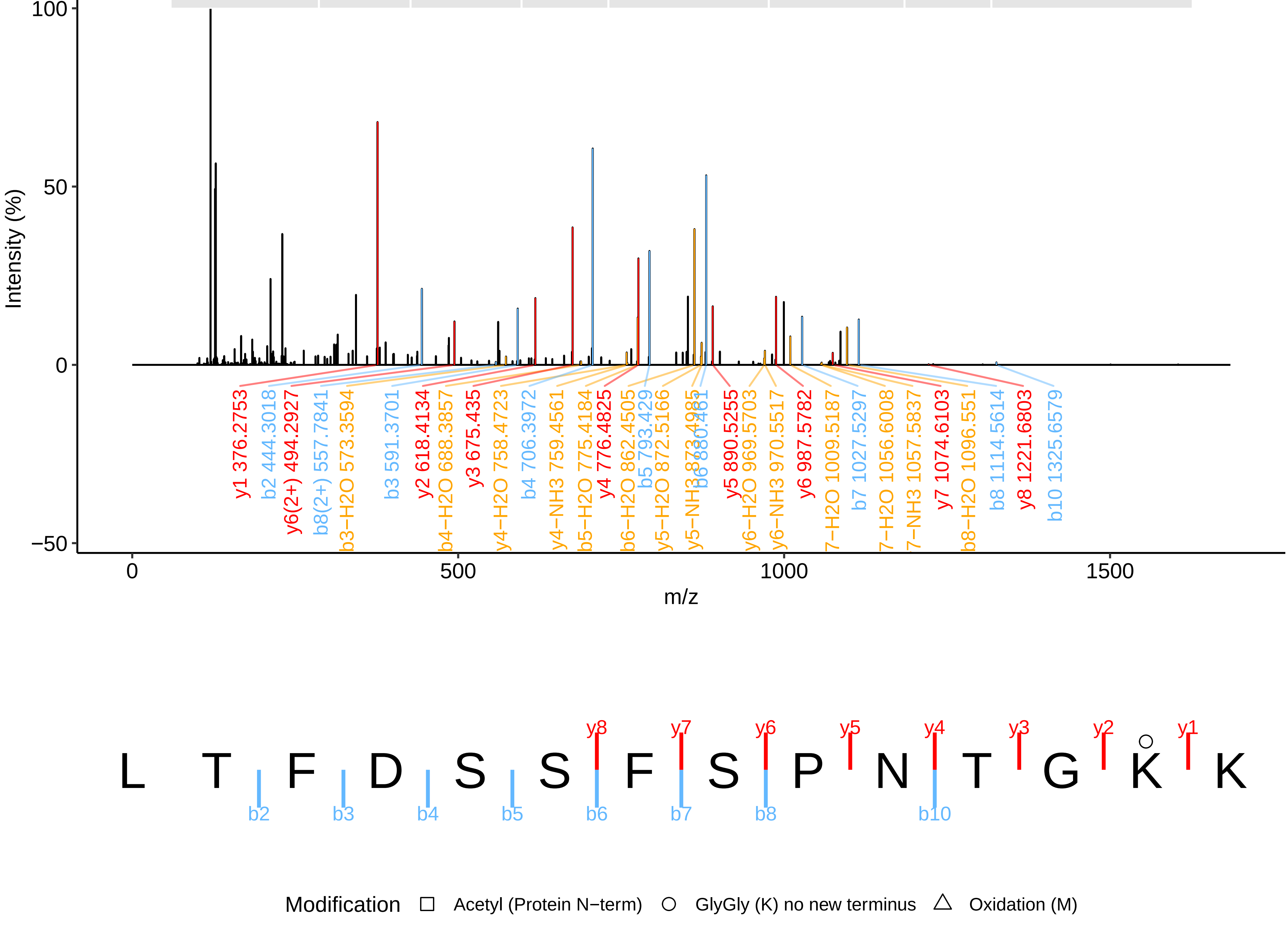

20200921_KIZ_MUb4_GlyGly_10plex_2 − Scan number: 16485

Gene Names	Charge	m/z	Mass	Retention time	PEP	Score	Precursor Intensity
VDAC1	3	548.2739	1641.8	64.358	9.4814e−13	143.42	8145892

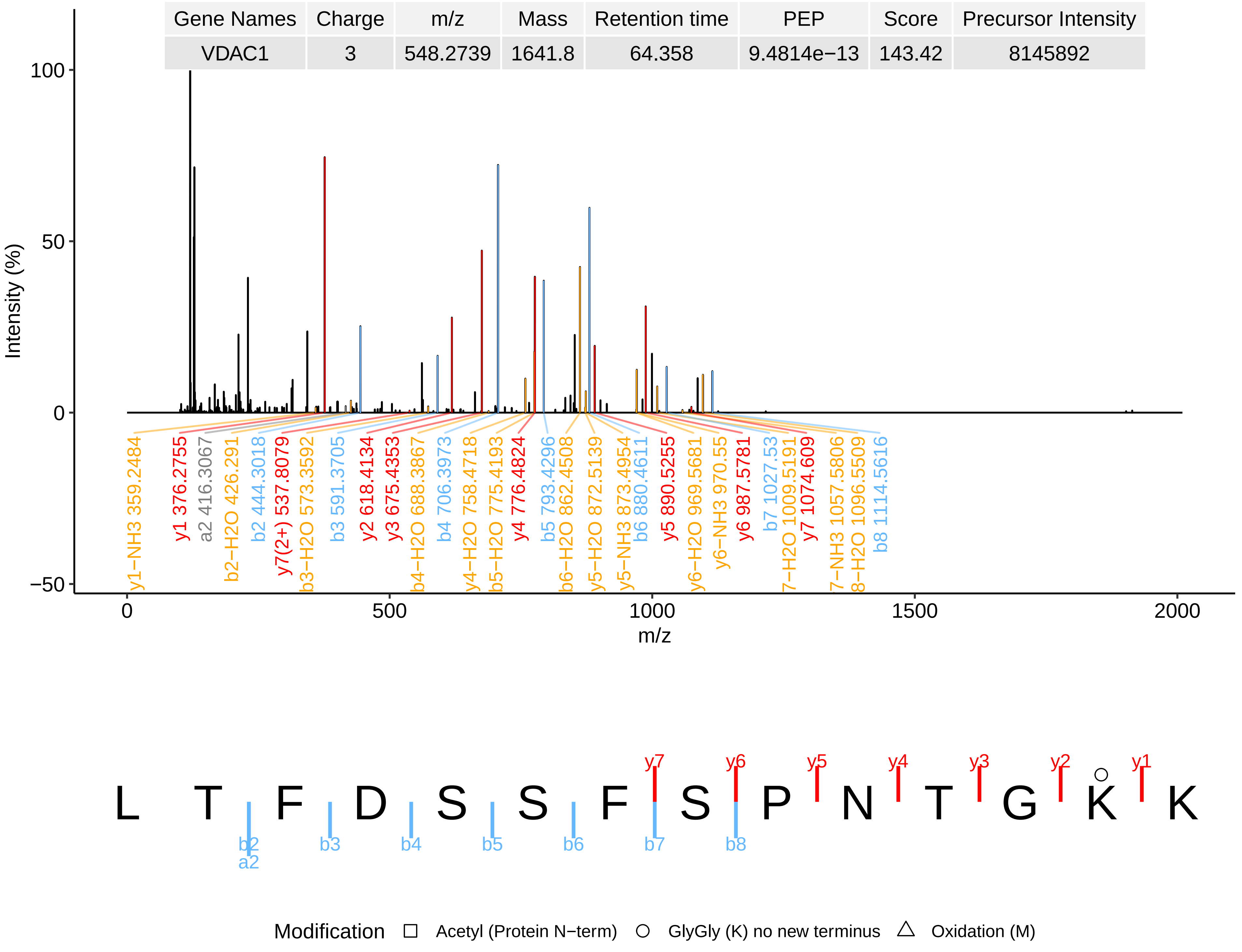

20200921_KIZ_MUb4_GlyGly_10plex_2 − Scan number: 16351

Gene Names	Charge	m/z	Mass	Retention time	PEP	Score	Precursor Intensity
VDAC1	4	411.4572	1641.8	63.832	4.8506e−06	109.84	NA

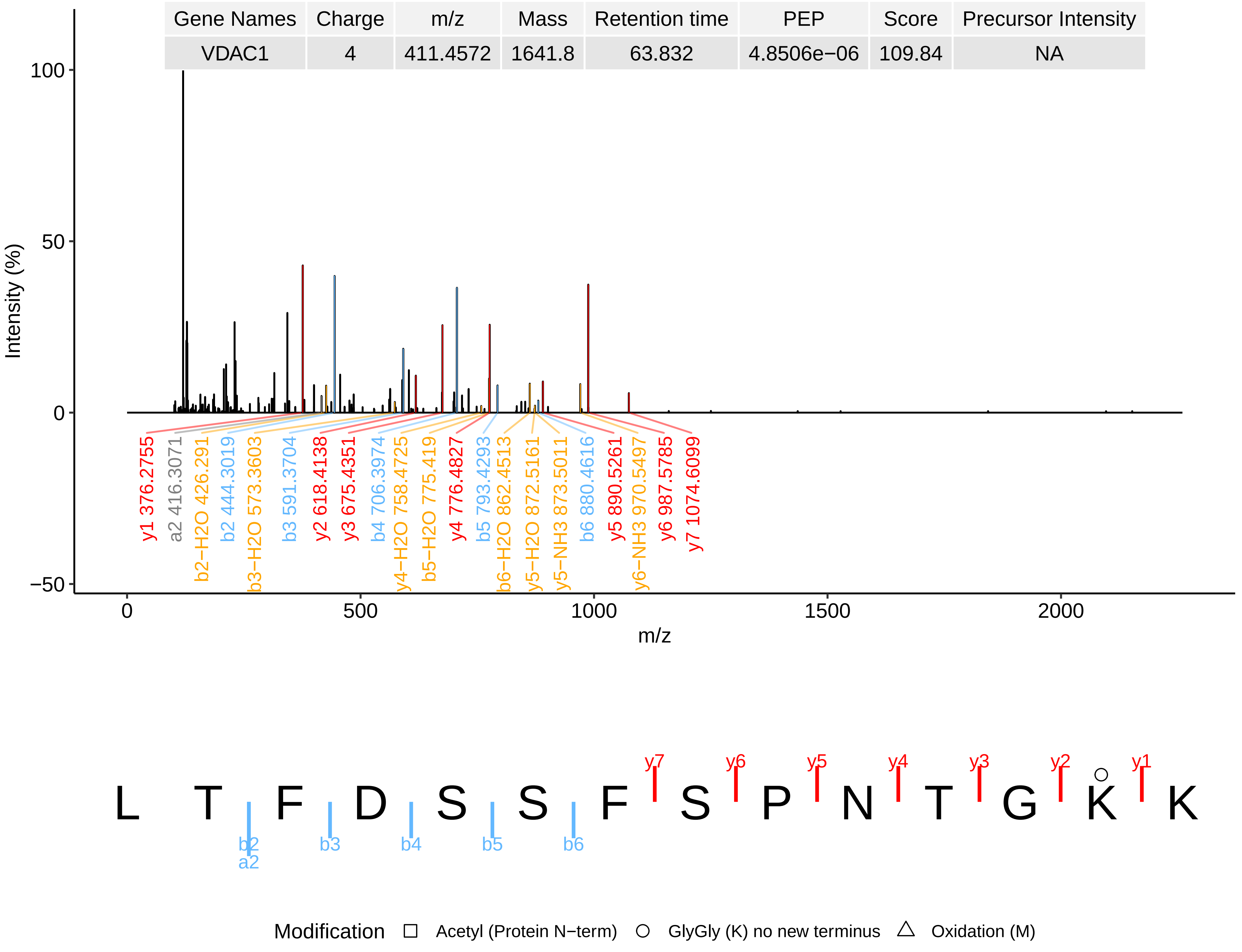

20200921_KIZ_MUb4_GlyGly_10plex_2 − Scan number: 16401

Gene Names	Charge	m/z	Mass	Retention time	PEP	Score	Precursor Intensity
VDAC1	2	821.9072	1641.8	64.033	4.3108e−21	172.34	2874664

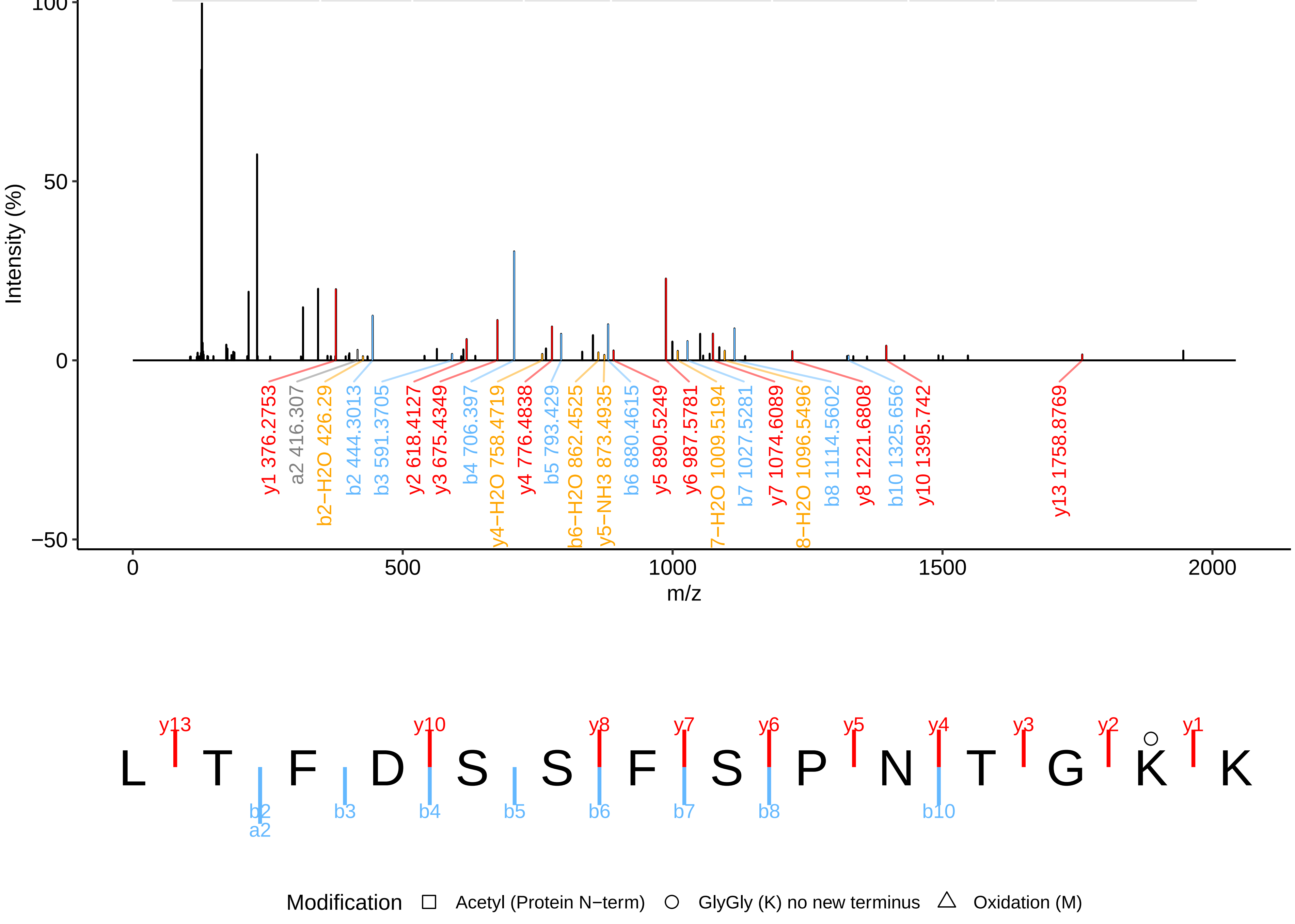

20200921_KIZ_MUb4_GlyGly_10plex_2 − Scan number: 16610

Gene Names	Charge	m/z	Mass	Retention time	PEP	Score	Precursor Intensity
VDAC1	3	548.2739	1641.8	64.864	0.00015367	101.53	727299.8

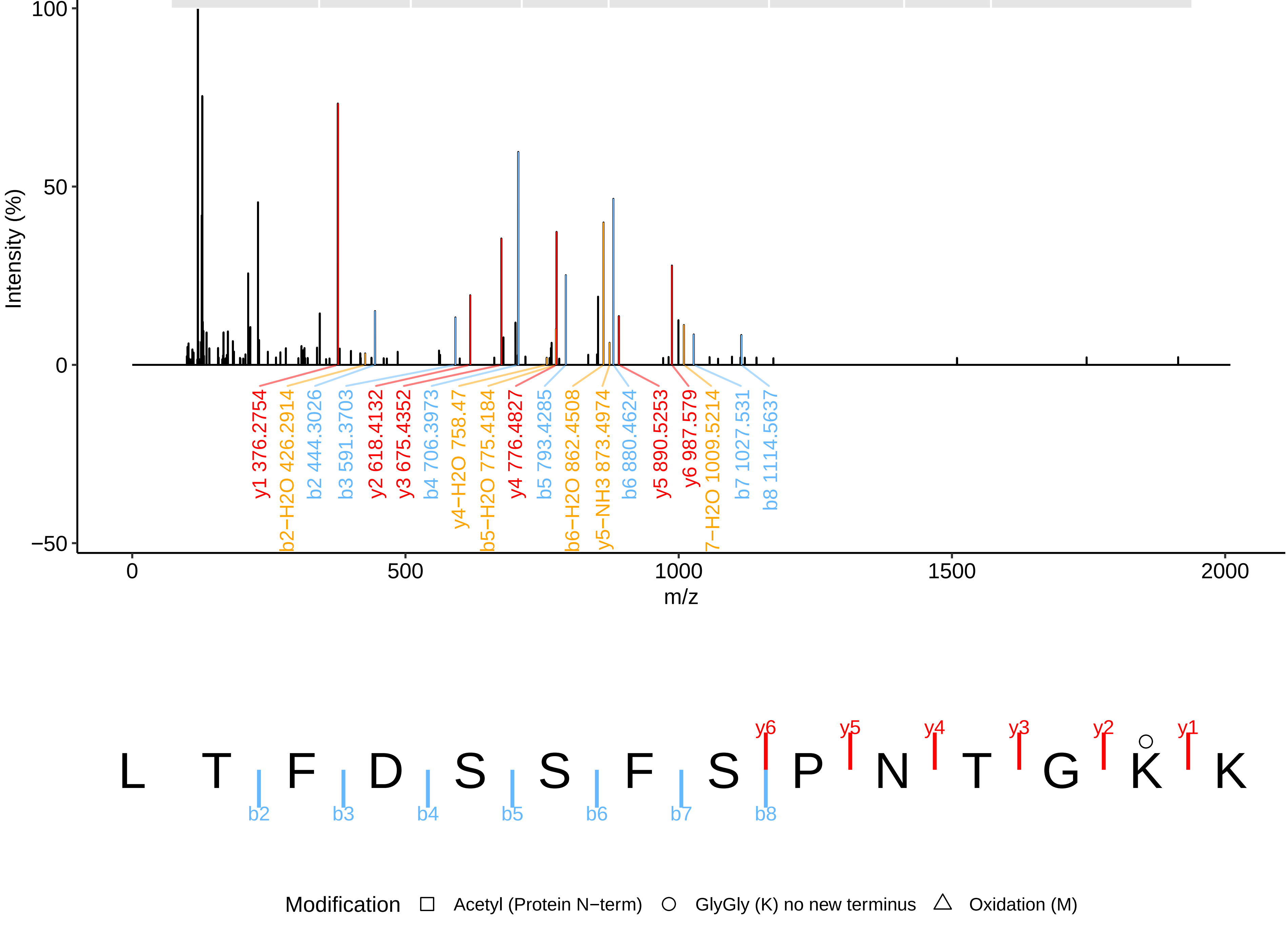

20200924_KIZ_MUb5_GlyGly_1 − Scan number: 16780

Gene Names	Charge	m/z	Mass	Retention time	PEP	Score	Precursor Intensity
VDAC1	3	548.2739	1641.8	54.258	2.4592e−10	132.79	25547968

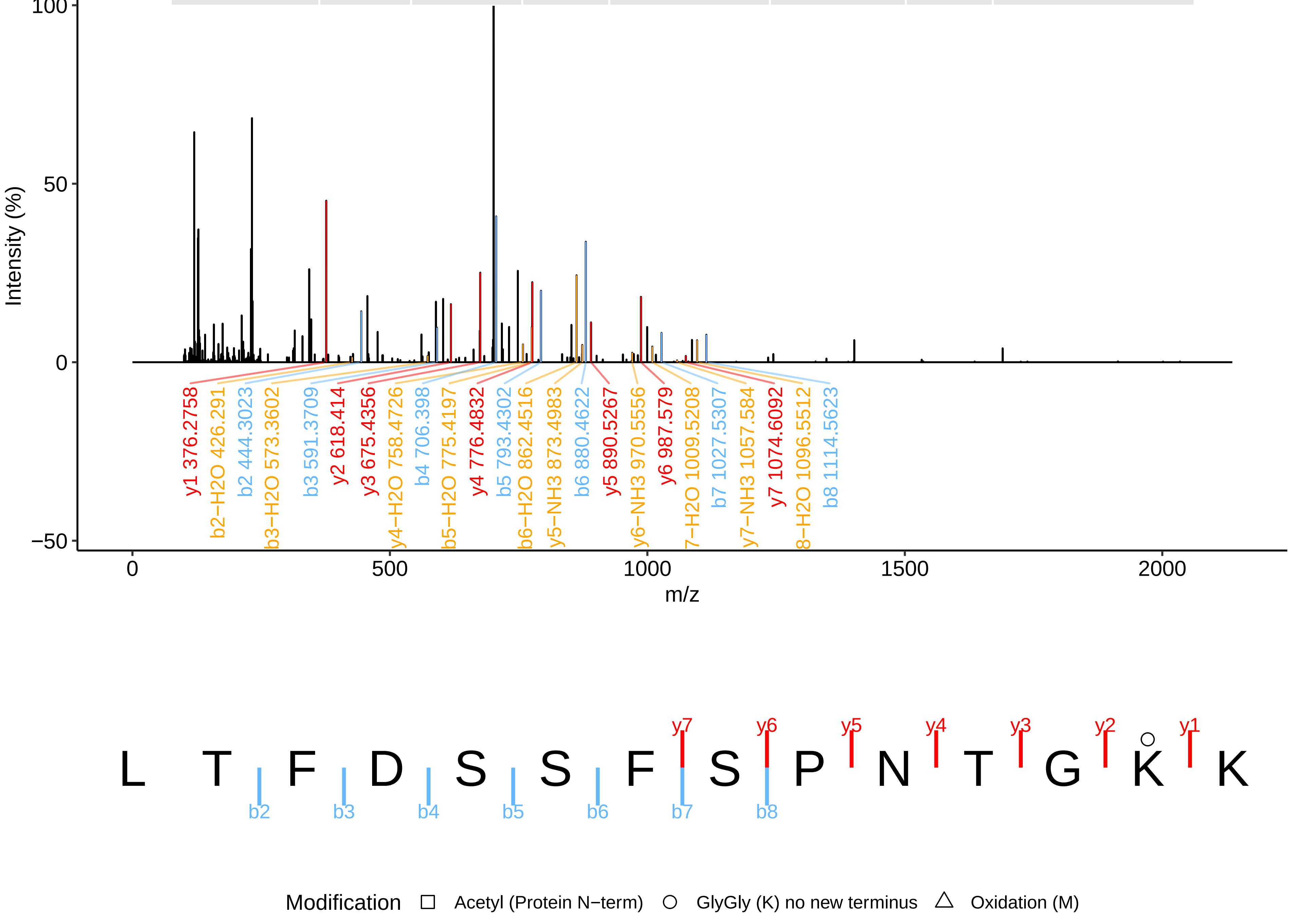

20200924_KIZ_MUb5_GlyGly_1 − Scan number: 16785

Gene Names	Charge	m/z	Mass	Retention time	PEP	Score	Precursor Intensity
VDAC1	3	548.2739	1641.8	54.272	0.0031889	86.262	14839898

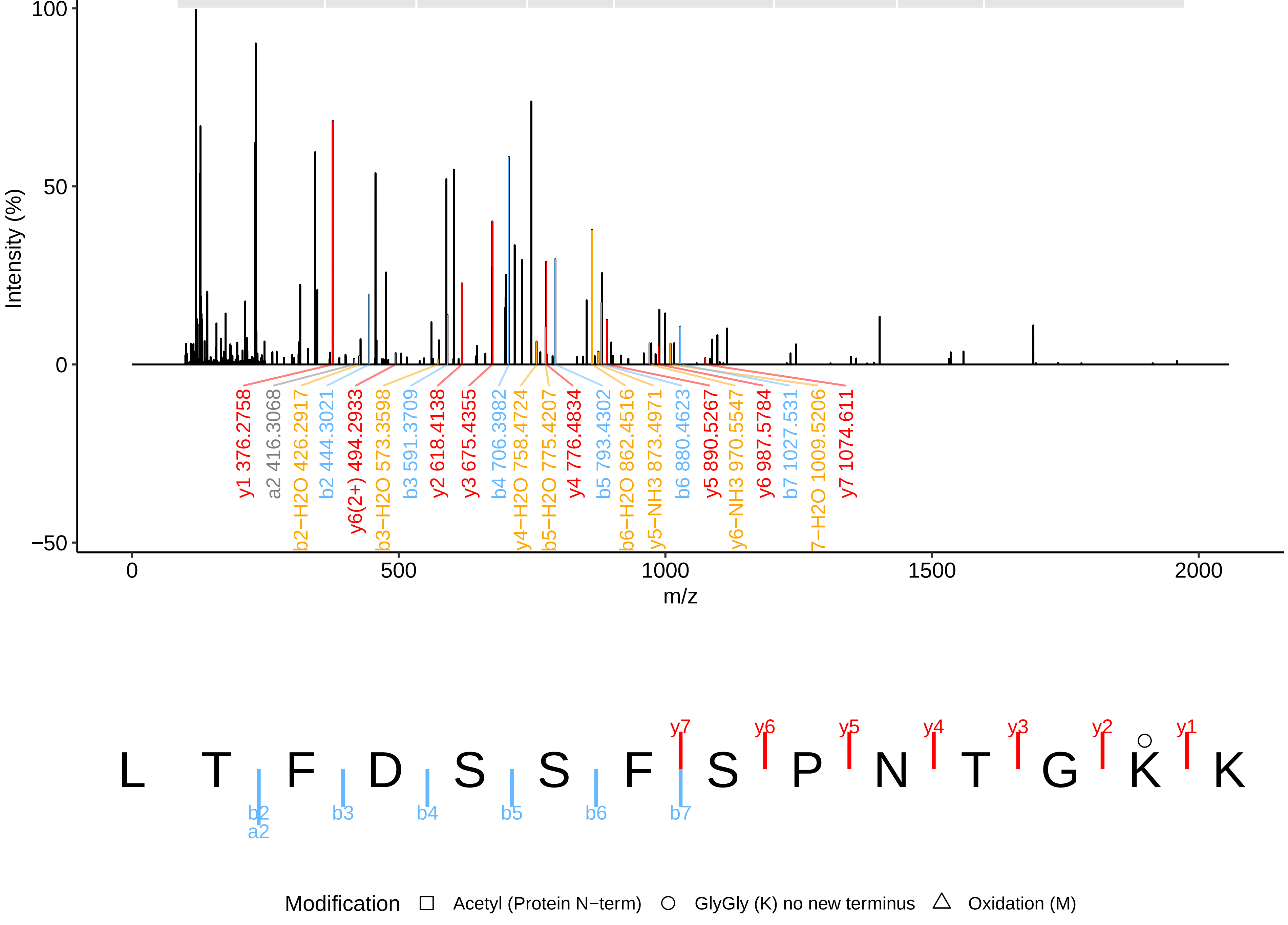

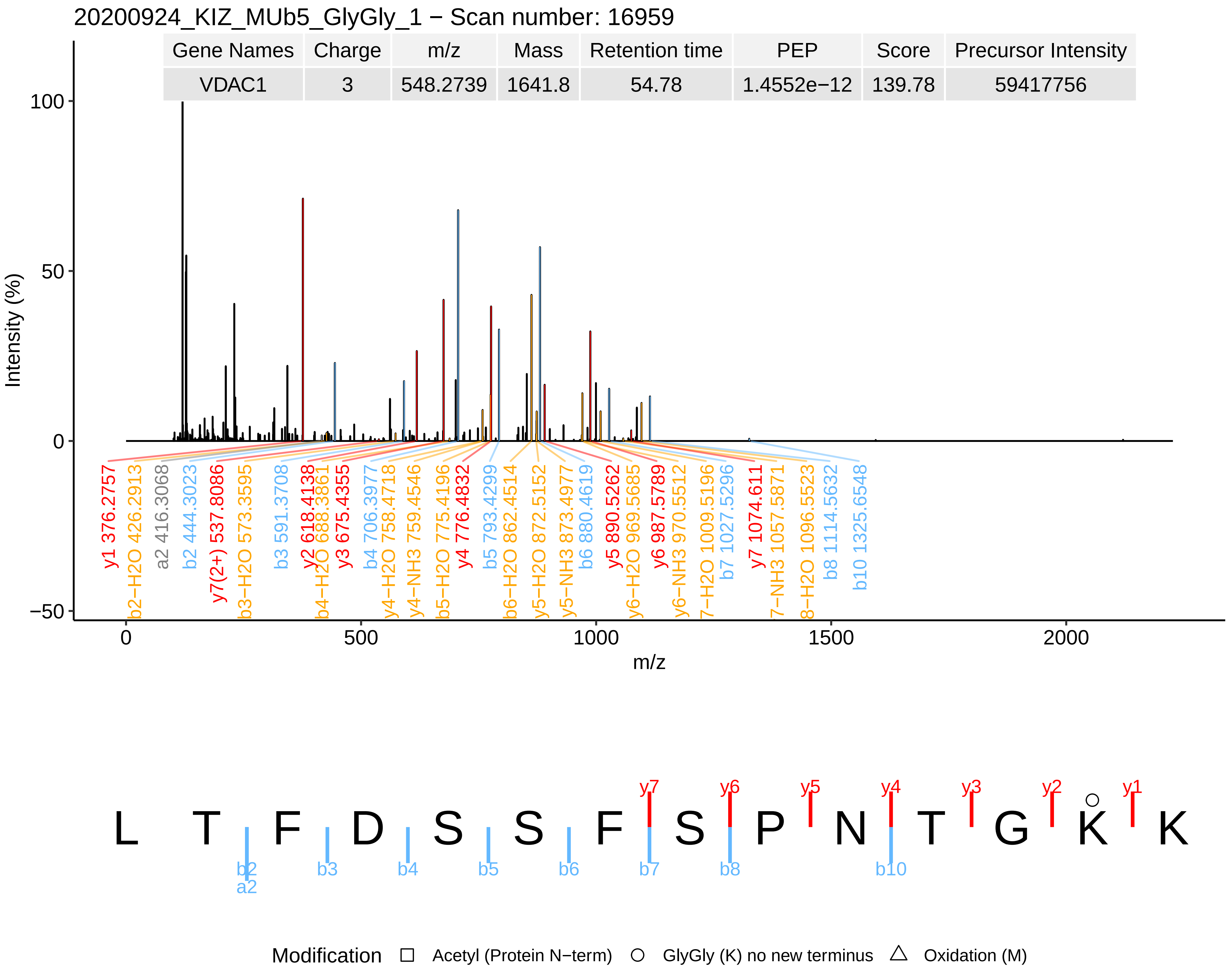

20200924_KIZ_MUb5_GlyGly_1 − Scan number: 16959
Gene Names | Charge | m/z | Mass | Retention time | PEP | Score | Precursor Intensity
VDAC1 | 3 | 548.2739 | 1641.8 | 54.78 | 1.4552e−12 | 139.78 | 59417756
Intensity (%)
100
50
0
−50
m/z
0
500
1000
1500
2000
y1 376.2757
b2−H2O 426.2913
a2 416.3068
b2 444.3023
y7(2+) 537.8086
b3−H2O 573.3595
b3 591.3708
y2 618.4138
b4−H2O 688.3861
y3 675.4355
b4 706.3977
y4−H2O 758.4718
y4−NH3 759.4546
b5−H2O 775.4196
y4 776.4832
b5 793.4299
b6−H2O 862.4514
y5−H2O 872.5152
y5−NH3 873.4977
b6 880.4619
y5 890.5262
y6−H2O 969.5685
y6 987.5789
y6−NH3 970.5512
7−H2O 1009.5196
b7 1027.5296
y7 1074.611
7−NH3 1057.5871
8−H2O 1096.5523
b8 1114.5632
b10 1325.6548
L T F D S S F S P N T G K K
y7 y6 y5 y4 y3 y2 y1
b2 b3 b4 b5 b6 b7 b8 b10
a2
Modification
Acetyl (Protein N−term)
GlyGly (K) no new terminus
Oxidation (M)

20200924_KIZ_MUb5_GlyGly_1 − Scan number: 17116

Gene Names	Charge	m/z	Mass	Retention time	PEP	Score	Precursor Intensity
VDAC1	3	548.2739	1641.8	55.282	1.8515e−12	136.93	237973232

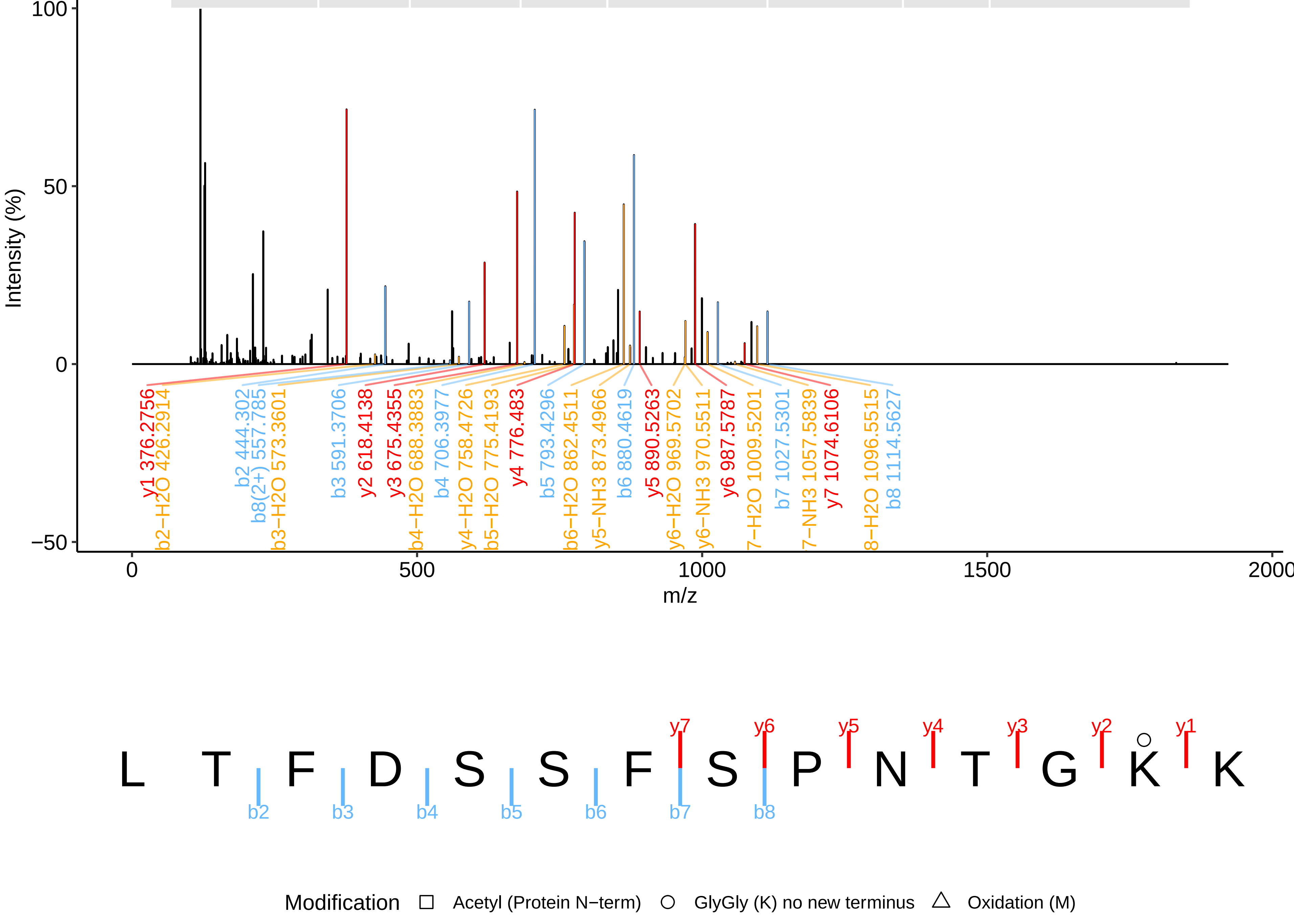

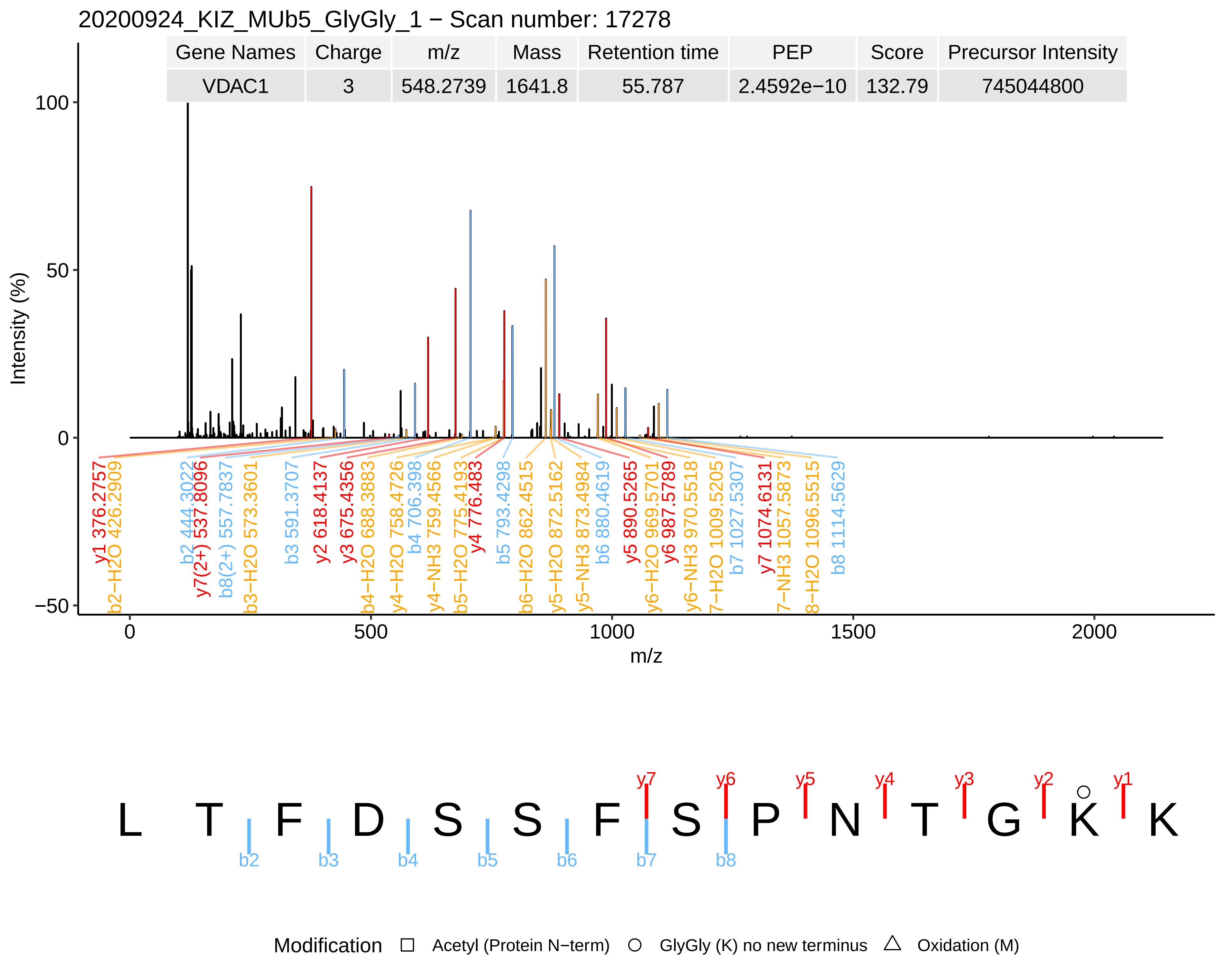
20200924_KIZ_MUb5_GlyGly_1 − Scan number: 17278
Gene Names | Charge | m/z | Mass | Retention time | PEP | Score | Precursor Intensity
VDAC1 | 3 | 548.2739 | 1641.8 | 55.787 | 2.4592e−10 | 132.79 | 745044800
Intensity (%)
m/z
y1 376.2757
b2−H2O 426.2909
b2 444.3022
y7(2+) 537.8096
b8(2+) 557.7837
b3−H2O 573.3601
b3 591.3707
y2 618.4137
y3 675.4356
b4−H2O 688.3883
y4−H2O 758.4726
b4 706.398
y4−NH3 759.4566
b5−H2O 775.4193
y4 776.483
b5 793.4298
b6−H2O 862.4515
y5−H2O 872.5162
y5−NH3 873.4984
b6 880.4619
y5 890.5265
y6−H2O 969.5701
y6 987.5789
y6−NH3 970.5518
7−H2O 1009.5205
b7 1027.5307
y7 1074.6131
7−NH3 1057.5873
8−H2O 1096.5515
b8 1114.5629
L T F D S S F S P N T G K K
y7 y6 y5 y4 y3 y2 y1
b2 b3 b4 b5 b6 b7 b8
Modification
Acetyl (Protein N−term)
GlyGly (K) no new terminus
Oxidation (M)

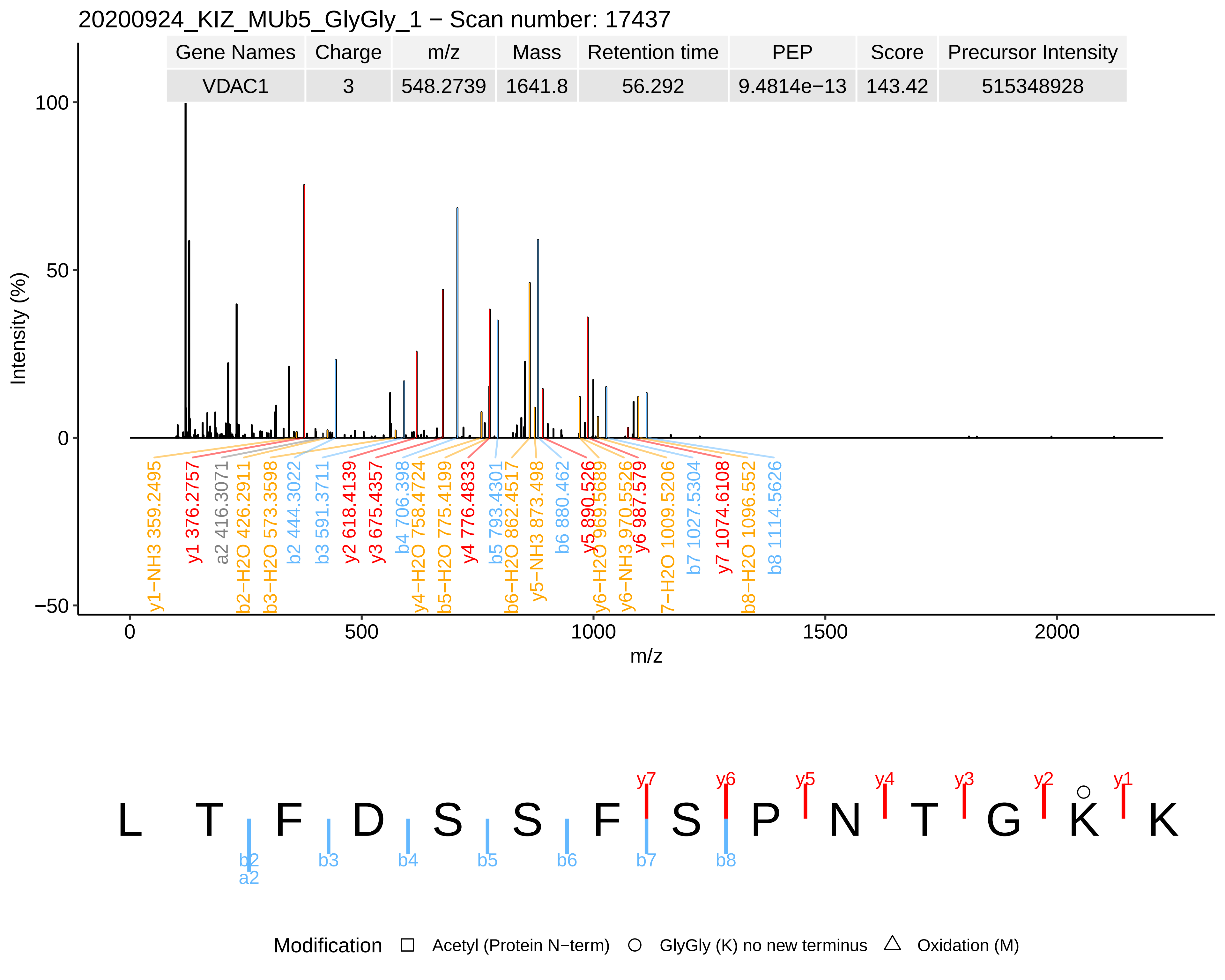
20200924_KIZ_MUb5_GlyGly_1 − Scan number: 17437
Gene Names | Charge | m/z | Mass | Retention time | PEP | Score | Precursor Intensity
VDAC1 | 3 | 548.2739 | 1641.8 | 56.292 | 9.4814e−13 | 143.42 | 515348928
Intensity (%)
100
50
0
−50
m/z
0
500
1000
1500
2000
y1−NH3 359.2495
y1 376.2757
a2 416.3071
b2−H2O 426.2911
b3−H2O 573.3598
b2 444.3022
b3 591.3711
y2 618.4139
y3 675.4357
b4 706.398
y4−H2O 758.4724
b5−H2O 775.4199
y4 776.4833
b5 793.4301
b6−H2O 862.4517
y5−NH3 873.498
b6 880.462
y5 890.526
y6−H2O 969.5689
y6−NH3 970.5526
y6 987.579
y7−H2O 1009.5206
b7 1027.5304
y7 1074.6108
b8−H2O 1096.552
b8 1114.5626
L T F D S S F S P N T G K K
y7 y6 y5 y4 y3 y2 y1
b2 a2 b3 b4 b5 b6 b7 b8
Modification
Acetyl (Protein N−term)
GlyGly (K) no new terminus
Oxidation (M)

20200924_KIZ_MUb5_GlyGly_1 − Scan number: 17589

Gene Names	Charge	m/z	Mass	Retention time	PEP	Score	Precursor Intensity
VDAC1	3	548.2739	1641.8	56.799	9.4814e−13	143.42	143041232

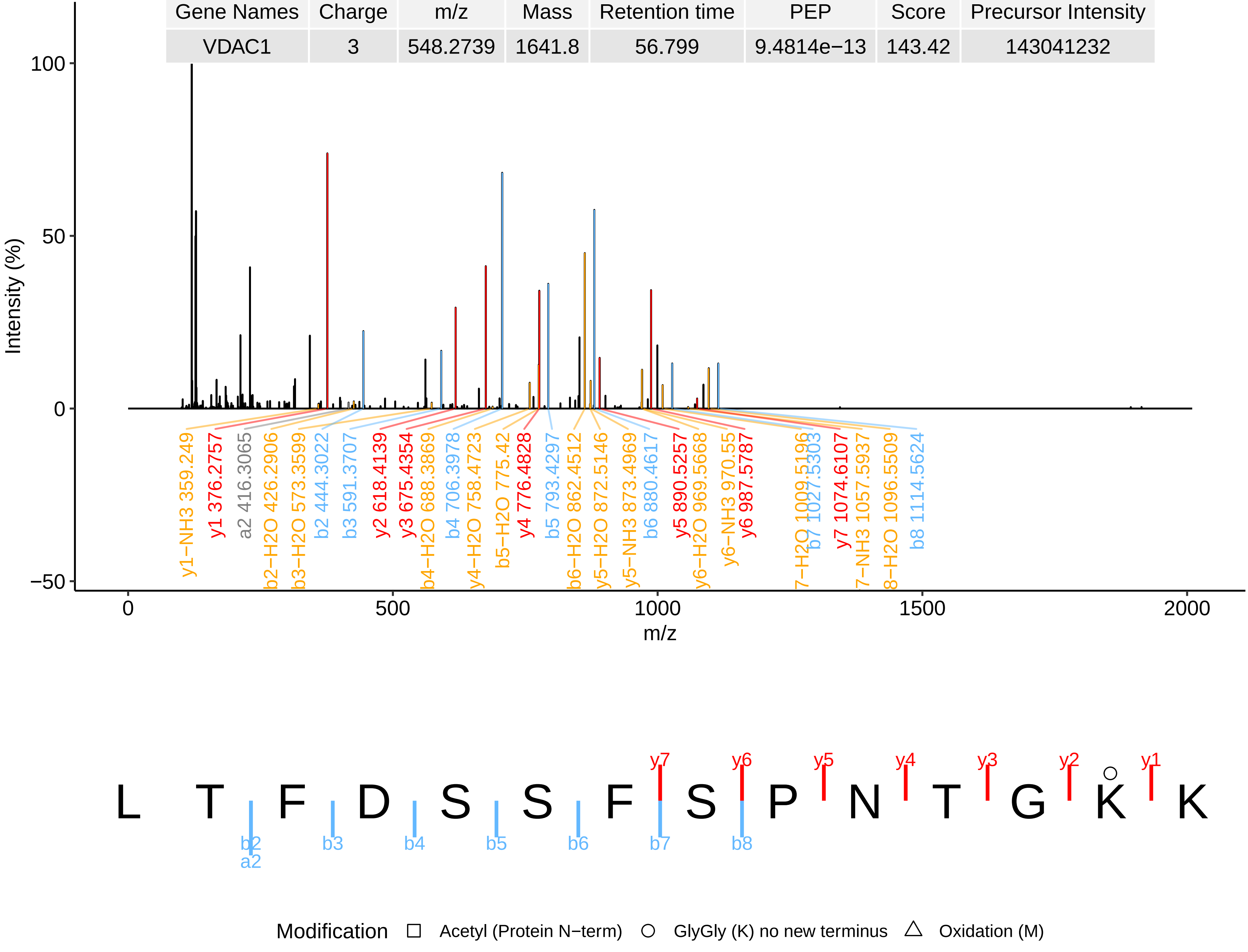

20200924_KIZ_MUb5_GlyGly_1 − Scan number: 17738

Gene Names	Charge	m/z	Mass	Retention time	PEP	Score	Precursor Intensity
VDAC1	3	548.2739	1641.8	57.304	9.4814e−13	143.42	28140392

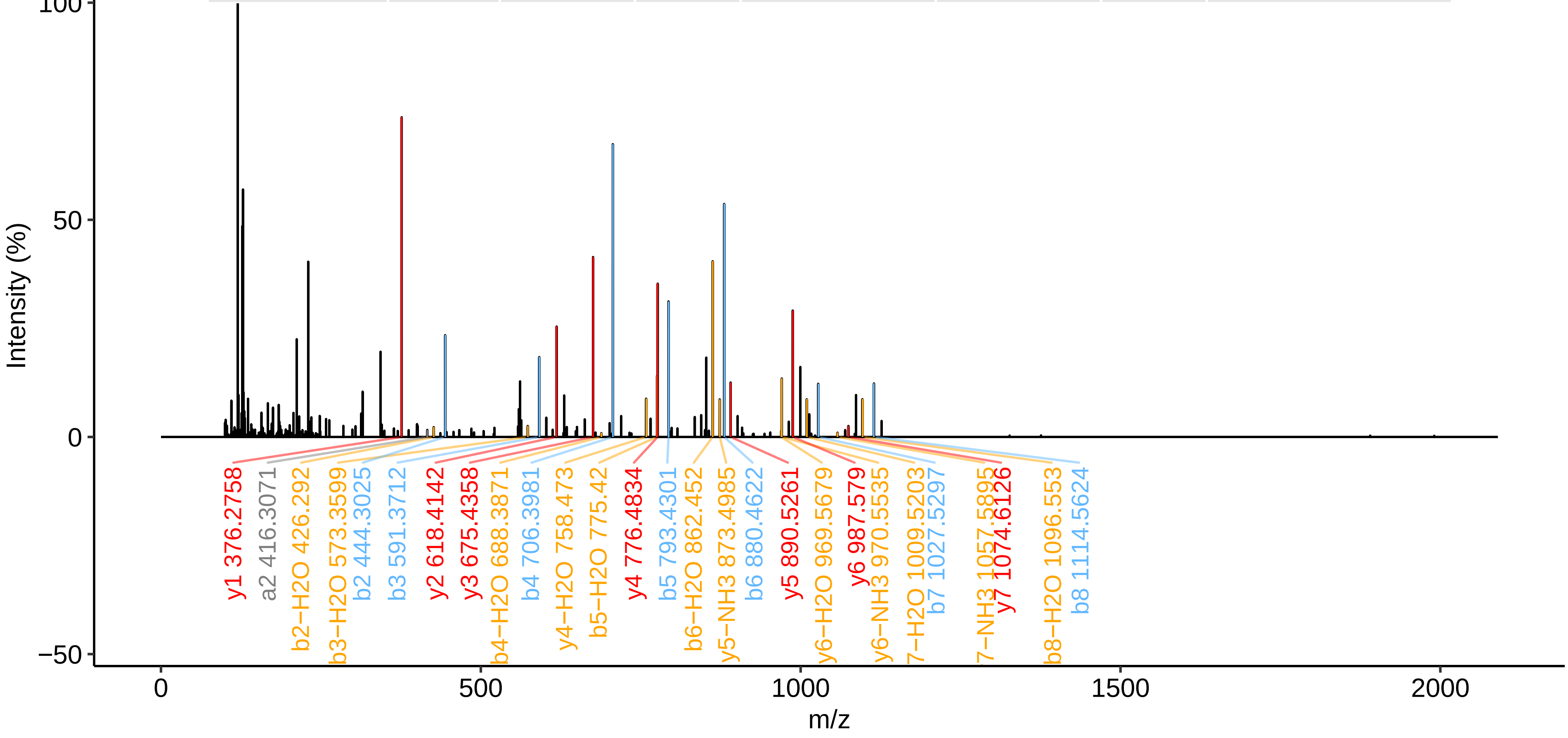

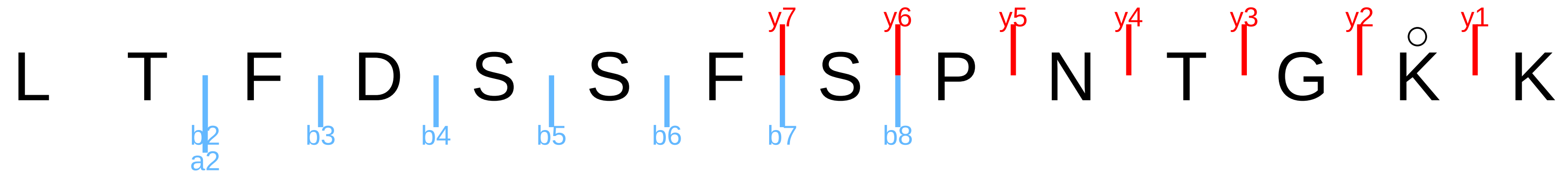

Modification □ Acetyl (Protein N−term) ○ GlyGly (K) no new terminus △ Oxidation (M)

20200924_KIZ_MUb5_GlyGly_1 − Scan number: 17196

Gene Names	Charge	m/z	Mass	Retention time	PEP	Score	Precursor Intensity
VDAC1	4	411.4572	1641.8	55.528	5.5886e−09	125.82	31162372

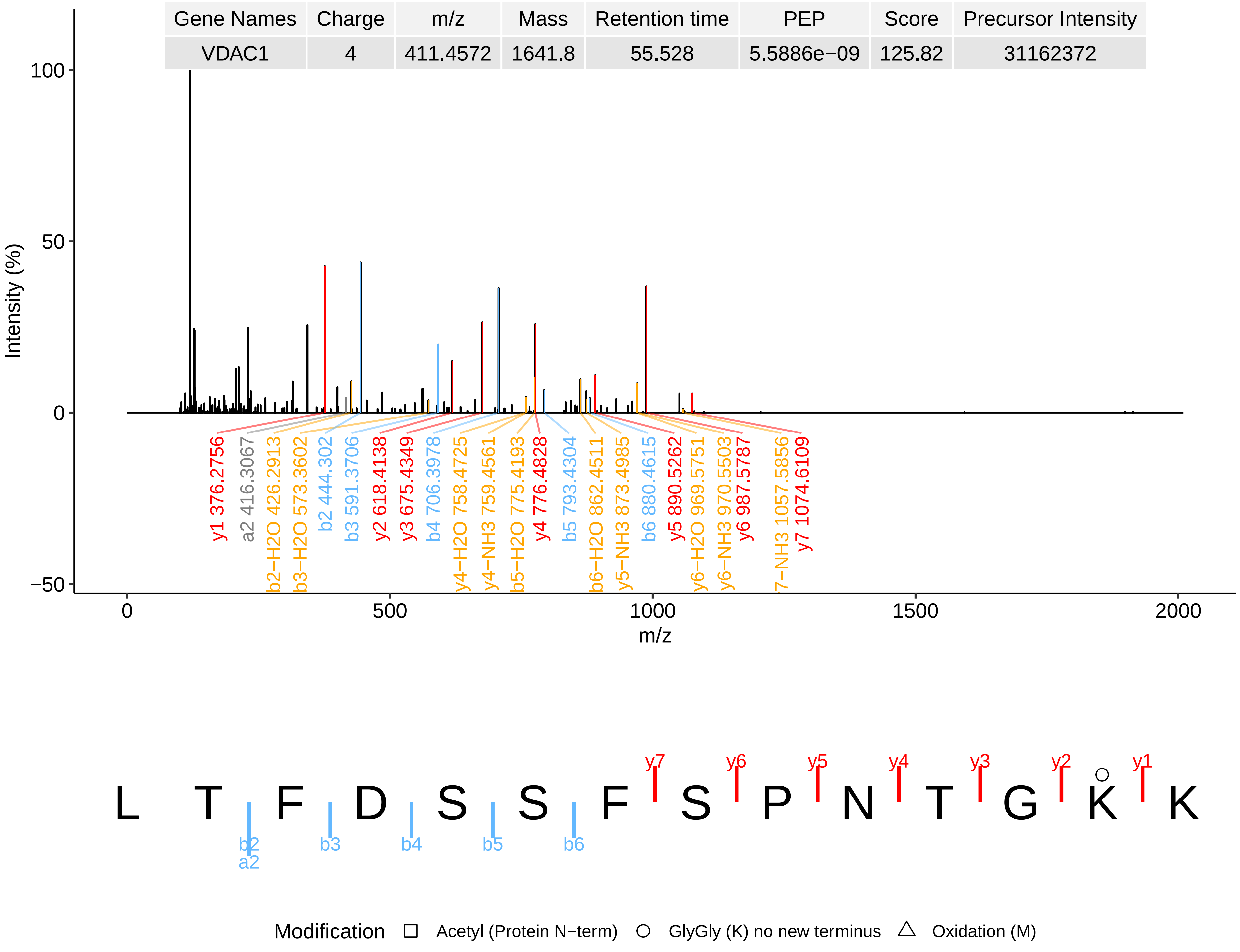

20200924_KIZ_MUb5_GlyGly_1 − Scan number: 17359

Gene Names	Charge	m/z	Mass	Retention time	PEP	Score	Precursor Intensity
VDAC1	4	411.4572	1641.8	56.039	7.3262e−09	123.64	NA

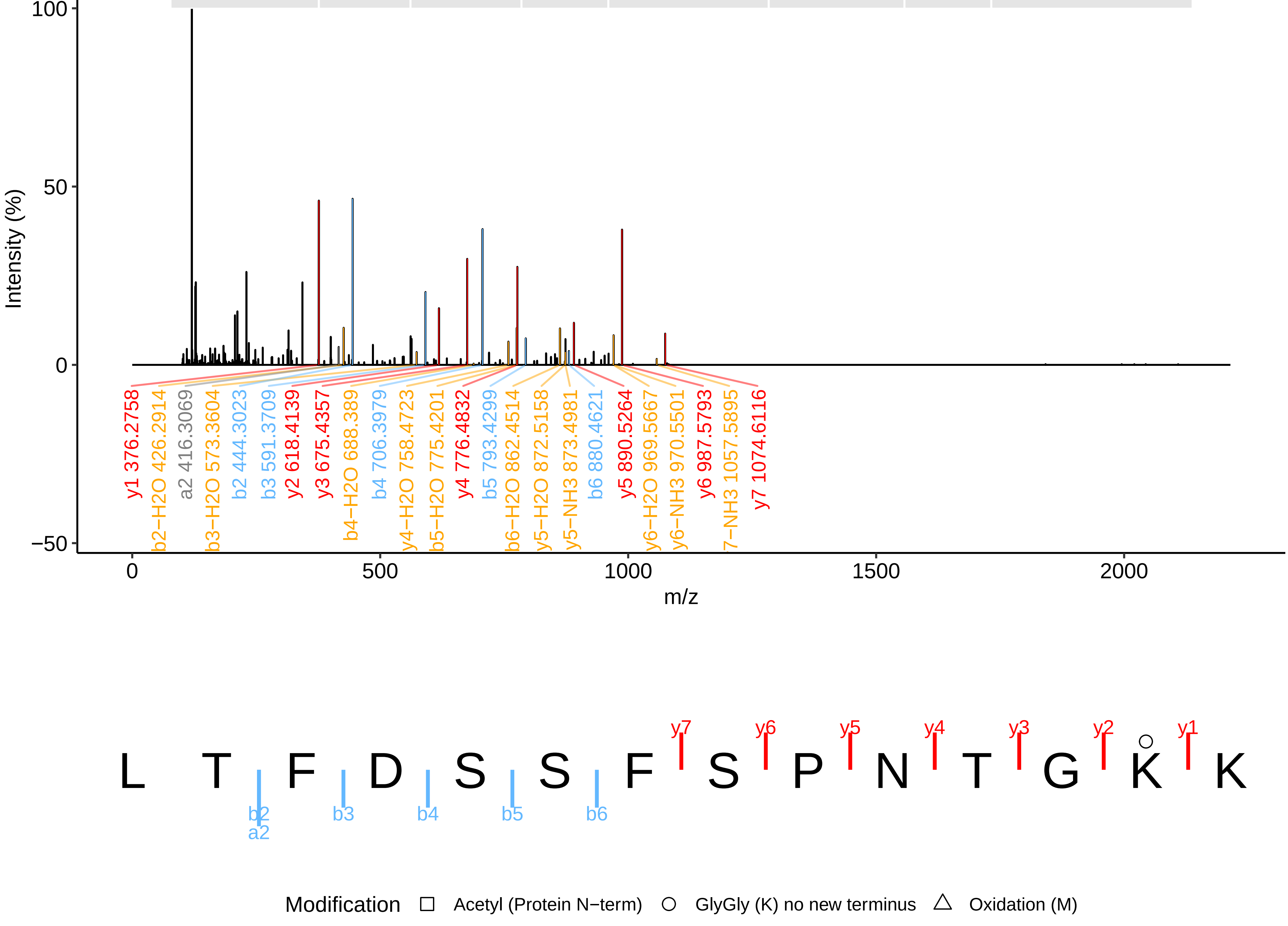

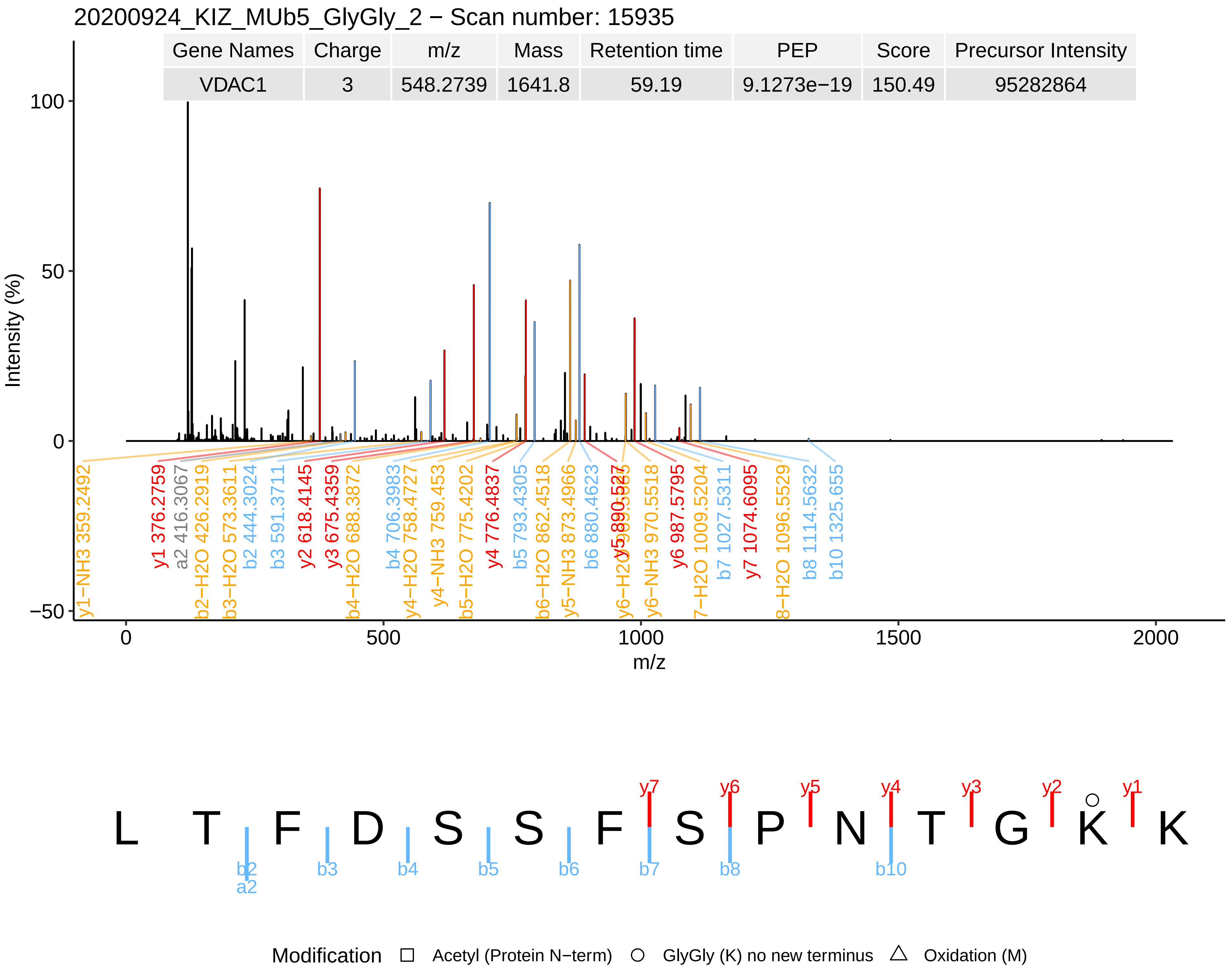
20200924_KIZ_MUb5_GlyGly_2 − Scan number: 15935
Gene Names
Charge
m/z
Mass
Retention time
PEP
Score
Precursor Intensity
VDAC1
3
548.2739
1641.8
59.19
9.1273e−19
150.49
95282864
Intensity (%)
100
50
0
−50
m/z
0
500
1000
1500
2000
y1−NH3 359.2492
y1 376.2759
a2 416.3067
b2−H2O 426.2919
b3−H2O 573.3611
b2 444.3024
b3 591.3711
y2 618.4145
y3 675.4359
b4−H2O 688.3872
b4 706.3983
y4−H2O 758.4727
y4−NH3 759.453
b5−H2O 775.4202
y4 776.4837
b5 793.4305
b6−H2O 862.4518
y5−NH3 873.4966
b6 880.4623
y5 890.527
b6−H2O 970.5518
y6 987.5795
b7 1027.5311
y7 1074.6095
b8 1114.5632
b10 1325.655
L T F D S S F S P N T G K K
y7 y6 y5 y4 y3 y2 y1
b2 a2 b3 b4 b5 b6 b7 b8 b10
Modification
Acetyl (Protein N−term)
GlyGly (K) no new terminus
Oxidation (M)

20200924_KIZ_MUb5_GlyGly_2 − Scan number: 16077

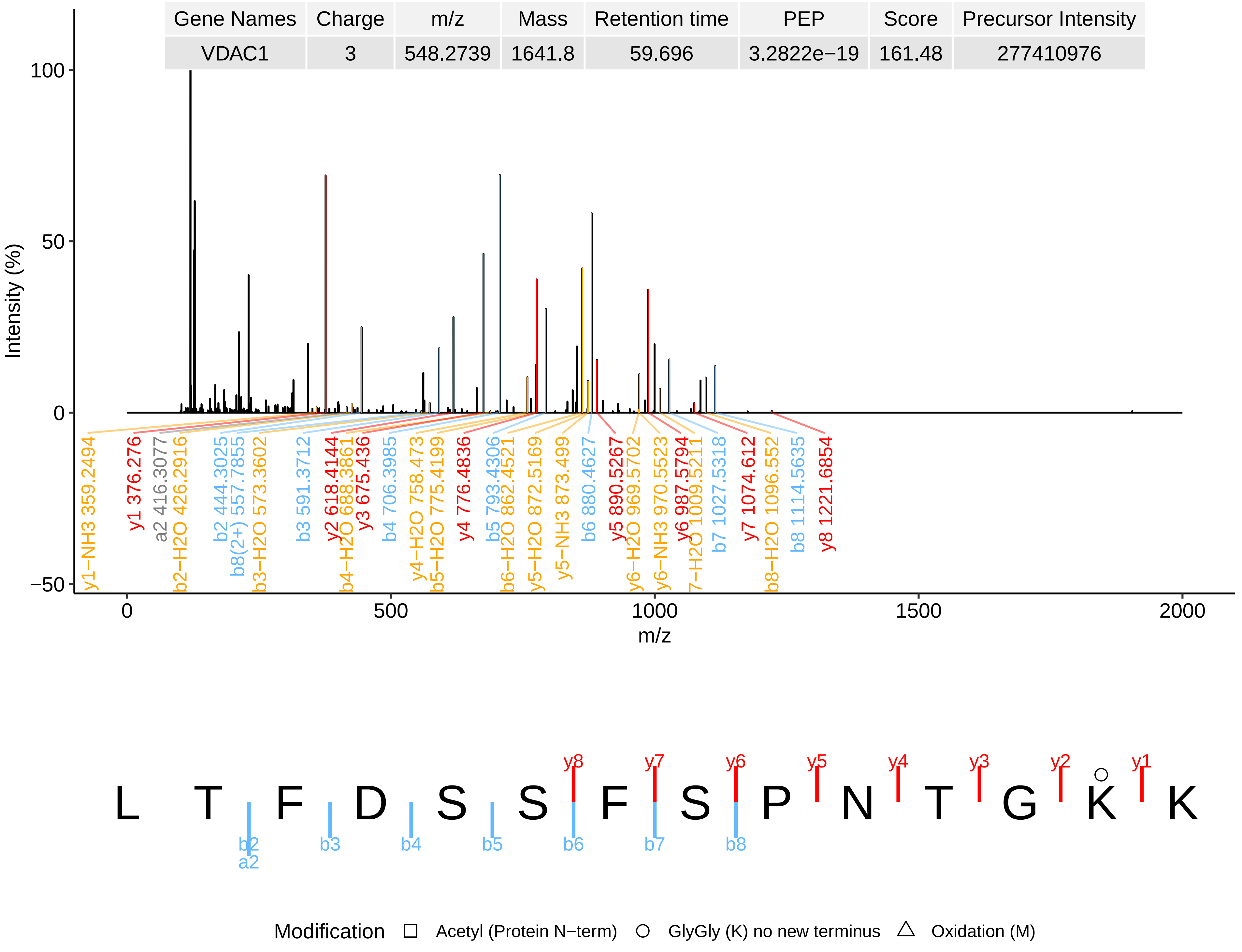

20200924_KIZ_MUb5_GlyGly_2 − Scan number: 16221

Gene Names	Charge	m/z	Mass	Retention time	PEP	Score	Precursor Intensity
VDAC1	3	548.2739	1641.8	60.201	7.0687e−19	154.36	133843376

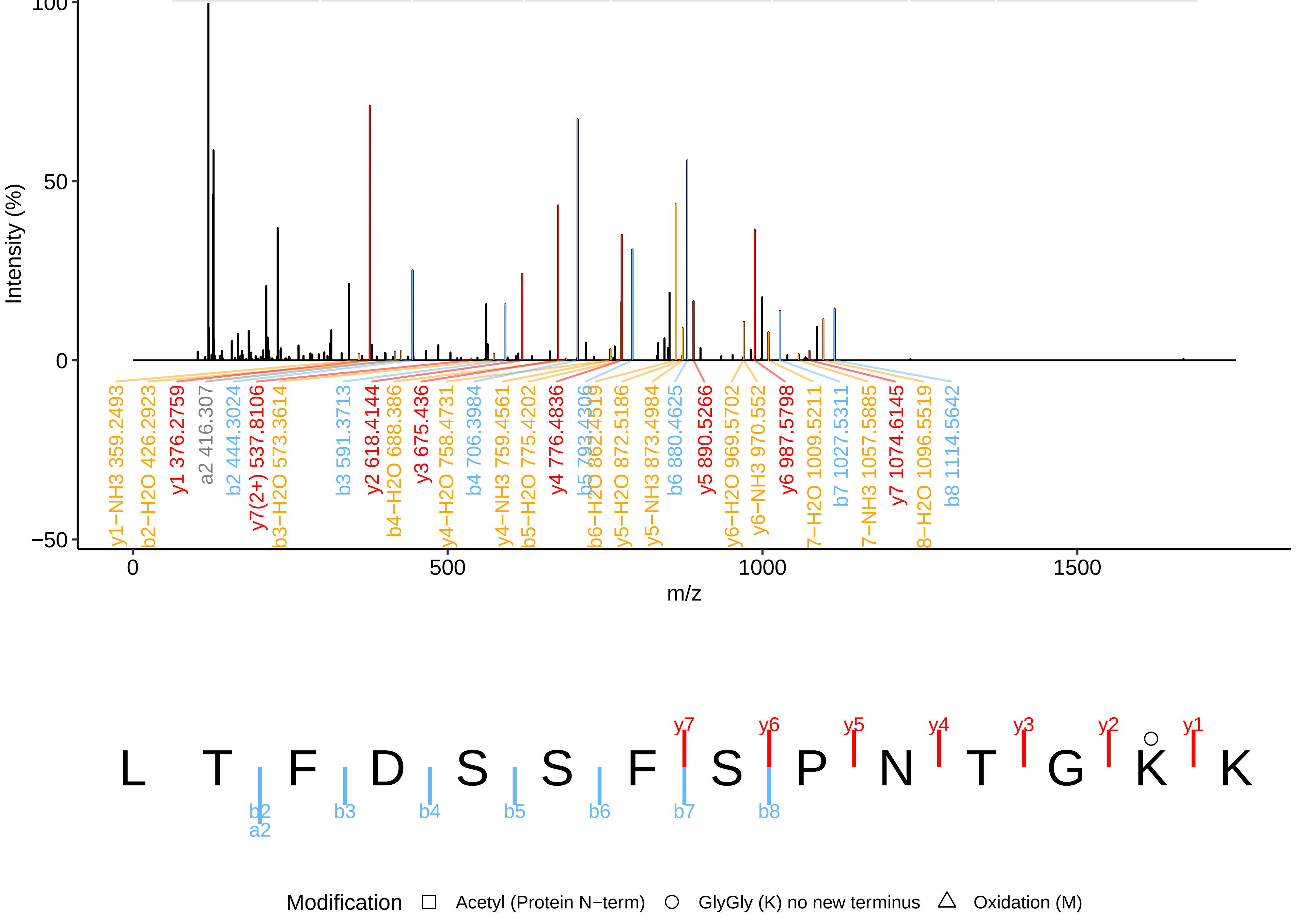

20200924_KIZ_MUb5_GlyGly_2 − Scan number: 16361

Gene Names	Charge	m/z	Mass	Retention time	PEP	Score	Precursor Intensity
VDAC1	3	548.2739	1641.8	60.706	7.3019e−08	122.46	19358238

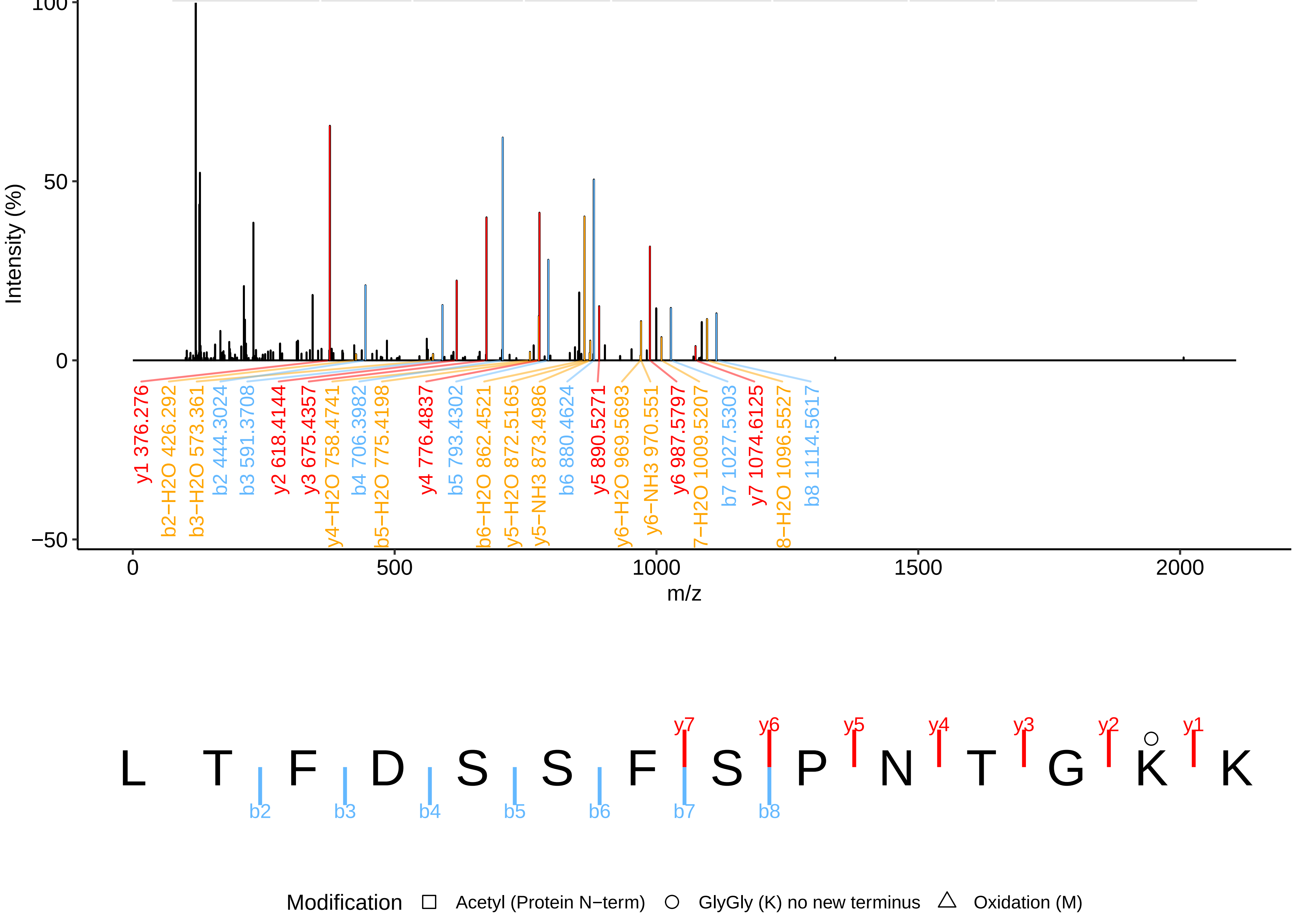

20200924_KIZ_MUb5_GlyGly_2 − Scan number: 15782

Gene Names	Charge	m/z	Mass	Retention time	PEP	Score	Precursor Intensity
VDAC1	3	548.2739	1641.8	58.647	4.8925e−08	129.37	7185441

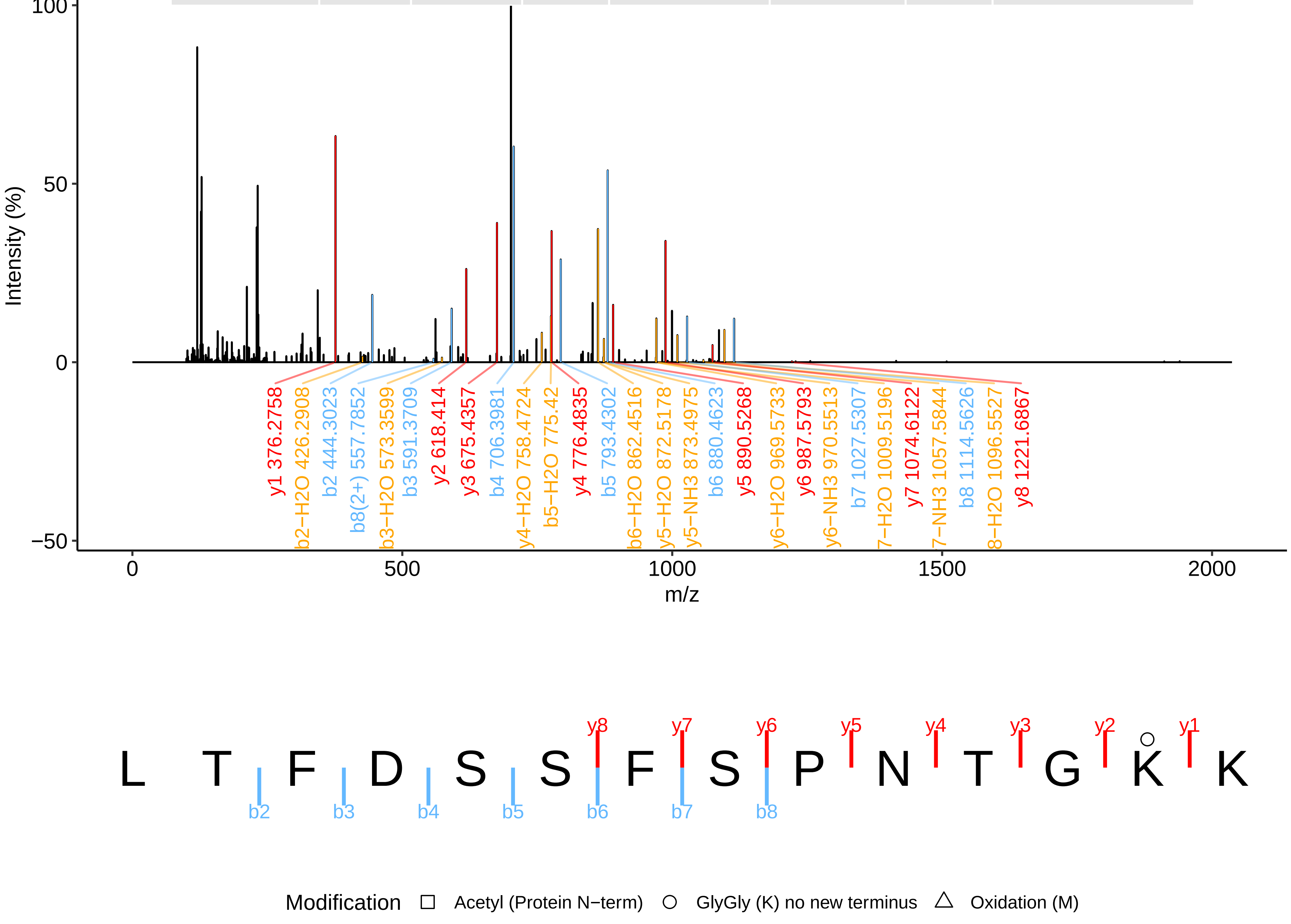

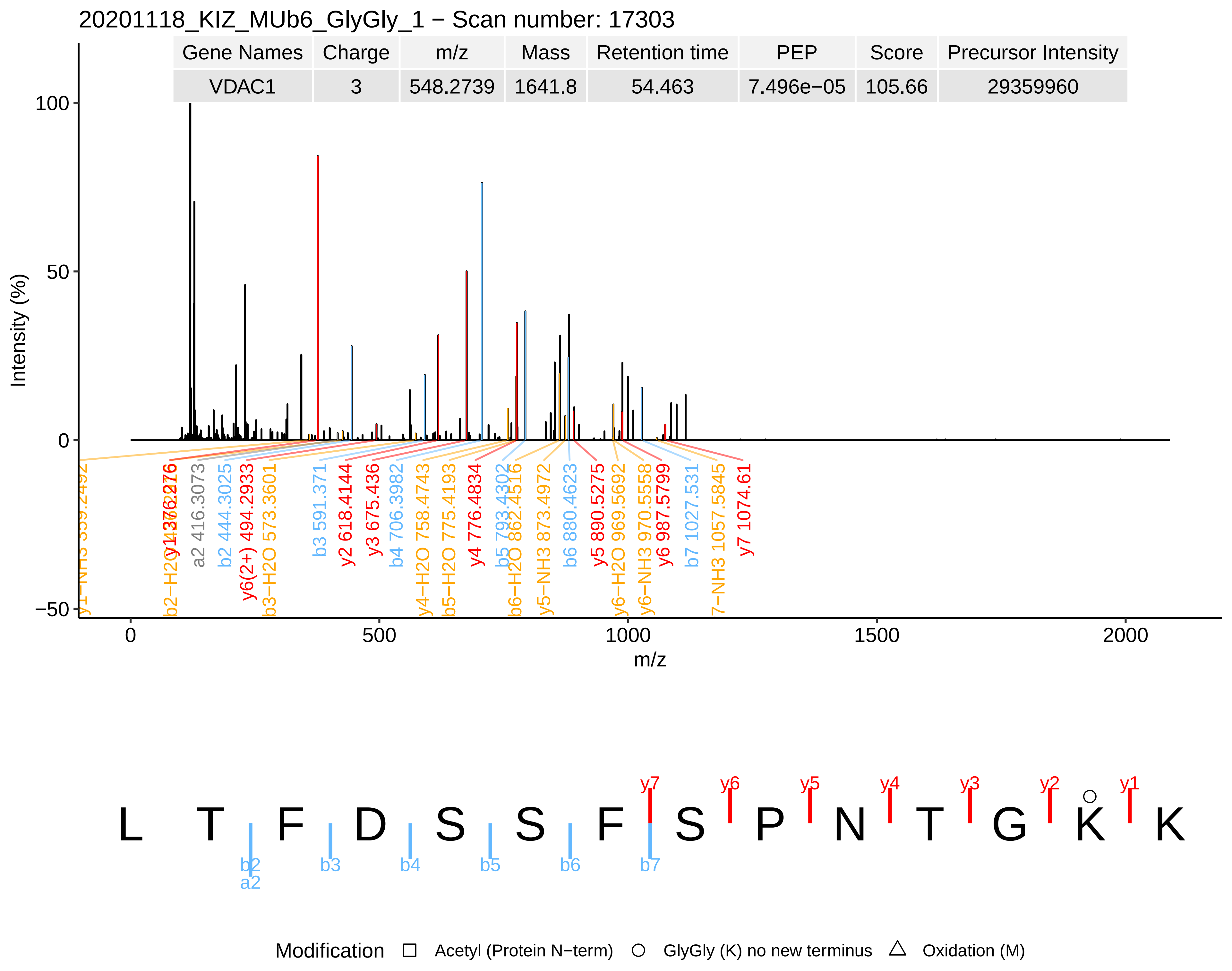

20201118_KIZ_MUb6_GlyGly_1 − Scan number: 17303
Gene Names | Charge | m/z | Mass | Retention time | PEP | Score | Precursor Intensity
VDAC1 | 3 | 548.2739 | 1641.8 | 54.463 | 7.496e−05 | 105.66 | 29359960
Intensity (%)
m/z
b2−H2O 426.2
a2 416.3073
b2 444.3025
y6(2+) 494.2933
b3−H2O 573.3601
b3 591.371
y2 618.4144
y3 675.436
b4 706.3982
y4−H2O 758.4743
b5−H2O 775.4193
y4 776.4834
b5 793.4302
b6−H2O 862.4516
y5−NH3 873.4972
b6 880.4623
y5 890.5275
y6−H2O 969.5692
y6−NH3 970.5558
y6 987.5799
b7 1027.531
y7−NH3 1057.5845
y7 1074.61
L T F D S S F S P N T G K K
y7 y6 y5 y4 y3 y2 y1
b2 a2 b3 b4 b5 b6 b7
Modification
Acetyl (Protein N−term)
GlyGly (K) no new terminus
Oxidation (M)

20201118_KIZ_MUb6_GlyGly_1 − Scan number: 17465

Gene Names	Charge	m/z	Mass	Retention time	PEP	Score	Precursor Intensity
VDAC1	3	548.2739	1641.8	54.962	7.0687e−19	154.36	330222912

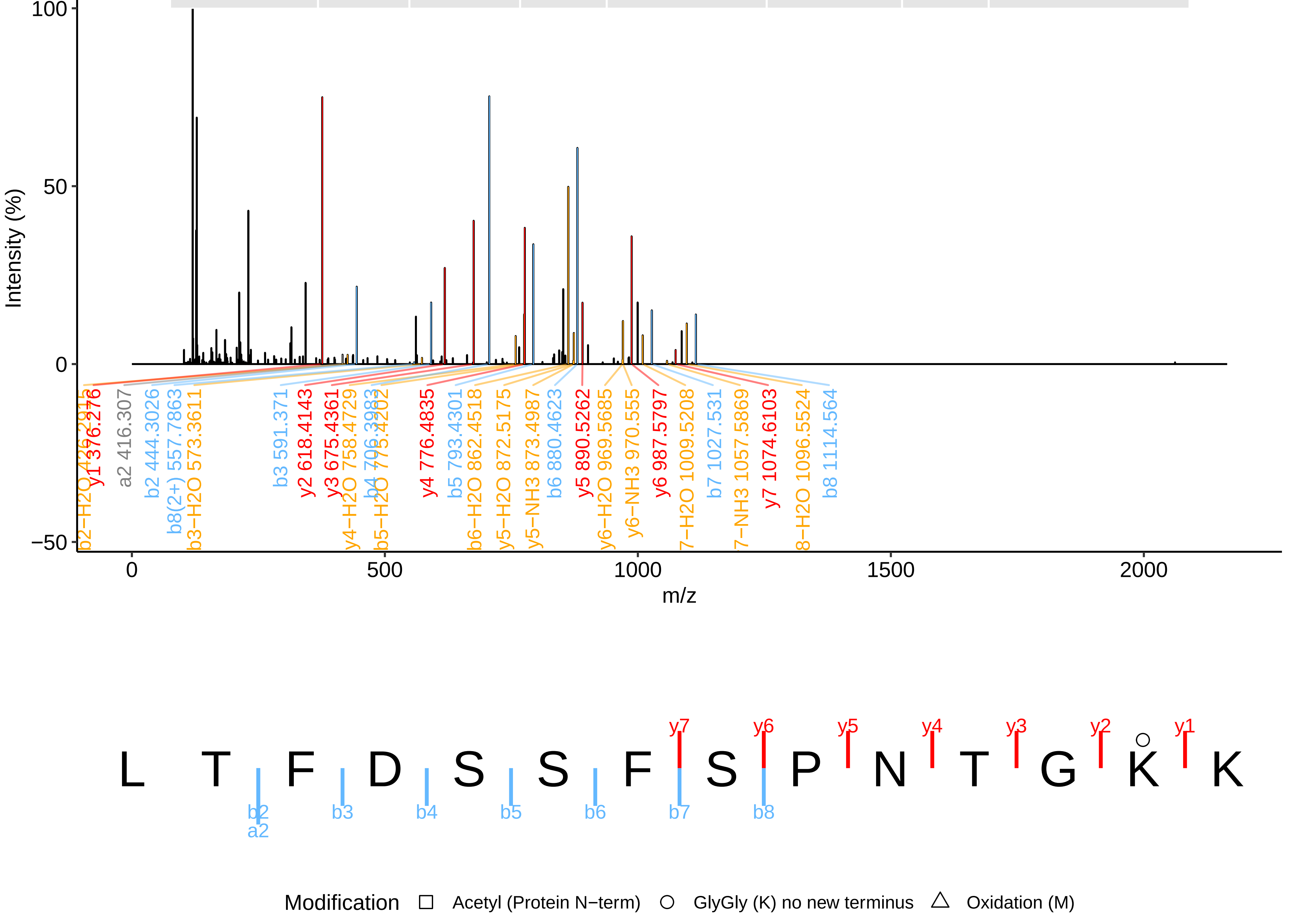

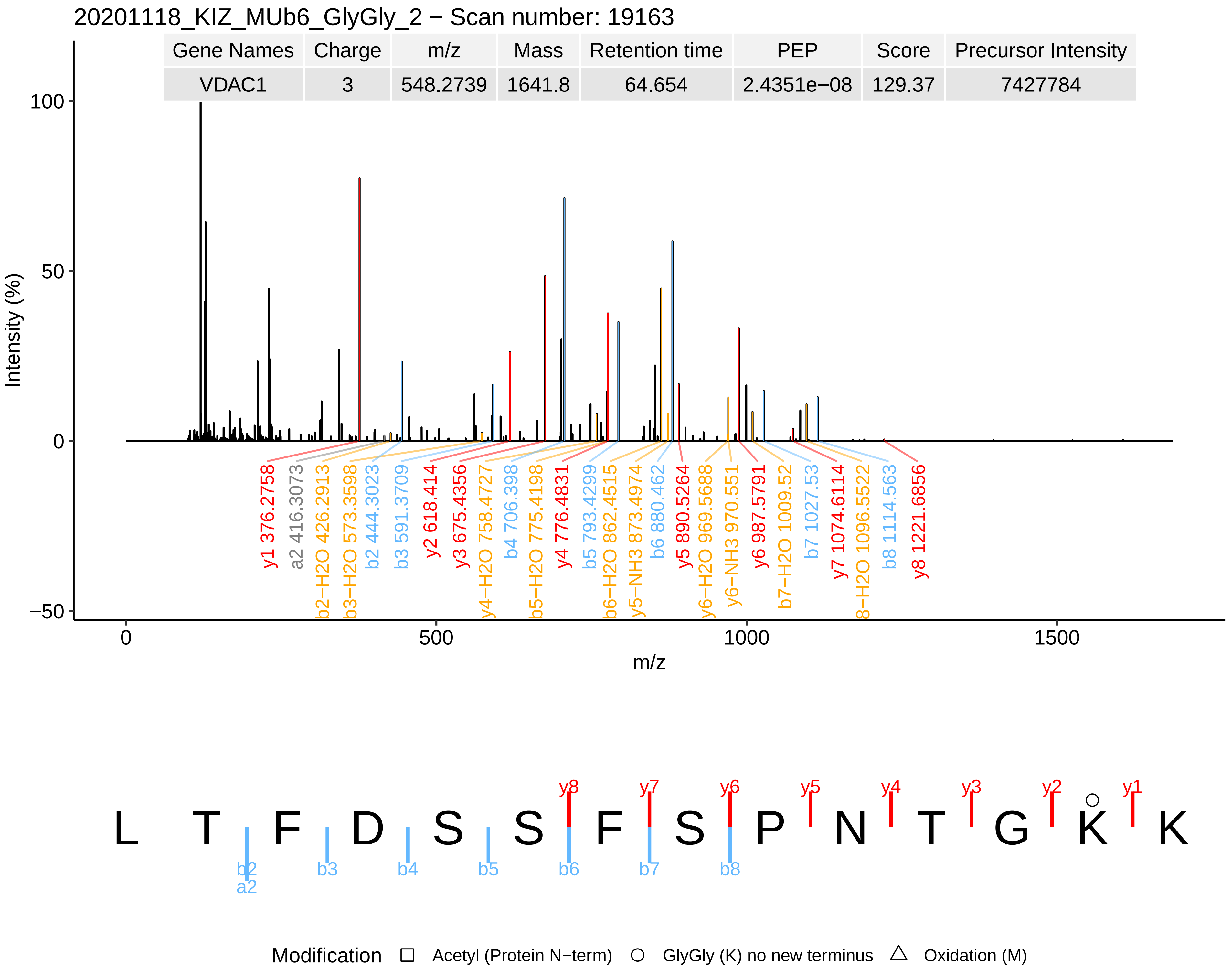
20201118_KIZ_MUb6_GlyGly_2 − Scan number: 19163
Gene Names
Charge
m/z
Mass
Retention time
PEP
Score
Precursor Intensity
VDAC1
3
548.2739
1641.8
64.654
2.4351e−08
129.37
7427784
Intensity (%)
m/z
y1 376.2758
a2 416.3073
b2−H2O 426.2913
b3−H2O 573.3598
b2 444.3023
b3 591.3709
y2 618.414
y3 675.4356
y4−H2O 758.4727
b4 706.398
b5−H2O 775.4198
y4 776.4831
b5 793.4299
b6−H2O 862.4515
y5−NH3 873.4974
b6 880.462
y5 890.5264
y6−H2O 969.5688
y6−NH3 970.551
y6 987.5791
b7−H2O 1009.52
b7 1027.53
y7 1074.6114
b8−H2O 1096.5522
b8 1114.563
y8 1221.6856
L T F D S S F S P N T G K K
Modification
Acetyl (Protein N−term)
GlyGly (K) no new terminus
Oxidation (M)

20201118_KIZ_MUb6_GlyGly_2 − Scan number: 19312

Gene Names	Charge	m/z	Mass	Retention time	PEP	Score	Precursor Intensity
VDAC1	3	548.2739	1641.8	65.165	4.2218e−08	126.83	102392520

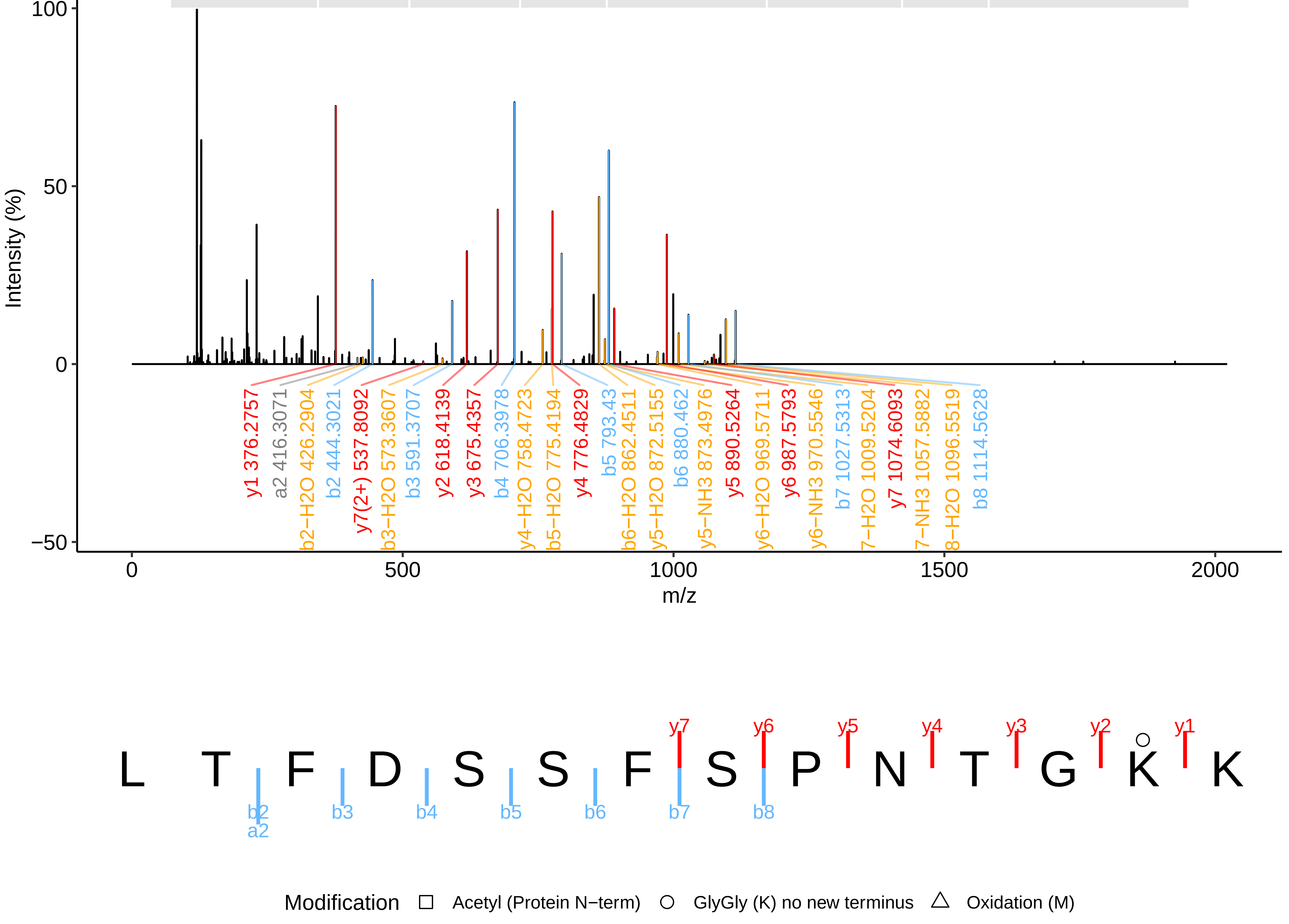

20201118_KIZ_MUb6_GlyGly_2 − Scan number: 19191

Gene Names	Charge	m/z	Mass	Retention time	PEP	Score	Precursor Intensity
VDAC1	4	411.4572	1641.8	64.753	0.00020313	91.62	9934601

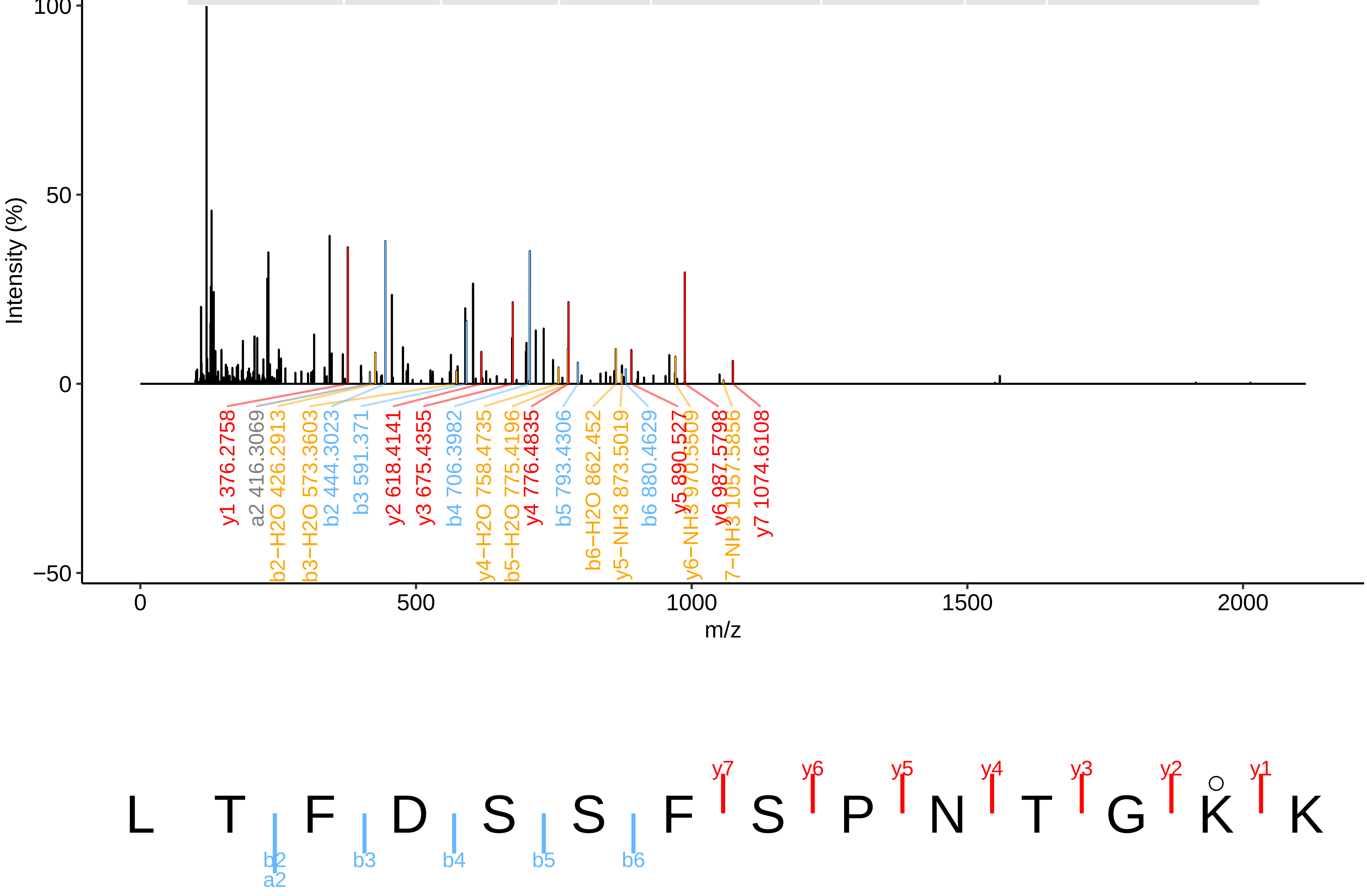

Modification □ Acetyl (Protein N−term) ○ GlyGly (K) no new terminus △ Oxidation (M)

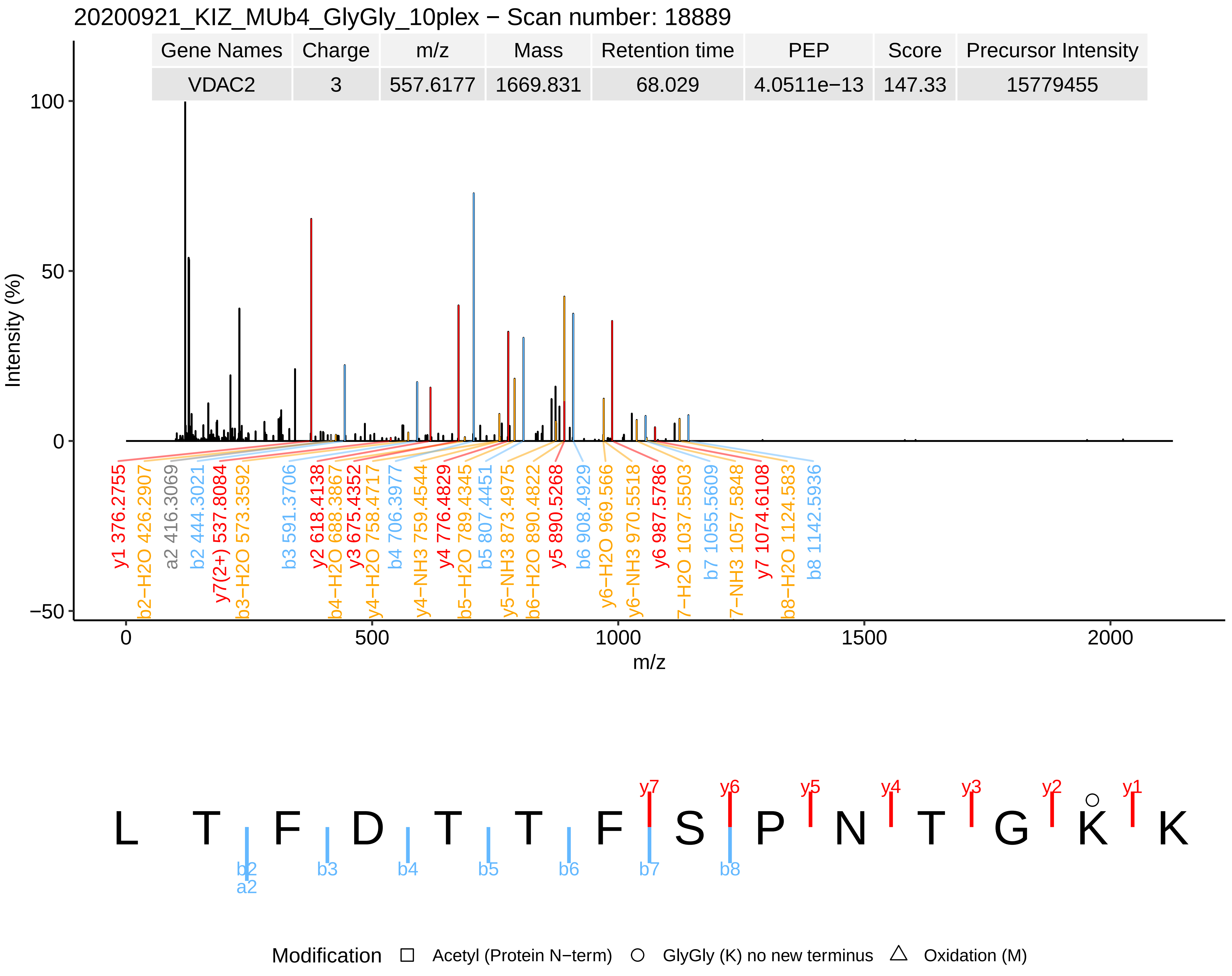
20200921_KIZ_MUb4_GlyGly_10plex − Scan number: 18889
Gene Names | Charge | m/z | Mass | Retention time | PEP | Score | Precursor Intensity
VDAC2 | 3 | 557.6177 | 1669.831 | 68.029 | 4.0511e−13 | 147.33 | 15779455
Intensity (%)
m/z
y1 376.2755
b2−H2O 426.2907
a2 416.3069
b2 444.3021
y7(2+) 537.8084
b3−H2O 573.3592
b3 591.3706
y2 618.4138
b4−H2O 688.3867
y3 675.4352
y4−H2O 758.4717
b4 706.3977
y4−NH3 759.4544
y4 776.4829
b5−H2O 789.4345
b5 807.4451
y5−NH3 873.4975
b6−H2O 890.4822
y5 890.5268
b6 908.4929
y6−H2O 969.566
y6−NH3 970.5518
y6 987.5786
7−H2O 1037.5503
b7 1055.5609
7−NH3 1057.5848
y7 1074.6108
b8−H2O 1124.583
b8 1142.5936
L T F D T T F S P N T G K K
Modification
Acetyl (Protein N−term)
GlyGly (K) no new terminus
Oxidation (M)

20200921_KIZ_MUb4_GlyGly_10plex − Scan number: 18949

Gene Names	Charge	m/z	Mass	Retention time	PEP	Score	Precursor Intensity
VDAC2	3	557.6177	1669.831	68.242	1.5781e−19	164.68	80687160

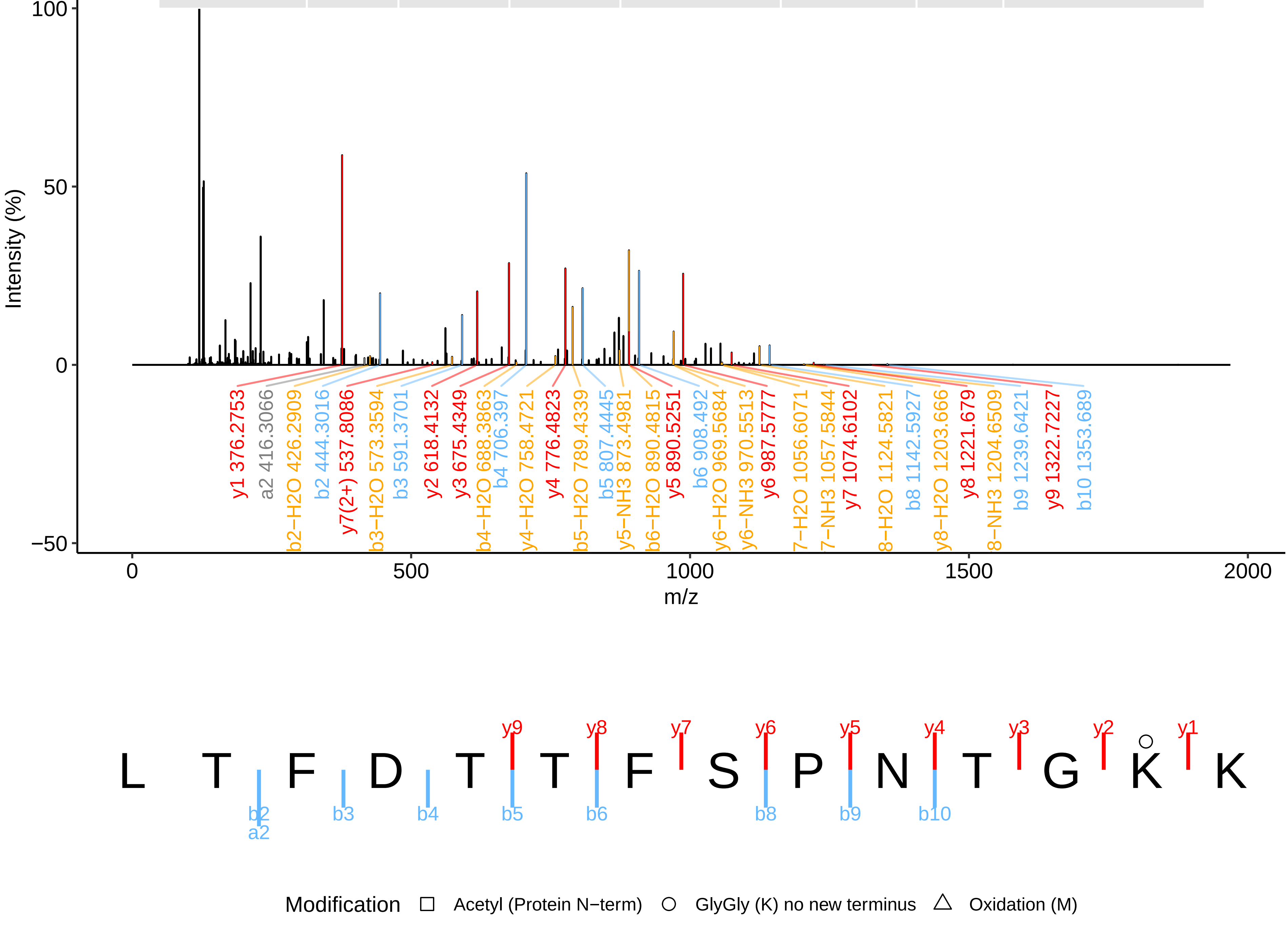

20200921_KIZ_MUb4_GlyGly_10plex − Scan number: 19091

Gene Names	Charge	m/z	Mass	Retention time	PEP	Score	Precursor Intensity
VDAC2	3	557.6177	1669.831	68.742	1.1127e−07	117.03	184270416

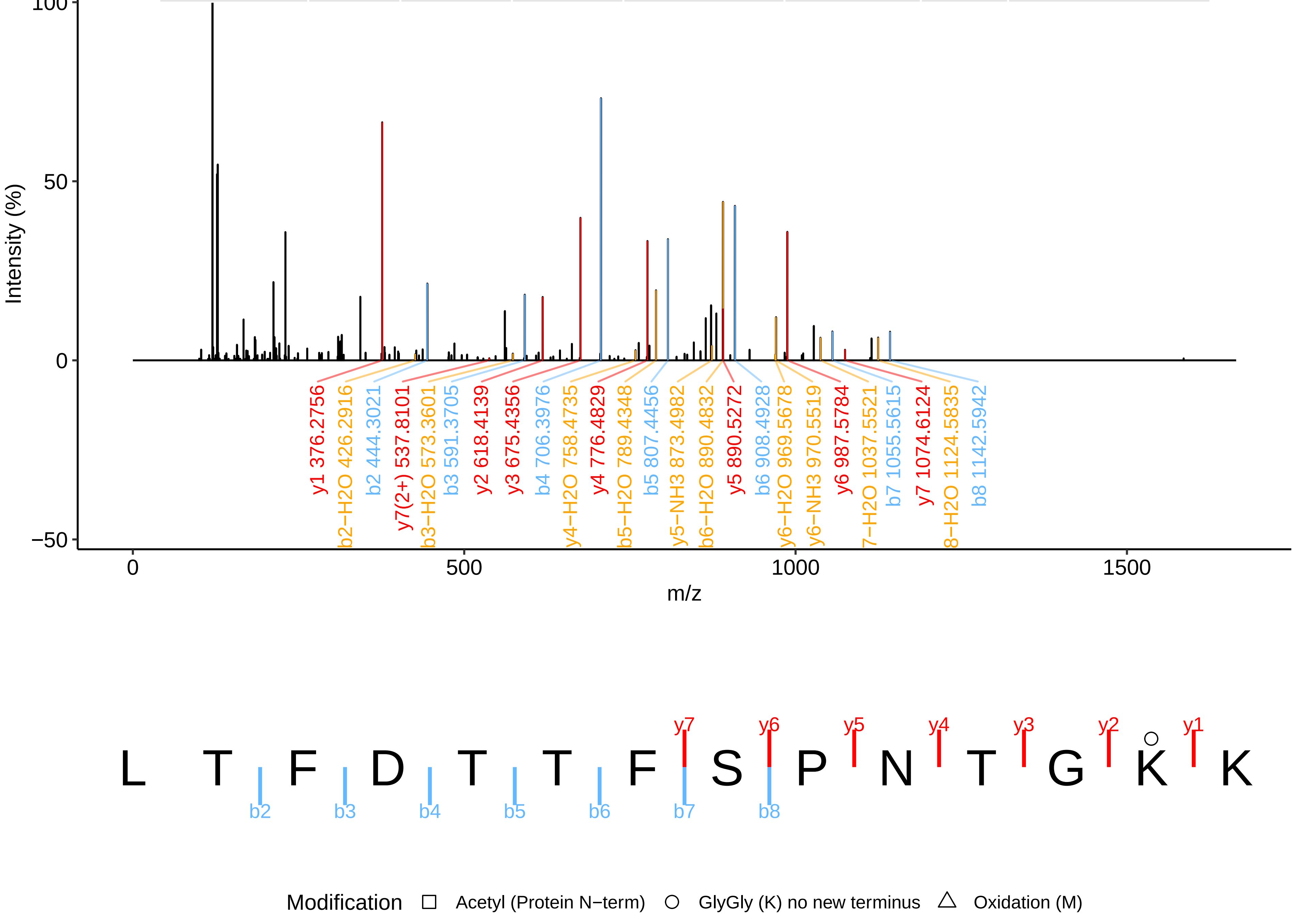

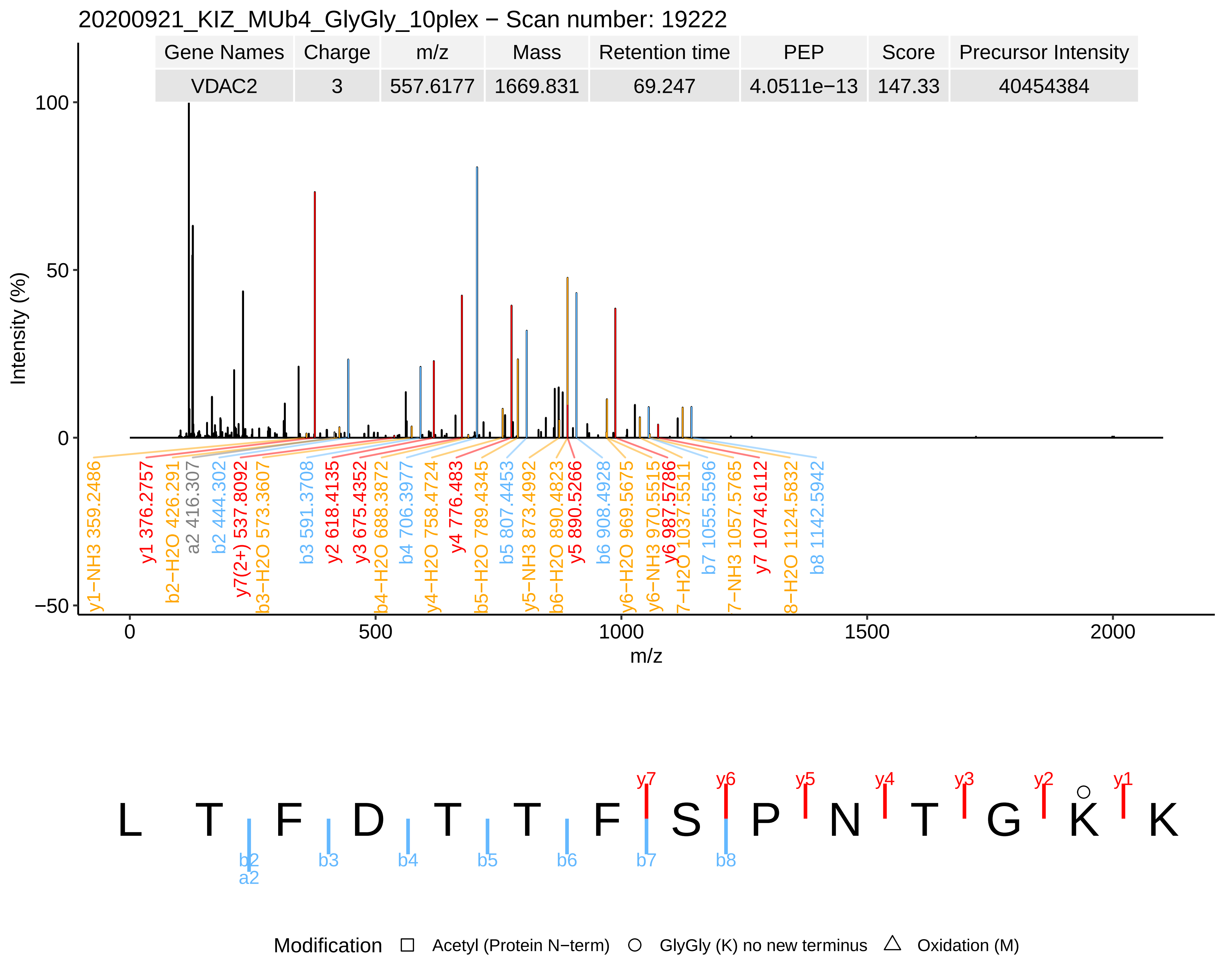

20200921_KIZ_MUb4_GlyGly_10plex − Scan number: 19222
Gene Names | Charge | m/z | Mass | Retention time | PEP | Score | Precursor Intensity
VDAC2 | 3 | 557.6177 | 1669.831 | 69.247 | 4.0511e−13 | 147.33 | 40454384
Intensity (%)
100
50
0
−50
0
500
1000
1500
2000
m/z
y1−NH3 359.2486
y1 376.2757
b2−H2O 426.291
a2 416.307
b2 444.302
y7(2+) 537.8092
b3−H2O 573.3607
b3 591.3708
y2 618.4135
y3 675.4352
b4−H2O 688.3872
b4 706.3977
y4−H2O 758.4724
y4 776.483
b5−H2O 789.4345
b5 807.4453
y5−NH3 873.4992
b6−H2O 890.4823
y5 890.5266
b6 908.4928
y6−H2O 969.5675
y6−NH3 970.5515
y6 987.5786
b7−H2O 1037.5511
b7 1055.5596
b7−NH3 1057.5765
y7 1074.6112
b8−H2O 1124.5832
b8 1142.5942
L T F D T T F S P N T G K K
y7 y6 y5 y4 y3 y2 y1
b2 a2 b3 b4 b5 b6 b7 b8
Modification
Acetyl (Protein N−term)
GlyGly (K) no new terminus
Oxidation (M)

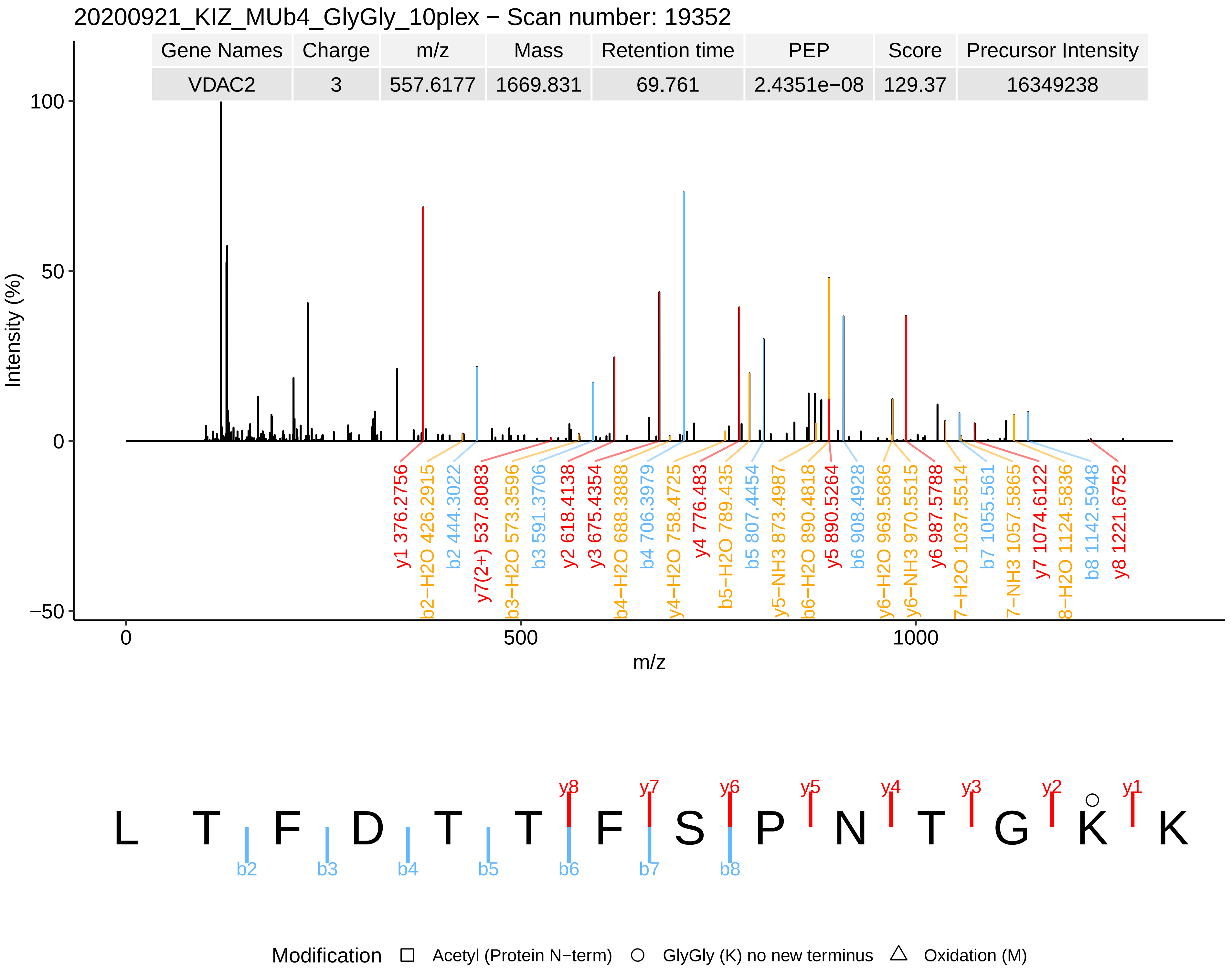
20200921_KIZ_MUb4_GlyGly_10plex − Scan number: 19352
Gene Names | Charge | m/z | Mass | Retention time | PEP | Score | Precursor Intensity
VDAC2 | 3 | 557.6177 | 1669.831 | 69.761 | 2.4351e−08 | 129.37 | 16349238
Intensity (%)
100
50
0
−50
0
500
1000
m/z
y1 376.2756
b2−H2O 426.2915
b2 444.3022
y7(2+) 537.8083
b3−H2O 573.3596
b3 591.3706
y2 618.4138
y3 675.4354
b4−H2O 688.3888
b4 706.3979
y4−H2O 758.4725
y4 776.483
b5−H2O 789.435
b5 807.4454
y5−NH3 873.4987
b6−H2O 890.4818
y5 890.5264
b6 908.4928
y6−H2O 969.5686
y6−NH3 970.5515
y6 987.5788
7−H2O 1037.5514
b7 1055.561
7−NH3 1057.5865
y7 1074.6122
8−H2O 1124.5836
b8 1142.5948
y8 1221.6752
L T F D T T F S P N T G K K
y8 y7 y6 y5 y4 y3 y2 y1
b2 b3 b4 b5 b6 b7 b8
Modification
Acetyl (Protein N−term)
GlyGly (K) no new terminus
Oxidation (M)

20200921_KIZ_MUb4_GlyGly_10plex − Scan number: 18910

Gene Names	Charge	m/z	Mass	Retention time	PEP	Score	Precursor Intensity
VDAC2	4	418.4651	1669.831	68.102	3.5098e−06	111.39	3765539

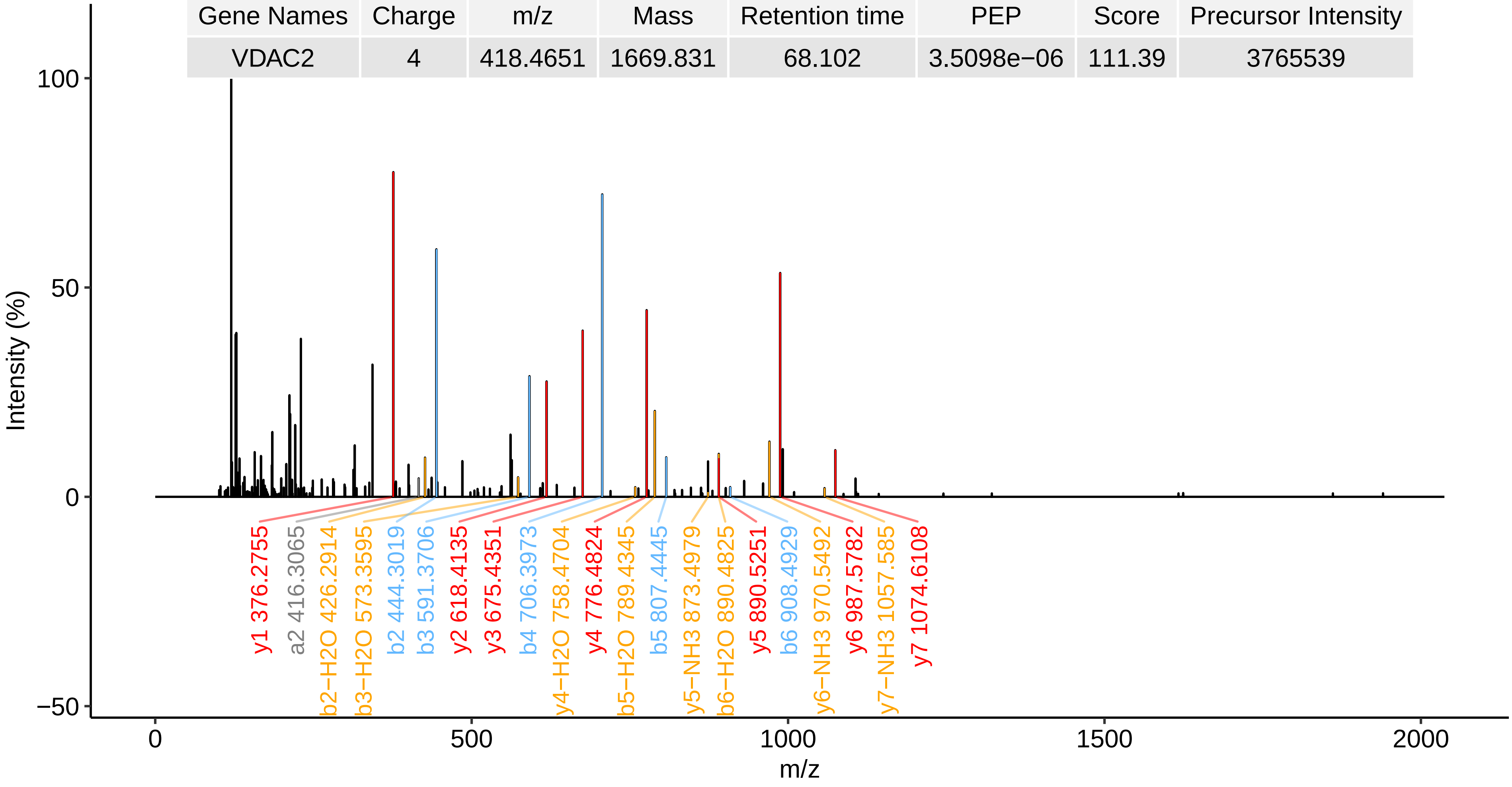

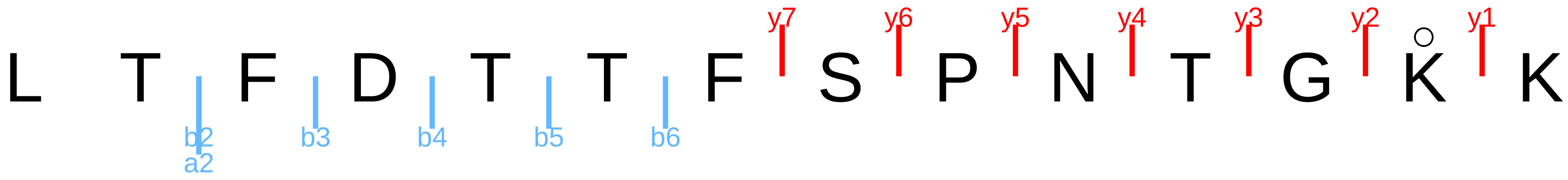

Modification □ Acetyl (Protein N−term) ○ GlyGly (K) no new terminus △ Oxidation (M)

20200921_KIZ_MUb4_GlyGly_10plex − Scan number: 19052

Gene Names	Charge	m/z	Mass	Retention time	PEP	Score	Precursor Intensity
VDAC2	4	418.4651	1669.831	68.605	3.1579e−06	111.79	16606680

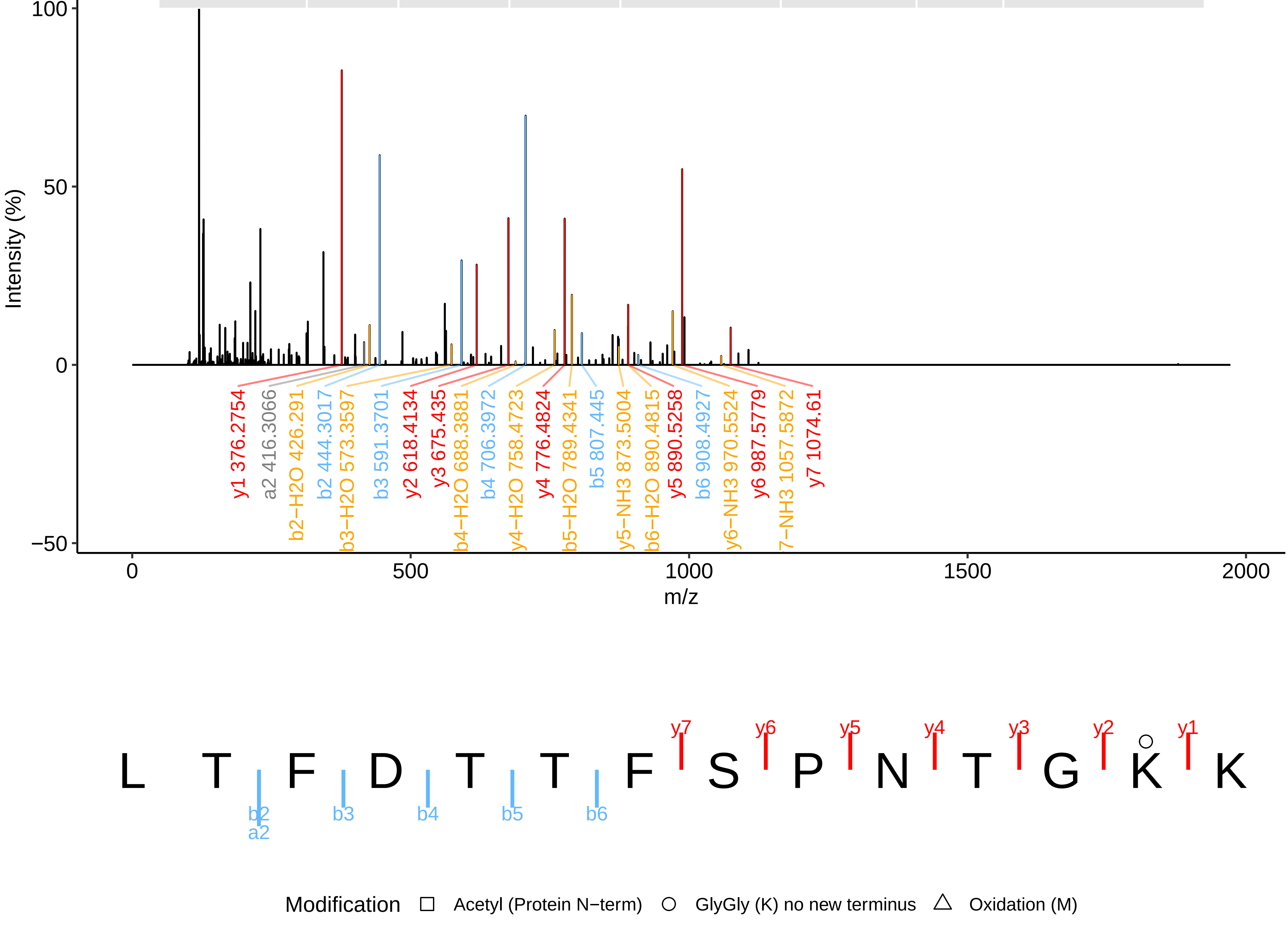

20200921_KIZ_MUb4_GlyGly_10plex − Scan number: 19192

Gene Names	Charge	m/z	Mass	Retention time	PEP	Score	Precursor Intensity
VDAC2	4	418.4651	1669.831	69.129	1.2198e−05	101.39	2682451

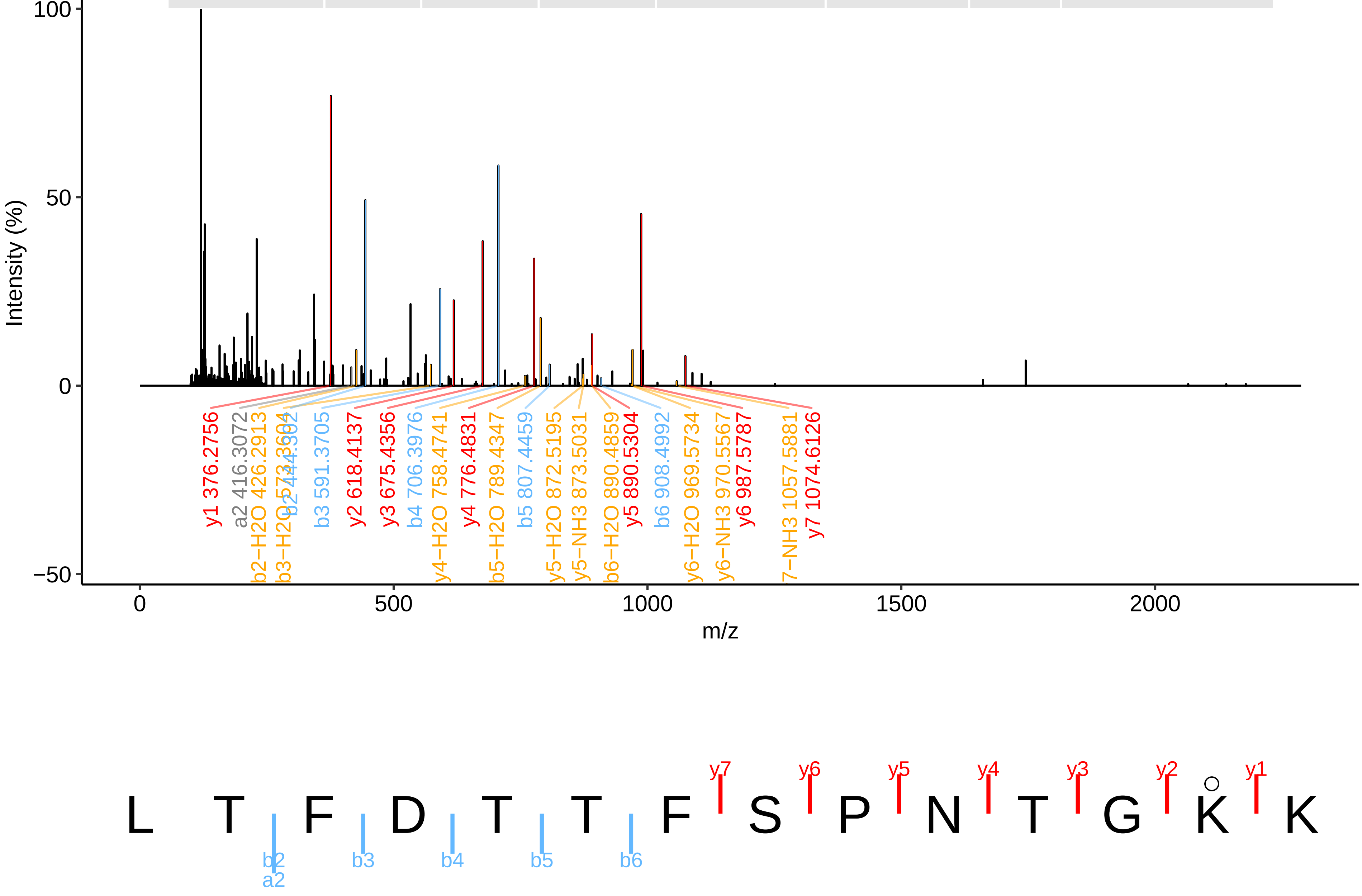

Modification □ Acetyl (Protein N−term) ○ GlyGly (K) no new terminus △ Oxidation (M)

20200921_KIZ_MUb4_GlyGly_10plex − Scan number: 19481

Gene Names	Charge	m/z	Mass	Retention time	PEP	Score	Precursor Intensity
VDAC2	3	557.6177	1669.831	70.265	4.9346e−05	109	9060403

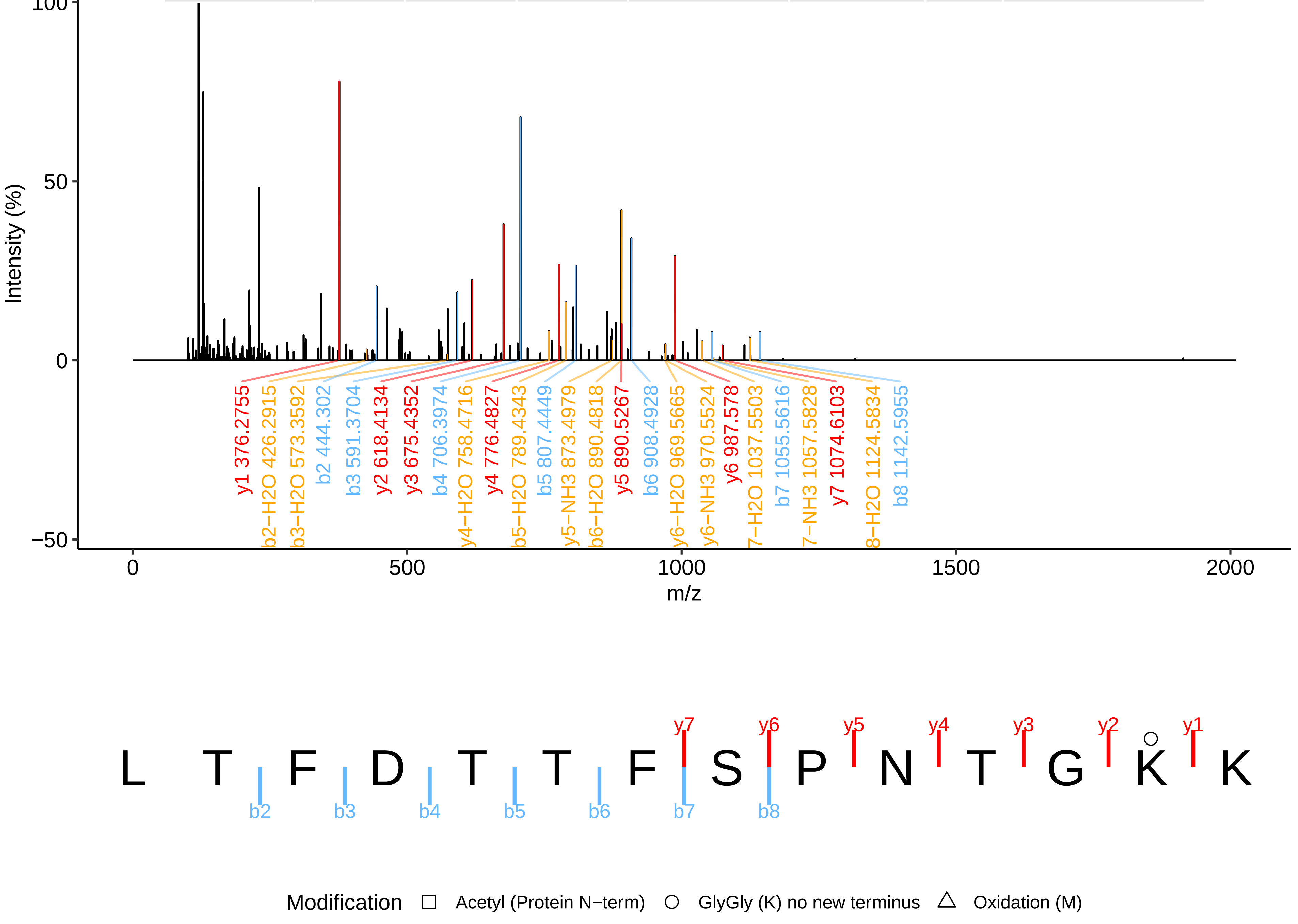

20200921_KIZ_MUb4_GlyGly_10plex − Scan number: 19614

Gene Names	Charge	m/z	Mass	Retention time	PEP	Score	Precursor Intensity
VDAC2	3	557.6177	1669.831	70.778	7.3019e−08	122.46	9483786

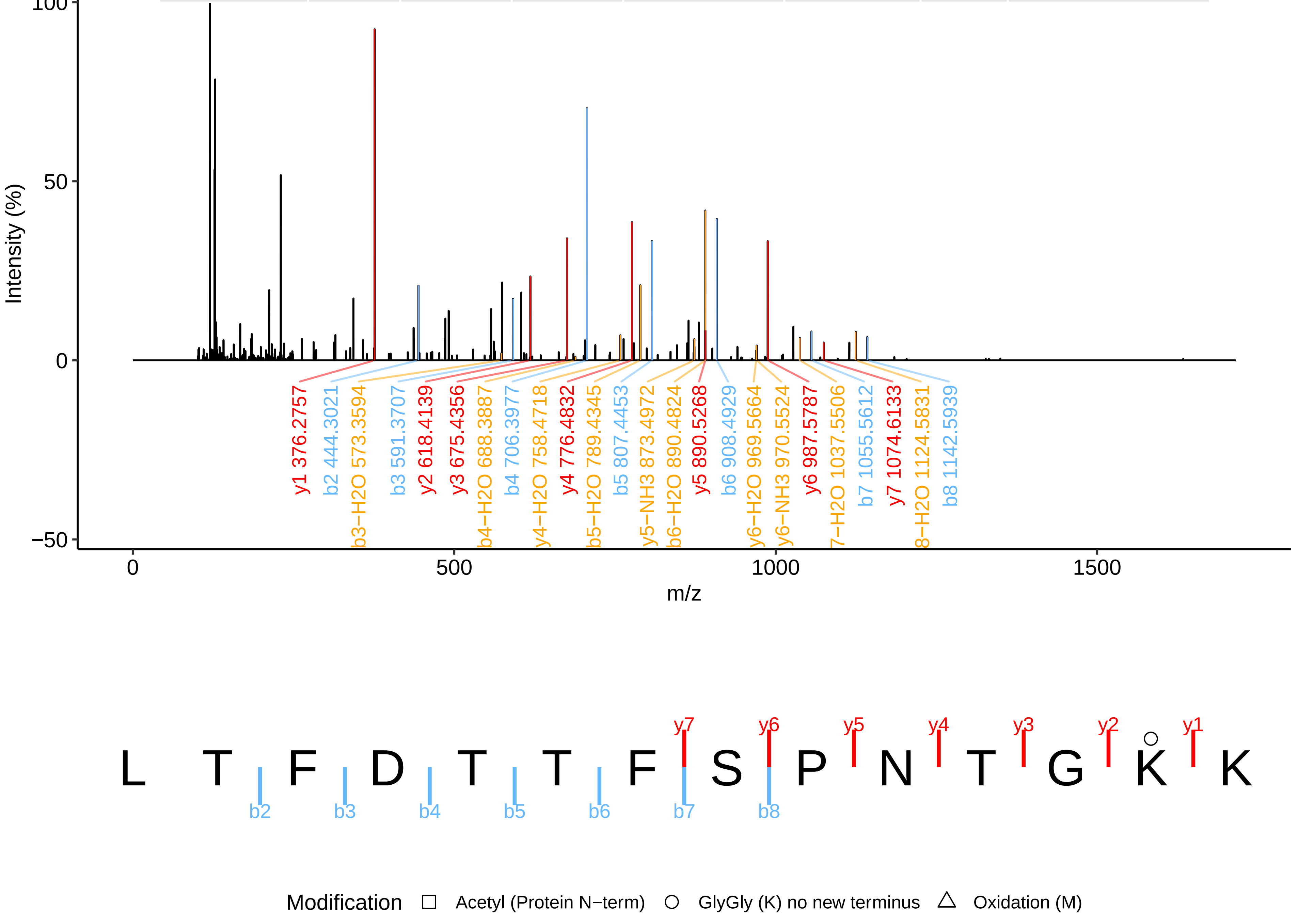

20200921_KIZ_MUb4_GlyGly_10plex − Scan number: 20029

Gene Names	Charge	m/z	Mass	Retention time	PEP	Score	Precursor Intensity
VDAC2	3	557.6177	1669.831	72.316	0.027579	70.628	3602946

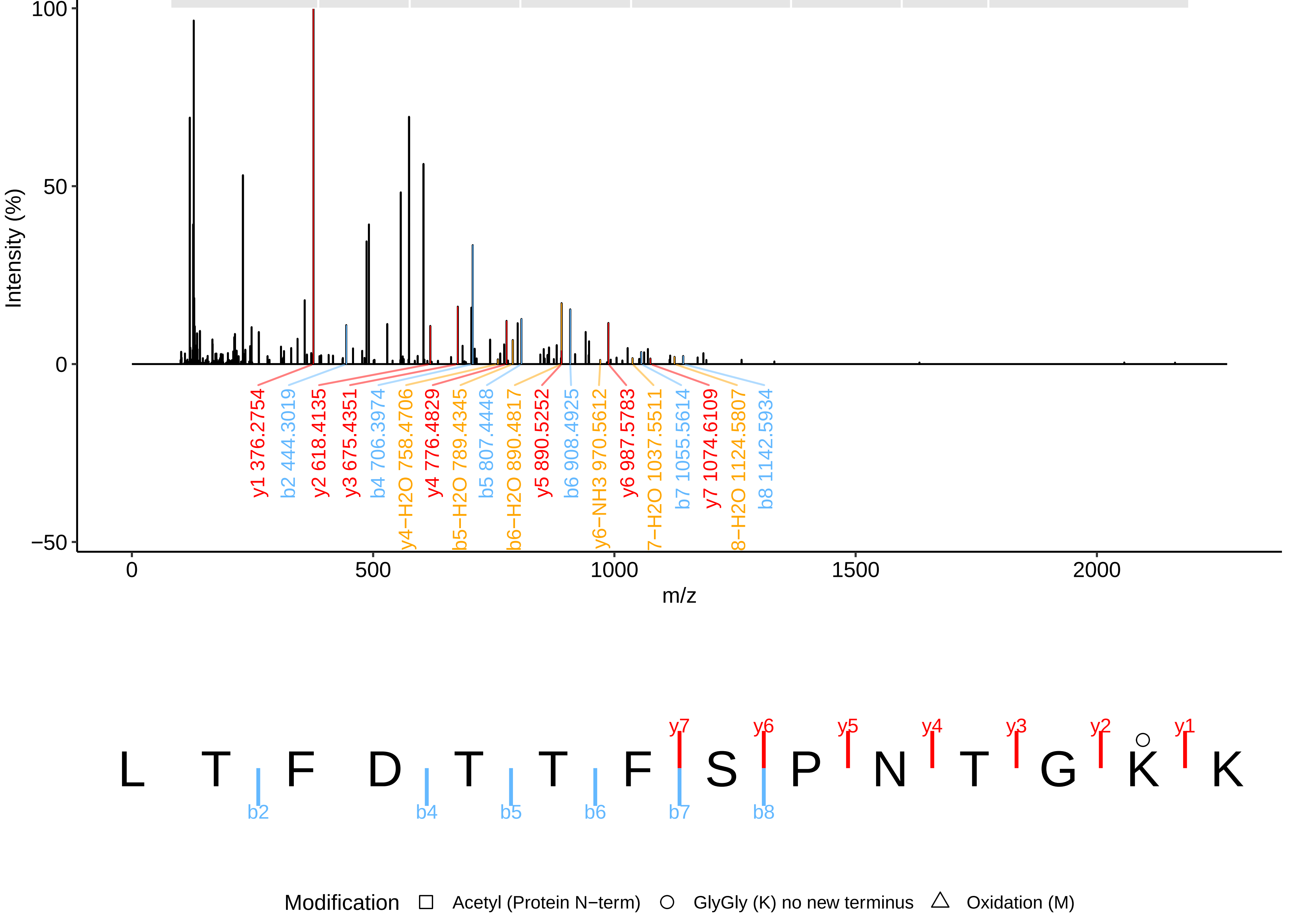

20200921_KIZ_MUb4_GlyGly_10plex − Scan number: 35379

Gene Names	Charge	m/z	Mass	Retention time	PEP	Score	Precursor Intensity
VDAC2	3	557.6177	1669.831	129.67	2.7892e−05	111.79	2840160

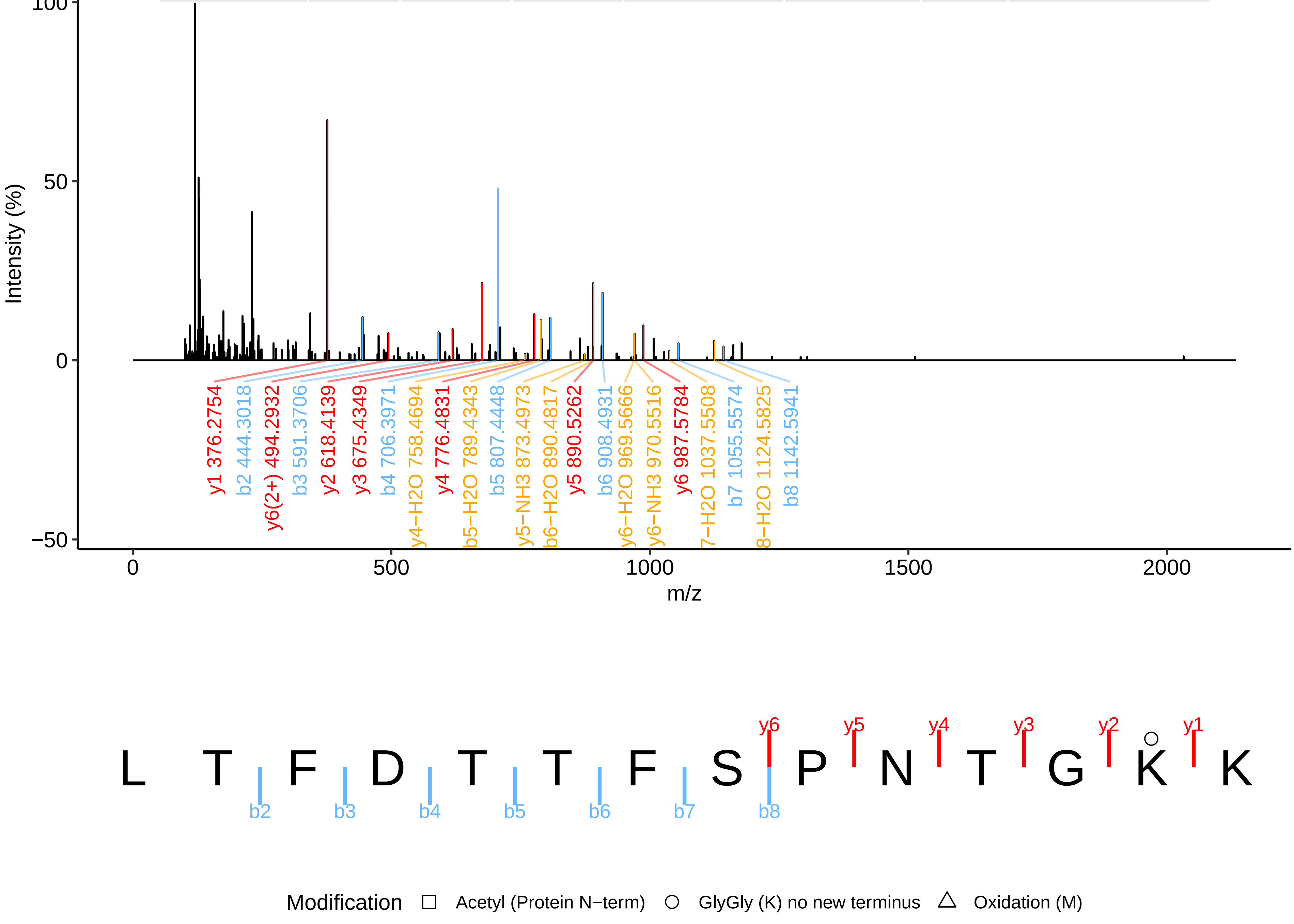

20200921_KIZ_MUb4_GlyGly_10plex − Scan number: 20250

Gene Names	Charge	m/z	Mass	Retention time	PEP	Score	Precursor Intensity
VDAC2	3	557.6177	1669.831	73.17	0.002941	83.358	4284060

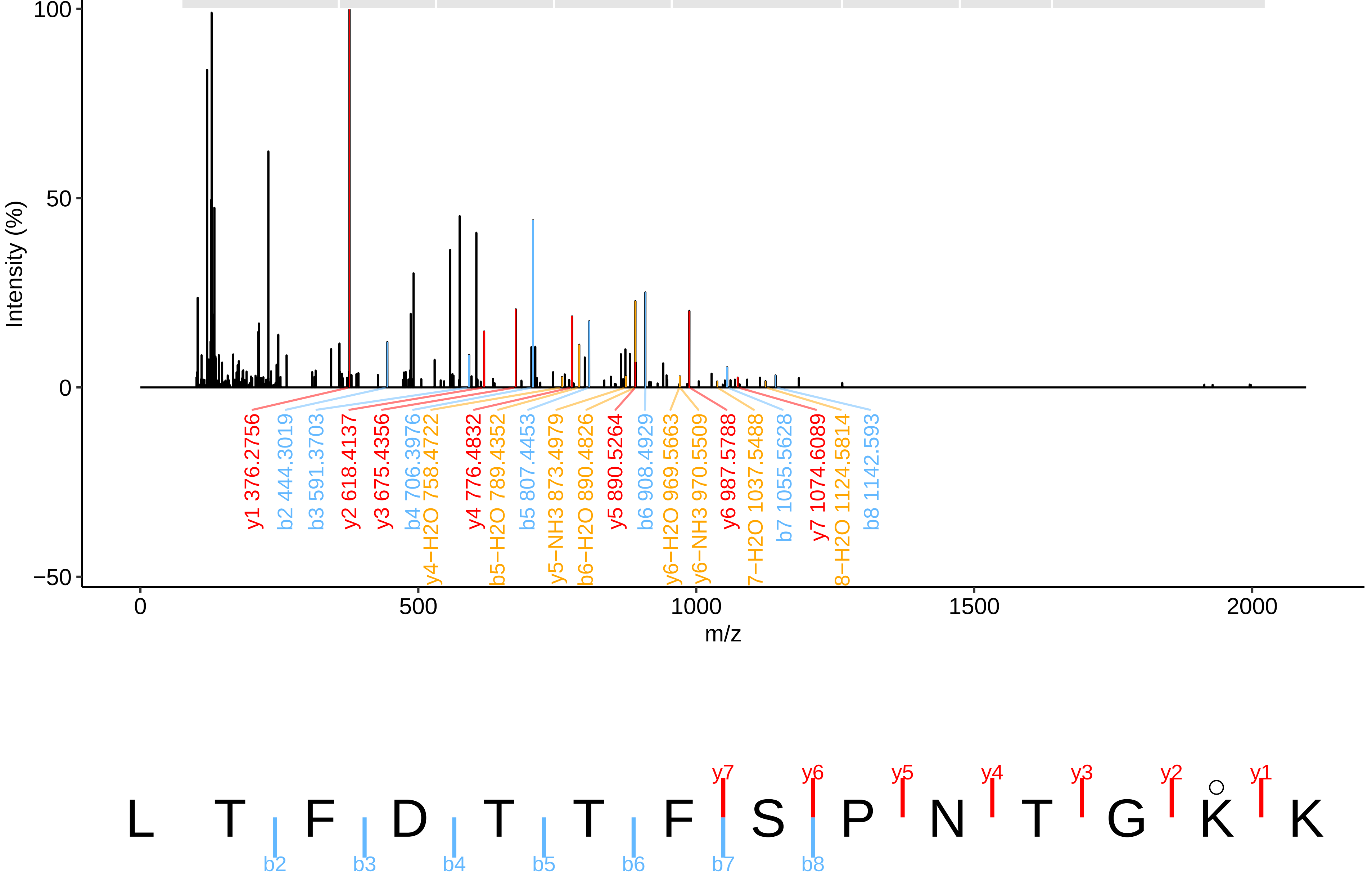

Modification □ Acetyl (Protein N−term) ○ GlyGly (K) no new terminus △ Oxidation (M)

20200921_KIZ_MUb4_GlyGly_10plex − Scan number: 21821

Gene Names	Charge	m/z	Mass	Retention time	PEP	Score	Precursor Intensity
VDAC2	3	557.6177	1669.831	79.085	0.00015367	101.53	NA

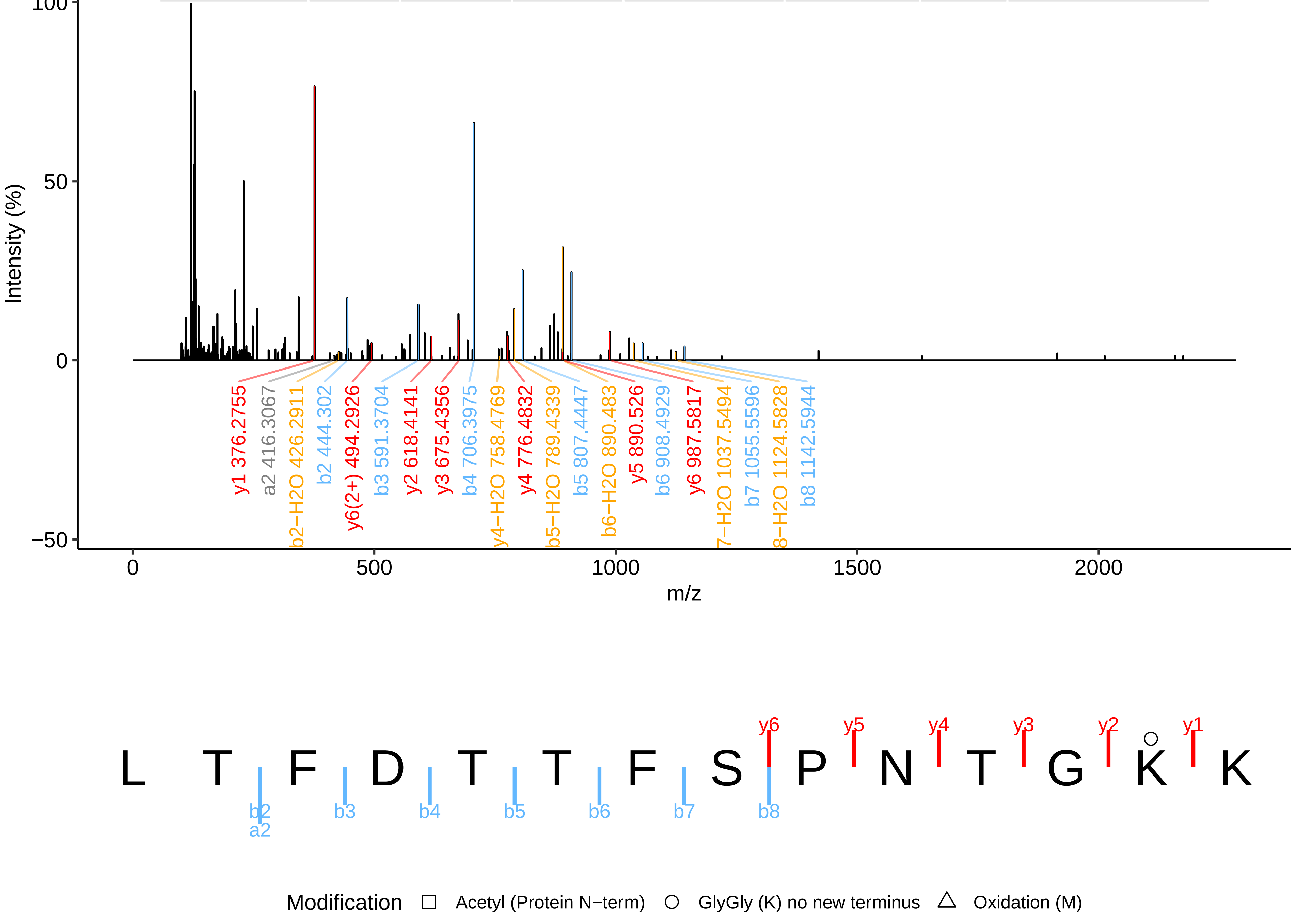

20200921_KIZ_MUb4_GlyGly_10plex_2 − Scan number: 17388

Gene Names	Charge	m/z	Mass	Retention time	PEP	Score	Precursor Intensity
VDAC2	3	557.6177	1669.831	67.998	1.6444e−12	138.42	3151163

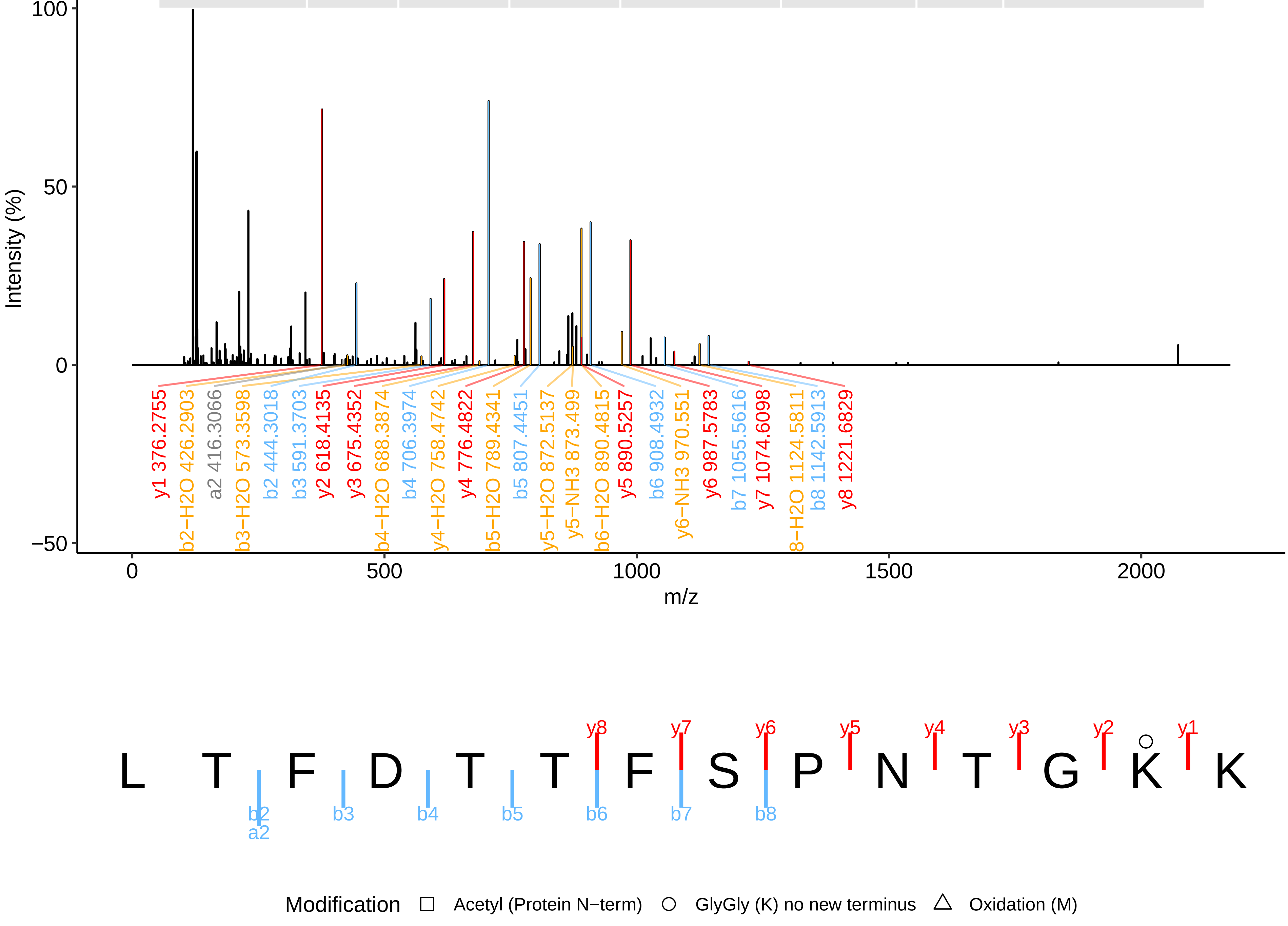

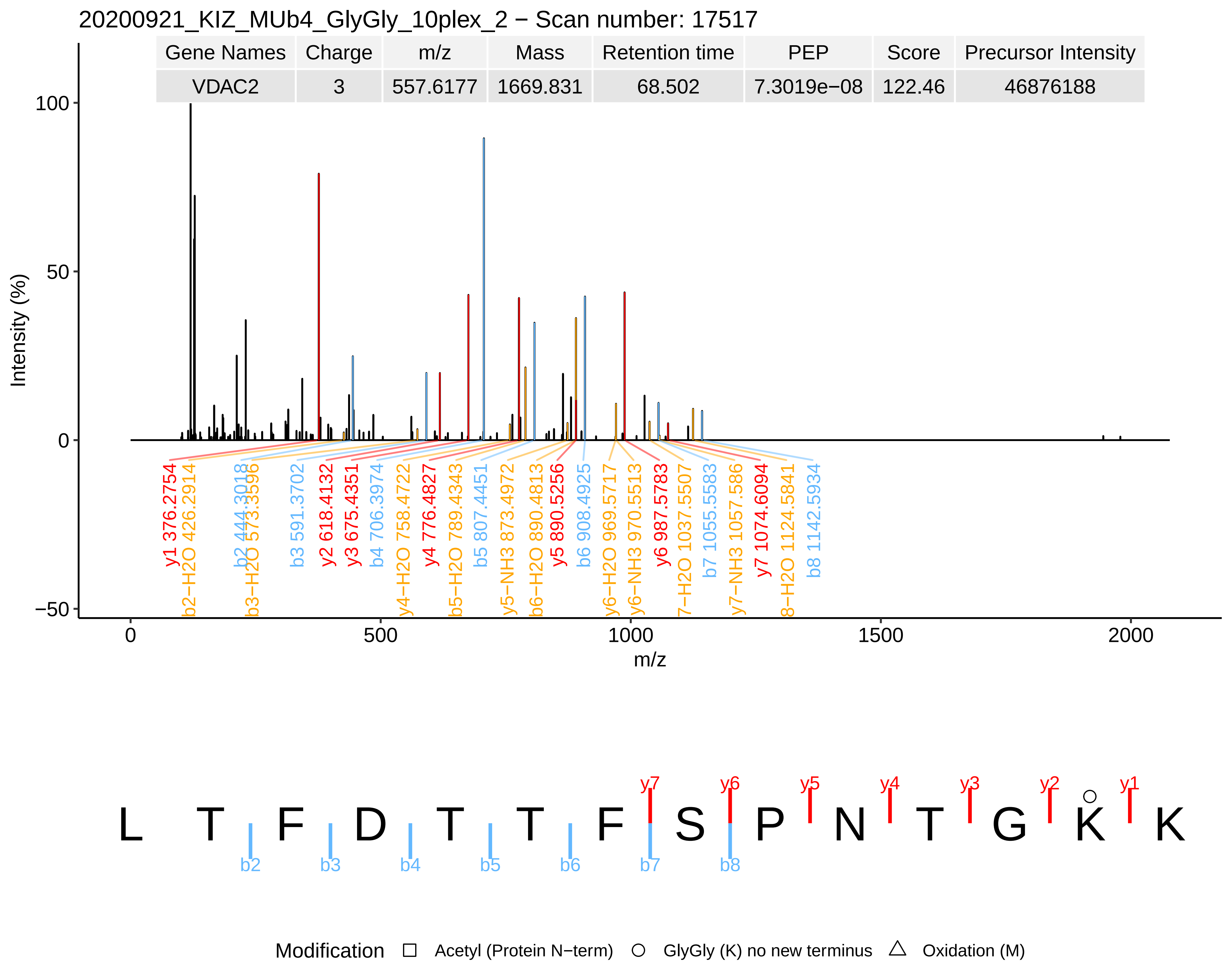

20200921_KIZ_MUb4_GlyGly_10plex_2 − Scan number: 17517
Gene Names | Charge | m/z | Mass | Retention time | PEP | Score | Precursor Intensity
VDAC2 | 3 | 557.6177 | 1669.831 | 68.502 | 7.3019e−08 | 122.46 | 46876188
Intensity (%)
100
50
0
−50
0
500
1000
1500
2000
m/z
y1 376.2754
b2−H2O 426.2914
b2 444.3018
b3−H2O 573.3596
b3 591.3702
y2 618.4132
y3 675.4351
b4 706.3974
y4−H2O 758.4722
y4 776.4827
b5−H2O 789.4343
b5 807.4451
y5−NH3 873.4972
b6−H2O 890.4813
y5 890.5256
b6 908.4925
y6−H2O 969.5717
y6−NH3 970.5513
y6 987.5783
7−H2O 1037.5507
b7 1055.5583
y7−NH3 1057.586
y7 1074.6094
8−H2O 1124.5841
b8 1142.5934
L T F D T T F S P N T G K K
y7 y6 y5 y4 y3 y2 y1
b2 b3 b4 b5 b6 b7 b8
Modification
Acetyl (Protein N−term)
GlyGly (K) no new terminus
Oxidation (M)

20200921_KIZ_MUb4_GlyGly_10plex_2 − Scan number: 17546

Gene Names	Charge	m/z	Mass	Retention time	PEP	Score	Precursor Intensity
VDAC2	4	418.4651	1669.831	68.622	0.038662	50.563	NA

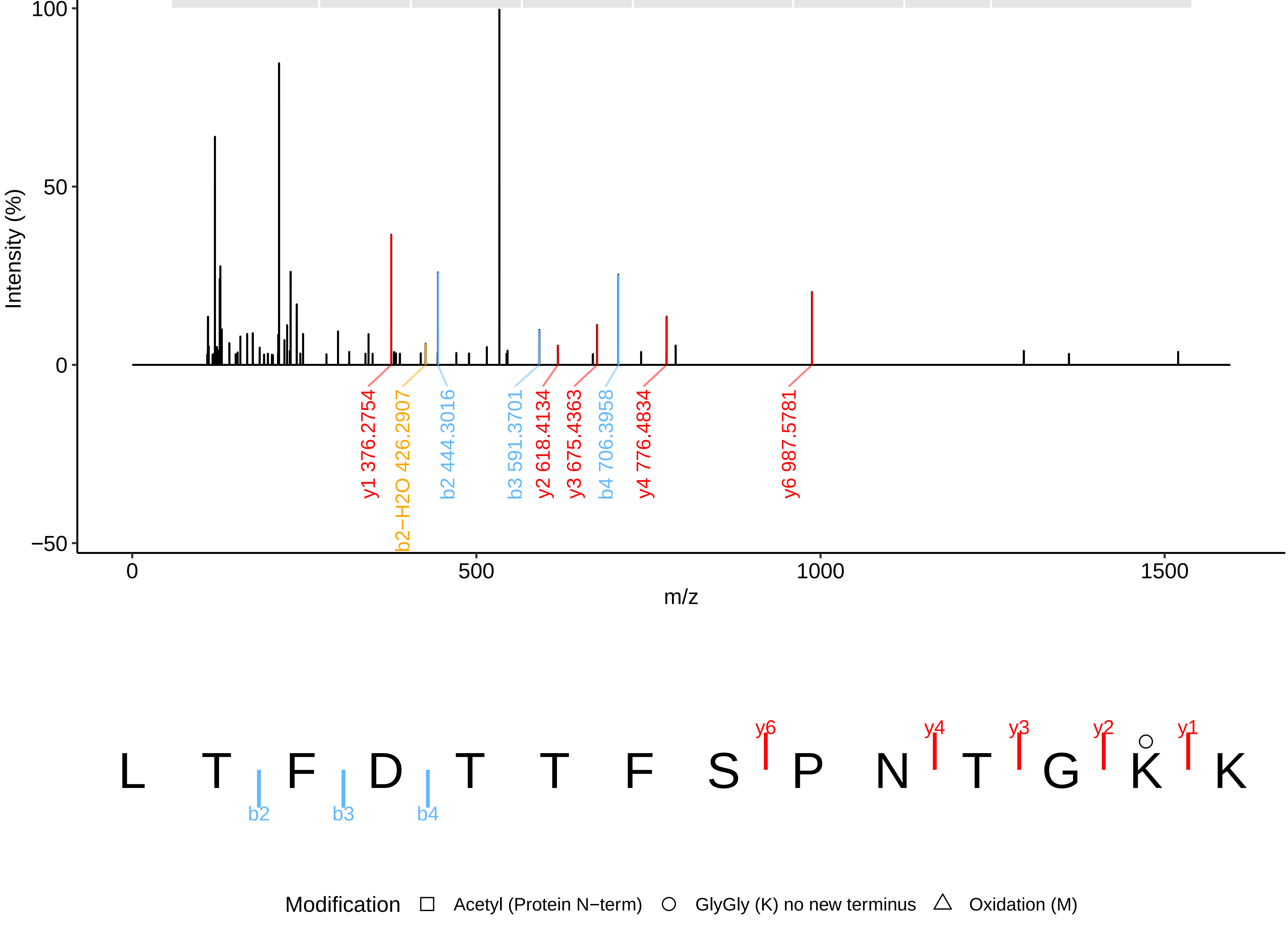

20200921_KIZ_MUb4_GlyGly_10plex_2 − Scan number: 17743

Gene Names	Charge	m/z	Mass	Retention time	PEP	Score	Precursor Intensity
VDAC2	3	557.6177	1669.831	69.415	0.029678	69.716	1054431

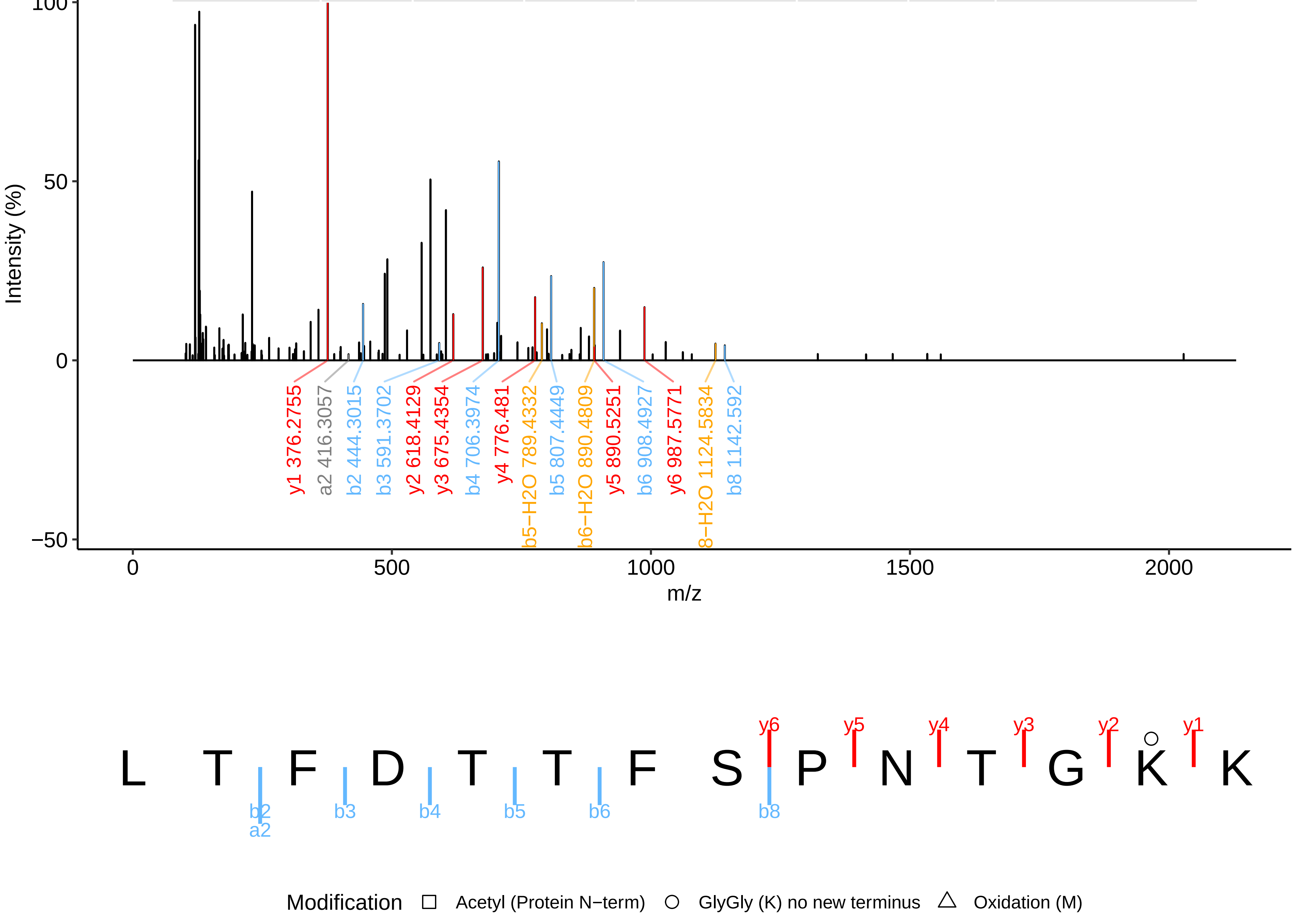

20200924_KIZ_MUb5_GlyGly_1 − Scan number: 18293

Gene Names	Charge	m/z	Mass	Retention time	PEP	Score	Precursor Intensity
VDAC2	3	557.6177	1669.831	59.149	4.0511e−13	147.33	231581856

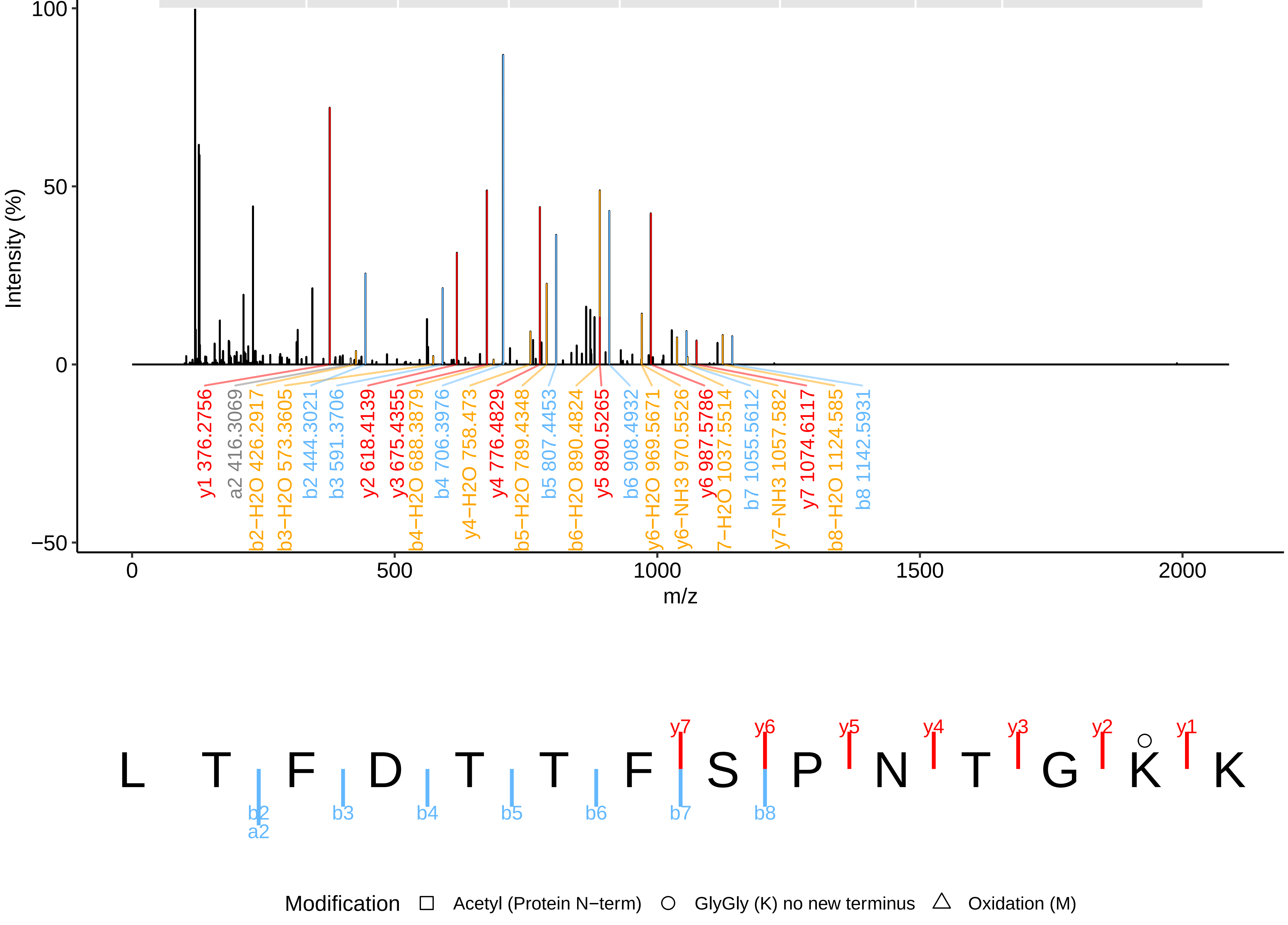

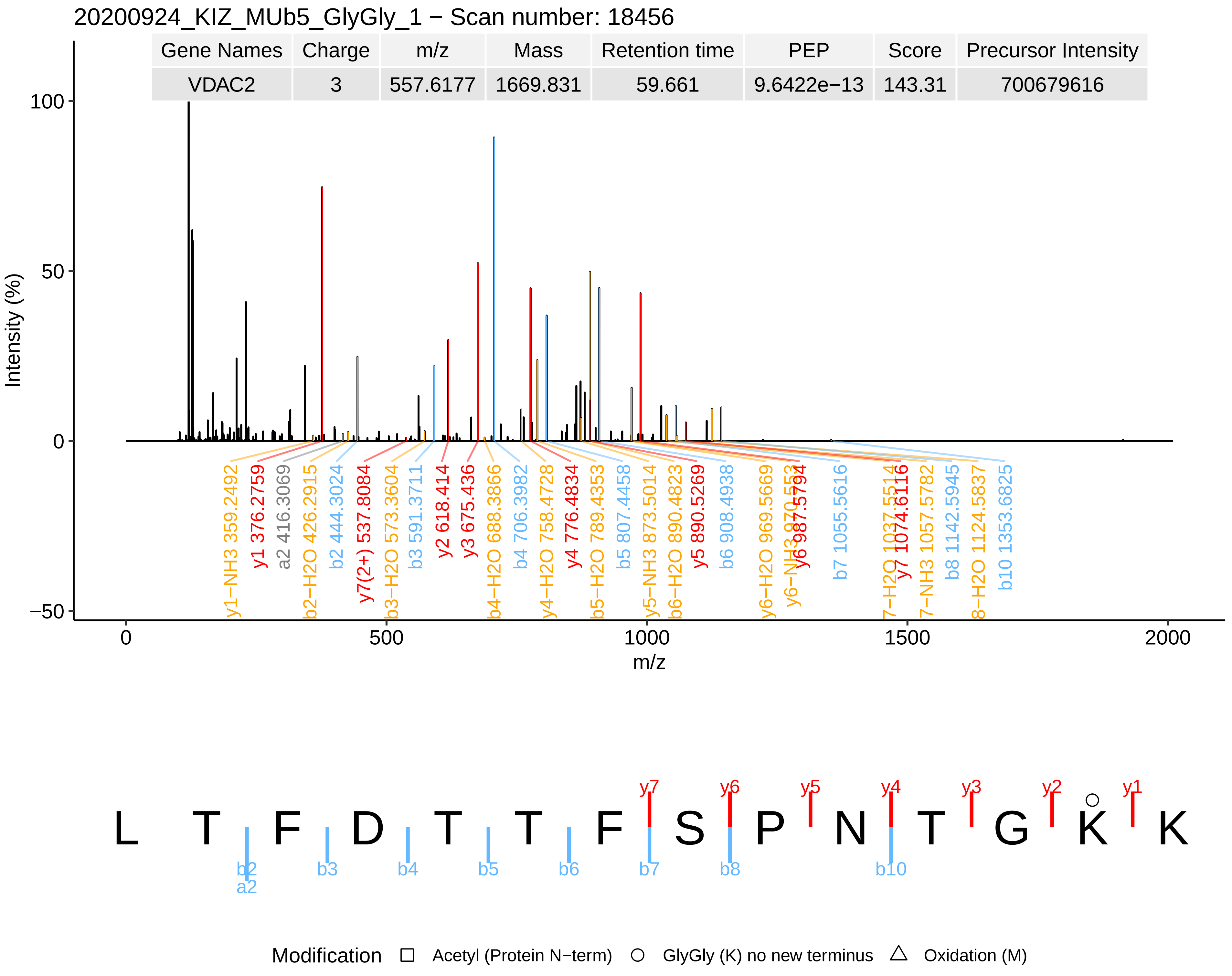
20200924_KIZ_MUb5_GlyGly_1 − Scan number: 18456
Gene Names | Charge | m/z | Mass | Retention time | PEP | Score | Precursor Intensity
VDAC2 | 3 | 557.6177 | 1669.831 | 59.661 | 9.6422e−13 | 143.31 | 700679616
Intensity (%)
m/z
y1−NH3 359.2492
y1 376.2759
a2 416.3069
b2−H2O 426.2915
b2 444.3024
y7(2+) 537.8084
b3−H2O 573.3604
b3 591.3711
y2 618.414
y3 675.436
b4−H2O 688.3866
b4 706.3982
y4−H2O 758.4728
y4 776.4834
b5−H2O 789.4353
b5 807.4458
y5−NH3 873.5014
b6−H2O 890.4823
y5 890.5269
b6 908.4938
y6−H2O 969.5669
y6−NH3 970.553
y6 987.5794
b7 1055.5616
y7−H2O 1057.5514
y7 1074.6116
y7−NH3 1057.5782
b8 1142.5945
b8−H2O 1124.5837
b10 1353.6825
L T F D T T F S P N T G K K
y7 y6 y5 y4 y3 y2 y1
b2 a2 b3 b4 b5 b6 b7 b8 b10
Modification
Acetyl (Protein N−term)
GlyGly (K) no new terminus
Oxidation (M)

20200924_KIZ_MUb5_GlyGly_1 − Scan number: 18619

Gene Names	Charge	m/z	Mass	Retention time	PEP	Score	Precursor Intensity
VDAC2	3	557.6177	1669.831	60.175	1.1301e−12	142.12	777468032

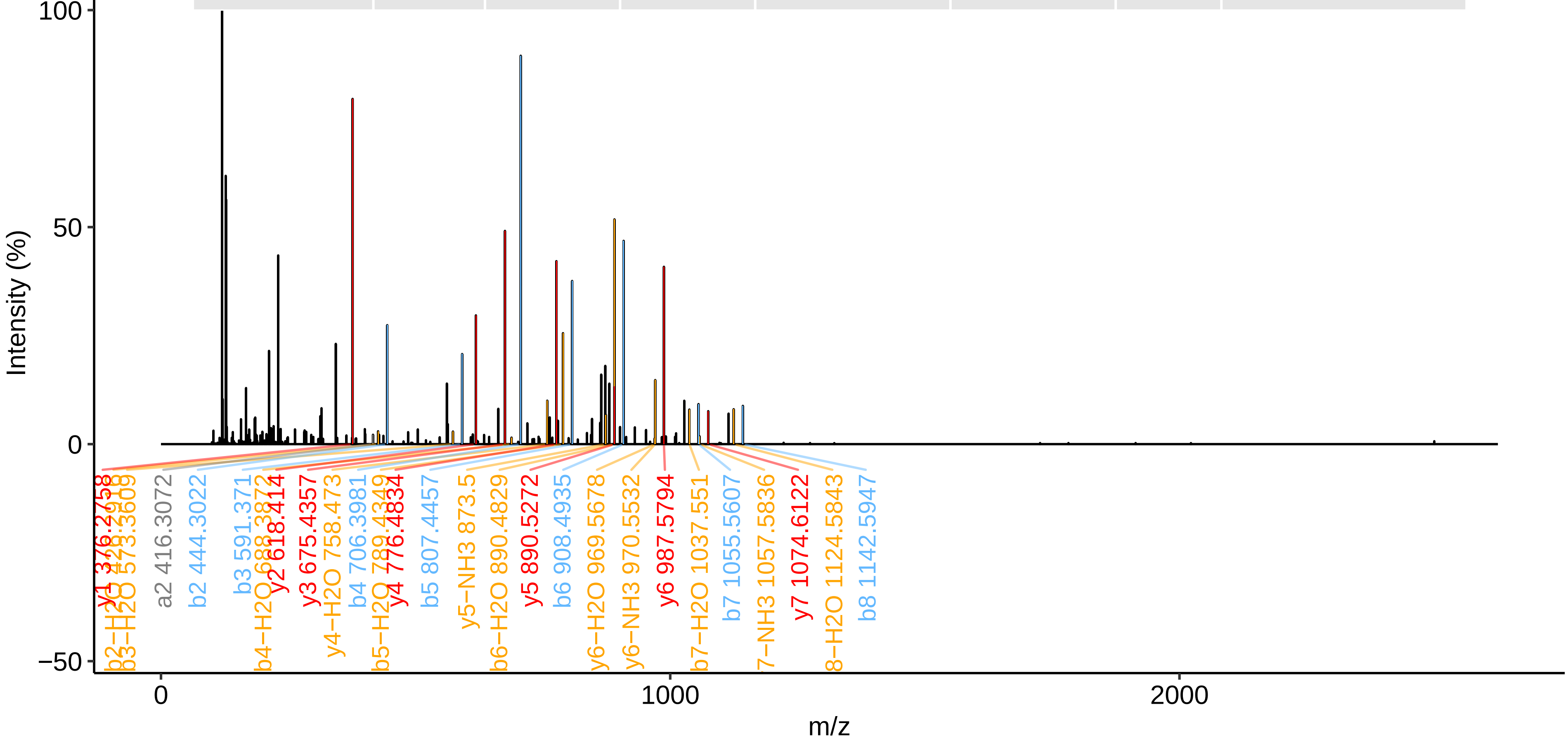

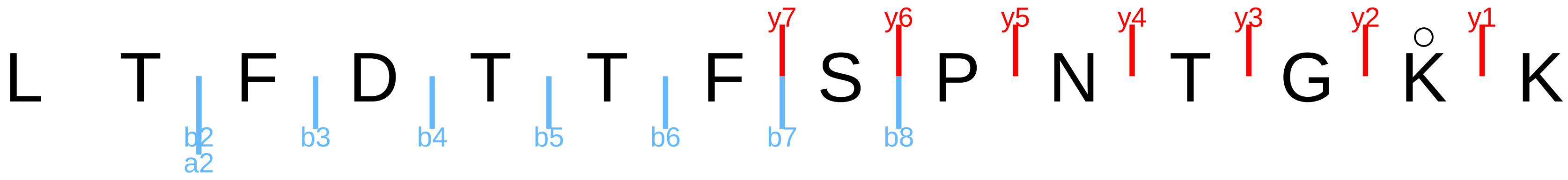

Modification □ Acetyl (Protein N−term) ○ GlyGly (K) no new terminus △ Oxidation (M)

20200924_KIZ_MUb5_GlyGly_1 − Scan number: 18778

Gene Names	Charge	m/z	Mass	Retention time	PEP	Score	Precursor Intensity
VDAC2	3	557.6177	1669.831	60.677	7.3897e−19	153.75	428182720

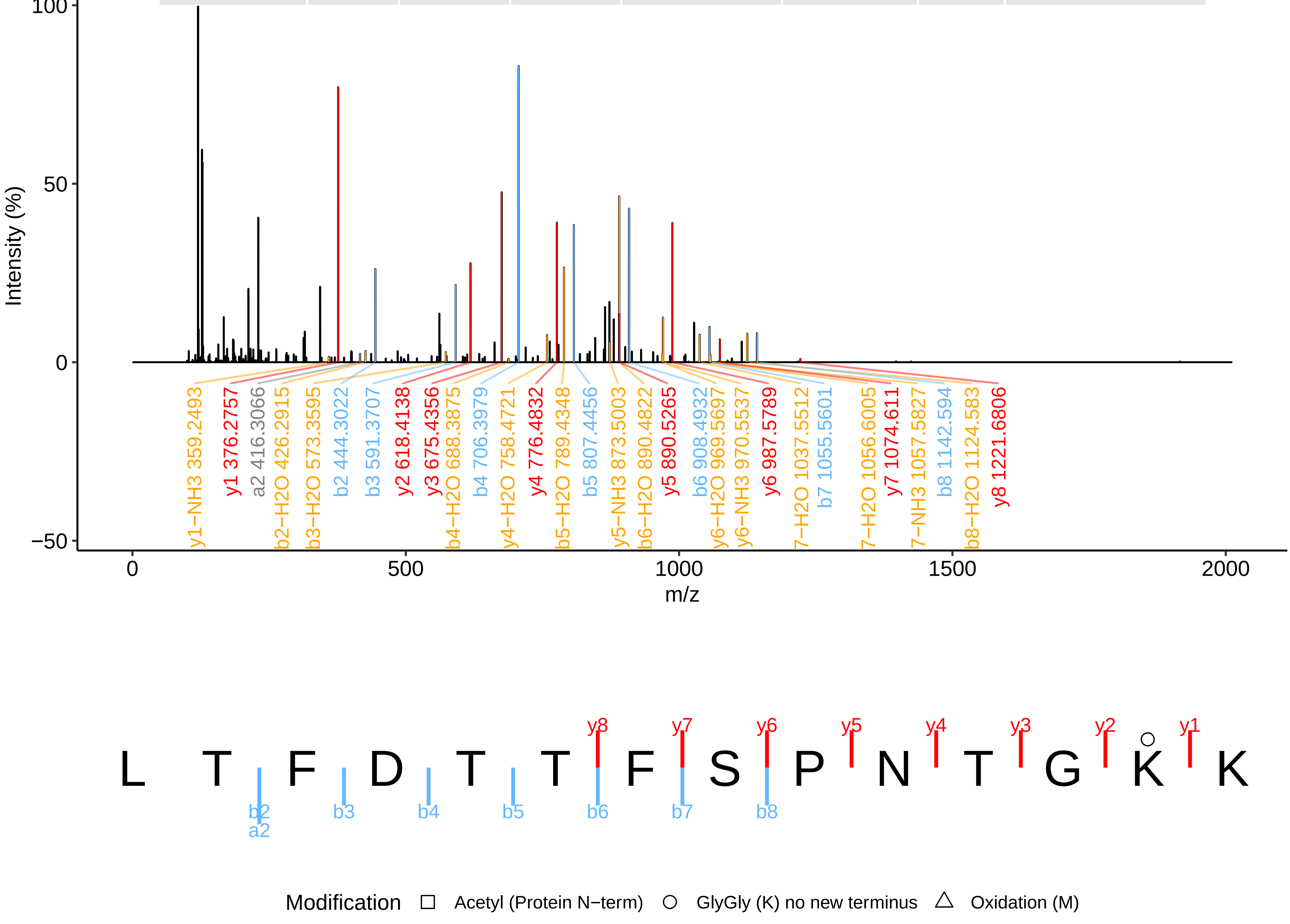

20200924_KIZ_MUb5_GlyGly_1 − Scan number: 18937

Gene Names	Charge	m/z	Mass	Retention time	PEP	Score	Precursor Intensity
VDAC2	3	557.6177	1669.831	61.187	1.4238e−12	140.01	64375104

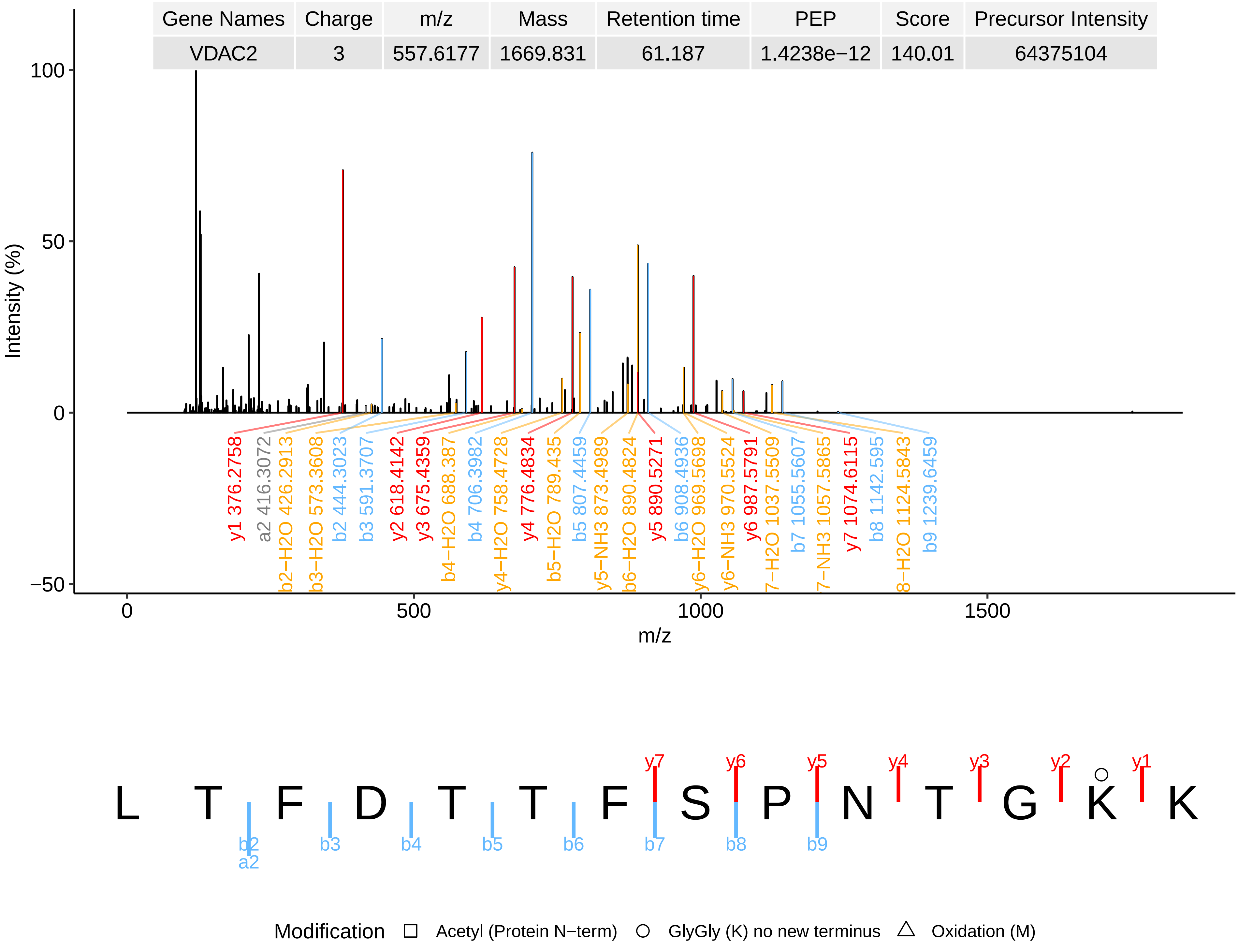

20200924_KIZ_MUb5_GlyGly_1 − Scan number: 18382

Gene Names	Charge	m/z	Mass	Retention time	PEP	Score	Precursor Intensity
VDAC2	4	418.4651	1669.831	59.433	6.9863e−06	107.39	NA

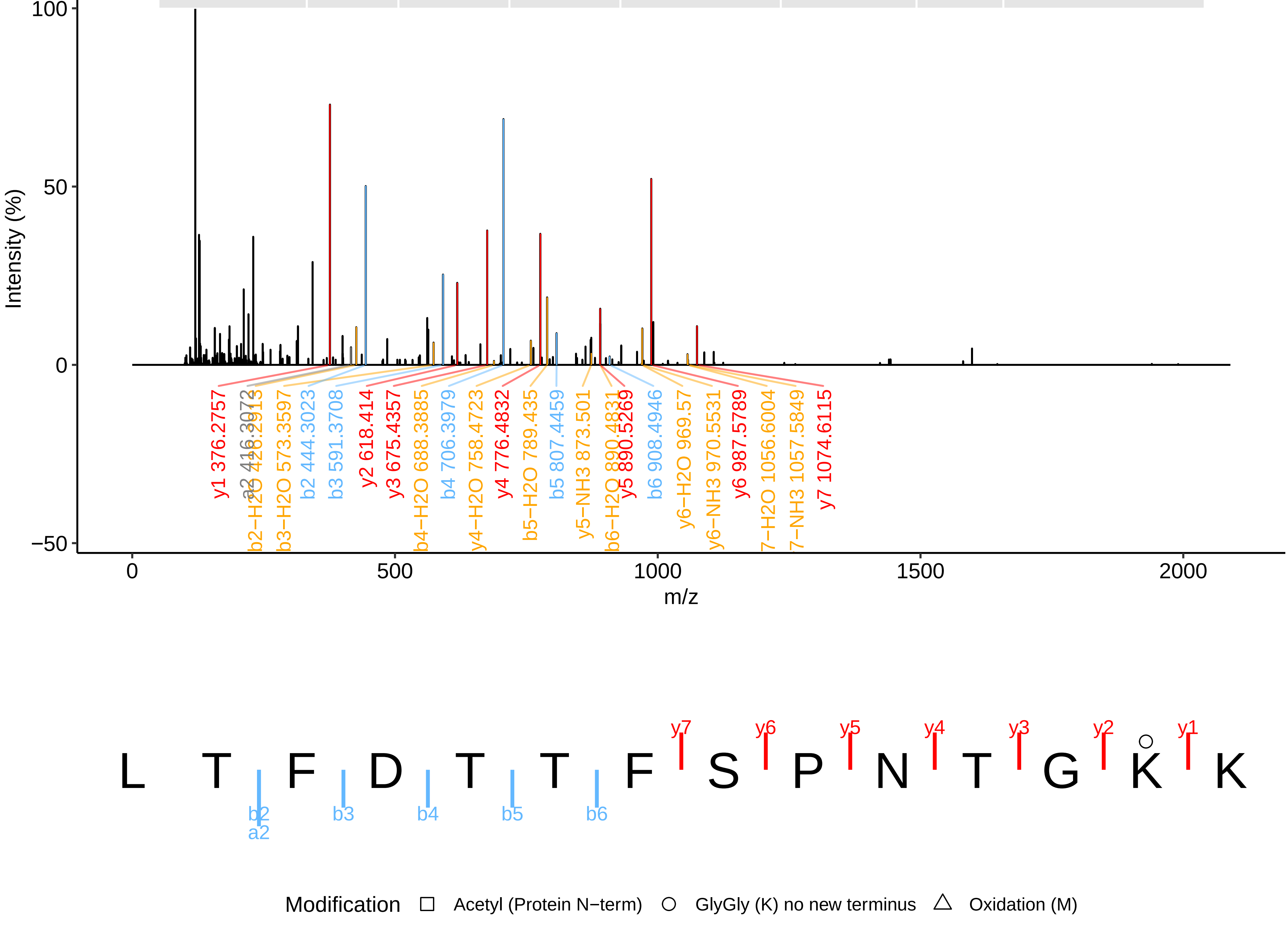

20200924_KIZ_MUb5_GlyGly_1 − Scan number: 18137

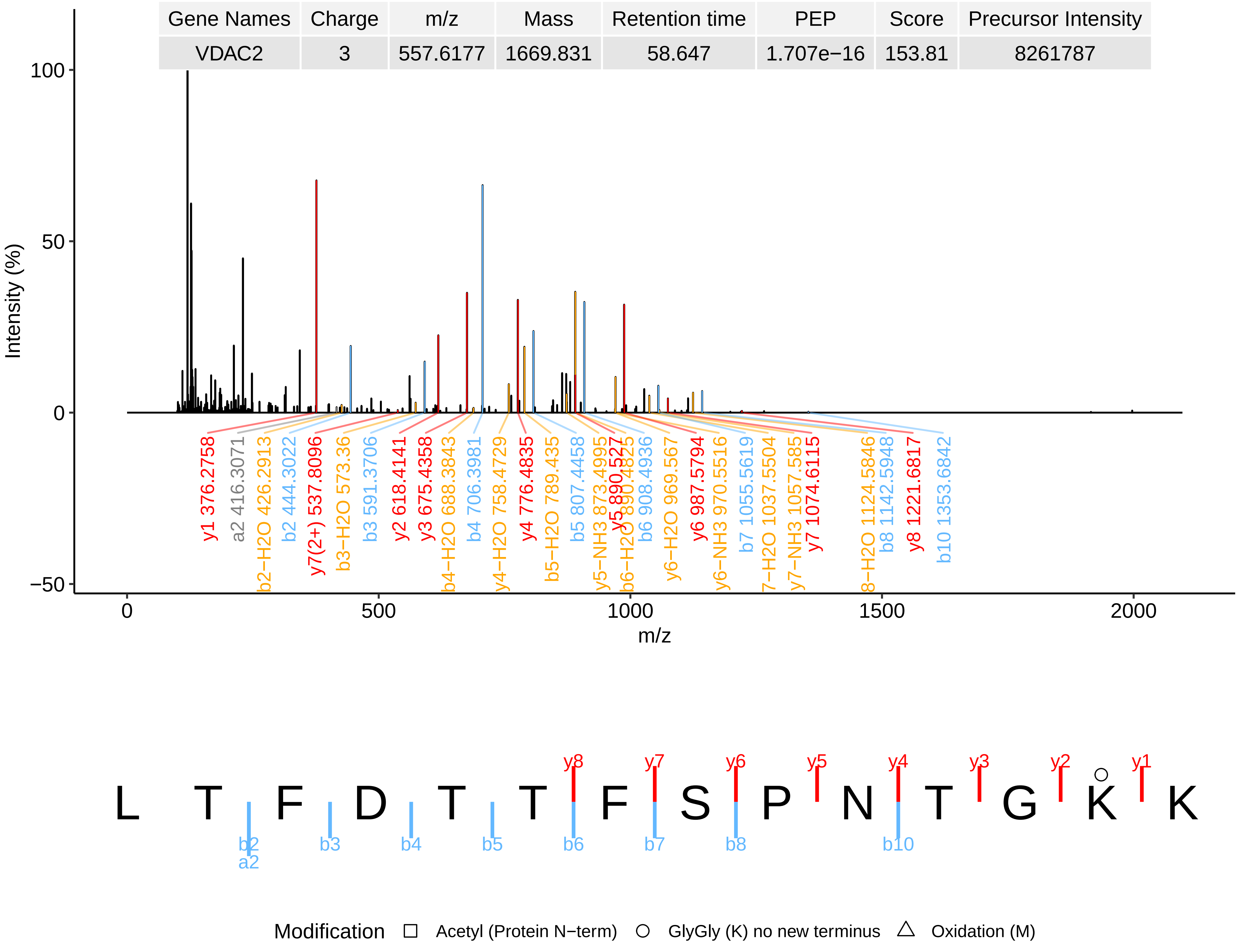

20200924_KIZ_MUb5_GlyGly_1 − Scan number: 18446

Gene Names	Charge	m/z	Mass	Retention time	PEP	Score	Precursor Intensity
VDAC2	2	835.9229	1669.831	59.629	2.9339e−88	312.95	NA

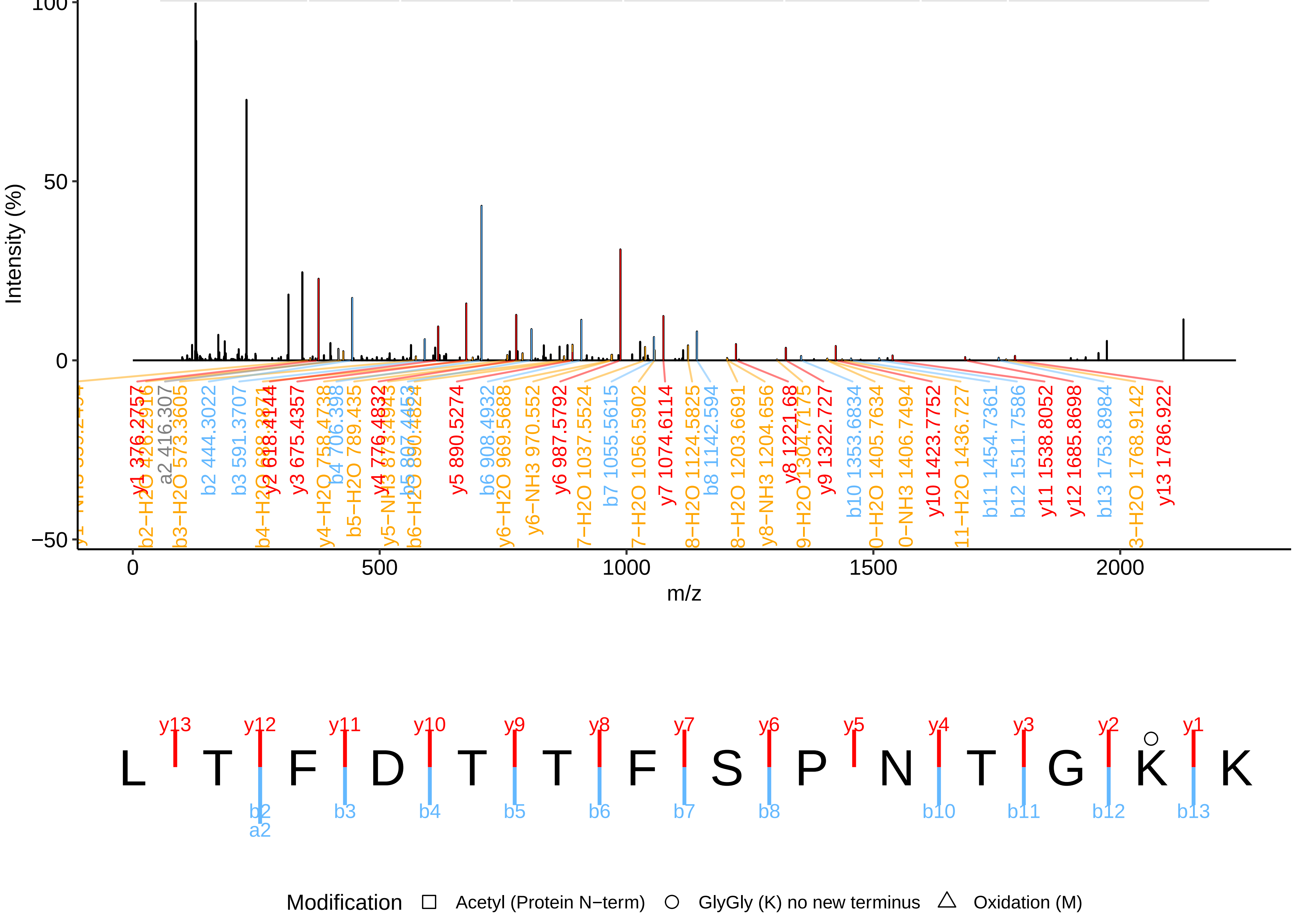

20200924_KIZ_MUb5_GlyGly_2 − Scan number: 16885

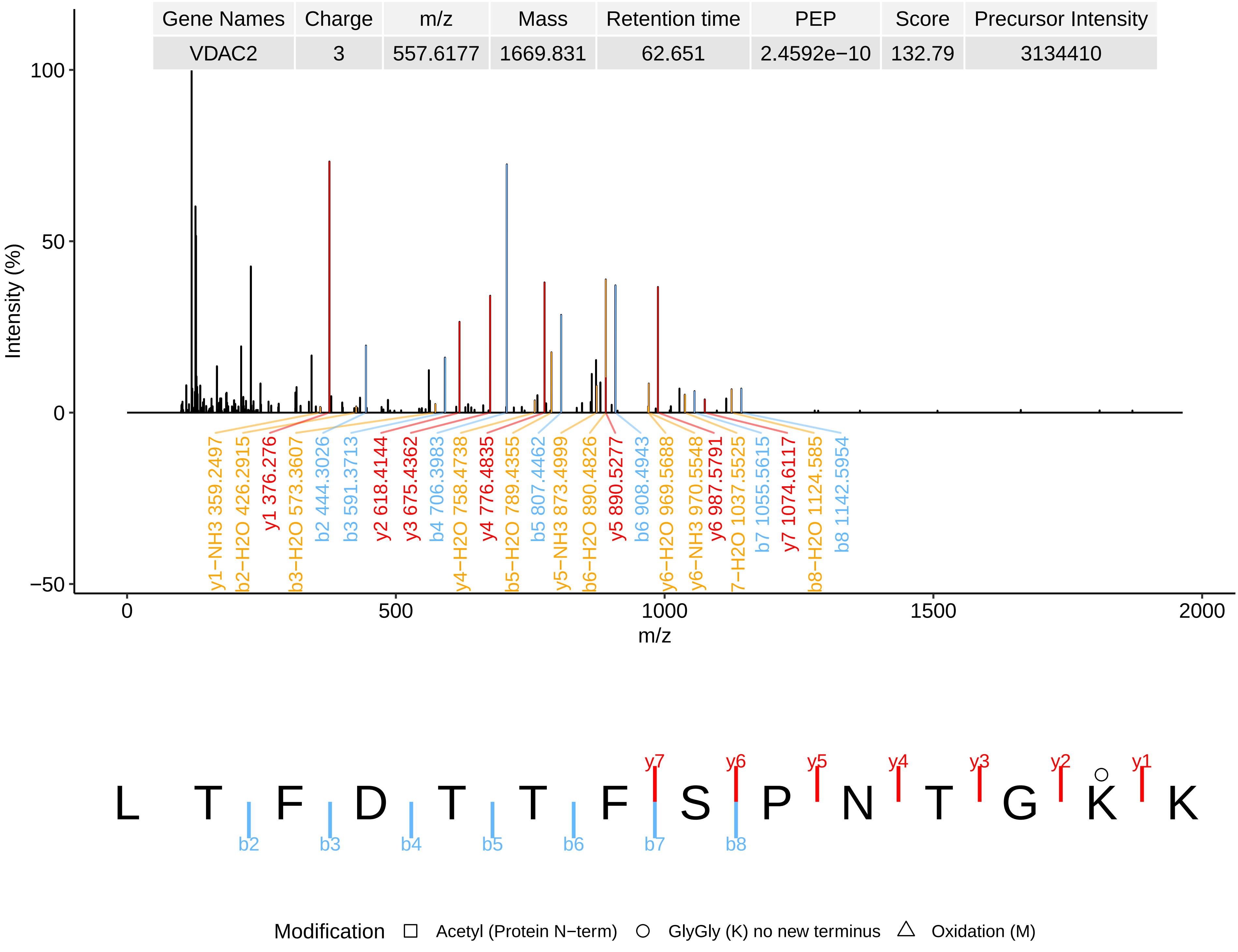

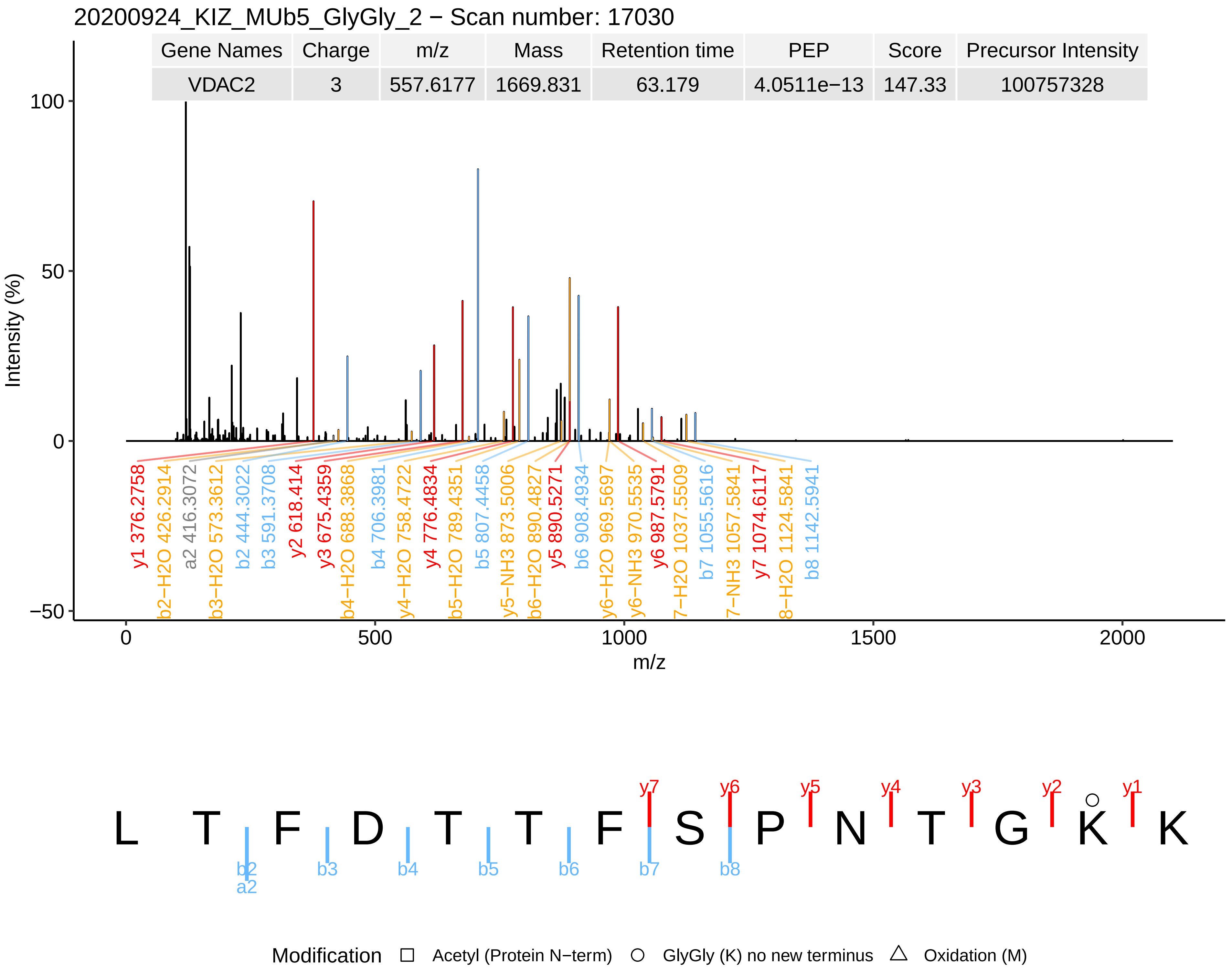

20200924_KIZ_MUb5_GlyGly_2 − Scan number: 17030
Gene Names | Charge | m/z | Mass | Retention time | PEP | Score | Precursor Intensity
VDAC2 | 3 | 557.6177 | 1669.831 | 63.179 | 4.0511e−13 | 147.33 | 100757328
Intensity (%)
100
50
0
−50
0
500
1000
1500
2000
m/z
y1 376.2758
b2−H2O 426.2914
a2 416.3072
b3−H2O 573.3612
b2 444.3022
b3 591.3708
y2 618.414
y3 675.4359
b4−H2O 688.3868
b4 706.3981
y4−H2O 758.4722
y4 776.4834
b5−H2O 789.4351
b5 807.4458
y5−NH3 873.5006
b6−H2O 890.4827
y5 890.5271
b6 908.4934
y6−H2O 969.5697
y6−NH3 970.5535
y6 987.5791
7−H2O 1037.5509
b7 1055.5616
7−NH3 1057.5841
y7 1074.6117
8−H2O 1124.5841
b8 1142.5941
L T F D T T F S P N T G K K
y7 y6 y5 y4 y3 y2 y1
b2 a2 b3 b4 b5 b6 b7 b8
Modification
Acetyl (Protein N−term)
GlyGly (K) no new terminus
Oxidation (M)

20200924_KIZ_MUb5_GlyGly_2 − Scan number: 17176

Gene Names	Charge	m/z	Mass	Retention time	PEP	Score	Precursor Intensity
VDAC2	3	557.6177	1669.831	63.69	9.467e−08	119.39	298354432

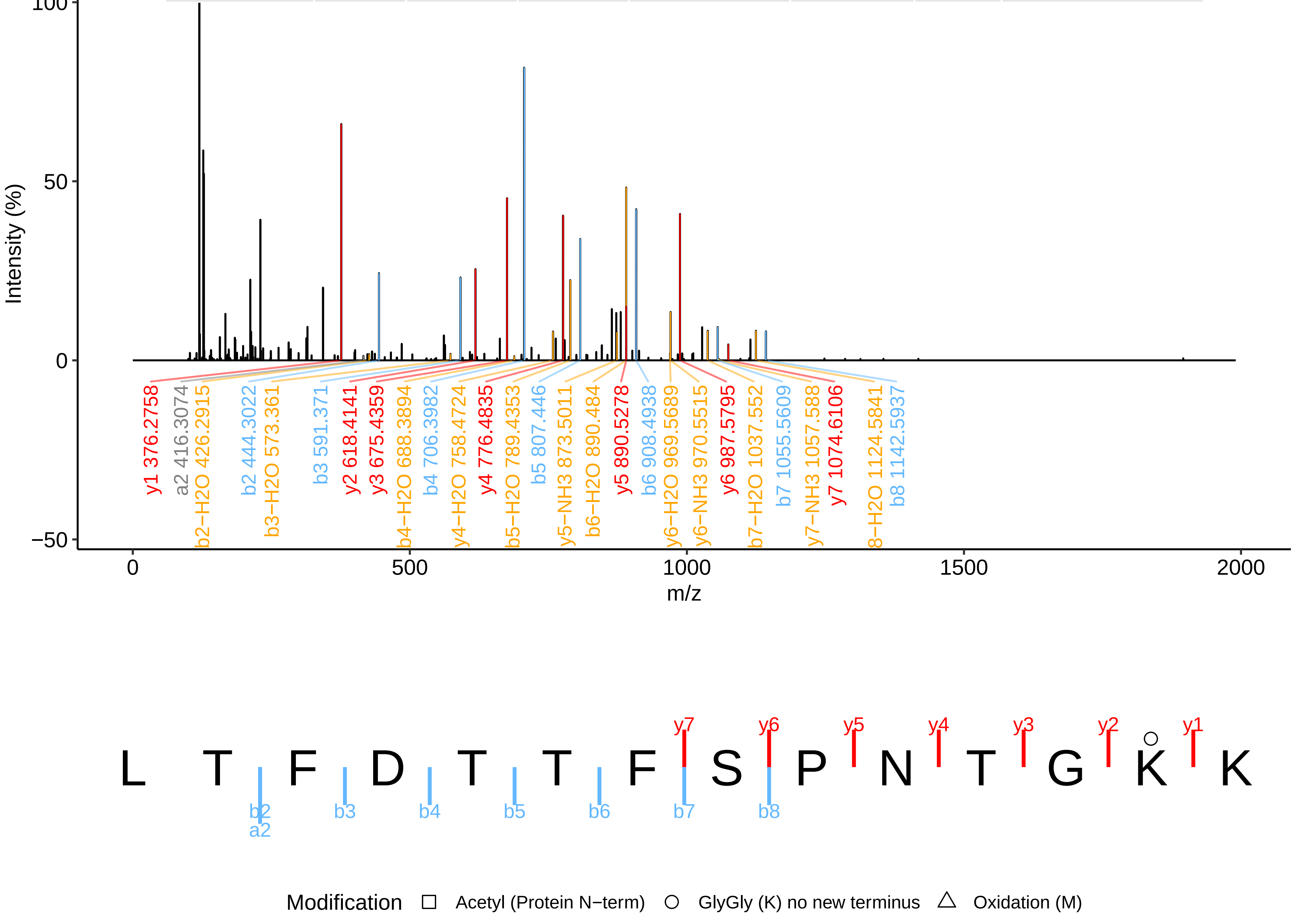

20200924_KIZ_MUb5_GlyGly_2 − Scan number: 17320

Gene Names	Charge	m/z	Mass	Retention time	PEP	Score	Precursor Intensity
VDAC2	3	557.6177	1669.831	64.198	7.3897e−19	153.75	198465104

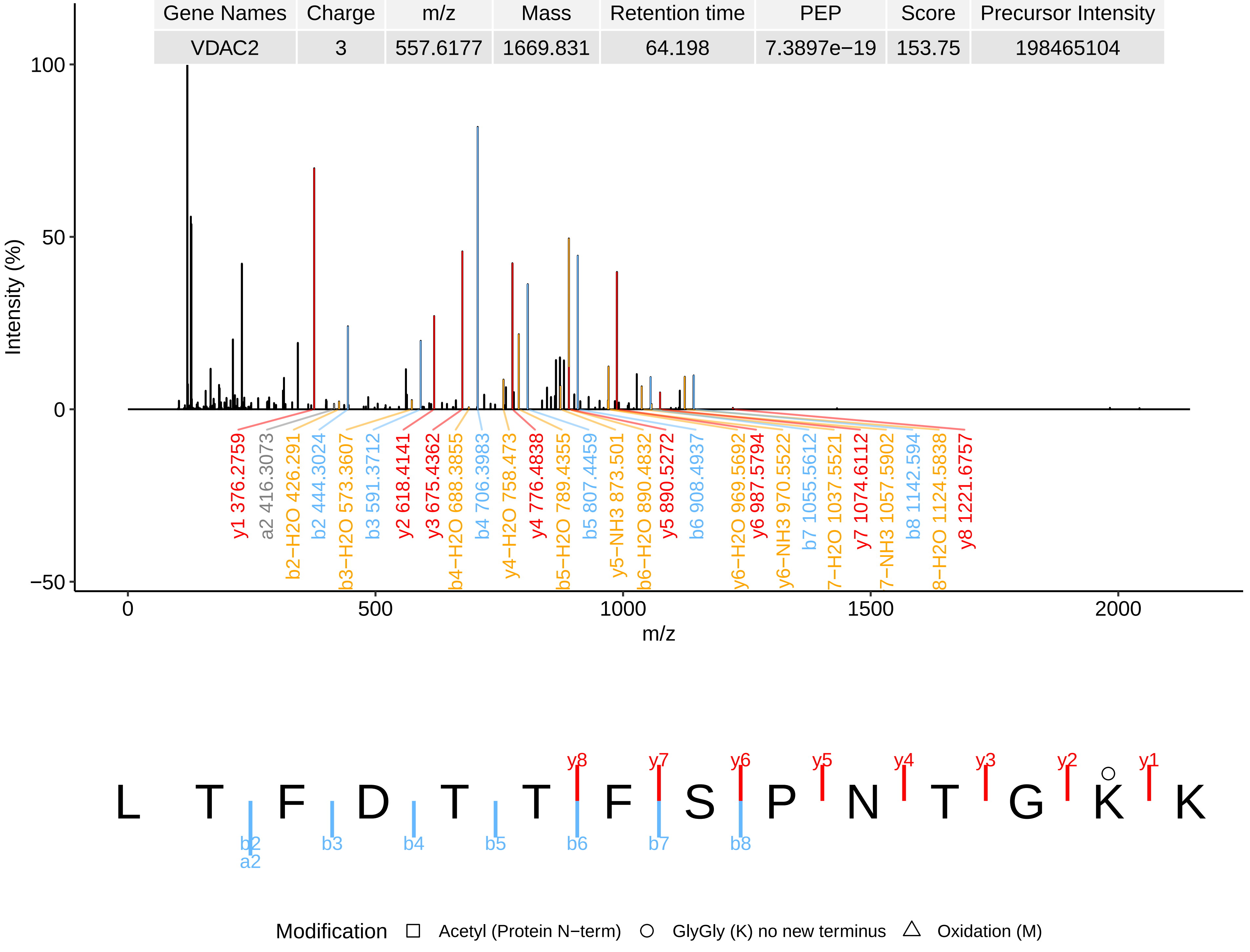

20200924_KIZ_MUb5_GlyGly_2 − Scan number: 17460

Gene Names	Charge	m/z	Mass	Retention time	PEP	Score	Precursor Intensity
VDAC2	3	557.6177	1669.831	64.707	1.1301e−12	142.12	37412420

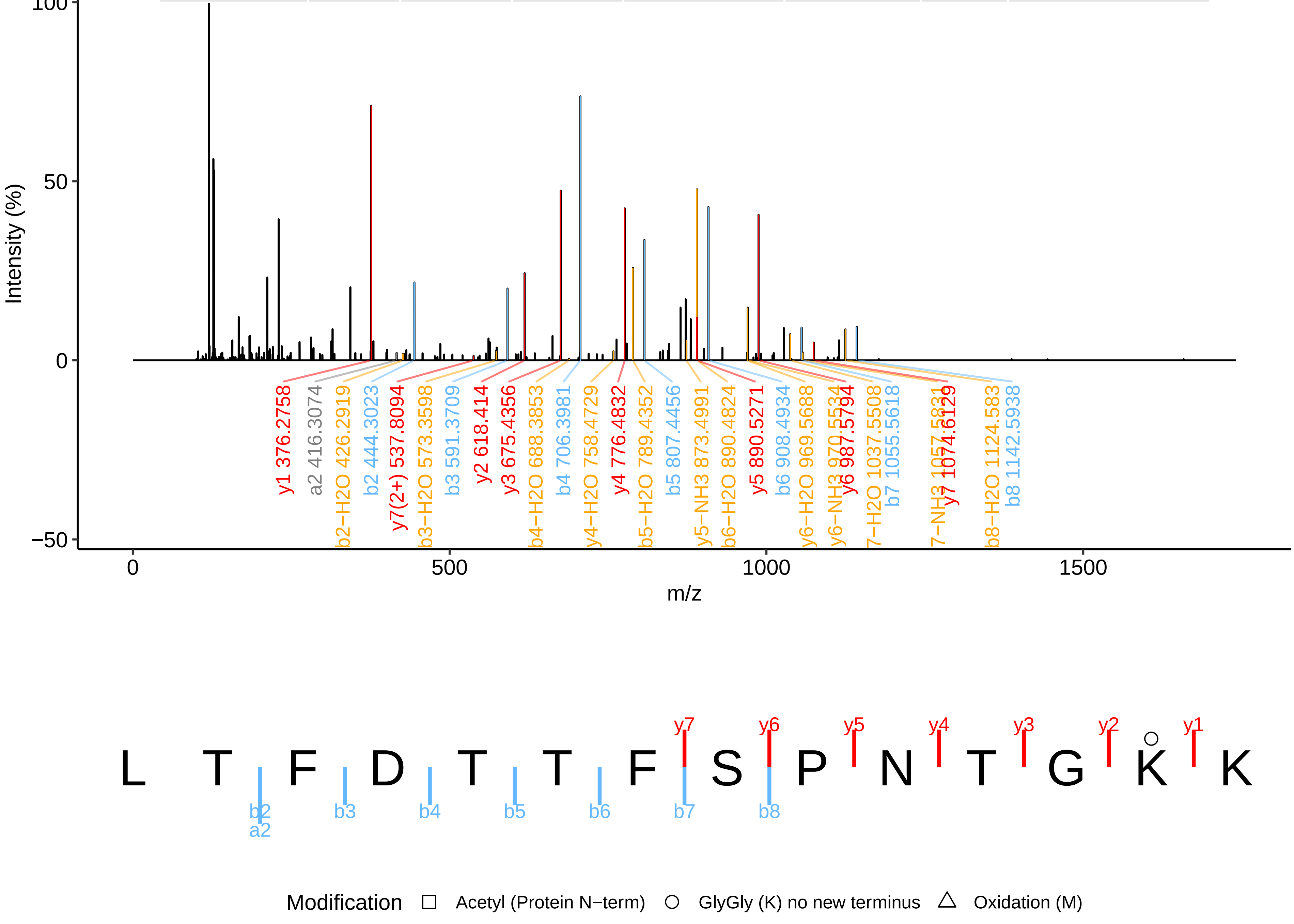

20200924_KIZ_MUb5_GlyGly_2 − Scan number: 16969

Gene Names	Charge	m/z	Mass	Retention time	PEP	Score	Precursor Intensity
VDAC2	4	418.4651	1669.831	62.965	0.00029356	88.552	3281288

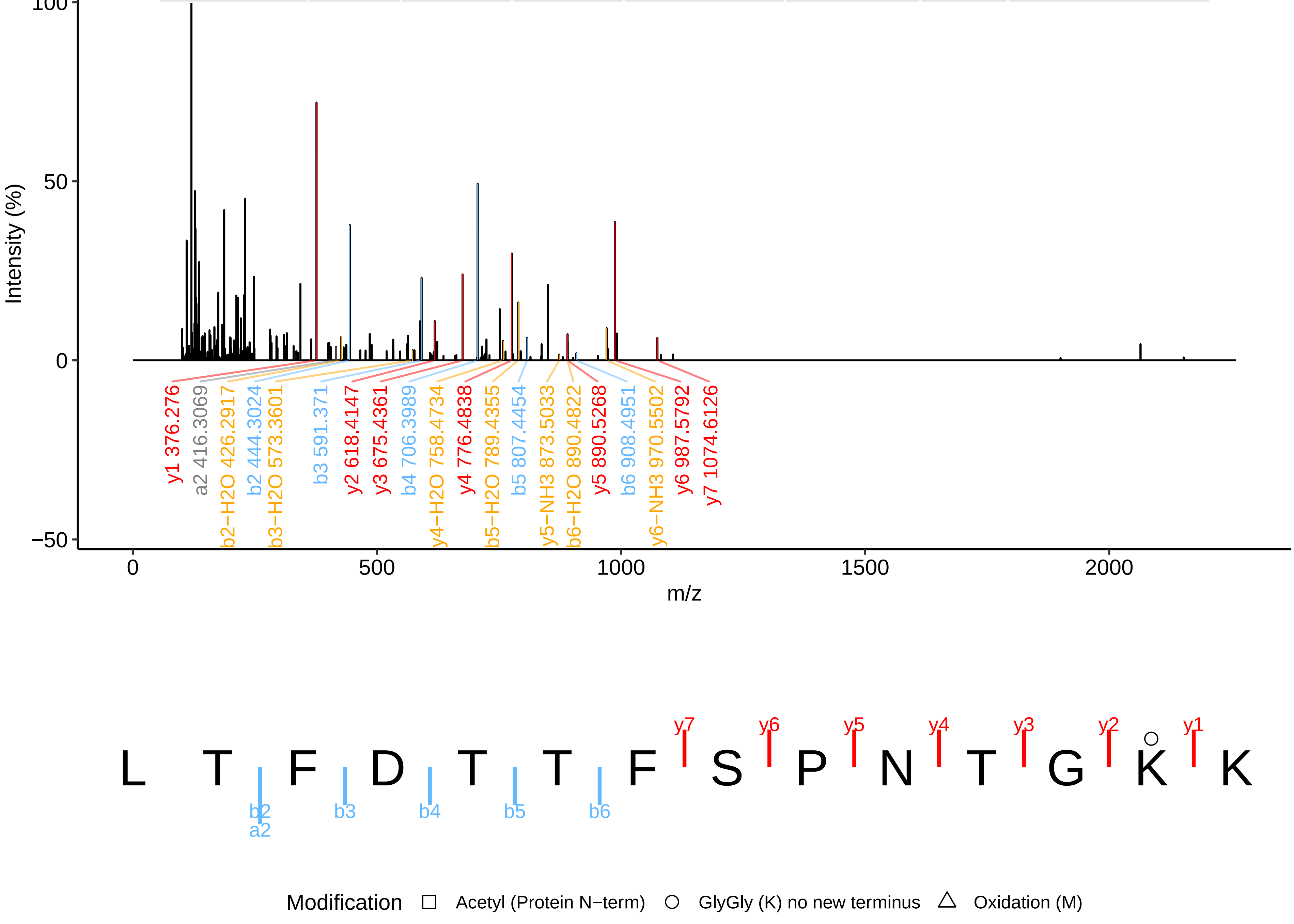

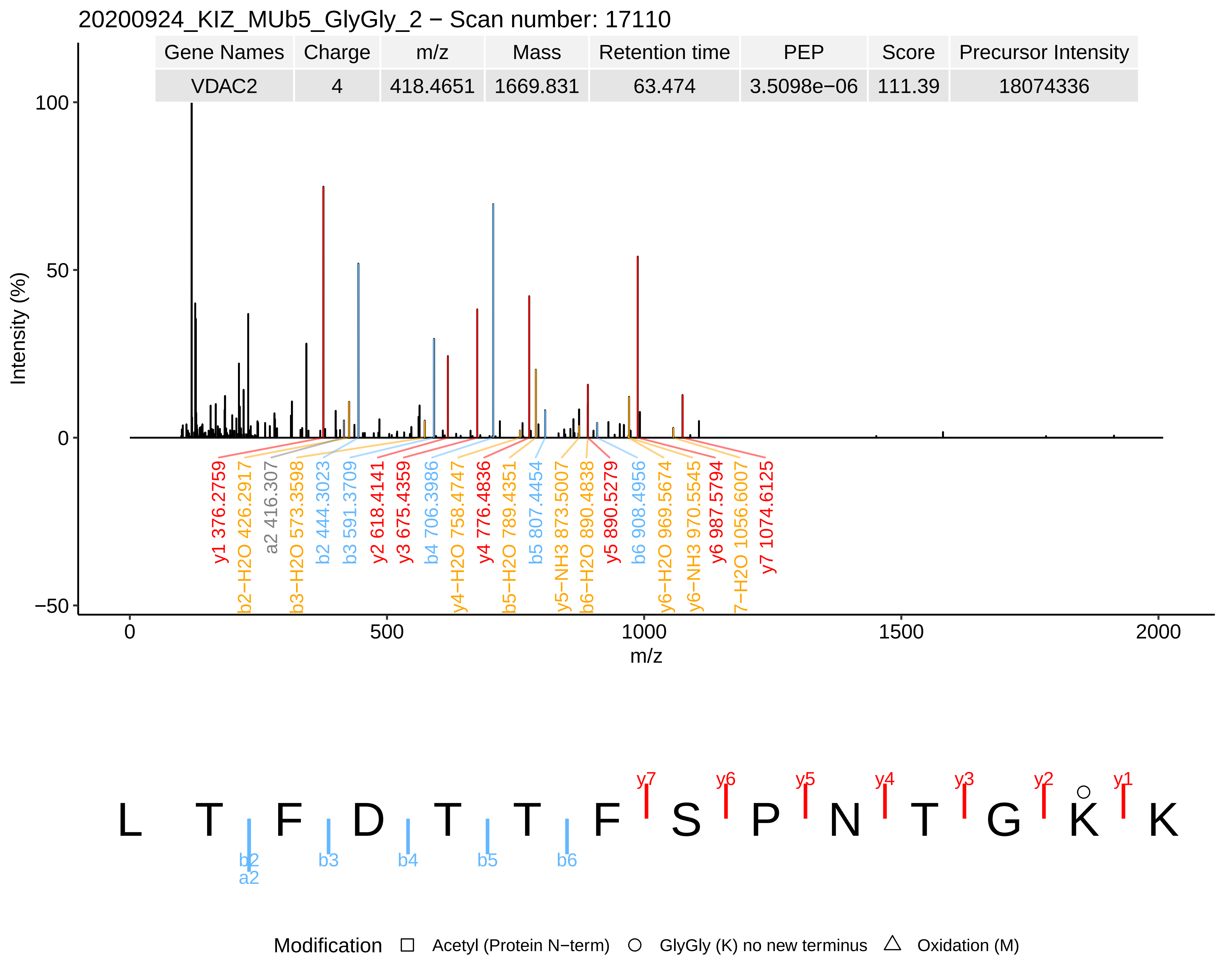
20200924_KIZ_MUb5_GlyGly_2 − Scan number: 17110
Gene Names | Charge | m/z | Mass | Retention time | PEP | Score | Precursor Intensity
VDAC2 | 4 | 418.4651 | 1669.831 | 63.474 | 3.5098e−06 | 111.39 | 18074336
Intensity (%)
100
50
0
−50
0
500
1000
1500
2000
m/z
y1 376.2759
b2−H2O 426.2917
a2 416.307
b3−H2O 573.3598
b2 444.3023
b3 591.3709
y2 618.4141
y3 675.4359
b4 706.3986
y4−H2O 758.4747
y4 776.4836
b5−H2O 789.4351
b5 807.4454
y5−NH3 873.5007
b6−H2O 890.4838
y5 890.5279
b6 908.4956
y6−H2O 969.5674
y6−NH3 970.5545
y6 987.5794
7−H2O 1056.6007
y7 1074.6125
L T F D T T F S P N T G K K
y7 y6 y5 y4 y3 y2 y1
b2 a2 b3 b4 b5 b6
Modification
Acetyl (Protein N−term)
GlyGly (K) no new terminus
Oxidation (M)

20201118_KIZ_MUb6_GlyGly_1 − Scan number: 18578

Gene Names	Charge	m/z	Mass	Retention time	PEP	Score	Precursor Intensity
VDAC2	3	557.6177	1669.831	58.406	4.2557e−13	147.18	19465484

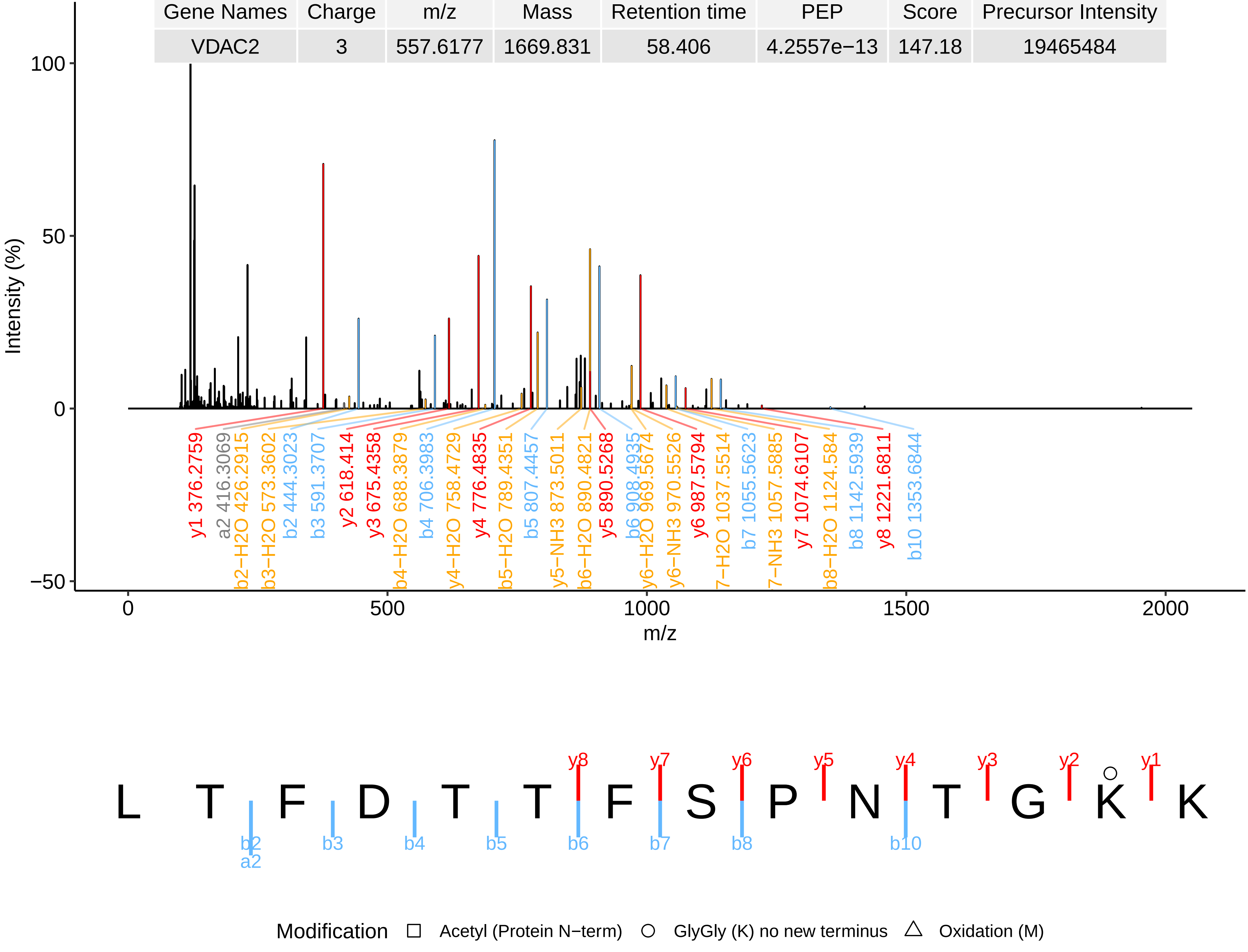

20201118_KIZ_MUb6_GlyGly_1 − Scan number: 18754

Gene Names	Charge	m/z	Mass	Retention time	PEP	Score	Precursor Intensity
VDAC2	3	557.6177	1669.831	58.915	9.4814e−13	143.42	691375808

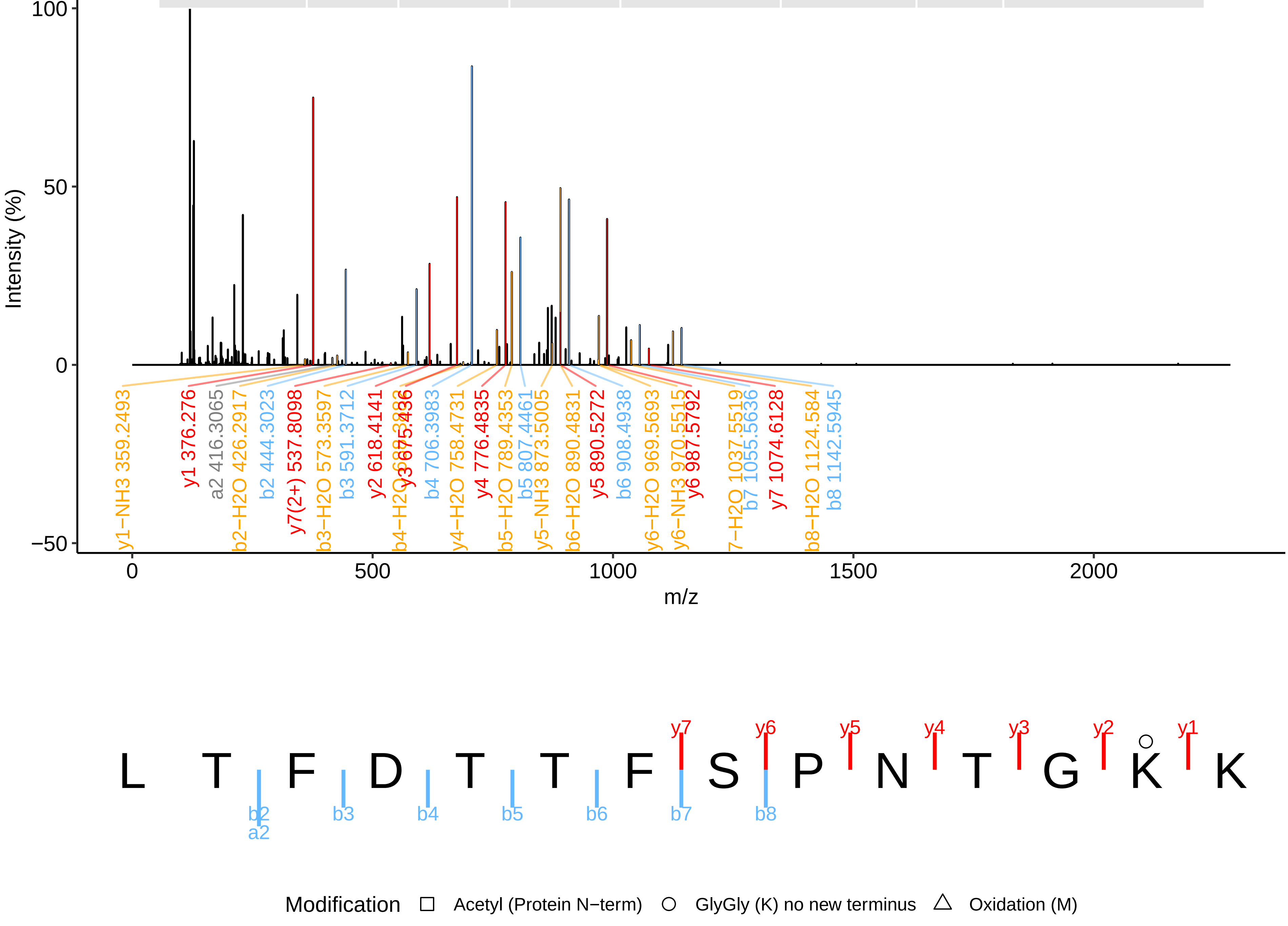

20201118_KIZ_MUb6_GlyGly_1 − Scan number: 18603

Gene Names	Charge	m/z	Mass	Retention time	PEP	Score	Precursor Intensity
VDAC2	4	418.4651	1669.831	58.471	0.0035964	68.809	61222856

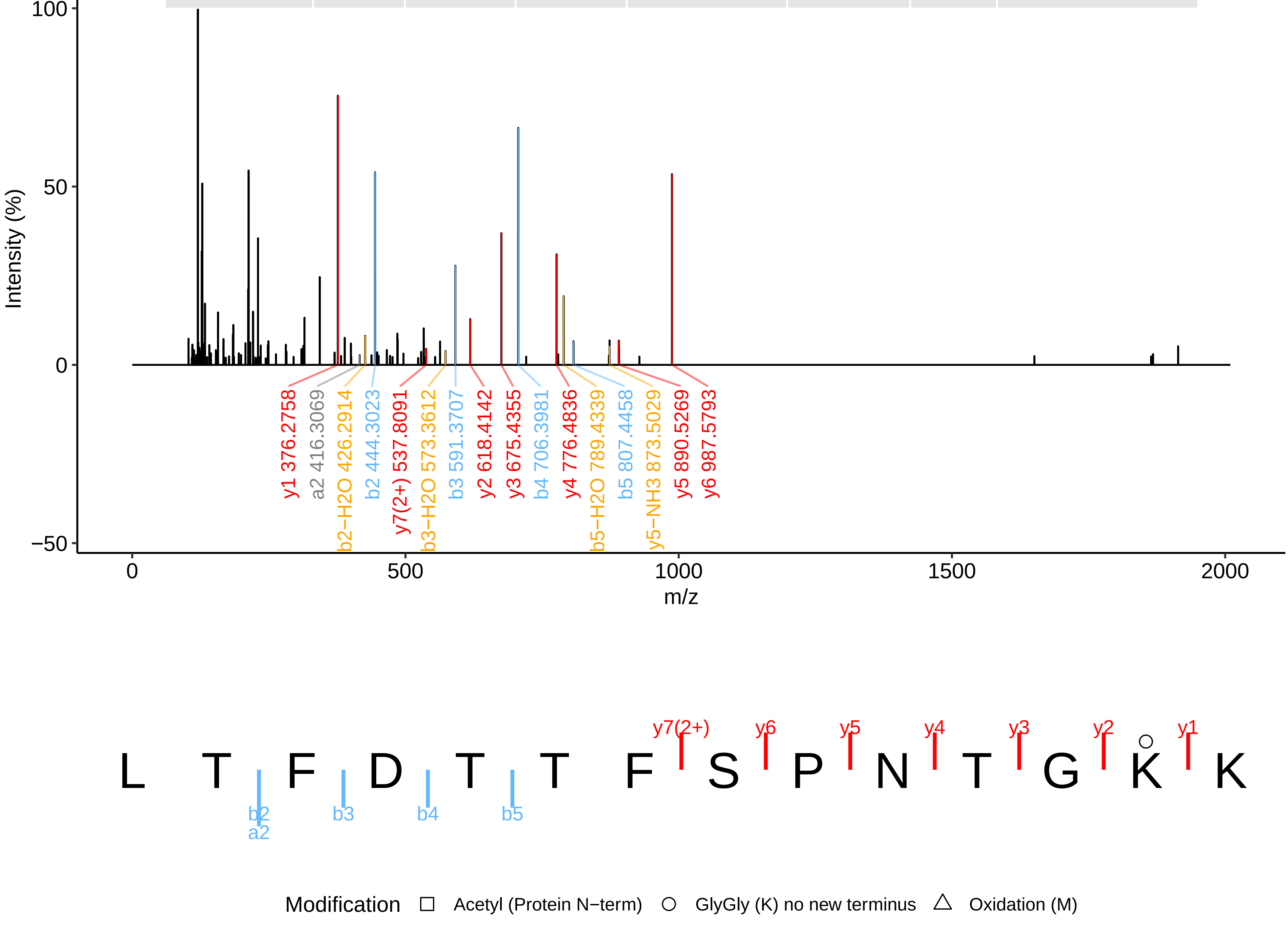

20201118_KIZ_MUb6_GlyGly_2 − Scan number: 20573

Gene Names	Charge	m/z	Mass	Retention time	PEP	Score	Precursor Intensity
VDAC2	3	557.6177	1669.831	69.309	3.0007e−08	128.57	10935432

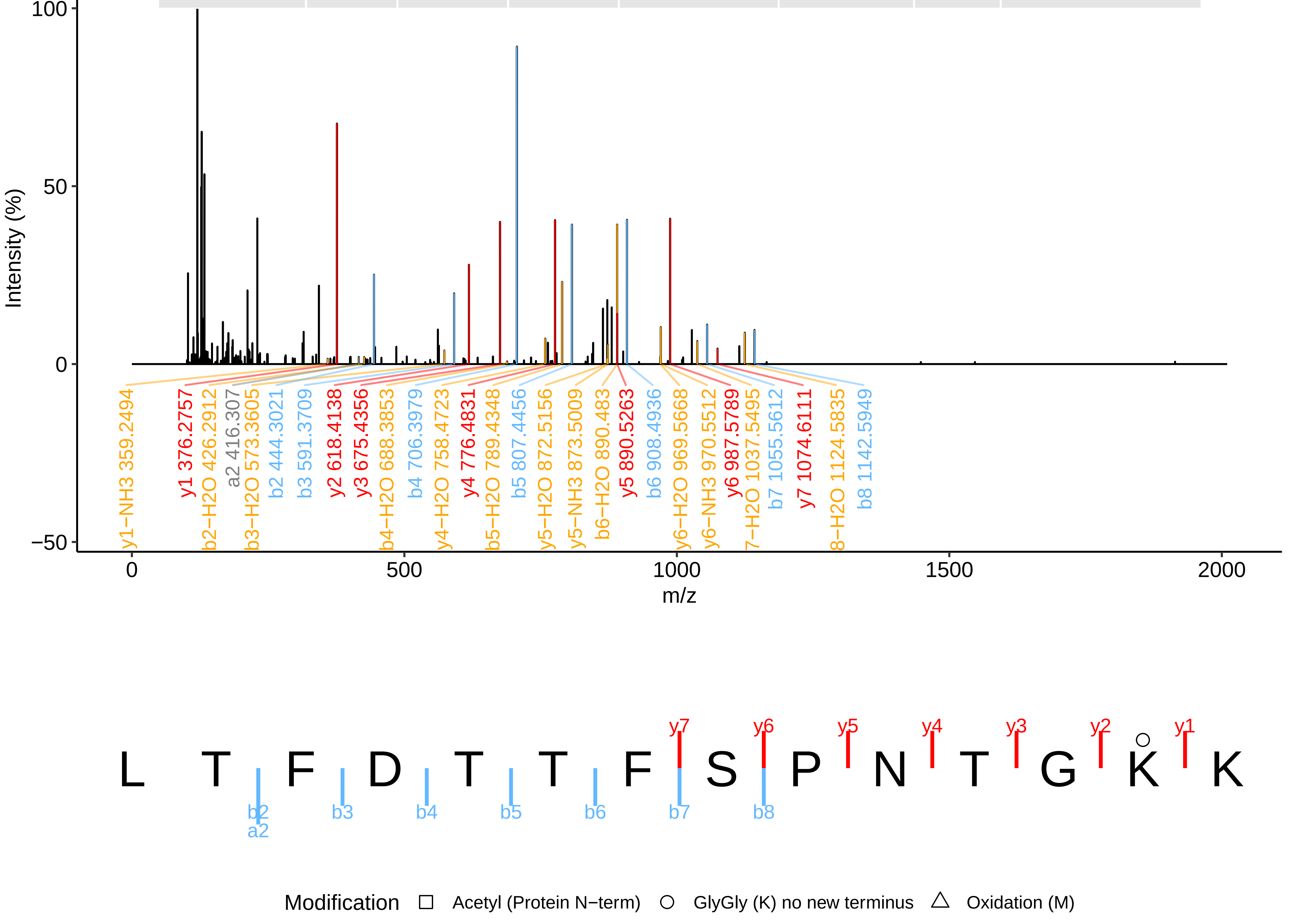

20201118_KIZ_MUb6_GlyGly_2 − Scan number: 20714

Gene Names	Charge	m/z	Mass	Retention time	PEP	Score	Precursor Intensity
VDAC2	3	557.6177	1669.831	69.816	9.4814e−13	143.42	189606144

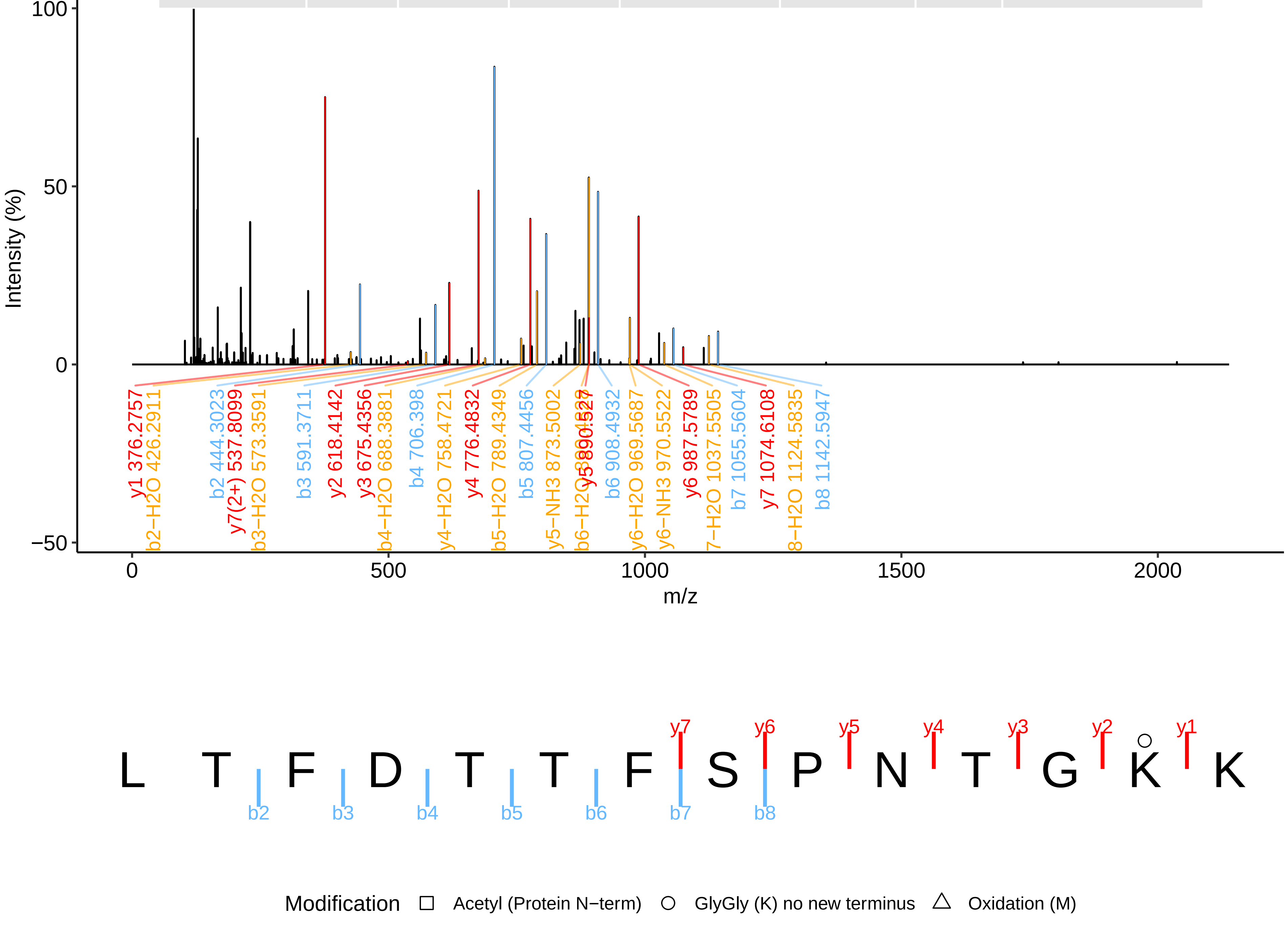

20201118_KIZ_MUb6_GlyGly_2 − Scan number: 20729

Gene Names	Charge	m/z	Mass	Retention time	PEP	Score	Precursor Intensity
VDAC2	4	418.4651	1669.831	69.864	0.00034512	86.803	12603212

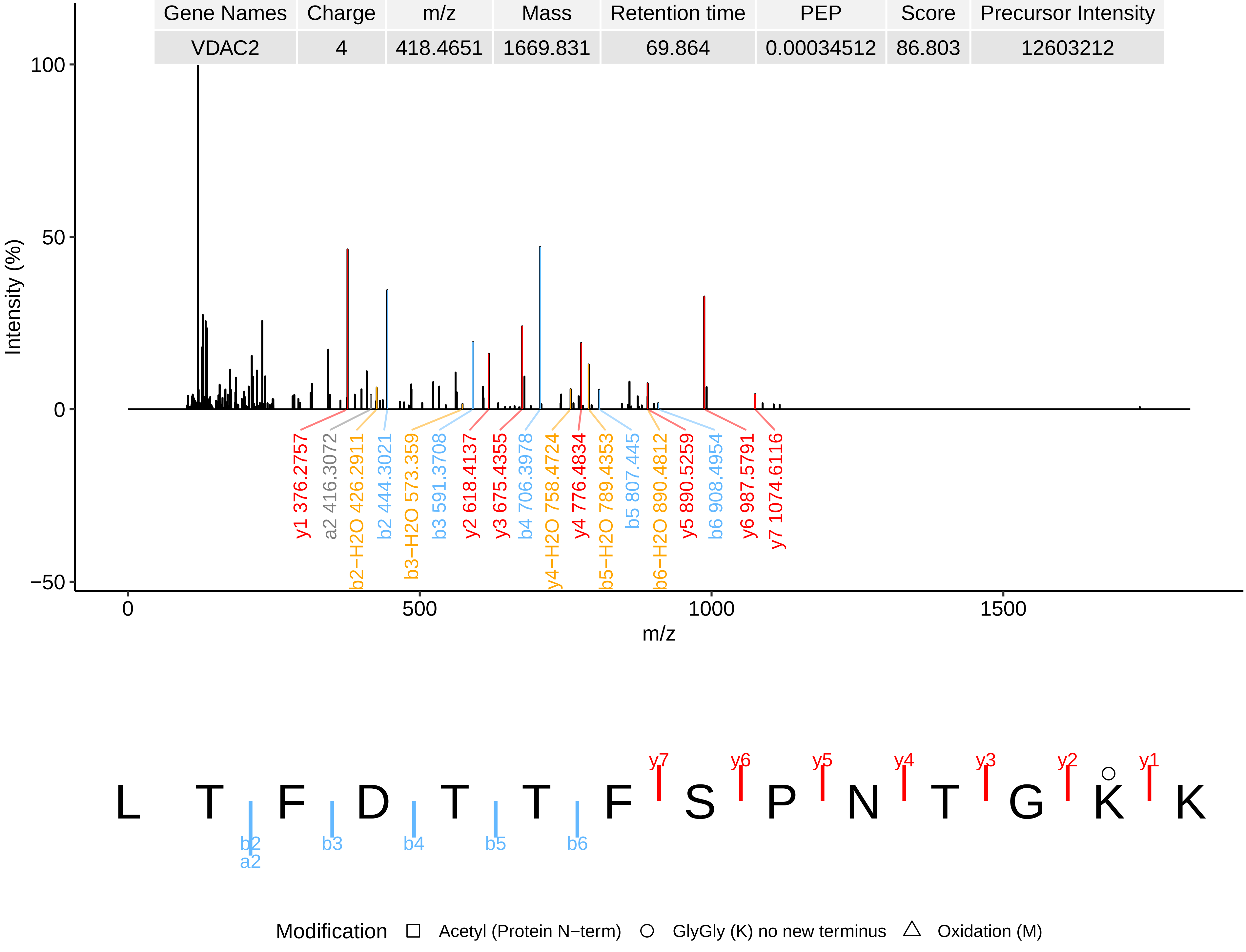

20200921_KIZ_MUb4_GlyGly_10plex − Scan number: 24220

Gene Names	Charge	m/z	Mass	Retention time	PEP	Score	Precursor Intensity
VDAC3	3	554.3138	1659.92	88.002	0.0025928	88.552	4204096

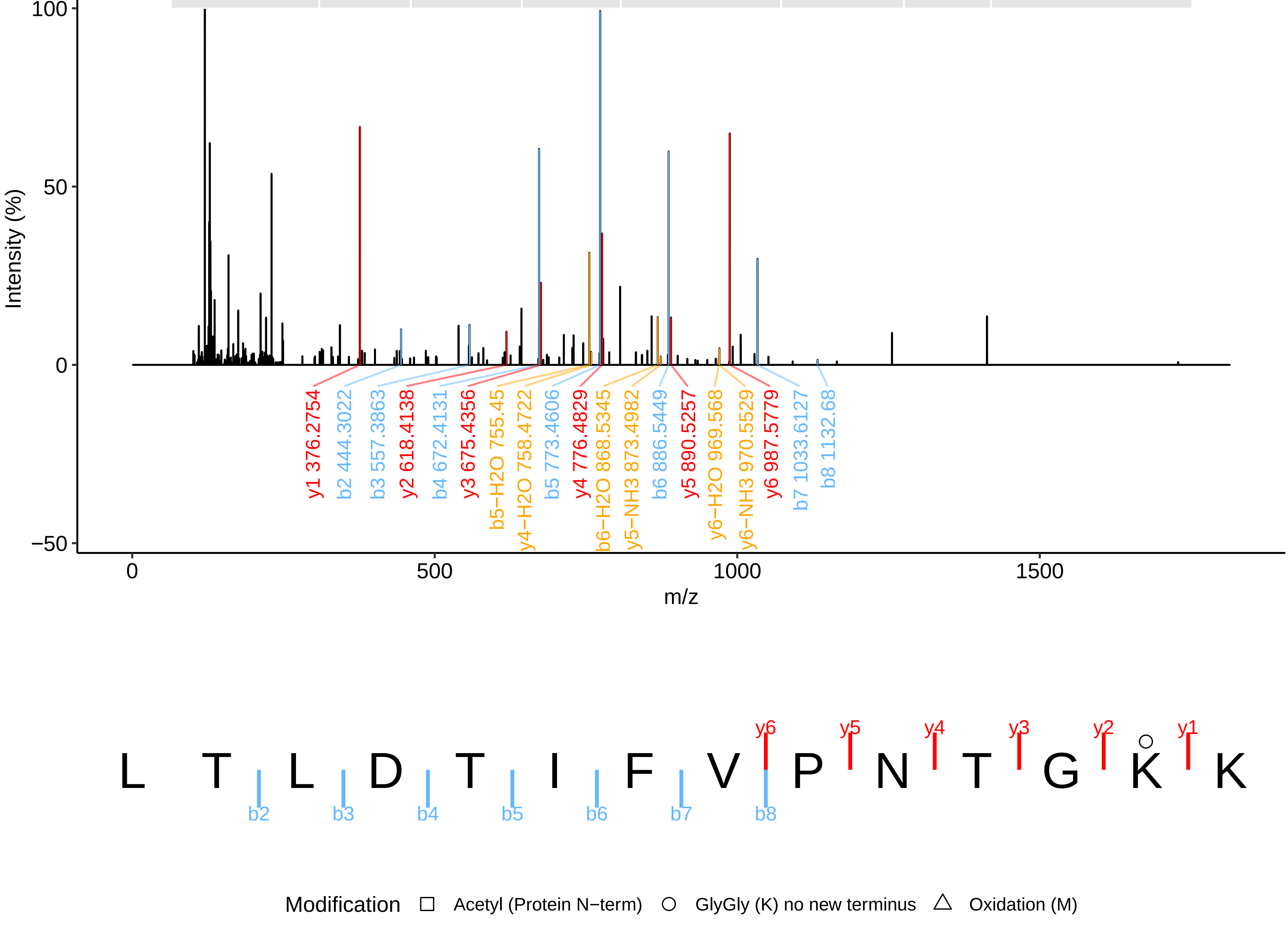

20200921_KIZ_MUb4_GlyGly_10plex − Scan number: 24351

Gene Names	Charge	m/z	Mass	Retention time	PEP	Score	Precursor Intensity
VDAC3	3	554.3138	1659.92	88.513	9.5602e−08	119.25	15040596

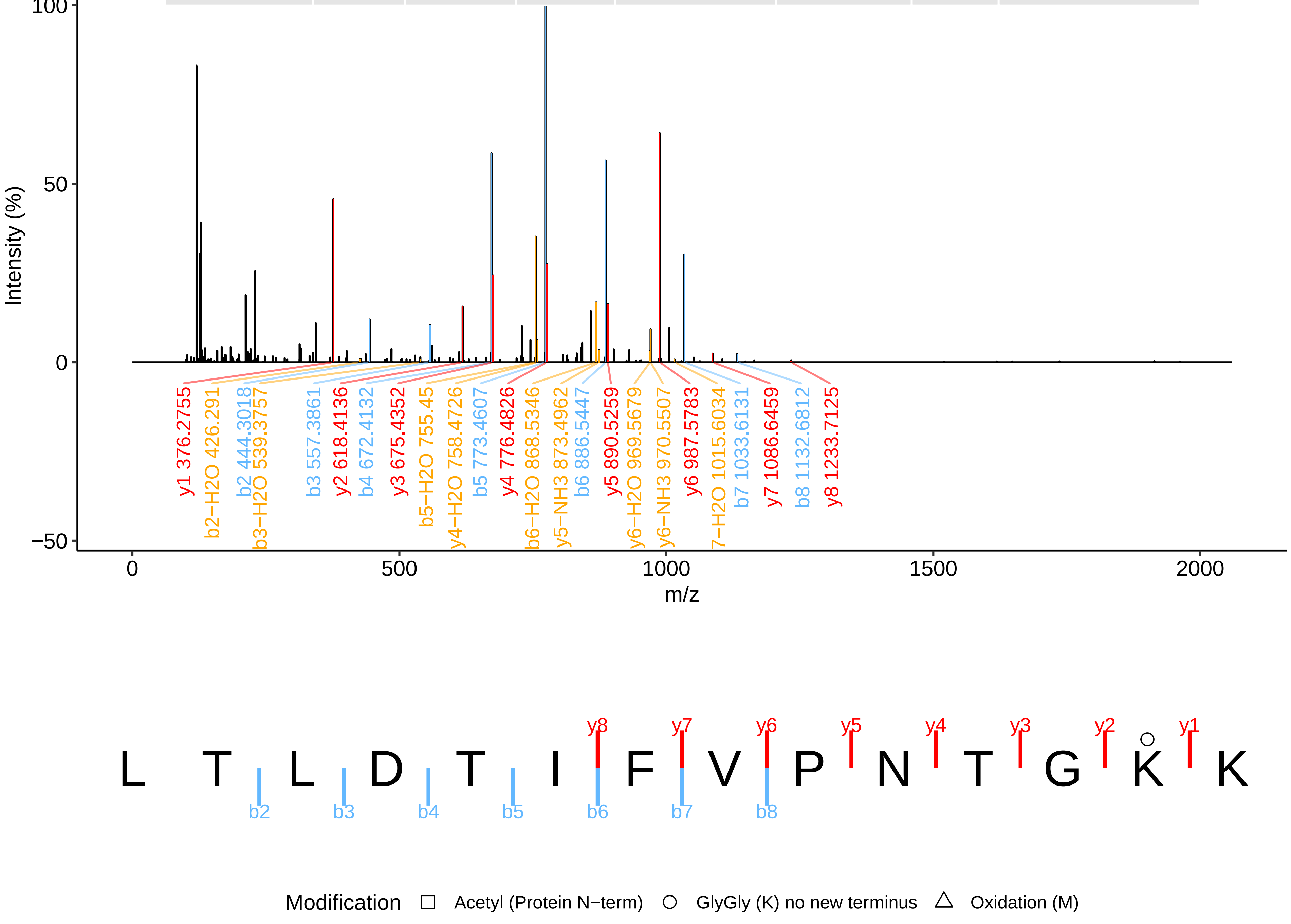

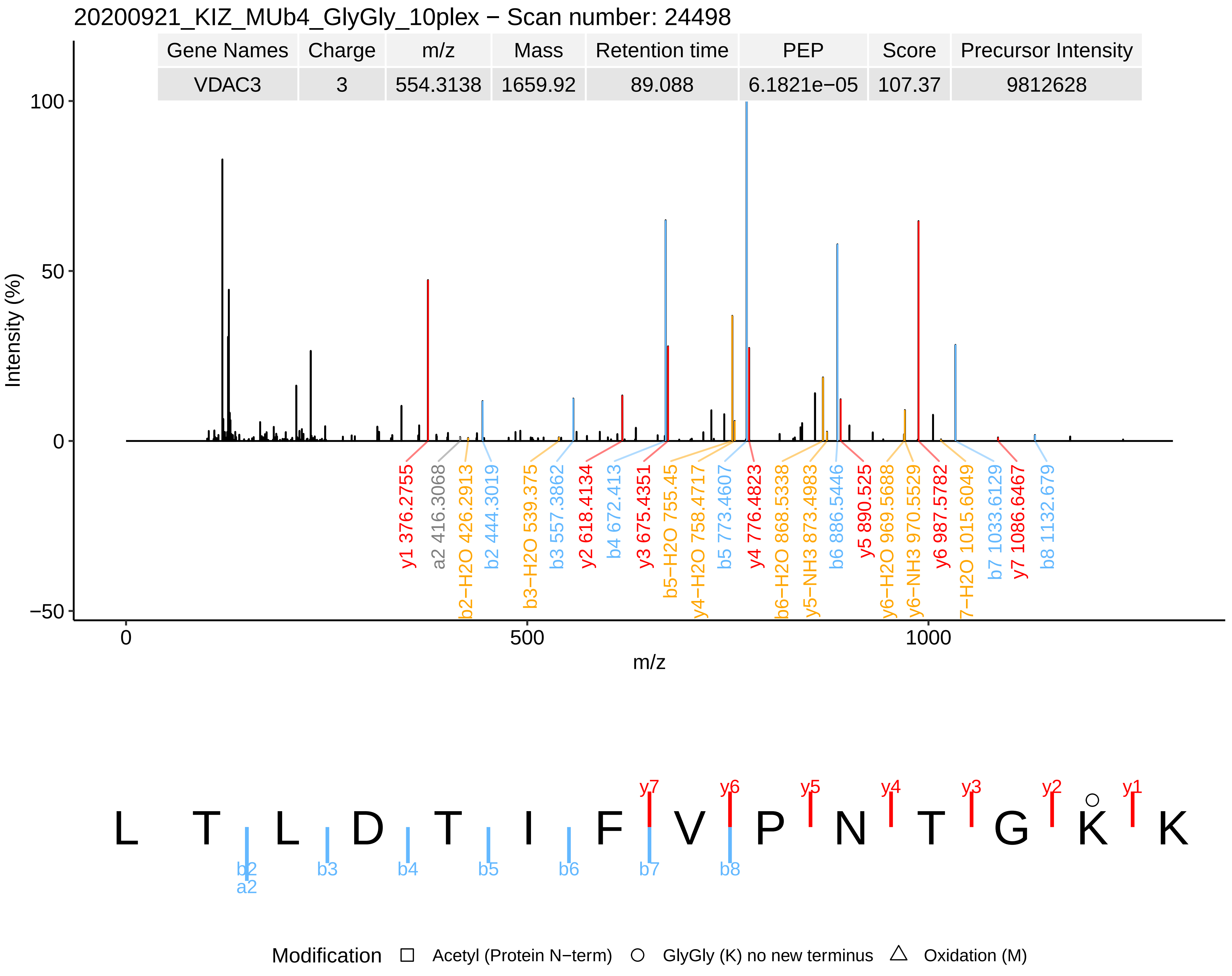
20200921_KIZ_MUb4_GlyGly_10plex − Scan number: 24498
Gene Names
Charge
m/z
Mass
Retention time
PEP
Score
Precursor Intensity
VDAC3
3
554.3138
1659.92
89.088
6.1821e−05
107.37
9812628
Intensity (%)
m/z
y1 376.2755
a2 416.3068
b2−H2O 426.2913
b2 444.3019
b3−H2O 539.375
b3 557.3862
y2 618.4134
b4 672.413
y3 675.4351
b5−H2O 755.45
y4−H2O 758.4717
b5 773.4607
y4 776.4823
b6−H2O 868.5338
y5−NH3 873.4983
b6 886.5446
y5 890.525
y6−H2O 969.5688
y6−NH3 970.5529
y6 987.5782
y7−H2O 1015.6049
b7 1033.6129
y7 1086.6467
b8 1132.679
L T L D T I F V P N T G K K
Modification
Acetyl (Protein N−term)
GlyGly (K) no new terminus
Oxidation (M)

20200921_KIZ_MUb4_GlyGly_10plex − Scan number: 25098

Gene Names	Charge	m/z	Mass	Retention time	PEP	Score	Precursor Intensity
VDAC3	3	554.3138	1659.92	91.287	0.00012651	98.942	4435657

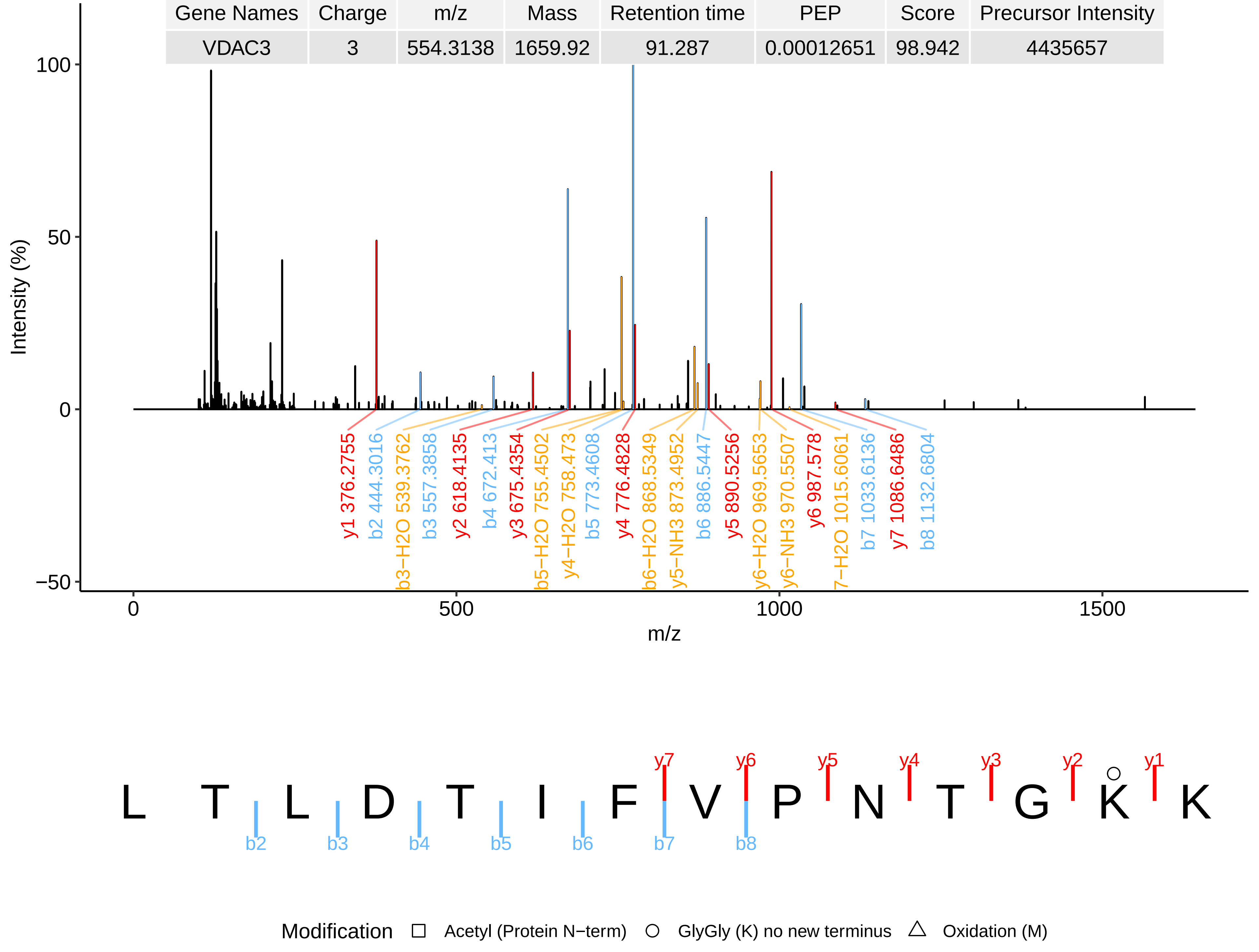

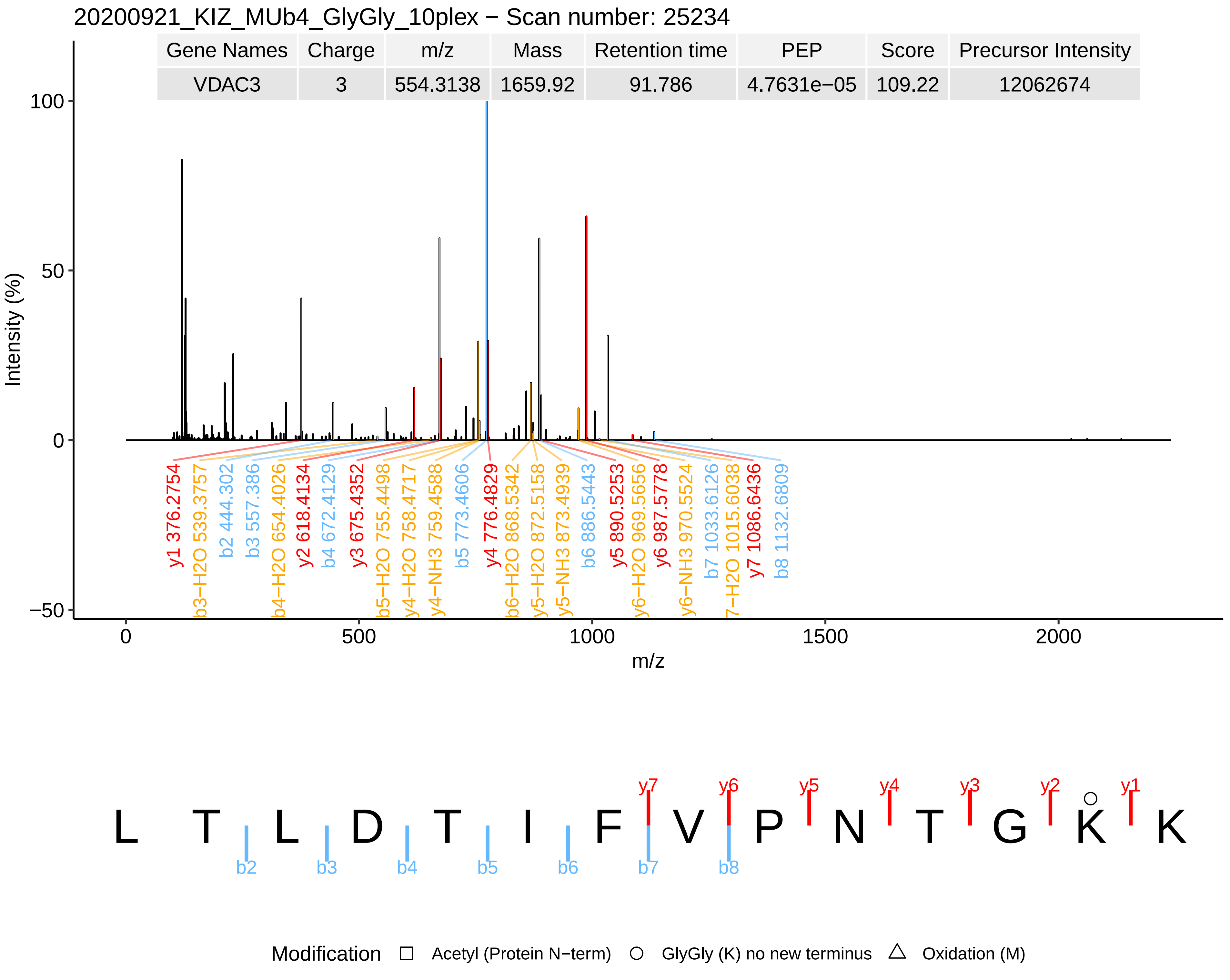
20200921_KIZ_MUb4_GlyGly_10plex − Scan number: 25234
Gene Names | Charge | m/z | Mass | Retention time | PEP | Score | Precursor Intensity
VDAC3 | 3 | 554.3138 | 1659.92 | 91.786 | 4.7631e−05 | 109.22 | 12062674
Intensity (%)
m/z
y1 376.2754
b3−H2O 539.3757
b2 444.302
b3 557.386
b4−H2O 654.4026
y2 618.4134
b4 672.4129
y3 675.4352
b5−H2O 755.4498
y4−H2O 758.4717
y4−NH3 759.4588
b5 773.4606
y4 776.4829
b6−H2O 868.5342
y5−H2O 872.5158
y5−NH3 873.4939
b6 886.5443
y5 890.5253
y6−H2O 969.5656
y6 987.5778
y6−NH3 970.5524
b7 1033.6126
7−H2O 1015.6038
y7 1086.6436
b8 1132.6809
L T L D T I F V P N T G K K
y7 y6 y5 y4 y3 y2 y1
b2 b3 b4 b5 b6 b7 b8
Modification
Acetyl (Protein N−term)
GlyGly (K) no new terminus
Oxidation (M)

20200921_KIZ_MUb4_GlyGly_10plex − Scan number: 25378

Gene Names	Charge	m/z	Mass	Retention time	PEP	Score	Precursor Intensity
VDAC3	3	554.3138	1659.92	92.301	1.6444e−12	138.42	56935444

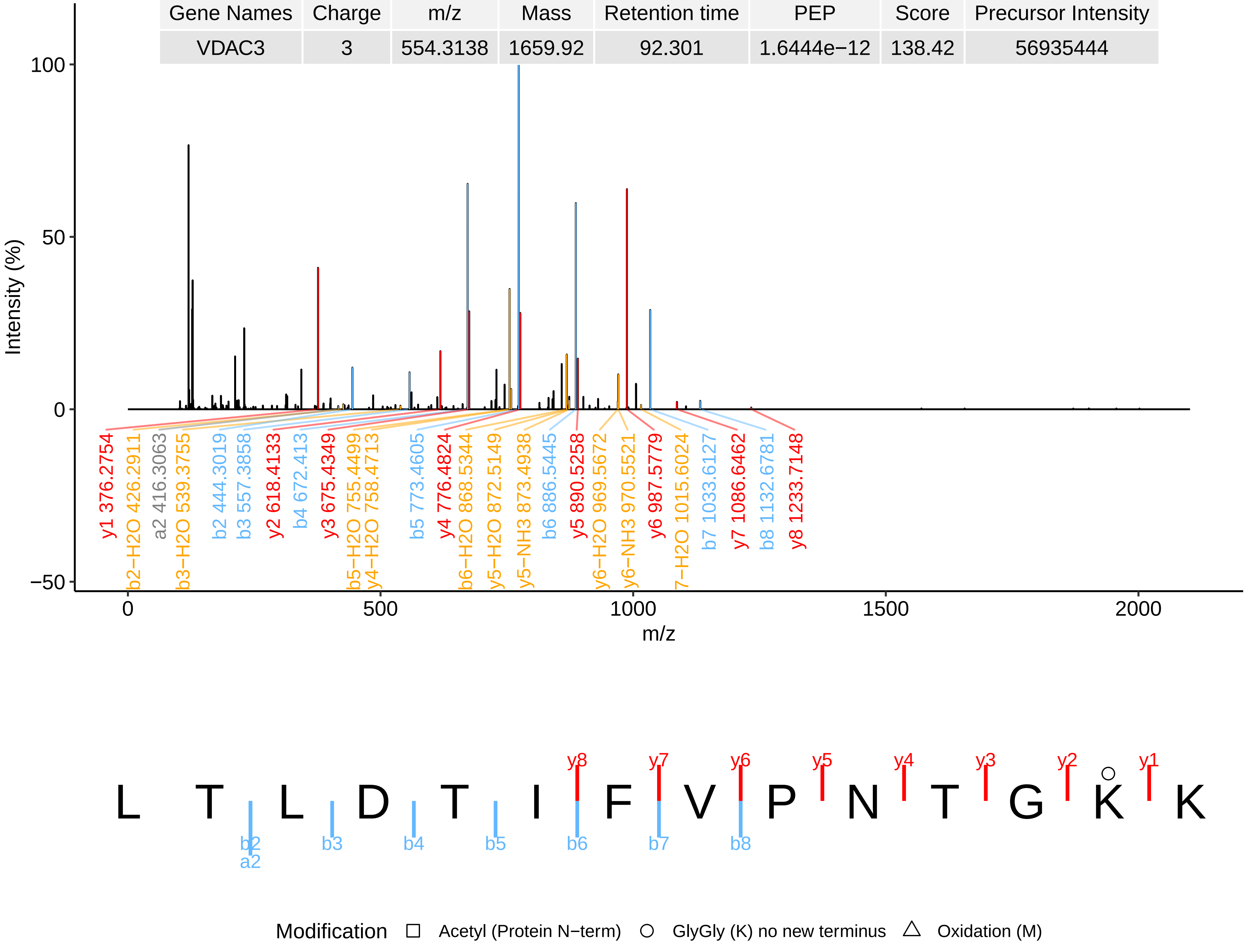

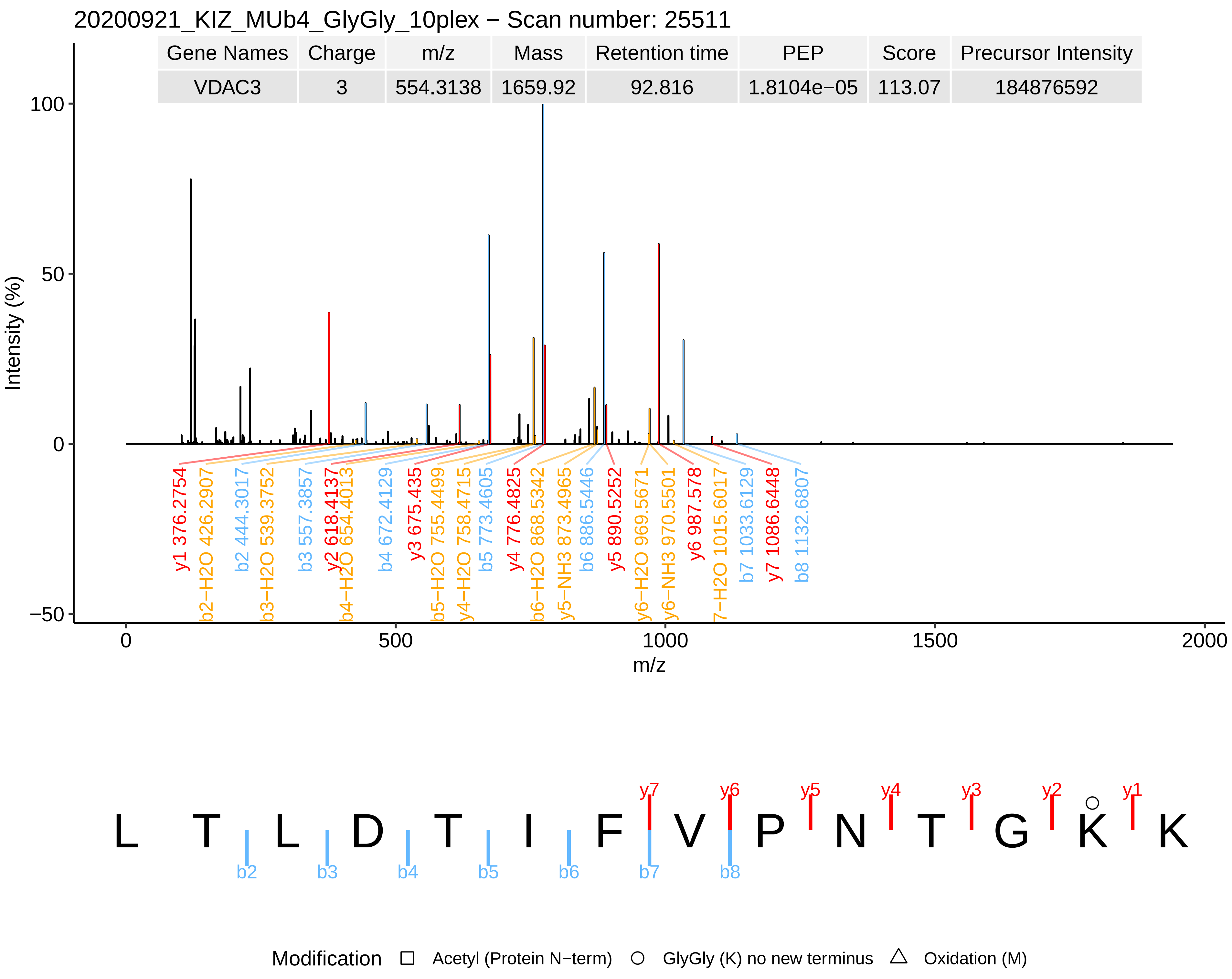
20200921_KIZ_MUb4_GlyGly_10plex − Scan number: 25511
Gene Names | Charge | m/z | Mass | Retention time | PEP | Score | Precursor Intensity
VDAC3 | 3 | 554.3138 | 1659.92 | 92.816 | 1.8104e−05 | 113.07 | 184876592
Intensity (%)
m/z
y1 376.2754
b2−H2O 426.2907
b2 444.3017
b3−H2O 539.3752
b3 557.3857
y2 618.4137
b4−H2O 654.4013
b4 672.4129
y3 675.435
b5−H2O 755.4499
y4−H2O 758.4715
b5 773.4605
y4 776.4825
b6−H2O 868.5342
y5−NH3 873.4965
b6 886.5446
y5 890.5252
y6−H2O 969.5671
y6−NH3 970.5501
y6 987.578
y7−H2O 1015.6017
b7 1033.6129
y7 1086.6448
b8 1132.6807
L T L D T I F V P N T G K K
Modification □ Acetyl (Protein N−term) ○ GlyGly (K) no new terminus △ Oxidation (M)

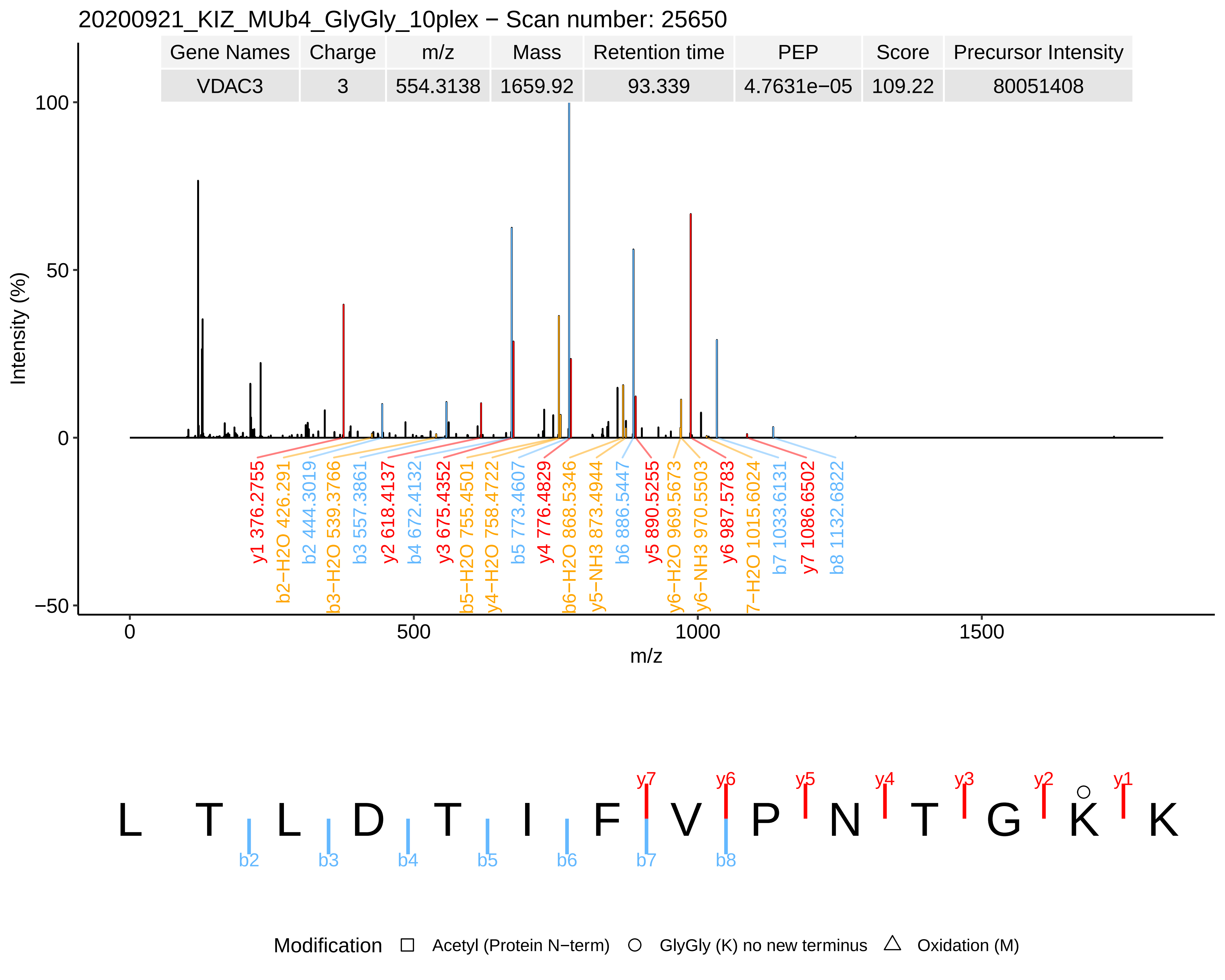

20200921_KIZ_MUb4_GlyGly_10plex − Scan number: 25650
Gene Names | Charge | m/z | Mass | Retention time | PEP | Score | Precursor Intensity
VDAC3 | 3 | 554.3138 | 1659.92 | 93.339 | 4.7631e−05 | 109.22 | 80051408
Intensity (%)
100
50
0
−50
0
500
1000
1500
m/z
y1 376.2755
b2−H2O 426.291
b2 444.3019
b3−H2O 539.3766
b3 557.3861
y2 618.4137
b4 672.4132
y3 675.4352
b5−H2O 755.4501
y4−H2O 758.4722
b5 773.4607
y4 776.4829
b6−H2O 868.5346
y5−NH3 873.4944
b6 886.5447
y5 890.5255
y6−H2O 969.5673
y6−NH3 970.5503
y6 987.5783
7−H2O 1015.6024
b7 1033.6131
y7 1086.6502
b8 1132.6822
L T L D T I F V P N T G K K
y7 y6 y5 y4 y3 y2 y1
b2 b3 b4 b5 b6 b7 b8
Modification
Acetyl (Protein N−term)
GlyGly (K) no new terminus
Oxidation (M)

20200921_KIZ_MUb4_GlyGly_10plex − Scan number: 25783

Gene Names	Charge	m/z	Mass	Retention time	PEP	Score	Precursor Intensity
VDAC3	3	554.3138	1659.92	93.851	2.2947e−05	112.44	14563708

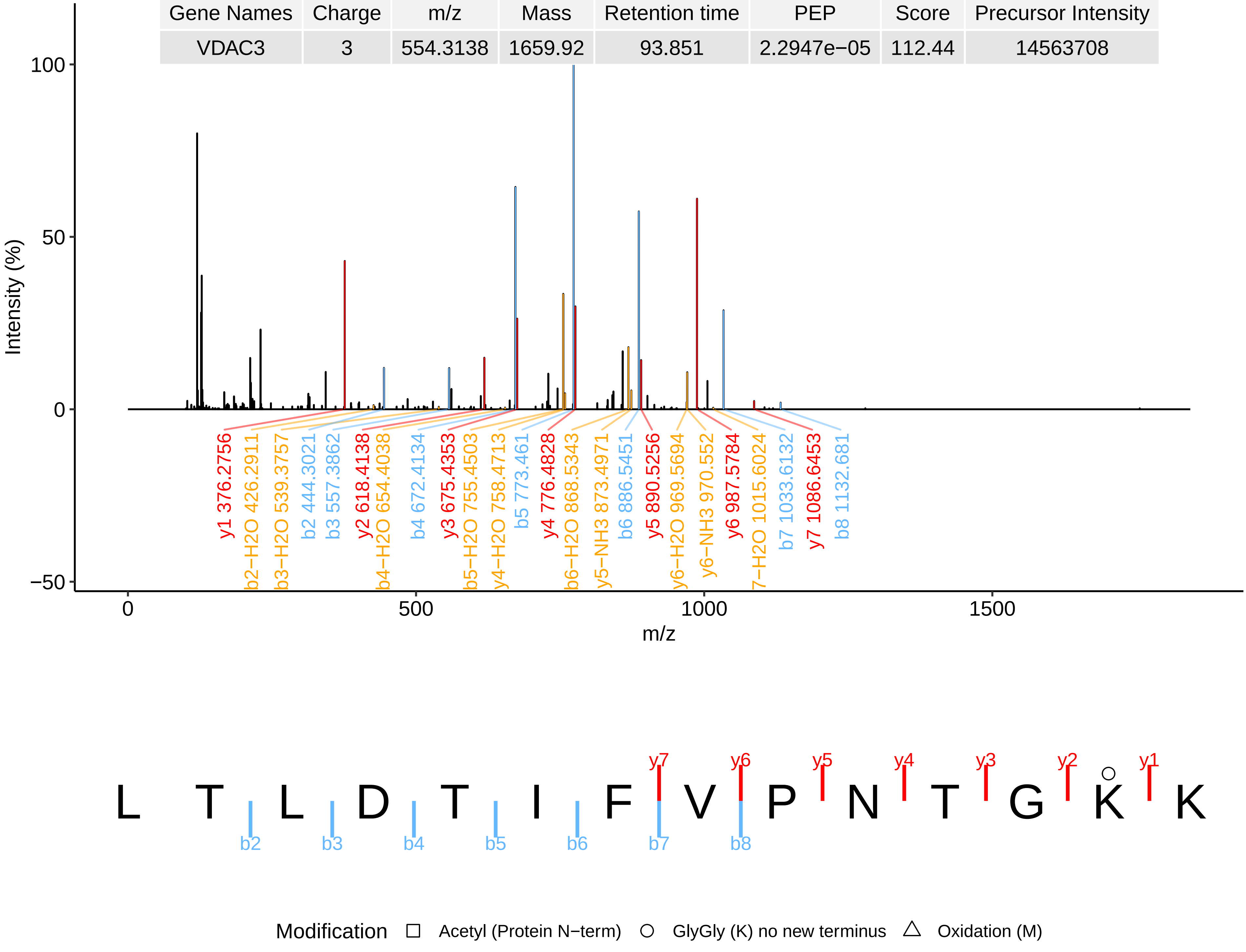

20200921_KIZ_MUb4_GlyGly_10plex − Scan number: 25325

Gene Names	Charge	m/z	Mass	Retention time	PEP	Score	Precursor Intensity
VDAC3	4	415.9872	1659.92	92.113	0.00022935	90.731	6117177

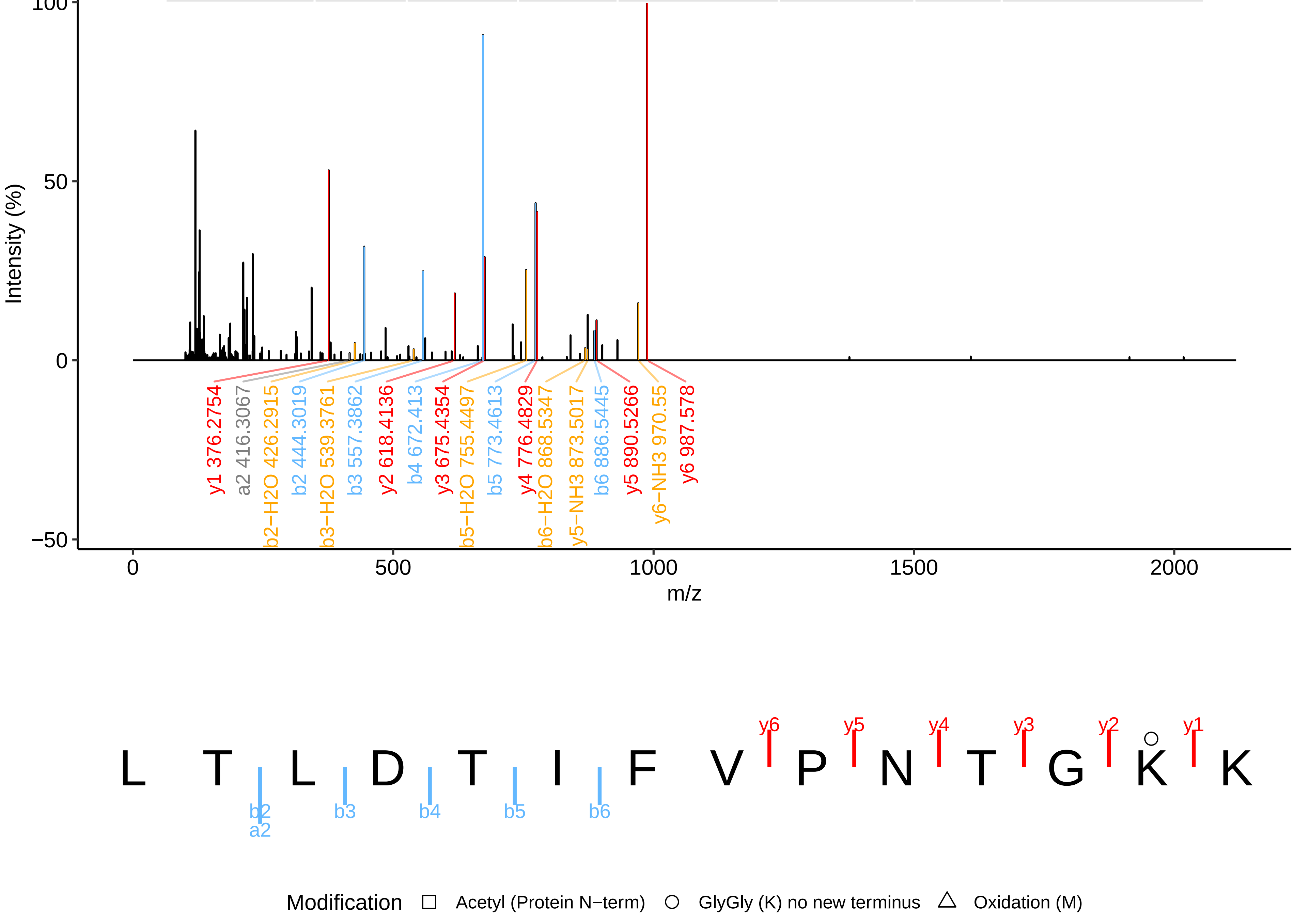

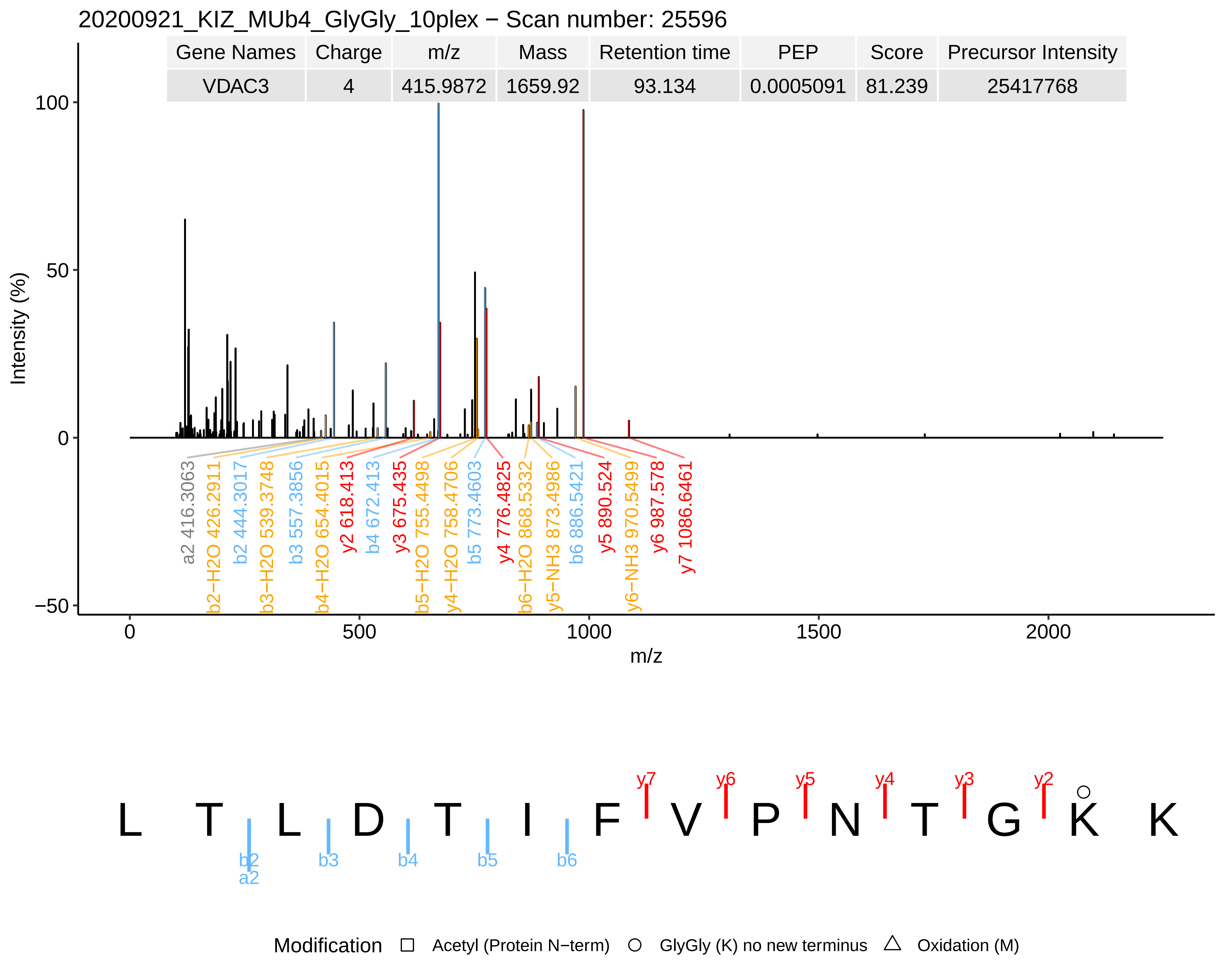

20200921_KIZ_MUb4_GlyGly_10plex − Scan number: 25596
Gene Names
Charge
m/z
Mass
Retention time
PEP
Score
Precursor Intensity
VDAC3
4
415.9872
1659.92
93.134
0.0005091
81.239
25417768
Intensity (%)
100
50
0
−50
0
500
1000
1500
2000
m/z
a2 416.3063
b2−H2O 426.2911
b2 444.3017
b3−H2O 539.3748
b3 557.3856
b4−H2O 654.4015
y2 618.413
b4 672.413
y3 675.435
b5−H2O 755.4498
y4−H2O 758.4706
b5 773.4603
y4 776.4825
b6−H2O 868.5332
y5−NH3 873.4986
b6 886.5421
y5 890.524
y6−NH3 970.5499
y6 987.578
y7 1086.6461
L T L D T I F V P N T G K K
y7 y6 y5 y4 y3 y2
b2 a2 b3 b4 b5 b6
Modification
Acetyl (Protein N−term)
GlyGly (K) no new terminus
Oxidation (M)

20200921_KIZ_MUb4_GlyGly_10plex_2 − Scan number: 22291

Gene Names	Charge	m/z	Mass	Retention time	PEP	Score	Precursor Intensity
VDAC3	3	554.3138	1659.92	87.856	4.2842e−05	109.84	2506450

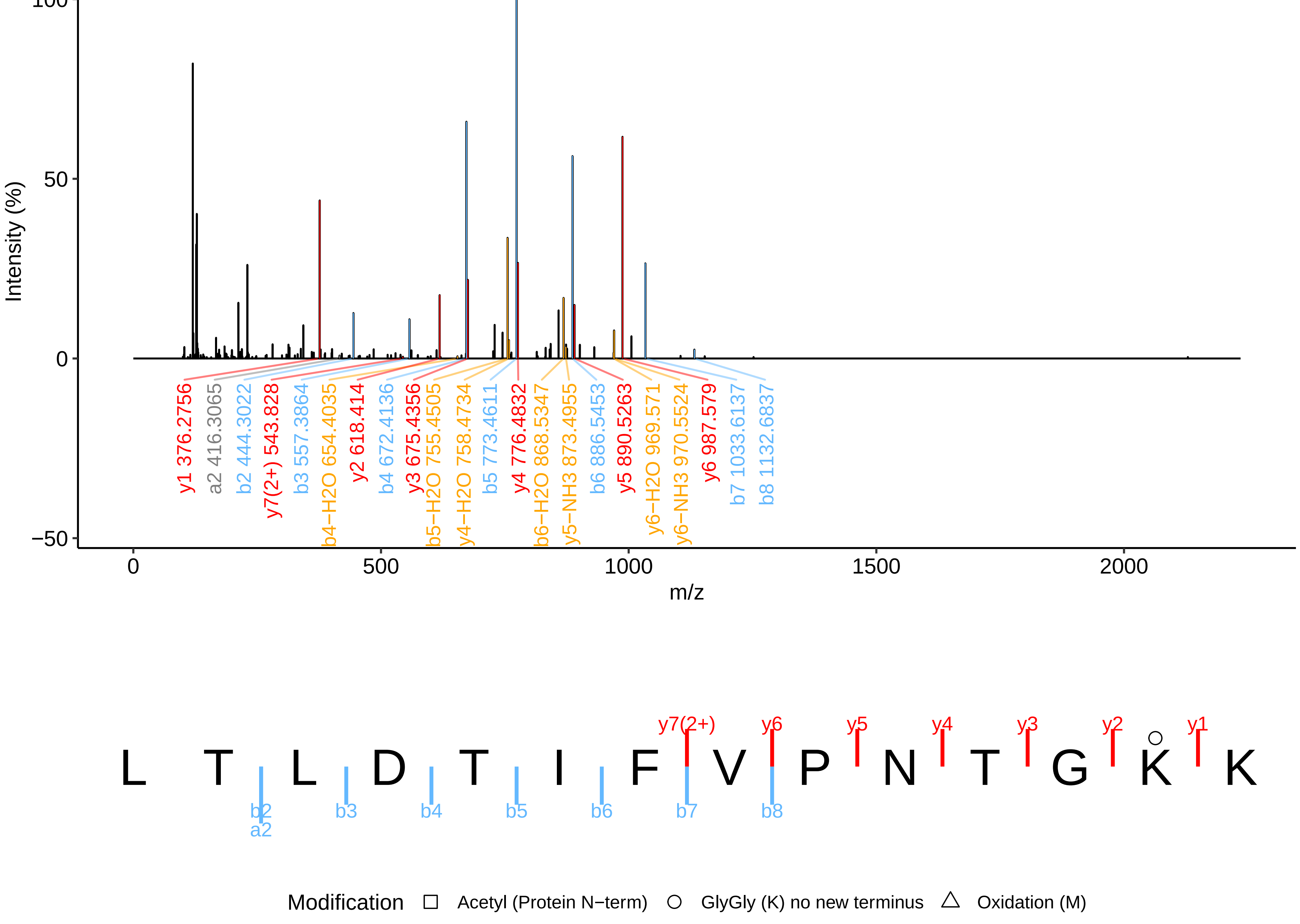

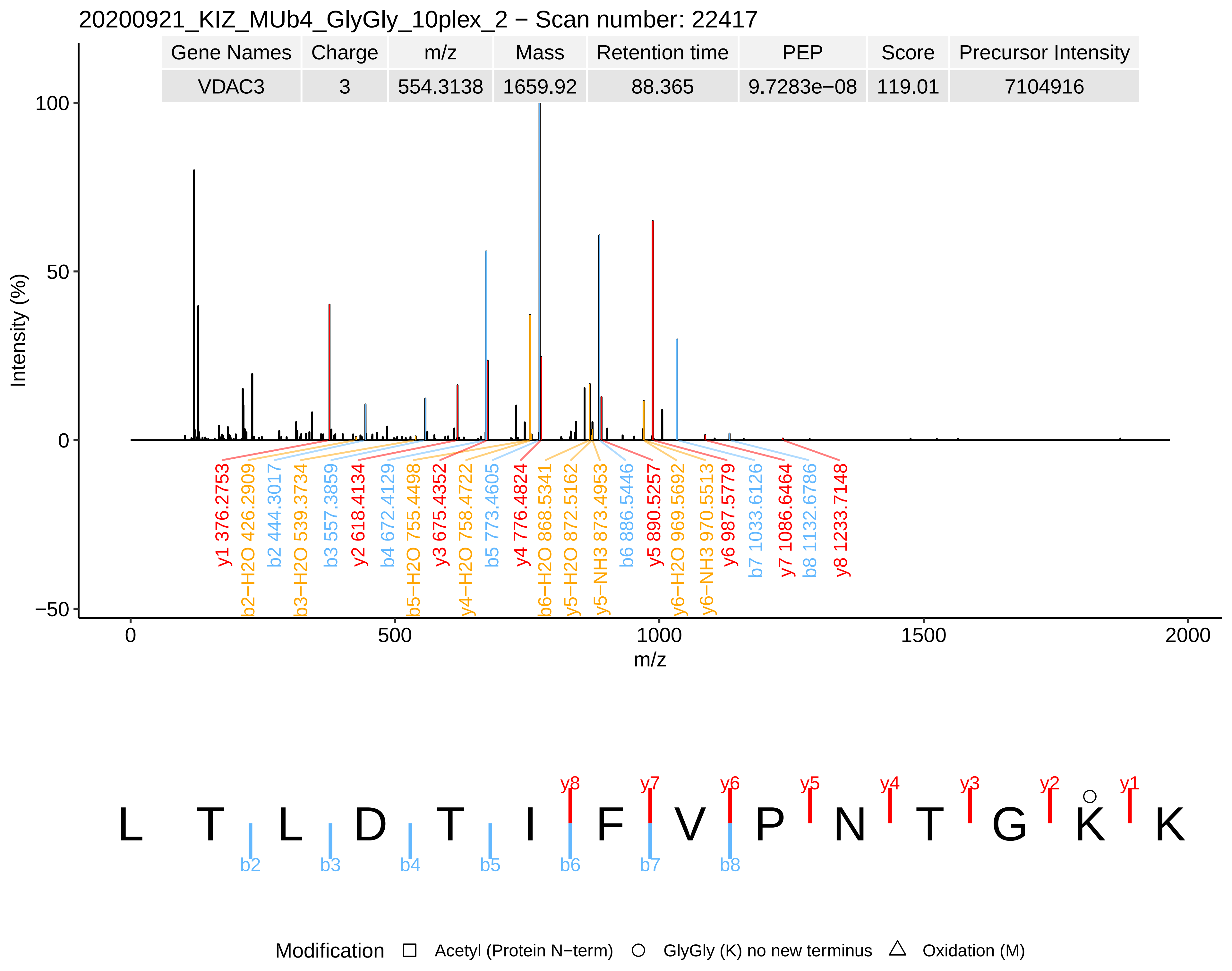
20200921_KIZ_MUb4_GlyGly_10plex_2 − Scan number: 22417
Gene Names | Charge | m/z | Mass | Retention time | PEP | Score | Precursor Intensity
VDAC3 | 3 | 554.3138 | 1659.92 | 88.365 | 9.7283e−08 | 119.01 | 7104916
Intensity (%)
m/z
y1 376.2753
b2−H2O 426.2909
b2 444.3017
b3−H2O 539.3734
b3 557.3859
y2 618.4134
b4 672.4129
b5−H2O 755.4498
y3 675.4352
y4−H2O 758.4722
b5 773.4605
y4 776.4824
b6−H2O 868.5341
y5−H2O 872.5162
y5−NH3 873.4953
b6 886.5446
y5 890.5257
y6−H2O 969.5692
y6−NH3 970.5513
y6 987.5779
b7 1033.6126
y7 1086.6464
b8 1132.6786
y8 1233.7148
L T L D T I F V P N T G K K
y8 y7 y6 y5 y4 y3 y2 y1
b2 b3 b4 b5 b6 b7 b8
Modification
Acetyl (Protein N−term)
GlyGly (K) no new terminus
Oxidation (M)

20200924_KIZ_MUb5_GlyGly_1 − Scan number: 24459

Gene Names	Charge	m/z	Mass	Retention time	PEP	Score	Precursor Intensity
VDAC3	3	554.3138	1659.92	78.564	4.1399e−08	126.95	93816056

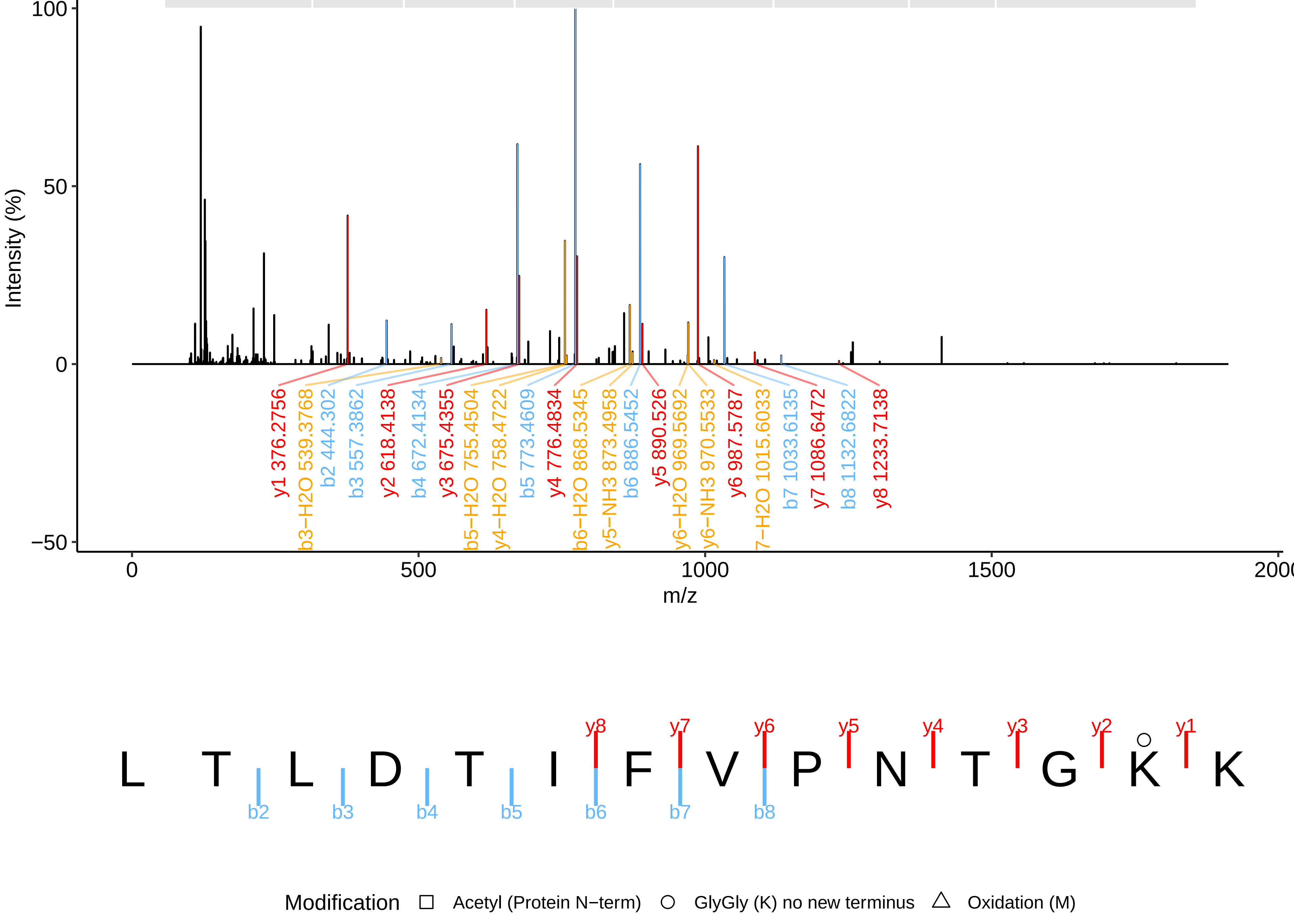

20200924_KIZ_MUb5_GlyGly_1 − Scan number: 24622

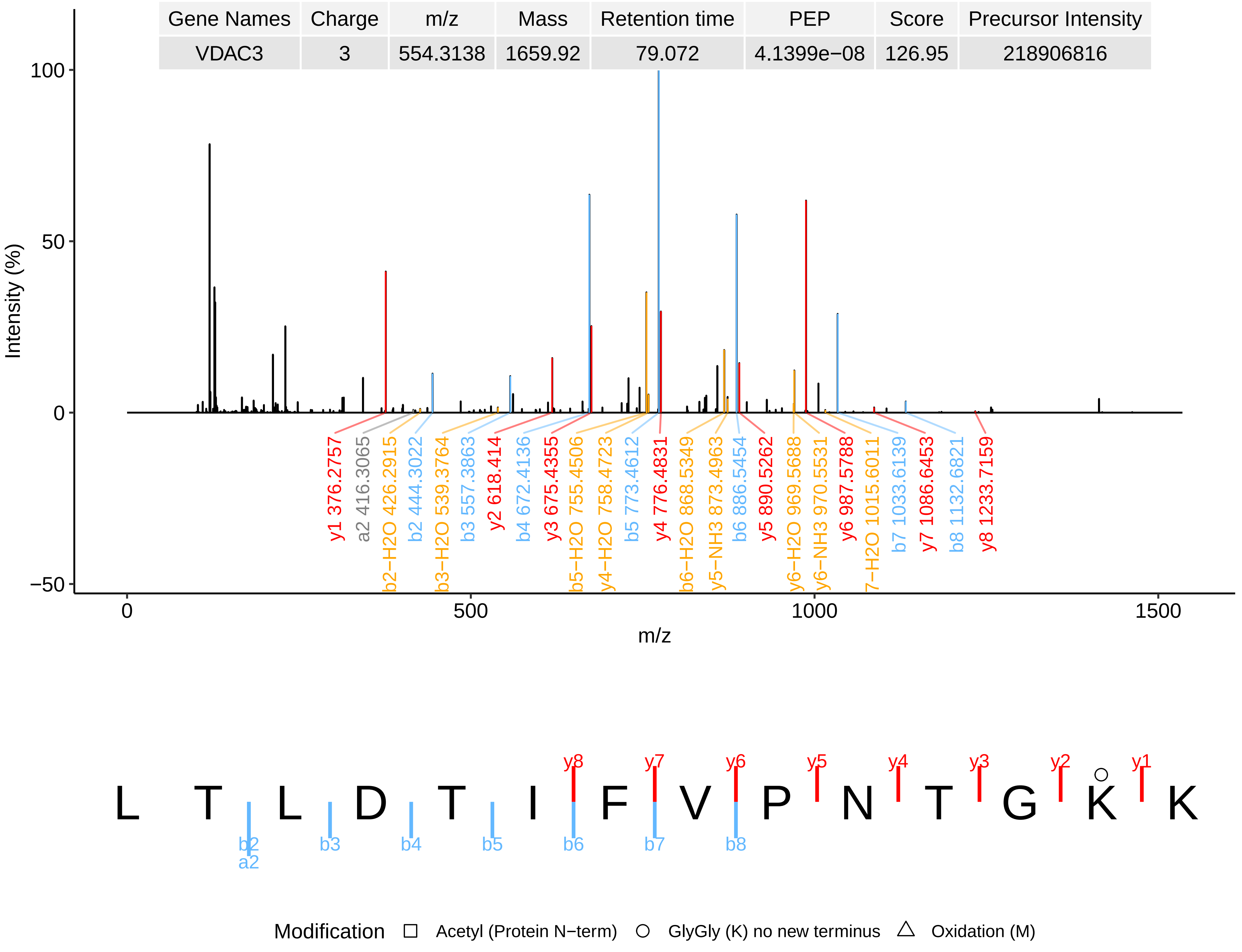

20200924_KIZ_MUb5_GlyGly_1 − Scan number: 24789

Gene Names	Charge	m/z	Mass	Retention time	PEP	Score	Precursor Intensity
VDAC3	3	554.3138	1659.92	79.581	4.1399e−08	126.95	207599184

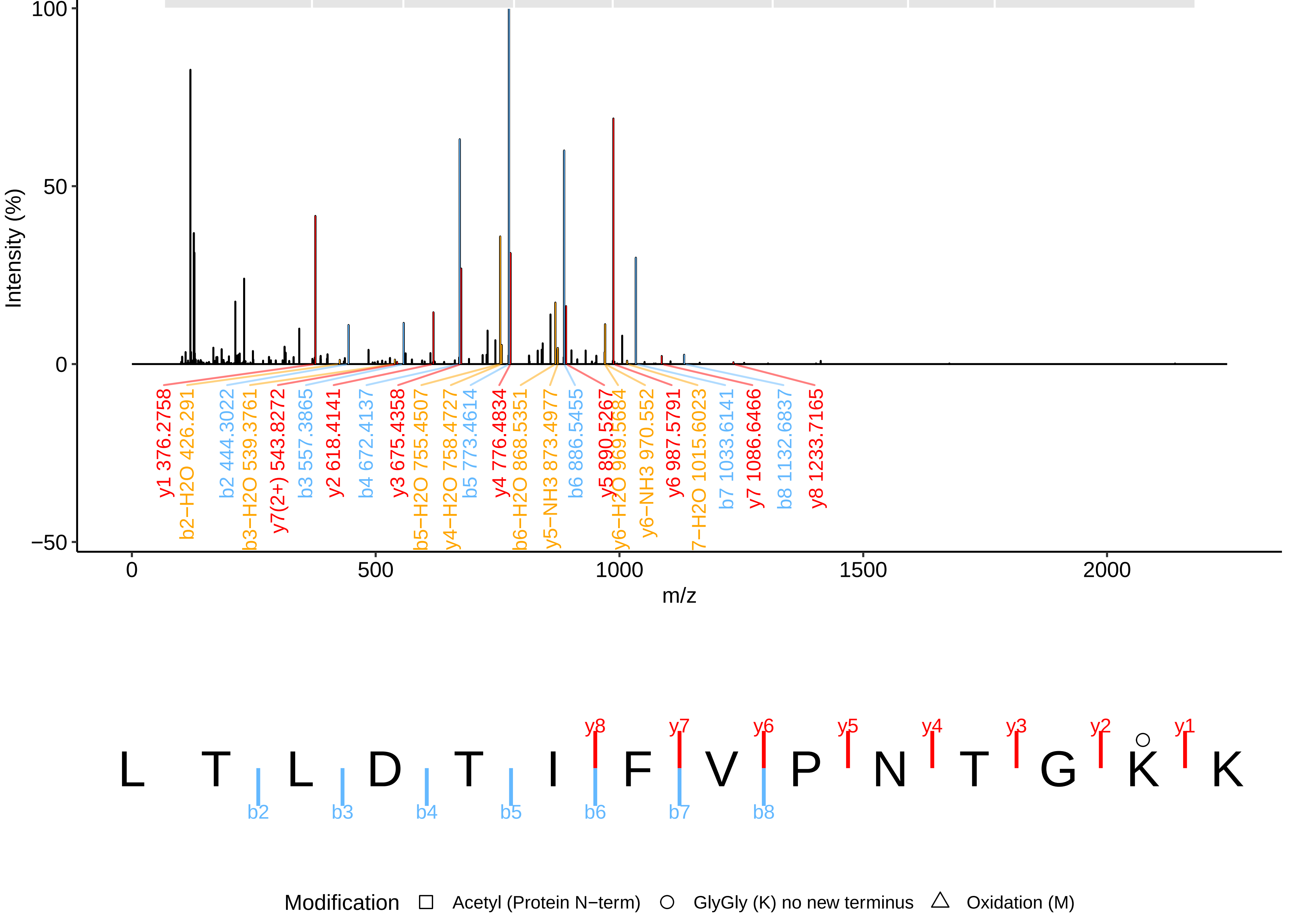

20200924_KIZ_MUb5_GlyGly_1 − Scan number: 24954

Gene Names	Charge	m/z	Mass	Retention time	PEP	Score	Precursor Intensity
VDAC3	3	554.3138	1659.92	80.096	4.1399e−08	126.95	40380252

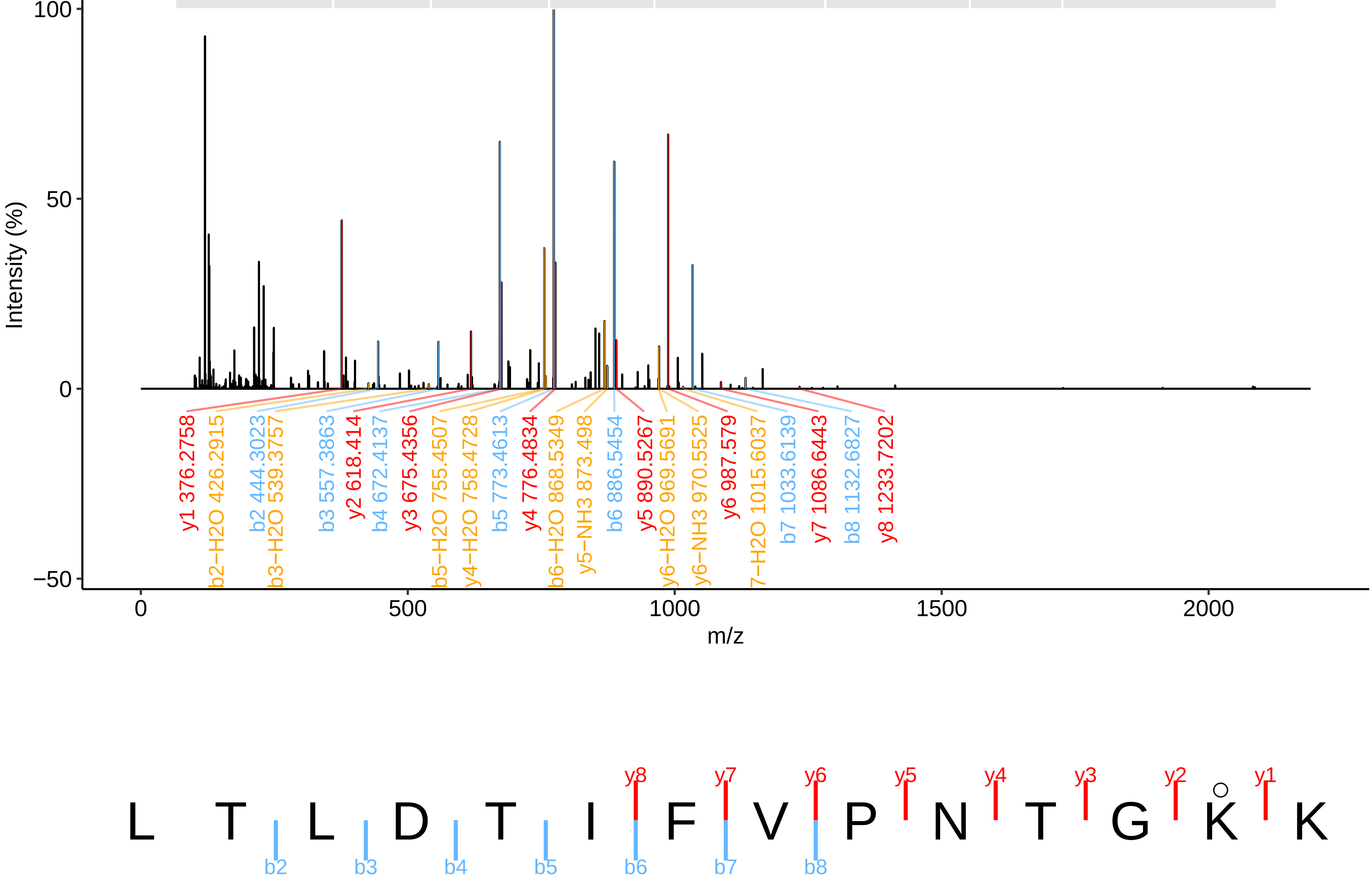

Modification □ Acetyl (Protein N−term) ○ GlyGly (K) no new terminus △ Oxidation (M)

20200924_KIZ_MUb5_GlyGly_1 − Scan number: 24624

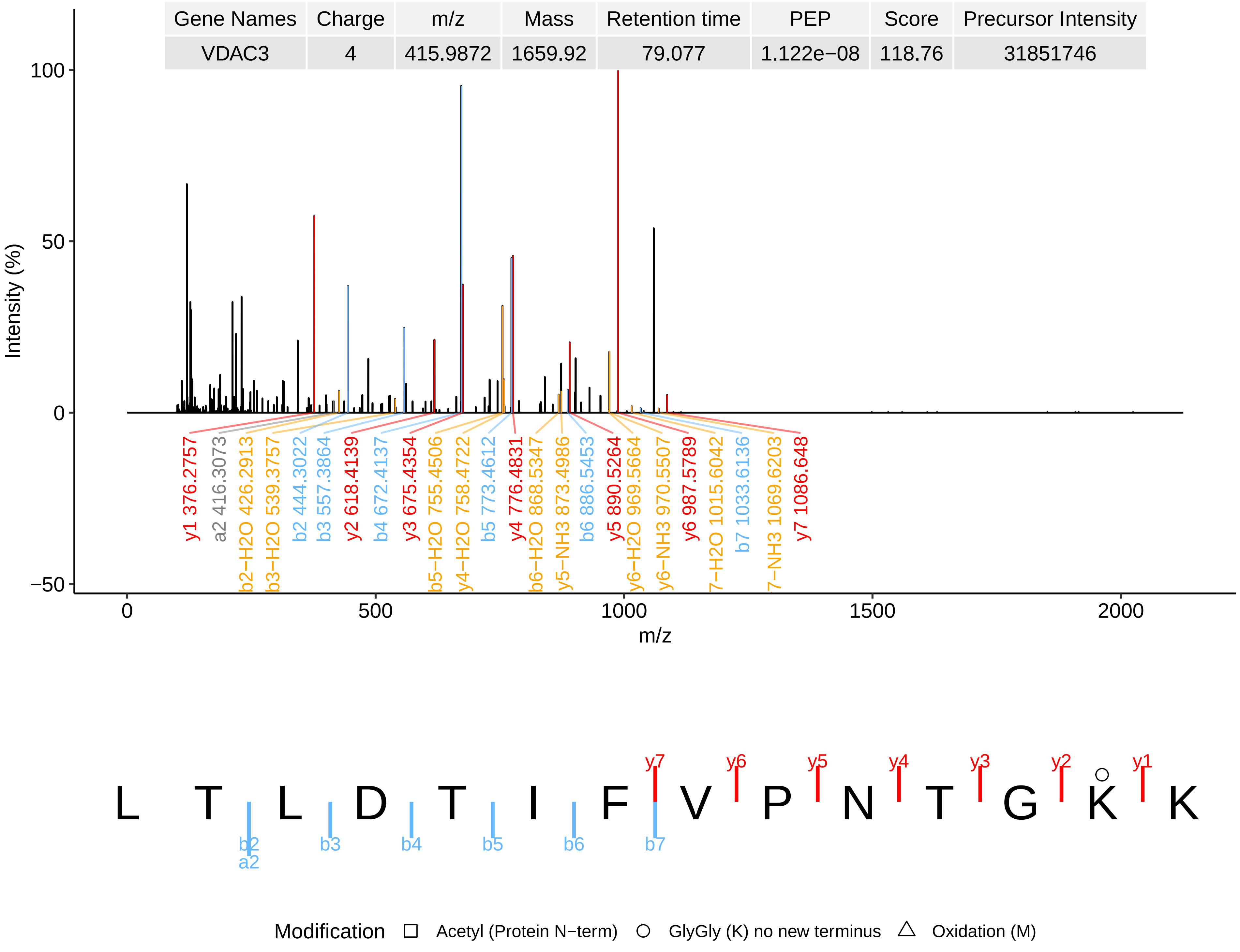

Gene Names	Charge	m/z	Mass	Retention time	PEP	Score	Precursor Intensity
VDAC3	4	415.9872	1659.92	79.077	1.122e−08	118.76	31851746

20200924_KIZ_MUb5_GlyGly_2 − Scan number: 22017

Gene Names	Charge	m/z	Mass	Retention time	PEP	Score	Precursor Intensity
VDAC3	3	554.3138	1659.92	81.547	9.7283e−08	119.01	16105359

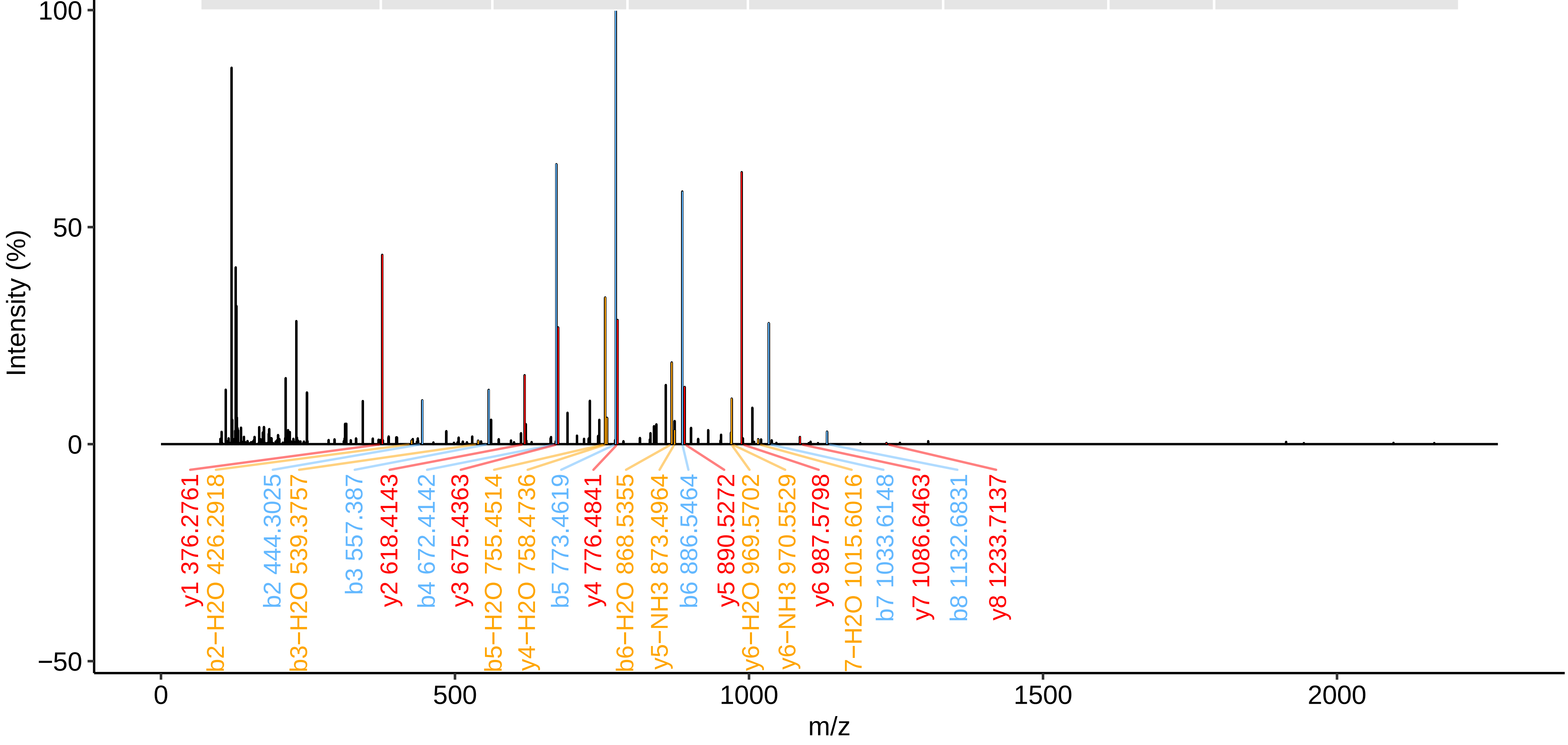

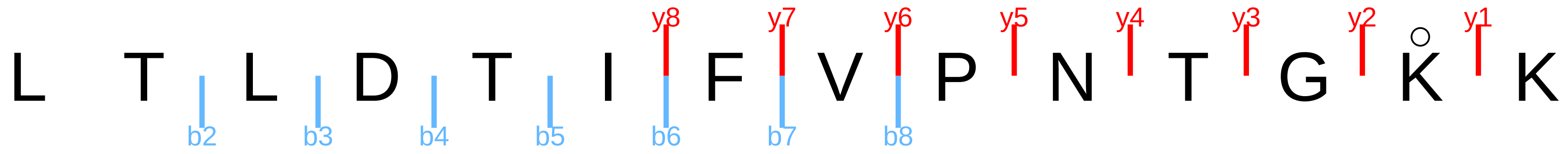

Modification □ Acetyl (Protein N−term) ○ GlyGly (K) no new terminus △ Oxidation (M)

20200924_KIZ_MUb5_GlyGly_2 − Scan number: 22153

Gene Names	Charge	m/z	Mass	Retention time	PEP	Score	Precursor Intensity
VDAC3	3	554.3138	1659.92	82.054	1.0185e−07	118.37	46936972

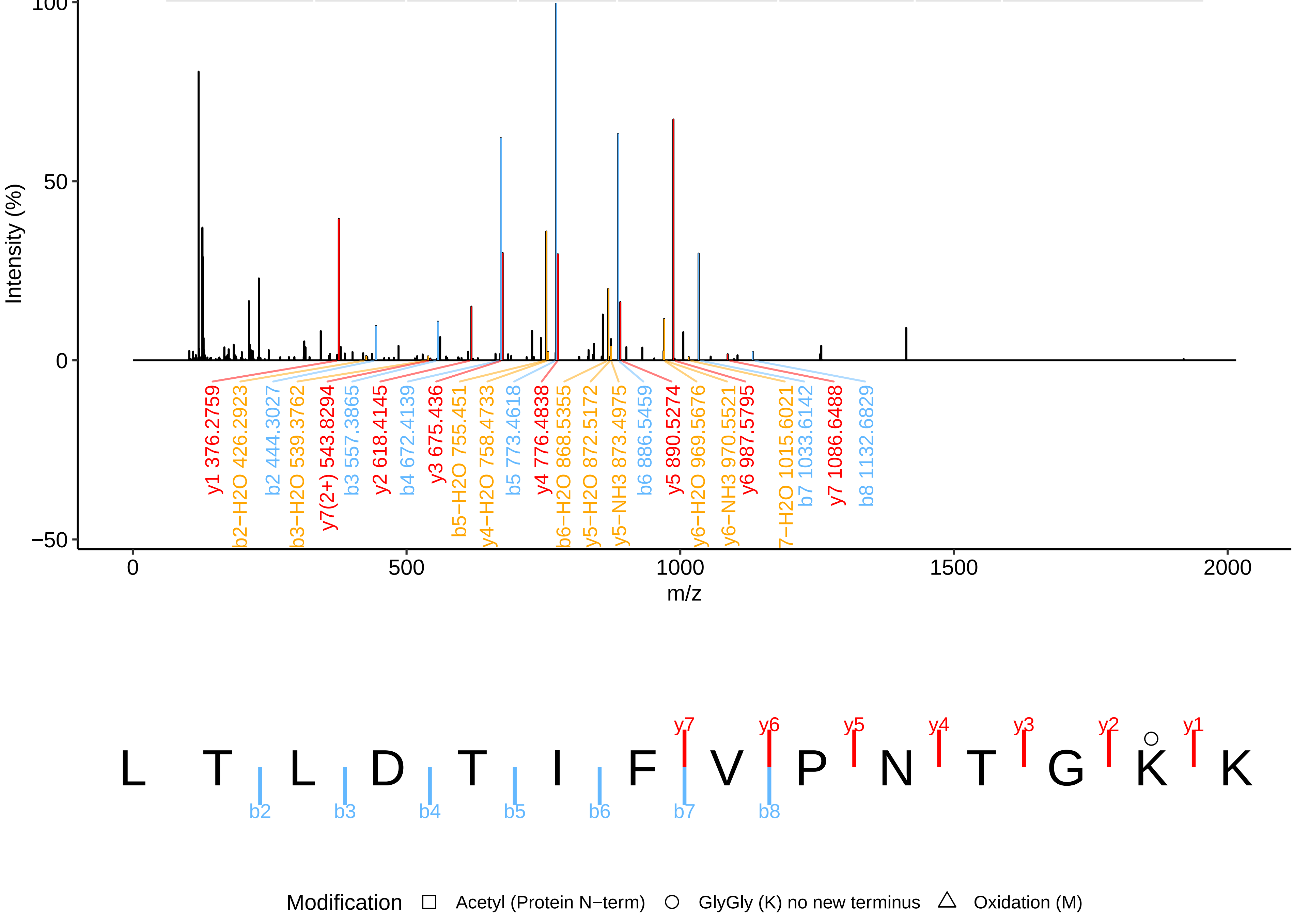

20200924_KIZ_MUb5_GlyGly_2 − Scan number: 22290

Gene Names	Charge	m/z	Mass	Retention time	PEP	Score	Precursor Intensity
VDAC3	3	554.3138	1659.92	82.565	4.7631e−05	109.22	72464368

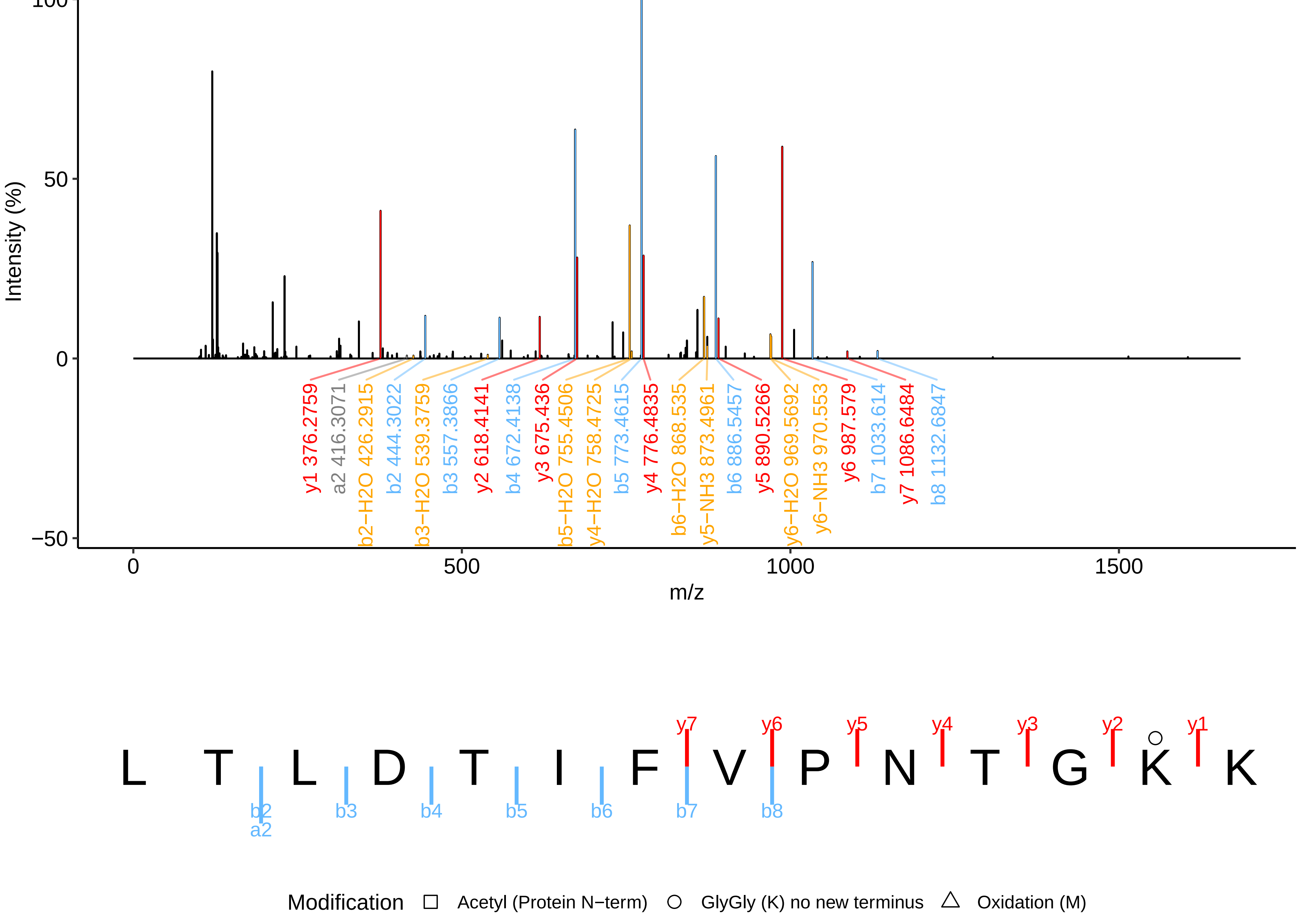

20200924_KIZ_MUb5_GlyGly_2 − Scan number: 22432

Gene Names	Charge	m/z	Mass	Retention time	PEP	Score	Precursor Intensity
VDAC3	3	554.3138	1659.92	83.07	1.199e−07	115.8	12192245

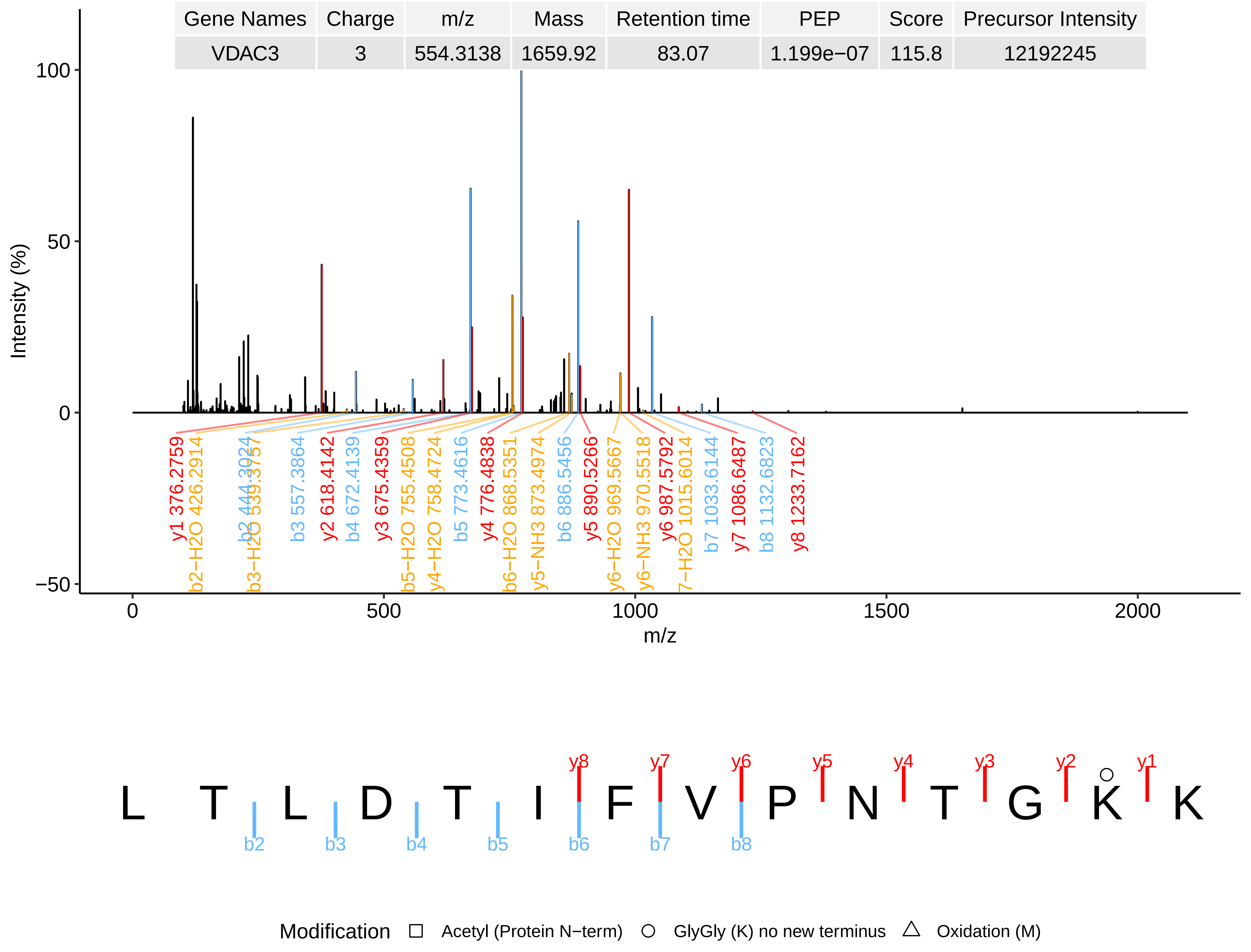

20200924_KIZ_MUb5_GlyGly_2 − Scan number: 22055

Gene Names	Charge	m/z	Mass	Retention time	PEP	Score	Precursor Intensity
VDAC3	4	415.9872	1659.92	81.681	0.00011273	94.688	4104283

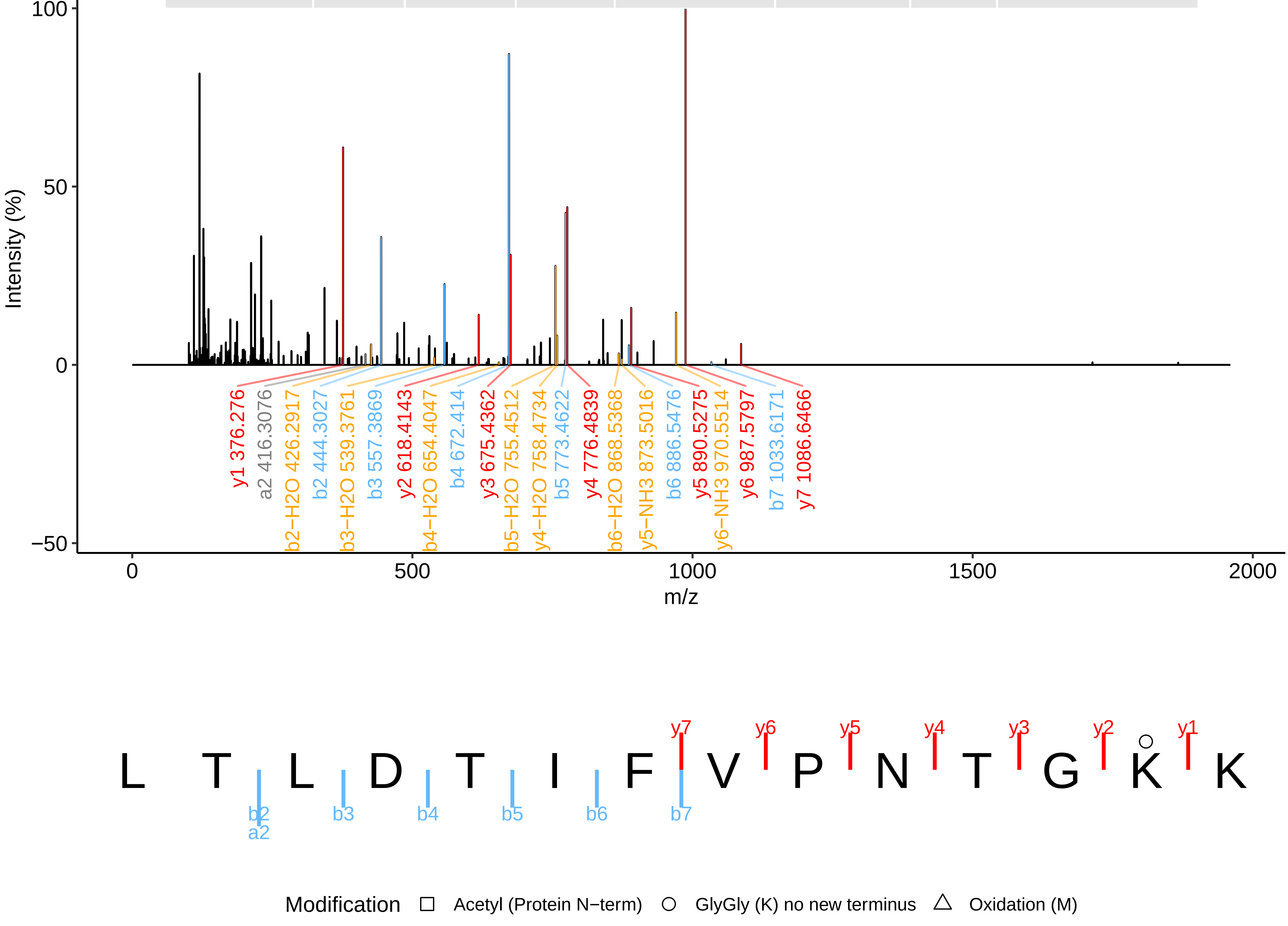

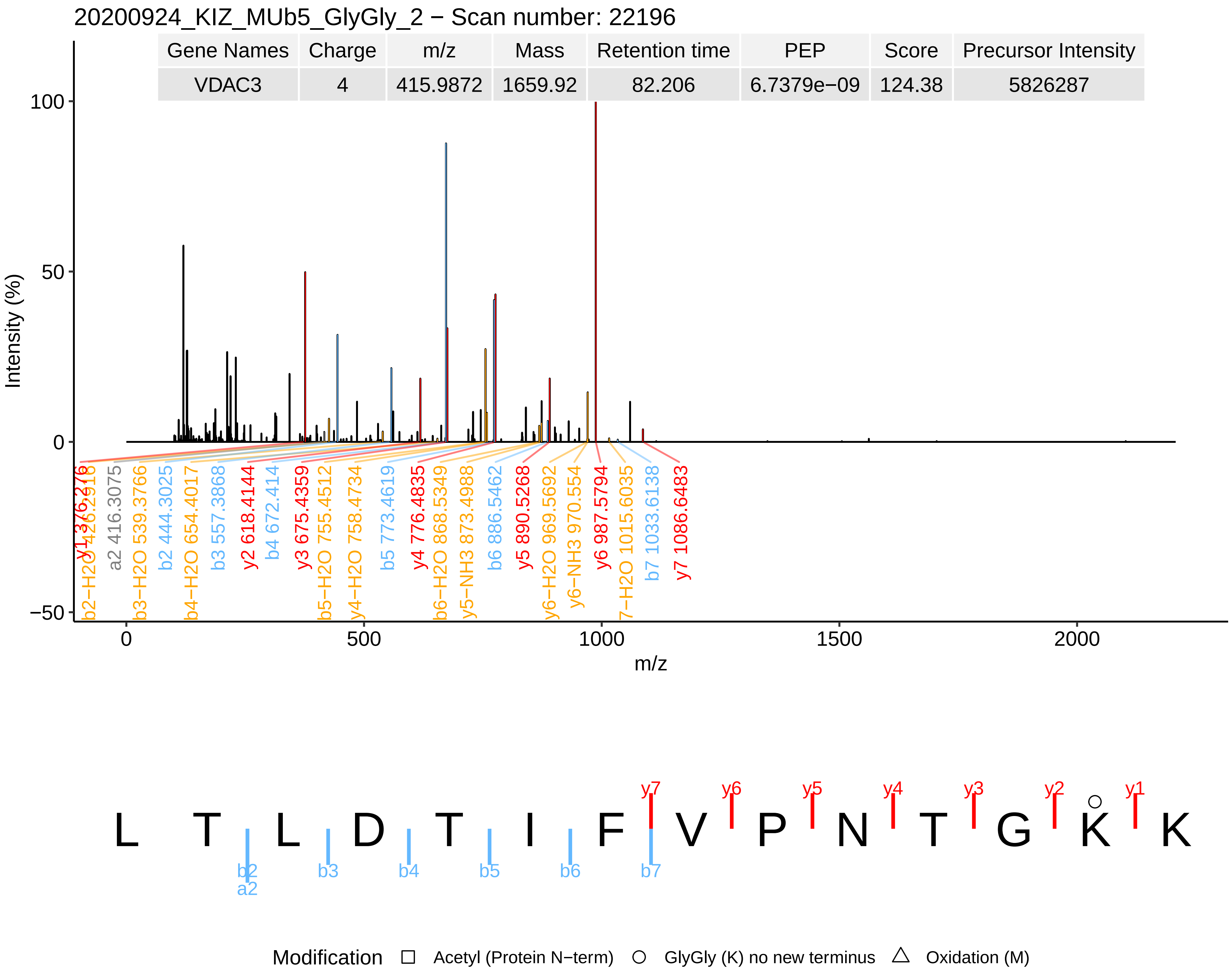
20200924_KIZ_MUb5_GlyGly_2 − Scan number: 22196
Gene Names | Charge | m/z | Mass | Retention time | PEP | Score | Precursor Intensity
VDAC3 | 4 | 415.9872 | 1659.92 | 82.206 | 6.7379e−09 | 124.38 | 5826287
Intensity (%)
m/z
y1 376.276
b2−H2O 426.2918
a2 416.3075
b3−H2O 539.3766
b2 444.3025
b4−H2O 654.4017
b3 557.3868
y2 618.4144
b4 672.414
y3 675.4359
b5−H2O 755.4512
y4−H2O 758.4734
b5 773.4619
y4 776.4835
b6−H2O 868.5349
y5−NH3 873.4988
b6 886.5462
y5 890.5268
y6−H2O 969.5692
y6−NH3 970.554
y6 987.5794
y7−H2O 1015.6035
b7 1033.6138
y7 1086.6483
L T L D T I F V P N T G K K
Modification
Acetyl (Protein N−term)
GlyGly (K) no new terminus
Oxidation (M)

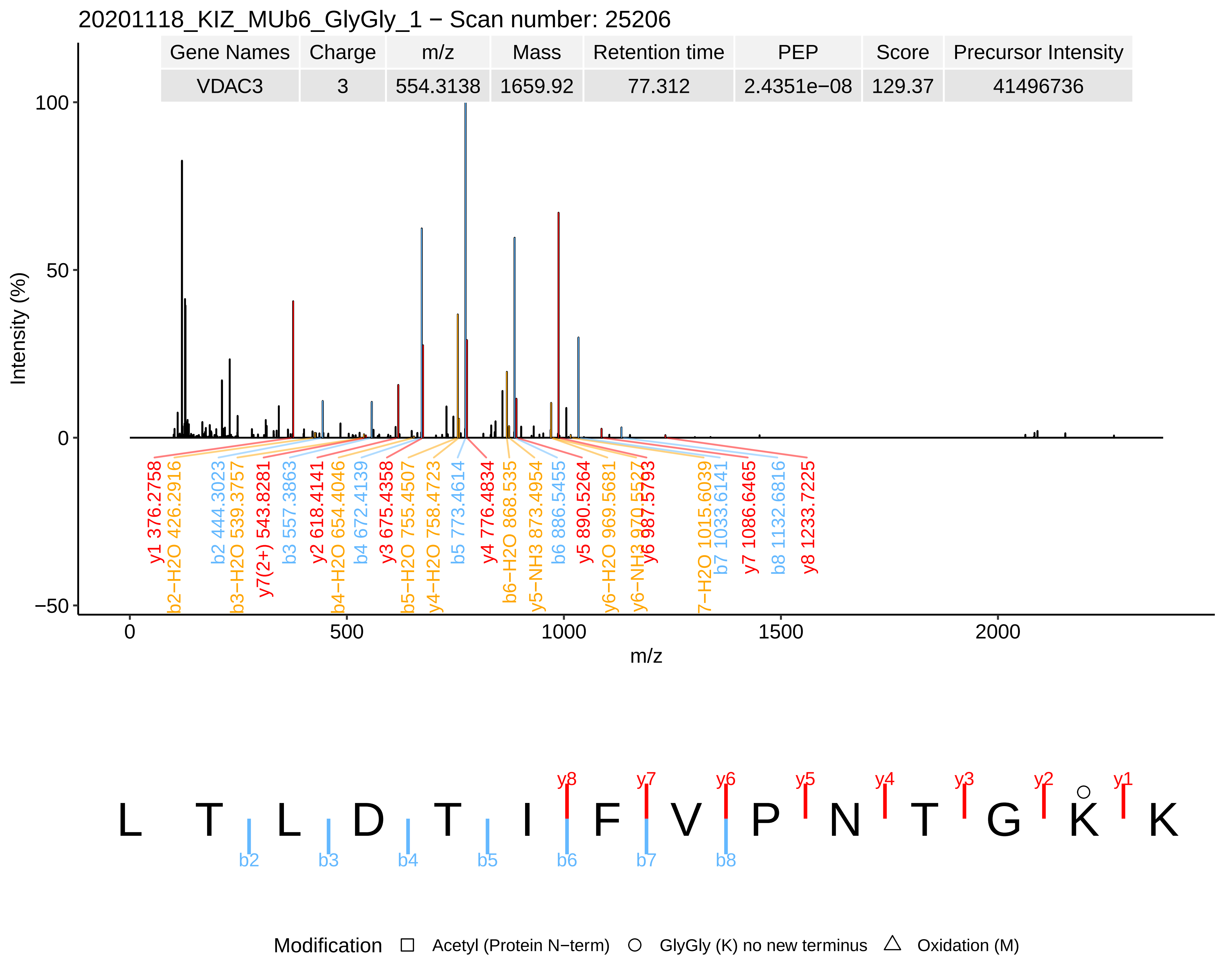

20201118_KIZ_MUb6_GlyGly_1 − Scan number: 25206
Gene Names | Charge | m/z | Mass | Retention time | PEP | Score | Precursor Intensity
VDAC3 | 3 | 554.3138 | 1659.92 | 77.312 | 2.4351e−08 | 129.37 | 41496736
Intensity (%)
m/z
y1 376.2758
b2−H2O 426.2916
b2 444.3023
b3−H2O 539.3757
y7(2+) 543.8281
b3 557.3863
y2 618.4141
b4−H2O 654.4046
b4 672.4139
y3 675.4358
b5−H2O 755.4507
y4−H2O 758.4723
b5 773.4614
y4 776.4834
b6−H2O 868.535
y5−NH3 873.4954
b6 886.5455
y5 890.5264
y6−H2O 969.5681
y6−NH3 970.5527
y6 987.5793
y7−H2O 1015.6039
b7 1033.6141
y7 1086.6465
b8 1132.6816
y8 1233.7225
L T L D T I F V P N T G K K
Modification
Acetyl (Protein N−term)
GlyGly (K) no new terminus
Oxidation (M)

20201118_KIZ_MUb6_GlyGly_2 − Scan number: 27282

Gene Names	Charge	m/z	Mass	Retention time	PEP	Score	Precursor Intensity
VDAC3	3	554.3138	1659.92	90.86	4.1399e−08	126.95	22140678

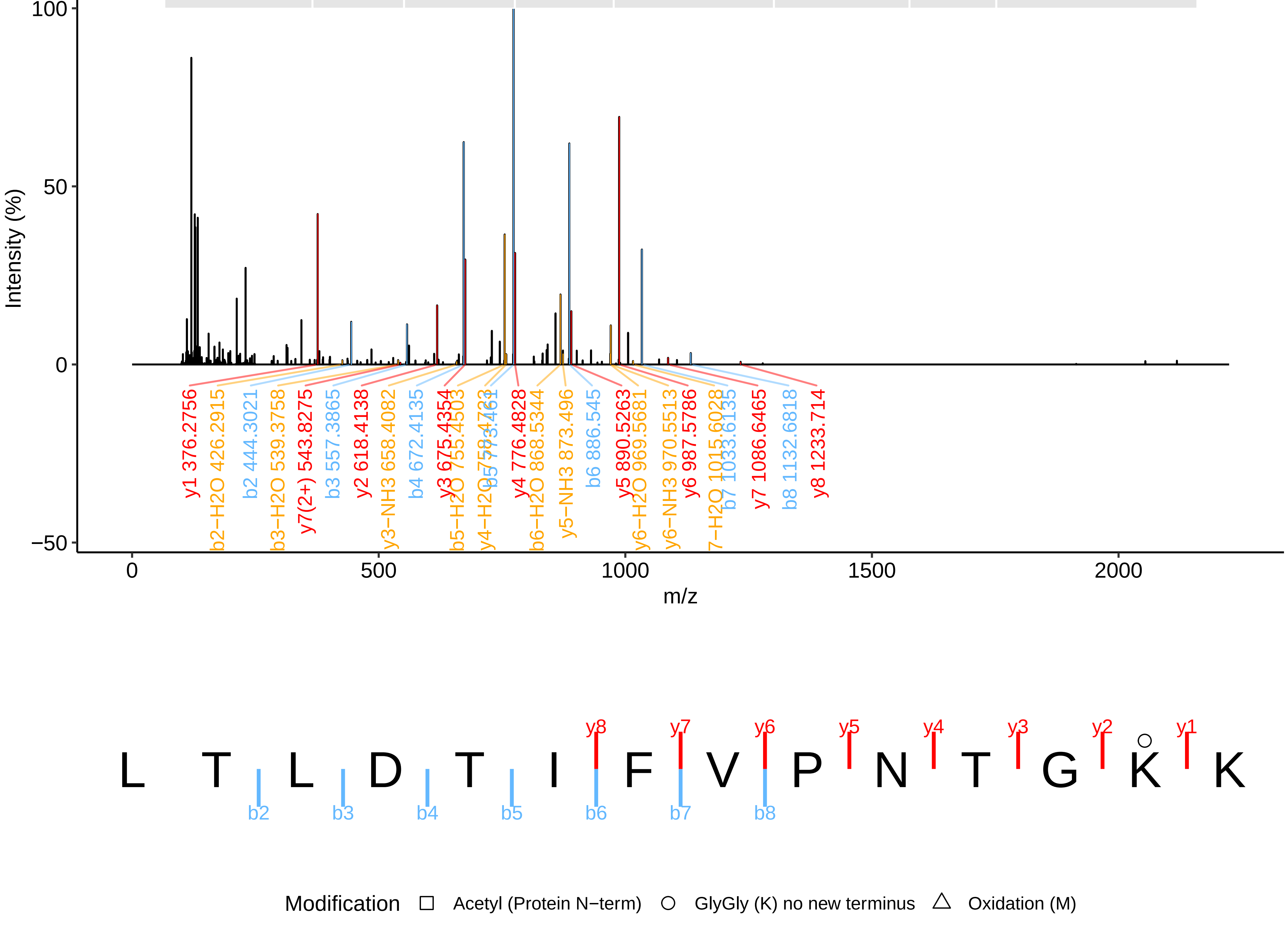

20201118_KIZ_MUb6_GlyGly_2 − Scan number: 27441

Gene Names	Charge	m/z	Mass	Retention time	PEP	Score	Precursor Intensity
VDAC3	3	554.3138	1659.92	91.361	6.7271e−08	123.28	34202440

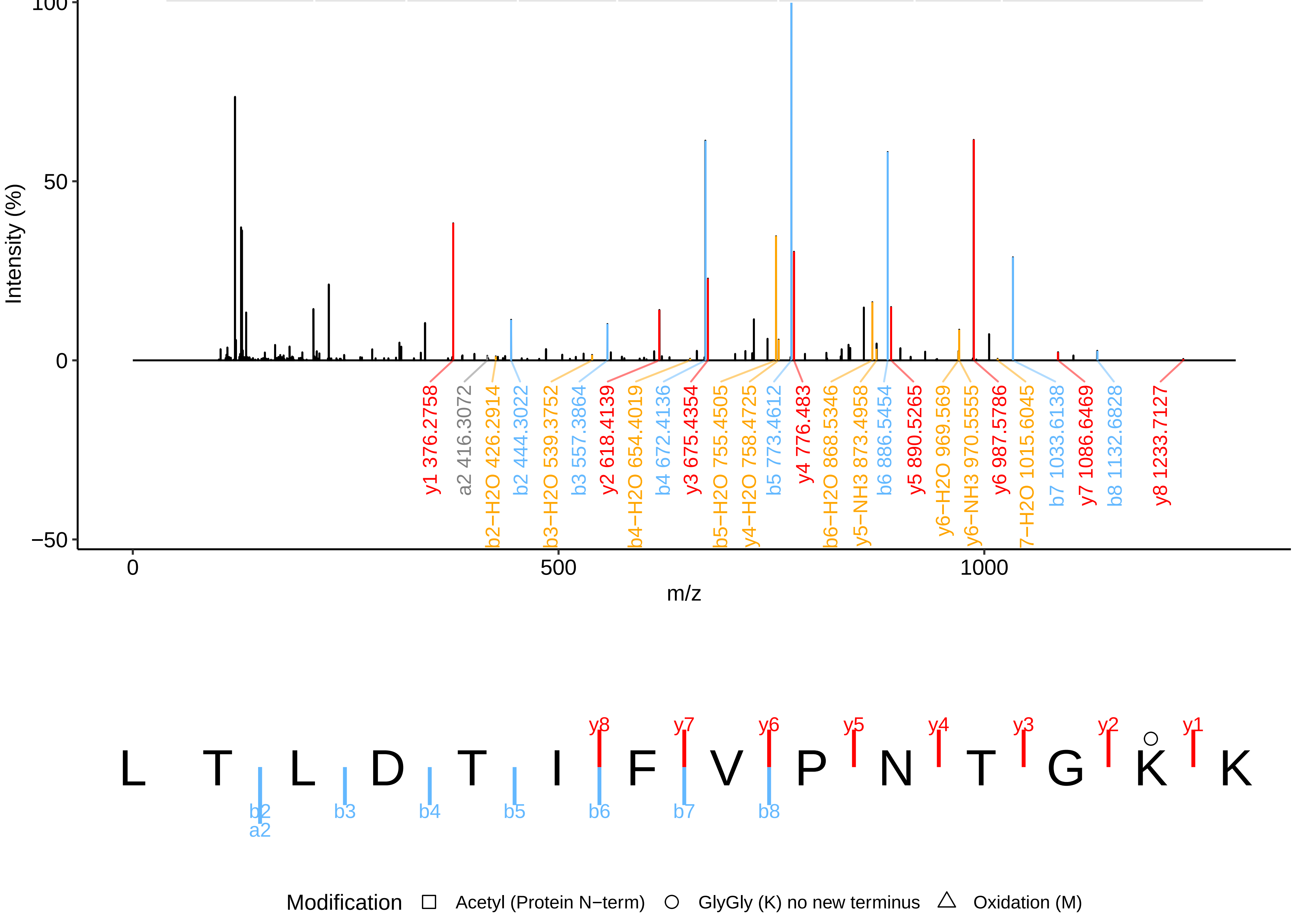

20201118_KIZ_MUb6_GlyGly_2 − Scan number: 27311

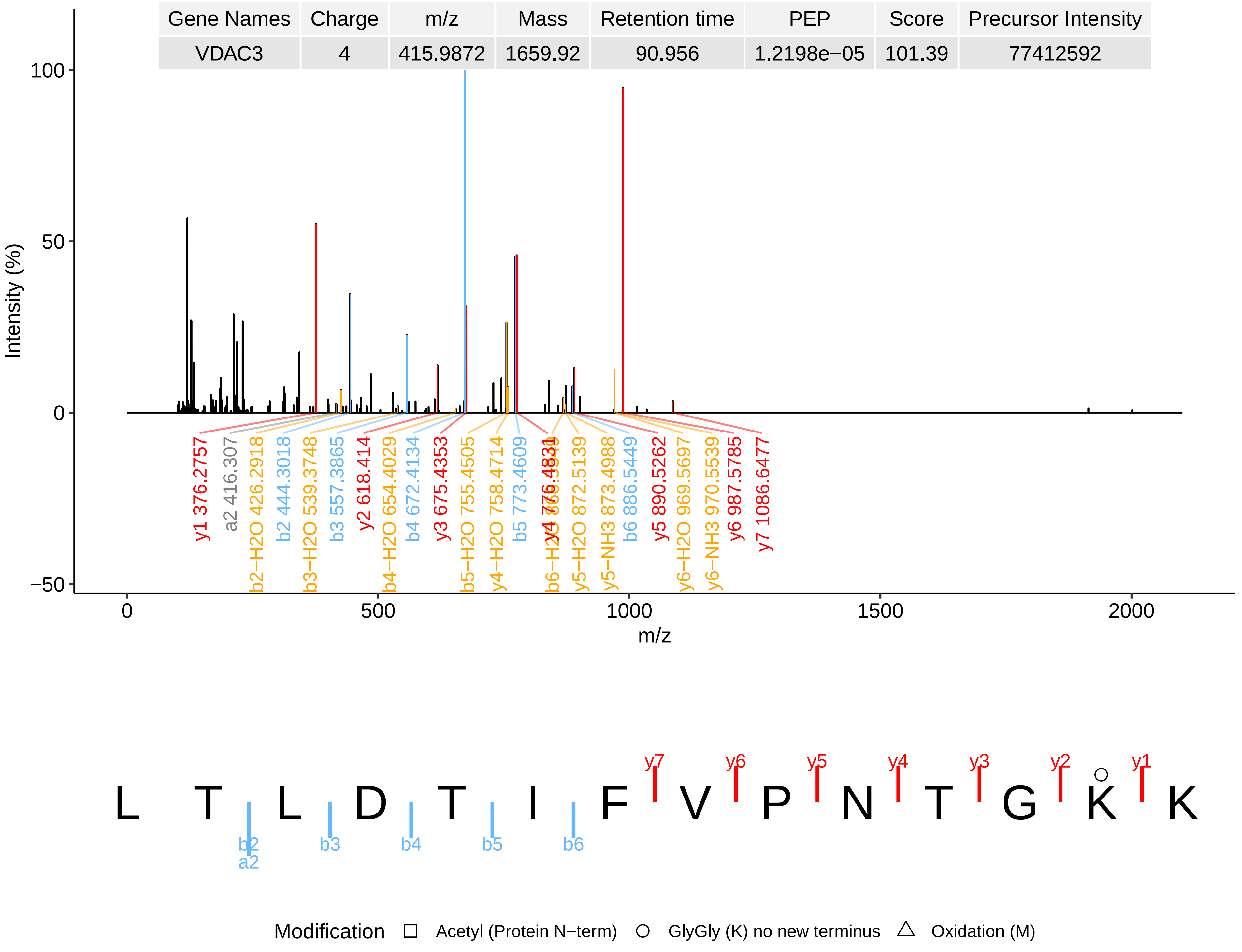

20201118_KIZ_MUb6_GlyGly_2 − Scan number: 27553

Gene Names	Charge	m/z	Mass	Retention time	PEP	Score	Precursor Intensity
GPC3	4	418.475	1669.871	91.737	0.088746	40.552	NA

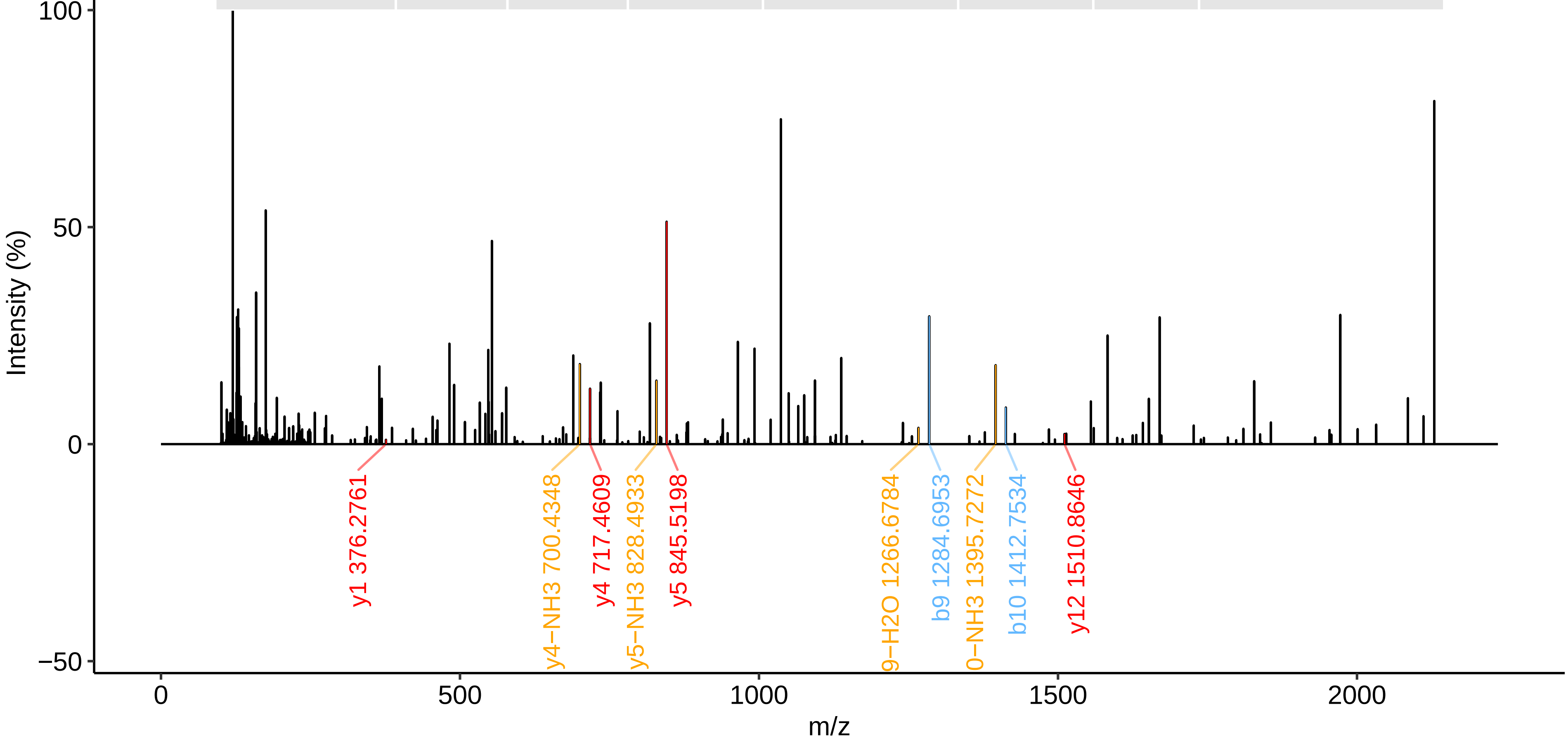

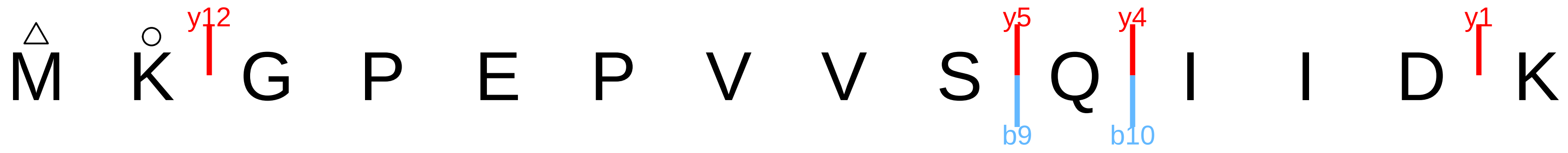

Modification □ Acetyl (Protein N−term) ○ GlyGly (K) no new terminus △ Oxidation (M)

20200921_KIZ_MUb4_GlyGly_10plex_2 − Scan number: 15145

Gene Names	Charge	m/z	Mass	Retention time	PEP	Score	Precursor Intensity
PHB2	3	550.9555	1649.845	59.165	0.06428	44.511	781837.7

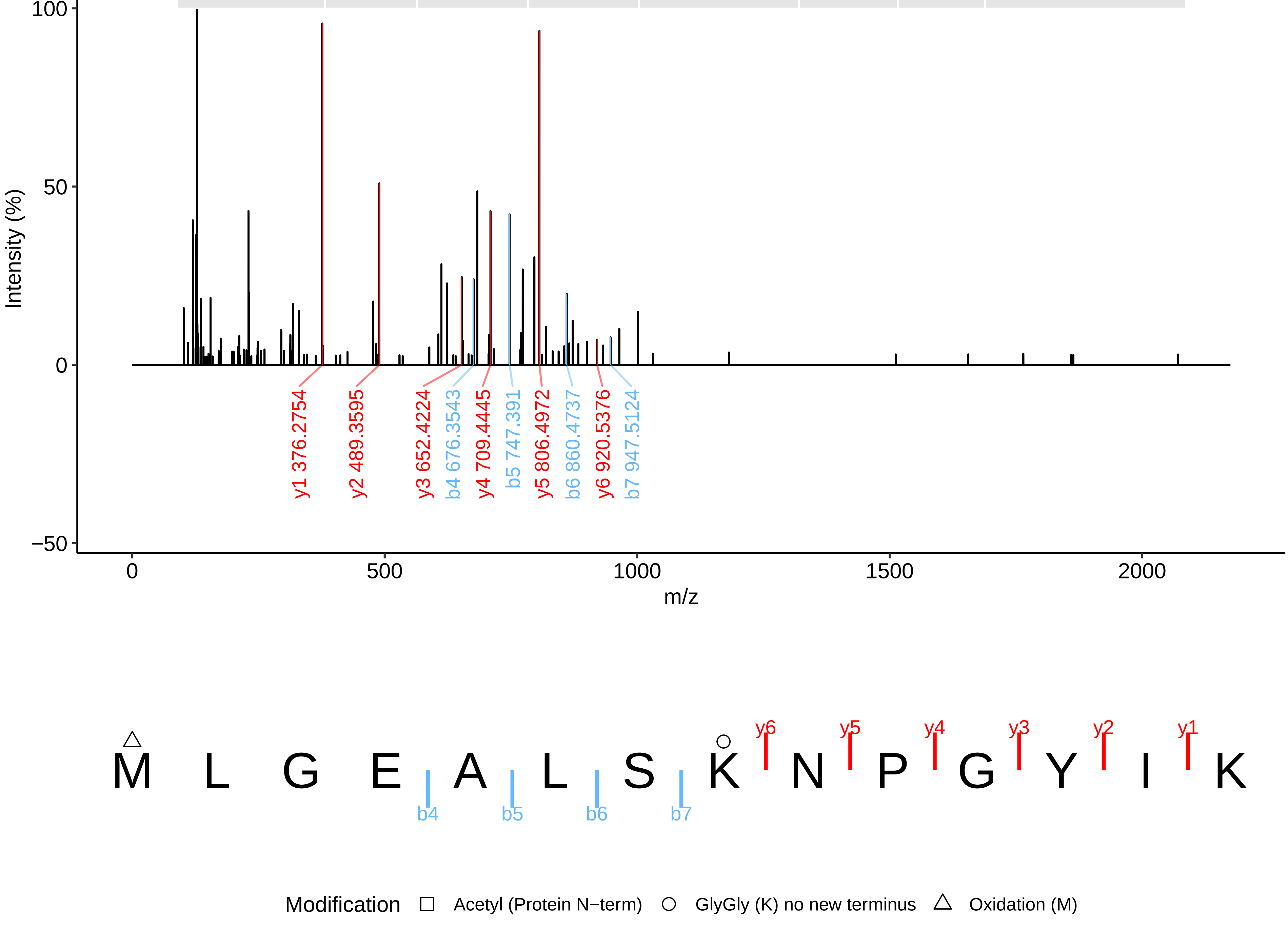

20200921_KIZ_MUb4_GlyGly_10plex_2 − Scan number: 17949

Gene Names	Charge	m/z	Mass	Retention time	PEP	Score	Precursor Intensity
PHB2	3	545.6239	1633.85	70.235	0.0030351	74.46	1828651

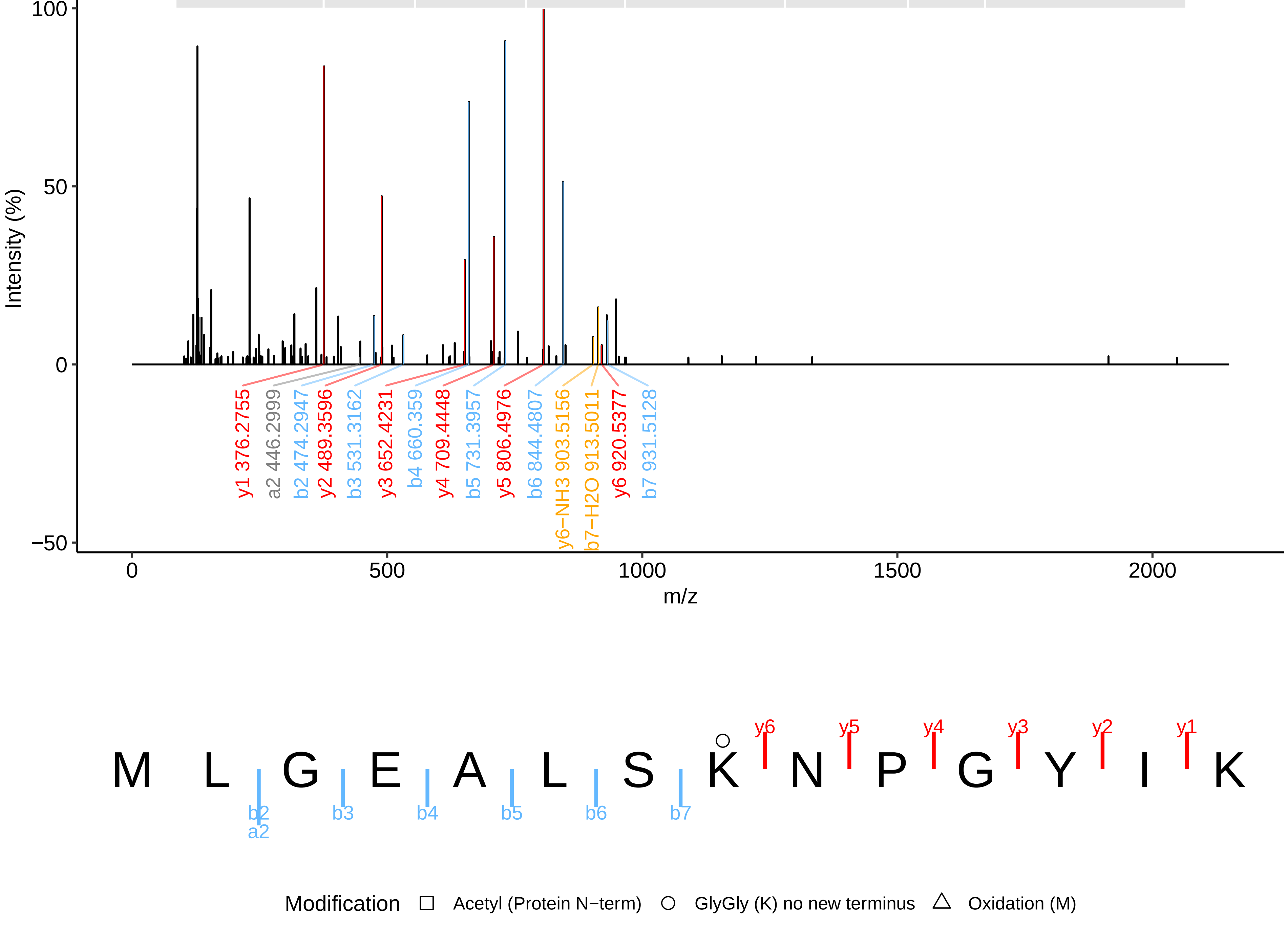

20200921_KIZ_MUb4_GlyGly_10plex − Scan number: 10876

Gene Names	Charge	m/z	Mass	Retention time	PEP	Score	Precursor Intensity
PTP4A1	4	480.7449	1918.951	37.792	4.1652e−13	141.46	6042757

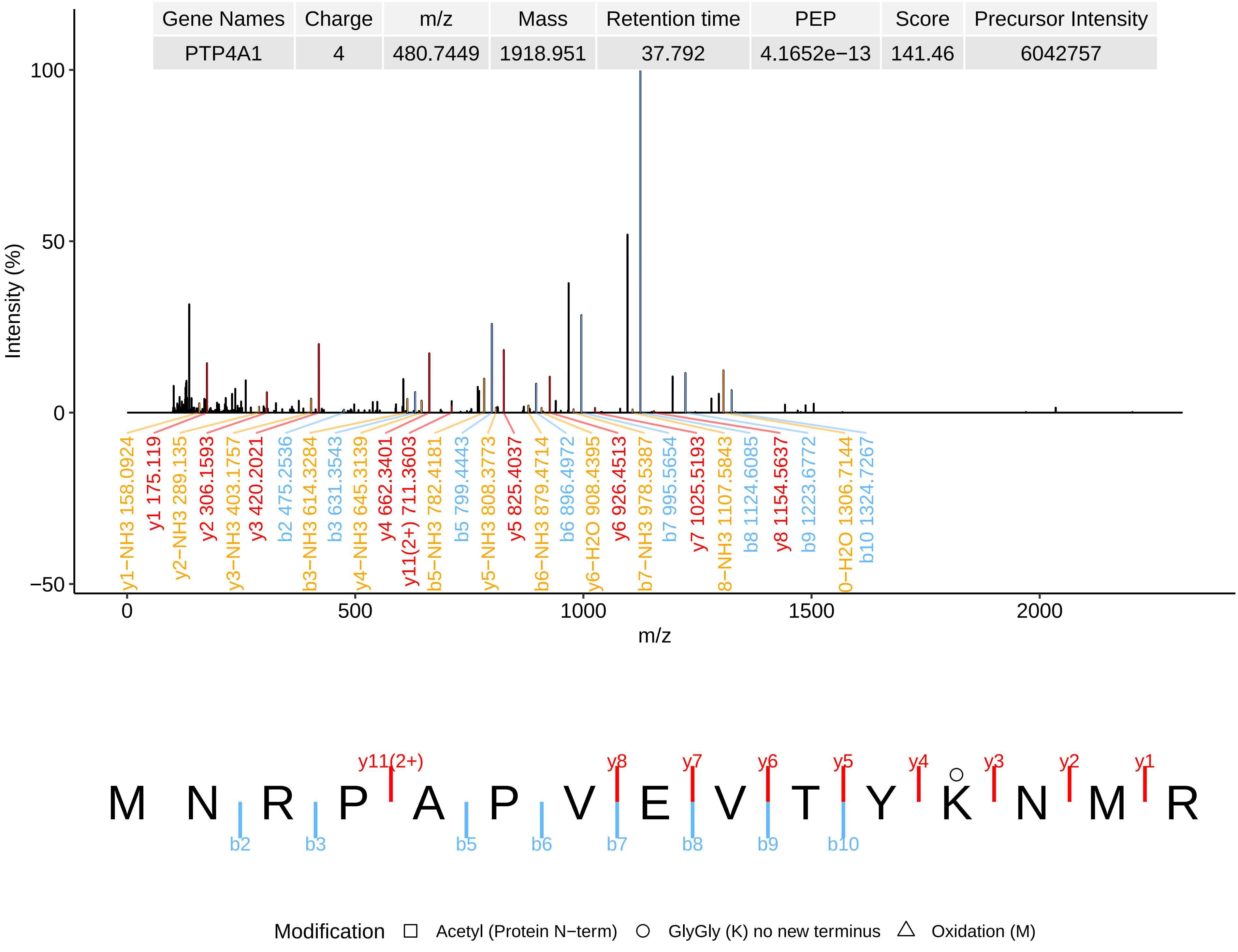

20200921_KIZ_MUb4_GlyGly_10plex_2 − Scan number: 10078

Gene Names	Charge	m/z	Mass	Retention time	PEP	Score	Precursor Intensity
PTP4A1	4	480.7449	1918.951	38.462	8.2513e−13	132.86	2110110

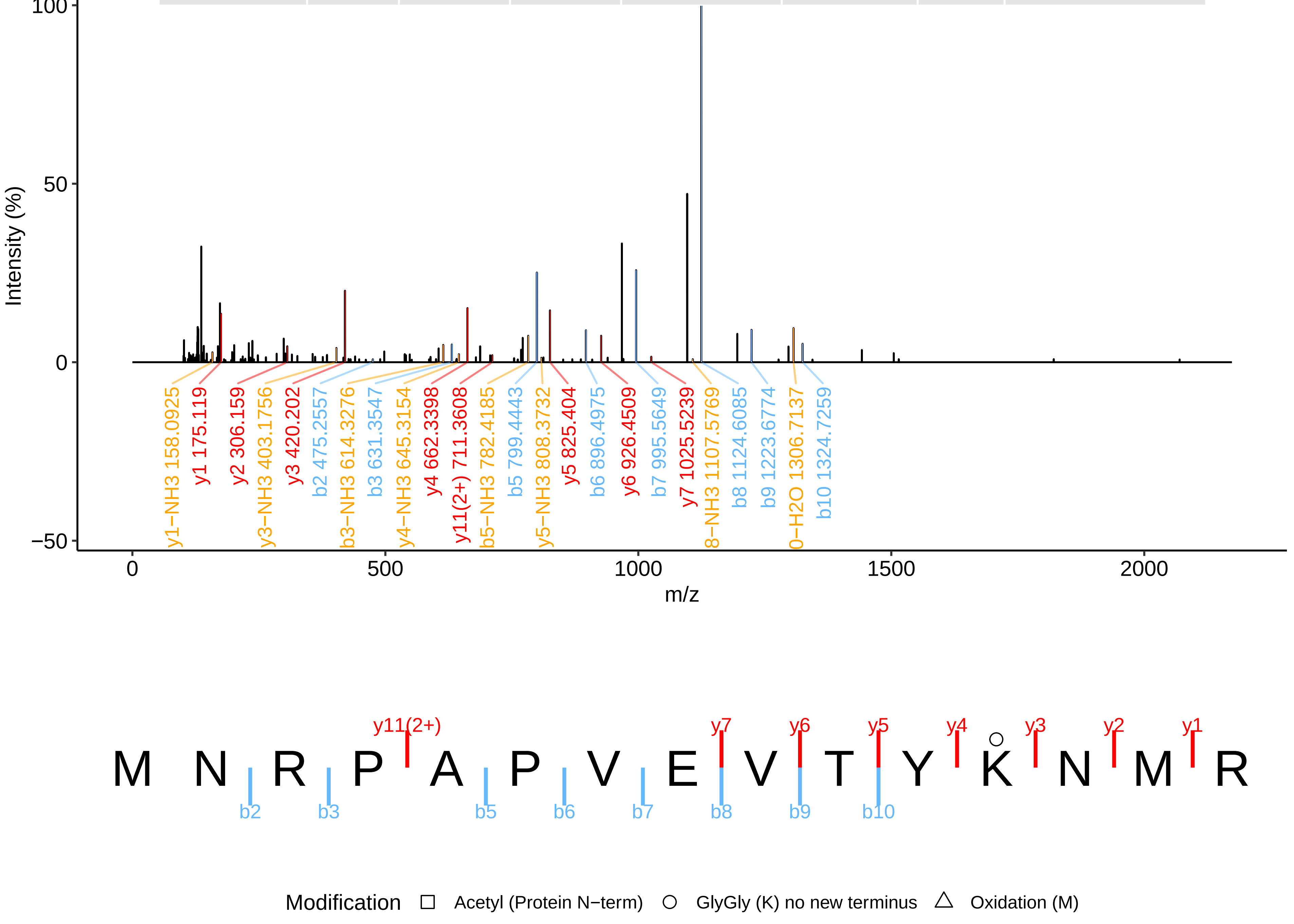

20200921_KIZ_MUb4_GlyGly_10plex − Scan number: 12213

Gene Names	Charge	m/z	Mass	Retention time	PEP	Score	Precursor Intensity
PDCD6IP	4	423.2149	1688.83	42.757	9.8473e−09	118.37	11647473

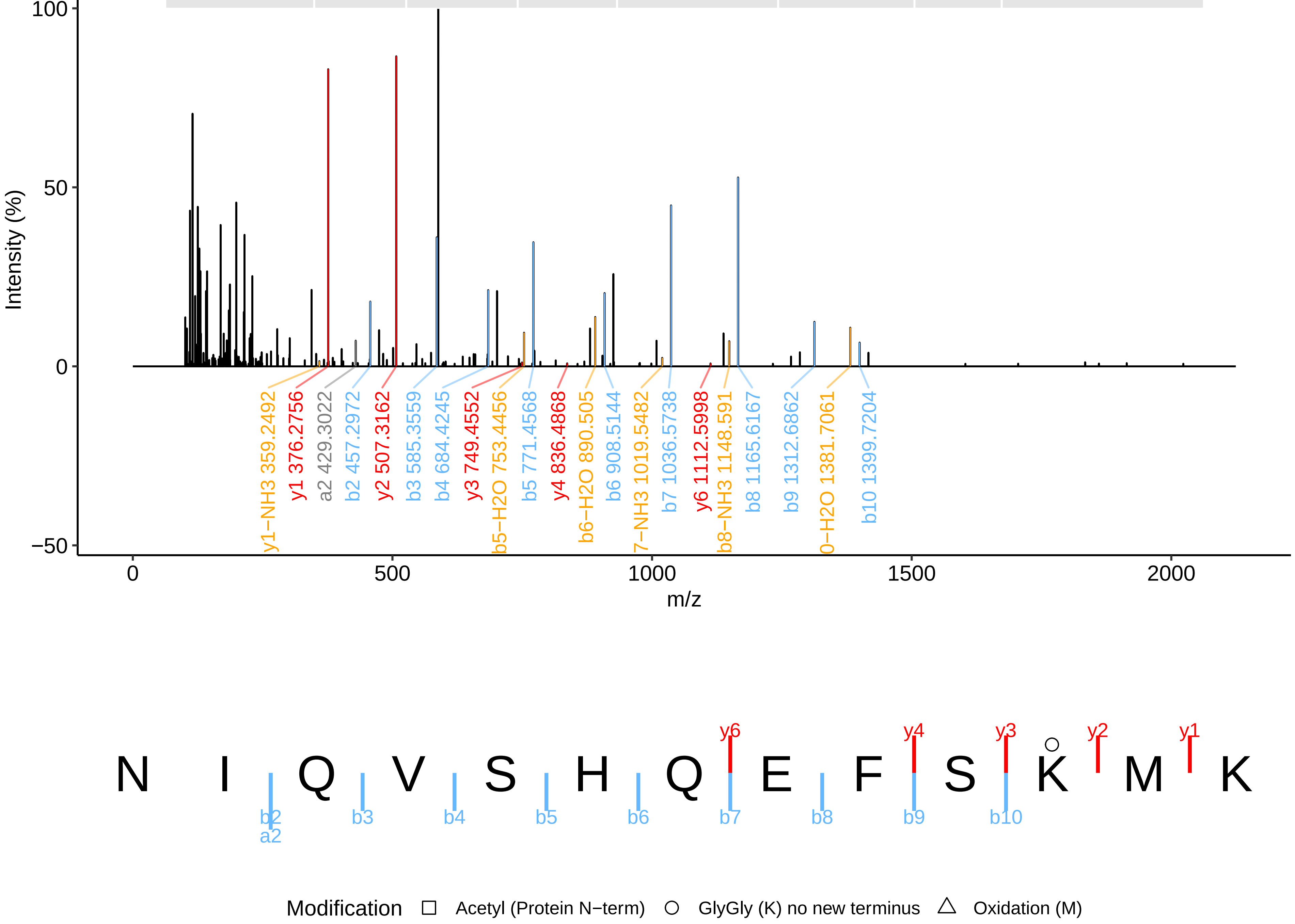

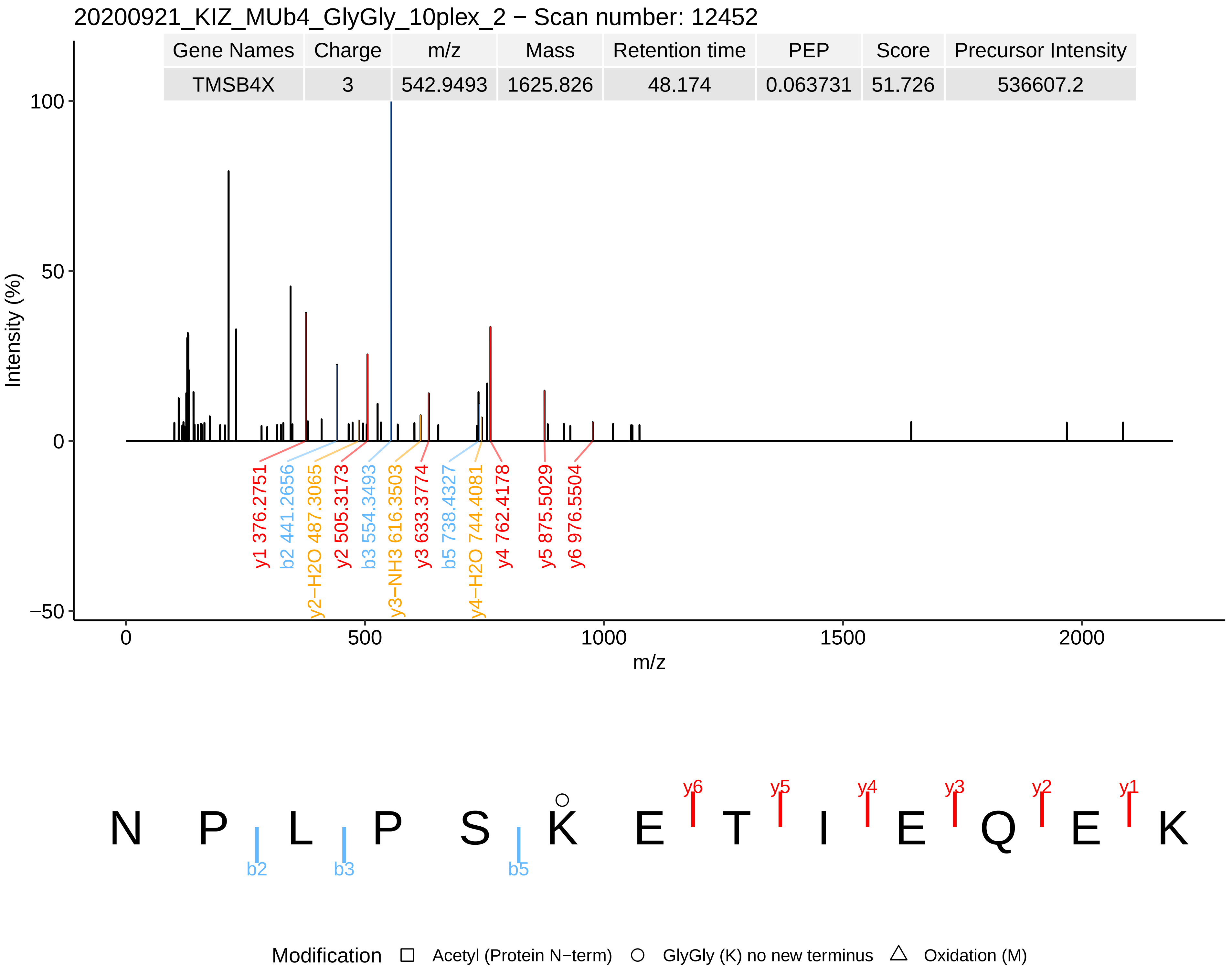
20200921_KIZ_MUb4_GlyGly_10plex_2 − Scan number: 12452
Gene Names
Charge
m/z
Mass
Retention time
PEP
Score
Precursor Intensity
TMSB4X
3
542.9493
1625.826
48.174
0.063731
51.726
536607.2
Intensity (%)
100
50
0
−50
0
500
1000
1500
2000
m/z
y1 376.2751
b2 441.2656
y2−H2O 487.3065
y2 505.3173
b3 554.3493
y3−NH3 616.3503
y3 633.3774
b5 738.4327
y4−H2O 744.4081
y4 762.4178
y5 875.5029
y6 976.5504
N P L P S K E T I E Q E K
b2
b3
b5
y6
y5
y4
y3
y2
y1
Modification
Acetyl (Protein N−term)
GlyGly (K) no new terminus
Oxidation (M)

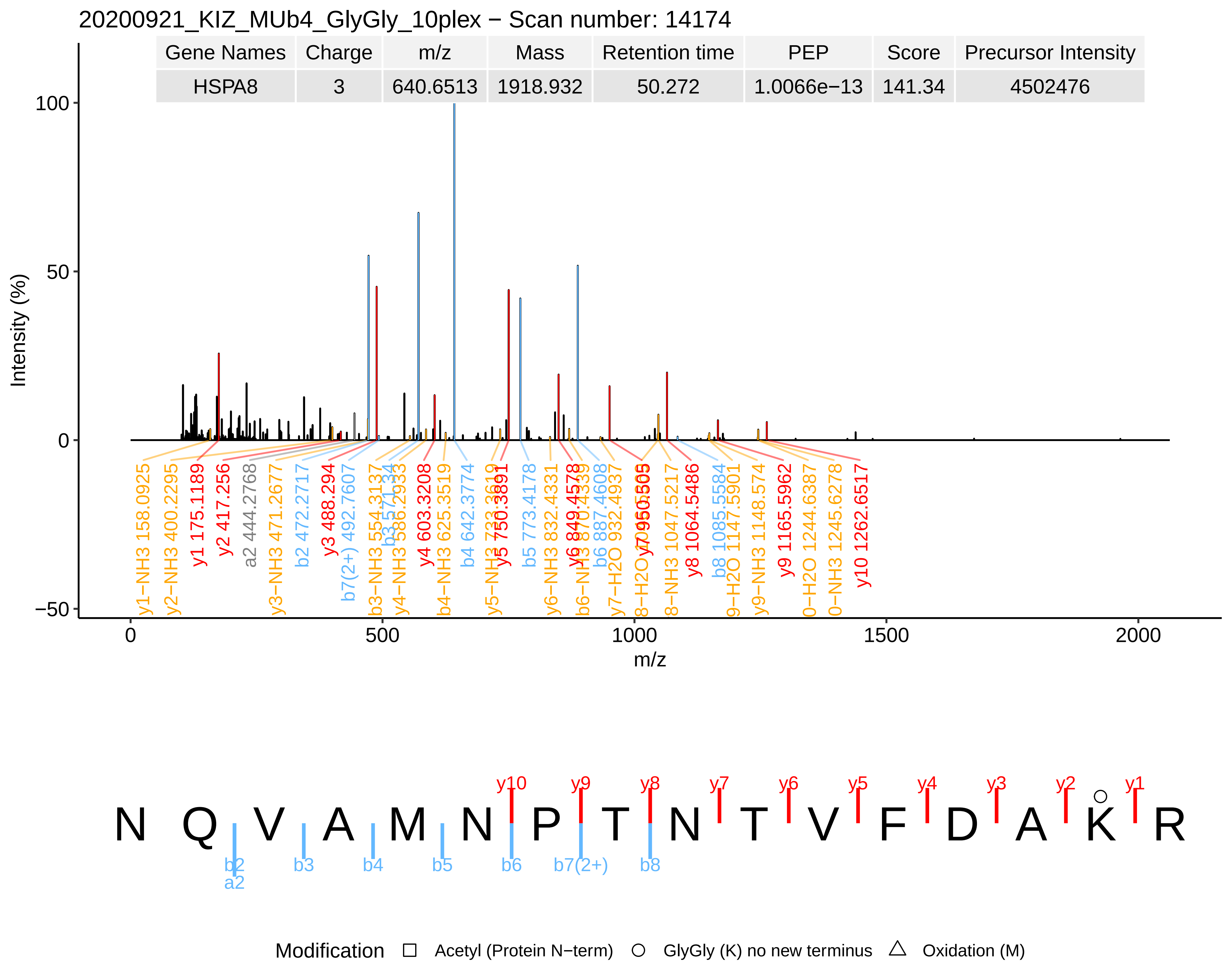

20200921_KIZ_MUb4_GlyGly_10plex − Scan number: 14174
Gene Names | Charge | m/z | Mass | Retention time | PEP | Score | Precursor Intensity
HSPA8 | 3 | 640.6513 | 1918.932 | 50.272 | 1.0066e−13 | 141.34 | 4502476
Intensity (%)
100
50
0
−50
0
500
1000
1500
2000
m/z
y1−NH3 158.0925
y2−NH3 400.2295
y1 175.1189
y2 417.256
a2 444.2768
y3−NH3 471.2677
b2 472.2717
y3 488.294
b7(2+) 492.7607
b3−NH3 554.3137
y4−NH3 586.2933
y4 603.3208
b4−NH3 625.3519
b4 642.3774
y5−NH3 733.3619
y5 750.3891
b5 773.4178
y6−NH3 832.4331
y6 849.4578
b6 887.4608
y7−H2O 932.4937
y8−NH3 1047.5217
y8 1064.5486
b8 1085.5584
y9−NH3 1148.574
y9 1165.5962
y10 1262.6517
N Q V A M N P T N T V F D A K R
y10 y9 y8 y7 y6 y5 y4 y3 y2 y1
b2 a2 b3 b4 b5 b6 b7(2+) b8
Modification
Acetyl (Protein N−term)
GlyGly (K) no new terminus
Oxidation (M)

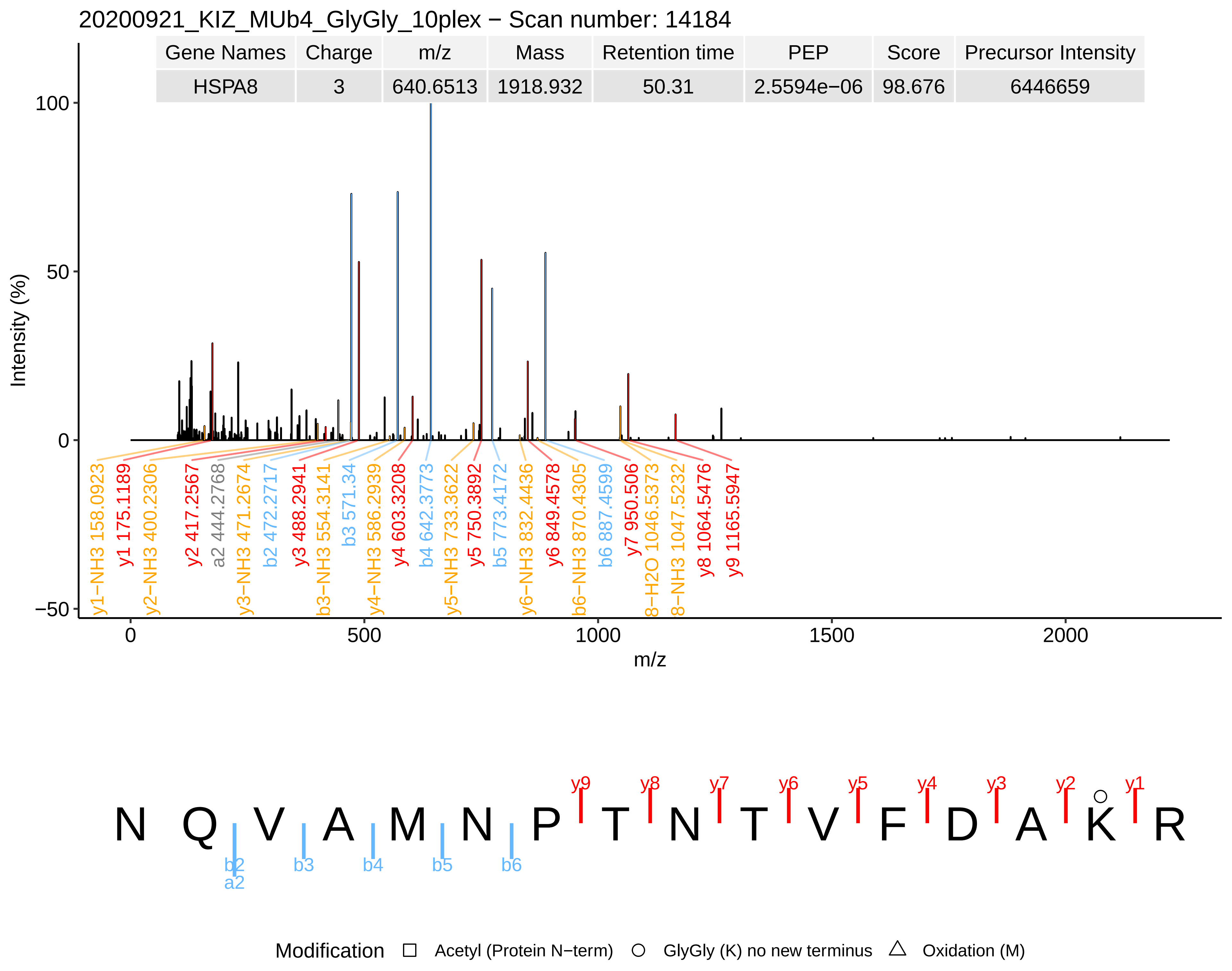

20200921_KIZ_MUb4_GlyGly_10plex − Scan number: 14184
Gene Names | Charge | m/z | Mass | Retention time | PEP | Score | Precursor Intensity
HSPA8 | 3 | 640.6513 | 1918.932 | 50.31 | 2.5594e−06 | 98.676 | 6446659
Intensity (%)
m/z
y1−NH3 158.0923
y1 175.1189
y2−NH3 400.2306
y2 417.2567
a2 444.2768
y3−NH3 471.2674
b2 472.2717
y3 488.2941
b3−NH3 554.3141
b3 571.34
y4−NH3 586.2939
y4 603.3208
b4 642.3773
y5−NH3 733.3622
y5 750.3892
b5 773.4172
y6−NH3 832.4436
y6 849.4578
b6−NH3 870.4305
b6 887.4599
y7 950.506
8−H2O 1046.5373
8−NH3 1047.5232
y8 1064.5476
y9 1165.5947
N Q V A M N P T N T V F D A K R
Modification
Acetyl (Protein N−term)
GlyGly (K) no new terminus
Oxidation (M)

20200921_KIZ_MUb4_GlyGly_10plex_2 − Scan number: 12991

Gene Names	Charge	m/z	Mass	Retention time	PEP	Score	Precursor Intensity
HSPA8	3	640.6513	1918.932	50.395	0.00099393	79.771	695697.6

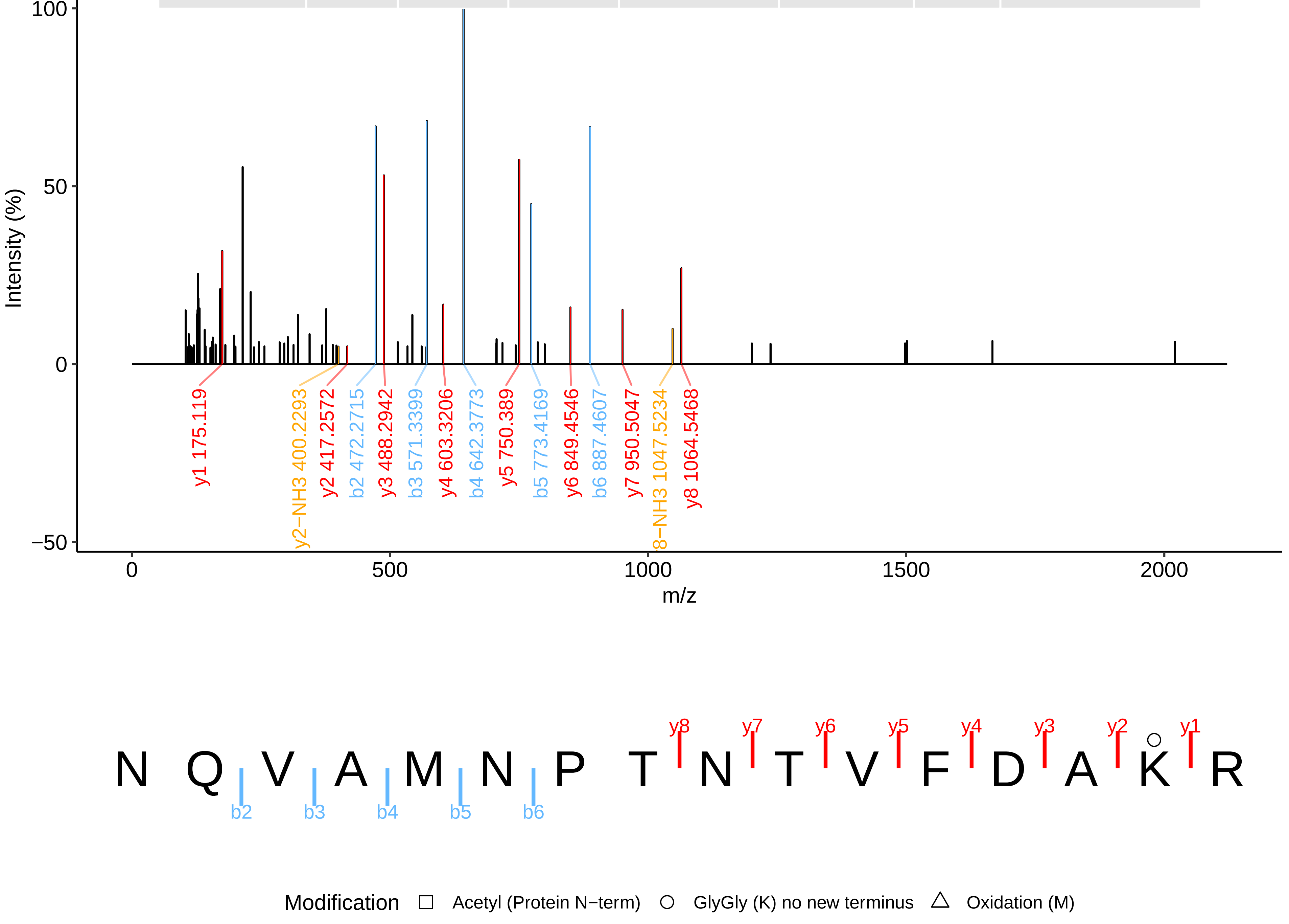

20200921_KIZ_MUb4_GlyGly_10plex − Scan number: 19507

Gene Names	Charge	m/z	Mass	Retention time	PEP	Score	Precursor Intensity
VDAC1	3	613.6551	1837.944	70.36	1.4049e−05	80.534	9031512

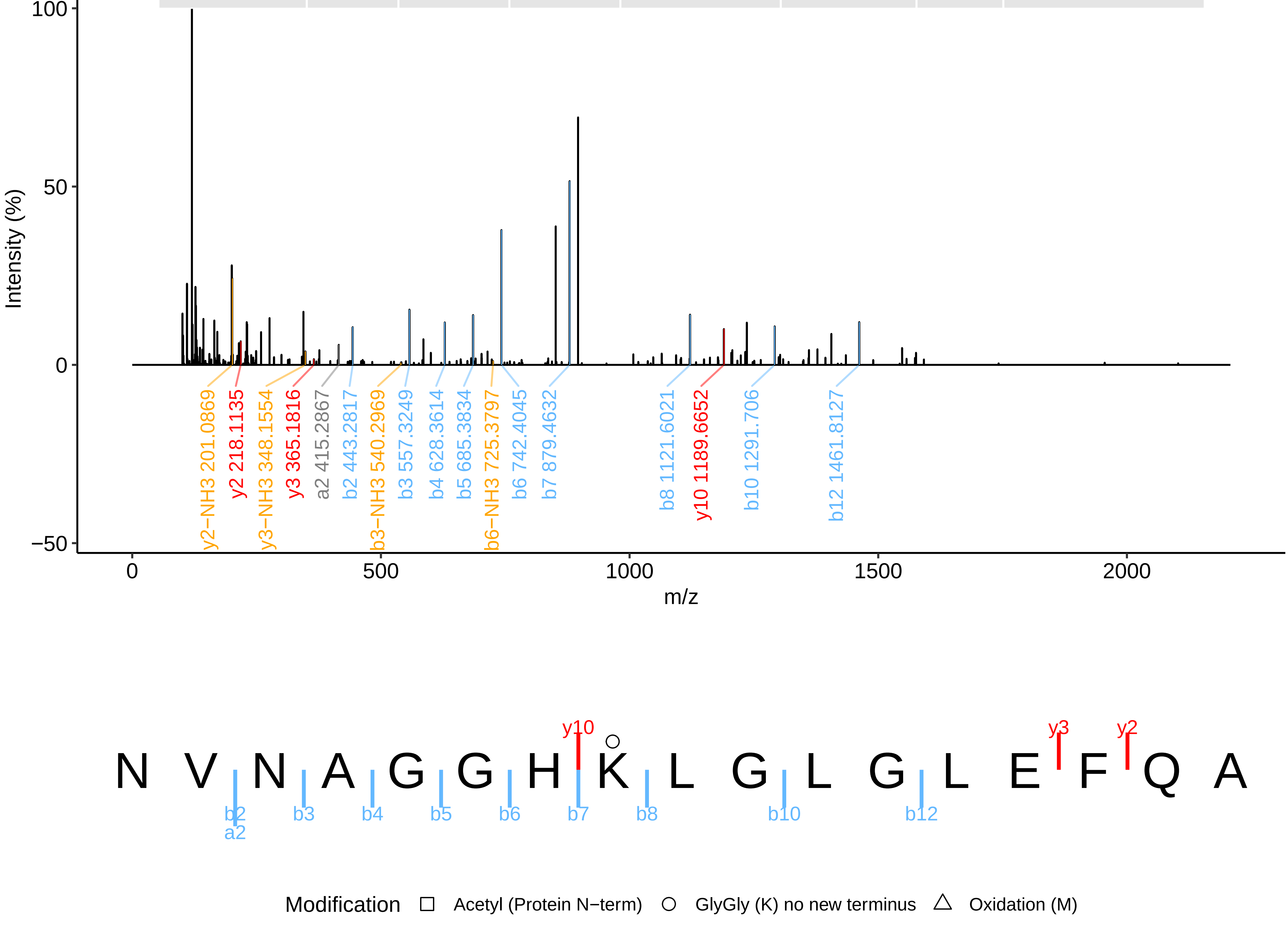

20200921_KIZ_MUb4_GlyGly_10plex − Scan number: 19639

Gene Names	Charge	m/z	Mass	Retention time	PEP	Score	Precursor Intensity
VDAC1	3	613.6551	1837.944	70.864	4.7984e−10	107.07	13883802

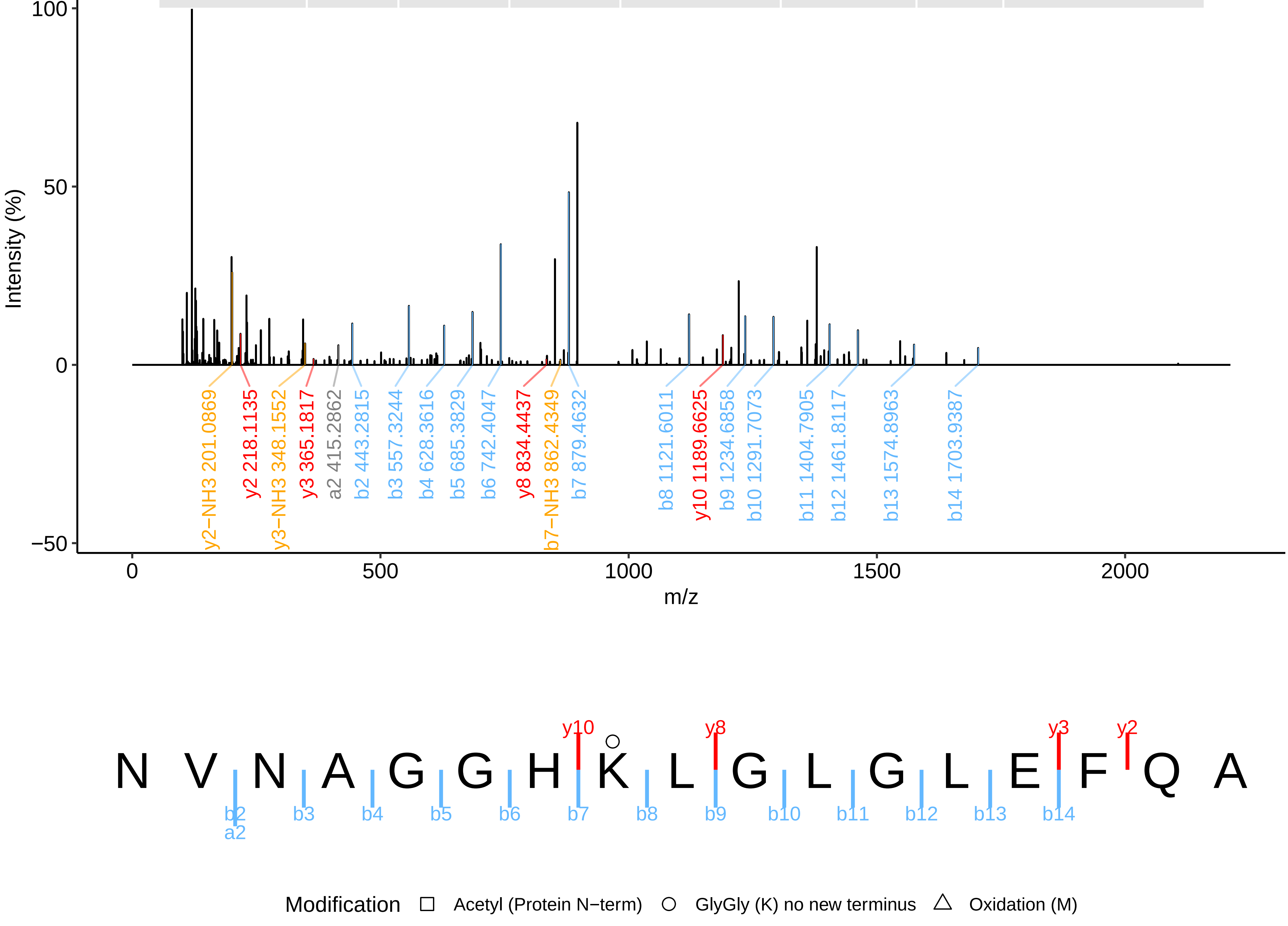

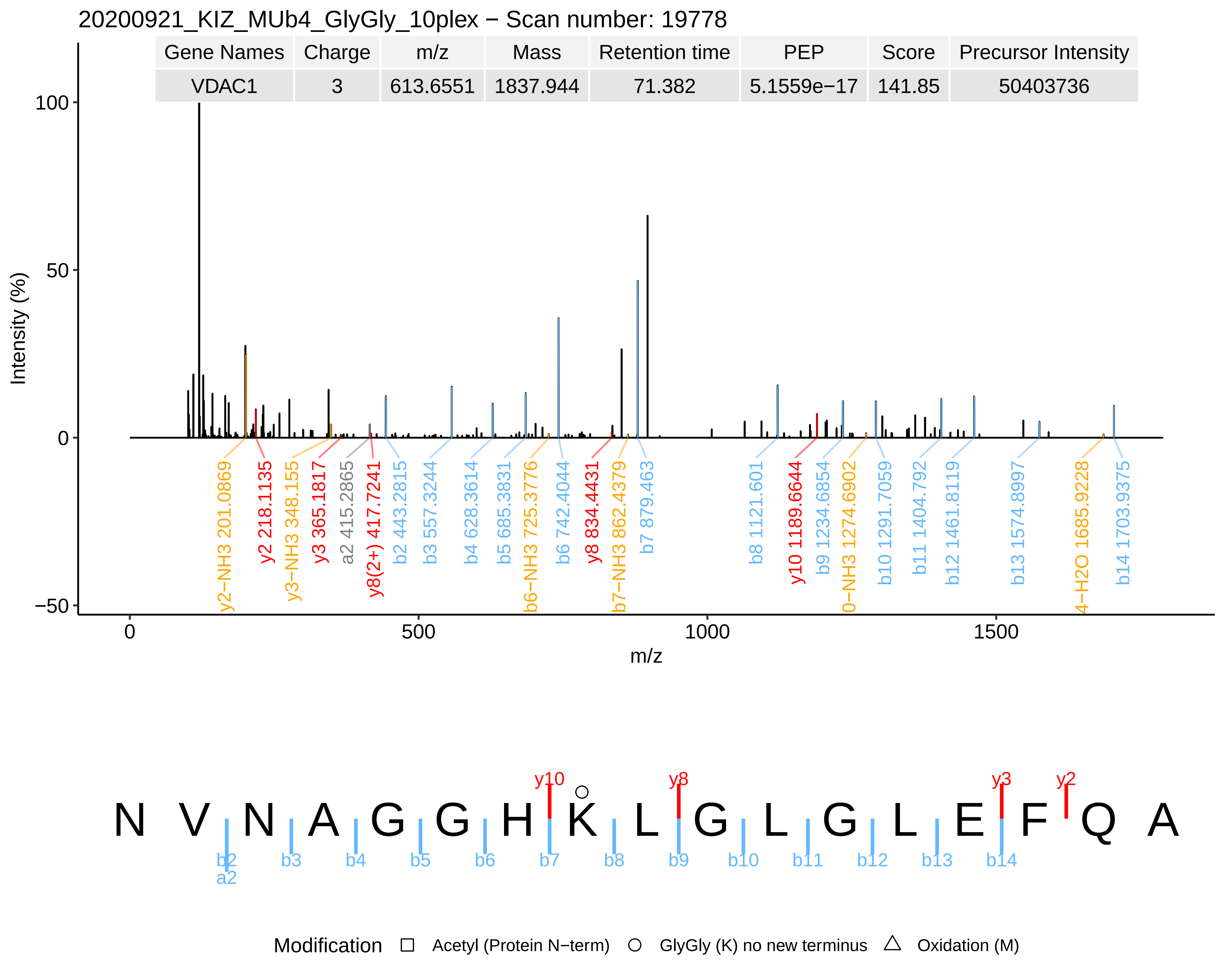
20200921_KIZ_MUb4_GlyGly_10plex − Scan number: 19778
Gene Names | Charge | m/z | Mass | Retention time | PEP | Score | Precursor Intensity
VDAC1 | 3 | 613.6551 | 1837.944 | 71.382 | 5.1559e−17 | 141.85 | 50403736
Intensity (%)
100
50
0
−50
y2−NH3 201.0869
y2 218.1135
y3−NH3 348.155
y3 365.1817
a2 415.2865
y8(2+) 417.7241
b2 443.2815
b3 557.3244
b4 628.3614
b5 685.3831
b6−NH3 725.3776
b6 742.4044
y8 834.4431
b7−NH3 862.4379
b7 879.463
b8 1121.601
y10 1189.6644
b9 1234.6854
0−NH3 1274.6902
b10 1291.7059
b11 1404.792
b12 1461.8119
b13 1574.8997
4−H2O 1685.9228
b14 1703.9375
0
500
1000
1500
m/z
N V N A G G H K L G L G L E F Q A
y10 y8 y3 y2
b2 a2 b3 b4 b5 b6 b7 b8 b9 b10 b11 b12 b13 b14
Modification
Acetyl (Protein N−term)
GlyGly (K) no new terminus
Oxidation (M)

20200921_KIZ_MUb4_GlyGly_10plex − Scan number: 19913

Gene Names	Charge	m/z	Mass	Retention time	PEP	Score	Precursor Intensity
VDAC1	3	613.6551	1837.944	71.882	1.0507e−16	133.14	31512534

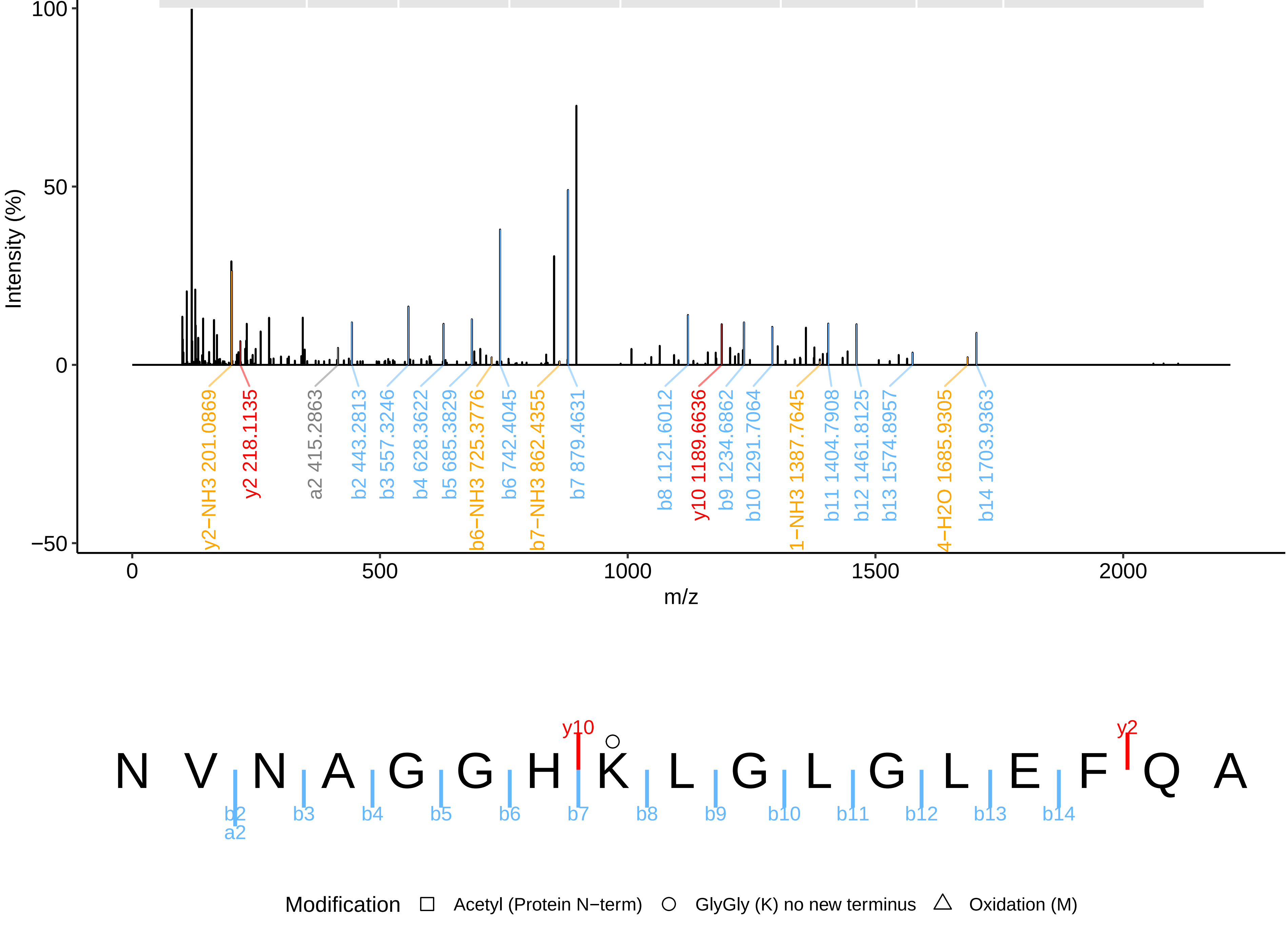

20200921_KIZ_MUb4_GlyGly_10plex − Scan number: 20050

Gene Names	Charge	m/z	Mass	Retention time	PEP	Score	Precursor Intensity
VDAC1	3	613.6551	1837.944	72.397	2.3253e−13	129.93	17043630

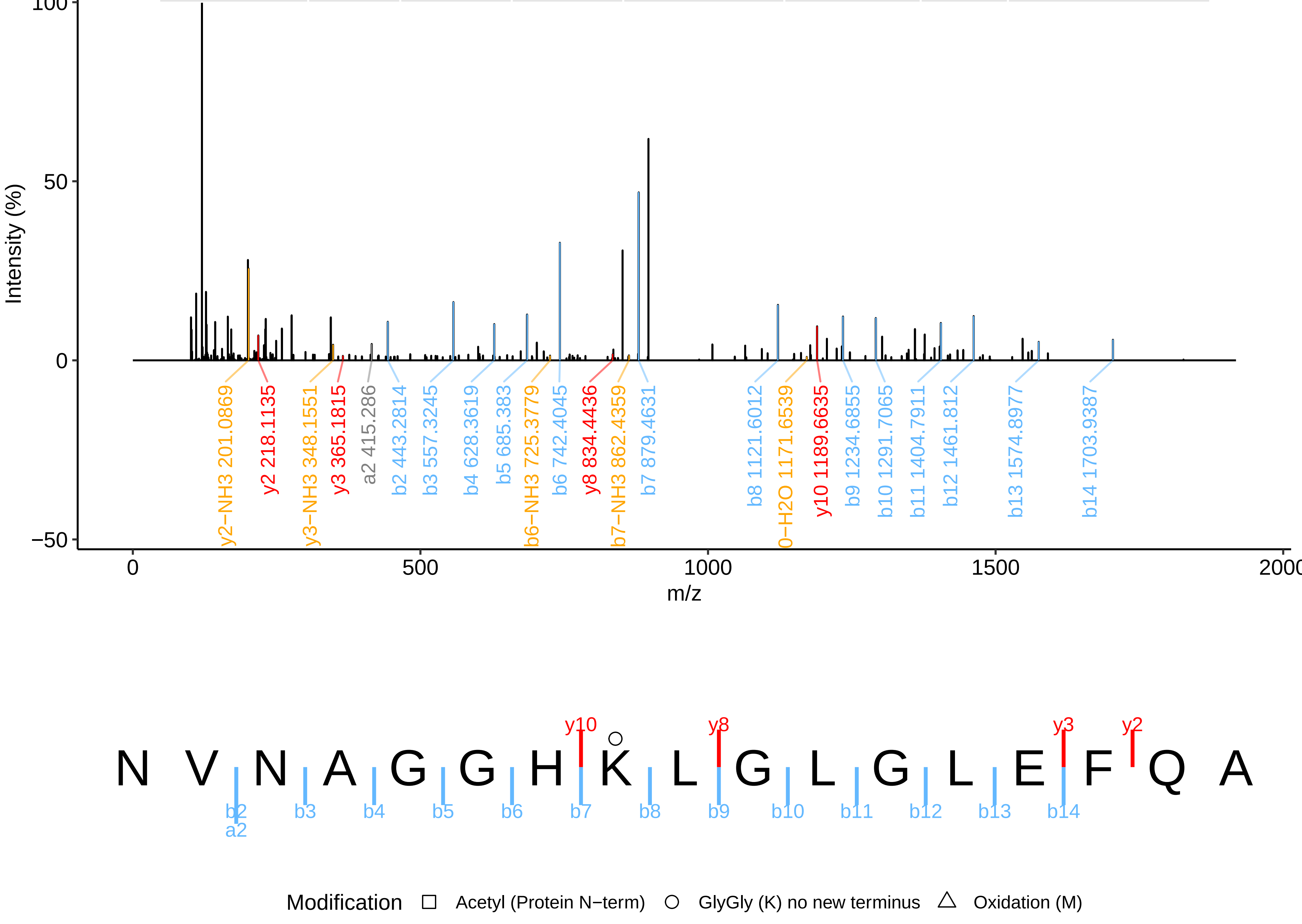

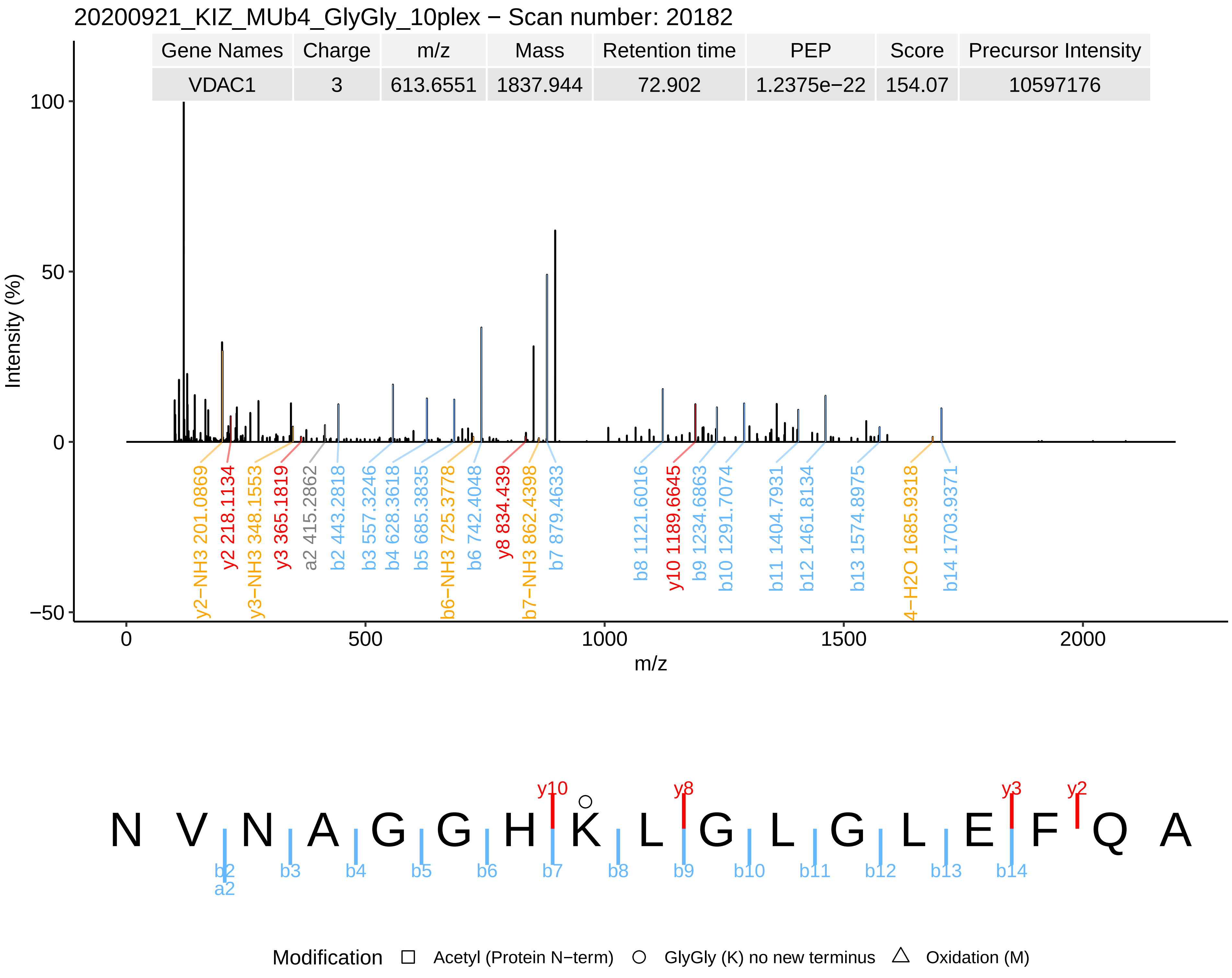

20200921_KIZ_MUb4_GlyGly_10plex − Scan number: 20182
Gene Names | Charge | m/z | Mass | Retention time | PEP | Score | Precursor Intensity
VDAC1 | 3 | 613.6551 | 1837.944 | 72.902 | 1.2375e−22 | 154.07 | 10597176
Intensity (%)
100
50
0
−50
0
500
1000
1500
2000
m/z
y2−NH3 201.0869
y2 218.1134
y3−NH3 348.1553
y3 365.1819
a2 415.2862
b2 443.2818
b3 557.3246
b4 628.3618
b5 685.3835
b6−NH3 725.3778
b6 742.4048
y8 834.439
b7−NH3 862.4398
b7 879.4633
b8 1121.6016
y10 1189.6645
b9 1234.6863
b10 1291.7074
b11 1404.7931
b12 1461.8134
b13 1574.8975
4−H2O 1685.9318
b14 1703.9371
N V N A G G H K L G L G L E F Q A
y10 y8 y3 y2
b2 a2 b3 b4 b5 b6 b7 b8 b9 b10 b11 b12 b13 b14
Modification
Acetyl (Protein N−term)
GlyGly (K) no new terminus
Oxidation (M)

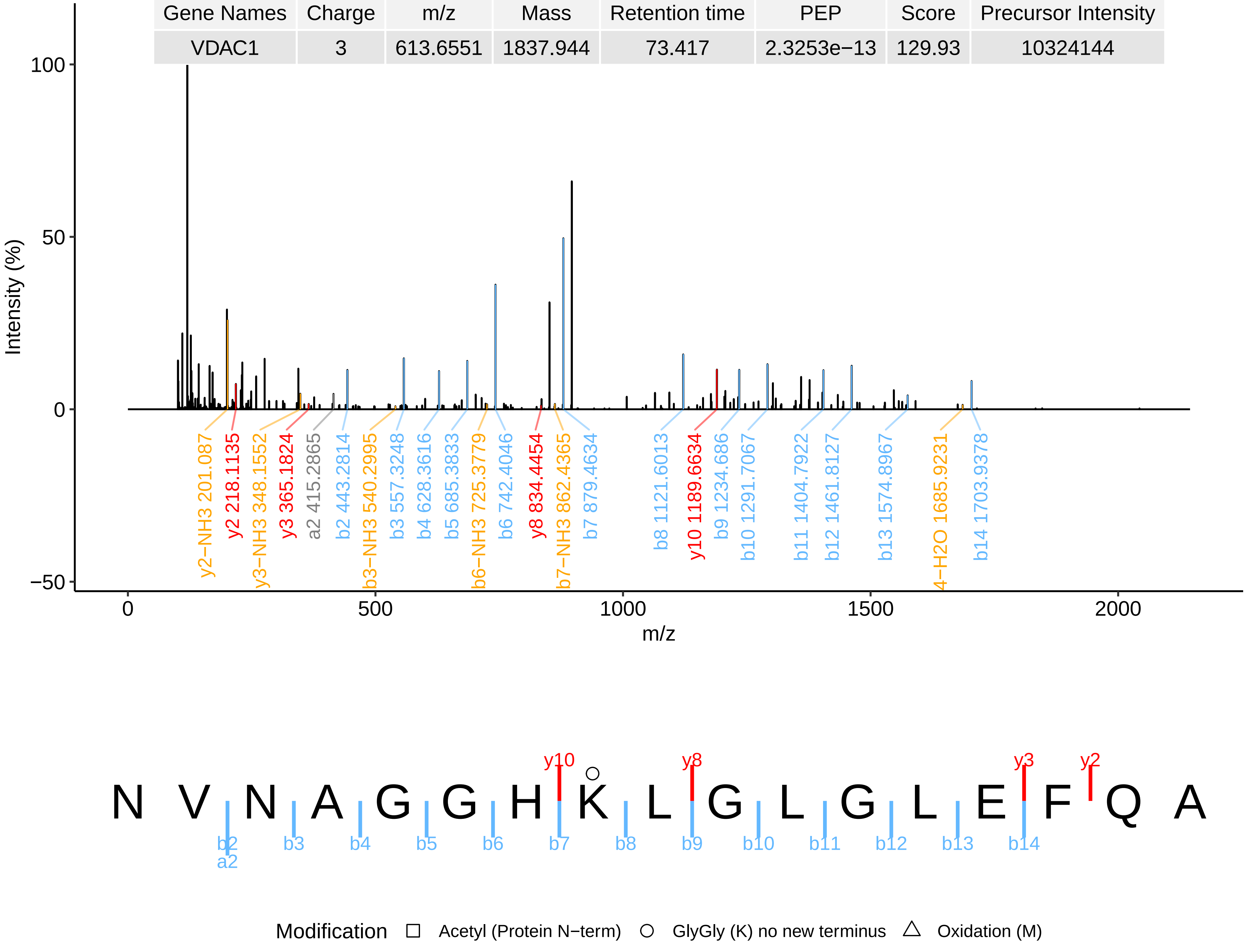

Gene Names	Charge	m/z	Mass	Retention time	PEP	Score	Precursor Intensity
VDAC1	3	613.6551	1837.944	73.417	2.3253e−13	129.93	10324144

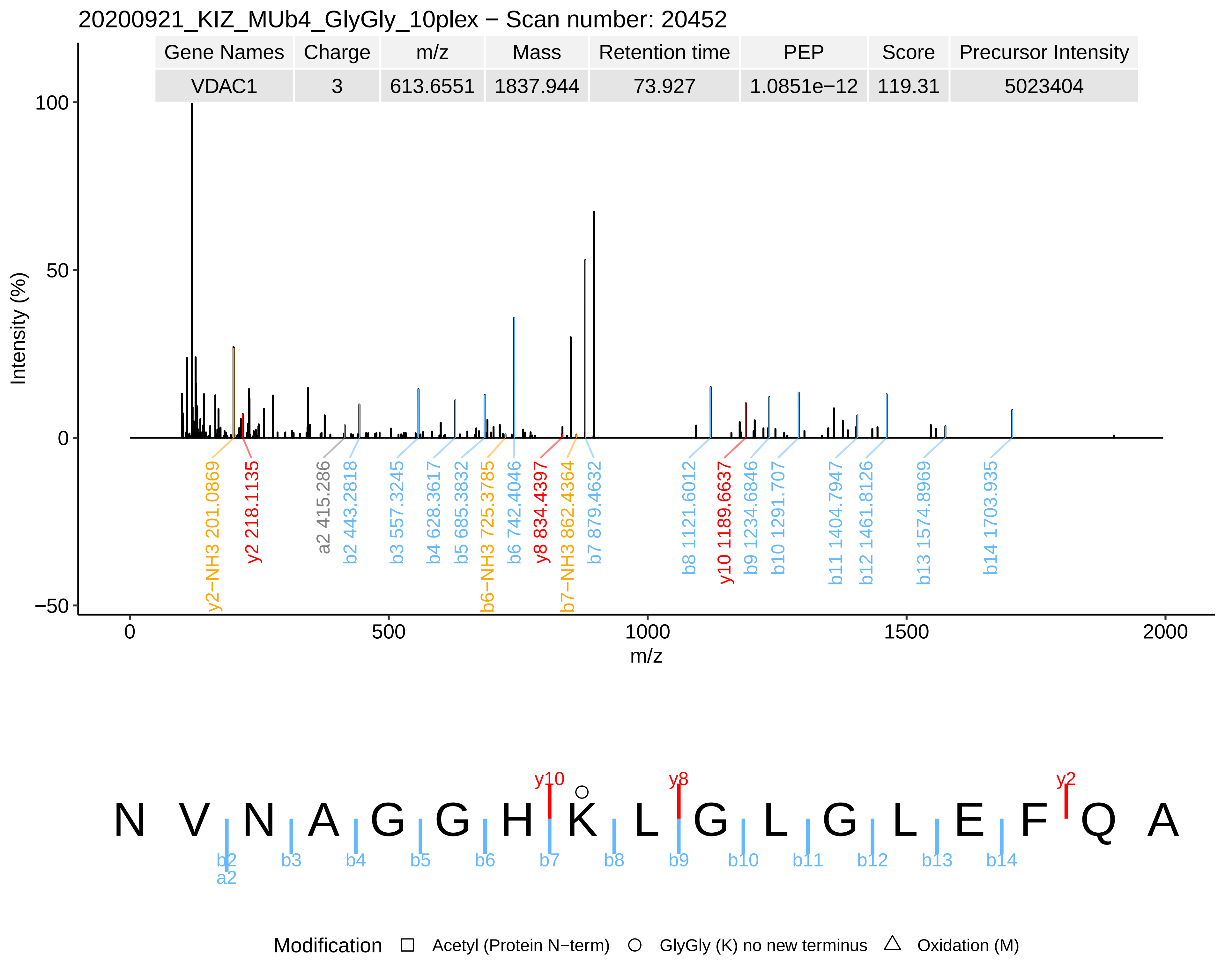
20200921_KIZ_MUb4_GlyGly_10plex − Scan number: 20452
Gene Names | Charge | m/z | Mass | Retention time | PEP | Score | Precursor Intensity
VDAC1 | 3 | 613.6551 | 1837.944 | 73.927 | 1.0851e−12 | 119.31 | 5023404
Intensity (%)
100
50
0
−50
0
500
1000
1500
2000
m/z
y2−NH3 201.0869
y2 218.1135
a2 415.286
b2 443.2818
b3 557.3245
b4 628.3617
b5 685.3832
b6−NH3 725.3785
b6 742.4046
y8 834.4397
b7−NH3 862.4364
b7 879.4632
b8 1121.6012
y10 1189.6637
b9 1234.6846
b10 1291.707
b11 1404.7947
b12 1461.8126
b13 1574.8969
b14 1703.935
N V N A G G H K L G L G L E F Q A
y10
y8
y2
b2
a2
b3
b4
b5
b6
b7
b8
b9
b10
b11
b12
b13
b14
Modification
Acetyl (Protein N−term)
GlyGly (K) no new terminus
Oxidation (M)

20200921_KIZ_MUb4_GlyGly_10plex − Scan number: 35374

Gene Names	Charge	m/z	Mass	Retention time	PEP	Score	Precursor Intensity
VDAC1	3	613.6551	1837.944	129.65	1.0055e−12	120.3	3534719

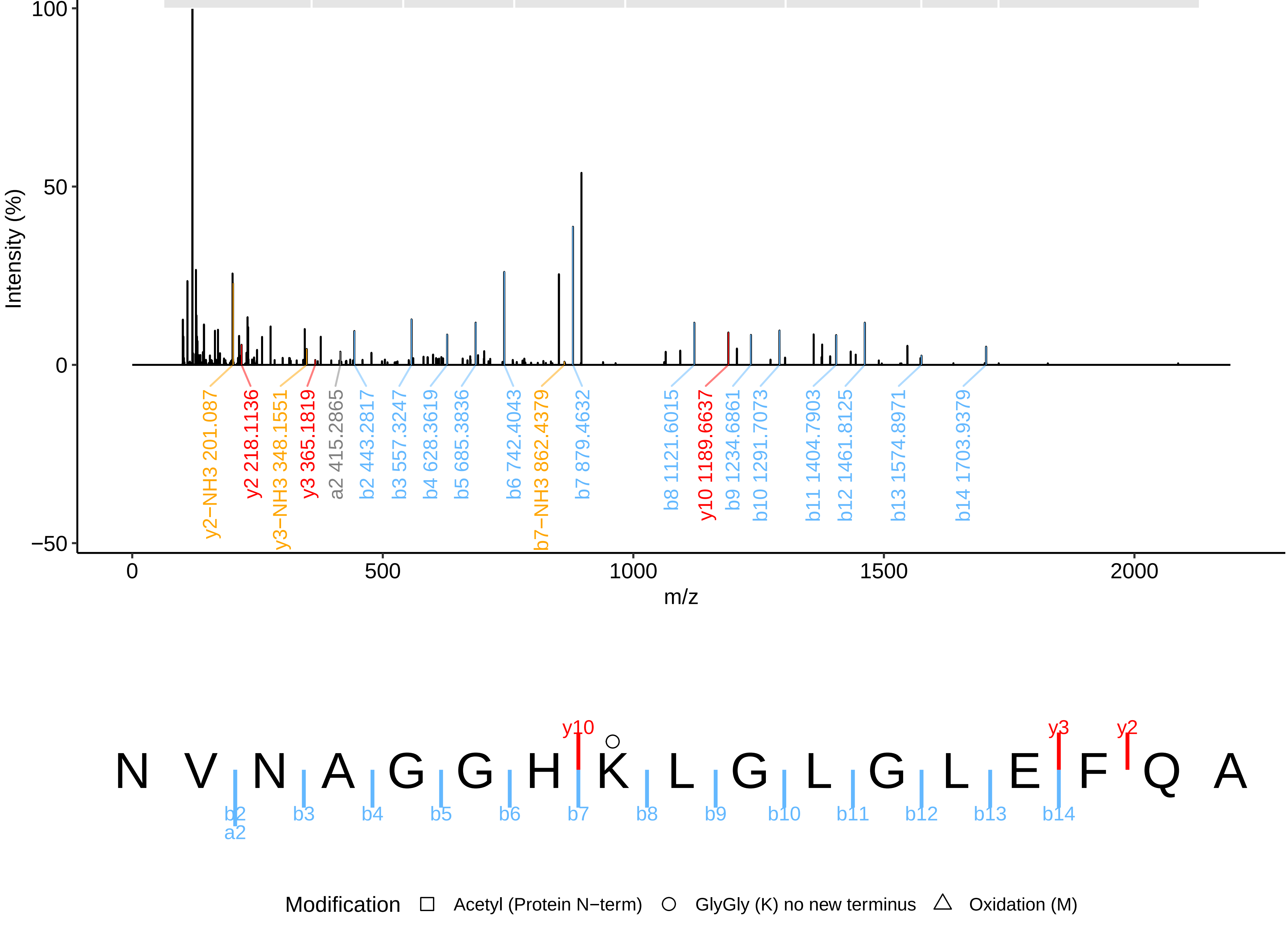

20200921_KIZ_MUb4_GlyGly_10plex − Scan number: 20868

Gene Names	Charge	m/z	Mass	Retention time	PEP	Score	Precursor Intensity
VDAC1	3	613.6551	1837.944	75.489	1.1243e−07	98.694	3408291

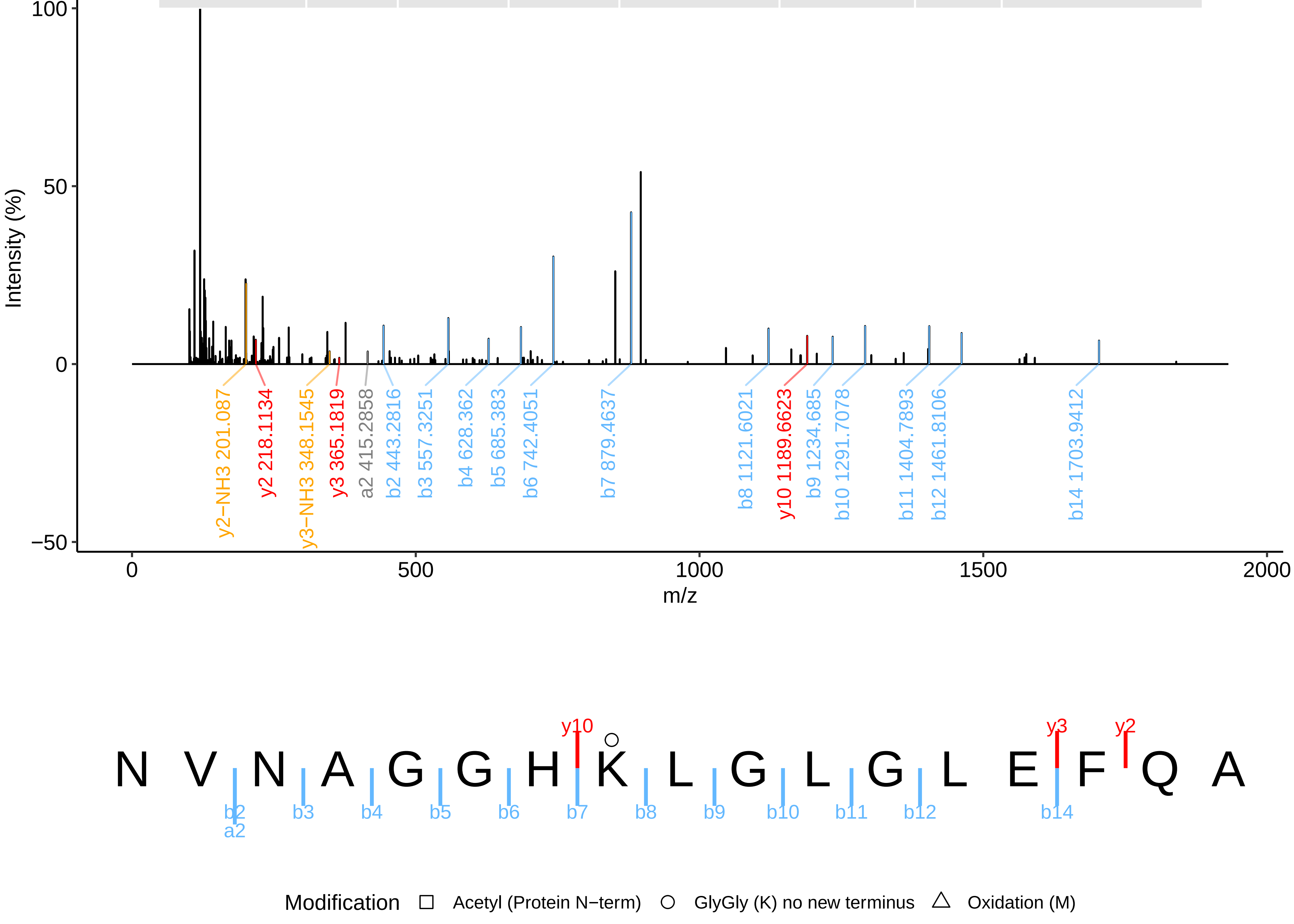

20200921_KIZ_MUb4_GlyGly_10plex − Scan number: 21855

Gene Names	Charge	m/z	Mass	Retention time	PEP	Score	Precursor Intensity
VDAC1	3	613.6551	1837.944	79.218	0.027397	57.53	NA

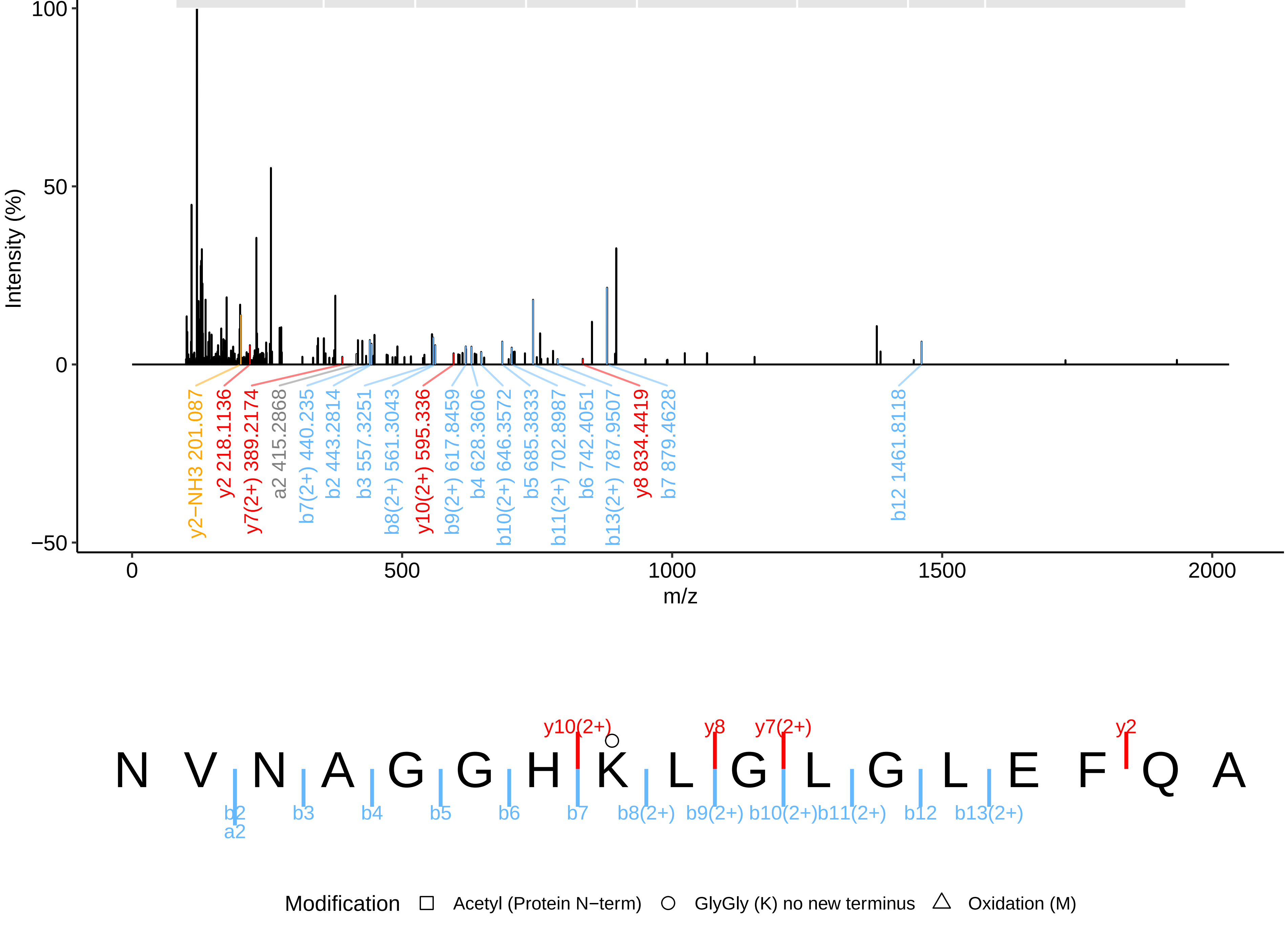

20200921_KIZ_MUb4_GlyGly_10plex_2 − Scan number: 17570

Gene Names	Charge	m/z	Mass	Retention time	PEP	Score	Precursor Intensity
VDAC1	3	613.6551	1837.944	68.715	4.7984e−10	107.07	10702038

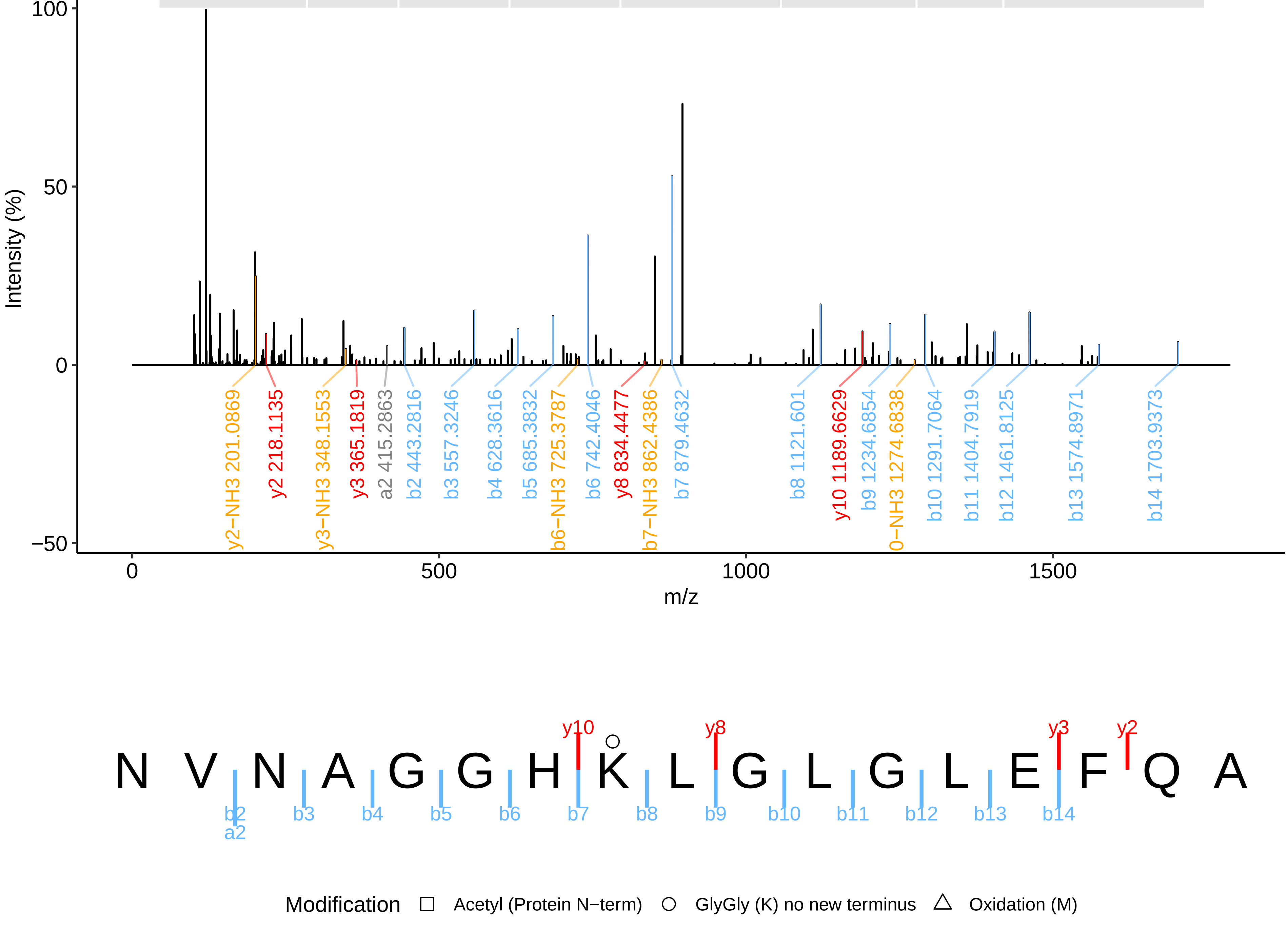

20200921_KIZ_MUb4_GlyGly_10plex_2 − Scan number: 17697

Gene Names	Charge	m/z	Mass	Retention time	PEP	Score	Precursor Intensity
VDAC1	3	613.6551	1837.944	69.225	1.2375e−22	154.07	15711686

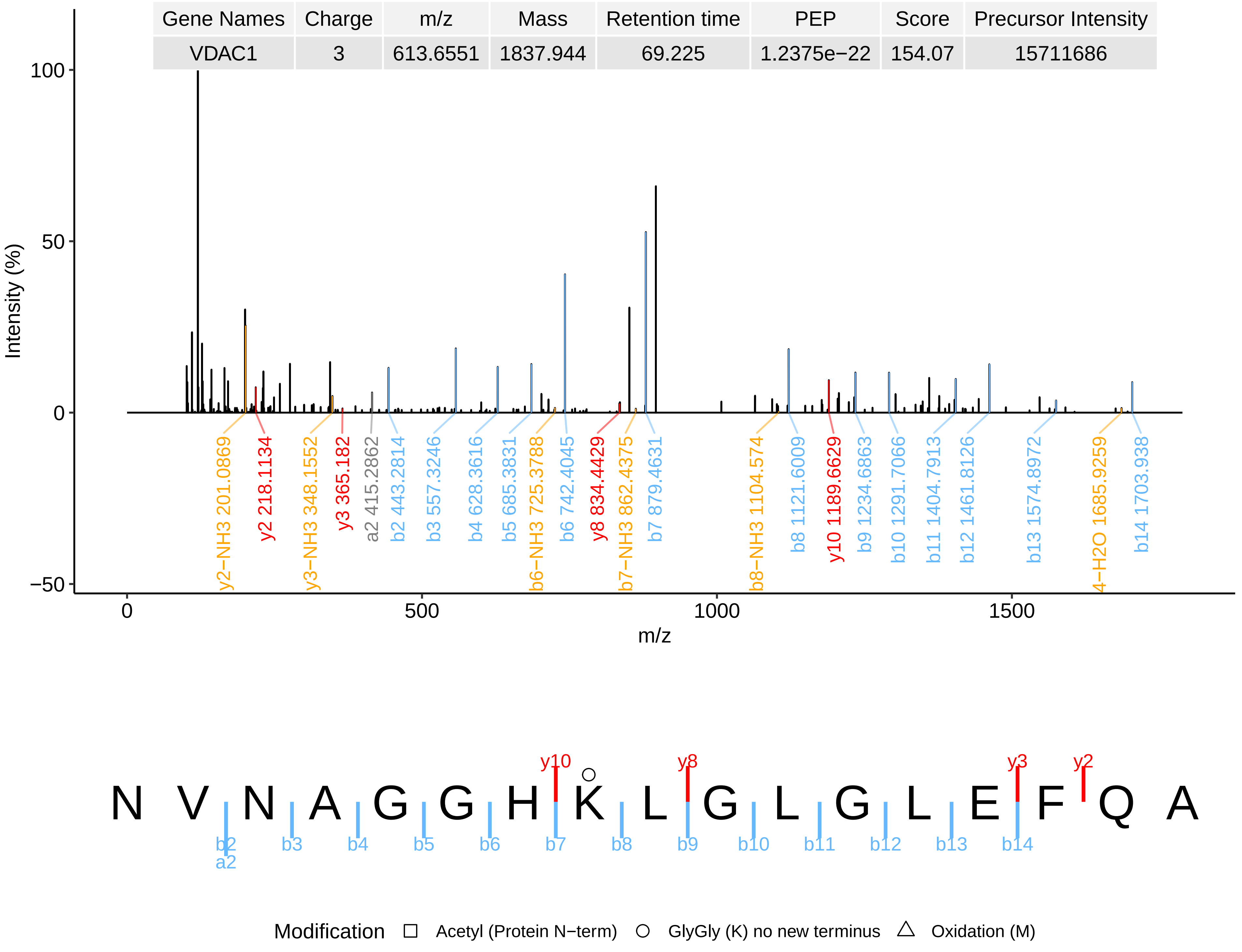

20200921_KIZ_MUb4_GlyGly_10plex_2 − Scan number: 17832

Gene Names	Charge	m/z	Mass	Retention time	PEP	Score	Precursor Intensity
VDAC1	3	613.6551	1837.944	69.77	1.0851e−12	119.31	2476454

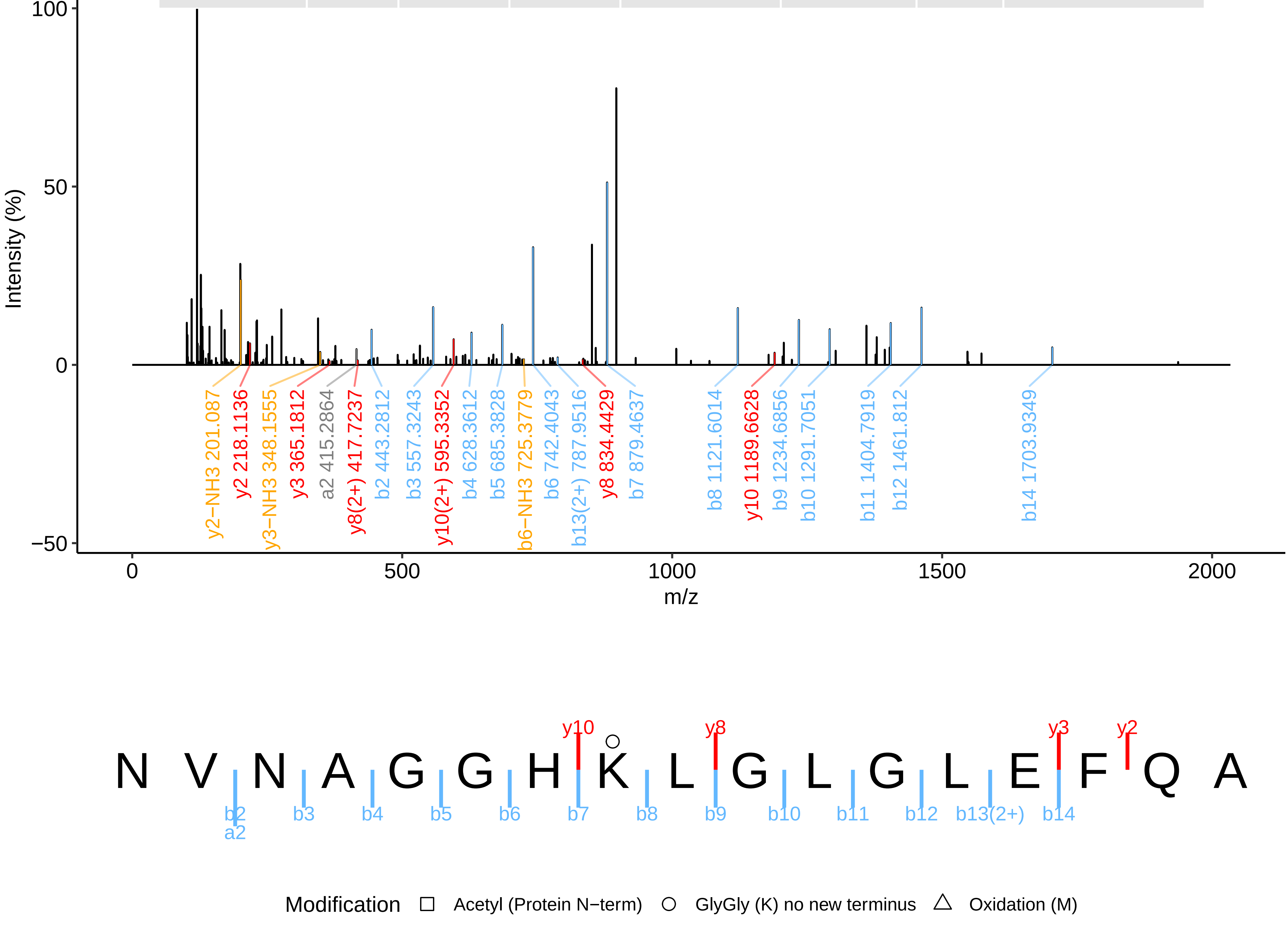

20200921_KIZ_MUb4_GlyGly_10plex_2 − Scan number: 18085

Gene Names	Charge	m/z	Mass	Retention time	PEP	Score	Precursor Intensity
VDAC1	3	613.6551	1837.944	70.779	0.003623	70.98	1033987

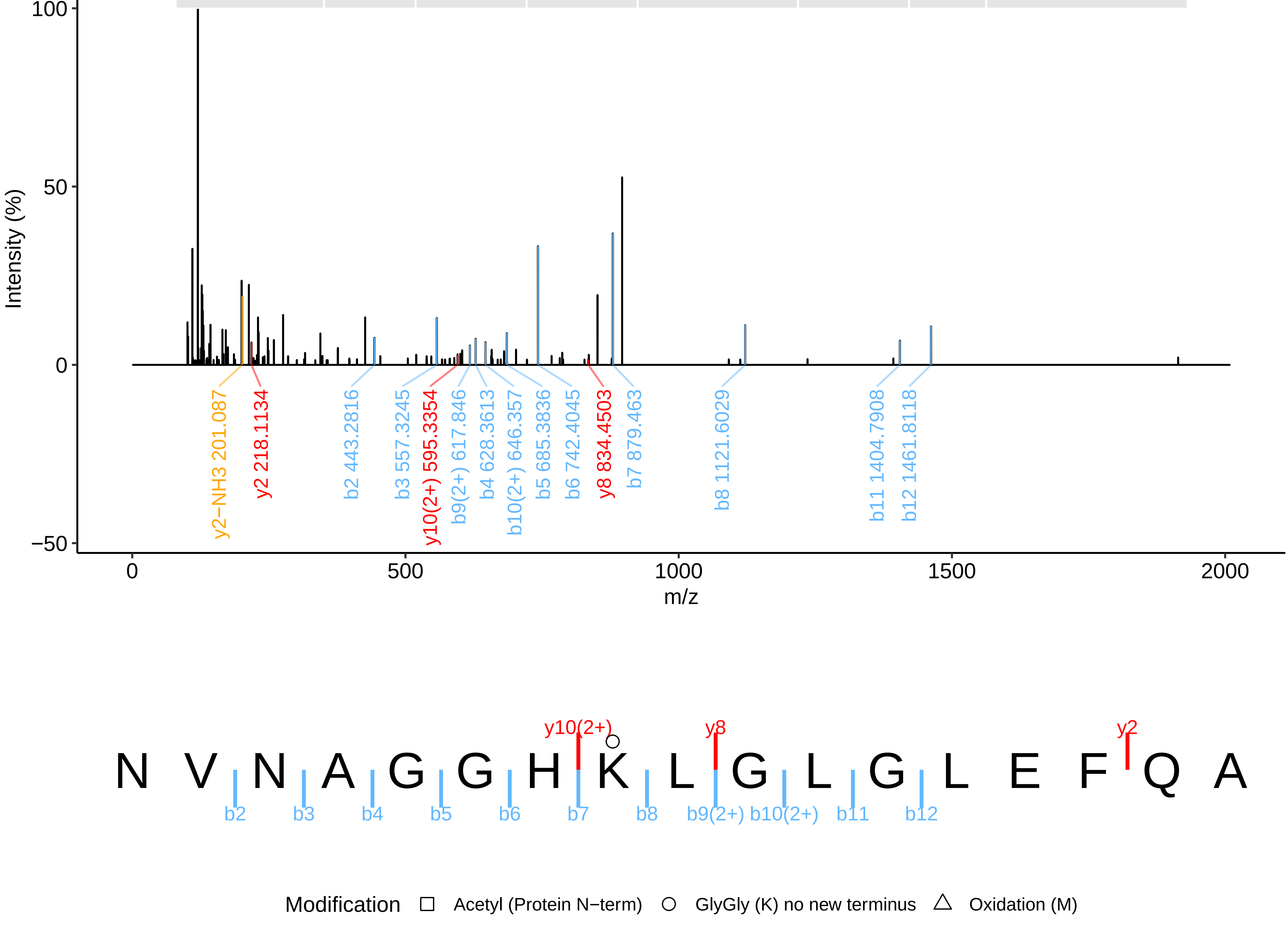

20201118_KIZ_MUb6_GlyGly_1 − Scan number: 18631

Gene Names	Charge	m/z	Mass	Retention time	PEP	Score	Precursor Intensity
VDAC1	3	613.6551	1837.944	58.553	5.1559e−17	141.85	NA

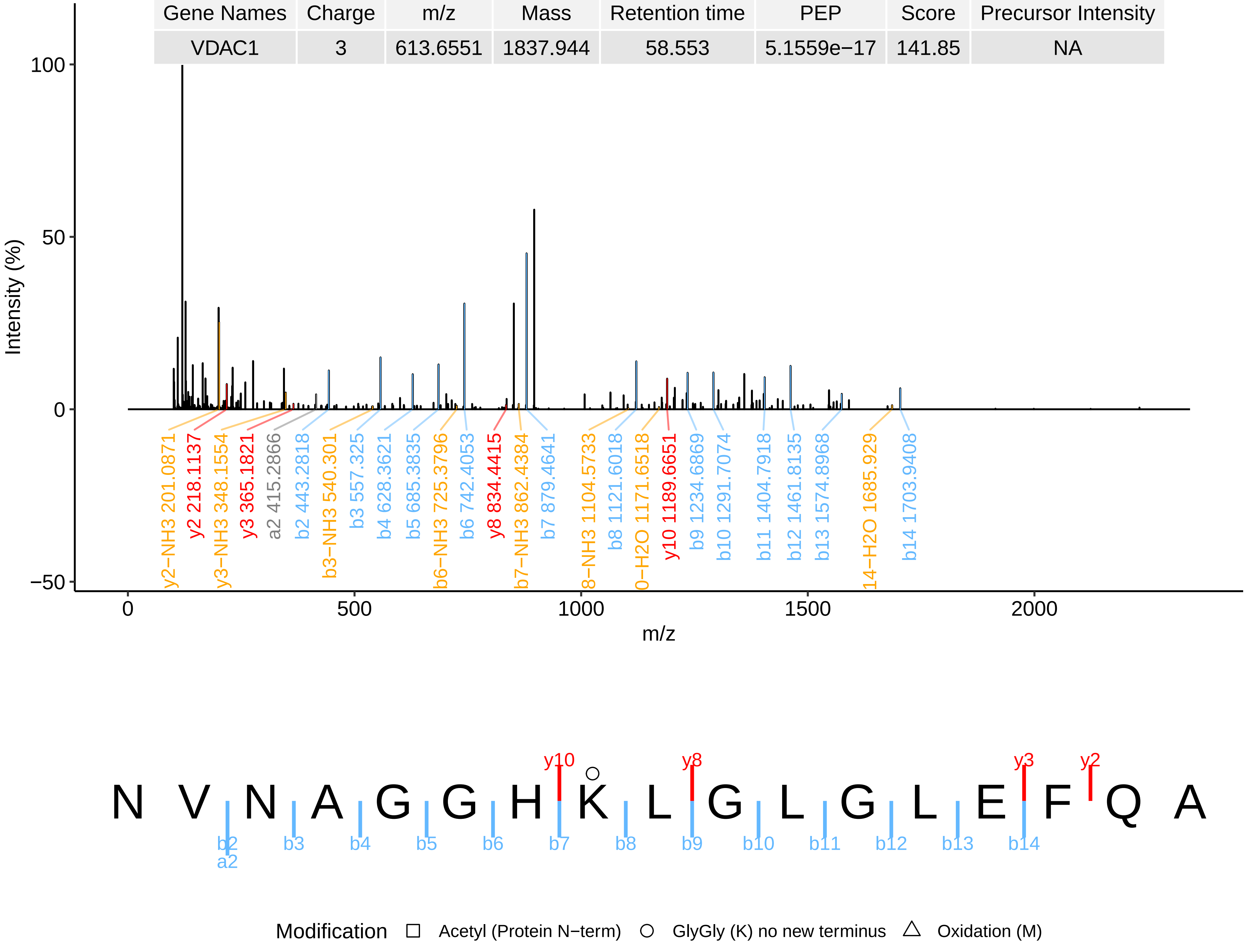

20201118_KIZ_MUb6_GlyGly_1 − Scan number: 18802

Gene Names	Charge	m/z	Mass	Retention time	PEP	Score	Precursor Intensity
VDAC1	3	613.6551	1837.944	59.057	5.1559e−17	141.85	189425520

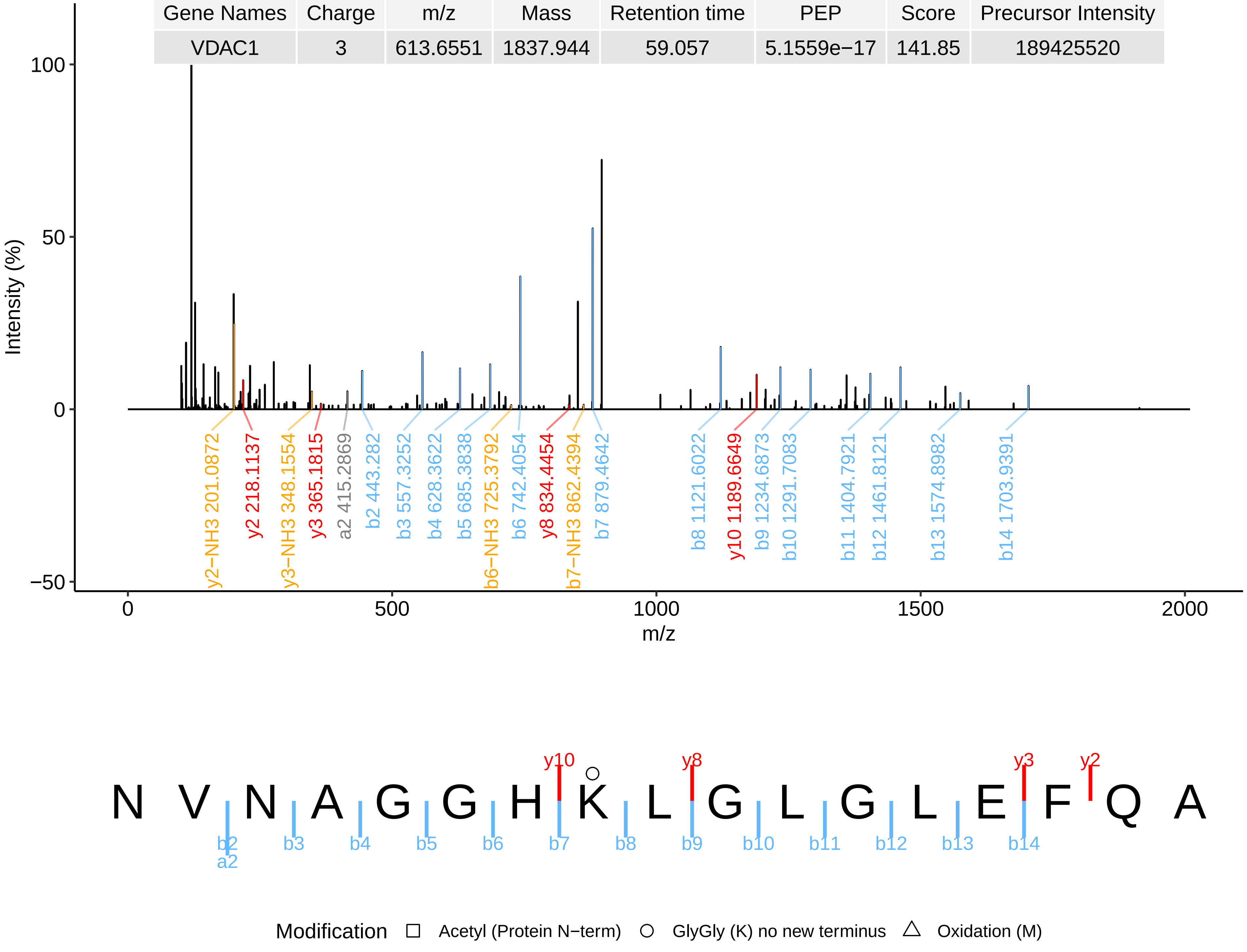

20201118_KIZ_MUb6_GlyGly_2 − Scan number: 20797

Gene Names	Charge	m/z	Mass	Retention time	PEP	Score	Precursor Intensity
VDAC1	3	613.6551	1837.944	70.084	2.3253e−13	129.93	13055960

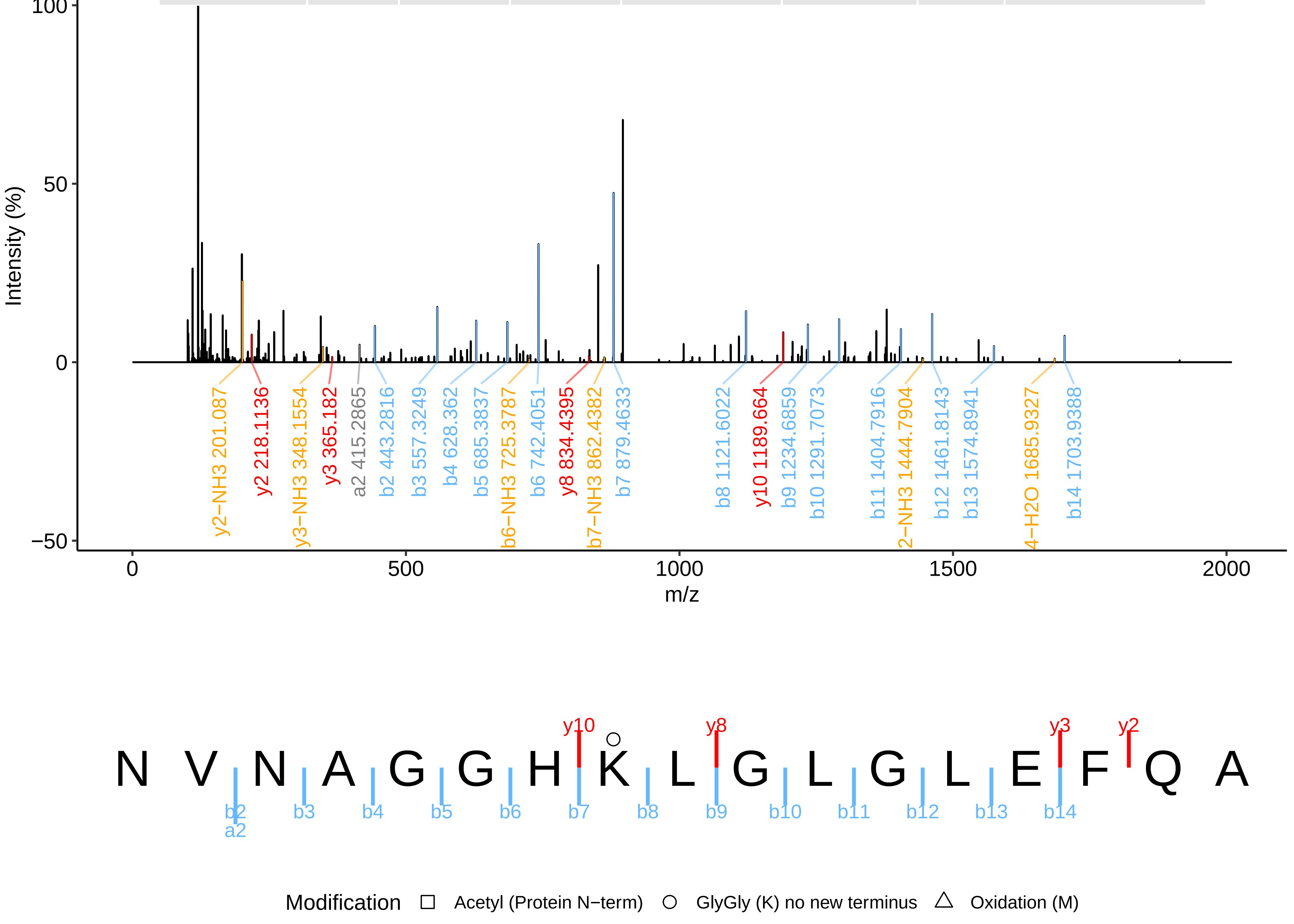

20201118_KIZ_MUb6_GlyGly_2 − Scan number: 20945

Gene Names	Charge	m/z	Mass	Retention time	PEP	Score	Precursor Intensity
VDAC1	3	613.6551	1837.944	70.588	3.57e−08	109.83	NA

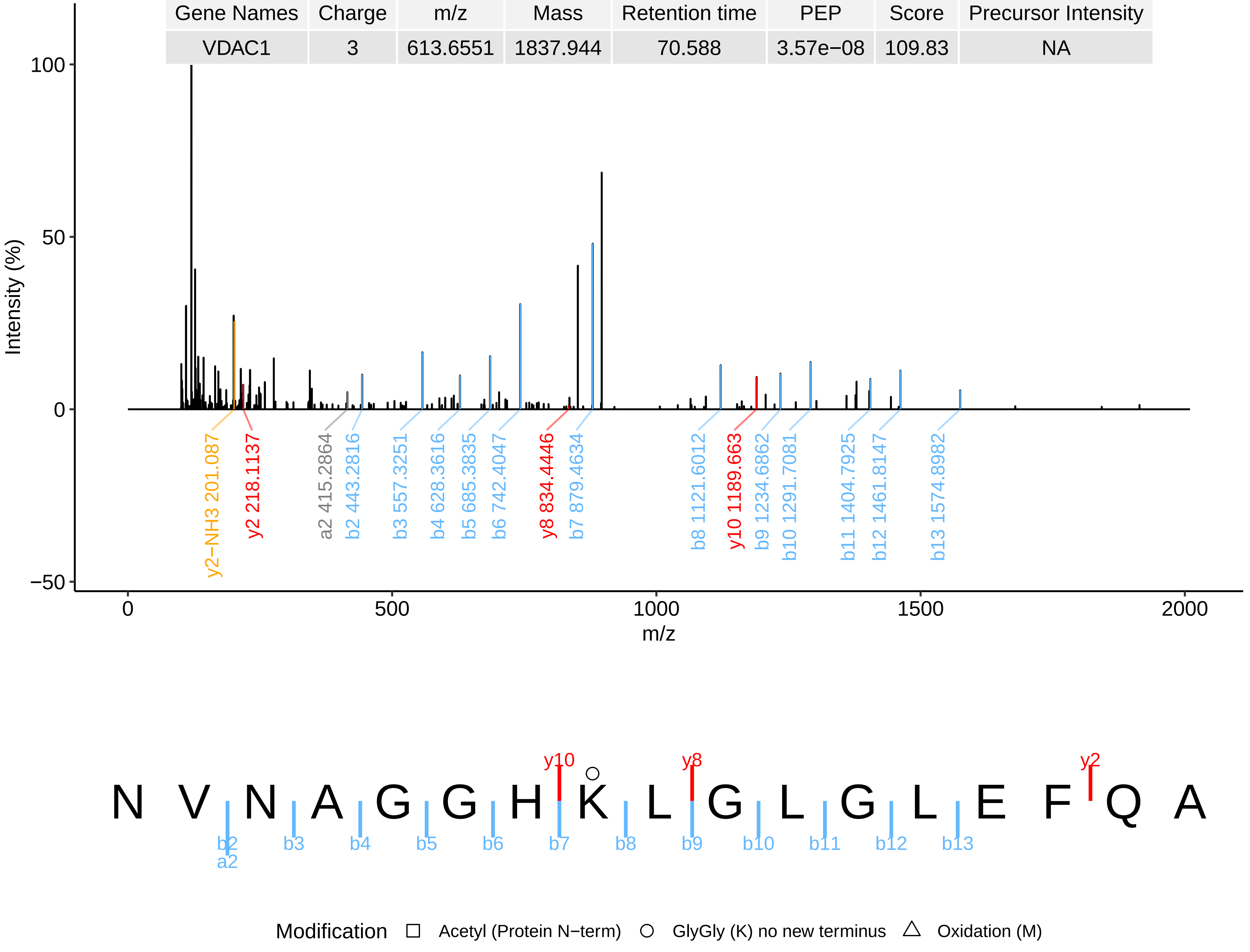

20200921_KIZ_MUb4_GlyGly_10plex_2 − Scan number: 19609

Gene Names	Charge	m/z	Mass	Retention time	PEP	Score	Precursor Intensity
PHGDH	3	546.9894	1637.947	76.986	0.0003422	92.457	513649.9

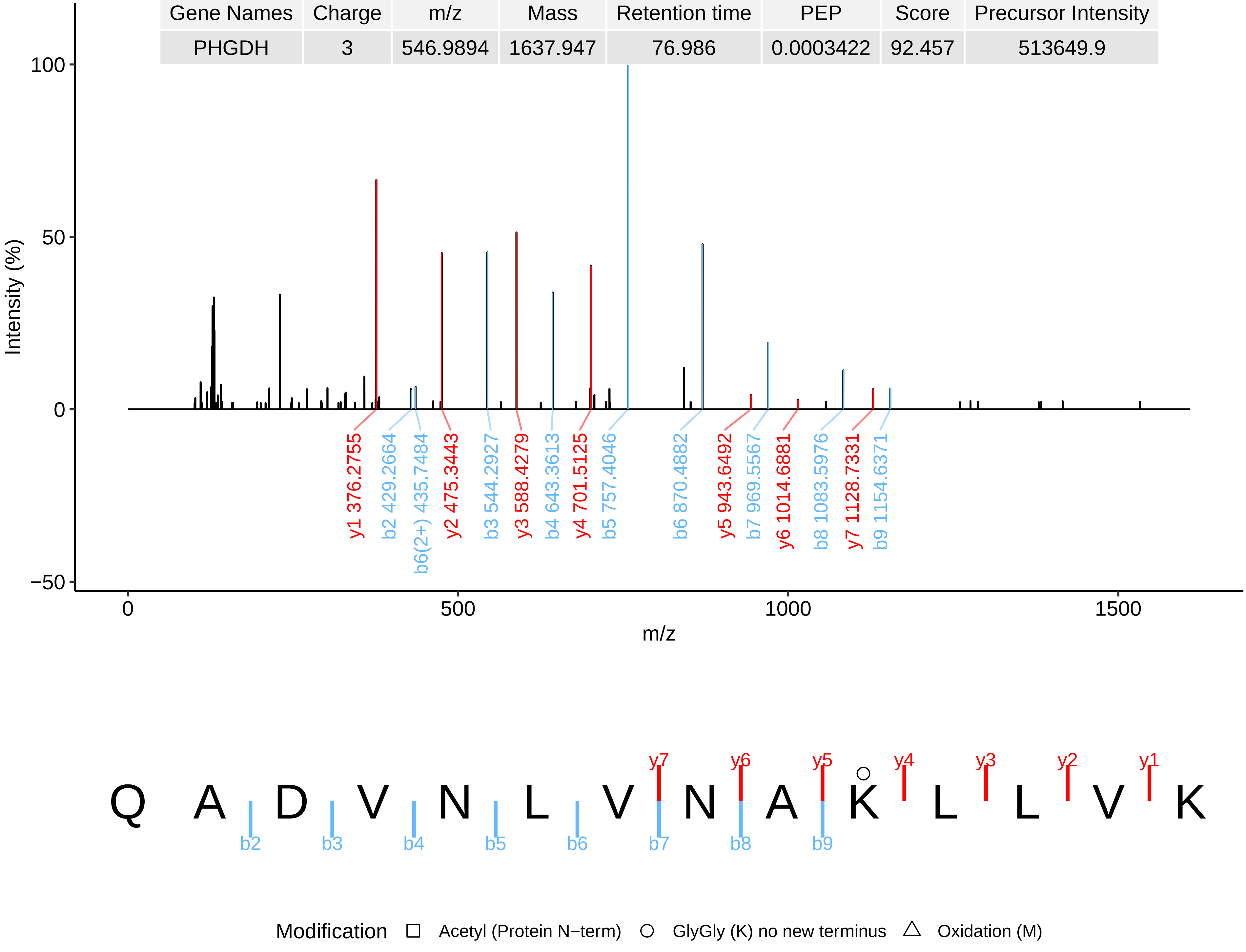

20200921_KIZ_MUb4_GlyGly_10plex − Scan number: 15181

Gene Names	Charge	m/z	Mass	Retention time	PEP	Score	Precursor Intensity
ZNF451	3	547.6031	1639.788	54.167	0.0083014	68.809	4376587

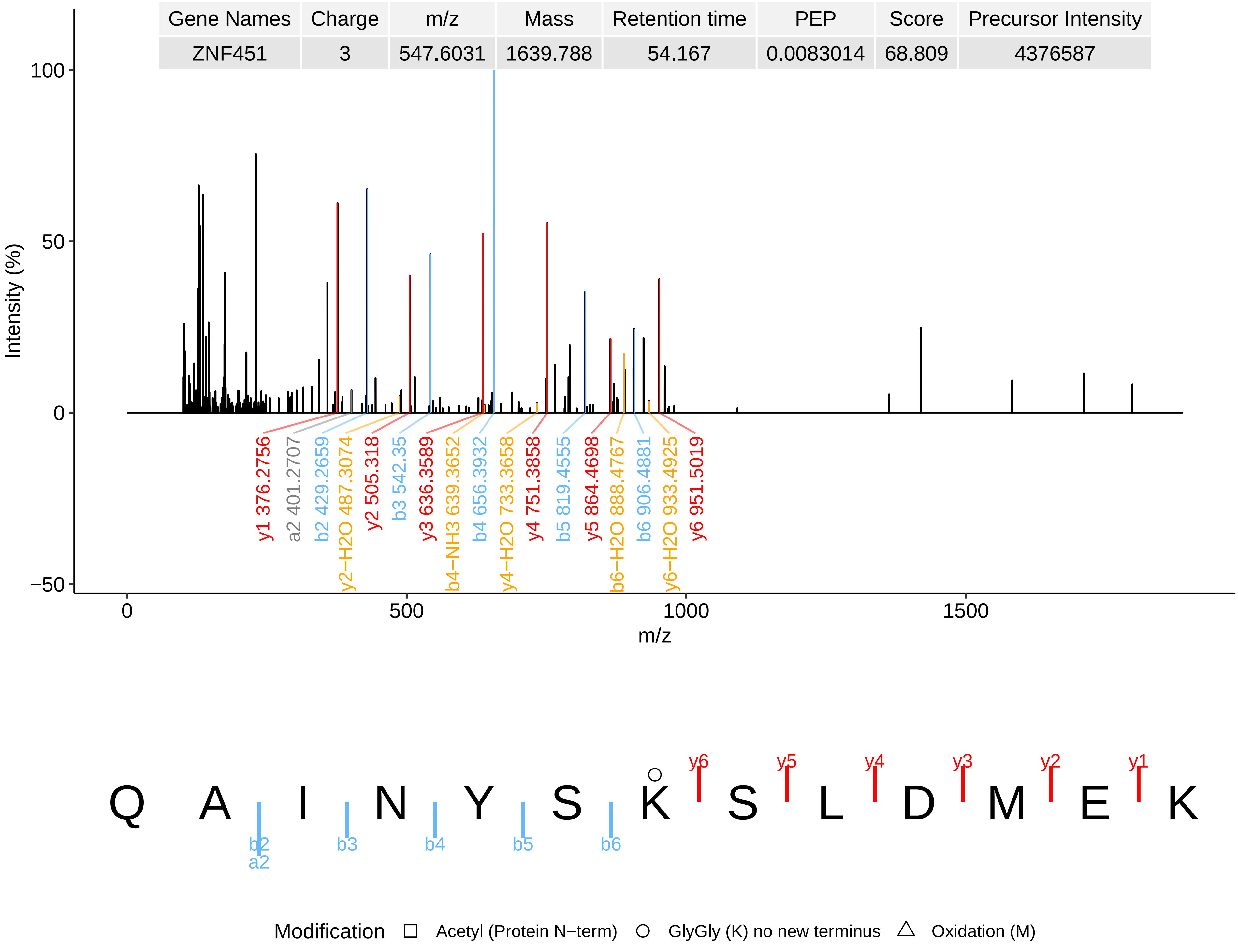

20200921_KIZ_MUb4_GlyGly_10plex_2 − Scan number: 15894

Gene Names	Charge	m/z	Mass	Retention time	PEP	Score	Precursor Intensity
OPTN	3	559.9481	1676.823	62.246	0.007841	69.602	180654.3

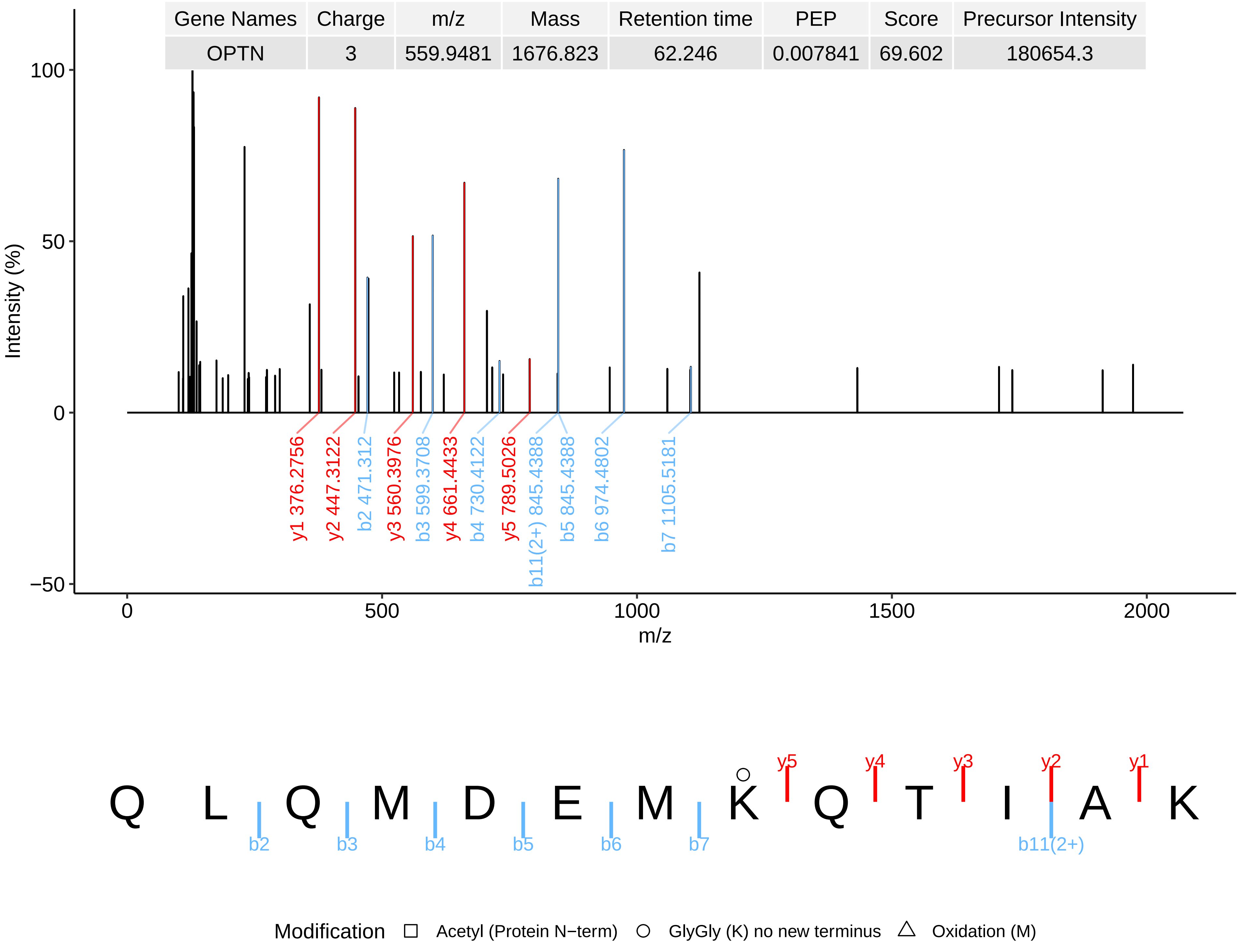

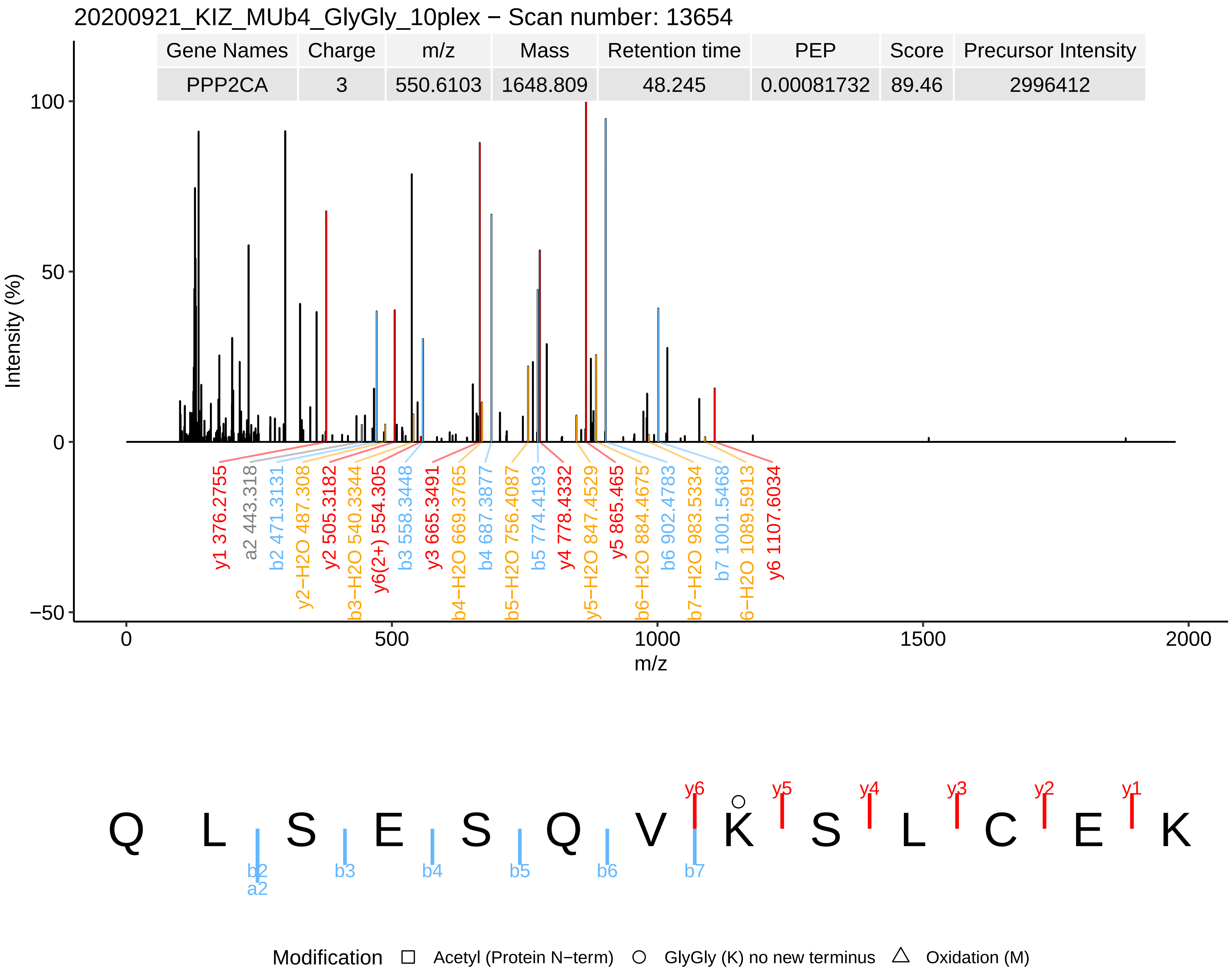
20200921_KIZ_MUb4_GlyGly_10plex − Scan number: 13654
Gene Names | Charge | m/z | Mass | Retention time | PEP | Score | Precursor Intensity
PPP2CA | 3 | 550.6103 | 1648.809 | 48.245 | 0.00081732 | 89.46 | 2996412
Intensity (%)
m/z
y1 376.2755
a2 443.318
b2 471.3131
y2−H2O 487.308
y2 505.3182
b3−H2O 540.3344
y6(2+) 554.305
b3 558.3448
y3 665.3491
b4−H2O 669.3765
b4 687.3877
b5−H2O 756.4087
b5 774.4193
y4 778.4332
y5−H2O 847.4529
y5 865.465
b6−H2O 884.4675
b6 902.4783
b7−H2O 983.5334
b7 1001.5468
y6−H2O 1089.5913
y6 1107.6034
Q L S E S Q V K S L C E K
Modification
Acetyl (Protein N−term)
GlyGly (K) no new terminus
Oxidation (M)

20200921_KIZ_MUb4_GlyGly_10plex_2 − Scan number: 12546

Gene Names	Charge	m/z	Mass	Retention time	PEP	Score	Precursor Intensity
PPP2CA	3	550.6103	1648.809	48.563	0.00077671	86.467	725707.8

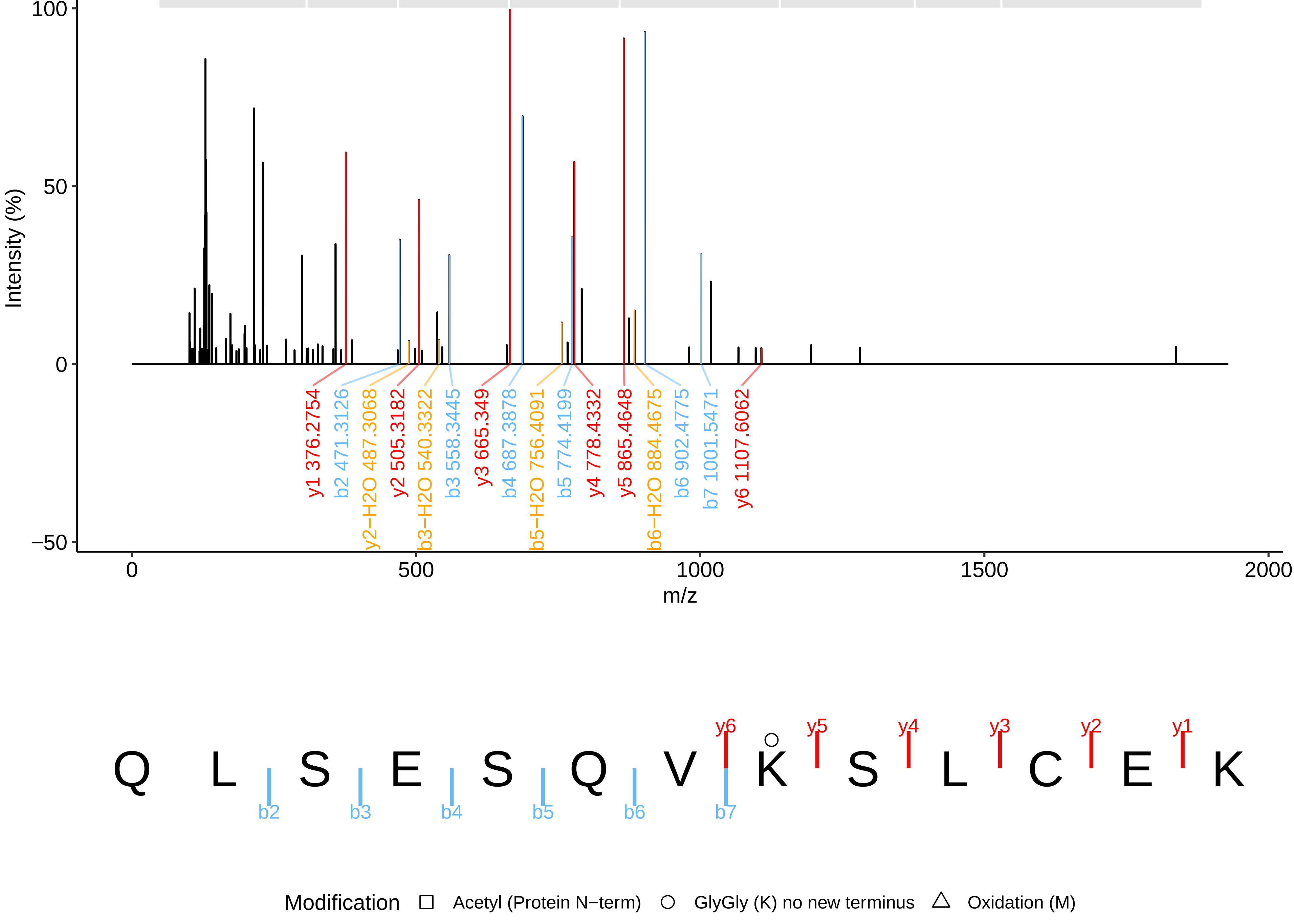

20200921_KIZ_MUb4_GlyGly_10plex − Scan number: 16204

Gene Names	Charge	m/z	Mass	Retention time	PEP	Score	Precursor Intensity
UCHL5	3	558.9312	1673.772	58.135	1.777e−20	163.51	5428939

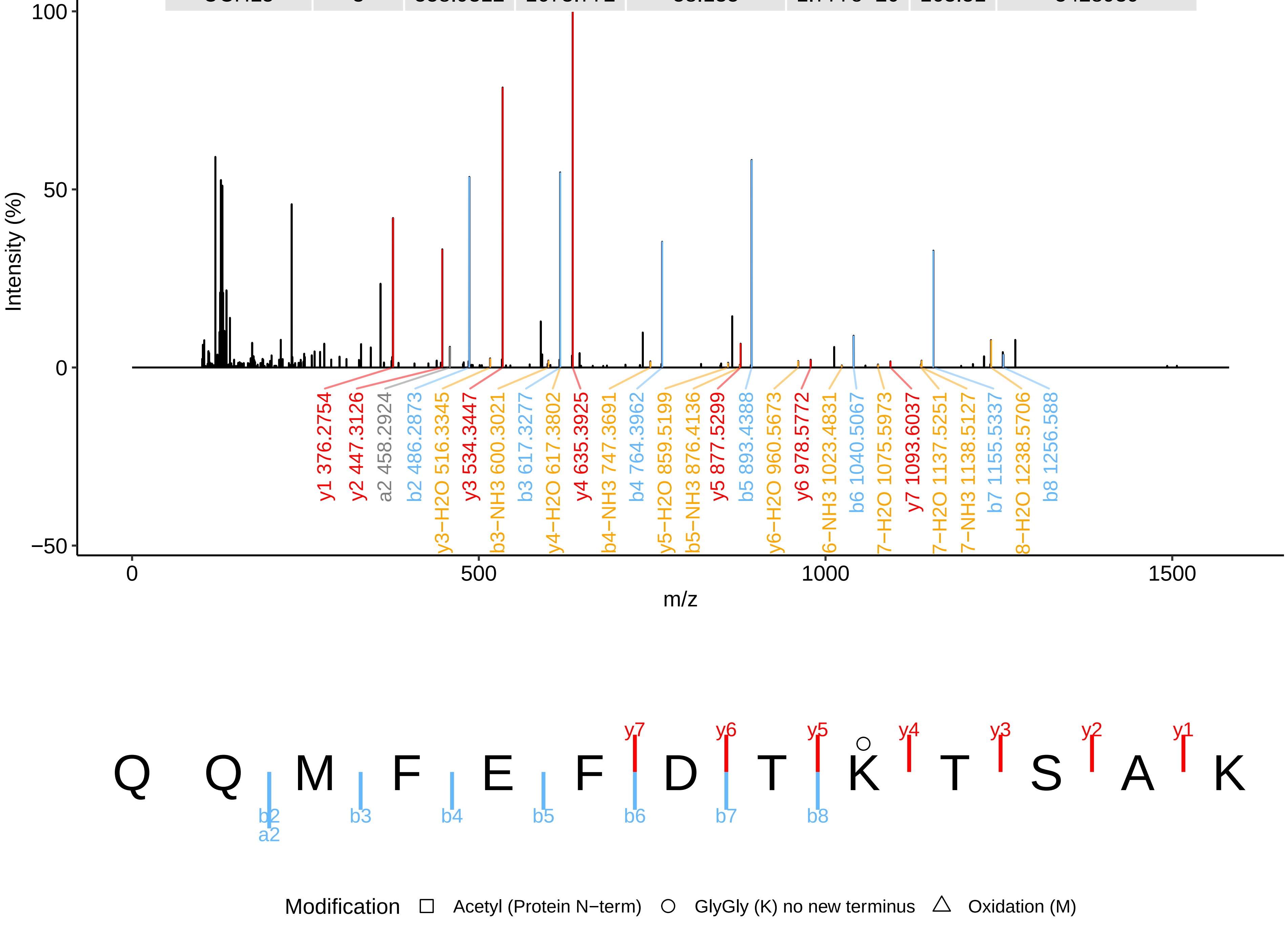

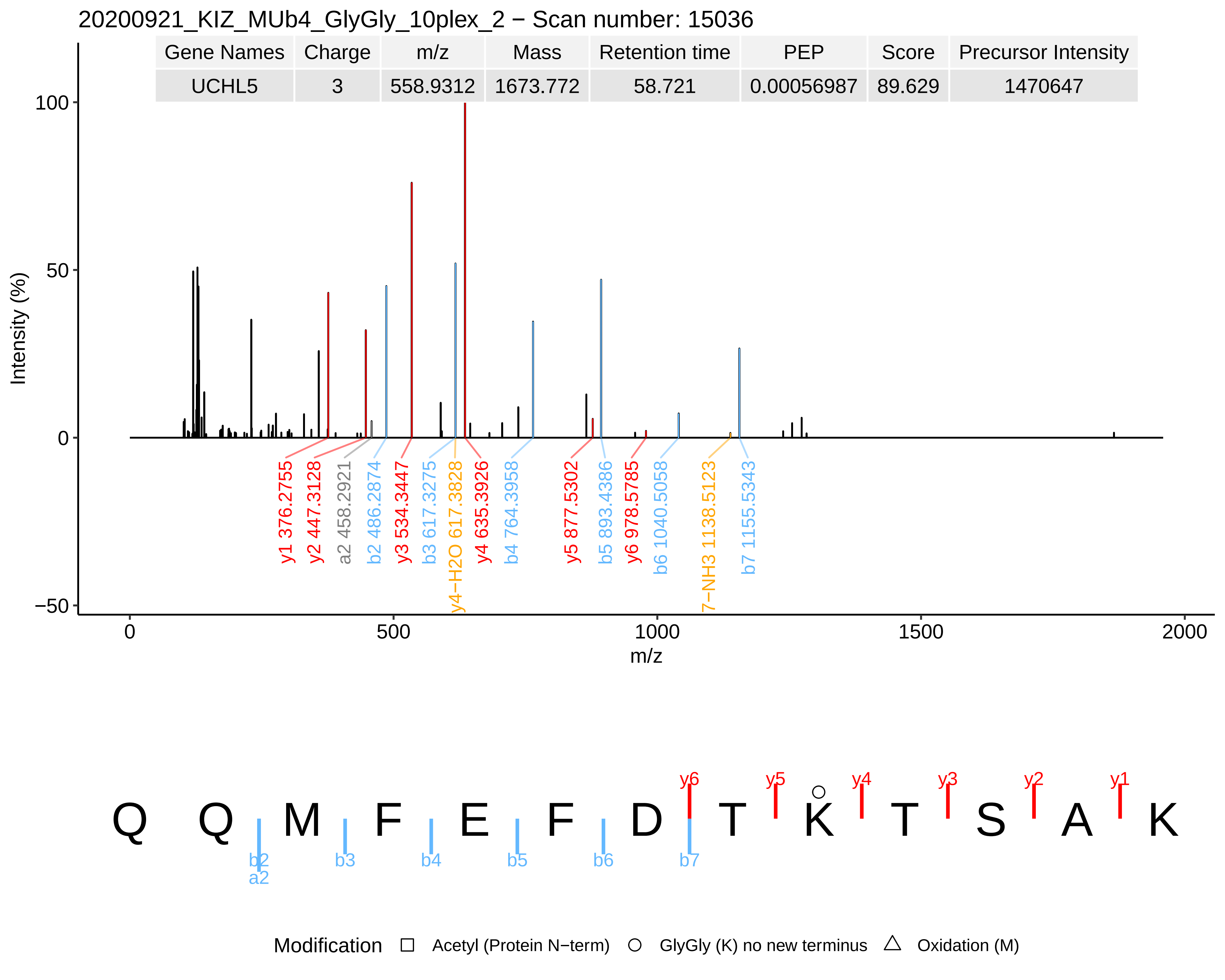

20200921_KIZ_MUb4_GlyGly_10plex_2 − Scan number: 15036
Gene Names | Charge | m/z | Mass | Retention time | PEP | Score | Precursor Intensity
UCHL5 | 3 | 558.9312 | 1673.772 | 58.721 | 0.00056987 | 89.629 | 1470647
Intensity (%)
100
50
0
−50
y1 376.2755
y2 447.3128
a2 458.2921
b2 486.2874
y3 534.3447
b3 617.3275
y4−H2O 617.3828
y4 635.3926
b4 764.3958
y5 877.5302
b5 893.4386
y6 978.5785
b6 1040.5058
7−NH3 1138.5123
b7 1155.5343
0
500
1000
1500
2000
m/z
Q Q M F E F D T K T S A K
y6 y5 y4 y3 y2 y1
b2 a2 b3 b4 b5 b6 b7
Modification
Acetyl (Protein N−term)
GlyGly (K) no new terminus
Oxidation (M)

20201118_KIZ_MUb6_GlyGly_2 − Scan number: 17552

Gene Names	Charge	m/z	Mass	Retention time	PEP	Score	Precursor Intensity
UCHL5	3	558.9312	1673.772	59.328	0.00035882	92.856	4228080

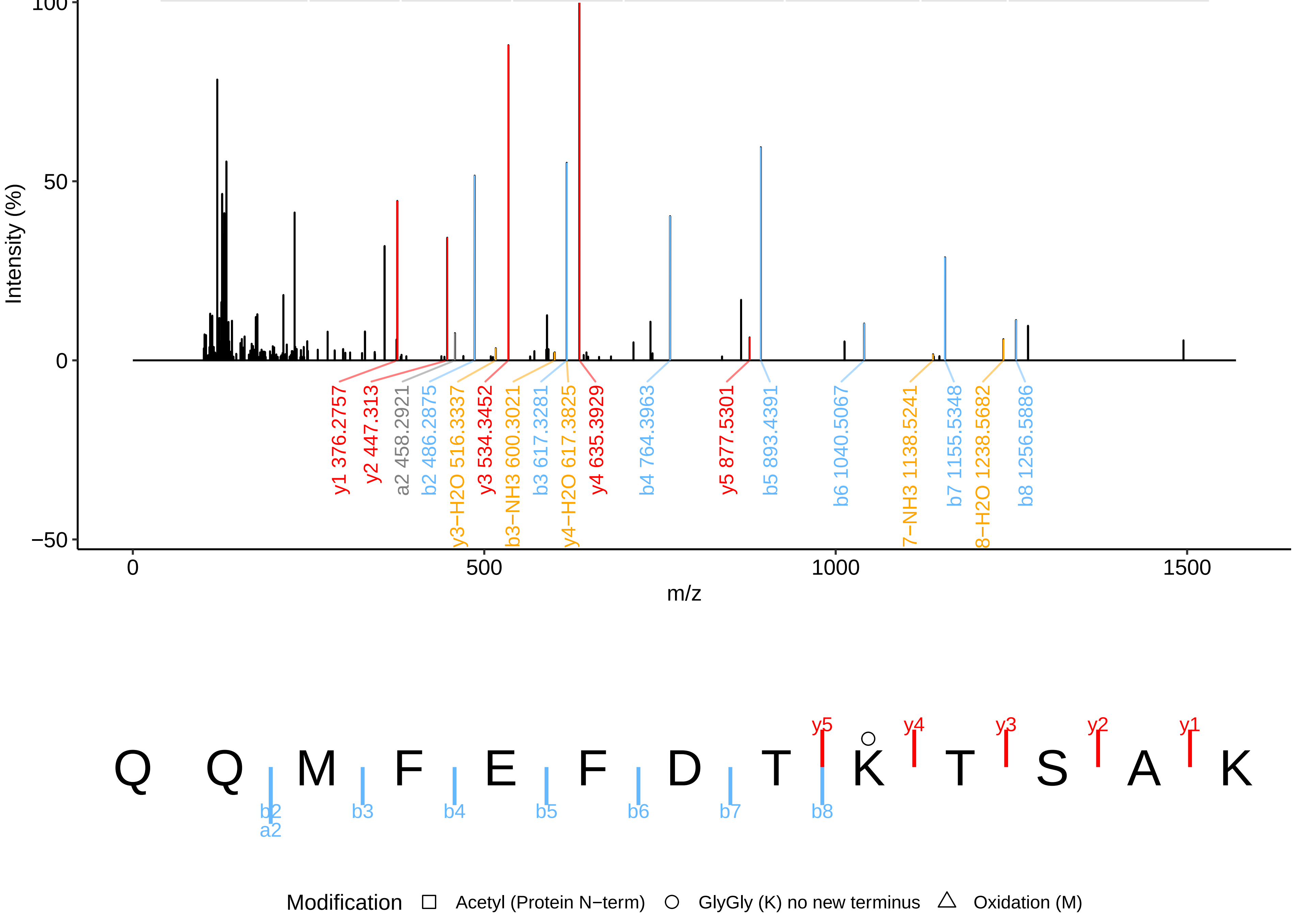

20200921_KIZ_MUb4_GlyGly_10plex − Scan number: 10525

Gene Names	Charge	m/z	Mass	Retention time	PEP	Score	Precursor Intensity
OSTM1	3	637.3169	1908.929	36.451	4.4966e−06	95.57	7530958

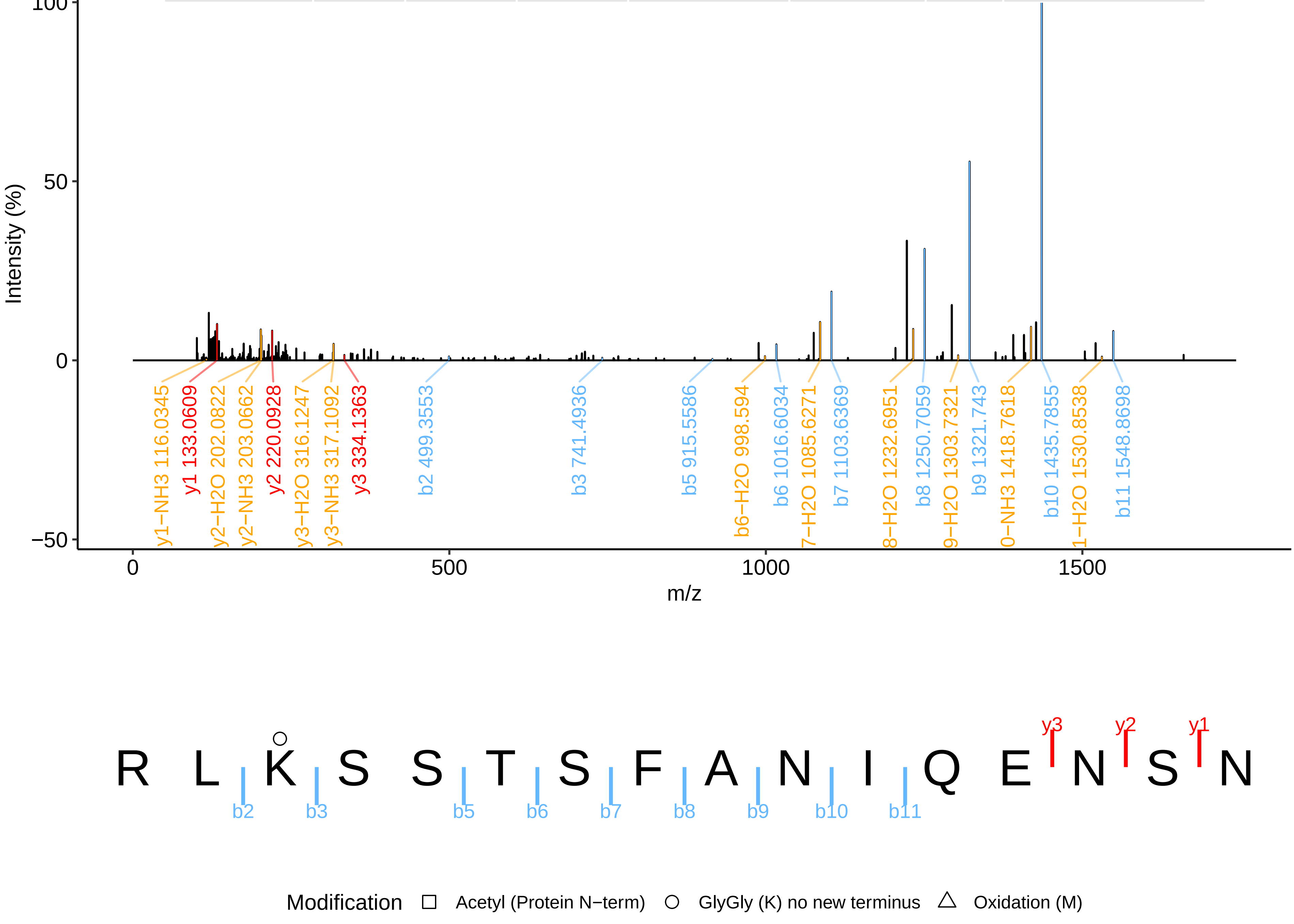

20200921_KIZ_MUb4_GlyGly_10plex − Scan number: 32362

Gene Names	Charge	m/z	Mass	Retention time	PEP	Score	Precursor Intensity
MYOF	3	544.6504	1630.929	119.6	1.175e−05	106.32	6995186

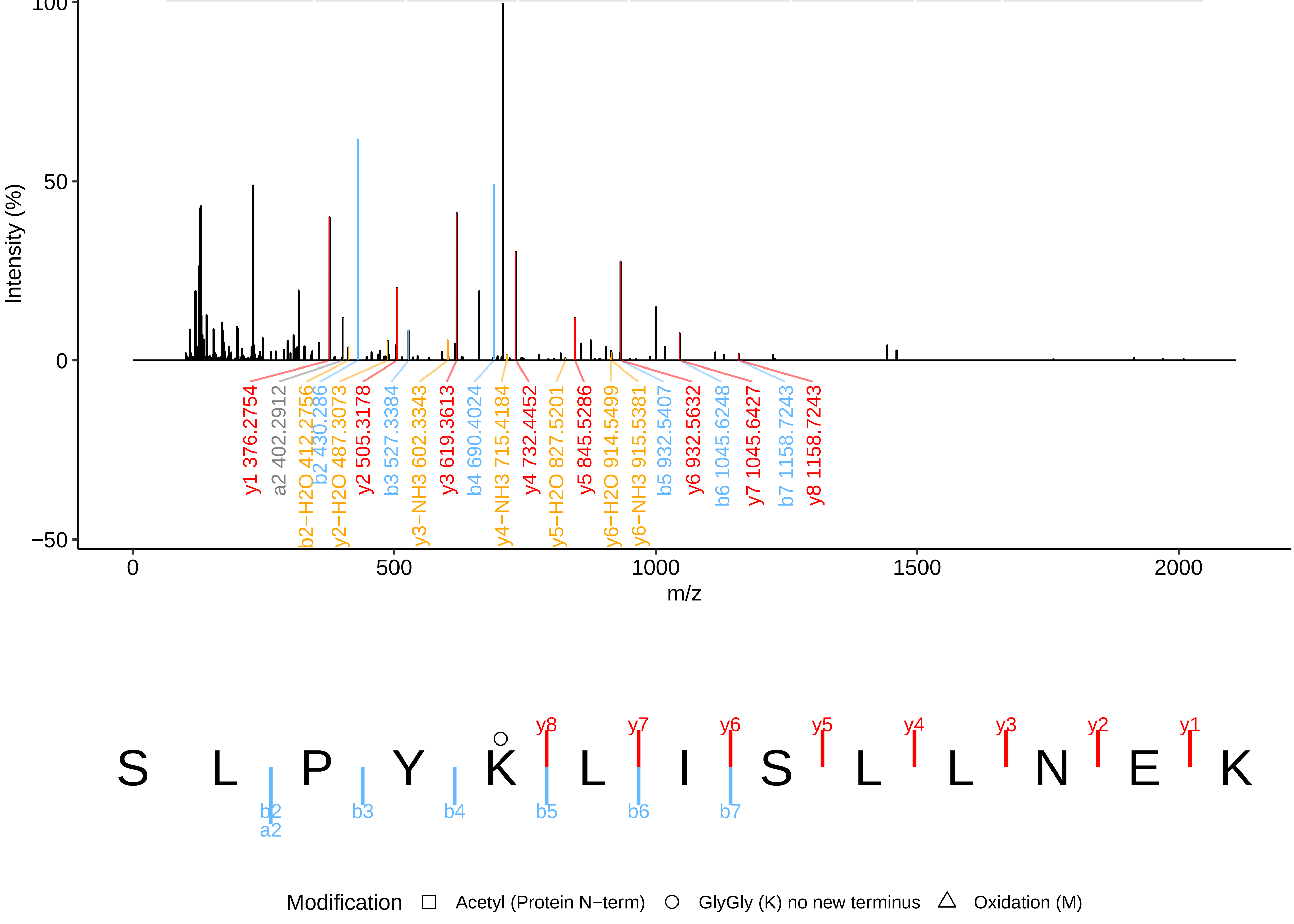

20200921_KIZ_MUb4_GlyGly_10plex_2 − Scan number: 24301

Gene Names	Charge	m/z	Mass	Retention time	PEP	Score	Precursor Intensity
NSFL1C	3	549.6177	1645.831	96.007	0.031998	58.752	NA

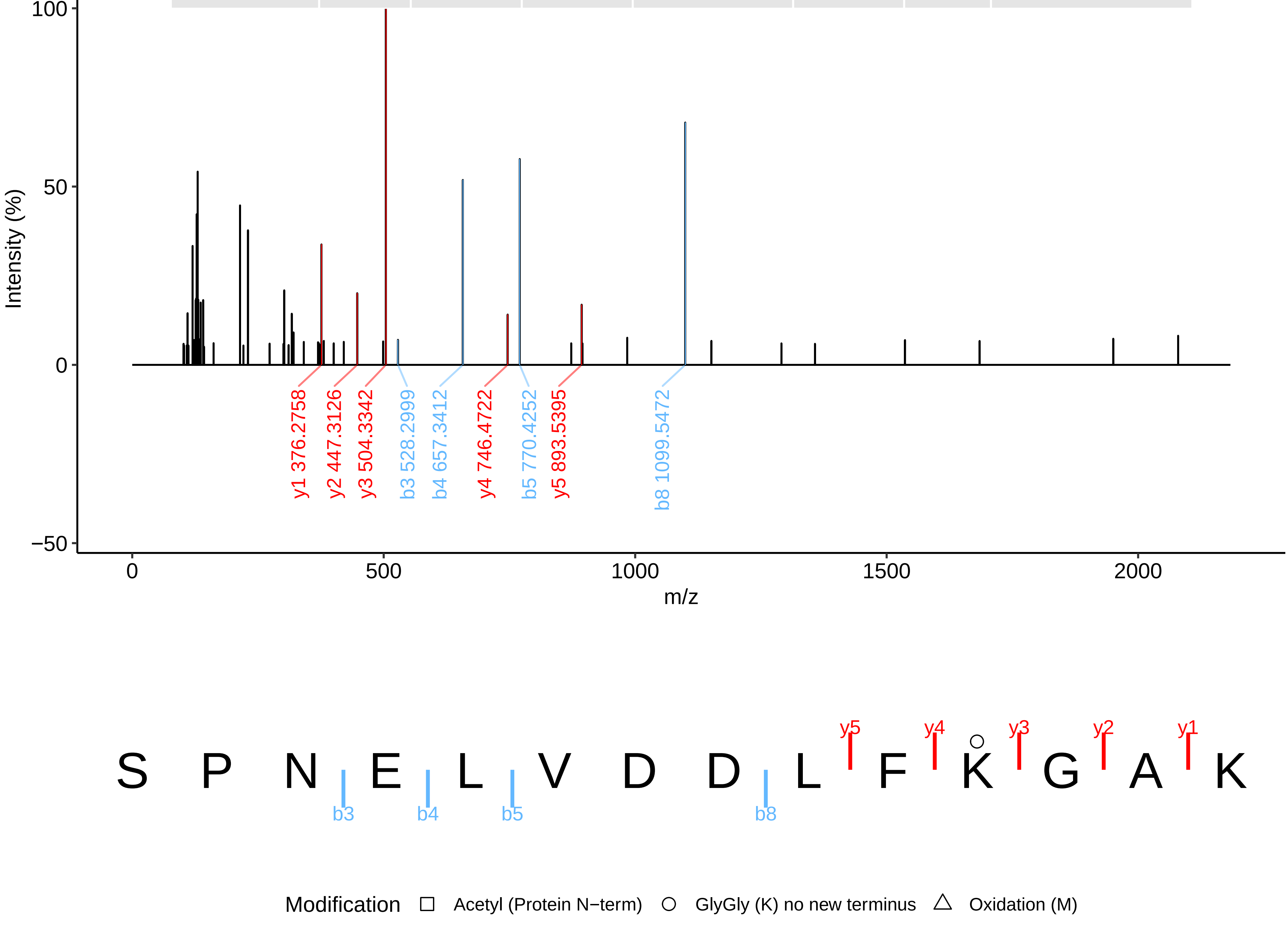

20200924_KIZ_MUb5_GlyGly_1 − Scan number: 27097

Gene Names	Charge	m/z	Mass	Retention time	PEP	Score	Precursor Intensity
NSFL1C	3	549.6177	1645.831	86.649	0.00034305	90.367	17444780

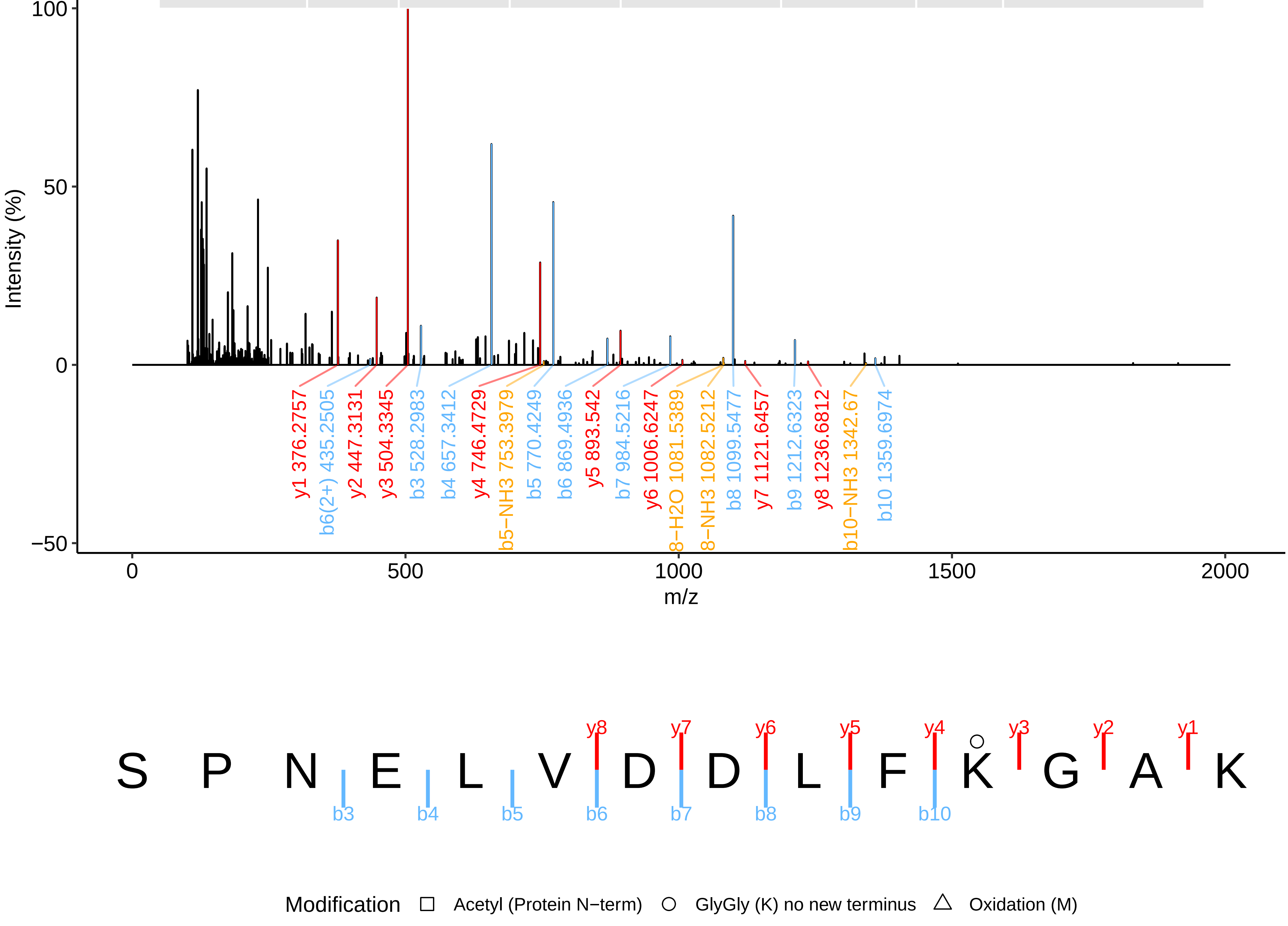

20200924_KIZ_MUb5_GlyGly_2 − Scan number: 24295

Gene Names	Charge	m/z	Mass	Retention time	PEP	Score	Precursor Intensity
NSFL1C	3	549.6177	1645.831	90.02	0.00088659	82.651	6515308

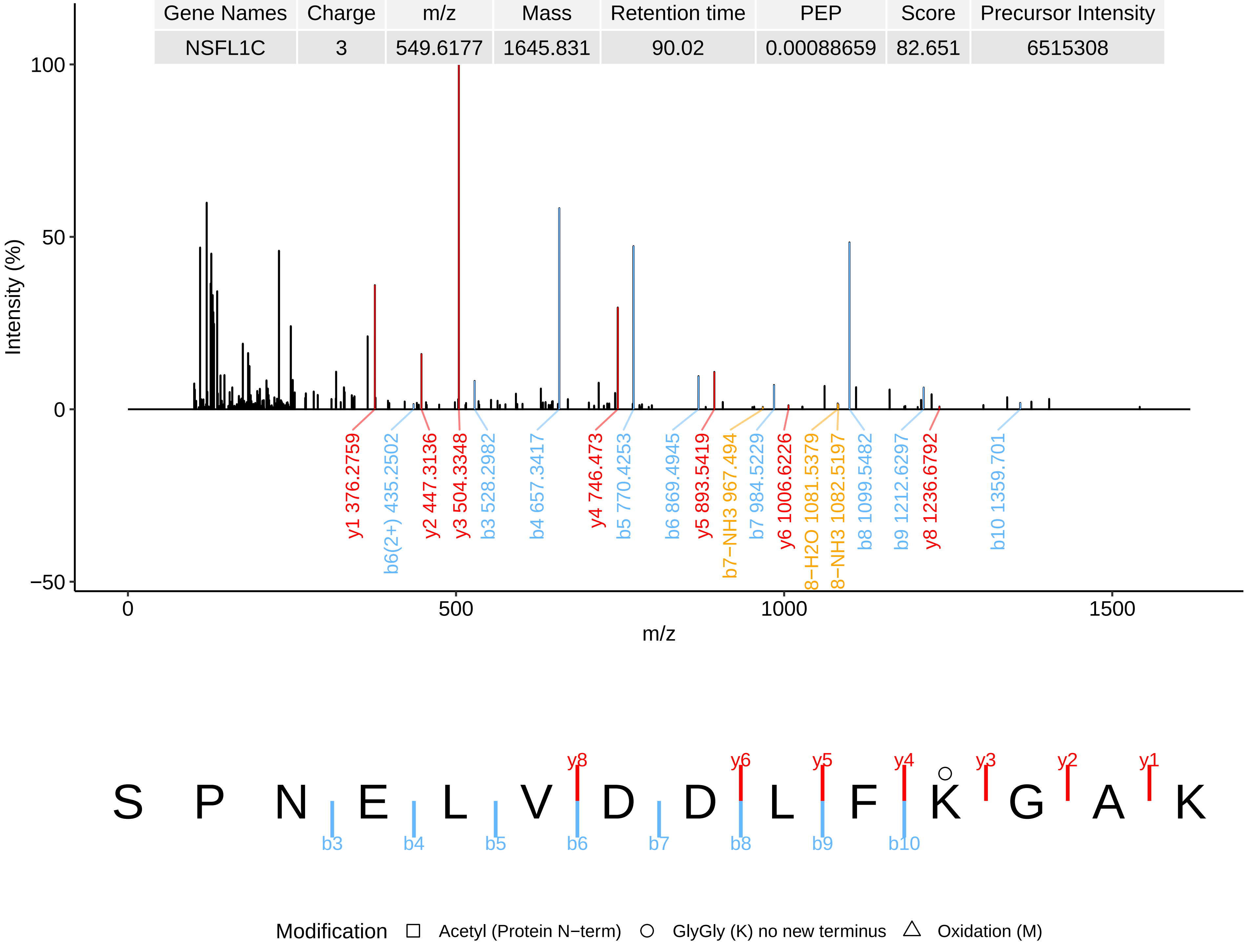

20200921_KIZ_MUb4_GlyGly_10plex − Scan number: 24443

Gene Names	Charge	m/z	Mass	Retention time	PEP	Score	Precursor Intensity
VCP	3	549.298	1644.872	88.883	0.0025497	75.764	5809857

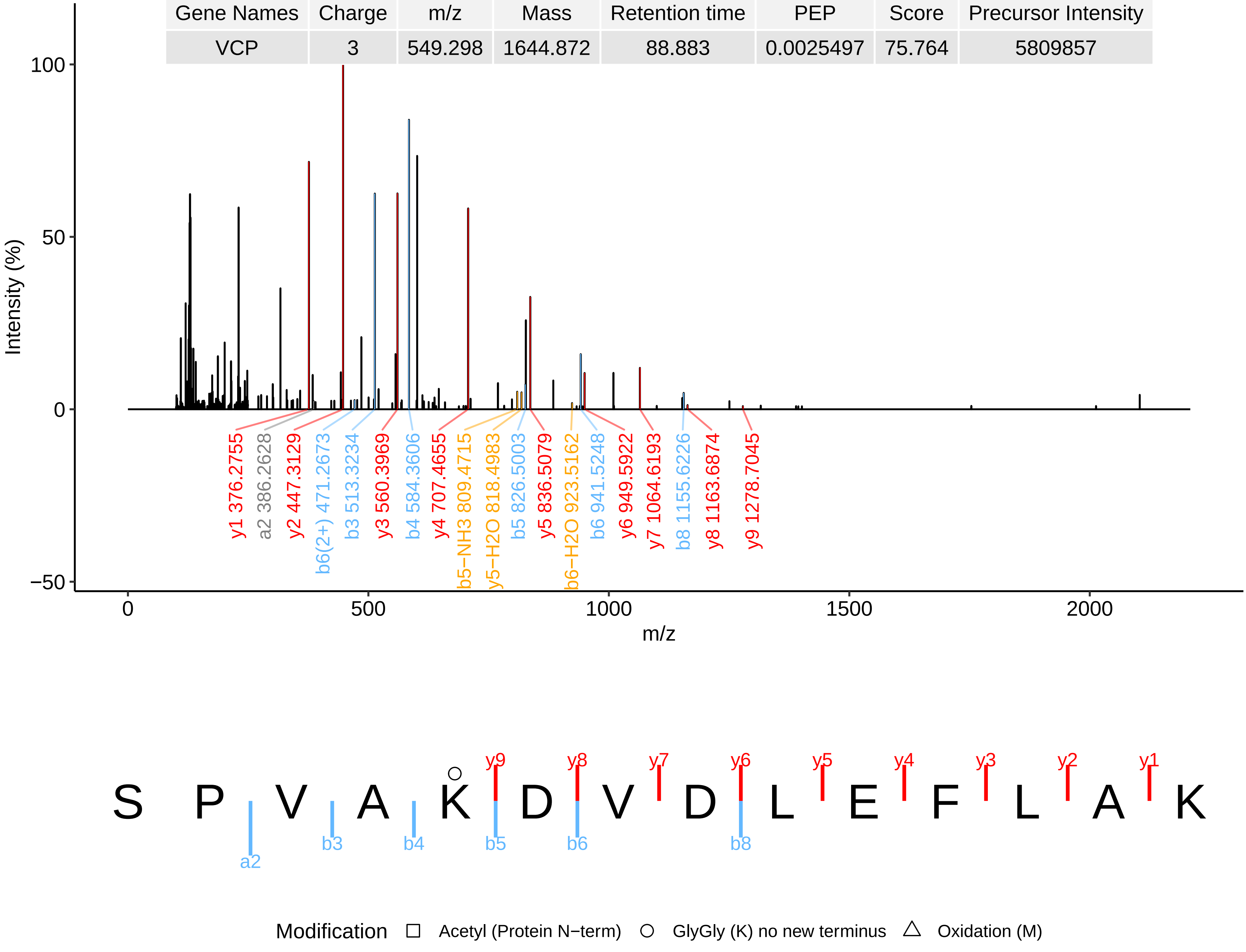

20200921_KIZ_MUb4_GlyGly_10plex − Scan number: 24603

Gene Names	Charge	m/z	Mass	Retention time	PEP	Score	Precursor Intensity
VCP	3	549.298	1644.872	89.483	0.0063041	63.709	4693270

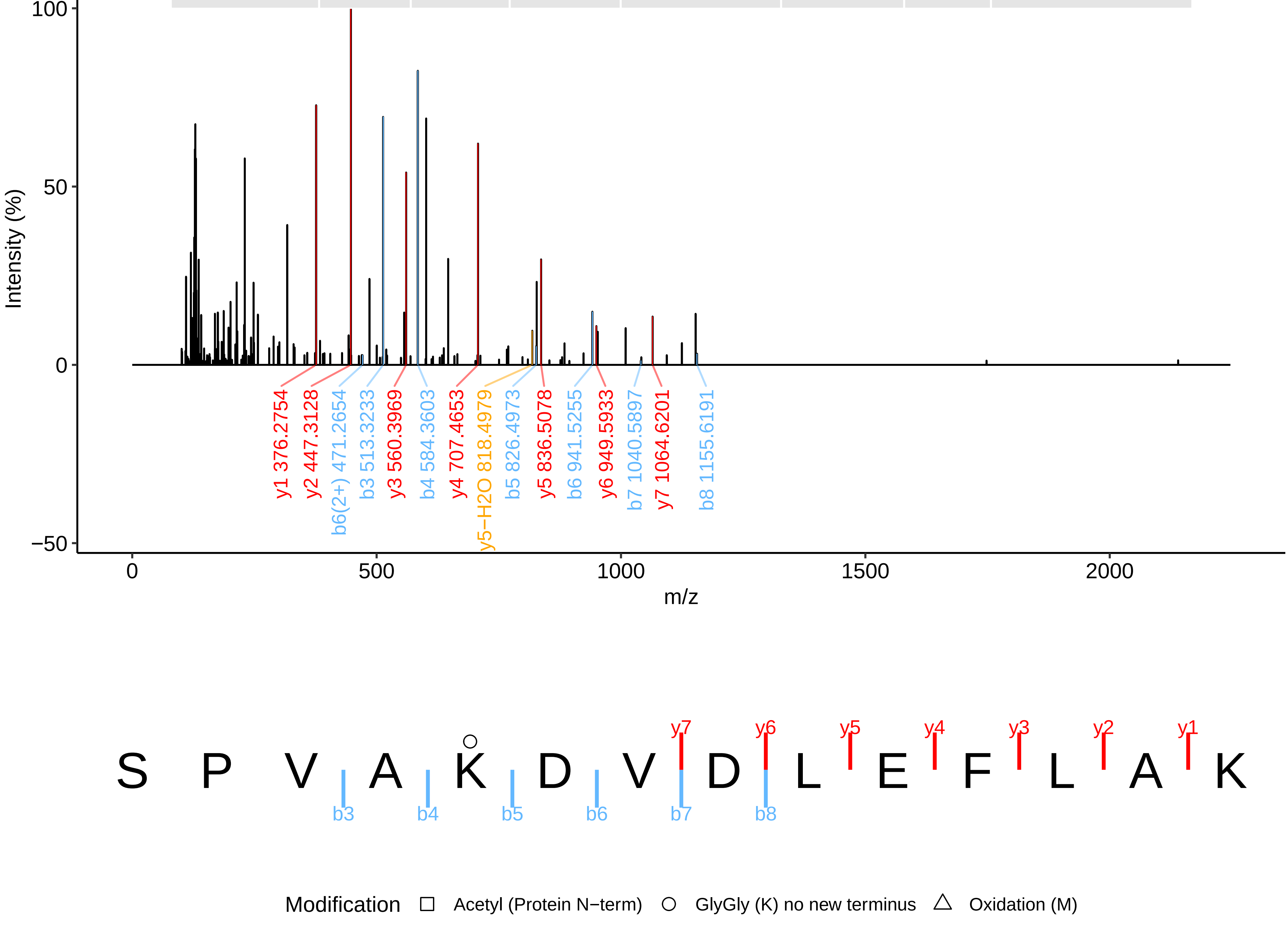

20200921_KIZ_MUb4_GlyGly_10plex_2 − Scan number: 21189

Gene Names	Charge	m/z	Mass	Retention time	PEP	Score	Precursor Intensity
VCP	3	549.298	1644.872	83.391	0.032709	56.482	1021839

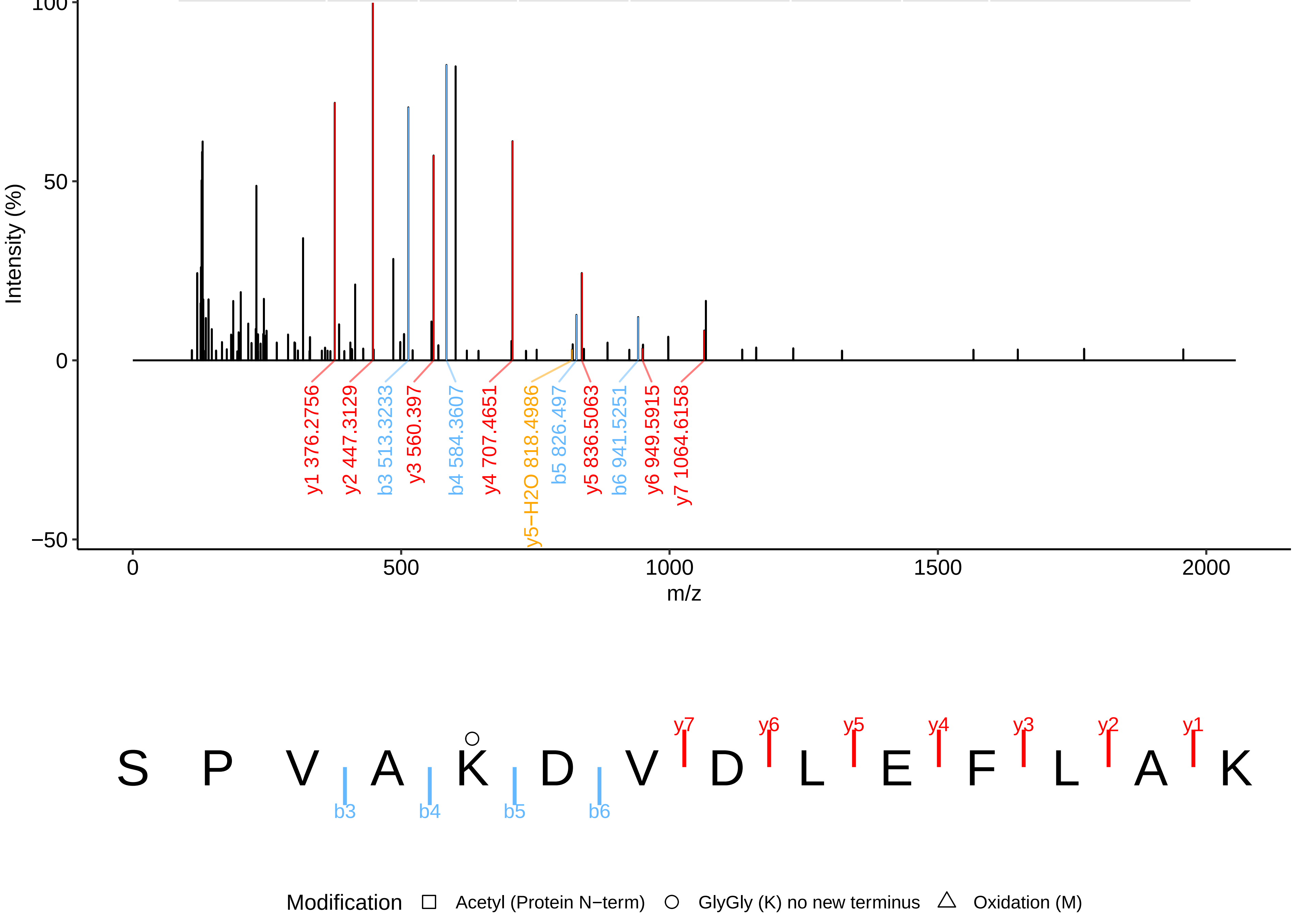

20200921_KIZ_MUb4_GlyGly_10plex_2 − Scan number: 21216

Gene Names	Charge	m/z	Mass	Retention time	PEP	Score	Precursor Intensity
VCP	3	549.298	1644.872	83.499	0.10289	40.477	1872086

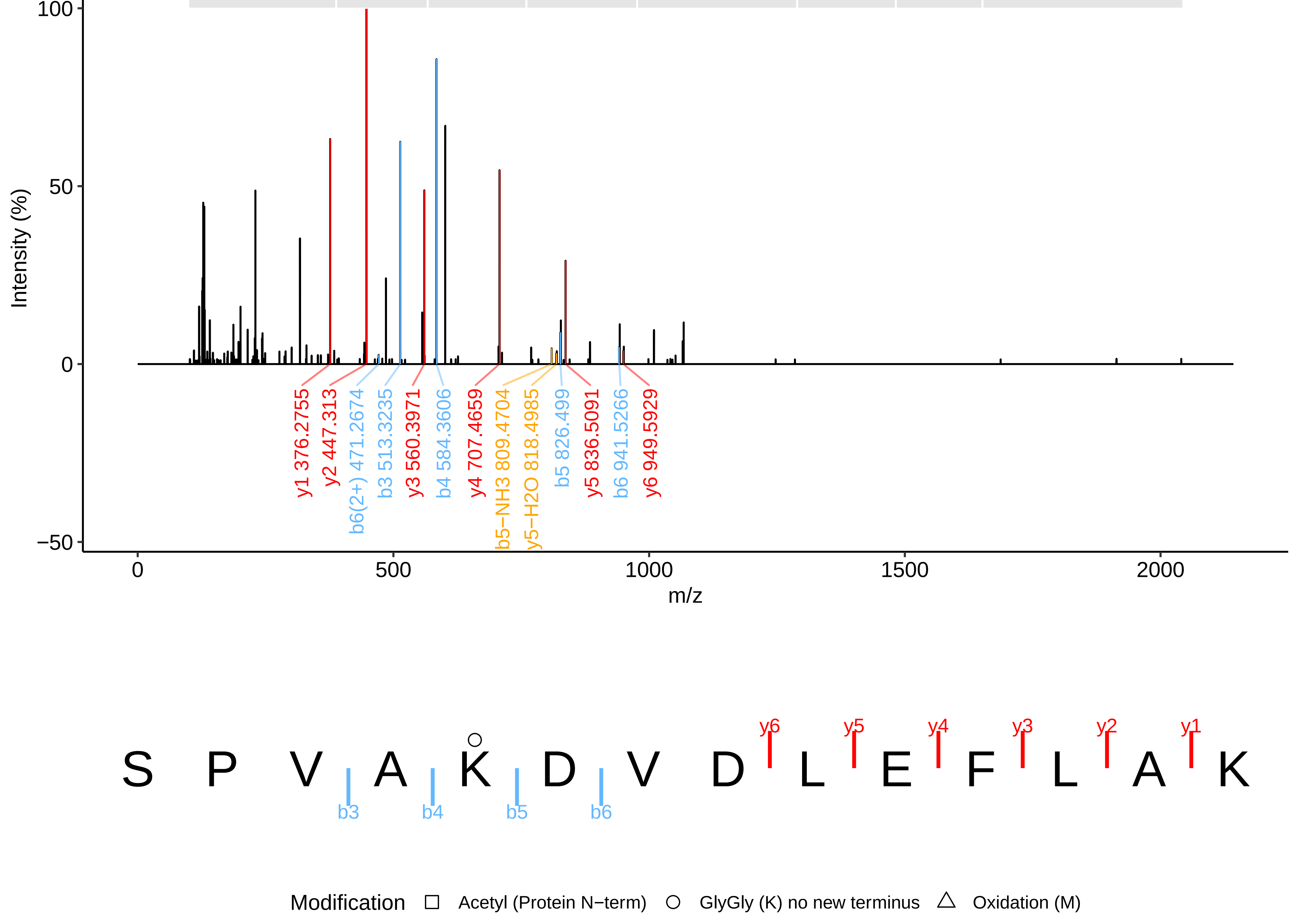

20200924_KIZ_MUb5_GlyGly_1 − Scan number: 23445

Gene Names	Charge	m/z	Mass	Retention time	PEP	Score	Precursor Intensity
VCP	3	549.298	1644.872	75.358	0.071403	46.318	7728097

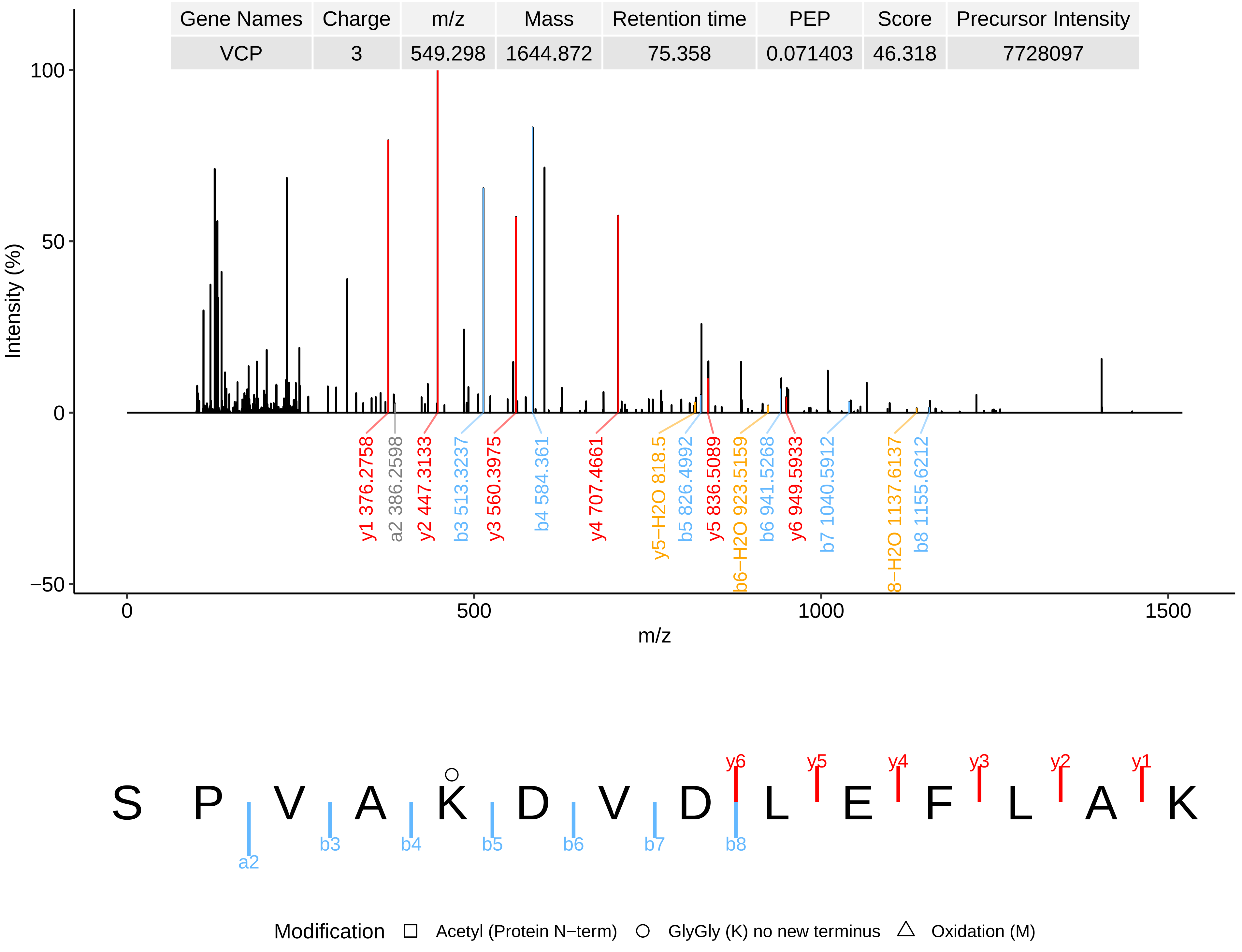

20200924_KIZ_MUb5_GlyGly_1 − Scan number: 23608

Gene Names	Charge	m/z	Mass	Retention time	PEP	Score	Precursor Intensity
VCP	3	549.298	1644.872	75.867	0.0014886	78.615	13374392

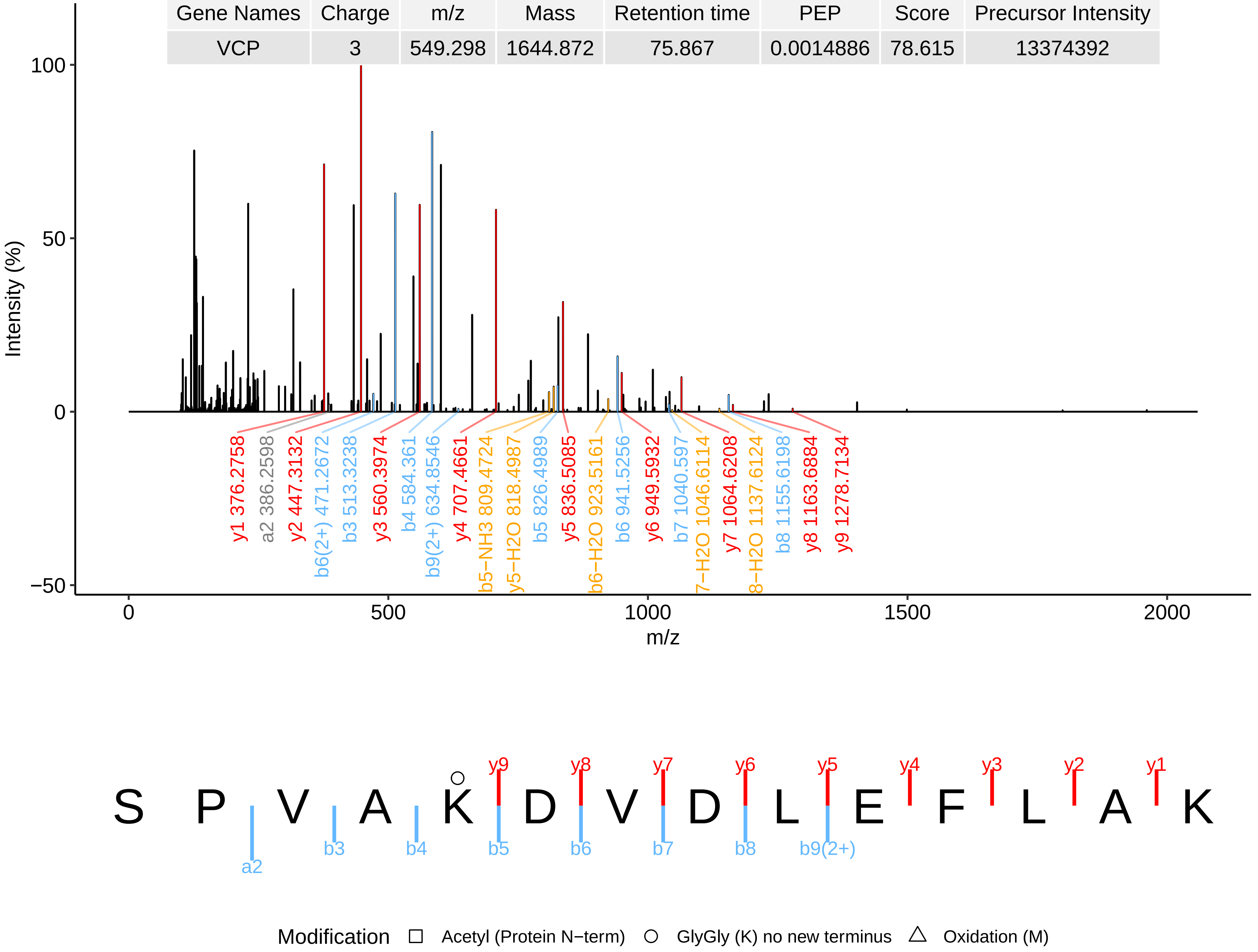

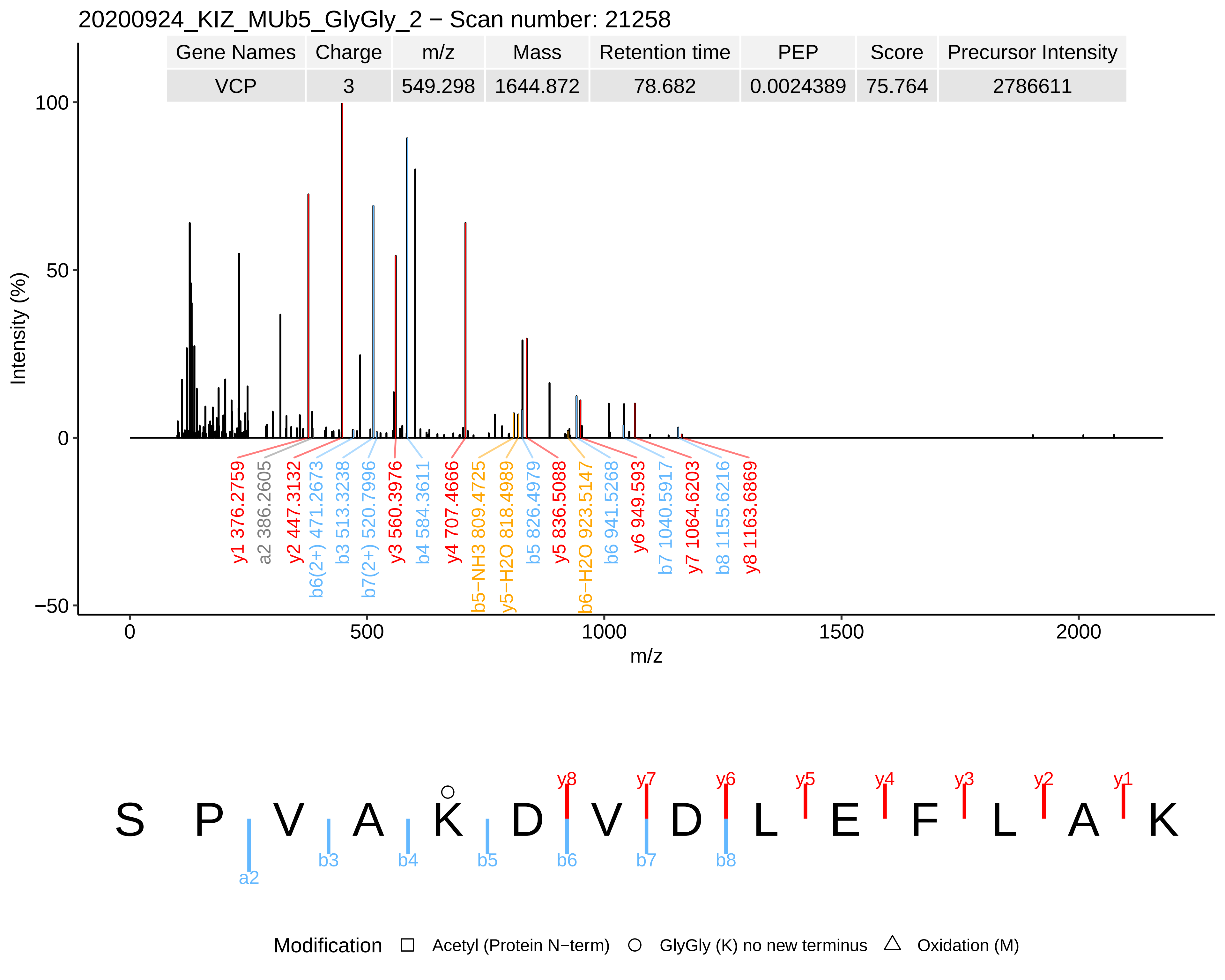

20200924_KIZ_MUb5_GlyGly_2 − Scan number: 21258
Gene Names | Charge | m/z | Mass | Retention time | PEP | Score | Precursor Intensity
VCP | 3 | 549.298 | 1644.872 | 78.682 | 0.0024389 | 75.764 | 2786611
Intensity (%)
100
50
0
−50
0
500
1000
1500
2000
m/z
y1 376.2759
a2 386.2605
y2 447.3132
b6(2+) 471.2673
b3 513.3238
b7(2+) 520.7996
y3 560.3976
b4 584.3611
y4 707.4666
b5−NH3 809.4725
y5−H2O 818.4989
b5 826.4979
y5 836.5088
b6−H2O 923.5147
b6 941.5268
y6 949.593
b7 1040.5917
y7 1064.6203
b8 1155.6216
y8 1163.6869
S P V A K D V D L E F L A K
y8 y7 y6 y5 y4 y3 y2 y1
a2 b3 b4 b5 b6 b7 b8
Modification
Acetyl (Protein N−term)
GlyGly (K) no new terminus
Oxidation (M)

20201118_KIZ_MUb6_GlyGly_1 − Scan number: 24177

Gene Names	Charge	m/z	Mass	Retention time	PEP	Score	Precursor Intensity
VCP	3	549.298	1644.872	74.513	0.0049705	69.261	26233808

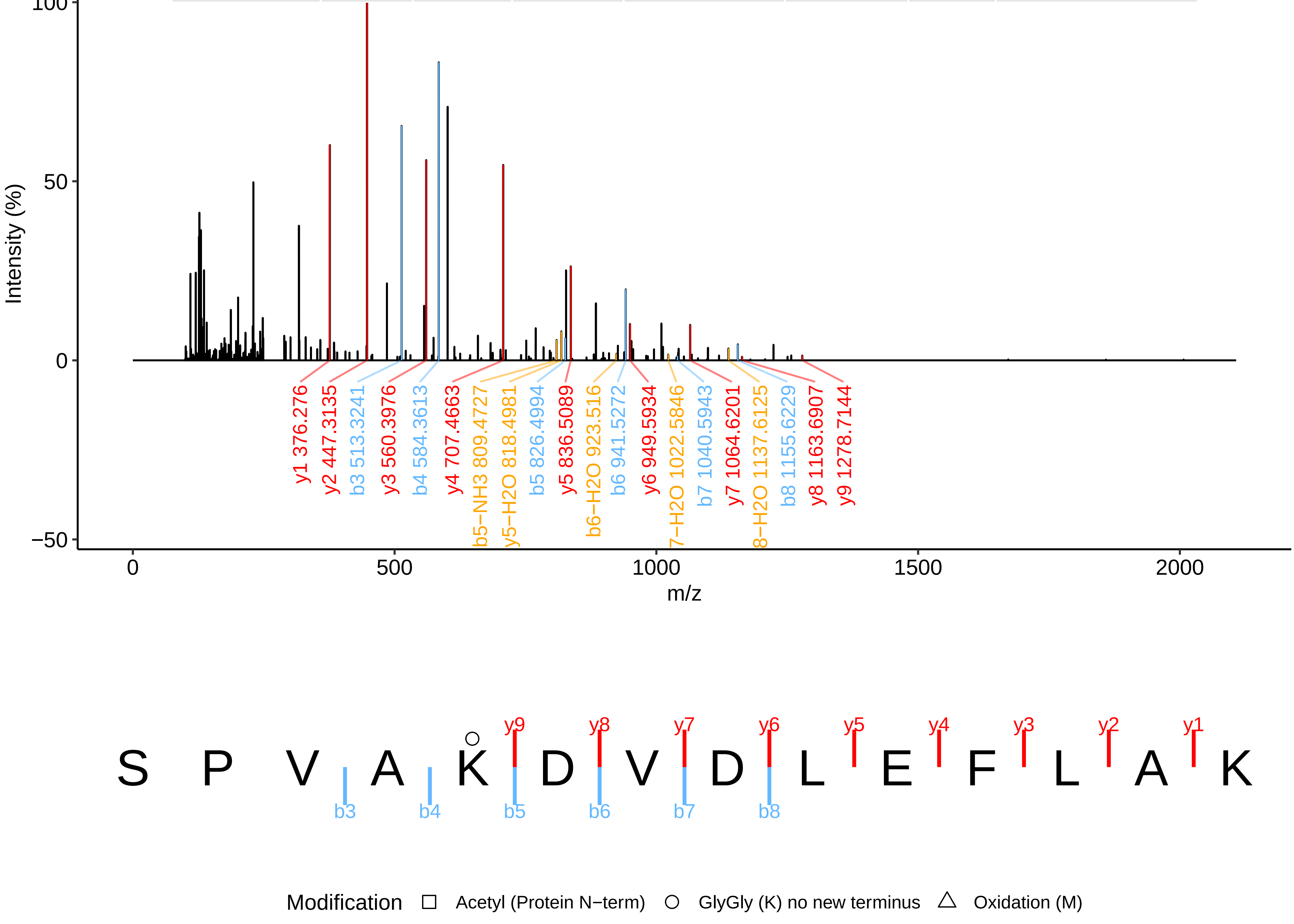

20201118_KIZ_MUb6_GlyGly_1 − Scan number: 24180

Gene Names	Charge	m/z	Mass	Retention time	PEP	Score	Precursor Intensity
VCP	3	549.298	1644.872	74.522	0.00066796	82.651	24404010

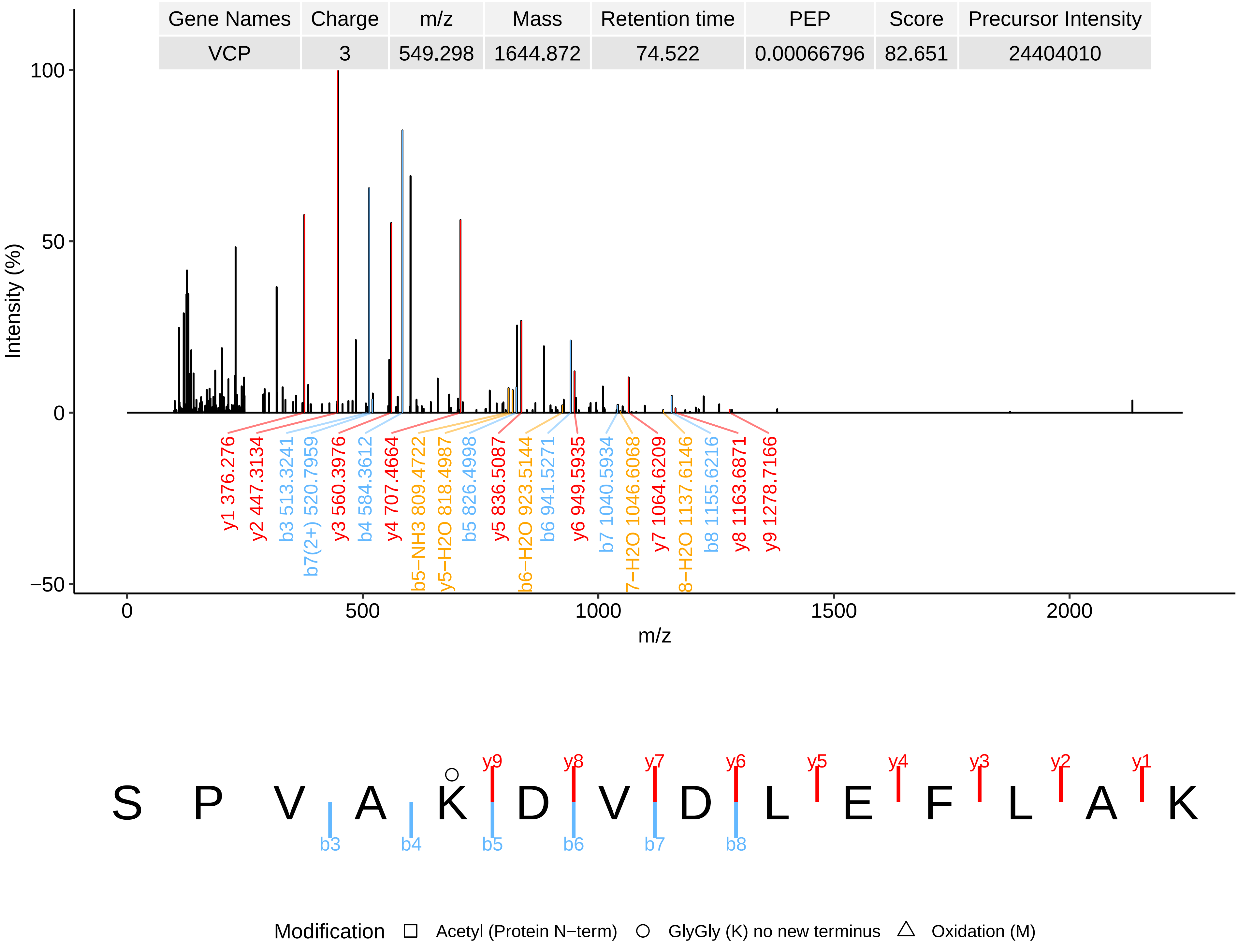

20200921_KIZ_MUb4_GlyGly_10plex − Scan number: 3432

Gene Names	Charge	m/z	Mass	Retention time	PEP	Score	Precursor Intensity
ADAM9	3	632.6208	1894.841	10.871	2.2042e−38	196.52	3725046

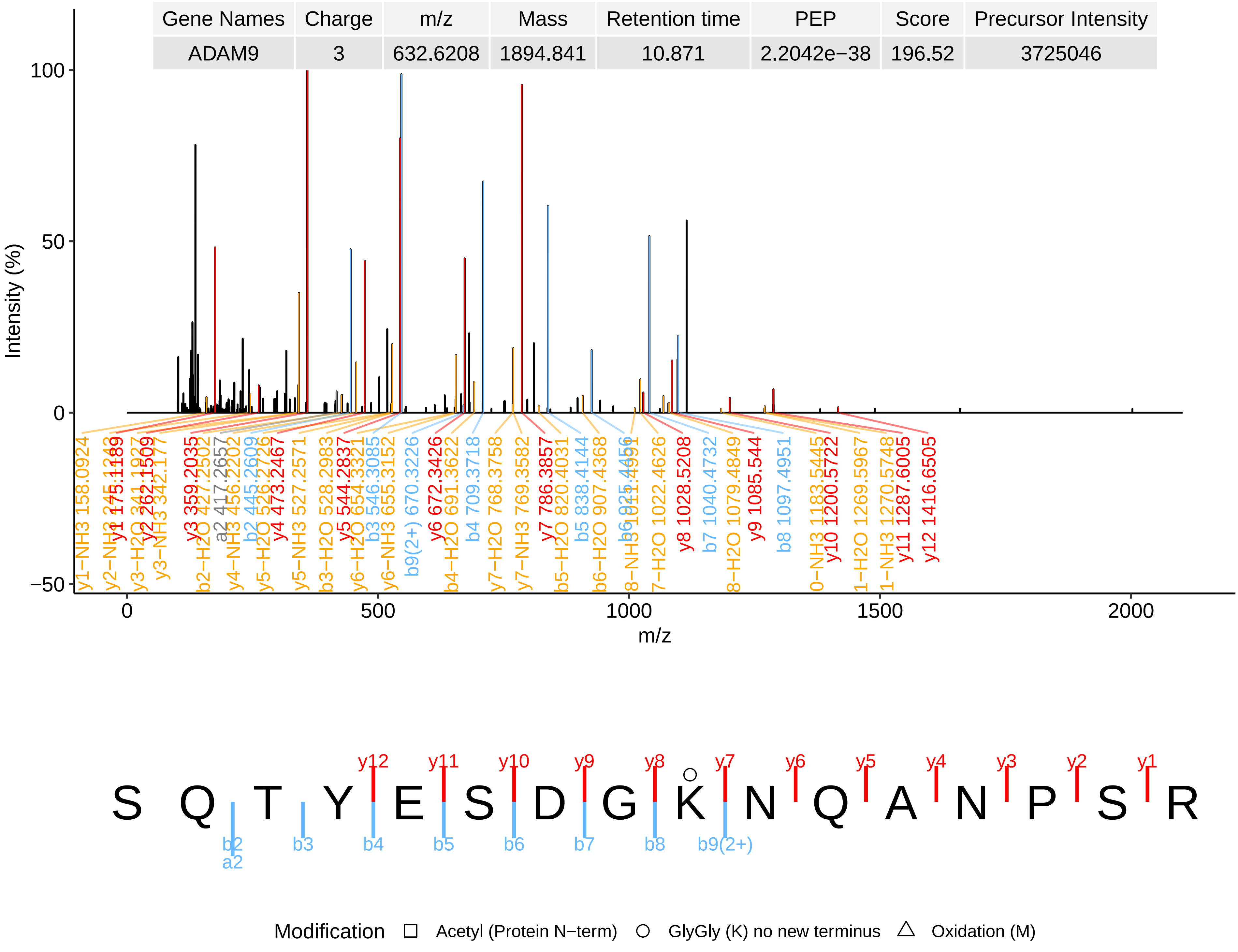

20200921_KIZ_MUb4_GlyGly_10plex_2 − Scan number: 3297

Gene Names	Charge	m/z	Mass	Retention time	PEP	Score	Precursor Intensity
ADAM9	3	632.6208	1894.841	11.01	7.5714e−15	146.1	3177062

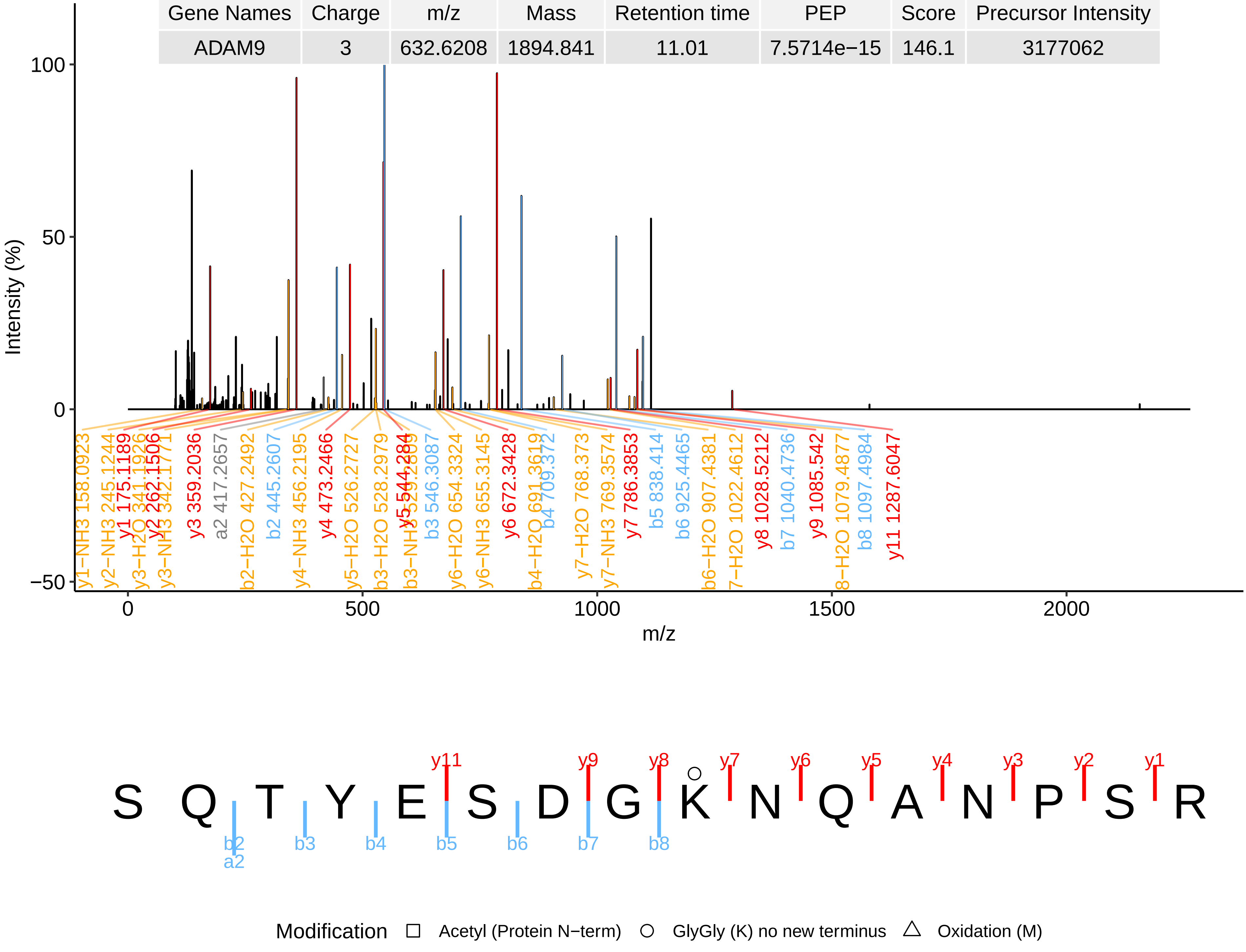

20200924_KIZ_MUb5_GlyGly_1 − Scan number: 3112

Gene Names	Charge	m/z	Mass	Retention time	PEP	Score	Precursor Intensity
ADAM9	3	632.6208	1894.841	9.6033	4.6301e−63	229.68	12604205

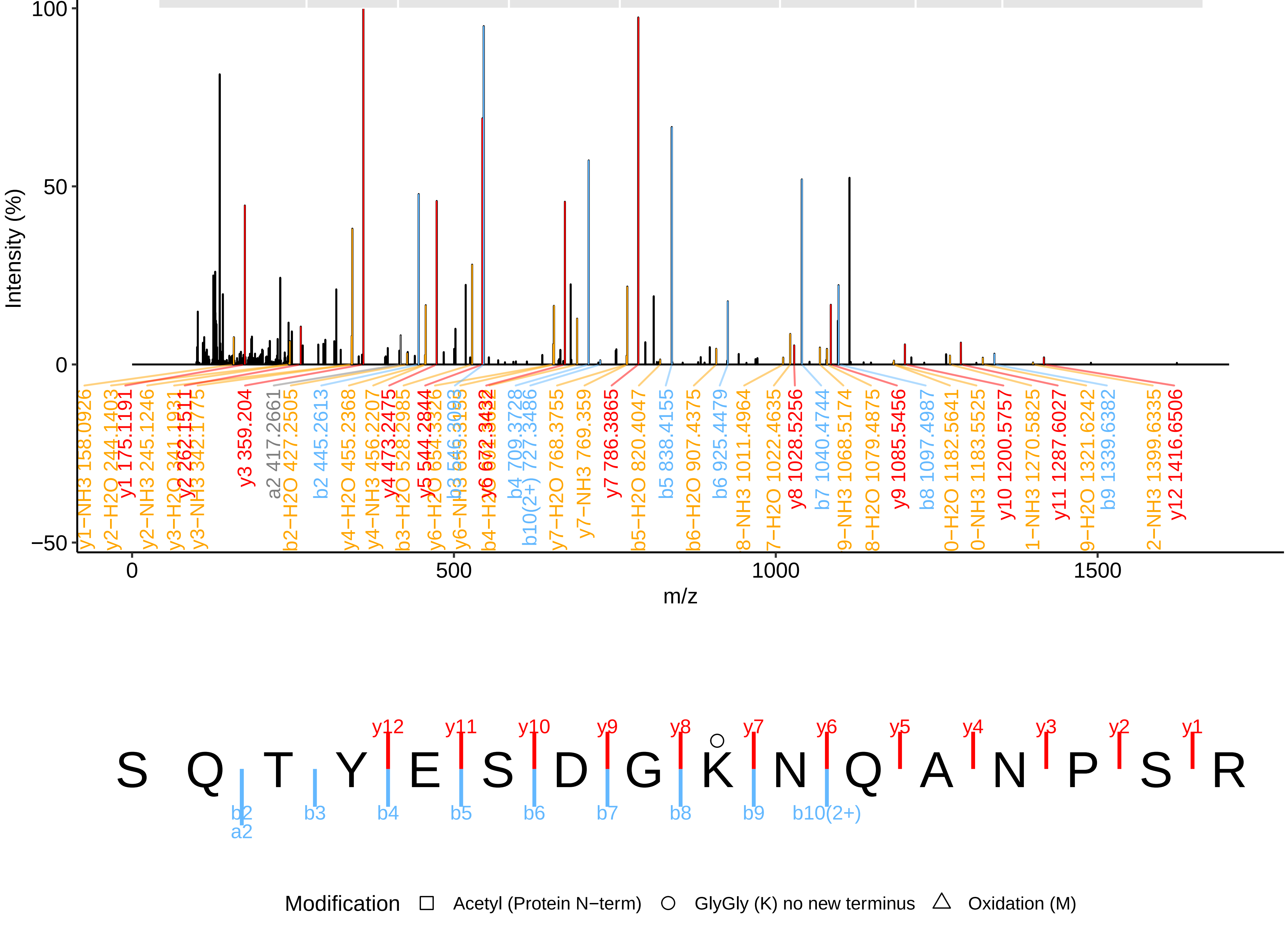

20201118_KIZ_MUb6_GlyGly_1 − Scan number: 3099

Gene Names	Charge	m/z	Mass	Retention time	PEP	Score	Precursor Intensity
ADAM9	3	632.6208	1894.841	9.5537	5.9833e−22	167.29	6674011

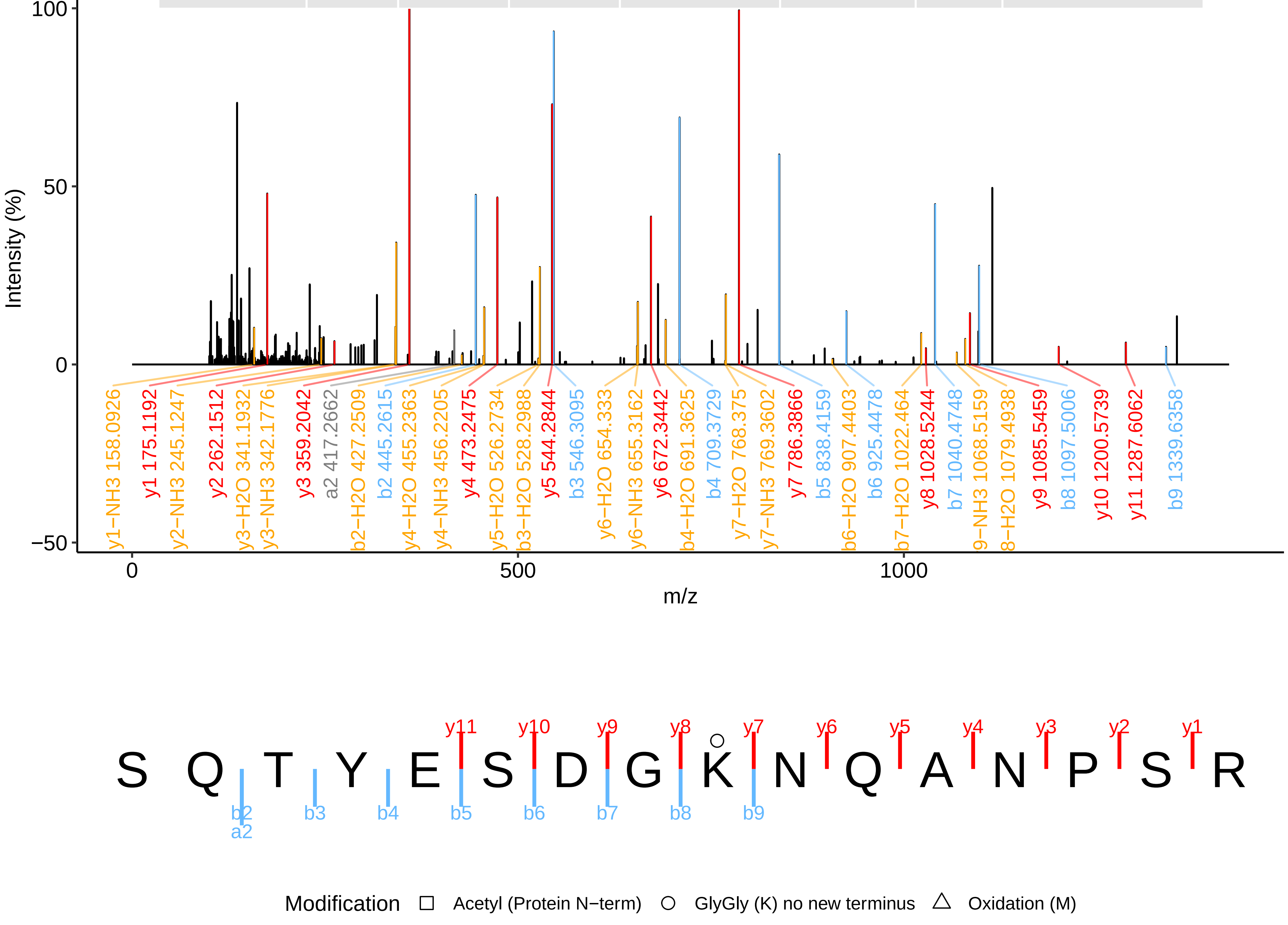

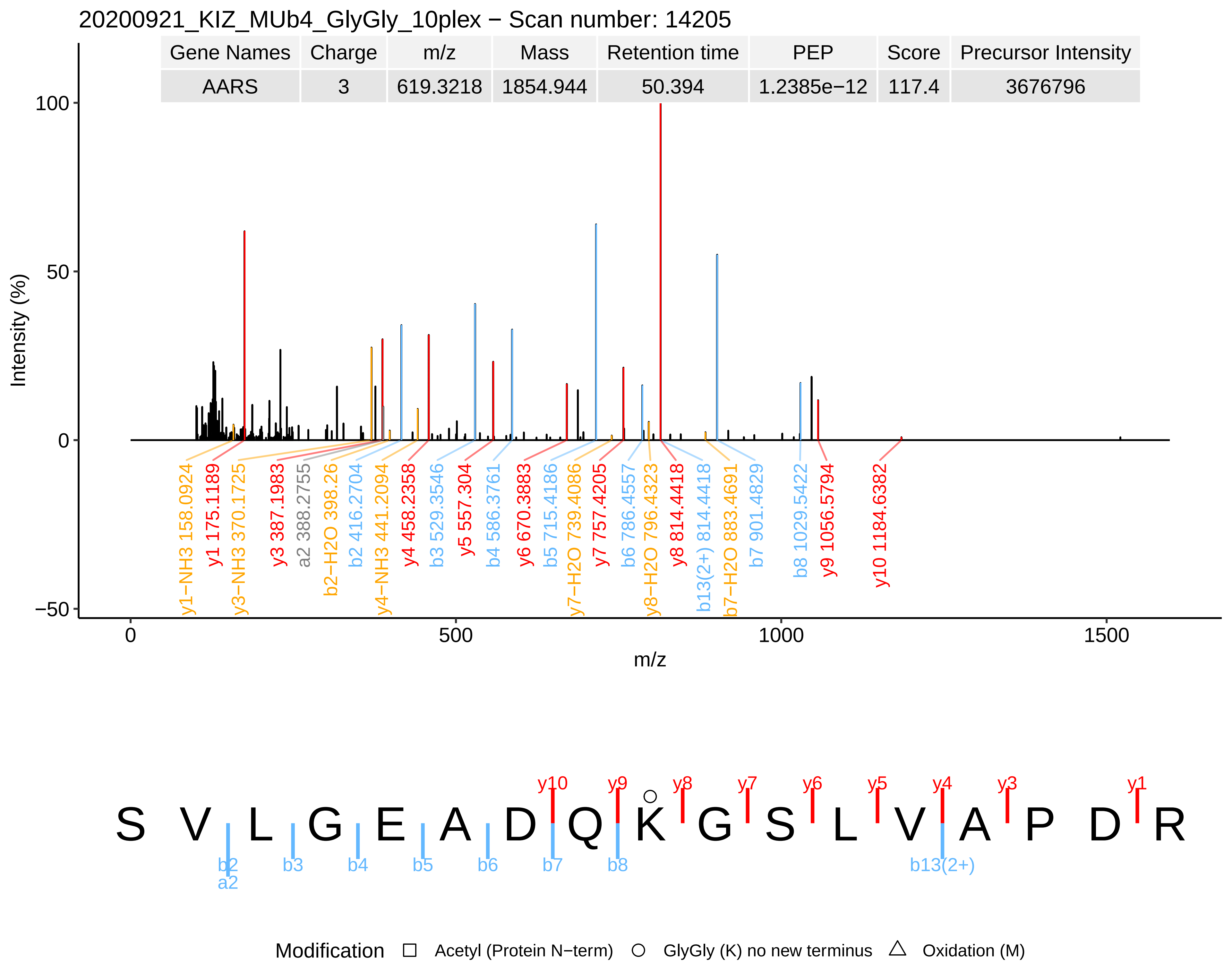
20200921_KIZ_MUb4_GlyGly_10plex − Scan number: 14205
Gene Names | Charge | m/z | Mass | Retention time | PEP | Score | Precursor Intensity
AARS | 3 | 619.3218 | 1854.944 | 50.394 | 1.2385e−12 | 117.4 | 3676796
Intensity (%)
m/z
y1−NH3 158.0924
y1 175.1189
y3−NH3 370.1725
y3 387.1983
a2 388.2755
b2−H2O 398.26
b2 416.2704
y4−NH3 441.2094
y4 458.2358
b3 529.3546
y5 557.304
b4 586.3761
y6 670.3883
b5 715.4186
y7−H2O 739.4086
y7 757.4205
b6 786.4557
y8−H2O 796.4323
y8 814.4418
b13(2+) 814.4418
b7−H2O 883.4691
b7 901.4829
b8 1029.5422
y9 1056.5794
y10 1184.6382
S V L G E A D Q K G S L V A P D R
y10 y9 y8 y7 y6 y5 y4 y3 y1
b2 a2 b3 b4 b5 b6 b7 b8 b13(2+)
Modification
Acetyl (Protein N−term)
GlyGly (K) no new terminus
Oxidation (M)

20200921_KIZ_MUb4_GlyGly_10plex − Scan number: 8864

Gene Names	Charge	m/z	Mass	Retention time	PEP	Score	Precursor Intensity
CLN3	3	616.3095	1845.907	30.216	4.9442e−10	106.82	7668142

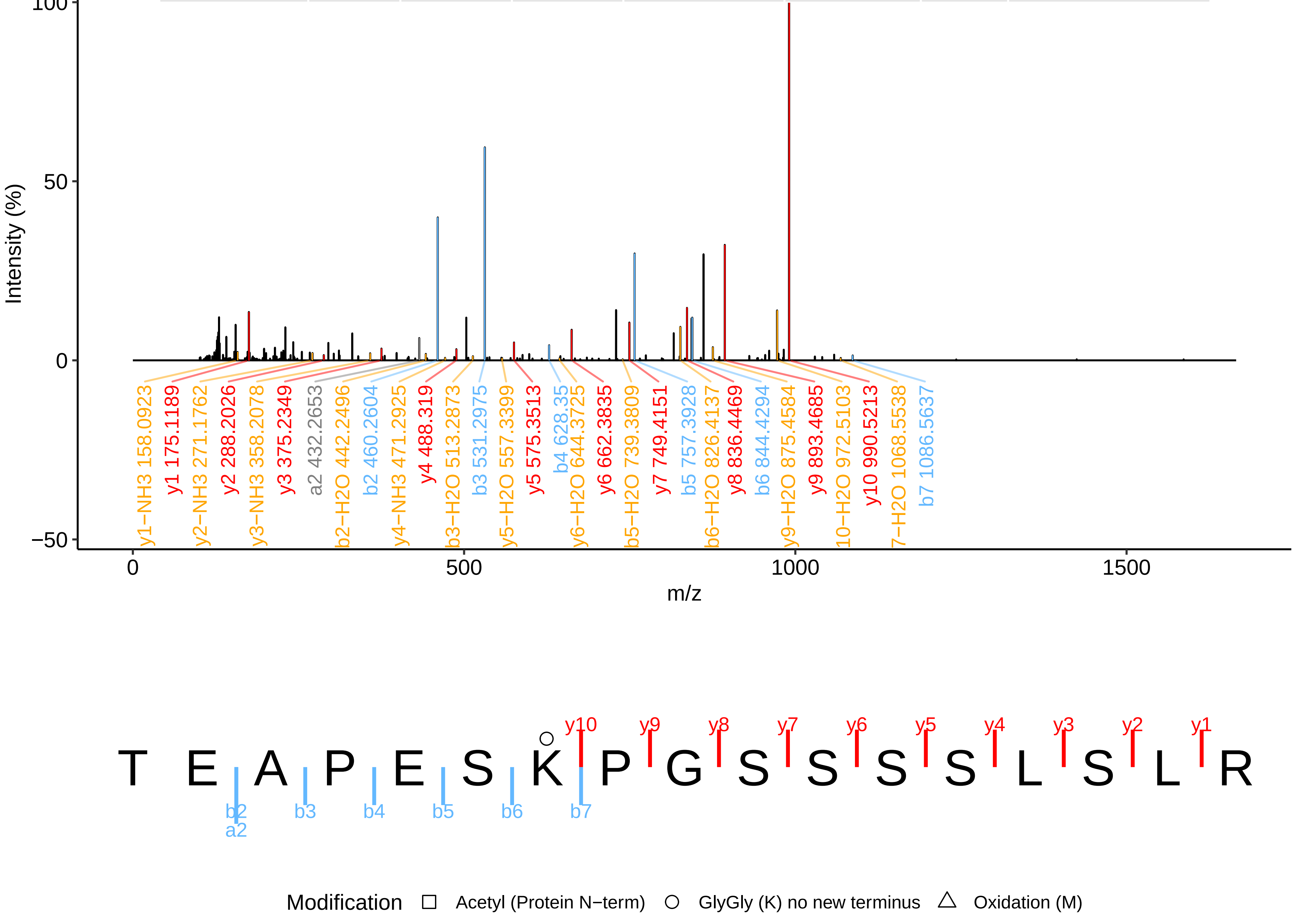

20200921_KIZ_MUb4_GlyGly_10plex_2 − Scan number: 8154

Gene Names	Charge	m/z	Mass	Retention time	PEP	Score	Precursor Intensity
CLN3	3	616.3095	1845.907	30.671	0.0023475	65.107	705594.6

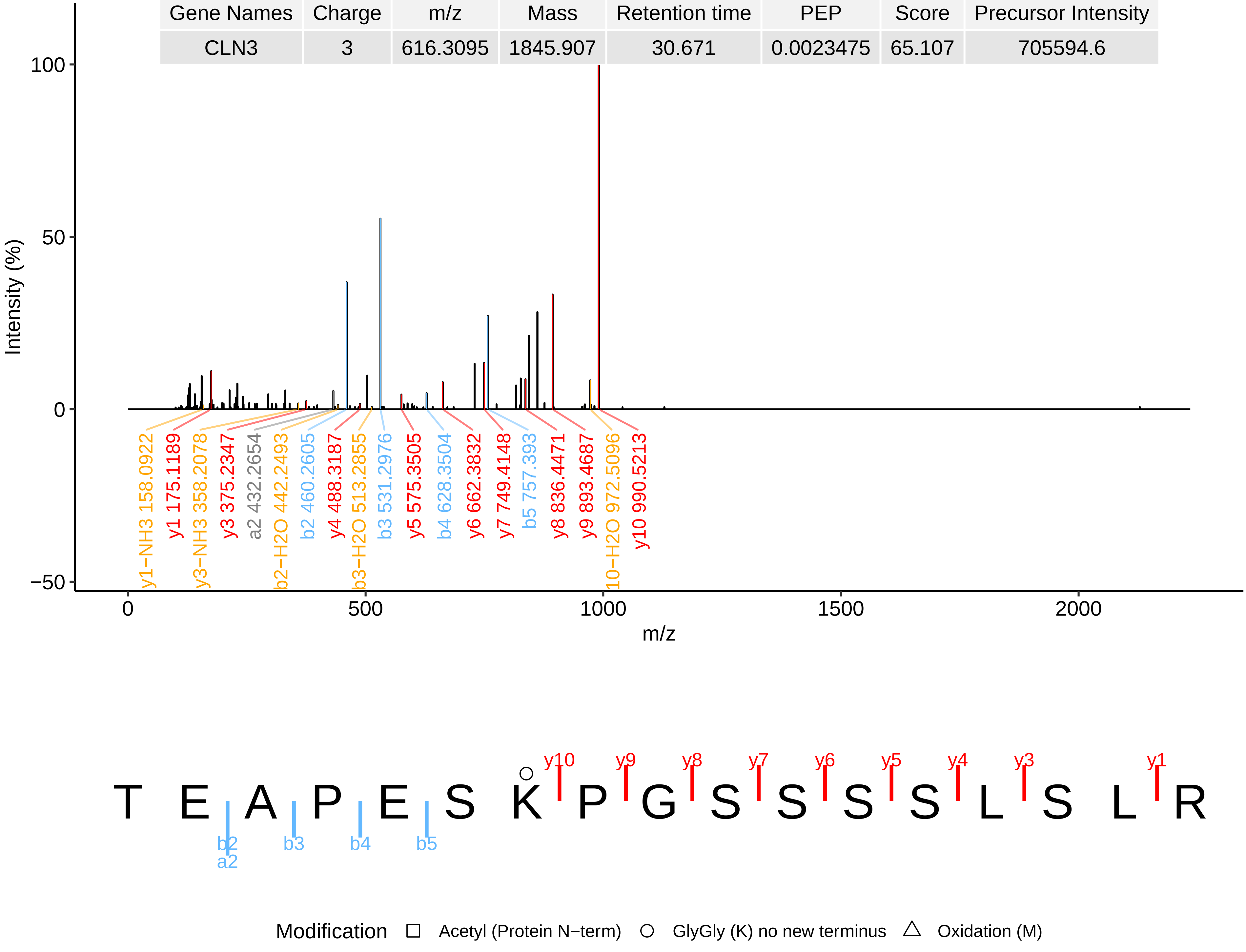

20200921_KIZ_MUb4_GlyGly_10plex_2 − Scan number: 8157

Gene Names	Charge	m/z	Mass	Retention time	PEP	Score	Precursor Intensity
CLN3	3	616.3095	1845.907	30.683	1.5166e−07	97.579	2979503

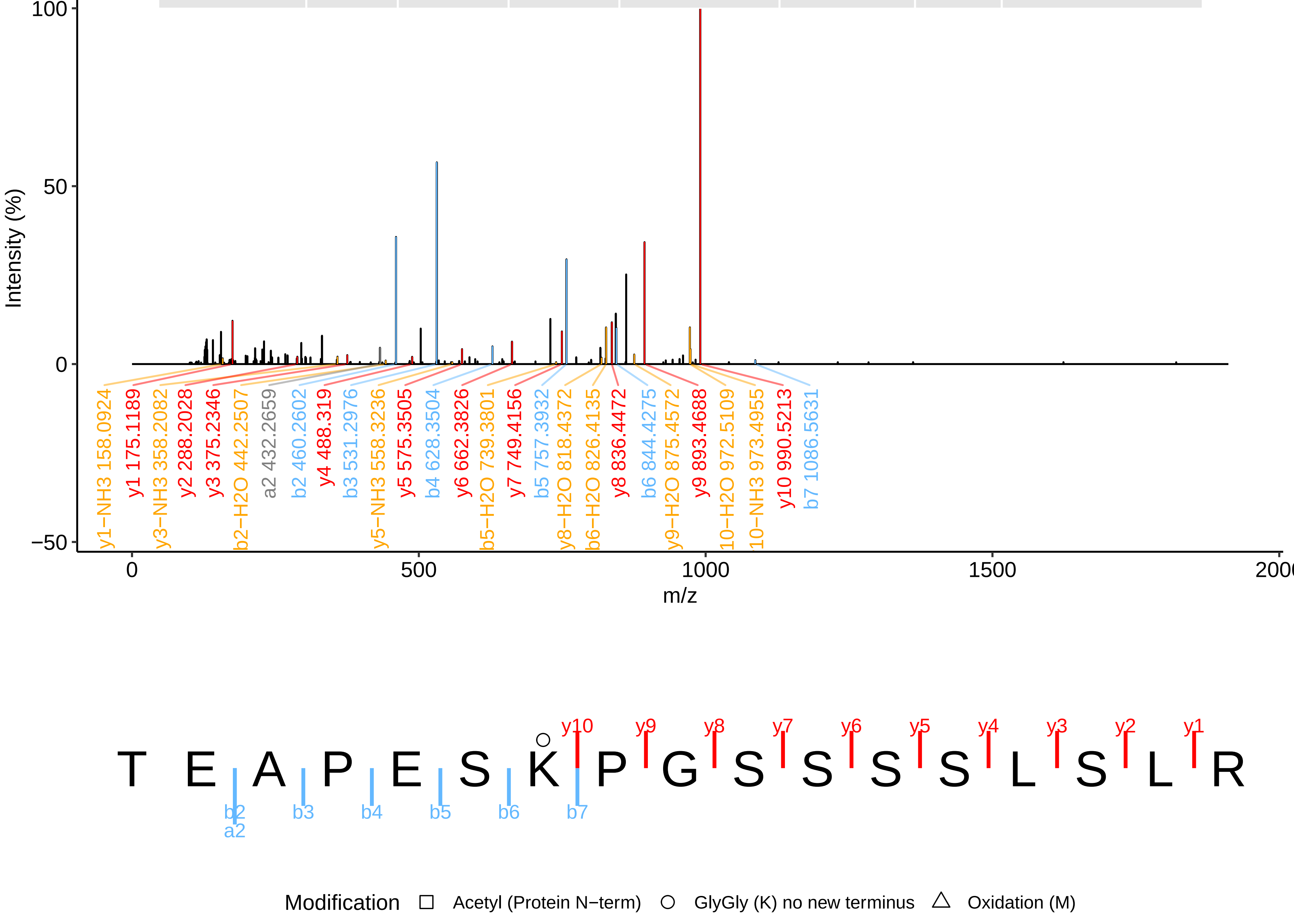

20200924_KIZ_MUb5_GlyGly_1 − Scan number: 7846

Gene Names	Charge	m/z	Mass	Retention time	PEP	Score	Precursor Intensity
CLN3	3	616.3095	1845.907	24.998	5.6862e−10	105.53	6708674

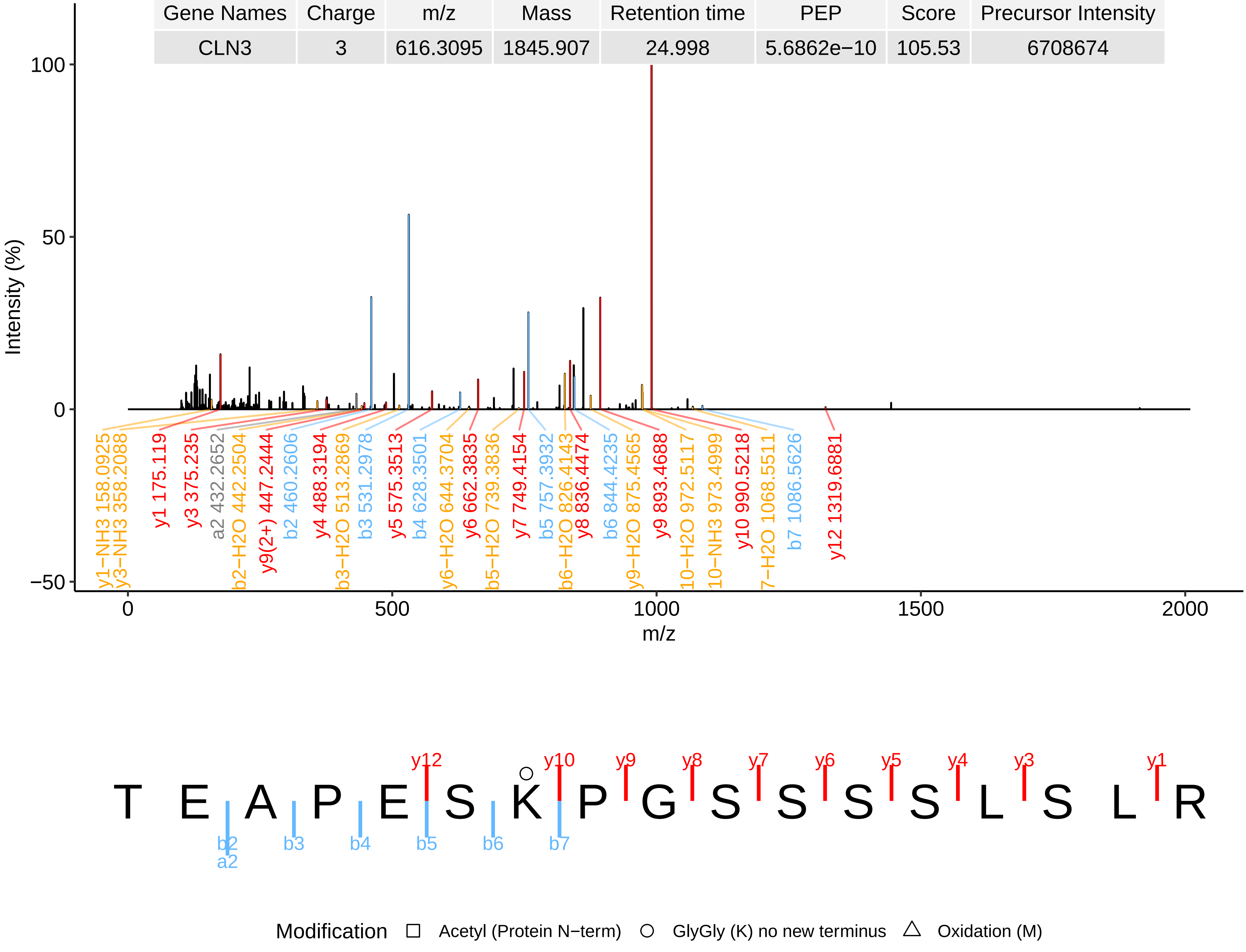

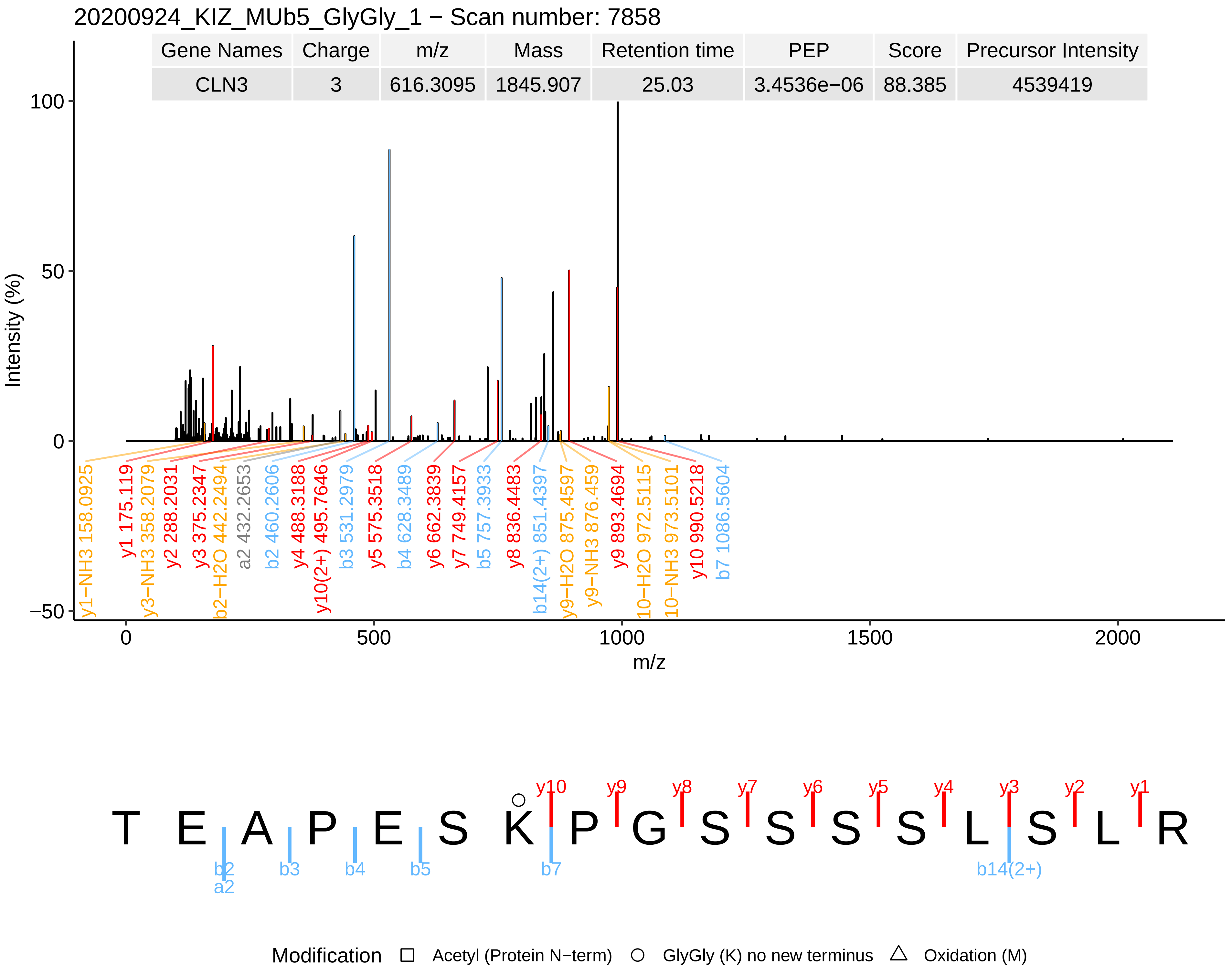

20200924_KIZ_MUb5_GlyGly_1 − Scan number: 7858
Gene Names | Charge | m/z | Mass | Retention time | PEP | Score | Precursor Intensity
CLN3 | 3 | 616.3095 | 1845.907 | 25.03 | 3.4536e−06 | 88.385 | 4539419
Intensity (%)
m/z
y1−NH3 158.0925
y1 175.119
y3−NH3 358.2079
y2 288.2031
y3 375.2347
b2−H2O 442.2494
a2 432.2653
b2 460.2606
y4 488.3188
y10(2+) 495.7646
b3 531.2979
y5 575.3518
b4 628.3489
y6 662.3839
y7 749.4157
b5 757.3933
y8 836.4483
b14(2+) 851.4397
y9−H2O 875.4597
y9−NH3 876.459
y9 893.4694
y10−H2O 972.5115
y10−NH3 973.5101
y10 990.5218
b7 1086.5604
T E A P E S K P G S S S S L S L R
Modification
Acetyl (Protein N−term)
GlyGly (K) no new terminus
Oxidation (M)

20200924_KIZ_MUb5_GlyGly_1 − Scan number: 8019

Gene Names	Charge	m/z	Mass	Retention time	PEP	Score	Precursor Intensity
CLN3	3	616.3095	1845.907	25.549	1.2565e−12	117.18	6988226

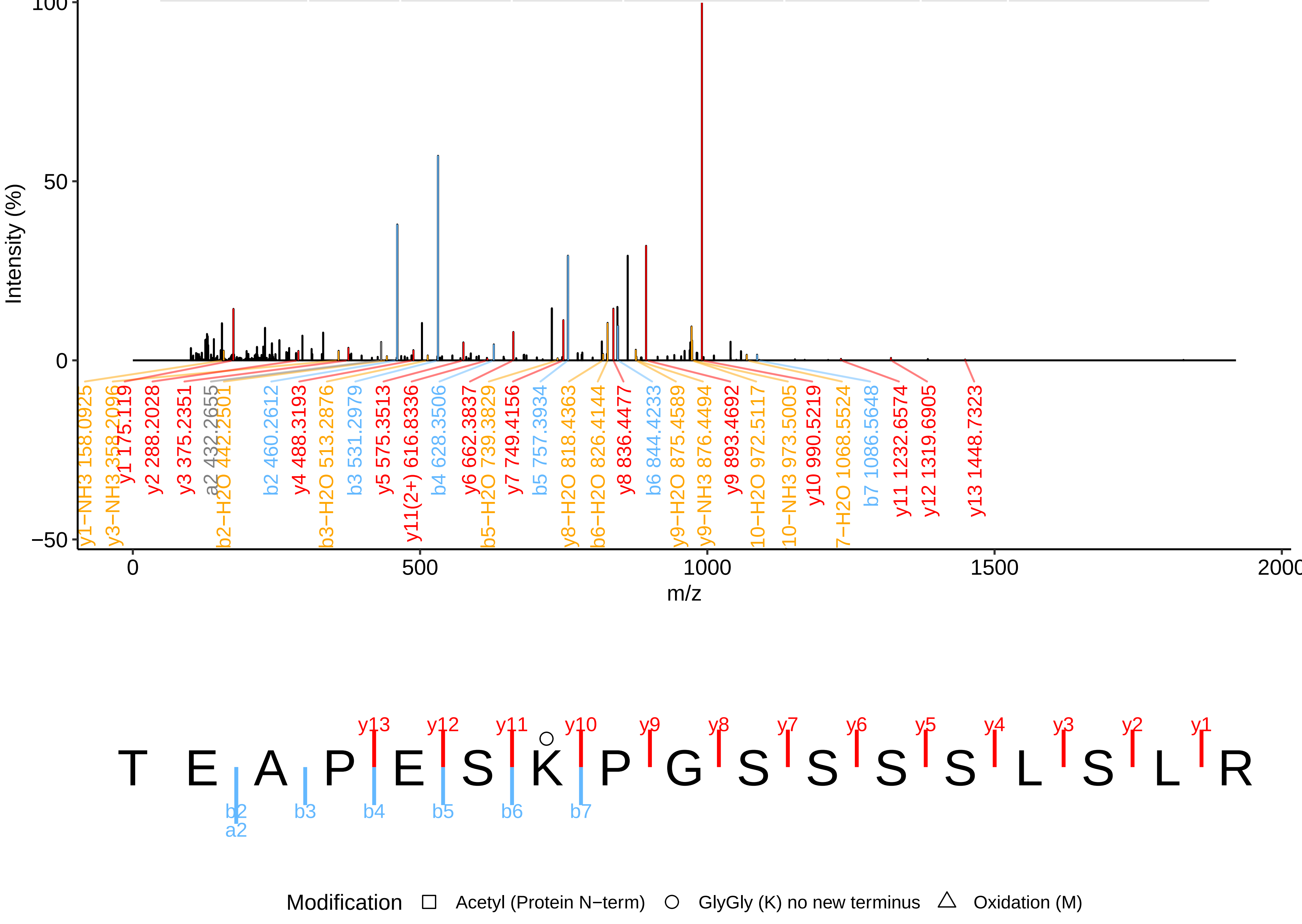

20200924_KIZ_MUb5_GlyGly_2 − Scan number: 7486

Gene Names	Charge	m/z	Mass	Retention time	PEP	Score	Precursor Intensity
CLN3	3	616.3095	1845.907	27.946	8.2465e−08	103.04	1480243

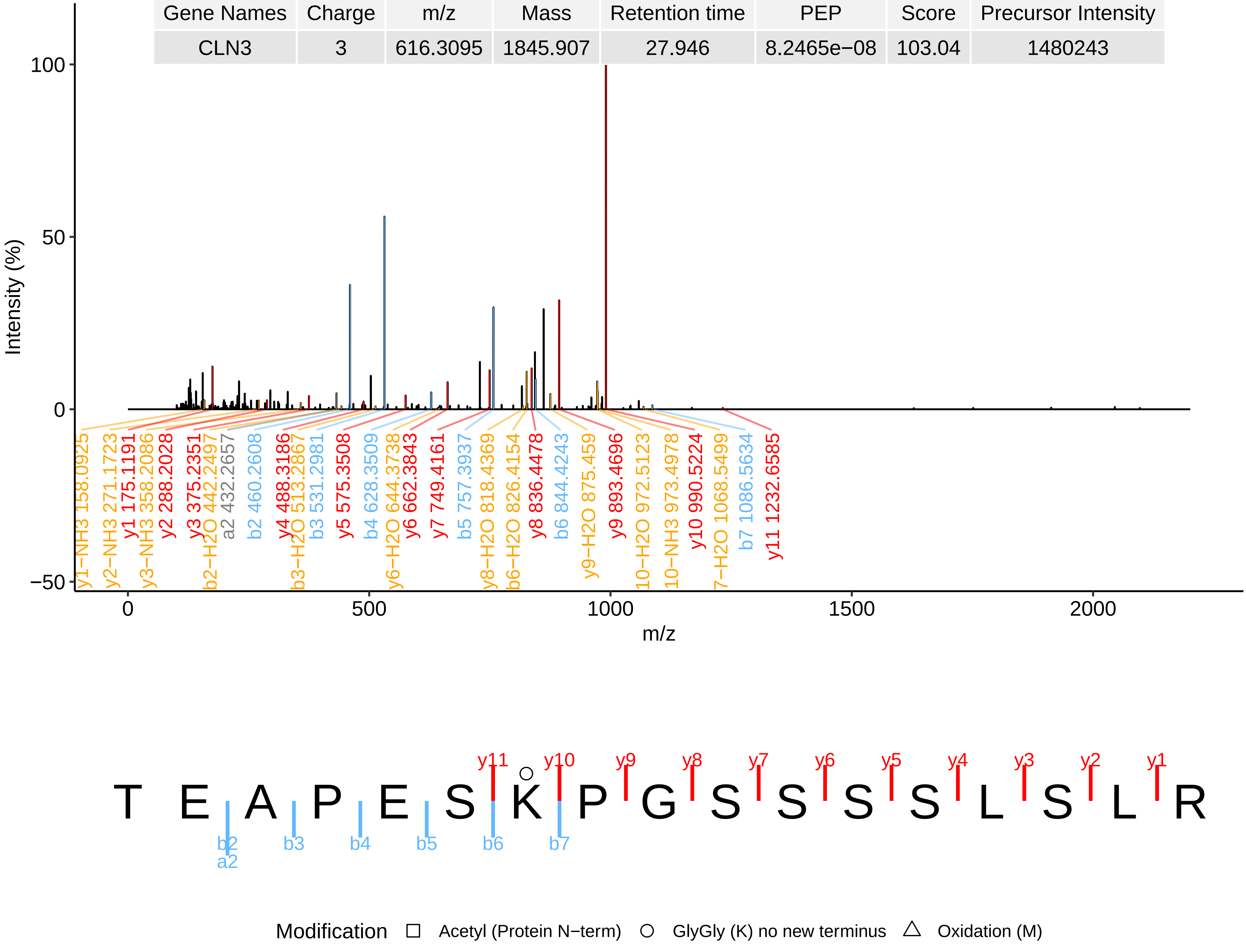

20201118_KIZ_MUb6_GlyGly_2 − Scan number: 9128

Gene Names	Charge	m/z	Mass	Retention time	PEP	Score	Precursor Intensity
CLN3	3	616.3095	1845.907	30.935	4.6405e−11	114.63	5498552

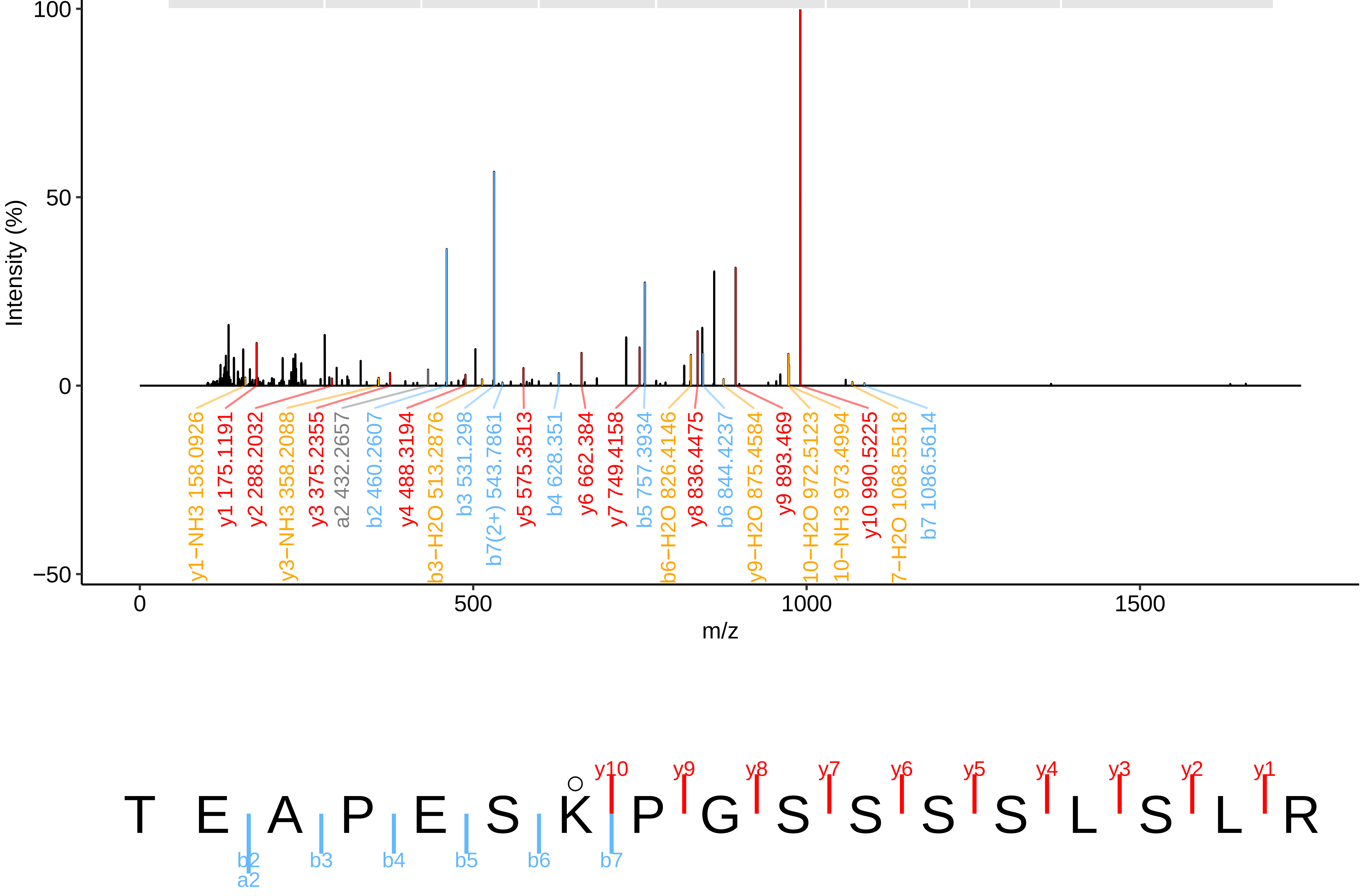

Modification □ Acetyl (Protein N−term) ○ GlyGly (K) no new terminus △ Oxidation (M)

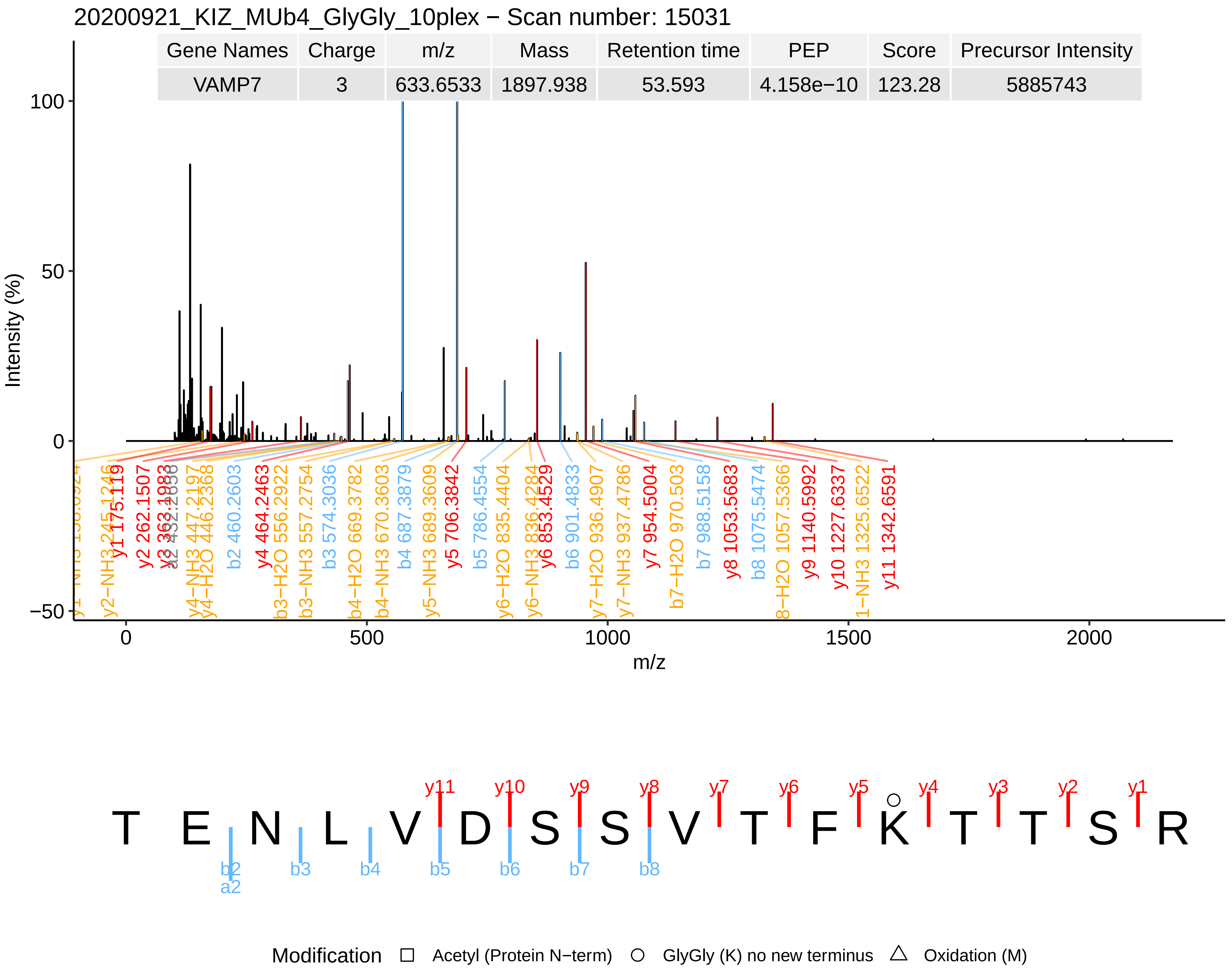
20200921_KIZ_MUb4_GlyGly_10plex − Scan number: 15031
Gene Names | Charge | m/z | Mass | Retention time | PEP | Score | Precursor Intensity
VAMP7 | 3 | 633.6533 | 1897.938 | 53.593 | 4.158e−10 | 123.28 | 5885743
Intensity (%)
m/z
y2 262.1507
b2 460.2603
y4 464.2463
b3−H2O 556.2922
b3−NH3 557.2754
b3 574.3036
b4−H2O 669.3782
b4−NH3 670.3603
b4 687.3879
y5−NH3 689.3609
y5 706.3842
b5 786.4554
y6−H2O 835.4404
y6 853.4529
b6 901.4833
y7−H2O 936.4907
y7−NH3 937.4786
y7 954.5004
b7−H2O 970.503
b7 988.5158
y8 1053.5683
b8 1075.5474
y9 1140.5992
y10 1227.6337
y11 1342.6591
T E N L V D S S V T F K T T S R
Modification
Acetyl (Protein N−term)
GlyGly (K) no new terminus
Oxidation (M)

20200921_KIZ_MUb4_GlyGly_10plex_2 − Scan number: 13857

Gene Names	Charge	m/z	Mass	Retention time	PEP	Score	Precursor Intensity
VAMP7	3	633.6533	1897.938	53.896	1.2092e−05	86.488	1070953

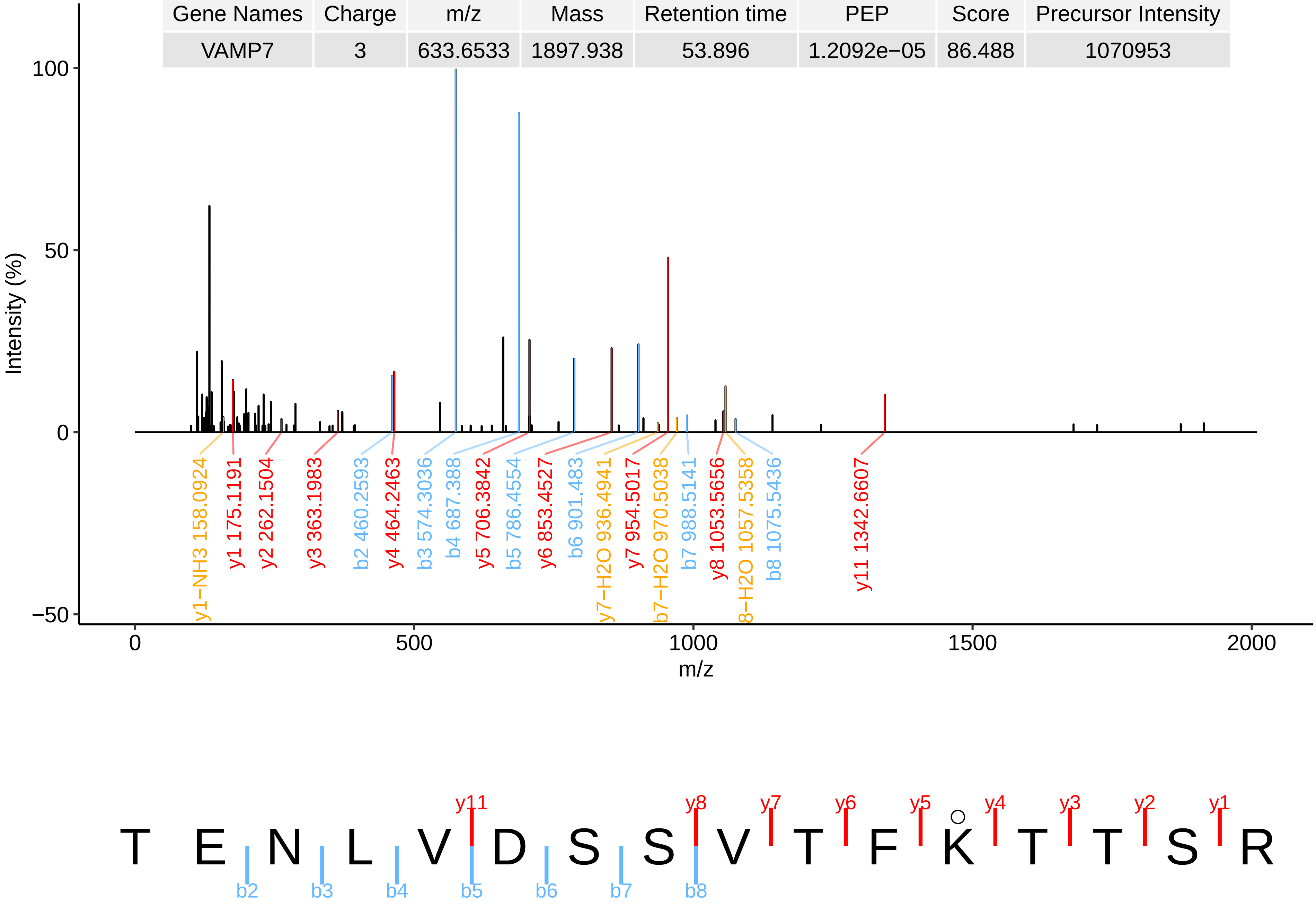

Modification □ Acetyl (Protein N−term) ○ GlyGly (K) no new terminus △ Oxidation (M)

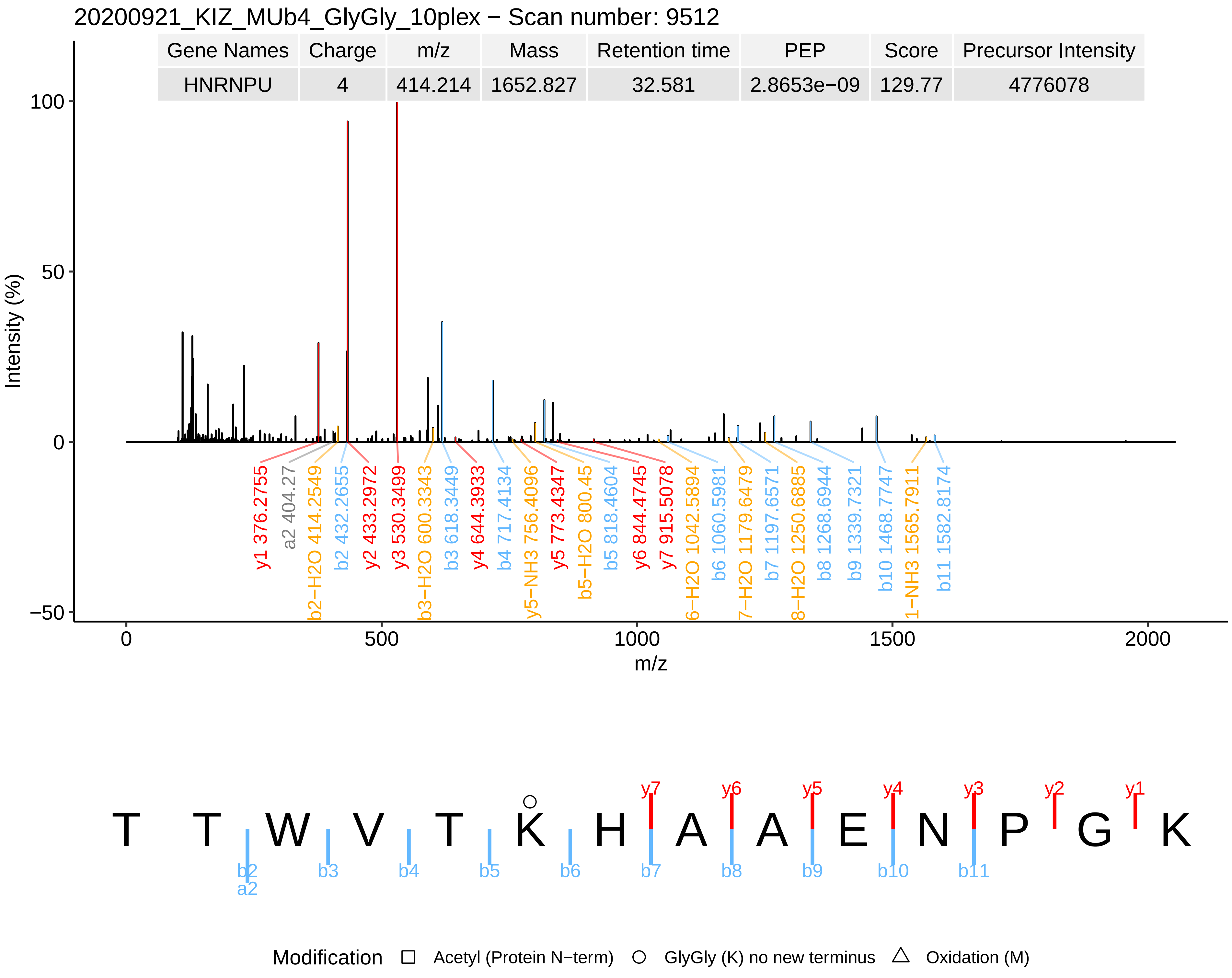
20200921_KIZ_MUb4_GlyGly_10plex − Scan number: 9512
Gene Names | Charge | m/z | Mass | Retention time | PEP | Score | Precursor Intensity
HNRNPU | 4 | 414.214 | 1652.827 | 32.581 | 2.8653e−09 | 129.77 | 4776078
Intensity (%)
100
50
0
−50
m/z
0
500
1000
1500
2000
y1 376.2755
a2 404.27
b2−H2O 414.2549
b2 432.2655
y2 433.2972
y3 530.3499
b3−H2O 600.3343
b3 618.3449
y4 644.3933
b4 717.4134
y5−NH3 756.4096
y5 773.4347
b5−H2O 800.45
b5 818.4604
y6 844.4745
y7 915.5078
6−H2O 1042.5894
b6 1060.5981
7−H2O 1179.6479
b7 1197.6571
8−H2O 1250.6885
b8 1268.6944
b9 1339.7321
b10 1468.7747
1−NH3 1565.7911
b11 1582.8174
T T W V T K H A A E N P G K
y7 y6 y5 y4 y3 y2 y1
b2 a2 b3 b4 b5 b6 b7 b8 b9 b10 b11
Modification
Acetyl (Protein N−term)
GlyGly (K) no new terminus
Oxidation (M)

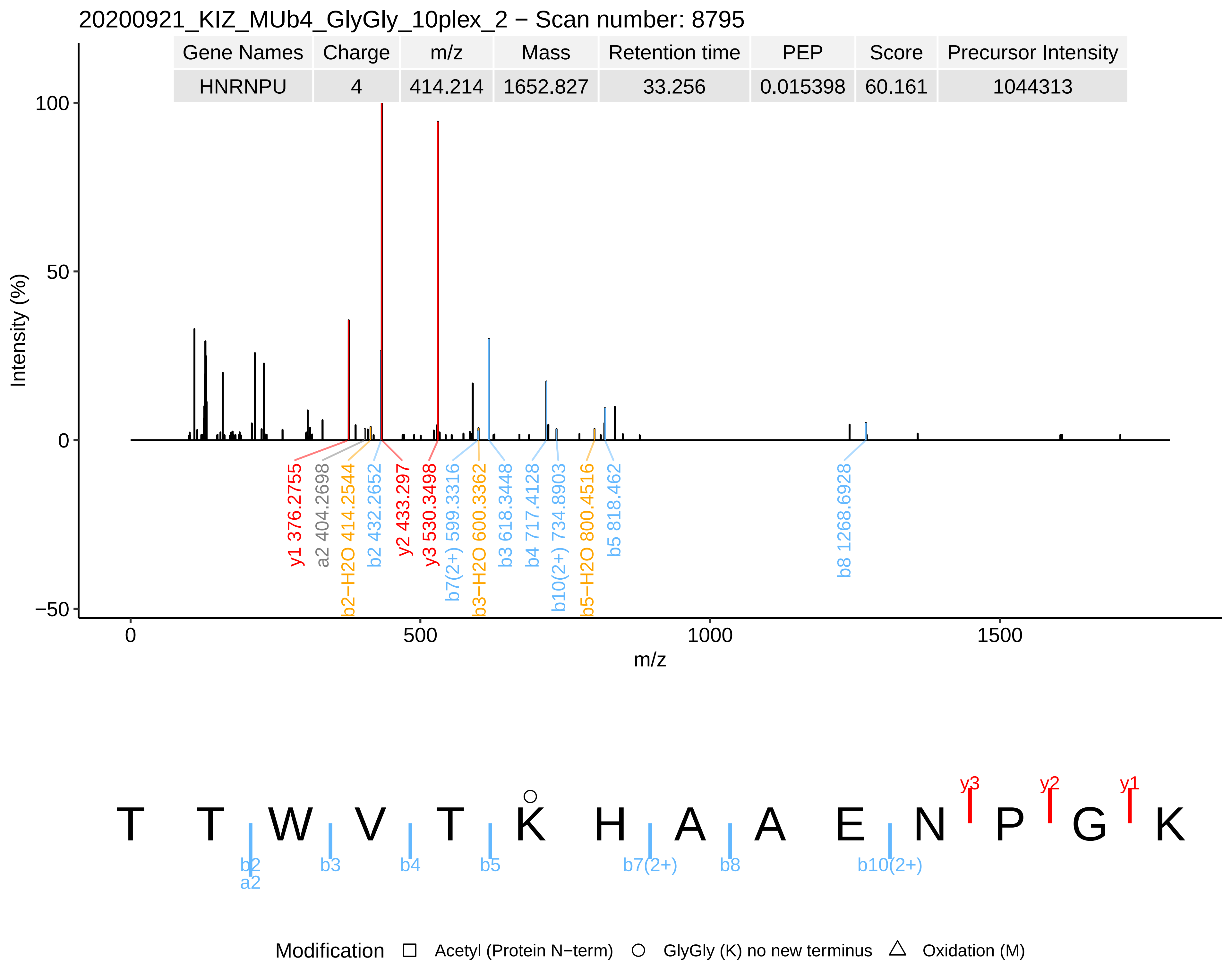
20200921_KIZ_MUb4_GlyGly_10plex_2 − Scan number: 8795
Gene Names | Charge | m/z | Mass | Retention time | PEP | Score | Precursor Intensity
HNRNPU | 4 | 414.214 | 1652.827 | 33.256 | 0.015398 | 60.161 | 1044313
Intensity (%)
m/z
y1 376.2755
a2 404.2698
b2−H2O 414.2544
b2 432.2652
y2 433.297
y3 530.3498
b7(2+) 599.3316
b3−H2O 600.3362
b3 618.3448
b4 717.4128
b10(2+) 734.8903
b5−H2O 800.4516
b5 818.462
b8 1268.6928
T T W V T K H A A E N P G K
y3 y2 y1
b2 a2 b3 b4 b5 b7(2+) b8 b10(2+)
Modification
Acetyl (Protein N−term)
GlyGly (K) no new terminus
Oxidation (M)

20200921_KIZ_MUb4_GlyGly_10plex_2 − Scan number: 17042

Gene Names	Charge	m/z	Mass	Retention time	PEP	Score	Precursor Intensity
CALM2;CALM1	3	623.6428	1867.906	66.588	0.0010345	74.786	1362578

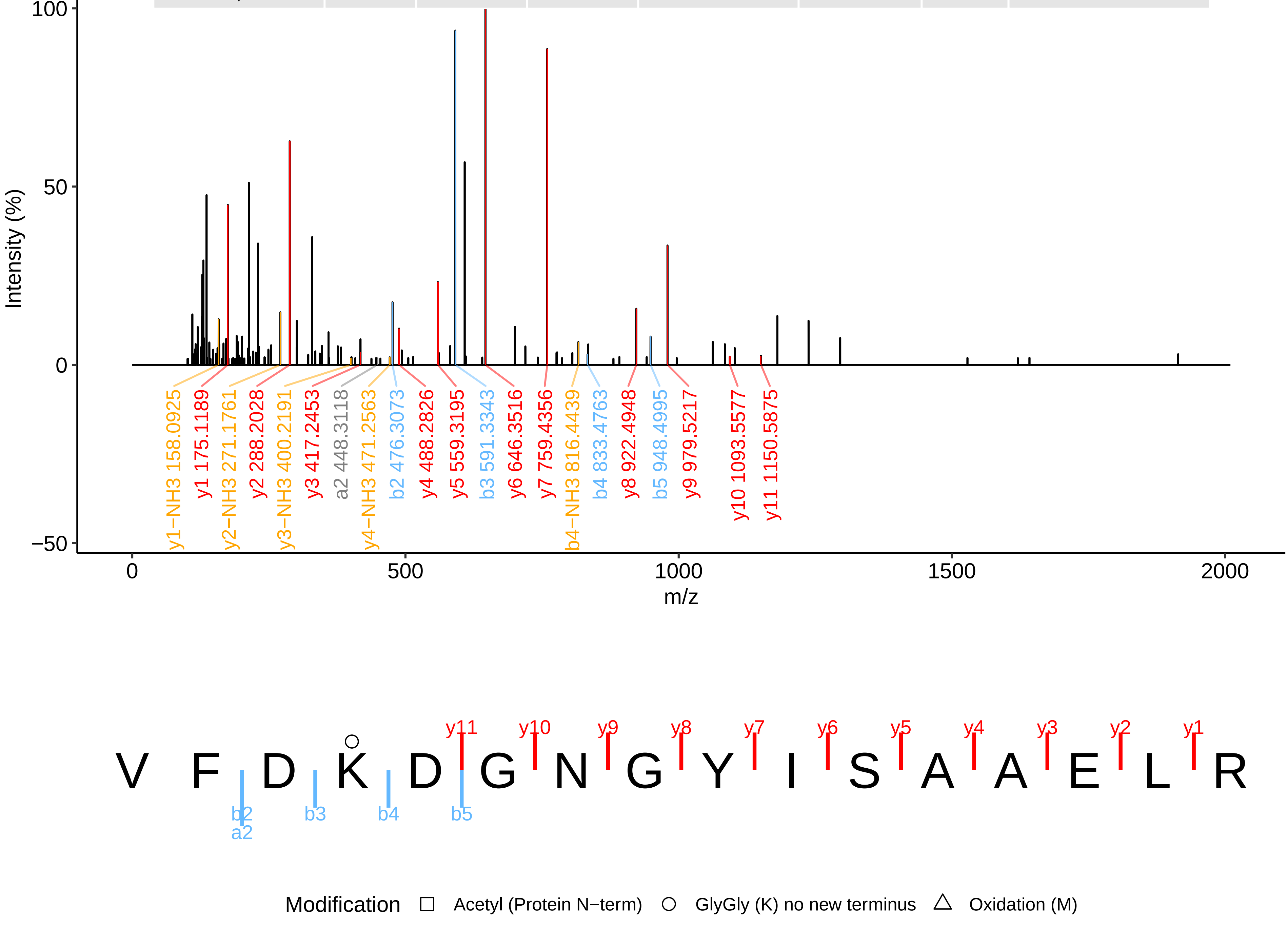

20200921_KIZ_MUb4_GlyGly_10plex_2 − Scan number: 16559

Gene Names	Charge	m/z	Mass	Retention time	PEP	Score	Precursor Intensity
MGST3	3	627.6441	1879.91	64.655	0.006825	76.588	1009528

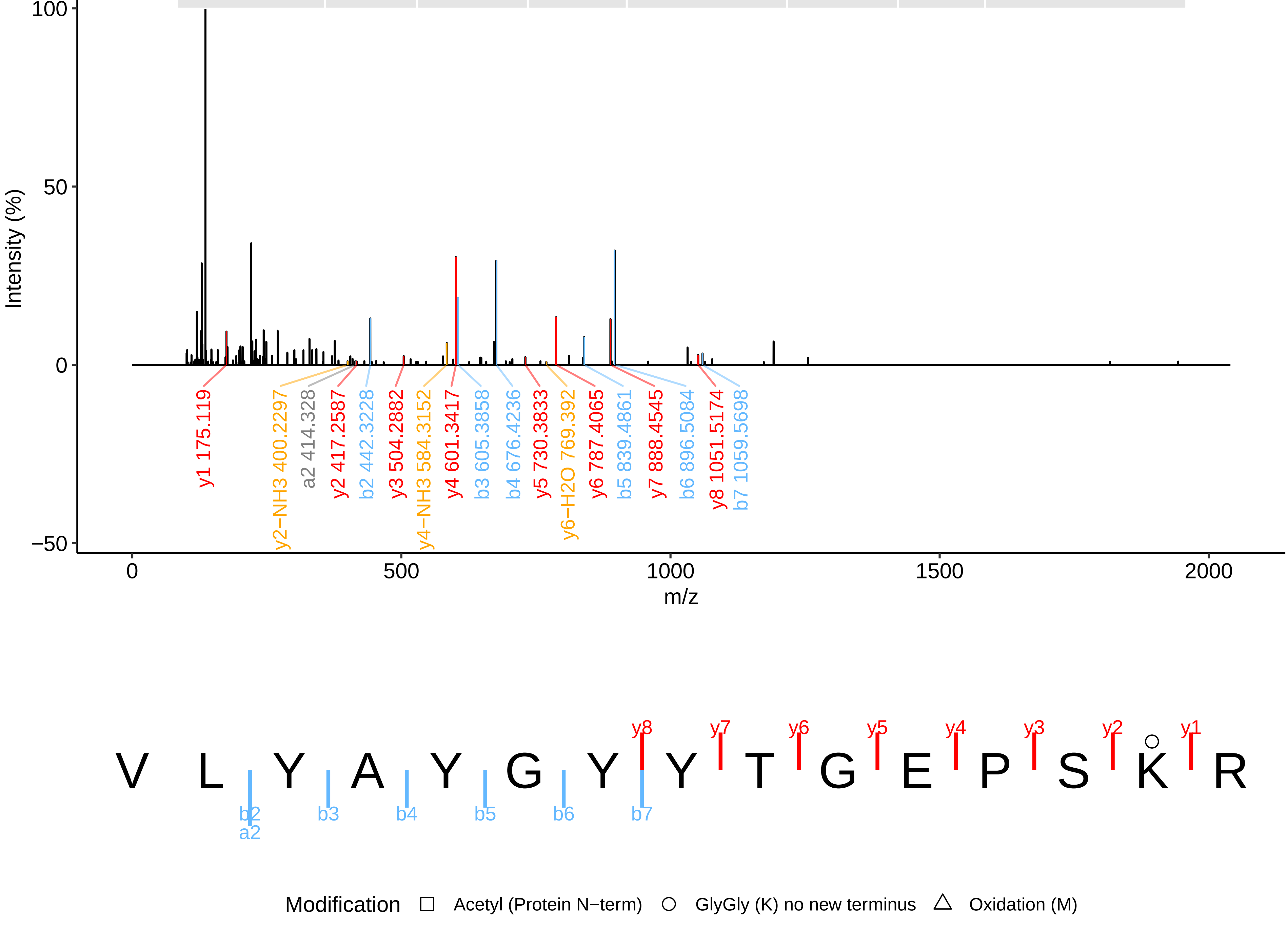

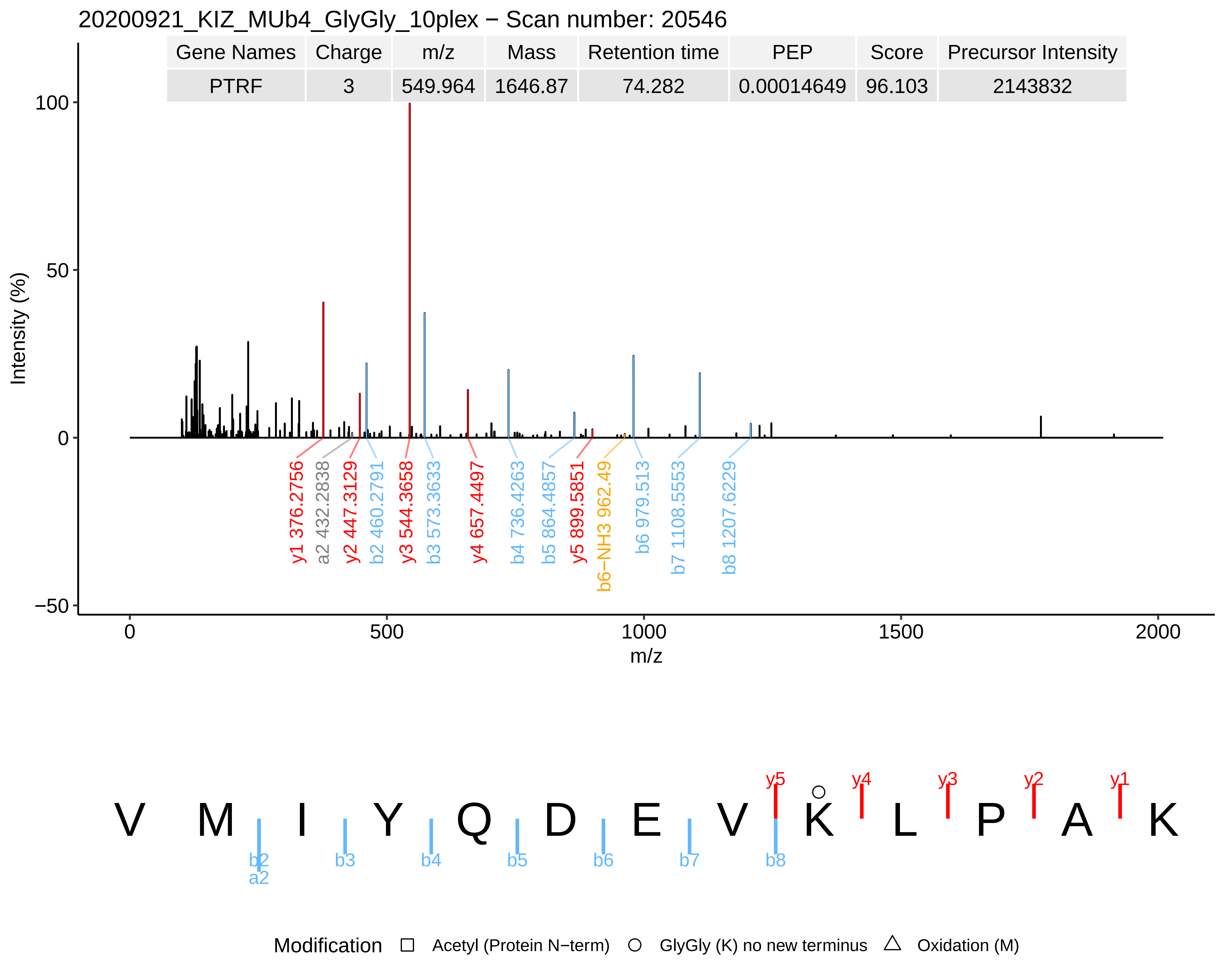
20200921_KIZ_MUb4_GlyGly_10plex − Scan number: 20546
Gene Names | Charge | m/z | Mass | Retention time | PEP | Score | Precursor Intensity
PTRF | 3 | 549.964 | 1646.87 | 74.282 | 0.00014649 | 96.103 | 2143832
Intensity (%)
100
50
0
−50
0
500
1000
1500
2000
m/z
y1 376.2756
a2 432.2838
y2 447.3129
b2 460.2791
y3 544.3658
b3 573.3633
y4 657.4497
b4 736.4263
b5 864.4857
y5 899.5851
b6−NH3 962.49
b6 979.513
b7 1108.5553
b8 1207.6229
V M I Y Q D E V K L P A K
y5 y4 y3 y2 y1
b2 a2 b3 b4 b5 b6 b7 b8
Modification
Acetyl (Protein N−term)
GlyGly (K) no new terminus
Oxidation (M)

20200921_KIZ_MUb4_GlyGly_10plex − Scan number: 13943

Gene Names	Charge	m/z	Mass	Retention time	PEP	Score	Precursor Intensit
NME1−NME2;NME2;NME2P1;NME1	3	633.9913	1898.952	49.37	1.1735e−12	118.21	3121928

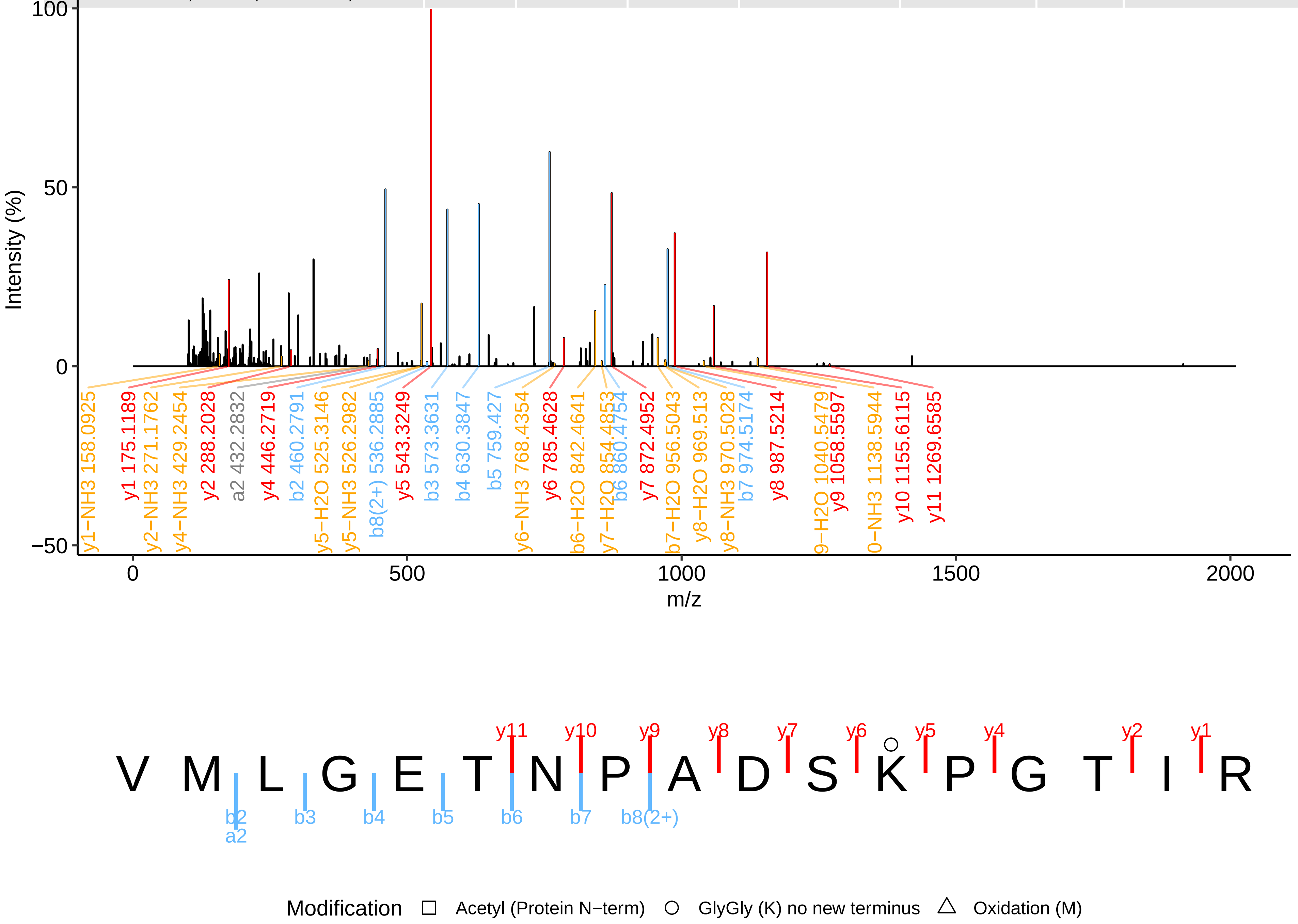

20200921_KIZ_MUb4_GlyGly_10plex_2 − Scan number: 12800

Gene Names	Charge	m/z	Mass	Retention time	PEP	Score	Precursor Intensity
NME1−NME2;NME2;NME2P1;NME1	3	633.9913	1898.952	49.603	6.914e−10	103.39	NA

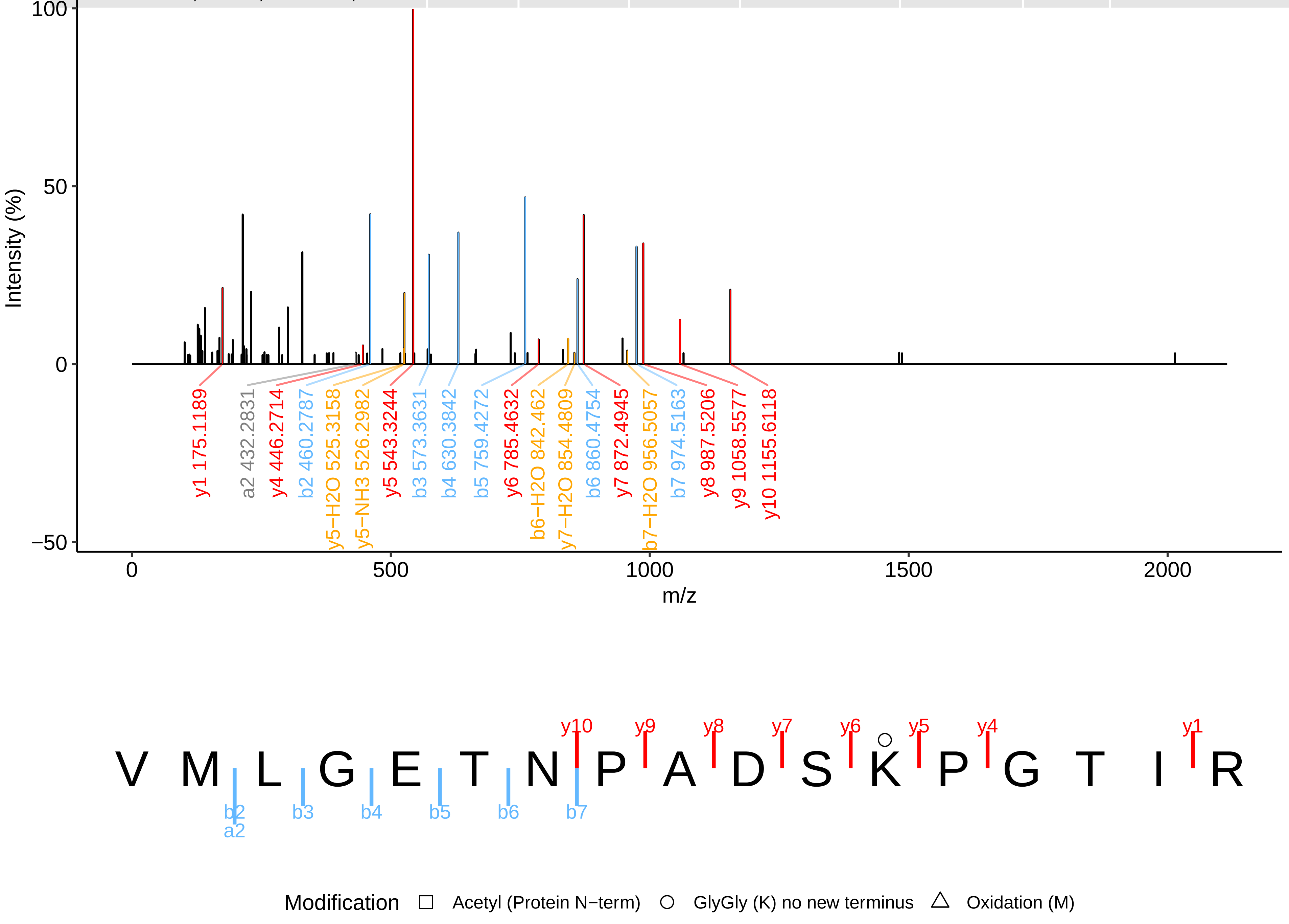

20200921_KIZ_MUb4_GlyGly_10plex_2 − Scan number: 12809

Gene Names	Charge	m/z	Mass	Retention time	PEP	Score	Precursor Intensity
NME1−NME2;NME2;NME2P1;NME1	3	633.9913	1898.952	49.641	0.0021106	66.673	1439238

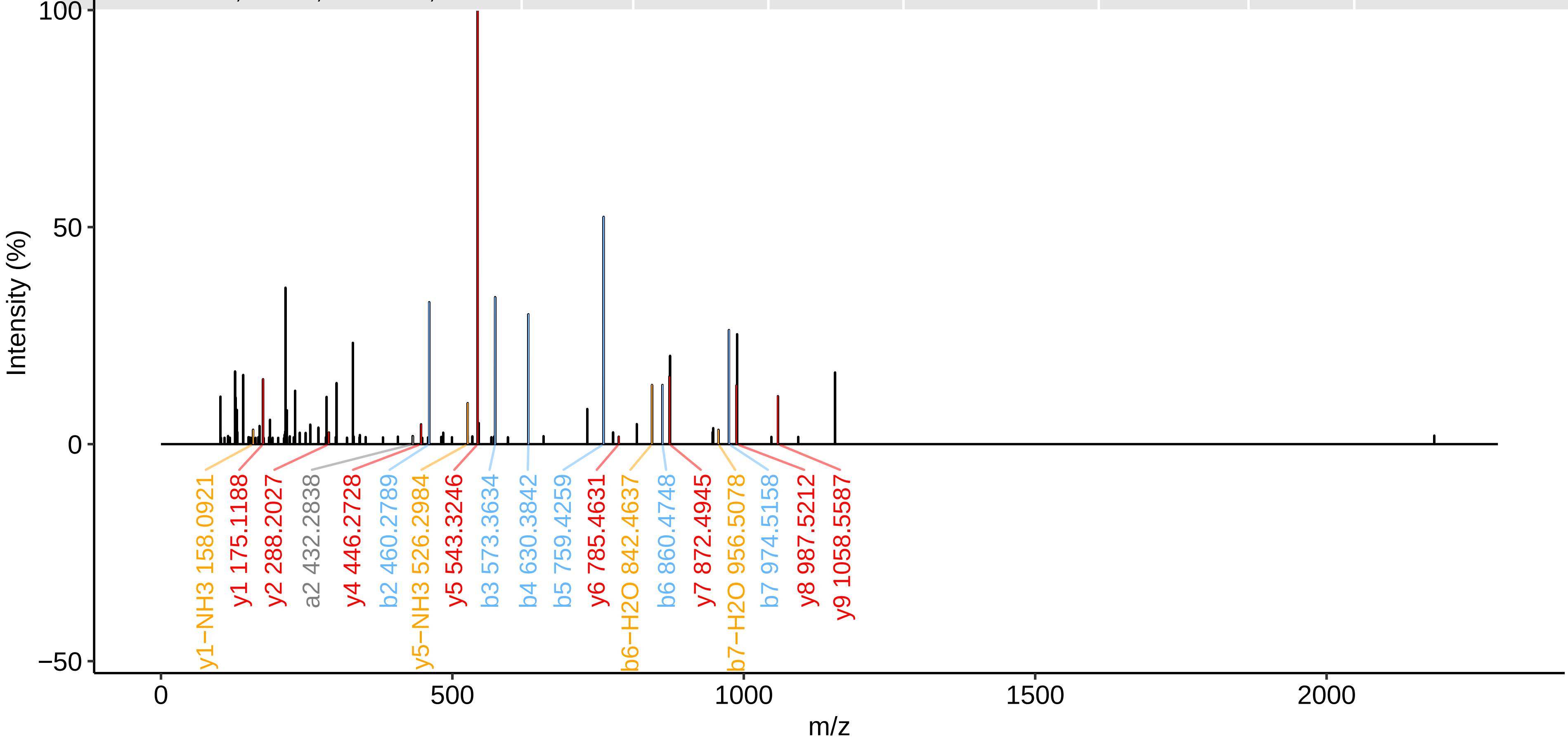

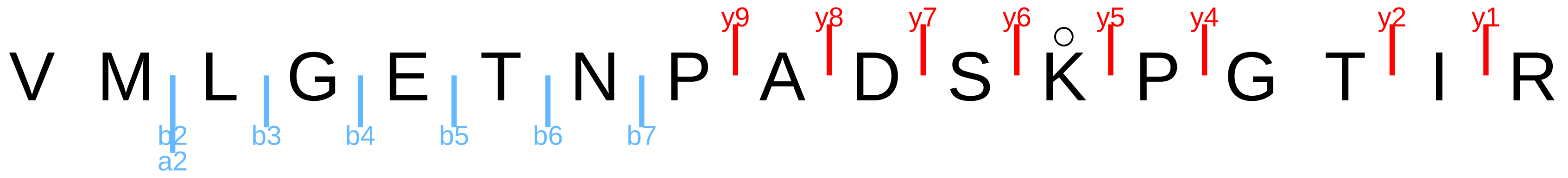

Modification □ Acetyl (Protein N−term) ○ GlyGly (K) no new terminus △ Oxidation (M)

20200924_KIZ_MUb5_GlyGly_1 − Scan number: 13007

Gene Names	Charge	m/z	Mass	Retention time	PEP	Score	Precursor Intensit
NME1−NME2;NME2;NME2P1;NME1	3	633.9913	1898.952	42.414	5.2103e−10	106.36	19007686

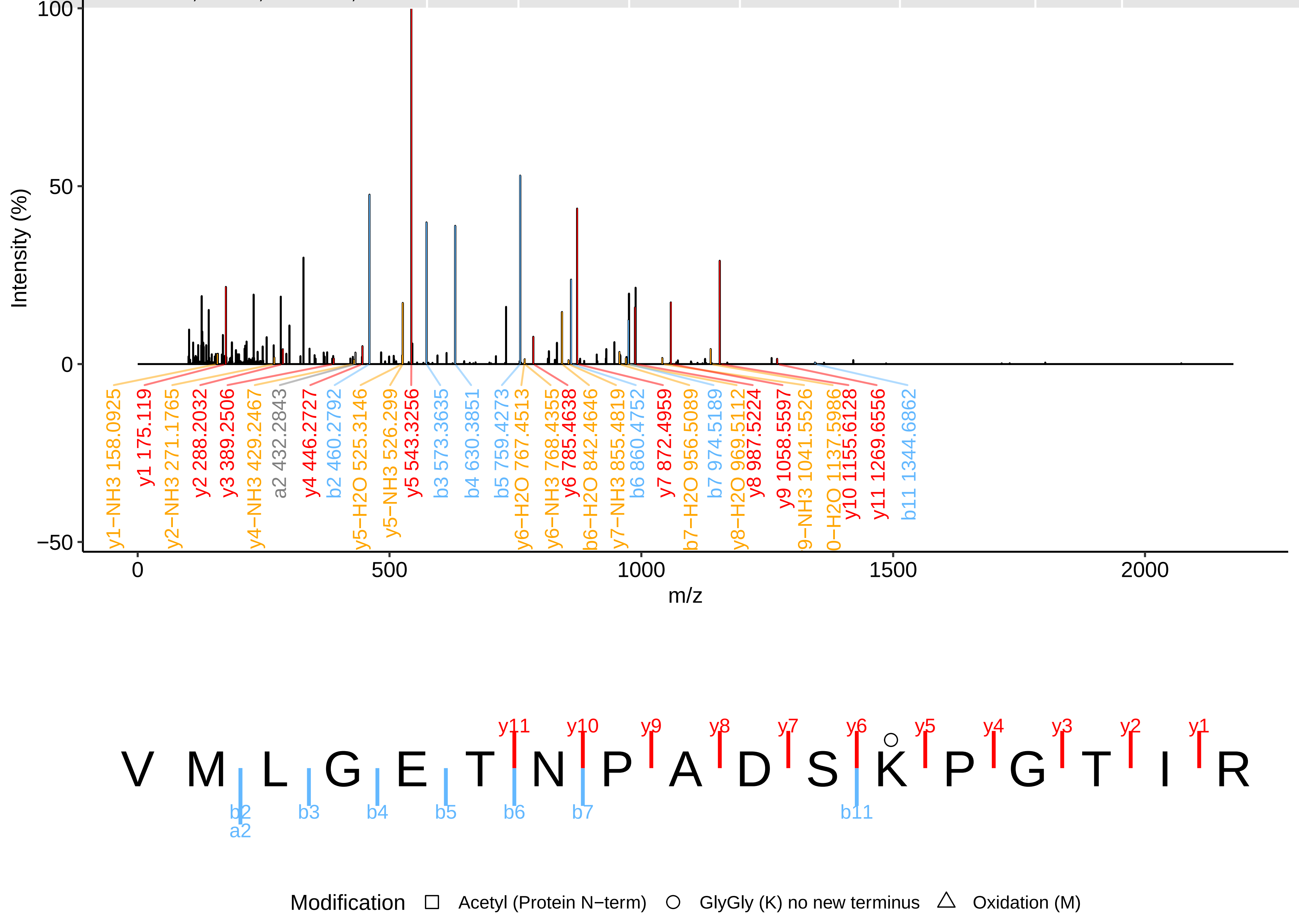

20200924_KIZ_MUb5_GlyGly_1 − Scan number: 13164

Gene Names	Charge	m/z	Mass	Retention time	PEP	Score	Precursor Intensit
NME1−NME2;NME2;NME2P1;NME1	3	633.9913	1898.952	42.919	4.3601e−17	143.14	22378646

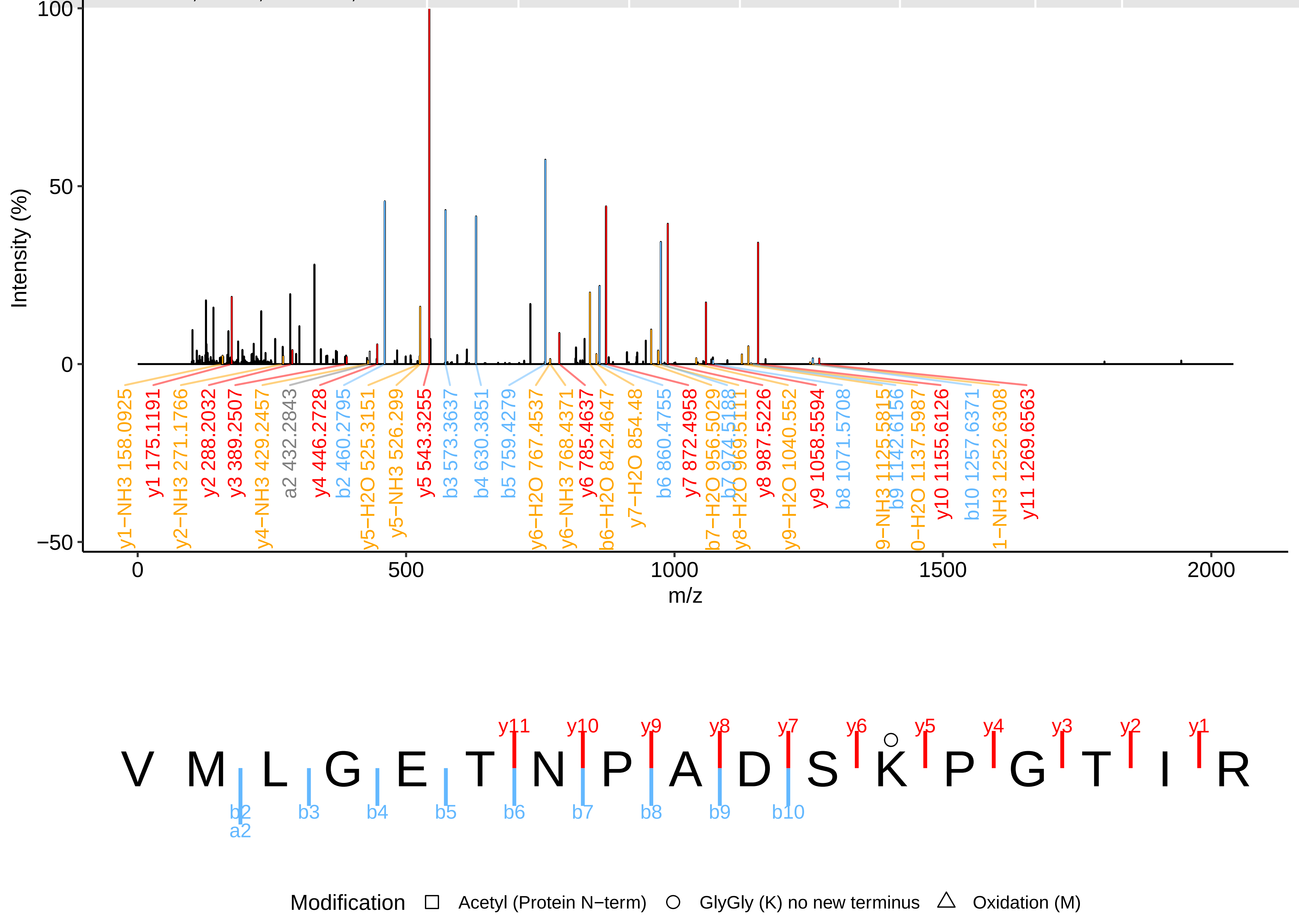

20200924_KIZ_MUb5_GlyGly_1 − Scan number: 12972

Gene Names	Charge	m/z	Mass	Retention time	PEP	Score	Precursor Intensit
NME1−NME2;NME2;NME2P1;NME1	3	633.9913	1898.952	42.287	5.3786e−16	137.19	3886254

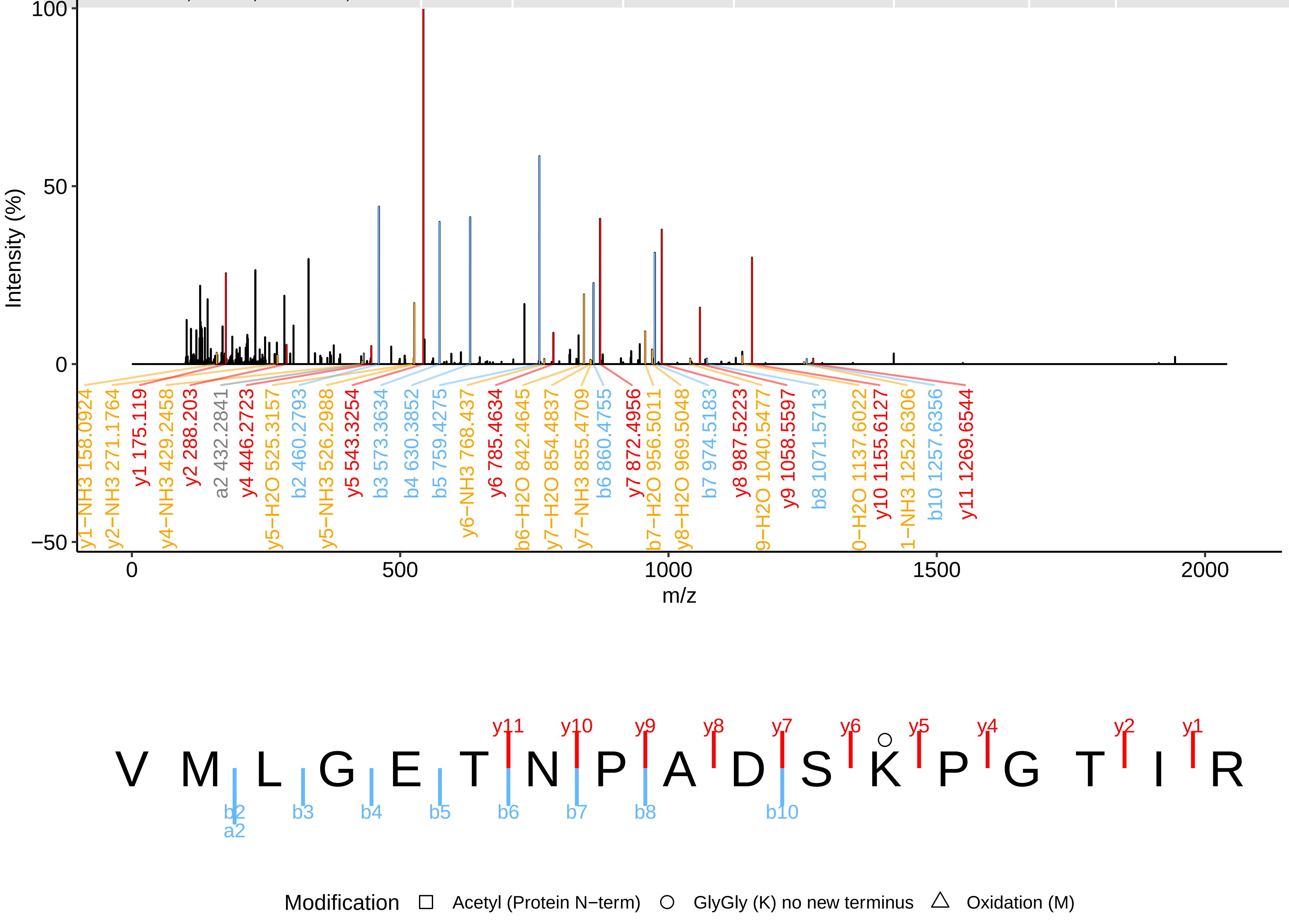

20200924_KIZ_MUb5_GlyGly_2 − Scan number: 12251

Gene Names	Charge	m/z	Mass	Retention time	PEP	Score	Precursor Intensity
NME1−NME2;NME2;NME2P1;NME1	3	633.9913	1898.952	45.797	5.701e−17	140.96	6738837

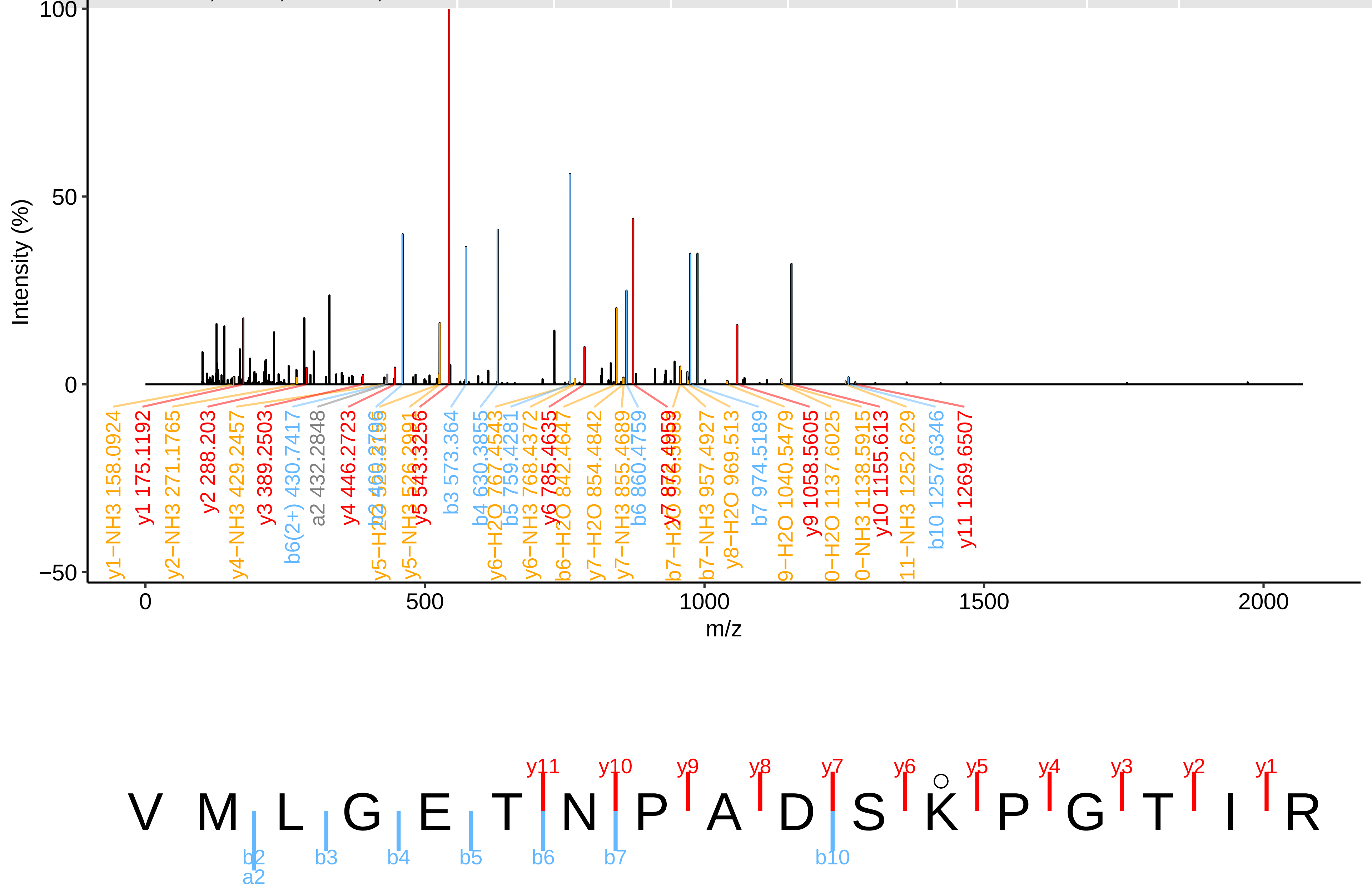

Modification □ Acetyl (Protein N−term) ○ GlyGly (K) no new terminus △ Oxidation (M)

20200924_KIZ_MUb5_GlyGly_2 − Scan number: 12114

Gene Names	Charge	m/z	Mass	Retention time	PEP	Score	Precursor Intensit
NME1−NME2;NME2;NME2P1;NME1	3	633.9913	1898.952	45.286	5.5211e−05	89.642	NA

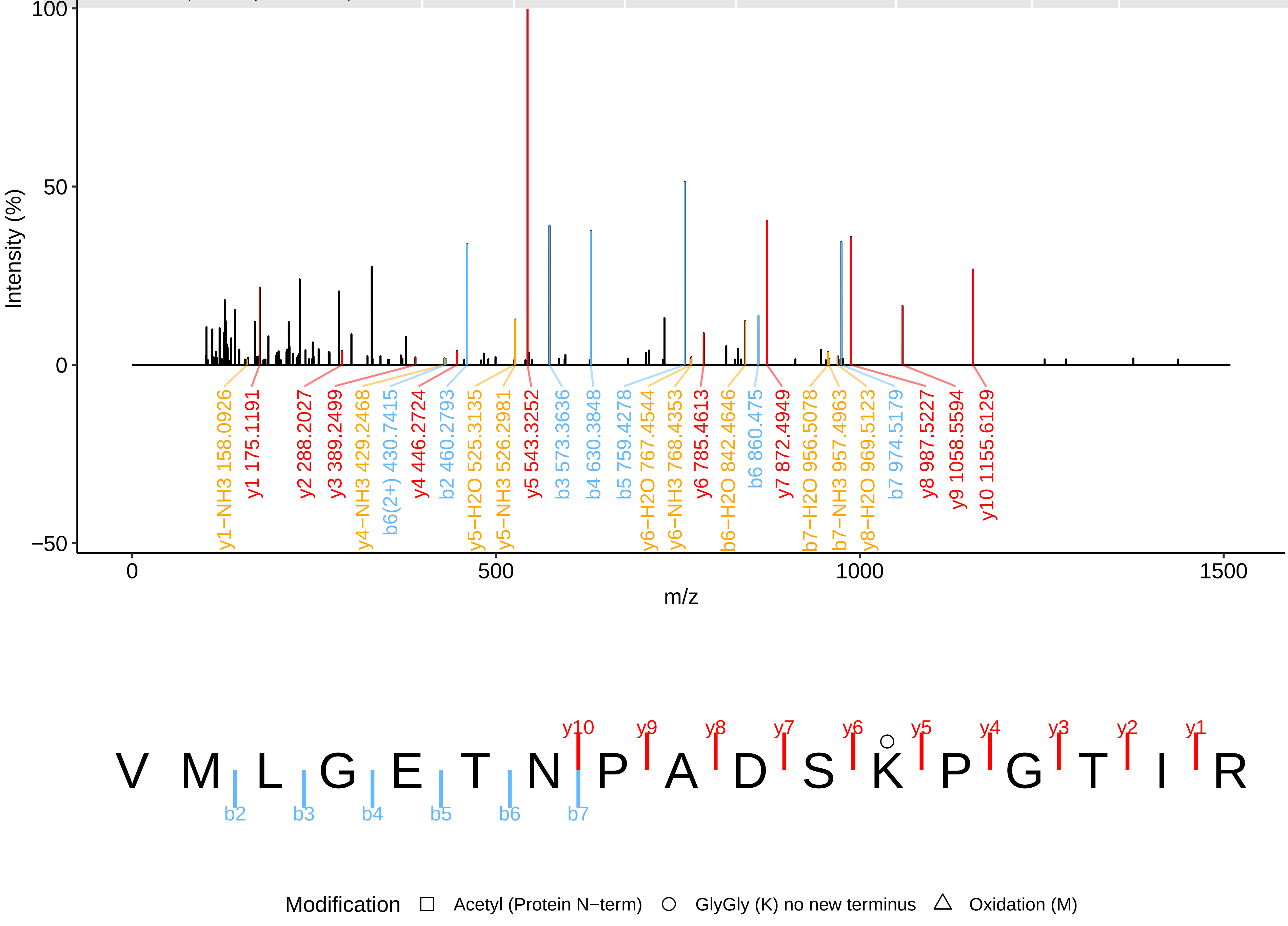

20200921_KIZ_MUb4_GlyGly_10plex_2 − Scan number: 11165

Gene Names	Charge	m/z	Mass	Retention time	PEP	Score	Precursor Intensity
NME1−NME2;NME2;NME2P1;NME1	3	639.3229	1914.947	42.907	0.0015733	67.605	1117806

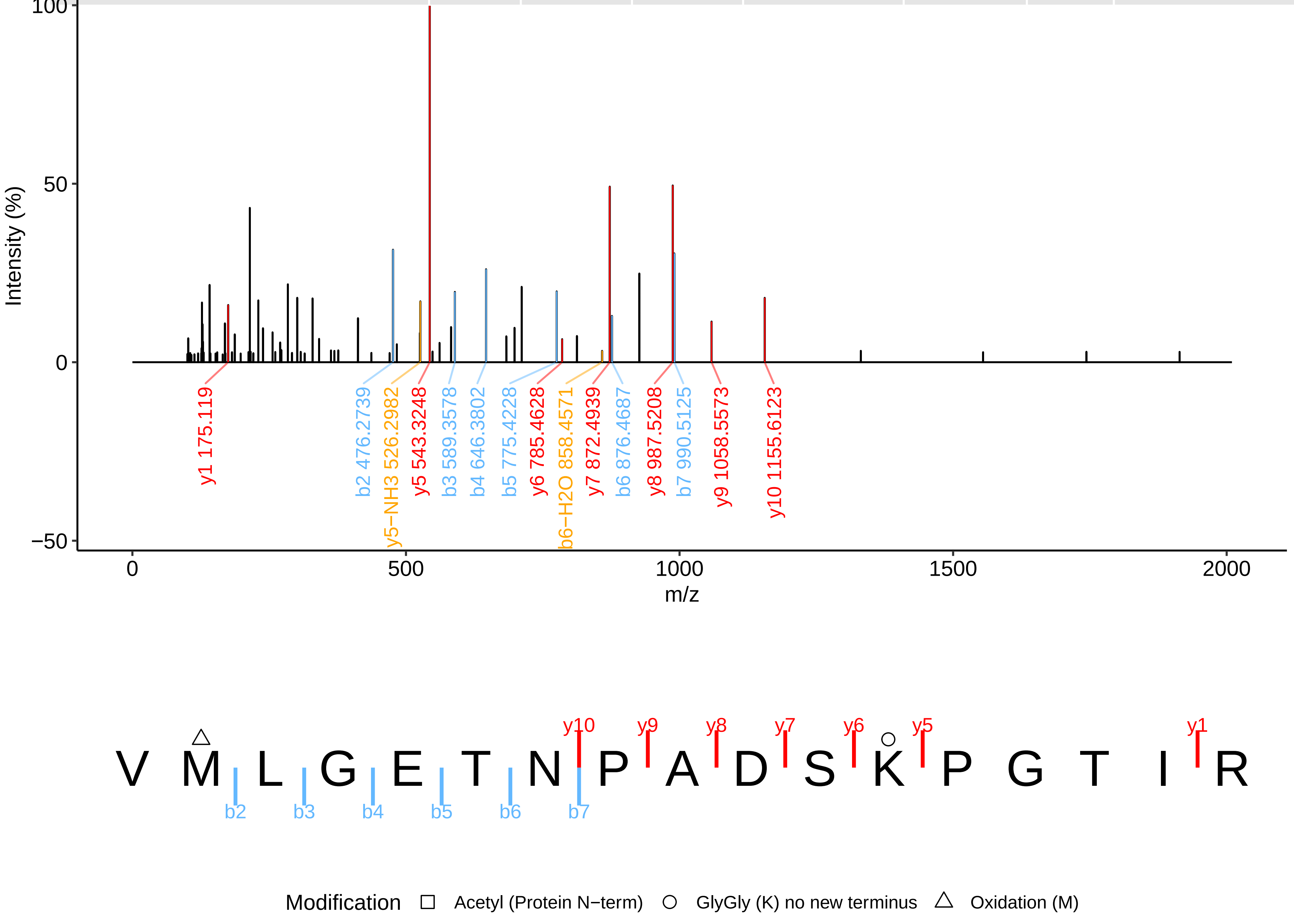

20200924_KIZ_MUb5_GlyGly_1 − Scan number: 11399

Gene Names	Charge	m/z	Mass	Retention time	PEP	Score	Precursor Intensit
NME1−NME2;NME2;NME2P1;NME1	3	639.3229	1914.947	36.926	5.9018e−08	108.34	3173178

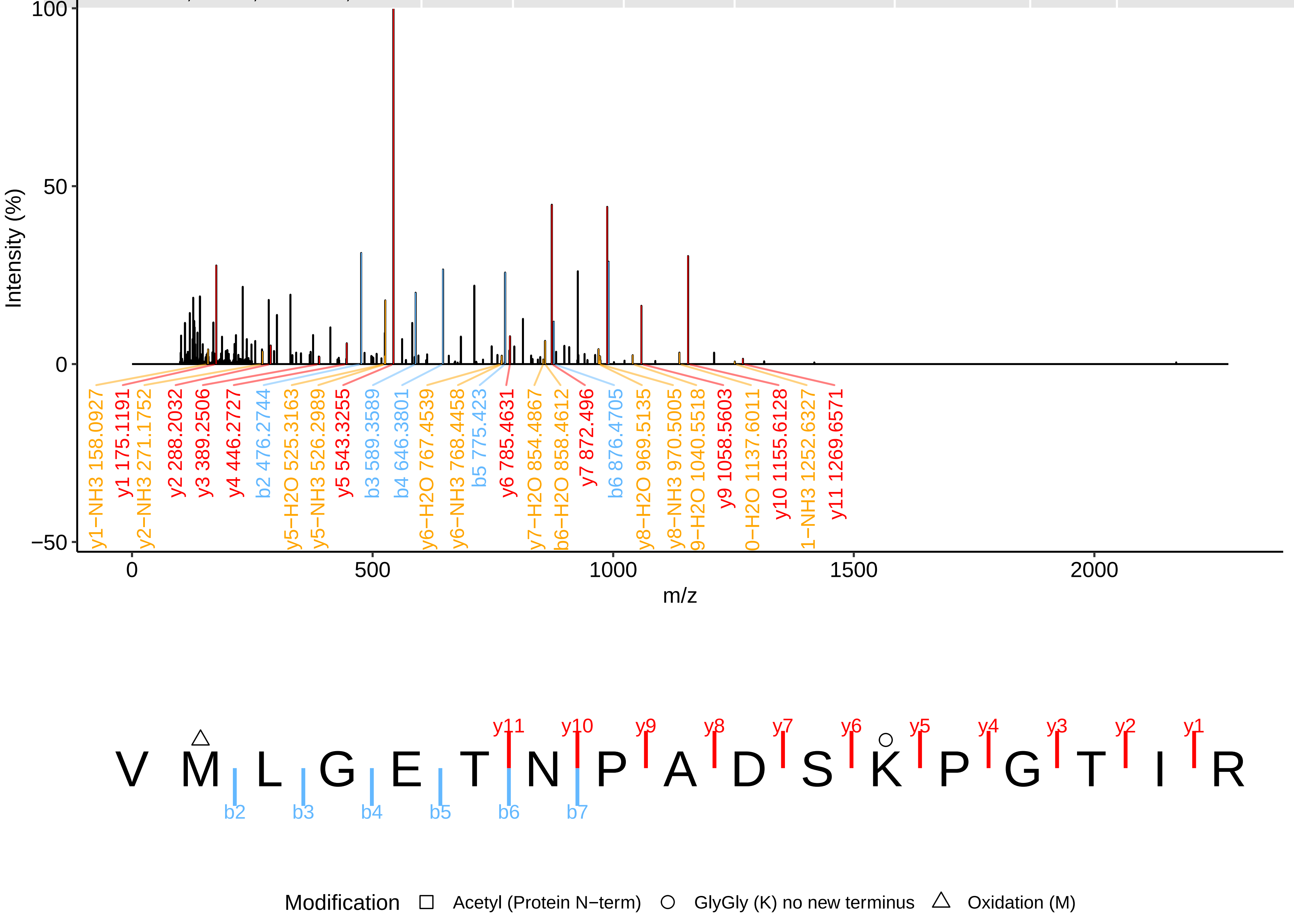

20200924_KIZ_MUb5_GlyGly_2 − Scan number: 10418

Gene Names	Charge	m/z	Mass	Retention time	PEP	Score	Precursor Intensit
NME1−NME2;NME2;NME2P1;NME1	3	639.3229	1914.947	38.913	1.2664e−11	118.21	4700501

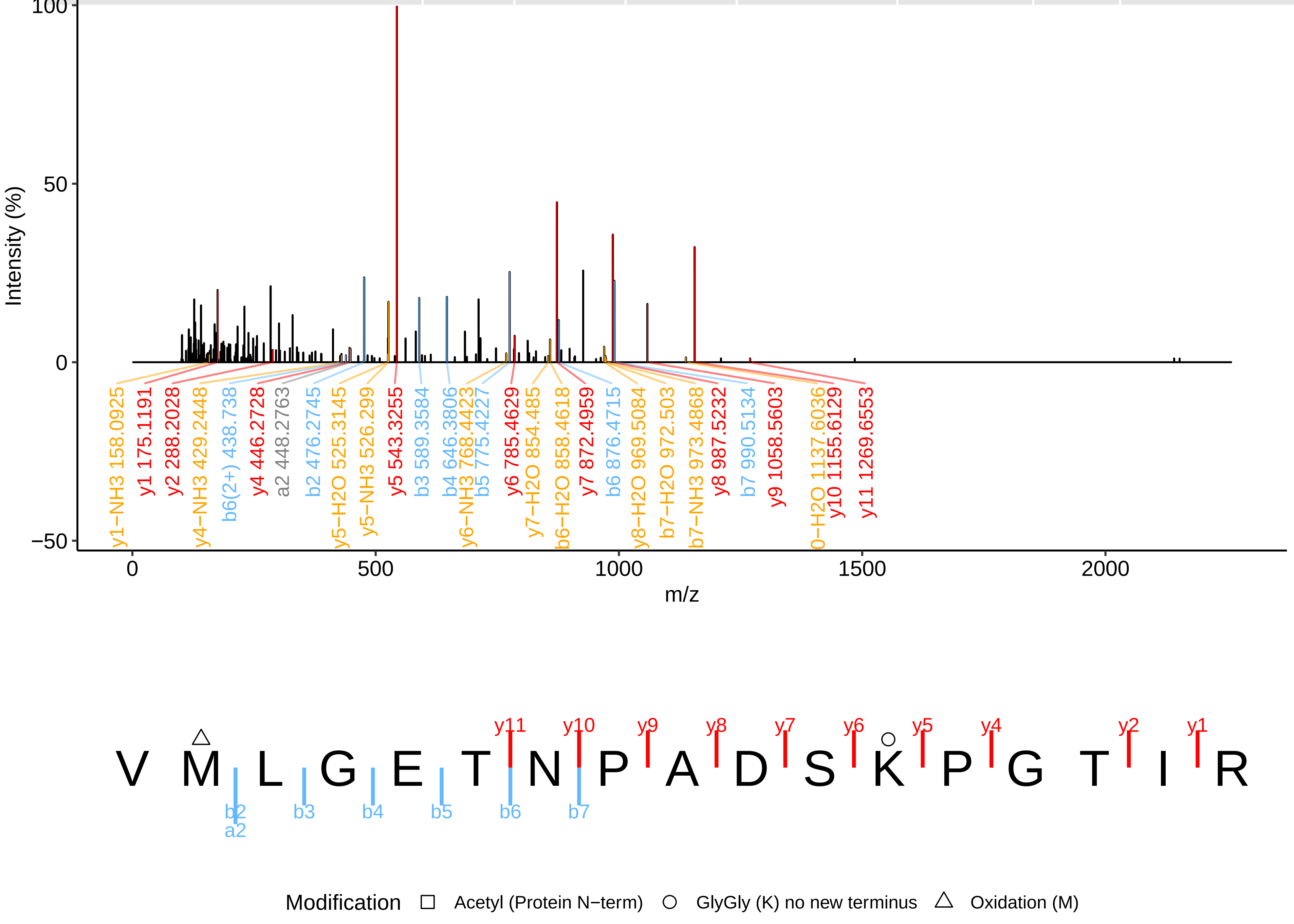

20200924_KIZ_MUb5_GlyGly_2 − Scan number: 10560

Gene Names	Charge	m/z	Mass	Retention time	PEP	Score	Precursor Intensit
NME1−NME2;NME2;NME2P1;NME1	3	639.3229	1914.947	39.419	5.6387e−08	108.63	2201813

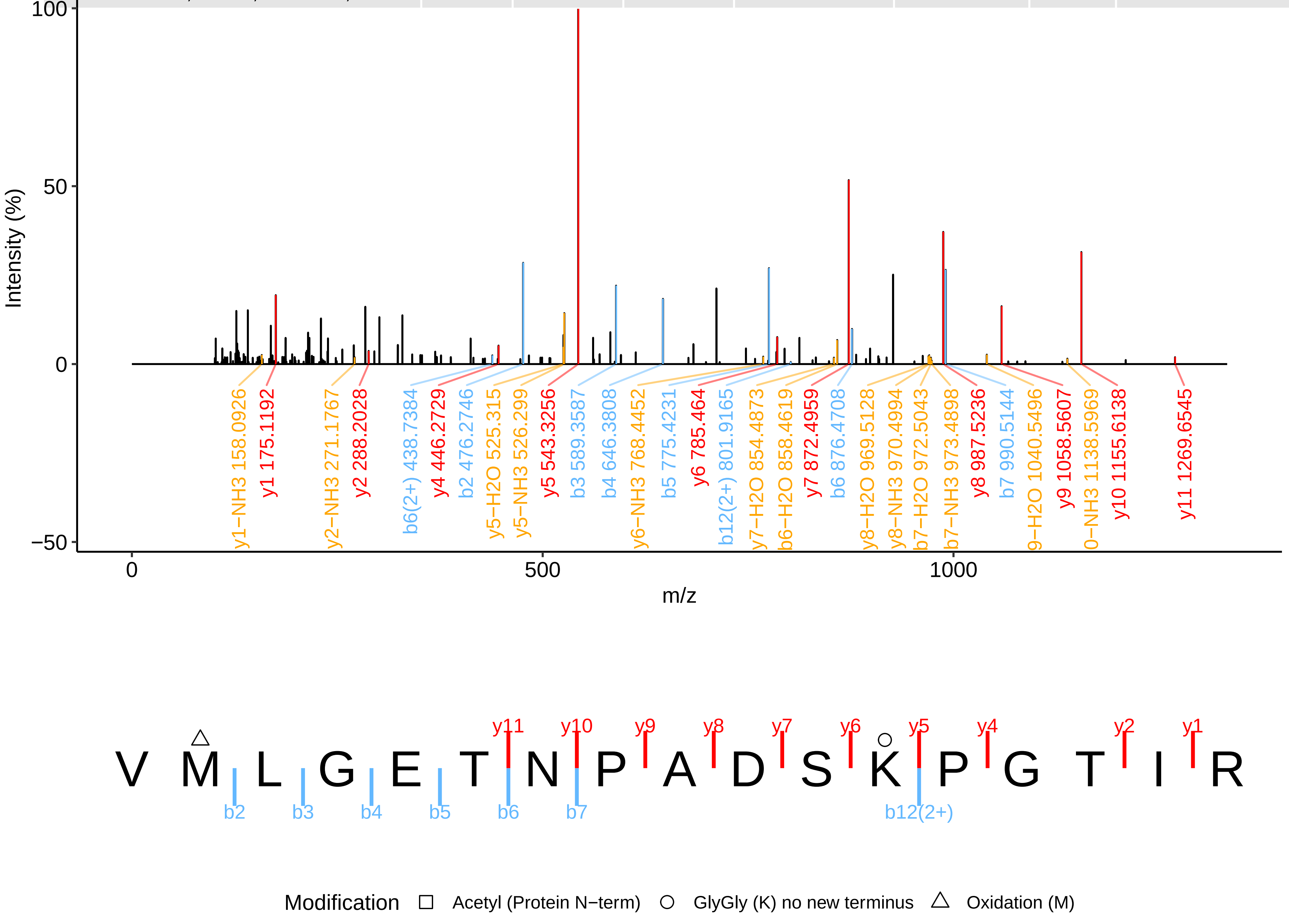

20200921_KIZ_MUb4_GlyGly_10plex − Scan number: 14739

Gene Names	Charge	m/z	Mass	Retention time	PEP	Score	Precursor Intensity
SRSF7	3	611.9866	1832.938	52.44	1.1061e−06	94.922	NA

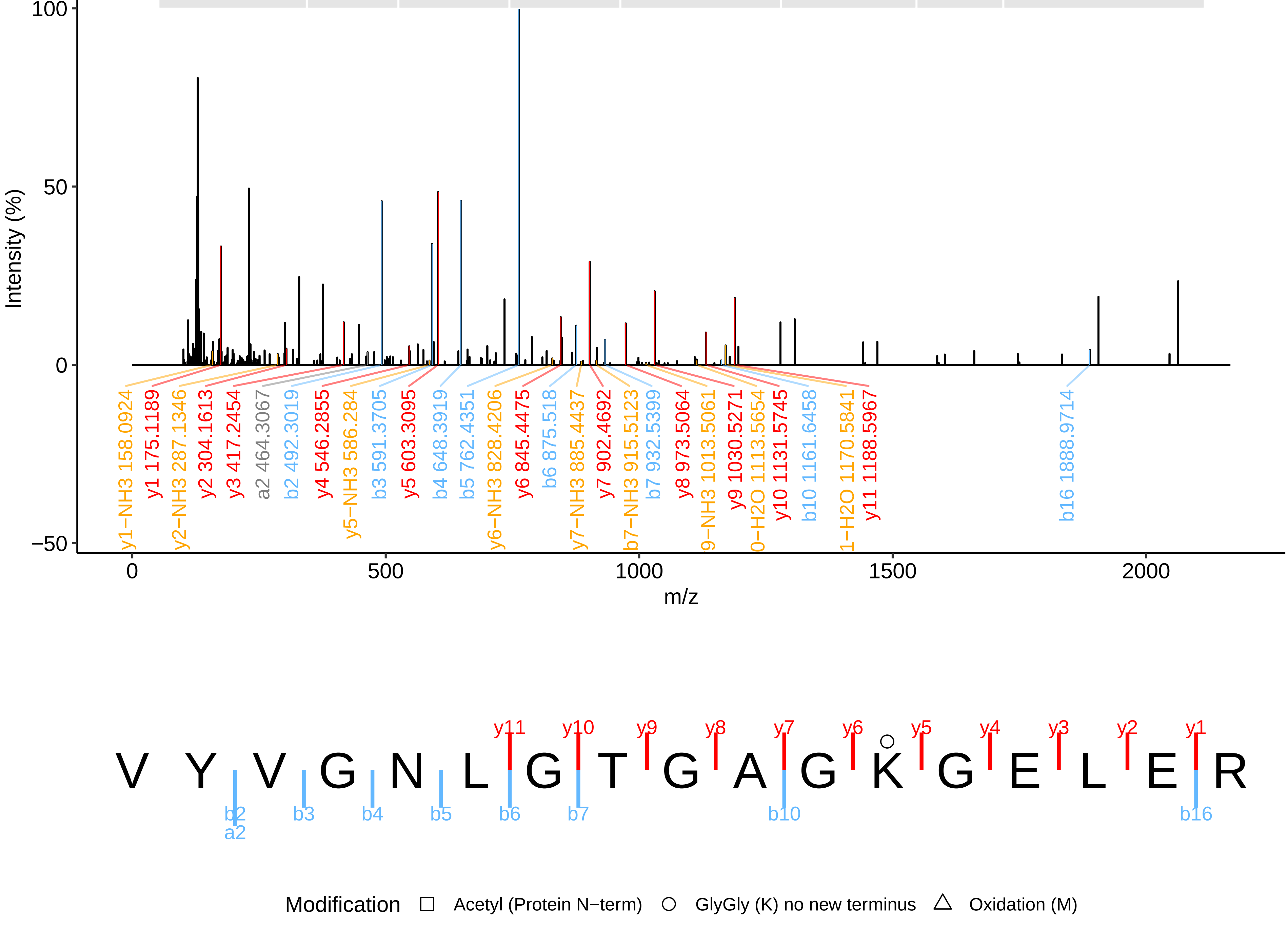

20200921_KIZ_MUb4_GlyGly_10plex_2 − Scan number: 13592

Gene Names	Charge	m/z	Mass	Retention time	PEP	Score	Precursor Intensity
SRSF7	3	611.9866	1832.938	52.81	0.0023475	65.107	961885.6

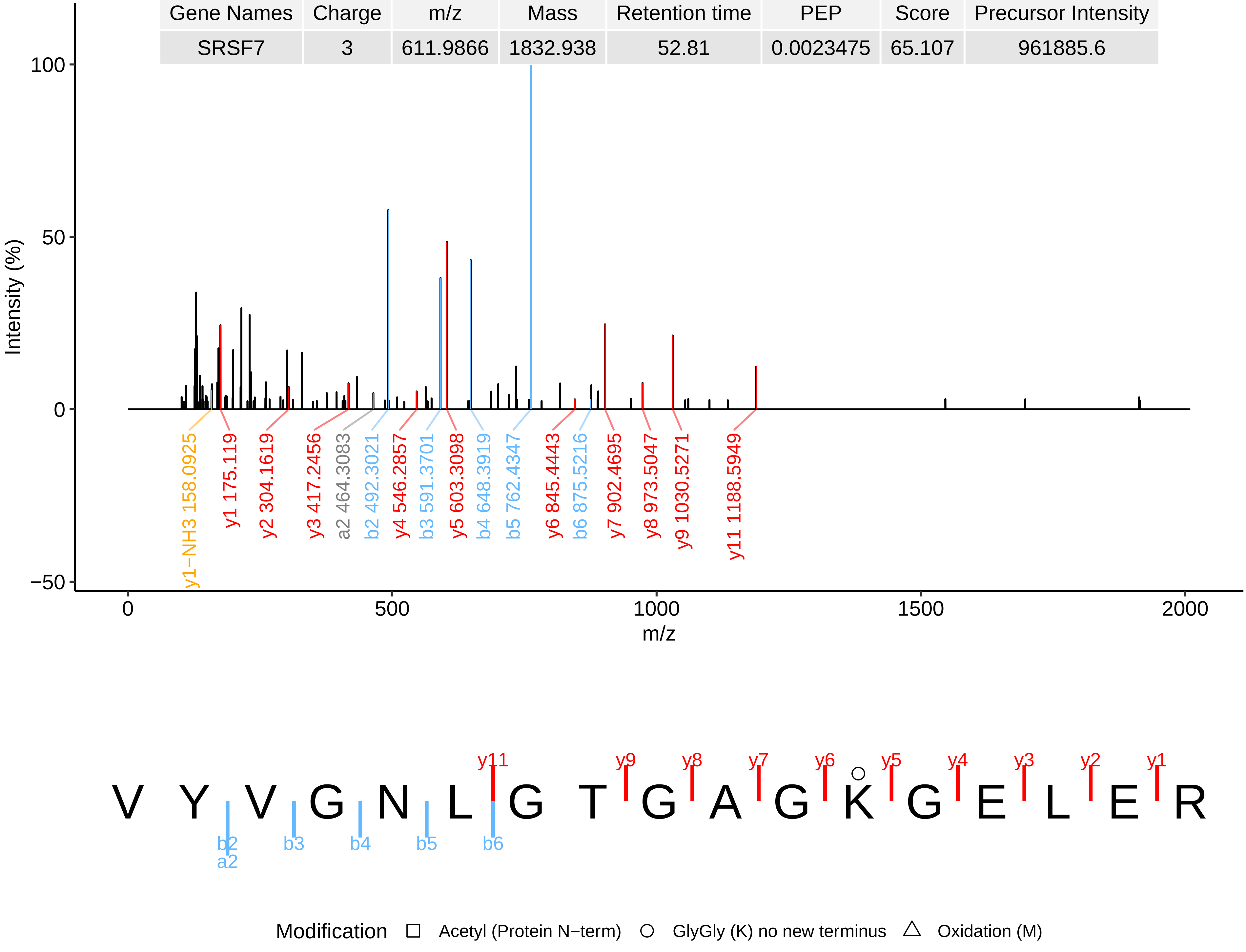

20200924_KIZ_MUb5_GlyGly_1 − Scan number: 13863

Gene Names	Charge	m/z	Mass	Retention time	PEP	Score	Precursor Intensity
SRSF7	3	611.9866	1832.938	45.225	0.0011113	73.279	17731012

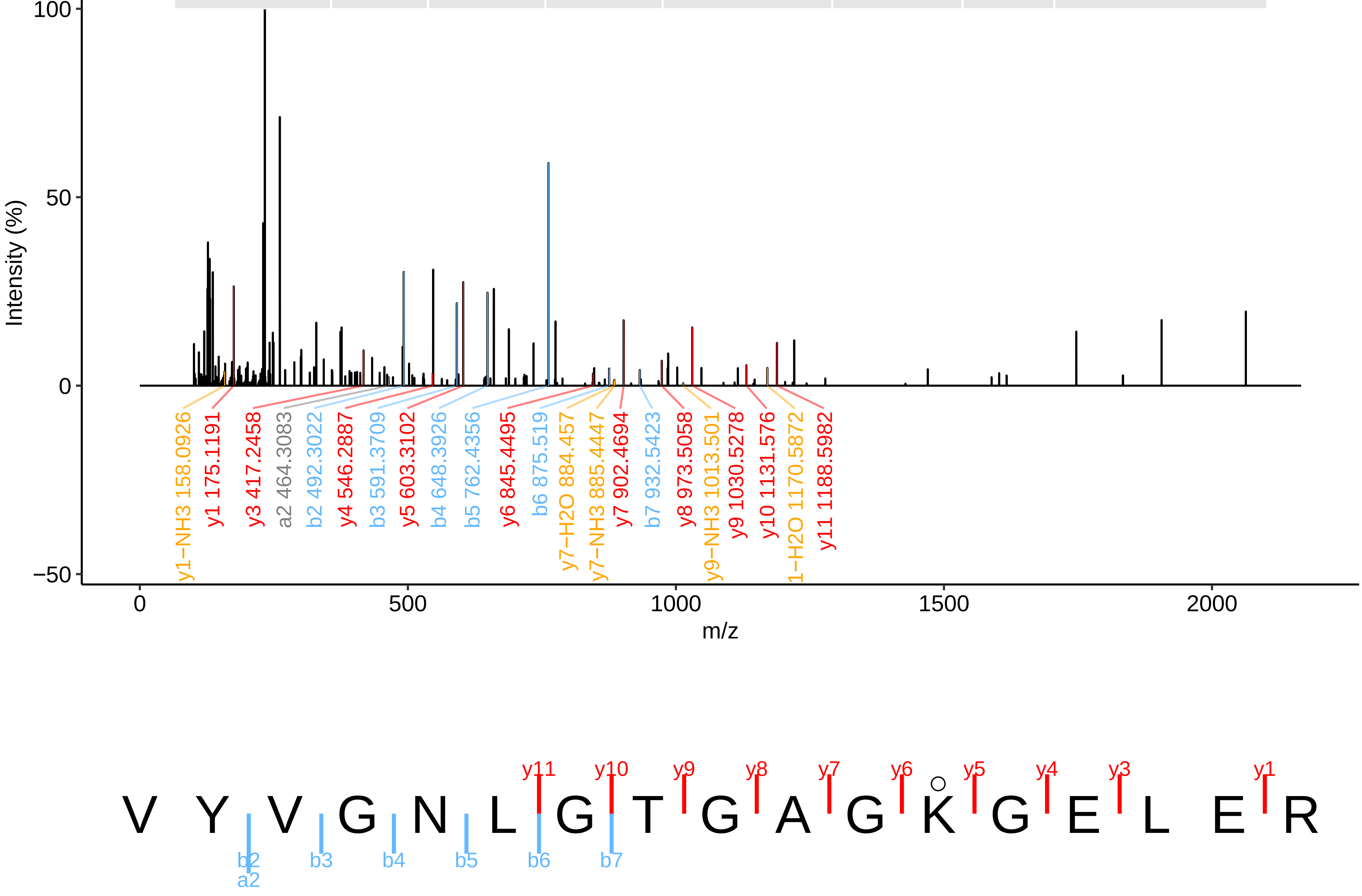

Modification □ Acetyl (Protein N−term) ○ GlyGly (K) no new terminus △ Oxidation (M)

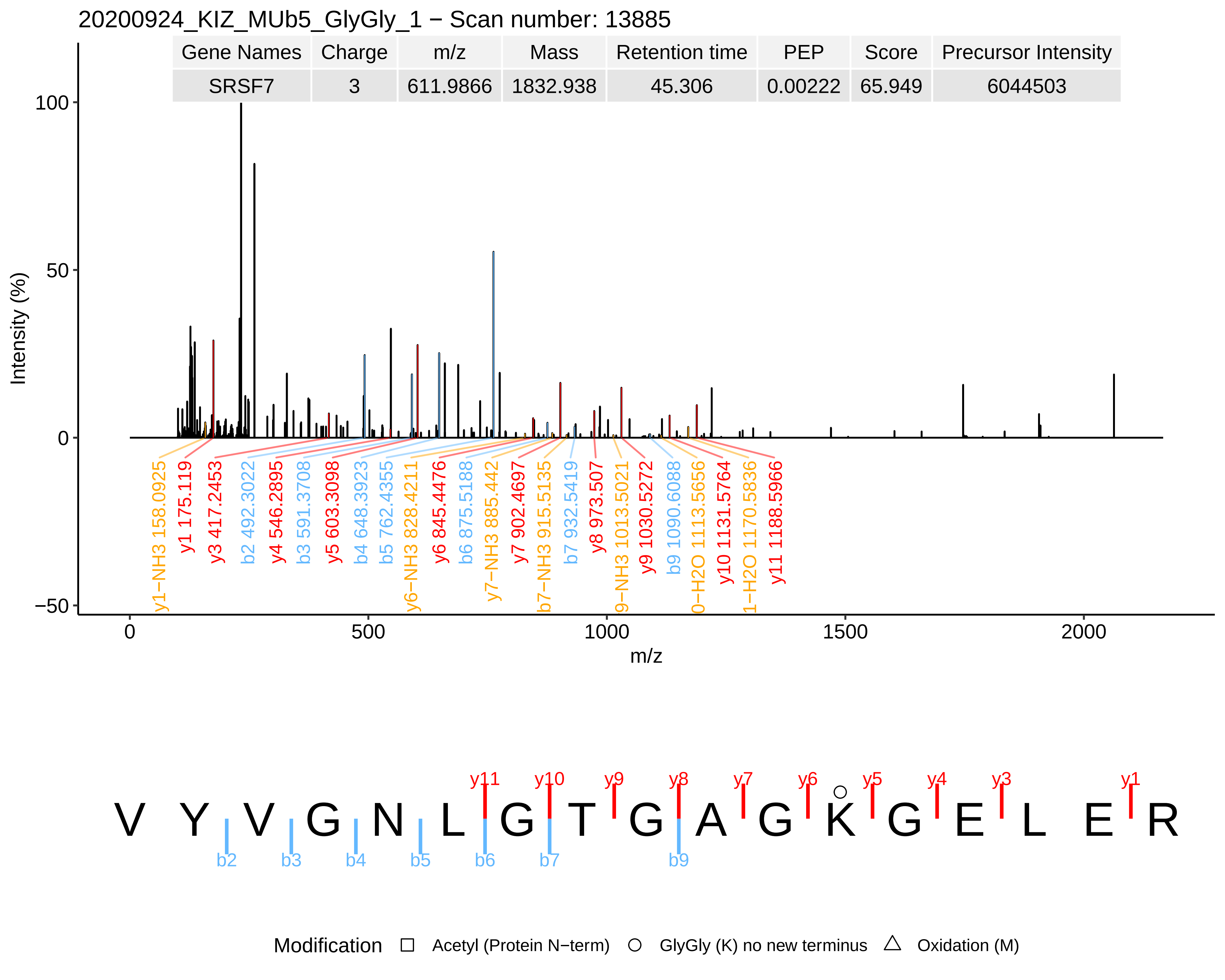
20200924_KIZ_MUb5_GlyGly_1 − Scan number: 13885
Gene Names | Charge | m/z | Mass | Retention time | PEP | Score | Precursor Intensity
SRSF7 | 3 | 611.9866 | 1832.938 | 45.306 | 0.00222 | 65.949 | 6044503
Intensity (%)
100
50
0
−50
0
500
1000
1500
2000
m/z
y1−NH3 158.0925
y1 175.119
y3 417.2453
b2 492.3022
y4 546.2895
b3 591.3708
y5 603.3098
b4 648.3923
b5 762.4355
y6−NH3 828.4211
y6 845.4476
b6 875.5188
y7−NH3 885.442
y7 902.4697
b7−NH3 915.5135
b7 932.5419
y8 973.507
9−NH3 1013.5021
y9 1030.5272
b9 1090.6088
0−H2O 1113.5656
y10 1131.5764
1−H2O 1170.5836
y11 1188.5966
V Y V G N L G T G A G K G E L E R
y11 y10 y9 y8 y7 y6 y5 y4 y3 y1
b2 b3 b4 b5 b6 b7 b9
Modification
Acetyl (Protein N−term)
GlyGly (K) no new terminus
Oxidation (M)

20200921_KIZ_MUb4_GlyGly_10plex_2 − Scan number: 10866

Gene Names	Charge	m/z	Mass	Retention time	PEP	Score	Precursor Intensity
CHCHD3	3	615.9586	1844.854	41.678	0.096173	60.549	1103128

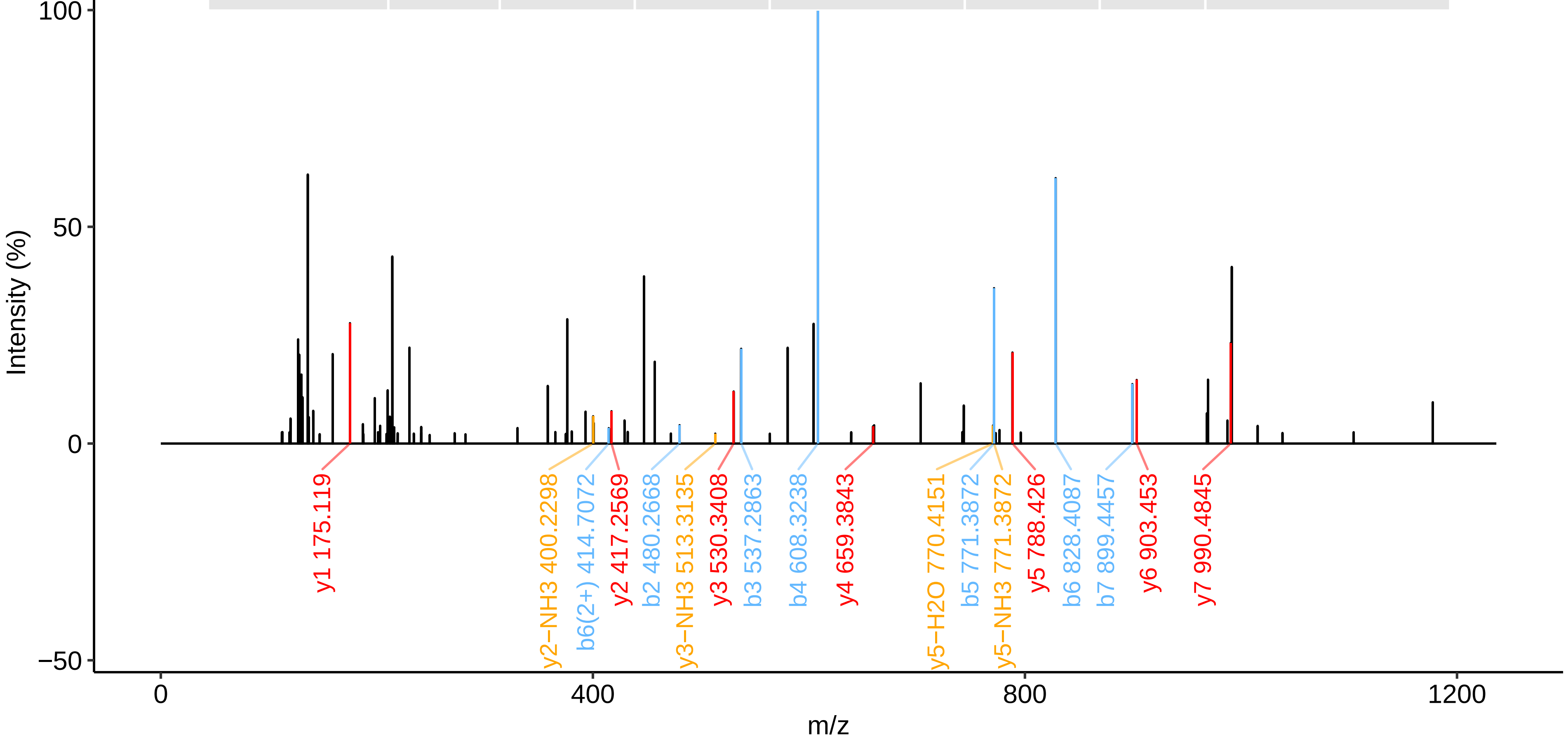

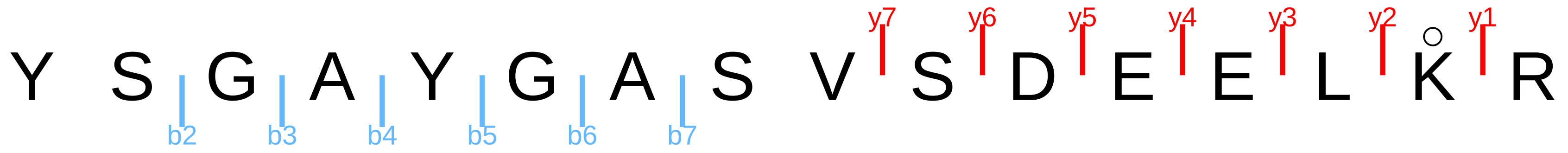

Modification □ Acetyl (Protein N−term) ○ GlyGly (K) no new terminus △ Oxidation (M)

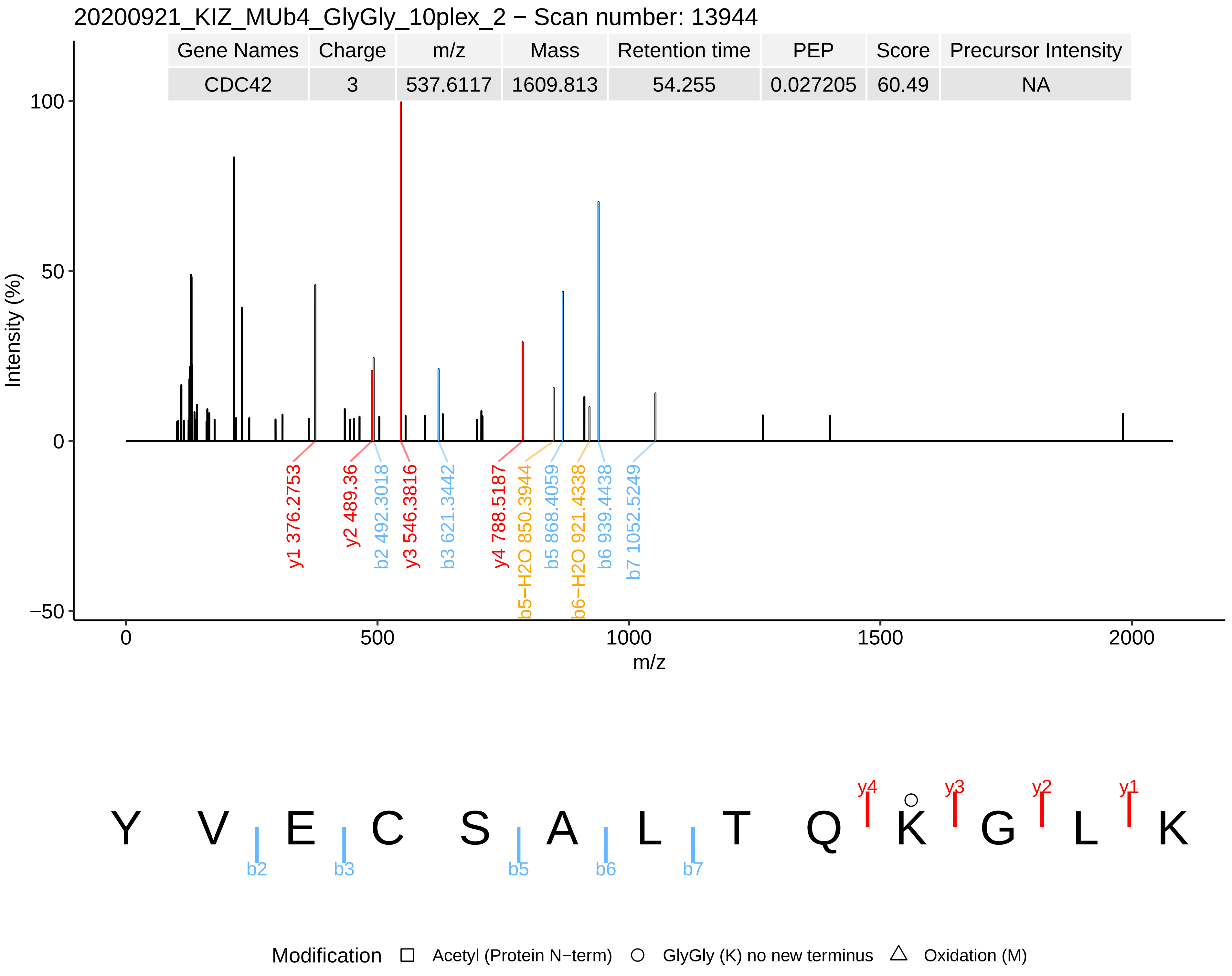
20200921_KIZ_MUb4_GlyGly_10plex_2 − Scan number: 13944
Gene Names
Charge
m/z
Mass
Retention time
PEP
Score
Precursor Intensity
CDC42
3
537.6117
1609.813
54.255
0.027205
60.49
NA
Intensity (%)
100
50
0
−50
0
500
1000
1500
2000
m/z
y1 376.2753
y2 489.36
b2 492.3018
y3 546.3816
b3 621.3442
y4 788.5187
b5−H2O 850.3944
b5 868.4059
b6−H2O 921.4338
b6 939.4438
b7 1052.5249
Y V E C S A L T Q K G L K
y4
y3
y2
y1
b2
b3
b5
b6
b7
Modification
Acetyl (Protein N−term)
GlyGly (K) no new terminus
Oxidation (M)

20200921_KIZ_MUb4_GlyGly_10plex − Scan number: 23275

Gene Names	Charge	m/z	Mass	Retention time	PEP	Score	Precursor Intensity
HSP90AA1;HSP90AA2P;HSP90AB1	3	577.3196	1728.937	84.476	3.654e−10	124.43	3254801

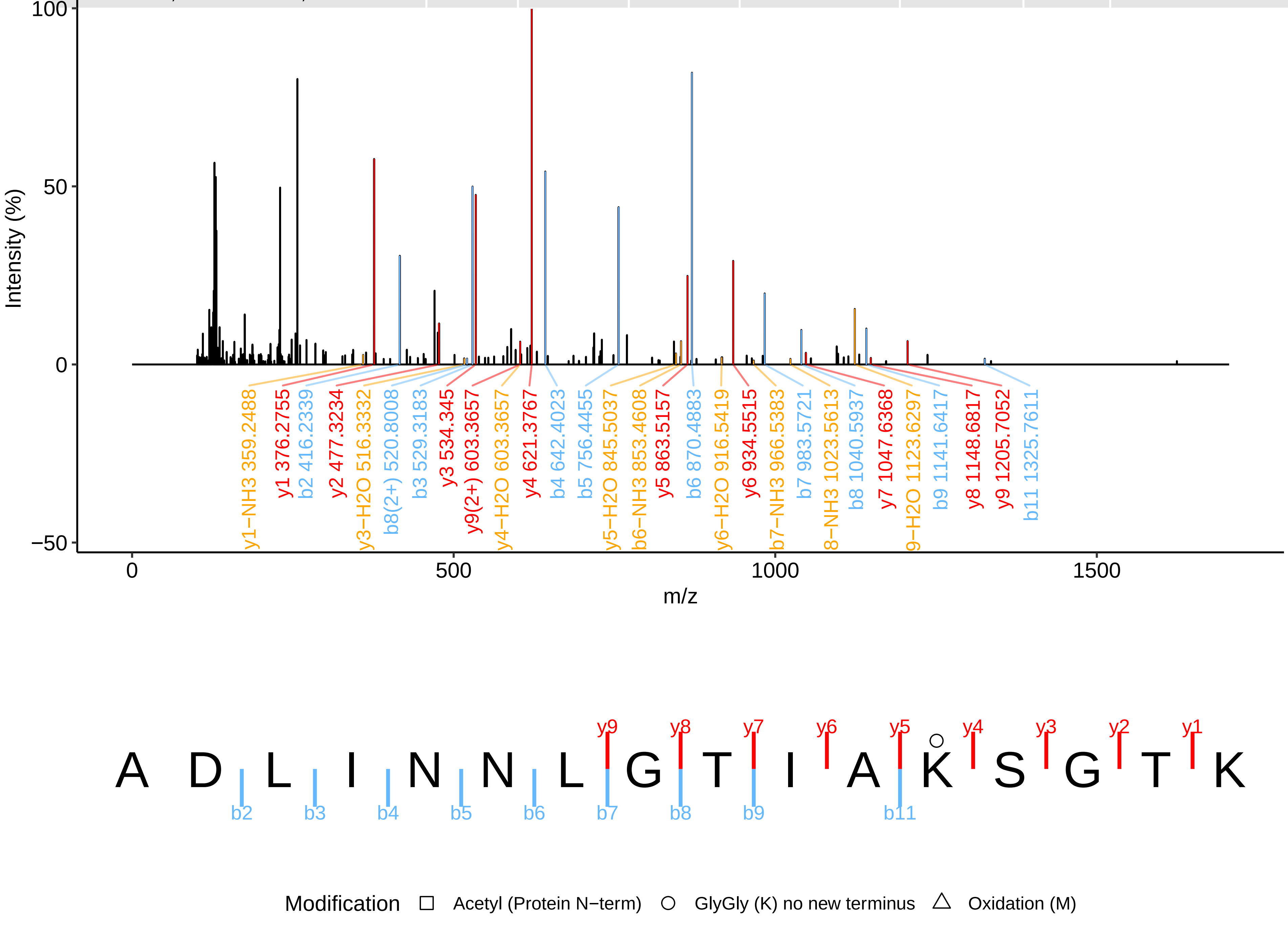

20200921_KIZ_MUb4_GlyGly_10plex_2 − Scan number: 20637

Gene Names	Charge	m/z	Mass	Retention time	PEP	Score	Precursor Intensit
HSP90AA1;HSP90AA2P;HSP90AB1	3	577.3196	1728.937	81.142	2.2902e−06	96.163	1113217

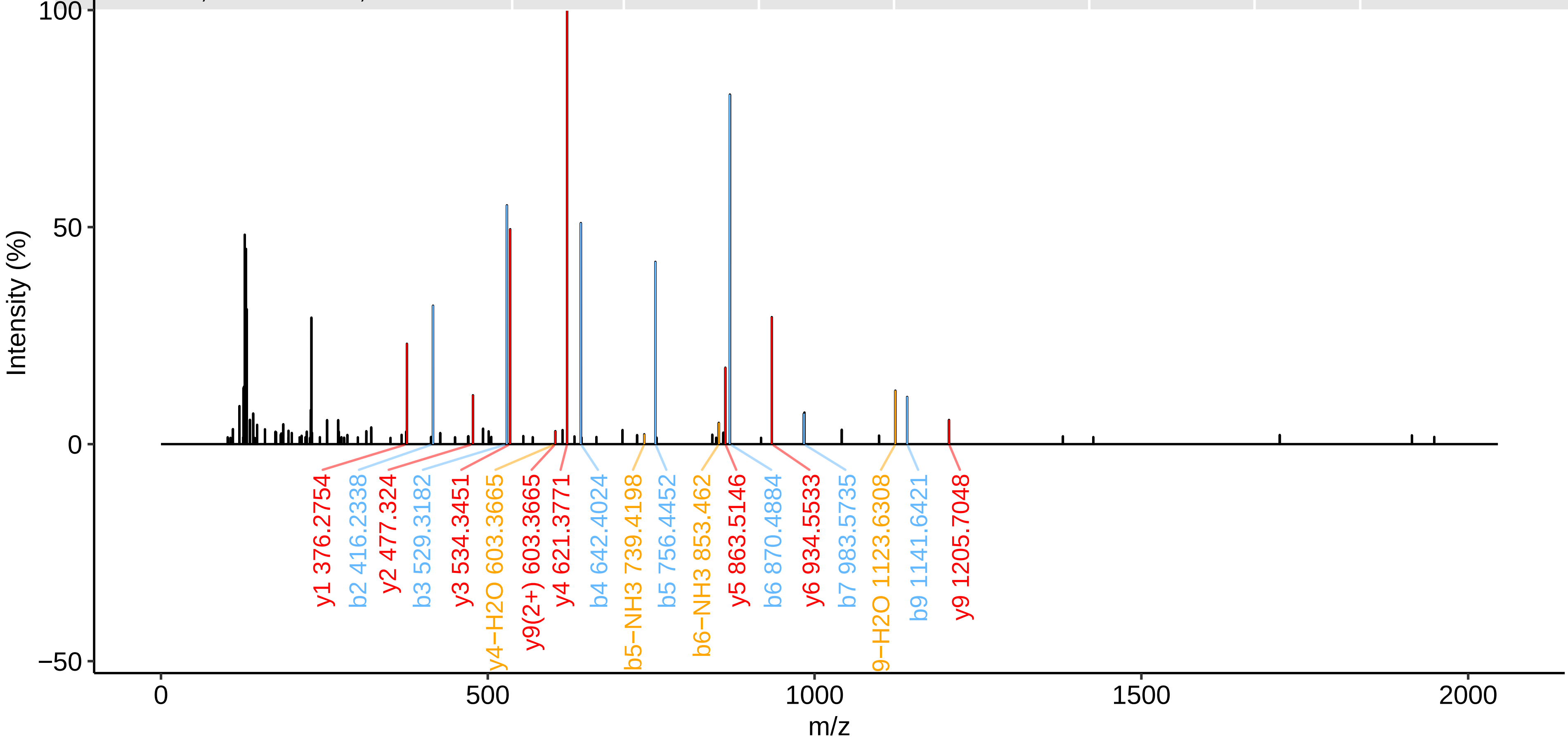

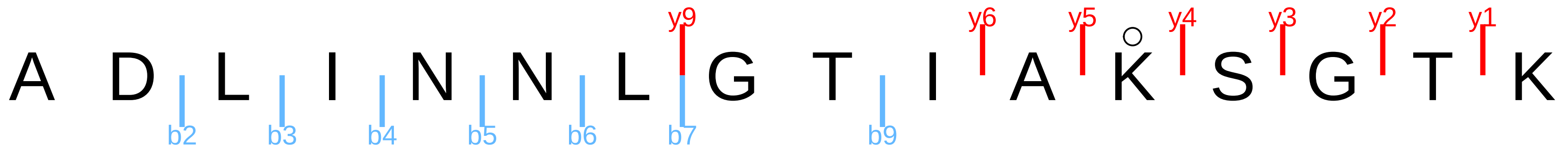

Modification □ Acetyl (Protein N−term) ○ GlyGly (K) no new terminus △ Oxidation (M)

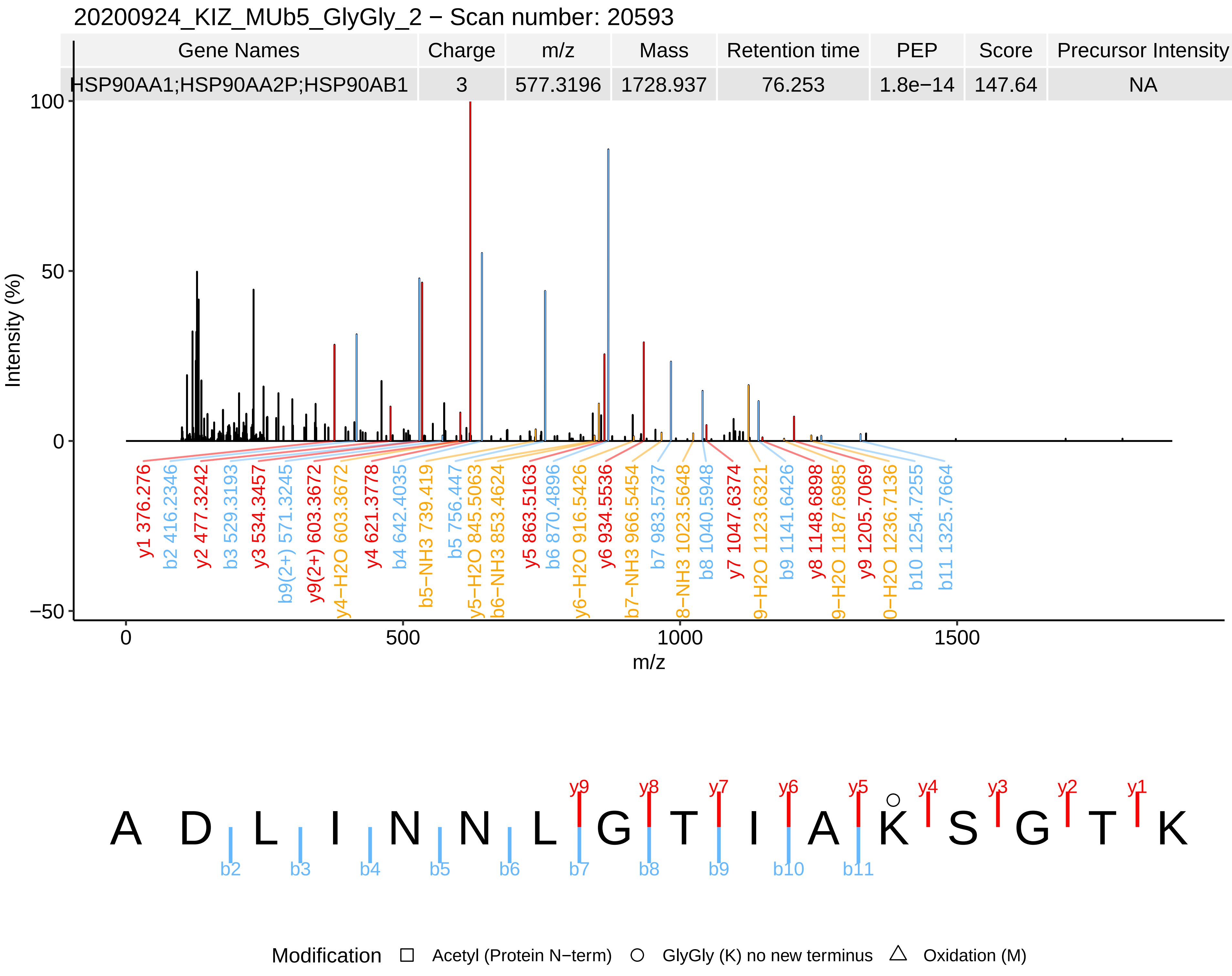
20200924_KIZ_MUb5_GlyGly_2 − Scan number: 20593
Gene Names | Charge | m/z | Mass | Retention time | PEP | Score | Precursor Intensity
HSP90AA1;HSP90AA2P;HSP90AB1 | 3 | 577.3196 | 1728.937 | 76.253 | 1.8e−14 | 147.64 | NA
Intensity (%)
100
50
0
−50
0
500
1000
1500
m/z
y1 376.276
b2 416.2346
y2 477.3242
b3 529.3193
y3 534.3457
b9(2+) 571.3245
y9(2+) 603.3672
y4−H2O 603.3672
y4 621.3778
b4 642.4035
b5−NH3 739.419
b5 756.447
y5−H2O 845.5063
b6−NH3 853.4624
y5 863.5163
b6 870.4896
y6−H2O 916.5426
y6 934.5536
b7−NH3 966.5454
b7 983.5737
8−NH3 1023.5648
b8 1040.5948
y7 1047.6374
9−H2O 1123.6321
b9 1141.6426
y8 1148.6898
9−H2O 1187.6985
y9 1205.7069
0−H2O 1236.7136
b10 1254.7255
b11 1325.7664
A D L I N N L G T I A K S G T K
y9 y8 y7 y6 y5 y4 y3 y2 y1
b2 b3 b4 b5 b6 b7 b8 b9 b10 b11
Modification
Acetyl (Protein N−term)
GlyGly (K) no new terminus
Oxidation (M)

20201118_KIZ_MUb6_GlyGly_1 − Scan number: 23707

Gene Names	Charge	m/z	Mass	Retention time	PEP	Score	Precursor Intensit
HSP90AA1;HSP90AA2P;HSP90AB1	3	577.3196	1728.937	73.233	6.3215e−10	118.31	24346250

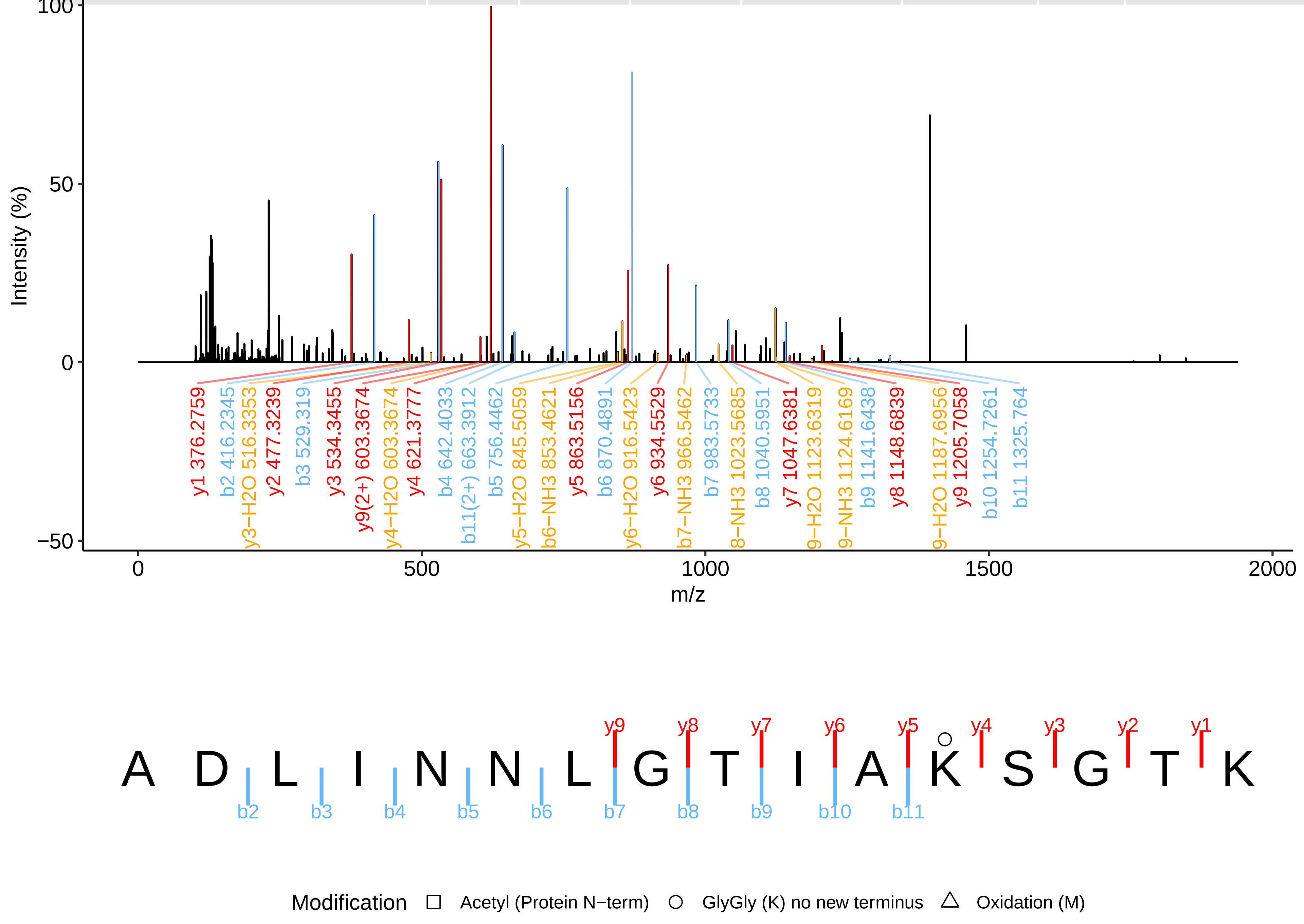

20201118_KIZ_MUb6_GlyGly_2 − Scan number: 25001

Gene Names	Charge	m/z	Mass	Retention time	PEP	Score	Precursor Intensit
HSP90AA1;HSP90AA2P;HSP90AB1	3	577.3196	1728.937	83.604	8.8377e−20	153.48	18413474

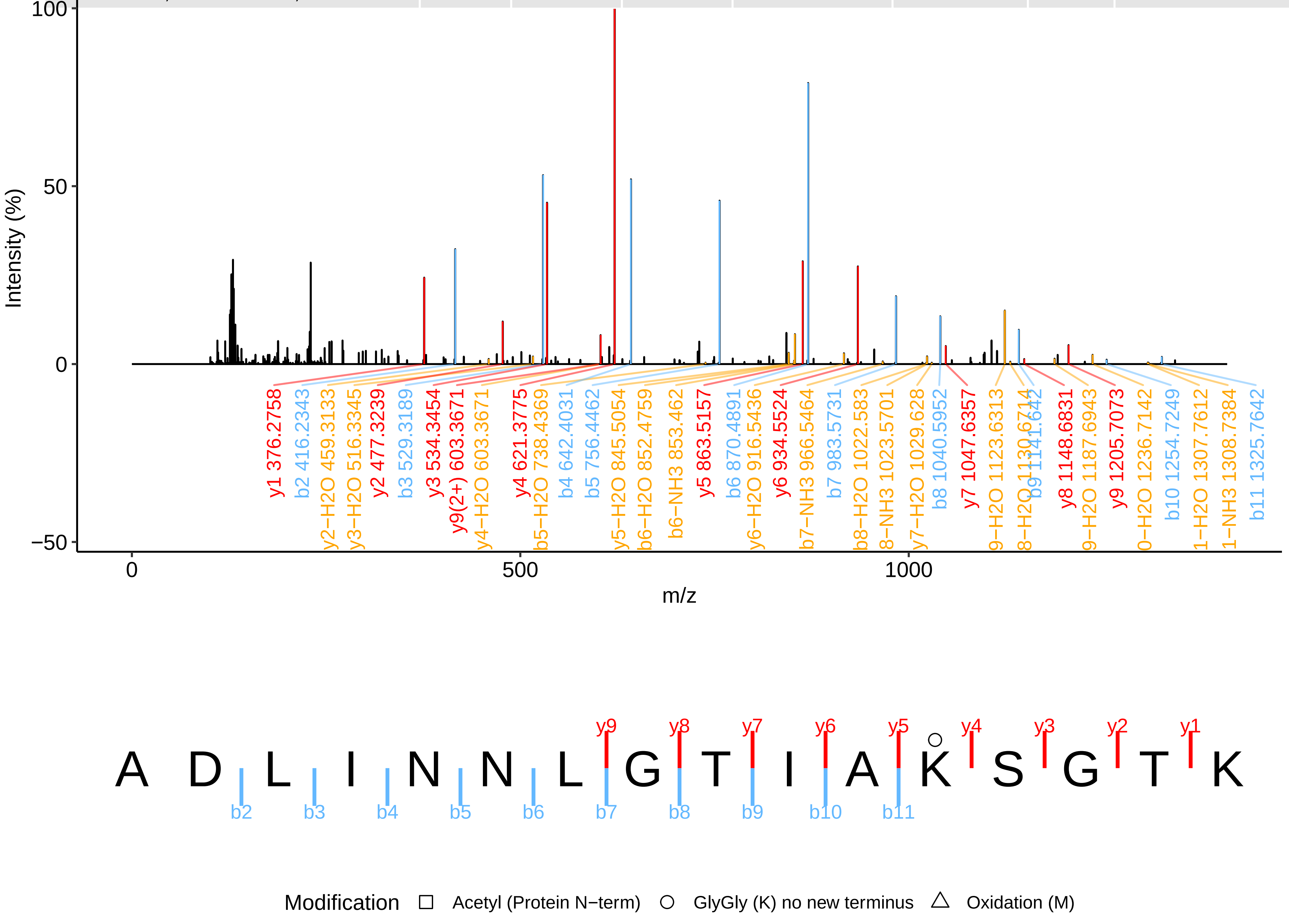

20200921_KIZ_MUb4_GlyGly_10plex − Scan number: 26440

Gene Names	Charge	m/z	Mass	Retention time	PEP	Score	Precursor Intensity
CUL2	3	587.9783	1760.913	96.343	0.00039088	89.231	20544106

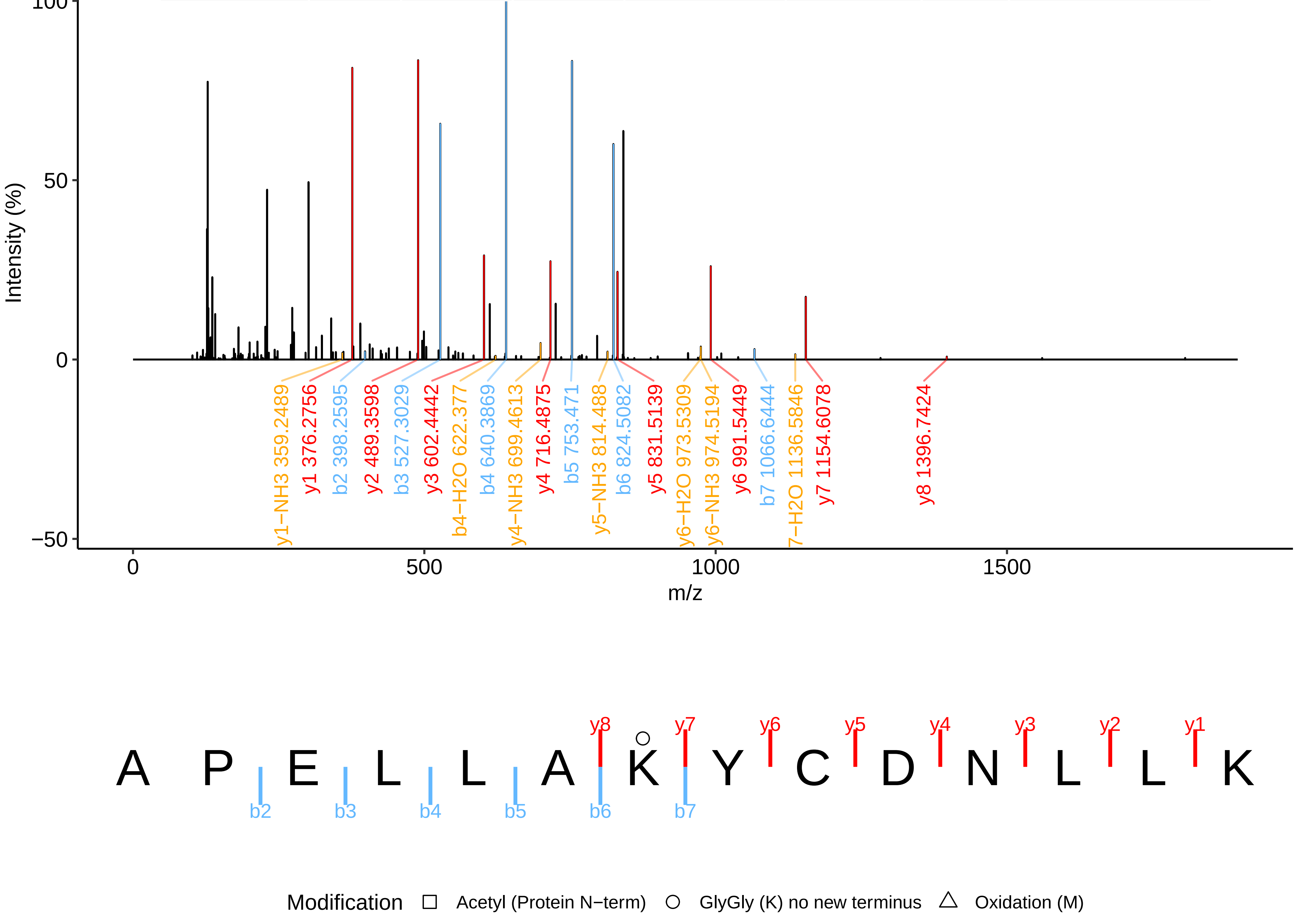

20200921_KIZ_MUb4_GlyGly_10plex_2 − Scan number: 22787

Gene Names	Charge	m/z	Mass	Retention time	PEP	Score	Precursor Intensity
CUL2	3	587.9783	1760.913	89.884	0.0054273	68.034	744234.9

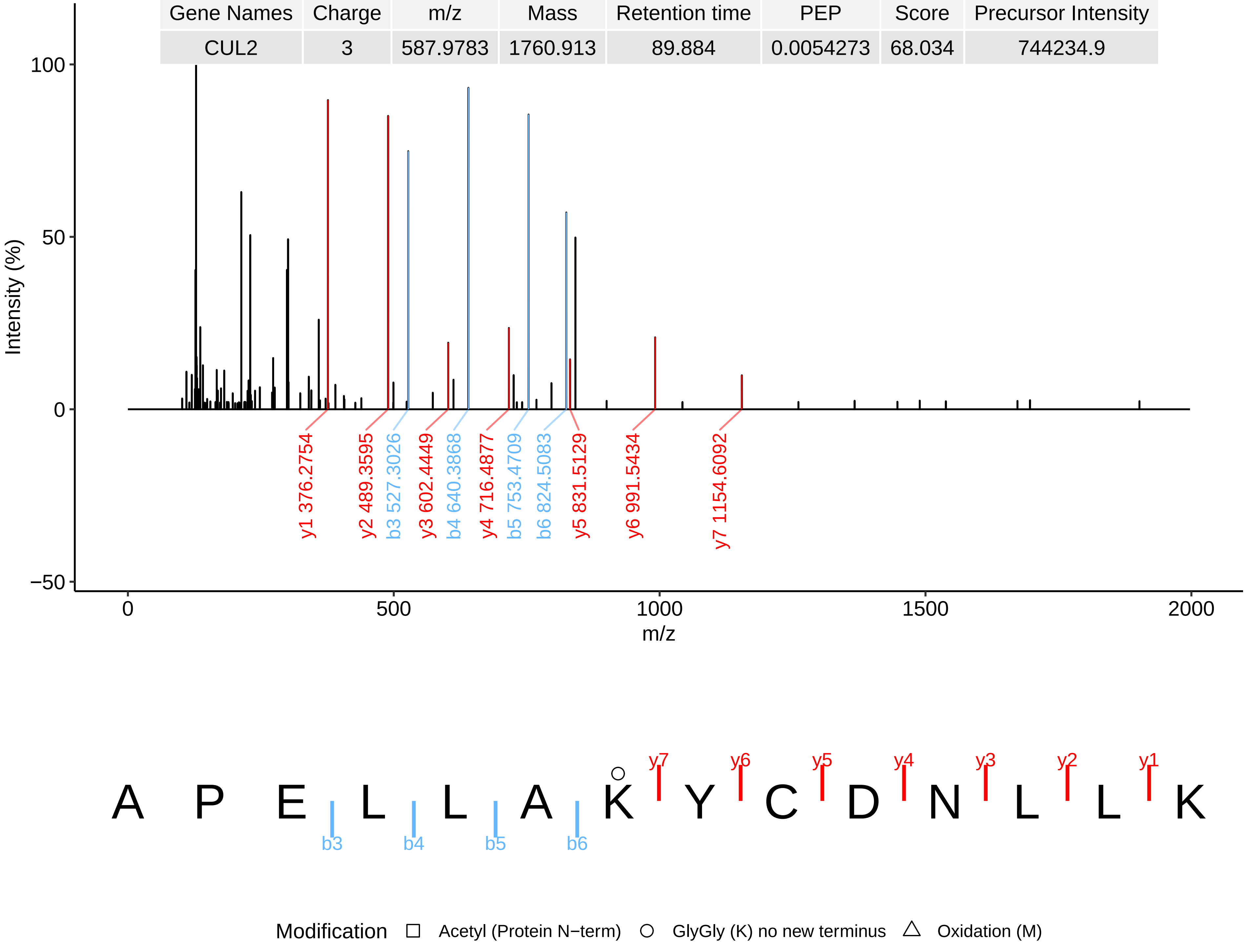

20200924_KIZ_MUb5_GlyGly_1 − Scan number: 25300

Gene Names	Charge	m/z	Mass	Retention time	PEP	Score	Precursor Intensity
CUL2	3	587.9783	1760.913	81.163	0.00068453	86.291	NA

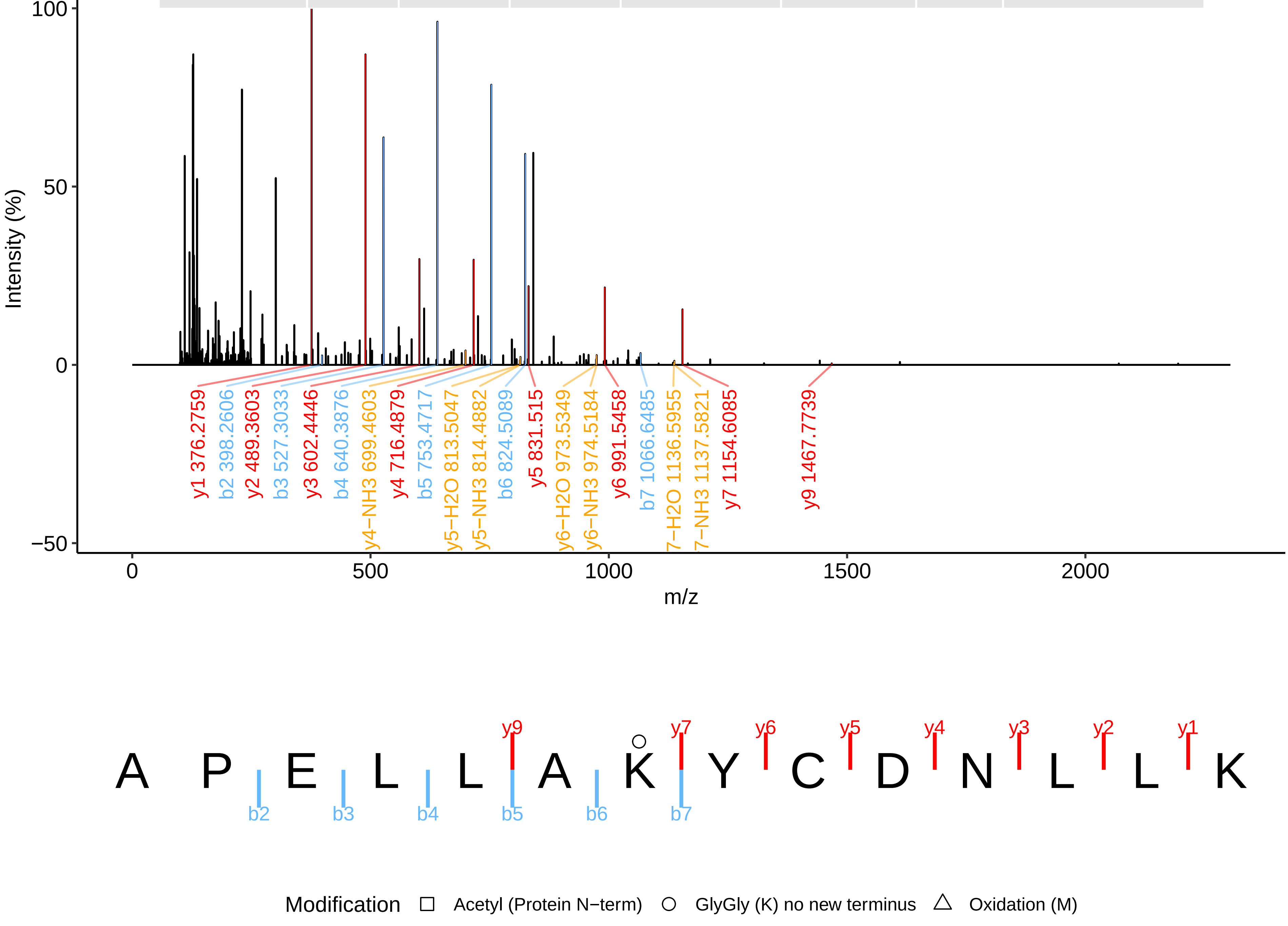

20200924_KIZ_MUb5_GlyGly_2 − Scan number: 22765

Gene Names	Charge	m/z	Mass	Retention time	PEP	Score	Precursor Intensity
CUL2	3	587.9783	1760.913	84.326	0.0049174	68.034	2482737

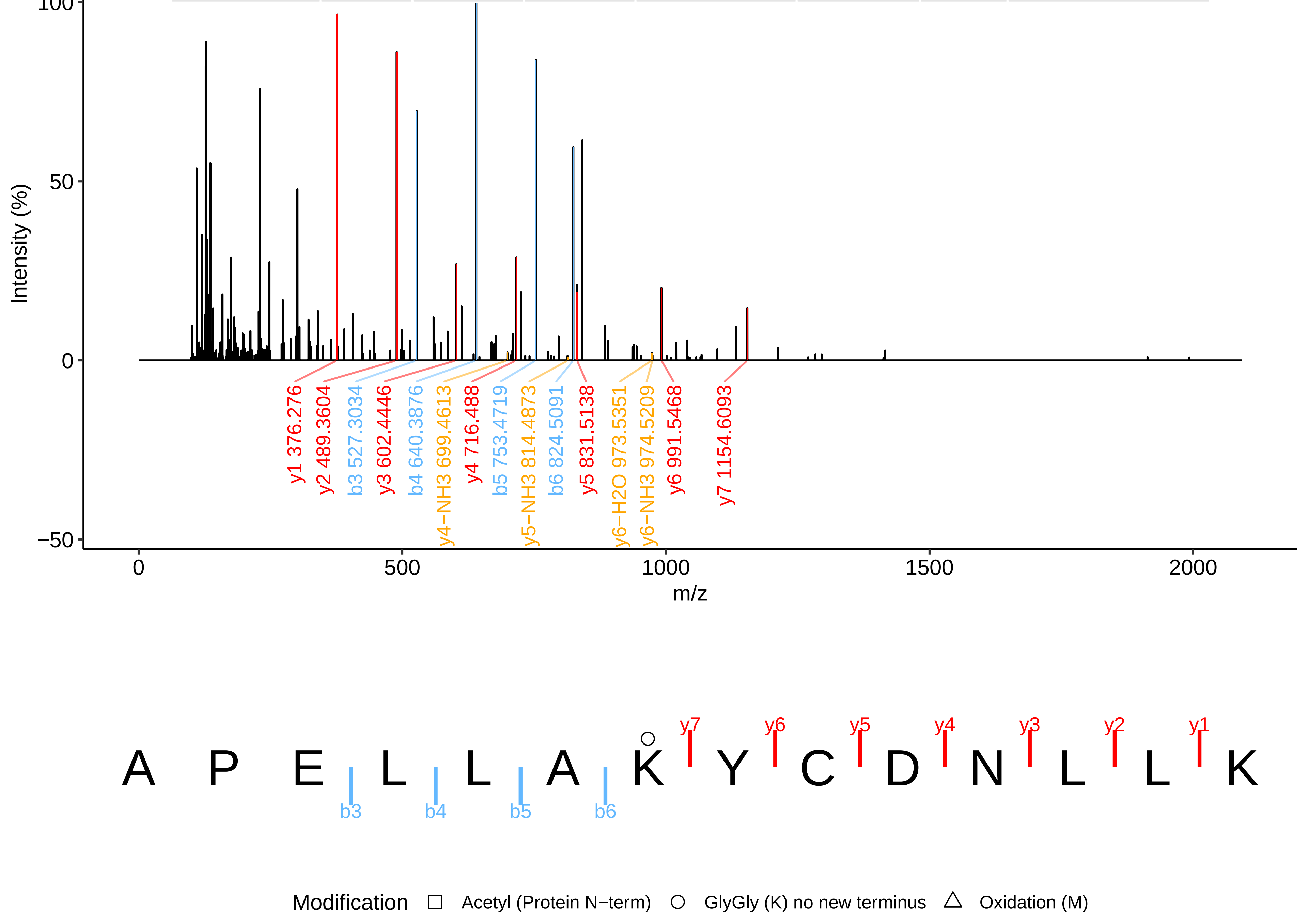

20201118_KIZ_MUb6_GlyGly_1 − Scan number: 26236

Gene Names	Charge	m/z	Mass	Retention time	PEP	Score	Precursor Intensity
CUL2	3	587.9783	1760.913	79.997	0.033681	58.487	17430776

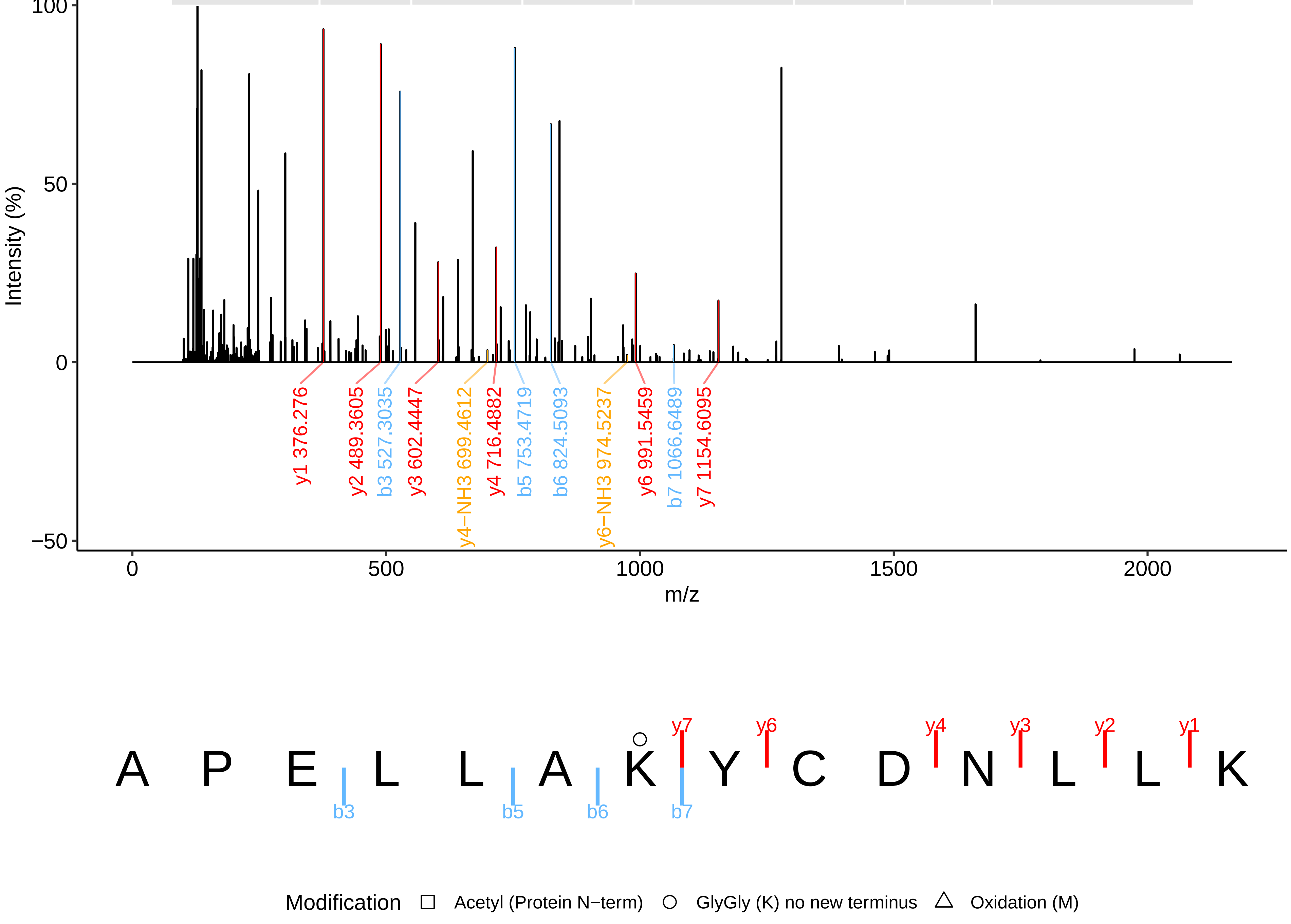

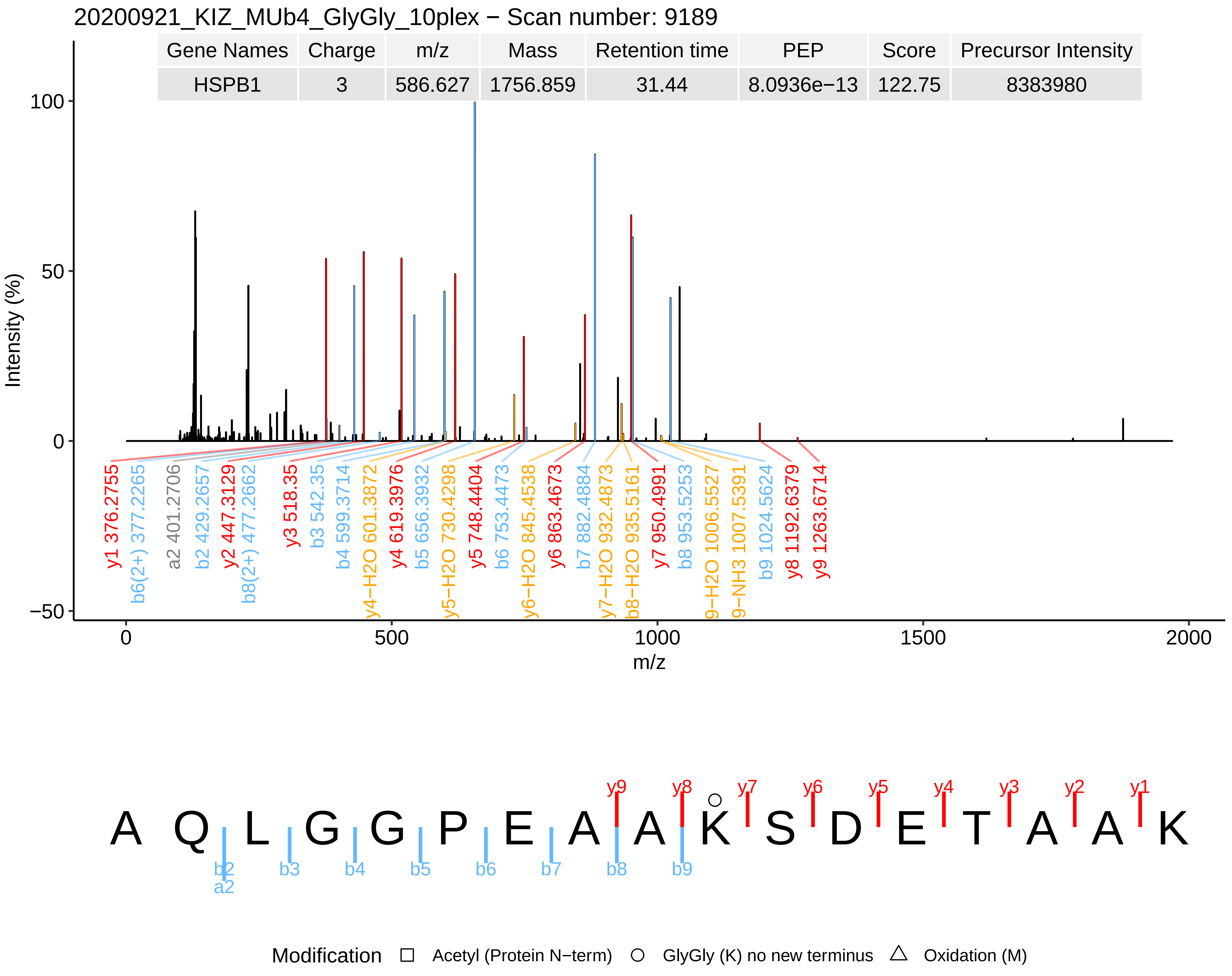

20200921_KIZ_MUb4_GlyGly_10plex − Scan number: 9189
Gene Names | Charge | m/z | Mass | Retention time | PEP | Score | Precursor Intensity
HSPB1 | 3 | 586.627 | 1756.859 | 31.44 | 8.0936e−13 | 122.75 | 8383980
Intensity (%)
100
50
0
−50
0
500
1000
1500
2000
m/z
y1 376.2755
b6(2+) 377.2265
a2 401.2706
b2 429.2657
y2 447.3129
b8(2+) 477.2662
y3 518.35
b3 542.35
b4 599.3714
y4−H2O 601.3872
y4 619.3976
b5 656.3932
y5−H2O 730.4298
y5 748.4404
b6 753.4473
y6−H2O 845.4538
y6 863.4673
b7 882.4884
y7−H2O 932.4873
b8−H2O 935.5161
y7 950.4991
b8 953.5253
9−H2O 1006.5527
9−NH3 1007.5391
b9 1024.5624
y8 1192.6379
y9 1263.6714
y9 y8 y7 y6 y5 y4 y3 y2 y1
A Q L G G P E A A K S D E T A A K
b2 a2 b3 b4 b5 b6 b7 b8 b9
Modification
Acetyl (Protein N−term)
GlyGly (K) no new terminus
Oxidation (M)

20200921_KIZ_MUb4_GlyGly_10plex_2 − Scan number: 8460

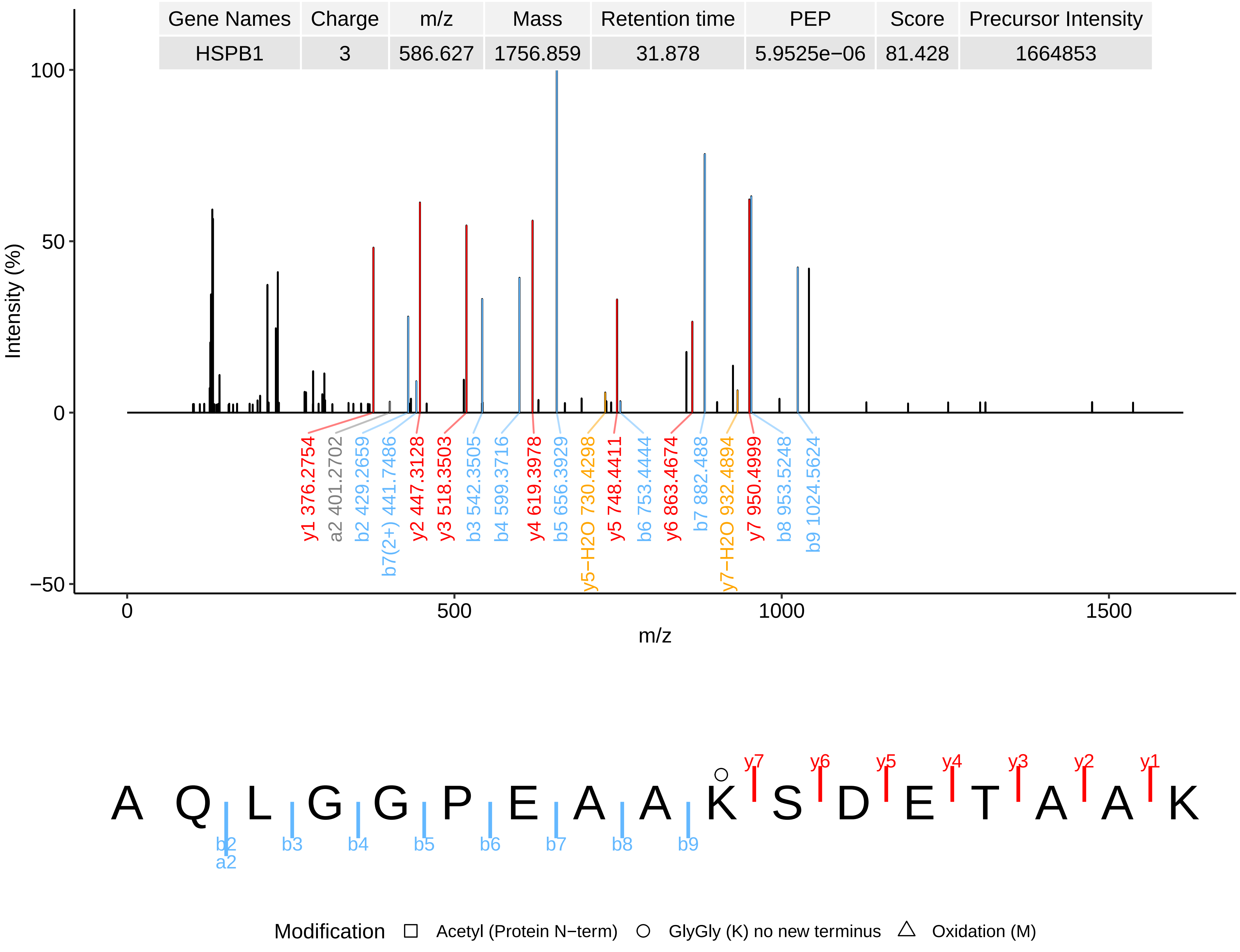

20200924_KIZ_MUb5_GlyGly_1 − Scan number: 8303

Gene Names	Charge	m/z	Mass	Retention time	PEP	Score	Precursor Intensity
HSPB1	3	586.627	1756.859	26.517	9.7143e−10	98.507	3805231

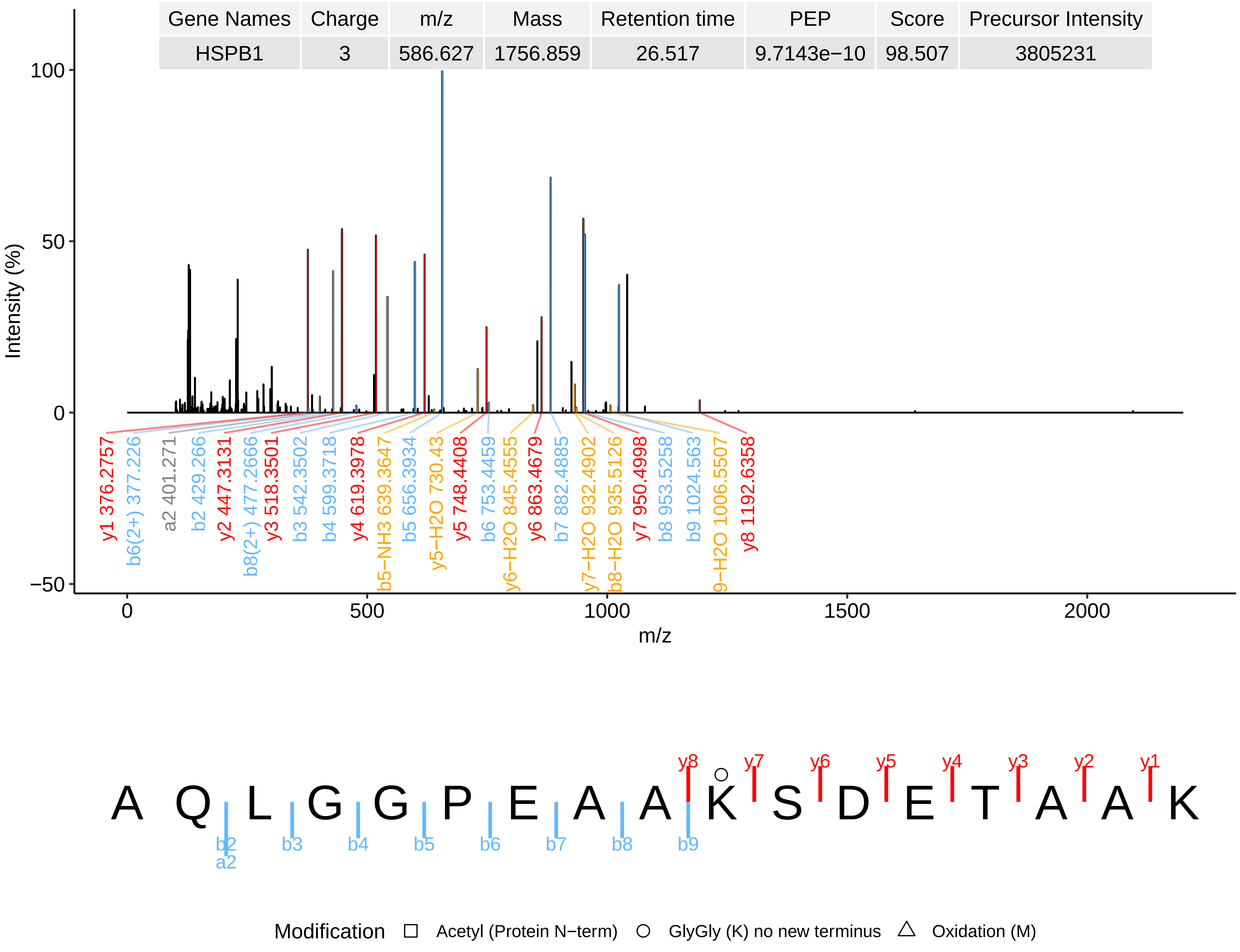

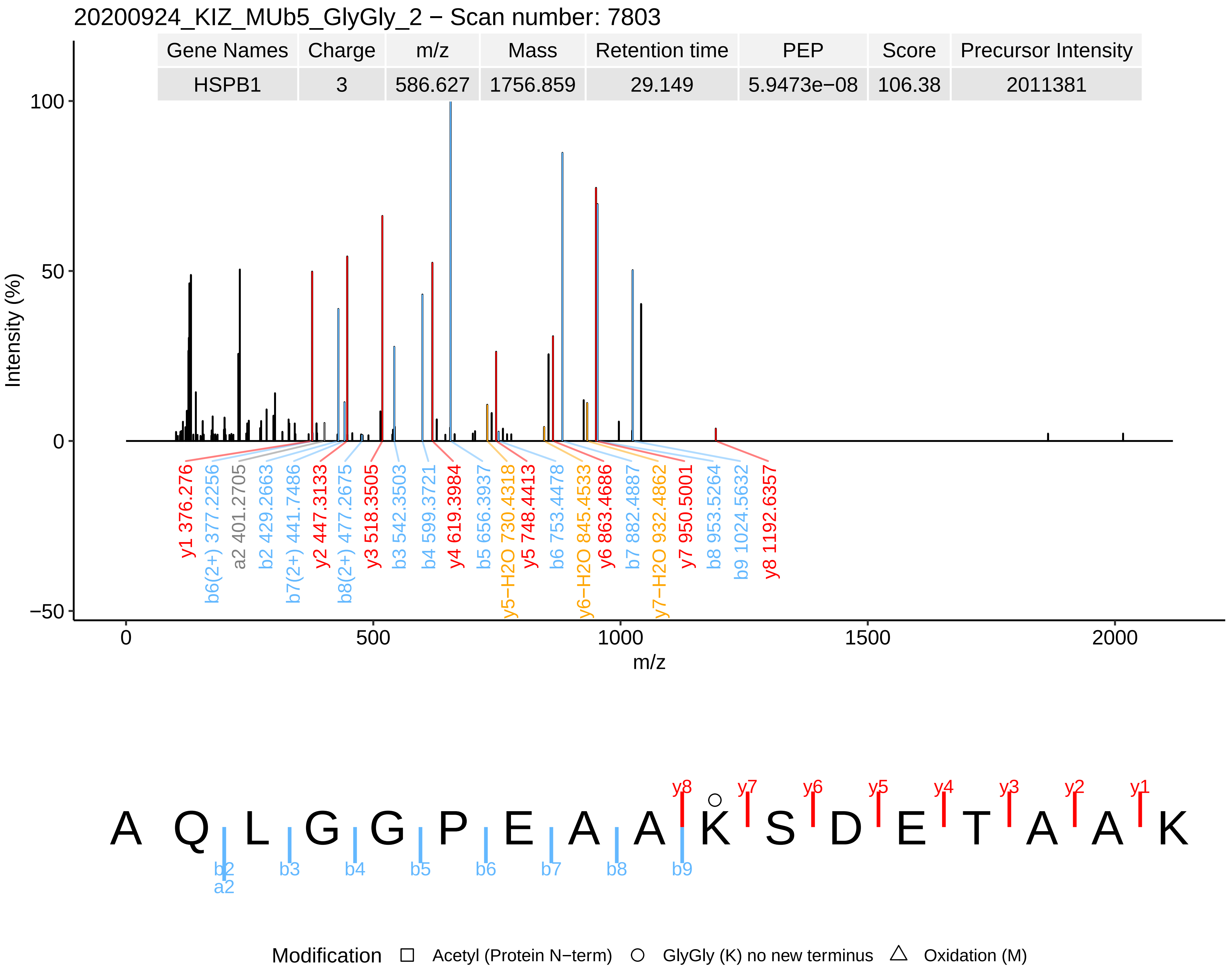

20200924_KIZ_MUb5_GlyGly_2 − Scan number: 7803
Gene Names | Charge | m/z | Mass | Retention time | PEP | Score | Precursor Intensity
HSPB1 | 3 | 586.627 | 1756.859 | 29.149 | 5.9473e−08 | 106.38 | 2011381
Intensity (%)
m/z
y1 376.276
b6(2+) 377.2256
a2 401.2705
b2 429.2663
b7(2+) 441.7486
y2 447.3133
b8(2+) 477.2675
y3 518.3505
b3 542.3503
b4 599.3721
y4 619.3984
b5 656.3937
y5−H2O 730.4318
y5 748.4413
b6 753.4478
y6−H2O 845.4533
y6 863.4686
b7 882.4887
y7−H2O 932.4862
y7 950.5001
b8 953.5264
b9 1024.5632
y8 1192.6357
A Q L G G P E A A K S D E T A A K
Modification
Acetyl (Protein N−term)
GlyGly (K) no new terminus
Oxidation (M)

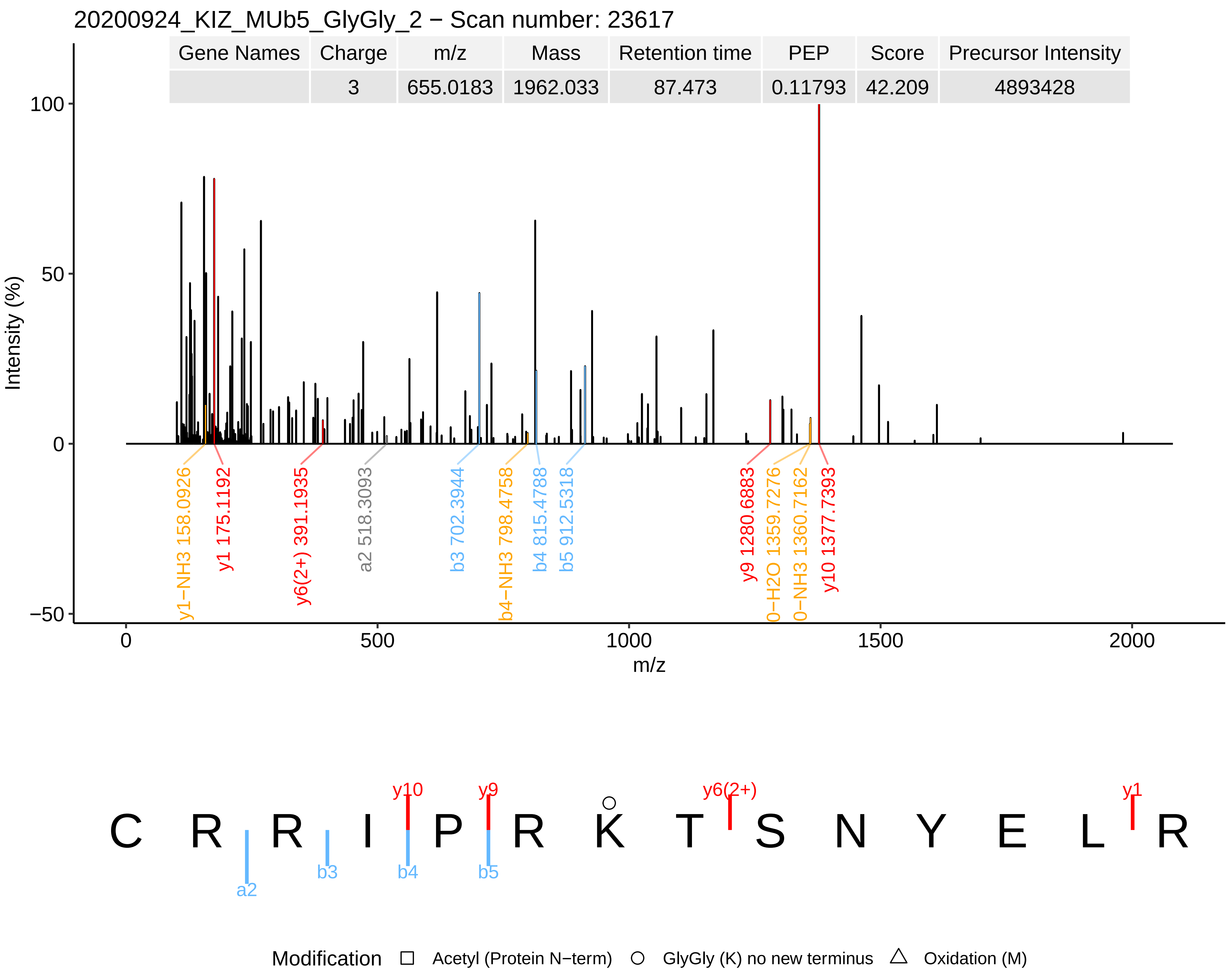

20200924_KIZ_MUb5_GlyGly_2 − Scan number: 23617
Gene Names | Charge | m/z | Mass | Retention time | PEP | Score | Precursor Intensity
3 | 655.0183 | 1962.033 | 87.473 | 0.11793 | 42.209 | 4893428
Intensity (%)
m/z
y1−NH3 158.0926
y1 175.1192
y6(2+) 391.1935
a2 518.3093
b3 702.3944
b4−NH3 798.4758
b4 815.4788
b5 912.5318
y9 1280.6883
0−H2O 1359.7276
0−NH3 1360.7162
y10 1377.7393
C R R I P R K T S N Y E L R
y10
y9
y6(2+)
y1
a2
b3
b4
b5
Modification
Acetyl (Protein N−term)
GlyGly (K) no new terminus
Oxidation (M)

20200921_KIZ_MUb4_GlyGly_10plex_2 − Scan number: 19103

Gene Names	Charge	m/z	Mass	Retention time	PEP	Score	Precursor Intensity
RAD23B;RAD23A	3	564.642	1690.904	74.928	0.00012027	86.291	1115167

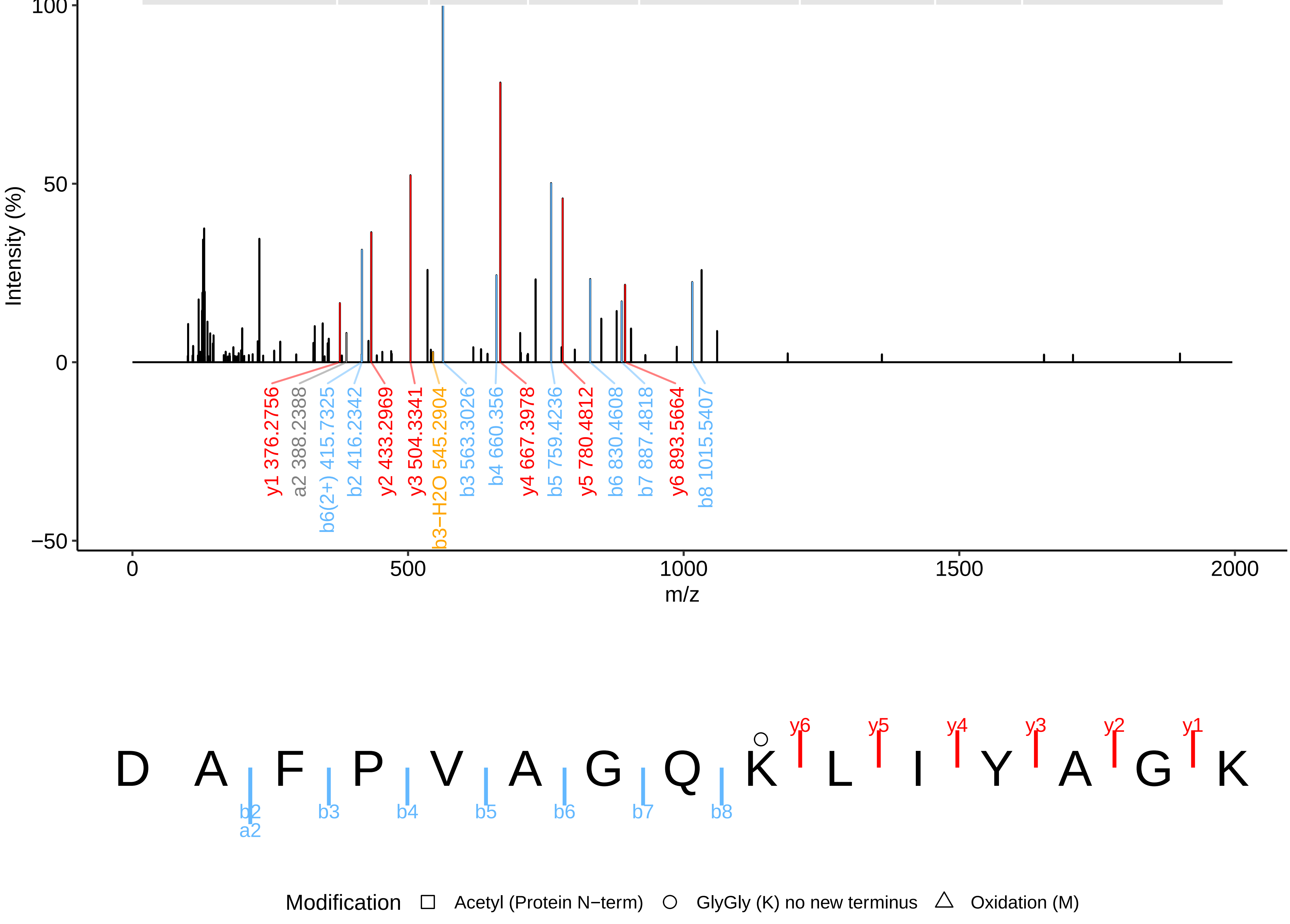

20200921_KIZ_MUb4_GlyGly_10plex − Scan number: 18065

Gene Names	Charge	m/z	Mass	Retention time	PEP	Score	Precursor Intensity
UBAP2L	3	666.9887	1997.944	64.889	3.5684e−12	117.18	2426608

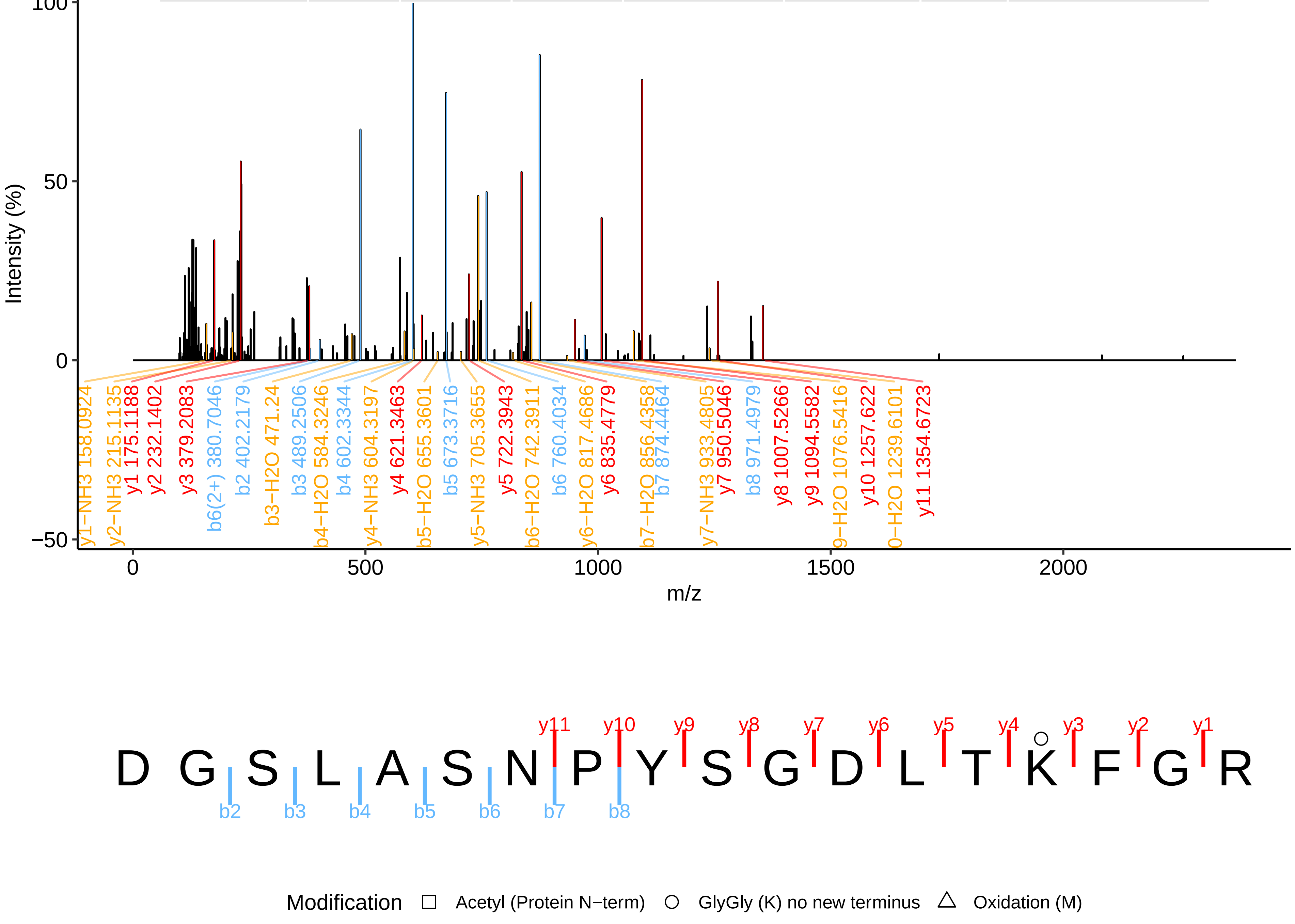

20200921_KIZ_MUb4_GlyGly_10plex_2 − Scan number: 16517

Gene Names	Charge	m/z	Mass	Retention time	PEP	Score	Precursor Intensity
UBAP2L	3	666.9887	1997.944	64.485	2.4298e−06	94.01	953145

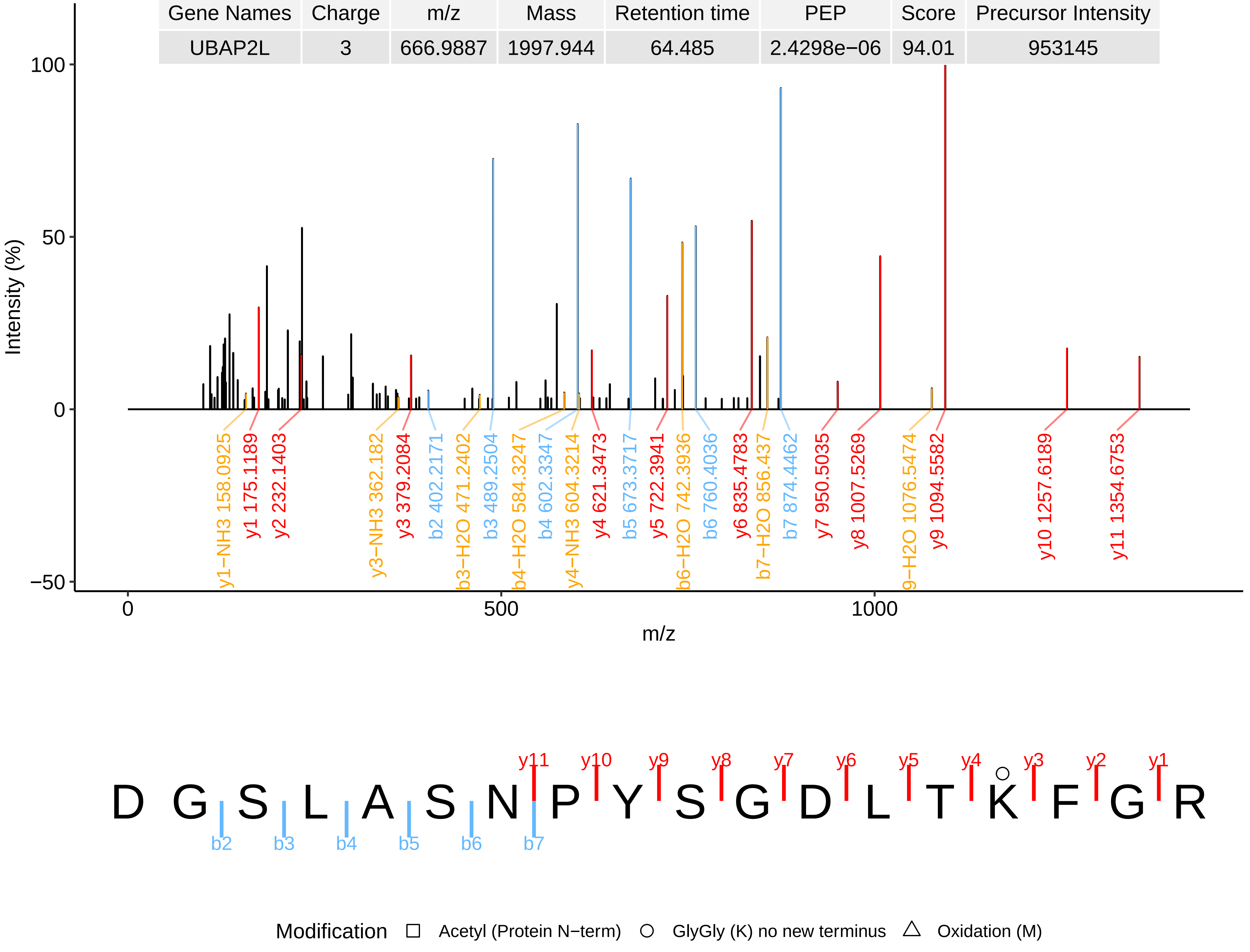

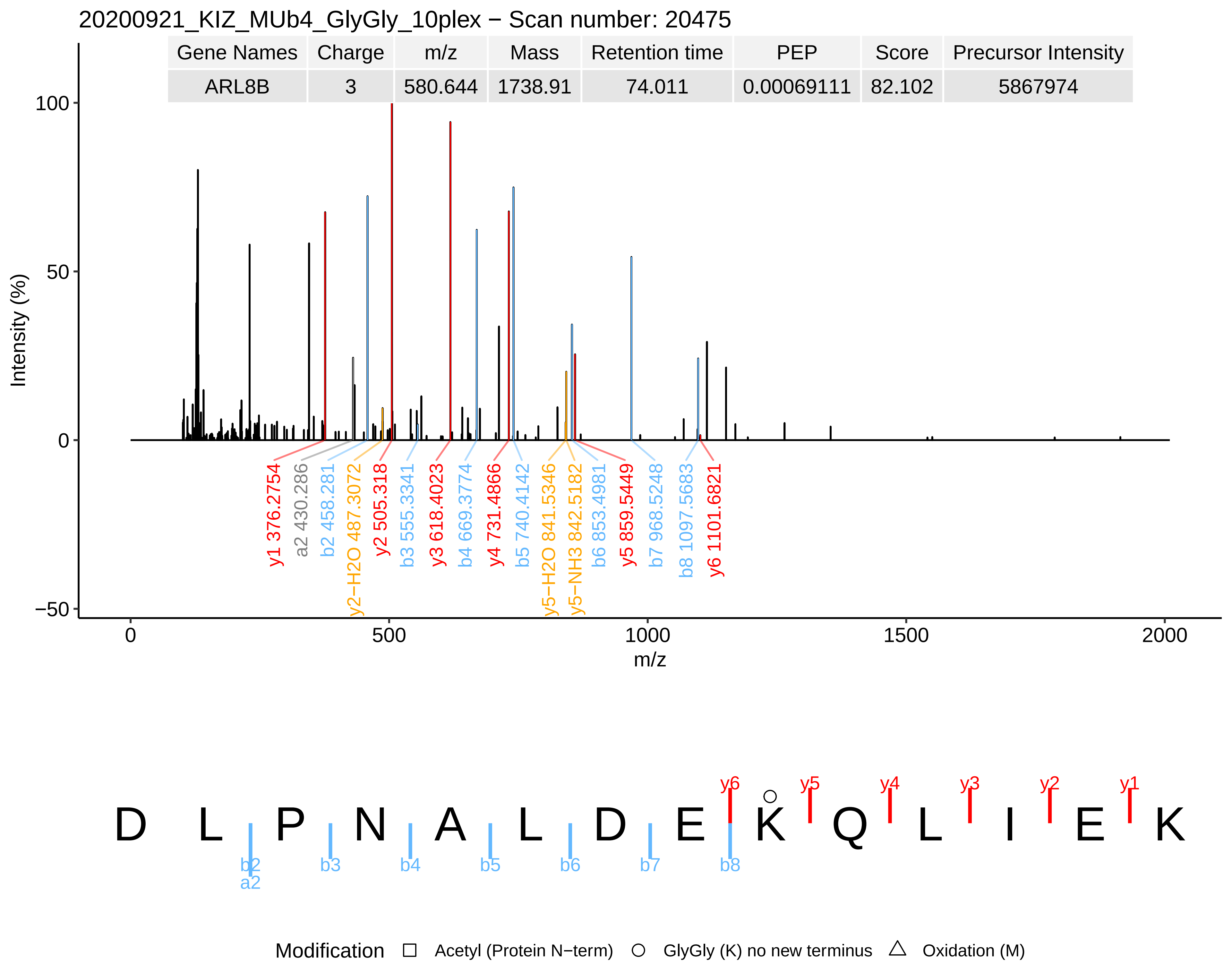
20200921_KIZ_MUb4_GlyGly_10plex − Scan number: 20475
Gene Names
ARL8B
Charge
3
m/z
580.644
Mass
1738.91
Retention time
74.011
PEP
0.00069111
Score
82.102
Precursor Intensity
5867974
Intensity (%)
100
50
0
−50
0
500
1000
1500
2000
m/z
y1 376.2754
a2 430.286
b2 458.281
y2−H2O 487.3072
y2 505.318
b3 555.3341
y3 618.4023
b4 669.3774
y4 731.4866
b5 740.4142
y5−H2O 841.5346
y5−NH3 842.5182
b6 853.4981
y5 859.5449
b7 968.5248
b8 1097.5683
y6 1101.6821
D L P N A L D E K Q L I E K
y6 y5 y4 y3 y2 y1
b2 a2 b3 b4 b5 b6 b7 b8
Modification
Acetyl (Protein N−term)
GlyGly (K) no new terminus
Oxidation (M)

20200921_KIZ_MUb4_GlyGly_10plex − Scan number: 20607

Gene Names	Charge	m/z	Mass	Retention time	PEP	Score	Precursor Intensity
ARL8B	3	580.644	1738.91	74.512	1.4288e−08	120.29	32100682

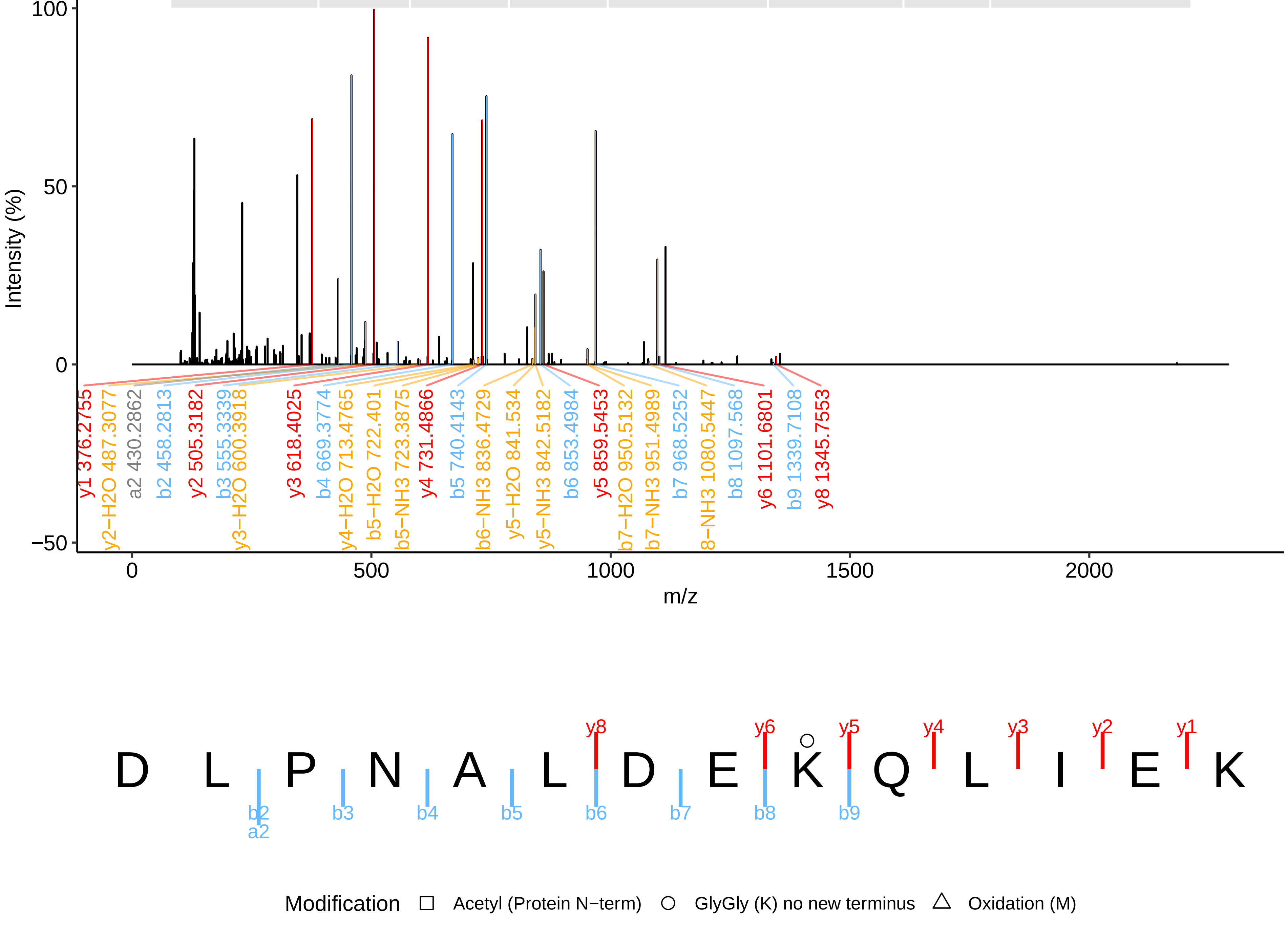

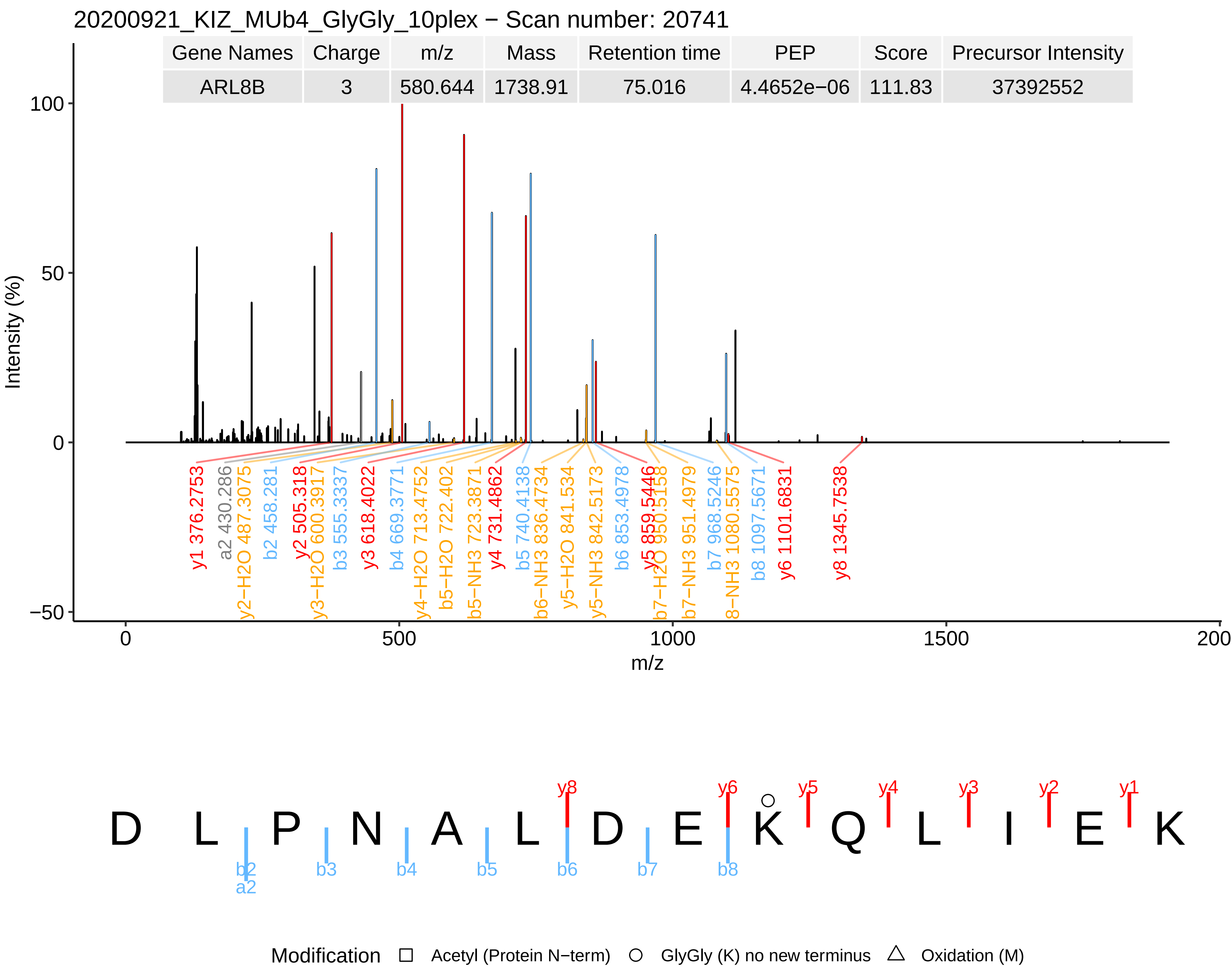

20200921_KIZ_MUb4_GlyGly_10plex − Scan number: 20741
Gene Names | Charge | m/z | Mass | Retention time | PEP | Score | Precursor Intensity
ARL8B | 3 | 580.644 | 1738.91 | 75.016 | 4.4652e−06 | 111.83 | 37392552
Intensity (%)
m/z
y1 376.2753
a2 430.286
y2−H2O 487.3075
b2 458.281
y2 505.318
y3−H2O 600.3917
b3 555.3337
y3 618.4022
b4 669.3771
y4−H2O 713.4752
b5−H2O 722.402
b5−NH3 723.3871
y4 731.4862
b5 740.4138
b6−NH3 836.4734
y5−H2O 841.534
y5−NH3 842.5173
b6 853.4978
y5 859.5446
b7−H2O 950.5158
b7−NH3 951.4979
b7 968.5246
b8−NH3 1080.5575
b8 1097.5671
y6 1101.6831
y8 1345.7538
D L P N A L D E K Q L I E K
y8 y6 y5 y4 y3 y2 y1
b2 a2 b3 b4 b5 b6 b7 b8
Modification
Acetyl (Protein N−term)
GlyGly (K) no new terminus
Oxidation (M)

20200921_KIZ_MUb4_GlyGly_10plex − Scan number: 20881

Gene Names	Charge	m/z	Mass	Retention time	PEP	Score	Precursor Intensity
ARL8B	3	580.644	1738.91	75.532	8.2211e−14	146.59	26151886

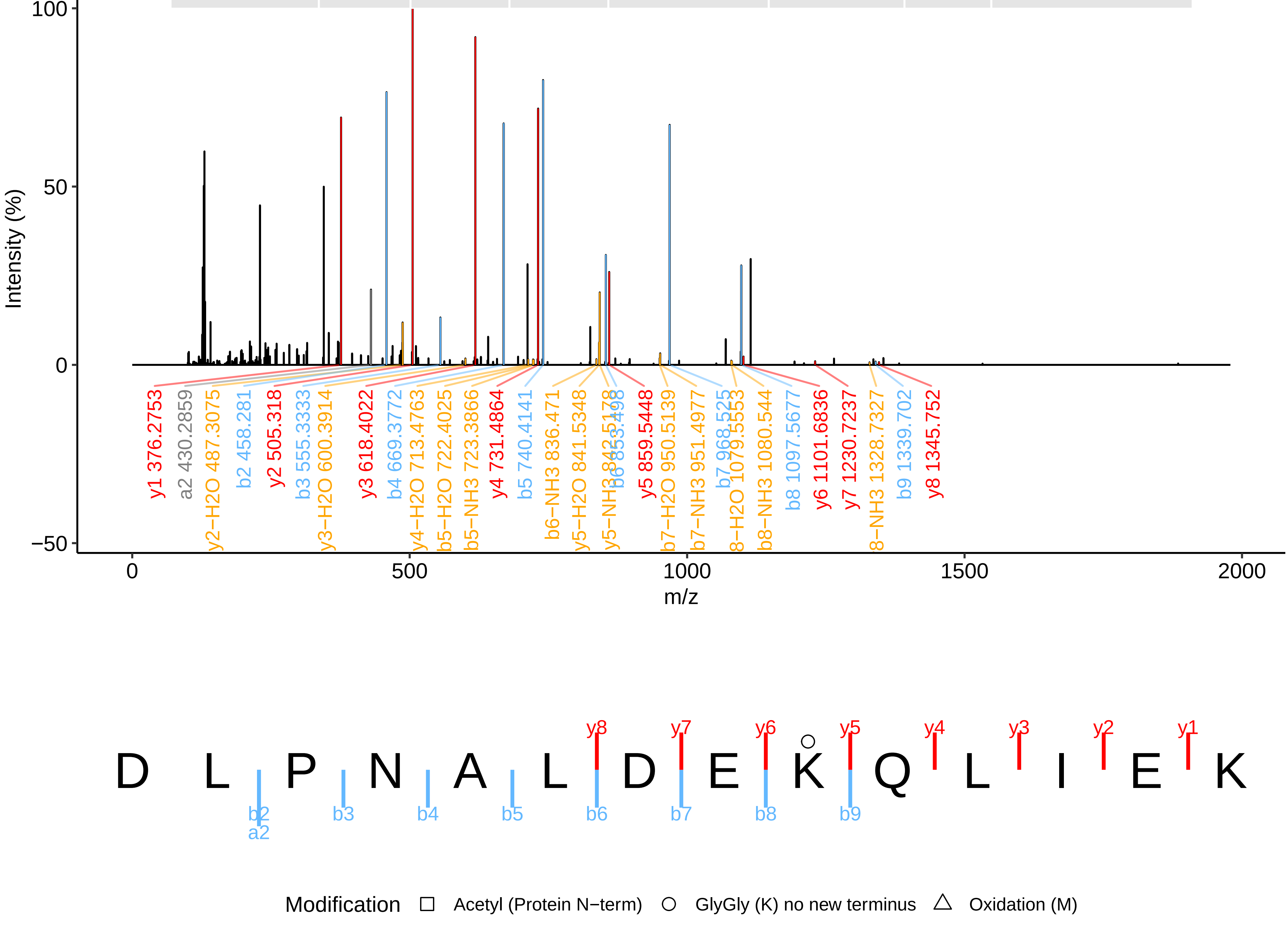

20200921_KIZ_MUb4_GlyGly_10plex − Scan number: 21017

Gene Names	Charge	m/z	Mass	Retention time	PEP	Score	Precursor Intensity
ARL8B	3	580.644	1738.91	76.046	7.8887e−06	109.07	7271193

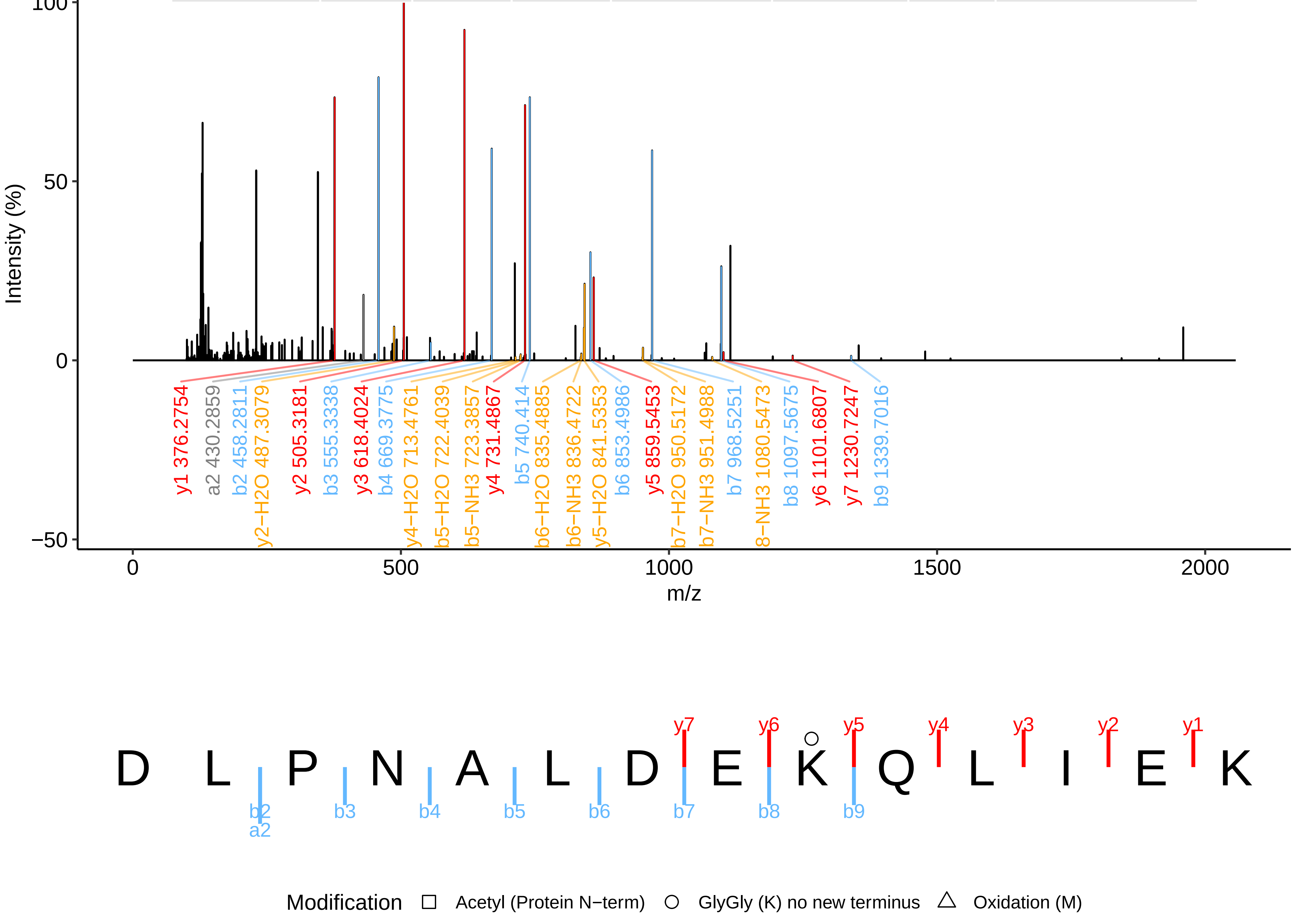

20200921_KIZ_MUb4_GlyGly_10plex − Scan number: 21155

Gene Names	Charge	m/z	Mass	Retention time	PEP	Score	Precursor Intensity
ARL8B	3	580.644	1738.91	76.558	0.00039862	91.441	NA

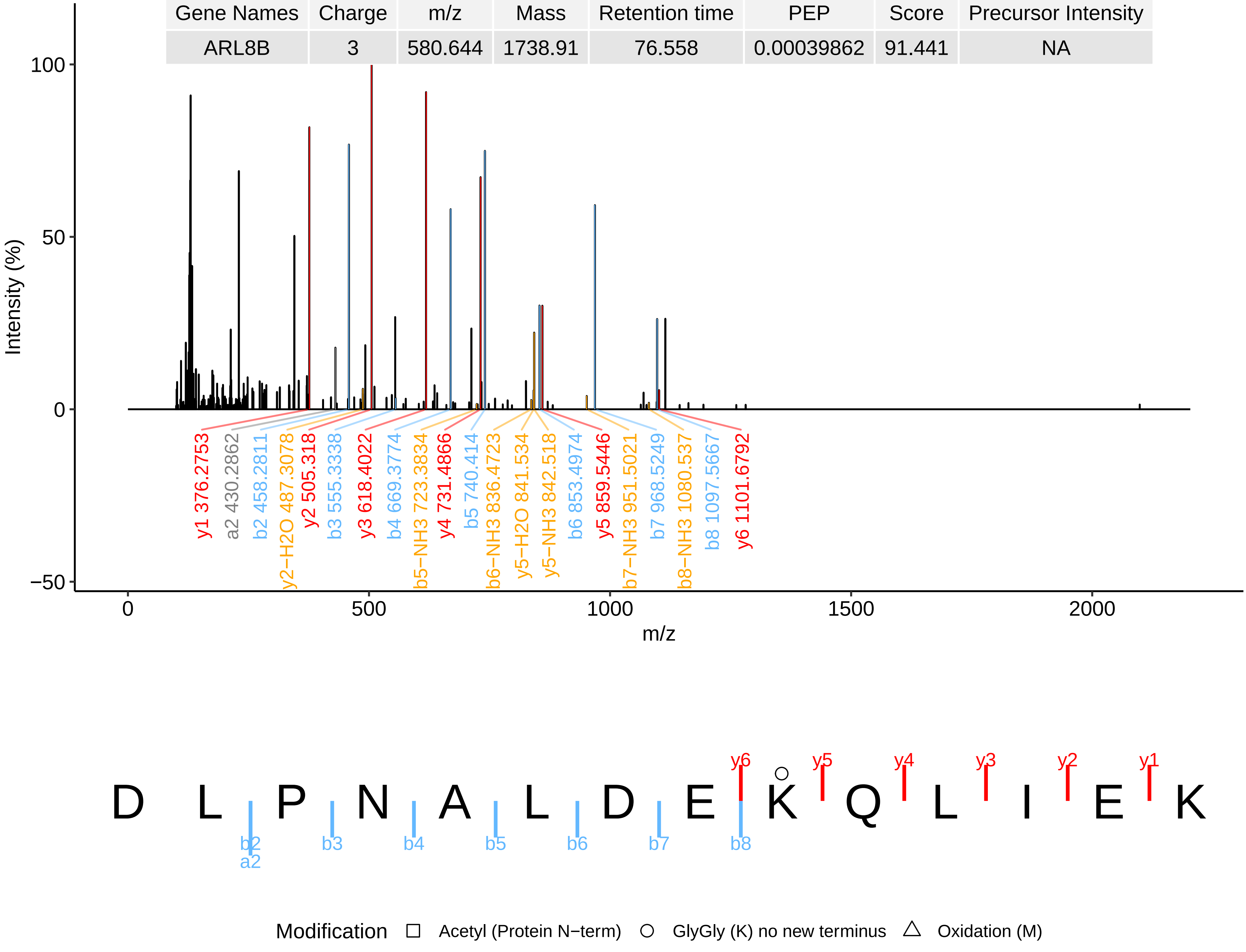

20200921_KIZ_MUb4_GlyGly_10plex_2 − Scan number: 18713

Gene Names	Charge	m/z	Mass	Retention time	PEP	Score	Precursor Intensity
ARL8B	3	580.644	1738.91	73.342	0.017221	60.55	1264743

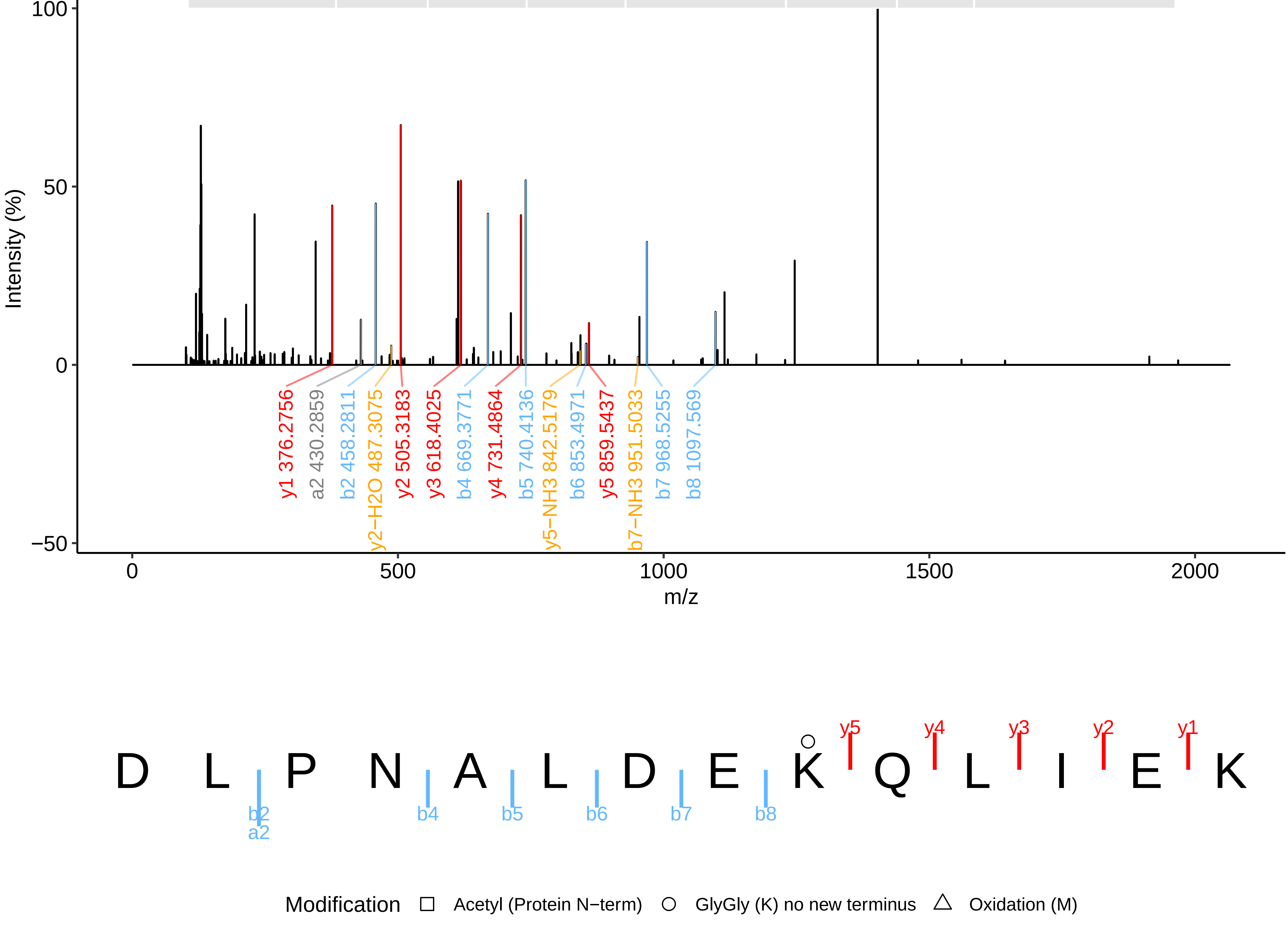

20200921_KIZ_MUb4_GlyGly_10plex_2 − Scan number: 18841

Gene Names	Charge	m/z	Mass	Retention time	PEP	Score	Precursor Intensity
ARL8B	3	580.644	1738.91	73.867	0.00064826	86.944	743699

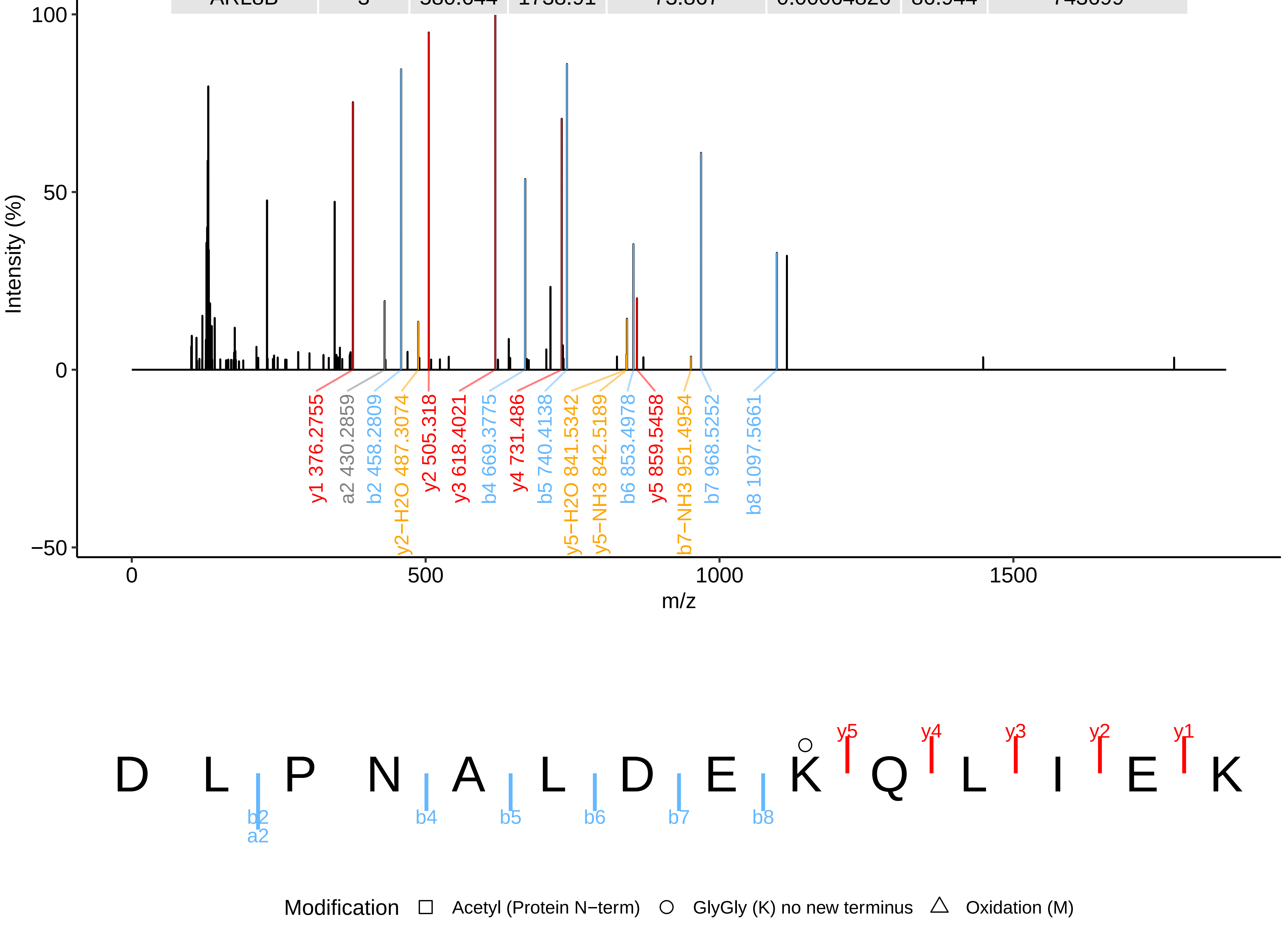

20200924_KIZ_MUb5_GlyGly_1 − Scan number: 20563

Gene Names	Charge	m/z	Mass	Retention time	PEP	Score	Precursor Intensity
ARL8B	3	580.644	1738.91	66.287	3.4563e−13	134.88	53594608

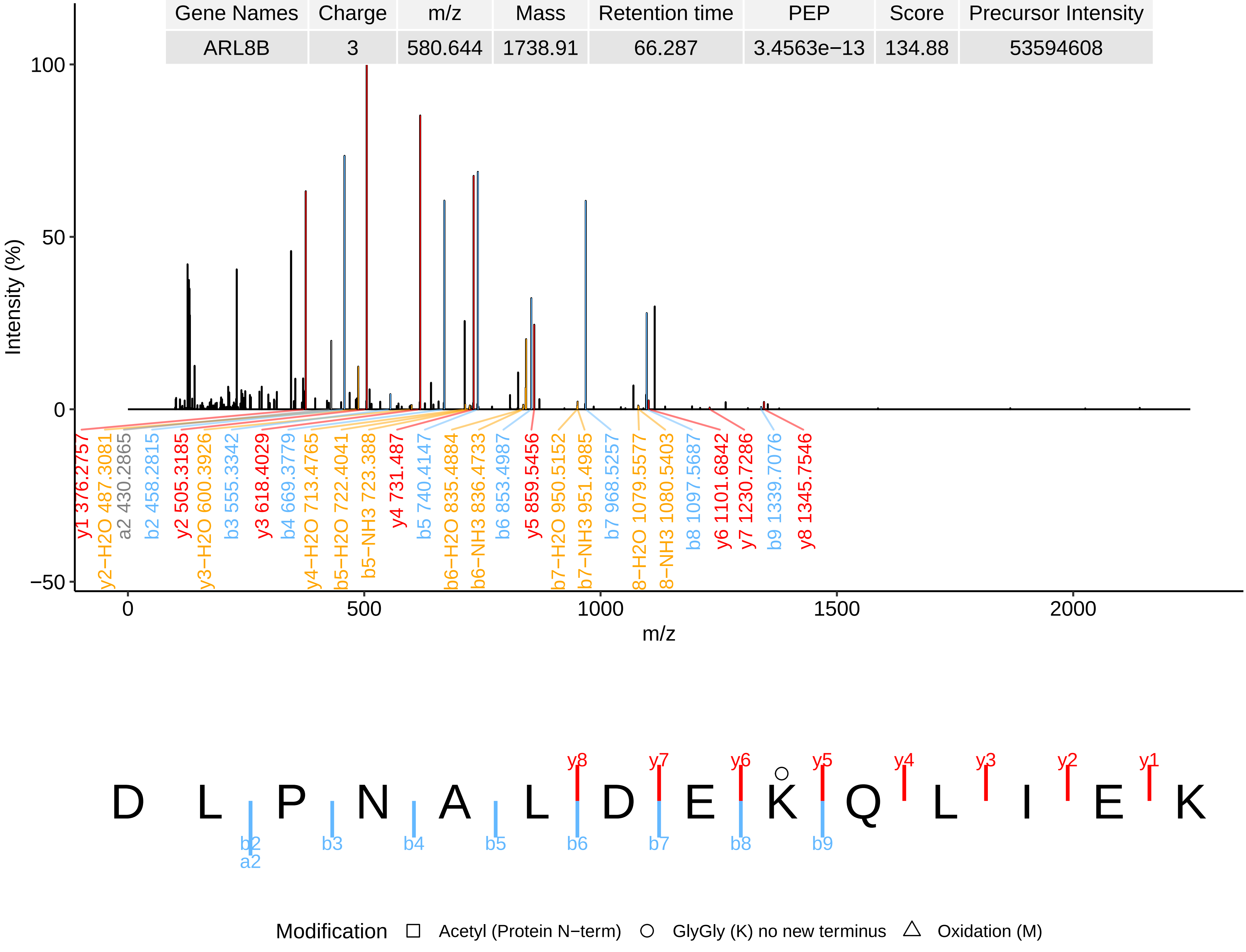

20200924_KIZ_MUb5_GlyGly_1 − Scan number: 20718

Gene Names	Charge	m/z	Mass	Retention time	PEP	Score	Precursor Intensity
ARL8B	3	580.644	1738.91	66.79	2.7068e−13	138.21	39389808

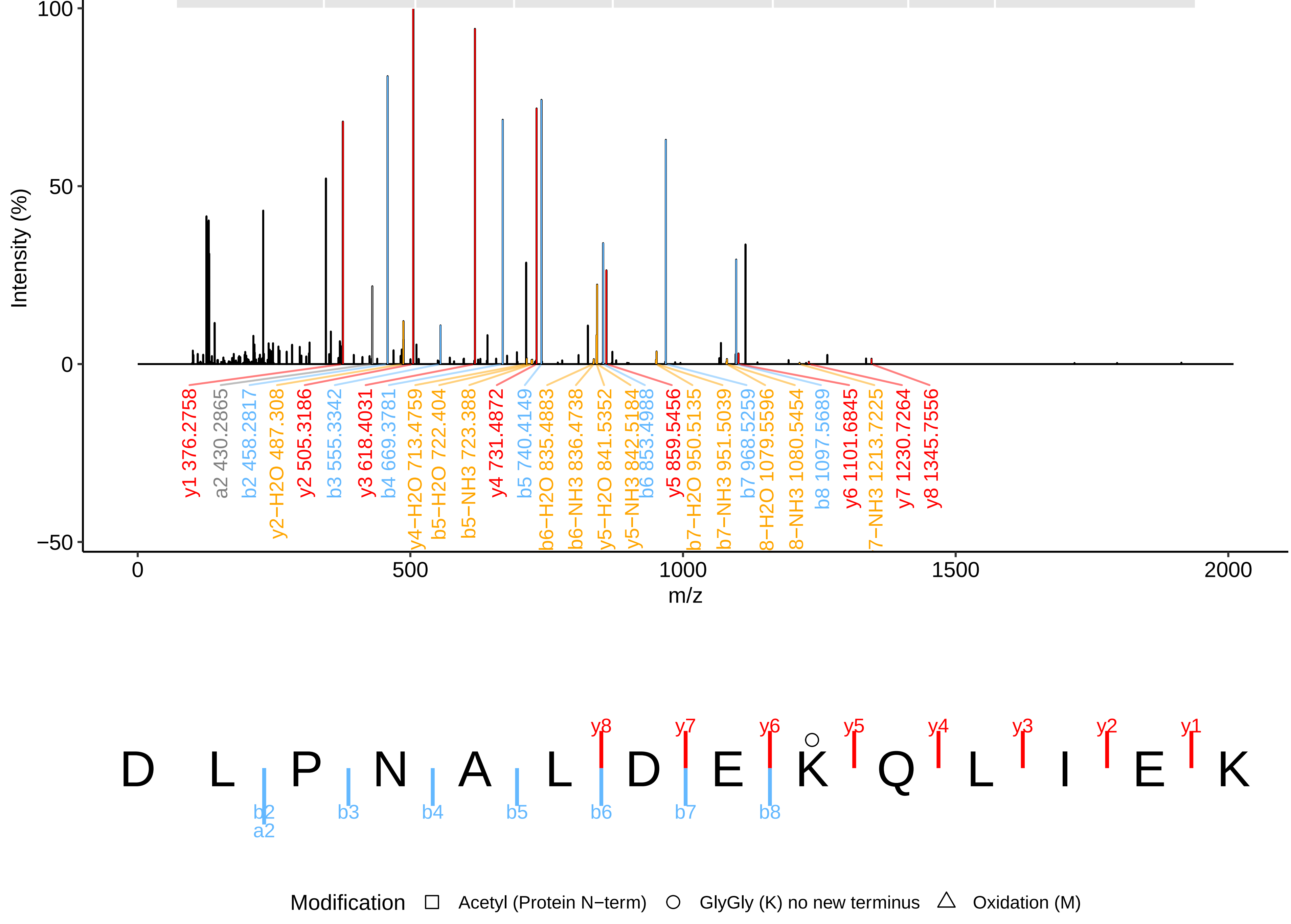

20200924_KIZ_MUb5_GlyGly_1 − Scan number: 20403

Gene Names	Charge	m/z	Mass	Retention time	PEP	Score	Precursor Intensity
ARL8B	3	580.644	1738.91	65.784	7.7377e−08	115.68	10122752

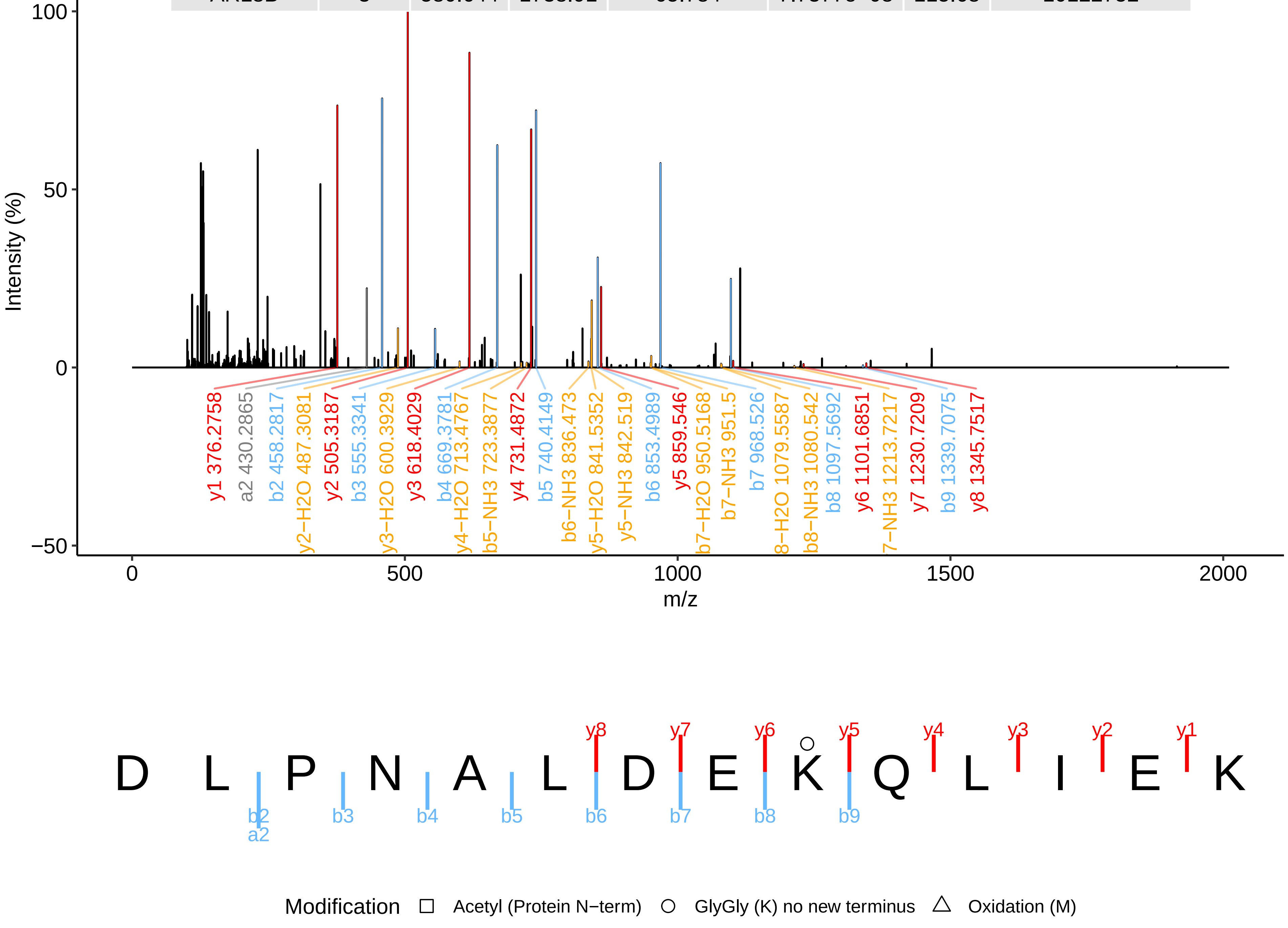

20200924_KIZ_MUb5_GlyGly_2 − Scan number: 18613

Gene Names	Charge	m/z	Mass	Retention time	PEP	Score	Precursor Intensity
ARL8B	3	580.644	1738.91	69.044	1.6621e−08	118.24	5179983

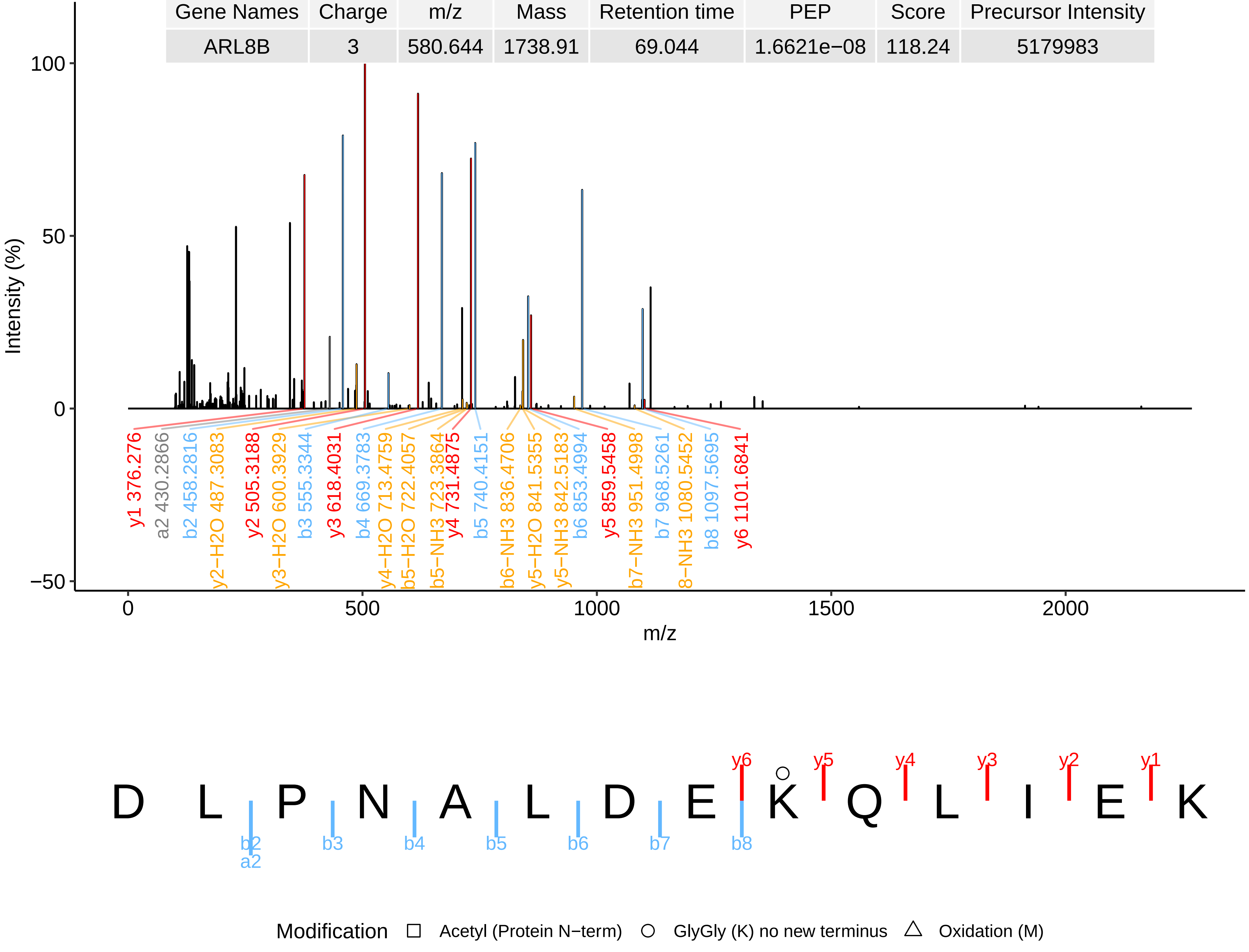

20200924_KIZ_MUb5_GlyGly_2 − Scan number: 18748

Gene Names	Charge	m/z	Mass	Retention time	PEP	Score	Precursor Intensity
ARL8B	3	580.644	1738.91	69.548	1.2964e−08	121.45	23428544

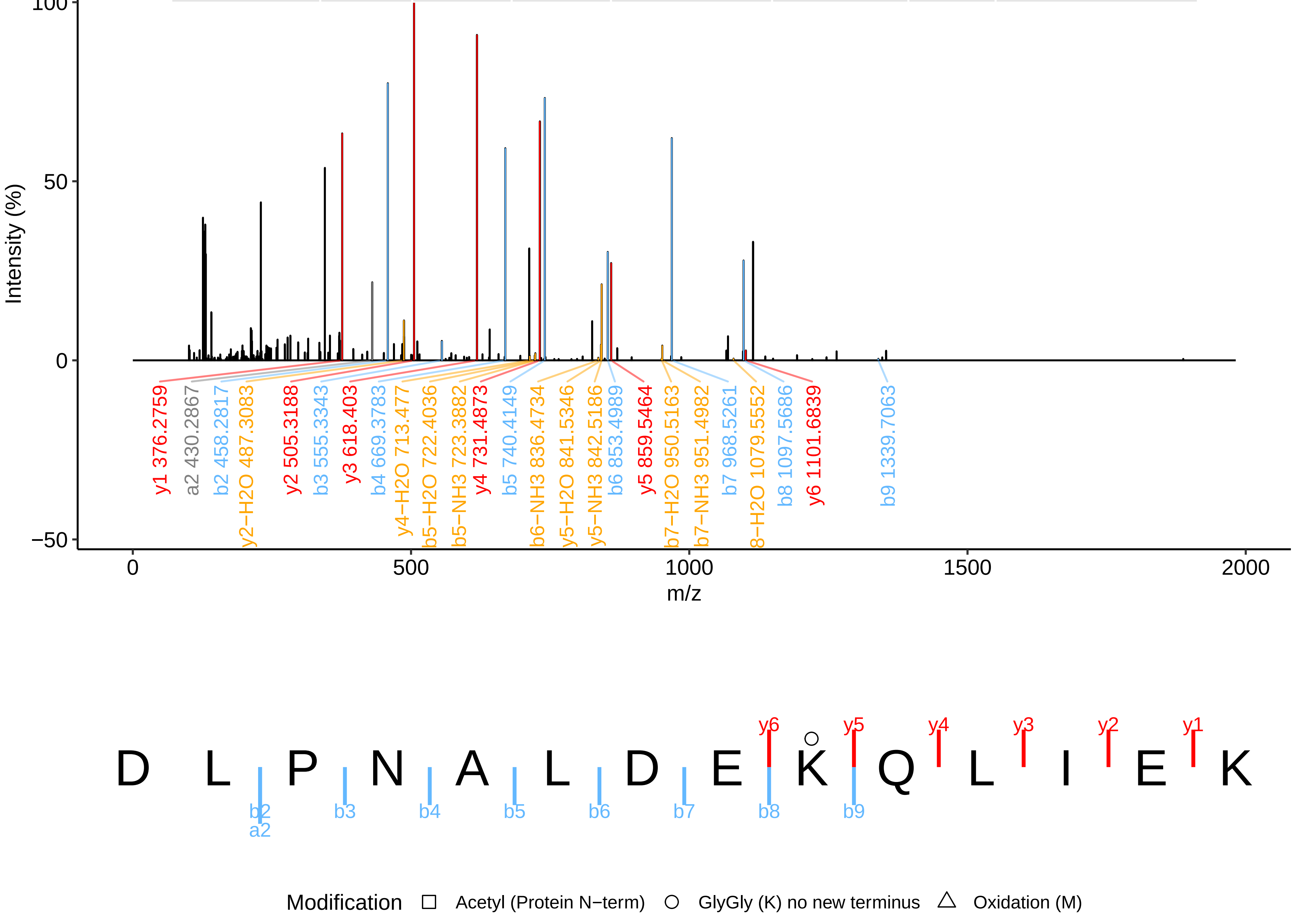

20200924_KIZ_MUb5_GlyGly_2 − Scan number: 18887

Gene Names	Charge	m/z	Mass	Retention time	PEP	Score	Precursor Intensity
ARL8B	3	580.644	1738.91	70.059	8.6696e−09	125.22	5355577

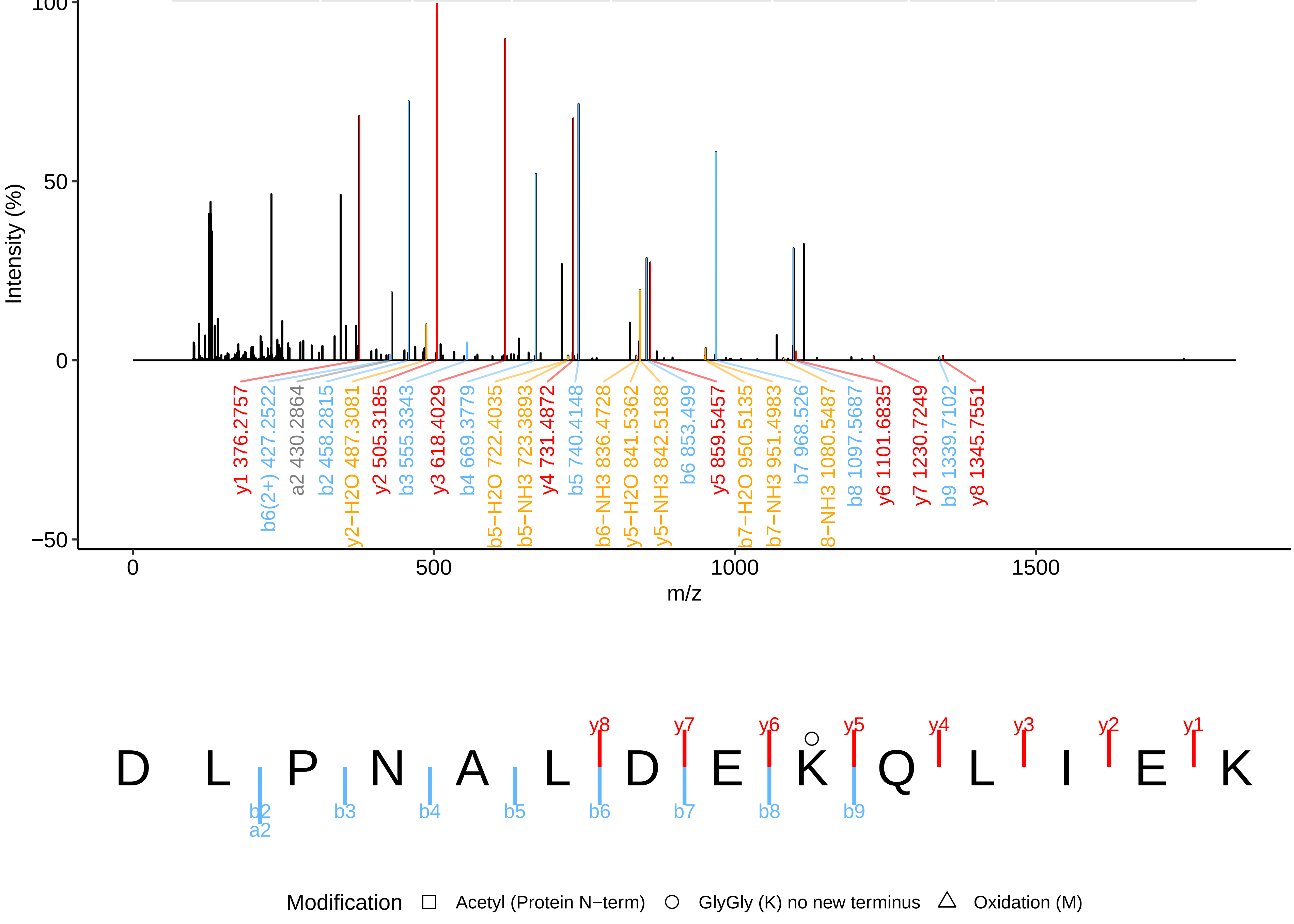

20201118_KIZ_MUb6_GlyGly_2 − Scan number: 22402

Gene Names	Charge	m/z	Mass	Retention time	PEP	Score	Precursor Intensity
ARL8B	3	580.644	1738.91	75.314	0.00082883	83.692	NA

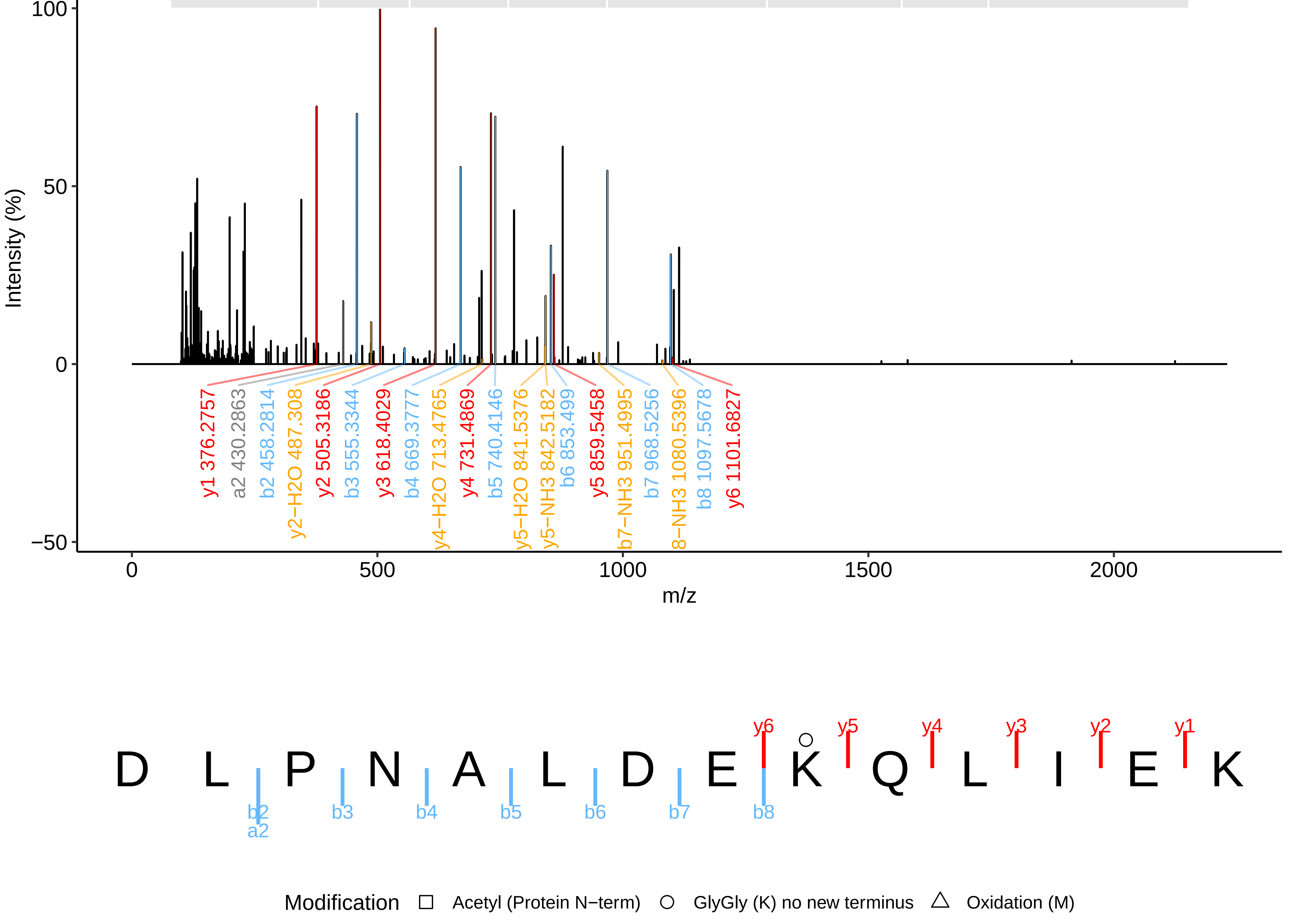

20200921_KIZ_MUb4_GlyGly_10plex − Scan number: 30156

Gene Names	Charge	m/z	Mass	Retention time	PEP	Score	Precursor Intensity
STAM2	3	593.9818	1778.924	110.78	6.9261e−14	140.84	2007600

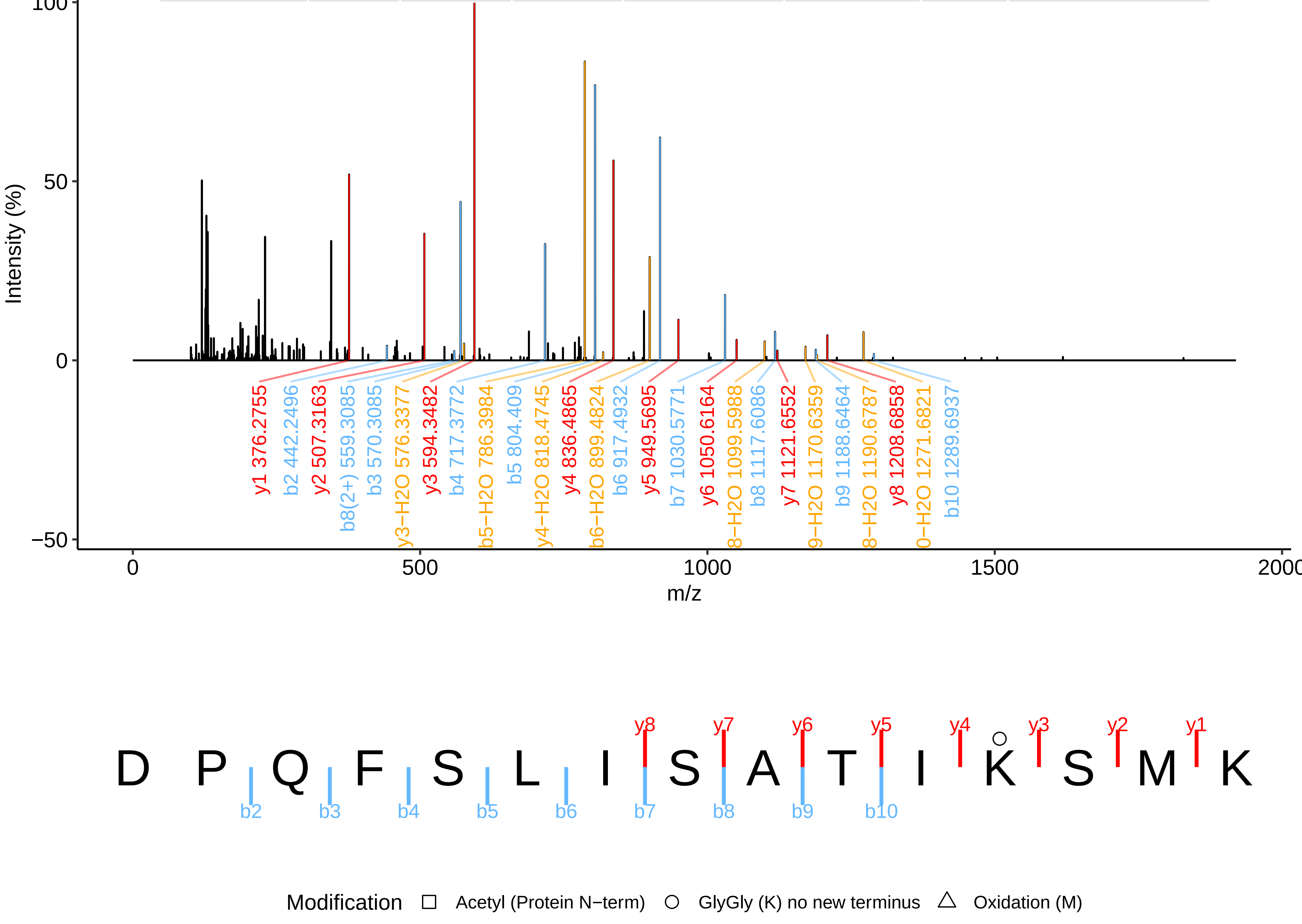

20200921_KIZ_MUb4_GlyGly_10plex_2 − Scan number: 20386

Gene Names	Charge	m/z	Mass	Retention time	PEP	Score	Precursor Intensity
PRMT1	3	584.3244	1749.951	80.125	0.064343	50.806	700228.1

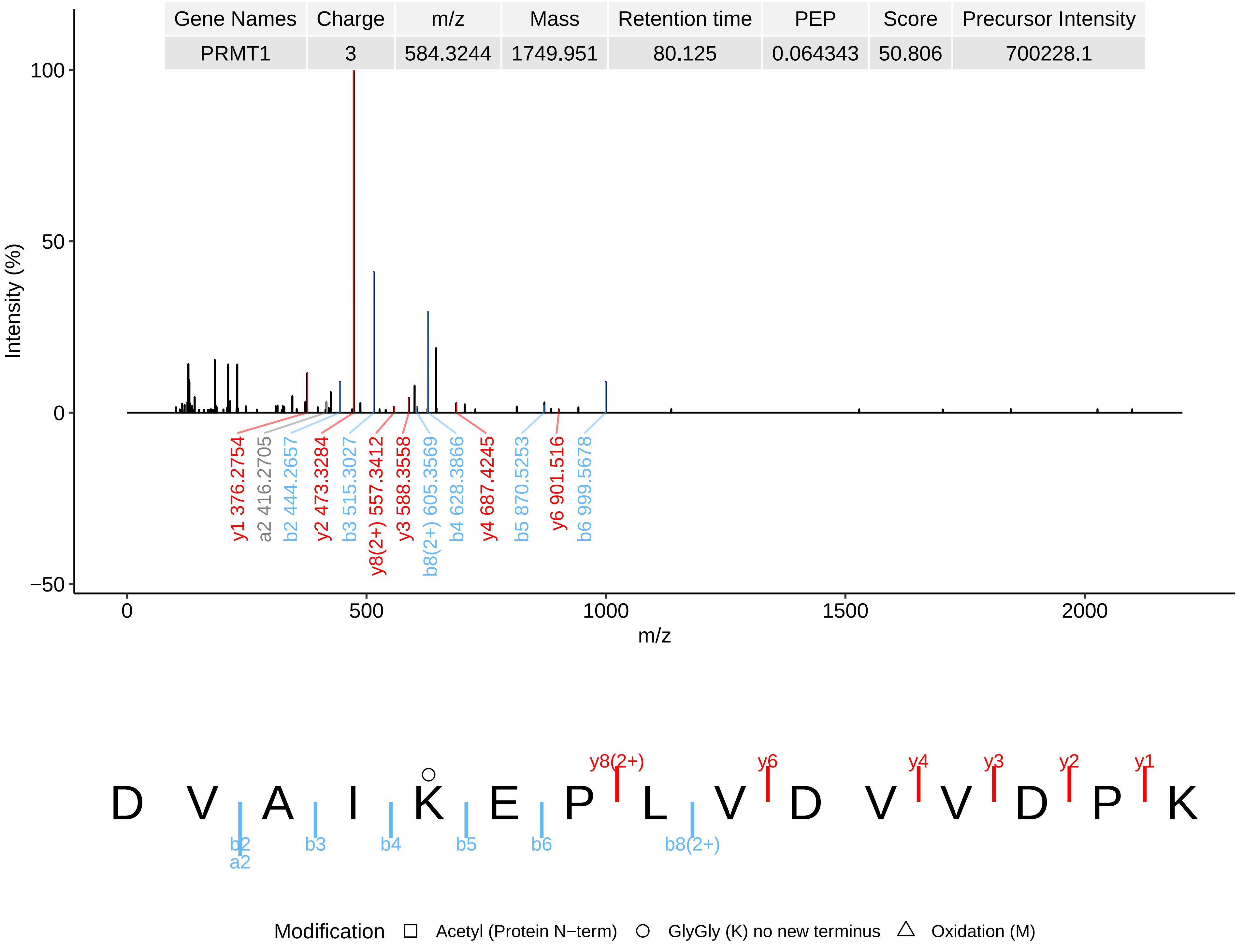

20200921_KIZ_MUb4_GlyGly_10plex − Scan number: 8922

Gene Names	Charge	m/z	Mass	Retention time	PEP	Score	Precursor Intensity
SPG20	3	568.6114	1702.812	30.433	3.0236e−09	122.66	50407640

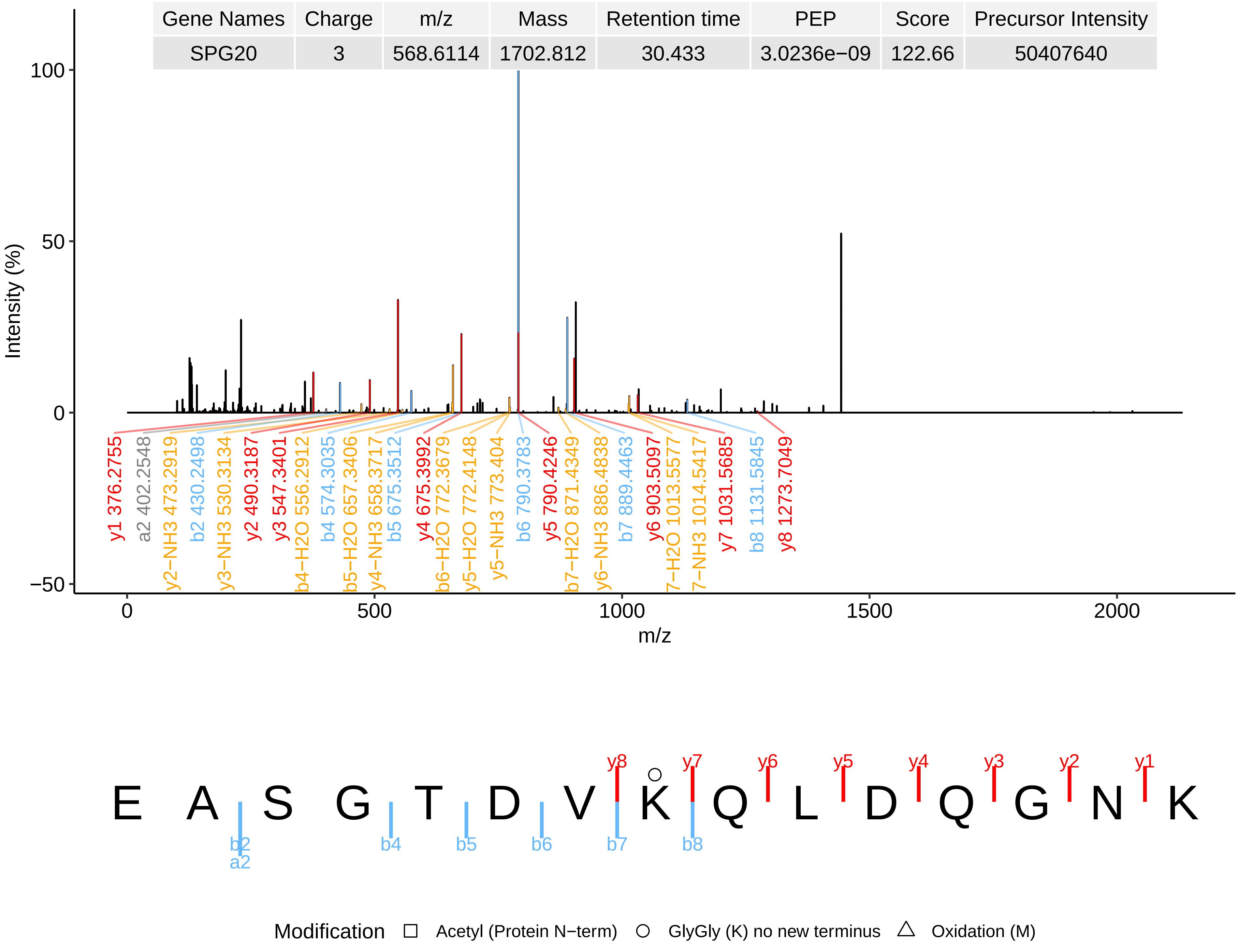

20200921_KIZ_MUb4_GlyGly_10plex_2 − Scan number: 8165

Gene Names	Charge	m/z	Mass	Retention time	PEP	Score	Precursor Intensity
SPG20	3	568.6114	1702.812	30.715	6.0208e−07	113.35	4154213

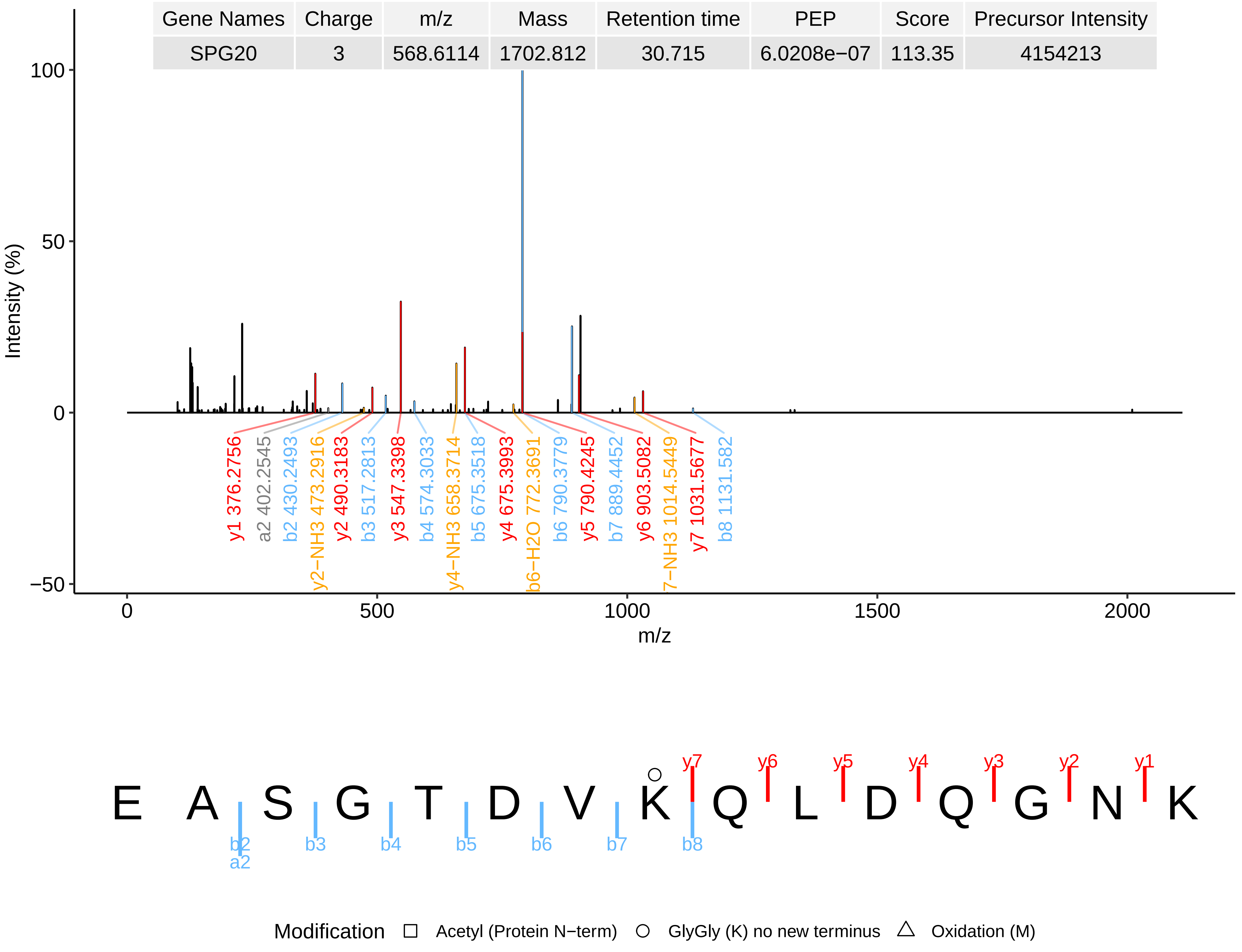

20200921_KIZ_MUb4_GlyGly_10plex_2 − Scan number: 8168

Gene Names	Charge	m/z	Mass	Retention time	PEP	Score	Precursor Intensity
SPG20	3	568.6114	1702.812	30.727	2.0016e−09	126.1	6854359

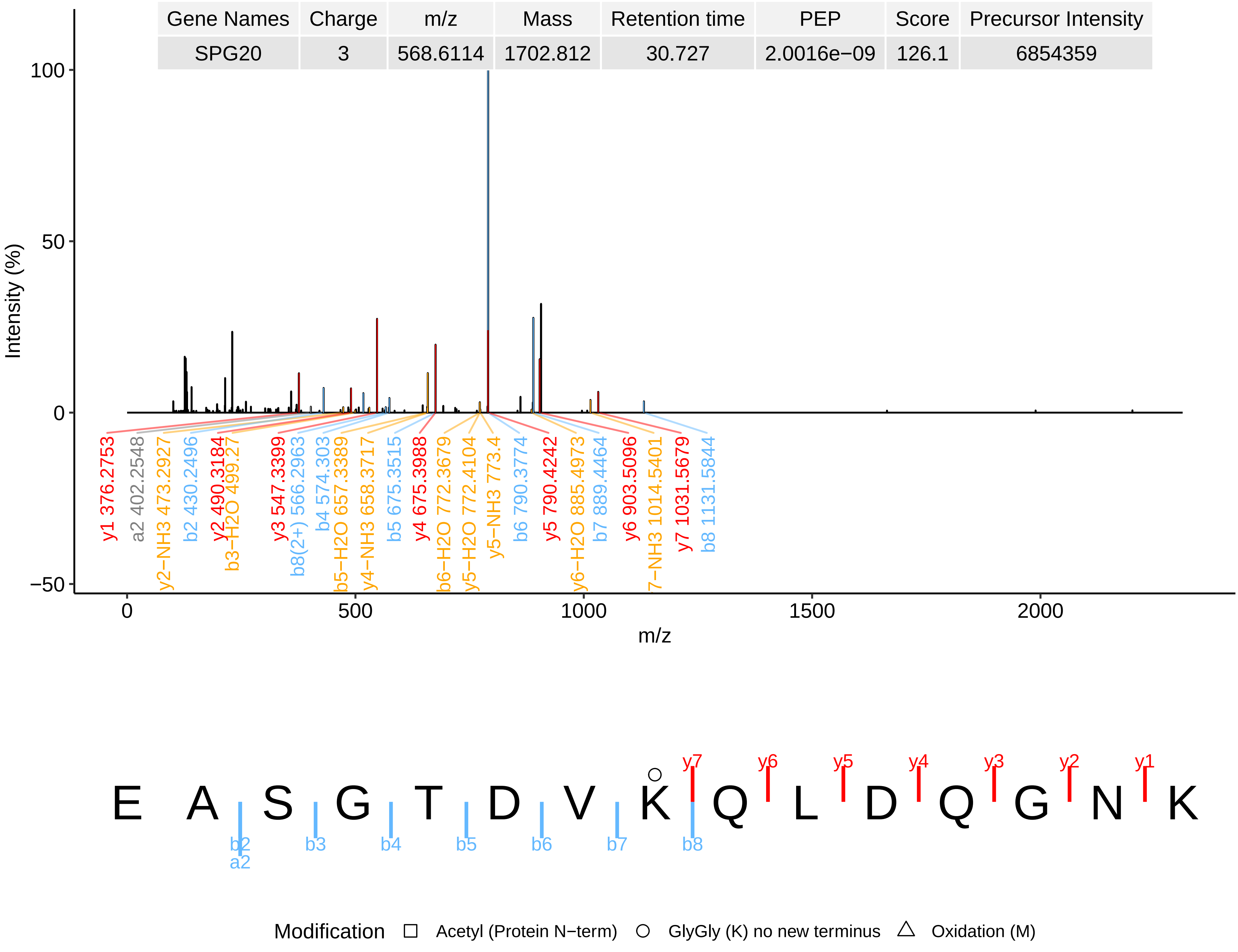

20200924_KIZ_MUb5_GlyGly_1 − Scan number: 7979

Gene Names	Charge	m/z	Mass	Retention time	PEP	Score	Precursor Intensity
SPG20	3	568.6114	1702.812	25.418	3.8416e−10	131.53	11181635

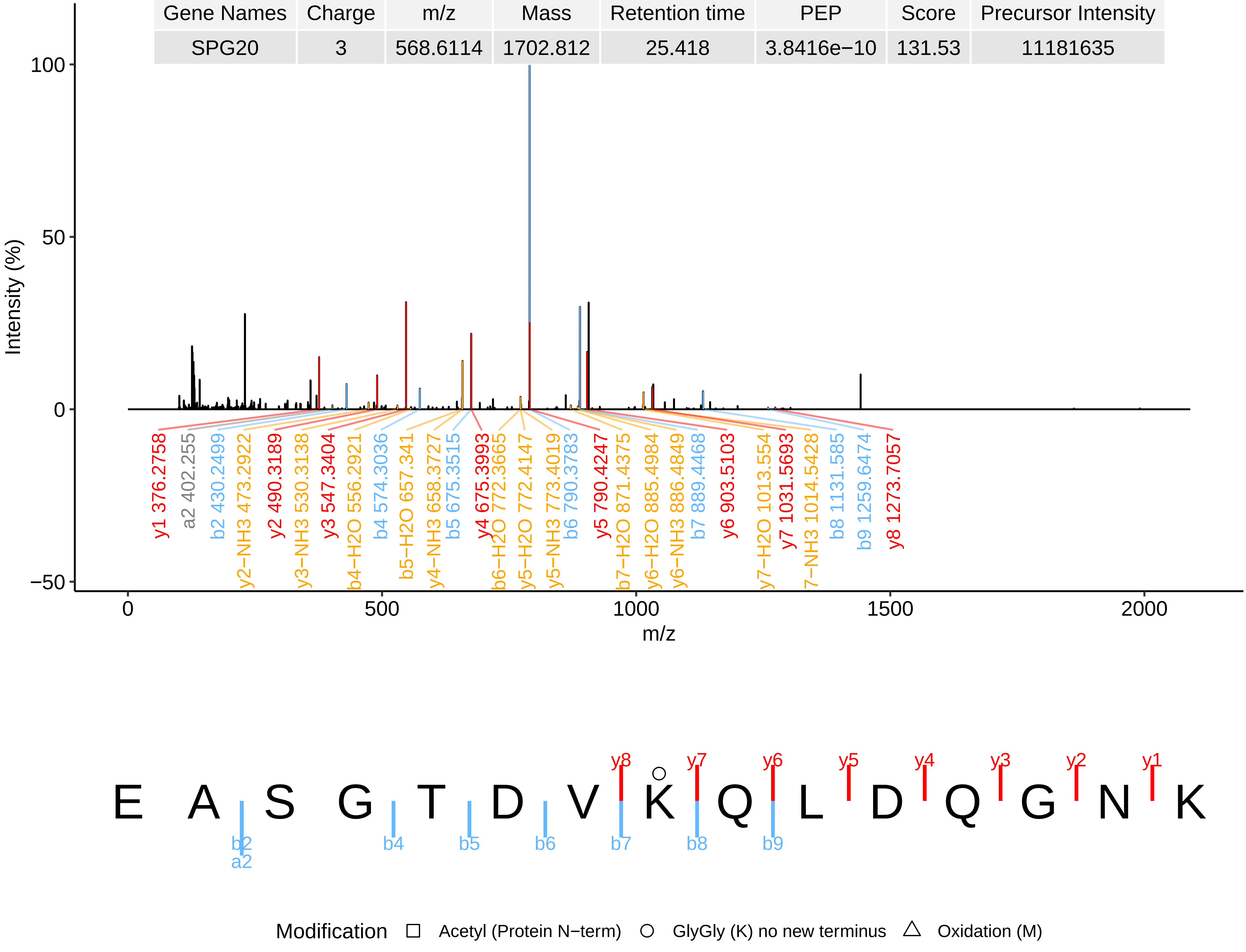

20200924_KIZ_MUb5_GlyGly_1 − Scan number: 8129

Gene Names	Charge	m/z	Mass	Retention time	PEP	Score	Precursor Intensity
SPG20	3	568.6114	1702.812	25.927	5.7891e−14	142.38	22662492

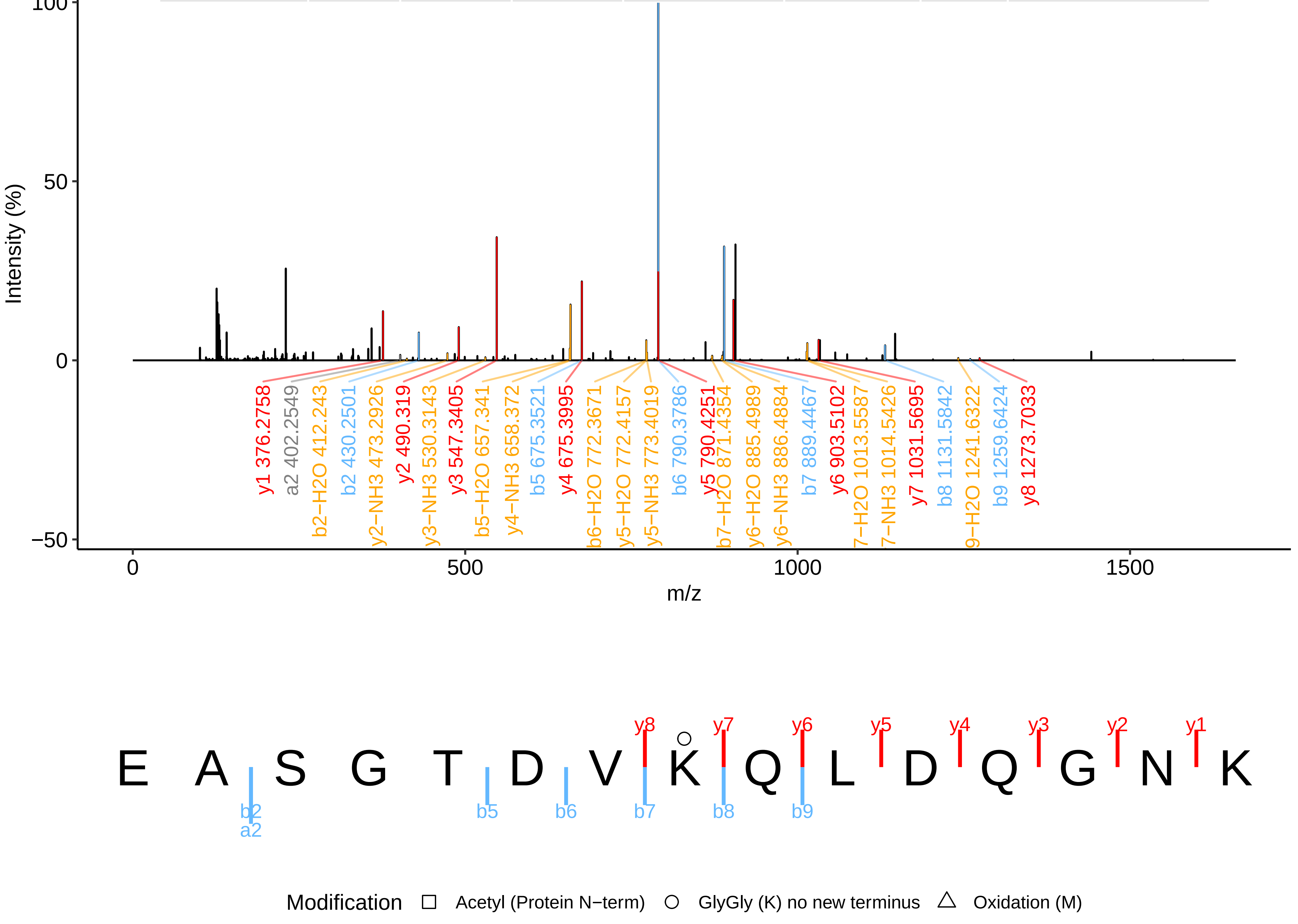

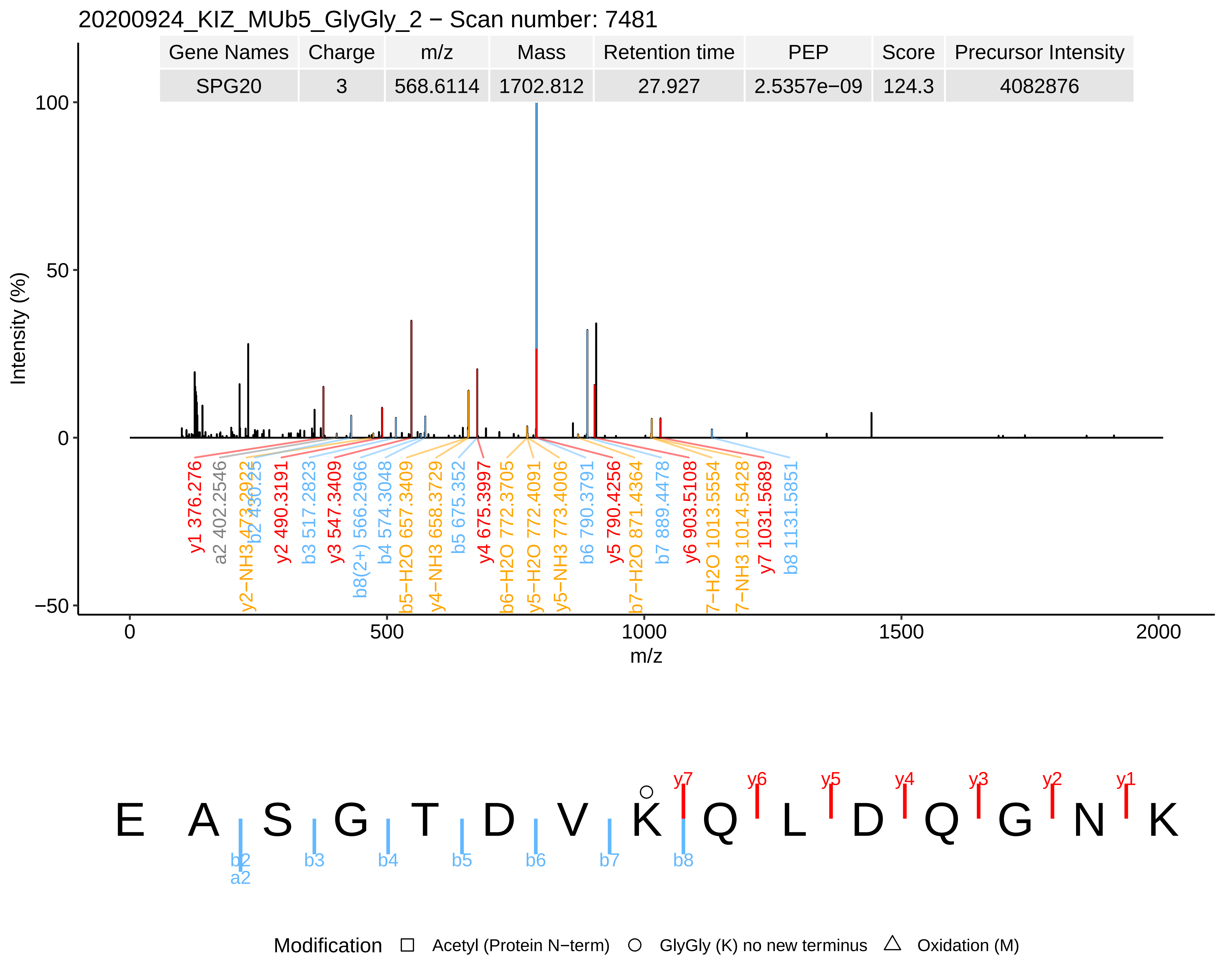
20200924_KIZ_MUb5_GlyGly_2 − Scan number: 7481
Gene Names | Charge | m/z | Mass | Retention time | PEP | Score | Precursor Intensity
SPG20 | 3 | 568.6114 | 1702.812 | 27.927 | 2.5357e−09 | 124.3 | 4082876
Intensity (%)
100
50
0
−50
y1 376.276
a2 402.2546
y2−NH3 473.2922
b2 430.25
y2 490.3191
b3 517.2823
y3 547.3409
b8(2+) 566.2966
b4 574.3048
b5−H2O 657.3409
y4−NH3 658.3729
b5 675.352
y4 675.3997
b6−H2O 772.3705
y5−H2O 772.4091
y5−NH3 773.4006
b6 790.3791
y5 790.4256
b7−H2O 871.4364
b7 889.4478
y6 903.5108
y7−H2O 1013.5554
y7−NH3 1014.5428
y7 1031.5689
b8 1131.5851
0
500
1000
1500
2000
m/z
E A S G T D V K Q L D Q G N K
y7 y6 y5 y4 y3 y2 y1
b2 a2 b3 b4 b5 b6 b7 b8
Modification
Acetyl (Protein N−term)
GlyGly (K) no new terminus
Oxidation (M)

20200924_KIZ_MUb5_GlyGly_2 − Scan number: 7612

Gene Names	Charge	m/z	Mass	Retention time	PEP	Score	Precursor Intensity
SPG20	3	568.6114	1702.812	28.427	3.526e−06	103.23	3307096

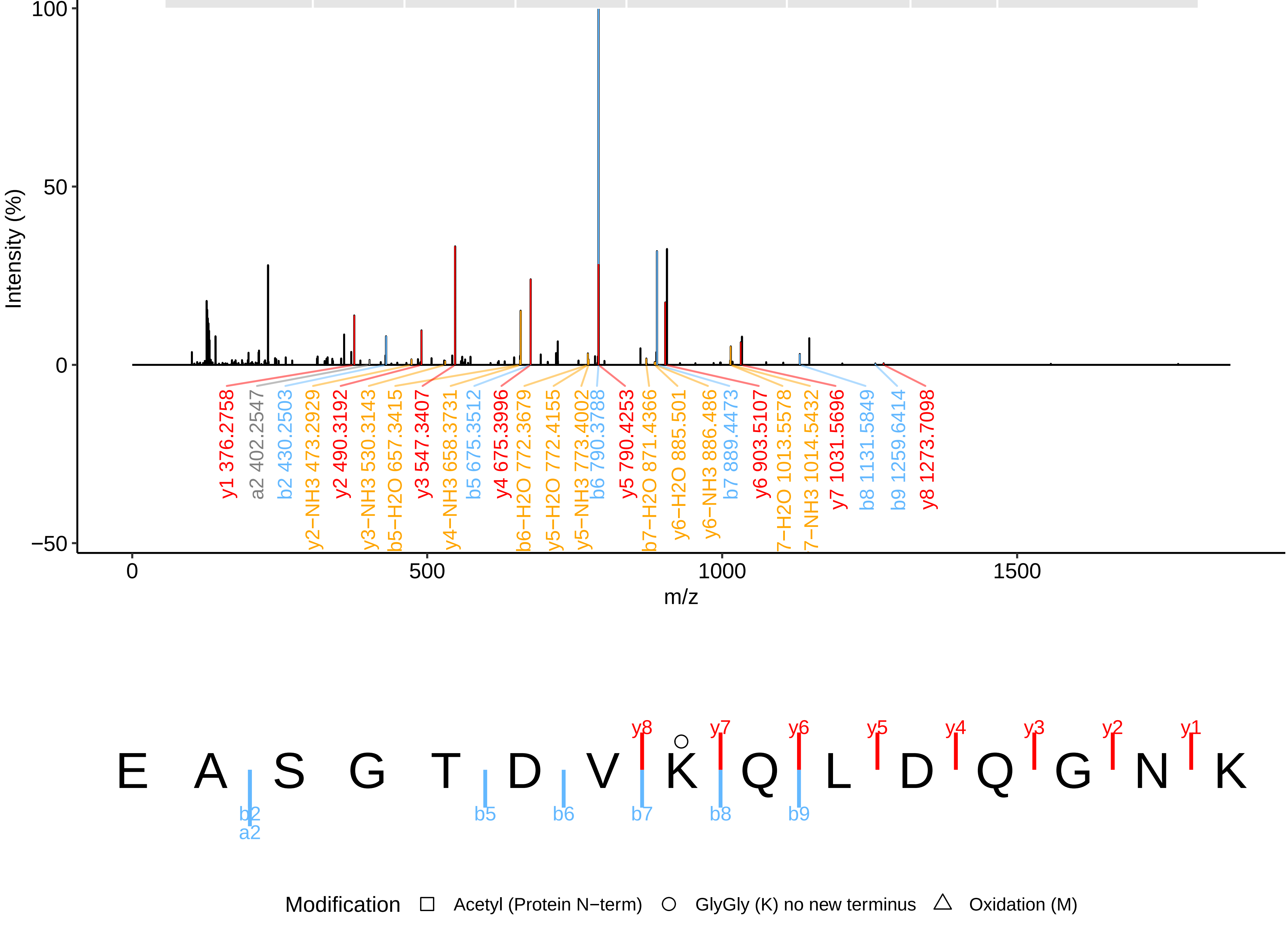

20201118_KIZ_MUb6_GlyGly_1 − Scan number: 7743

Gene Names	Charge	m/z	Mass	Retention time	PEP	Score	Precursor Intensity
SPG20	3	568.6114	1702.812	24.537	1.0373e−17	152	NA

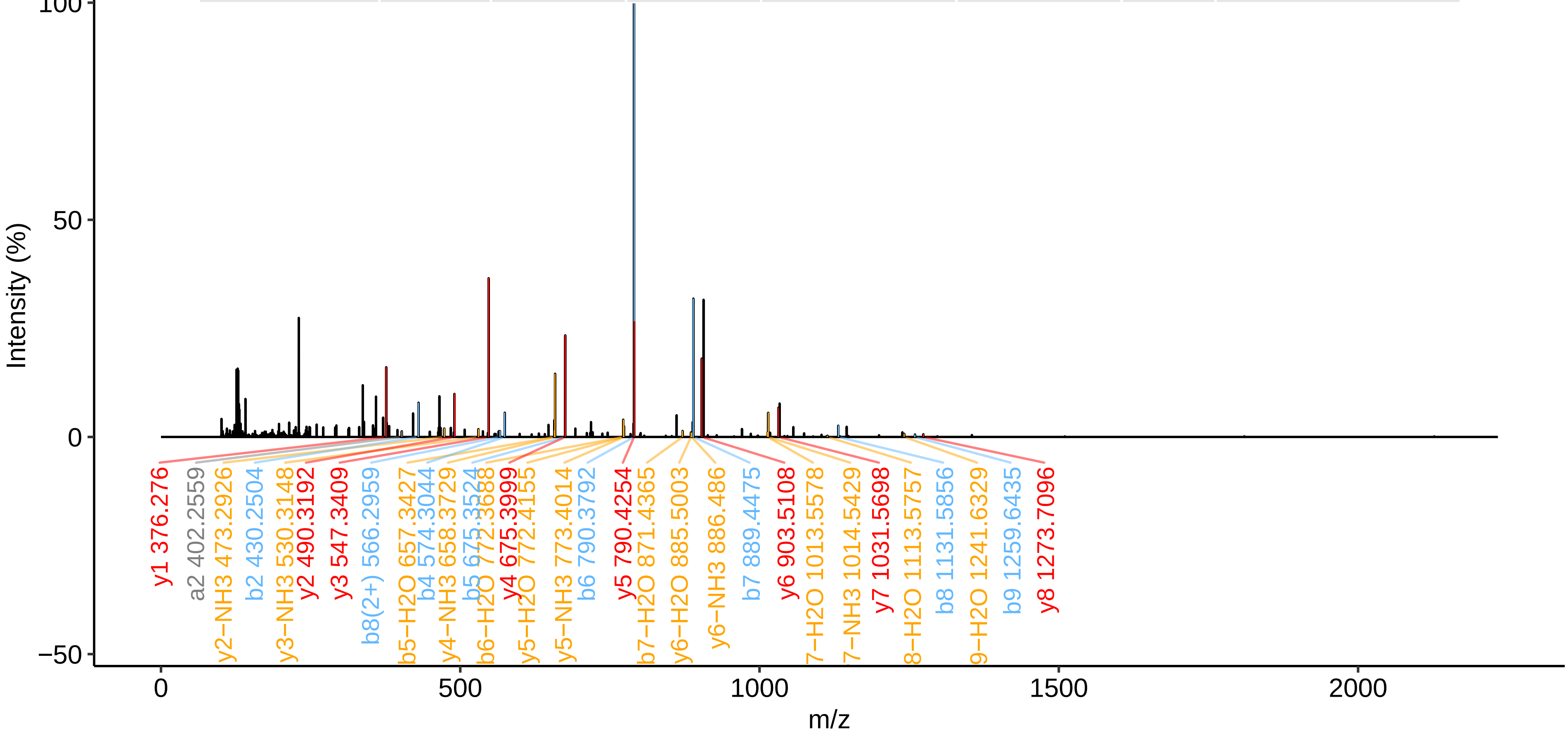

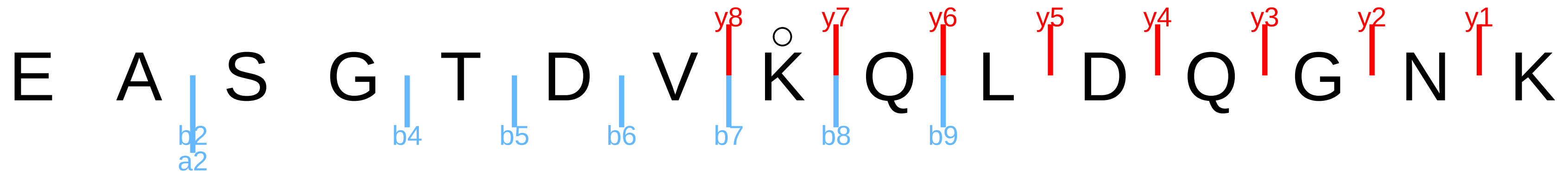

Modification □ Acetyl (Protein N−term) ○ GlyGly (K) no new terminus △ Oxidation (M)

20201118_KIZ_MUb6_GlyGly_2 − Scan number: 9092

Gene Names	Charge	m/z	Mass	Retention time	PEP	Score	Precursor Intensity
SPG20	3	568.6114	1702.812	30.814	6.0208e−07	113.35	2253028

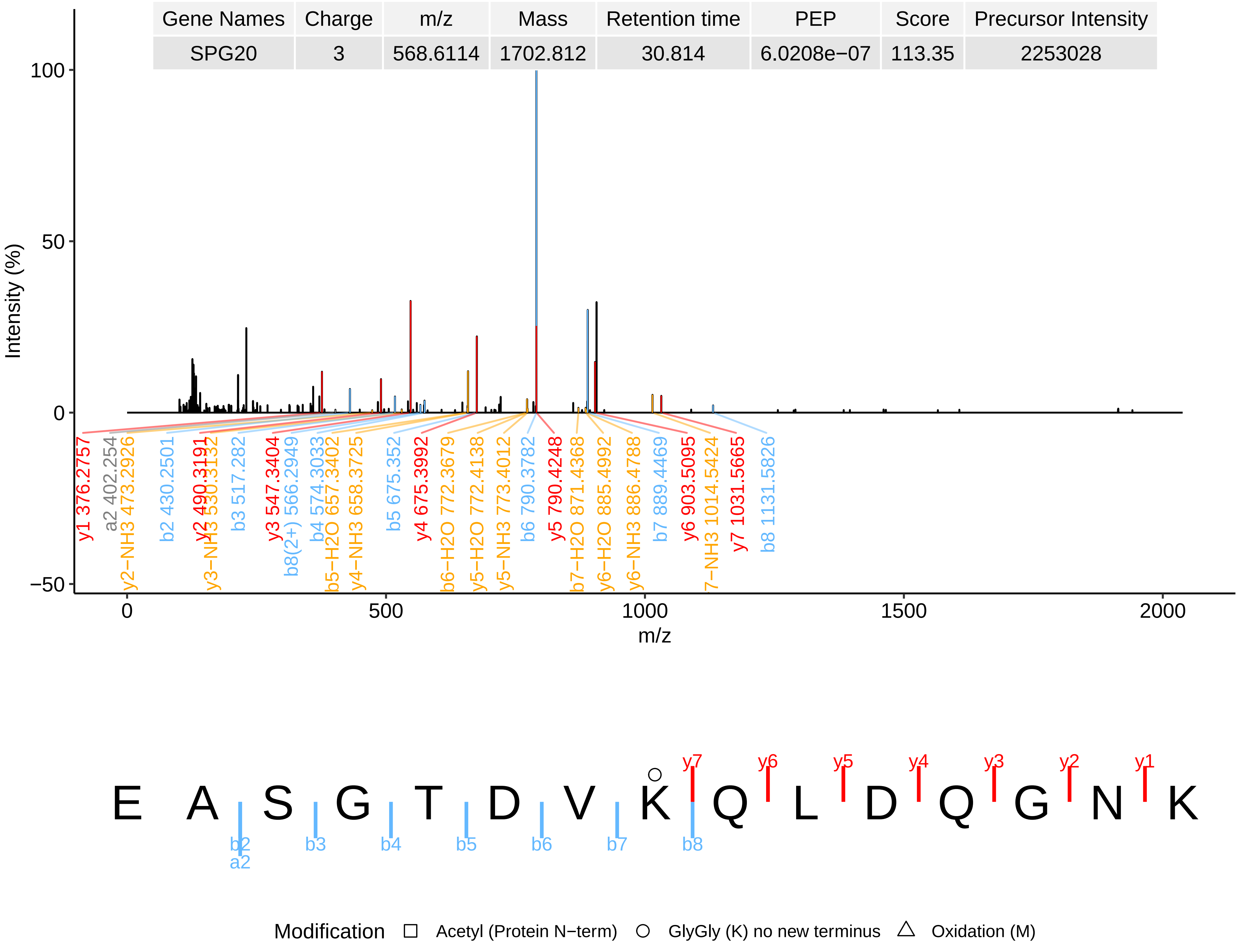

20200921_KIZ_MUb4_GlyGly_10plex − Scan number: 13236

Gene Names	Charge	m/z	Mass	Retention time	PEP	Score	Precursor Intensity
SLC43A3	3	569.2846	1704.832	46.629	1.4427e−06	110.44	5303031

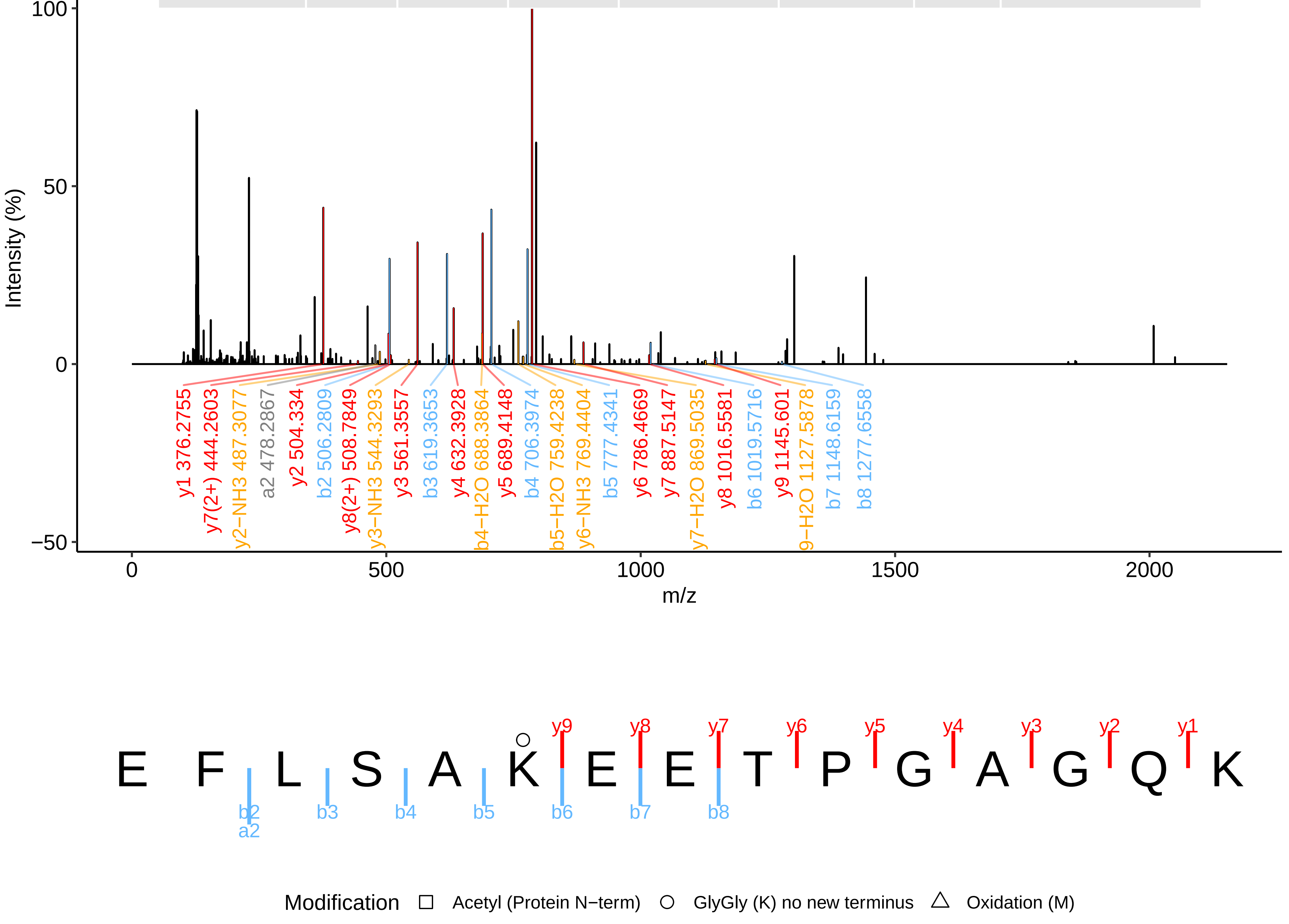

20200921_KIZ_MUb4_GlyGly_10plex − Scan number: 13368

Gene Names	Charge	m/z	Mass	Retention time	PEP	Score	Precursor Intensity
SLC43A3	3	569.2846	1704.832	47.135	8.6157e−14	138.55	33719100

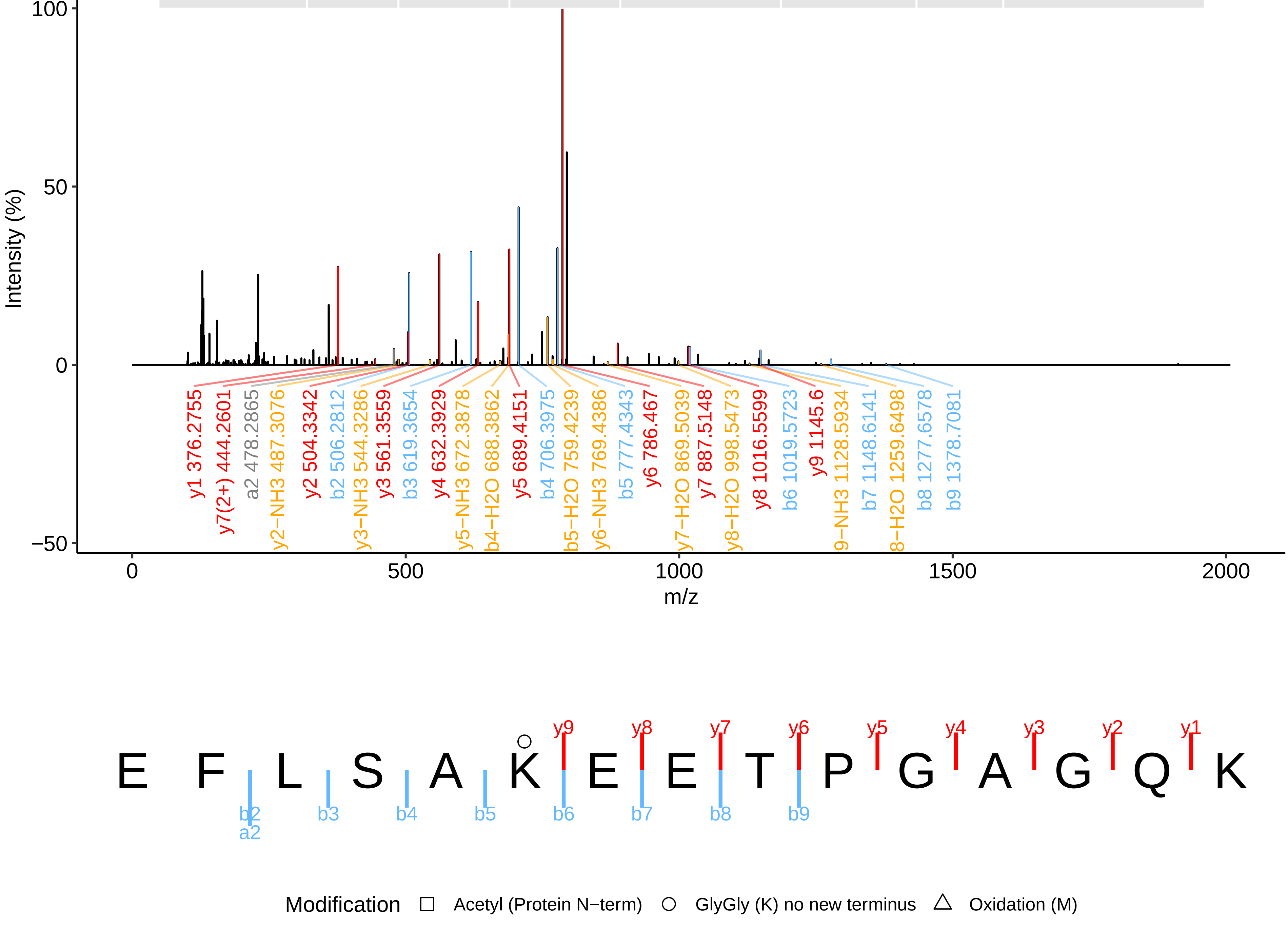

20200921_KIZ_MUb4_GlyGly_10plex − Scan number: 13335

Gene Names	Charge	m/z	Mass	Retention time	PEP	Score	Precursor Intensity
SLC43A3	4	427.2153	1704.832	47.012	9.7606e−05	86.467	58037740

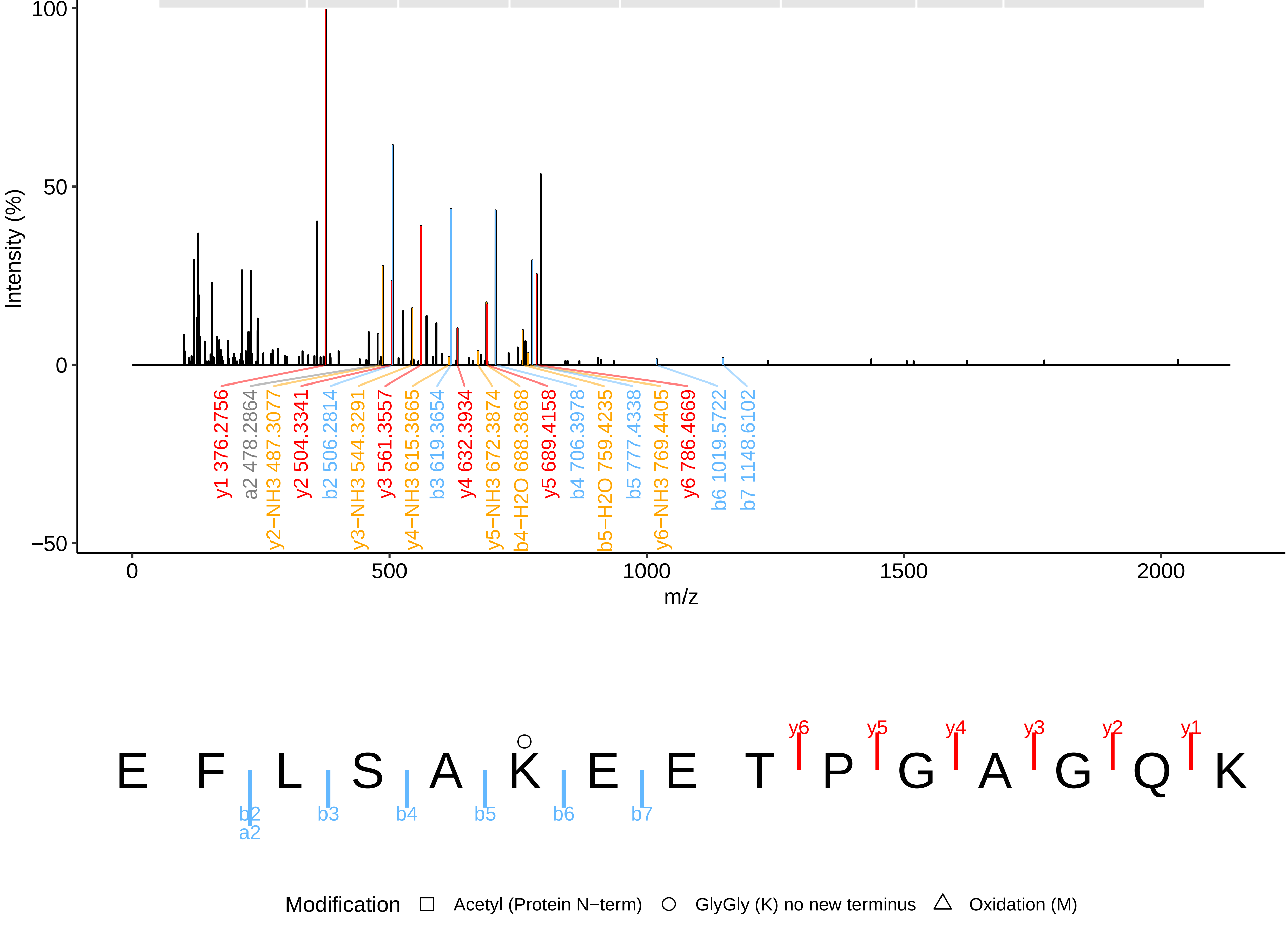

20200921_KIZ_MUb4_GlyGly_10plex_2 − Scan number: 12202

Gene Names	Charge	m/z	Mass	Retention time	PEP	Score	Precursor Intensity
SLC43A3	3	569.2846	1704.832	47.16	0.00011384	86.944	799692.5

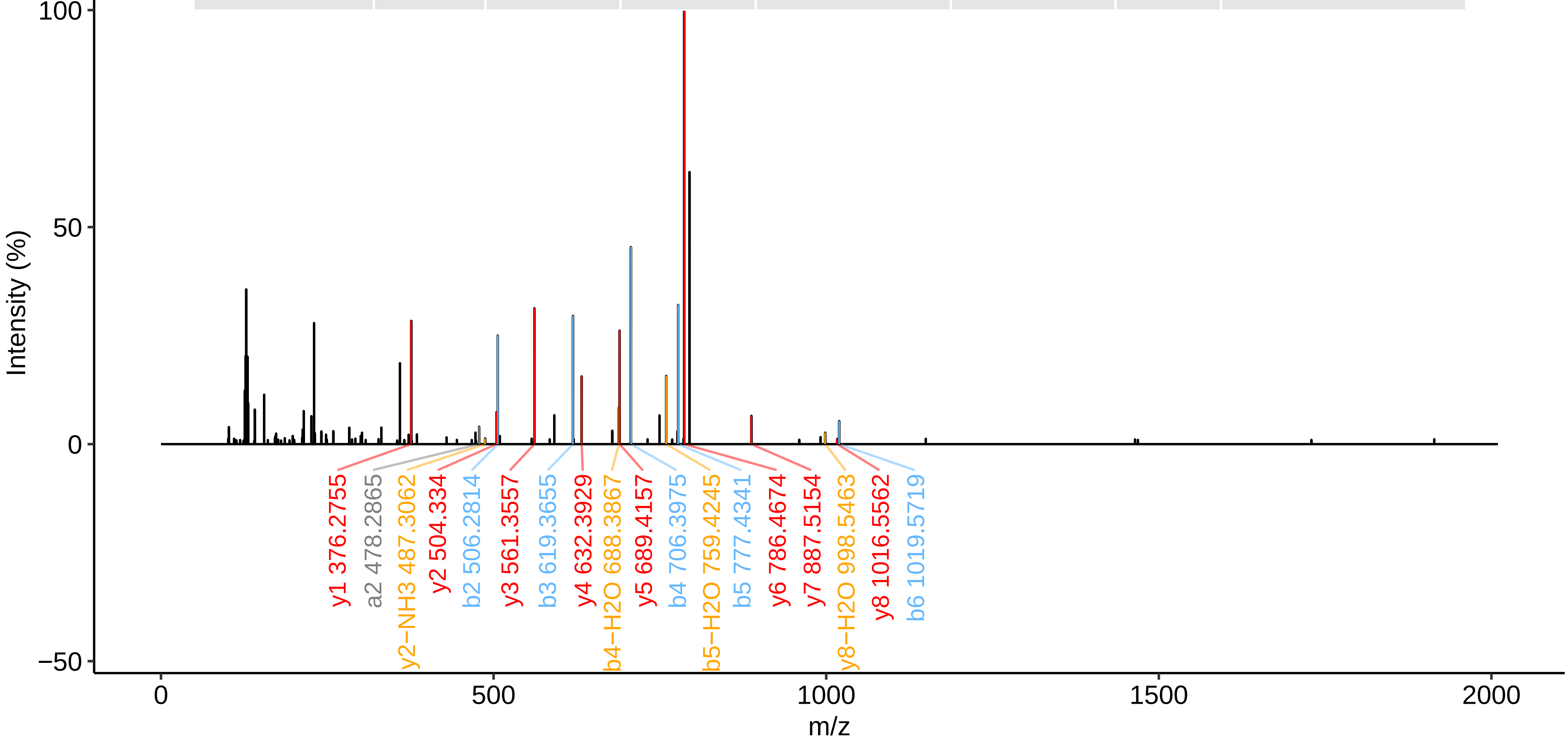

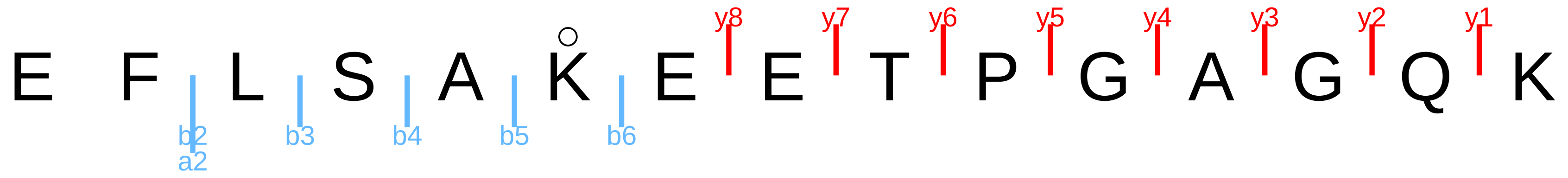

Modification □ Acetyl (Protein N−term) ○ GlyGly (K) no new terminus △ Oxidation (M)

20200921_KIZ_MUb4_GlyGly_10plex_2 − Scan number: 12329

Gene Names	Charge	m/z	Mass	Retention time	PEP	Score	Precursor Intensity
SLC43A3	3	569.2846	1704.832	47.675	0.0032748	67.234	861962.1

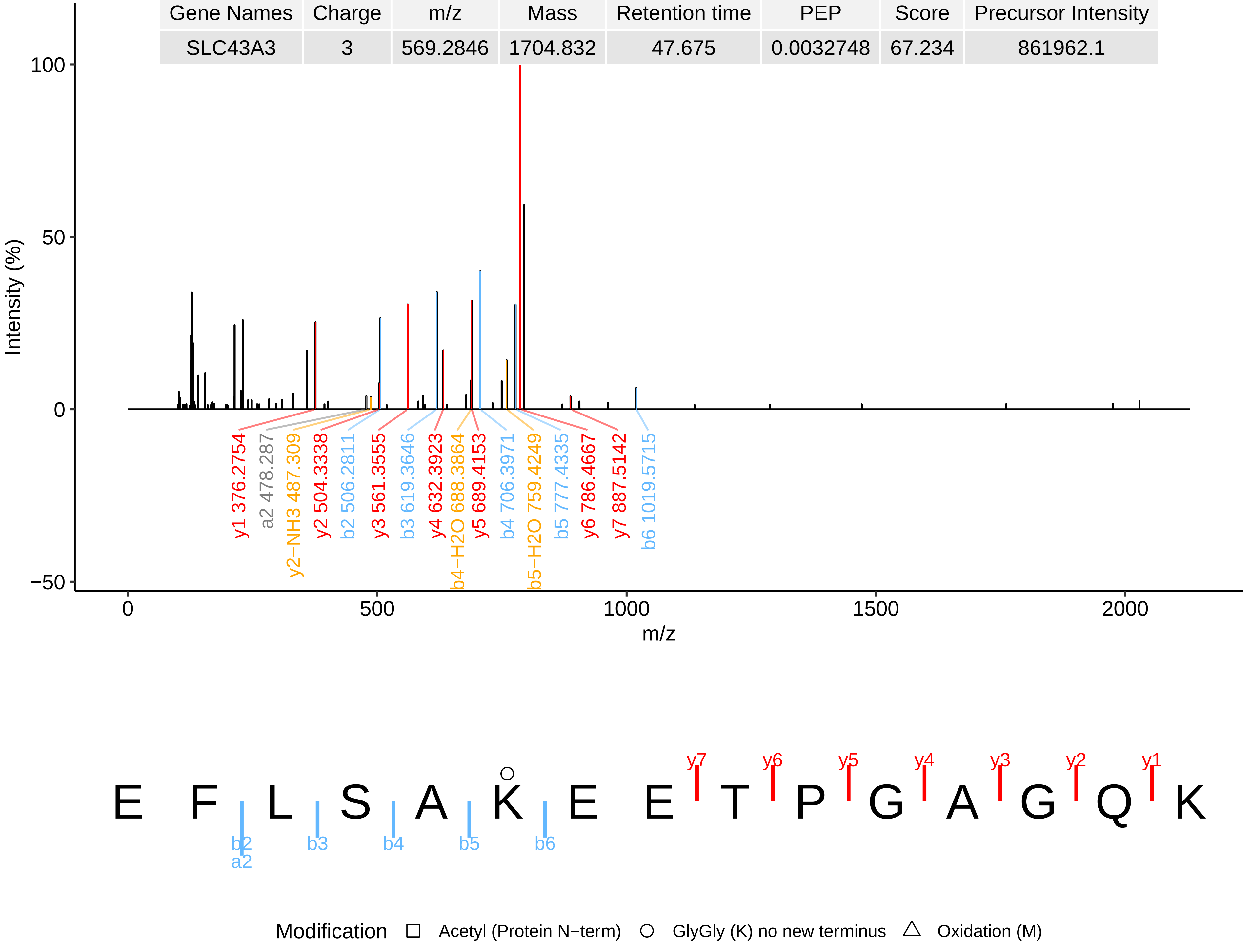

20200924_KIZ_MUb5_GlyGly_1 − Scan number: 12145

Gene Names	Charge	m/z	Mass	Retention time	PEP	Score	Precursor Intensity
SLC43A3	3	569.2846	1704.832	39.471	0.0021735	71.98	11847889

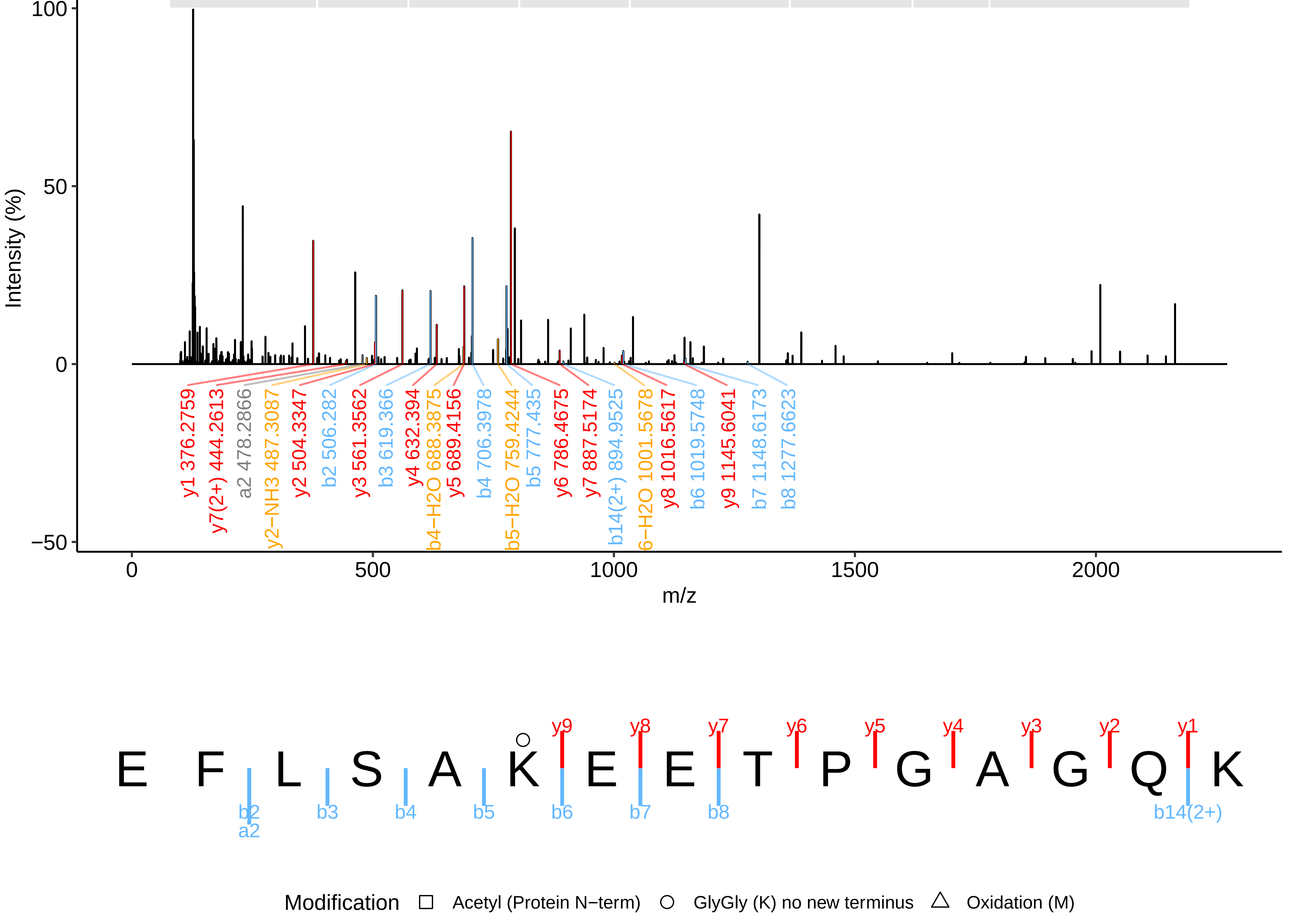

20200924_KIZ_MUb5_GlyGly_1 − Scan number: 12293

Gene Names	Charge	m/z	Mass	Retention time	PEP	Score	Precursor Intensity
SLC43A3	3	569.2846	1704.832	39.98	1.3501e−14	148.41	201232704

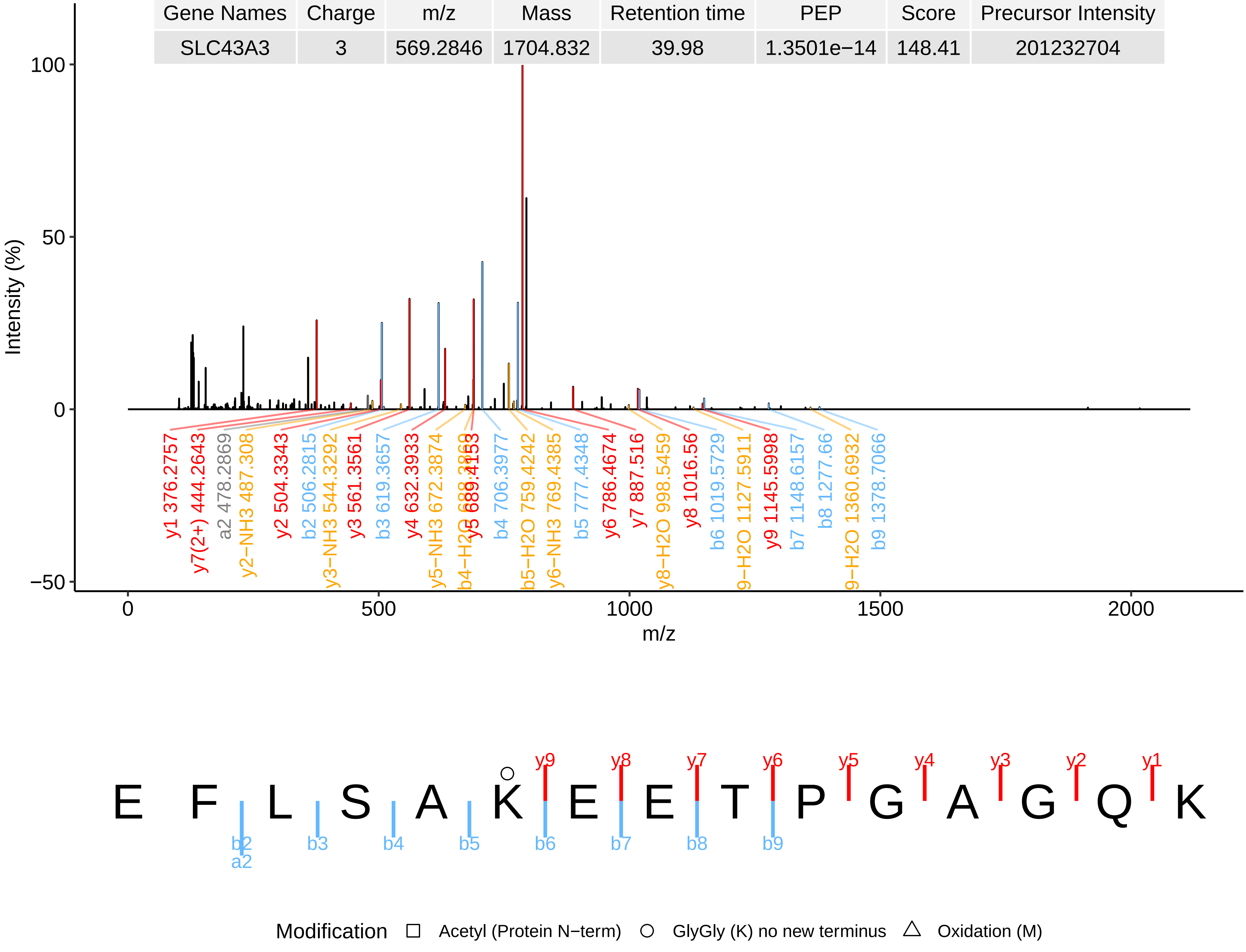

20200924_KIZ_MUb5_GlyGly_1 − Scan number: 12443

Gene Names	Charge	m/z	Mass	Retention time	PEP	Score	Precursor Intensity
SLC43A3	3	569.2846	1704.832	40.485	8.6157e−14	138.55	294388768

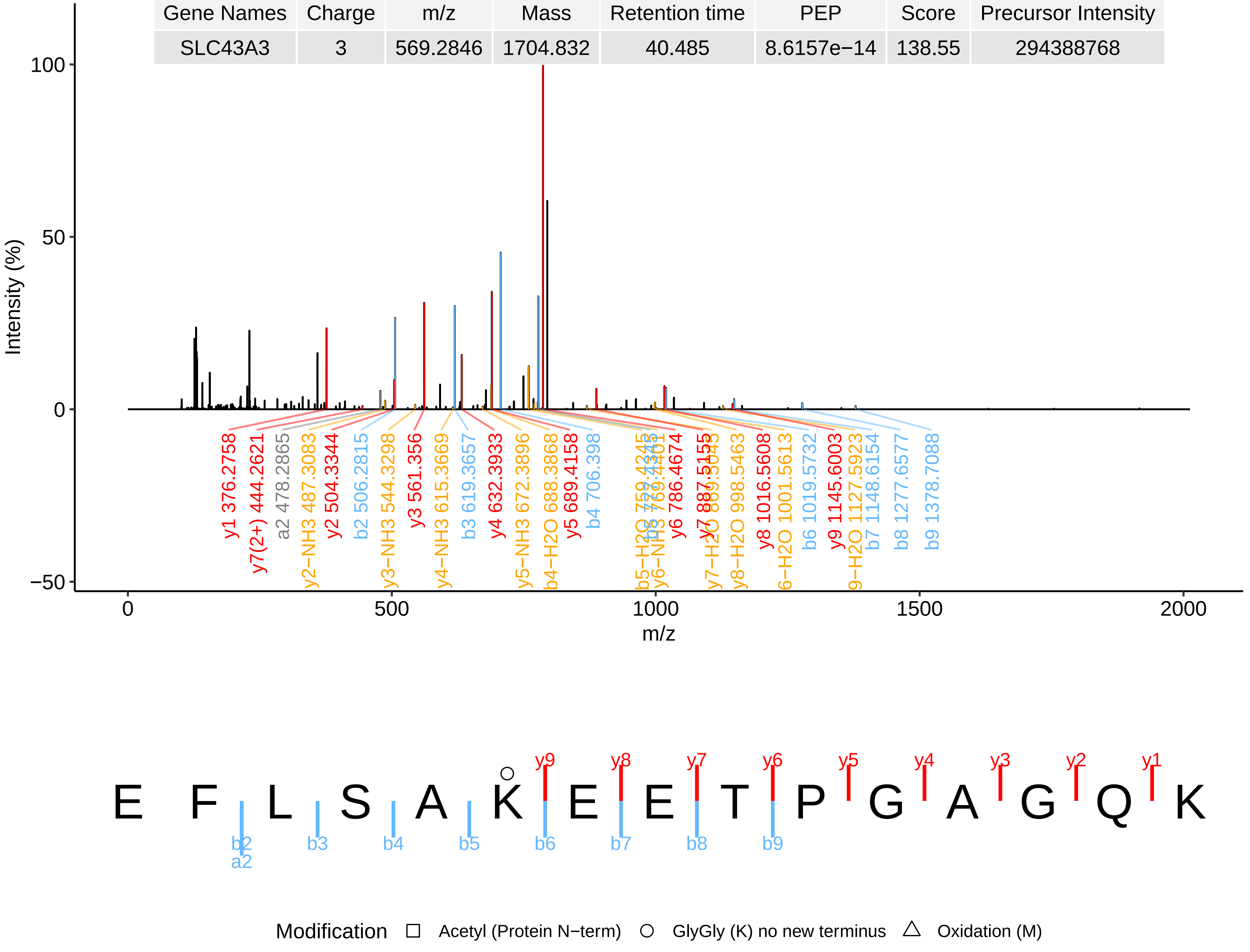

20200924_KIZ_MUb5_GlyGly_1 − Scan number: 12593

Gene Names	Charge	m/z	Mass	Retention time	PEP	Score	Precursor Intensity
SLC43A3	3	569.2846	1704.832	40.999	4.2553e−09	118.52	10799931

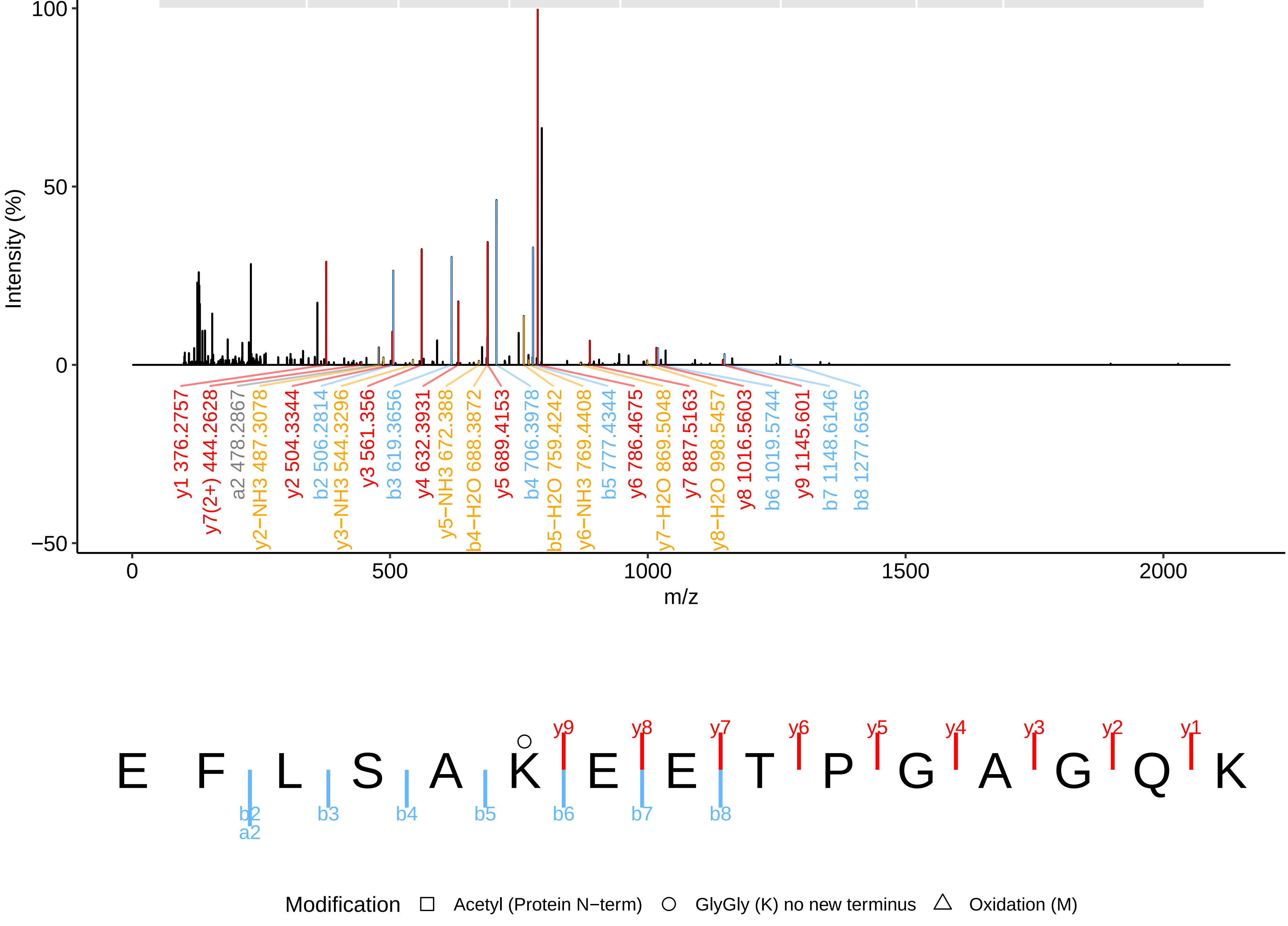

20200924_KIZ_MUb5_GlyGly_1 − Scan number: 12366

Gene Names	Charge	m/z	Mass	Retention time	PEP	Score	Precursor Intensity
SLC43A3	4	427.2153	1704.832	40.225	0.00013967	81.278	67077952

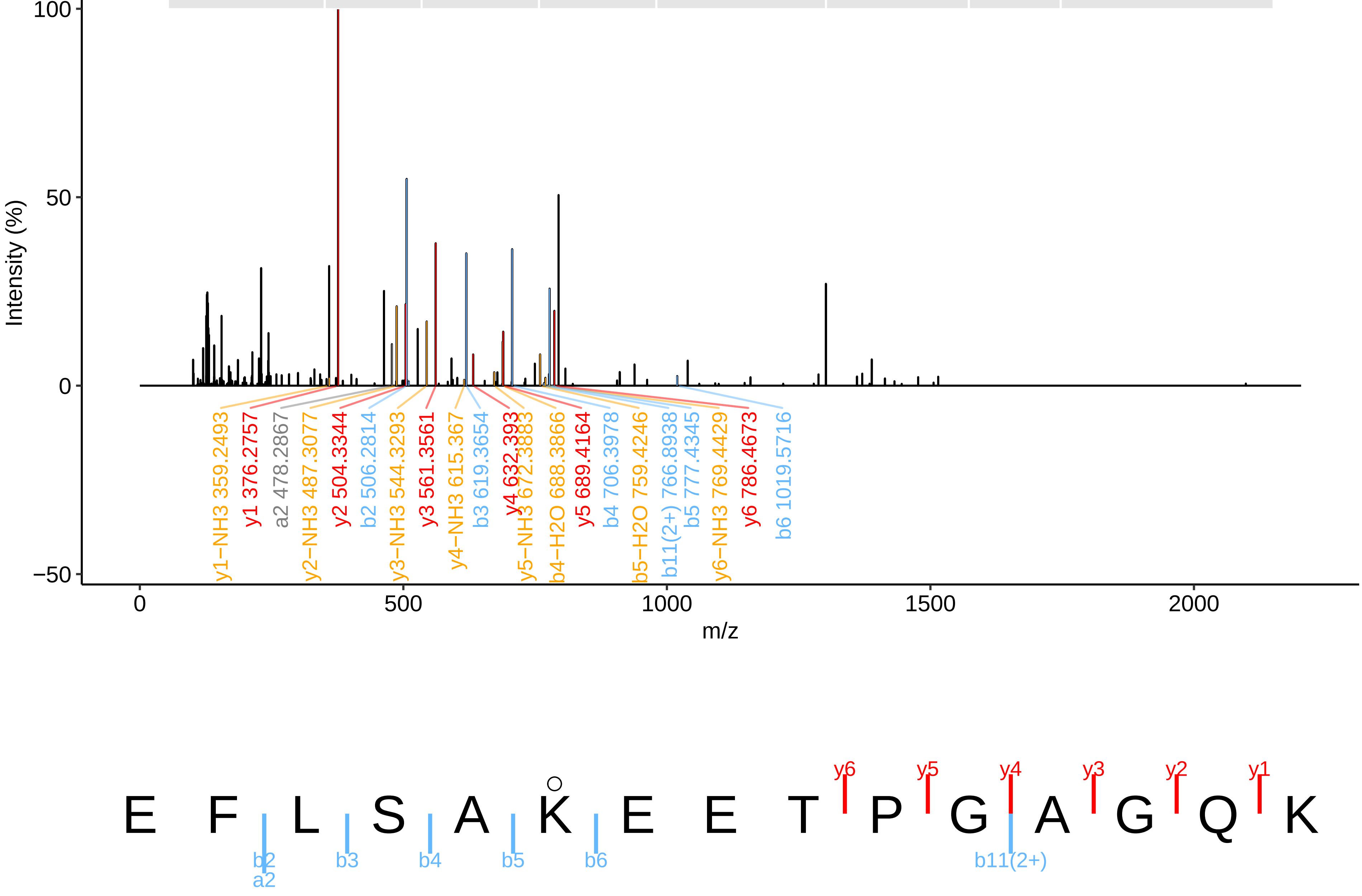

Modification □ Acetyl (Protein N−term) ○ GlyGly (K) no new terminus △ Oxidation (M)

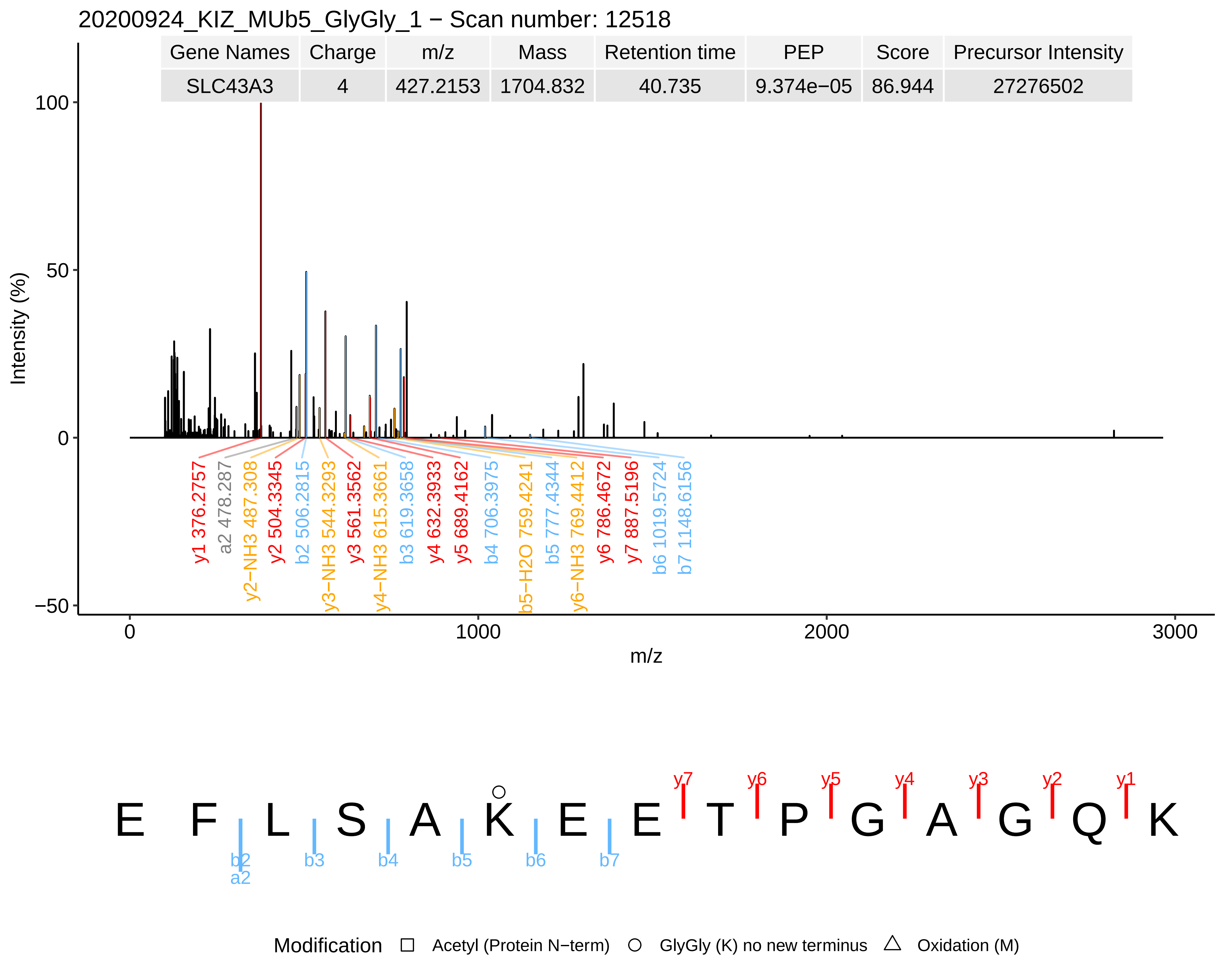

20200924_KIZ_MUb5_GlyGly_1 − Scan number: 12518
Gene Names | Charge | m/z | Mass | Retention time | PEP | Score | Precursor Intensity
SLC43A3 | 4 | 427.2153 | 1704.832 | 40.735 | 9.374e−05 | 86.944 | 27276502
Intensity (%)
m/z
y1 376.2757
a2 478.287
y2−NH3 487.308
y2 504.3345
b2 506.2815
y3−NH3 544.3293
y3 561.3562
y4−NH3 615.3661
b3 619.3658
y4 632.3933
y5 689.4162
b4 706.3975
b5−H2O 759.4241
b5 777.4344
y6−NH3 769.4412
y6 786.4672
y7 887.5196
b6 1019.5724
b7 1148.6156
E F L S A K E E T P G A G Q K
y7 y6 y5 y4 y3 y2 y1
b2 a2 b3 b4 b5 b6 b7
Modification □ Acetyl (Protein N−term) ○ GlyGly (K) no new terminus △ Oxidation (M)

20200924_KIZ_MUb5_GlyGly_2 − Scan number: 11462

Gene Names	Charge	m/z	Mass	Retention time	PEP	Score	Precursor Intensity
SLC43A3	4	427.2153	1704.832	42.852	0.0020969	70.373	5185048

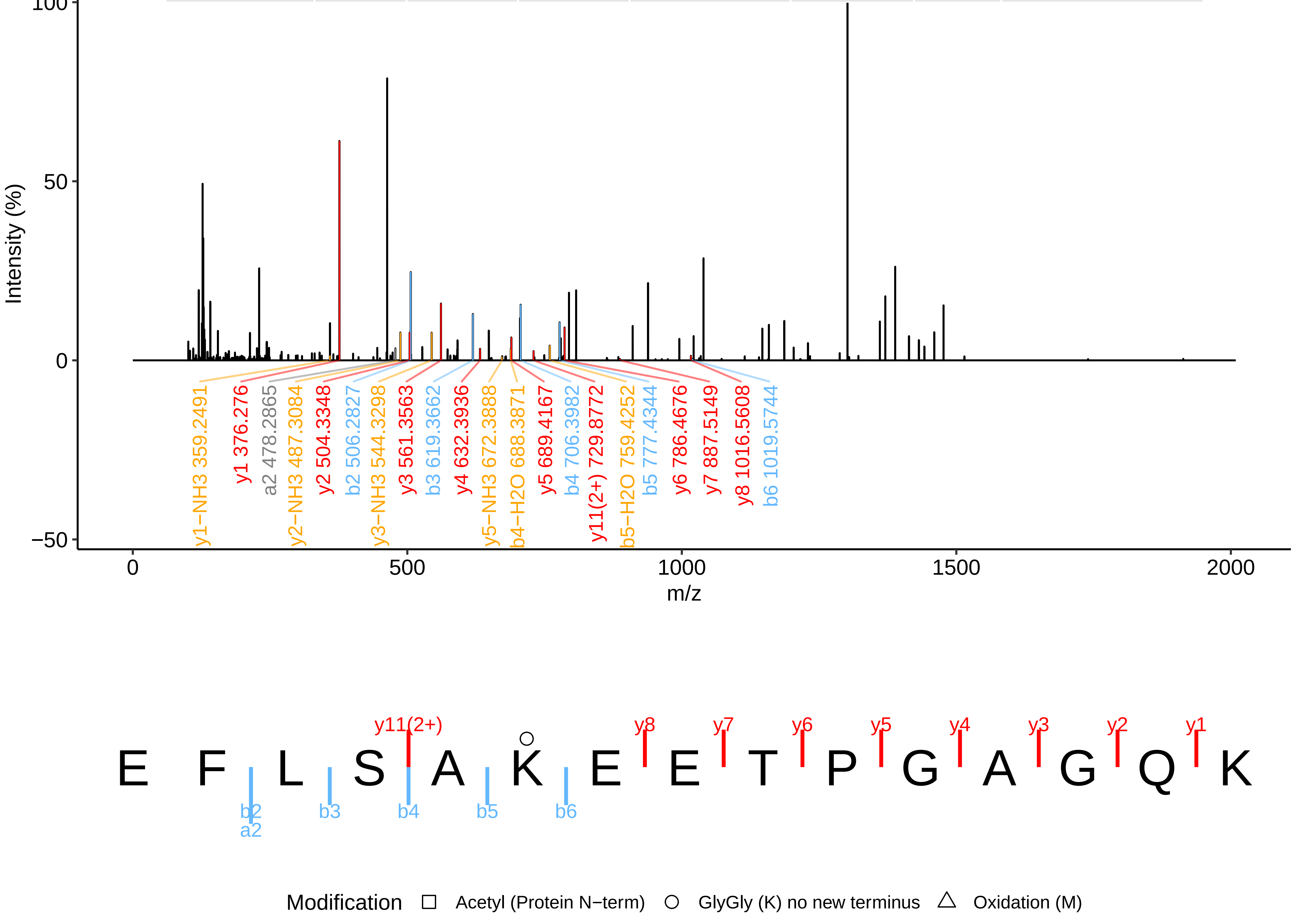

20200924_KIZ_MUb5_GlyGly_2 − Scan number: 11596

Gene Names	Charge	m/z	Mass	Retention time	PEP	Score	Precursor Intensity
SLC43A3	4	427.2153	1704.832	43.355	0.00011519	84.297	11627290

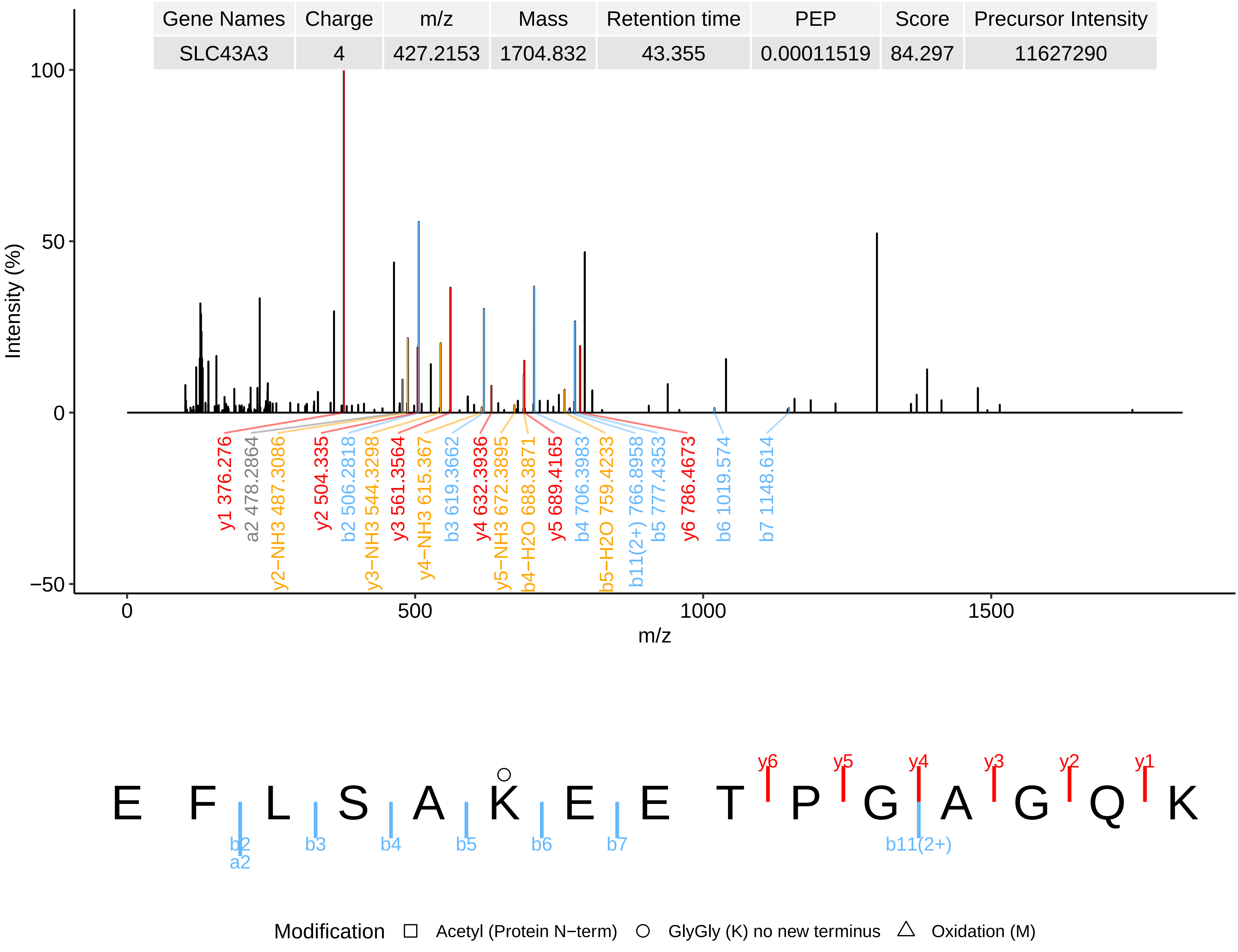

20200924_KIZ_MUb5_GlyGly_2 − Scan number: 11497

Gene Names	Charge	m/z	Mass	Retention time	PEP	Score	Precursor Intensity
SLC43A3	3	569.2846	1704.832	42.978	8.6157e−14	138.55	38080156

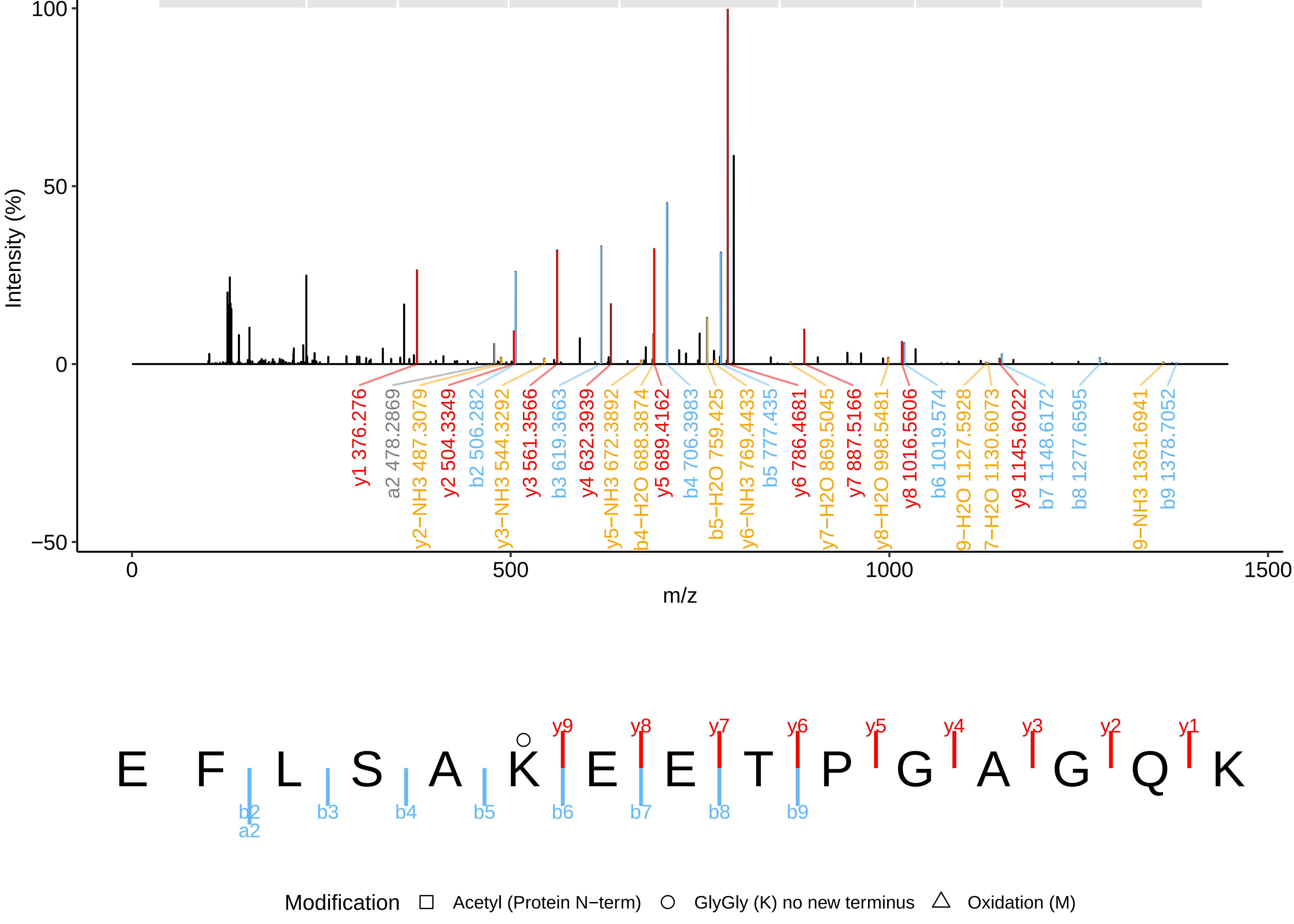

20200924_KIZ_MUb5_GlyGly_2 − Scan number: 11632

Gene Names	Charge	m/z	Mass	Retention time	PEP	Score	Precursor Intensity
SLC43A3	3	569.2846	1704.832	43.488	9.0845e−10	129.77	50992800

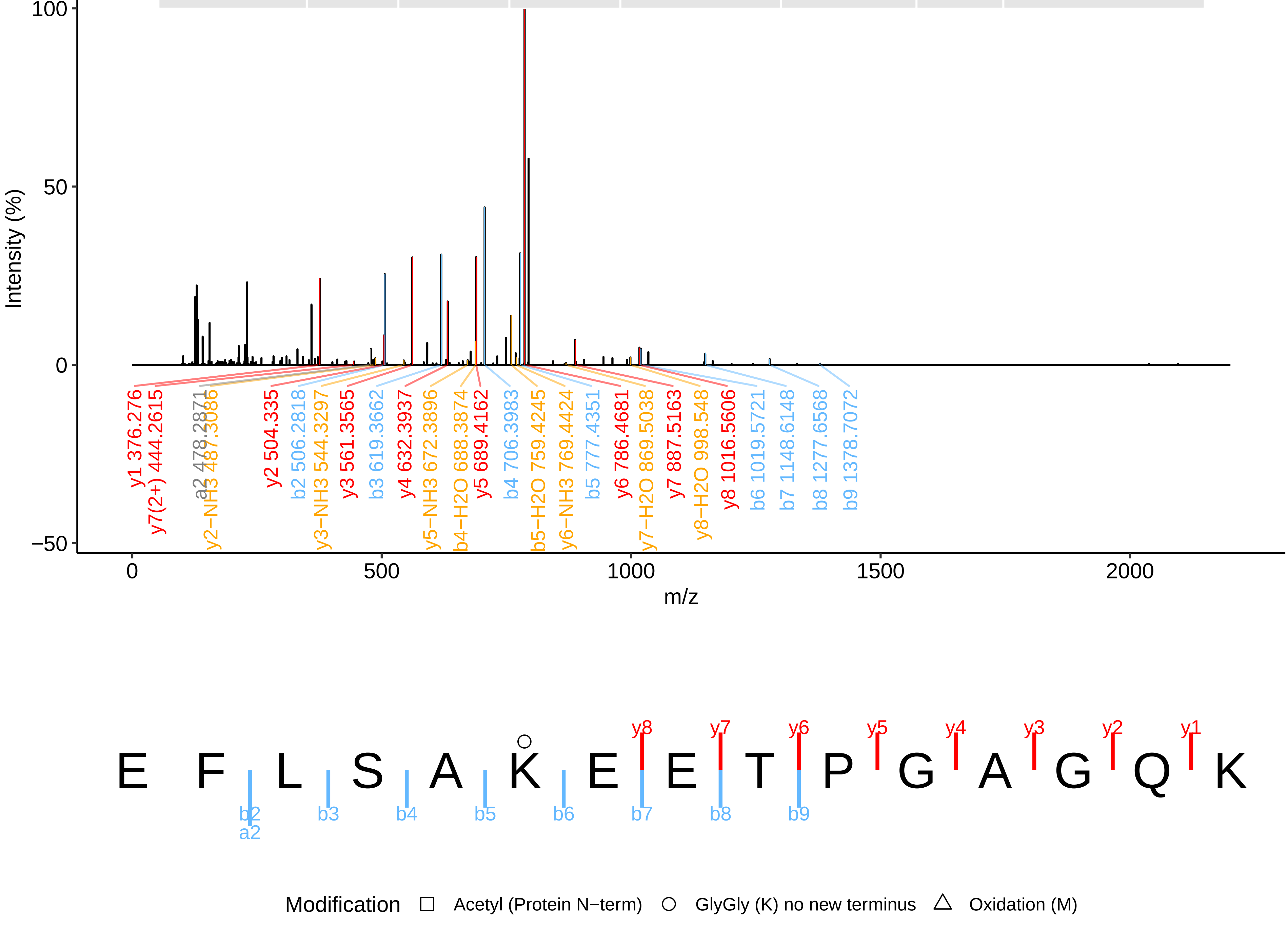

20200924_KIZ_MUb5_GlyGly_2 − Scan number: 11772

Gene Names	Charge	m/z	Mass	Retention time	PEP	Score	Precursor Intensity
SLC43A3	3	569.2846	1704.832	44.005	1.2085e−09	128.76	17726210

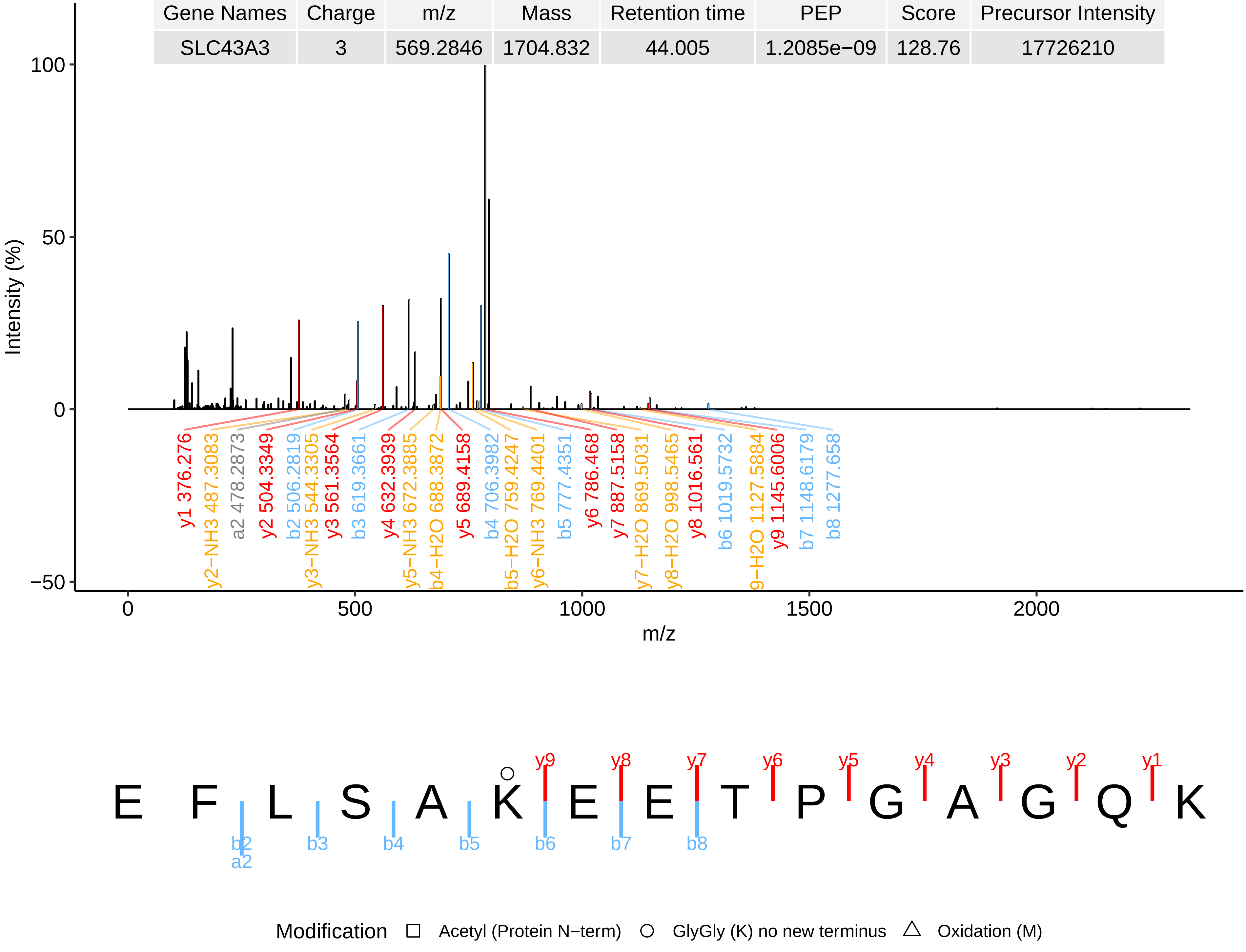

20200924_KIZ_MUb5_GlyGly_2 − Scan number: 11365

Gene Names	Charge	m/z	Mass	Retention time	PEP	Score	Precursor Intensity
SLC43A3	3	569.2846	1704.832	42.472	7.3252e−05	94.717	1832794

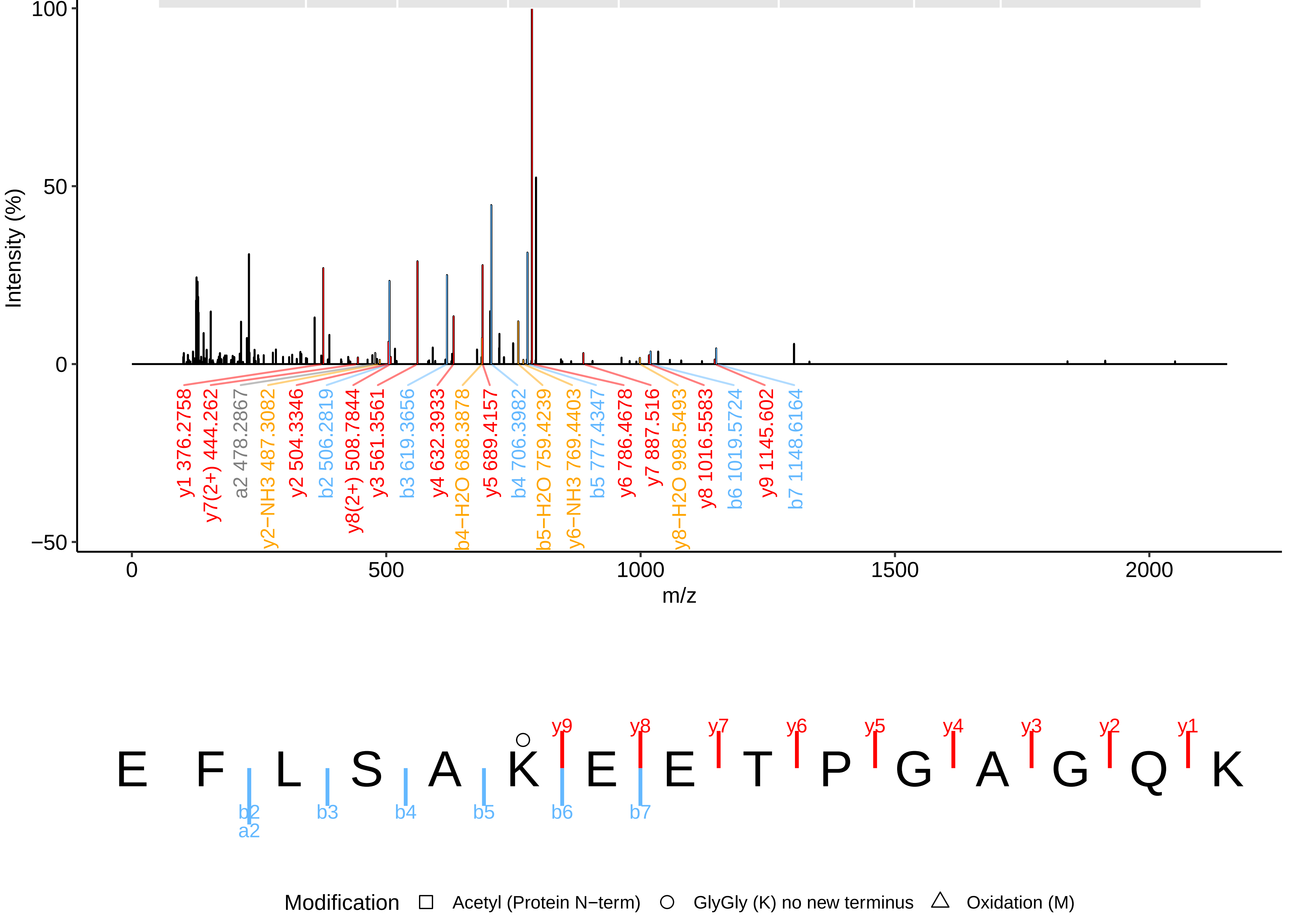

20201118_KIZ_MUb6_GlyGly_1 − Scan number: 12248

Gene Names	Charge	m/z	Mass	Retention time	PEP	Score	Precursor Intensity
SLC43A3	3	569.2846	1704.832	38.649	6.593e−05	91.812	10453218

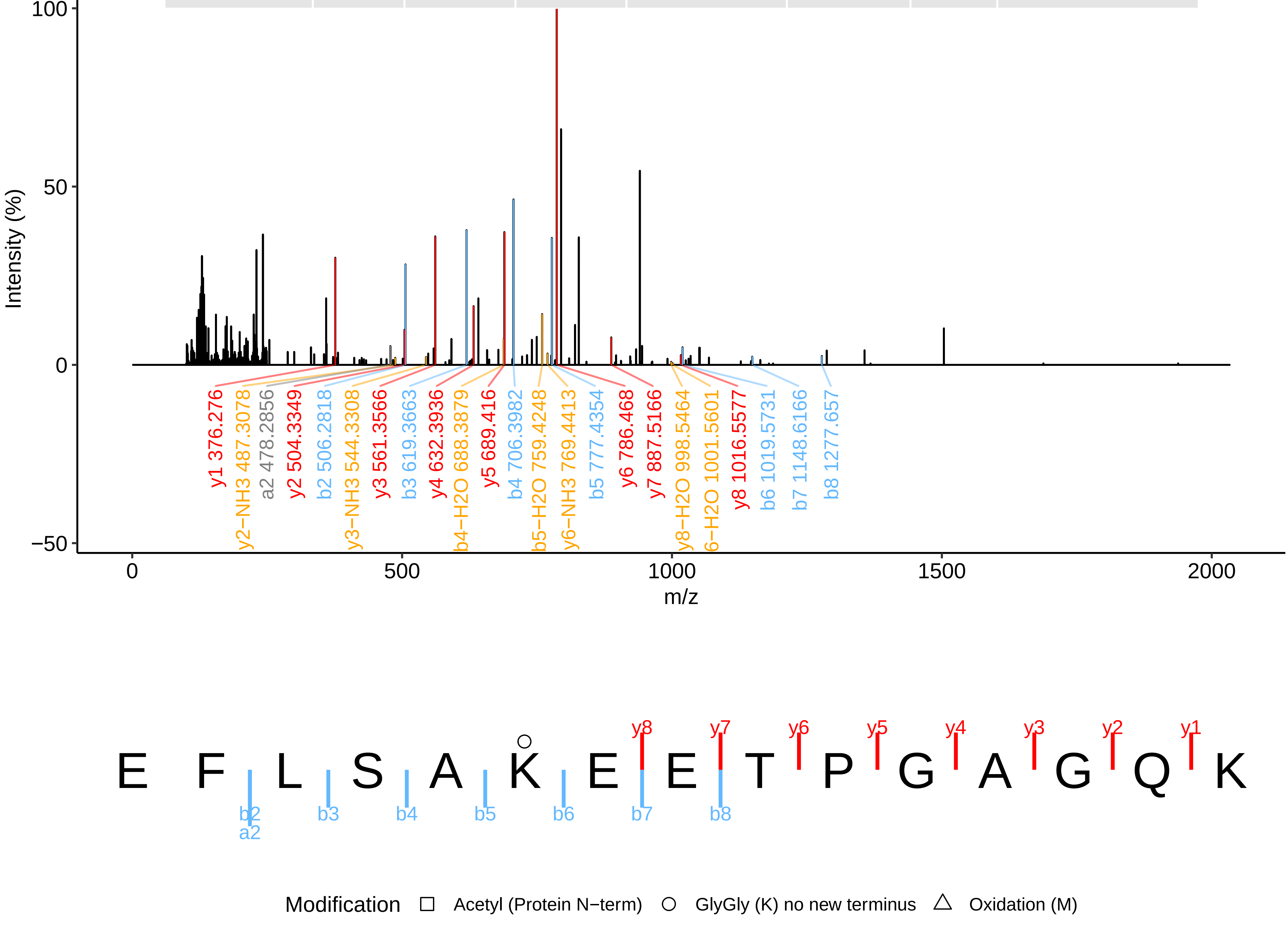

20201118_KIZ_MUb6_GlyGly_1 − Scan number: 12253

Gene Names	Charge	m/z	Mass	Retention time	PEP	Score	Precursor Intensity
SLC43A3	3	569.2846	1704.832	38.667	8.6157e−14	138.55	12838234

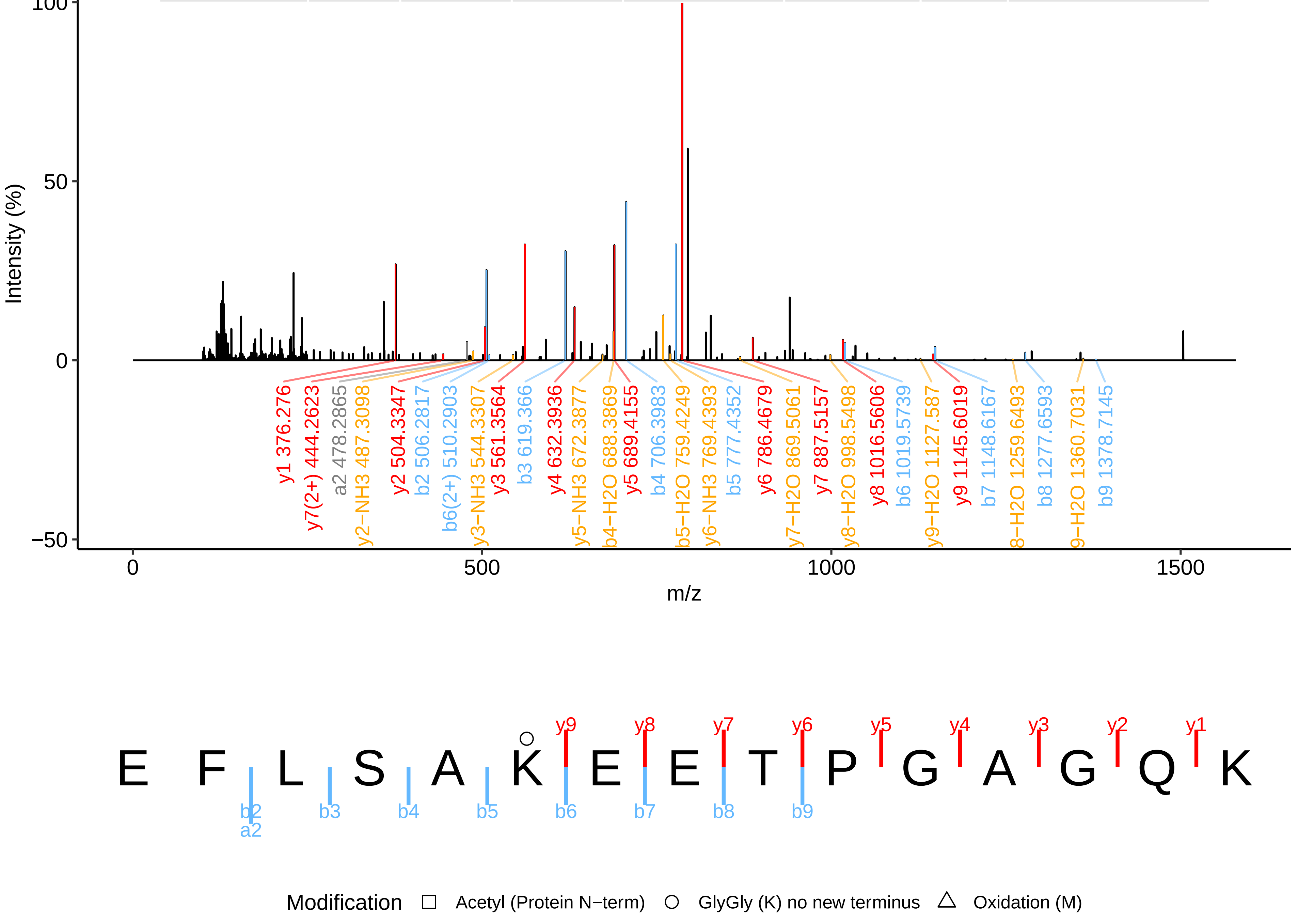

20201118_KIZ_MUb6_GlyGly_1 − Scan number: 12405

Gene Names	Charge	m/z	Mass	Retention time	PEP	Score	Precursor Intensity
SLC43A3	3	569.2846	1704.832	39.168	8.6157e−14	138.55	18399648

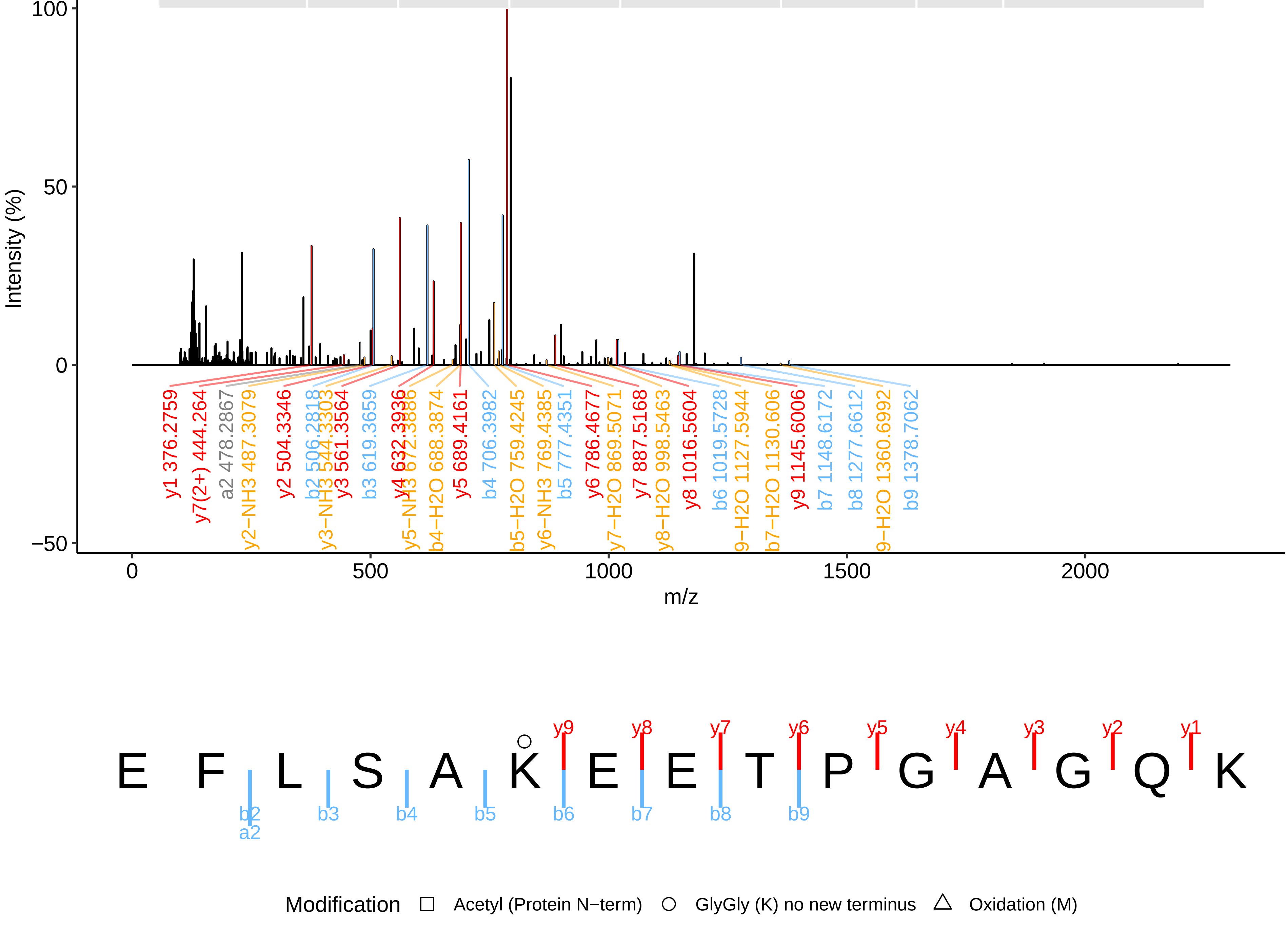

20201118_KIZ_MUb6_GlyGly_1 − Scan number: 12305

Gene Names	Charge	m/z	Mass	Retention time	PEP	Score	Precursor Intensity
SLC43A3	4	427.2153	1704.832	38.843	0.00011519	84.297	54921728

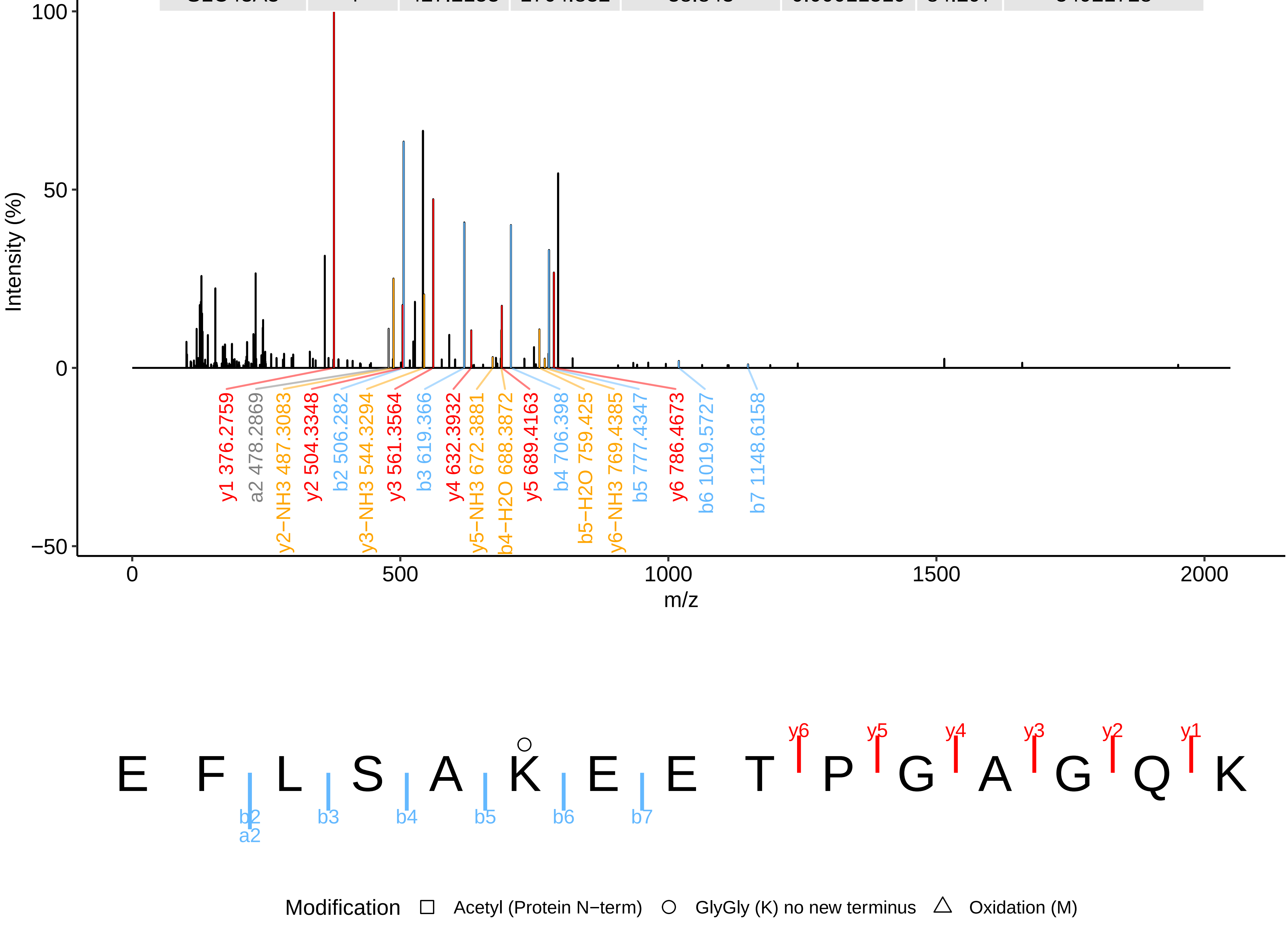

20201118_KIZ_MUb6_GlyGly_2 − Scan number: 14198

Gene Names	Charge	m/z	Mass	Retention time	PEP	Score	Precursor Intensity
SLC43A3	3	569.2846	1704.832	47.827	7.6509e−14	139.86	7868914

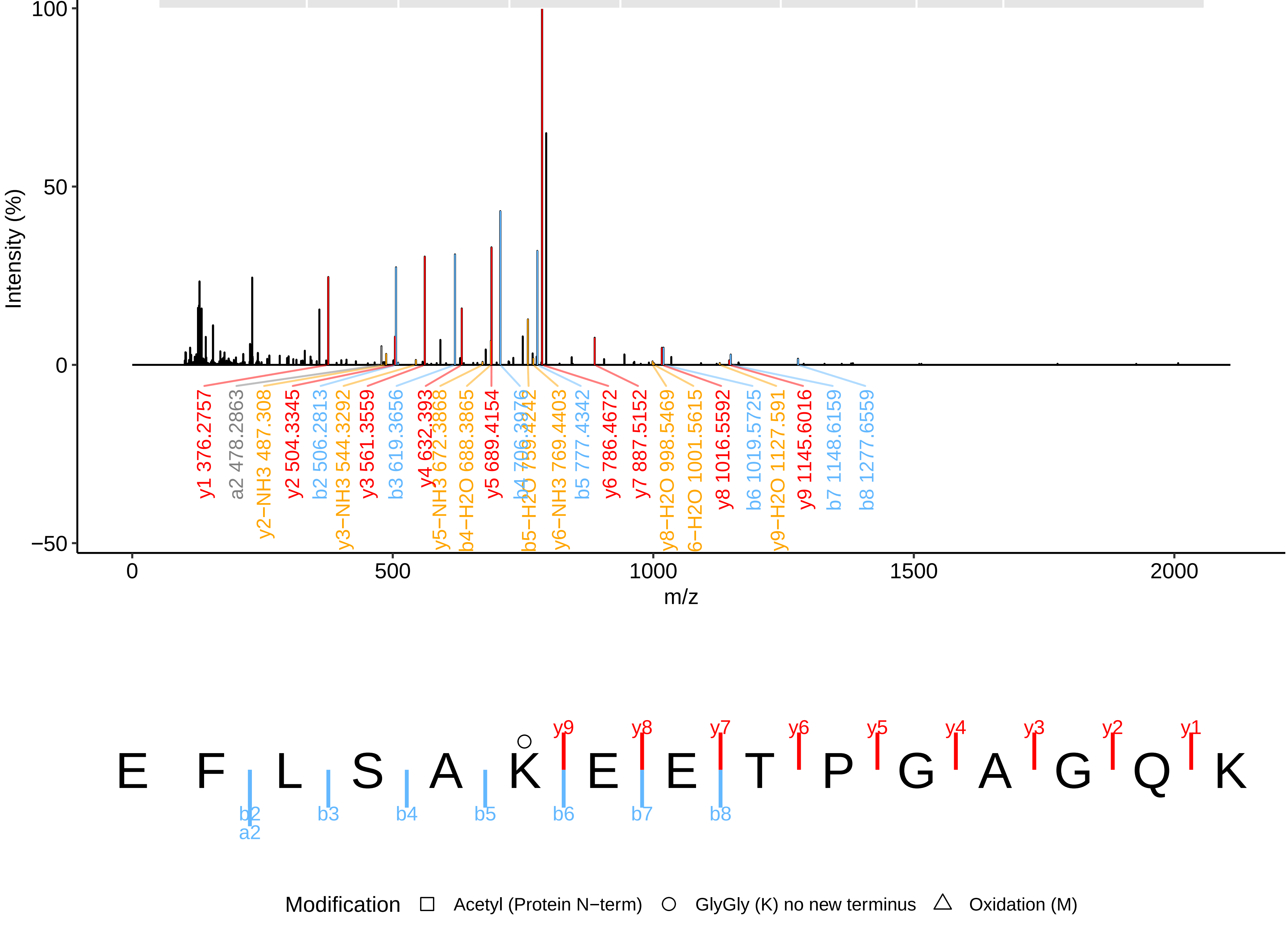

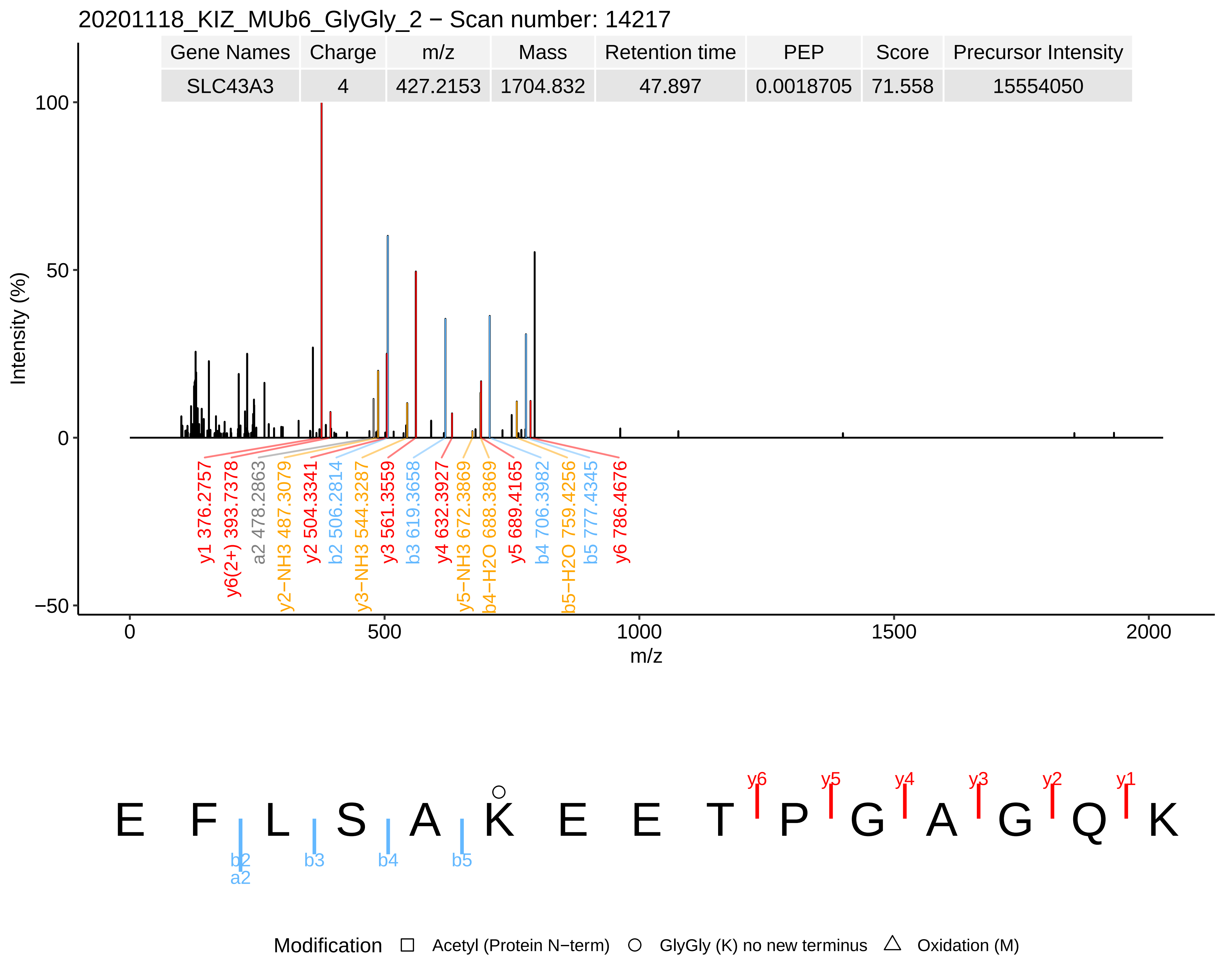

20201118_KIZ_MUb6_GlyGly_2 − Scan number: 14217
Gene Names
Charge
m/z
Mass
Retention time
PEP
Score
Precursor Intensity
SLC43A3
4
427.2153
1704.832
47.897
0.0018705
71.558
15554050
Intensity (%)
m/z
y1 376.2757
y6(2+) 393.7378
a2 478.2863
y2−NH3 487.3079
y2 504.3341
b2 506.2814
y3−NH3 544.3287
y3 561.3559
b3 619.3658
y4 632.3927
y5−NH3 672.3869
b4−H2O 688.3869
y5 689.4165
b4 706.3982
b5−H2O 759.4256
b5 777.4345
y6 786.4676
E F L S A K E E T P G A G Q K
Modification
Acetyl (Protein N−term)
GlyGly (K) no new terminus
Oxidation (M)

20200921_KIZ_MUb4_GlyGly_10plex_2 − Scan number: 14209

Gene Names	Charge	m/z	Mass	Retention time	PEP	Score	Precursor Intensity
TOMM70A	3	593.9635	1778.869	55.354	0.0028716	68.972	997725.5

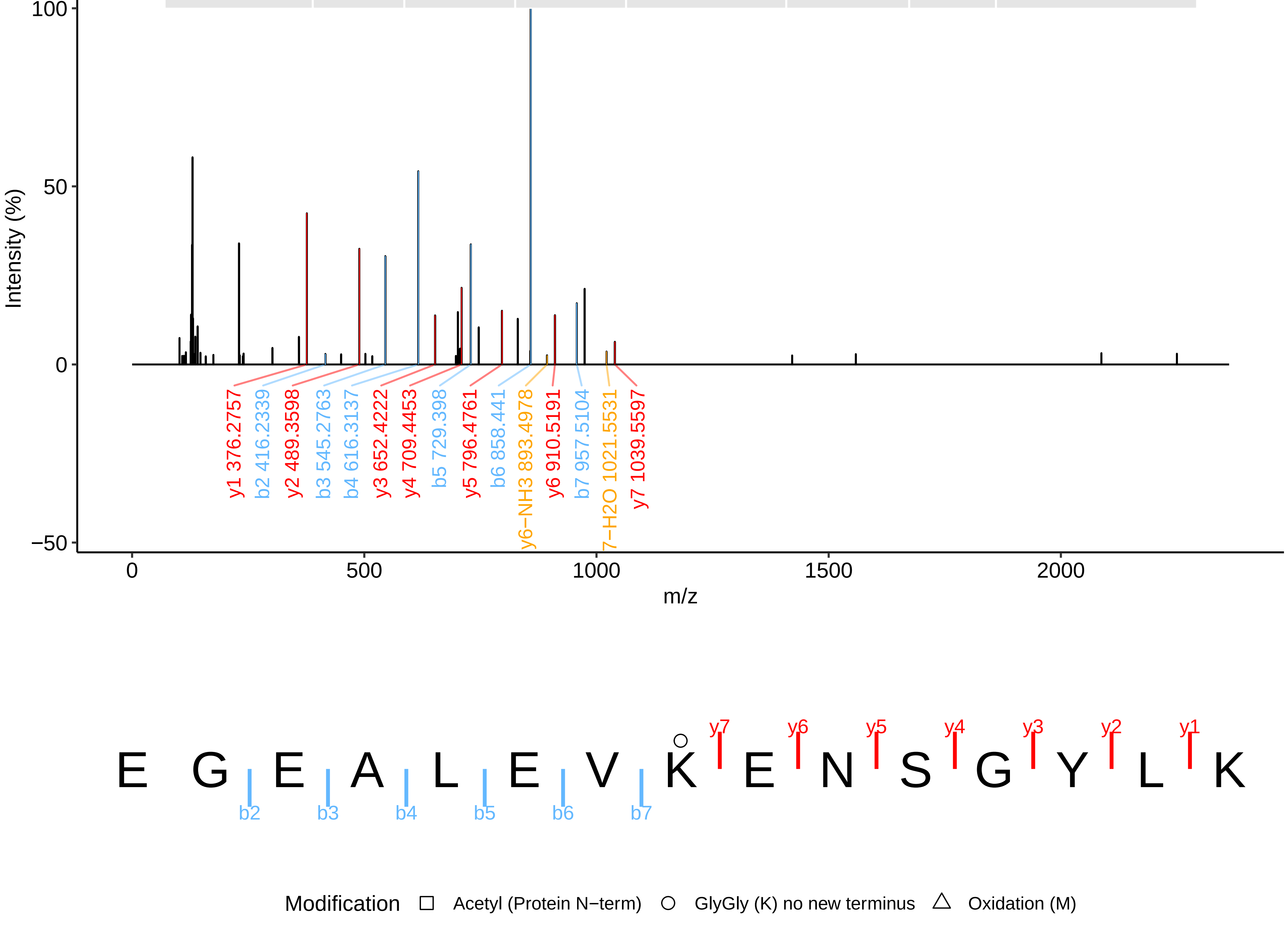

20200924_KIZ_MUb5_GlyGly_2 − Scan number: 4001

Gene Names	Charge	m/z	Mass	Retention time	PEP	Score	Precursor Intensity
BASP1	4	386.9351	1543.711	14.621	0.0054281	85.457	7695246

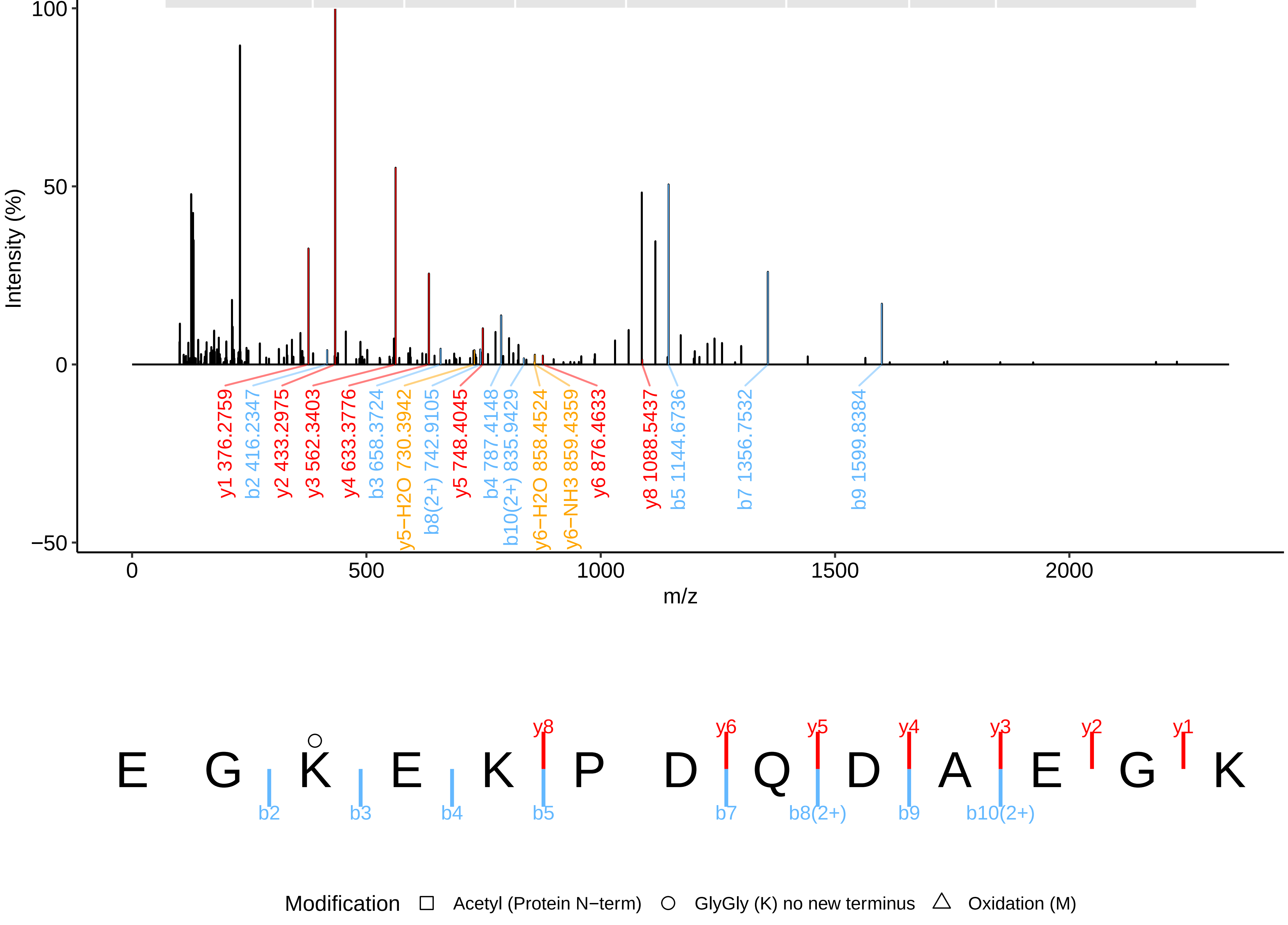

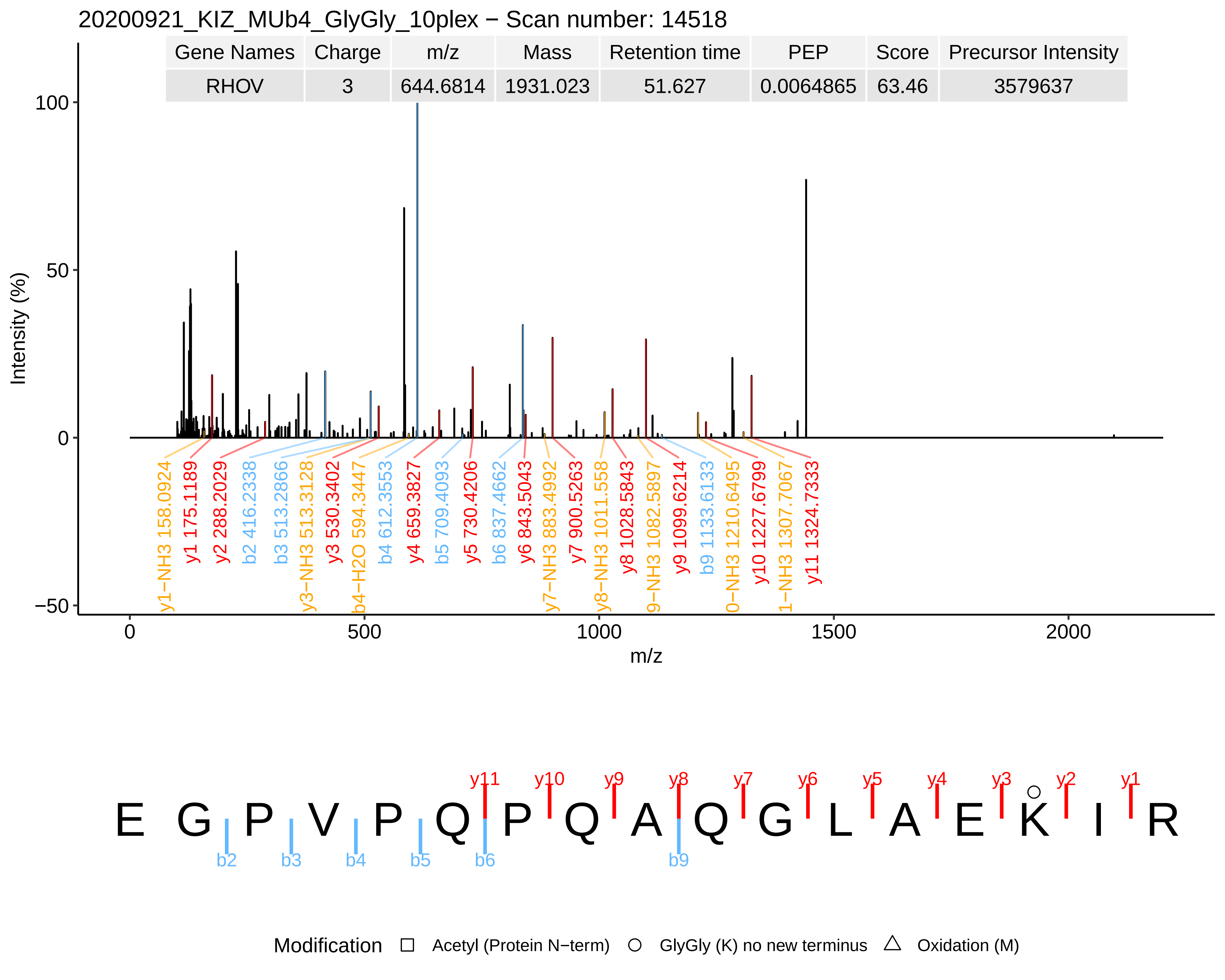
20200921_KIZ_MUb4_GlyGly_10plex − Scan number: 14518
Gene Names | Charge | m/z | Mass | Retention time | PEP | Score | Precursor Intensity
RHOV | 3 | 644.6814 | 1931.023 | 51.627 | 0.0064865 | 63.46 | 3579637
Intensity (%)
100
50
0
−50
0
500
1000
1500
2000
m/z
y1−NH3 158.0924
y1 175.1189
y2 288.2029
b2 416.2338
b3 513.2866
y3−NH3 513.3128
y3 530.3402
b4−H2O 594.3447
b4 612.3553
y4 659.3827
b5 709.4093
y5 730.4206
b6 837.4662
y6 843.5043
y7−NH3 883.4992
y7 900.5263
y8−NH3 1011.558
y8 1028.5843
9−NH3 1082.5897
y9 1099.6214
b9 1133.6133
0−NH3 1210.6495
y10 1227.6799
1−NH3 1307.7067
y11 1324.7333
E G P V P Q P Q A Q G L A E K I R
y11 y10 y9 y8 y7 y6 y5 y4 y3 y2 y1
b2 b3 b4 b5 b6 b9
Modification
Acetyl (Protein N−term)
GlyGly (K) no new terminus
Oxidation (M)

20200921_KIZ_MUb4_GlyGly_10plex − Scan number: 8817

Gene Names	Charge	m/z	Mass	Retention time	PEP	Score	Precursor Intensity
SUMO2	4	431.9695	1723.849	30.045	0.00039299	87.18	7407824

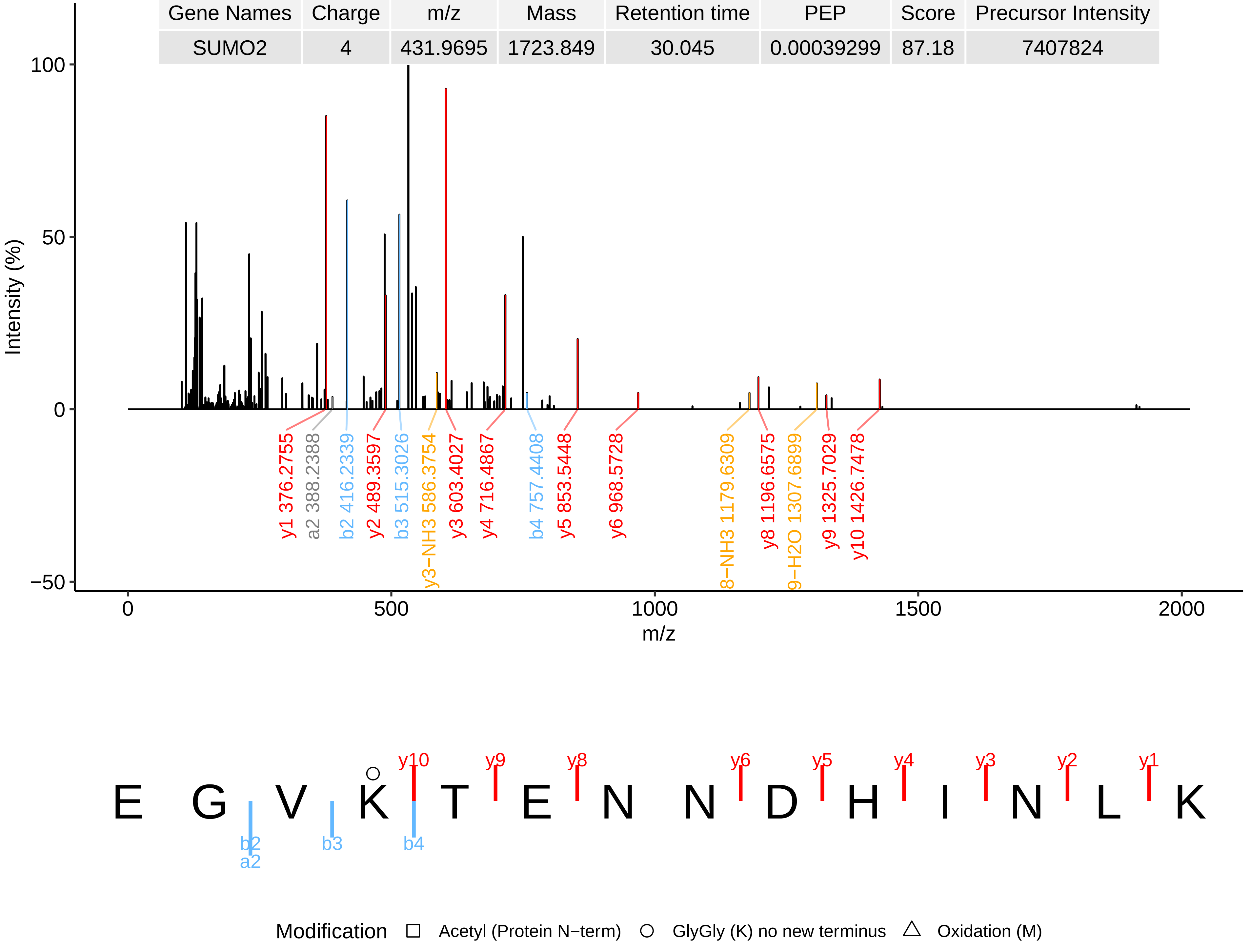

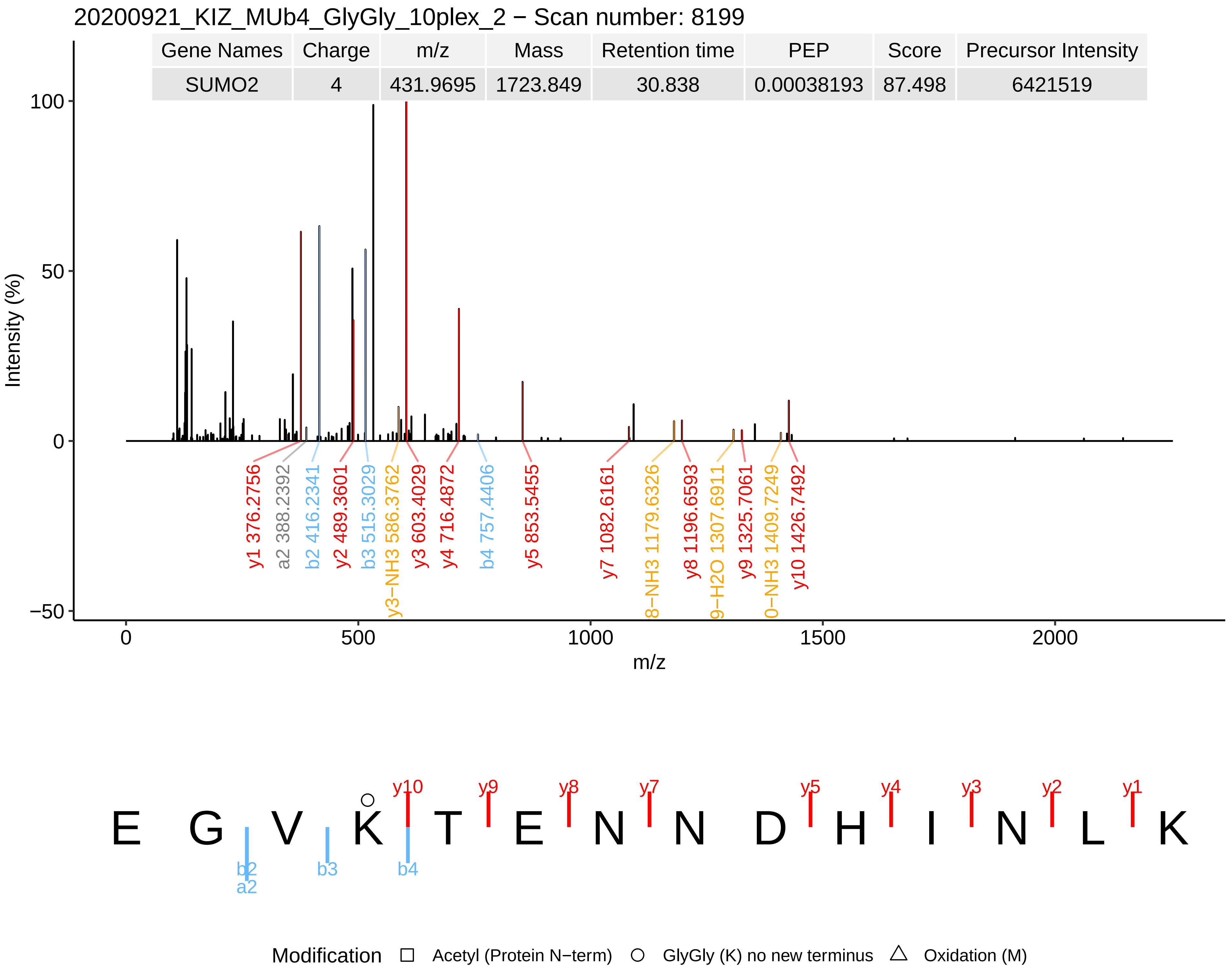

Gene Names	Charge	m/z	Mass	Retention time	PEP	Score	Precursor Intensity
SUMO2	4	431.9695	1723.849	30.838	0.00038193	87.498	6421519

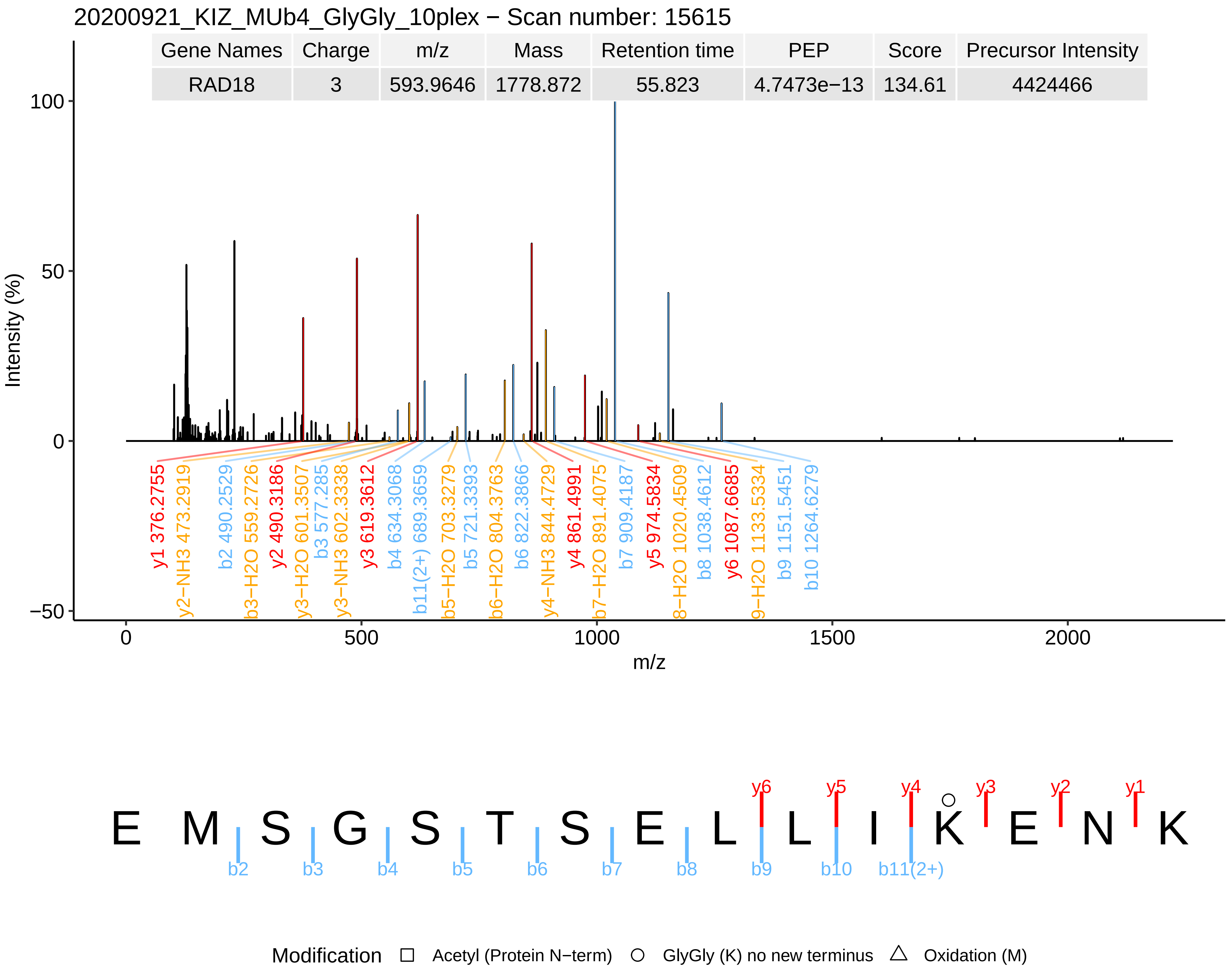

20200921_KIZ_MUb4_GlyGly_10plex − Scan number: 15615
Gene Names: RAD18
Charge: 3
m/z: 593.9646
Mass: 1778.872
Retention time: 55.823
PEP: 4.7473e−13
Score: 134.61
Precursor Intensity: 4424466
Intensity (%)
m/z
y1 376.2755
y2−NH3 473.2919
b2 490.2529
b3−H2O 559.2726
y2 490.3186
y3−H2O 601.3507
b3 577.285
y3−NH3 602.3338
y3 619.3612
b4 634.3068
b11(2+) 689.3659
b5−H2O 703.3279
b5 721.3393
b6−H2O 804.3763
b6 822.3866
y4−NH3 844.4729
y4 861.4991
b7−H2O 891.4075
b7 909.4187
y5 974.5834
8−H2O 1020.4509
b8 1038.4612
y6 1087.6685
9−H2O 1133.5334
b9 1151.5451
b10 1264.6279
E M S G S T S E L L I K E N K
y6 y5 y4 y3 y2 y1
b2 b3 b4 b5 b6 b7 b8 b9 b10 b11(2+)
Modification
Acetyl (Protein N−term)
GlyGly (K) no new terminus
Oxidation (M)

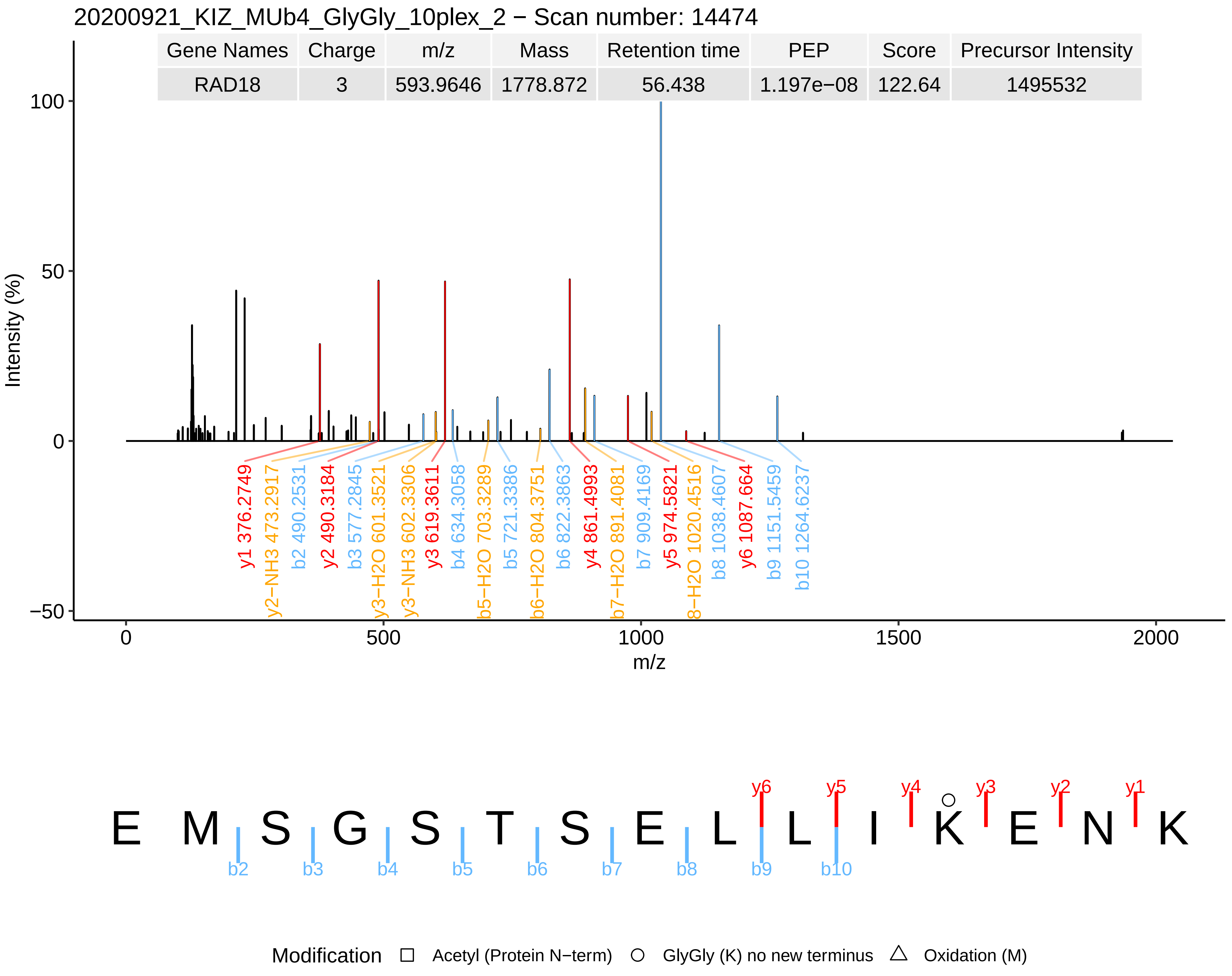
20200921_KIZ_MUb4_GlyGly_10plex_2 − Scan number: 14474
Gene Names
Charge
m/z
Mass
Retention time
PEP
Score
Precursor Intensity
RAD18
3
593.9646
1778.872
56.438
1.197e−08
122.64
1495532
Intensity (%)
100
50
0
−50
0
500
1000
1500
2000
m/z
y1 376.2749
y2−NH3 473.2917
b2 490.2531
y2 490.3184
b3 577.2845
y3−H2O 601.3521
y3−NH3 602.3306
y3 619.3611
b4 634.3058
b5−H2O 703.3289
b5 721.3386
b6−H2O 804.3751
b6 822.3863
y4 861.4993
b7−H2O 891.4081
b7 909.4169
y5 974.5821
b8−H2O 1020.4516
b8 1038.4607
y6 1087.664
b9 1151.5459
b10 1264.6237
E M S G S T S E L L I K E N K
y6 y5 y4 y3 y2 y1
b2 b3 b4 b5 b6 b7 b8 b9 b10
Modification
Acetyl (Protein N−term)
GlyGly (K) no new terminus
Oxidation (M)

20200921_KIZ_MUb4_GlyGly_10plex − Scan number: 15202

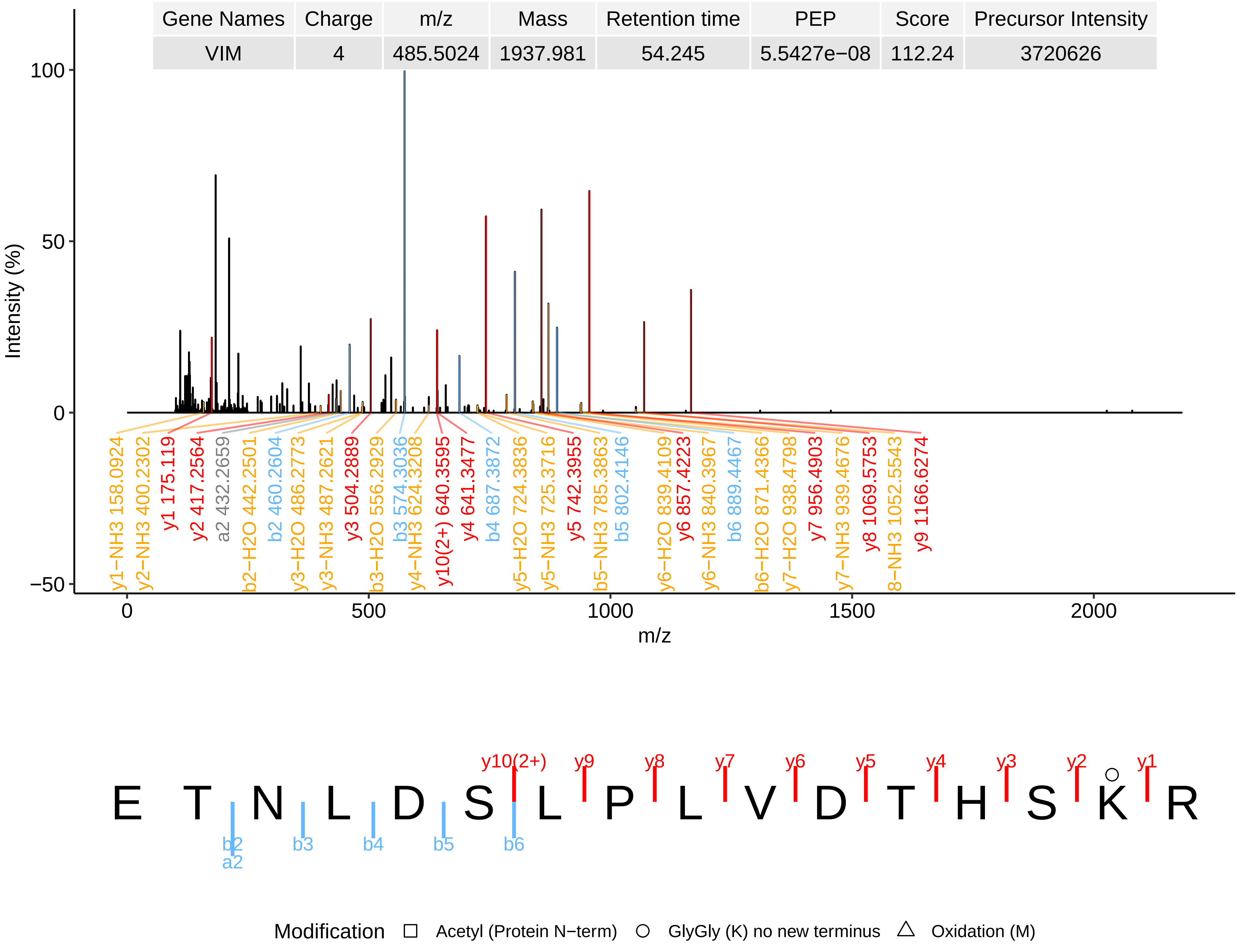

20200921_KIZ_MUb4_GlyGly_10plex_2 − Scan number: 14060

Gene Names	Charge	m/z	Mass	Retention time	PEP	Score	Precursor Intensity
VIM	4	485.5024	1937.981	54.734	0.045859	50.053	1000657

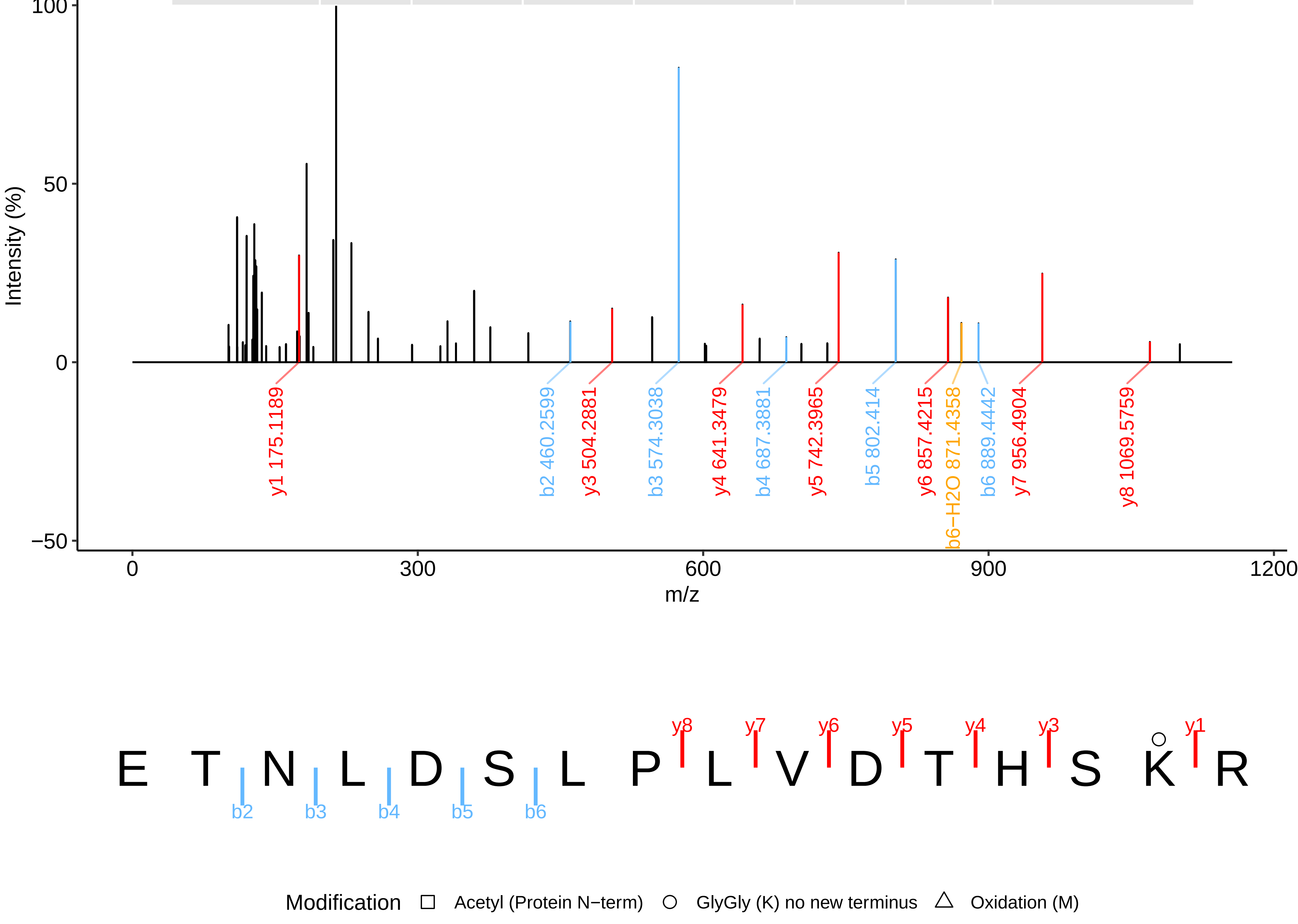

20200921_KIZ_MUb4_GlyGly_10plex_2 − Scan number: 14083

Gene Names	Charge	m/z	Mass	Retention time	PEP	Score	Precursor Intensity
VIM	4	485.5024	1937.981	54.833	9.9366e−06	84.41	1359070

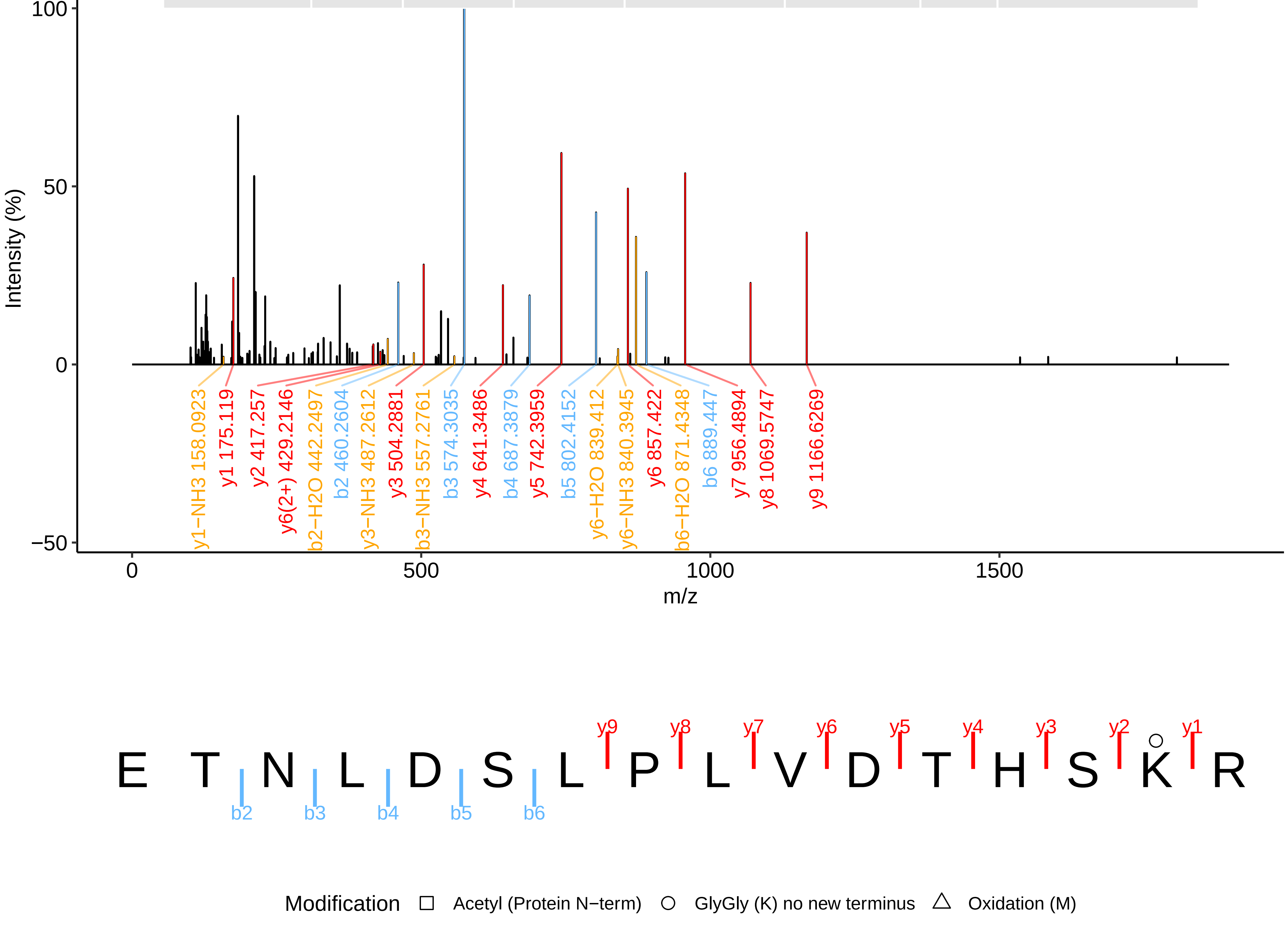

20200921_KIZ_MUb4_GlyGly_10plex − Scan number: 13606

Gene Names	Charge	m/z	Mass	Retention time	PEP	Score	Precursor Intensity
BCAP31	3	571.9637	1712.869	48.059	0.00064824	76.341	3516797

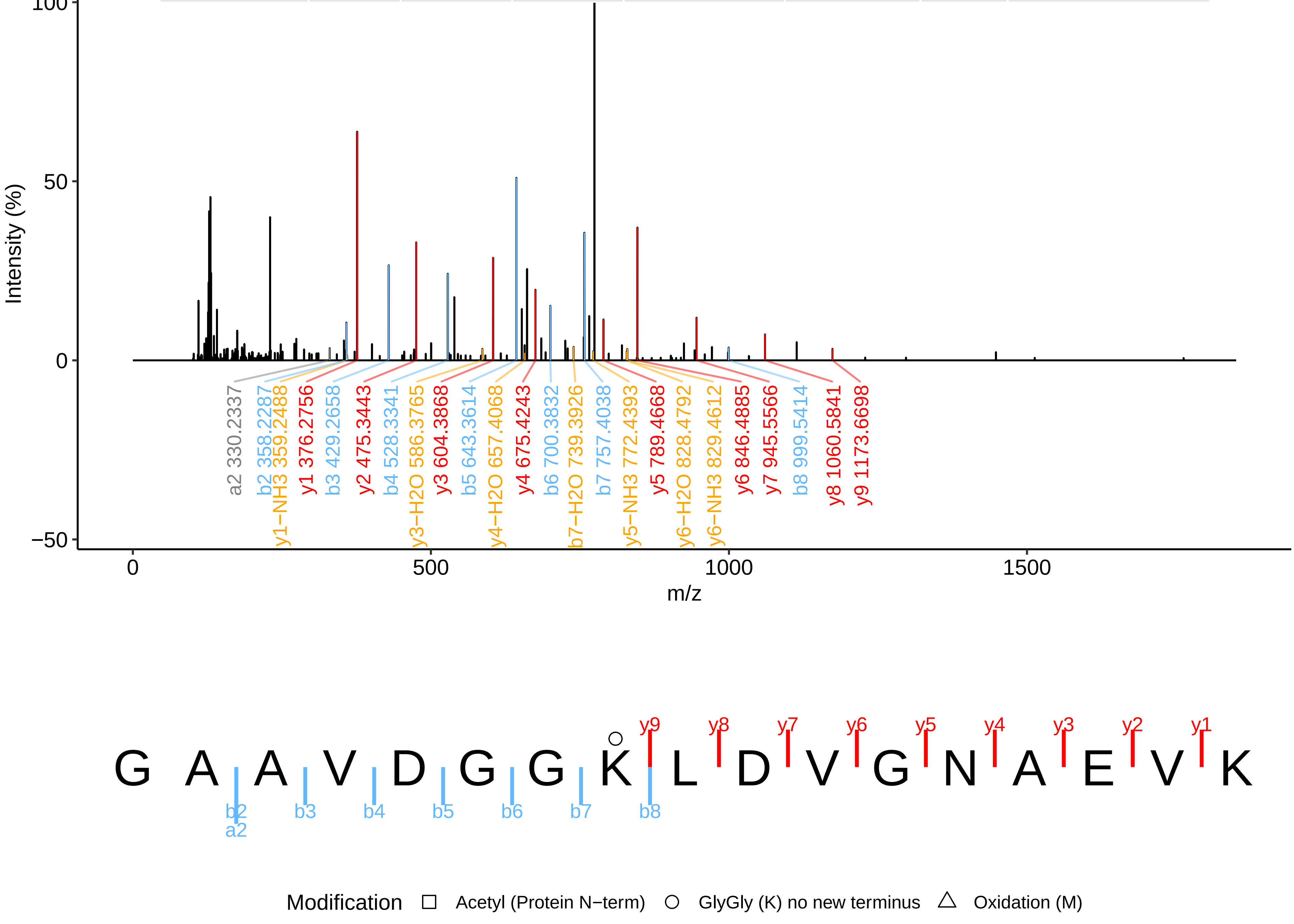

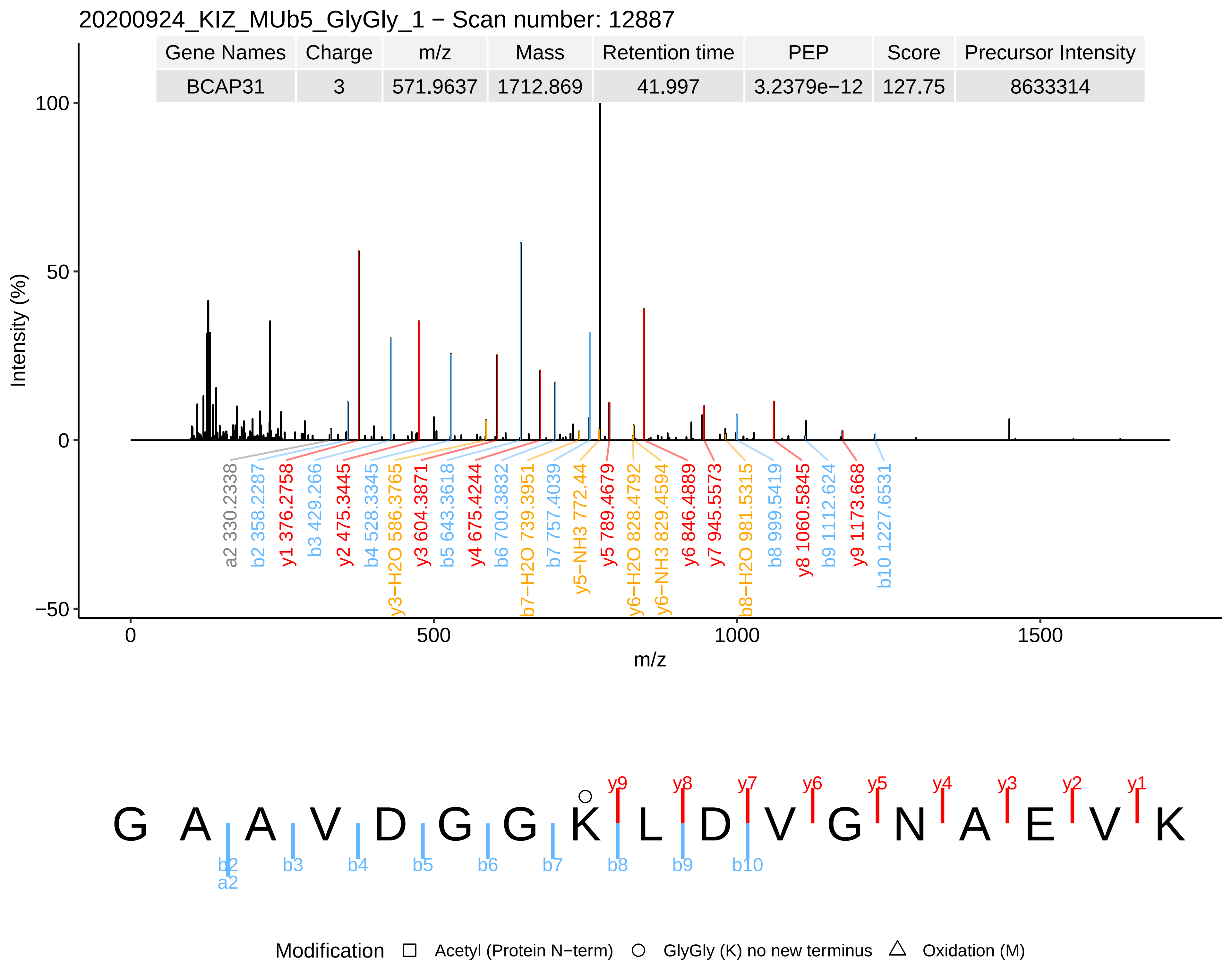
20200924_KIZ_MUb5_GlyGly_1 − Scan number: 12887
Gene Names | Charge | m/z | Mass | Retention time | PEP | Score | Precursor Intensity
BCAP31 | 3 | 571.9637 | 1712.869 | 41.997 | 3.2379e−12 | 127.75 | 8633314
Intensity (%)
m/z
a2 330.2338
b2 358.2287
y1 376.2758
b3 429.266
y2 475.3445
b4 528.3345
y3−H2O 586.3765
y3 604.3871
b5 643.3618
y4 675.4244
b6 700.3832
b7−H2O 739.3951
b7 757.4039
y5−NH3 772.44
y5 789.4679
y6−H2O 828.4792
y6−NH3 829.4594
y6 846.4889
y7 945.5573
b8−H2O 981.5315
b8 999.5419
y8 1060.5845
b9 1112.624
y9 1173.668
b10 1227.6531
G A A V D G G K L D V G N A E V K
y9 y8 y7 y6 y5 y4 y3 y2 y1
b2 b3 b4 b5 b6 b7 b8 b9 b10
a2
Modification
Acetyl (Protein N−term)
GlyGly (K) no new terminus
Oxidation (M)

20200921_KIZ_MUb4_GlyGly_10plex_2 − Scan number: 21043

Gene Names	Charge	m/z	Mass	Retention time	PEP	Score	Precursor Intensity
TUBA1C;TUBA1B	3	575.6562	1723.947	82.8	0.015915	60.474	827604.6

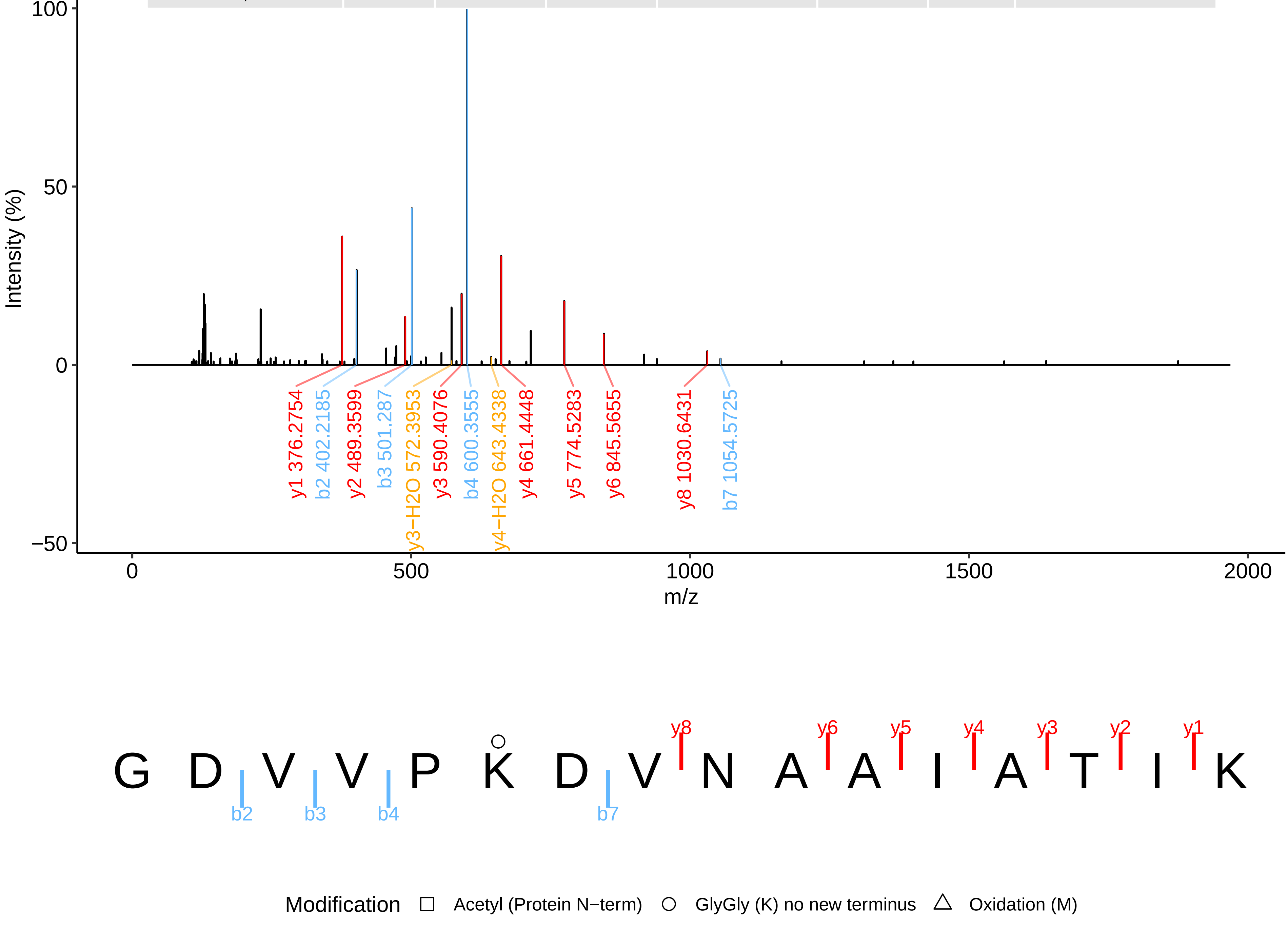

20200924_KIZ_MUb5_GlyGly_1 − Scan number: 23400

Gene Names	Charge	m/z	Mass	Retention time	PEP	Score	Precursor Intensity
TUBA1C;TUBA1B	3	575.6562	1723.947	75.209	0.0044752	70.942	NA

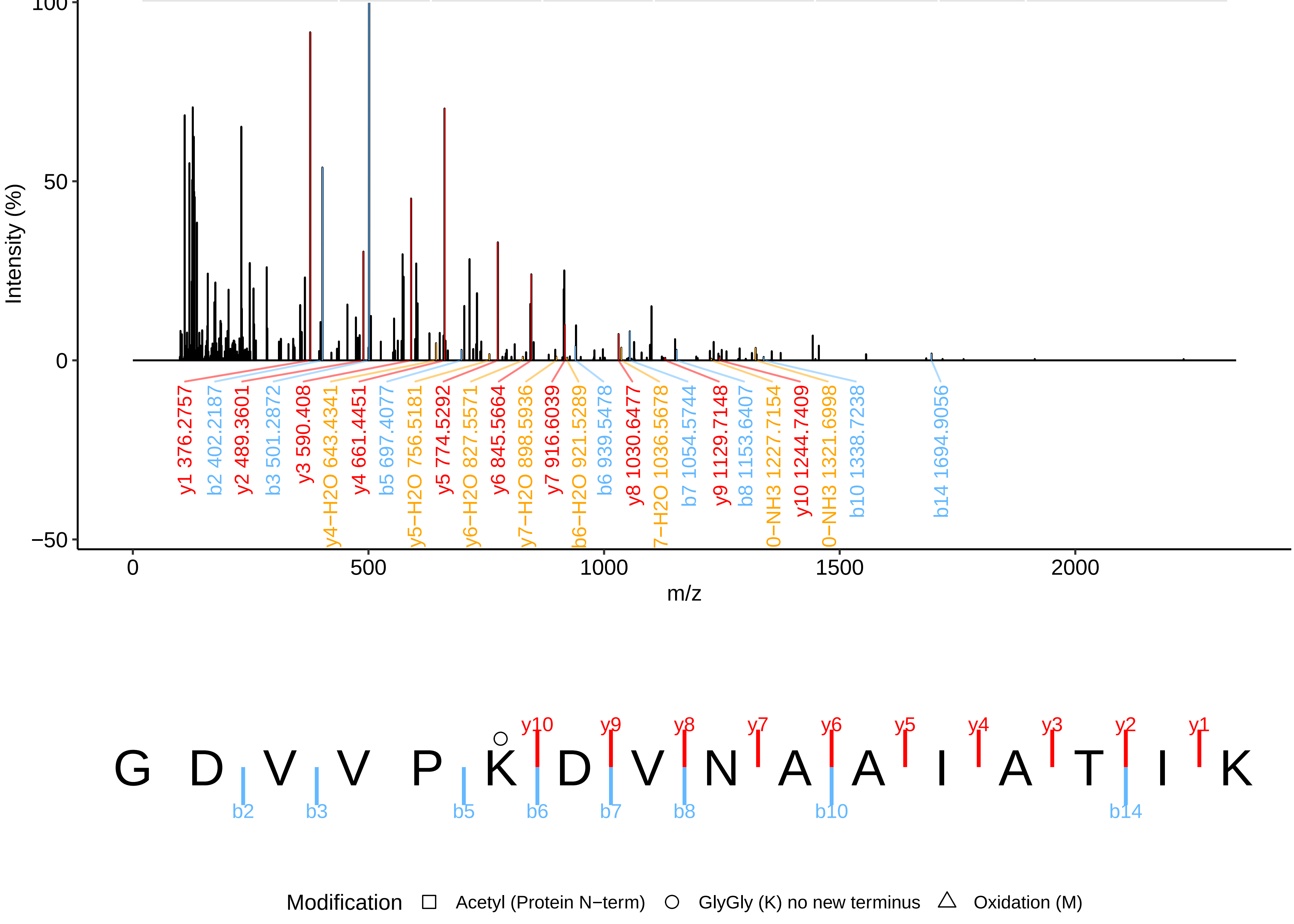

20200921_KIZ_MUb4_GlyGly_10plex − Scan number: 27688

Gene Names	Charge	m/z	Mass	Retention time	PEP	Score	Precursor Intensity
HPD	3	672.3581	2014.052	101.11	6.6985e−06	91.812	3743795

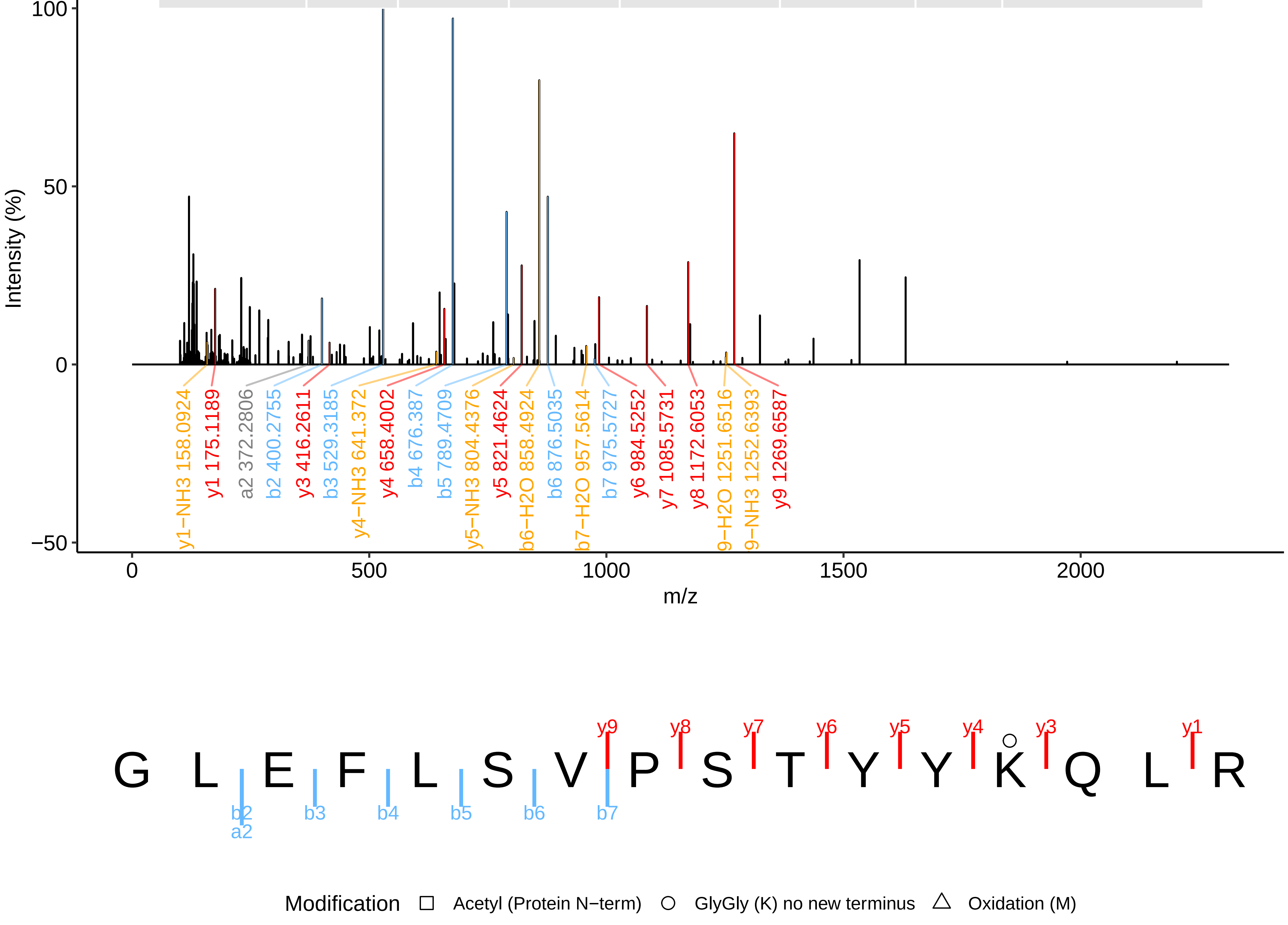

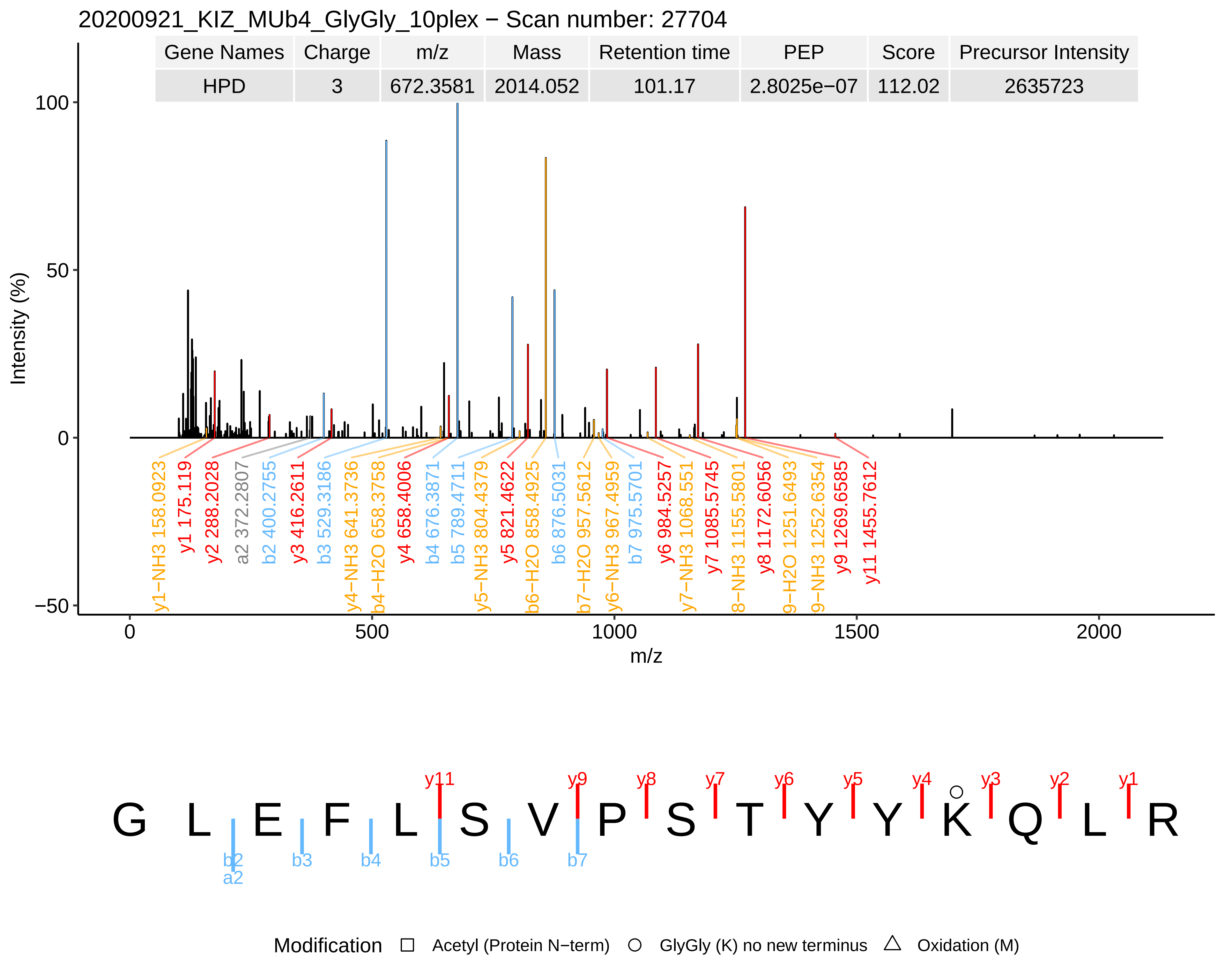
20200921_KIZ_MUb4_GlyGly_10plex − Scan number: 27704
Gene Names | Charge | m/z | Mass | Retention time | PEP | Score | Precursor Intensity
HPD | 3 | 672.3581 | 2014.052 | 101.17 | 2.8025e−07 | 112.02 | 2635723
Intensity (%)
100
50
0
−50
y1−NH3 158.0923
y1 175.119
y2 288.2028
a2 372.2807
b2 400.2755
y3 416.2611
b3 529.3186
y4−NH3 641.3736
b4−H2O 658.3758
y4 658.4006
b4 676.3871
b5 789.4711
y5−NH3 804.4379
y5 821.4622
b6−H2O 858.4925
b6 876.5031
b7−H2O 957.5612
y6−NH3 967.4959
b7 975.5701
y6 984.5257
y7−NH3 1068.551
y7 1085.5745
y8−NH3 1155.5801
y8 1172.6056
y9−H2O 1251.6493
y9−NH3 1252.6354
y9 1269.6585
y11 1455.7612
0
500
1000
1500
2000
m/z
G L E F L S V P S T Y Y K Q L R
y11 y9 y8 y7 y6 y5 y4 y3 y2 y1
b2 a2 b3 b4 b5 b6 b7
Modification
Acetyl (Protein N−term)
GlyGly (K) no new terminus
Oxidation (M)

20200921_KIZ_MUb4_GlyGly_10plex − Scan number: 24469

Gene Names	Charge	m/z	Mass	Retention time	PEP	Score	Precursor Intensity
PCNA	4	434.2315	1732.897	88.984	0.00056005	82.362	5643834

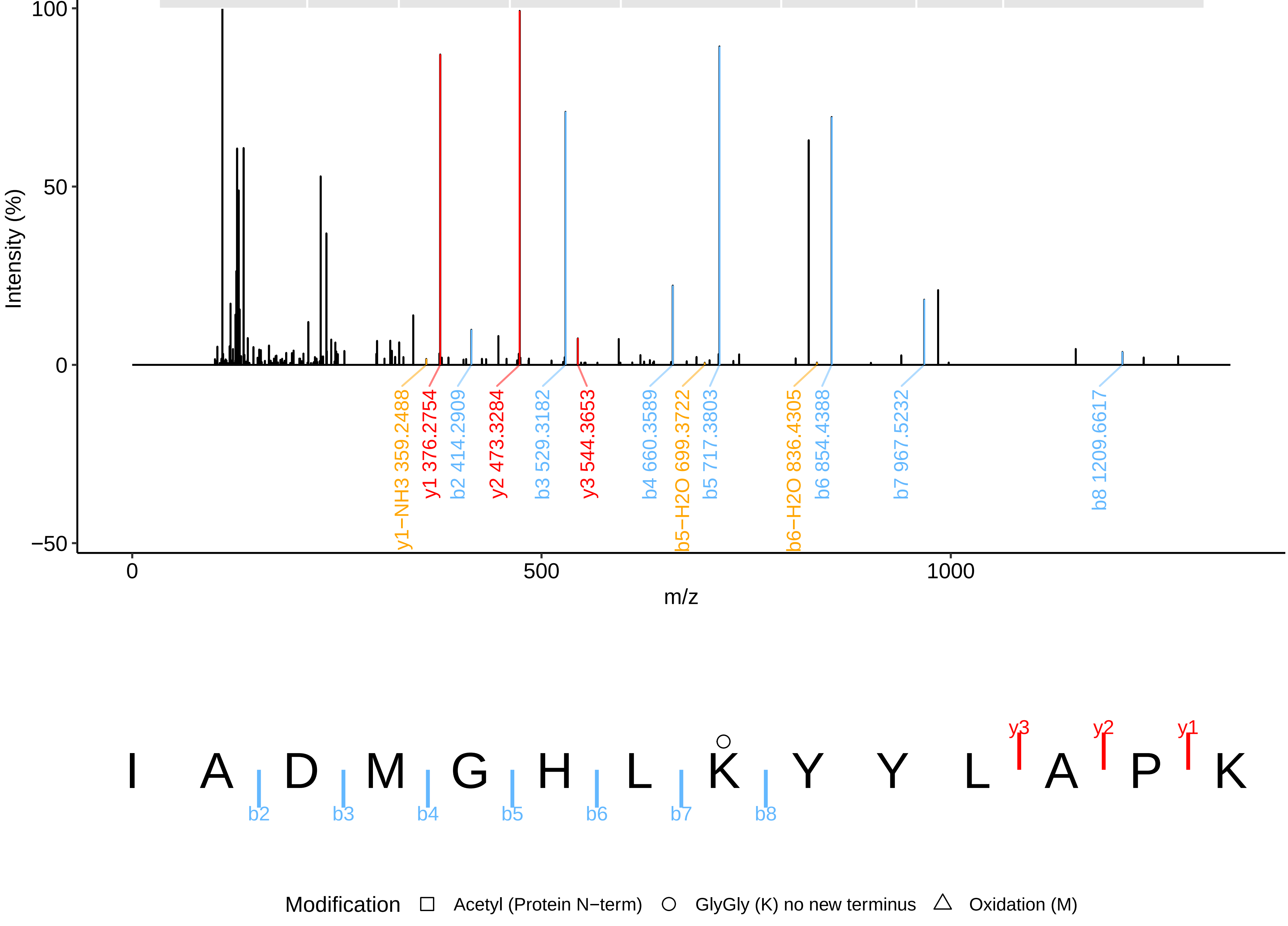

20200921_KIZ_MUb4_GlyGly_10plex − Scan number: 24482

Gene Names	Charge	m/z	Mass	Retention time	PEP	Score	Precursor Intensity
PCNA	4	434.2315	1732.897	89.032	0.0011803	78.763	5960404

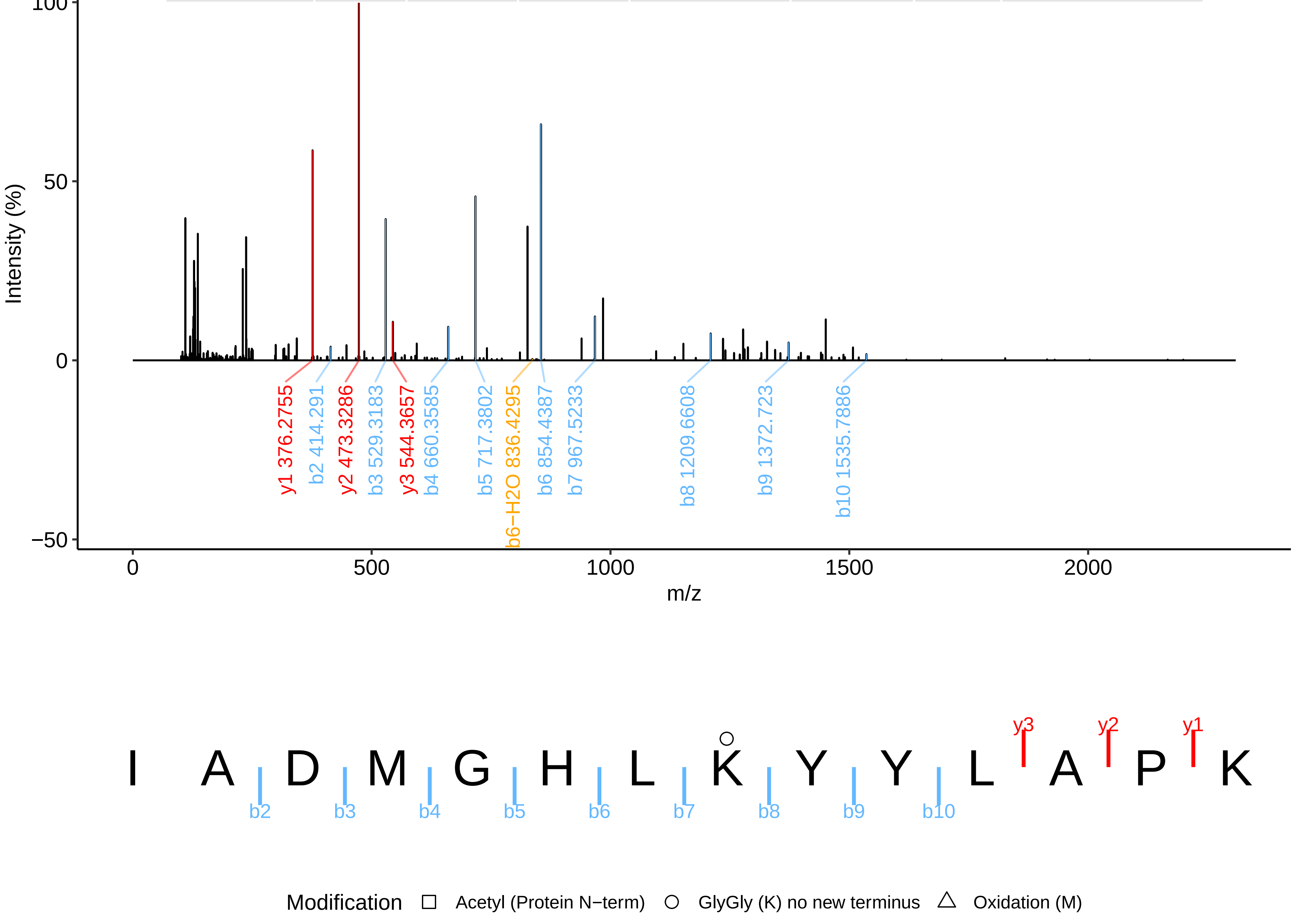

20200921_KIZ_MUb4_GlyGly_10plex − Scan number: 24623

Gene Names	Charge	m/z	Mass	Retention time	PEP	Score	Precursor Intensity
PCNA	4	434.2315	1732.897	89.555	0.020648	58.487	NA

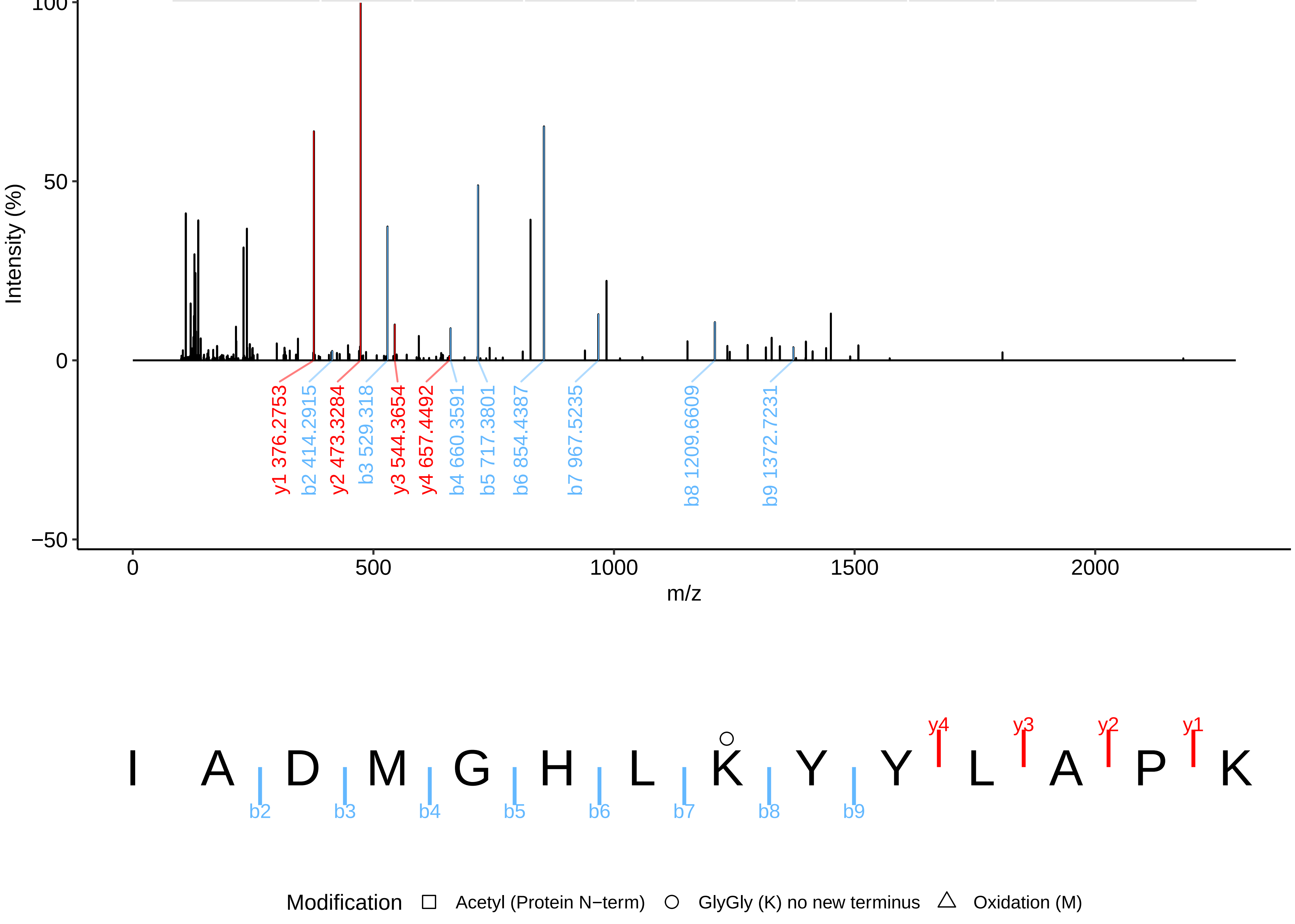

20200921_KIZ_MUb4_GlyGly_10plex_2 − Scan number: 15625

Gene Names	Charge	m/z	Mass	Retention time	PEP	Score	Precursor Intensity
PCNA	4	438.2303	1748.892	61.143	0.0613	46.481	1871744

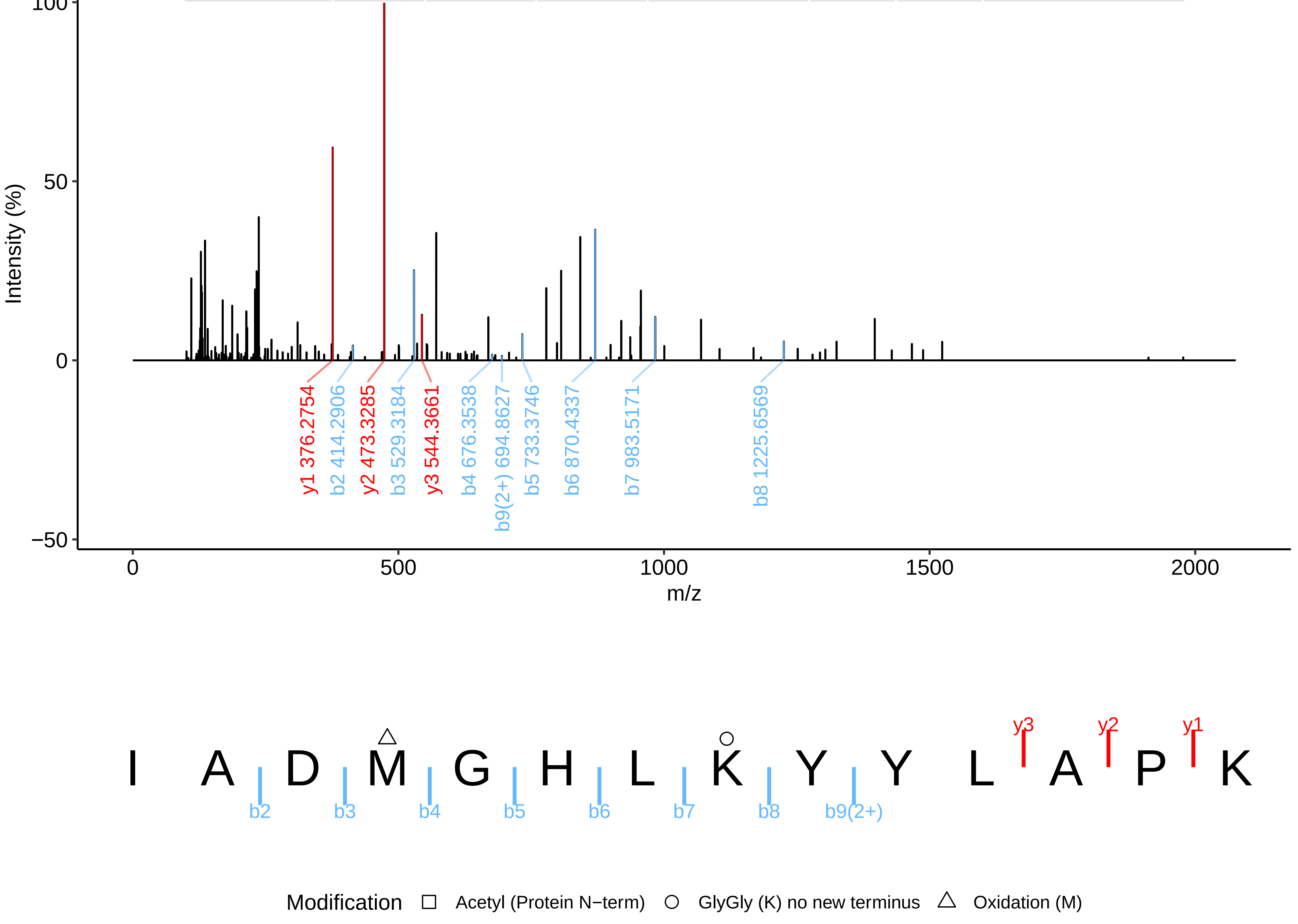

20200921_KIZ_MUb4_GlyGly_10plex_2 − Scan number: 18021

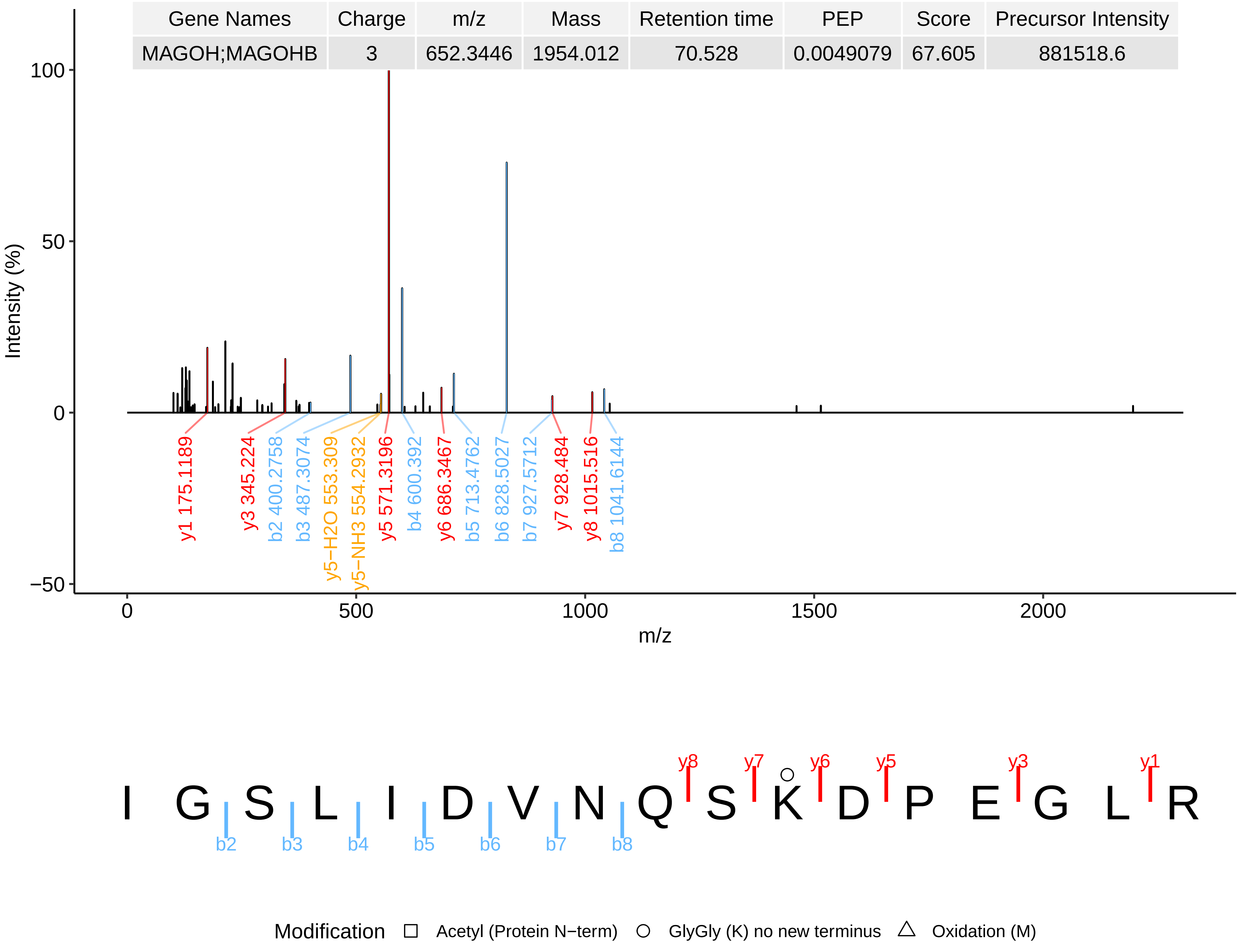

20200921_KIZ_MUb4_GlyGly_10plex − Scan number: 15816

Gene Names	Charge	m/z	Mass	Retention time	PEP	Score	Precursor Intensity
TOM1L2	3	593.3317	1776.973	56.623	4.6841e−09	117.08	5182094

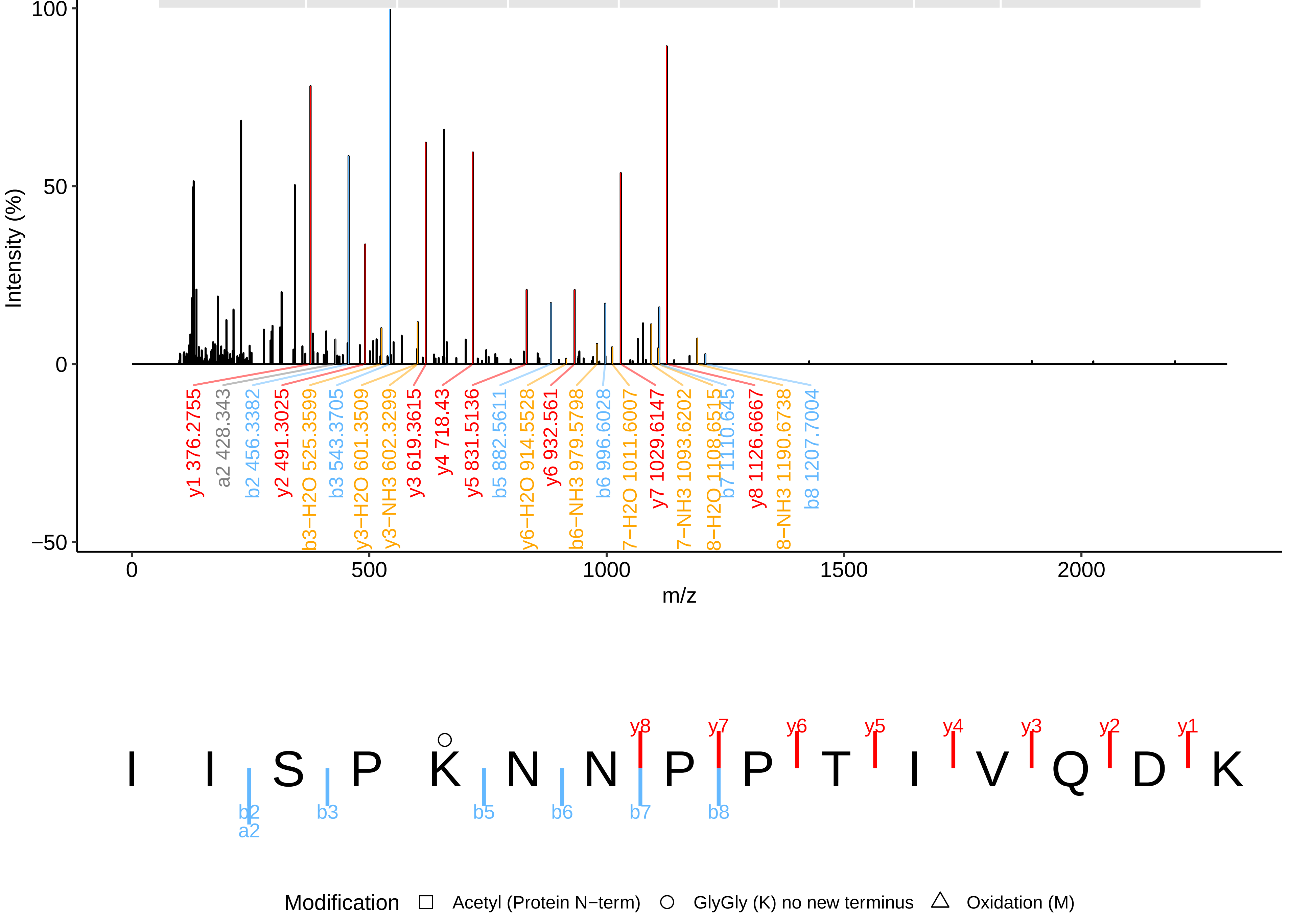

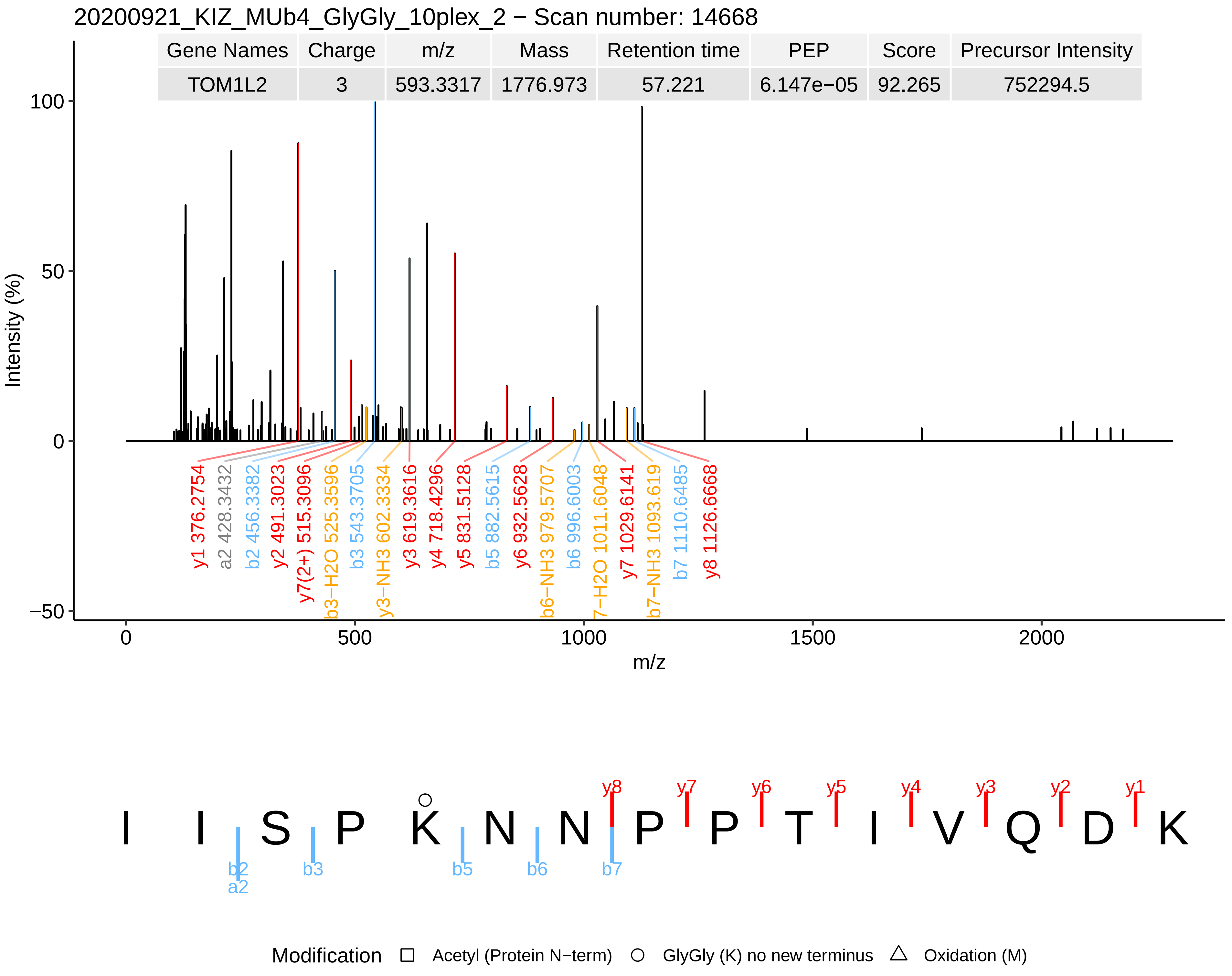

20200921_KIZ_MUb4_GlyGly_10plex_2 − Scan number: 14668

Gene Names	Charge	m/z	Mass	Retention time	PEP	Score	Precursor Intensity
TOM1L2	3	593.3317	1776.973	57.221	6.147e−05	92.265	752294.5

Intensity (%)
m/z
y1 376.2754
a2 428.3432
b2 456.3382
y2 491.3023
y7(2+) 515.3096
b3−H2O 525.3596
b3 543.3705
y3−NH3 602.3334
y3 619.3616
y4 718.4296
y5 831.5128
b5 882.5615
y6 932.5628
b6−NH3 979.5707
b6 996.6003
y7−H2O 1011.6048
y7 1029.6141
b7−NH3 1093.619
b7 1110.6485
y8 1126.6668
I I S P K N N P P T I V Q D K
y8 y7 y6 y5 y4 y3 y2 y1
b2 a2 b3 b5 b6 b7
Modification
Acetyl (Protein N−term)
GlyGly (K) no new terminus
Oxidation (M)

20200921_KIZ_MUb4_GlyGly_10plex − Scan number: 27653

Gene Names	Charge	m/z	Mass	Retention time	PEP	Score	Precursor Intensity
SLC27A2	3	576.3136	1725.919	100.98	1.4905e−05	103.88	6416751

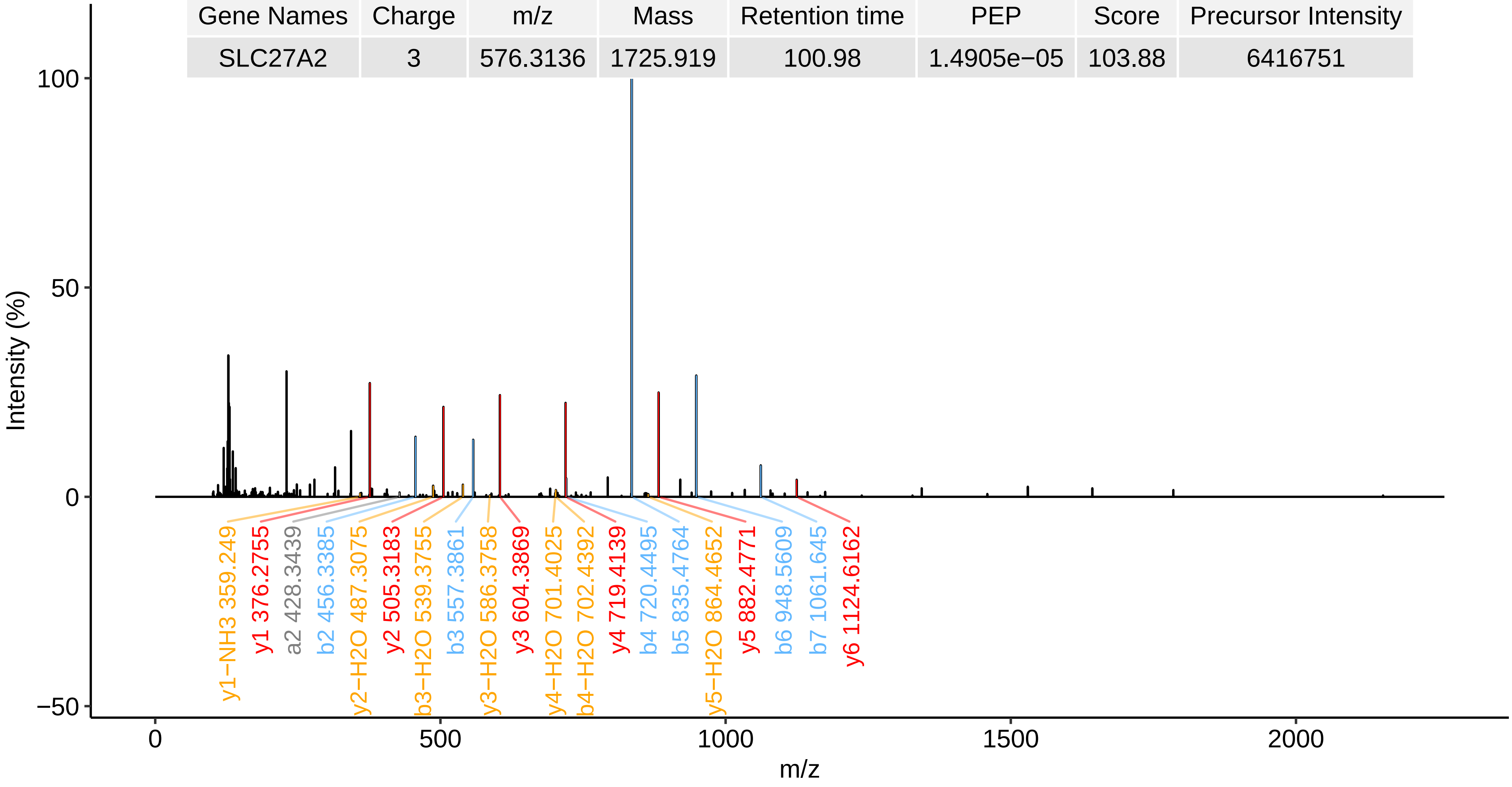

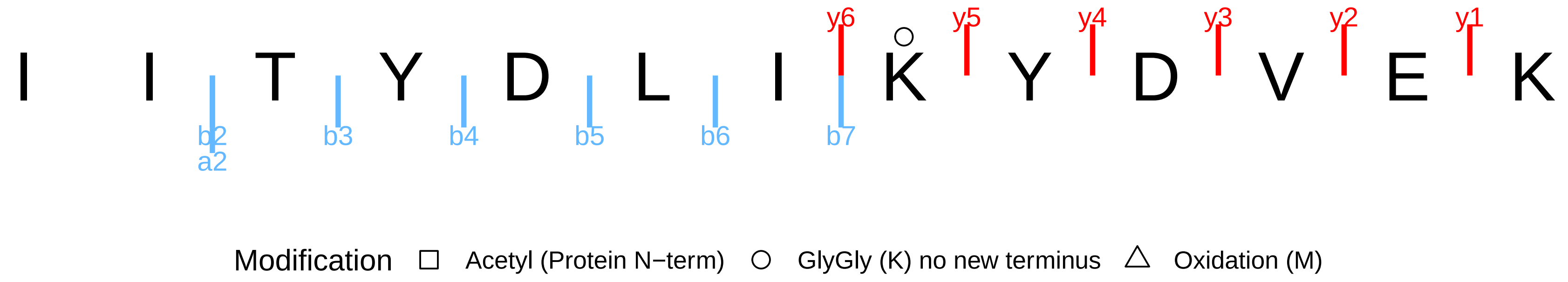

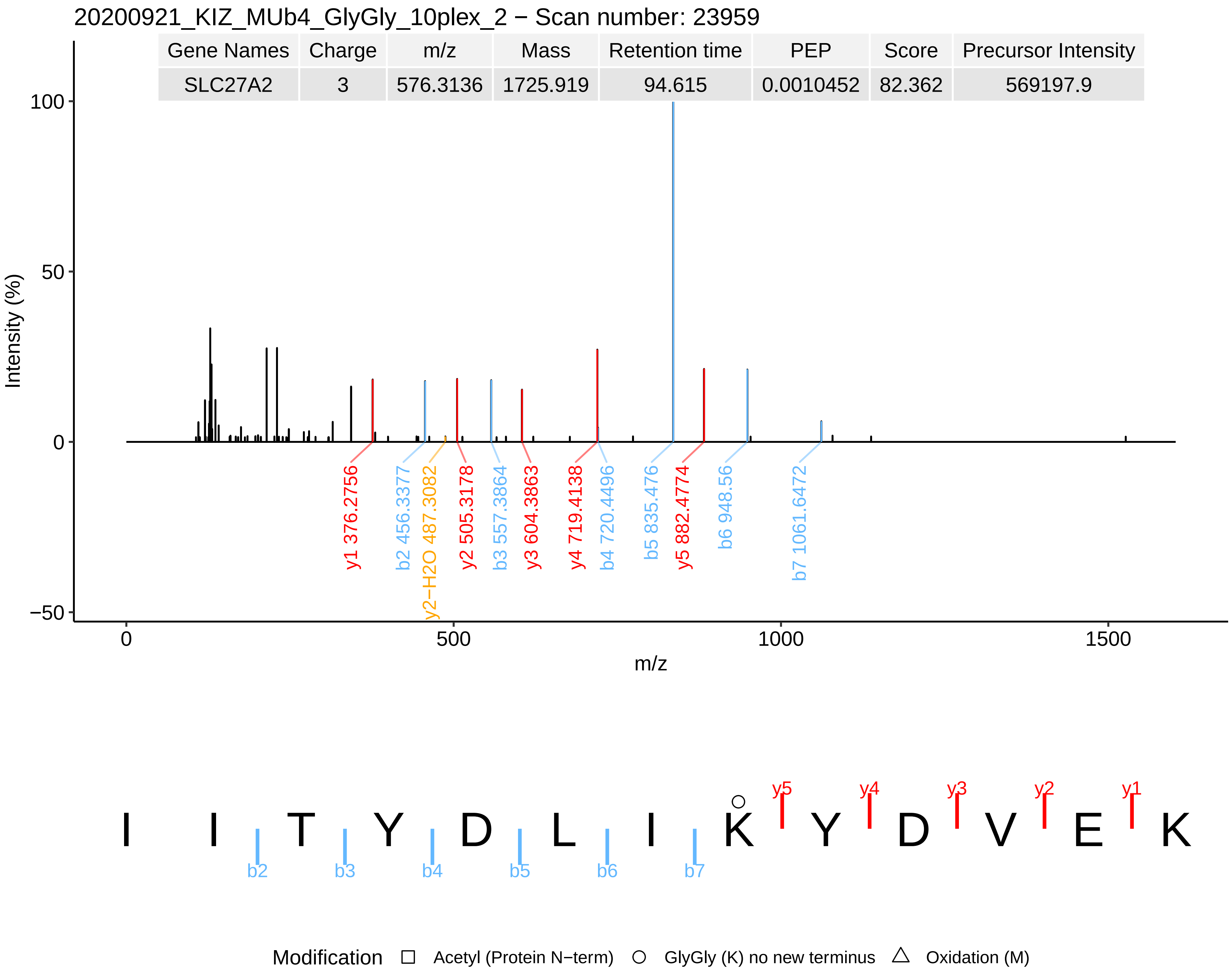
20200921_KIZ_MUb4_GlyGly_10plex_2 − Scan number: 23959
Gene Names | Charge | m/z | Mass | Retention time | PEP | Score | Precursor Intensity
SLC27A2 | 3 | 576.3136 | 1725.919 | 94.615 | 0.0010452 | 82.362 | 569197.9
Intensity (%)
100
50
0
−50
0
500
1000
1500
m/z
y1 376.2756
b2 456.3377
y2−H2O 487.3082
y2 505.3178
b3 557.3864
y3 604.3863
y4 719.4138
b4 720.4496
b5 835.476
y5 882.4774
b6 948.56
b7 1061.6472
I I T Y D L I K Y D V E K
b2 b3 b4 b5 b6 b7
y5 y4 y3 y2 y1
Modification
Acetyl (Protein N−term)
GlyGly (K) no new terminus
Oxidation (M)

20200921_KIZ_MUb4_GlyGly_10plex_2 − Scan number: 16482

Gene Names	Charge	m/z	Mass	Retention time	PEP	Score	Precursor Intensity
TUBB4B	3	653.3304	1956.969	64.344	0.0021788	68.257	1101467

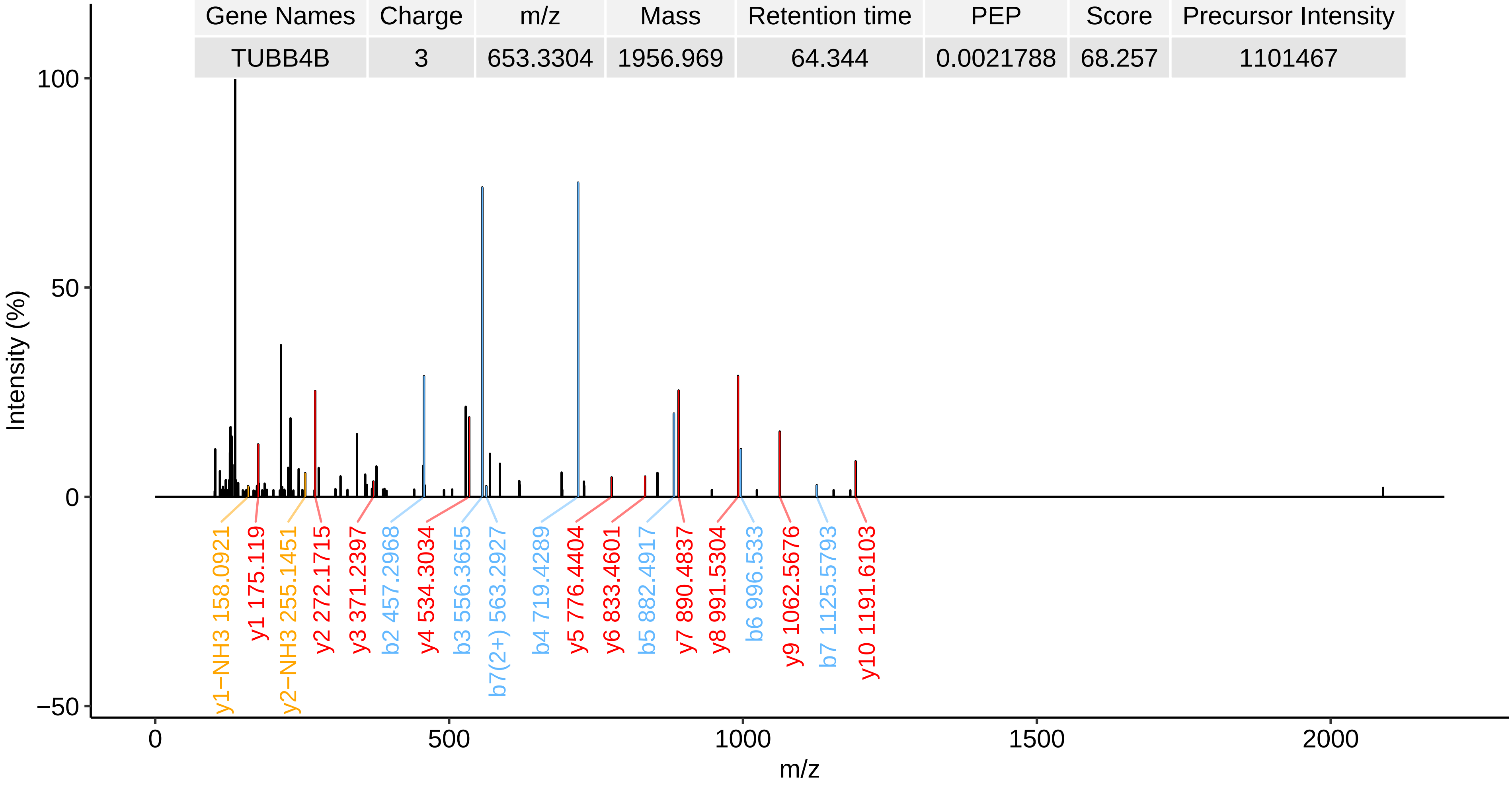

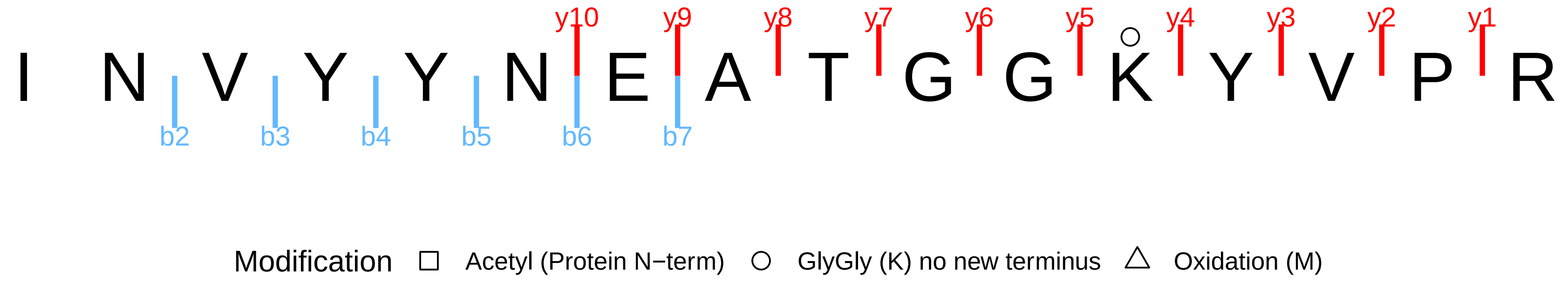

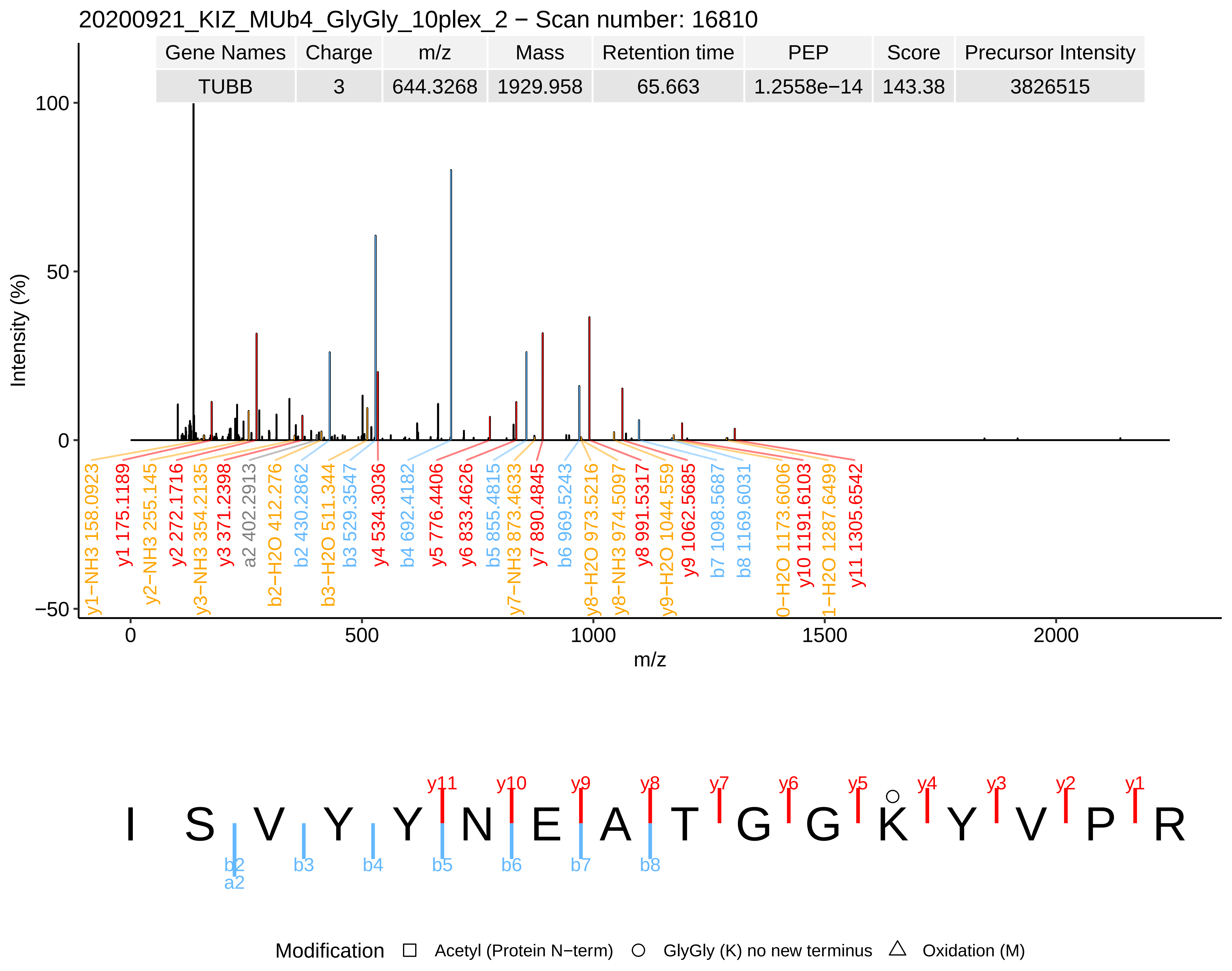

20200921_KIZ_MUb4_GlyGly_10plex_2 − Scan number: 16810
Gene Names | Charge | m/z | Mass | Retention time | PEP | Score | Precursor Intensity
TUBB | 3 | 644.3268 | 1929.958 | 65.663 | 1.2558e−14 | 143.38 | 3826515
Intensity (%)
m/z
y1−NH3 158.0923
y1 175.1189
y2−NH3 255.145
y2 272.1716
y3−NH3 354.2135
y3 371.2398
a2 402.2913
b2−H2O 412.276
b2 430.2862
b3−H2O 511.344
b3 529.3547
y4 534.3036
b4 692.4182
y5 776.4406
y6 833.4626
b5 855.4815
y7−NH3 873.4633
y7 890.4845
b6 969.5243
y8−H2O 973.5216
y8−NH3 974.5097
y8 991.5317
y9−H2O 1044.559
y9 1062.5685
b7 1098.5687
b8 1169.6031
0−H2O 1173.6006
y10 1191.6103
1−H2O 1287.6499
y11 1305.6542
I S V Y Y N E A T G G K Y V P R
Modification
Acetyl (Protein N−term)
GlyGly (K) no new terminus
Oxidation (M)

20200924_KIZ_MUb5_GlyGly_1 − Scan number: 17929

Gene Names	Charge	m/z	Mass	Retention time	PEP	Score	Precursor Intensity
TUBB	3	644.3268	1929.958	57.948	1.8085e−28	176.23	6353021

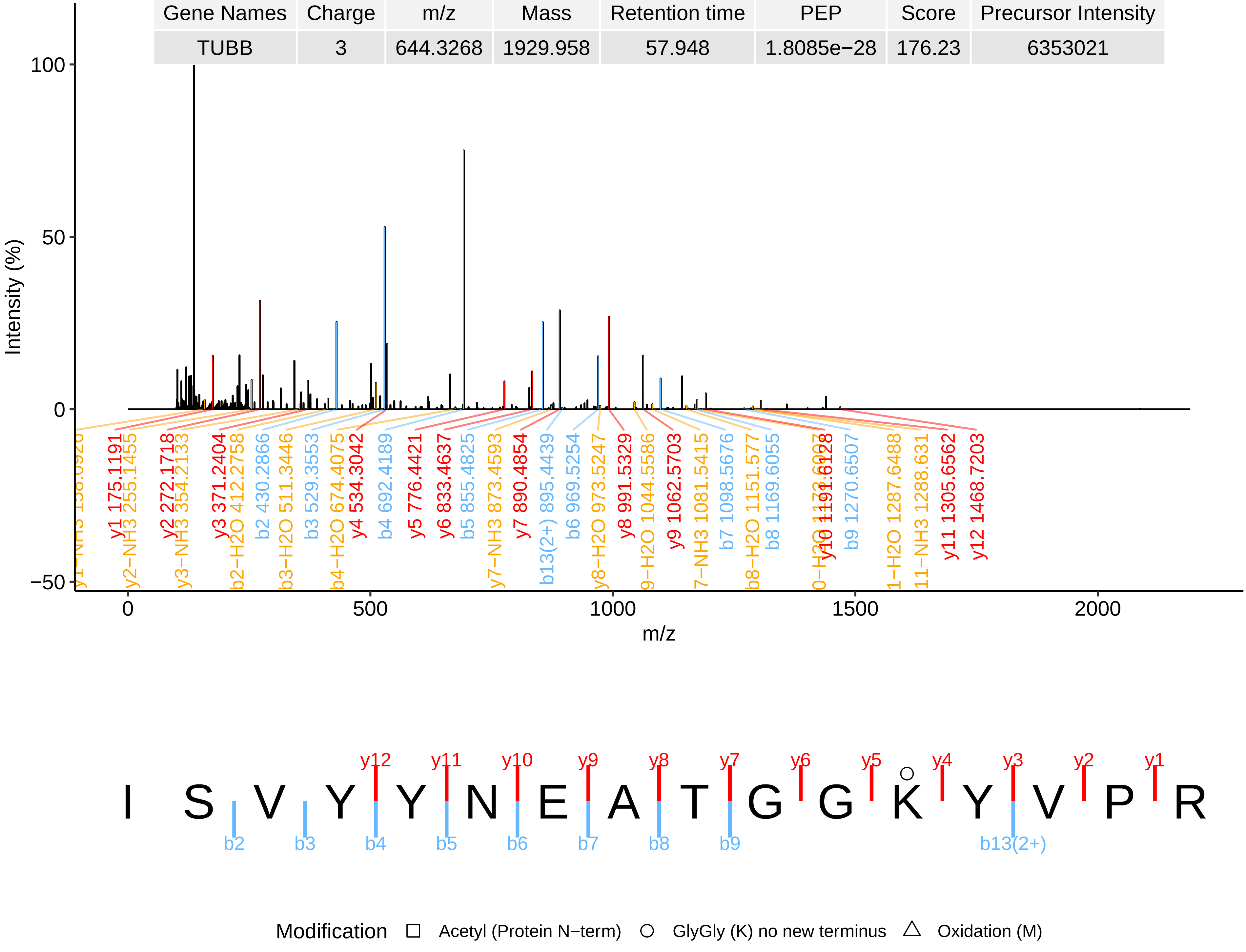

20200924_KIZ_MUb5_GlyGly_2 − Scan number: 16503

Gene Names	Charge	m/z	Mass	Retention time	PEP	Score	Precursor Intensity
TUBB	3	644.3268	1929.958	61.232	2.2733e−05	95.57	2061954

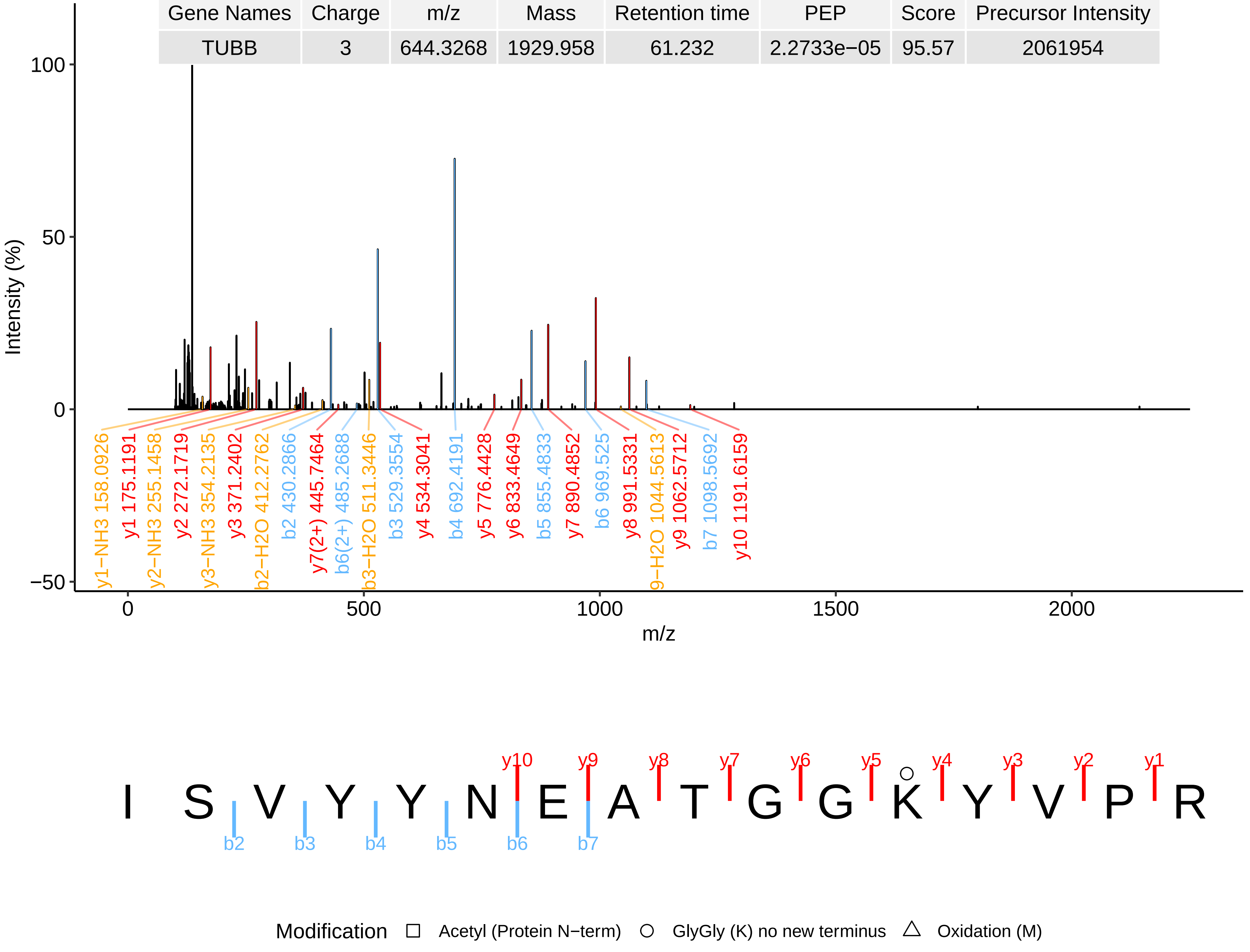

20200924_KIZ_MUb5_GlyGly_2 − Scan number: 16751

Gene Names	Charge	m/z	Mass	Retention time	PEP	Score	Precursor Intensity
TUBB	3	644.3268	1929.958	62.145	2.4797e−27	174.83	1905841

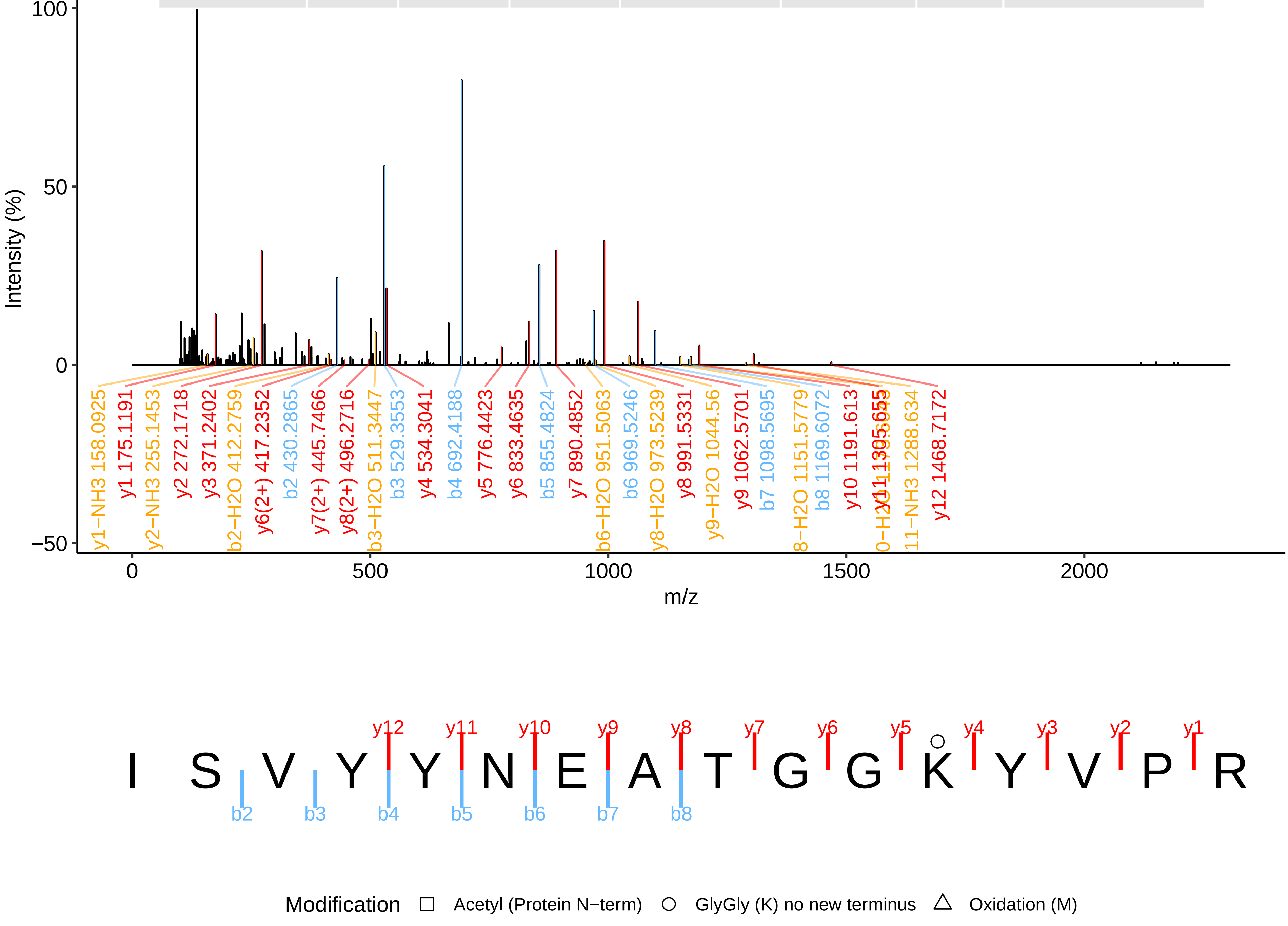

20201118_KIZ_MUb6_GlyGly_2 − Scan number: 19461

Gene Names	Charge	m/z	Mass	Retention time	PEP	Score	Precursor Intensity
TUBB	3	644.3268	1929.958	65.67	3.8017e−10	124.09	6021506

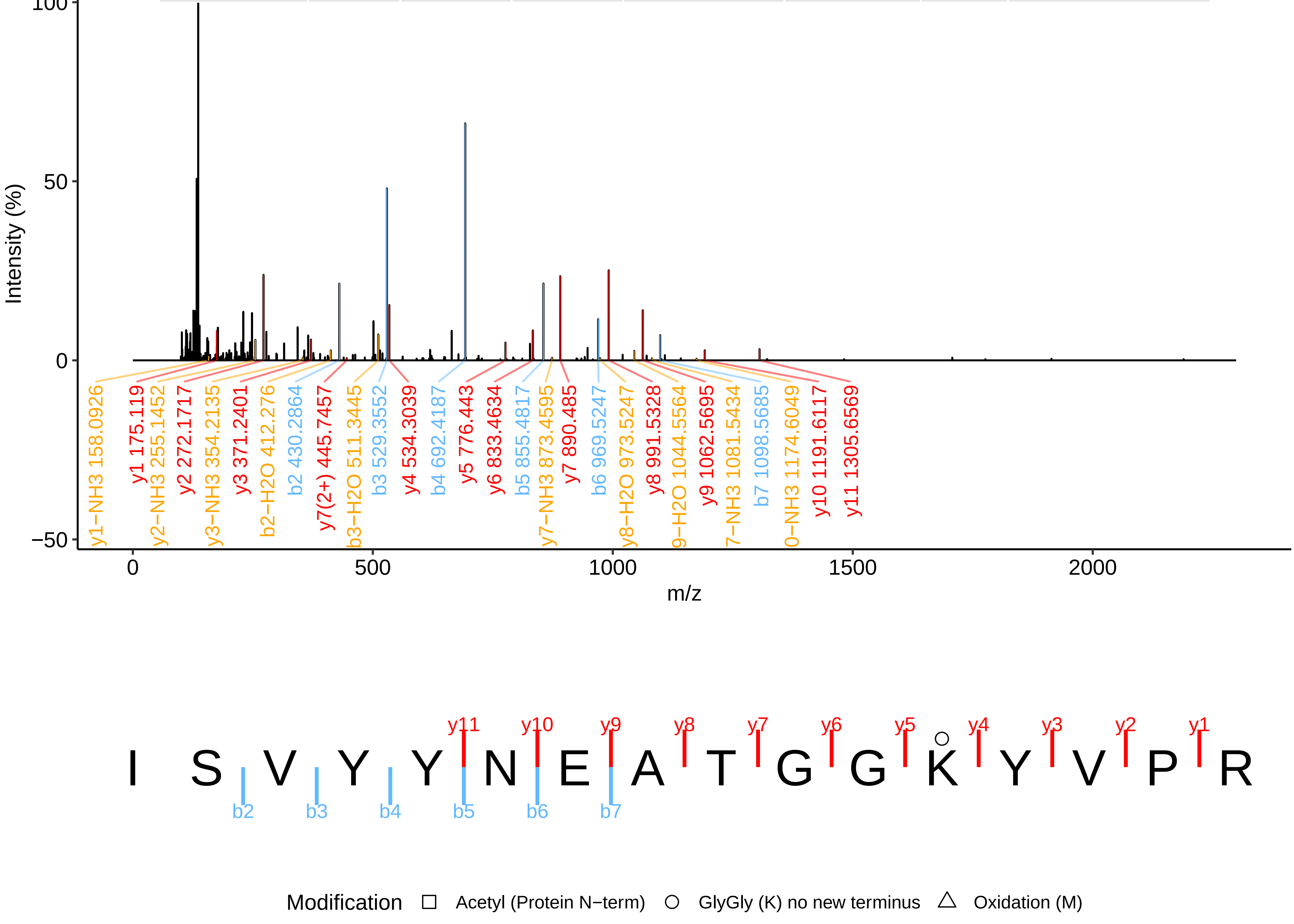

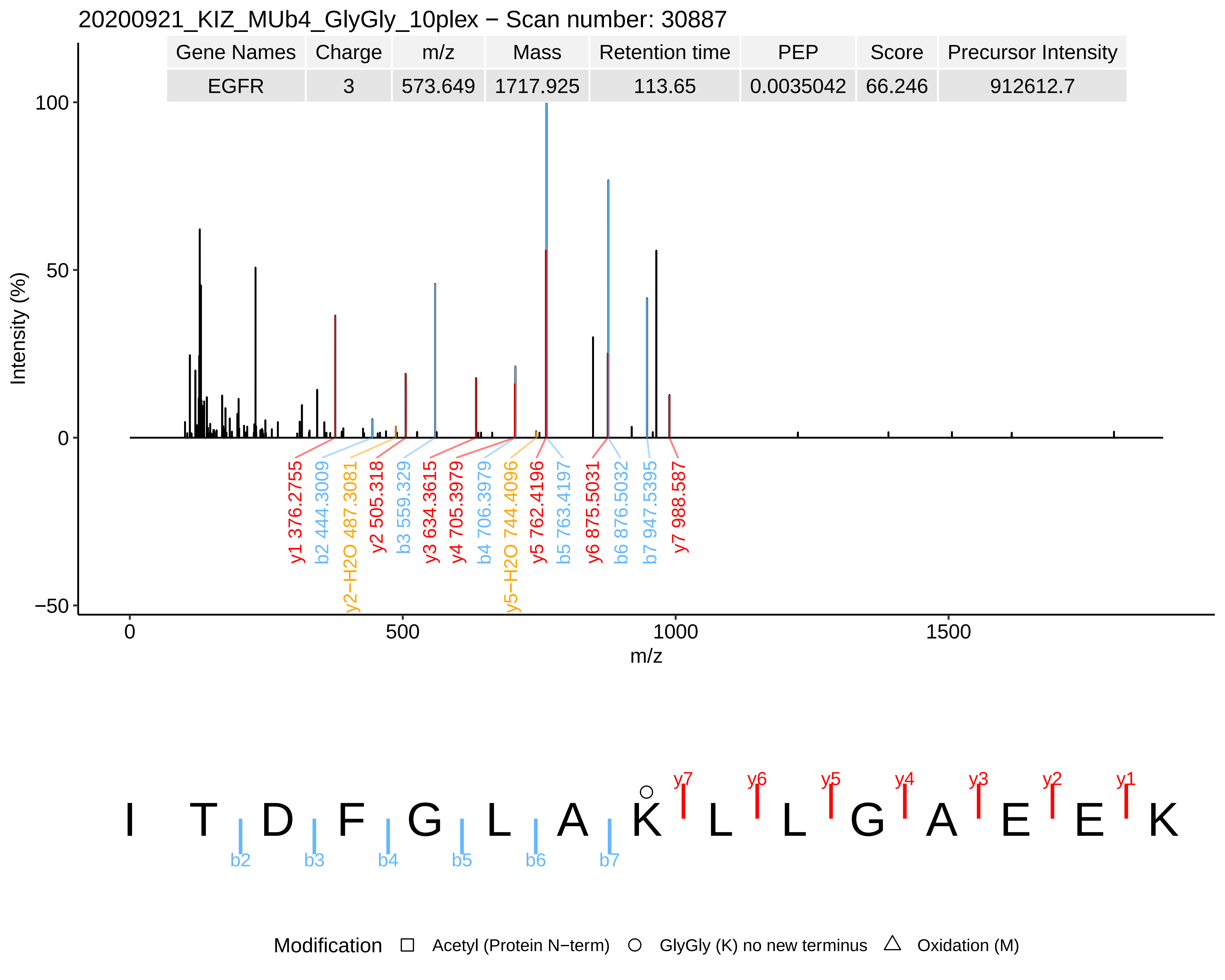
20200921_KIZ_MUb4_GlyGly_10plex − Scan number: 30887
Gene Names | Charge | m/z | Mass | Retention time | PEP | Score | Precursor Intensity
EGFR | 3 | 573.649 | 1717.925 | 113.65 | 0.0035042 | 66.246 | 912612.7
Intensity (%)
m/z
y1 376.2755
b2 444.3009
y2−H2O 487.3081
y2 505.318
b3 559.329
y3 634.3615
y4 705.3979
b4 706.3979
y5−H2O 744.4096
y5 762.4196
b5 763.4197
y6 875.5031
b6 876.5032
b7 947.5395
y7 988.587
I T D F G L A K L L G A E E K
Modification
Acetyl (Protein N−term)
GlyGly (K) no new terminus
Oxidation (M)

20200921_KIZ_MUb4_GlyGly_10plex − Scan number: 8336

Gene Names	Charge	m/z	Mass	Retention time	PEP	Score	Precursor Intensity
FIS1	4	370.9493	1479.768	28.258	2.7186e−05	112.15	5421620

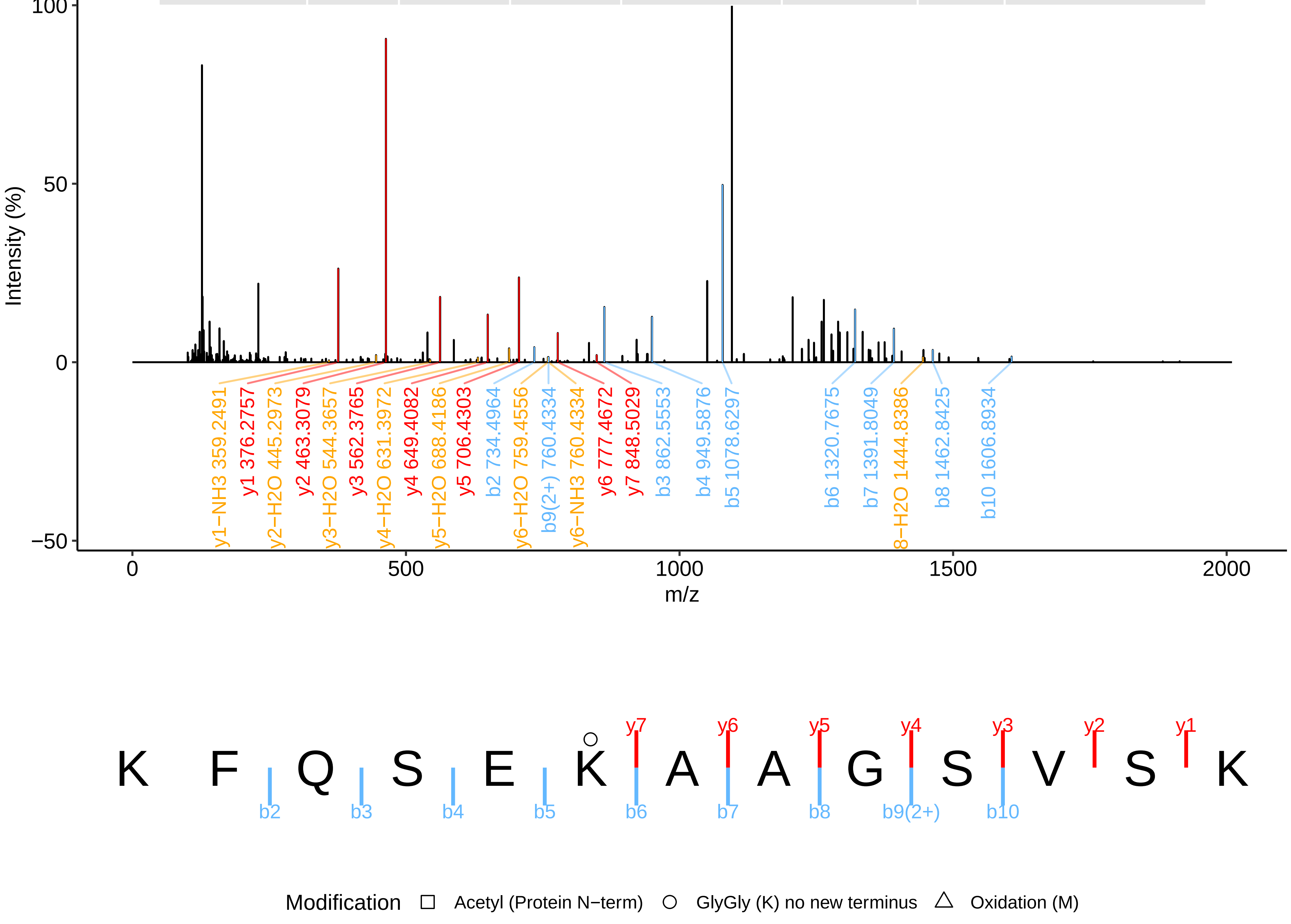

20201118_KIZ_MUb6_GlyGly_2 − Scan number: 9062

Gene Names	Charge	m/z	Mass	Retention time	PEP	Score	Precursor Intensity
FIS1	4	370.9493	1479.768	30.699	0.0015228	94.728	7892712

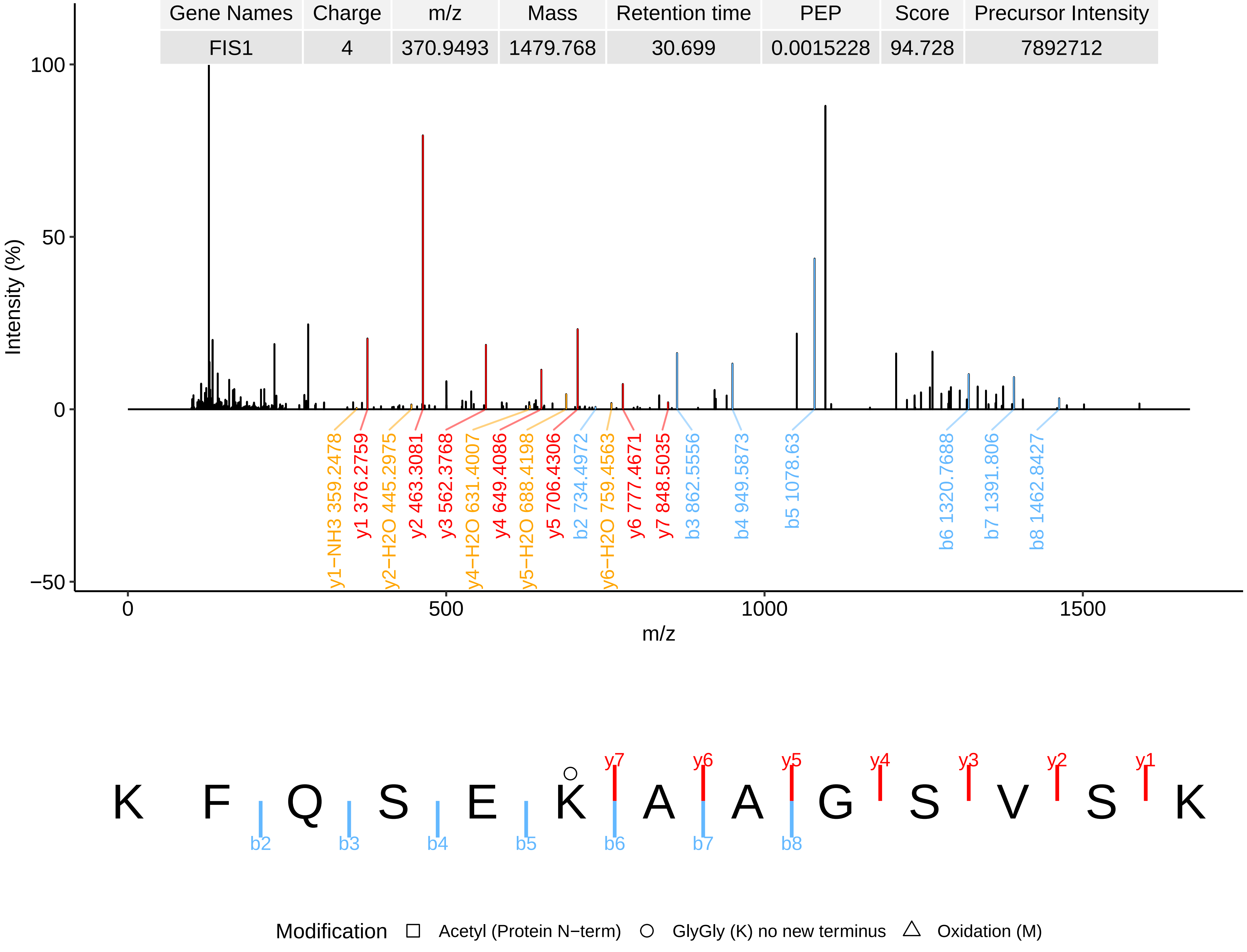

20200921_KIZ_MUb4_GlyGly_10plex_2 − Scan number: 9003

Gene Names	Charge	m/z	Mass	Retention time	PEP	Score	Precursor Intensity
SLC39A6	4	386.6954	1542.753	34.099	0.081292	63.537	1379047

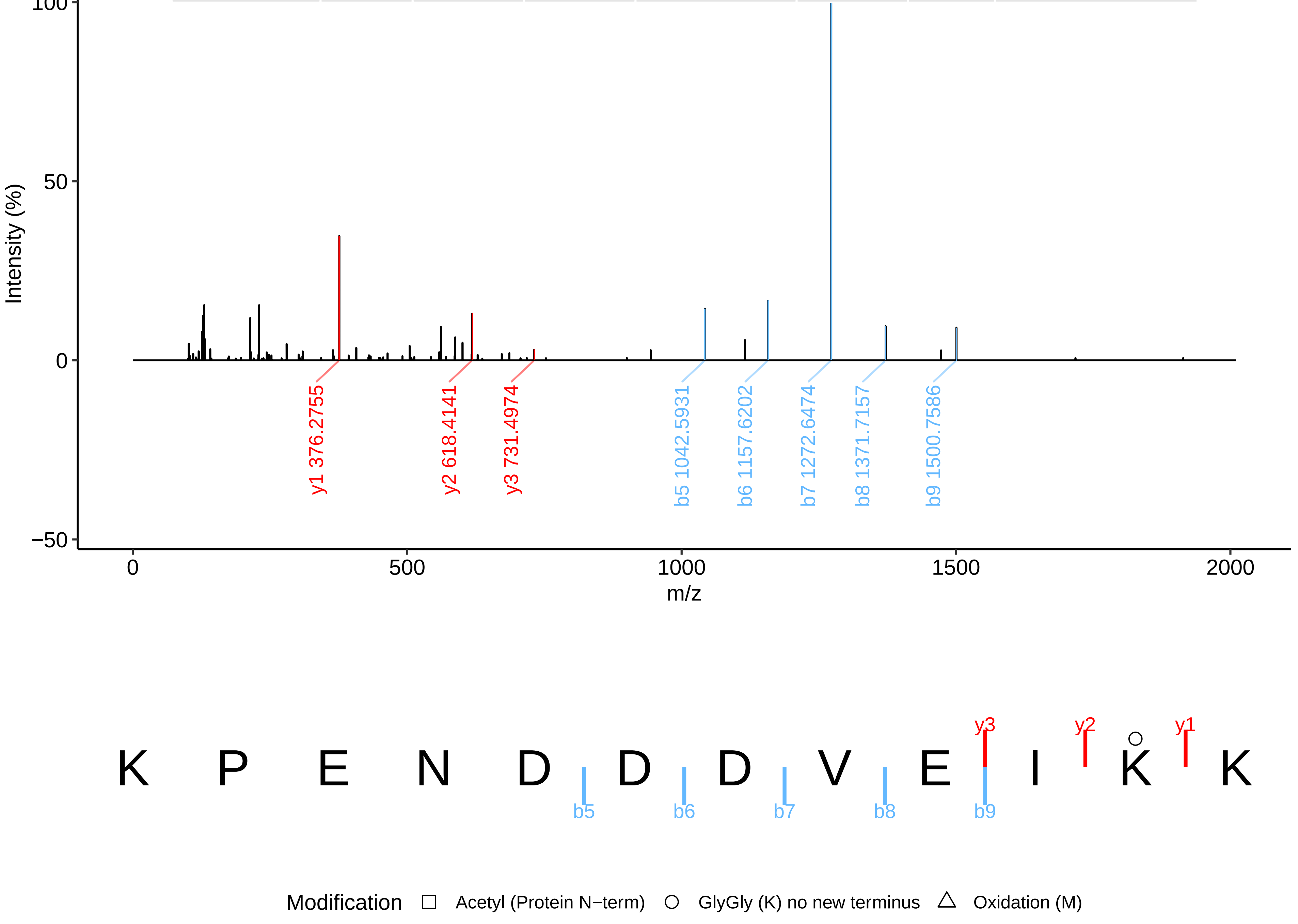

20200921_KIZ_MUb4_GlyGly_10plex − Scan number: 7214

Gene Names	Charge	m/z	Mass	Retention time	PEP	Score	Precursor Intensity
SDC2	4	373.4545	1489.789	24.134	0.010753	79.646	10426598

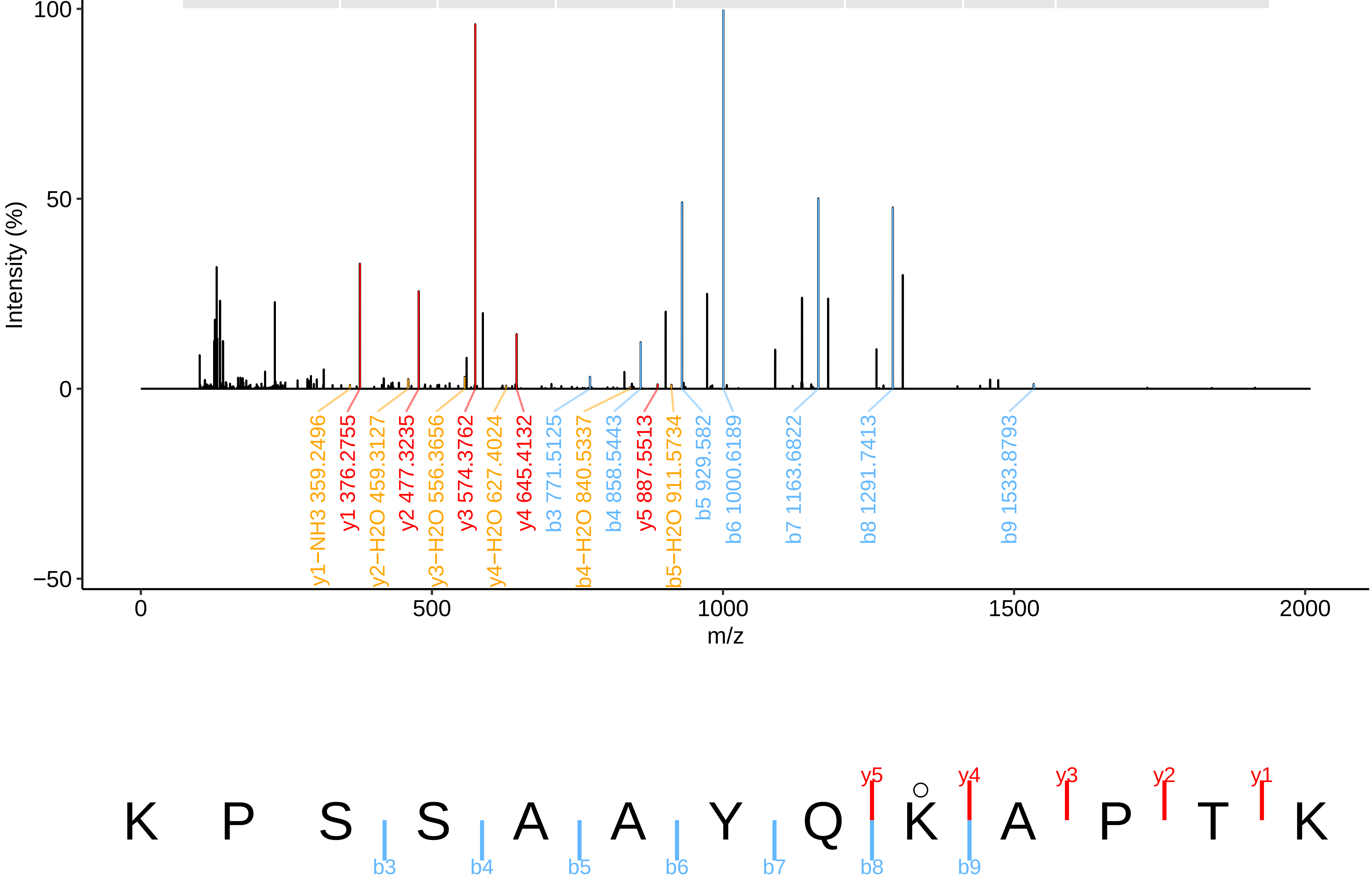

Modification □ Acetyl (Protein N−term) ○ GlyGly (K) no new terminus △ Oxidation (M)

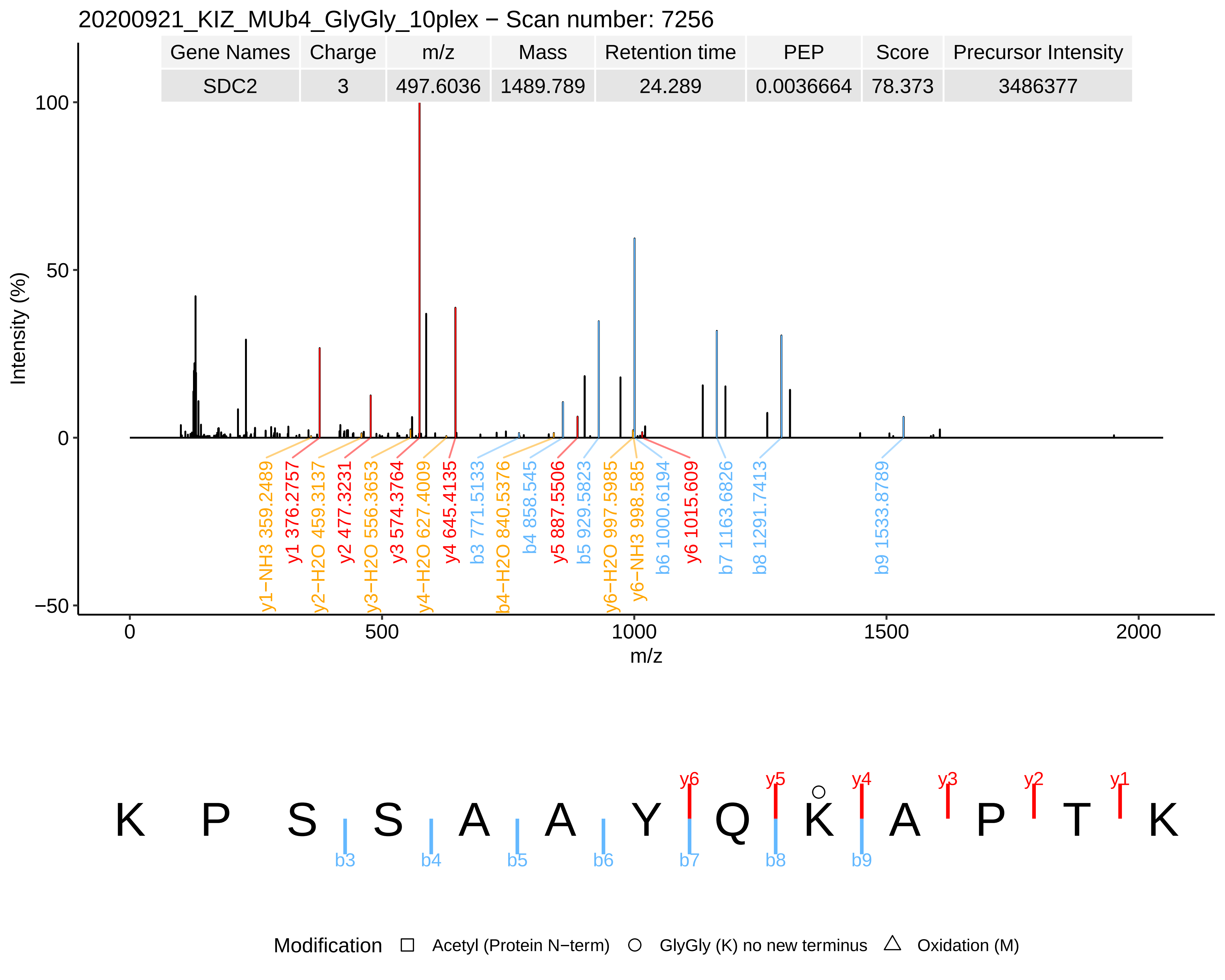

20200921_KIZ_MUb4_GlyGly_10plex − Scan number: 7256
Gene Names | Charge | m/z | Mass | Retention time | PEP | Score | Precursor Intensity
SDC2 | 3 | 497.6036 | 1489.789 | 24.289 | 0.0036664 | 78.373 | 3486377
Intensity (%)
m/z
y1−NH3 359.2489
y1 376.2757
y2−H2O 459.3137
y2 477.3231
y3−H2O 556.3653
y3 574.3764
y4−H2O 627.4009
y4 645.4135
b3 771.5133
b4−H2O 840.5376
b4 858.545
y5 887.5506
b5 929.5823
y6−H2O 997.5985
y6−NH3 998.585
b6 1000.6194
y6 1015.609
b7 1163.6826
b8 1291.7413
b9 1533.8789
K P S S A A Y Q K A P T K
y6 y5 y4 y3 y2 y1
b3 b4 b5 b6 b7 b8 b9
Modification
Acetyl (Protein N−term)
GlyGly (K) no new terminus
Oxidation (M)

20200921_KIZ_MUb4_GlyGly_10plex_2 − Scan number: 6696

Gene Names	Charge	m/z	Mass	Retention time	PEP	Score	Precursor Intensity
SDC2	4	373.4545	1489.789	24.728	0.040994	70.963	750368.9

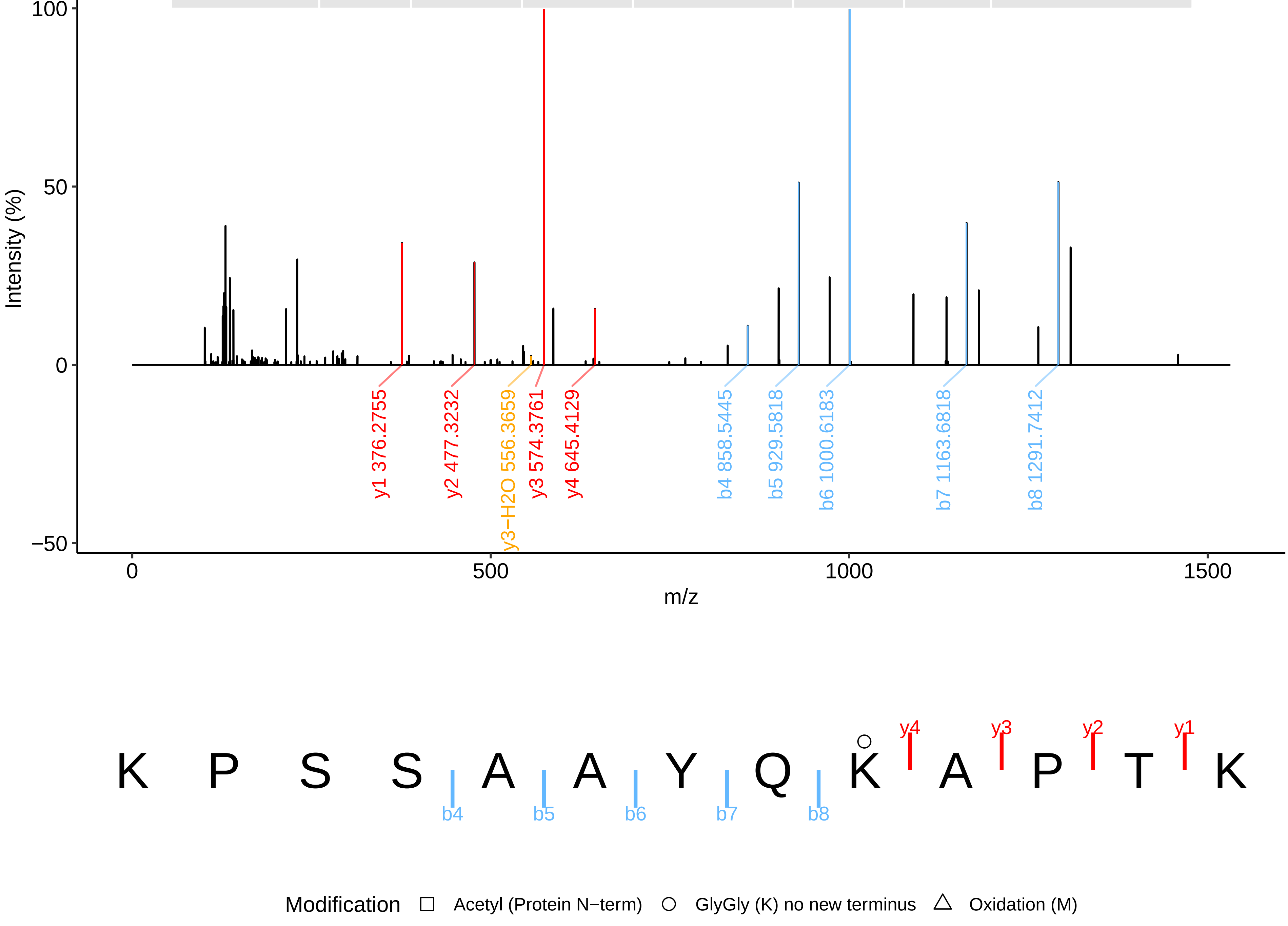

20200921_KIZ_MUb4_GlyGly_10plex_2 − Scan number: 6729

Gene Names	Charge	m/z	Mass	Retention time	PEP	Score	Precursor Intensity
SDC2	3	497.6036	1489.789	24.863	0.015666	64.104	848856.4

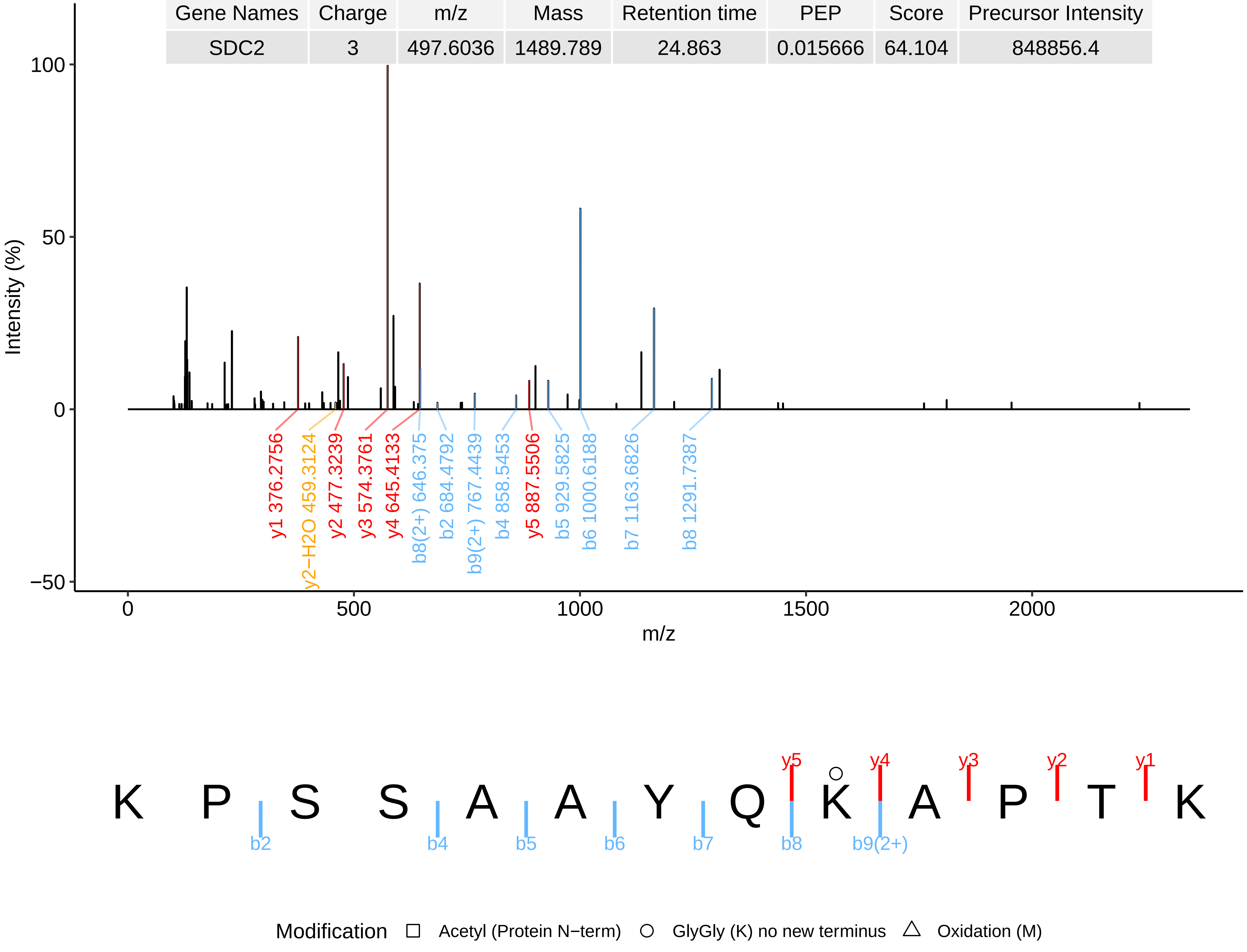

20200924_KIZ_MUb5_GlyGly_1 − Scan number: 6213

Gene Names	Charge	m/z	Mass	Retention time	PEP	Score	Precursor Intensity
SDC2	4	373.4545	1489.789	19.491	0.045345	69.714	8810838

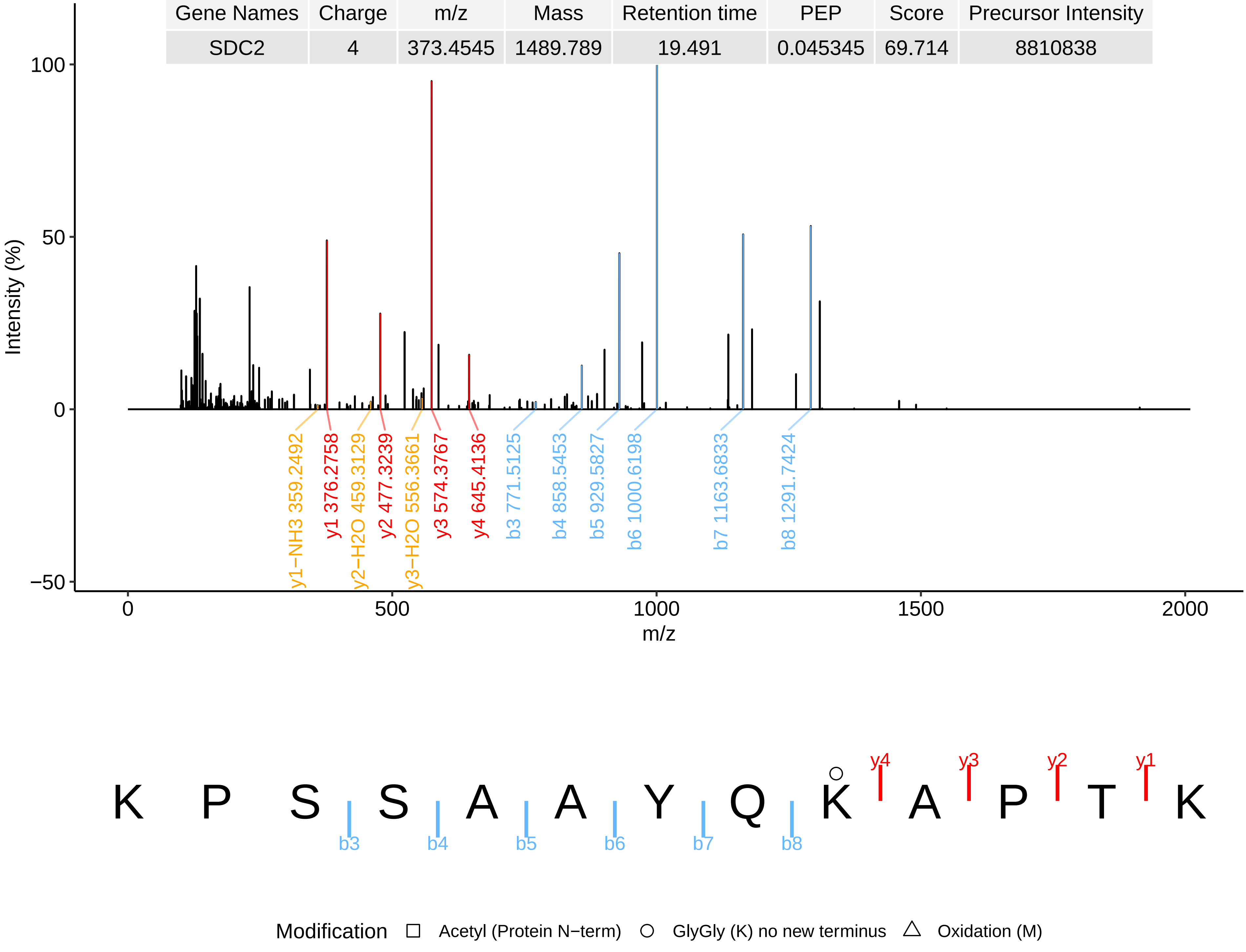

20201118_KIZ_MUb6_GlyGly_2 − Scan number: 7688

Gene Names	Charge	m/z	Mass	Retention time	PEP	Score	Precursor Intensity
SDC2	4	373.4545	1489.789	26.24	0.040994	70.963	2971179

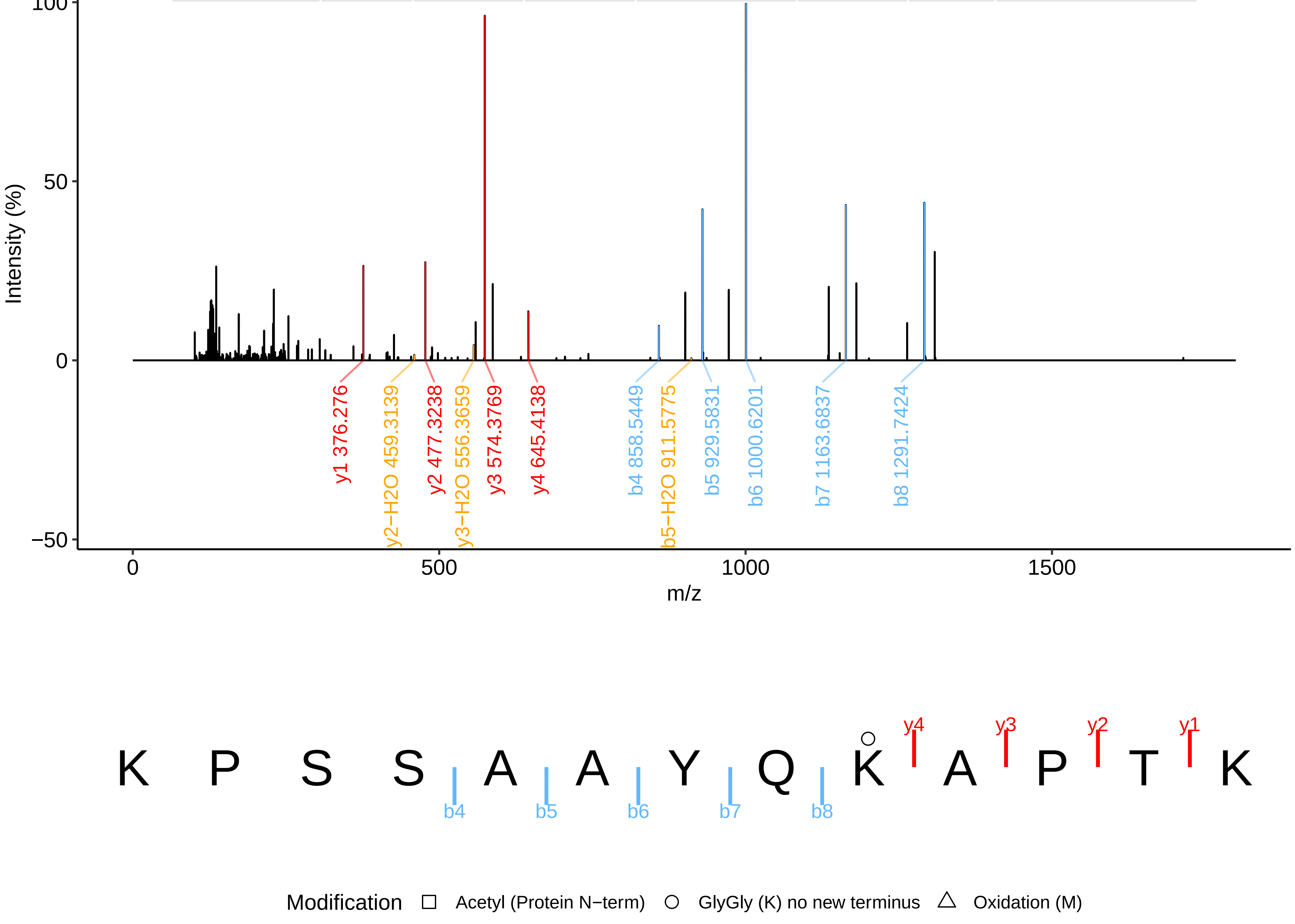

20200921_KIZ_MUb4_GlyGly_10plex − Scan number: 4796

Gene Names	Charge	m/z	Mass	Retention time	PEP	Score	Precursor Intensity
ATAD3B;ATAD3A	4	426.9638	1703.826	15.64	1.8555e−10	132.79	9893400

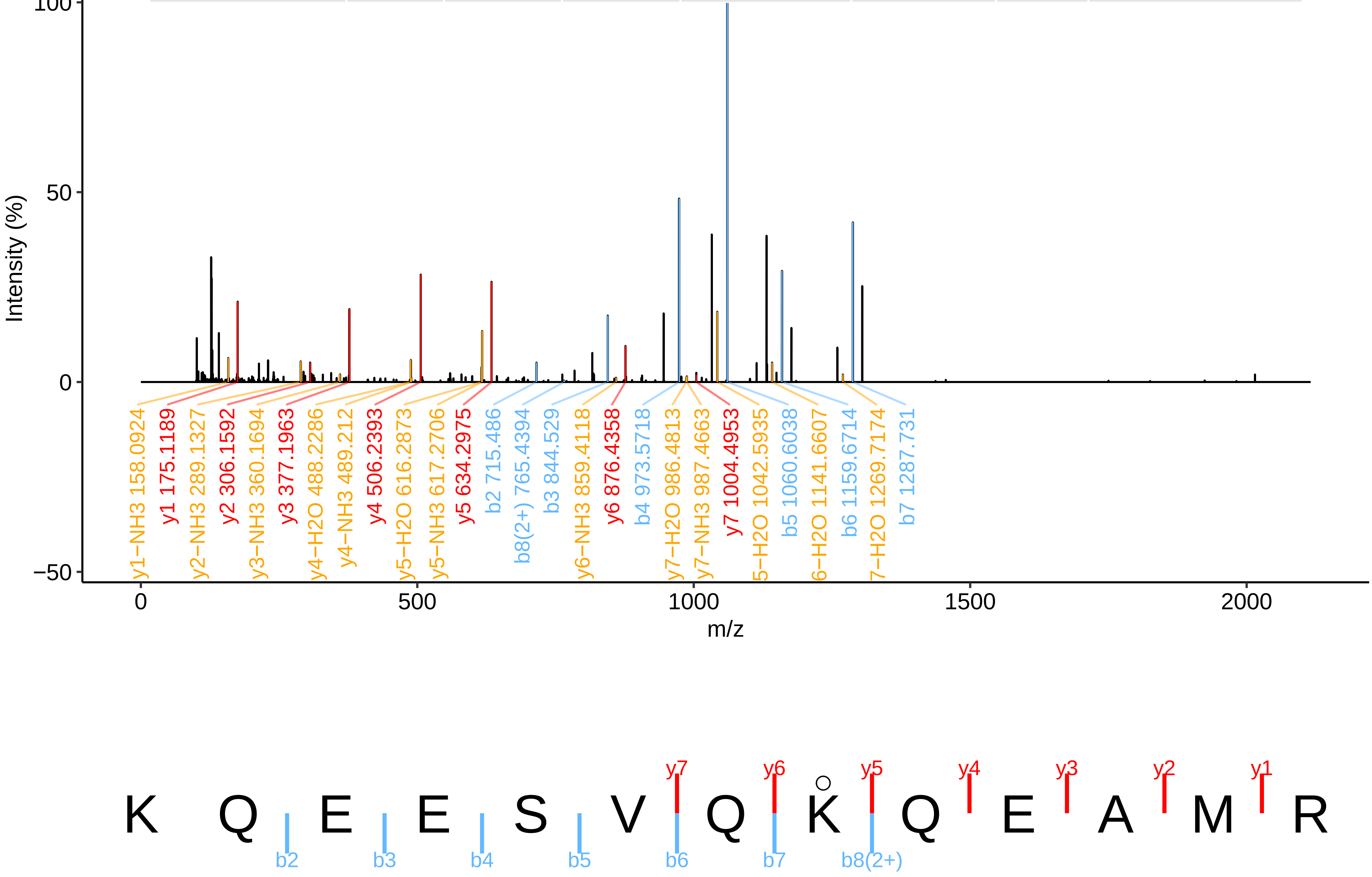

Modification □ Acetyl (Protein N−term) ○ GlyGly (K) no new terminus △ Oxidation (M)

20200921_KIZ_MUb4_GlyGly_10plex_2 − Scan number: 19541

Gene Names	Charge	m/z	Mass	Retention time	PEP	Score	Precursor Intensity
STK26;STK25;STK24	3	649.6813	1946.022	76.711	4.1952e−09	103.61	280504.1

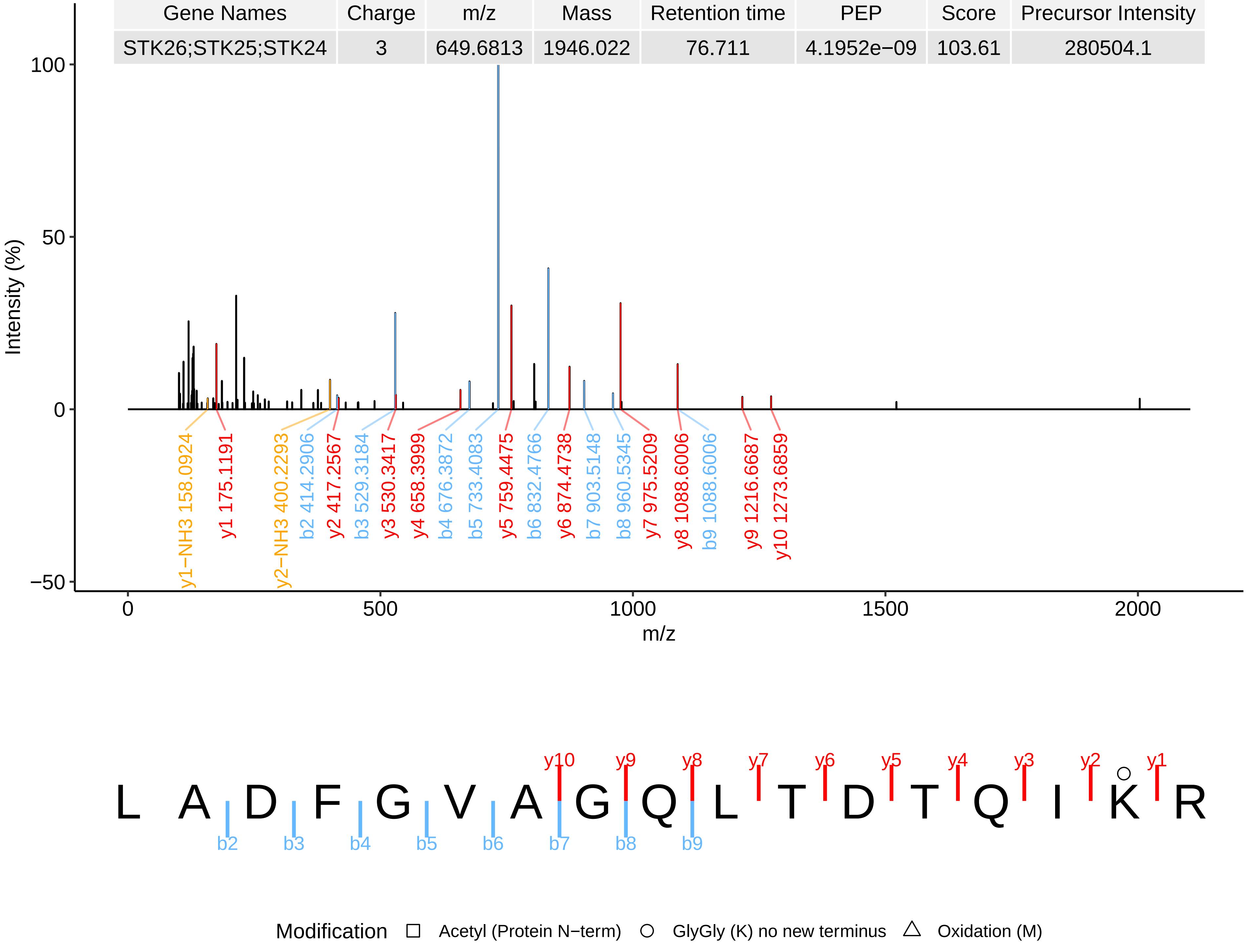

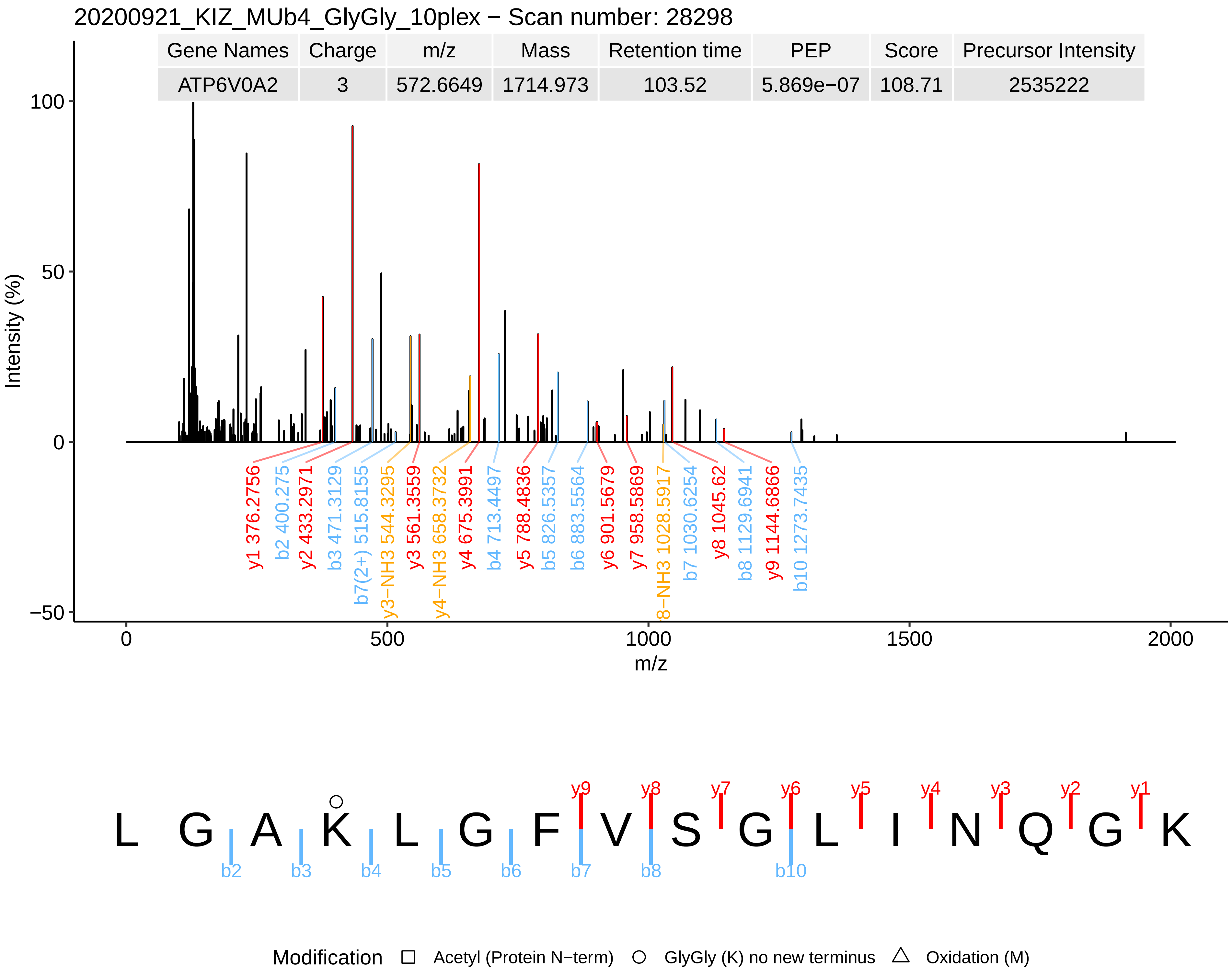
20200921_KIZ_MUb4_GlyGly_10plex − Scan number: 28298
Gene Names | Charge | m/z | Mass | Retention time | PEP | Score | Precursor Intensity
ATP6V0A2 | 3 | 572.6649 | 1714.973 | 103.52 | 5.869e−07 | 108.71 | 2535222
Intensity (%)
m/z
y1 376.2756
b2 400.275
y2 433.2971
b3 471.3129
b7(2+) 515.8155
y3−NH3 544.3295
y3 561.3559
y4−NH3 658.3732
y4 675.3991
b4 713.4497
y5 788.4836
b5 826.5357
b6 883.5564
y6 901.5679
y7 958.5869
8−NH3 1028.5917
b7 1030.6254
y8 1045.62
b8 1129.6941
y9 1144.6866
b10 1273.7435
L G A K L G F V S G L I N Q G K
Modification
Acetyl (Protein N−term)
GlyGly (K) no new terminus
Oxidation (M)

20200921_KIZ_MUb4_GlyGly_10plex − Scan number: 25884

Gene Names	Charge	m/z	Mass	Retention time	PEP	Score	Precursor Intensity
RAD23B	3	587.9964	1760.967	94.233	4.0781e−06	101.32	3631001

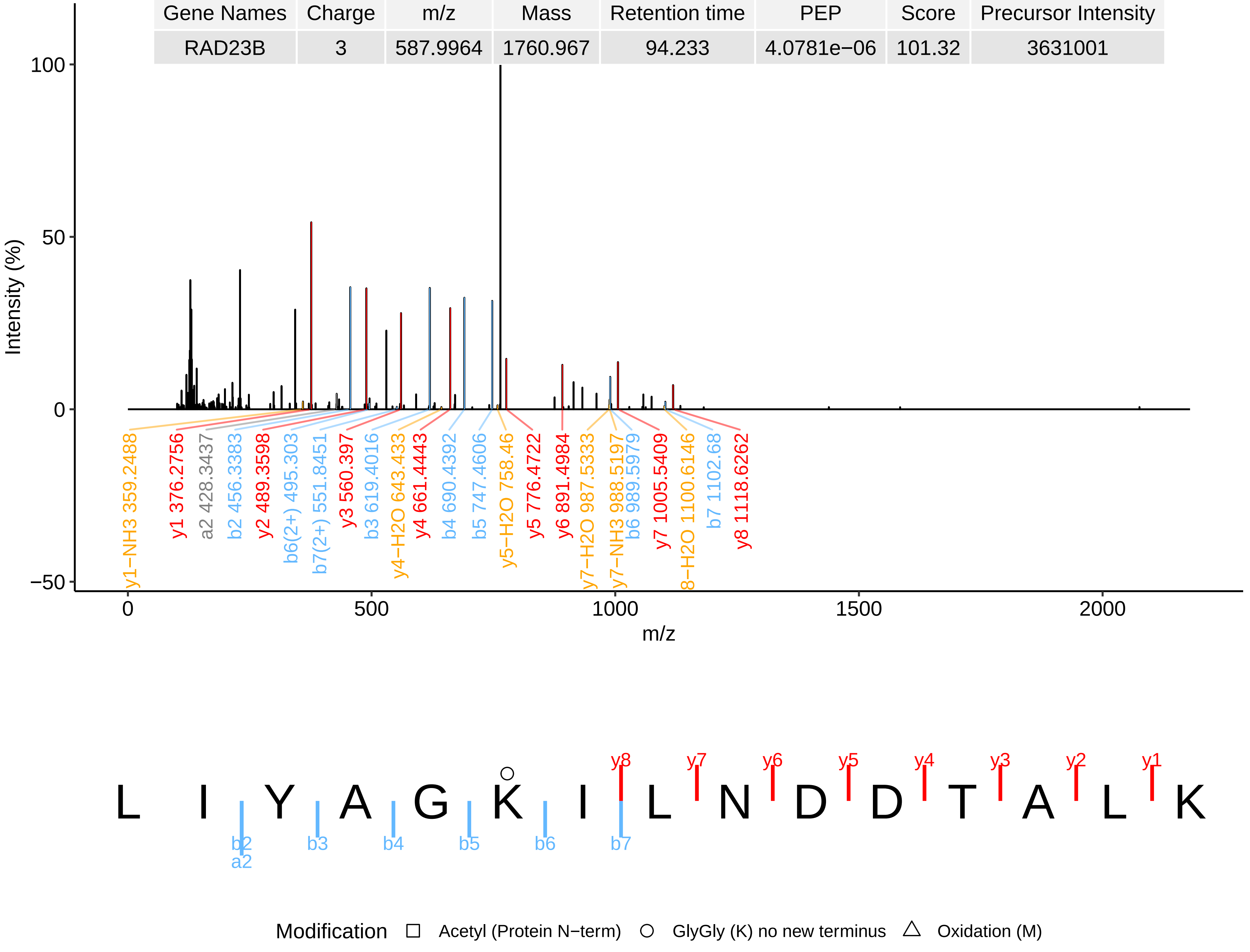

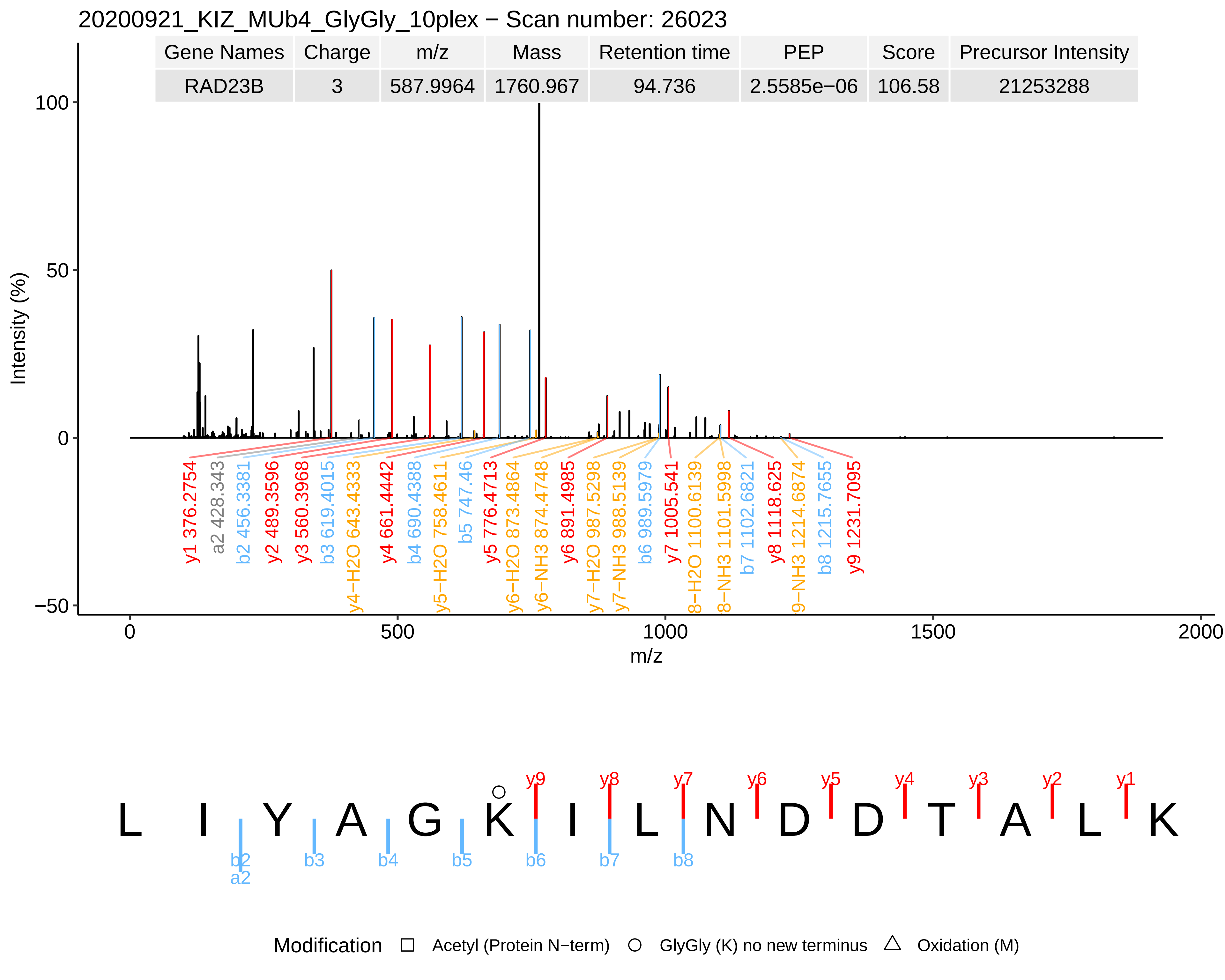
20200921_KIZ_MUb4_GlyGly_10plex − Scan number: 26023
Gene Names | Charge | m/z | Mass | Retention time | PEP | Score | Precursor Intensity
RAD23B | 3 | 587.9964 | 1760.967 | 94.736 | 2.5585e−06 | 106.58 | 21253288
Intensity (%)
m/z
y1 376.2754
a2 428.343
b2 456.3381
y2 489.3596
y3 560.3968
b3 619.4015
y4−H2O 643.4333
y4 661.4442
b4 690.4388
y5−H2O 758.4611
b5 747.46
y5 776.4713
y6−H2O 873.4864
y6−NH3 874.4748
y6 891.4985
y7−H2O 987.5298
y7−NH3 988.5139
b6 989.5979
y7 1005.541
8−H2O 1100.6139
8−NH3 1101.5998
b7 1102.6821
y8 1118.625
9−NH3 1214.6874
b8 1215.7655
y9 1231.7095
L I Y A G K I L N D D T A L K
Modification
Acetyl (Protein N−term)
GlyGly (K) no new terminus
Oxidation (M)

20200921_KIZ_MUb4_GlyGly_10plex_2 − Scan number: 22310

Gene Names	Charge	m/z	Mass	Retention time	PEP	Score	Precursor Intensity
RAD23B	3	587.9964	1760.967	87.935	9.4401e−06	97.551	1239865

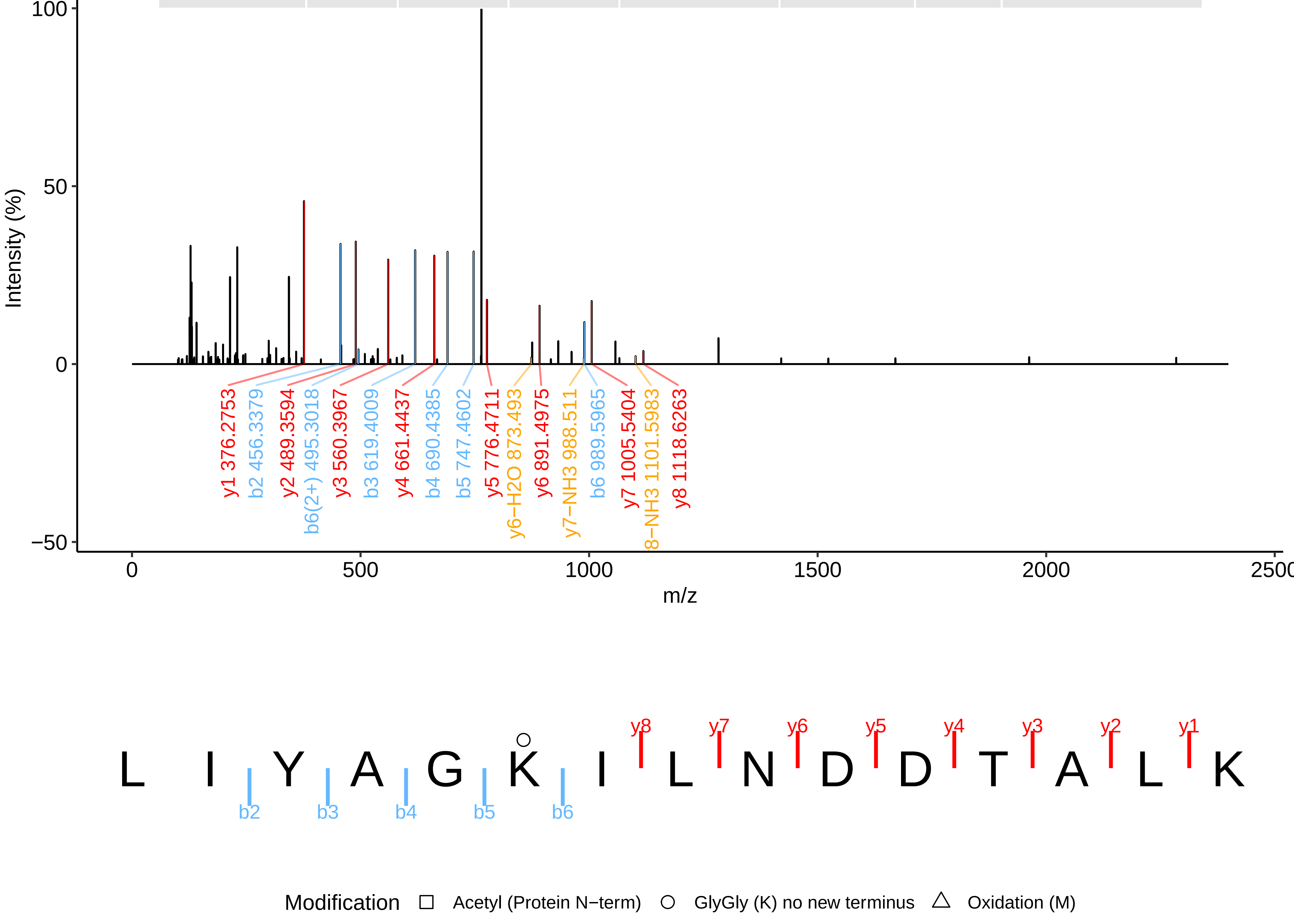

20200924_KIZ_MUb5_GlyGly_2 − Scan number: 22275

Gene Names	Charge	m/z	Mass	Retention time	PEP	Score	Precursor Intensity
RAD23B	3	587.9964	1760.967	82.506	2.1491e−06	107.99	9149040

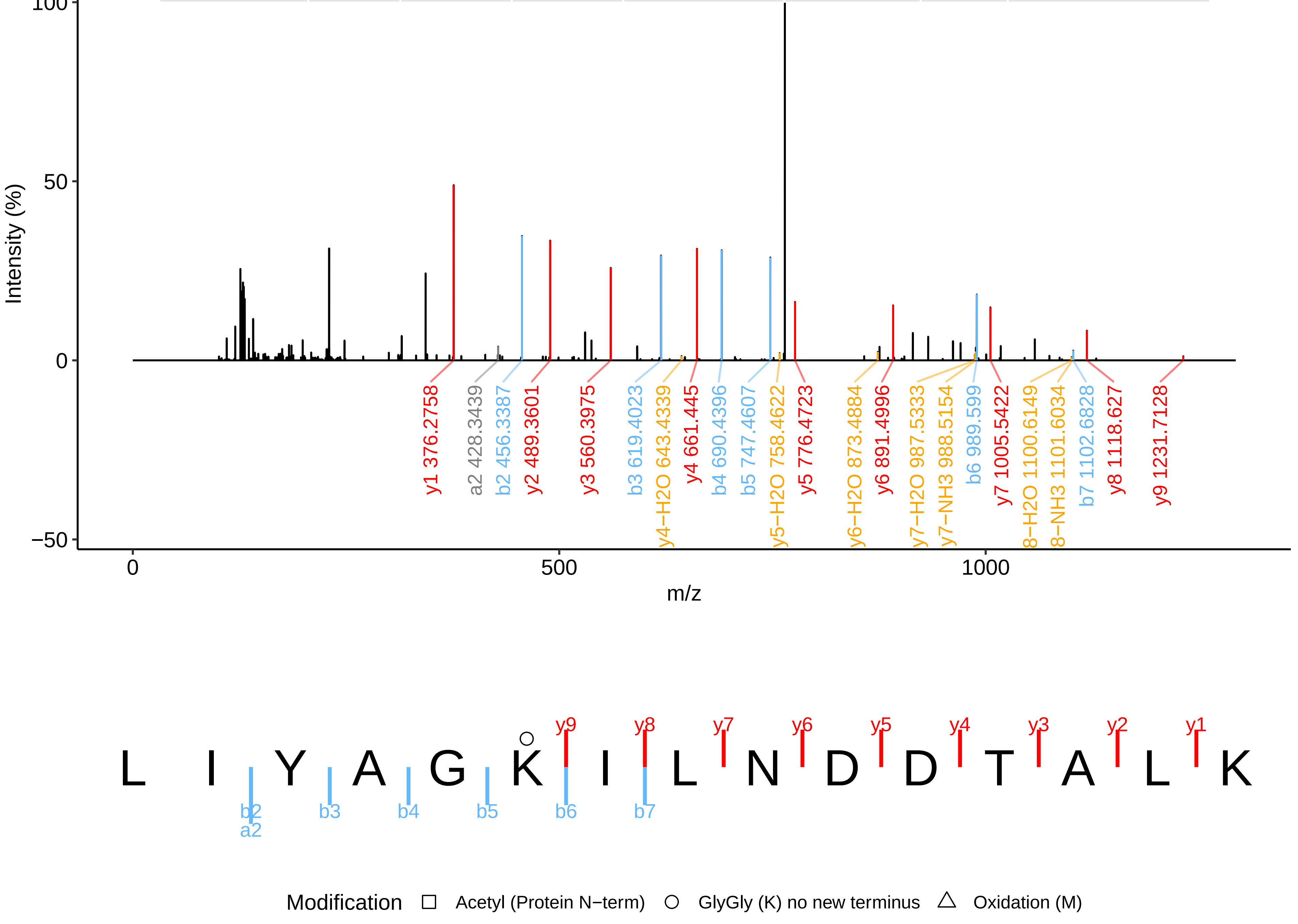

20201118_KIZ_MUb6_GlyGly_1 − Scan number: 25482

Gene Names	Charge	m/z	Mass	Retention time	PEP	Score	Precursor Intensity
RAD23B	3	587.9964	1760.967	78.034	0.00014775	83.499	19878664

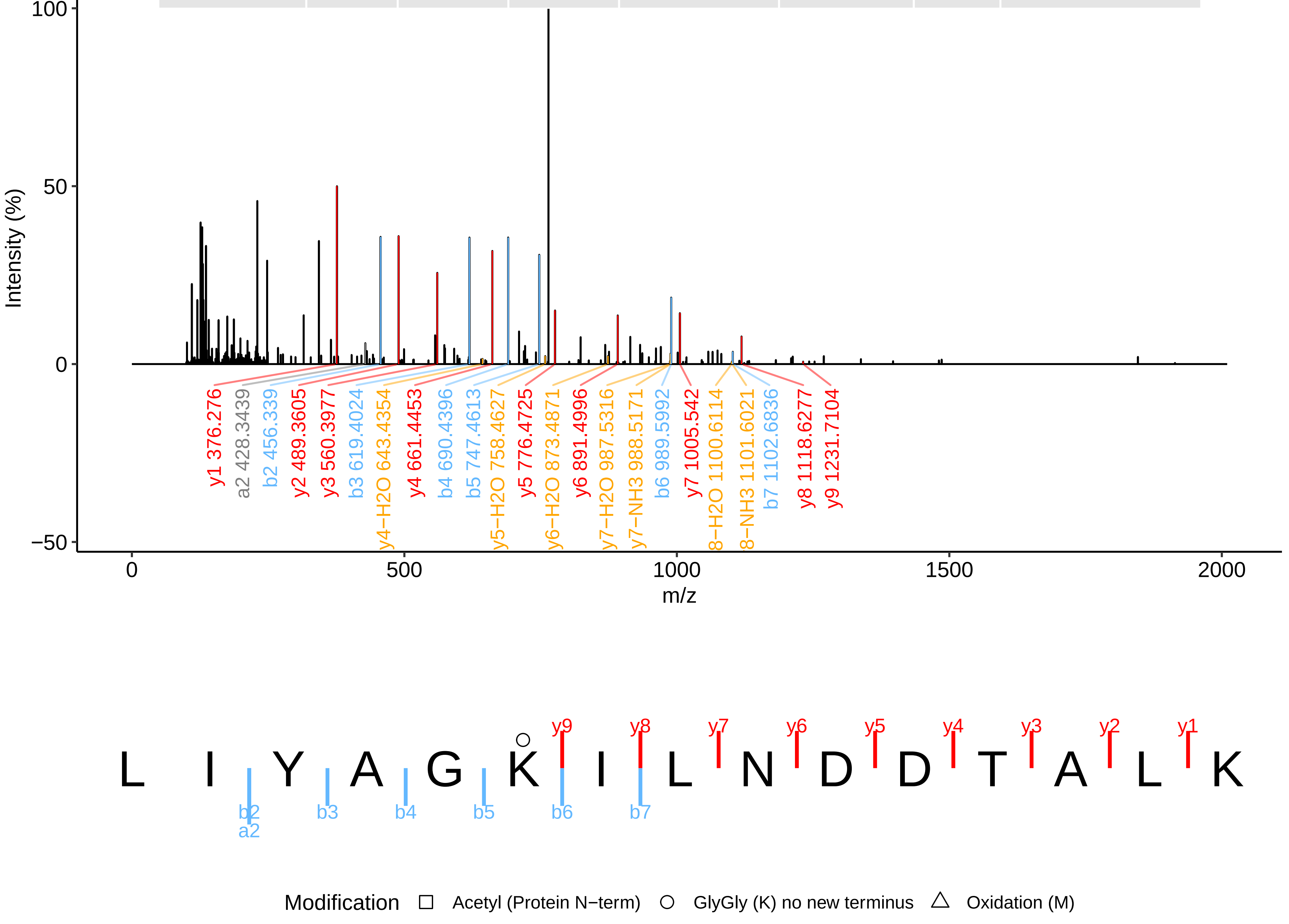

20200921_KIZ_MUb4_GlyGly_10plex_2 − Scan number: 14376

Gene Names	Charge	m/z	Mass	Retention time	PEP	Score	Precursor Intensity
DNAJC13	3	658.992	1973.954	56.04	0.022953	63.037	1084710

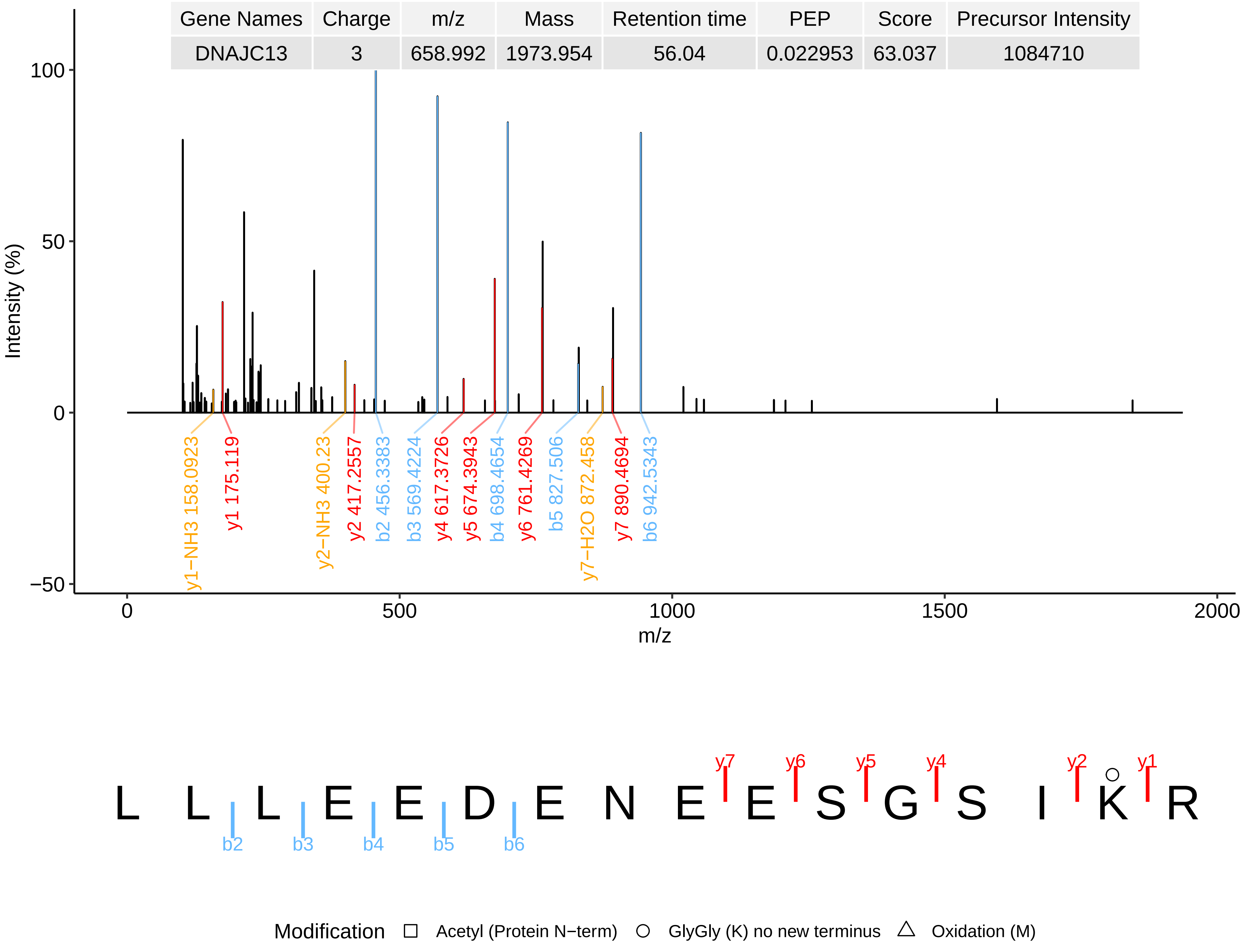

20200921_KIZ_MUb4_GlyGly_10plex − Scan number: 16769

Gene Names	Charge	m/z	Mass	Retention time	PEP	Score	Precursor Intensity
SLC4A2	3	580.316	1737.926	60.346	1.0198e−06	97.417	3999659

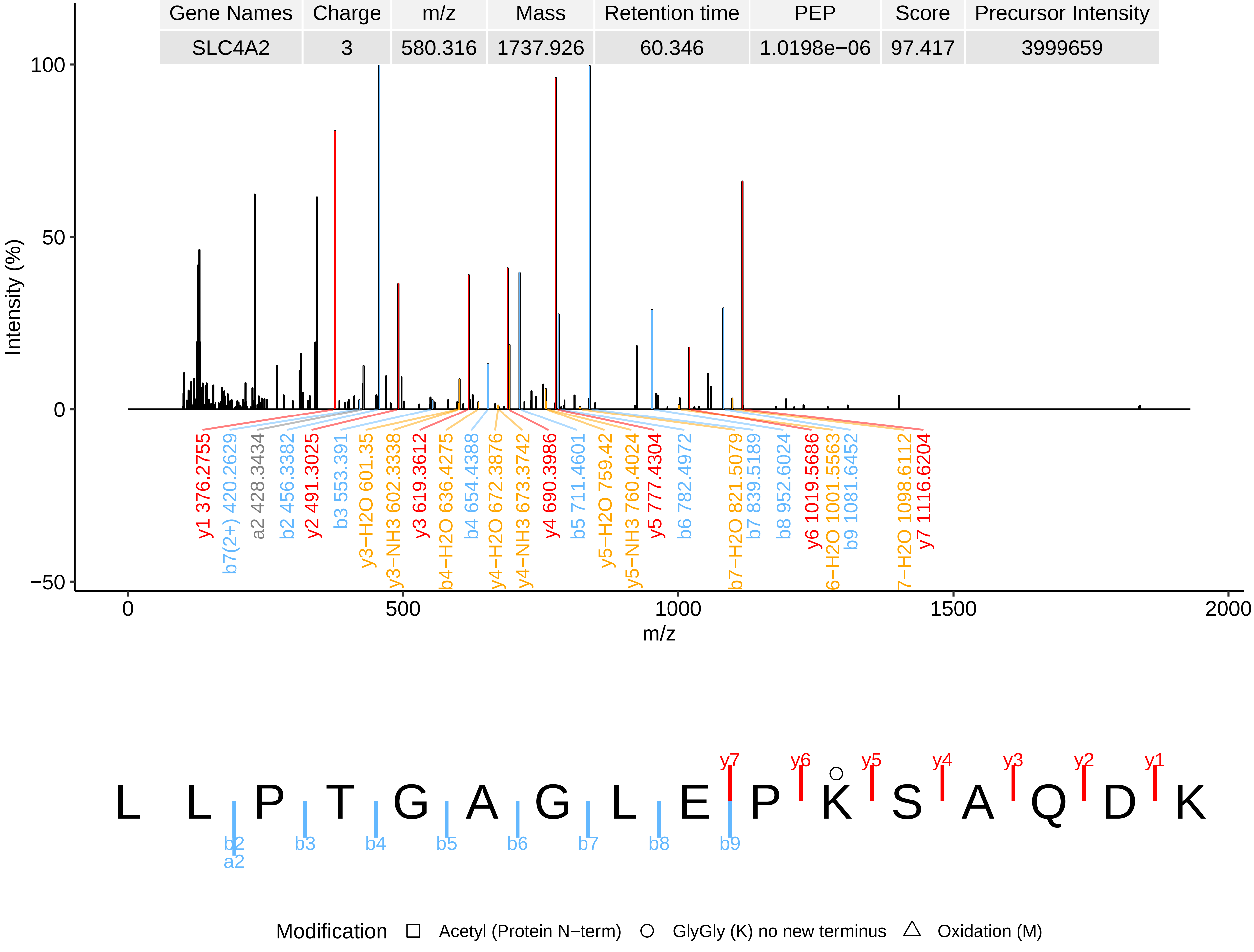

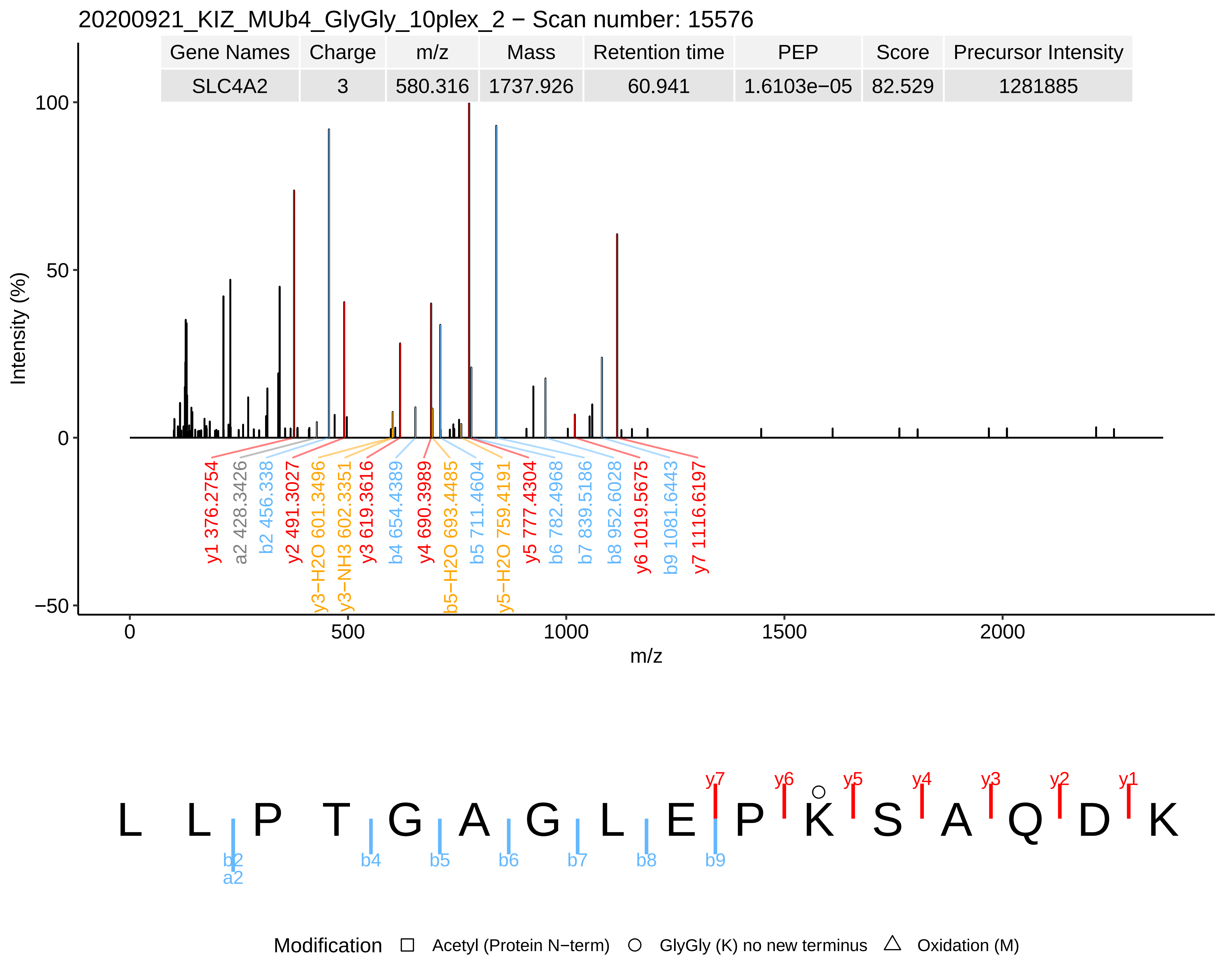

20200921_KIZ_MUb4_GlyGly_10plex_2 − Scan number: 15576
Gene Names | Charge | m/z | Mass | Retention time | PEP | Score | Precursor Intensity
SLC4A2 | 3 | 580.316 | 1737.926 | 60.941 | 1.6103e−05 | 82.529 | 1281885
Intensity (%)
m/z
y1 376.2754
a2 428.3426
b2 456.338
y2 491.3027
y3−H2O 601.3496
y3−NH3 602.3351
y3 619.3616
b4 654.4389
y4 690.3989
b5−H2O 693.4485
b5 711.4604
y5−H2O 759.4191
y5 777.4304
b6 782.4968
b7 839.5186
b8 952.6028
y6 1019.5675
b9 1081.6443
y7 1116.6197
L L P T G A G L E P K S A Q D K
Modification
Acetyl (Protein N−term)
GlyGly (K) no new terminus
Oxidation (M)

20200921_KIZ_MUb4_GlyGly_10plex_2 − Scan number: 15606

Gene Names	Charge	m/z	Mass	Retention time	PEP	Score	Precursor Intensity
SLC4A2	3	580.316	1737.926	61.068	1.0047e−05	88.507	1724276

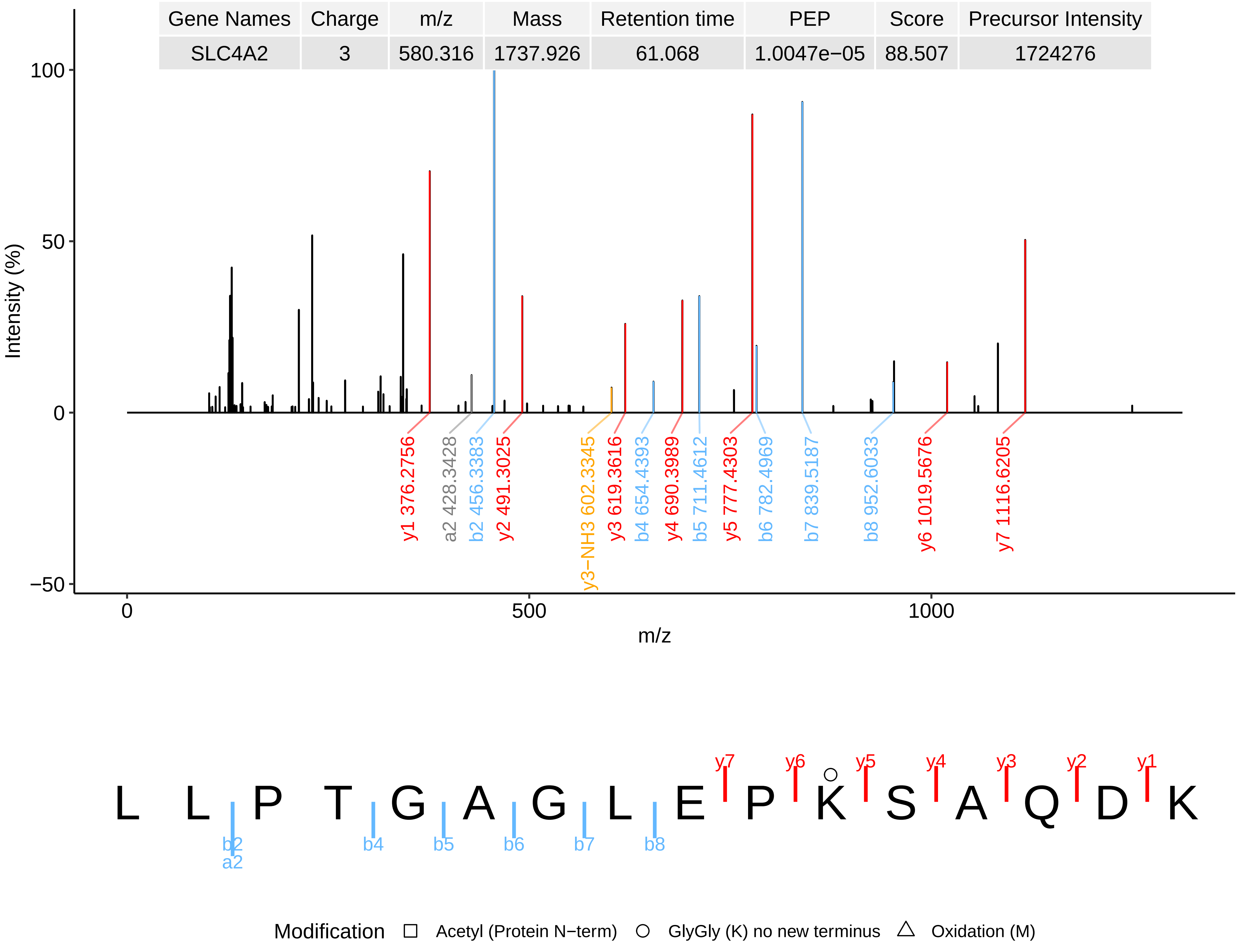

20200921_KIZ_MUb4_GlyGly_10plex_2 − Scan number: 15975

Gene Names	Charge	m/z	Mass	Retention time	PEP	Score	Precursor Intensity
ITGB1	3	594.2823	1779.825	62.576	0.011179	62.042	804237.2

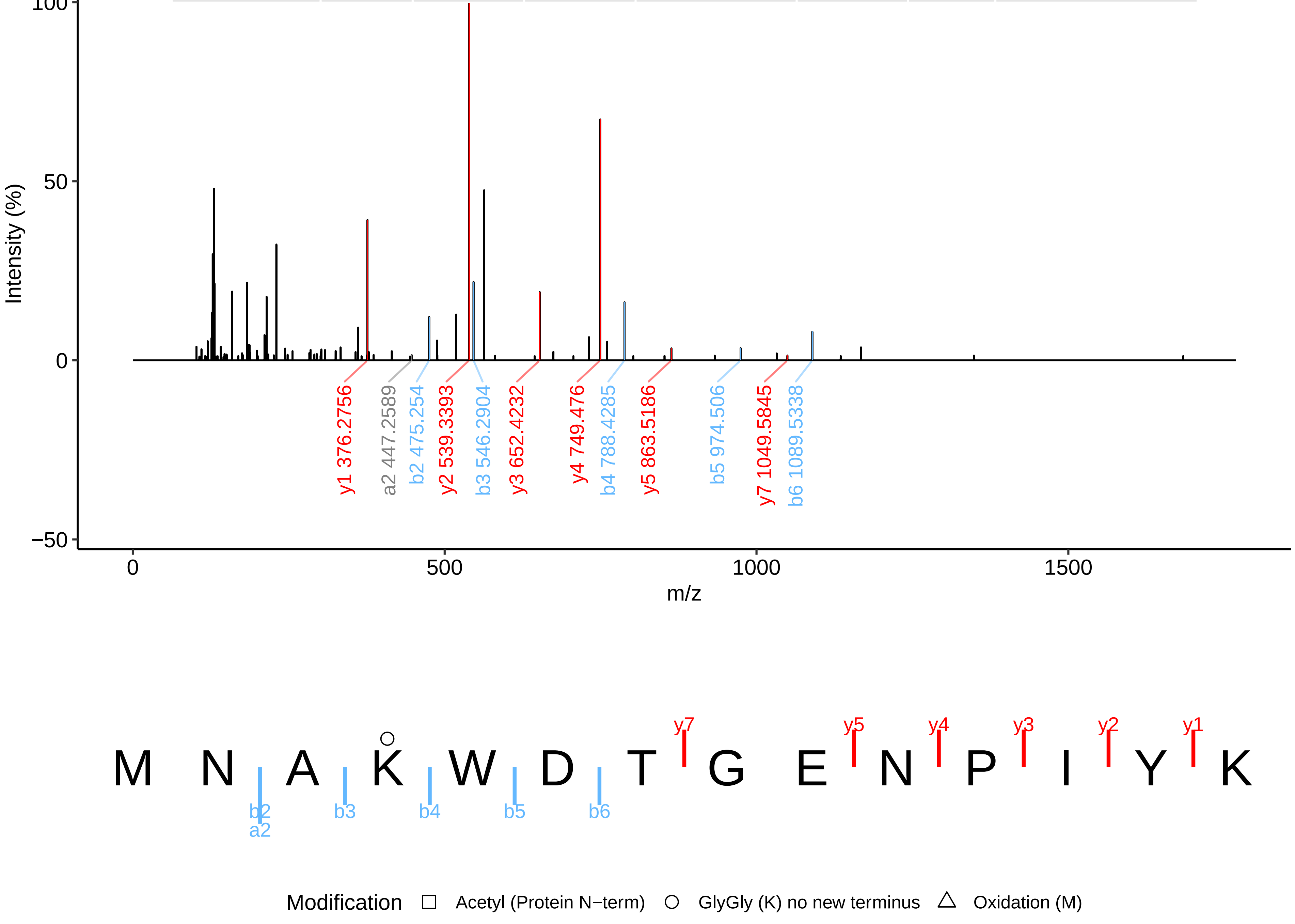

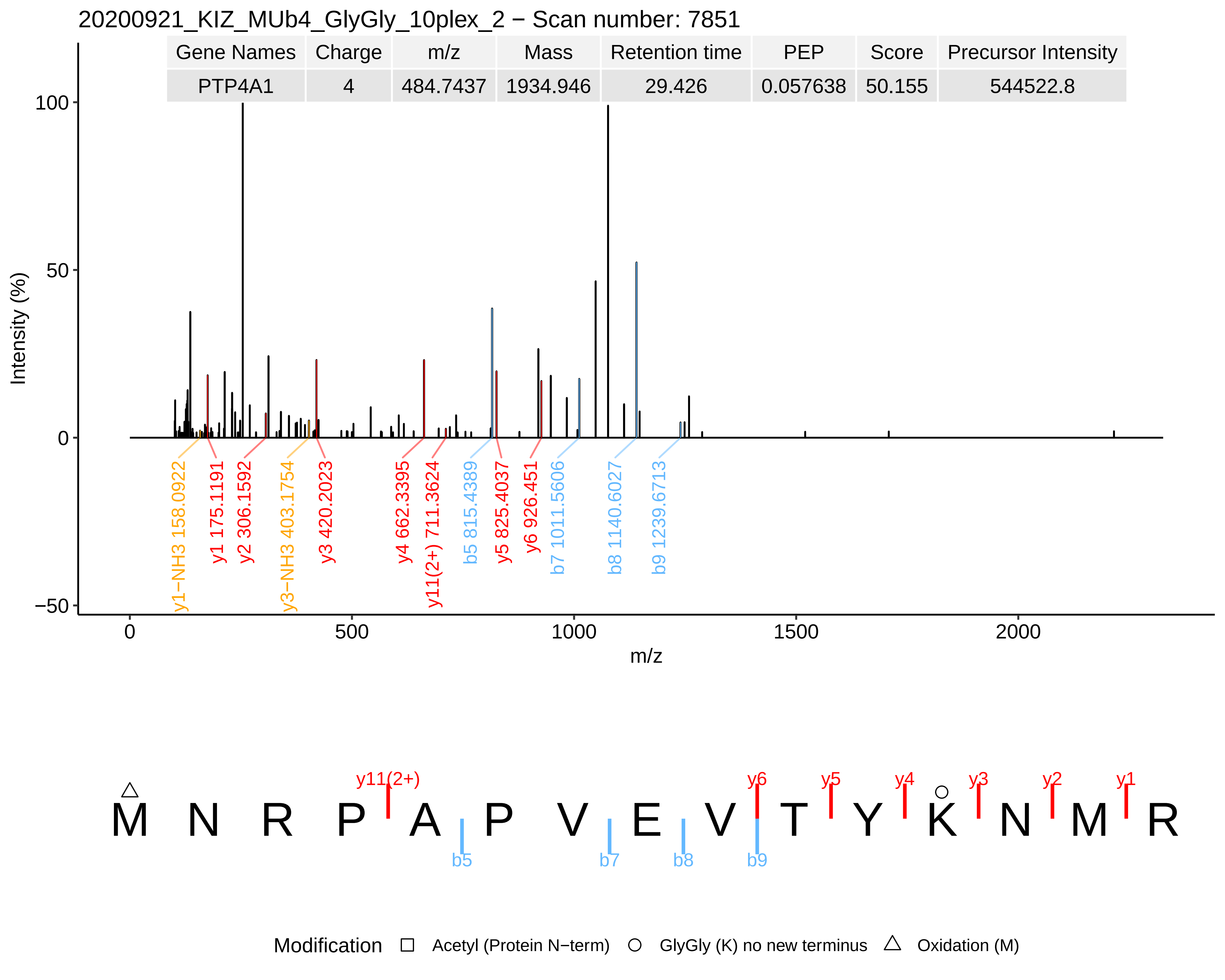
20200921_KIZ_MUb4_GlyGly_10plex_2 − Scan number: 7851
Gene Names | Charge | m/z | Mass | Retention time | PEP | Score | Precursor Intensity
PTP4A1 | 4 | 484.7437 | 1934.946 | 29.426 | 0.057638 | 50.155 | 544522.8
Intensity (%)
m/z
y1−NH3 158.0922
y1 175.1191
y2 306.1592
y3−NH3 403.1754
y3 420.2023
y4 662.3395
y11(2+) 711.3624
b5 815.4389
y5 825.4037
y6 926.451
b7 1011.5606
b8 1140.6027
b9 1239.6713
M N R P A P V E V T Y K N M R
y11(2+) y6 y5 y4 y3 y2 y1
b5 b7 b8 b9
Modification
Acetyl (Protein N−term)
GlyGly (K) no new terminus
Oxidation (M)

20200921_KIZ_MUb4_GlyGly_10plex − Scan number: 18873

Gene Names	Charge	m/z	Mass	Retention time	PEP	Score	Precursor Intensity
NAA15	3	575.9459	1724.816	67.978	0.058661	55.841	NA

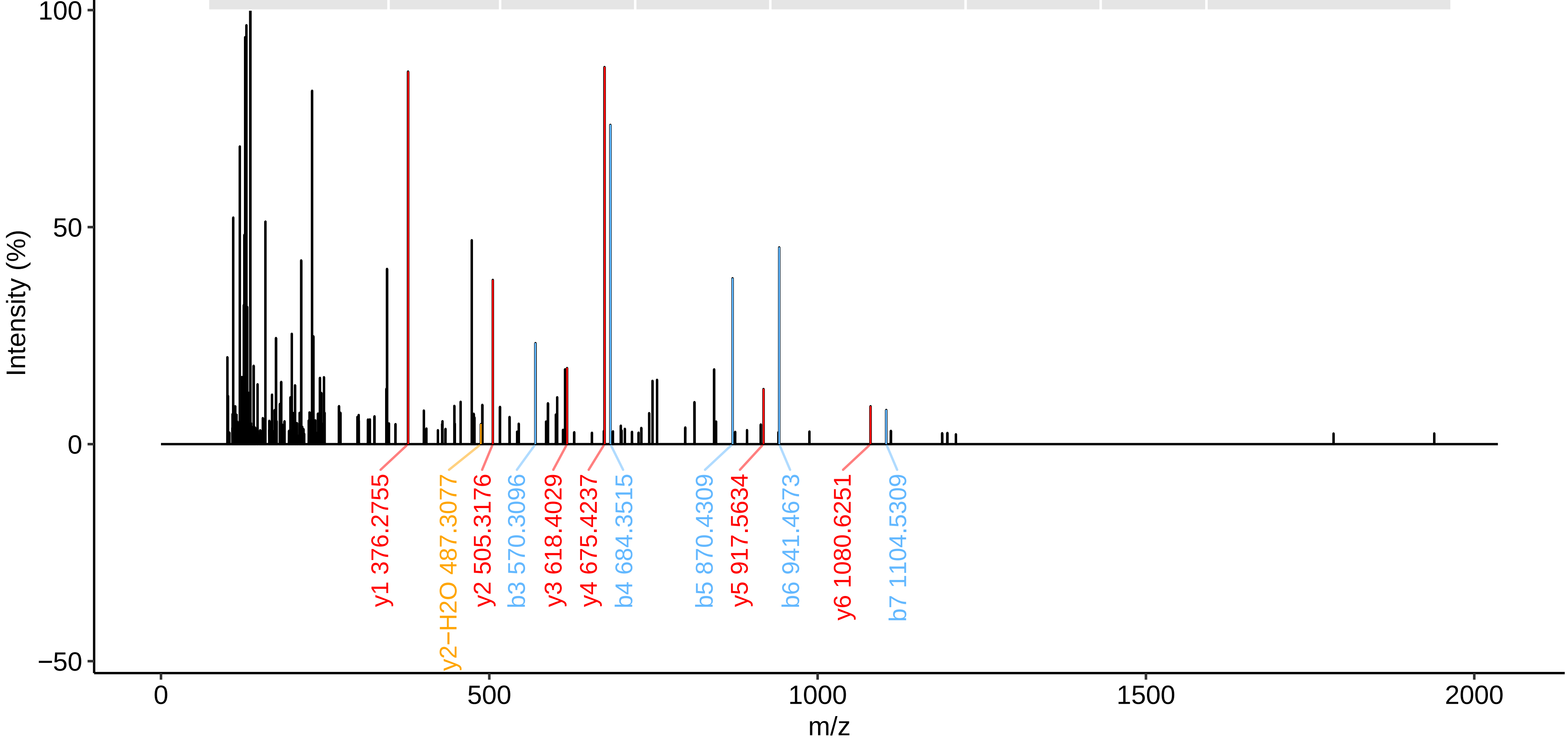

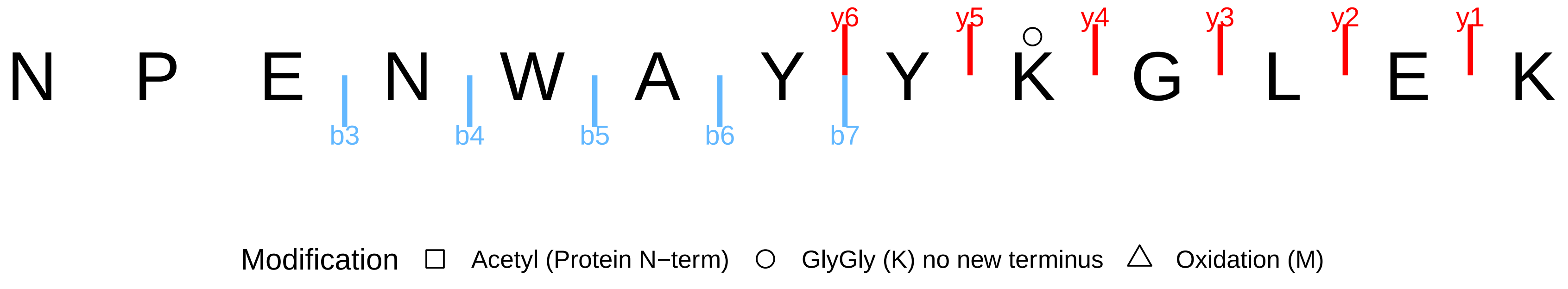

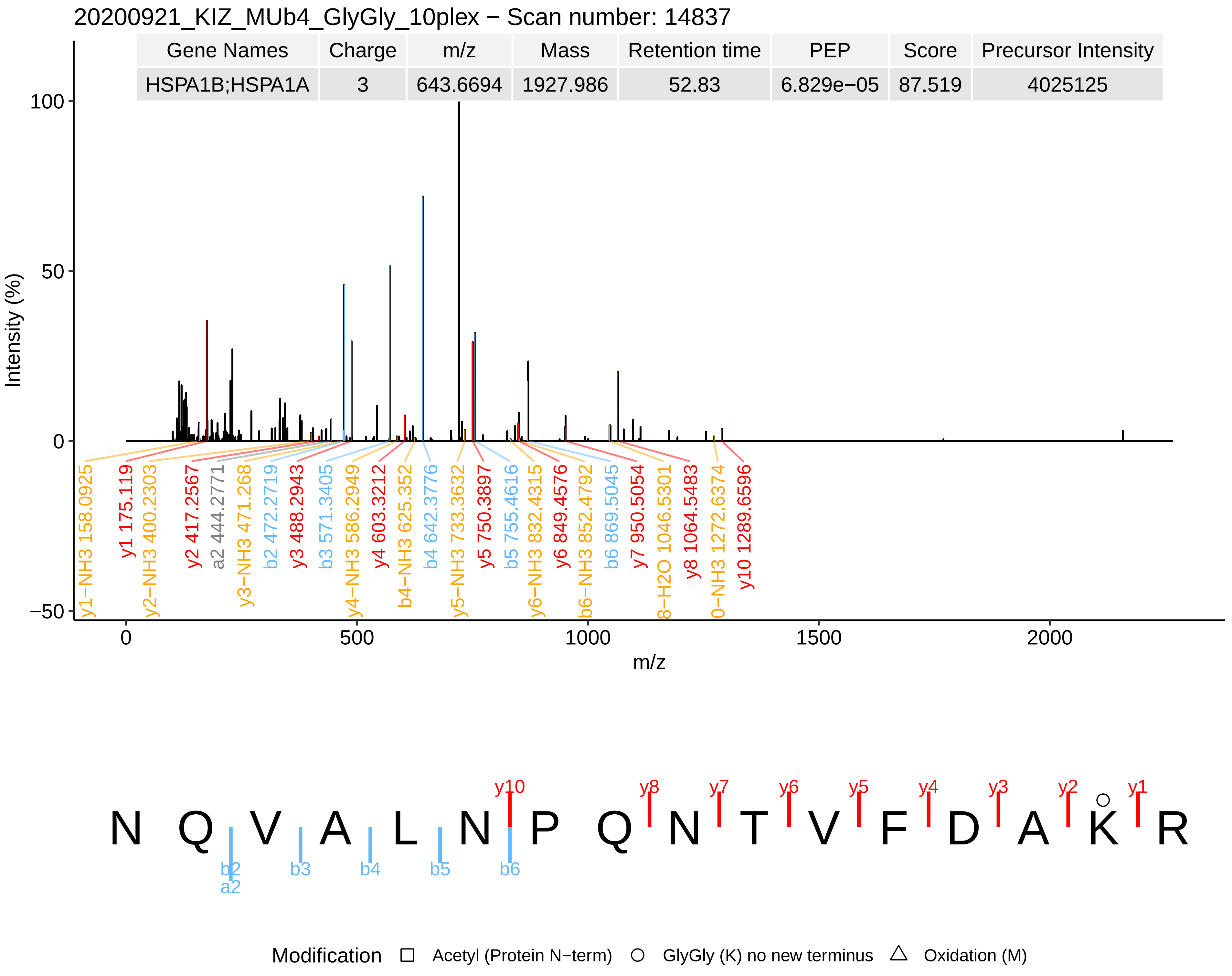

20200921_KIZ_MUb4_GlyGly_10plex − Scan number: 14837
Gene Names
HSPA1B;HSPA1A
Charge
3
m/z
643.6694
Mass
1927.986
Retention time
52.83
PEP
6.829e−05
Score
87.519
Precursor Intensity
4025125
Intensity (%)
100
50
0
−50
0
500
1000
1500
2000
m/z
y1−NH3 158.0925
y1 175.119
y2−NH3 400.2303
y2 417.2567
a2 444.2771
y3−NH3 471.268
b2 472.2719
y3 488.2943
b3 571.3405
y4−NH3 586.2949
y4 603.3212
b4−NH3 625.352
b4 642.3776
y5−NH3 733.3632
y5 750.3897
b5 755.4616
y6−NH3 832.4315
y6 849.4576
b6−NH3 852.4792
b6 869.5045
y7 950.5054
8−H2O 1046.5301
y8 1064.5483
0−NH3 1272.6374
y10 1289.6596
N Q V A L N P Q N T V F D A K R
y10
y8
y7
y6
y5
y4
y3
y2
y1
b2
a2
b3
b4
b5
b6
Modification
Acetyl (Protein N−term)
GlyGly (K) no new terminus
Oxidation (M)

20200921_KIZ_MUb4_GlyGly_10plex_2 − Scan number: 19855

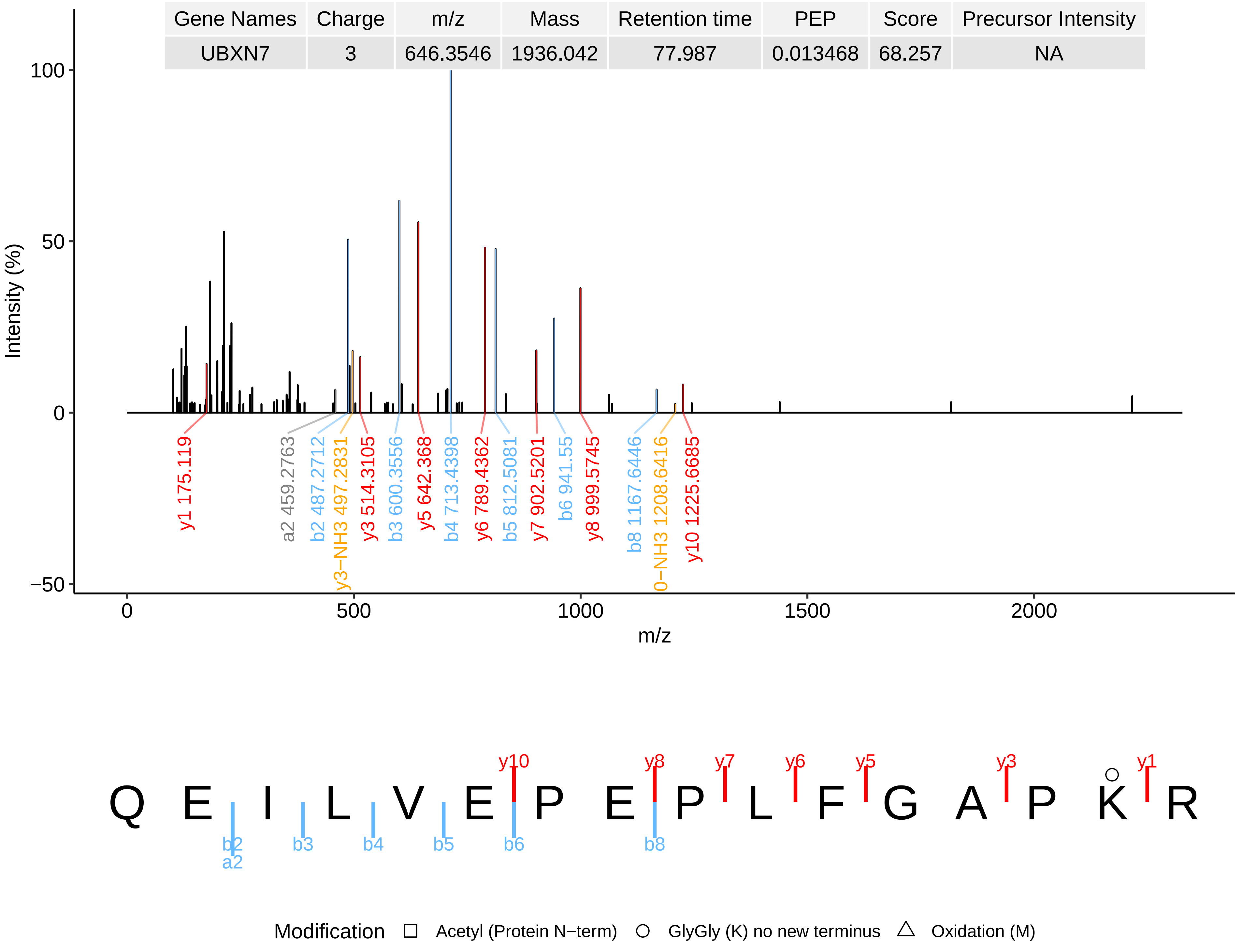

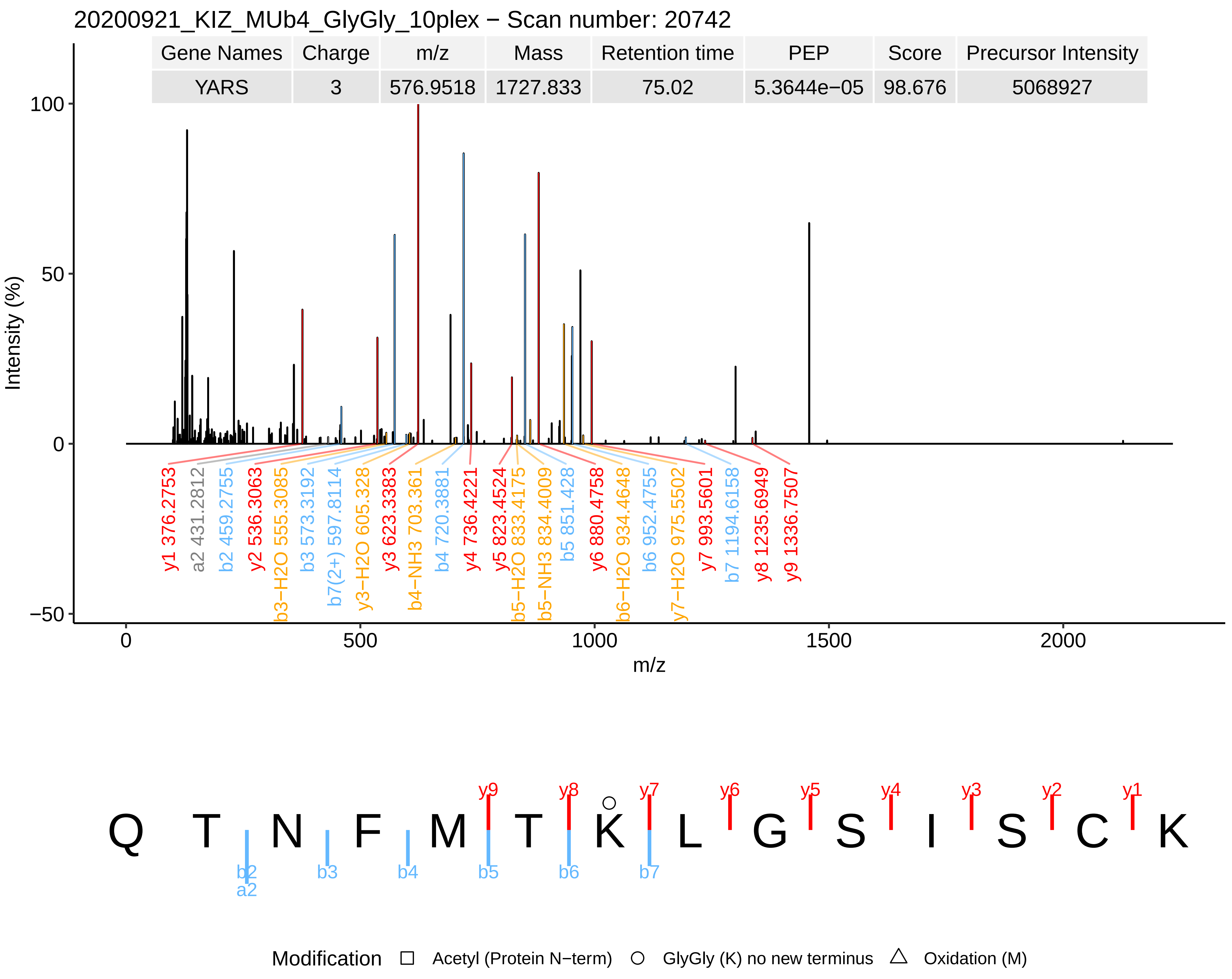

20200921_KIZ_MUb4_GlyGly_10plex − Scan number: 20742
Gene Names
Charge
m/z
Mass
Retention time
PEP
Score
Precursor Intensity
YARS
3
576.9518
1727.833
75.02
5.3644e−05
98.676
5068927
Intensity (%)
m/z
y1 376.2753
a2 431.2812
b2 459.2755
y2 536.3063
b3−H2O 555.3085
b3 573.3192
b7(2+) 597.8114
y3−H2O 605.328
y3 623.3383
b4−NH3 703.361
b4 720.3881
y4 736.4221
y5 823.4524
b5−H2O 833.4175
b5−NH3 834.4009
b5 851.428
y6 880.4758
b6−H2O 934.4648
b6 952.4755
y7−H2O 975.5502
y7 993.5601
b7 1194.6158
y8 1235.6949
y9 1336.7507
Q T N F M T K L G S I S C K
Modification
Acetyl (Protein N−term)
GlyGly (K) no new terminus
Oxidation (M)

20200921_KIZ_MUb4_GlyGly_10plex_2 − Scan number: 18845

Gene Names	Charge	m/z	Mass	Retention time	PEP	Score	Precursor Intensity
YARS	3	576.9518	1727.833	73.884	0.10422	40.237	1029899

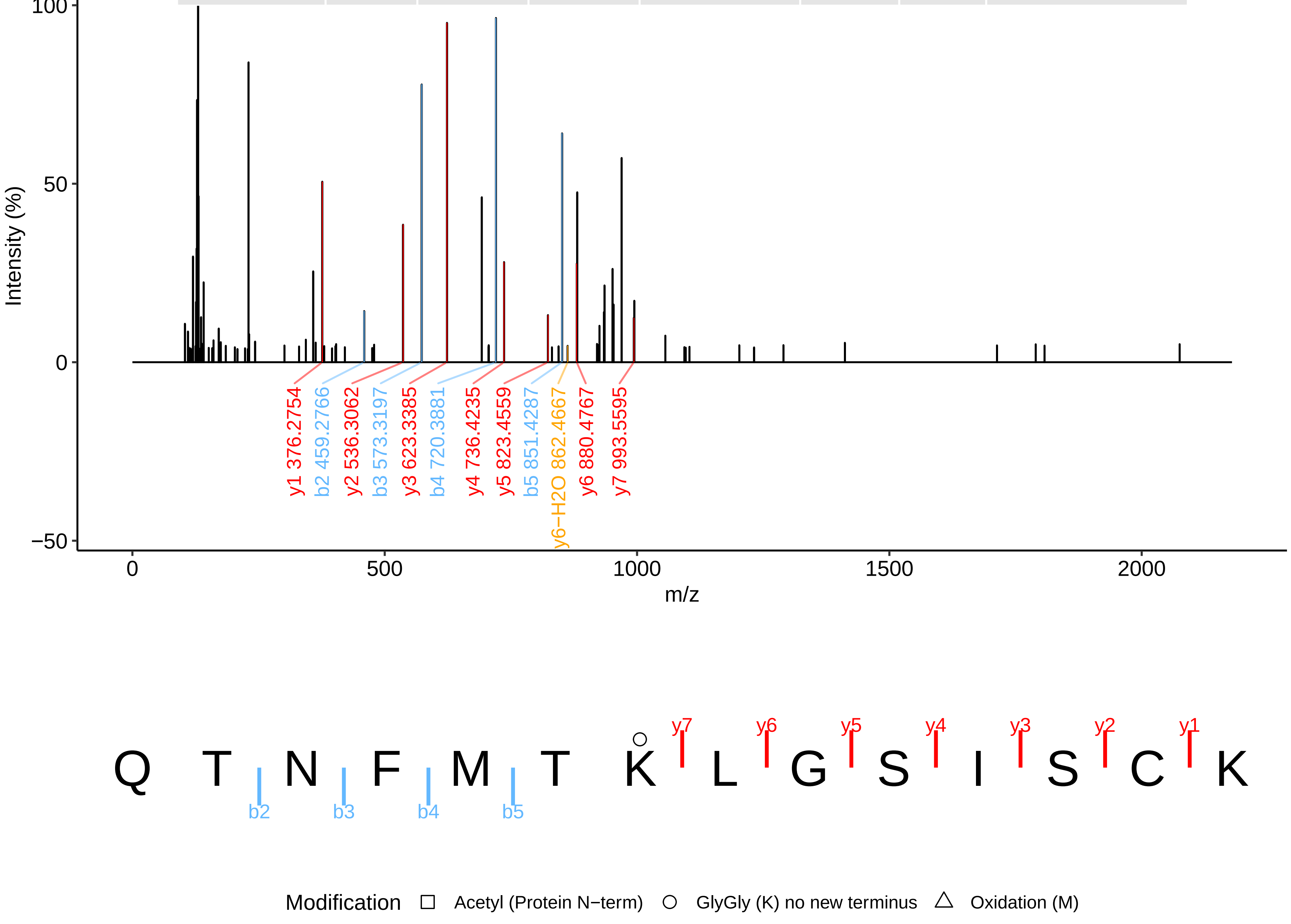

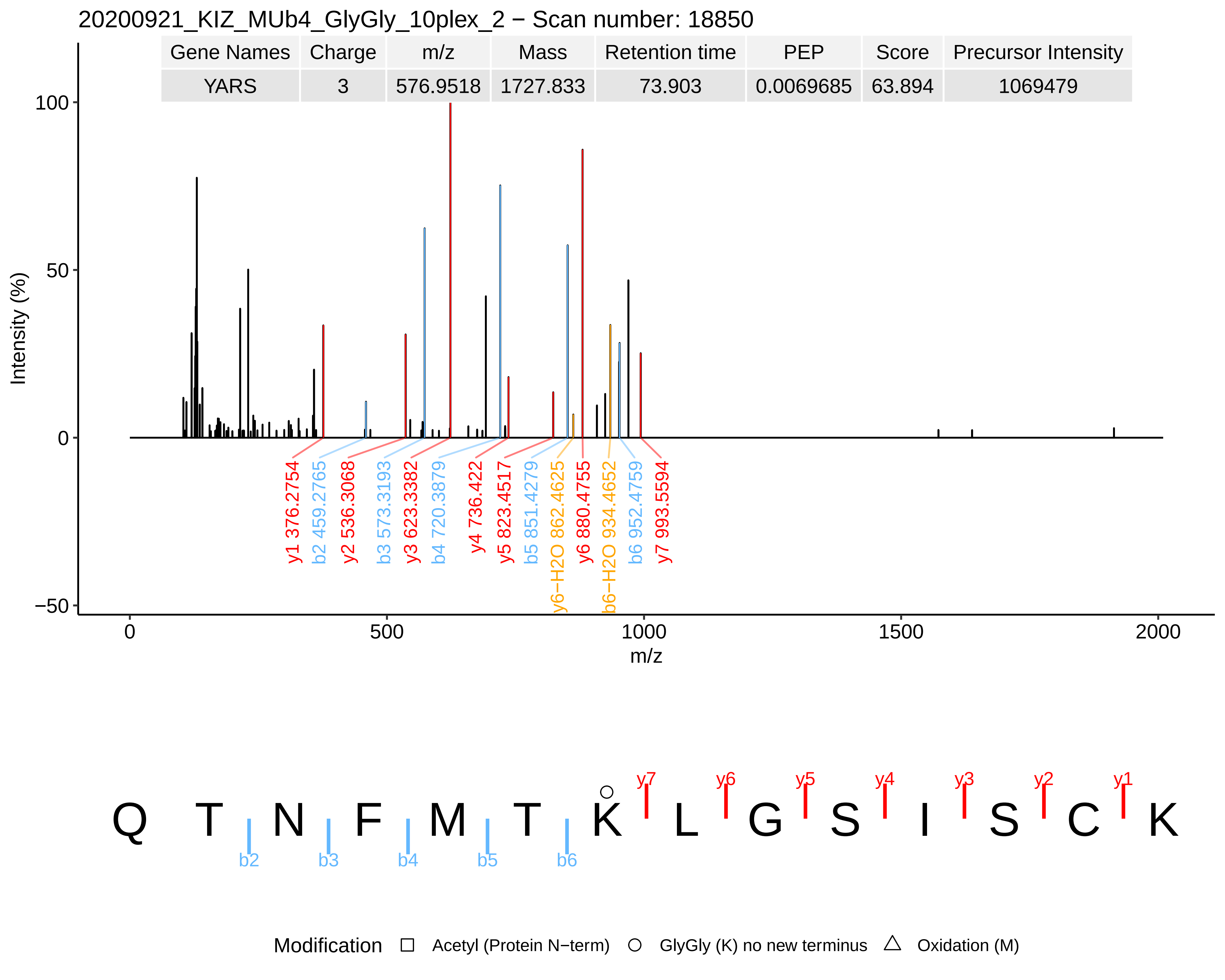
20200921_KIZ_MUb4_GlyGly_10plex_2 − Scan number: 18850
Gene Names | Charge | m/z | Mass | Retention time | PEP | Score | Precursor Intensity
YARS | 3 | 576.9518 | 1727.833 | 73.903 | 0.0069685 | 63.894 | 1069479
Intensity (%)
m/z
y1 376.2754
b2 459.2765
y2 536.3068
b3 573.3193
y3 623.3382
b4 720.3879
y4 736.422
y5 823.4517
b5 851.4279
y6−H2O 862.4625
y6 880.4755
b6−H2O 934.4652
b6 952.4759
y7 993.5594
Q T N F M T K L G S I S C K
b2 b3 b4 b5 b6
y7 y6 y5 y4 y3 y2 y1
Modification
Acetyl (Protein N−term)
GlyGly (K) no new terminus
Oxidation (M)

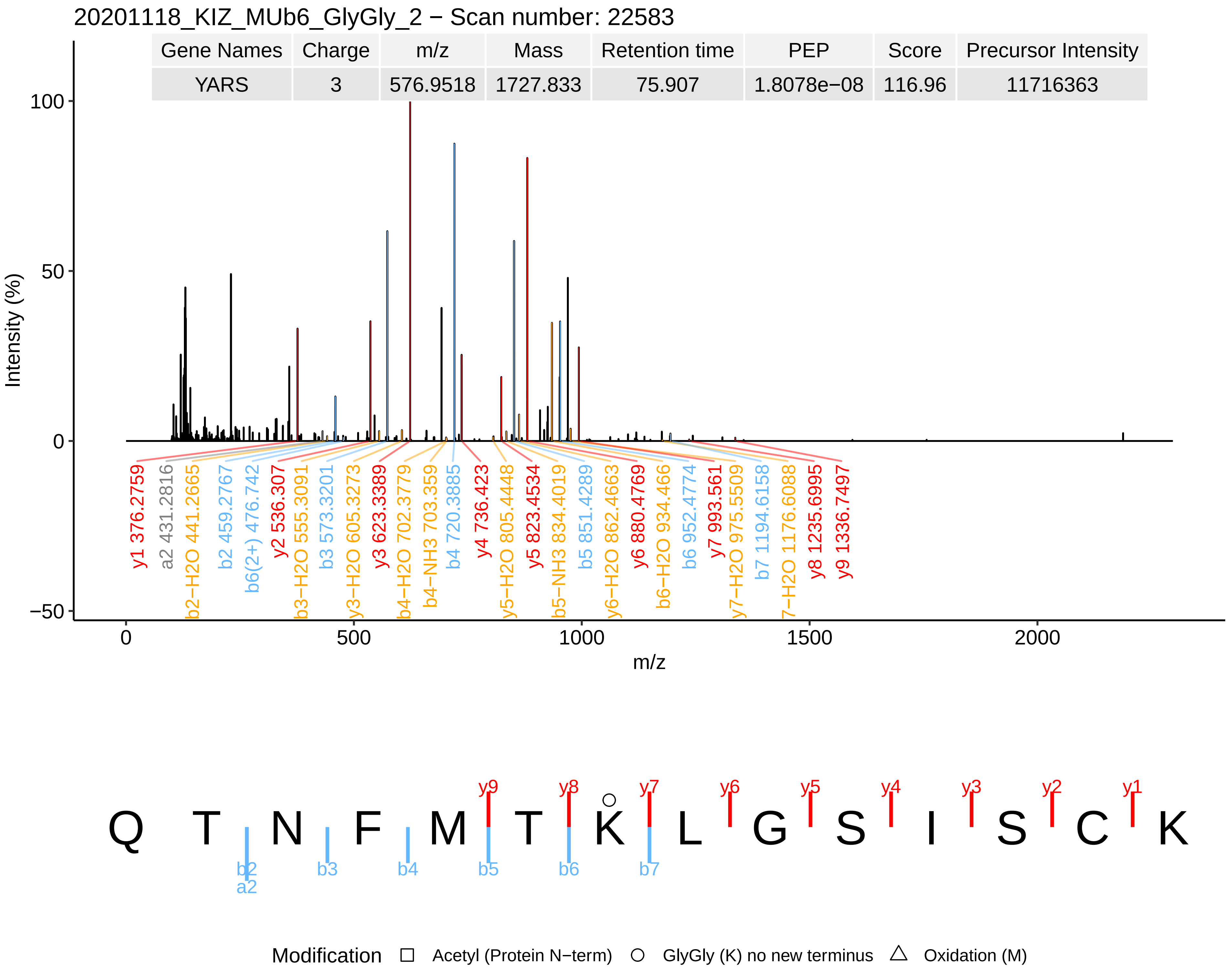

20201118_KIZ_MUb6_GlyGly_2 − Scan number: 22583
Gene Names | Charge | m/z | Mass | Retention time | PEP | Score | Precursor Intensity
YARS | 3 | 576.9518 | 1727.833 | 75.907 | 1.8078e−08 | 116.96 | 11716363
Intensity (%)
m/z
y1 376.2759
a2 431.2816
b2−H2O 441.2665
b2 459.2767
b6(2+) 476.742
y2 536.307
b3−H2O 555.3091
b3 573.3201
y3−H2O 605.3273
y3 623.3389
b4−H2O 702.3779
b4−NH3 703.359
b4 720.3885
y4 736.423
y5−H2O 805.4448
y5 823.4534
b5−NH3 834.4019
b5 851.4289
y6−H2O 862.4663
y6 880.4769
b6−H2O 934.466
b6 952.4774
y7 993.561
y7−H2O 975.5509
b7 1194.6158
y7−H2O 1176.6088
y8 1235.6995
y9 1336.7497
Q T N F M T K L G S I S C K
Modification □ Acetyl (Protein N−term) ○ GlyGly (K) no new terminus △ Oxidation (M)

20200921_KIZ_MUb4_GlyGly_10plex_2 − Scan number: 14622

Gene Names	Charge	m/z	Mass	Retention time	PEP	Score	Precursor Intensity
YARS	3	582.2834	1743.828	57.034	0.037254	52.823	962704.8

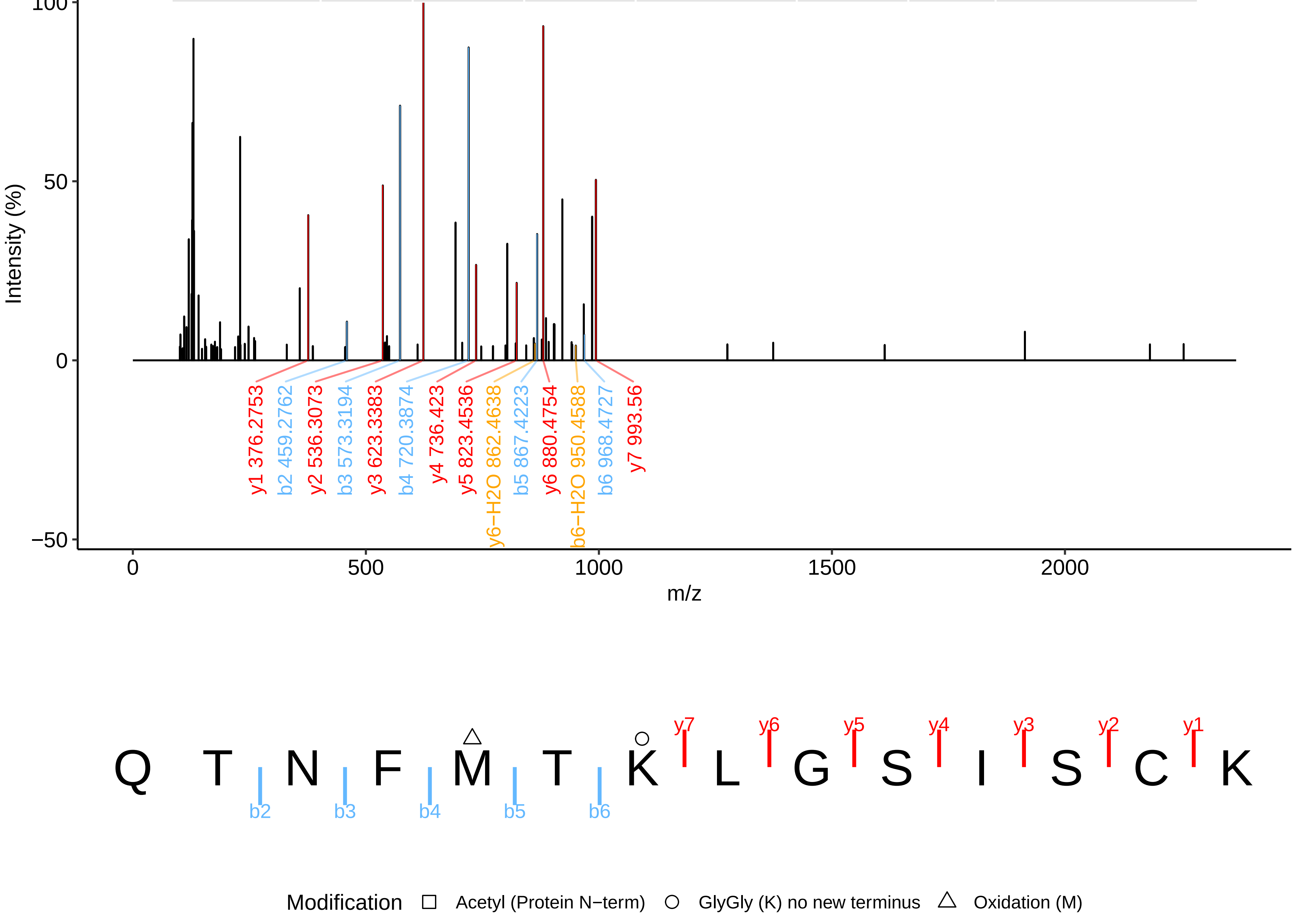

20200921_KIZ_MUb4_GlyGly_10plex − Scan number: 10993

Gene Names	Charge	m/z	Mass	Retention time	PEP	Score	Precursor Intensity
RAD18	4	446.4982	1781.964	38.225	2.0846e−06	114.31	14492477

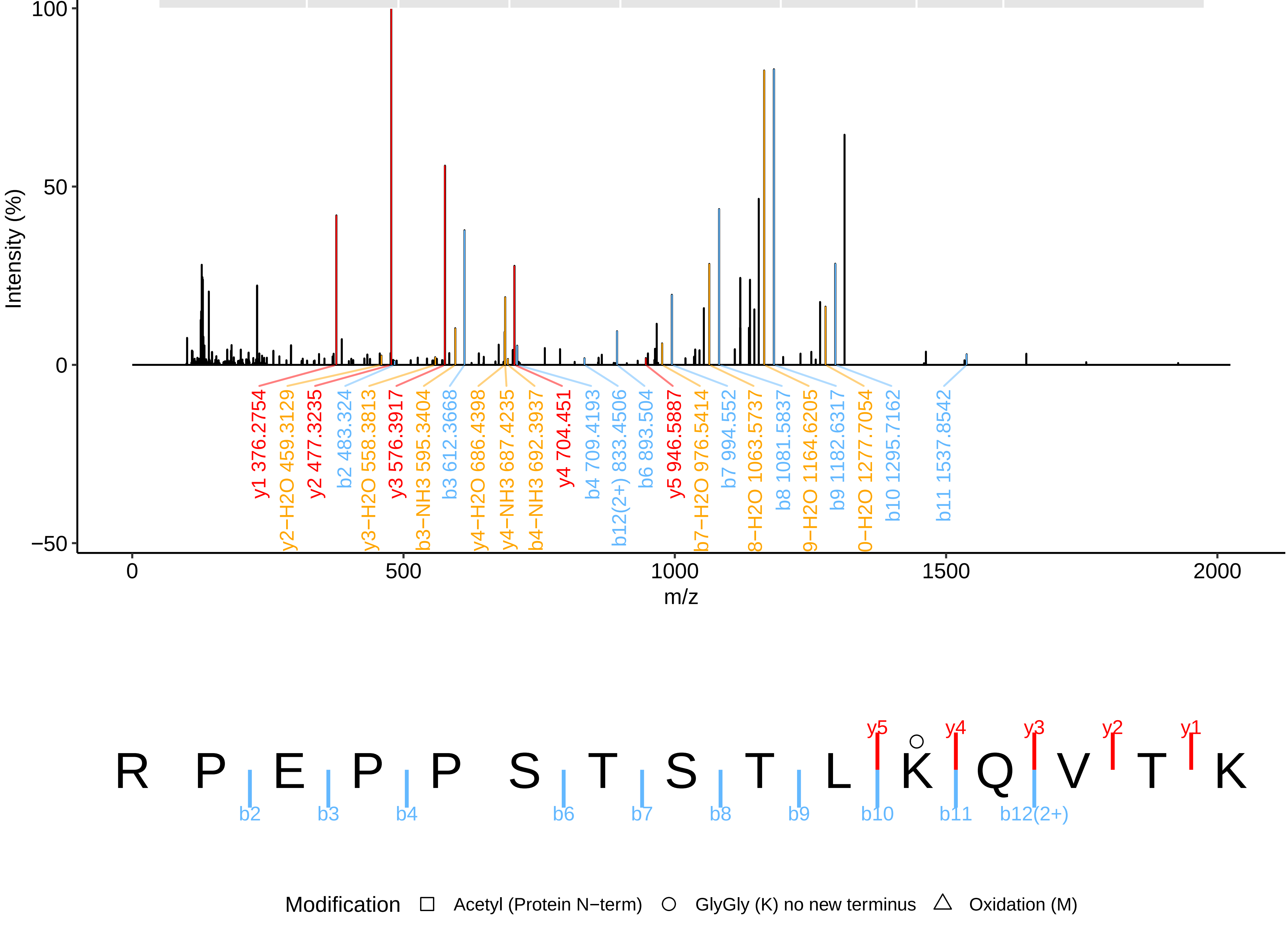

20200921_KIZ_MUb4_GlyGly_10plex − Scan number: 11130

Gene Names	Charge	m/z	Mass	Retention time	PEP	Score	Precursor Intensity
RAD18	4	446.4982	1781.964	38.732	4.2757e−05	97.836	28189538

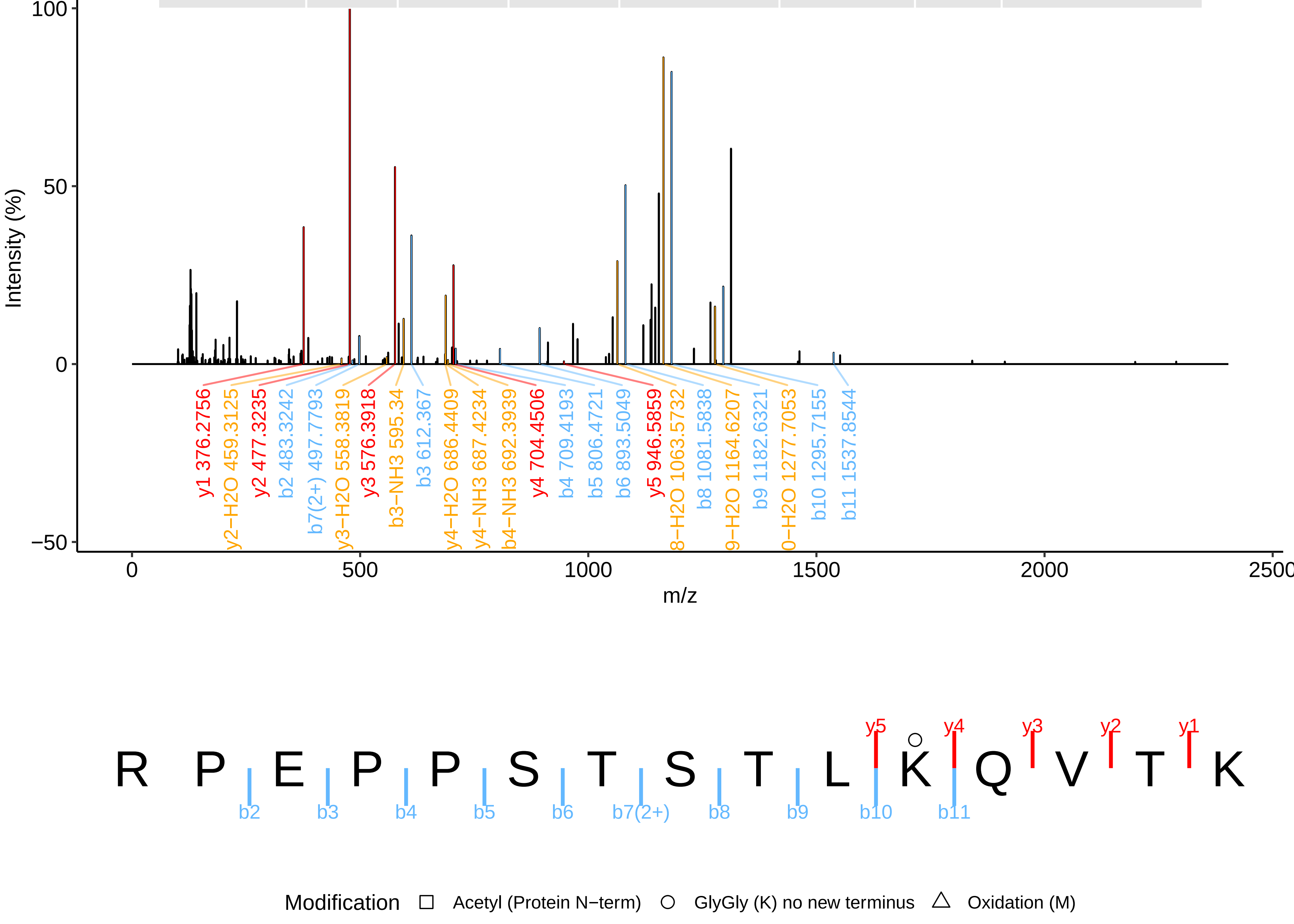

20200921_KIZ_MUb4_GlyGly_10plex − Scan number: 11040

Gene Names	Charge	m/z	Mass	Retention time	PEP	Score	Precursor Intensity
RAD18	3	594.9951	1781.964	38.401	1.8447e−05	99.481	NA

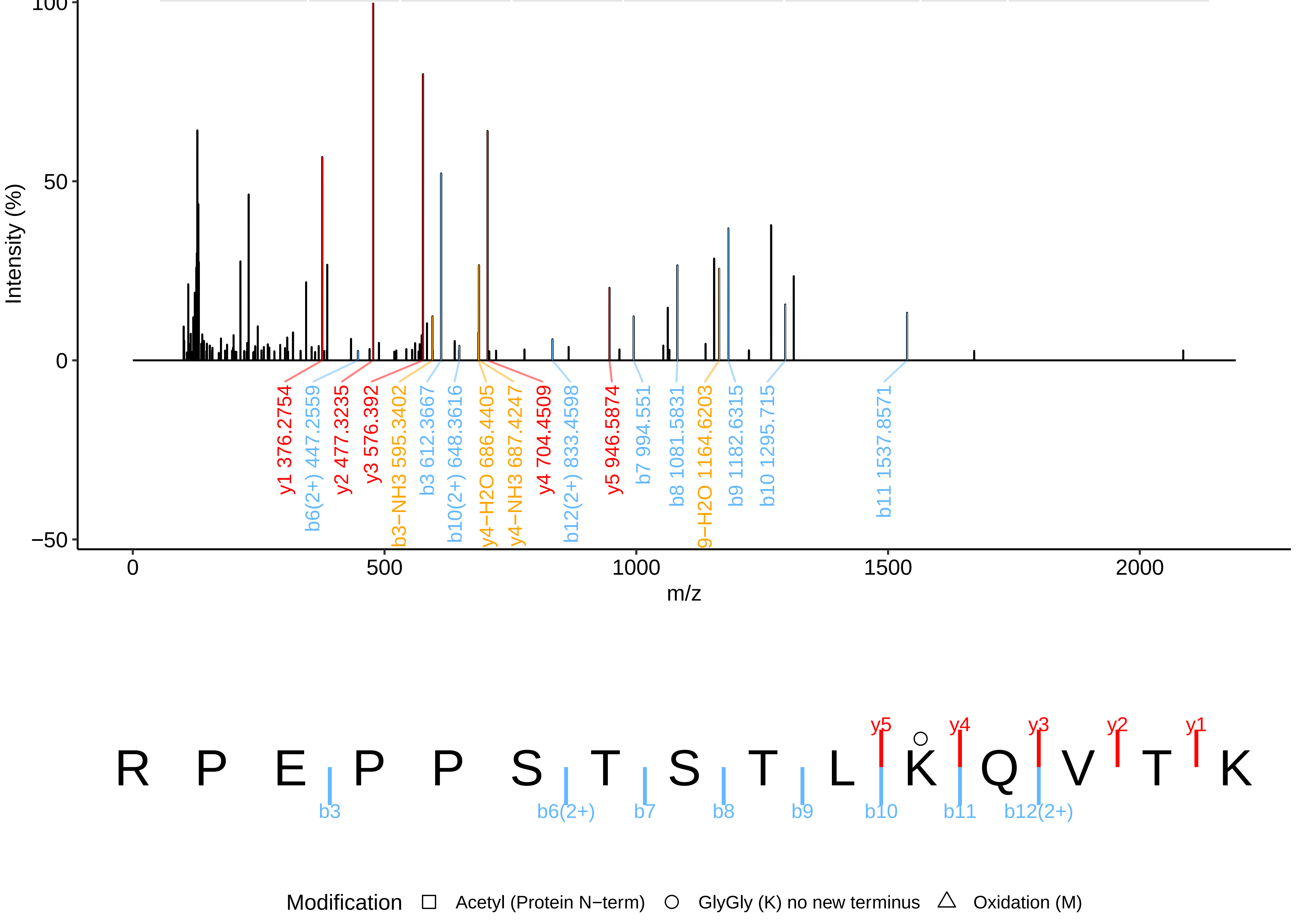

20200921_KIZ_MUb4_GlyGly_10plex_2 − Scan number: 10141

Gene Names	Charge	m/z	Mass	Retention time	PEP	Score	Precursor Intensity
RAD18	4	446.4982	1781.964	38.725	2.5701e−05	101.62	1810925

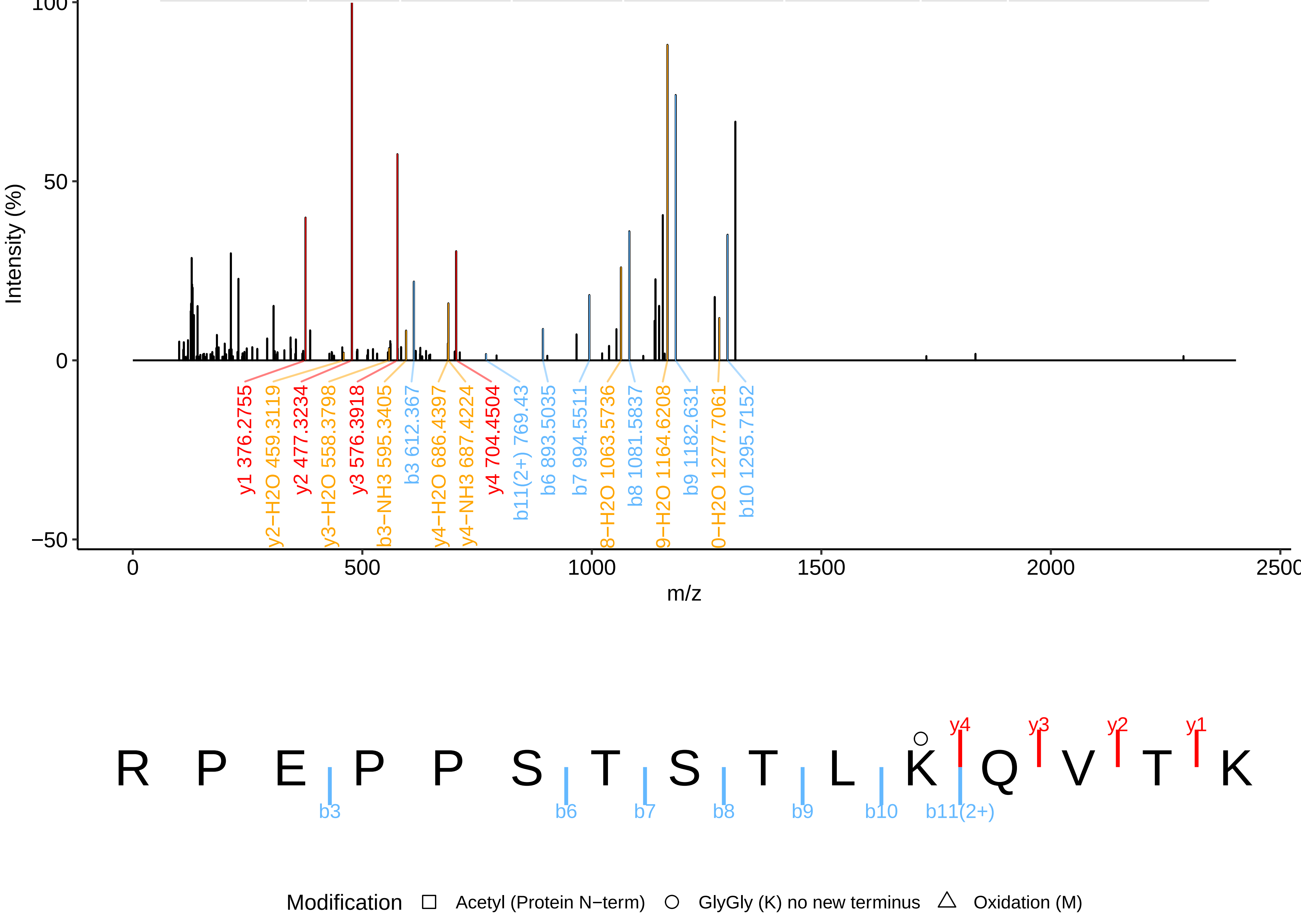

20200921_KIZ_MUb4_GlyGly_10plex_2 − Scan number: 10267

Gene Names	Charge	m/z	Mass	Retention time	PEP	Score	Precursor Intensity
RAD18	4	446.4982	1781.964	39.229	3.2413e−08	115.91	10819582

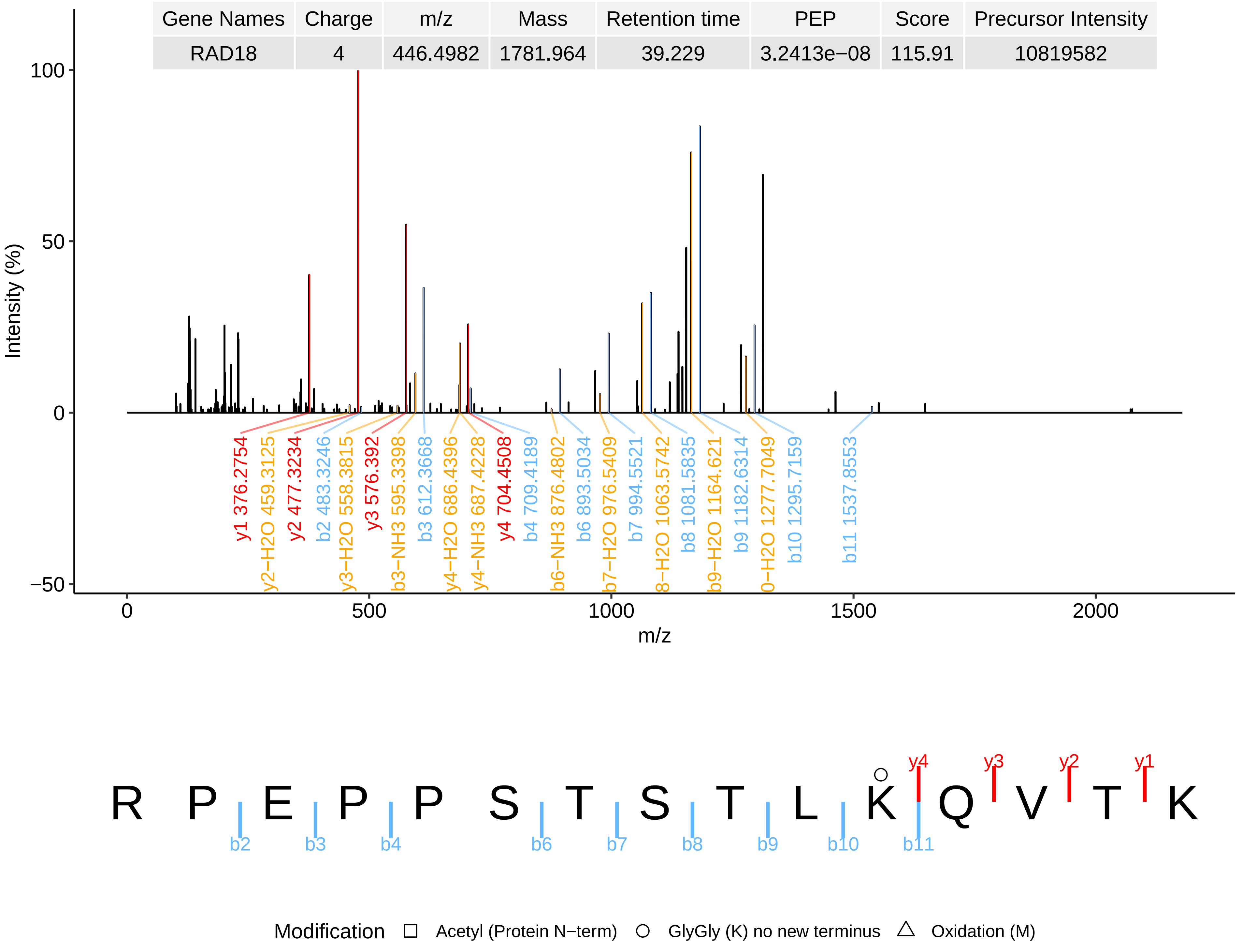

20200924_KIZ_MUb5_GlyGly_1 − Scan number: 9824

Gene Names	Charge	m/z	Mass	Retention time	PEP	Score	Precursor Intensity
RAD18	4	446.4982	1781.964	31.644	1.6729e−08	124.09	12259562

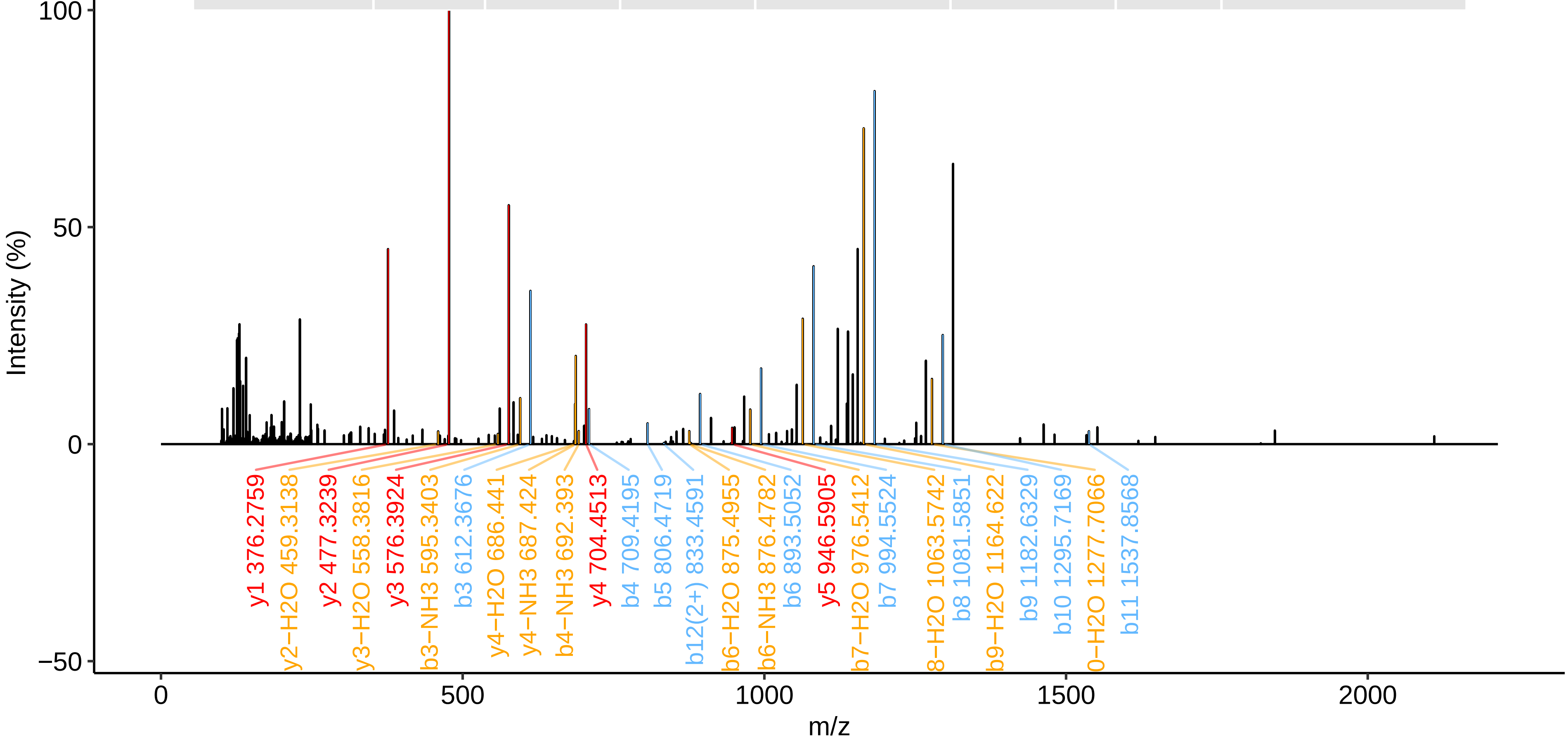

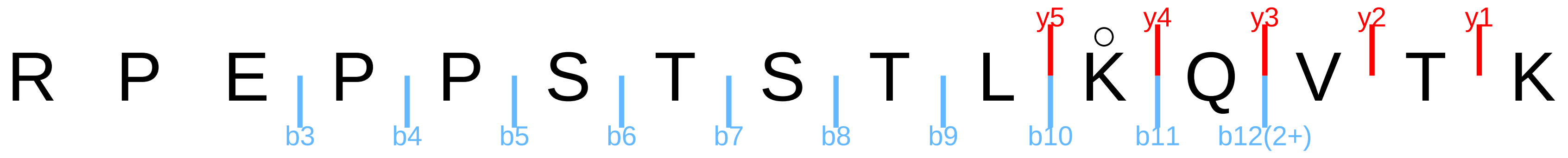

Modification □ Acetyl (Protein N−term) ○ GlyGly (K) no new terminus △ Oxidation (M)

20200924_KIZ_MUb5_GlyGly_1 − Scan number: 9978

Gene Names	Charge	m/z	Mass	Retention time	PEP	Score	Precursor Intensity
RAD18	4	446.4982	1781.964	32.155	7.0486e−06	111.64	17282482

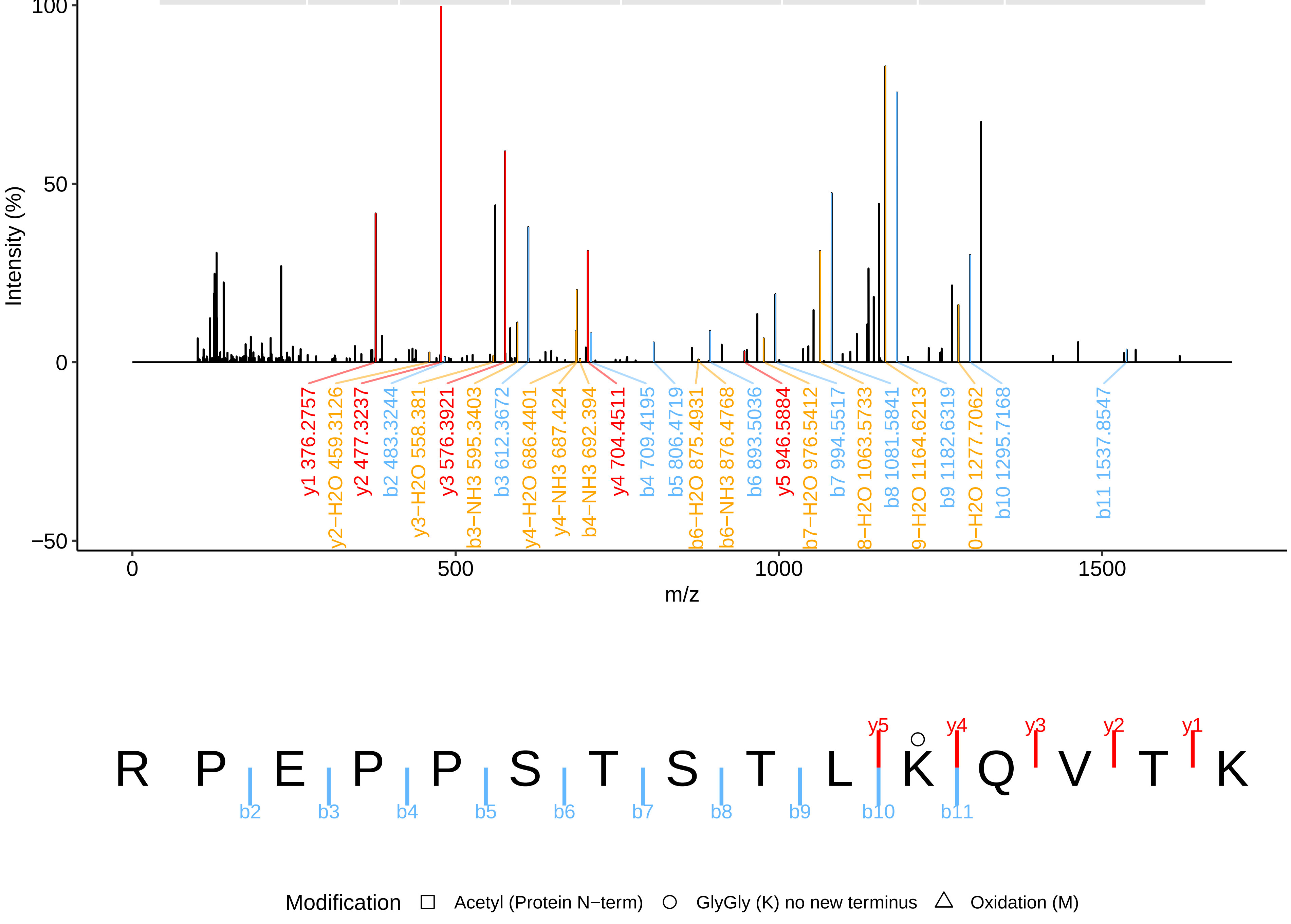

20200924_KIZ_MUb5_GlyGly_1 − Scan number: 10133

Gene Names	Charge	m/z	Mass	Retention time	PEP	Score	Precursor Intensity
RAD18	4	446.4982	1781.964	32.662	1.0069e−08	127.57	NA

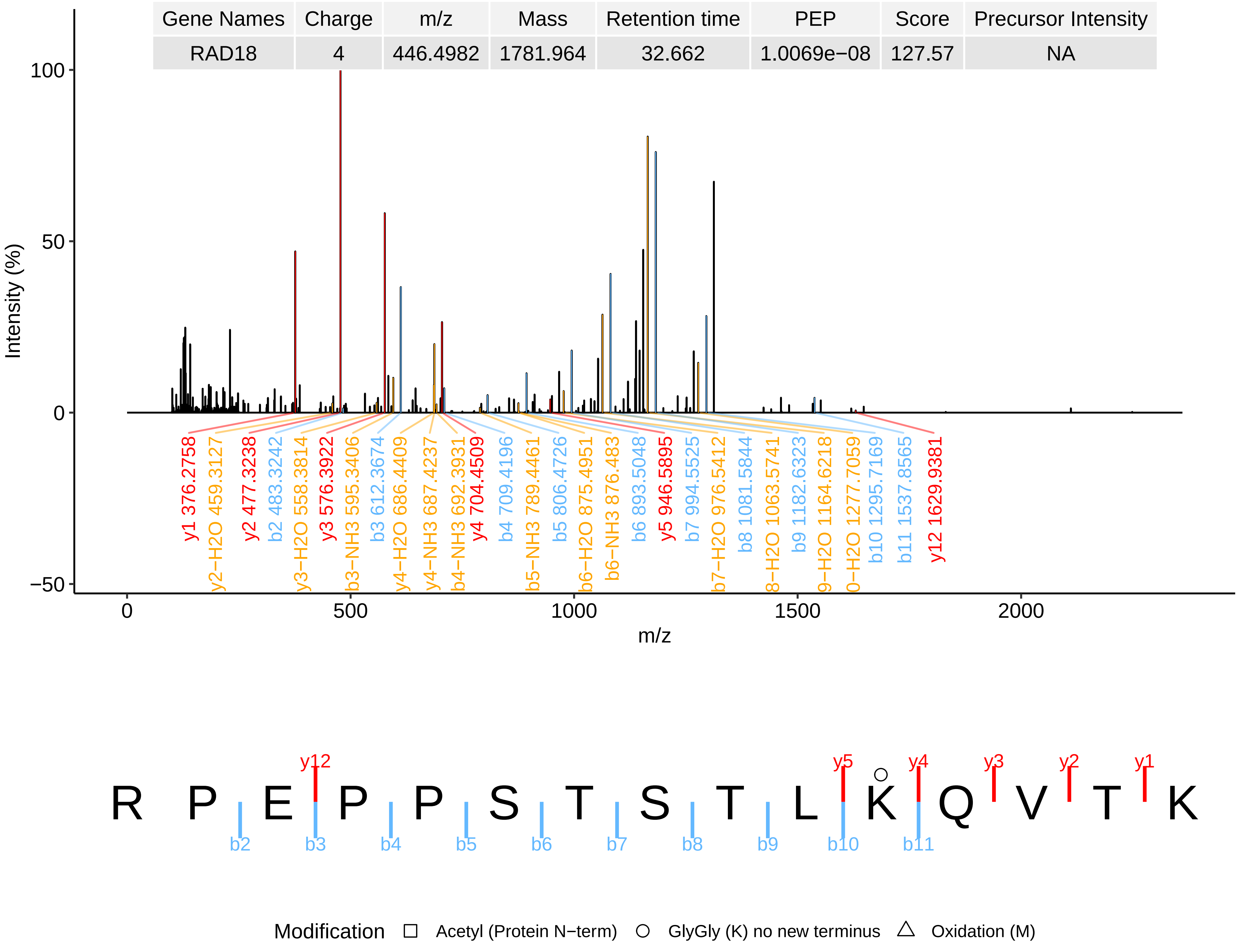

20200924_KIZ_MUb5_GlyGly_2 − Scan number: 9106

Gene Names	Charge	m/z	Mass	Retention time	PEP	Score	Precursor Intensity
RAD18	4	446.4982	1781.964	34.034	4.0389e−09	130.72	6549907

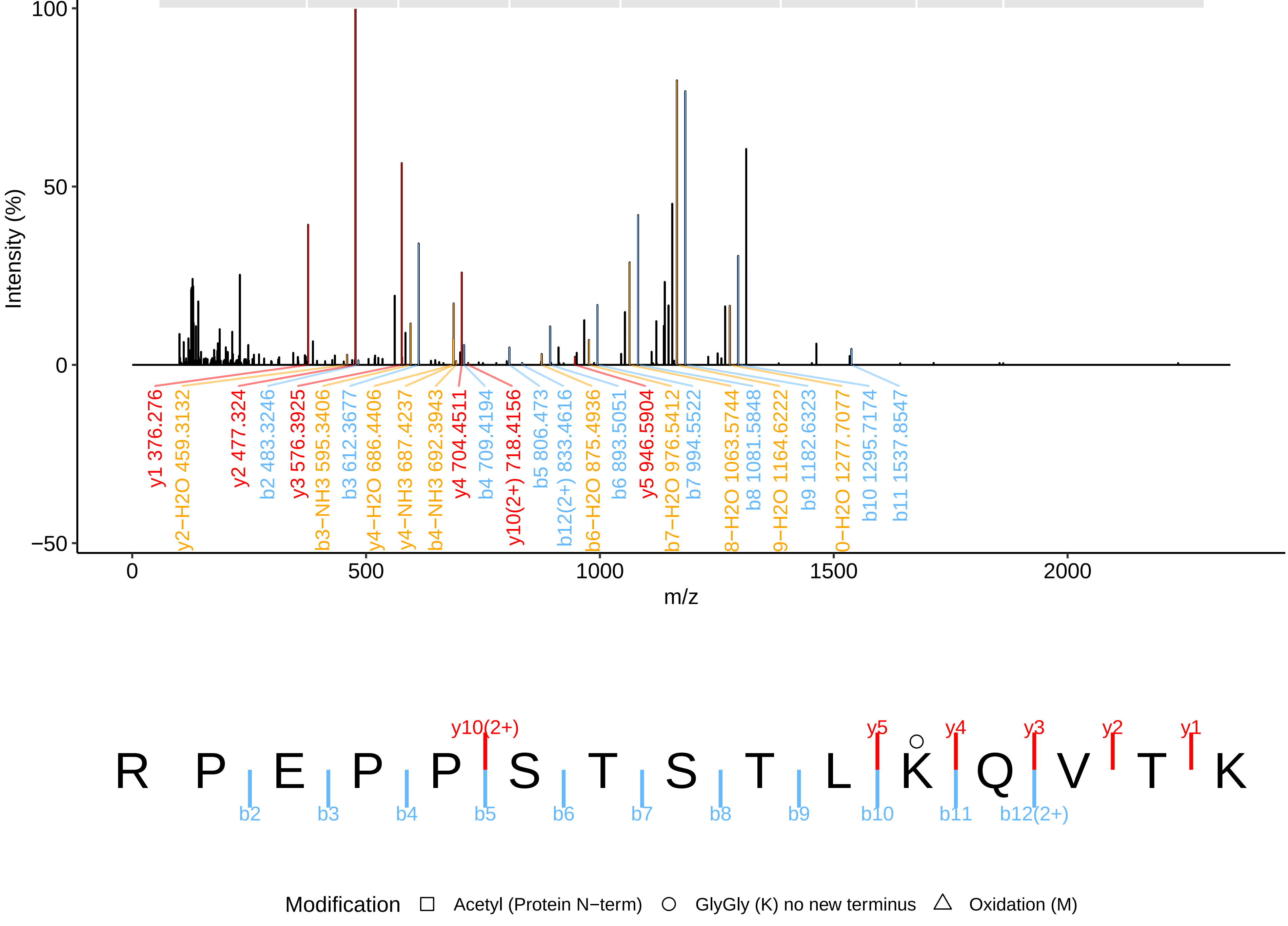

20200924_KIZ_MUb5_GlyGly_2 − Scan number: 9391

Gene Names	Charge	m/z	Mass	Retention time	PEP	Score	Precursor Intensity
RAD18	4	446.4982	1781.964	35.095	2.151e−05	103.88	4986290

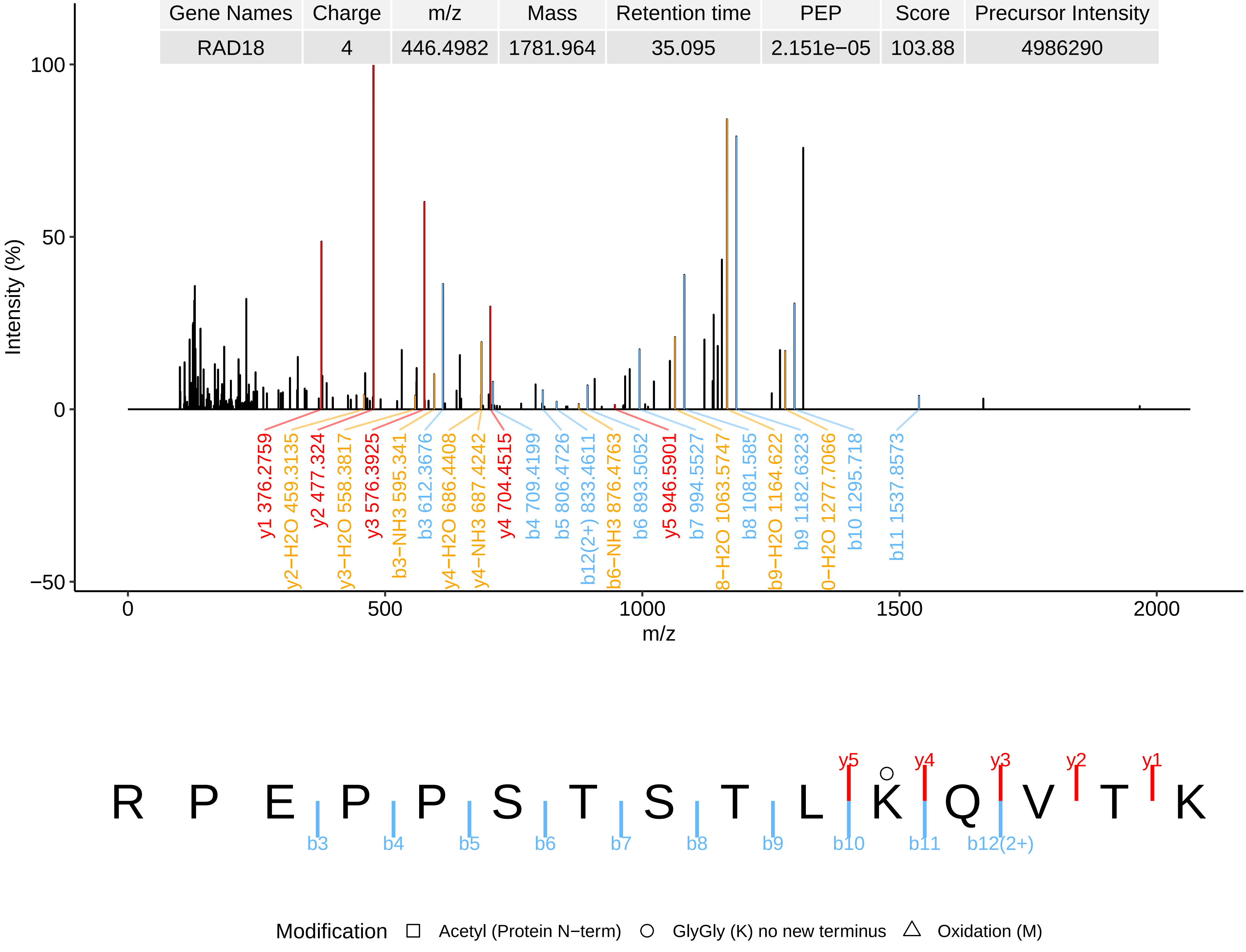

20200921_KIZ_MUb4_GlyGly_10plex − Scan number: 15170

Gene Names	Charge	m/z	Mass	Retention time	PEP	Score	Precursor Intensity
SLC19A1	3	587.2989	1758.875	54.123	6.8557e−06	91.657	3230070

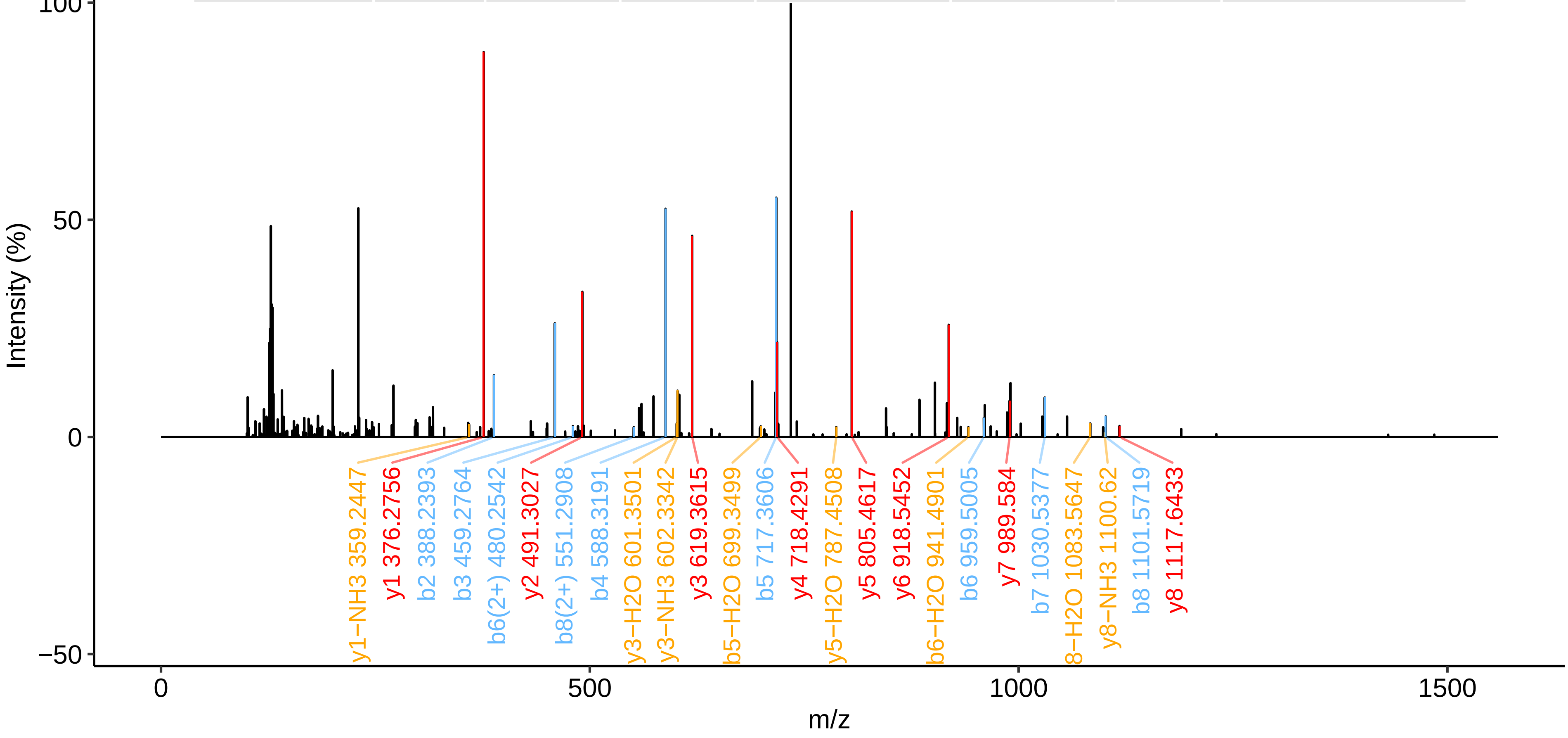

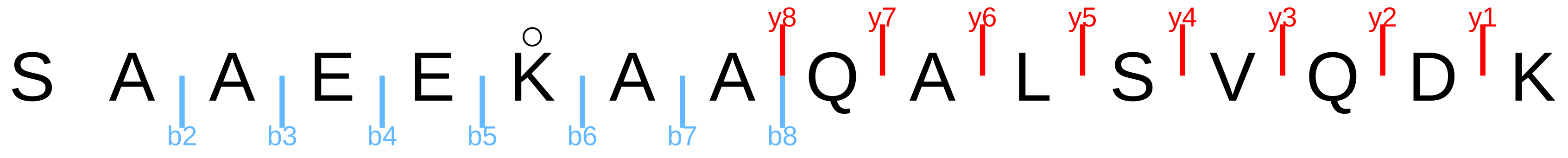

Modification □ Acetyl (Protein N−term) ○ GlyGly (K) no new terminus △ Oxidation (M)

20200921_KIZ_MUb4_GlyGly_10plex − Scan number: 15301

Gene Names	Charge	m/z	Mass	Retention time	PEP	Score	Precursor Intensity
SLC19A1	3	587.2989	1758.875	54.627	0.0025705	66.022	7133782

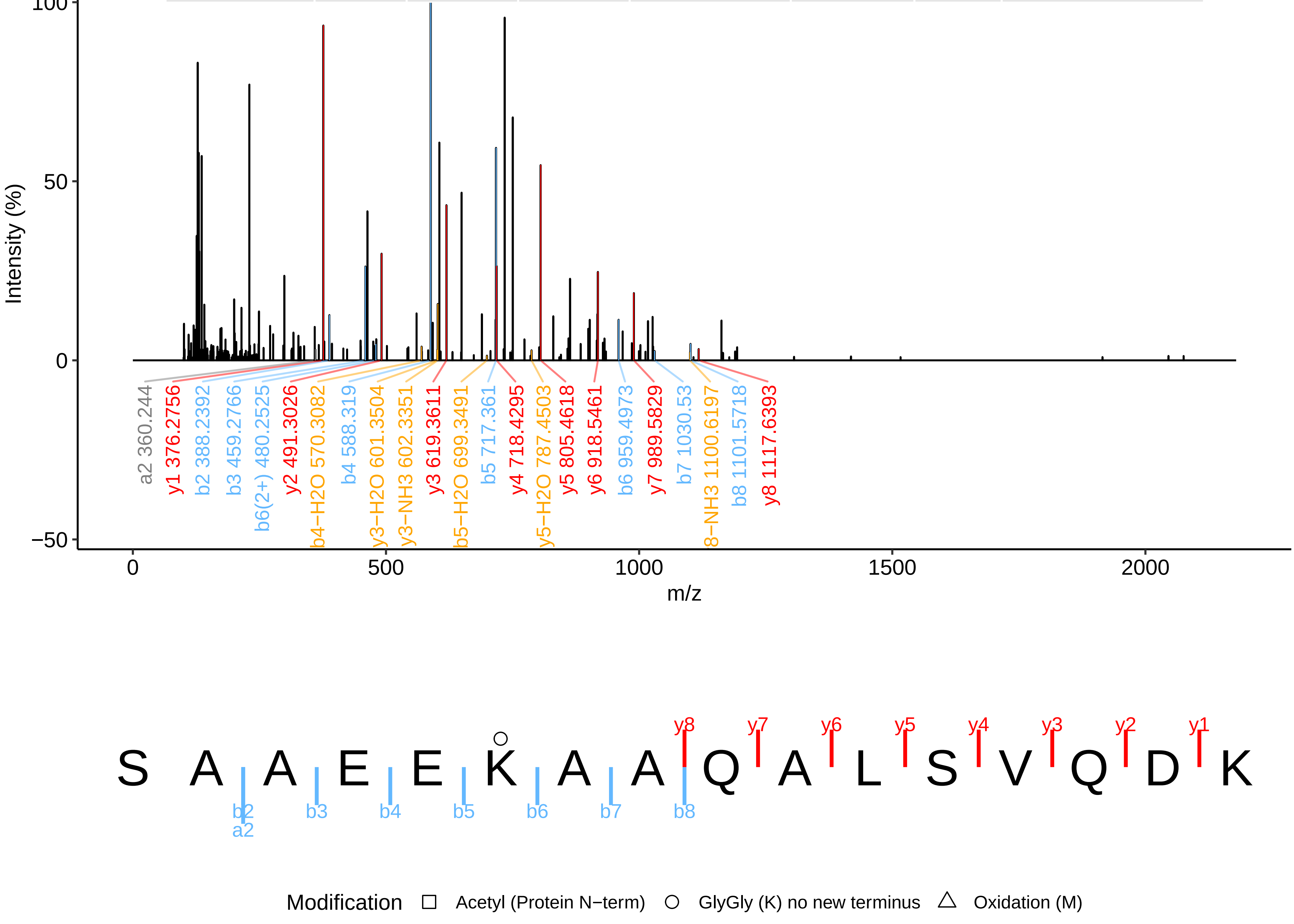

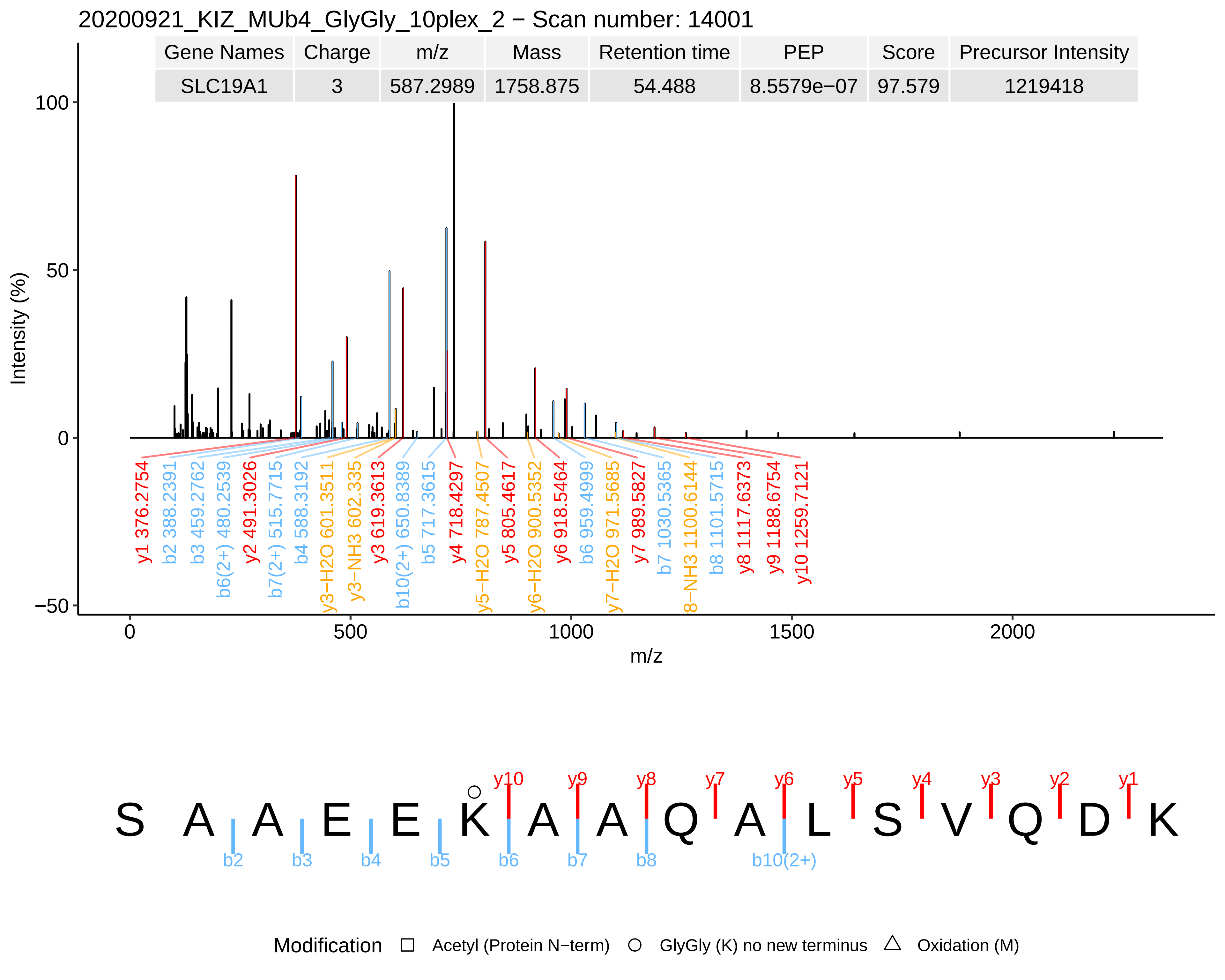

20200921_KIZ_MUb4_GlyGly_10plex_2 − Scan number: 14001
Gene Names | Charge | m/z | Mass | Retention time | PEP | Score | Precursor Intensity
SLC19A1 | 3 | 587.2989 | 1758.875 | 54.488 | 8.5579e−07 | 97.579 | 1219418
Intensity (%)
m/z
y1 376.2754
b2 388.2391
b3 459.2762
b6(2+) 480.2539
y2 491.3026
b7(2+) 515.7715
b4 588.3192
y3−H2O 601.3511
y3−NH3 602.335
y3 619.3613
b10(2+) 650.8389
b5 717.3615
y4 718.4297
y5−H2O 787.4507
y5 805.4617
y6−H2O 900.5352
y6 918.5464
b6 959.4999
y7−H2O 971.5685
y7 989.5827
b7 1030.5365
8−NH3 1100.6144
b8 1101.5715
y8 1117.6373
y9 1188.6754
y10 1259.7121
S A A E E K A A Q A L S V Q D K
Modification
Acetyl (Protein N−term)
GlyGly (K) no new terminus
Oxidation (M)

20200921_KIZ_MUb4_GlyGly_10plex_2 − Scan number: 14041

Gene Names	Charge	m/z	Mass	Retention time	PEP	Score	Precursor Intensity
SLC19A1	4	440.726	1758.875	54.656	0.03652	54.501	1538981

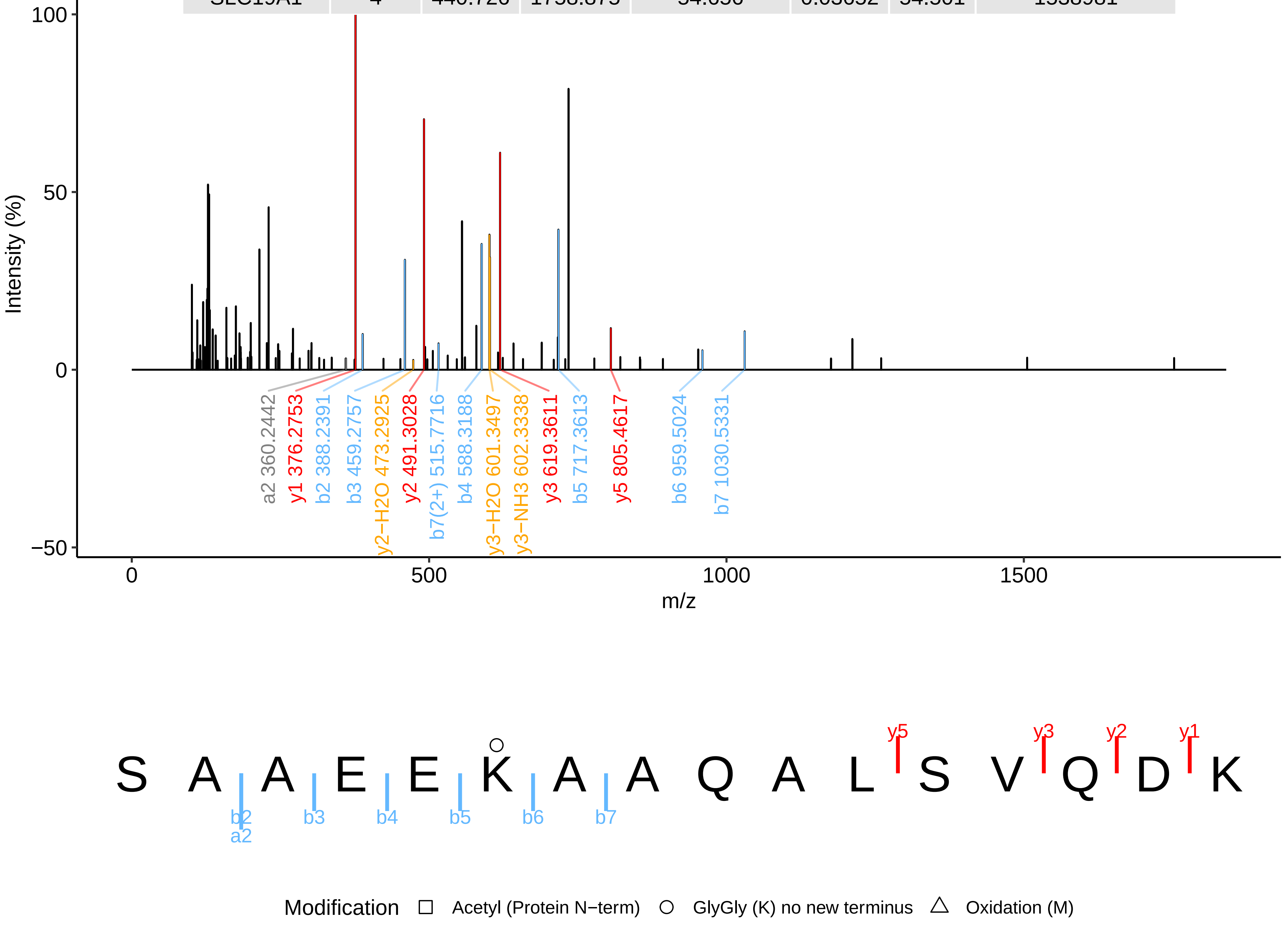

20200924_KIZ_MUb5_GlyGly_2 − Scan number: 13486

Gene Names	Charge	m/z	Mass	Retention time	PEP	Score	Precursor Intensity
SLC19A1	3	587.2989	1758.875	50.435	3.2312e−09	115.31	6076445

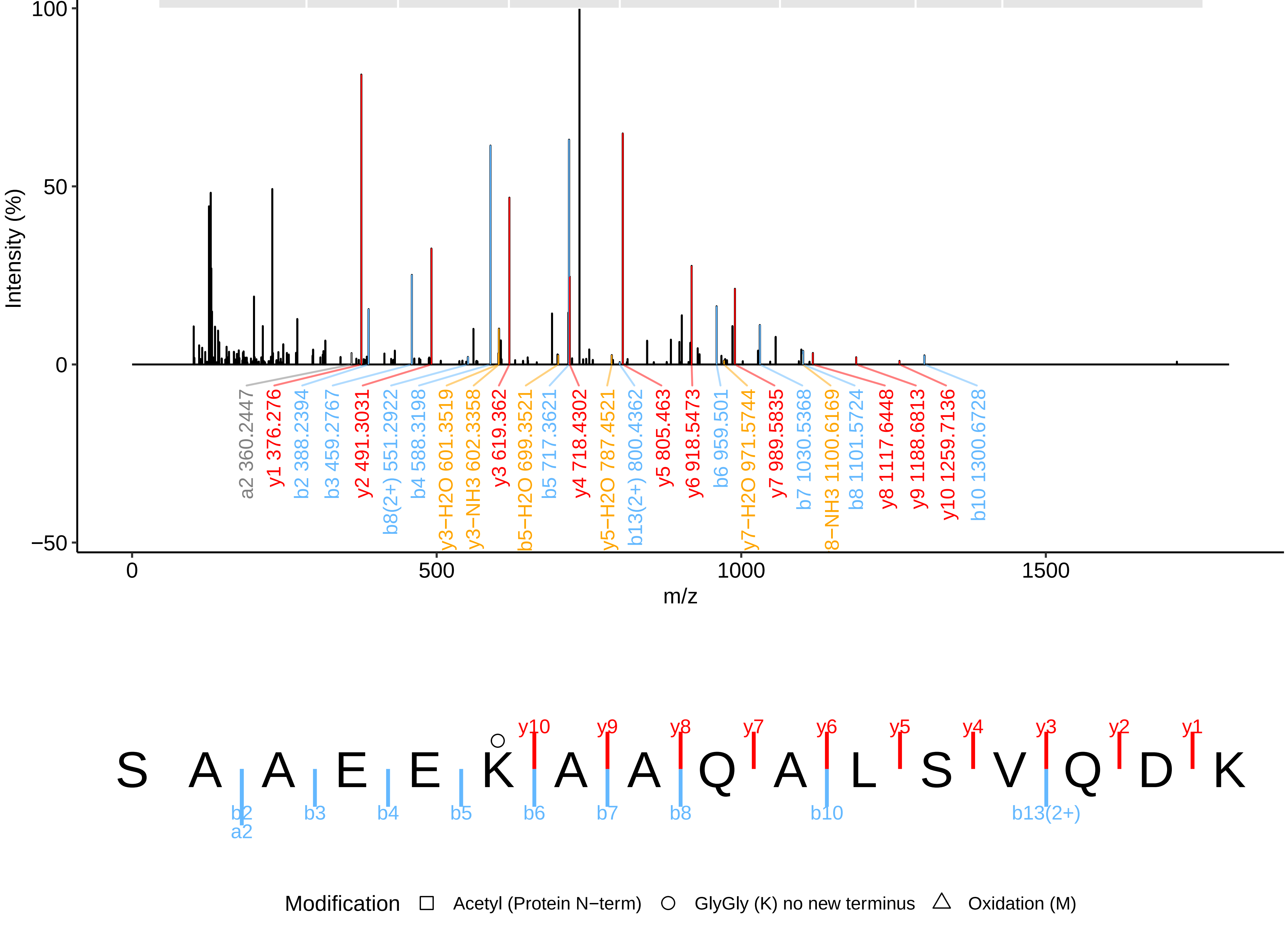

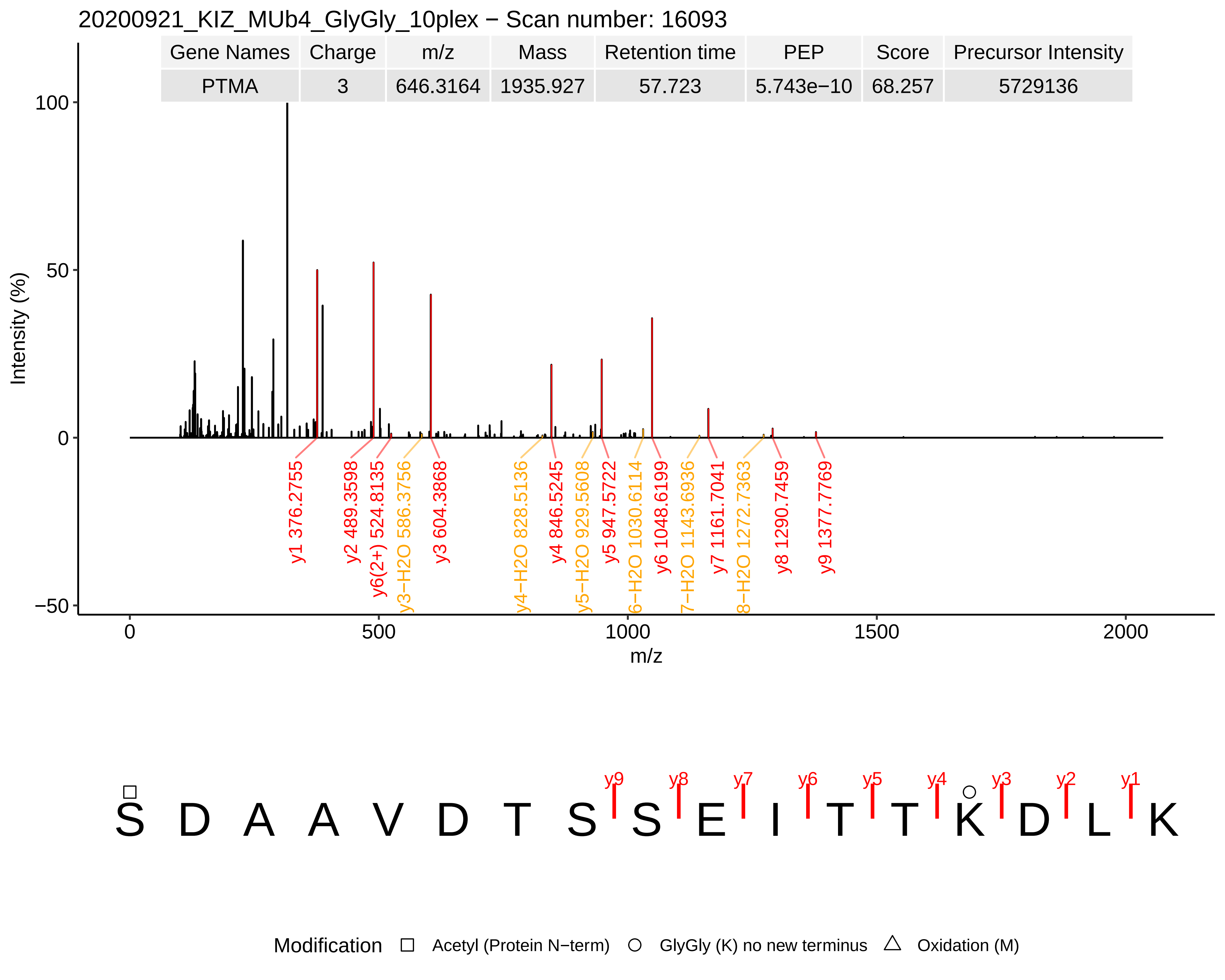

20200921_KIZ_MUb4_GlyGly_10plex − Scan number: 16093
Gene Names | Charge | m/z | Mass | Retention time | PEP | Score | Precursor Intensity
PTMA | 3 | 646.3164 | 1935.927 | 57.723 | 5.743e−10 | 68.257 | 5729136
Intensity (%)
100
50
0
−50
0
500
1000
1500
2000
m/z
y1 376.2755
y2 489.3598
y6(2+) 524.8135
y3−H2O 586.3756
y3 604.3868
y4−H2O 828.5136
y4 846.5245
y5−H2O 929.5608
y5 947.5722
y6−H2O 1030.6114
y6 1048.6199
y7−H2O 1143.6936
y7 1161.7041
y8−H2O 1272.7363
y8 1290.7459
y9 1377.7769
y9
y8
y7
y6
y5
y4
y3
y2
y1
S D A A V D T S S E I T T K D L K
Modification
Acetyl (Protein N−term)
GlyGly (K) no new terminus
Oxidation (M)

20200921_KIZ_MUb4_GlyGly_10plex − Scan number: 16105

Gene Names	Charge	m/z	Mass	Retention time	PEP	Score	Precursor Intensity
PTMA	2	968.9709	1935.927	57.765	3.8782e−40	97.911	6278801

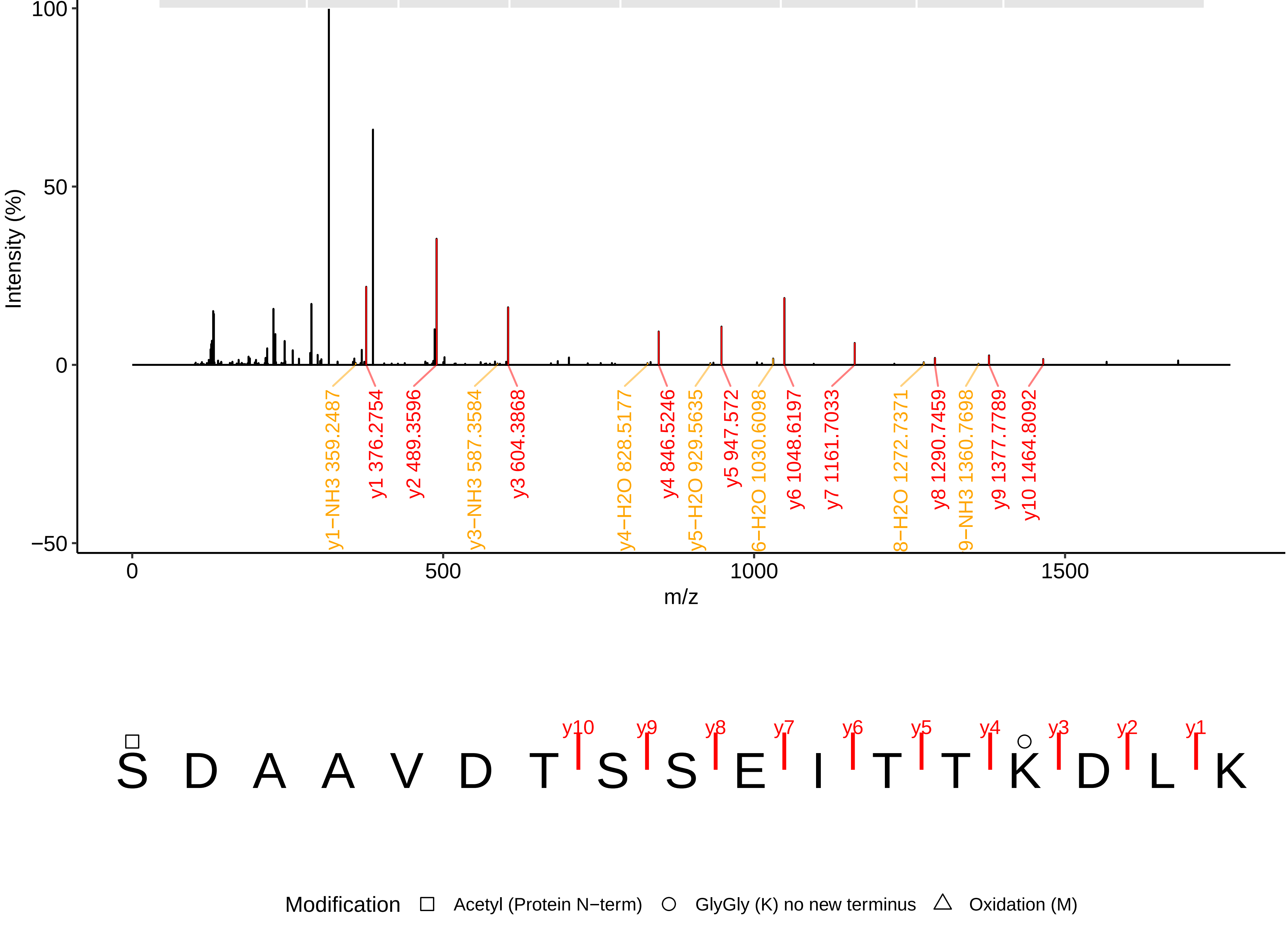

20200921_KIZ_MUb4_GlyGly_10plex_2 − Scan number: 14825

Gene Names	Charge	m/z	Mass	Retention time	PEP	Score	Precursor Intensity
PTMA	2	968.9709	1935.927	57.866	0.00063363	52.928	842515.6

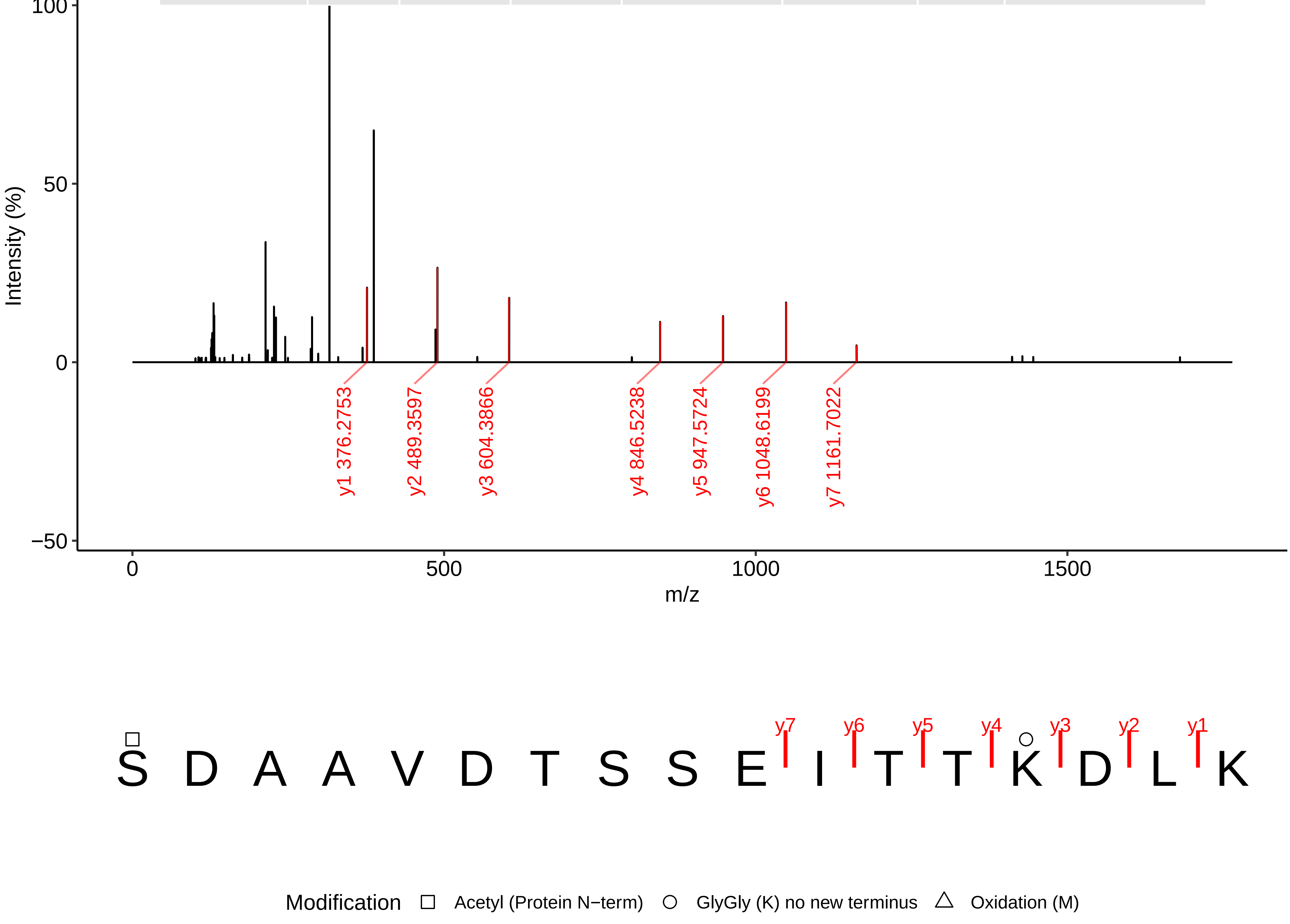

20200924_KIZ_MUb5_GlyGly_2 − Scan number: 14687

Gene Names	Charge	m/z	Mass	Retention time	PEP	Score	Precursor Intensity
PTMA	3	646.3164	1935.927	54.881	0.00054573	52.453	2530259

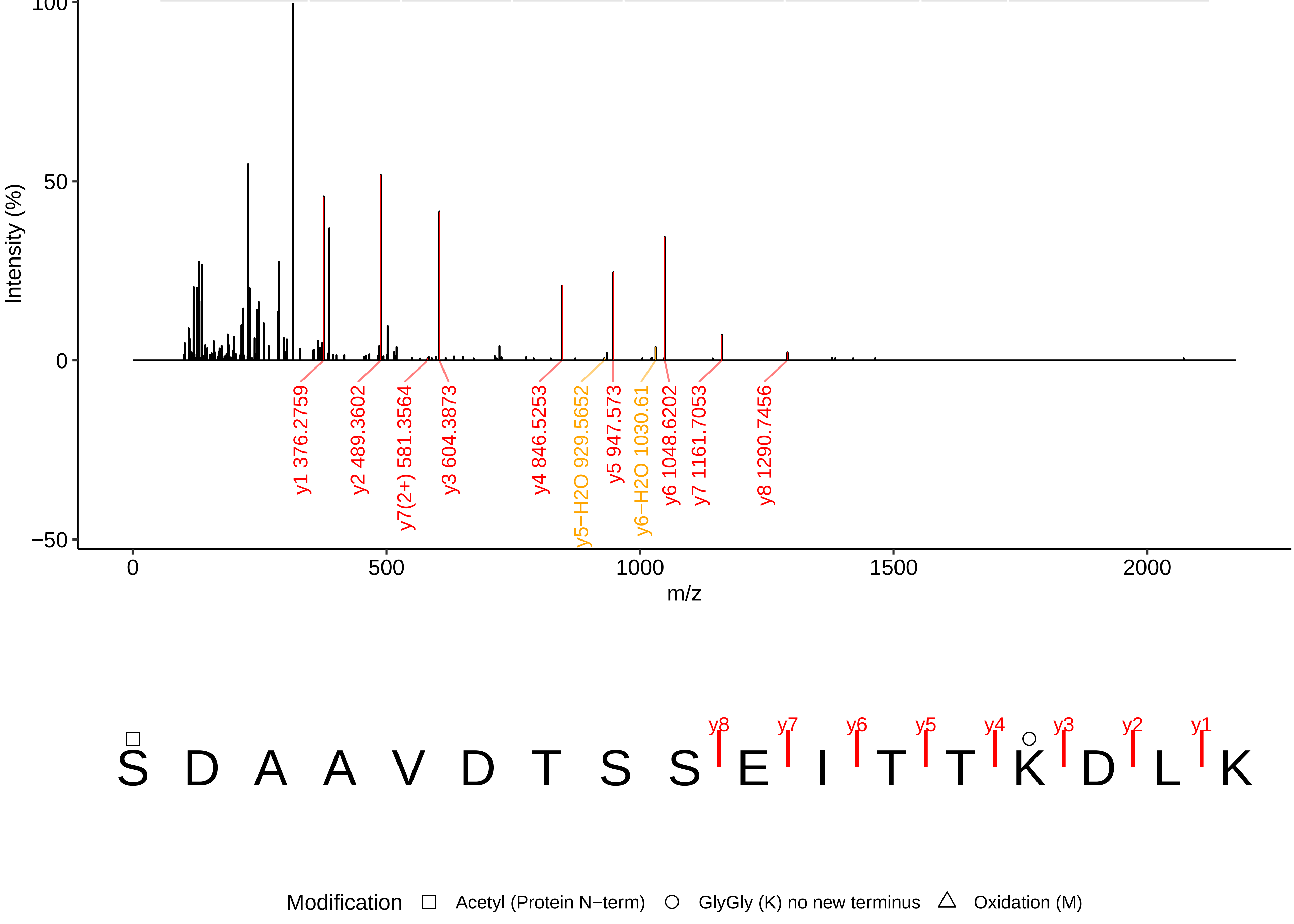

20200924_KIZ_MUb5_GlyGly_1 − Scan number: 34747

Gene Names	Charge	m/z	Mass	Retention time	PEP	Score	Precursor Intensity
LRGUK	2	979.9748	1957.935	110.57	0.092093	42.001	10518973

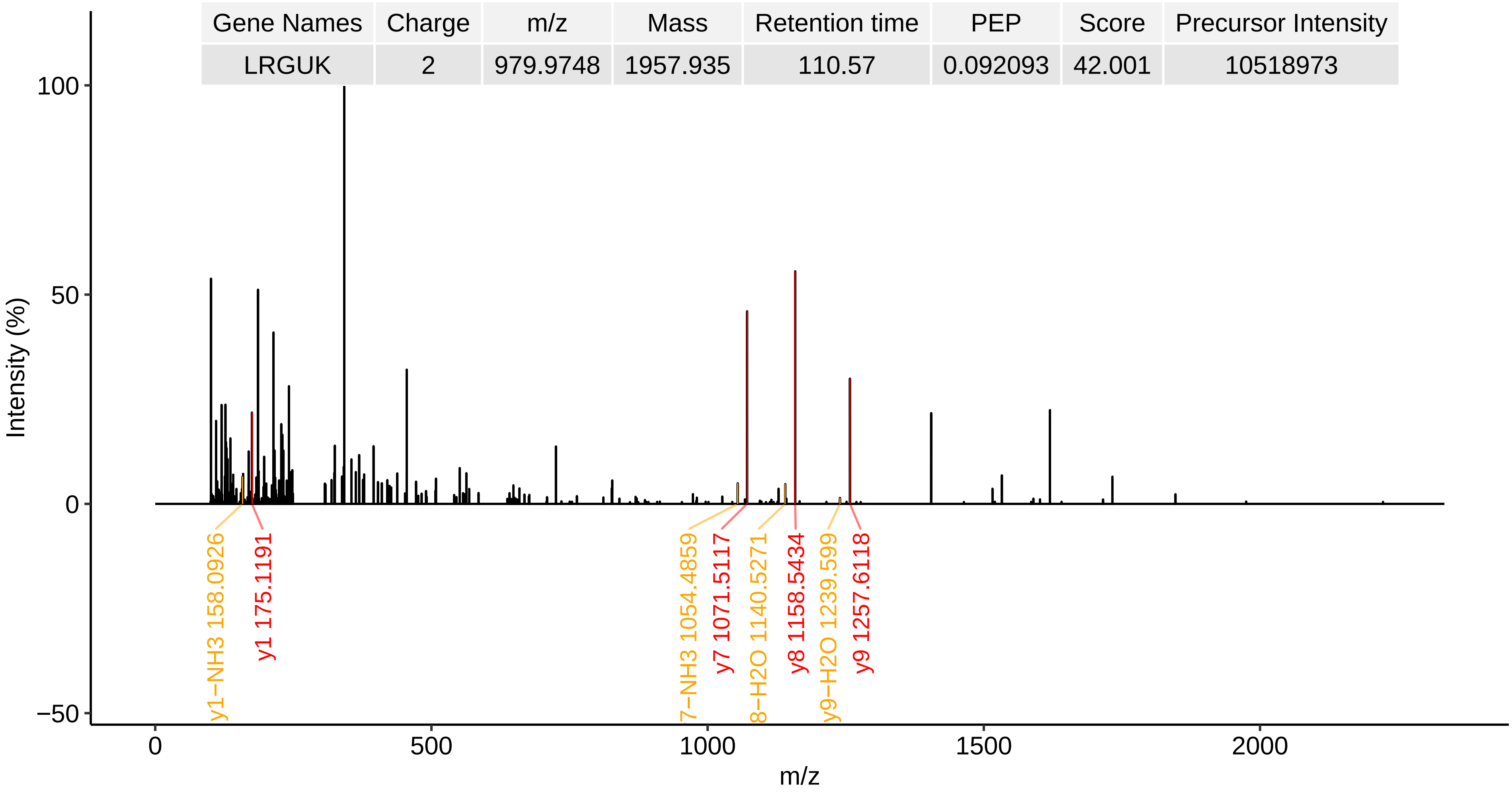

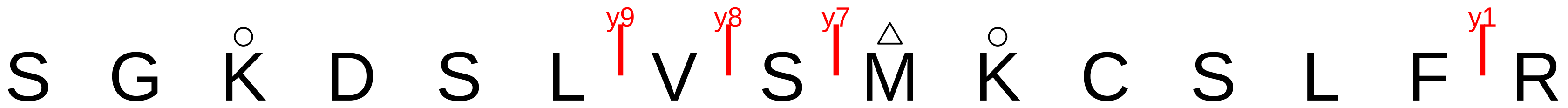

Modification □ Acetyl (Protein N−term) ○ GlyGly (K) no new terminus △ Oxidation (M)

20200921_KIZ_MUb4_GlyGly_10plex − Scan number: 7337

Gene Names	Charge	m/z	Mass	Retention time	PEP	Score	Precursor Intensity
TMEM59	4	438.9654	1751.833	24.606	4.1668e−09	115.82	4154392

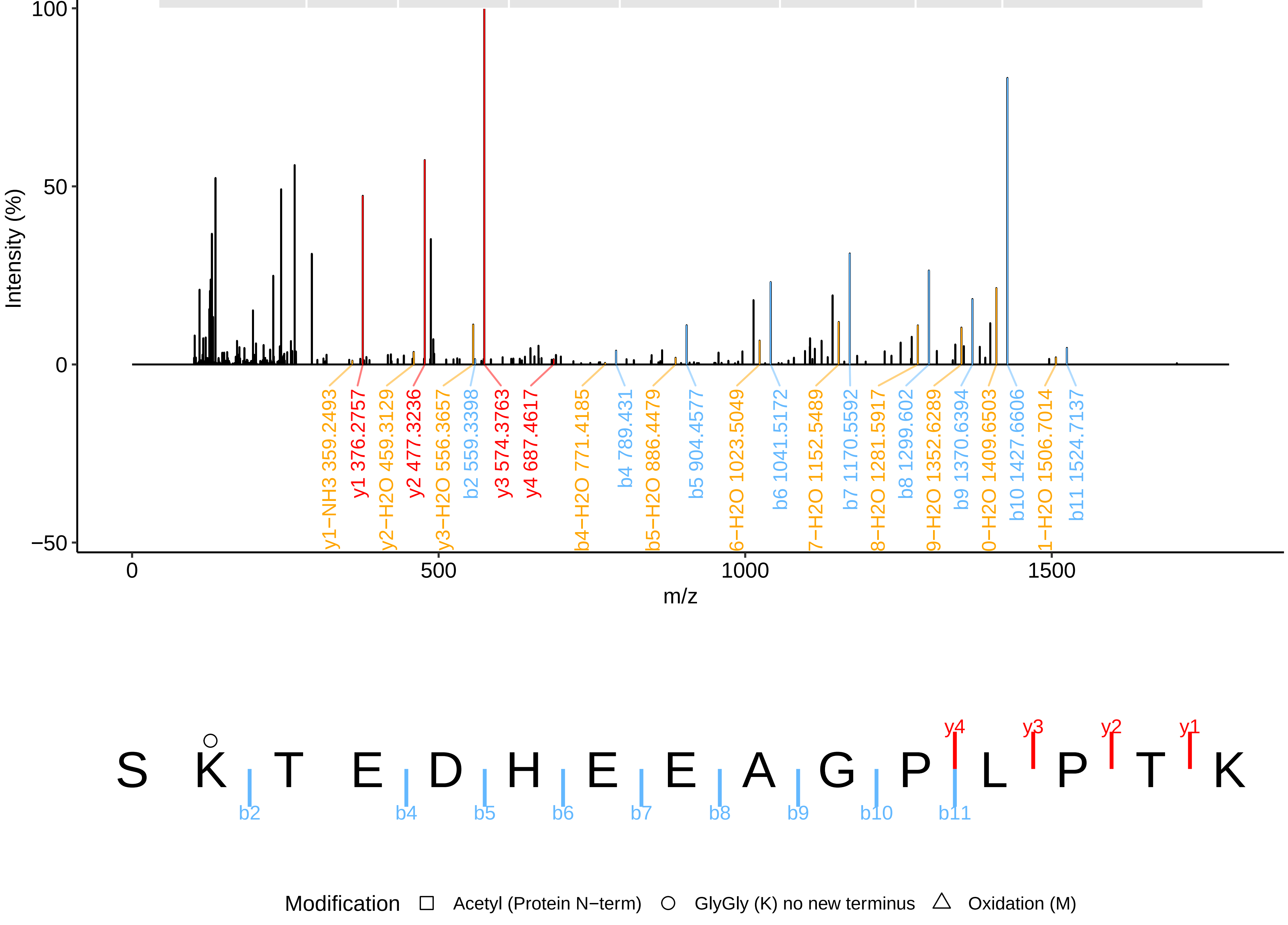

20200921_KIZ_MUb4_GlyGly_10plex_2 − Scan number: 6749

Gene Names	Charge	m/z	Mass	Retention time	PEP	Score	Precursor Intensity
TMEM59	4	438.9654	1751.833	24.942	7.3298e−05	89.466	775432.1

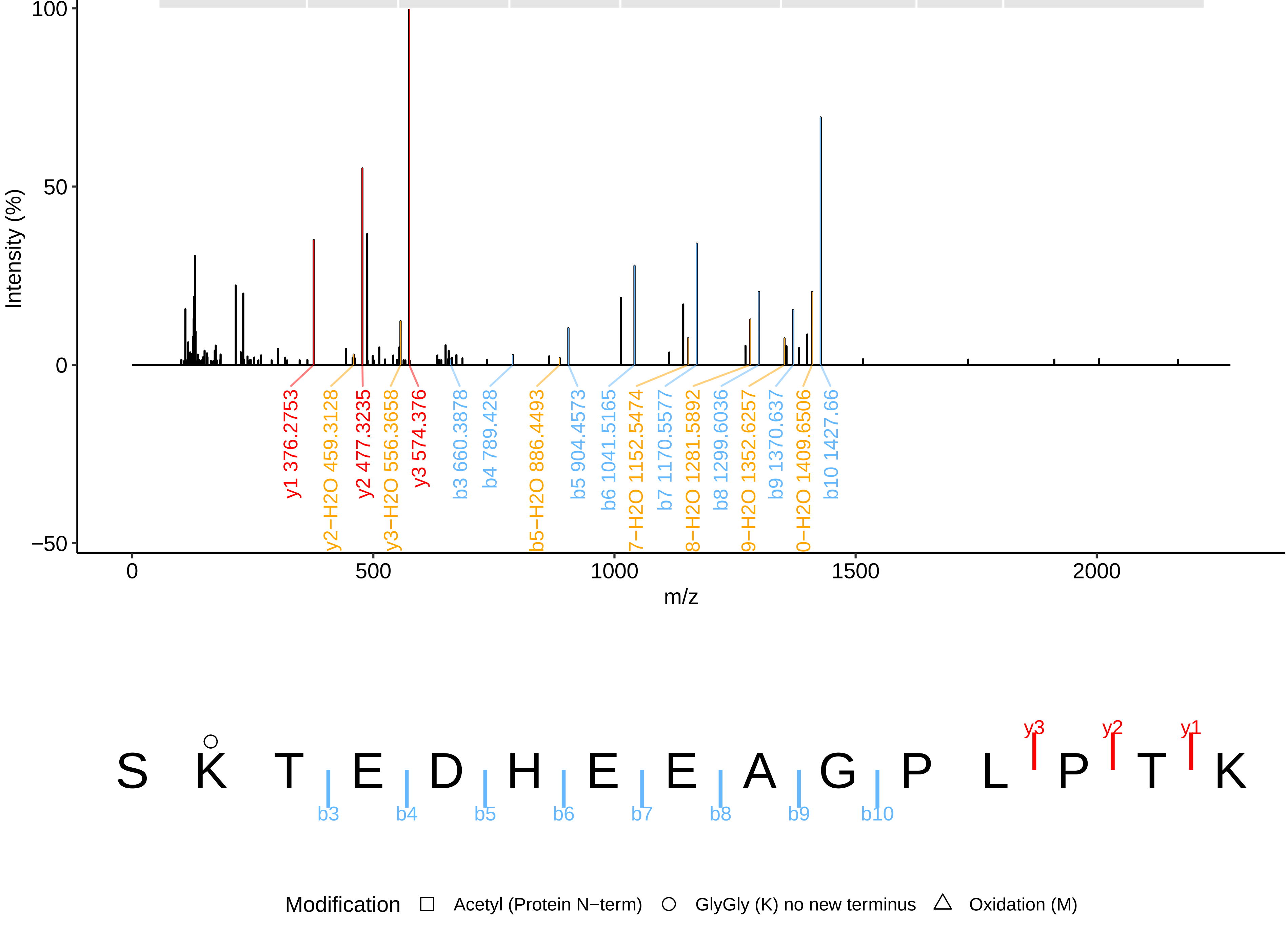

20200921_KIZ_MUb4_GlyGly_10plex_2 − Scan number: 18626

Gene Names	Charge	m/z	Mass	Retention time	PEP	Score	Precursor Intensity
SND1	3	587.6282	1759.863	72.988	0.004639	71.344	551178.8

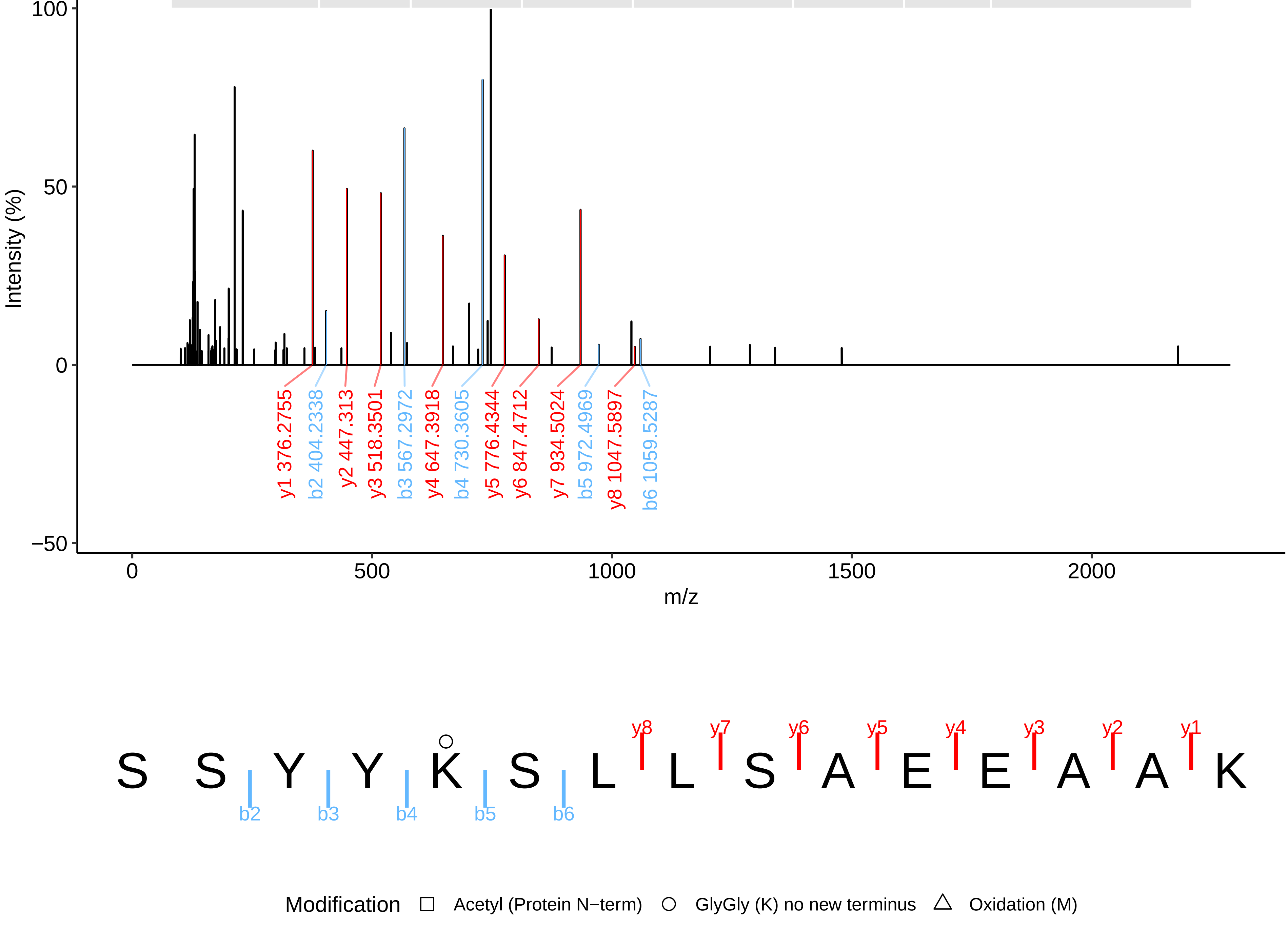

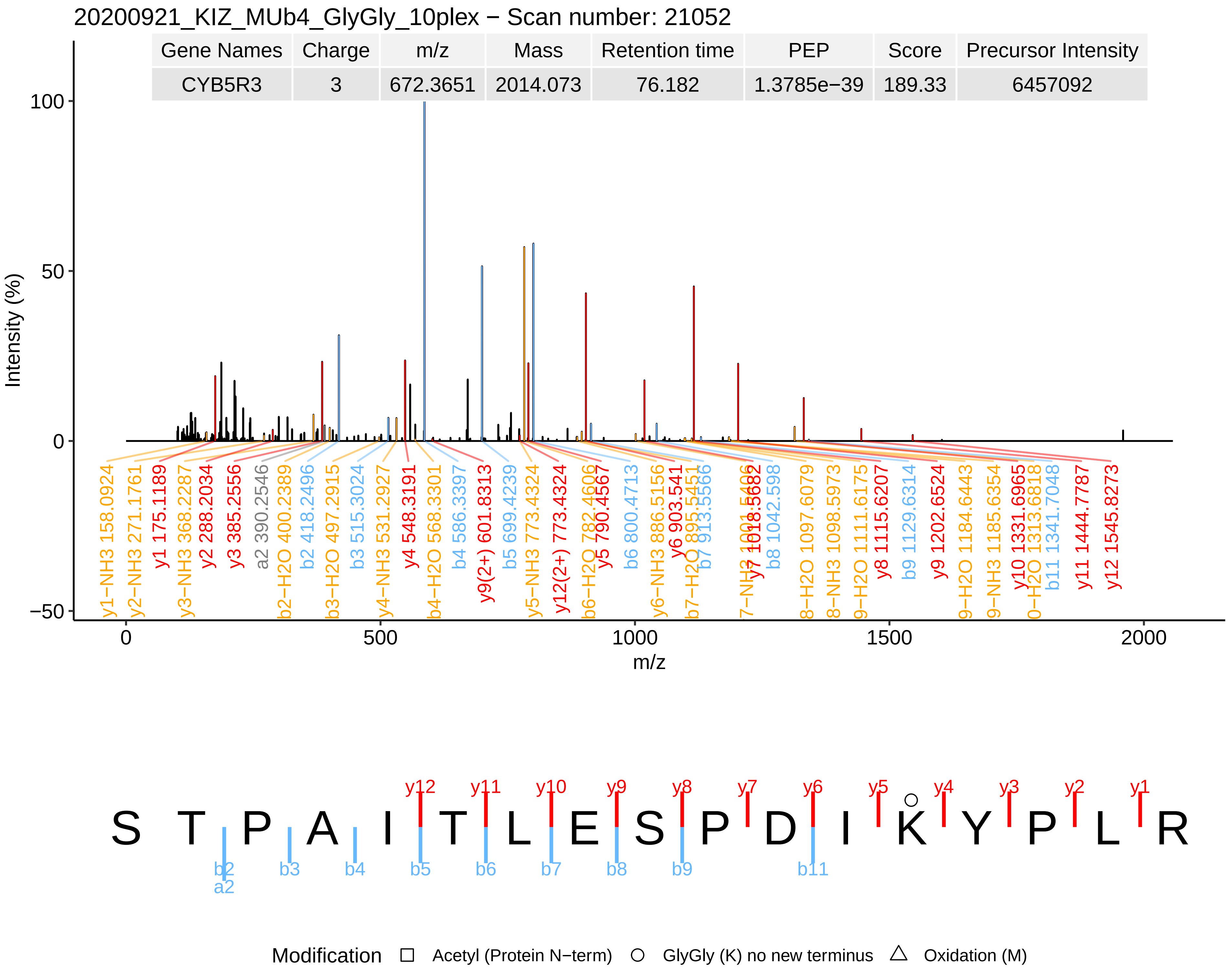
20200921_KIZ_MUb4_GlyGly_10plex − Scan number: 21052
Gene Names | Charge | m/z | Mass | Retention time | PEP | Score | Precursor Intensity
CYB5R3 | 3 | 672.3651 | 2014.073 | 76.182 | 1.3785e−39 | 189.33 | 6457092
Intensity (%)
m/z
S T P A I T L E S P D I K Y P L R
y12 y11 y10 y9 y8 y7 y6 y5 y4 y3 y2 y1
b2 a2 b3 b4 b5 b6 b7 b8 b9 b11
Modification
Acetyl (Protein N−term)
GlyGly (K) no new terminus
Oxidation (M)

20200921_KIZ_MUb4_GlyGly_10plex_2 − Scan number: 19314

Gene Names	Charge	m/z	Mass	Retention time	PEP	Score	Precursor Intensity
CYB5R3	3	672.3651	2014.073	75.796	1.693e−13	130.72	1553267

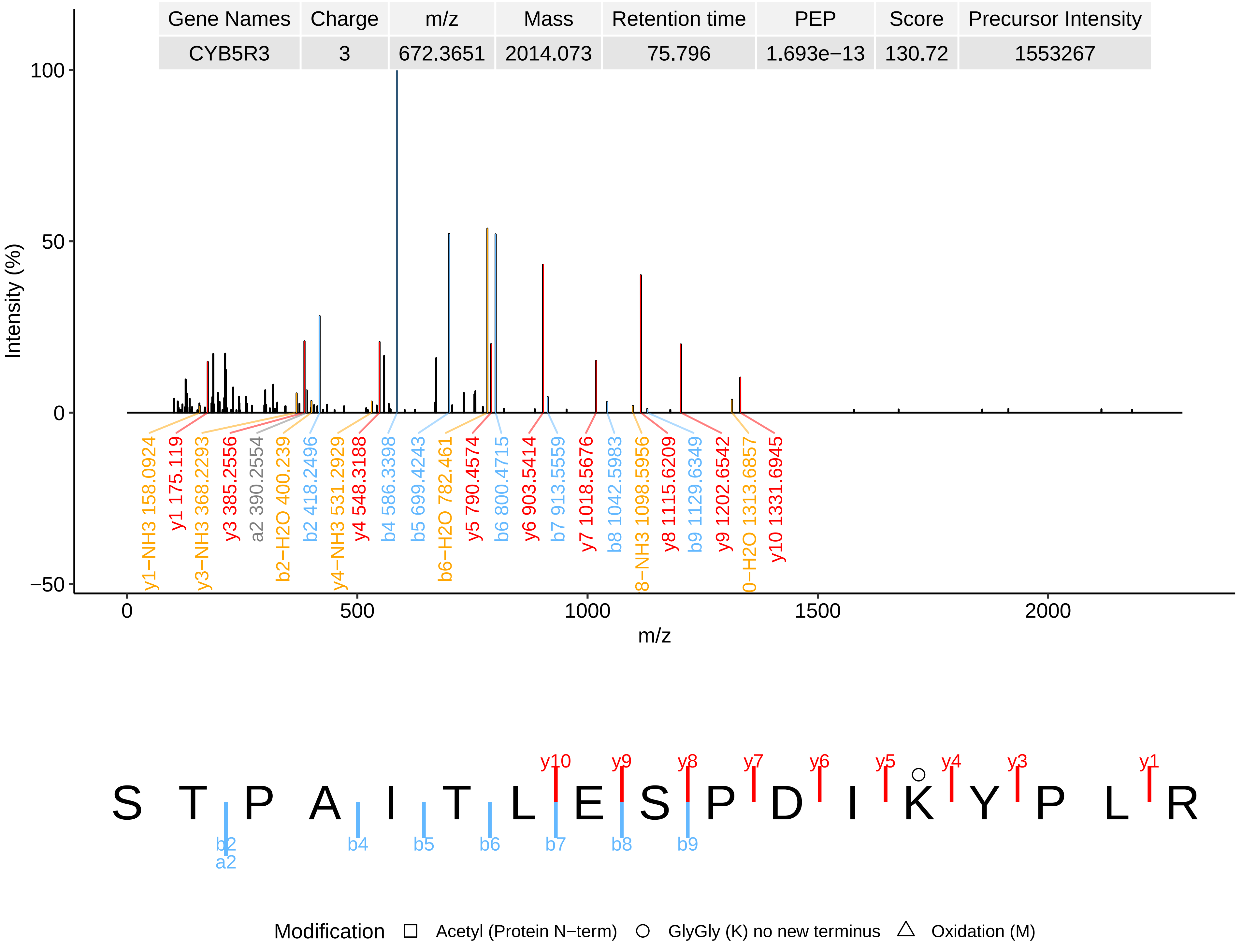

20200924_KIZ_MUb5_GlyGly_2 − Scan number: 19271

Gene Names	Charge	m/z	Mass	Retention time	PEP	Score	Precursor Intensity
CYB5R3	3	672.3651	2014.073	71.453	2.2652e−17	149.3	NA

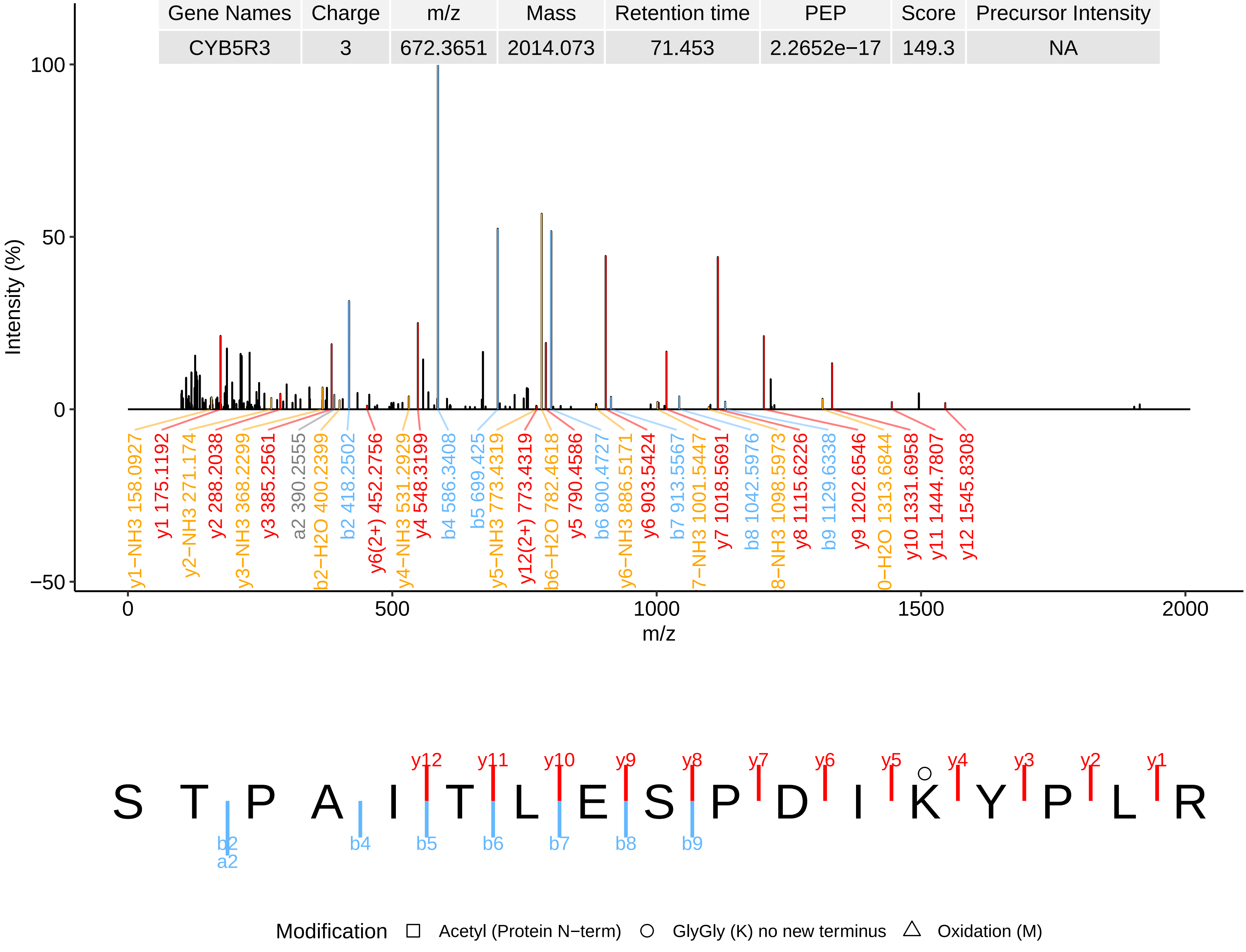

20201118_KIZ_MUb6_GlyGly_2 − Scan number: 23117

Gene Names	Charge	m/z	Mass	Retention time	PEP	Score	Precursor Intensity
CYB5R3	3	672.3651	2014.073	77.623	7.517e−48	205.27	9265192

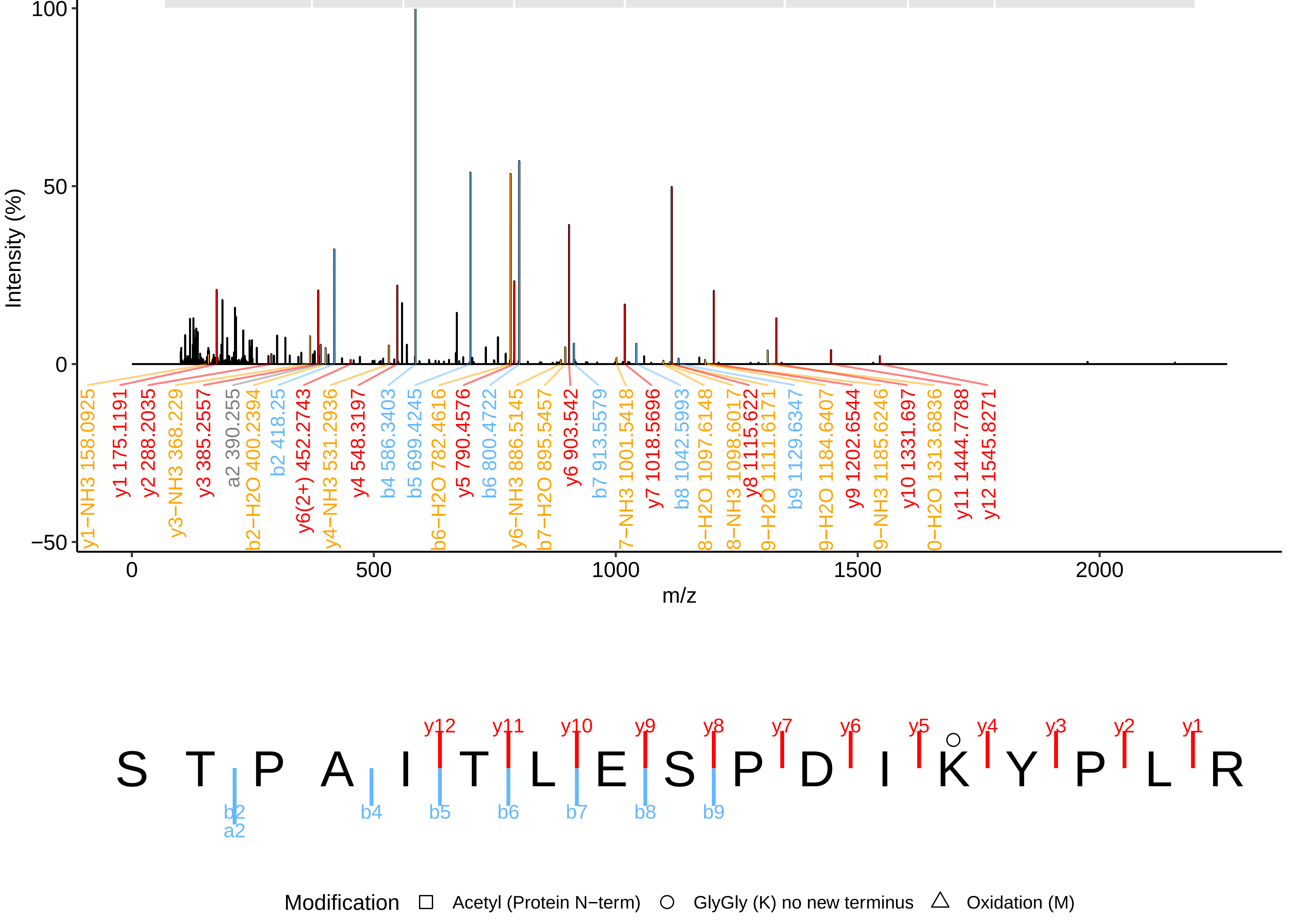

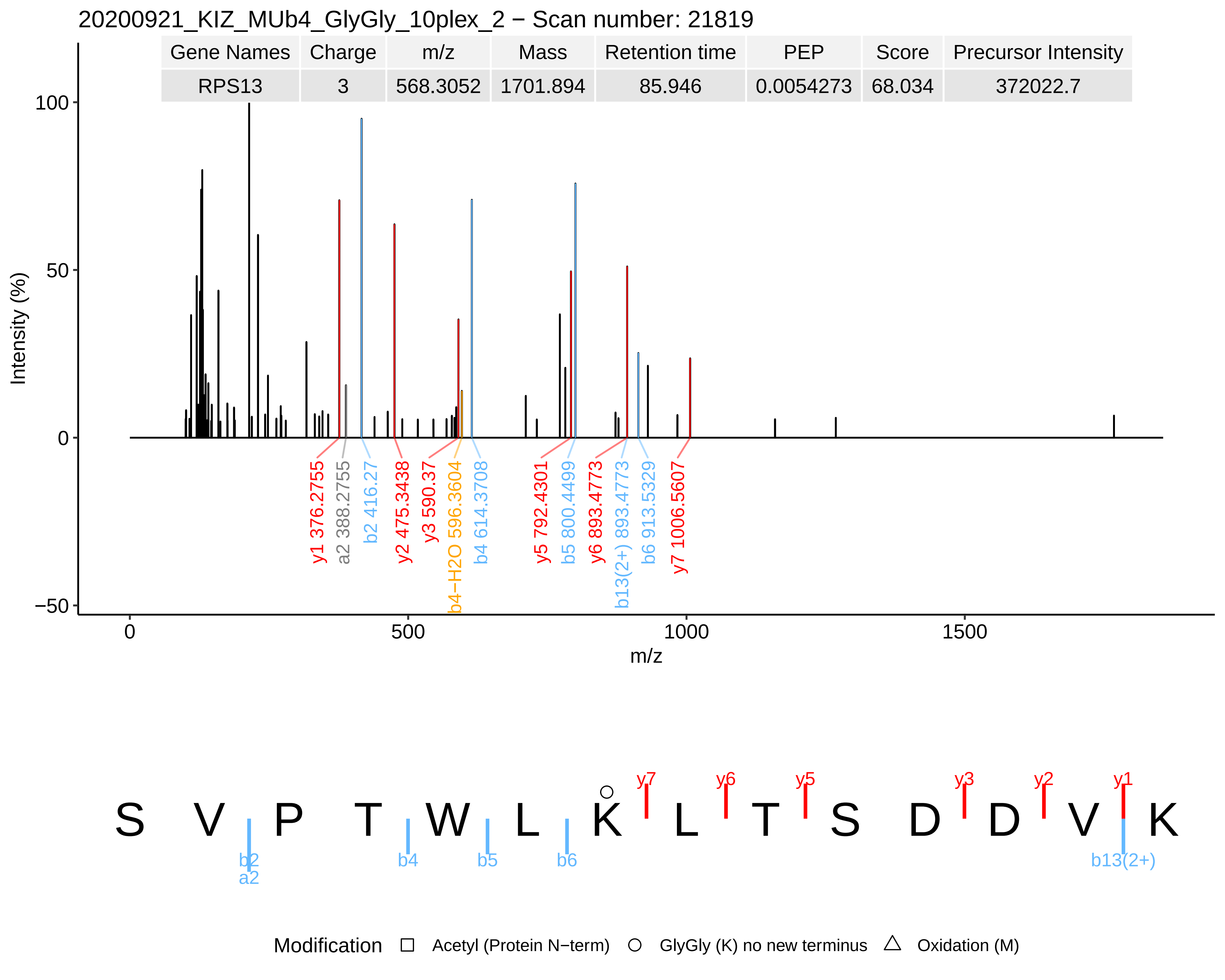
20200921_KIZ_MUb4_GlyGly_10plex_2 − Scan number: 21819
Gene Names
Charge
m/z
Mass
Retention time
PEP
Score
Precursor Intensity
RPS13
3
568.3052
1701.894
85.946
0.0054273
68.034
372022.7
Intensity (%)
100
50
0
−50
0
500
1000
1500
m/z
y1 376.2755
a2 388.2755
b2 416.27
y2 475.3438
y3 590.37
b4−H2O 596.3604
b4 614.3708
y5 792.4301
b5 800.4499
y6 893.4773
b13(2+) 893.4773
b6 913.5329
y7 1006.5607
S V P T W L K L T S D D V K
y7
y6
y5
y3
y2
y1
b2
a2
b4
b5
b6
b13(2+)
Modification
Acetyl (Protein N−term)
GlyGly (K) no new terminus
Oxidation (M)

20200921_KIZ_MUb4_GlyGly_10plex_2 − Scan number: 16491

Gene Names	Charge	m/z	Mass	Retention time	PEP	Score	Precursor Intensity
XRCC6	3	641.3374	1920.991	64.381	0.00069386	79.97	1020548

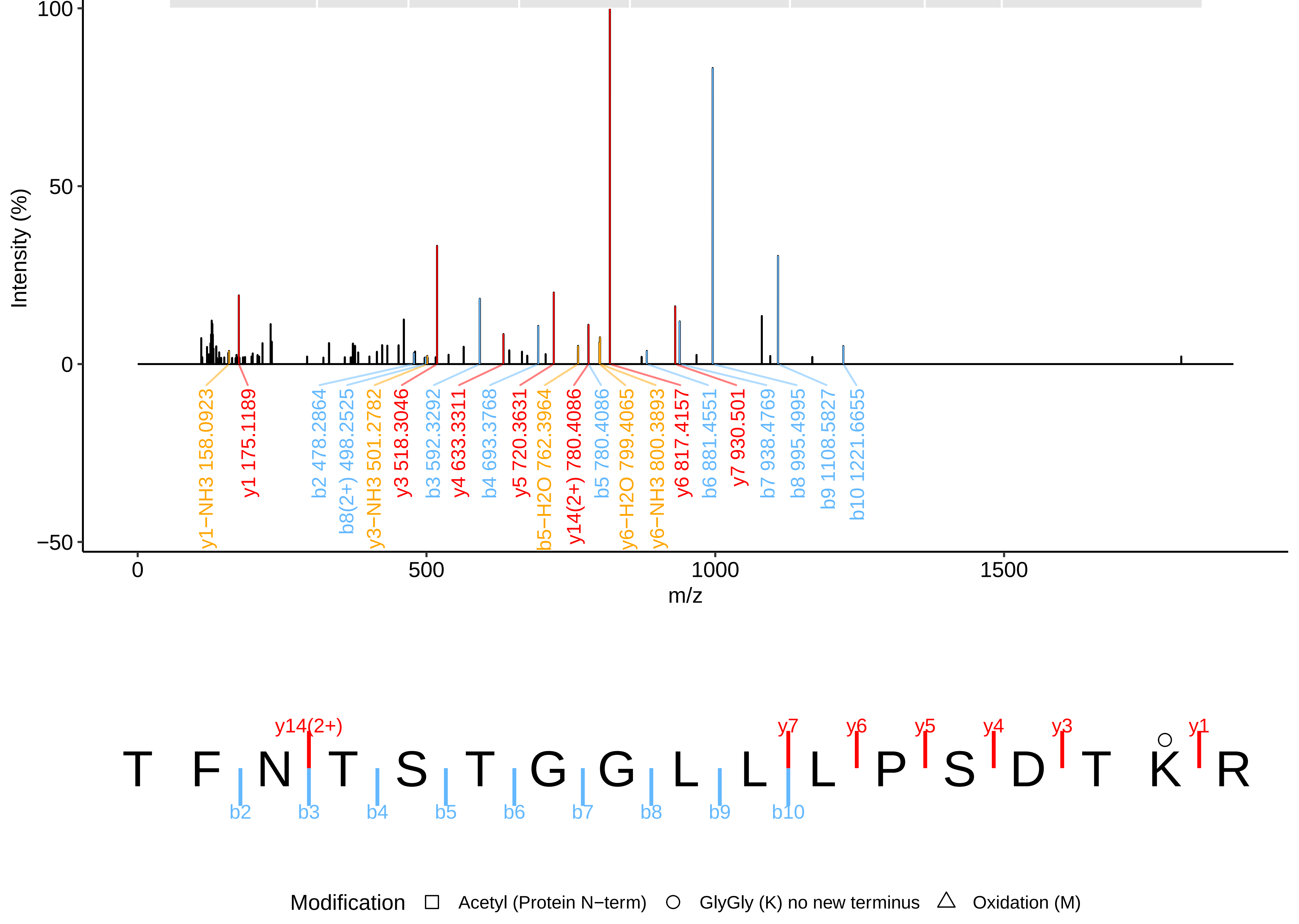

20200921_KIZ_MUb4_GlyGly_10plex − Scan number: 8146

Gene Names	Charge	m/z	Mass	Retention time	PEP	Score	Precursor Intensity
CYCS	3	595.6236	1783.849	27.565	1.1433e−05	87.138	6038648

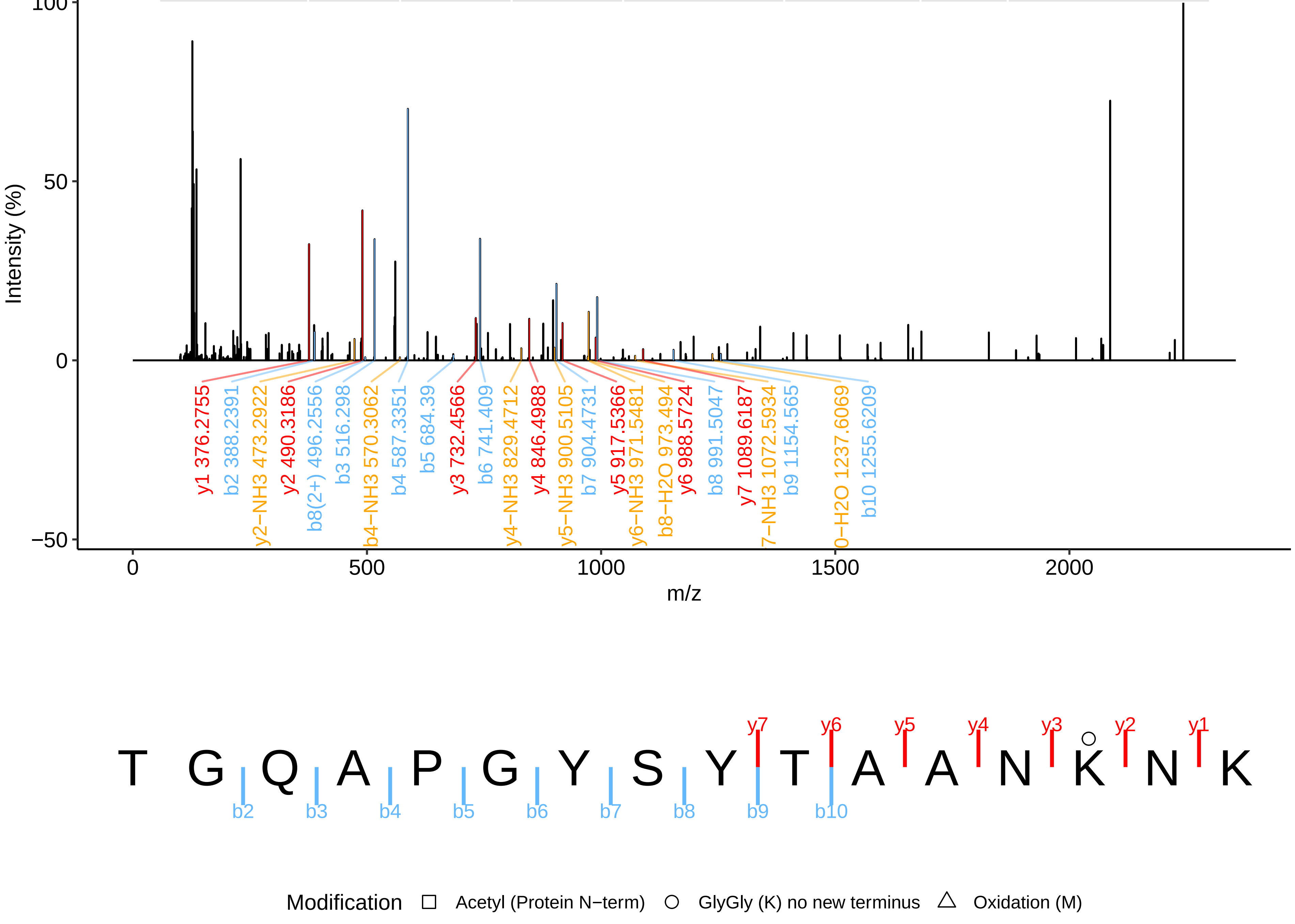

20200921_KIZ_MUb4_GlyGly_10plex − Scan number: 8167

Gene Names	Charge	m/z	Mass	Retention time	PEP	Score	Precursor Intensity
CYCS	3	595.6236	1783.849	27.642	0.0022508	67.846	15009697

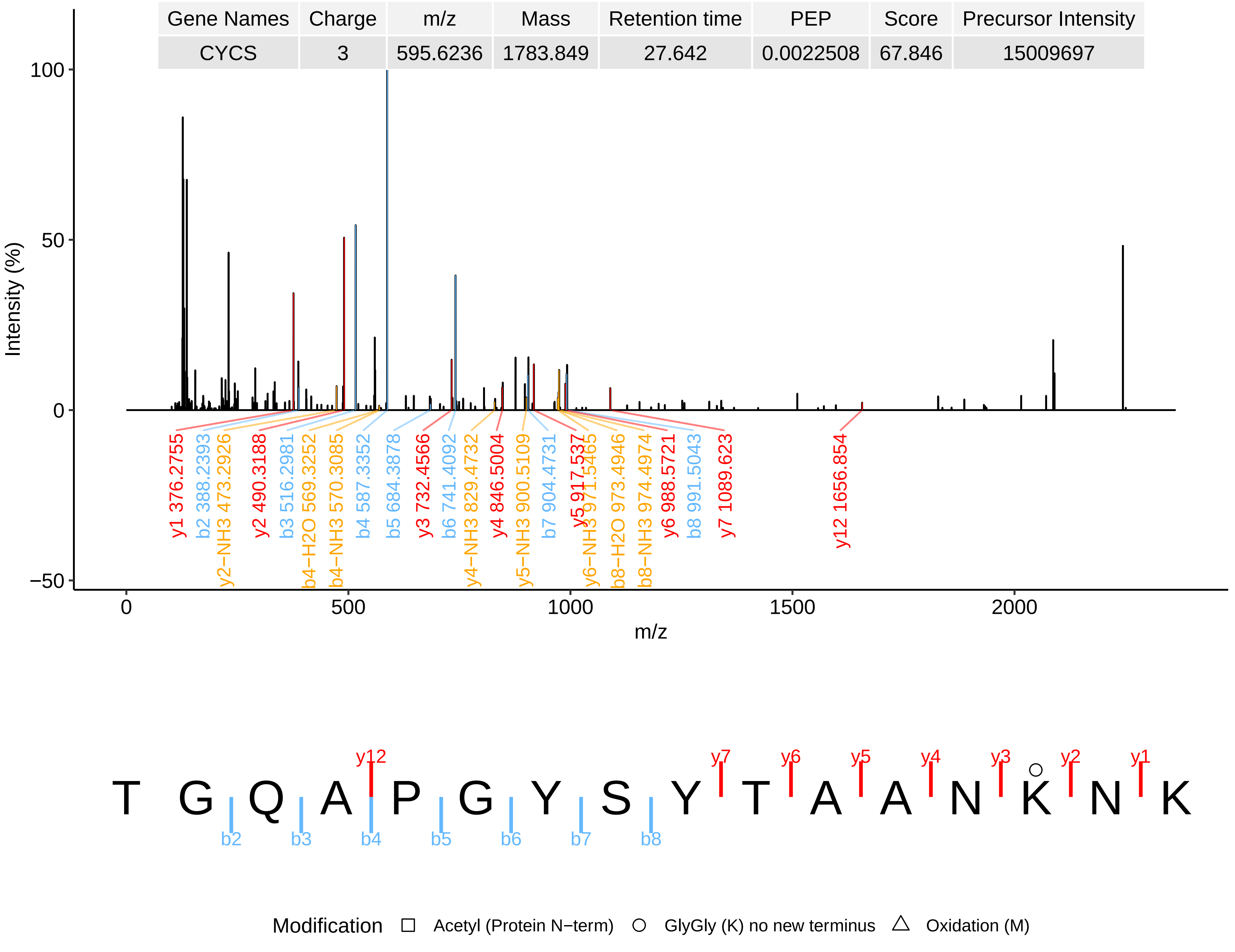

20200921_KIZ_MUb4_GlyGly_10plex_2 − Scan number: 7572

Gene Names	Charge	m/z	Mass	Retention time	PEP	Score	Precursor Intensity
CYCS	3	595.6236	1783.849	28.301	0.012949	61.097	1133076

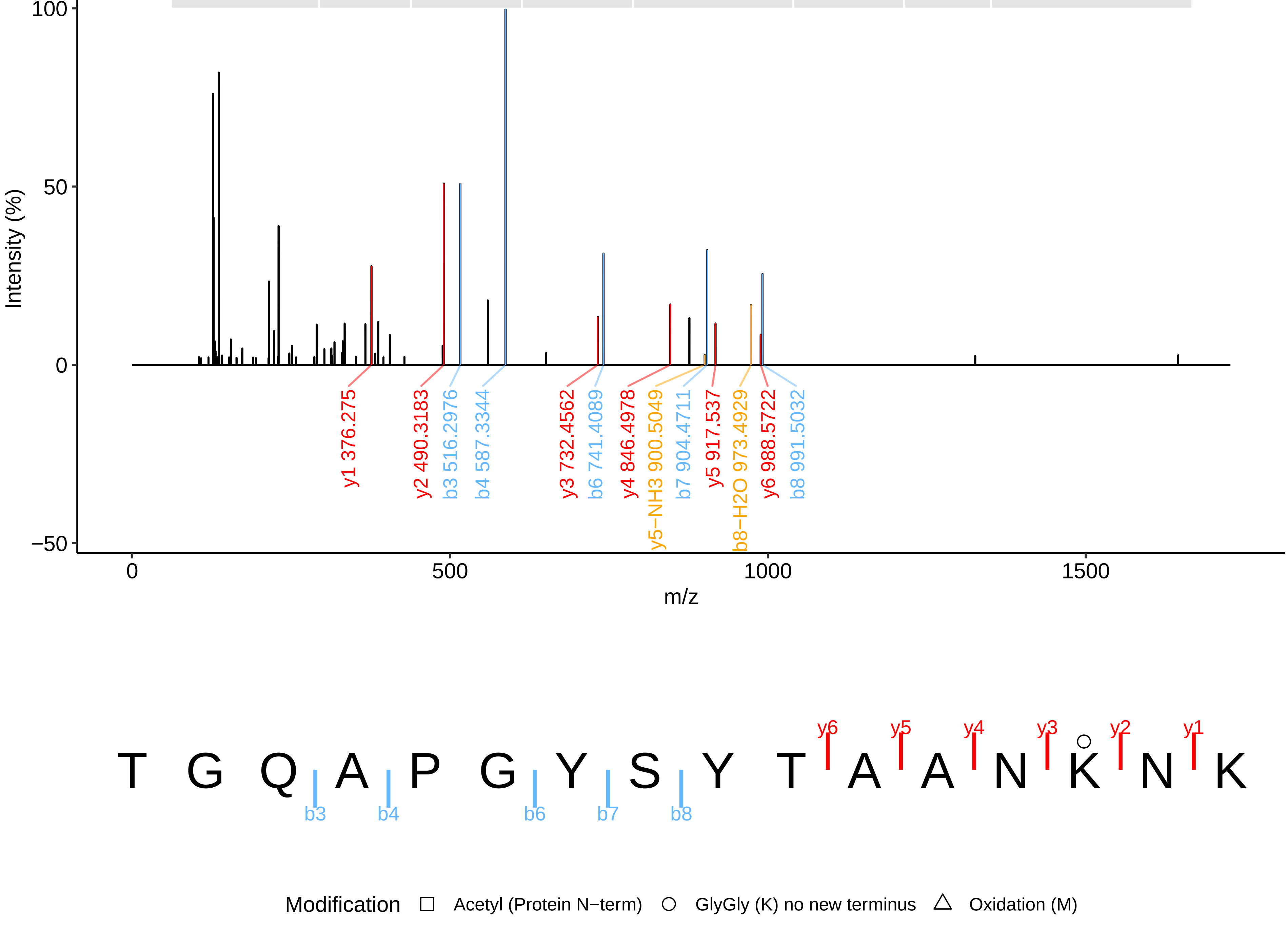

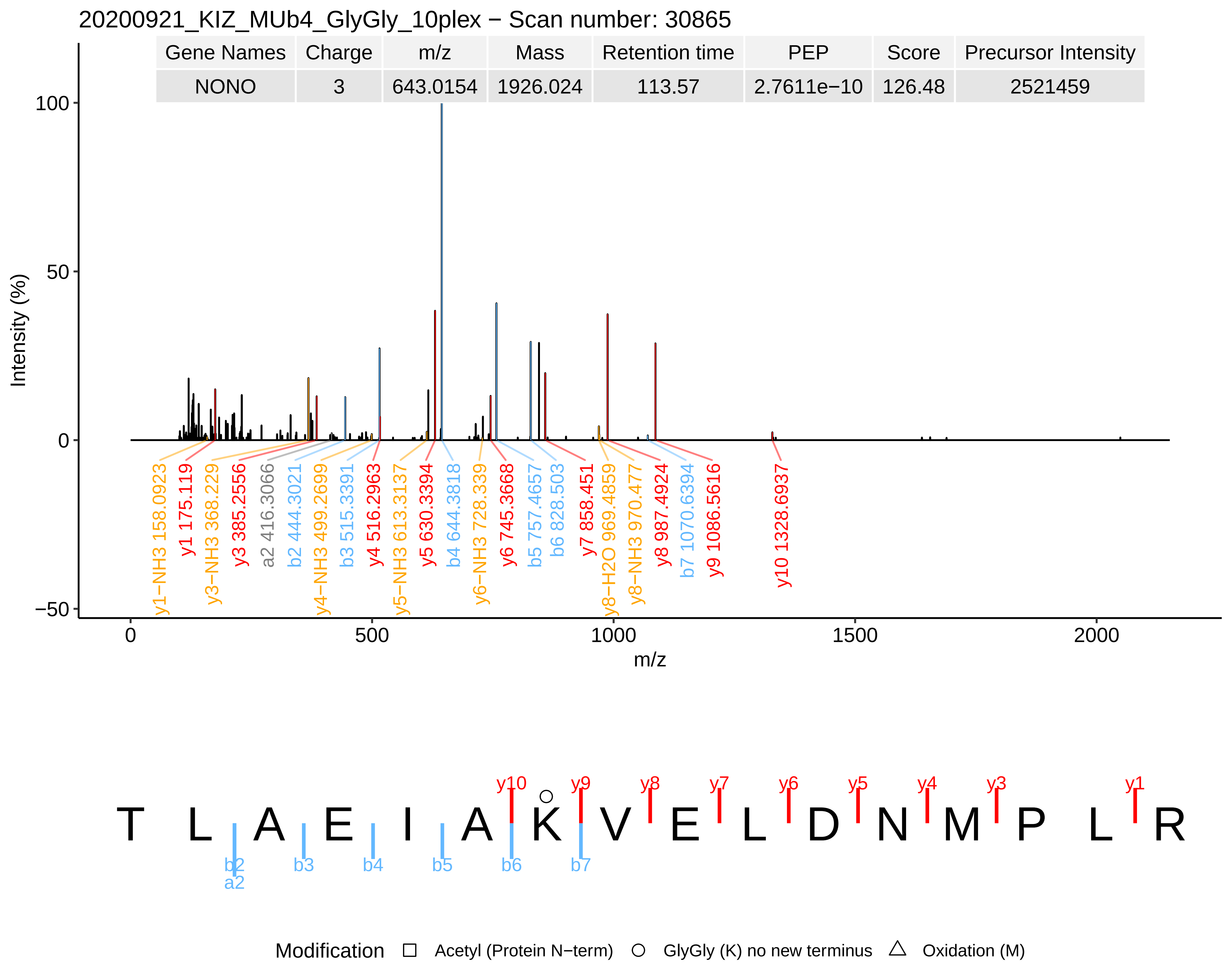

20200921_KIZ_MUb4_GlyGly_10plex − Scan number: 30865
Gene Names | Charge | m/z | Mass | Retention time | PEP | Score | Precursor Intensity
NONO | 3 | 643.0154 | 1926.024 | 113.57 | 2.7611e−10 | 126.48 | 2521459
Intensity (%)
m/z
y1−NH3 158.0923
y1 175.119
y3−NH3 368.229
y3 385.2556
a2 416.3066
b2 444.3021
y4−NH3 499.2699
b3 515.3391
y4 516.2963
y5−NH3 613.3137
y5 630.3394
b4 644.3818
y6−NH3 728.339
y6 745.3668
b5 757.4657
b6 828.503
y7 858.451
y8−H2O 969.4859
y8−NH3 970.477
y8 987.4924
b7 1070.6394
y9 1086.5616
y10 1328.6937
T L A E I A K V E L D N M P L R
Modification
Acetyl (Protein N−term)
GlyGly (K) no new terminus
Oxidation (M)

20200921_KIZ_MUb4_GlyGly_10plex_2 − Scan number: 14181

Gene Names	Charge	m/z	Mass	Retention time	PEP	Score	Precursor Intensity
UBE4B	3	568.9444	1703.812	55.235	0.0082607	67.456	713837.6

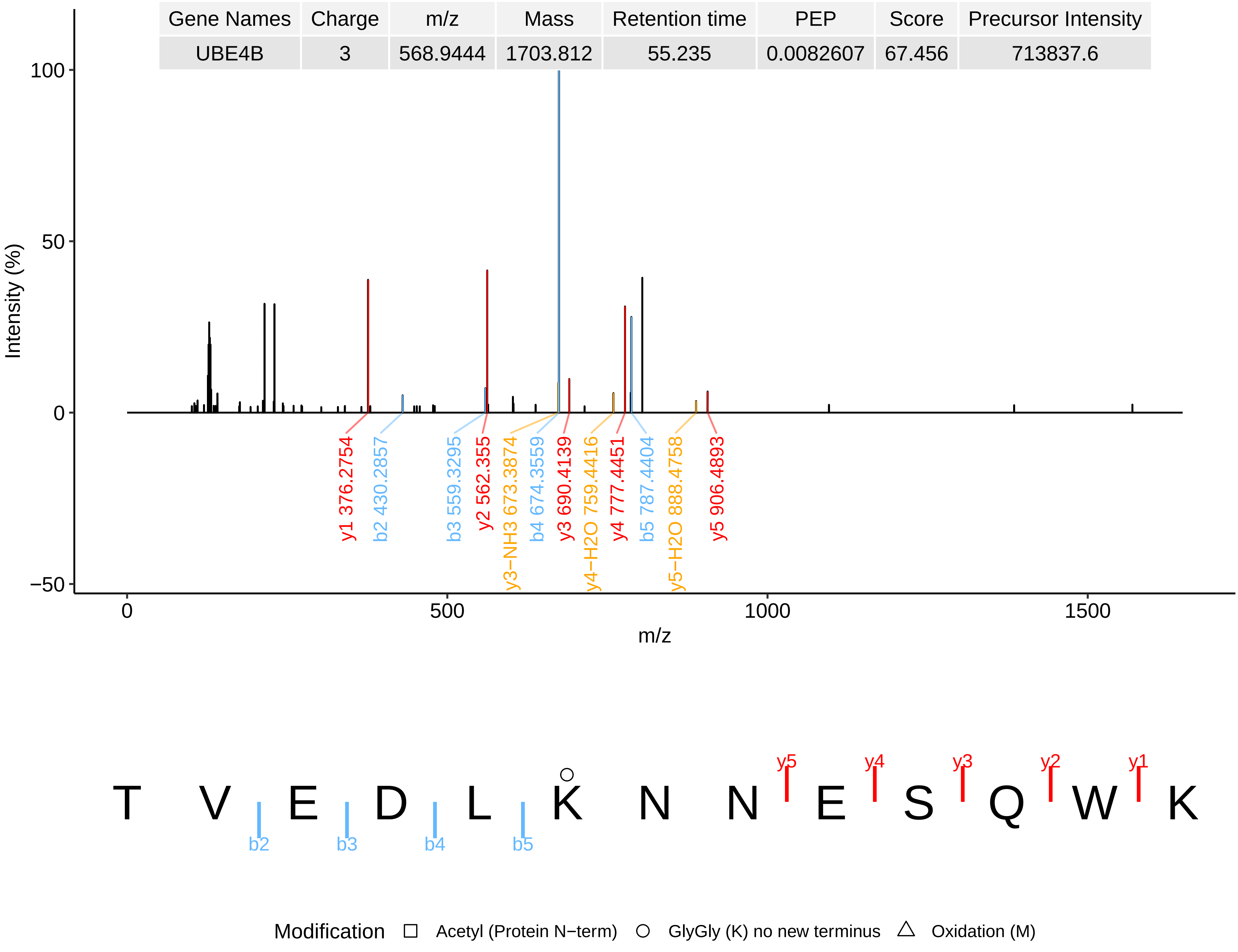

20200921_KIZ_MUb4_GlyGly_10plex − Scan number: 12559

Gene Names	Charge	m/z	Mass	Retention time	PEP	Score	Precursor Intensity
PSMB2	4	446.9703	1783.852	44.085	4.0437e−06	98.439	8047896

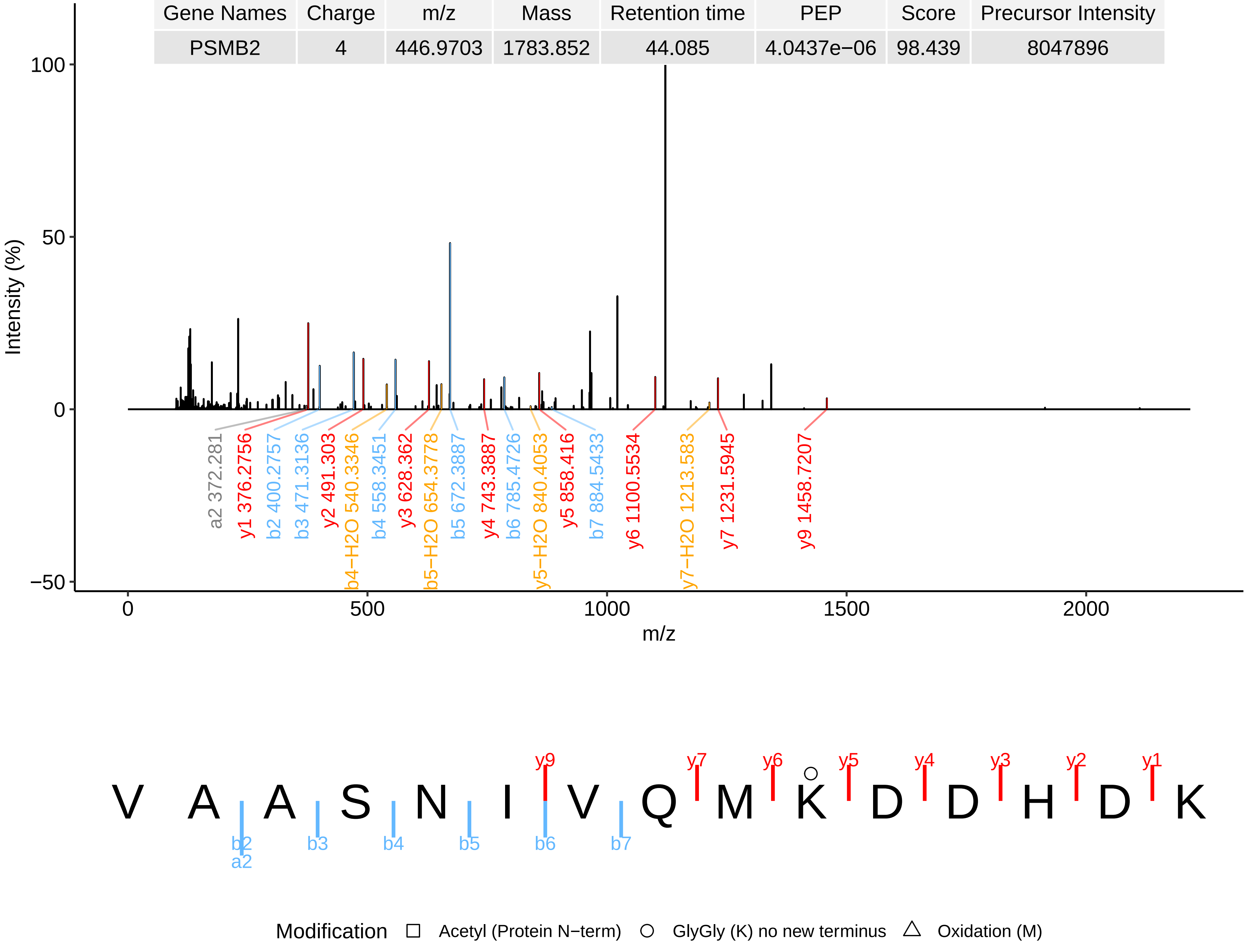

20200921_KIZ_MUb4_GlyGly_10plex_2 − Scan number: 12075

Gene Names	Charge	m/z	Mass	Retention time	PEP	Score	Precursor Intensity
TRIM28	3	643.3326	1926.976	46.639	5.9735e−06	92.528	1857141

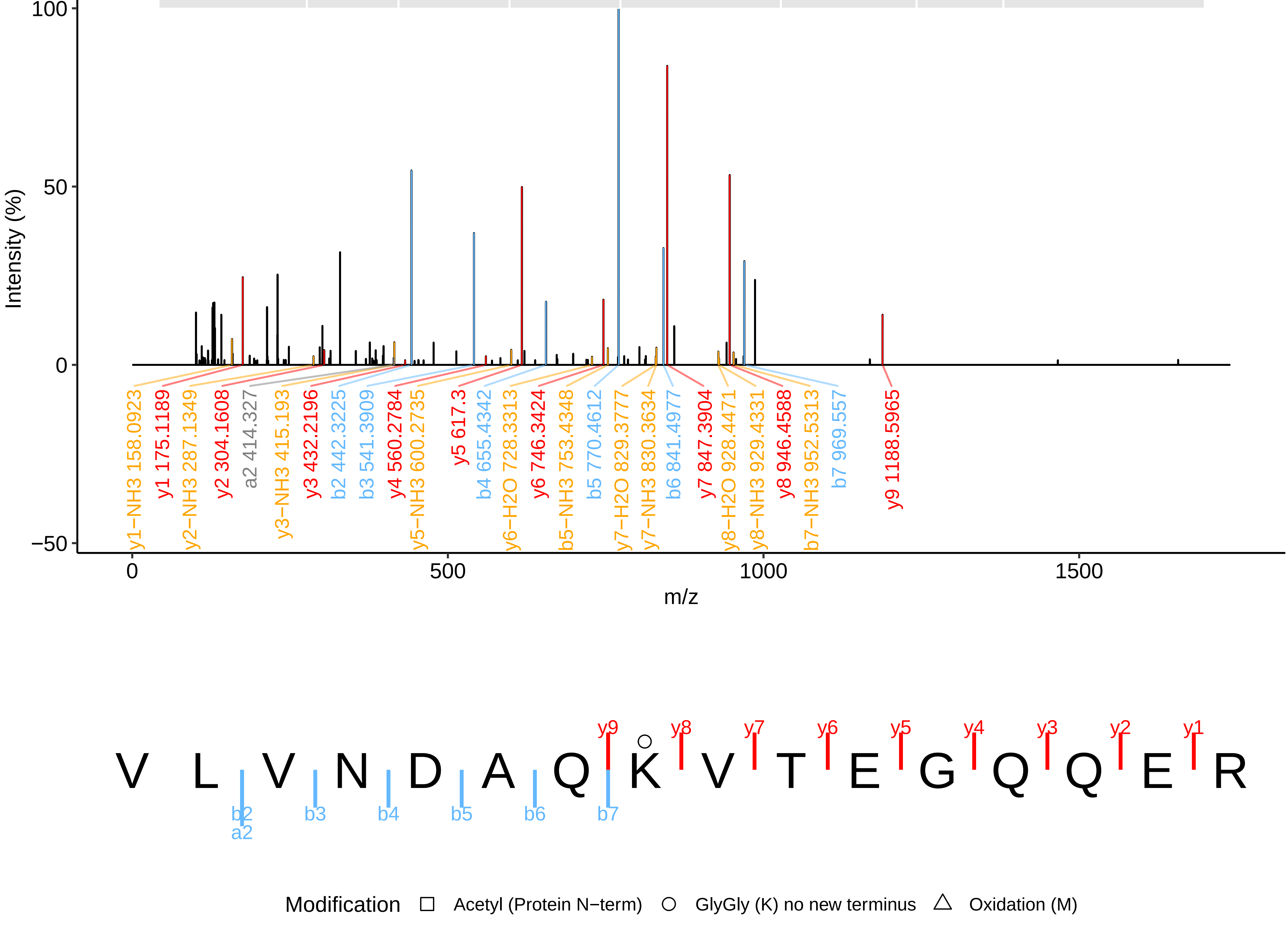

20200924_KIZ_MUb5_GlyGly_2 − Scan number: 11300

Gene Names	Charge	m/z	Mass	Retention time	PEP	Score	Precursor Intensity
TRIM28	3	643.3326	1926.976	42.218	0.0057231	68.257	1053623

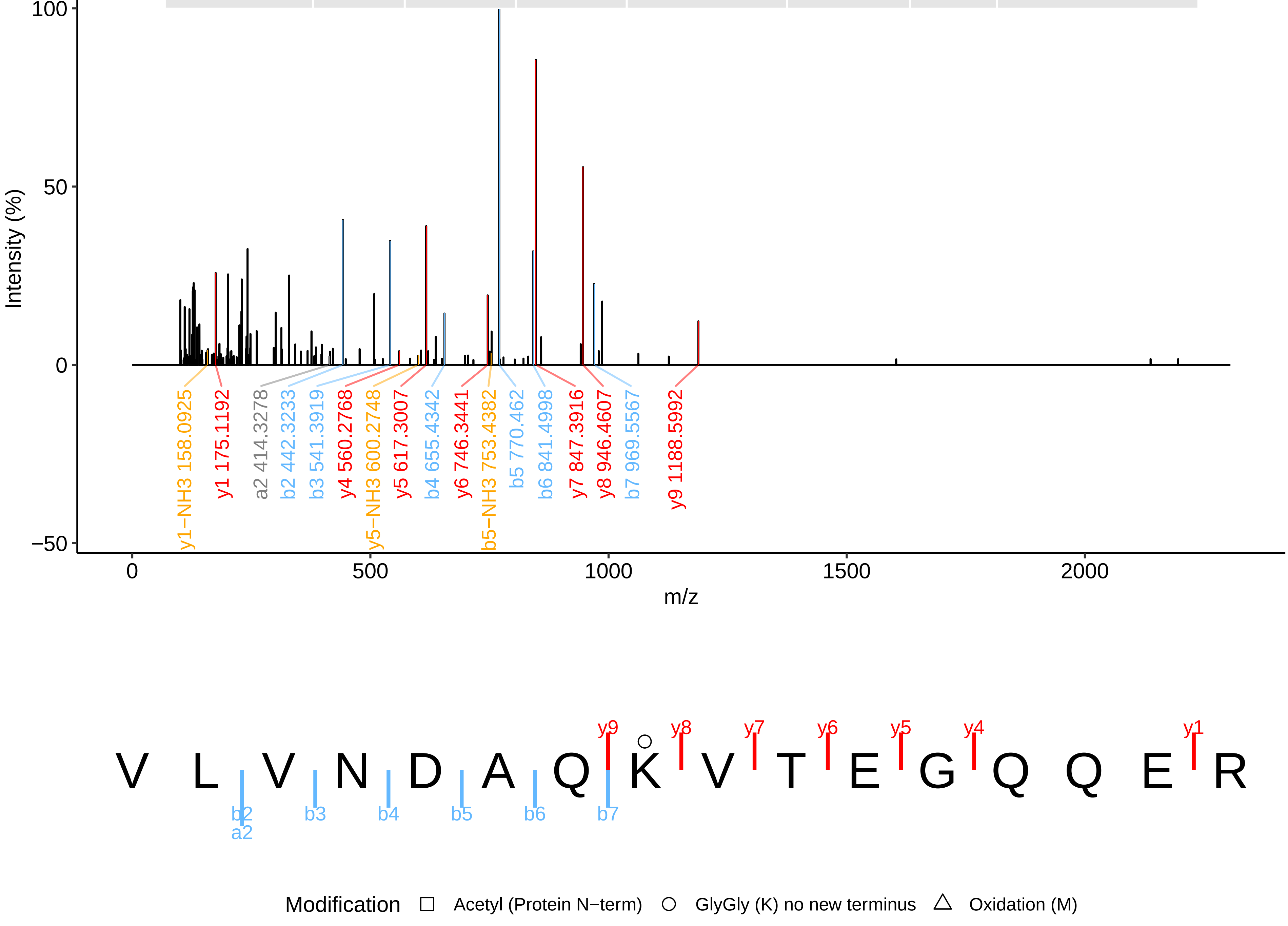

20200921_KIZ_MUb4_GlyGly_10plex_2 − Scan number: 20985

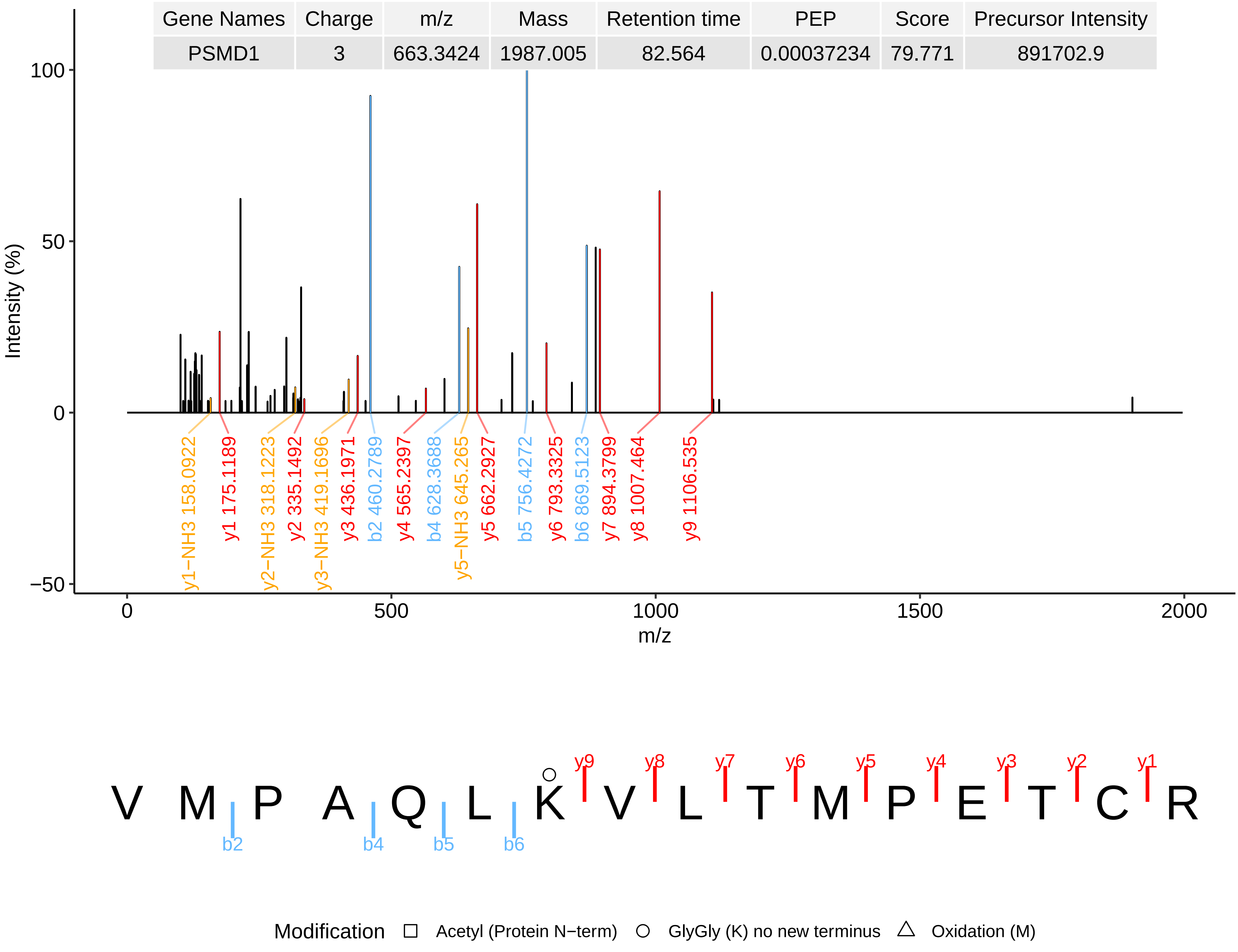

20200921_KIZ_MUb4_GlyGly_10plex_2 − Scan number: 15614

Gene Names	Charge	m/z	Mass	Retention time	PEP	Score	Precursor Intensity
KIDINS220	3	564.9577	1691.851	61.099	0.10112	46.319	644645.1

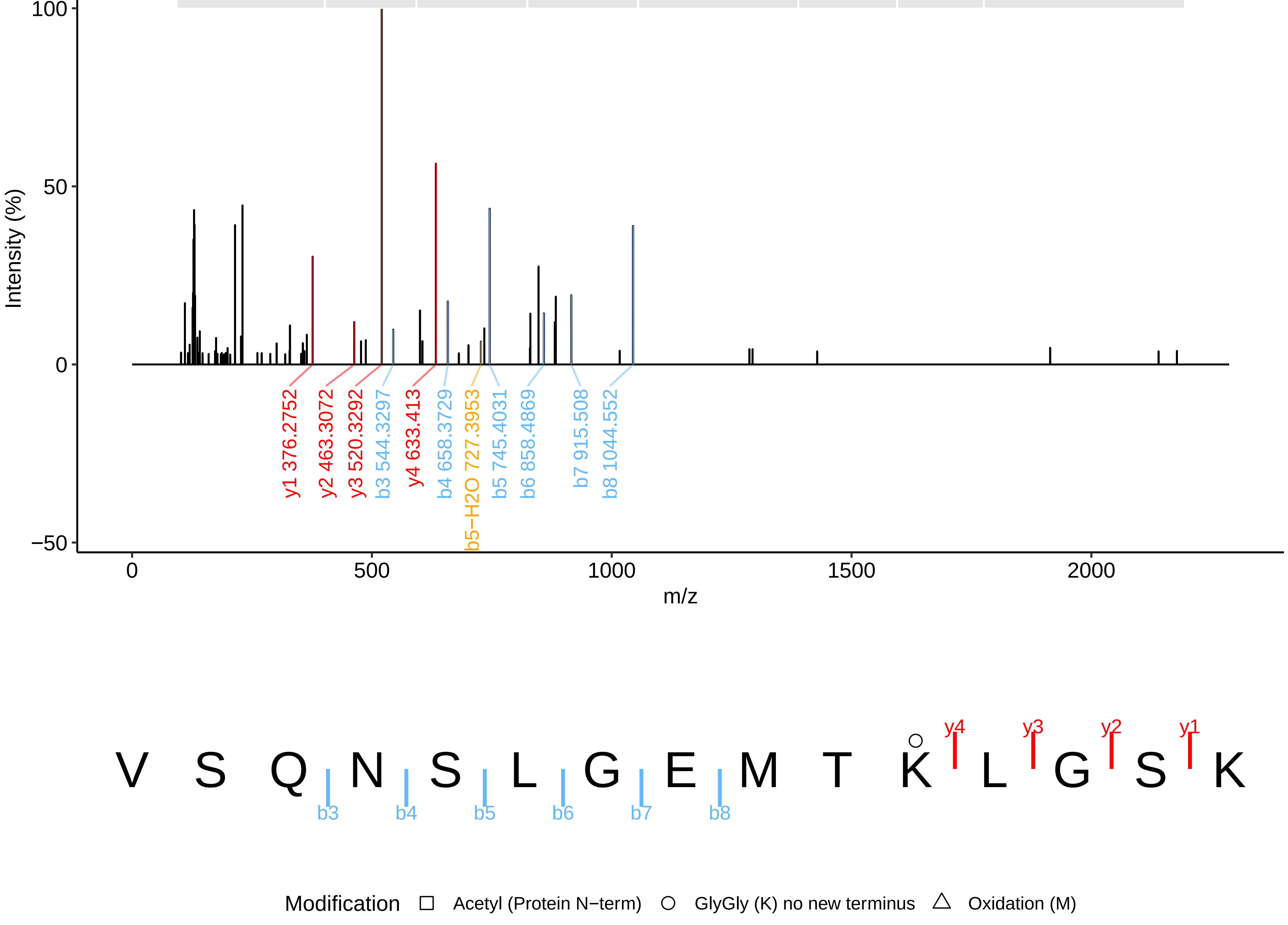

20200921_KIZ_MUb4_GlyGly_10plex_2 − Scan number: 19717

Gene Names	Charge	m/z	Mass	Retention time	PEP	Score	Precursor Intensity
TXNDC17	3	577.3331	1728.977	77.422	0.021551	59.607	746877

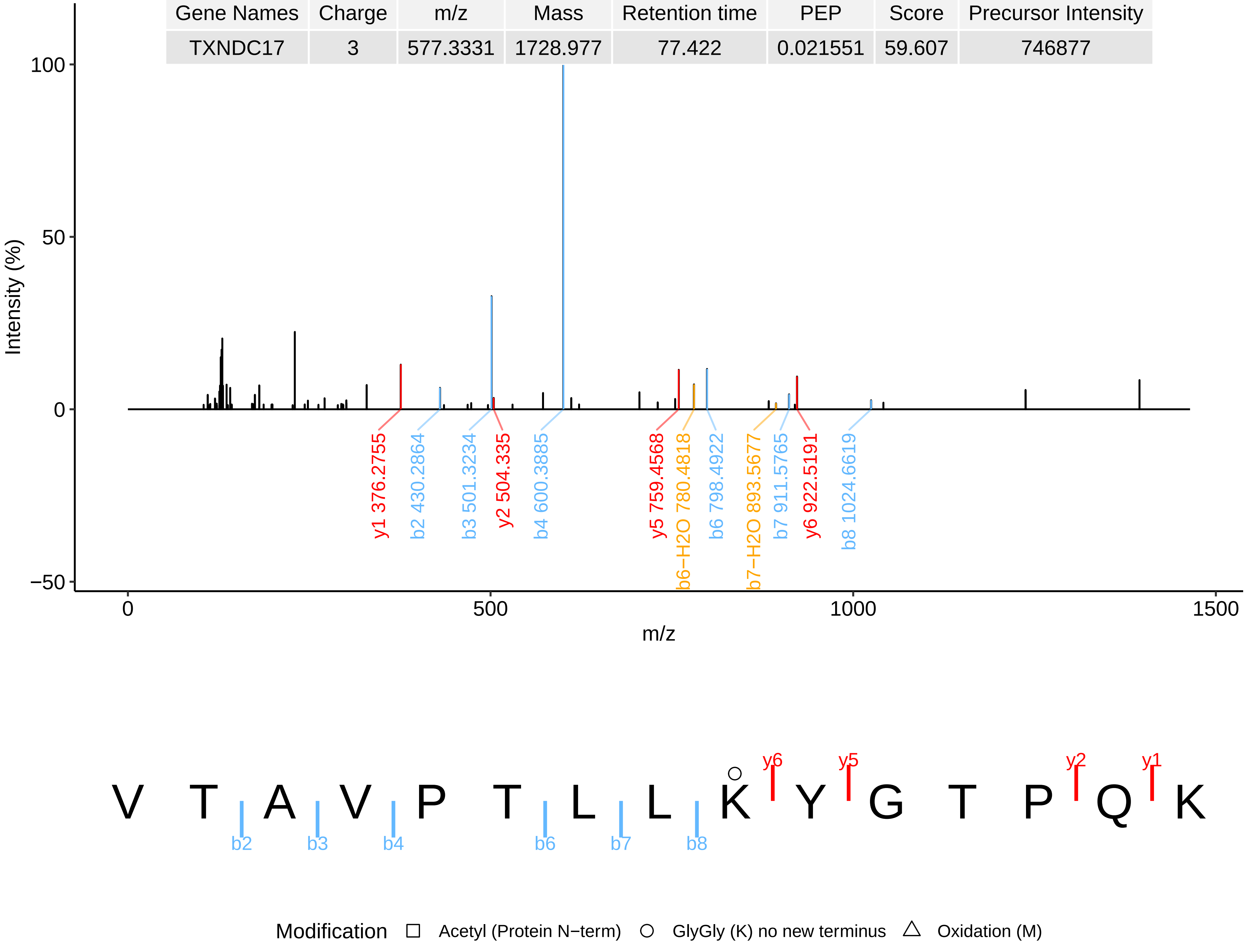

20200921_KIZ_MUb4_GlyGly_10plex − Scan number: 13474

Gene Names	Charge	m/z	Mass	Retention time	PEP	Score	Precursor Intensity
SRSF3	3	664.3362	1989.987	47.557	3.4363e−30	171.81	4549838

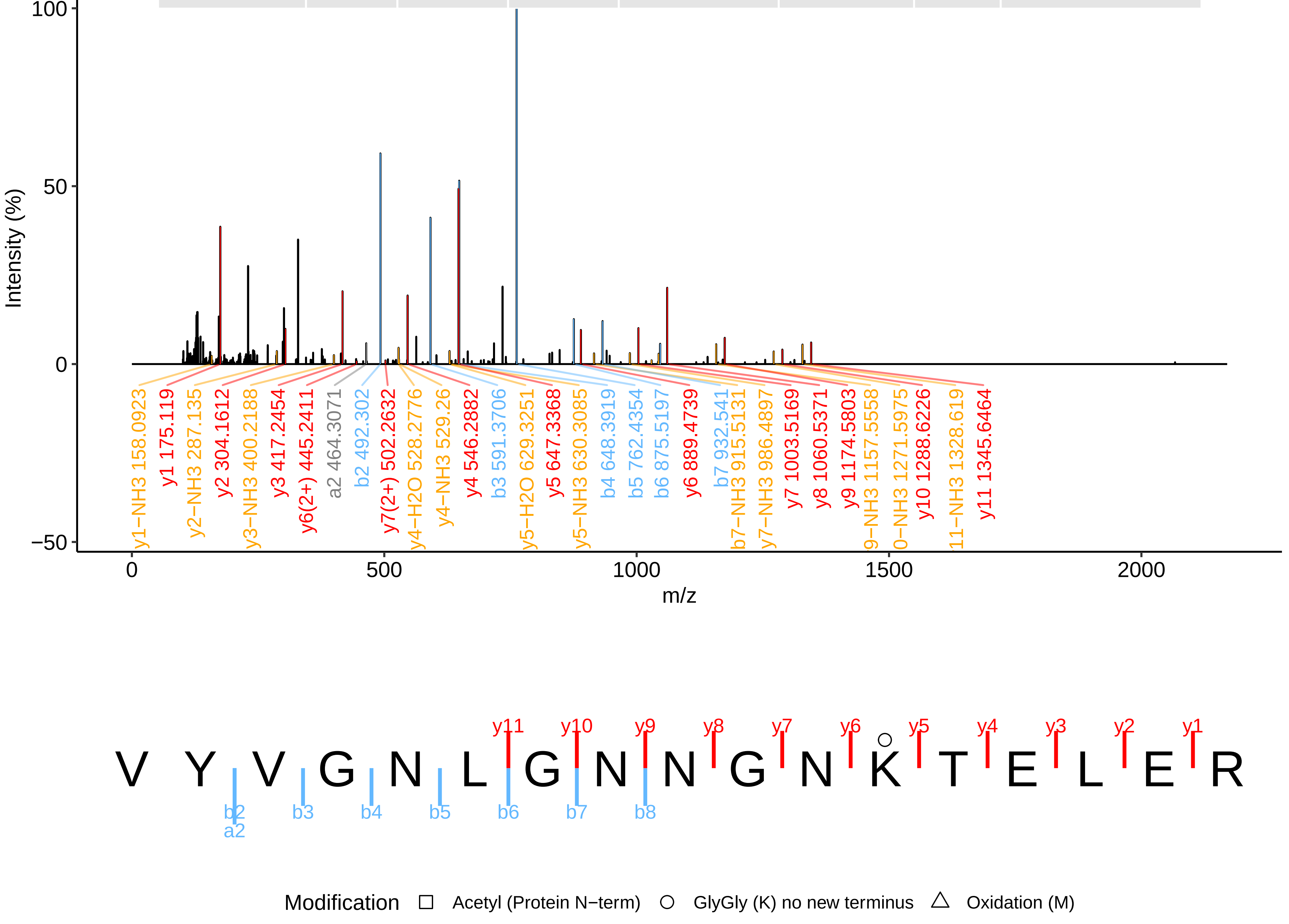

20200921_KIZ_MUb4_GlyGly_10plex_2 − Scan number: 12397

Gene Names	Charge	m/z	Mass	Retention time	PEP	Score	Precursor Intensity
SRSF3	3	664.3362	1989.987	47.952	1.2405e−12	117.37	936384.4

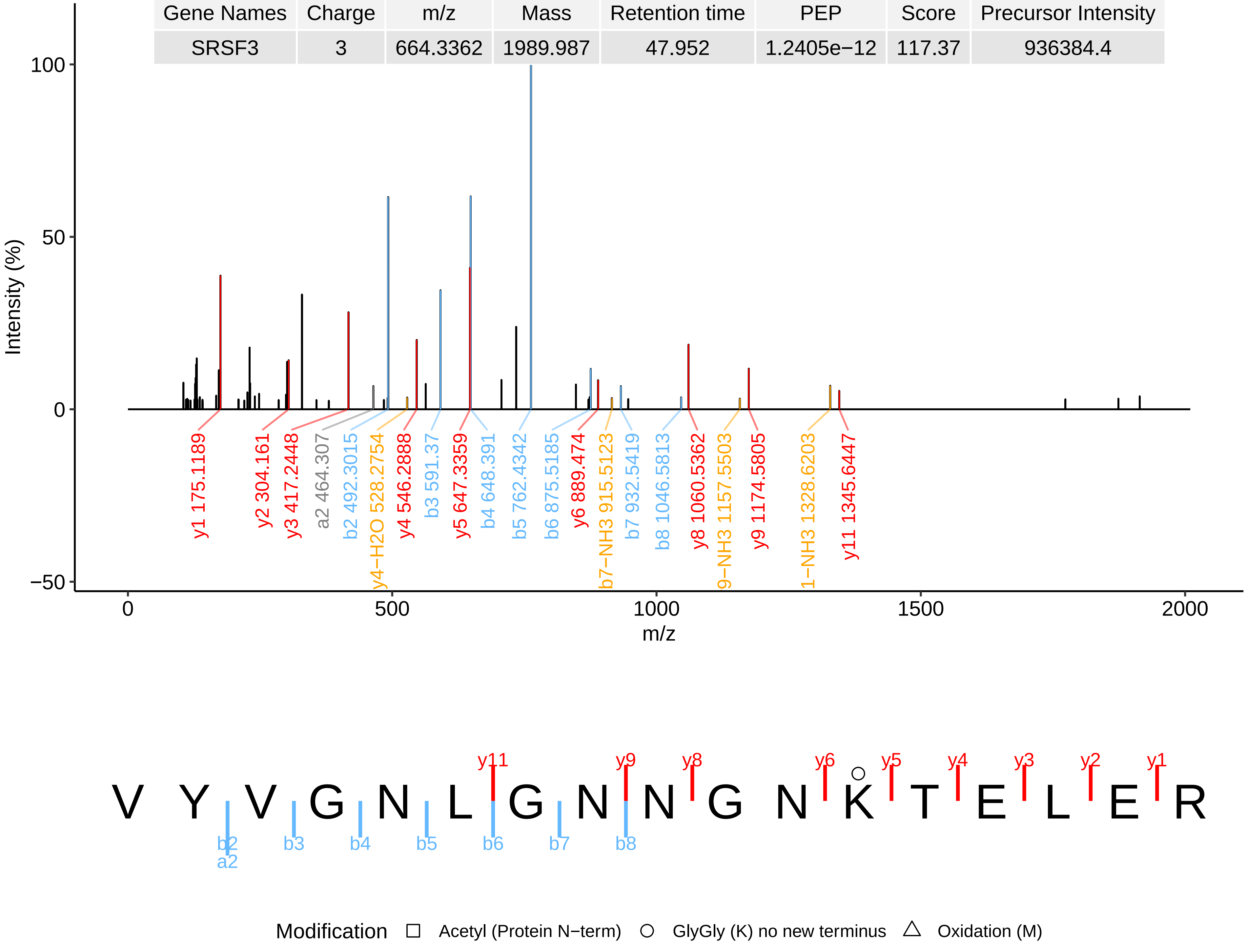

20200921_KIZ_MUb4_GlyGly_10plex − Scan number: 26669

Gene Names	Charge	m/z	Mass	Retention time	PEP	Score	Precursor Intensity
PRPF8	3	621.9977	1862.971	97.196	0.00024277	79.022	4139455

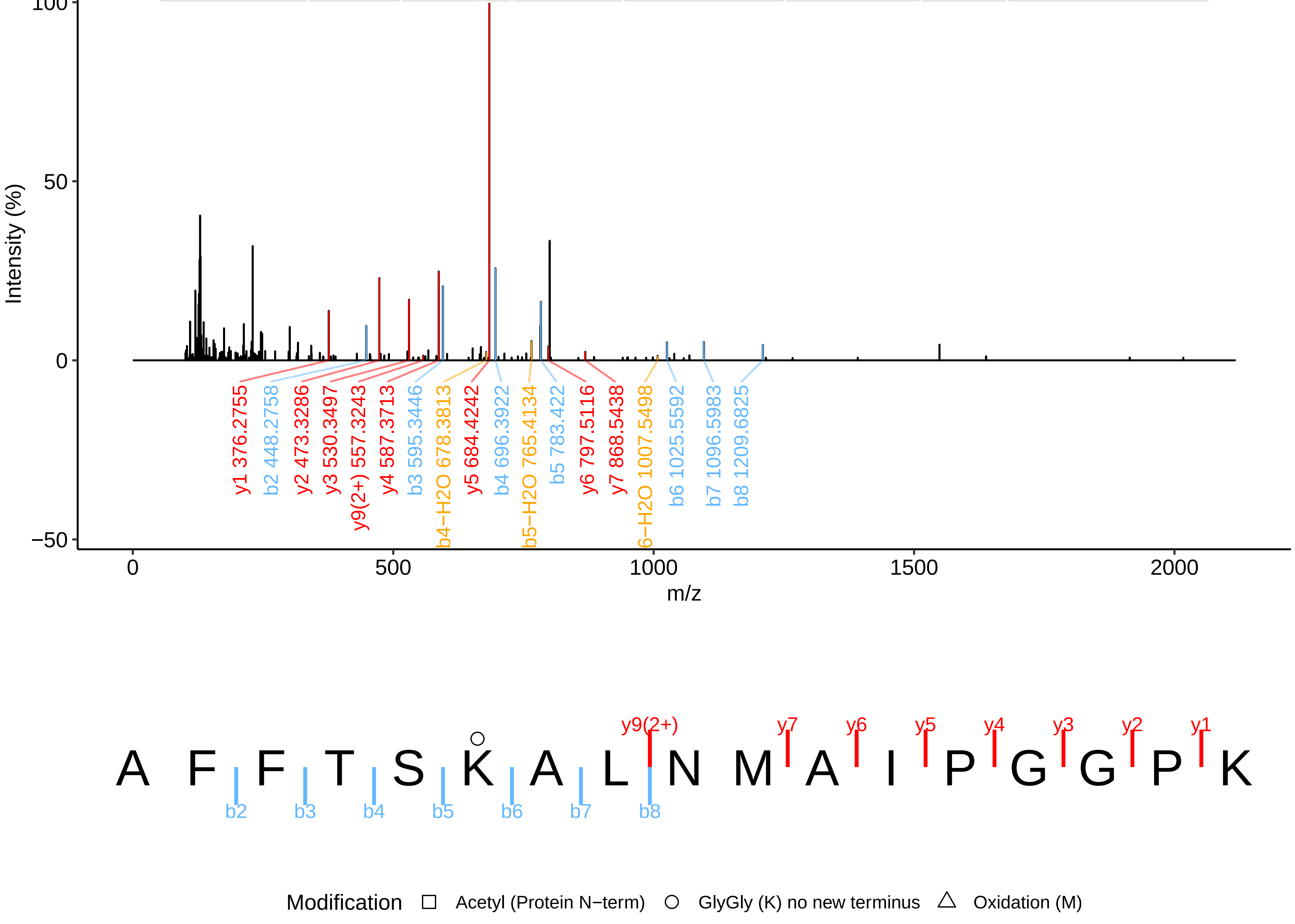

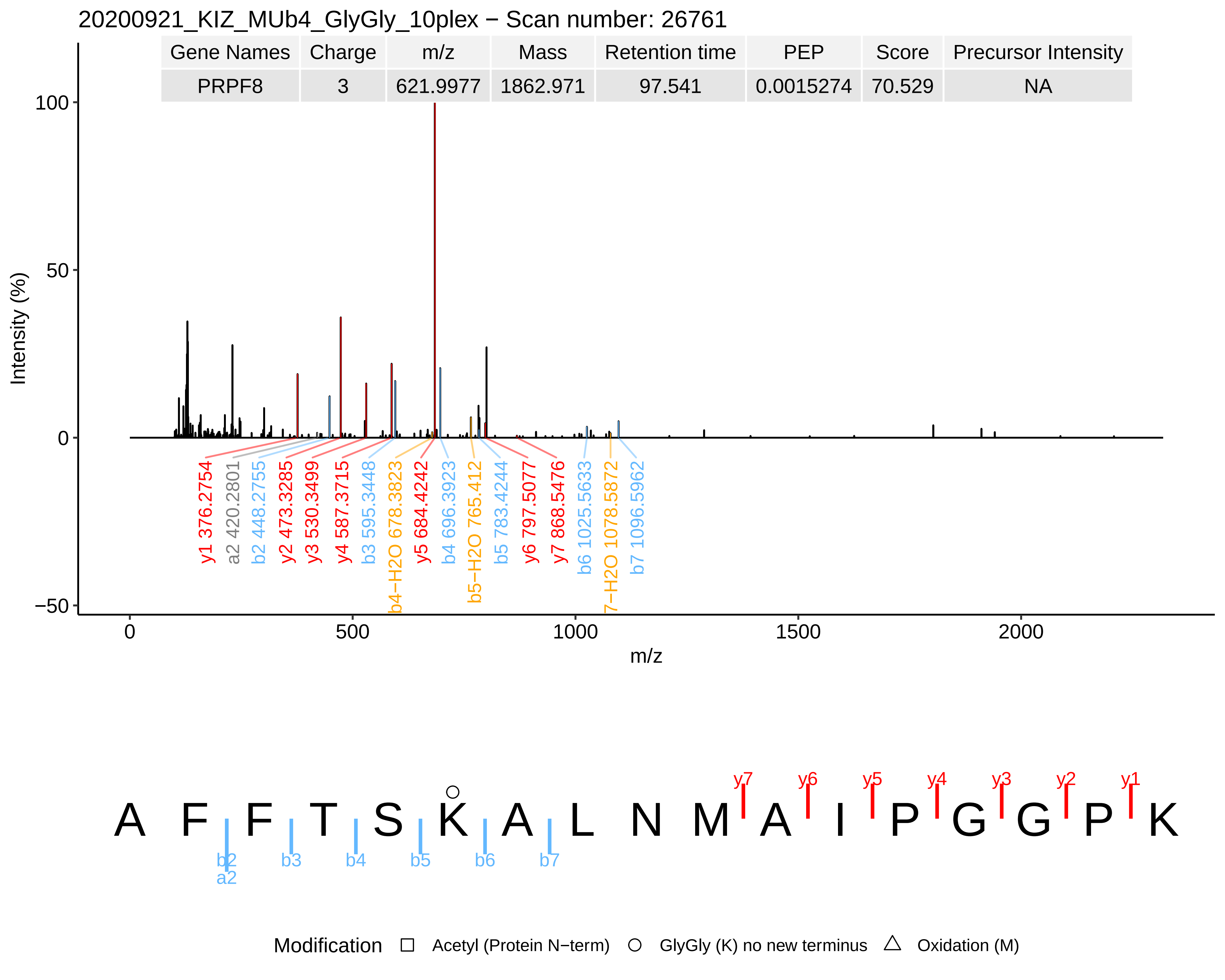
20200921_KIZ_MUb4_GlyGly_10plex − Scan number: 26761
Gene Names | Charge | m/z | Mass | Retention time | PEP | Score | Precursor Intensity
PRPF8 | 3 | 621.9977 | 1862.971 | 97.541 | 0.0015274 | 70.529 | NA
Intensity (%)
m/z
y1 376.2754
a2 420.2801
b2 448.2755
y2 473.3285
y3 530.3499
y4 587.3715
b3 595.3448
b4−H2O 678.3823
y5 684.4242
b4 696.3923
b5−H2O 765.412
b5 783.4244
y6 797.5077
y7 868.5476
b6 1025.5633
7−H2O 1078.5872
b7 1096.5962
A F F T S K A L N M A I P G G P K
Modification
Acetyl (Protein N−term)
GlyGly (K) no new terminus
Oxidation (M)

20200921_KIZ_MUb4_GlyGly_10plex_2 − Scan number: 11399

Gene Names	Charge	m/z	Mass	Retention time	PEP	Score	Precursor Intensity
RPS20	4	567.046	2264.155	43.868	0.029454	57.499	544143.8

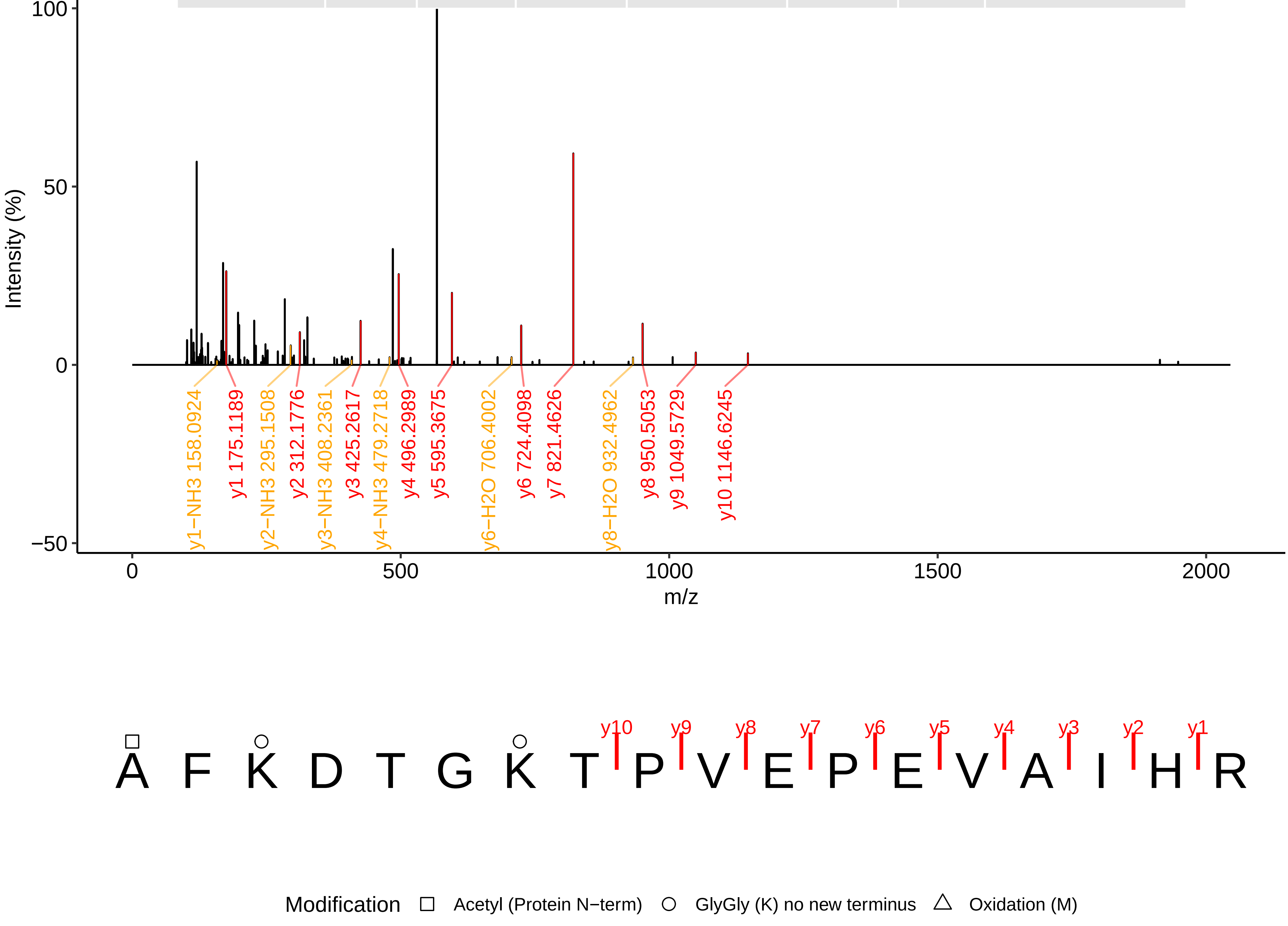

20200921_KIZ_MUb4_GlyGly_10plex − Scan number: 9725

Gene Names	Charge	m/z	Mass	Retention time	PEP	Score	Precursor Intensity
PPAP2B	3	683.6872	2048.04	33.4	0.0011306	71.685	3816625

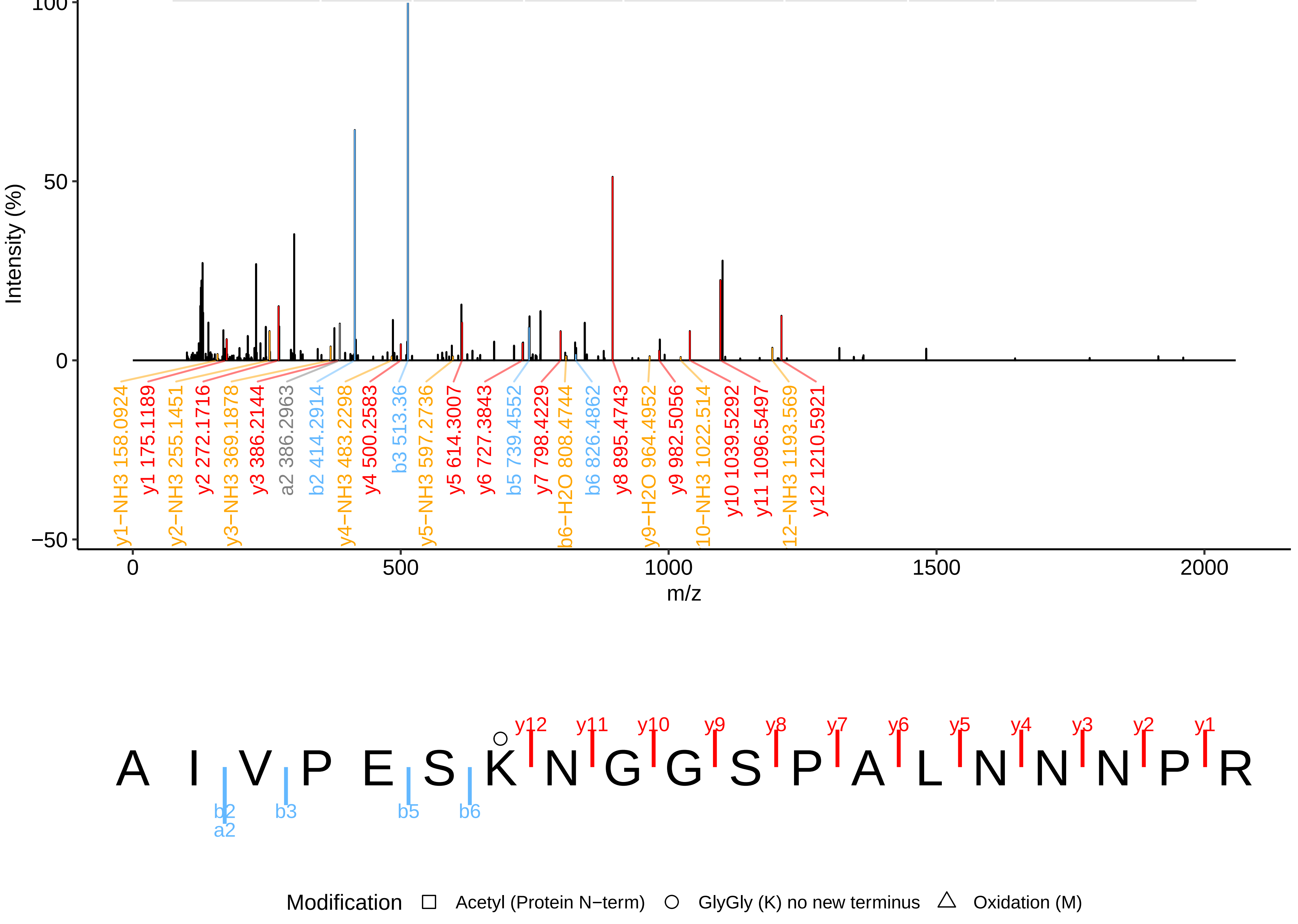

20200921_KIZ_MUb4_GlyGly_10plex − Scan number: 9737

Gene Names	Charge	m/z	Mass	Retention time	PEP	Score	Precursor Intensity
PPAP2B	3	683.6872	2048.04	33.449	9.9823e−20	135.23	NA

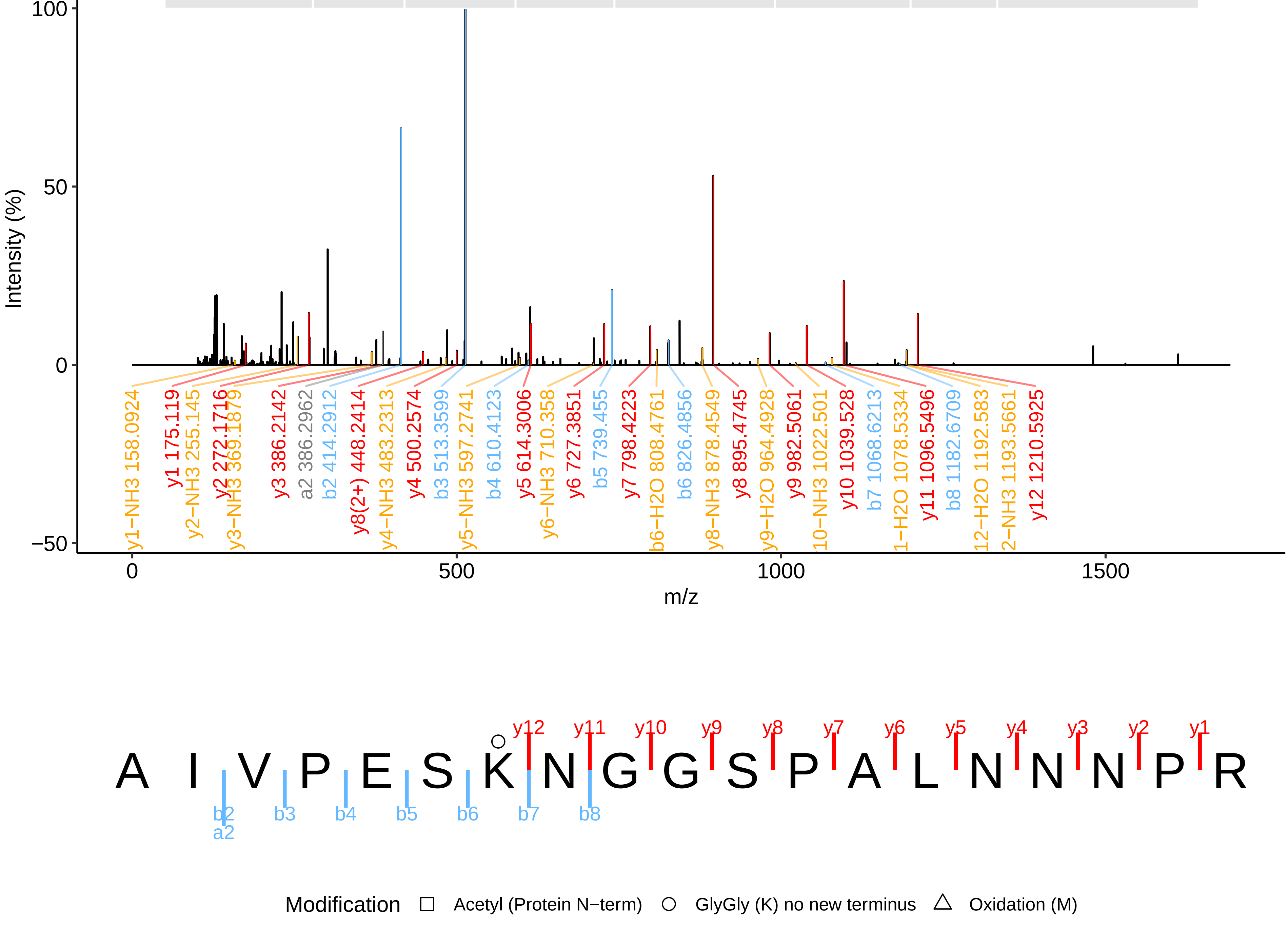

20200921_KIZ_MUb4_GlyGly_10plex_2 − Scan number: 8918

Gene Names	Charge	m/z	Mass	Retention time	PEP	Score	Precursor Intensity
PPAP2B	3	683.6872	2048.04	33.756	0.0086223	60.949	799253.8

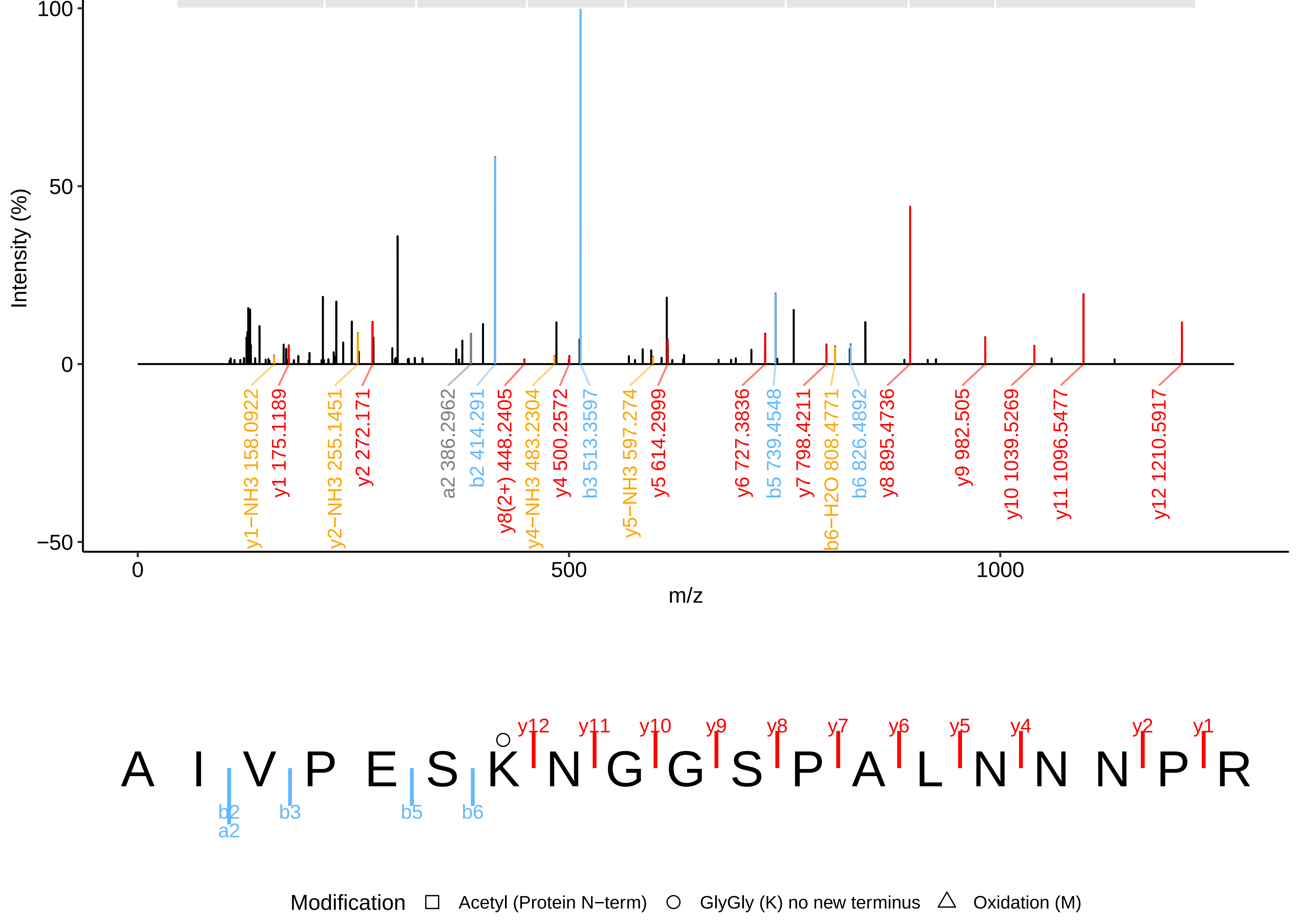

20200921_KIZ_MUb4_GlyGly_10plex − Scan number: 9271

Gene Names	Charge	m/z	Mass	Retention time	PEP	Score	Precursor Intensity
SLC7A5	4	457.4865	1825.917	31.739	9.3265e−20	158.81	4535417

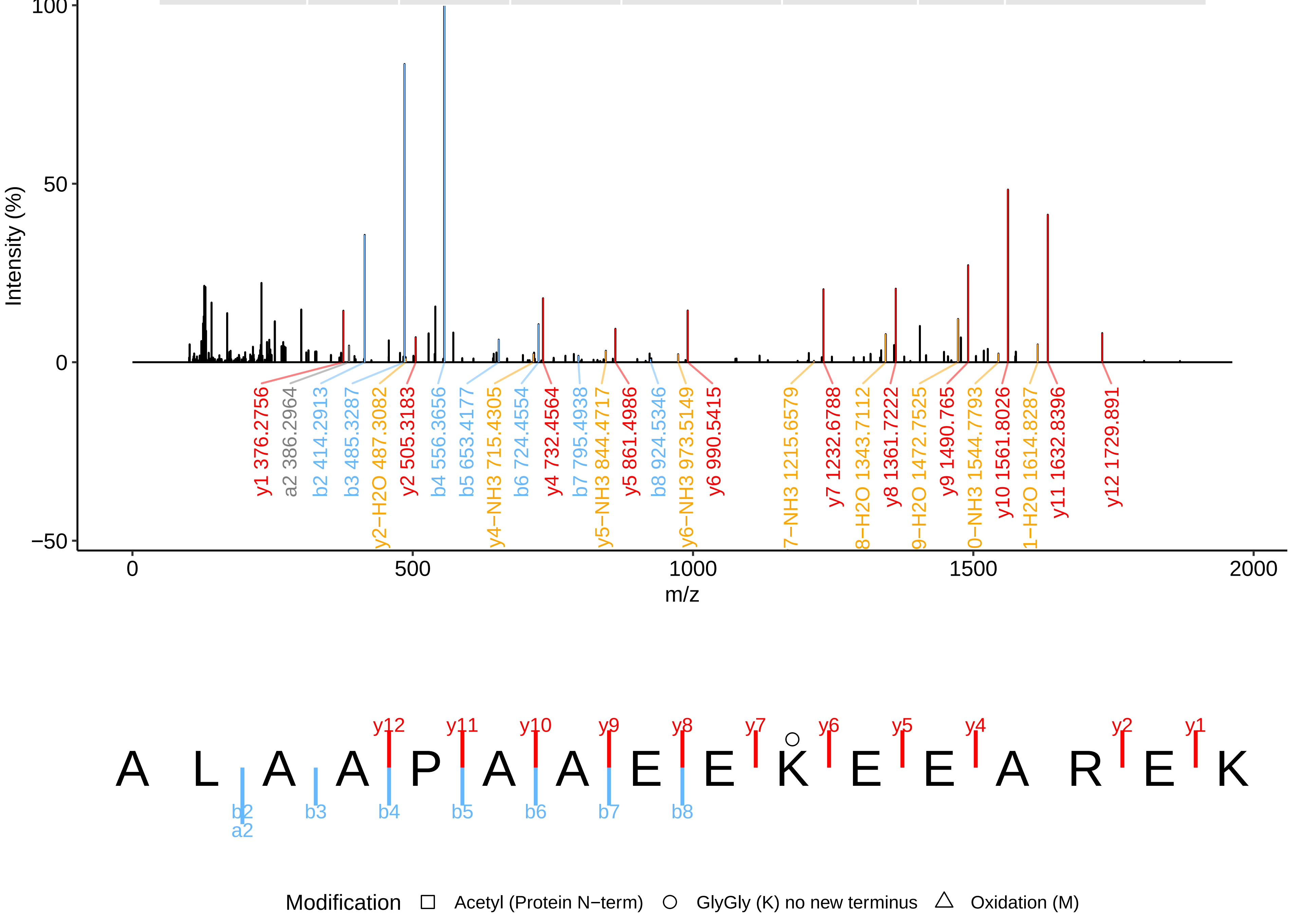

20200921_KIZ_MUb4_GlyGly_10plex_2 − Scan number: 8578

Gene Names	Charge	m/z	Mass	Retention time	PEP	Score	Precursor Intensity
SLC7A5	4	457.4865	1825.917	32.363	5.8366e−14	145.29	1911324

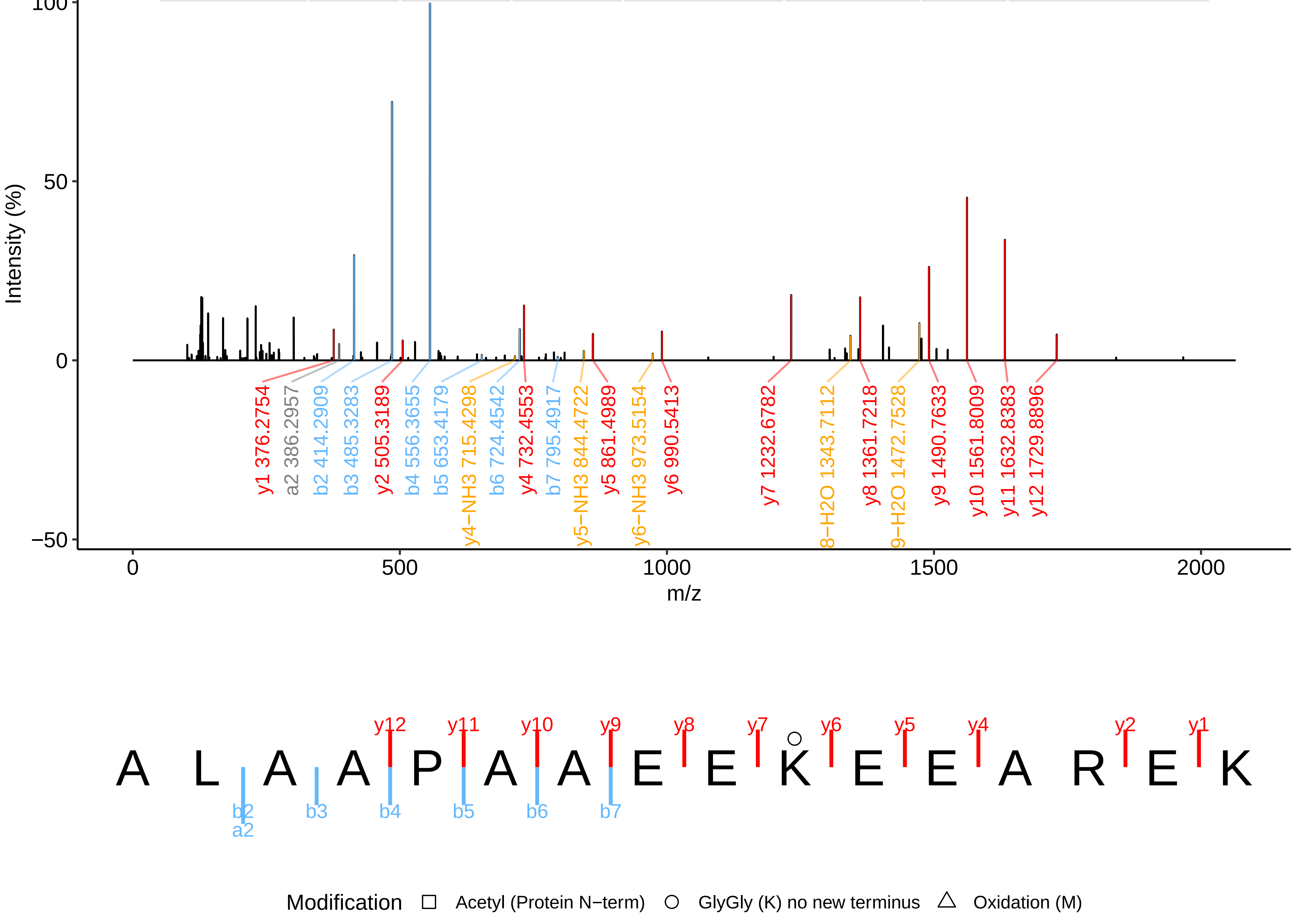

20200924_KIZ_MUb5_GlyGly_1 − Scan number: 8022

Gene Names	Charge	m/z	Mass	Retention time	PEP	Score	Precursor Intensity
SLC7A5	4	457.4865	1825.917	25.561	1.7882e−14	148.72	4641035

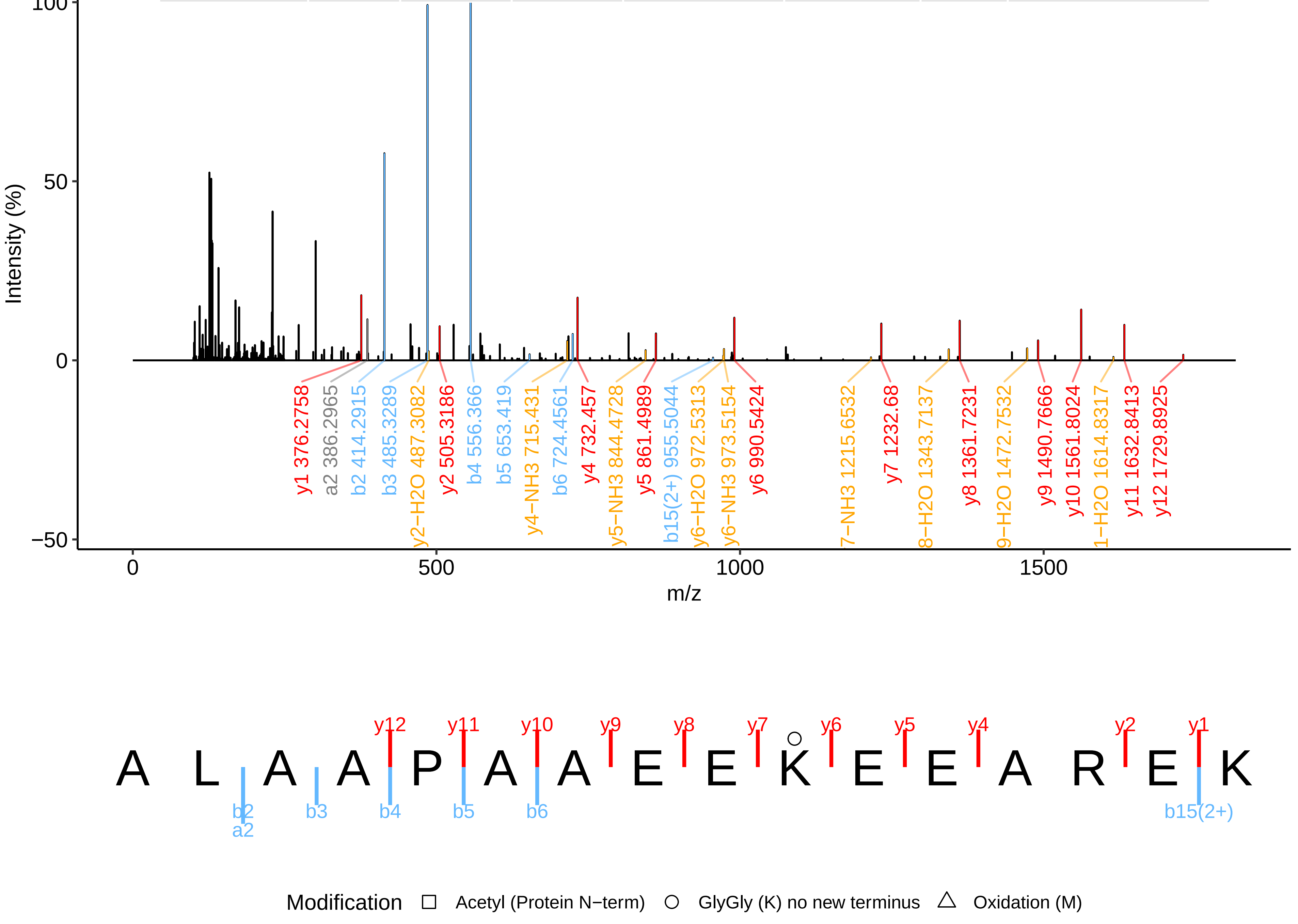

20200924_KIZ_MUb5_GlyGly_1 − Scan number: 8211

Gene Names	Charge	m/z	Mass	Retention time	PEP	Score	Precursor Intensity
SLC7A5	4	457.4865	1825.917	26.205	3.863e−20	163.99	9207441

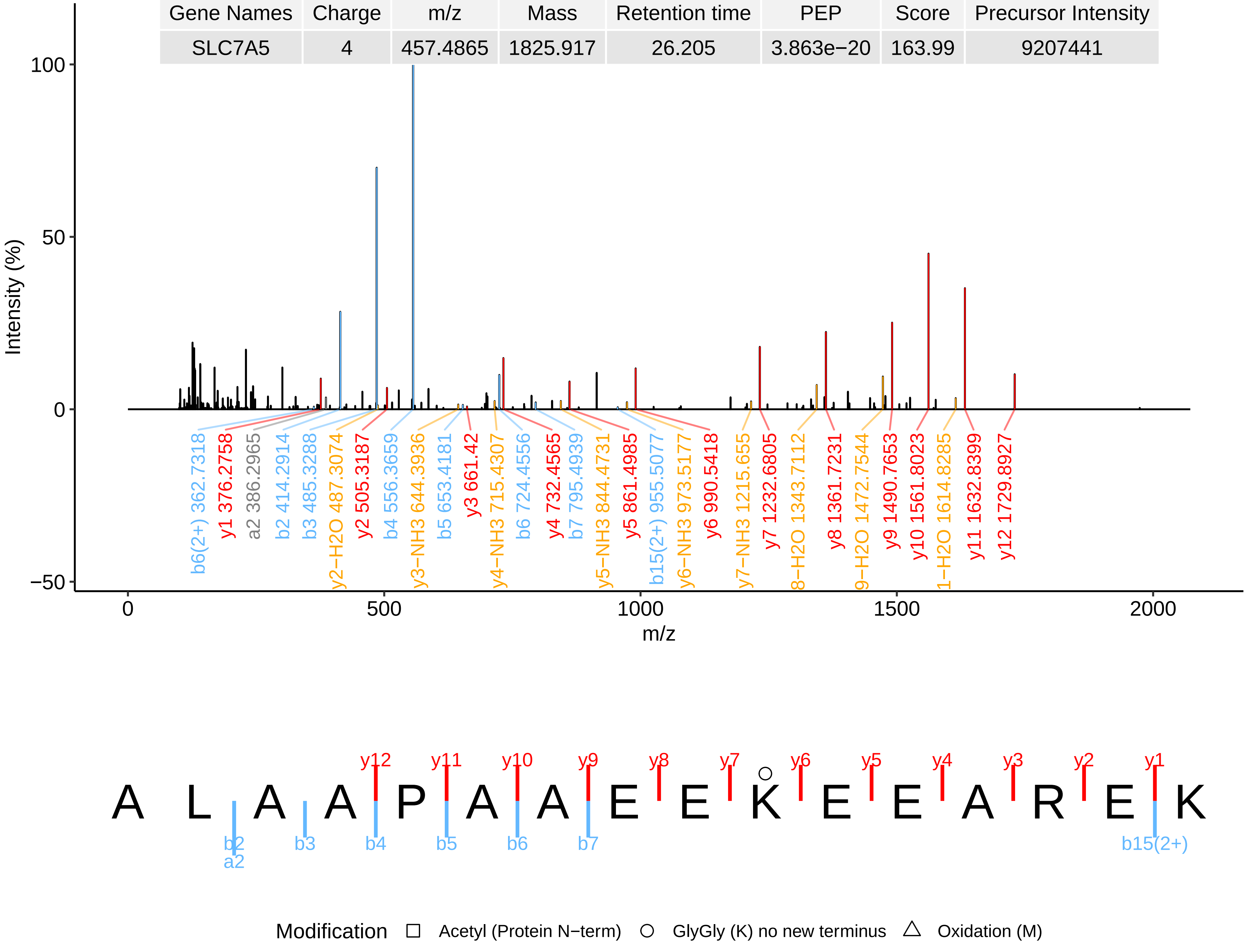

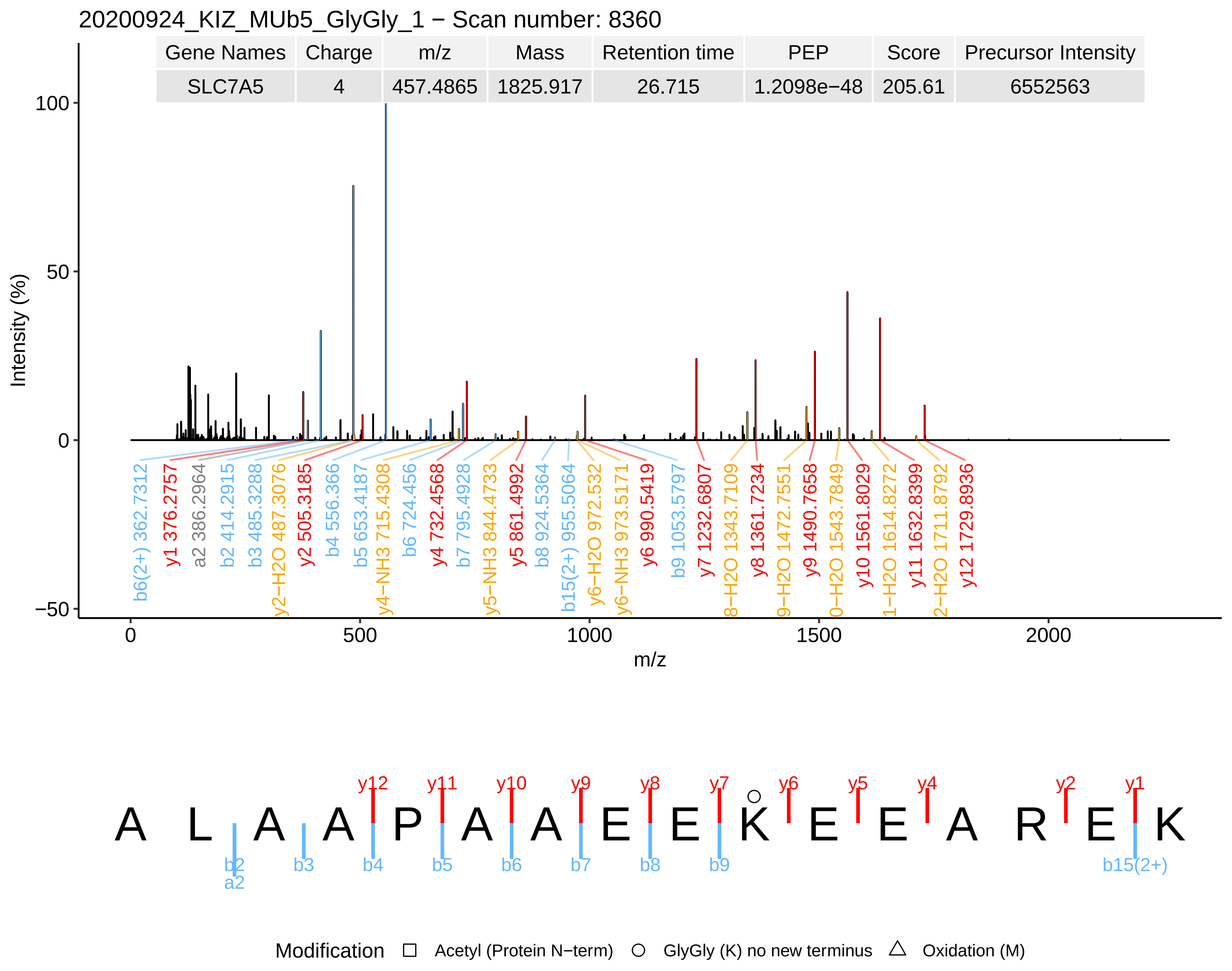

20200924_KIZ_MUb5_GlyGly_1 − Scan number: 8360
Gene Names | Charge | m/z | Mass | Retention time | PEP | Score | Precursor Intensity
SLC7A5 | 4 | 457.4865 | 1825.917 | 26.715 | 1.2098e−48 | 205.61 | 6552563
Intensity (%)
m/z
b6(2+) 362.7312
y1 376.2757
a2 386.2964
b2 414.2915
b3 485.3288
y2−H2O 487.3076
y2 505.3185
b4 556.366
b5 653.4187
y4−NH3 715.4308
b6 724.456
y4 732.4568
b7 795.4928
y5−NH3 844.4733
y5 861.4992
b8 924.5364
b15(2+) 955.5064
y6−H2O 972.532
y6−NH3 973.5171
y6 990.5419
b9 1053.5797
y7 1232.6807
8−H2O 1343.7109
y8 1361.7234
9−H2O 1472.7551
y9 1490.7658
0−H2O 1543.7849
y10 1561.8029
1−H2O 1614.8272
y11 1632.8399
2−H2O 1711.8792
y12 1729.8936
A L A A P A A E E K E E A R E K
y12 y11 y10 y9 y8 y7 y6 y5 y4 y2 y1
b2 a2 b3 b4 b5 b6 b7 b8 b9 b15(2+)
Modification
Acetyl (Protein N−term)
GlyGly (K) no new terminus
Oxidation (M)

20200924_KIZ_MUb5_GlyGly_2 − Scan number: 7859

Gene Names	Charge	m/z	Mass	Retention time	PEP	Score	Precursor Intensity
SLC7A5	4	457.4865	1825.917	29.372	1.2461e−06	107.61	3970549

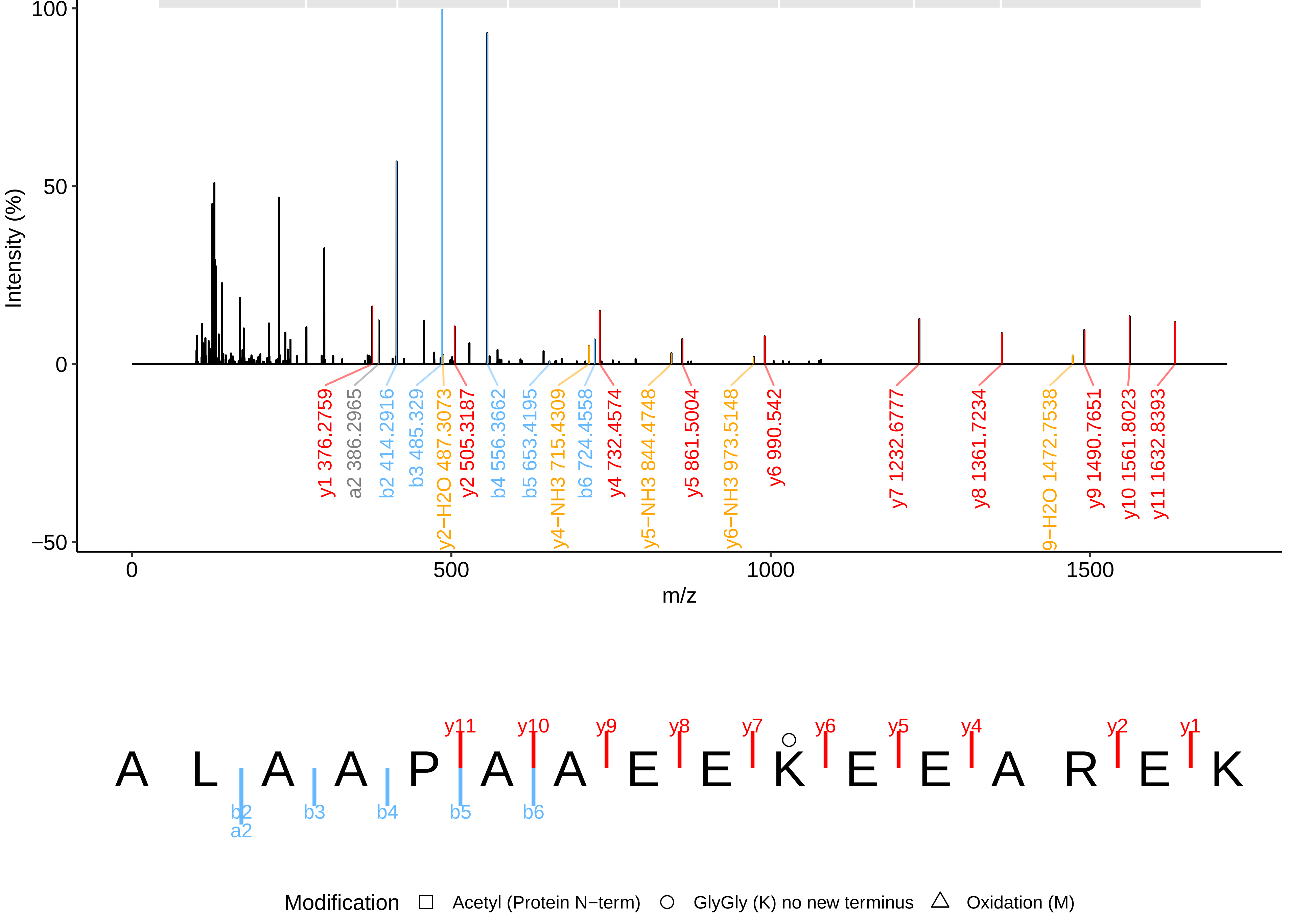

20200921_KIZ_MUb4_GlyGly_10plex − Scan number: 25566

Gene Names	Charge	m/z	Mass	Retention time	PEP	Score	Precursor Intensity
CCDC51	3	684.06	2049.158	93.027	5.2906e−06	89.311	4551411

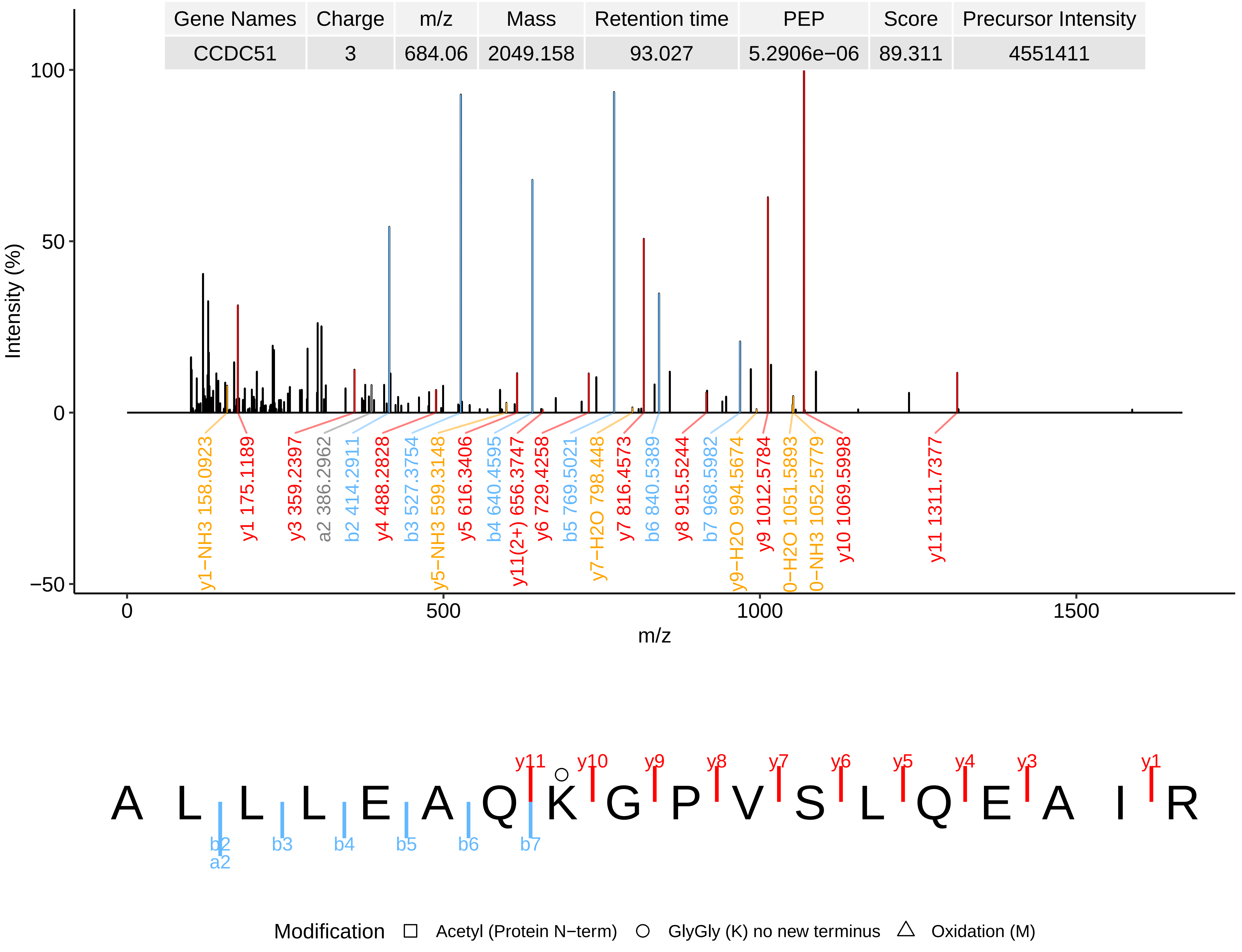

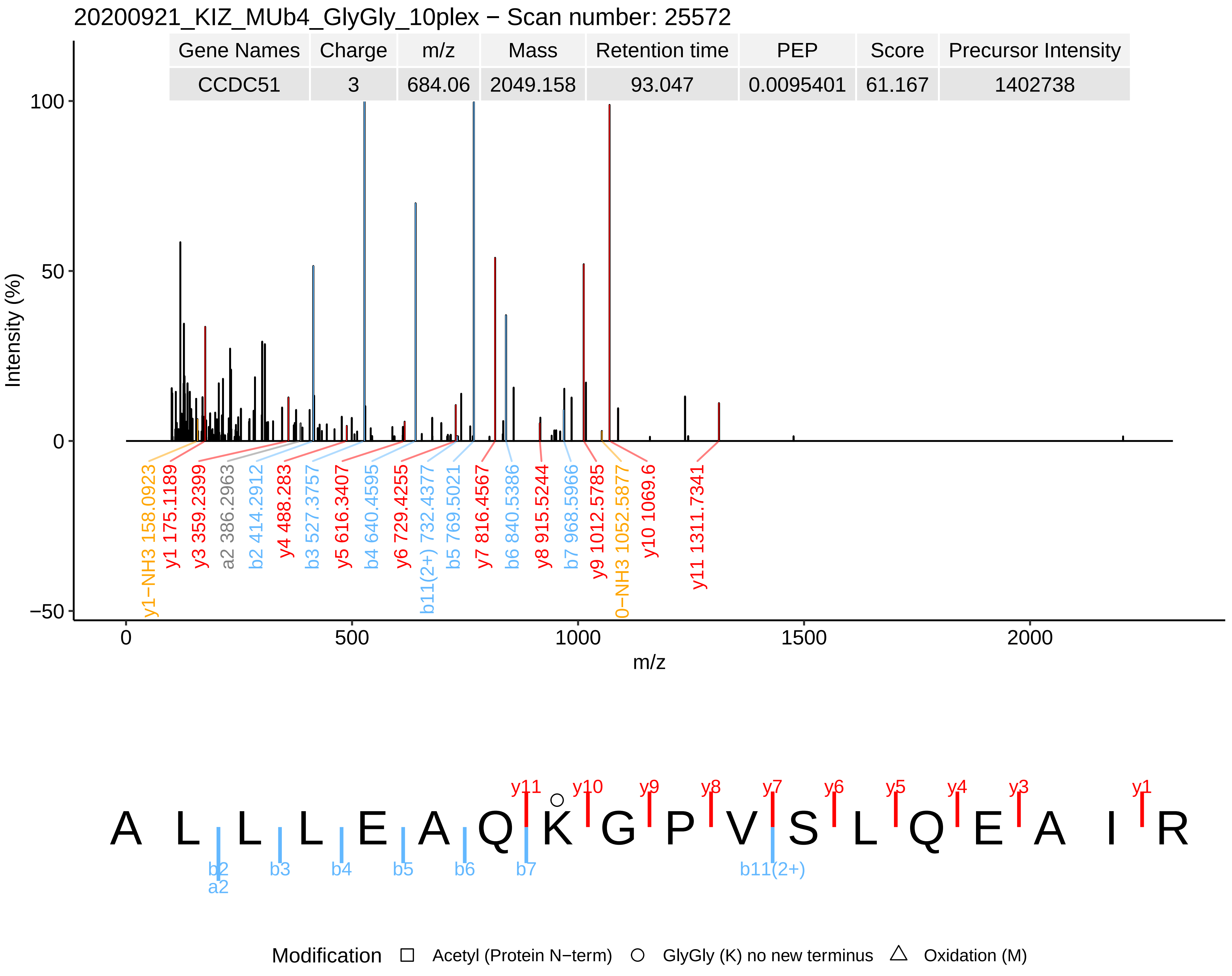
20200921_KIZ_MUb4_GlyGly_10plex − Scan number: 25572
Gene Names | Charge | m/z | Mass | Retention time | PEP | Score | Precursor Intensity
CCDC51 | 3 | 684.06 | 2049.158 | 93.047 | 0.0095401 | 61.167 | 1402738
Intensity (%)
m/z
y1−NH3 158.0923
y1 175.1189
y3 359.2399
a2 386.2963
b2 414.2912
y4 488.283
b3 527.3757
y5 616.3407
b4 640.4595
y6 729.4255
b11(2+) 732.4377
b5 769.5021
y7 816.4567
b6 840.5386
y8 915.5244
b7 968.5966
y9 1012.5785
0−NH3 1052.5877
y10 1069.6
y11 1311.7341
A L L L E A Q K G P V S L Q E A I R
y11 y10 y9 y8 y7 y6 y5 y4 y3 y1
b2 b3 b4 b5 b6 b7 b11(2+)
a2
Modification □ Acetyl (Protein N−term) ○ GlyGly (K) no new terminus △ Oxidation (M)

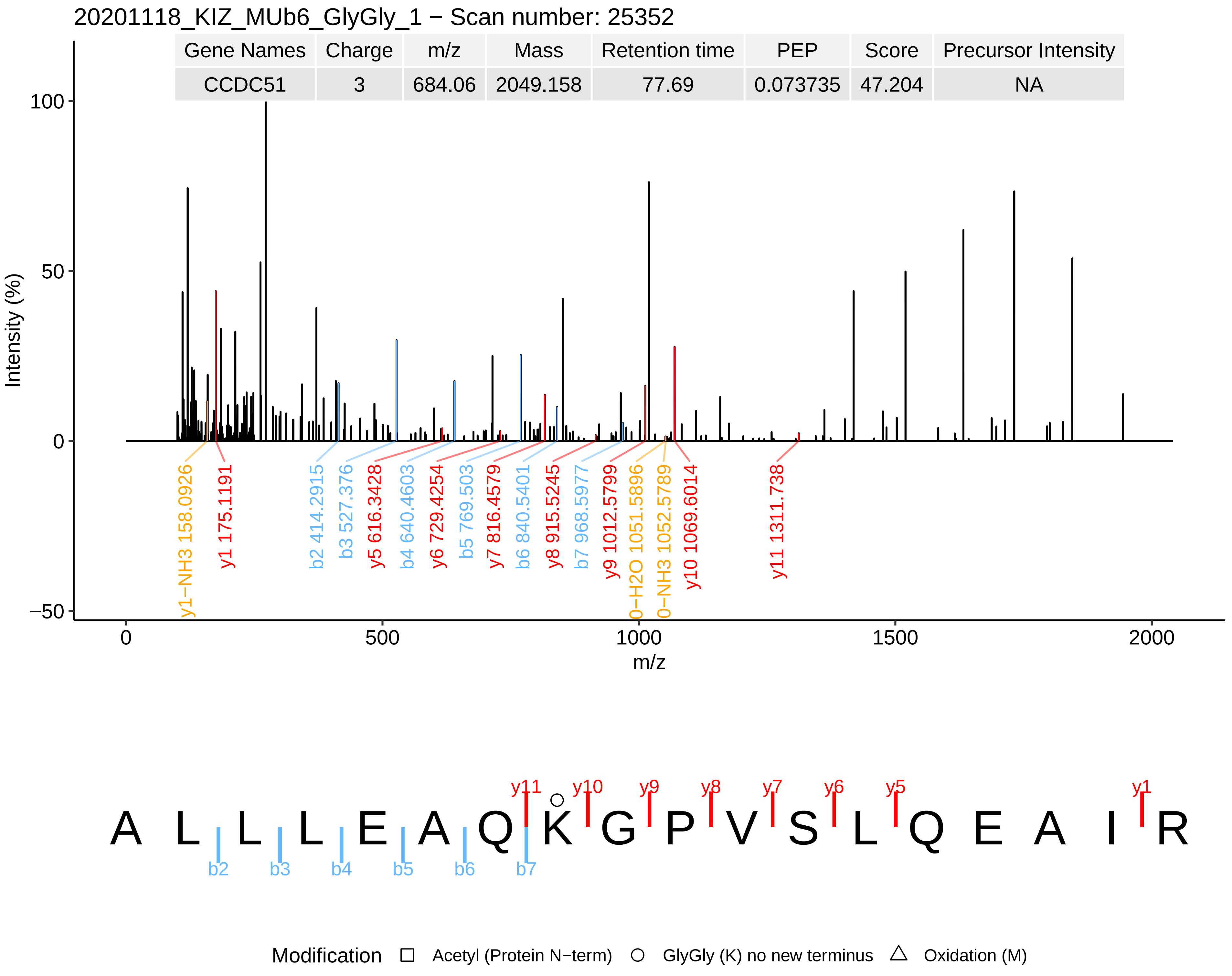
20201118_KIZ_MUb6_GlyGly_1 − Scan number: 25352
Gene Names | Charge | m/z | Mass | Retention time | PEP | Score | Precursor Intensity
CCDC51 | 3 | 684.06 | 2049.158 | 77.69 | 0.073735 | 47.204 | NA
Intensity (%)
100
50
0
−50
m/z
0
500
1000
1500
2000
y1−NH3 158.0926
y1 175.1191
b2 414.2915
b3 527.376
y5 616.3428
b4 640.4603
y6 729.4254
b5 769.503
y7 816.4579
b6 840.5401
y8 915.5245
b7 968.5977
y9 1012.5799
0−H2O 1051.5896
0−NH3 1052.5789
y10 1069.6014
y11 1311.738
A L L L E A Q K G P V S L Q E A I R
y11 y10 y9 y8 y7 y6 y5 y1
b2 b3 b4 b5 b6 b7
Modification
Acetyl (Protein N−term)
GlyGly (K) no new terminus
Oxidation (M)

20200921_KIZ_MUb4_GlyGly_10plex − Scan number: 27853

Gene Names	Charge	m/z	Mass	Retention time	PEP	Score	Precursor Intensity
HIST1H1E;HIST1H1D;HIST1H1C	3	614.0107	1839.01	101.77	3.7362e−14	131.48	2230084

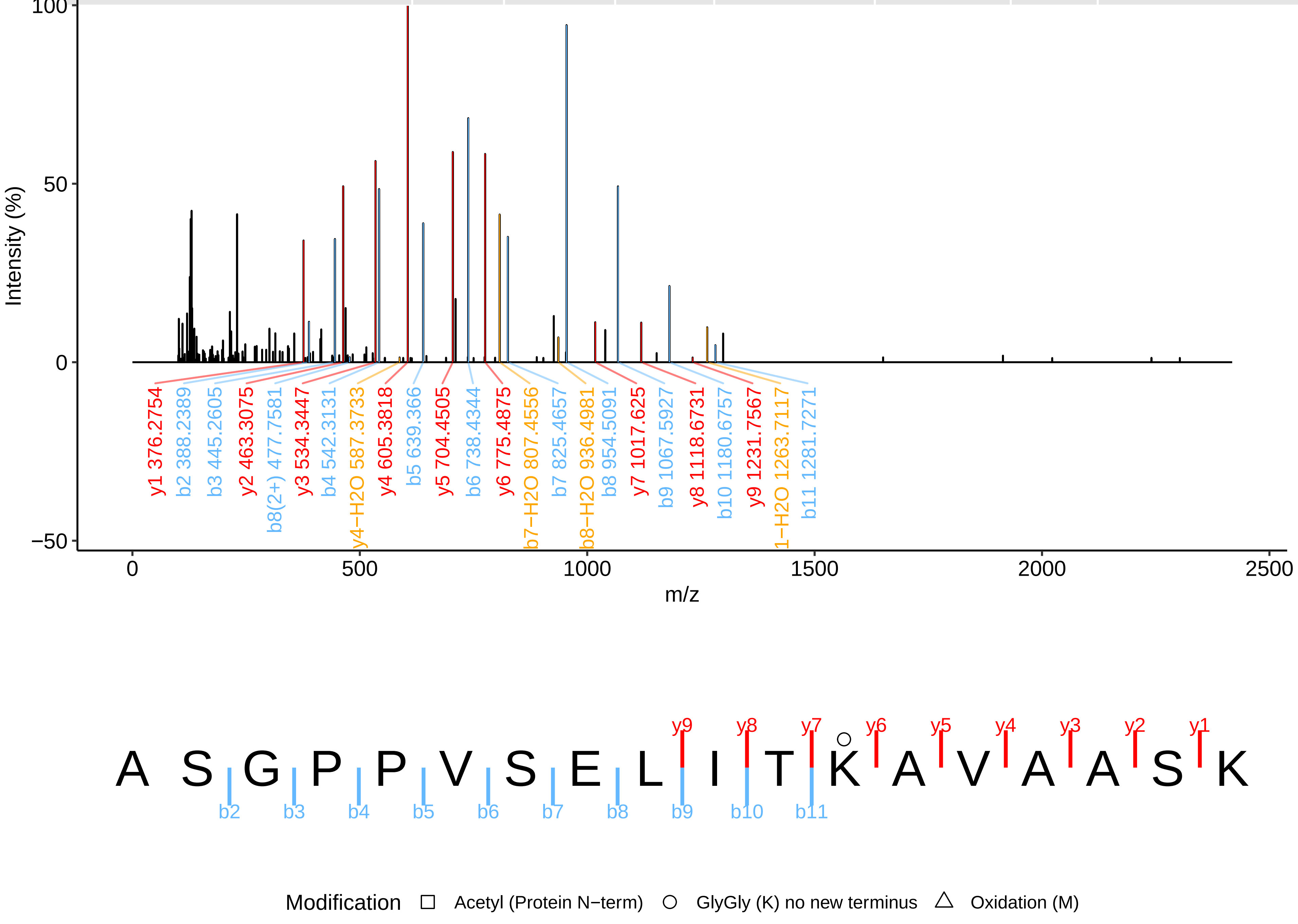

20200921_KIZ_MUb4_GlyGly_10plex_2 − Scan number: 24417

Gene Names	Charge	m/z	Mass	Retention time	PEP	Score	Precursor Intensity
HIST1H1E;HIST1H1D;HIST1H1C	3	614.0107	1839.01	96.481	7.5284e−06	85.636	1100307

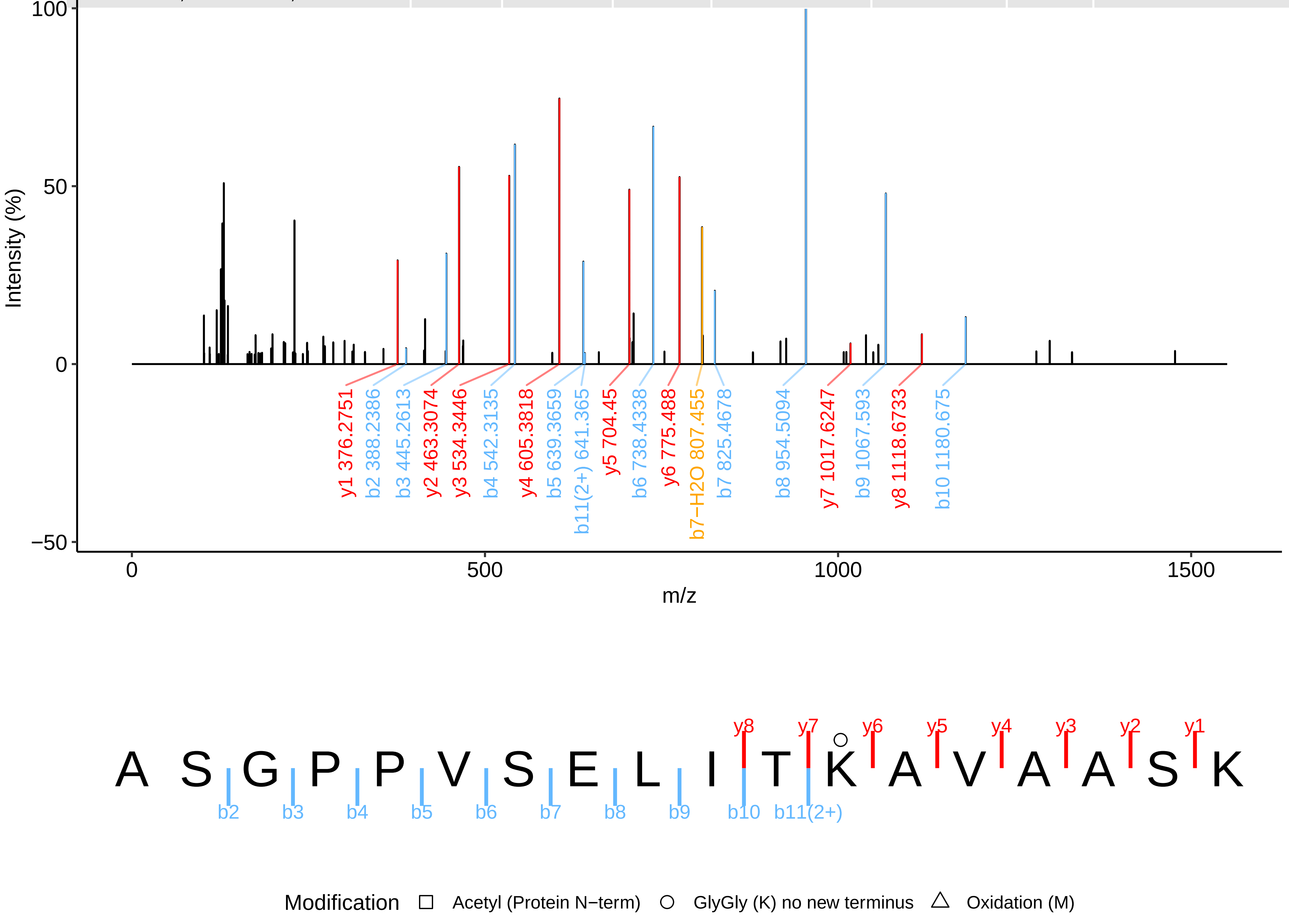

20200921_KIZ_MUb4_GlyGly_10plex_2 − Scan number: 24455

Gene Names	Charge	m/z	Mass	Retention time	PEP	Score	Precursor Intensity
HIST1H1E;HIST1H1D;HIST1H1C	3	614.0107	1839.01	96.636	0.0014017	70.96	513776.6

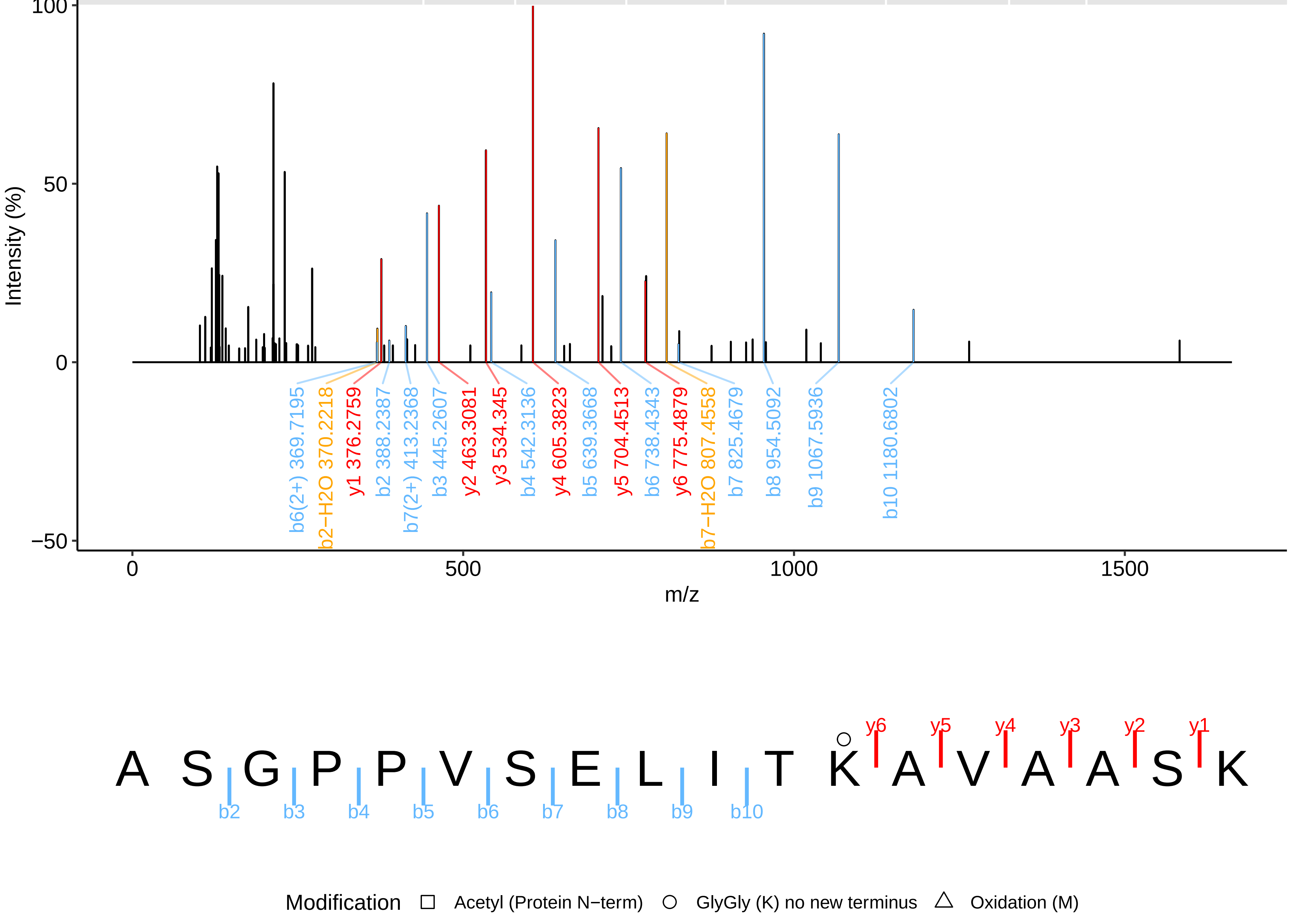

20200921_KIZ_MUb4_GlyGly_10plex_2 − Scan number: 5107

Gene Names	Charge	m/z	Mass	Retention time	PEP	Score	Precursor Intensity
AIFM1	3	682.6664	2044.977	18.207	0.030275	53.453	383681.4

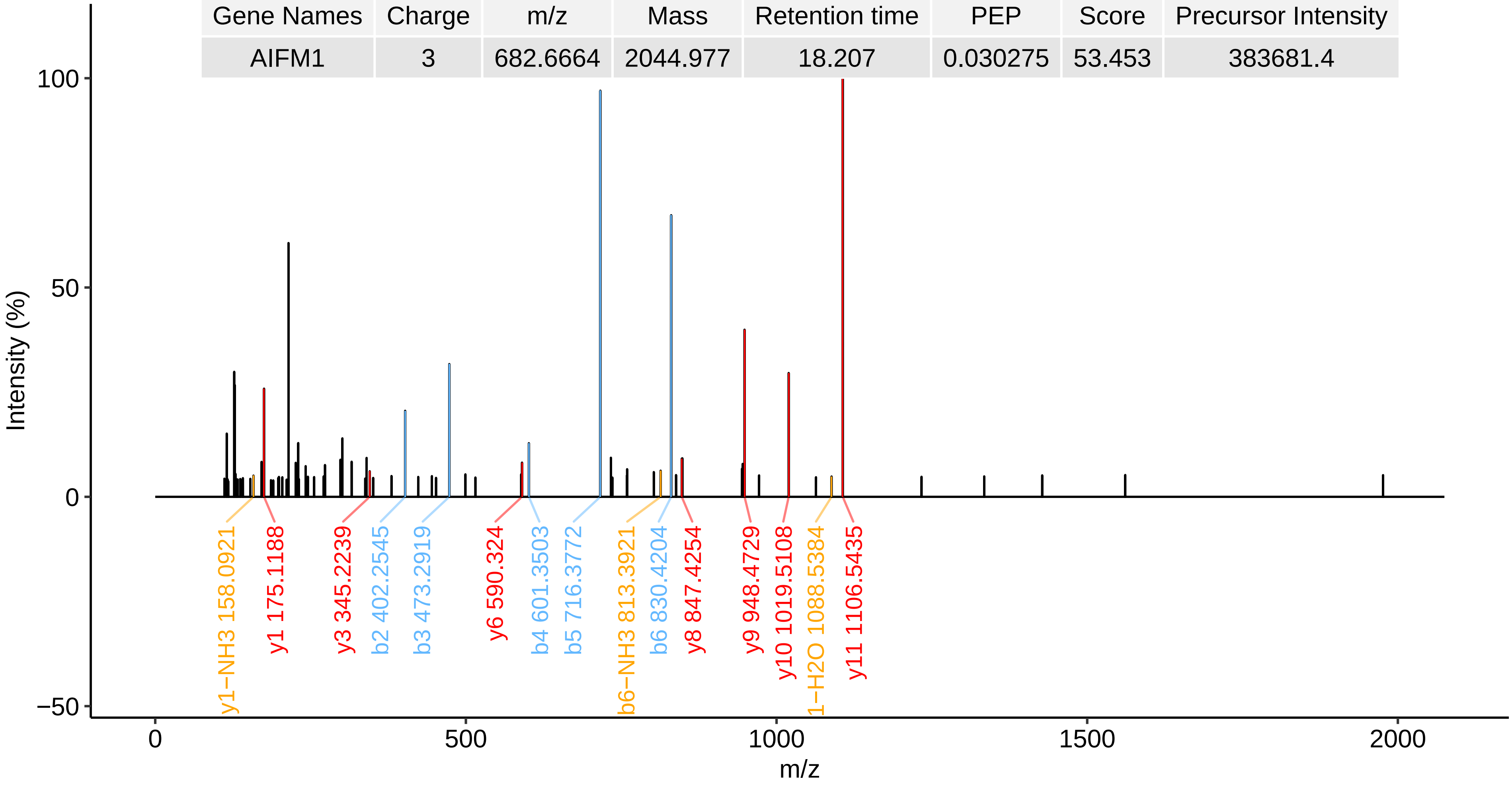

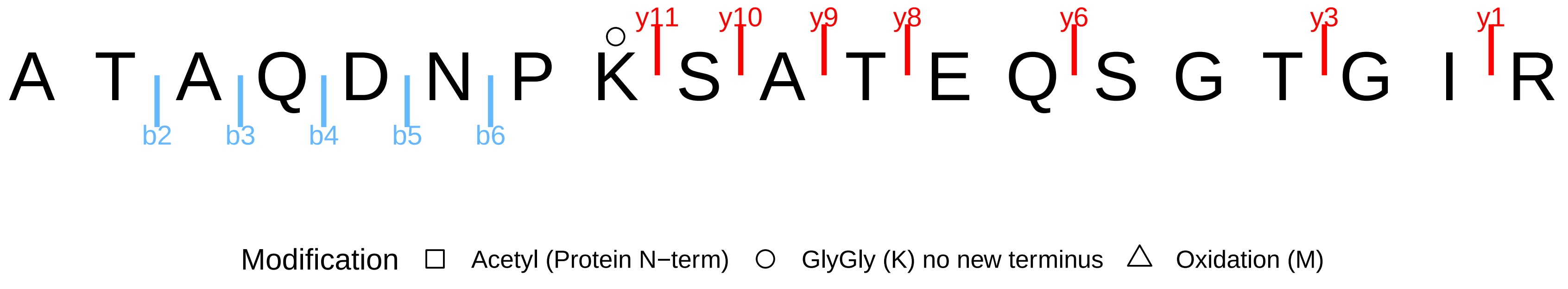

20200924_KIZ_MUb5_GlyGly_1 − Scan number: 29501

Gene Names	Charge	m/z	Mass	Retention time	PEP	Score	Precursor Intensity
SUPT16H	3	604.6636	1810.969	94.132	0.01321	68.809	5805046

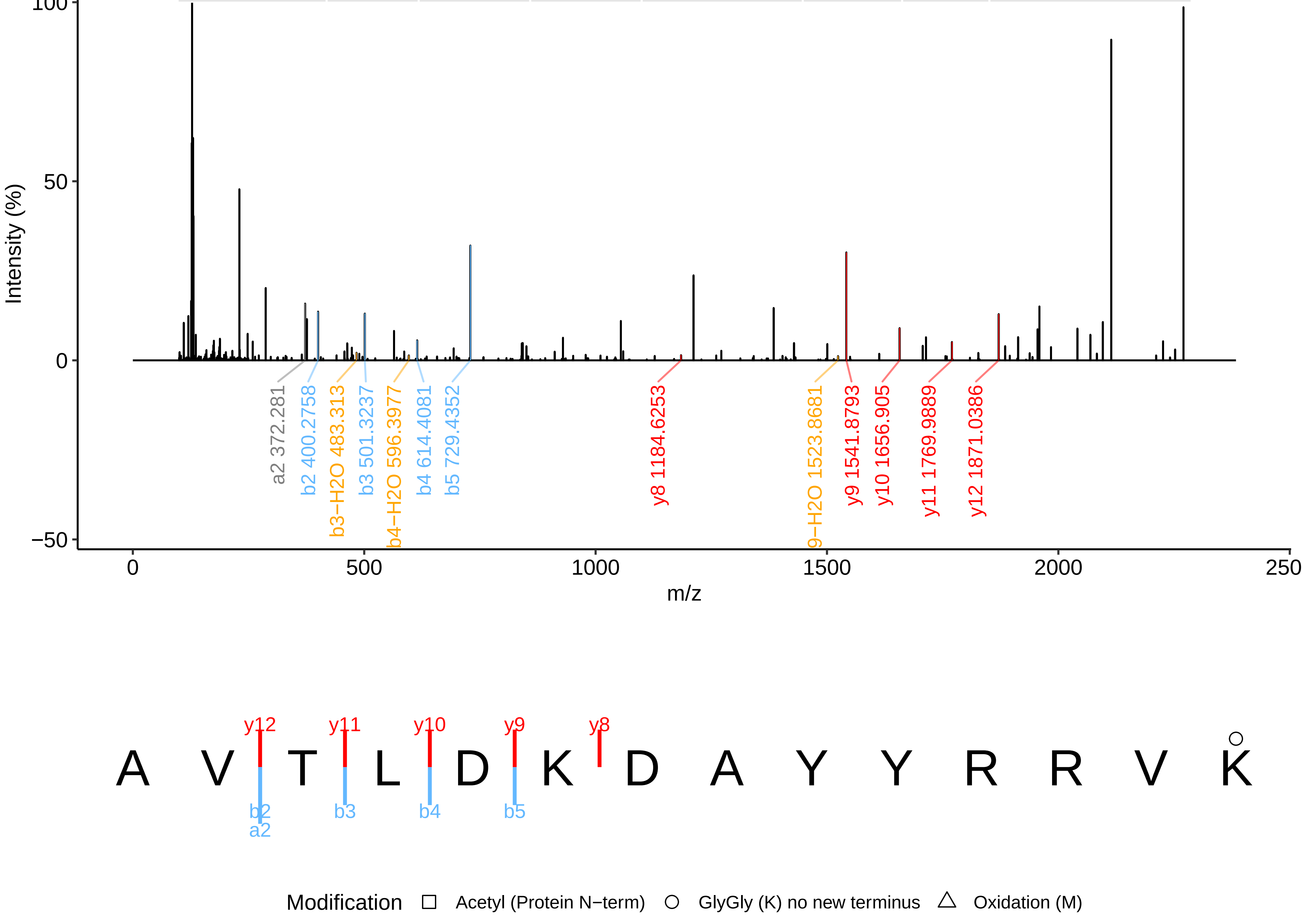

20200924_KIZ_MUb5_GlyGly_1 − Scan number: 29674

Gene Names	Charge	m/z	Mass	Retention time	PEP	Score	Precursor Intensity
SUPT16H	3	604.6636	1810.969	94.674	0.016836	61.212	NA

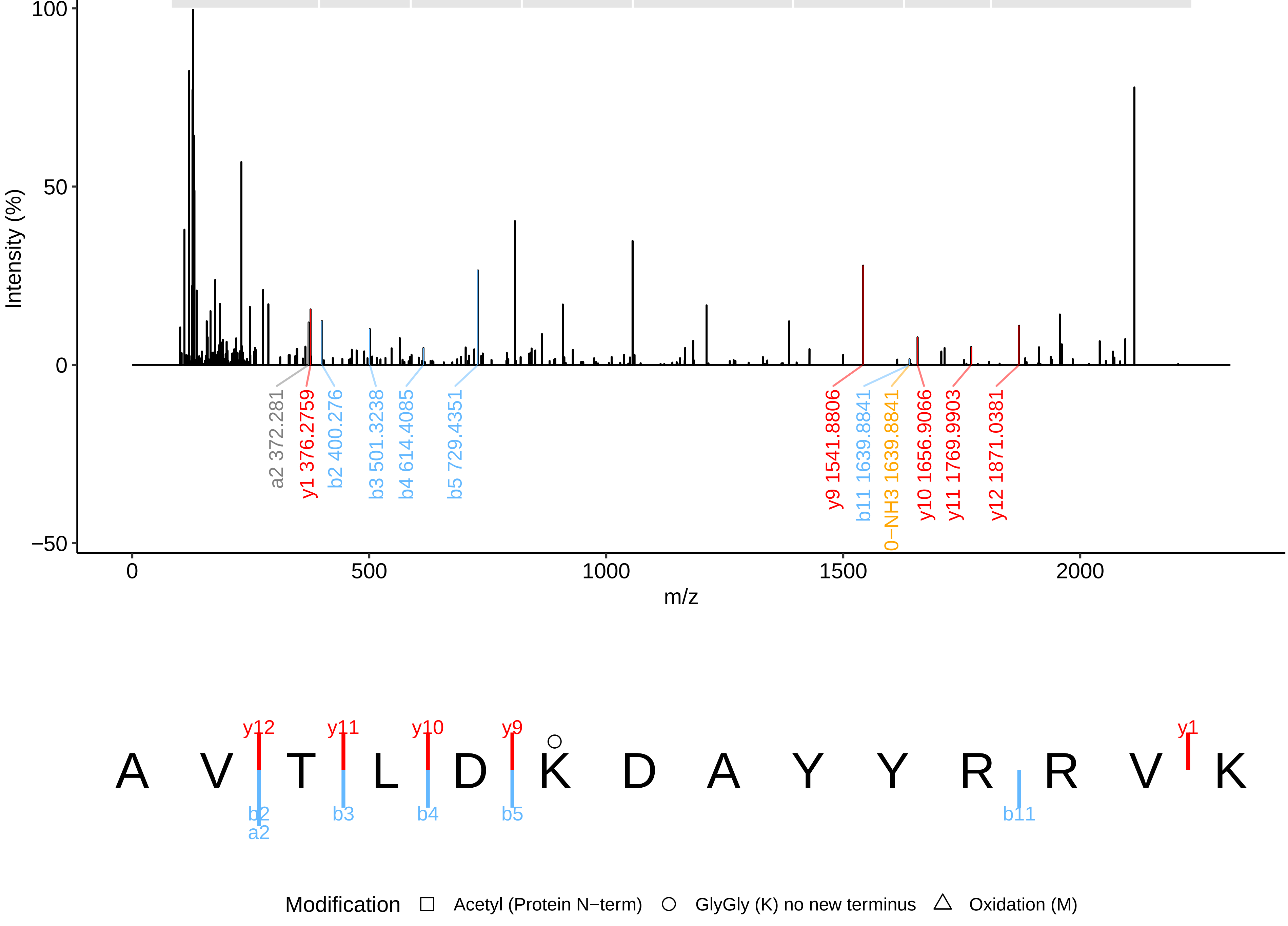

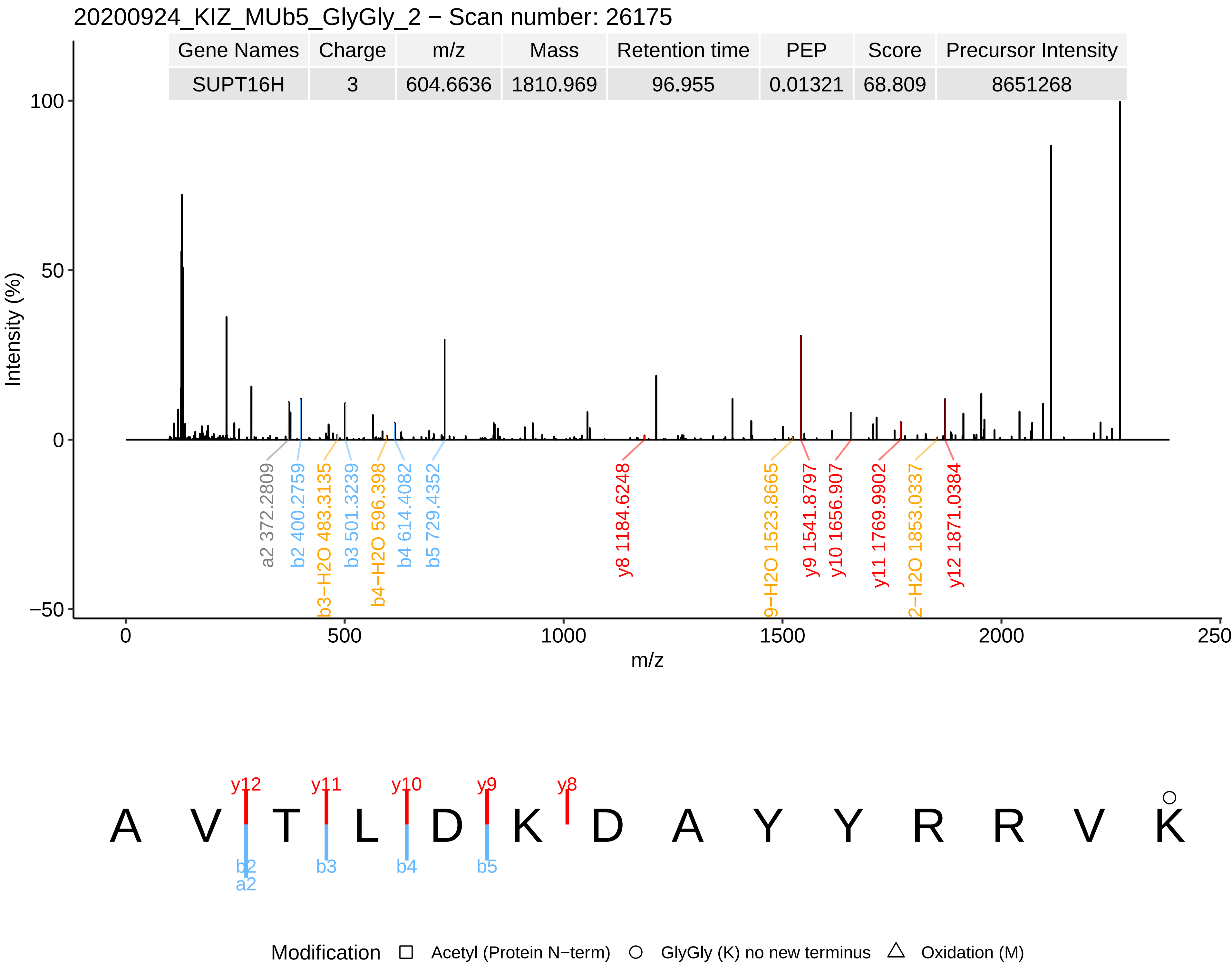
20200924_KIZ_MUb5_GlyGly_2 − Scan number: 26175
Gene Names | Charge | m/z | Mass | Retention time | PEP | Score | Precursor Intensity
SUPT16H | 3 | 604.6636 | 1810.969 | 96.955 | 0.01321 | 68.809 | 8651268
Intensity (%)
100
50
0
−50
0
500
1000
1500
2000
250
m/z
a2 372.2809
b2 400.2759
b3−H2O 483.3135
b3 501.3239
b4−H2O 596.398
b4 614.4082
b5 729.4352
y8 1184.6248
9−H2O 1523.8665
y9 1541.8797
y10 1656.907
y11 1769.9902
2−H2O 1853.0337
y12 1871.0384
y12 y11 y10 y9 y8
A V T L D K D A Y Y R R V K
b2 b3 b4 b5
a2
Modification
Acetyl (Protein N−term)
GlyGly (K) no new terminus
Oxidation (M)

20200924_KIZ_MUb5_GlyGly_2 − Scan number: 26324

Gene Names	Charge	m/z	Mass	Retention time	PEP	Score	Precursor Intensity
SUPT16H	3	604.6636	1810.969	97.484	0.06186	58.752	4862094

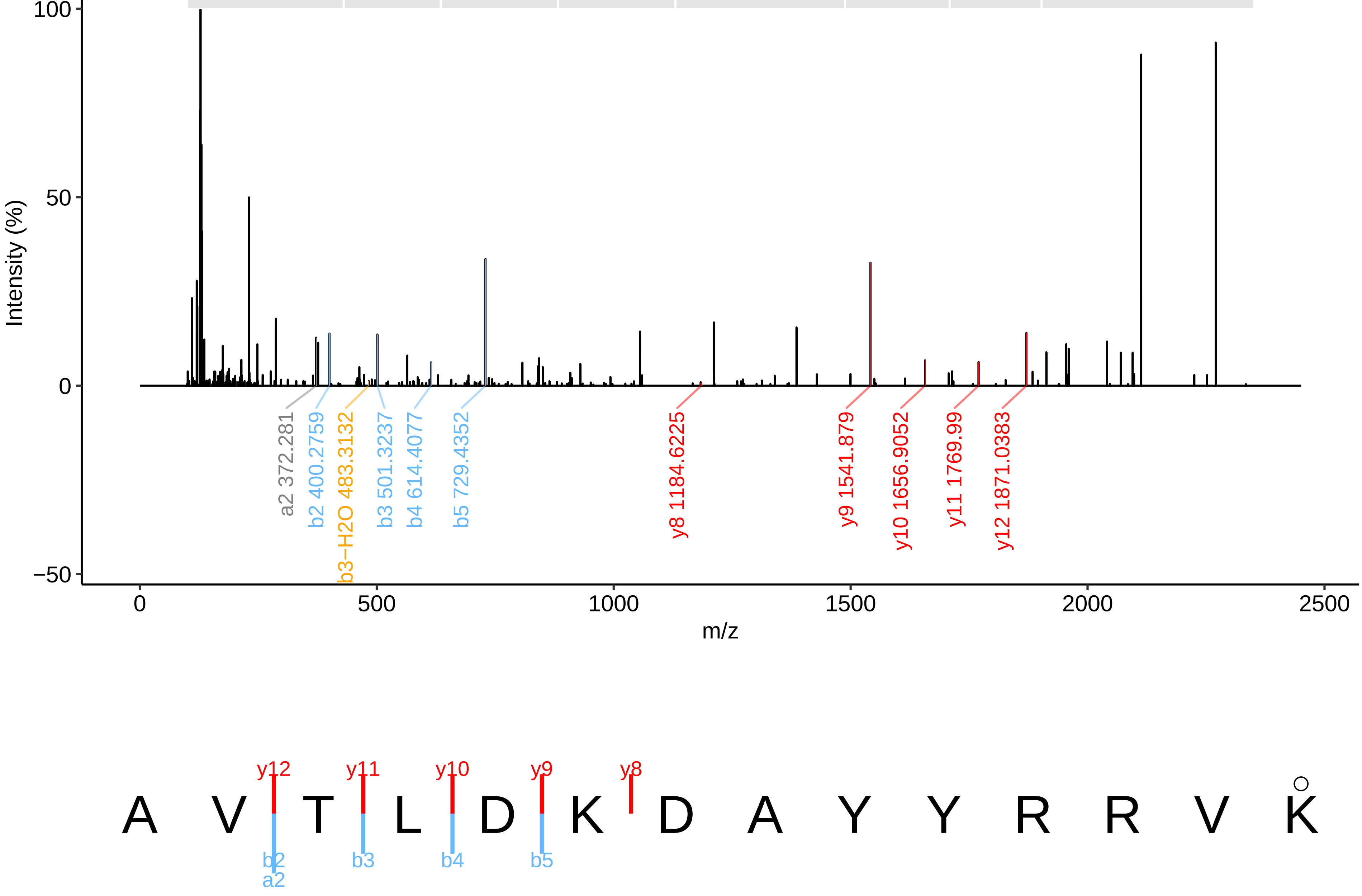

Modification □ Acetyl (Protein N−term) ○ GlyGly (K) no new terminus △ Oxidation (M)

20201118_KIZ_MUb6_GlyGly_1 − Scan number: 30911

Gene Names	Charge	m/z	Mass	Retention time	PEP	Score	Precursor Intensity
SUPT16H	3	604.6636	1810.969	92.149	0.0040763	70.963	37662988

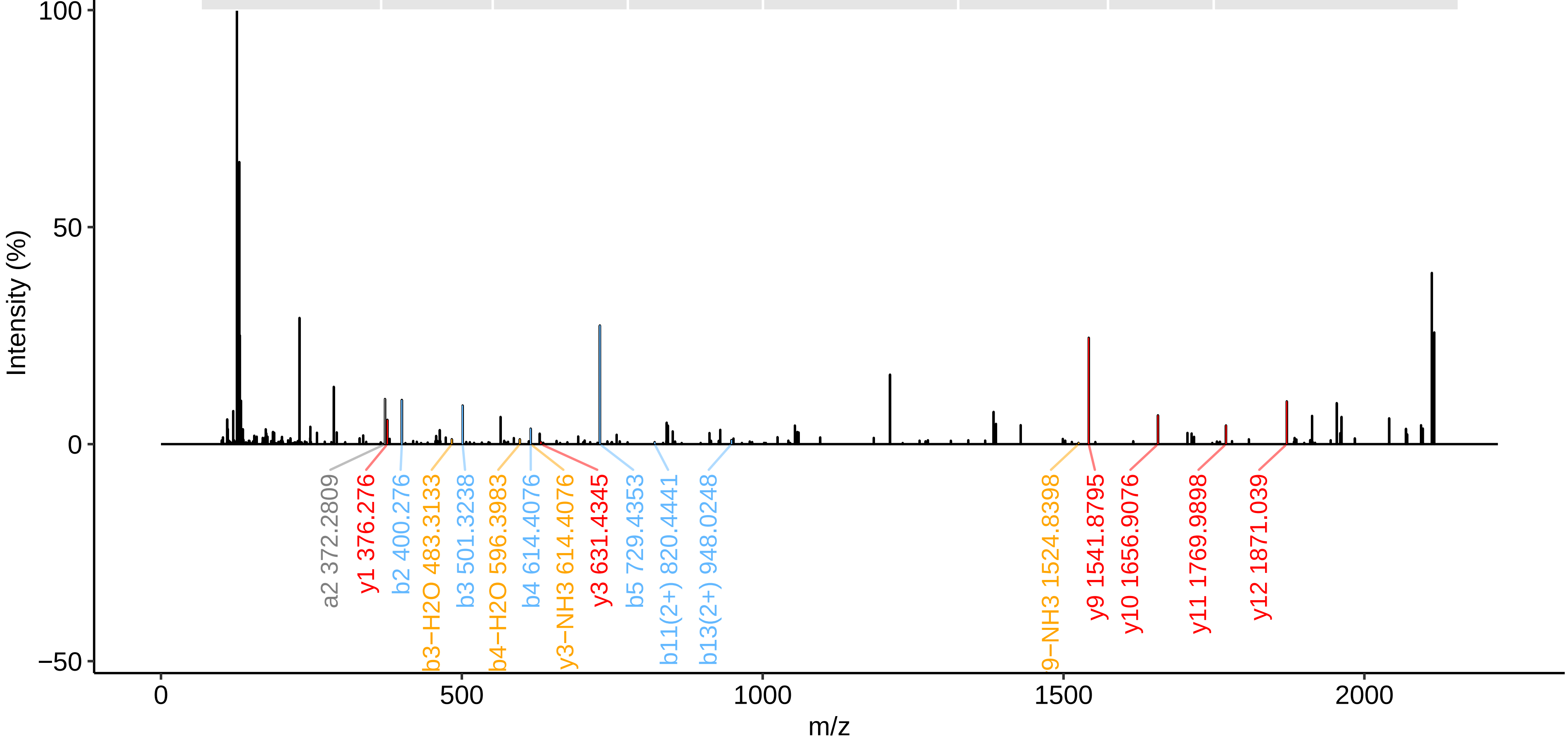

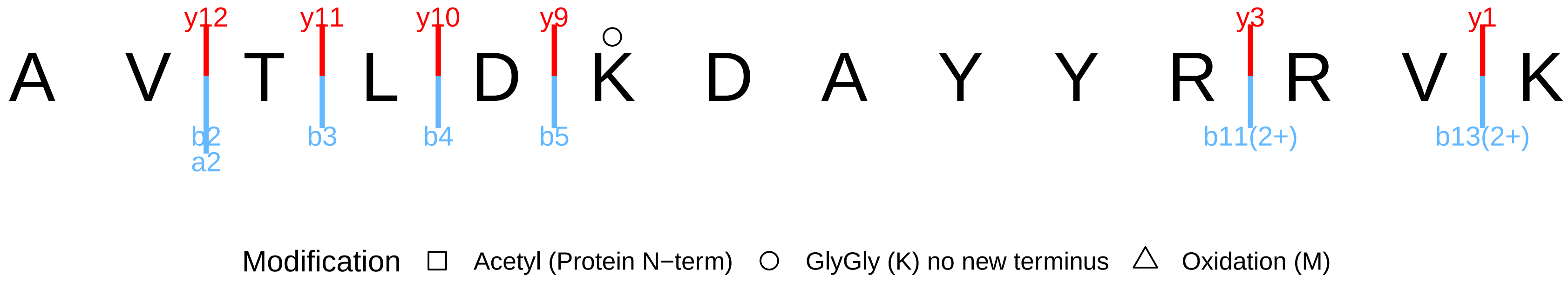

20200921_KIZ_MUb4_GlyGly_10plex_2 − Scan number: 20687

Gene Names	Charge	m/z	Mass	Retention time	PEP	Score	Precursor Intensity
FASN	3	614.3328	1839.976	81.354	0.0039585	62.861	1298490

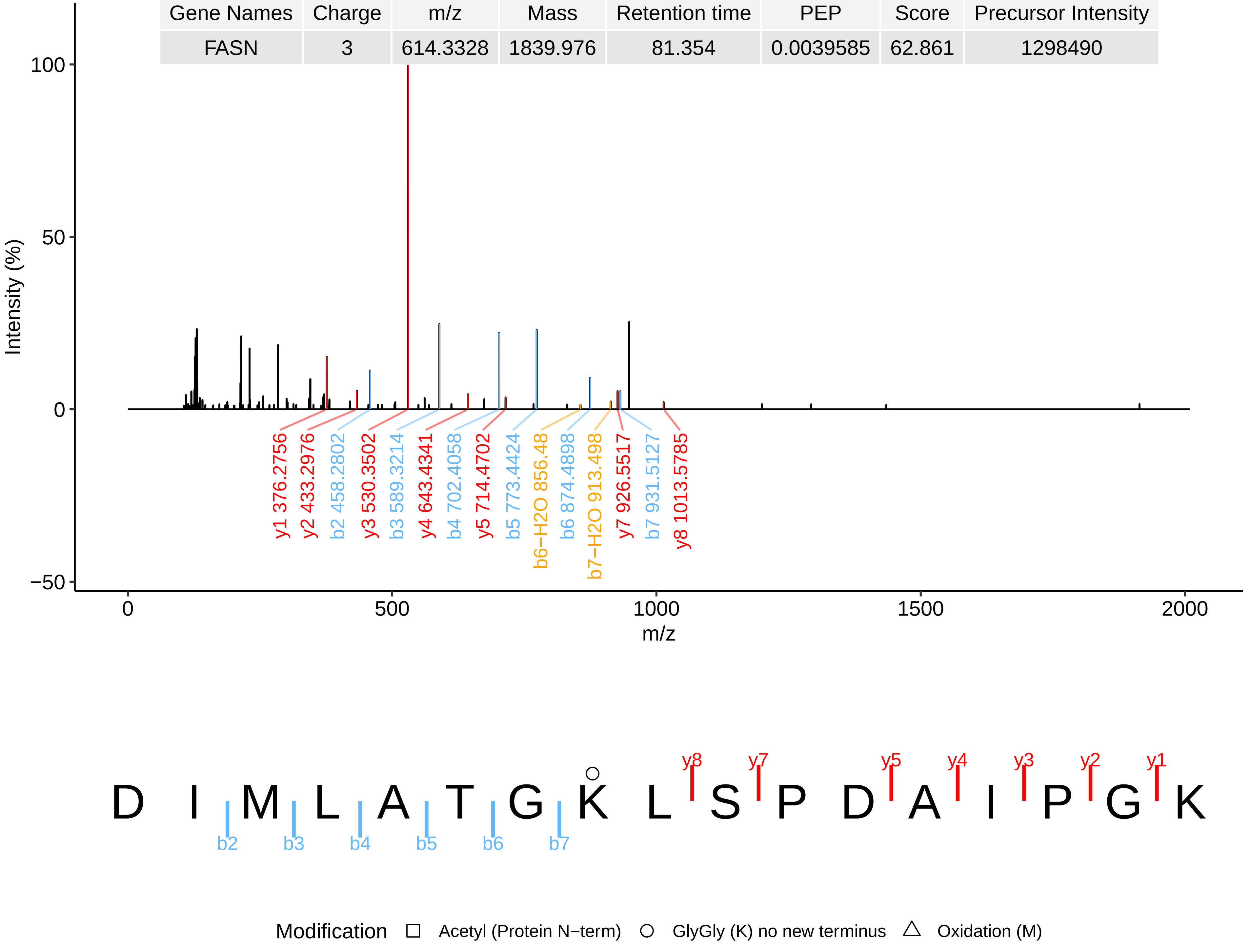

20200921_KIZ_MUb4_GlyGly_10plex − Scan number: 27230

Gene Names	Charge	m/z	Mass	Retention time	PEP	Score	Precursor Intensity
ANP32E	3	600.6669	1798.979	99.342	2.0883e−28	180.28	2418608

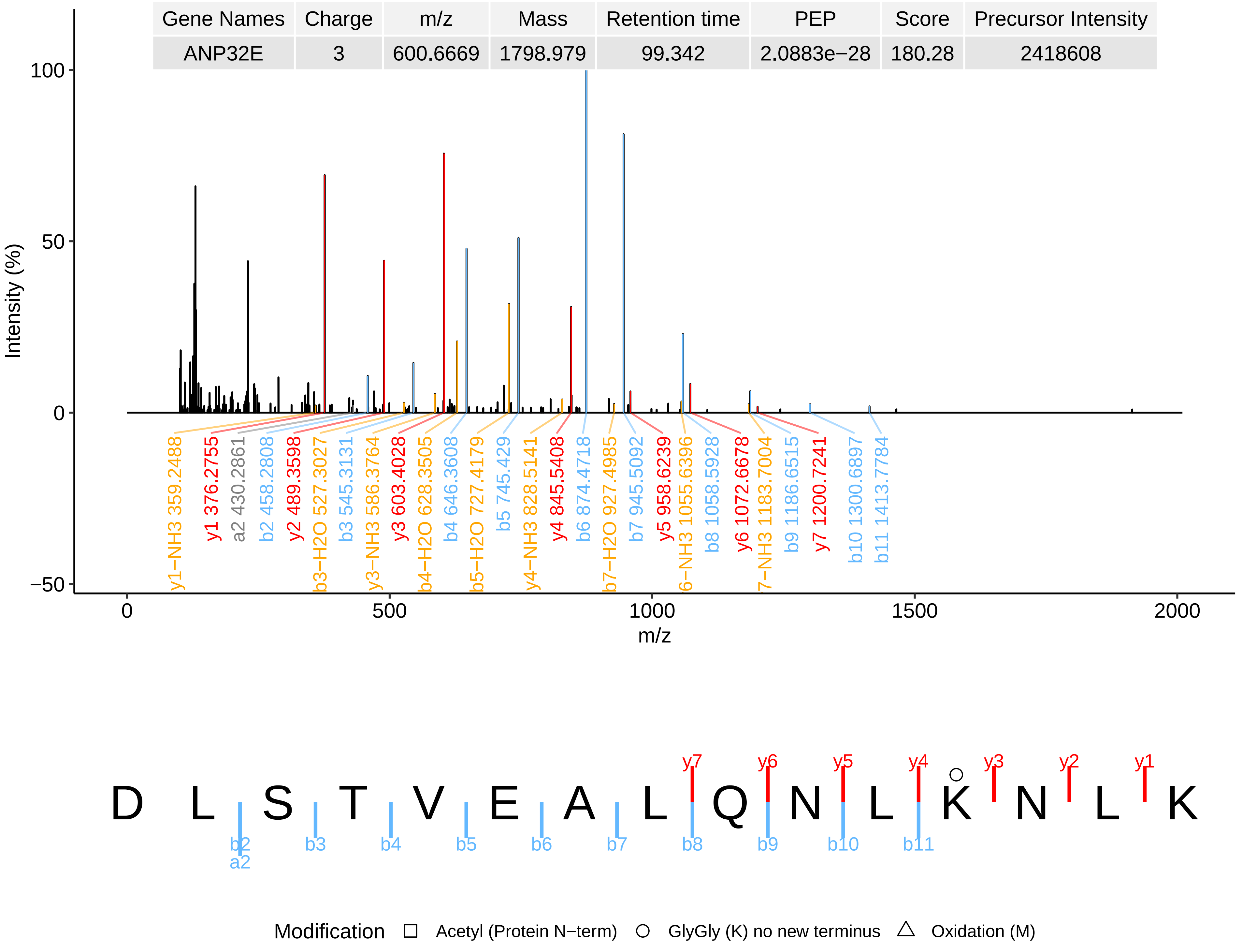

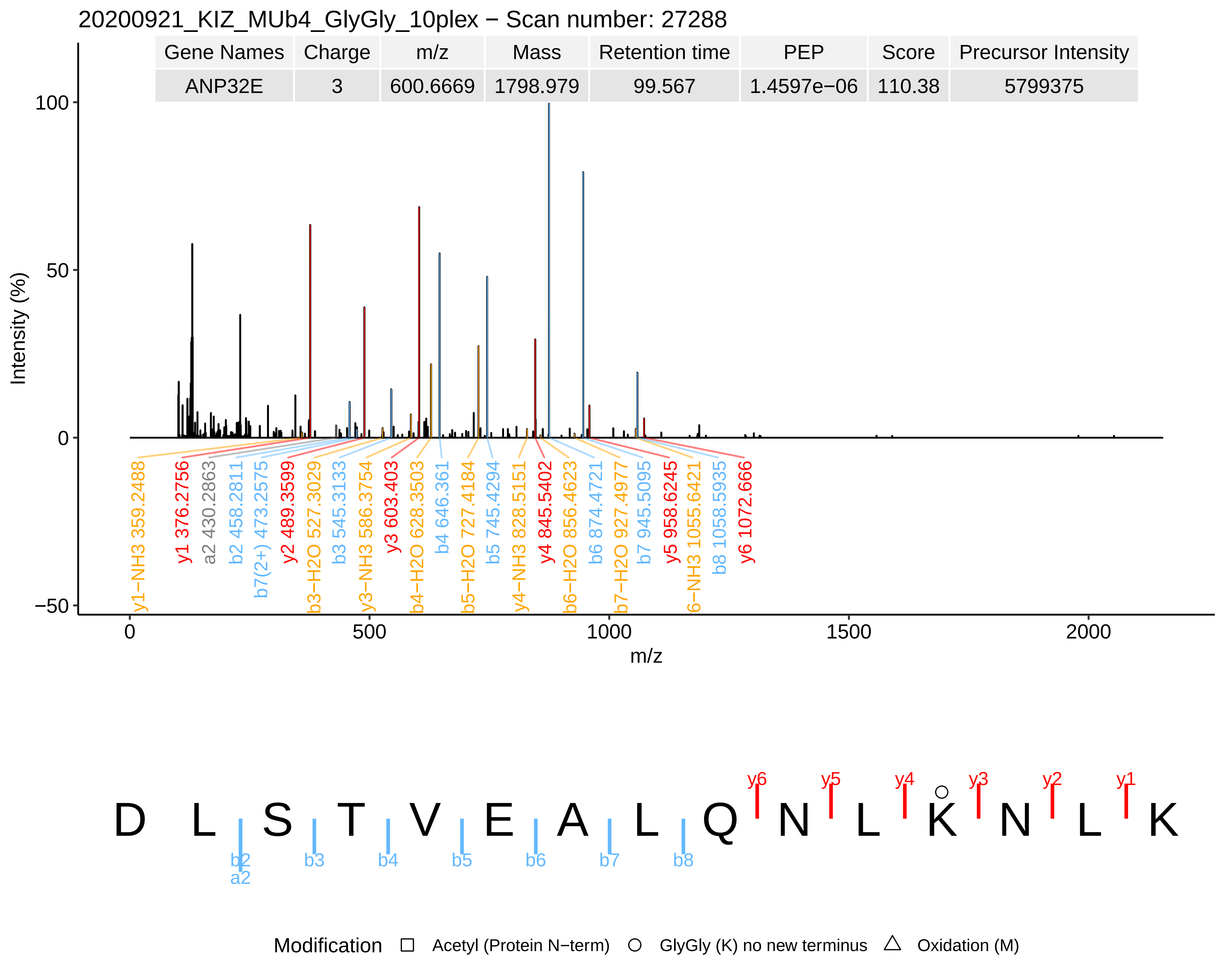

20200921_KIZ_MUb4_GlyGly_10plex − Scan number: 27288
Gene Names | Charge | m/z | Mass | Retention time | PEP | Score | Precursor Intensity
ANP32E | 3 | 600.6669 | 1798.979 | 99.567 | 1.4597e−06 | 110.38 | 5799375
Intensity (%)
m/z
y1−NH3 359.2488
y1 376.2756
a2 430.2863
b2 458.2811
b7(2+) 473.2575
y2 489.3599
b3−H2O 527.3029
b3 545.3133
y3−NH3 586.3754
y3 603.403
b4−H2O 628.3503
b4 646.361
b5−H2O 727.4184
b5 745.4294
y4−NH3 828.5151
y4 845.5402
b6−H2O 856.4623
b6 874.4721
b7−H2O 927.4977
b7 945.5095
y5 958.6245
6−NH3 1055.6421
b8 1058.5935
y6 1072.666
D L S T V E A L Q N L K N L K
Modification
Acetyl (Protein N−term)
GlyGly (K) no new terminus
Oxidation (M)

20200921_KIZ_MUb4_GlyGly_10plex_2 − Scan number: 17608

Gene Names	Charge	m/z	Mass	Retention time	PEP	Score	Precursor Intensity
CBX3	3	621.6315	1861.873	68.87	6.8324e−05	91.207	873969.3

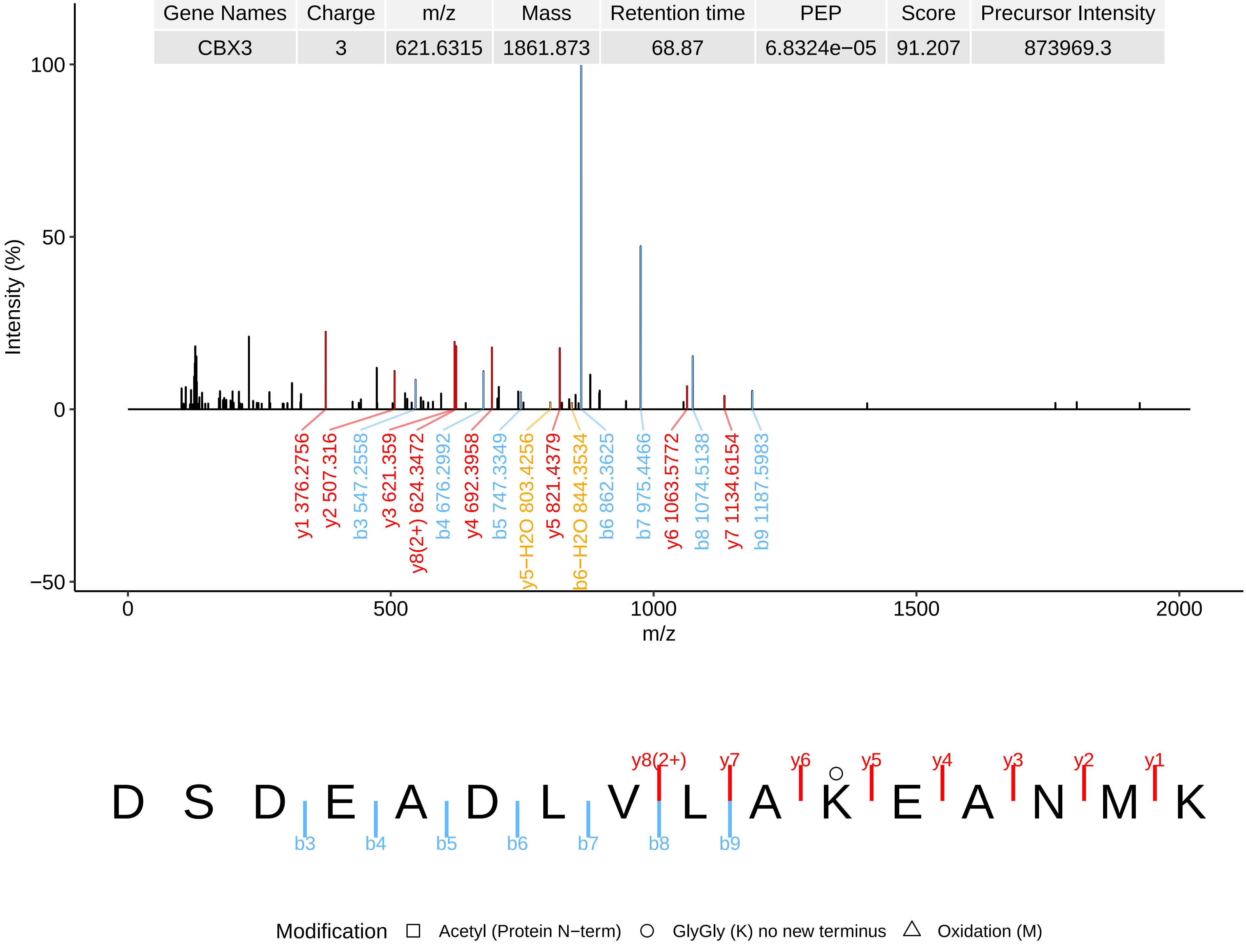

20200924_KIZ_MUb5_GlyGly_1 − Scan number: 19163

Gene Names	Charge	m/z	Mass	Retention time	PEP	Score	Precursor Intensity
CBX3	3	621.6315	1861.873	61.933	8.376e−14	139.64	NA

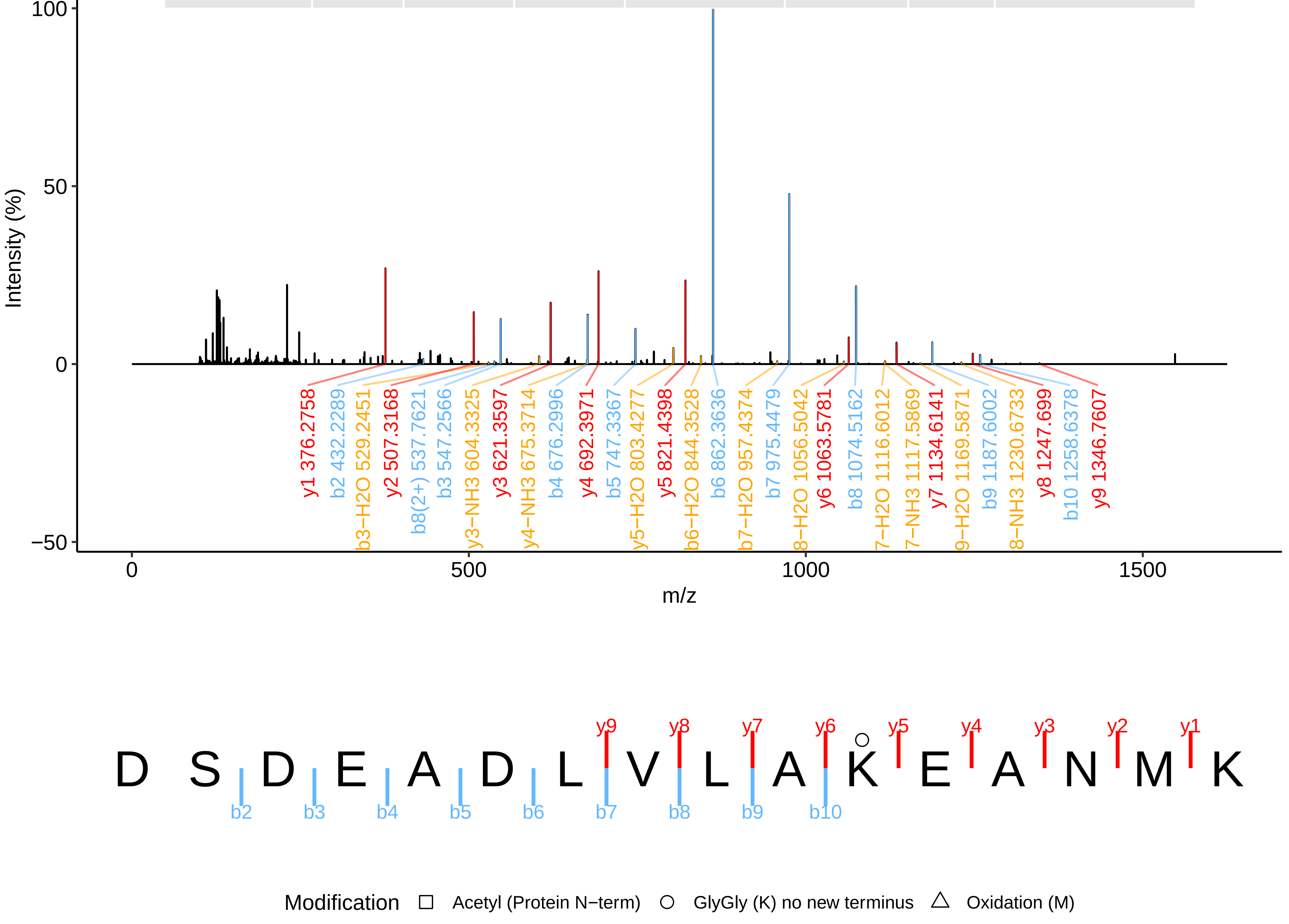

20200921_KIZ_MUb4_GlyGly_10plex_2 − Scan number: 19274

Gene Names	Charge	m/z	Mass	Retention time	PEP	Score	Precursor Intensity
RABEP2	3	685.0114	2052.012	75.632	9.7943e−13	120.63	1161647

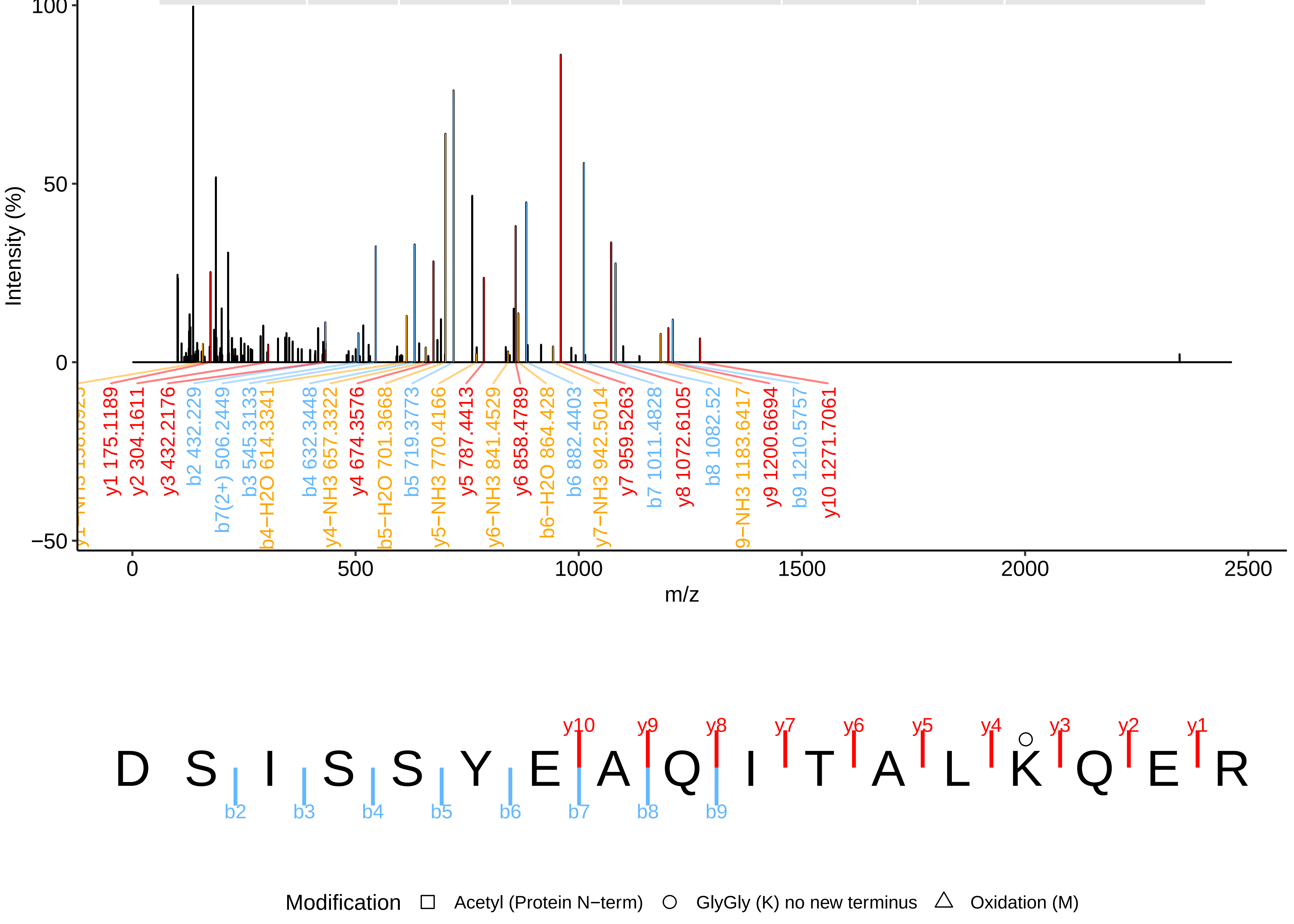

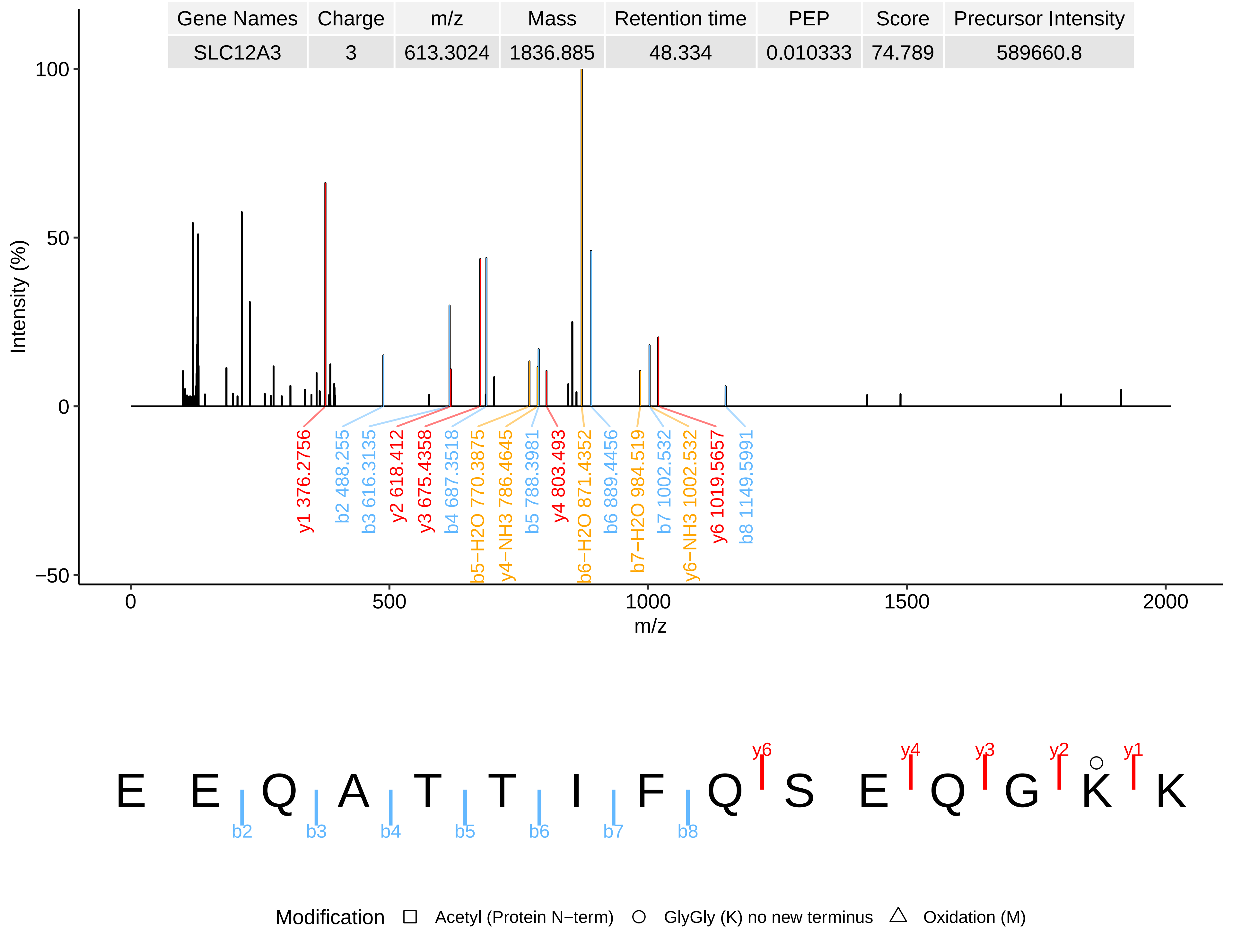

Gene Names	Charge	m/z	Mass	Retention time	PEP	Score	Precursor Intensity
SLC12A3	3	613.3024	1836.885	48.334	0.010333	74.789	589660.8

20200921_KIZ_MUb4_GlyGly_10plex − Scan number: 7599

Gene Names	Charge	m/z	Mass	Retention time	PEP	Score	Precursor Intensity
BASP1	3	619.2938	1854.86	25.561	1.6868e−17	147.49	65738460

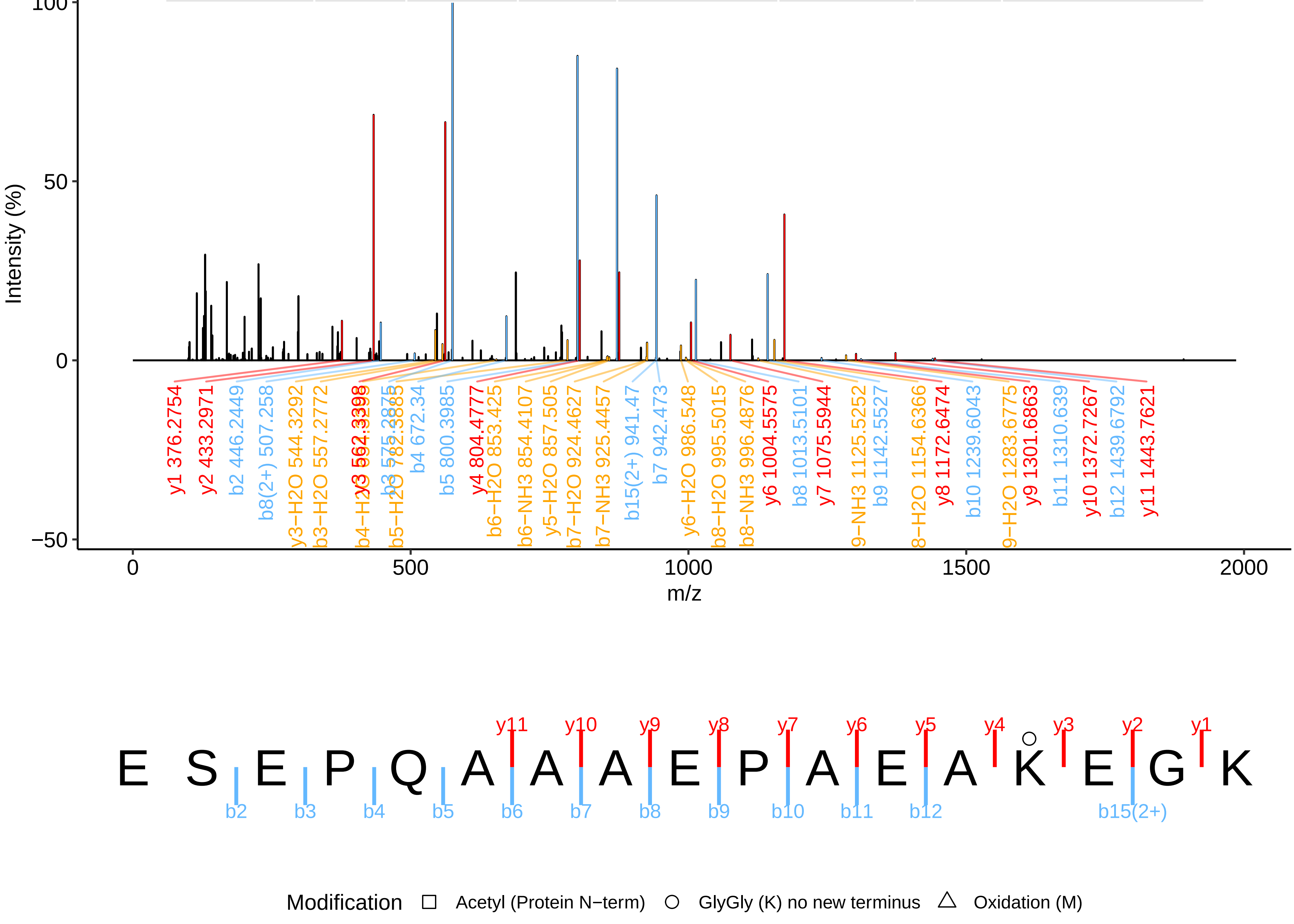

20200921_KIZ_MUb4_GlyGly_10plex − Scan number: 7739

Gene Names	Charge	m/z	Mass	Retention time	PEP	Score	Precursor Intensity
BASP1	3	619.2938	1854.86	26.086	1.2565e−12	117.18	5911962

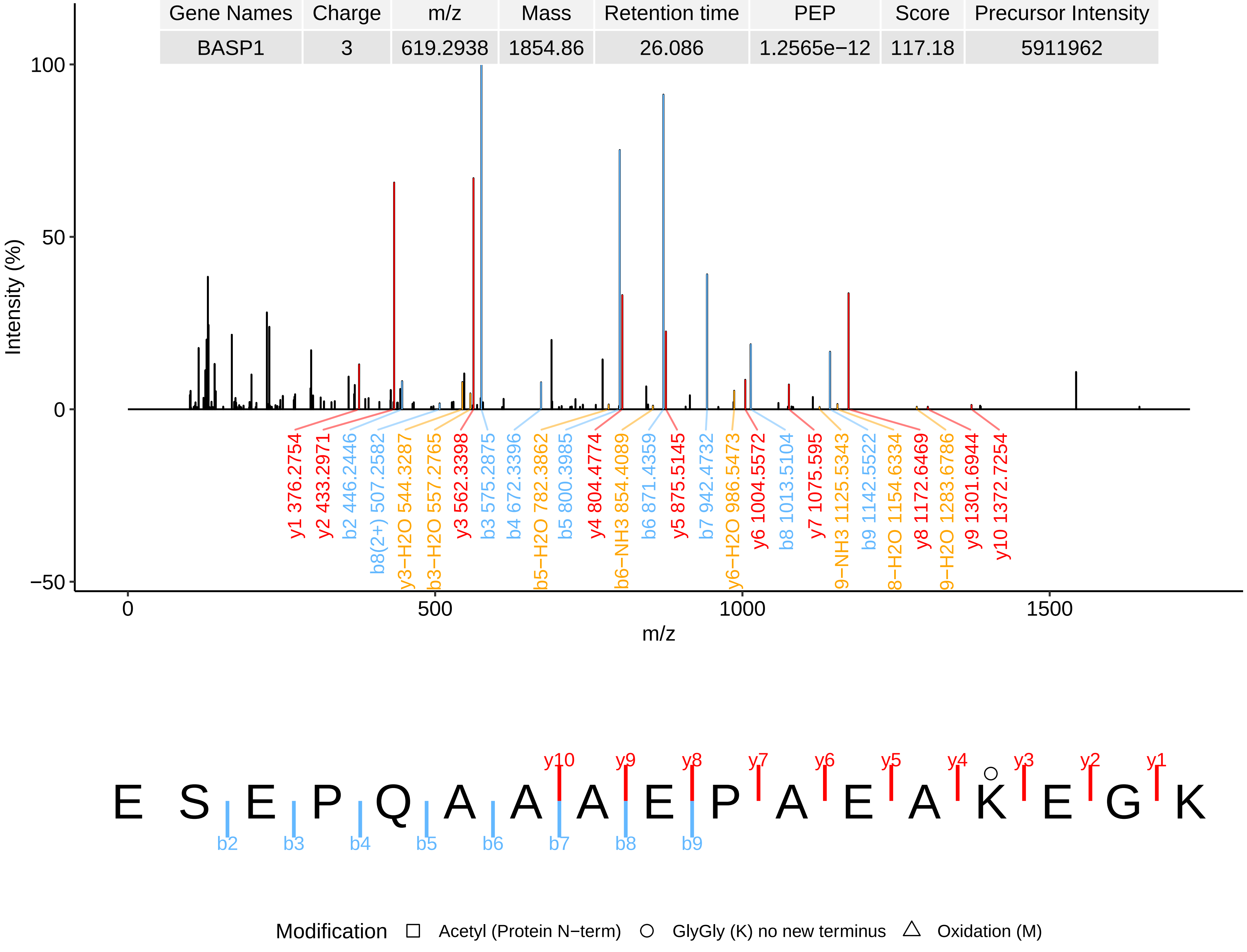

20200921_KIZ_MUb4_GlyGly_10plex_2 − Scan number: 6915

Gene Names	Charge	m/z	Mass	Retention time	PEP	Score	Precursor Intensity
BASP1	3	619.2938	1854.86	25.615	0.0014889	70.783	705724.2

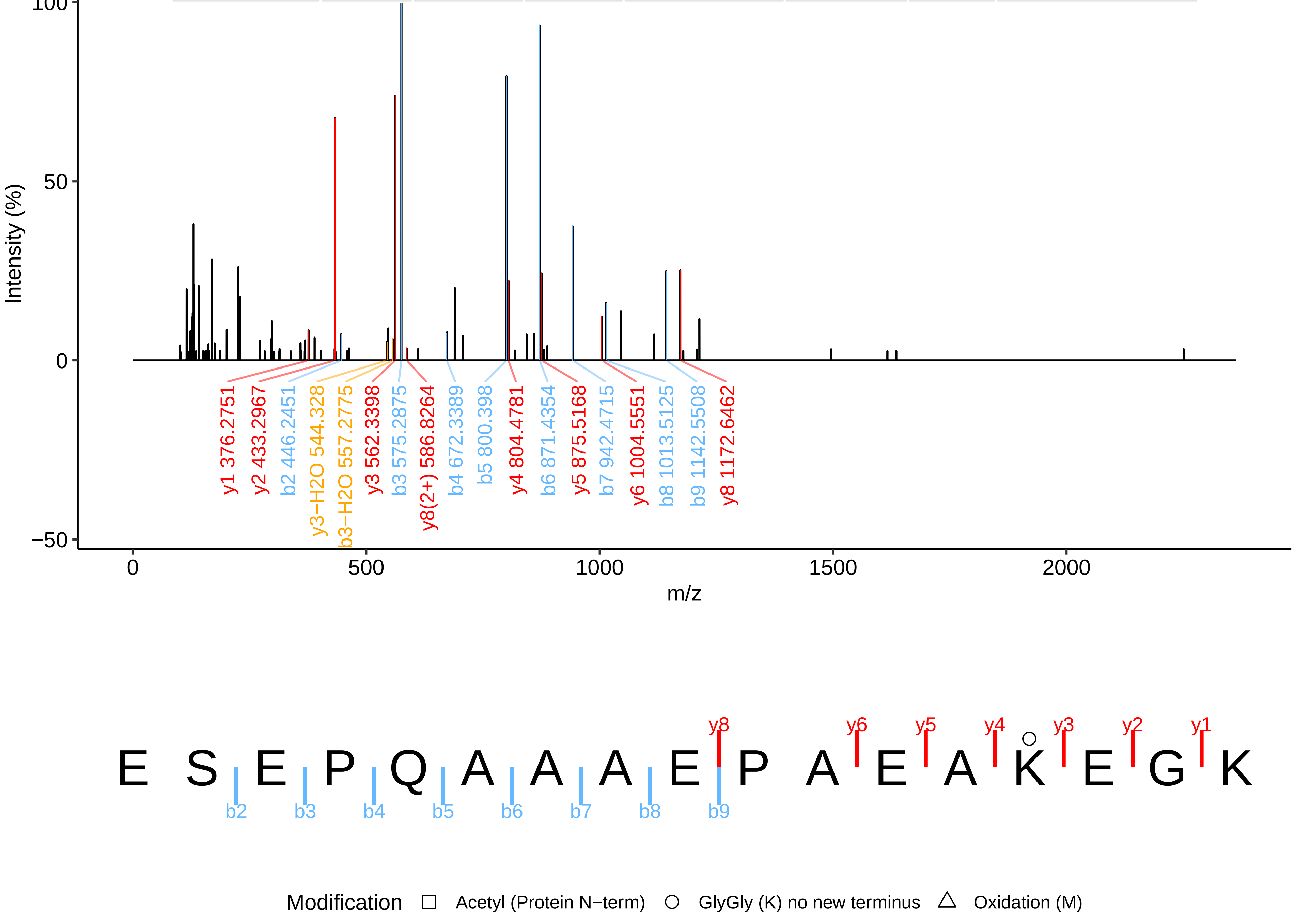

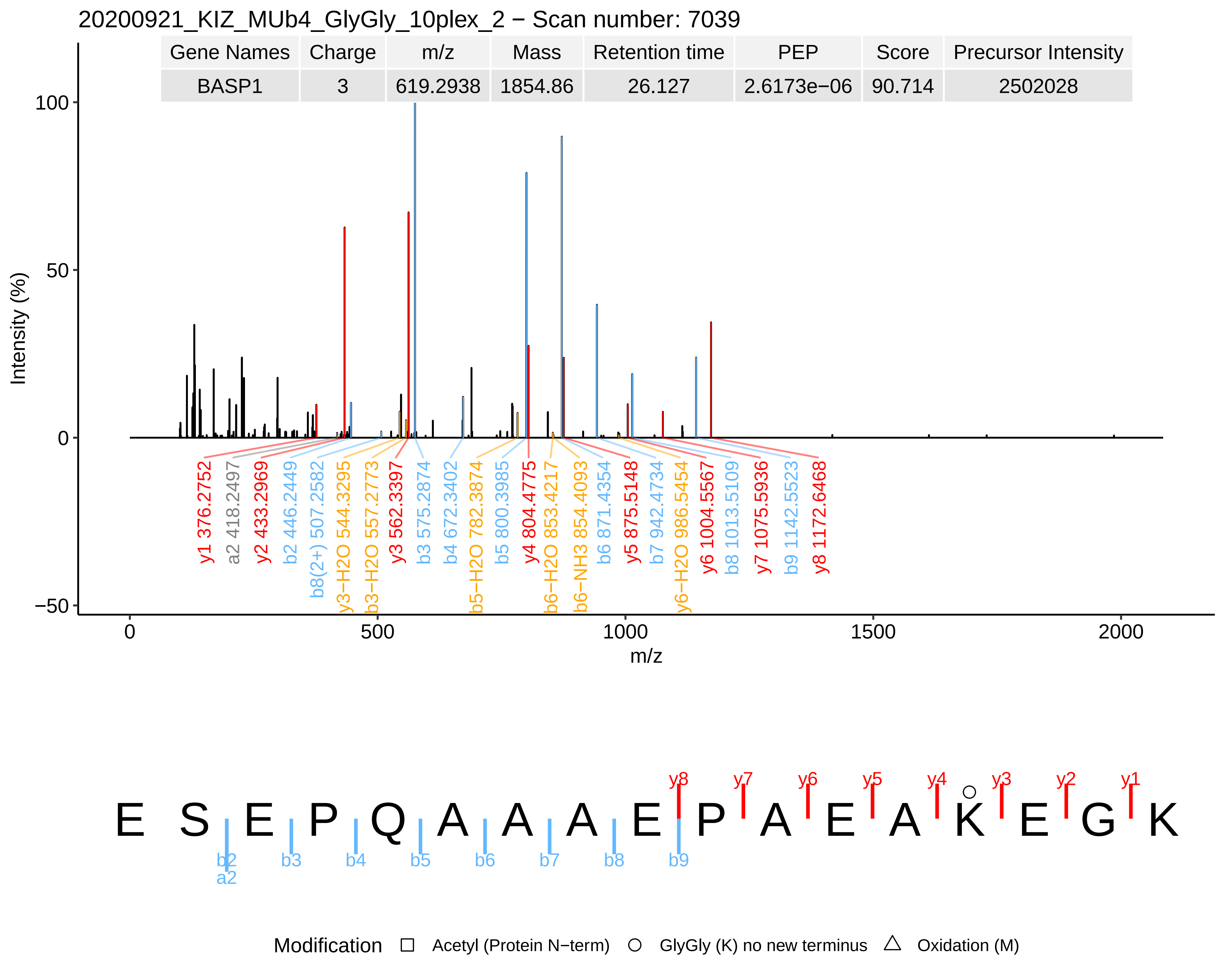

20200921_KIZ_MUb4_GlyGly_10plex_2 − Scan number: 7039
Gene Names | Charge | m/z | Mass | Retention time | PEP | Score | Precursor Intensity
BASP1 | 3 | 619.2938 | 1854.86 | 26.127 | 2.6173e−06 | 90.714 | 2502028
Intensity (%)
m/z
y1 376.2752
a2 418.2497
y2 433.2969
b2 446.2449
b8(2+) 507.2582
y3−H2O 544.3295
b3−H2O 557.2773
y3 562.3397
b3 575.2874
b4 672.3402
b5−H2O 782.3874
b5 800.3985
y4 804.4775
b6−H2O 853.4217
b6−NH3 854.4093
b6 871.4354
y5 875.5148
b7 942.4734
y6−H2O 986.5454
y6 1004.5567
b8 1013.5109
y7 1075.5936
b9 1142.5523
y8 1172.6468
E S E P Q A A A E P A E A K E G K
Modification
Acetyl (Protein N−term)
GlyGly (K) no new terminus
Oxidation (M)

20200924_KIZ_MUb5_GlyGly_1 − Scan number: 6654

Gene Names	Charge	m/z	Mass	Retention time	PEP	Score	Precursor Intensity
BASP1	3	619.2938	1854.86	20.983	6.007e−17	140.46	10790371

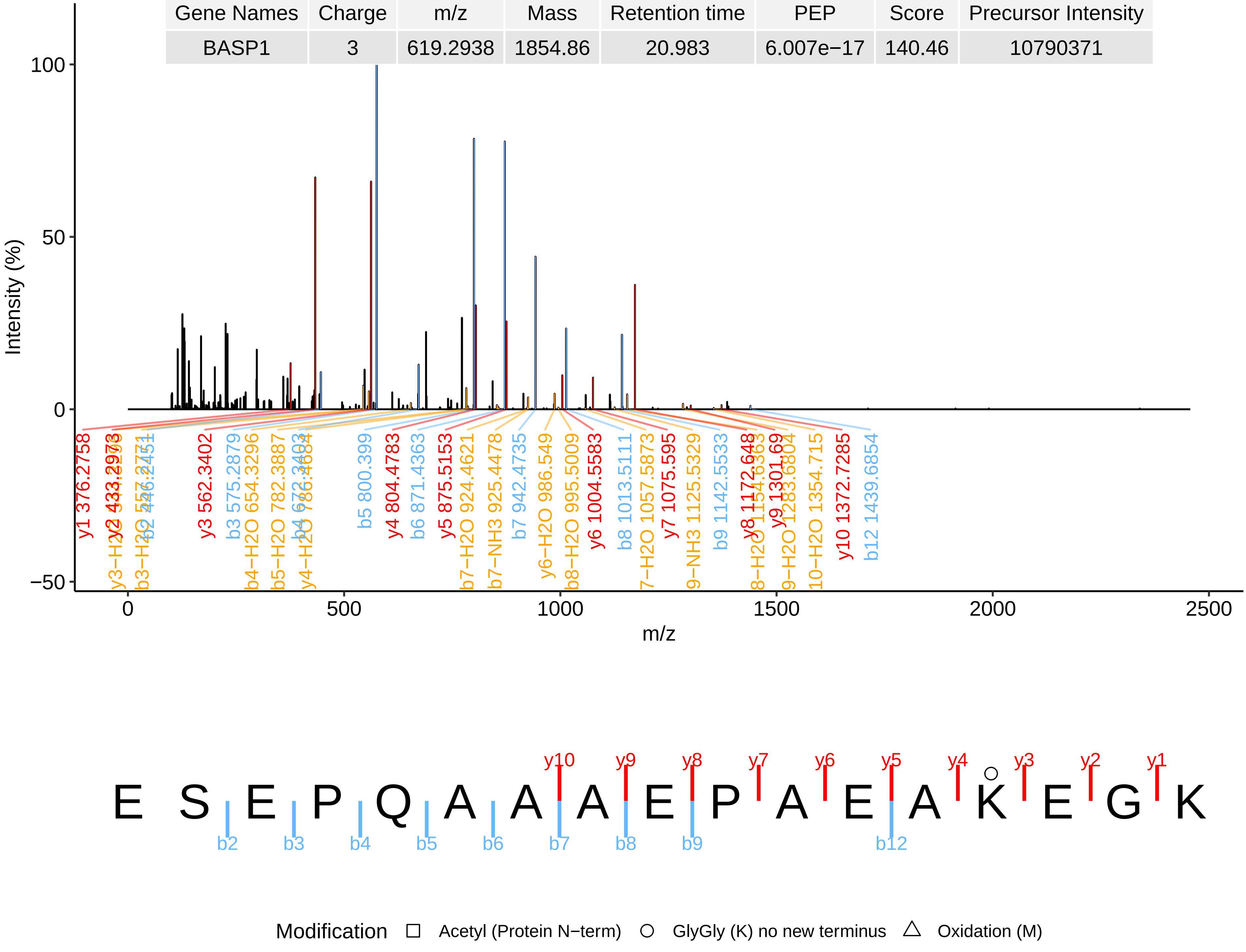

20200924_KIZ_MUb5_GlyGly_1 − Scan number: 6803

Gene Names	Charge	m/z	Mass	Retention time	PEP	Score	Precursor Intensity
BASP1	3	619.2938	1854.86	21.486	6.857e−13	124.29	81834792

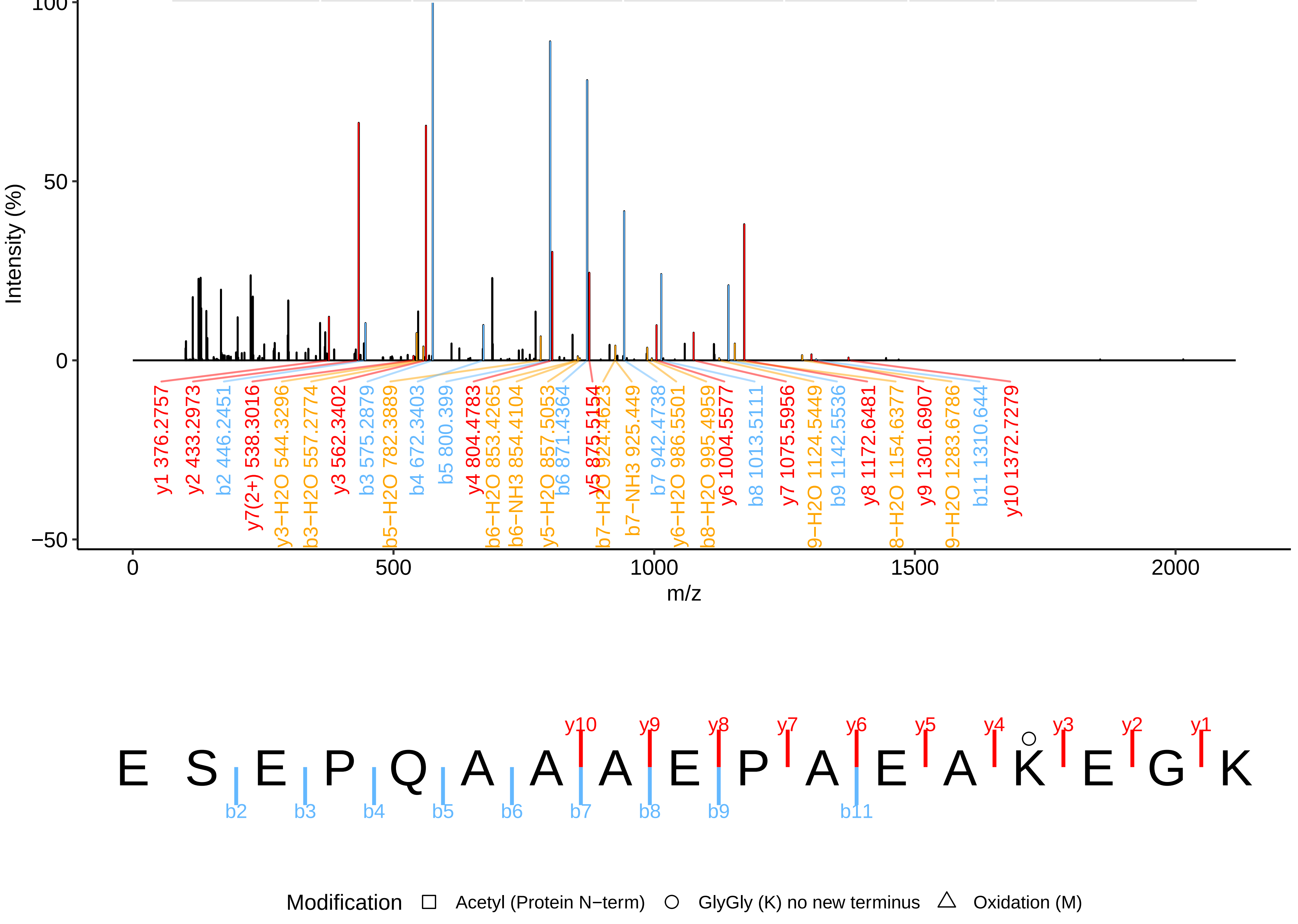

20200924_KIZ_MUb5_GlyGly_2 − Scan number: 6179

Gene Names	Charge	m/z	Mass	Retention time	PEP	Score	Precursor Intensity
BASP1	3	619.2938	1854.86	23.025	7.1125e−10	103.04	1893940

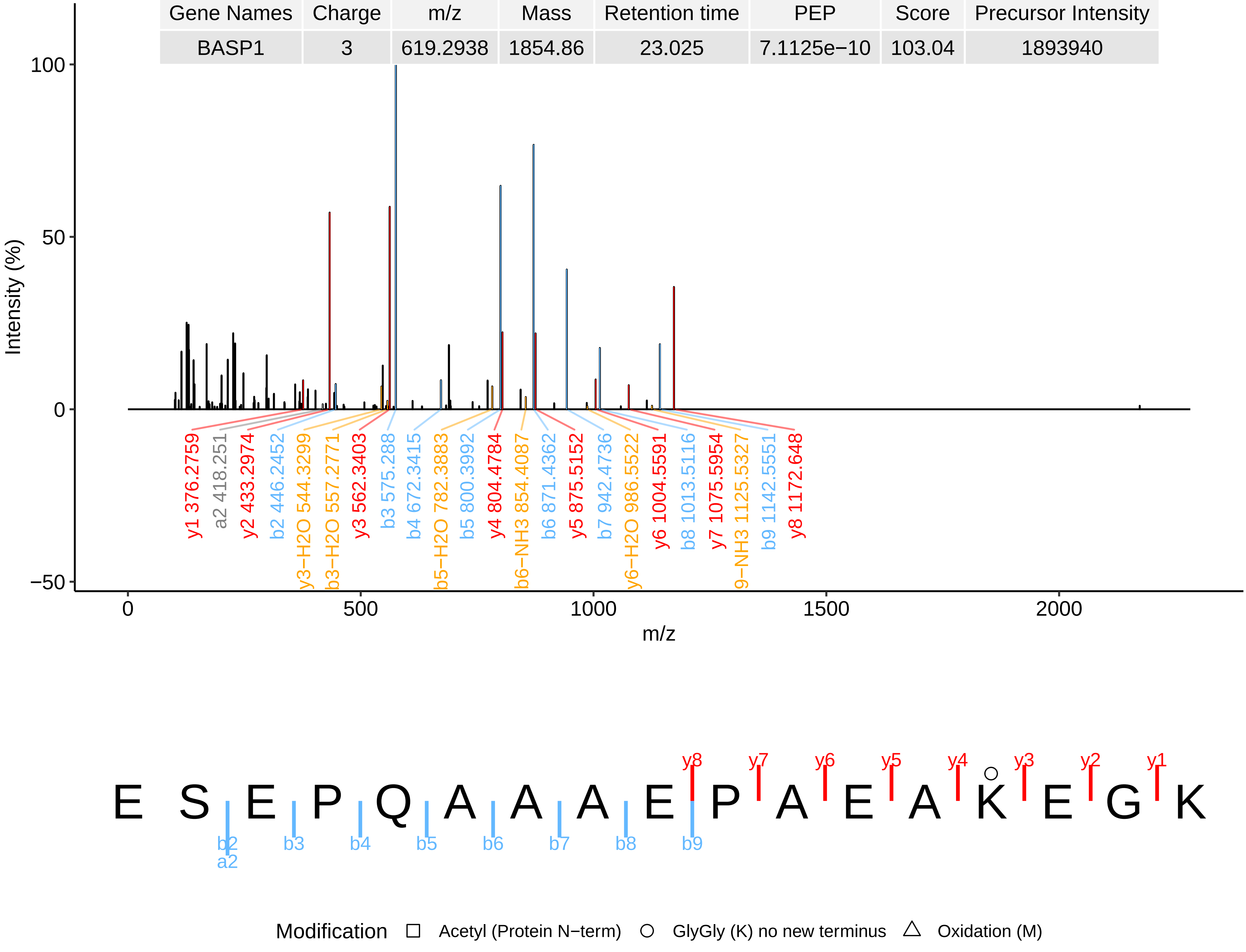

20200924_KIZ_MUb5_GlyGly_2 − Scan number: 6189

Gene Names	Charge	m/z	Mass	Retention time	PEP	Score	Precursor Intensity
BASP1	3	619.2938	1854.86	23.062	8.026e−07	95.767	1414716

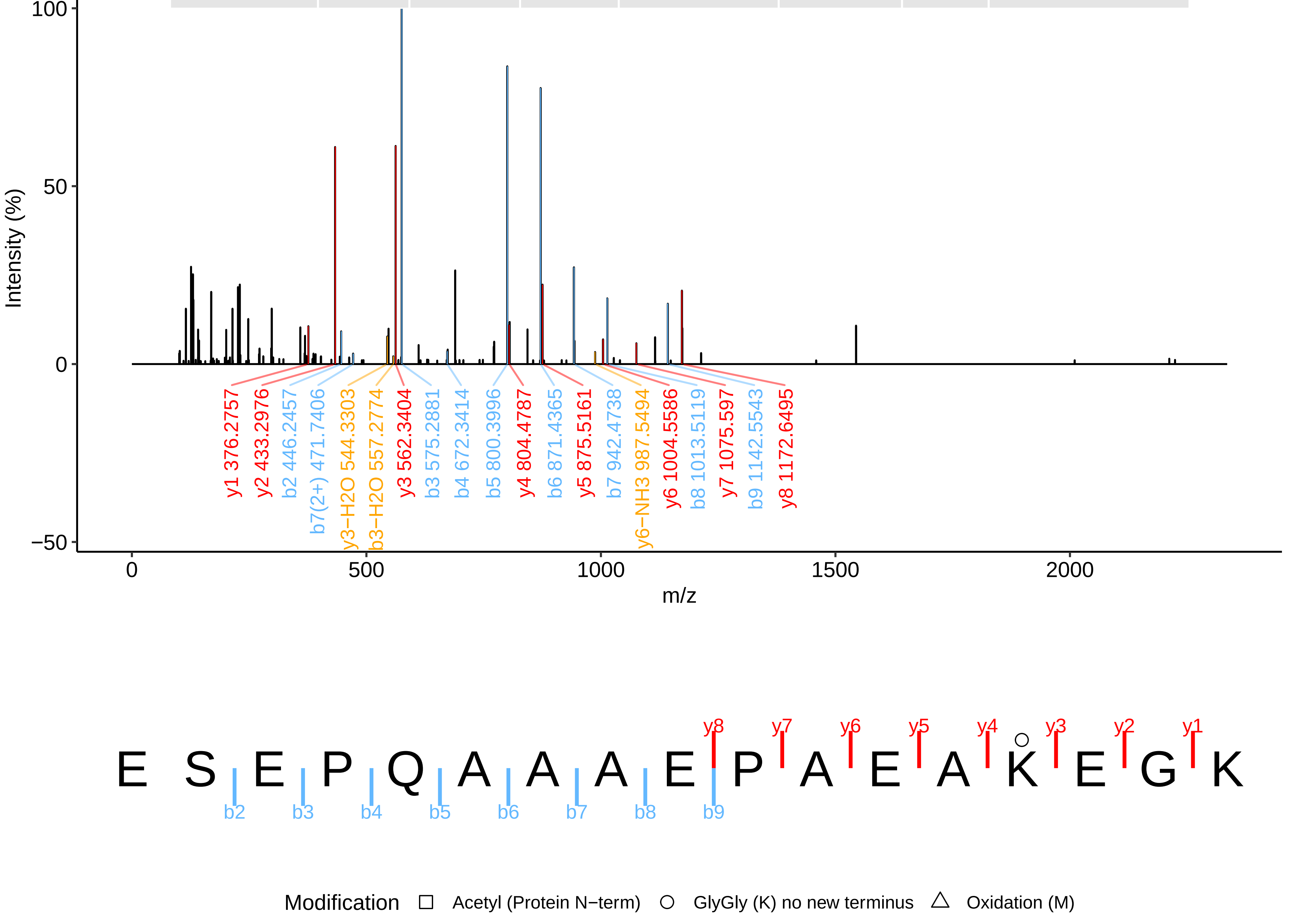

20200924_KIZ_MUb5_GlyGly_2 − Scan number: 6459

Gene Names	Charge	m/z	Mass	Retention time	PEP	Score	Precursor Intensity
BASP1	3	619.2938	1854.86	24.087	1.1532e−17	148.36	5142448

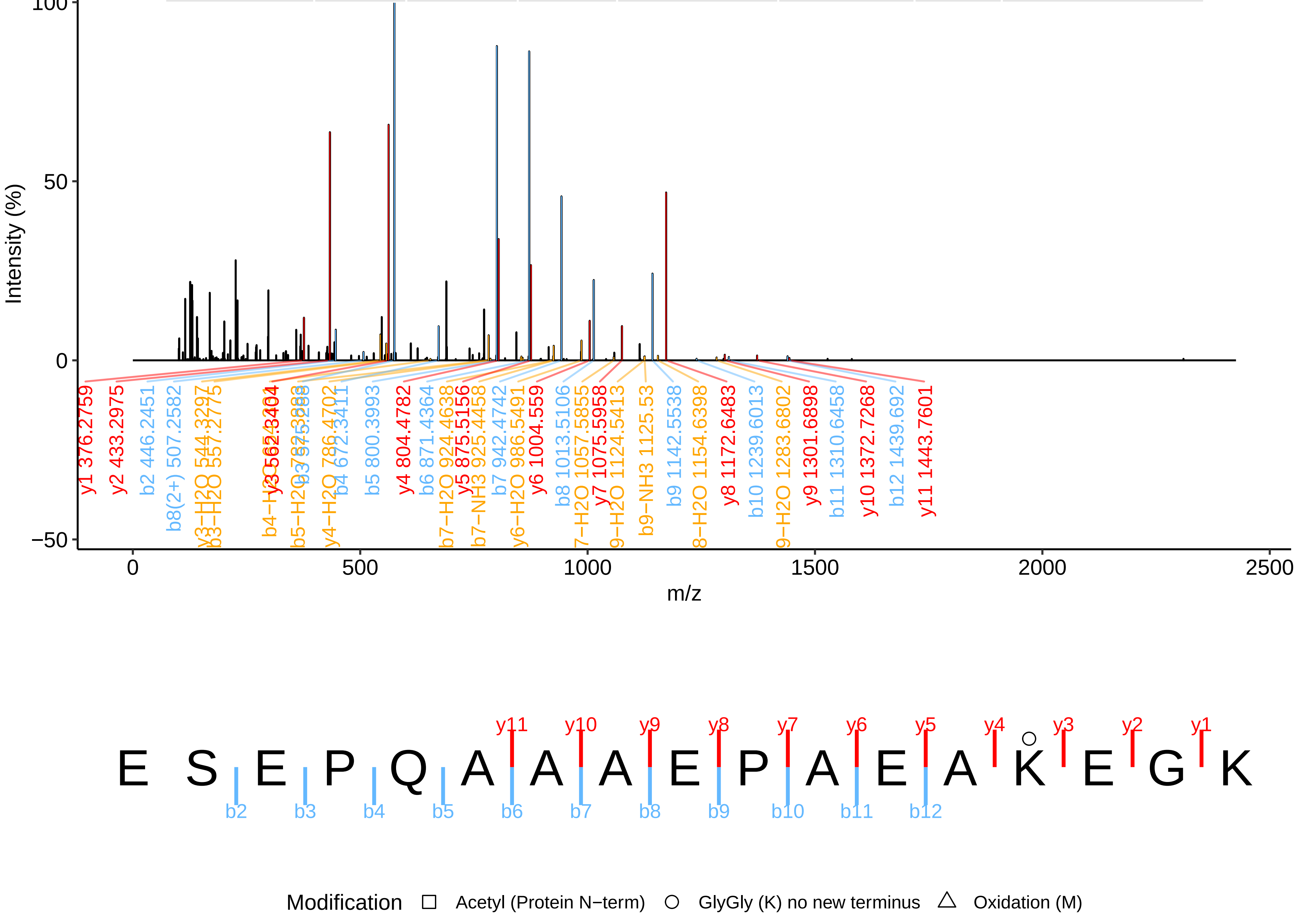

20201118_KIZ_MUb6_GlyGly_1 − Scan number: 6313

Gene Names	Charge	m/z	Mass	Retention time	PEP	Score	Precursor Intensity
BASP1	3	619.2938	1854.86	19.959	1.0778e−12	119.4	11366933

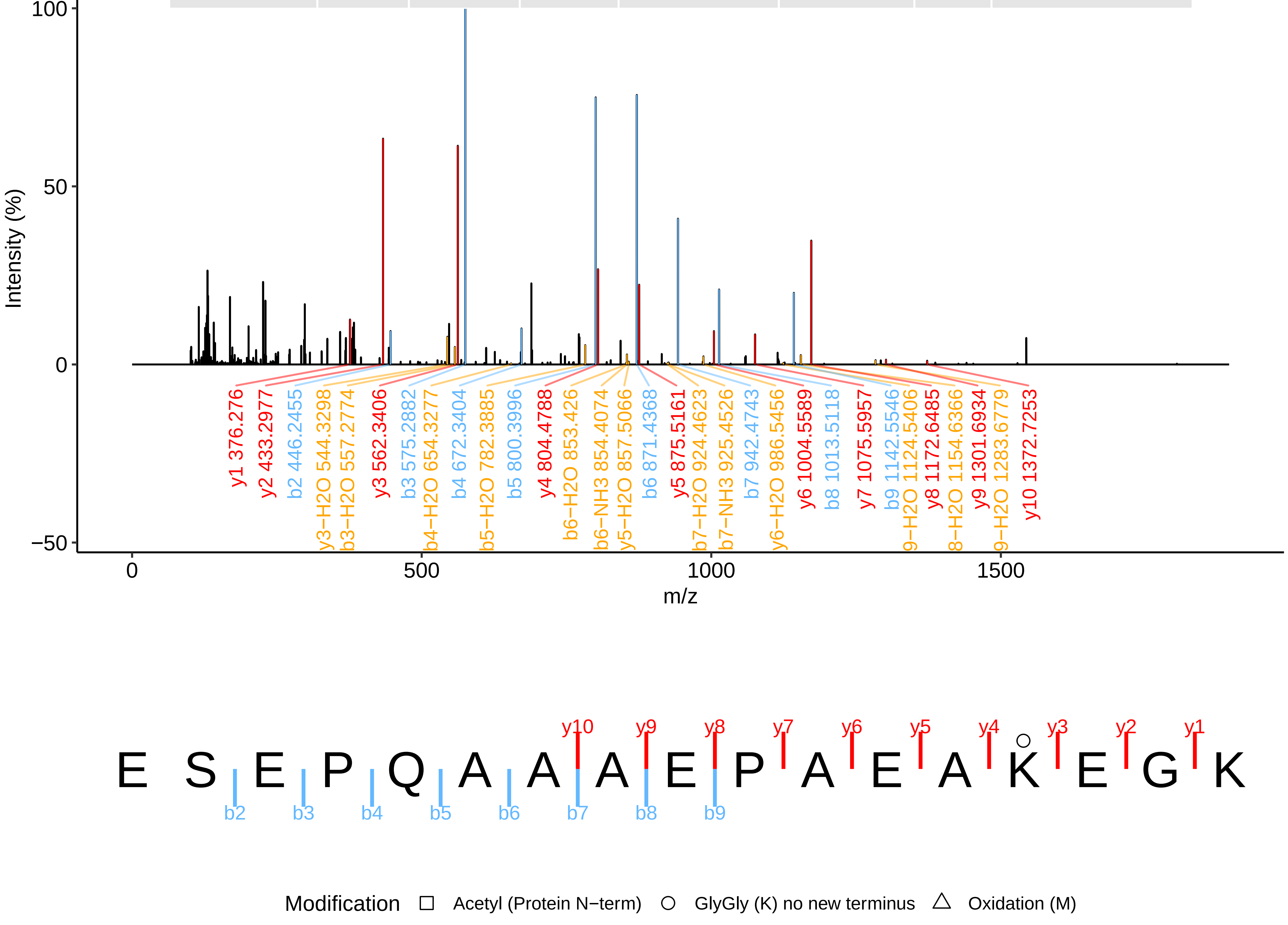

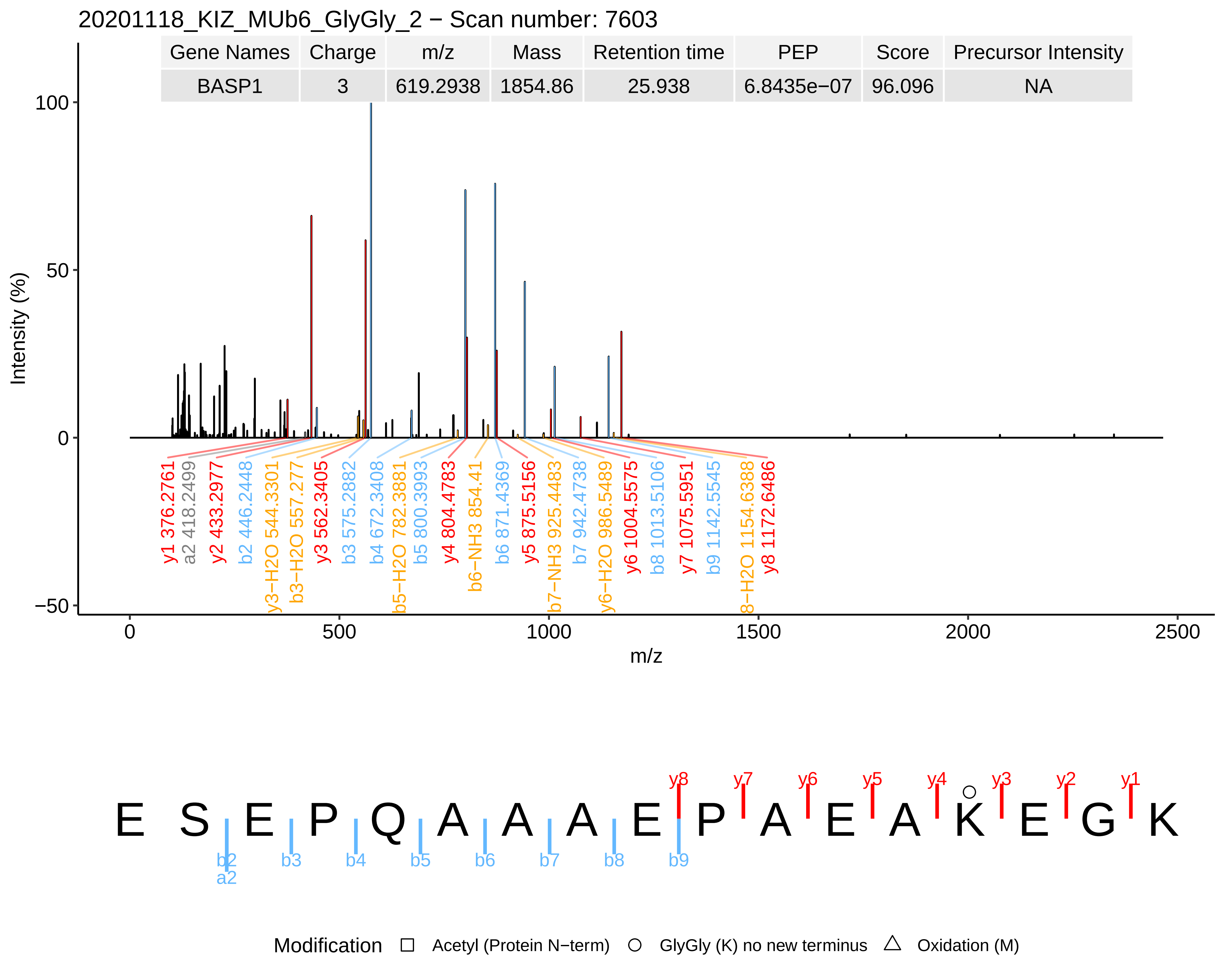

20201118_KIZ_MUb6_GlyGly_2 − Scan number: 7603

Gene Names	Charge	m/z	Mass	Retention time	PEP	Score	Precursor Intensity
BASP1	3	619.2938	1854.86	25.938	6.8435e−07	96.096	NA

Intensity (%)
m/z
y1 376.2761
a2 418.2499
y2 433.2977
b2 446.2448
y3−H2O 544.3301
b3−H2O 557.277
y3 562.3405
b3 575.2882
b4 672.3408
b5−H2O 782.3881
b5 800.3993
y4 804.4783
b6−NH3 854.41
b6 871.4369
y5 875.5156
b7−NH3 925.4483
b7 942.4738
y6−H2O 986.5489
y6 1004.5575
b8 1013.5106
y7 1075.5951
b9 1142.5545
8−H2O 1154.6388
y8 1172.6486
E S E P Q A A A E P A E A K E G K
Modification
Acetyl (Protein N−term)
GlyGly (K) no new terminus
Oxidation (M)

20201118_KIZ_MUb6_GlyGly_2 − Scan number: 7618

Gene Names	Charge	m/z	Mass	Retention time	PEP	Score	Precursor Intensity
BASP1	3	619.2938	1854.86	25.989	1.2172e−12	117.67	7018352

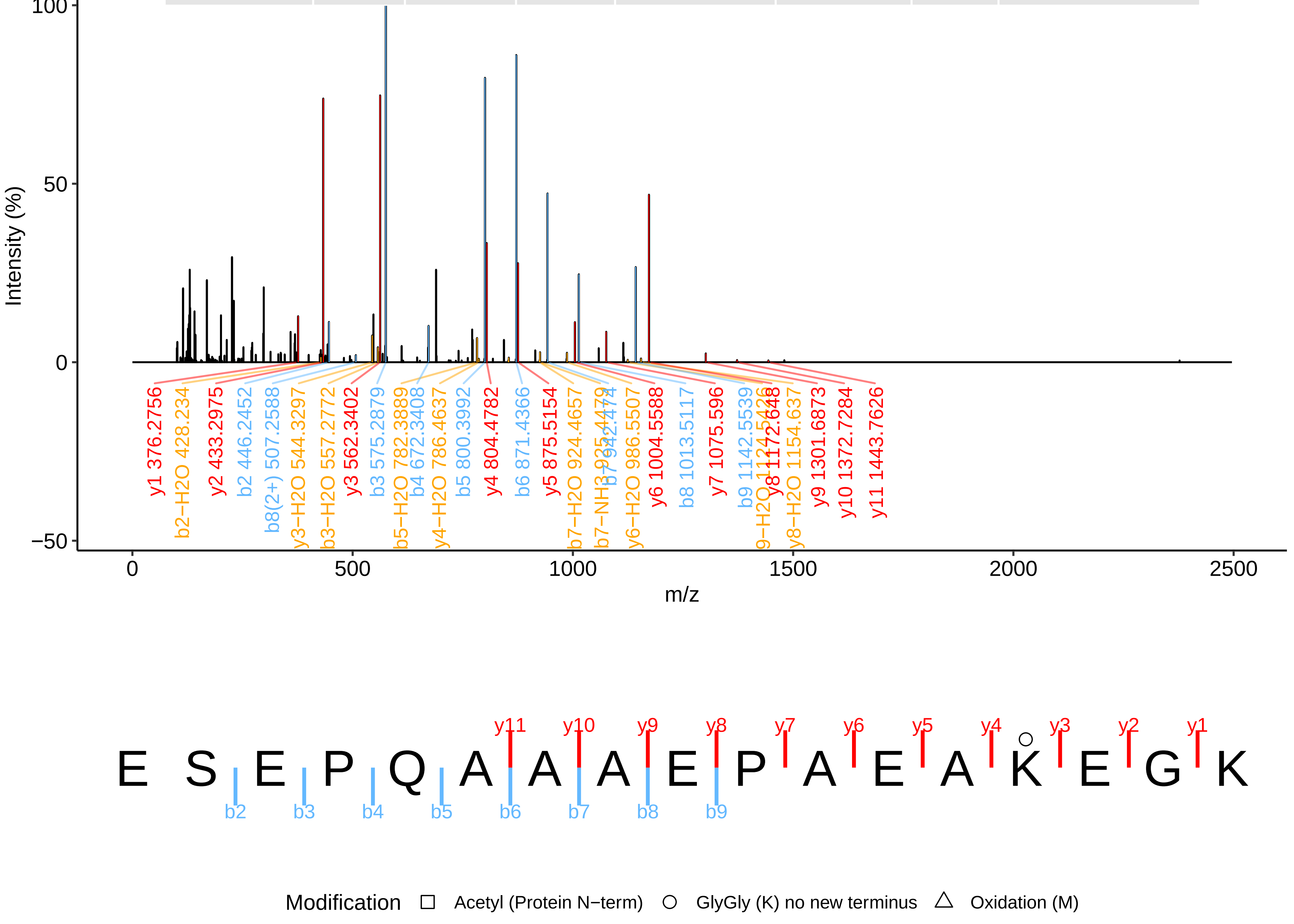

20200921_KIZ_MUb4_GlyGly_10plex − Scan number: 12646

Gene Names	Charge	m/z	Mass	Retention time	PEP	Score	Precursor Intensity
TOMM70A	3	605.9684	1814.883	44.421	6.3461e−05	92.063	7373187

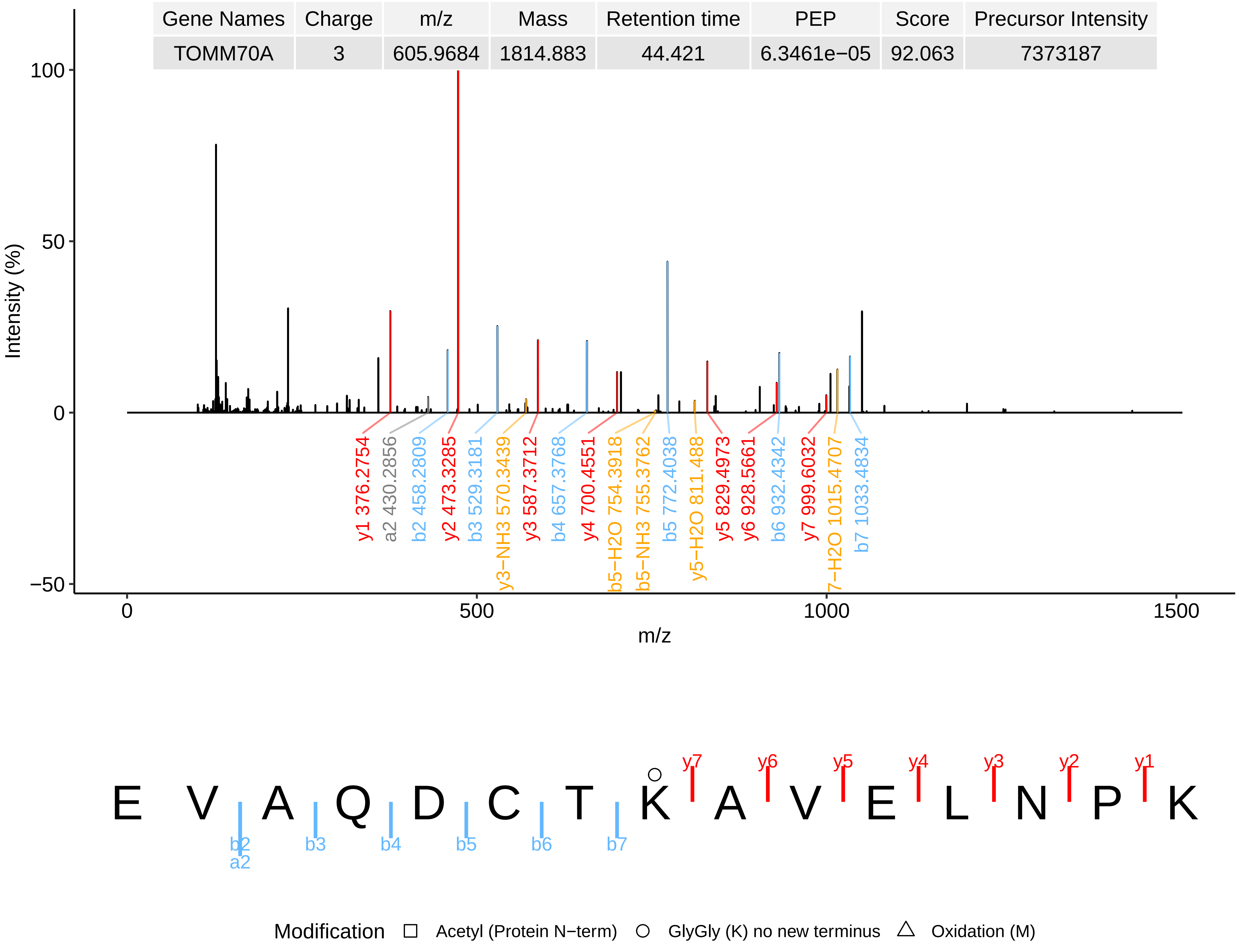

20200921_KIZ_MUb4_GlyGly_10plex_2 − Scan number: 11541

Gene Names	Charge	m/z	Mass	Retention time	PEP	Score	Precursor Intensity
TOMM70A	3	605.9684	1814.883	44.426	0.00032613	82.651	1025545

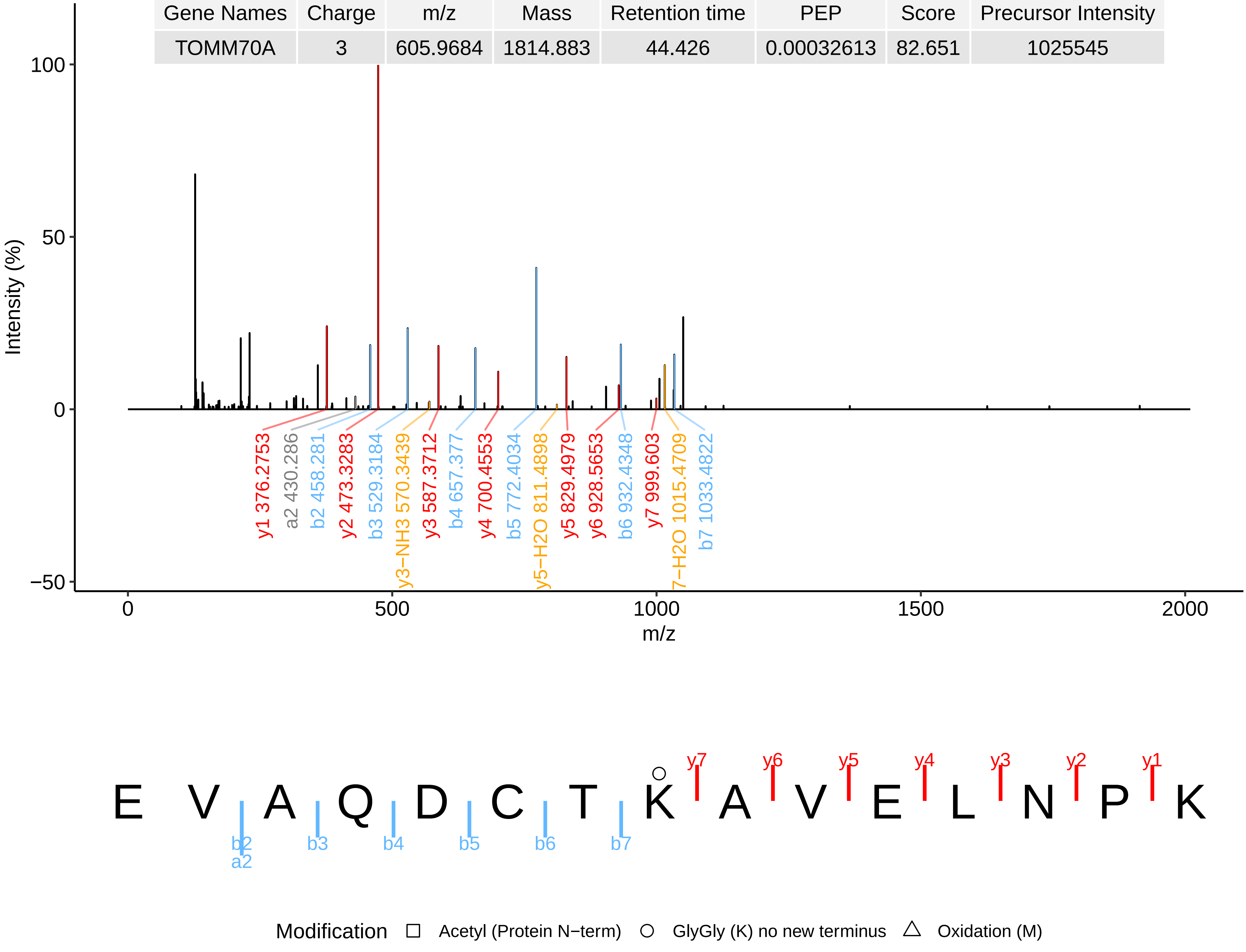

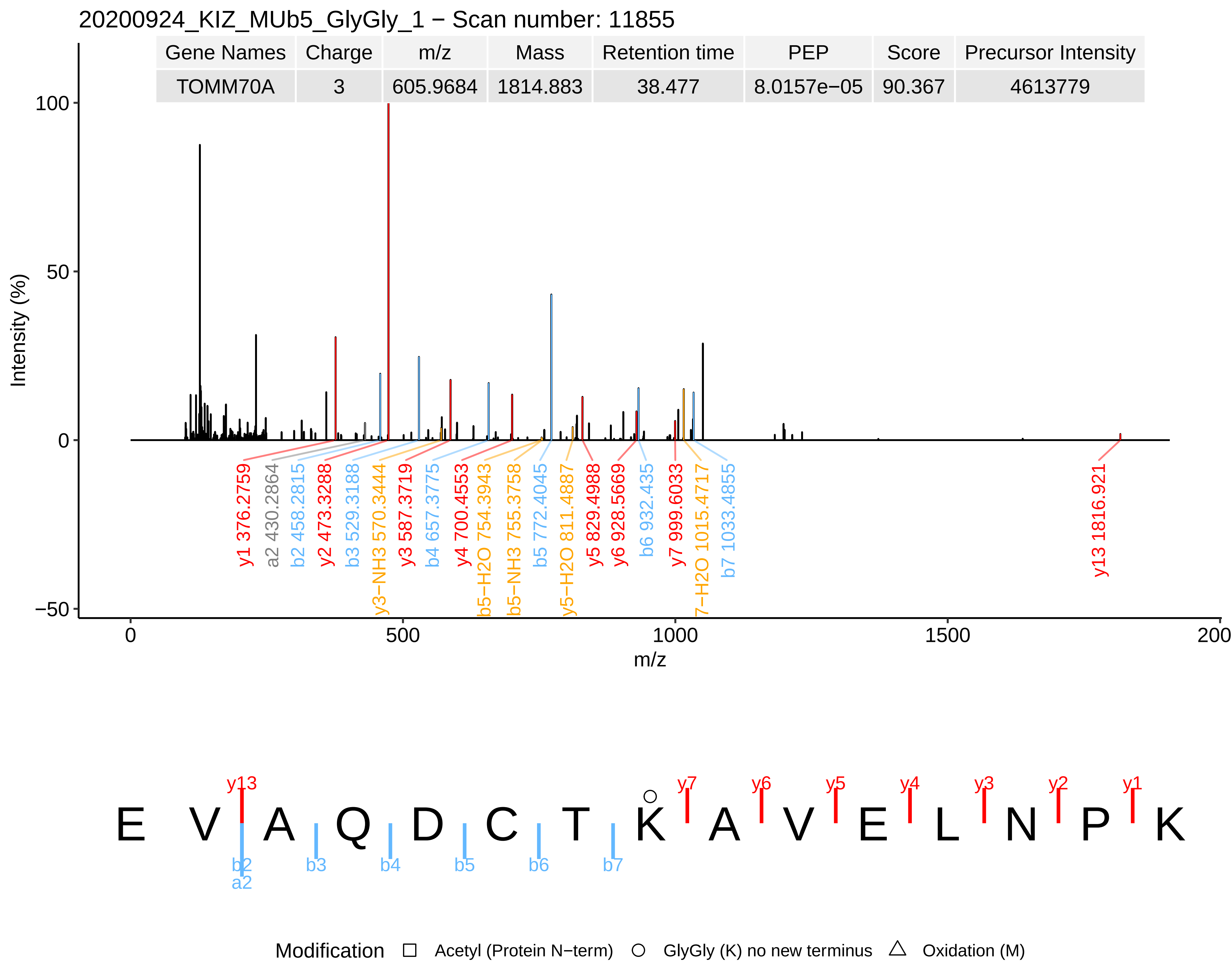
20200924_KIZ_MUb5_GlyGly_1 − Scan number: 11855
Gene Names | Charge | m/z | Mass | Retention time | PEP | Score | Precursor Intensity
TOMM70A | 3 | 605.9684 | 1814.883 | 38.477 | 8.0157e−05 | 90.367 | 4613779
Intensity (%)
100
50
0
−50
m/z
0
500
1000
1500
200
y1 376.2759
a2 430.2864
b2 458.2815
y2 473.3288
b3 529.3188
y3−NH3 570.3444
y3 587.3719
b4 657.3775
y4 700.4553
b5−H2O 754.3943
b5−NH3 755.3758
b5 772.4045
y5−H2O 811.4887
y5 829.4988
y6 928.5669
b6 932.435
y7 999.6033
7−H2O 1015.4717
b7 1033.4855
y13 1816.921
E V A Q D C T K A V E L N P K
y13 y7 y6 y5 y4 y3 y2 y1
b2 b3 b4 b5 b6 b7
a2
Modification
Acetyl (Protein N−term)
GlyGly (K) no new terminus
Oxidation (M)

20200924_KIZ_MUb5_GlyGly_2 − Scan number: 11010

Gene Names	Charge	m/z	Mass	Retention time	PEP	Score	Precursor Intensity
TOMM70A	3	605.9684	1814.883	41.114	0.00017524	80.706	4024061

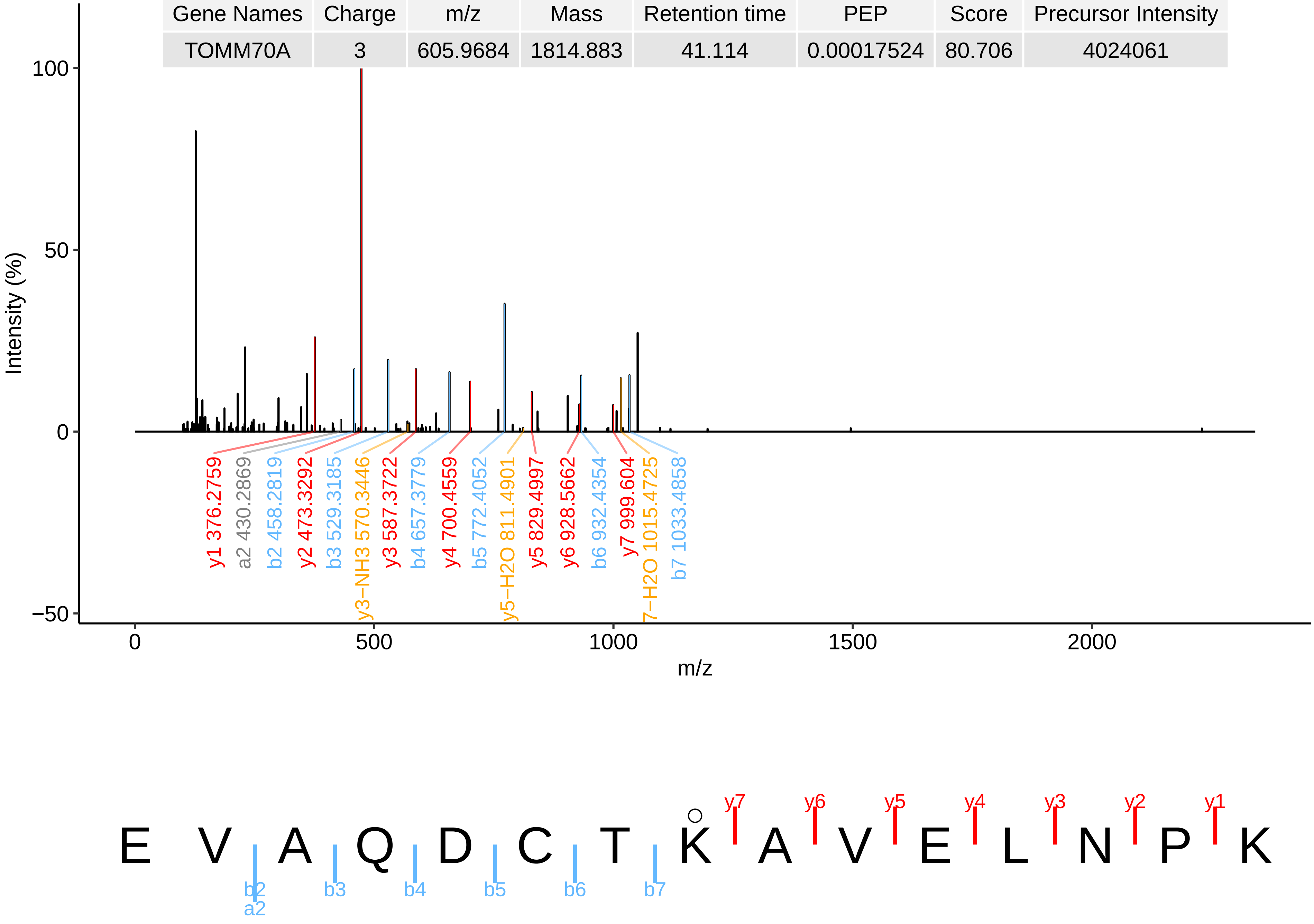

Modification □ Acetyl (Protein N−term) ○ GlyGly (K) no new terminus △ Oxidation (M)

20200921_KIZ_MUb4_GlyGly_10plex − Scan number: 24712

Gene Names	Charge	m/z	Mass	Retention time	PEP	Score	Precursor Intensity
LDHA	3	598.3187	1791.934	89.886	0.0003195	90.926	1341569

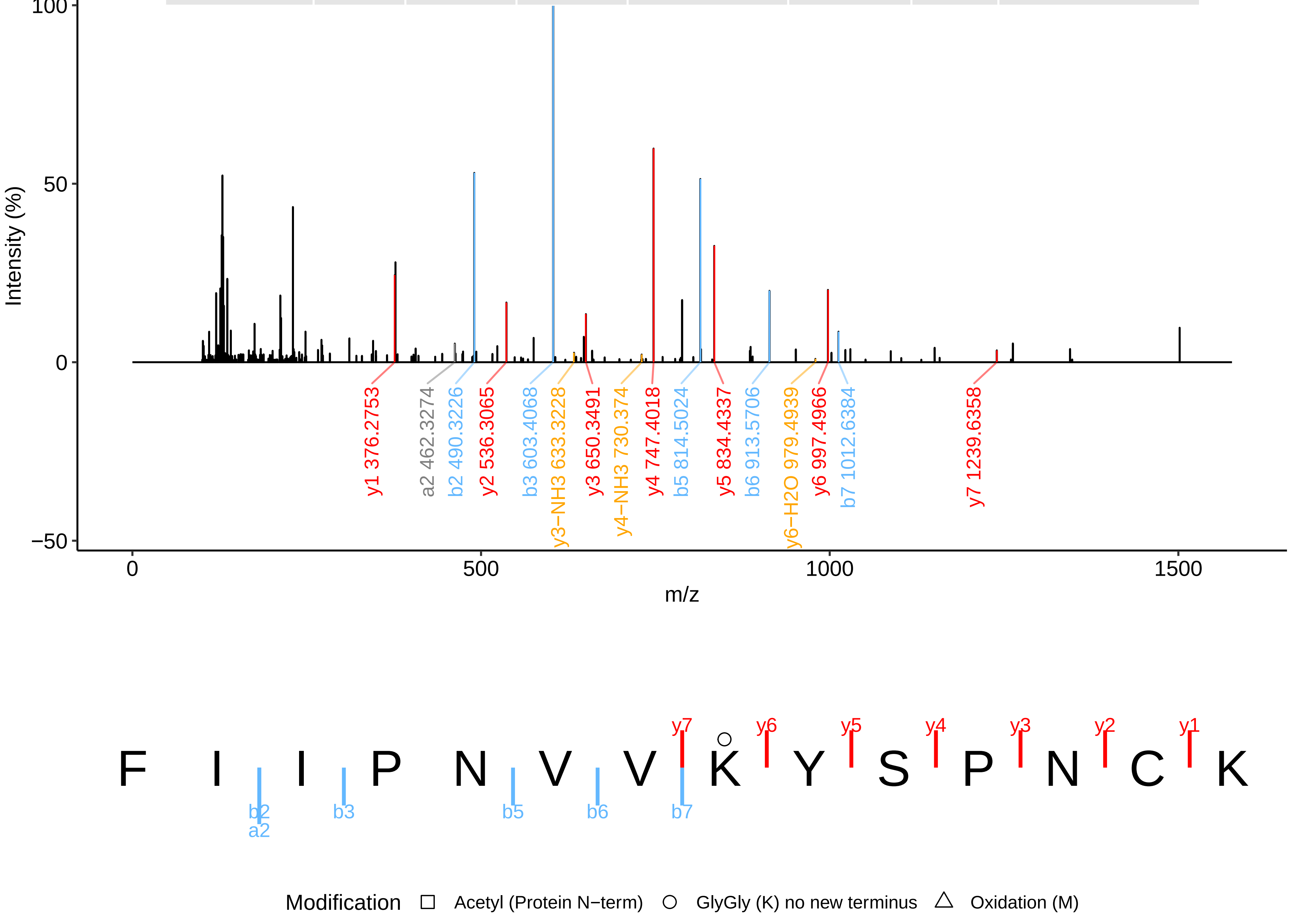

20200921_KIZ_MUb4_GlyGly_10plex − Scan number: 24785

Gene Names	Charge	m/z	Mass	Retention time	PEP	Score	Precursor Intensity
LDHA	3	598.3187	1791.934	90.17	0.00060406	84.169	3993022

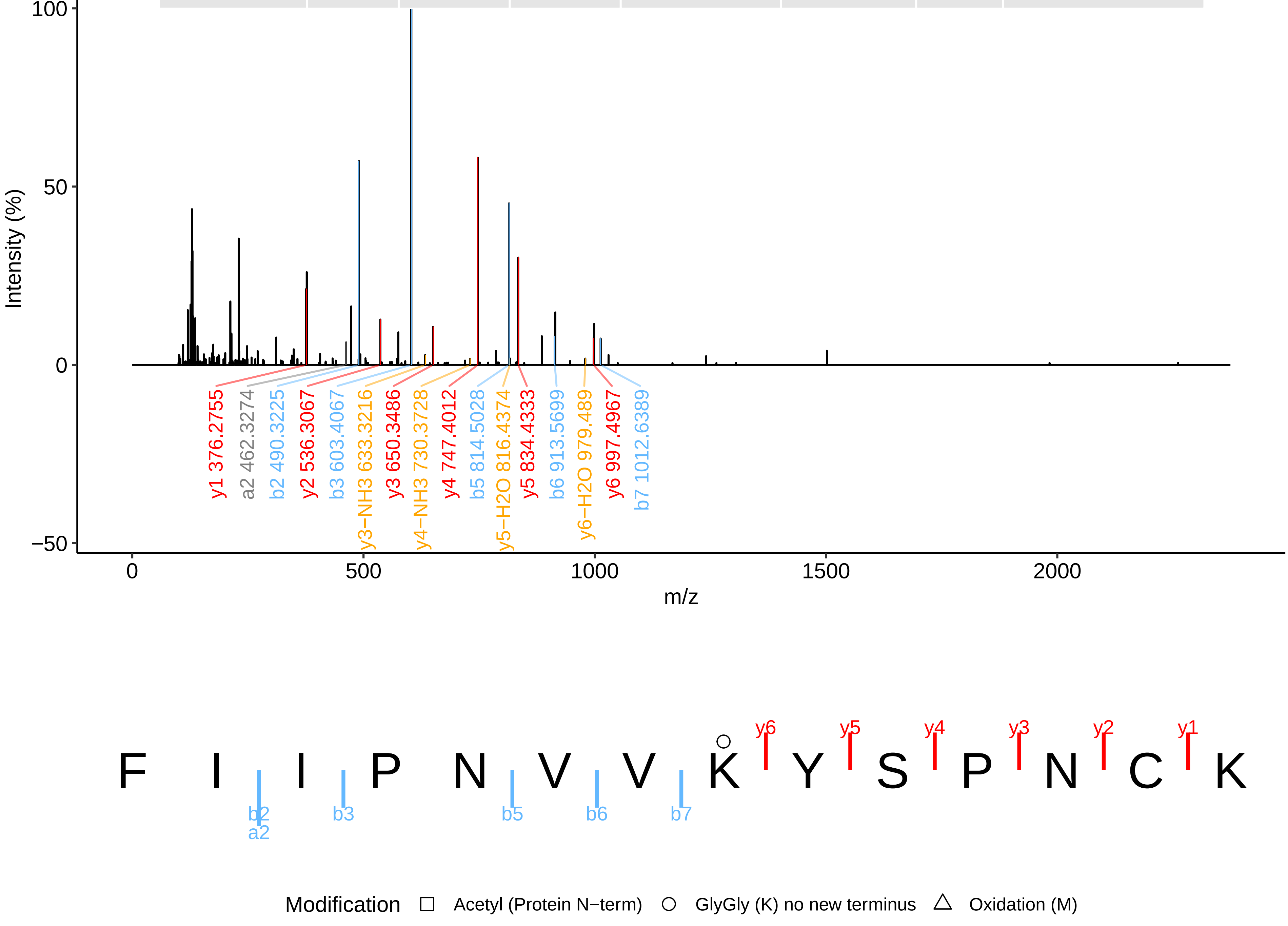

20200921_KIZ_MUb4_GlyGly_10plex_2 − Scan number: 21669

Gene Names	Charge	m/z	Mass	Retention time	PEP	Score	Precursor Intensity
LDHA	3	598.3187	1791.934	85.335	0.0034051	73.466	792935.1

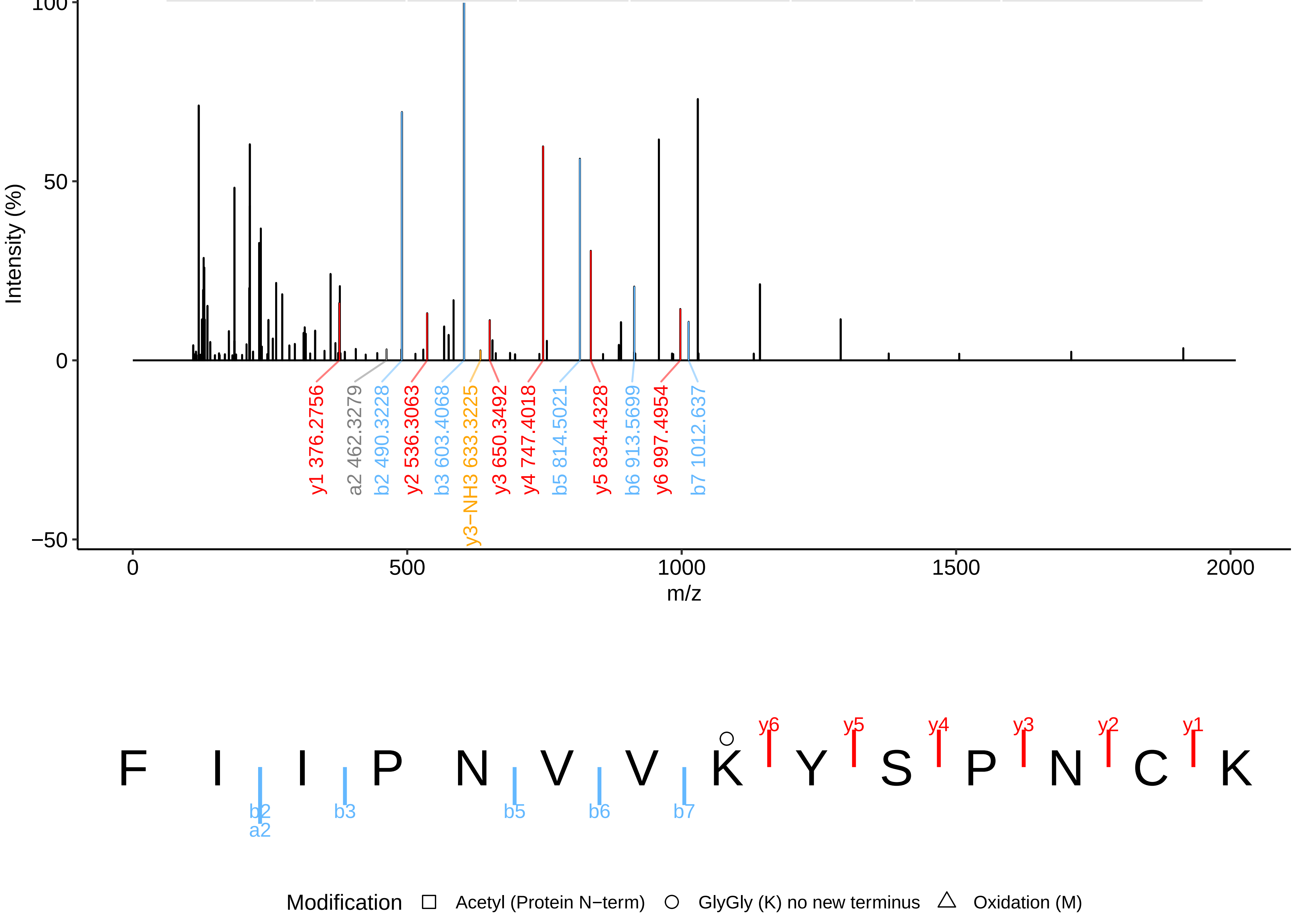

20201118_KIZ_MUb6_GlyGly_2 − Scan number: 26514

Gene Names	Charge	m/z	Mass	Retention time	PEP	Score	Precursor Intensity
LDHA	3	598.3187	1791.934	88.472	4.8067e−05	100.38	NA

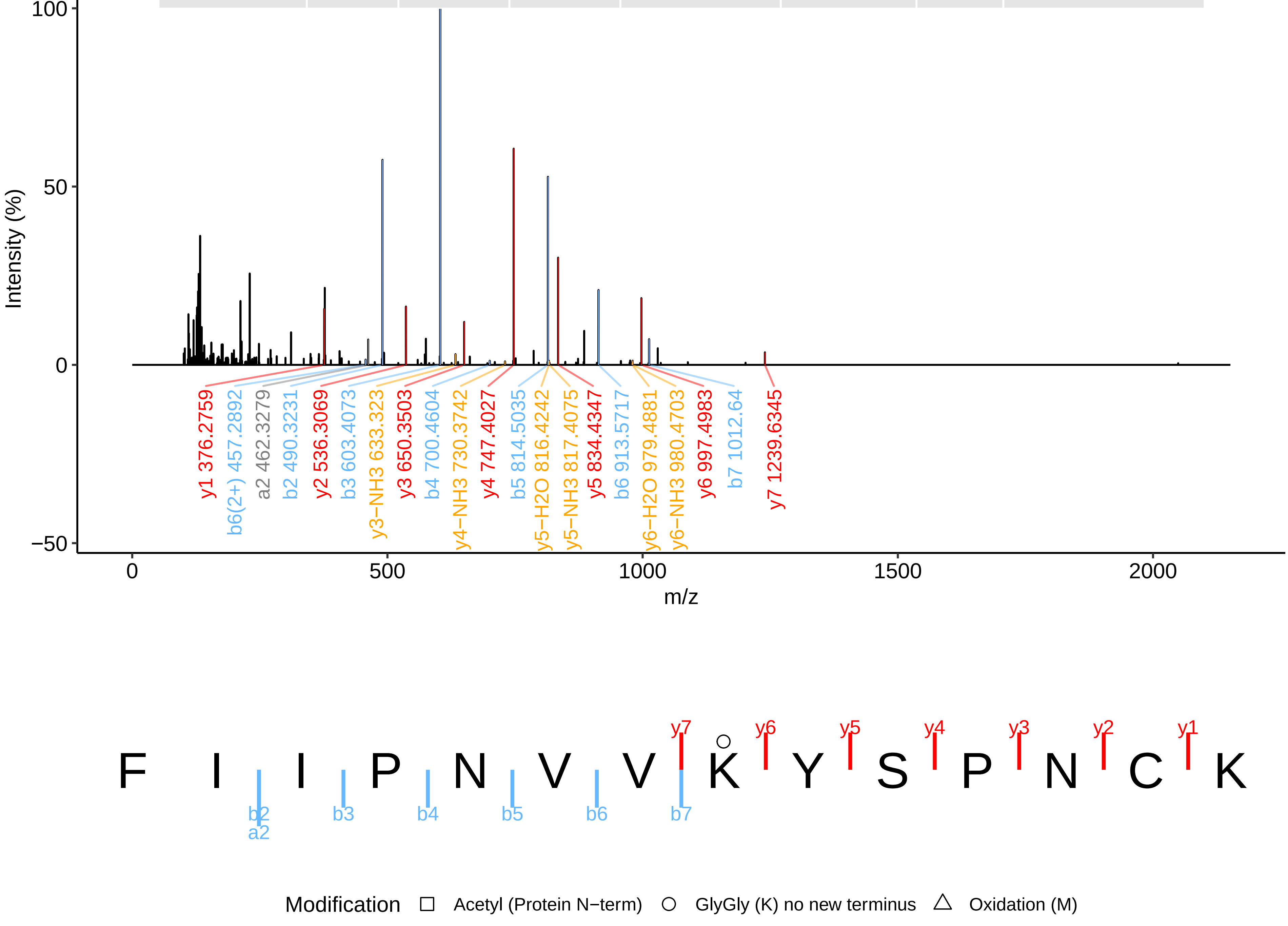

20200921_KIZ_MUb4_GlyGly_10plex_2 − Scan number: 15870

Gene Names	Charge	m/z	Mass	Retention time	PEP	Score	Precursor Intensity
CUL1	3	618.644	1852.91	62.149	3.286e−10	125.28	582773.1

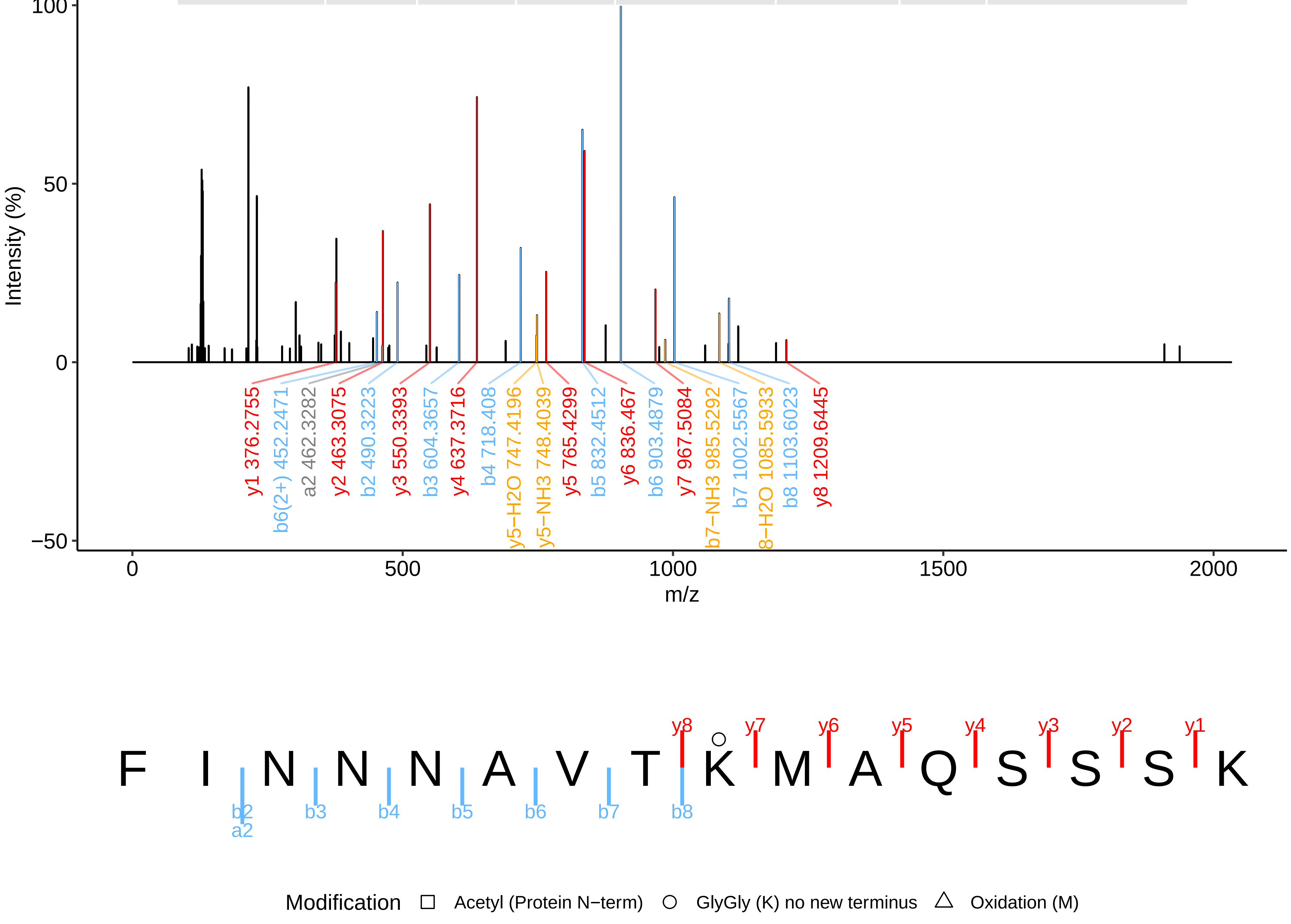

20200921_KIZ_MUb4_GlyGly_10plex_2 − Scan number: 15879

Gene Names	Charge	m/z	Mass	Retention time	PEP	Score	Precursor Intensity
CUL1	3	618.644	1852.91	62.186	1.1937e−05	86.641	410552.3

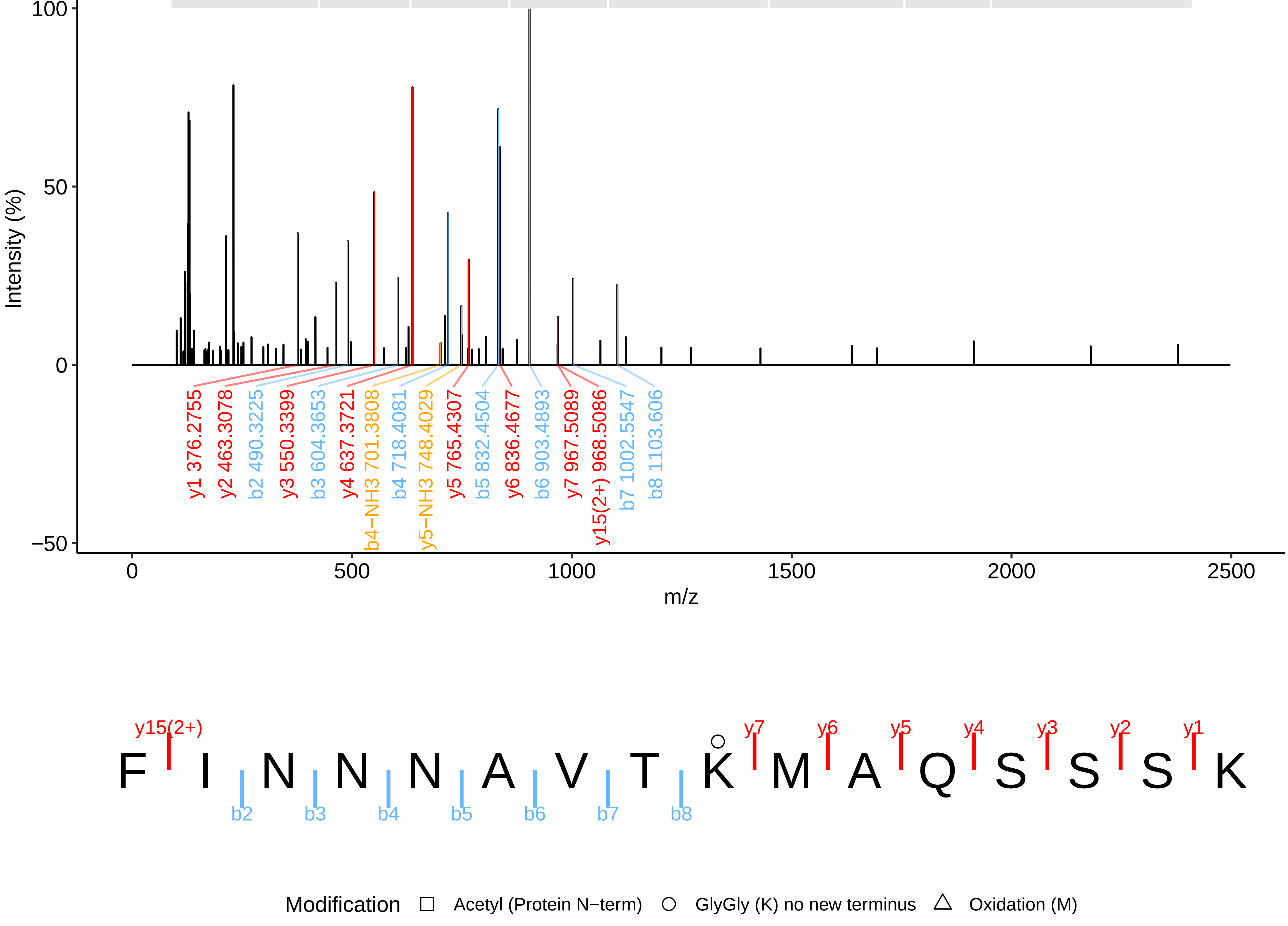

20200921_KIZ_MUb4_GlyGly_10plex − Scan number: 26469

Gene Names	Charge	m/z	Mass	Retention time	PEP	Score	Precursor Intensity
CYB5R1	3	605.2978	1812.872	96.456	0.089683	45.614	2669741

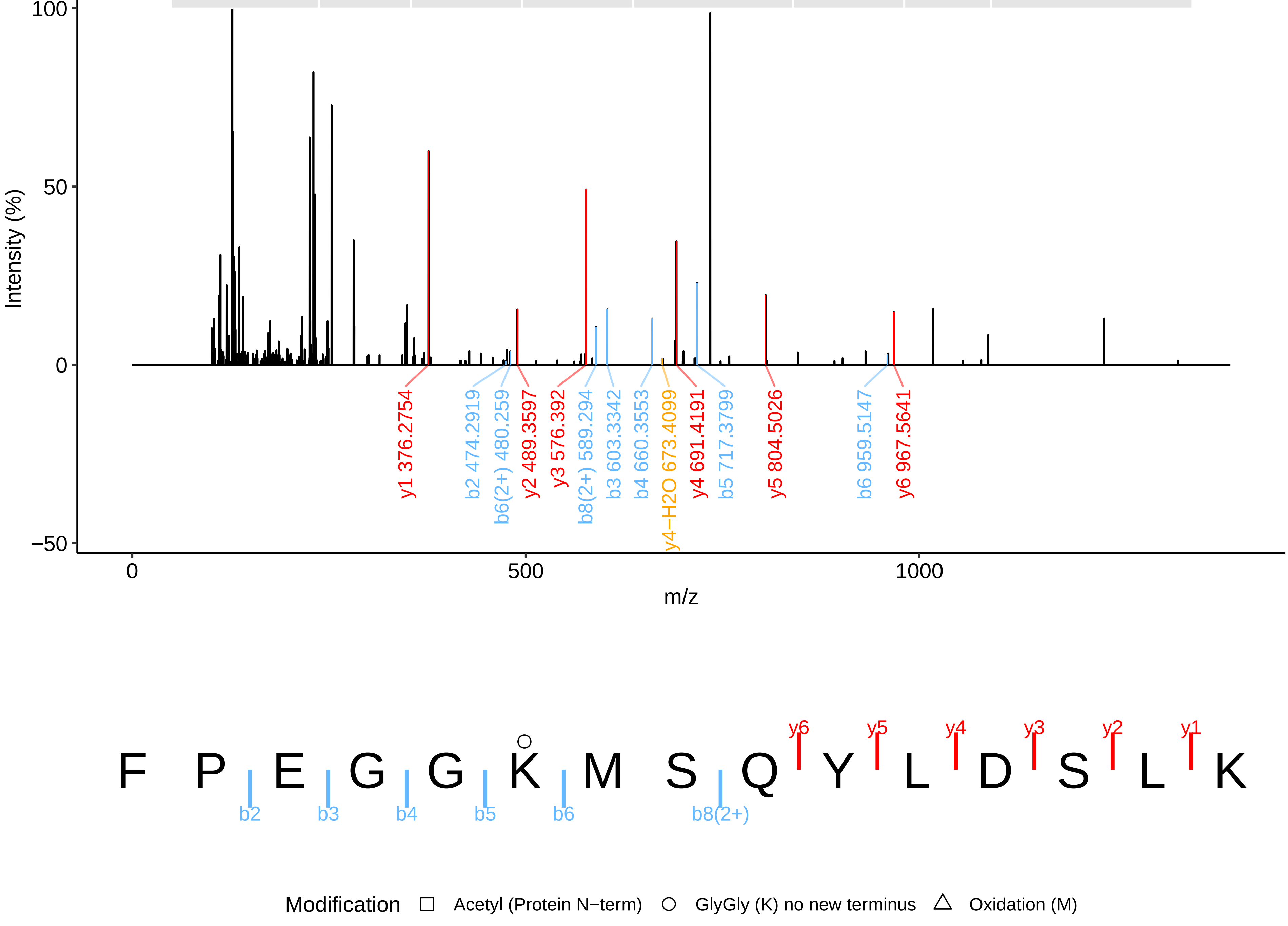

20200921_KIZ_MUb4_GlyGly_10plex − Scan number: 26576

Gene Names	Charge	m/z	Mass	Retention time	PEP	Score	Precursor Intensity
CYB5R1	3	605.2978	1812.872	96.849	0.0011499	76.391	3192299

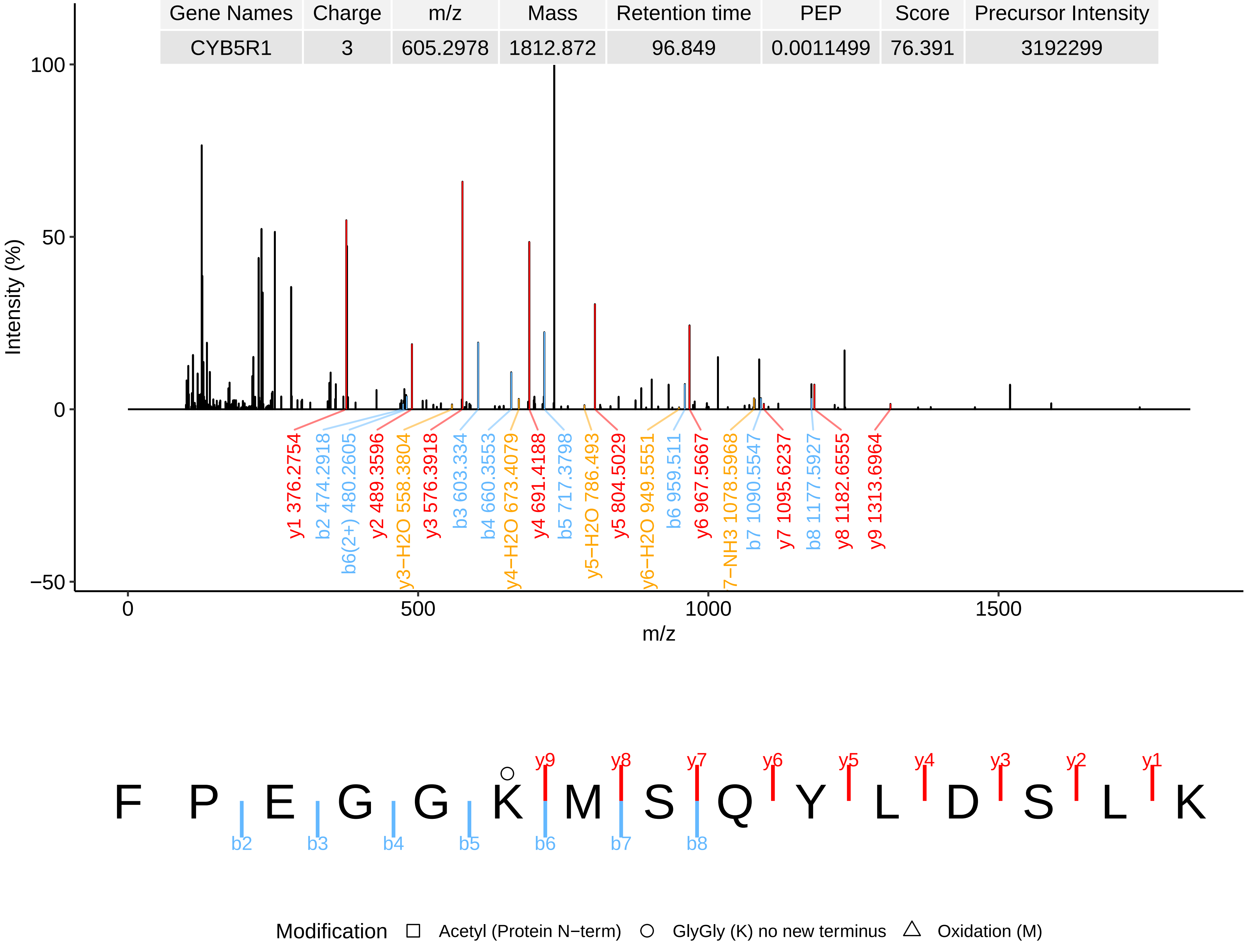

20201118_KIZ_MUb6_GlyGly_1 − Scan number: 26311

Gene Names	Charge	m/z	Mass	Retention time	PEP	Score	Precursor Intensity
CYB5R1	3	605.2978	1812.872	80.203	0.0050322	70.529	NA

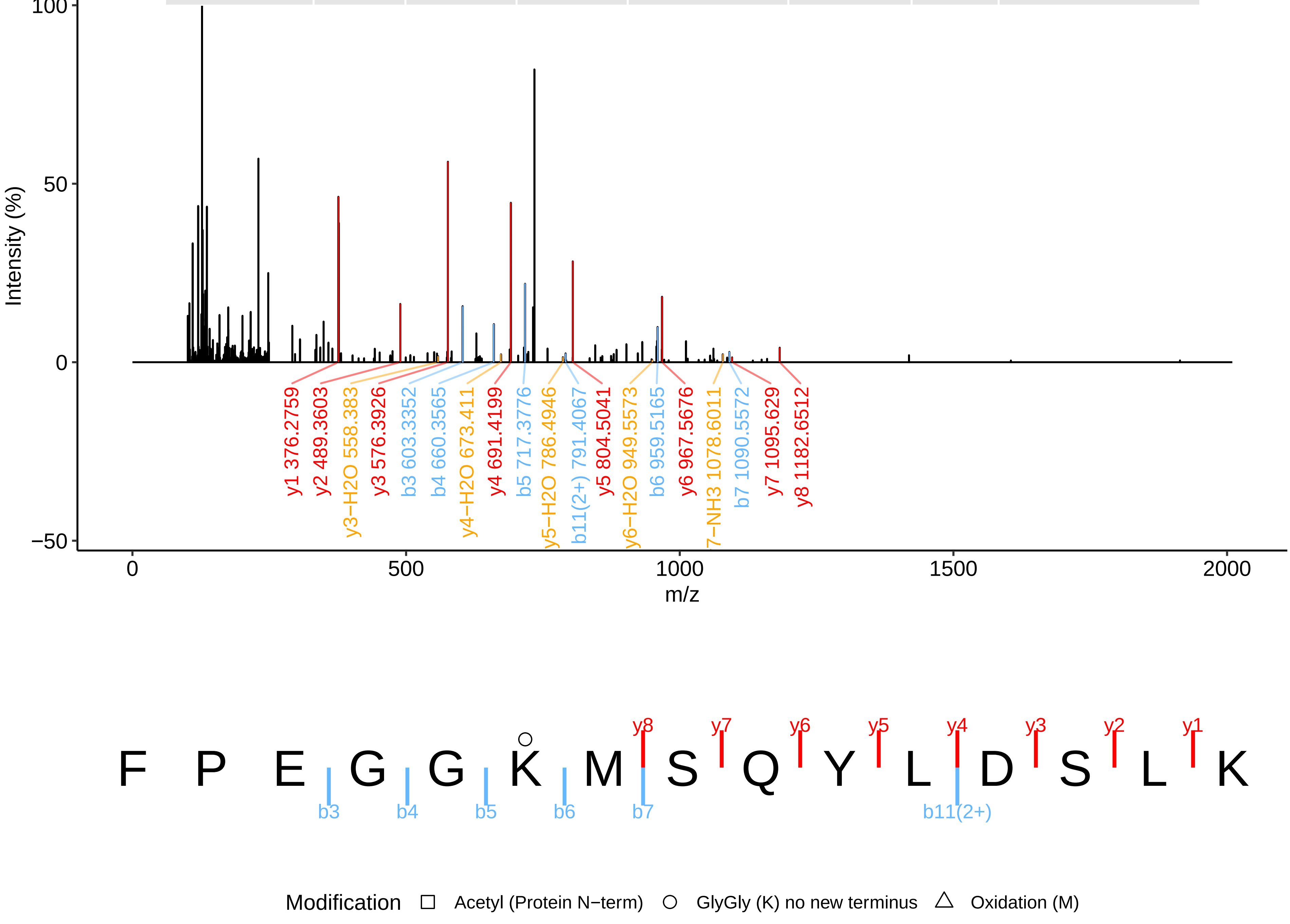

20200921_KIZ_MUb4_GlyGly_10plex − Scan number: 20743

Gene Names	Charge	m/z	Mass	Retention time	PEP	Score	Precursor Intensity
GAPDH	3	627.6899	1880.048	75.024	9.0438e−27	150.28	4503924

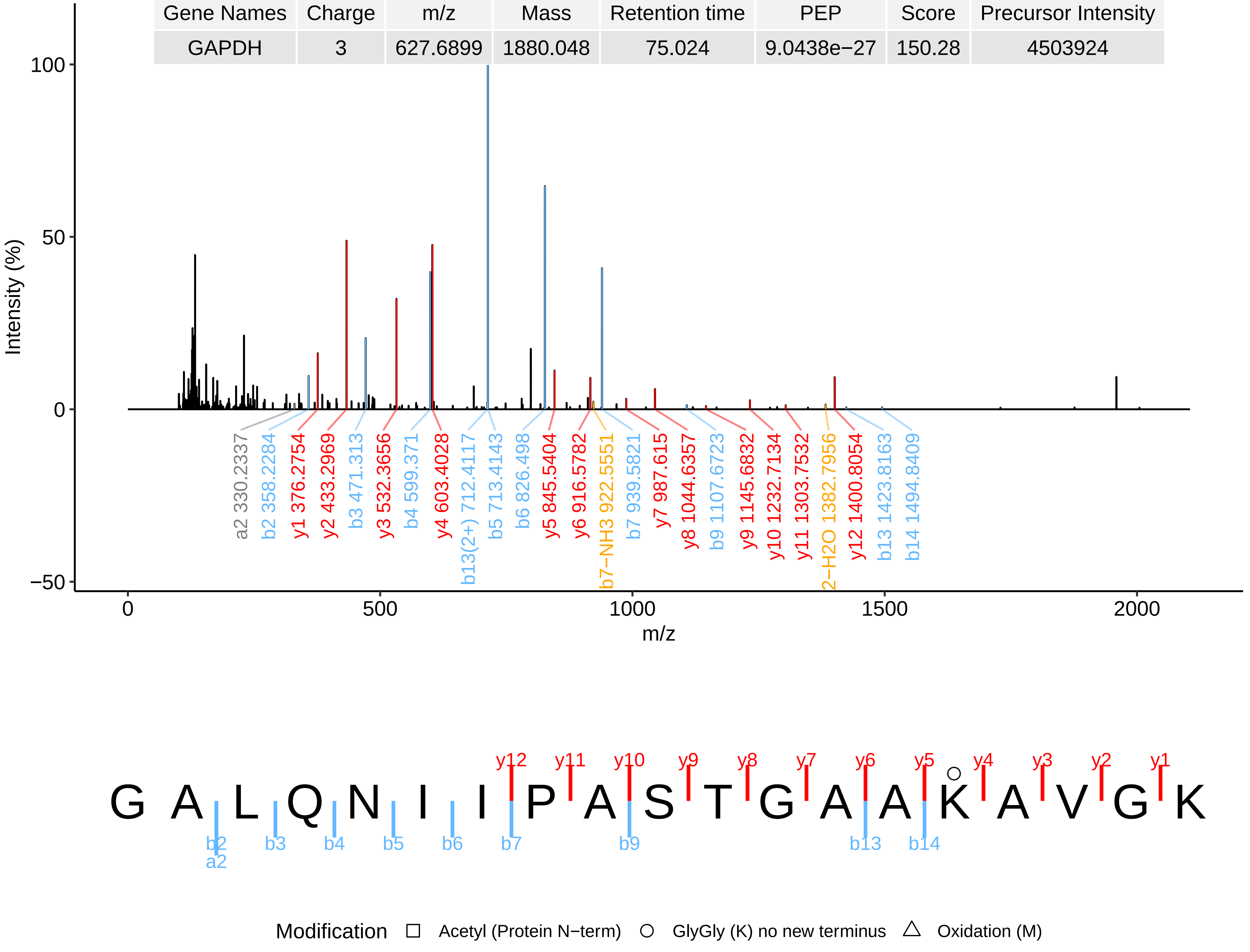

20200921_KIZ_MUb4_GlyGly_10plex_2 − Scan number: 18620

Gene Names	Charge	m/z	Mass	Retention time	PEP	Score	Precursor Intensity
GAPDH	3	627.6899	1880.048	72.963	0.00084213	73.983	1441832

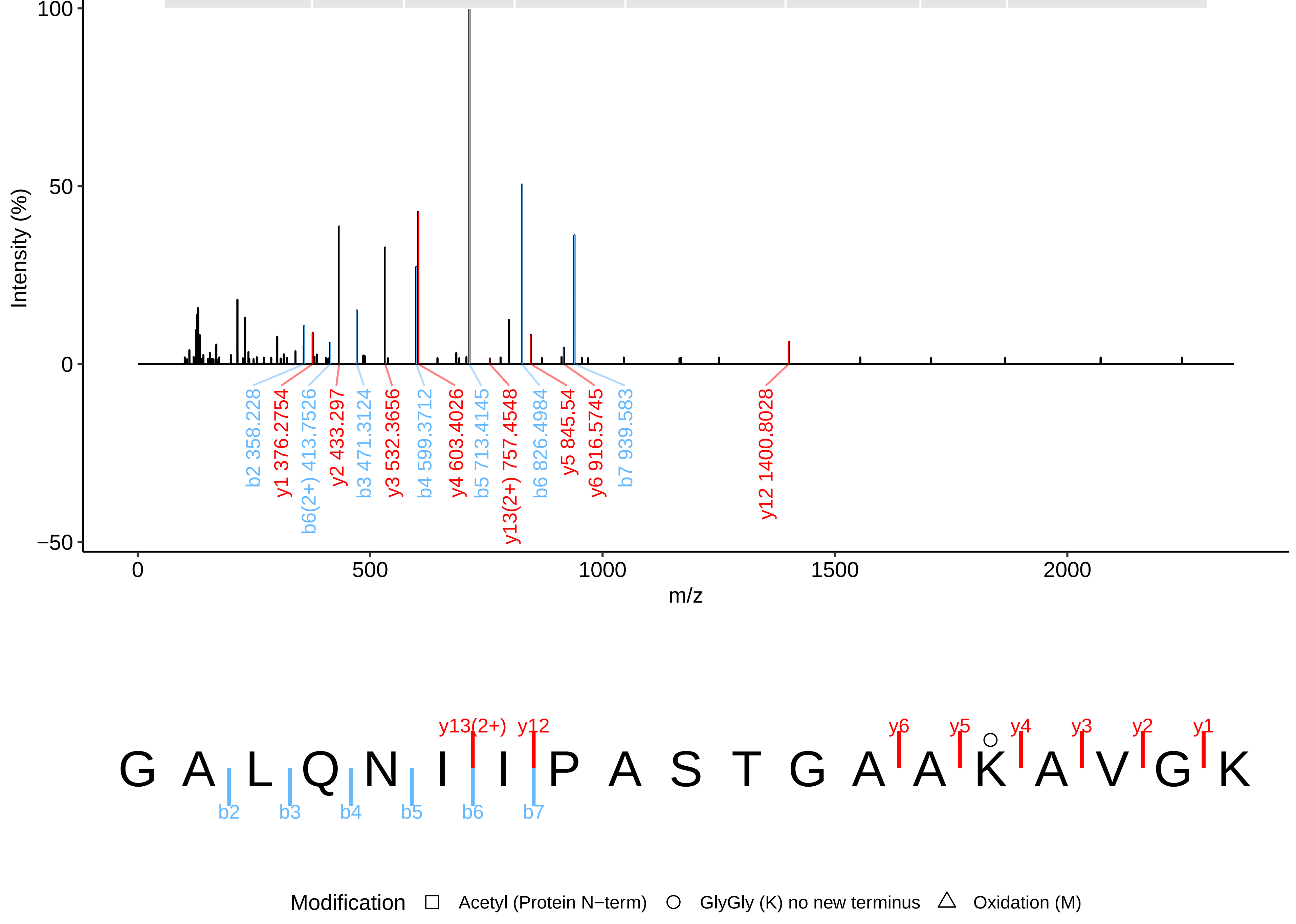

20200921_KIZ_MUb4_GlyGly_10plex_2 − Scan number: 18744

Gene Names	Charge	m/z	Mass	Retention time	PEP	Score	Precursor Intensity
GAPDH	3	627.6899	1880.048	73.471	1.4903e−09	105.39	1408474

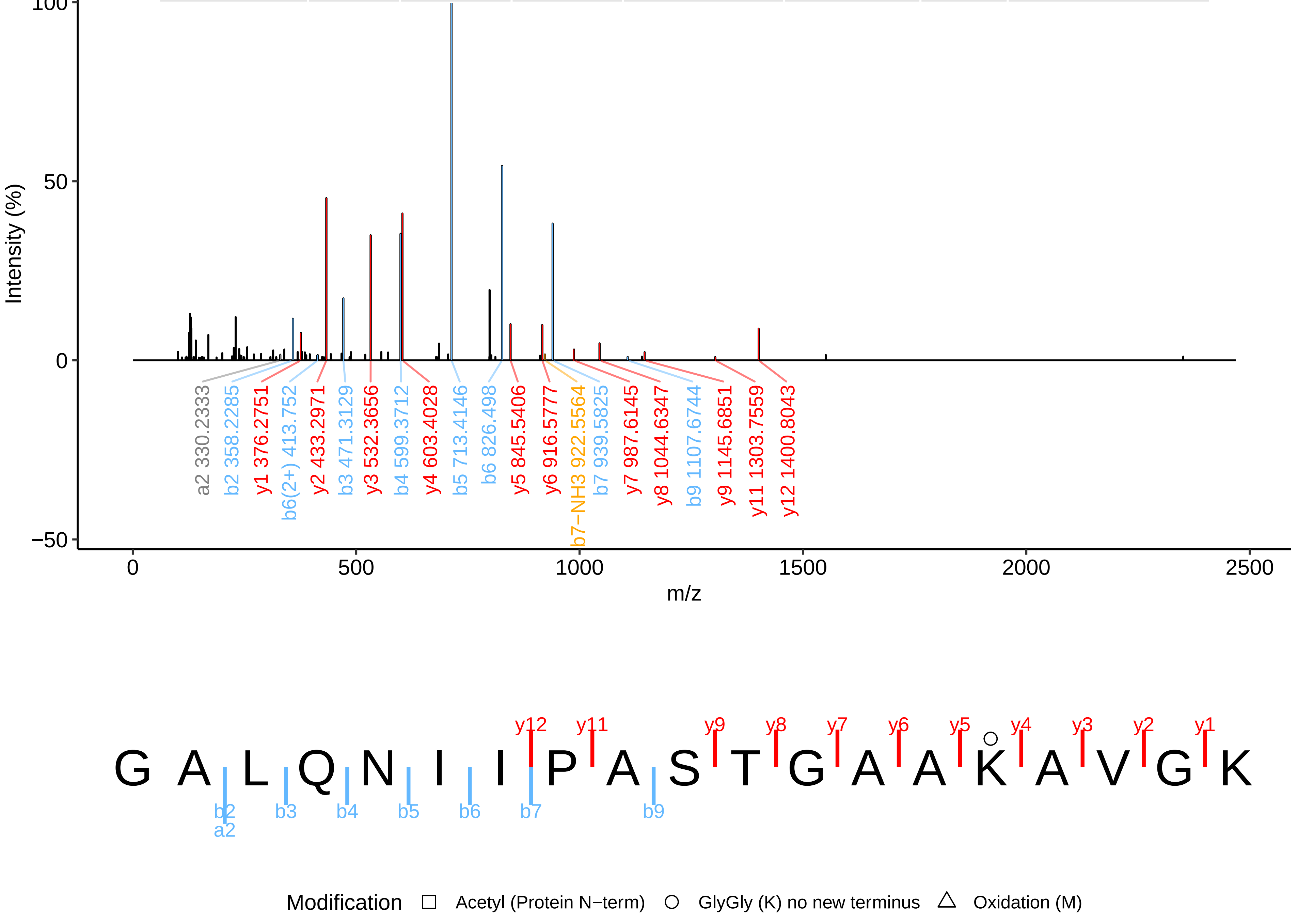

20200924_KIZ_MUb5_GlyGly_1 − Scan number: 20133

Gene Names	Charge	m/z	Mass	Retention time	PEP	Score	Precursor Intensity
GAPDH	3	627.6899	1880.048	64.952	8.368e−26	153.21	NA

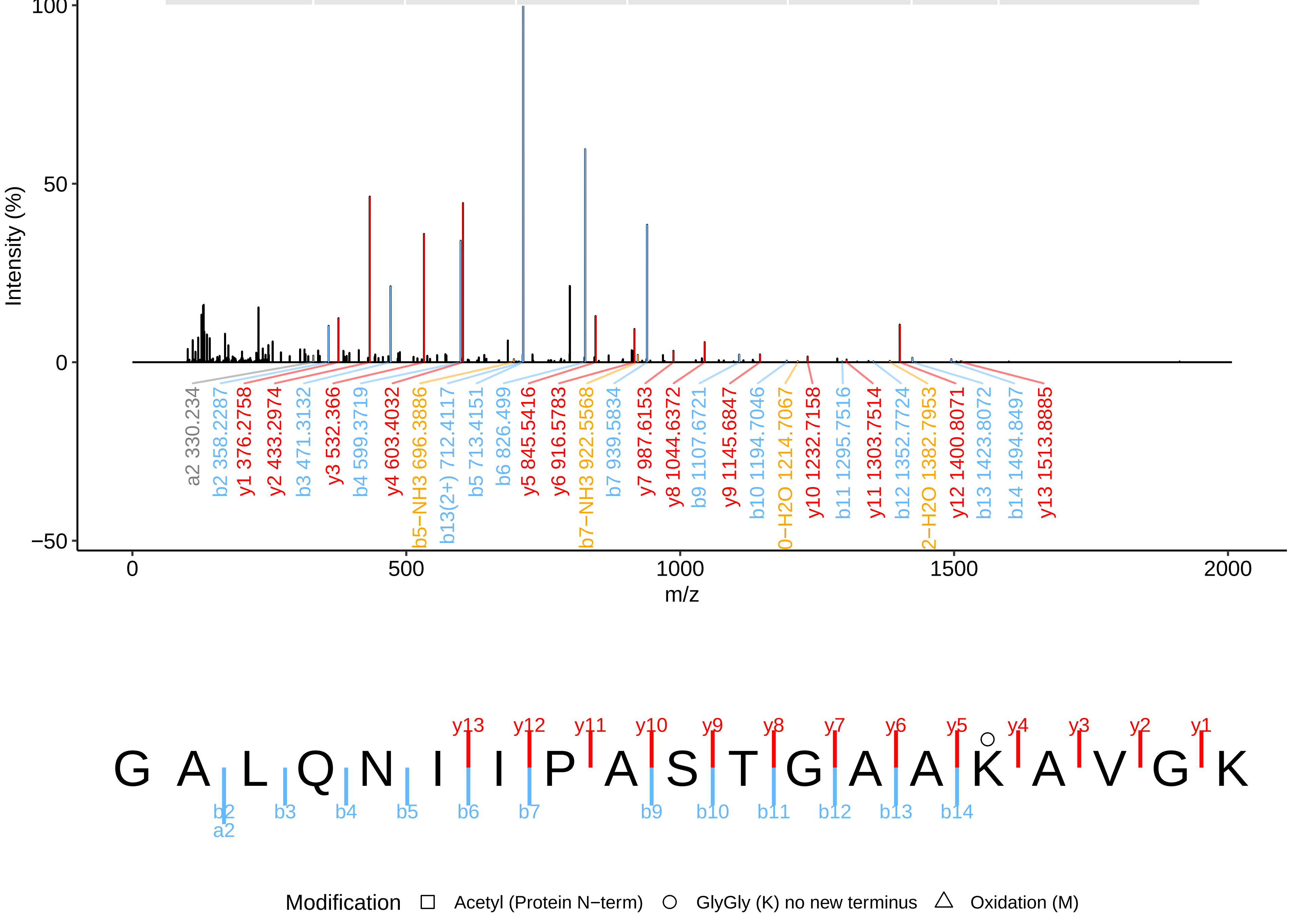

20200924_KIZ_MUb5_GlyGly_2 − Scan number: 18345

Gene Names	Charge	m/z	Mass	Retention time	PEP	Score	Precursor Intensity
GAPDH	3	627.6899	1880.048	68.04	6.4977e−06	91.457	2844342

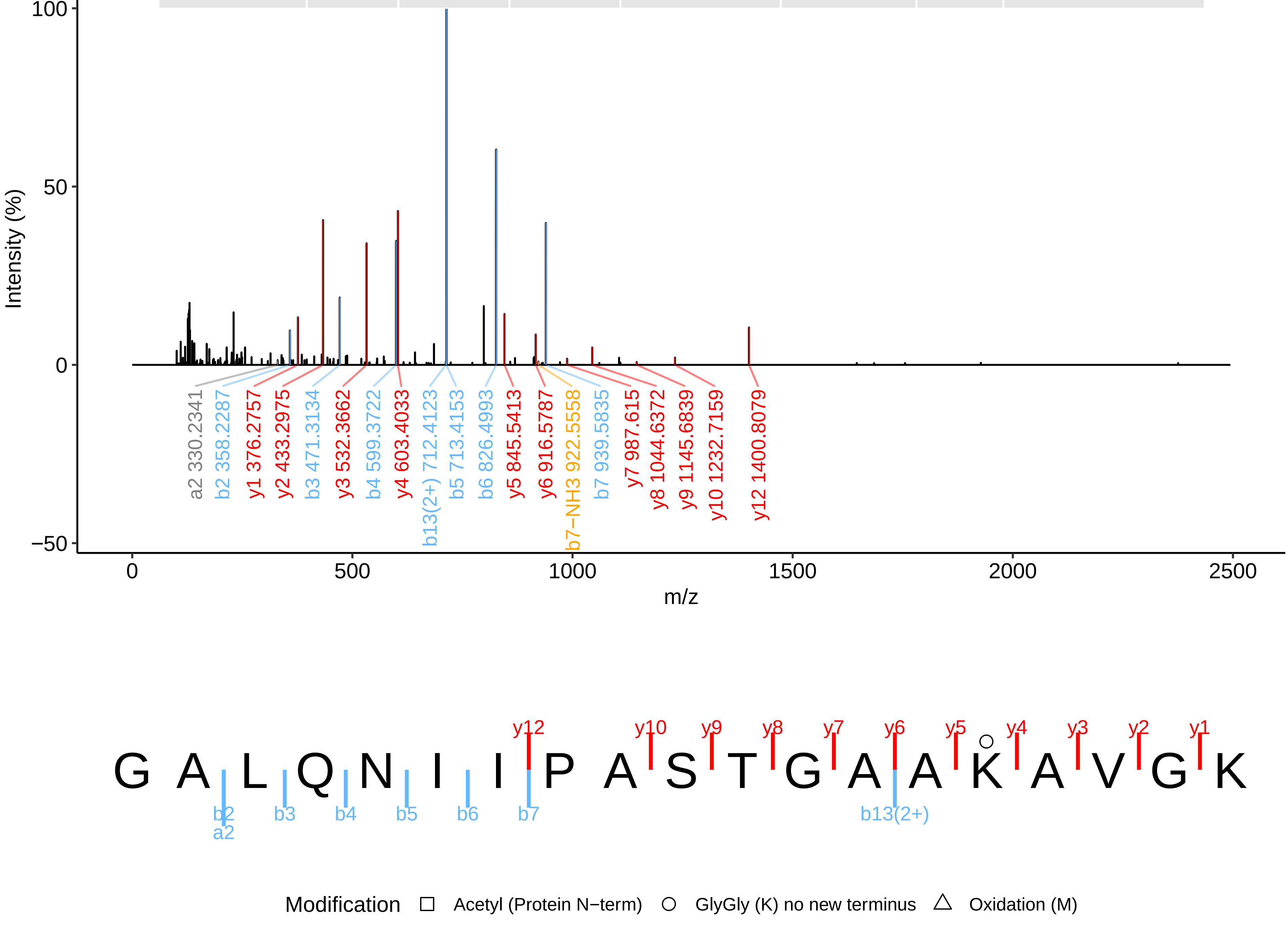

20200921_KIZ_MUb4_GlyGly_10plex − Scan number: 23300

Gene Names	Charge	m/z	Mass	Retention time	PEP	Score	Precursor Intensity
MBLAC2	4	522.0274	2084.08	84.568	6.8904e−10	106.36	3993695

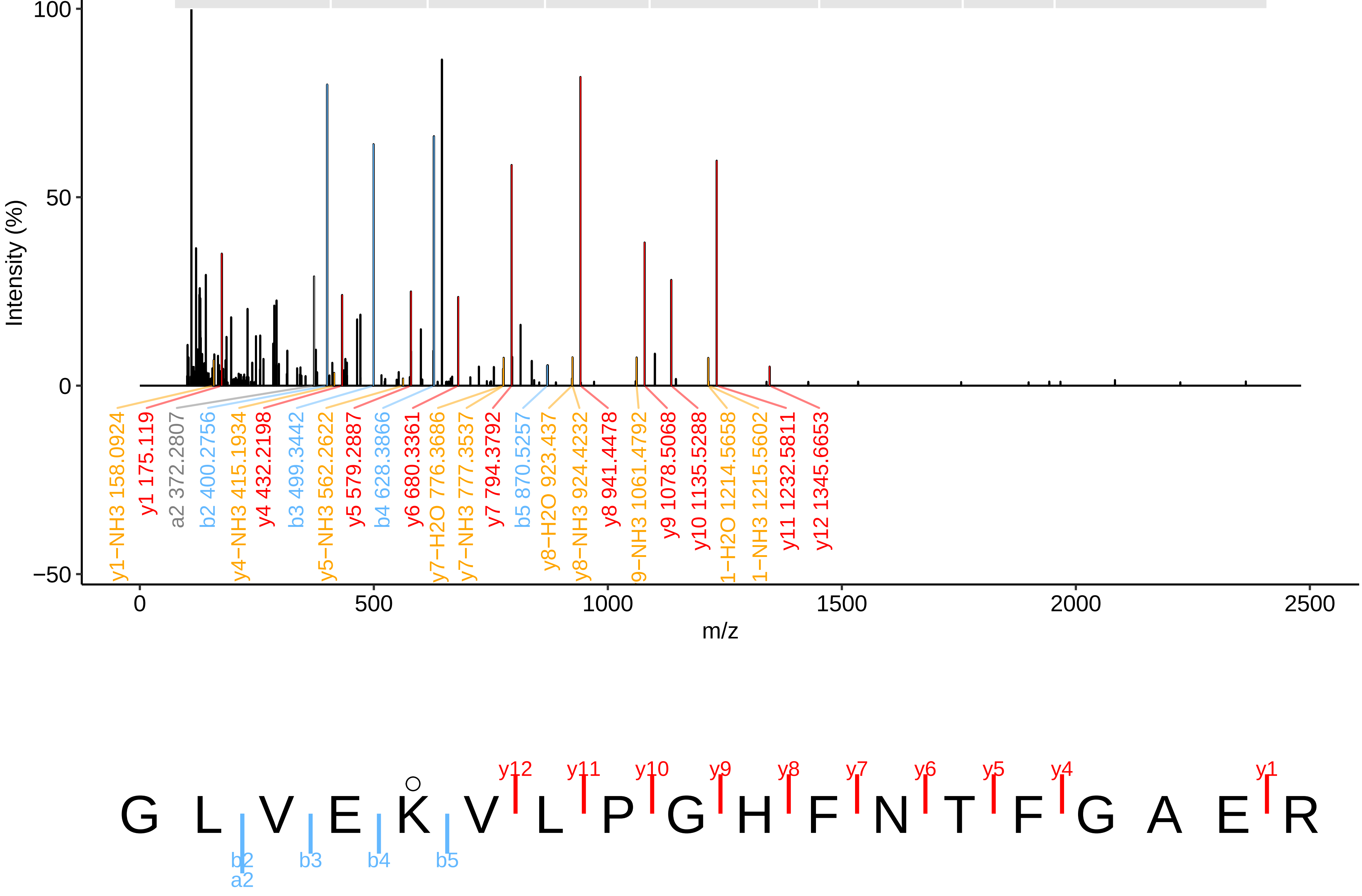

Modification □ Acetyl (Protein N−term) ○ GlyGly (K) no new terminus △ Oxidation (M)

20200921_KIZ_MUb4_GlyGly_10plex − Scan number: 25008

Gene Names	Charge	m/z	Mass	Retention time	PEP	Score	Precursor Intensity
FASN	3	609.3547	1825.042	90.953	3.4961e−09	117.2	NA

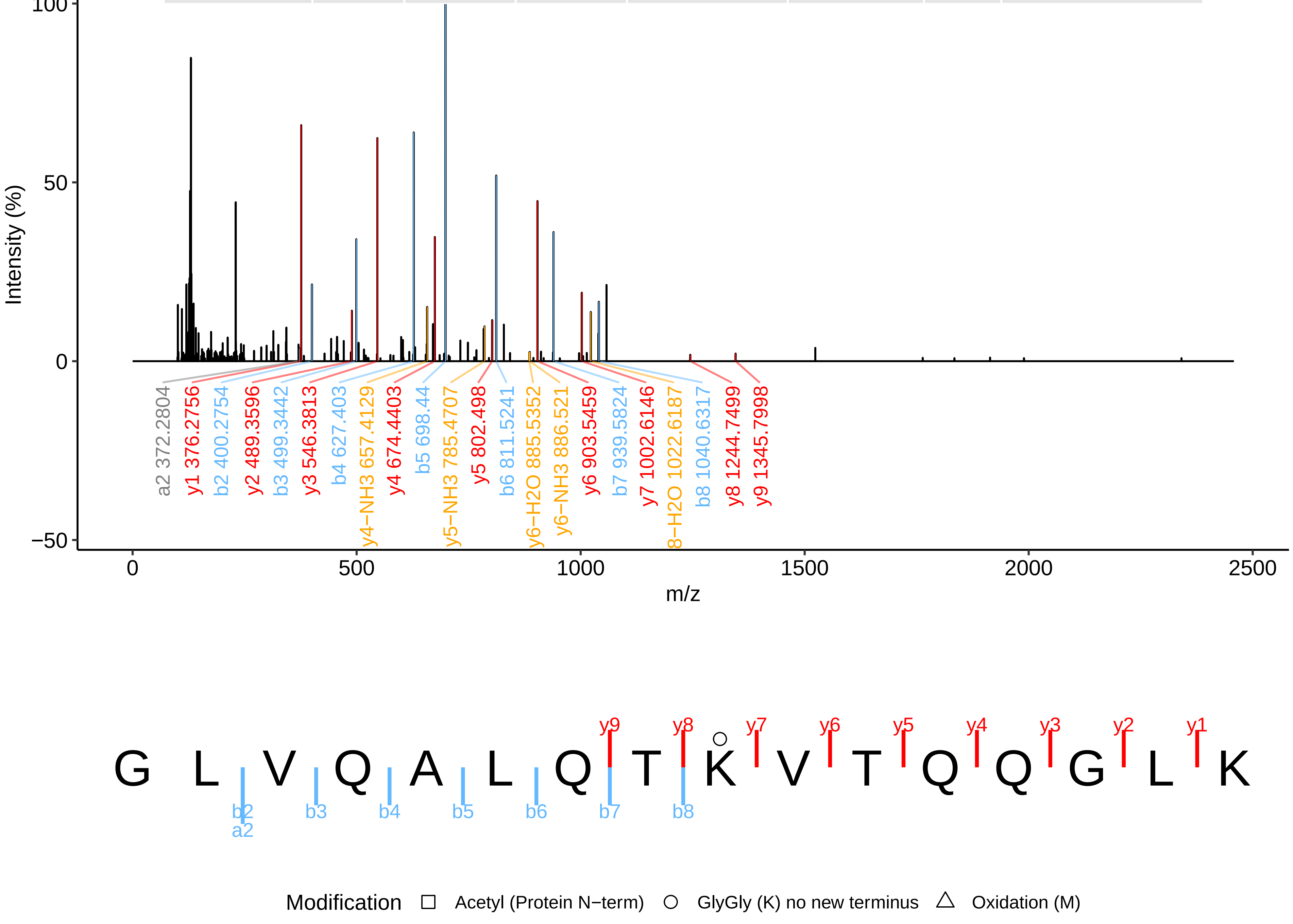

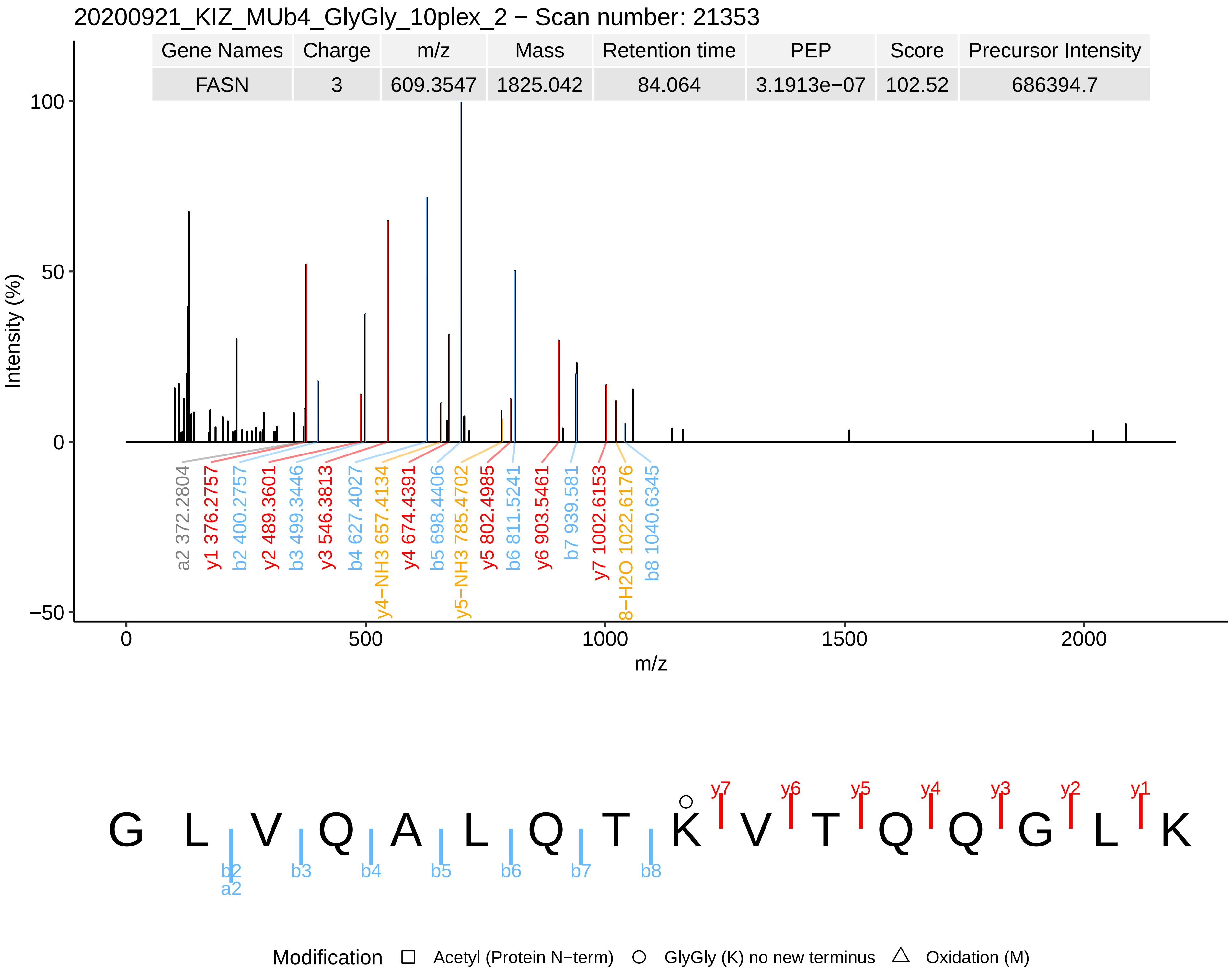
20200921_KIZ_MUb4_GlyGly_10plex_2 − Scan number: 21353
Gene Names | Charge | m/z | Mass | Retention time | PEP | Score | Precursor Intensity
FASN | 3 | 609.3547 | 1825.042 | 84.064 | 3.1913e−07 | 102.52 | 686394.7
Intensity (%)
m/z
a2 372.2804
y1 376.2757
b2 400.2757
y2 489.3601
b3 499.3446
y3 546.3813
b4 627.4027
y4−NH3 657.4134
y4 674.4391
b5 698.4406
y5−NH3 785.4702
y5 802.4985
b6 811.5241
y6 903.5461
b7 939.581
y7 1002.6153
8−H2O 1022.6176
b8 1040.6345
G L V Q A L Q T K V T Q Q G L K
Modification
Acetyl (Protein N−term)
GlyGly (K) no new terminus
Oxidation (M)

20200921_KIZ_MUb4_GlyGly_10plex_2 − Scan number: 21363

Gene Names	Charge	m/z	Mass	Retention time	PEP	Score	Precursor Intensity
FASN	3	609.3547	1825.042	84.105	2.5316e−07	105.19	865920.9

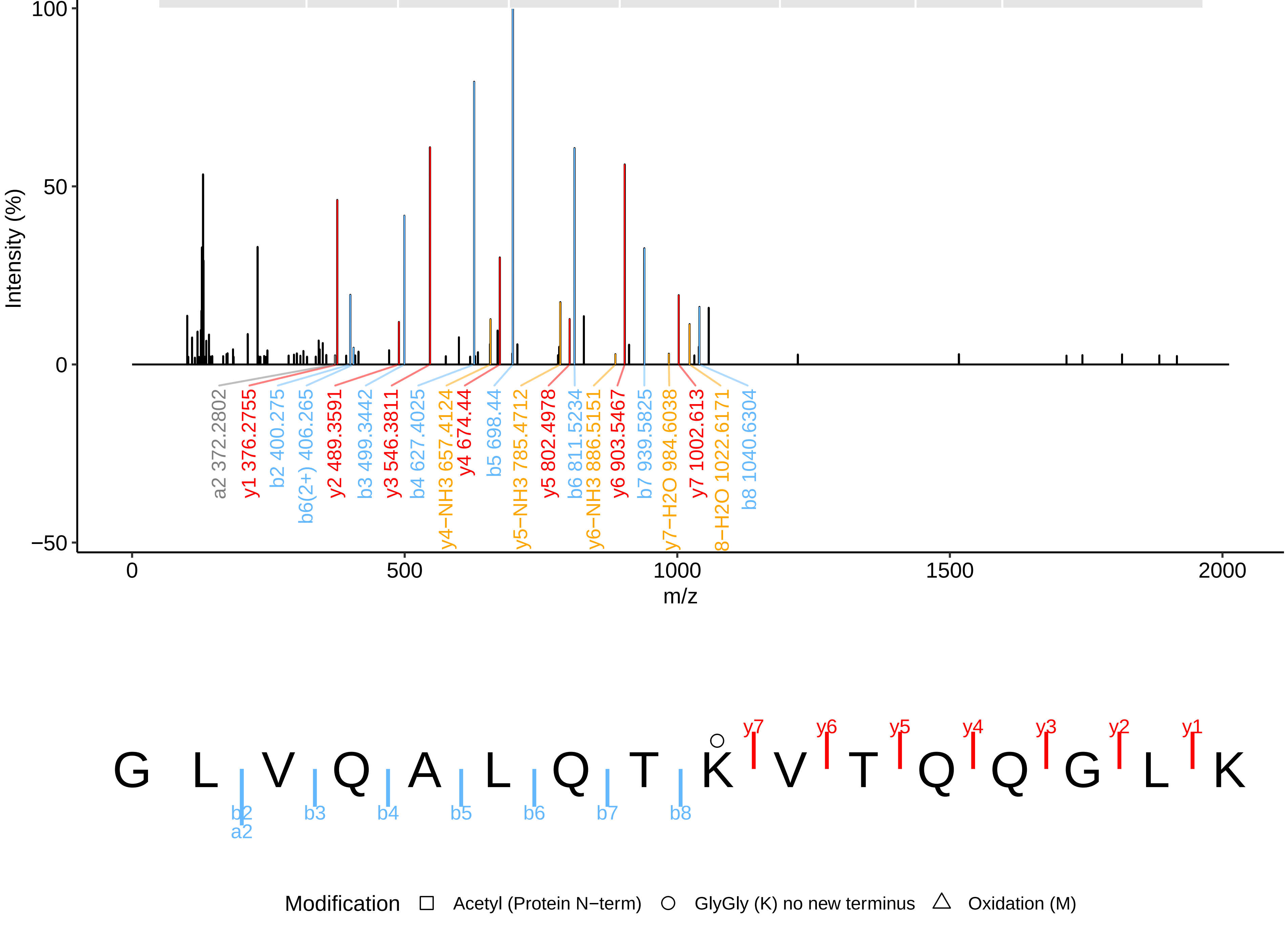

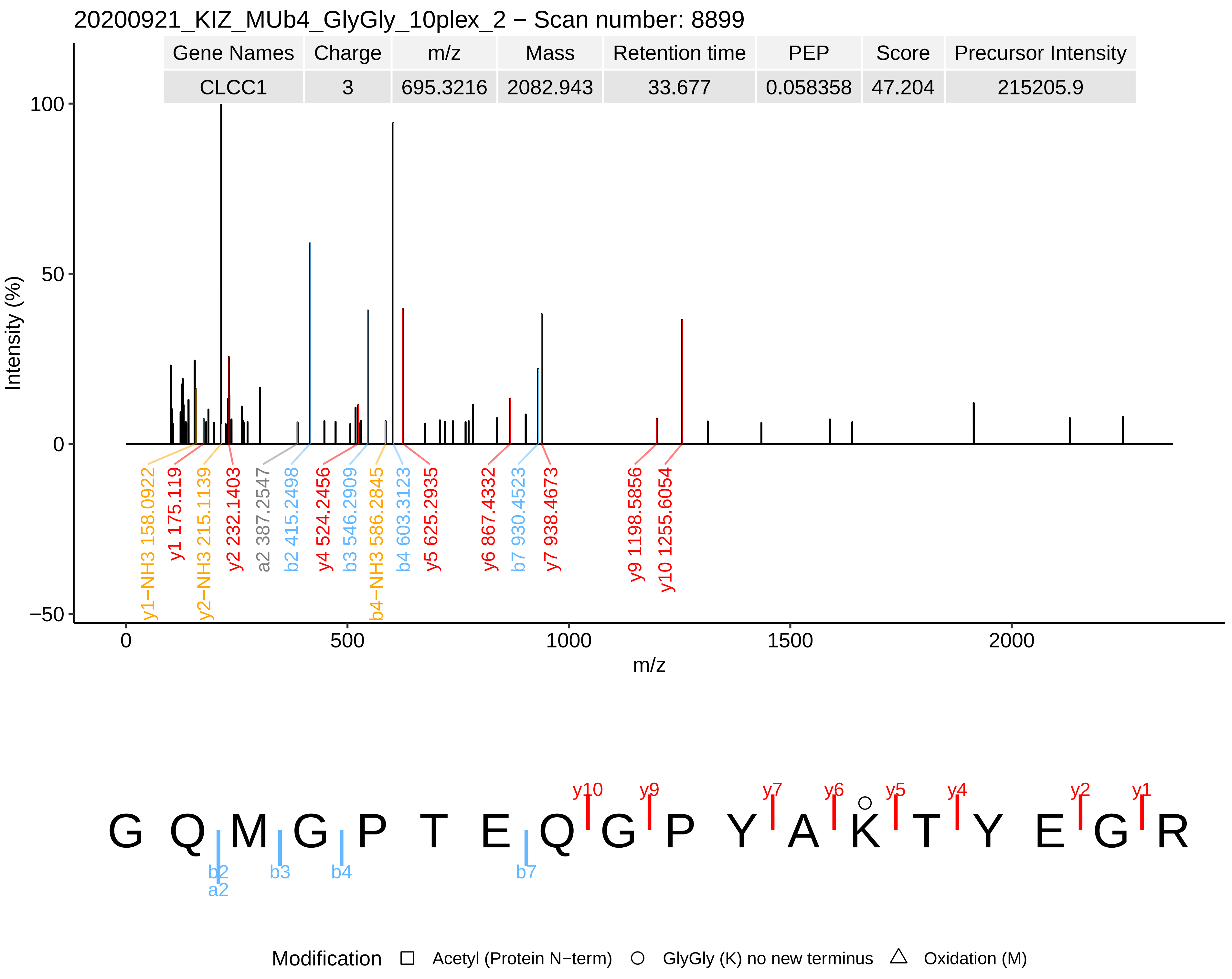

20200921_KIZ_MUb4_GlyGly_10plex_2 − Scan number: 8899
Gene Names: CLCC1
Charge: 3
m/z: 695.3216
Mass: 2082.943
Retention time: 33.677
PEP: 0.058358
Score: 47.204
Precursor Intensity: 215205.9
Intensity (%)
m/z
y1−NH3 158.0922
y1 175.119
y2−NH3 215.1139
y2 232.1403
a2 387.2547
b2 415.2498
y4 524.2456
b3 546.2909
b4−NH3 586.2845
b4 603.3123
y5 625.2935
y6 867.4332
b7 930.4523
y7 938.4673
y9 1198.5856
y10 1255.6054
G Q M G P T E Q G P Y A K T Y E G R
Modification
Acetyl (Protein N−term)
GlyGly (K) no new terminus
Oxidation (M)

20200921_KIZ_MUb4_GlyGly_10plex_2 − Scan number: 8923

Gene Names	Charge	m/z	Mass	Retention time	PEP	Score	Precursor Intensity
CLCC1	3	695.3216	2082.943	33.778	0.054568	48.288	855742.4

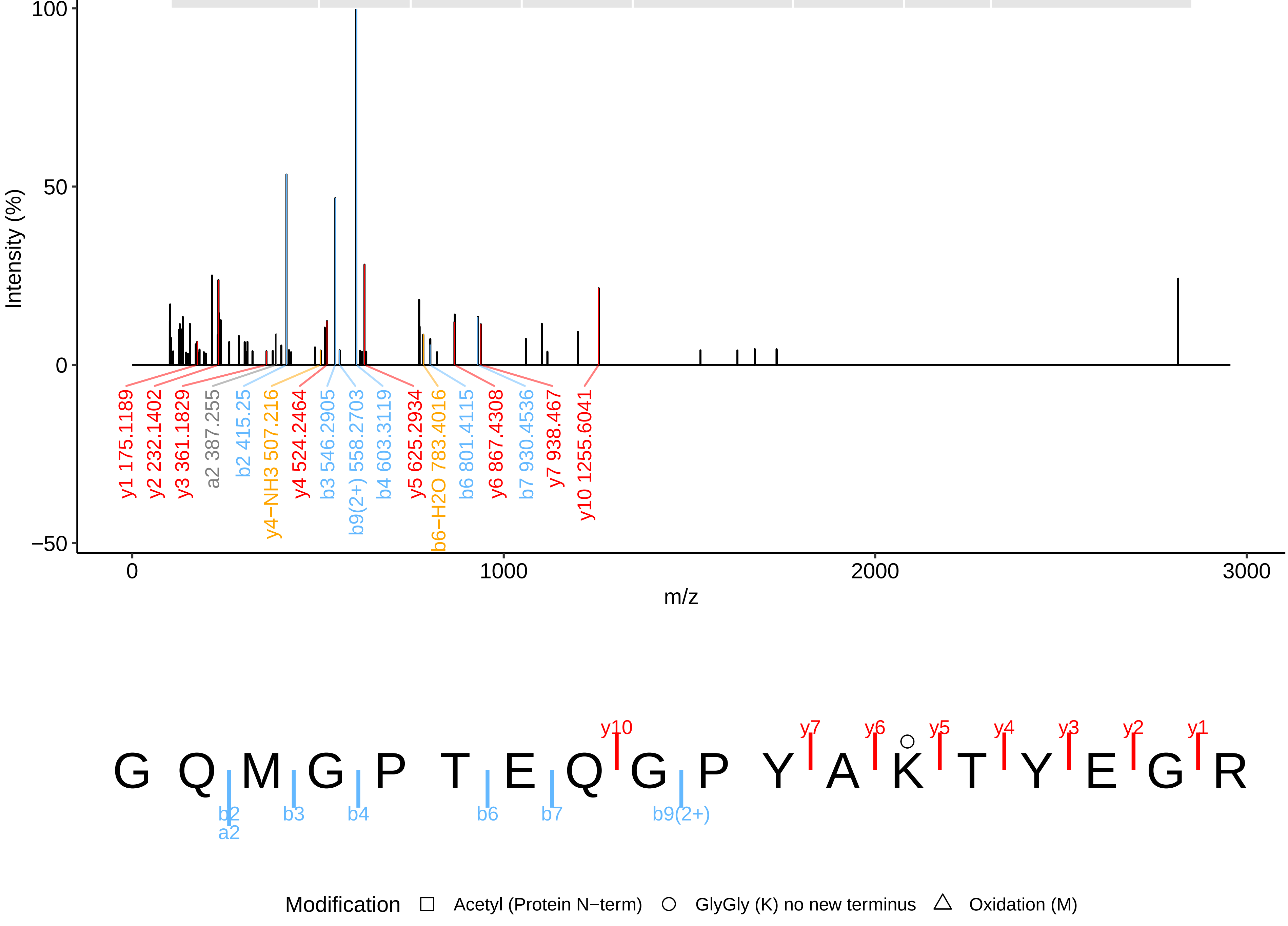

20200921_KIZ_MUb4_GlyGly_10plex_2 − Scan number: 14336

Gene Names	Charge	m/z	Mass	Retention time	PEP	Score	Precursor Intensity
SLC2A1	4	515.7392	2058.928	55.879	5.8829e−12	115.31	6993132

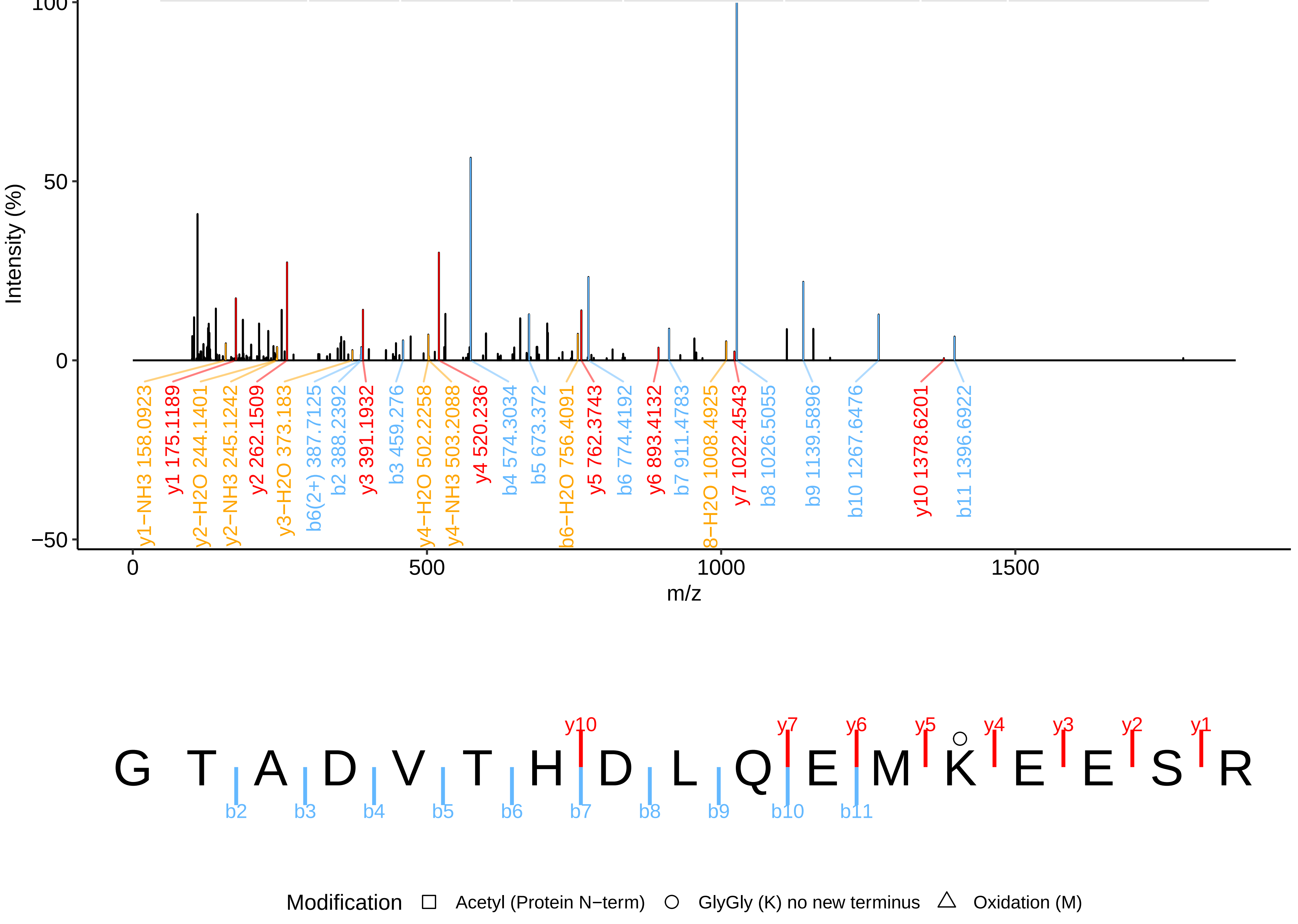

20200921_KIZ_MUb4_GlyGly_10plex_2 − Scan number: 17187

Gene Names	Charge	m/z	Mass	Retention time	PEP	Score	Precursor Intensity
HSPD1	3	606.3125	1815.916	67.173	0.00024616	86.467	NA

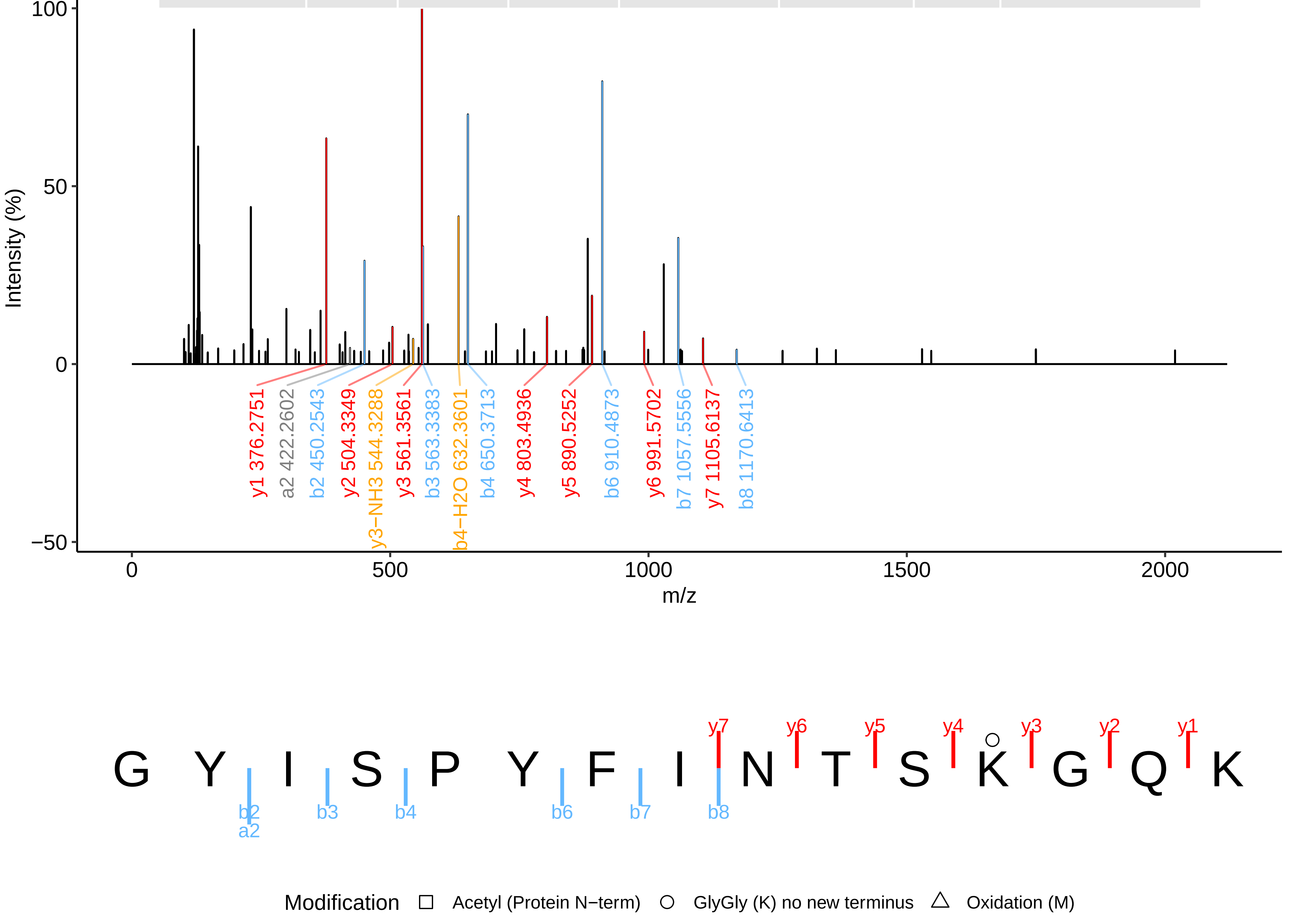

20200921_KIZ_MUb4_GlyGly_10plex − Scan number: 13447

Gene Names	Charge	m/z	Mass	Retention time	PEP	Score	Precursor Intensity
CISD1	4	470.2484	1876.964	47.448	4.0249e−15	147.1	4627326

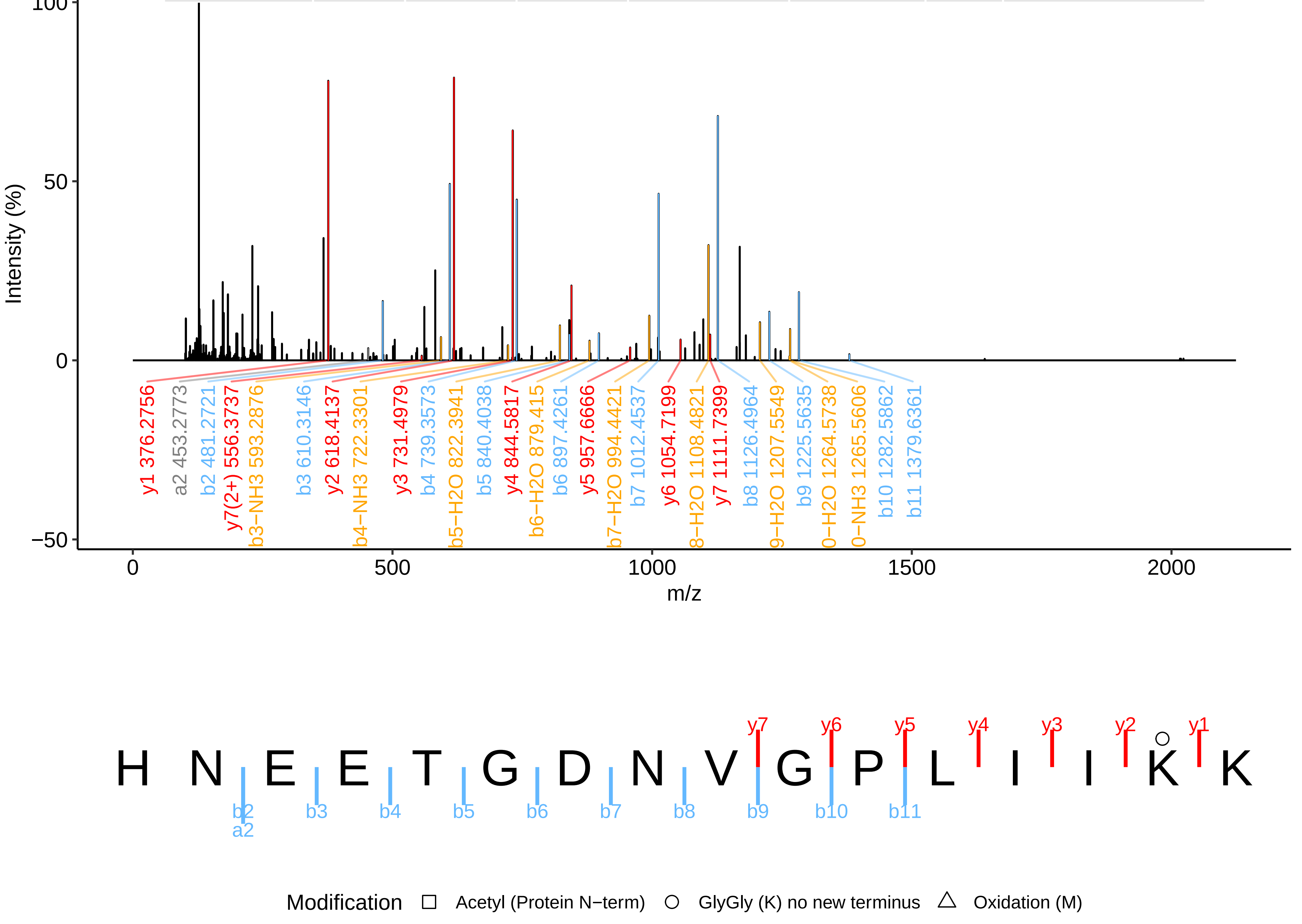

20200921_KIZ_MUb4_GlyGly_10plex − Scan number: 13496

Gene Names	Charge	m/z	Mass	Retention time	PEP	Score	Precursor Intensity
CISD1	3	626.662	1876.964	47.64	1.8846e−14	148.57	5227841

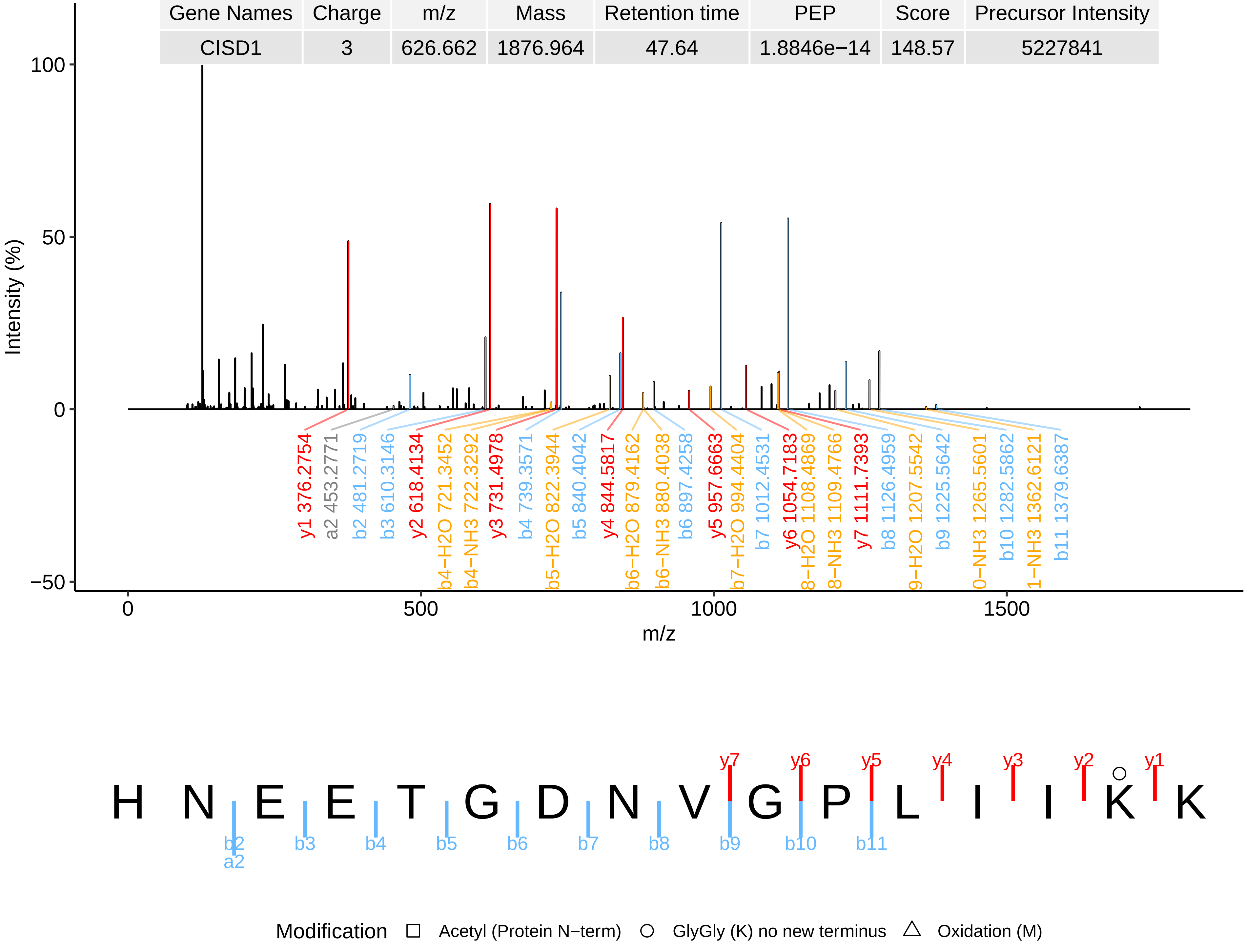

20200921_KIZ_MUb4_GlyGly_10plex_2 − Scan number: 12437

Gene Names	Charge	m/z	Mass	Retention time	PEP	Score	Precursor Intensity
CISD1	4	470.2484	1876.964	48.115	1.183e−14	141	1776073

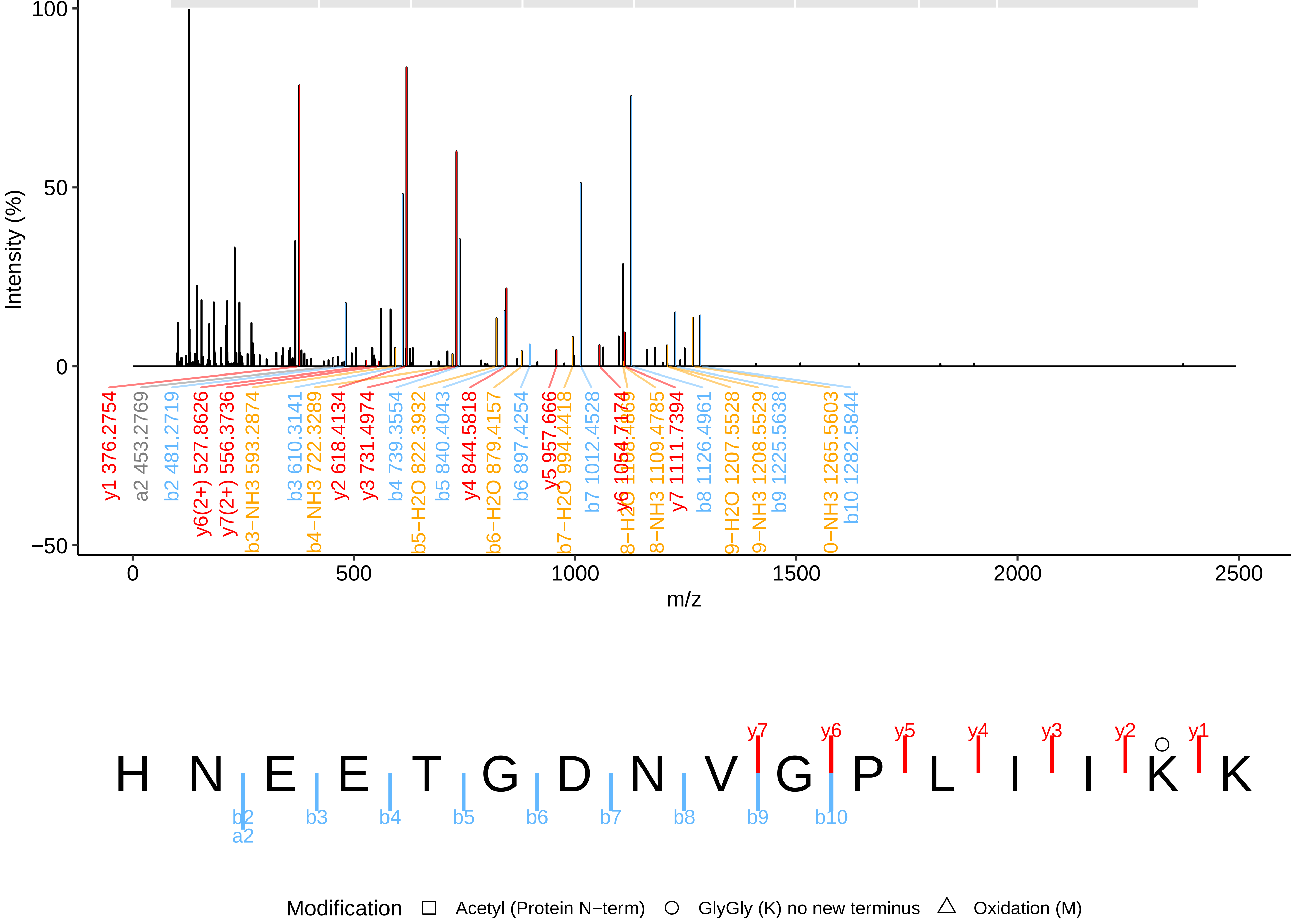

20200924_KIZ_MUb5_GlyGly_1 − Scan number: 12201

Gene Names	Charge	m/z	Mass	Retention time	PEP	Score	Precursor Intensity
CISD1	4	470.2484	1876.964	39.656	1.8514e−07	104.73	NA

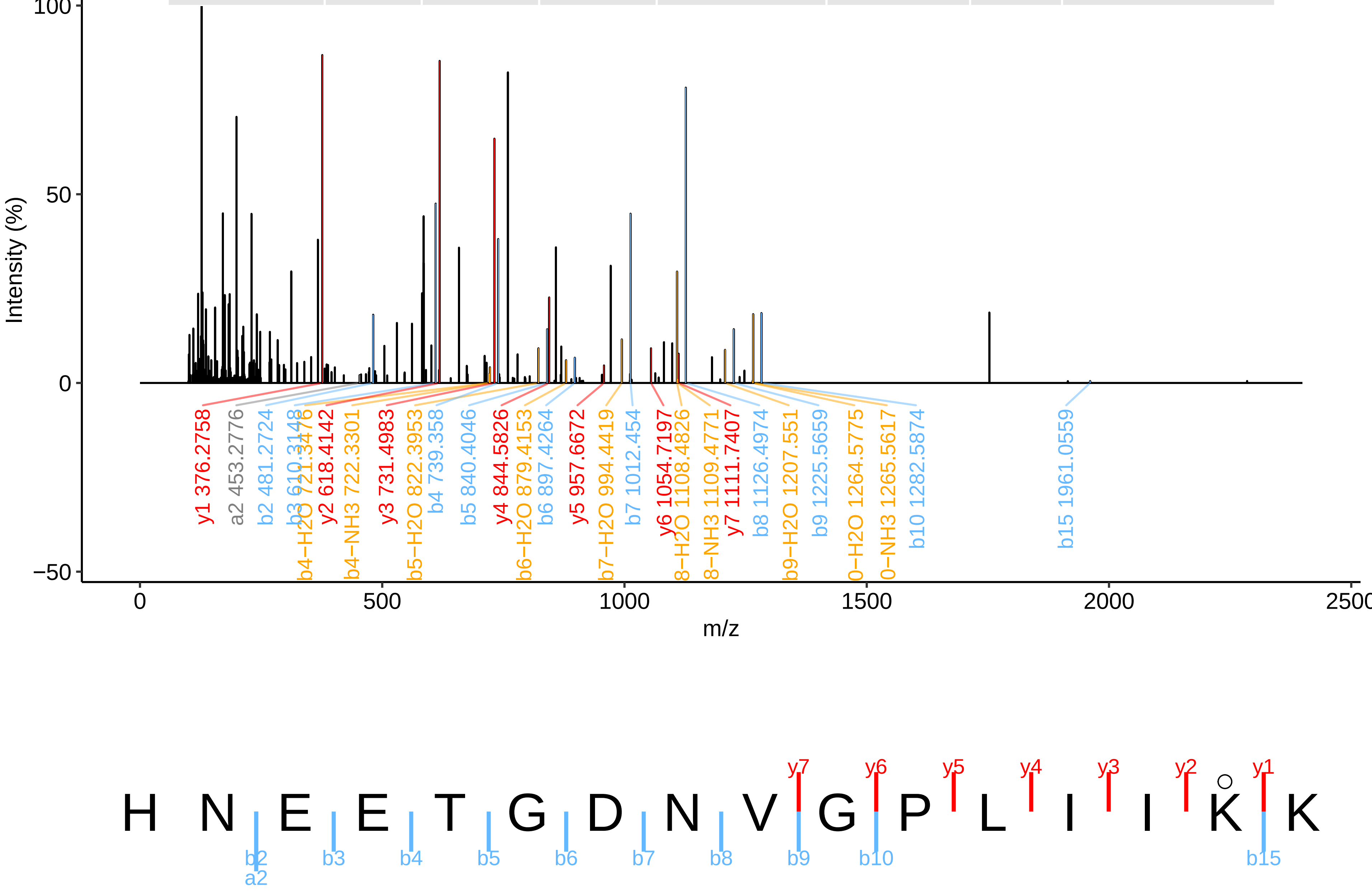

Modification □ Acetyl (Protein N−term) ○ GlyGly (K) no new terminus △ Oxidation (M)

20200924_KIZ_MUb5_GlyGly_2 − Scan number: 11574

Gene Names	Charge	m/z	Mass	Retention time	PEP	Score	Precursor Intensity
CISD1	4	470.2484	1876.964	43.276	1.2122e−05	81.327	5055299

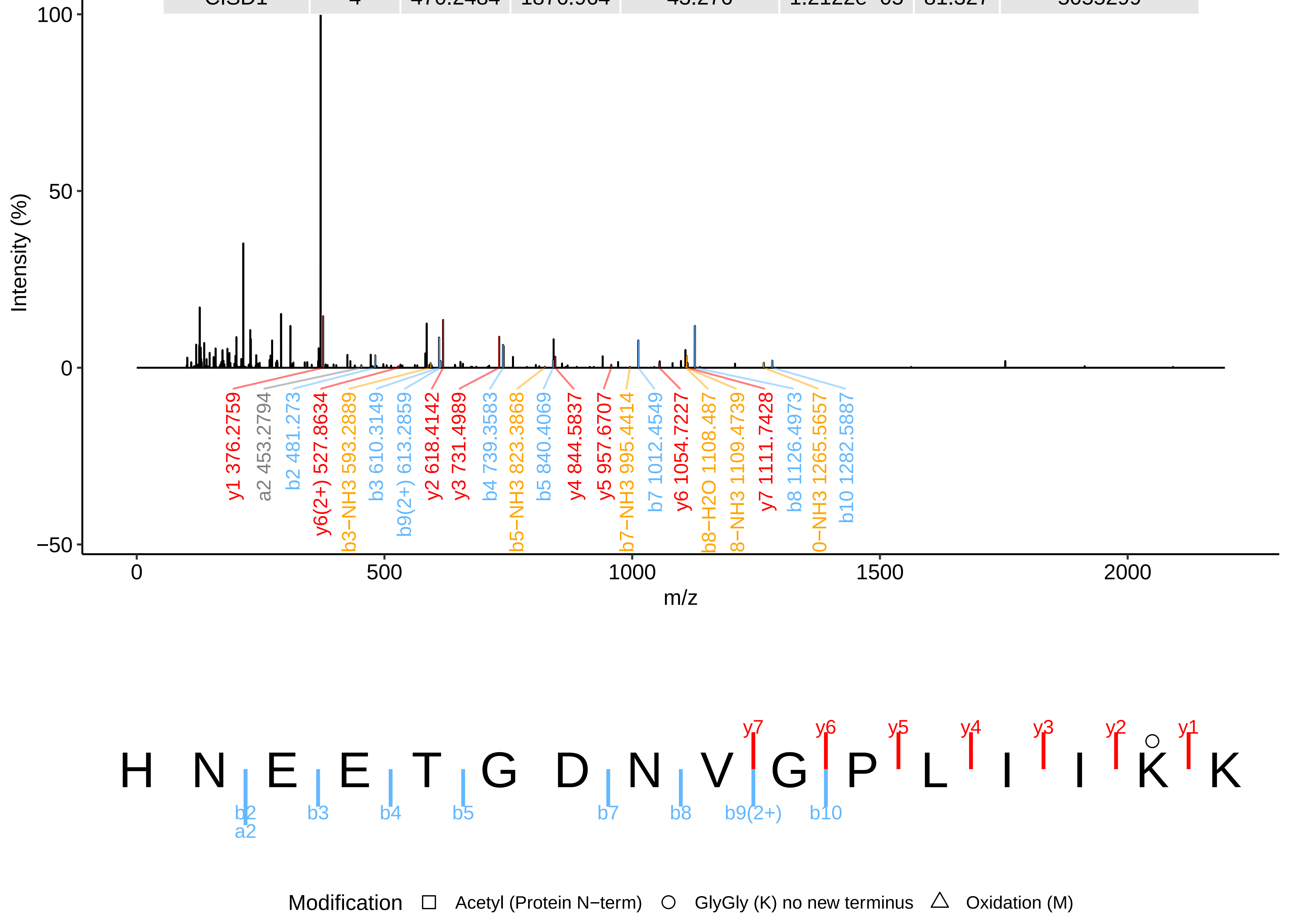

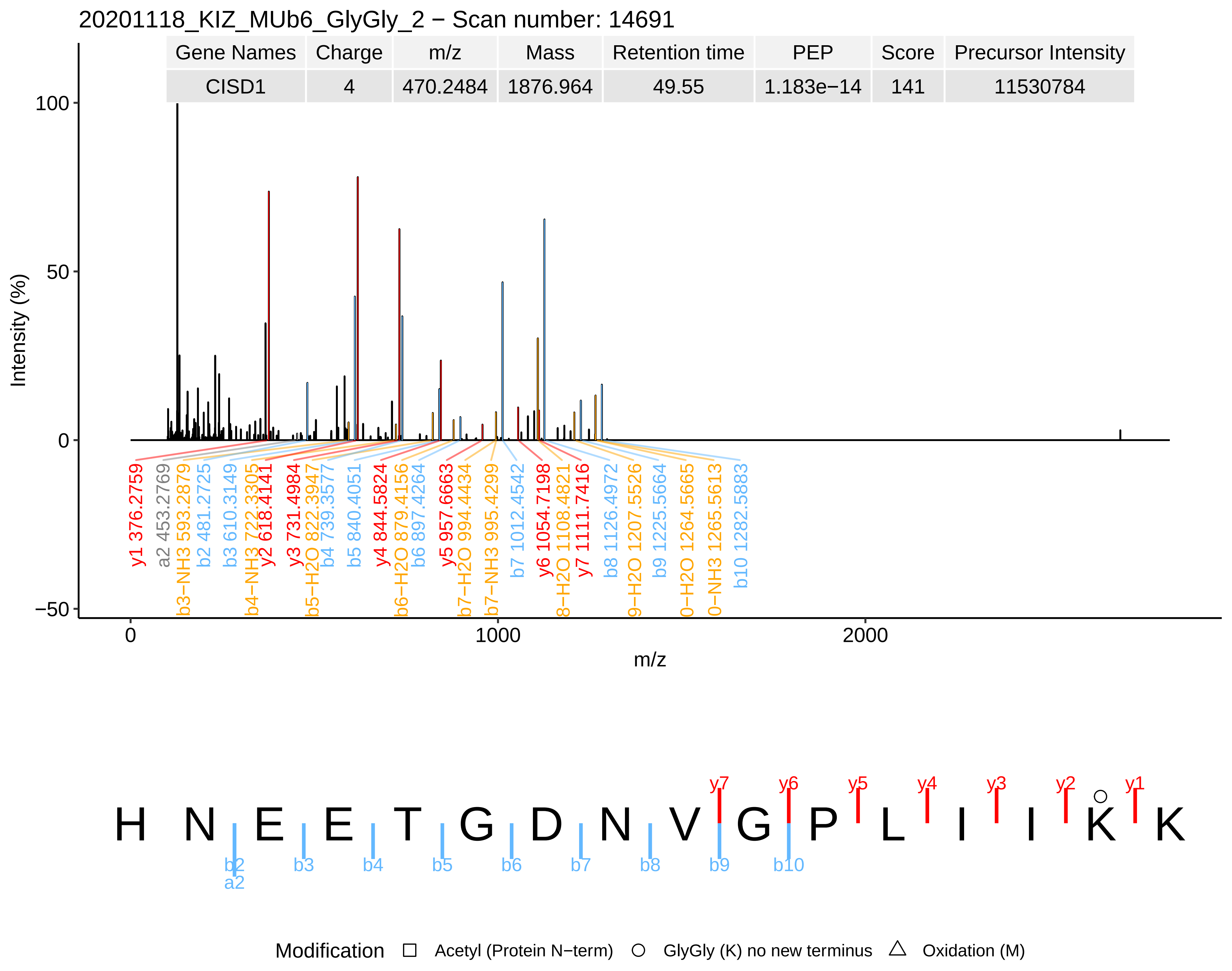

20201118_KIZ_MUb6_GlyGly_2 − Scan number: 14691
Gene Names | Charge | m/z | Mass | Retention time | PEP | Score | Precursor Intensity
CISD1 | 4 | 470.2484 | 1876.964 | 49.55 | 1.183e−14 | 141 | 11530784
Intensity (%)
100
50
0
−50
0
1000
2000
m/z
y1 376.2759
a2 453.2769
b3−NH3 593.2879
b2 481.2725
b3 610.3149
b4−NH3 722.3305
y2 618.4141
y3 731.4984
b5−H2O 822.3947
b4 739.3577
b5 840.4051
y4 844.5824
b6−H2O 879.4156
b6 897.4264
y5 957.6663
b7−H2O 994.4434
b7−NH3 995.4299
b7 1012.4542
y6 1054.7198
8−H2O 1108.4821
y7 1111.7416
b8 1126.4972
9−H2O 1207.5526
b9 1225.5664
0−H2O 1264.5665
0−NH3 1265.5613
b10 1282.5883
y7 y6 y5 y4 y3 y2 y1
H N E E T G D N V G P L I I K K
b2 a2 b3 b4 b5 b6 b7 b8 b9 b10
Modification
Acetyl (Protein N−term)
GlyGly (K) no new terminus
Oxidation (M)

20200921_KIZ_MUb4_GlyGly_10plex_2 − Scan number: 20079

Gene Names	Charge	m/z	Mass	Retention time	PEP	Score	Precursor Intensity
FH	3	627.9792	1880.916	78.889	0.0002681	85.42	882312.4

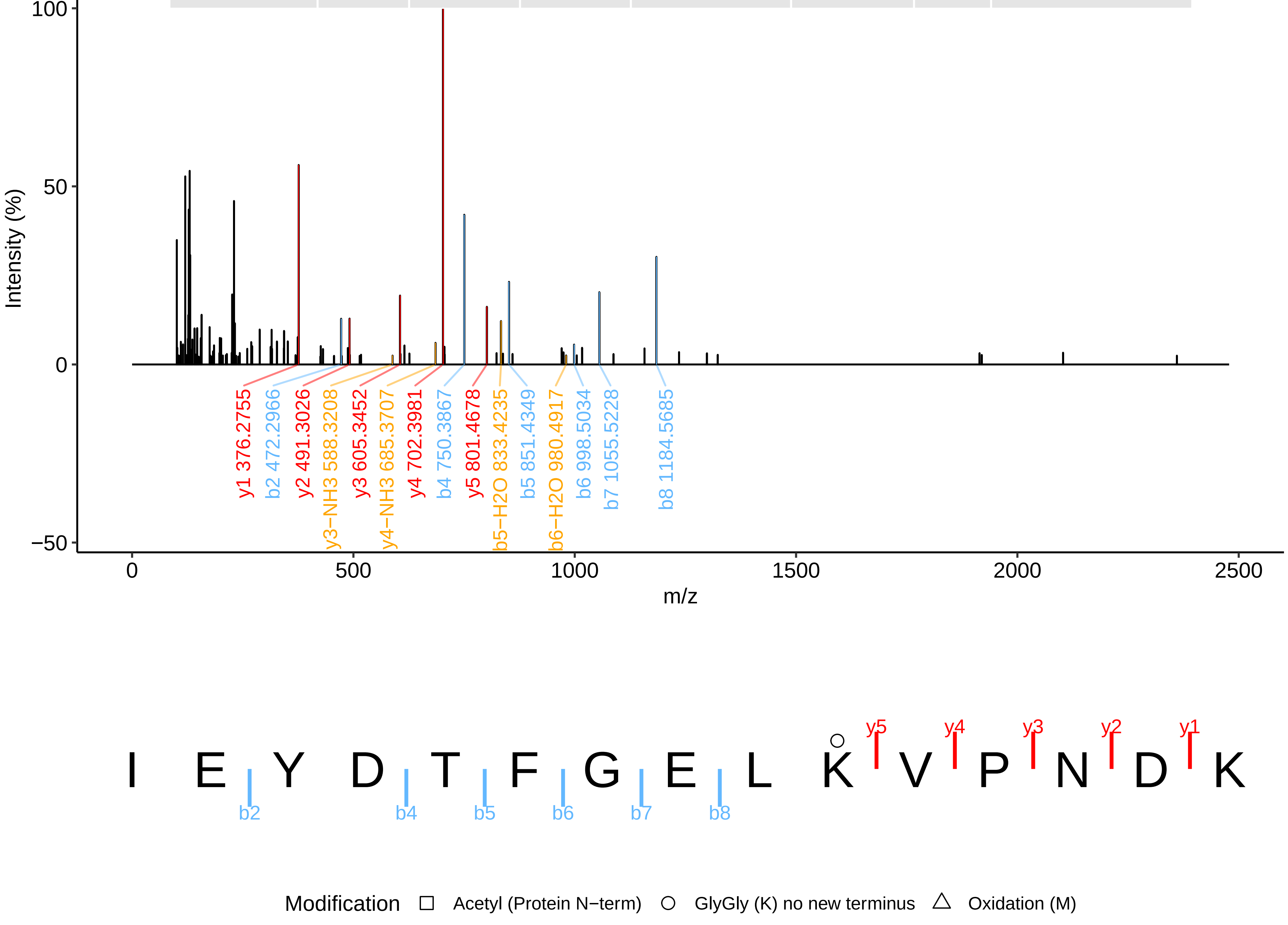

20200921_KIZ_MUb4_GlyGly_10plex − Scan number: 14756

Gene Names	Charge	m/z	Mass	Retention time	PEP	Score	Precursor Intensity
SPG20	3	622.3387	1863.994	52.509	0.0014445	72.446	3481094

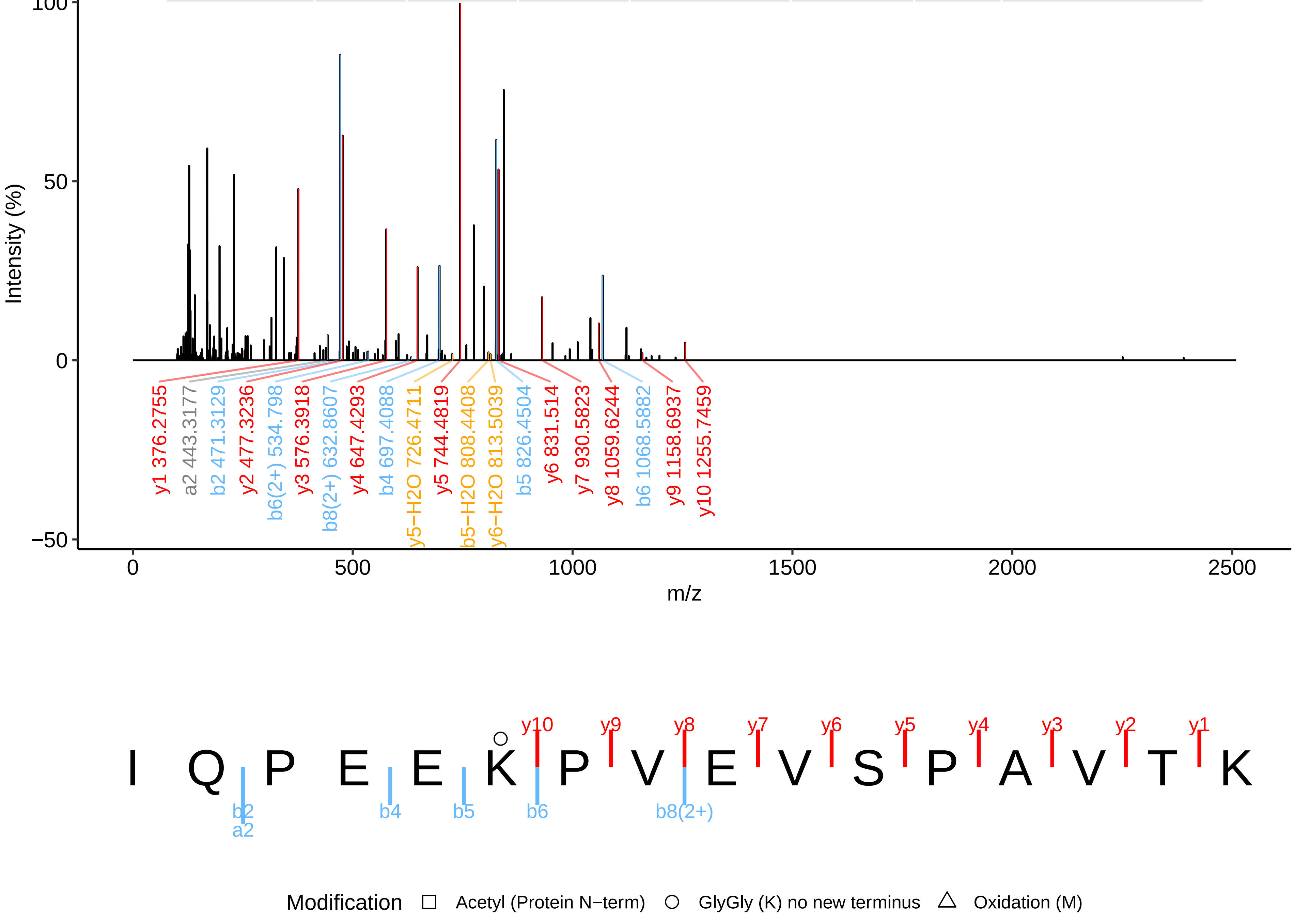

20200921_KIZ_MUb4_GlyGly_10plex − Scan number: 26989

Gene Names	Charge	m/z	Mass	Retention time	PEP	Score	Precursor Intensity
ATXN10	3	691.7003	2072.079	98.421	1.1488e−18	150.05	5866403

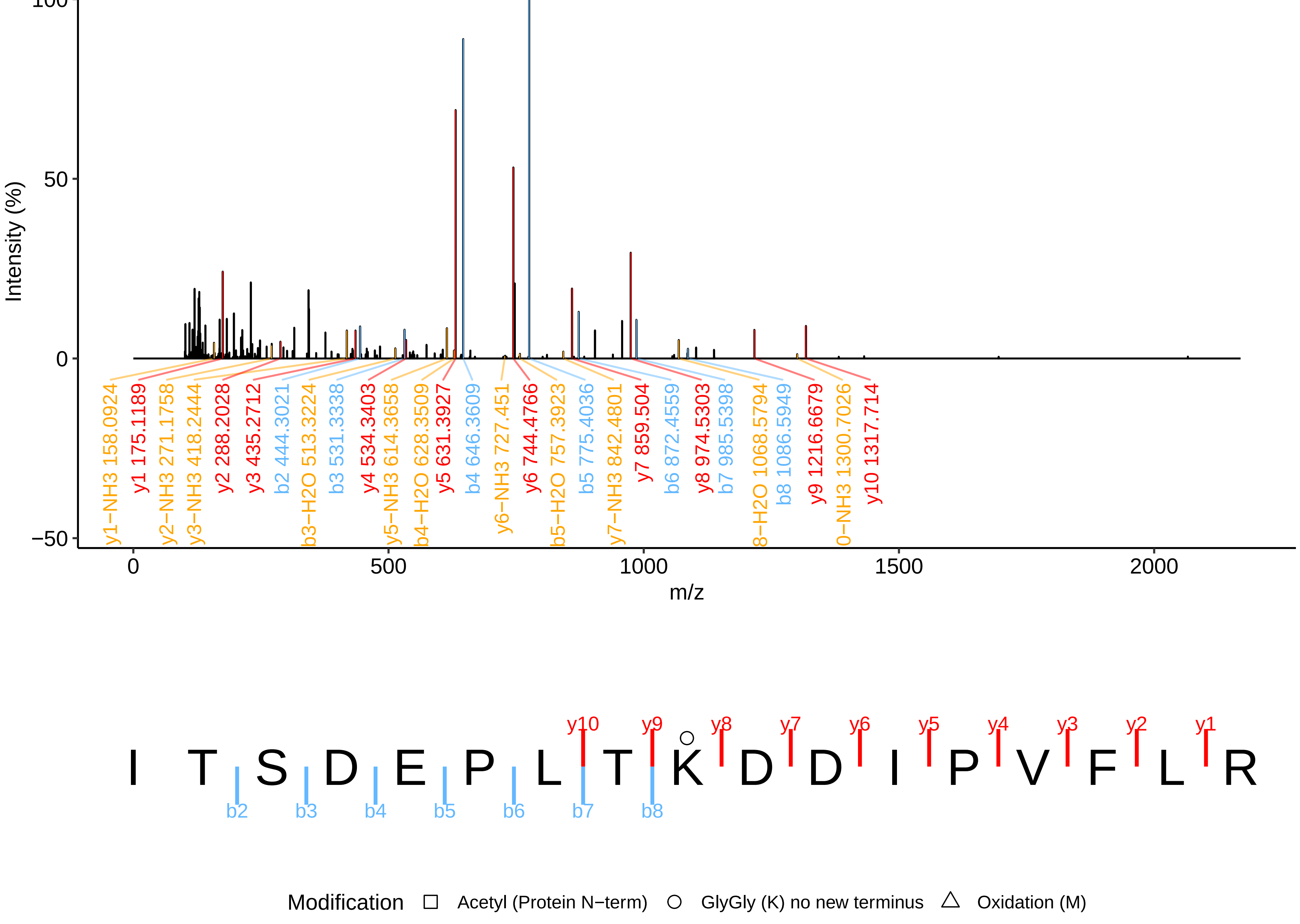

20200921_KIZ_MUb4_GlyGly_10plex − Scan number: 27120

Gene Names	Charge	m/z	Mass	Retention time	PEP	Score	Precursor Intensity
ATXN10	3	691.7003	2072.079	98.928	6.8044e−24	166.9	25489818

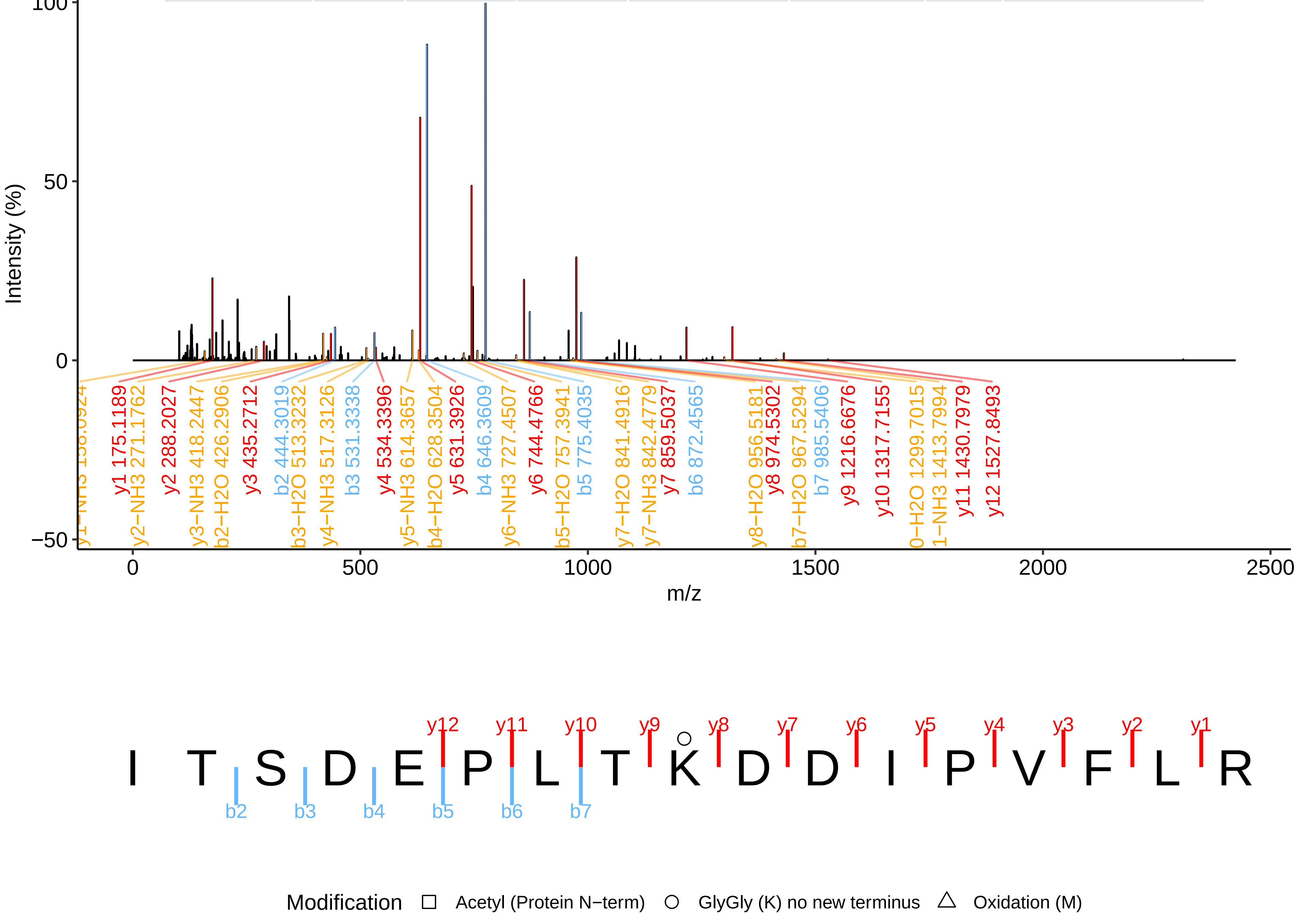

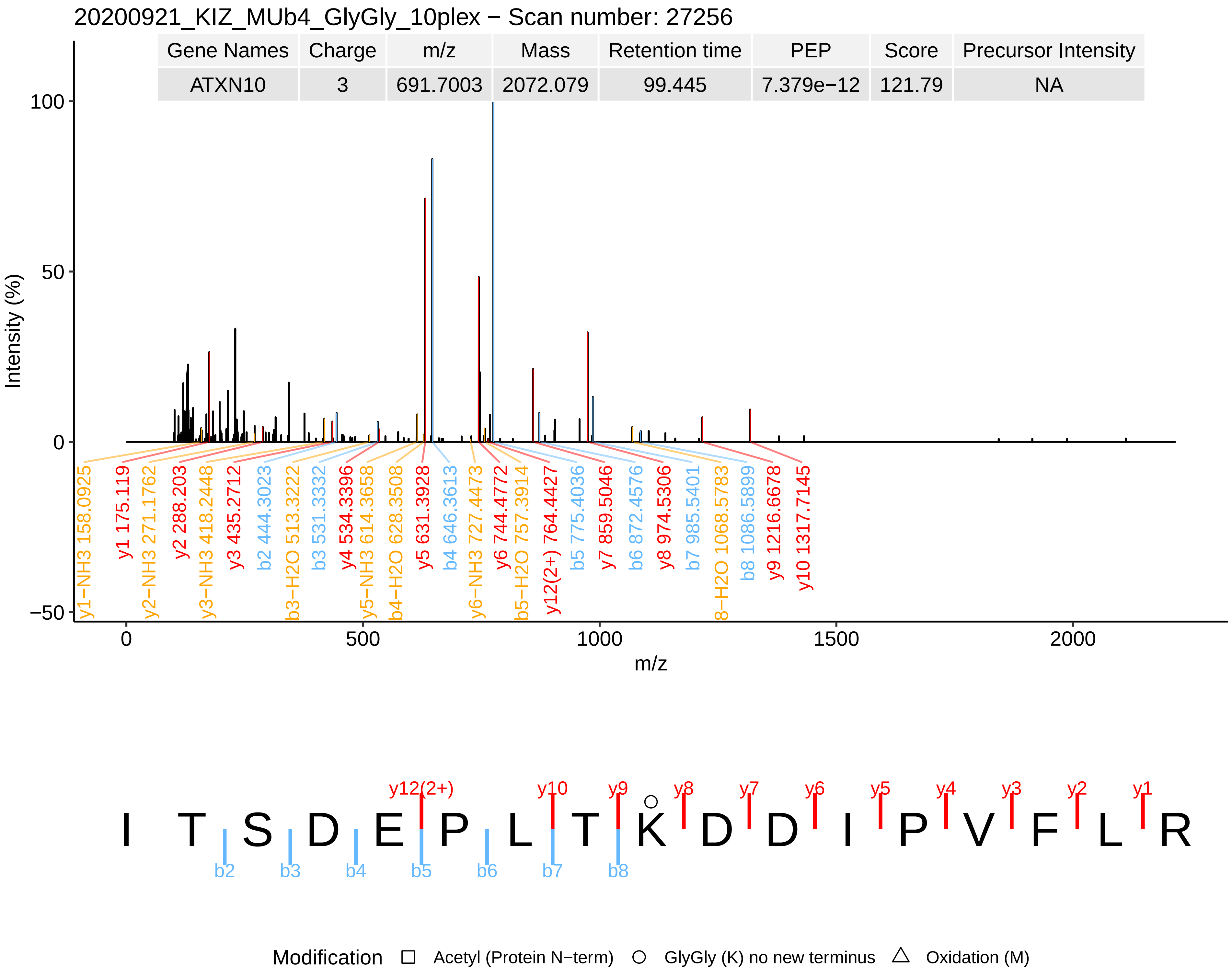
20200921_KIZ_MUb4_GlyGly_10plex − Scan number: 27256
Gene Names | Charge | m/z | Mass | Retention time | PEP | Score | Precursor Intensity
ATXN10 | 3 | 691.7003 | 2072.079 | 99.445 | 7.379e−12 | 121.79 | NA
Intensity (%)
100
50
0
−50
0
500
1000
1500
2000
m/z
y1−NH3 158.0925
y1 175.119
y2−NH3 271.1762
y2 288.203
y3−NH3 418.2448
y3 435.2712
b2 444.3023
b3−H2O 513.3222
b3 531.3332
y4 534.3396
y5−NH3 614.3658
b4−H2O 628.3508
y5 631.3928
b4 646.3613
y6−NH3 727.4473
y6 744.4772
b5−H2O 757.3914
y12(2+) 764.4427
b5 775.4036
y7 859.5046
b6 872.4576
y8 974.5306
b7 985.5401
8−H2O 1068.5783
b8 1086.5899
y9 1216.6678
y10 1317.7145
y12(2+)
y10
y9
y8
y7
y6
y5
y4
y3
y2
y1
I T S D E P L T K D D I P V F L R
b2
b3
b4
b5
b6
b7
b8
Modification
Acetyl (Protein N−term)
GlyGly (K) no new terminus
Oxidation (M)

20200921_KIZ_MUb4_GlyGly_10plex − Scan number: 27396

Gene Names	Charge	m/z	Mass	Retention time	PEP	Score	Precursor Intensity
ATXN10	3	691.7003	2072.079	99.984	0.0067327	62.813	NA

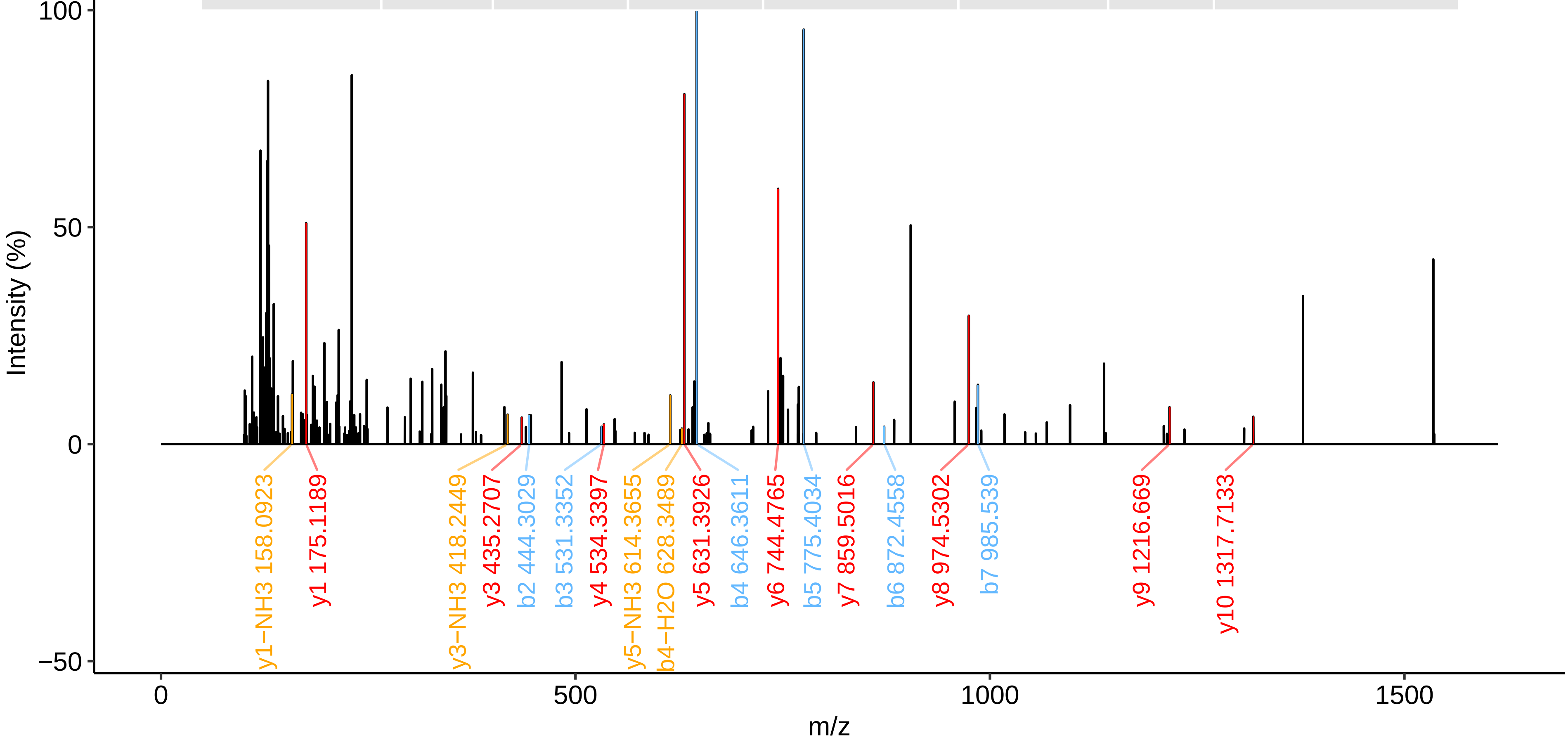

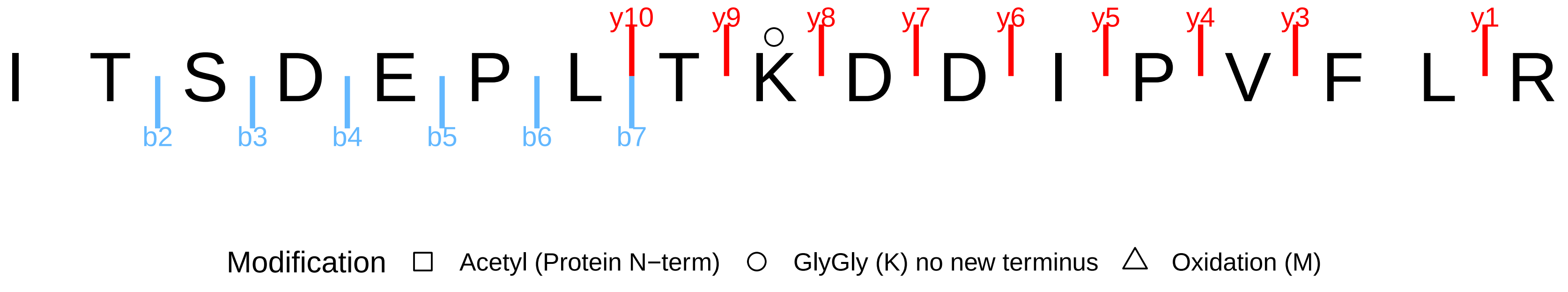

20200921_KIZ_MUb4_GlyGly_10plex_2 − Scan number: 23729

Gene Names	Charge	m/z	Mass	Retention time	PEP	Score	Precursor Intensity
ATXN10	3	691.7003	2072.079	93.686	1.6131e−06	93.51	NA

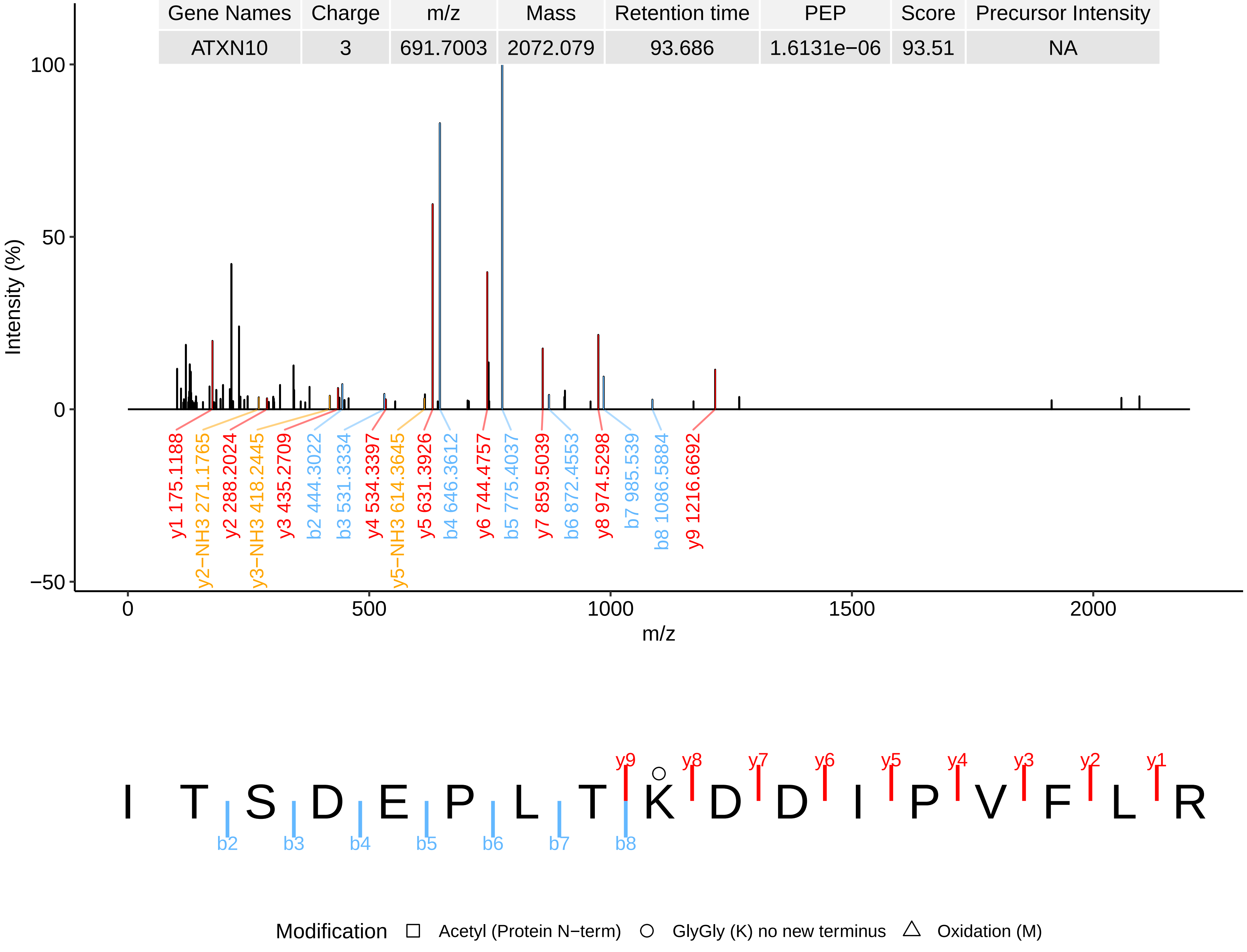

20200921_KIZ_MUb4_GlyGly_10plex_2 − Scan number: 23858

Gene Names	Charge	m/z	Mass	Retention time	PEP	Score	Precursor Intensity
ATXN10	3	691.7003	2072.079	94.208	5.9453e−06	81.448	789079.4

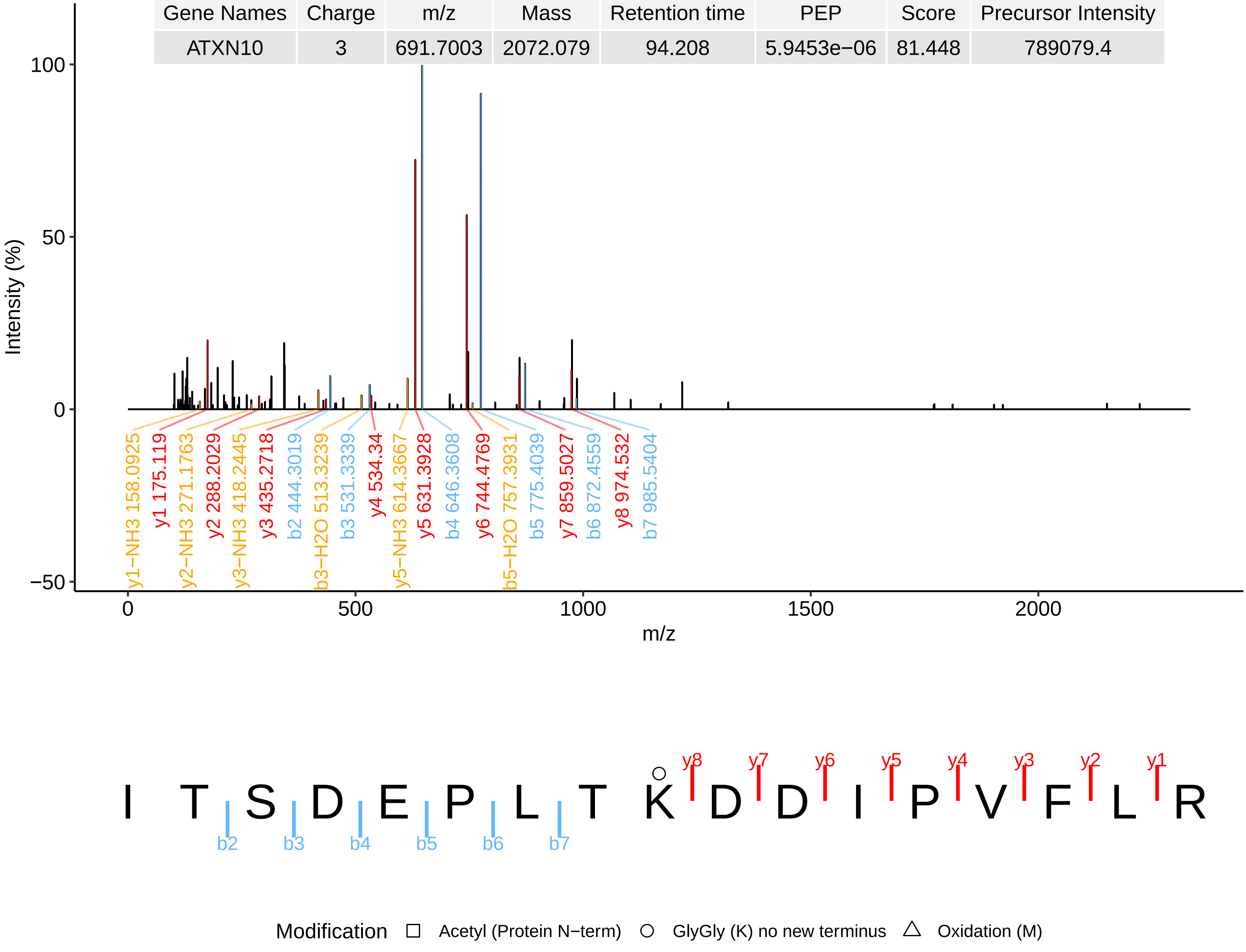

20200924_KIZ_MUb5_GlyGly_1 − Scan number: 26706

Gene Names	Charge	m/z	Mass	Retention time	PEP	Score	Precursor Intensity
ATXN10	3	691.7003	2072.079	85.459	1.3135e−22	153.24	81331768

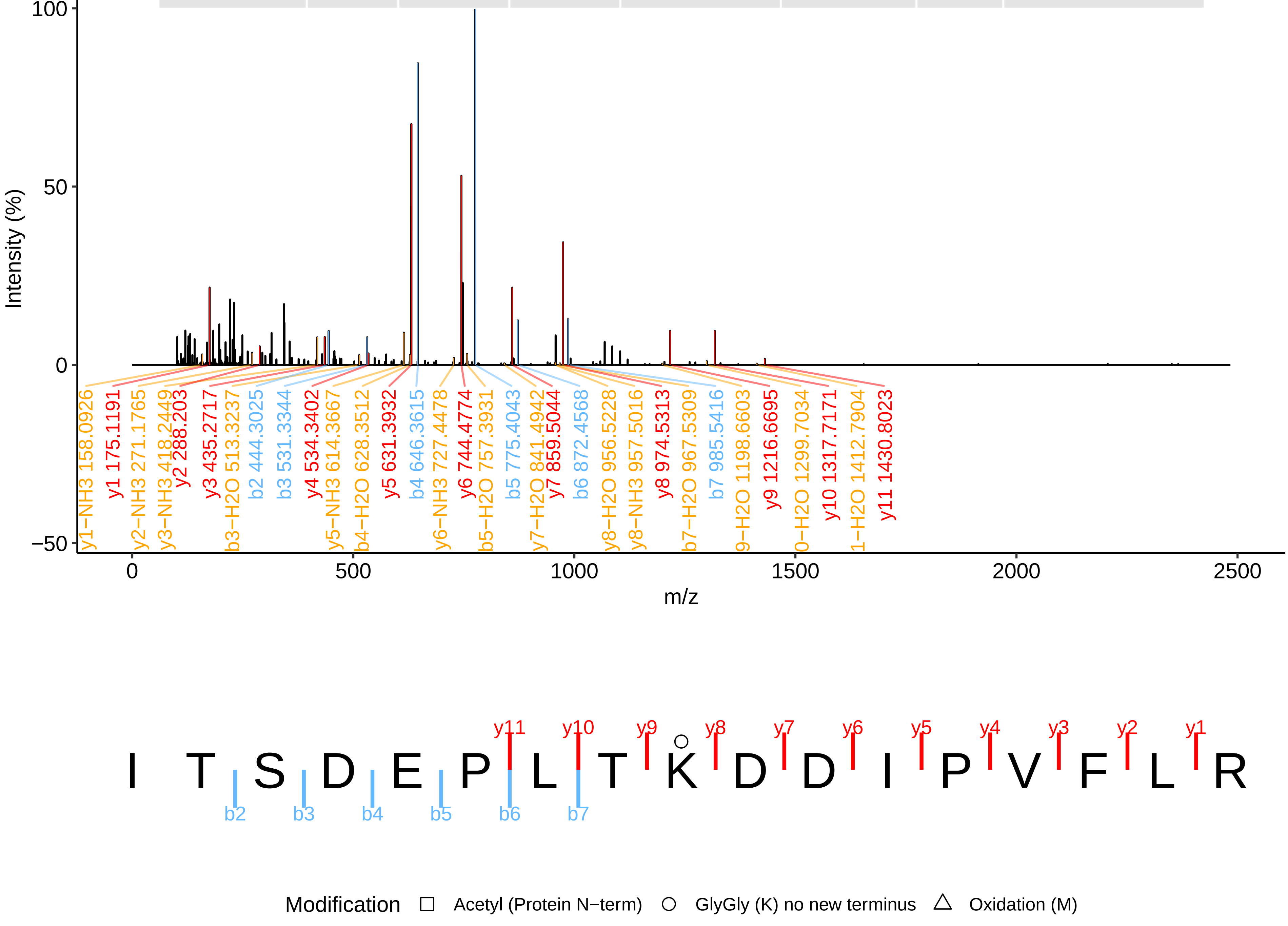

20200924_KIZ_MUb5_GlyGly_1 − Scan number: 26875

Gene Names	Charge	m/z	Mass	Retention time	PEP	Score	Precursor Intensity
ATXN10	3	691.7003	2072.079	85.962	1.3004e−63	222.16	33246372

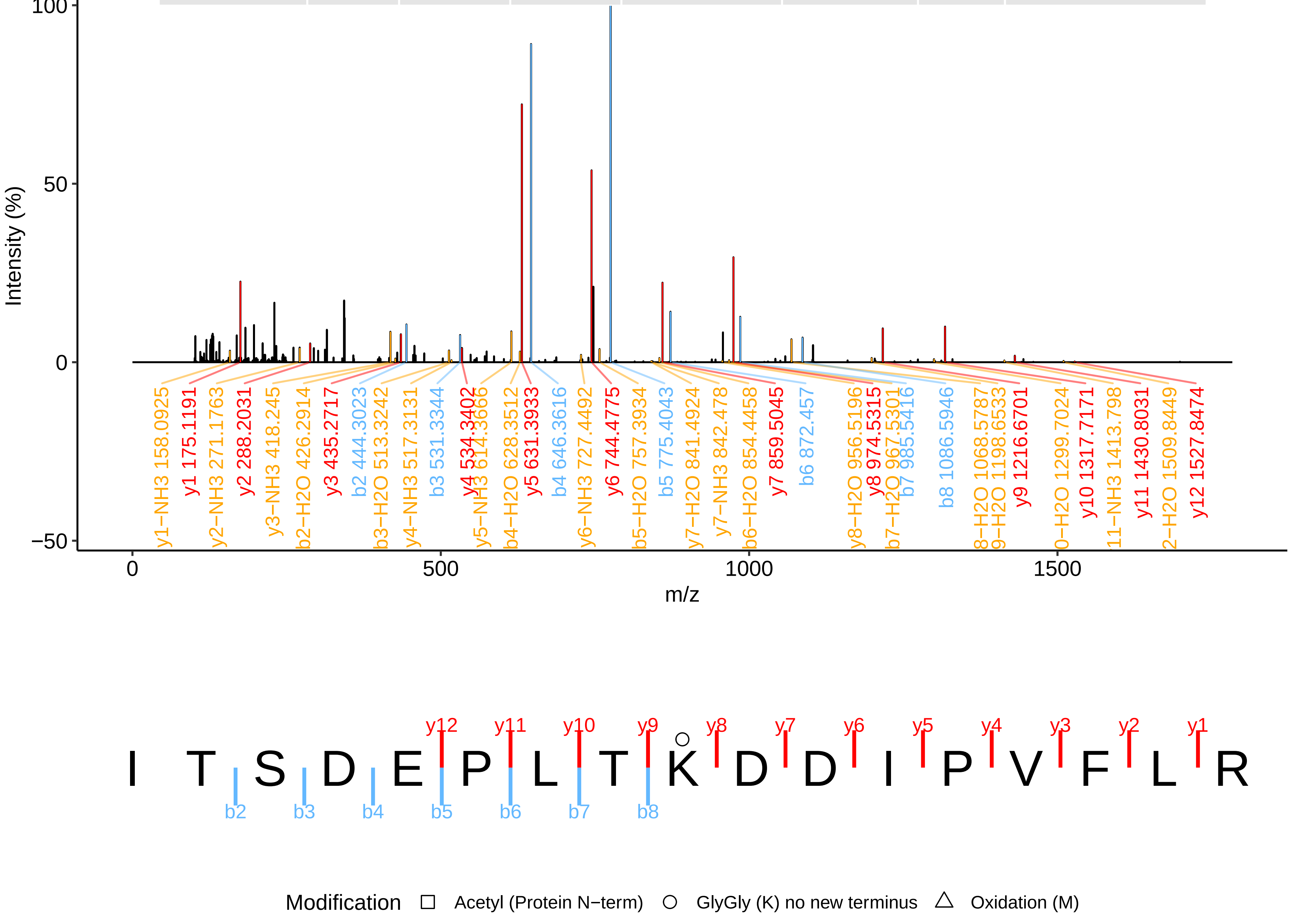

20200924_KIZ_MUb5_GlyGly_2 − Scan number: 23854

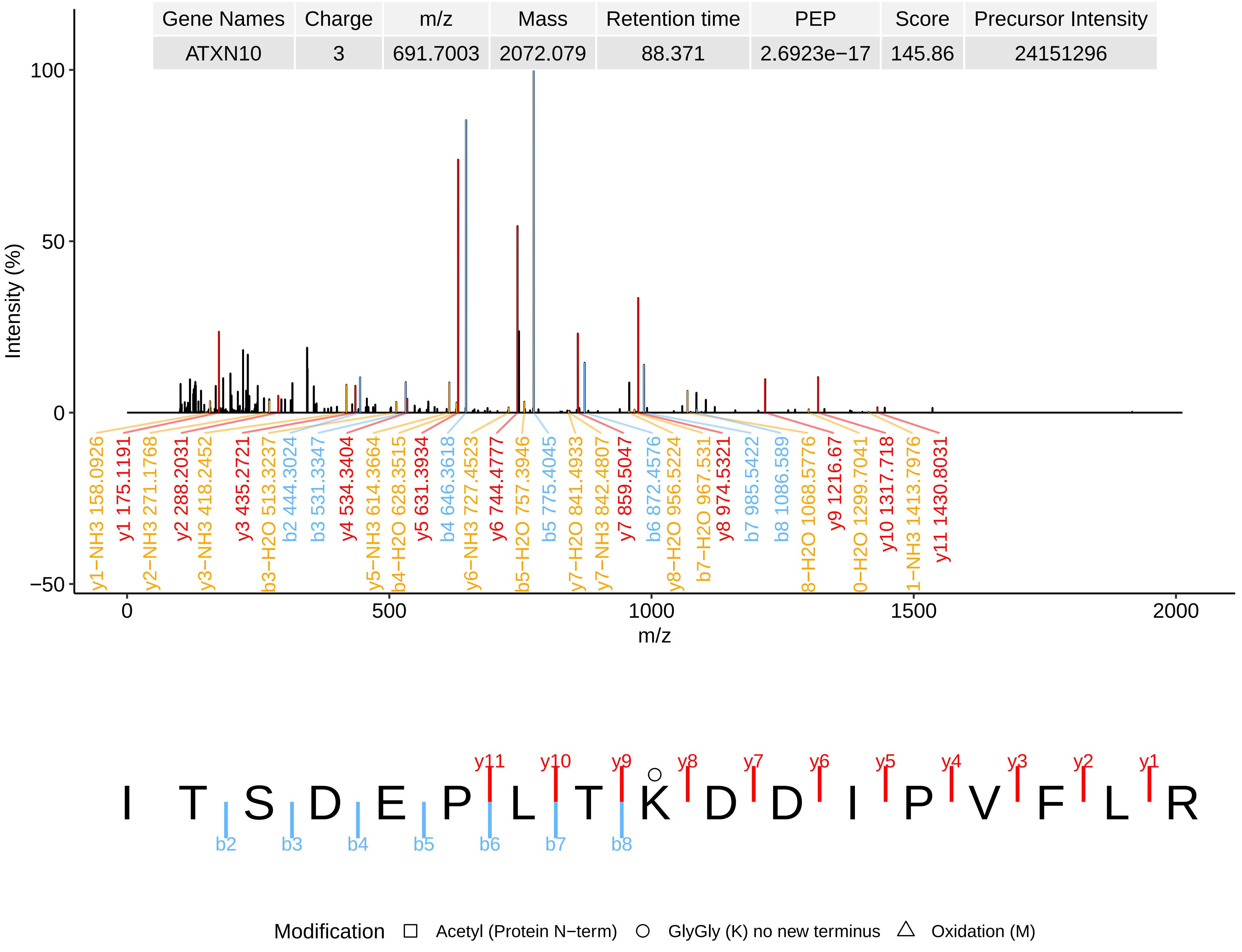

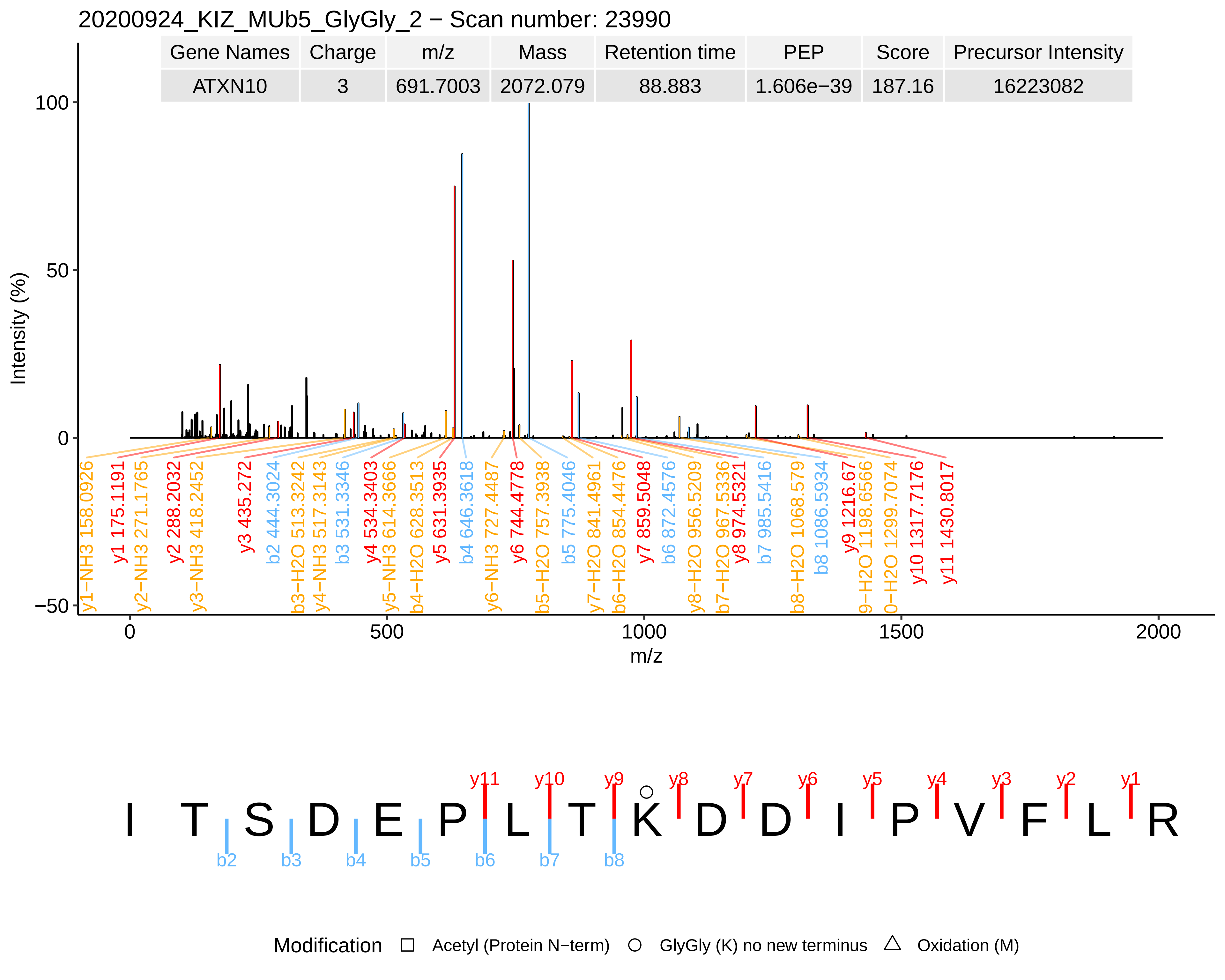
20200924_KIZ_MUb5_GlyGly_2 − Scan number: 23990
Gene Names | Charge | m/z | Mass | Retention time | PEP | Score | Precursor Intensity
ATXN10 | 3 | 691.7003 | 2072.079 | 88.883 | 1.606e−39 | 187.16 | 16223082
Intensity (%)
100
50
0
−50
m/z
0
500
1000
1500
2000
y1−NH3 158.0926
y1 175.1191
y2−NH3 271.1765
y2 288.2032
y3−NH3 418.2452
y3 435.272
b2 444.3024
b3−H2O 513.3242
y4−NH3 517.3143
b3 531.3346
y4 534.3403
y5−NH3 614.3666
b4−H2O 628.3513
y5 631.3935
b4 646.3618
y6−NH3 727.4487
y6 744.4778
b5−H2O 757.3938
b5 775.4046
y7−H2O 841.4961
b6−H2O 854.4476
y7 859.5048
b6 872.4576
y8−H2O 956.5209
b7−H2O 967.5336
y8 974.5321
b7 985.5416
b8−H2O 1068.579
b8 1086.5934
y9 1216.67
9−H2O 1198.6566
0−H2O 1299.7074
y10 1317.7176
y11 1430.8017
I T S D E P L T K D D I P V F L R
y11 y10 y9 y8 y7 y6 y5 y4 y3 y2 y1
b2 b3 b4 b5 b6 b7 b8
Modification
Acetyl (Protein N−term)
GlyGly (K) no new terminus
Oxidation (M)

20201118_KIZ_MUb6_GlyGly_1 − Scan number: 27636

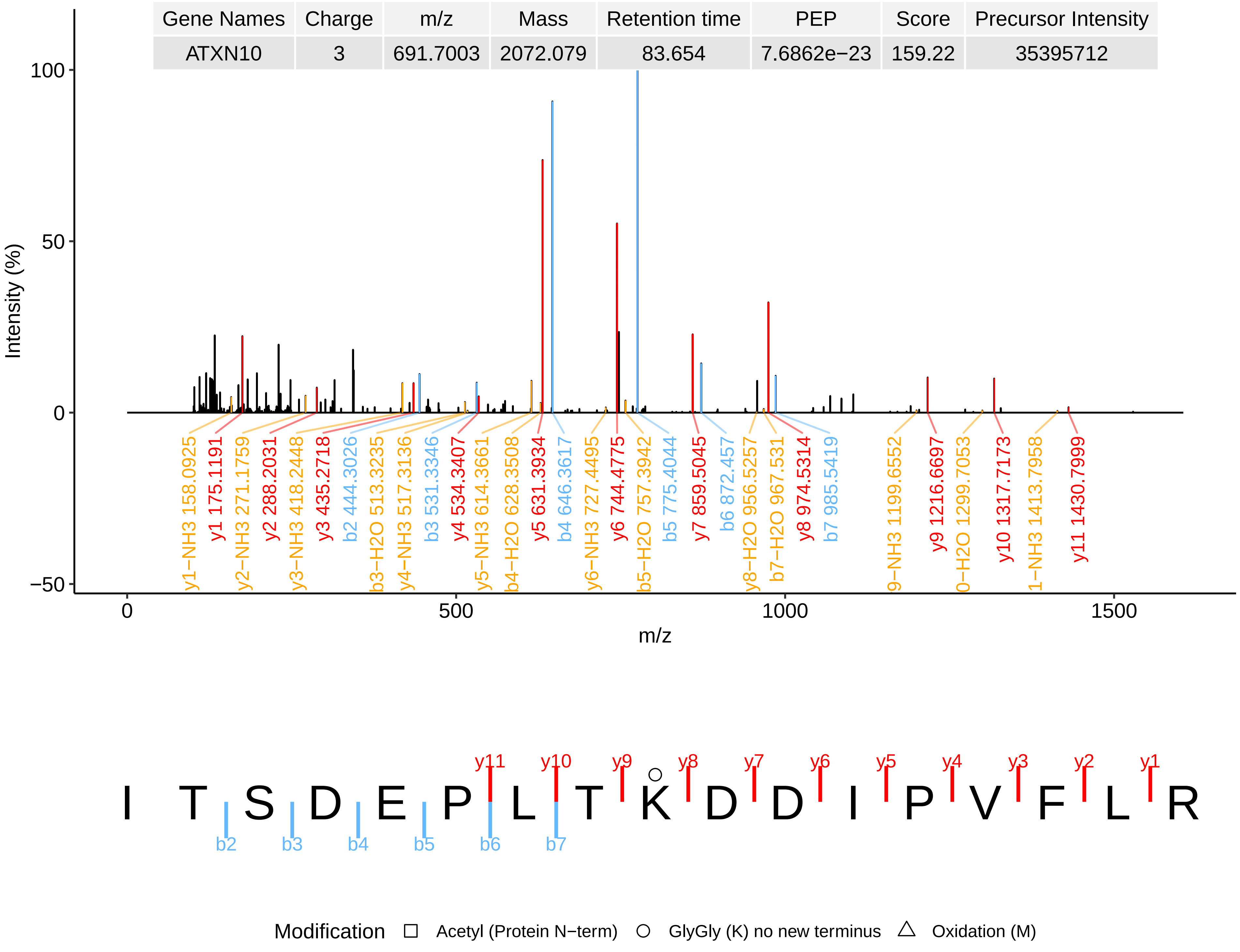

20201118_KIZ_MUb6_GlyGly_2 − Scan number: 28880

Gene Names	Charge	m/z	Mass	Retention time	PEP	Score	Precursor Intensity
ATXN10	3	691.7003	2072.079	95.805	8.4382e−51	209.39	11541984

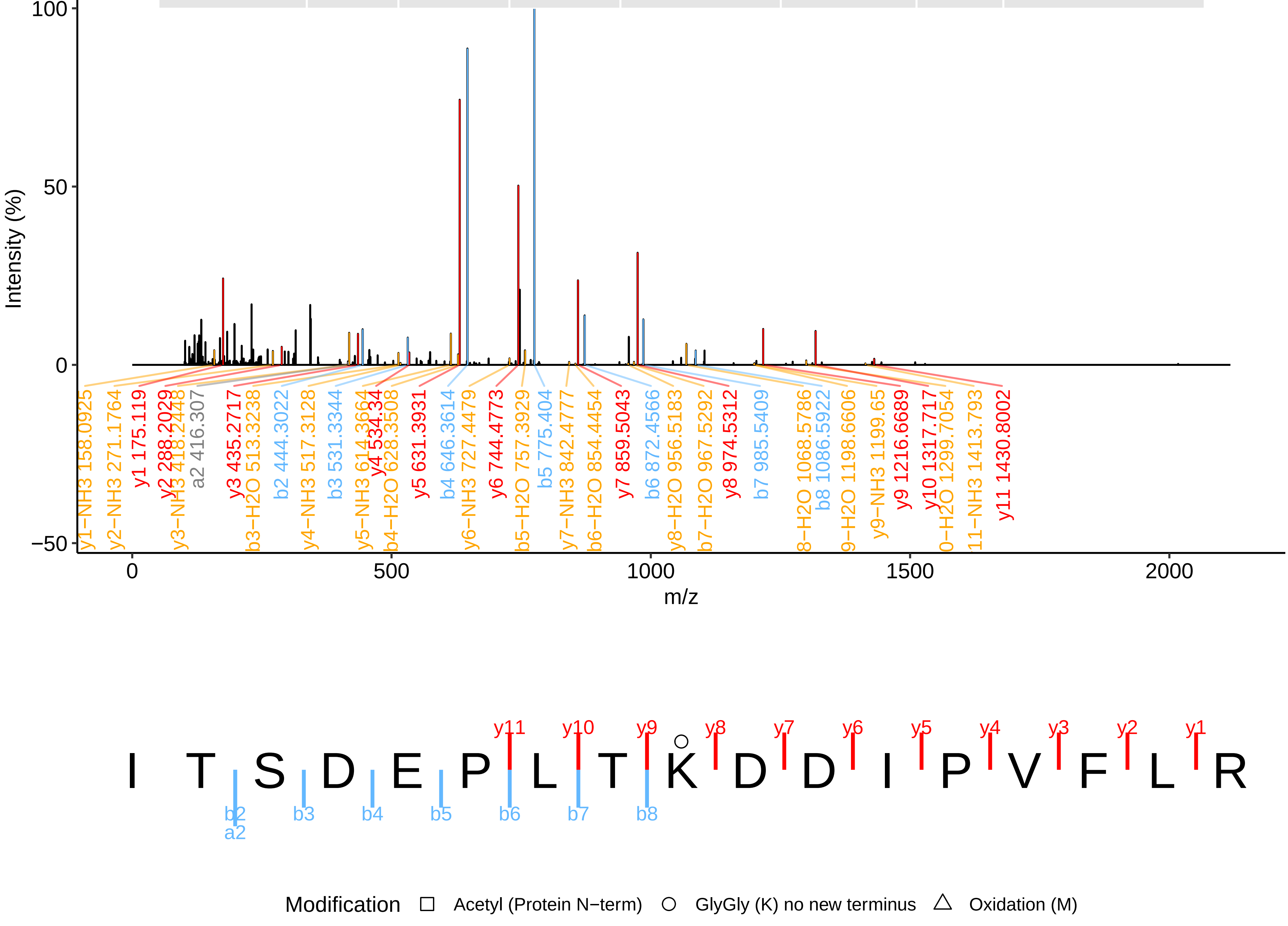

20200921_KIZ_MUb4_GlyGly_10plex_2 − Scan number: 15316

Gene Names	Charge	m/z	Mass	Retention time	PEP	Score	Precursor Intensity
HUWE1	3	617.6584	1849.953	59.866	0.015184	60.209	491940.9

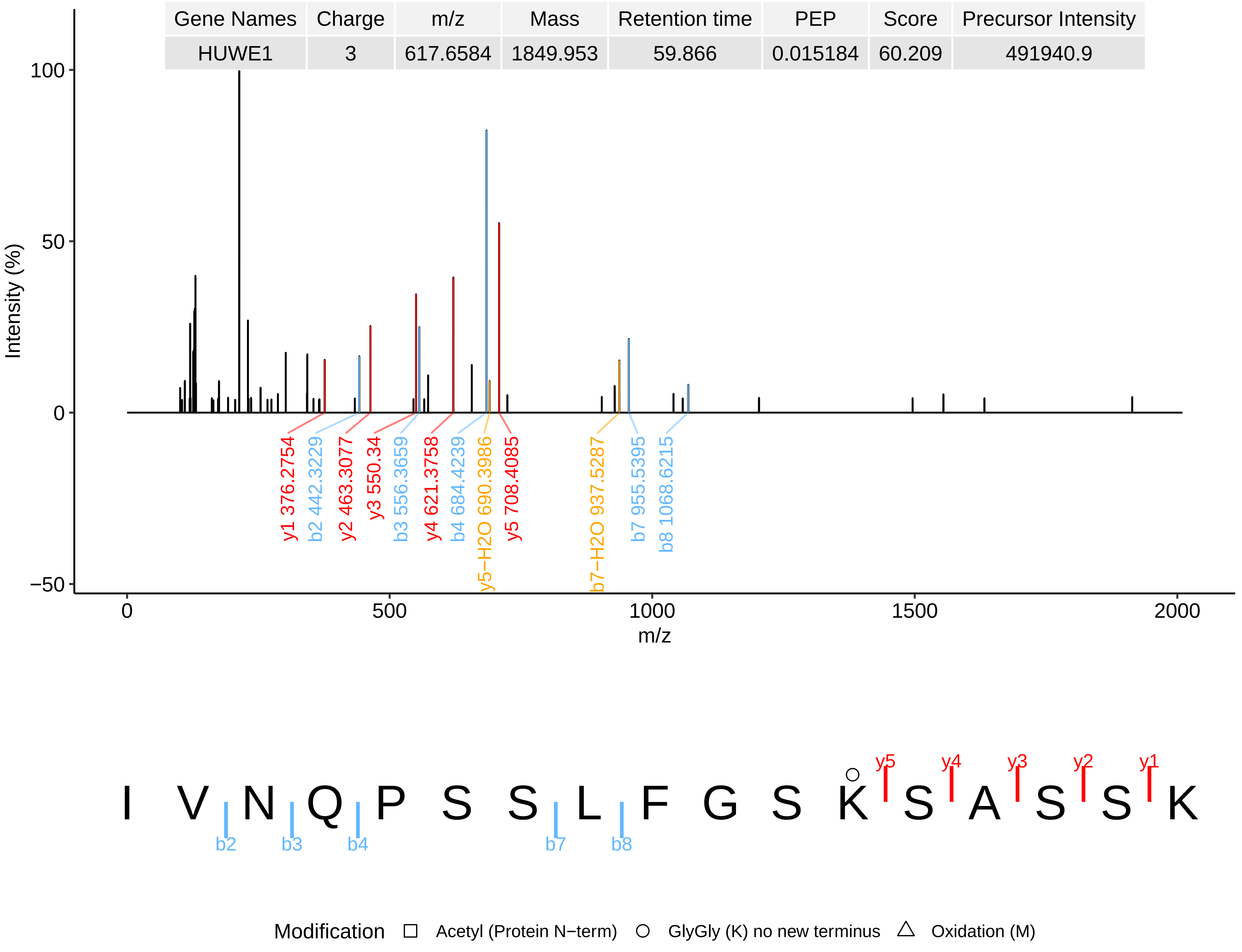

20200921_KIZ_MUb4_GlyGly_10plex_2 − Scan number: 18068

Gene Names	Charge	m/z	Mass	Retention time	PEP	Score	Precursor Intensity
SLC29A1	3	624.3301	1869.968	70.712	9.3819e−08	111.64	1793554

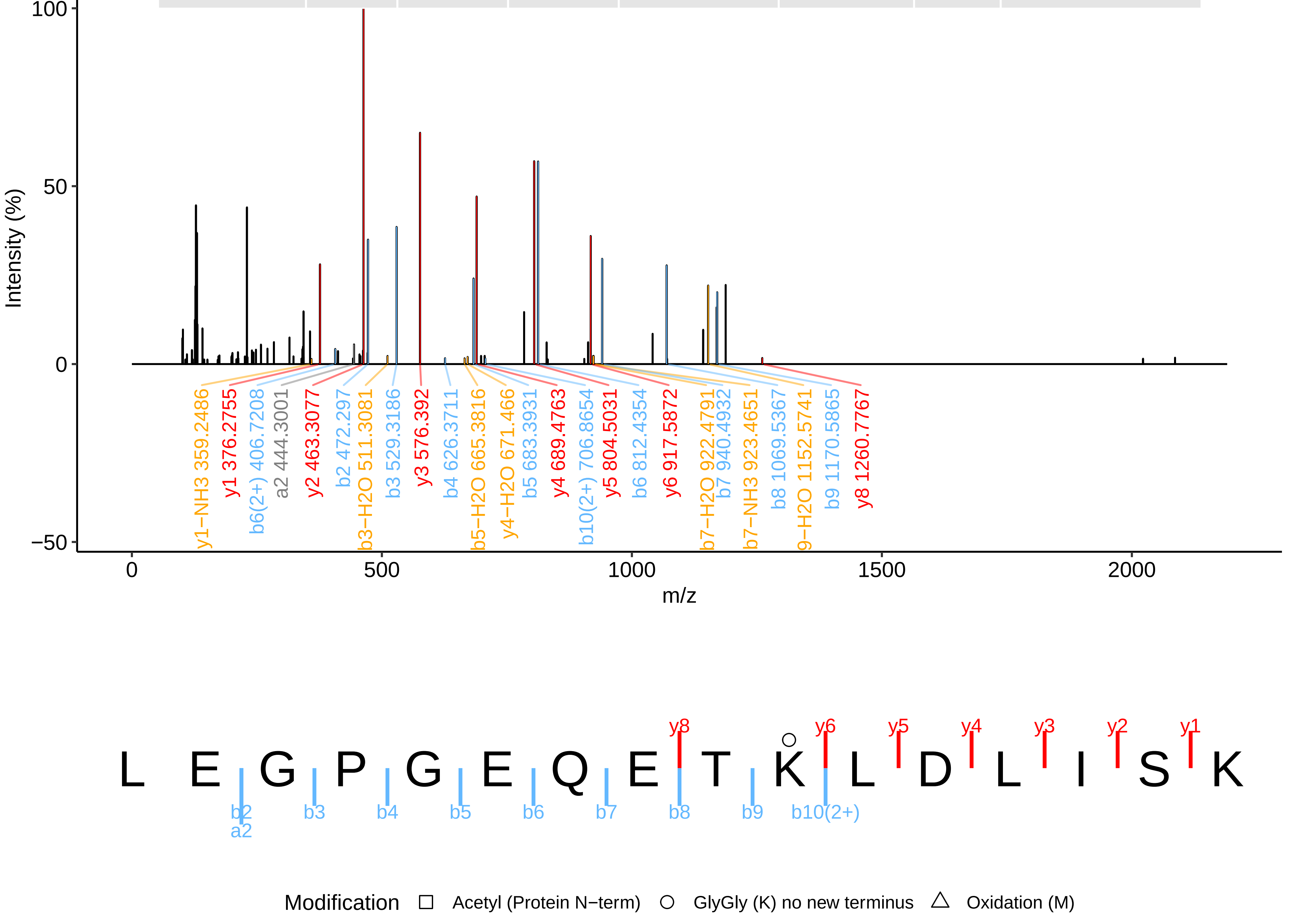

20200921_KIZ_MUb4_GlyGly_10plex_2 − Scan number: 18197

Gene Names	Charge	m/z	Mass	Retention time	PEP	Score	Precursor Intensity
SLC29A1	3	624.3301	1869.968	71.232	0.00054801	77.562	747407.6

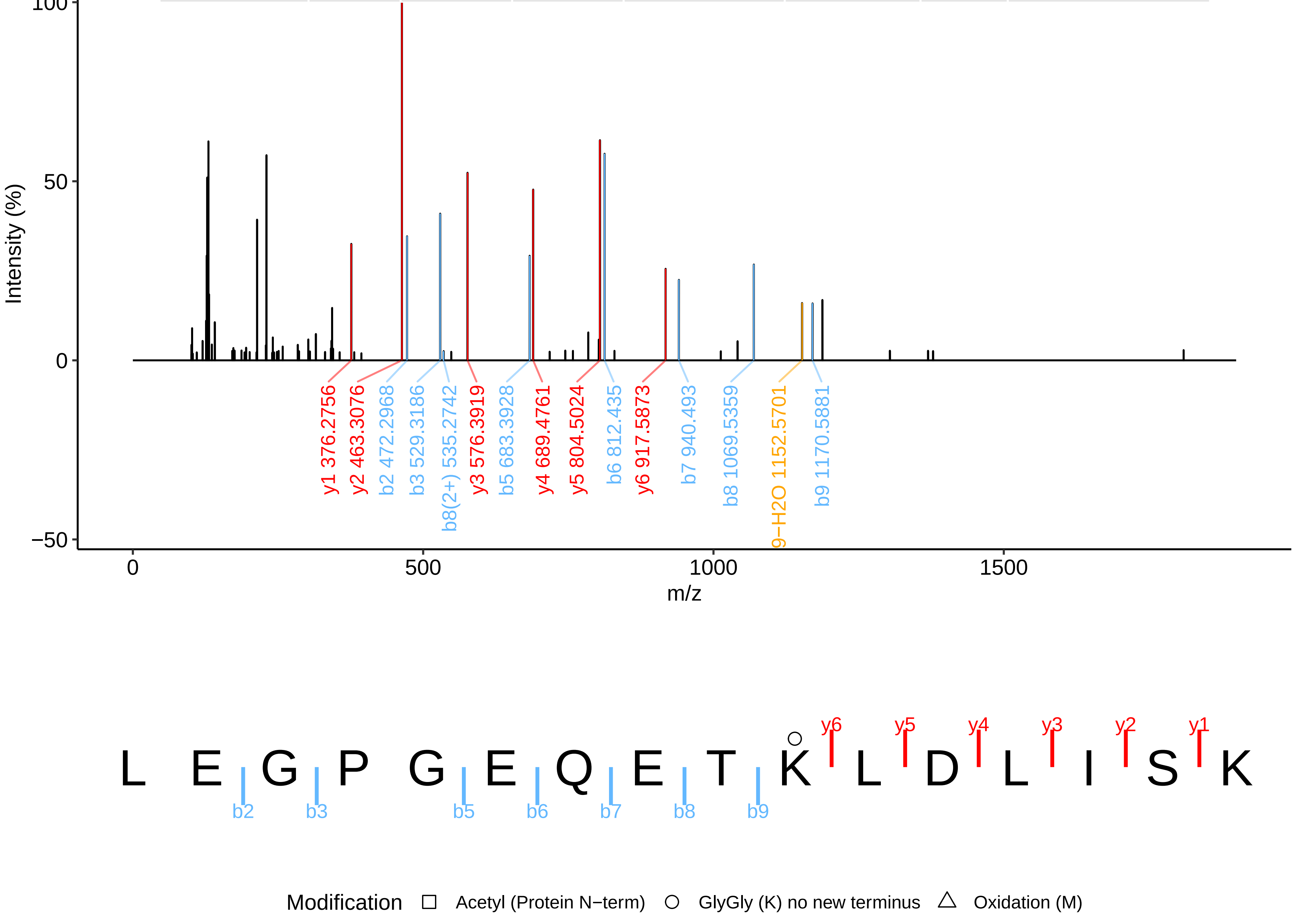

20200924_KIZ_MUb5_GlyGly_1 − Scan number: 19516

Gene Names	Charge	m/z	Mass	Retention time	PEP	Score	Precursor Intensity
SLC29A1	3	624.3301	1869.968	63.094	3.4363e−07	101.53	13248916

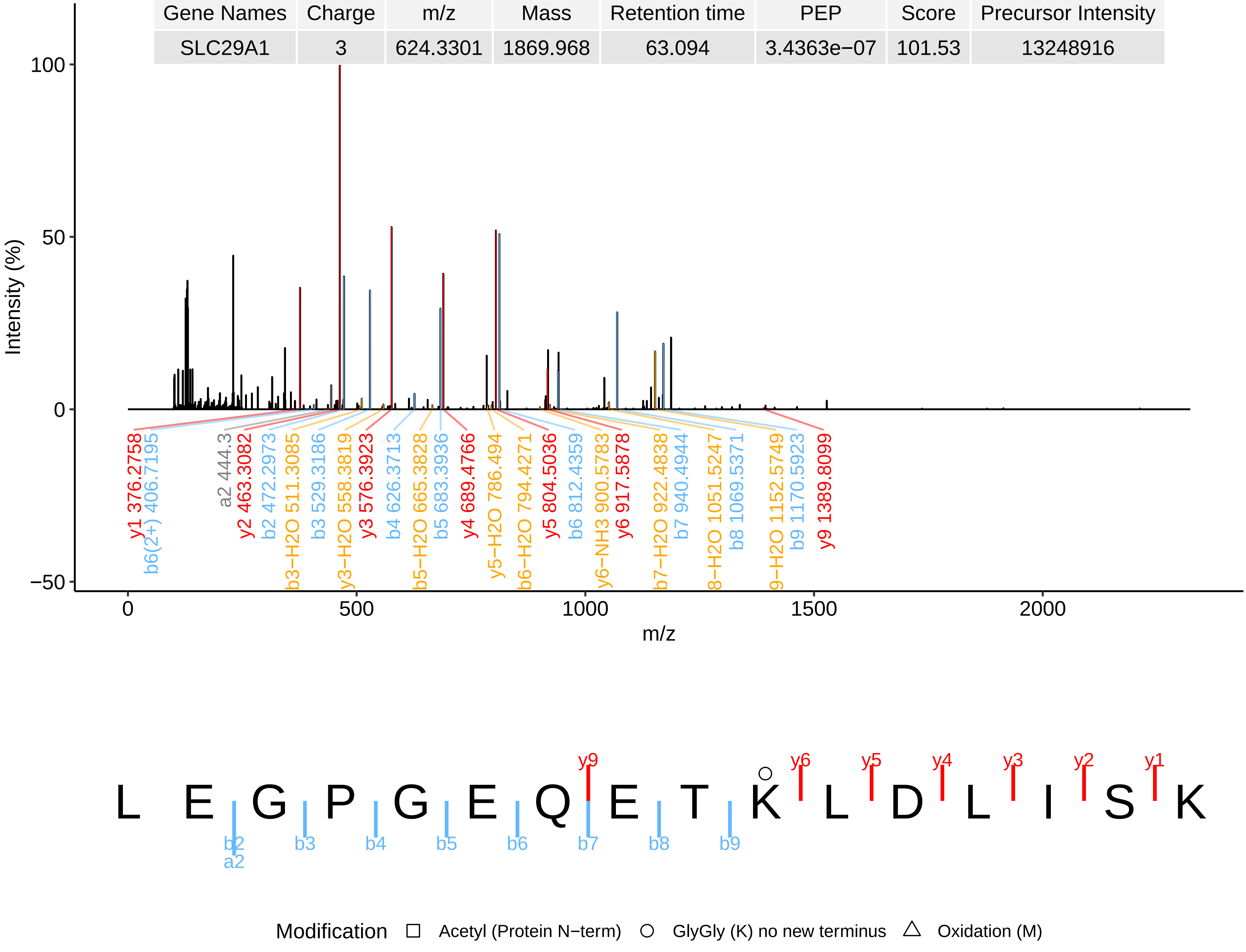

20200924_KIZ_MUb5_GlyGly_1 − Scan number: 19596

Gene Names	Charge	m/z	Mass	Retention time	PEP	Score	Precursor Intensity
SLC29A1	3	624.3301	1869.968	63.345	1.6984e−14	140.96	17623764

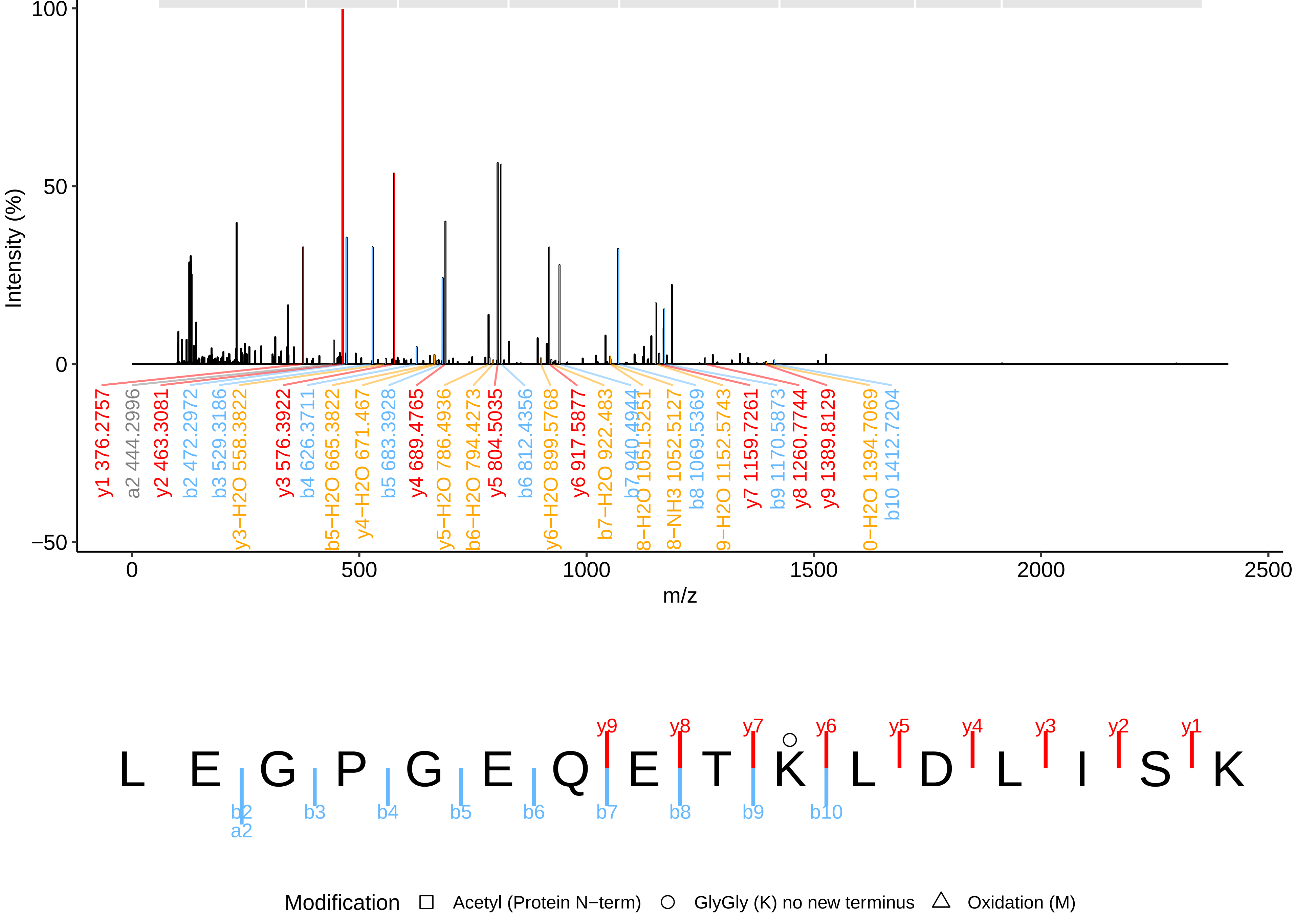

20200924_KIZ_MUb5_GlyGly_2 − Scan number: 17904

Gene Names	Charge	m/z	Mass	Retention time	PEP	Score	Precursor Intensity
SLC29A1	3	624.3301	1869.968	66.364	1.6813e−07	108.63	NA

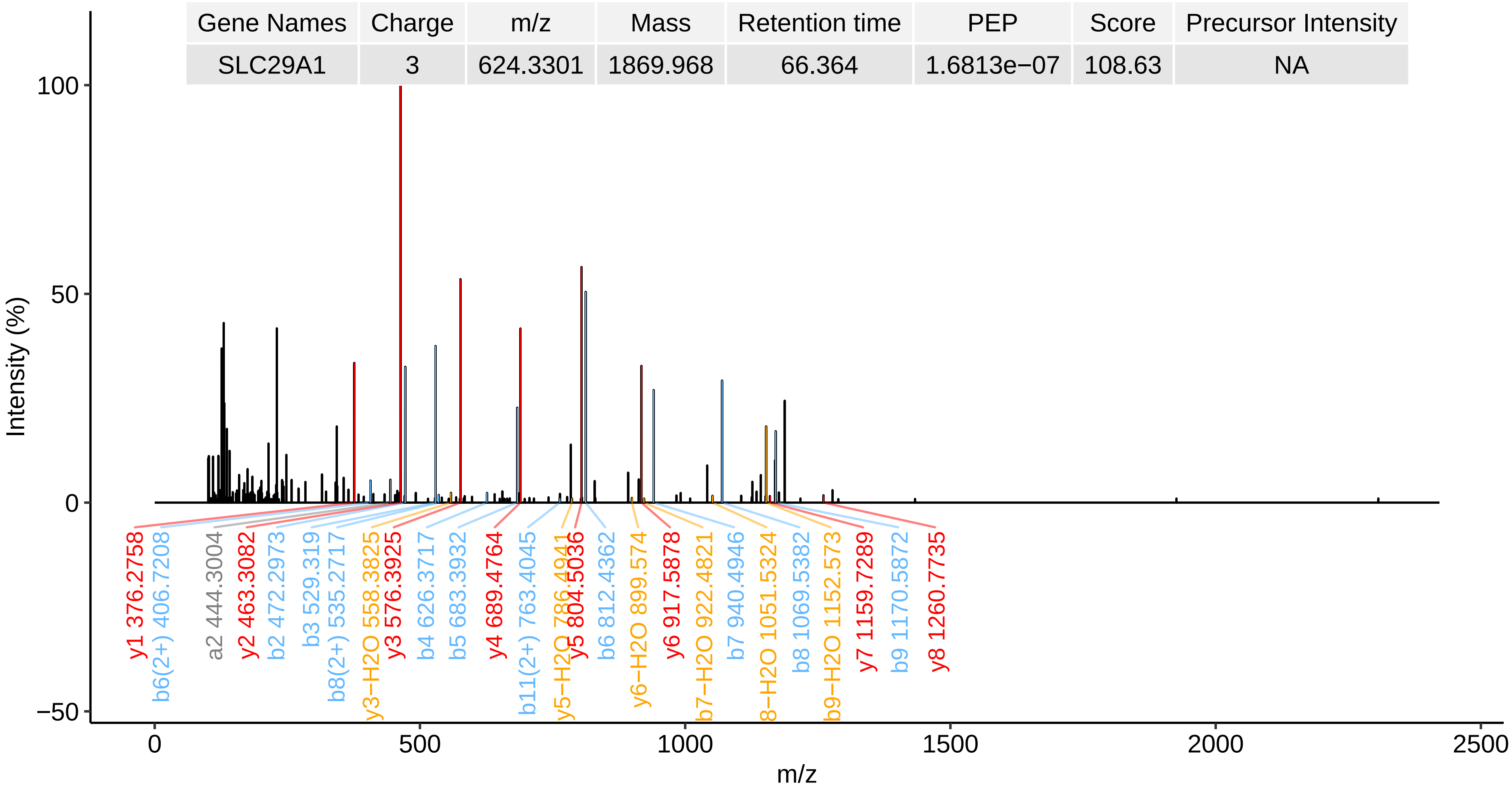

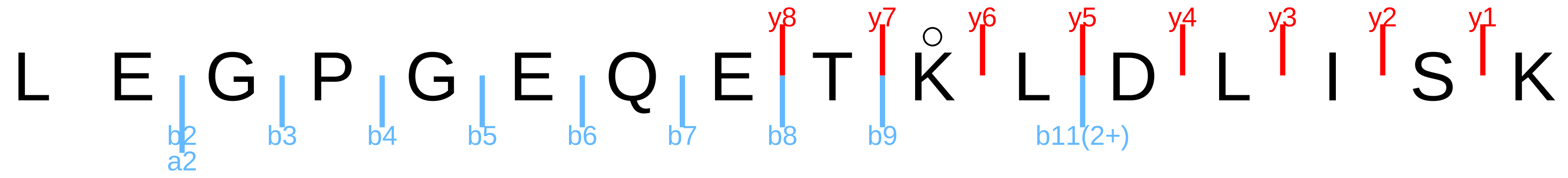

Modification □ Acetyl (Protein N−term) ○ GlyGly (K) no new terminus △ Oxidation (M)

20200924_KIZ_MUb5_GlyGly_2 − Scan number: 17918

Gene Names	Charge	m/z	Mass	Retention time	PEP	Score	Precursor Intensity
SLC29A1	3	624.3301	1869.968	66.413	3.7123e−07	100.41	4640377

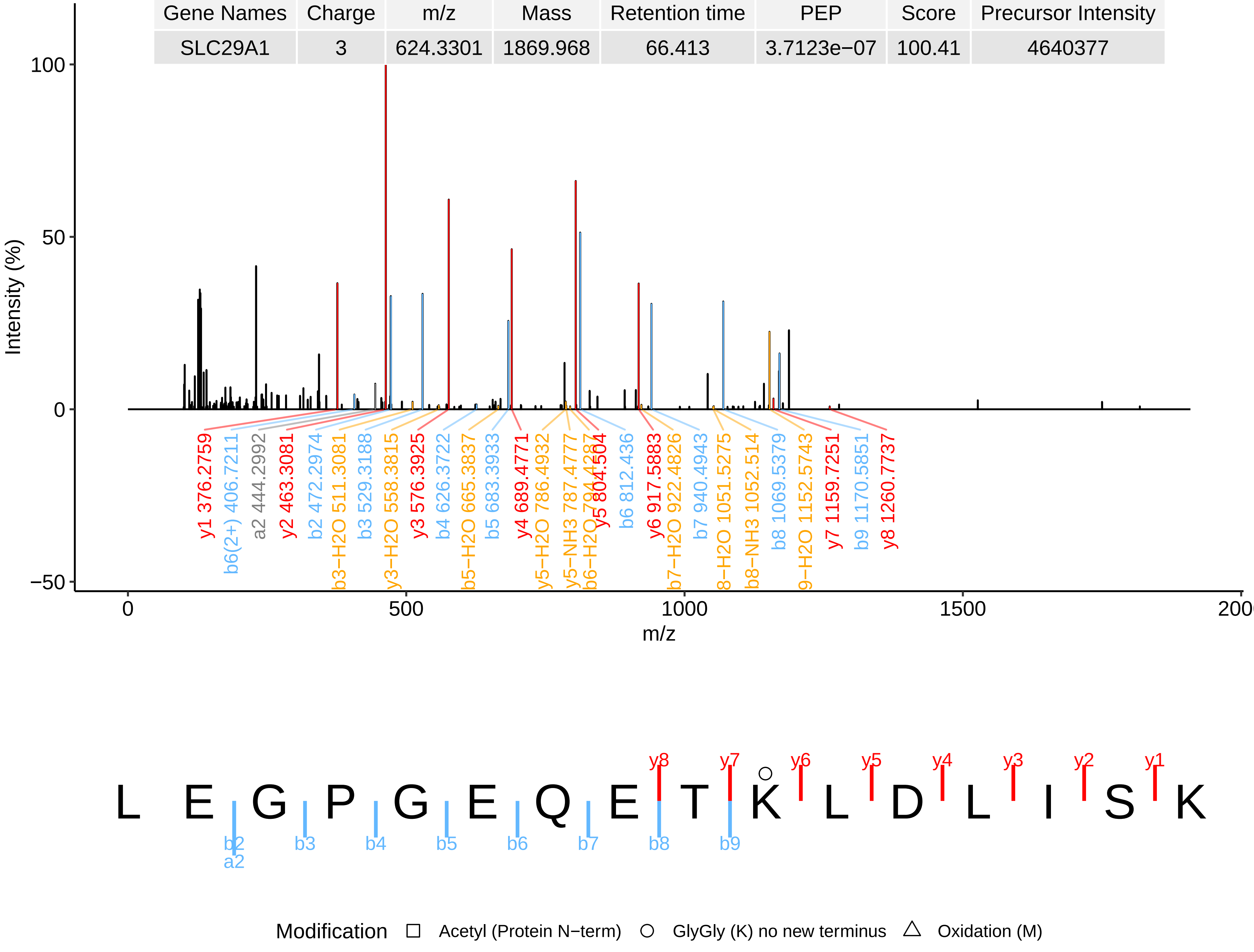

20200924_KIZ_MUb5_GlyGly_2 − Scan number: 18056

Gene Names	Charge	m/z	Mass	Retention time	PEP	Score	Precursor Intensity
SLC29A1	3	624.3301	1869.968	66.942	1.8224e−07	108.06	7166230

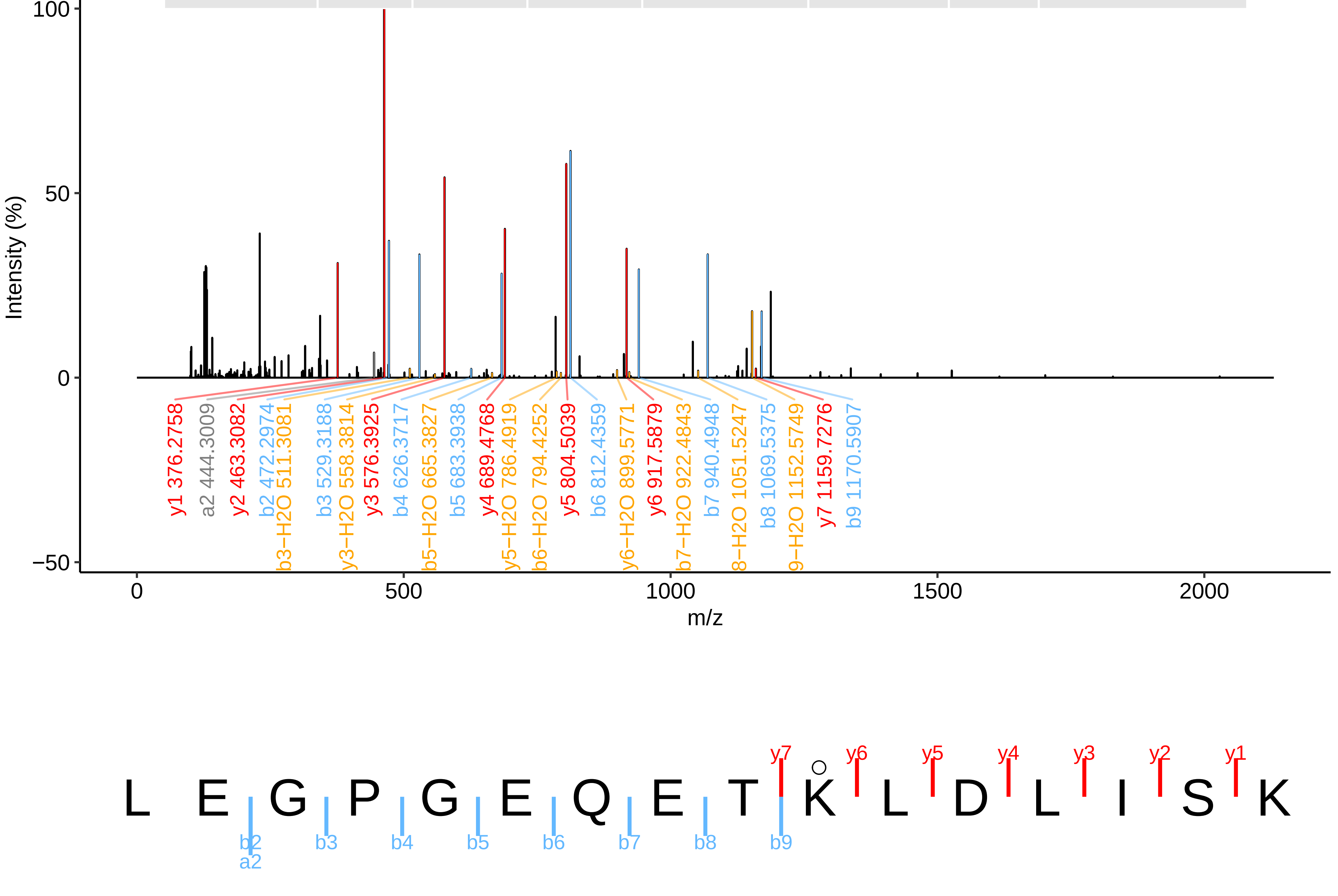

Modification □ Acetyl (Protein N−term) ○ GlyGly (K) no new terminus △ Oxidation (M)

20201118_KIZ_MUb6_GlyGly_2 − Scan number: 21666

Gene Names	Charge	m/z	Mass	Retention time	PEP	Score	Precursor Intensity
SLC29A1	3	624.3301	1869.968	72.906	1.2801e−13	134.25	18240868

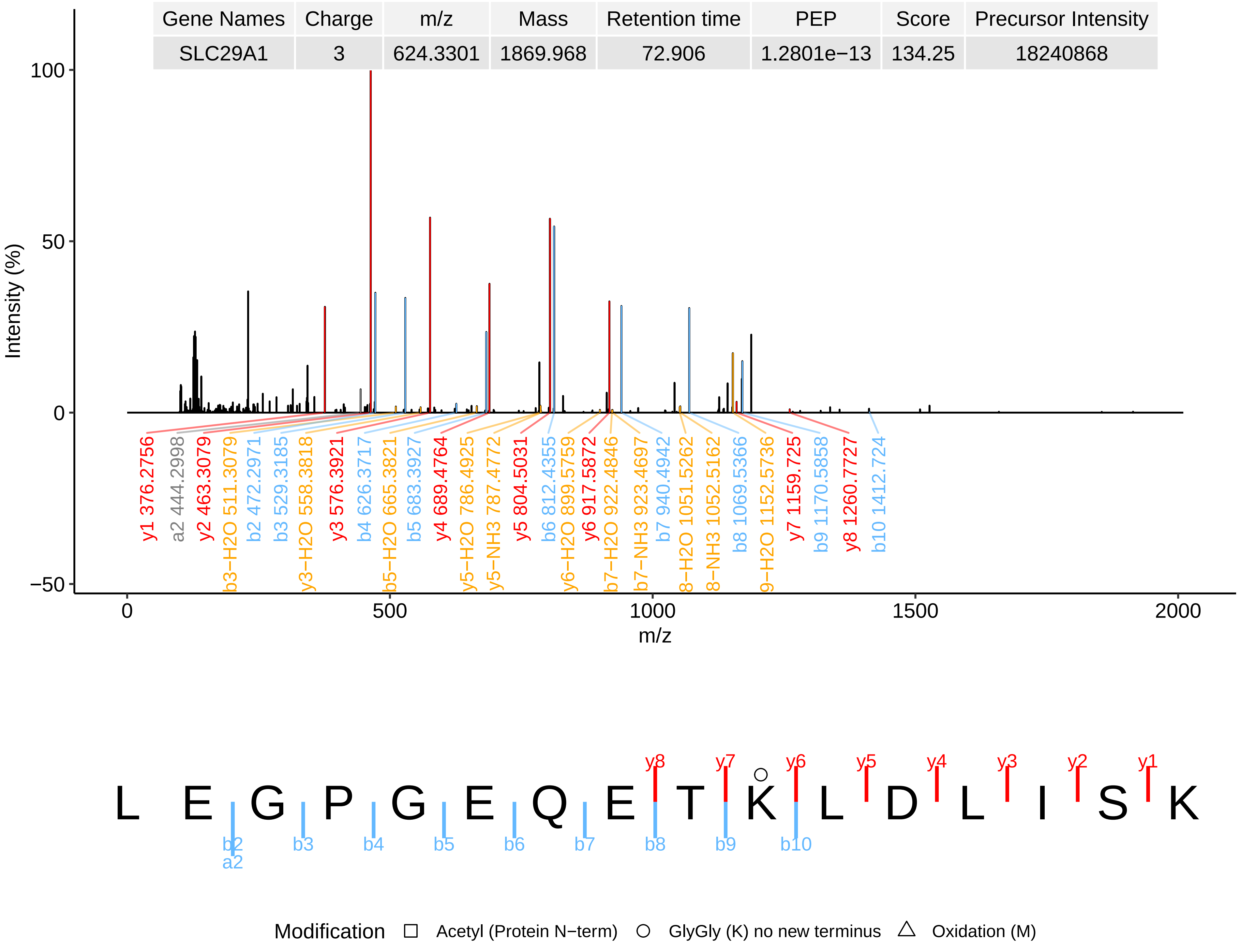

20200921_KIZ_MUb4_GlyGly_10plex − Scan number: 16736

Gene Names	Charge	m/z	Mass	Retention time	PEP	Score	Precursor Intensity
PRKRA	4	411.4581	1641.803	60.217	0.053463	48.625	9575892

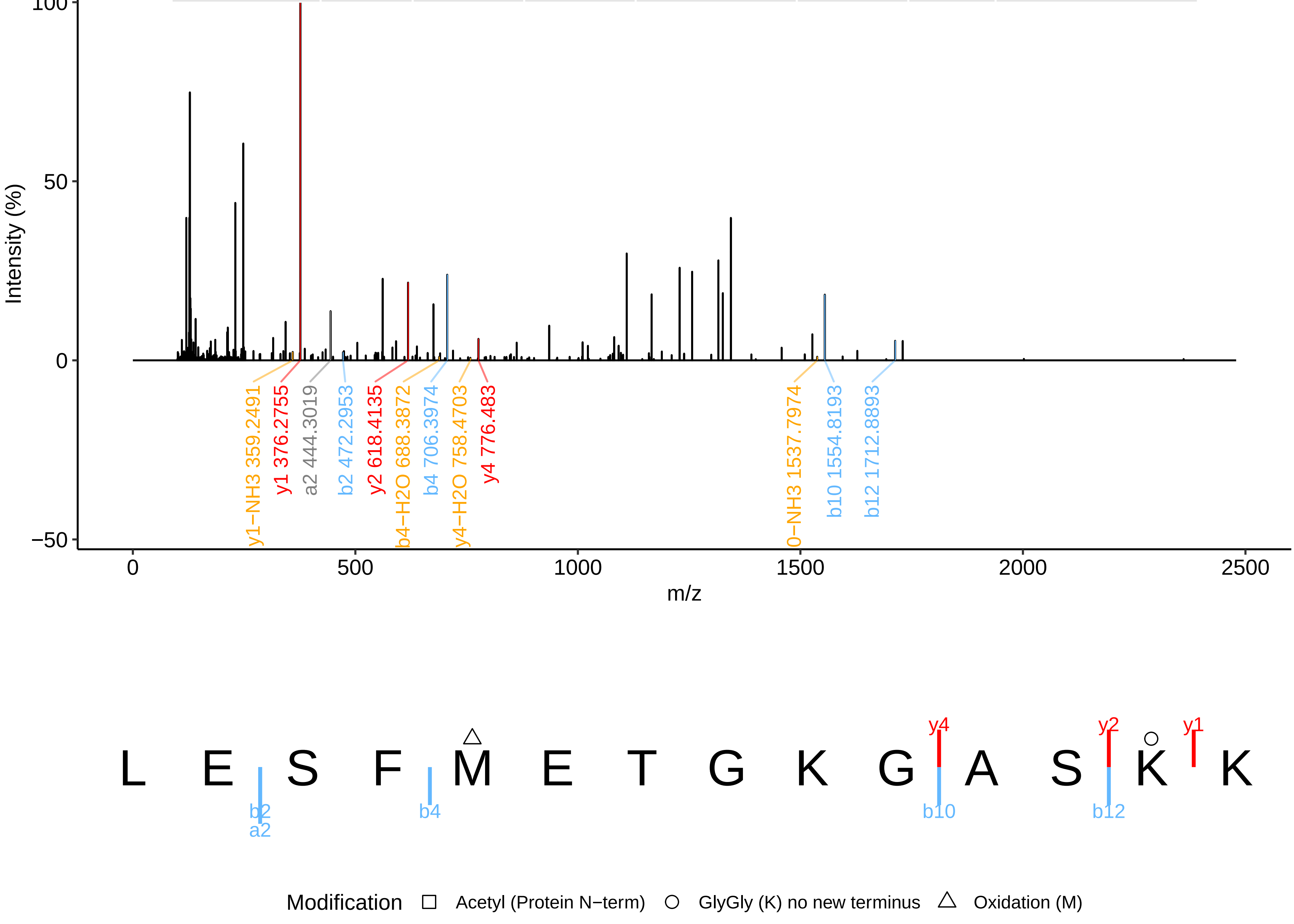

20200921_KIZ_MUb4_GlyGly_10plex_2 − Scan number: 15782

Gene Names	Charge	m/z	Mass	Retention time	PEP	Score	Precursor Intensity
PRKRA	4	411.4581	1641.803	61.787	0.0091496	62.258	1397391

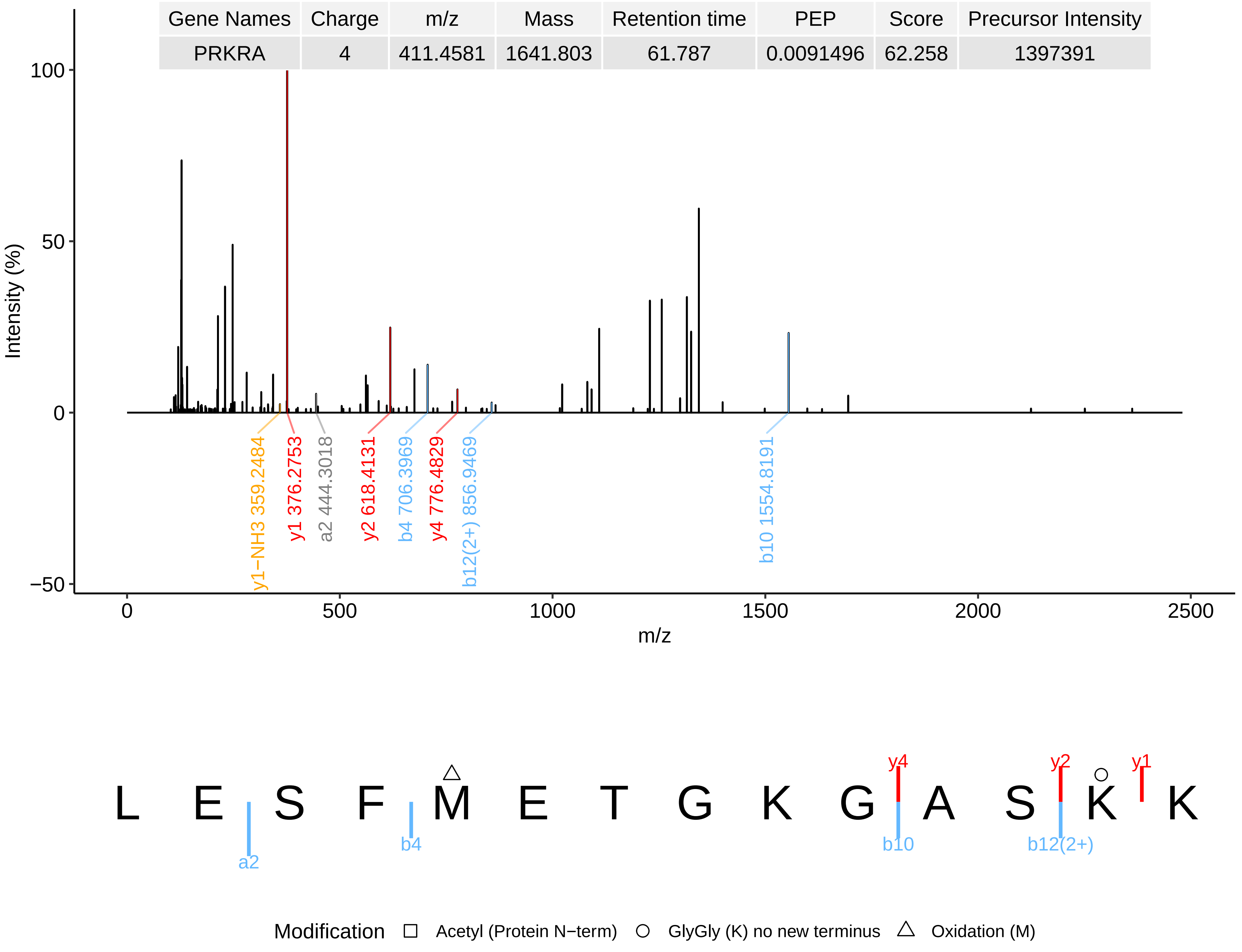

20200924_KIZ_MUb5_GlyGly_1 − Scan number: 15536

Gene Names	Charge	m/z	Mass	Retention time	PEP	Score	Precursor Intensity
PRKRA	4	411.4581	1641.803	50.733	0.051853	49.12	18360660

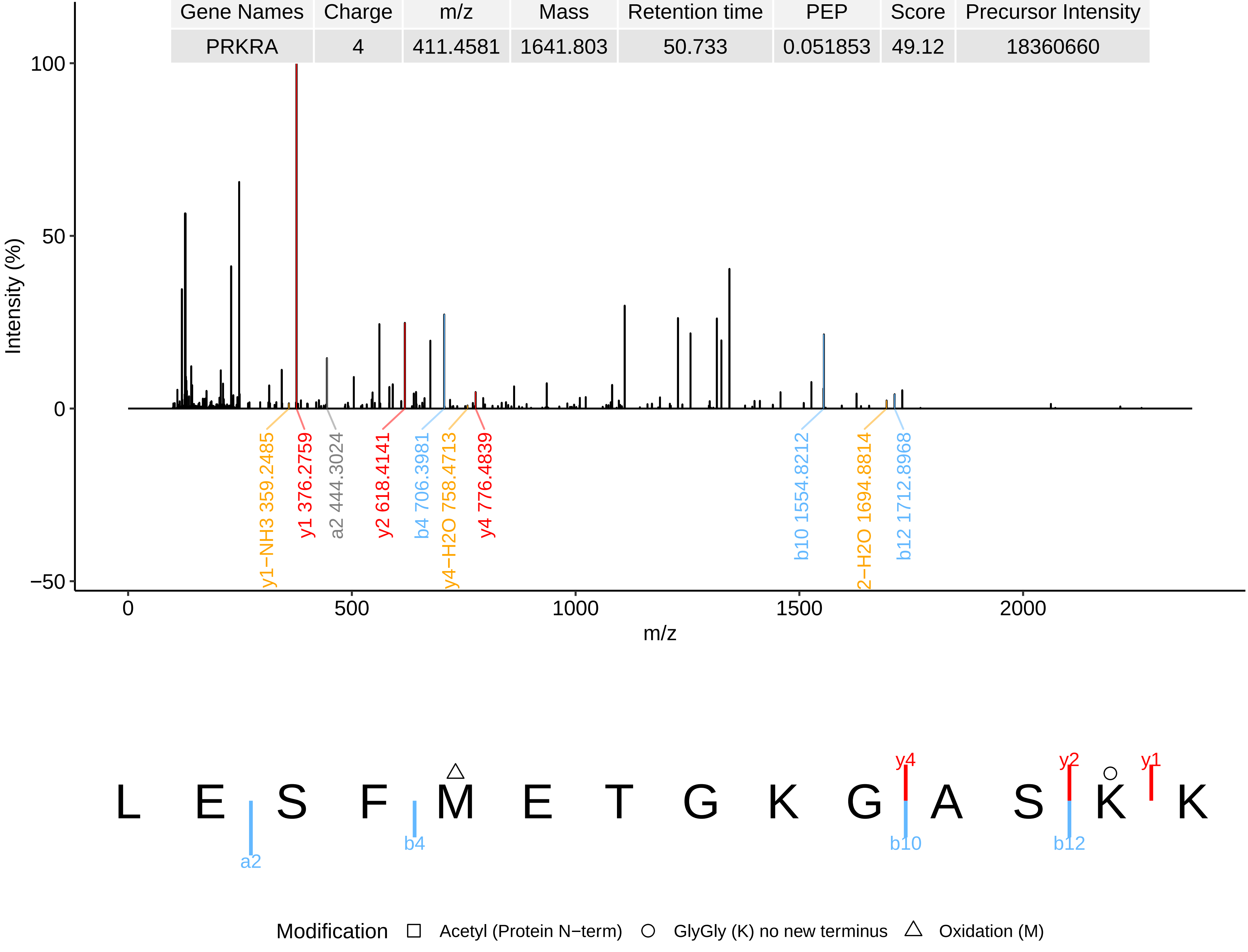

20200924_KIZ_MUb5_GlyGly_2 − Scan number: 14848

Gene Names	Charge	m/z	Mass	Retention time	PEP	Score	Precursor Intensity
PRKRA	4	411.4581	1641.803	55.478	0.082308	41.644	3543186

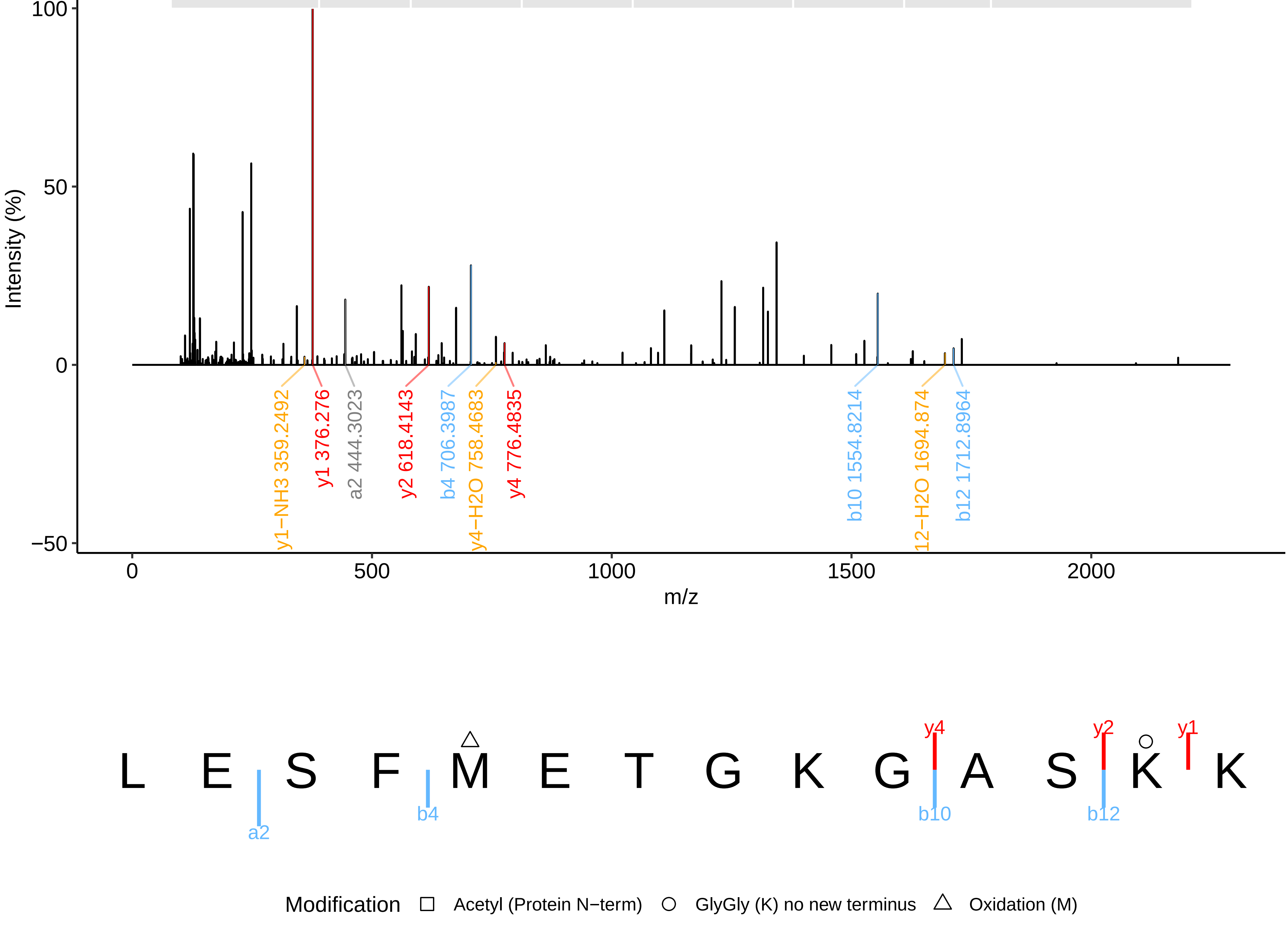

20201118_KIZ_MUb6_GlyGly_2 − Scan number: 18187

Gene Names	Charge	m/z	Mass	Retention time	PEP	Score	Precursor Intensity
PRKRA	4	411.4581	1641.803	61.513	0.082308	41.644	7024969

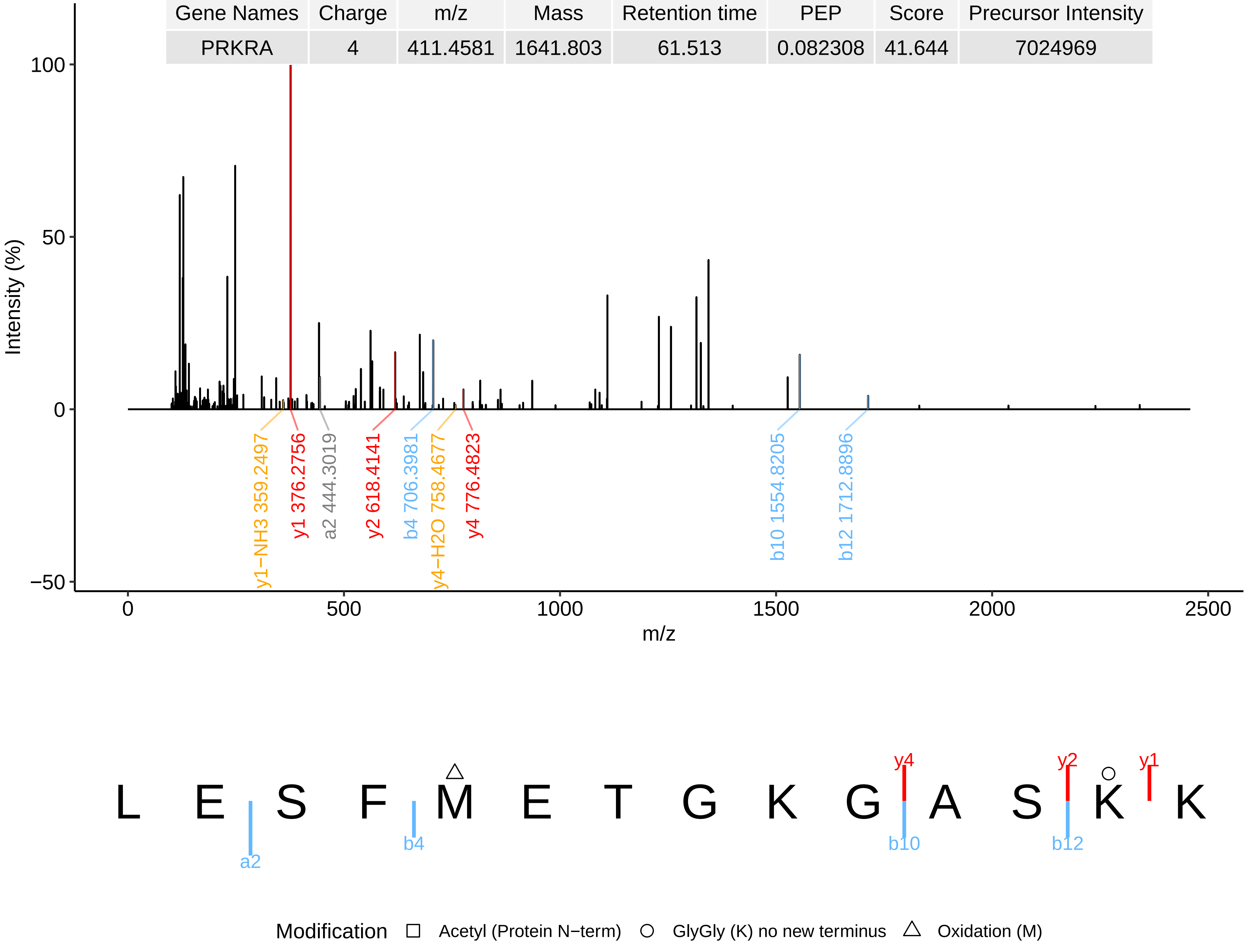

20200921_KIZ_MUb4_GlyGly_10plex_2 − Scan number: 21730

Gene Names	Charge	m/z	Mass	Retention time	PEP	Score	Precursor Intensity
CYC1	3	676.9986	2027.974	85.581	0.072575	48.899	547174.9

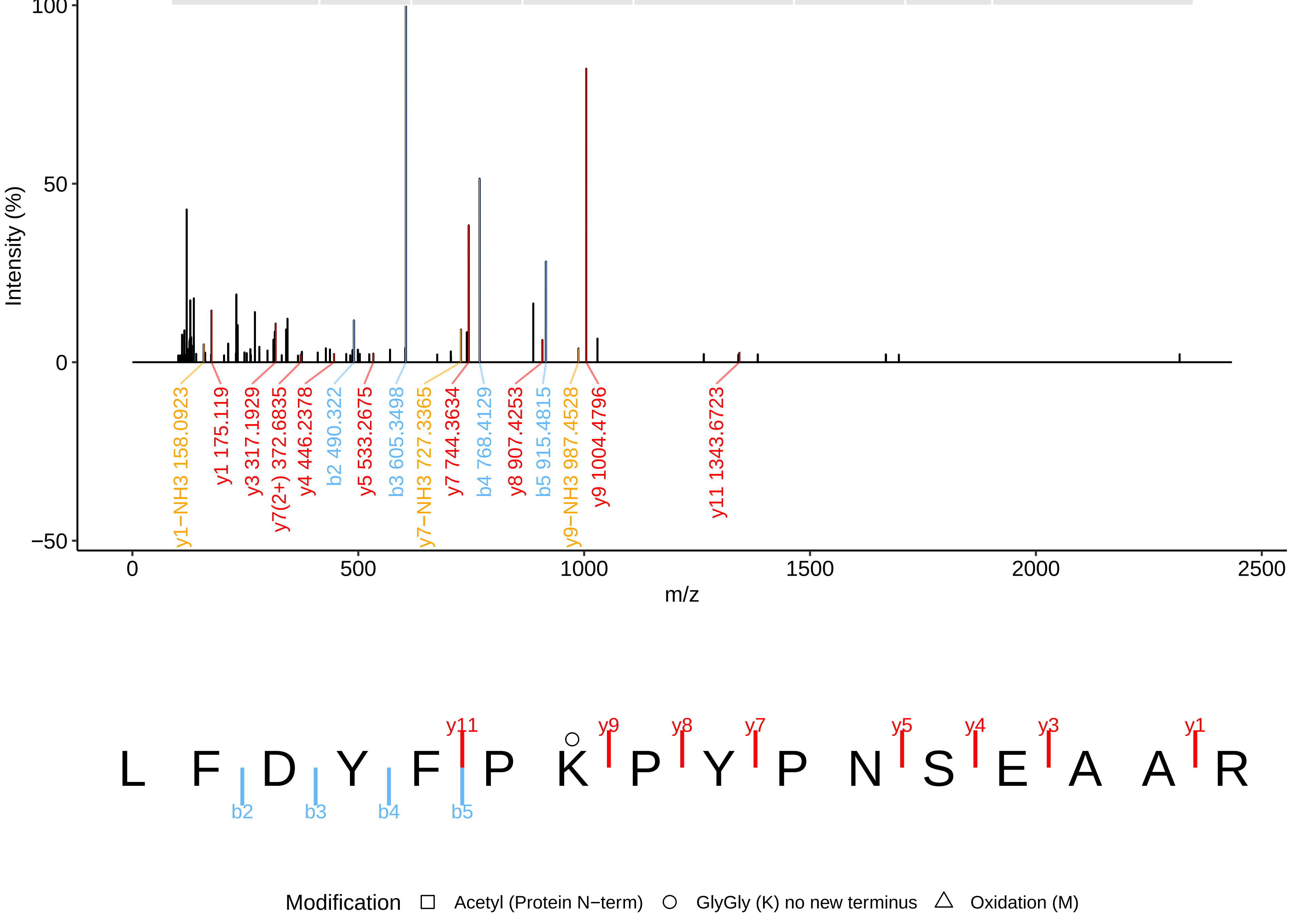

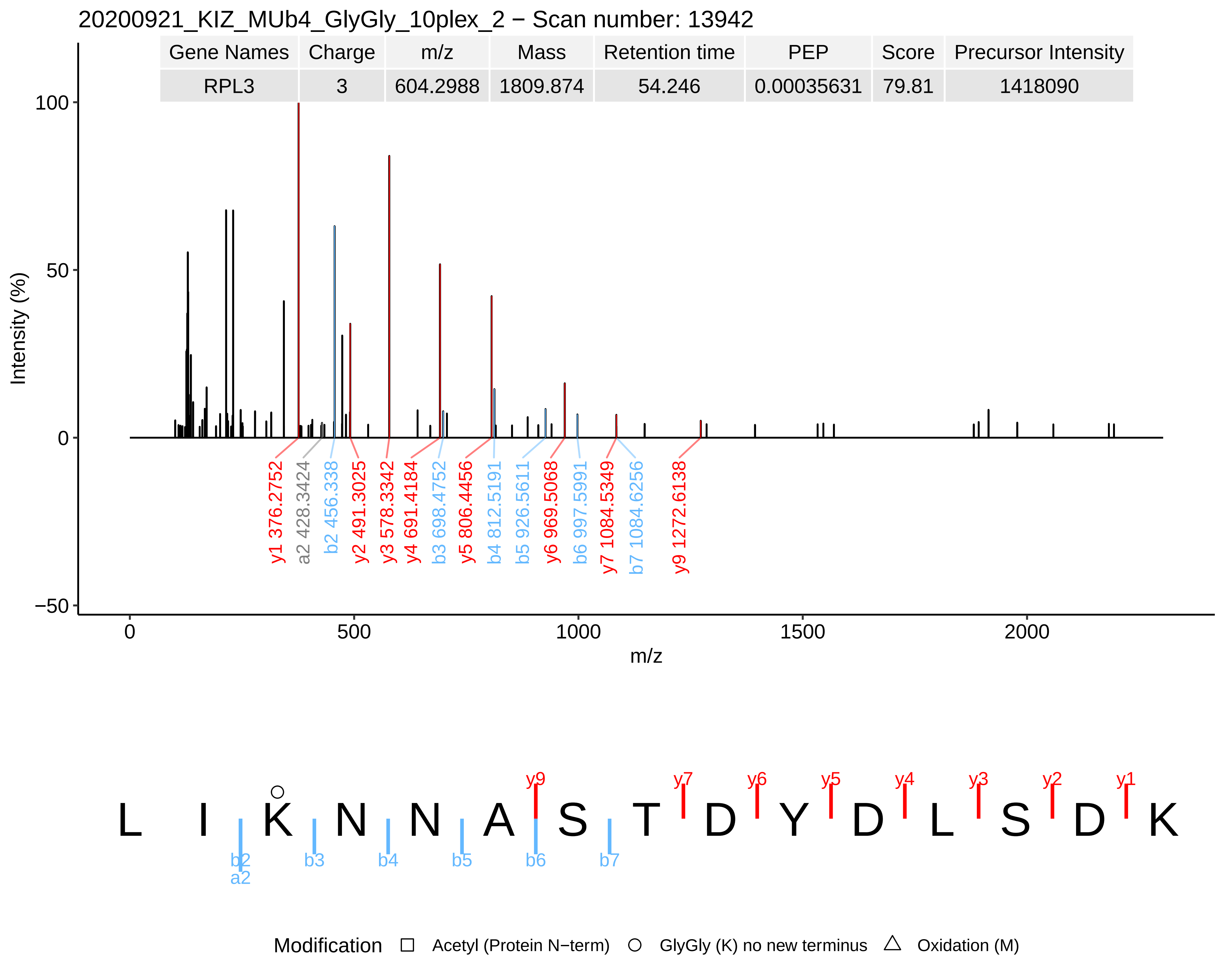
20200921_KIZ_MUb4_GlyGly_10plex_2 − Scan number: 13942
Gene Names | Charge | m/z | Mass | Retention time | PEP | Score | Precursor Intensity
RPL3 | 3 | 604.2988 | 1809.874 | 54.246 | 0.00035631 | 79.81 | 1418090
Intensity (%)
100
50
0
−50
y1 376.2752
a2 428.3424
b2 456.338
y2 491.3025
y3 578.3342
y4 691.4184
b3 698.4752
y5 806.4456
b4 812.5191
b5 926.5611
y6 969.5068
b6 997.5991
y7 1084.5349
b7 1084.6256
y9 1272.6138
0
500
1000
1500
2000
m/z
L I K N N A S T D Y D L S D K
y9 y7 y6 y5 y4 y3 y2 y1
b2 a2 b3 b4 b5 b6 b7
Modification
Acetyl (Protein N−term)
GlyGly (K) no new terminus
Oxidation (M)

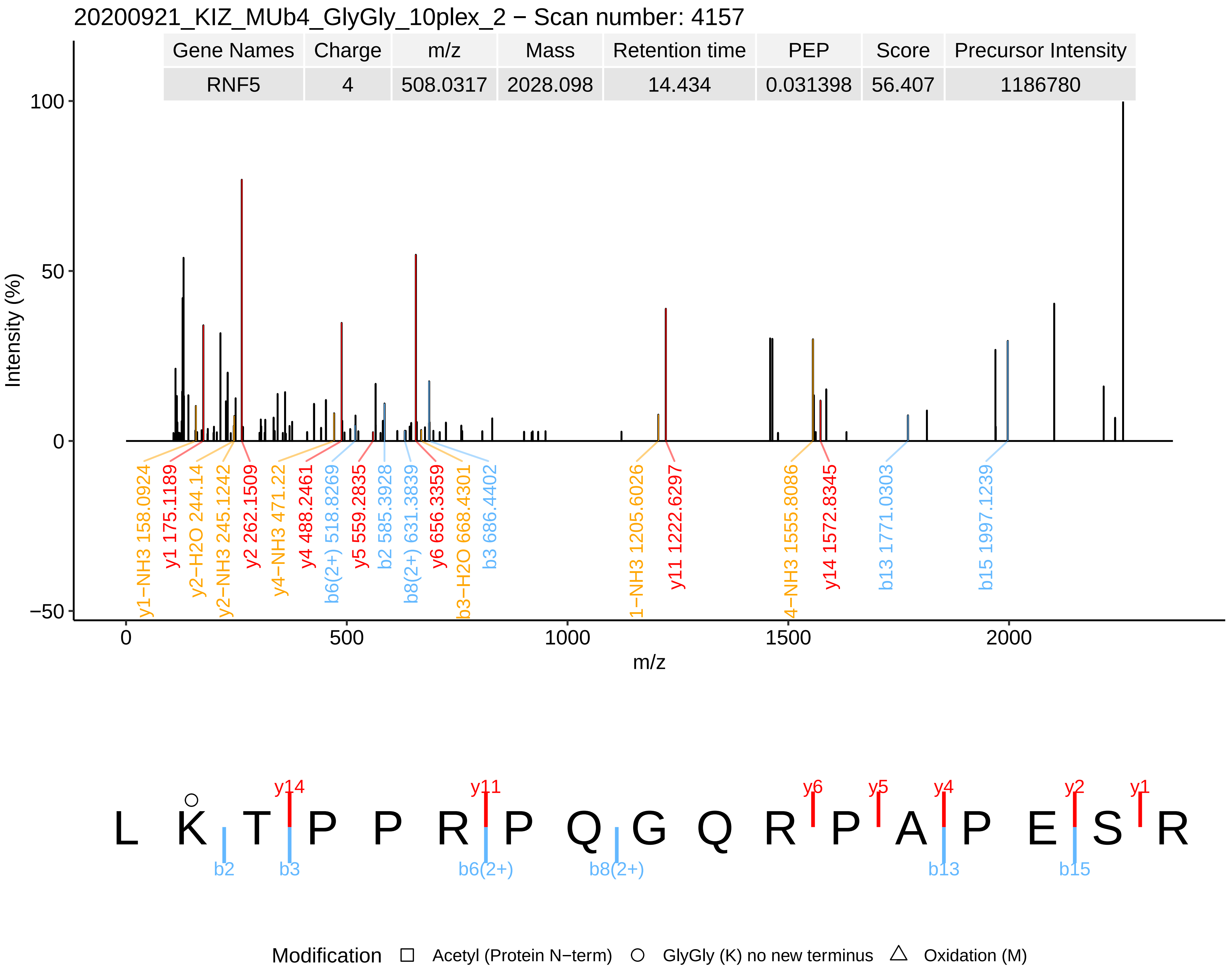
20200921_KIZ_MUb4_GlyGly_10plex_2 − Scan number: 4157
Gene Names | Charge | m/z | Mass | Retention time | PEP | Score | Precursor Intensity
RNF5 | 4 | 508.0317 | 2028.098 | 14.434 | 0.031398 | 56.407 | 1186780
Intensity (%)
m/z
y1−NH3 158.0924
y1 175.1189
y2−H2O 244.14
y2−NH3 245.1242
y2 262.1509
y4−NH3 471.22
y4 488.2461
b6(2+) 518.8269
y5 559.2835
b2 585.3928
b8(2+) 631.3839
y6 656.3359
b3−H2O 668.4301
b3 686.4402
1−NH3 1205.6026
y11 1222.6297
4−NH3 1555.8086
y14 1572.8345
b13 1771.0303
b15 1997.1239
L K T P P R P Q G Q R P A P E S R
y14 y11 y6 y5 y4 y2 y1
b2 b3 b6(2+) b8(2+) b13 b15
Modification
Acetyl (Protein N−term)
GlyGly (K) no new terminus
Oxidation (M)

20201118_KIZ_MUb6_GlyGly_1 − Scan number: 3502

Gene Names	Charge	m/z	Mass	Retention time	PEP	Score	Precursor Intensity
RNF5	5	406.6268	2028.098	10.745	0.0012391	71.601	14411641

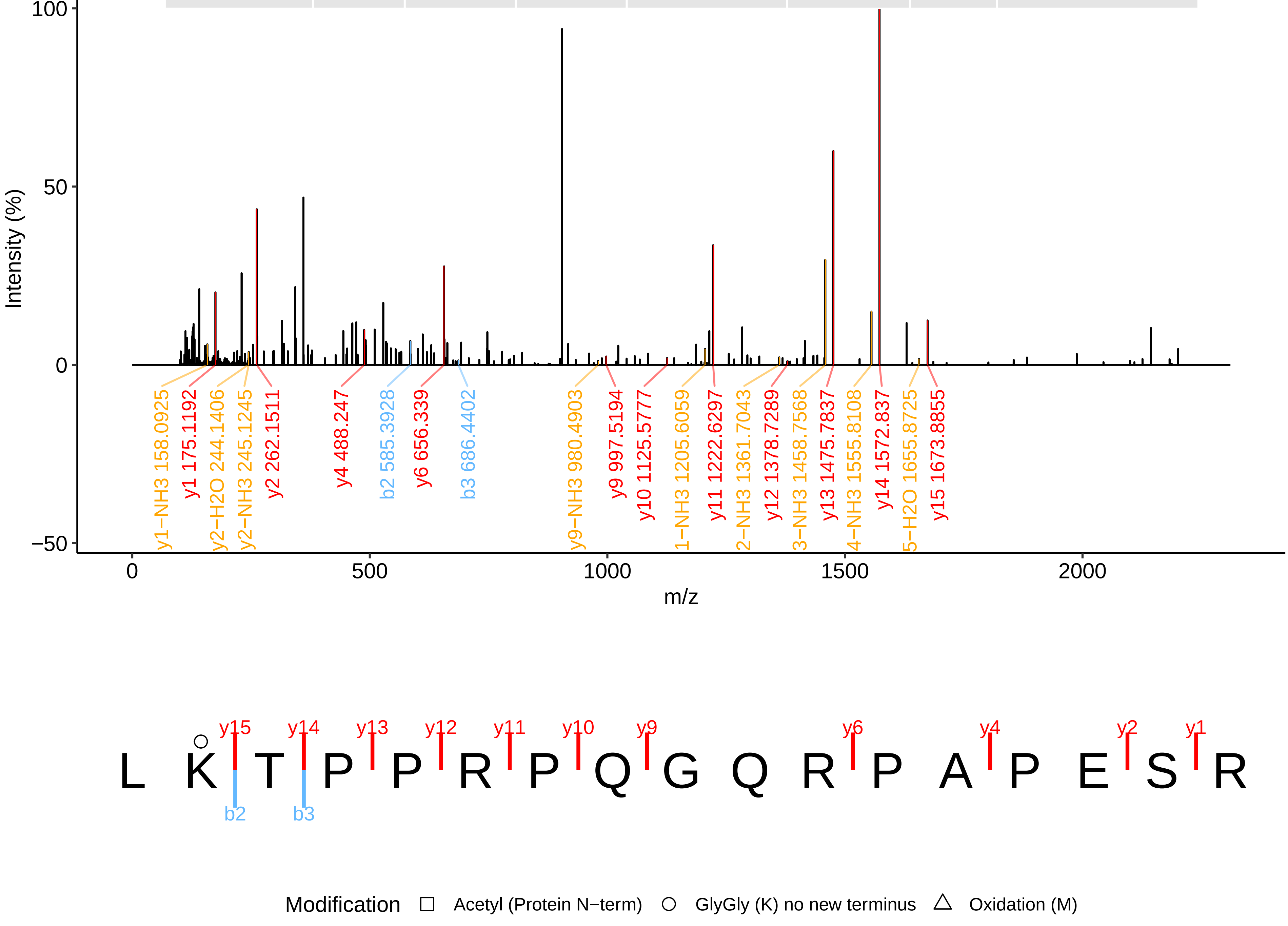

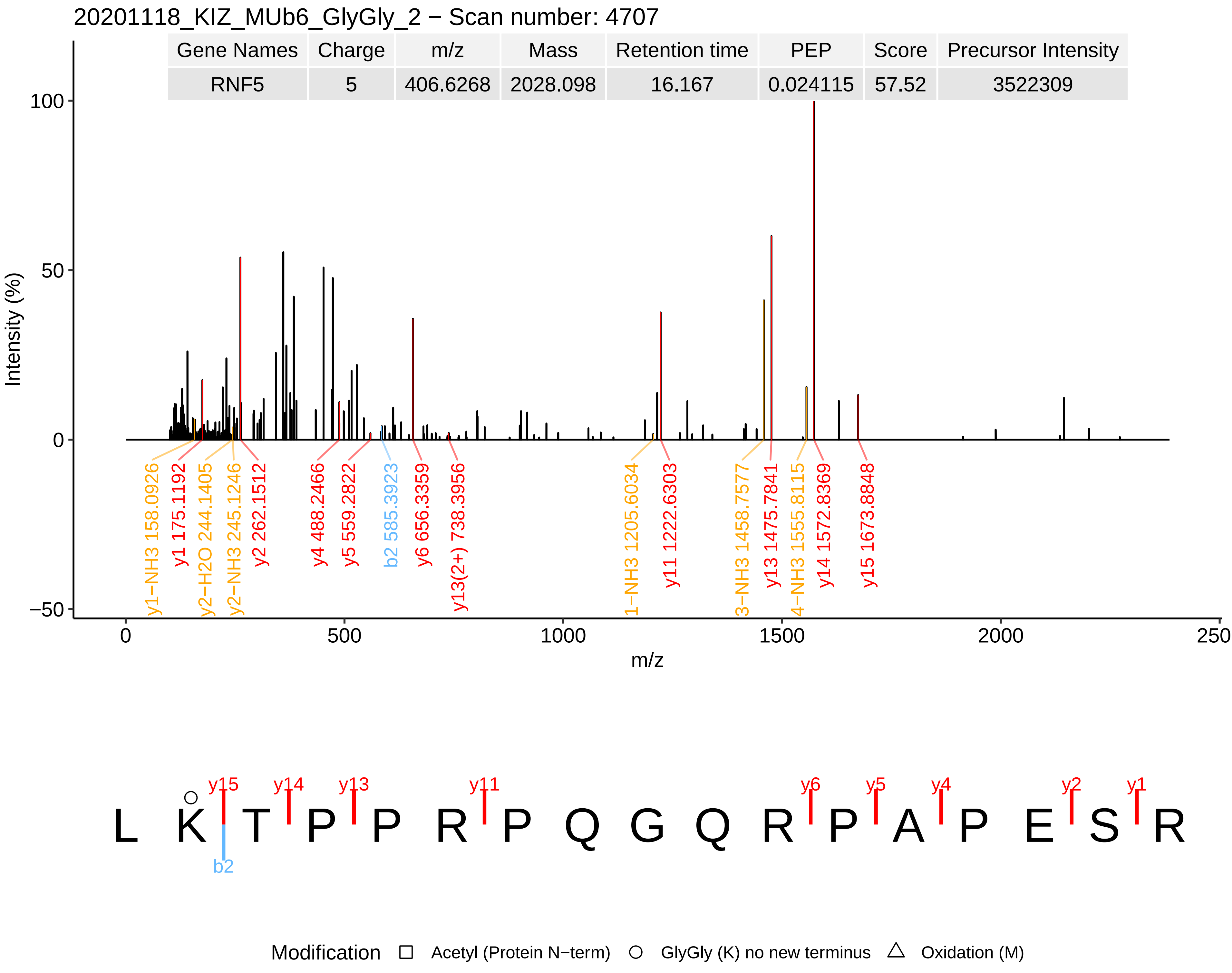
20201118_KIZ_MUb6_GlyGly_2 − Scan number: 4707
Gene Names | Charge | m/z | Mass | Retention time | PEP | Score | Precursor Intensity
RNF5 | 5 | 406.6268 | 2028.098 | 16.167 | 0.024115 | 57.52 | 3522309
Intensity (%)
100
50
0
−50
y1−NH3 158.0926
y1 175.1192
y2−H2O 244.1405
y2−NH3 245.1246
y2 262.1512
y4 488.2466
y5 559.2822
b2 585.3923
y6 656.3359
y13(2+) 738.3956
1−NH3 1205.6034
y11 1222.6303
3−NH3 1458.7577
y13 1475.7841
4−NH3 1555.8115
y14 1572.8369
y15 1673.8848
0
500
1000
1500
2000
250
m/z
L K T P P R P Q G Q R P A P E S R
y15 y14 y13 y11 y6 y5 y4 y2 y1
b2
Modification
Acetyl (Protein N−term)
GlyGly (K) no new terminus
Oxidation (M)

20200921_KIZ_MUb4_GlyGly_10plex_2 − Scan number: 25184

Gene Names	Charge	m/z	Mass	Retention time	PEP	Score	Precursor Intensity
YIPF4	3	699.704	2096.09	99.589	0.00067758	75.97	620649.6

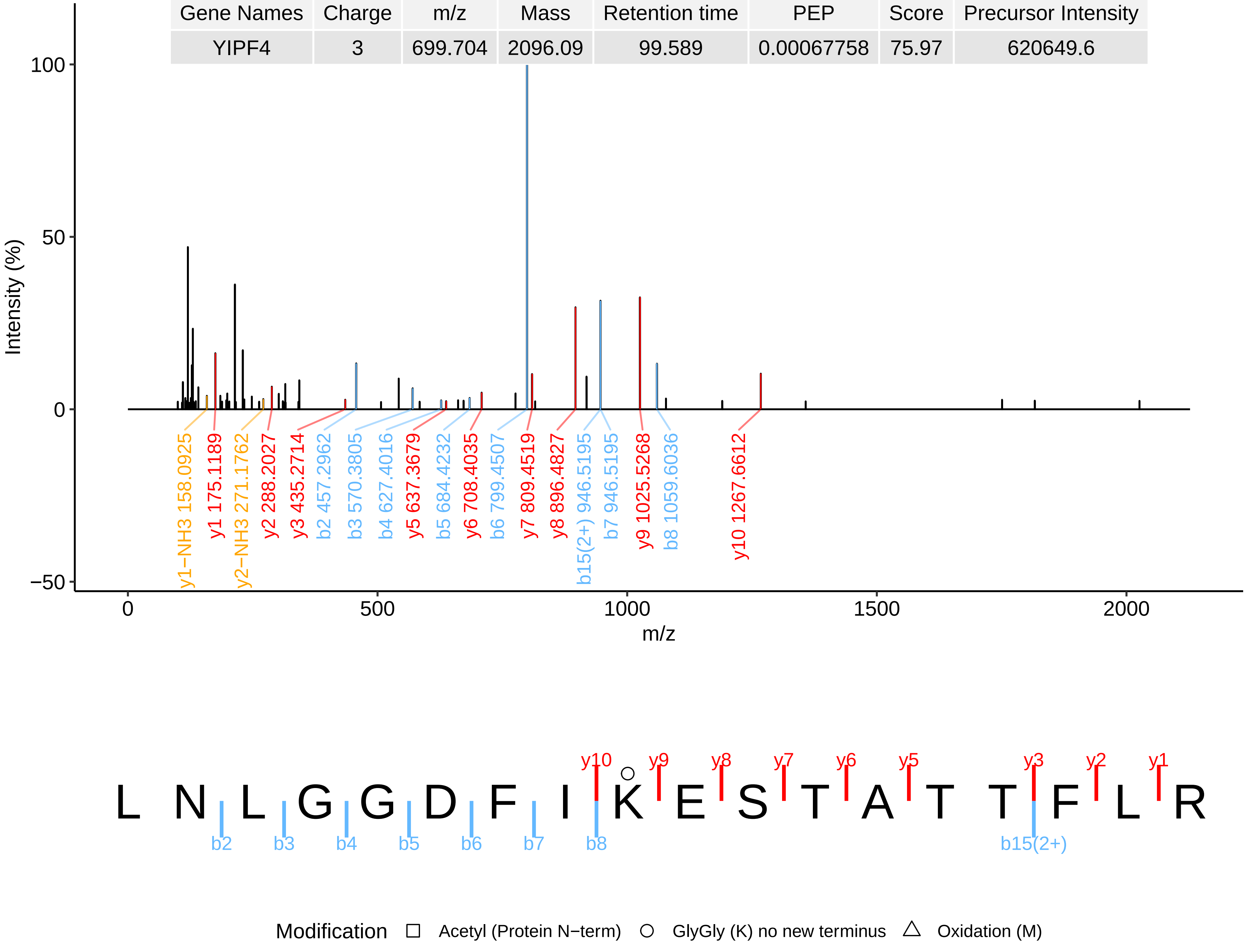

20200921_KIZ_MUb4_GlyGly_10plex − Scan number: 27699

Gene Names	Charge	m/z	Mass	Retention time	PEP	Score	Precursor Intensity
EEF1A1;EEF1A1P5;EEF1A2	3	699.4003	2095.179	101.16	7.4721e−06	88.385	3822587

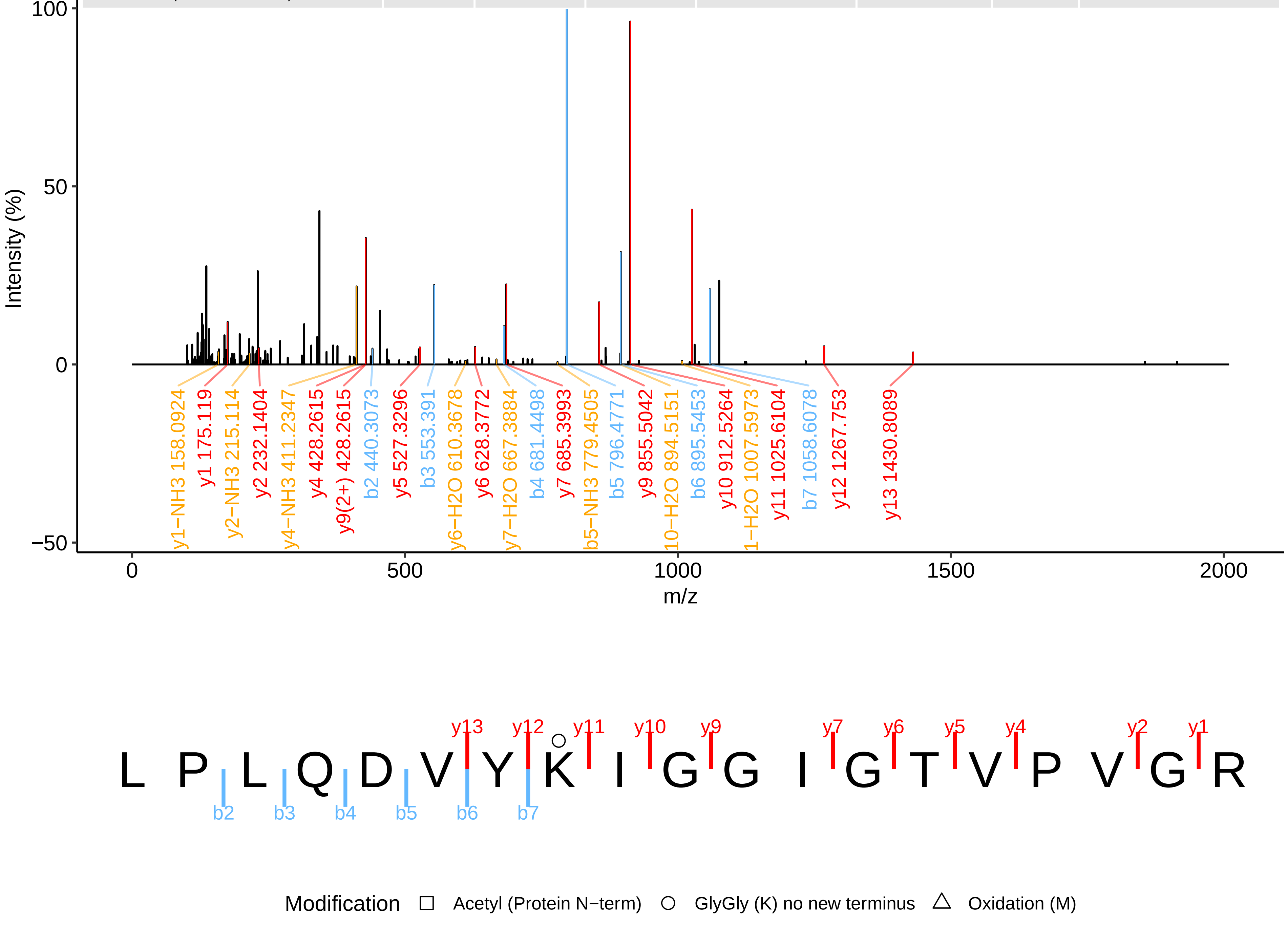

20200924_KIZ_MUb5_GlyGly_2 − Scan number: 24808

Gene Names	Charge	m/z	Mass	Retention time	PEP	Score	Precursor Intensity
EEF1A1;EEF1A1P5;EEF1A2	3	699.4003	2095.179	91.873	0.05654	50.883	NA

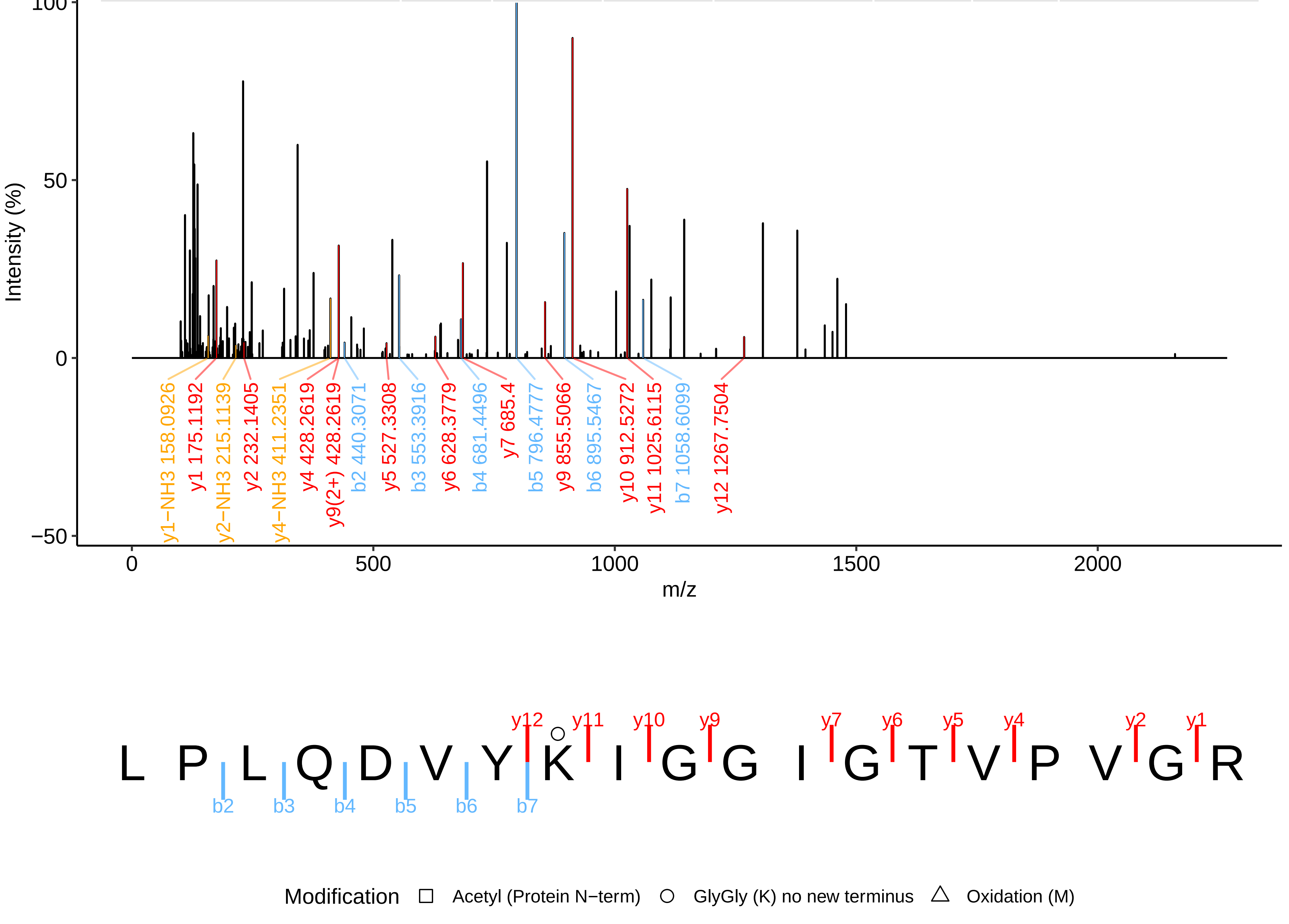

20200921_KIZ_MUb4_GlyGly_10plex − Scan number: 11758

Gene Names	Charge	m/z	Mass	Retention time	PEP	Score	Precursor Intensity
VANGL1	3	680.6653	2038.974	41.061	0.057125	49.993	NA

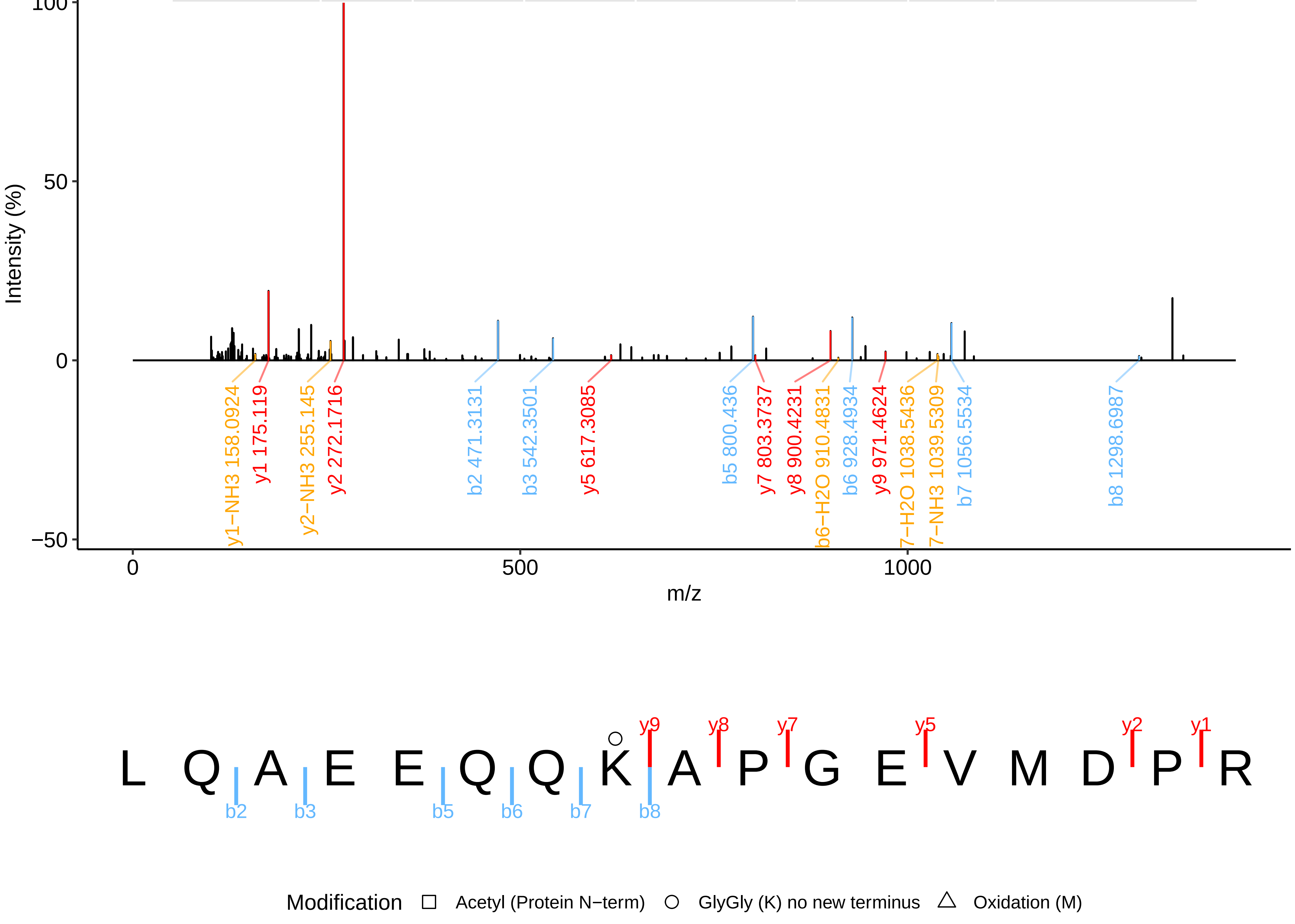

20200921_KIZ_MUb4_GlyGly_10plex_2 − Scan number: 10754

Gene Names	Charge	m/z	Mass	Retention time	PEP	Score	Precursor Intensity
VANGL1	3	680.6653	2038.974	41.211	0.0048692	62.646	NA

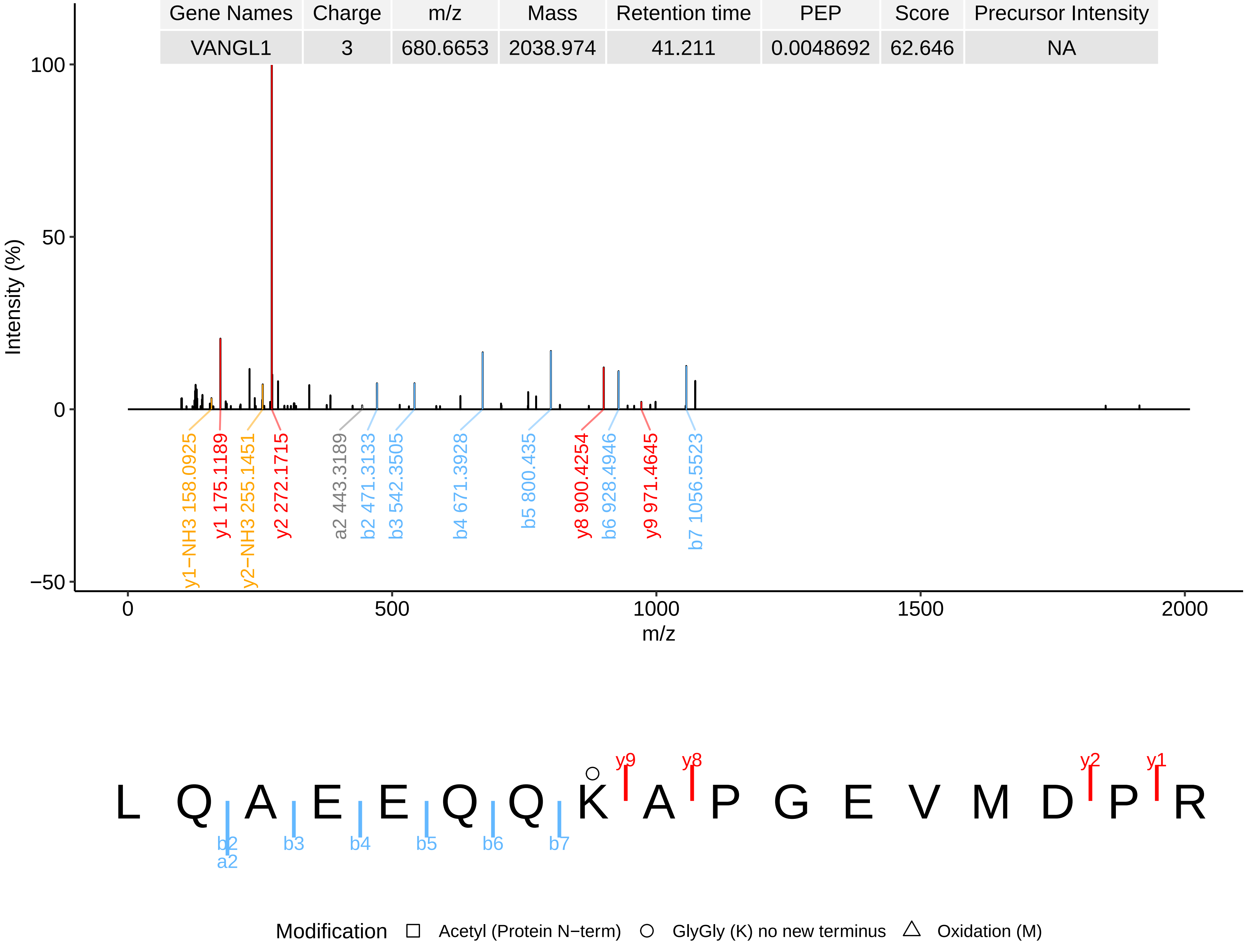

20200921_KIZ_MUb4_GlyGly_10plex − Scan number: 13294

Gene Names	Charge	m/z	Mass	Retention time	PEP	Score	Precursor Intensity
PSMD4	4	521.2682	2081.044	46.854	4.6821e−10	105.53	10288371

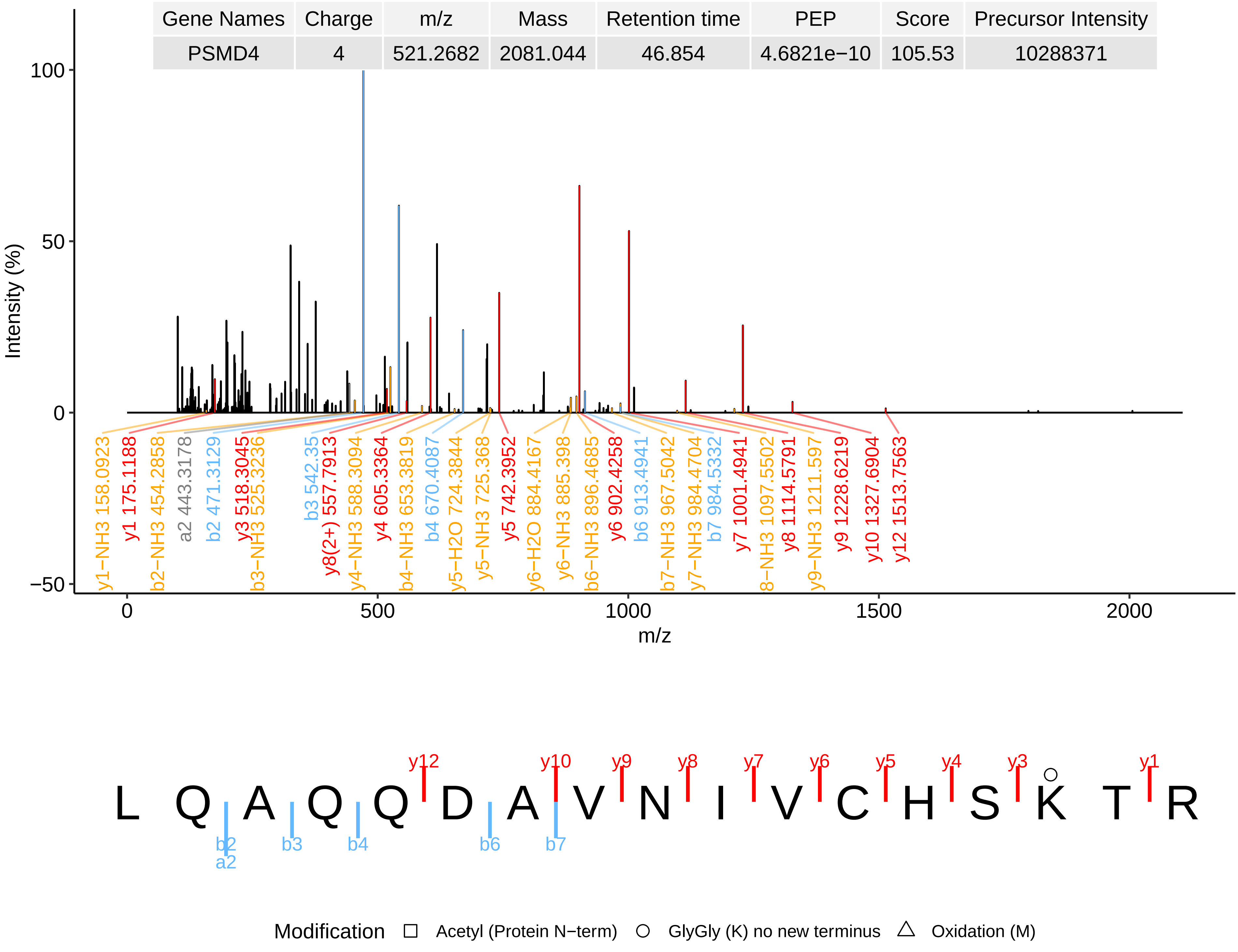

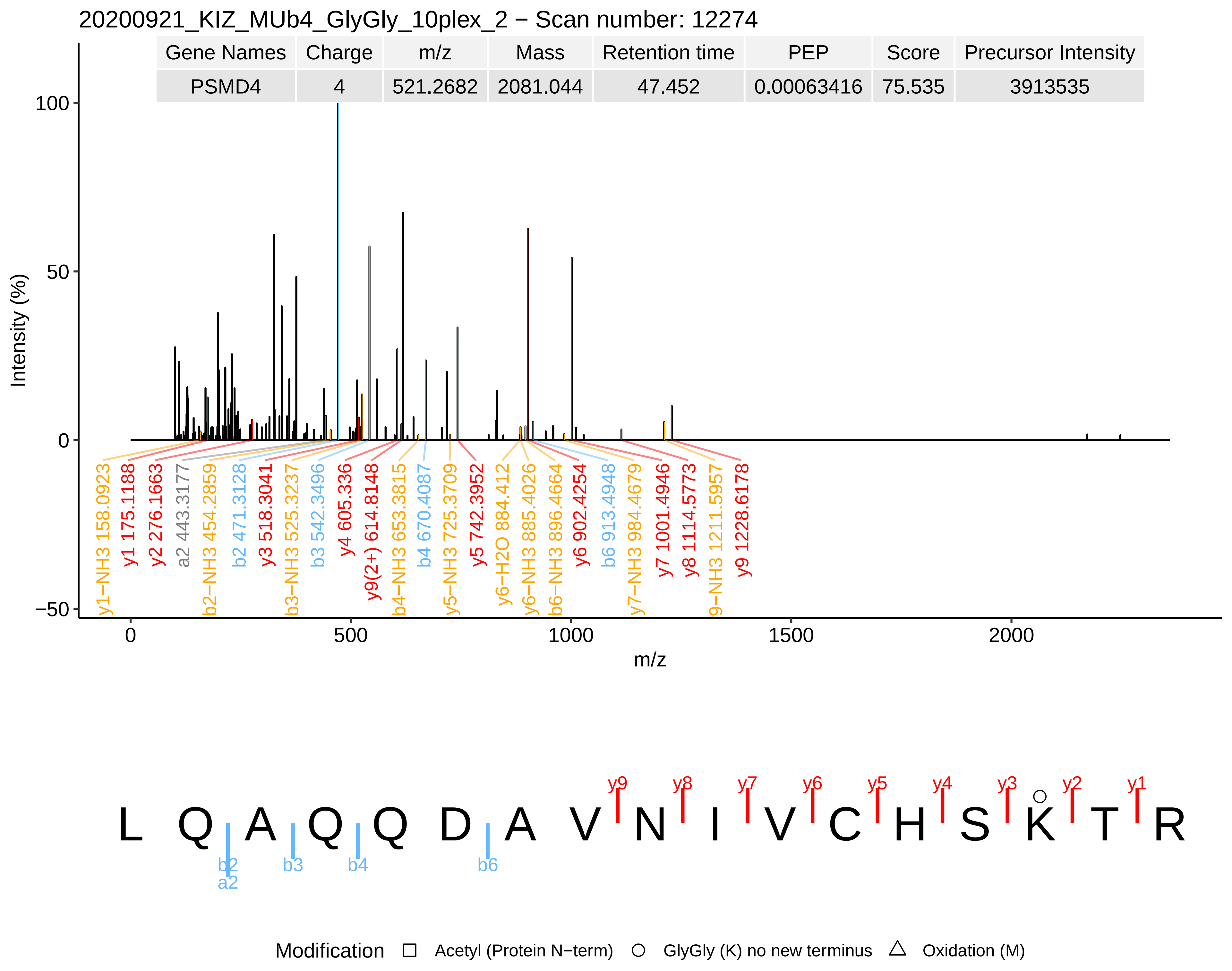

20200921_KIZ_MUb4_GlyGly_10plex_2 − Scan number: 12274
Gene Names | Charge | m/z | Mass | Retention time | PEP | Score | Precursor Intensity
PSMD4 | 4 | 521.2682 | 2081.044 | 47.452 | 0.00063416 | 75.535 | 3913535
Intensity (%)
m/z
y1−NH3 158.0923
y1 175.1188
y2 276.1663
a2 443.3177
b2−NH3 454.2859
b2 471.3128
y3 518.3041
b3−NH3 525.3237
b3 542.3496
y4 605.336
y9(2+) 614.8148
b4−NH3 653.3815
b4 670.4087
y5−NH3 725.3709
y5 742.3952
y6−H2O 884.412
y6−NH3 885.4026
b6−NH3 896.4664
y6 902.4254
b6 913.4948
y7−NH3 984.4679
y7 1001.4946
y8 1114.5773
y9−NH3 1211.5957
y9 1228.6178
L Q A Q Q D A V N I V C H S K T R
y9 y8 y7 y6 y5 y4 y3 y2 y1
b2 a2 b3 b4 b6
Modification
Acetyl (Protein N−term)
GlyGly (K) no new terminus
Oxidation (M)

20200921_KIZ_MUb4_GlyGly_10plex − Scan number: 16245

Gene Names	Charge	m/z	Mass	Retention time	PEP	Score	Precursor Intensity
LSS	3	699.6727	2095.996	58.292	2.2272e−07	106.42	1246994

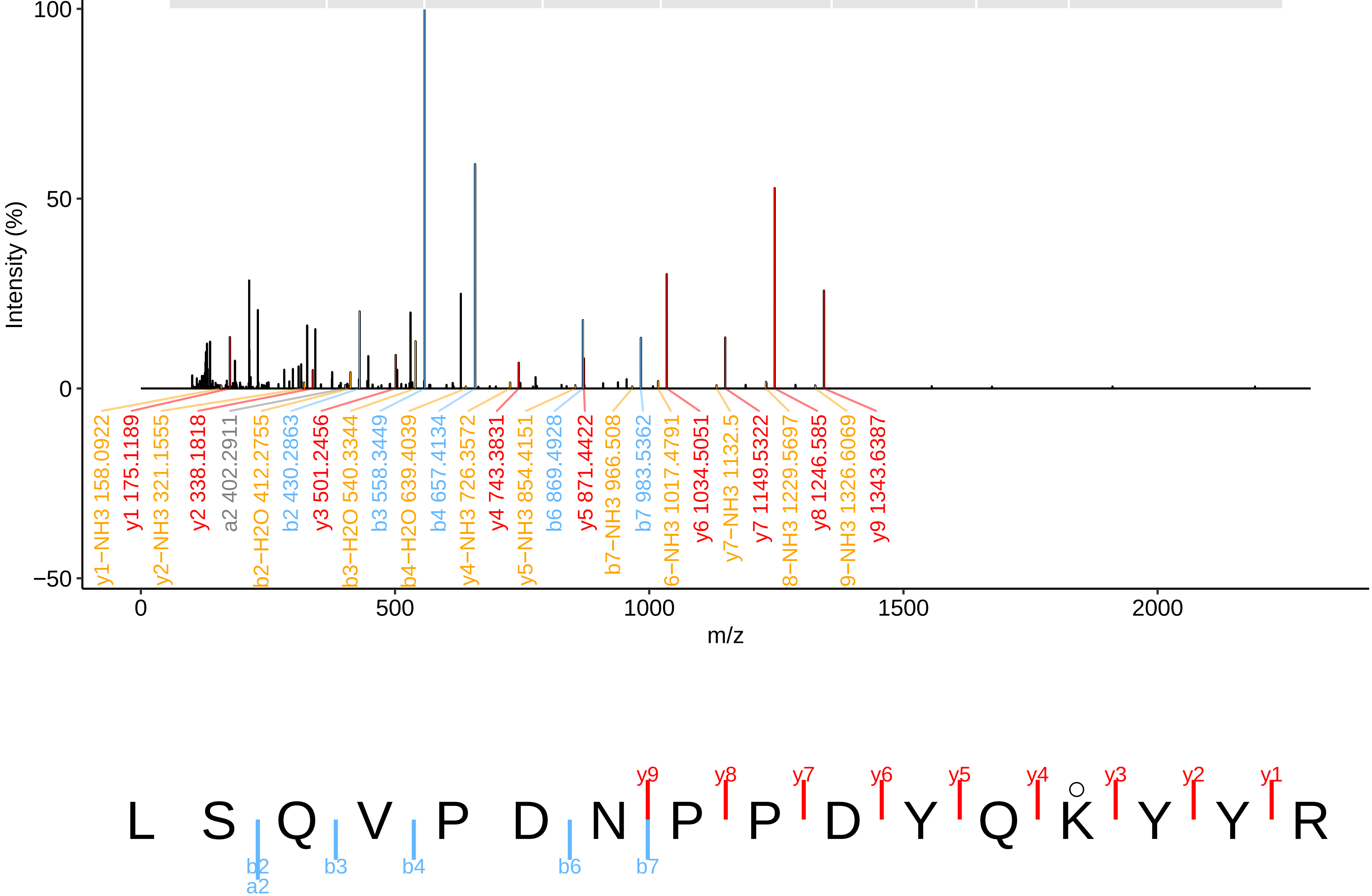

Modification □ Acetyl (Protein N−term) ○ GlyGly (K) no new terminus △ Oxidation (M)

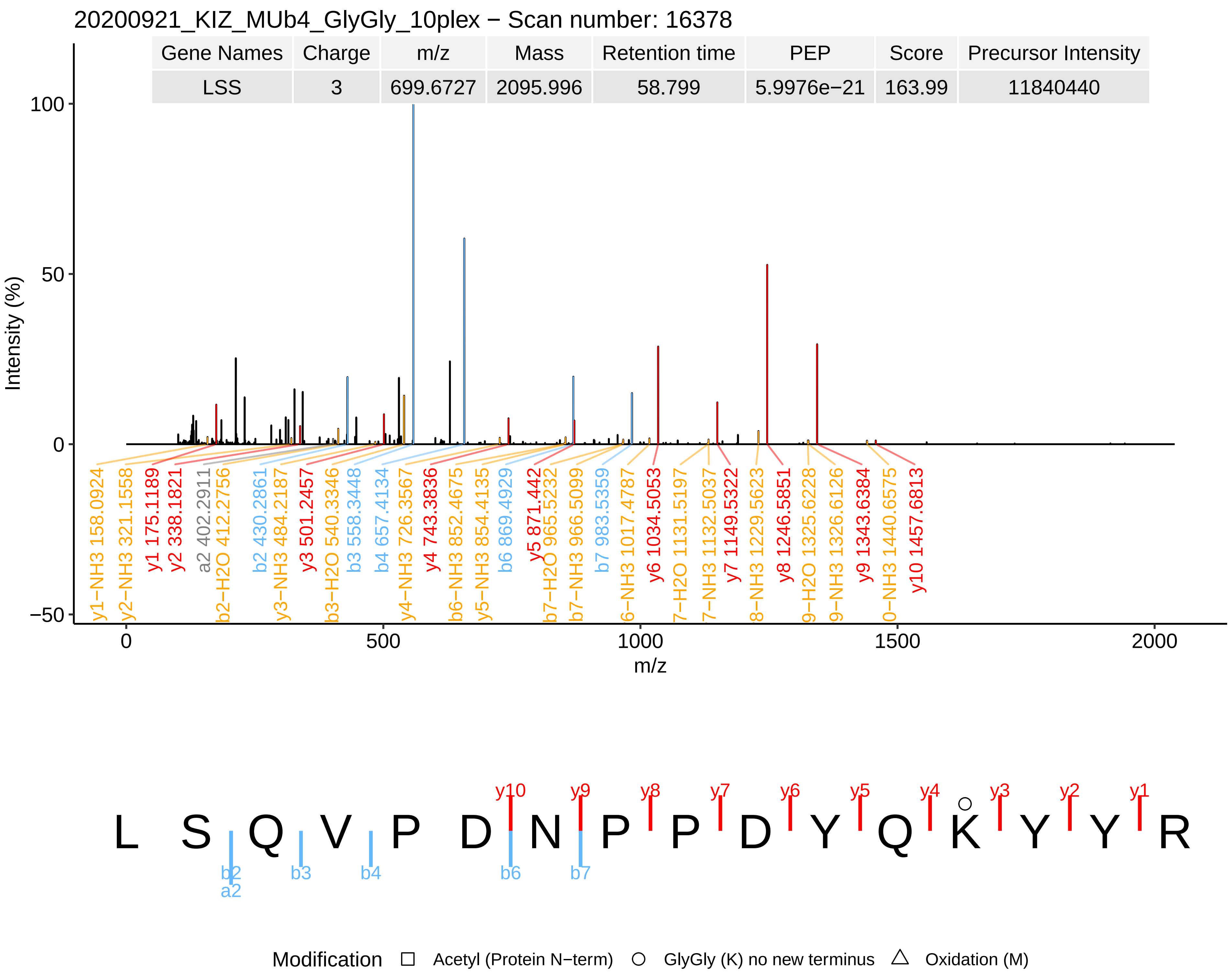
20200921_KIZ_MUb4_GlyGly_10plex − Scan number: 16378
Gene Names | Charge | m/z | Mass | Retention time | PEP | Score | Precursor Intensity
LSS | 3 | 699.6727 | 2095.996 | 58.799 | 5.9976e−21 | 163.99 | 11840440
Intensity (%)
100
50
0
−50
0
500
1000
1500
2000
m/z
y1−NH3 158.0924
y2−NH3 321.1558
y1 175.1189
y2 338.1821
a2 402.2911
b2−H2O 412.2756
b2 430.2861
y3−NH3 484.2187
y3 501.2457
b3−H2O 540.3346
b3 558.3448
b4 657.4134
y4−NH3 726.3567
y4 743.3836
b6−NH3 852.4675
y5−NH3 854.4135
b6 869.4929
y5 871.442
b7−H2O 965.5232
b7−NH3 966.5099
b7 983.5359
6−NH3 1017.4787
y6 1034.5053
7−H2O 1131.5197
7−NH3 1132.5037
y7 1149.5322
8−NH3 1229.5623
y8 1246.5851
9−H2O 1325.6228
9−NH3 1326.6126
y9 1343.6384
0−NH3 1440.6575
y10 1457.6813
L S Q V P D N P P D Y Q K Y Y R
y10 y9 y8 y7 y6 y5 y4 y3 y2 y1
b2 a2 b3 b4 b6 b7
Modification
Acetyl (Protein N−term)
GlyGly (K) no new terminus
Oxidation (M)

20200921_KIZ_MUb4_GlyGly_10plex_2 − Scan number: 14942

Gene Names	Charge	m/z	Mass	Retention time	PEP	Score	Precursor Intensity
LSS	3	699.6727	2095.996	58.334	0.00054801	77.562	635761.8

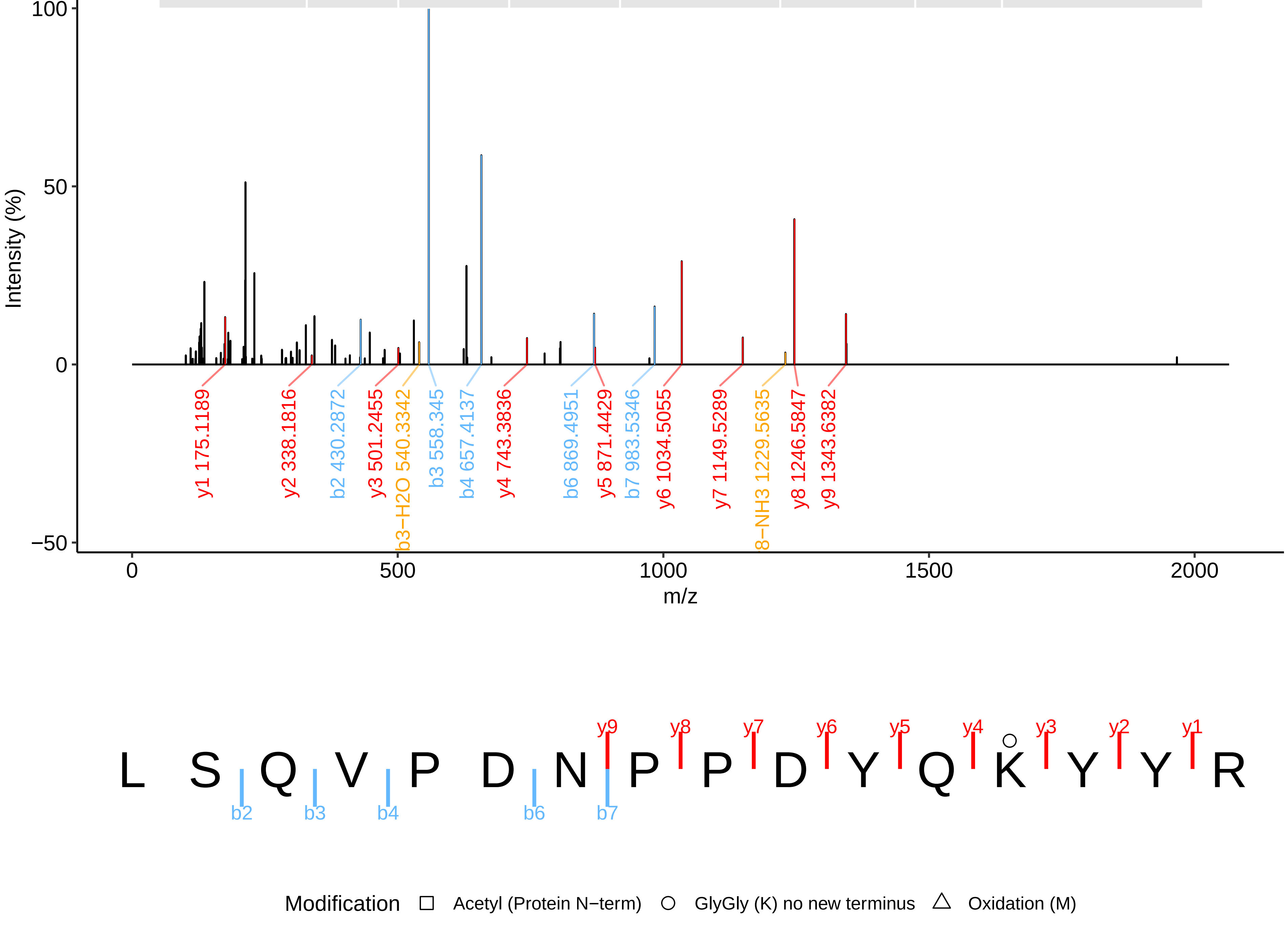

20200921_KIZ_MUb4_GlyGly_10plex_2 − Scan number: 15008

Gene Names	Charge	m/z	Mass	Retention time	PEP	Score	Precursor Intensity
LSS	3	699.6727	2095.996	58.606	9.9918e−06	88.561	2863328

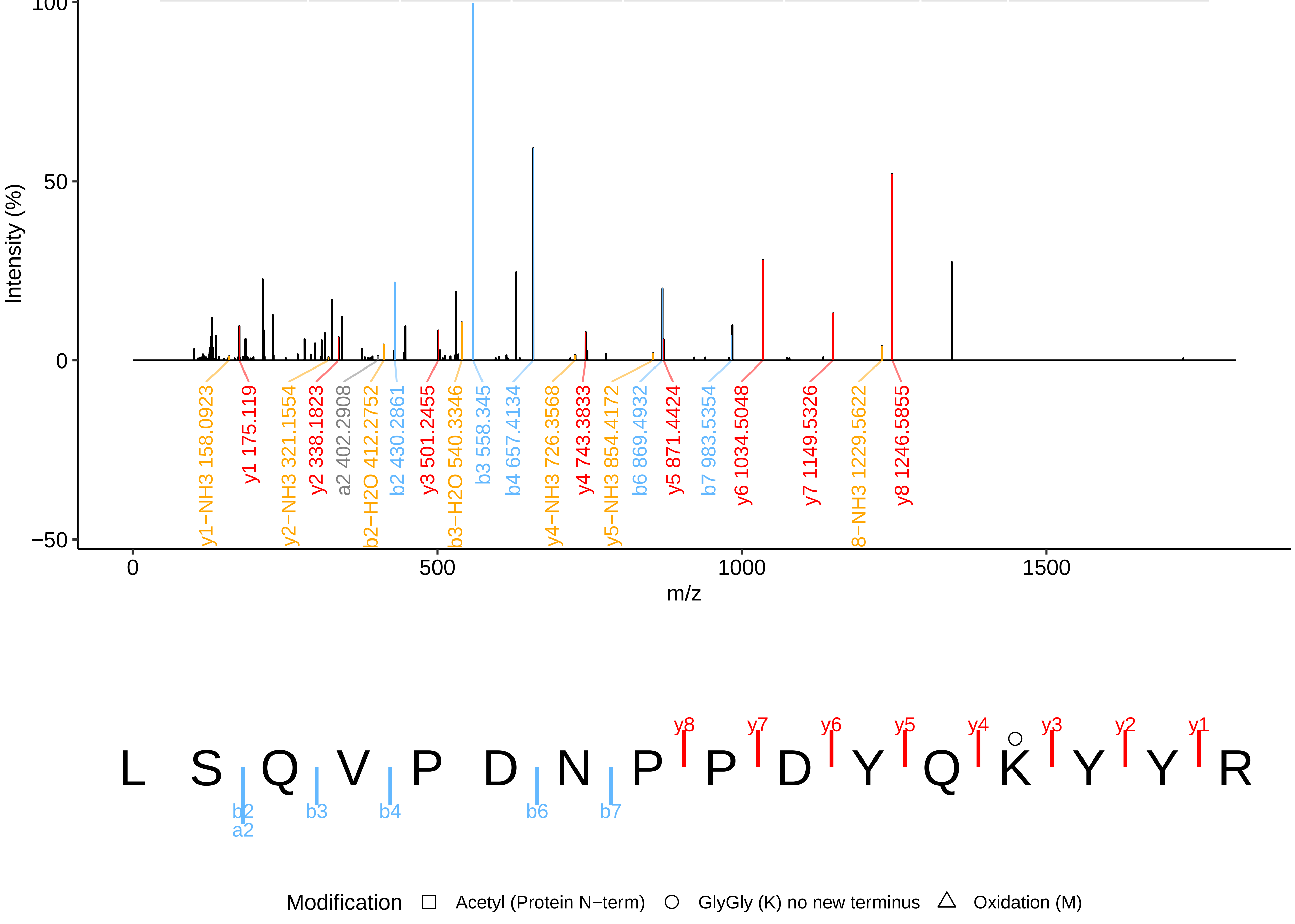

20200924_KIZ_MUb5_GlyGly_2 − Scan number: 14716

Gene Names	Charge	m/z	Mass	Retention time	PEP	Score	Precursor Intensity
LSS	3	699.6727	2095.996	54.992	7.4066e−10	115.82	2567271

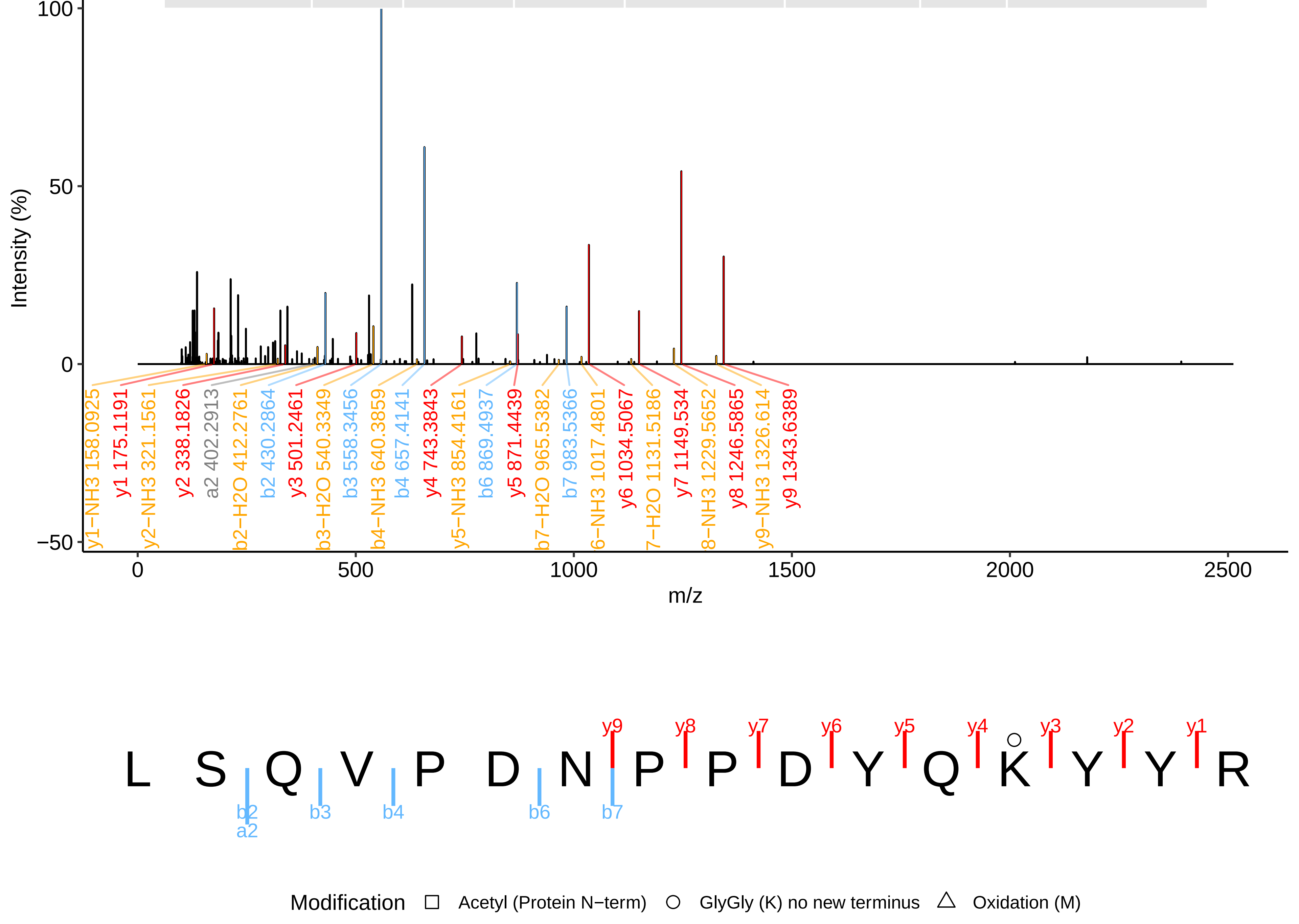

20200924_KIZ_MUb5_GlyGly_2 − Scan number: 14854

Gene Names	Charge	m/z	Mass	Retention time	PEP	Score	Precursor Intensity
LSS	3	699.6727	2095.996	55.501	1.72e−14	140.84	2399886

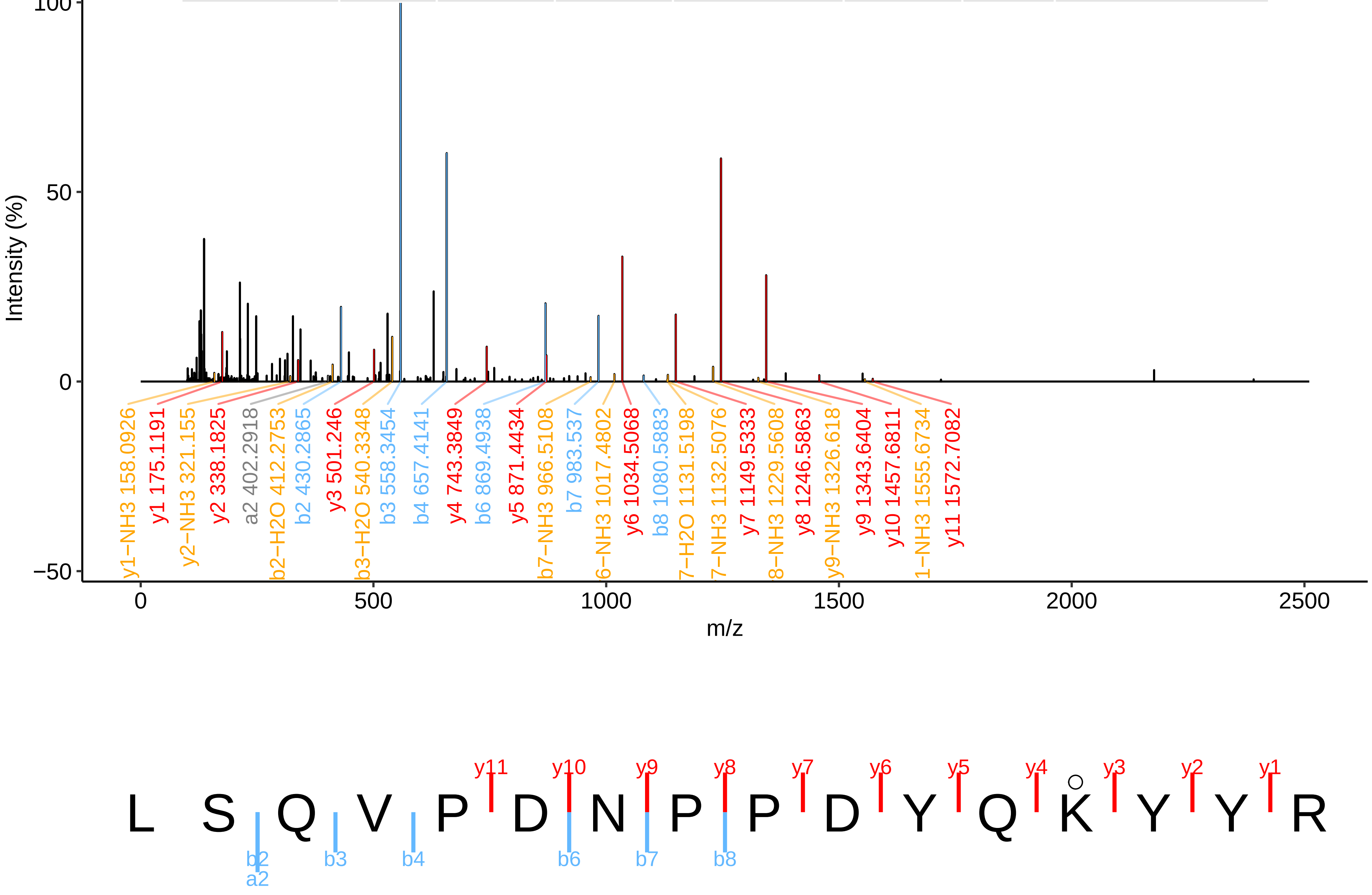

Modification □ Acetyl (Protein N−term) ○ GlyGly (K) no new terminus △ Oxidation (M)

20200921_KIZ_MUb4_GlyGly_10plex_2 − Scan number: 9758

Gene Names	Charge	m/z	Mass	Retention time	PEP	Score	Precursor Intensity
EAPP	3	688.0223	2061.045	37.196	2.9954e−09	100.21	634636.2

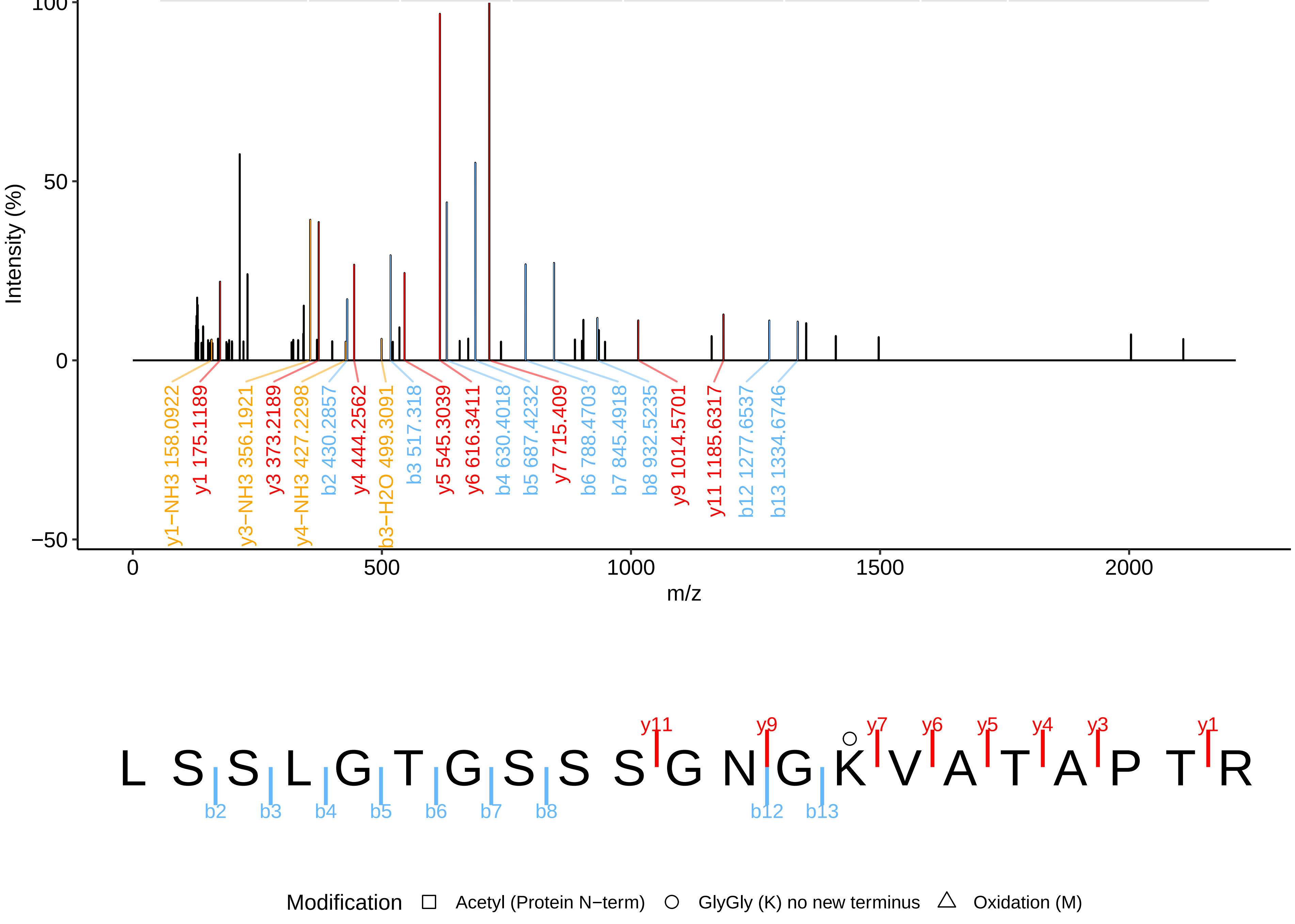

20200921_KIZ_MUb4_GlyGly_10plex − Scan number: 16559

Gene Names	Charge	m/z	Mass	Retention time	PEP	Score	Precursor Intensity
TXNL1	3	627.6368	1879.889	59.509	1.6619e−07	108.71	NA

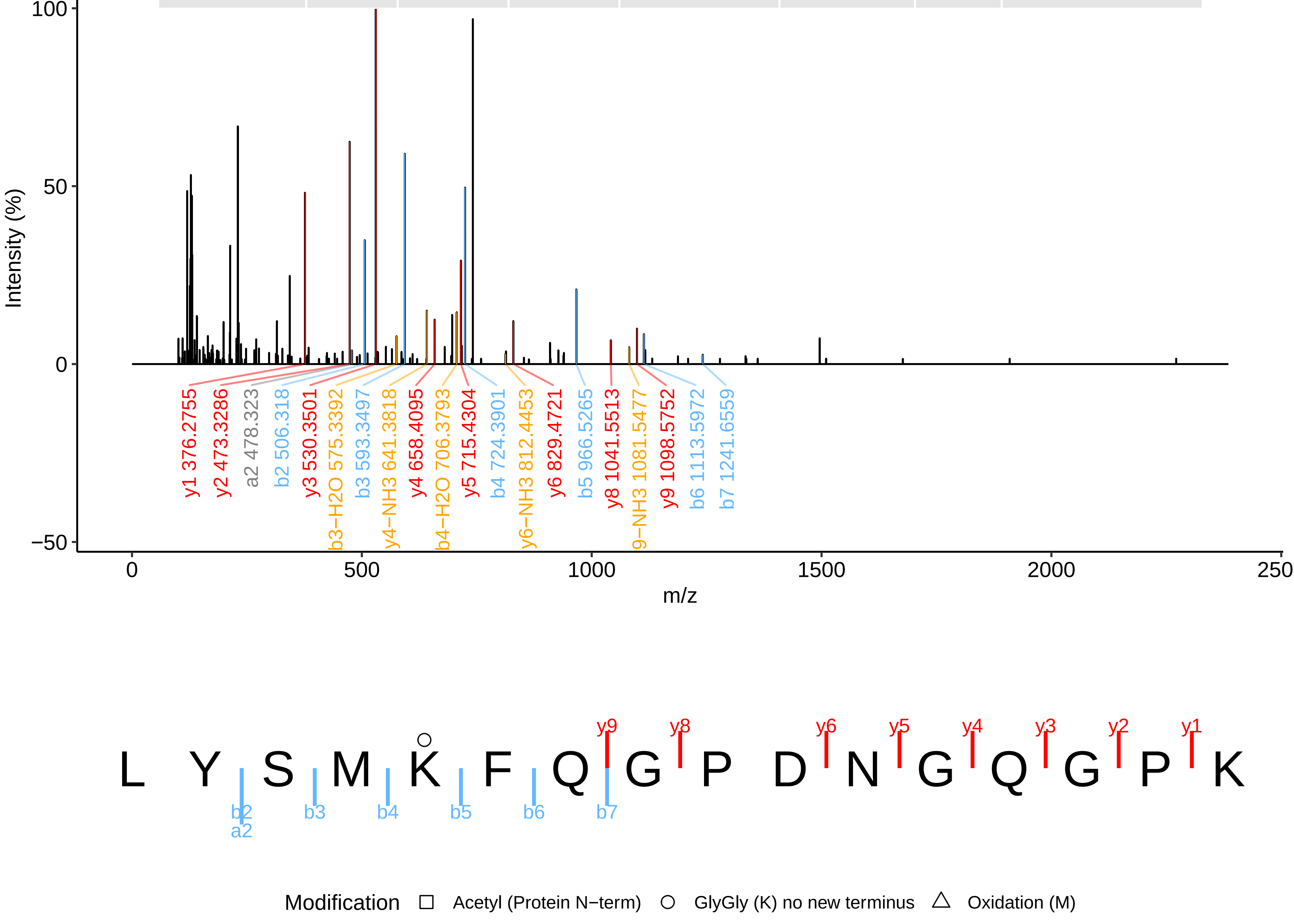

20200921_KIZ_MUb4_GlyGly_10plex_2 − Scan number: 15355

Gene Names	Charge	m/z	Mass	Retention time	PEP	Score	Precursor Intensity
TXNL1	3	627.6368	1879.889	60.029	0.063557	50.843	NA

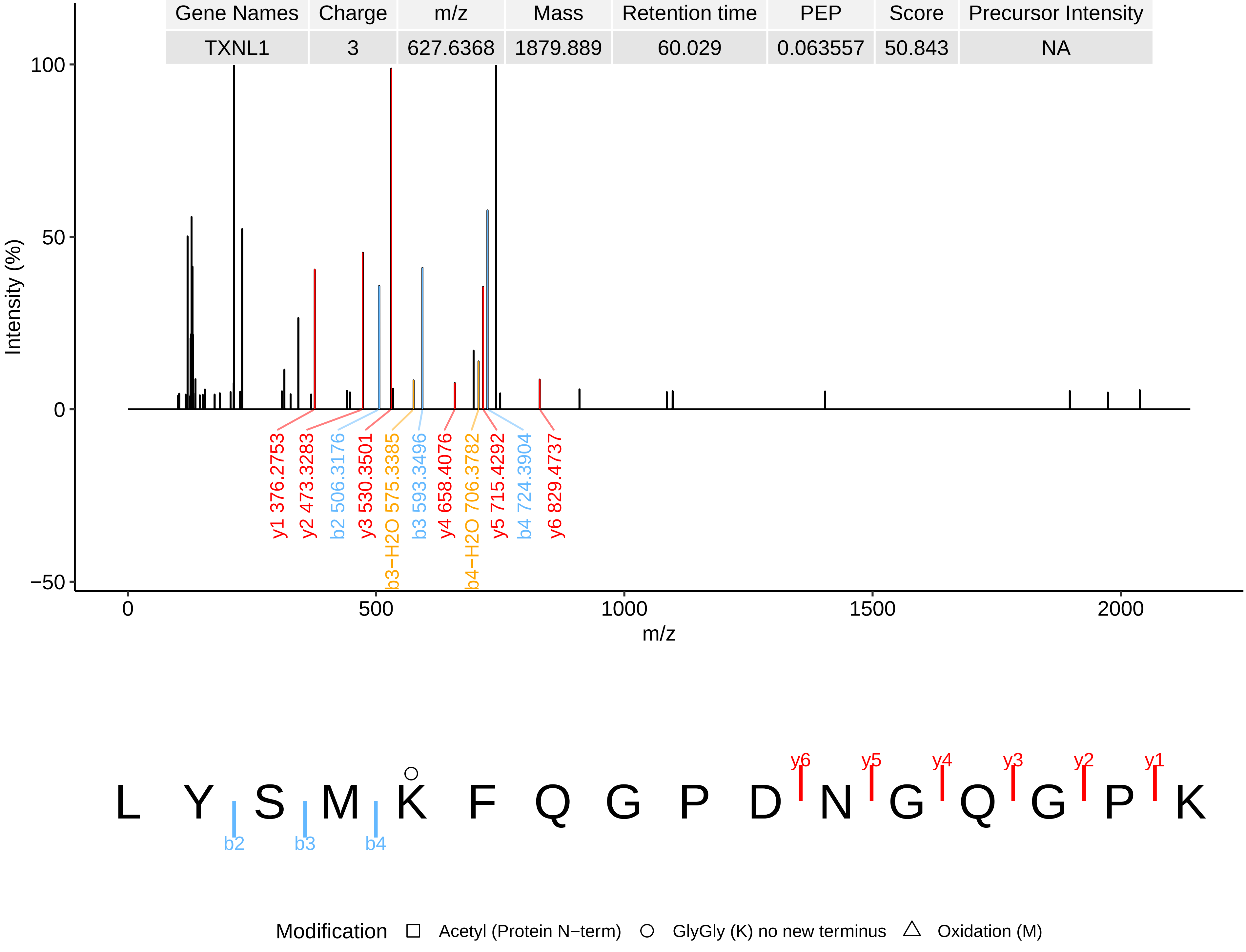

20200921_KIZ_MUb4_GlyGly_10plex − Scan number: 12821

Gene Names	Charge	m/z	Mass	Retention time	PEP	Score	Precursor Intensity
CBX1	3	605.3024	1812.885	45.094	7.7707e−06	90.754	2460968

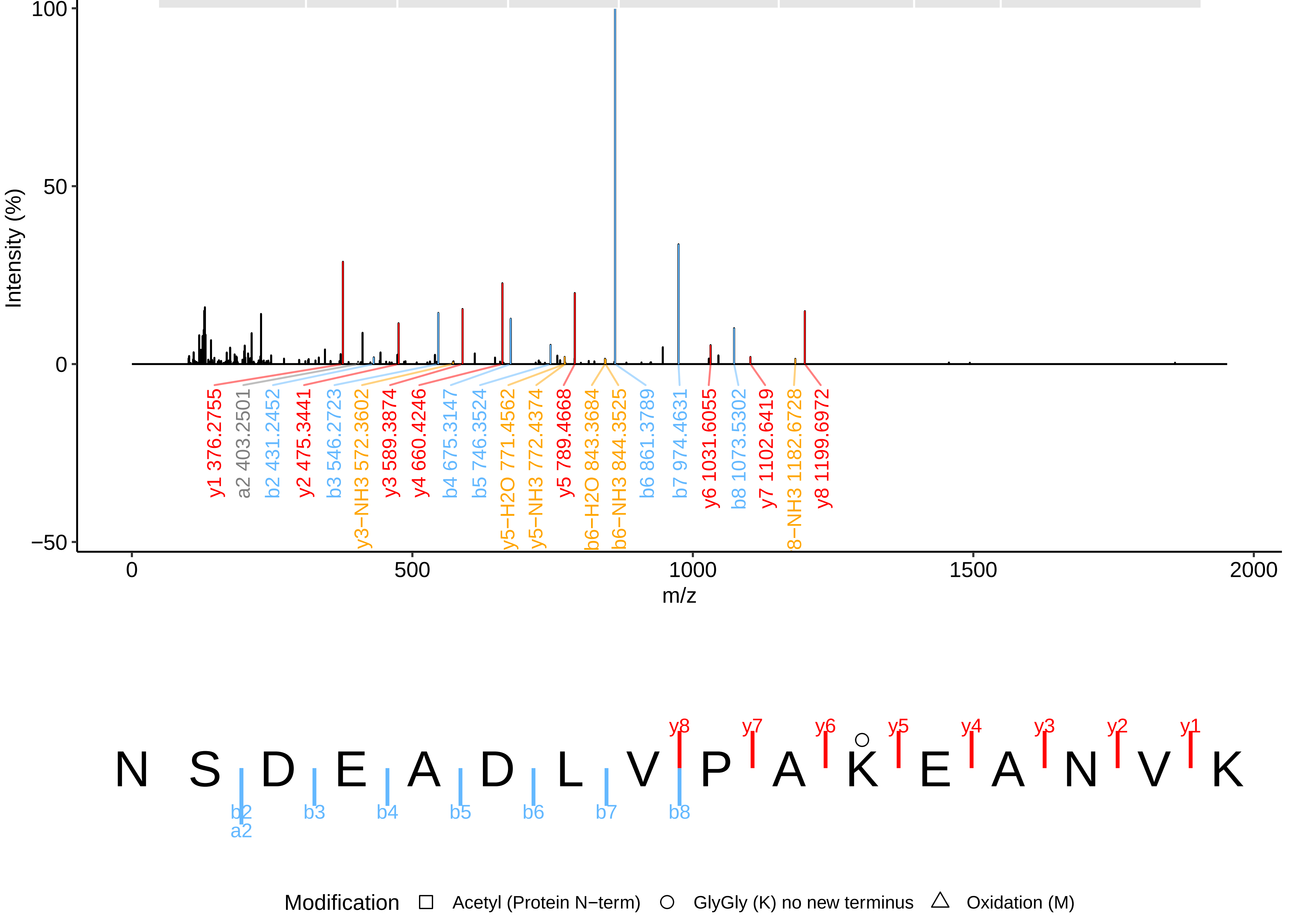

20200921_KIZ_MUb4_GlyGly_10plex − Scan number: 12839

Gene Names	Charge	m/z	Mass	Retention time	PEP	Score	Precursor Intensity
CBX1	3	605.3024	1812.885	45.161	1.2492e−07	110.38	3081339

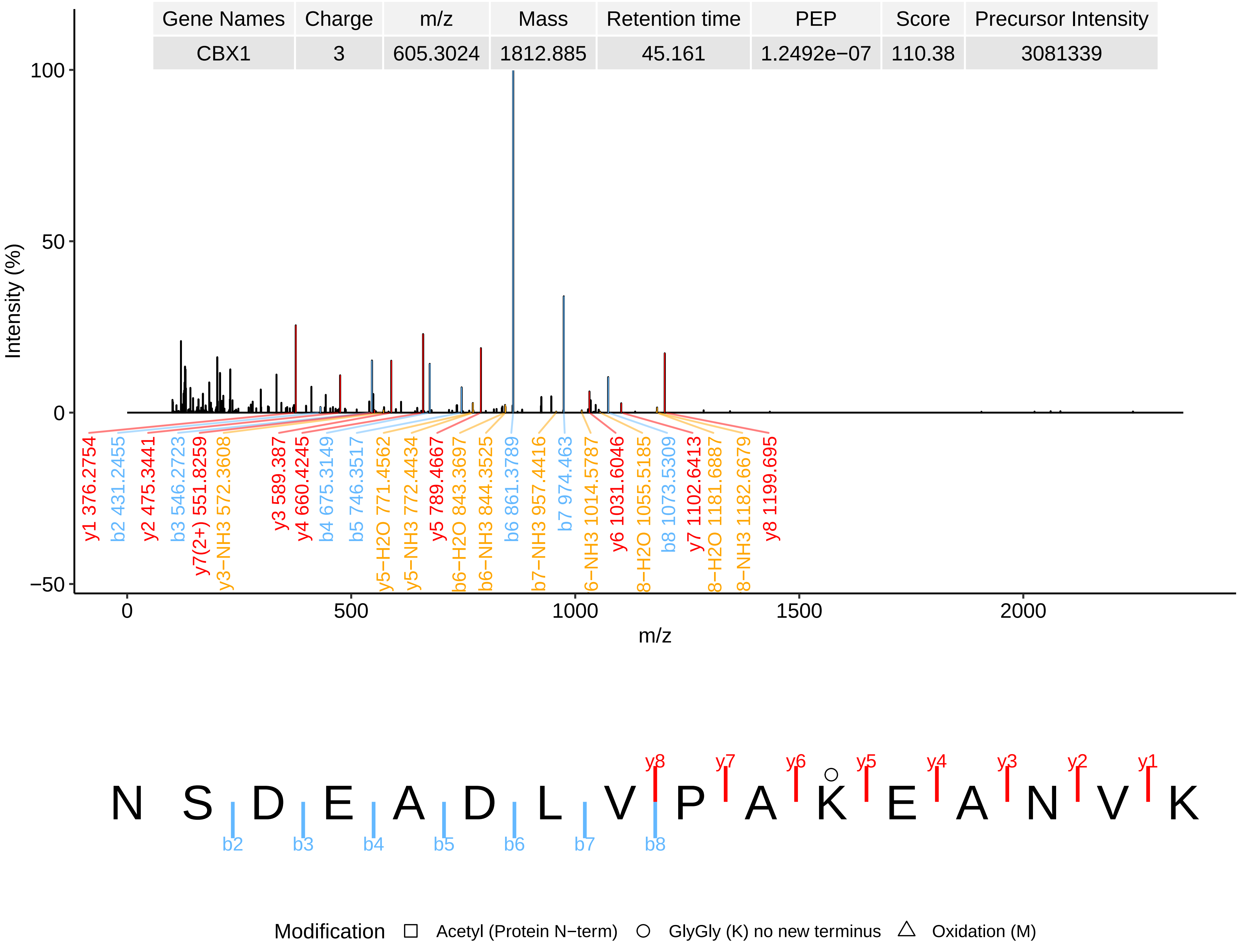

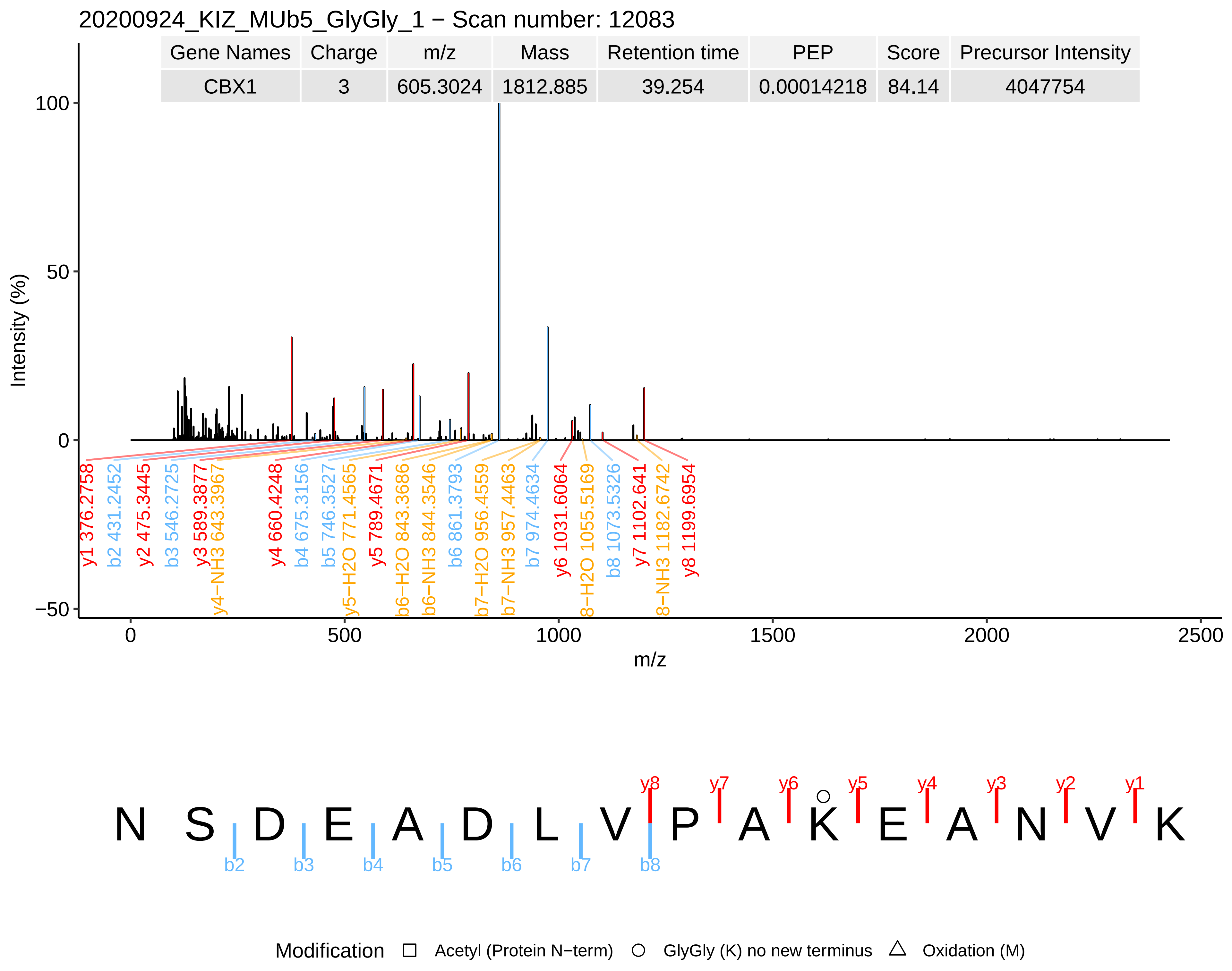

20200924_KIZ_MUb5_GlyGly_1 − Scan number: 12083
Gene Names | Charge | m/z | Mass | Retention time | PEP | Score | Precursor Intensity
CBX1 | 3 | 605.3024 | 1812.885 | 39.254 | 0.00014218 | 84.14 | 4047754
Intensity (%)
100
50
0
−50
y1 376.2758
b2 431.2452
y2 475.3445
b3 546.2725
y3 589.3877
y4−NH3 643.3967
y4 660.4248
b4 675.3156
b5 746.3527
y5−H2O 771.4565
y5 789.4671
b6−H2O 843.3686
b6−NH3 844.3546
b6 861.3793
b7−H2O 956.4559
b7−NH3 957.4463
b7 974.4634
y6 1031.6064
8−H2O 1055.5169
b8 1073.5326
y7 1102.641
8−NH3 1182.6742
y8 1199.6954
0
500
1000
1500
2000
2500
m/z
y8
y7
y6
y5
y4
y3
y2
y1
N S D E A D L V P A K E A N V K
b2
b3
b4
b5
b6
b7
b8
Modification
Acetyl (Protein N−term)
GlyGly (K) no new terminus
Oxidation (M)

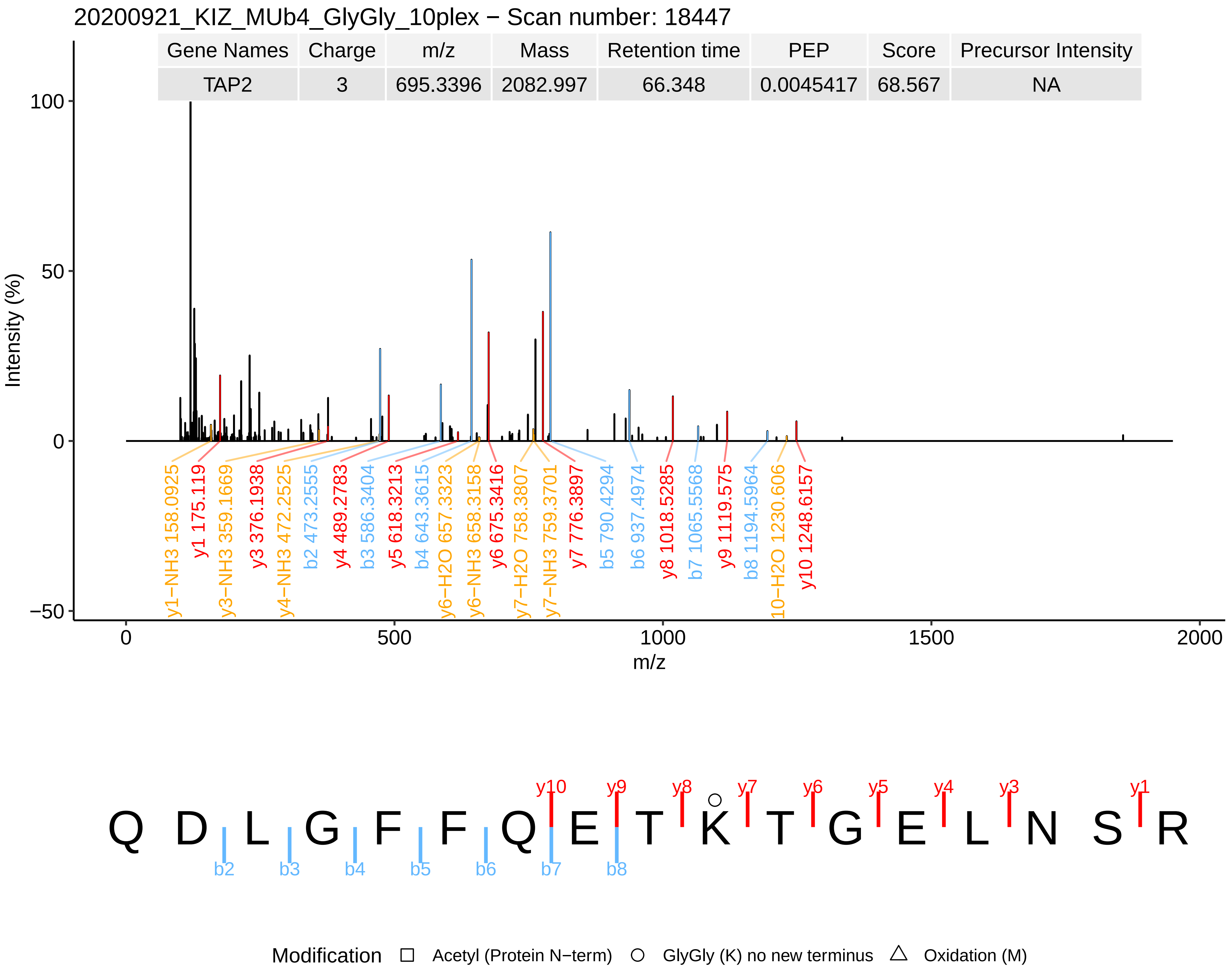
20200921_KIZ_MUb4_GlyGly_10plex − Scan number: 18447
Gene Names
Charge
m/z
Mass
Retention time
PEP
Score
Precursor Intensity
TAP2
3
695.3396
2082.997
66.348
0.0045417
68.567
NA
Intensity (%)
100
50
0
−50
0
500
1000
1500
2000
m/z
y1−NH3 158.0925
y1 175.119
y3−NH3 359.1669
y3 376.1938
y4−NH3 472.2525
b2 473.2555
y4 489.2783
b3 586.3404
y5 618.3213
b4 643.3615
y6−H2O 657.3323
y6−NH3 658.3158
y6 675.3416
y7−H2O 758.3807
y7−NH3 759.3701
y7 776.3897
b5 790.4294
b6 937.4974
y8 1018.5285
b7 1065.5568
y9 1119.575
b8 1194.5964
10−H2O 1230.606
y10 1248.6157
y10
y9
y8
y7
y6
y5
y4
y3
y1
Q D L G F F Q E T K T G E L N S R
b2
b3
b4
b5
b6
b7
b8
Modification
Acetyl (Protein N−term)
GlyGly (K) no new terminus
Oxidation (M)

20200921_KIZ_MUb4_GlyGly_10plex_2 − Scan number: 16749

Gene Names	Charge	m/z	Mass	Retention time	PEP	Score	Precursor Intensity
TAP2	3	695.3396	2082.997	65.418	5.4736e−10	105.9	1258864

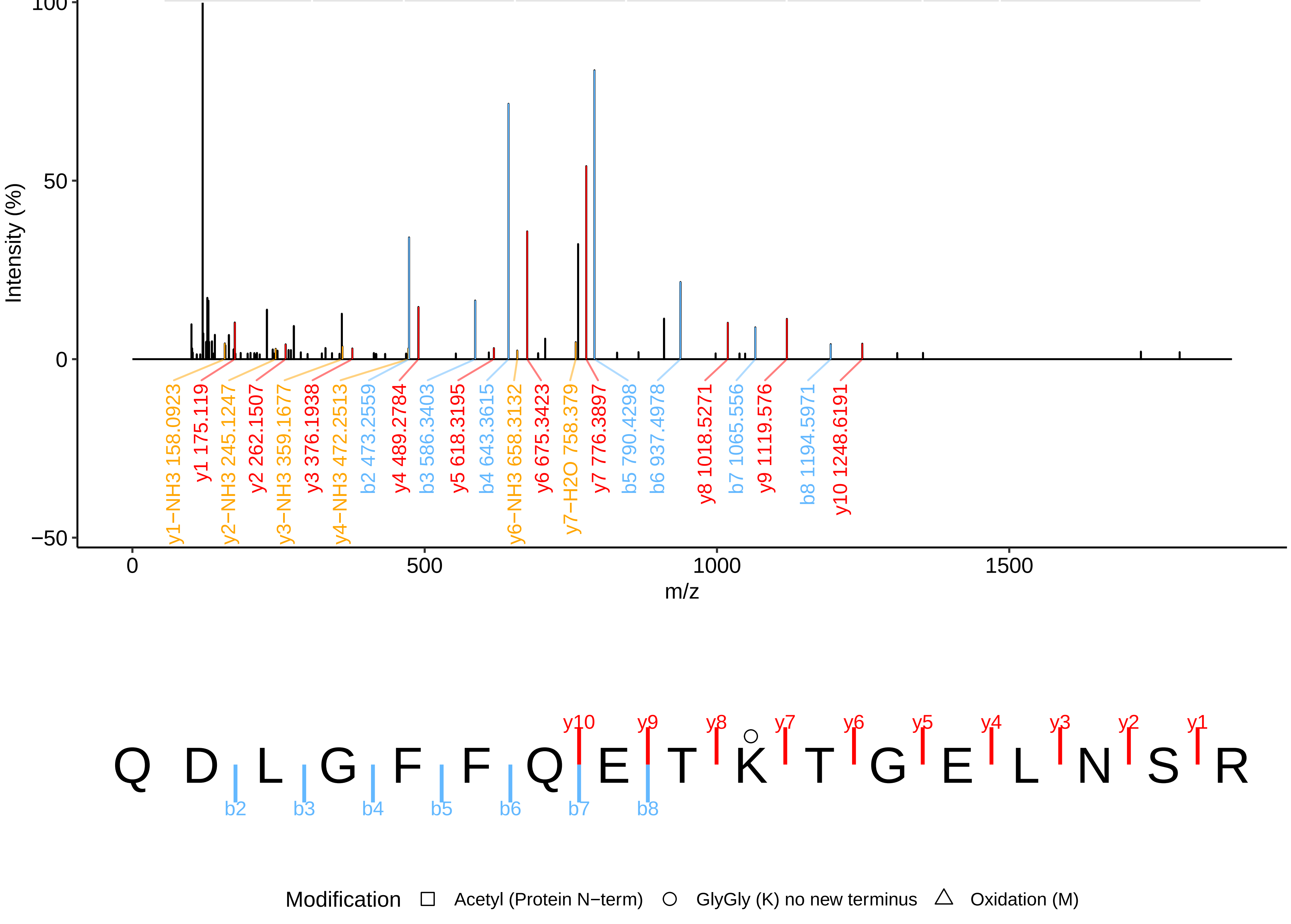

20201118_KIZ_MUb6_GlyGly_2 − Scan number: 19474

Gene Names	Charge	m/z	Mass	Retention time	PEP	Score	Precursor Intensity
TAP2	3	695.3396	2082.997	65.716	6.2933e−13	124.99	5920475

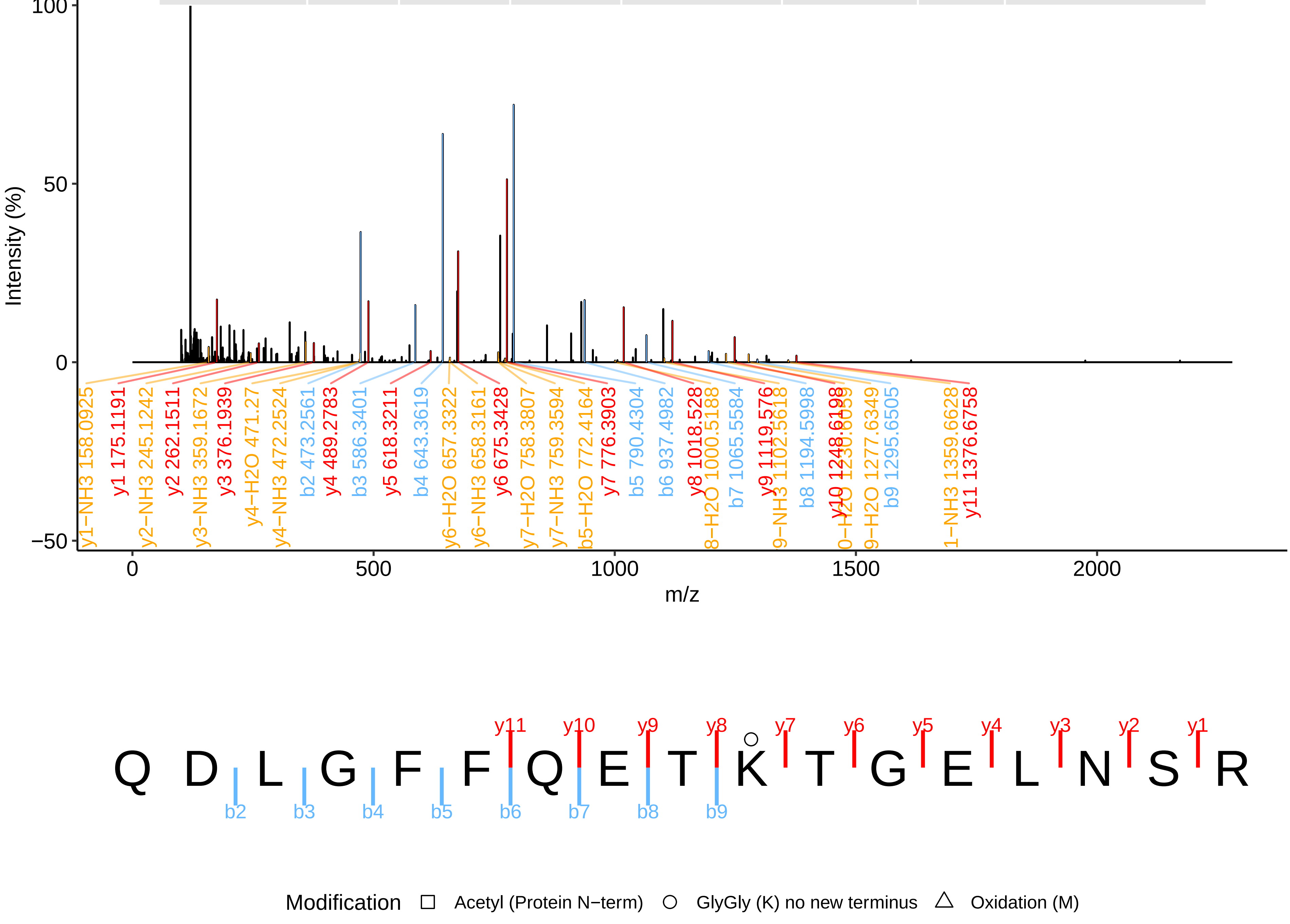

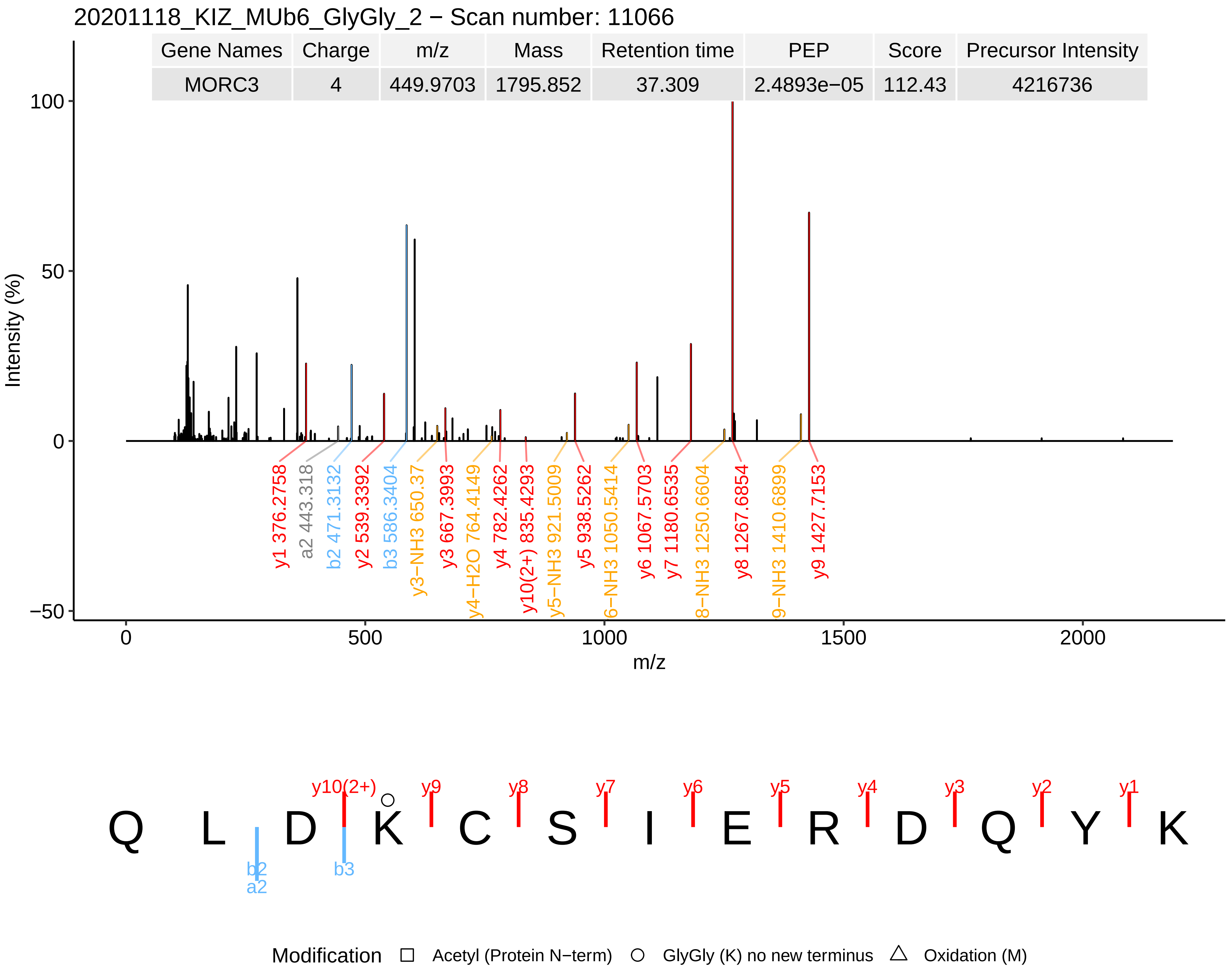
20201118_KIZ_MUb6_GlyGly_2 − Scan number: 11066
Gene Names | Charge | m/z | Mass | Retention time | PEP | Score | Precursor Intensity
MORC3 | 4 | 449.9703 | 1795.852 | 37.309 | 2.4893e−05 | 112.43 | 4216736
Intensity (%)
100
50
0
−50
0
500
1000
1500
2000
m/z
y1 376.2758
a2 443.318
b2 471.3132
y2 539.3392
b3 586.3404
y3−NH3 650.37
y3 667.3993
y4−H2O 764.4149
y4 782.4262
y10(2+) 835.4293
y5−NH3 921.5009
y5 938.5262
6−NH3 1050.5414
y6 1067.5703
y7 1180.6535
8−NH3 1250.6604
y8 1267.6854
9−NH3 1410.6899
y9 1427.7153
y10(2+)
y9
y8
y7
y6
y5
y4
y3
y2
y1
Q L D K C S I E R D Q Y K
b2
a2
b3
Modification
Acetyl (Protein N−term)
GlyGly (K) no new terminus
Oxidation (M)

20200921_KIZ_MUb4_GlyGly_10plex − Scan number: 14566

Gene Names	Charge	m/z	Mass	Retention time	PEP	Score	Precursor Intensity
ANKRD13A	3	689.687	2066.039	51.813	0.018465	59.434	2439493

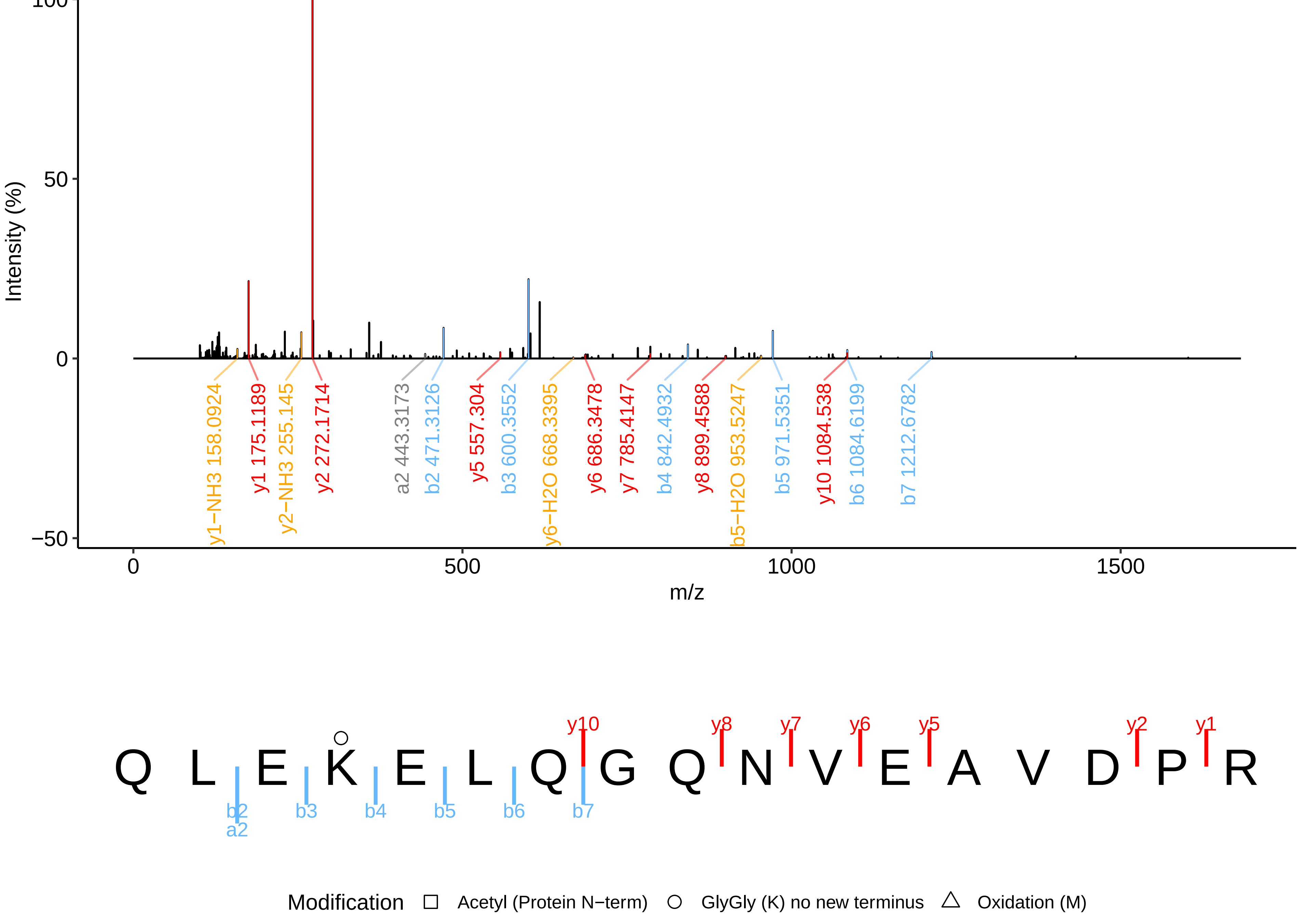

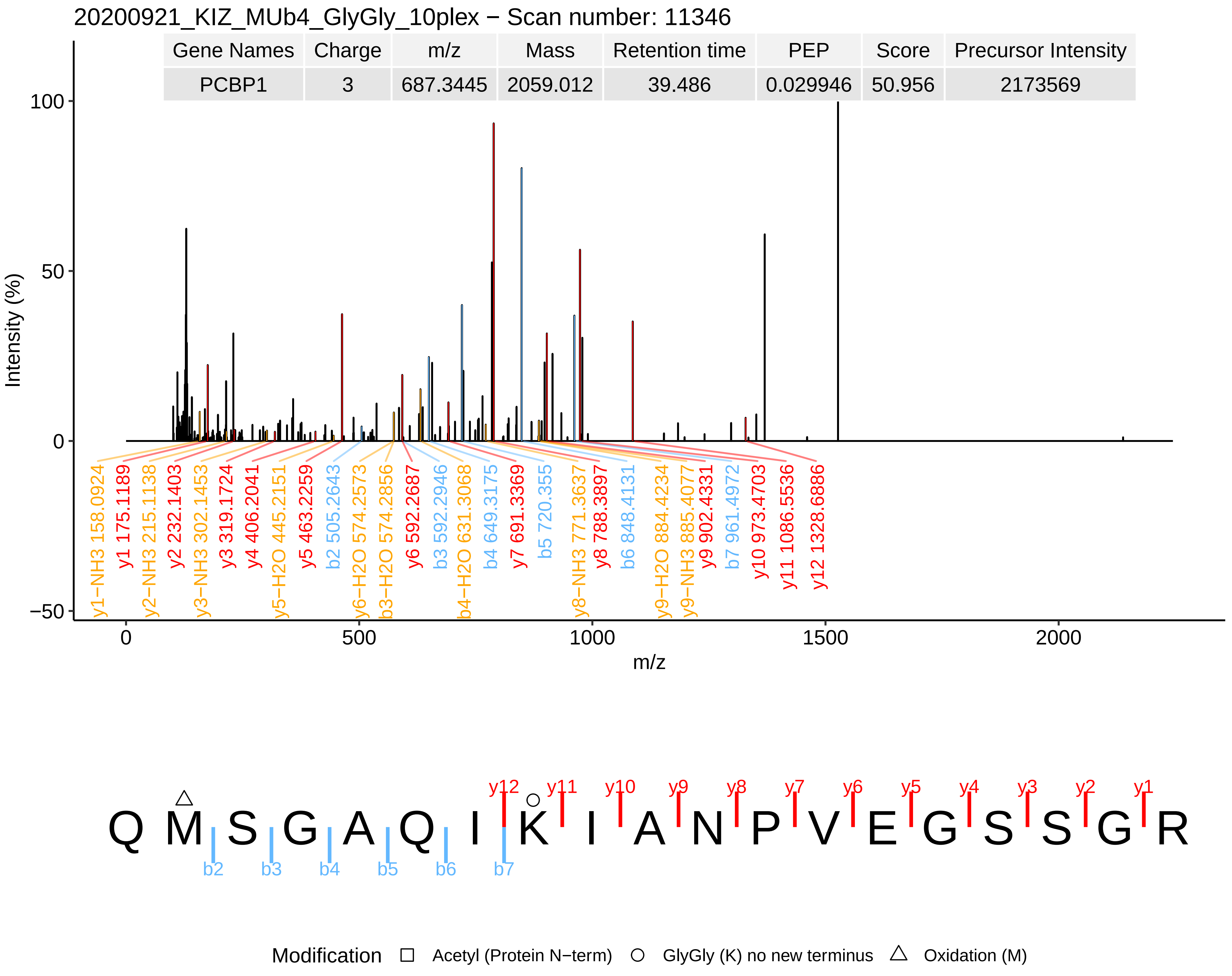

20200921_KIZ_MUb4_GlyGly_10plex − Scan number: 11346
Gene Names | Charge | m/z | Mass | Retention time | PEP | Score | Precursor Intensity
PCBP1 | 3 | 687.3445 | 2059.012 | 39.486 | 0.029946 | 50.956 | 2173569
Intensity (%)
m/z
y1−NH3 158.0924
y1 175.1189
y2−NH3 215.1138
y2 232.1403
y3−NH3 302.1453
y3 319.1724
y4 406.2041
y5−H2O 445.2151
y5 463.2259
b2 505.2643
y6−H2O 574.2573
b3−H2O 574.2856
y6 592.2687
b3 592.2946
b4−H2O 631.3068
b4 649.3175
y7 691.3369
b5 720.355
y8−NH3 771.3637
y8 788.3897
b6 848.4131
y9−H2O 884.4234
y9−NH3 885.4077
y9 902.4331
b7 961.4972
y10 973.4703
y11 1086.5536
y12 1328.6886
Q M S G A Q I K I A N P V E G S S G R
y12 y11 y10 y9 y8 y7 y6 y5 y4 y3 y2 y1
b2 b3 b4 b5 b6 b7
Modification
Acetyl (Protein N−term)
GlyGly (K) no new terminus
Oxidation (M)

20200921_KIZ_MUb4_GlyGly_10plex − Scan number: 11351

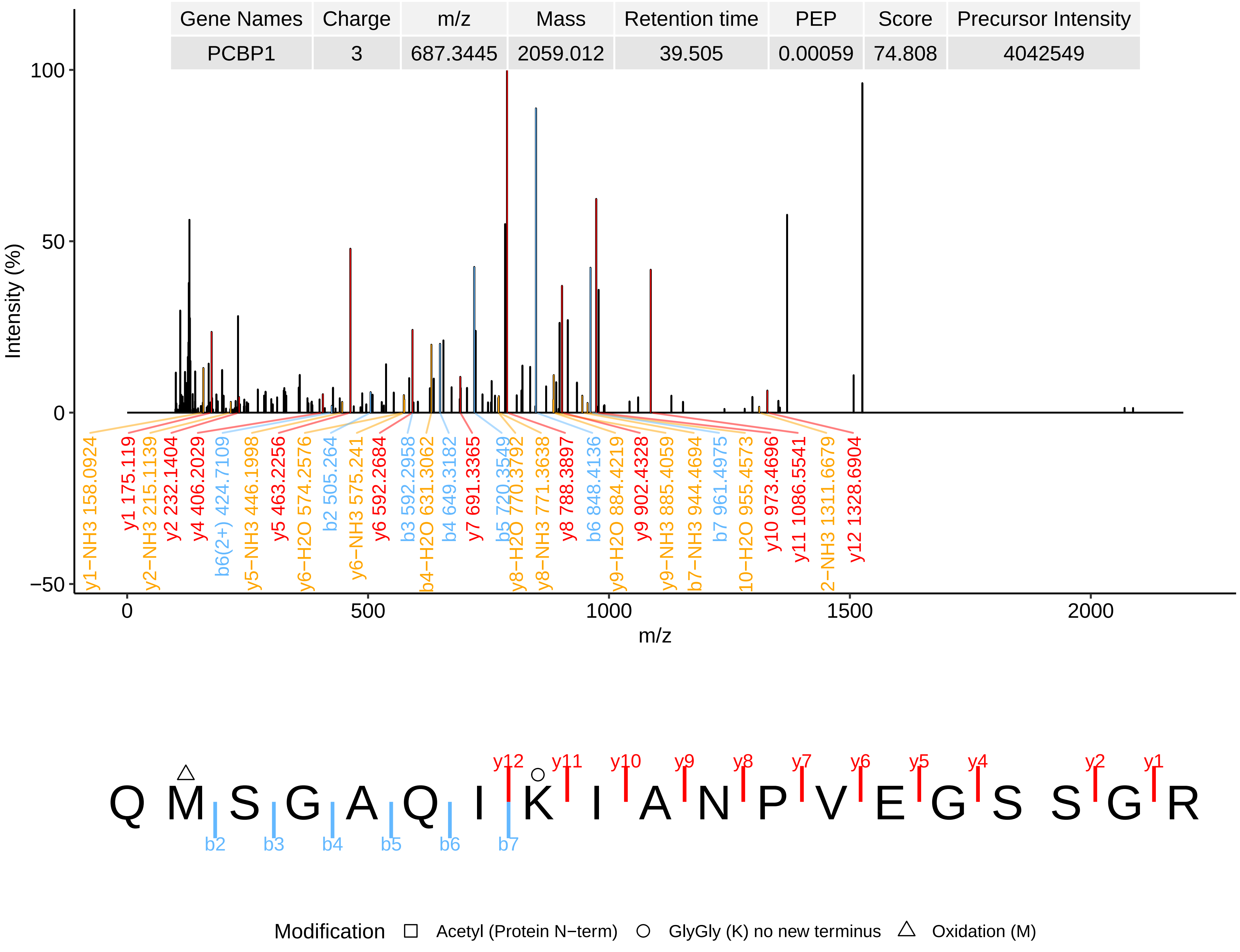

20200921_KIZ_MUb4_GlyGly_10plex − Scan number: 14115

Gene Names	Charge	m/z	Mass	Retention time	PEP	Score	Precursor Intensity
PCBP1	3	682.0129	2043.017	50.044	4.1488e−21	133.95	5175106

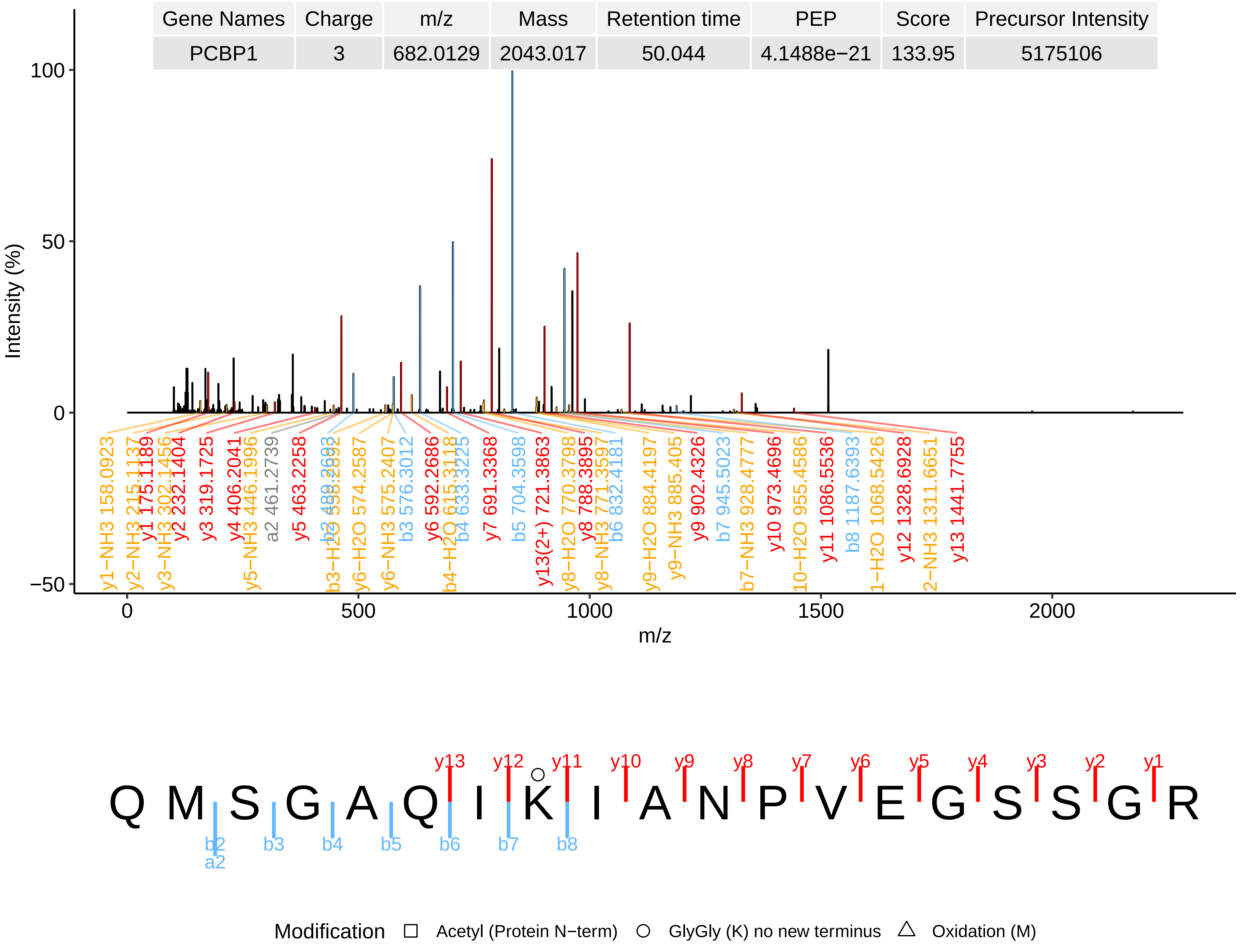

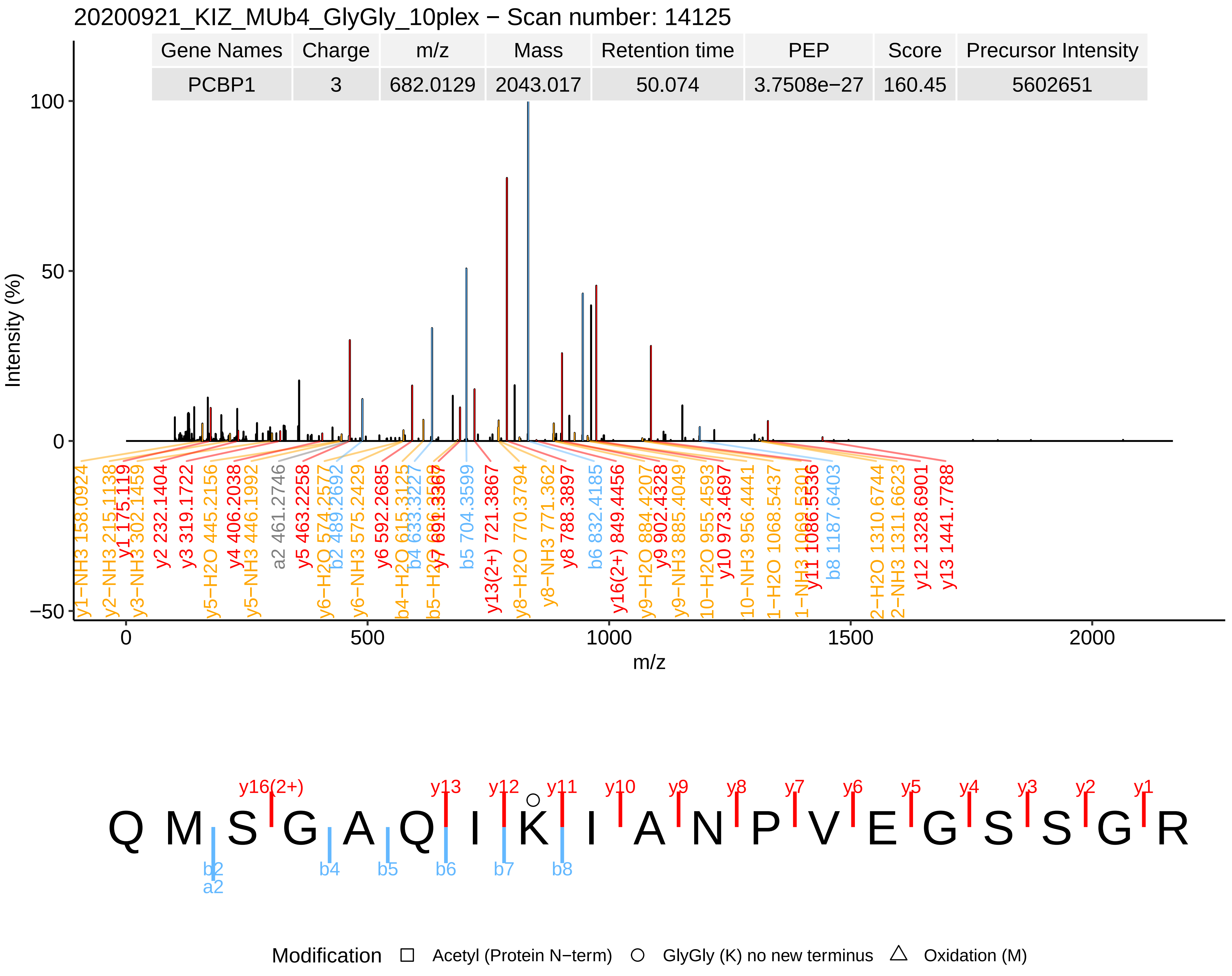

20200921_KIZ_MUb4_GlyGly_10plex − Scan number: 14125
Gene Names | Charge | m/z | Mass | Retention time | PEP | Score | Precursor Intensity
PCBP1 | 3 | 682.0129 | 2043.017 | 50.074 | 3.7508e−27 | 160.45 | 5602651
Intensity (%)
100
50
0
−50
0
500
1000
1500
2000
m/z
y1−NH3 158.0924
y2−NH3 215.1138
y1 175.119
y3−NH3 302.1459
y2 232.1404
y3 319.1722
y5−H2O 445.2156
y4 406.2038
y5−NH3 446.1992
a2 461.2746
y5 463.2258
y6−H2O 574.2577
b2 489.2692
y6−NH3 575.2429
y6 592.2685
b4−H2O 615.3125
b4 633.3227
b5−H2O 686.3367
y7 691.3367
b5 704.3599
y13(2+) 721.3867
y8−H2O 770.3794
y8−NH3 771.362
y8 788.3897
b6 832.4185
y16(2+) 849.4456
y9−H2O 884.4207
y9 902.4328
y9−NH3 885.4049
y10 973.4697
y11 1086.5536
b8 1187.6403
y12 1328.6901
y13 1441.7788
Q M S G A Q I K I A N P V E G S S G R
y16(2+) y13 y12 y11 y10 y9 y8 y7 y6 y5 y4 y3 y2 y1
b2 a2 b4 b5 b6 b7 b8
Modification
Acetyl (Protein N−term)
GlyGly (K) no new terminus
Oxidation (M)

20200921_KIZ_MUb4_GlyGly_10plex_2 − Scan number: 12925

Gene Names	Charge	m/z	Mass	Retention time	PEP	Score	Precursor Intensity
PCBP1	3	682.0129	2043.017	50.122	0.01426	58.997	295569.9

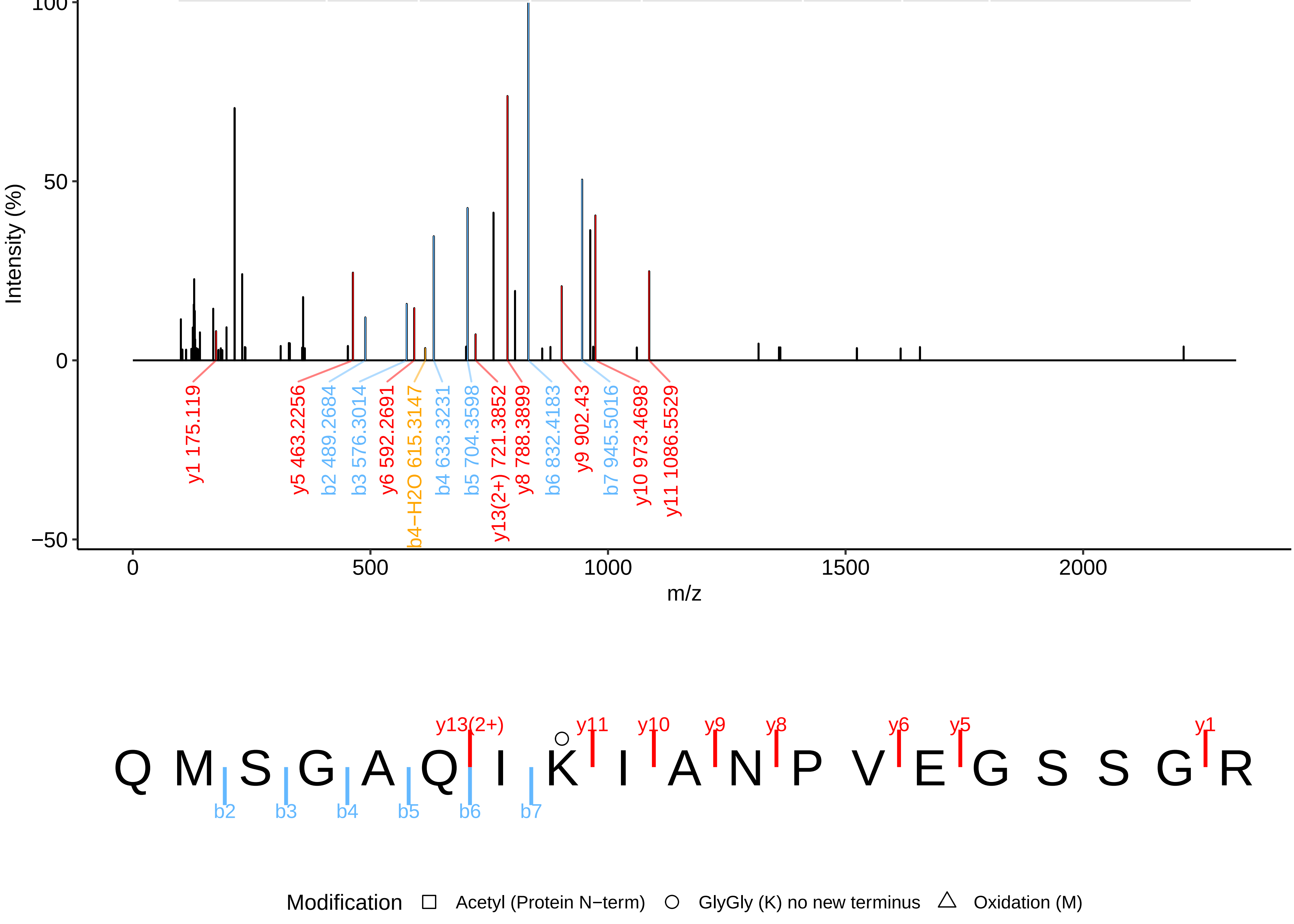

20200921_KIZ_MUb4_GlyGly_10plex_2 − Scan number: 12932

Gene Names	Charge	m/z	Mass	Retention time	PEP	Score	Precursor Intensity
PCBP1	3	682.0129	2043.017	50.15	1.1478e−05	83.229	591400.5

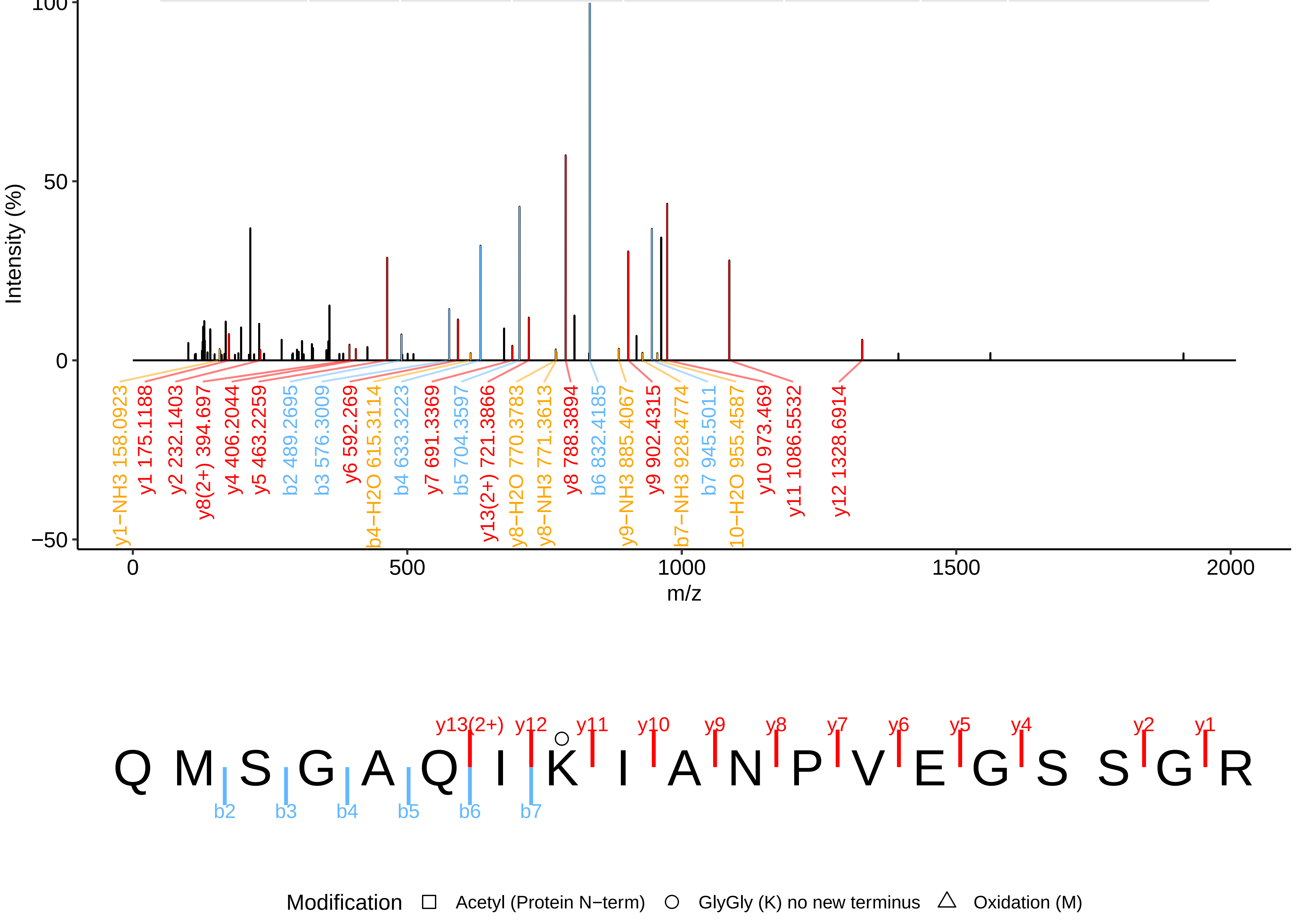

20200921_KIZ_MUb4_GlyGly_10plex_2 − Scan number: 13416

Gene Names	Charge	m/z	Mass	Retention time	PEP	Score	Precursor Intensity
PCBP2	3	706.0199	2115.038	52.085	0.00207	64.2	631141

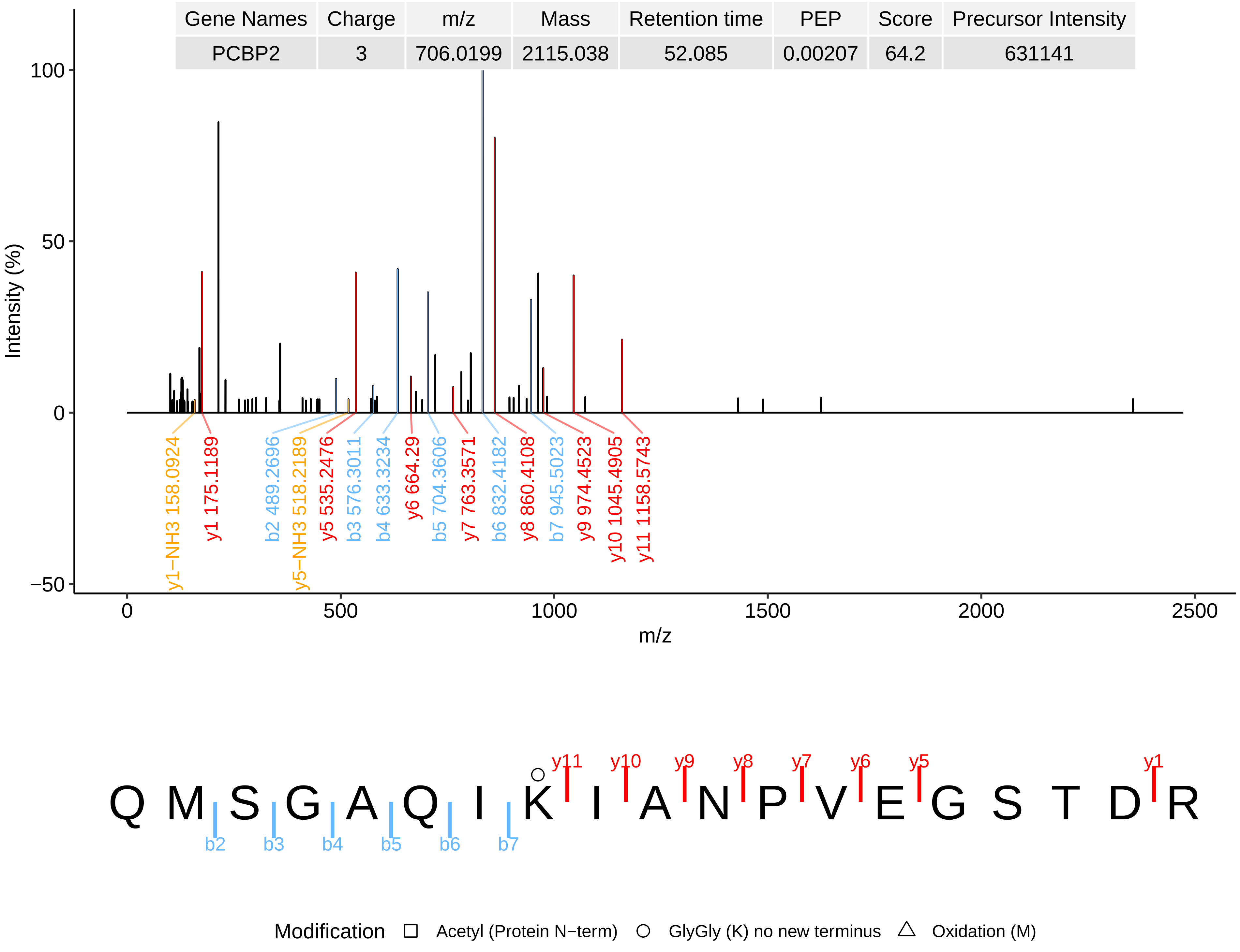

20200921_KIZ_MUb4_GlyGly_10plex_2 − Scan number: 13455

Gene Names	Charge	m/z	Mass	Retention time	PEP	Score	Precursor Intensity
PCBP2	3	706.0199	2115.038	52.246	0.039023	50.425	783220.9

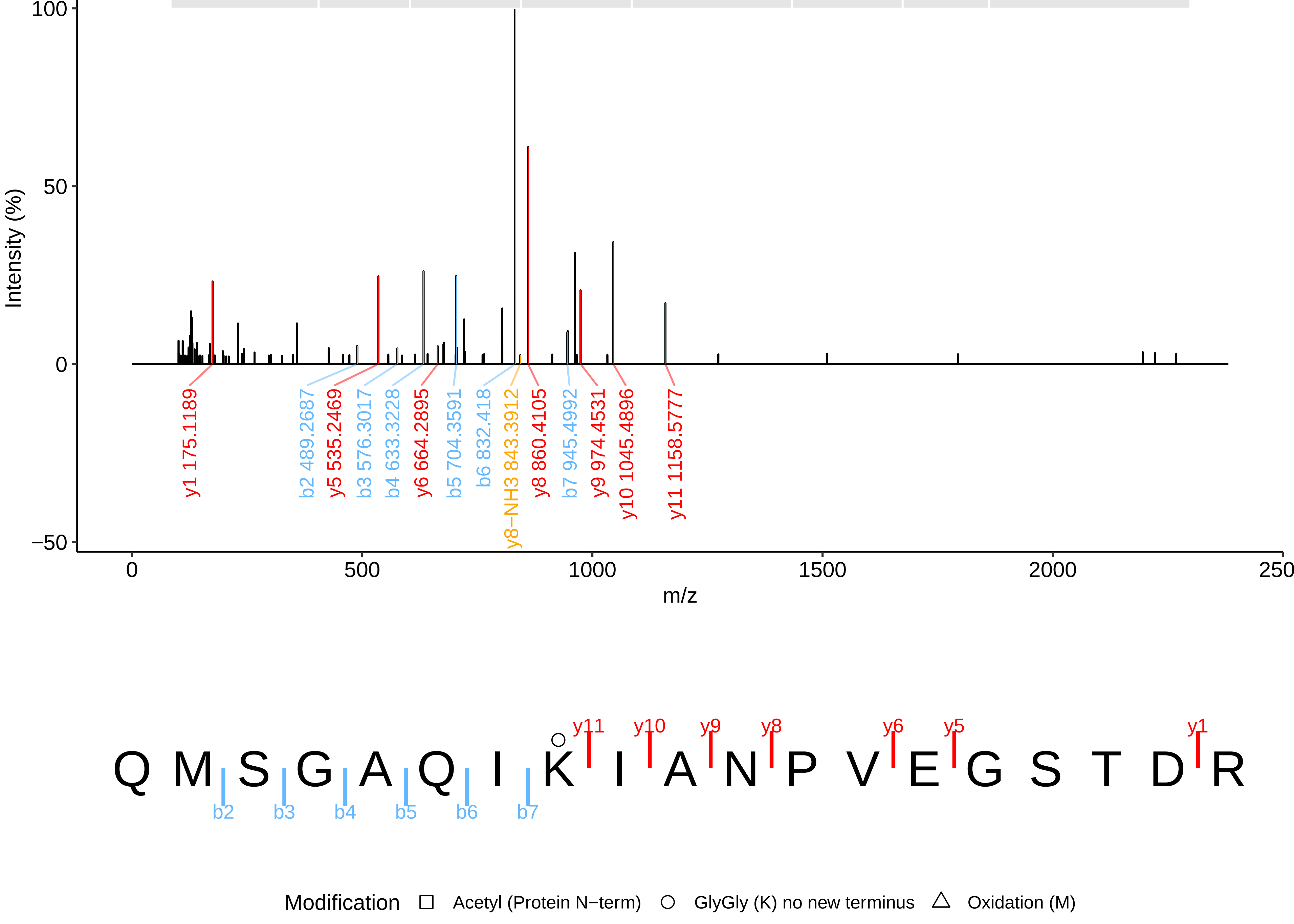

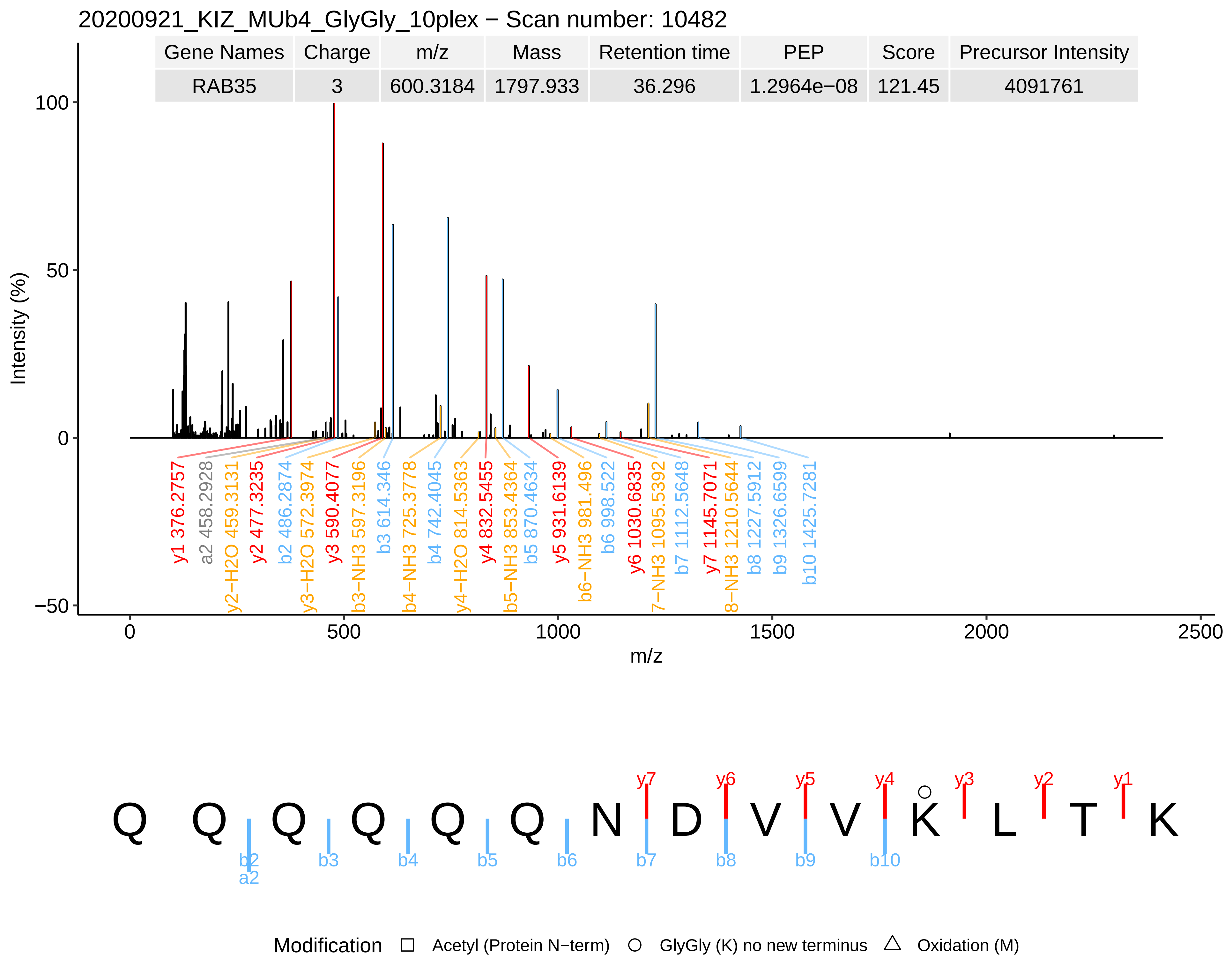
20200921_KIZ_MUb4_GlyGly_10plex − Scan number: 10482
Gene Names | Charge | m/z | Mass | Retention time | PEP | Score | Precursor Intensity
RAB35 | 3 | 600.3184 | 1797.933 | 36.296 | 1.2964e−08 | 121.45 | 4091761
Intensity (%)
100
50
0
−50
y1 376.2757
a2 458.2928
y2−H2O 459.3131
y2 477.3235
b2 486.2874
y3−H2O 572.3974
y3 590.4077
b3−NH3 597.3196
b3 614.346
b4−NH3 725.3778
b4 742.4045
y4−H2O 814.5363
y4 832.5455
b5−NH3 853.4364
b5 870.4634
y5 931.6139
b6−NH3 981.496
b6 998.522
y6 1030.6835
7−NH3 1095.5392
b7 1112.5648
y7 1145.7071
8−NH3 1210.5644
b8 1227.5912
b9 1326.6599
b10 1425.7281
0
500
1000
1500
2000
2500
m/z
y7
y6
y5
y4
y3
y2
y1
Q Q Q Q Q Q N D V V K L T K
b2
a2
b3
b4
b5
b6
b7
b8
b9
b10
Modification
Acetyl (Protein N−term)
GlyGly (K) no new terminus
Oxidation (M)

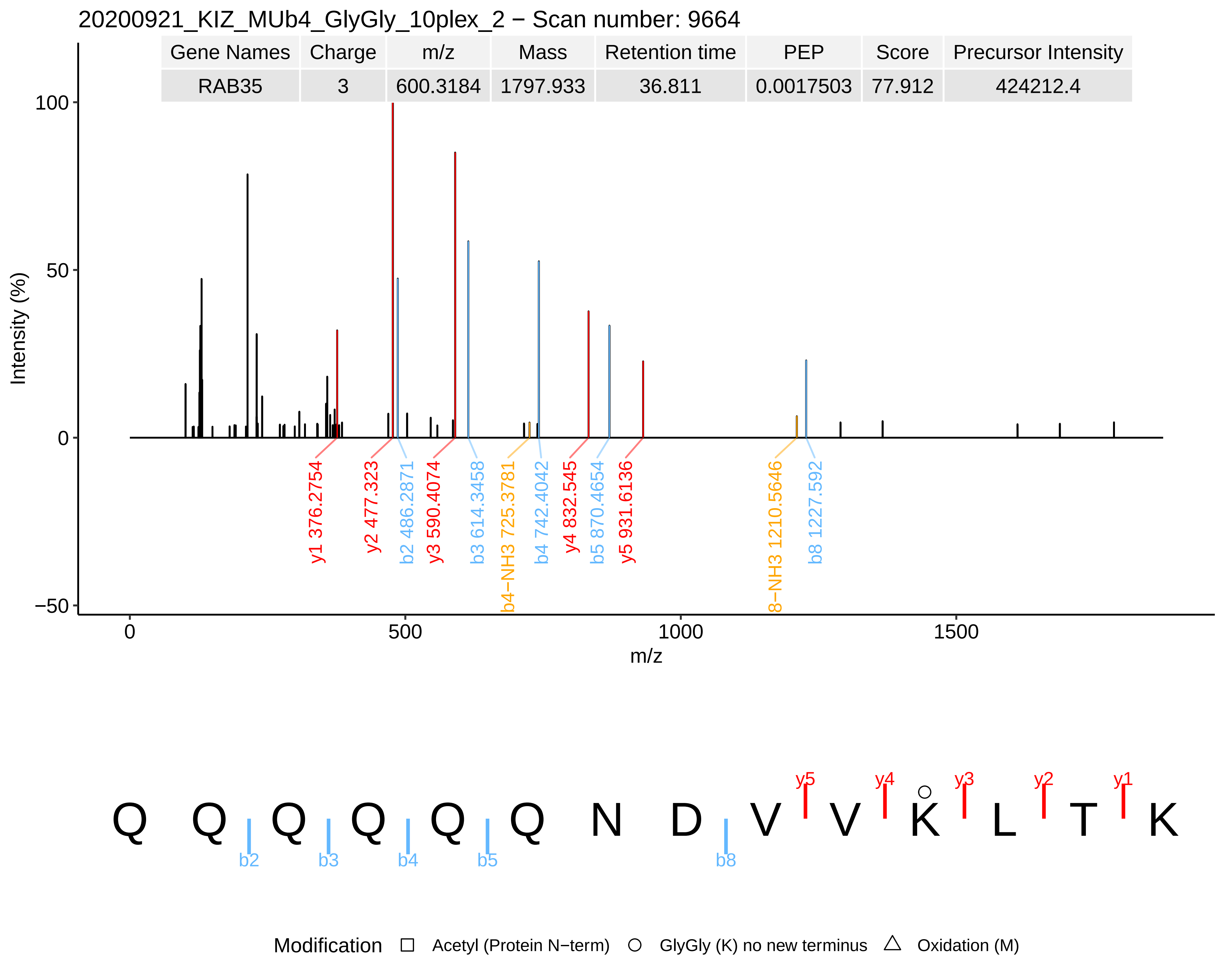

20200921_KIZ_MUb4_GlyGly_10plex_2 − Scan number: 9664
Gene Names | Charge | m/z | Mass | Retention time | PEP | Score | Precursor Intensity
RAB35 | 3 | 600.3184 | 1797.933 | 36.811 | 0.0017503 | 77.912 | 424212.4
Intensity (%)
m/z
y1 376.2754
y2 477.323
b2 486.2871
y3 590.4074
b3 614.3458
b4−NH3 725.3781
b4 742.4042
y4 832.545
b5 870.4654
y5 931.6136
8−NH3 1210.5646
b8 1227.592
Q Q Q Q Q Q N D V V K L T K
Modification
Acetyl (Protein N−term)
GlyGly (K) no new terminus
Oxidation (M)

20200921_KIZ_MUb4_GlyGly_10plex − Scan number: 15219

Gene Names	Charge	m/z	Mass	Retention time	PEP	Score	Precursor Intensity
RARS	3	692.3274	2073.96	54.317	2.6377e−51	210.37	6800884

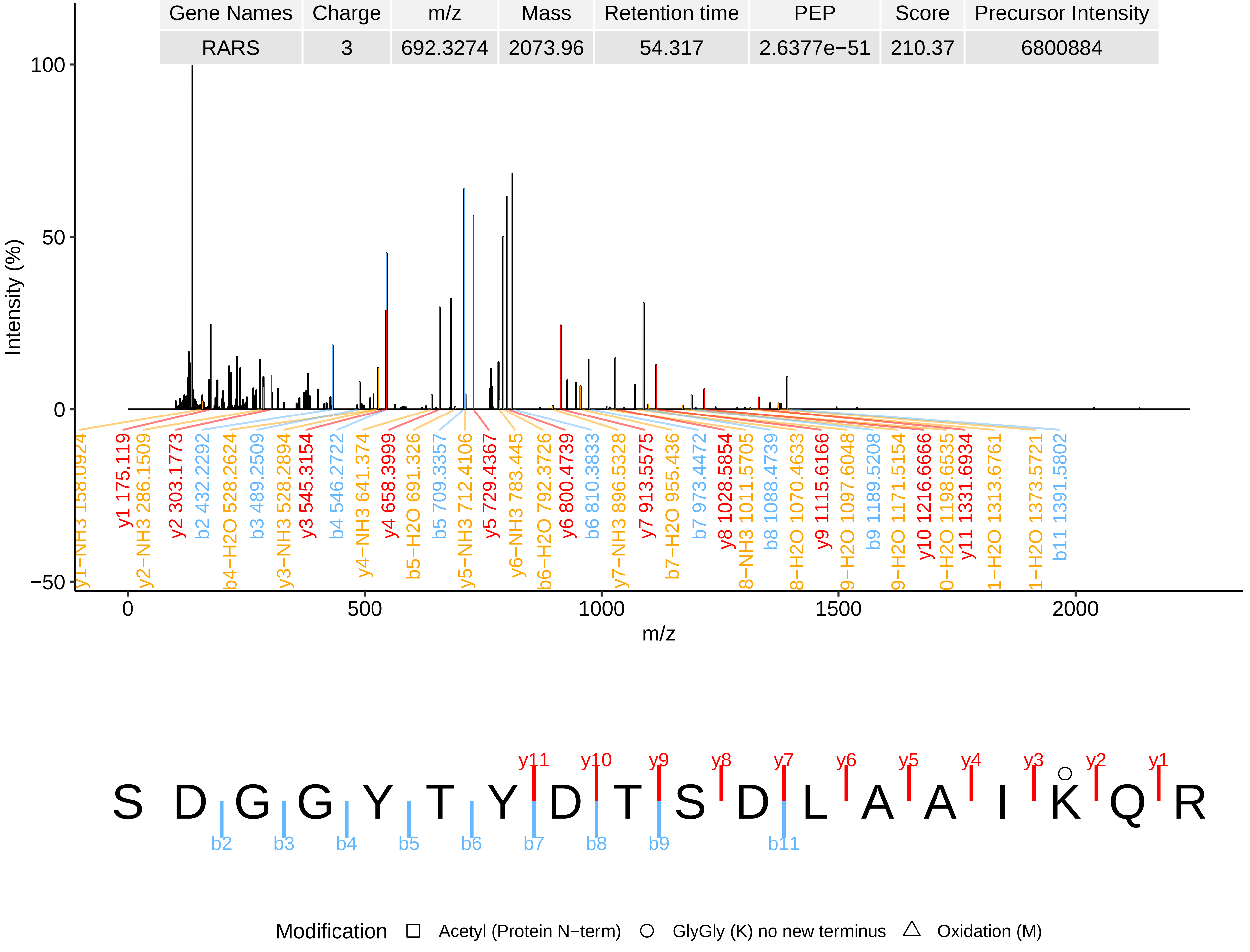

20200921_KIZ_MUb4_GlyGly_10plex_2 − Scan number: 14038

Gene Names	Charge	m/z	Mass	Retention time	PEP	Score	Precursor Intensity
RARS	3	692.3274	2073.96	54.643	4.4043e−13	117	1673588

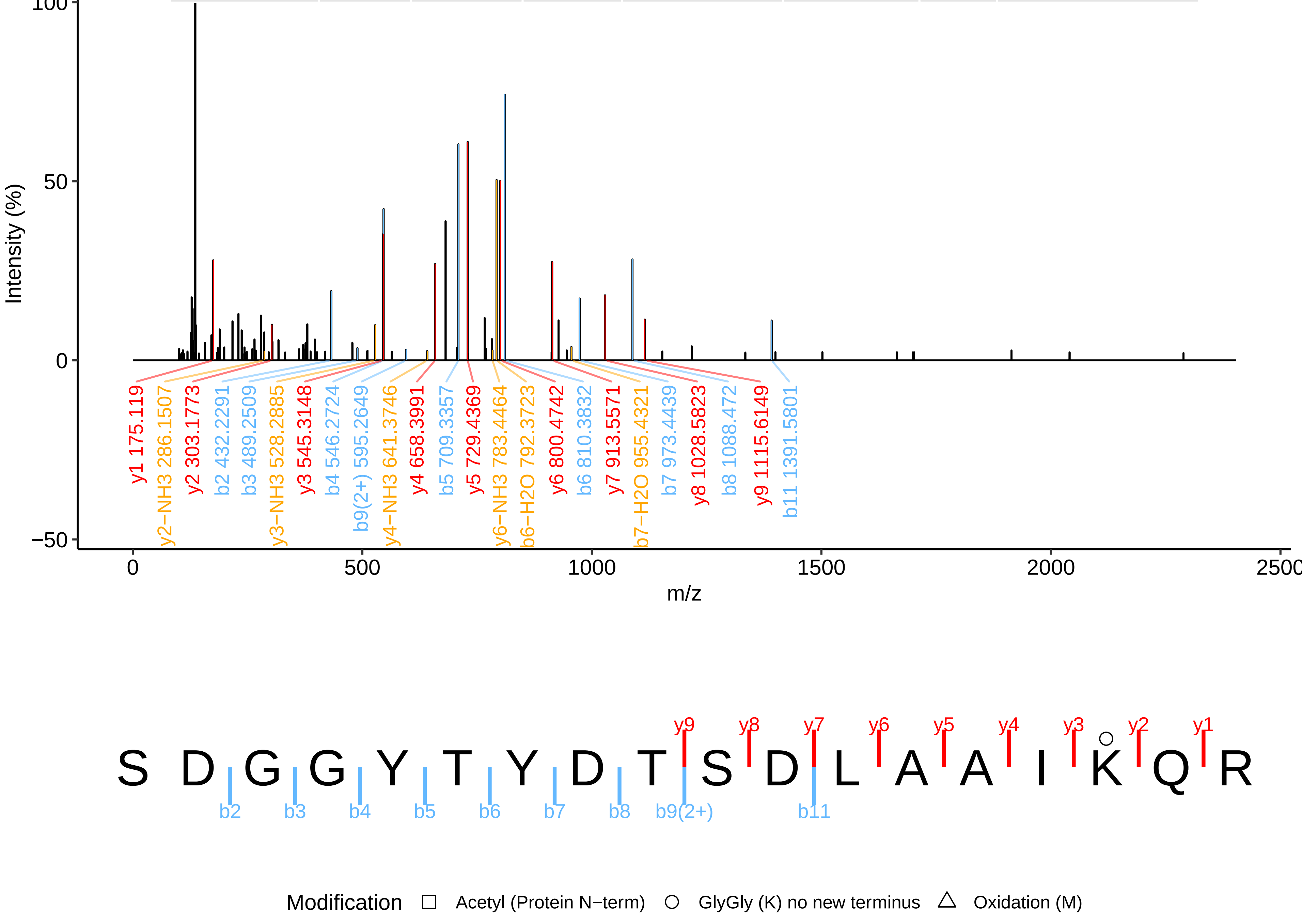

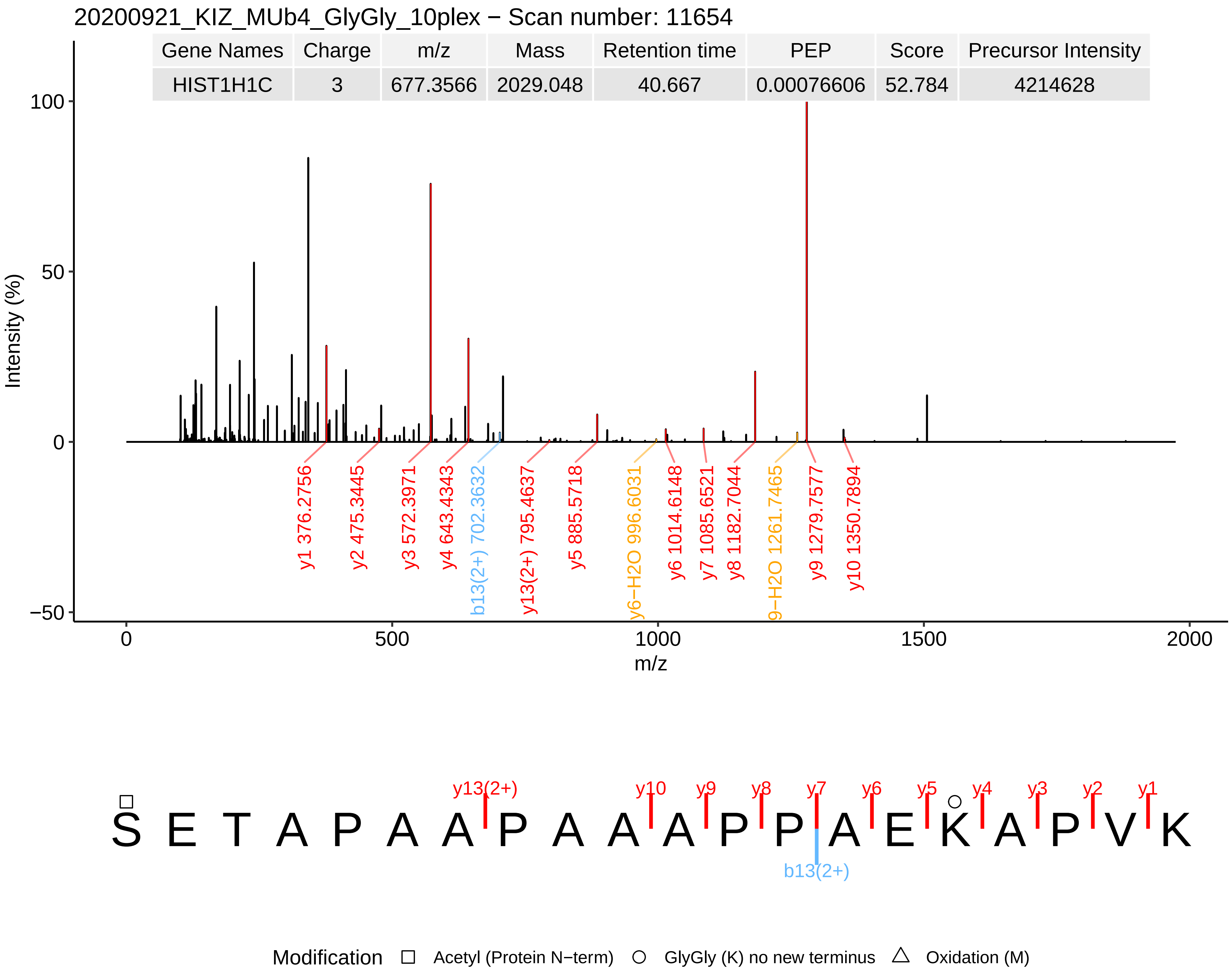

20200921_KIZ_MUb4_GlyGly_10plex − Scan number: 11654
Gene Names | Charge | m/z | Mass | Retention time | PEP | Score | Precursor Intensity
HIST1H1C | 3 | 677.3566 | 2029.048 | 40.667 | 0.00076606 | 52.784 | 4214628
Intensity (%)
m/z
y1 376.2756
y2 475.3445
y3 572.3971
y4 643.4343
b13(2+) 702.3632
y13(2+) 795.4637
y5 885.5718
y6−H2O 996.6031
y6 1014.6148
y7 1085.6521
y8 1182.7044
9−H2O 1261.7465
y9 1279.7577
y10 1350.7894
S E T A P A A P A A A P P A E K A P V K
y13(2+) y10 y9 y8 y7 y6 y5 y4 y3 y2 y1
b13(2+)
Modification
Acetyl (Protein N−term)
GlyGly (K) no new terminus
Oxidation (M)

20200924_KIZ_MUb5_GlyGly_1 − Scan number: 11270

Gene Names	Charge	m/z	Mass	Retention time	PEP	Score	Precursor Intensity
HIST1H1C	3	677.3566	2029.048	36.489	0.00026277	60.564	7493960

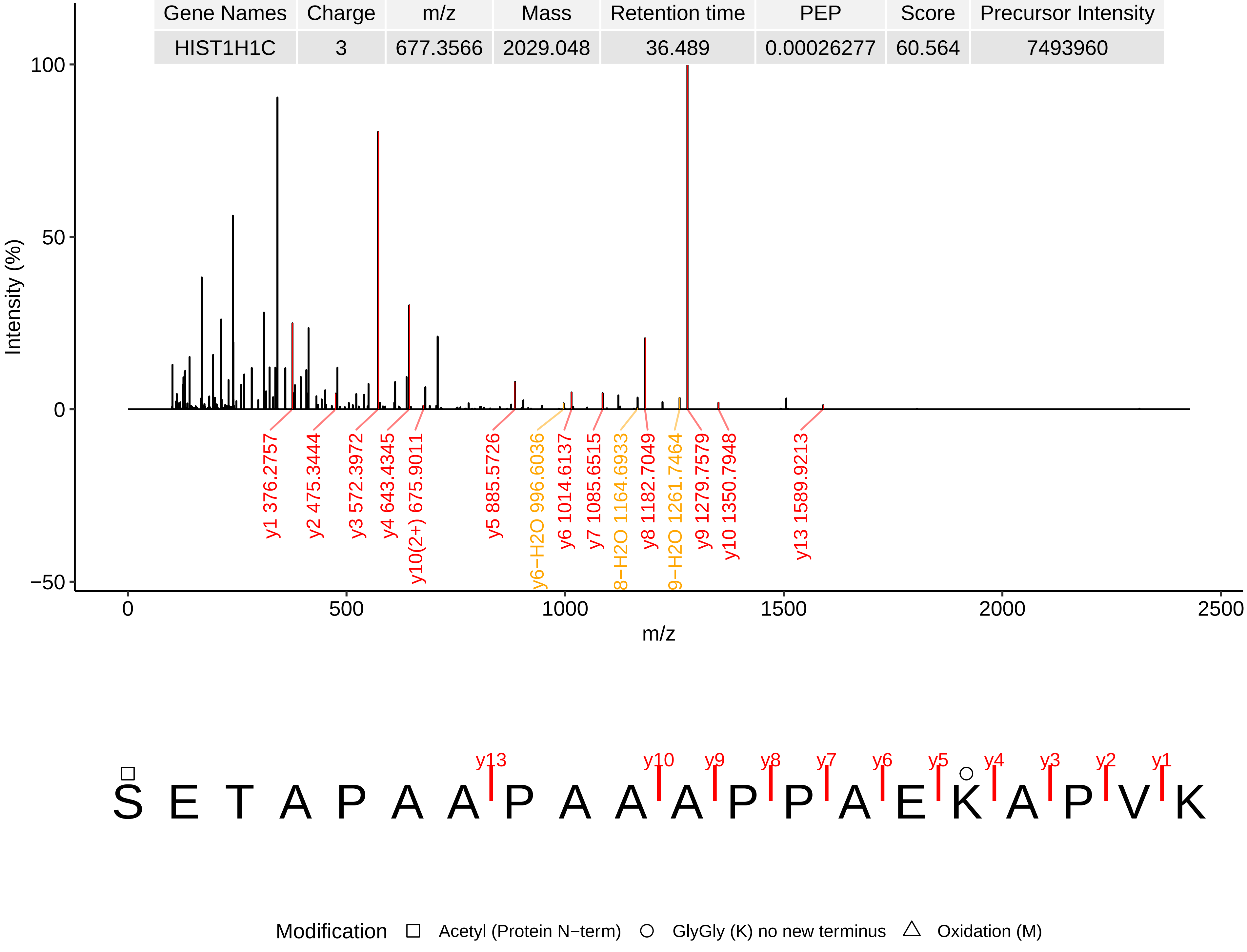

20200921_KIZ_MUb4_GlyGly_10plex − Scan number: 11761

Gene Names	Charge	m/z	Mass	Retention time	PEP	Score	Precursor Intensity
HIST1H1E	3	687.3601	2059.059	41.072	2.8255e−06	57.366	1188284

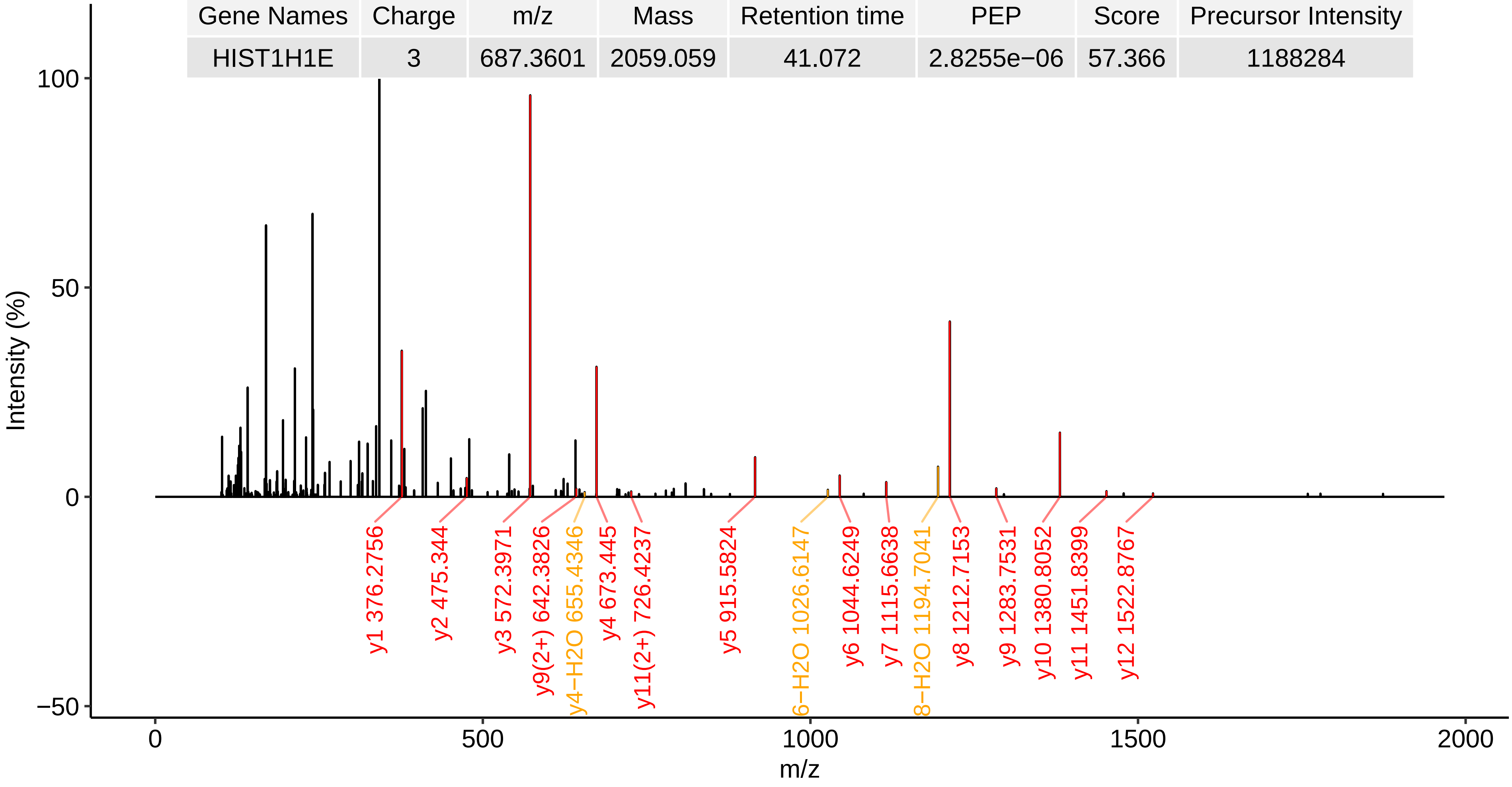

S E T A P A A P A A P A P A E K T P V K

y12 y11 y10 y9 y8 y7 y6 y5 y4 y3 y2 y1

Modification □ Acetyl (Protein N−term) ○ GlyGly (K) no new terminus △ Oxidation (M)

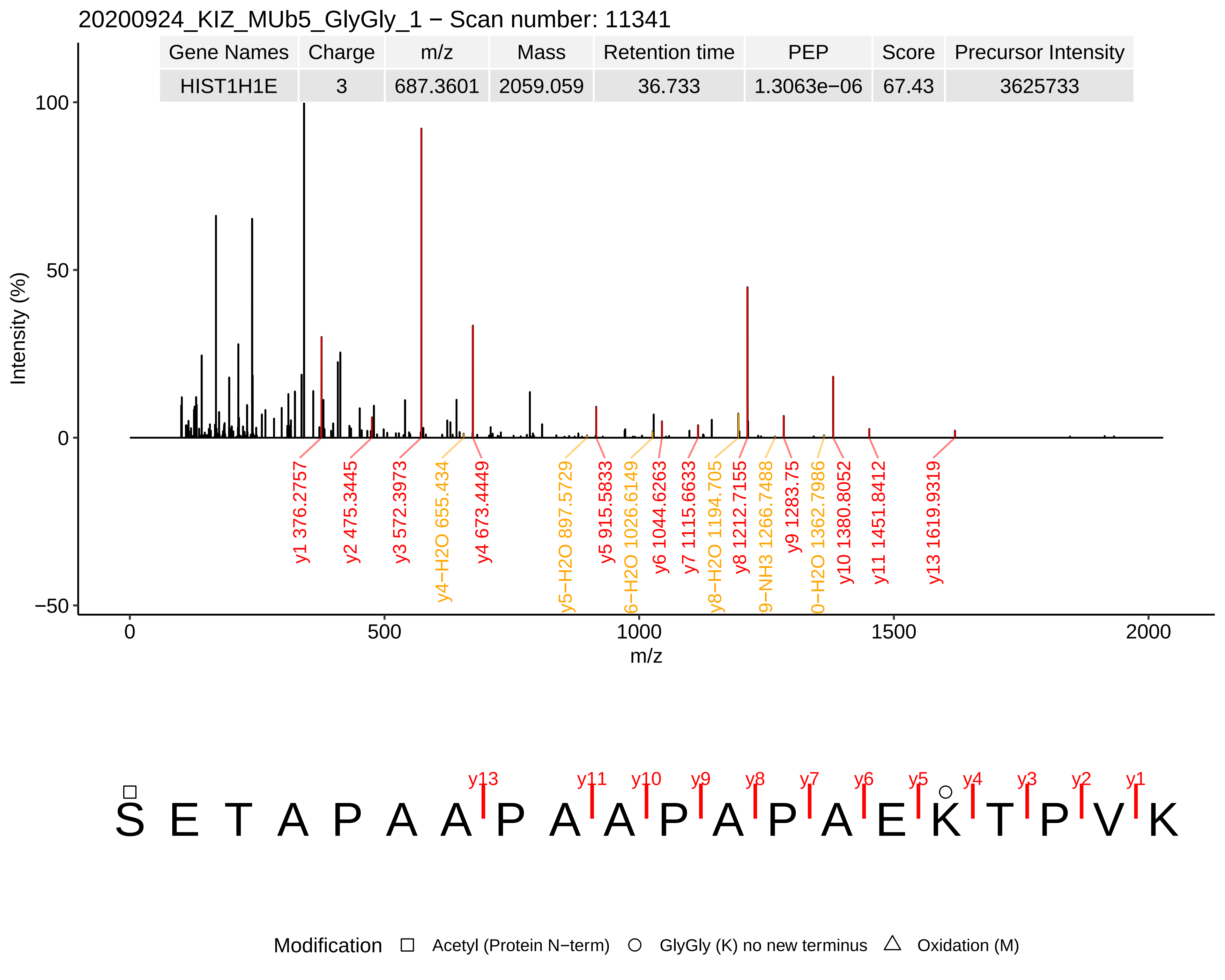

20200924_KIZ_MUb5_GlyGly_1 − Scan number: 11341
Gene Names | Charge | m/z | Mass | Retention time | PEP | Score | Precursor Intensity
HIST1H1E | 3 | 687.3601 | 2059.059 | 36.733 | 1.3063e−06 | 67.43 | 3625733
Intensity (%)
m/z
y1 376.2757
y2 475.3445
y3 572.3973
y4−H2O 655.434
y4 673.4449
y5−H2O 897.5729
y5 915.5833
6−H2O 1026.6149
y6 1044.6263
y7 1115.6633
y8−H2O 1194.705
y8 1212.7155
9−NH3 1266.7488
y9 1283.75
0−H2O 1362.7986
y10 1380.8052
y11 1451.8412
y13 1619.9319
S E T A P A A P A A P A P A E K T P V K
y13 y11 y10 y9 y8 y7 y6 y5 y4 y3 y2 y1
Modification
Acetyl (Protein N−term)
GlyGly (K) no new terminus
Oxidation (M)

20200921_KIZ_MUb4_GlyGly_10plex − Scan number: 14794

Gene Names	Charge	m/z	Mass	Retention time	PEP	Score	Precursor Intensity
SLC2A3	3	602.9502	1805.829	52.662	0.0011752	73.983	4638892

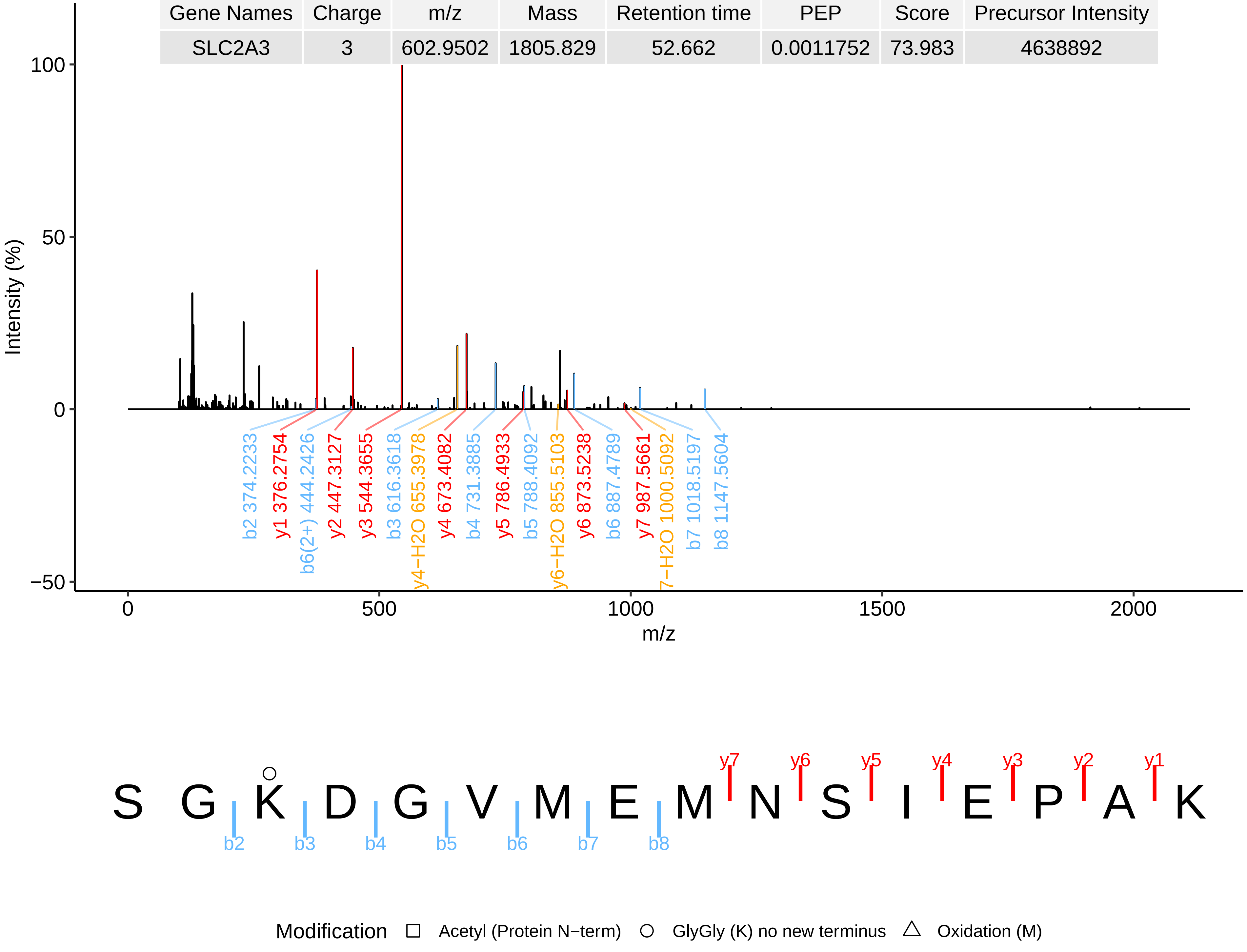

20200921_KIZ_MUb4_GlyGly_10plex_2 − Scan number: 13616

Gene Names	Charge	m/z	Mass	Retention time	PEP	Score	Precursor Intensity
SLC2A3	3	602.9502	1805.829	52.909	0.0541	52.453	709228.4

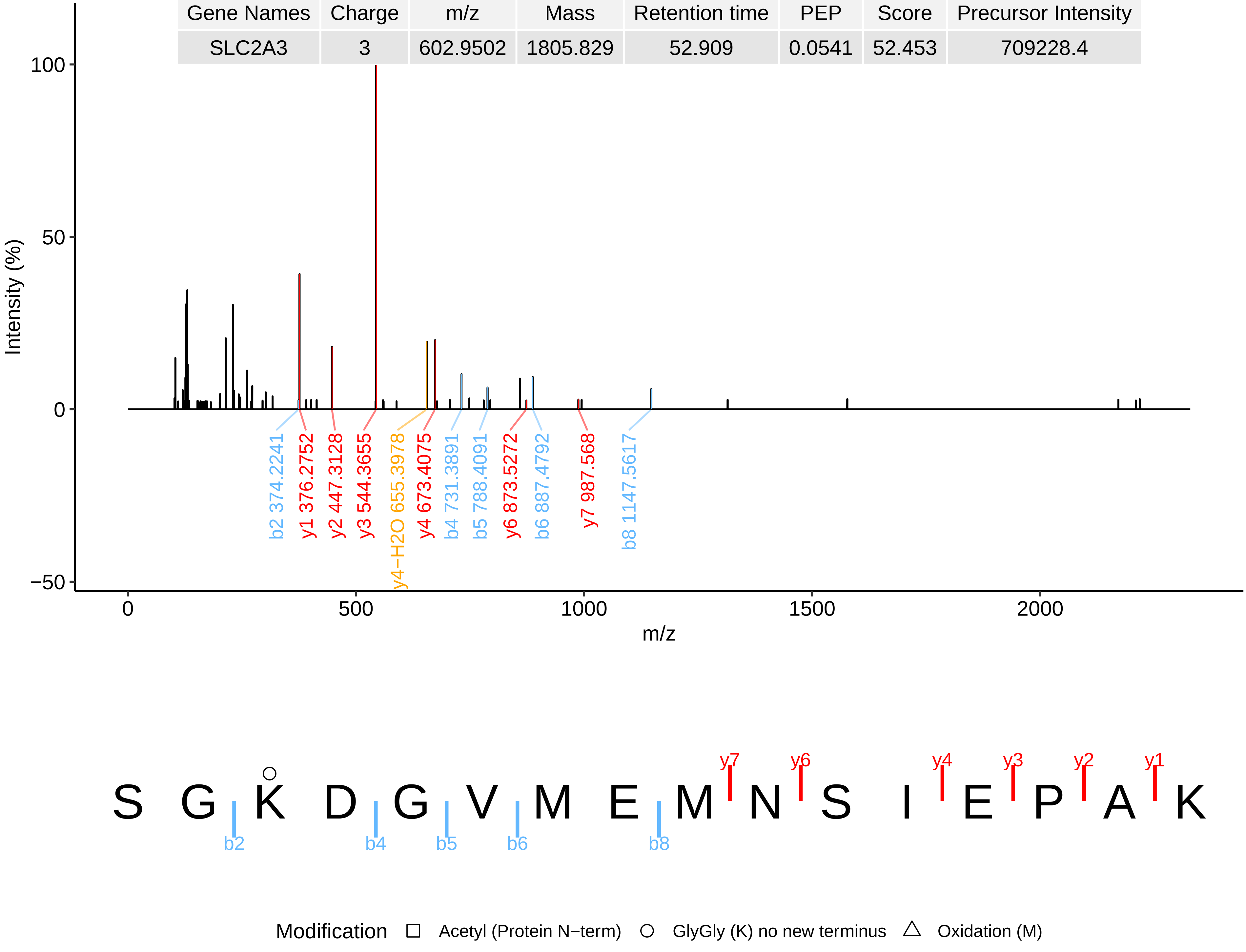

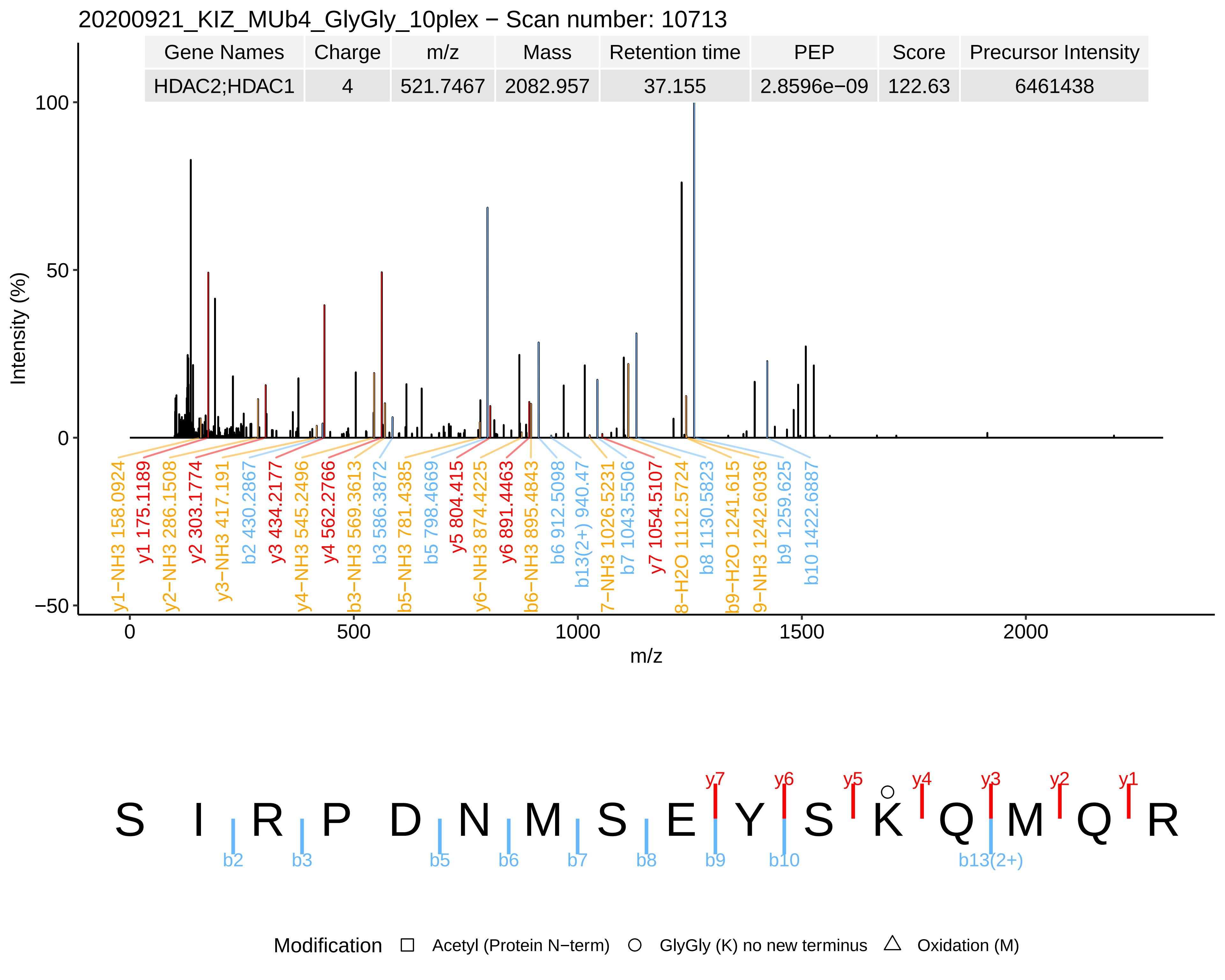
20200921_KIZ_MUb4_GlyGly_10plex − Scan number: 10713
Gene Names | Charge | m/z | Mass | Retention time | PEP | Score | Precursor Intensity
HDAC2;HDAC1 | 4 | 521.7467 | 2082.957 | 37.155 | 2.8596e−09 | 122.63 | 6461438
Intensity (%)
100
50
0
−50
0
500
1000
1500
2000
m/z
y1−NH3 158.0924
y1 175.1189
y2−NH3 286.1508
y2 303.1774
y3−NH3 417.191
b2 430.2867
y3 434.2177
y4−NH3 545.2496
y4 562.2766
b3−NH3 569.3613
b3 586.3872
b5−NH3 781.4385
b5 798.4669
y5 804.415
y6−NH3 874.4225
y6 891.4463
b6−NH3 895.4843
b6 912.5098
b13(2+) 940.47
y7−NH3 1026.5231
b7 1043.5506
y7 1054.5107
b8−H2O 1112.5724
b8 1130.5823
b9−H2O 1241.615
b9−NH3 1242.6036
b9 1259.625
b10 1422.6887
S I R P D N M S E Y S K Q M Q R
y7 y6 y5 y4 y3 y2 y1
b2 b3 b5 b6 b7 b8 b9 b10 b13(2+)
Modification
Acetyl (Protein N−term)
GlyGly (K) no new terminus
Oxidation (M)

20200921_KIZ_MUb4_GlyGly_10plex_2 − Scan number: 9910

Gene Names	Charge	m/z	Mass	Retention time	PEP	Score	Precursor Intensity
HDAC2;HDAC1	4	521.7467	2082.957	37.79	2.6244e−06	98.943	6794771

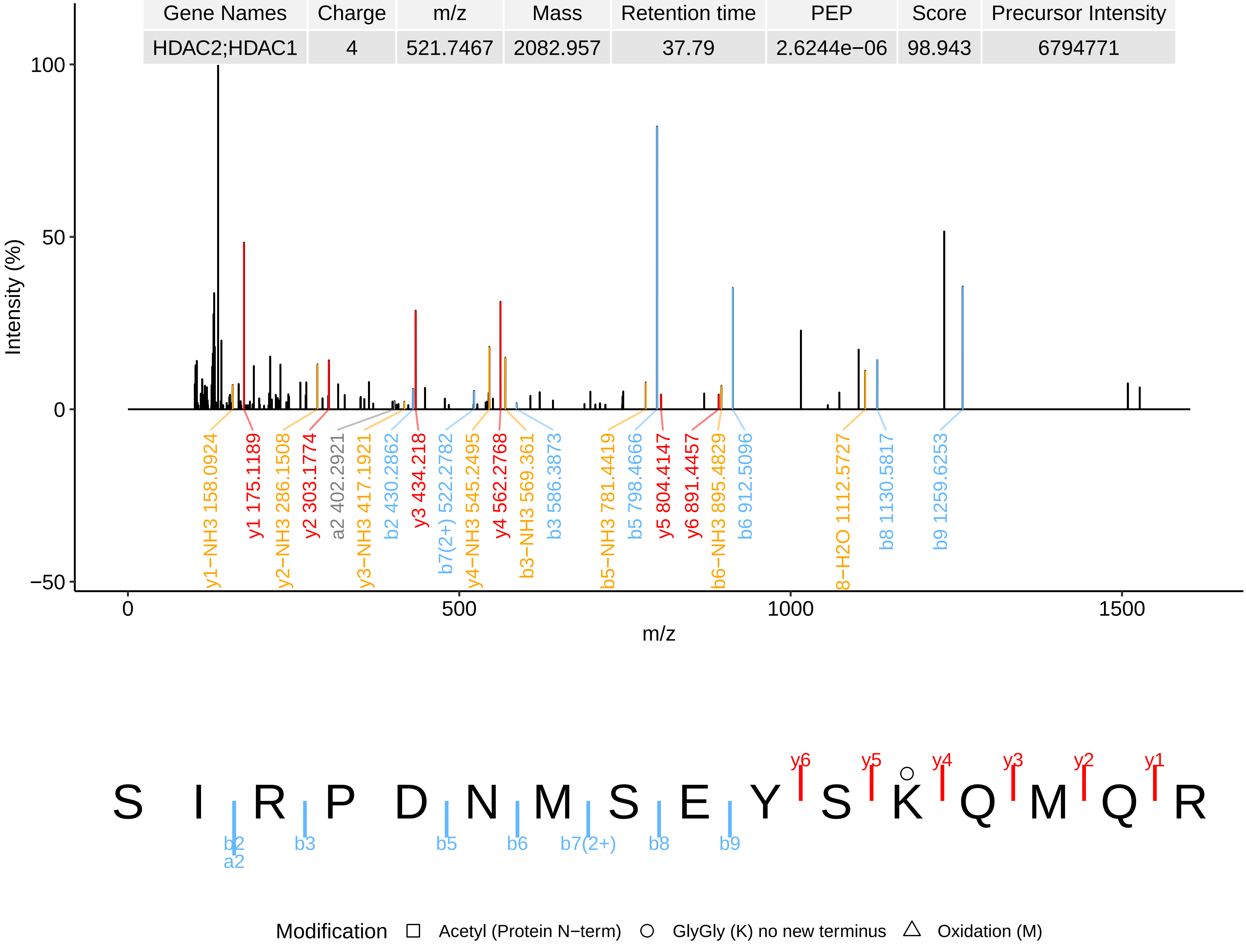

20200921_KIZ_MUb4_GlyGly_10plex_2 − Scan number: 6866

Gene Names	Charge	m/z	Mass	Retention time	PEP	Score	Precursor Intensity
ITFG3	3	629.6328	1885.877	25.415	0.0022929	67.605	1515449

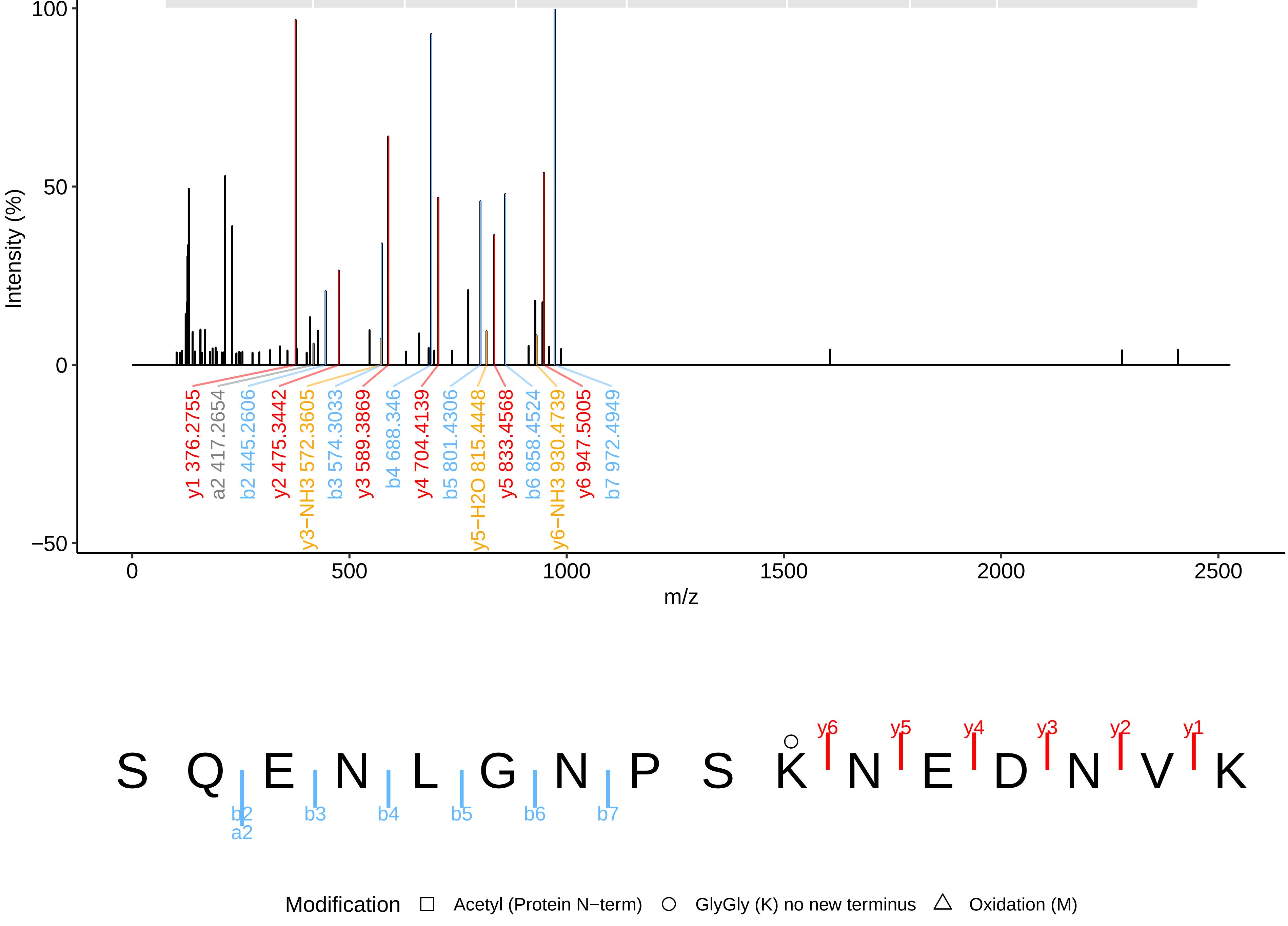

20200921_KIZ_MUb4_GlyGly_10plex_2 − Scan number: 6932

Gene Names	Charge	m/z	Mass	Retention time	PEP	Score	Precursor Intensity
ITFG3	3	629.6328	1885.877	25.684	0.067874	49.559	917596.9

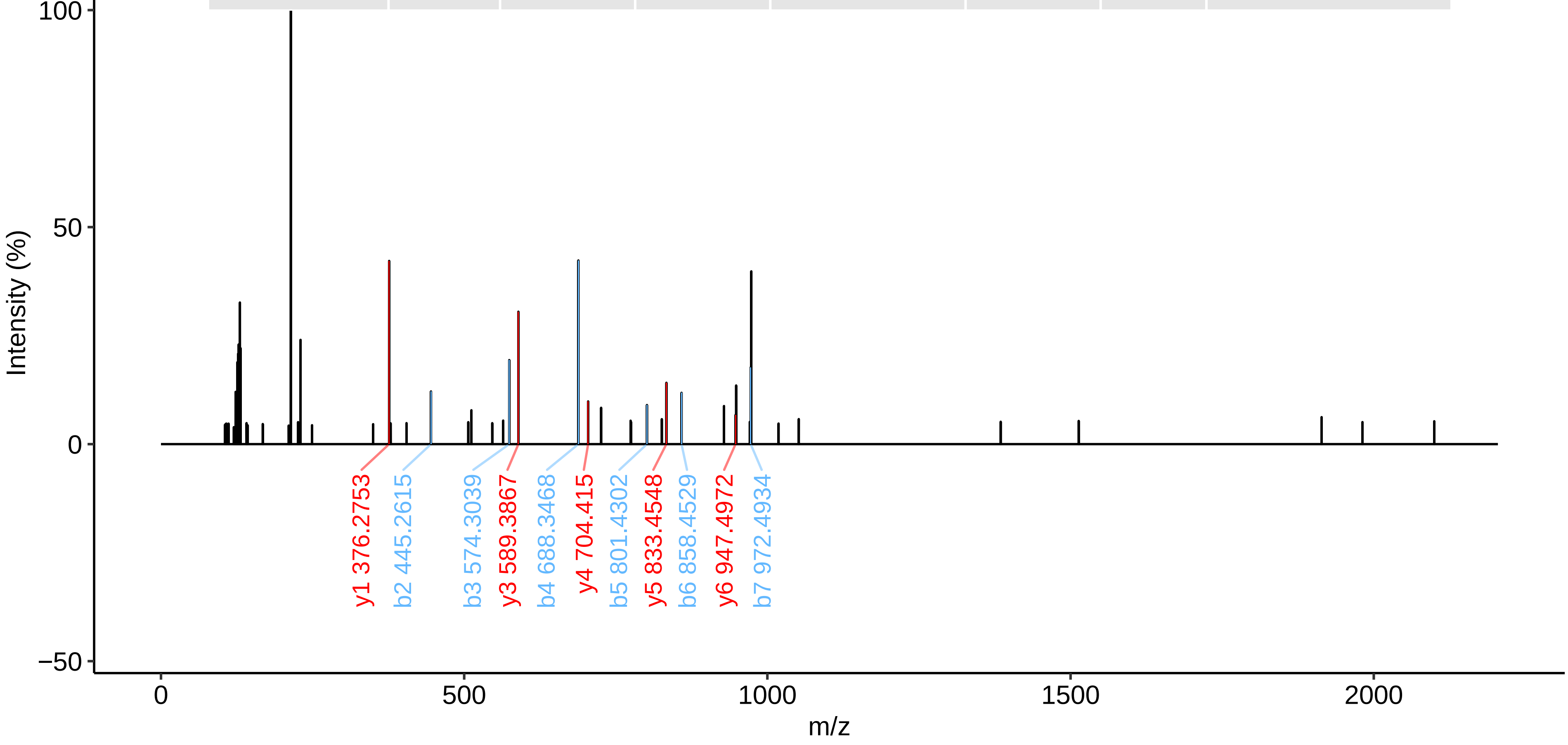

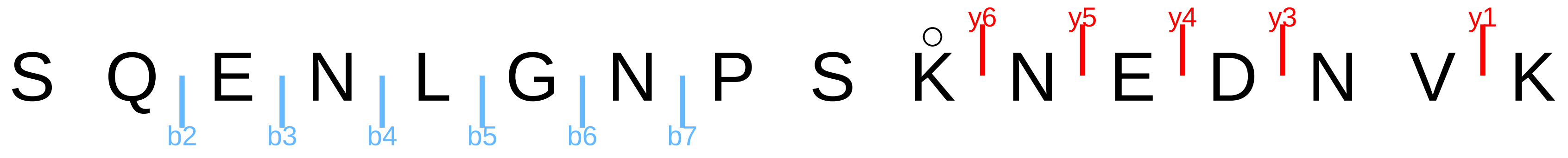

Modification □ Acetyl (Protein N−term) ○ GlyGly (K) no new terminus △ Oxidation (M)

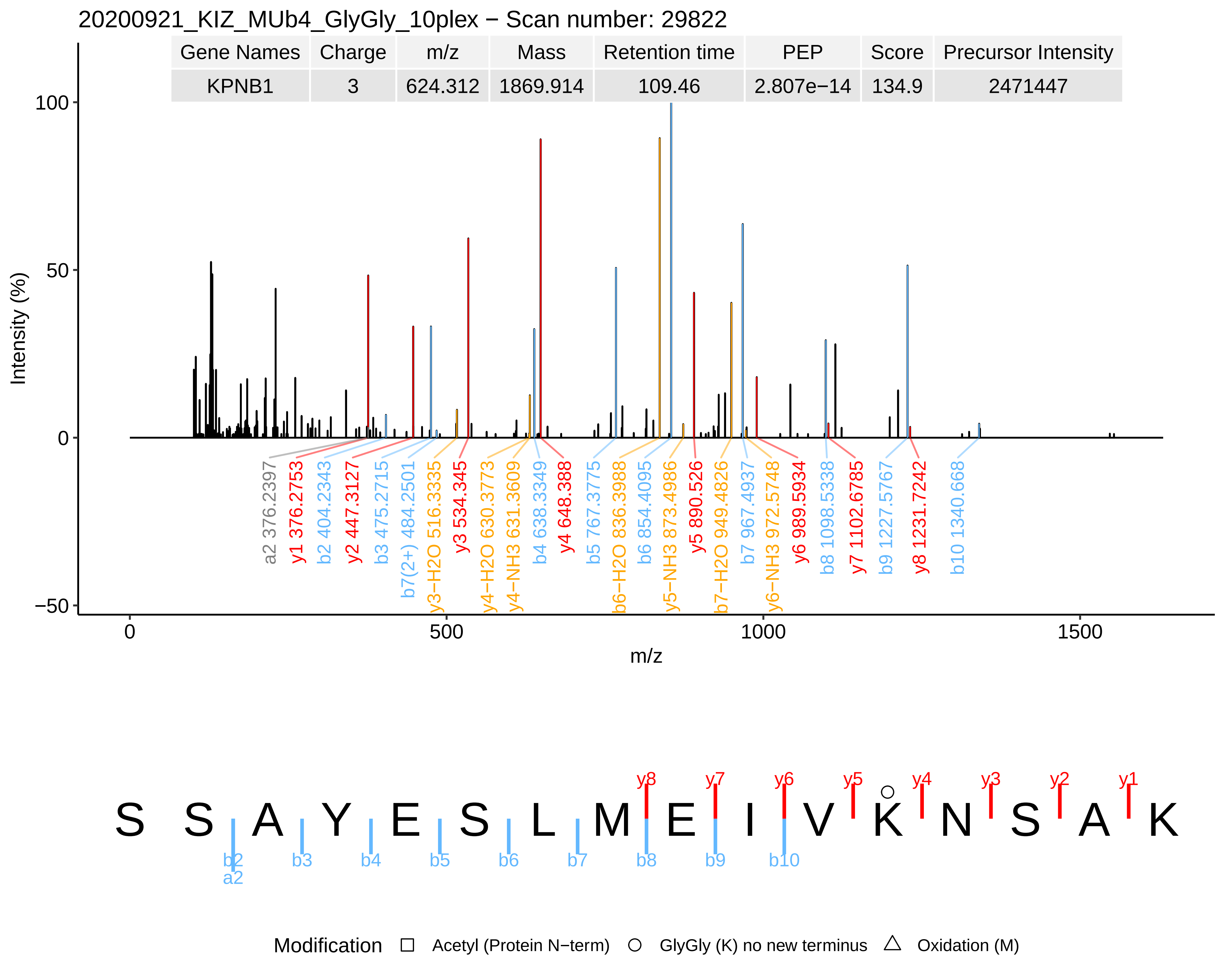
20200921_KIZ_MUb4_GlyGly_10plex − Scan number: 29822
Gene Names | Charge | m/z | Mass | Retention time | PEP | Score | Precursor Intensity
KPNB1 | 3 | 624.312 | 1869.914 | 109.46 | 2.807e−14 | 134.9 | 2471447
Intensity (%)
m/z
a2 376.2397
y1 376.2753
b2 404.2343
y2 447.3127
b3 475.2715
b7(2+) 484.2501
y3−H2O 516.3335
y3 534.345
y4−H2O 630.3773
y4−NH3 631.3609
b4 638.3349
y4 648.388
b5 767.3775
b6−H2O 836.3988
b6 854.4095
y5−NH3 873.4986
y5 890.526
b7−H2O 949.4826
b7 967.4937
y6−NH3 972.5748
y6 989.5934
b8 1098.5338
y7 1102.6785
b9 1227.5767
y8 1231.7242
b10 1340.668
S S A Y E S L M E I V K N S A K
Modification
Acetyl (Protein N−term)
GlyGly (K) no new terminus
Oxidation (M)

20200921_KIZ_MUb4_GlyGly_10plex − Scan number: 14396

Gene Names	Charge	m/z	Mass	Retention time	PEP	Score	Precursor Intensity
PFN1	3	613.3145	1836.922	51.156	6.1564e−17	140.22	7492598

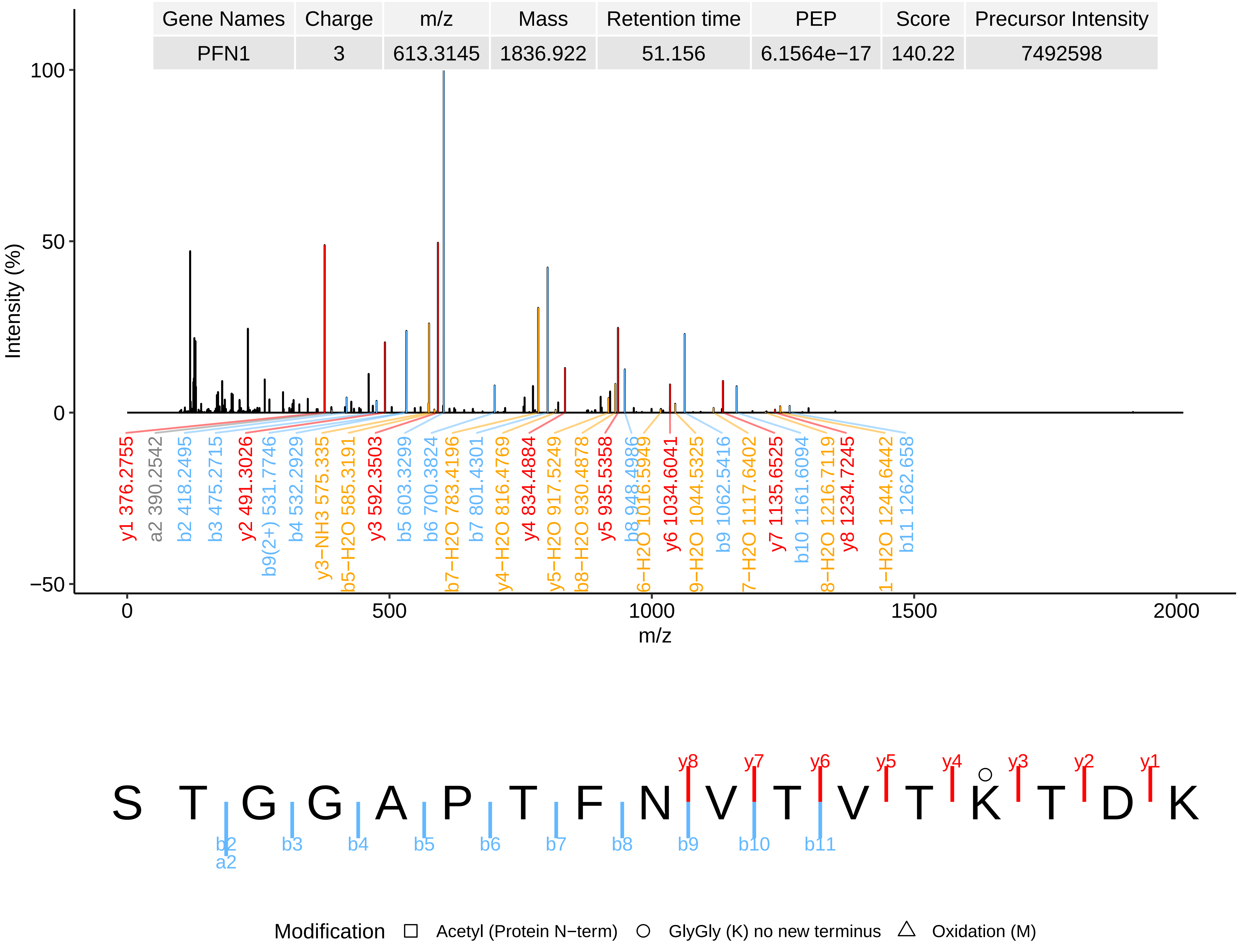

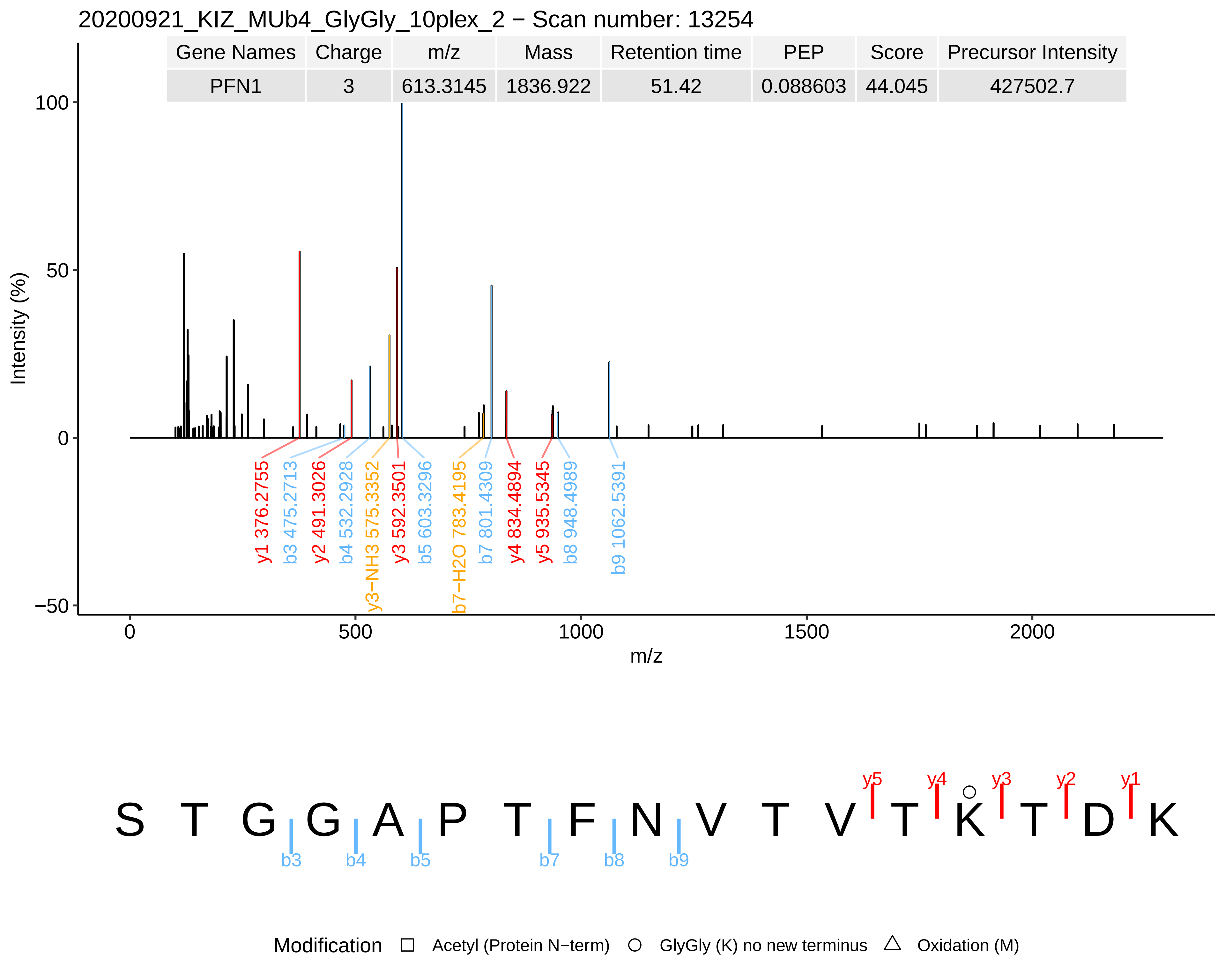
20200921_KIZ_MUb4_GlyGly_10plex_2 − Scan number: 13254
Gene Names
Charge
m/z
Mass
Retention time
PEP
Score
Precursor Intensity
PFN1
3
613.3145
1836.922
51.42
0.088603
44.045
427502.7
Intensity (%)
100
50
0
−50
0
500
1000
1500
2000
m/z
y1 376.2755
b3 475.2713
y2 491.3026
b4 532.2928
y3−NH3 575.3352
y3 592.3501
b5 603.3296
b7−H2O 783.4195
b7 801.4309
y4 834.4894
y5 935.5345
b8 948.4989
b9 1062.5391
S T G G A P T F N V T V T K T D K
y5
y4
y3
y2
y1
b3
b4
b5
b7
b8
b9
Modification
Acetyl (Protein N−term)
GlyGly (K) no new terminus
Oxidation (M)

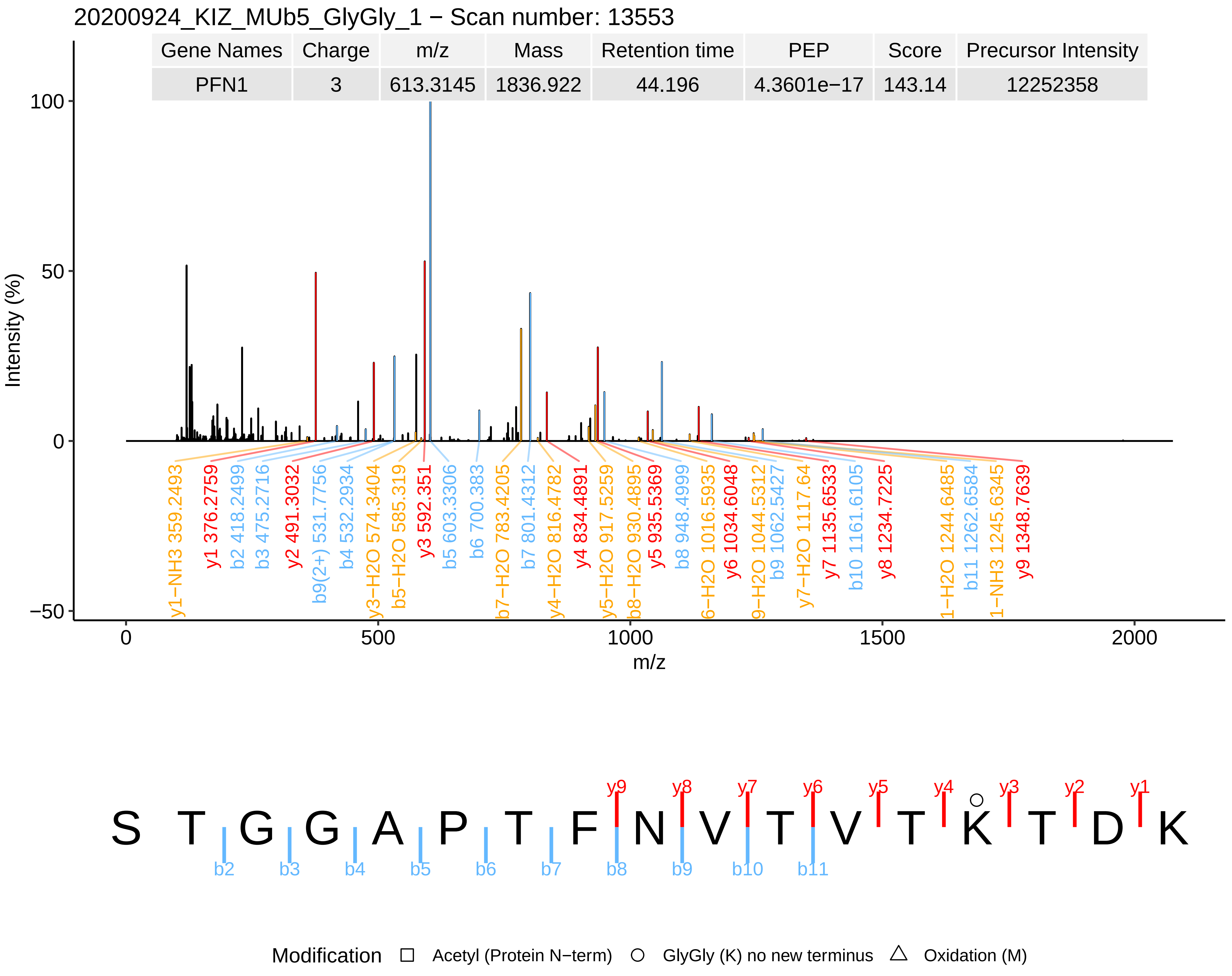

20200924_KIZ_MUb5_GlyGly_1 − Scan number: 13553
Gene Names | Charge | m/z | Mass | Retention time | PEP | Score | Precursor Intensity
PFN1 | 3 | 613.3145 | 1836.922 | 44.196 | 4.3601e−17 | 143.14 | 12252358
Intensity (%)
100
50
0
−50
m/z
0
500
1000
1500
2000
y1−NH3 359.2493
y1 376.2759
b2 418.2499
b3 475.2716
y2 491.3032
b9(2+) 531.7756
b4 532.2934
y3−H2O 574.3404
b5−H2O 585.319
y3 592.351
b5 603.3306
b6 700.383
b7−H2O 783.4205
b7 801.4312
y4−H2O 816.4782
y4 834.4891
y5−H2O 917.5259
b8−H2O 930.4895
y5 935.5369
b8 948.4999
6−H2O 1016.5935
y6 1034.6048
9−H2O 1044.5312
b9 1062.5427
y7−H2O 1117.64
y7 1135.6533
b10 1161.6105
y8 1234.7225
1−H2O 1244.6485
b11 1262.6584
1−NH3 1245.6345
y9 1348.7639
S T G G A P T F N V T V T K T D K
y9 y8 y7 y6 y5 y4 y3 y2 y1
b2 b3 b4 b5 b6 b7 b8 b9 b10 b11
Modification
Acetyl (Protein N−term)
GlyGly (K) no new terminus
Oxidation (M)

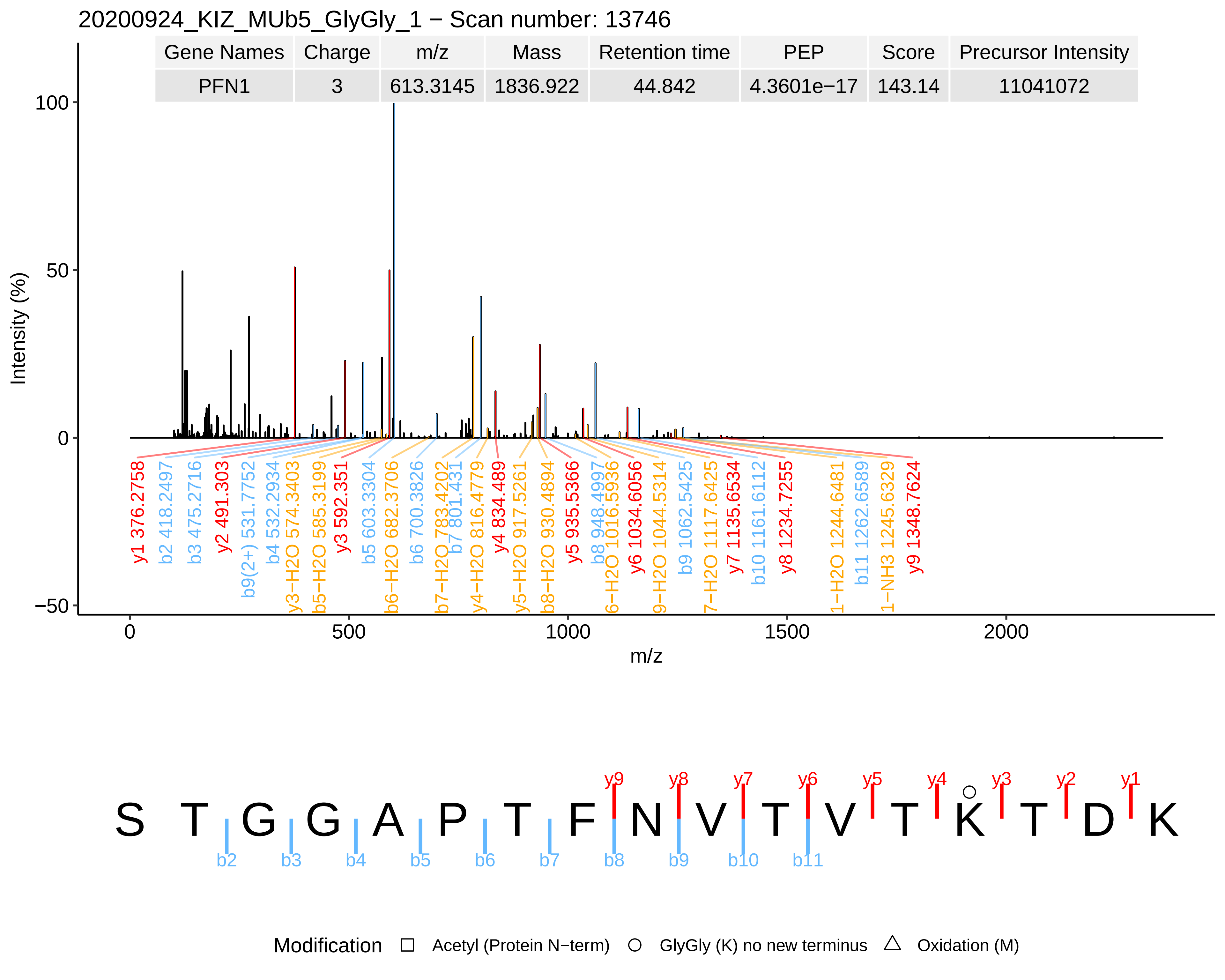
20200924_KIZ_MUb5_GlyGly_1 − Scan number: 13746
Gene Names | Charge | m/z | Mass | Retention time | PEP | Score | Precursor Intensity
PFN1 | 3 | 613.3145 | 1836.922 | 44.842 | 4.3601e−17 | 143.14 | 11041072
Intensity (%)
100
50
0
−50
m/z
0
500
1000
1500
2000
y1 376.2758
b2 418.2497
b3 475.2716
y2 491.303
b9(2+) 531.7752
b4 532.2934
y3−H2O 574.3403
b5−H2O 585.3199
y3 592.351
b5 603.3304
b6−H2O 682.3706
b6 700.3826
b7−H2O 783.4202
b7 801.431
y4−H2O 816.4779
y4 834.489
y5−H2O 917.5261
b8−H2O 930.4894
y5 935.5366
b8 948.4997
y6−H2O 1016.5936
y6 1034.6056
y9−H2O 1044.5314
b9 1062.5425
y7−H2O 1117.6425
y7 1135.6534
b10 1161.6112
y8 1234.7255
b11−H2O 1244.6481
b11 1262.6589
b11−NH3 1245.6329
y9 1348.7624
S T G G A P T F N V T V T K T D K
y9 y8 y7 y6 y5 y4 y3 y2 y1
b2 b3 b4 b5 b6 b7 b8 b9 b10 b11
Modification
Acetyl (Protein N−term)
GlyGly (K) no new terminus
Oxidation (M)

20200924_KIZ_MUb5_GlyGly_1 − Scan number: 13596

Gene Names	Charge	m/z	Mass	Retention time	PEP	Score	Precursor Intensity
PFN1	3	613.3145	1836.922	44.338	9.0834e−17	147.7	23095146

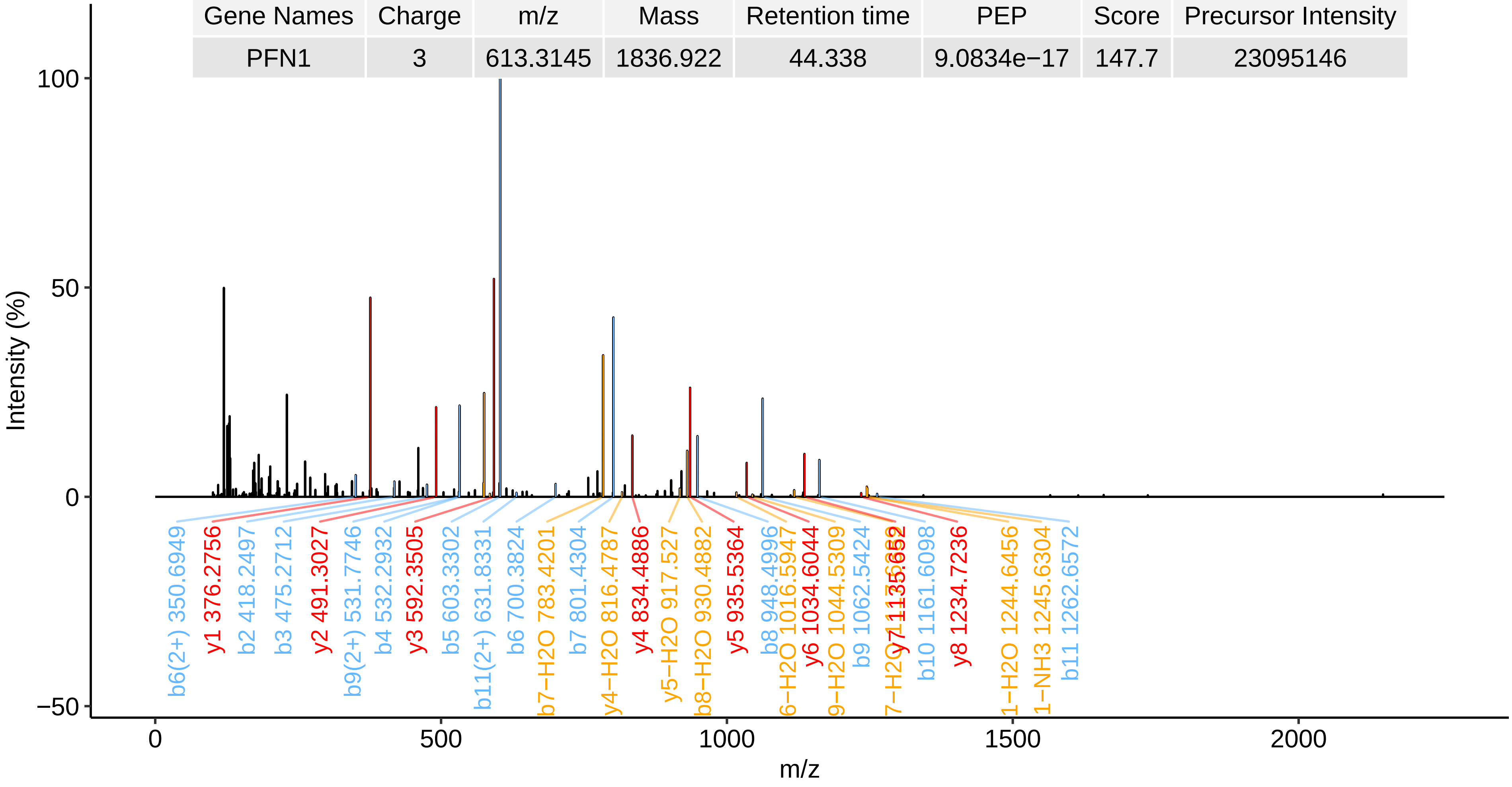

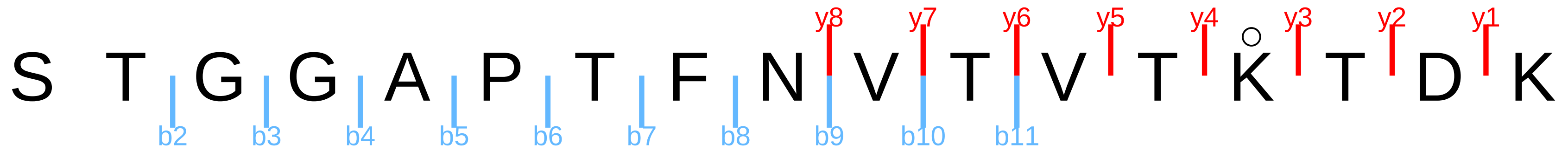

Modification □ Acetyl (Protein N−term) ○ GlyGly (K) no new terminus △ Oxidation (M)

20200924_KIZ_MUb5_GlyGly_2 − Scan number: 12613

Gene Names	Charge	m/z	Mass	Retention time	PEP	Score	Precursor Intensity
PFN1	3	613.3145	1836.922	47.153	9.7143e−10	98.507	2511556

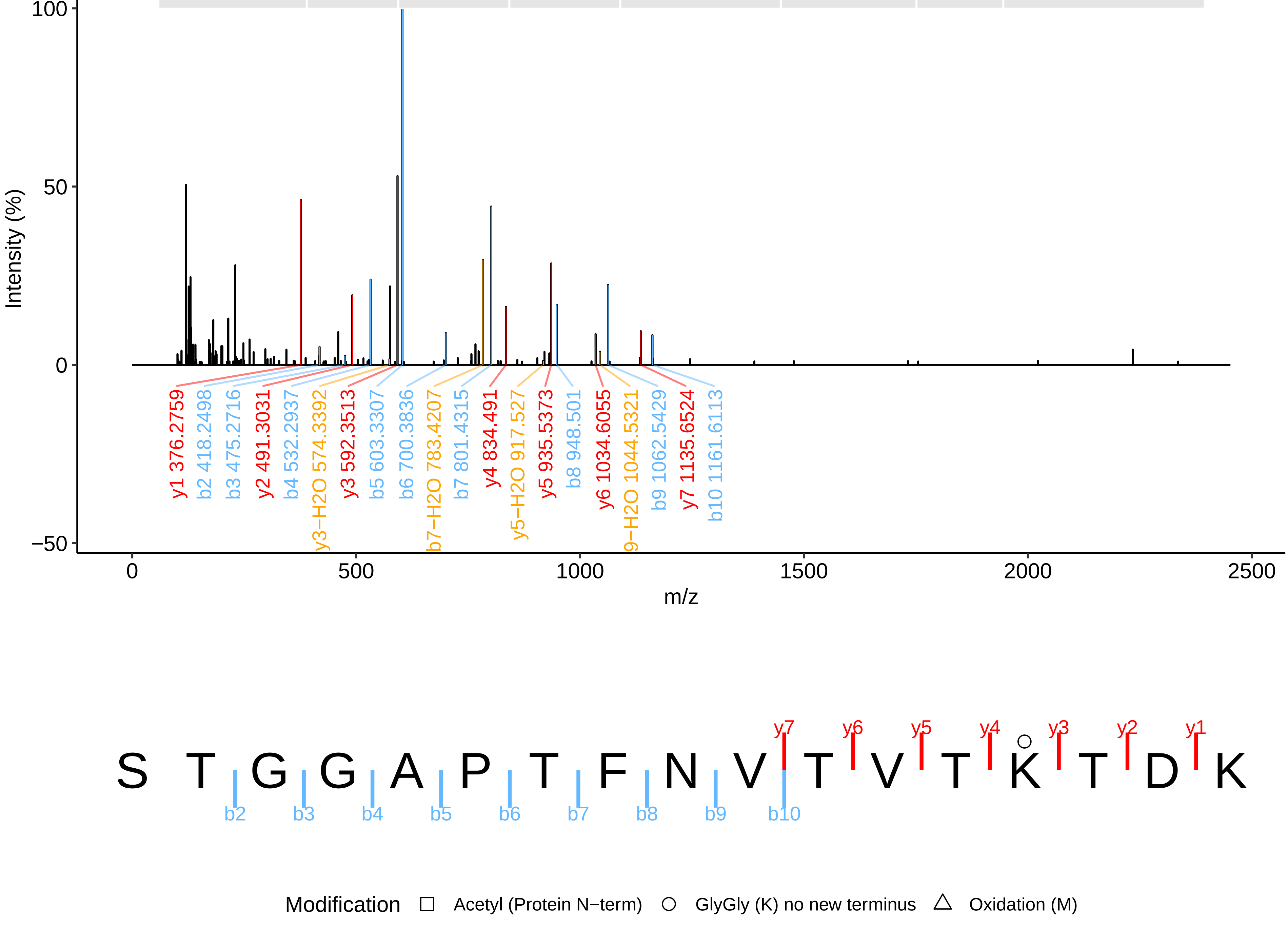

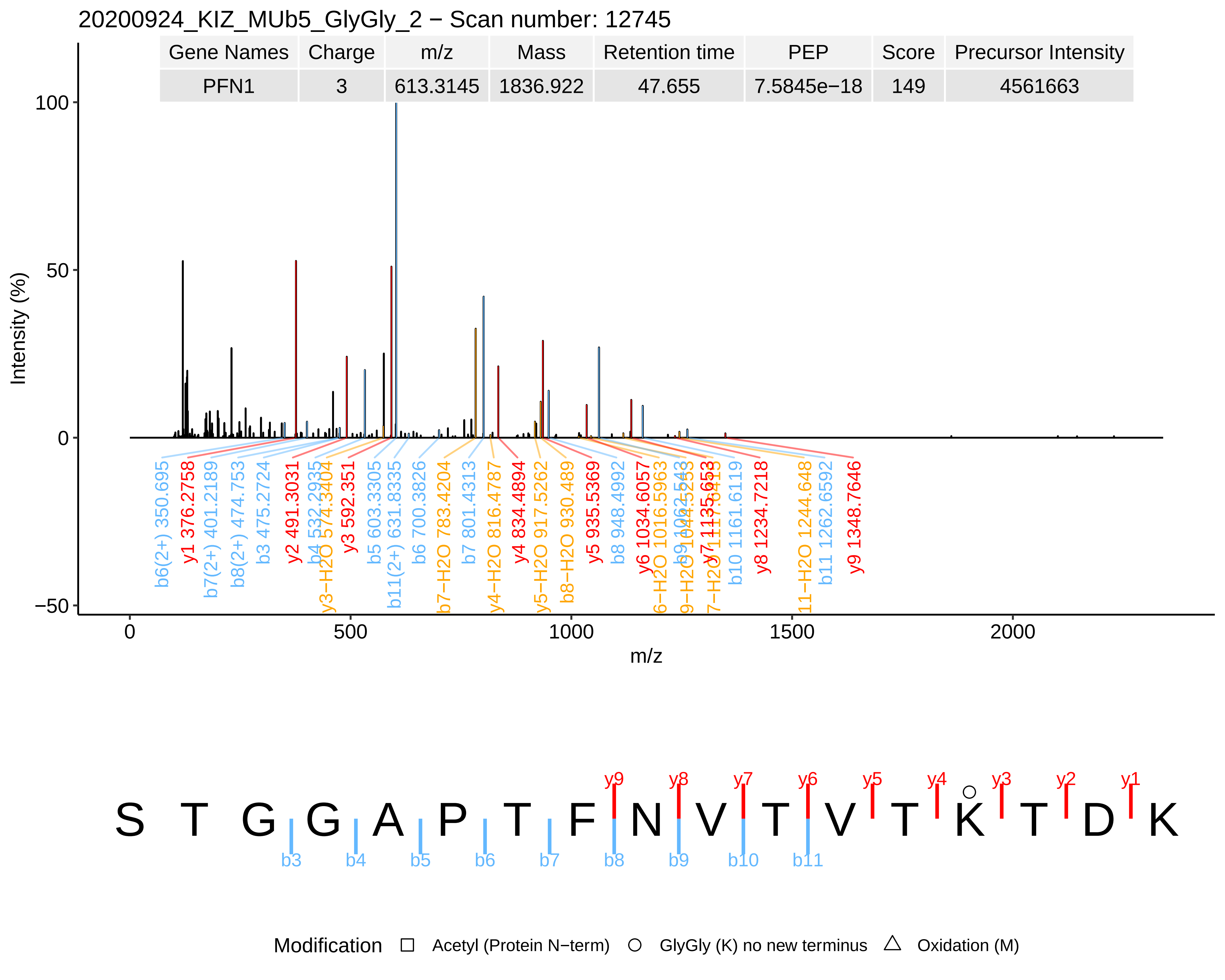
20200924_KIZ_MUb5_GlyGly_2 − Scan number: 12745
Gene Names | Charge | m/z | Mass | Retention time | PEP | Score | Precursor Intensity
PFN1 | 3 | 613.3145 | 1836.922 | 47.655 | 7.5845e−18 | 149 | 4561663
Intensity (%)
100
50
0
−50
0
500
1000
1500
2000
m/z
b6(2+) 350.695
y1 376.2758
b7(2+) 401.2189
b8(2+) 474.753
b3 475.2724
y2 491.3031
b4 532.2935
y3−H2O 574.3404
y3 592.351
b5 603.3305
b11(2+) 631.8335
b6 700.3826
b7−H2O 783.4204
b7 801.4313
y4−H2O 816.4787
y4 834.4894
y5−H2O 917.5262
b8−H2O 930.489
y5 935.5369
b8 948.4992
y6 1034.6057
y6−H2O 1016.5963
b10 1161.6119
y8 1234.7218
y11−H2O 1244.648
b11 1262.6592
y9 1348.7646
S T G G A P T F N V T V T K T D K
y9 y8 y7 y6 y5 y4 y3 y2 y1
b3 b4 b5 b6 b7 b8 b9 b10 b11
Modification
Acetyl (Protein N−term)
GlyGly (K) no new terminus
Oxidation (M)

20200924_KIZ_MUb5_GlyGly_2 − Scan number: 12582

Gene Names	Charge	m/z	Mass	Retention time	PEP	Score	Precursor Intensity
PFN1	3	613.3145	1836.922	47.037	5.9616e−08	106.36	NA

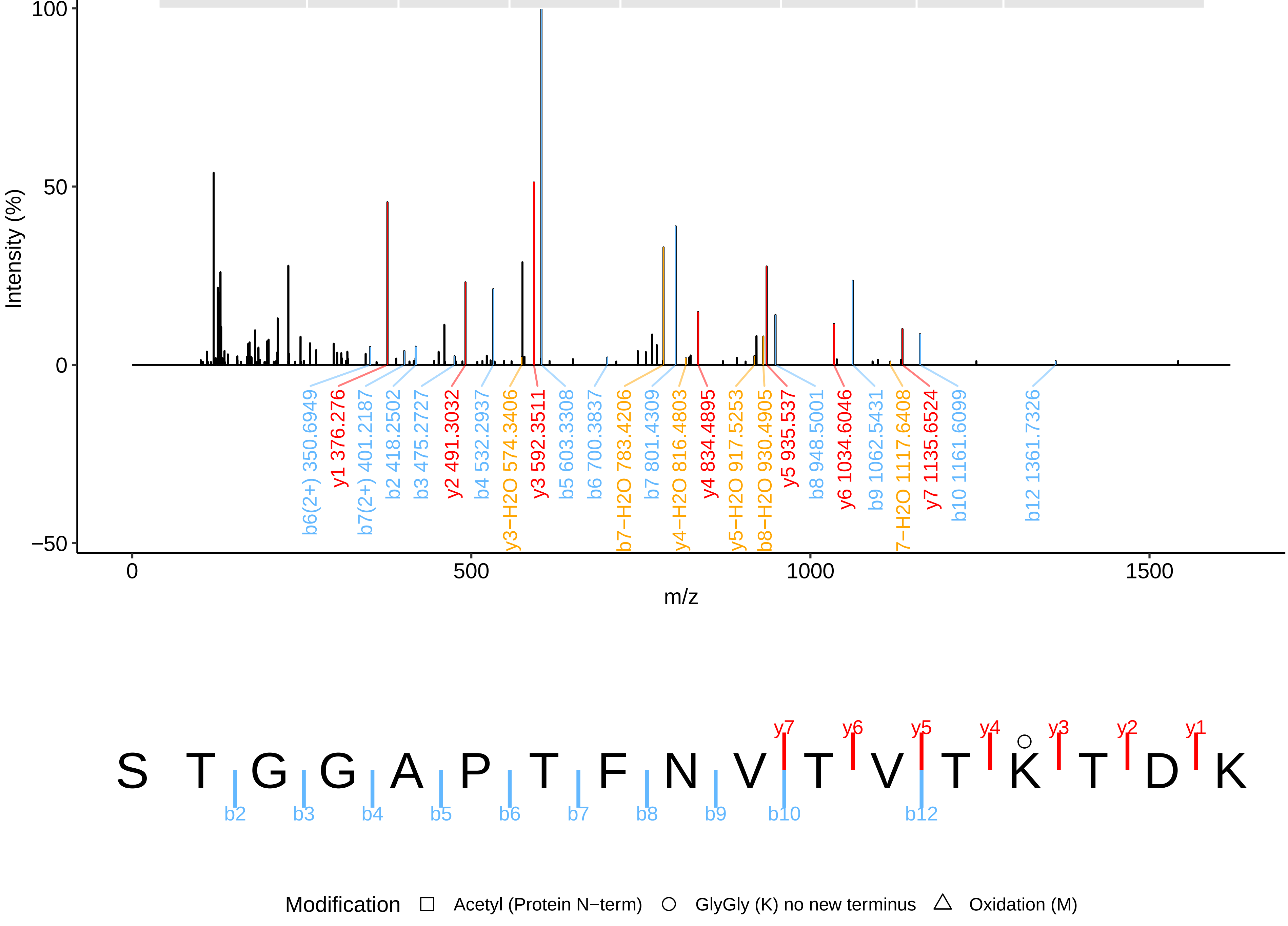

20201118_KIZ_MUb6_GlyGly_1 − Scan number: 13833

Gene Names	Charge	m/z	Mass	Retention time	PEP	Score	Precursor Intensity
PFN1	3	613.3145	1836.922	43.752	4.9183e−09	114.29	NA

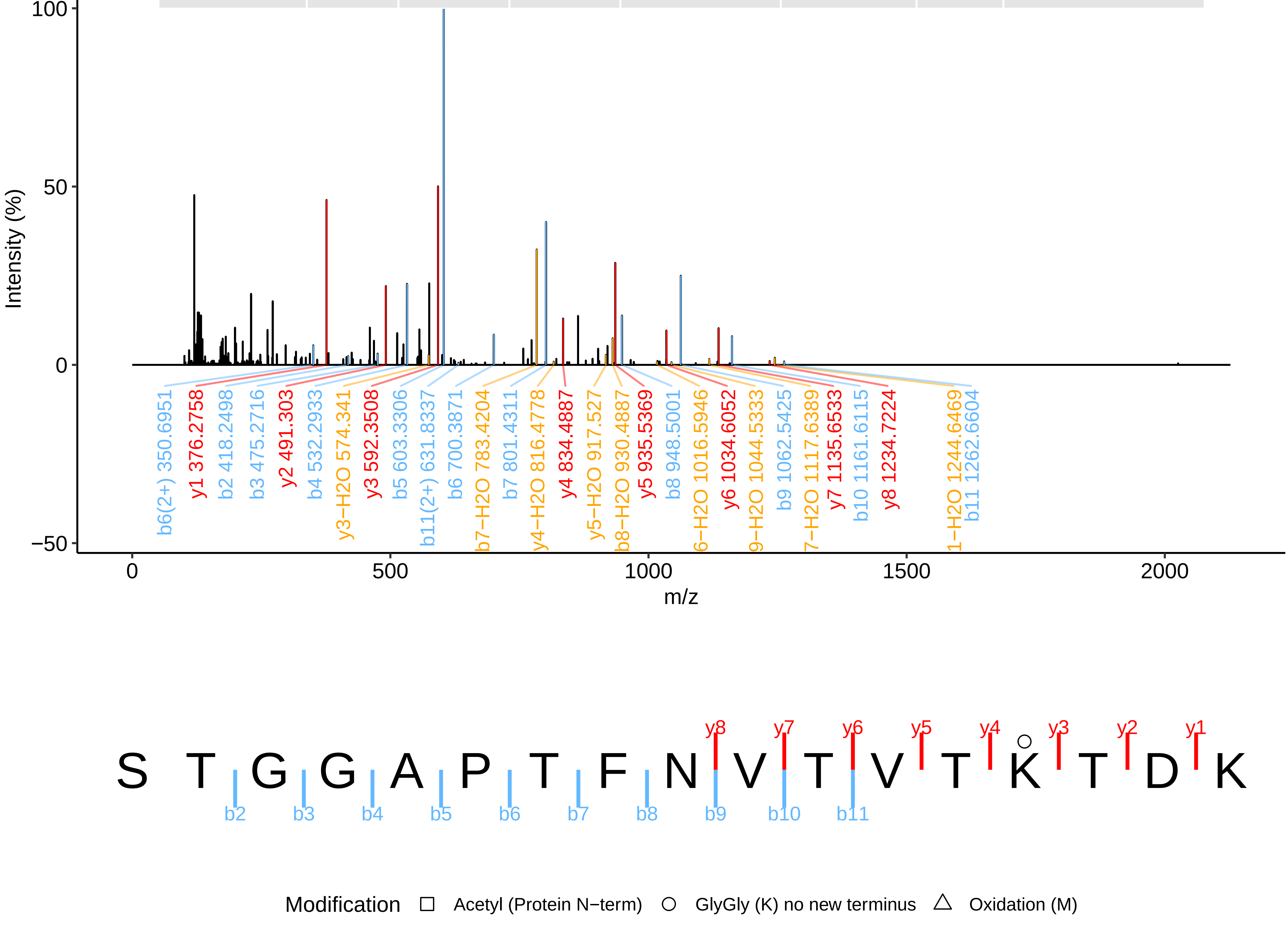

20201118_KIZ_MUb6_GlyGly_2 − Scan number: 15459

Gene Names	Charge	m/z	Mass	Retention time	PEP	Score	Precursor Intensity
PFN1	3	613.3145	1836.922	52.146	9.181e−23	157.58	11517786

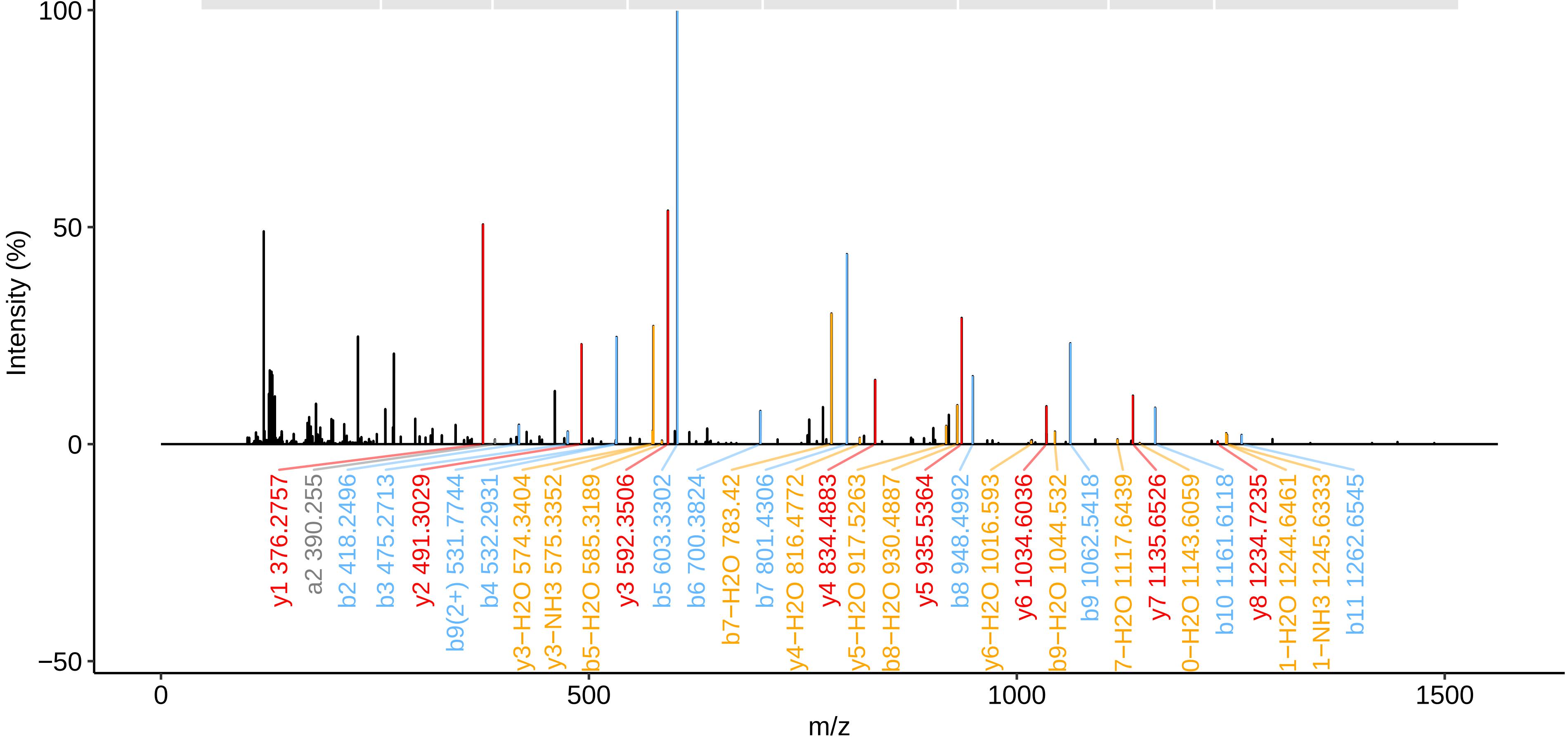

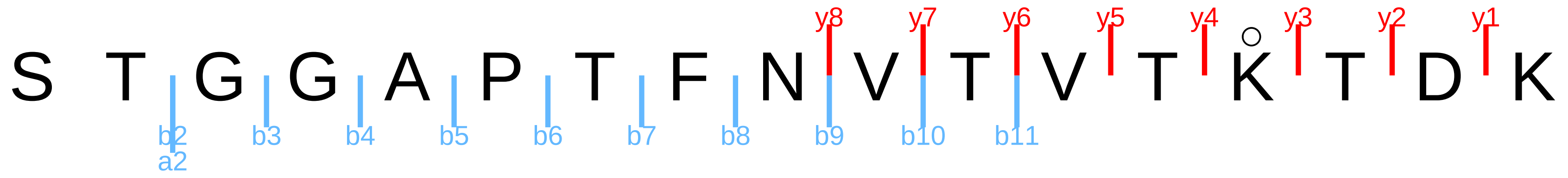

Modification □ Acetyl (Protein N−term) ○ GlyGly (K) no new terminus △ Oxidation (M)

20200921_KIZ_MUb4_GlyGly_10plex_2 − Scan number: 17542

Gene Names	Charge	m/z	Mass	Retention time	PEP	Score	Precursor Intensity
RMDN3	3	613.2987	1836.874	68.604	0.0083878	62.055	810625.8

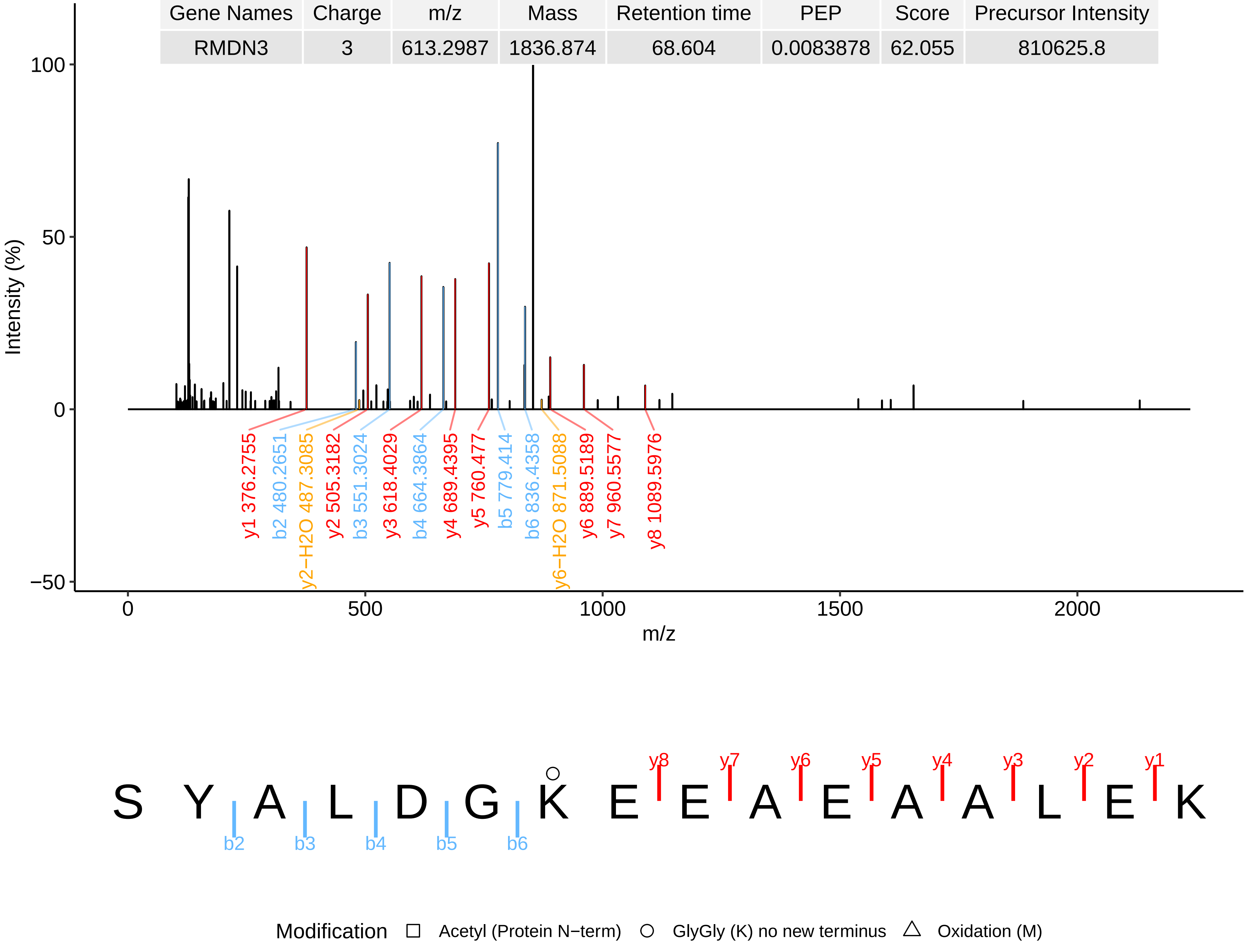

20200921_KIZ_MUb4_GlyGly_10plex_2 − Scan number: 17582

Gene Names	Charge	m/z	Mass	Retention time	PEP	Score	Precursor Intensity
RMDN3	3	613.2987	1836.874	68.762	0.00067801	76.82	1431968

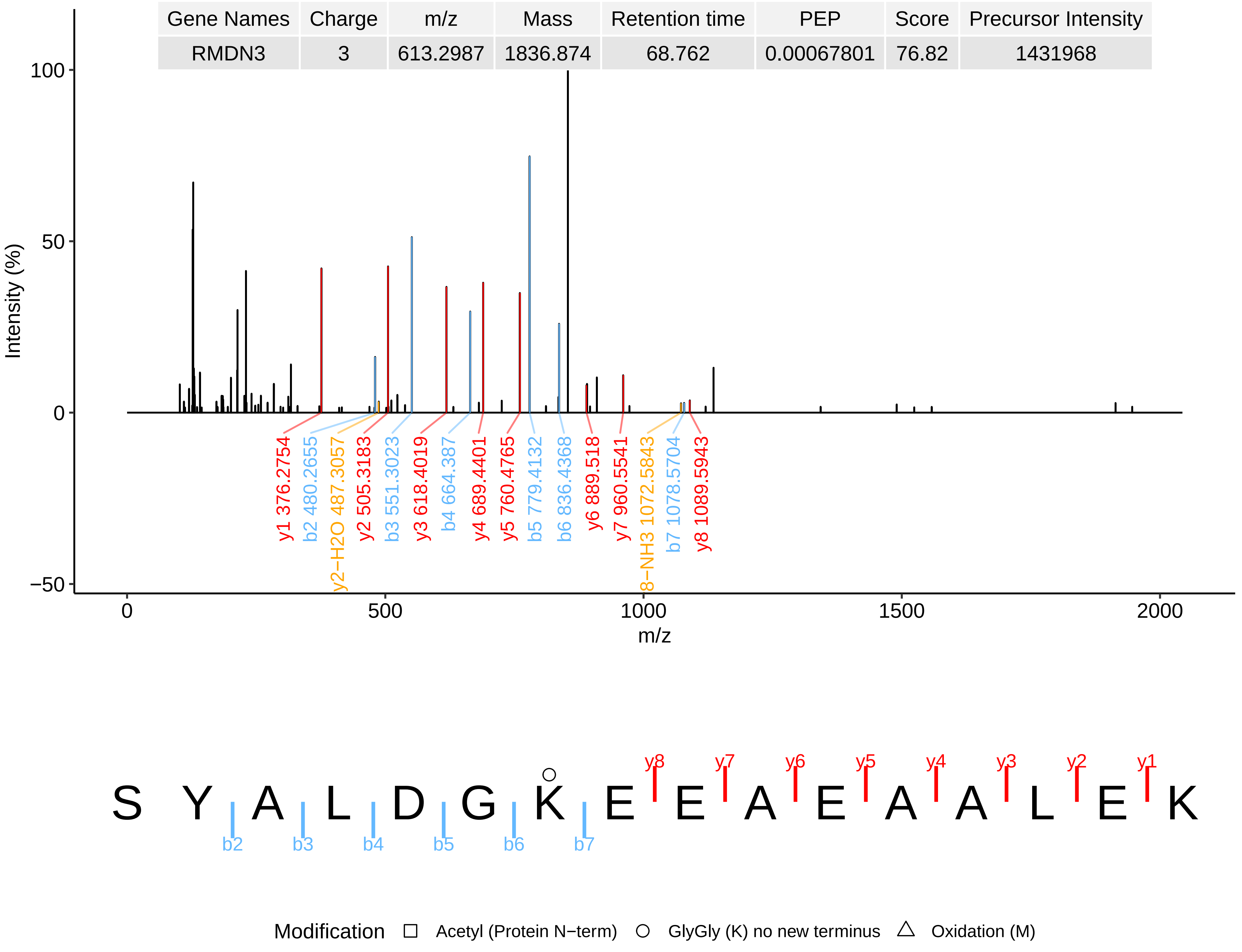

20200921_KIZ_MUb4_GlyGly_10plex − Scan number: 25418

Gene Names	Charge	m/z	Mass	Retention time	PEP	Score	Precursor Intensity
XRCC5	3	691.3601	2071.059	92.452	1.6846e−10	112.5	NA

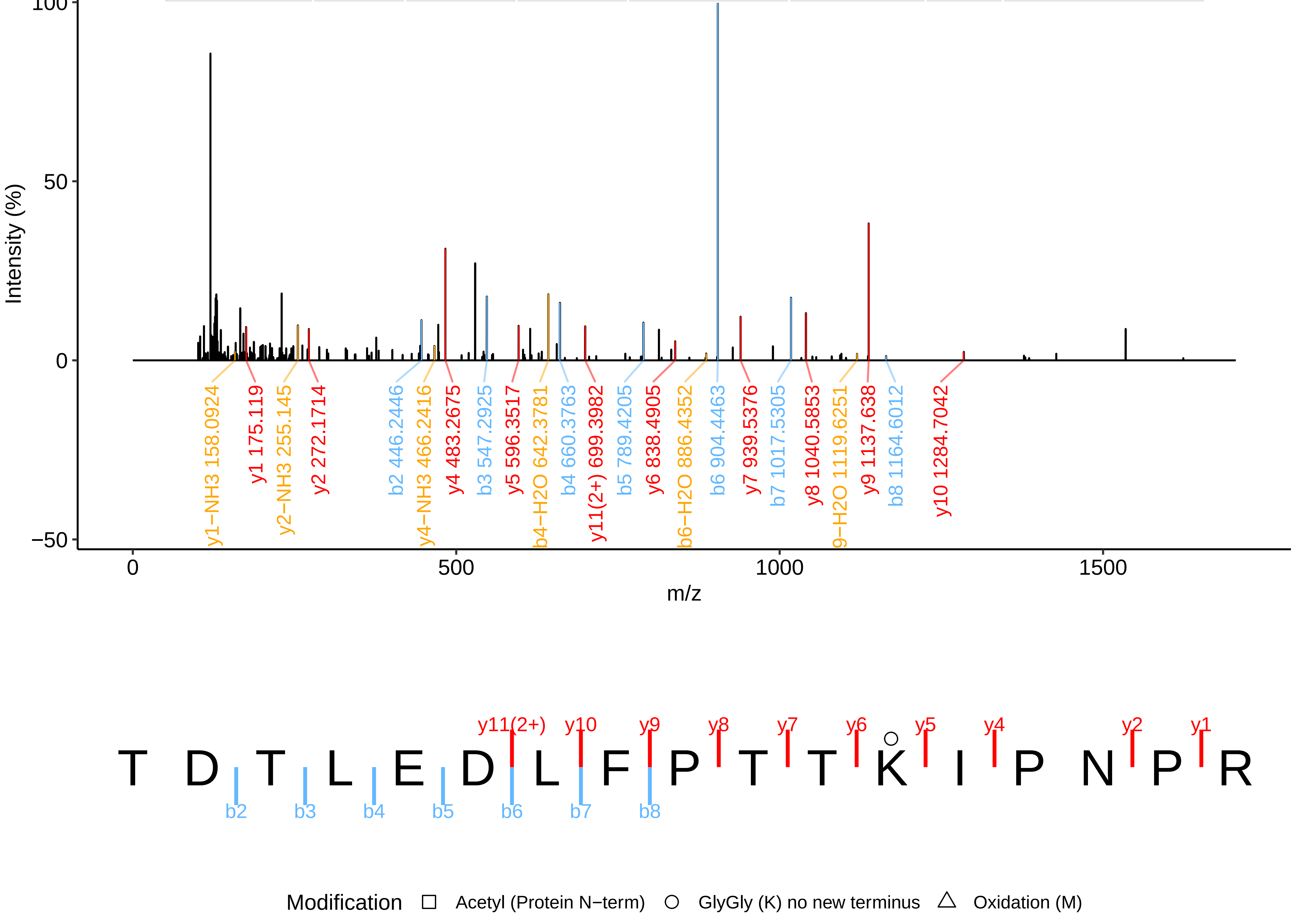

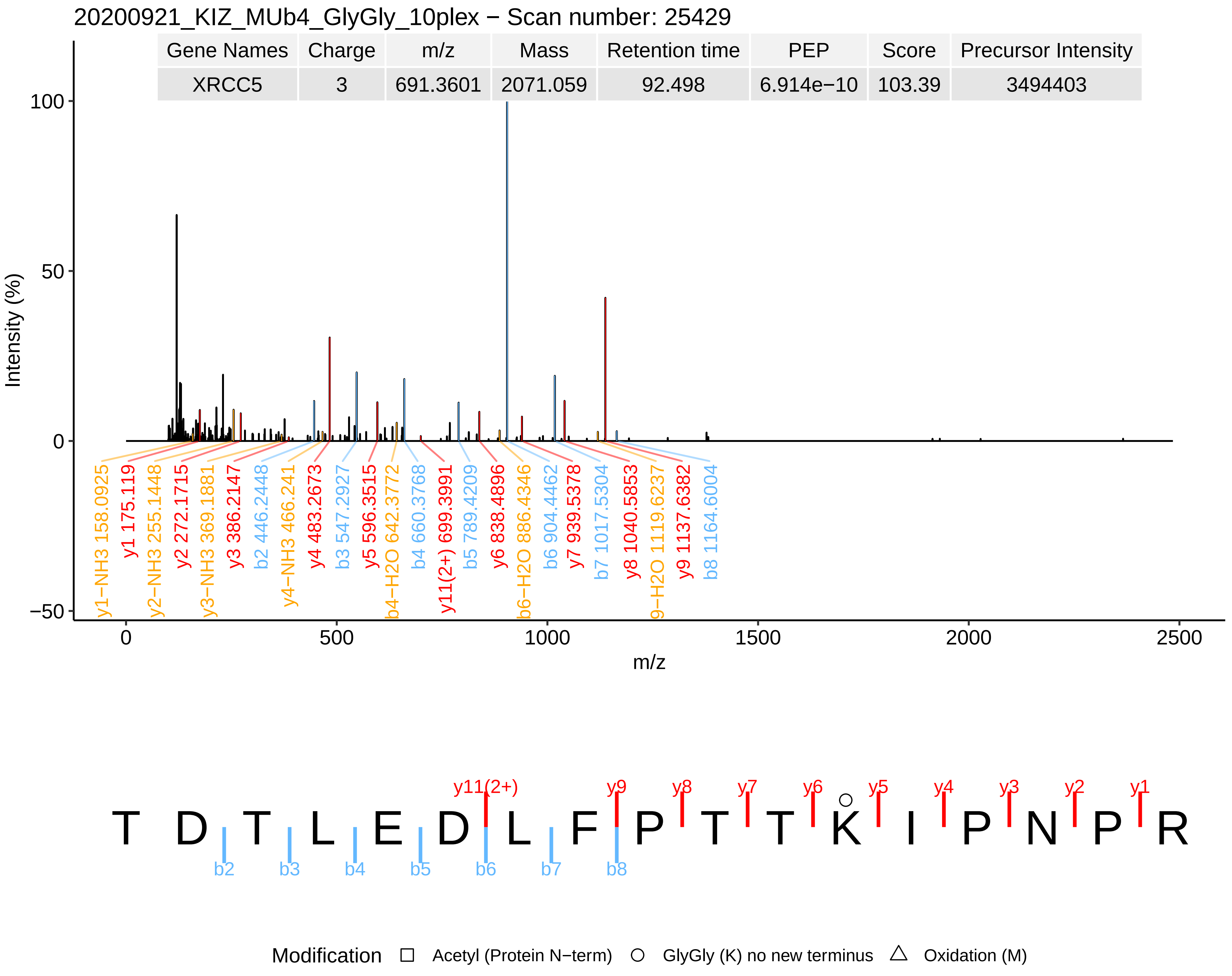

20200921_KIZ_MUb4_GlyGly_10plex − Scan number: 25429
Gene Names | Charge | m/z | Mass | Retention time | PEP | Score | Precursor Intensity
XRCC5 | 3 | 691.3601 | 2071.059 | 92.498 | 6.914e−10 | 103.39 | 3494403
Intensity (%)
m/z
y1−NH3 158.0925
y1 175.119
y2−NH3 255.1448
y2 272.1715
y3−NH3 369.1881
y3 386.2147
b2 446.2448
y4−NH3 466.241
y4 483.2673
b3 547.2927
y5 596.3515
b4−H2O 642.3772
b4 660.3768
y11(2+) 699.3991
b5 789.4209
y6 838.4896
b6−H2O 886.4346
b6 904.4462
y7 939.5378
b7 1017.5304
y8 1040.5853
9−H2O 1119.6237
y9 1137.6382
b8 1164.6004
T D T L E D L F P T T K I P N P R
Modification
Acetyl (Protein N−term)
GlyGly (K) no new terminus
Oxidation (M)

20200921_KIZ_MUb4_GlyGly_10plex − Scan number: 25445

Gene Names	Charge	m/z	Mass	Retention time	PEP	Score	Precursor Intensity
XRCC5	3	691.3601	2071.059	92.564	8.8628e−13	121.79	4101655

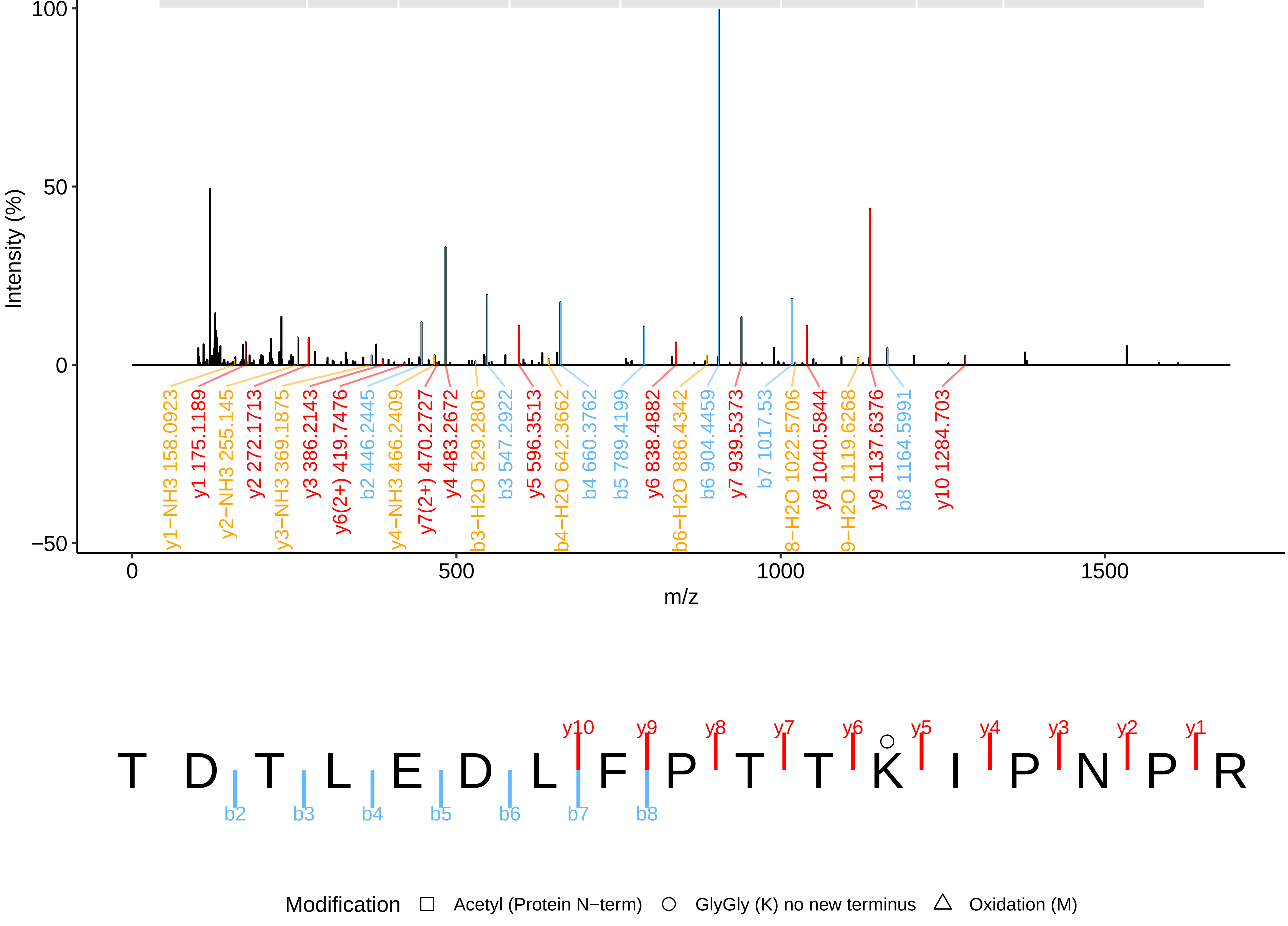

20200921_KIZ_MUb4_GlyGly_10plex − Scan number: 25697

Gene Names	Charge	m/z	Mass	Retention time	PEP	Score	Precursor Intensity
XRCC5	3	691.3601	2071.059	93.517	3.1127e−14	132.44	8996281

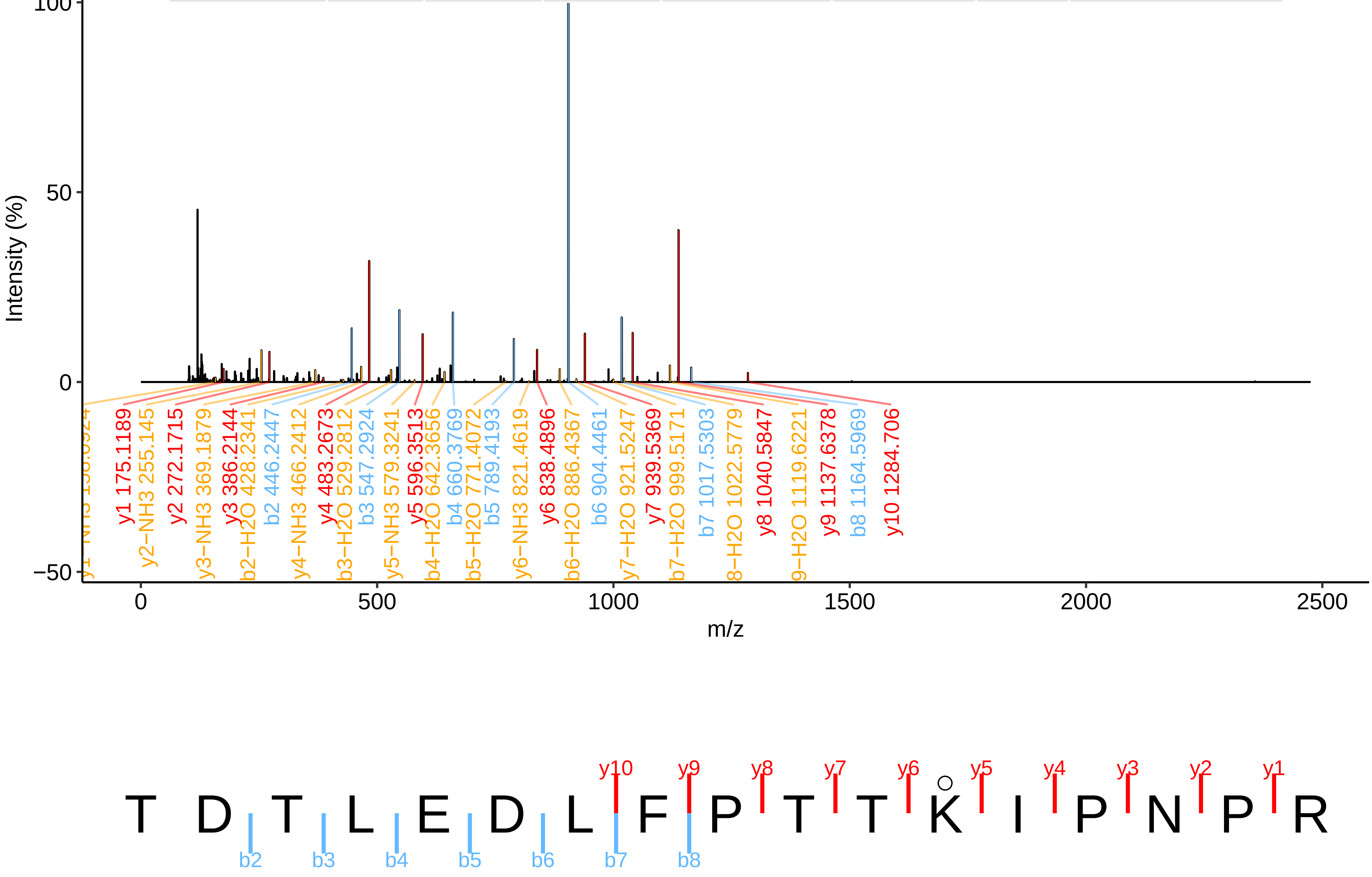

Modification □ Acetyl (Protein N−term) ○ GlyGly (K) no new terminus △ Oxidation (M)

20200921_KIZ_MUb4_GlyGly_10plex_2 − Scan number: 22493

Gene Names	Charge	m/z	Mass	Retention time	PEP	Score	Precursor Intensity
XRCC5	3	691.3601	2071.059	88.682	5.9392e−08	97.836	614059.2

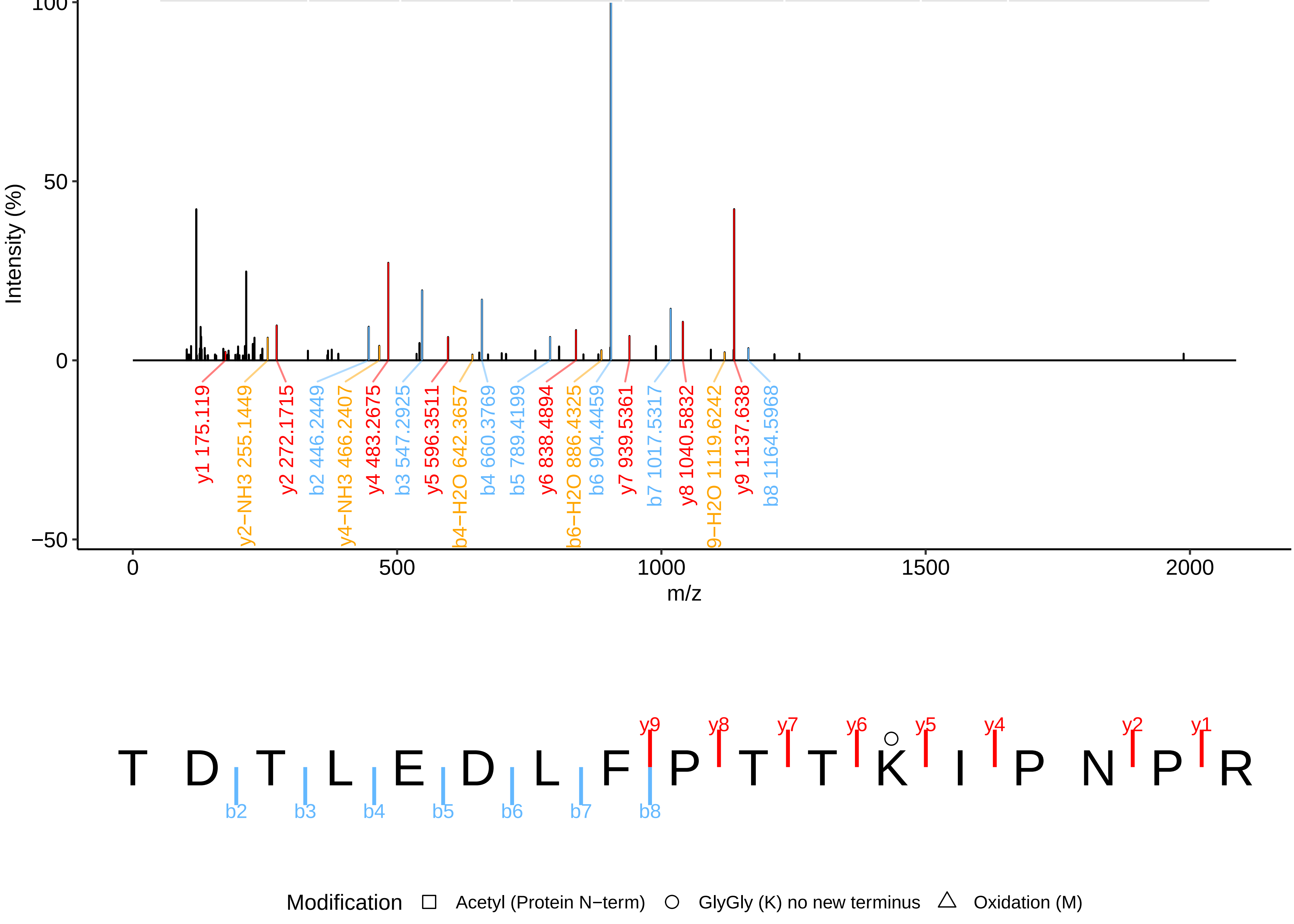

20200921_KIZ_MUb4_GlyGly_10plex_2 − Scan number: 22617

Gene Names	Charge	m/z	Mass	Retention time	PEP	Score	Precursor Intensity
XRCC5	3	691.3601	2071.059	89.194	3.0673e−13	129.01	5102432

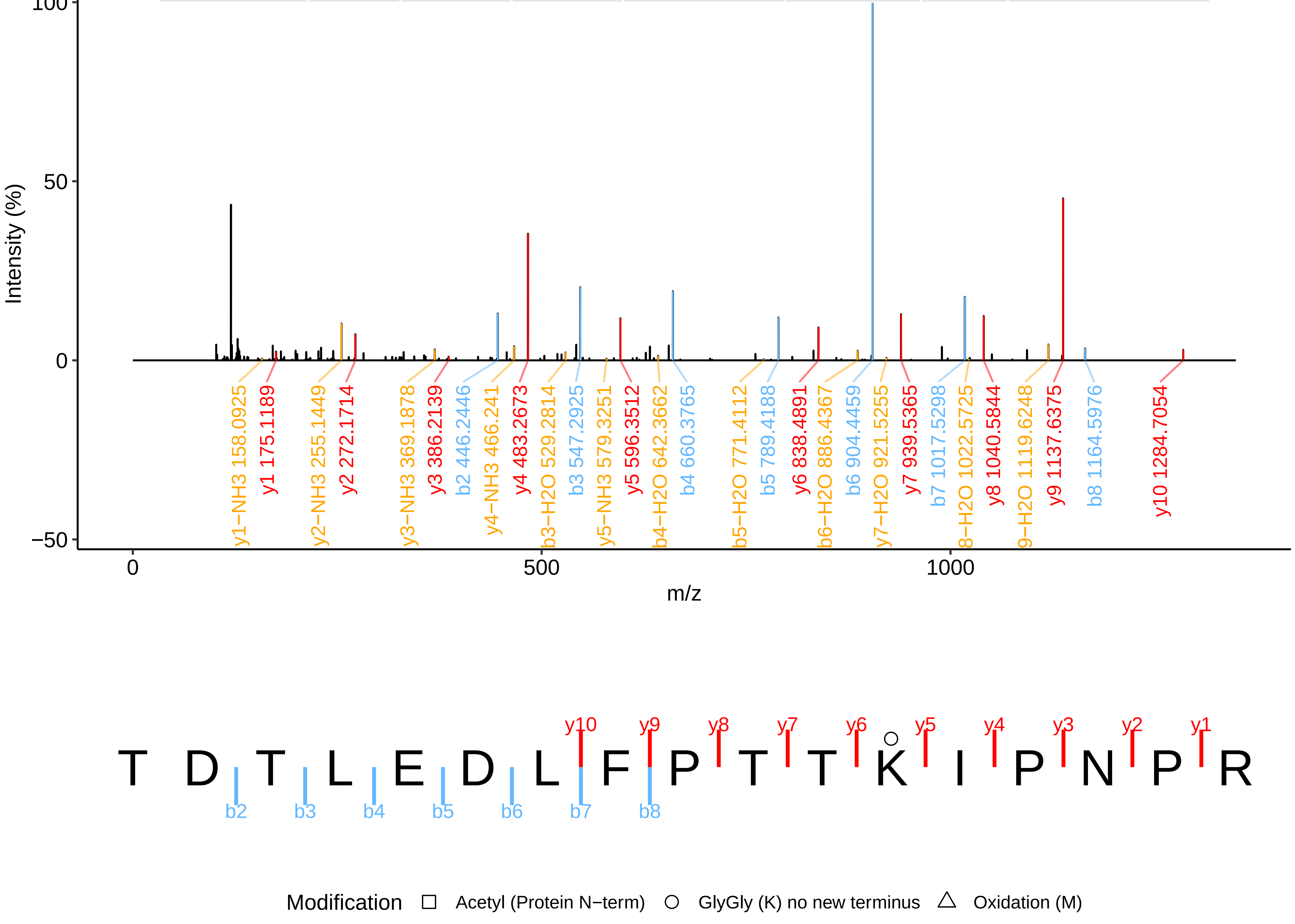

20200921_KIZ_MUb4_GlyGly_10plex_2 − Scan number: 22743

Gene Names	Charge	m/z	Mass	Retention time	PEP	Score	Precursor Intensity
XRCC5	3	691.3601	2071.059	89.706	4.2745e−11	114.69	526135.6

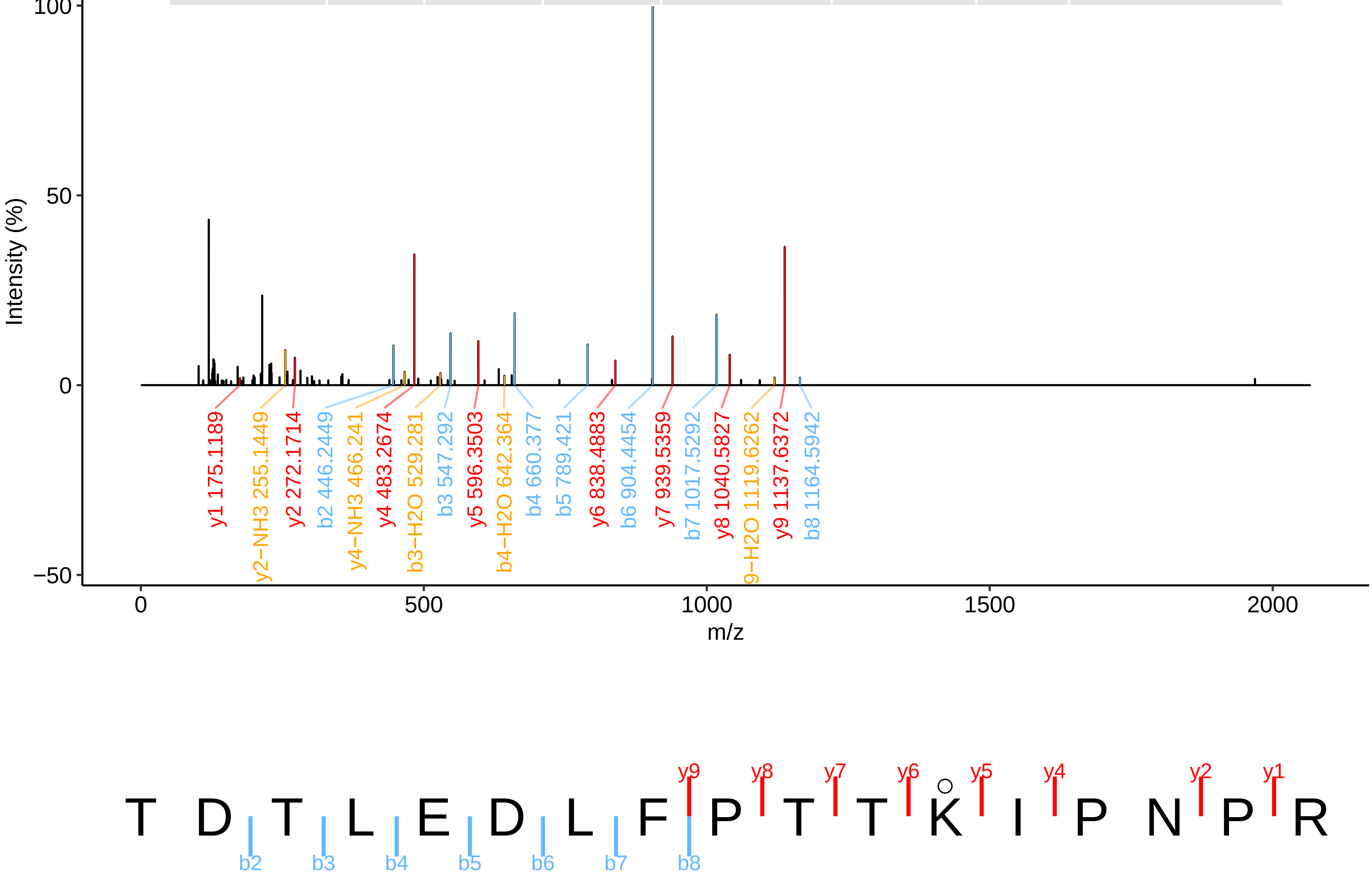

Modification □ Acetyl (Protein N−term) ○ GlyGly (K) no new terminus △ Oxidation (M)

20200924_KIZ_MUb5_GlyGly_1 − Scan number: 25373

Gene Names	Charge	m/z	Mass	Retention time	PEP	Score	Precursor Intensity
XRCC5	3	691.3601	2071.059	81.379	2.7682e−13	129.38	77049968

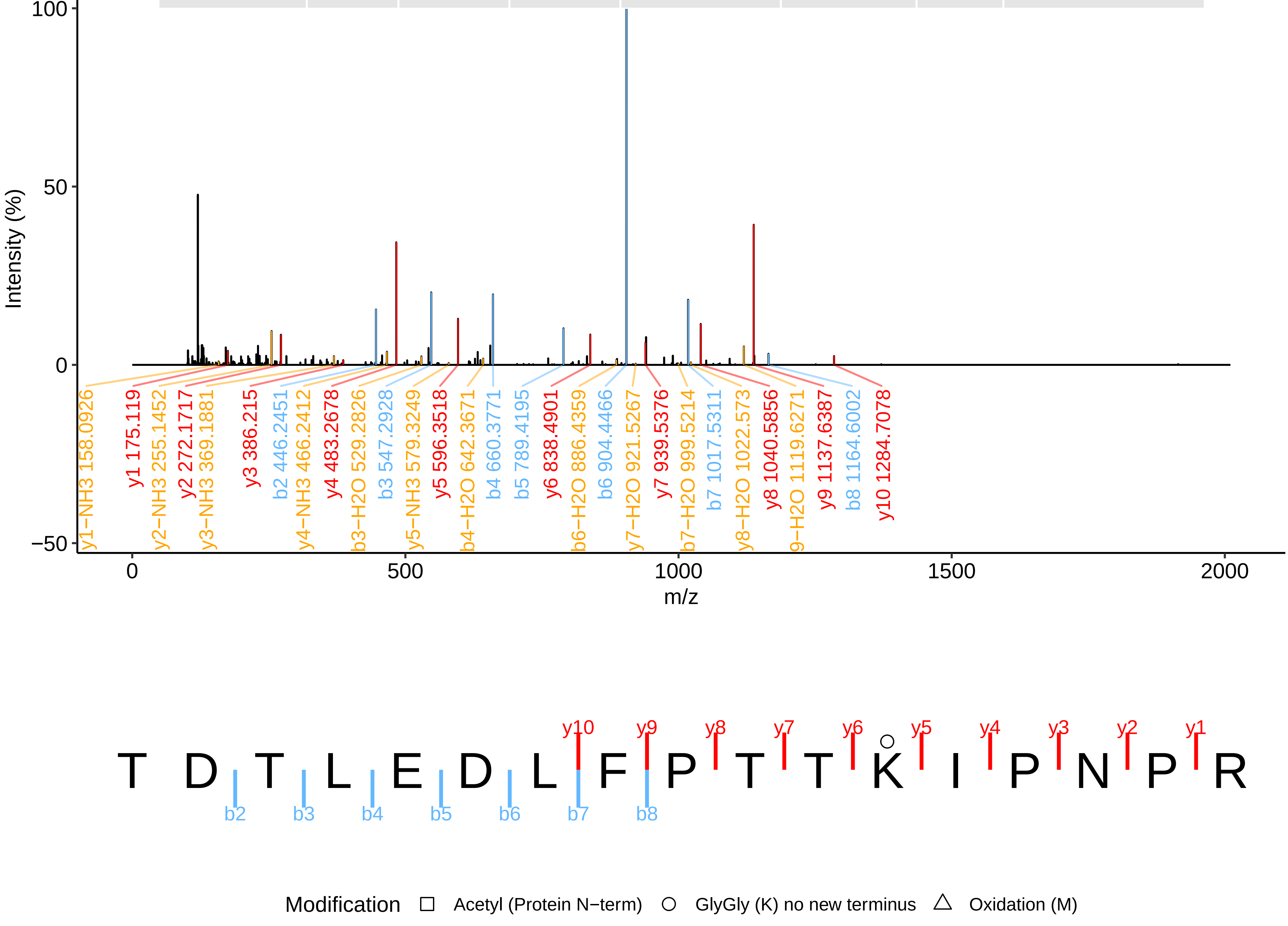

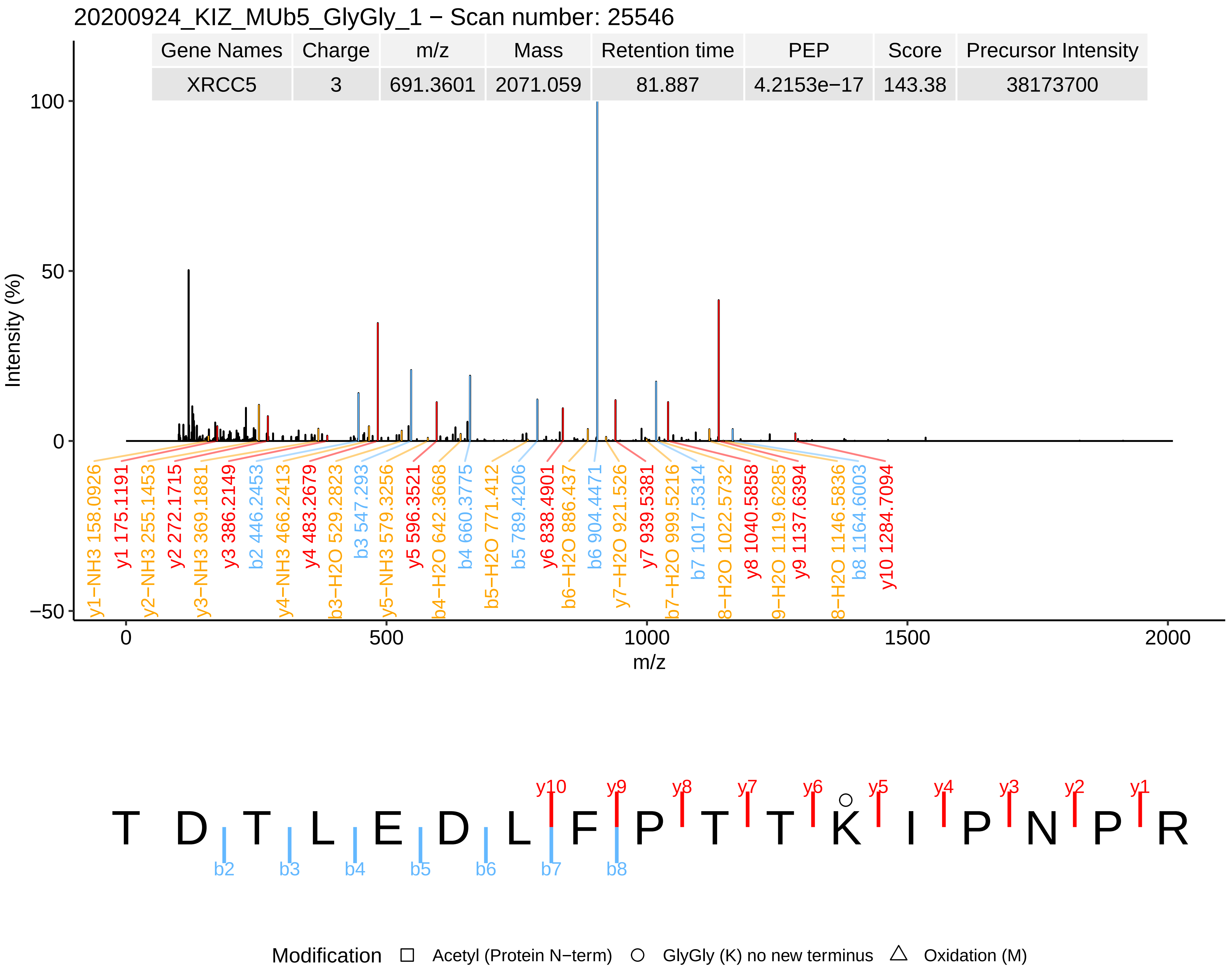
20200924_KIZ_MUb5_GlyGly_1 − Scan number: 25546
Gene Names
Charge
m/z
Mass
Retention time
PEP
Score
Precursor Intensity
XRCC5
3
691.3601
2071.059
81.887
4.2153e−17
143.38
38173700
Intensity (%)
100
50
0
−50
0
500
1000
1500
2000
m/z
y1−NH3 158.0926
y1 175.1191
y2−NH3 255.1453
y2 272.1715
y3−NH3 369.1881
y3 386.2149
b2 446.2453
y4−NH3 466.2413
y4 483.2679
b3−H2O 529.2823
b3 547.293
y5−NH3 579.3256
y5 596.3521
b4−H2O 642.3668
b4 660.3775
b5−H2O 771.412
b5 789.4206
y6 838.4901
b6−H2O 886.437
b6 904.4471
y7−H2O 921.526
y7 939.5381
b7−H2O 999.5216
b7 1017.5314
8−H2O 1022.5732
y8 1040.5858
9−H2O 1119.6285
y9 1137.6394
8−H2O 1146.5836
b8 1164.6003
y10 1284.7094
T D T L E D L F P T T K I P N P R
y10
y9
y8
y7
y6
y5
y4
y3
y2
y1
b2
b3
b4
b5
b6
b7
b8
Modification
Acetyl (Protein N−term)
GlyGly (K) no new terminus
Oxidation (M)

20200924_KIZ_MUb5_GlyGly_1 − Scan number: 25218

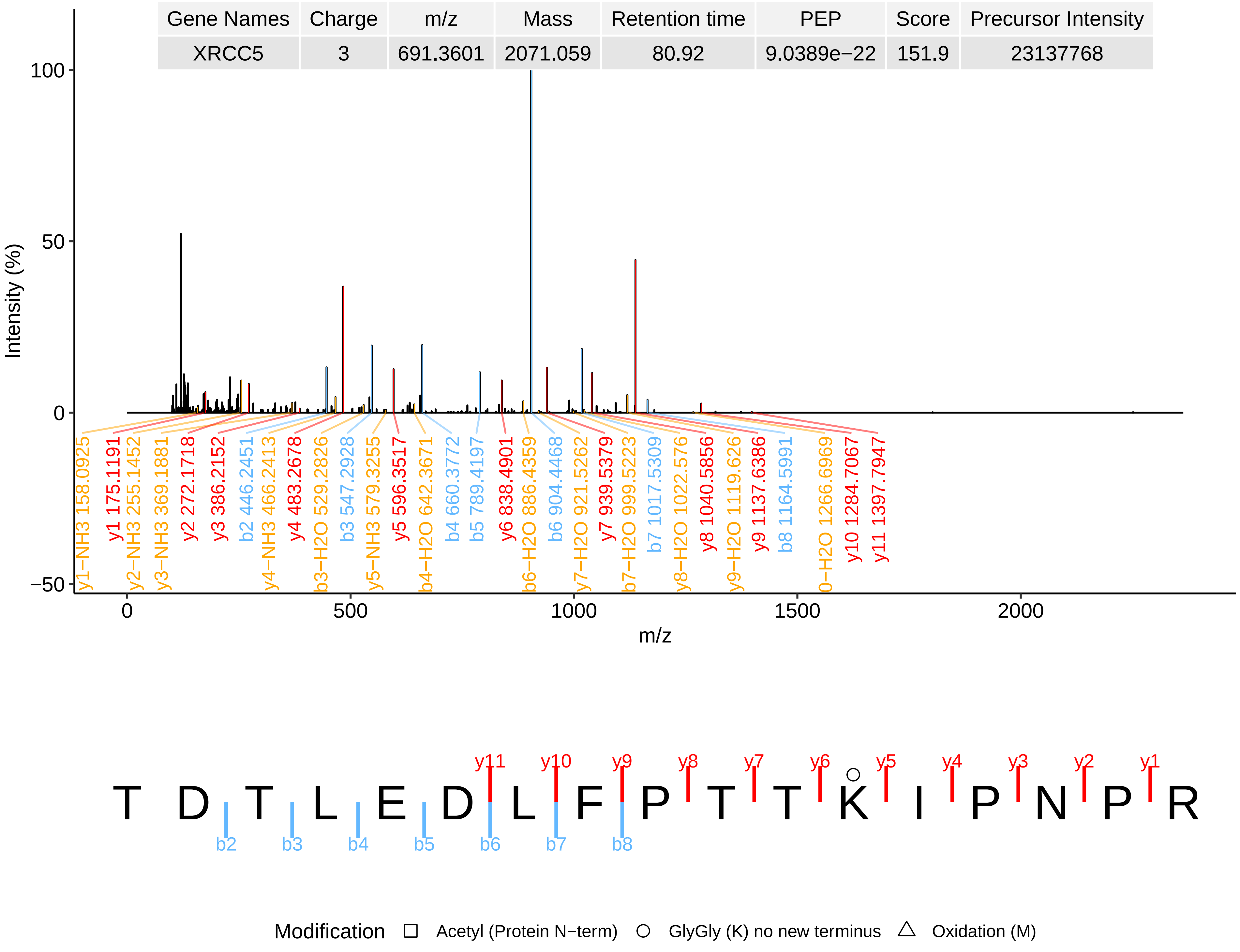

20200924_KIZ_MUb5_GlyGly_2 − Scan number: 22530

Gene Names	Charge	m/z	Mass	Retention time	PEP	Score	Precursor Intensity
XRCC5	3	691.3601	2071.059	83.442	5.0407e−06	83.966	3493898

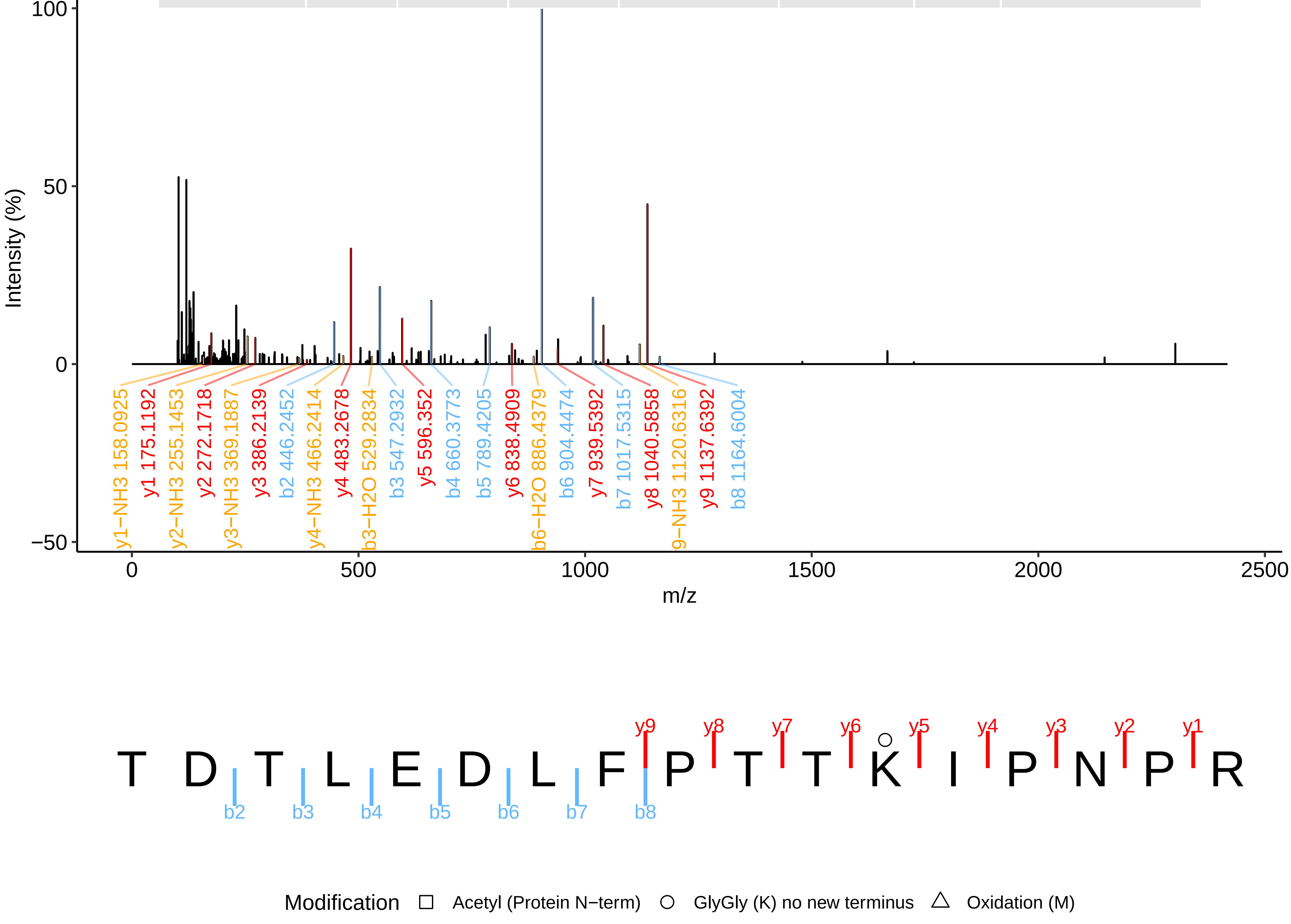

20200924_KIZ_MUb5_GlyGly_2 − Scan number: 22666

Gene Names	Charge	m/z	Mass	Retention time	PEP	Score	Precursor Intensity
XRCC5	3	691.3601	2071.059	83.955	1.1488e−18	150.05	15619490

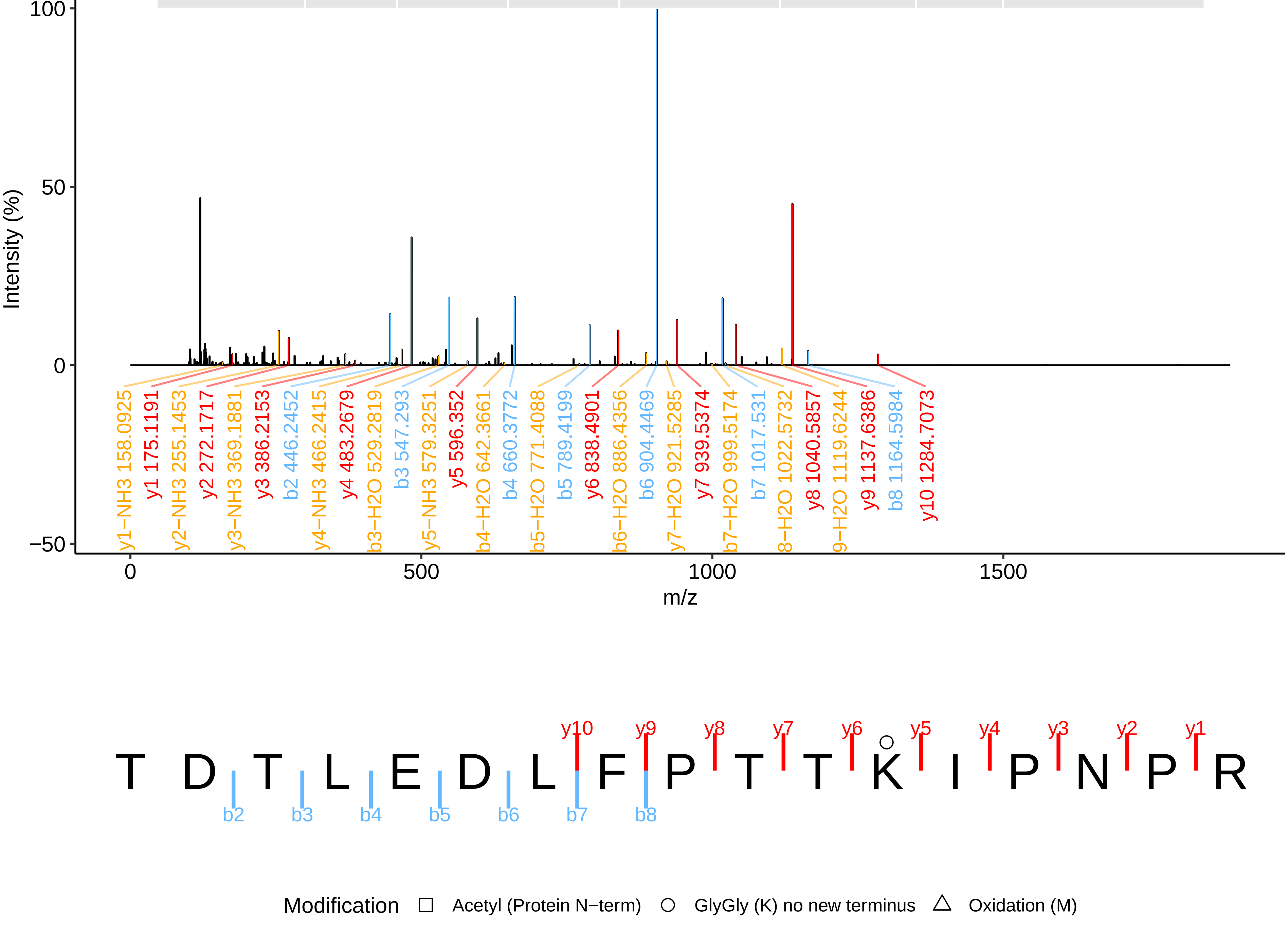

20200924_KIZ_MUb5_GlyGly_2 − Scan number: 22805

Gene Names	Charge	m/z	Mass	Retention time	PEP	Score	Precursor Intensity
XRCC5	3	691.3601	2071.059	84.471	4.9852e−17	148.66	6695437

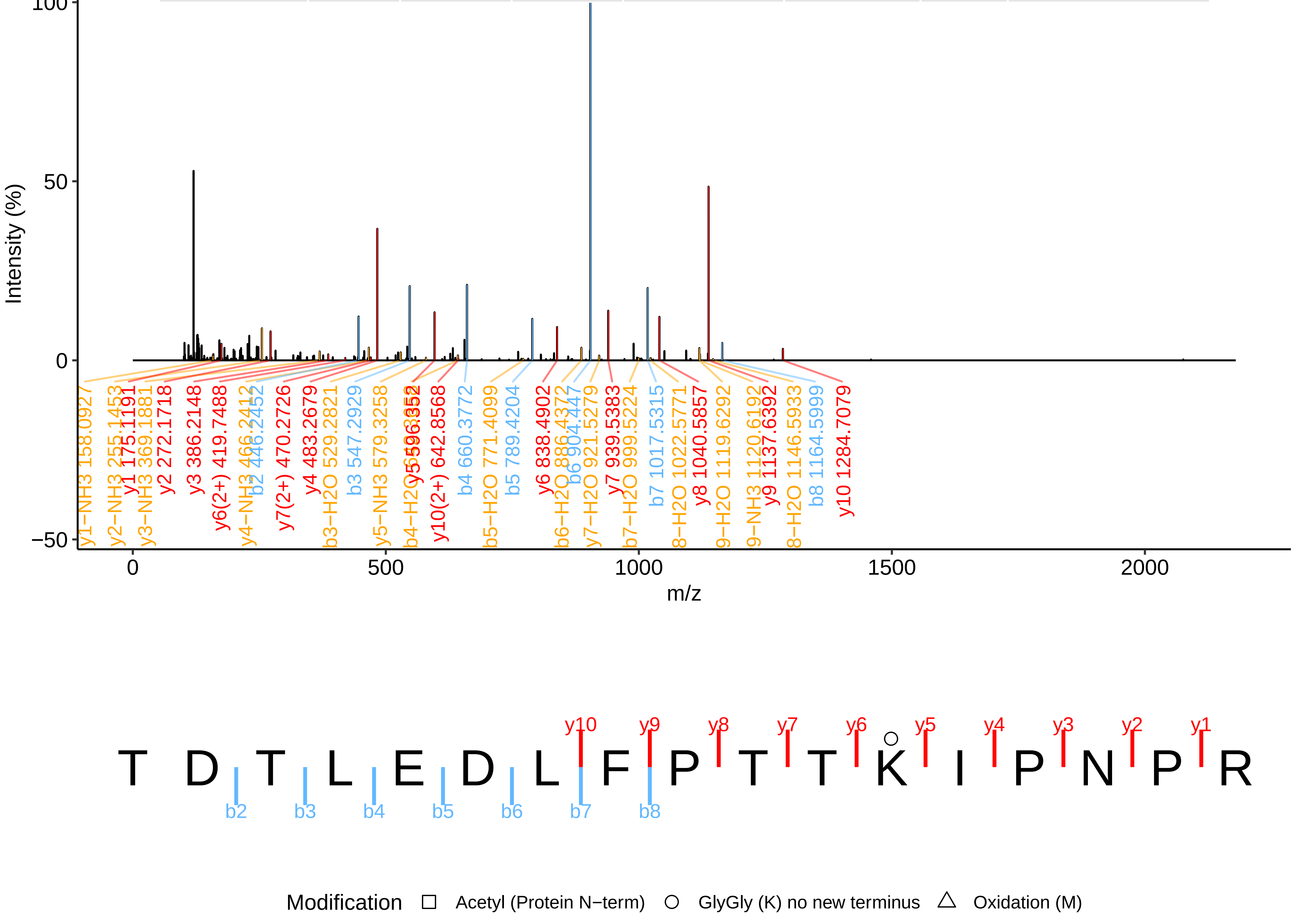

20200921_KIZ_MUb4_GlyGly_10plex − Scan number: 27581

Gene Names	Charge	m/z	Mass	Retention time	PEP	Score	Precursor Intensity
PFN1	3	677.0407	2028.1	100.69	1.6767e−13	126.8	4287924

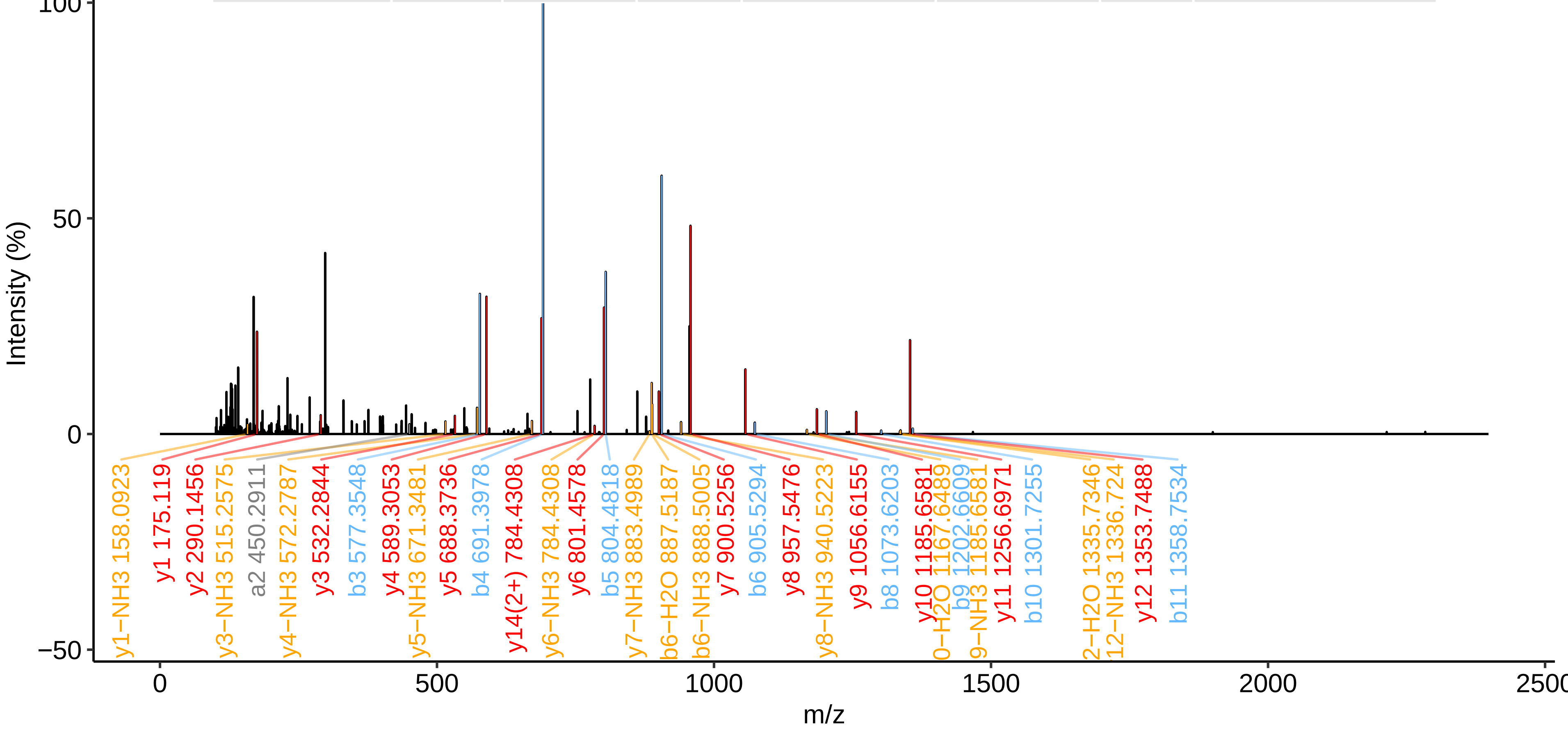

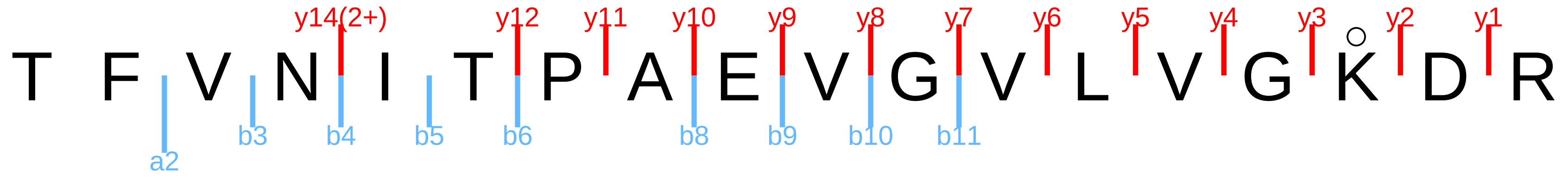

Modification □ Acetyl (Protein N−term) ○ GlyGly (K) no new terminus △ Oxidation (M)

20200921_KIZ_MUb4_GlyGly_10plex_2 − Scan number: 24408

Gene Names	Charge	m/z	Mass	Retention time	PEP	Score	Precursor Intensity
PFN1	3	677.0407	2028.1	96.448	7.8265e−06	85.146	1663102

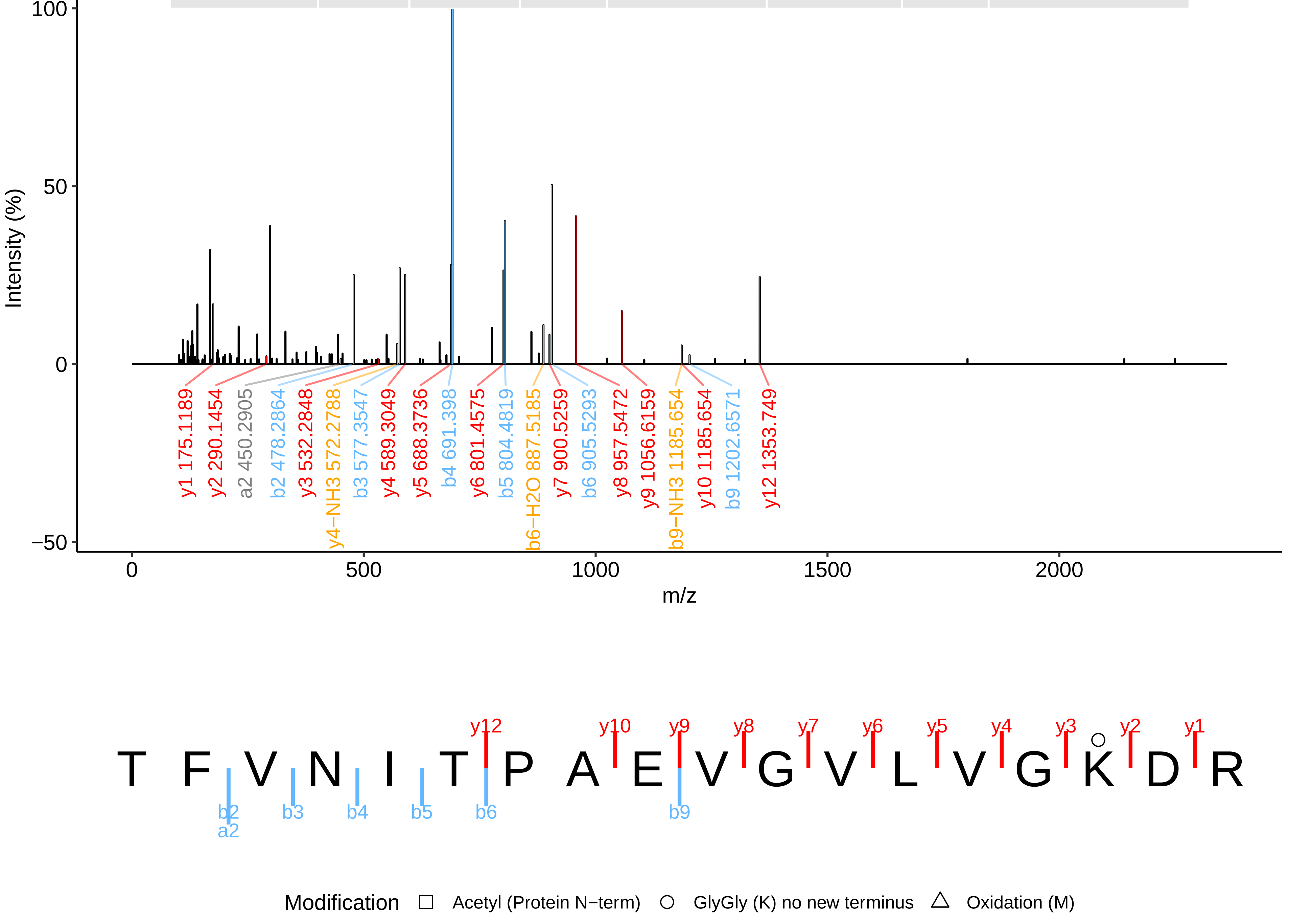

20200921_KIZ_MUb4_GlyGly_10plex − Scan number: 13731

Gene Names	Charge	m/z	Mass	Retention time	PEP	Score	Precursor Intensity
POLR2A	3	625.6341	1873.881	48.542	6.5552e−14	141.85	7201986

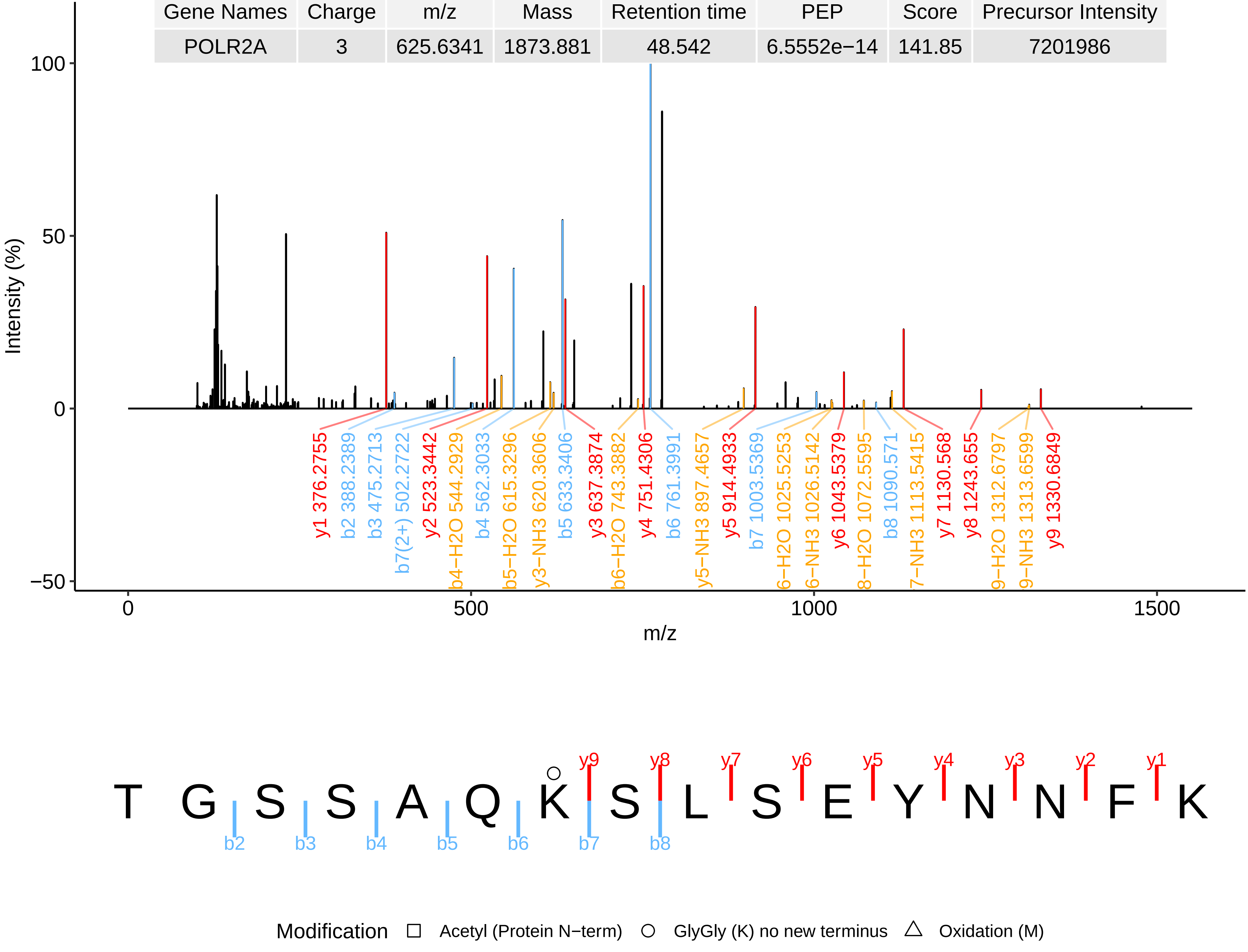

20200921_KIZ_MUb4_GlyGly_10plex_2 − Scan number: 12630

Gene Names	Charge	m/z	Mass	Retention time	PEP	Score	Precursor Intensity
POLR2A	3	625.6341	1873.881	48.903	0.034362	56.599	782166

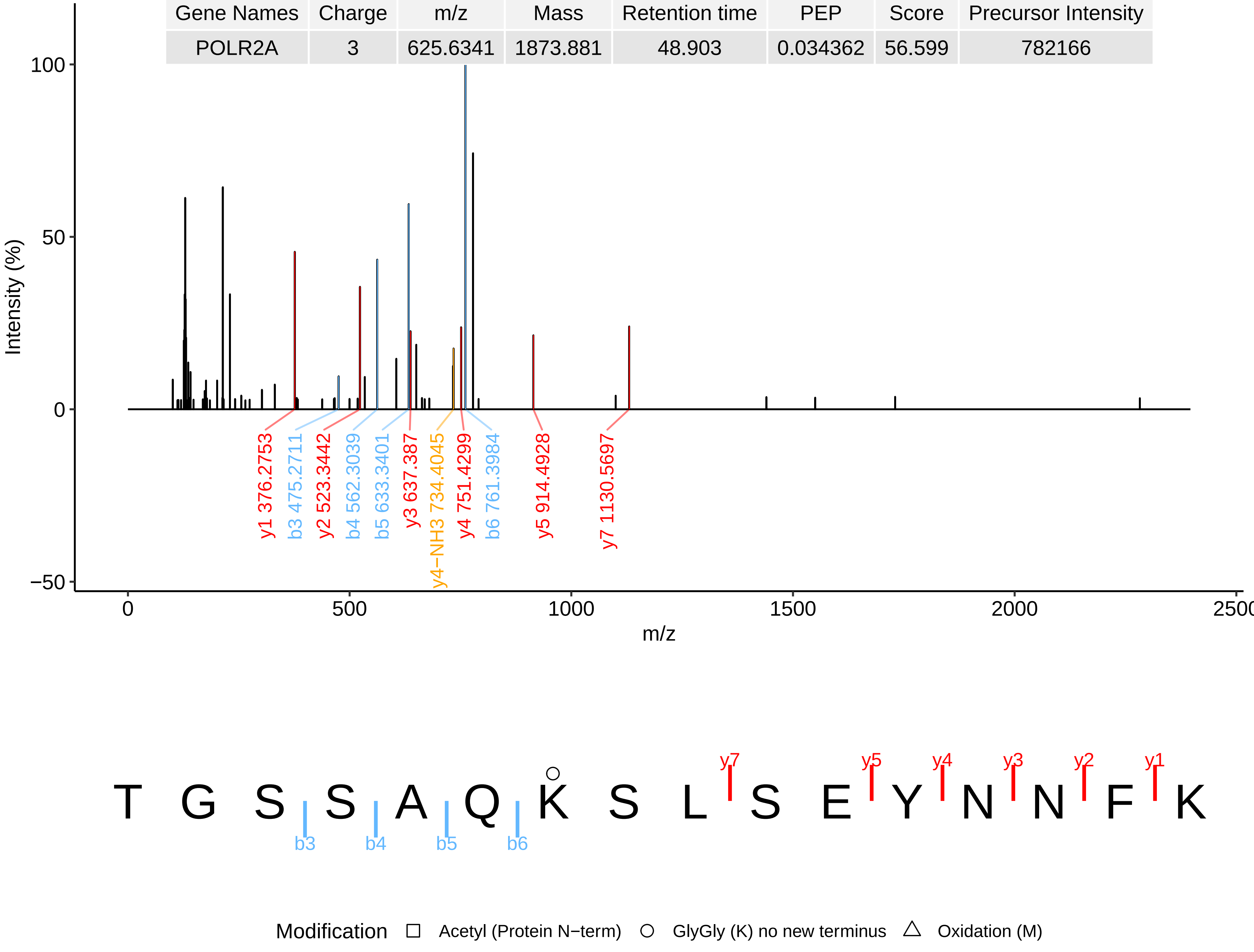

20200921_KIZ_MUb4_GlyGly_10plex − Scan number: 13380

Gene Names	Charge	m/z	Mass	Retention time	PEP	Score	Precursor Intensity
TOM1	4	451.0046	1799.989	47.182	0.0036499	63.128	7181389

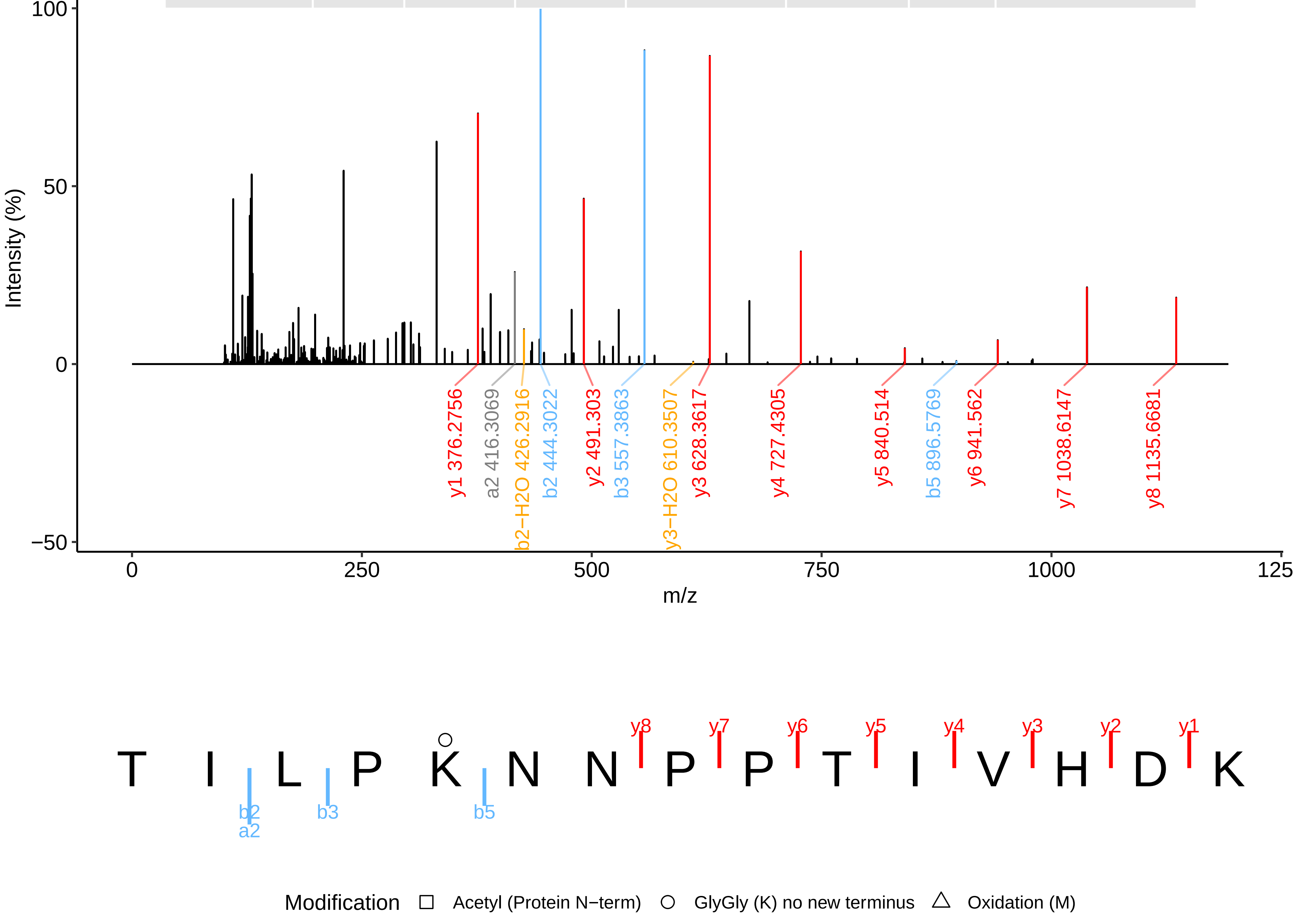

20200921_KIZ_MUb4_GlyGly_10plex − Scan number: 13389

Gene Names	Charge	m/z	Mass	Retention time	PEP	Score	Precursor Intensity
TOM1	4	451.0046	1799.989	47.216	7.927e−05	88.73	5624512

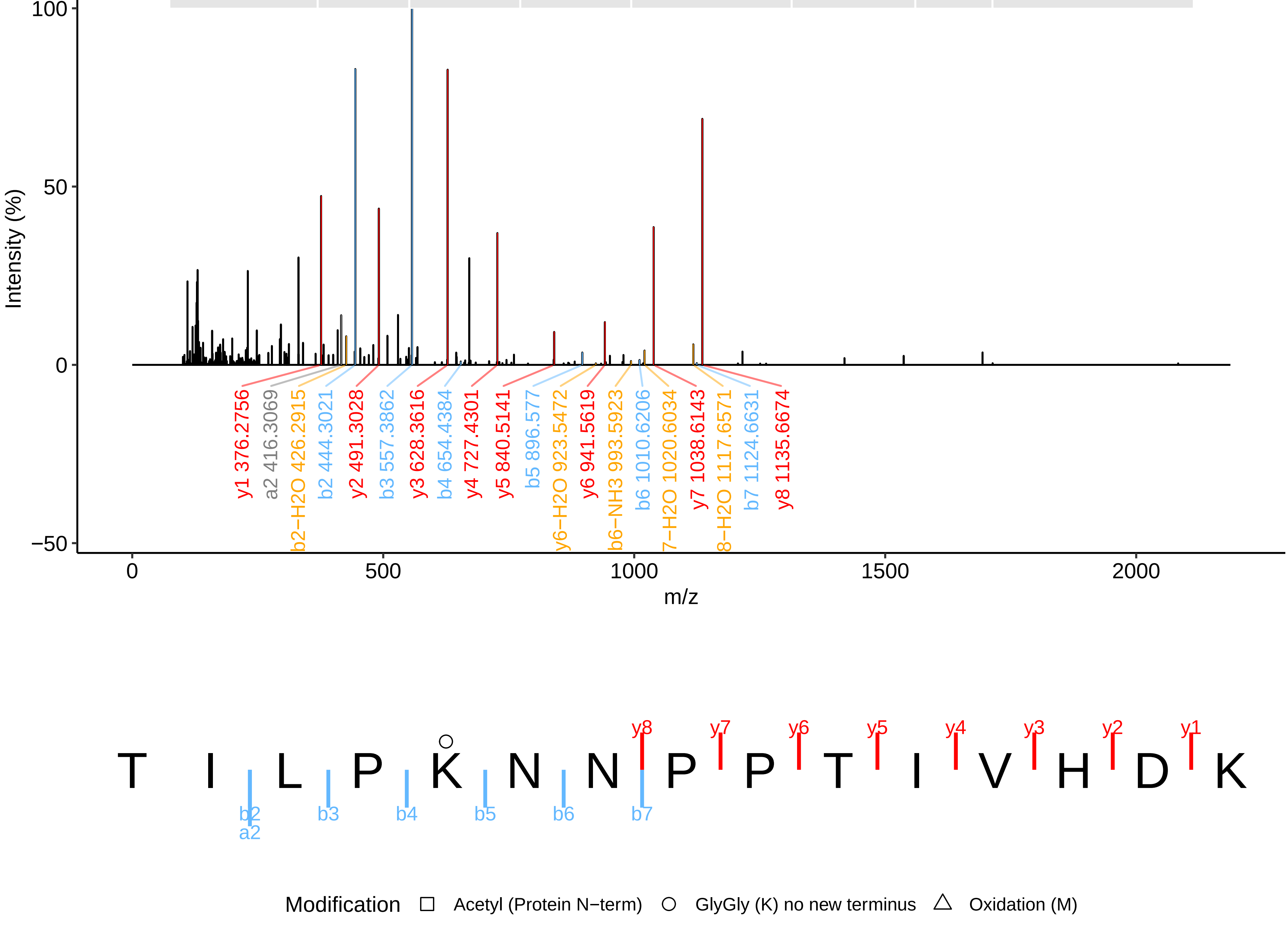

20200921_KIZ_MUb4_GlyGly_10plex_2 − Scan number: 12362

Gene Names	Charge	m/z	Mass	Retention time	PEP	Score	Precursor Intensity
TOM1	4	451.0046	1799.989	47.811	0.0036499	63.128	1722597

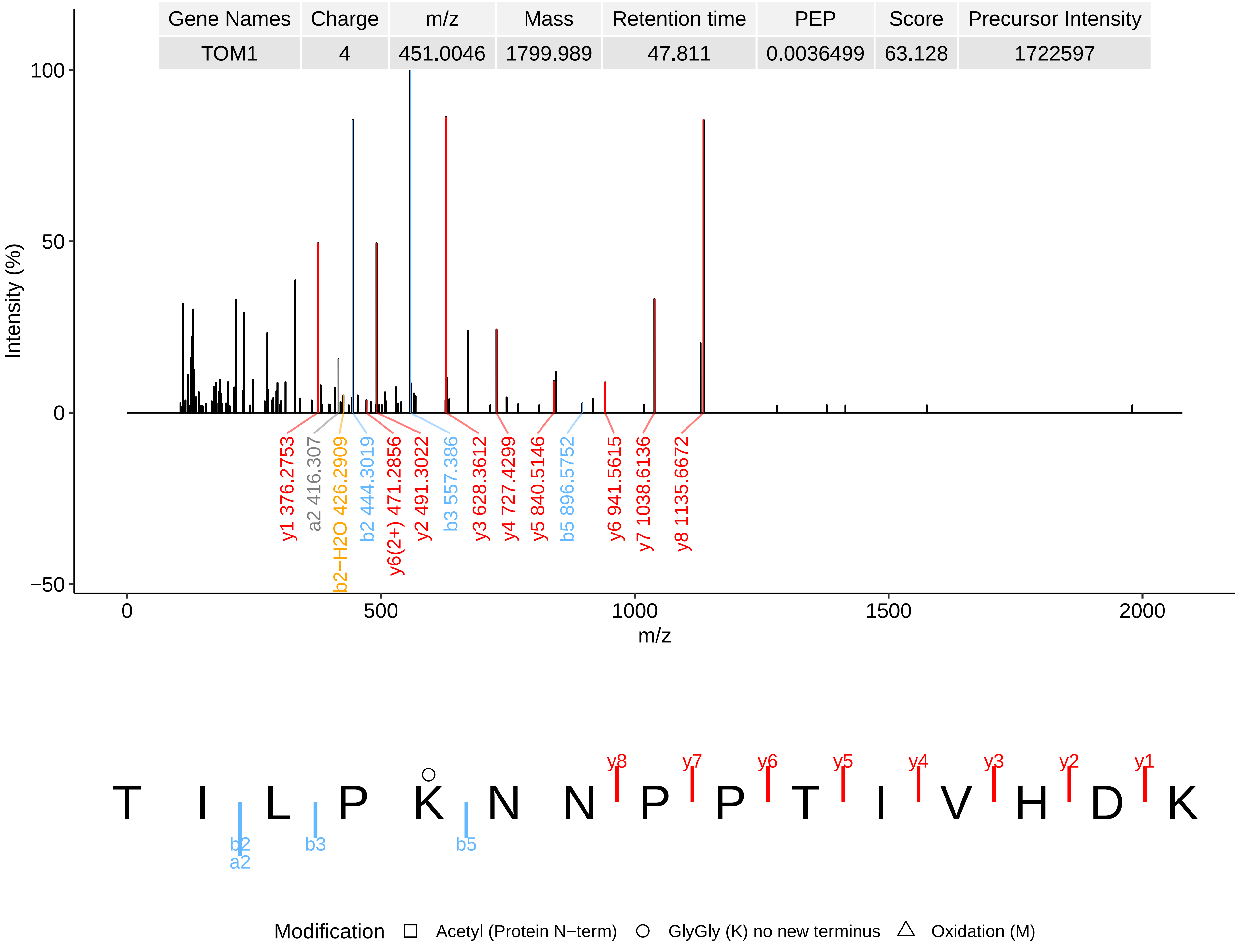

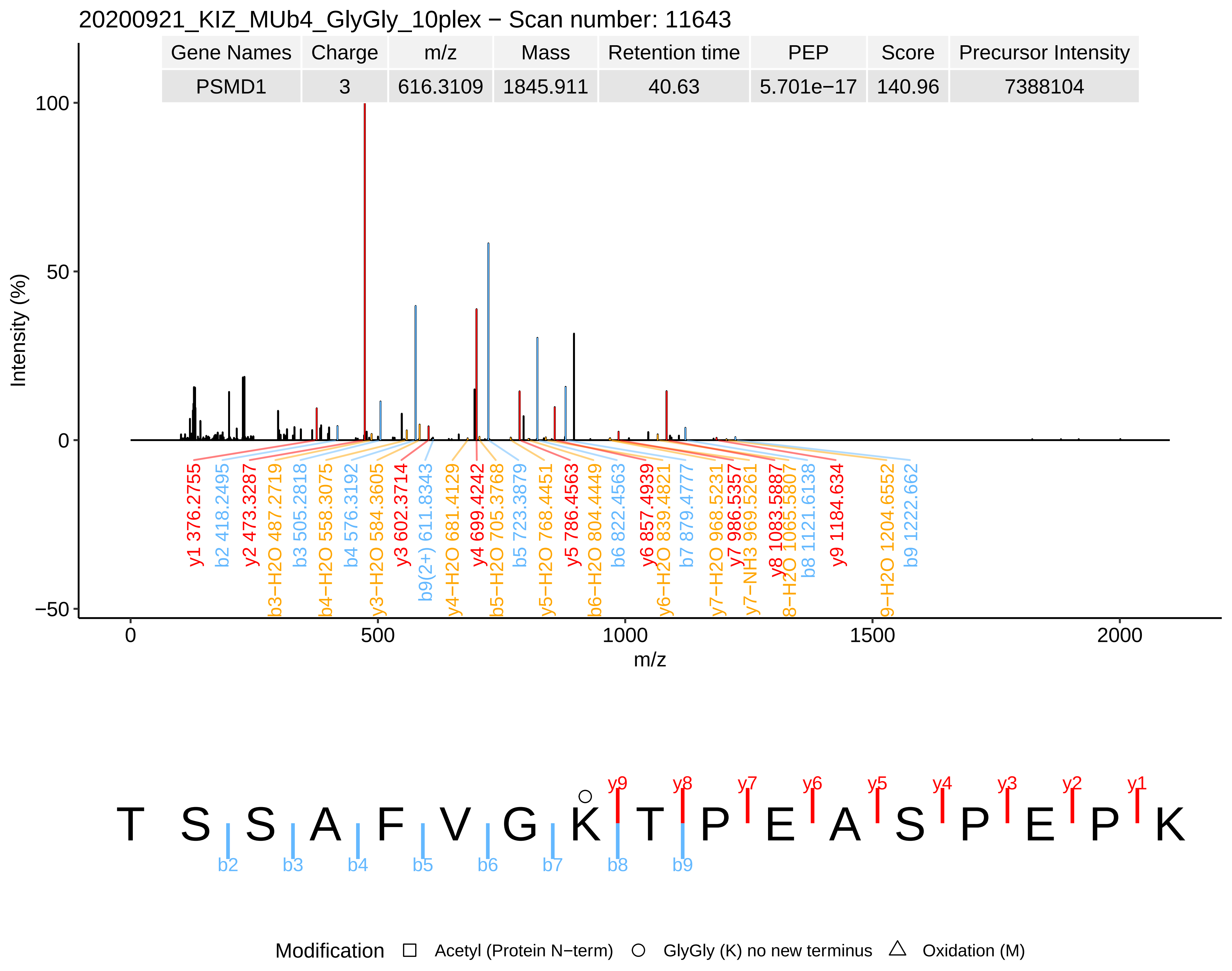

20200921_KIZ_MUb4_GlyGly_10plex − Scan number: 11643
Gene Names | Charge | m/z | Mass | Retention time | PEP | Score | Precursor Intensity
PSMD1 | 3 | 616.3109 | 1845.911 | 40.63 | 5.701e−17 | 140.96 | 7388104
Intensity (%)
100
50
0
−50
0
500
1000
1500
2000
m/z
y1 376.2755
b2 418.2495
y2 473.3287
b3−H2O 487.2719
b3 505.2818
b4−H2O 558.3075
b4 576.3192
y3−H2O 584.3605
y3 602.3714
b9(2+) 611.8343
y4−H2O 681.4129
y4 699.4242
b5−H2O 705.3768
b5 723.3879
y5−H2O 768.4451
y5 786.4563
b6−H2O 804.4449
b6 822.4563
y6 857.4939
y6−H2O 839.4821
b7 879.4777
y7−H2O 968.5231
y7 986.5357
y7−NH3 969.5261
y8 1083.5887
8−H2O 1065.5807
b8 1121.6138
y9 1184.634
9−H2O 1204.6552
b9 1222.662
T S S A F V G K T P E A S P E P K
y9 y8 y7 y6 y5 y4 y3 y2 y1
b2 b3 b4 b5 b6 b7 b8 b9
Modification
Acetyl (Protein N−term)
GlyGly (K) no new terminus
Oxidation (M)

20200921_KIZ_MUb4_GlyGly_10plex − Scan number: 11674

Gene Names	Charge	m/z	Mass	Retention time	PEP	Score	Precursor Intensity
PSMD1	3	616.3109	1845.911	40.742	7.8708e−13	123.02	12532620

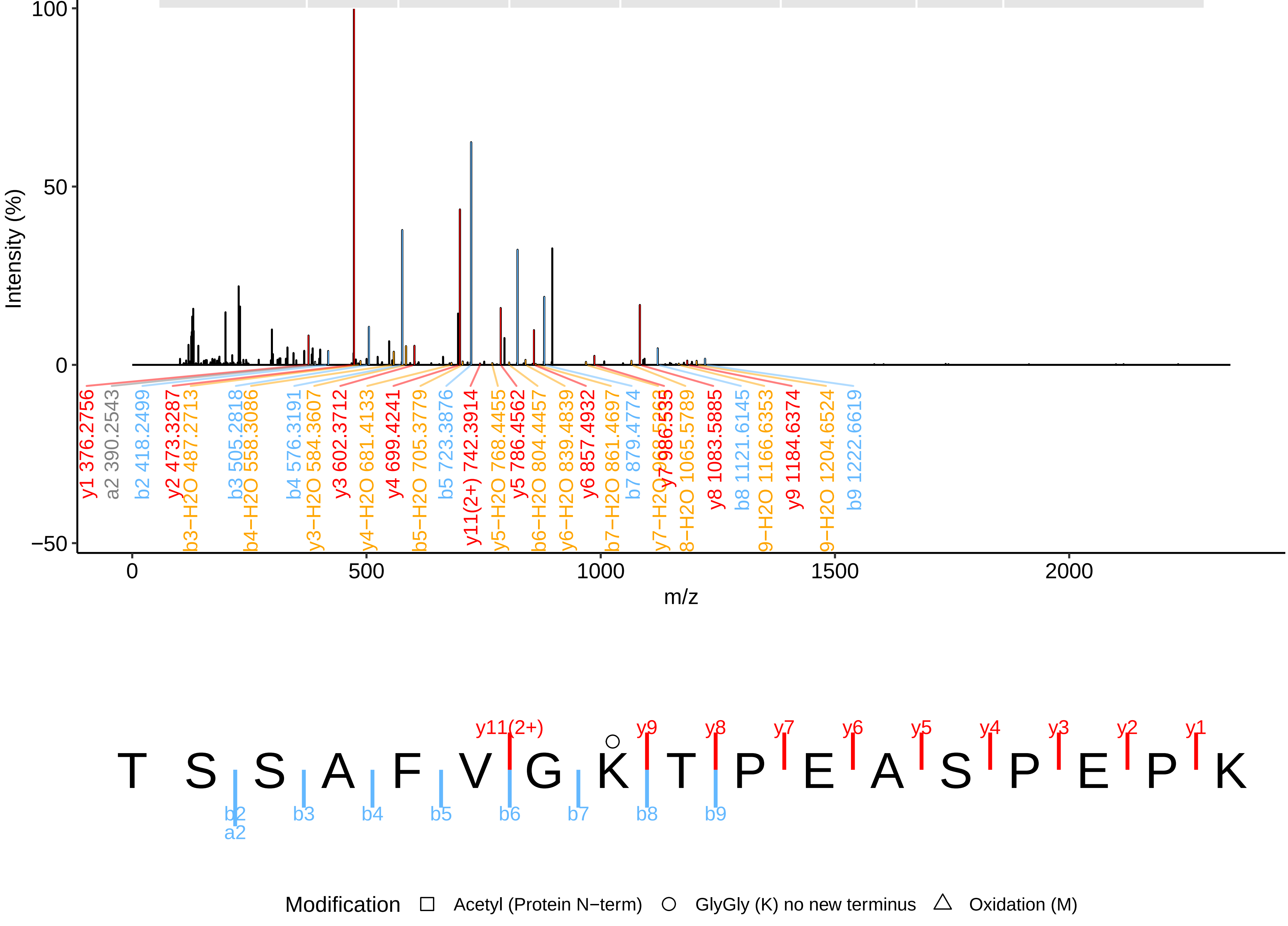

20200921_KIZ_MUb4_GlyGly_10plex − Scan number: 11675

Gene Names	Charge	m/z	Mass	Retention time	PEP	Score	Precursor Intensity
PSMD1	4	462.485	1845.911	40.747	0.075977	43.532	5533893

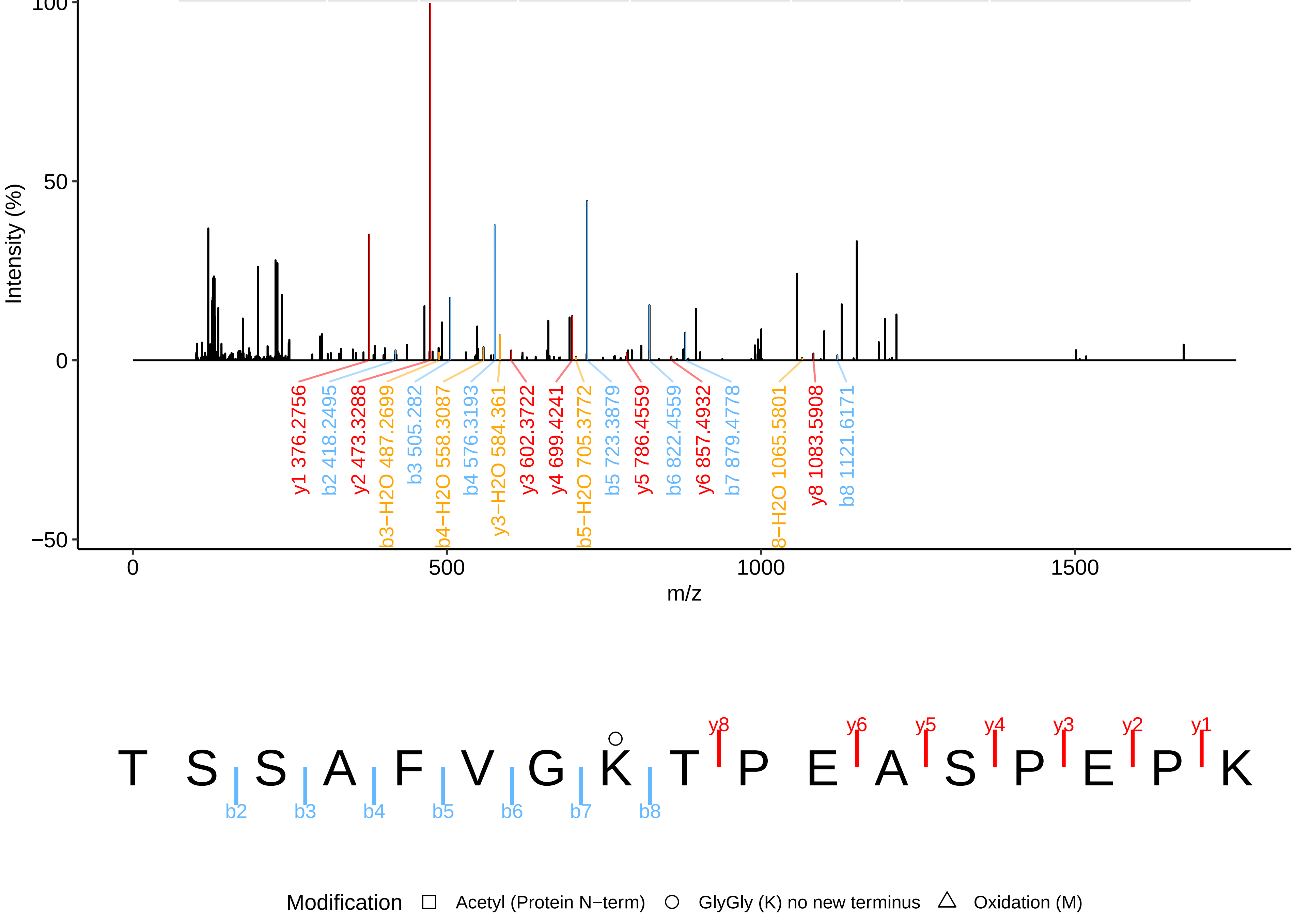

20200921_KIZ_MUb4_GlyGly_10plex_2 − Scan number: 10691

Gene Names	Charge	m/z	Mass	Retention time	PEP	Score	Precursor Intensity
PSMD1	3	616.3109	1845.911	40.957	0.060269	49.559	518543.6

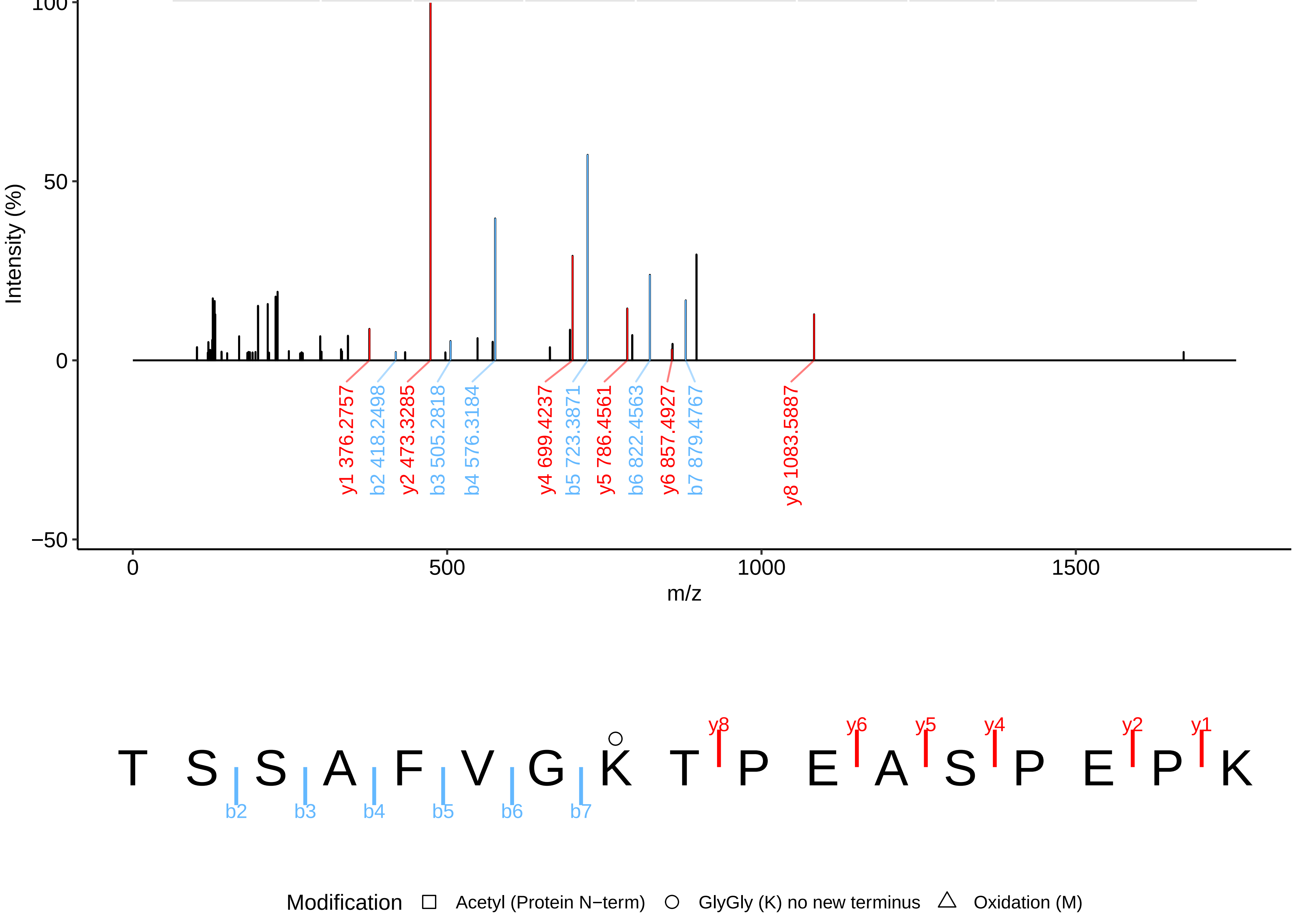

20200921_KIZ_MUb4_GlyGly_10plex_2 − Scan number: 10703

Gene Names	Charge	m/z	Mass	Retention time	PEP	Score	Precursor Intensity
PSMD1	3	616.3109	1845.911	41.003	0.0010049	73.983	532748.4

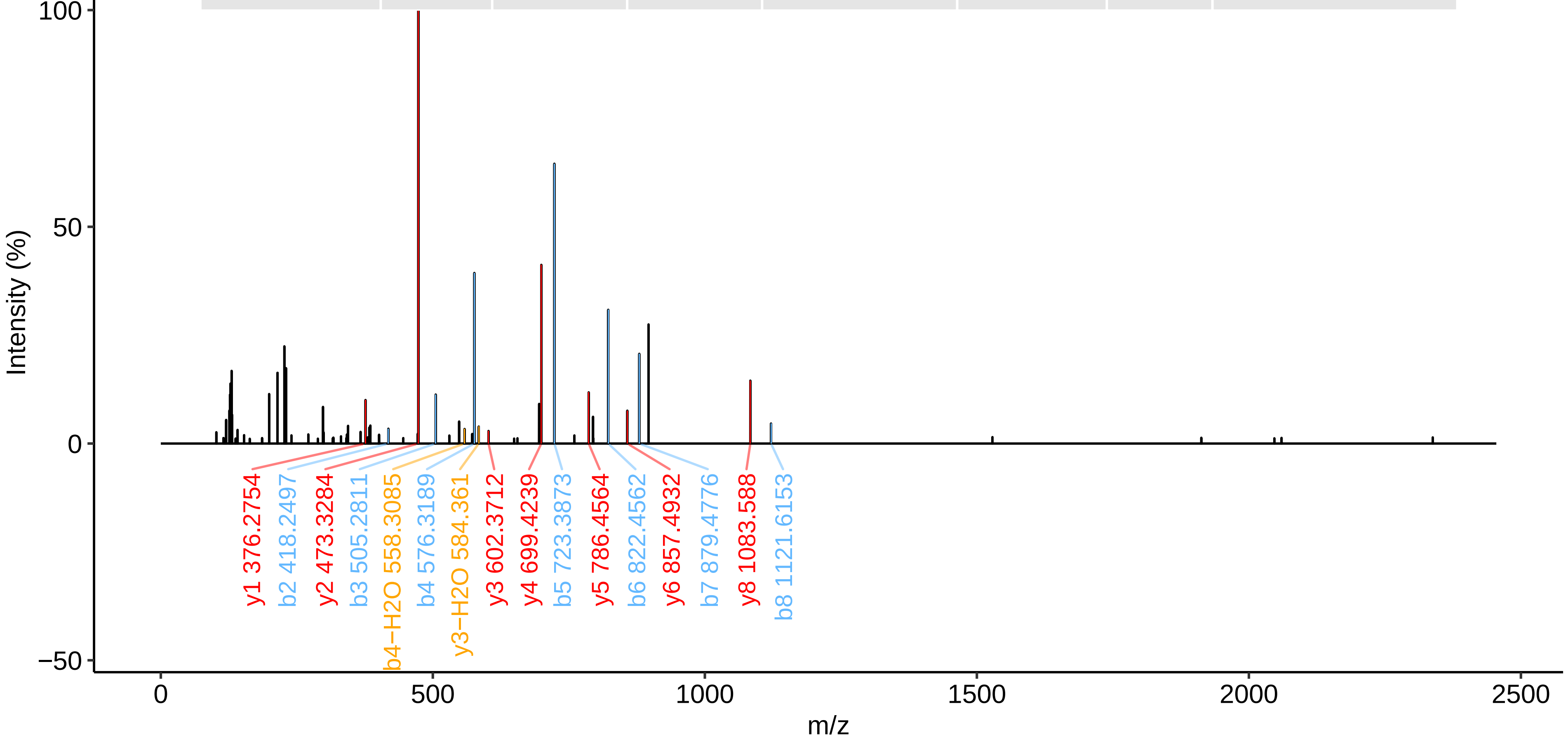

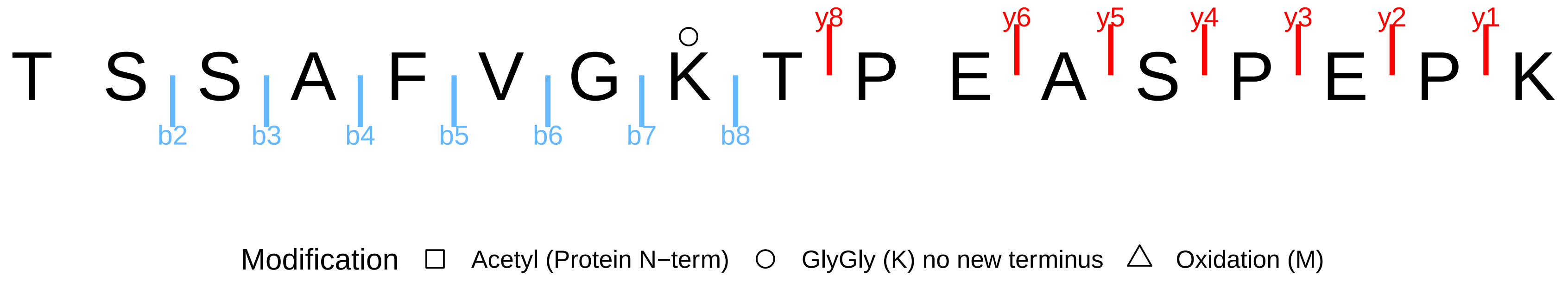

20200921_KIZ_MUb4_GlyGly_10plex_2 − Scan number: 16515

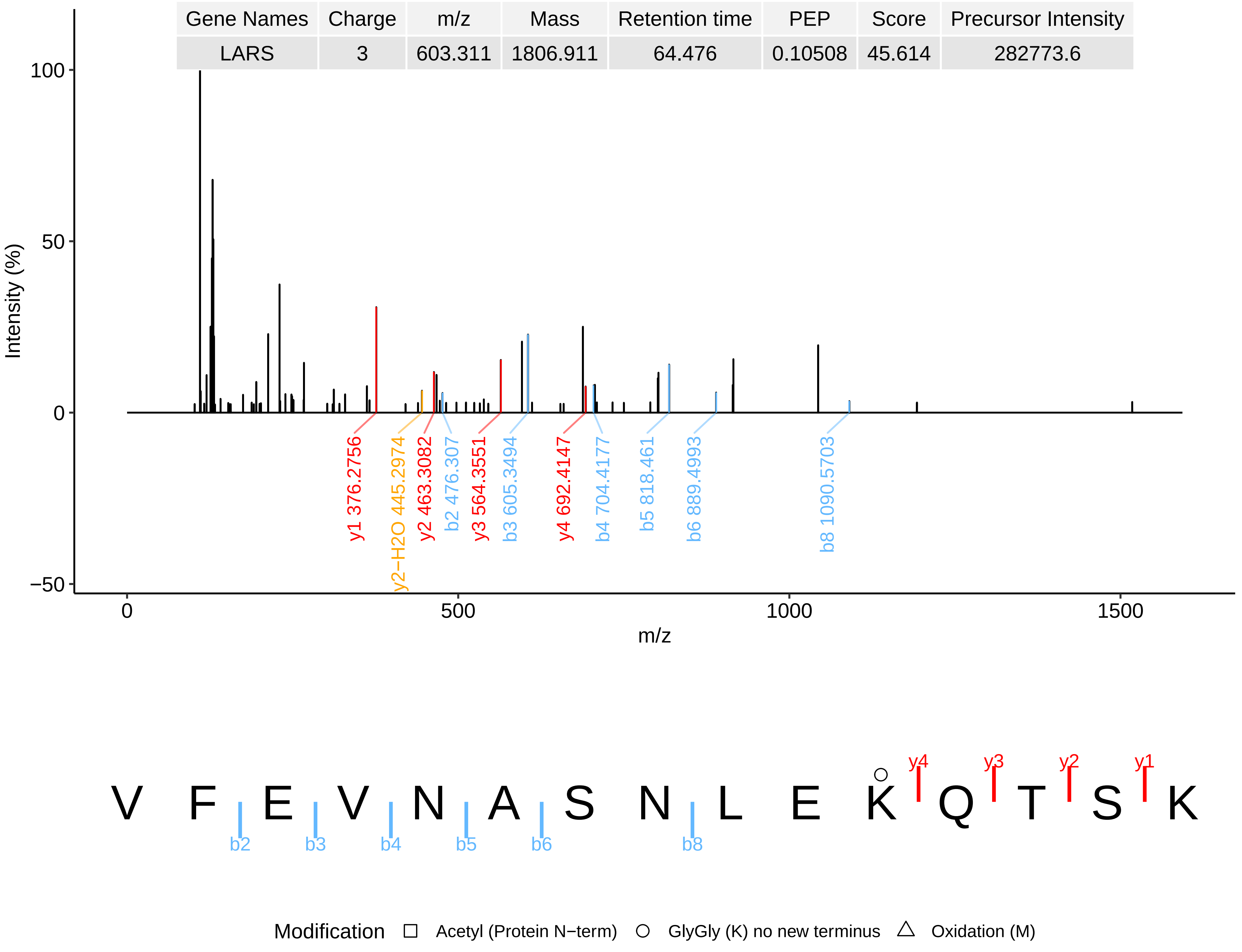

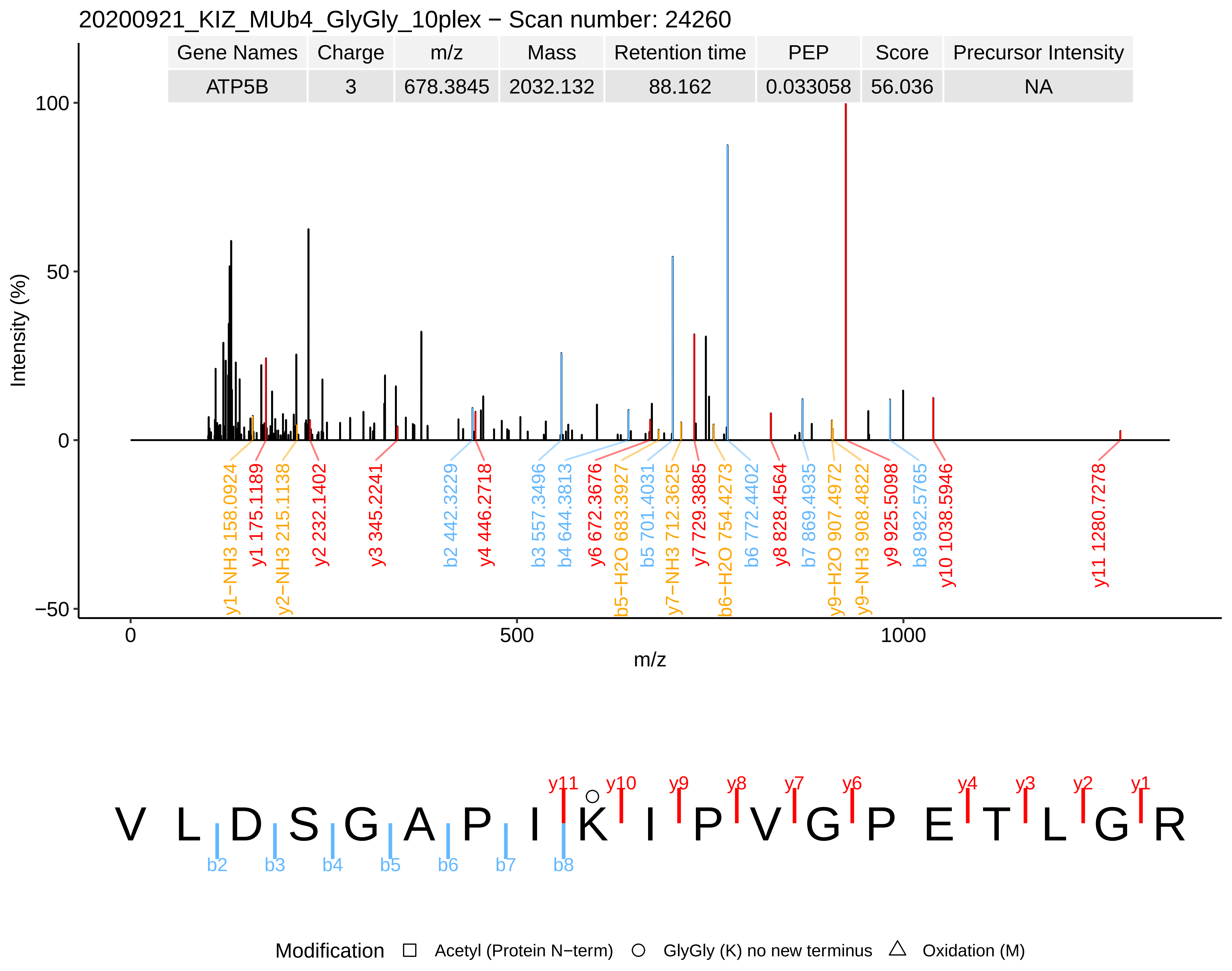

20200921_KIZ_MUb4_GlyGly_10plex − Scan number: 24260
Gene Names | Charge | m/z | Mass | Retention time | PEP | Score | Precursor Intensity
ATP5B | 3 | 678.3845 | 2032.132 | 88.162 | 0.033058 | 56.036 | NA
Intensity (%)
m/z
y1−NH3 158.0924
y1 175.1189
y2−NH3 215.1138
y2 232.1402
y3 345.2241
b2 442.3229
y4 446.2718
b3 557.3496
b4 644.3813
y6 672.3676
b5−H2O 683.3927
b5 701.4031
y7−NH3 712.3625
y7 729.3885
b6−H2O 754.4273
b6 772.4402
y8 828.4564
b7 869.4935
y9−H2O 907.4972
y9−NH3 908.4822
y9 925.5098
b8 982.5765
y10 1038.5946
y11 1280.7278
V L D S G A P I K I P V G P E T L G R
Modification
Acetyl (Protein N−term)
GlyGly (K) no new terminus
Oxidation (M)

20200921_KIZ_MUb4_GlyGly_10plex_2 − Scan number: 18412

Gene Names	Charge	m/z	Mass	Retention time	PEP	Score	Precursor Intensity
HSPA8	3	606.9612	1817.862	72.099	0.003684	65.472	803758.8

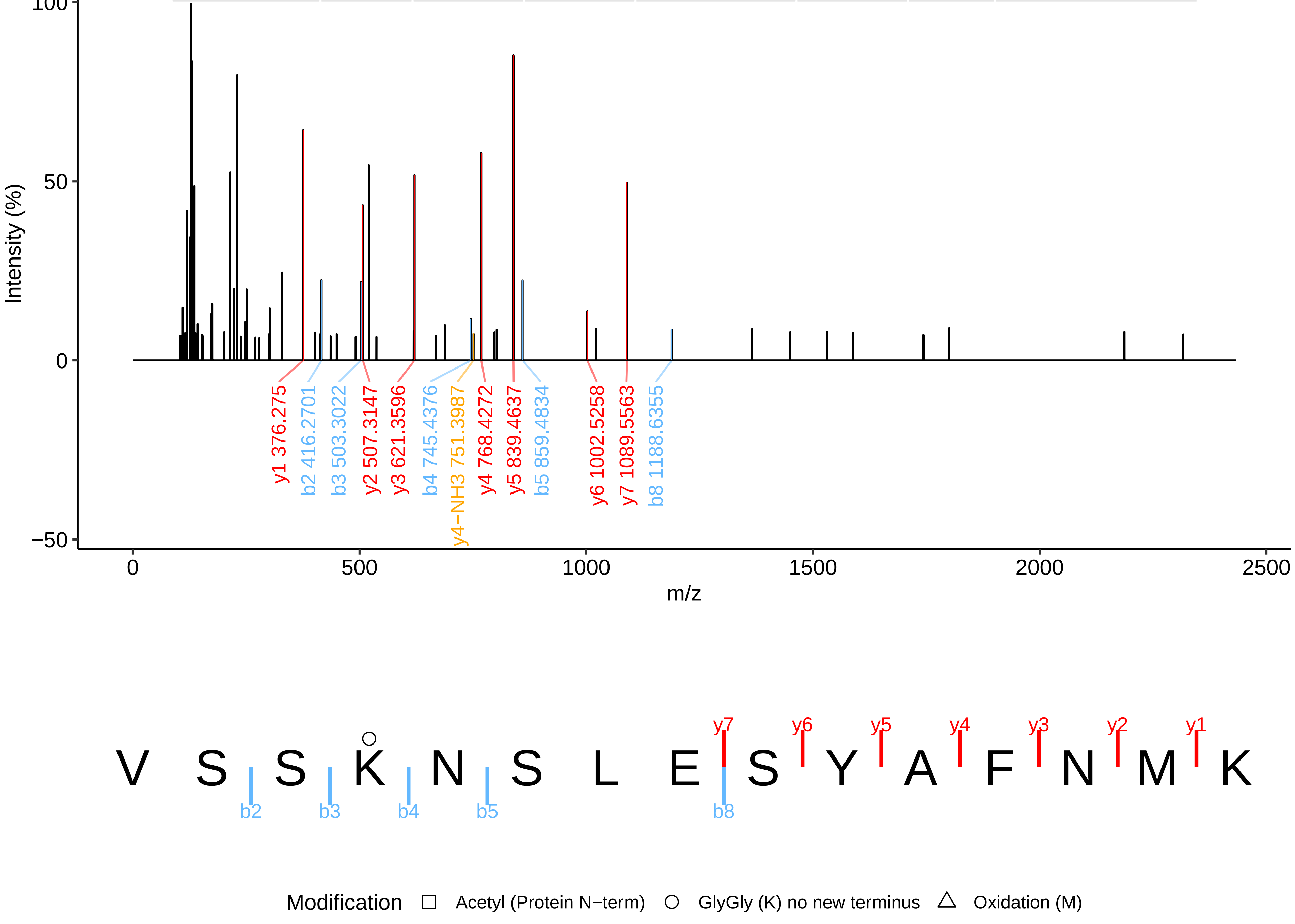

20200921_KIZ_MUb4_GlyGly_10plex_2 − Scan number: 14599

Gene Names	Charge	m/z	Mass	Retention time	PEP	Score	Precursor Intensity
CFL1;CFL2	3	599.2844	1794.831	56.939	0.00067252	82.543	765821.2

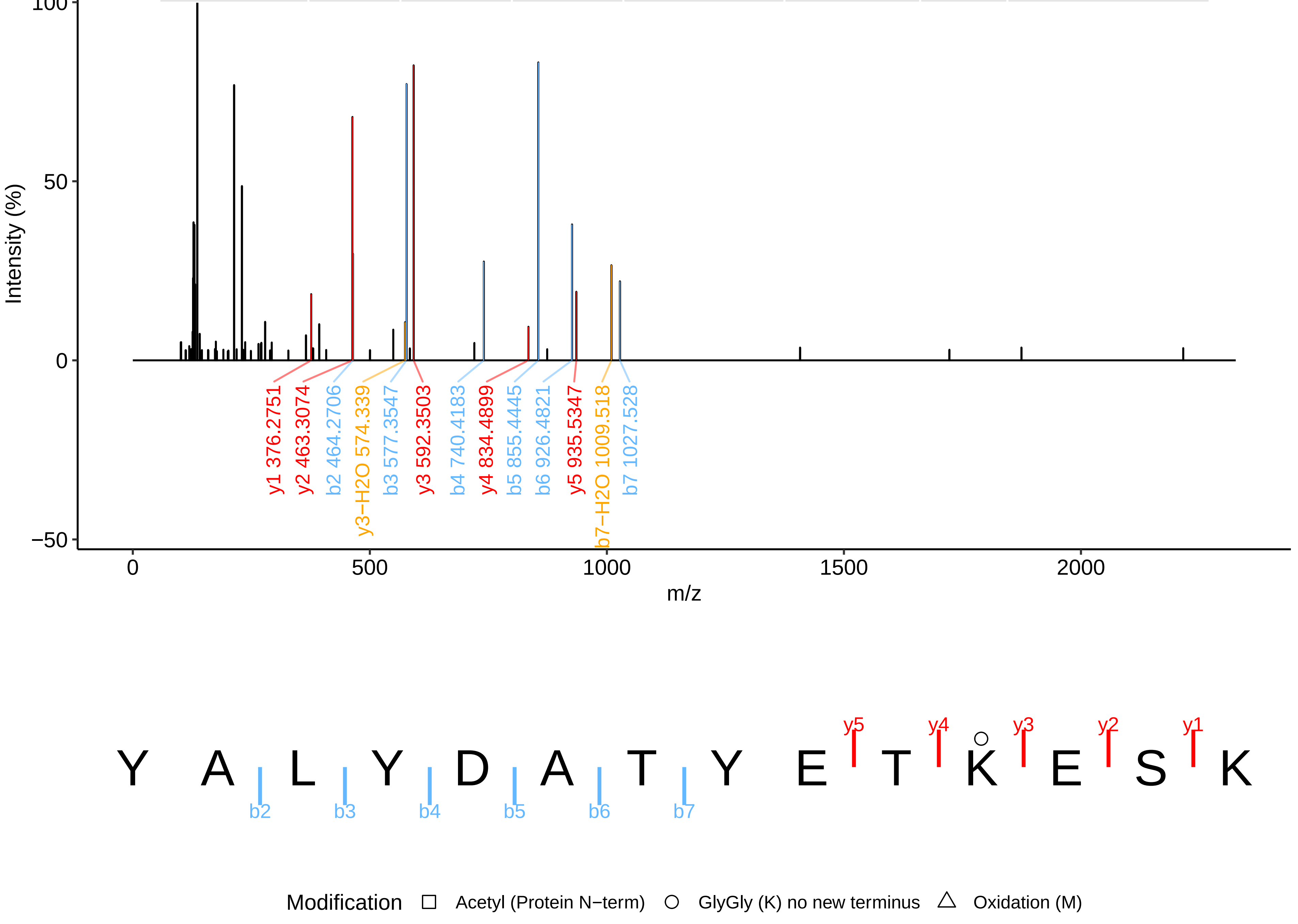

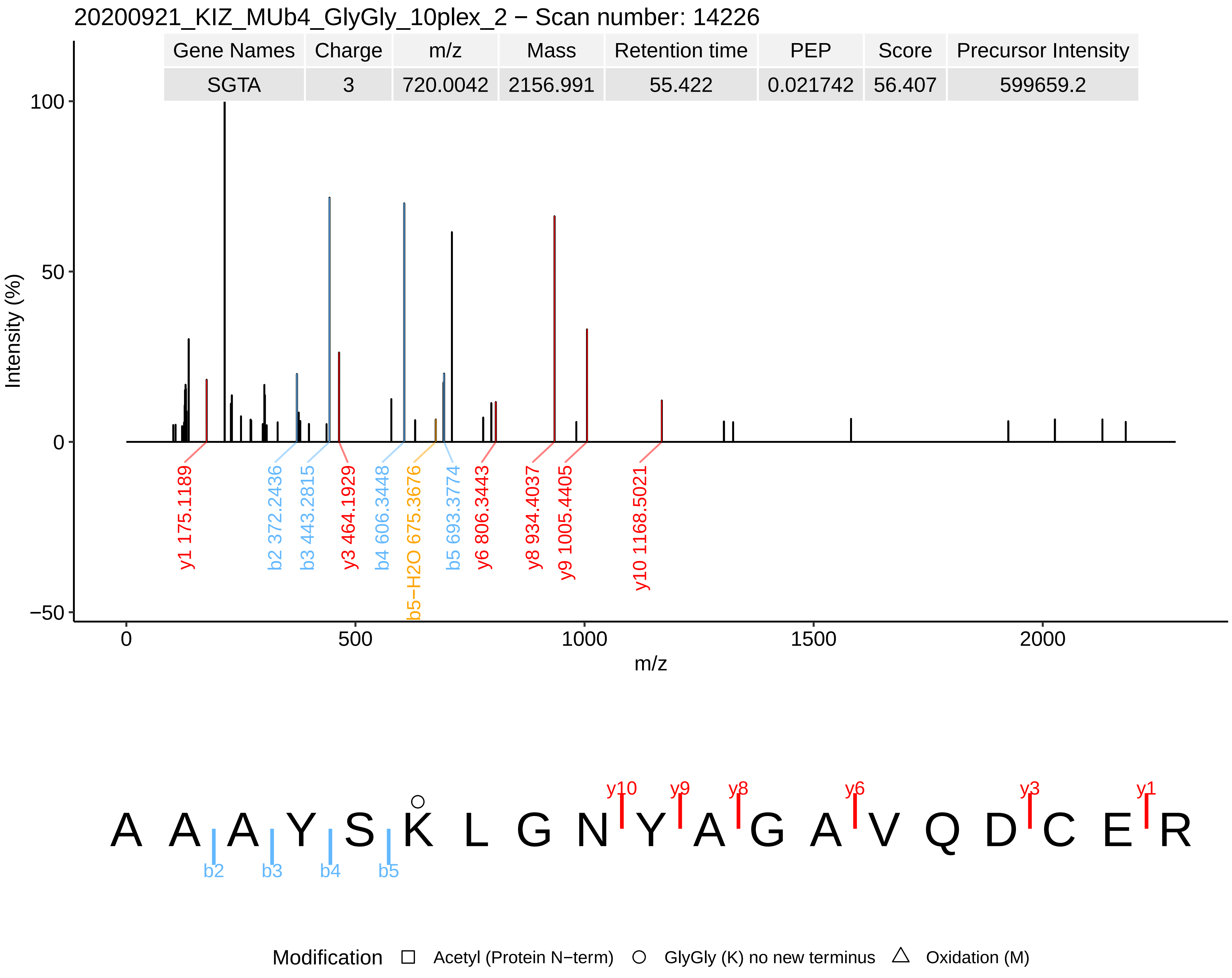
20200921_KIZ_MUb4_GlyGly_10plex_2 − Scan number: 14226
Gene Names | Charge | m/z | Mass | Retention time | PEP | Score | Precursor Intensity
SGTA | 3 | 720.0042 | 2156.991 | 55.422 | 0.021742 | 56.407 | 599659.2
Intensity (%)
100
50
0
−50
0
500
1000
1500
2000
m/z
y1 175.1189
b2 372.2436
b3 443.2815
y3 464.1929
b4 606.3448
b5−H2O 675.3676
b5 693.3774
y6 806.3443
y8 934.4037
y9 1005.4405
y10 1168.5021
A A A Y S K L G N Y A G A V Q D C E R
y10
y9
y8
y6
y3
y1
b2
b3
b4
b5
Modification
Acetyl (Protein N−term)
GlyGly (K) no new terminus
Oxidation (M)

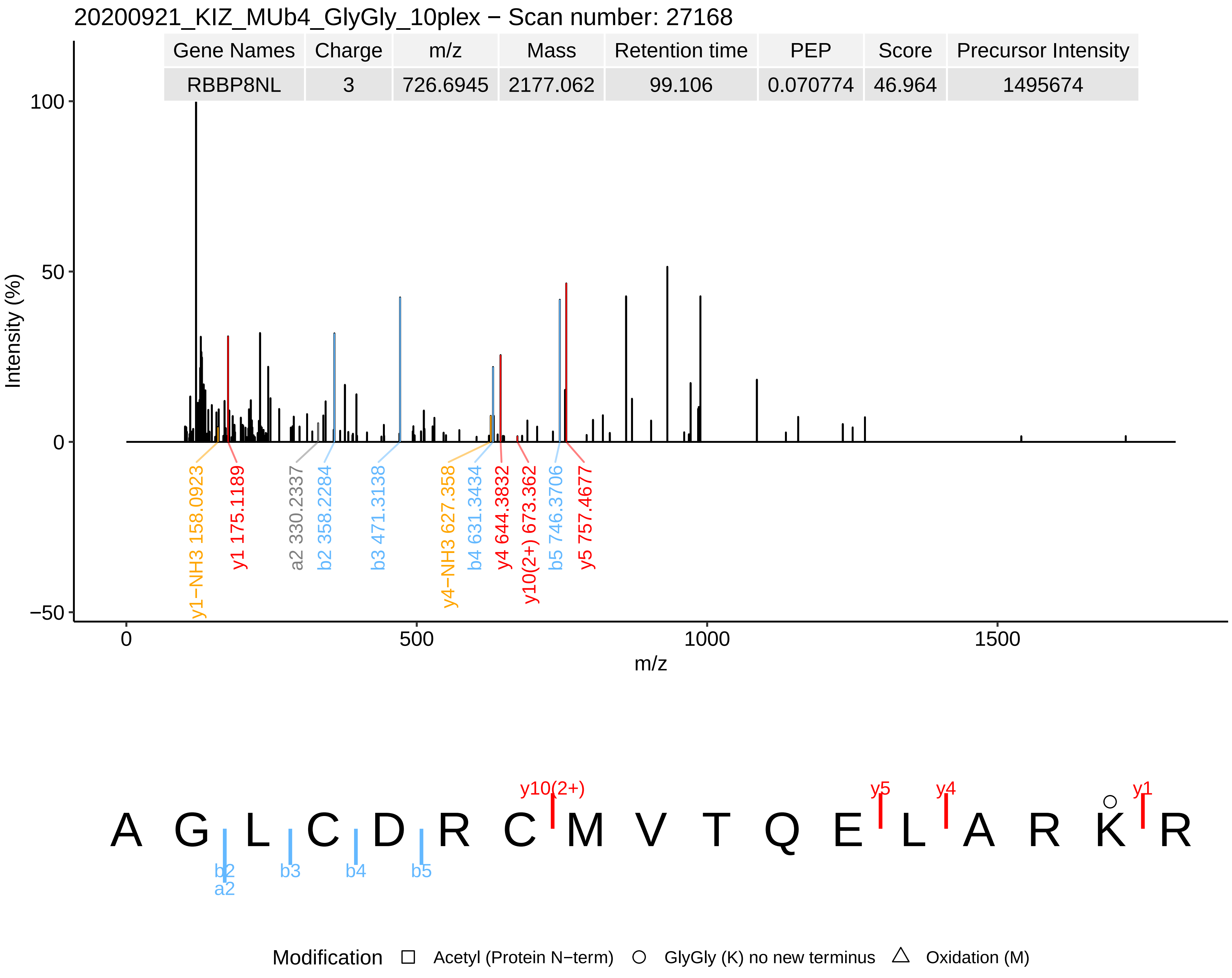

20200921_KIZ_MUb4_GlyGly_10plex − Scan number: 27168
Gene Names | Charge | m/z | Mass | Retention time | PEP | Score | Precursor Intensity
RBBP8NL | 3 | 726.6945 | 2177.062 | 99.106 | 0.070774 | 46.964 | 1495674
Intensity (%)
100
50
0
−50
0
500
1000
1500
m/z
y1−NH3 158.0923
y1 175.1189
a2 330.2337
b2 358.2284
b3 471.3138
y4−NH3 627.358
b4 631.3434
y4 644.3832
y10(2+) 673.362
b5 746.3706
y5 757.4677
A G L C D R C M V T Q E L A R K R
y10(2+)
y5
y4
y1
b2
a2
b3
b4
b5
Modification
Acetyl (Protein N−term)
GlyGly (K) no new terminus
Oxidation (M)

20200924_KIZ_MUb5_GlyGly_1 − Scan number: 17494

Gene Names	Charge	m/z	Mass	Retention time	PEP	Score	Precursor Intensity
SGTA	3	655.3251	1962.954	56.475	6.4265e−20	157.23	12362590

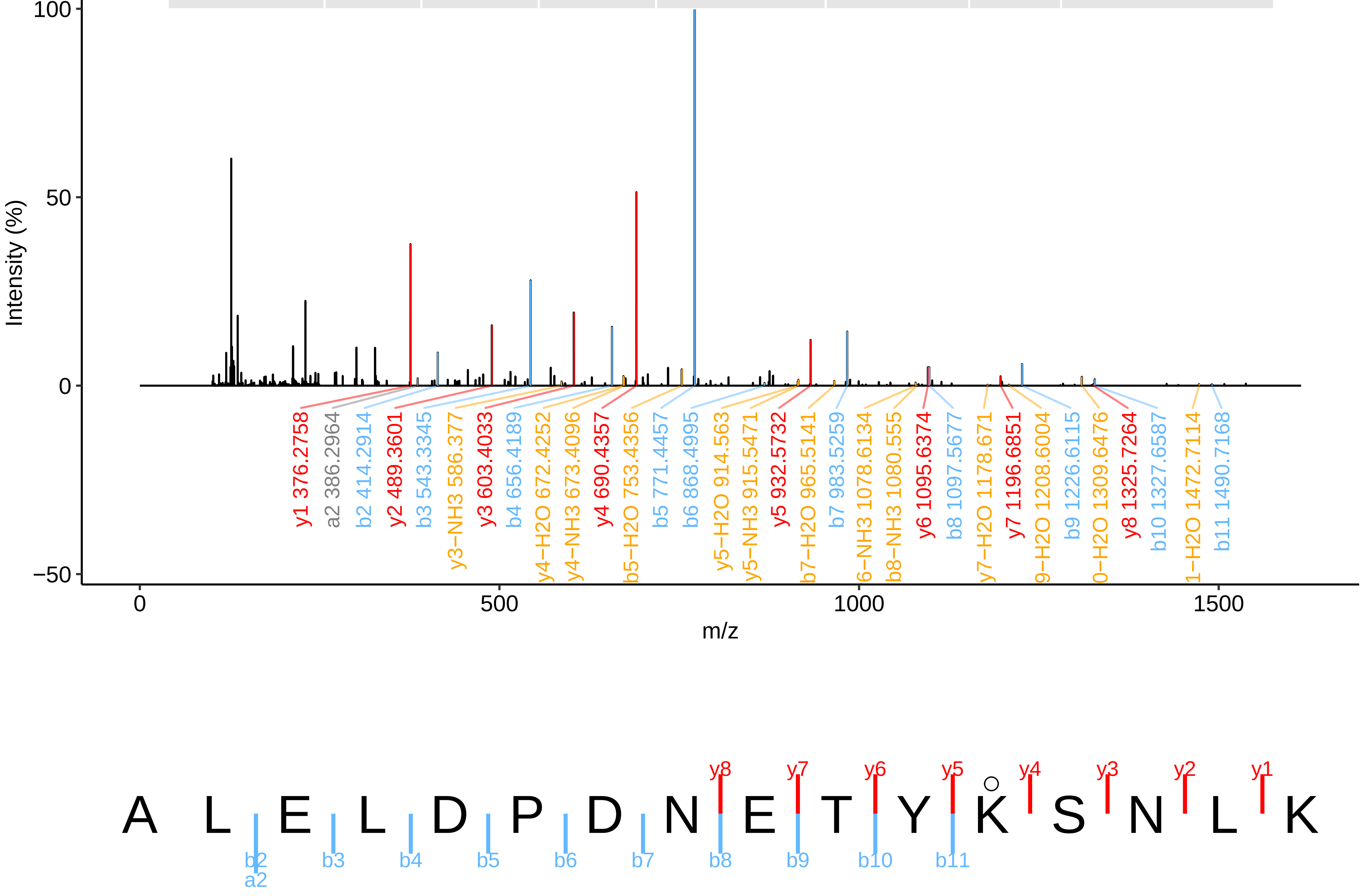

Modification □ Acetyl (Protein N−term) ○ GlyGly (K) no new terminus △ Oxidation (M)

20200924_KIZ_MUb5_GlyGly_2 − Scan number: 16051

Gene Names	Charge	m/z	Mass	Retention time	PEP	Score	Precursor Intensity
SGTA	3	655.3251	1962.954	59.616	1.3715e−13	133.14	NA

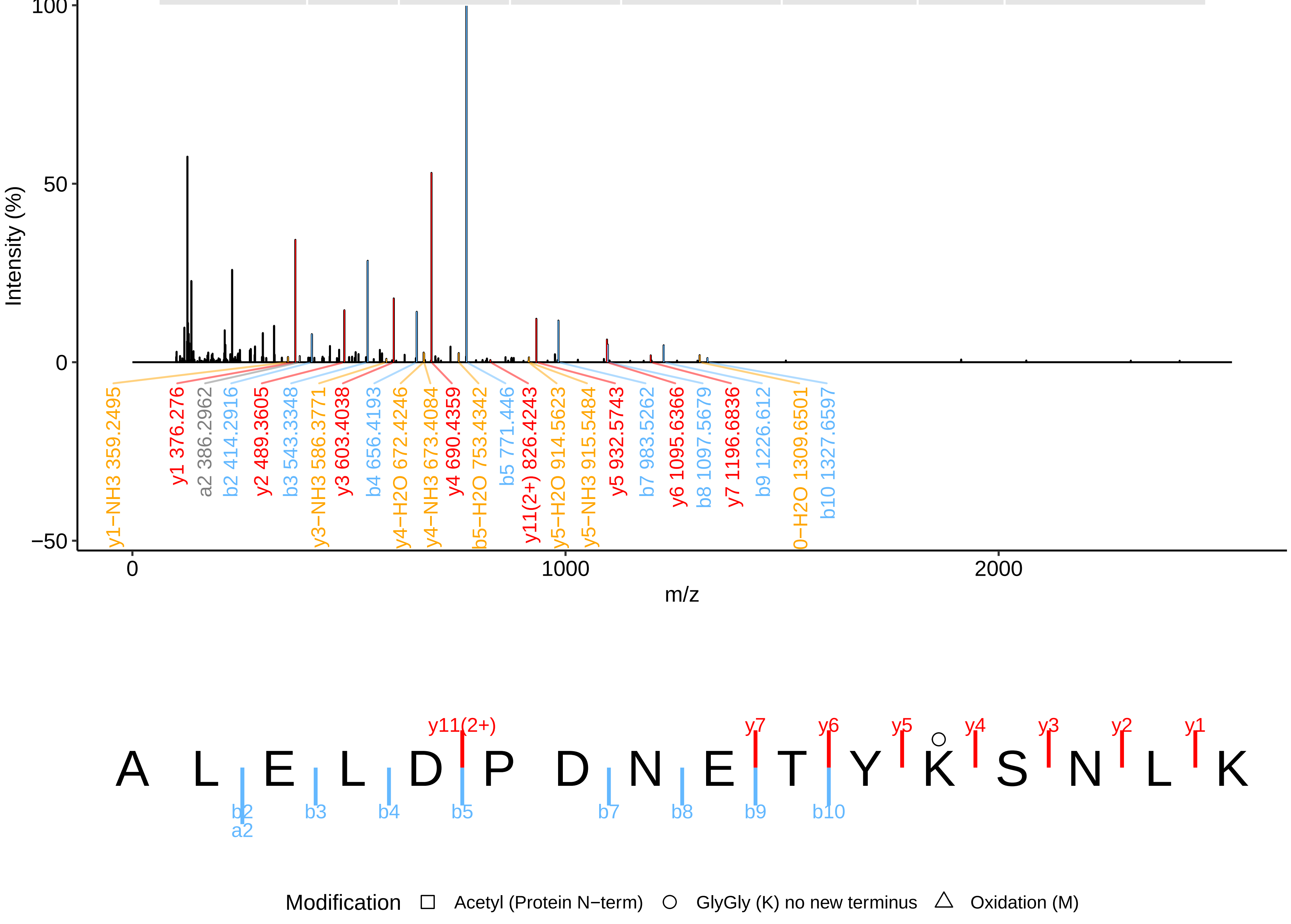

20200921_KIZ_MUb4_GlyGly_10plex_2 − Scan number: 7546

Gene Names	Charge	m/z	Mass	Retention time	PEP	Score	Precursor Intensity
BASP1	4	442.9733	1767.864	28.195	0.016303	66.246	330771.5

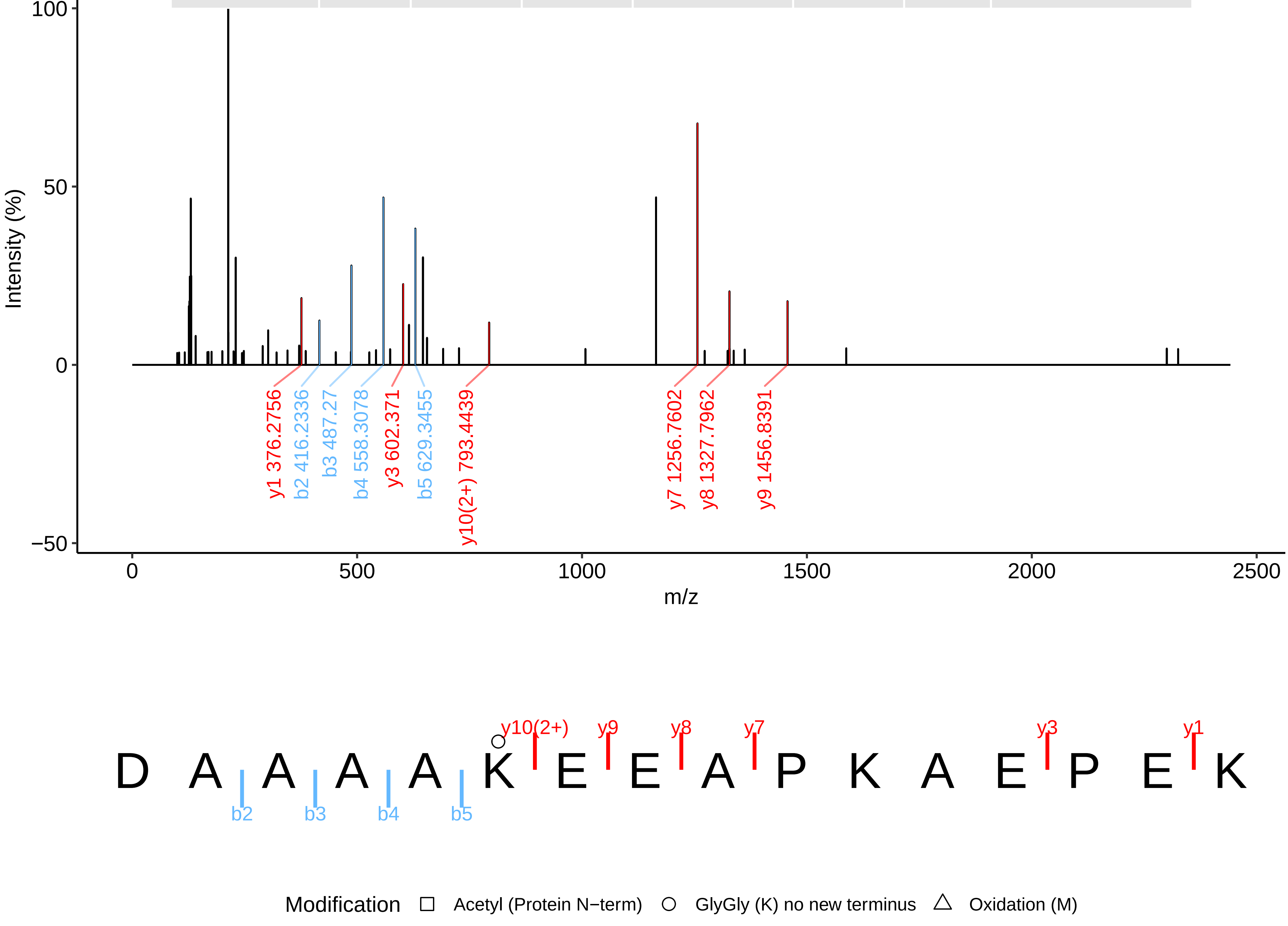

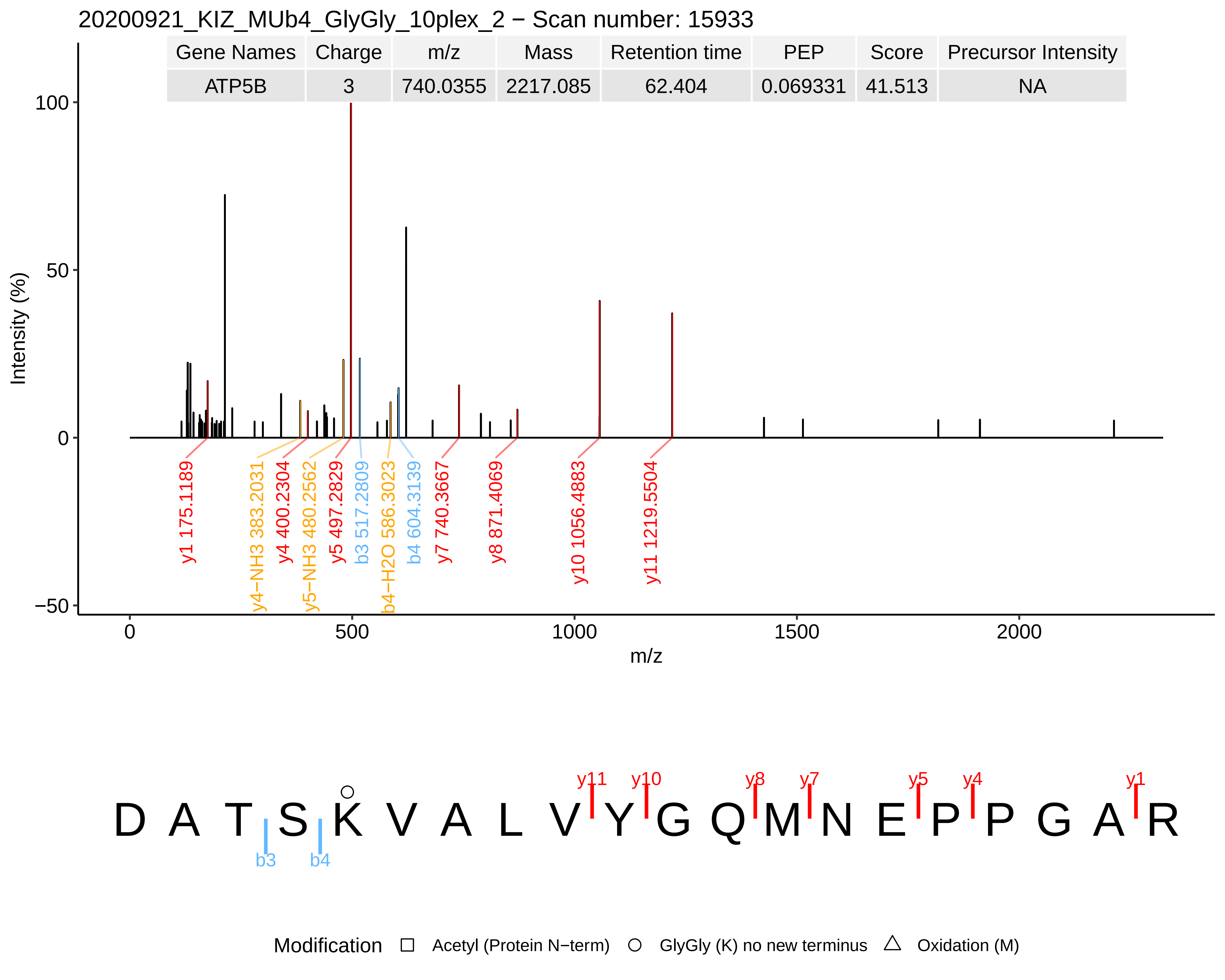
20200921_KIZ_MUb4_GlyGly_10plex_2 − Scan number: 15933
Gene Names | Charge | m/z | Mass | Retention time | PEP | Score | Precursor Intensity
ATP5B | 3 | 740.0355 | 2217.085 | 62.404 | 0.069331 | 41.513 | NA
Intensity (%)
100
50
0
−50
y1 175.1189
y4−NH3 383.2031
y4 400.2304
y5−NH3 480.2562
y5 497.2829
b3 517.2809
b4−H2O 586.3023
b4 604.3139
y7 740.3667
y8 871.4069
y10 1056.4883
y11 1219.5504
0
500
1000
1500
2000
m/z
y11
y10
y8
y7
y5
y4
y1
D A T S K V A L V Y G Q M N E P P G A R
b3
b4
Modification
Acetyl (Protein N−term)
GlyGly (K) no new terminus
Oxidation (M)

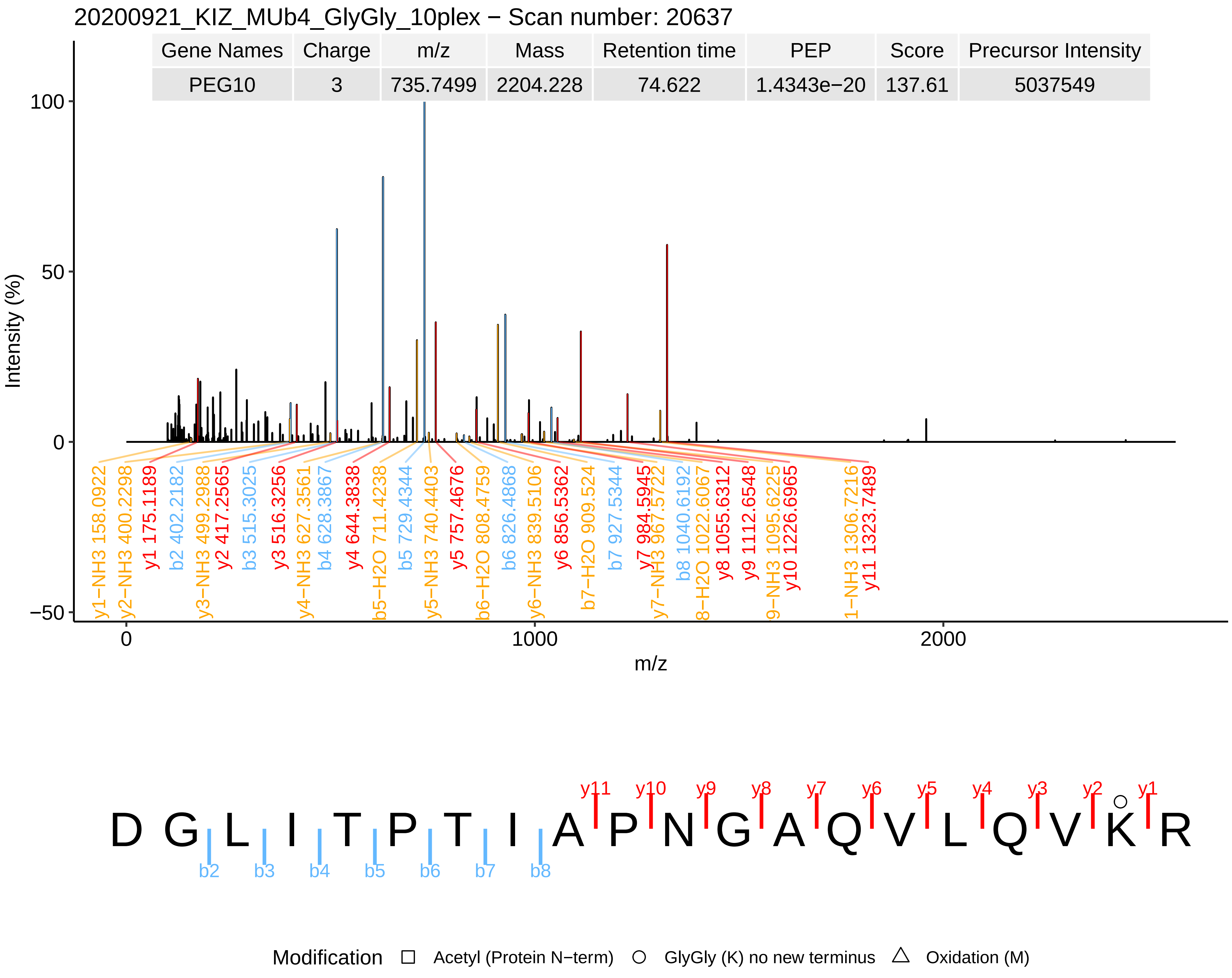
20200921_KIZ_MUb4_GlyGly_10plex − Scan number: 20637
Gene Names | Charge | m/z | Mass | Retention time | PEP | Score | Precursor Intensity
PEG10 | 3 | 735.7499 | 2204.228 | 74.622 | 1.4343e−20 | 137.61 | 5037549
Intensity (%)
100
50
0
−50
0
1000
2000
m/z
y1−NH3 158.0922
y2−NH3 400.2298
y1 175.1189
b2 402.2182
y3−NH3 499.2988
y2 417.2565
b3 515.3025
y3 516.3256
y4−NH3 627.3561
b4 628.3867
y4 644.3838
b5−H2O 711.4238
b5 729.4344
y5−NH3 740.4403
y5 757.4676
b6−H2O 808.4759
b6 826.4868
y6−NH3 839.5106
y6 856.5362
b7−H2O 909.524
b7 927.5344
y7 984.5945
y7−NH3 967.5722
b8 1040.6192
8−H2O 1022.6067
y8 1055.6312
y9 1112.6548
9−NH3 1095.6225
y10 1226.6965
1−NH3 1306.7216
y11 1323.7489
D G L I T P T I A P N G A Q V L Q V K R
y11 y10 y9 y8 y7 y6 y5 y4 y3 y2 y1
b2 b3 b4 b5 b6 b7 b8
Modification
Acetyl (Protein N−term)
GlyGly (K) no new terminus
Oxidation (M)

20200921_KIZ_MUb4_GlyGly_10plex − Scan number: 20773

Gene Names	Charge	m/z	Mass	Retention time	PEP	Score	Precursor Intensity
PEG10	3	735.7499	2204.228	75.132	5.0771e−27	162.41	NA

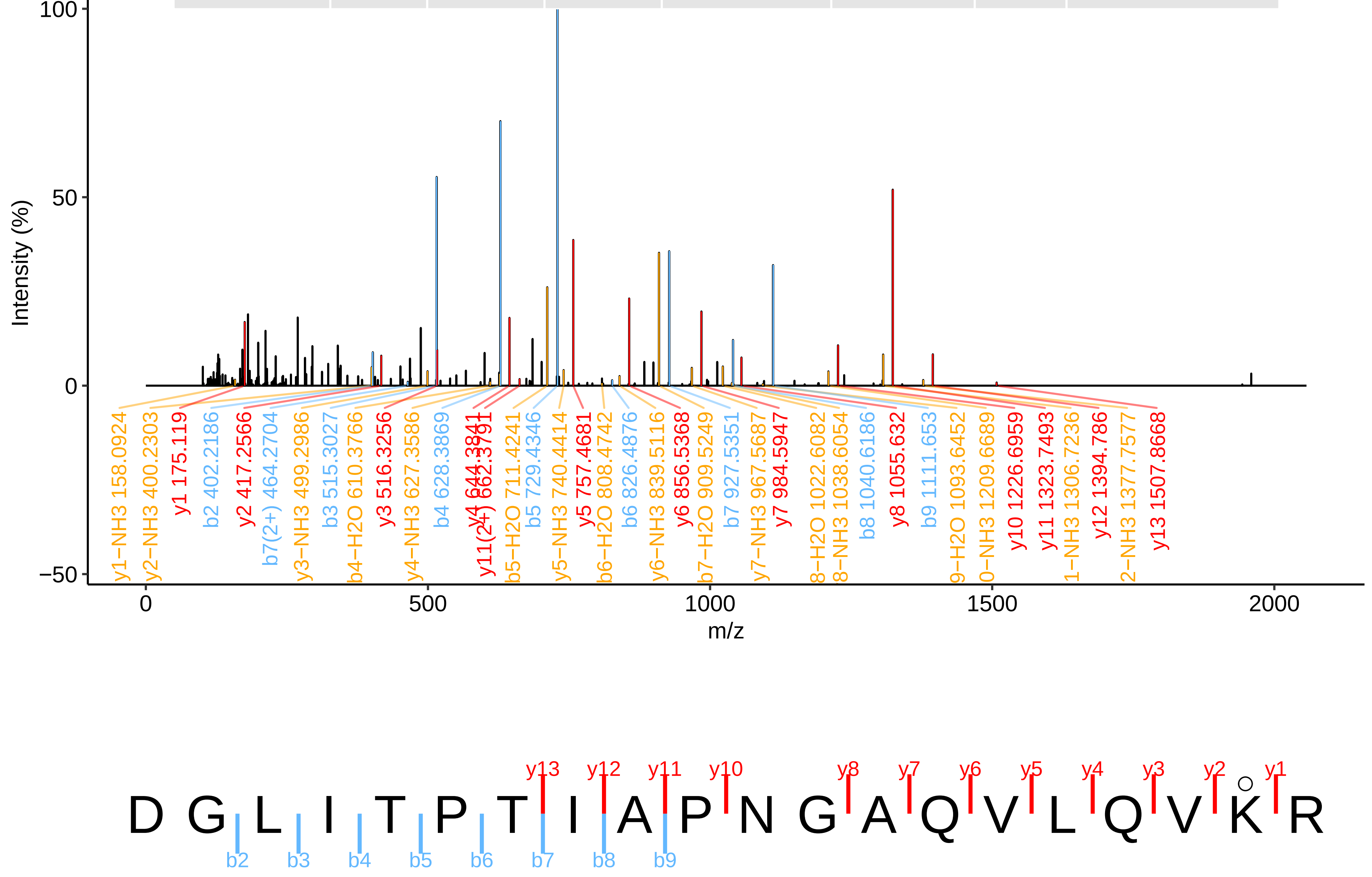

Modification □ Acetyl (Protein N−term) ○ GlyGly (K) no new terminus △ Oxidation (M)

20200921_KIZ_MUb4_GlyGly_10plex_2 − Scan number: 18942

Gene Names	Charge	m/z	Mass	Retention time	PEP	Score	Precursor Intensity
PEG10	3	735.7499	2204.228	74.273	1.5579e−08	104.44	893357.9

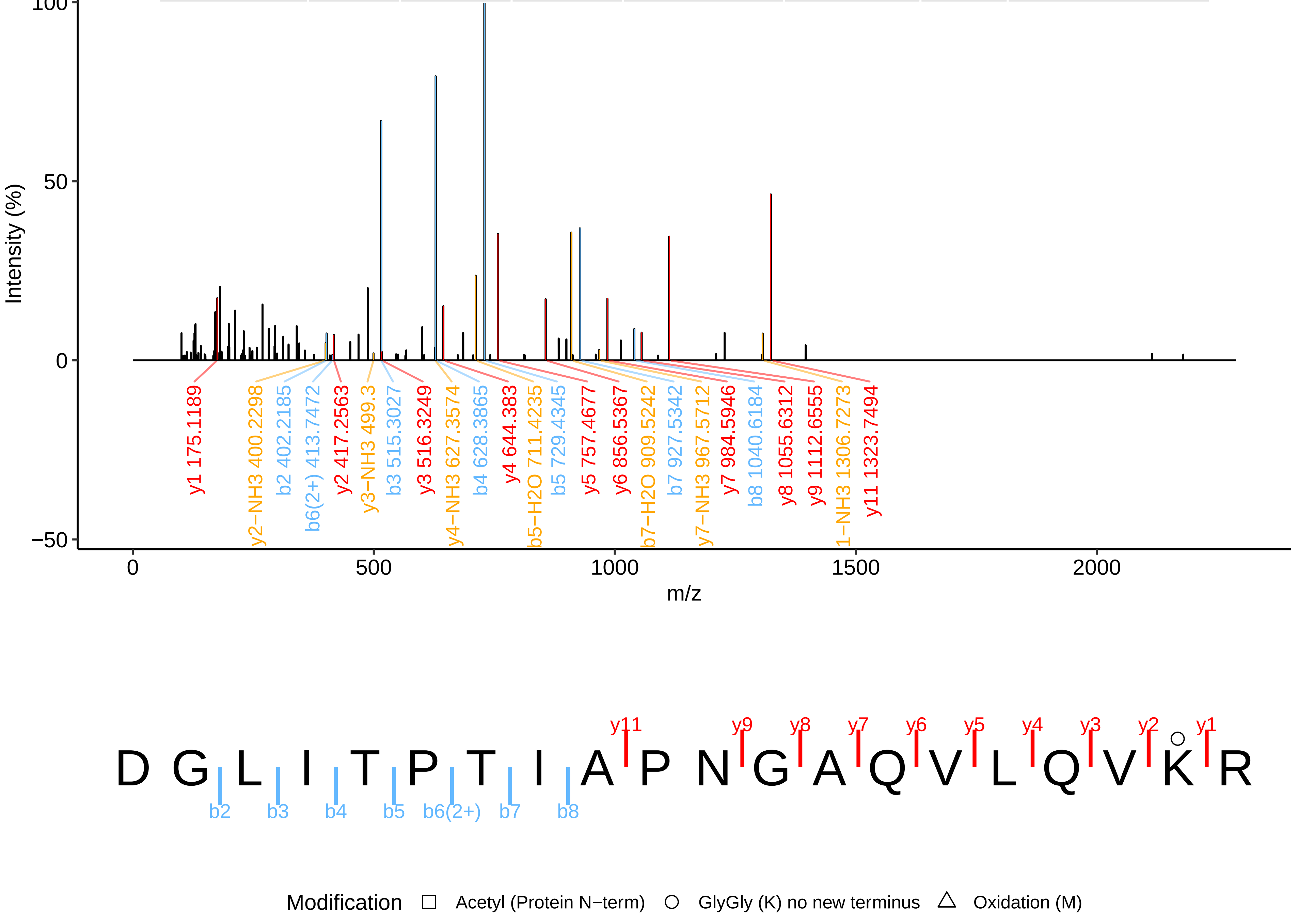

20200921_KIZ_MUb4_GlyGly_10plex_2 − Scan number: 18944

Gene Names	Charge	m/z	Mass	Retention time	PEP	Score	Precursor Intensity
PEG10	3	735.7499	2204.228	74.281	9.3967e−21	141.97	1526995

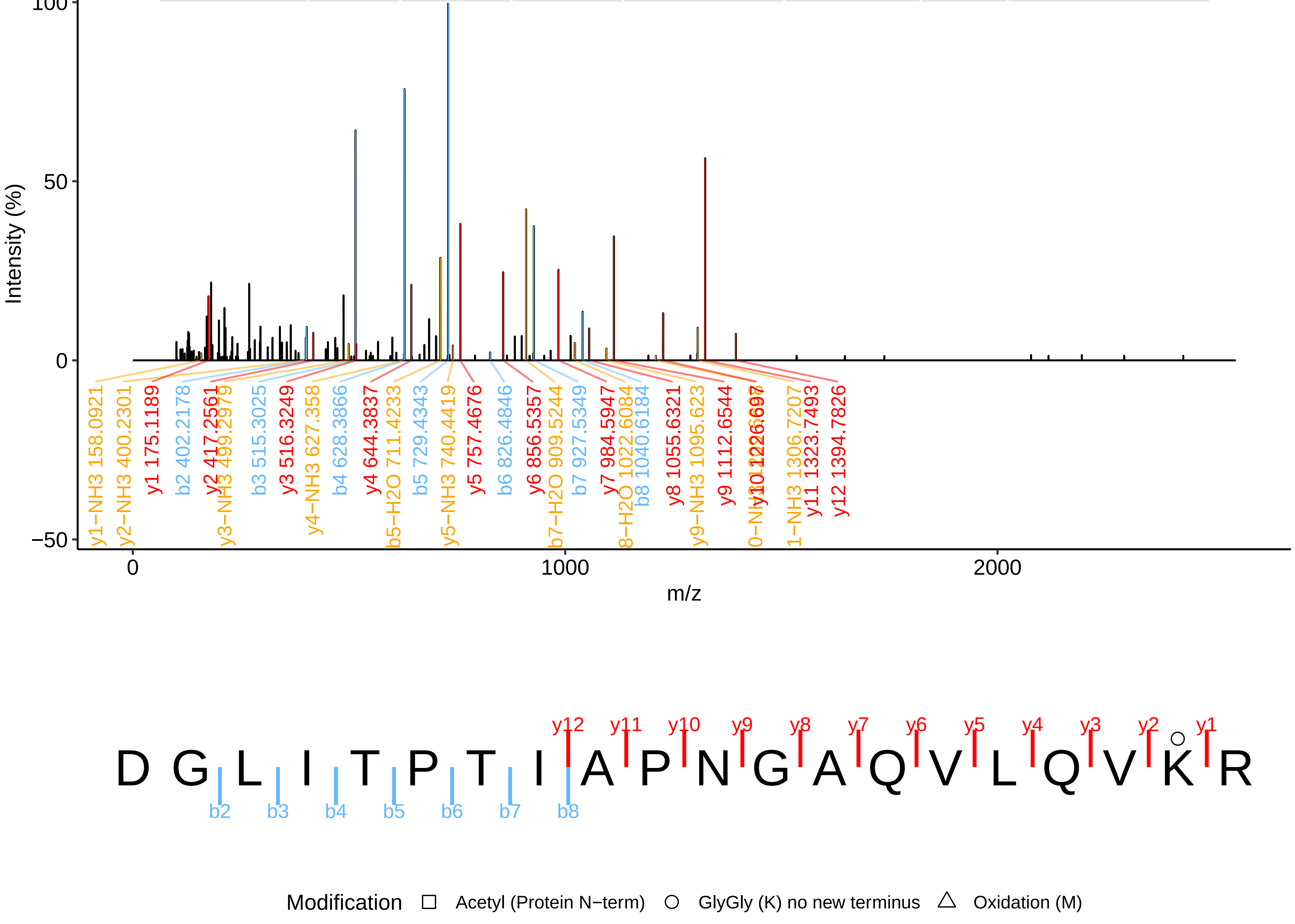

20200924_KIZ_MUb5_GlyGly_2 − Scan number: 18829

Gene Names	Charge	m/z	Mass	Retention time	PEP	Score	Precursor Intensity
PEG10	3	735.7499	2204.228	69.849	1.1834e−05	95.767	2079550

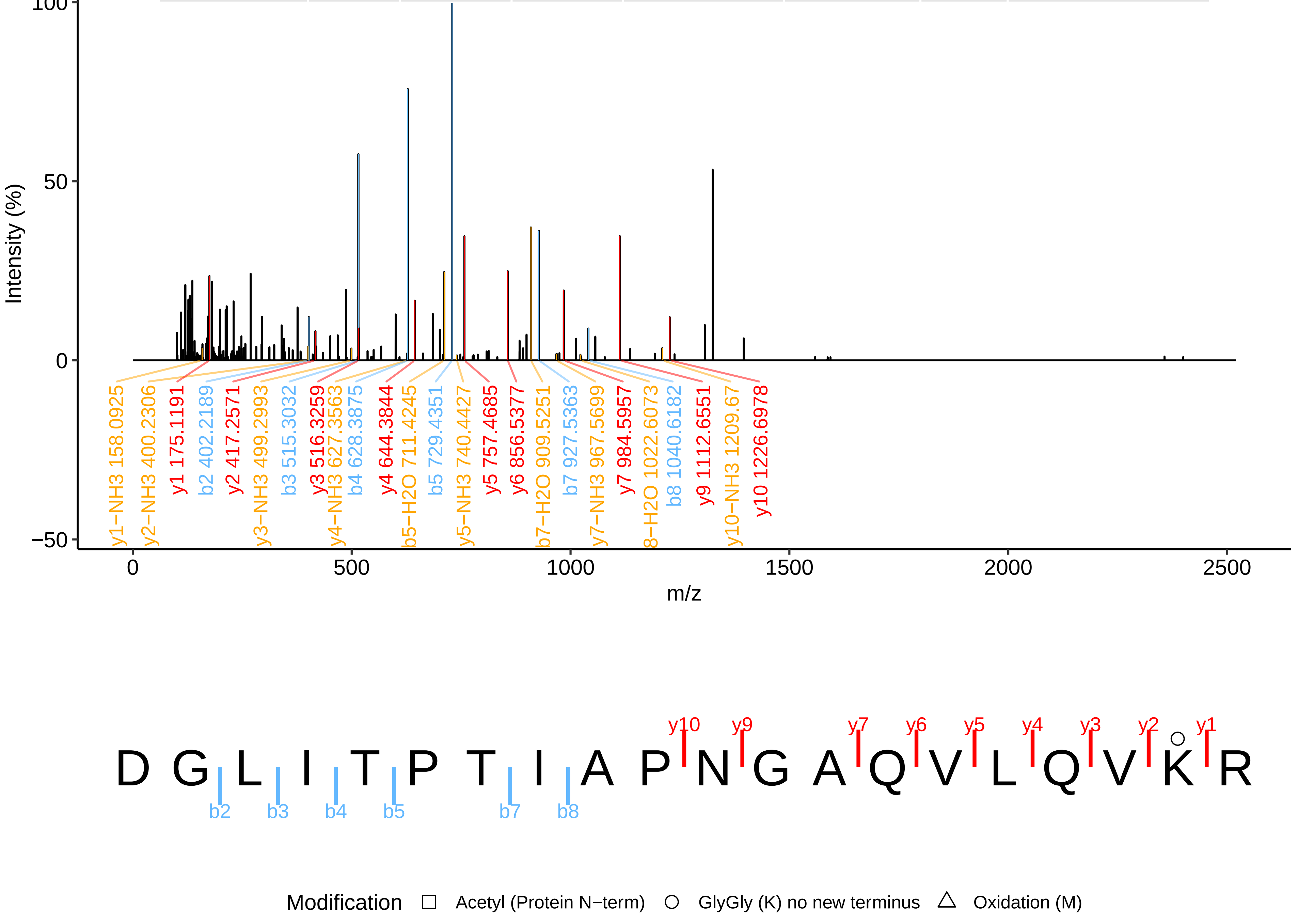

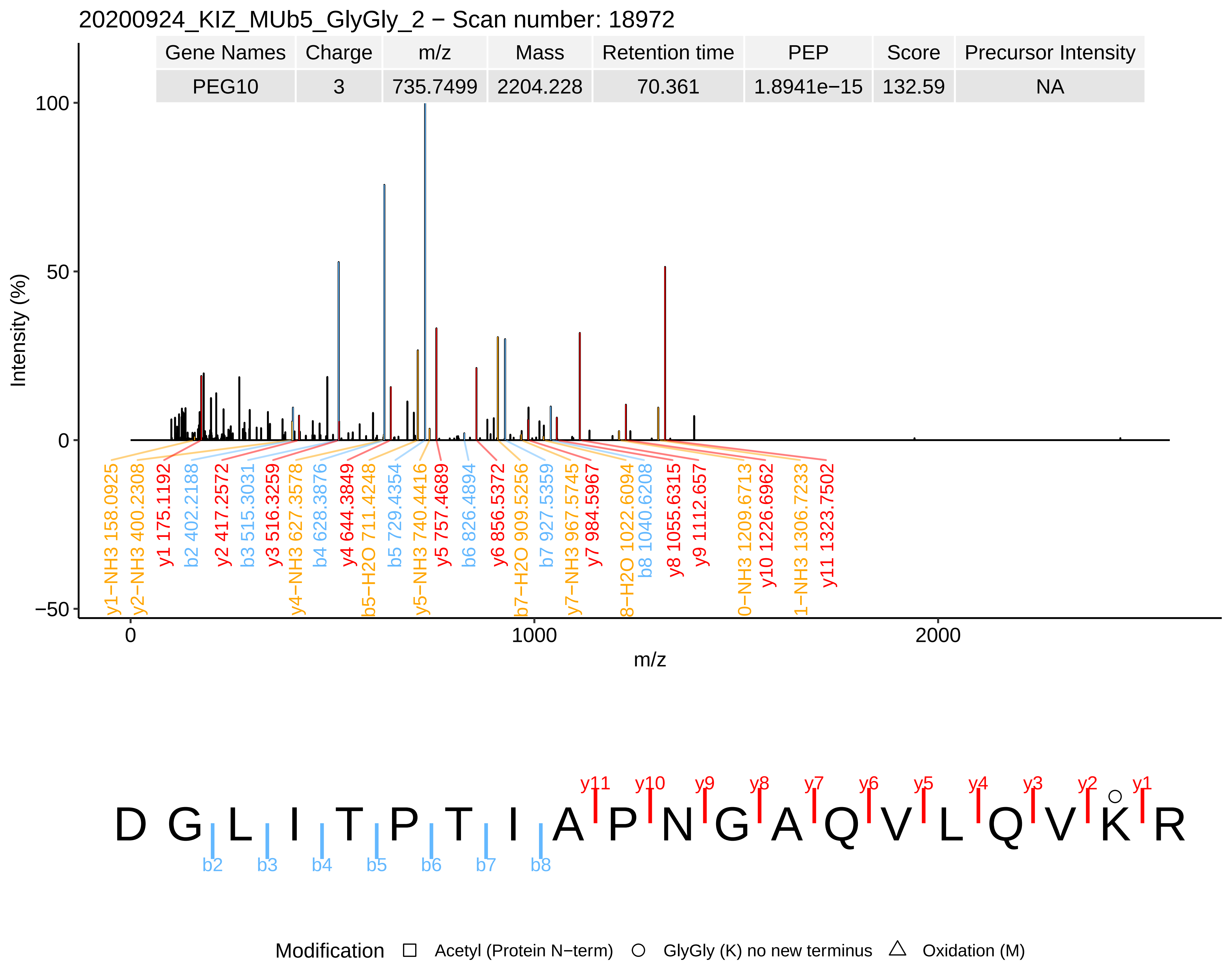
20200924_KIZ_MUb5_GlyGly_2 − Scan number: 18972
Gene Names | Charge | m/z | Mass | Retention time | PEP | Score | Precursor Intensity
PEG10 | 3 | 735.7499 | 2204.228 | 70.361 | 1.8941e−15 | 132.59 | NA
Intensity (%)
100
50
0
−50
m/z
0
1000
2000
y1−NH3 158.0925
y2−NH3 400.2308
y1 175.1192
b2 402.2188
y2 417.2572
b3 515.3031
y3 516.3259
y4−NH3 627.3578
b4 628.3876
y4 644.3849
b5−H2O 711.4248
b5 729.4354
y5−NH3 740.4416
y5 757.4689
b6 826.4894
y6 856.5372
b7−H2O 909.5256
b7 927.5359
y7−NH3 967.5745
y7 984.5967
8−H2O 1022.6094
b8 1040.6208
y8 1055.6315
y9 1112.657
0−NH3 1209.6713
y10 1226.6962
1−NH3 1306.7233
y11 1323.7502
D G L I T P T I A P N G A Q V L Q V K R
y11 y10 y9 y8 y7 y6 y5 y4 y3 y2 y1
b2 b3 b4 b5 b6 b7 b8
Modification
Acetyl (Protein N−term)
GlyGly (K) no new terminus
Oxidation (M)

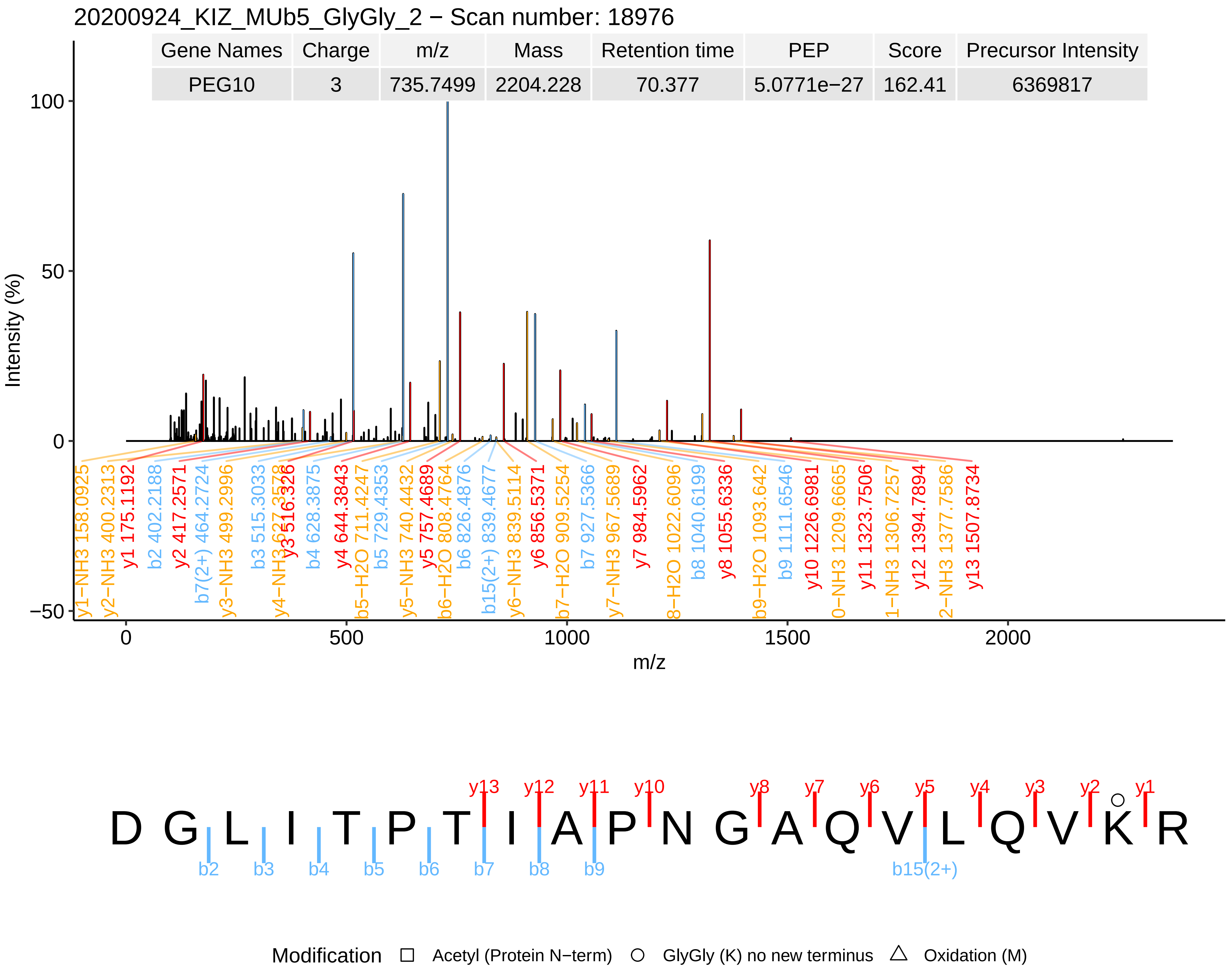
20200924_KIZ_MUb5_GlyGly_2 − Scan number: 18976
Gene Names | Charge | m/z | Mass | Retention time | PEP | Score | Precursor Intensity
PEG10 | 3 | 735.7499 | 2204.228 | 70.377 | 5.0771e−27 | 162.41 | 6369817
Intensity (%)
100
50
0
−50
0
500
1000
1500
2000
m/z
y1−NH3 158.0925
y2−NH3 400.2313
y1 175.1192
b2 402.2188
y2 417.2571
b7(2+) 464.2724
y3−NH3 499.2996
b3 515.3033
y4−NH3 627.3578
y3 516.326
b4 628.3875
y4 644.3843
b5−H2O 711.4247
b5 729.4353
y5−NH3 740.4432
y5 757.4689
b6−H2O 808.4764
b6 826.4876
b15(2+) 839.4677
y6−NH3 839.5114
y6 856.5371
b7−H2O 909.5254
b7 927.5366
y7−NH3 967.5689
y7 984.5962
8−H2O 1022.6096
b8 1040.6199
y8 1055.6336
b9−H2O 1093.642
b9 1111.6546
y10 1226.6981
0−NH3 1209.6665
y11 1323.7506
1−NH3 1306.7257
y12 1394.7894
2−NH3 1377.7586
y13 1507.8734
y13 y12 y11 y10 y8 y7 y6 y5 y4 y3 y2 y1
D G L I T P T I A P N G A Q V L Q V K R
b2 b3 b4 b5 b6 b7 b8 b9 b15(2+)
Modification
Acetyl (Protein N−term)
GlyGly (K) no new terminus
Oxidation (M)

20201118_KIZ_MUb6_GlyGly_2 − Scan number: 22841

Gene Names	Charge	m/z	Mass	Retention time	PEP	Score	Precursor Intensity
PEG10	3	735.7499	2204.228	76.748	8.7628e−43	186.85	10608238

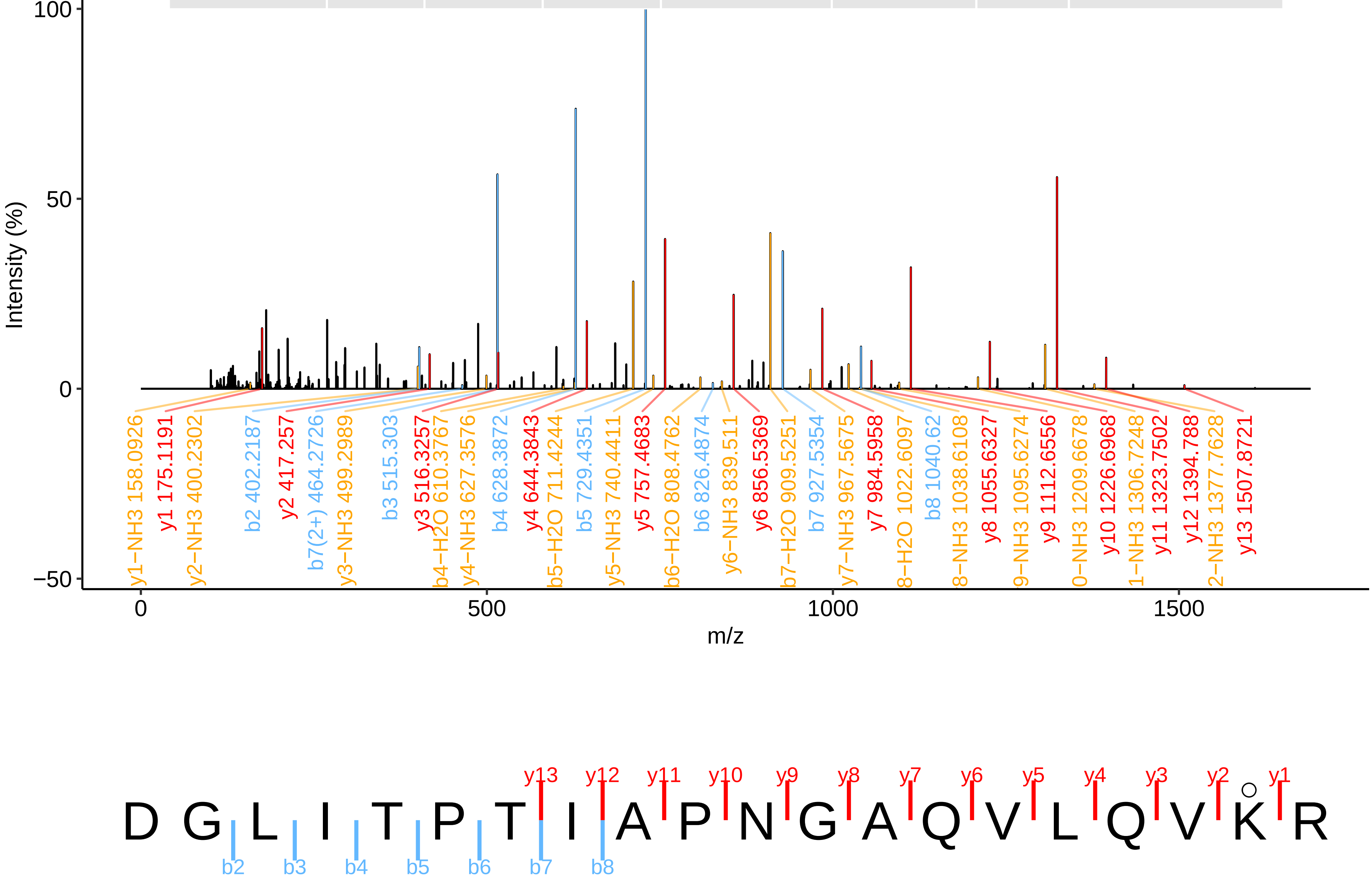

Modification □ Acetyl (Protein N−term) ○ GlyGly (K) no new terminus △ Oxidation (M)

20201118_KIZ_MUb6_GlyGly_2 − Scan number: 22874

Gene Names	Charge	m/z	Mass	Retention time	PEP	Score	Precursor Intensity
PEG10	3	735.7499	2204.228	76.849	9.3967e−21	141.97	8143460

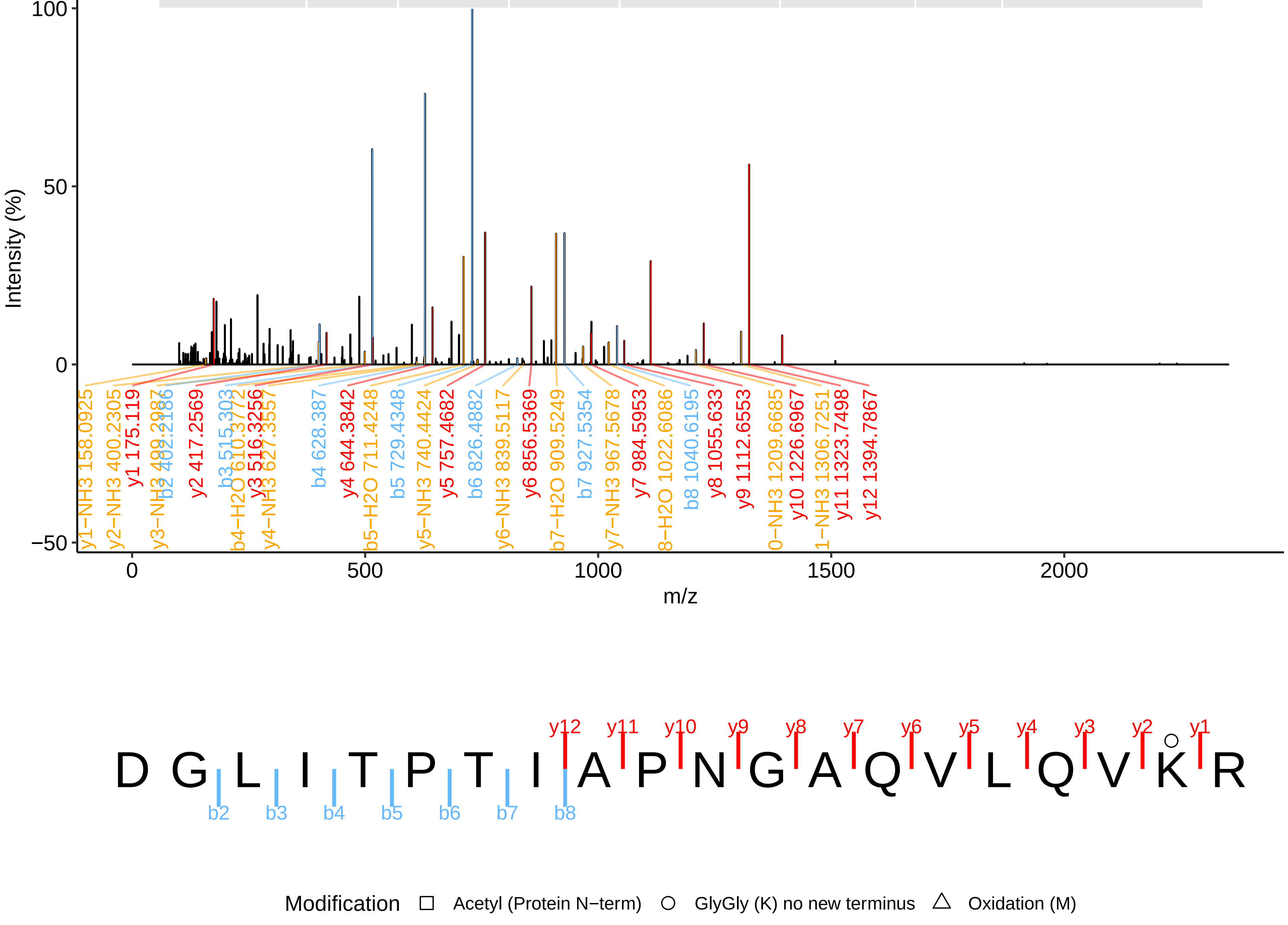

20200921_KIZ_MUb4_GlyGly_10plex − Scan number: 16024

Gene Names	Charge	m/z	Mass	Retention time	PEP	Score	Precursor Intensity
SLC1A3	3	655.9679	1964.882	57.442	4.476e−20	163.23	1237434

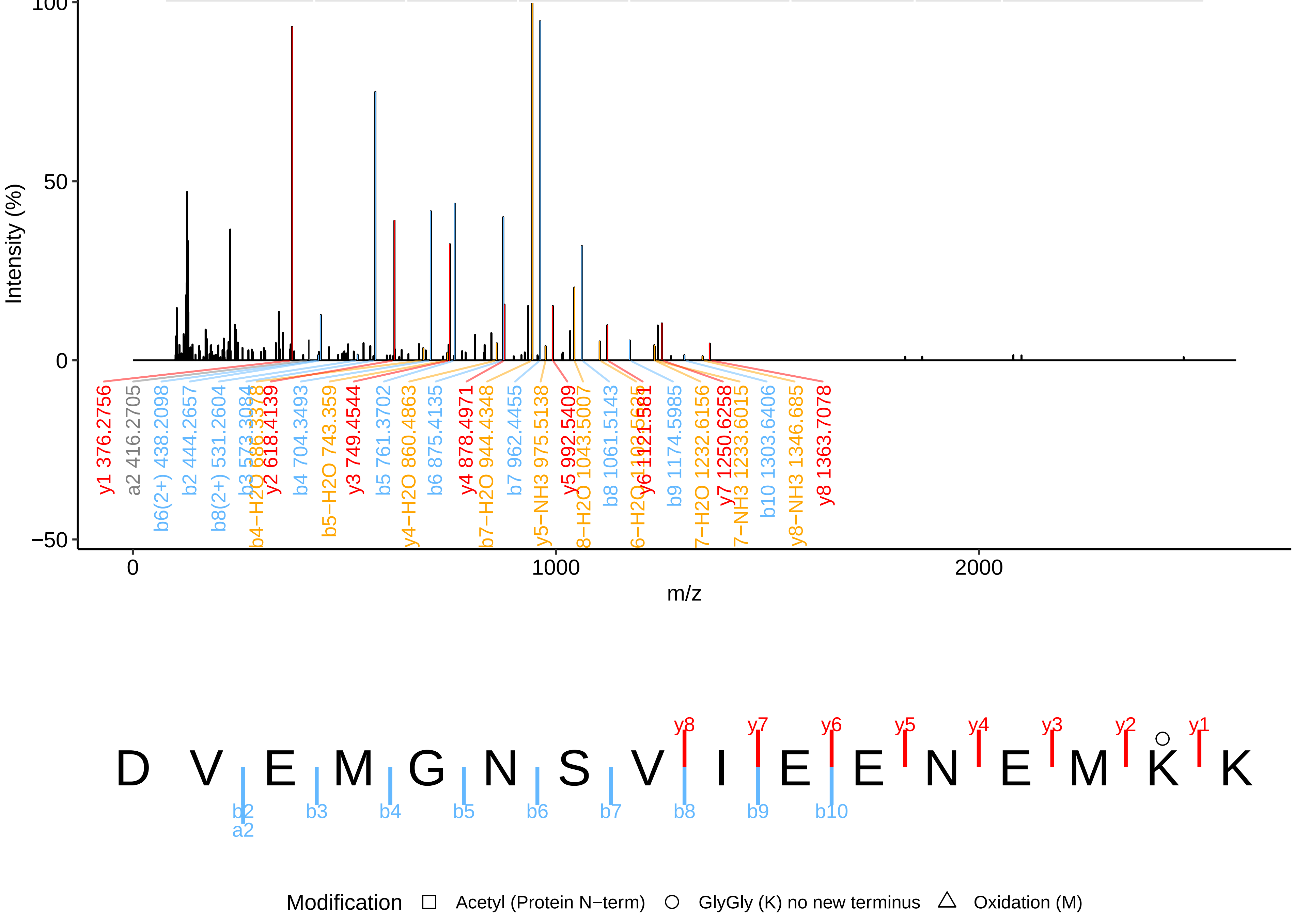

20200921_KIZ_MUb4_GlyGly_10plex_2 − Scan number: 14791

Gene Names	Charge	m/z	Mass	Retention time	PEP	Score	Precursor Intensity
SLC1A3	3	655.9679	1964.882	57.732	1.8402e−06	103.39	859357.2

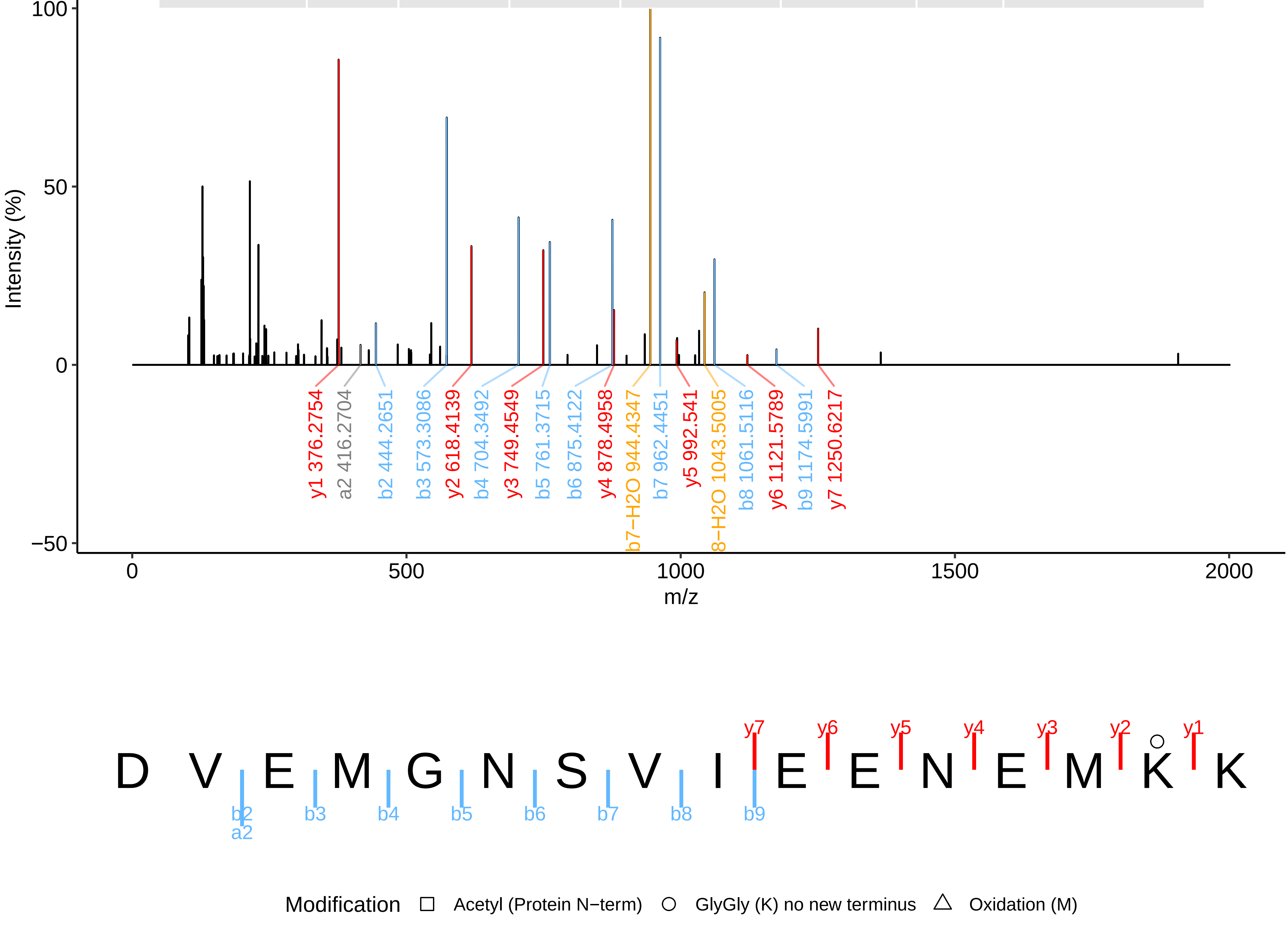

20200924_KIZ_MUb5_GlyGly_1 − Scan number: 15444

Gene Names	Charge	m/z	Mass	Retention time	PEP	Score	Precursor Intensity
SLC1A3	3	655.9679	1964.882	50.43	2.3401e−20	165.34	12669872

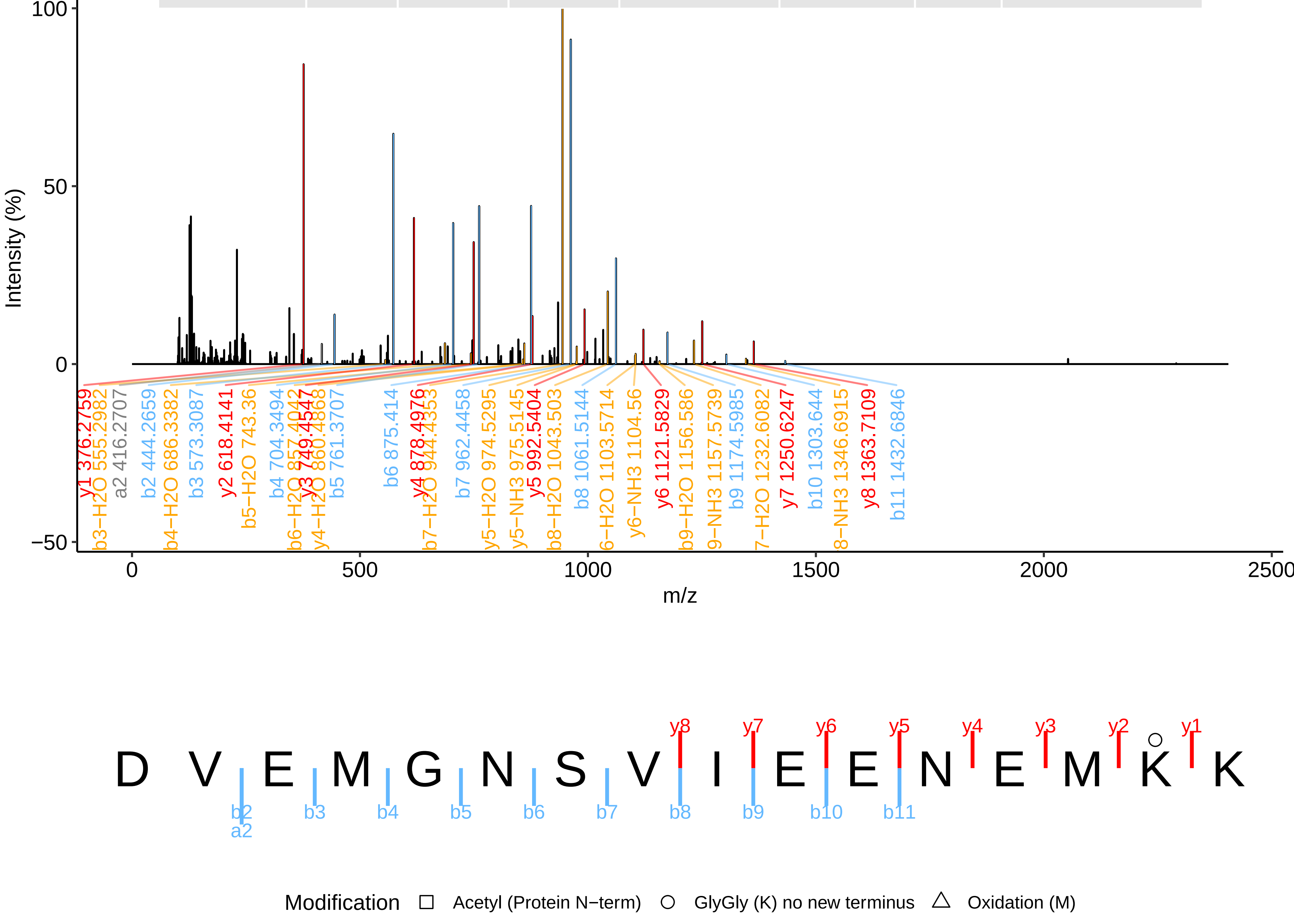

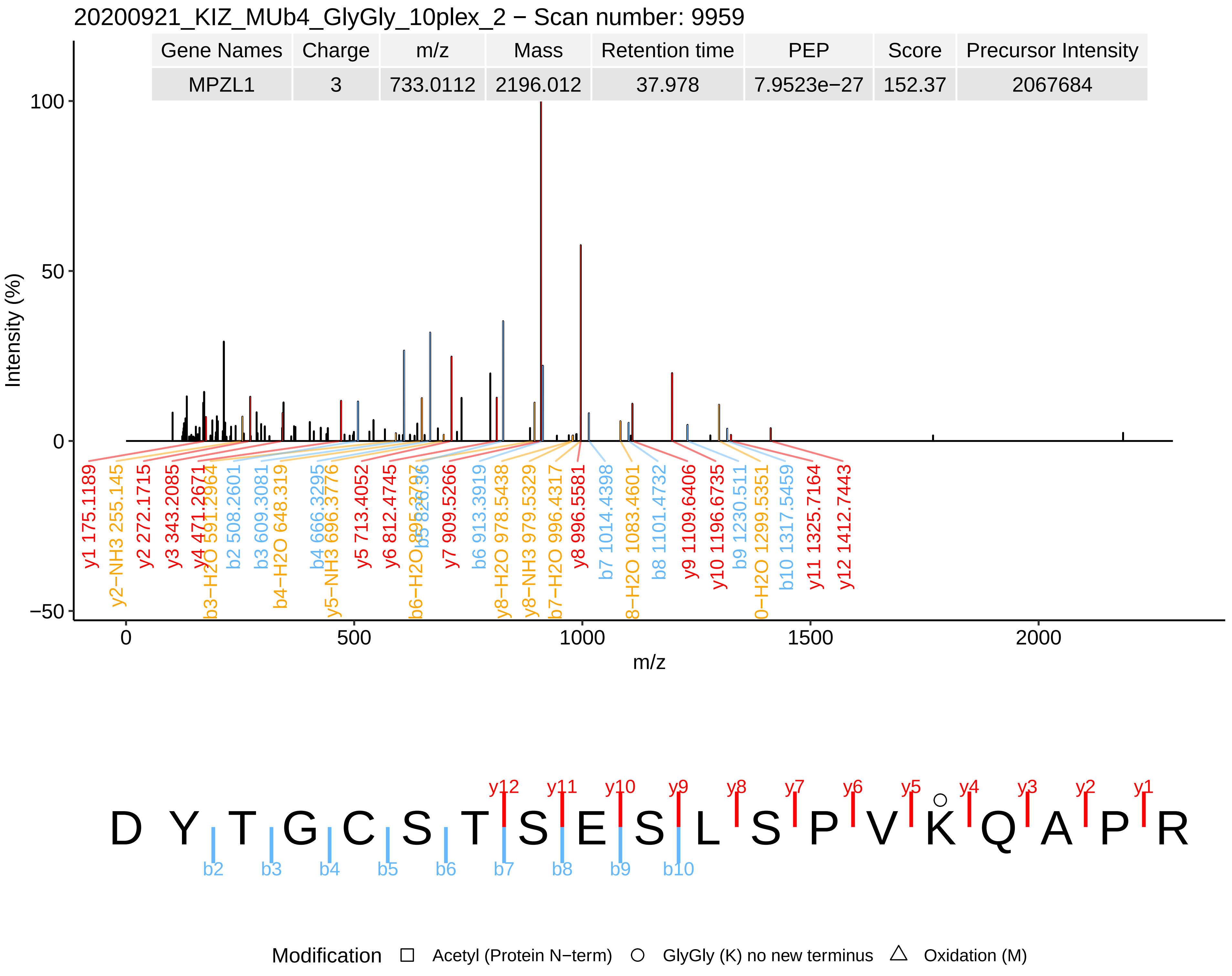
20200921_KIZ_MUb4_GlyGly_10plex_2 − Scan number: 9959
Gene Names | Charge | m/z | Mass | Retention time | PEP | Score | Precursor Intensity
MPZL1 | 3 | 733.0112 | 2196.012 | 37.978 | 7.9523e−27 | 152.37 | 2067684
Intensity (%)
m/z
y1 175.1189
y2−NH3 255.145
y2 272.1715
y3 343.2085
y4 471.2671
b3−H2O 591.2964
b2 508.2601
b3 609.3081
b4−H2O 648.319
b4 666.3295
y5−NH3 696.3776
y5 713.4052
y6 812.4745
b6−H2O 895.3797
b5 826.36
y7 909.5266
b6 913.3919
y8−H2O 978.5438
y8−NH3 979.5329
b7−H2O 996.4317
y8 996.5581
b7 1014.4398
b8−H2O 1083.4601
b8 1101.4732
y9 1109.6406
y10 1196.6735
b9 1230.511
b10−H2O 1299.5351
b10 1317.5459
y11 1325.7164
y12 1412.7443
D Y T G C S T S E S L S P V K Q A P R
Modification
Acetyl (Protein N−term)
GlyGly (K) no new terminus
Oxidation (M)

20201118_KIZ_MUb6_GlyGly_2 − Scan number: 11154

Gene Names	Charge	m/z	Mass	Retention time	PEP	Score	Precursor Intensity
MPZL1	3	733.0112	2196.012	37.616	1.0671e−26	165.45	2546115

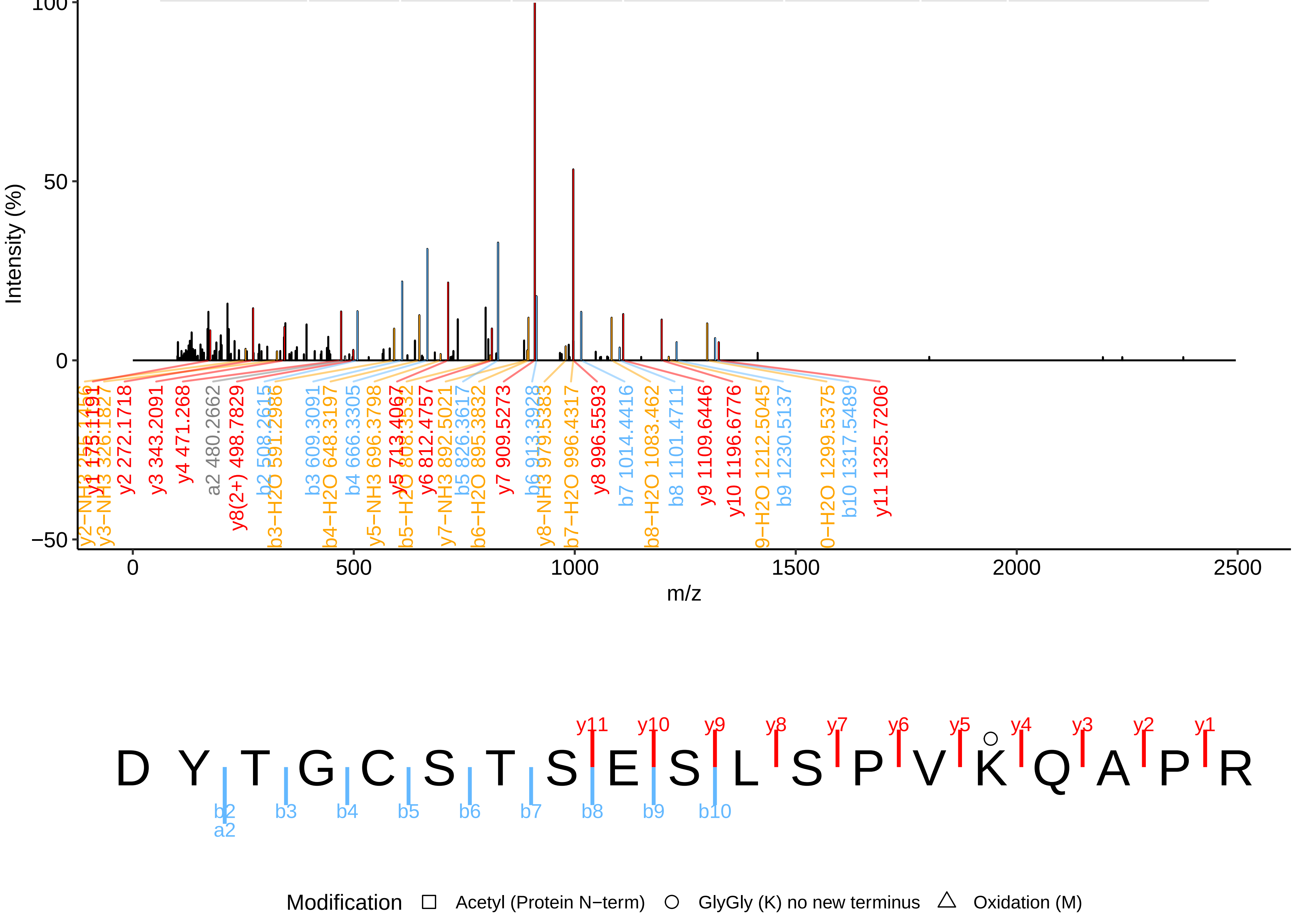

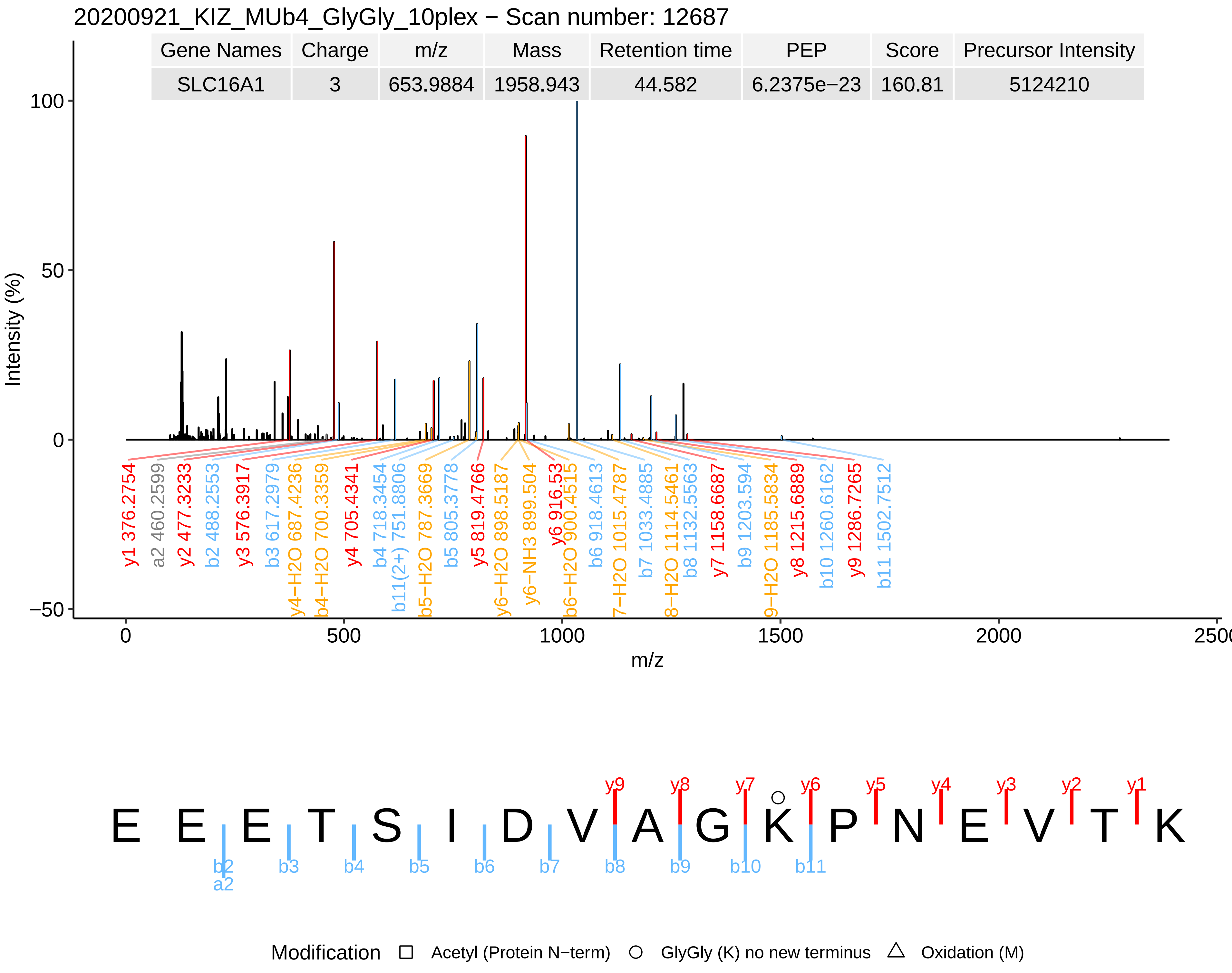
20200921_KIZ_MUb4_GlyGly_10plex − Scan number: 12687
Gene Names
Charge
m/z
Mass
Retention time
PEP
Score
Precursor Intensity
SLC16A1
3
653.9884
1958.943
44.582
6.2375e−23
160.81
5124210
Intensity (%)
m/z
y1 376.2754
a2 460.2599
y2 477.3233
b2 488.2553
y3 576.3917
b3 617.2979
y4−H2O 687.4236
b4−H2O 700.3359
y4 705.4341
b4 718.3454
b11(2+) 751.8806
b5−H2O 787.3669
b5 805.3778
y5 819.4766
y6−H2O 898.5187
y6−NH3 899.504
y6 916.53
b6−H2O 900.4515
b6 918.4613
b7 1033.4885
b8 1132.5563
y7 1158.6687
b9 1203.594
y8 1215.6889
b10 1260.6162
y9 1286.7265
b11 1502.7512
E E E T S I D V A G K P N E V T K
Modification
Acetyl (Protein N−term)
GlyGly (K) no new terminus
Oxidation (M)

20200921_KIZ_MUb4_GlyGly_10plex_2 − Scan number: 11543

Gene Names	Charge	m/z	Mass	Retention time	PEP	Score	Precursor Intensity
SLC16A1	3	653.9884	1958.943	44.435	7.5116e−17	138.01	3314716

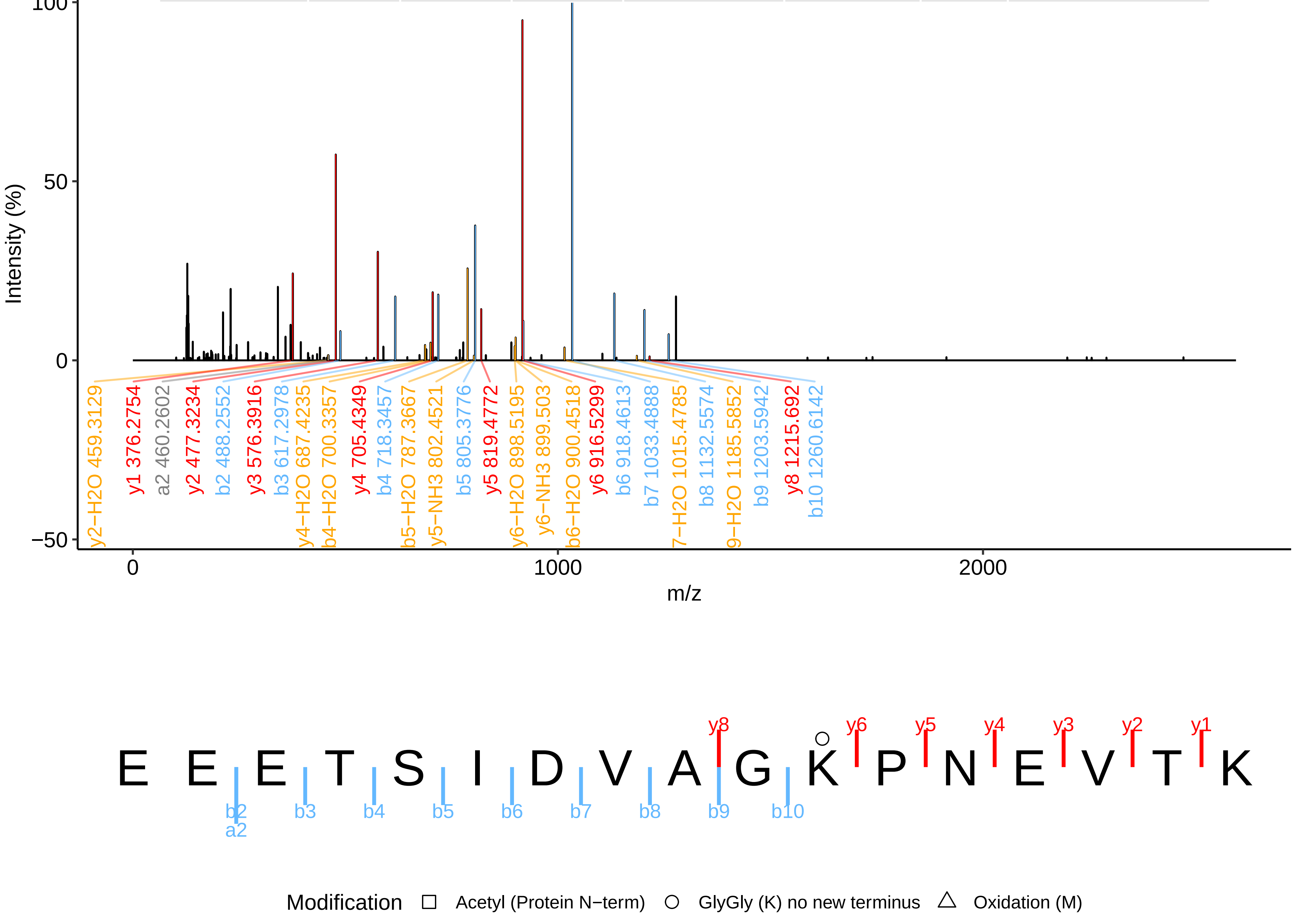

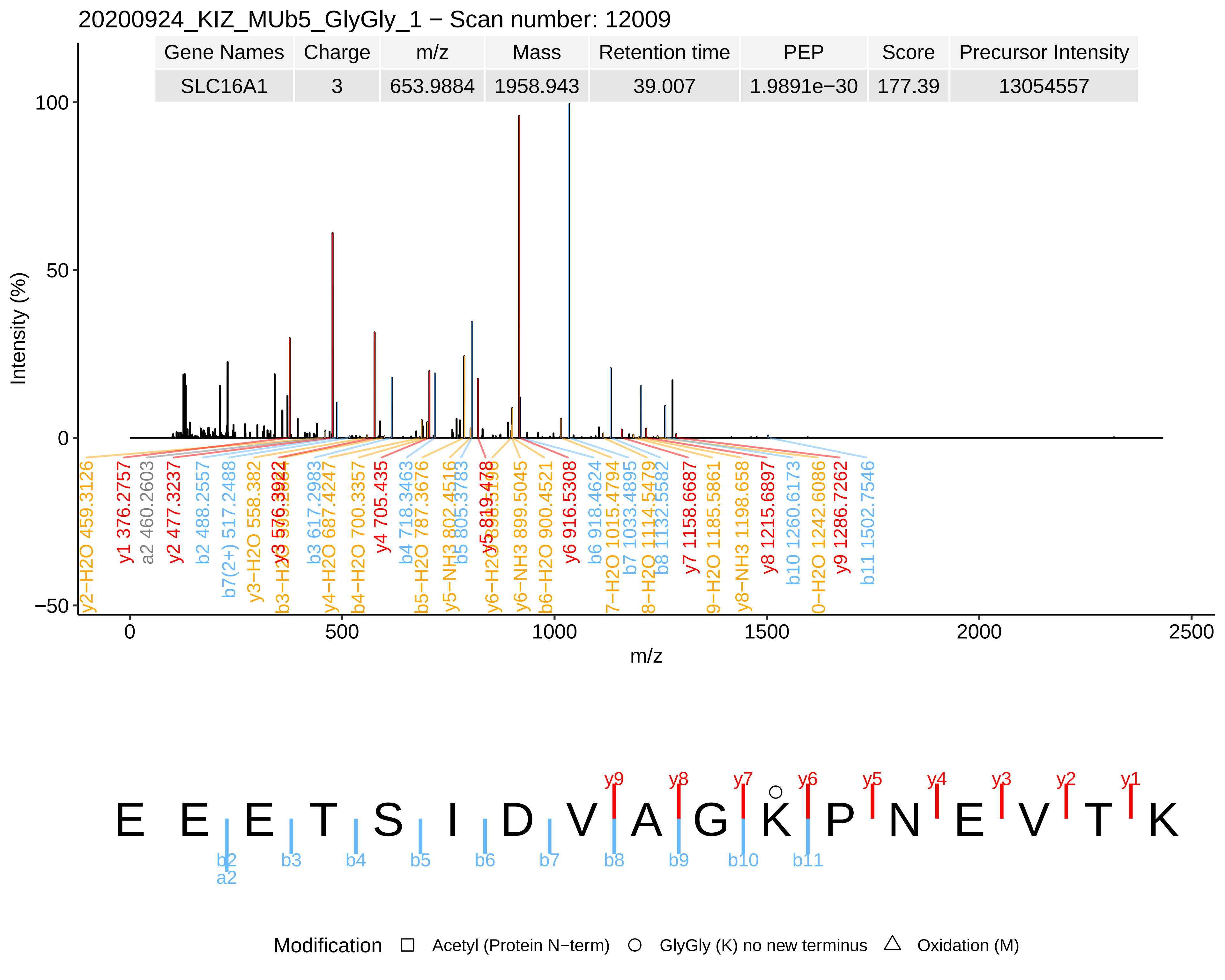

20200924_KIZ_MUb5_GlyGly_1 − Scan number: 12009
Gene Names | Charge | m/z | Mass | Retention time | PEP | Score | Precursor Intensity
SLC16A1 | 3 | 653.9884 | 1958.943 | 39.007 | 1.9891e−30 | 177.39 | 13054557
Intensity (%)
m/z
y2−H2O 459.3126
y1 376.2757
a2 460.2603
y2 477.3237
b2 488.2557
b7(2+) 517.2488
y3−H2O 558.382
y3 576.3922
b3 617.2983
y4−H2O 687.4247
b4−H2O 700.3357
y4 705.435
b4 718.3463
b5−H2O 787.3676
y5−NH3 802.4516
b5 805.3783
y5 819.478
y6−NH3 899.5045
b6−H2O 900.4521
y6 916.5308
b6 918.4624
b7 1033.4895
b8 1132.5582
y7 1158.6687
y8−NH3 1198.658
y8 1215.6897
b10 1260.6173
y9 1286.7262
b11 1502.7546
E E E T S I D V A G K P N E V T K
Modification
Acetyl (Protein N−term)
GlyGly (K) no new terminus
Oxidation (M)

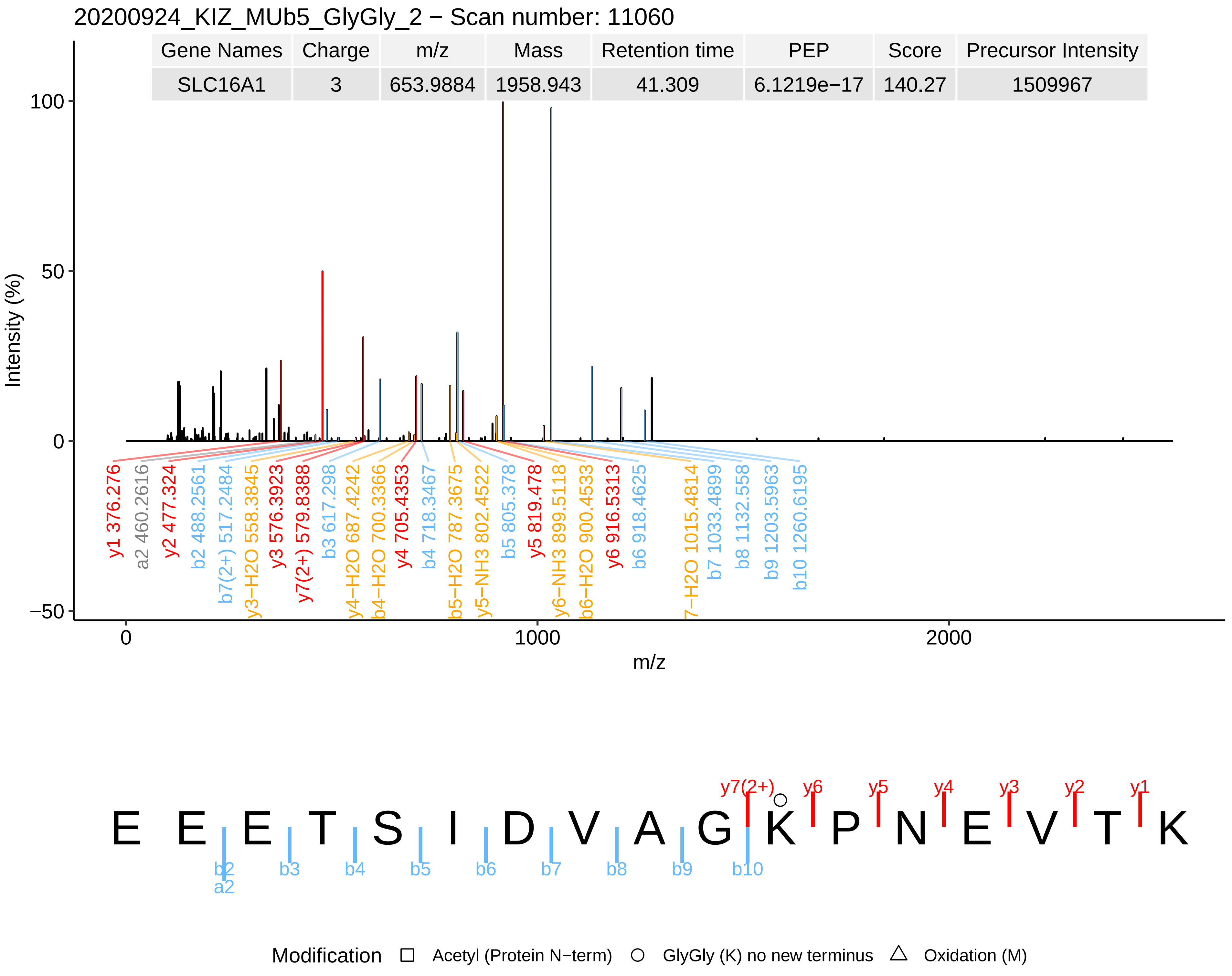

20200924_KIZ_MUb5_GlyGly_2 − Scan number: 11060
Gene Names | Charge | m/z | Mass | Retention time | PEP | Score | Precursor Intensity
SLC16A1 | 3 | 653.9884 | 1958.943 | 41.309 | 6.1219e−17 | 140.27 | 1509967
Intensity (%)
100
50
0
−50
y1 376.276
a2 460.2616
y2 477.324
b2 488.2561
b7(2+) 517.2484
y3−H2O 558.3845
y3 576.3923
y7(2+) 579.8388
b3 617.298
y4−H2O 687.4242
b4−H2O 700.3366
y4 705.4353
b4 718.3467
b5−H2O 787.3675
y5−NH3 802.4522
b5 805.378
y5 819.478
y6−NH3 899.5118
b6−H2O 900.4533
y6 916.5313
b6 918.4625
7−H2O 1015.4814
b7 1033.4899
b8 1132.558
b9 1203.5963
b10 1260.6195
0
1000
2000
m/z
E E E T S I D V A G K P N E V T K
y7(2+) y6 y5 y4 y3 y2 y1
b2 a2 b3 b4 b5 b6 b7 b8 b9 b10
Modification
Acetyl (Protein N−term)
GlyGly (K) no new terminus
Oxidation (M)

20200921_KIZ_MUb4_GlyGly_10plex − Scan number: 17983

Gene Names	Charge	m/z	Mass	Retention time	PEP	Score	Precursor Intensity
PHB	3	726.3538	2176.04	64.583	1.067e−13	128.99	4517477

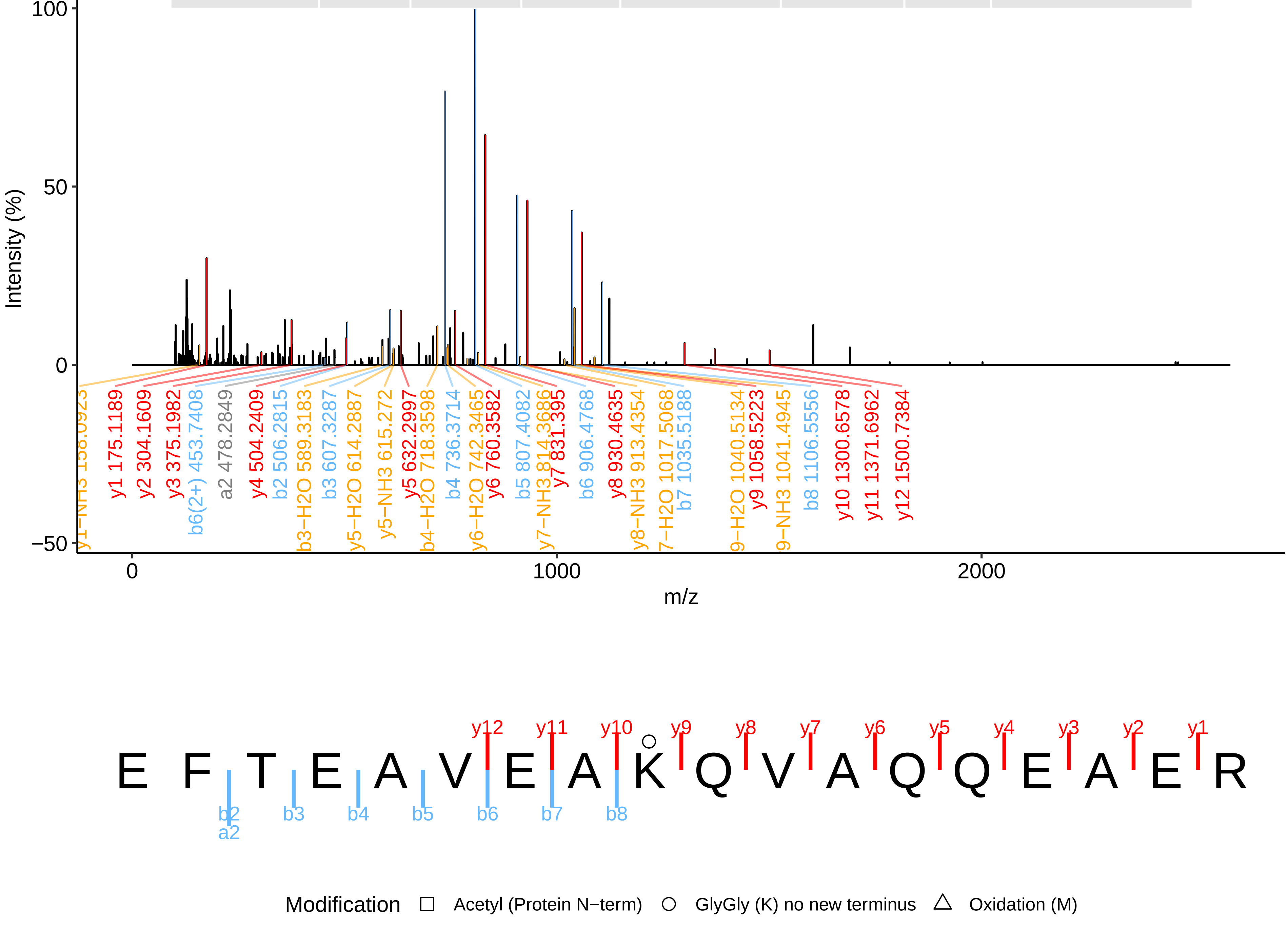

20200921_KIZ_MUb4_GlyGly_10plex_2 − Scan number: 15687

Gene Names	Charge	m/z	Mass	Retention time	PEP	Score	Precursor Intensity
PHB	3	726.3538	2176.04	61.401	3.0087e−10	112.16	1214715

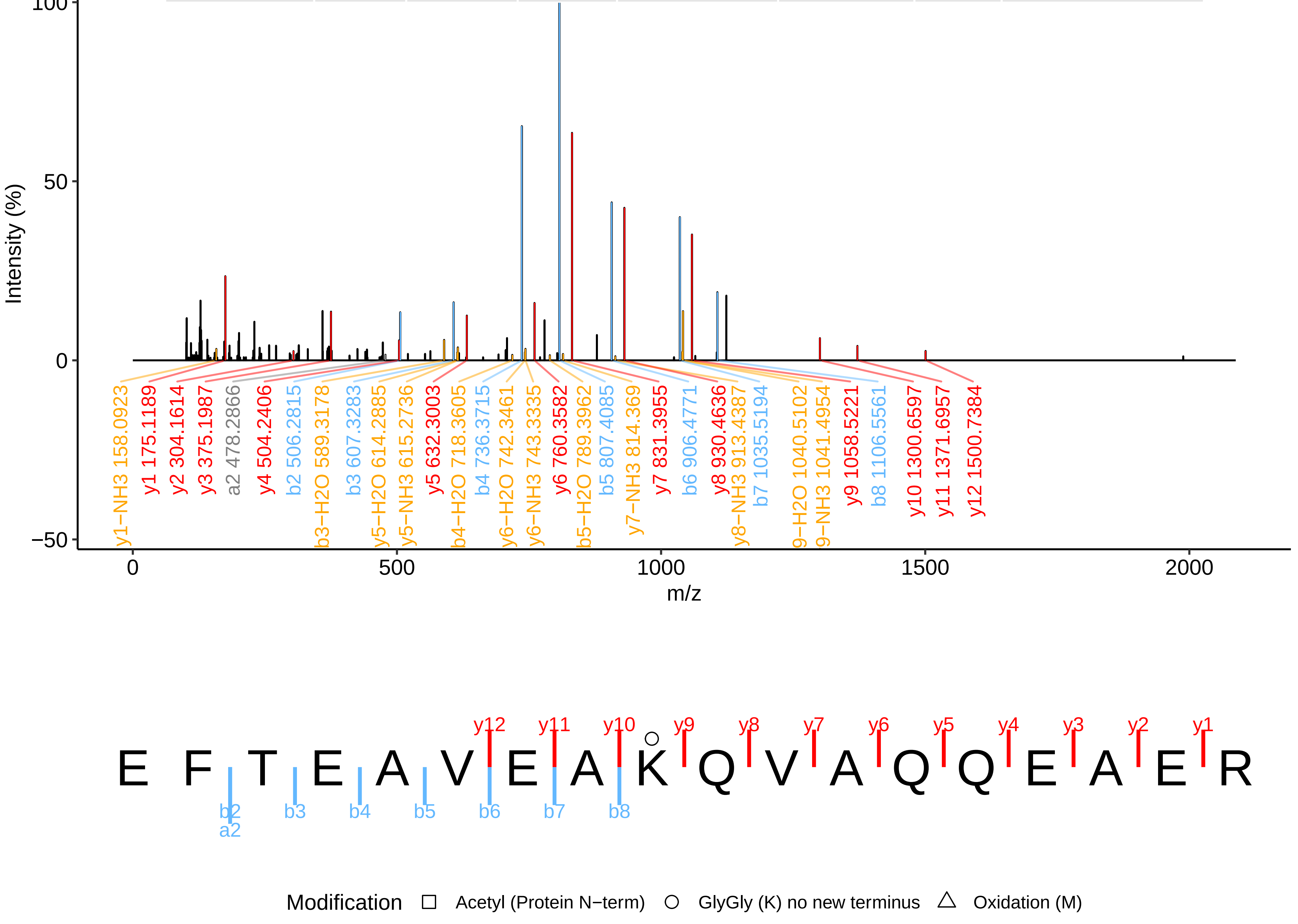

20201118_KIZ_MUb6_GlyGly_2 − Scan number: 18060

Gene Names	Charge	m/z	Mass	Retention time	PEP	Score	Precursor Intensity
PHB	3	726.3538	2176.04	61.093	5.0789e−15	132.64	20948878

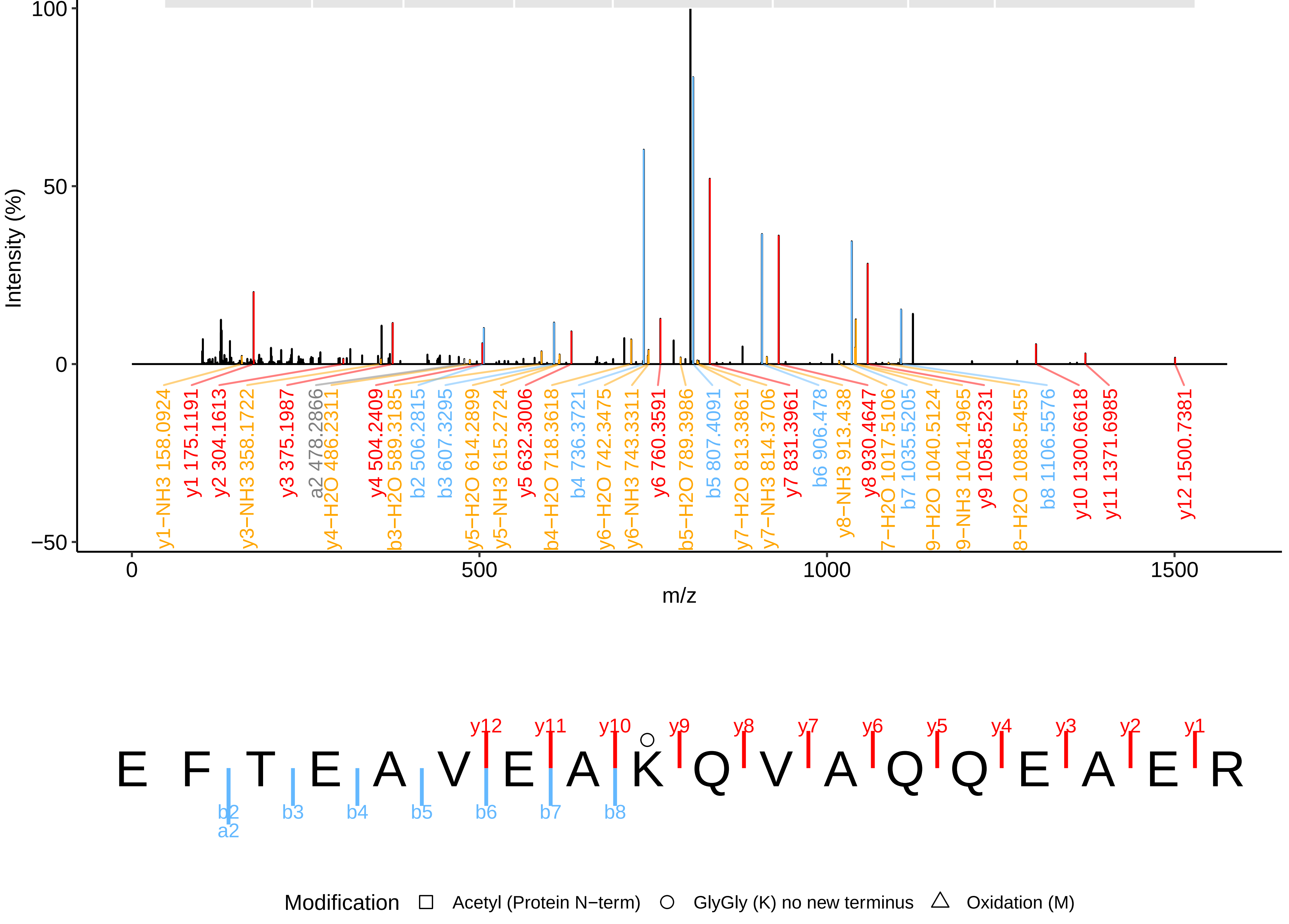

20200924_KIZ_MUb5_GlyGly_1 − Scan number: 5295

Gene Names	Charge	m/z	Mass	Retention time	PEP	Score	Precursor Intensity
BASP1	4	422.6997	1686.77	16.431	0.001513	92.935	8477299

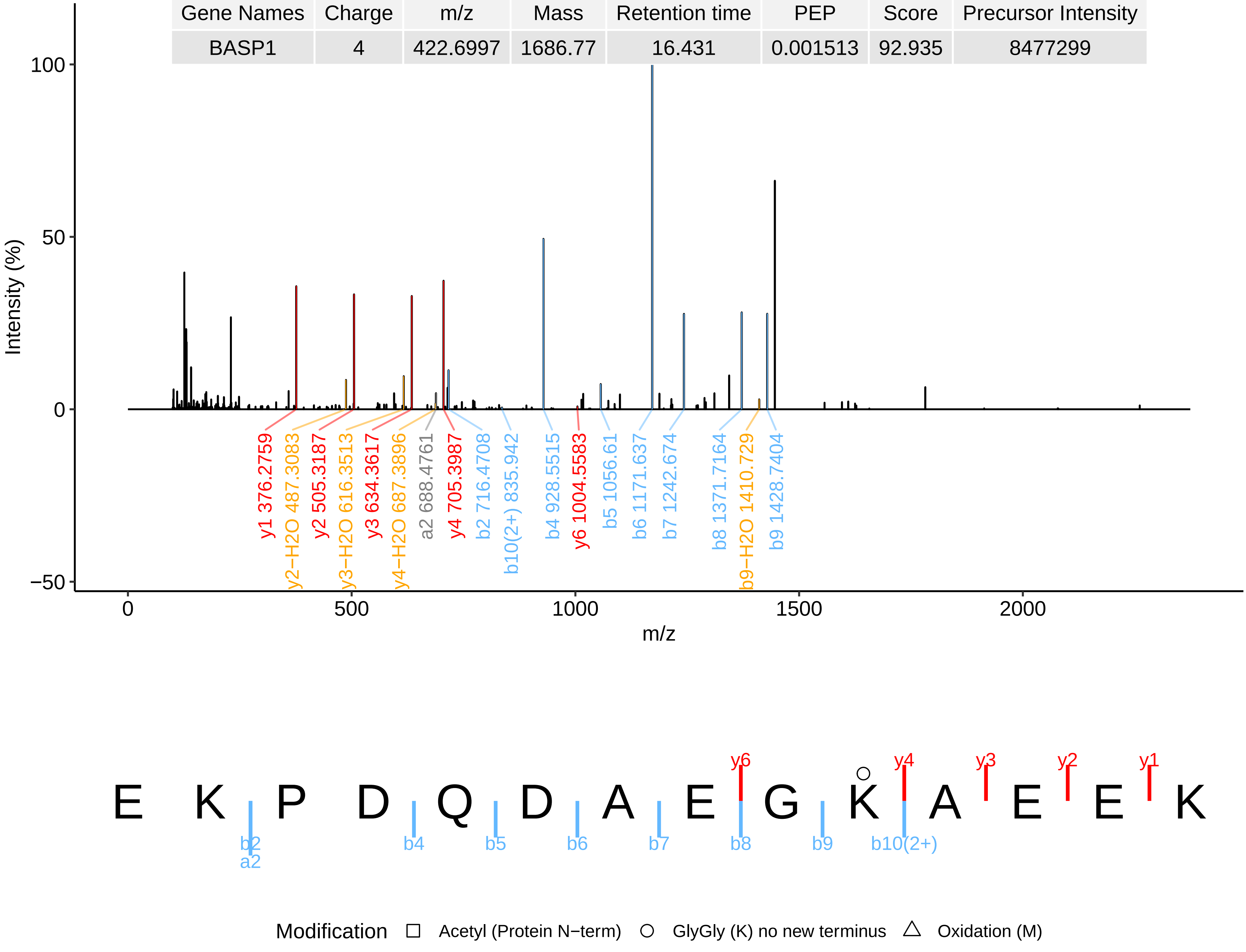

20200924_KIZ_MUb5_GlyGly_1 − Scan number: 5449

Gene Names	Charge	m/z	Mass	Retention time	PEP	Score	Precursor Intensity
BASP1	4	422.6997	1686.77	16.954	0.001513	92.935	18716596

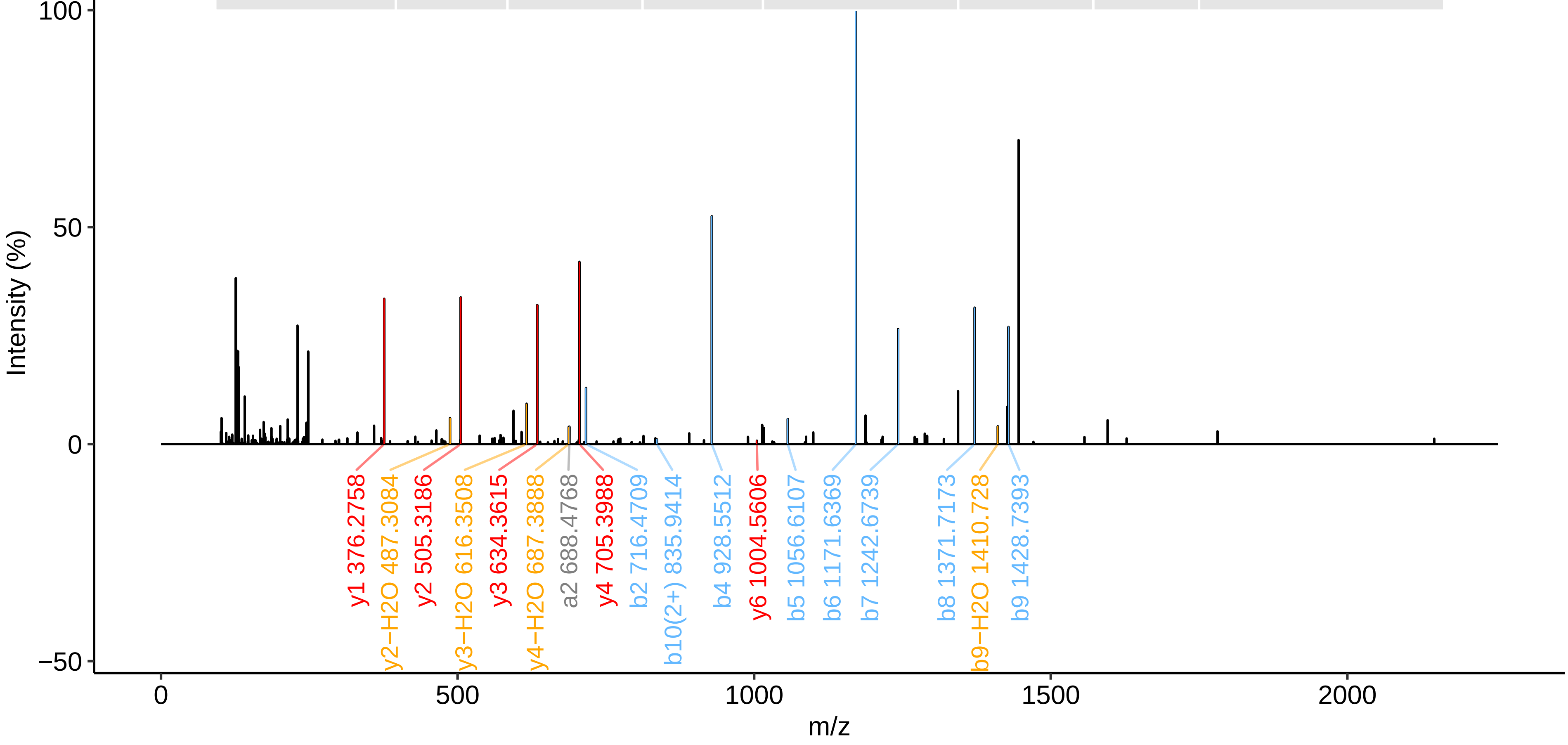

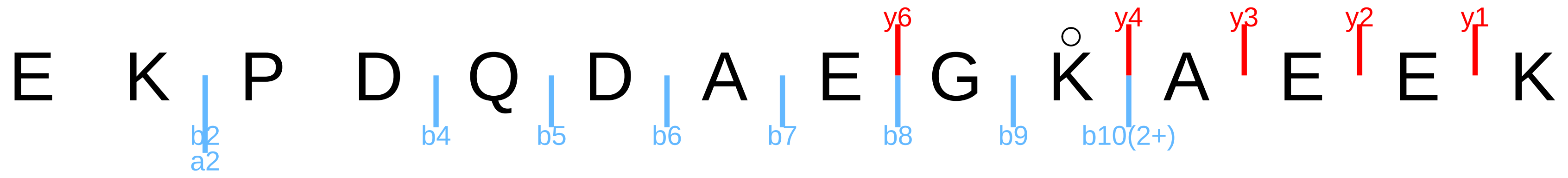

Modification □ Acetyl (Protein N−term) ○ GlyGly (K) no new terminus △ Oxidation (M)

20200924_KIZ_MUb5_GlyGly_2 − Scan number: 5110

Gene Names	Charge	m/z	Mass	Retention time	PEP	Score	Precursor Intensity
BASP1	4	422.6997	1686.77	18.918	0.020924	73.887	1061757

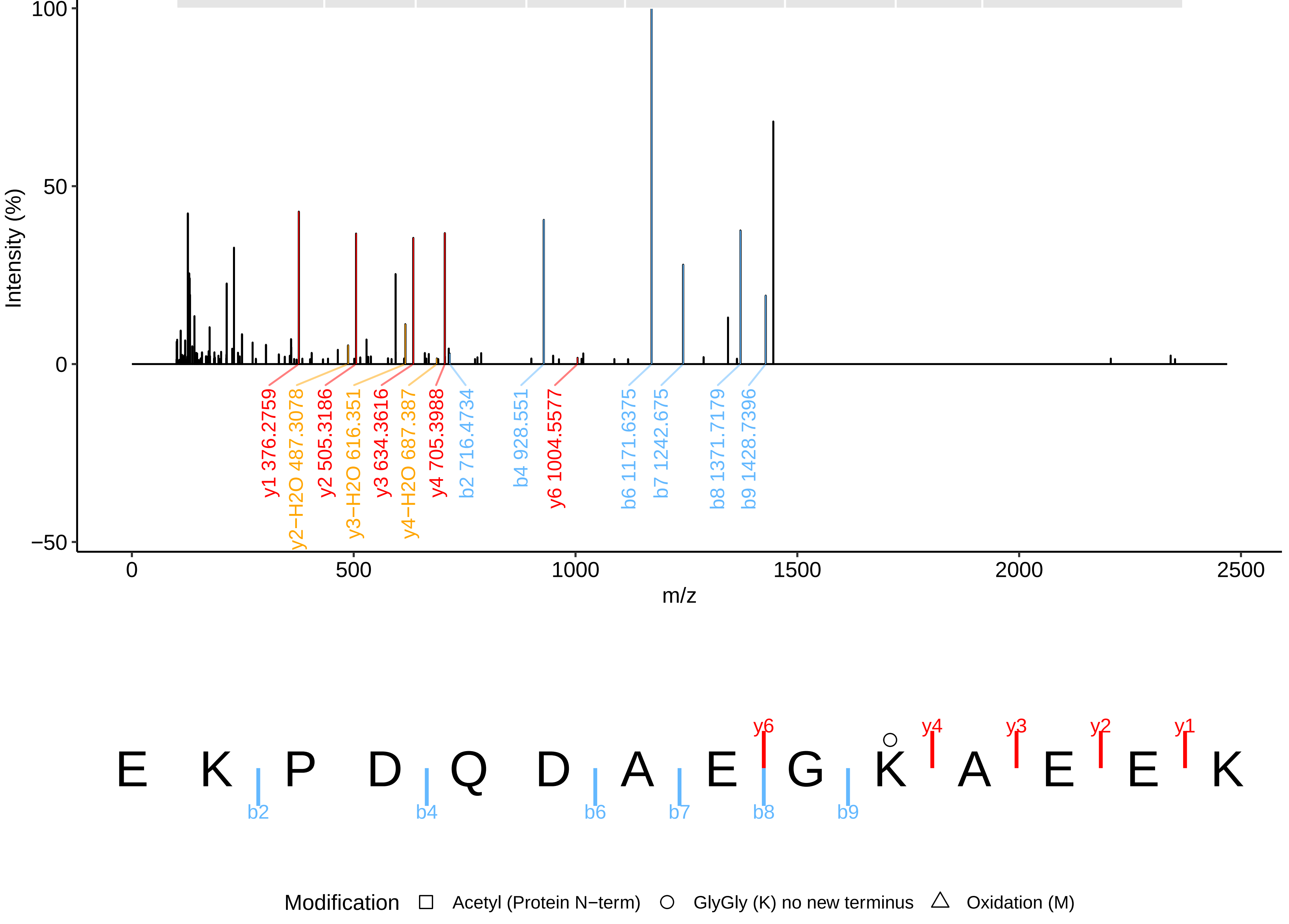

20200924_KIZ_MUb5_GlyGly_2 − Scan number: 5246

Gene Names	Charge	m/z	Mass	Retention time	PEP	Score	Precursor Intensity
BASP1	4	422.6997	1686.77	19.446	0.019026	74.678	2346089

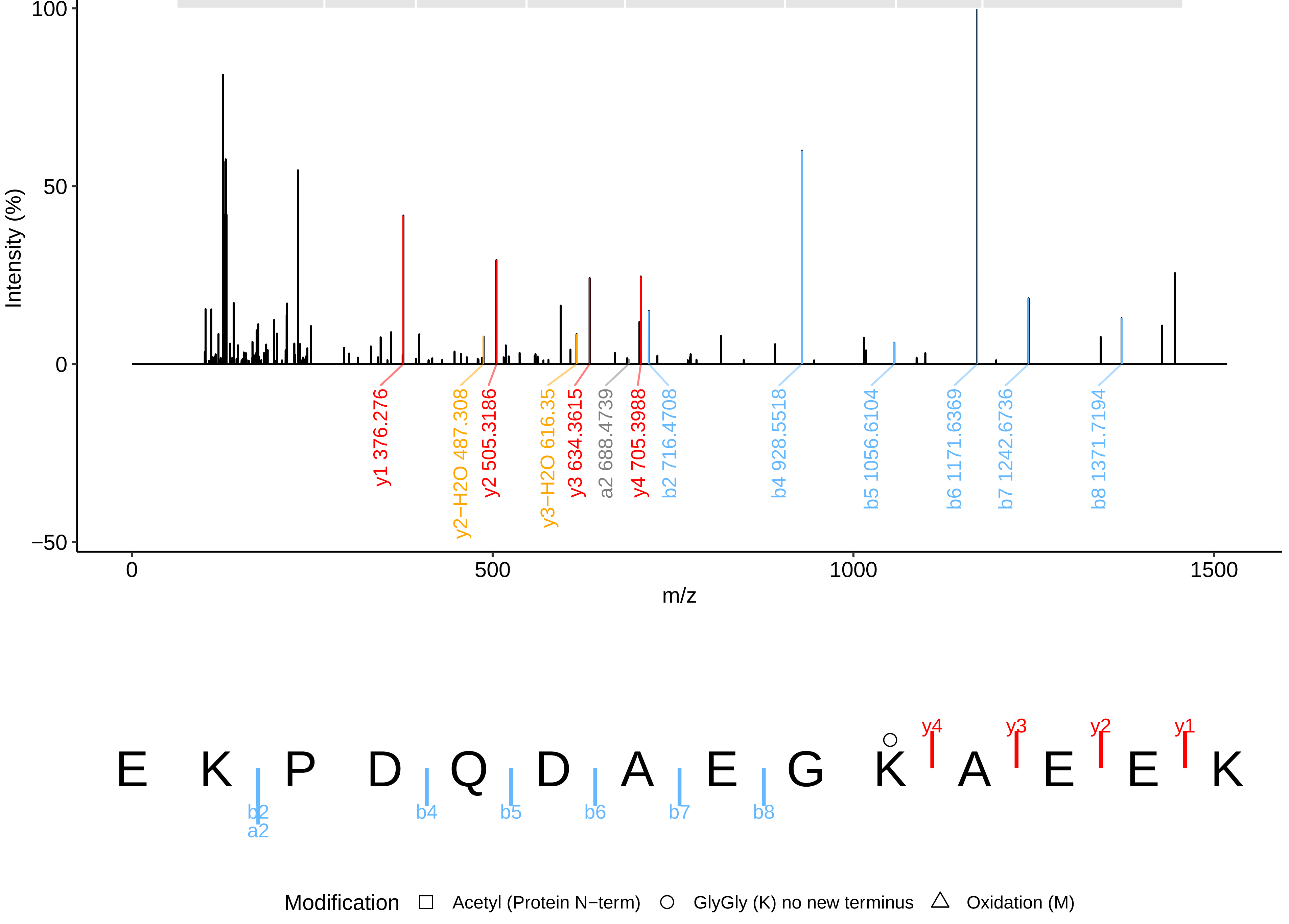

20200924_KIZ_MUb5_GlyGly_2 − Scan number: 5249

Gene Names	Charge	m/z	Mass	Retention time	PEP	Score	Precursor Intensity
BASP1	4	422.6997	1686.77	19.458	0.0040099	83.729	3127448

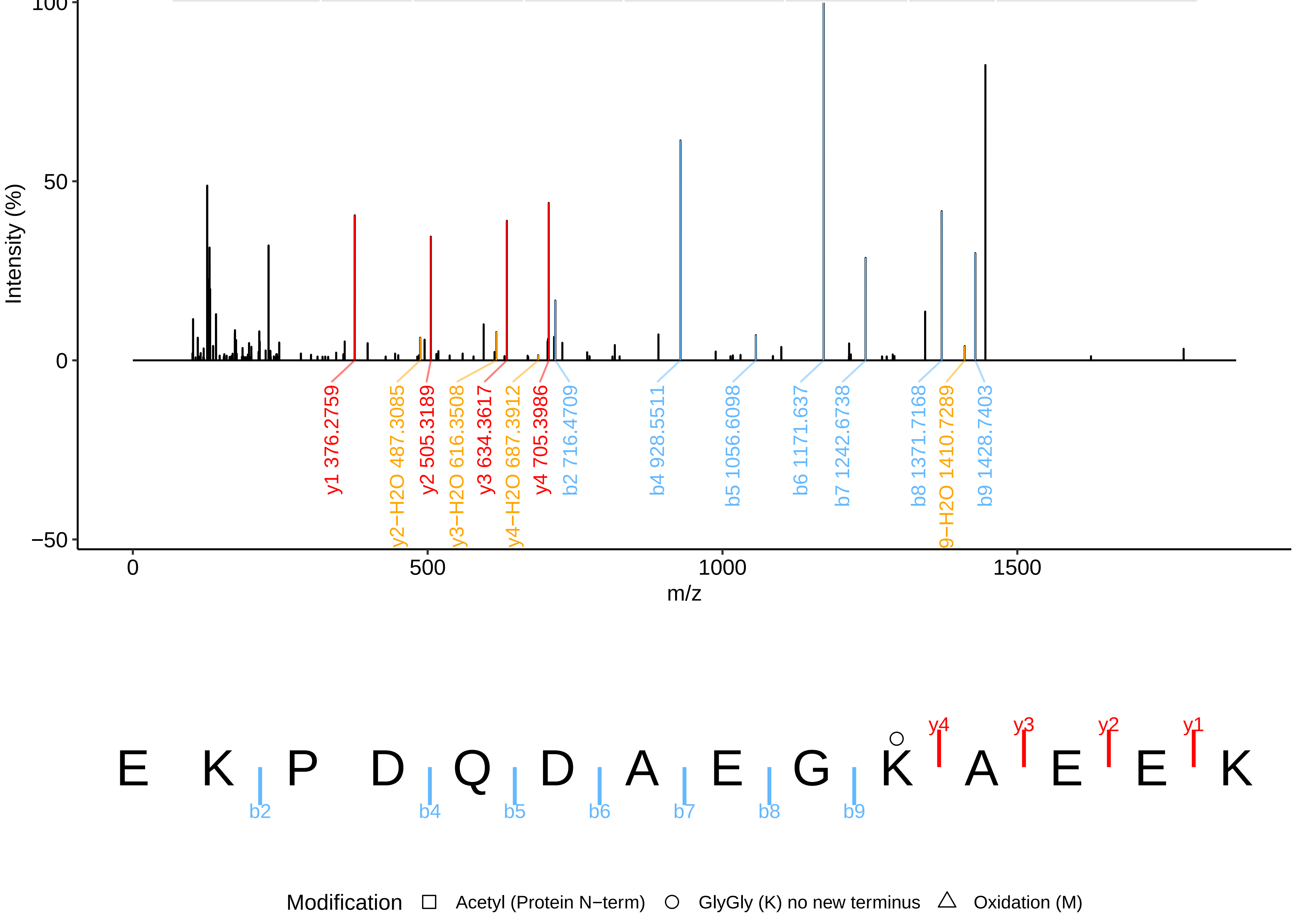

20200921_KIZ_MUb4_GlyGly_10plex_2 − Scan number: 16481

Gene Names	Charge	m/z	Mass	Retention time	PEP	Score	Precursor Intensity
TOM1	3	730.3659	2188.076	64.34	3.1401e−06	88.755	1781523

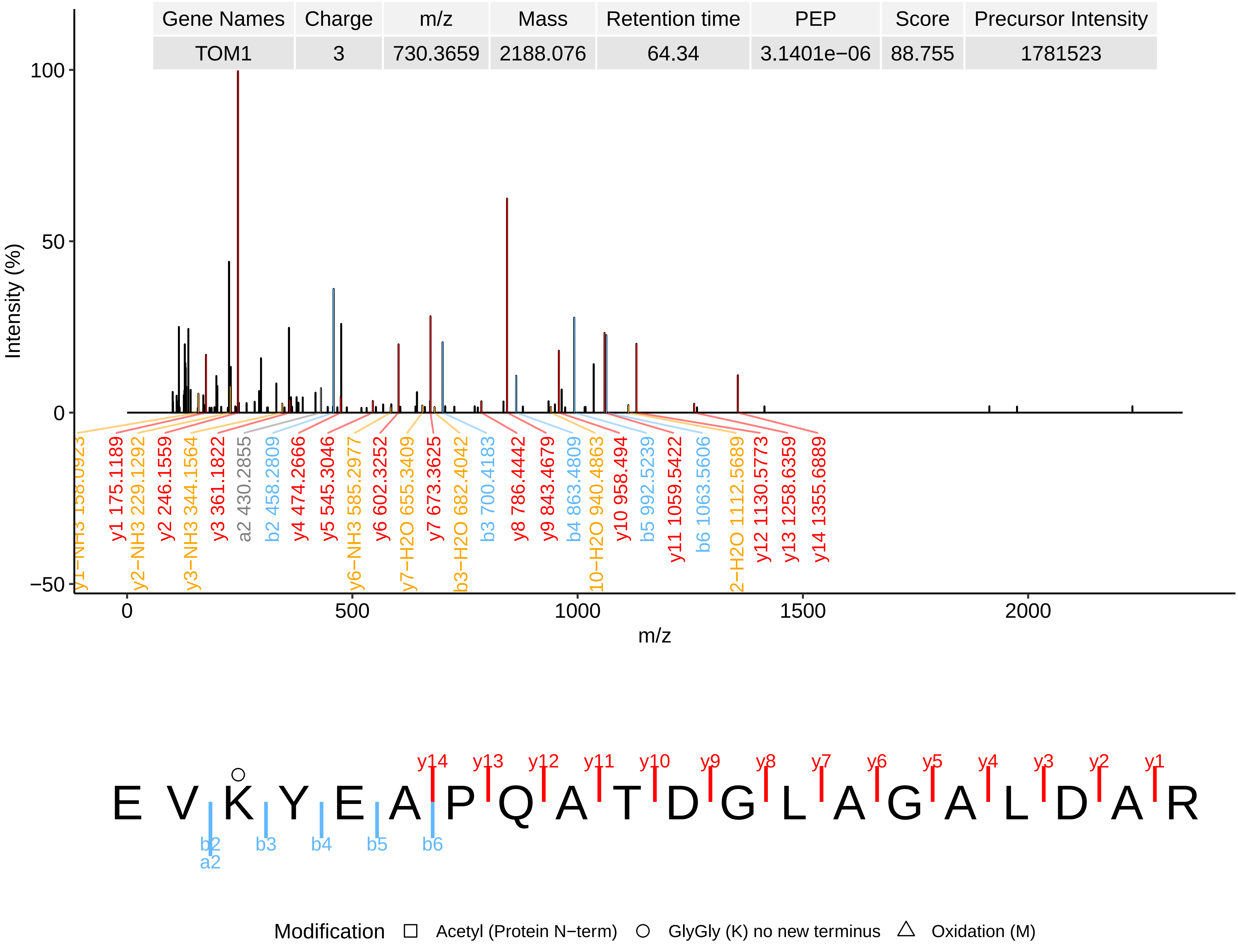

20200924_KIZ_MUb5_GlyGly_1 − Scan number: 17752

Gene Names	Charge	m/z	Mass	Retention time	PEP	Score	Precursor Intensity
TOM1	3	730.3659	2188.076	57.355	7.9166e−06	88.755	8493928

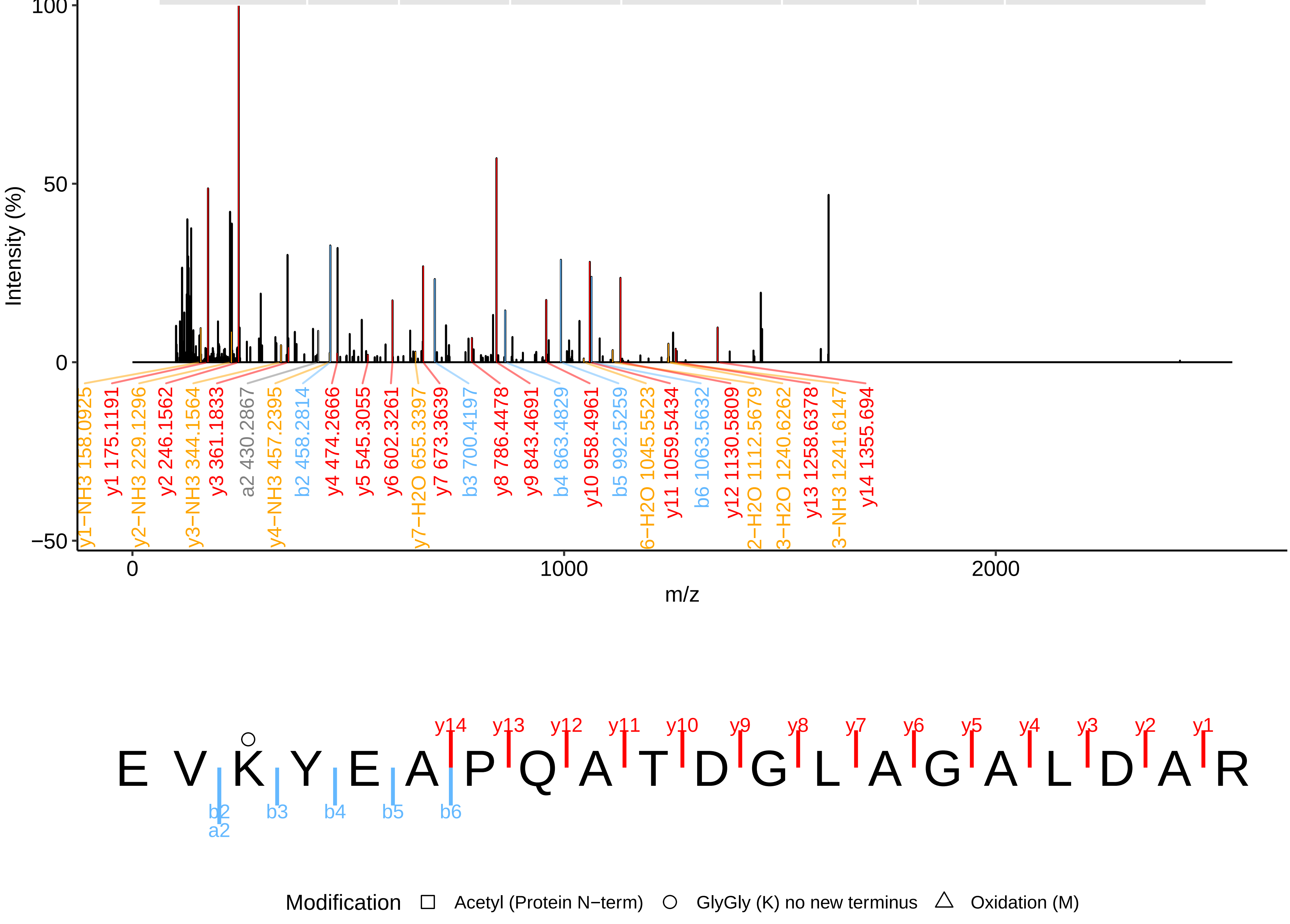

20200921_KIZ_MUb4_GlyGly_10plex − Scan number: 4491

Gene Names	Charge	m/z	Mass	Retention time	PEP	Score	Precursor Intensity
GNAS	4	495.4775	1977.881	14.636	1.5447e−27	173.36	12695613

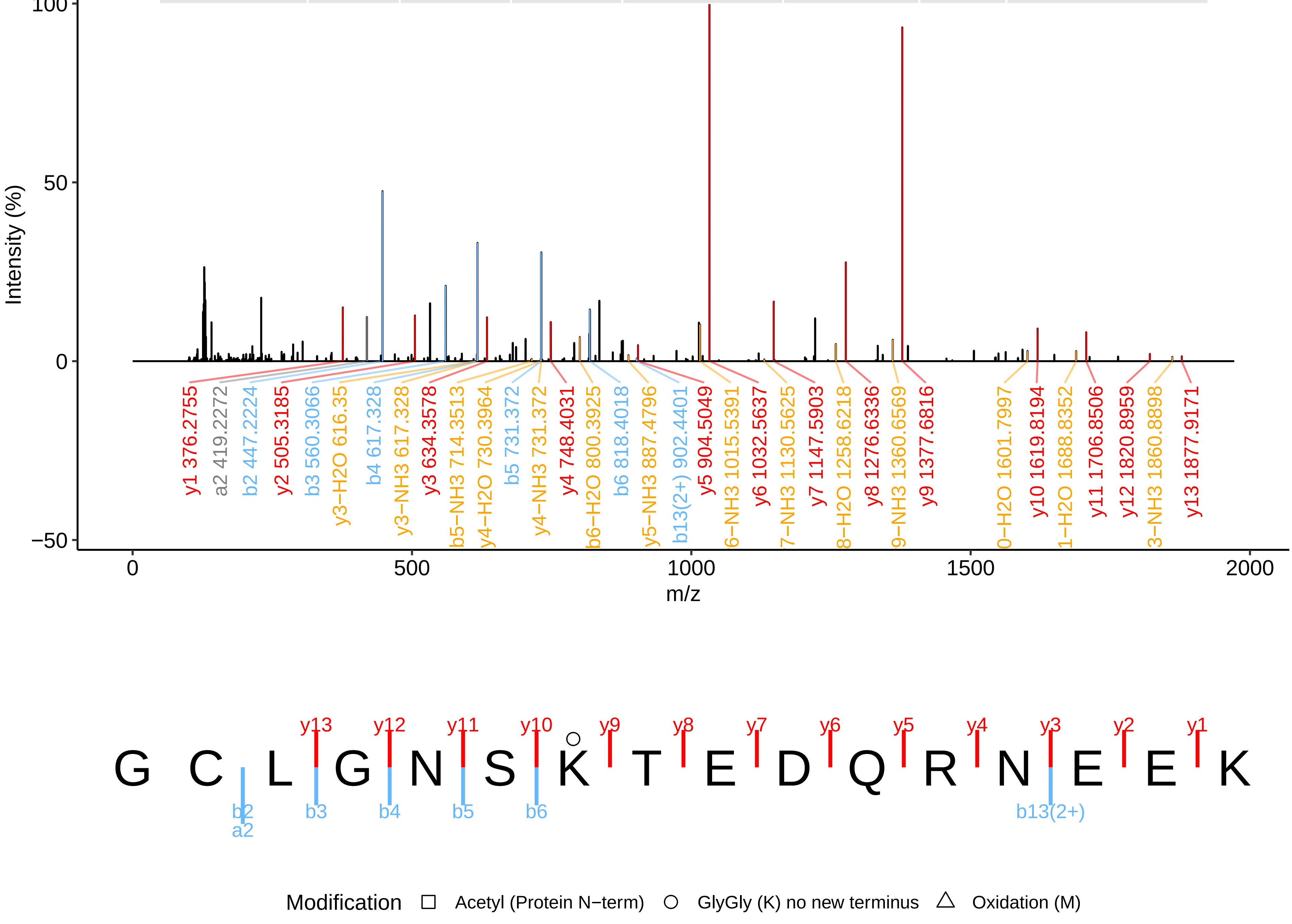

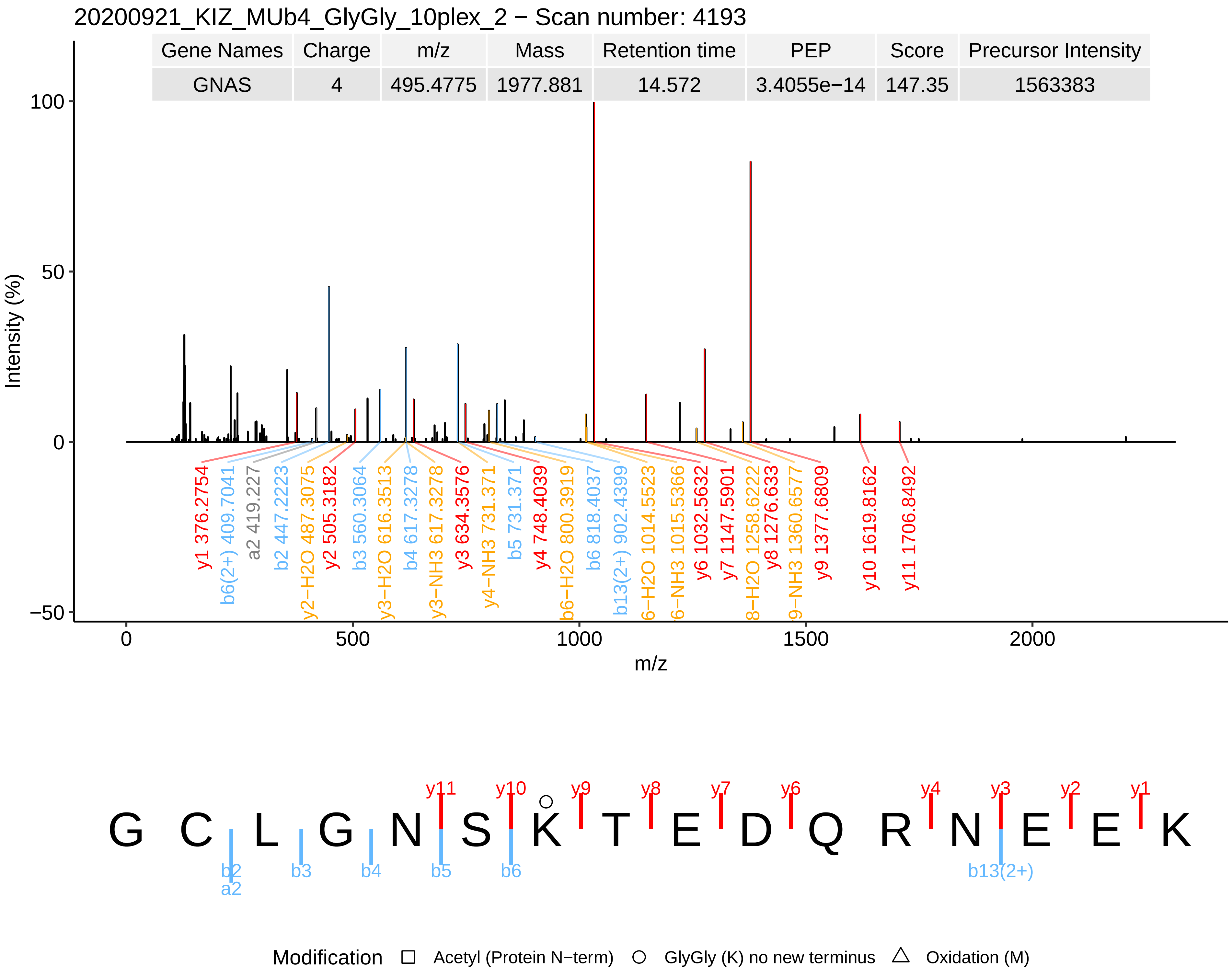
20200921_KIZ_MUb4_GlyGly_10plex_2 − Scan number: 4193
Gene Names | Charge | m/z | Mass | Retention time | PEP | Score | Precursor Intensity
GNAS | 4 | 495.4775 | 1977.881 | 14.572 | 3.4055e−14 | 147.35 | 1563383
Intensity (%)
100
50
0
−50
0
500
1000
1500
2000
m/z
y1 376.2754
b6(2+) 409.7041
a2 419.227
b2 447.2223
y2−H2O 487.3075
y2 505.3182
b3 560.3064
y3−H2O 616.3513
b4 617.3278
y3−NH3 617.3278
y3 634.3576
y4−NH3 731.371
b5 731.371
y4 748.4039
b6−H2O 800.3919
b6 818.4037
b13(2+) 902.4399
6−H2O 1014.5523
6−NH3 1015.5366
y6 1032.5632
y7 1147.5901
8−H2O 1258.6222
y8 1276.633
9−NH3 1360.6577
y9 1377.6809
y10 1619.8162
y11 1706.8492
y11 y10 y9 y8 y7 y6 y4 y3 y2 y1
G C L G N S K T E D Q R N E E K
b2 a2 b3 b4 b5 b6 b13(2+)
Modification
Acetyl (Protein N−term)
GlyGly (K) no new terminus
Oxidation (M)

20201118_KIZ_MUb6_GlyGly_1 − Scan number: 3552

Gene Names	Charge	m/z	Mass	Retention time	PEP	Score	Precursor Intensity
GNAS	4	495.4775	1977.881	10.902	6.1321e−20	161.84	23803574

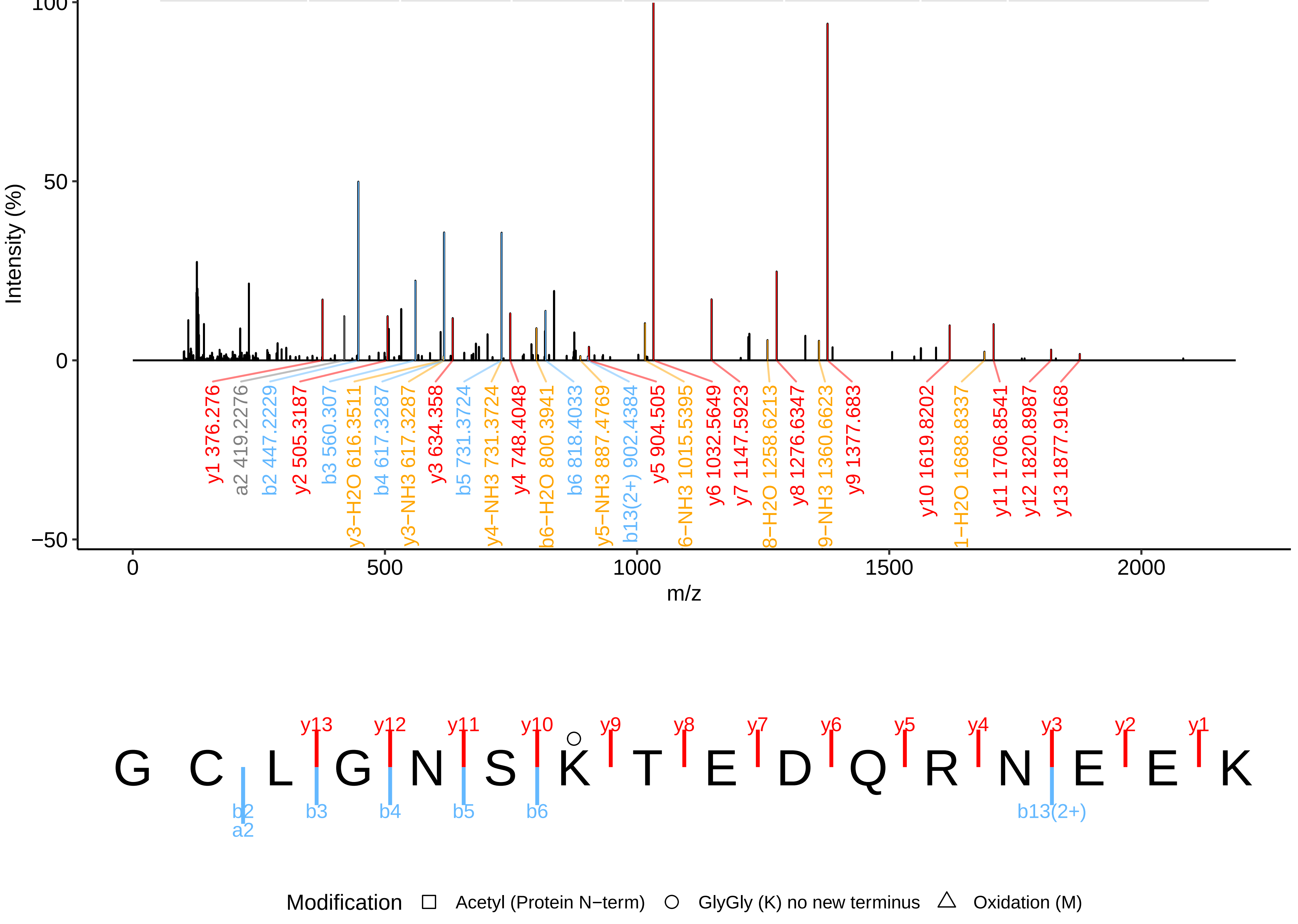

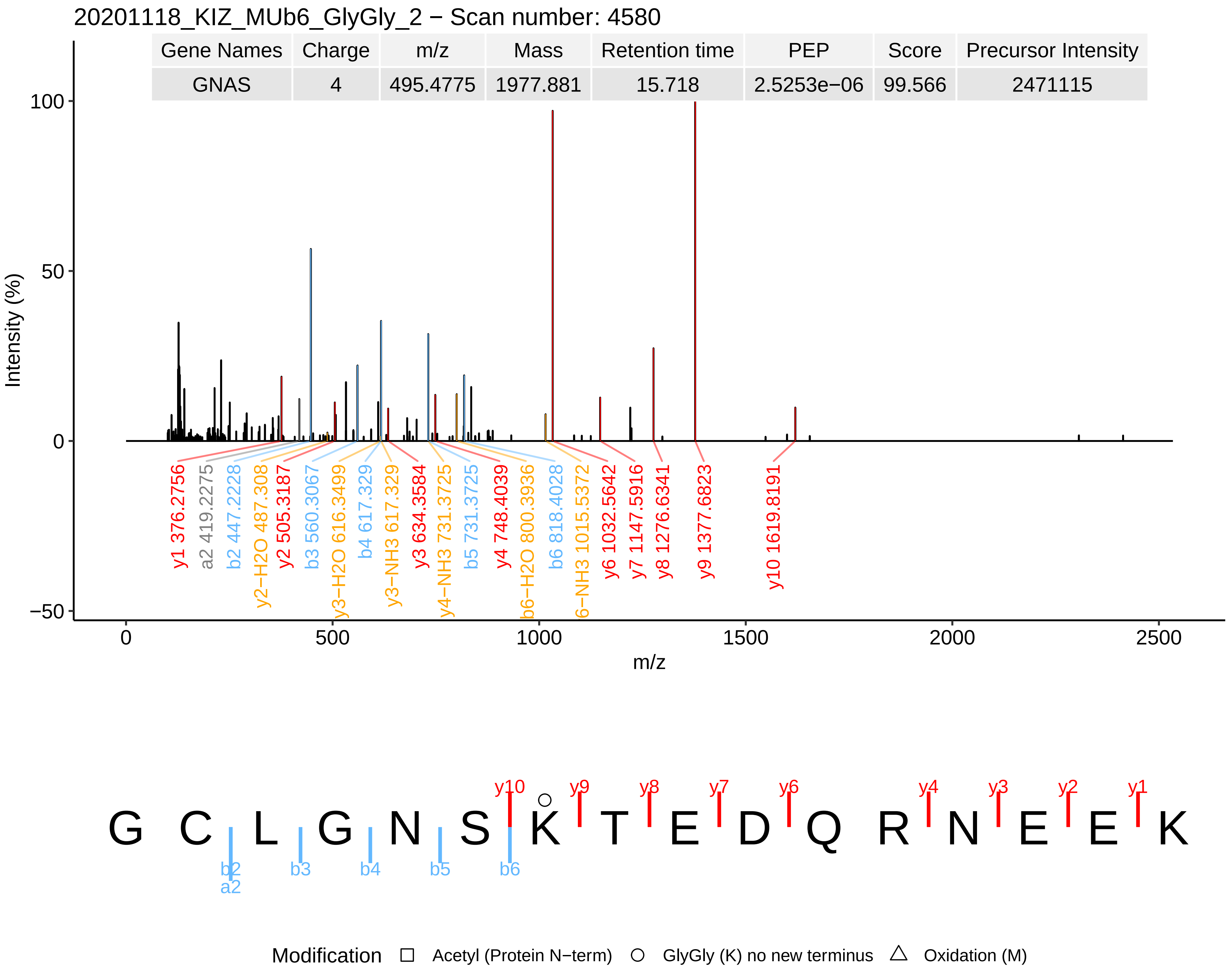

20201118_KIZ_MUb6_GlyGly_2 − Scan number: 4580
Gene Names | Charge | m/z | Mass | Retention time | PEP | Score | Precursor Intensity
GNAS | 4 | 495.4775 | 1977.881 | 15.718 | 2.5253e−06 | 99.566 | 2471115
Intensity (%)
m/z
y1 376.2756
a2 419.2275
b2 447.2228
y2−H2O 487.308
y2 505.3187
b3 560.3067
y3−H2O 616.3499
b4 617.329
y3−NH3 617.329
y3 634.3584
y4−NH3 731.3725
b5 731.3725
y4 748.4039
b6−H2O 800.3936
b6 818.4028
6−NH3 1015.5372
y6 1032.5642
y7 1147.5916
y8 1276.6341
y9 1377.6823
y10 1619.8191
G C L G N S K T E D Q R N E E K
Modification
Acetyl (Protein N−term)
GlyGly (K) no new terminus
Oxidation (M)

20200921_KIZ_MUb4_GlyGly_10plex − Scan number: 28261

Gene Names	Charge	m/z	Mass	Retention time	PEP	Score	Precursor Intensity
ERBB2	3	655.0386	1962.094	103.37	0.00011834	80.534	1212080

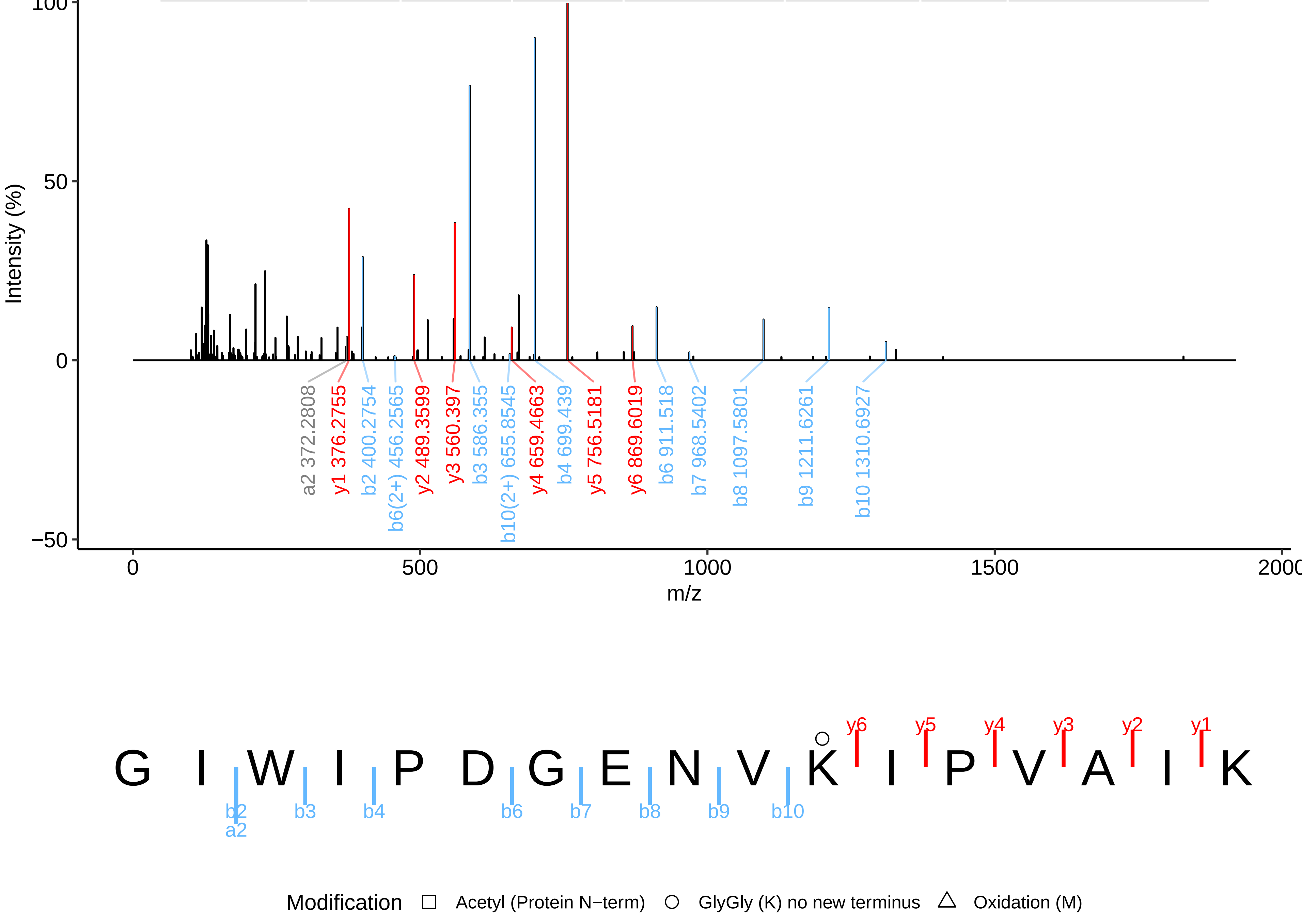

20200921_KIZ_MUb4_GlyGly_10plex_2 − Scan number: 21336

Gene Names	Charge	m/z	Mass	Retention time	PEP	Score	Precursor Intensity
GNAS	3	734.7182	2201.133	83.997	9.738e−06	82.007	951073.6

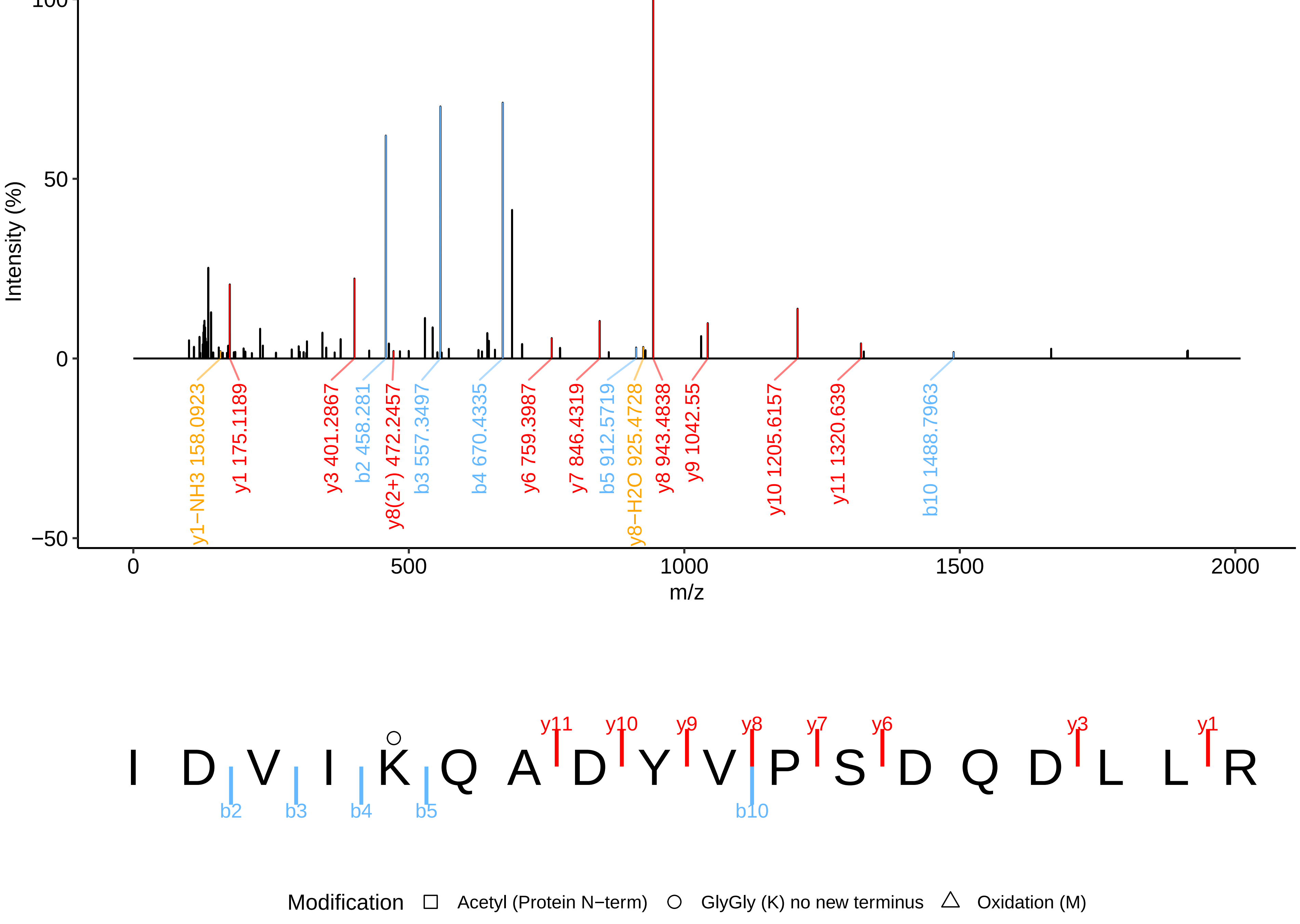

20200921_KIZ_MUb4_GlyGly_10plex − Scan number: 24493

Gene Names	Charge	m/z	Mass	Retention time	PEP	Score	Precursor Intensity
NACA	3	668.6847	2003.032	89.071	4.3999e−16	139.49	NA

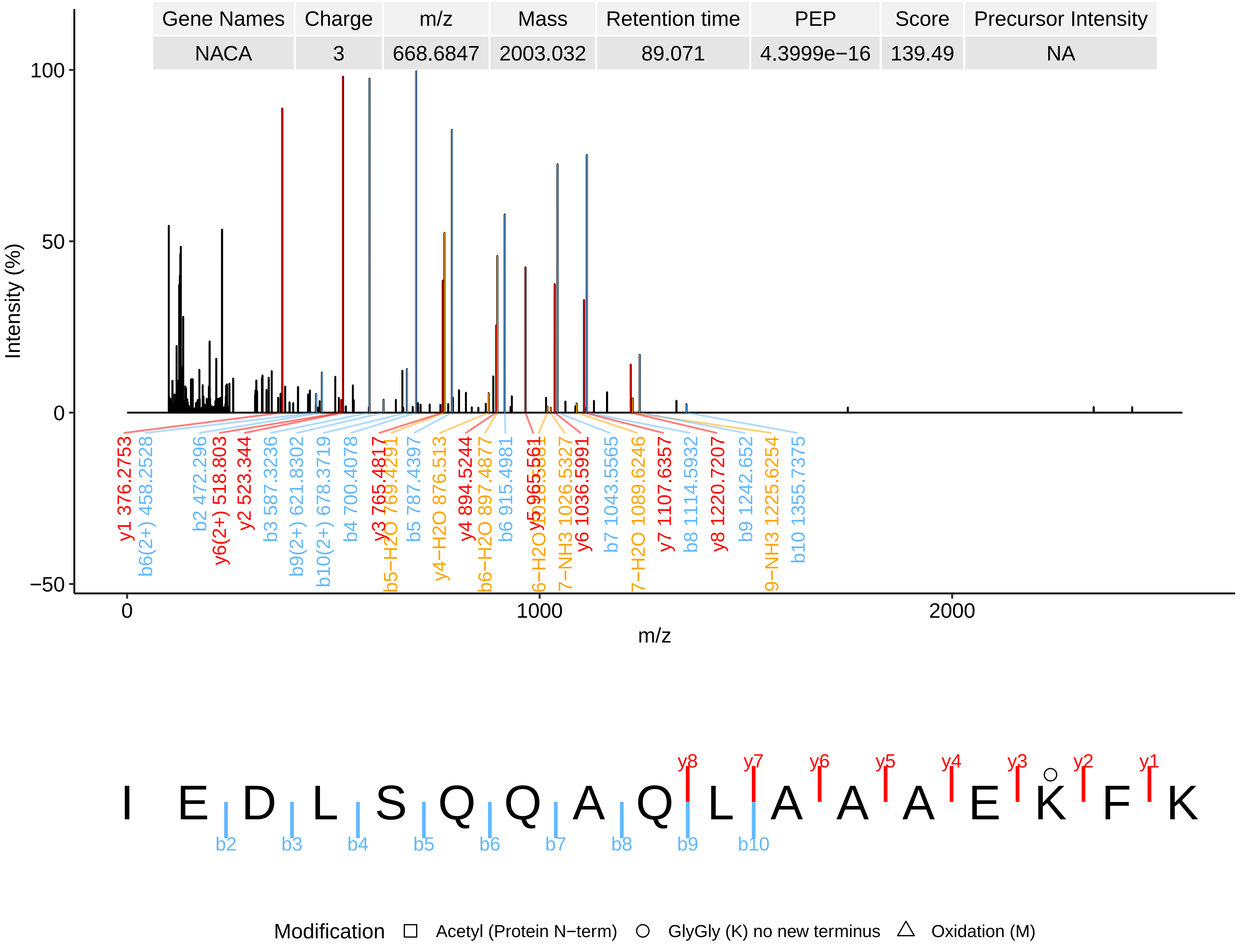

20200921_KIZ_MUb4_GlyGly_10plex − Scan number: 24630

Gene Names	Charge	m/z	Mass	Retention time	PEP	Score	Precursor Intensity
NACA	3	668.6847	2003.032	89.581	1.1874e−11	115.31	NA

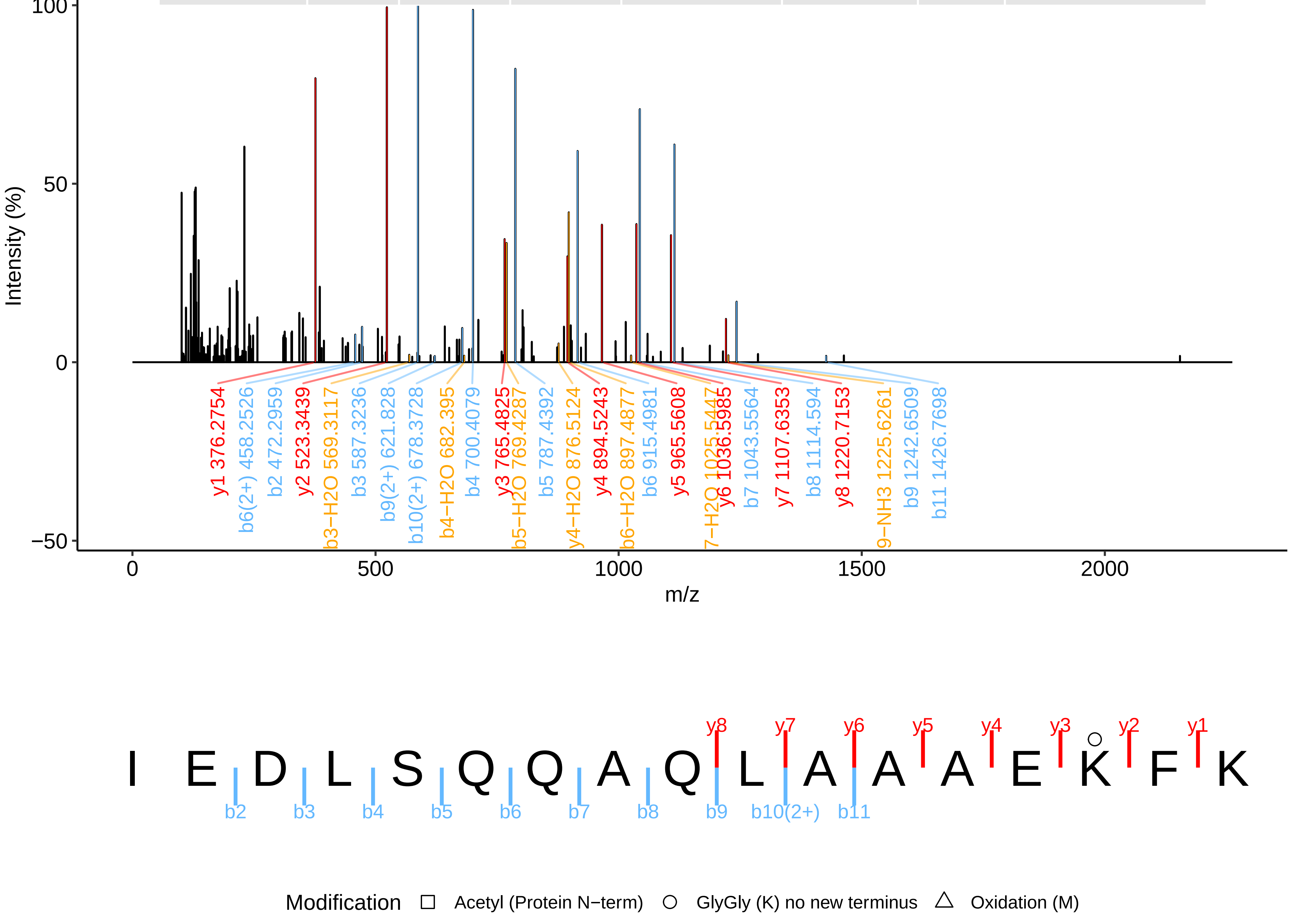

20200921_KIZ_MUb4_GlyGly_10plex − Scan number: 19286

Gene Names	Charge	m/z	Mass	Retention time	PEP	Score	Precursor Intensity
ACSL4	3	646.6775	1937.011	69.498	3.6312e−05	80.387	1852424

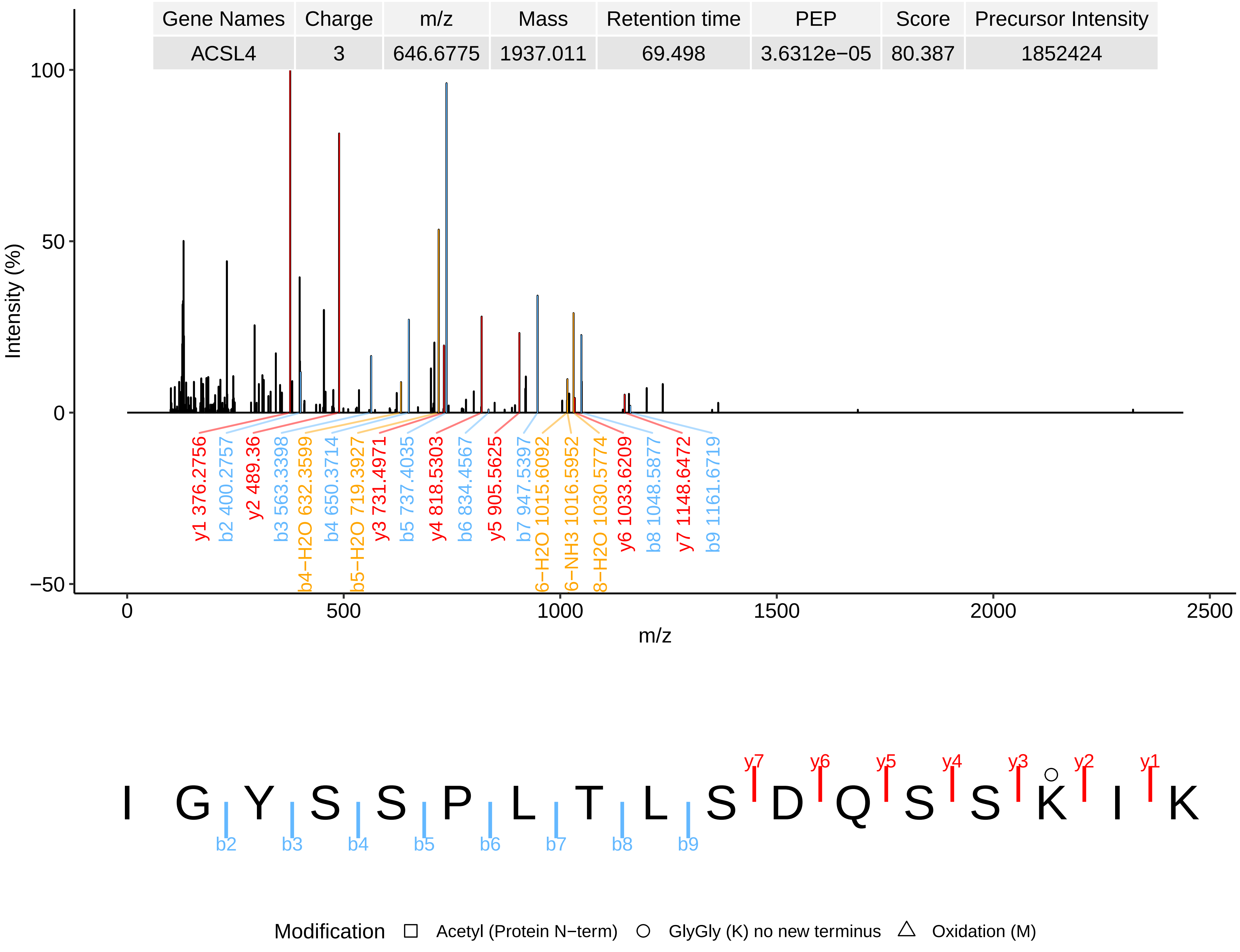

20200921_KIZ_MUb4_GlyGly_10plex_2 − Scan number: 17611

Gene Names	Charge	m/z	Mass	Retention time	PEP	Score	Precursor Intensity
ACSL4	3	646.6775	1937.011	68.882	5.7018e−07	96.414	1354663

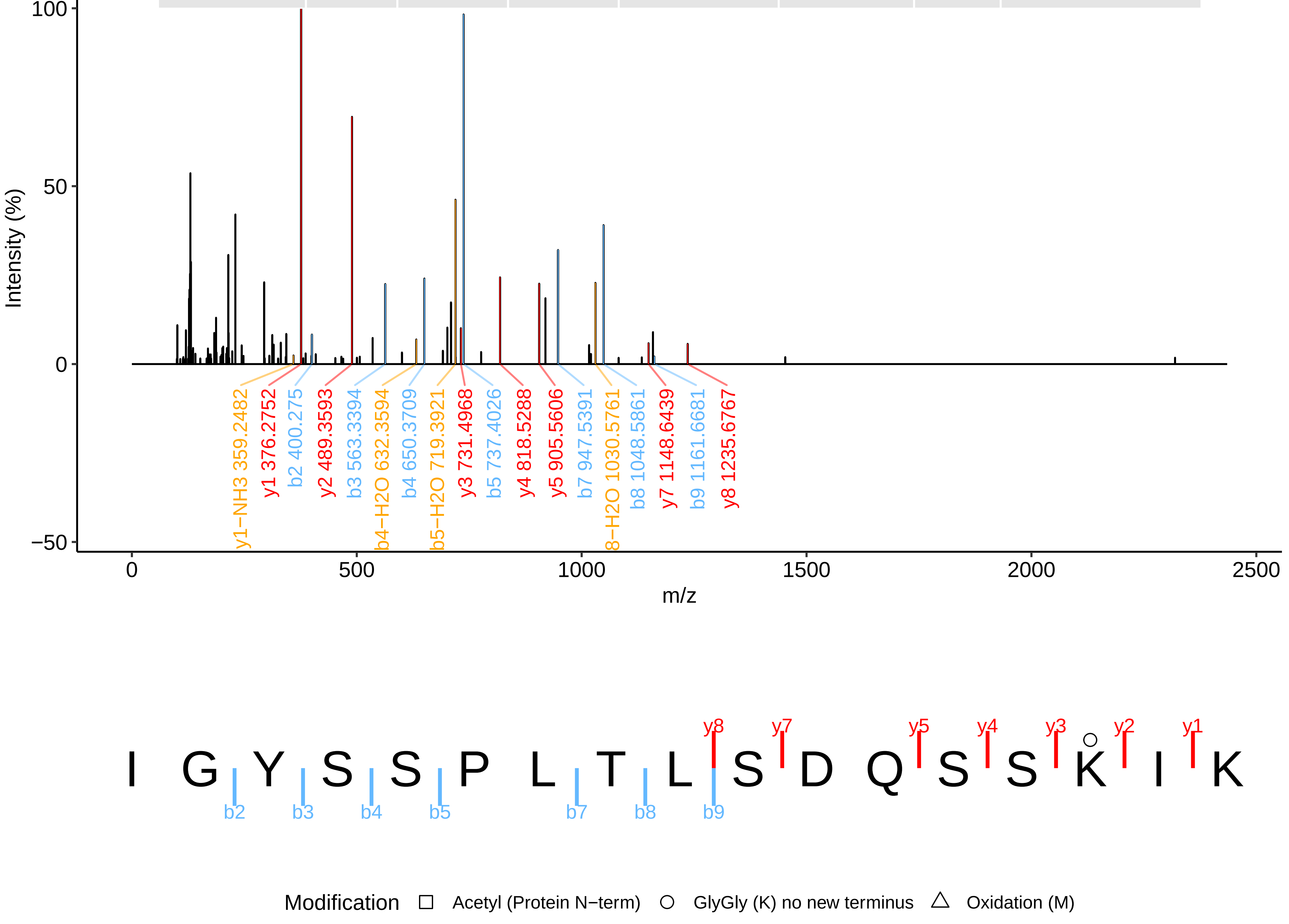

20200921_KIZ_MUb4_GlyGly_10plex_2 − Scan number: 17617

Gene Names	Charge	m/z	Mass	Retention time	PEP	Score	Precursor Intensity
ACSL4	3	646.6775	1937.011	68.906	1.0615e−10	113.59	2371778

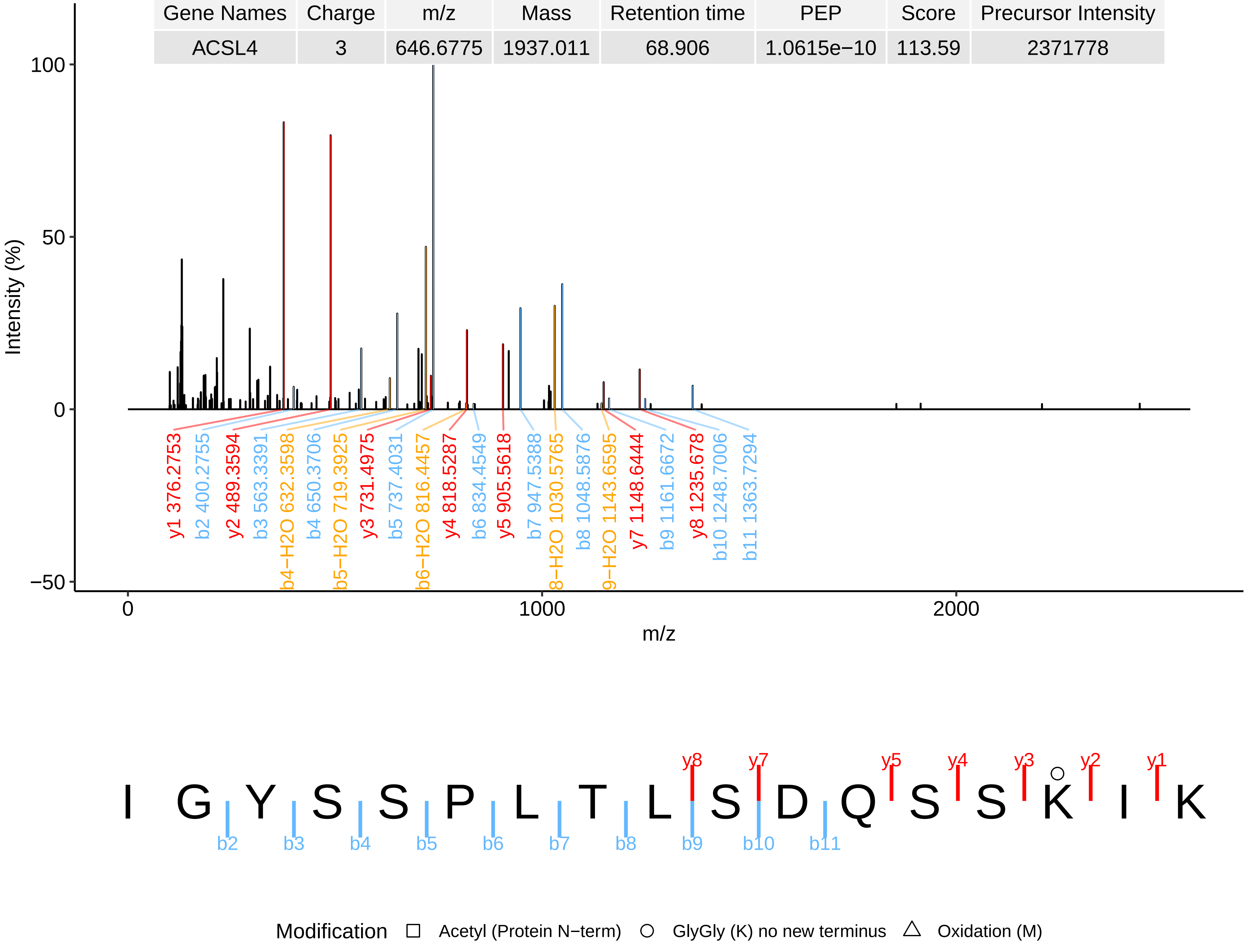

20200921_KIZ_MUb4_GlyGly_10plex_2 − Scan number: 22594

Gene Names	Charge	m/z	Mass	Retention time	PEP	Score	Precursor Intensity
HSPA8;HSPA2	3	634.6825	1901.026	89.103	0.00036127	82.59	NA

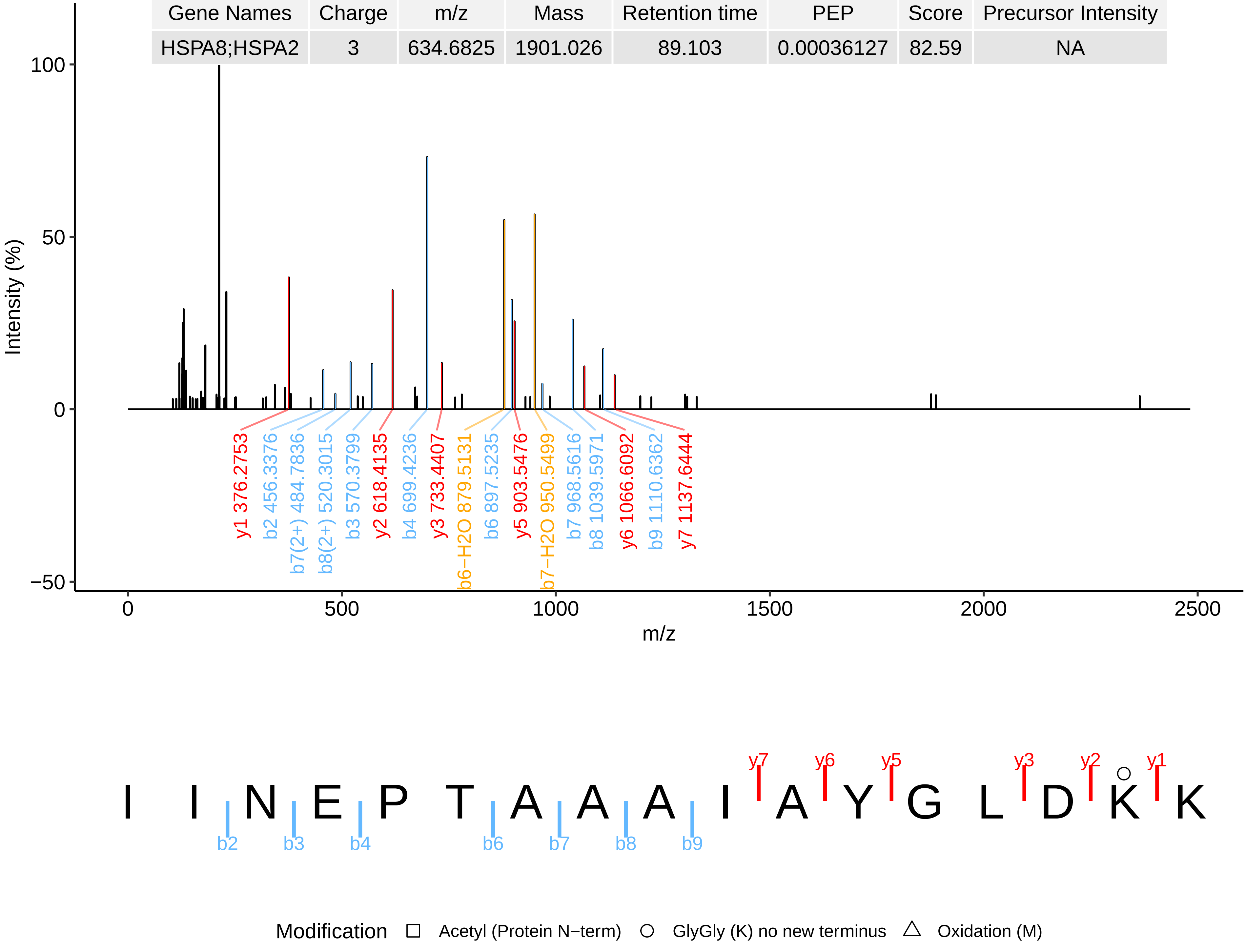

20200921_KIZ_MUb4_GlyGly_10plex − Scan number: 13644

Gene Names	Charge	m/z	Mass	Retention time	PEP	Score	Precursor Intensity
NDUFA8	4	431.4674	1721.841	48.206	0.045345	69.714	3316313

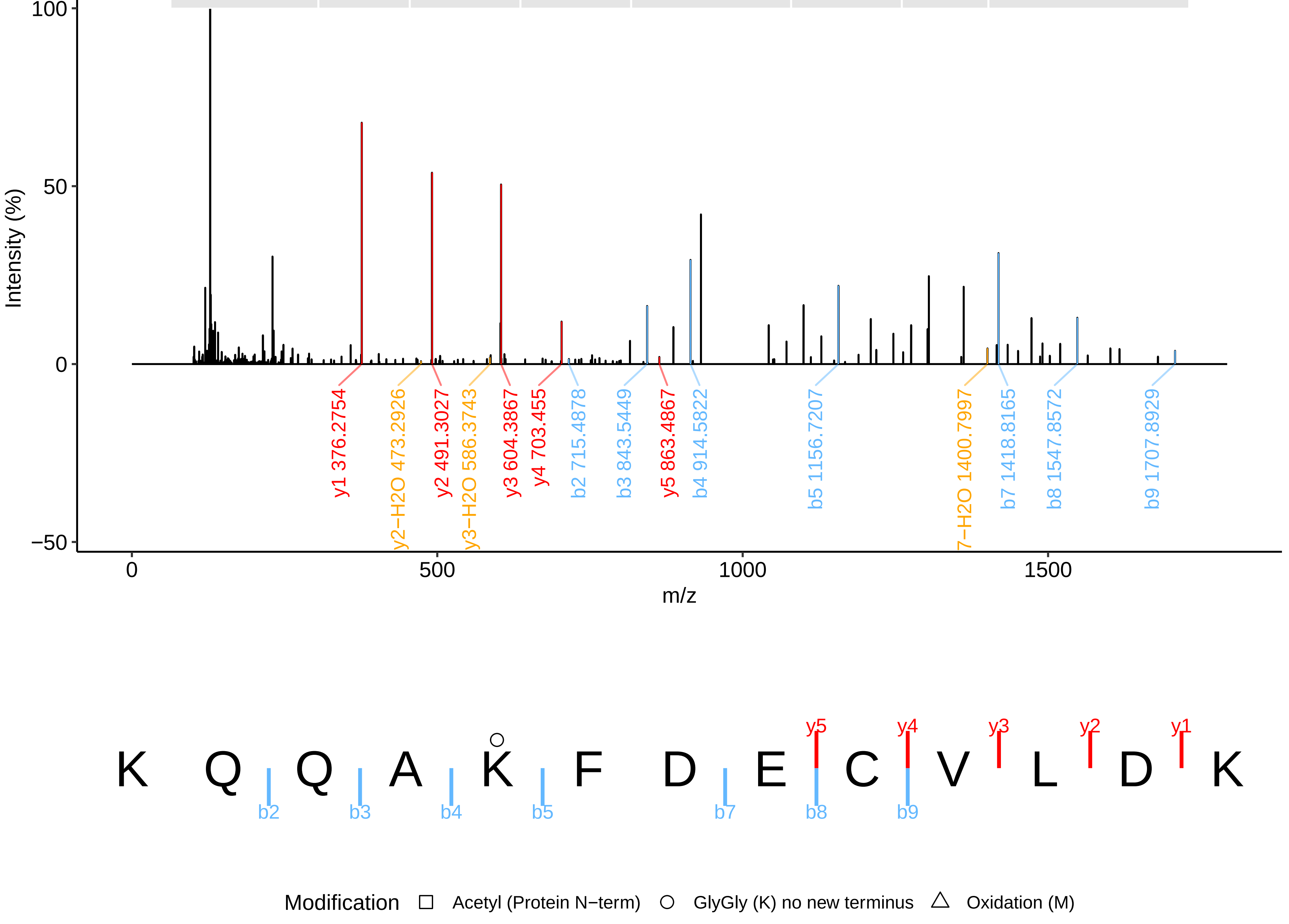

20200921_KIZ_MUb4_GlyGly_10plex_2 − Scan number: 12602

Gene Names	Charge	m/z	Mass	Retention time	PEP	Score	Precursor Intensity
NDUFA8	4	431.4674	1721.841	48.79	0.031727	73.624	2528043

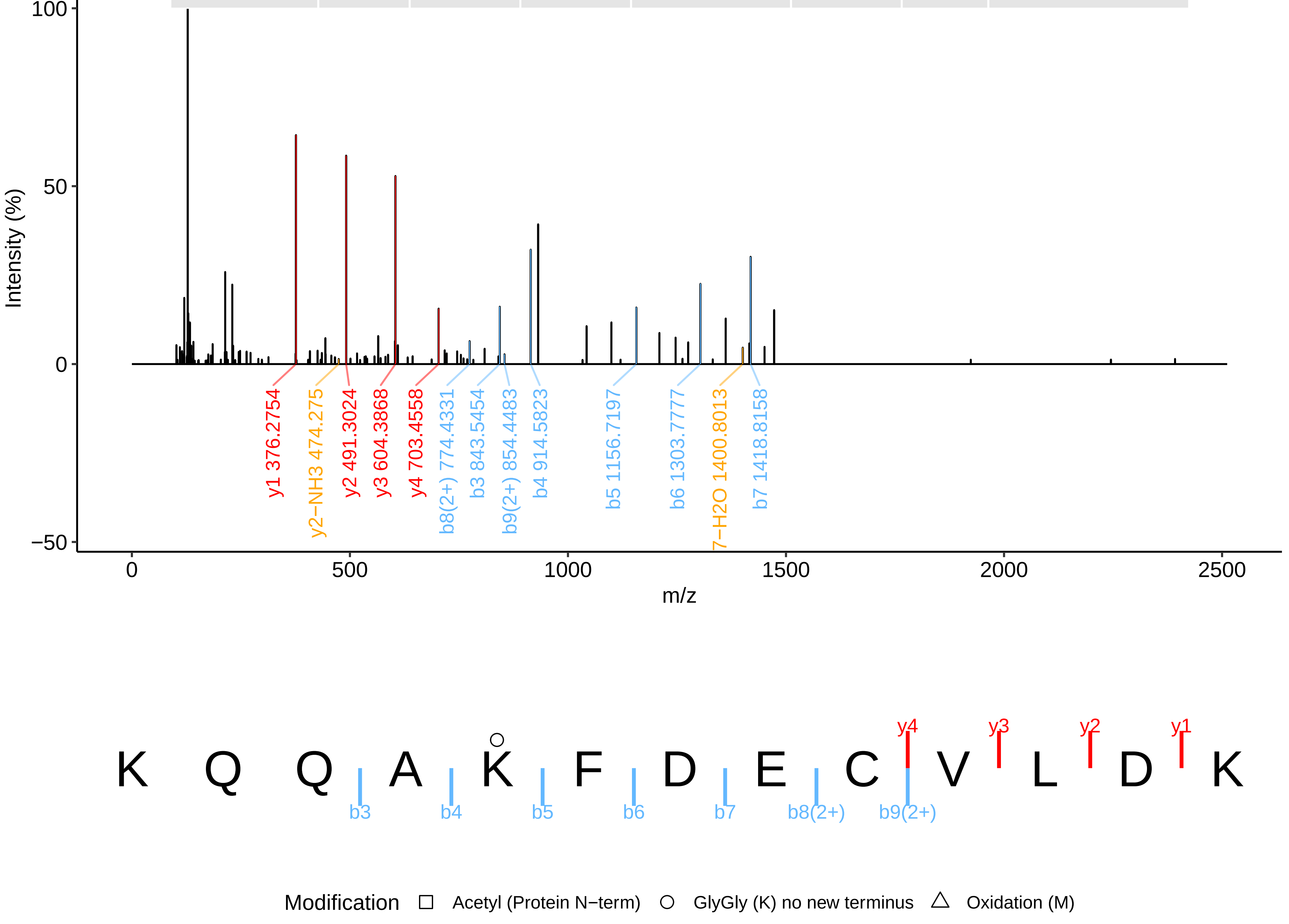

20200921_KIZ_MUb4_GlyGly_10plex − Scan number: 22214

Gene Names	Charge	m/z	Mass	Retention time	PEP	Score	Precursor Intensity
VCP	3	668.3471	2002.019	80.557	3.1855e−08	110.38	NA

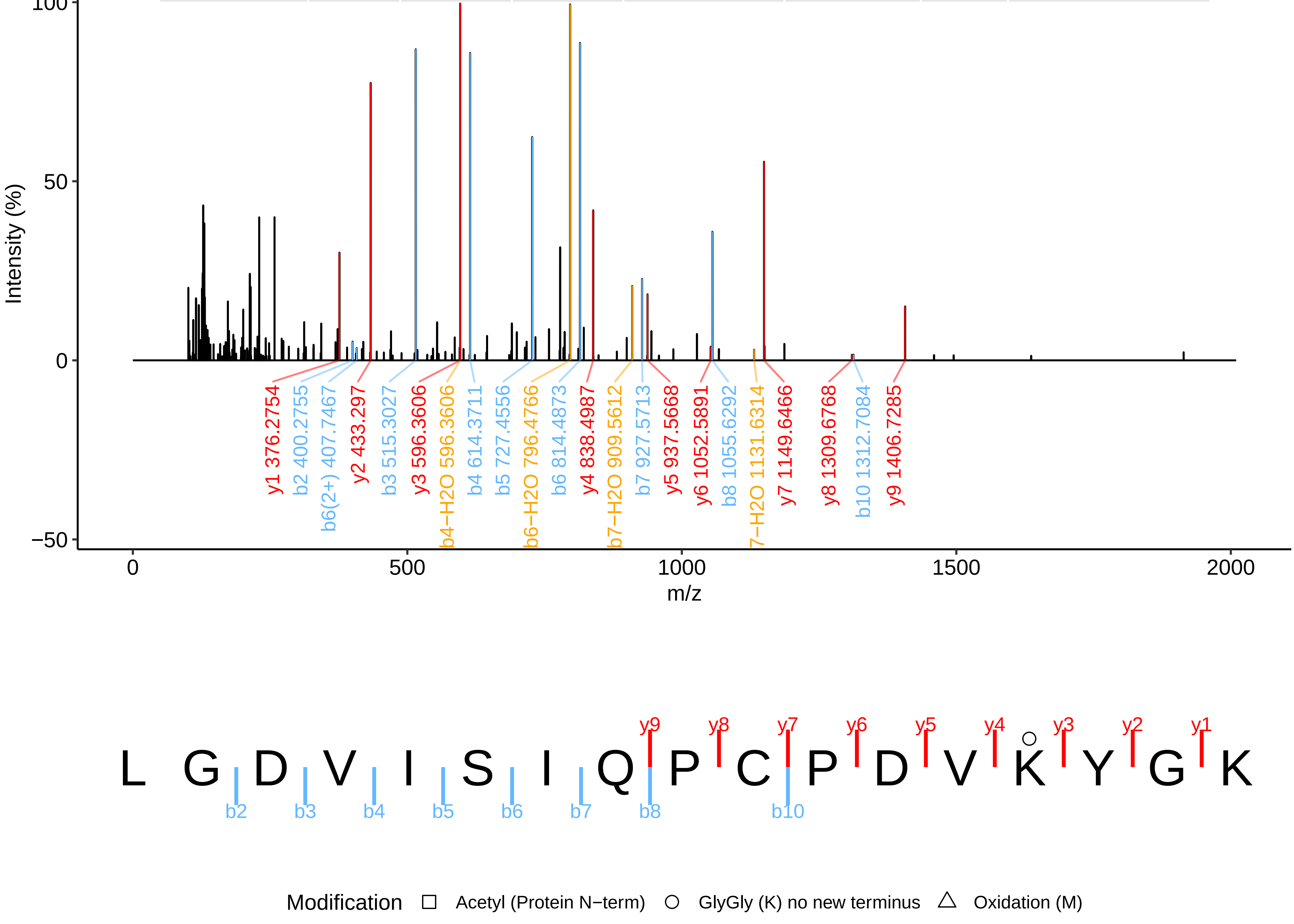

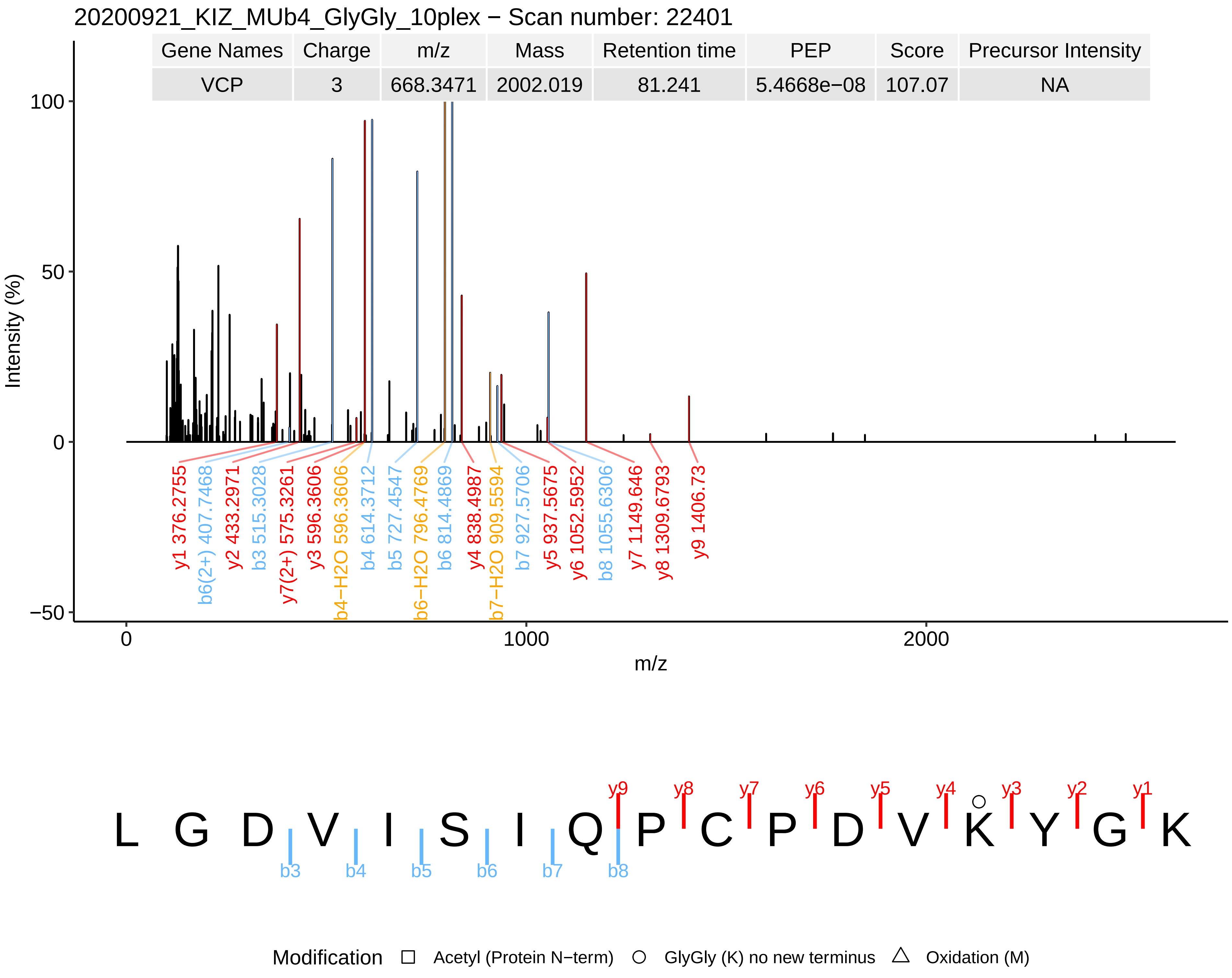
20200921_KIZ_MUb4_GlyGly_10plex − Scan number: 22401
Gene Names | Charge | m/z | Mass | Retention time | PEP | Score | Precursor Intensity
VCP | 3 | 668.3471 | 2002.019 | 81.241 | 5.4668e−08 | 107.07 | NA
Intensity (%)
100
50
0
−50
0
1000
2000
m/z
y1 376.2755
b6(2+) 407.7468
y2 433.2971
b3 515.3028
y7(2+) 575.3261
y3 596.3606
b4−H2O 596.3606
b4 614.3712
b5 727.4547
b6−H2O 796.4769
b6 814.4869
y4 838.4987
b7−H2O 909.5594
b7 927.5706
y5 937.5675
y6 1052.5952
b8 1055.6306
y7 1149.646
y8 1309.6793
y9 1406.73
L G D V I S I Q P C P D V K Y G K
y9 y8 y7 y6 y5 y4 y3 y2 y1
b3 b4 b5 b6 b7 b8
Modification
Acetyl (Protein N−term)
GlyGly (K) no new terminus
Oxidation (M)

20200921_KIZ_MUb4_GlyGly_10plex_2 − Scan number: 20007

Gene Names	Charge	m/z	Mass	Retention time	PEP	Score	Precursor Intensity
VCP	3	668.3471	2002.019	78.591	1.1635e−12	118.33	1717617

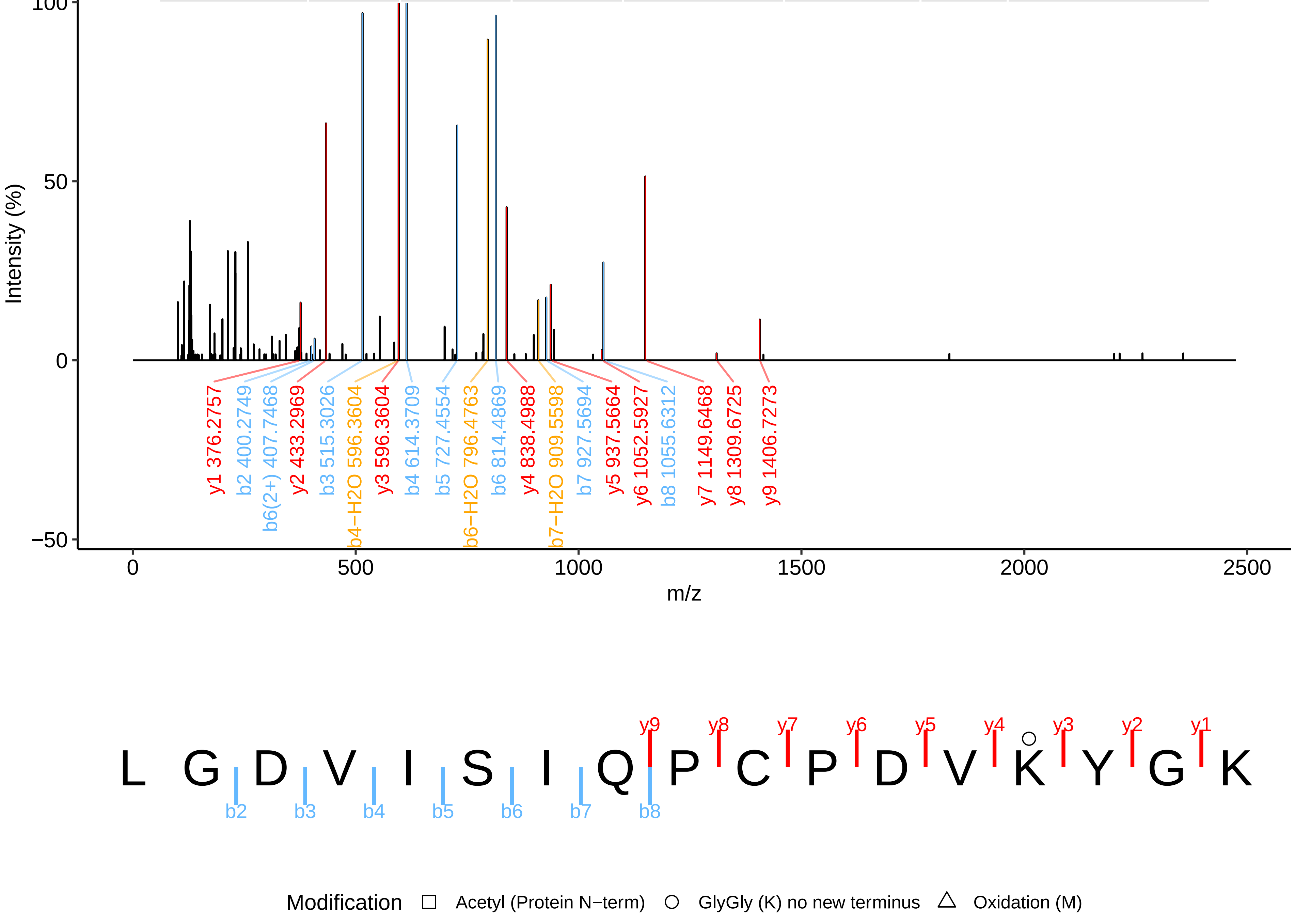

20200924_KIZ_MUb5_GlyGly_1 − Scan number: 22370

Gene Names	Charge	m/z	Mass	Retention time	PEP	Score	Precursor Intensity
VCP	3	668.3471	2002.019	71.981	8.1595e−12	120.66	18728998

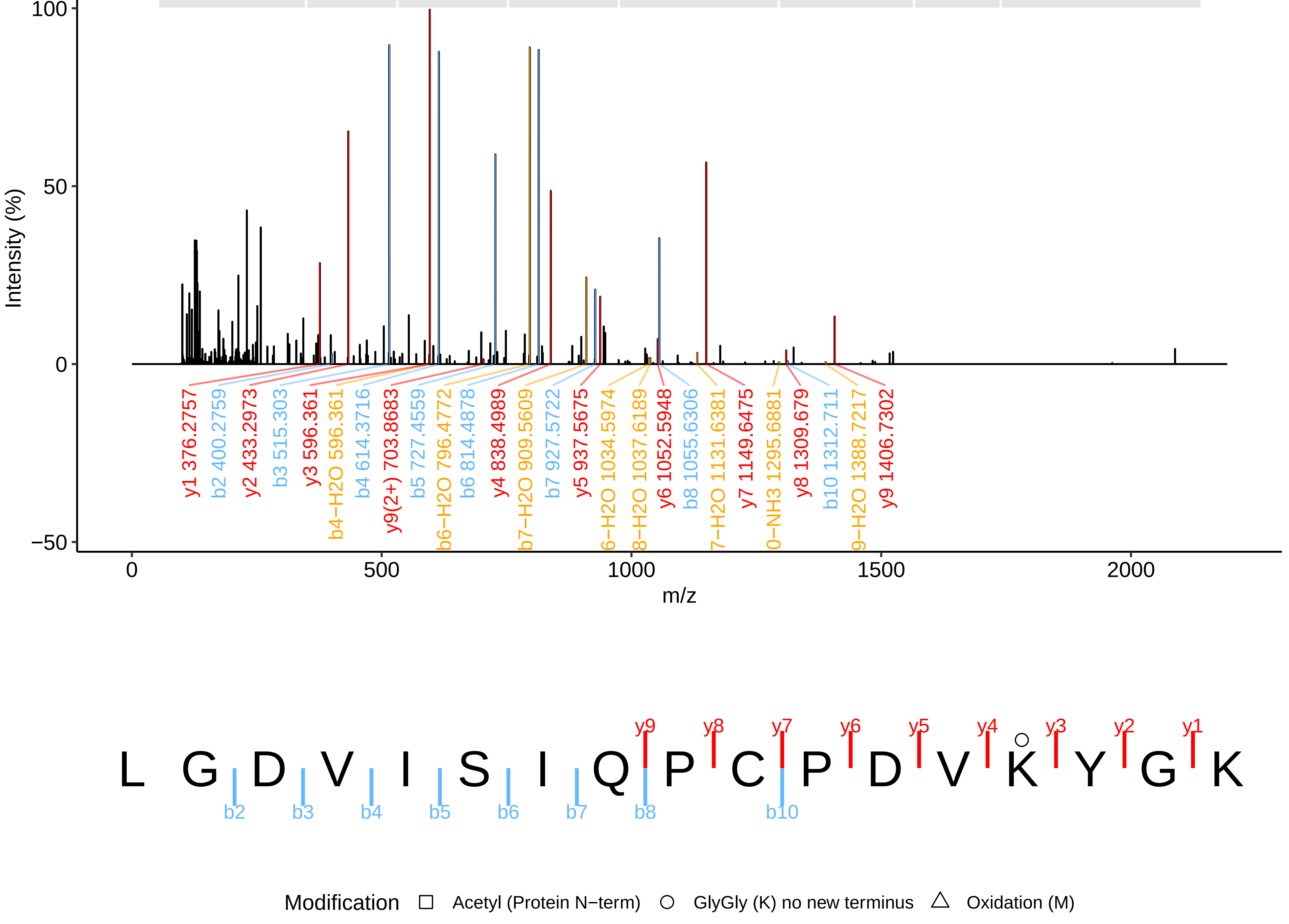

20200924_KIZ_MUb5_GlyGly_2 − Scan number: 19972

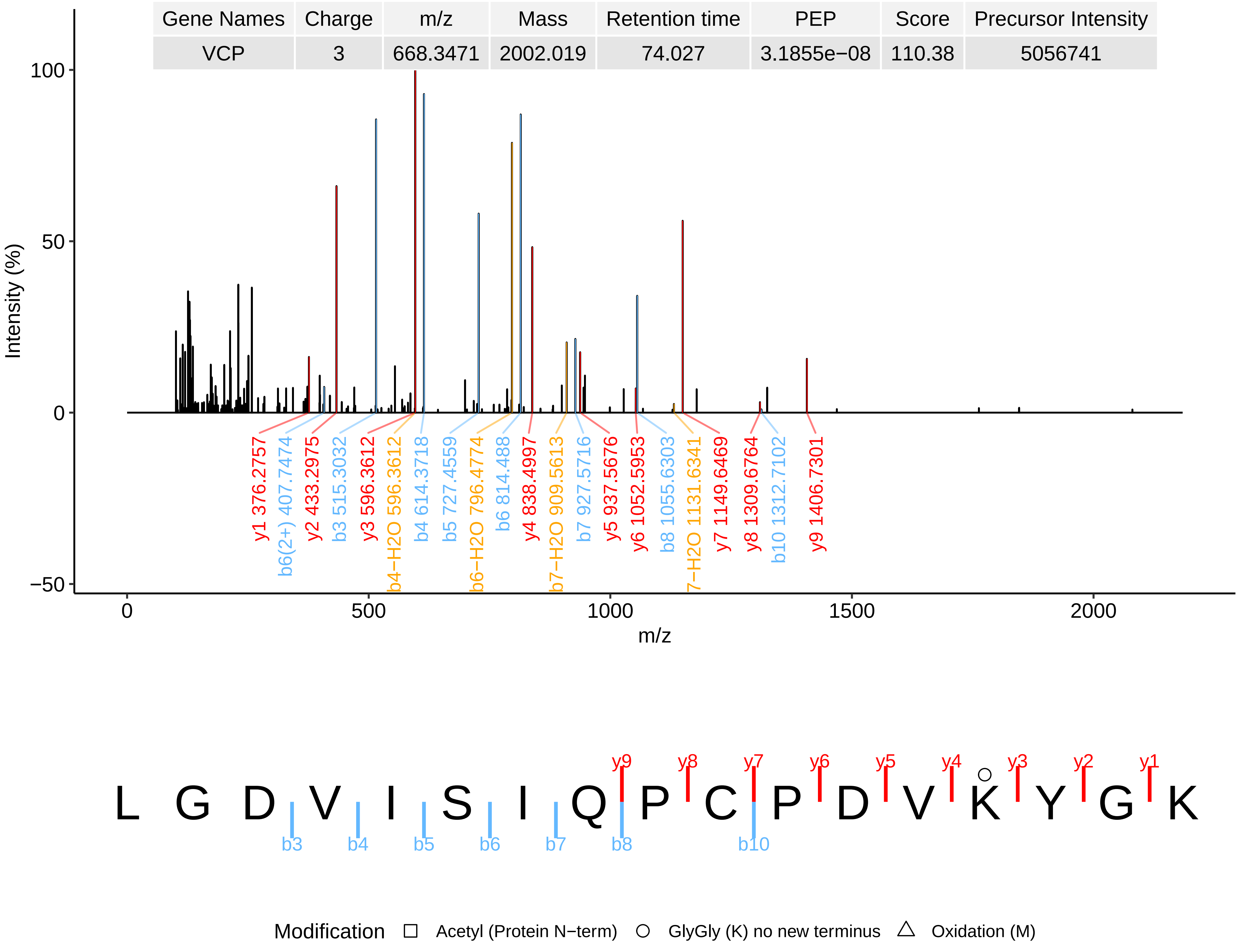

20201118_KIZ_MUb6_GlyGly_2 − Scan number: 24215

Gene Names	Charge	m/z	Mass	Retention time	PEP	Score	Precursor Intensity
VCP	3	668.3471	2002.019	81.136	1.2754e−13	131.24	12592531

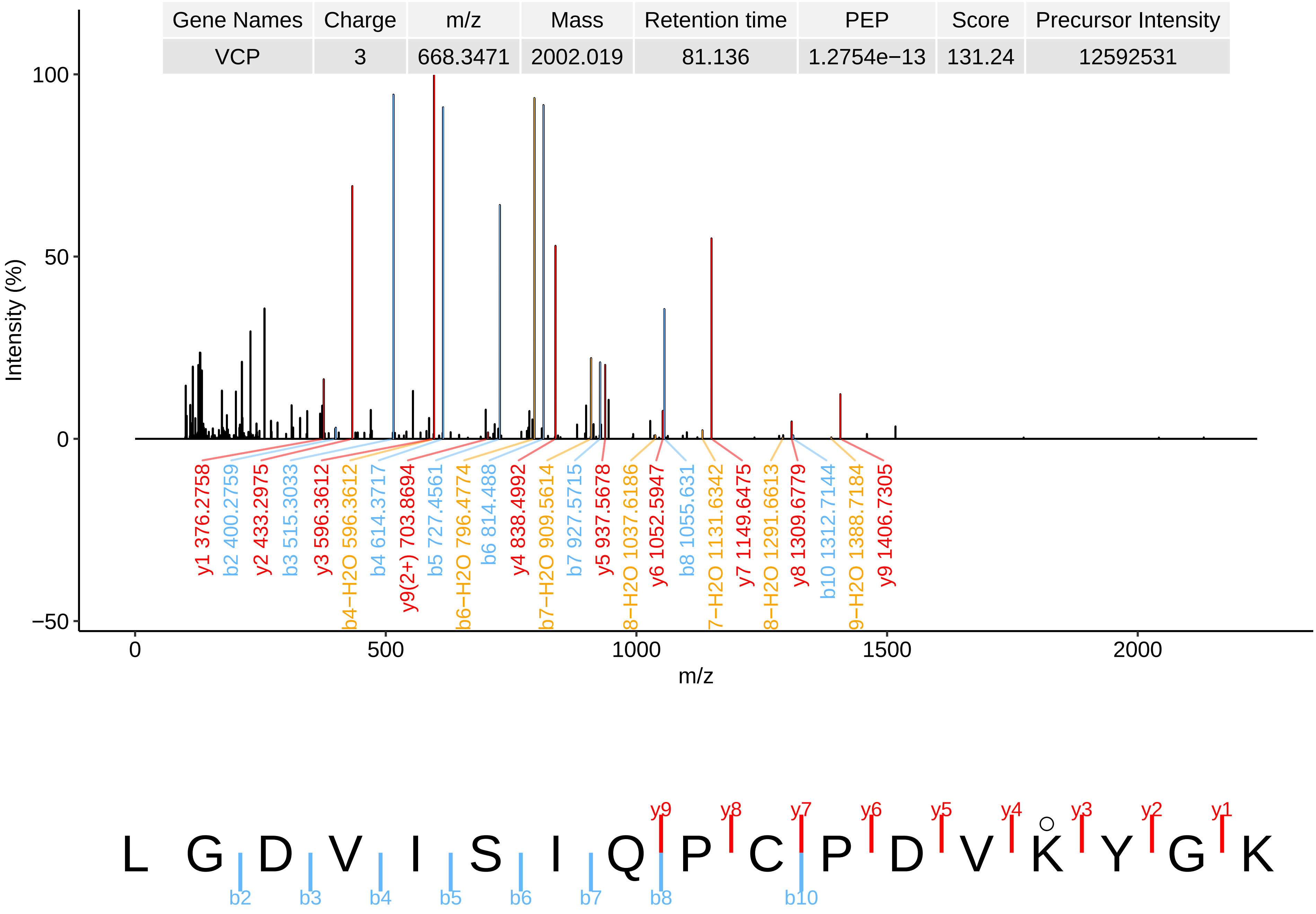

Modification □ Acetyl (Protein N−term) ○ GlyGly (K) no new terminus △ Oxidation (M)

20201118_KIZ_MUb6_GlyGly_2 − Scan number: 24230

Gene Names	Charge	m/z	Mass	Retention time	PEP	Score	Precursor Intensity
VCP	3	668.3471	2002.019	81.178	3.9719e−13	127.88	18583186

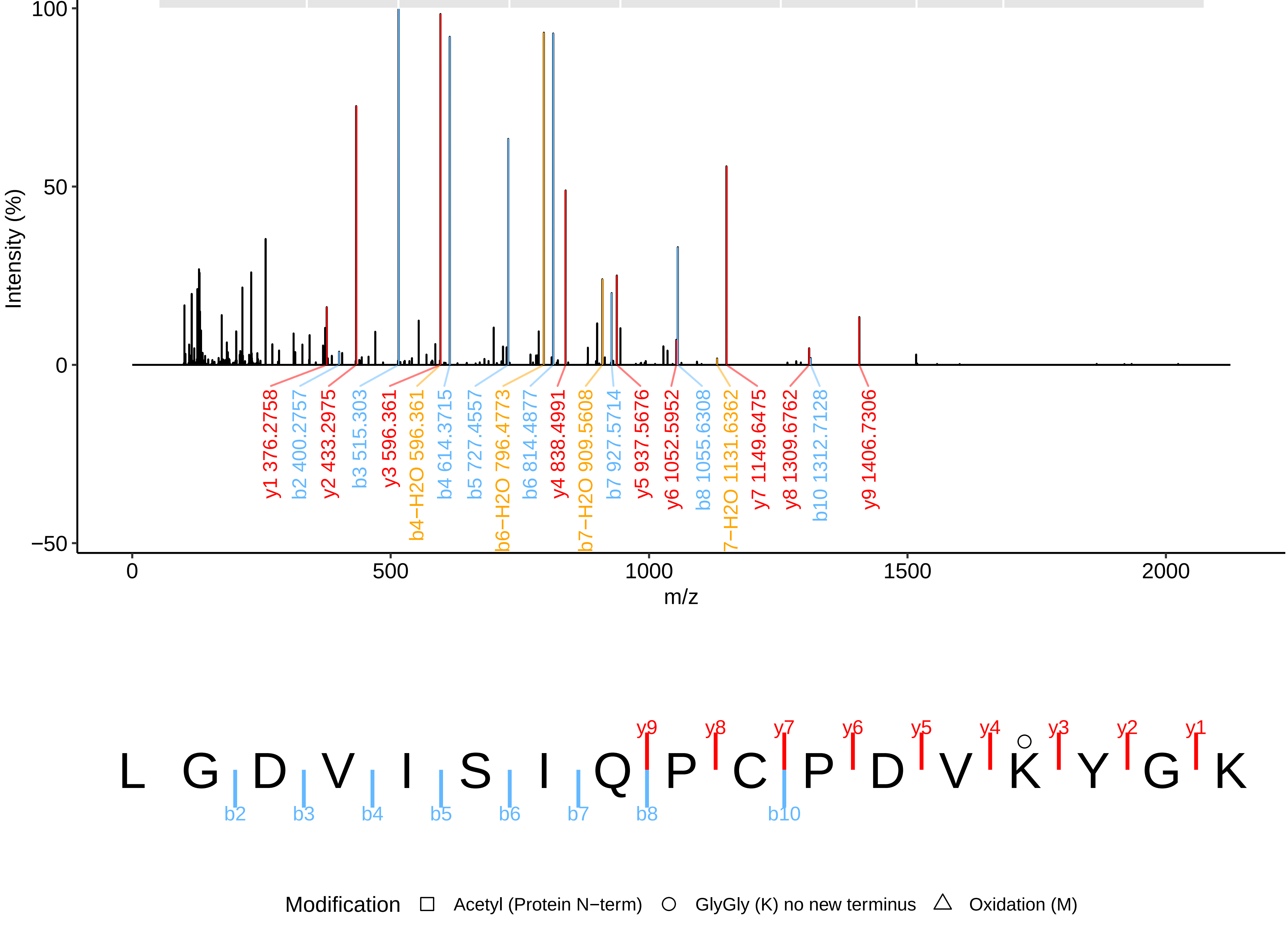

20200921_KIZ_MUb4_GlyGly_10plex_2 − Scan number: 22559

Gene Names	Charge	m/z	Mass	Retention time	PEP	Score	Precursor Intensity
RAD23A	4	556.0548	2220.19	88.954	4.8105e−05	85.734	1127828

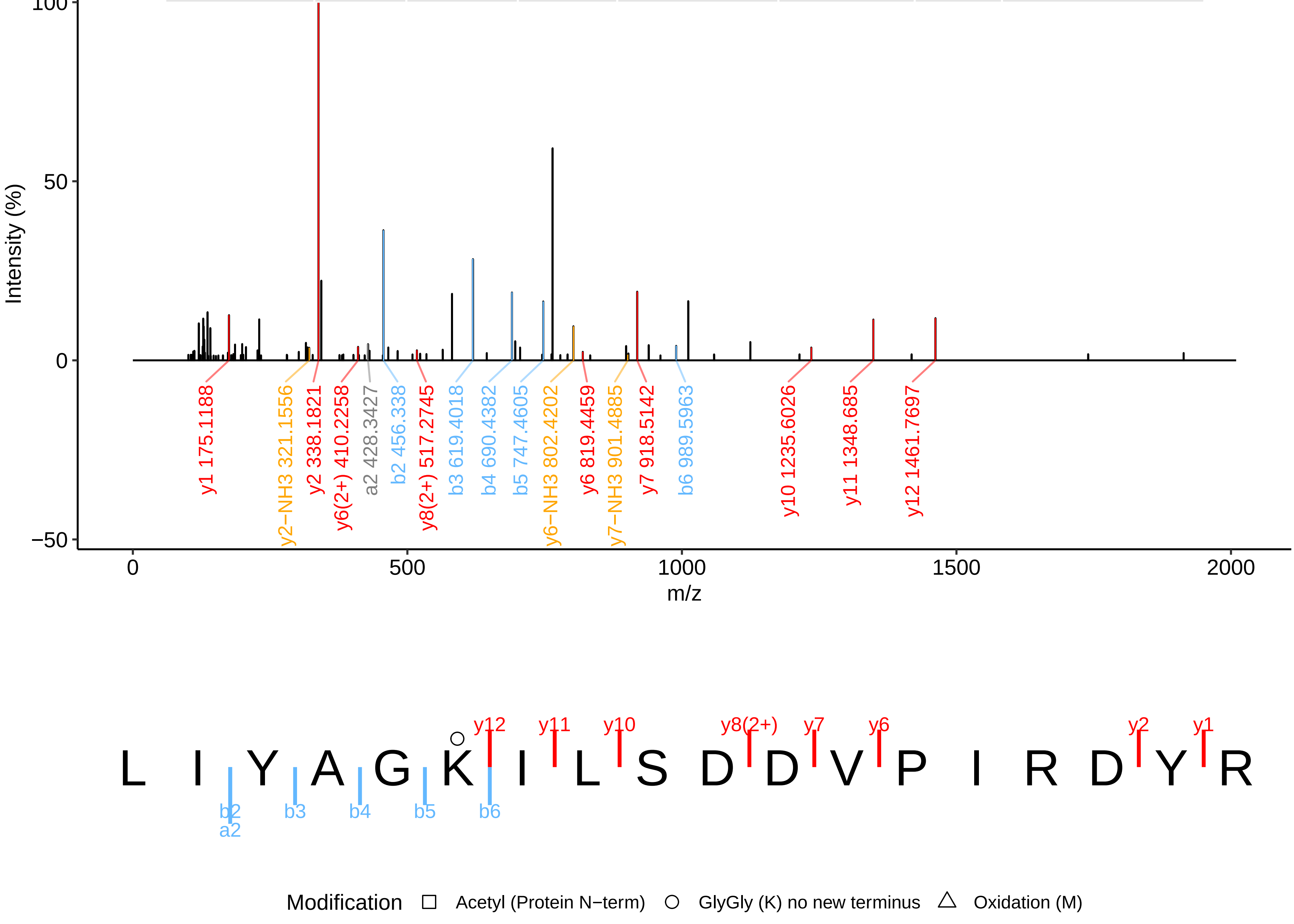

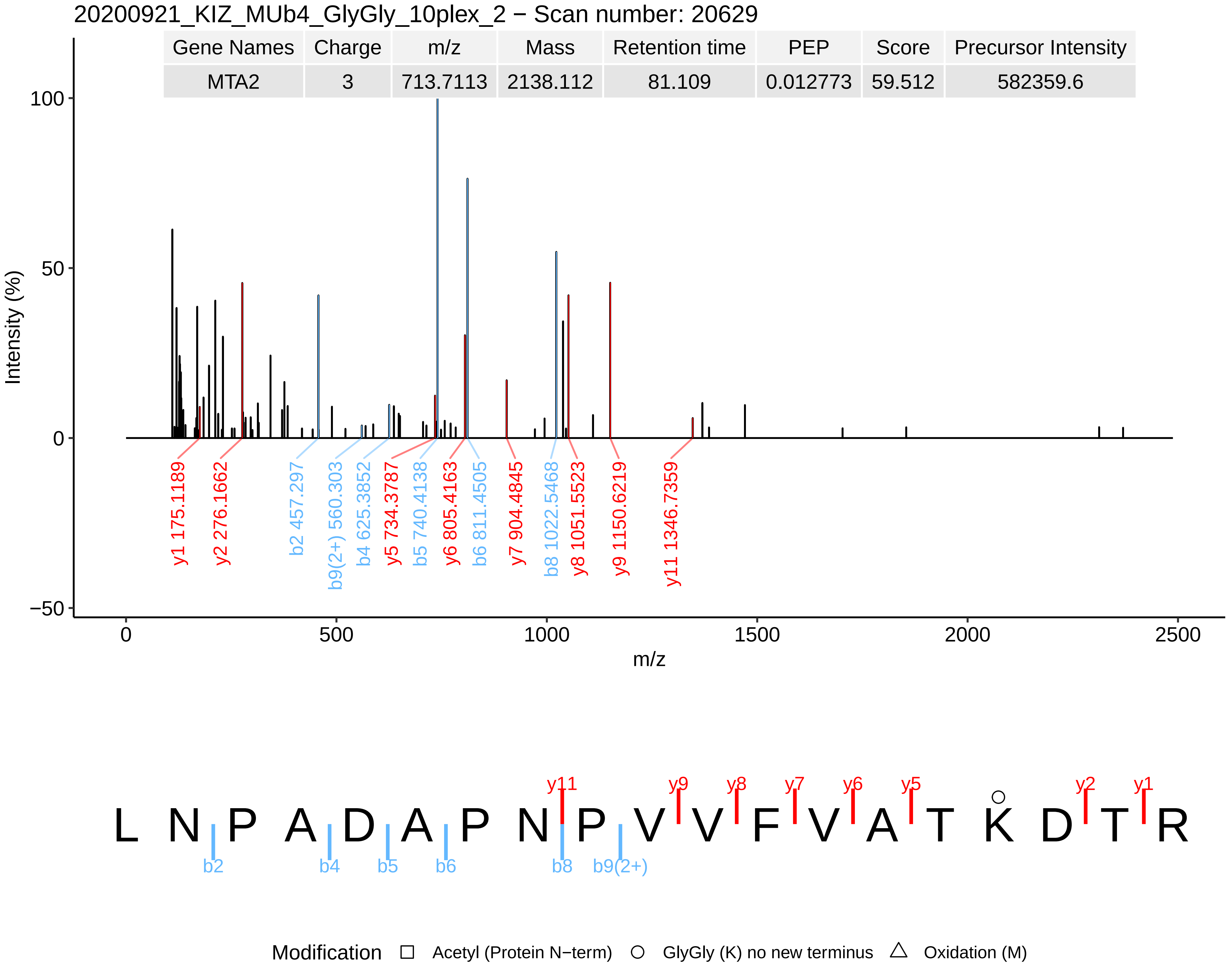
20200921_KIZ_MUb4_GlyGly_10plex_2 − Scan number: 20629
Gene Names | Charge | m/z | Mass | Retention time | PEP | Score | Precursor Intensity
MTA2 | 3 | 713.7113 | 2138.112 | 81.109 | 0.012773 | 59.512 | 582359.6
Intensity (%)
m/z
y1 175.1189
y2 276.1662
b2 457.297
b9(2+) 560.303
b4 625.3852
y5 734.3787
b5 740.4138
y6 805.4163
b6 811.4505
y7 904.4845
b8 1022.5468
y8 1051.5523
y9 1150.6219
y11 1346.7359
L N P A D A P N P V V F V A T K D T R
y11 y9 y8 y7 y6 y5 y2 y1
b2 b4 b5 b6 b8 b9(2+)
Modification
Acetyl (Protein N−term)
GlyGly (K) no new terminus
Oxidation (M)

20200924_KIZ_MUb5_GlyGly_1 − Scan number: 23090

Gene Names	Charge	m/z	Mass	Retention time	PEP	Score	Precursor Intensity
MTA2	3	713.7113	2138.112	74.25	0.0027699	62.684	14173311

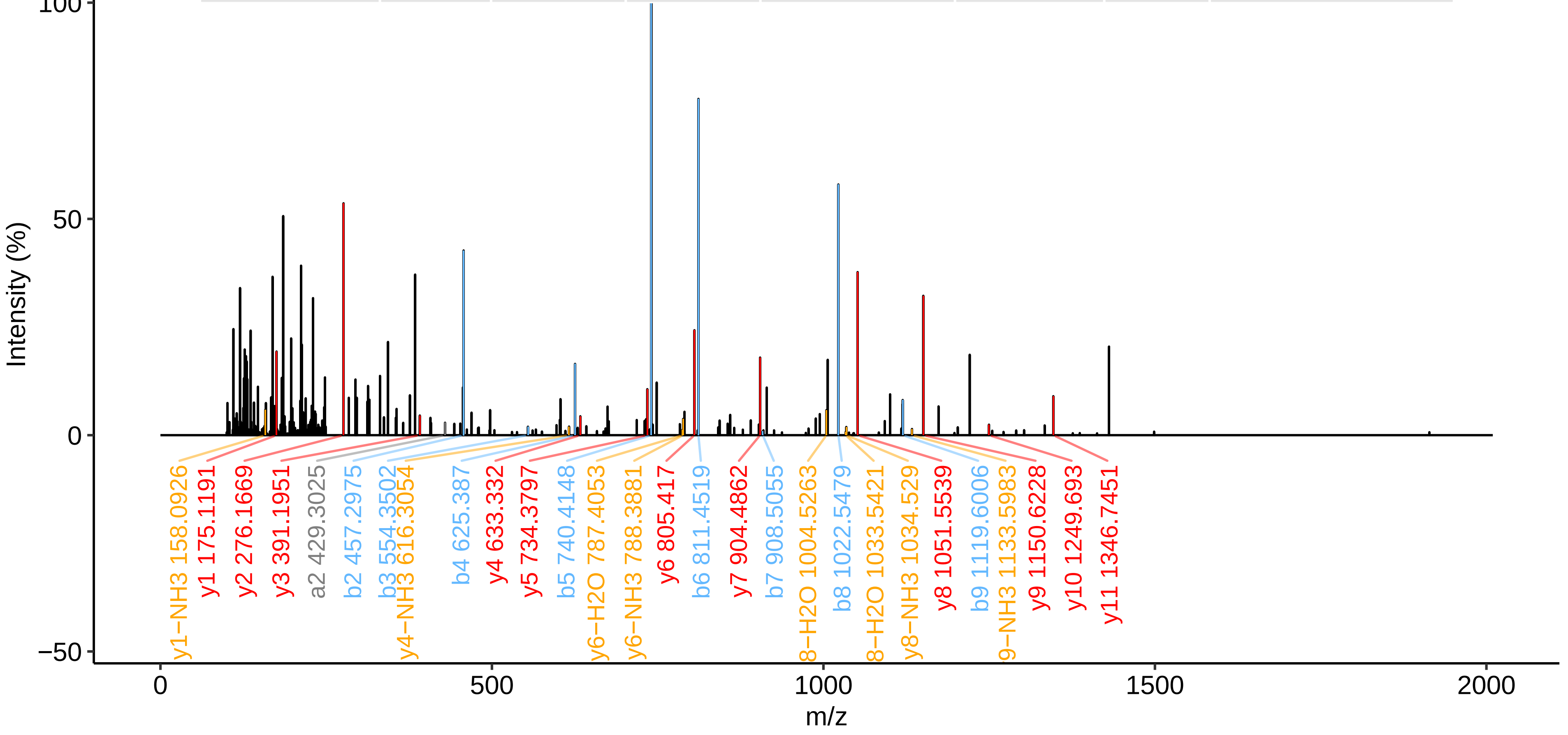

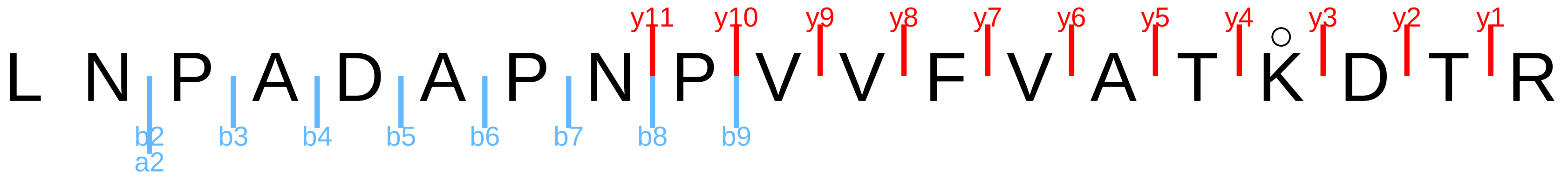

Modification □ Acetyl (Protein N−term) ○ GlyGly (K) no new terminus △ Oxidation (M)

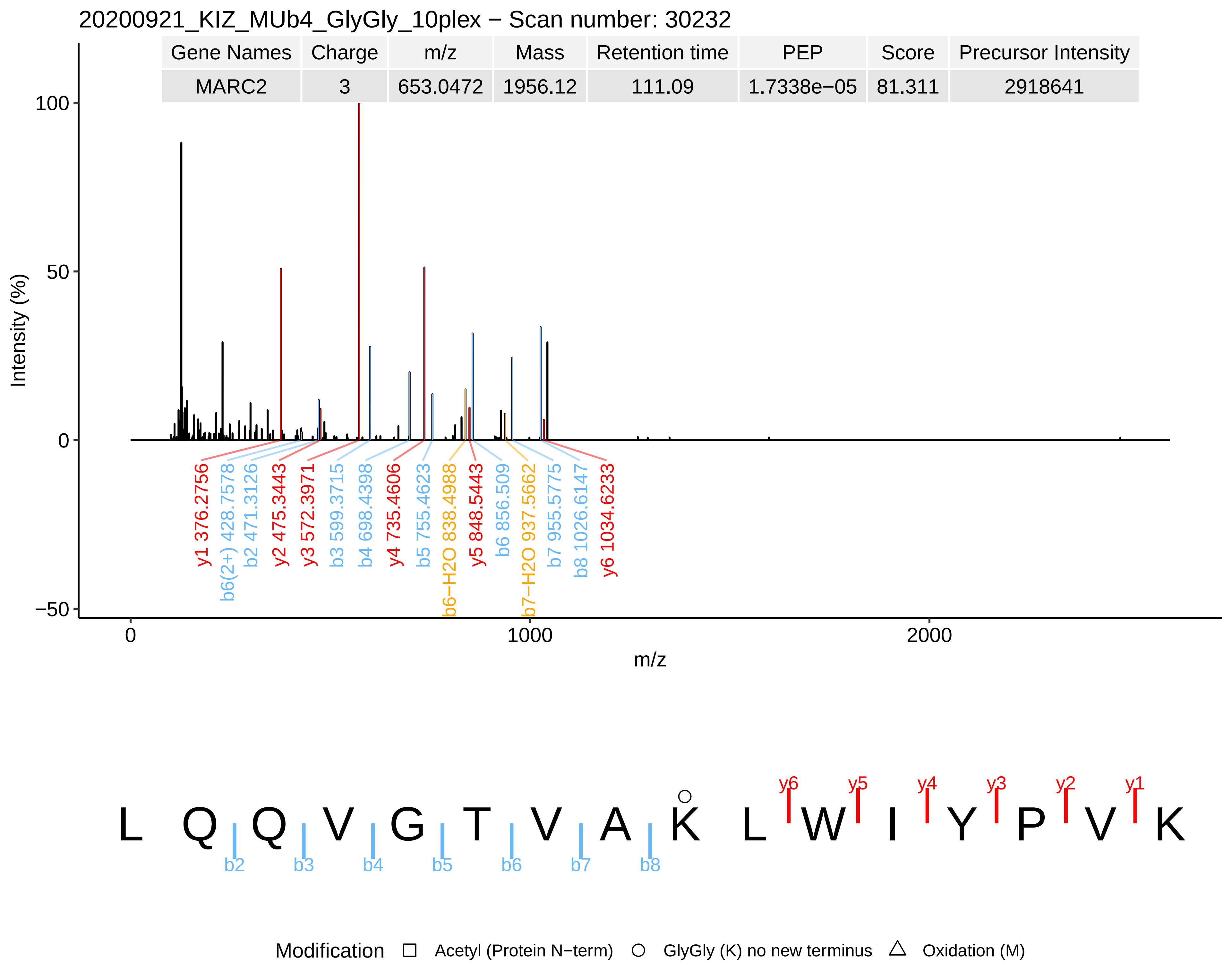
20200921_KIZ_MUb4_GlyGly_10plex − Scan number: 30232
Gene Names | Charge | m/z | Mass | Retention time | PEP | Score | Precursor Intensity
MARC2 | 3 | 653.0472 | 1956.12 | 111.09 | 1.7338e−05 | 81.311 | 2918641
Intensity (%)
100
50
0
−50
y1 376.2756
b6(2+) 428.7578
b2 471.3126
y2 475.3443
y3 572.3971
b3 599.3715
b4 698.4398
y4 735.4606
b5 755.4623
b6−H2O 838.4988
y5 848.5443
b6 856.509
b7−H2O 937.5662
b7 955.5775
b8 1026.6147
y6 1034.6233
0
1000
2000
m/z
L Q Q V G T V A K L W I Y P V K
y6 y5 y4 y3 y2 y1
b2 b3 b4 b5 b6 b7 b8
Modification
Acetyl (Protein N−term)
GlyGly (K) no new terminus
Oxidation (M)

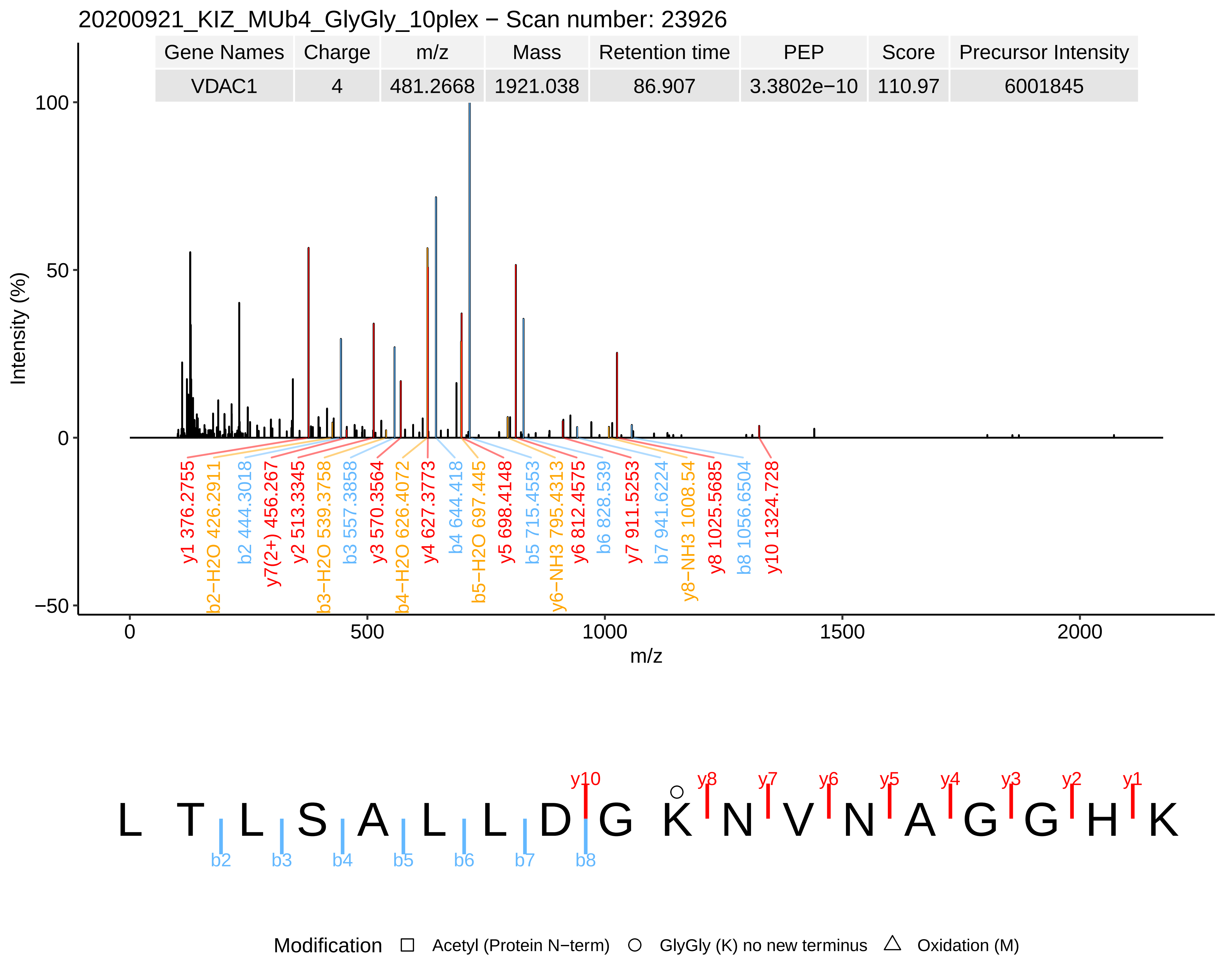
20200921_KIZ_MUb4_GlyGly_10plex − Scan number: 23926
Gene Names | Charge | m/z | Mass | Retention time | PEP | Score | Precursor Intensity
VDAC1 | 4 | 481.2668 | 1921.038 | 86.907 | 3.3802e−10 | 110.97 | 6001845
Intensity (%)
100
50
0
−50
0
500
1000
1500
2000
m/z
y1 376.2755
b2−H2O 426.2911
b2 444.3018
y7(2+) 456.267
y2 513.3345
b3−H2O 539.3758
b3 557.3858
y3 570.3564
b4−H2O 626.4072
y4 627.3773
b4 644.418
b5−H2O 697.445
y5 698.4148
b5 715.4553
y6−NH3 795.4313
y6 812.4575
b6 828.539
y7 911.5253
b7 941.6224
y8−NH3 1008.54
y8 1025.5685
b8 1056.6504
y10 1324.728
L T L S A L L D G K N V N A G G H K
y10 y8 y7 y6 y5 y4 y3 y2 y1
b2 b3 b4 b5 b6 b7 b8
Modification
Acetyl (Protein N−term)
GlyGly (K) no new terminus
Oxidation (M)

20200921_KIZ_MUb4_GlyGly_10plex − Scan number: 24068

Gene Names	Charge	m/z	Mass	Retention time	PEP	Score	Precursor Intensity
VDAC1	4	481.2668	1921.038	87.419	2.338e−17	136.81	17183142

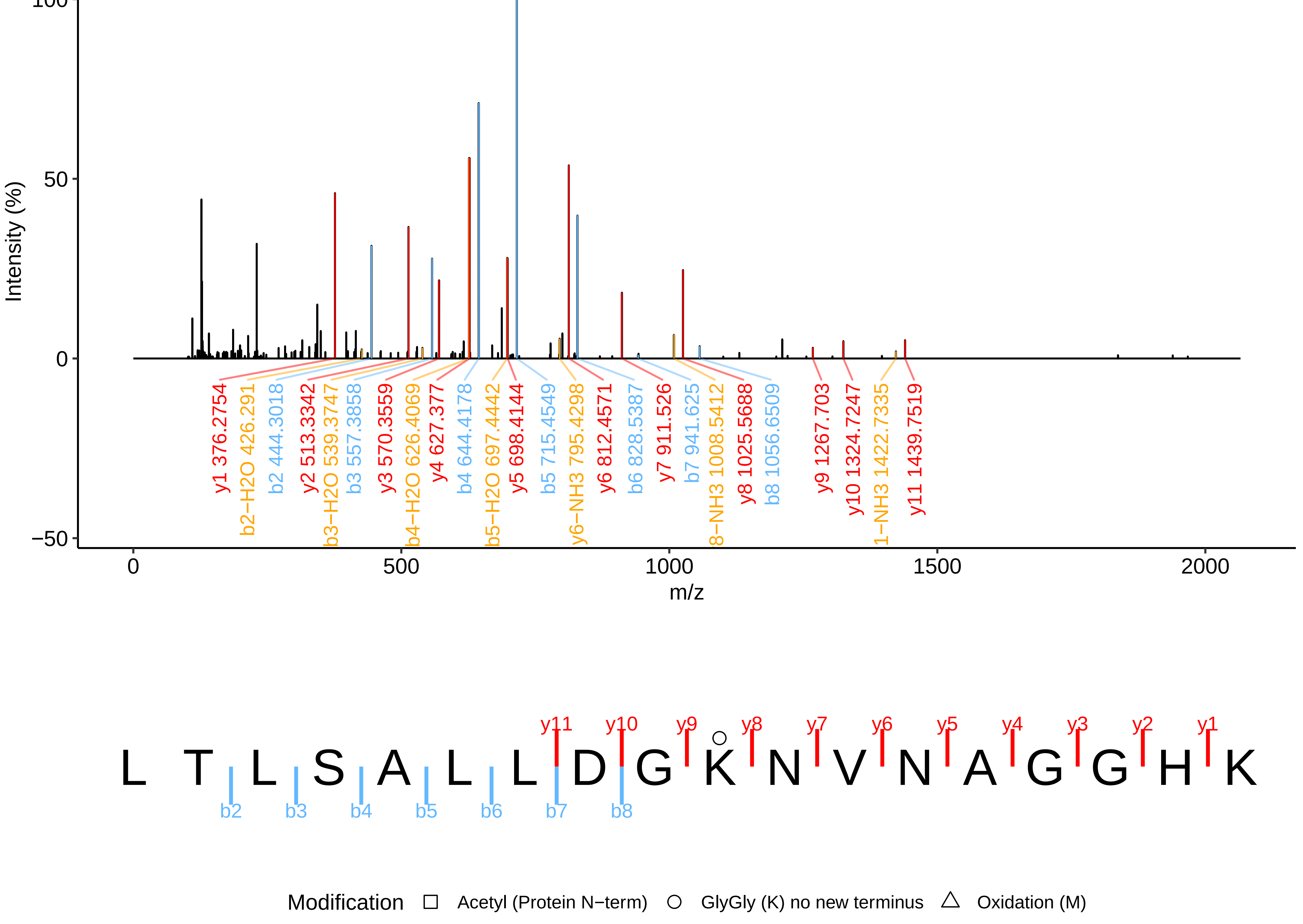

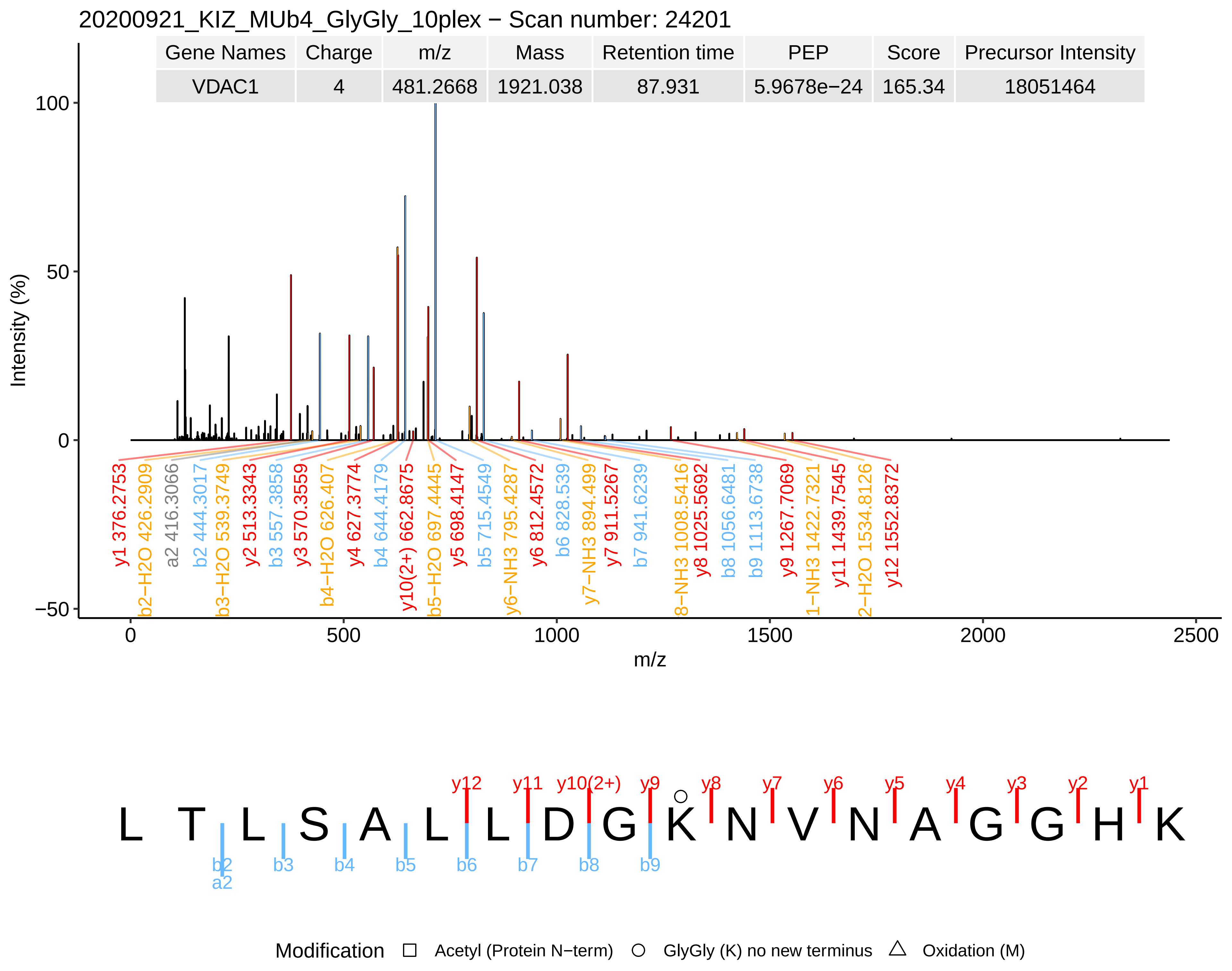
20200921_KIZ_MUb4_GlyGly_10plex − Scan number: 24201
Gene Names | Charge | m/z | Mass | Retention time | PEP | Score | Precursor Intensity
VDAC1 | 4 | 481.2668 | 1921.038 | 87.931 | 5.9678e−24 | 165.34 | 18051464
Intensity (%)
100
50
0
−50
m/z
0
500
1000
1500
2000
2500
y1 376.2753
b2−H2O 426.2909
a2 416.3066
b2 444.3017
b3−H2O 539.3749
y2 513.3343
b3 557.3858
y3 570.3559
b4−H2O 626.407
y4 627.3774
b4 644.4179
y10(2+) 662.8675
b5−H2O 697.4445
y5 698.4147
b5 715.4549
y6−NH3 795.4287
y6 812.4572
b6 828.539
y7−NH3 894.499
y7 911.5267
b7 941.6239
8−NH3 1008.5416
y8 1025.5692
b8 1056.6481
b9 1113.6738
y9 1267.7069
1−NH3 1422.7321
y11 1439.7545
2−H2O 1534.8126
y12 1552.8372
y12 y11 y10(2+) y9 y8 y7 y6 y5 y4 y3 y2 y1
L T L S A L L D G K N V N A G G H K
b2 b3 b4 b5 b6 b7 b8 b9
a2
Modification
Acetyl (Protein N−term)
GlyGly (K) no new terminus
Oxidation (M)

20200921_KIZ_MUb4_GlyGly_10plex − Scan number: 24333

Gene Names	Charge	m/z	Mass	Retention time	PEP	Score	Precursor Intensity
VDAC1	4	481.2668	1921.038	88.445	1.9289e−24	166.9	26516788

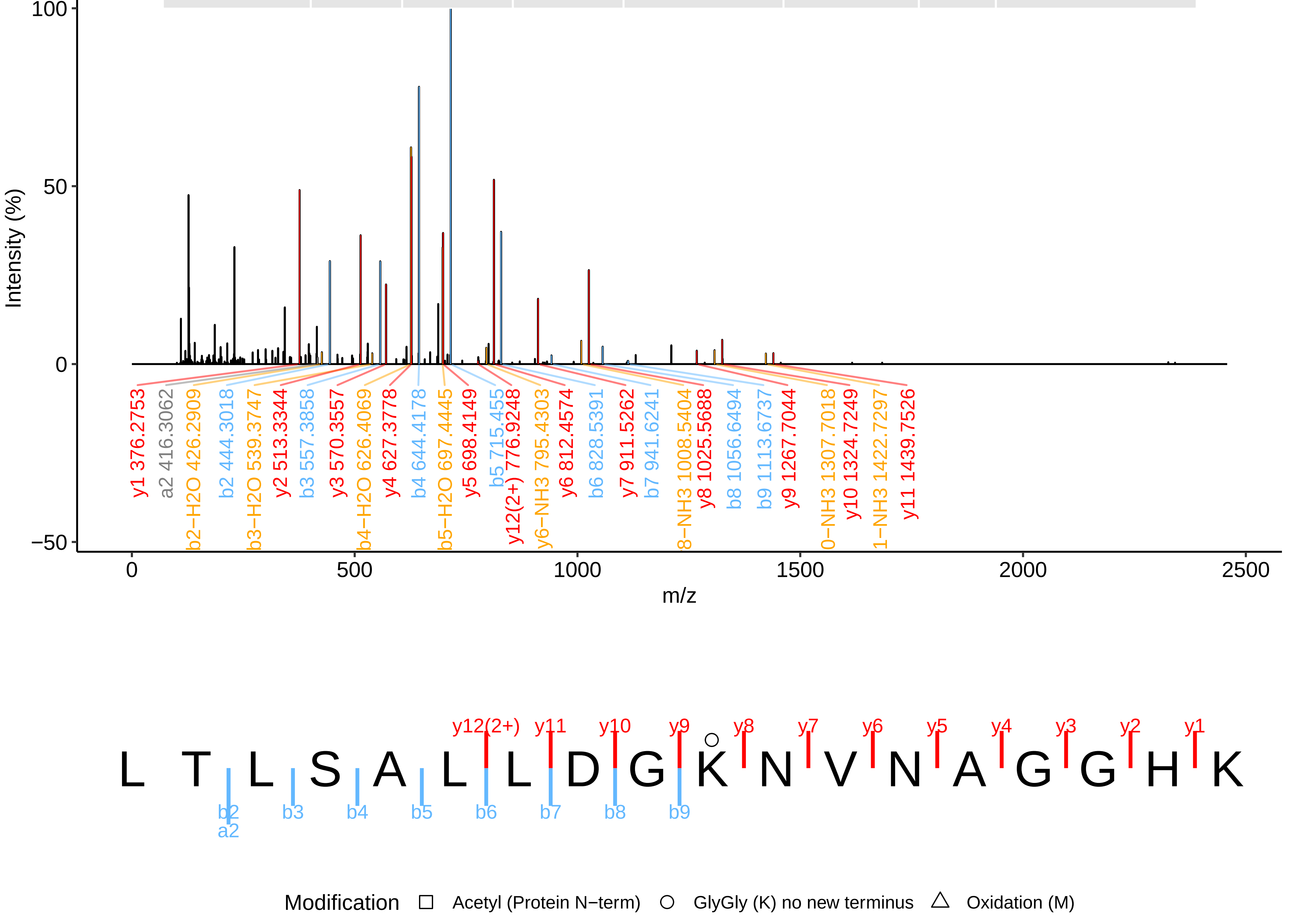

20200921_KIZ_MUb4_GlyGly_10plex_2 − Scan number: 19955

Gene Names	Charge	m/z	Mass	Retention time	PEP	Score	Precursor Intensity
VDAC1	4	481.2668	1921.038	78.386	8.8411e−10	103.79	516143.4

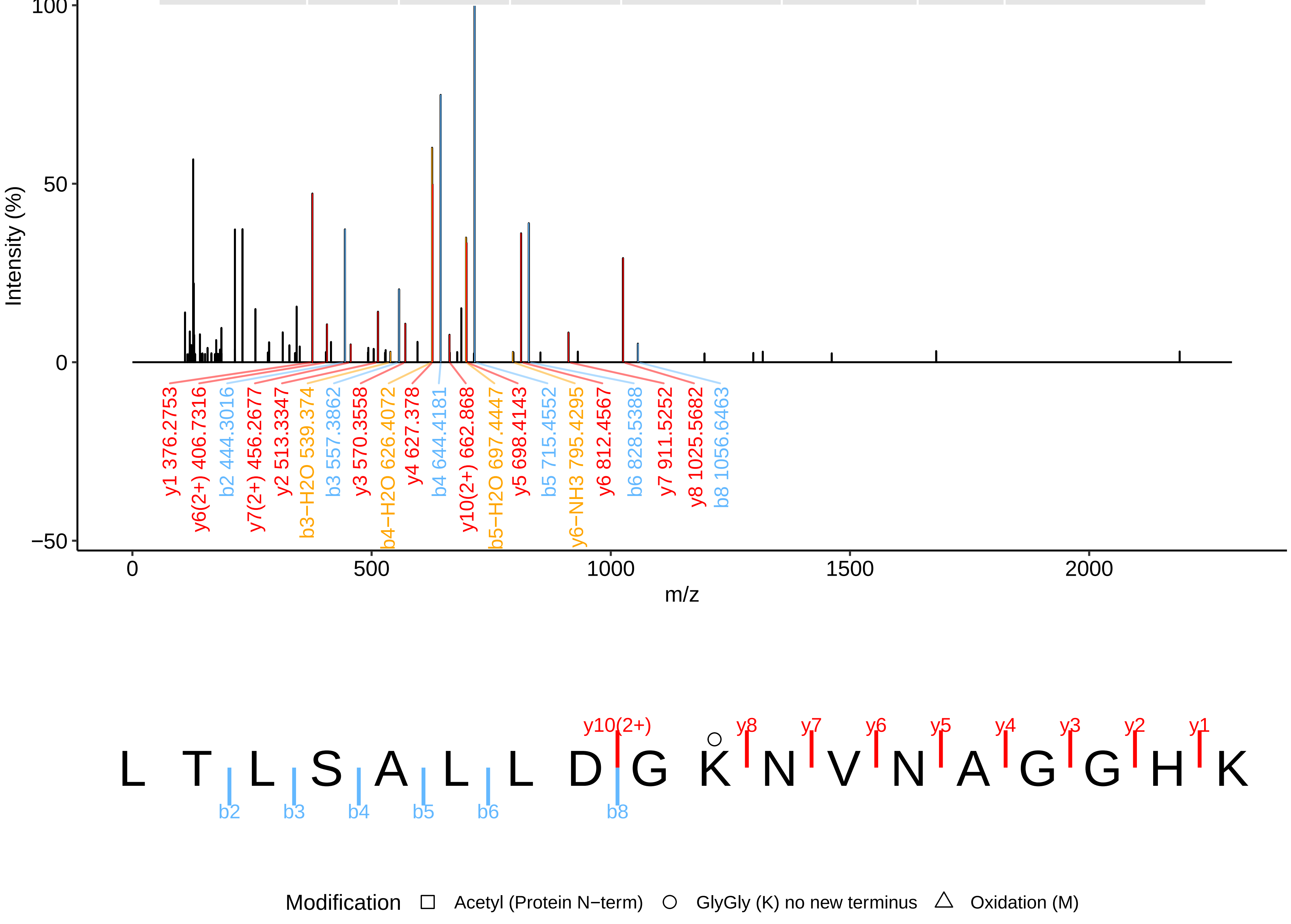

20200921_KIZ_MUb4_GlyGly_10plex_2 − Scan number: 20090

Gene Names	Charge	m/z	Mass	Retention time	PEP	Score	Precursor Intensity
VDAC1	4	481.2668	1921.038	78.938	0.0029776	62.861	1176677

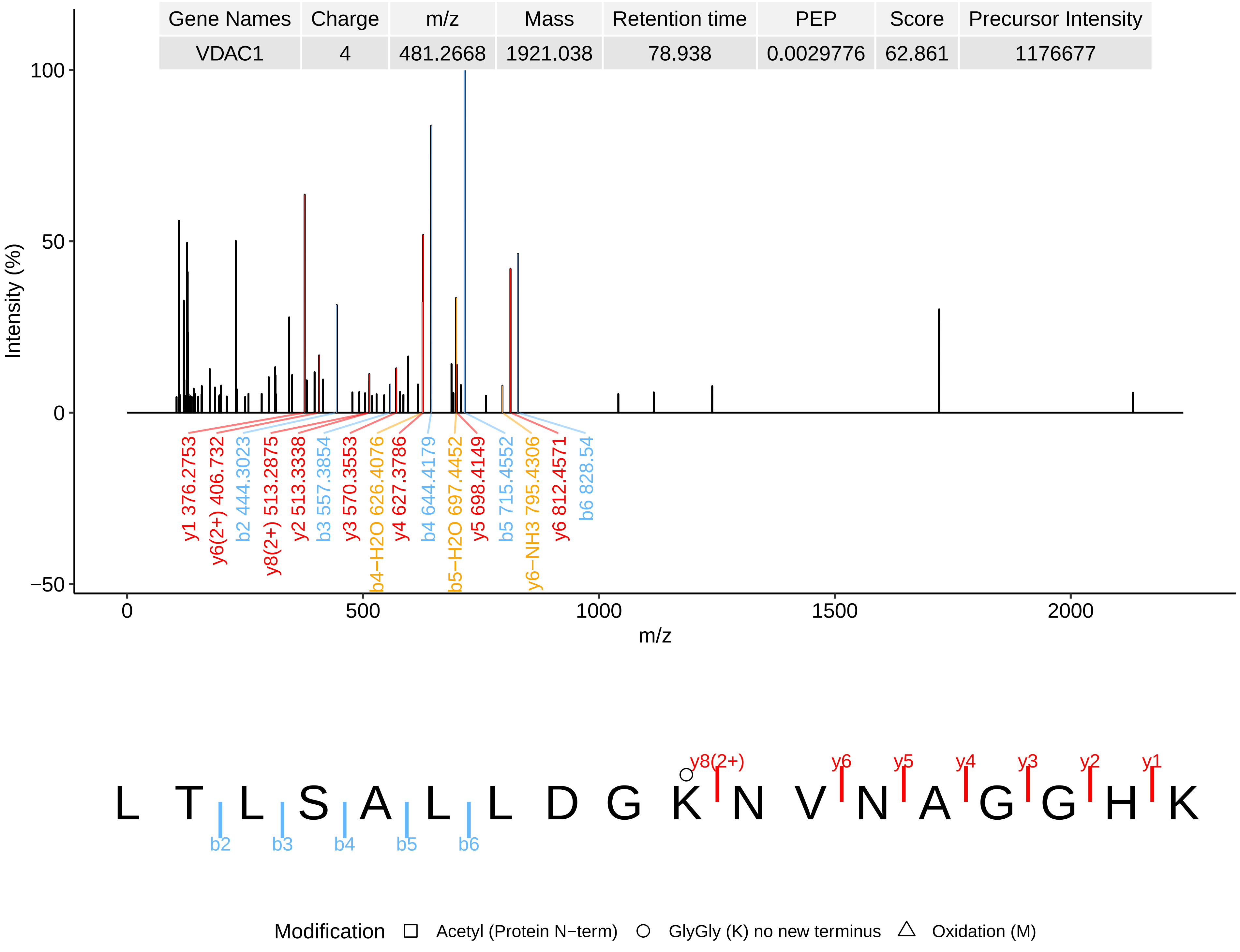

20200924_KIZ_MUb5_GlyGly_2 − Scan number: 19656

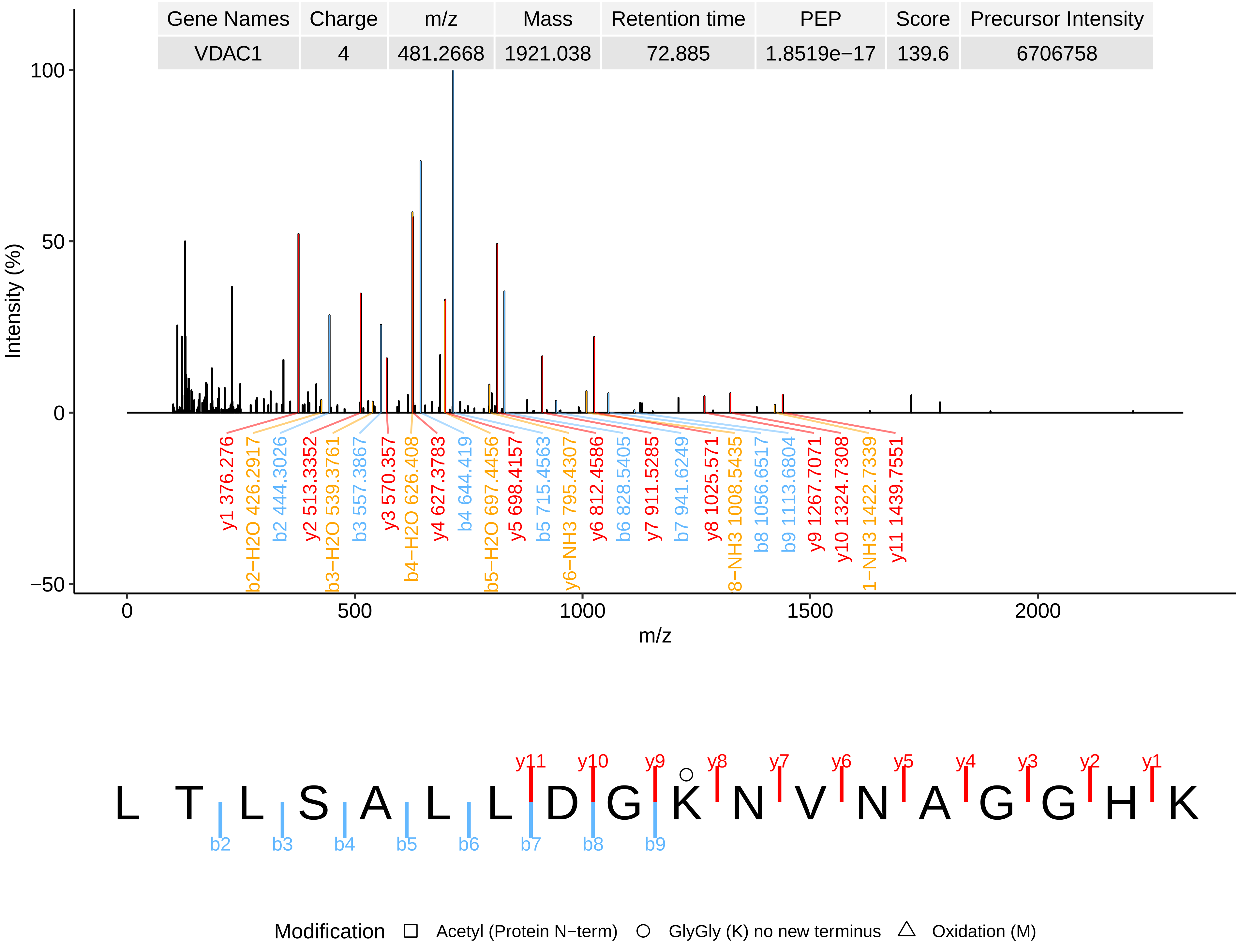

20201118_KIZ_MUb6_GlyGly_1 − Scan number: 21974

Gene Names	Charge	m/z	Mass	Retention time	PEP	Score	Precursor Intensity
VDAC1	4	481.2668	1921.038	68.403	7.8983e−14	129.38	57896800

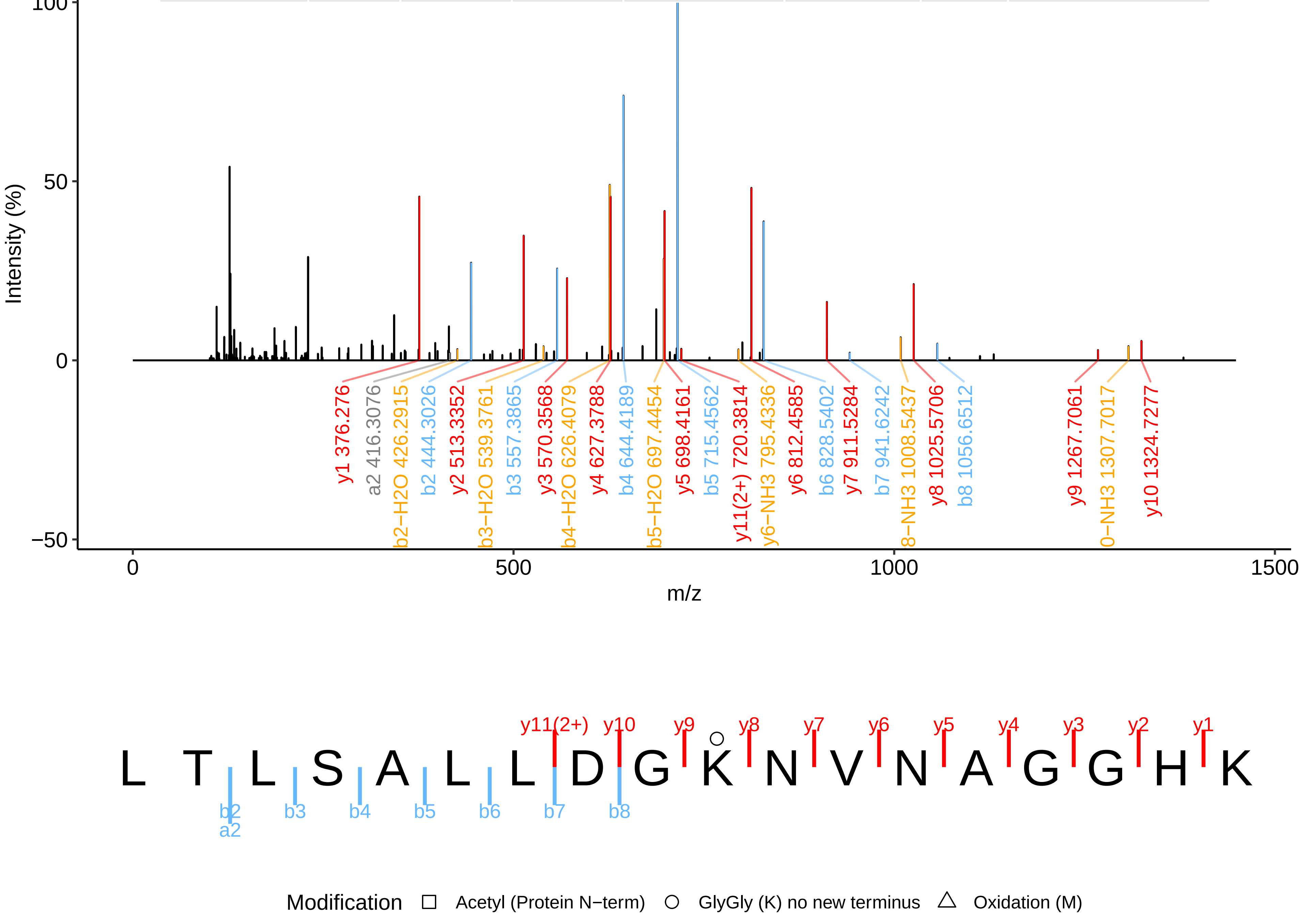

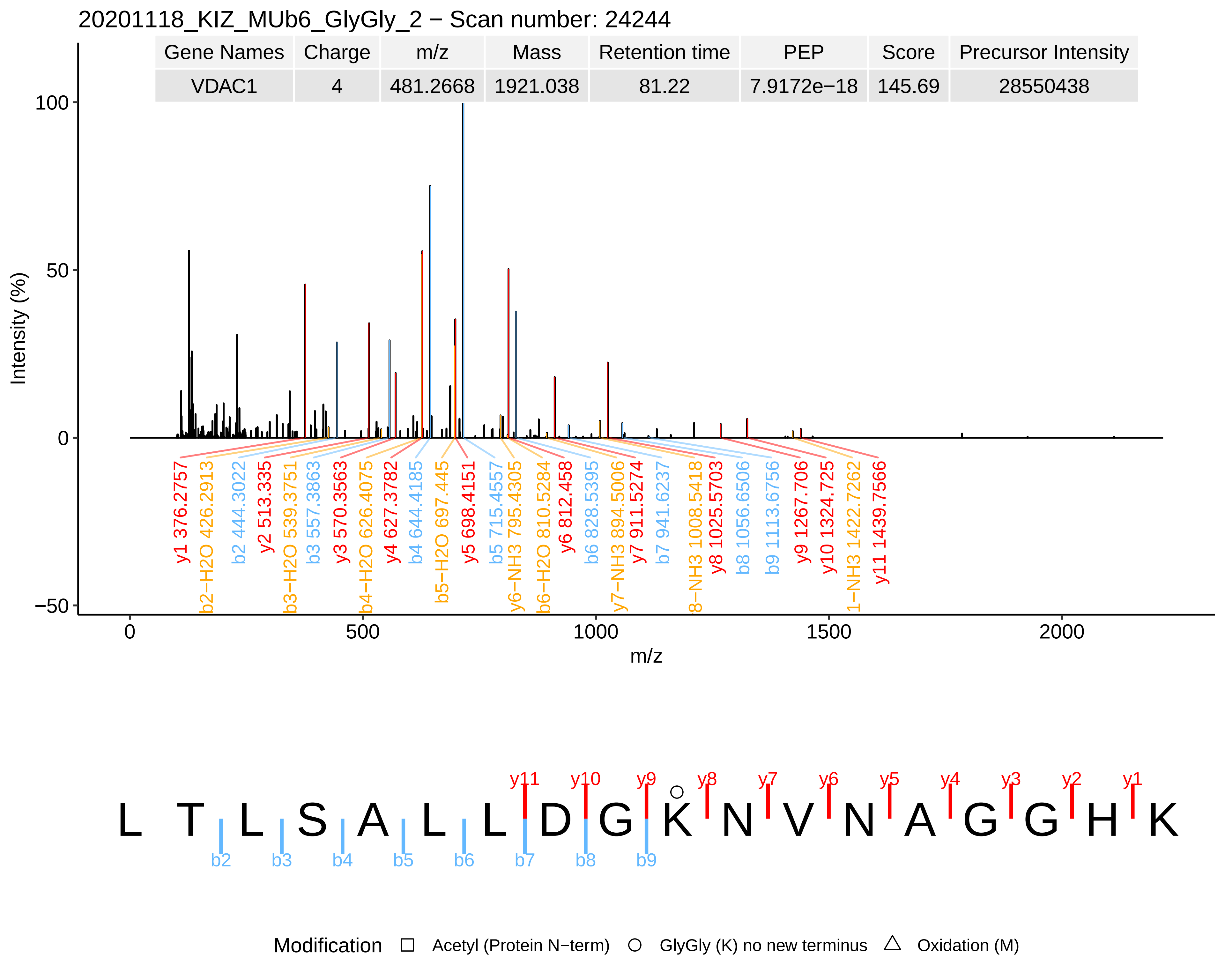
20201118_KIZ_MUb6_GlyGly_2 − Scan number: 24244
Gene Names | Charge | m/z | Mass | Retention time | PEP | Score | Precursor Intensity
VDAC1 | 4 | 481.2668 | 1921.038 | 81.22 | 7.9172e−18 | 145.69 | 28550438
Intensity (%)
100
50
0
−50
y1 376.2757
b2−H2O 426.2913
b2 444.3022
y2 513.335
b3−H2O 539.3751
b3 557.3863
y3 570.3563
b4−H2O 626.4075
y4 627.3782
b4 644.4185
b5−H2O 697.445
y5 698.4151
b5 715.4557
y6−NH3 795.4305
b6−H2O 810.5284
y6 812.458
b6 828.5395
y7−NH3 894.5006
y7 911.5274
b7 941.6237
8−NH3 1008.5418
y8 1025.5703
b8 1056.6506
b9 1113.6756
y9 1267.706
y10 1324.725
1−NH3 1422.7262
y11 1439.7566
0
500
1000
1500
2000
m/z
y11 y10 y9 y8 y7 y6 y5 y4 y3 y2 y1
L T L S A L L D G K N V N A G G H K
b2 b3 b4 b5 b6 b7 b8 b9
Modification
Acetyl (Protein N−term)
GlyGly (K) no new terminus
Oxidation (M)

20200921_KIZ_MUb4_GlyGly_10plex − Scan number: 22645

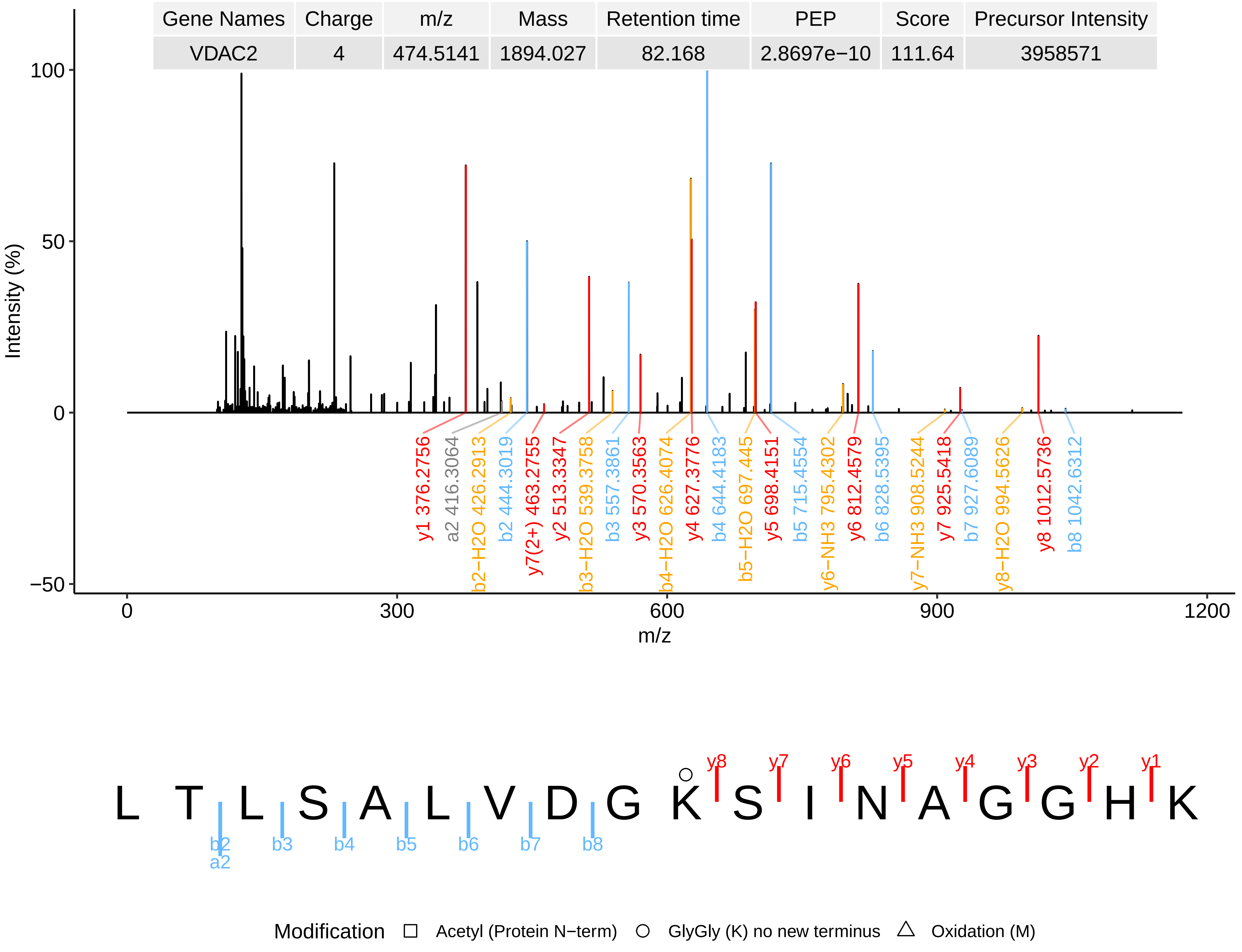

20200921_KIZ_MUb4_GlyGly_10plex − Scan number: 22766

Gene Names	Charge	m/z	Mass	Retention time	PEP	Score	Precursor Intensity
VDAC2	4	474.5141	1894.027	82.621	1.7794e−18	149.22	18332120

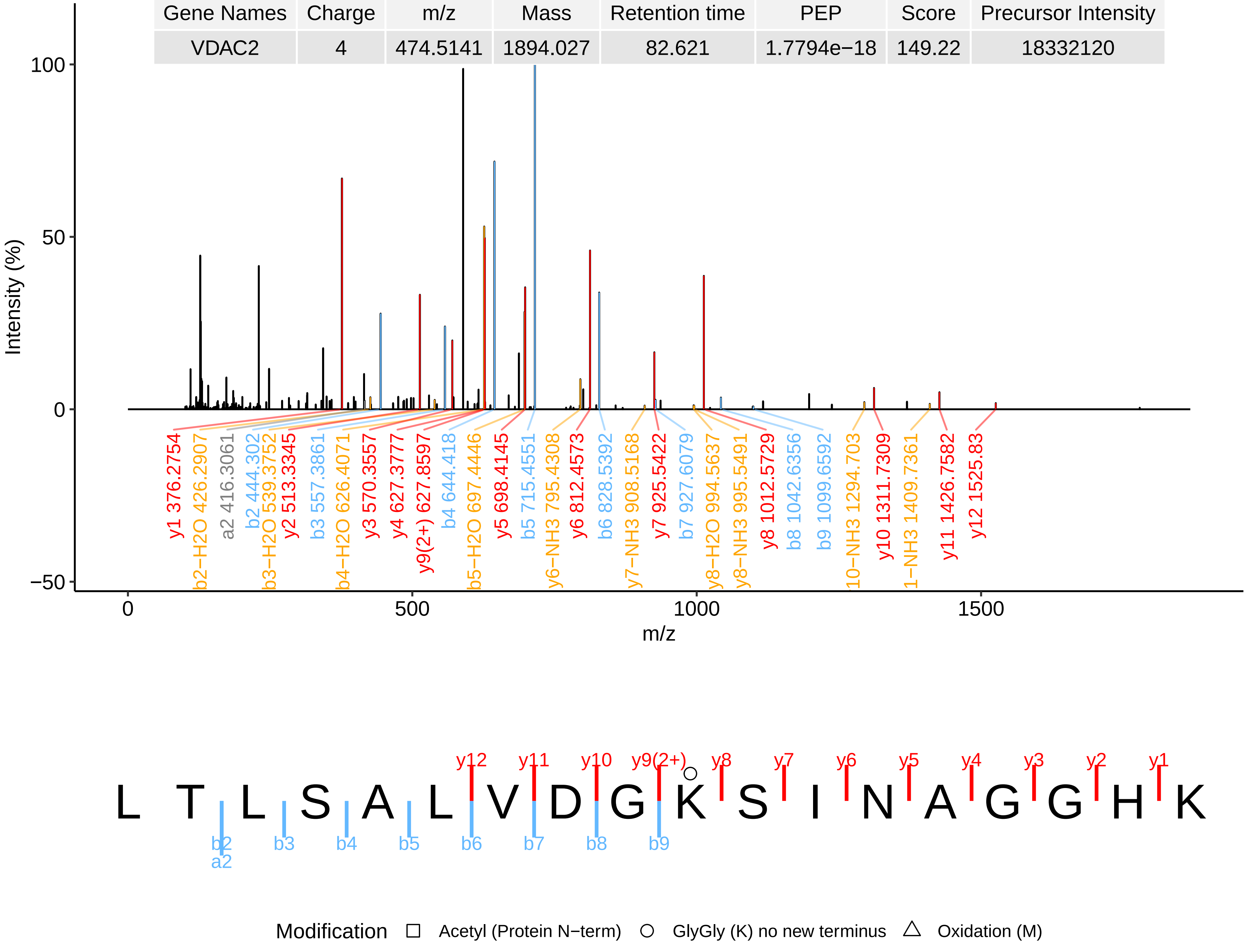

20200921_KIZ_MUb4_GlyGly_10plex − Scan number: 22851

Gene Names	Charge	m/z	Mass	Retention time	PEP	Score	Precursor Intensity
VDAC2	4	474.5141	1894.027	82.932	0.0014493	68.481	8551209

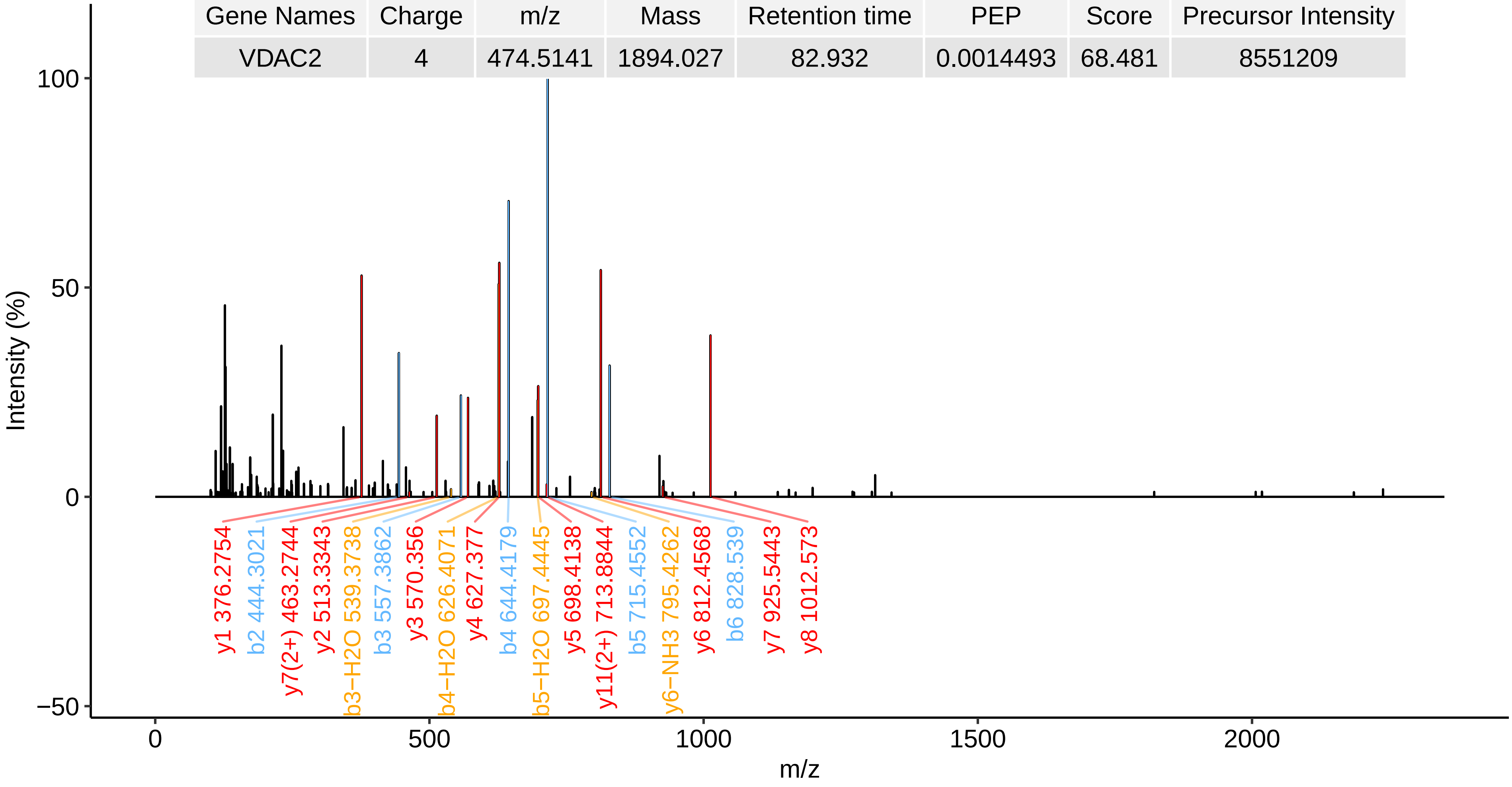

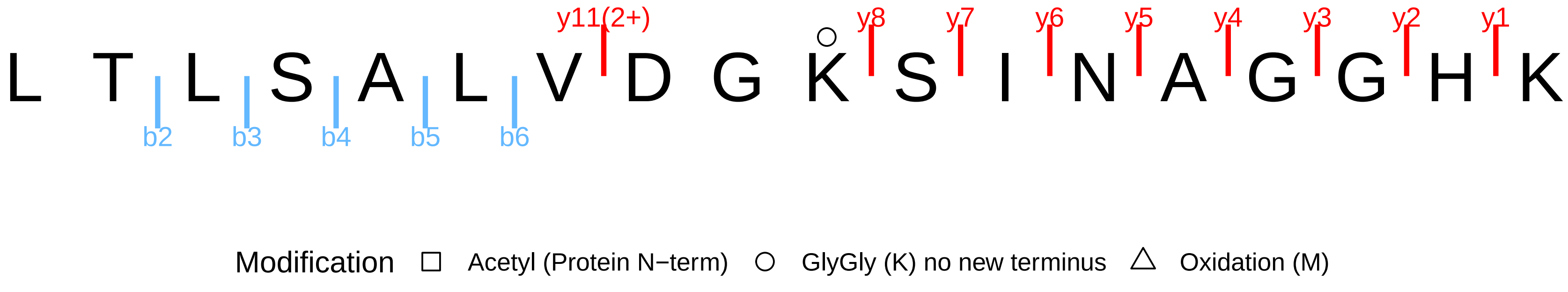

20200921_KIZ_MUb4_GlyGly_10plex_2 − Scan number: 18932

Gene Names	Charge	m/z	Mass	Retention time	PEP	Score	Precursor Intensity
VDAC2	4	474.5141	1894.027	74.233	5.158e−10	108.63	2907522

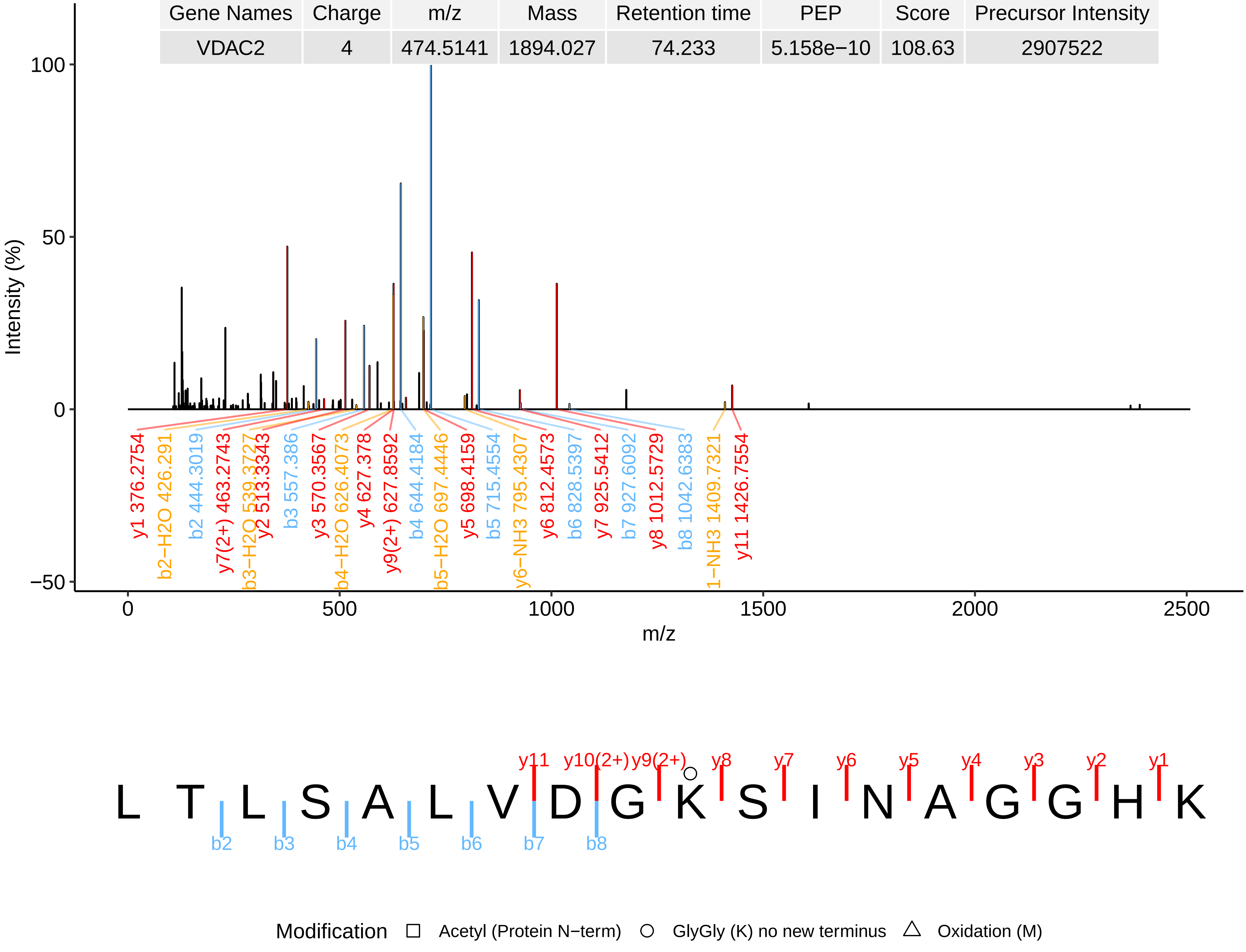

20201118_KIZ_MUb6_GlyGly_1 − Scan number: 20721

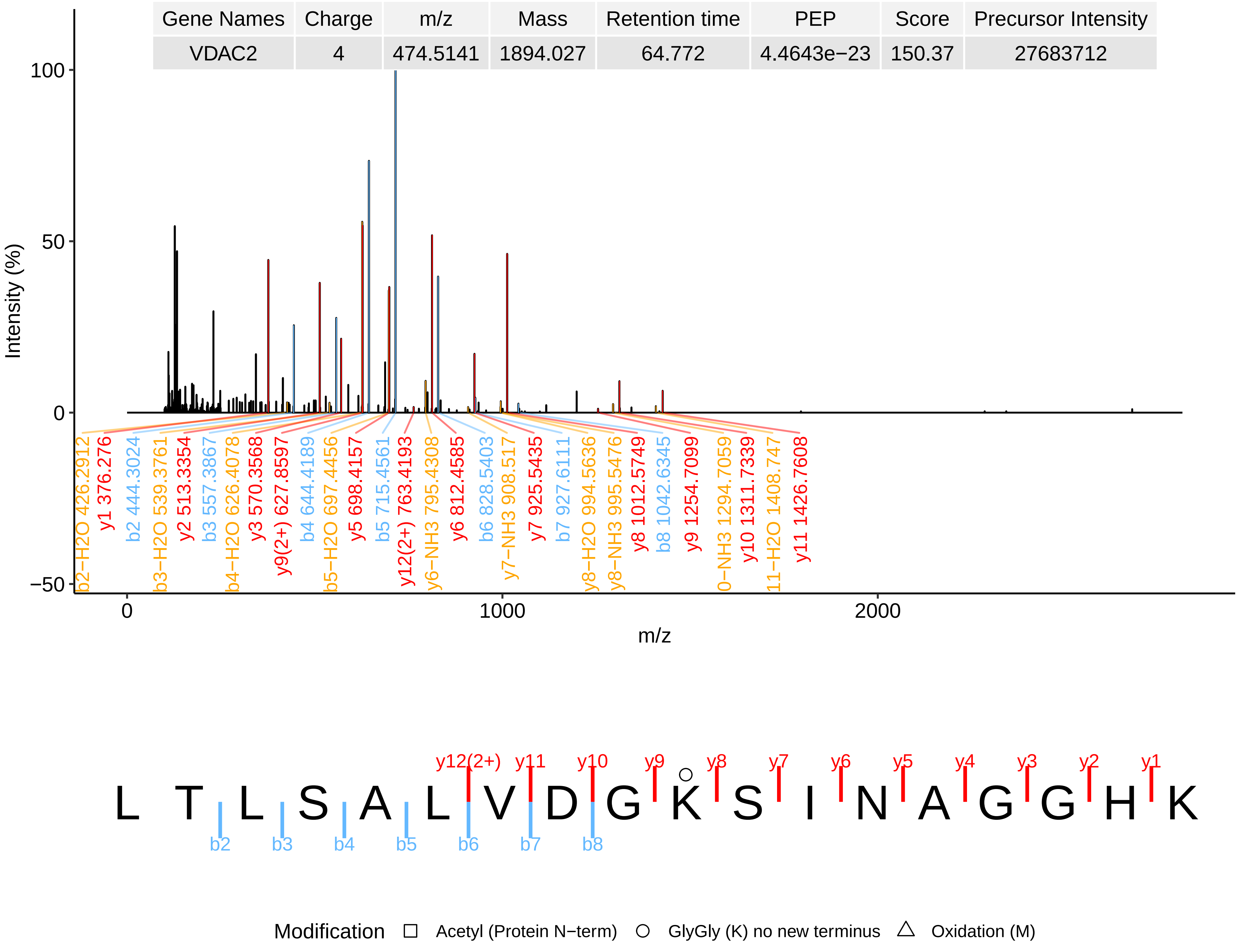

20200921_KIZ_MUb4_GlyGly_10plex − Scan number: 24340

Gene Names	Charge	m/z	Mass	Retention time	PEP	Score	Precursor Intensity
BIRC2	3	718.332	2151.974	88.473	0.021207	42.947	2568043

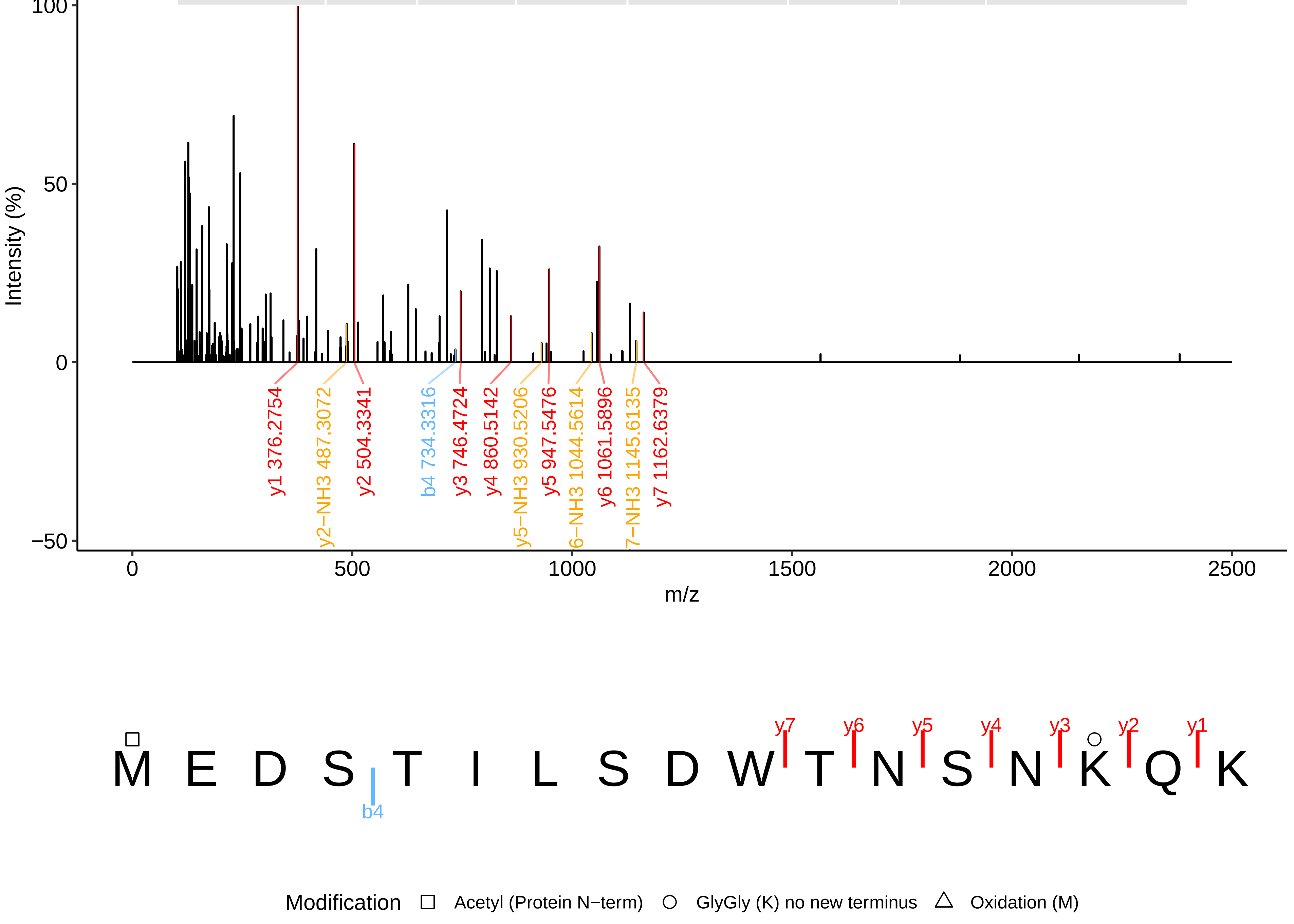

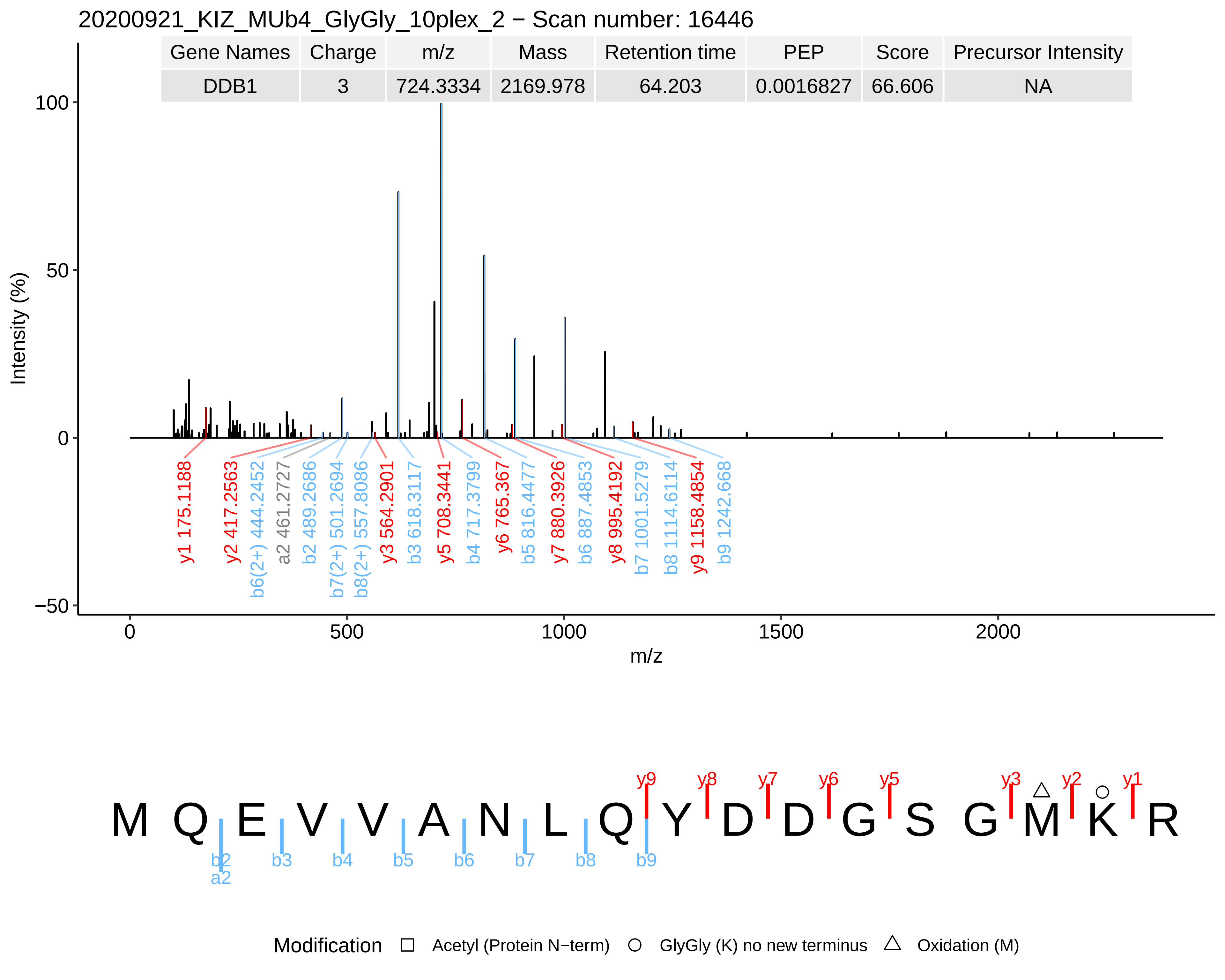

20200921_KIZ_MUb4_GlyGly_10plex_2 − Scan number: 16446
Gene Names | Charge | m/z | Mass | Retention time | PEP | Score | Precursor Intensity
DDB1 | 3 | 724.3334 | 2169.978 | 64.203 | 0.0016827 | 66.606 | NA
Intensity (%)
m/z
y1 175.1188
y2 417.2563
b6(2+) 444.2452
a2 461.2727
b2 489.2686
b7(2+) 501.2694
b8(2+) 557.8086
y3 564.2901
b3 618.3117
y5 708.3441
b4 717.3799
y6 765.367
b5 816.4477
y7 880.3926
b6 887.4853
y8 995.4192
b7 1001.5279
b8 1114.6114
y9 1158.4854
b9 1242.668
M Q E V V A N L Q Y D D G S G M K R
Modification
Acetyl (Protein N−term)
GlyGly (K) no new terminus
Oxidation (M)

20200921_KIZ_MUb4_GlyGly_10plex_2 − Scan number: 14593

Gene Names	Charge	m/z	Mass	Retention time	PEP	Score	Precursor Intensity
MFN1	4	419.4856	1673.913	56.913	3.8759e−06	111.91	894530.3

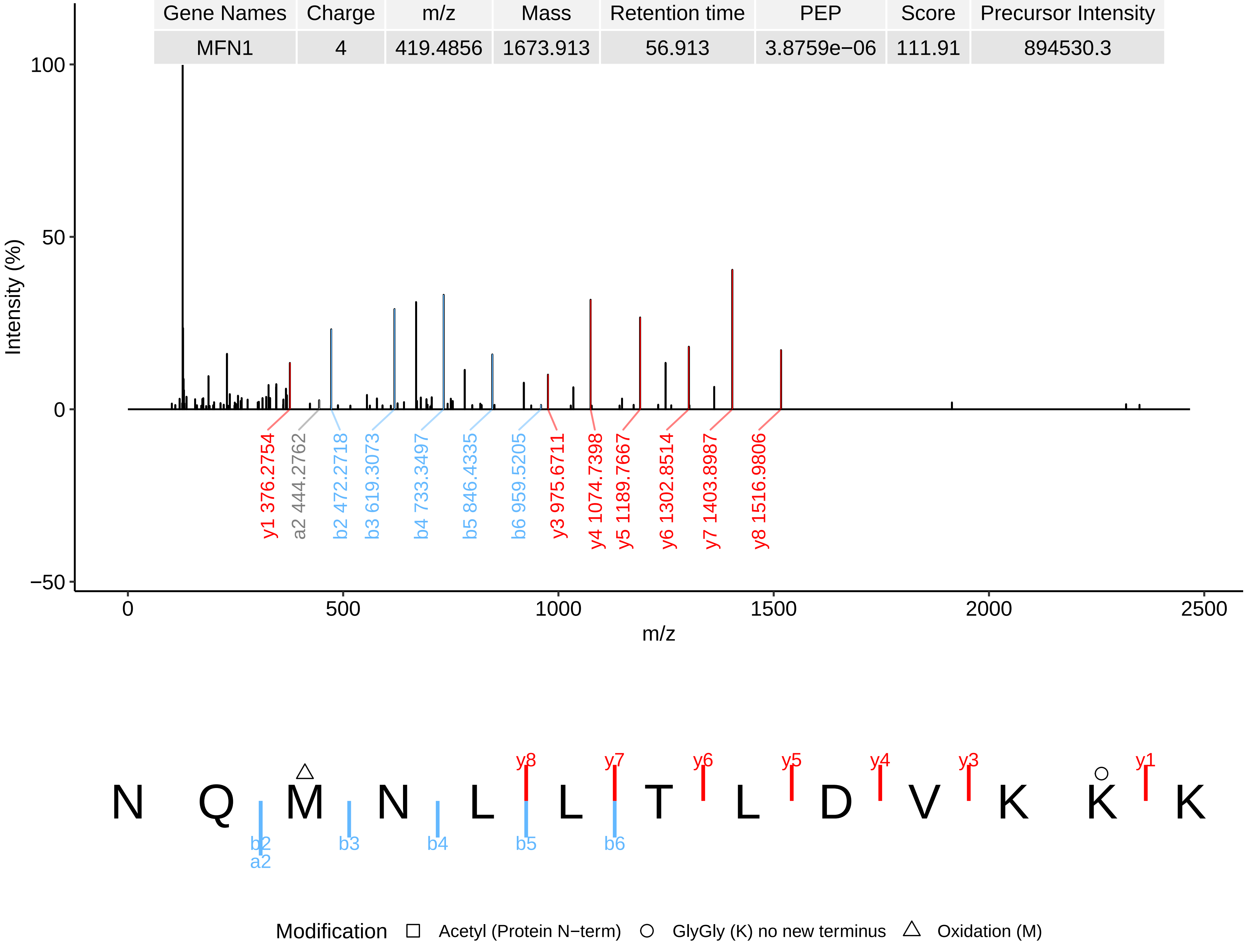

20200921_KIZ_MUb4_GlyGly_10plex_2 − Scan number: 19546

Gene Names	Charge	m/z	Mass	Retention time	PEP	Score	Precursor Intensity
PHB	3	732.4096	2194.207	76.73	0.0097325	60.564	697454.2

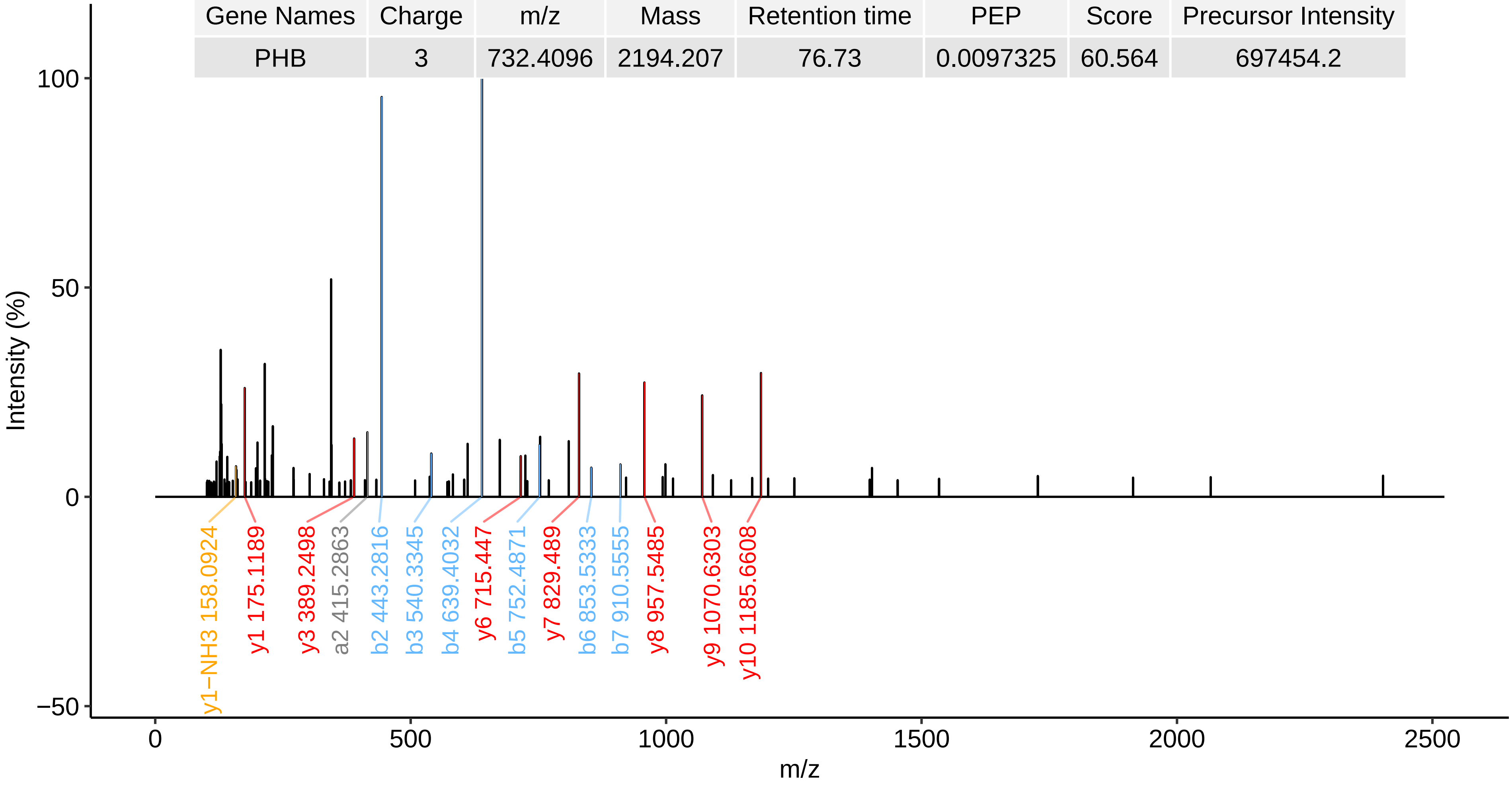

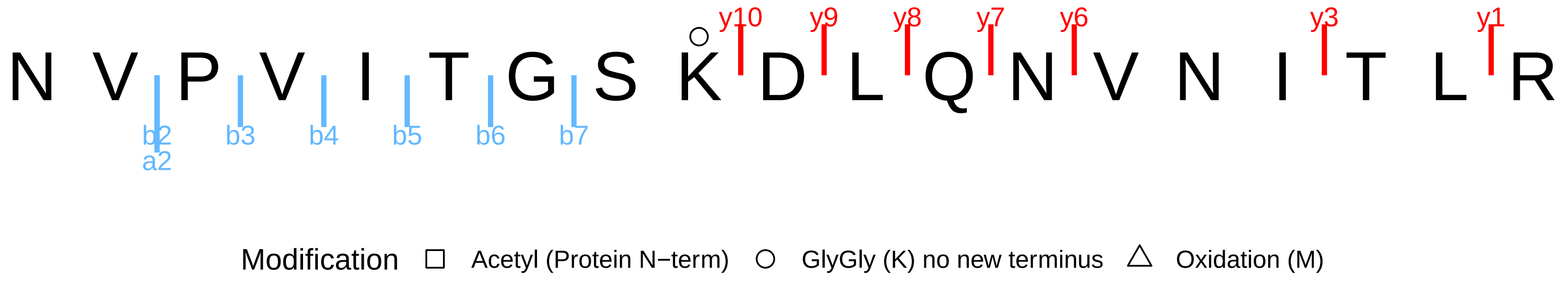

20200921_KIZ_MUb4_GlyGly_10plex − Scan number: 26506

Gene Names	Charge	m/z	Mass	Retention time	PEP	Score	Precursor Intensity
COX7A2	3	665.0141	1992.02	96.586	0.00063434	79.646	NA

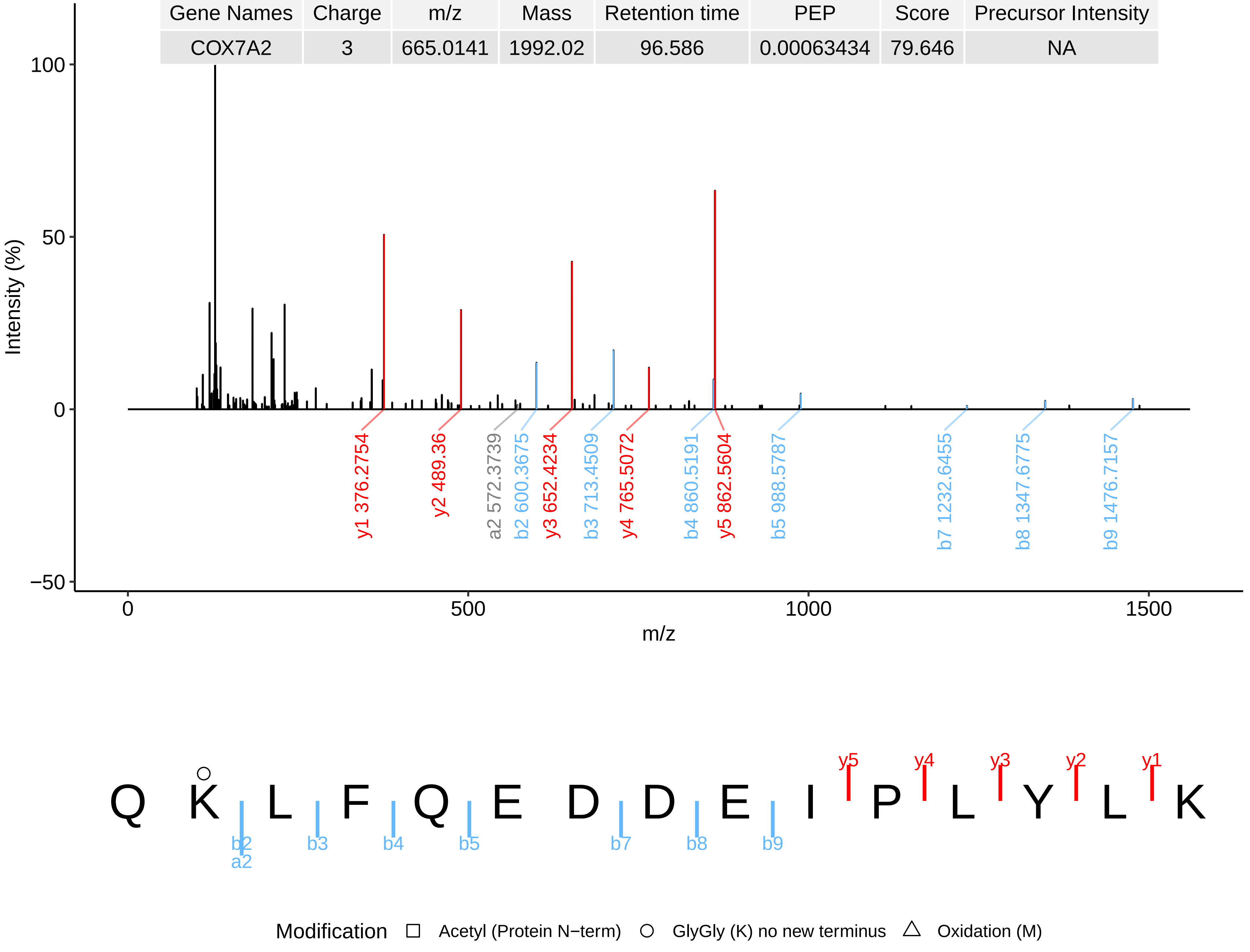

20200921_KIZ_MUb4_GlyGly_10plex − Scan number: 14036

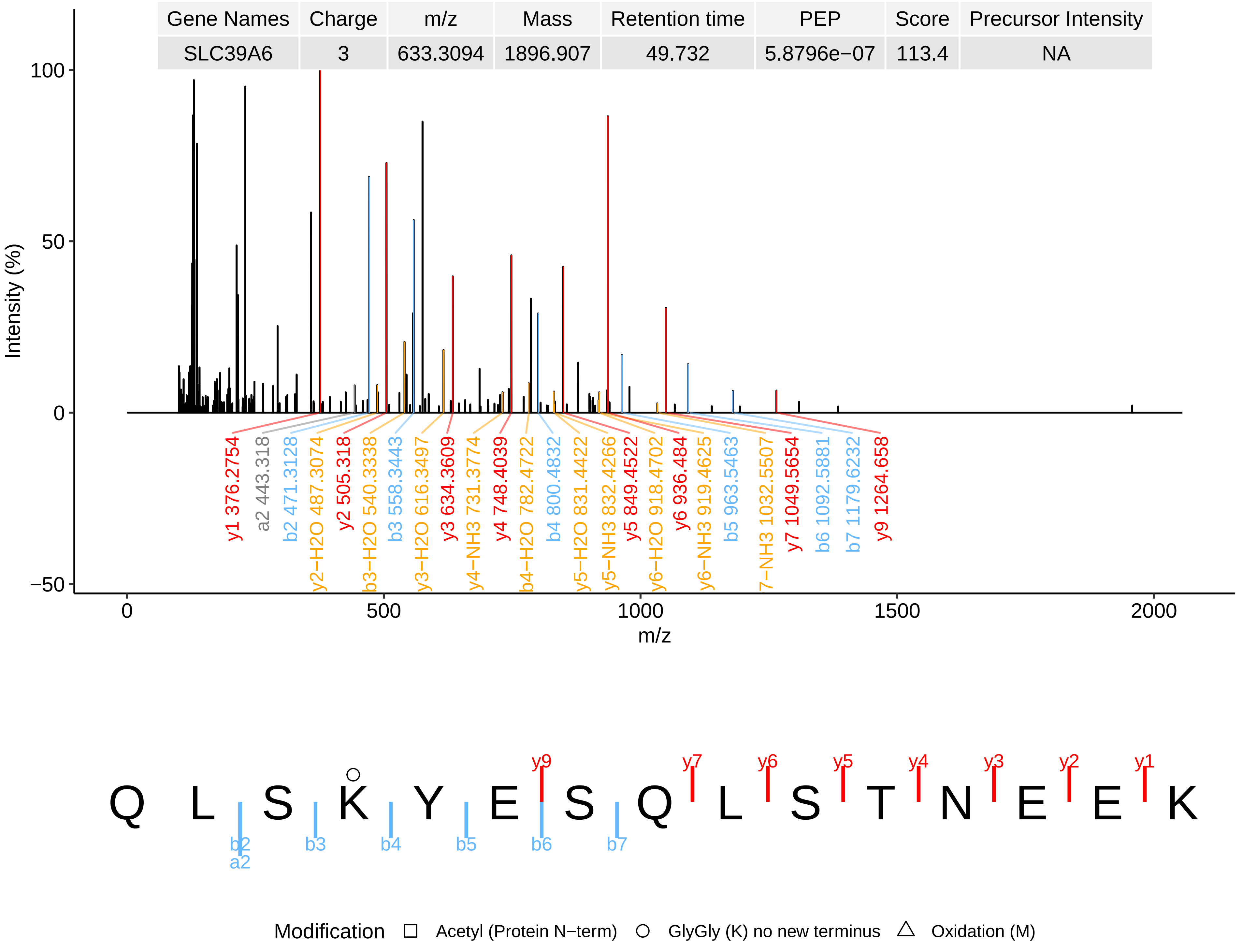

20200921_KIZ_MUb4_GlyGly_10plex − Scan number: 17341

Gene Names	Charge	m/z	Mass	Retention time	PEP	Score	Precursor Intensity
EPHA2	3	633.6425	1897.906	62.369	0.00013085	91.969	NA

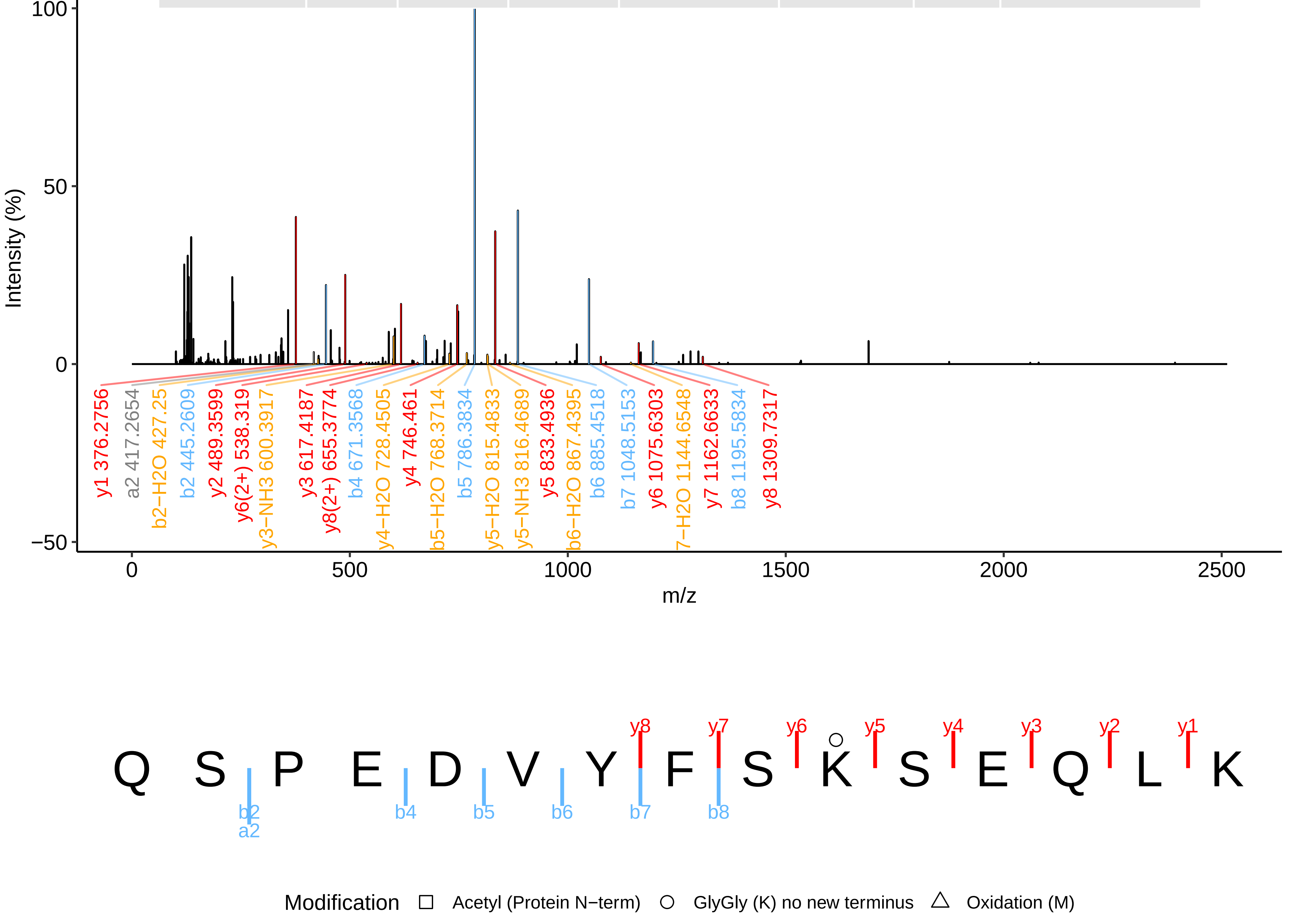

20200921_KIZ_MUb4_GlyGly_10plex_2 − Scan number: 15804

Gene Names	Charge	m/z	Mass	Retention time	PEP	Score	Precursor Intensity
EPHA2	3	633.6425	1897.906	61.882	0.0022404	71.692	1457905

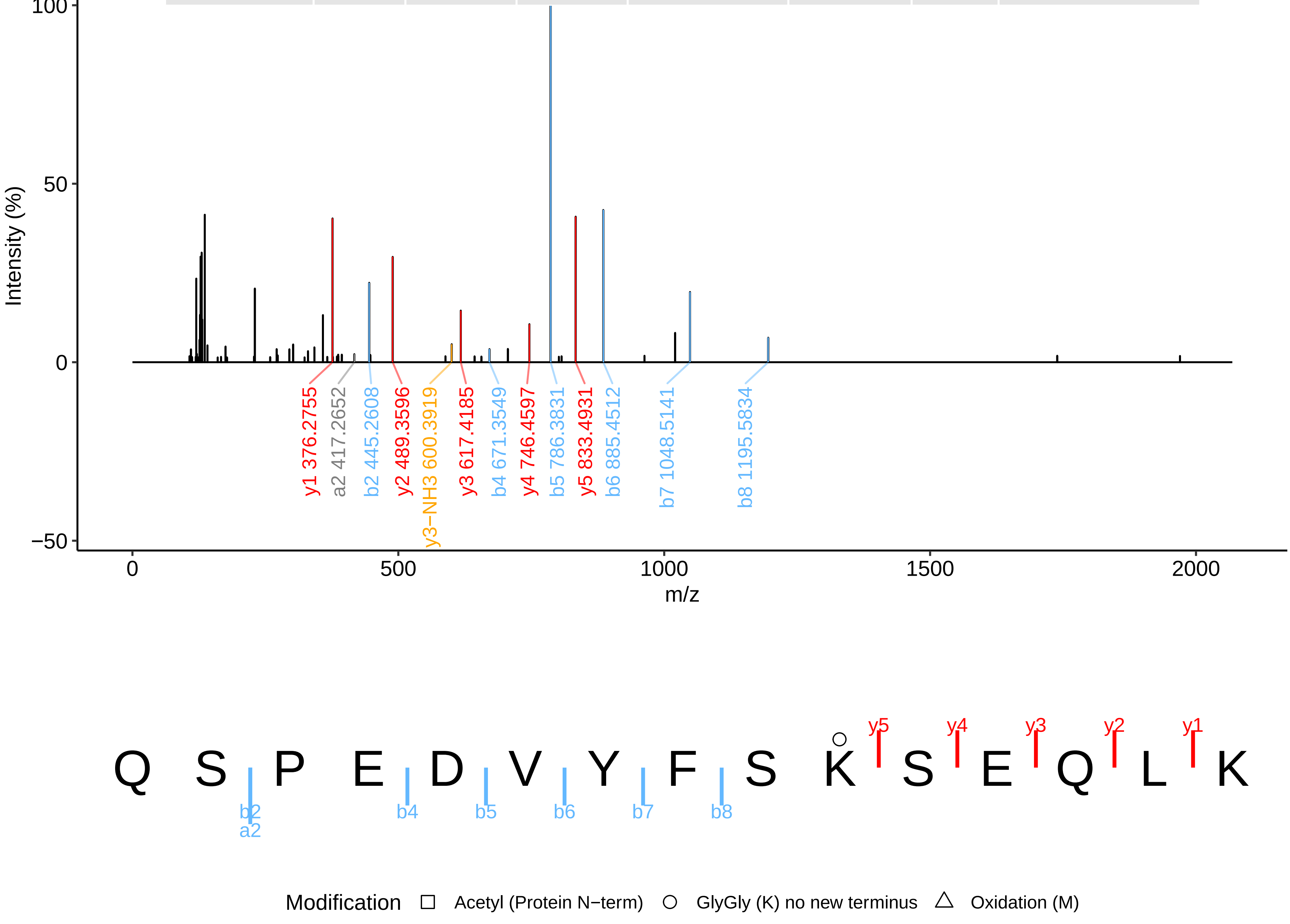

20200921_KIZ_MUb4_GlyGly_10plex_2 − Scan number: 15865

Gene Names	Charge	m/z	Mass	Retention time	PEP	Score	Precursor Intensity
EPHA2	3	633.6425	1897.906	62.127	0.0048747	63.037	1506705

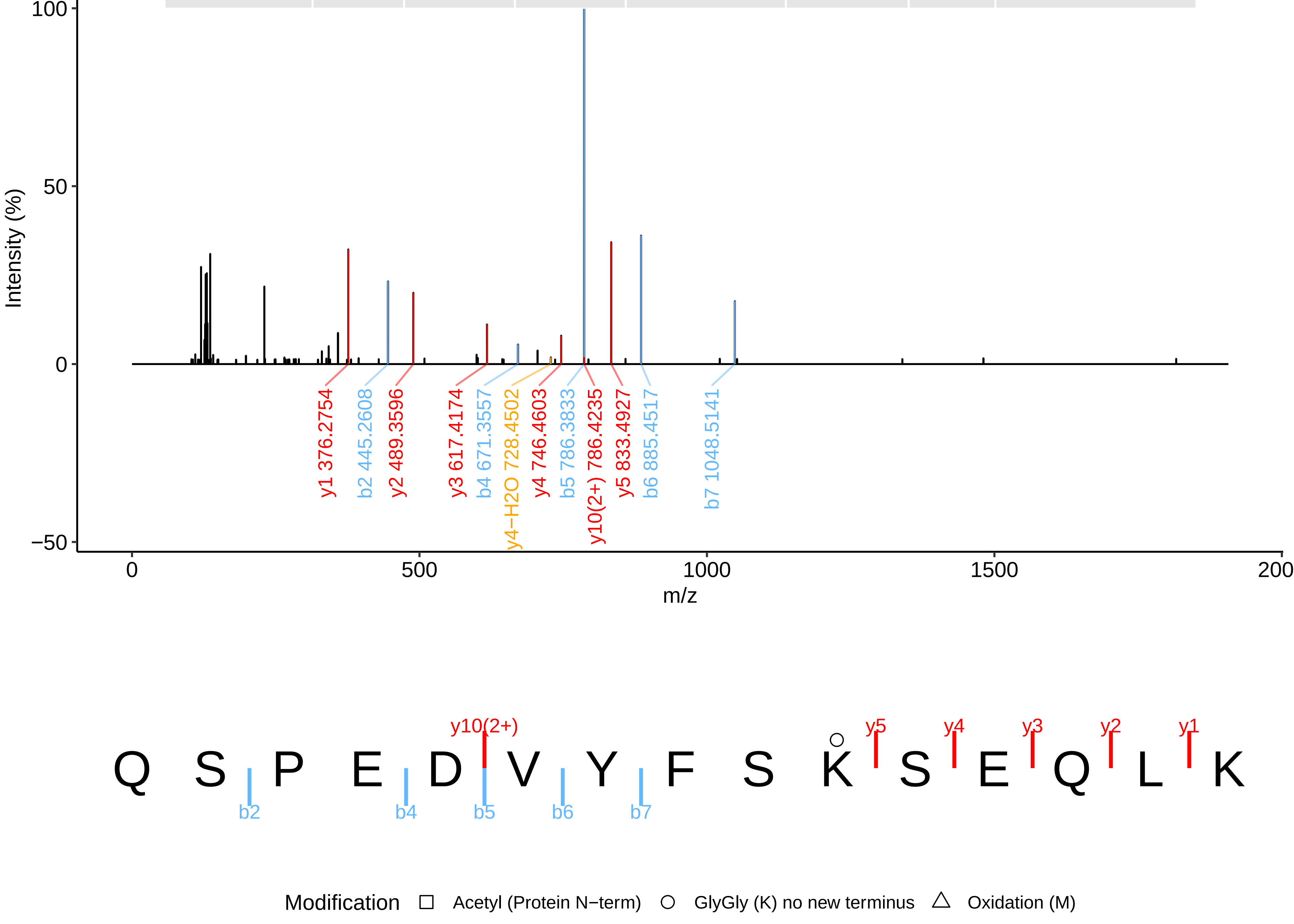

20200924_KIZ_MUb5_GlyGly_1 − Scan number: 5417

Gene Names	Charge	m/z	Mass	Retention time	PEP	Score	Precursor Intensity
MLLT4	3	639.984	1916.93	16.845	0.030322	58.487	NA

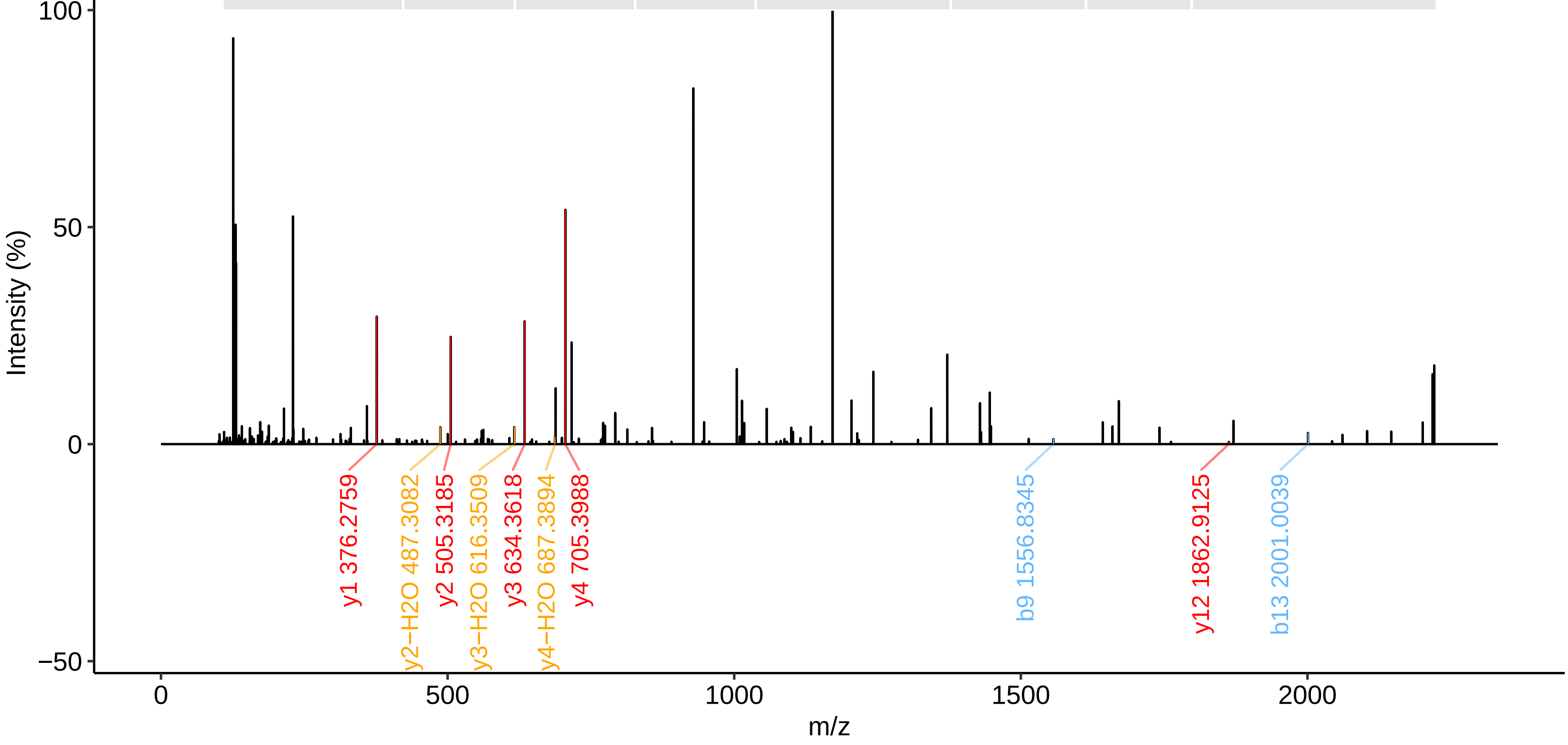

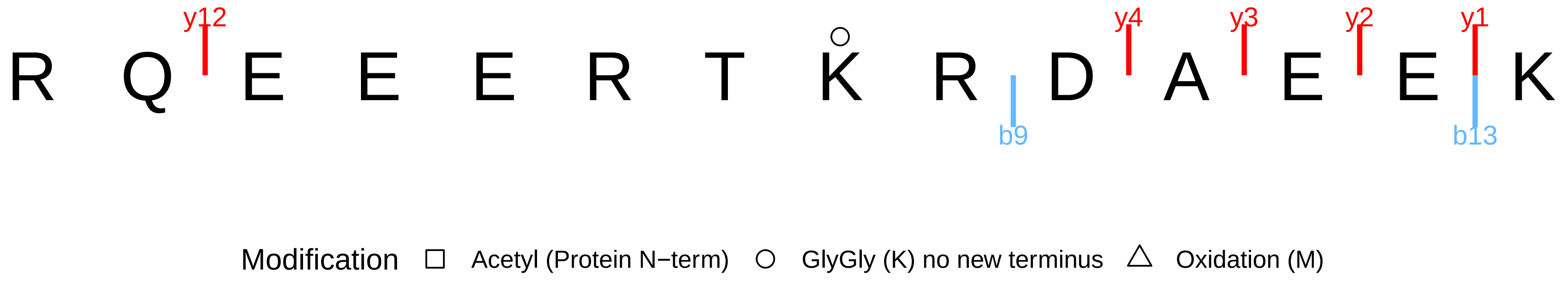

20200921_KIZ_MUb4_GlyGly_10plex_2 − Scan number: 18729

Gene Names	Charge	m/z	Mass	Retention time	PEP	Score	Precursor Intensity
NEDD8;NEDD8−MDP1	3	639.3335	1914.979	73.407	0.0029805	63.682	1279507

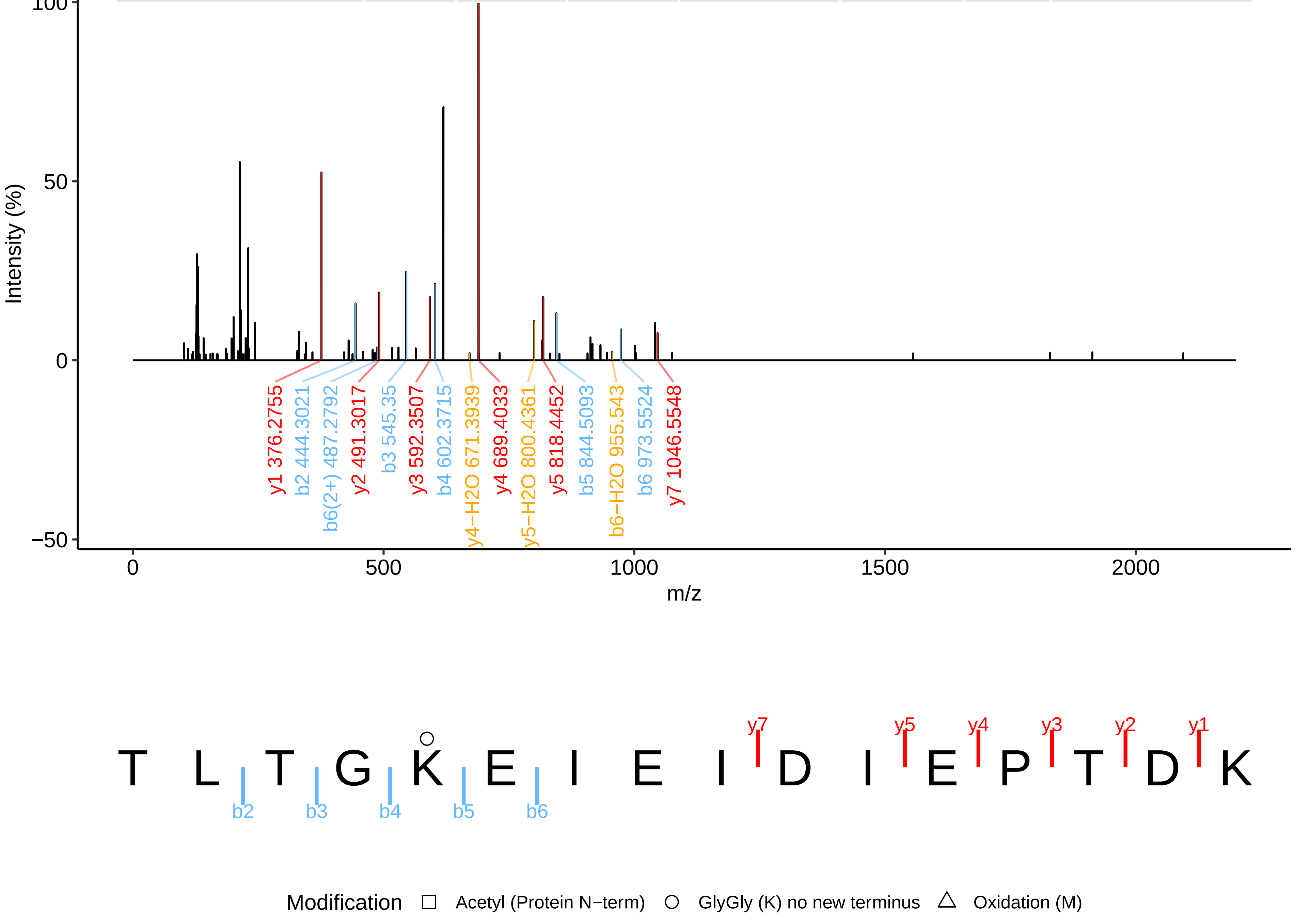

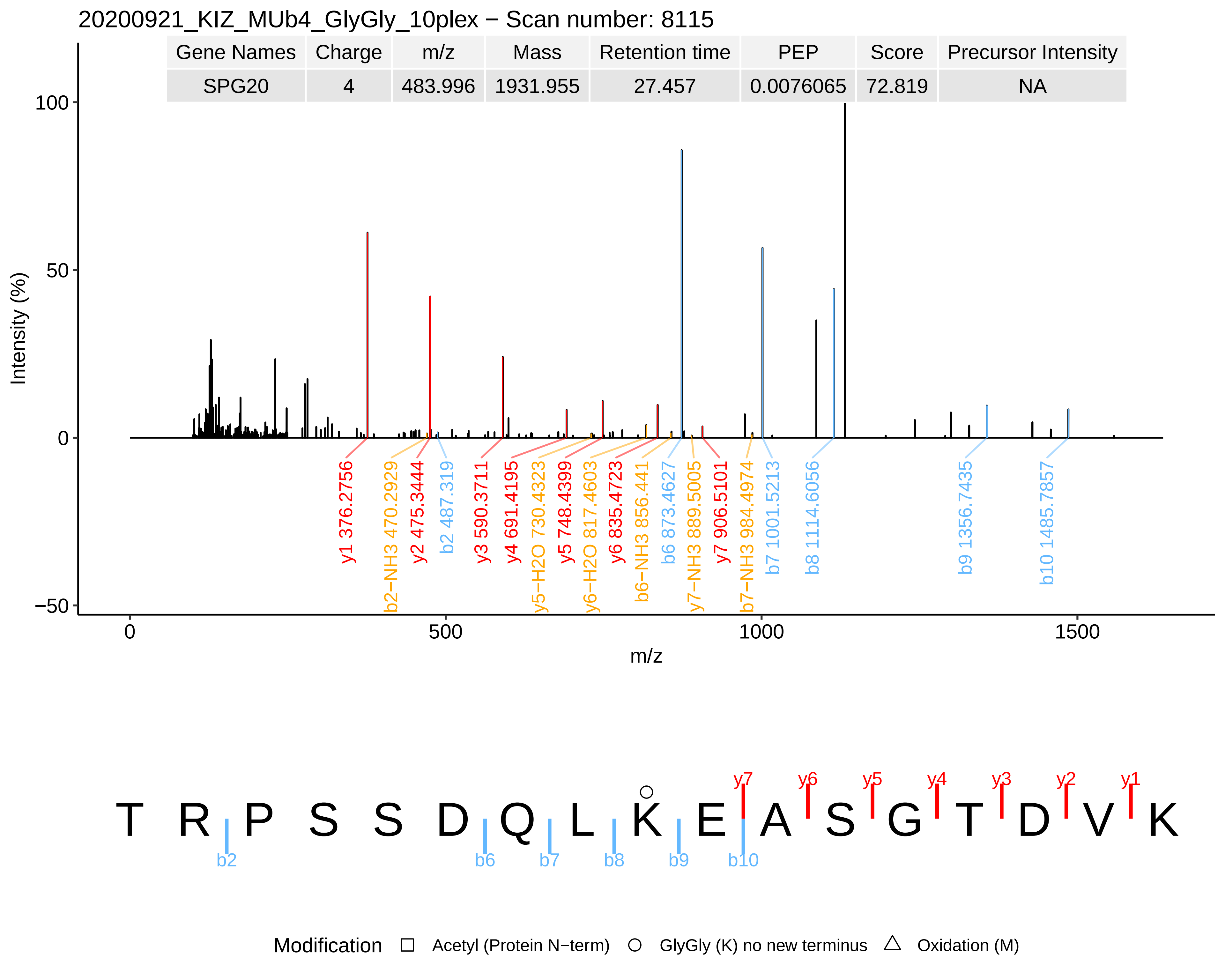
20200921_KIZ_MUb4_GlyGly_10plex − Scan number: 8115
Gene Names | Charge | m/z | Mass | Retention time | PEP | Score | Precursor Intensity
SPG20 | 4 | 483.996 | 1931.955 | 27.457 | 0.0076065 | 72.819 | NA
Intensity (%)
100
50
0
−50
0
500
1000
1500
m/z
y1 376.2756
b2−NH3 470.2929
y2 475.3444
b2 487.319
y3 590.3711
y4 691.4195
y5−H2O 730.4323
y5 748.4399
y6−H2O 817.4603
y6 835.4723
b6−NH3 856.441
b6 873.4627
y7−NH3 889.5005
y7 906.5101
b7−NH3 984.4974
b7 1001.5213
b8 1114.6056
b9 1356.7435
b10 1485.7857
T R P S S D Q L K E A S G T D V K
y7 y6 y5 y4 y3 y2 y1
b2 b6 b7 b8 b9 b10
Modification
Acetyl (Protein N−term)
GlyGly (K) no new terminus
Oxidation (M)

20200921_KIZ_MUb4_GlyGly_10plex − Scan number: 8263

Gene Names	Charge	m/z	Mass	Retention time	PEP	Score	Precursor Intensity
SPG20	4	483.996	1931.955	27.989	6.3084e−12	120.63	4074576

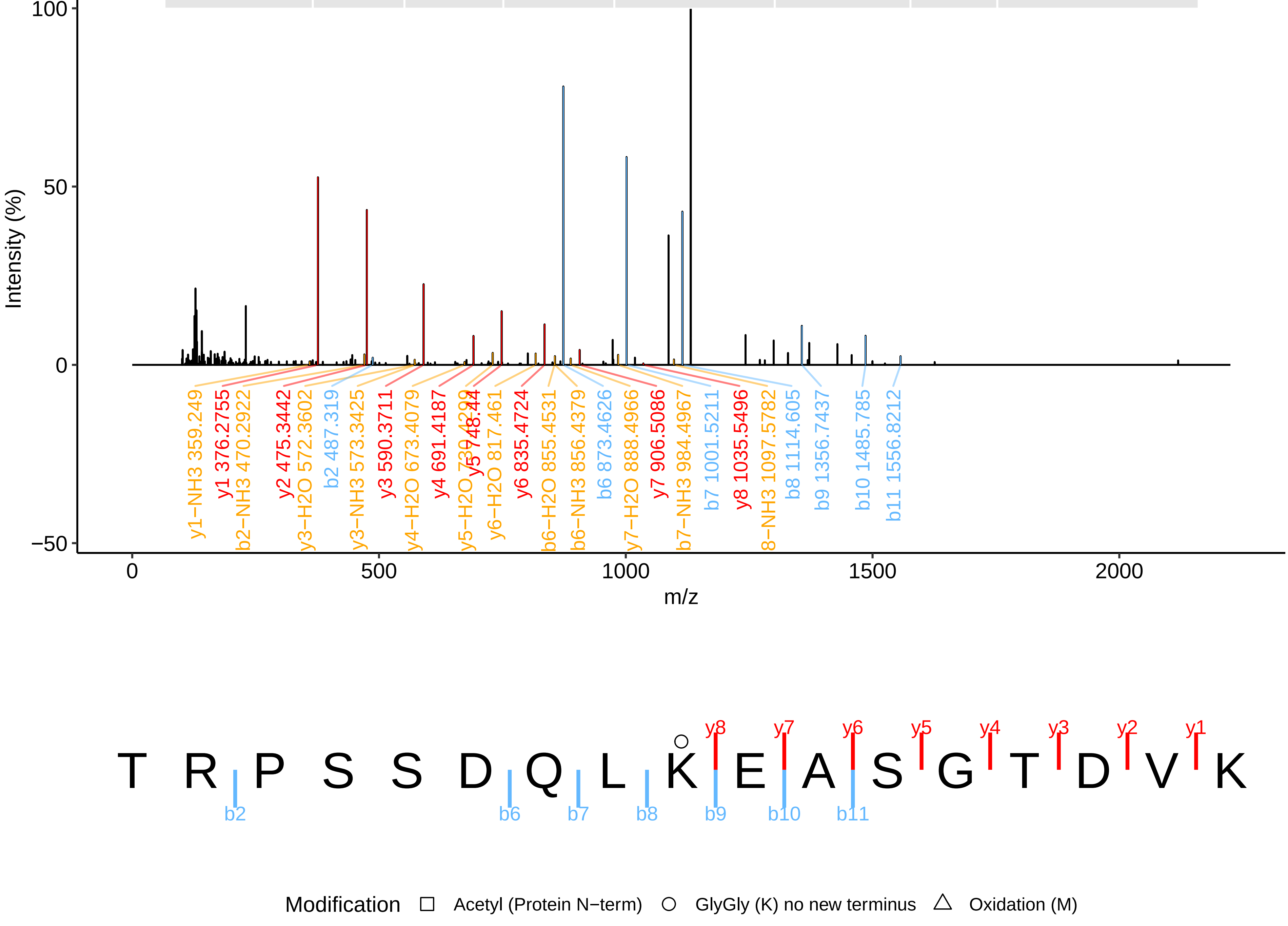

20200924_KIZ_MUb5_GlyGly_1 − Scan number: 7202

Gene Names	Charge	m/z	Mass	Retention time	PEP	Score	Precursor Intensity
SPG20	4	483.996	1931.955	22.832	4.5382e−09	103.16	10805619

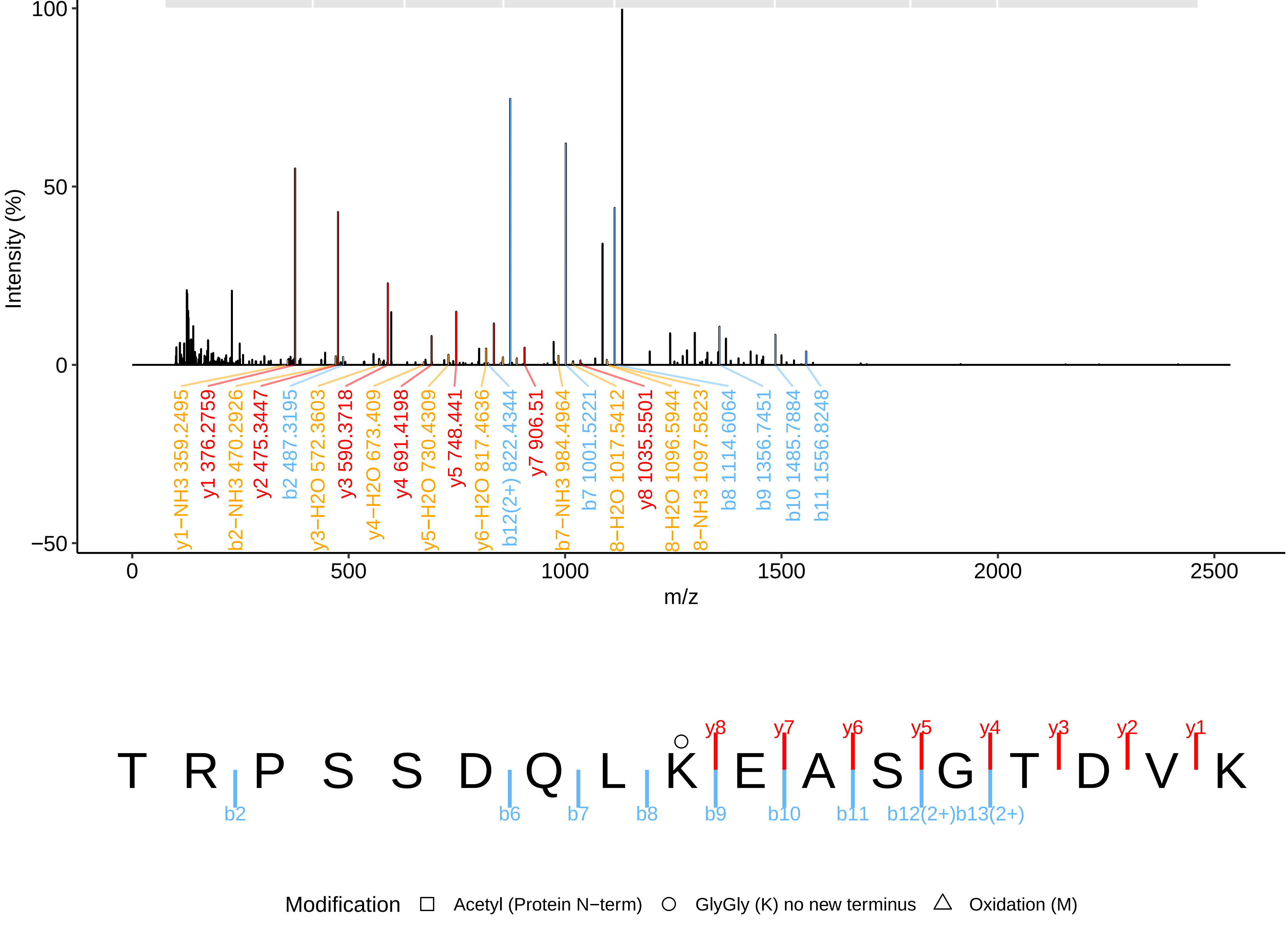

20201118_KIZ_MUb6_GlyGly_2 − Scan number: 8423

Gene Names	Charge	m/z	Mass	Retention time	PEP	Score	Precursor Intensity
SPG20	4	483.996	1931.955	28.606	2.3635e−09	109.04	7187553

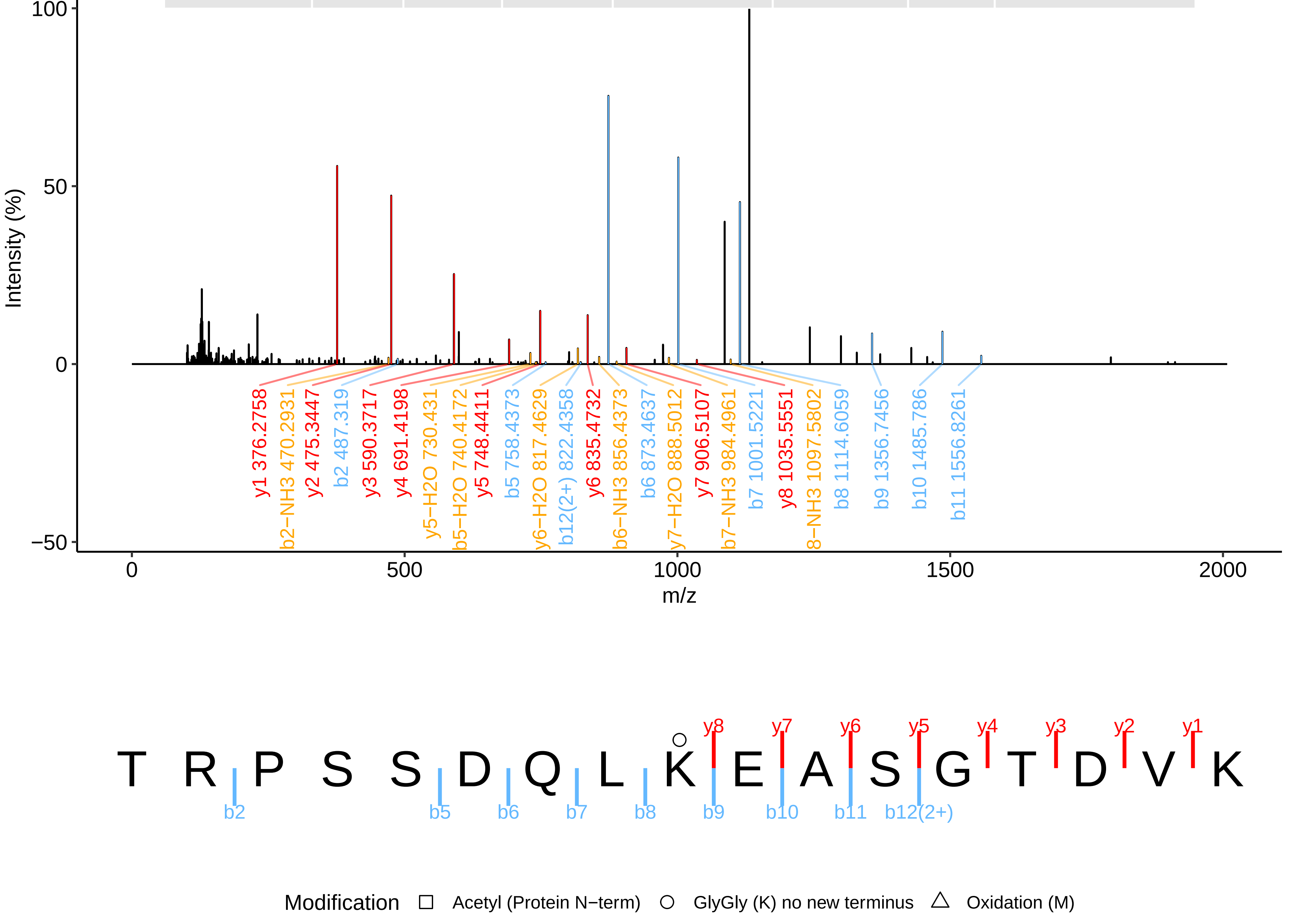

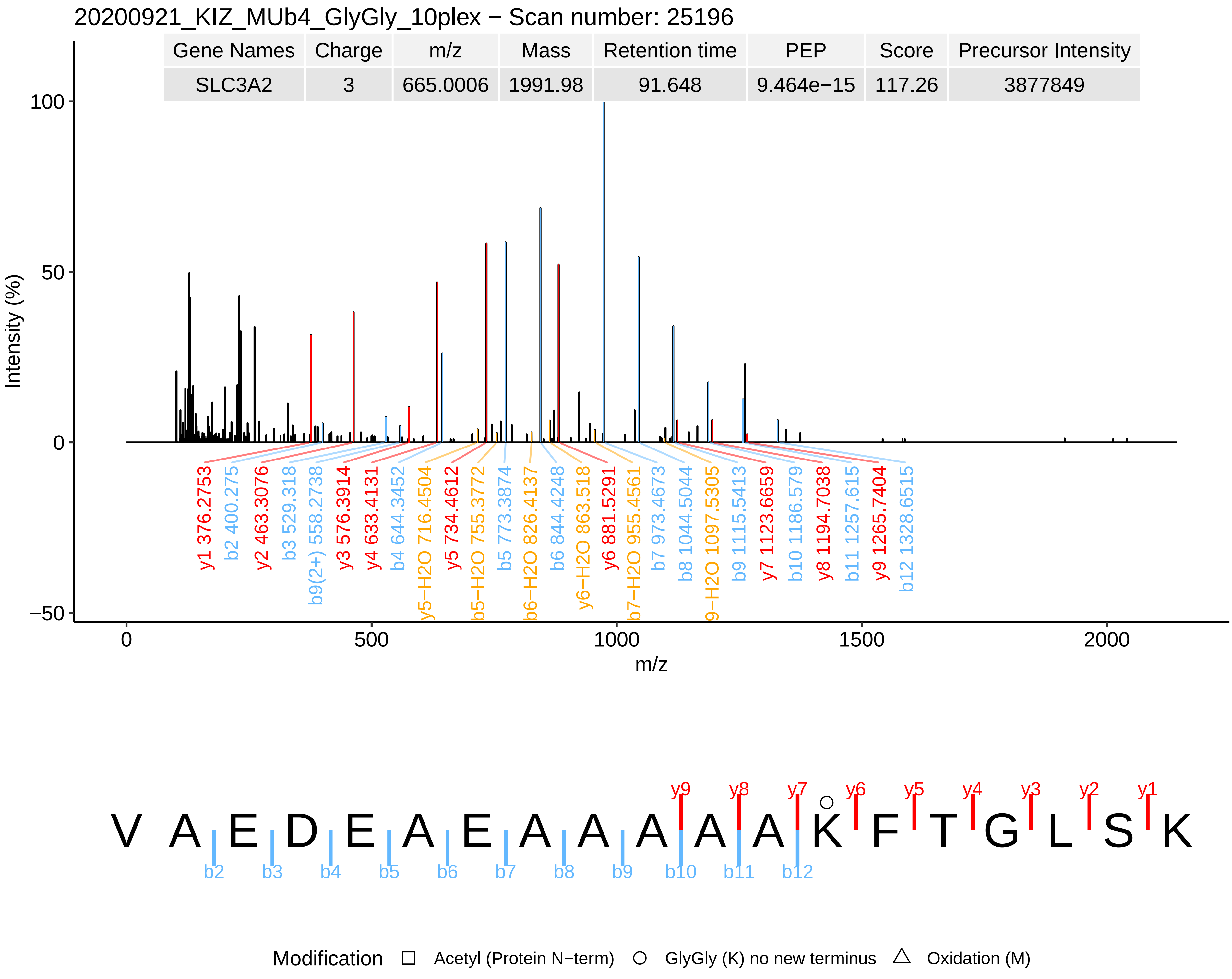
20200921_KIZ_MUb4_GlyGly_10plex − Scan number: 25196
Gene Names | Charge | m/z | Mass | Retention time | PEP | Score | Precursor Intensity
SLC3A2 | 3 | 665.0006 | 1991.98 | 91.648 | 9.464e−15 | 117.26 | 3877849
Intensity (%)
100
50
0
−50
0
500
1000
1500
2000
m/z
y1 376.2753
b2 400.275
y2 463.3076
b3 529.318
b9(2+) 558.2738
y3 576.3914
y4 633.4131
b4 644.3452
y5−H2O 716.4504
y5 734.4612
b5−H2O 755.3772
b5 773.3874
b6−H2O 826.4137
b6 844.4248
y6−H2O 863.518
y6 881.5291
b7−H2O 955.4561
b7 973.4673
b8 1044.5044
9−H2O 1097.5305
b9 1115.5413
y7 1123.6659
b10 1186.579
y8 1194.7038
b11 1257.615
y9 1265.7404
b12 1328.6515
V A E D E A E A A A A A K F T G L S K
y9 y8 y7 y6 y5 y4 y3 y2 y1
b2 b3 b4 b5 b6 b7 b8 b9 b10 b11 b12
Modification
Acetyl (Protein N−term)
GlyGly (K) no new terminus
Oxidation (M)

20200921_KIZ_MUb4_GlyGly_10plex_2 − Scan number: 21557

Gene Names	Charge	m/z	Mass	Retention time	PEP	Score	Precursor Intensity
SLC3A2	3	665.0006	1991.98	84.882	8.1608e−15	119.4	817119.2

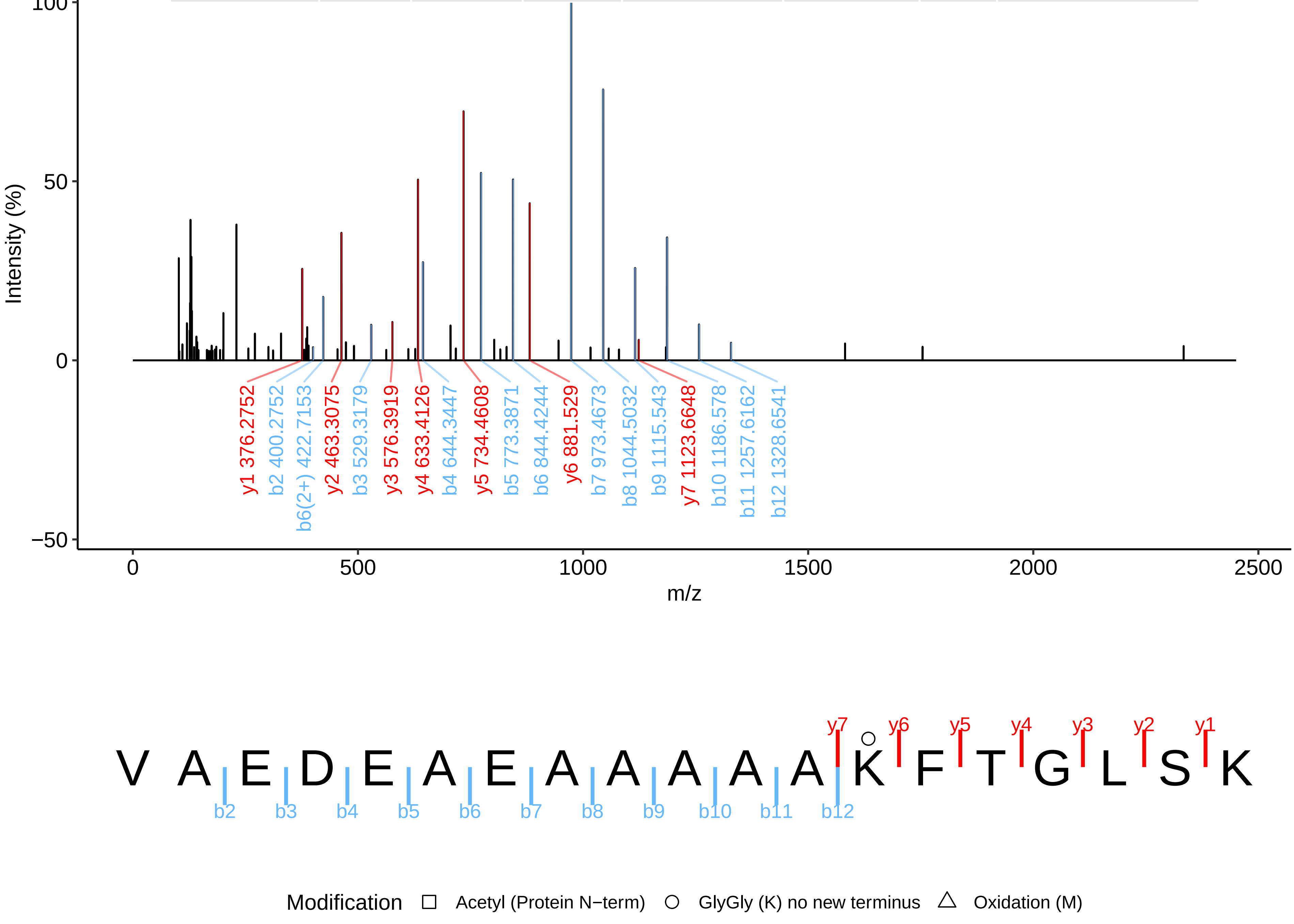

20200924_KIZ_MUb5_GlyGly_1 − Scan number: 24135

Gene Names	Charge	m/z	Mass	Retention time	PEP	Score	Precursor Intensity
SLC3A2	3	665.0006	1991.98	77.55	5.8938e−14	123.41	NA

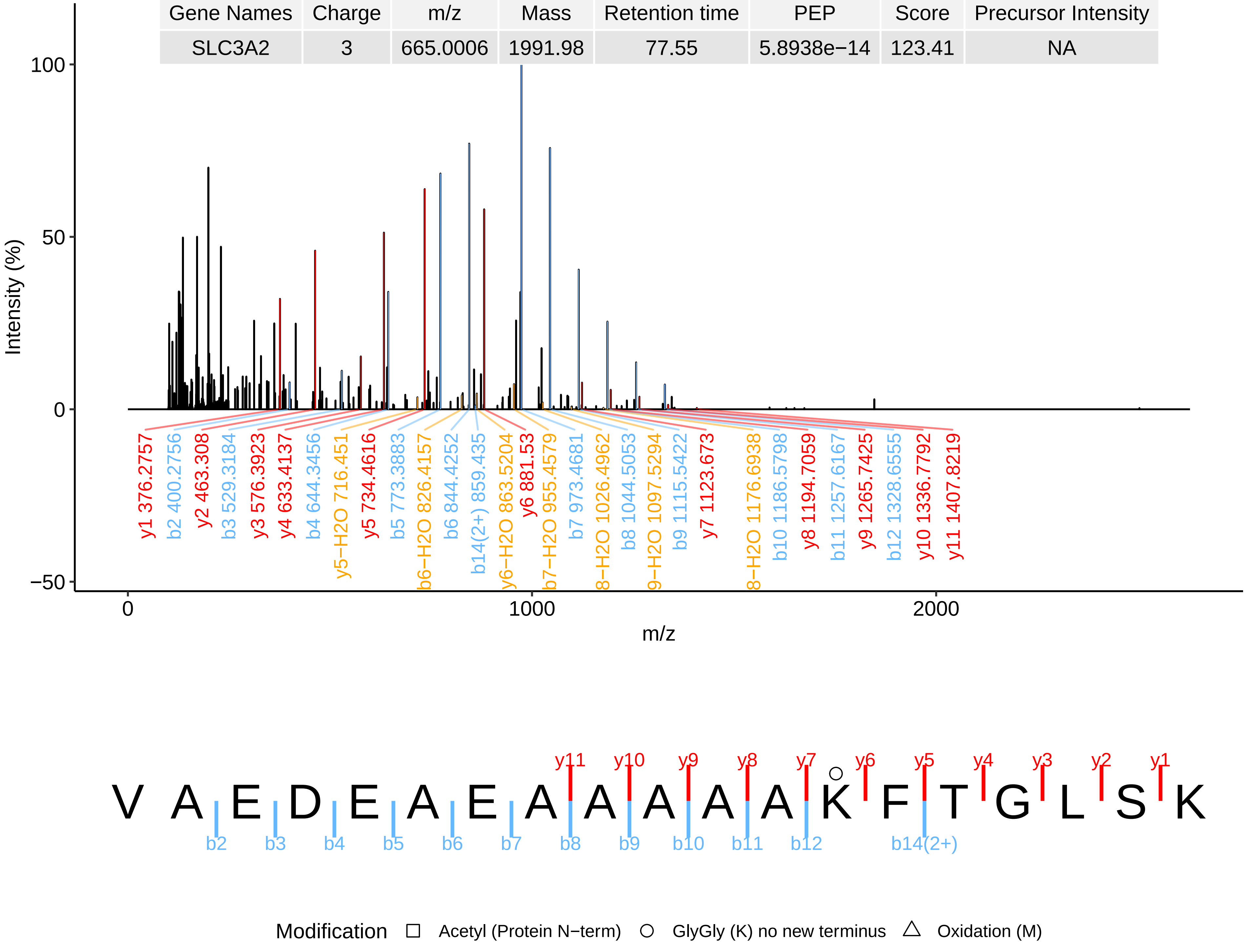

20201118_KIZ_MUb6_GlyGly_1 − Scan number: 24870

Gene Names	Charge	m/z	Mass	Retention time	PEP	Score	Precursor Intensity
SLC3A2	3	665.0006	1991.98	76.421	3.4022e−14	127.22	NA

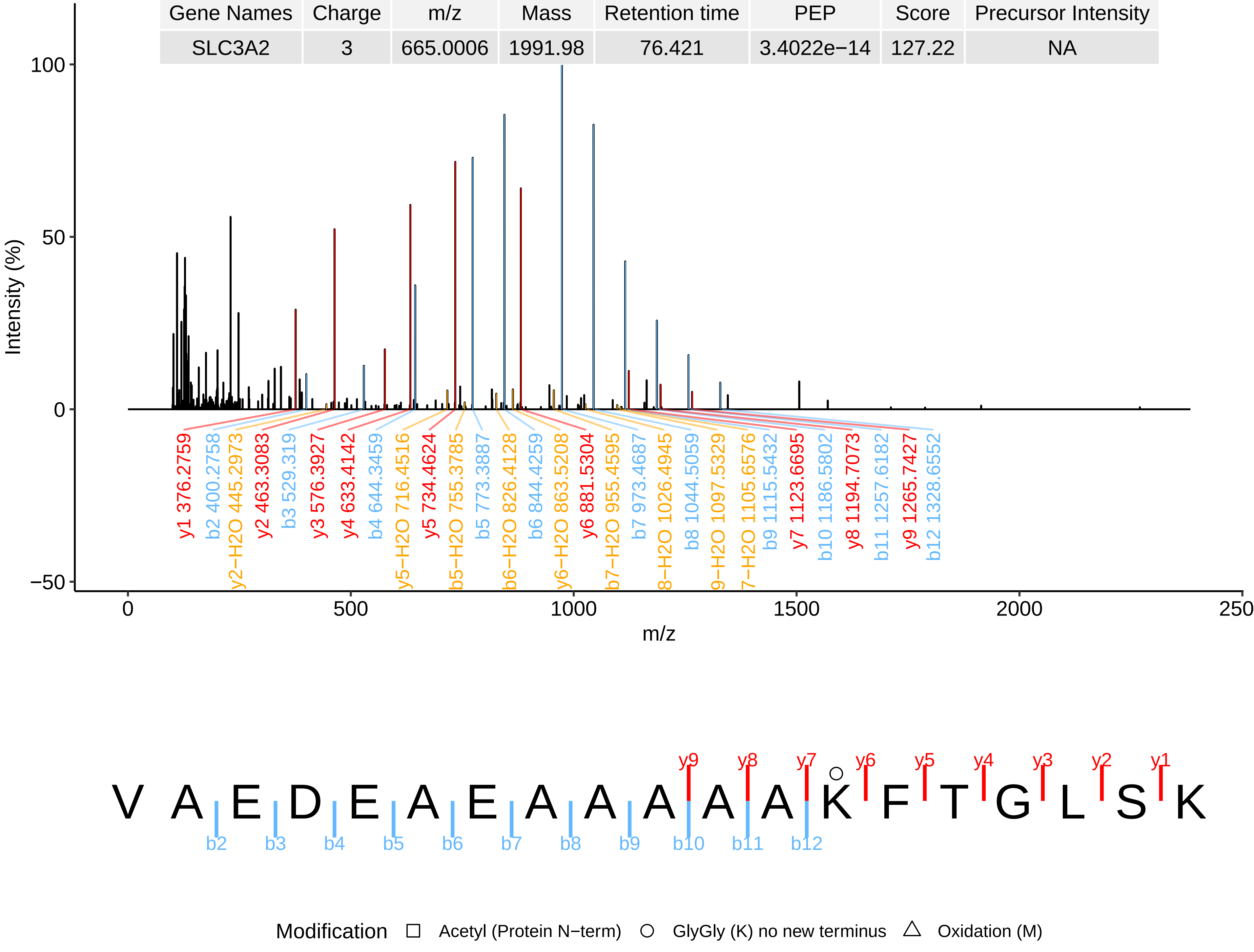

20200921_KIZ_MUb4_GlyGly_10plex − Scan number: 16232

Gene Names	Charge	m/z	Mass	Retention time	PEP	Score	Precursor Intensity
PGK1	3	716.0201	2145.039	58.242	1.5346e−30	179.14	5340866

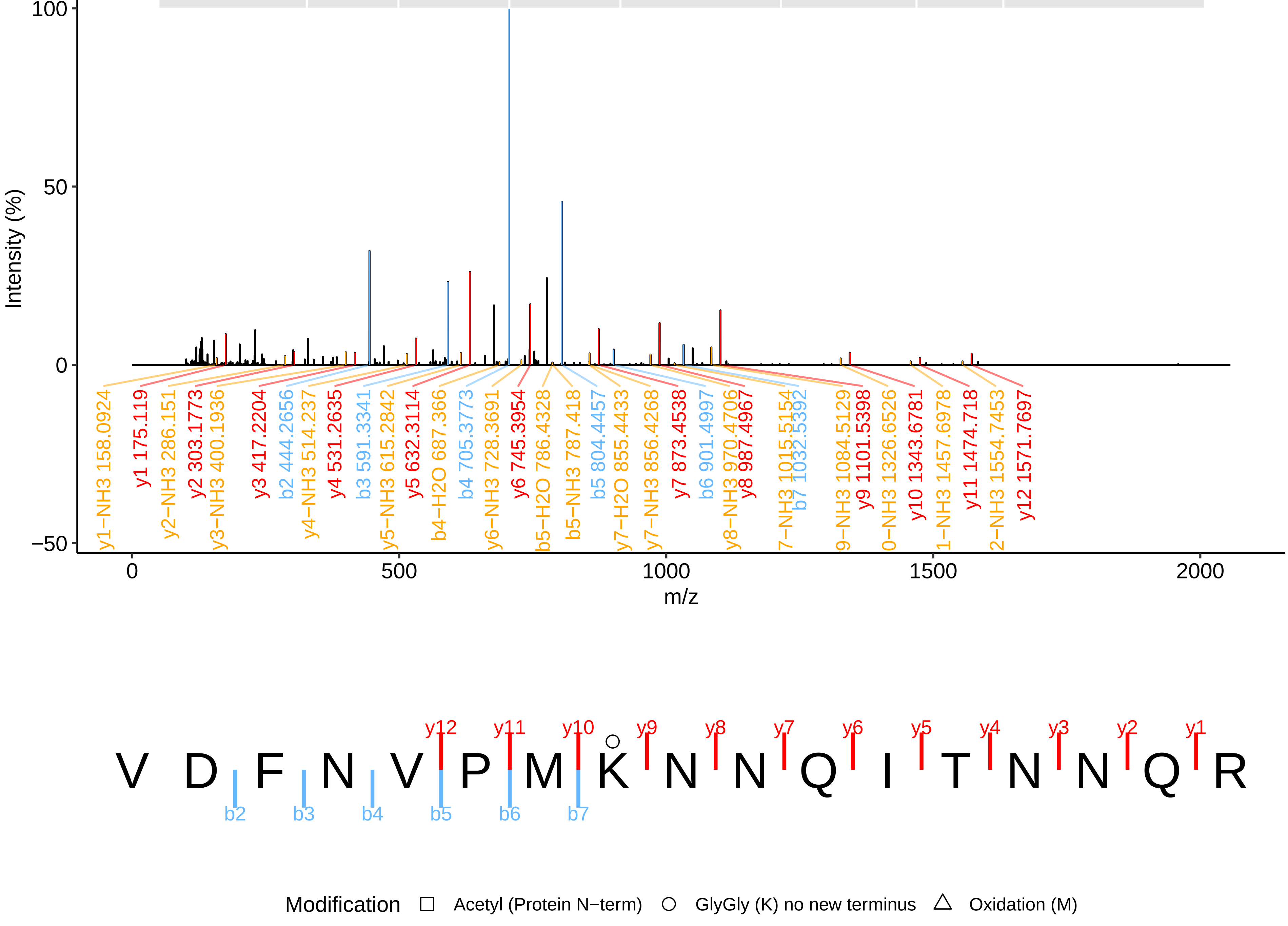

20200921_KIZ_MUb4_GlyGly_10plex_2 − Scan number: 15022

Gene Names	Charge	m/z	Mass	Retention time	PEP	Score	Precursor Intensity
PGK1	3	716.0201	2145.039	58.662	2.551e−07	97.291	1353869

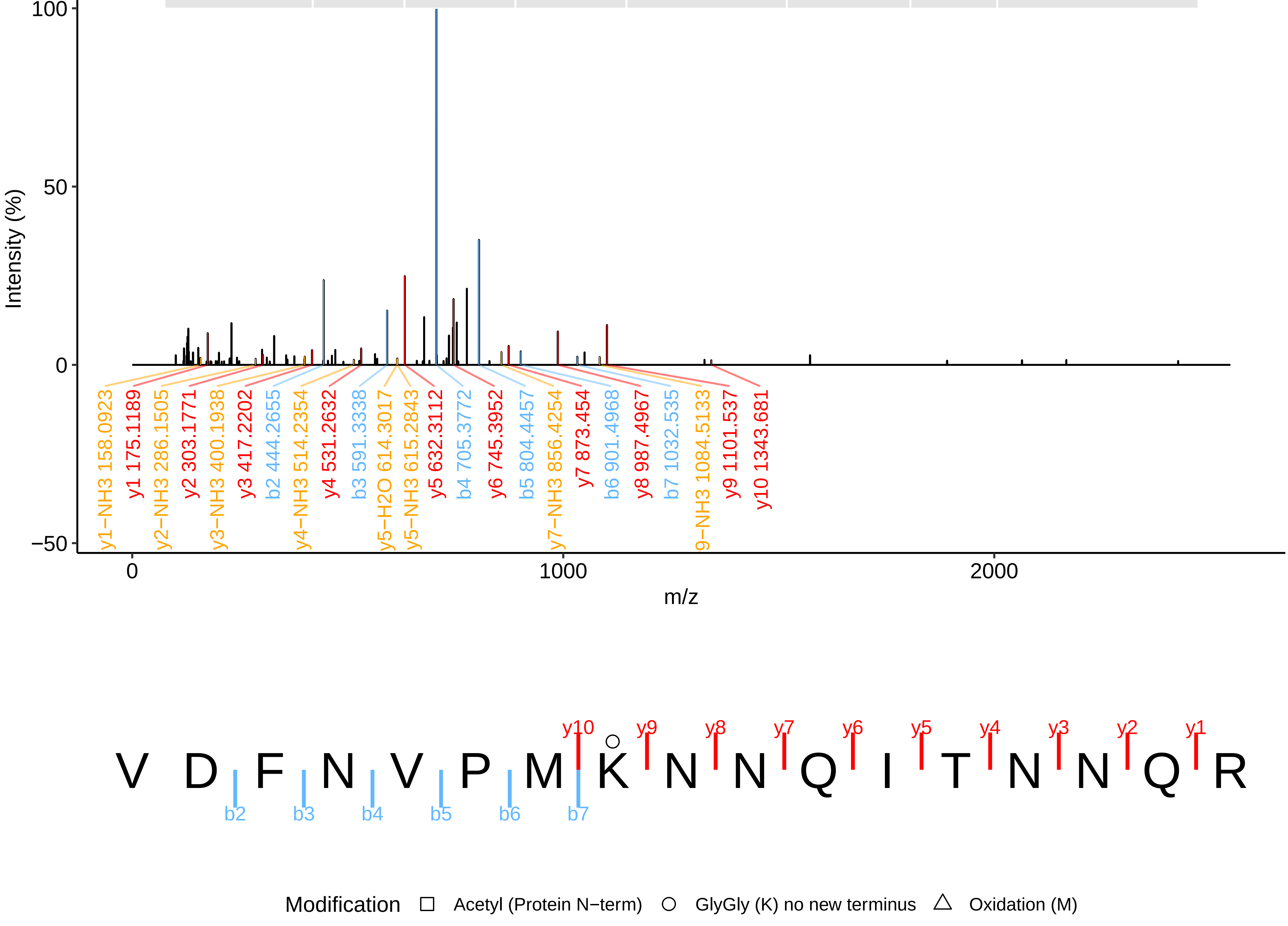

20200924_KIZ_MUb5_GlyGly_1 − Scan number: 15524

Gene Names	Charge	m/z	Mass	Retention time	PEP	Score	Precursor Intensity
PGK1	3	716.0201	2145.039	50.695	1.5346e−30	179.14	14275452

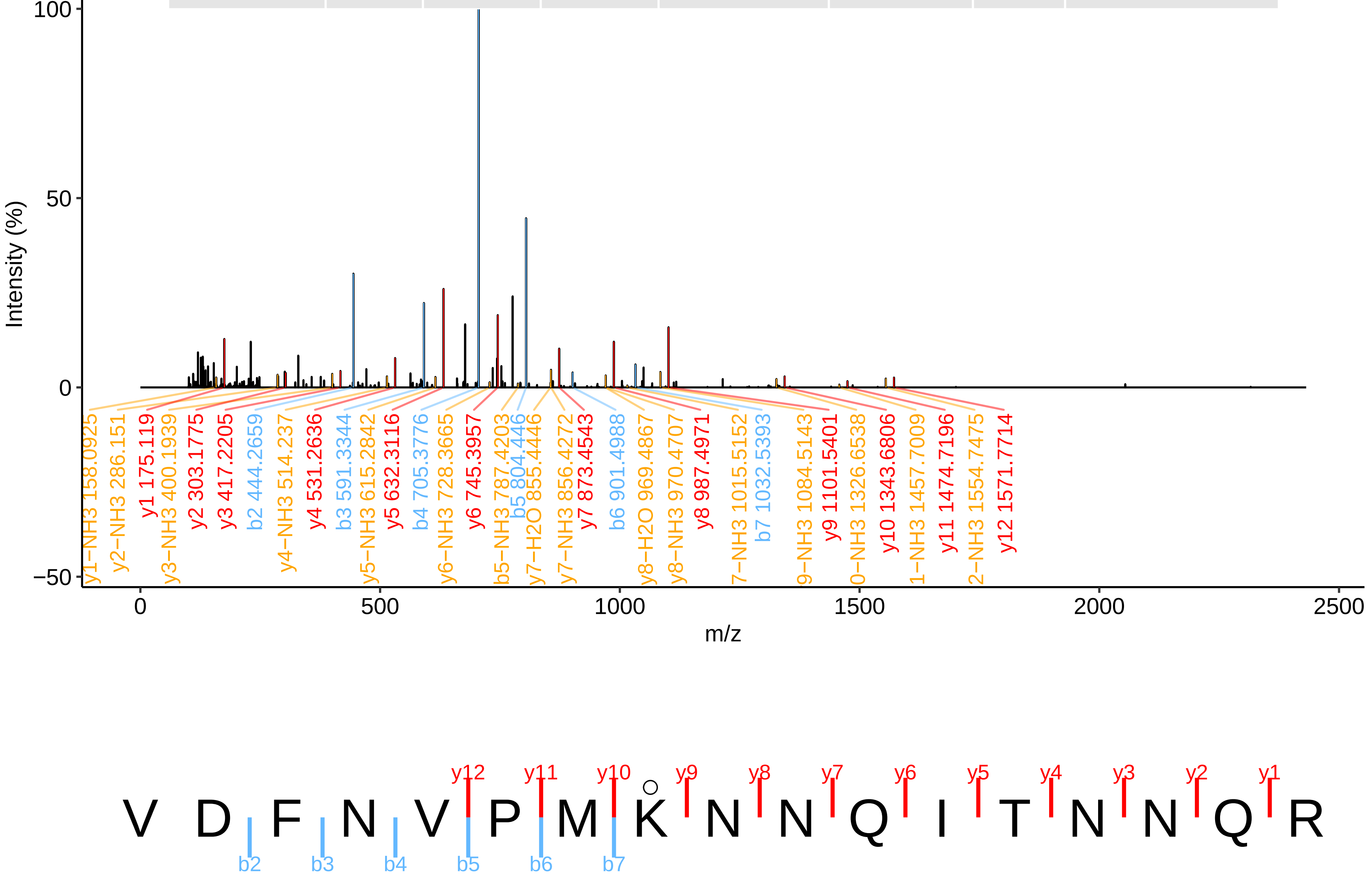

Modification □ Acetyl (Protein N−term) ○ GlyGly (K) no new terminus △ Oxidation (M)

20200921_KIZ_MUb4_GlyGly_10plex − Scan number: 26055

Gene Names	Charge	m/z	Mass	Retention time	PEP	Score	Precursor Intensity
GINM1	3	737.3987	2209.174	94.864	1.8406e−20	138.55	2685994

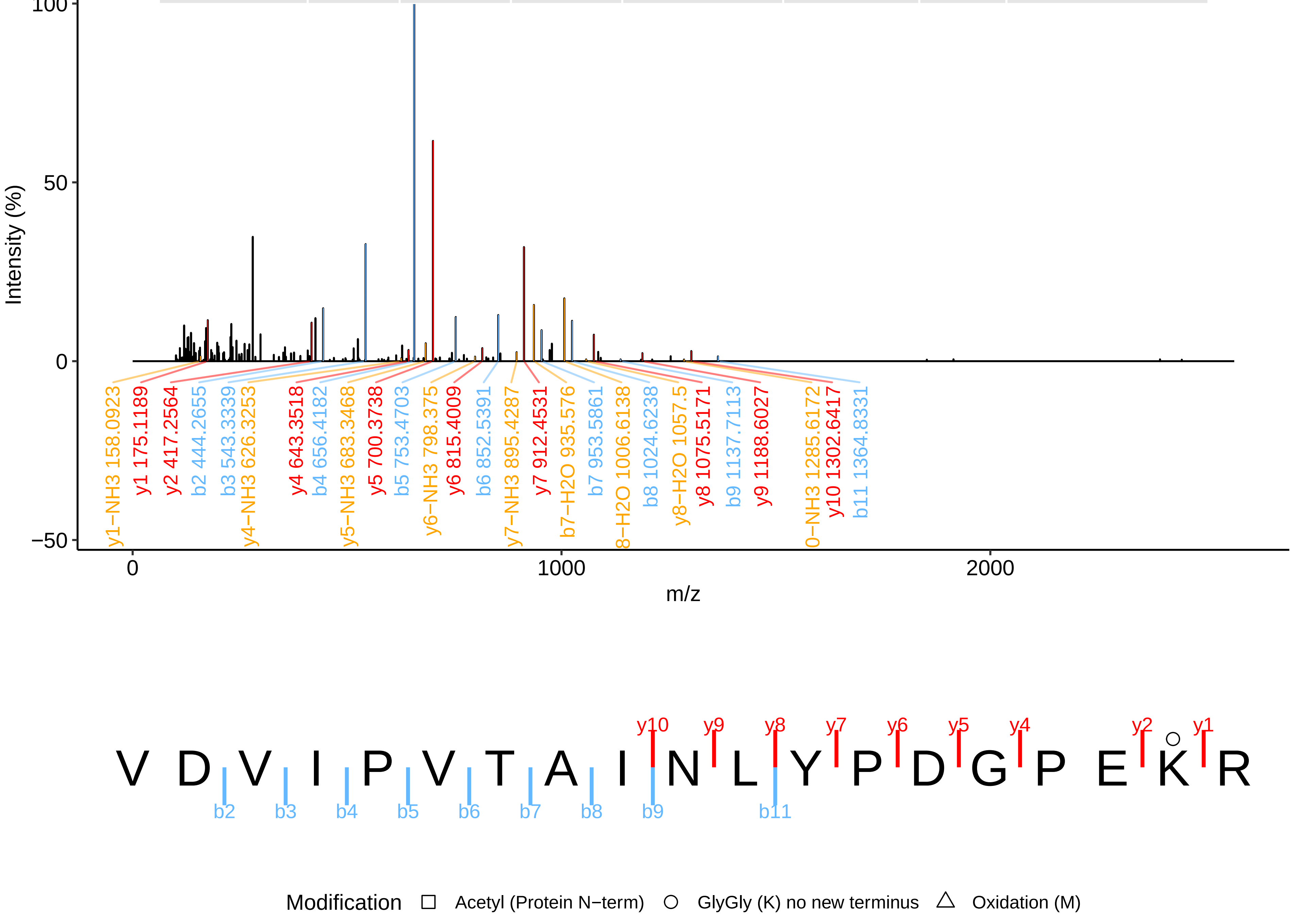

20200921_KIZ_MUb4_GlyGly_10plex − Scan number: 26078

Gene Names	Charge	m/z	Mass	Retention time	PEP	Score	Precursor Intensity
GINM1	3	737.3987	2209.174	94.957	1.8406e−20	138.55	5613742

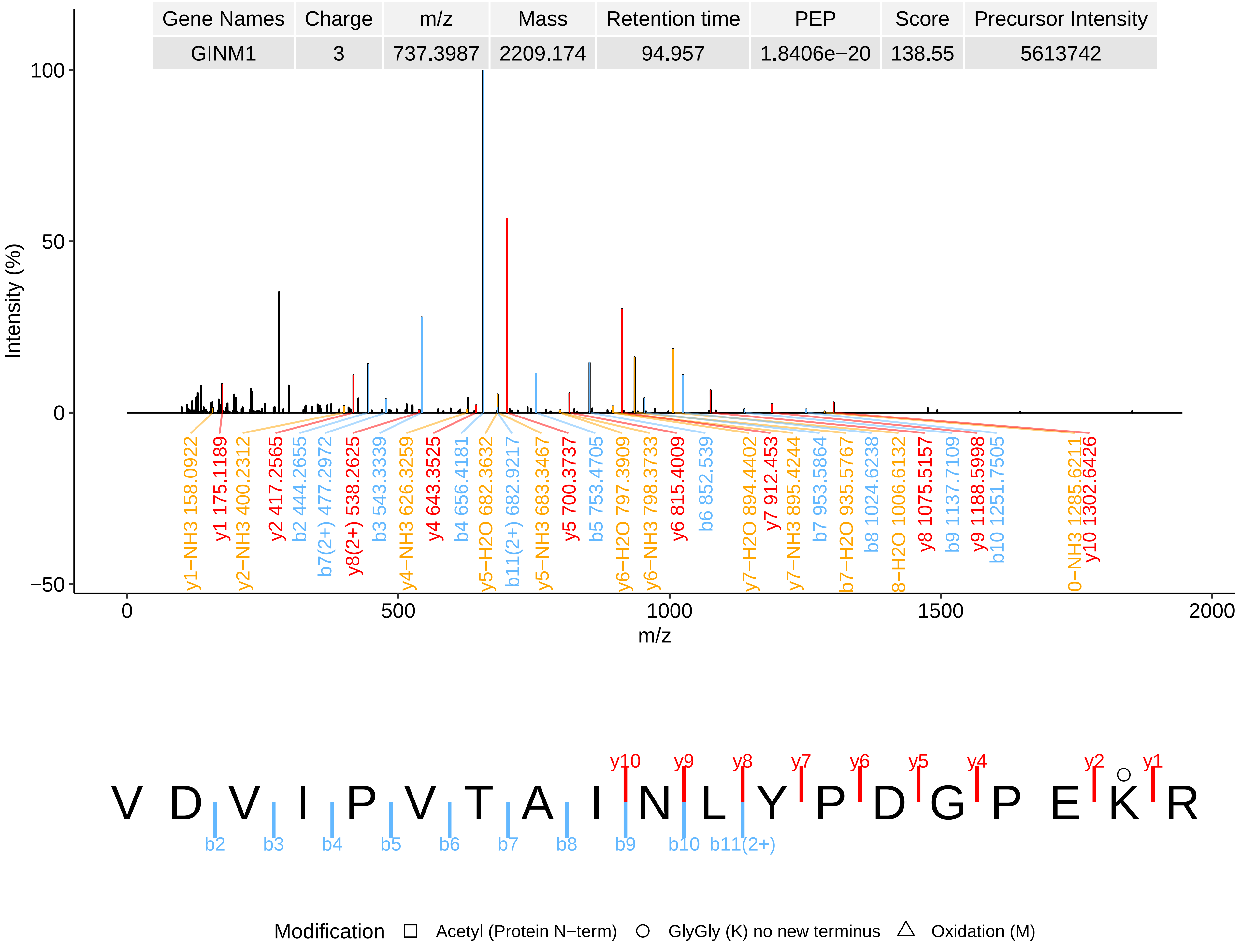

20200921_KIZ_MUb4_GlyGly_10plex_2 − Scan number: 23312

Gene Names	Charge	m/z	Mass	Retention time	PEP	Score	Precursor Intensity
GINM1	3	737.3987	2209.174	91.999	4.121e−10	114.96	741866.9

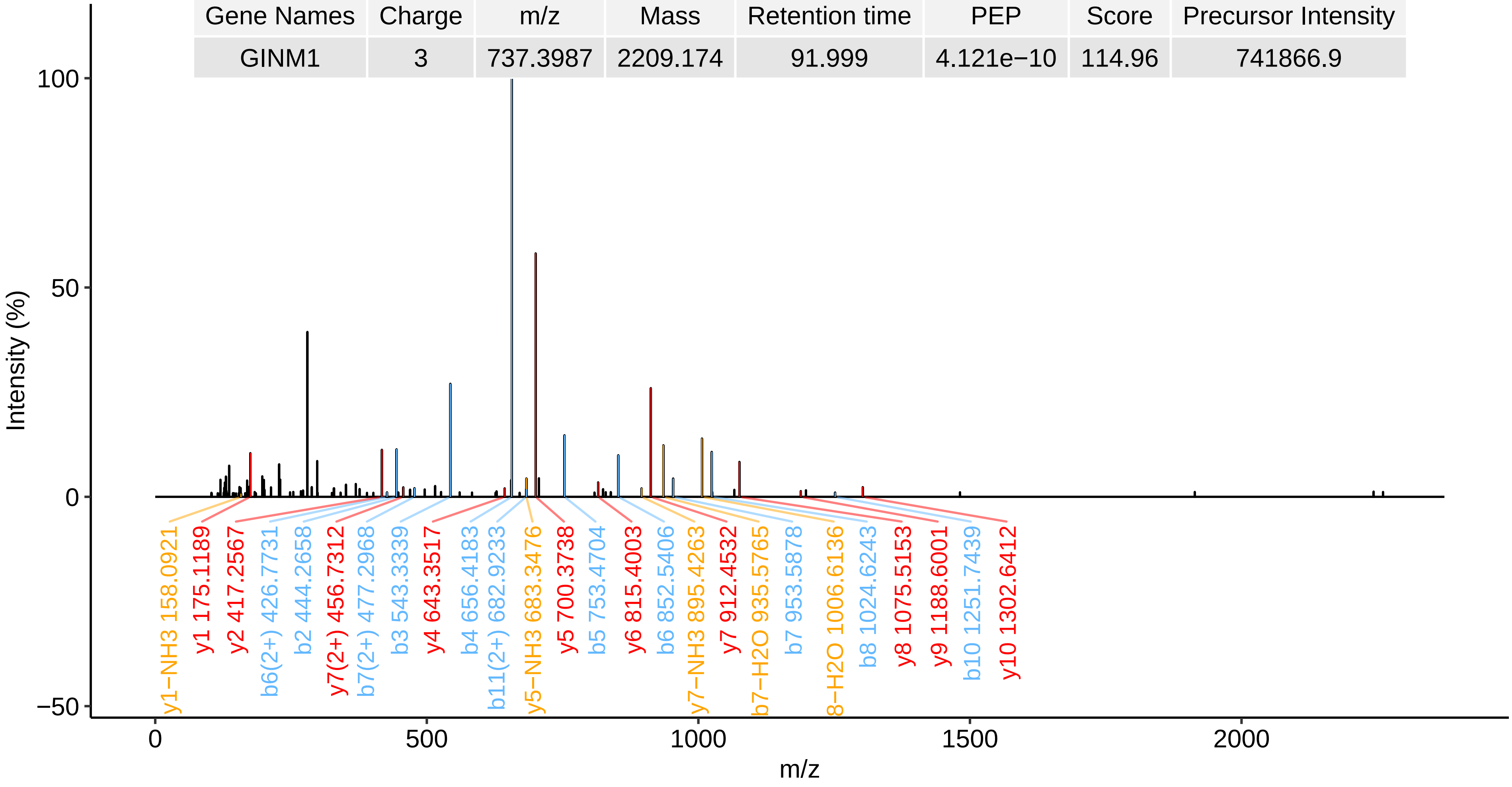

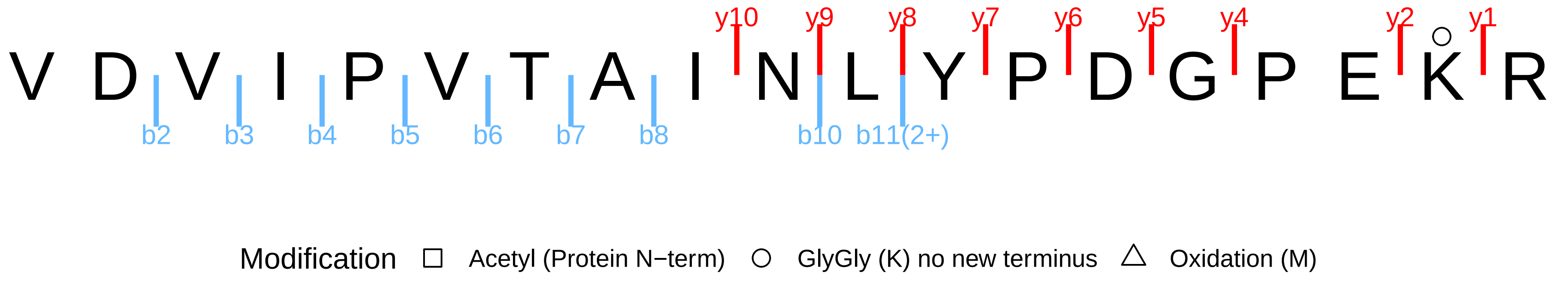

20200921_KIZ_MUb4_GlyGly_10plex_2 − Scan number: 14395

Gene Names	Charge	m/z	Mass	Retention time	PEP	Score	Precursor Intensity
SET	4	497.7484	1986.965	56.121	0.00081044	74.12	1306315

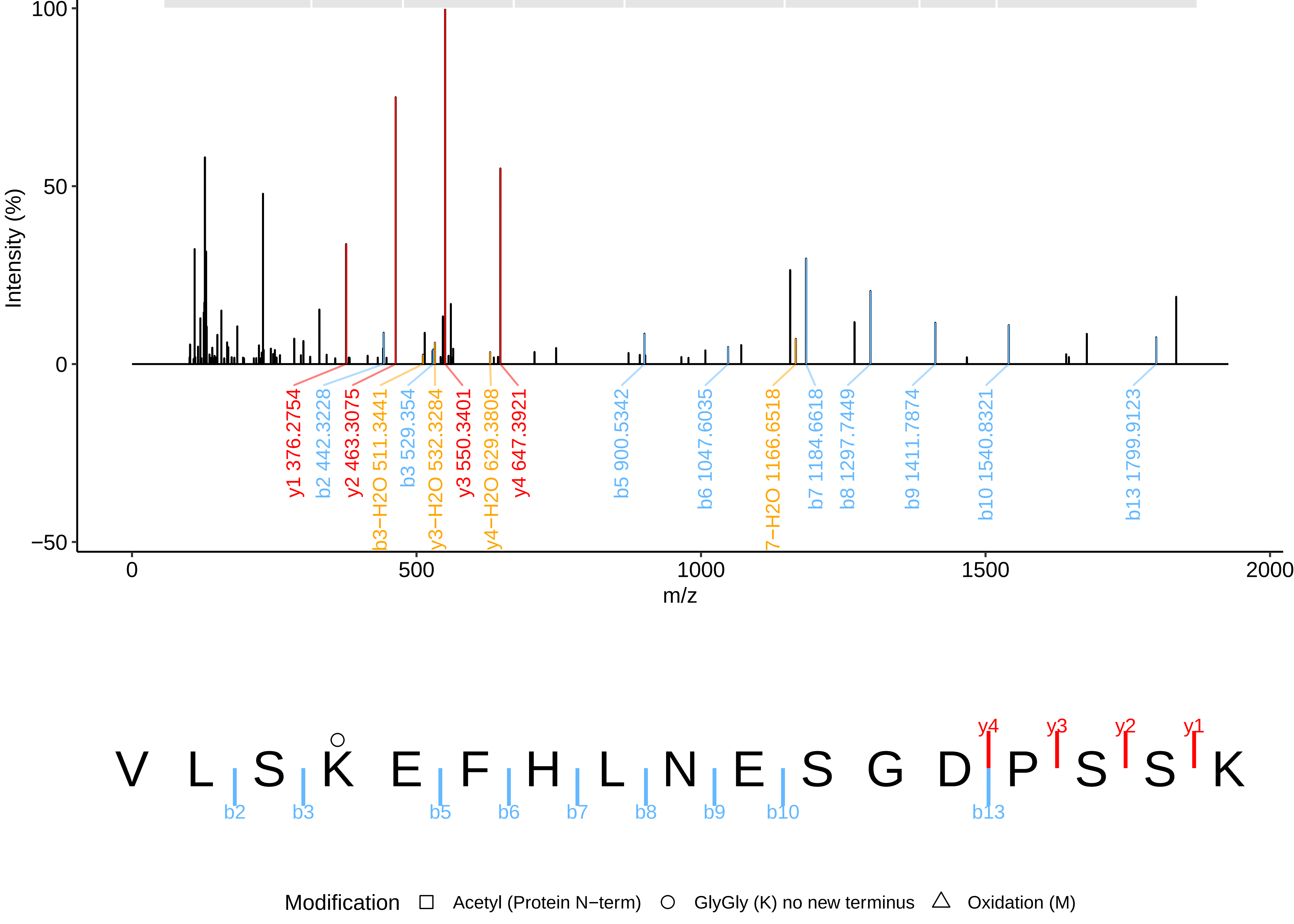

20200921_KIZ_MUb4_GlyGly_10plex_2 − Scan number: 15745

Gene Names	Charge	m/z	Mass	Retention time	PEP	Score	Precursor Intensity
CHCHD3	3	646.6532	1936.938	61.628	1.4647e−05	83.966	742409.2

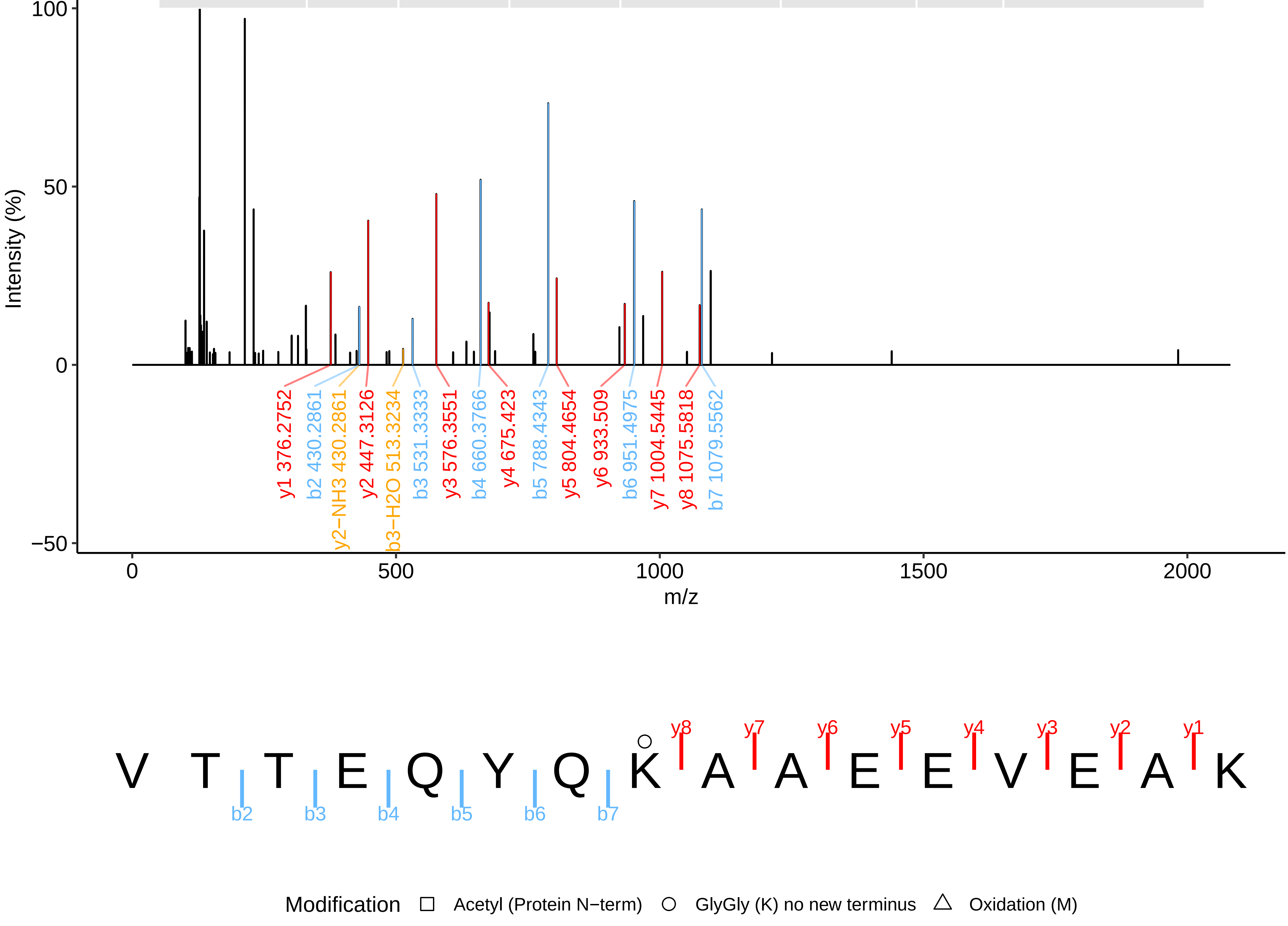

20200921_KIZ_MUb4_GlyGly_10plex_2 − Scan number: 15776

Gene Names	Charge	m/z	Mass	Retention time	PEP	Score	Precursor Intensity
CHCHD3	3	646.6532	1936.938	61.763	0.0019905	69.331	1443157

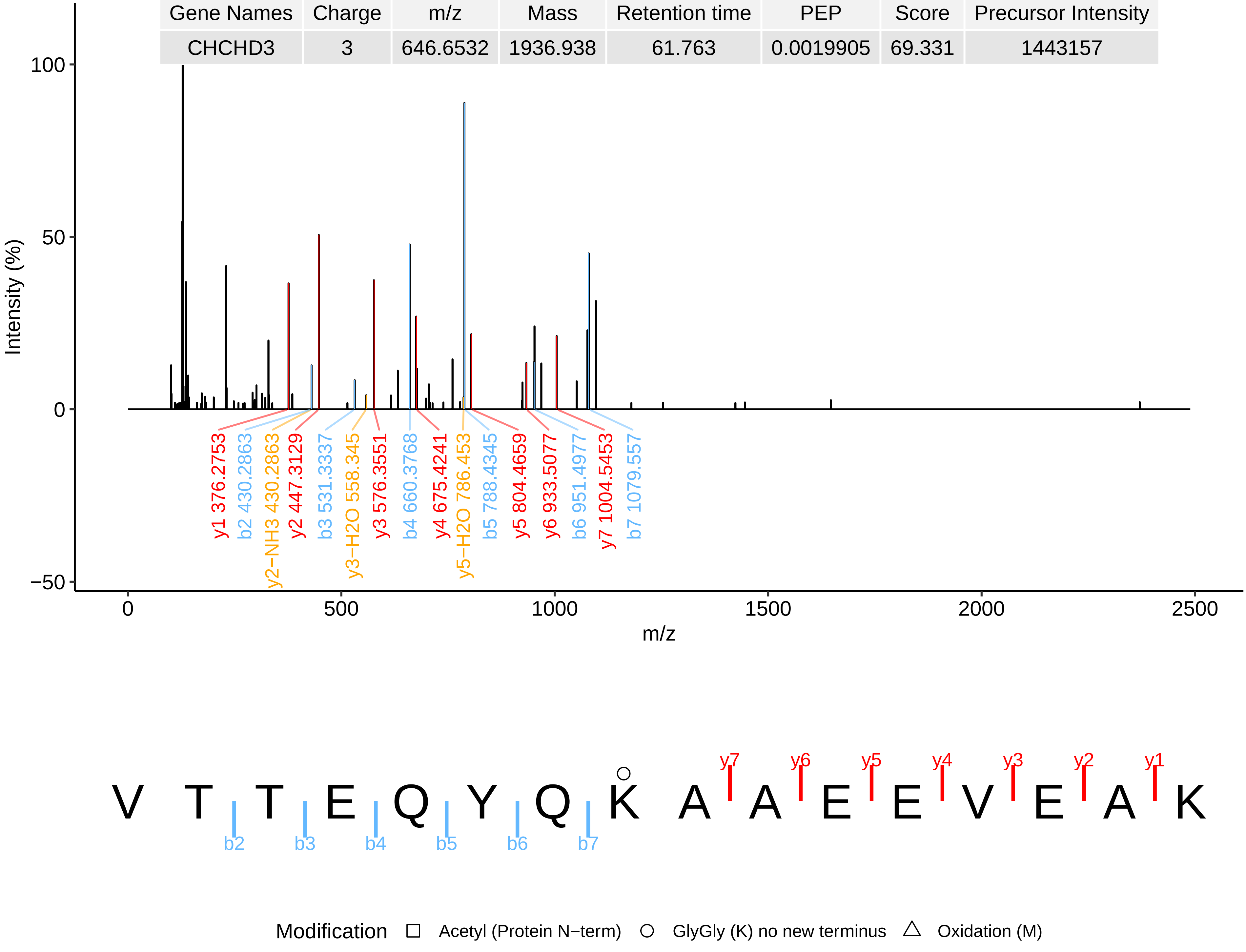

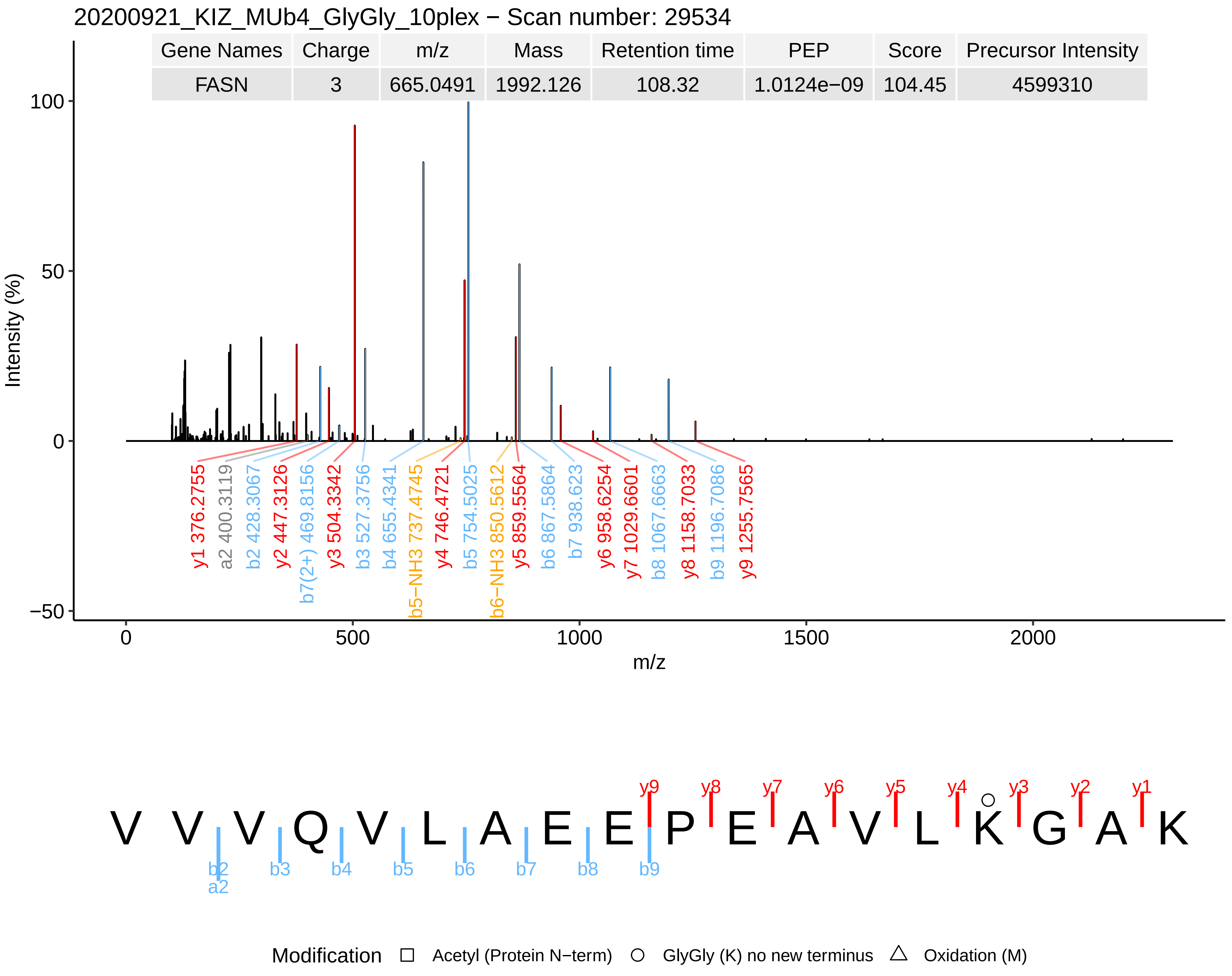

20200921_KIZ_MUb4_GlyGly_10plex − Scan number: 29534
Gene Names
Charge
m/z
Mass
Retention time
PEP
Score
Precursor Intensity
FASN
3
665.0491
1992.126
108.32
1.0124e−09
104.45
4599310
Intensity (%)
100
50
0
−50
m/z
0
500
1000
1500
2000
y1 376.2755
a2 400.3119
b2 428.3067
y2 447.3126
b7(2+) 469.8156
y3 504.3342
b3 527.3756
b4 655.4341
b5−NH3 737.4745
y4 746.4721
b5 754.5025
b6−NH3 850.5612
y5 859.5564
b6 867.5864
b7 938.623
y6 958.6254
y7 1029.6601
b8 1067.6663
y8 1158.7033
b9 1196.7086
y9 1255.7565
V V V Q V L A E E P E A V L K G A K
y9 y8 y7 y6 y5 y4 y3 y2 y1
b2 b3 b4 b5 b6 b7 b8 b9
a2
Modification
Acetyl (Protein N−term)
GlyGly (K) no new terminus
Oxidation (M)

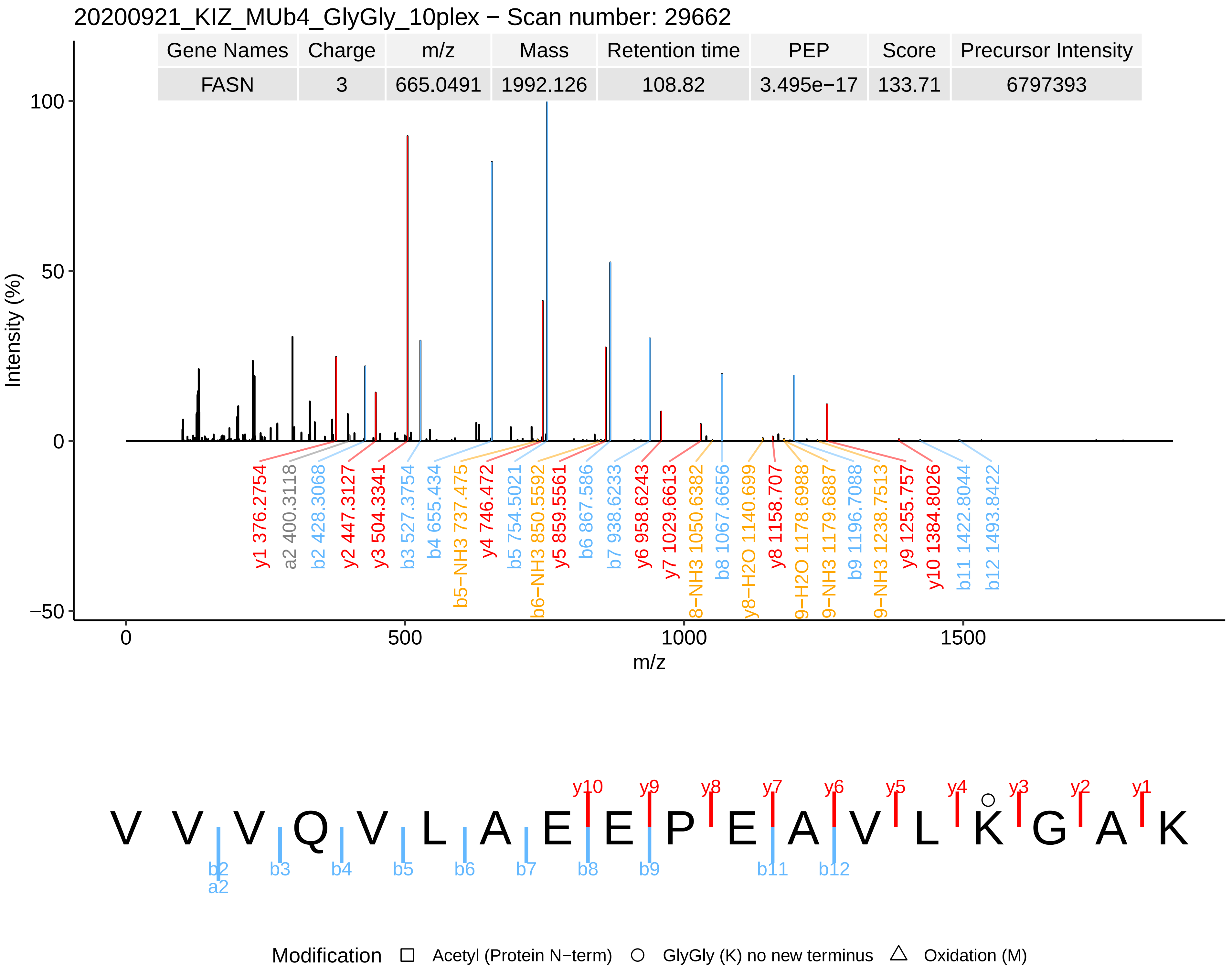
20200921_KIZ_MUb4_GlyGly_10plex − Scan number: 29662
Gene Names | Charge | m/z | Mass | Retention time | PEP | Score | Precursor Intensity
FASN | 3 | 665.0491 | 1992.126 | 108.82 | 3.495e−17 | 133.71 | 6797393
Intensity (%)
100
50
0
−50
0
500
1000
1500
m/z
y1 376.2754
a2 400.3118
b2 428.3068
y2 447.3127
y3 504.3341
b3 527.3754
b4 655.434
b5−NH3 737.475
y4 746.472
b5 754.5021
b6−NH3 850.5592
y5 859.5561
b6 867.586
b7 938.6233
y6 958.6243
y7 1029.6613
8−NH3 1050.6382
b8 1067.6656
y8−H2O 1140.699
y8 1158.707
9−H2O 1178.6988
9−NH3 1179.6887
b9 1196.7088
9−NH3 1238.7513
y9 1255.757
y10 1384.8026
b11 1422.8044
b12 1493.8422
y10 y9 y8 y7 y6 y5 y4 y3 y2 y1
V V V Q V L A E E P E A V L K G A K
b2 b3 b4 b5 b6 b7 b8 b9 b11 b12
a2
Modification
Acetyl (Protein N−term)
GlyGly (K) no new terminus
Oxidation (M)

20200921_KIZ_MUb4_GlyGly_10plex − Scan number: 29569

Gene Names	Charge	m/z	Mass	Retention time	PEP	Score	Precursor Intensity
FASN	4	499.0387	1992.126	108.45	0.00045324	76.85	4448338

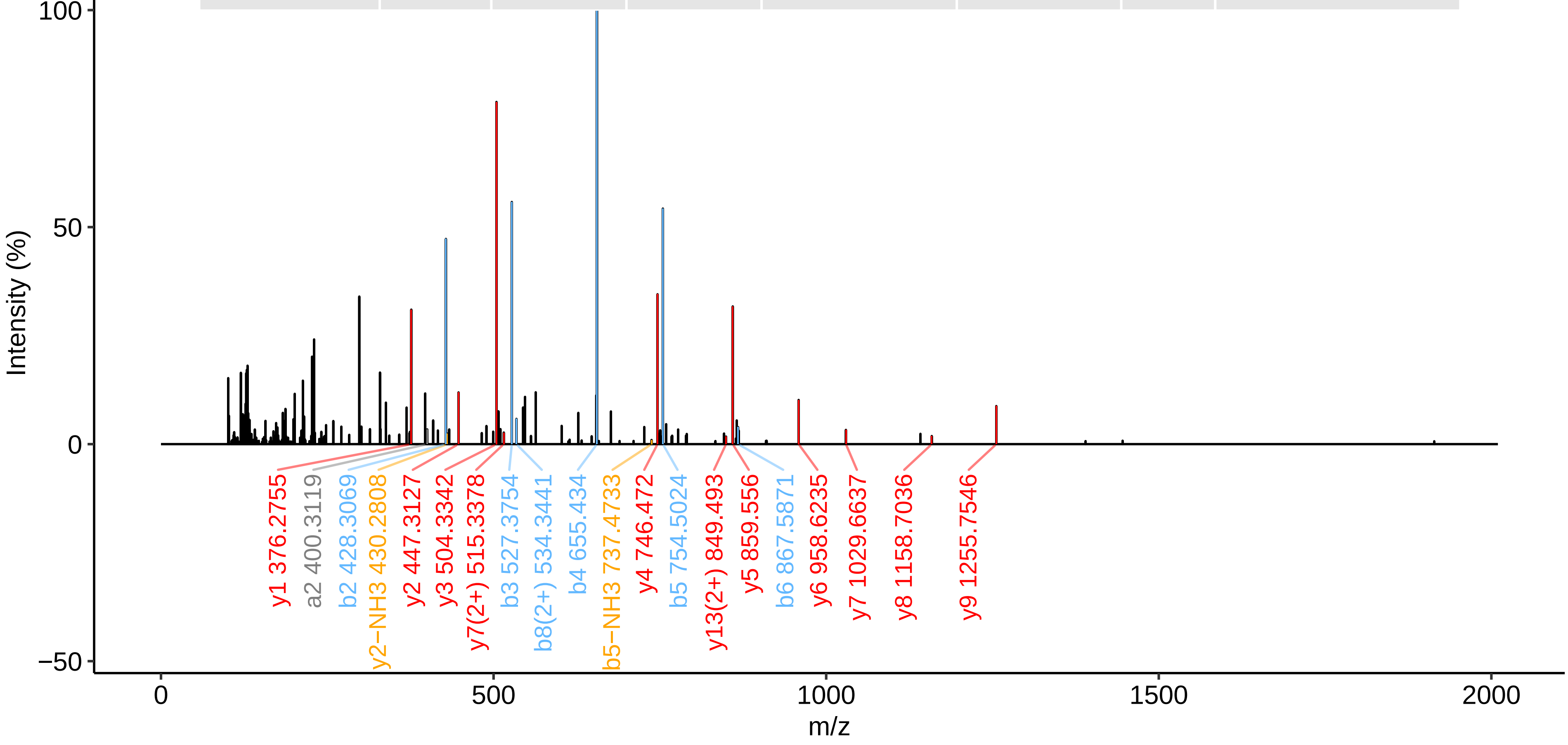

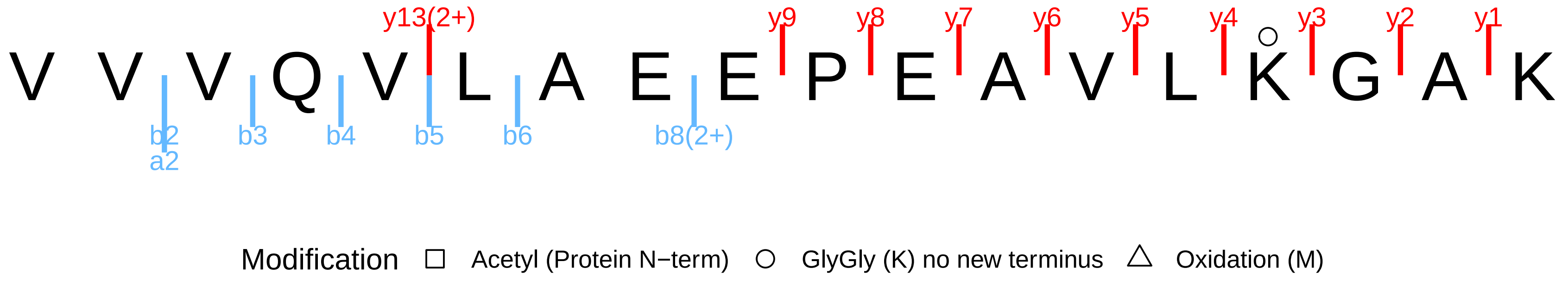

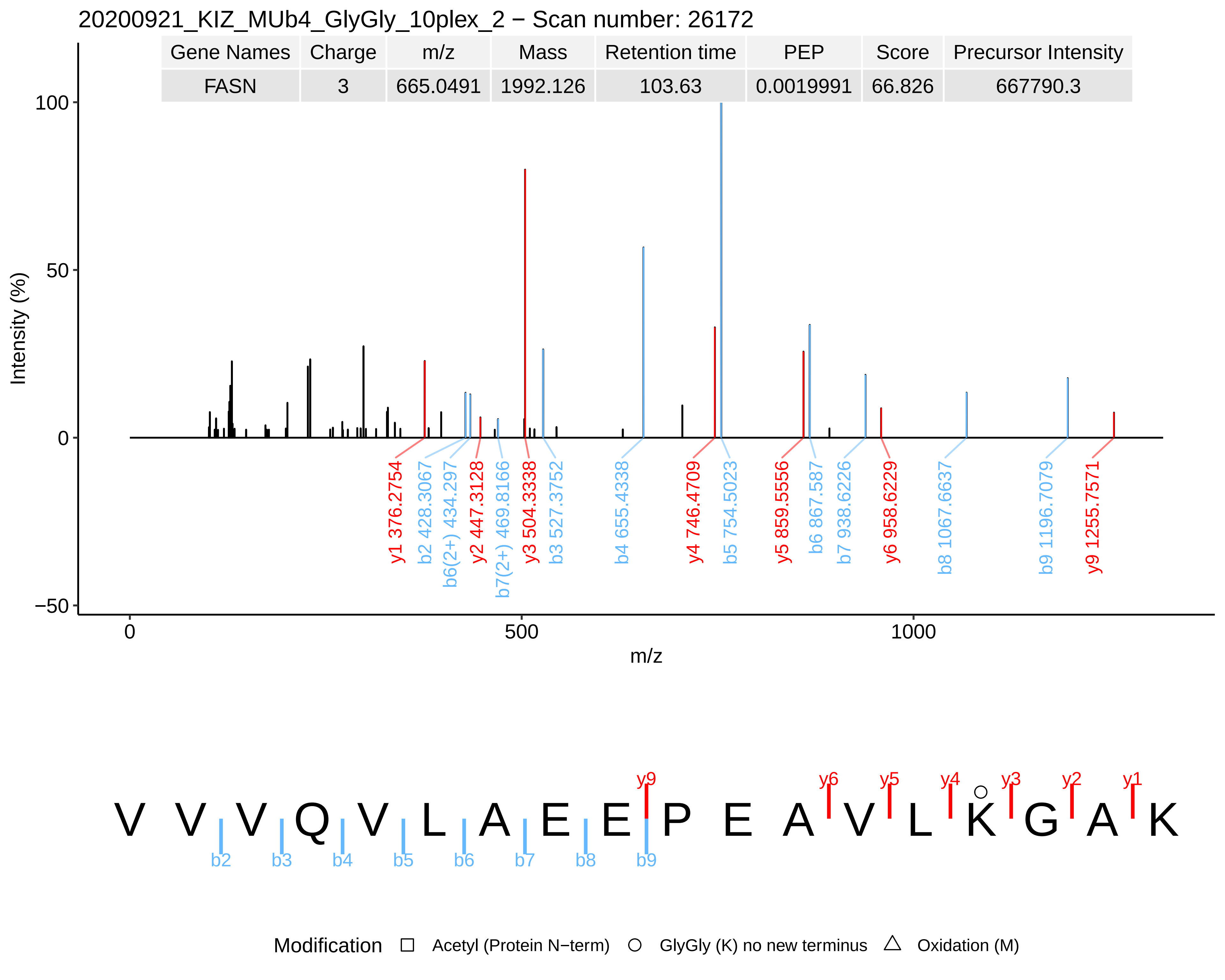
20200921_KIZ_MUb4_GlyGly_10plex_2 − Scan number: 26172
Gene Names | Charge | m/z | Mass | Retention time | PEP | Score | Precursor Intensity
FASN | 3 | 665.0491 | 1992.126 | 103.63 | 0.0019991 | 66.826 | 667790.3
Intensity (%)
m/z
y1 376.2754
b2 428.3067
b6(2+) 434.297
y2 447.3128
b7(2+) 469.8166
y3 504.3338
b3 527.3752
b4 655.4338
y4 746.4709
b5 754.5023
y5 859.5556
b6 867.587
b7 938.6226
y6 958.6229
b8 1067.6637
b9 1196.7079
y9 1255.7571
V V V Q V L A E E P E A V L K G A K
y9 y6 y5 y4 y3 y2 y1
b2 b3 b4 b5 b6 b7 b8 b9
Modification
Acetyl (Protein N−term)
GlyGly (K) no new terminus
Oxidation (M)

20200921_KIZ_MUb4_GlyGly_10plex_2 − Scan number: 26190

Gene Names	Charge	m/z	Mass	Retention time	PEP	Score	Precursor Intensity
FASN	4	499.0387	1992.126	103.71	0.035123	51.695	437482.8

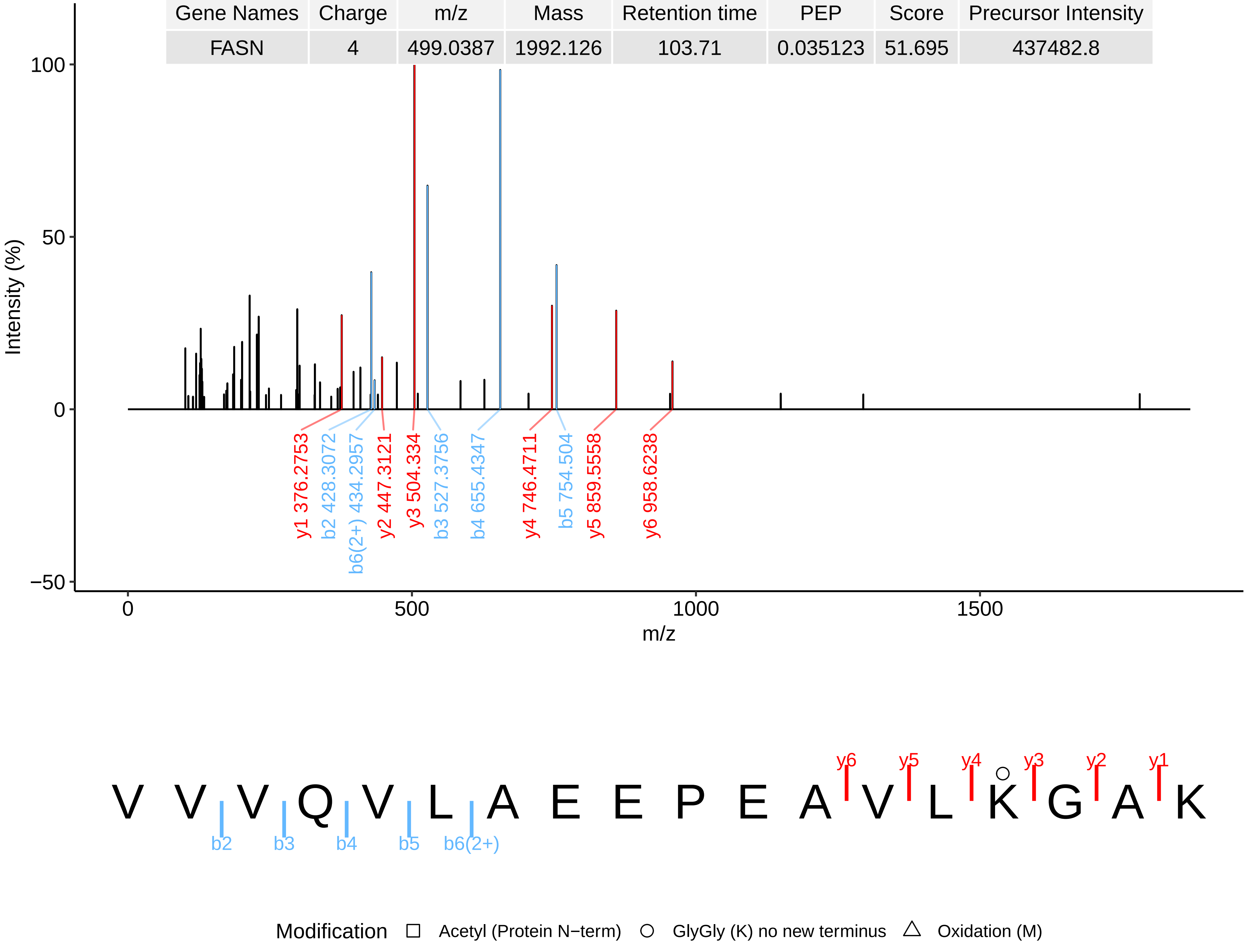

20200924_KIZ_MUb5_GlyGly_1 − Scan number: 29349

Gene Names	Charge	m/z	Mass	Retention time	PEP	Score	Precursor Intensity
FASN	3	665.0491	1992.126	93.666	3.3408e−17	134.44	16200359

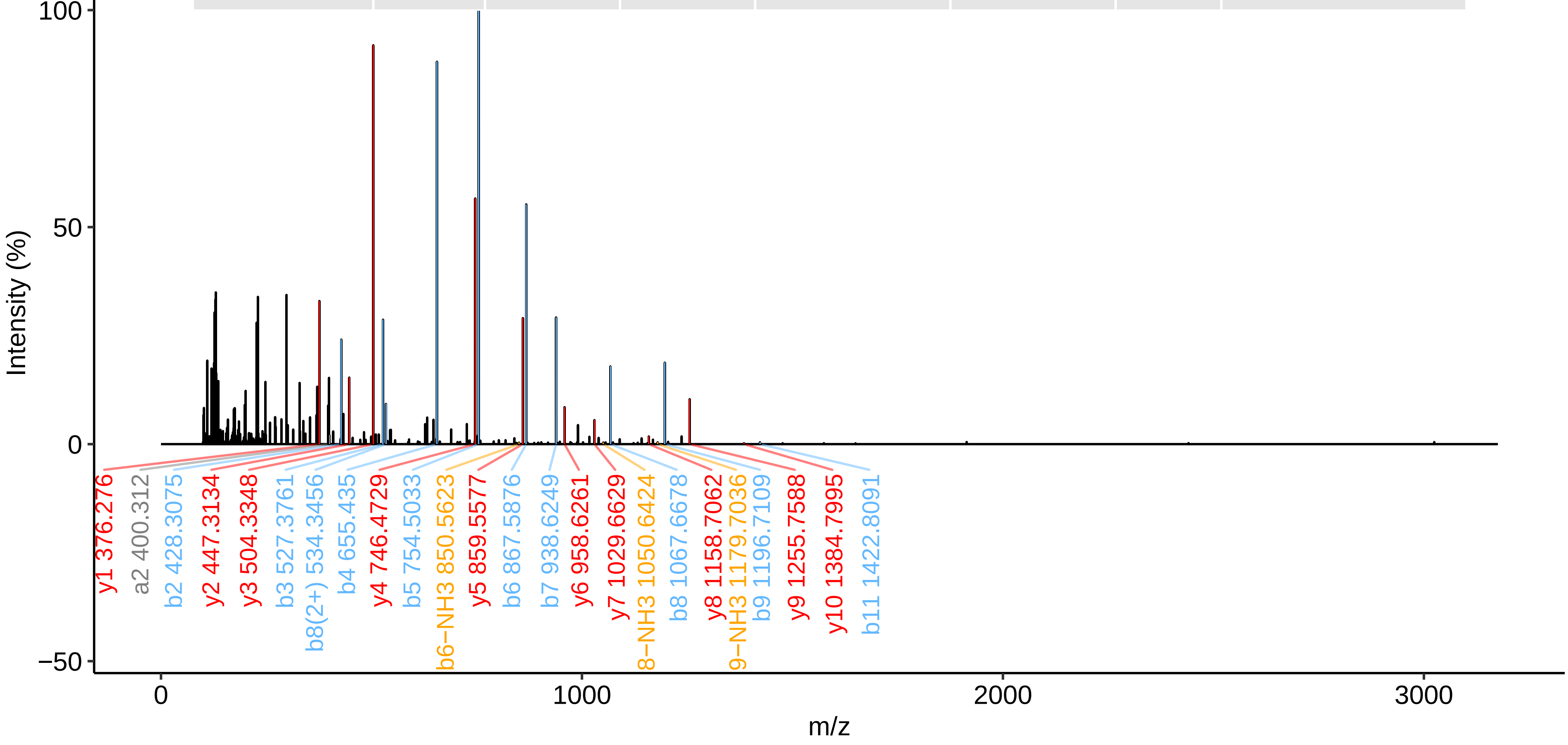

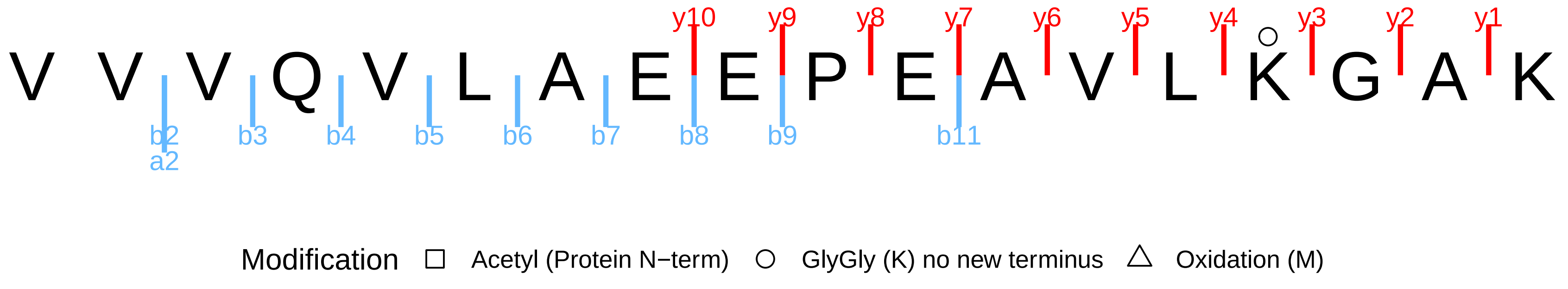

20200921_KIZ_MUb4_GlyGly_10plex − Scan number: 23293

Gene Names	Charge	m/z	Mass	Retention time	PEP	Score	Precursor Intensity
CPT1A	4	500.0095	1996.009	84.541	6.3434e−06	112.02	6222661

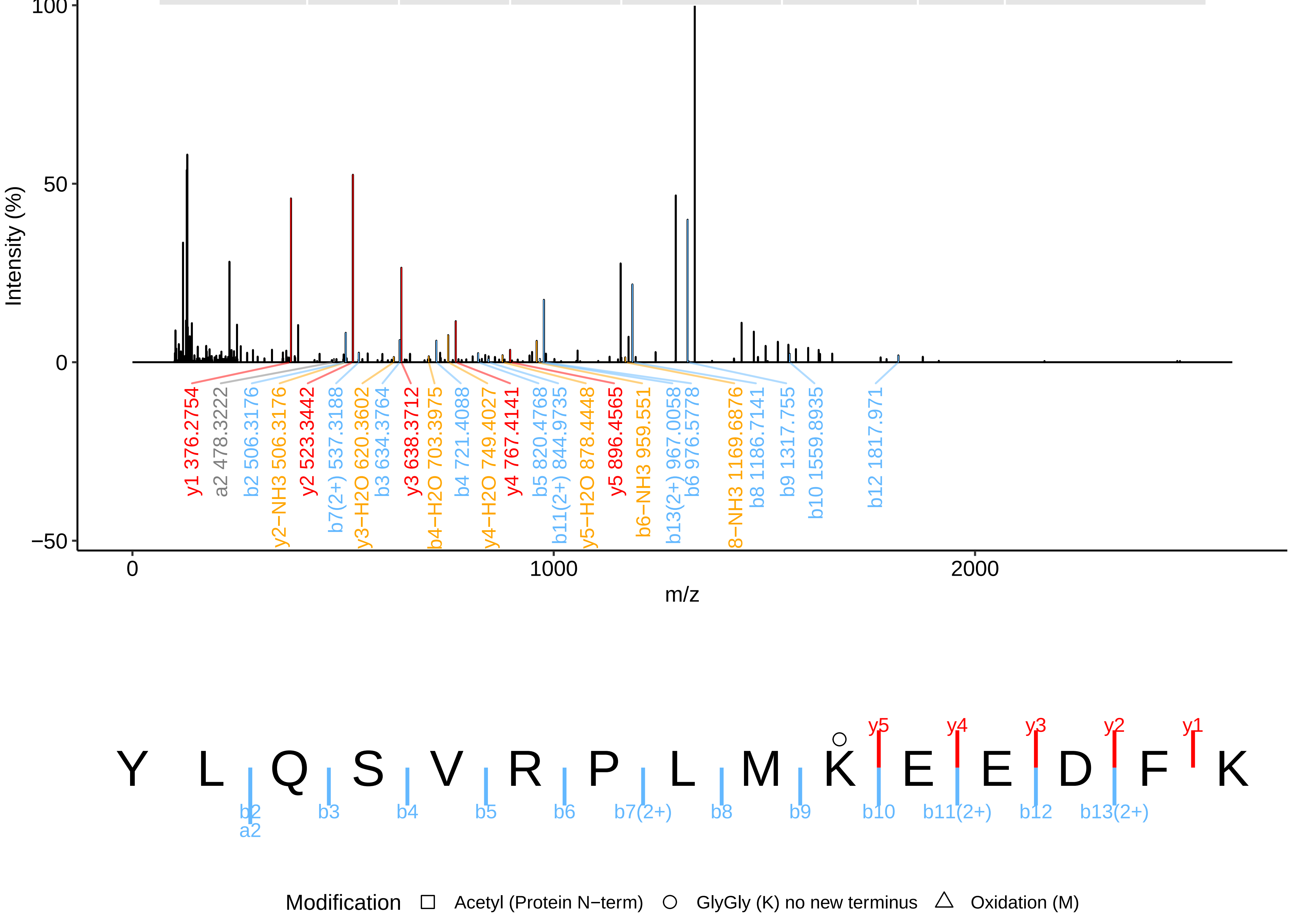

20201118_KIZ_MUb6_GlyGly_1 − Scan number: 19782

Gene Names	Charge	m/z	Mass	Retention time	PEP	Score	Precursor Intensity
CPT1A	4	500.0095	1996.009	61.985	1.6766e−05	106.42	45051040

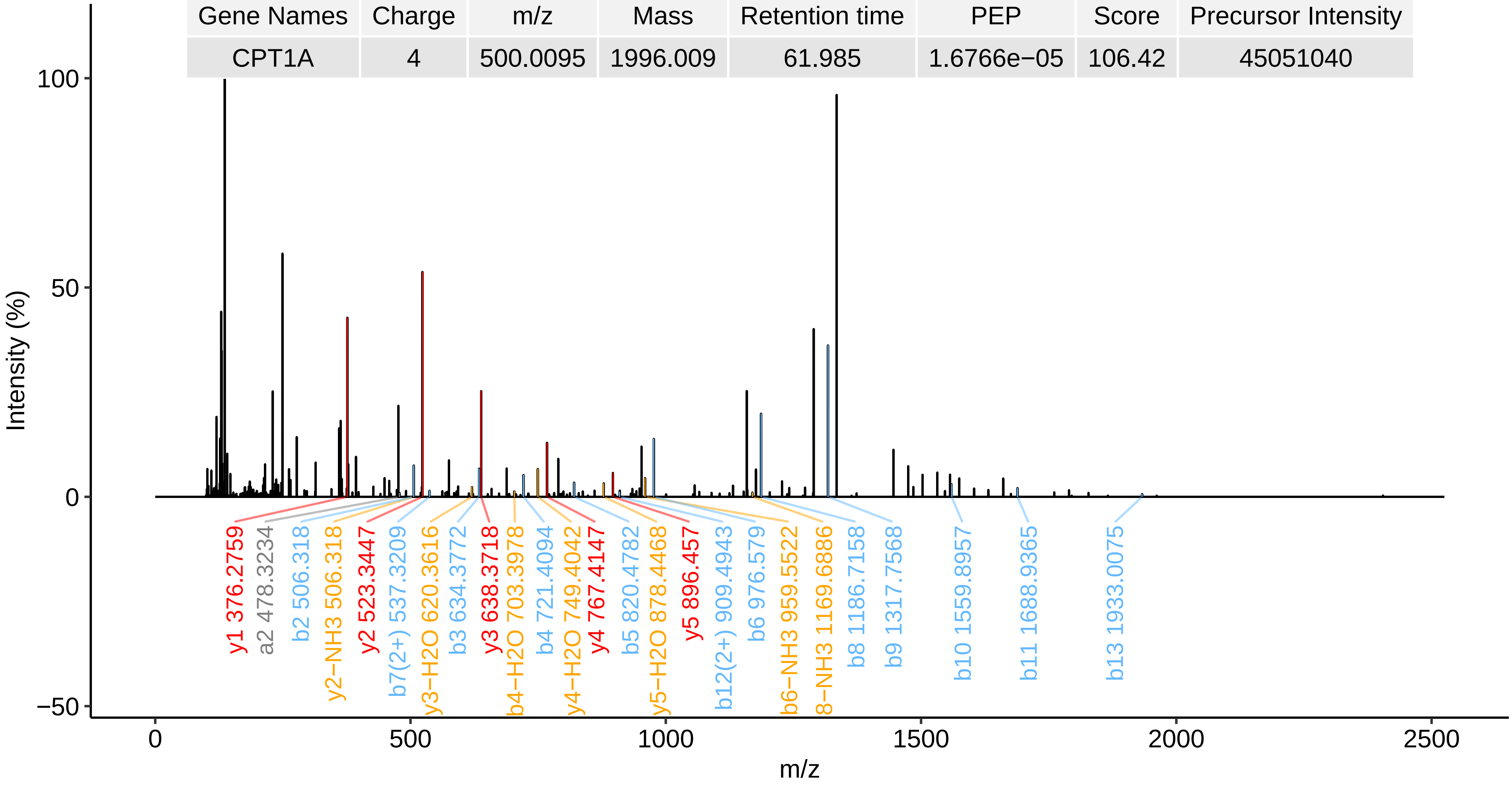

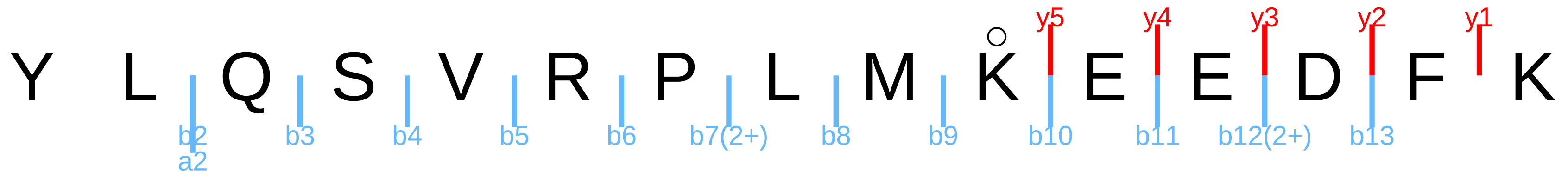

Modification □ Acetyl (Protein N−term) ○ GlyGly (K) no new terminus △ Oxidation (M)

20201118_KIZ_MUb6_GlyGly_2 − Scan number: 22131

Gene Names	Charge	m/z	Mass	Retention time	PEP	Score	Precursor Intensity
CPT1A	4	500.0095	1996.009	74.404	3.534e−09	130.98	18214826

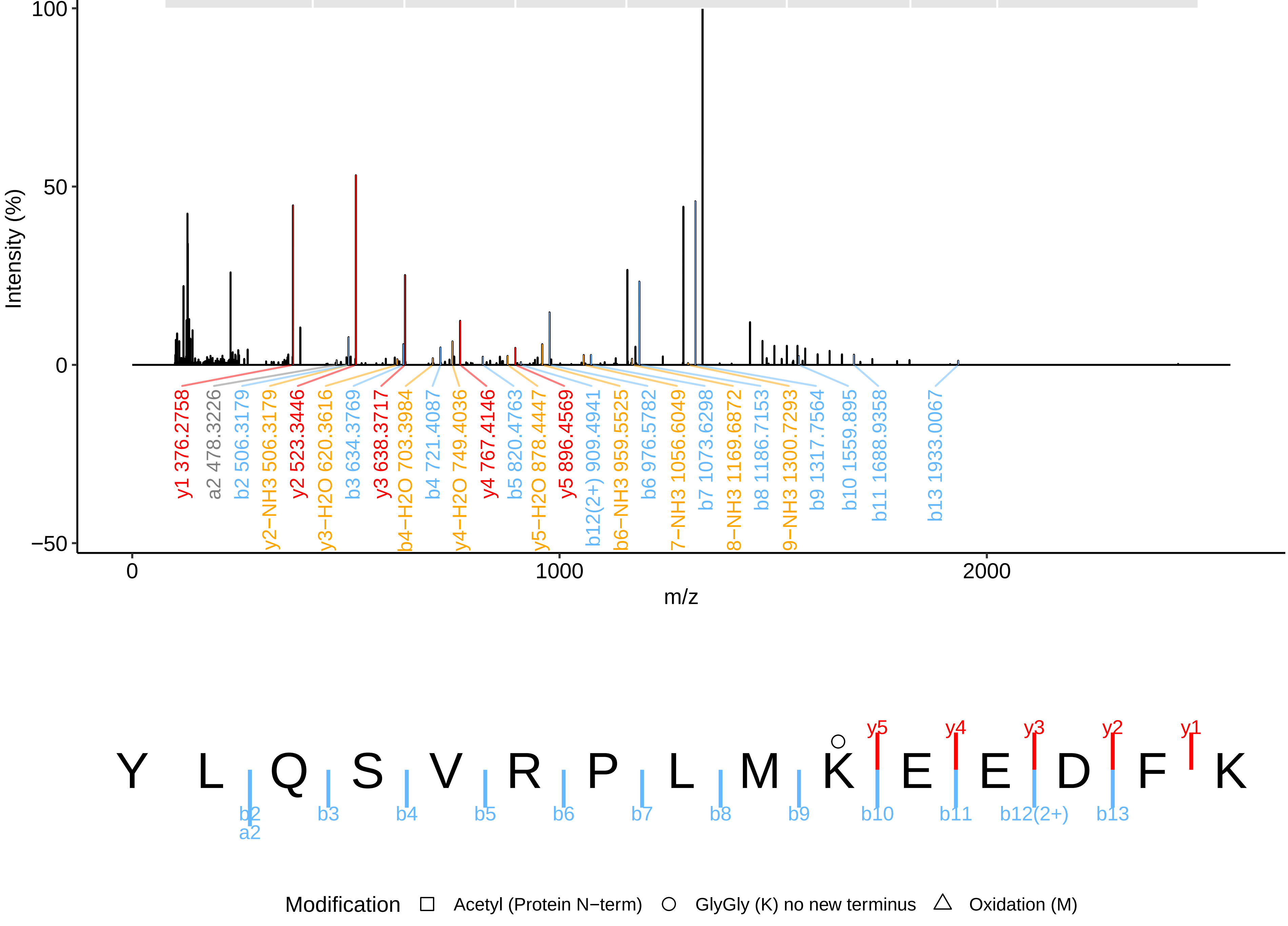

20200921_KIZ_MUb4_GlyGly_10plex_2 − Scan number: 11472

Gene Names	Charge	m/z	Mass	Retention time	PEP	Score	Precursor Intensity
S100P	3	633.9813	1898.922	44.167	7.379e−12	121.79	1523233

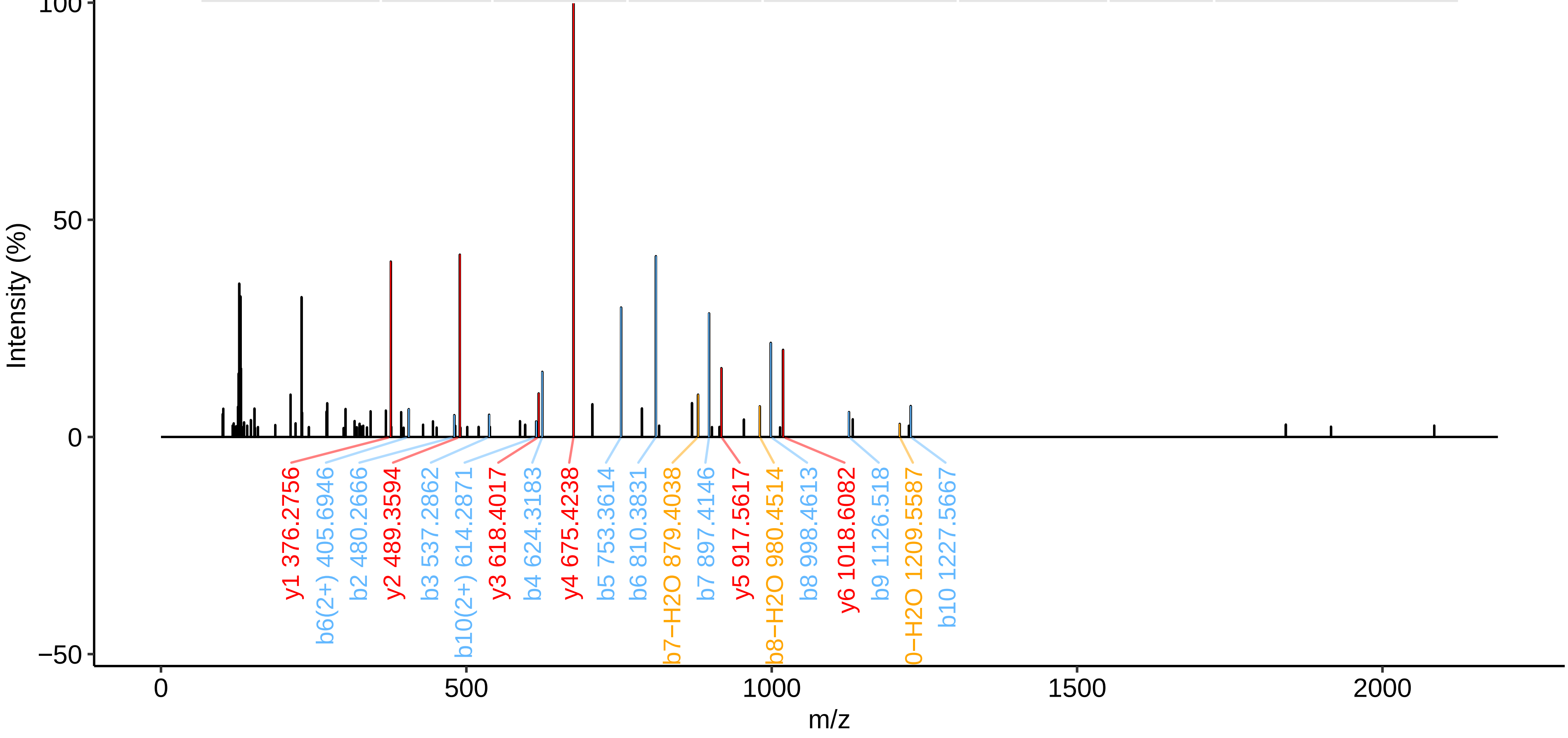

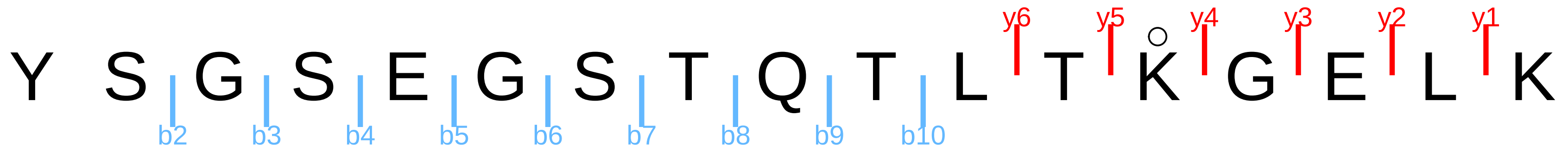

Modification □ Acetyl (Protein N−term) ○ GlyGly (K) no new terminus △ Oxidation (M)

20200921_KIZ_MUb4_GlyGly_10plex_2 − Scan number: 13128

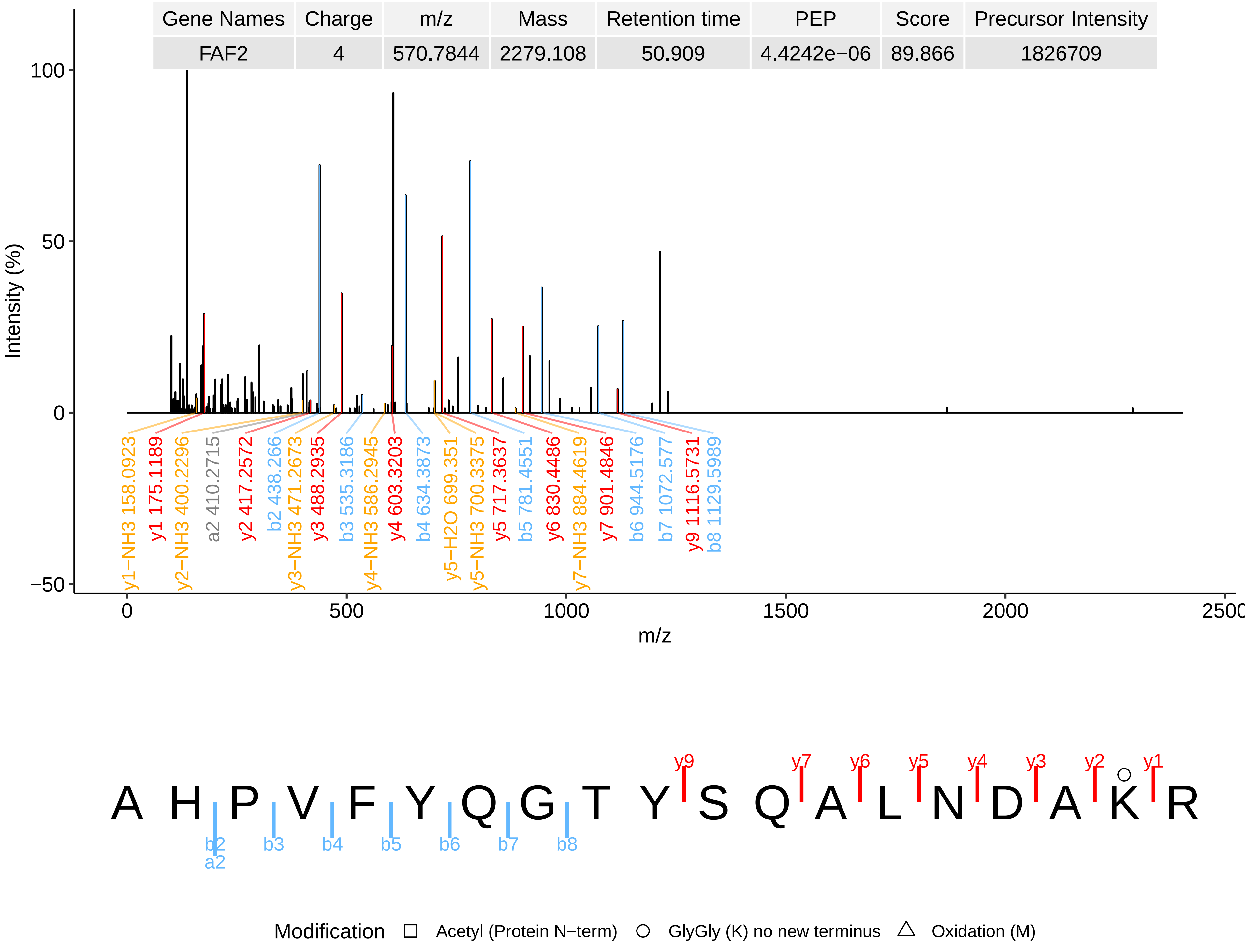

20201118_KIZ_MUb6_GlyGly_1 − Scan number: 13247

Gene Names	Charge	m/z	Mass	Retention time	PEP	Score	Precursor Intensity
FAF2	4	570.7844	2279.108	41.922	2.33e−21	137.17	8672887

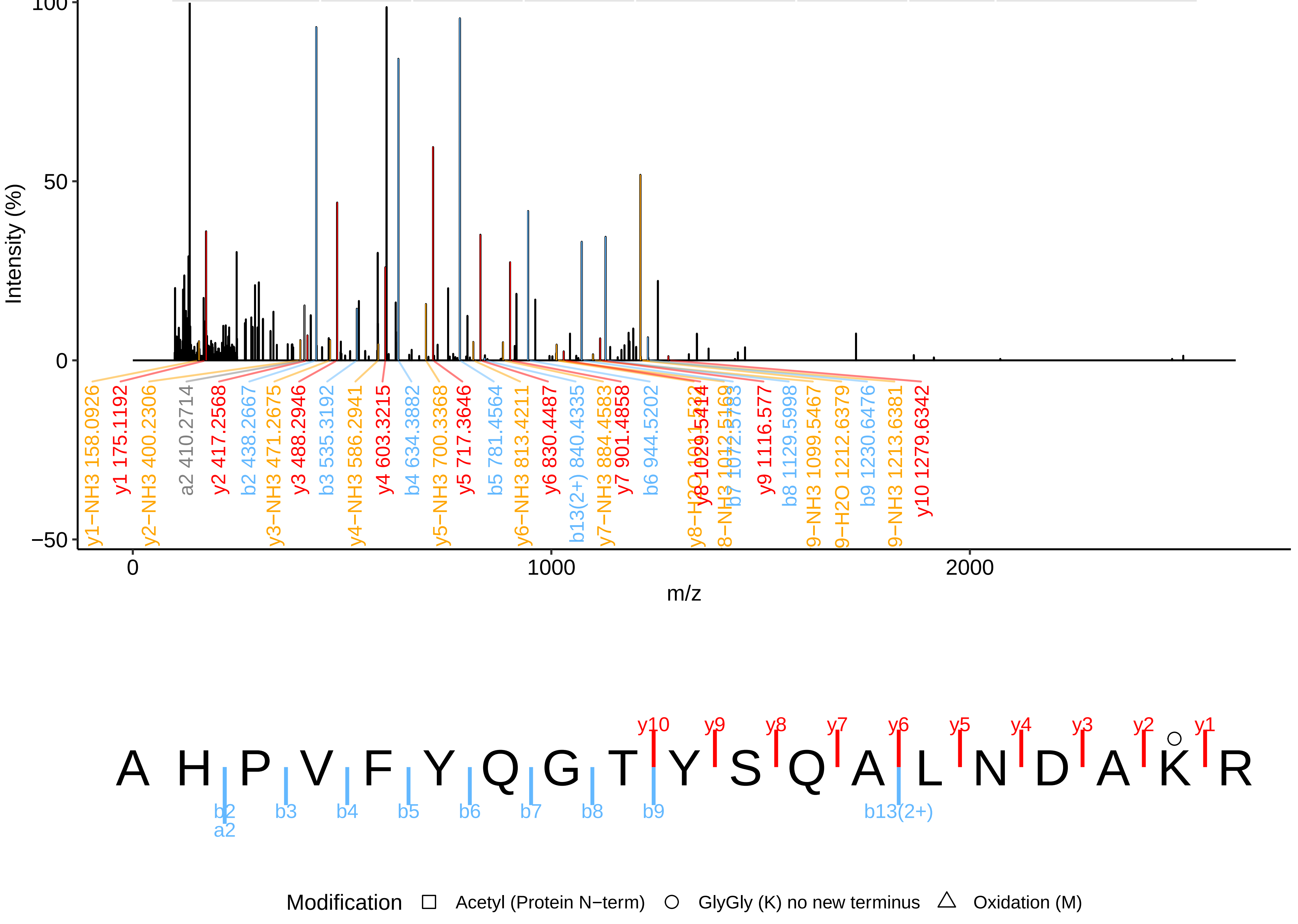

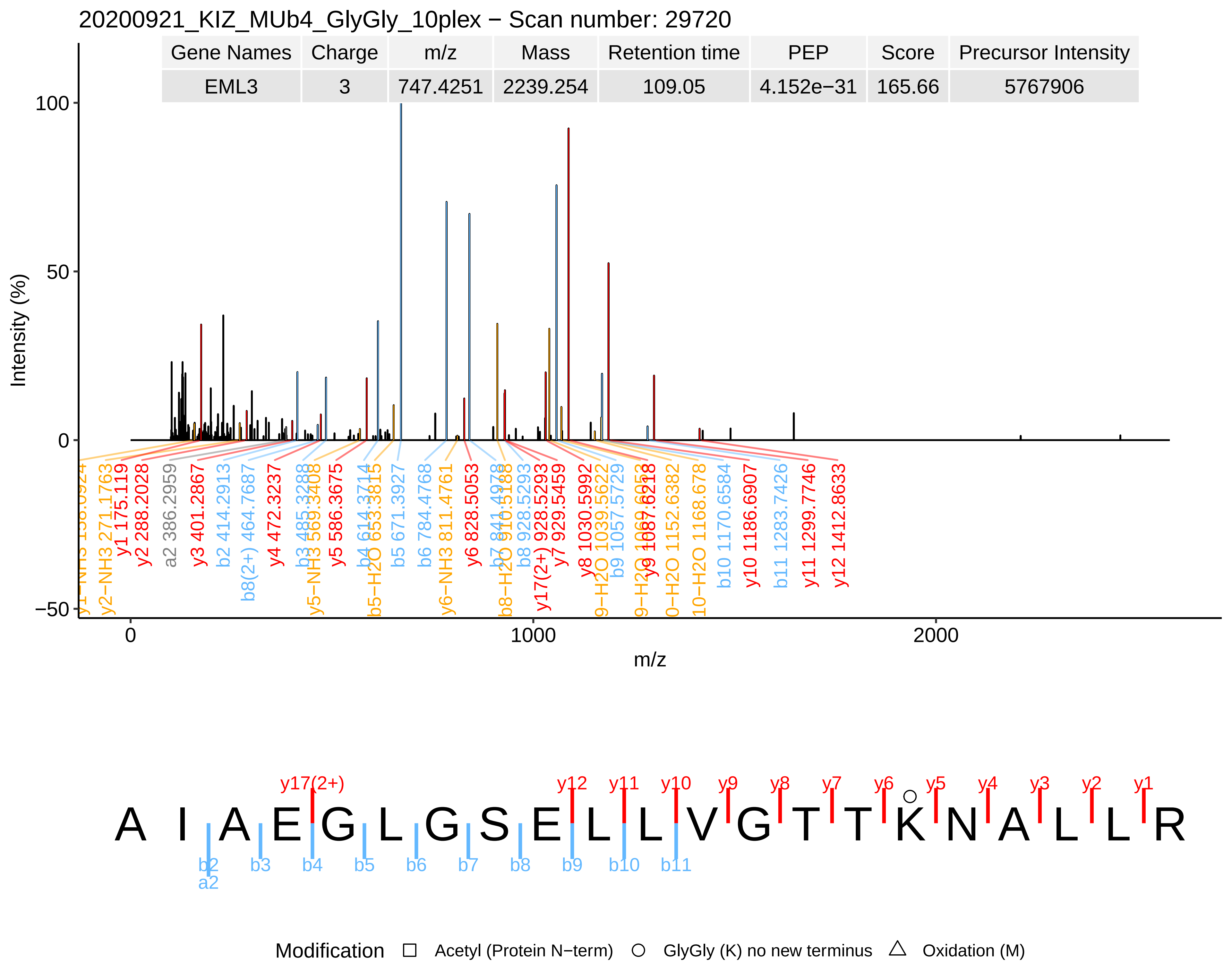

20200921_KIZ_MUb4_GlyGly_10plex − Scan number: 29720
Gene Names | Charge | m/z | Mass | Retention time | PEP | Score | Precursor Intensity
EML3 | 3 | 747.4251 | 2239.254 | 109.05 | 4.152e−31 | 165.66 | 5767906
Intensity (%)
m/z
A I A E G L G S E L L V G T T K N A L L R
Modification
Acetyl (Protein N−term)
GlyGly (K) no new terminus
Oxidation (M)

20200921_KIZ_MUb4_GlyGly_10plex_2 − Scan number: 26319

Gene Names	Charge	m/z	Mass	Retention time	PEP	Score	Precursor Intensity
EML3	3	747.4251	2239.254	104.24	4.7633e−07	97.284	808981.3

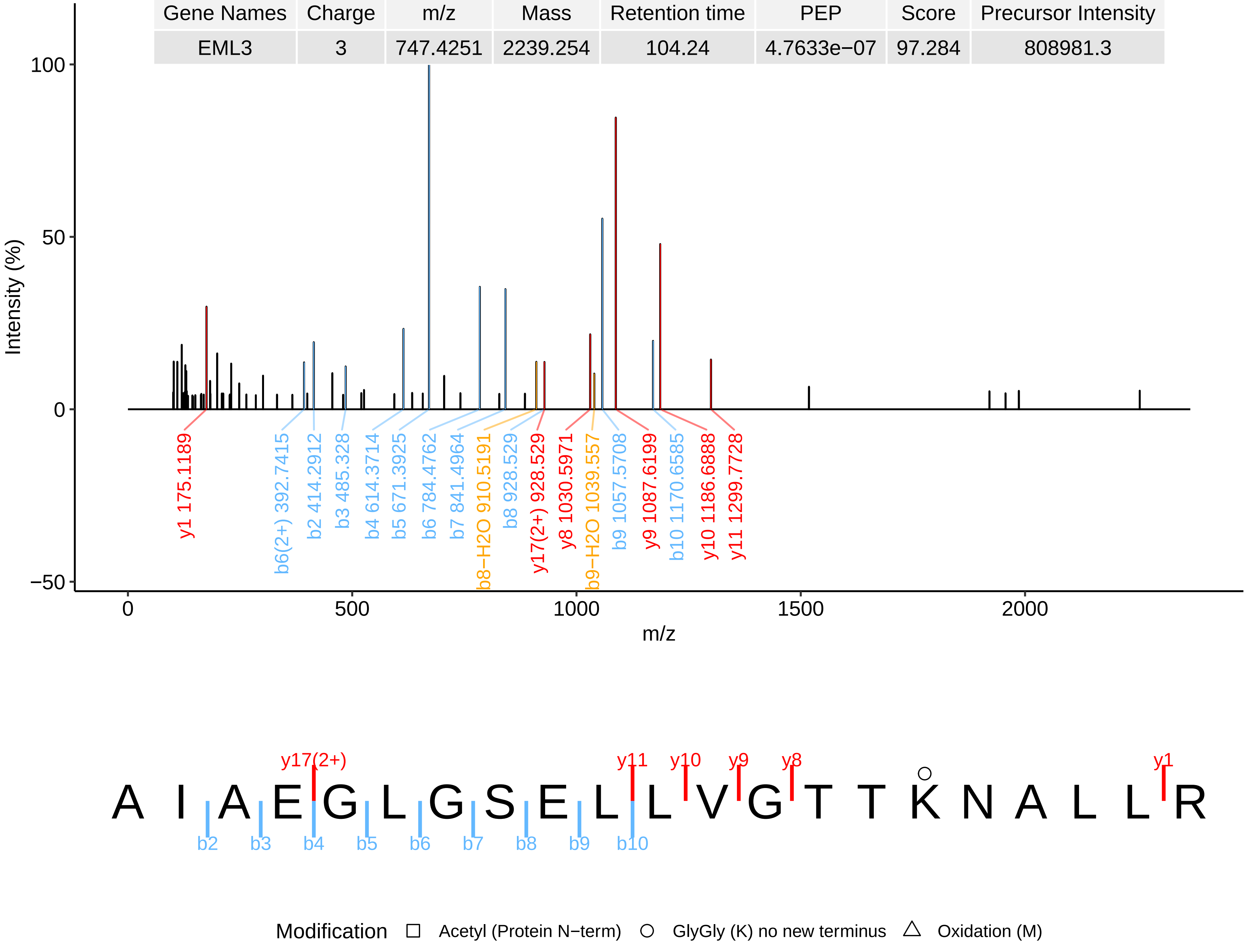

20200924_KIZ_MUb5_GlyGly_2 − Scan number: 16712

Gene Names	Charge	m/z	Mass	Retention time	PEP	Score	Precursor Intensity
VCP	3	687.6868	2060.039	62.002	1.0794e−21	40.515	2691080

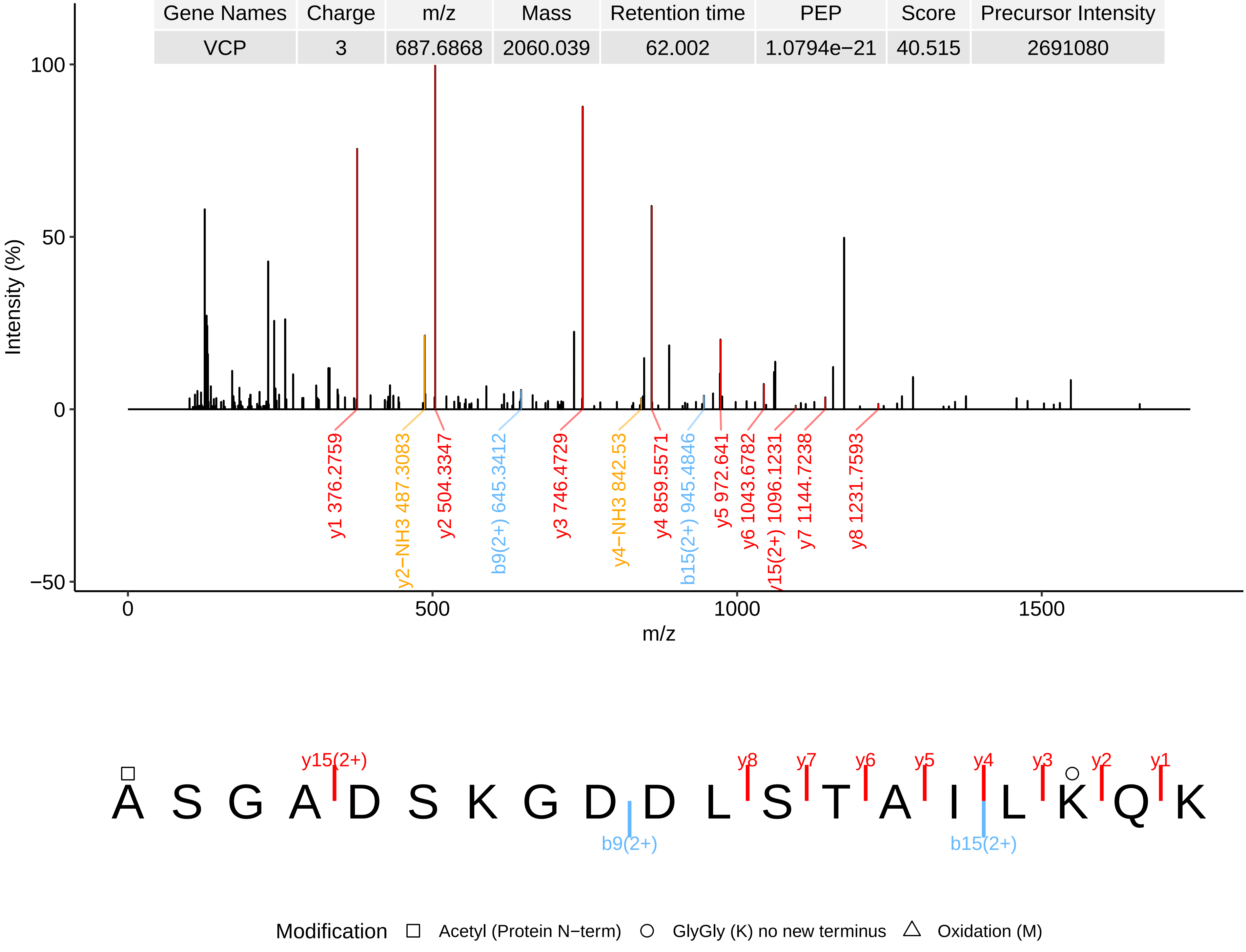

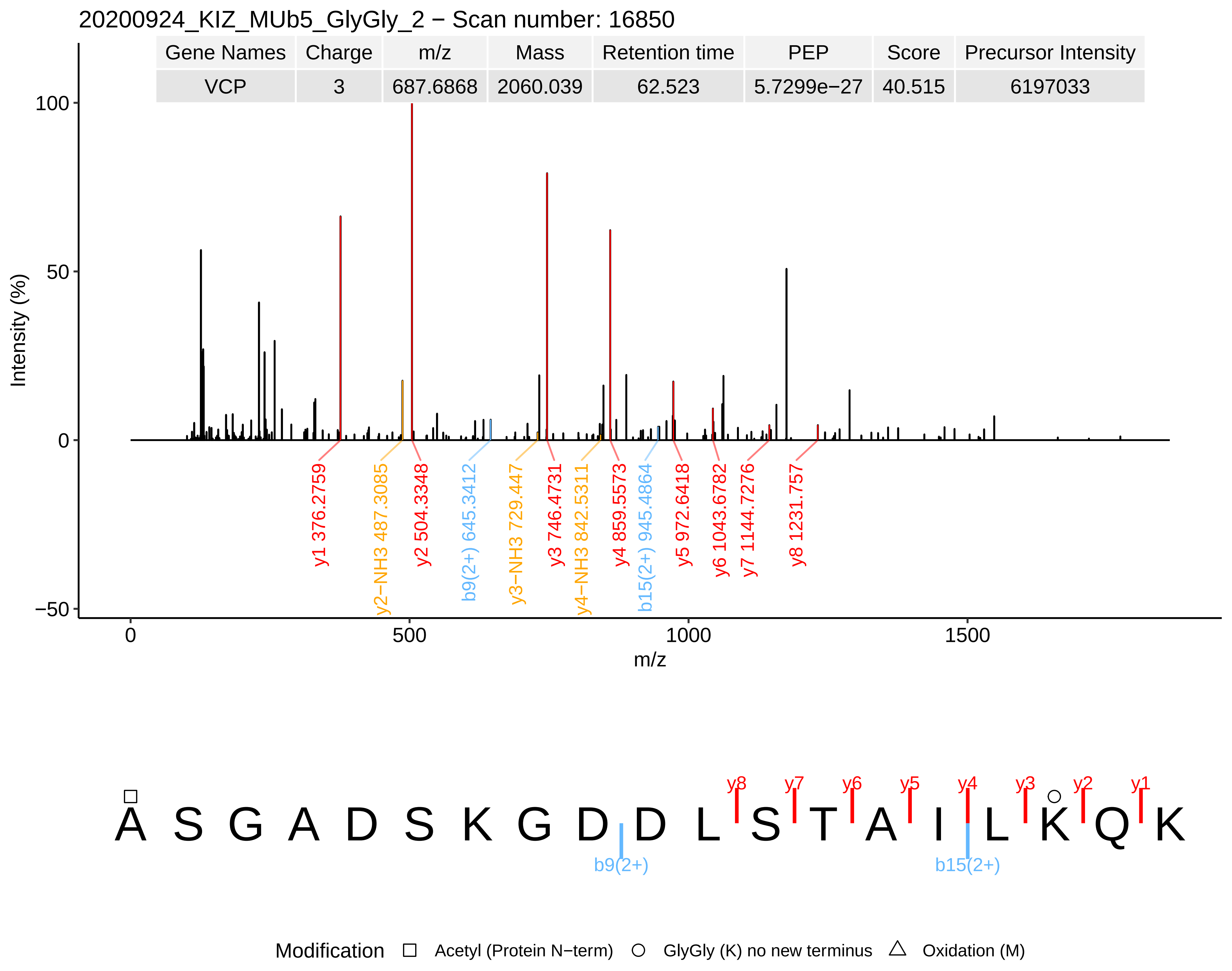
20200924_KIZ_MUb5_GlyGly_2 − Scan number: 16850
Gene Names | Charge | m/z | Mass | Retention time | PEP | Score | Precursor Intensity
VCP | 3 | 687.6868 | 2060.039 | 62.523 | 5.7299e−27 | 40.515 | 6197033
Intensity (%)
100
50
0
−50
0
500
1000
1500
m/z
y1 376.2759
y2−NH3 487.3085
y2 504.3348
b9(2+) 645.3412
y3−NH3 729.447
y3 746.4731
y4−NH3 842.5311
y4 859.5573
b15(2+) 945.4864
y5 972.6418
y6 1043.6782
y7 1144.7276
y8 1231.757
A S G A D S K G D D L S T A I L K Q K
y8 y7 y6 y5 y4 y3 y2 y1
b9(2+)
b15(2+)
Modification
Acetyl (Protein N−term)
GlyGly (K) no new terminus
Oxidation (M)

20200921_KIZ_MUb4_GlyGly_10plex_2 − Scan number: 11410

Gene Names	Charge	m/z	Mass	Retention time	PEP	Score	Precursor Intensity
STAM	4	446.0034	1779.984	43.913	0.011406	68.919	895737.8

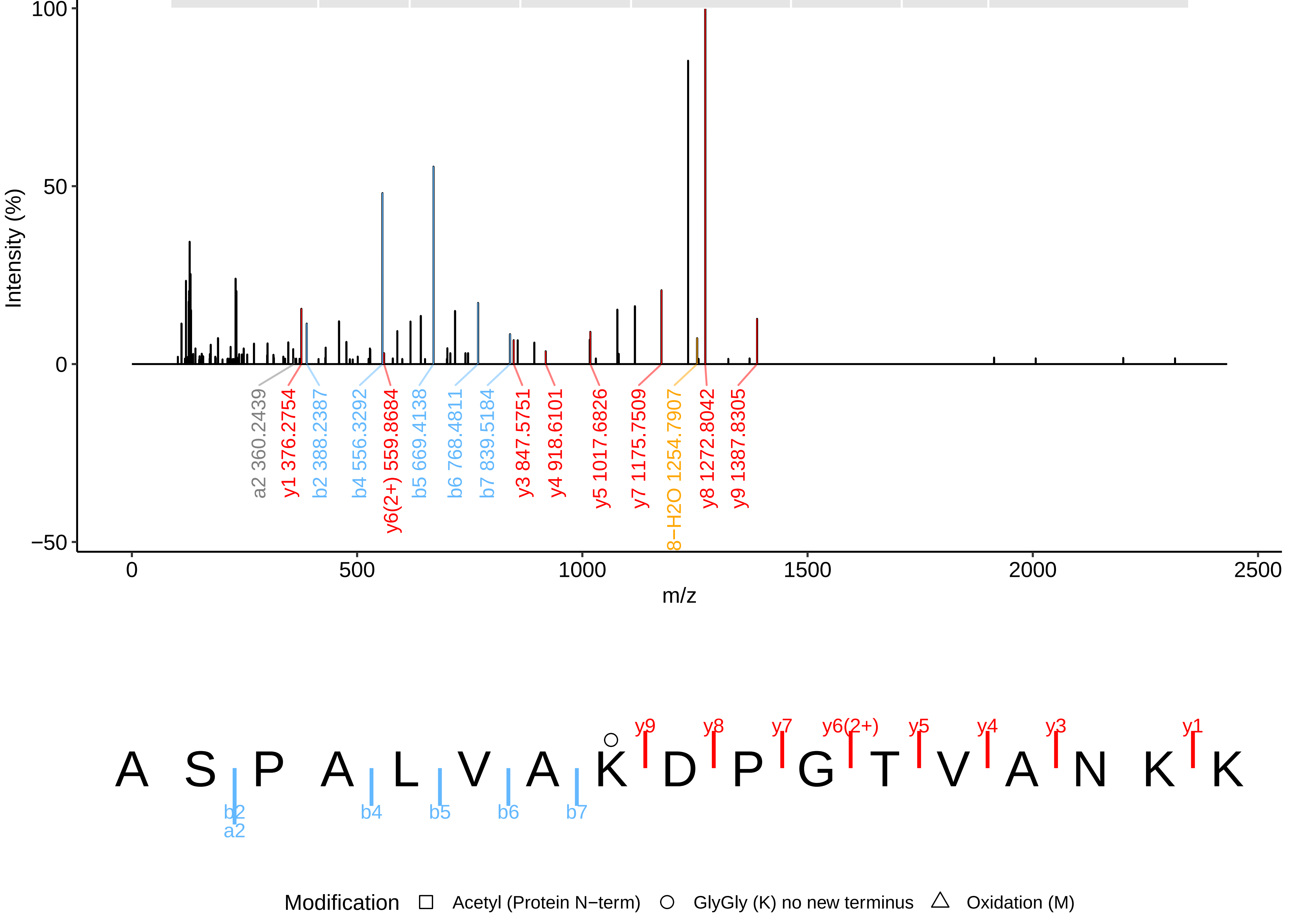

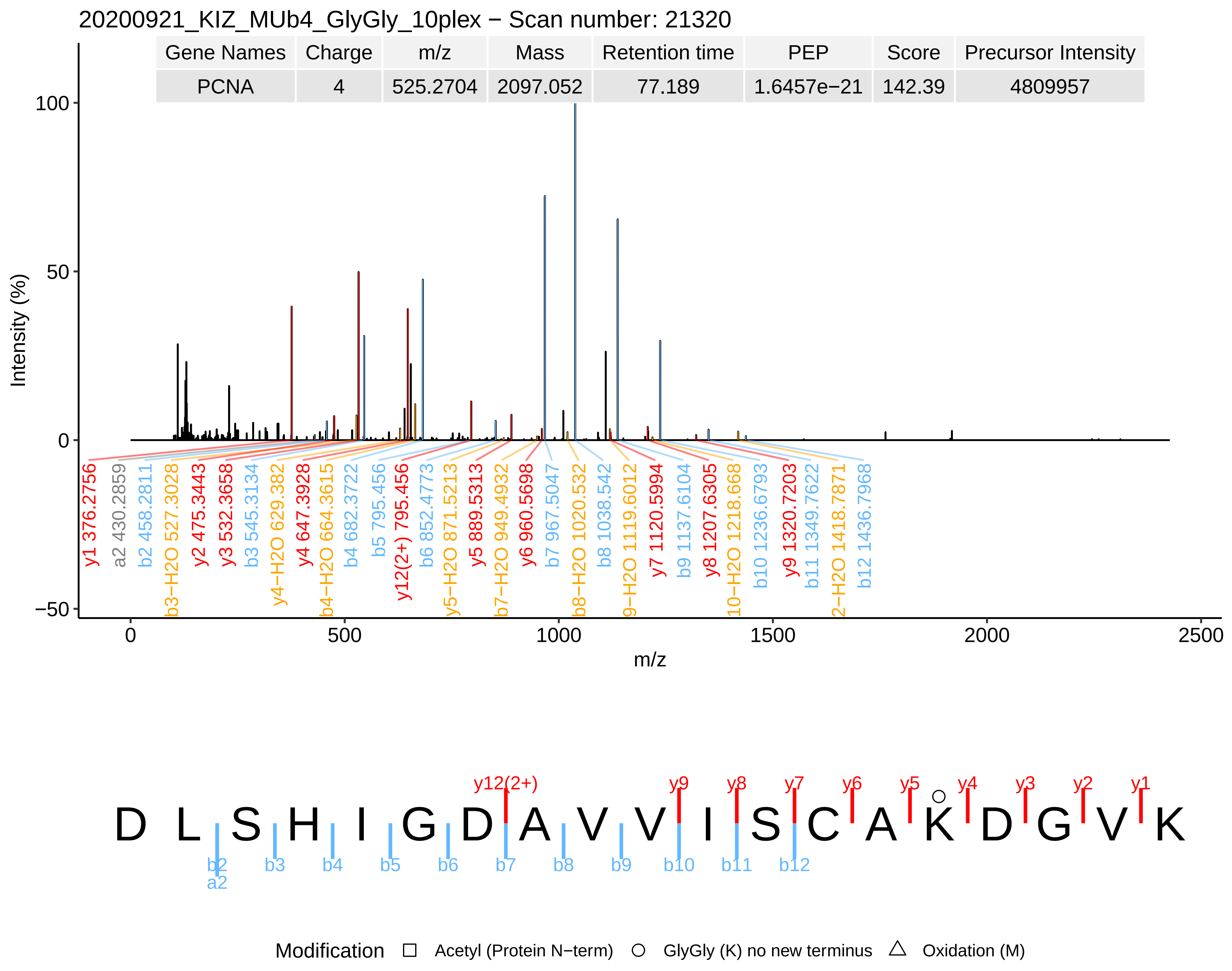
20200921_KIZ_MUb4_GlyGly_10plex − Scan number: 21320
Gene Names | Charge | m/z | Mass | Retention time | PEP | Score | Precursor Intensity
PCNA | 4 | 525.2704 | 2097.052 | 77.189 | 1.6457e−21 | 142.39 | 4809957
Intensity (%)
100
50
0
−50
m/z
0
500
1000
1500
2000
2500
y1 376.2756
a2 430.2859
b2 458.2811
b3−H2O 527.3028
y2 475.3443
y3 532.3658
b3 545.3134
y4−H2O 629.382
y4 647.3928
b4−H2O 664.3615
b4 682.3722
b5 795.456
y12(2+) 795.456
b6 852.4773
y5−H2O 871.5213
y5 889.5313
b7−H2O 949.4932
y6 960.5698
b7 967.5047
b8−H2O 1020.532
b8 1038.542
9−H2O 1119.6012
y7 1120.5994
b9 1137.6104
y8 1207.6305
10−H2O 1218.668
b10 1236.6793
y9 1320.7203
b11 1349.7622
2−H2O 1418.7871
b12 1436.7968
y12(2+) y9 y8 y7 y6 y5 y4 y3 y2 y1
D L S H I G D A V V I S C A K D G V K
b2 b3 b4 b5 b6 b7 b8 b9 b10 b11 b12
a2
Modification
Acetyl (Protein N−term)
GlyGly (K) no new terminus
Oxidation (M)

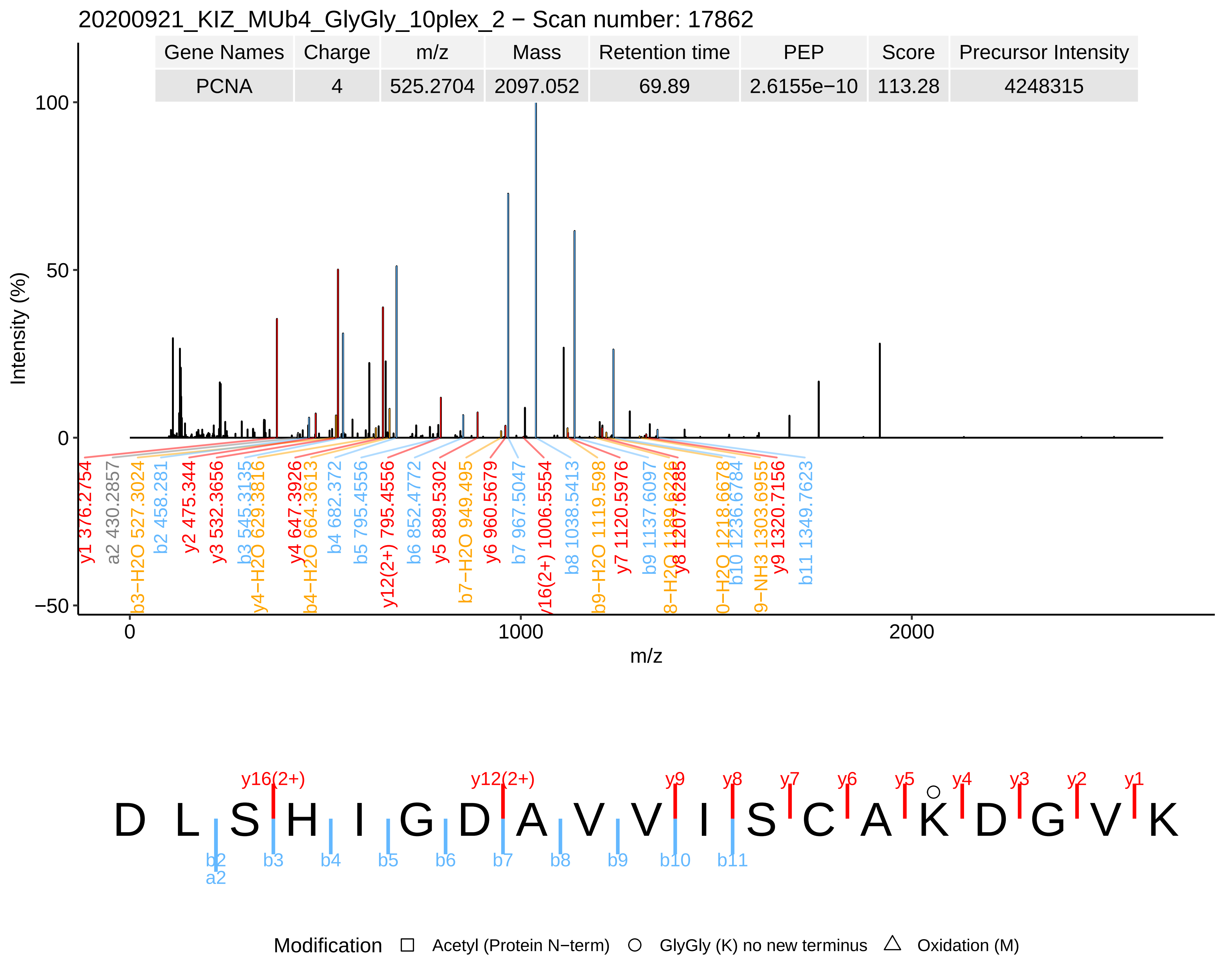

20200921_KIZ_MUb4_GlyGly_10plex_2 − Scan number: 17862
Gene Names | Charge | m/z | Mass | Retention time | PEP | Score | Precursor Intensity
PCNA | 4 | 525.2704 | 2097.052 | 69.89 | 2.6155e−10 | 113.28 | 4248315
Intensity (%)
100
50
0
−50
m/z
0
1000
2000
y1 376.2754
a2 430.2857
b3−H2O 527.3024
b2 458.281
y2 475.344
y3 532.3656
b3 545.3135
y4−H2O 629.3816
y4 647.3926
b4−H2O 664.3613
b4 682.372
b5 795.4556
y12(2+) 795.4556
b6 852.4772
y5 889.5302
b7−H2O 949.495
y6 960.5679
b7 967.5047
y16(2+) 1006.5554
b8 1038.5413
b9−H2O 1119.598
y7 1120.5976
b9 1137.6097
y8 1207.6285
b10 1236.6784
y9 1320.7156
b11 1349.7623
D L S H I G D A V V I S C A K D G V K
y16(2+) y12(2+) y9 y8 y7 y6 y5 y4 y3 y2 y1
b2 a2 b3 b4 b5 b6 b7 b8 b9 b10 b11
Modification
Acetyl (Protein N−term)
GlyGly (K) no new terminus
Oxidation (M)

20200921_KIZ_MUb4_GlyGly_10plex_2 − Scan number: 17867

Gene Names	Charge	m/z	Mass	Retention time	PEP	Score	Precursor Intensity
PCNA	3	700.0248	2097.052	69.91	1.0235e−05	84.829	775021.5

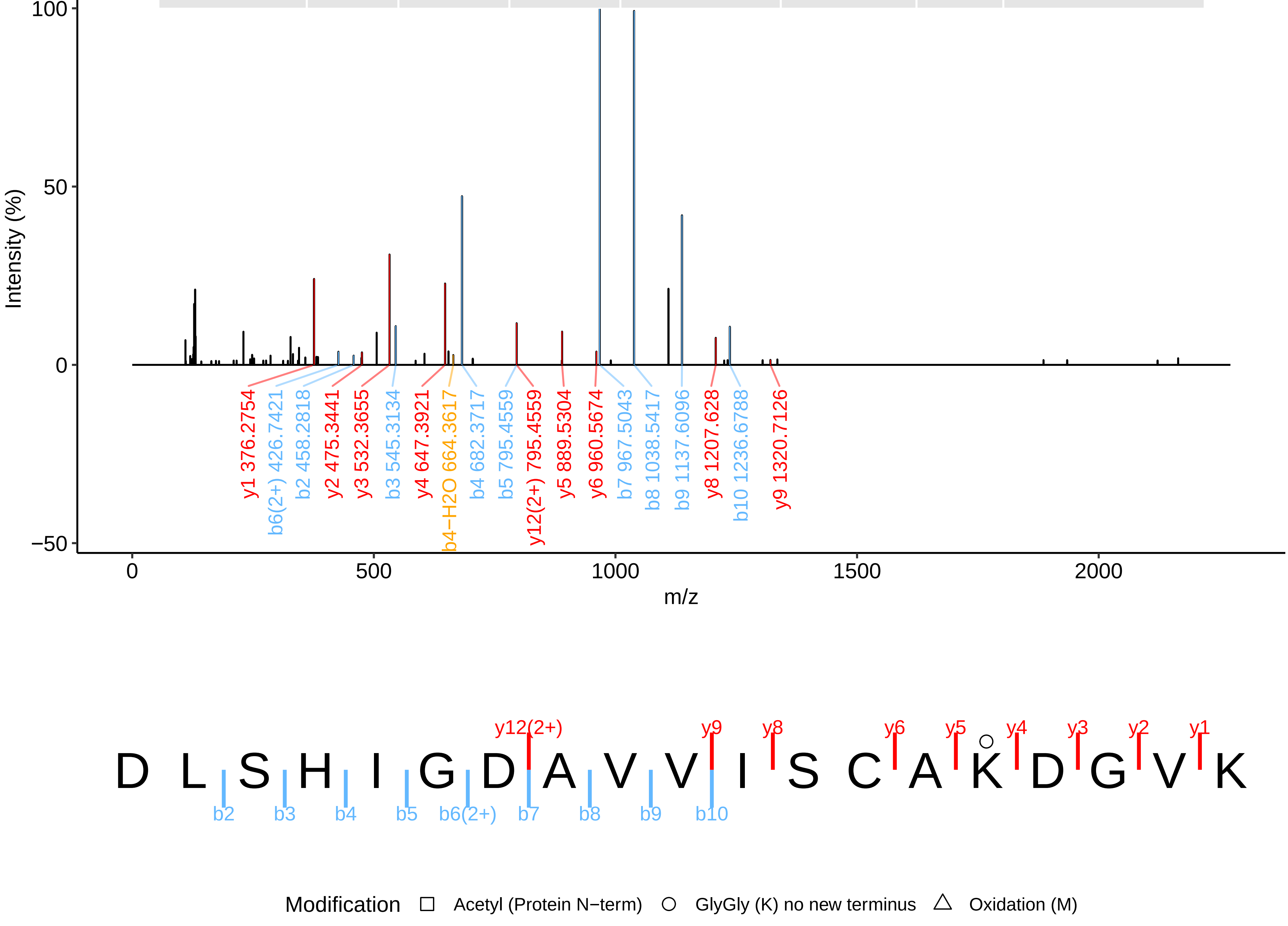

20200921_KIZ_MUb4_GlyGly_10plex_2 − Scan number: 17888

Gene Names	Charge	m/z	Mass	Retention time	PEP	Score	Precursor Intensity
PCNA	3	700.0248	2097.052	69.997	1.1751e−05	82.877	1102518

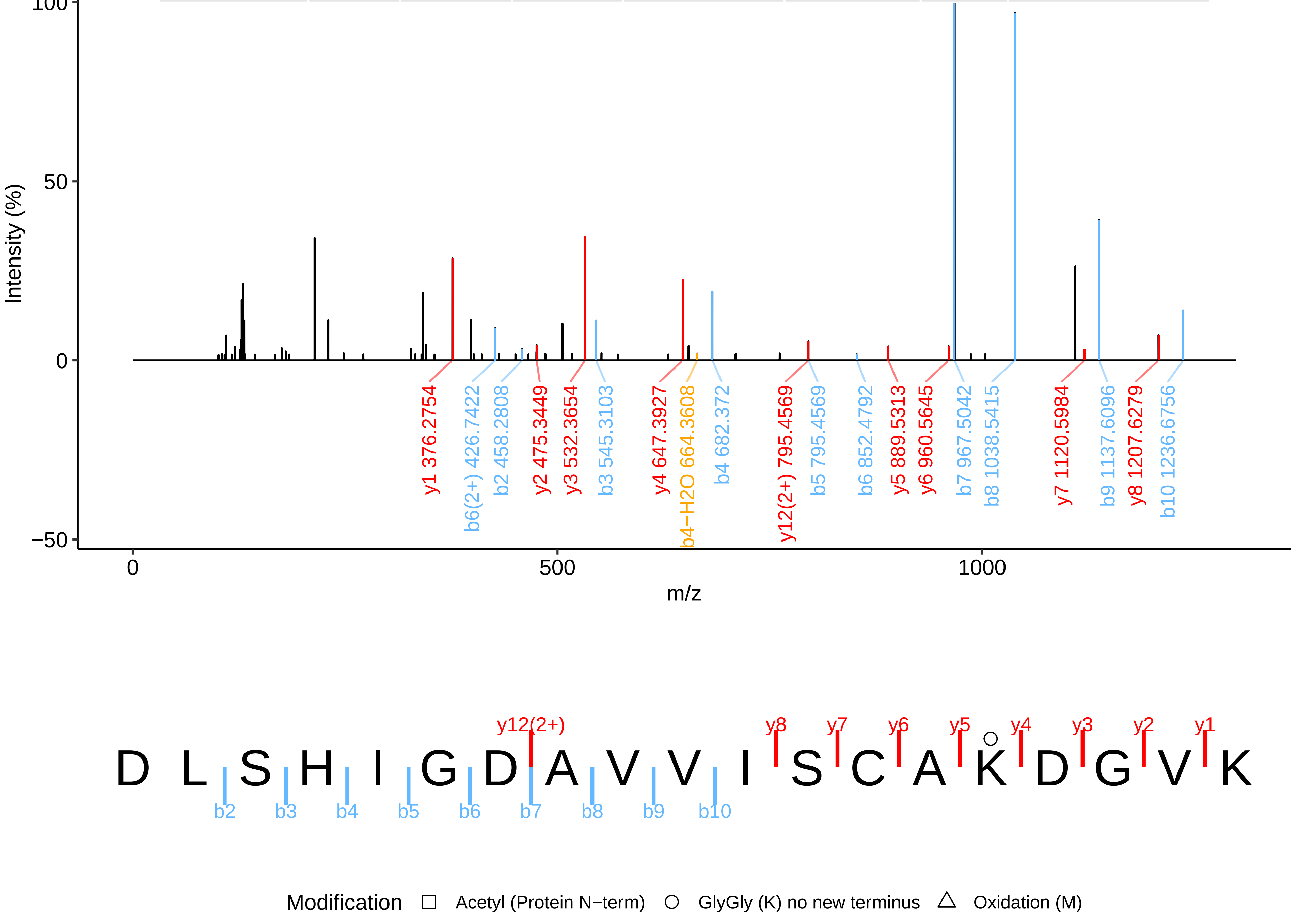

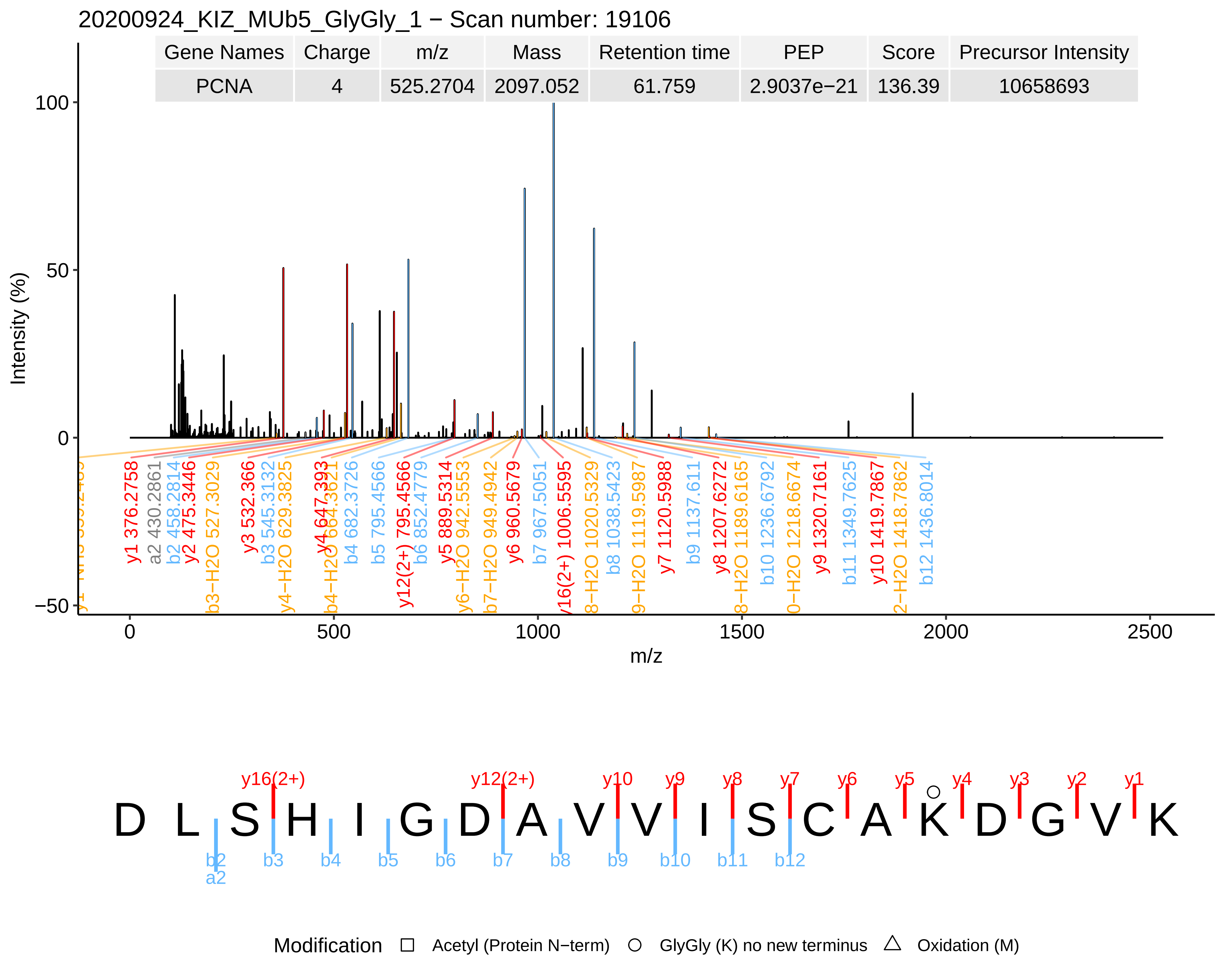
20200924_KIZ_MUb5_GlyGly_1 − Scan number: 19106
Gene Names | Charge | m/z | Mass | Retention time | PEP | Score | Precursor Intensity
PCNA | 4 | 525.2704 | 2097.052 | 61.759 | 2.9037e−21 | 136.39 | 10658693
Intensity (%)
m/z
y1 376.2758
a2 430.2861
b2 458.2814
y2 475.3446
b3−H2O 527.3029
y3 532.366
b3 545.3132
y4−H2O 629.3825
y4 647.393
b4−H2O 664.3621
b4 682.3726
b5 795.4566
y12(2+) 795.4566
b6 852.4779
y5 889.5314
y6−H2O 942.5553
b7−H2O 949.4942
y6 960.5679
b7 967.5051
y16(2+) 1006.5595
b8−H2O 1020.5329
b8 1038.5423
b9−H2O 1119.5987
y7 1120.5988
b9 1137.611
y8 1207.6272
b10 1236.6792
y9 1320.7161
b11 1349.7625
y10 1419.7867
b12 1436.8014
D L S H I G D A V V I S C A K D G V K
Modification Acetyl (Protein N−term) GlyGly (K) no new terminus Oxidation (M)

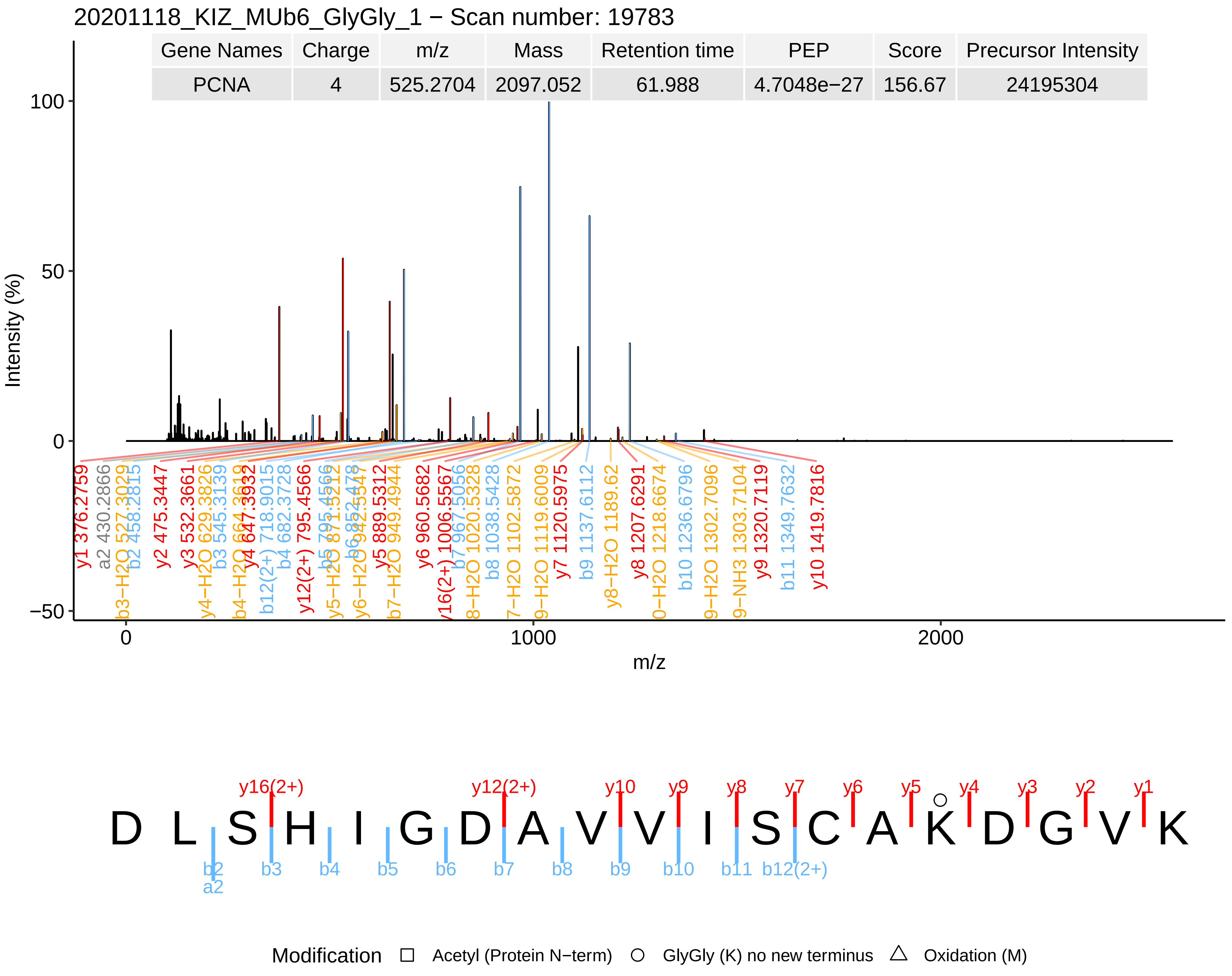
20201118_KIZ_MUb6_GlyGly_1 − Scan number: 19783
Gene Names | Charge | m/z | Mass | Retention time | PEP | Score | Precursor Intensity
PCNA | 4 | 525.2704 | 2097.052 | 61.988 | 4.7048e−27 | 156.67 | 24195304
Intensity (%)
m/z
y1 376.2759
a2 430.2866
b2 458.2815
y2 475.3447
y3 532.3661
b3 545.3139
y4 647.3932
b12(2+) 718.9015
b4 682.3728
y12(2+) 795.4566
b5 795.4566
y5 889.5312
y7−H2O 949.4944
y6 960.5682
b7 967.5056
b8 1038.5428
y7 1120.5975
b9 1137.6112
y8−H2O 1189.62
y8 1207.6291
b10 1236.6796
y9 1320.7119
b11 1349.7632
y10 1419.7816
D L S H I G D A V V I S C A K D G V K
Modification
Acetyl (Protein N−term)
GlyGly (K) no new terminus
Oxidation (M)

20201118_KIZ_MUb6_GlyGly_2 − Scan number: 21383

Gene Names	Charge	m/z	Mass	Retention time	PEP	Score	Precursor Intensity
PCNA	4	525.2704	2097.052	72.027	2.7552e−15	127.32	5294510

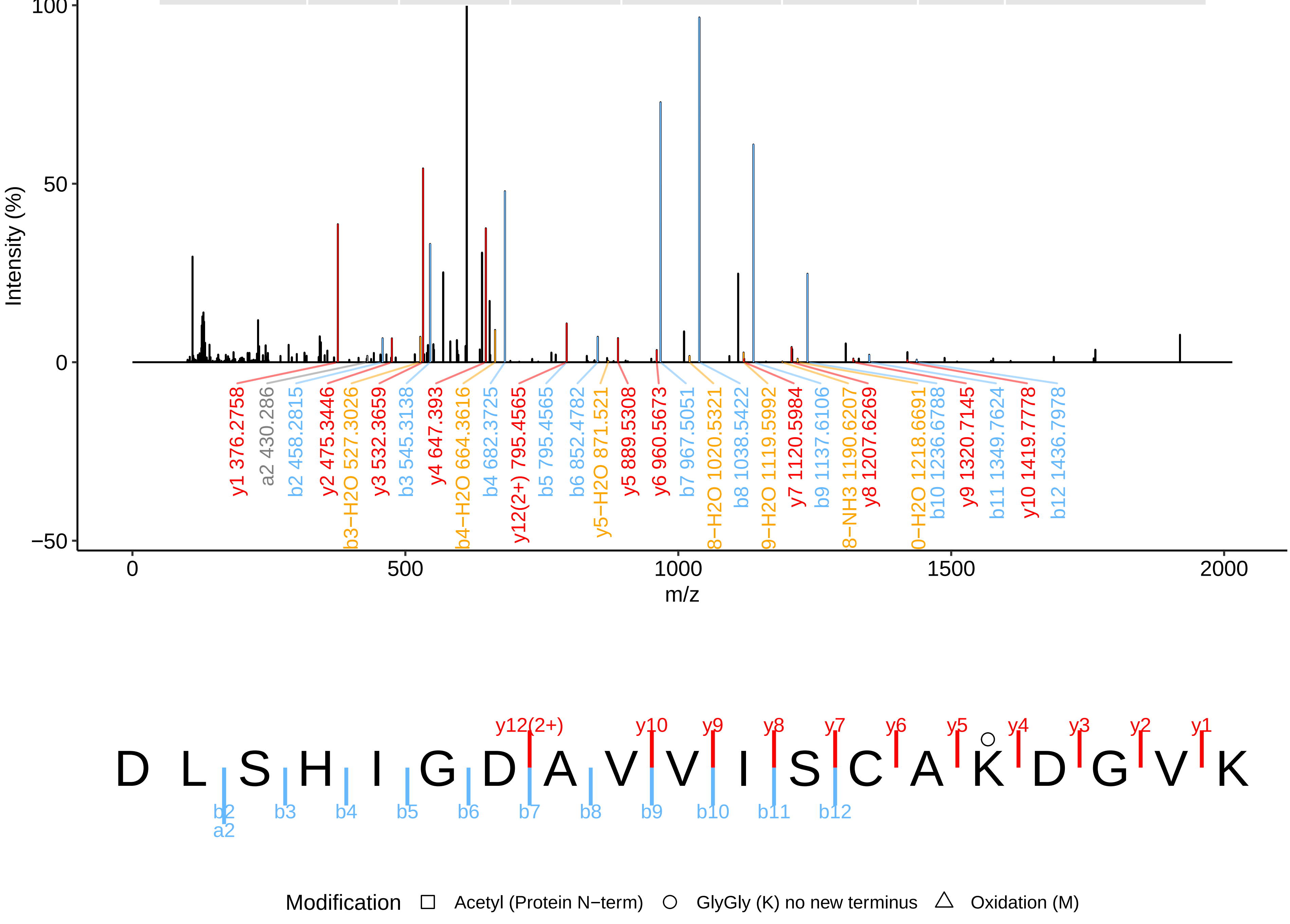

20201118_KIZ_MUb6_GlyGly_2 − Scan number: 21401

Gene Names	Charge	m/z	Mass	Retention time	PEP	Score	Precursor Intensity
PCNA	4	525.2704	2097.052	72.087	2.6116e−15	127.61	22361816

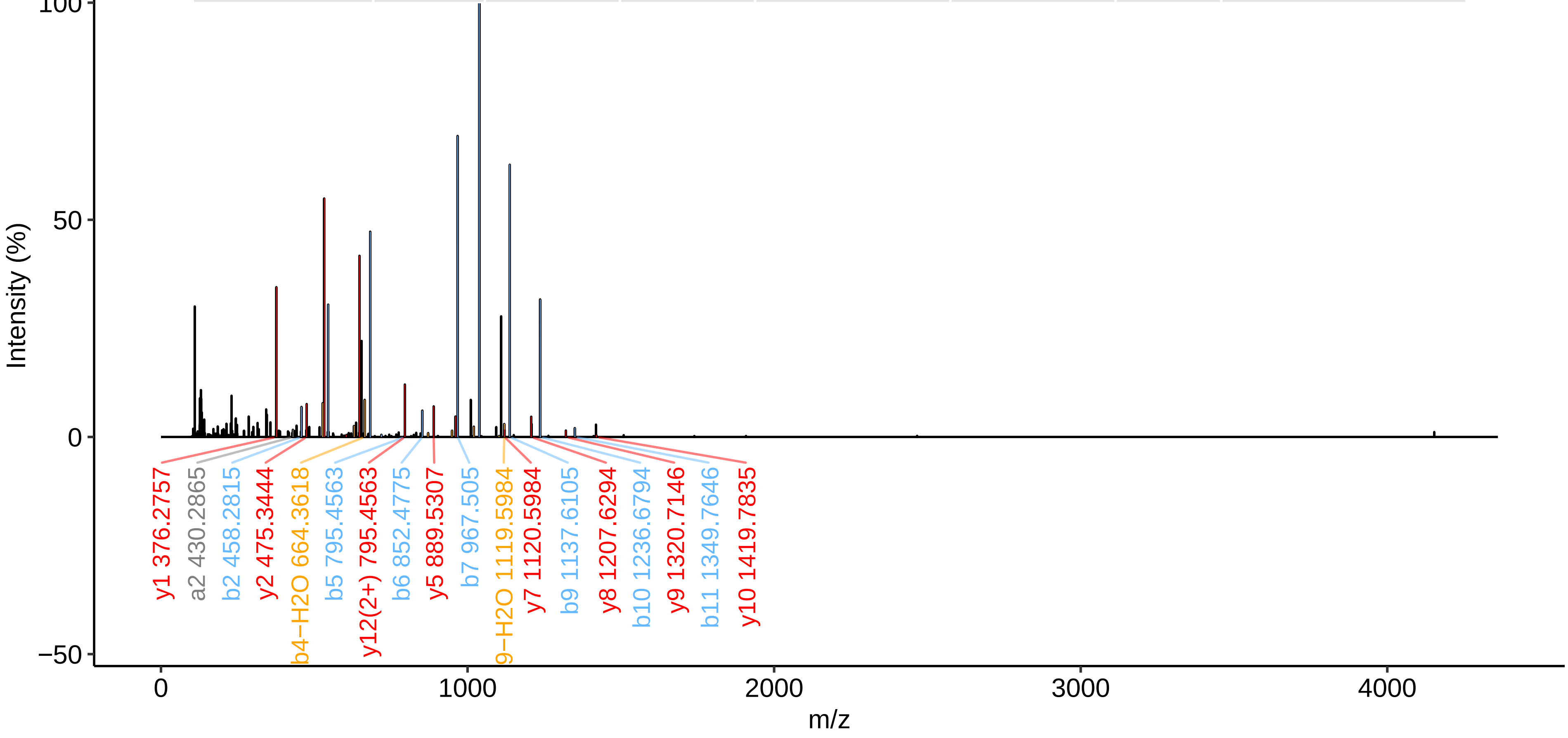

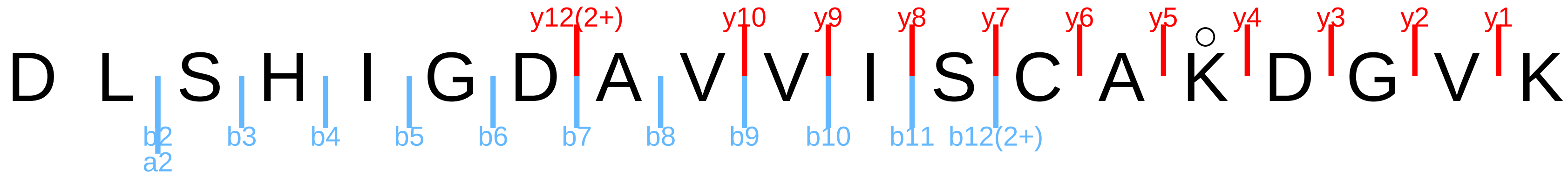

Modification □ Acetyl (Protein N−term) ○ GlyGly (K) no new terminus △ Oxidation (M)

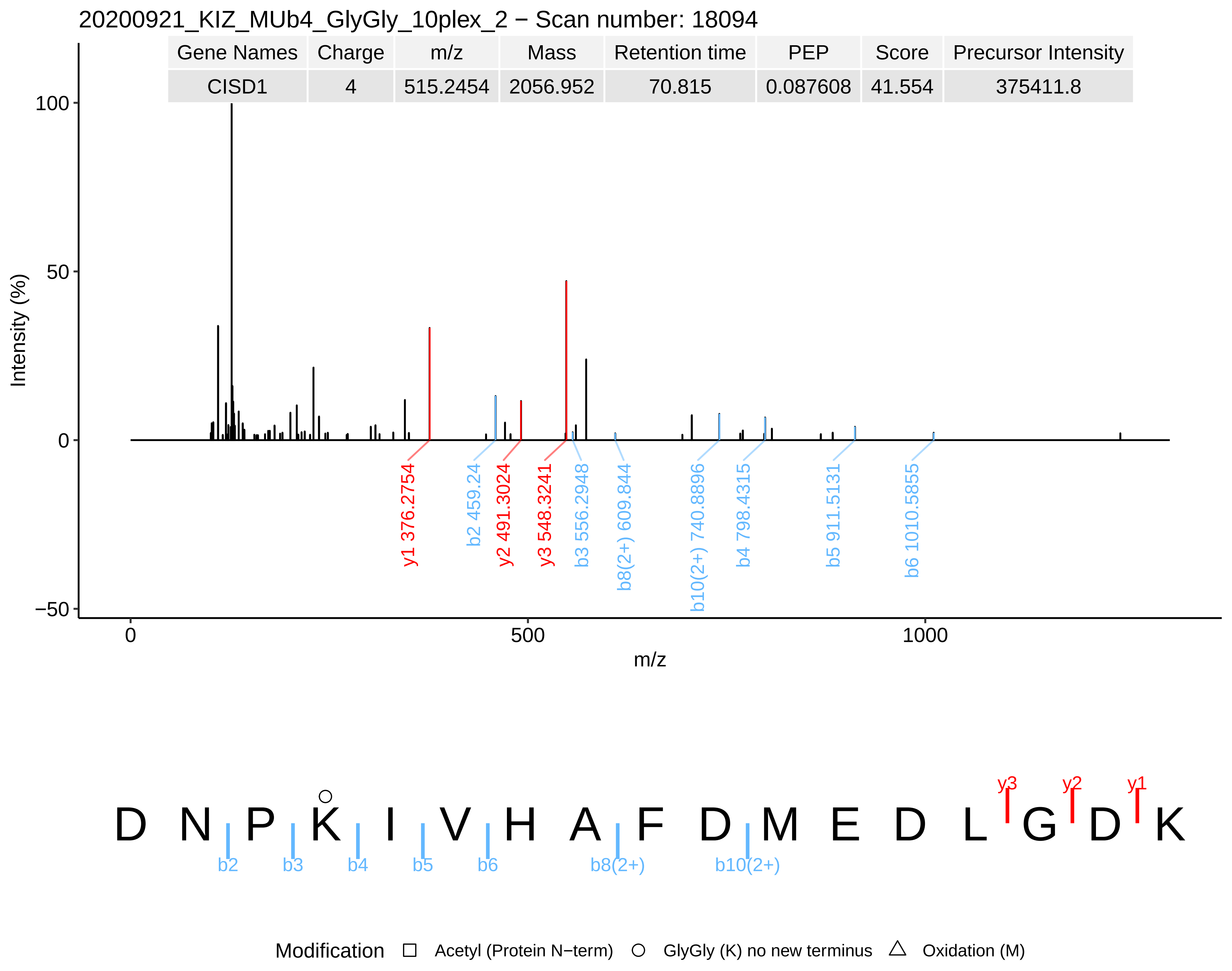

20200921_KIZ_MUb4_GlyGly_10plex_2 − Scan number: 18094
Gene Names | Charge | m/z | Mass | Retention time | PEP | Score | Precursor Intensity
CISD1 | 4 | 515.2454 | 2056.952 | 70.815 | 0.087608 | 41.554 | 375411.8
Intensity (%)
m/z
y1 376.2754
b2 459.24
y2 491.3024
y3 548.3241
b3 556.2948
b8(2+) 609.844
b10(2+) 740.8896
b4 798.4315
b5 911.5131
b6 1010.5855
D N P K I V H A F D M E D L G D K
Modification
Acetyl (Protein N−term)
GlyGly (K) no new terminus
Oxidation (M)

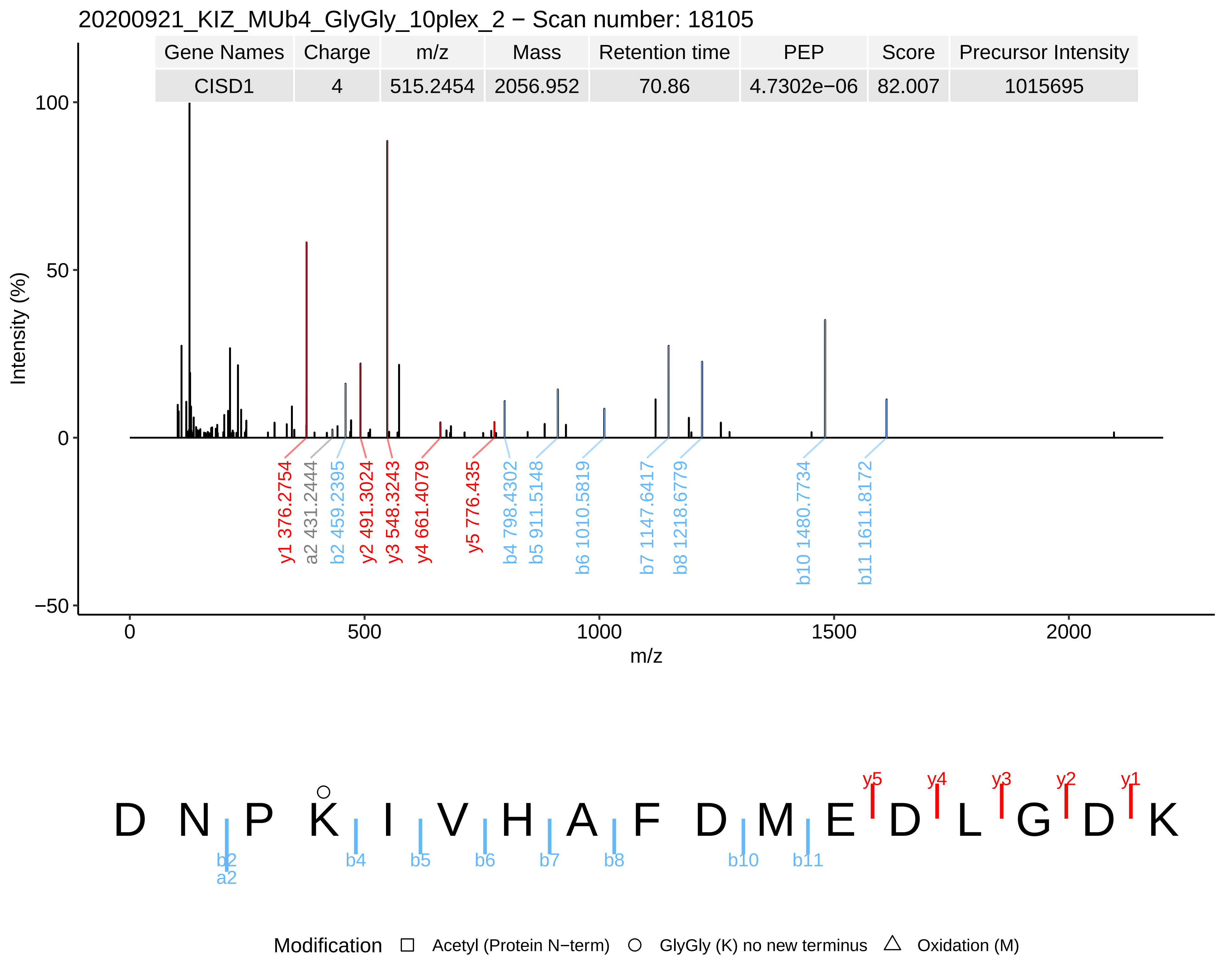
20200921_KIZ_MUb4_GlyGly_10plex_2 − Scan number: 18105
Gene Names | Charge | m/z | Mass | Retention time | PEP | Score | Precursor Intensity
CISD1 | 4 | 515.2454 | 2056.952 | 70.86 | 4.7302e−06 | 82.007 | 1015695
Intensity (%)
100
50
0
−50
0
500
1000
1500
2000
m/z
y1 376.2754
a2 431.2444
b2 459.2395
y2 491.3024
y3 548.3243
y4 661.4079
y5 776.435
b4 798.4302
b5 911.5148
b6 1010.5819
b7 1147.6417
b8 1218.6779
b10 1480.7734
b11 1611.8172
D N P K I V H A F D M E D L G D K
y5 y4 y3 y2 y1
b2 a2 b4 b5 b6 b7 b8 b10 b11
Modification
Acetyl (Protein N−term)
GlyGly (K) no new terminus
Oxidation (M)

20200921_KIZ_MUb4_GlyGly_10plex − Scan number: 12327

Gene Names	Charge	m/z	Mass	Retention time	PEP	Score	Precursor Intensity
STAM	4	465.2352	1856.912	43.18	1.1425e−05	96.673	5167593

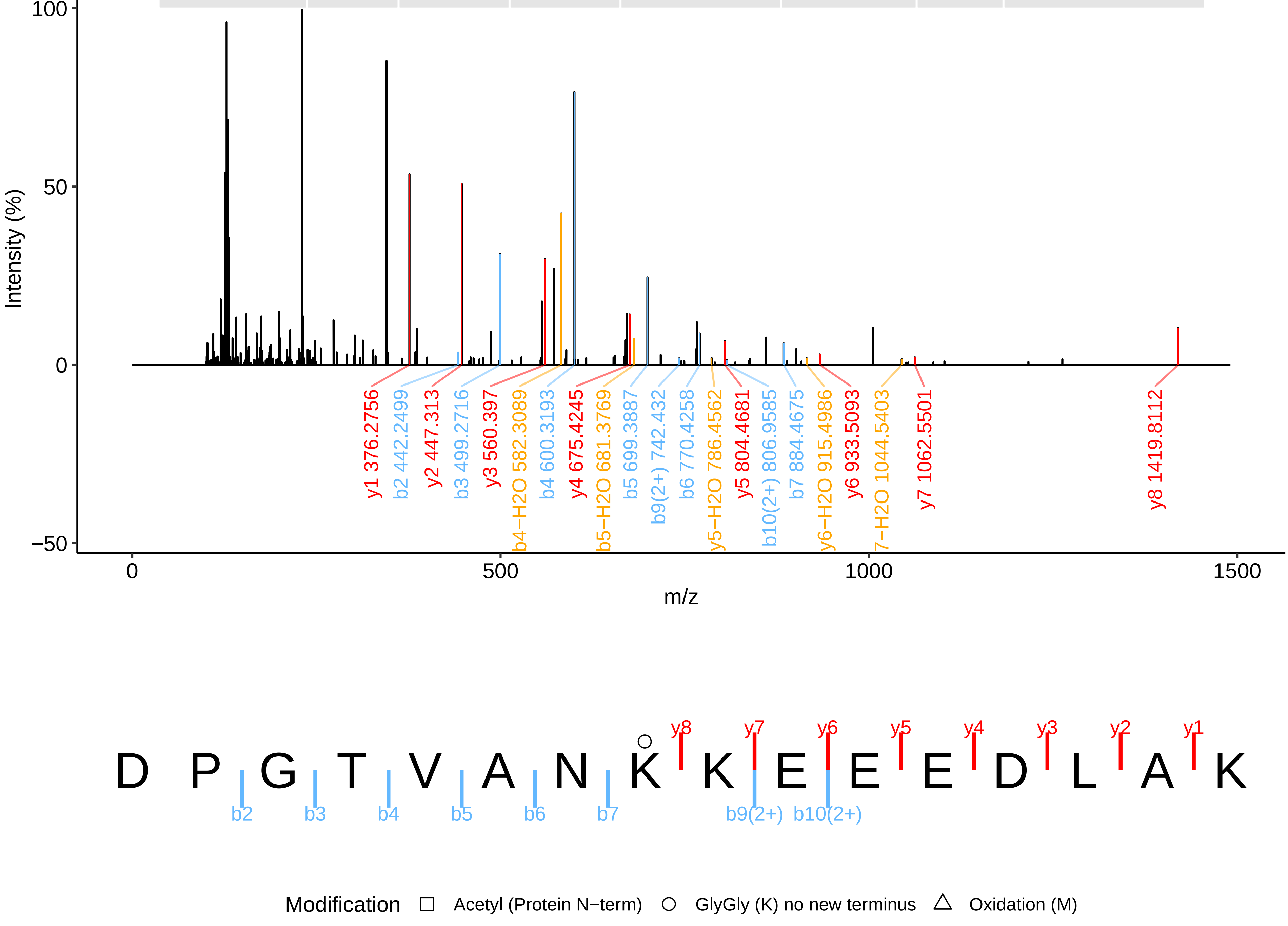

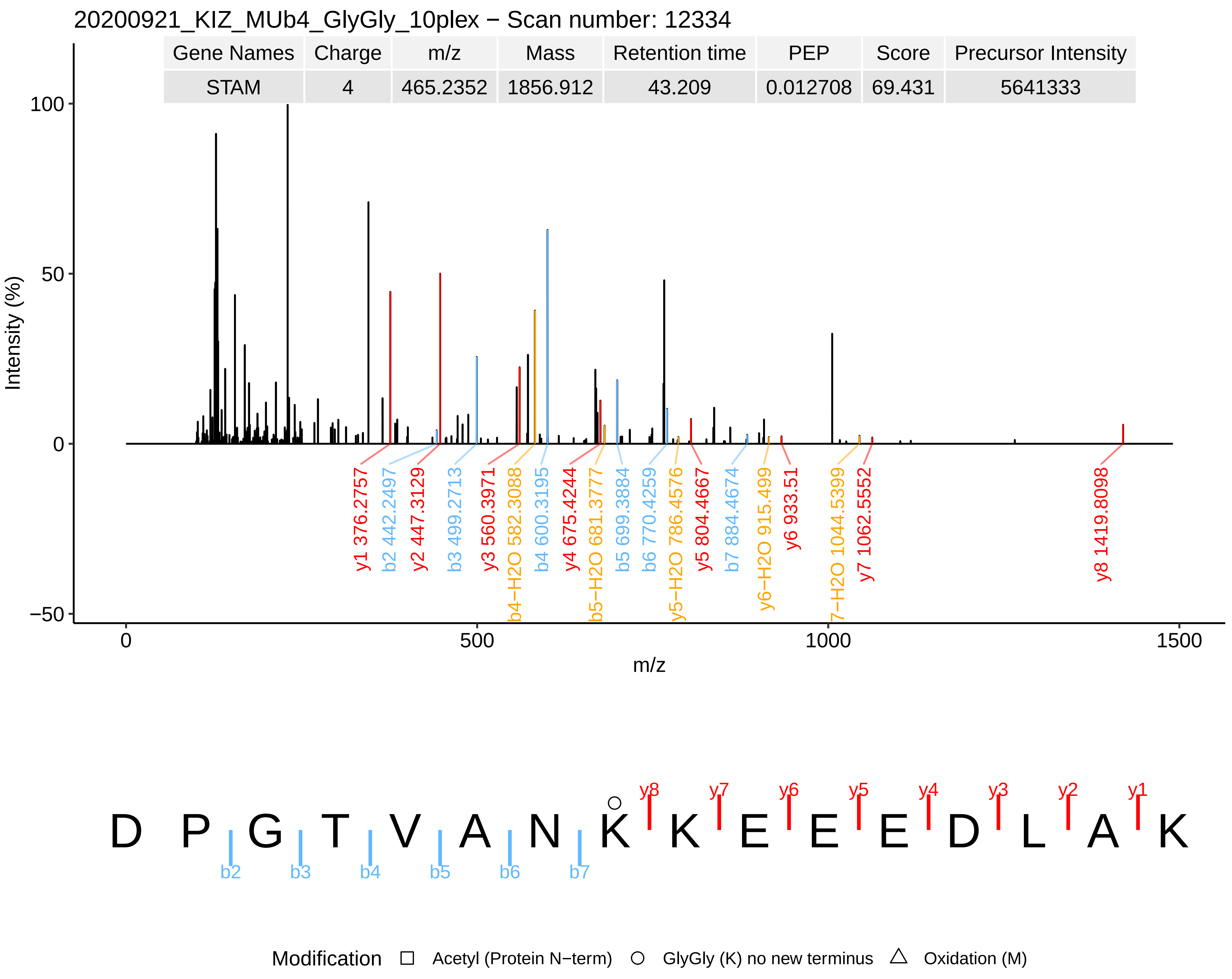
20200921_KIZ_MUb4_GlyGly_10plex − Scan number: 12334
Gene Names | Charge | m/z | Mass | Retention time | PEP | Score | Precursor Intensity
STAM | 4 | 465.2352 | 1856.912 | 43.209 | 0.012708 | 69.431 | 5641333
Intensity (%)
m/z
y1 376.2757
b2 442.2497
y2 447.3129
b3 499.2713
y3 560.3971
b4−H2O 582.3088
b4 600.3195
y4 675.4244
b5−H2O 681.3777
b5 699.3884
b6 770.4259
y5−H2O 786.4576
y5 804.4667
b7 884.4674
y6−H2O 915.499
y6 933.51
y7−H2O 1044.5399
y7 1062.5552
y8 1419.8098
D P G T V A N K K E E E D L A K
Modification
Acetyl (Protein N−term)
GlyGly (K) no new terminus
Oxidation (M)

20200921_KIZ_MUb4_GlyGly_10plex_2 − Scan number: 10556

Gene Names	Charge	m/z	Mass	Retention time	PEP	Score	Precursor Intensity
STAM	4	465.2352	1856.912	40.406	1.1425e−05	96.673	1339968

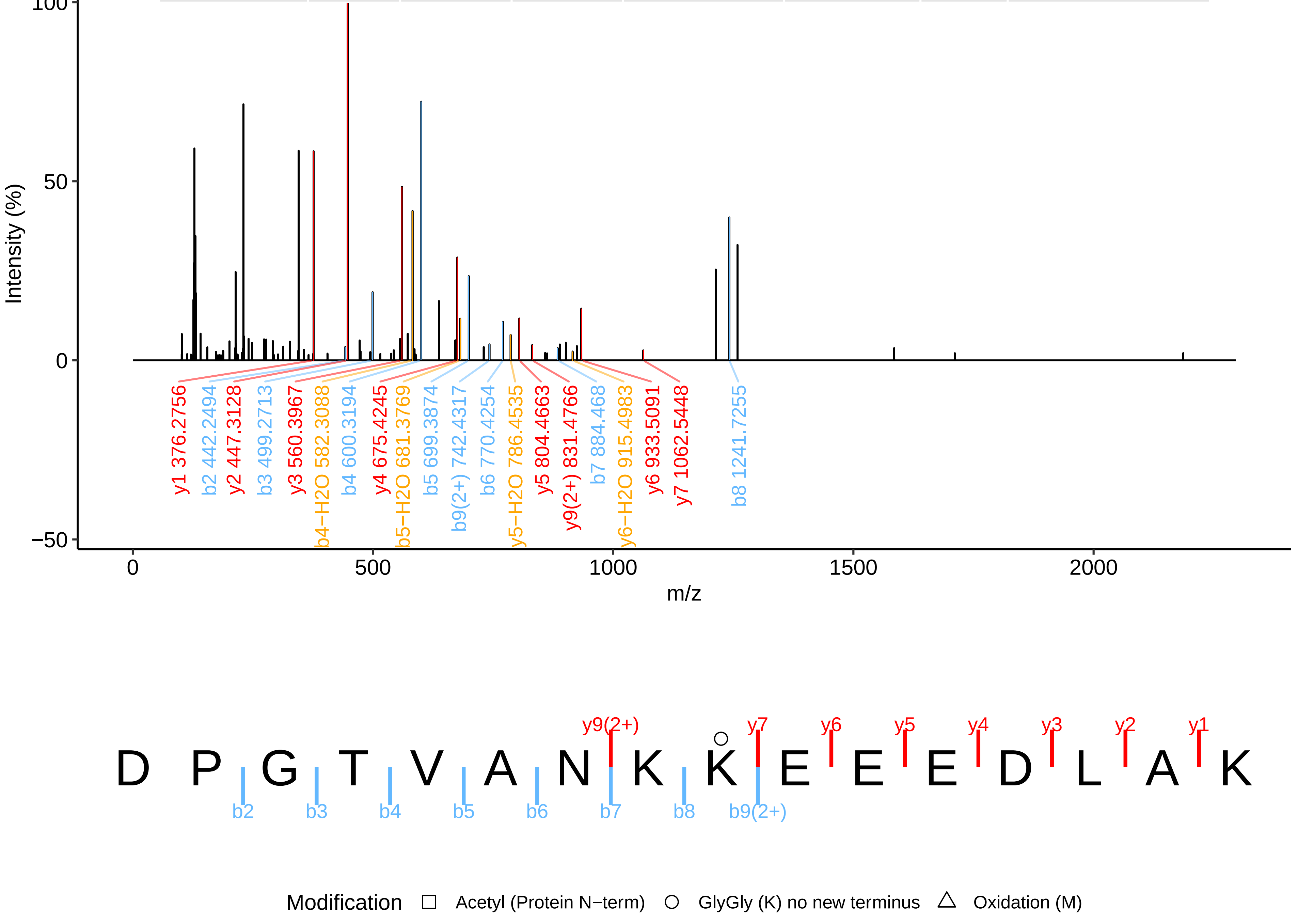

20200921_KIZ_MUb4_GlyGly_10plex_2 − Scan number: 13495

Gene Names	Charge	m/z	Mass	Retention time	PEP	Score	Precursor Intensity
PSMD1	3	687.9688	2060.884	52.407	3.6914e−13	119.56	1083645

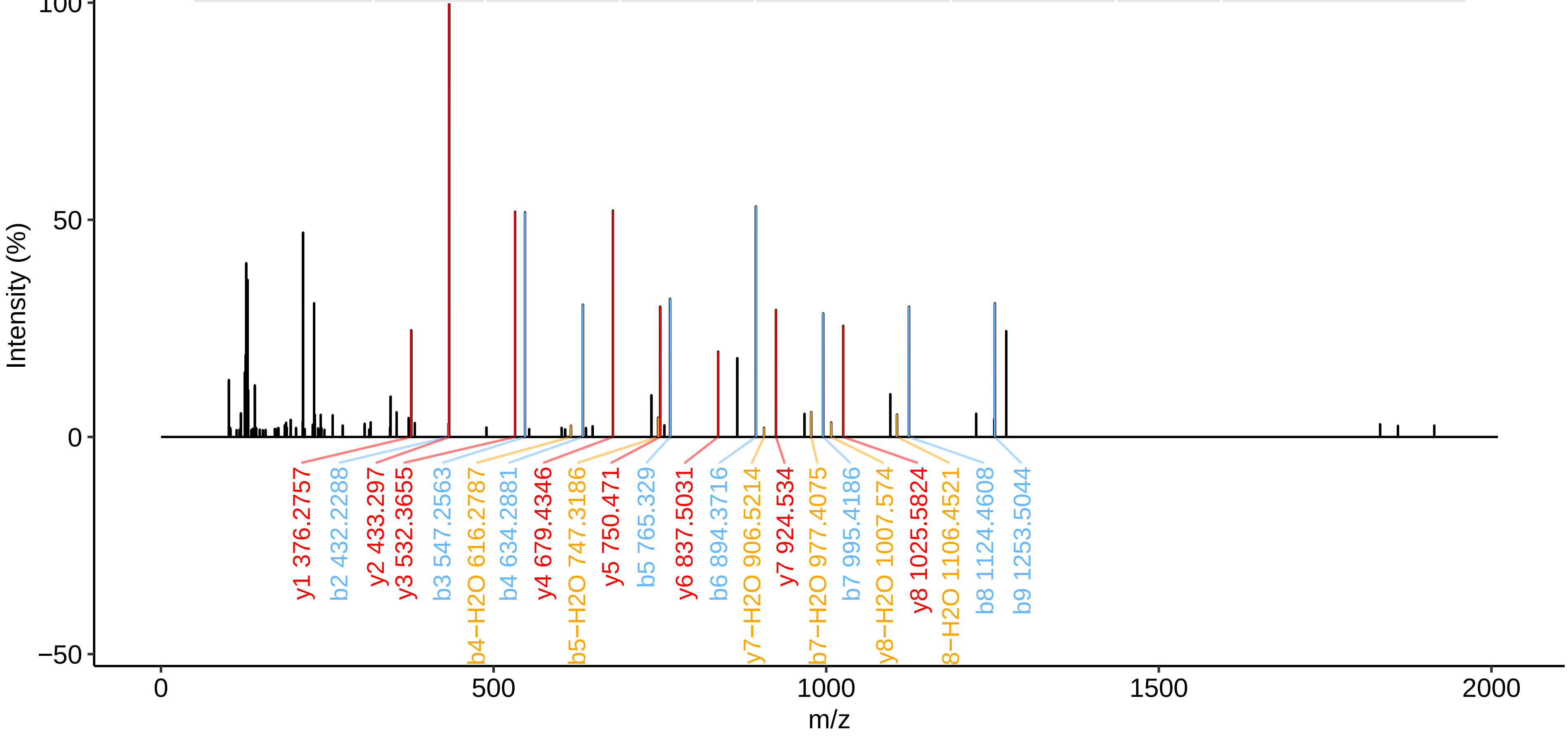

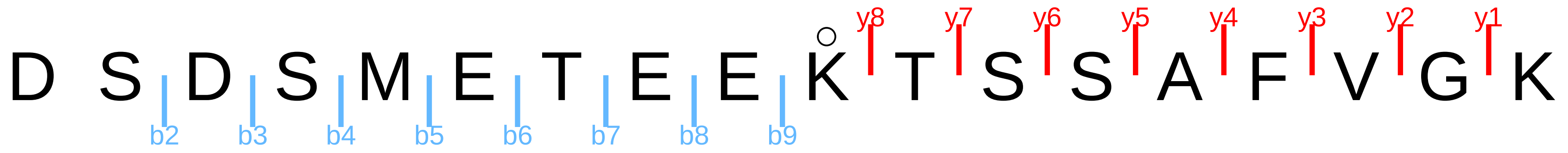

Modification □ Acetyl (Protein N−term) ○ GlyGly (K) no new terminus △ Oxidation (M)

20200921_KIZ_MUb4_GlyGly_10plex − Scan number: 11738

Gene Names	Charge	m/z	Mass	Retention time	PEP	Score	Precursor Intensity
STOM	4	505.9947	2019.95	40.986	1.0285e−16	147.64	4713316

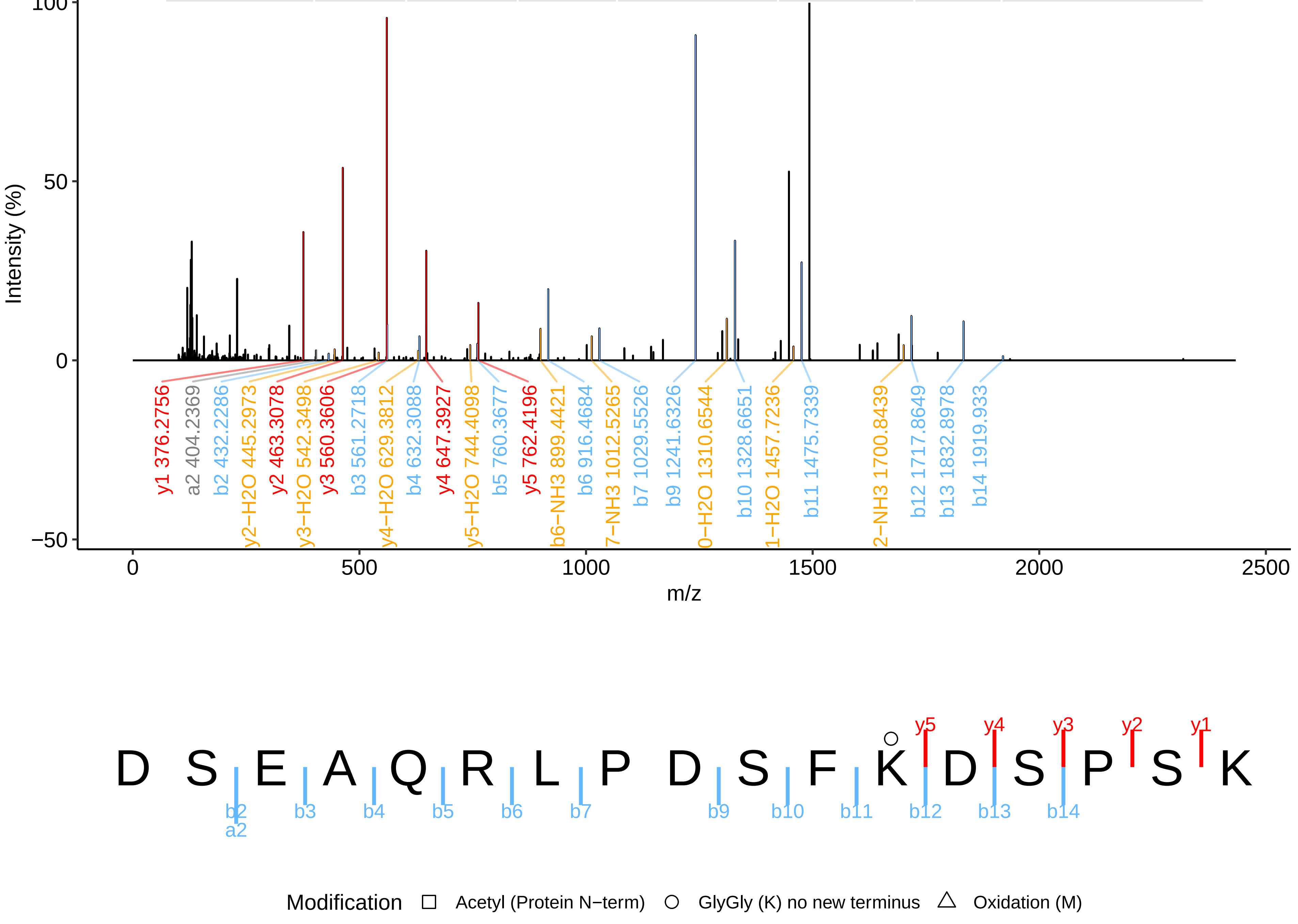

20200921_KIZ_MUb4_GlyGly_10plex_2 − Scan number: 10799

Gene Names	Charge	m/z	Mass	Retention time	PEP	Score	Precursor Intensity
STOM	4	505.9947	2019.95	41.398	4.3398e−12	124.43	1032312

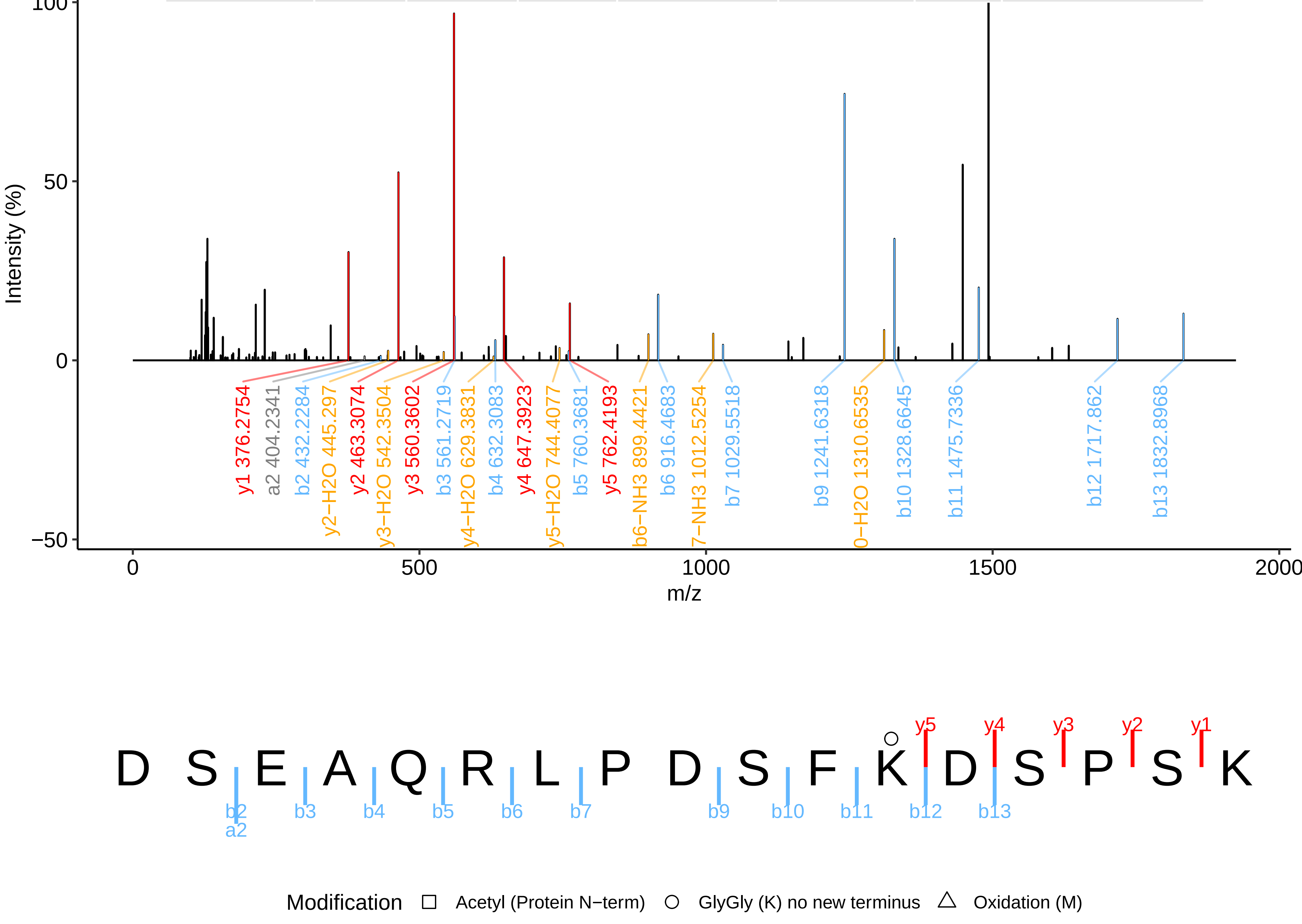

20200921_KIZ_MUb4_GlyGly_10plex_2 − Scan number: 15668

Gene Names	Charge	m/z	Mass	Retention time	PEP	Score	Precursor Intensity
UBQLN2;UBQLN1	3	704.0078	2109.001	61.321	0.0069041	62.165	243795.7

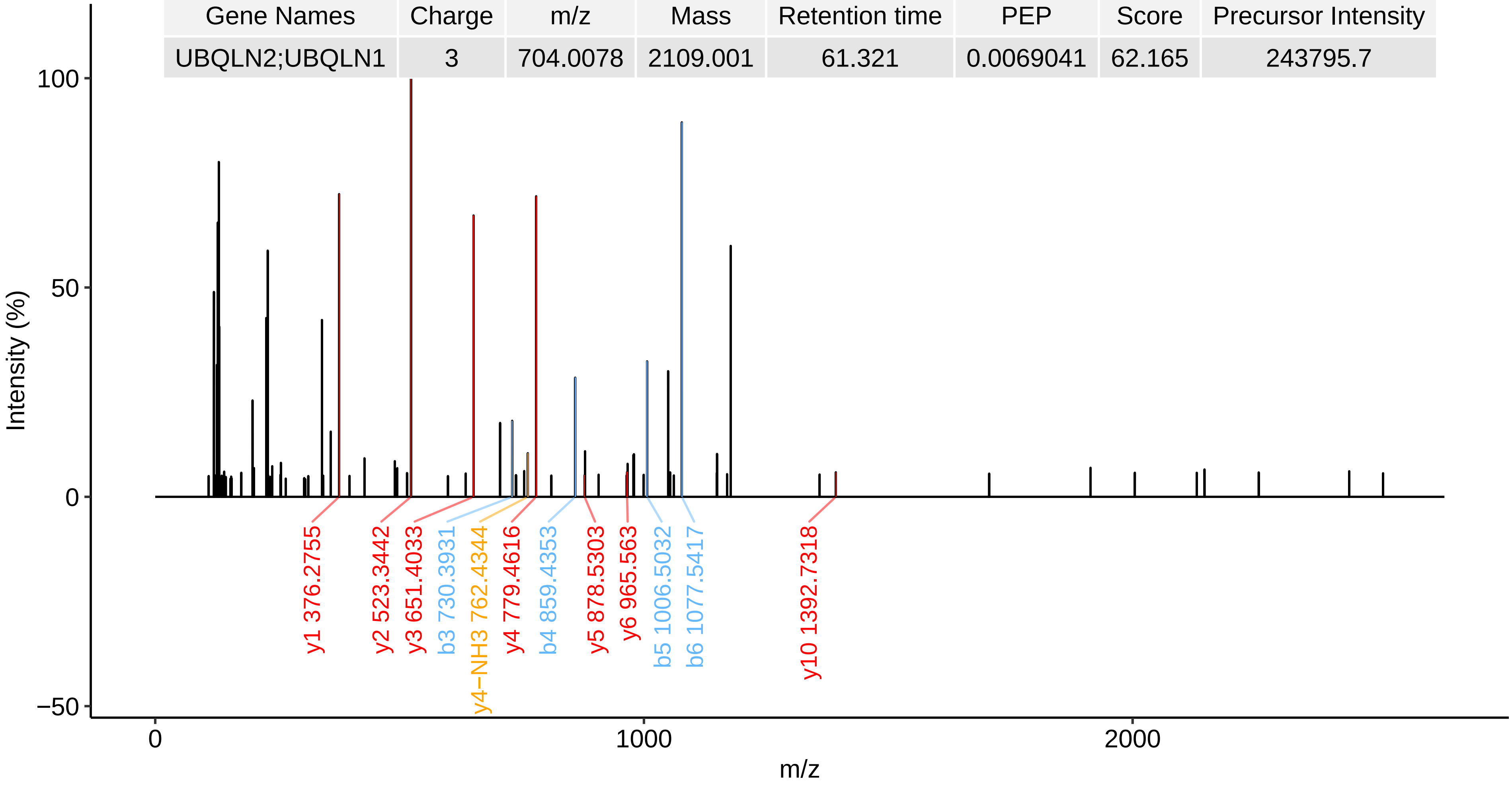

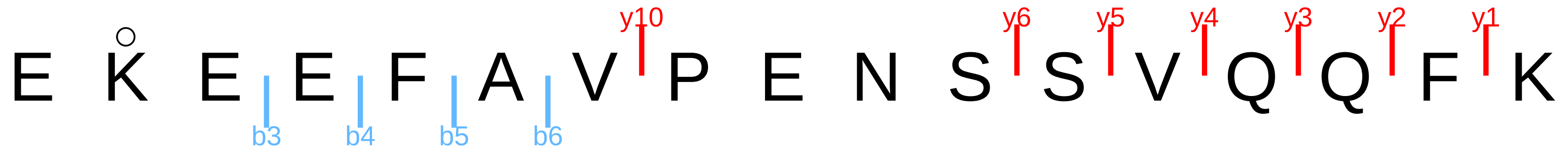

20201118_KIZ_MUb6_GlyGly_1 − Scan number: 26477

Gene Names	Charge	m/z	Mass	Retention time	PEP	Score	Precursor Intensity
	4	452.9976	1807.961	80.645	0.11793	59.542	24475610

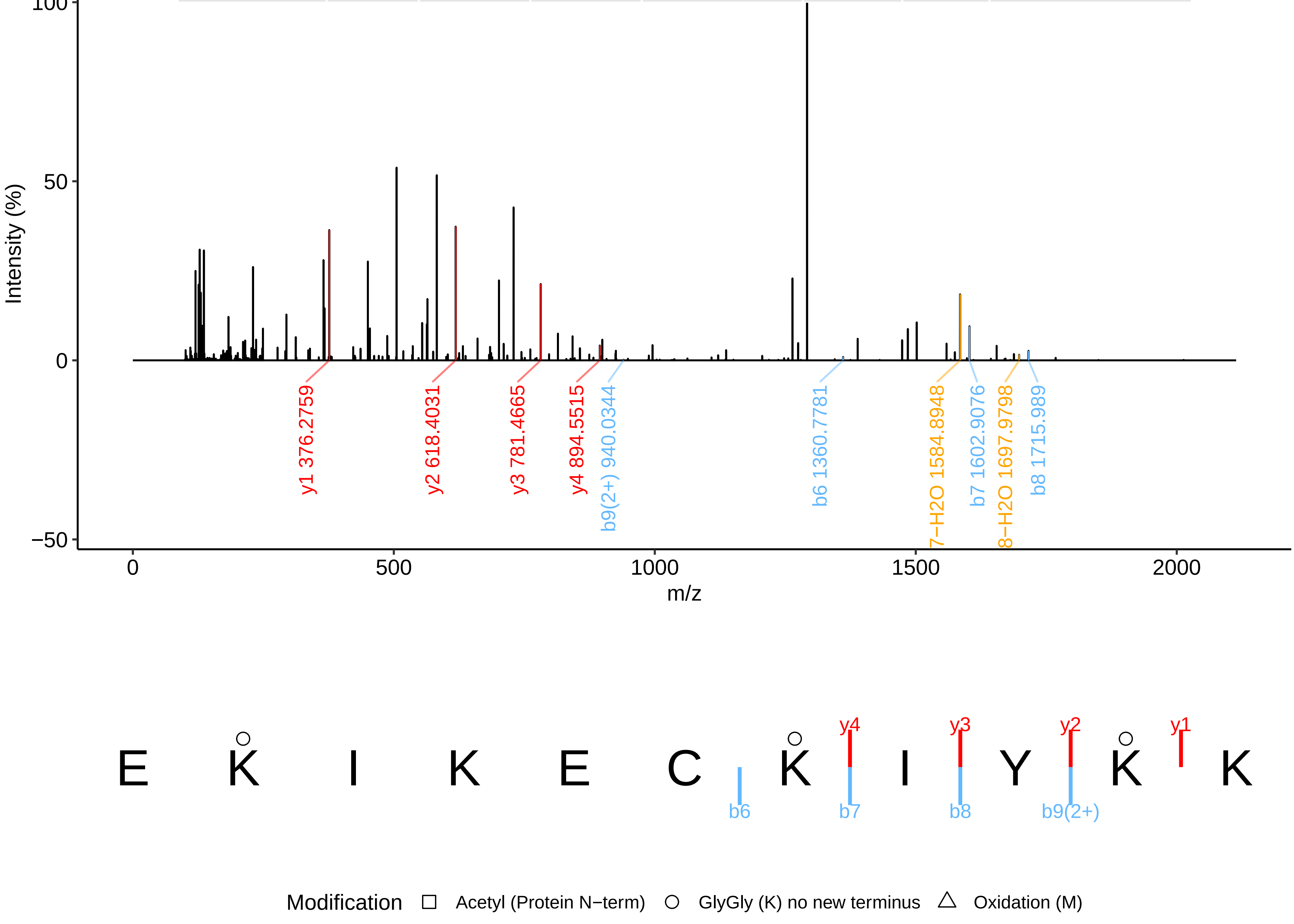

20200921_KIZ_MUb4_GlyGly_10plex − Scan number: 11408

Gene Names	Charge	m/z	Mass	Retention time	PEP	Score	Precursor Intensity
CDK5RAP3	4	583.2709	2329.055	39.733	4.4878e−06	89.049	1354296

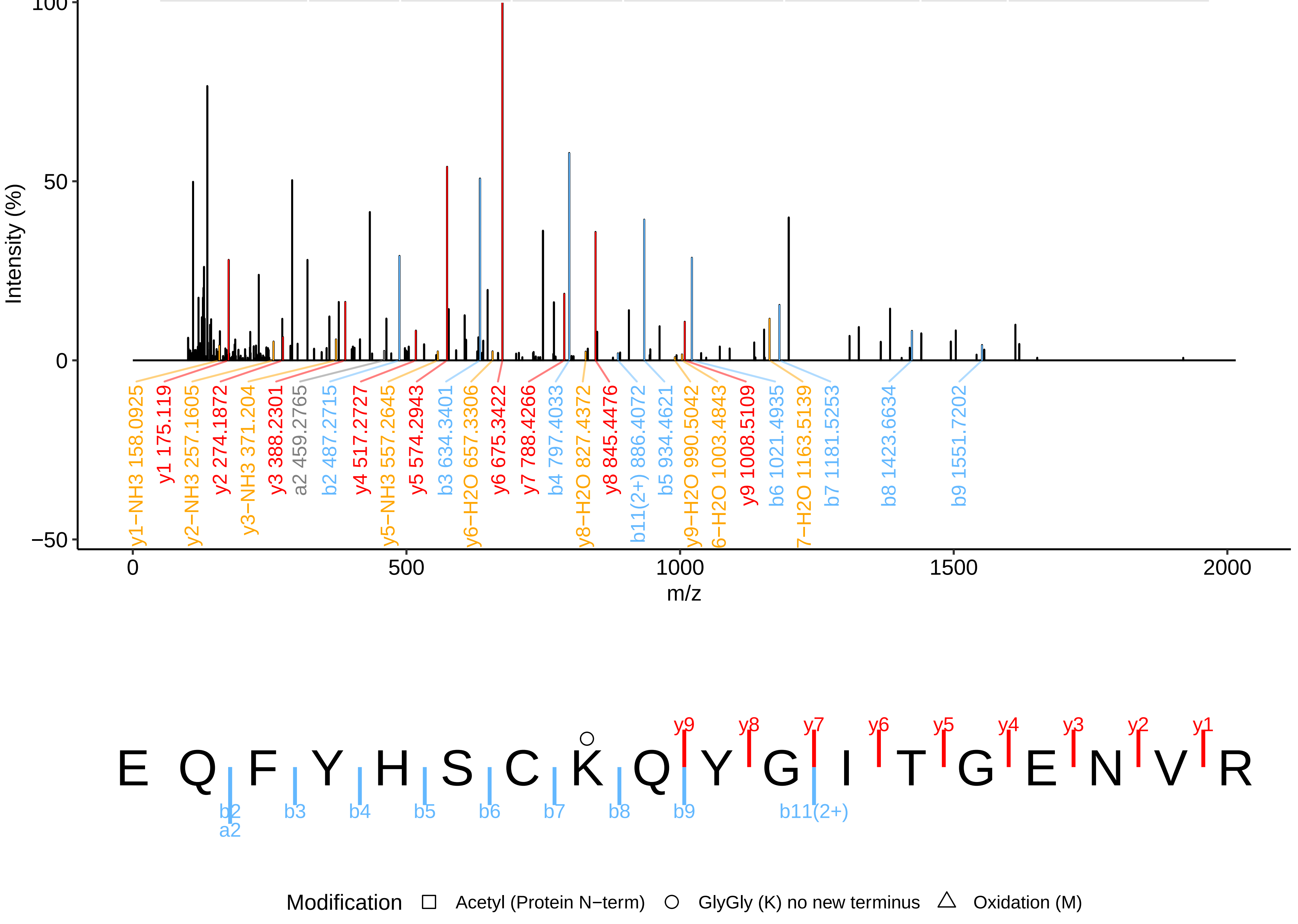

20200921_KIZ_MUb4_GlyGly_10plex − Scan number: 24227

Gene Names	Charge	m/z	Mass	Retention time	PEP	Score	Precursor Intensity
PKM	3	674.6761	2021.006	88.032	6.2132e−14	116.29	3491547

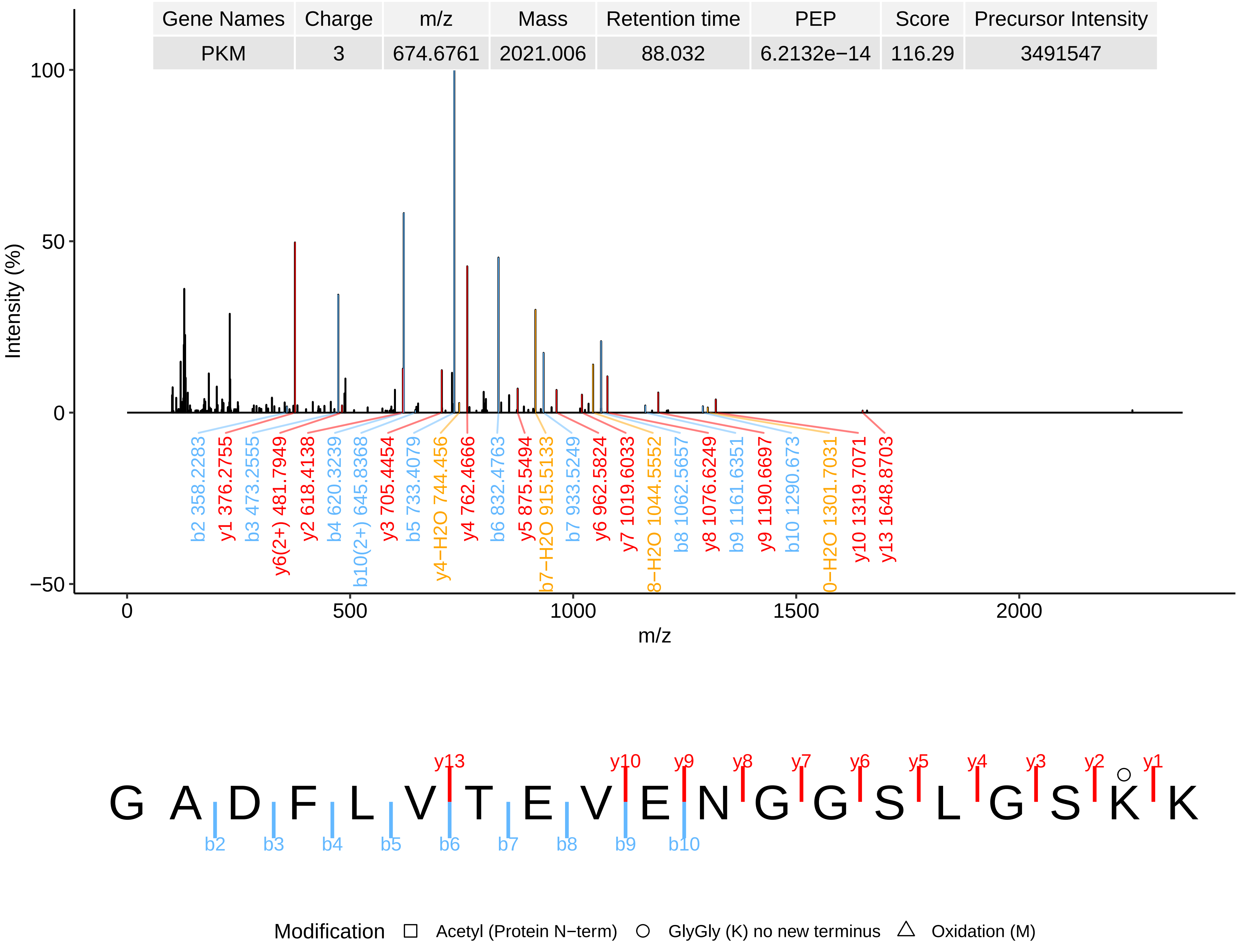

20200921_KIZ_MUb4_GlyGly_10plex − Scan number: 9646

Gene Names	Charge	m/z	Mass	Retention time	PEP	Score	Precursor Intensity
APP	4	513.7455	2050.953	33.1	2.8003e−06	95.067	4266879

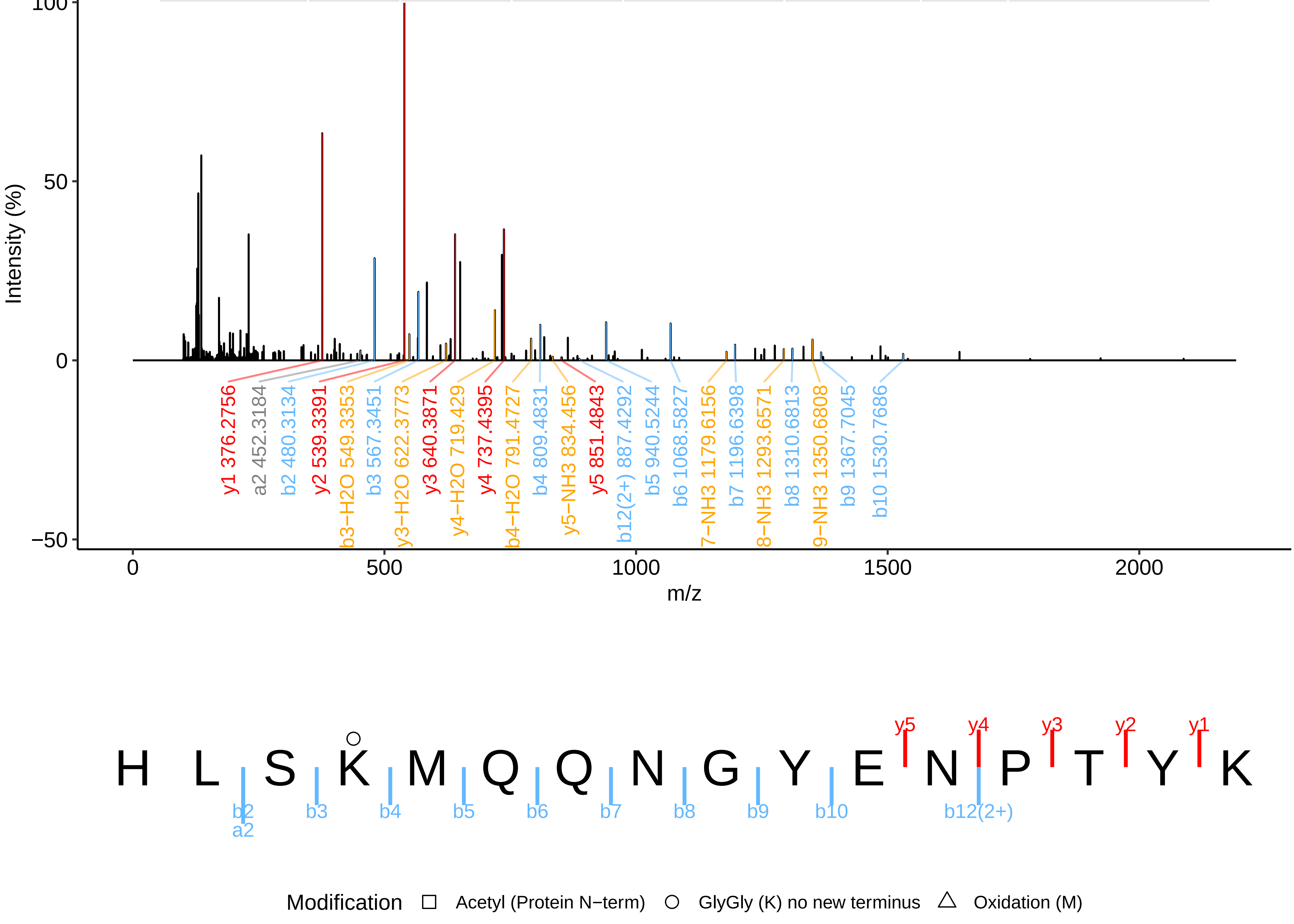

20200921_KIZ_MUb4_GlyGly_10plex_2 − Scan number: 8884

Gene Names	Charge	m/z	Mass	Retention time	PEP	Score	Precursor Intensity
APP	4	513.7455	2050.953	33.616	0.047221	51.771	670469.4

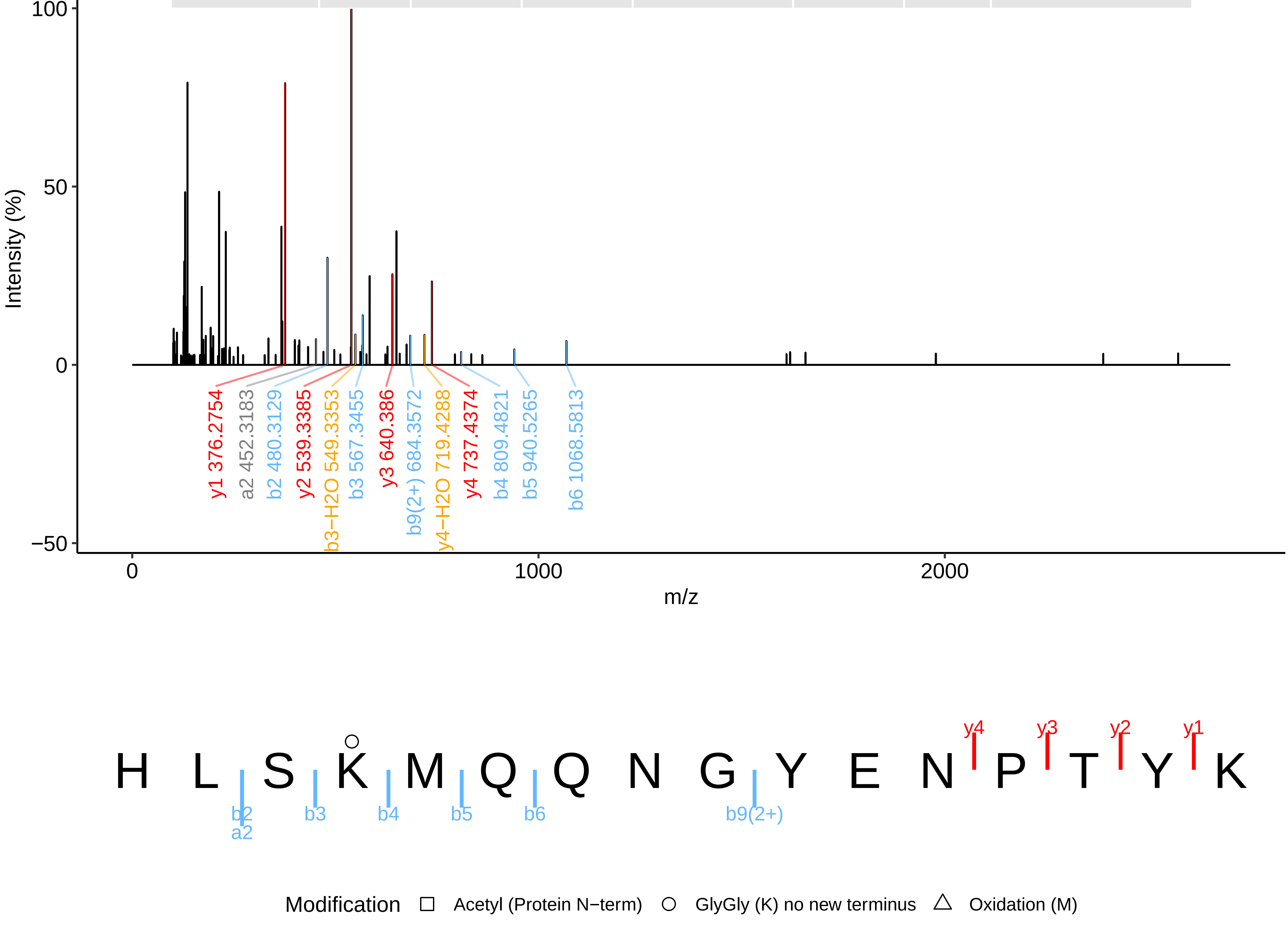

20200921_KIZ_MUb4_GlyGly_10plex − Scan number: 23686

Gene Names	Charge	m/z	Mass	Retention time	PEP	Score	Precursor Intensity
CISD1	4	564.0161	2252.035	86.015	3.639e−14	131.24	5566176

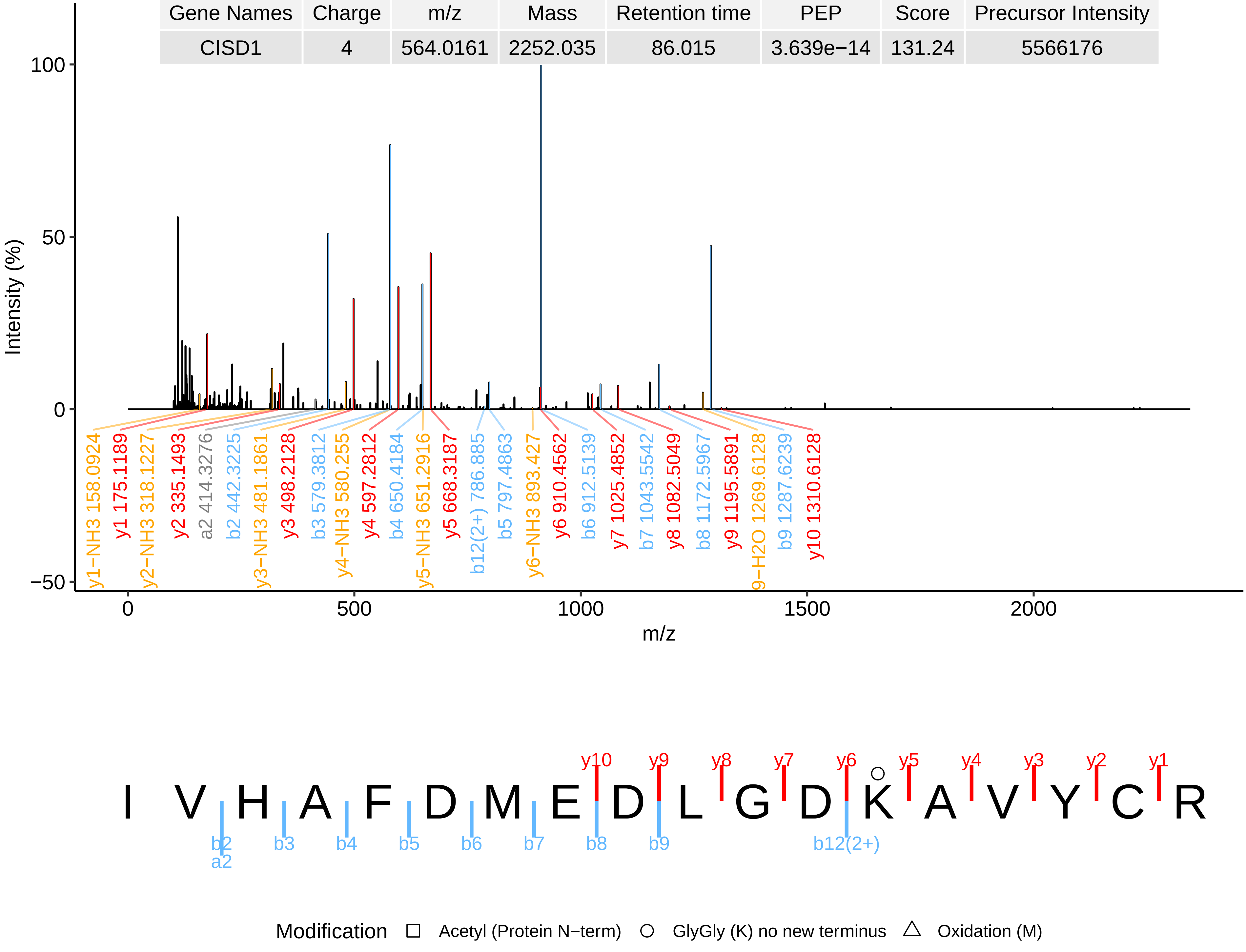

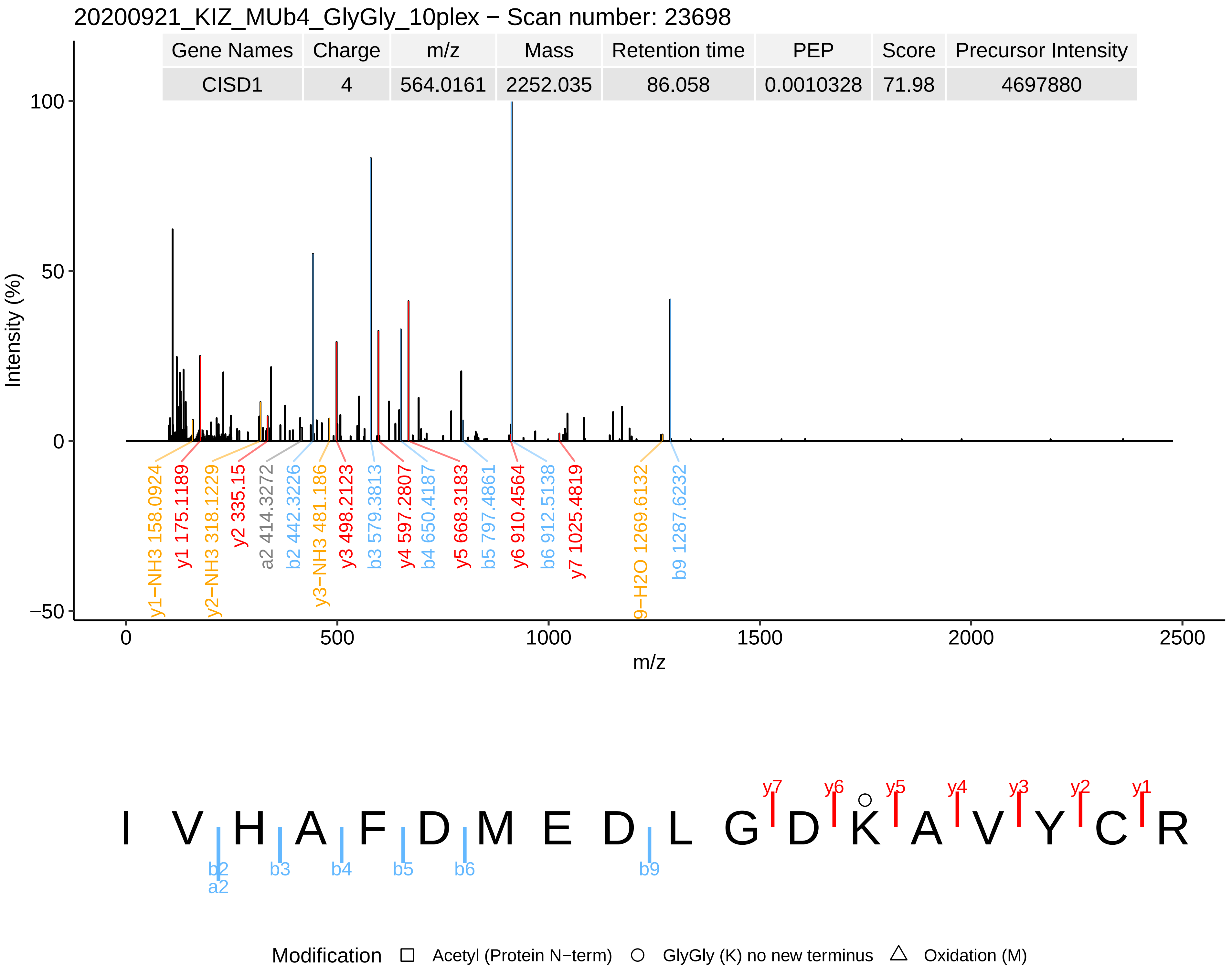

20200921_KIZ_MUb4_GlyGly_10plex − Scan number: 23698

Gene Names	Charge	m/z	Mass	Retention time	PEP	Score	Precursor Intensity
CISD1	4	564.0161	2252.035	86.058	0.0010328	71.98	4697880

Intensity (%)
m/z
y1−NH3 158.0924
y1 175.1189
y2−NH3 318.1229
y2 335.15
a2 414.3272
b2 442.3226
y3−NH3 481.186
y3 498.2123
b3 579.3813
y4 597.2807
b4 650.4187
y5 668.3183
b5 797.4861
y6 910.4564
b6 912.5138
y7 1025.4819
9−H2O 1269.6132
b9 1287.6232
I V H A F D M E D L G D K A V Y C R
y7 y6 y5 y4 y3 y2 y1
b2 a2 b3 b4 b5 b6 b9
Modification
Acetyl (Protein N−term)
GlyGly (K) no new terminus
Oxidation (M)

20200921_KIZ_MUb4_GlyGly_10plex − Scan number: 23823

Gene Names	Charge	m/z	Mass	Retention time	PEP	Score	Precursor Intensity
CISD1	4	564.0161	2252.035	86.522	4.4077e−23	150.59	10675489

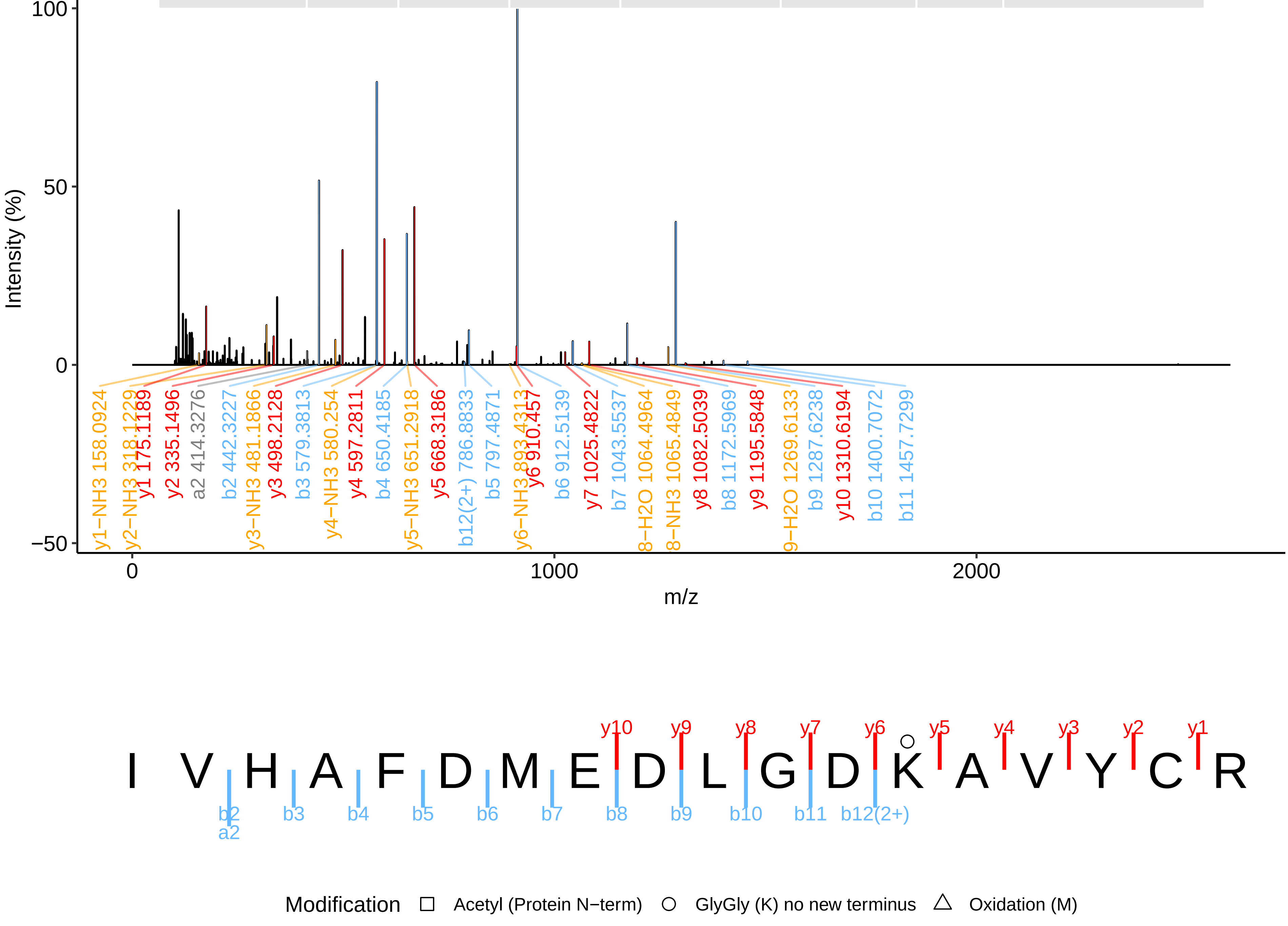

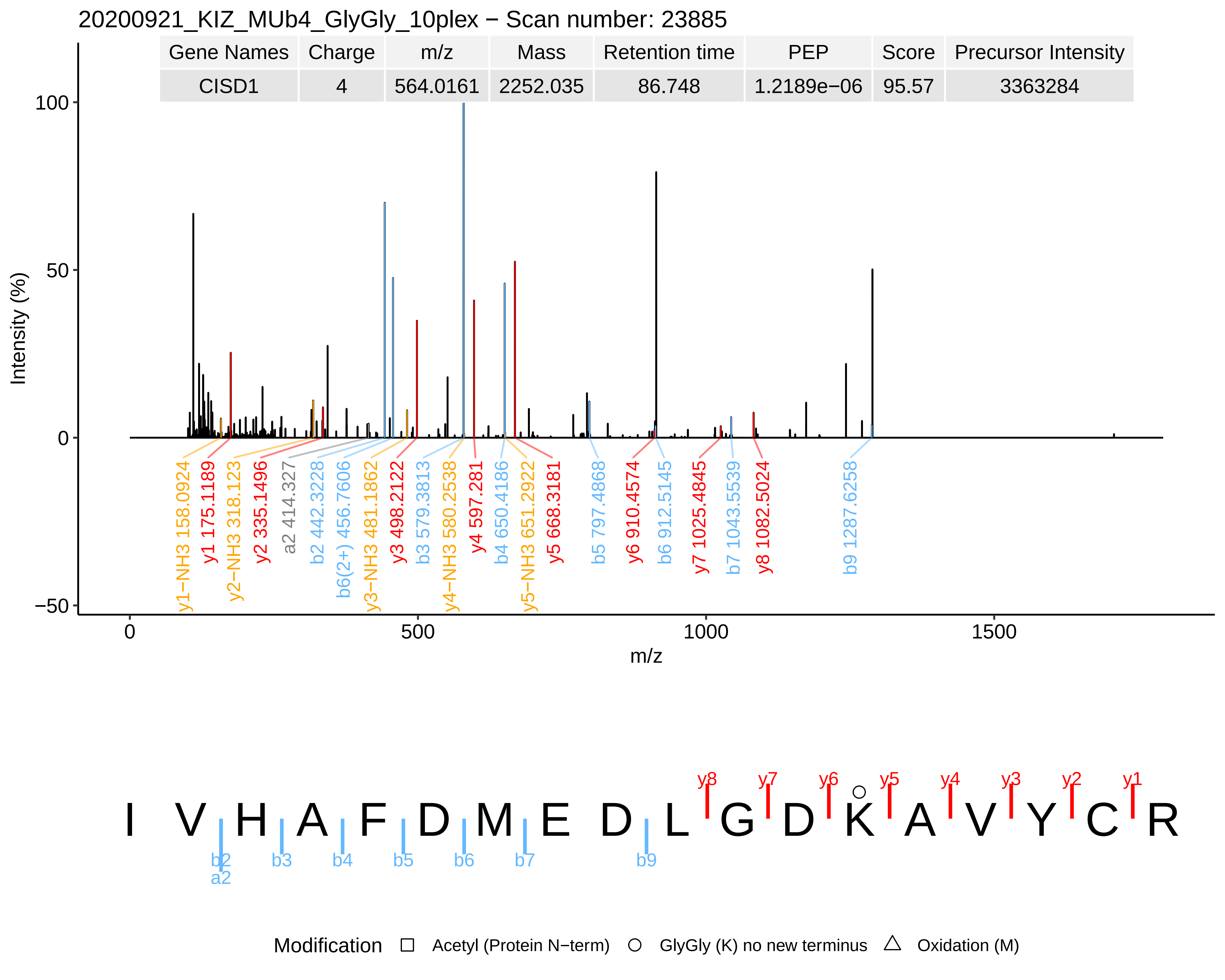

20200921_KIZ_MUb4_GlyGly_10plex − Scan number: 23885
Gene Names | Charge | m/z | Mass | Retention time | PEP | Score | Precursor Intensity
CISD1 | 4 | 564.0161 | 2252.035 | 86.748 | 1.2189e−06 | 95.57 | 3363284
Intensity (%)
m/z
y1−NH3 158.0924
y1 175.1189
y2−NH3 318.123
y2 335.1496
a2 414.327
b2 442.3228
b6(2+) 456.7606
y3−NH3 481.1862
y3 498.2122
b3 579.3813
y4−NH3 580.2538
y4 597.281
b4 650.4186
y5−NH3 651.2922
y5 668.3181
b5 797.4868
y6 910.4574
b6 912.5145
y7 1025.4845
b7 1043.5539
y8 1082.5024
b9 1287.6258
I V H A F D M E D L G D K A V Y C R
Modification
Acetyl (Protein N−term)
GlyGly (K) no new terminus
Oxidation (M)

20200921_KIZ_MUb4_GlyGly_10plex − Scan number: 24023

Gene Names	Charge	m/z	Mass	Retention time	PEP	Score	Precursor Intensity
CISD1	4	564.0161	2252.035	87.251	7.6794e−31	174.62	1495187

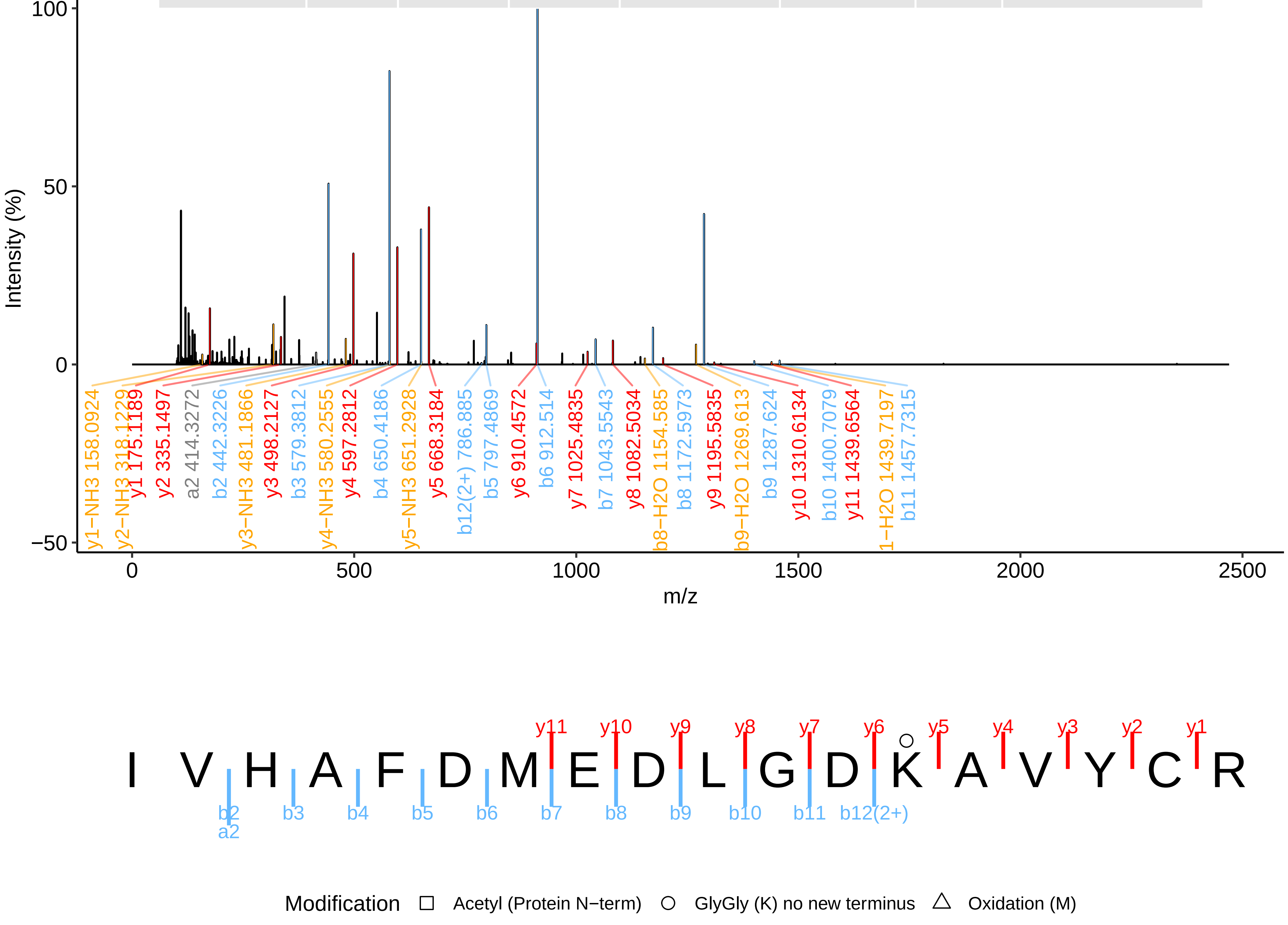

20200921_KIZ_MUb4_GlyGly_10plex − Scan number: 24024

Gene Names	Charge	m/z	Mass	Retention time	PEP	Score	Precursor Intensity
CISD1	4	564.0161	2252.035	87.255	0.0013007	69.729	3532006

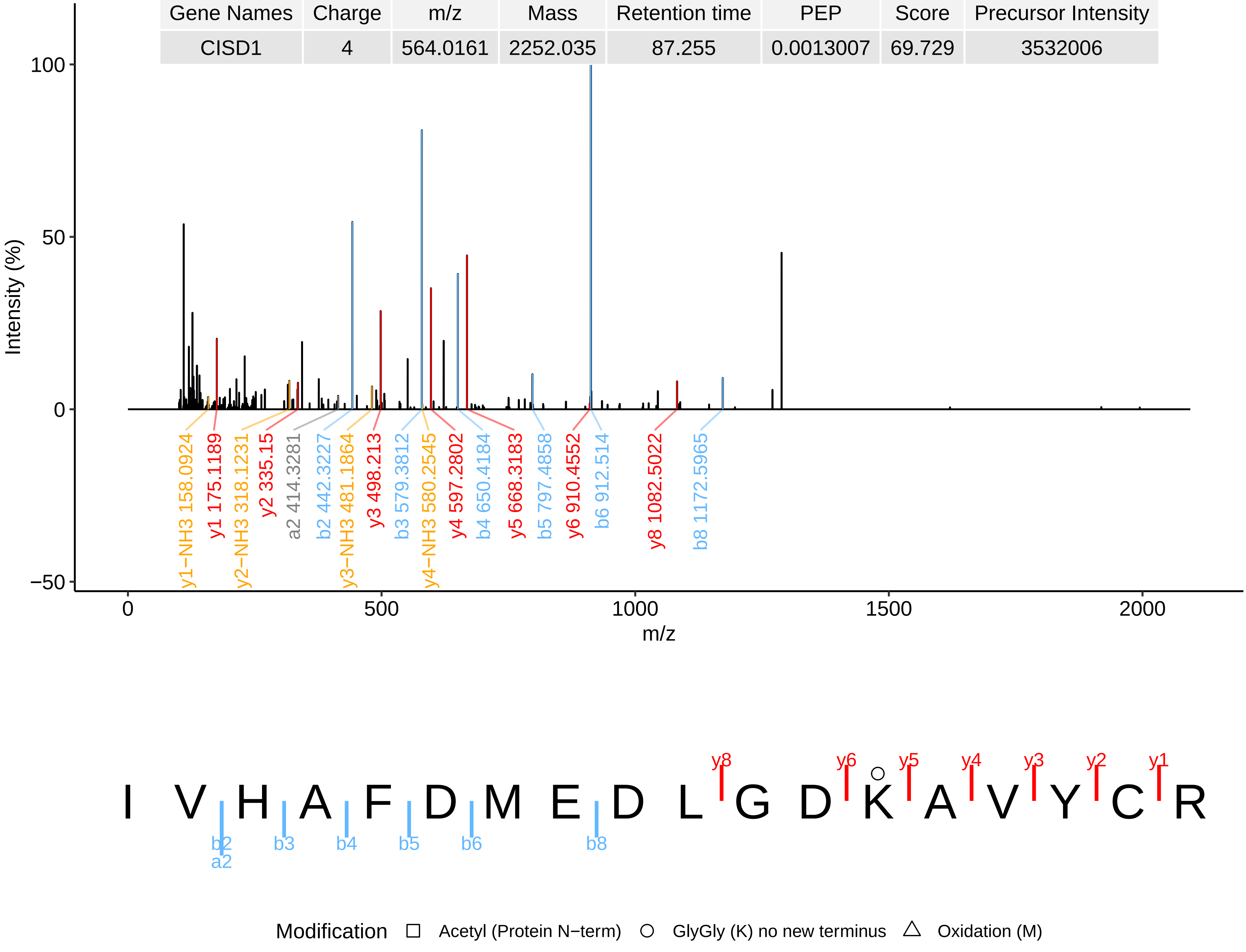

20200921_KIZ_MUb4_GlyGly_10plex_2 − Scan number: 19550

Gene Names	Charge	m/z	Mass	Retention time	PEP	Score	Precursor Intensity
CISD1	4	564.0161	2252.035	76.746	0.016746	58.079	837268.1

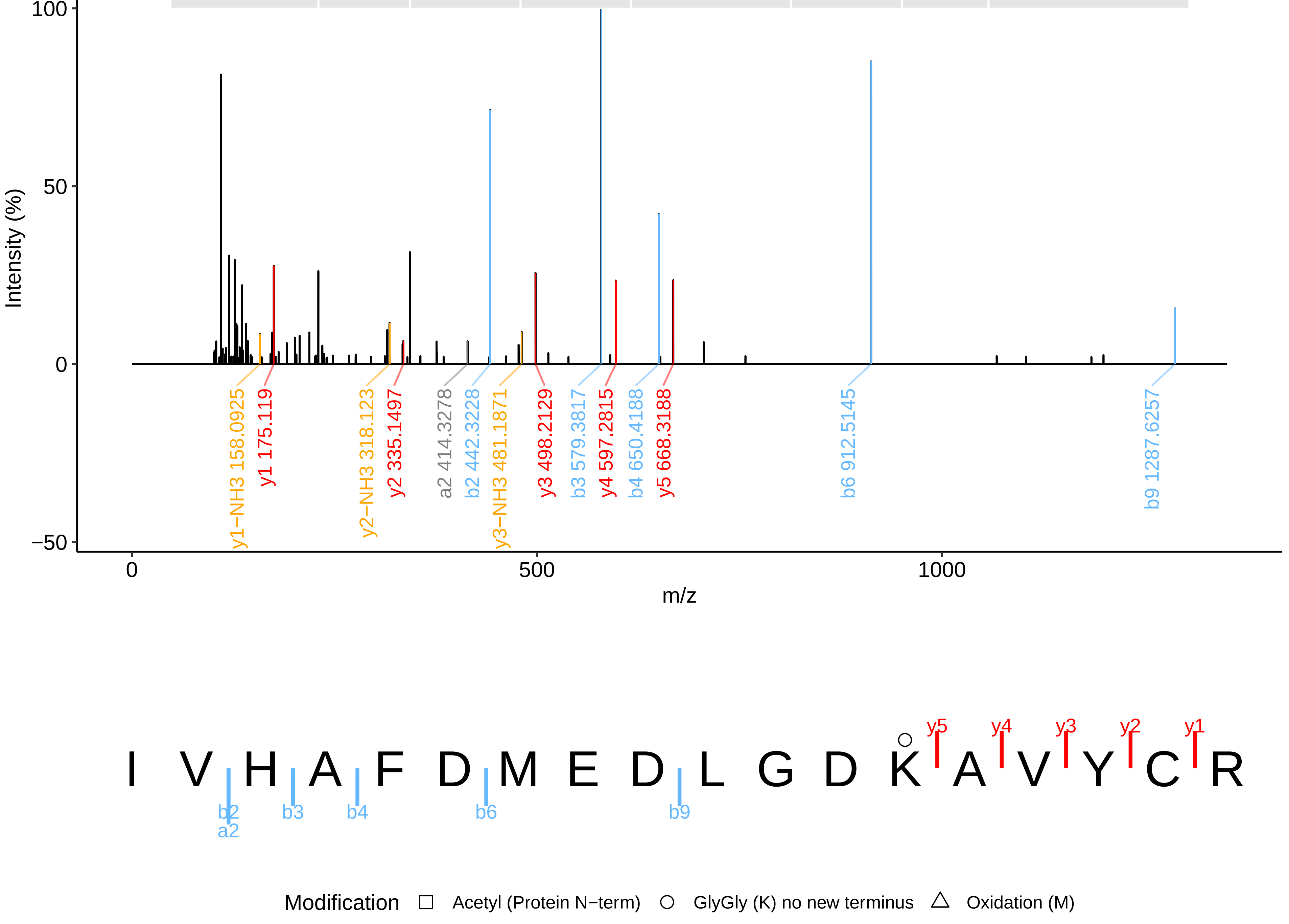

20200921_KIZ_MUb4_GlyGly_10plex_2 − Scan number: 19586

Gene Names	Charge	m/z	Mass	Retention time	PEP	Score	Precursor Intensity
CISD1	4	564.0161	2252.035	76.893	2.7201e−06	92.575	1163617

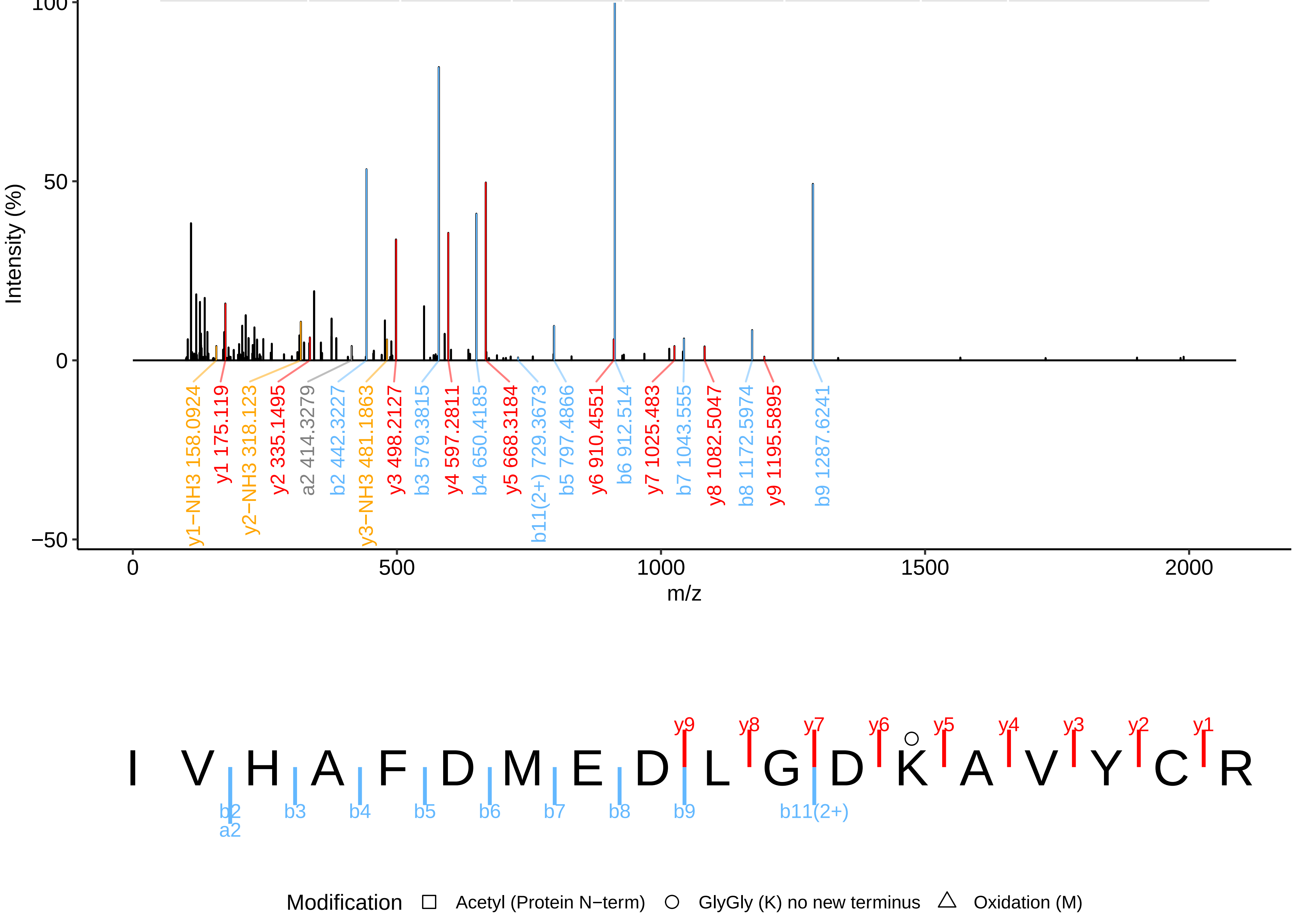

20200921_KIZ_MUb4_GlyGly_10plex_2 − Scan number: 19713

Gene Names	Charge	m/z	Mass	Retention time	PEP	Score	Precursor Intensity
CISD1	4	564.0161	2252.035	77.407	3.3483e−13	118.21	3611952

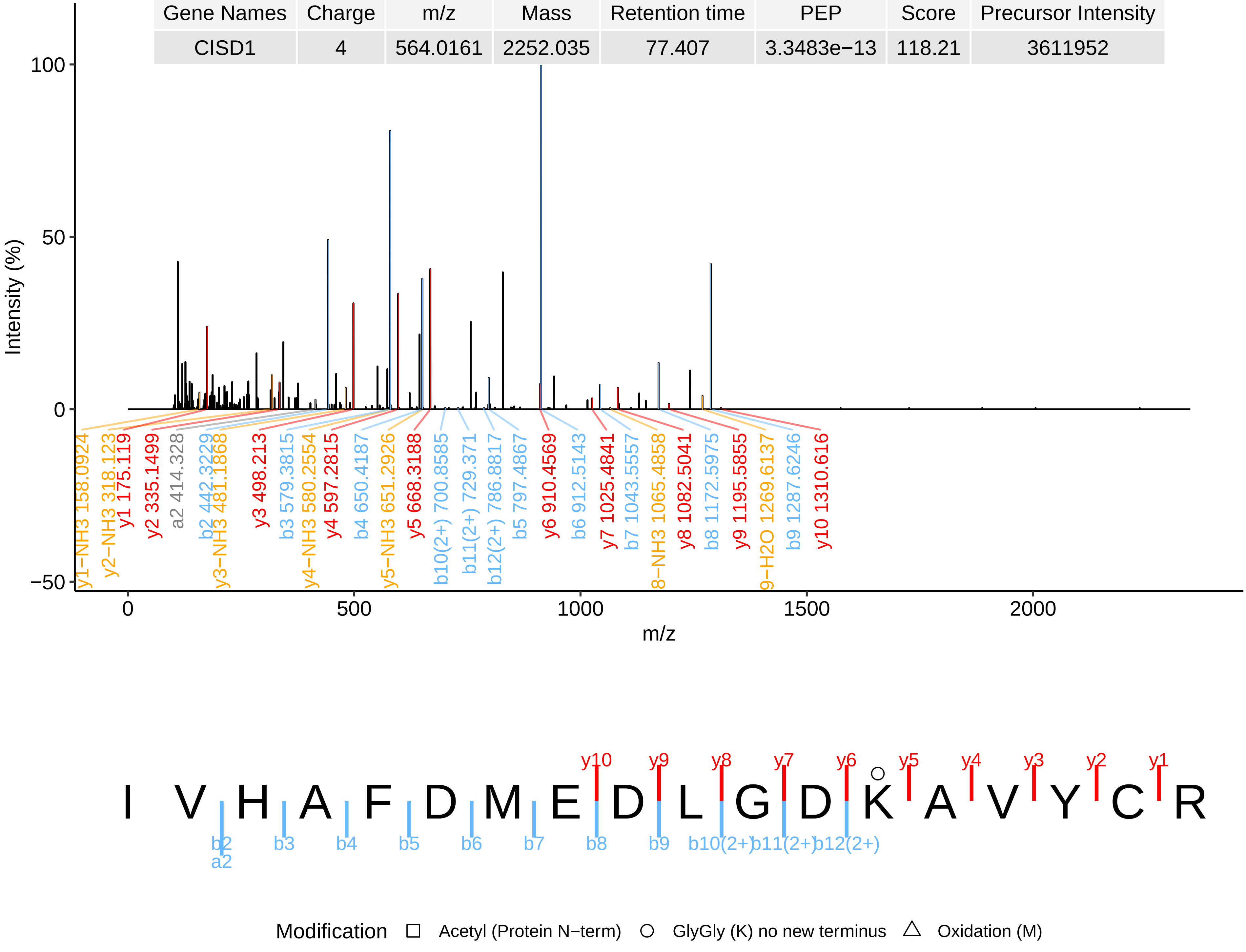

20200921_KIZ_MUb4_GlyGly_10plex_2 − Scan number: 19653

Gene Names	Charge	m/z	Mass	Retention time	PEP	Score	Precursor Intensity
CISD1	3	751.6857	2252.035	77.159	0.010892	61.265	NA

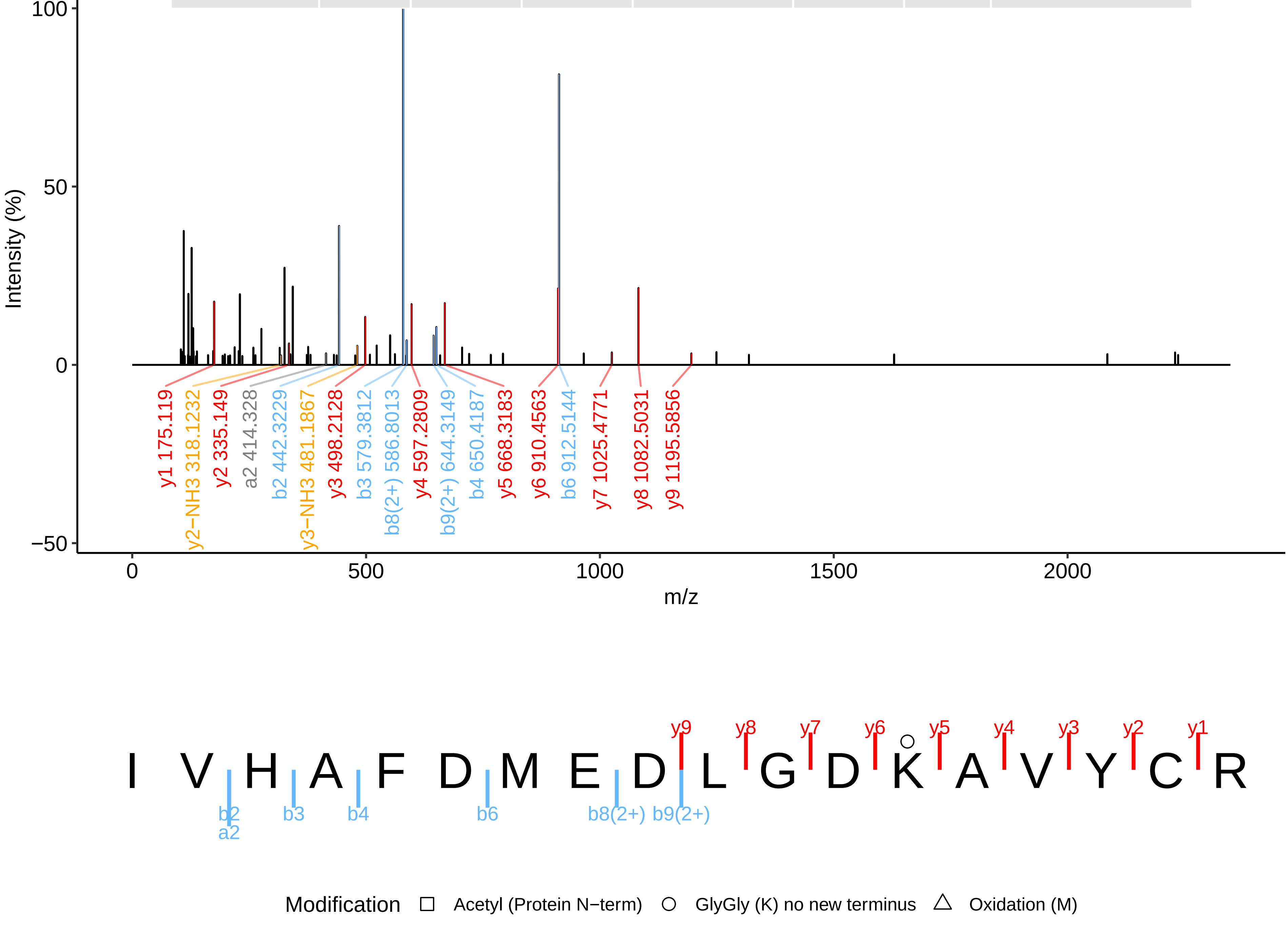

20200924_KIZ_MUb5_GlyGly_1 − Scan number: 21071

Gene Names	Charge	m/z	Mass	Retention time	PEP	Score	Precursor Intensity
CISD1	4	564.0161	2252.035	67.87	5.4309e−18	147.12	15051566

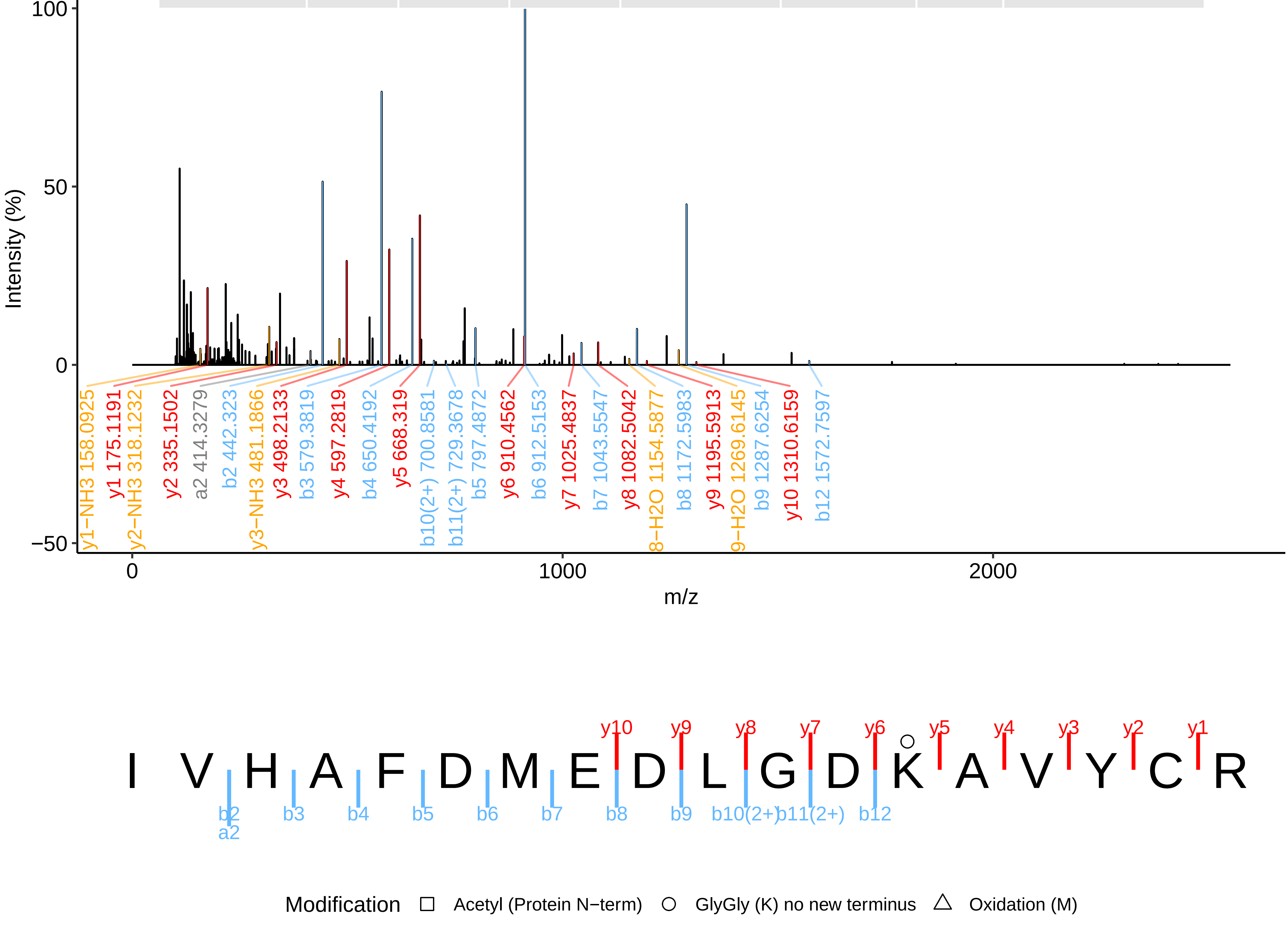

20200924_KIZ_MUb5_GlyGly_1 − Scan number: 21388

Gene Names	Charge	m/z	Mass	Retention time	PEP	Score	Precursor Intensity
CISD1	4	564.0161	2252.035	68.888	1.9289e−24	166.9	NA

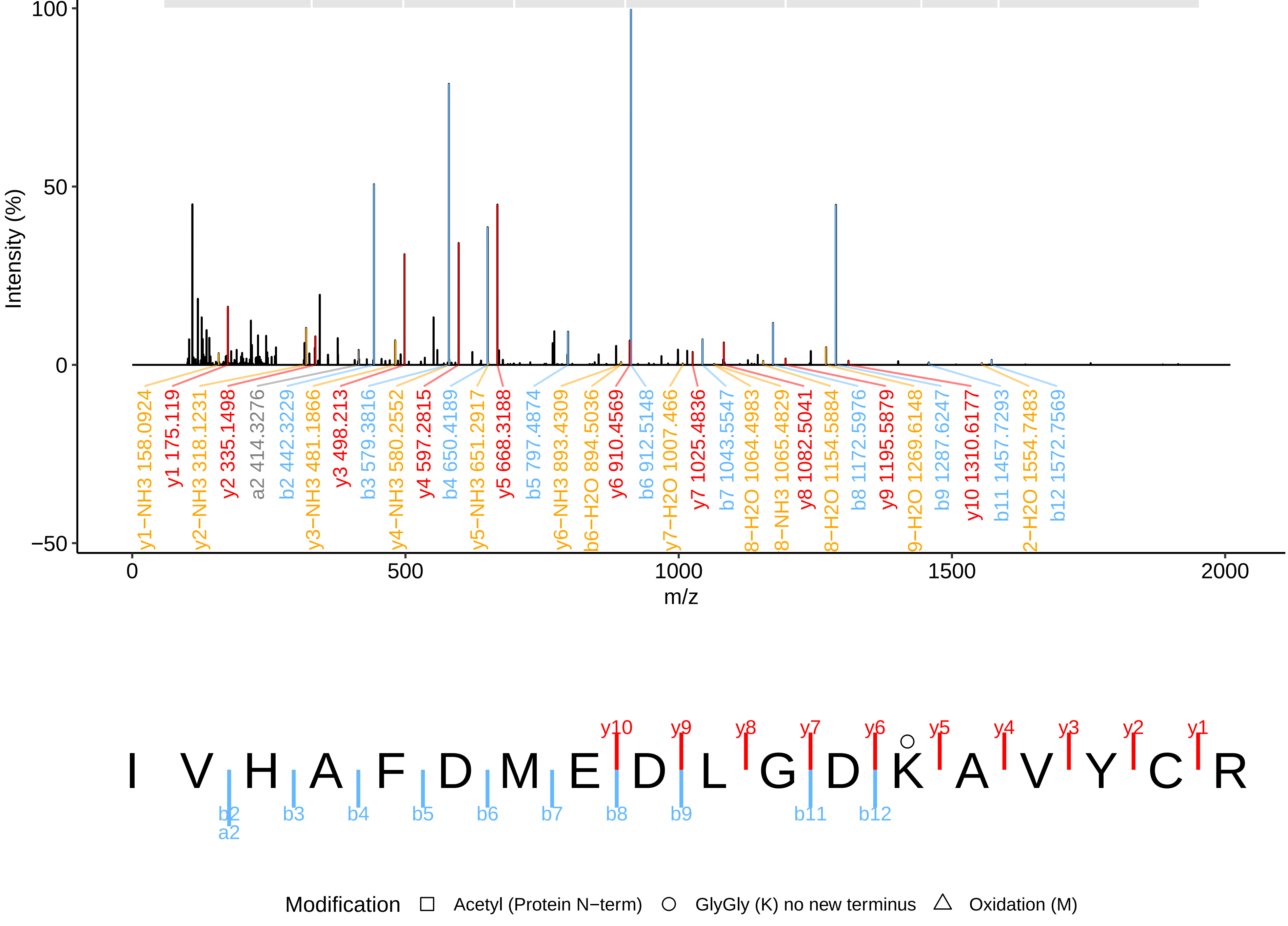

20200924_KIZ_MUb5_GlyGly_1 − Scan number: 21545

Gene Names	Charge	m/z	Mass	Retention time	PEP	Score	Precursor Intensity
CISD1	4	564.0161	2252.035	69.397	4.4077e−23	150.59	23956608

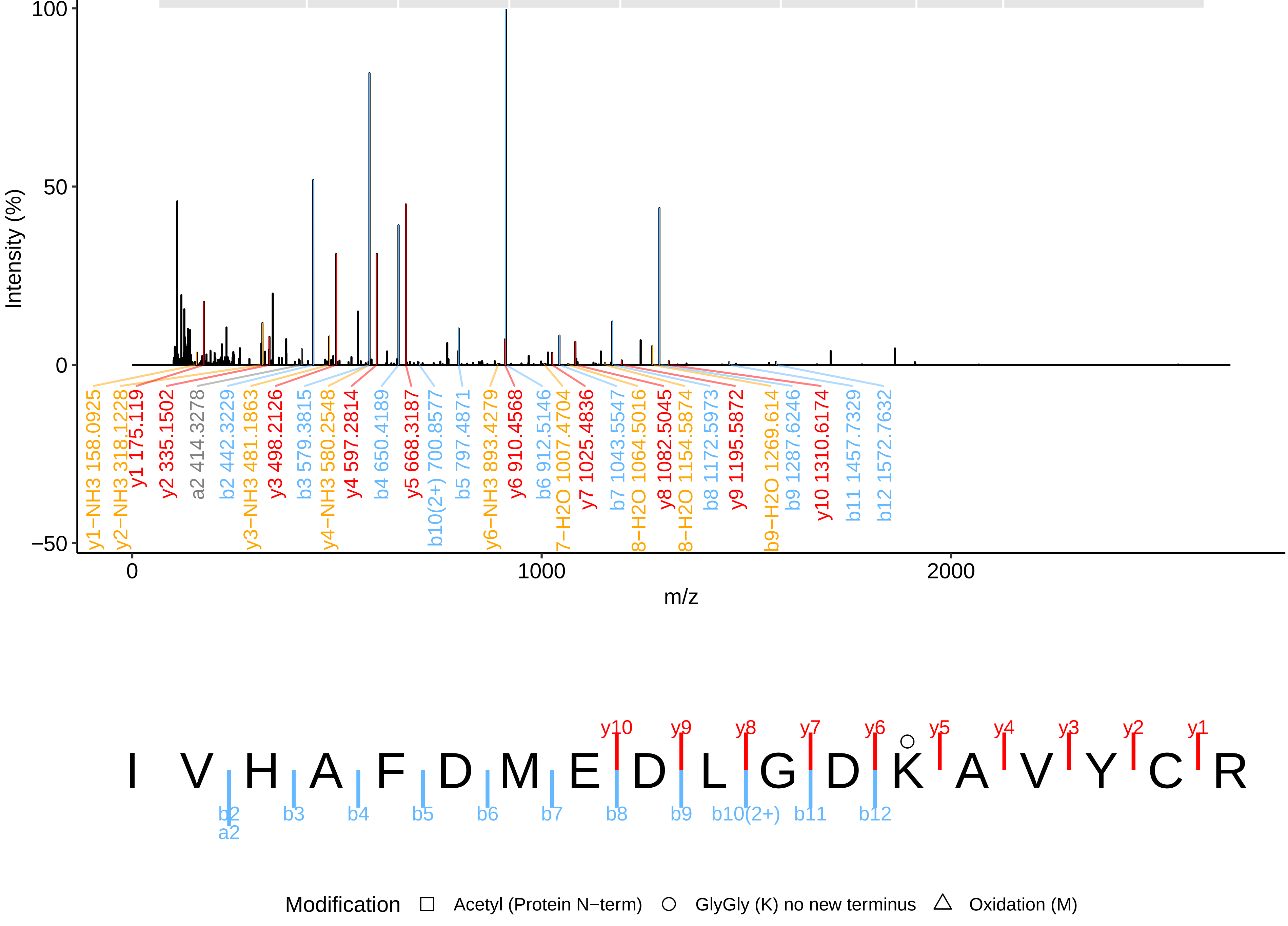

20200924_KIZ_MUb5_GlyGly_2 − Scan number: 19507

Gene Names	Charge	m/z	Mass	Retention time	PEP	Score	Precursor Intensity
CISD1	4	564.0161	2252.035	72.311	1.6378e−18	149.3	7089253

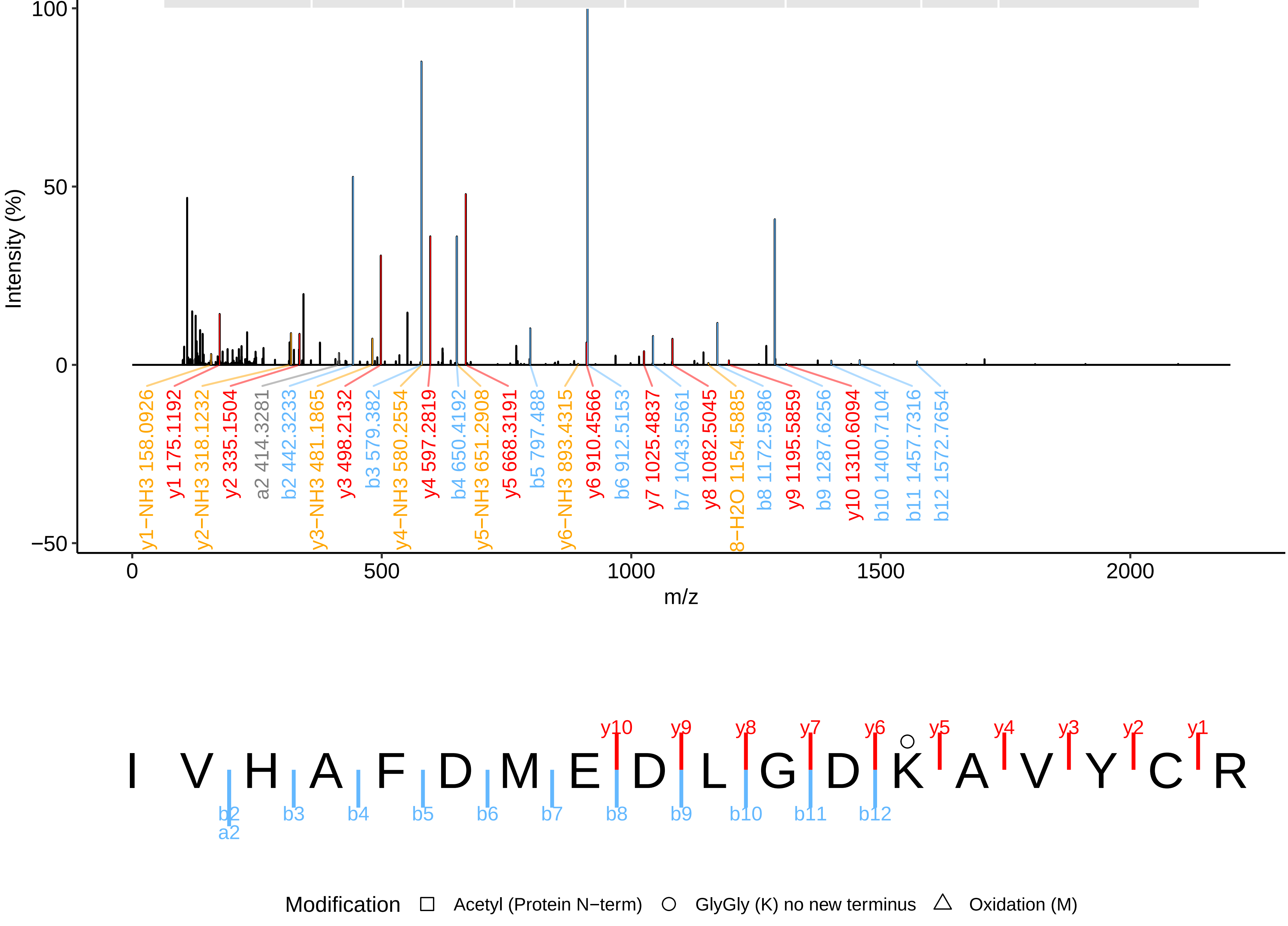

20201118_KIZ_MUb6_GlyGly_1 − Scan number: 21700

Gene Names	Charge	m/z	Mass	Retention time	PEP	Score	Precursor Intensity
CISD1	4	564.0161	2252.035	67.66	1.1415e−23	163.23	24820154

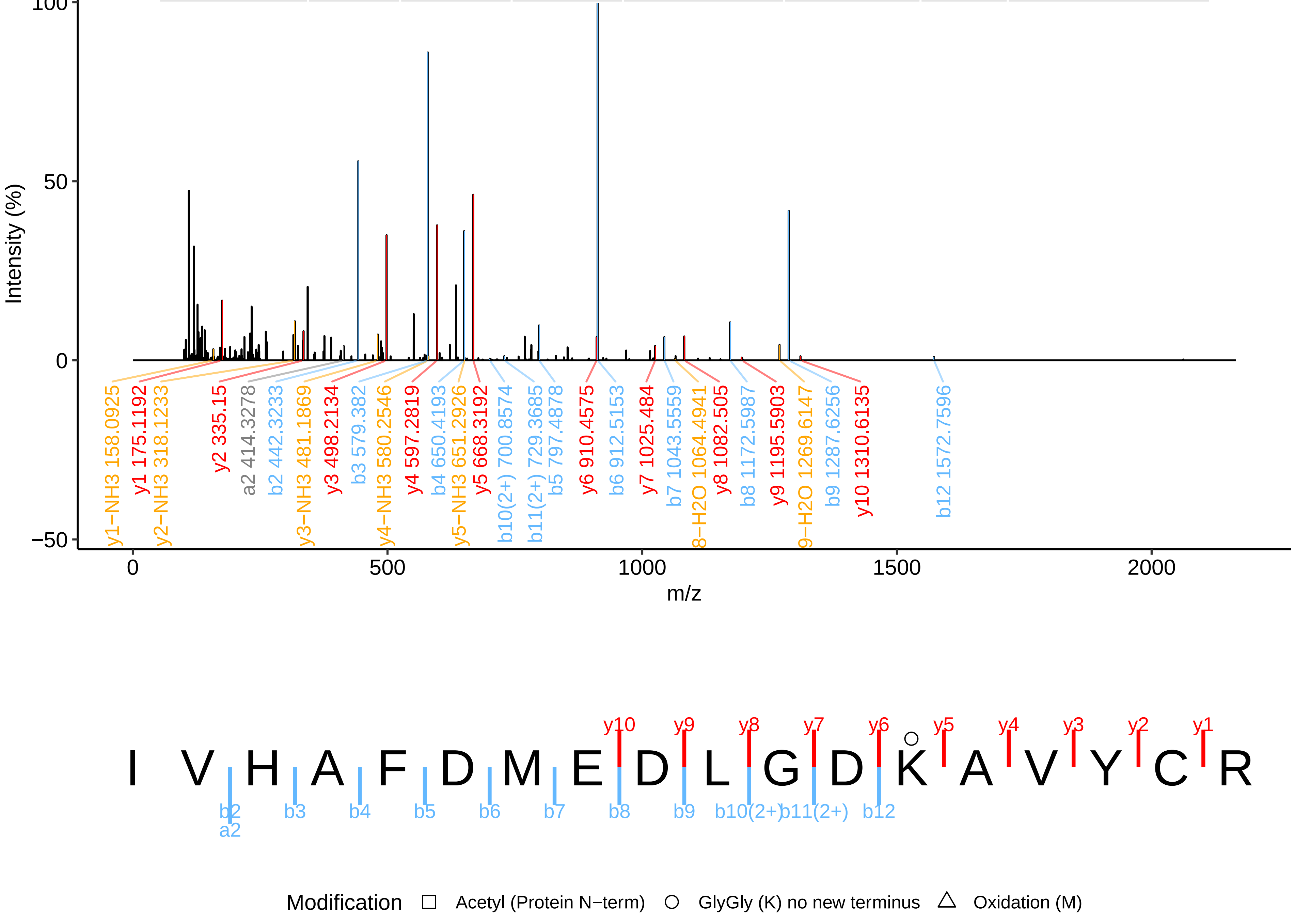

20201118_KIZ_MUb6_GlyGly_2 − Scan number: 23452

Gene Names	Charge	m/z	Mass	Retention time	PEP	Score	Precursor Intensity
CISD1	4	564.0161	2252.035	78.647	1.4176e−17	142.1	59954628

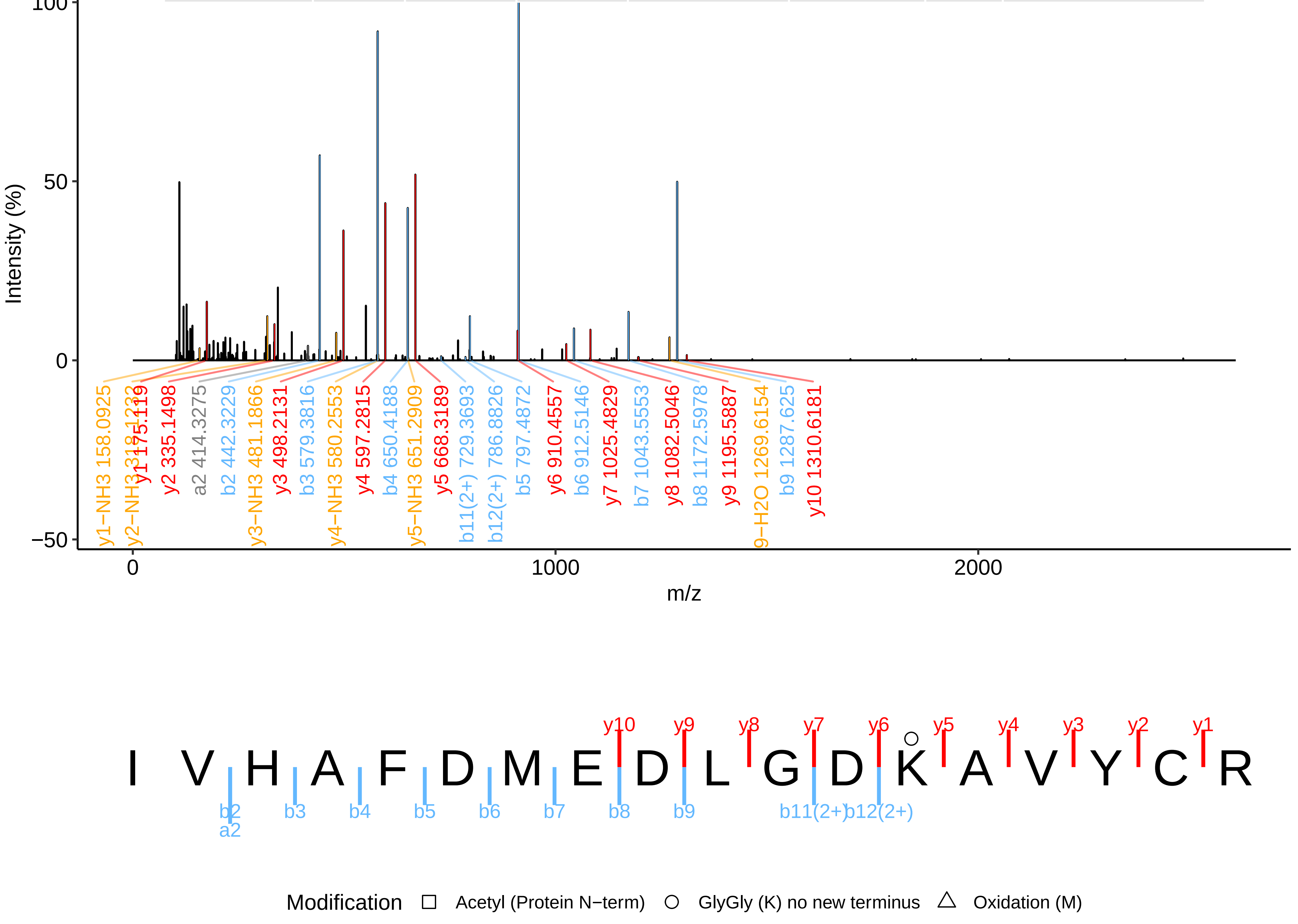

20201118_KIZ_MUb6_GlyGly_2 − Scan number: 23500

Gene Names	Charge	m/z	Mass	Retention time	PEP	Score	Precursor Intensity
CISD1	3	751.6857	2252.035	78.799	1.345e−16	140.22	8427053

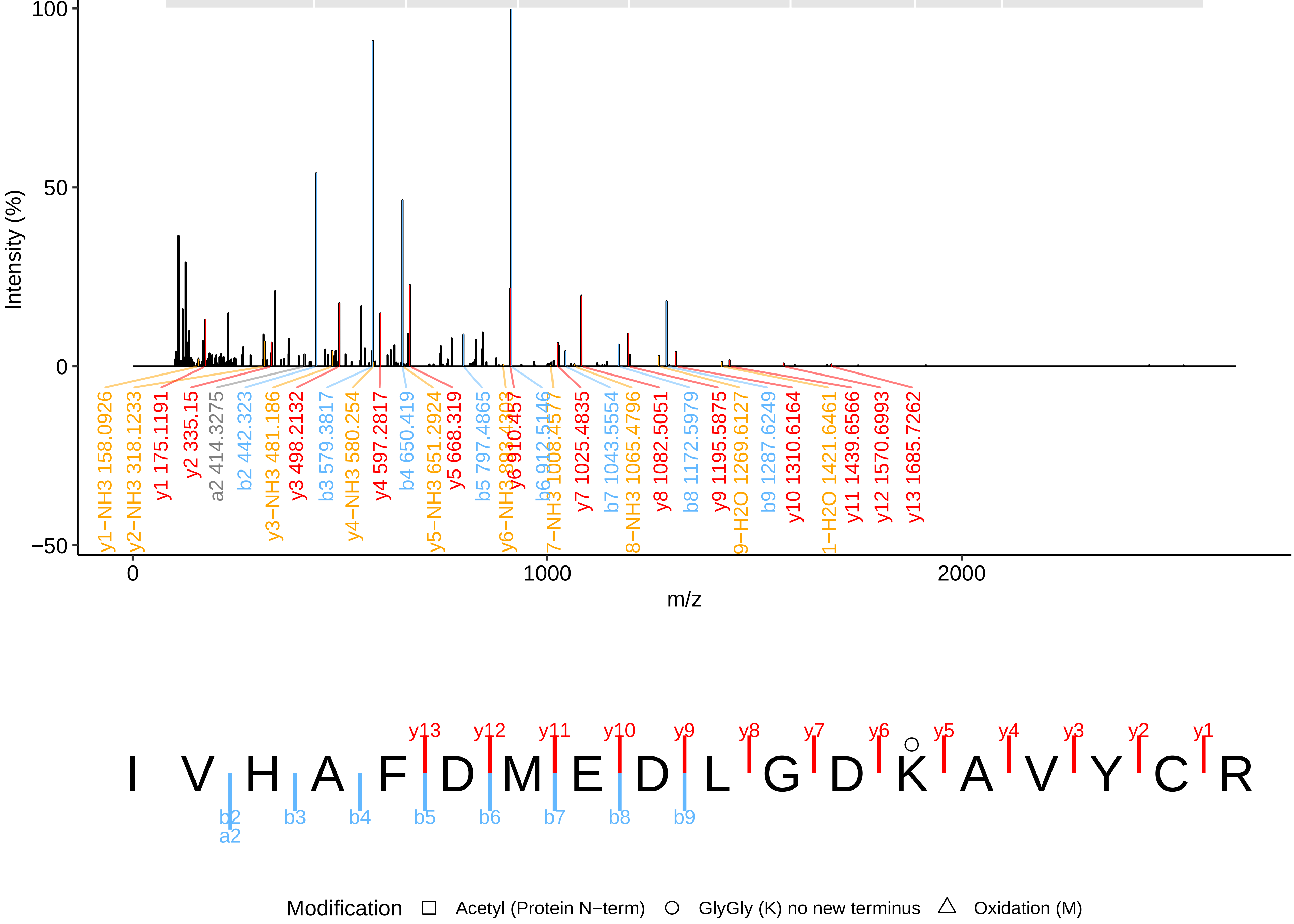

20200921_KIZ_MUb4_GlyGly_10plex_2 − Scan number: 15927

Gene Names	Charge	m/z	Mass	Retention time	PEP	Score	Precursor Intensity
CISD1	4	568.0149	2268.03	62.38	8.3071e−14	129.38	1157344

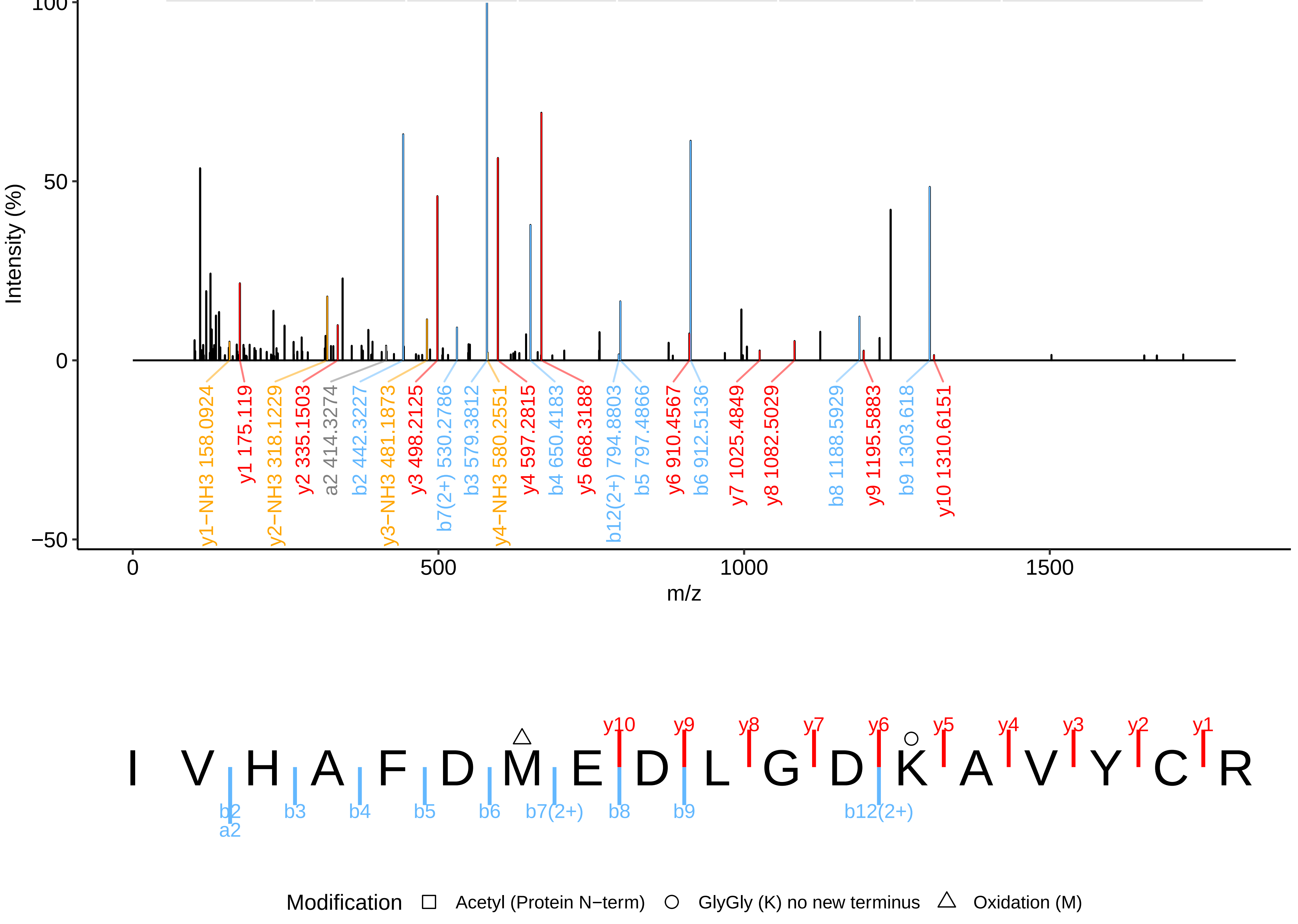

20200921_KIZ_MUb4_GlyGly_10plex − Scan number: 13417

Gene Names	Charge	m/z	Mass	Retention time	PEP	Score	Precursor Intensity
SLC7A5	3	777.385	2329.133	47.332	1.2772e−30	154.42	5129088

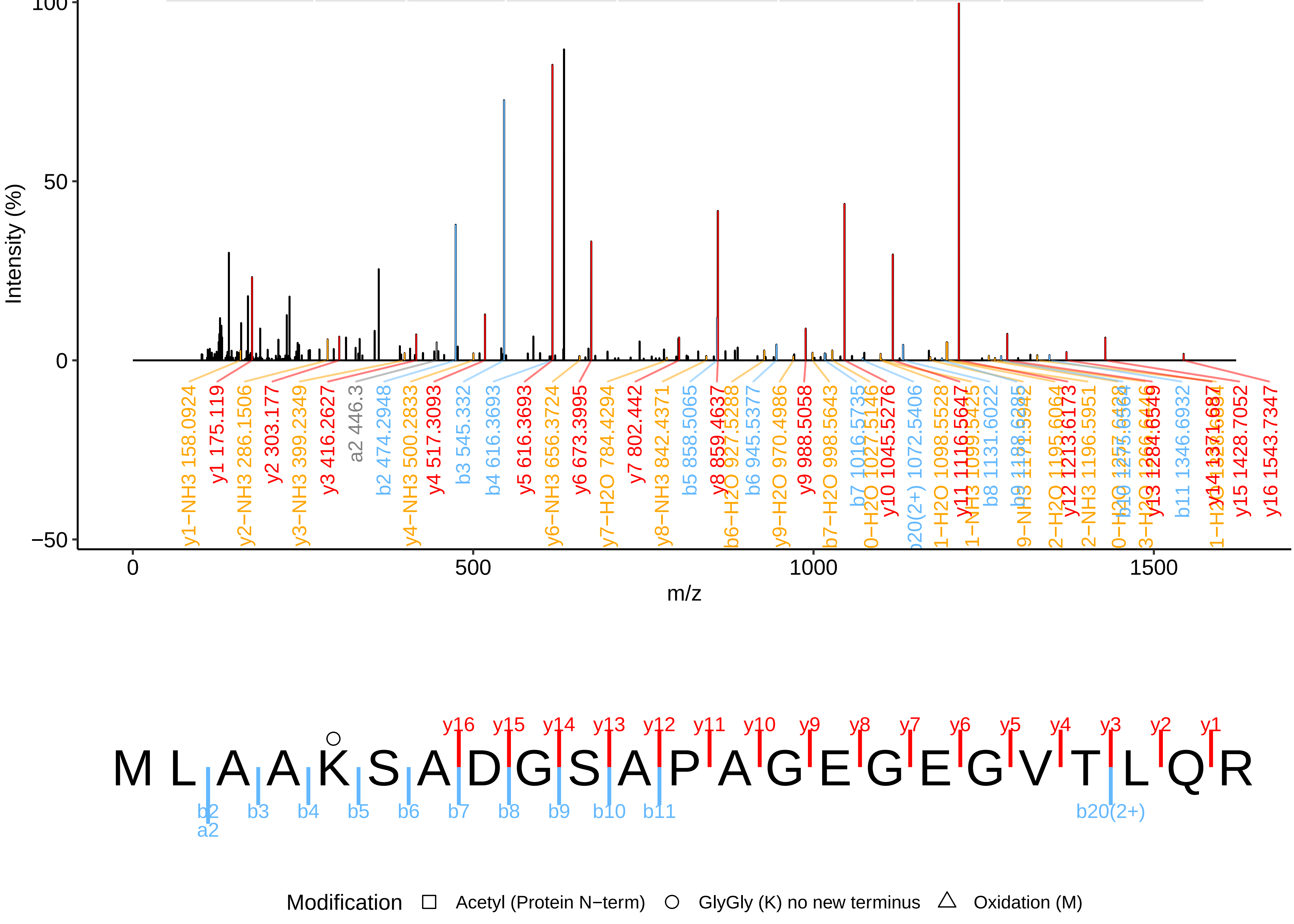

20200921_KIZ_MUb4_GlyGly_10plex − Scan number: 13434

Gene Names	Charge	m/z	Mass	Retention time	PEP	Score	Precursor Intensity
SLC7A5	4	583.2906	2329.133	47.395	8.75e−07	81.144	6888728

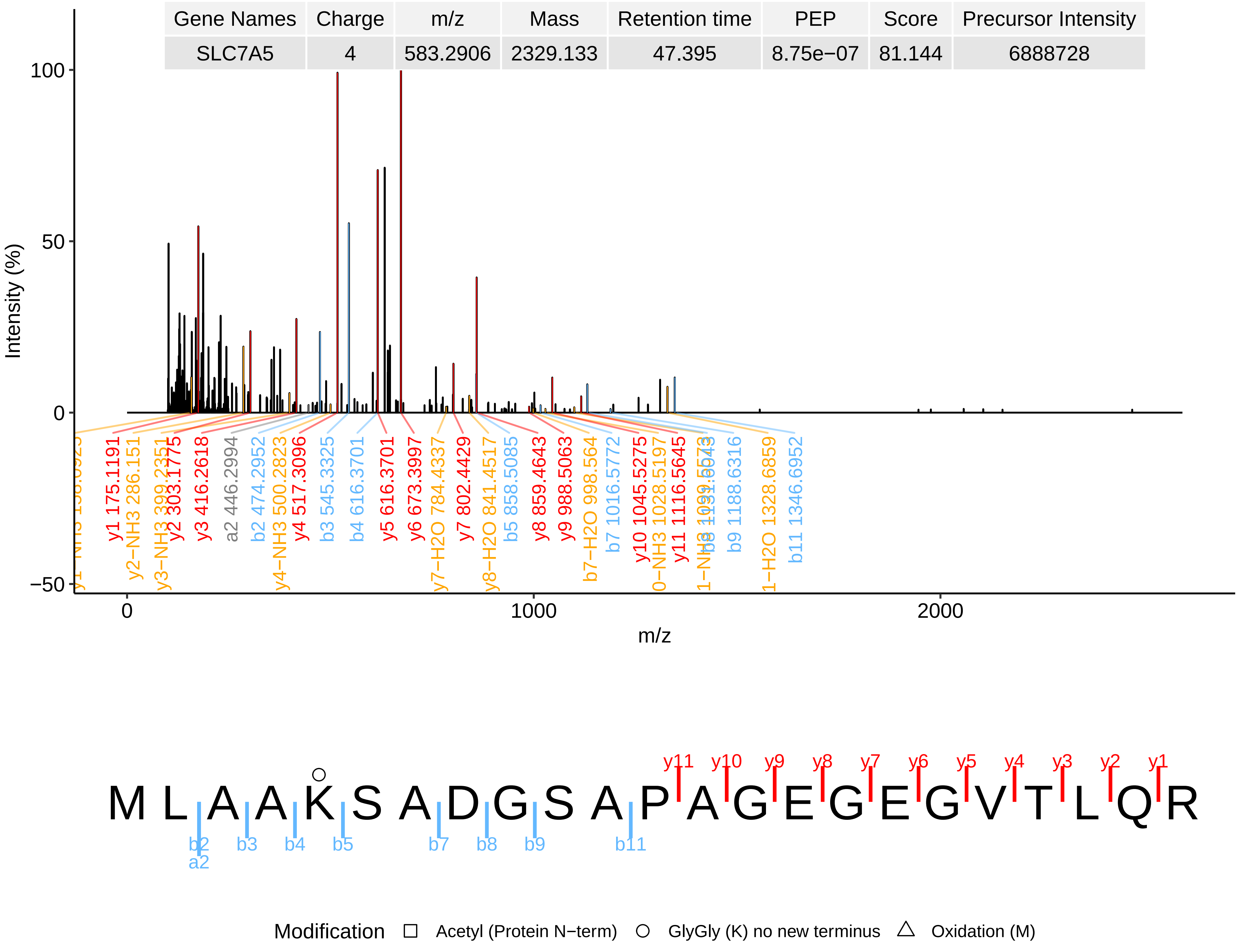

20200921_KIZ_MUb4_GlyGly_10plex_2 − Scan number: 12319

Gene Names	Charge	m/z	Mass	Retention time	PEP	Score	Precursor Intensity
SLC7A5	3	777.385	2329.133	47.634	0.00082064	64.295	503377.9

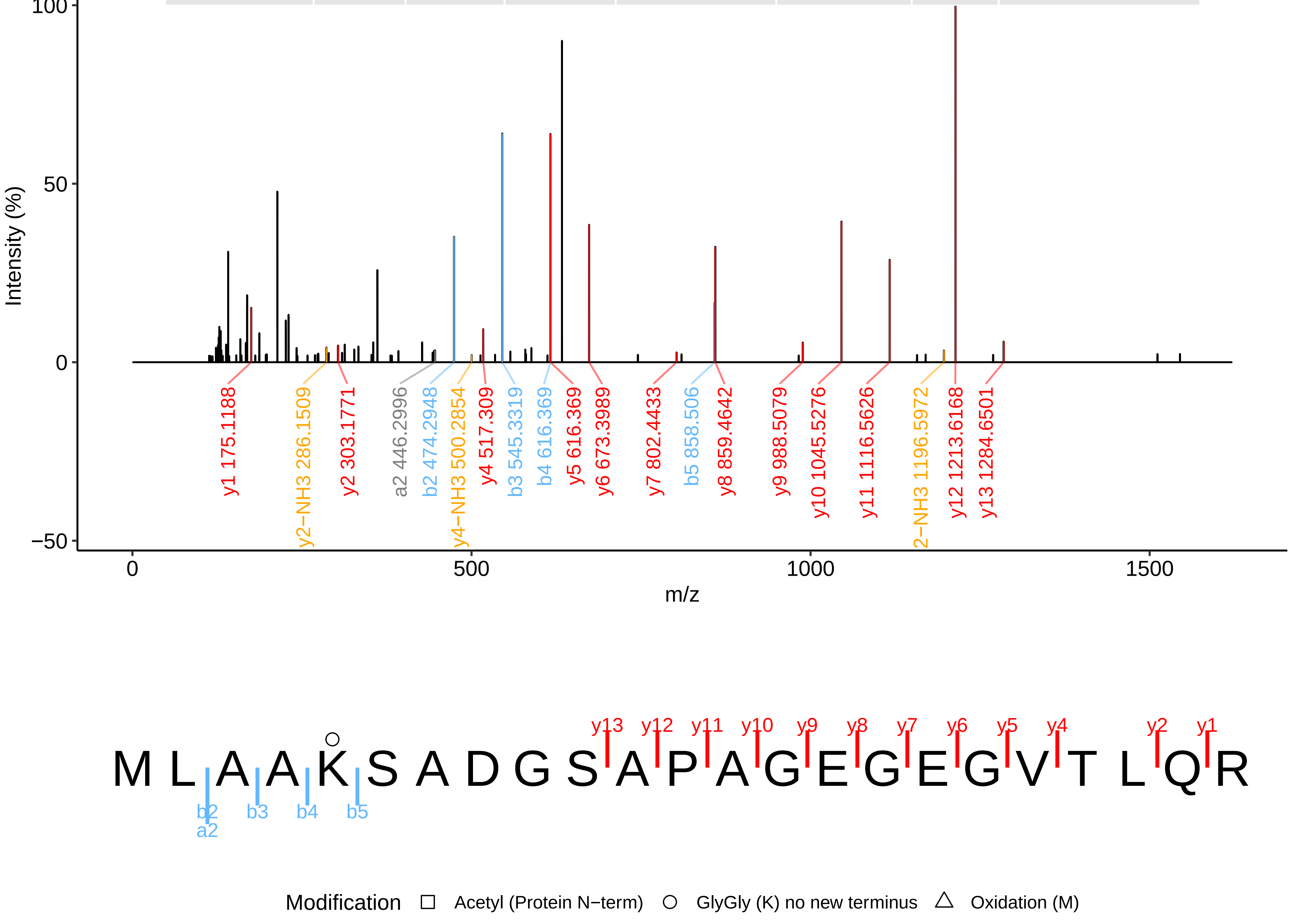

20200924_KIZ_MUb5_GlyGly_1 − Scan number: 12586

Gene Names	Charge	m/z	Mass	Retention time	PEP	Score	Precursor Intensity
SLC7A5	3	777.385	2329.133	40.977	4.1359e−22	134.66	7100221

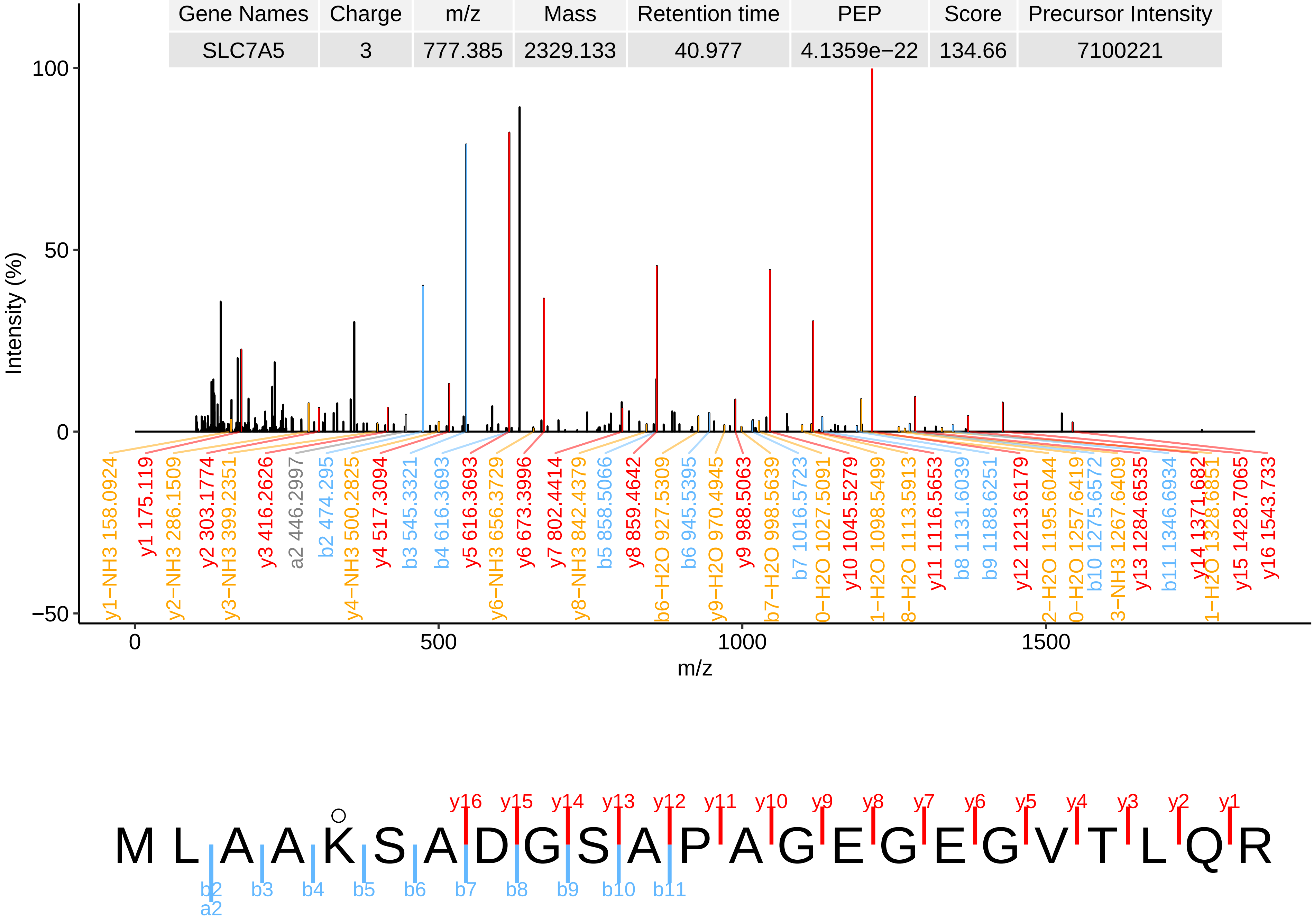

Modification □ Acetyl (Protein N−term) ○ GlyGly (K) no new terminus △ Oxidation (M)

20200924_KIZ_MUb5_GlyGly_1 − Scan number: 12597

Gene Names	Charge	m/z	Mass	Retention time	PEP	Score	Precursor Intensity
SLC7A5	3	777.385	2329.133	41.014	5.1819e−23	148.29	11332820

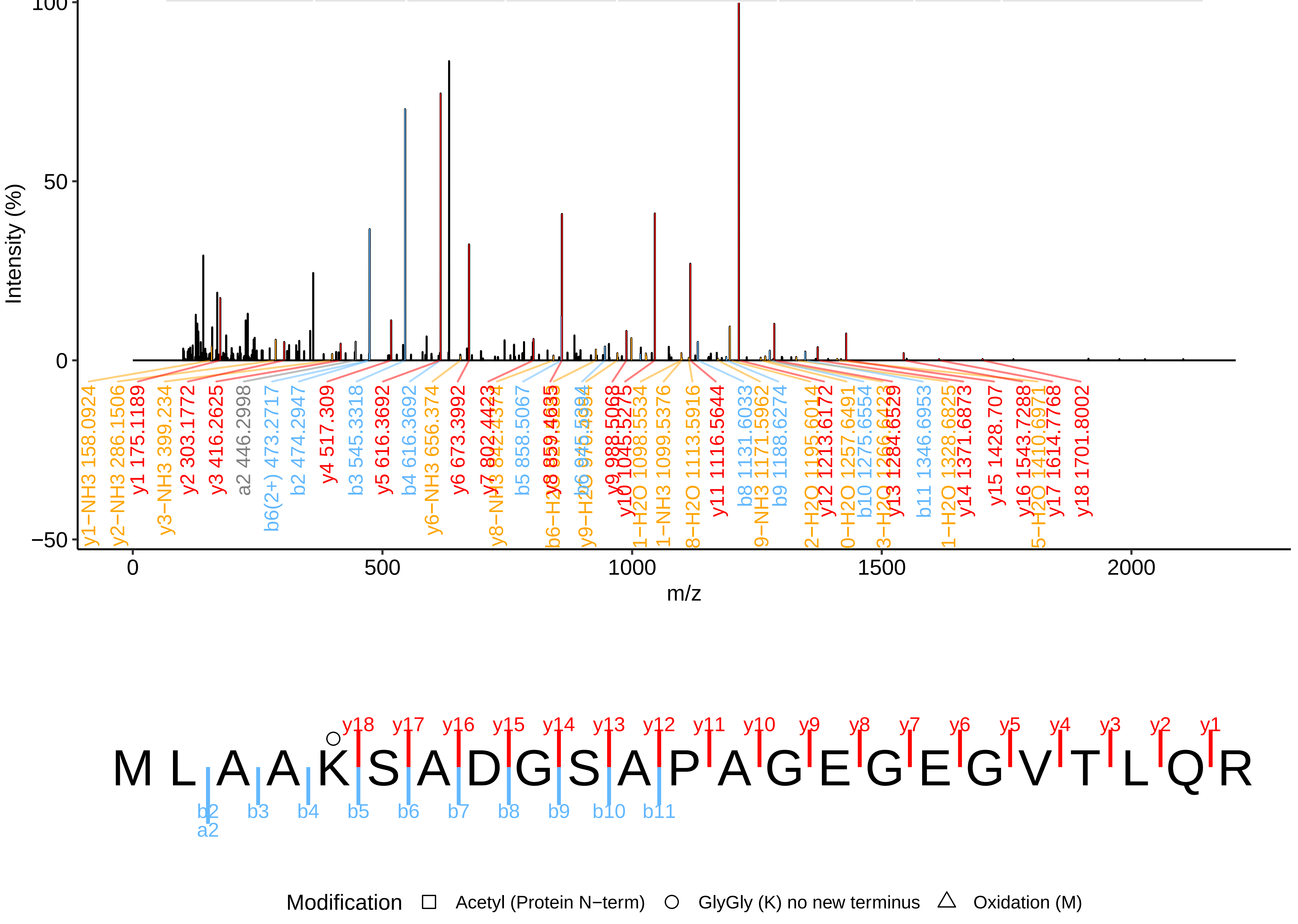

20200924_KIZ_MUb5_GlyGly_1 − Scan number: 12751

Gene Names	Charge	m/z	Mass	Retention time	PEP	Score	Precursor Intensity
SLC7A5	3	777.385	2329.133	41.532	4.6488e−31	162.83	136324864

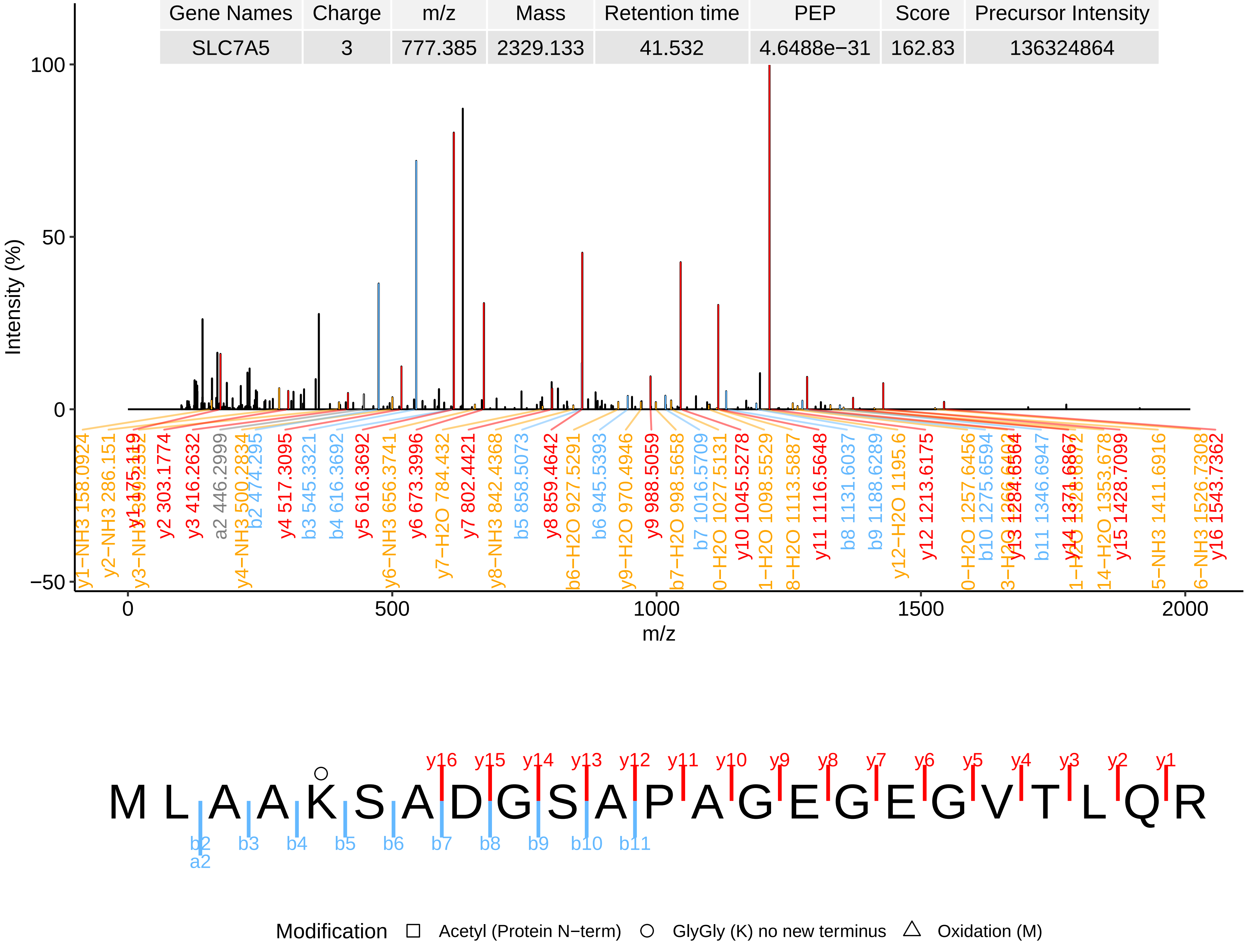

20200924_KIZ_MUb5_GlyGly_1 − Scan number: 12684

Gene Names	Charge	m/z	Mass	Retention time	PEP	Score	Precursor Intensity
SLC7A5	4	583.2906	2329.133	41.308	1.7342e−15	118.05	20475022

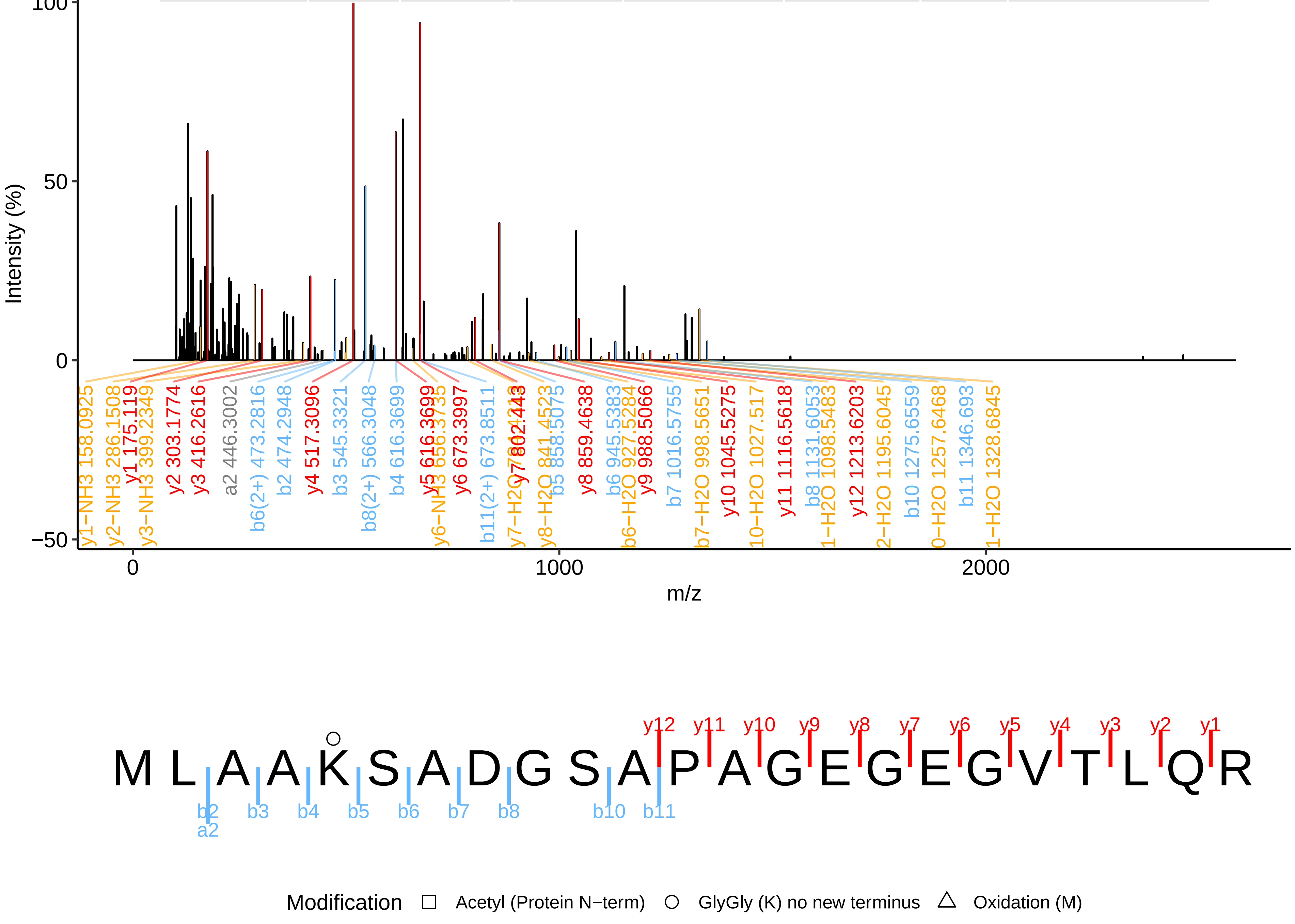

20200924_KIZ_MUb5_GlyGly_2 − Scan number: 11747

Gene Names	Charge	m/z	Mass	Retention time	PEP	Score	Precursor Intensity
SLC7A5	3	777.385	2329.133	43.911	7.9031e−16	127.28	11165423

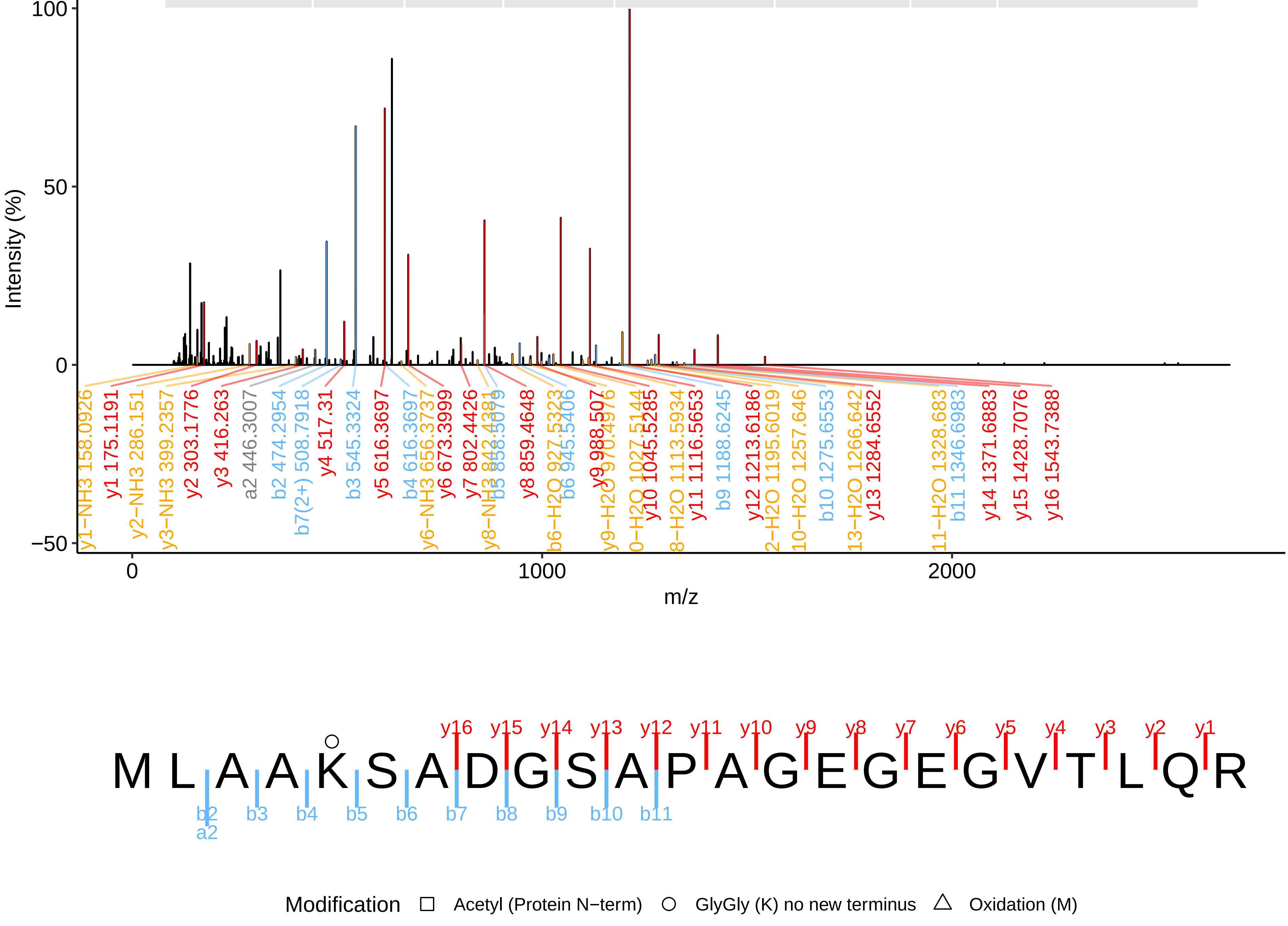

20200924_KIZ_MUb5_GlyGly_2 − Scan number: 11878

Gene Names	Charge	m/z	Mass	Retention time	PEP	Score	Precursor Intensity
SLC7A5	3	777.385	2329.133	44.41	1.0797e−22	146.17	23025316

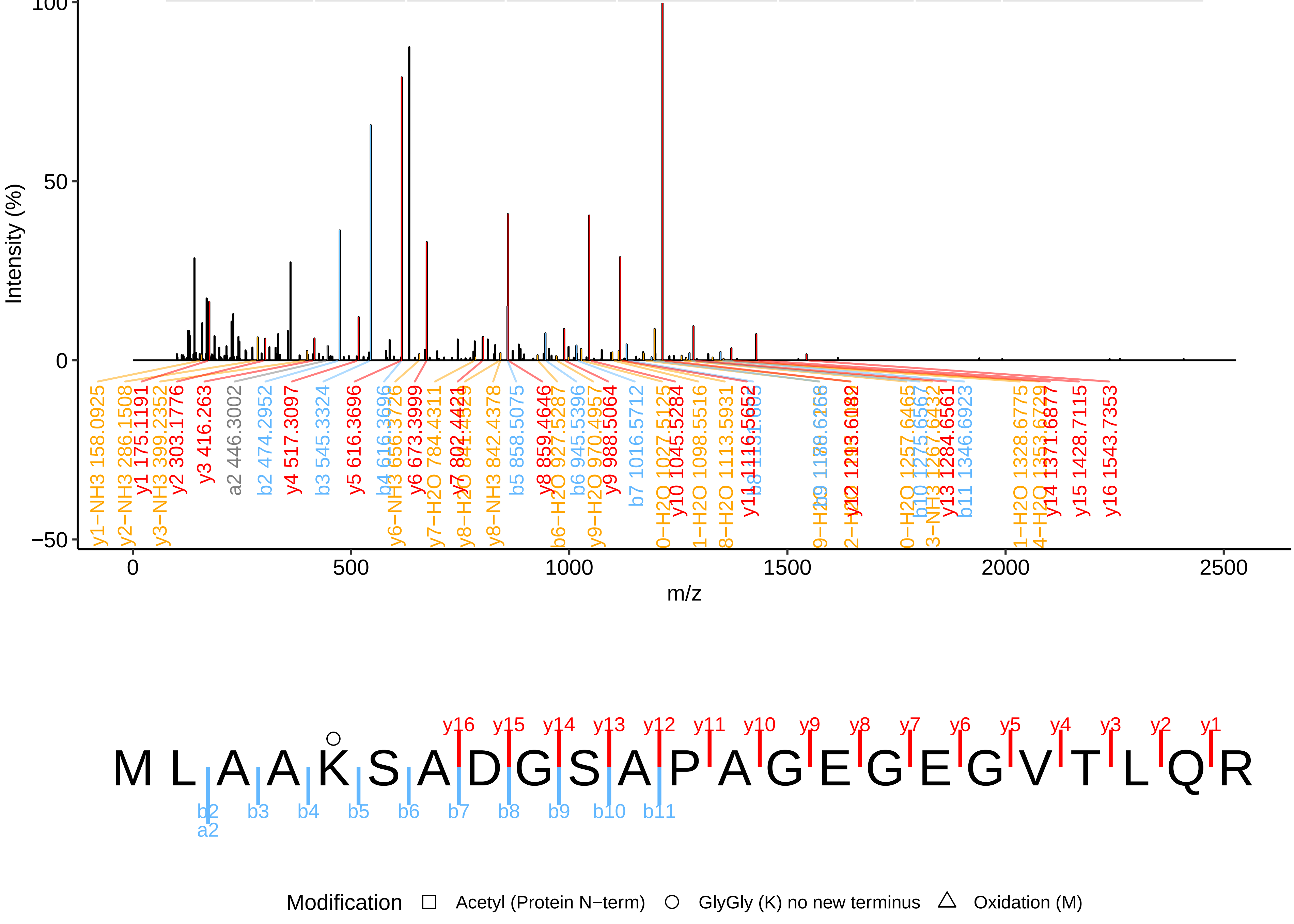

20200924_KIZ_MUb5_GlyGly_2 − Scan number: 12019

Gene Names	Charge	m/z	Mass	Retention time	PEP	Score	Precursor Intensity
SLC7A5	3	777.385	2329.133	44.937	6.734e−07	87.319	939176.6

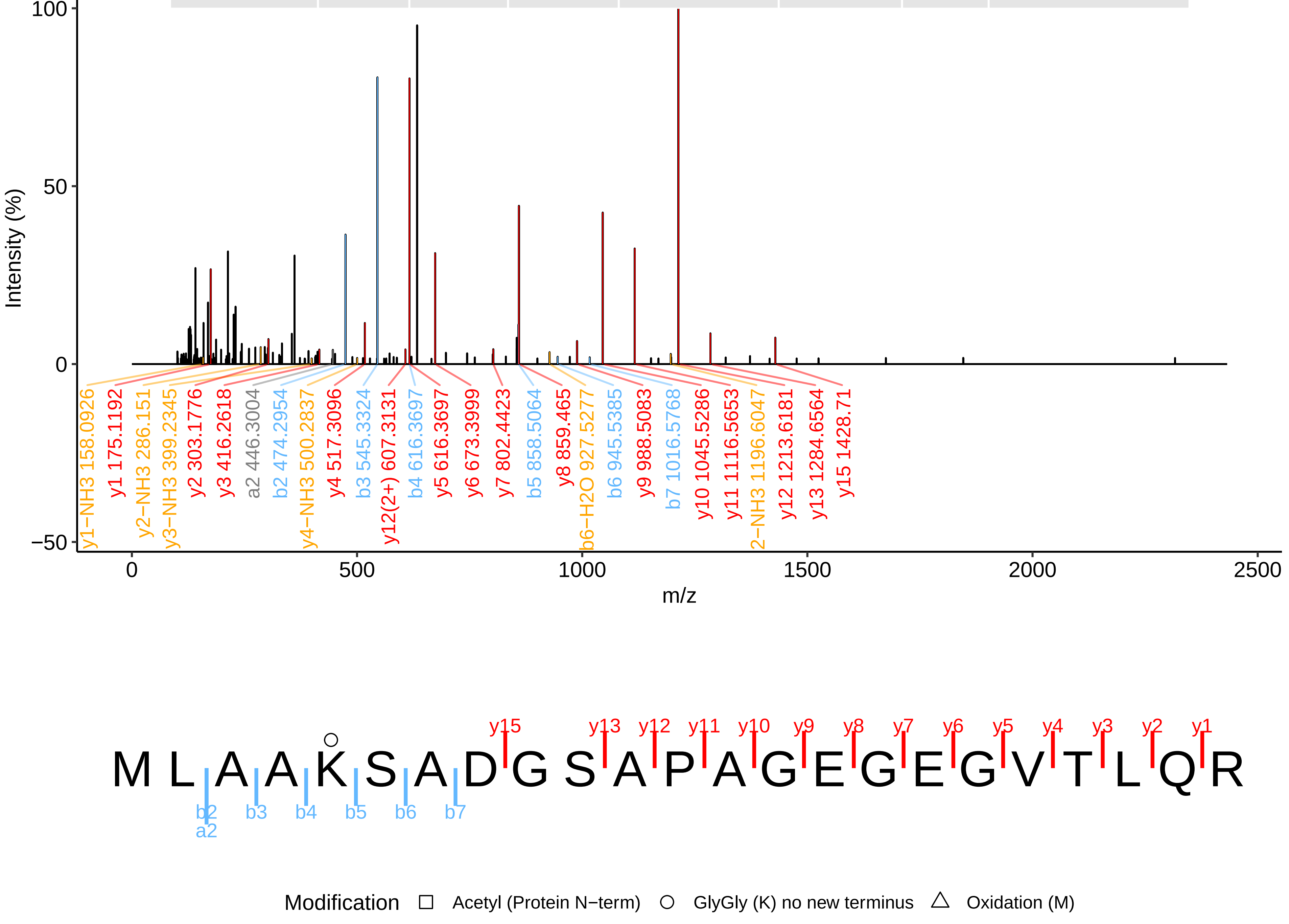

20201118_KIZ_MUb6_GlyGly_1 − Scan number: 12605

Gene Names	Charge	m/z	Mass	Retention time	PEP	Score	Precursor Intensity
SLC7A5	3	777.385	2329.133	39.84	1.5216e−30	151.89	NA

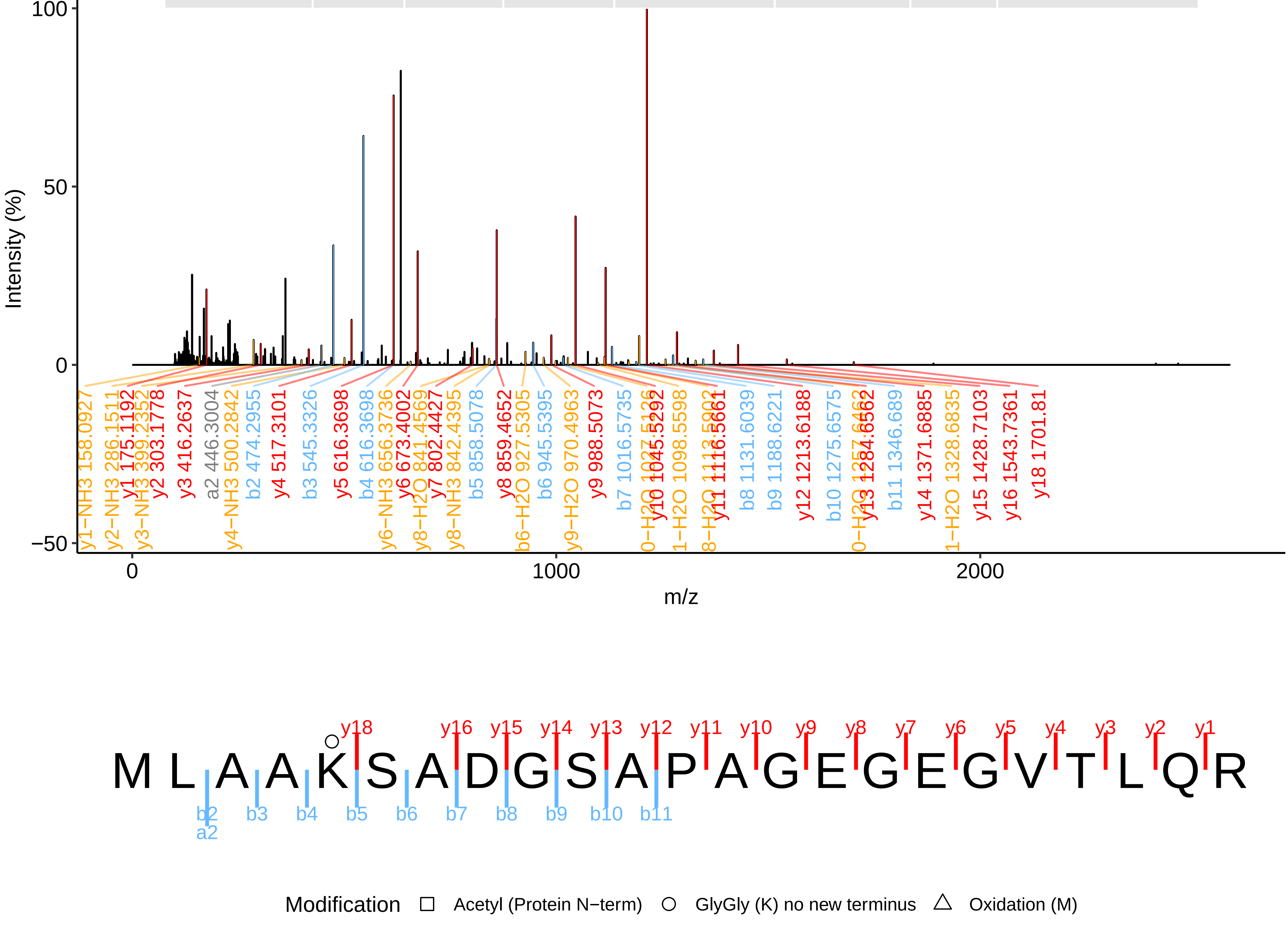

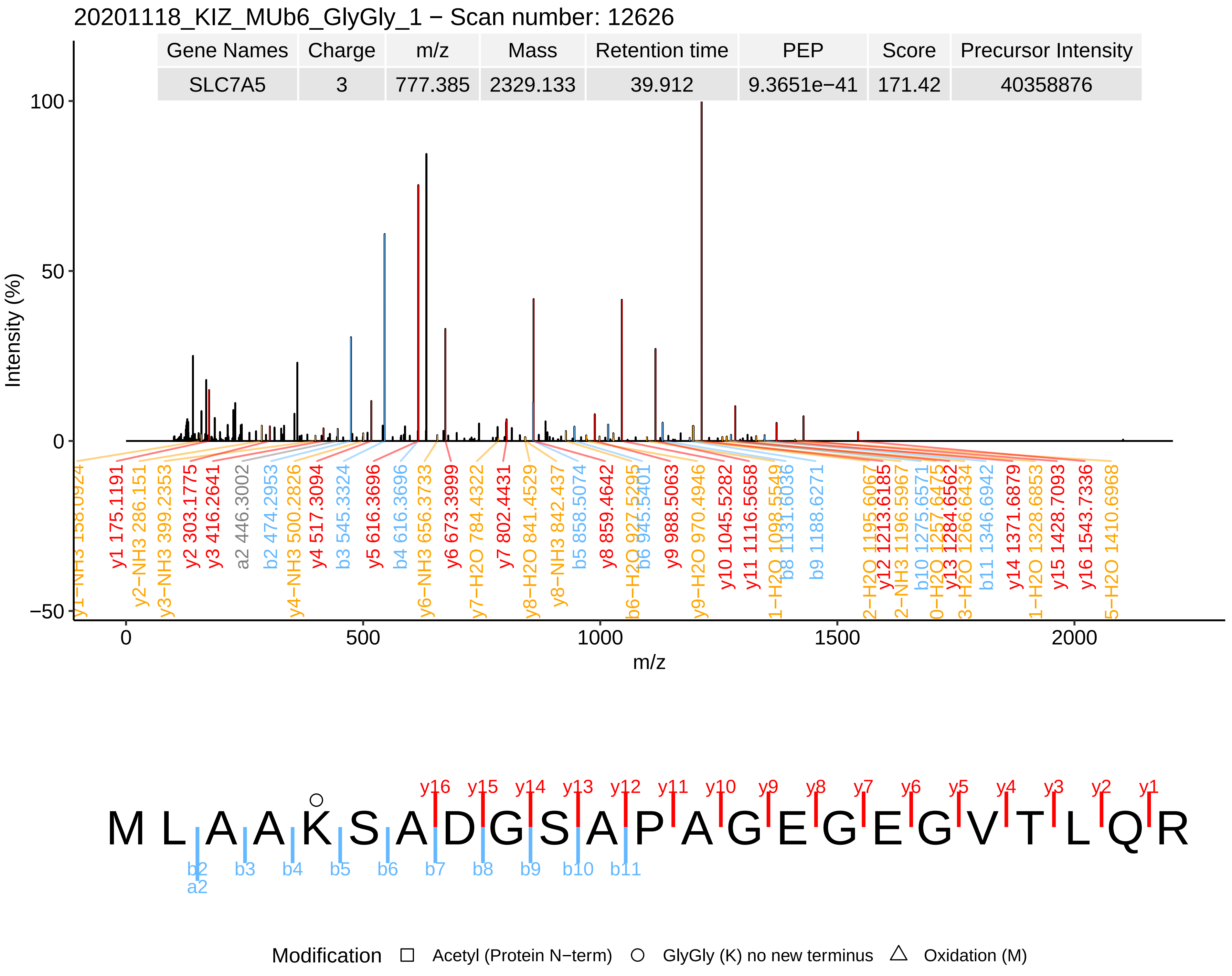
20201118_KIZ_MUb6_GlyGly_1 − Scan number: 12626
Gene Names | Charge | m/z | Mass | Retention time | PEP | Score | Precursor Intensity
SLC7A5 | 3 | 777.385 | 2329.133 | 39.912 | 9.3651e−41 | 171.42 | 40358876
Intensity (%)
m/z
y1 175.1191
y2−NH3 286.151
y3−NH3 399.2353
y2 303.1775
y3 416.2641
a2 446.3002
b2 474.2953
y4−NH3 500.2826
y4 517.3094
b3 545.3324
y5 616.3696
b4 616.3696
y6−NH3 656.3733
y6 673.3999
y7−H2O 784.4322
y7 802.4431
y8−H2O 841.4529
y8−NH3 842.437
b5 858.5074
y8 859.4642
b6 945.5401
y9 988.5063
y9−H2O 970.4946
y10 1045.5282
y11 1116.5658
b8 1131.6036
b9 1188.6271
y12 1213.6185
y12−NH3 1196.5967
b10 1275.6571
y13 1284.6562
b11 1346.6942
y14 1371.6879
y15 1428.7093
y16 1543.7336
M L A A K S A D G S A P A G E G E G V T L Q R
Modification
Acetyl (Protein N−term)
GlyGly (K) no new terminus
Oxidation (M)

20201118_KIZ_MUb6_GlyGly_2 − Scan number: 14248

Gene Names	Charge	m/z	Mass	Retention time	PEP	Score	Precursor Intensity
SLC7A5	3	777.385	2329.133	48.018	1.2545e−30	154.65	5447301

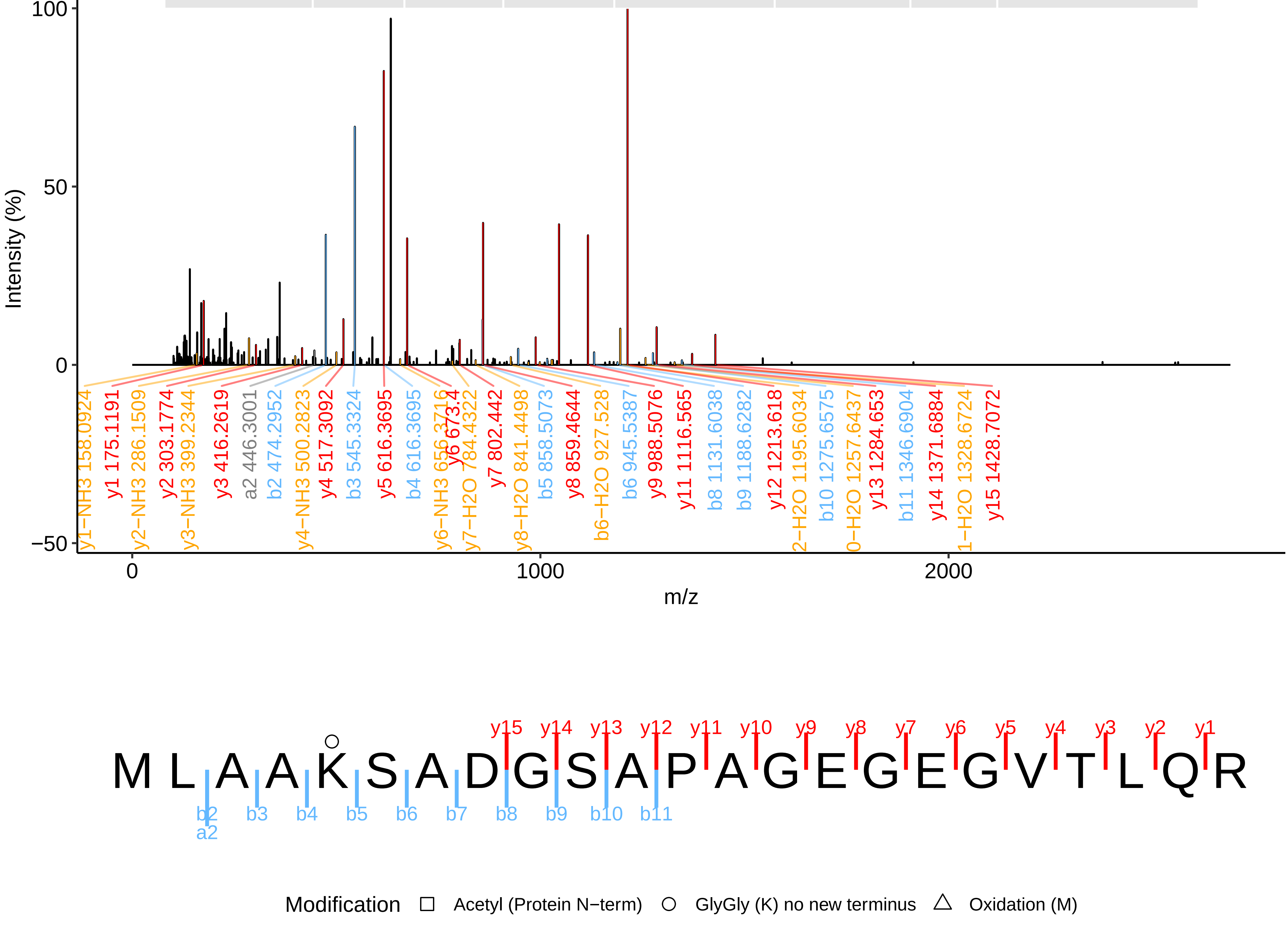

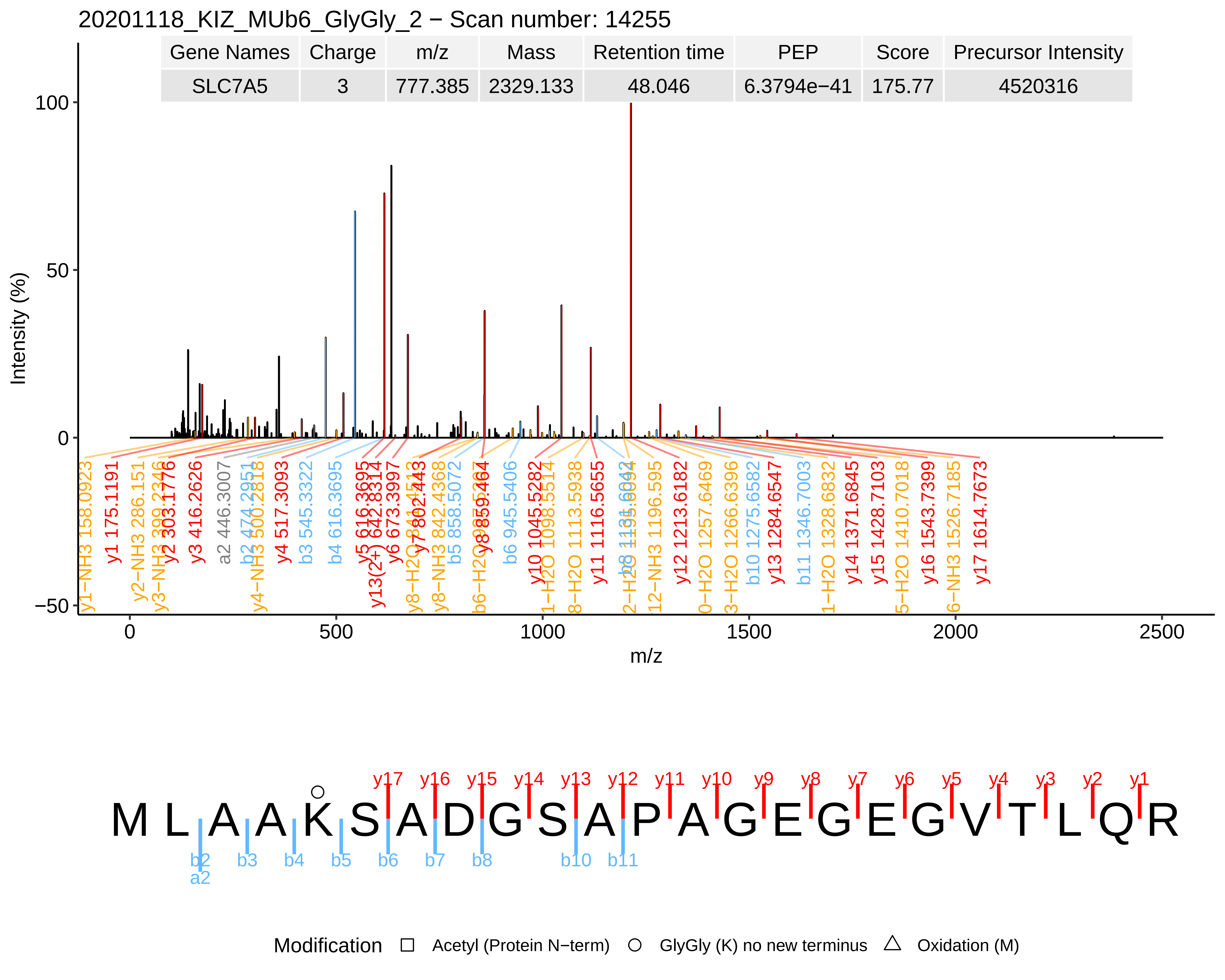

20201118_KIZ_MUb6_GlyGly_2 − Scan number: 14255
Gene Names | Charge | m/z | Mass | Retention time | PEP | Score | Precursor Intensity
SLC7A5 | 3 | 777.385 | 2329.133 | 48.046 | 6.3794e−41 | 175.77 | 4520316
Intensity (%)
m/z
y1−NH3 158.0923
y1 175.1191
y2−NH3 286.151
y3 416.2626
a2 446.3007
y4 517.3093
b3 545.3322
b4 616.3695
y6 673.3997
y8−NH3 842.4368
b5 858.5072
b6 945.5406
y10 1045.5282
y11 1116.5655
y12 1213.6182
b10 1275.6582
y13 1284.6547
b11 1346.7003
y14 1371.6845
y15 1428.7103
y16 1543.7399
y17 1614.7673
M L A A K S A D G S A P A G E G E G V T L Q R
y17 y16 y15 y14 y13 y12 y11 y10 y9 y8 y7 y6 y5 y4 y3 y2 y1
b2 b3 b4 b5 b6 b7 b8 b10 b11
a2
Modification
Acetyl (Protein N−term)
GlyGly (K) no new terminus
Oxidation (M)

20201118_KIZ_MUb6_GlyGly_2 − Scan number: 14263

Gene Names	Charge	m/z	Mass	Retention time	PEP	Score	Precursor Intensity
SLC7A5	4	583.2906	2329.133	48.076	6.155e−16	127.58	4051140

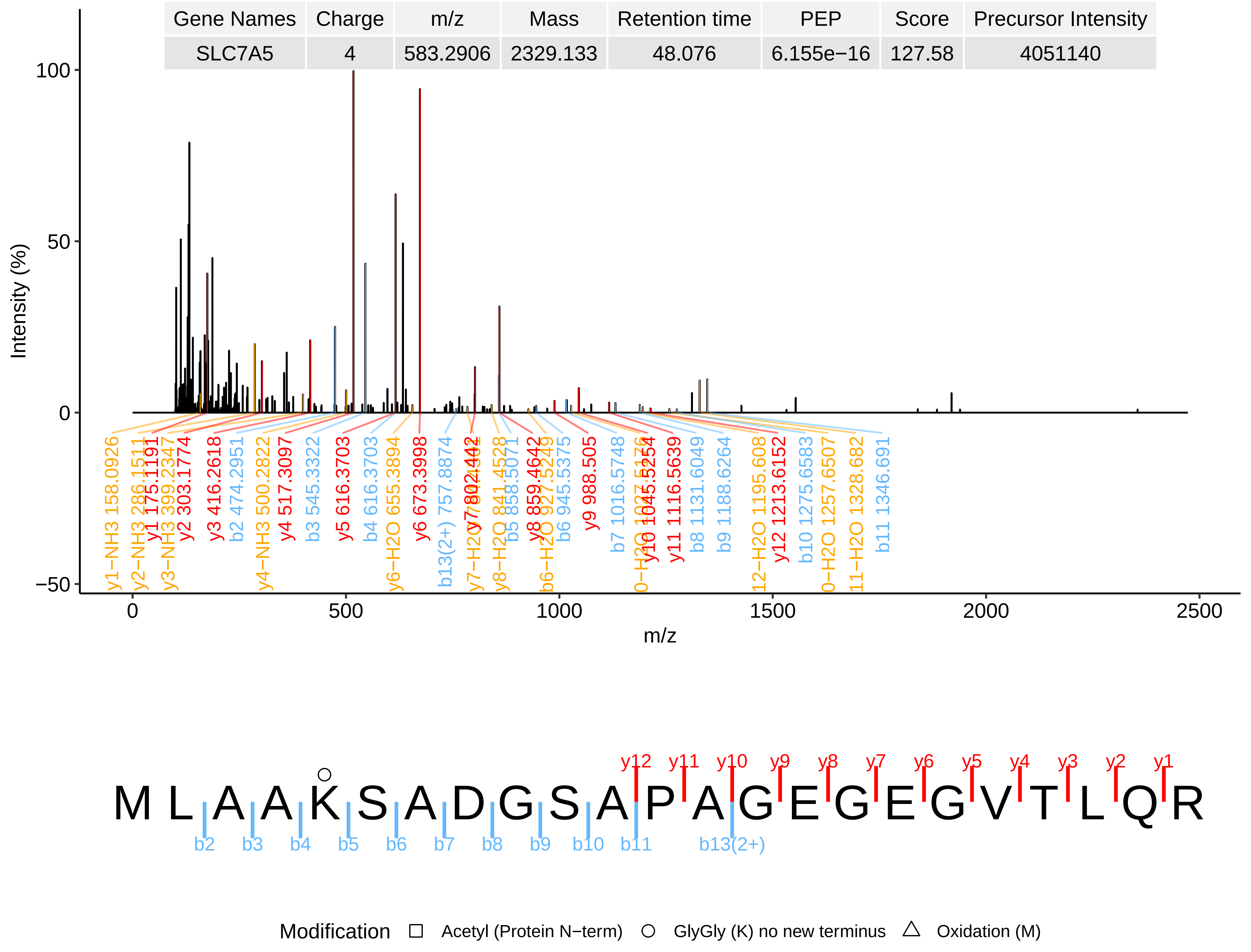

20200921_KIZ_MUb4_GlyGly_10plex_2 − Scan number: 18914

Gene Names	Charge	m/z	Mass	Retention time	PEP	Score	Precursor Intensity
TUBB;TUBB4B;TUBB2A;TUBB2B	3	679.9849	2036.933	74.16	0.085467	45.864	1111012

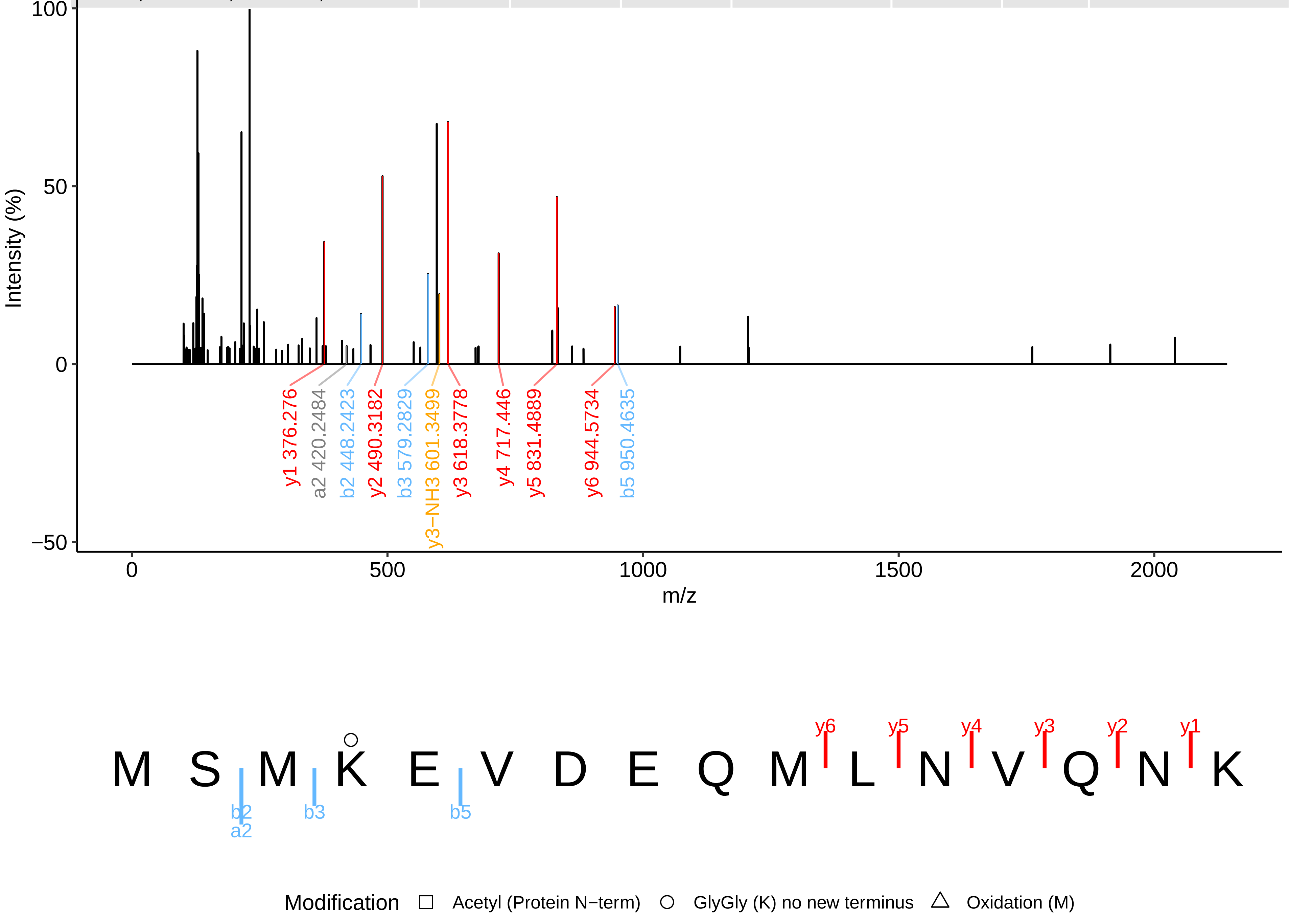

20200921_KIZ_MUb4_GlyGly_10plex_2 − Scan number: 18921

Gene Names	Charge	m/z	Mass	Retention time	PEP	Score	Precursor Intensity
TUBB;TUBB4B;TUBB2A;TUBB2B	3	679.9849	2036.933	74.191	1.0198e−06	97.417	1376090

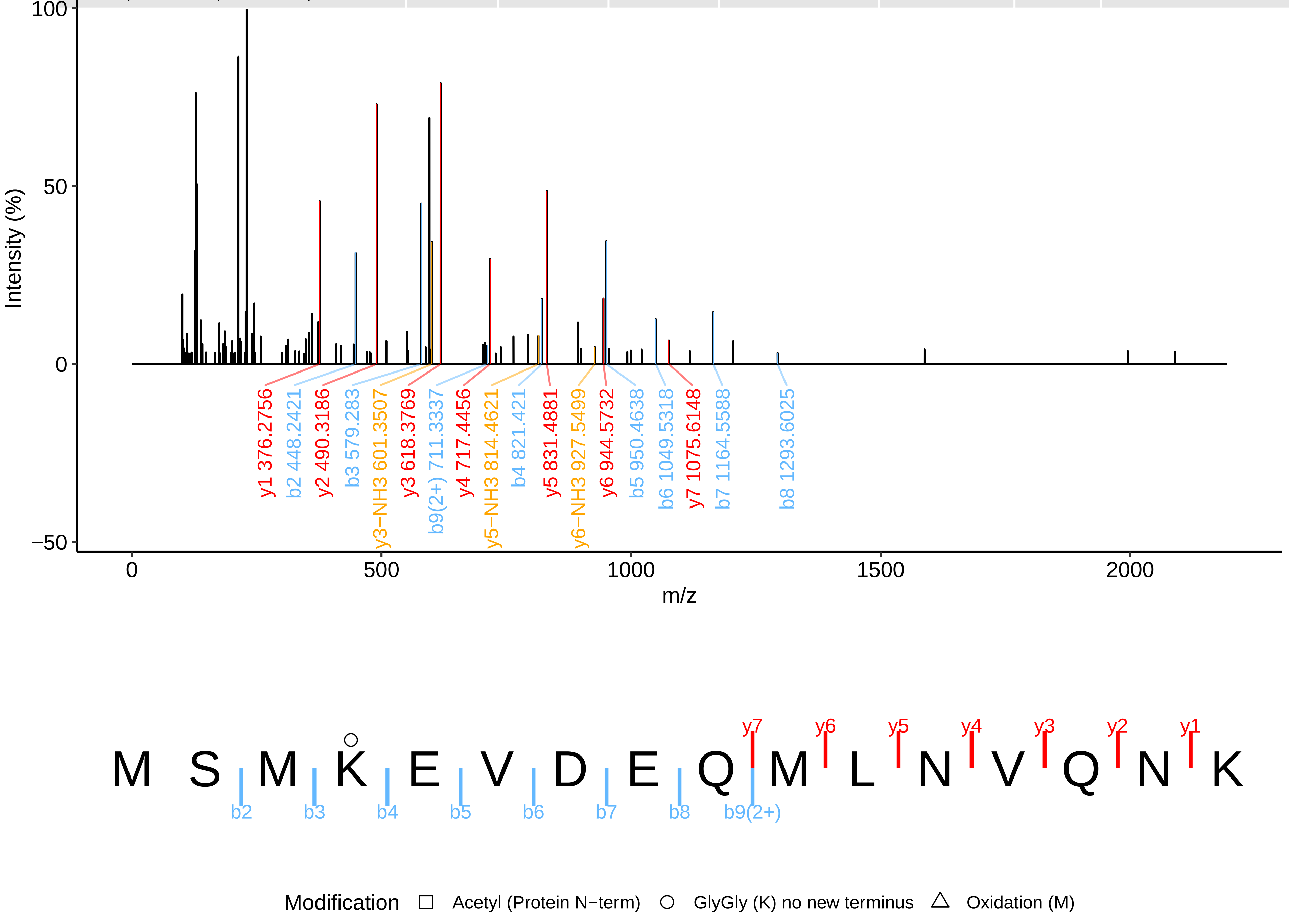

20200921_KIZ_MUb4_GlyGly_10plex_2 − Scan number: 13436

Gene Names	Charge	m/z	Mass	Retention time	PEP	Score	Precursor Intensity
HSPA8	4	453.7175	1810.841	52.167	0.0037828	84.566	454243.7

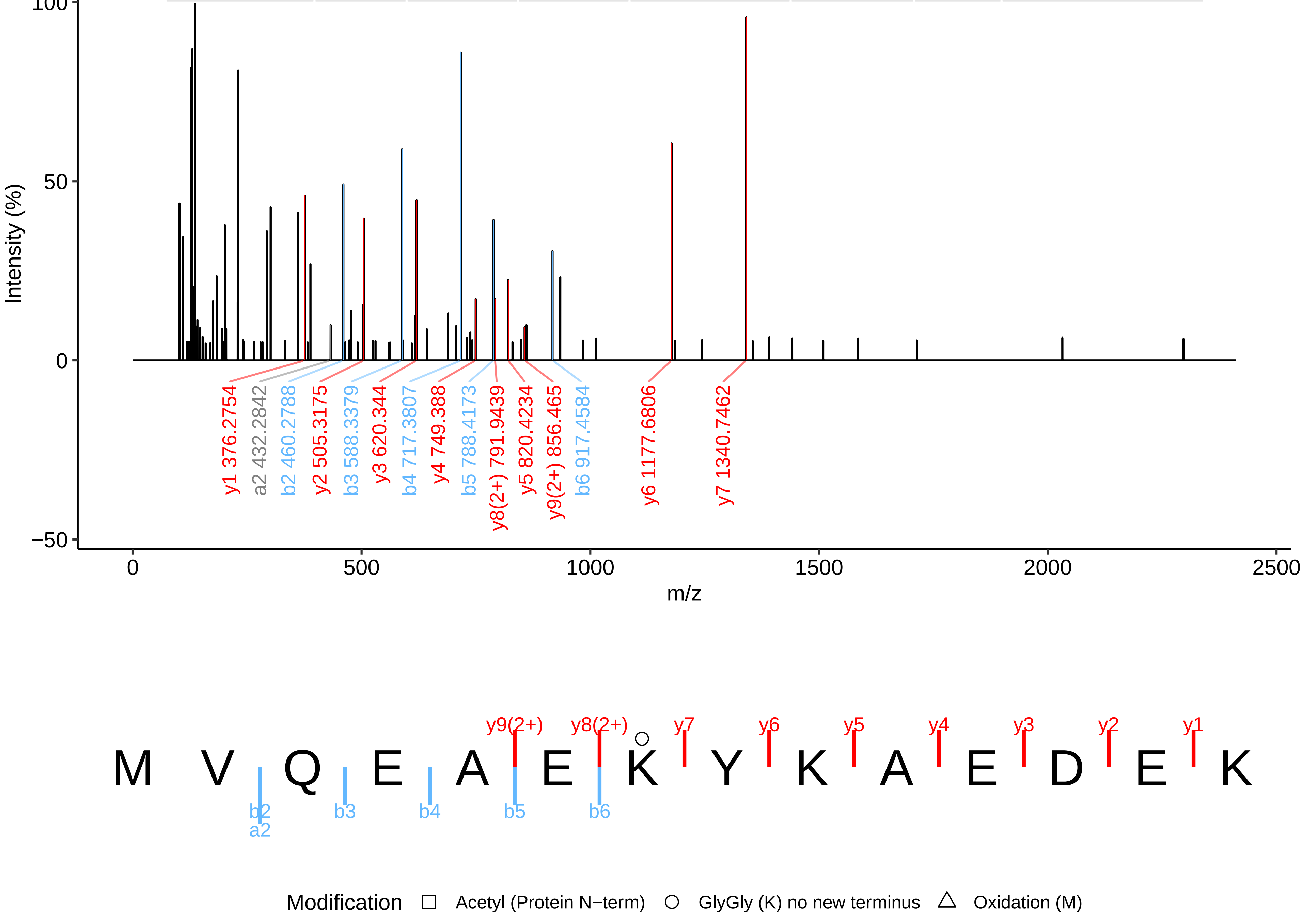

20200921_KIZ_MUb4_GlyGly_10plex_2 − Scan number: 13450

Gene Names	Charge	m/z	Mass	Retention time	PEP	Score	Precursor Intensity
HSPA8	4	453.7175	1810.841	52.226	0.0022095	90.367	591096.9

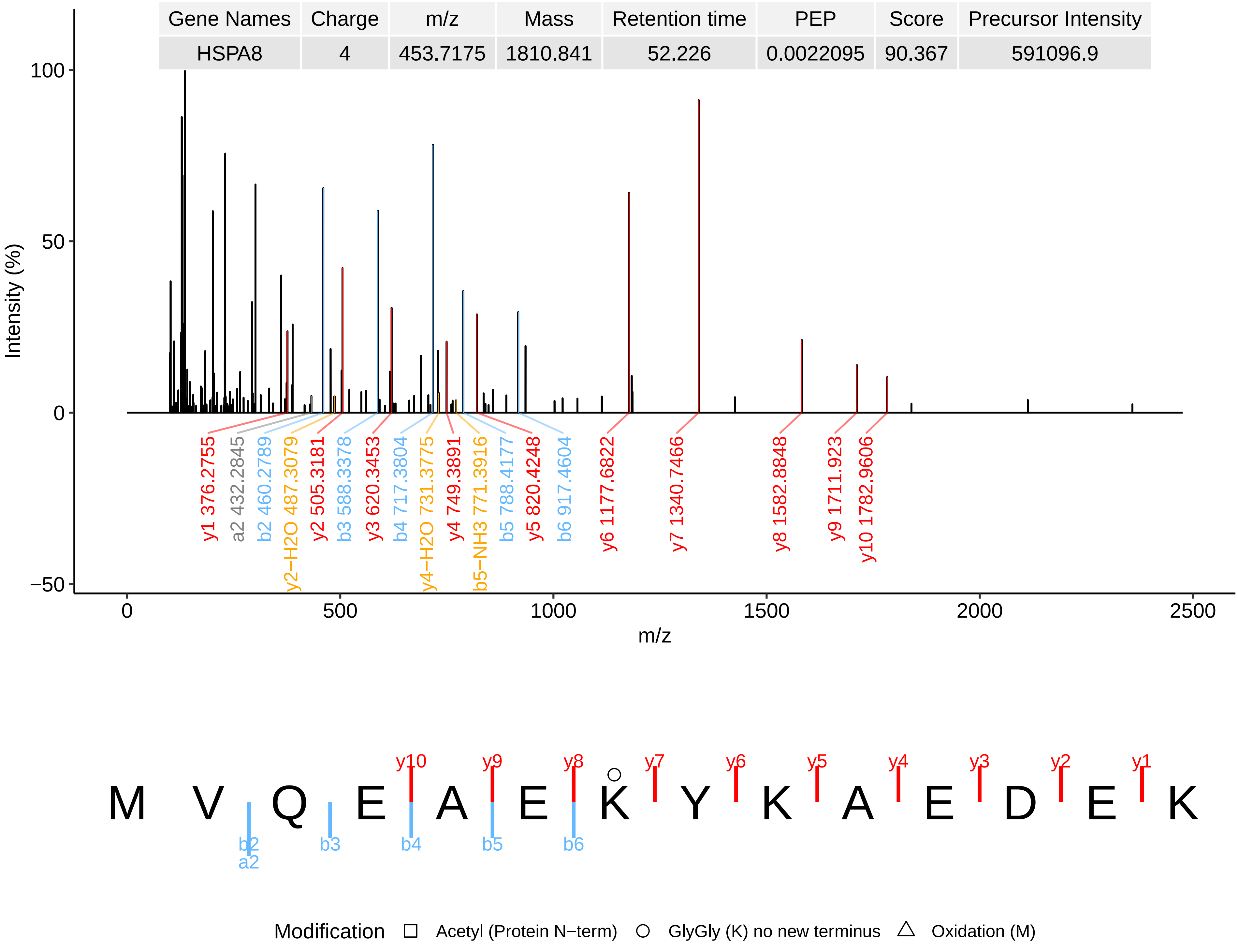

20200921_KIZ_MUb4_GlyGly_10plex − Scan number: 27368

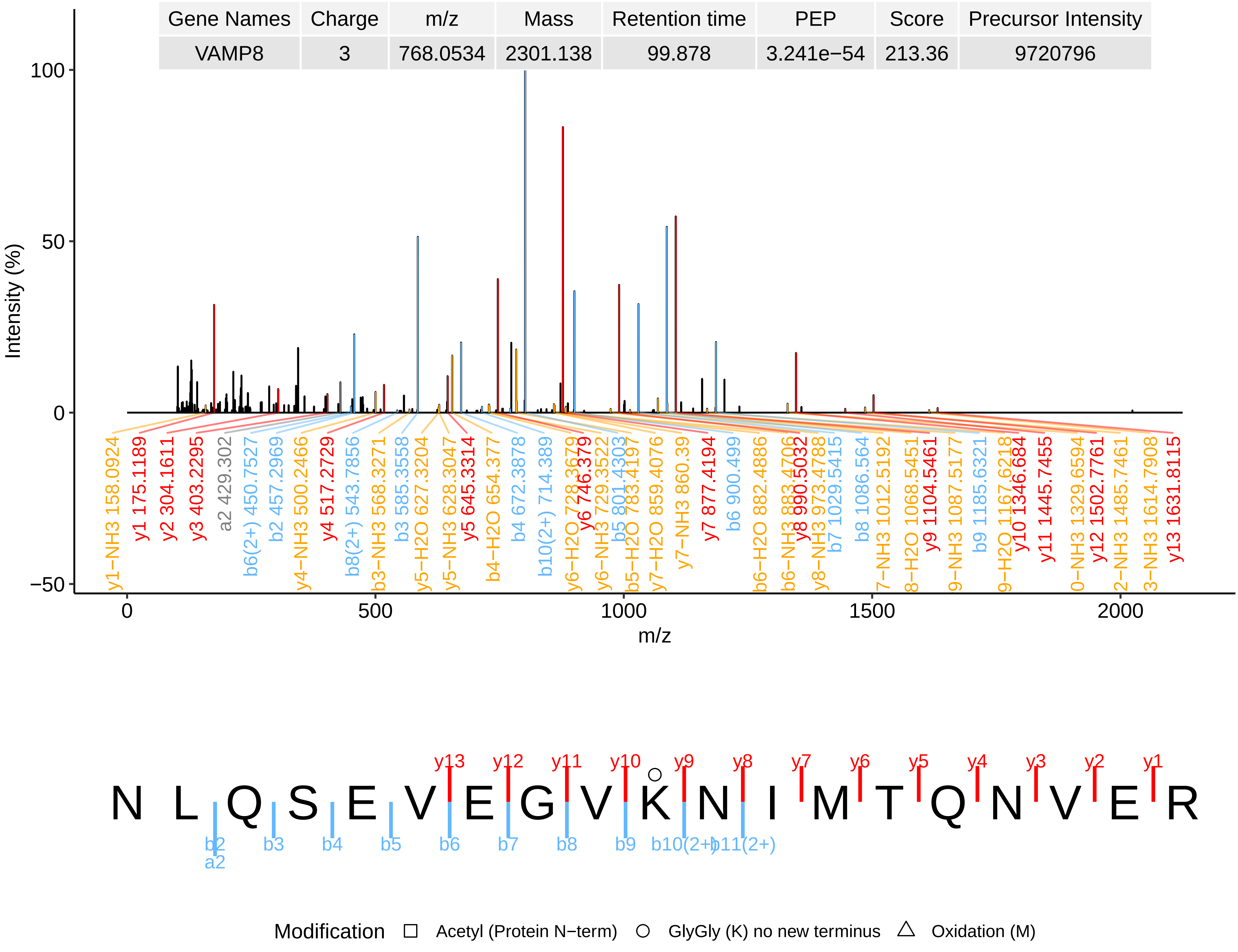

20200921_KIZ_MUb4_GlyGly_10plex_2 − Scan number: 23685

Gene Names	Charge	m/z	Mass	Retention time	PEP	Score	Precursor Intensity
VAMP8	3	768.0534	2301.138	93.503	1.2883e−09	106.75	778244.3

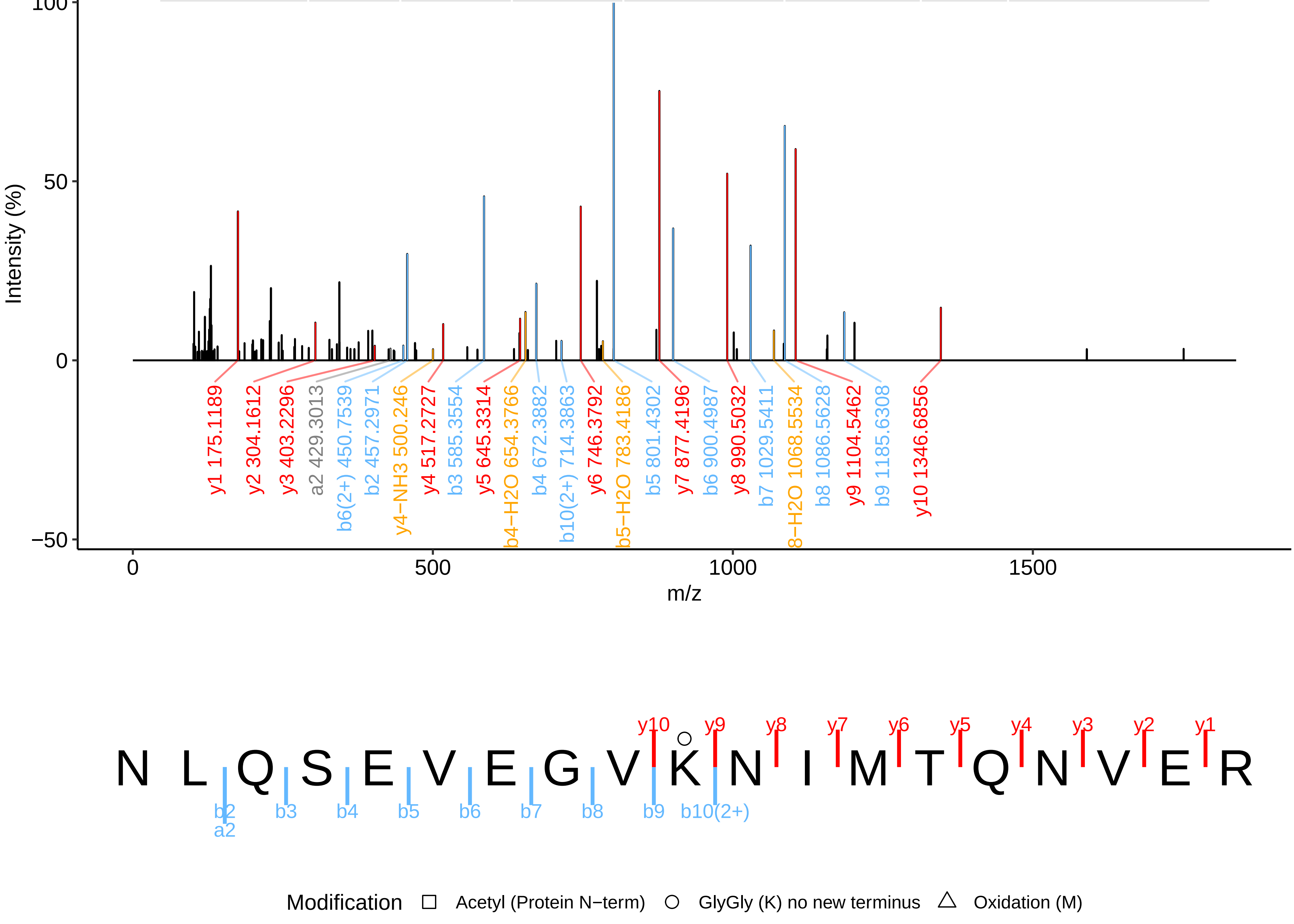

20200921_KIZ_MUb4_GlyGly_10plex − Scan number: 11581

Gene Names	Charge	m/z	Mass	Retention time	PEP	Score	Precursor Intensity
NUP155	3	767.0561	2298.146	40.403	5.4999e−16	122.19	2373259

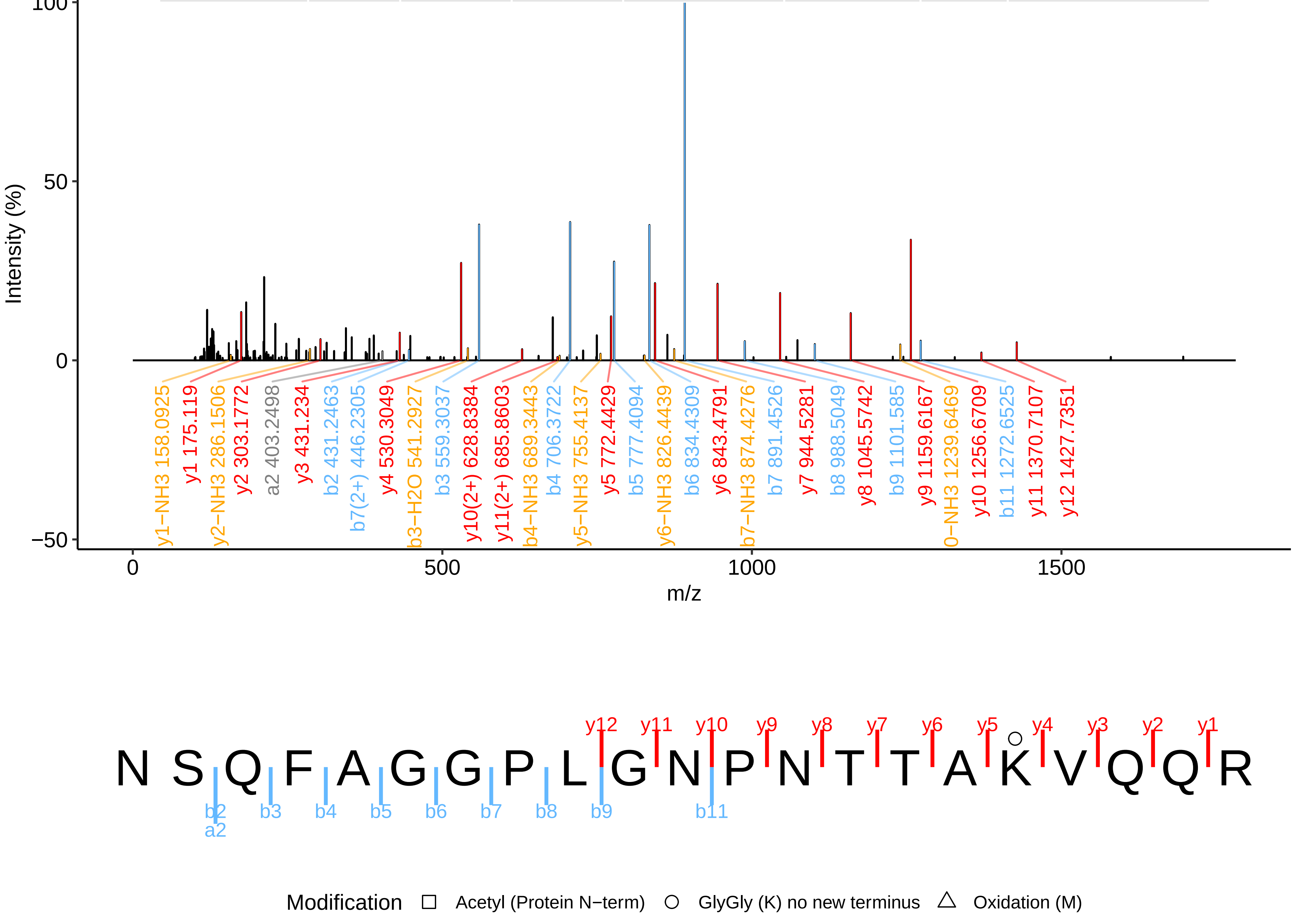

20200921_KIZ_MUb4_GlyGly_10plex − Scan number: 26707

Gene Names	Charge	m/z	Mass	Retention time	PEP	Score	Precursor Intensity
SQSTM1	3	776.3937	2326.159	97.342	9.8643e−06	86.479	4303819

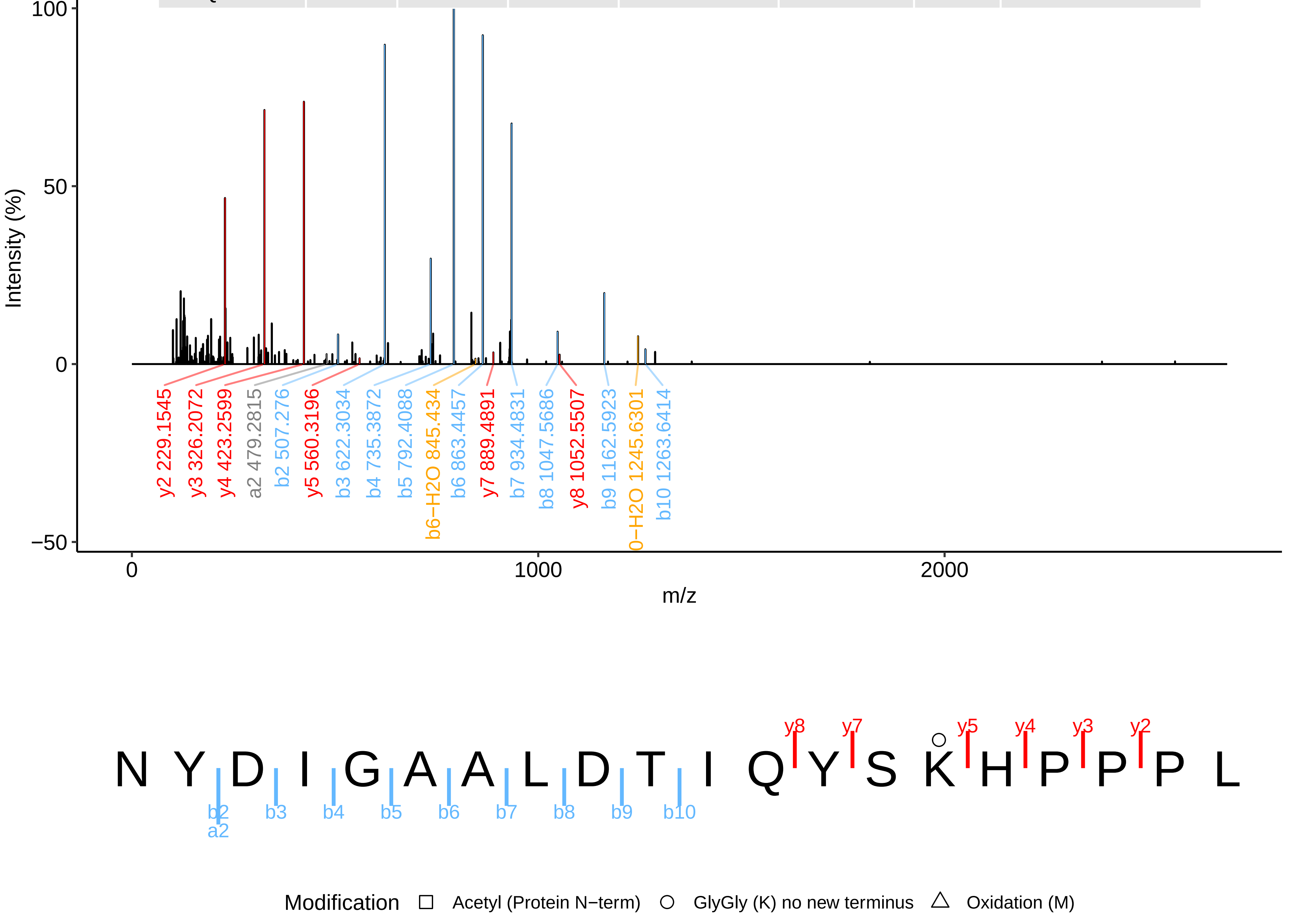

20200921_KIZ_MUb4_GlyGly_10plex − Scan number: 26711

Gene Names	Charge	m/z	Mass	Retention time	PEP	Score	Precursor Intensity
SQSTM1	3	776.3937	2326.159	97.36	0.00025209	78.021	3290374

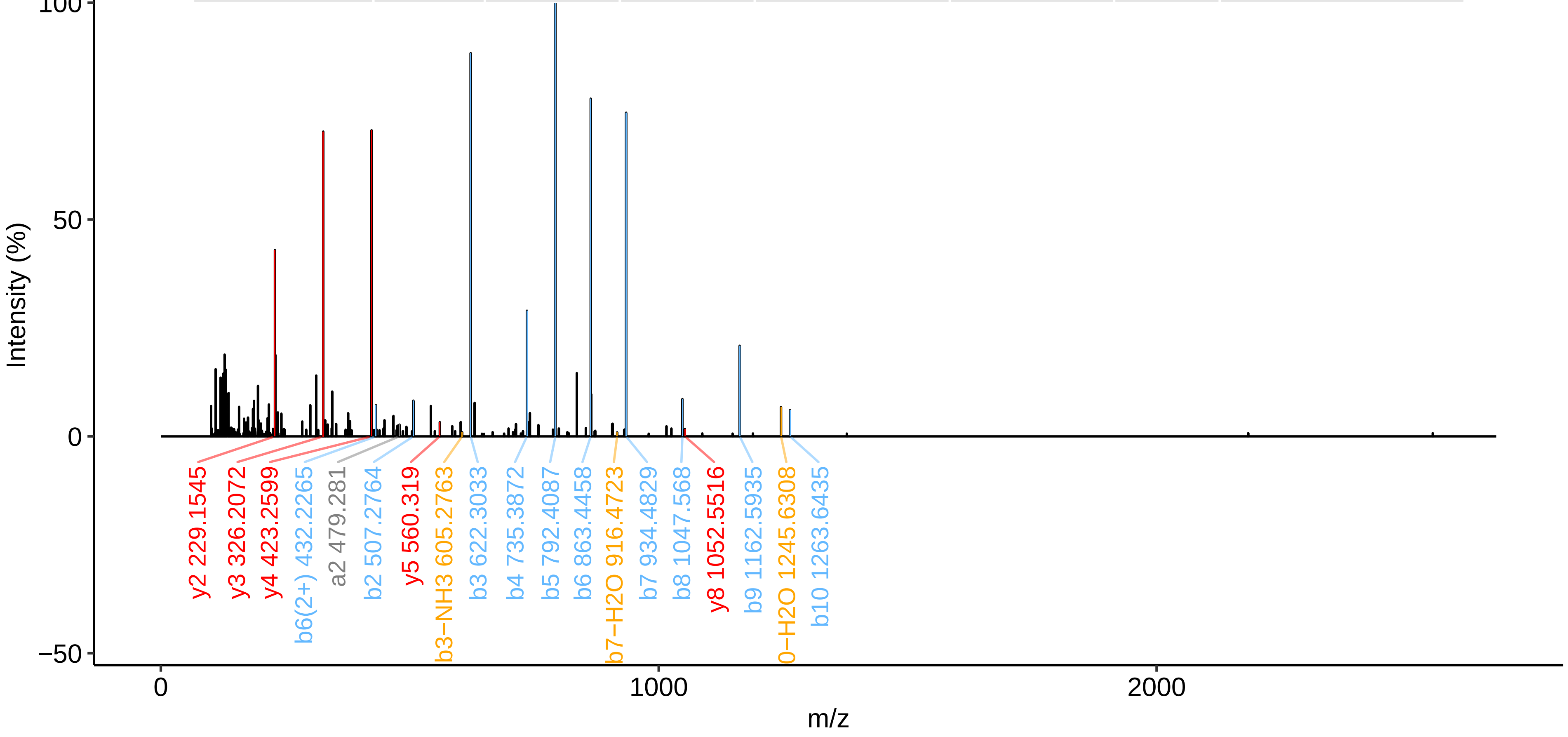

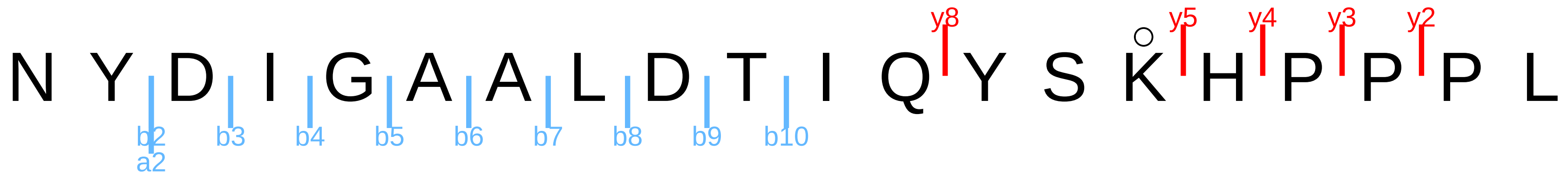

Modification □ Acetyl (Protein N−term) ○ GlyGly (K) no new terminus △ Oxidation (M)

20200921_KIZ_MUb4_GlyGly_10plex − Scan number: 26751

Gene Names	Charge	m/z	Mass	Retention time	PEP	Score	Precursor Intensity
SQSTM1	3	776.3937	2326.159	97.507	0.0010183	69.737	4423981

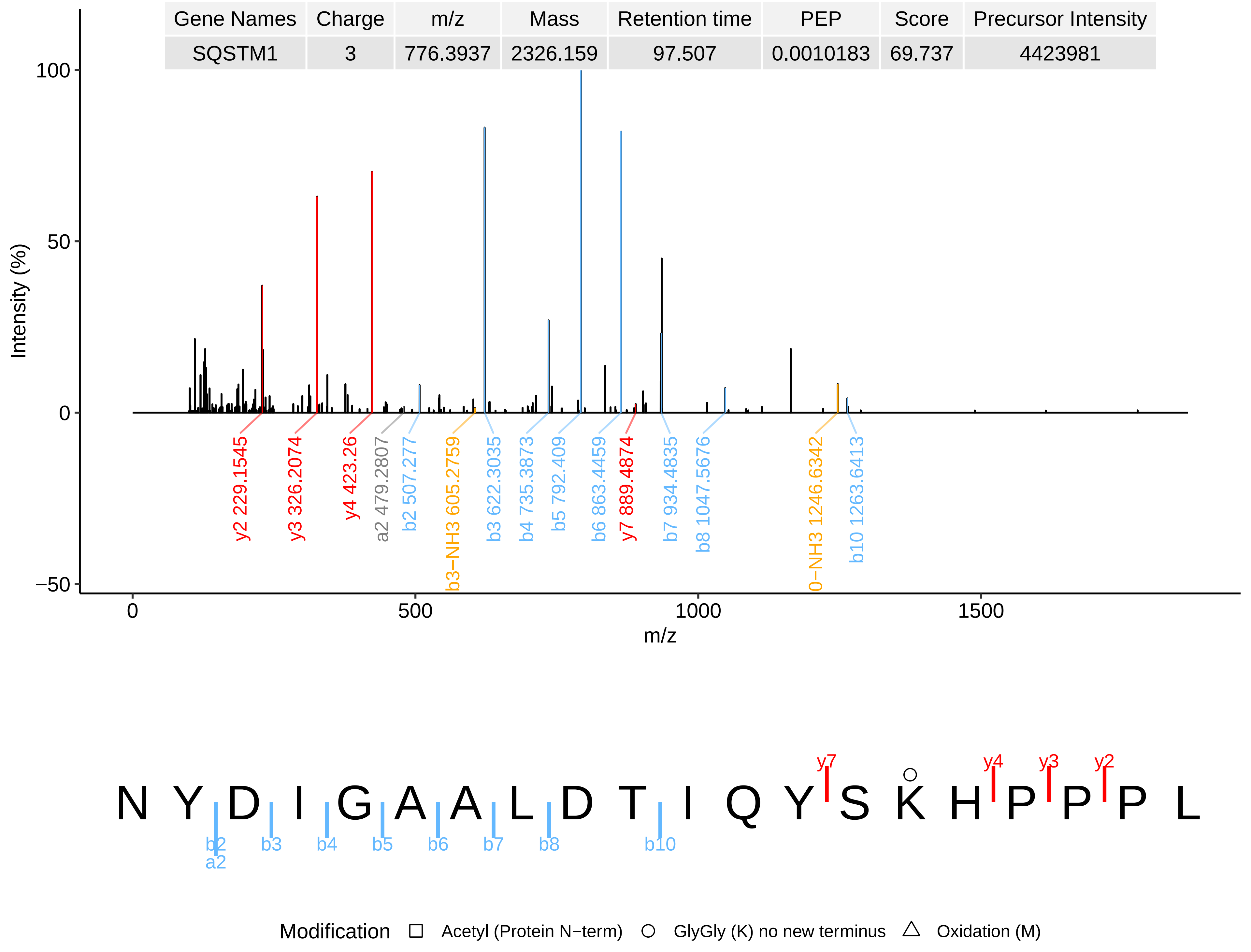

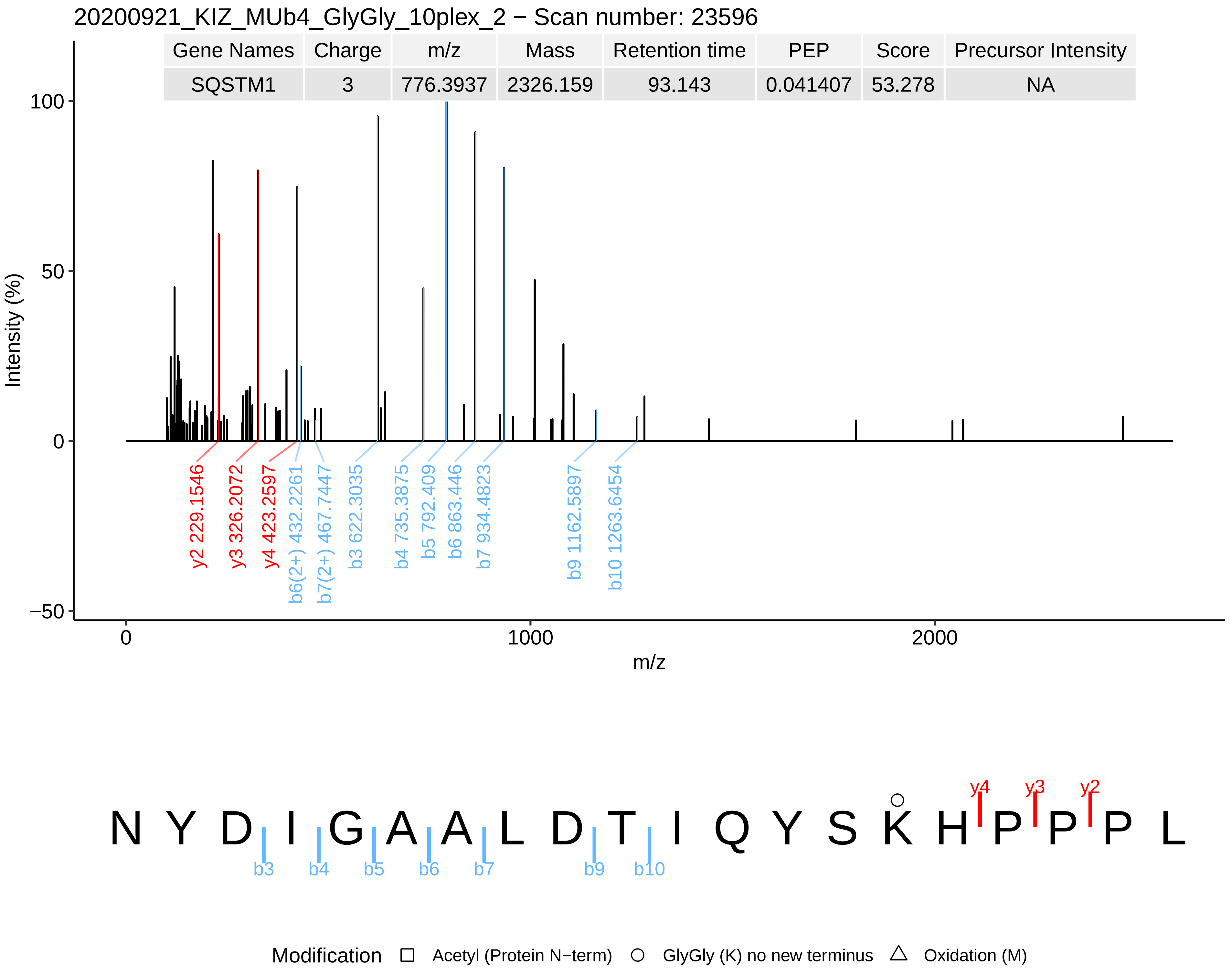

20200921_KIZ_MUb4_GlyGly_10plex_2 − Scan number: 23596
Gene Names | Charge | m/z | Mass | Retention time | PEP | Score | Precursor Intensity
SQSTM1 | 3 | 776.3937 | 2326.159 | 93.143 | 0.041407 | 53.278 | NA
Intensity (%)
100
50
0
−50
0
1000
2000
m/z
y2 229.1546
y3 326.2072
y4 423.2597
b6(2+) 432.2261
b7(2+) 467.7447
b3 622.3035
b4 735.3875
b5 792.409
b6 863.446
b7 934.4823
b9 1162.5897
b10 1263.6454
N Y D I G A A L D T I Q Y S K H P P P L
y4
y3
y2
b3
b4
b5
b6
b7
b9
b10
Modification
Acetyl (Protein N−term)
GlyGly (K) no new terminus
Oxidation (M)

20200921_KIZ_MUb4_GlyGly_10plex − Scan number: 32049

Gene Names	Charge	m/z	Mass	Retention time	PEP	Score	Precursor Intensity
RPL12	3	698.4158	2092.226	118.34	1.2963e−05	81.317	2089148

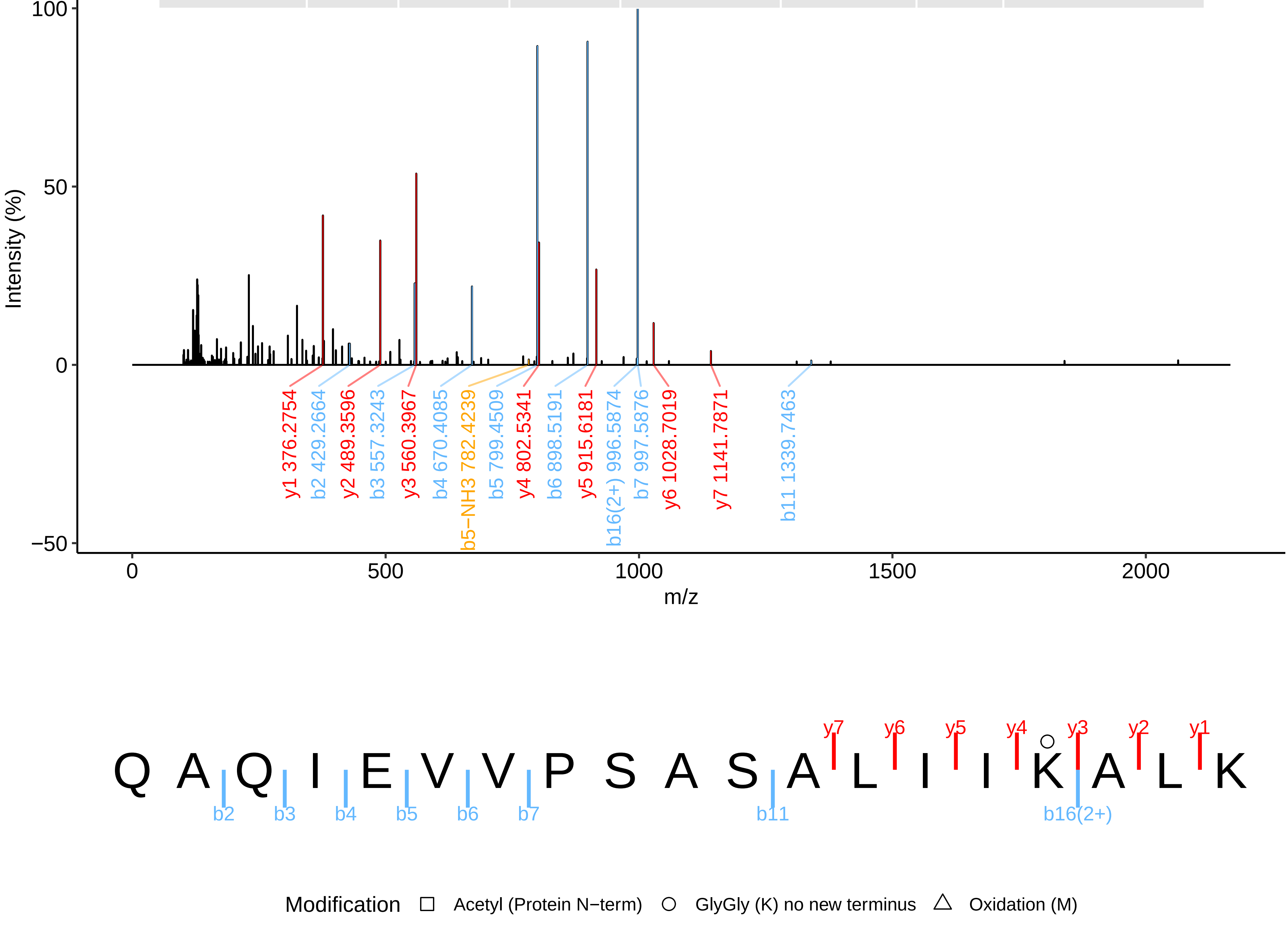

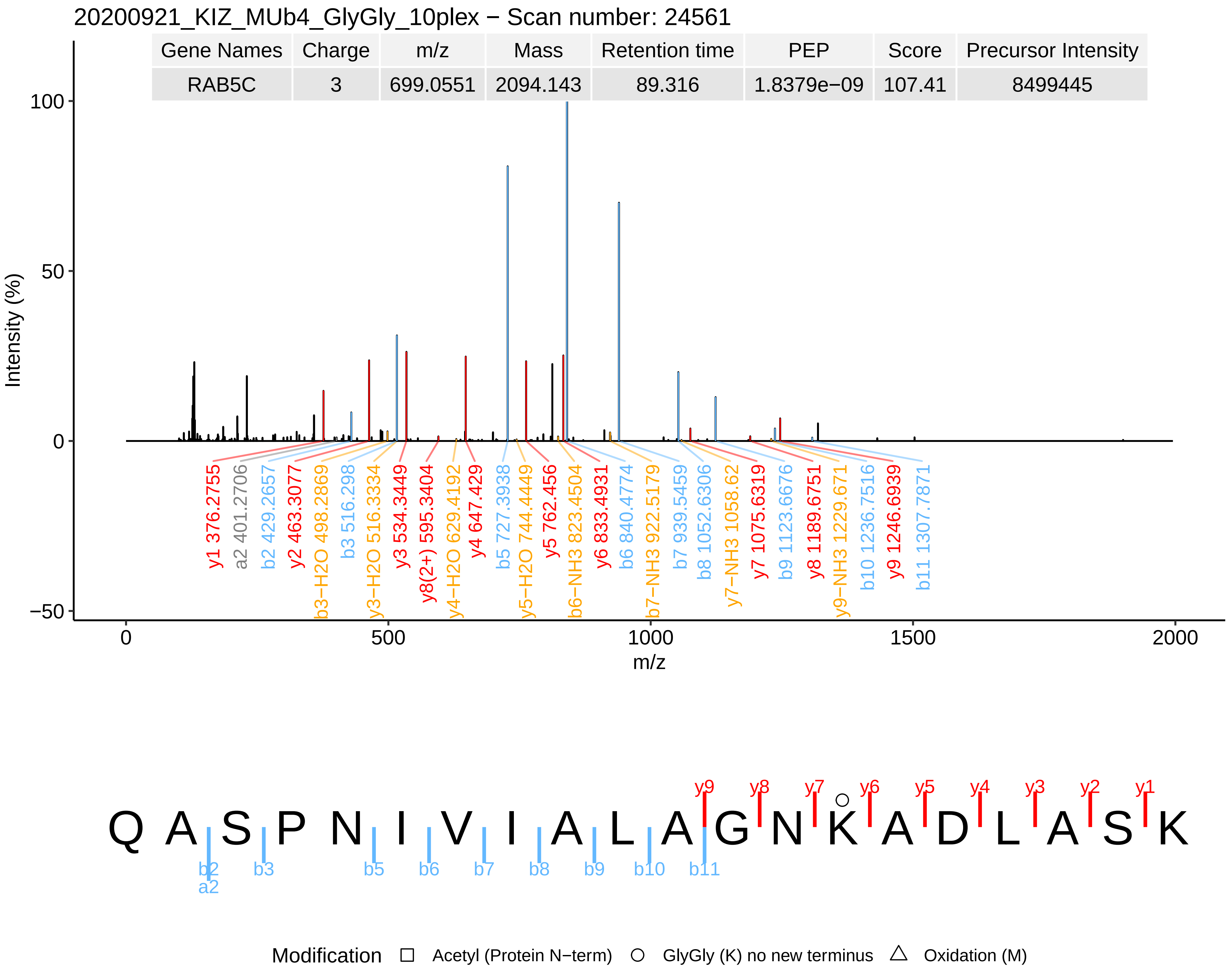
20200921_KIZ_MUb4_GlyGly_10plex − Scan number: 24561
Gene Names | Charge | m/z | Mass | Retention time | PEP | Score | Precursor Intensity
RAB5C | 3 | 699.0551 | 2094.143 | 89.316 | 1.8379e−09 | 107.41 | 8499445
Intensity (%)
100
50
0
−50
0
500
1000
1500
2000
m/z
y1 376.2755
a2 401.2706
b2 429.2657
y2 463.3077
b3−H2O 498.2869
b3 516.298
y3−H2O 516.3334
y3 534.3449
y8(2+) 595.3404
y4−H2O 629.4192
y4 647.429
b5 727.3938
y5−H2O 744.4449
y5 762.456
b6−NH3 823.4504
y6 833.4931
b6 840.4774
b7−NH3 922.5179
b7 939.5459
b8 1052.6306
y7−NH3 1058.62
y7 1075.6319
b9 1123.6676
y8 1189.6751
y9−NH3 1229.671
b10 1236.7516
y9 1246.6939
b11 1307.7871
y9 y8 y7 y6 y5 y4 y3 y2 y1
Q A S P N I V I A L A G N K A D L A S K
b2 b3 b5 b6 b7 b8 b9 b10 b11
a2
Modification
Acetyl (Protein N−term)
GlyGly (K) no new terminus
Oxidation (M)

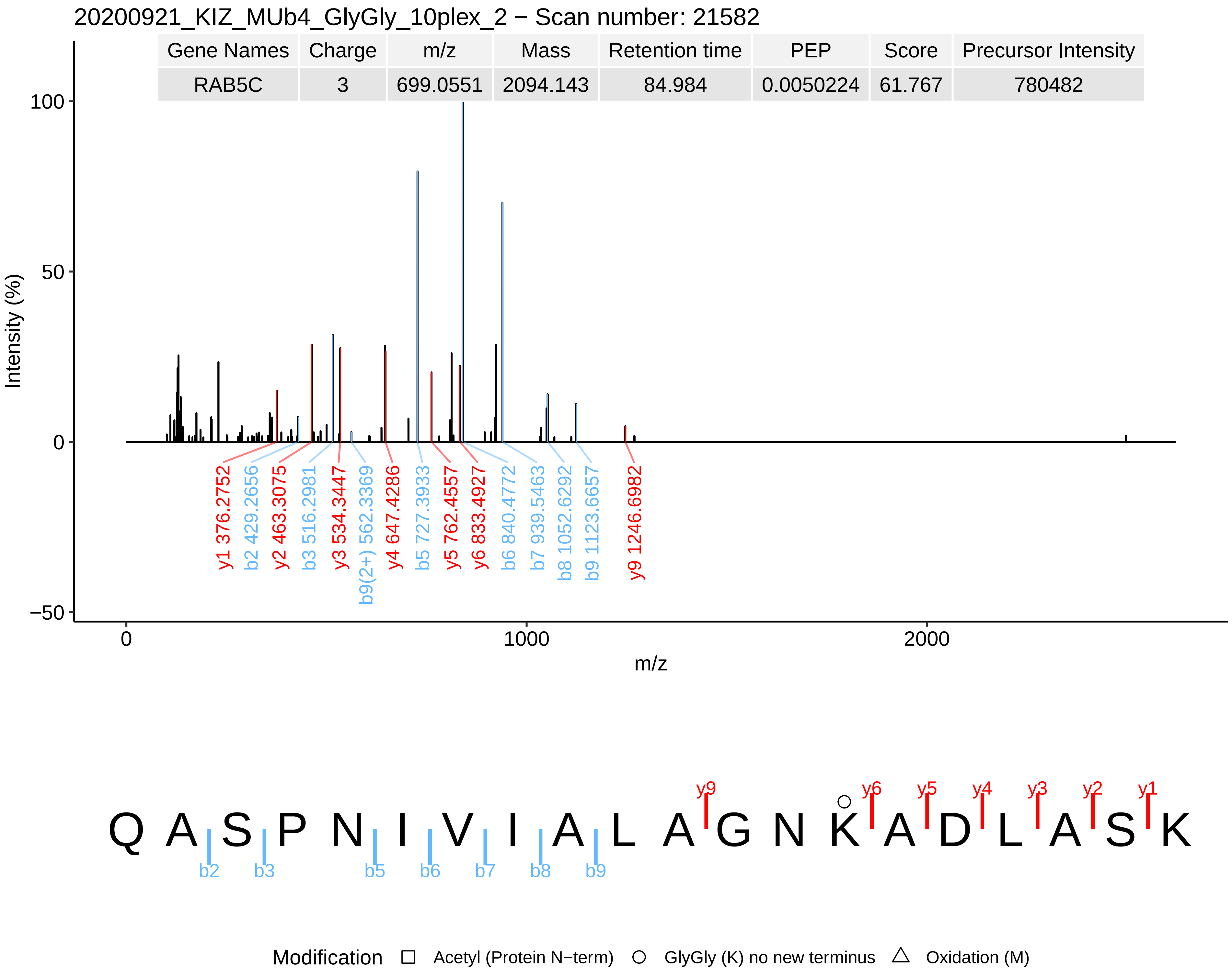

20200921_KIZ_MUb4_GlyGly_10plex_2 − Scan number: 21582
Gene Names | Charge | m/z | Mass | Retention time | PEP | Score | Precursor Intensity
RAB5C | 3 | 699.0551 | 2094.143 | 84.984 | 0.0050224 | 61.767 | 780482
Intensity (%)
100
50
0
−50
0
1000
2000
m/z
y1 376.2752
b2 429.2656
y2 463.3075
b3 516.2981
y3 534.3447
b9(2+) 562.3369
y4 647.4286
b5 727.3933
y5 762.4557
y6 833.4927
b6 840.4772
b7 939.5463
b8 1052.6292
b9 1123.6657
y9 1246.6982
Q A S P N I V I A L A G N K A D L A S K
y9 y6 y5 y4 y3 y2 y1
b2 b3 b5 b6 b7 b8 b9
Modification
Acetyl (Protein N−term)
GlyGly (K) no new terminus
Oxidation (M)

20200921_KIZ_MUb4_GlyGly_10plex − Scan number: 5410

Gene Names	Charge	m/z	Mass	Retention time	PEP	Score	Precursor Intensity
BASP1	3	682.9785	2045.914	17.719	0.00048021	74.047	4892097

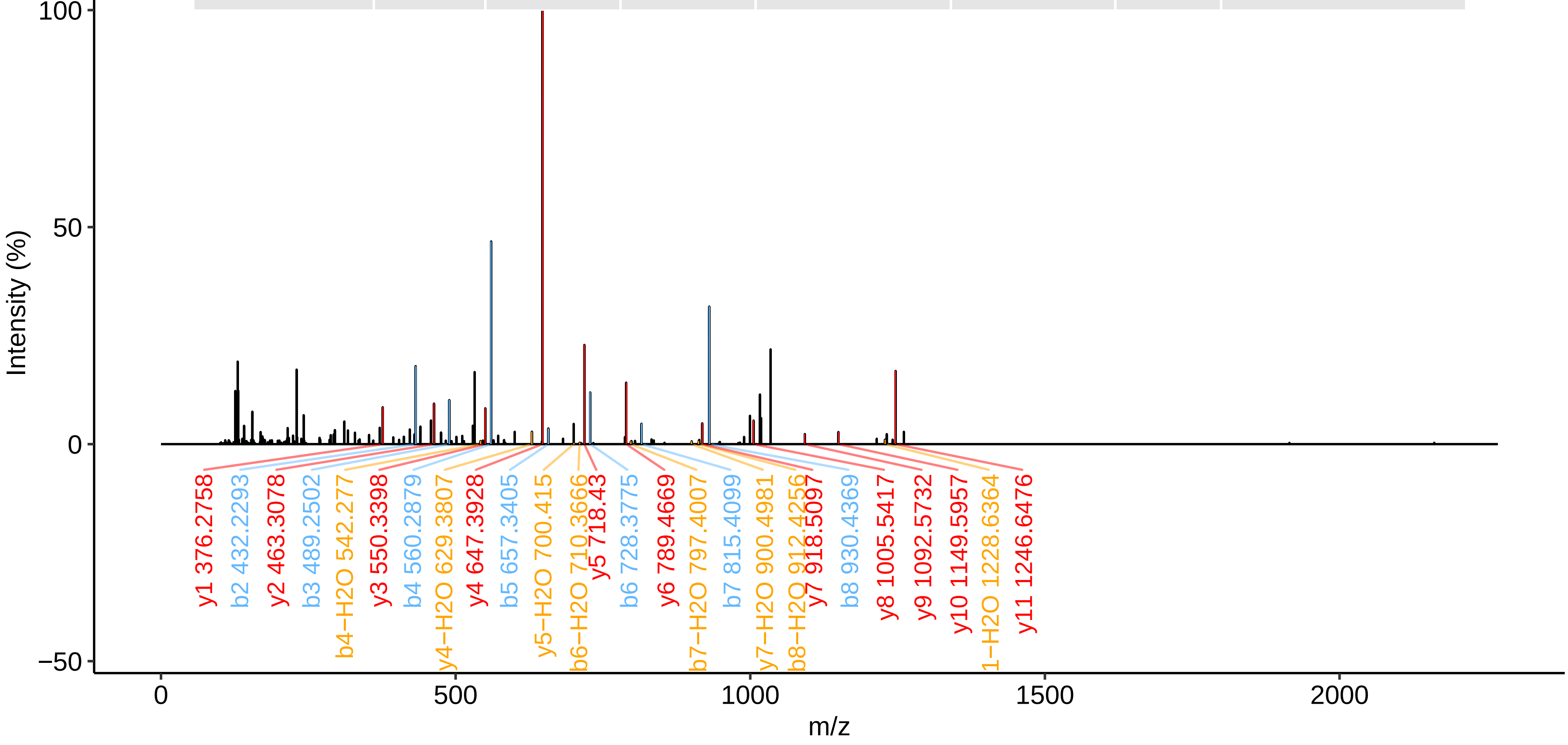

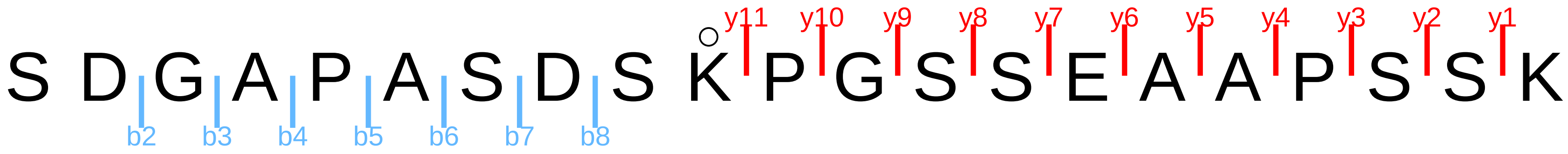

Modification □ Acetyl (Protein N−term) ○ GlyGly (K) no new terminus △ Oxidation (M)

20200921_KIZ_MUb4_GlyGly_10plex_2 − Scan number: 4935

Gene Names	Charge	m/z	Mass	Retention time	PEP	Score	Precursor Intensity
BASP1	3	682.9785	2045.914	17.514	2.8063e−09	101.17	2372275

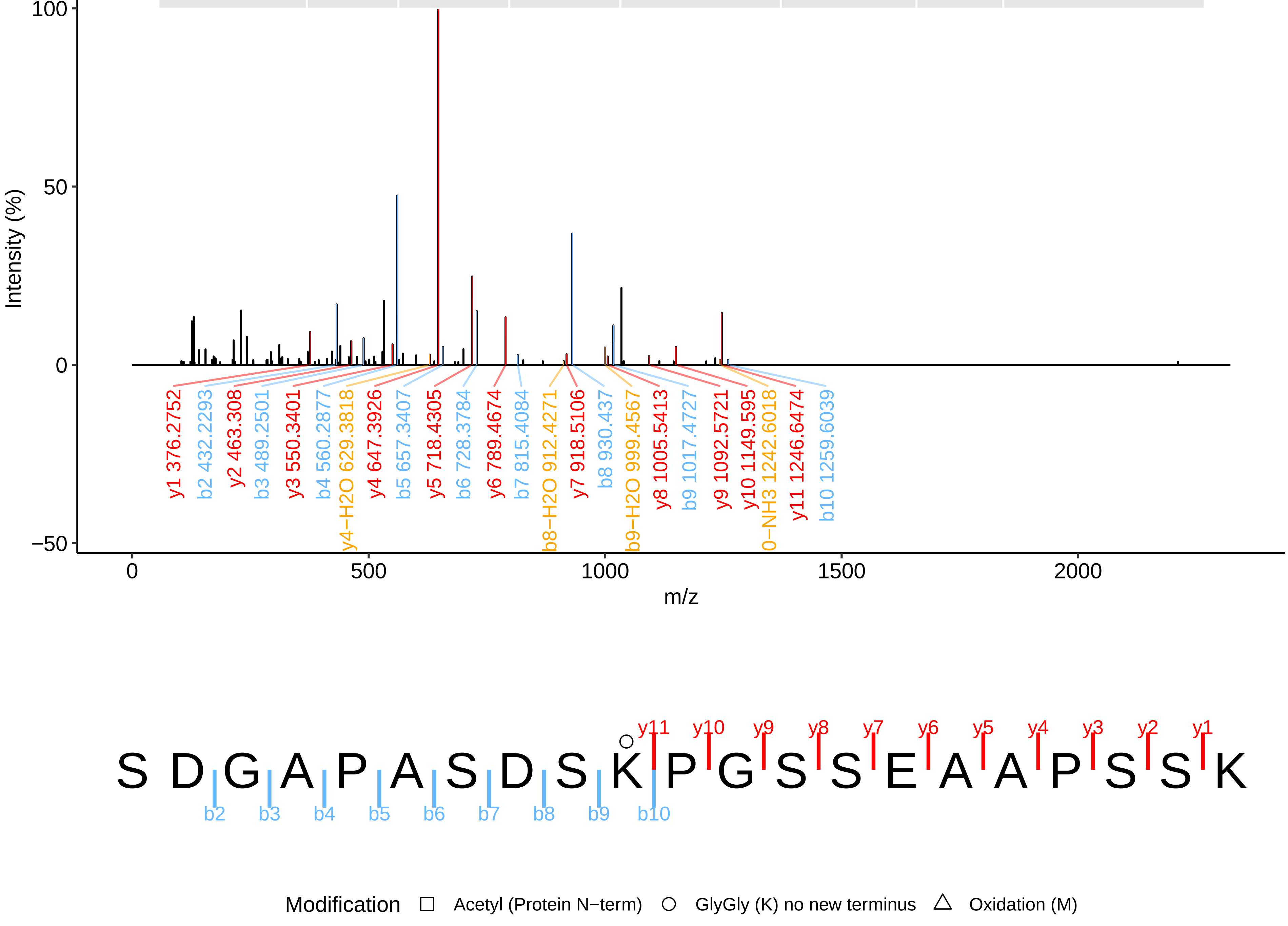

20200924_KIZ_MUb5_GlyGly_1 − Scan number: 4777

Gene Names	Charge	m/z	Mass	Retention time	PEP	Score	Precursor Intensity
BASP1	3	682.9785	2045.914	14.78	9.4739e−10	110.6	8813032

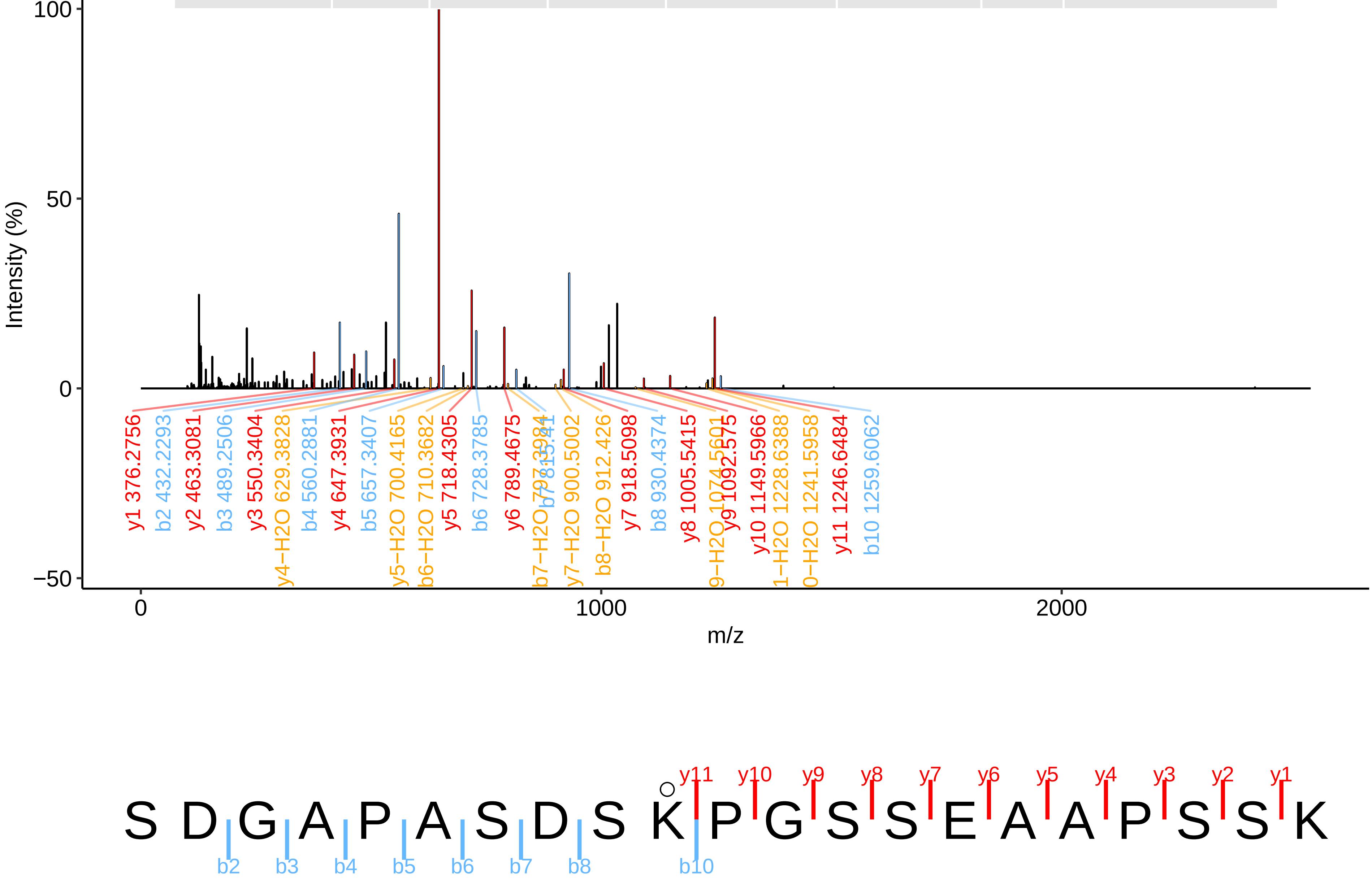

Modification □ Acetyl (Protein N−term) ○ GlyGly (K) no new terminus △ Oxidation (M)

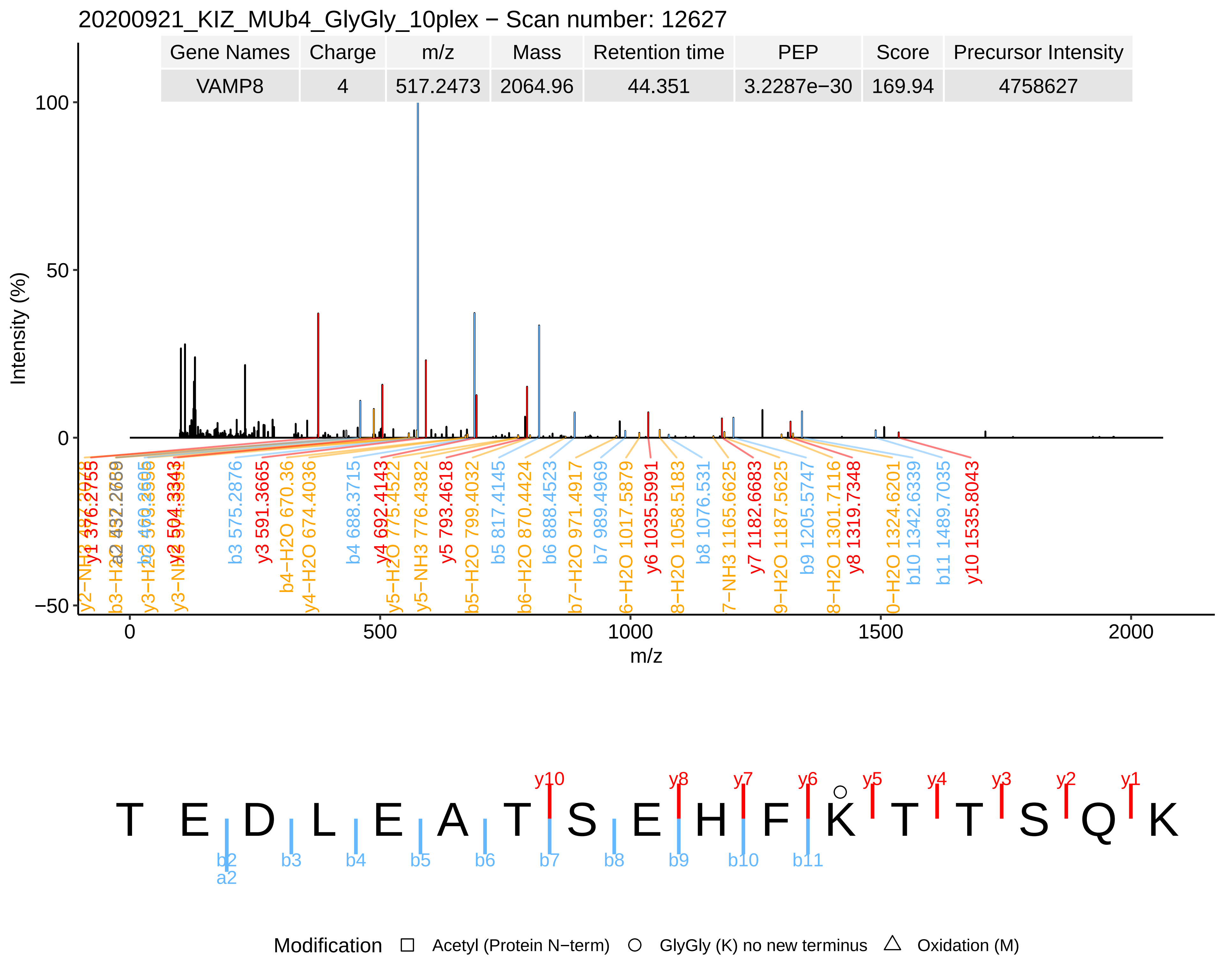

20200921_KIZ_MUb4_GlyGly_10plex − Scan number: 12627
Gene Names | Charge | m/z | Mass | Retention time | PEP | Score | Precursor Intensity
VAMP8 | 4 | 517.2473 | 2064.96 | 44.351 | 3.2287e−30 | 169.94 | 4758627
Intensity (%)
100
50
0
−50
m/z
0
500
1000
1500
2000
y1 376.2755
b3 575.2876
y3 591.3665
b4−H2O 670.36
y4−H2O 674.4036
b4 688.3715
y4 692.4143
y5−NH3 776.4382
y5 793.4618
b5−H2O 799.4032
b5 817.4145
b6−H2O 870.4424
b6 888.4523
b7−H2O 971.4917
b7 989.4969
y6 1035.5991
b8 1076.531
y7 1182.6683
b9 1205.5747
y8 1319.7348
b10 1342.6339
b11 1489.7035
y10 1535.8043
T E D L E A T S E H F K T T S Q K
y10 y8 y7 y6 y5 y4 y3 y2 y1
b2 a2 b3 b4 b5 b6 b7 b8 b9 b10 b11
Modification
Acetyl (Protein N−term)
GlyGly (K) no new terminus
Oxidation (M)

20200921_KIZ_MUb4_GlyGly_10plex − Scan number: 20909

Gene Names	Charge	m/z	Mass	Retention time	PEP	Score	Precursor Intensity
	3	701.7108	2102.111	75.646	0.0027292	68.919	5582258

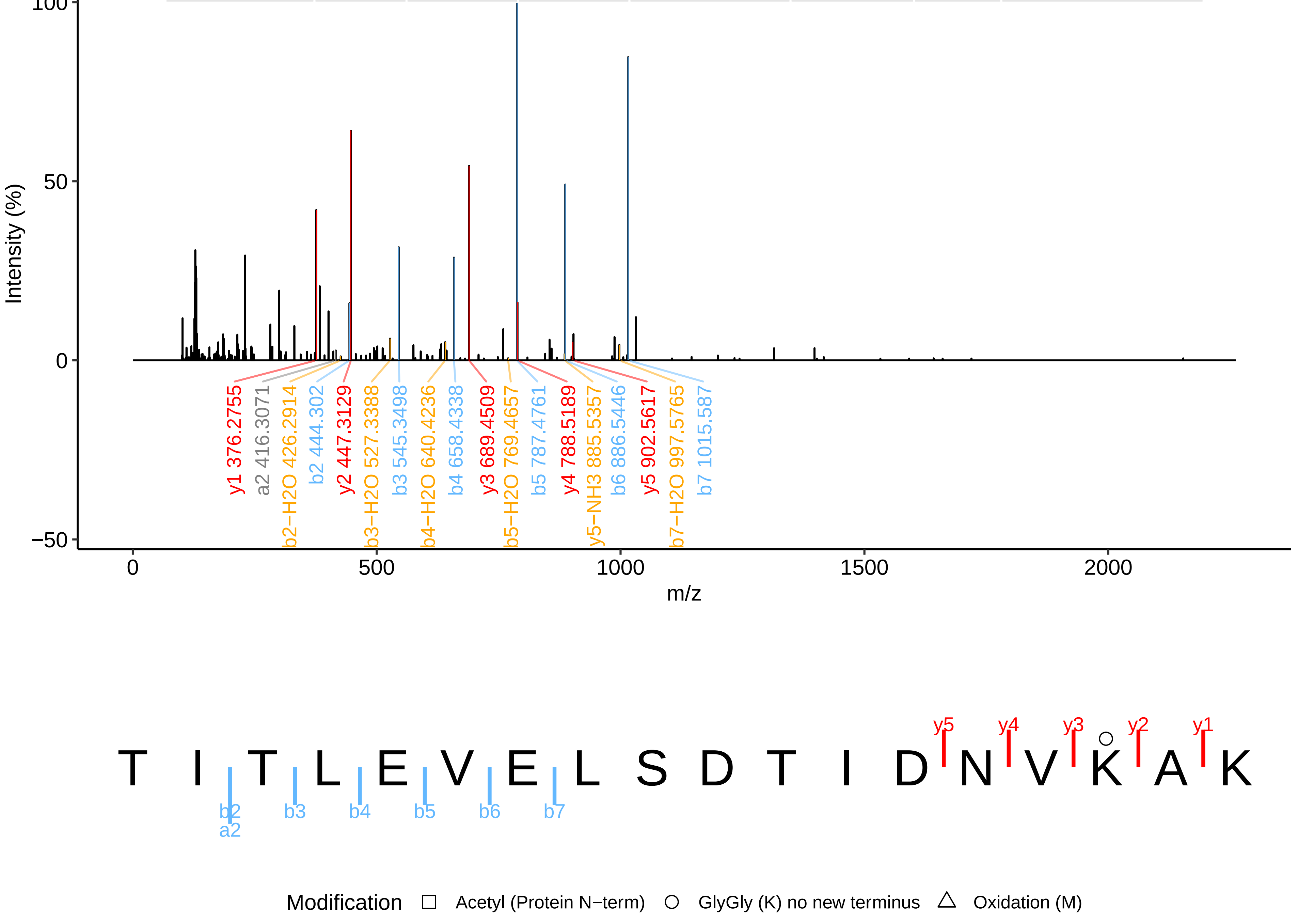

20200924_KIZ_MUb5_GlyGly_1 − Scan number: 20696

Gene Names	Charge	m/z	Mass	Retention time	PEP	Score	Precursor Intensity
	3	701.7108	2102.111	66.728	6.5693e−06	95.57	14441956

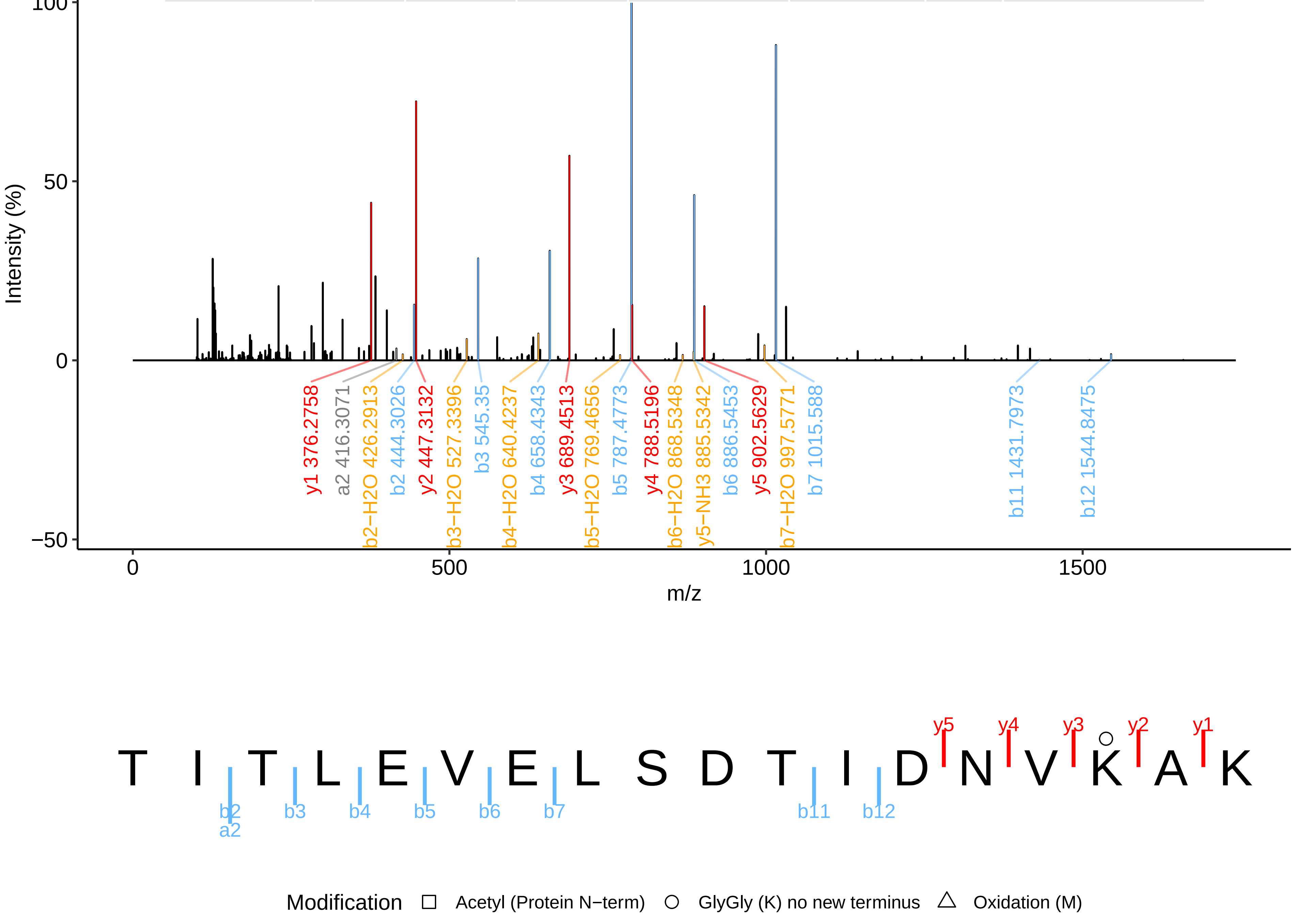

20200921_KIZ_MUb4_GlyGly_10plex − Scan number: 20948

Gene Names	Charge	m/z	Mass	Retention time	PEP	Score	Precursor Intensity
RPS27A;UBA52;UBC;UBB	4	526.031	2100.095	75.79	0.00097733	72.446	9580560

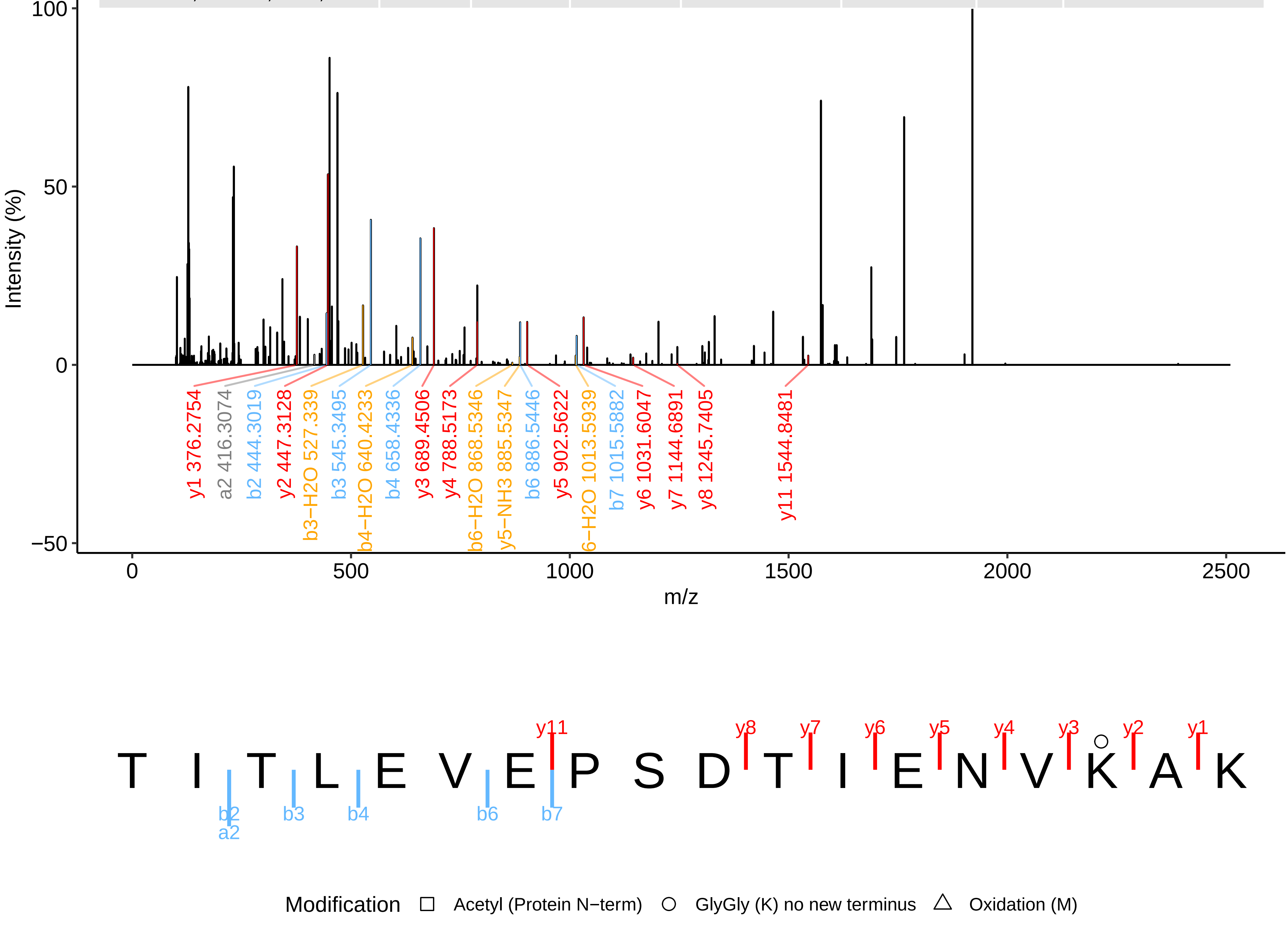

20200921_KIZ_MUb4_GlyGly_10plex − Scan number: 21047

Gene Names	Charge	m/z	Mass	Retention time	PEP	Score	Precursor Intensity
RPS27A;UBA52;UBC;UBB	3	701.0389	2100.095	76.16	9.54e−32	184	24720018

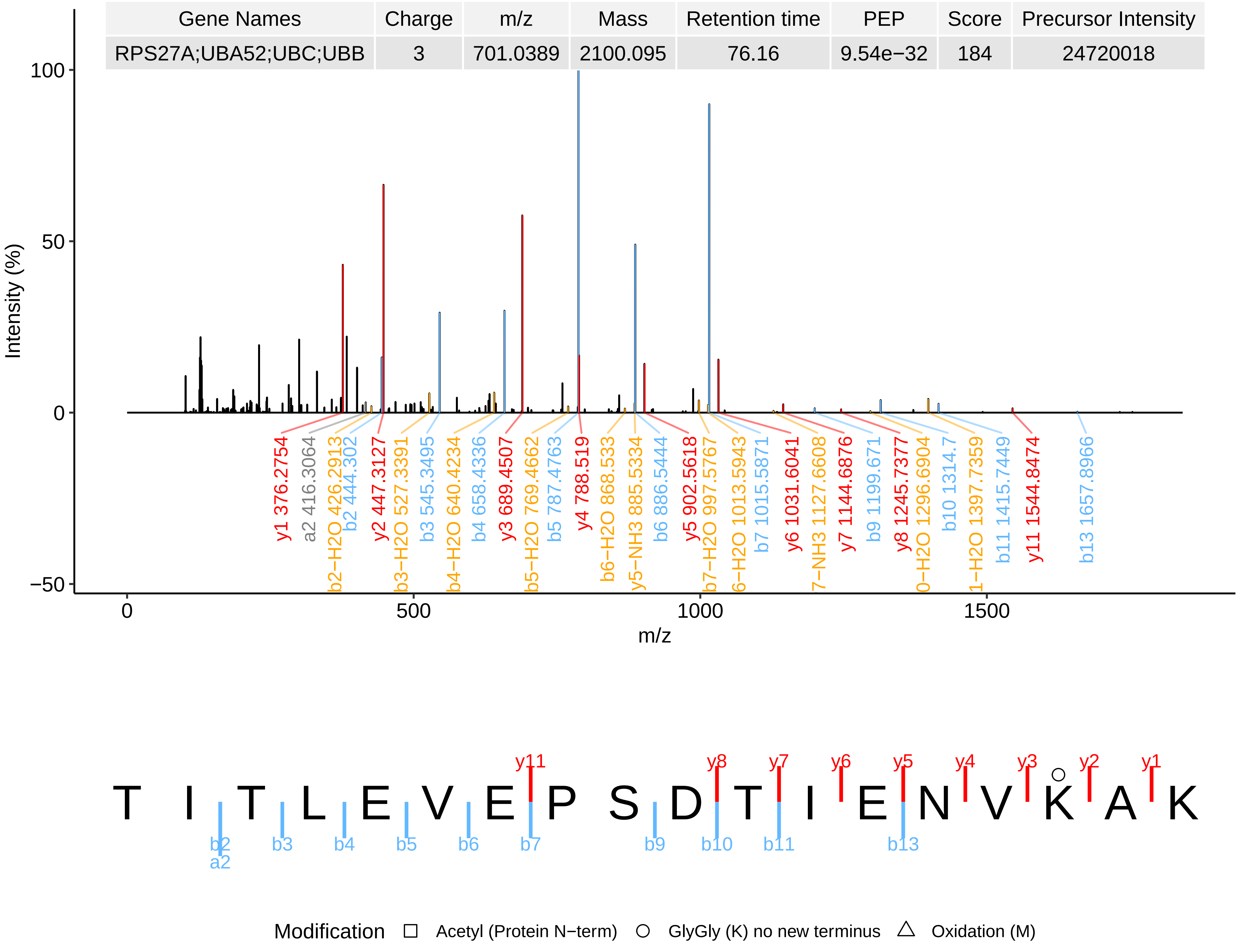

20200921_KIZ_MUb4_GlyGly_10plex − Scan number: 21184

Gene Names	Charge	m/z	Mass	Retention time	PEP	Score	Precursor Intensity
RPS27A;UBA52;UBC;UBB	3	701.0389	2100.095	76.662	4.372e−64	222.57	19247078

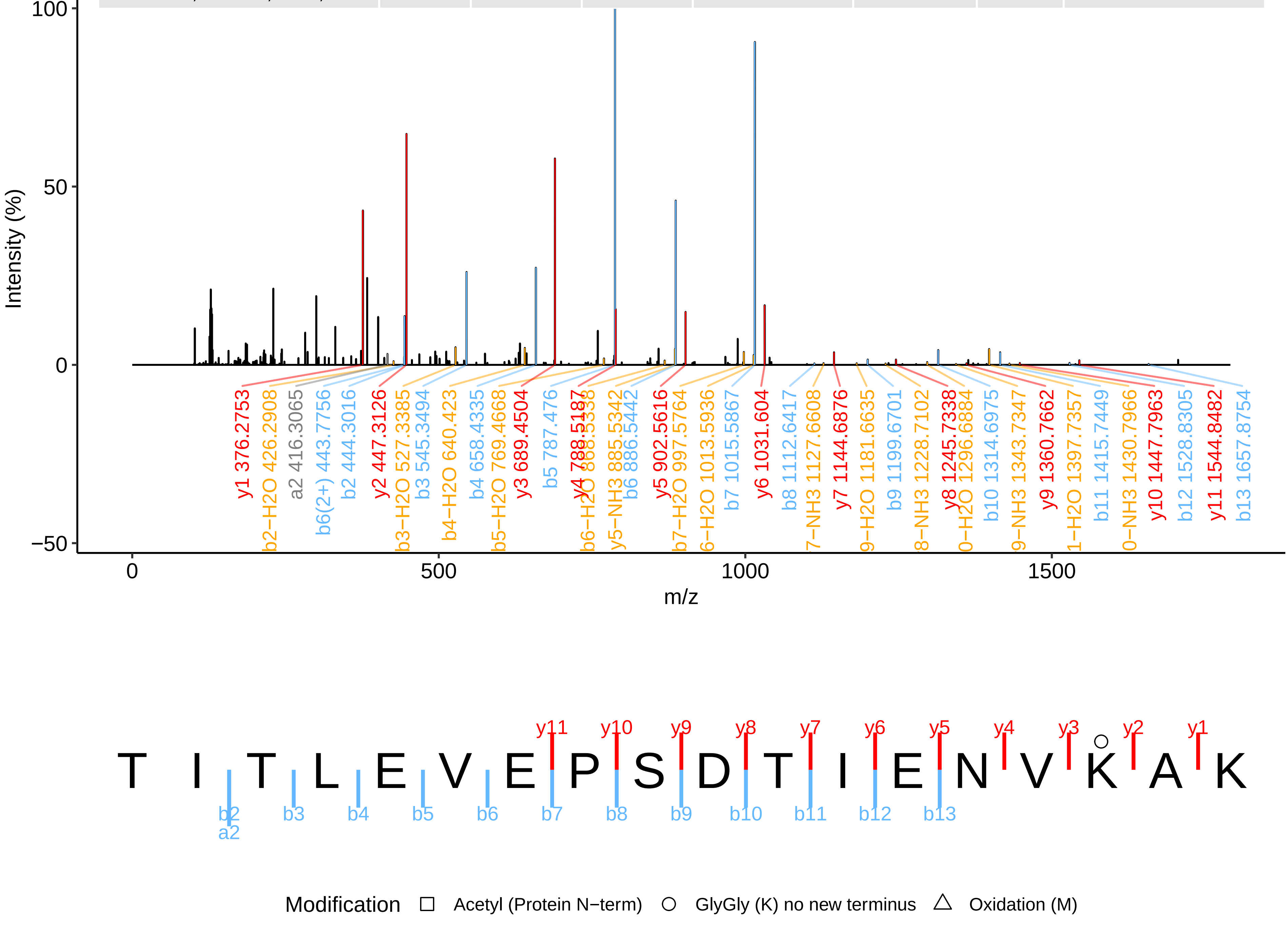

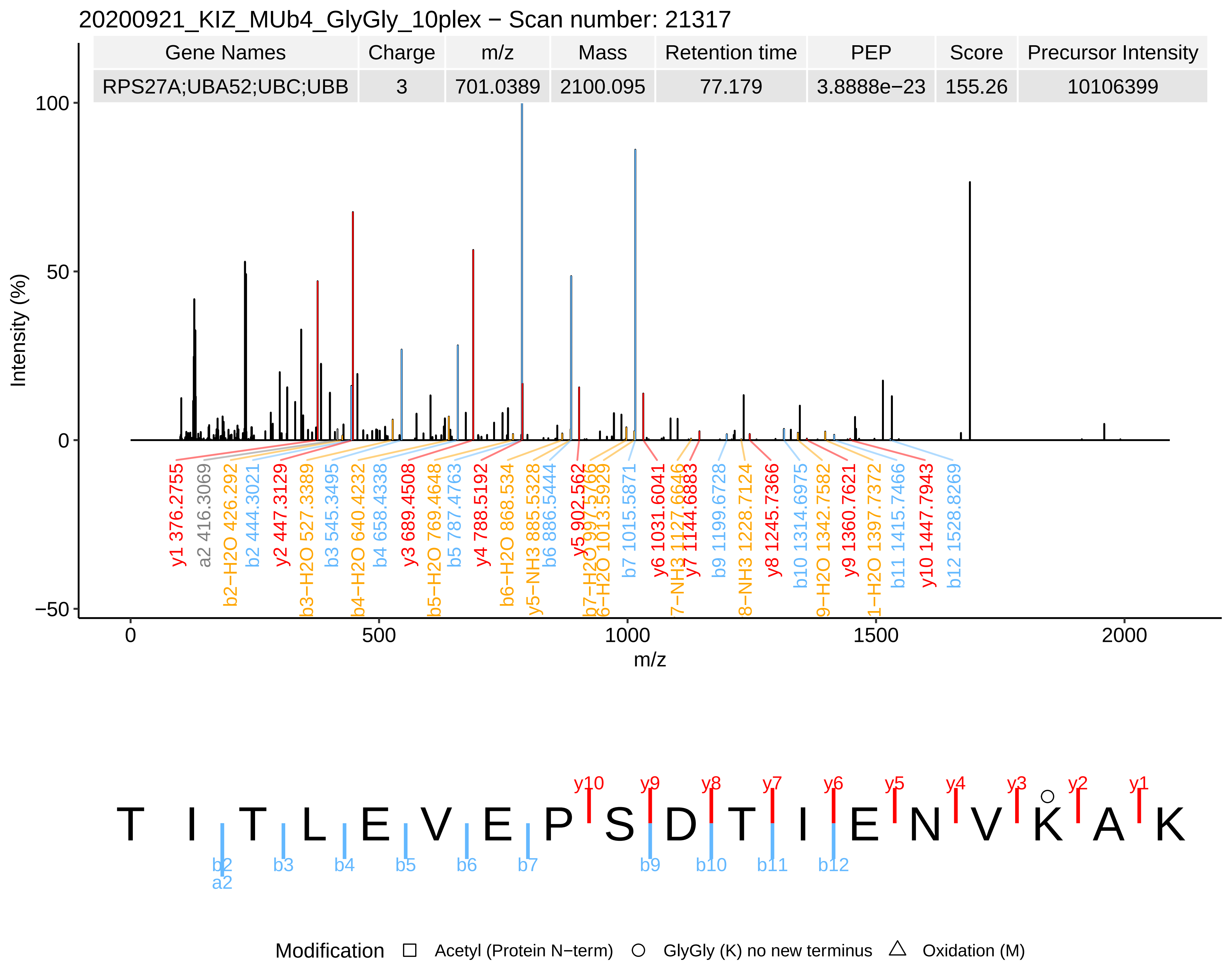
20200921_KIZ_MUb4_GlyGly_10plex − Scan number: 21317
Gene Names | Charge | m/z | Mass | Retention time | PEP | Score | Precursor Intensity
RPS27A;UBA52;UBC;UBB | 3 | 701.0389 | 2100.095 | 77.179 | 3.8888e−23 | 155.26 | 10106399
Intensity (%)
100
50
0
−50
m/z
0
500
1000
1500
2000
y1 376.2755
a2 416.3069
b2−H2O 426.292
b2 444.3021
y2 447.3129
b3−H2O 527.3389
b3 545.3495
b4−H2O 640.4232
b4 658.4338
y3 689.4508
b5−H2O 769.4648
b5 787.4763
y4 788.5192
b6−H2O 868.534
y5−NH3 885.5328
b6 886.5444
y5 902.562
b7 1015.5871
y6 1031.6041
y7 1144.6883
b9 1199.6728
y8 1245.7366
b10 1314.6975
y9 1360.7621
b11 1415.7466
y10 1447.7943
b12 1528.8269
T I T L E V E P S D T I E N V K A K
y10 y9 y8 y7 y6 y5 y4 y3 y2 y1
b2 b3 b4 b5 b6 b7 b9 b10 b11 b12
a2
Modification
Acetyl (Protein N−term)
GlyGly (K) no new terminus
Oxidation (M)

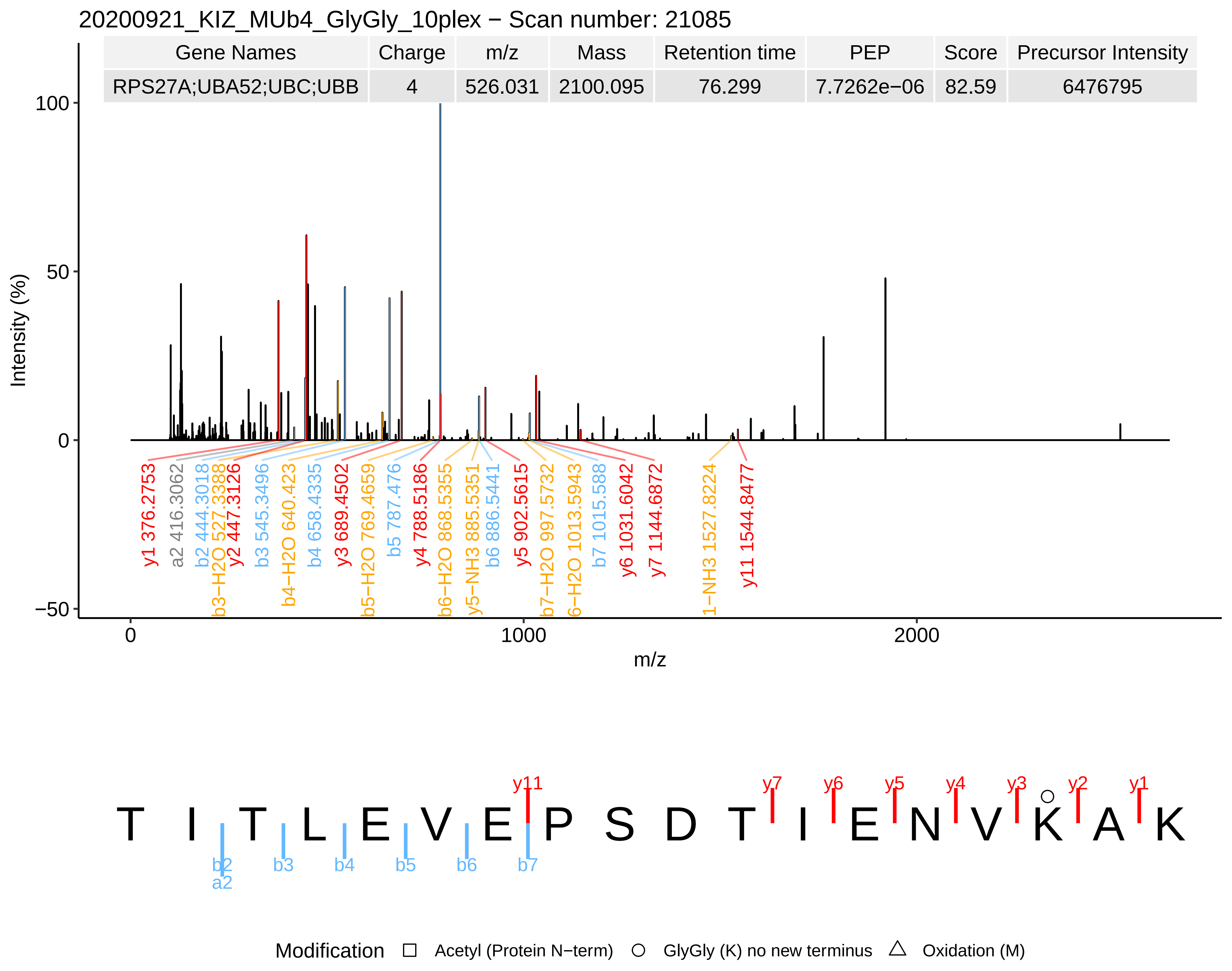
20200921_KIZ_MUb4_GlyGly_10plex − Scan number: 21085
Gene Names | Charge | m/z | Mass | Retention time | PEP | Score | Precursor Intensity
RPS27A;UBA52;UBC;UBB | 4 | 526.031 | 2100.095 | 76.299 | 7.7262e−06 | 82.59 | 6476795
Intensity (%)
100
50
0
−50
0
1000
2000
m/z
y1 376.2753
a2 416.3062
b2 444.3018
b3−H2O 527.3388
y2 447.3126
b3 545.3496
b4−H2O 640.423
b4 658.4335
y3 689.4502
b5−H2O 769.4659
b5 787.476
y4 788.5186
b6−H2O 868.5355
y5−NH3 885.5351
b6 886.5441
y5 902.5615
b7−H2O 997.5732
6−H2O 1013.5943
b7 1015.588
y6 1031.6042
y7 1144.6872
1−NH3 1527.8224
y11 1544.8477
T I T L E V E P S D T I E N V K A K
y11 y7 y6 y5 y4 y3 y2 y1
b2 a2 b3 b4 b5 b6 b7
Modification
Acetyl (Protein N−term)
GlyGly (K) no new terminus
Oxidation (M)

20200921_KIZ_MUb4_GlyGly_10plex − Scan number: 21321

Gene Names	Charge	m/z	Mass	Retention time	PEP	Score	Precursor Intensity
RPS27A;UBA52;UBC;UBB	4	526.031	2100.095	77.192	6.5812e−06	84.874	11465413

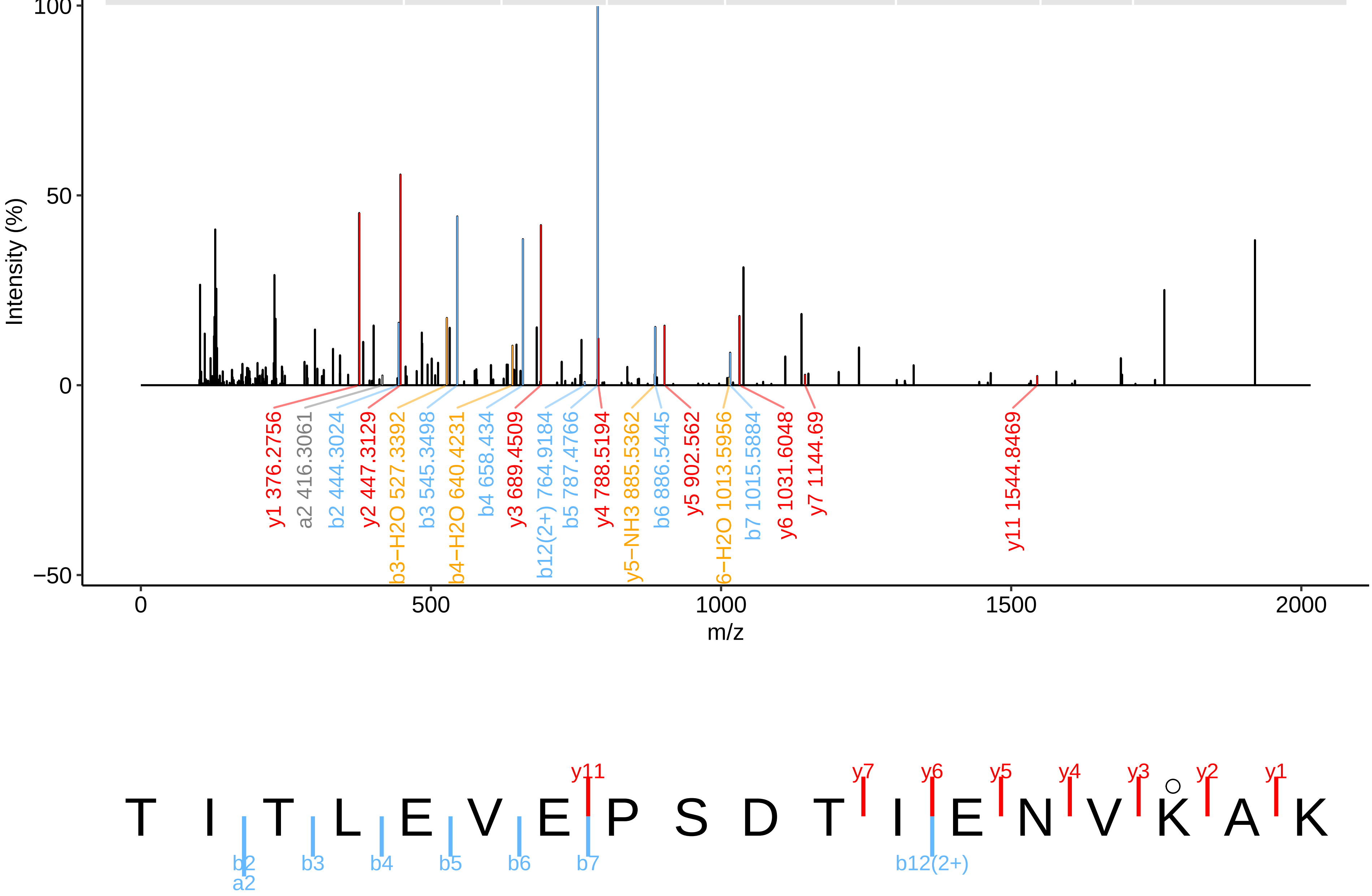

Modification □ Acetyl (Protein N−term) ○ GlyGly (K) no new terminus △ Oxidation (M)

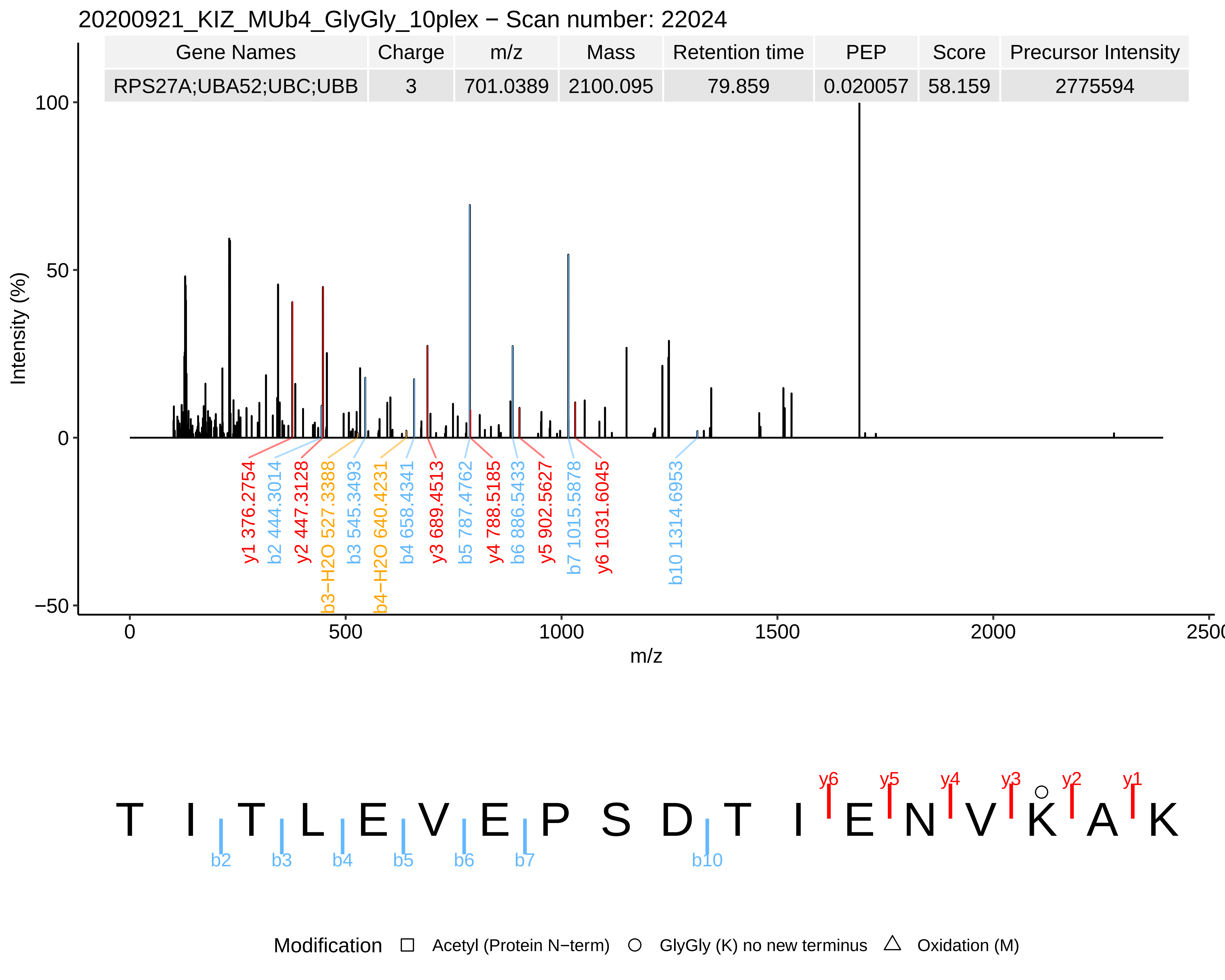
20200921_KIZ_MUb4_GlyGly_10plex − Scan number: 22024
Gene Names | Charge | m/z | Mass | Retention time | PEP | Score | Precursor Intensity
RPS27A;UBA52;UBC;UBB | 3 | 701.0389 | 2100.095 | 79.859 | 0.020057 | 58.159 | 2775594
Intensity (%)
m/z
y1 376.2754
b2 444.3014
y2 447.3128
b3−H2O 527.3388
b3 545.3493
b4−H2O 640.4231
b4 658.4341
y3 689.4513
b5 787.4762
y4 788.5185
b6 886.5433
y5 902.5627
b7 1015.5878
y6 1031.6045
b10 1314.6953
T I T L E V E P S D T I E N V K A K
Modification
Acetyl (Protein N−term)
GlyGly (K) no new terminus
Oxidation (M)

20200921_KIZ_MUb4_GlyGly_10plex_2 − Scan number: 18855

Gene Names	Charge	m/z	Mass	Retention time	PEP	Score	Precursor Intensity
RPS27A;UBA52;UBC;UBB	3	701.0389	2100.095	73.923	0.00044761	77.562	1496945

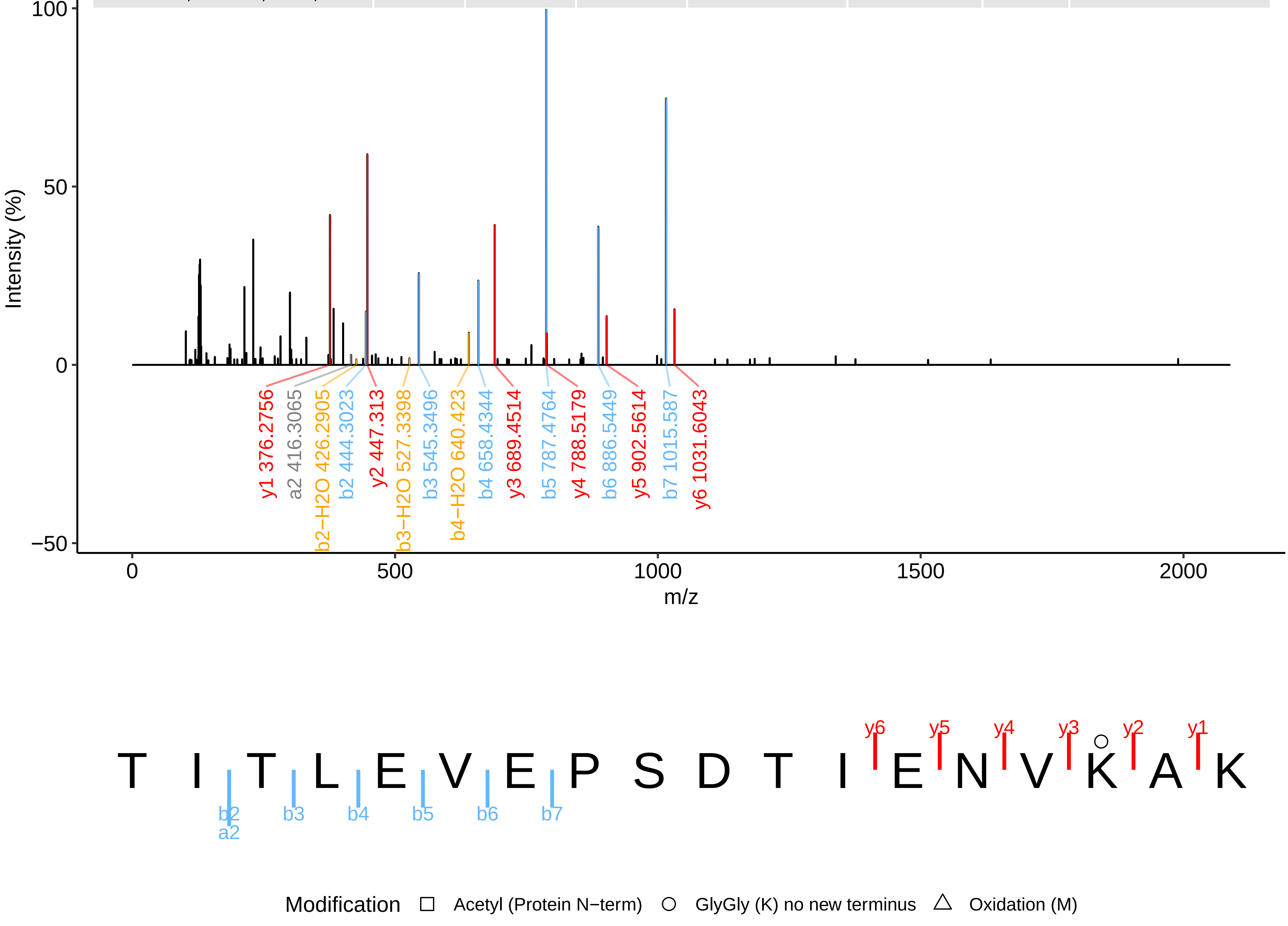

20200921_KIZ_MUb4_GlyGly_10plex_2 − Scan number: 18981

Gene Names	Charge	m/z	Mass	Retention time	PEP	Score	Precursor Intensity
RPS27A;UBA52;UBC;UBB	3	701.0389	2100.095	74.433	9.3307e−10	105.31	5090629

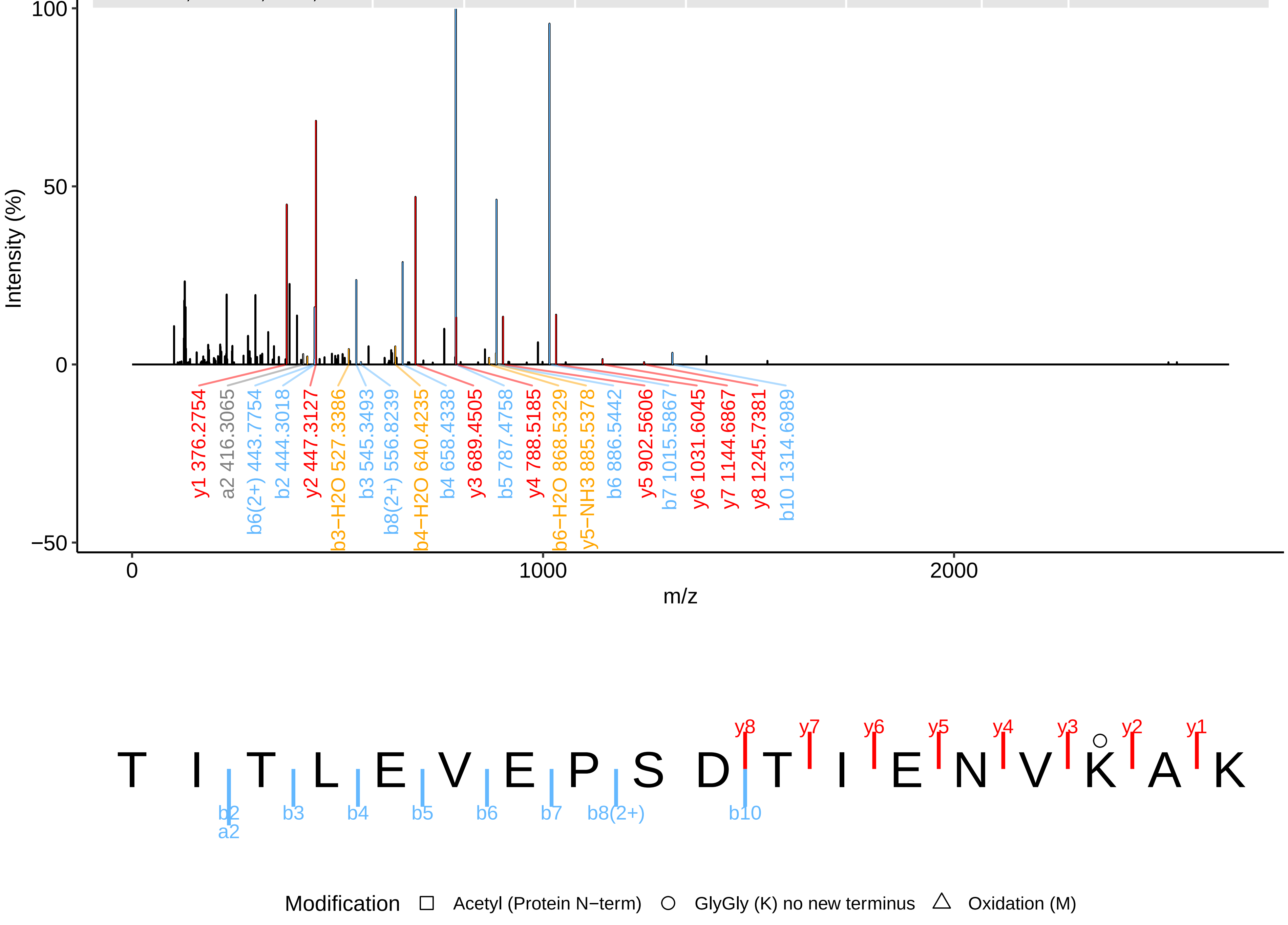

20200921_KIZ_MUb4_GlyGly_10plex_2 − Scan number: 18867

Gene Names	Charge	m/z	Mass	Retention time	PEP	Score	Precursor Intensity
RPS27A;UBA52;UBC;UBB	4	526.031	2100.095	73.971	8.2832e−08	97.836	3426595

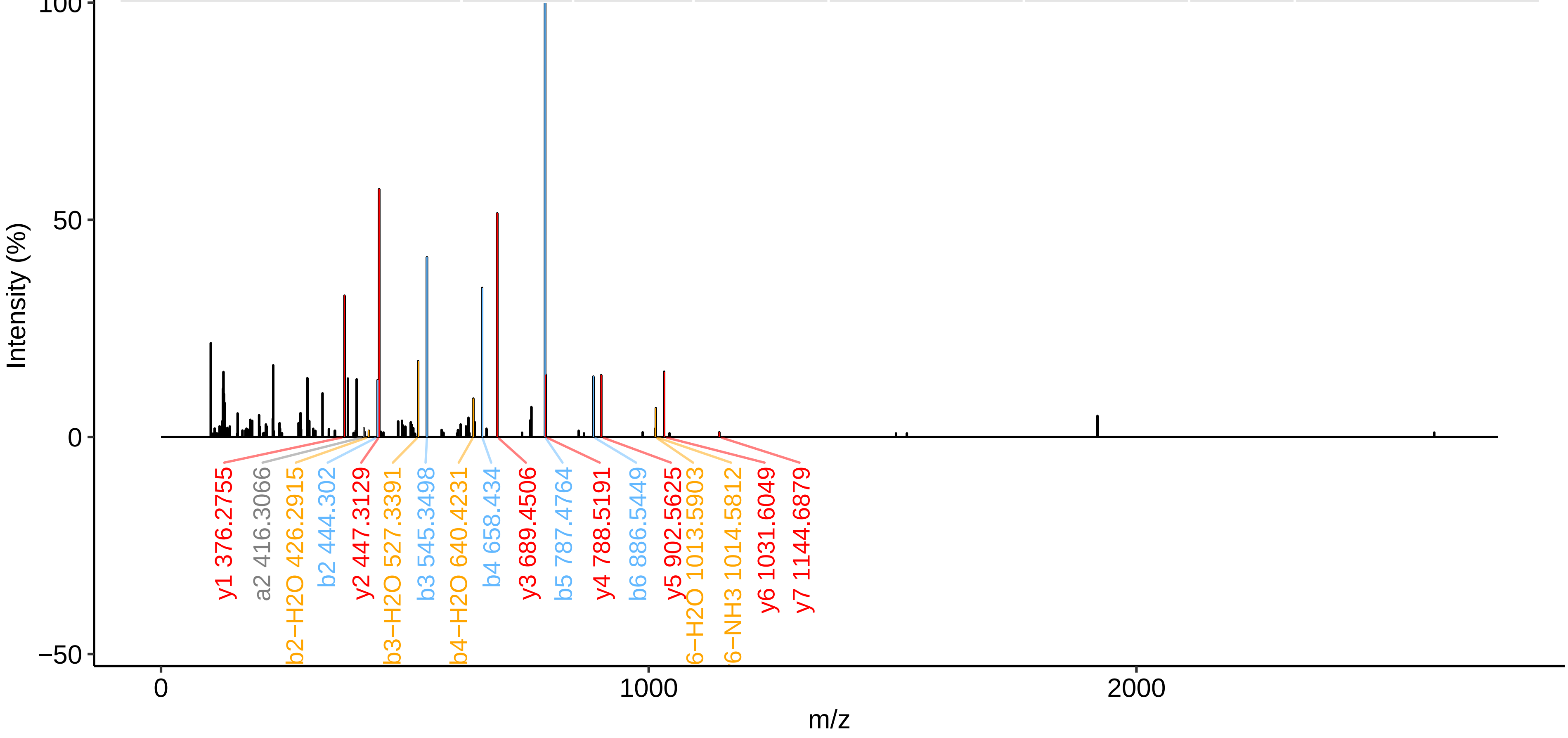

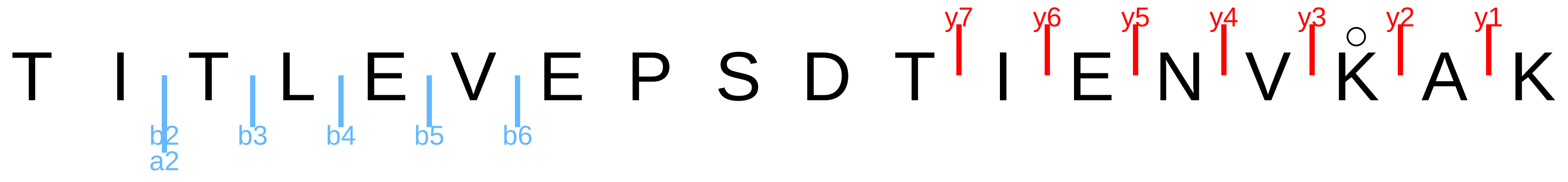

Modification □ Acetyl (Protein N−term) ○ GlyGly (K) no new terminus △ Oxidation (M)

20200921_KIZ_MUb4_GlyGly_10plex_2 − Scan number: 19115

Gene Names	Charge	m/z	Mass	Retention time	PEP	Score	Precursor Intensity
RPS27A;UBA52;UBC;UBB	3	701.0389	2100.095	74.978	0.00067728	77.562	NA

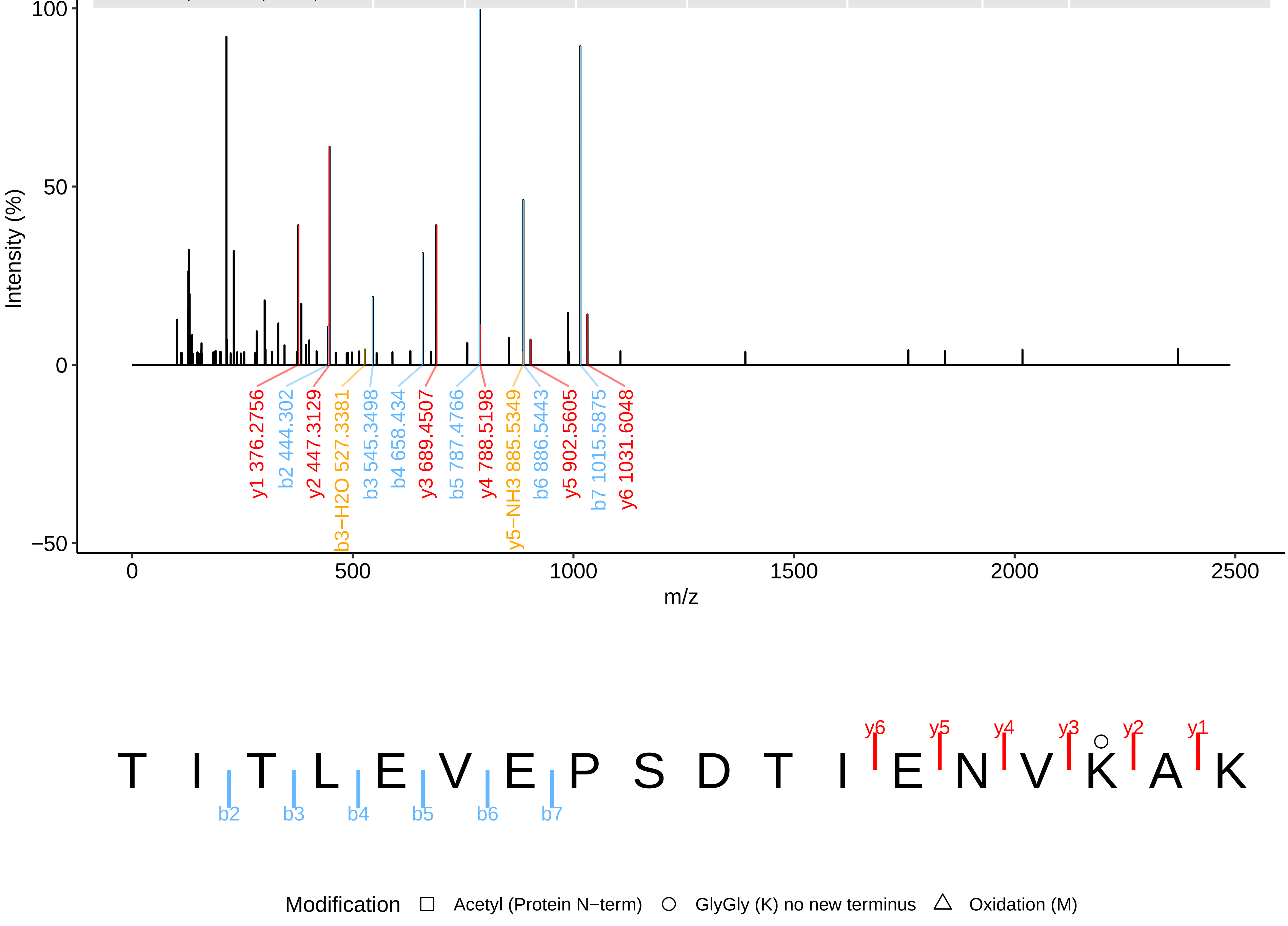

20200924_KIZ_MUb5_GlyGly_1 − Scan number: 20649

Gene Names	Charge	m/z	Mass	Retention time	PEP	Score	Precursor Intensity
RPS27A;UBA52;UBC;UBB	3	701.0389	2100.095	66.572	8.0786e−79	238.02	NA

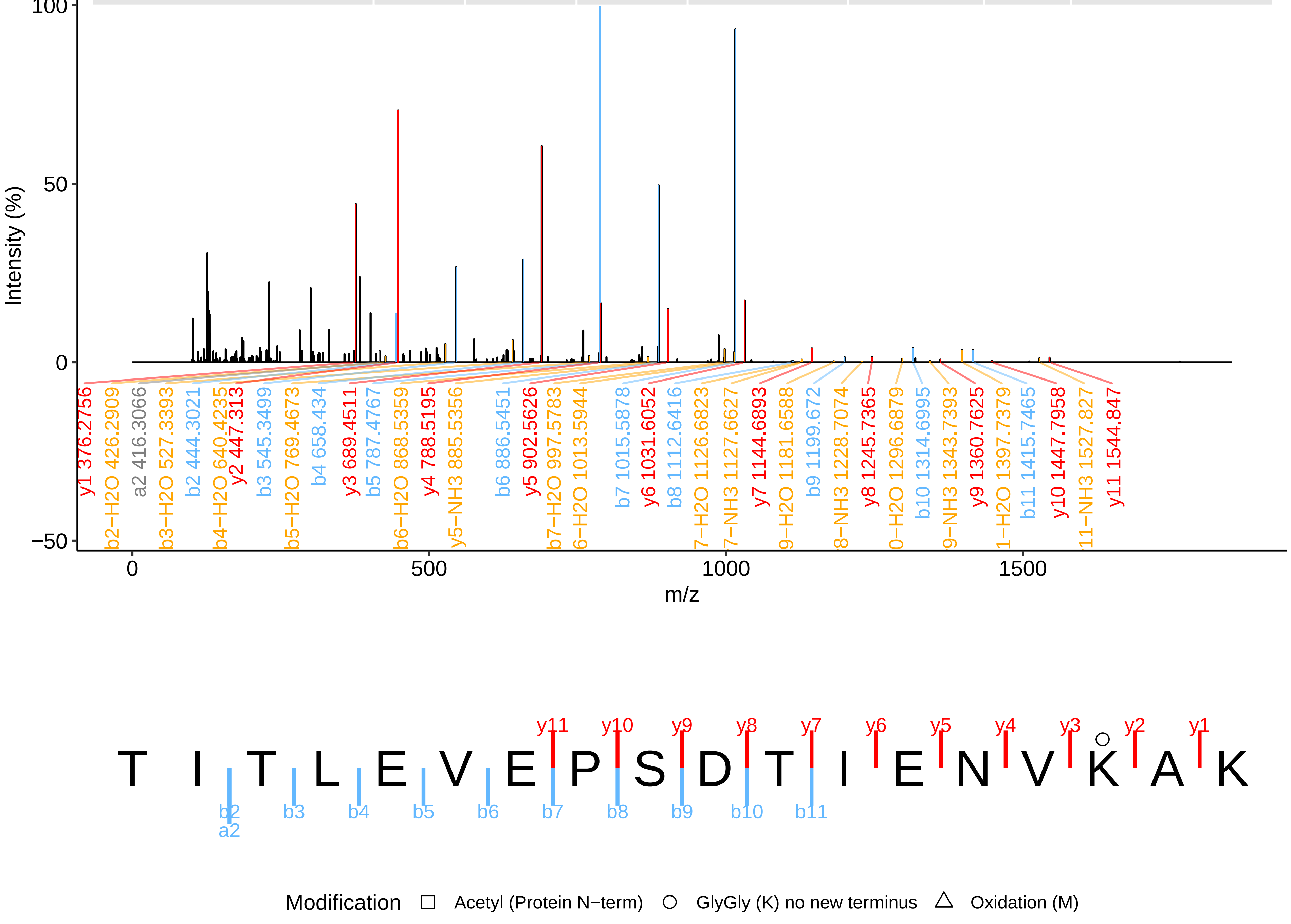

20200924_KIZ_MUb5_GlyGly_1 − Scan number: 20848

Gene Names	Charge	m/z	Mass	Retention time	PEP	Score	Precursor Intensity
RPS27A;UBA52;UBC;UBB	4	526.031	2100.095	67.186	1.3168e−10	113.69	46347252

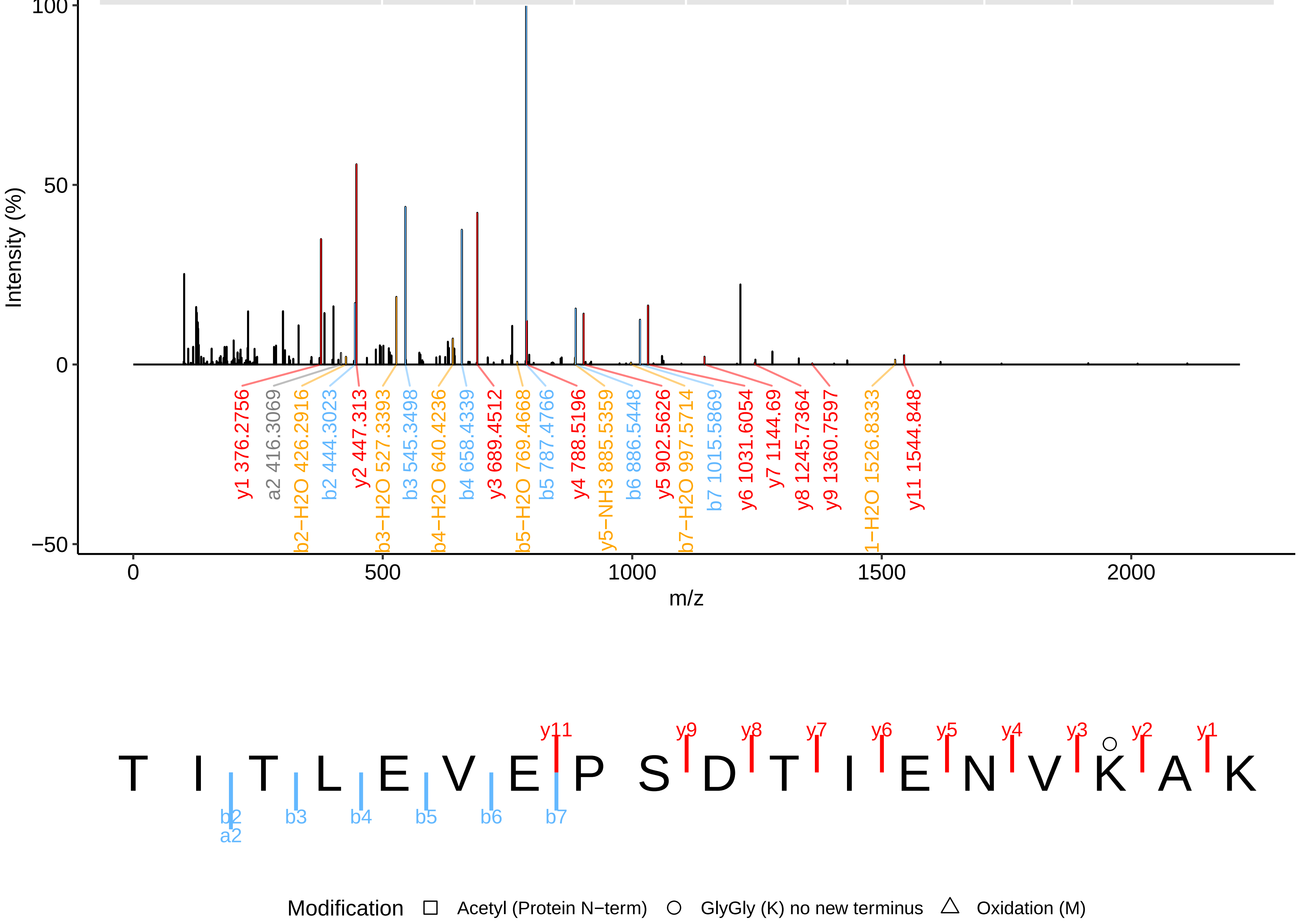

20200924_KIZ_MUb5_GlyGly_1 − Scan number: 20862

Gene Names	Charge	m/z	Mass	Retention time	PEP	Score	Precursor Intensity
RPS27A;UBA52;UBC;UBB	3	701.0389	2100.095	67.231	4.6685e−51	203.04	68723912

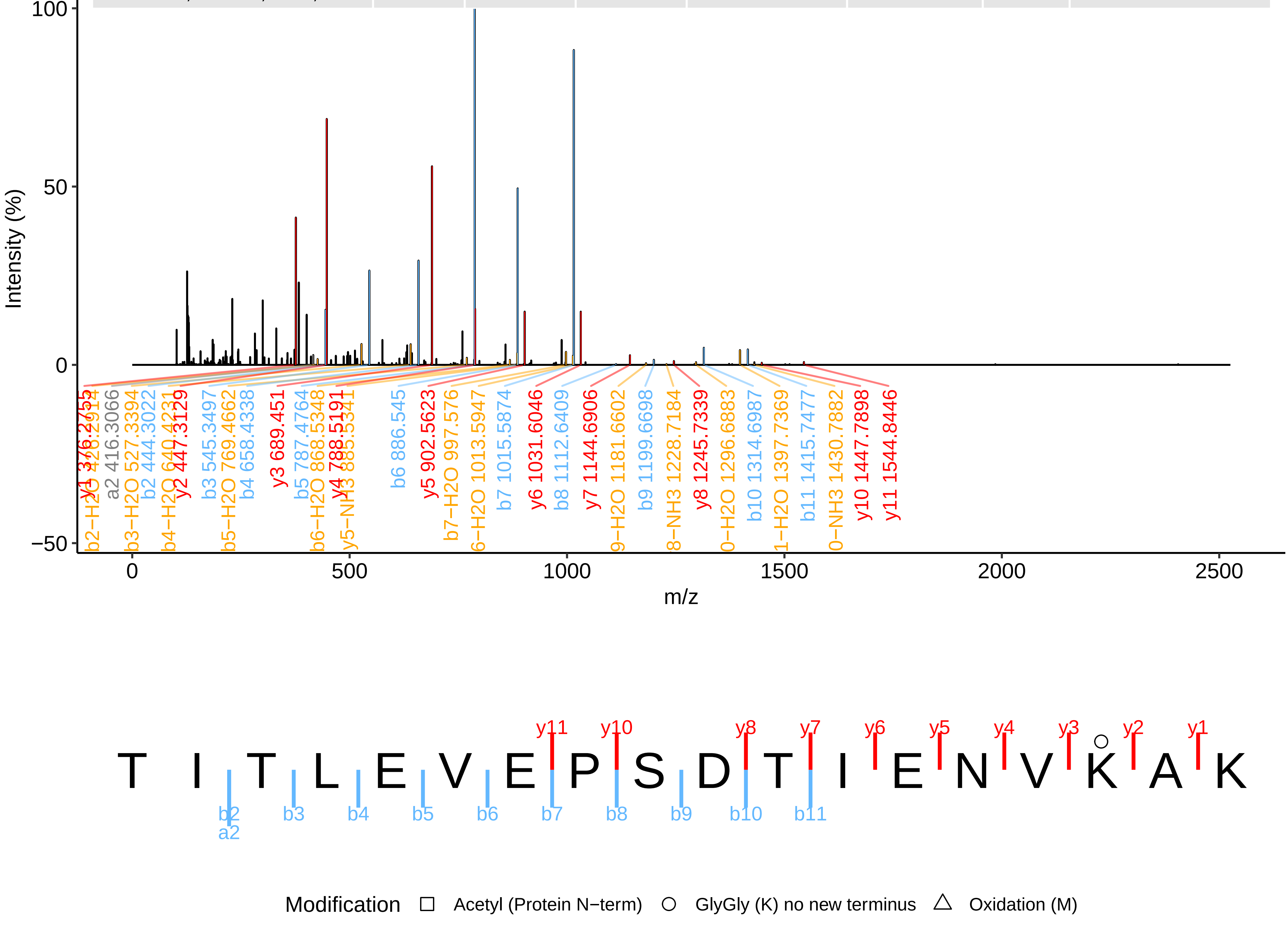

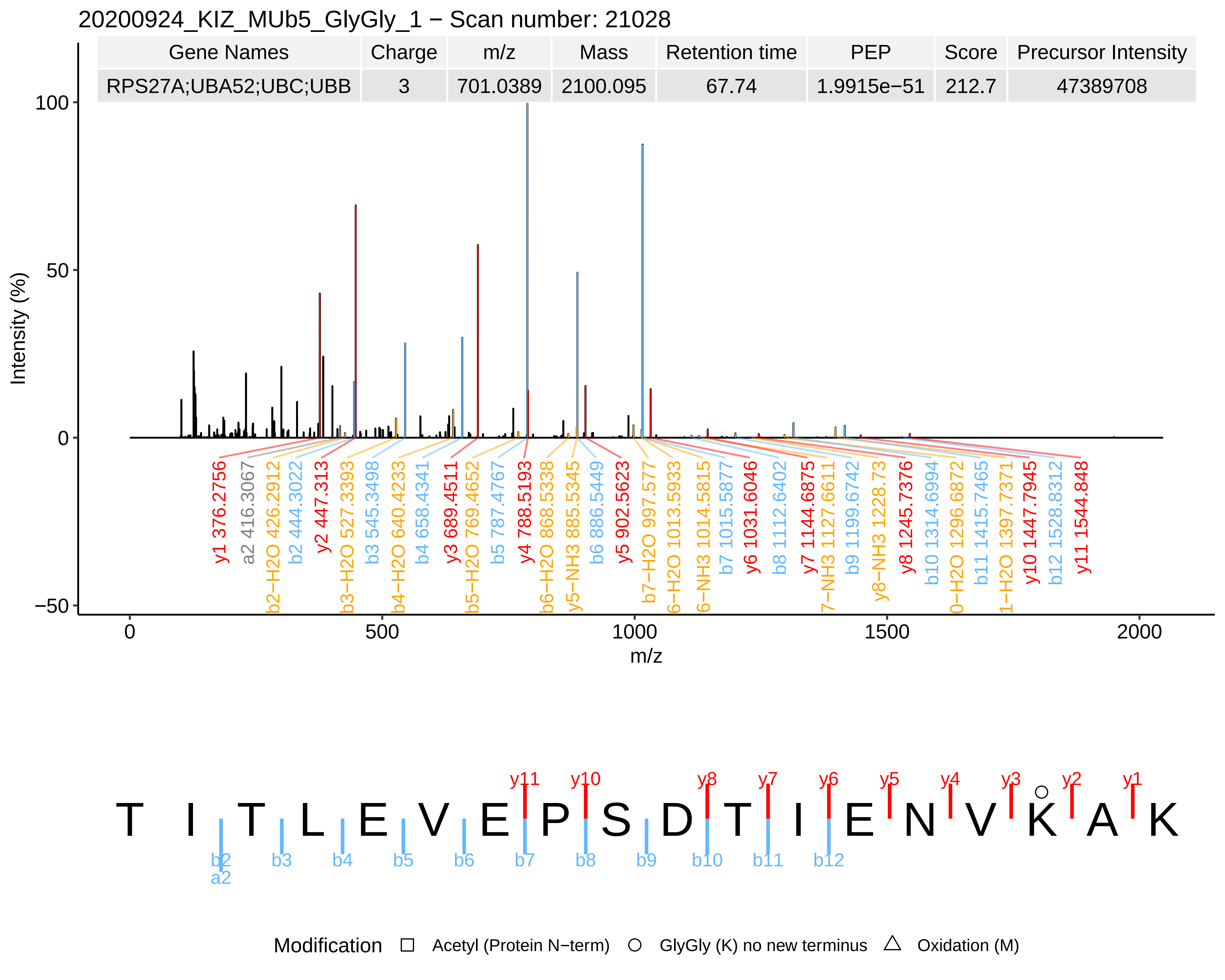

20200924_KIZ_MUb5_GlyGly_1 − Scan number: 21028
Gene Names | Charge | m/z | Mass | Retention time | PEP | Score | Precursor Intensity
RPS27A;UBA52;UBC;UBB | 3 | 701.0389 | 2100.095 | 67.74 | 1.9915e−51 | 212.7 | 47389708
Intensity (%)
m/z
y1 376.2756
a2 416.3067
b2−H2O 426.2912
b2 444.3022
y2 447.313
b3−H2O 527.3393
b3 545.3498
b4−H2O 640.4233
b4 658.4341
y3 689.4511
b5−H2O 769.4652
b5 787.4767
y4 788.5193
b6−H2O 868.5338
y5−NH3 885.5345
b6 886.5449
y5 902.5623
b7−H2O 997.577
b8−H2O 1013.5933
y6−NH3 1014.5815
b7 1015.5877
y6 1031.6046
b8 1112.6402
y7 1144.6875
y7−NH3 1127.6611
b9 1199.6742
y8−NH3 1228.73
y8 1245.7376
b10 1314.6994
b10−H2O 1296.6872
b11 1415.7465
b11−H2O 1397.7371
y10 1447.7945
b12 1528.8312
y11 1544.848
T I T L E V E P S D T I E N V K A K
Modification
Acetyl (Protein N−term)
GlyGly (K) no new terminus
Oxidation (M)

20200924_KIZ_MUb5_GlyGly_1 − Scan number: 21187

Gene Names	Charge	m/z	Mass	Retention time	PEP	Score	Precursor Intensity
RPS27A;UBA52;UBC;UBB	3	701.0389	2100.095	68.246	3.1767e−31	181.51	9891402

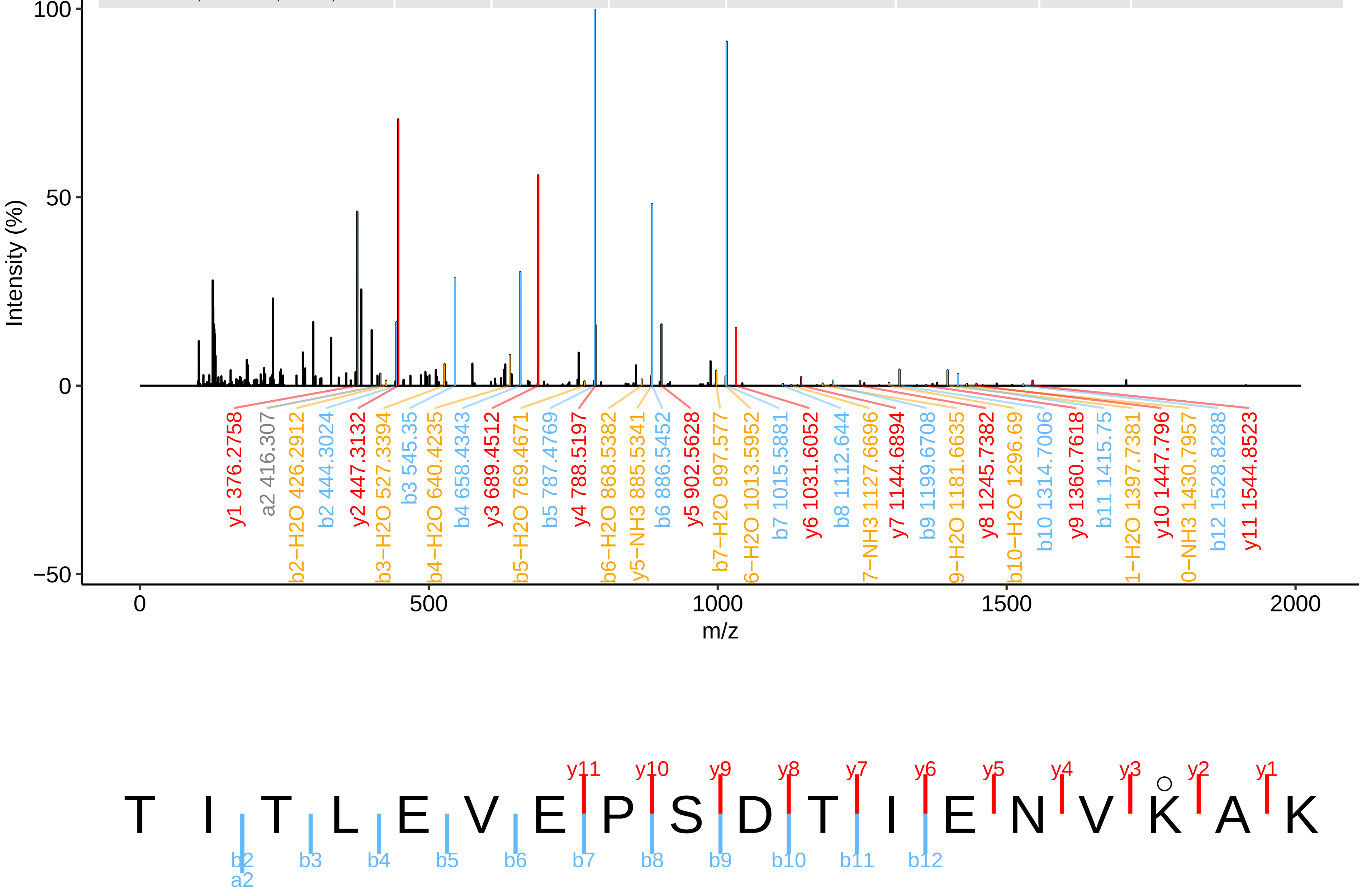

Modification □ Acetyl (Protein N−term) ○ GlyGly (K) no new terminus △ Oxidation (M)

20200924_KIZ_MUb5_GlyGly_2 − Scan number: 18723

Gene Names	Charge	m/z	Mass	Retention time	PEP	Score	Precursor Intensity
RPS27A;UBA52;UBC;UBB	3	701.0389	2100.095	69.454	3.6951e−13	119.54	NA

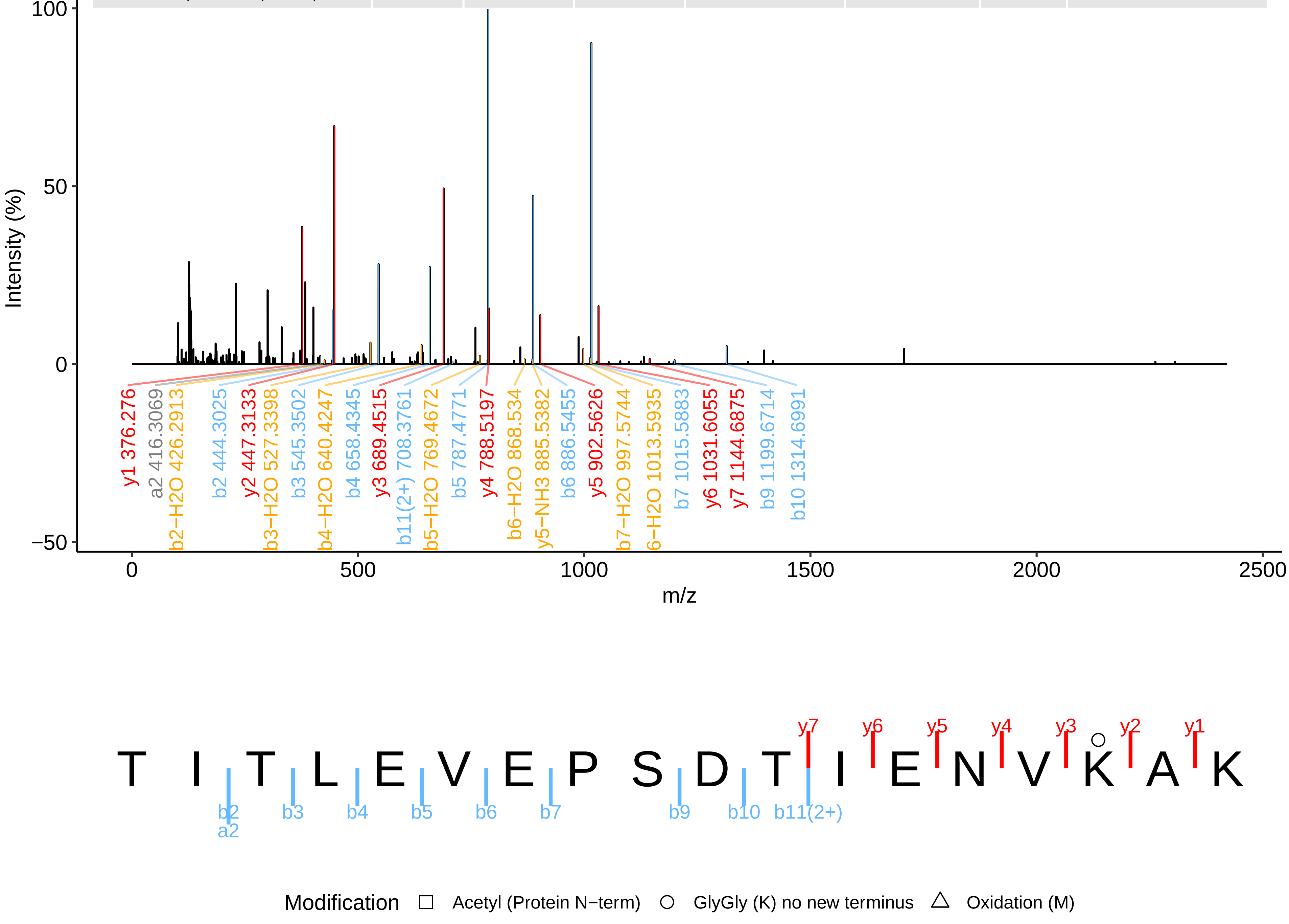

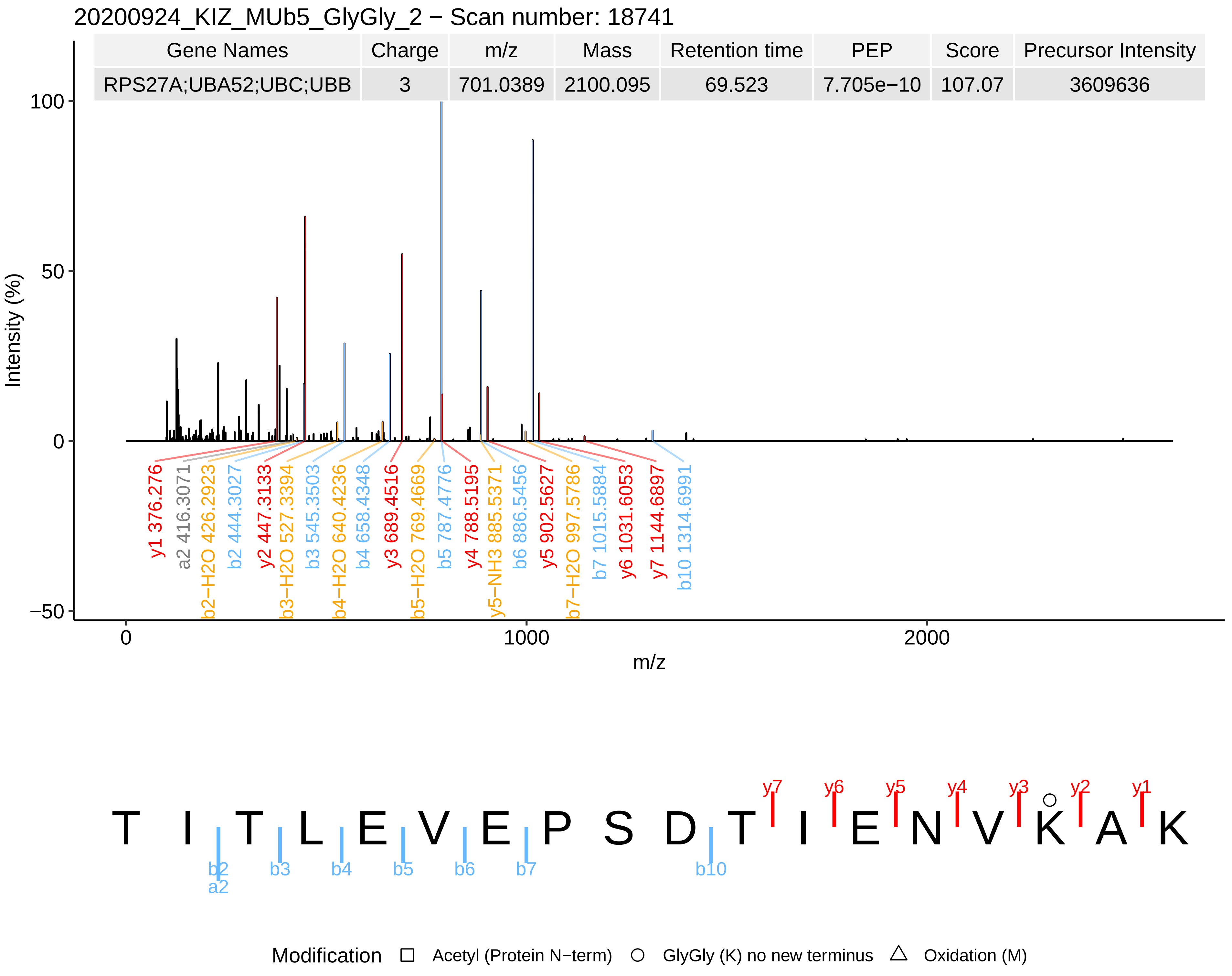

20200924_KIZ_MUb5_GlyGly_2 − Scan number: 18741
Gene Names | Charge | m/z | Mass | Retention time | PEP | Score | Precursor Intensity
RPS27A;UBA52;UBC;UBB | 3 | 701.0389 | 2100.095 | 69.523 | 7.705e−10 | 107.07 | 3609636
Intensity (%)
m/z
y1 376.276
a2 416.3071
b2−H2O 426.2923
b2 444.3027
y2 447.3133
b3−H2O 527.3394
b3 545.3503
b4−H2O 640.4236
b4 658.4348
y3 689.4516
b5−H2O 769.4669
b5 787.4776
y4 788.5195
y5−NH3 885.5371
b6 886.5456
y5 902.5627
b7−H2O 997.5786
b7 1015.5884
y6 1031.6053
y7 1144.6897
b10 1314.6991
T I T L E V E P S D T I E N V K A K
y7 y6 y5 y4 y3 y2 y1
b2 a2 b3 b4 b5 b6 b7 b10
Modification
Acetyl (Protein N−term)
GlyGly (K) no new terminus
Oxidation (M)

20200924_KIZ_MUb5_GlyGly_2 − Scan number: 18877

Gene Names	Charge	m/z	Mass	Retention time	PEP	Score	Precursor Intensity
RPS27A;UBA52;UBC;UBB	3	701.0389	2100.095	70.023	3.8643e−31	180.74	37661100

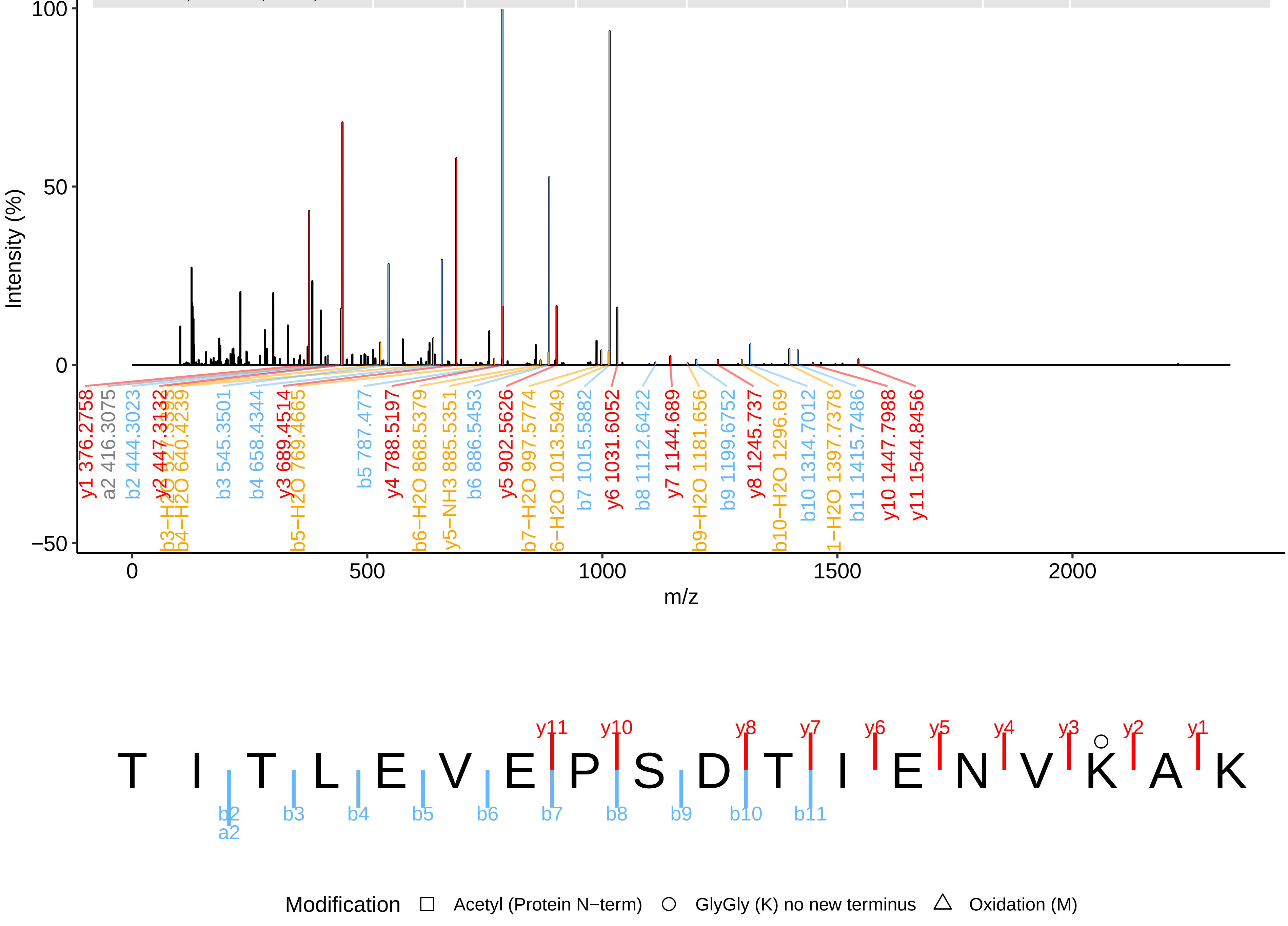

20200924_KIZ_MUb5_GlyGly_2 − Scan number: 19018

Gene Names	Charge	m/z	Mass	Retention time	PEP	Score	Precursor Intensity
RPS27A;UBA52;UBC;UBB	3	701.0389	2100.095	70.533	5.0615e−40	188.47	12283499

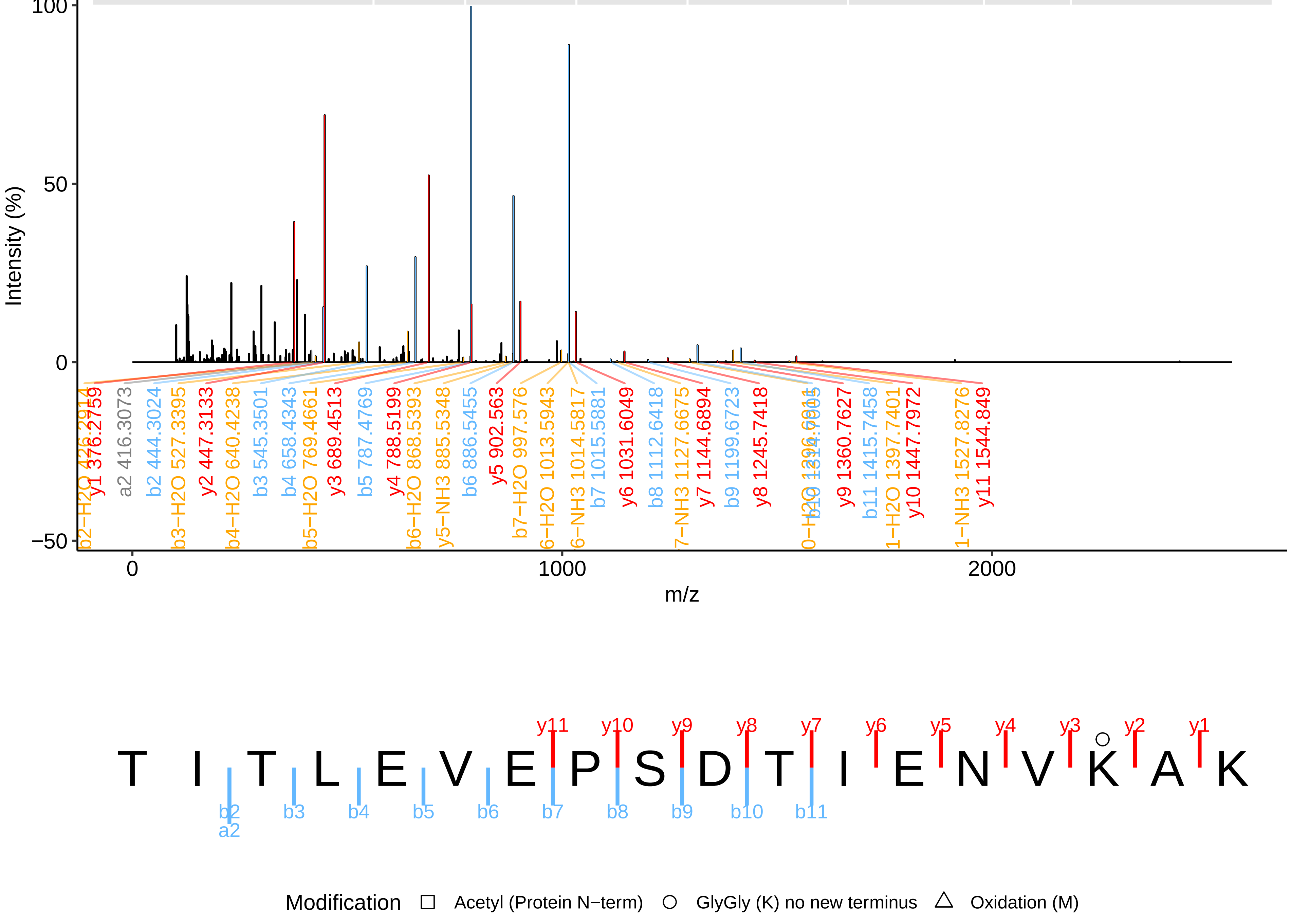

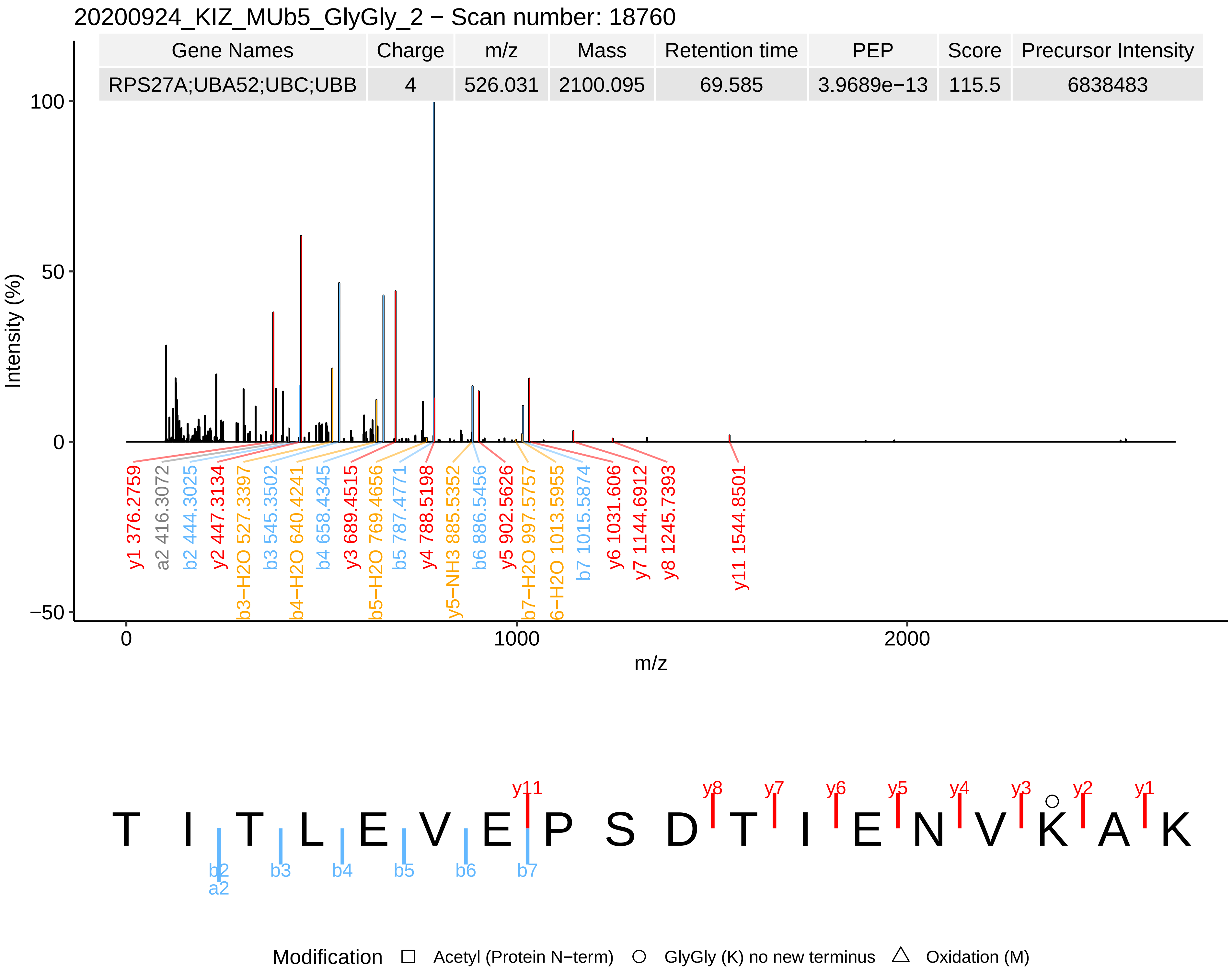
20200924_KIZ_MUb5_GlyGly_2 − Scan number: 18760
Gene Names | Charge | m/z | Mass | Retention time | PEP | Score | Precursor Intensity
RPS27A;UBA52;UBC;UBB | 4 | 526.031 | 2100.095 | 69.585 | 3.9689e−13 | 115.5 | 6838483
Intensity (%)
m/z
y1 376.2759
a2 416.3072
b2 444.3025
y2 447.3134
b3−H2O 527.3397
b3 545.3502
b4−H2O 640.4241
b4 658.4345
y3 689.4515
b5−H2O 769.4656
b5 787.4771
y4 788.5198
y5−NH3 885.5352
b6 886.5456
y5 902.5626
b7−H2O 997.5757
6−H2O 1013.5955
b7 1015.5874
y6 1031.606
y7 1144.6912
y8 1245.7393
y11 1544.8501
T I T L E V E P S D T I E N V K A K
y11 y8 y7 y6 y5 y4 y3 y2 y1
b2 a2 b3 b4 b5 b6 b7
Modification
Acetyl (Protein N−term)
GlyGly (K) no new terminus
Oxidation (M)

20200924_KIZ_MUb5_GlyGly_2 − Scan number: 18898

Gene Names	Charge	m/z	Mass	Retention time	PEP	Score	Precursor Intensity
RPS27A;UBA52;UBC;UBB	4	526.031	2100.095	70.089	1.1621e−13	127.75	13355849

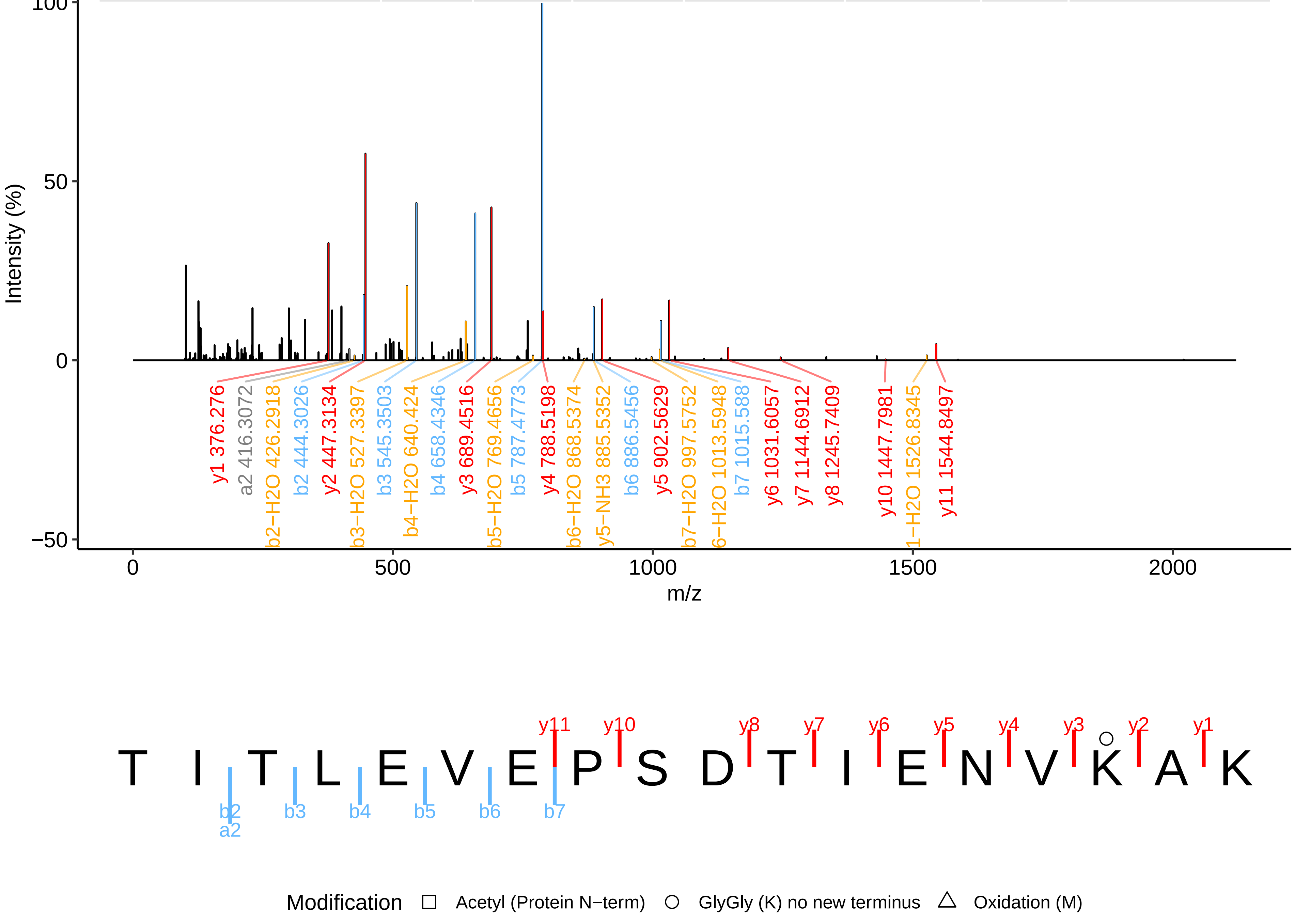

20201118_KIZ_MUb6_GlyGly_1 − Scan number: 21451

Gene Names	Charge	m/z	Mass	Retention time	PEP	Score	Precursor Intensity
RPS27A;UBA52;UBC;UBB	3	701.0389	2100.095	66.938	2.3056e−51	211.57	15449524

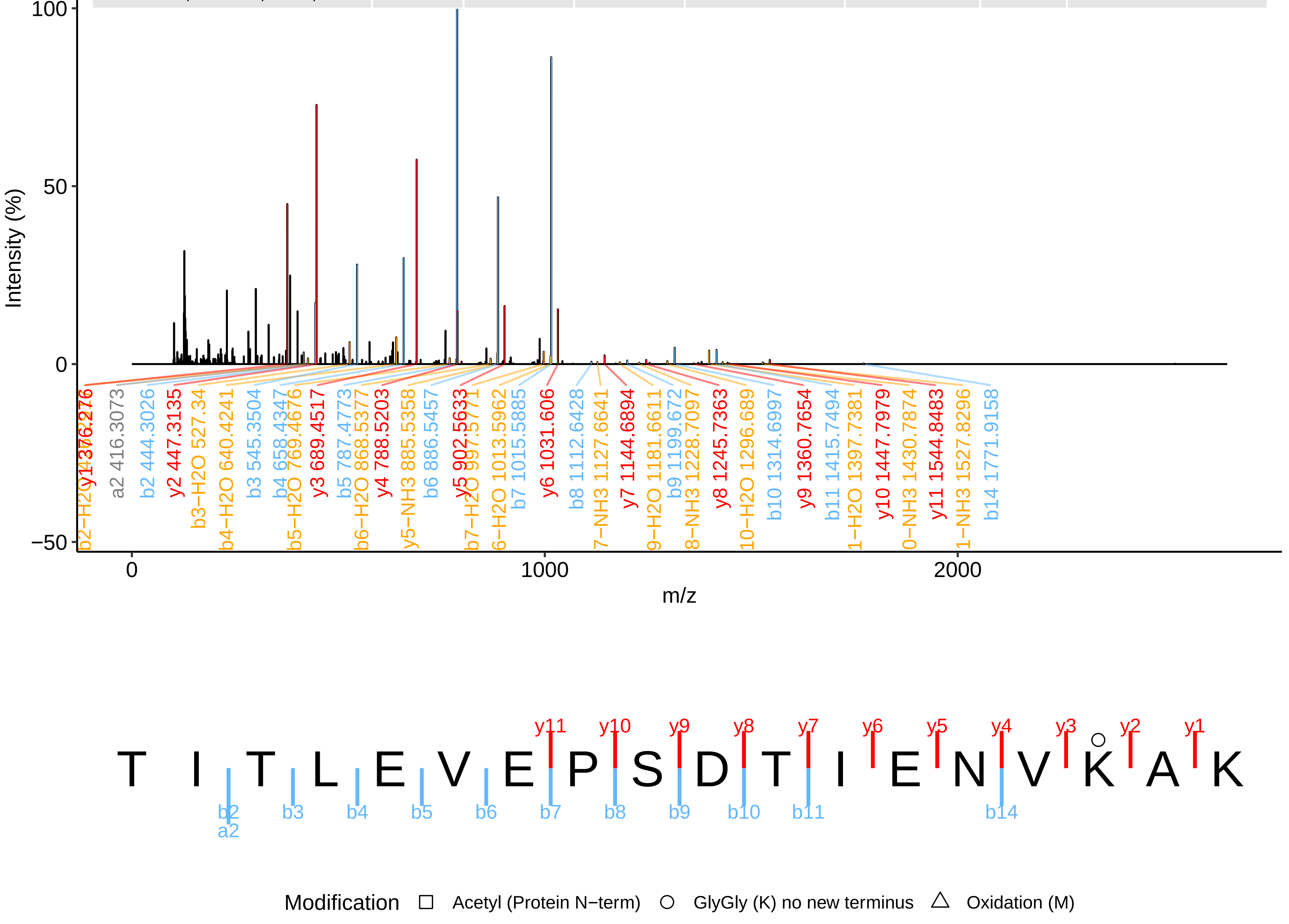

20201118_KIZ_MUb6_GlyGly_1 − Scan number: 21457

Gene Names	Charge	m/z	Mass	Retention time	PEP	Score	Precursor Intensity
RPS27A;UBA52;UBC;UBB	3	701.0389	2100.095	66.955	4.0661e−23	154.69	39999020

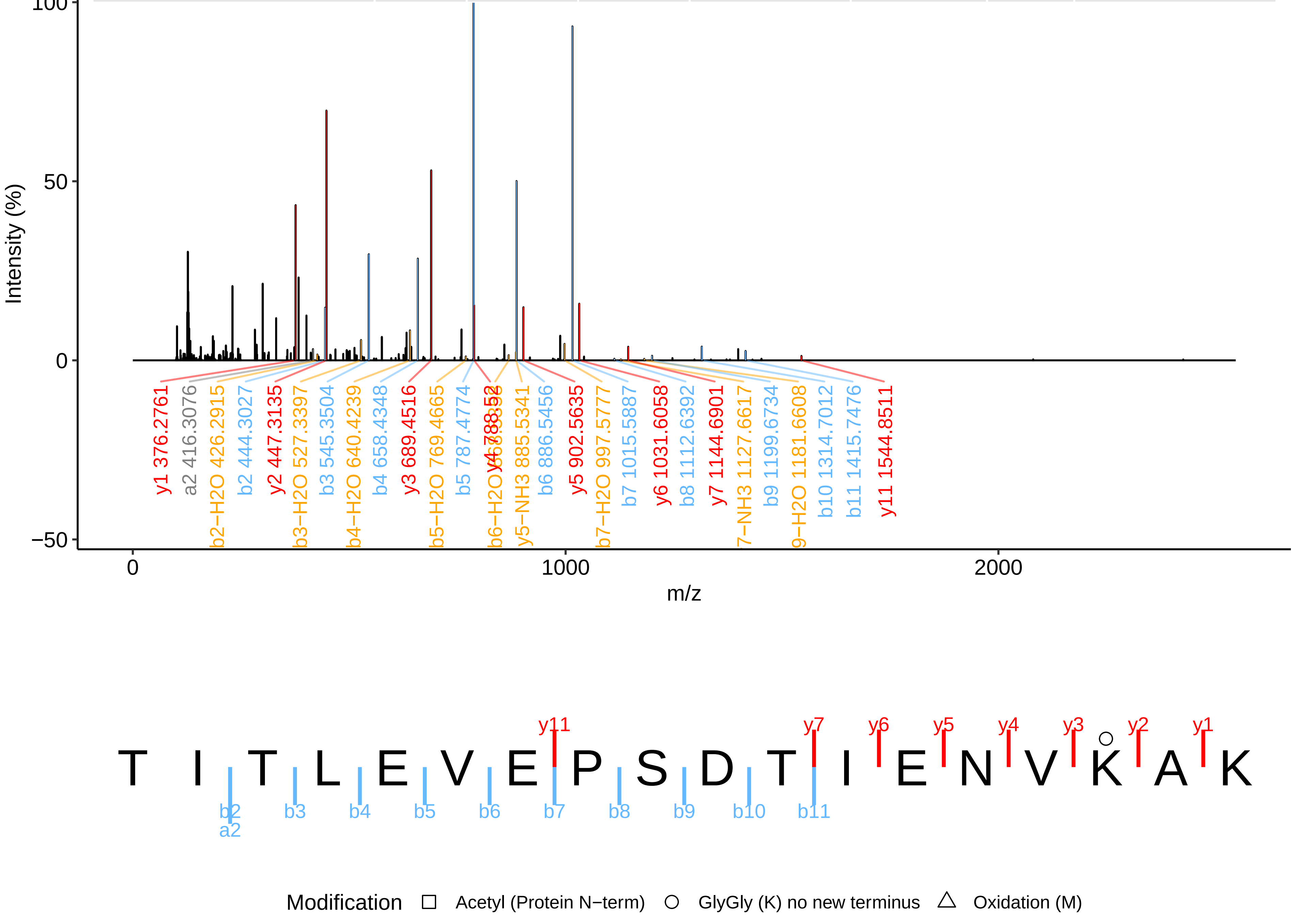

20201118_KIZ_MUb6_GlyGly_1 − Scan number: 21461

Gene Names	Charge	m/z	Mass	Retention time	PEP	Score	Precursor Intensity
RPS27A;UBA52;UBC;UBB	4	526.031	2100.095	66.967	2.7863e−13	120.66	37789984

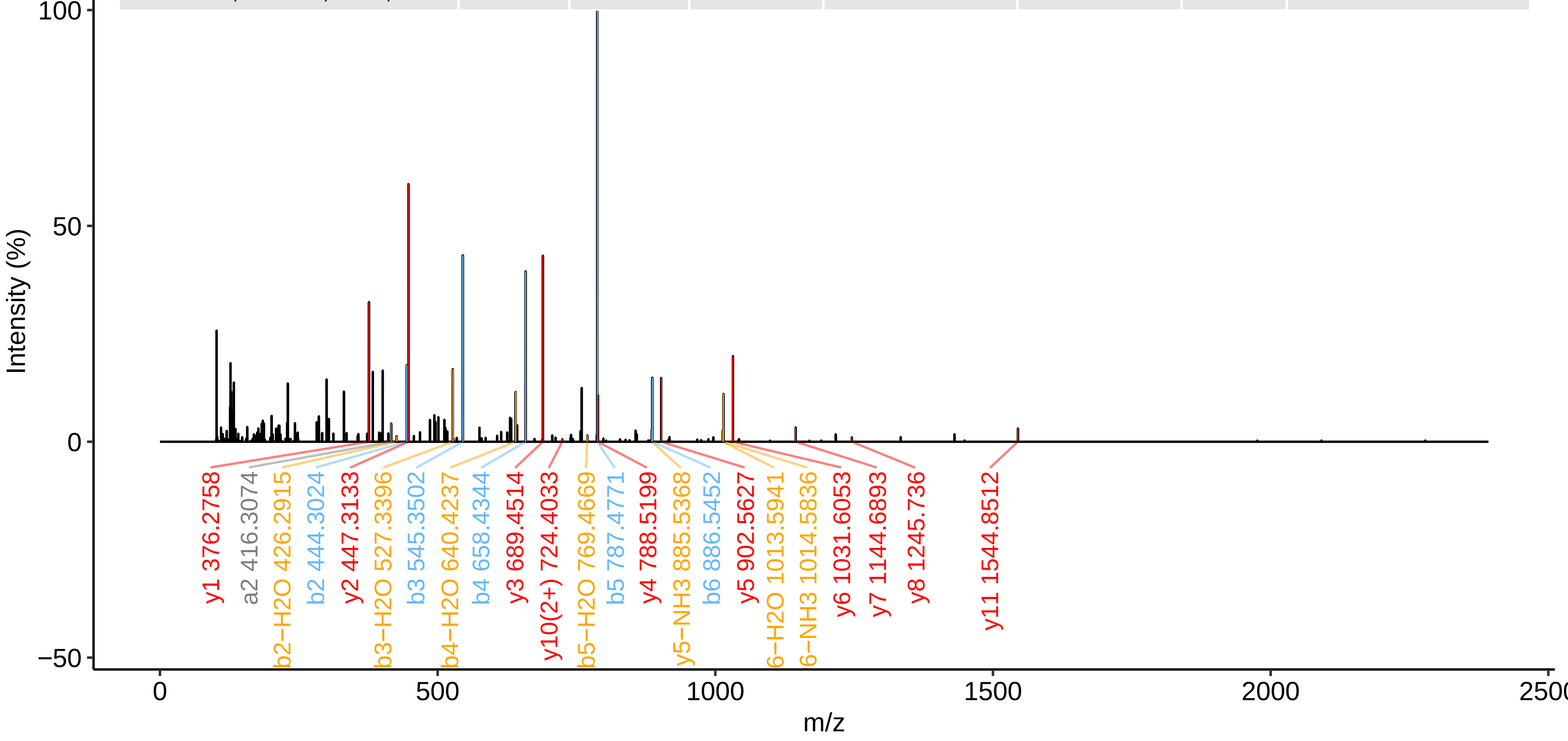

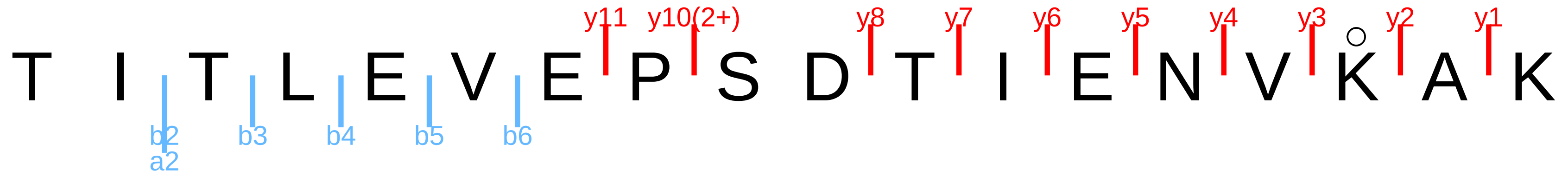

Modification □ Acetyl (Protein N−term) ○ GlyGly (K) no new terminus △ Oxidation (M)

20201118_KIZ_MUb6_GlyGly_2 − Scan number: 22604

Gene Names	Charge	m/z	Mass	Retention time	PEP	Score	Precursor Intensity
RPS27A;UBA52;UBC;UBB	3	701.0389	2100.095	75.98	4.6685e−51	203.04	13505124

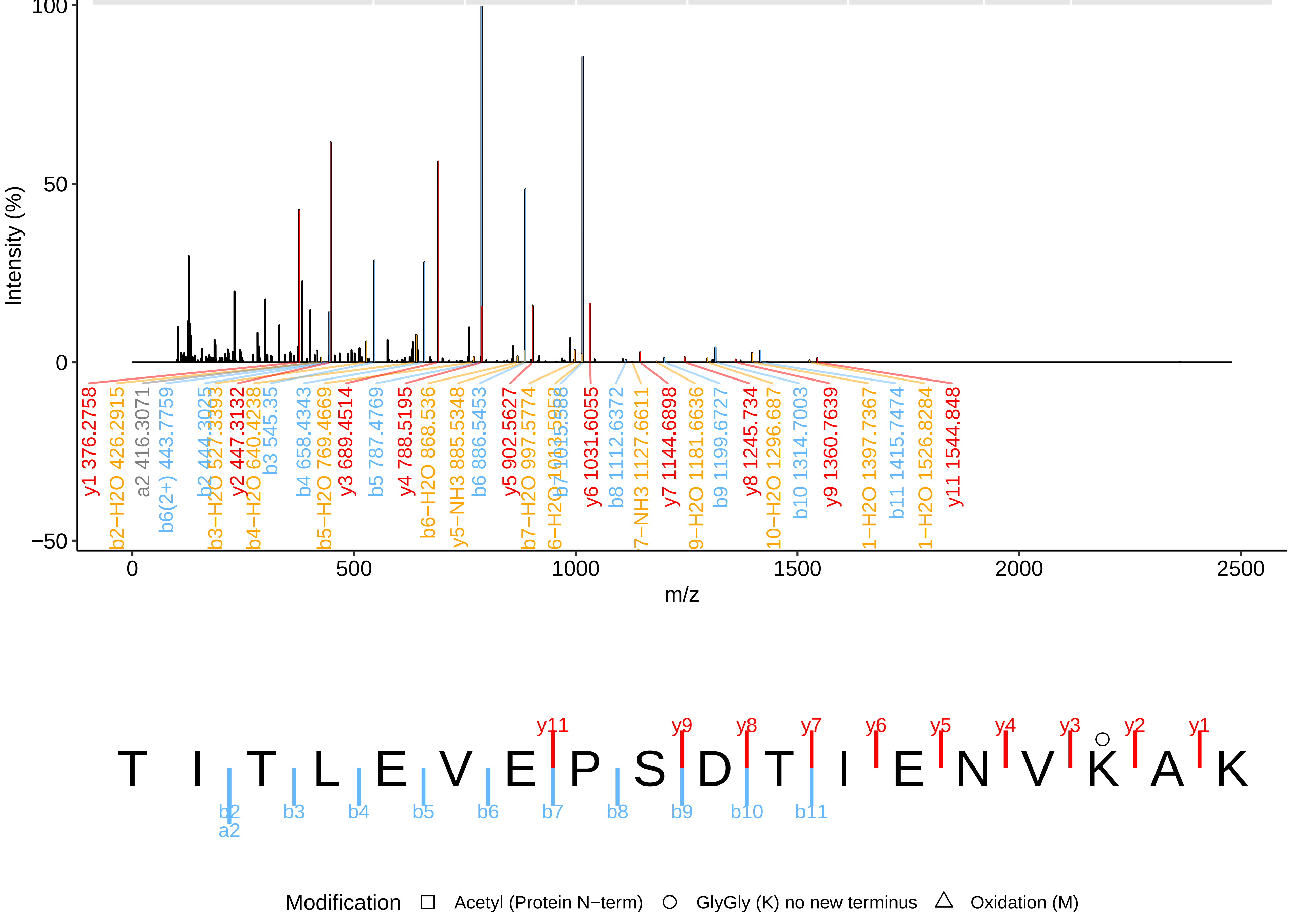

20201118_KIZ_MUb6_GlyGly_2 − Scan number: 22611

Gene Names	Charge	m/z	Mass	Retention time	PEP	Score	Precursor Intensity
RPS27A;UBA52;UBC;UBB	3	701.0389	2100.095	76.002	4.9574e−64	220.6	19096112

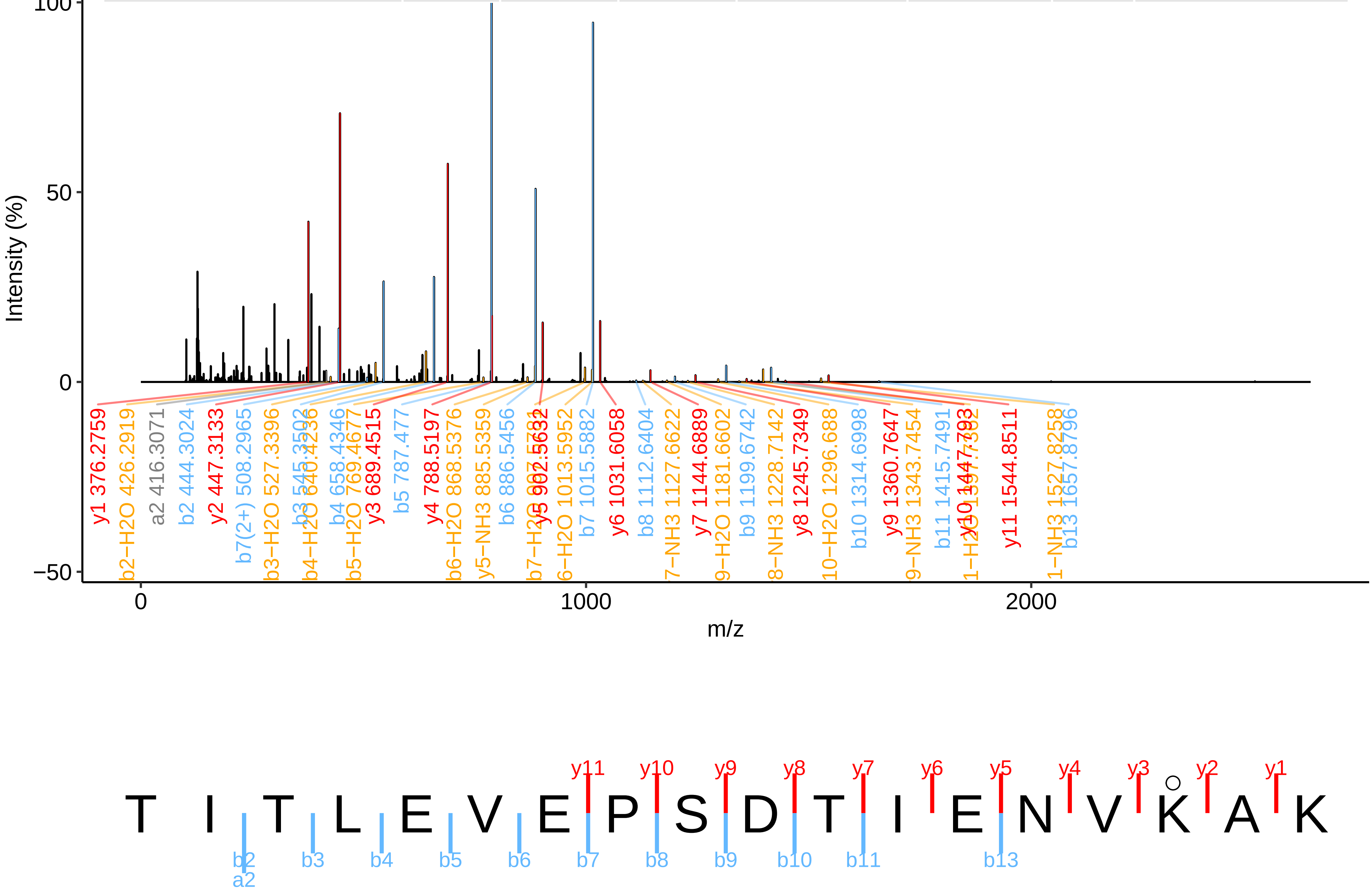

Modification □ Acetyl (Protein N−term) ○ GlyGly (K) no new terminus △ Oxidation (M)

20201118_KIZ_MUb6_GlyGly_2 − Scan number: 22615

Gene Names	Charge	m/z	Mass	Retention time	PEP	Score	Precursor Intensity
RPS27A;UBA52;UBC;UBB	4	526.031	2100.095	76.015	1.6366e−17	140.84	23817580

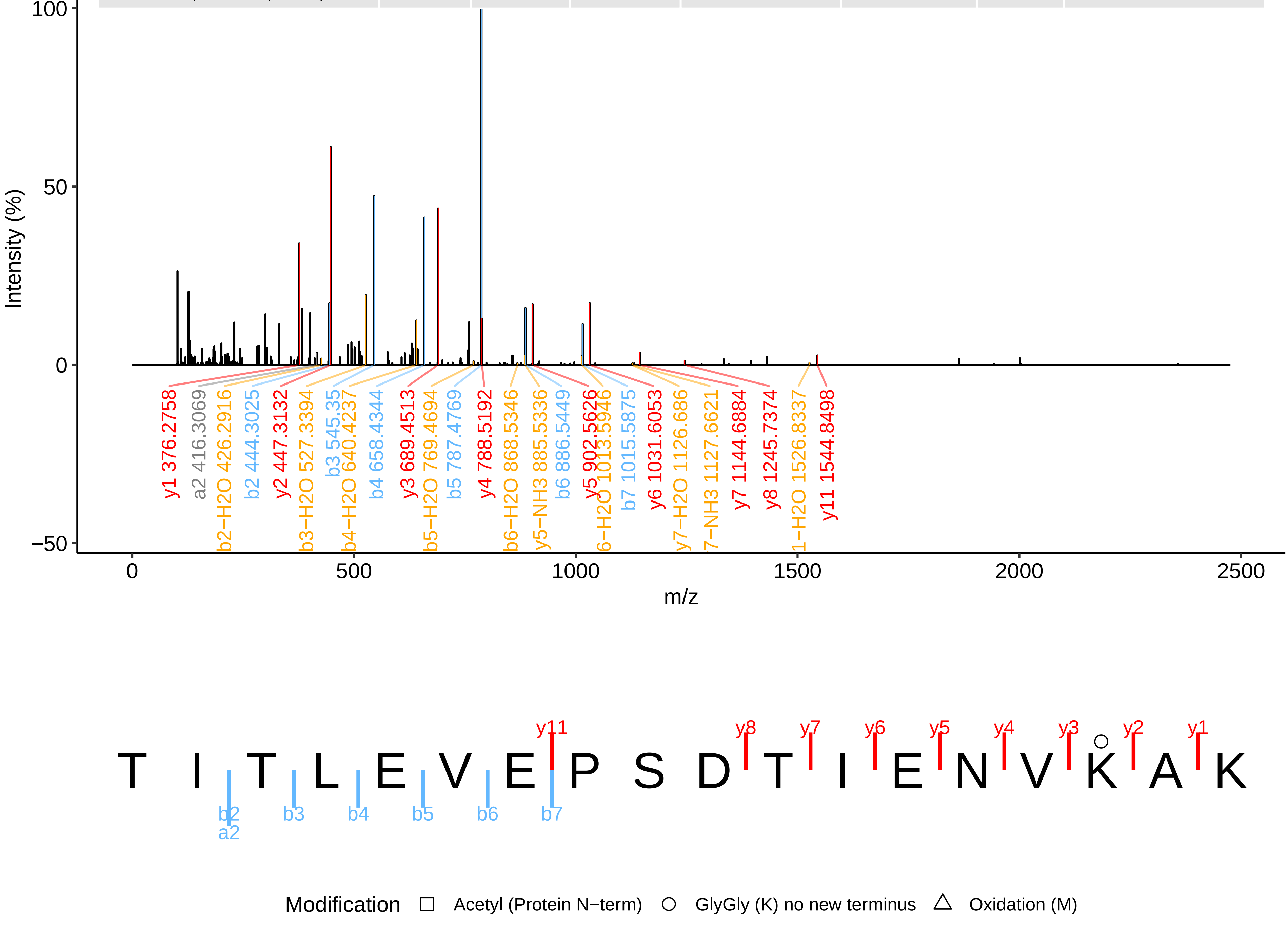

20200921_KIZ_MUb4_GlyGly_10plex − Scan number: 21439

Gene Names	Charge	m/z	Mass	Retention time	PEP	Score	Precursor Intensity
RPS27A;UBA52;UBB;UBC	5	449.6455	2243.191	77.646	0.074193	41.202	4221067

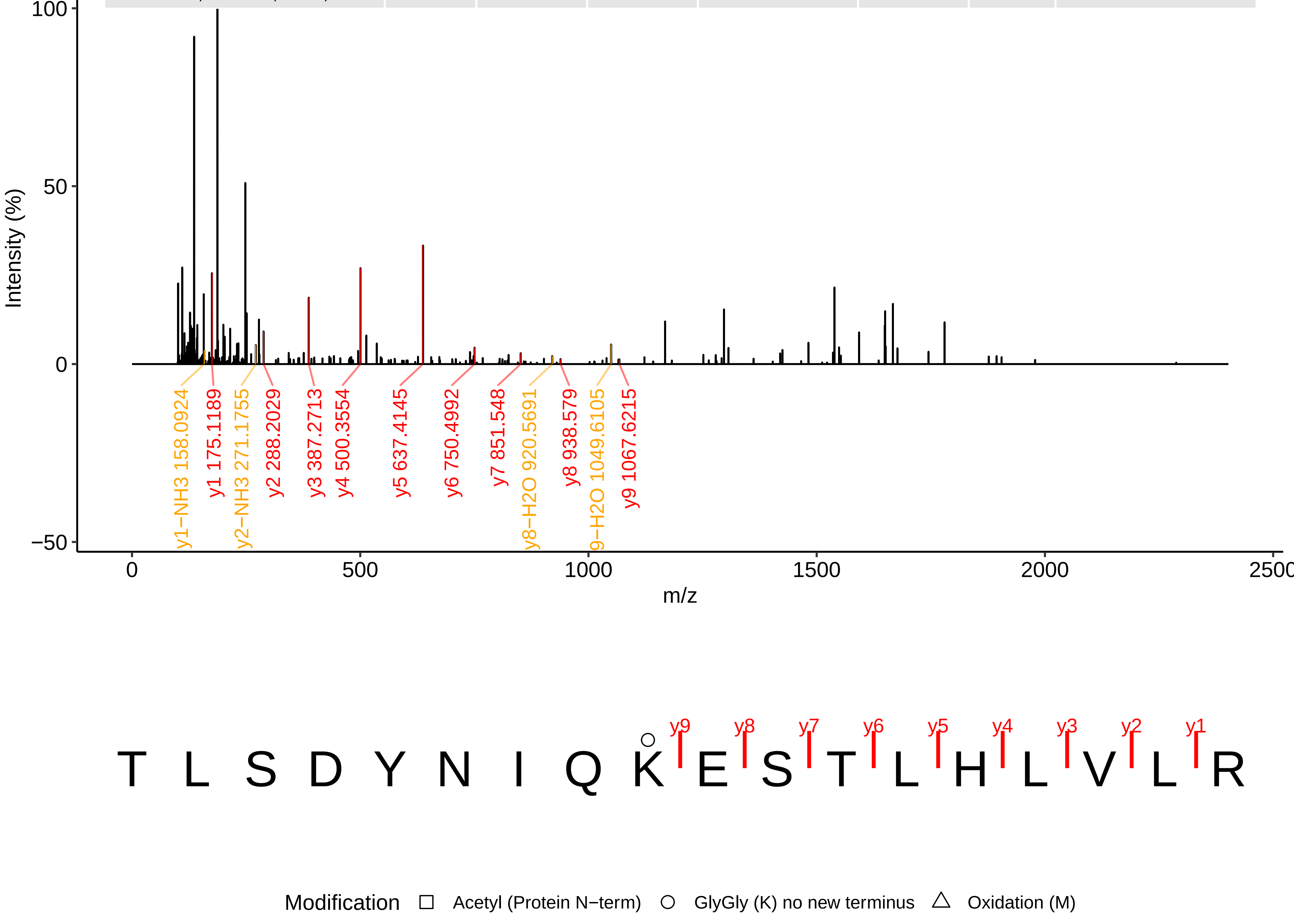

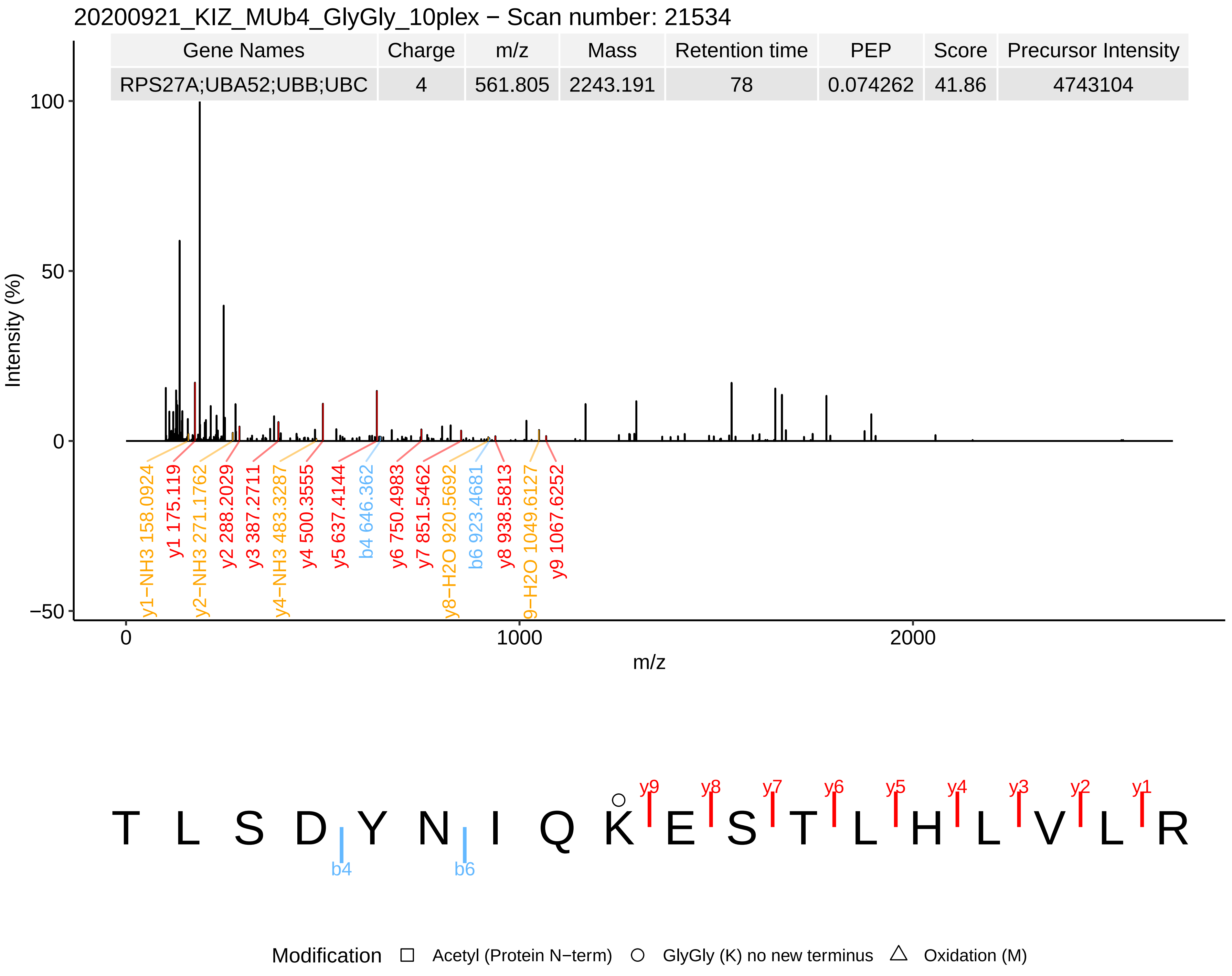
20200921_KIZ_MUb4_GlyGly_10plex − Scan number: 21534
Gene Names
Charge
m/z
Mass
Retention time
PEP
Score
Precursor Intensity
RPS27A;UBA52;UBB;UBC
4
561.805
2243.191
78
0.074262
41.86
4743104
Intensity (%)
100
50
0
−50
0
1000
2000
m/z
y1−NH3 158.0924
y1 175.119
y2−NH3 271.1762
y2 288.2029
y3 387.2711
y4−NH3 483.3287
y4 500.3555
y5 637.4144
b4 646.362
y6 750.4983
y7 851.5462
y8−H2O 920.5692
b6 923.4681
y8 938.5813
9−H2O 1049.6127
y9 1067.6252
T L S D Y N I Q K E S T L H L V L R
y9
y8
y7
y6
y5
y4
y3
y2
y1
b4
b6
Modification
Acetyl (Protein N−term)
GlyGly (K) no new terminus
Oxidation (M)

20200921_KIZ_MUb4_GlyGly_10plex − Scan number: 21576

Gene Names	Charge	m/z	Mass	Retention time	PEP	Score	Precursor Intensity
RPS27A;UBA52;UBB;UBC	5	449.6455	2243.191	78.16	0.033964	51.495	4015816

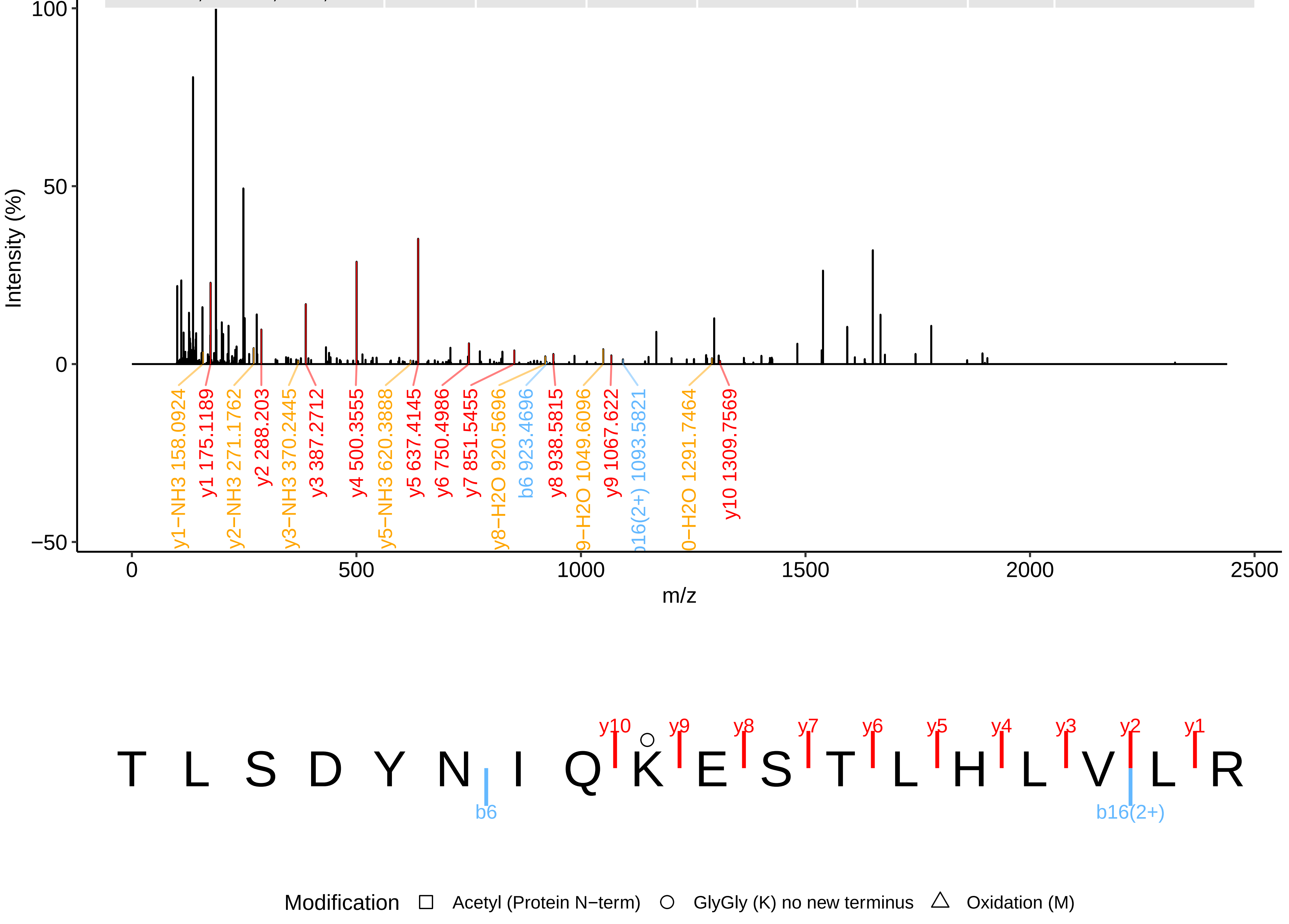

20200921_KIZ_MUb4_GlyGly_10plex − Scan number: 21844

Gene Names	Charge	m/z	Mass	Retention time	PEP	Score	Precursor Intensity
RPS27A;UBA52;UBB;UBC	5	449.6455	2243.191	79.174	0.017463	57.52	9100616

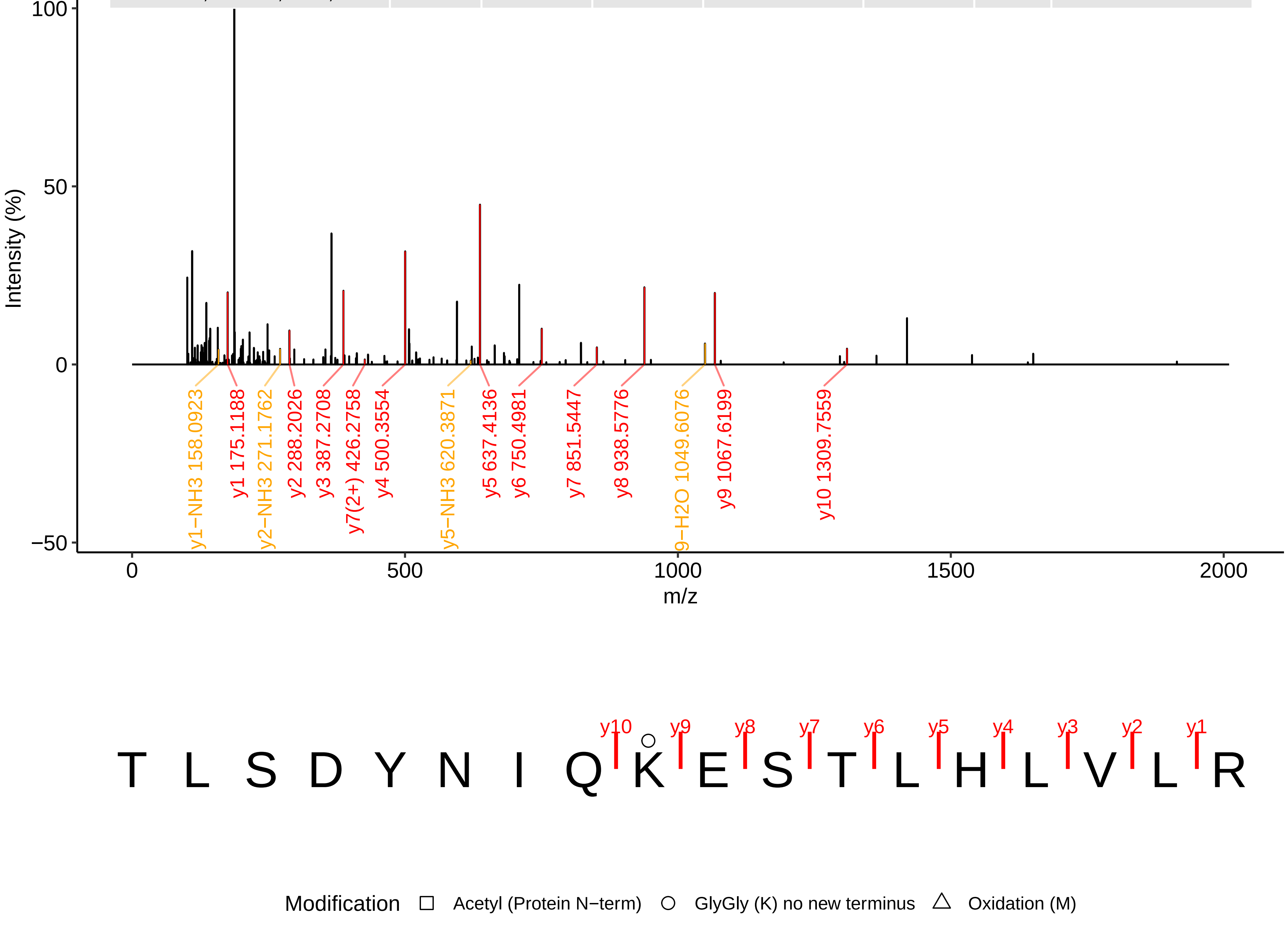

20200921_KIZ_MUb4_GlyGly_10plex − Scan number: 21936

Gene Names	Charge	m/z	Mass	Retention time	PEP	Score	Precursor Intensity
RPS27A;UBA52;UBB;UBC	4	561.805	2243.191	79.516	4.9472e−06	88.133	3695184

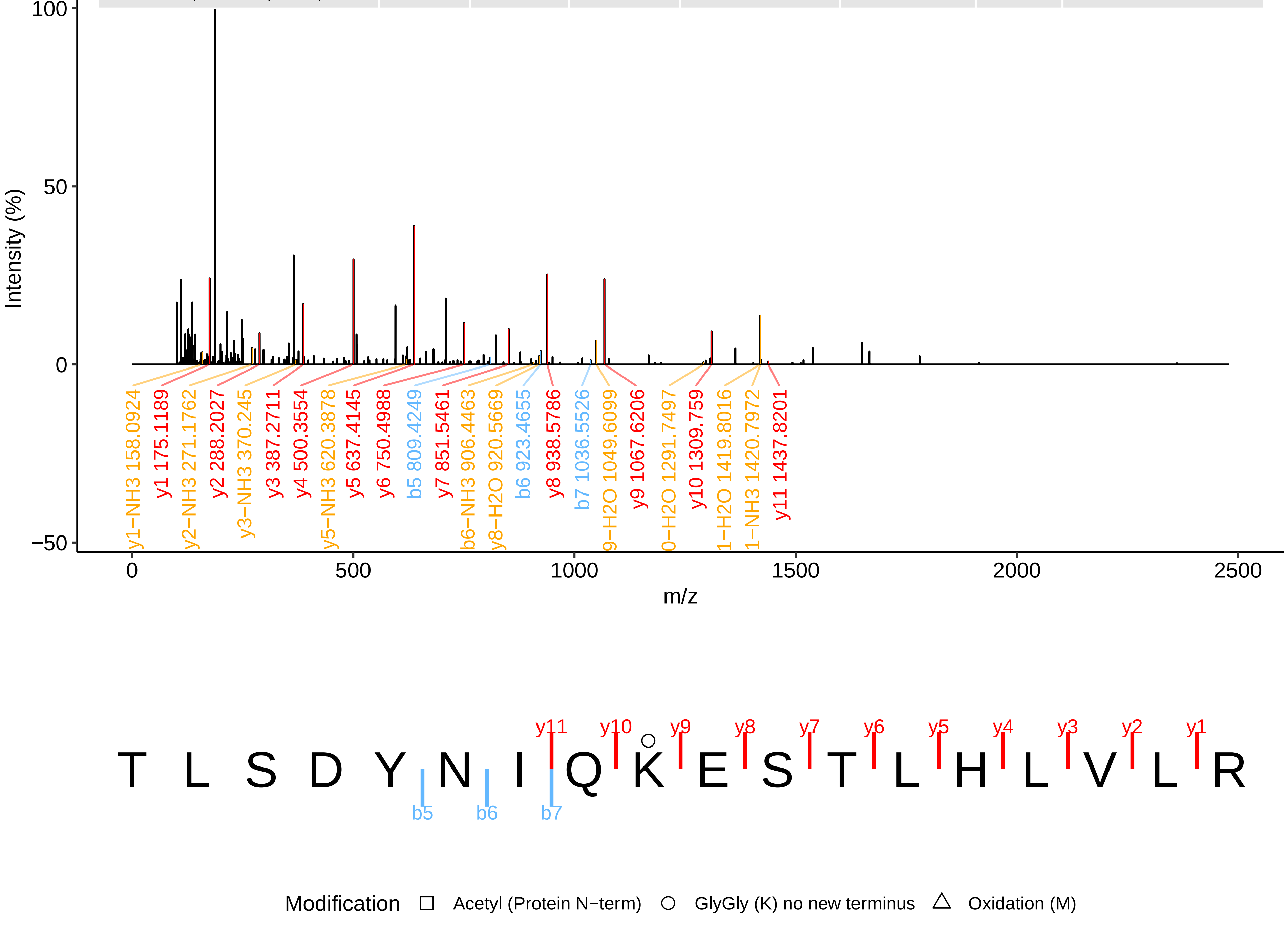

20200921_KIZ_MUb4_GlyGly_10plex − Scan number: 21982

Gene Names	Charge	m/z	Mass	Retention time	PEP	Score	Precursor Intensity
RPS27A;UBA52;UBB;UBC	5	449.6455	2243.191	79.699	0.017463	57.52	11189842

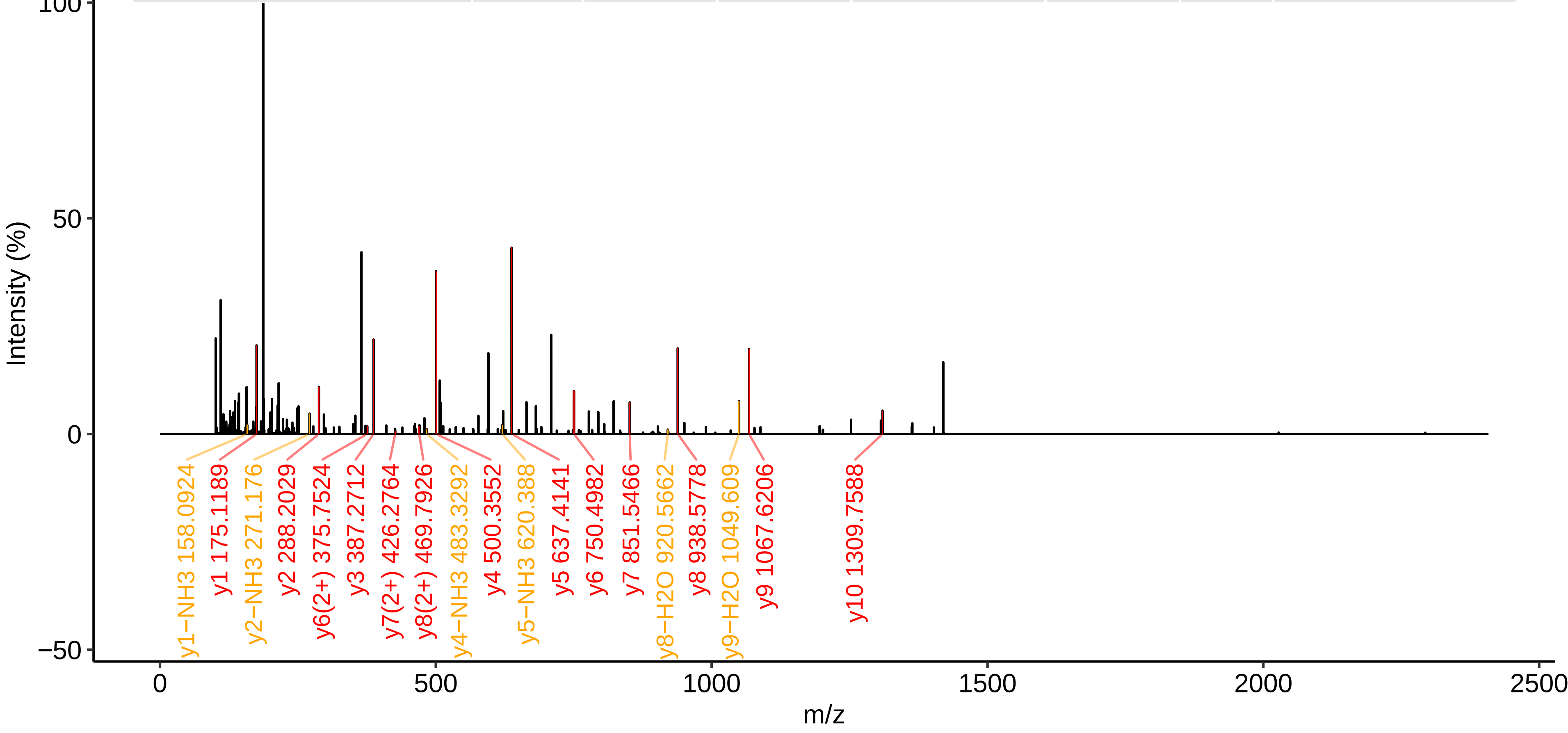

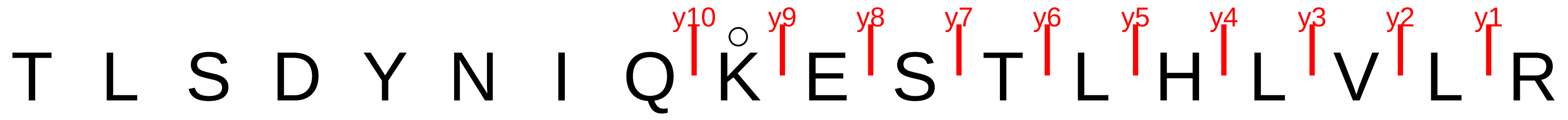

Modification □ Acetyl (Protein N−term) ○ GlyGly (K) no new terminus △ Oxidation (M)

20200921_KIZ_MUb4_GlyGly_10plex − Scan number: 22485

Gene Names	Charge	m/z	Mass	Retention time	PEP	Score	Precursor Intensity
RPS27A;UBA52;UBB;UBC	4	561.805	2243.191	81.557	8.2252e−06	81.594	2539365

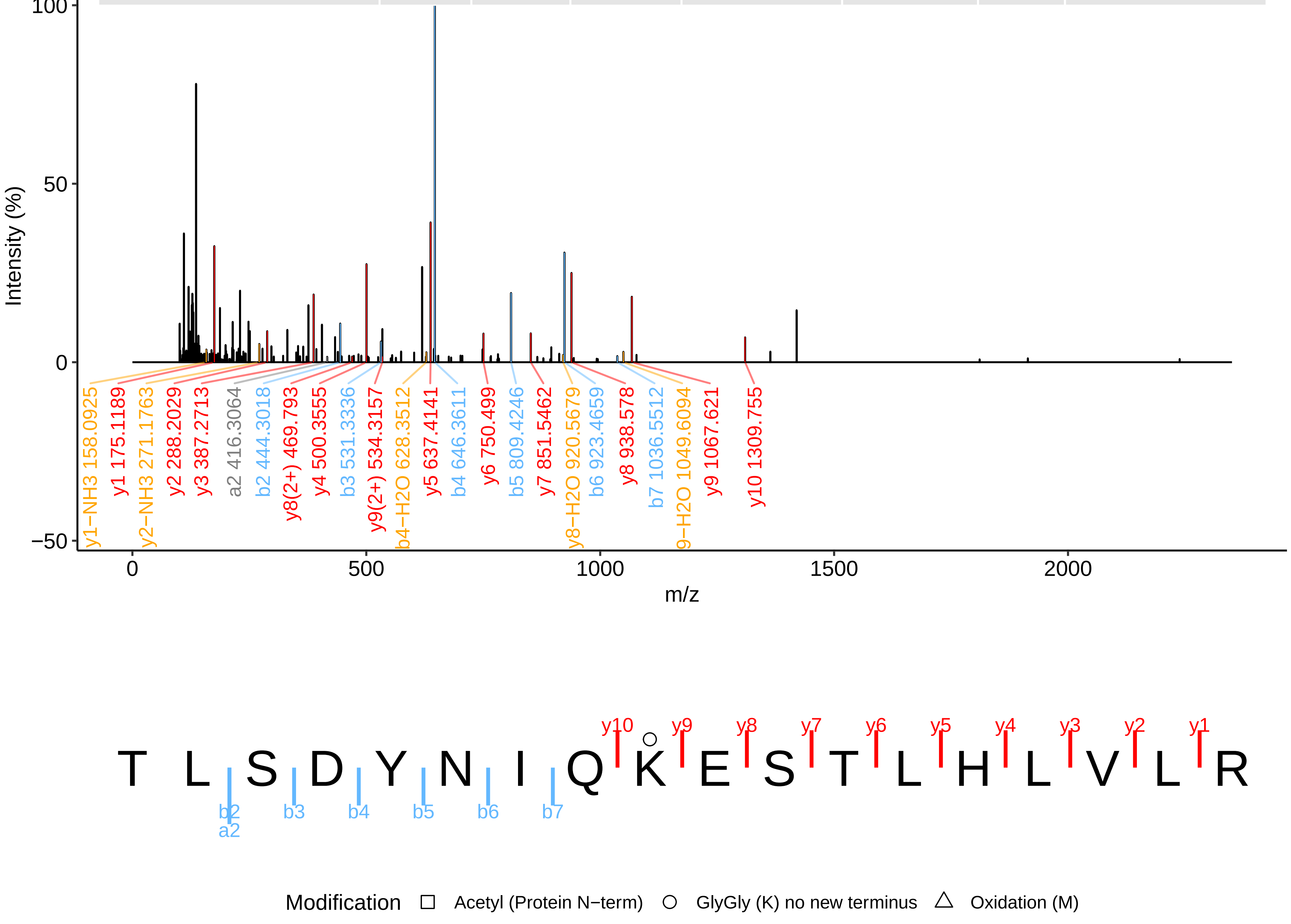

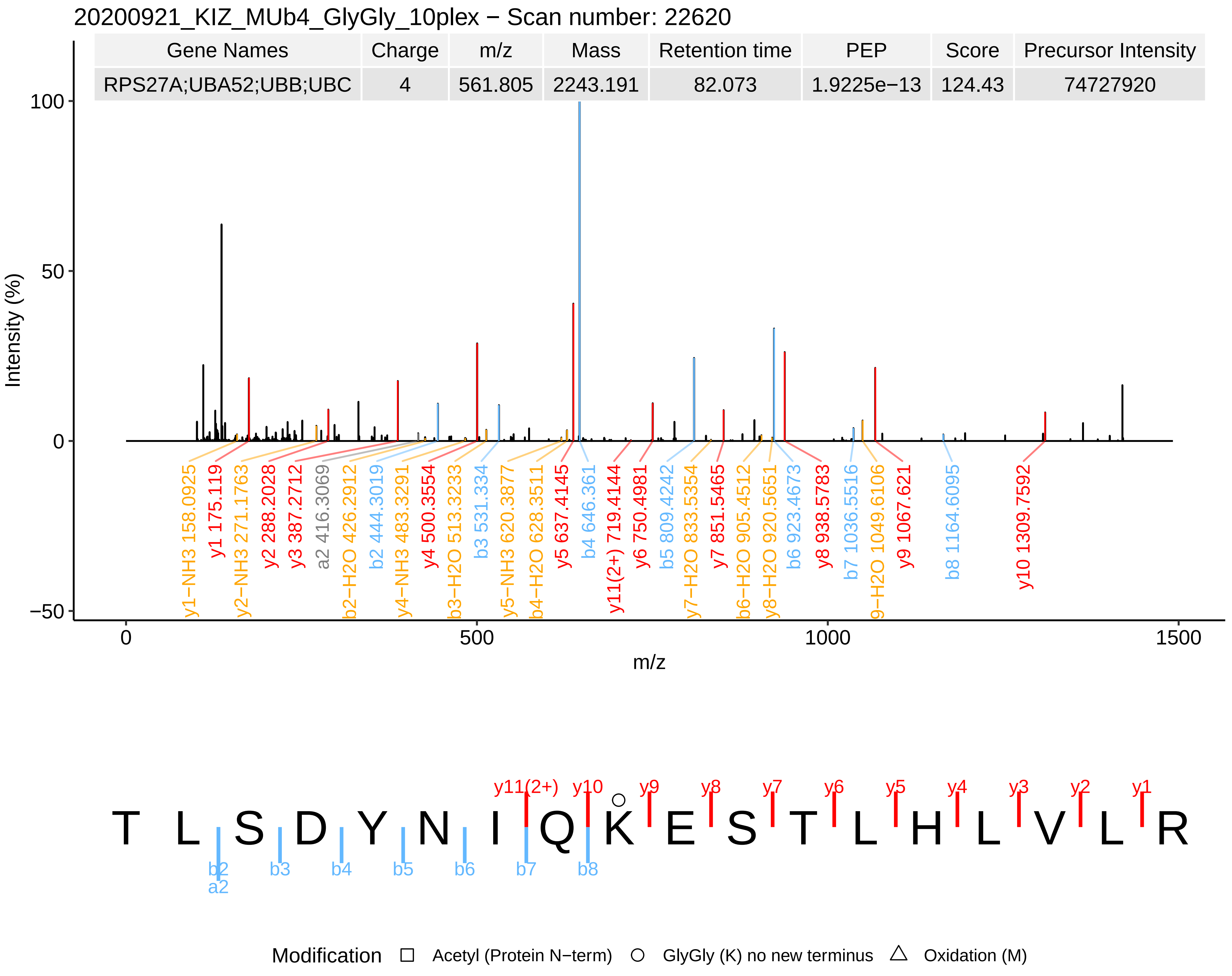
20200921_KIZ_MUb4_GlyGly_10plex − Scan number: 22620
Gene Names | Charge | m/z | Mass | Retention time | PEP | Score | Precursor Intensity
RPS27A;UBA52;UBB;UBC | 4 | 561.805 | 2243.191 | 82.073 | 1.9225e−13 | 124.43 | 74727920
Intensity (%)
100
50
0
−50
m/z
0
500
1000
1500
y1−NH3 158.0925
y1 175.119
y2−NH3 271.1763
y2 288.2028
y3 387.2712
a2 416.3069
b2−H2O 426.2912
b2 444.3019
y4−NH3 483.3291
y4 500.3554
b3−H2O 513.3233
b3 531.334
y5−NH3 620.3877
b4−H2O 628.3511
y5 637.4145
b4 646.361
y11(2+) 719.4144
y6 750.4981
b5 809.4242
y7−H2O 833.5354
y7 851.5465
b6−H2O 905.4512
y8−H2O 920.5651
b6 923.4673
y8 938.5783
b7 1036.5516
9−H2O 1049.6106
y9 1067.621
b8 1164.6095
y10 1309.7592
T L S D Y N I Q K E S T L H L V L R
y11(2+) y10 y9 y8 y7 y6 y5 y4 y3 y2 y1
b2 a2 b3 b4 b5 b6 b7 b8
Modification
Acetyl (Protein N−term)
GlyGly (K) no new terminus
Oxidation (M)

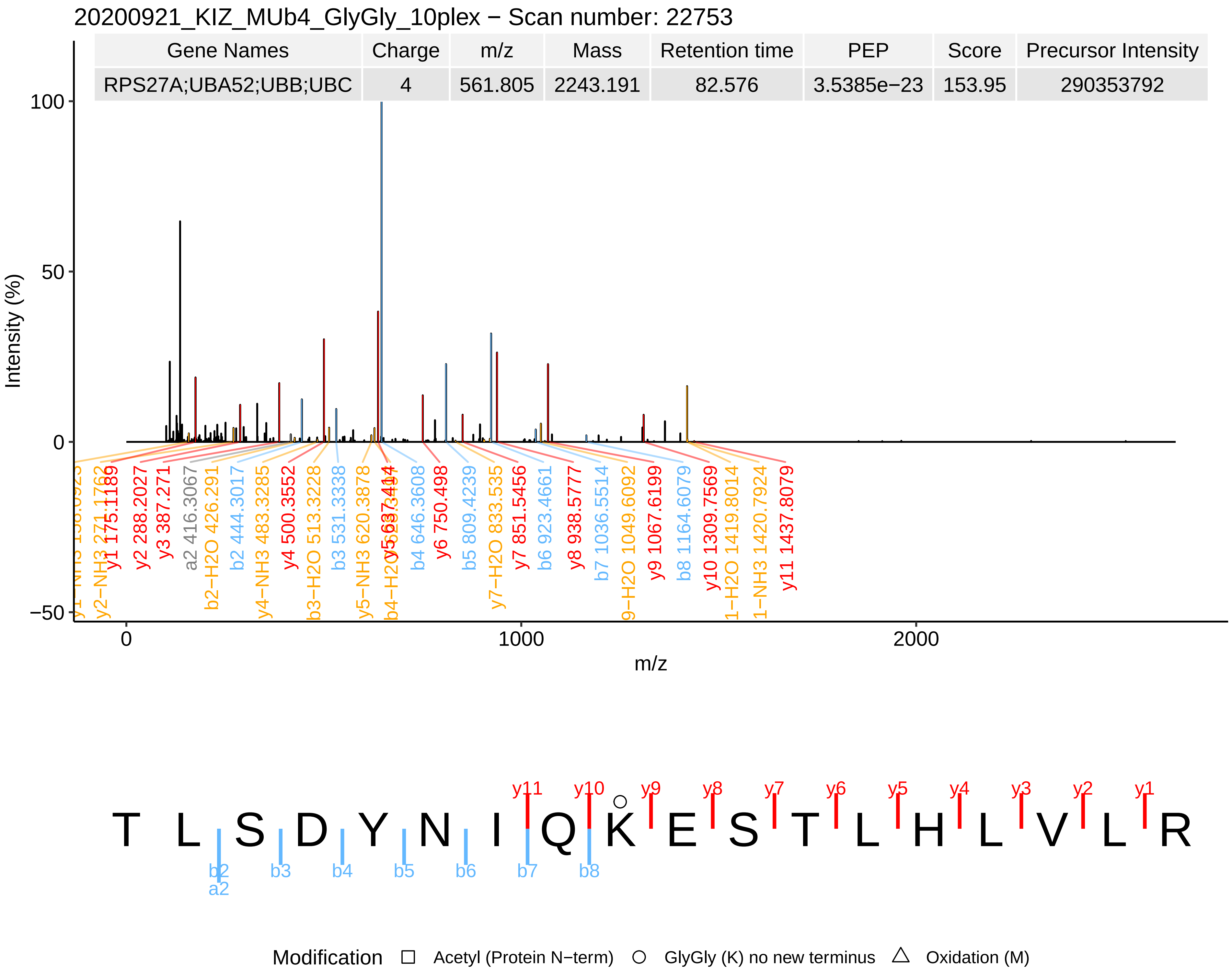

20200921_KIZ_MUb4_GlyGly_10plex − Scan number: 22753
Gene Names | Charge | m/z | Mass | Retention time | PEP | Score | Precursor Intensity
RPS27A;UBA52;UBB;UBC | 4 | 561.805 | 2243.191 | 82.576 | 3.5385e−23 | 153.95 | 290353792
Intensity (%)
100
50
0
−50
0
1000
2000
m/z
y2−NH3 271.1762
y1 175.1189
y2 288.2027
y3 387.271
a2 416.3067
b2−H2O 426.291
b2 444.3017
y4−NH3 483.3285
y4 500.3552
b3−H2O 513.3228
b3 531.3338
y5−NH3 620.3878
b4 646.3608
y6 750.498
b5 809.4239
y7−H2O 833.535
y7 851.5456
b6 923.4661
y8 938.5777
b7 1036.5514
y9 1067.6199
b8 1164.6079
y10 1309.7569
y11 1437.8079
T L S D Y N I Q K E S T L H L V L R
y11 y10 y9 y8 y7 y6 y5 y4 y3 y2 y1
b2 a2 b3 b4 b5 b6 b7 b8
Modification
Acetyl (Protein N−term)
GlyGly (K) no new terminus
Oxidation (M)

20200921_KIZ_MUb4_GlyGly_10plex − Scan number: 22892

Gene Names	Charge	m/z	Mass	Retention time	PEP	Score	Precursor Intensity
RPS27A;UBA52;UBB;UBC	4	561.805	2243.191	83.079	6.4182e−31	176.33	587007232

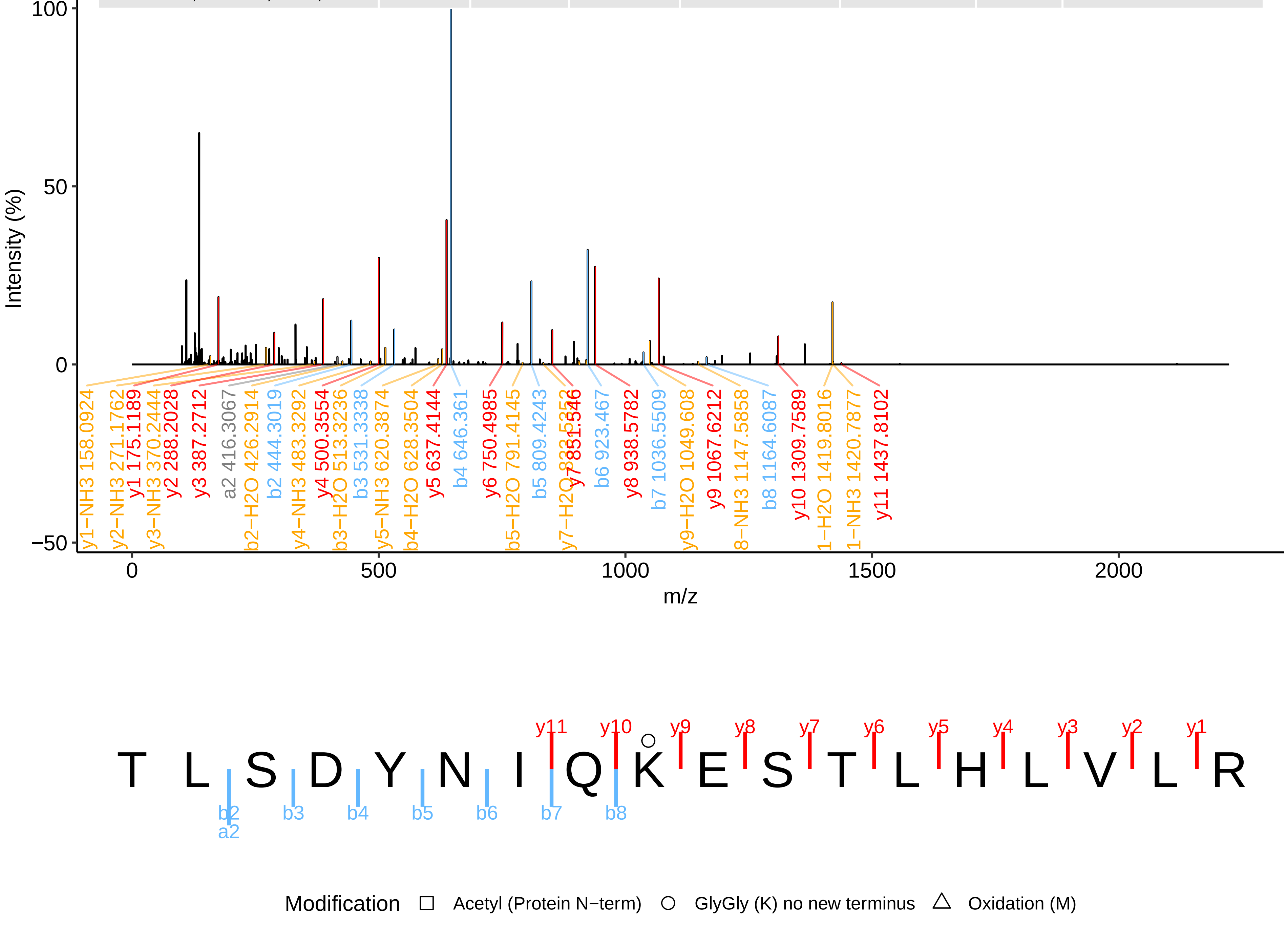

20200921_KIZ_MUb4_GlyGly_10plex − Scan number: 22983

Gene Names	Charge	m/z	Mass	Retention time	PEP	Score	Precursor Intensity
RPS27A;UBA52;UBB;UBC	3	748.7376	2243.191	83.396	1.4209e−30	169.15	20516470

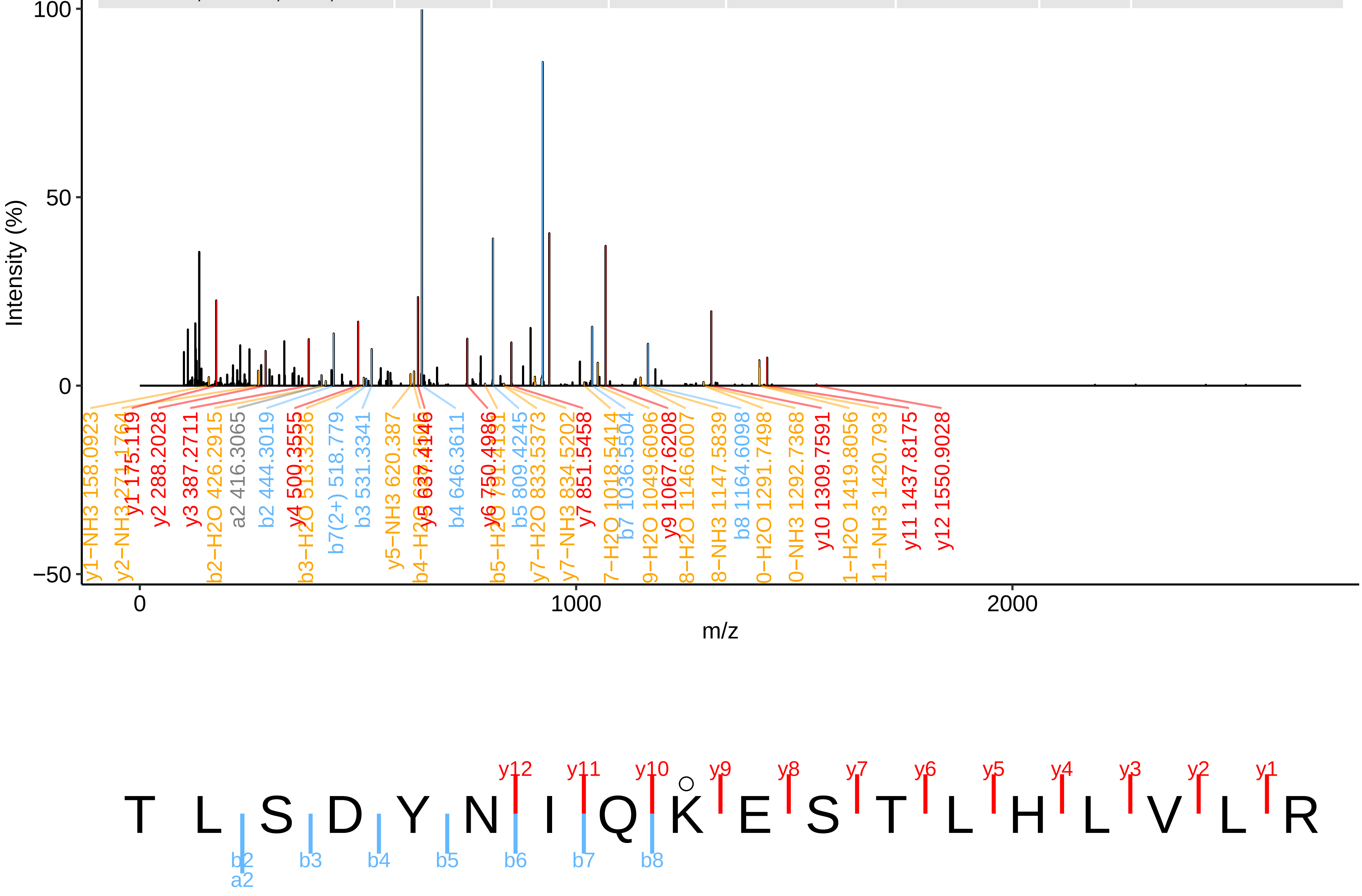

Modification □ Acetyl (Protein N−term) ○ GlyGly (K) no new terminus △ Oxidation (M)

20200921_KIZ_MUb4_GlyGly_10plex − Scan number: 23035

Gene Names	Charge	m/z	Mass	Retention time	PEP	Score	Precursor Intensity
RPS27A;UBA52;UBB;UBC	4	561.805	2243.191	83.587	1.2242e−30	168.41	520270176

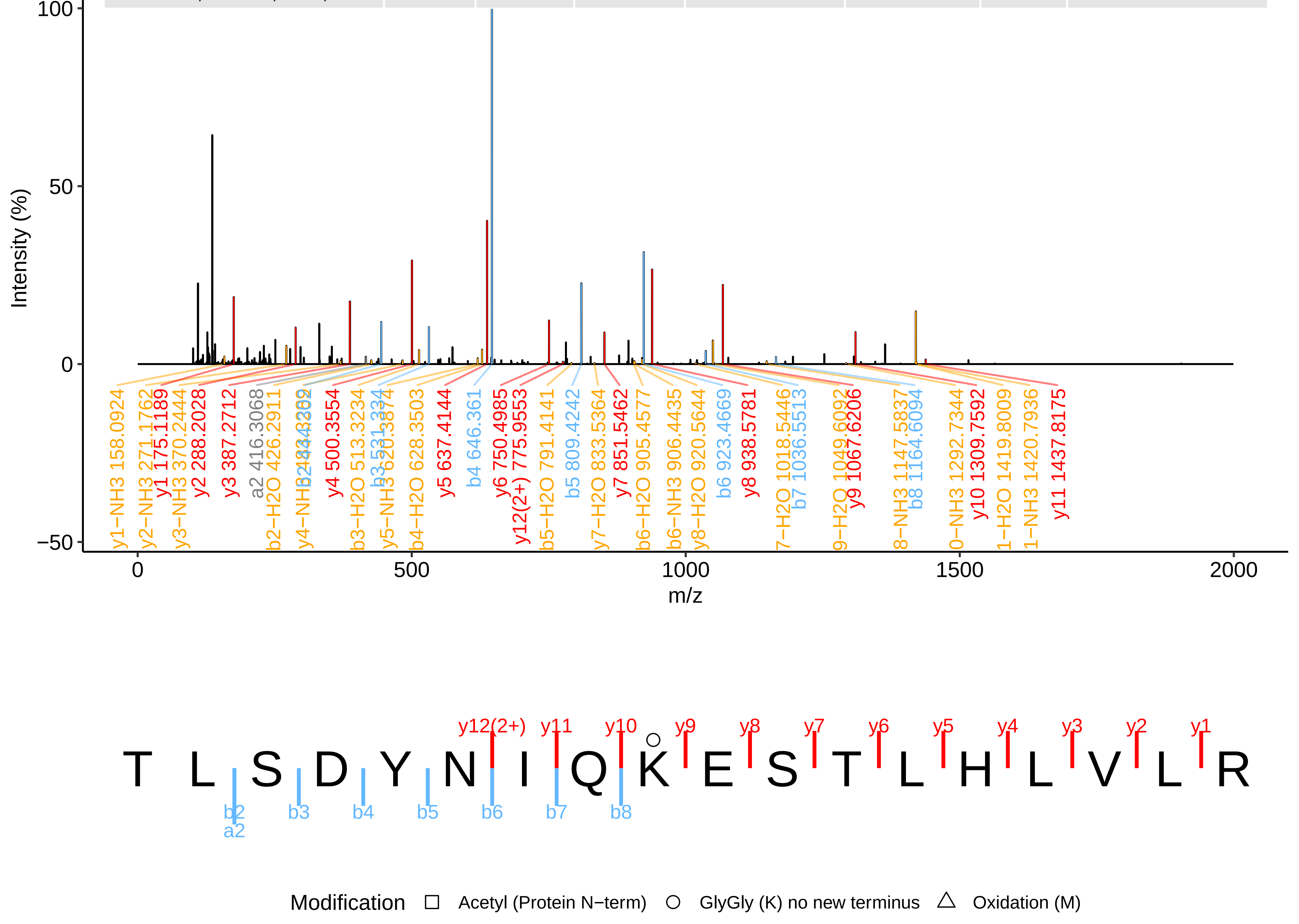

20200921_KIZ_MUb4_GlyGly_10plex − Scan number: 23069

Gene Names	Charge	m/z	Mass	Retention time	PEP	Score	Precursor Intensity
RPS27A;UBA52;UBB;UBC	4	561.805	2243.191	83.711	1.4196e−13	126.63	15580999

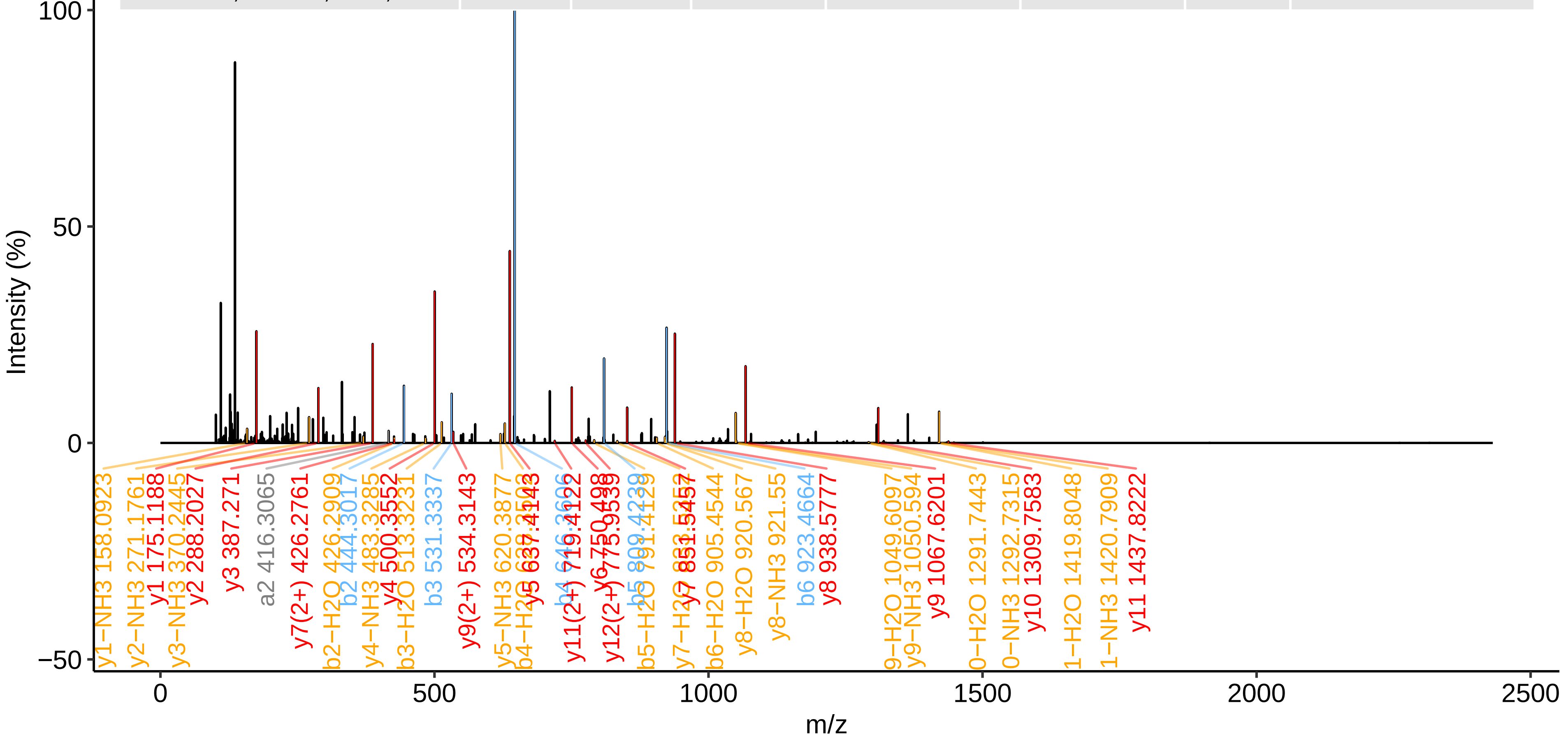

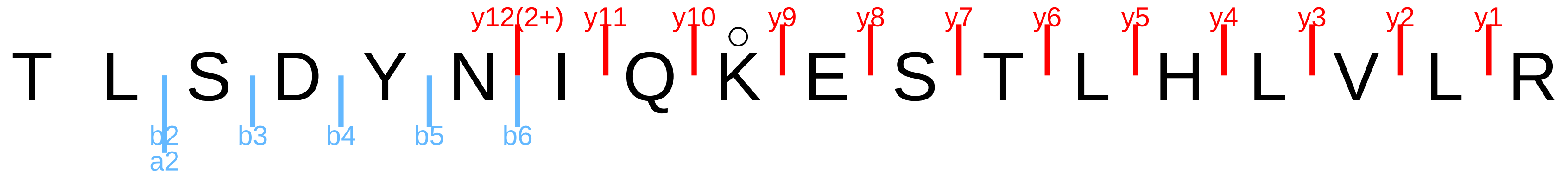

Modification □ Acetyl (Protein N−term) ○ GlyGly (K) no new terminus △ Oxidation (M)

20200921_KIZ_MUb4_GlyGly_10plex − Scan number: 23124

Gene Names	Charge	m/z	Mass	Retention time	PEP	Score	Precursor Intensity
RPS27A;UBA52;UBB;UBC	3	748.7376	2243.191	83.919	1.4324e−30	169.02	2181161

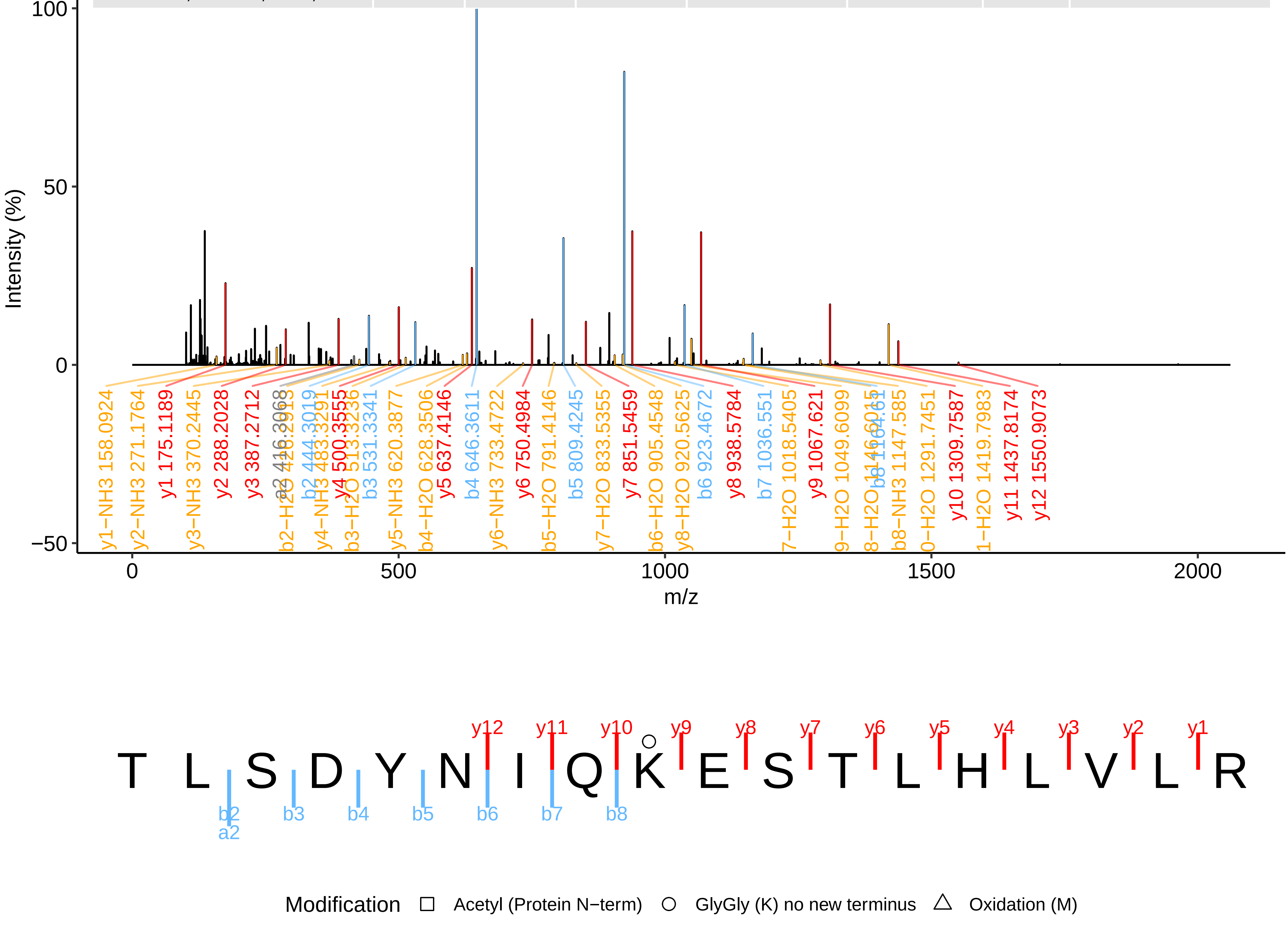

20200921_KIZ_MUb4_GlyGly_10plex − Scan number: 23205

Gene Names	Charge	m/z	Mass	Retention time	PEP	Score	Precursor Intensity
RPS27A;UBA52;UBB;UBC	4	561.805	2243.191	84.209	2.6791e−17	134.85	54213840

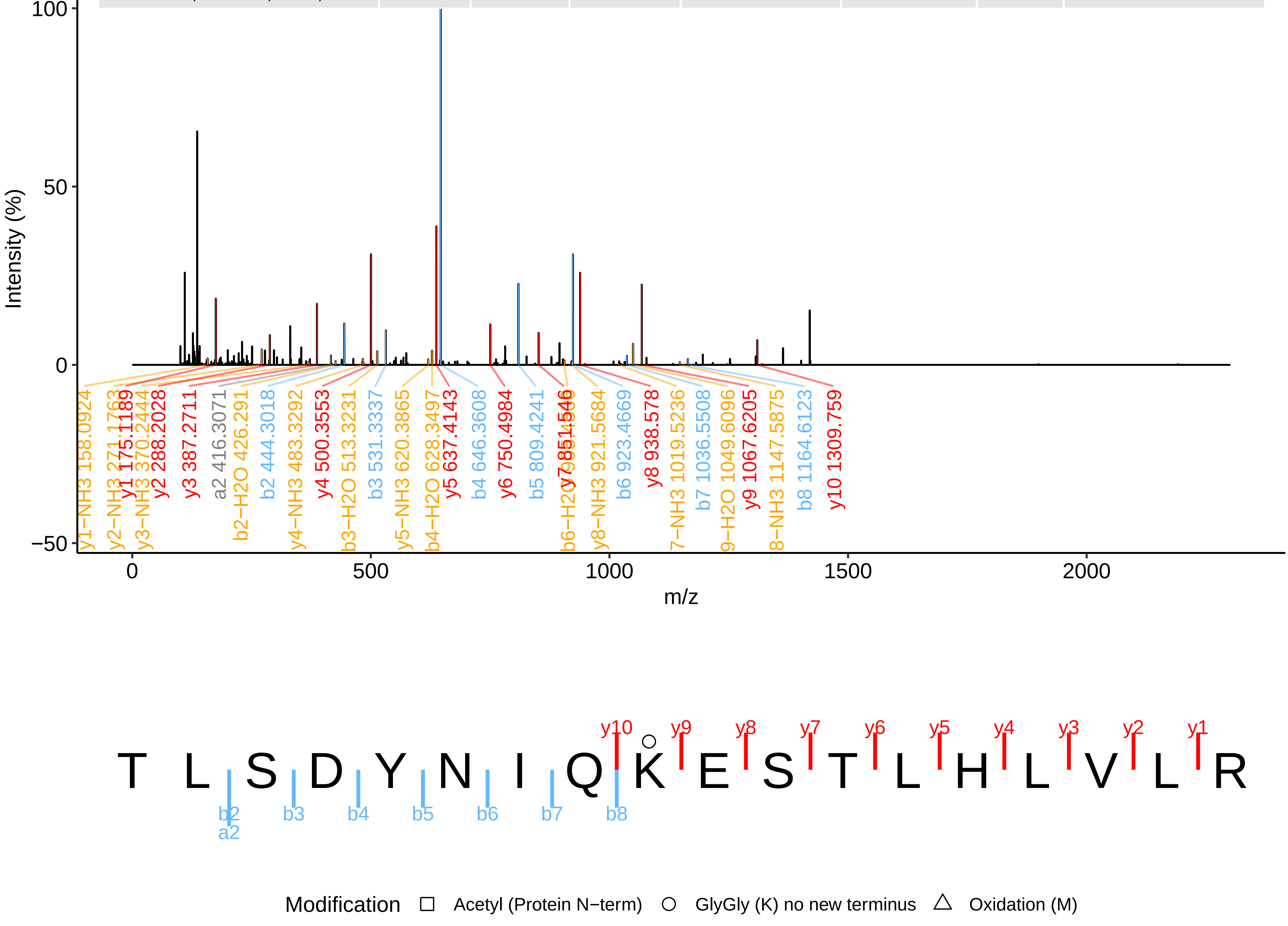

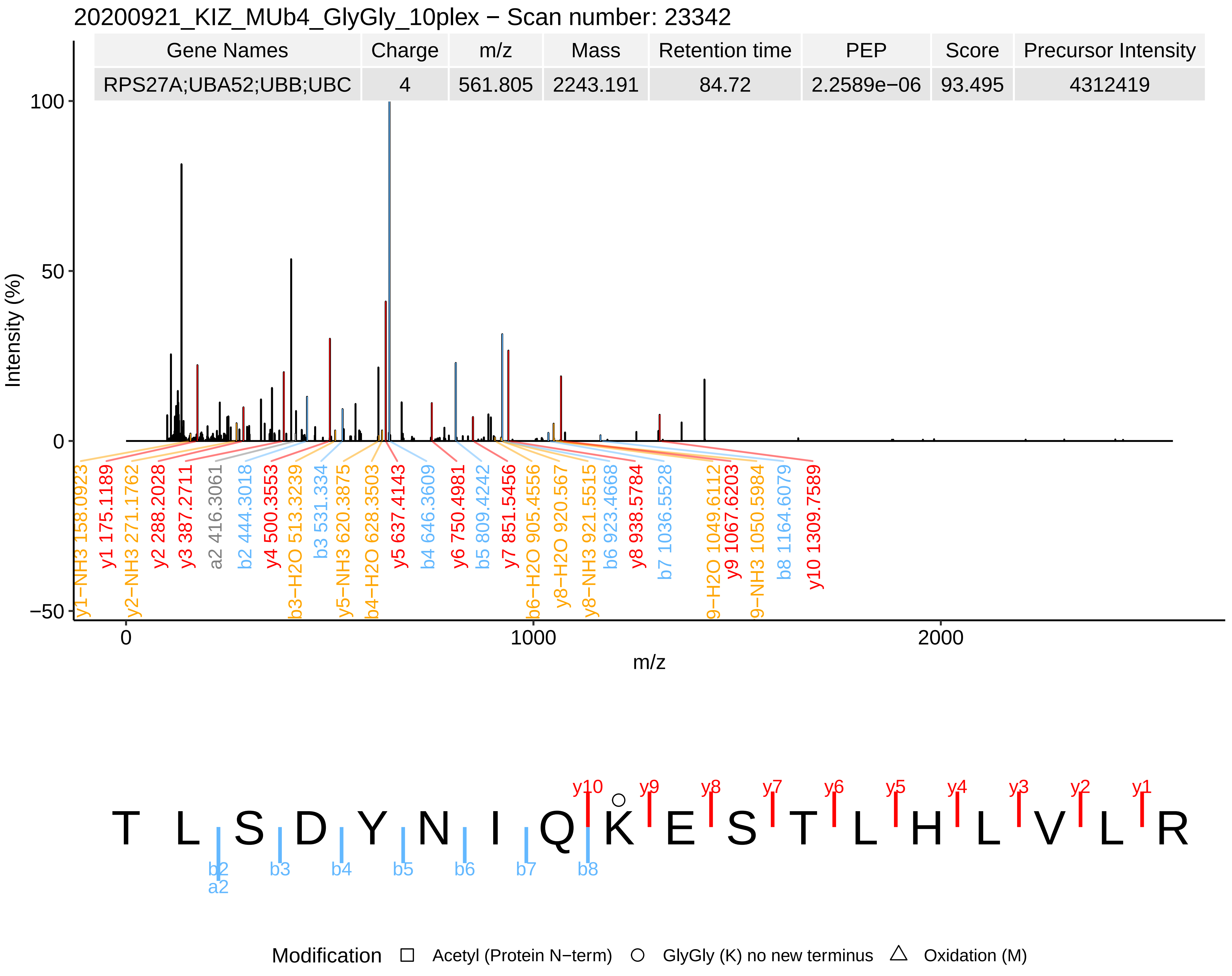
20200921_KIZ_MUb4_GlyGly_10plex − Scan number: 23342
Gene Names
Charge
m/z
Mass
Retention time
PEP
Score
Precursor Intensity
RPS27A;UBA52;UBB;UBC
4
561.805
2243.191
84.72
2.2589e−06
93.495
4312419
Intensity (%)
100
50
0
−50
0
1000
2000
m/z
y1−NH3 158.0923
y1 175.1189
y2−NH3 271.1762
y2 288.2028
y3 387.2711
a2 416.3061
b2 444.3018
y4 500.3553
b3−H2O 513.3239
b3 531.334
y5−NH3 620.3875
b4−H2O 628.3503
y5 637.4143
b4 646.3609
y6 750.4981
b5 809.4242
y7 851.5456
b6−H2O 905.4556
y8−H2O 920.567
y8−NH3 921.5515
b6 923.4668
y8 938.5784
b7 1036.5528
9−H2O 1049.6112
y9 1067.6203
9−NH3 1050.5984
b8 1164.6079
y10 1309.7589
y10
y9
y8
y7
y6
y5
y4
y3
y2
y1
T L S D Y N I Q K E S T L H L V L R
b2
a2
b3
b4
b5
b6
b7
b8
Modification
Acetyl (Protein N−term)
GlyGly (K) no new terminus
Oxidation (M)

20200921_KIZ_MUb4_GlyGly_10plex − Scan number: 23657

Gene Names	Charge	m/z	Mass	Retention time	PEP	Score	Precursor Intensity
RPS27A;UBA52;UBB;UBC	4	561.805	2243.191	85.895	6.3361e−06	85.362	7320675

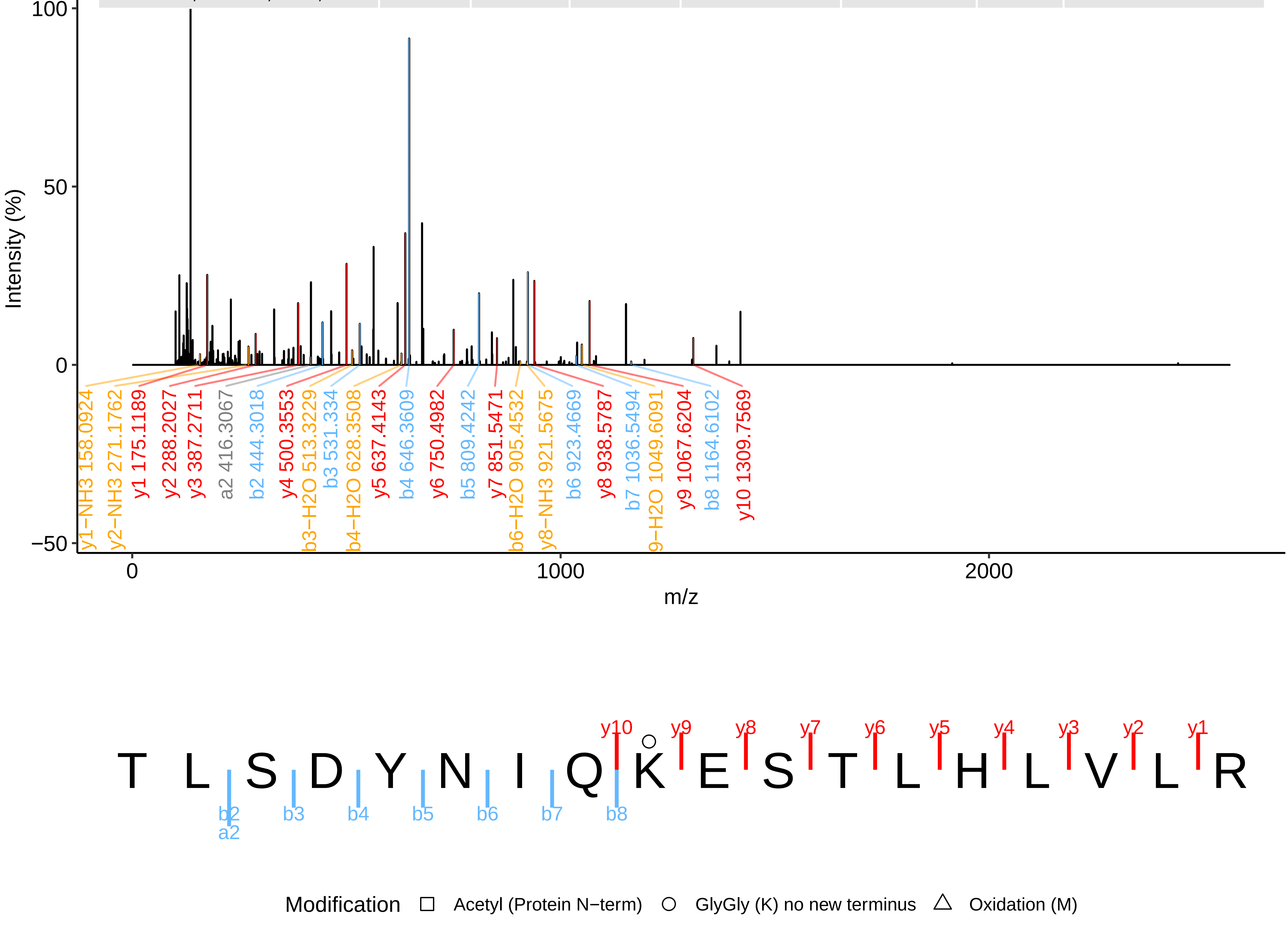

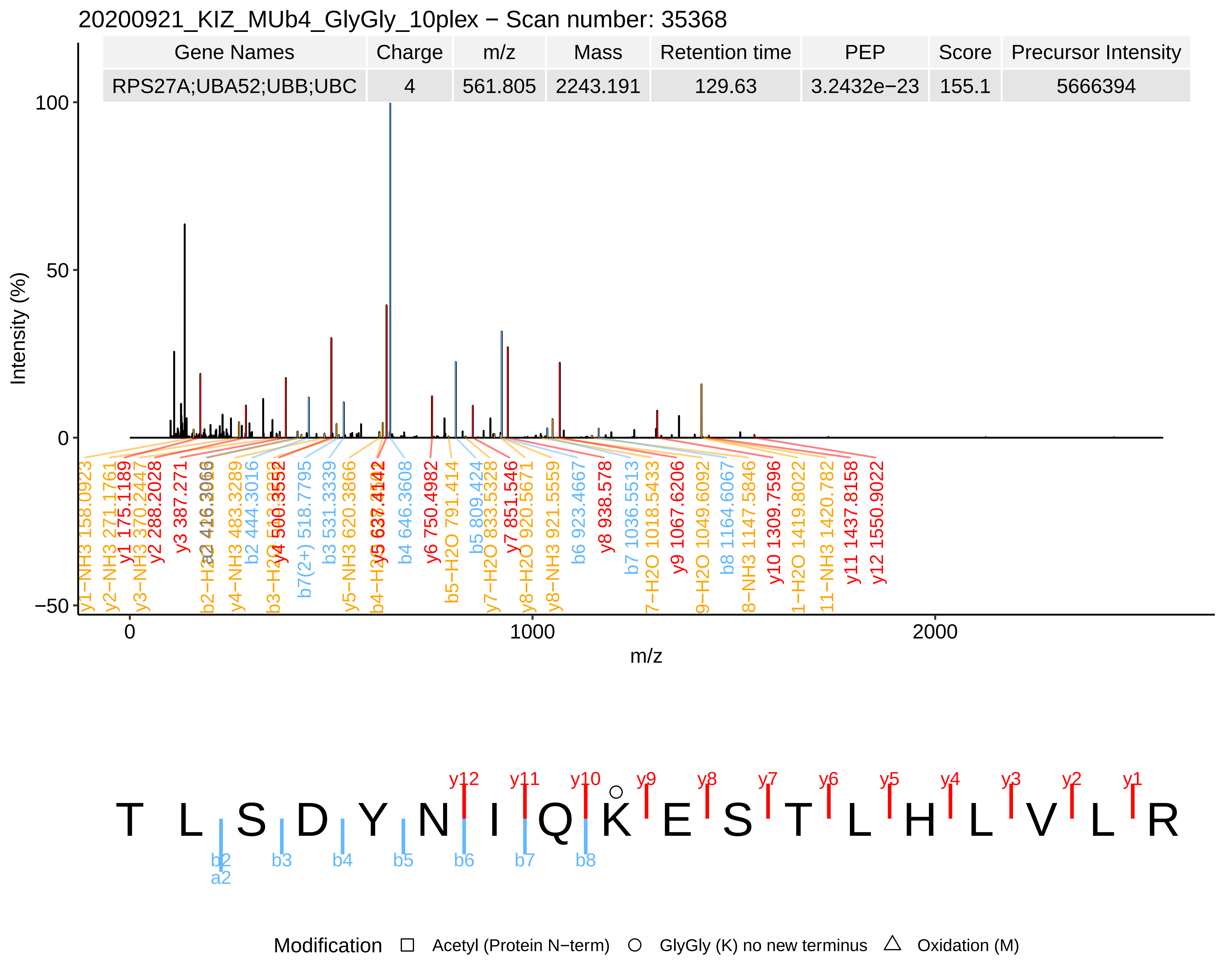
20200921_KIZ_MUb4_GlyGly_10plex − Scan number: 35368
Gene Names
Charge
m/z
Mass
Retention time
PEP
Score
Precursor Intensity
RPS27A;UBA52;UBB;UBC
4
561.805
2243.191
129.63
3.2432e−23
155.1
5666394
Intensity (%)
100
50
0
−50
0
1000
2000
m/z
y1−NH3 158.0923
y2−NH3 271.1761
y1 175.1189
y3−NH3 370.2447
y2 288.2028
y3 387.271
y4−NH3 483.3289
b2 444.3016
b7(2+) 518.7795
b3 531.3339
y5−NH3 620.3866
b4 646.3608
y6 750.4982
b5−H2O 791.414
b5 809.424
y7−H2O 833.5328
y7 851.546
y8−H2O 920.5671
y8−NH3 921.5559
b6 923.4667
y8 938.578
b7 1036.5513
y9 1067.6206
b8 1164.6067
y10 1309.7596
y11 1437.8158
y12 1550.9022
T L S D Y N I Q K E S T L H L V L R
y12 y11 y10 y9 y8 y7 y6 y5 y4 y3 y2 y1
b2 b3 b4 b5 b6 b7 b8
a2
Modification
Acetyl (Protein N−term)
GlyGly (K) no new terminus
Oxidation (M)

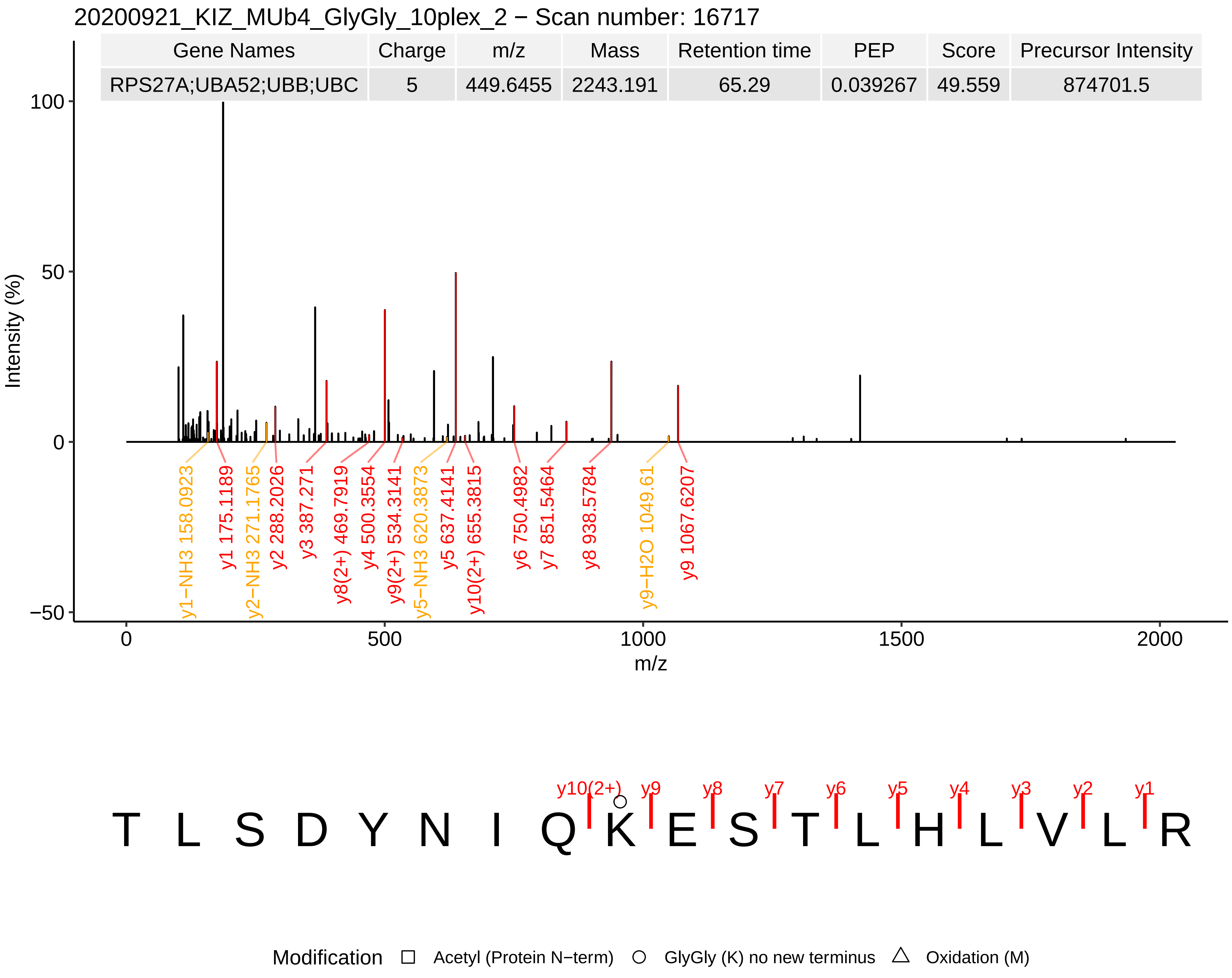
20200921_KIZ_MUb4_GlyGly_10plex_2 − Scan number: 16717
Gene Names | Charge | m/z | Mass | Retention time | PEP | Score | Precursor Intensity
RPS27A;UBA52;UBB;UBC | 5 | 449.6455 | 2243.191 | 65.29 | 0.039267 | 49.559 | 874701.5
Intensity (%)
m/z
y1−NH3 158.0923
y1 175.1189
y2−NH3 271.1765
y2 288.2026
y3 387.271
y8(2+) 469.7919
y4 500.3554
y9(2+) 534.3141
y5−NH3 620.3873
y5 637.4141
y10(2+) 655.3815
y6 750.4982
y7 851.5464
y8 938.5784
y9−H2O 1049.61
y9 1067.6207
y10(2+) y9 y8 y7 y6 y5 y4 y3 y2 y1
T L S D Y N I Q K E S T L H L V L R
Modification
Acetyl (Protein N−term)
GlyGly (K) no new terminus
Oxidation (M)

20200921_KIZ_MUb4_GlyGly_10plex_2 − Scan number: 16731

Gene Names	Charge	m/z	Mass	Retention time	PEP	Score	Precursor Intensity
RPS27A;UBA52;UBB;UBC	4	561.805	2243.191	65.346	0.0019284	64.454	1720067

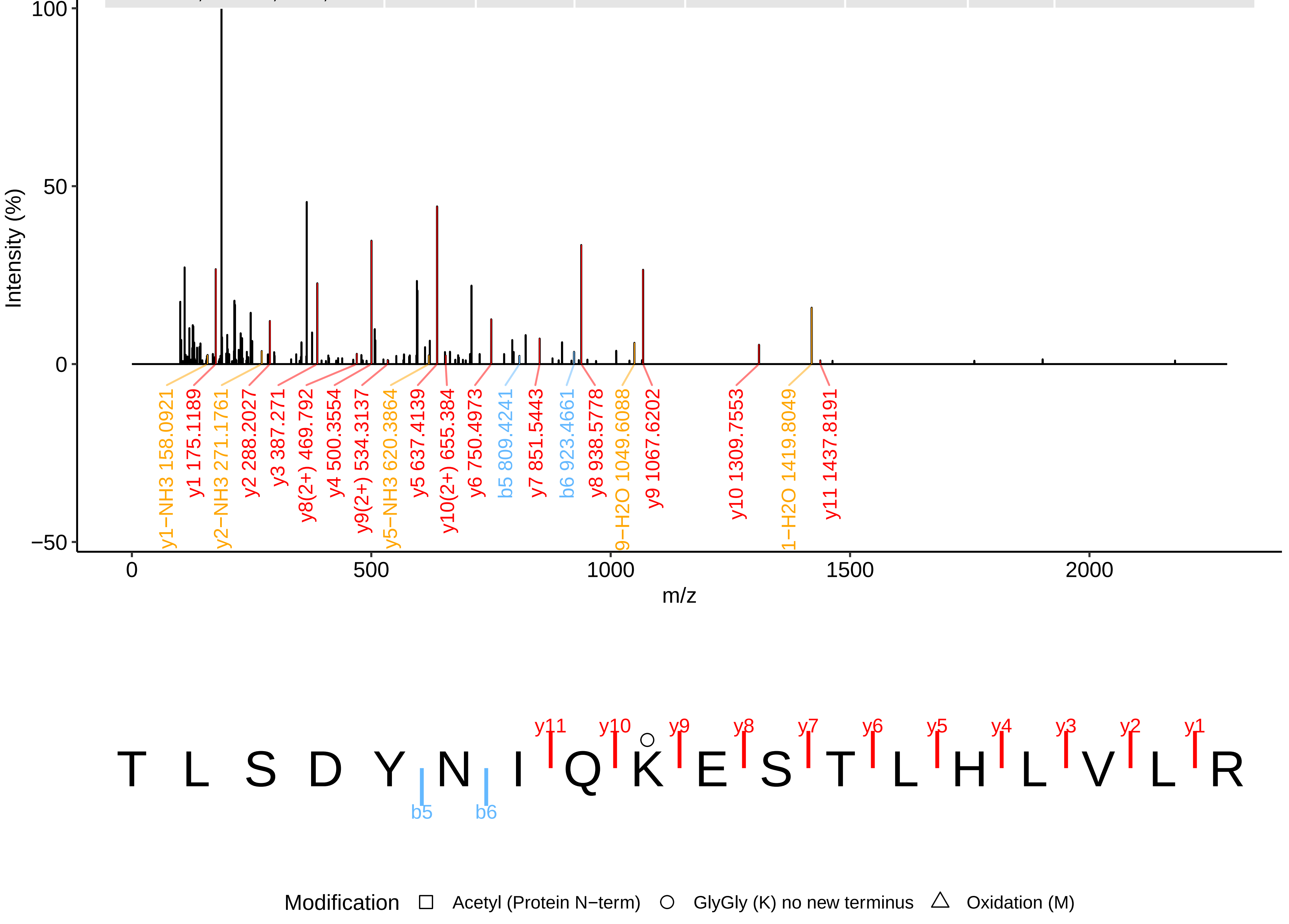

20200921_KIZ_MUb4_GlyGly_10plex_2 − Scan number: 18882

Gene Names	Charge	m/z	Mass	Retention time	PEP	Score	Precursor Intensity
RPS27A;UBA52;UBB;UBC	4	561.805	2243.191	74.033	0.0017783	65.716	527139.6

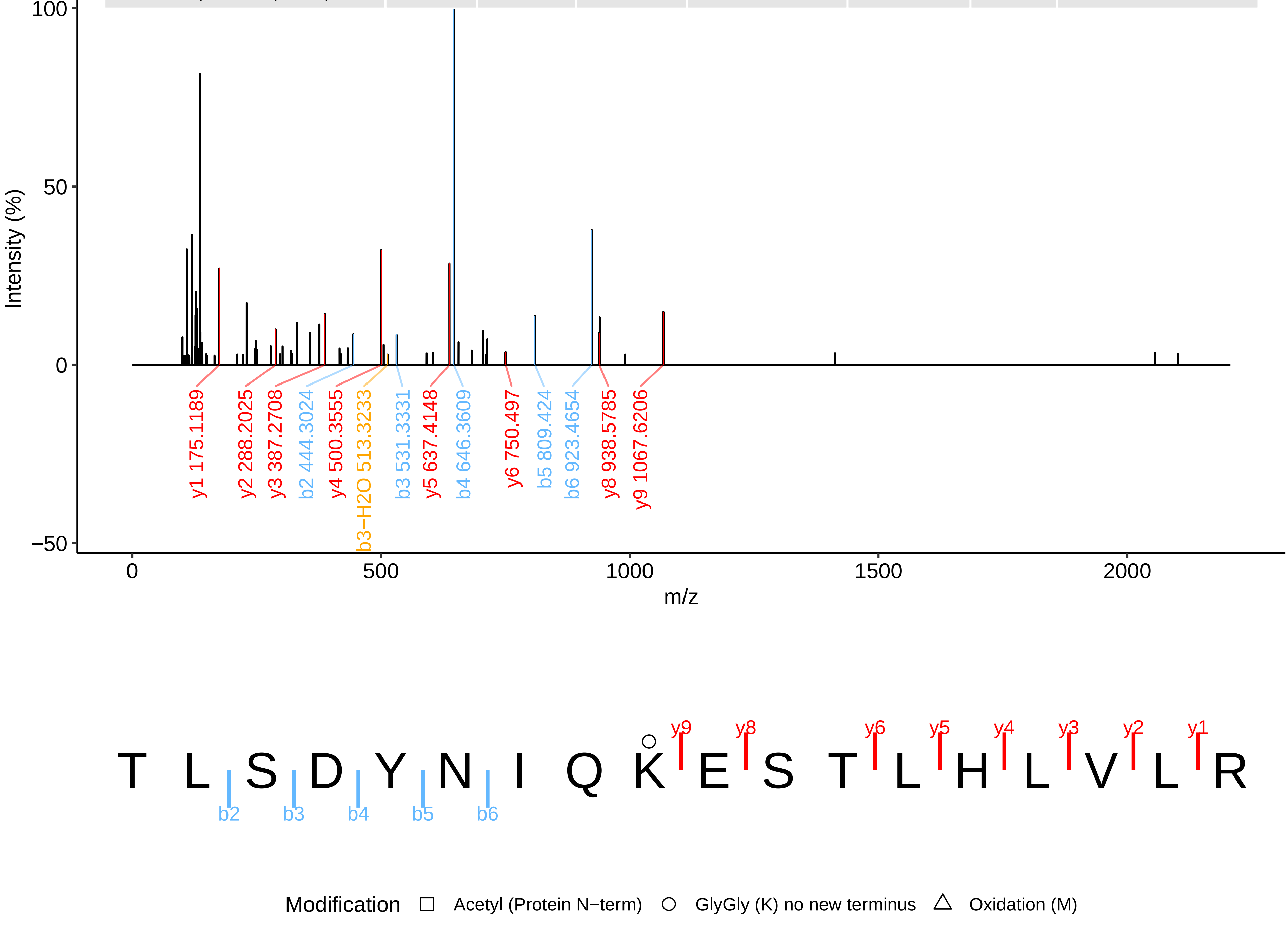

20200921_KIZ_MUb4_GlyGly_10plex_2 − Scan number: 19006

Gene Names	Charge	m/z	Mass	Retention time	PEP	Score	Precursor Intensity
RPS27A;UBA52;UBB;UBC	4	561.805	2243.191	74.536	5.4112e−15	132.59	182875456

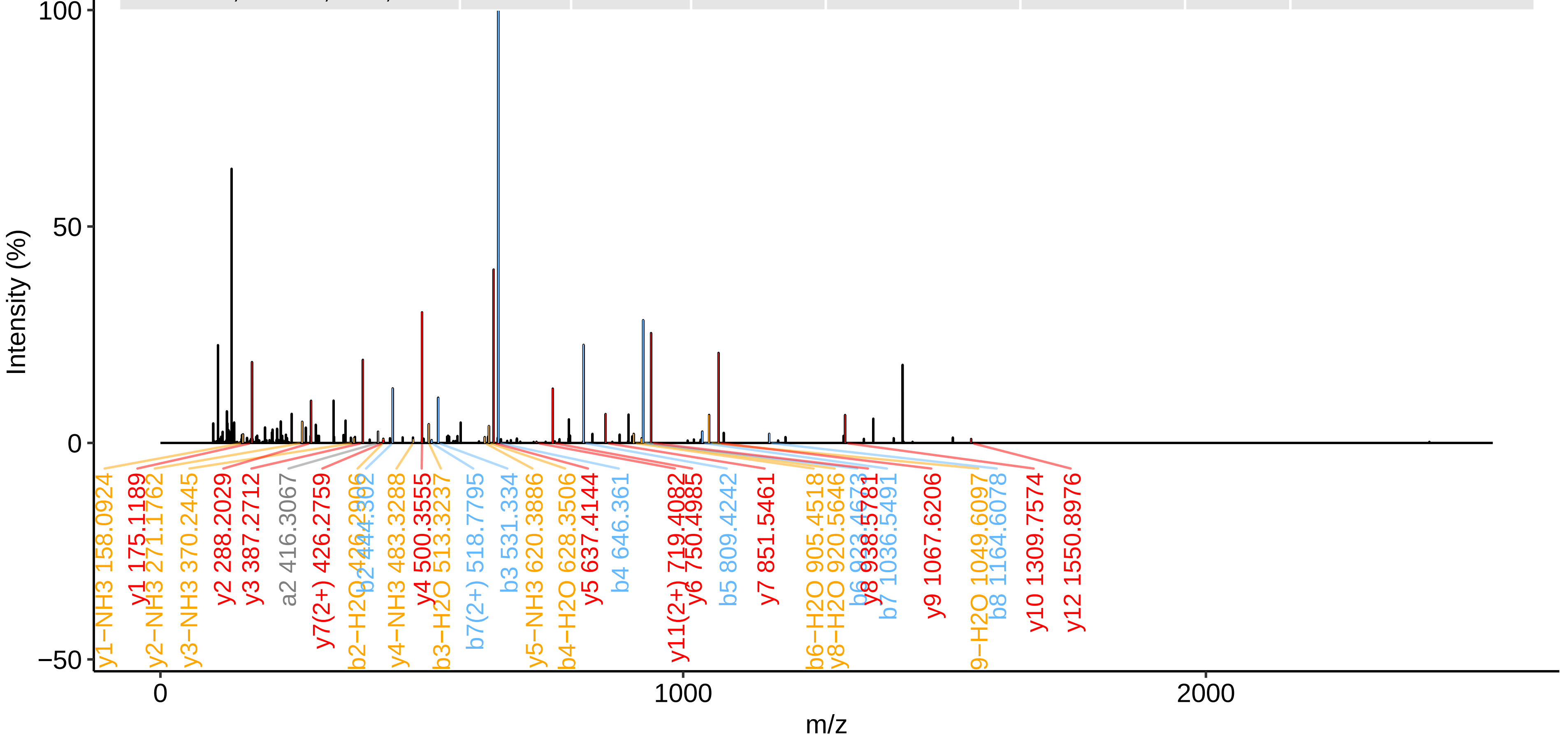

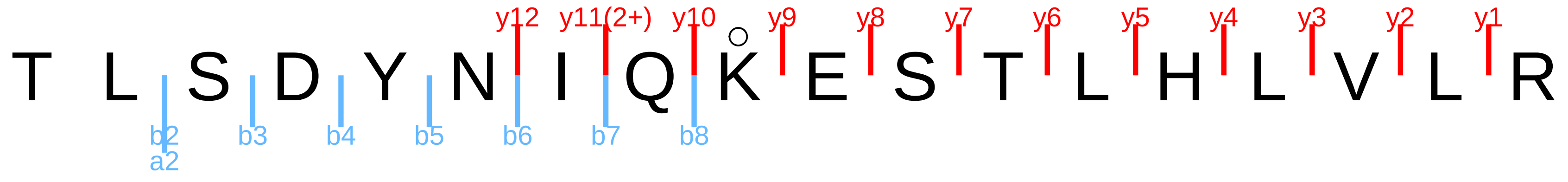

Modification □ Acetyl (Protein N−term) ○ GlyGly (K) no new terminus △ Oxidation (M)

20200921_KIZ_MUb4_GlyGly_10plex_2 − Scan number: 19056

Gene Names	Charge	m/z	Mass	Retention time	PEP	Score	Precursor Intensity
RPS27A;UBA52;UBB;UBC	4	561.805	2243.191	74.74	4.0201e−06	89.982	608949.9

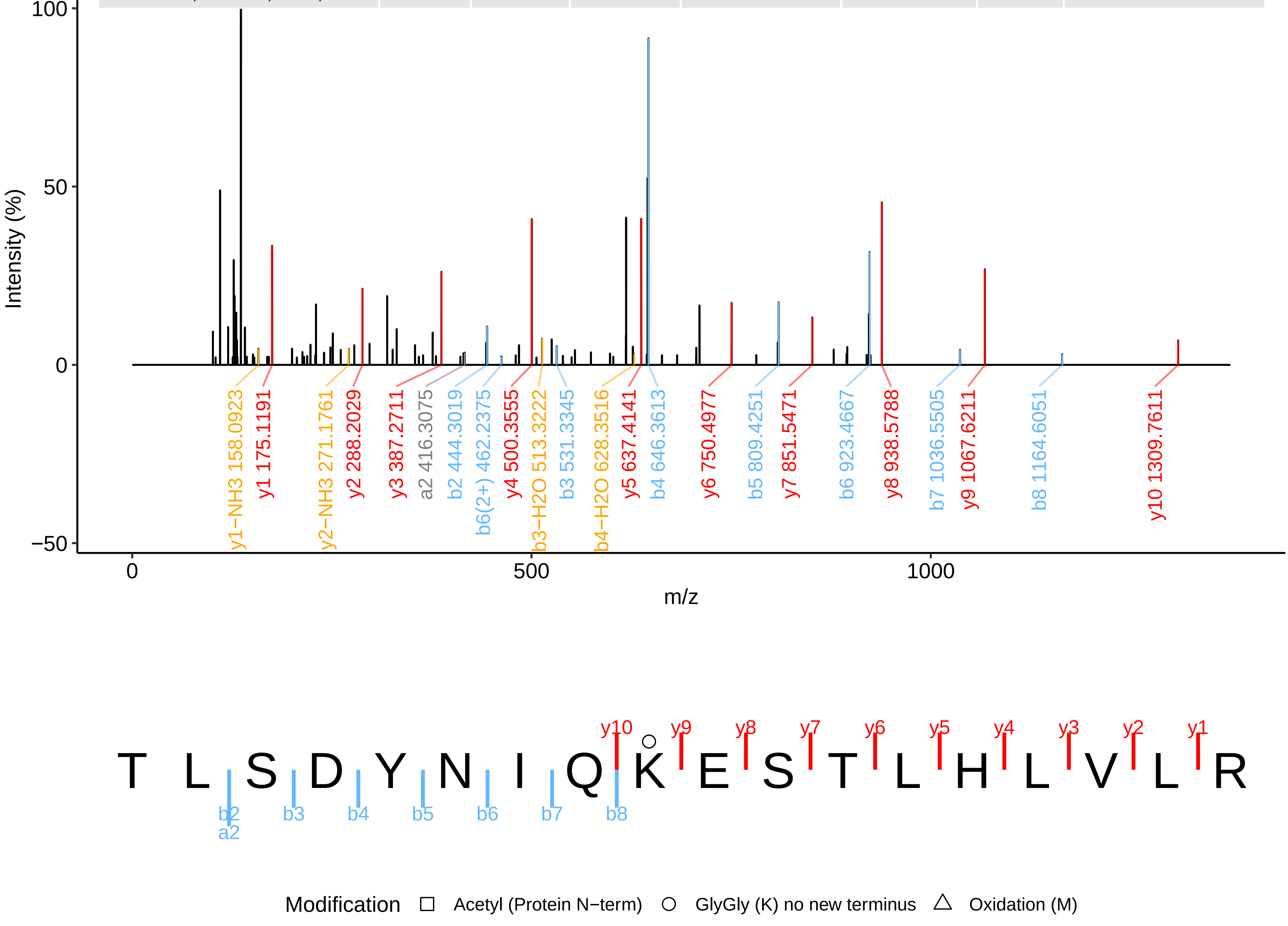

20200921_KIZ_MUb4_GlyGly_10plex_2 − Scan number: 19180

Gene Names	Charge	m/z	Mass	Retention time	PEP	Score	Precursor Intensity
RPS27A;UBA52;UBB;UBC	4	561.805	2243.191	75.25	1.9225e−13	124.43	4957455

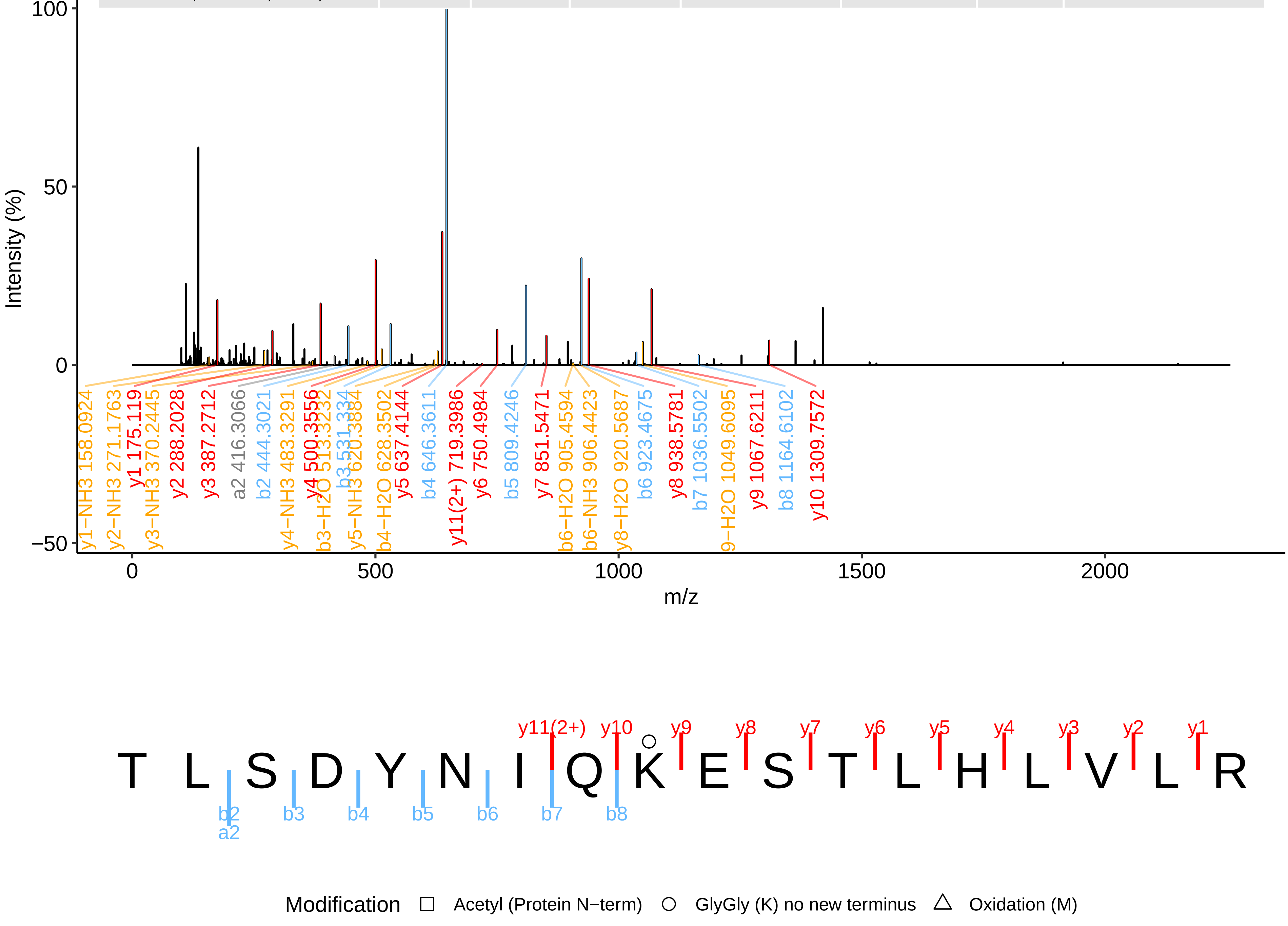

20200921_KIZ_MUb4_GlyGly_10plex_2 − Scan number: 18933

Gene Names	Charge	m/z	Mass	Retention time	PEP	Score	Precursor Intensity
RPS27A;UBA52;UBB;UBC	3	748.7376	2243.191	74.238	3.6728e−14	131.51	2285884

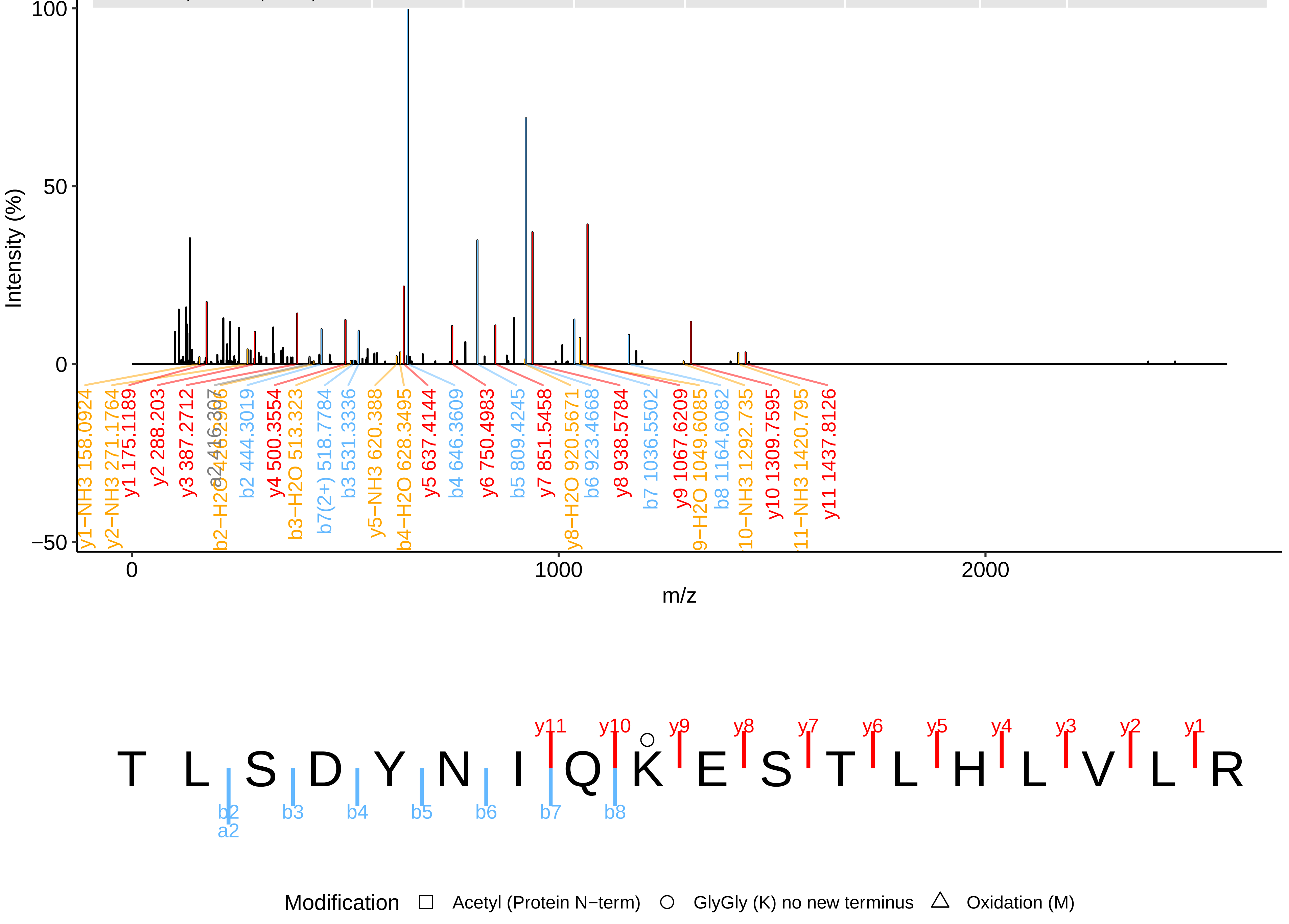

20200921_KIZ_MUb4_GlyGly_10plex_2 − Scan number: 19061

Gene Names	Charge	m/z	Mass	Retention time	PEP	Score	Precursor Intensity
RPS27A;UBA52;UBB;UBC	3	748.7376	2243.191	74.76	1.5009e−17	143.14	983728.6

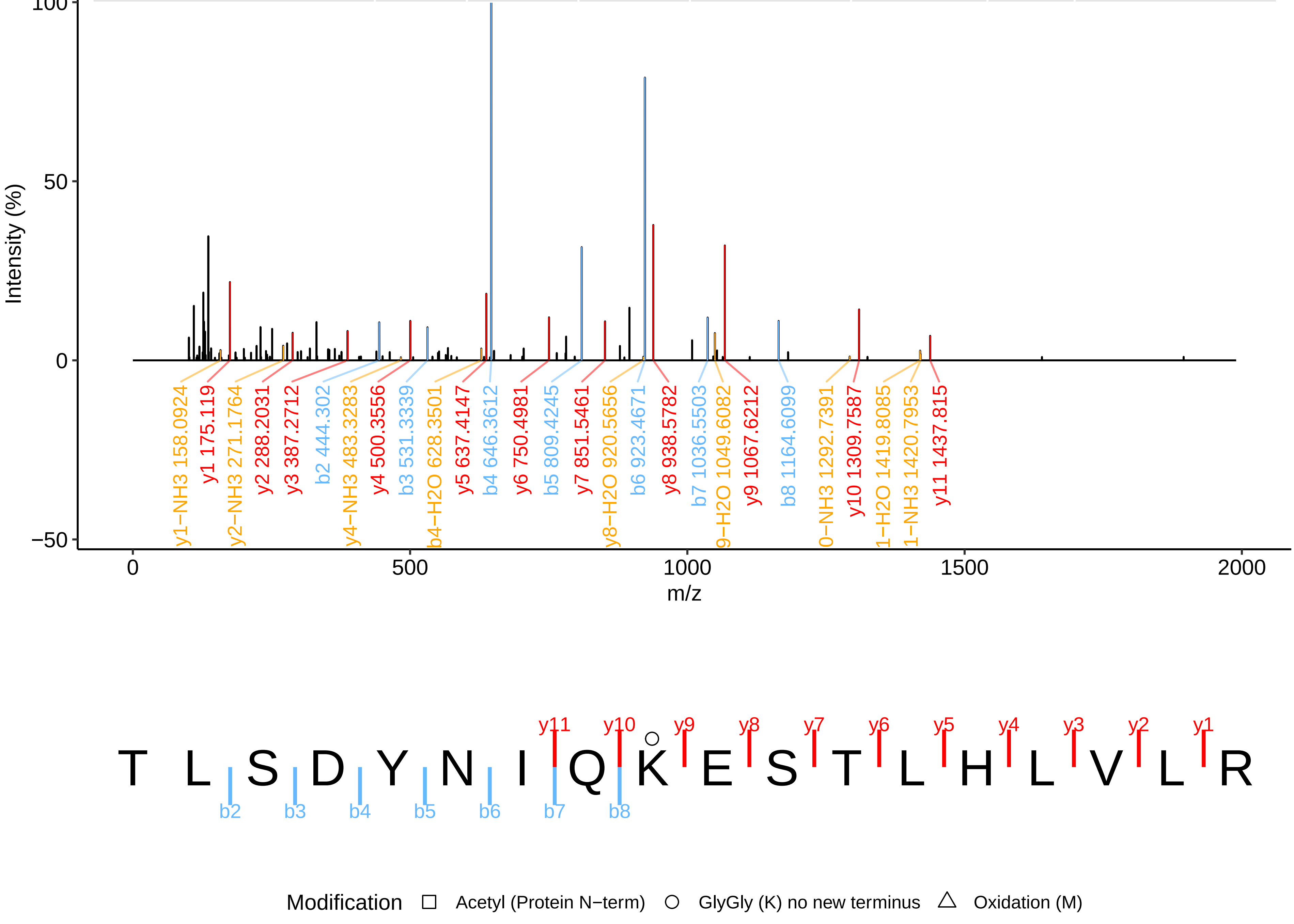

20200921_KIZ_MUb4_GlyGly_10plex_2 − Scan number: 19304

Gene Names	Charge	m/z	Mass	Retention time	PEP	Score	Precursor Intensity
RPS27A;UBA52;UBB;UBC	4	561.805	2243.191	75.755	3.1907e−10	111.22	2862747

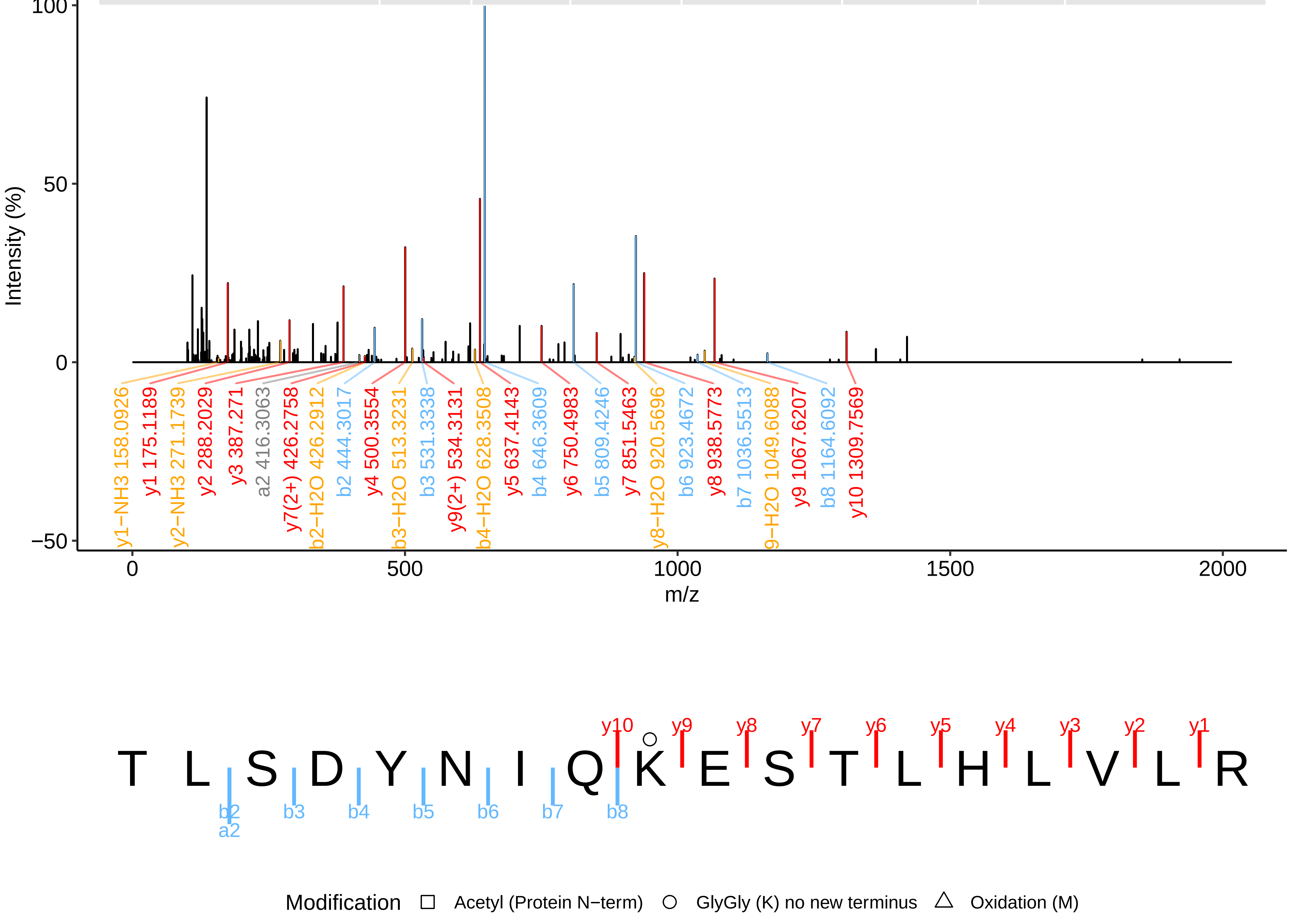

20200921_KIZ_MUb4_GlyGly_10plex_2 − Scan number: 20243

Gene Names	Charge	m/z	Mass	Retention time	PEP	Score	Precursor Intensity
RPS27A;UBA52;UBB;UBC	4	561.805	2243.191	79.552	0.039492	50.178	1577410

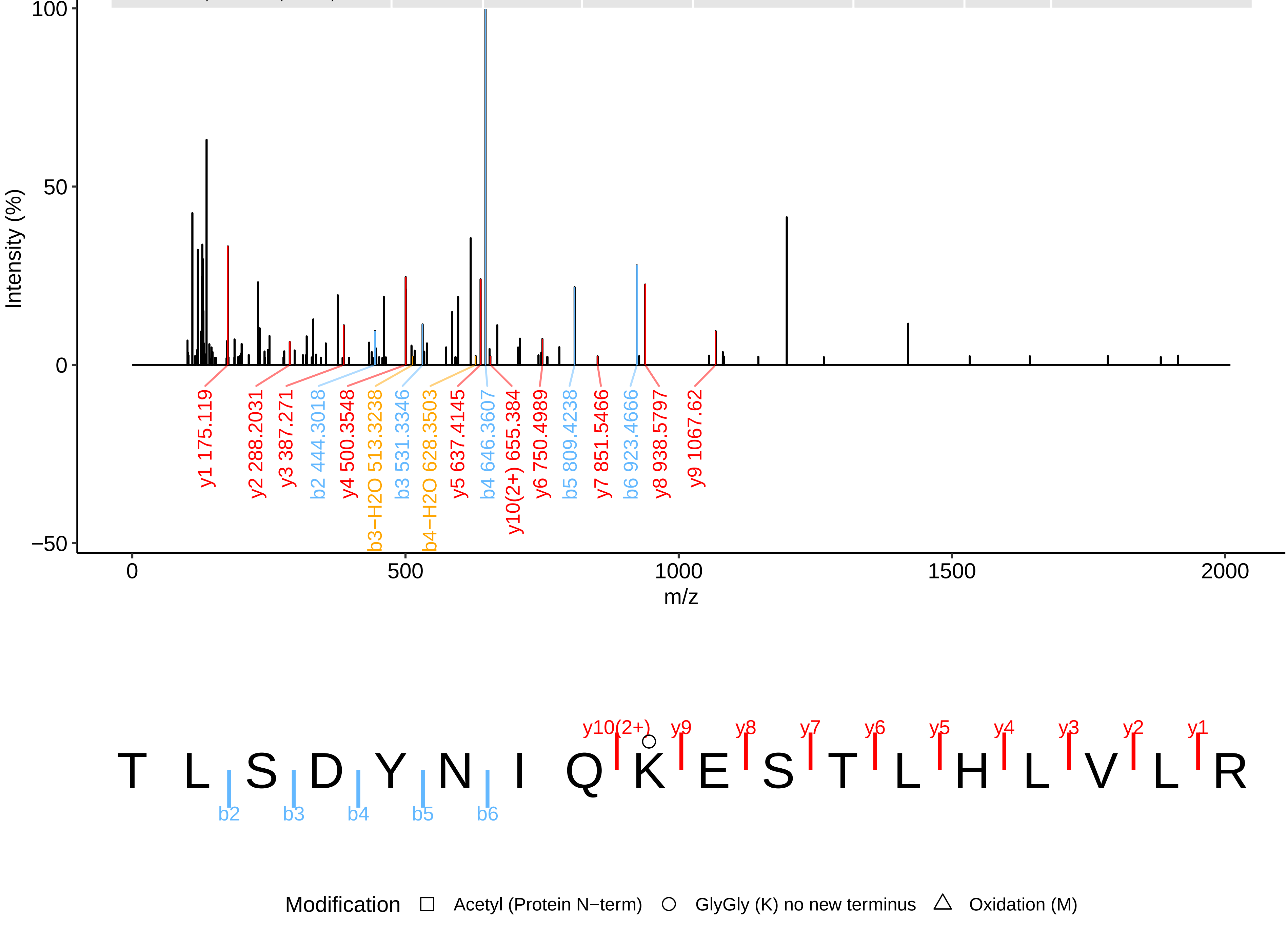

20200924_KIZ_MUb5_GlyGly_1 − Scan number: 15790

Gene Names	Charge	m/z	Mass	Retention time	PEP	Score	Precursor Intensity
RPS27A;UBA52;UBB;UBC	5	449.6455	2243.191	51.422	0.030221	52.862	96749160

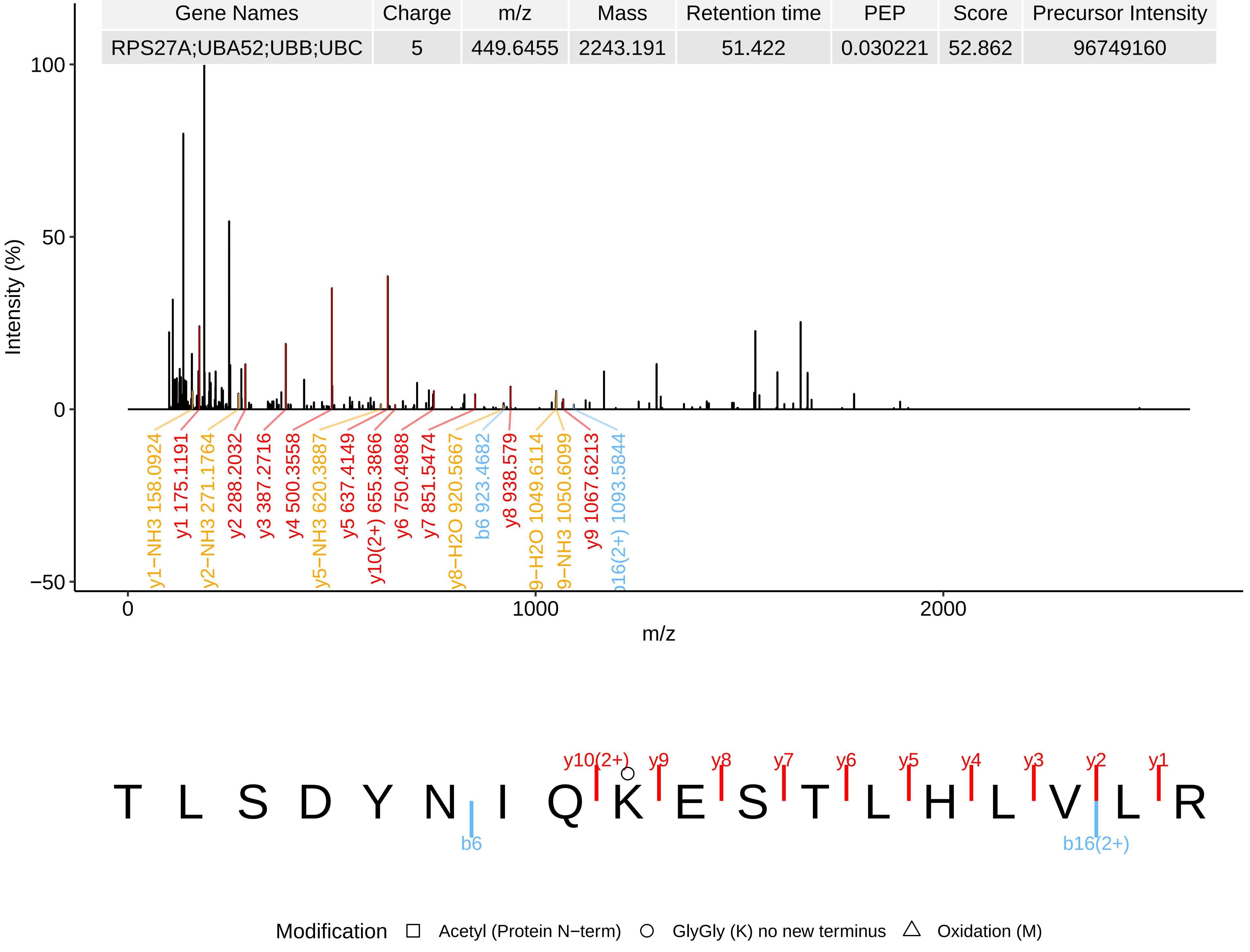

20200924_KIZ_MUb5_GlyGly_1 − Scan number: 19644

Gene Names	Charge	m/z	Mass	Retention time	PEP	Score	Precursor Intensity
RPS27A;UBA52;UBB;UBC	4	561.805	2243.191	63.492	1.2359e−17	143.14	18787348

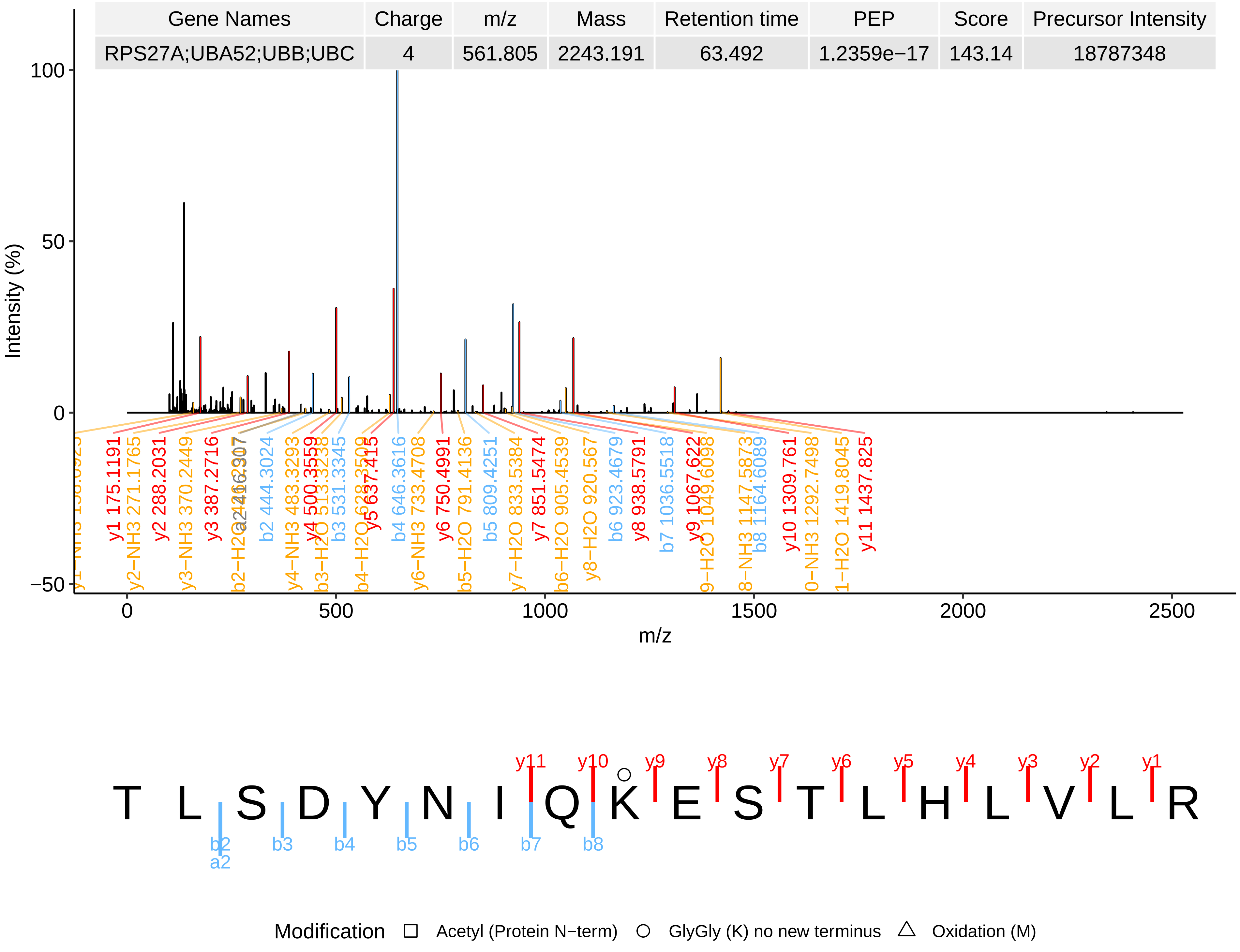

20200924_KIZ_MUb5_GlyGly_1 − Scan number: 19817

Gene Names	Charge	m/z	Mass	Retention time	PEP	Score	Precursor Intensity
RPS27A;UBA52;UBB;UBC	4	561.805	2243.191	64.01	3.5385e−23	153.95	143295664

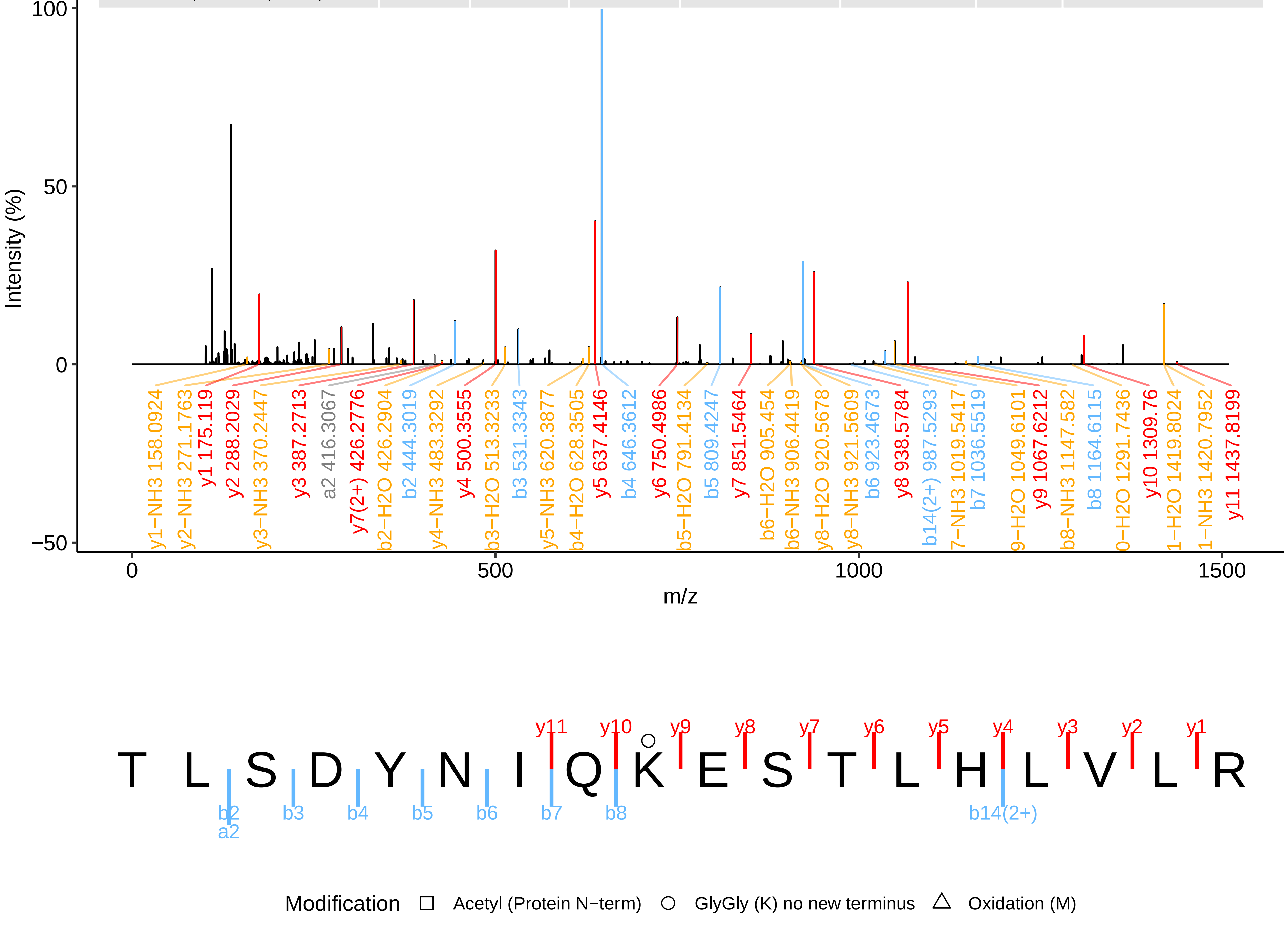

20200924_KIZ_MUb5_GlyGly_1 − Scan number: 19989

Gene Names	Charge	m/z	Mass	Retention time	PEP	Score	Precursor Intensity
RPS27A;UBA52;UBB;UBC	4	561.805	2243.191	64.514	3.5385e−23	153.95	129373304

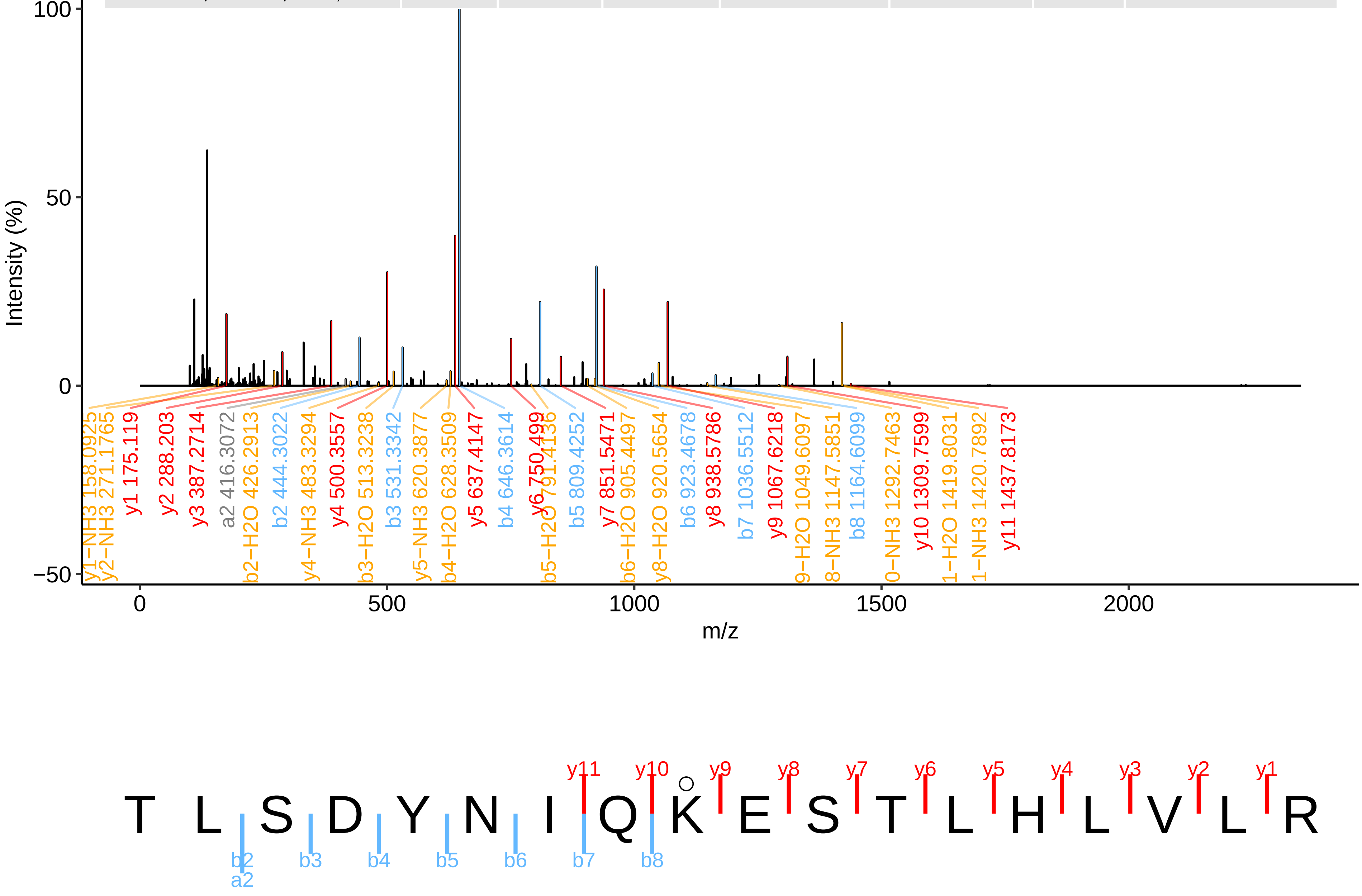

Modification □ Acetyl (Protein N−term) ○ GlyGly (K) no new terminus △ Oxidation (M)

20200924_KIZ_MUb5_GlyGly_1 − Scan number: 20153

Gene Names	Charge	m/z	Mass	Retention time	PEP	Score	Precursor Intensity
RPS27A;UBA52;UBB;UBC	4	561.805	2243.191	65.014	5.4112e−15	132.59	223617632

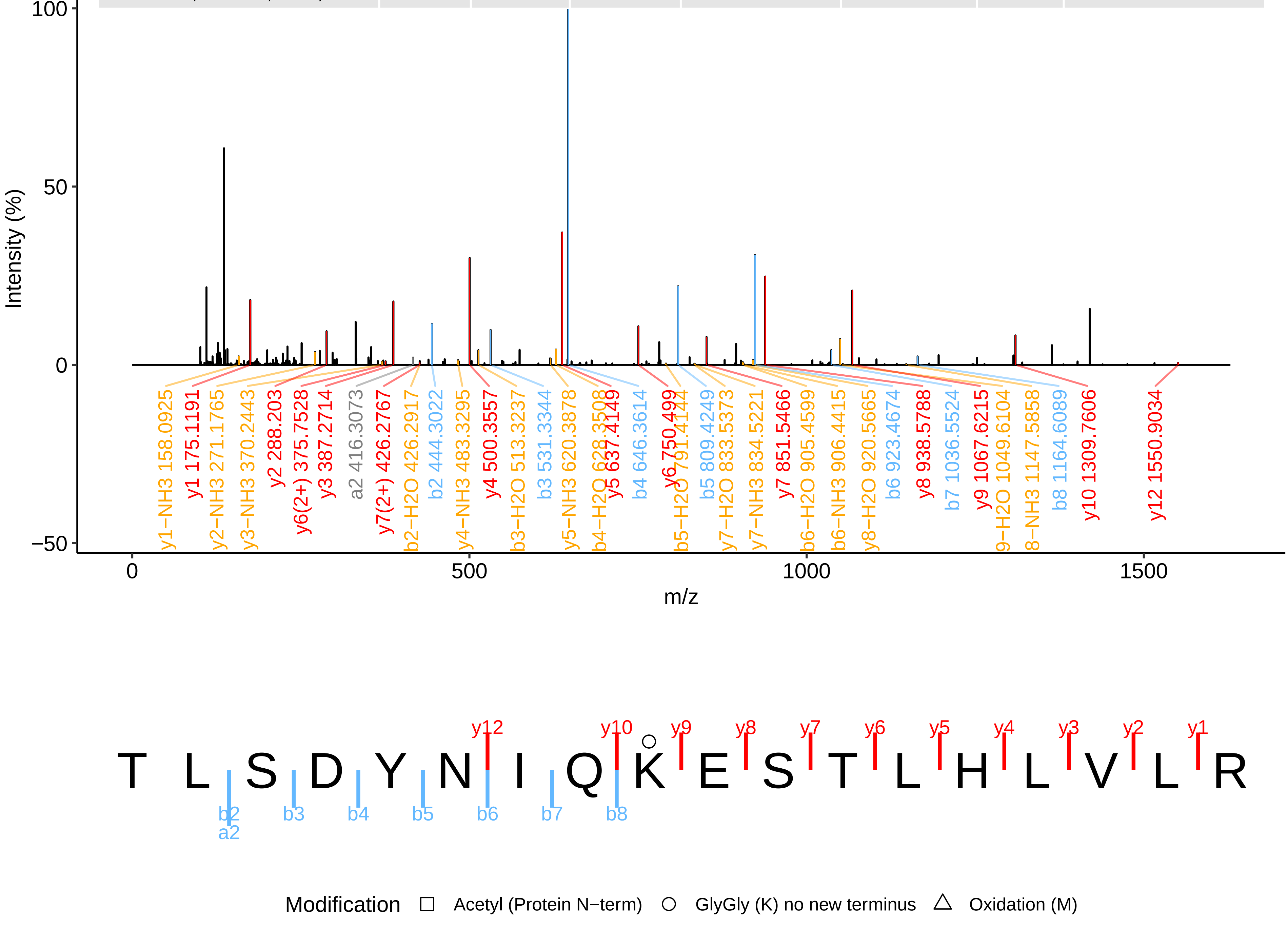

20200924_KIZ_MUb5_GlyGly_1 − Scan number: 20318

Gene Names	Charge	m/z	Mass	Retention time	PEP	Score	Precursor Intensity
RPS27A;UBA52;UBB;UBC	4	561.805	2243.191	65.515	3.4086e−23	154.46	277303232

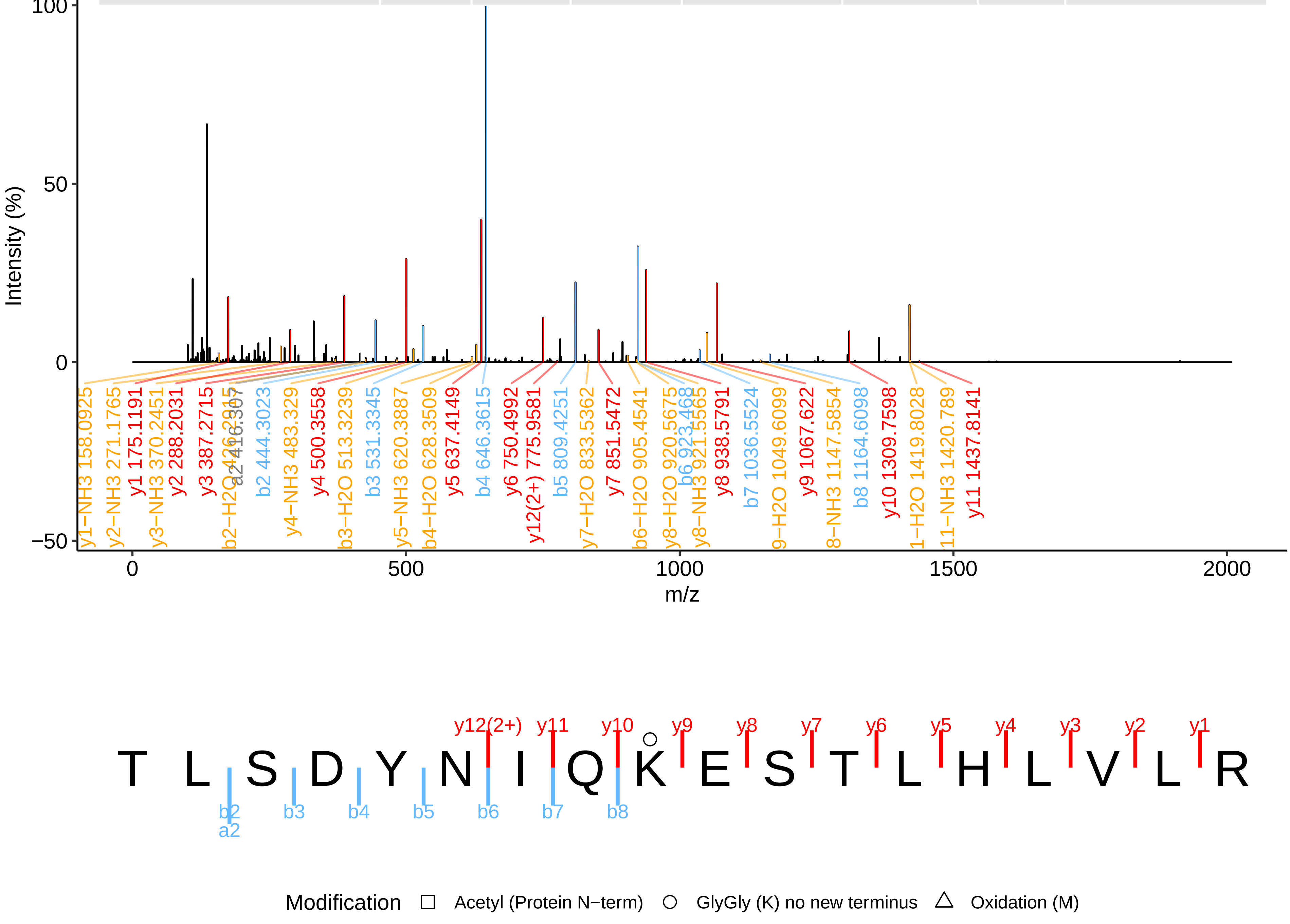

20200924_KIZ_MUb5_GlyGly_1 − Scan number: 20482

Gene Names	Charge	m/z	Mass	Retention time	PEP	Score	Precursor Intensity
RPS27A;UBA52;UBB;UBC	4	561.805	2243.191	66.017	4.1882e−23	151.44	179809600

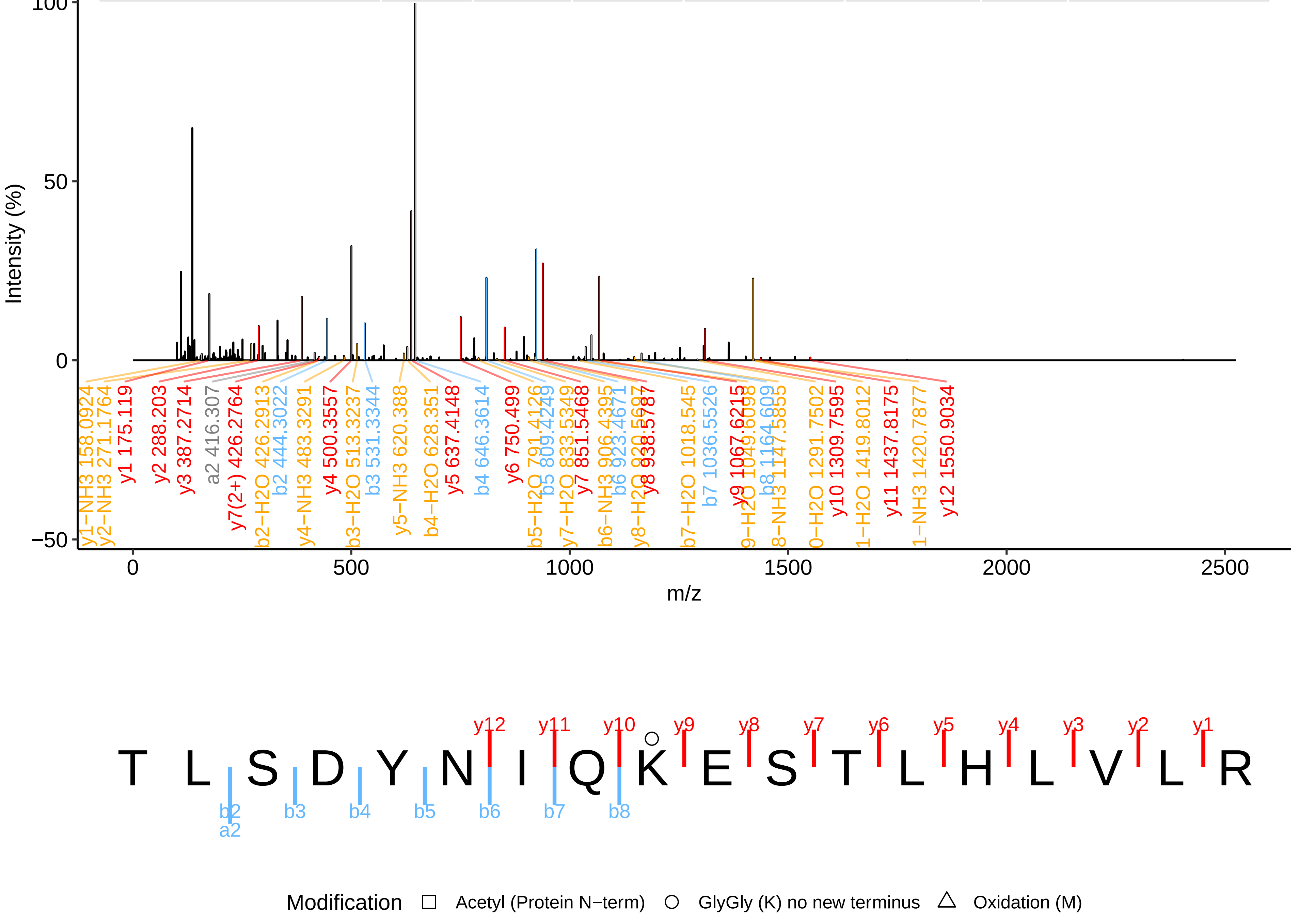

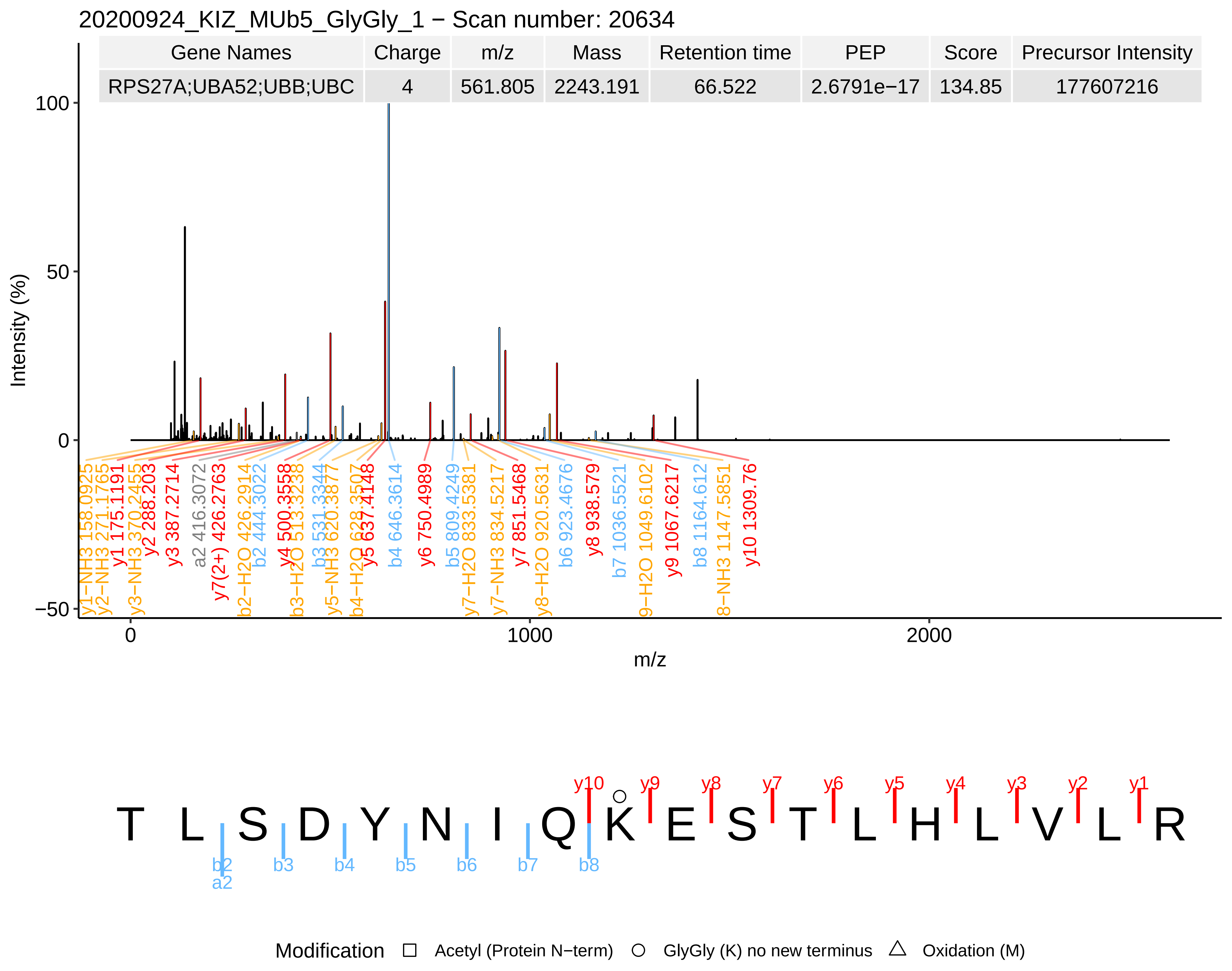

20200924_KIZ_MUb5_GlyGly_1 − Scan number: 20634
Gene Names | Charge | m/z | Mass | Retention time | PEP | Score | Precursor Intensity
RPS27A;UBA52;UBB;UBC | 4 | 561.805 | 2243.191 | 66.522 | 2.6791e−17 | 134.85 | 177607216
Intensity (%)
m/z
y1−NH3 158.0925
y2−NH3 271.1765
y1 175.1191
y3−NH3 370.2455
y2 288.203
y3 387.2714
a2 416.3072
y7(2+) 426.2763
b2−H2O 426.2914
b2 444.3022
y4 500.3558
b3−H2O 513.3238
b3 531.3344
y5−NH3 620.3877
b4−H2O 628.3507
y5 637.4148
b4 646.3614
y6 750.4989
b5 809.4249
y7−H2O 833.5381
y7−NH3 834.5217
y7 851.5468
y8−H2O 920.5631
b6 923.4676
y8 938.579
b7 1036.5521
y9−H2O 1049.6102
y9 1067.6217
b8 1164.612
y8−NH3 1147.5851
y10 1309.76
T L S D Y N I Q K E S T L H L V L R
Modification
Acetyl (Protein N−term)
GlyGly (K) no new terminus
Oxidation (M)

20200924_KIZ_MUb5_GlyGly_1 − Scan number: 20793

Gene Names	Charge	m/z	Mass	Retention time	PEP	Score	Precursor Intensity
RPS27A;UBA52;UBB;UBC	4	561.805	2243.191	67.026	4.5265e−18	147.64	136208784

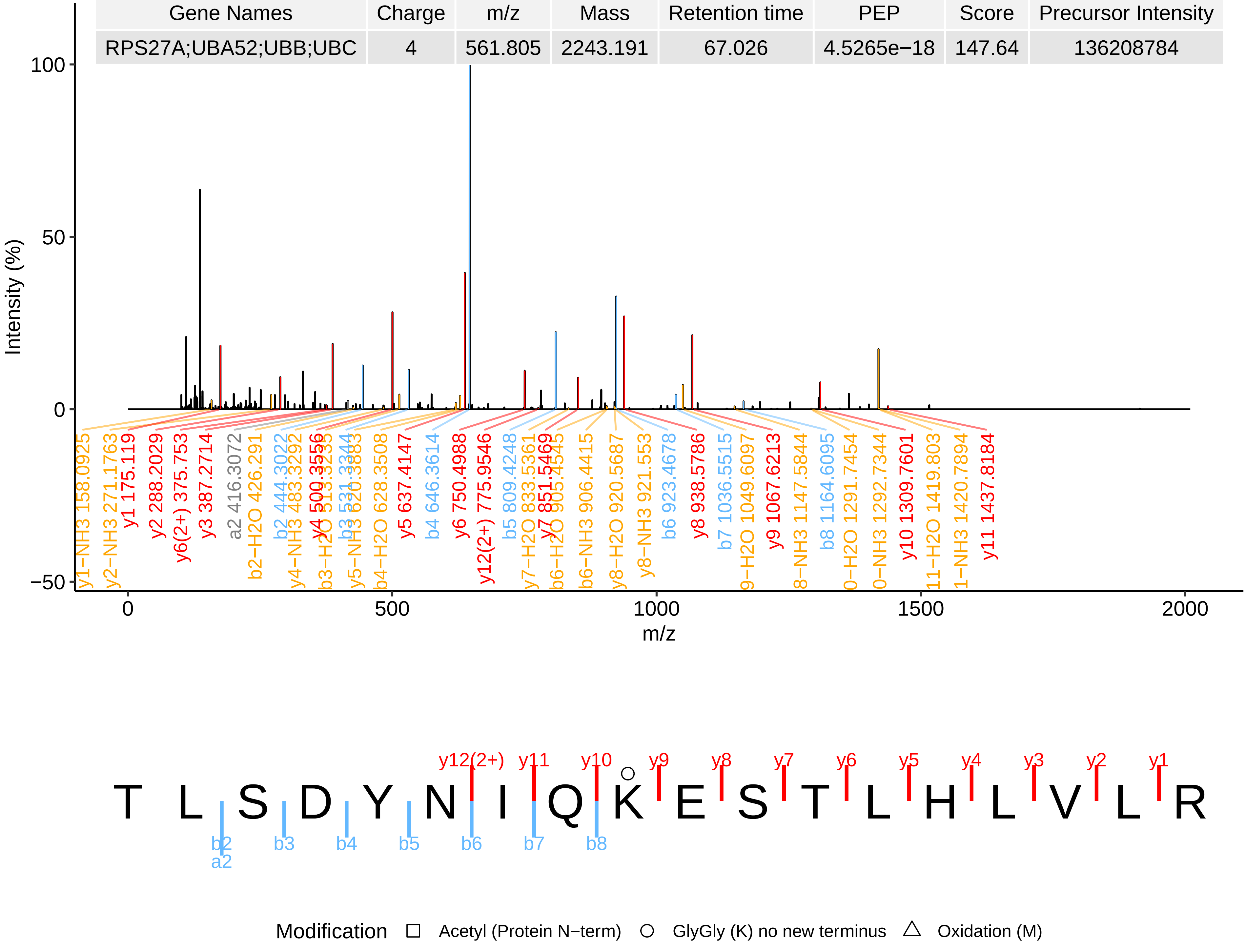

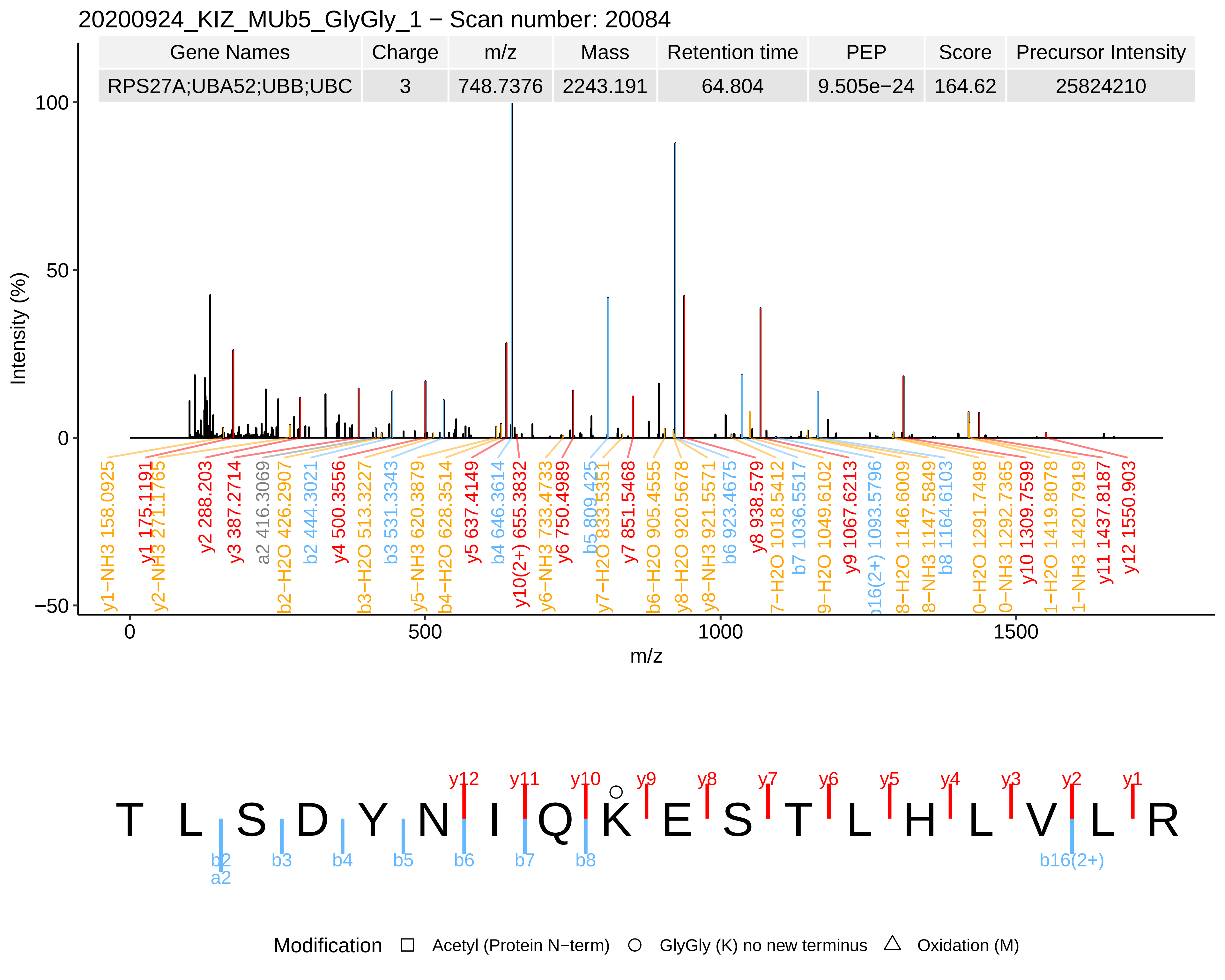
20200924_KIZ_MUb5_GlyGly_1 − Scan number: 20084
Gene Names | Charge | m/z | Mass | Retention time | PEP | Score | Precursor Intensity
RPS27A;UBA52;UBB;UBC | 3 | 748.7376 | 2243.191 | 64.804 | 9.505e−24 | 164.62 | 25824210
Intensity (%)
m/z
y1−NH3 158.0925
y1 175.1191
y2−NH3 271.1765
y2 288.203
y3 387.2714
a2 416.3069
b2−H2O 426.2907
b2 444.3021
y4 500.3556
b3−H2O 513.3227
b3 531.3343
y5−NH3 620.3879
b4−H2O 628.3514
y5 637.4149
b4 646.3614
y10(2+) 655.3832
y6−NH3 733.4733
y6 750.4989
b5 809.425
y7−H2O 833.5351
y7 851.5468
b6−H2O 905.4555
y8−H2O 920.5678
y8−NH3 921.5571
b6 923.4675
y8 938.579
b7 1036.5517
y9 1067.6213
b8 1164.6103
y10 1309.7599
y11 1437.8187
y12 1550.903
T L S D Y N I Q K E S T L H L V L R
y12 y11 y10 y9 y8 y7 y6 y5 y4 y3 y2 y1
b2 a2 b3 b4 b5 b6 b7 b8 b16(2+)
Modification
Acetyl (Protein N−term)
GlyGly (K) no new terminus
Oxidation (M)

20200924_KIZ_MUb5_GlyGly_1 − Scan number: 20419

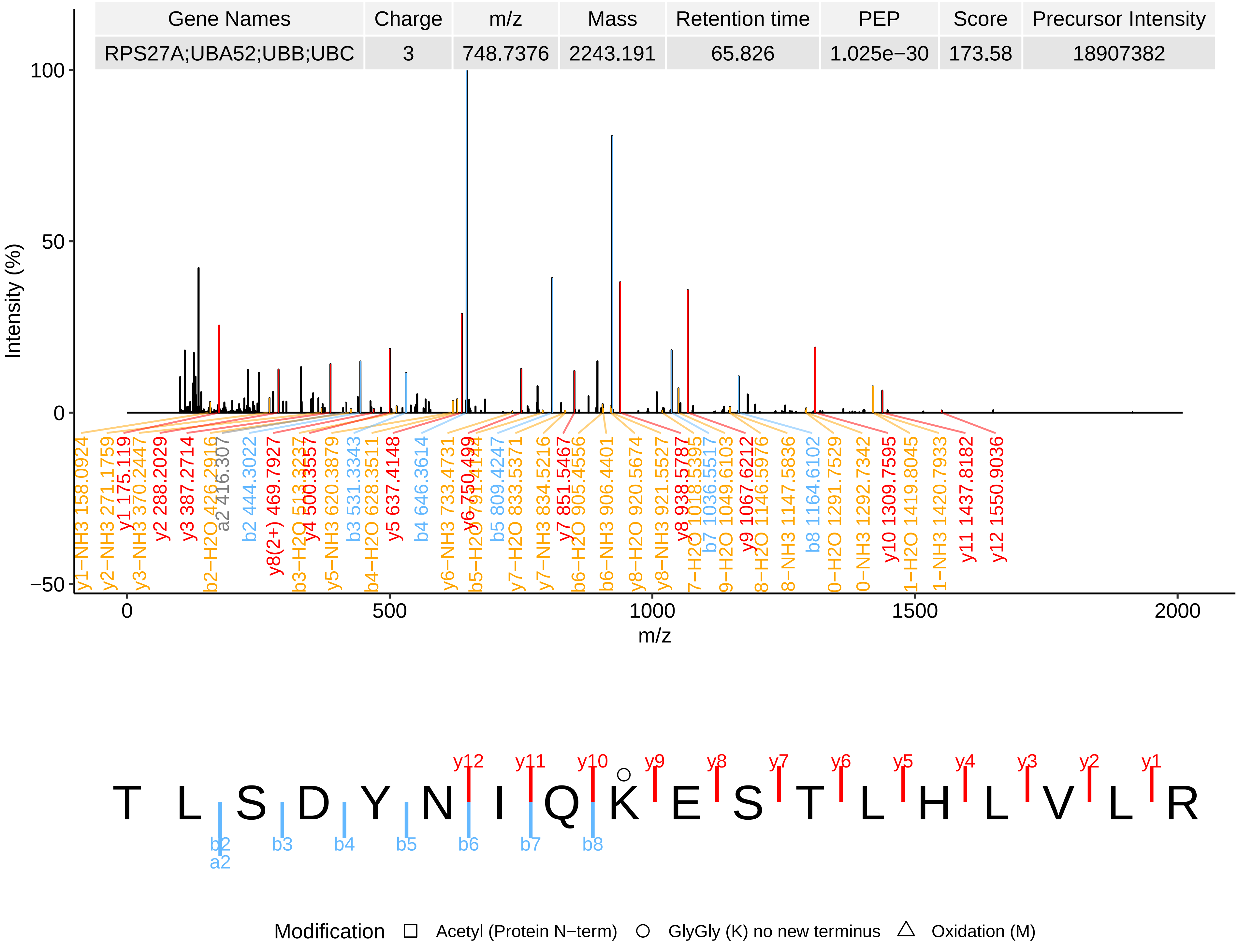

20200924_KIZ_MUb5_GlyGly_1 − Scan number: 20424

Gene Names	Charge	m/z	Mass	Retention time	PEP	Score	Precursor Intensity
RPS27A;UBA52;UBB;UBC	3	748.7376	2243.191	65.84	0.017185	58.98	NA

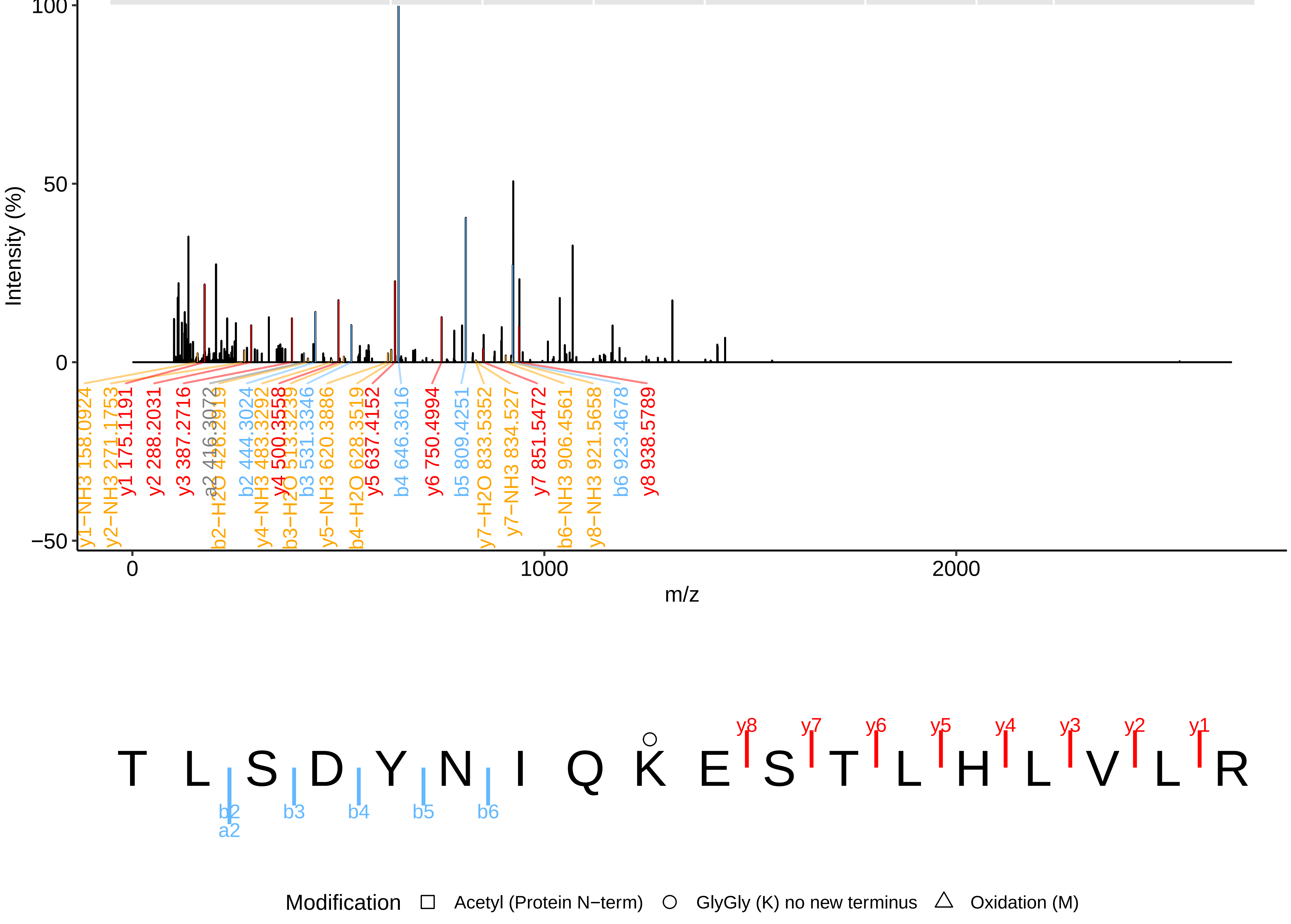

20200924_KIZ_MUb5_GlyGly_1 − Scan number: 20581

Gene Names	Charge	m/z	Mass	Retention time	PEP	Score	Precursor Intensity
RPS27A;UBA52;UBB;UBC	3	748.7376	2243.191	66.342	1.6442e−23	162.41	NA

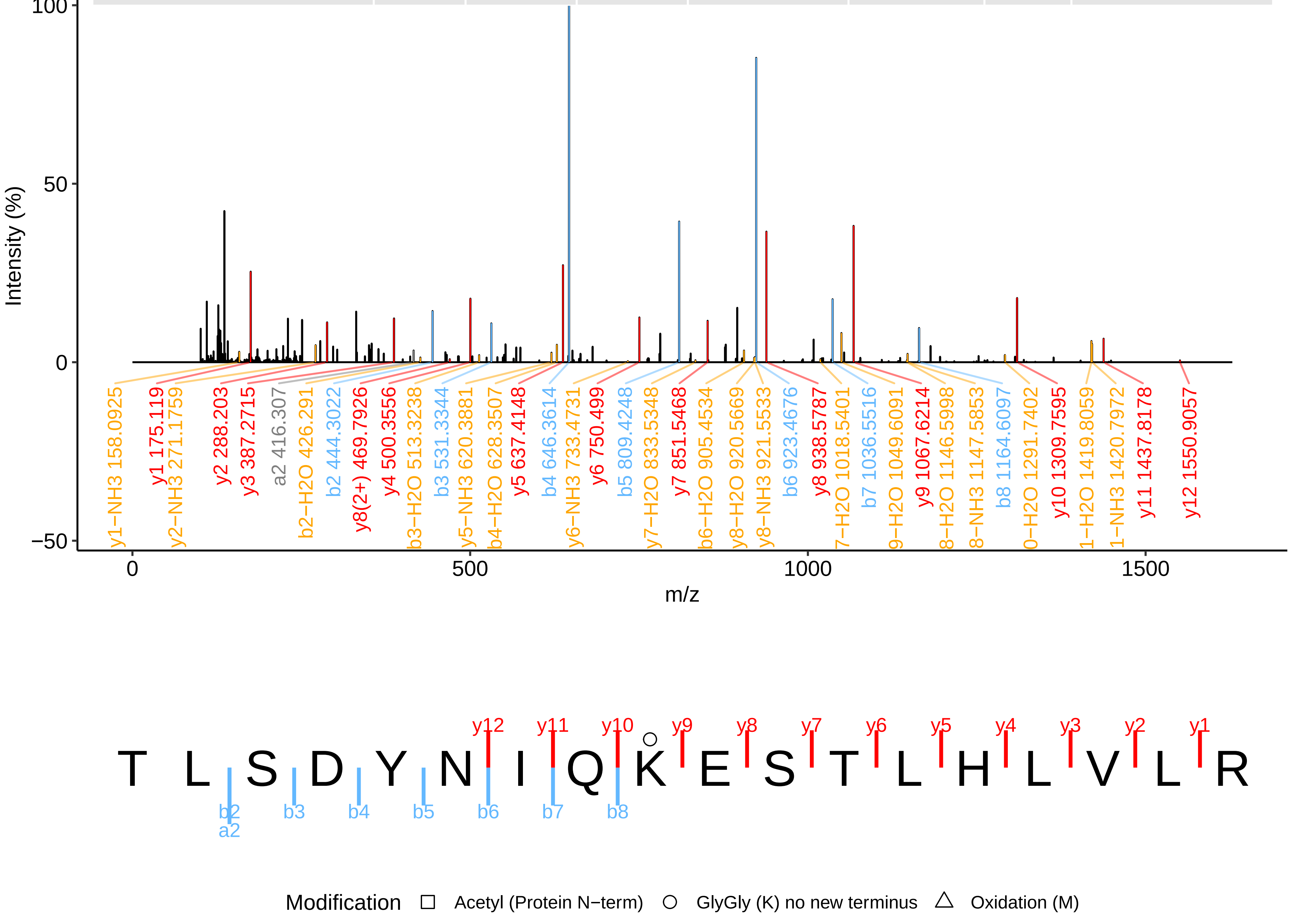

20200924_KIZ_MUb5_GlyGly_1 − Scan number: 20904

Gene Names	Charge	m/z	Mass	Retention time	PEP	Score	Precursor Intensity
RPS27A;UBA52;UBB;UBC	3	748.7376	2243.191	67.365	4.2973e−23	153.95	13793822

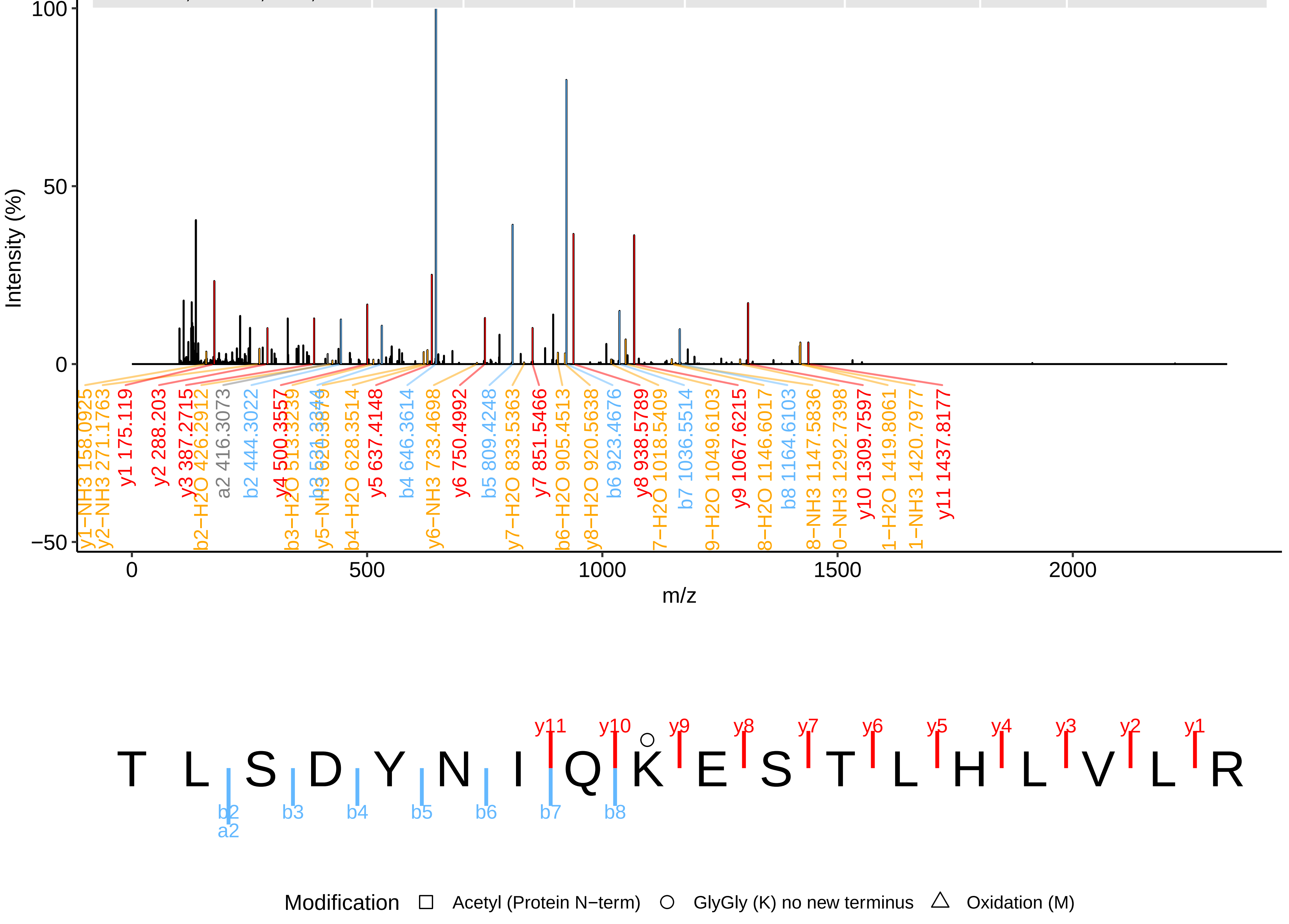

20200924_KIZ_MUb5_GlyGly_1 − Scan number: 20955

Gene Names	Charge	m/z	Mass	Retention time	PEP	Score	Precursor Intensity
RPS27A;UBA52;UBB;UBC	4	561.805	2243.191	67.53	4.5265e−18	147.64	132235640

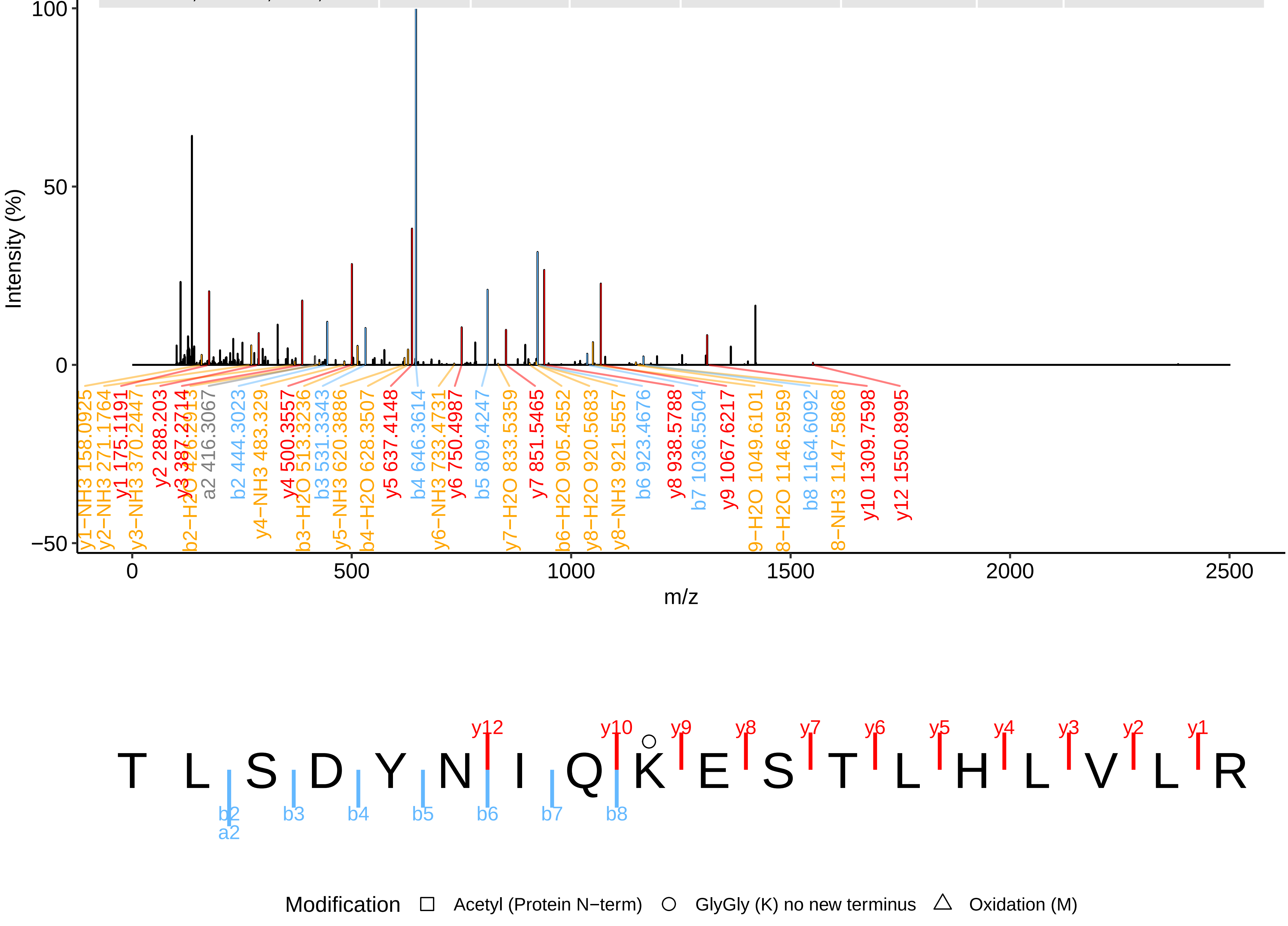

20200924_KIZ_MUb5_GlyGly_1 − Scan number: 19691

Gene Names	Charge	m/z	Mass	Retention time	PEP	Score	Precursor Intensity
RPS27A;UBA52;UBB;UBC	3	748.7376	2243.191	63.642	2.4124e−16	132.59	NA

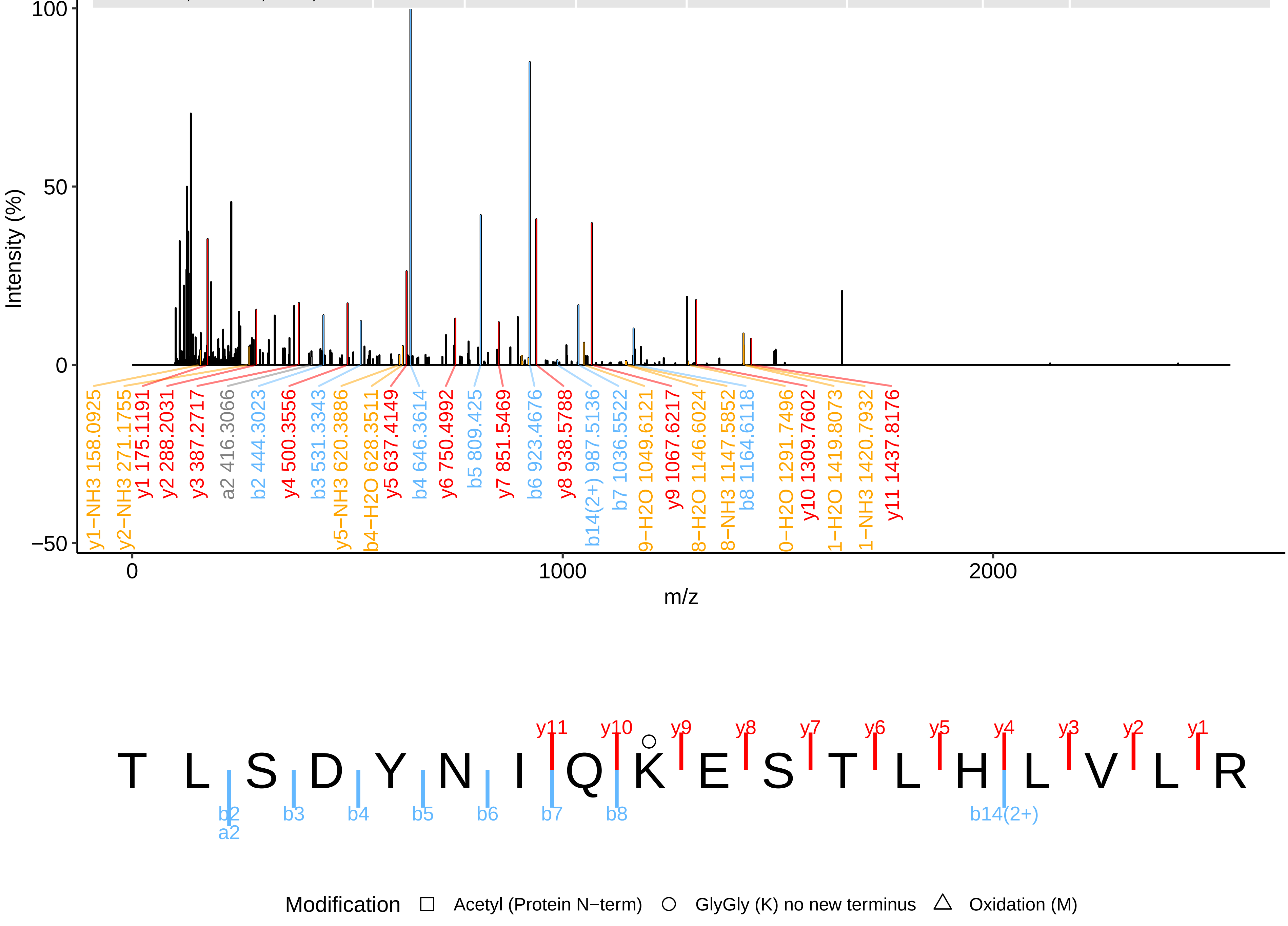

20200924_KIZ_MUb5_GlyGly_1 − Scan number: 19895

Gene Names	Charge	m/z	Mass	Retention time	PEP	Score	Precursor Intensity
RPS27A;UBA52;UBB;UBC	3	748.7376	2243.191	64.24	6.795e−17	144.97	17712756

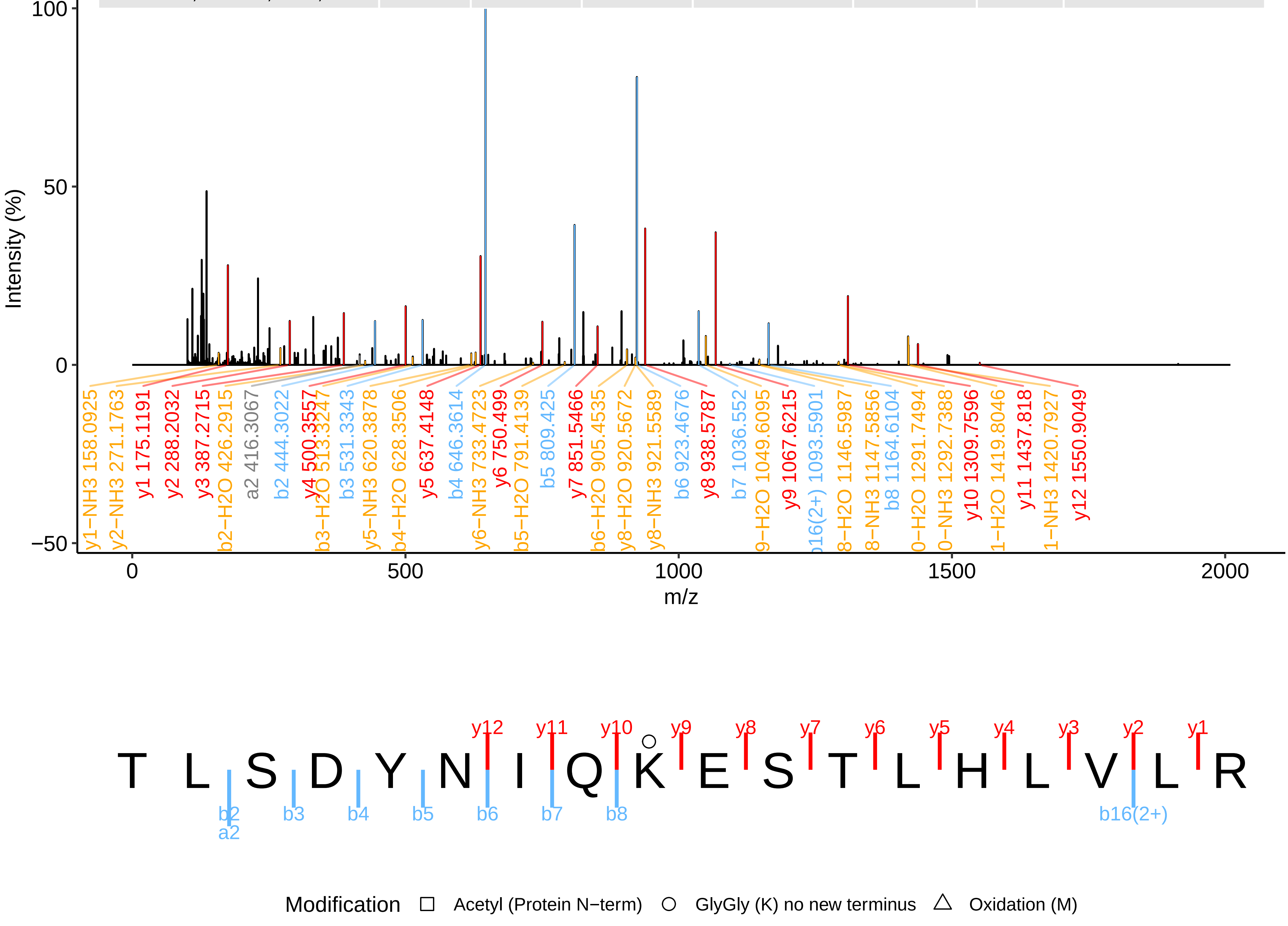

20200924_KIZ_MUb5_GlyGly_1 − Scan number: 20252

Gene Names	Charge	m/z	Mass	Retention time	PEP	Score	Precursor Intensity
RPS27A;UBA52;UBB;UBC	3	748.7376	2243.191	65.322	5.9789e−30	174.49	40473704

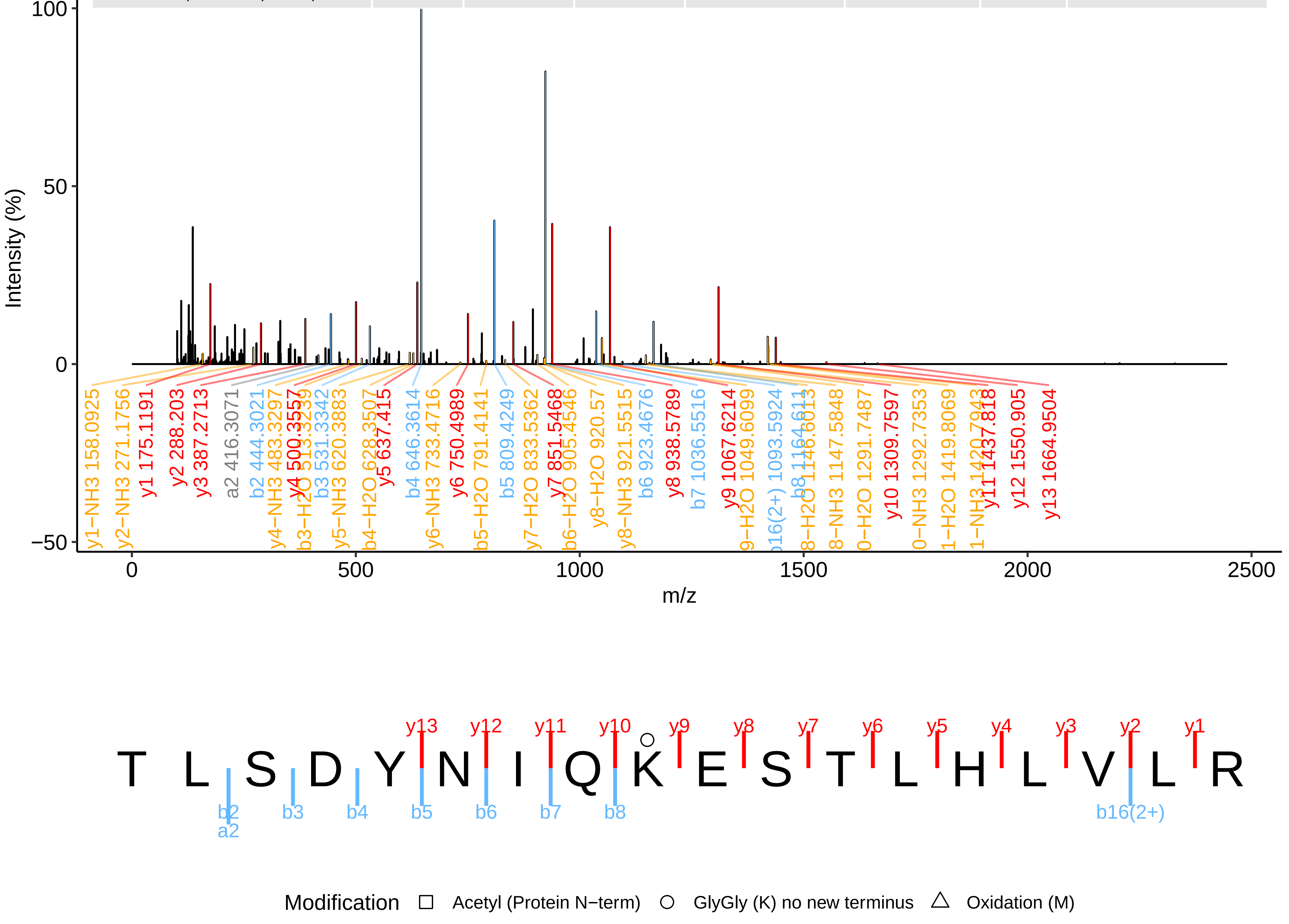

20200924_KIZ_MUb5_GlyGly_2 − Scan number: 18340

Gene Names	Charge	m/z	Mass	Retention time	PEP	Score	Precursor Intensity
RPS27A;UBA52;UBB;UBC	4	561.805	2243.191	68.02	2.9239e−17	133.45	22502288

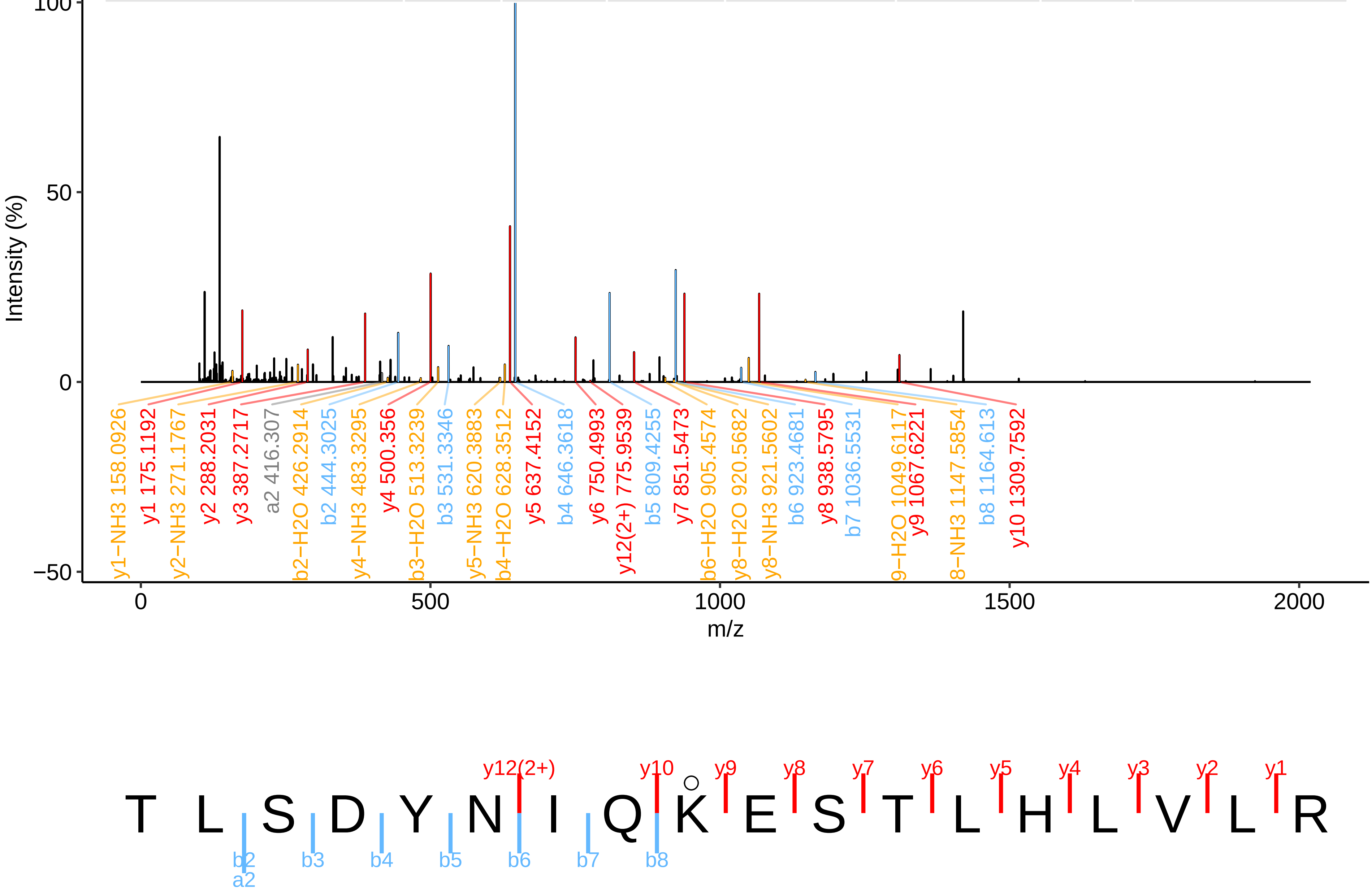

Modification □ Acetyl (Protein N−term) ○ GlyGly (K) no new terminus △ Oxidation (M)

20200924_KIZ_MUb5_GlyGly_2 − Scan number: 18474

Gene Names	Charge	m/z	Mass	Retention time	PEP	Score	Precursor Intensity
RPS27A;UBA52;UBB;UBC	4	561.805	2243.191	68.525	1.7451e−17	140.22	56761588

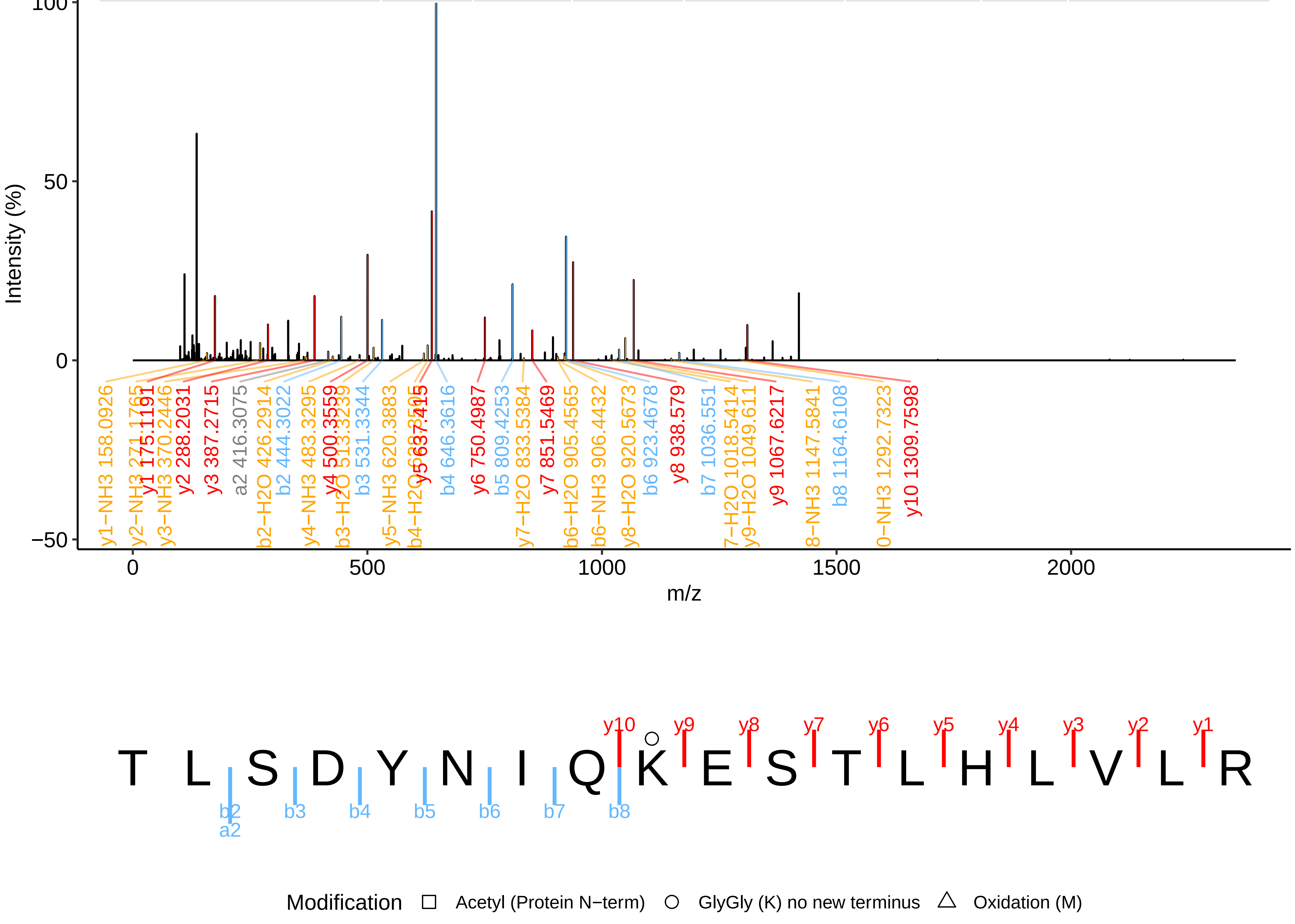

20200924_KIZ_MUb5_GlyGly_2 − Scan number: 18617

Gene Names	Charge	m/z	Mass	Retention time	PEP	Score	Precursor Intensity
RPS27A;UBA52;UBB;UBC	4	561.805	2243.191	69.057	2.6791e−17	134.85	120379736

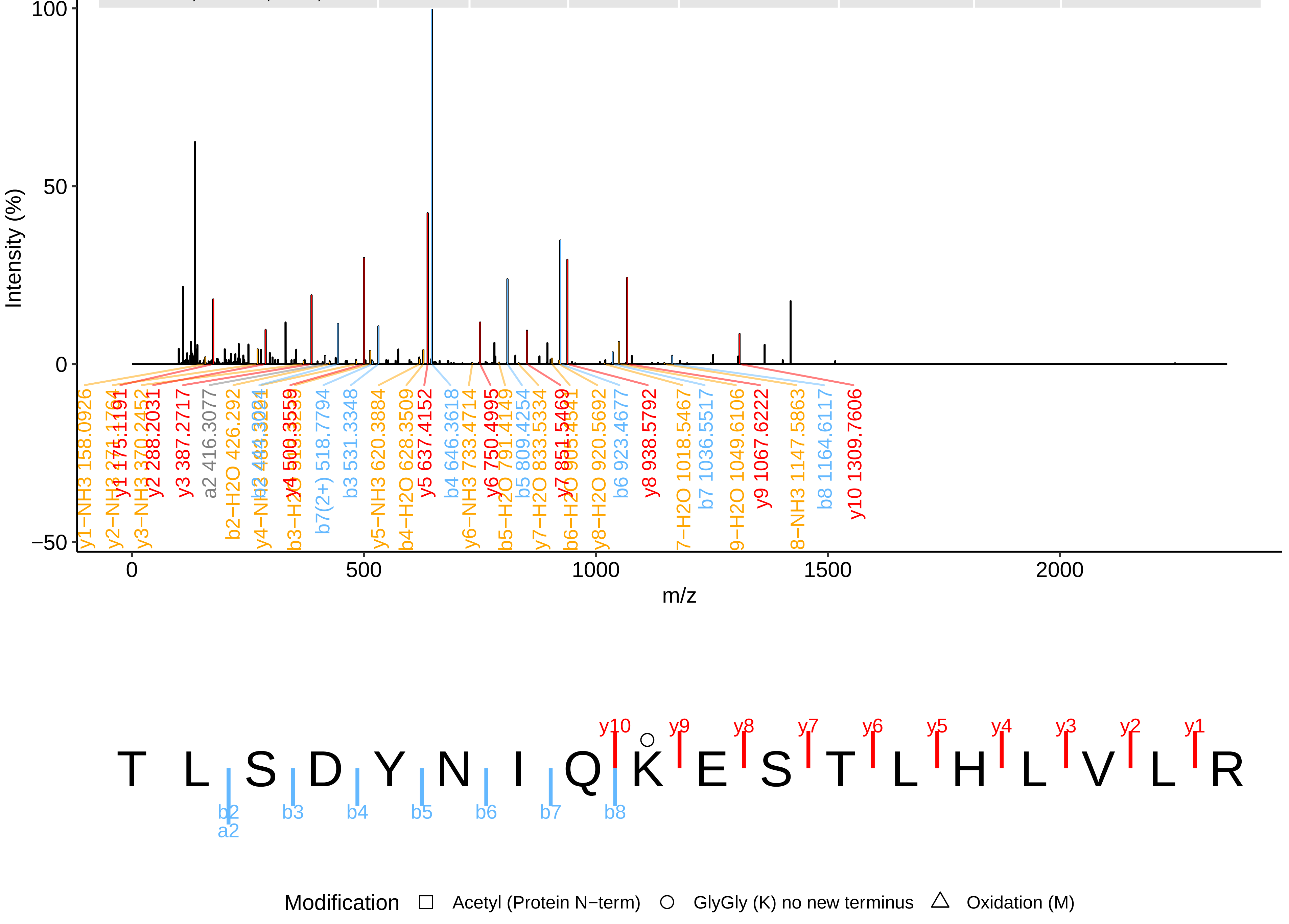

20200924_KIZ_MUb5_GlyGly_2 − Scan number: 18756

Gene Names	Charge	m/z	Mass	Retention time	PEP	Score	Precursor Intensity
RPS27A;UBA52;UBB;UBC	4	561.805	2243.191	69.572	1.2359e−17	143.14	139301376

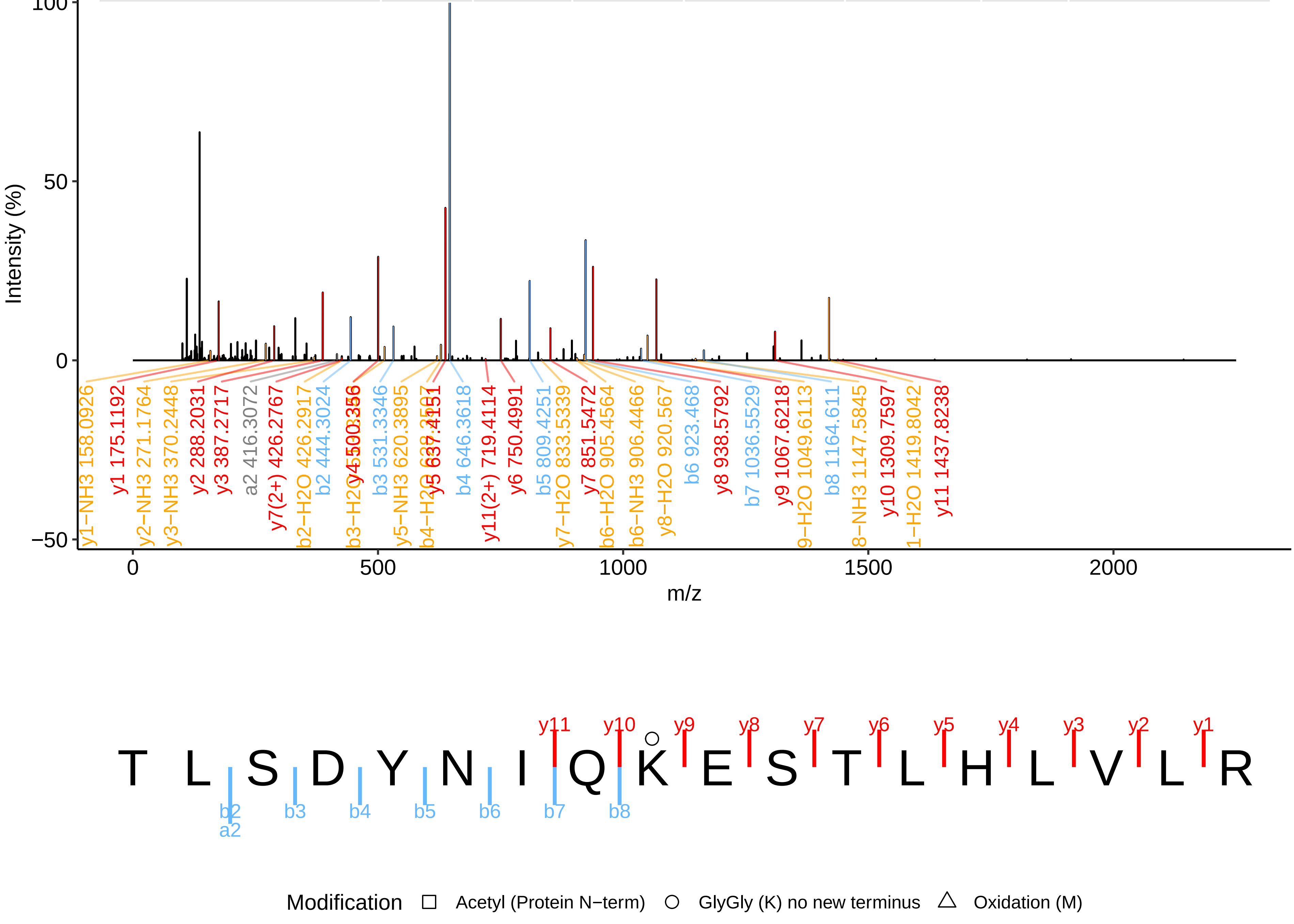

20200924_KIZ_MUb5_GlyGly_2 − Scan number: 18893

Gene Names	Charge	m/z	Mass	Retention time	PEP	Score	Precursor Intensity
RPS27A;UBA52;UBB;UBC	4	561.805	2243.191	70.075	2.5175e−23	157.91	110493712

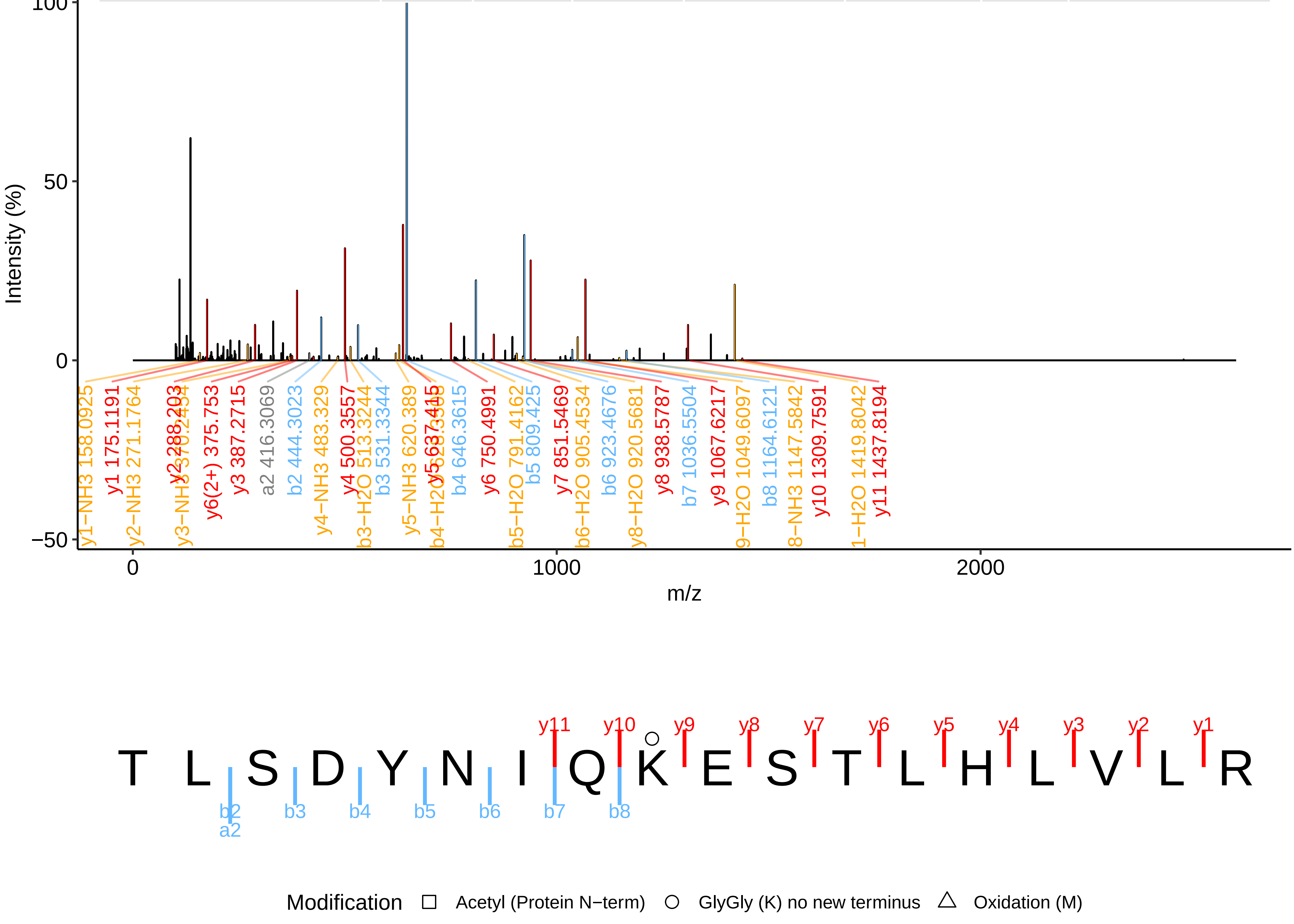

20200924_KIZ_MUb5_GlyGly_2 − Scan number: 19035

Gene Names	Charge	m/z	Mass	Retention time	PEP	Score	Precursor Intensity
RPS27A;UBA52;UBB;UBC	4	561.805	2243.191	70.591	4.5265e−18	147.64	64820872

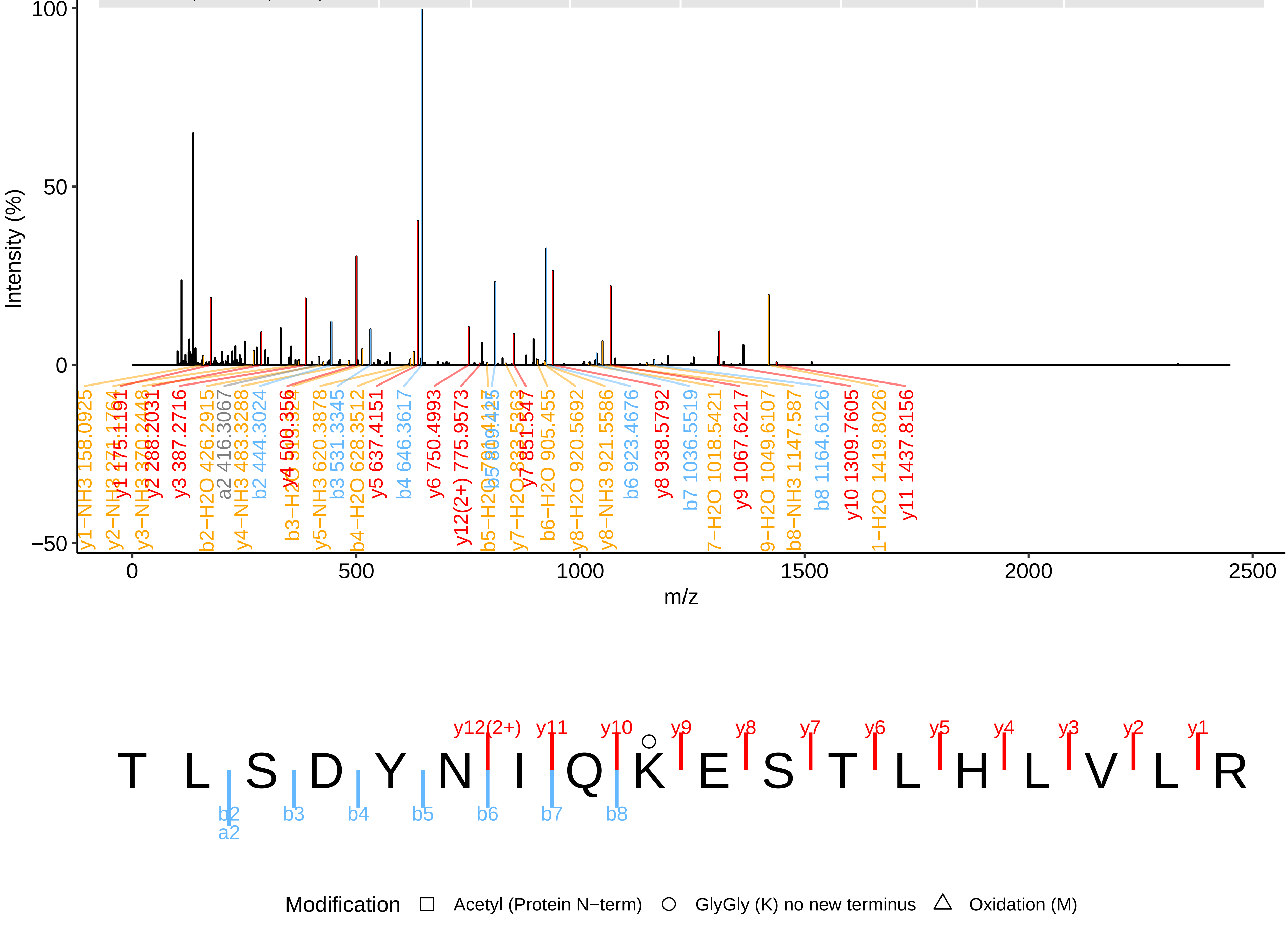

20200924_KIZ_MUb5_GlyGly_2 − Scan number: 19175

Gene Names	Charge	m/z	Mass	Retention time	PEP	Score	Precursor Intensity
RPS27A;UBA52;UBB;UBC	4	561.805	2243.191	71.097	1.2359e−17	143.14	13724698

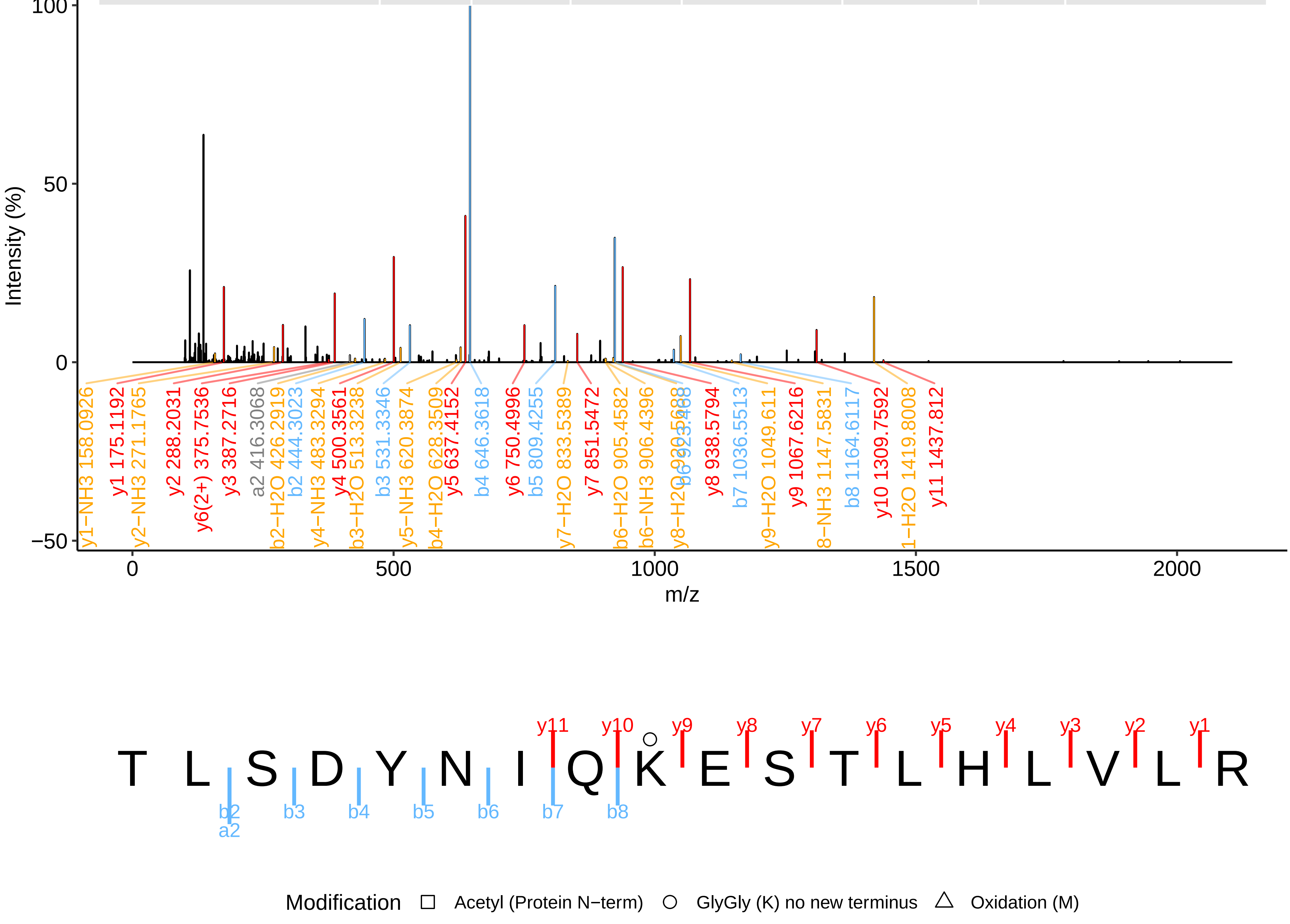

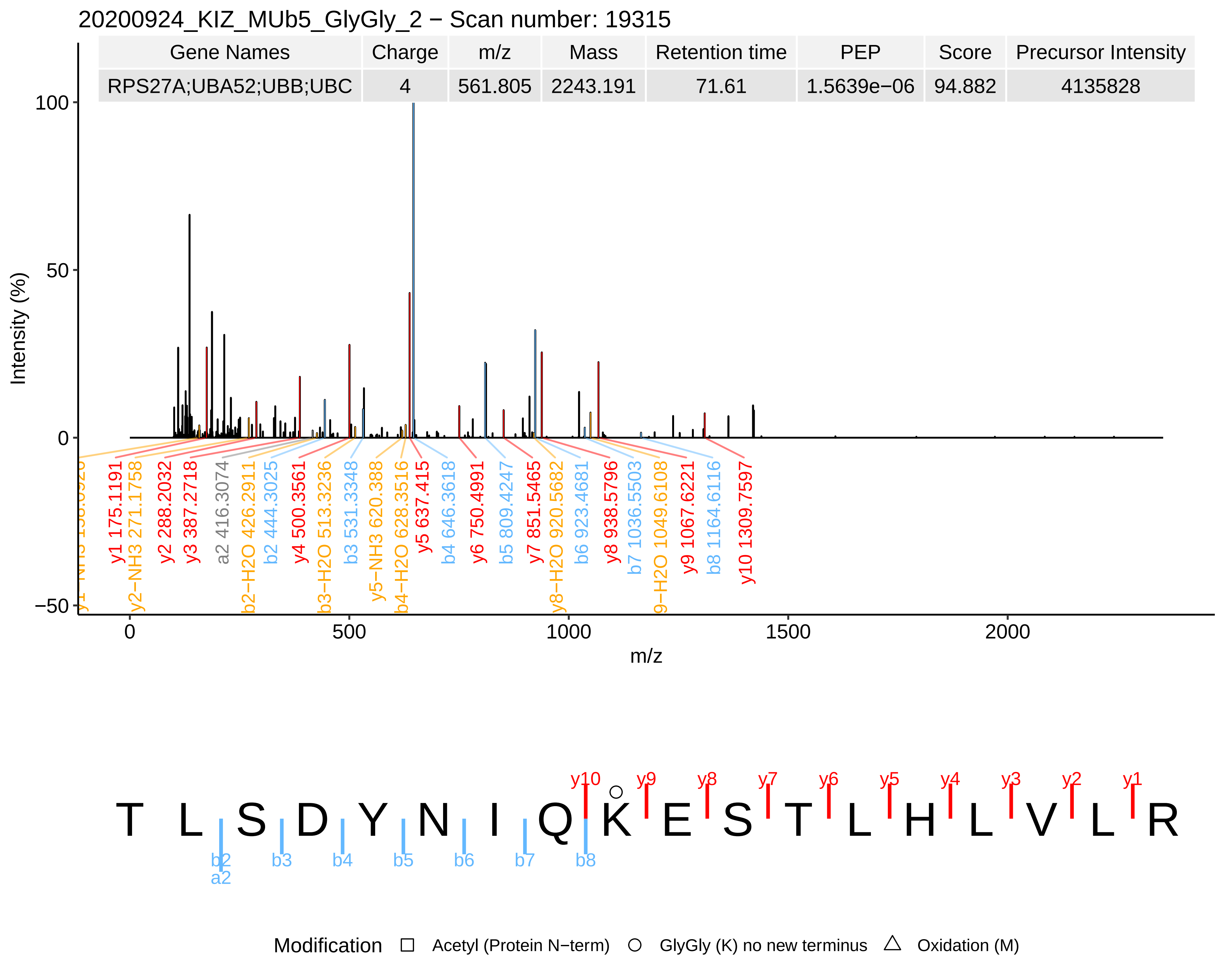
20200924_KIZ_MUb5_GlyGly_2 − Scan number: 19315
Gene Names
RPS27A;UBA52;UBB;UBC
Charge
4
m/z
561.805
Mass
2243.191
Retention time
71.61
PEP
1.5639e−06
Score
94.882
Precursor Intensity
4135828
Intensity (%)
m/z
y1 175.1191
y2−NH3 271.1758
y2 288.2032
y3 387.2718
a2 416.3074
b2−H2O 426.2911
b2 444.3025
y4 500.3561
b3−H2O 513.3236
b3 531.3348
y5−NH3 620.388
b4−H2O 628.3516
y5 637.415
b4 646.3618
y6 750.4991
b5 809.4247
y7 851.5465
y8−H2O 920.5682
b6 923.4681
y8 938.5796
b7 1036.5503
y9−H2O 1049.6108
y9 1067.6221
b8 1164.6116
y10 1309.7597
T L S D Y N I Q K E S T L H L V L R
Modification
Acetyl (Protein N−term)
GlyGly (K) no new terminus
Oxidation (M)

20200924_KIZ_MUb5_GlyGly_2 − Scan number: 18729

Gene Names	Charge	m/z	Mass	Retention time	PEP	Score	Precursor Intensity
RPS27A;UBA52;UBB;UBC	3	748.7376	2243.191	69.474	6.8937e−24	165.45	5570152

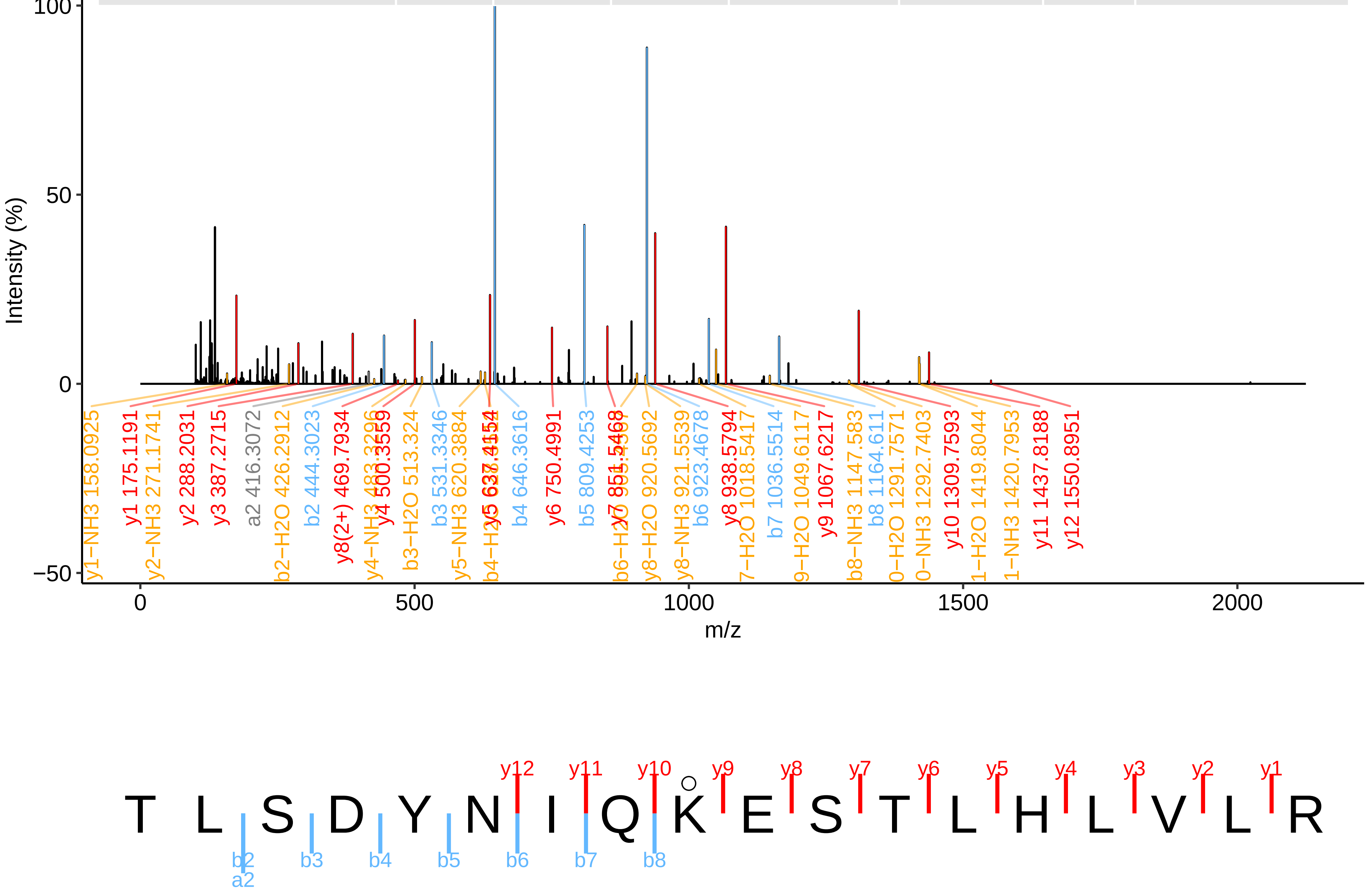

Modification □ Acetyl (Protein N−term) ○ GlyGly (K) no new terminus △ Oxidation (M)

20200924_KIZ_MUb5_GlyGly_2 − Scan number: 18867

Gene Names	Charge	m/z	Mass	Retention time	PEP	Score	Precursor Intensity
RPS27A;UBA52;UBB;UBC	3	748.7376	2243.191	69.989	1.9326e−17	141.1	NA

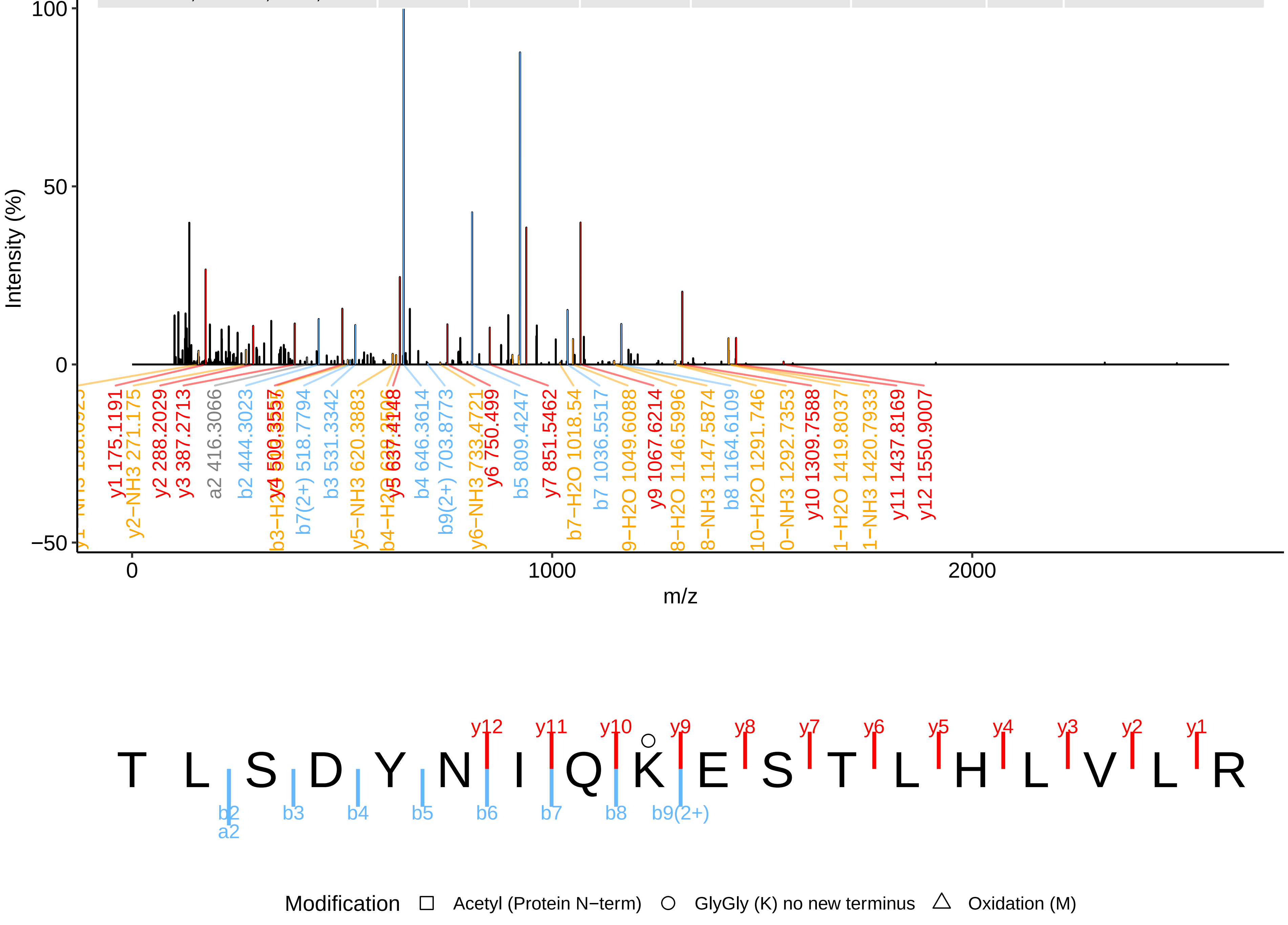

20200924_KIZ_MUb5_GlyGly_2 − Scan number: 18973

Gene Names	Charge	m/z	Mass	Retention time	PEP	Score	Precursor Intensity
RPS27A;UBA52;UBB;UBC	3	748.7376	2243.191	70.365	4.0245e−23	154.82	6112927

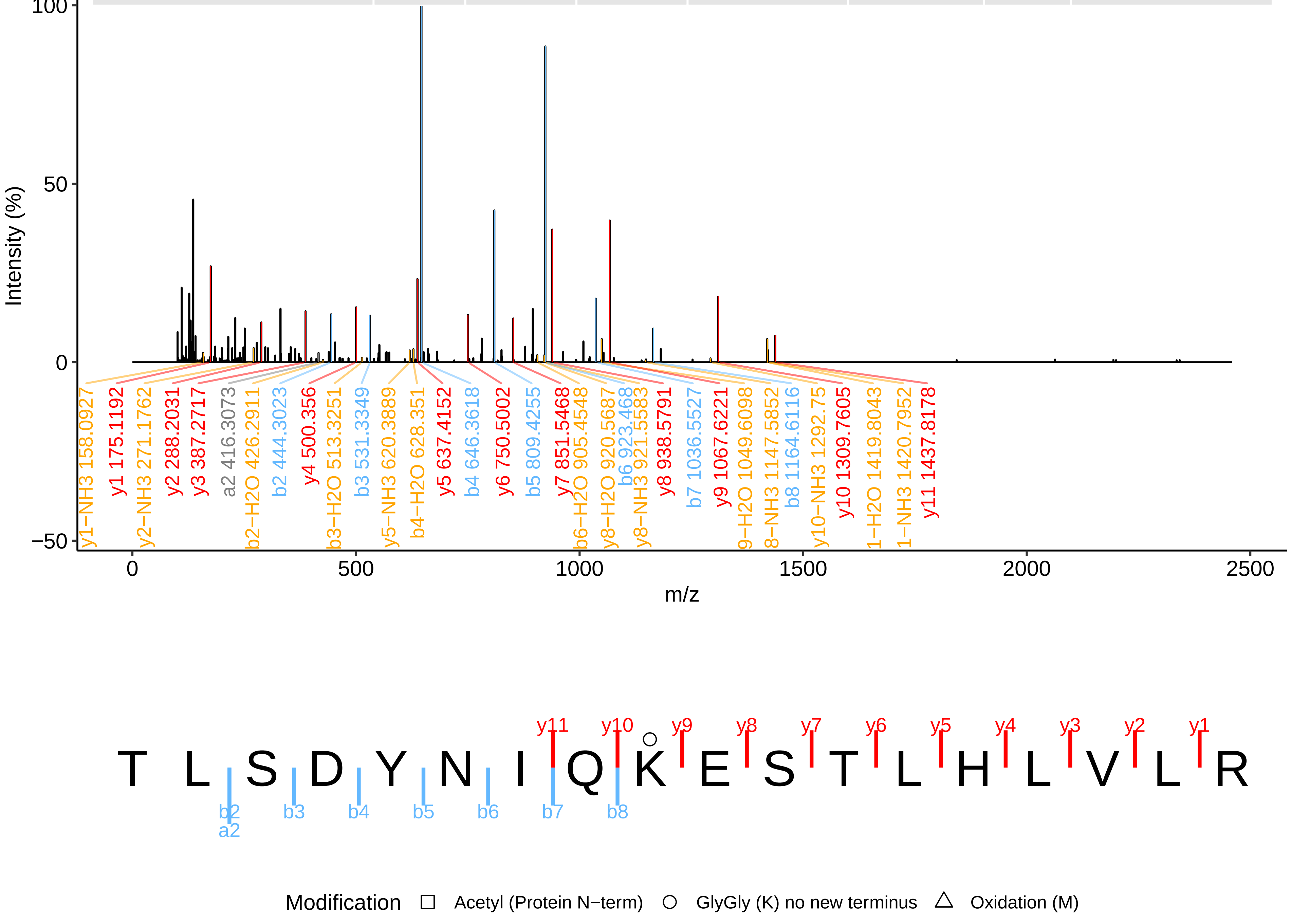

20200924_KIZ_MUb5_GlyGly_2 − Scan number: 19327

Gene Names	Charge	m/z	Mass	Retention time	PEP	Score	Precursor Intensity
RPS27A;UBA52;UBB;UBC	4	561.805	2243.191	71.655	1.0139e−09	102.08	6176551

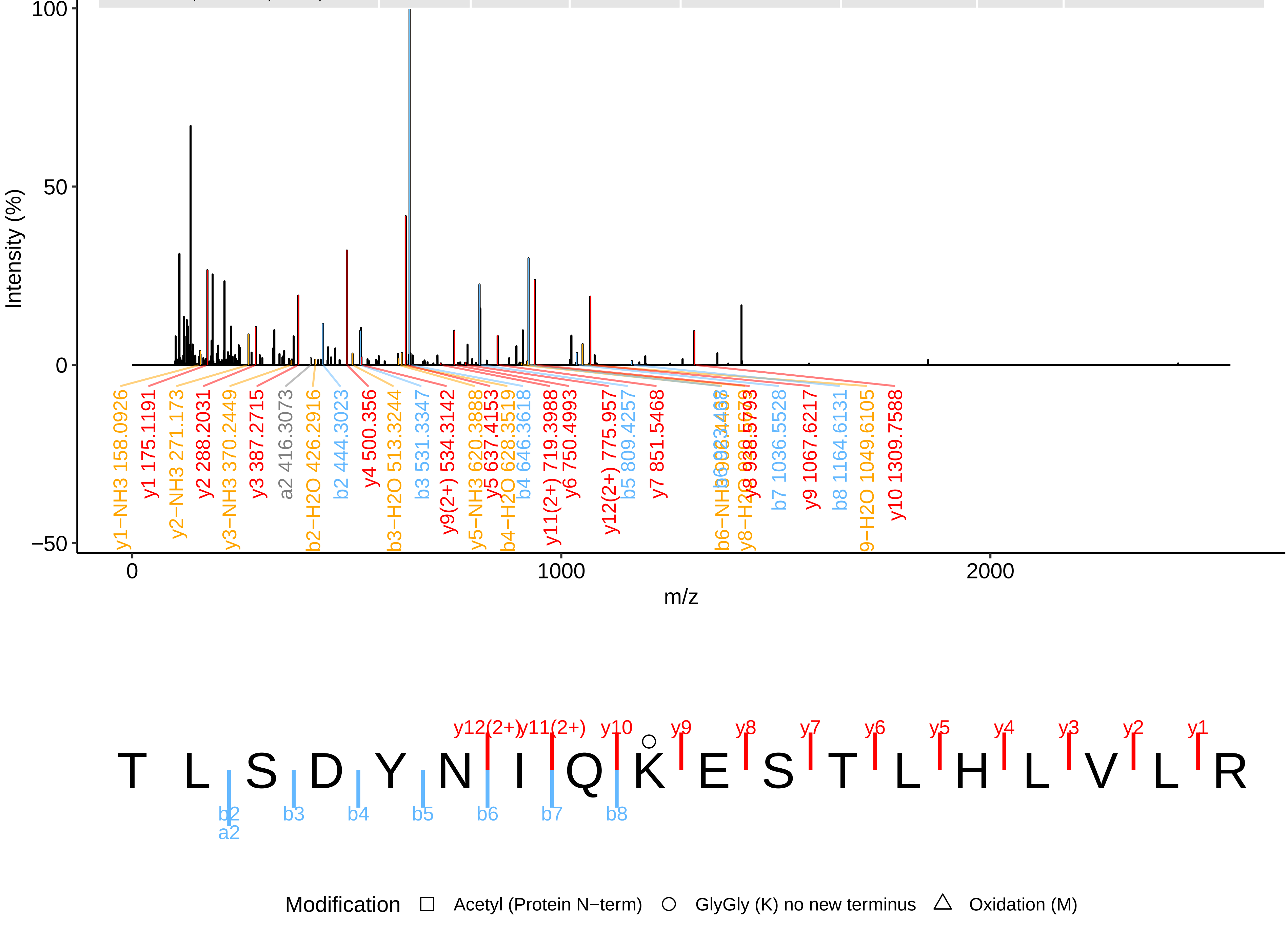

20201118_KIZ_MUb6_GlyGly_1 − Scan number: 17804

Gene Names	Charge	m/z	Mass	Retention time	PEP	Score	Precursor Intensity
RPS27A;UBA52;UBB;UBC	5	449.6455	2243.191	56.007	0.00096793	72.11	22277328

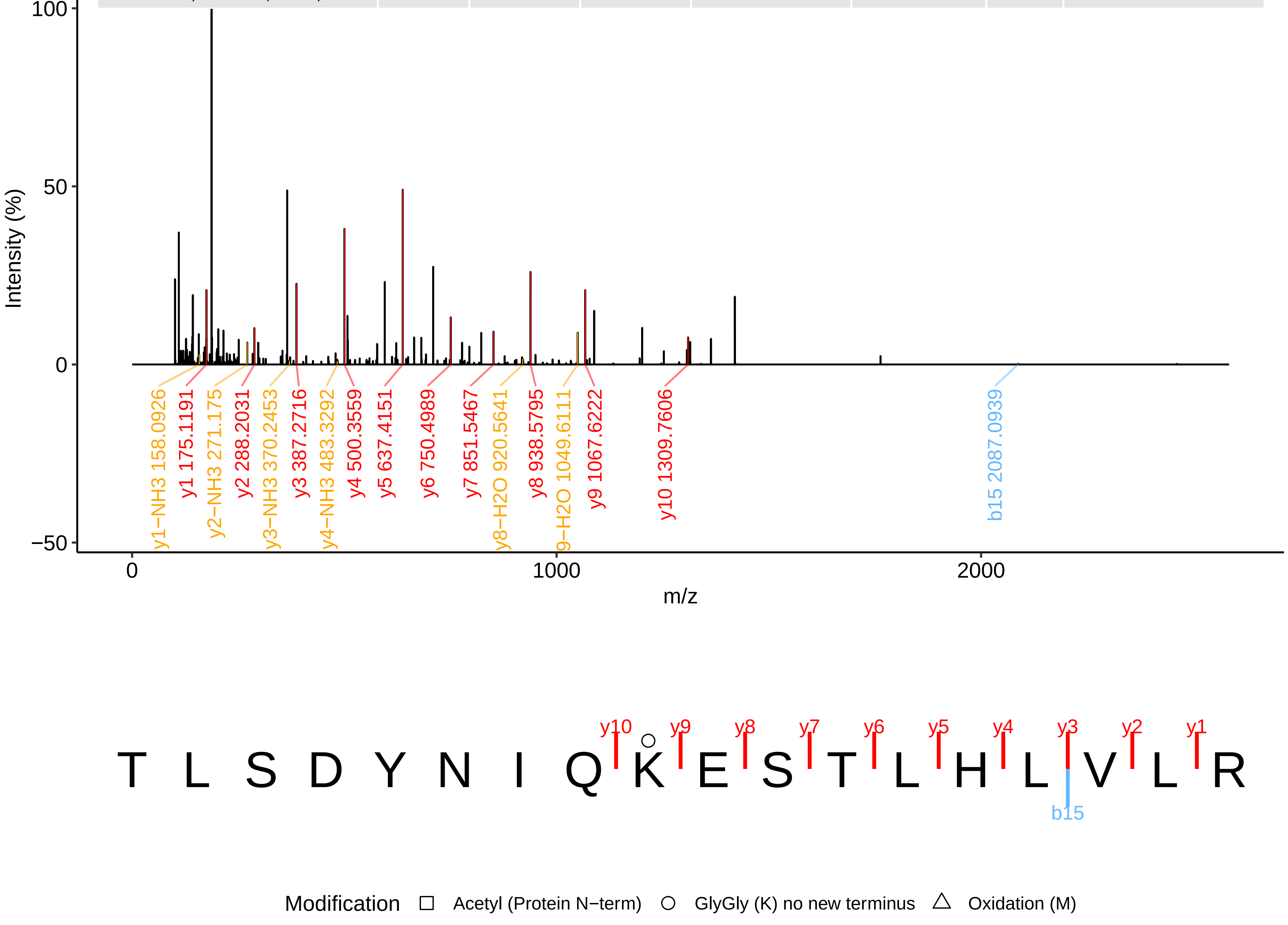

20201118_KIZ_MUb6_GlyGly_1 − Scan number: 17855

Gene Names	Charge	m/z	Mass	Retention time	PEP	Score	Precursor Intensity
RPS27A;UBA52;UBB;UBC	5	449.6455	2243.191	56.162	0.0015504	66.965	23554028

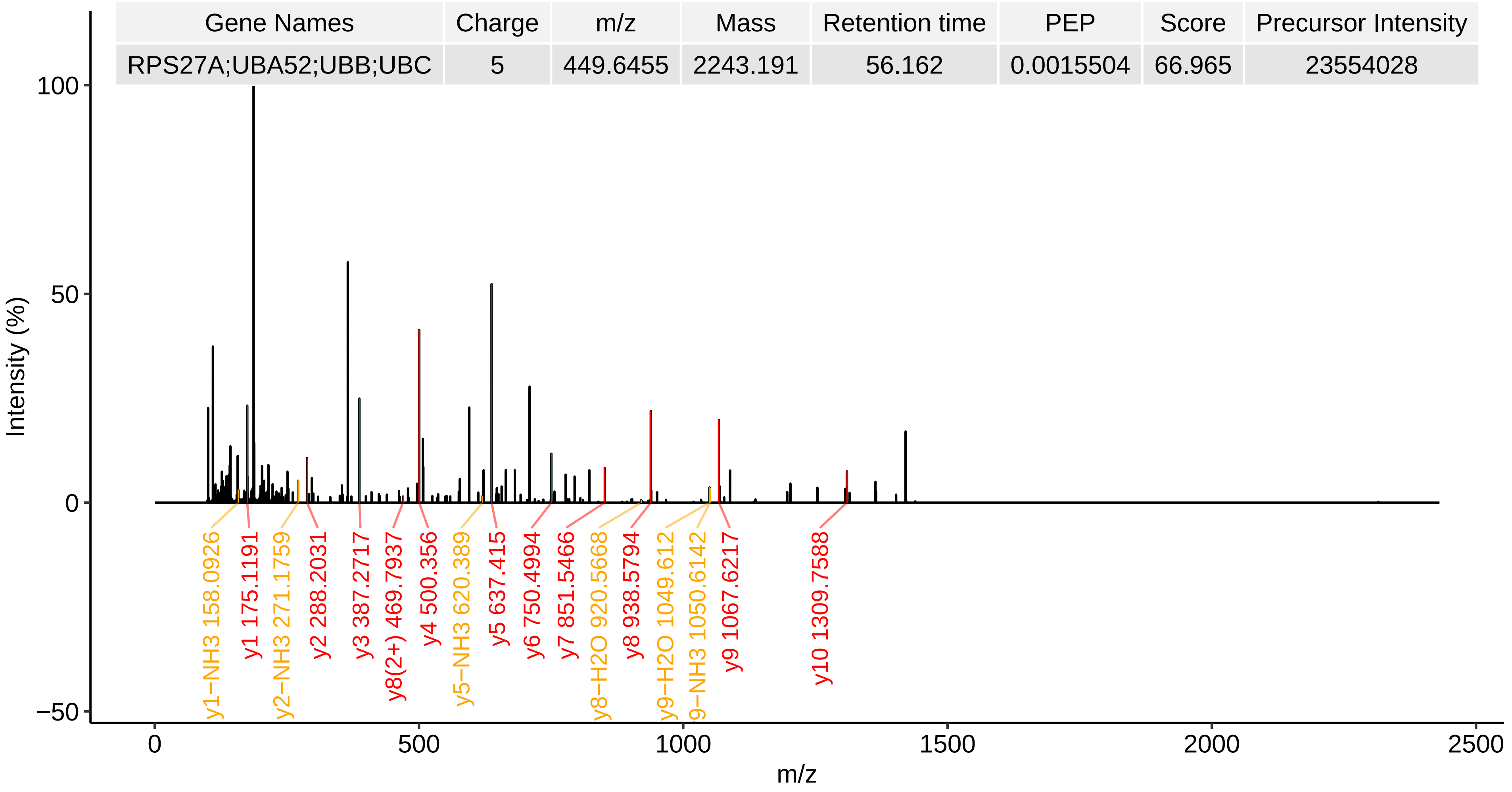

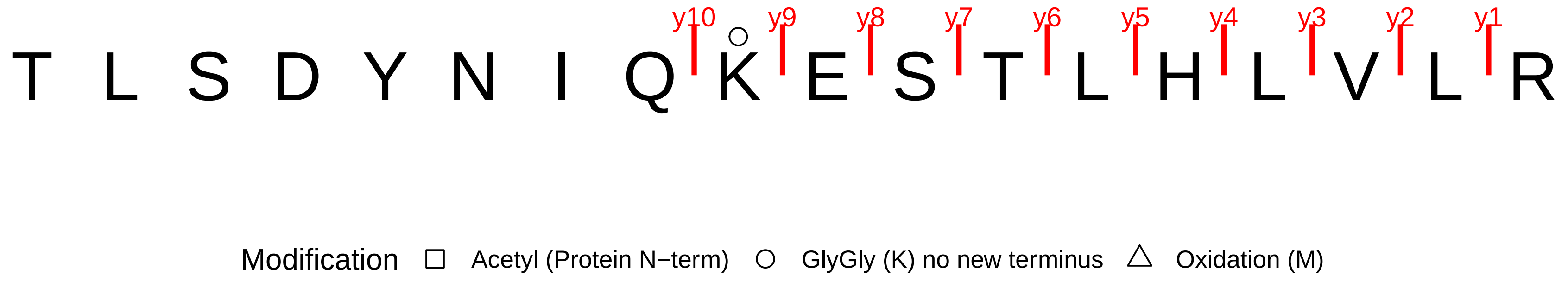

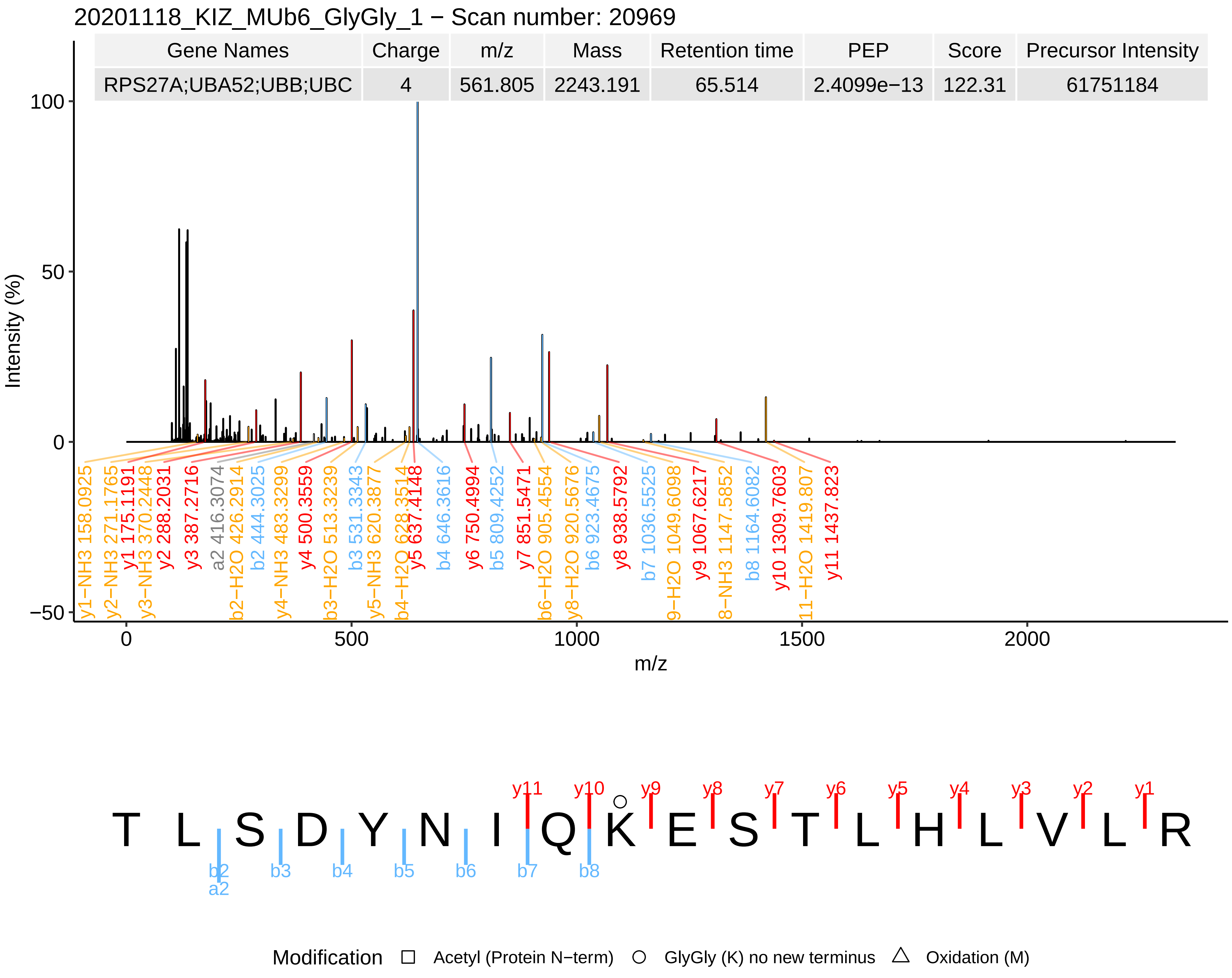
20201118_KIZ_MUb6_GlyGly_1 − Scan number: 20969
Gene Names
RPS27A;UBA52;UBB;UBC
Charge
4
m/z
561.805
Mass
2243.191
Retention time
65.514
PEP
2.4099e−13
Score
122.31
Precursor Intensity
61751184
Intensity (%)
m/z
y1−NH3 158.0925
y2−NH3 271.1765
y1 175.1191
y3−NH3 370.2448
y2 288.2031
y3 387.2716
a2 416.3074
b2−H2O 426.2914
b2 444.3025
y4−NH3 483.3299
y4 500.3559
b3−H2O 513.3239
b3 531.3343
y5−NH3 620.3877
b4−H2O 628.3514
y5 637.4148
b4 646.3616
y6 750.4994
b5 809.4252
y7 851.5471
b6−H2O 905.4554
y8−H2O 920.5676
b6 923.4675
y8 938.5792
b7 1036.5525
y9−H2O 1049.6098
y9 1067.6217
y8−NH3 1147.5852
b8 1164.6082
y10 1309.7603
y11−H2O 1419.807
y11 1437.823
T L S D Y N I Q K E S T L H L V L R
Modification
Acetyl (Protein N−term)
GlyGly (K) no new terminus
Oxidation (M)

20201118_KIZ_MUb6_GlyGly_1 − Scan number: 20974

Gene Names	Charge	m/z	Mass	Retention time	PEP	Score	Precursor Intensity
RPS27A;UBA52;UBB;UBC	4	561.805	2243.191	65.529	1.9225e−13	124.43	123917608

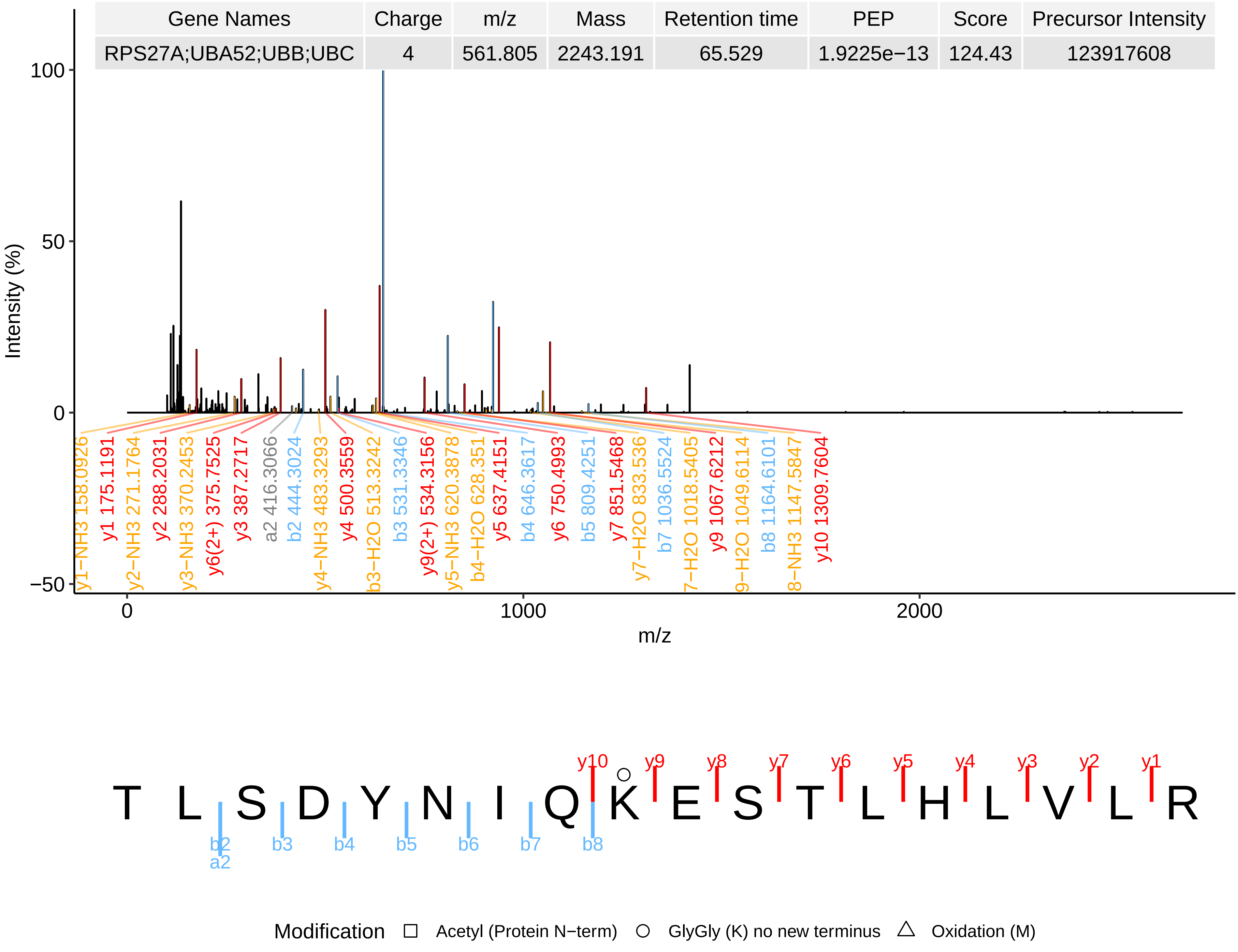

20201118_KIZ_MUb6_GlyGly_1 − Scan number: 21150

Gene Names	Charge	m/z	Mass	Retention time	PEP	Score	Precursor Intensity
RPS27A;UBA52;UBB;UBC	4	561.805	2243.191	66.035	5.7986e−14	130.29	28296502

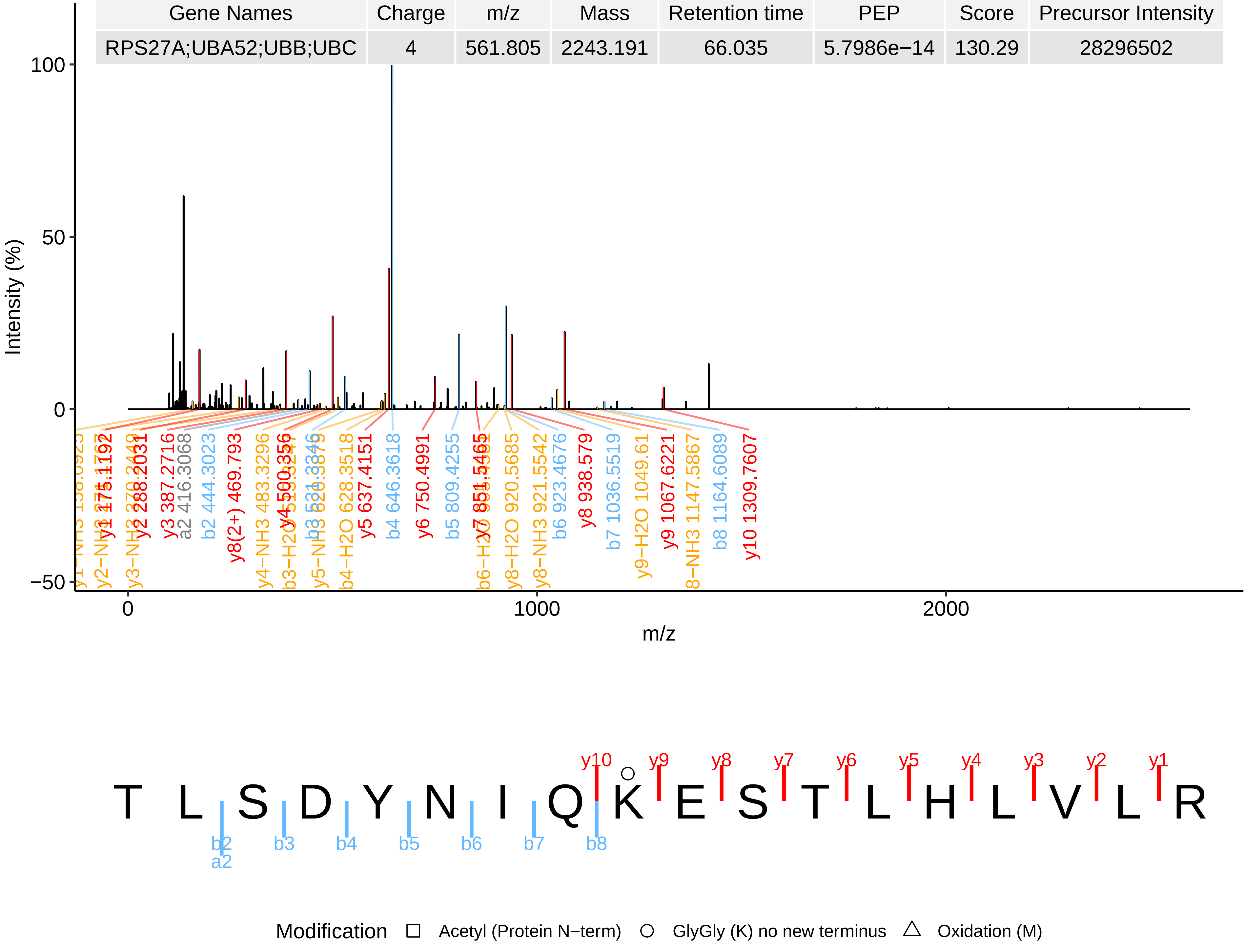

20201118_KIZ_MUb6_GlyGly_2 − Scan number: 19281

Gene Names	Charge	m/z	Mass	Retention time	PEP	Score	Precursor Intensity
RPS27A;UBA52;UBB;UBC	4	561.805	2243.191	65.053	0.0011563	70.942	12391796

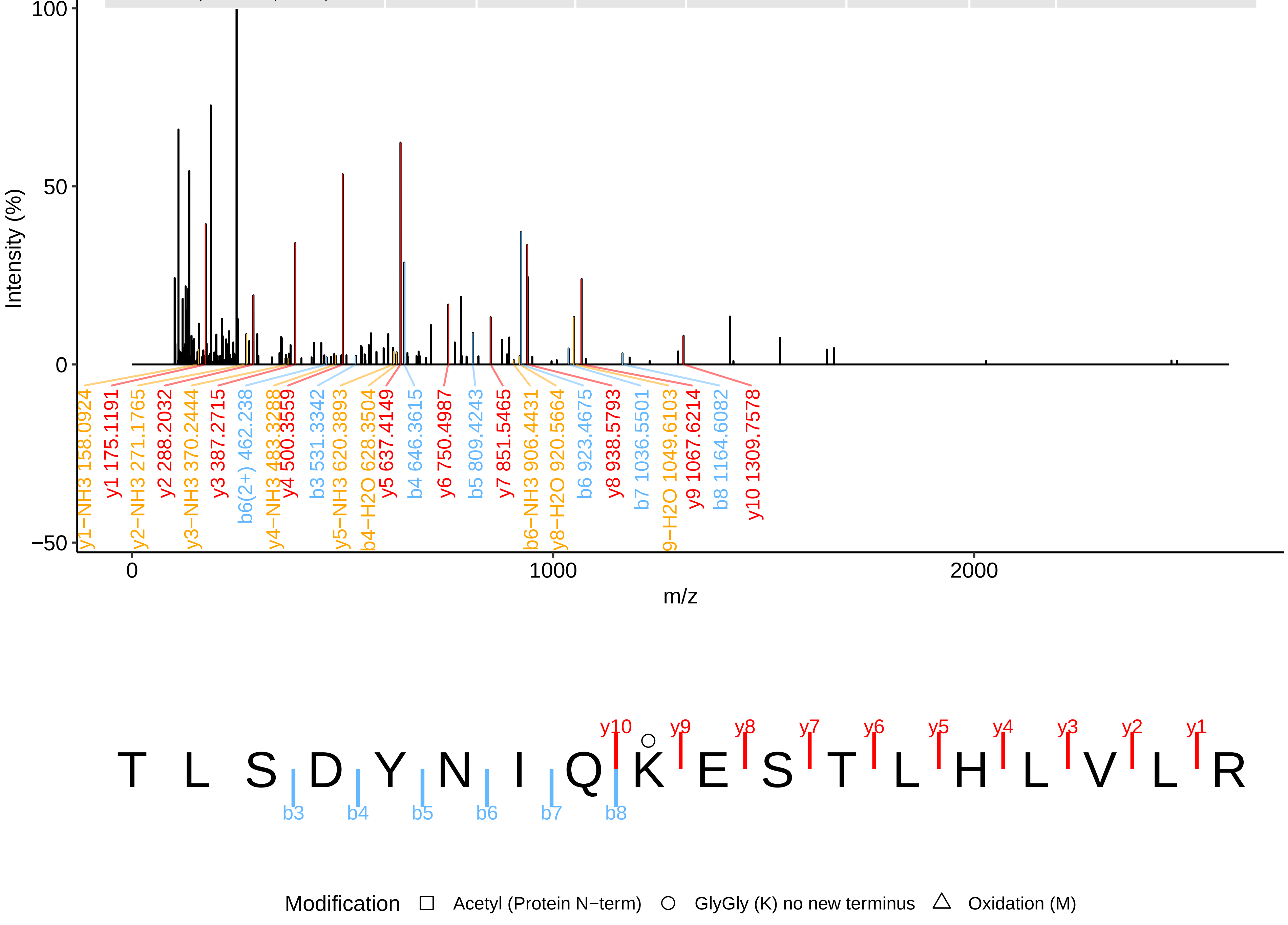

20201118_KIZ_MUb6_GlyGly_2 − Scan number: 19855

Gene Names	Charge	m/z	Mass	Retention time	PEP	Score	Precursor Intensity
RPS27A;UBA52;UBB;UBC	5	449.6455	2243.191	66.987	0.017463	57.52	14272926

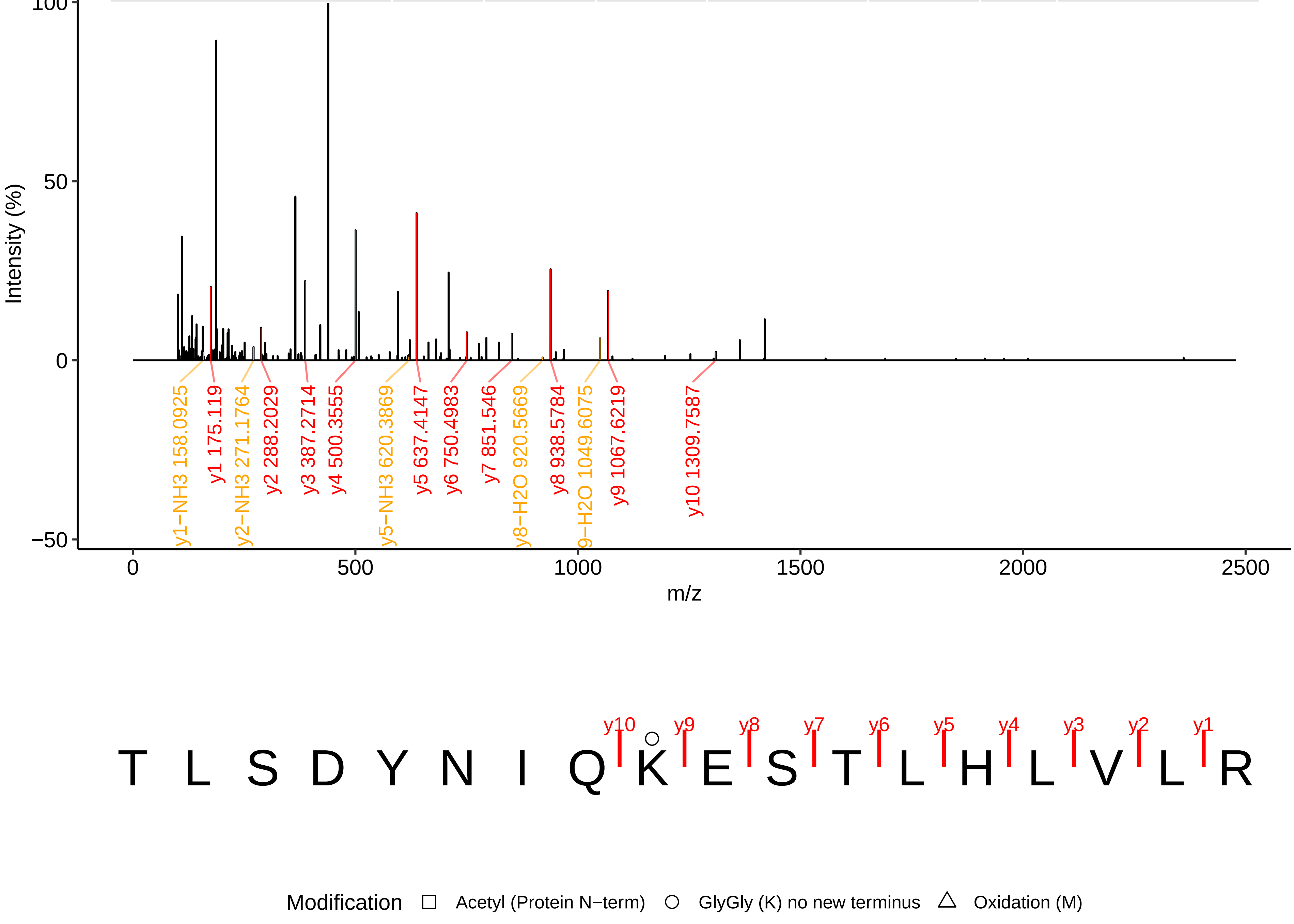

20201118_KIZ_MUb6_GlyGly_2 − Scan number: 19861

Gene Names	Charge	m/z	Mass	Retention time	PEP	Score	Precursor Intensity
RPS27A;UBA52;UBB;UBC	4	561.805	2243.191	67.007	0.0010086	72.184	11616208

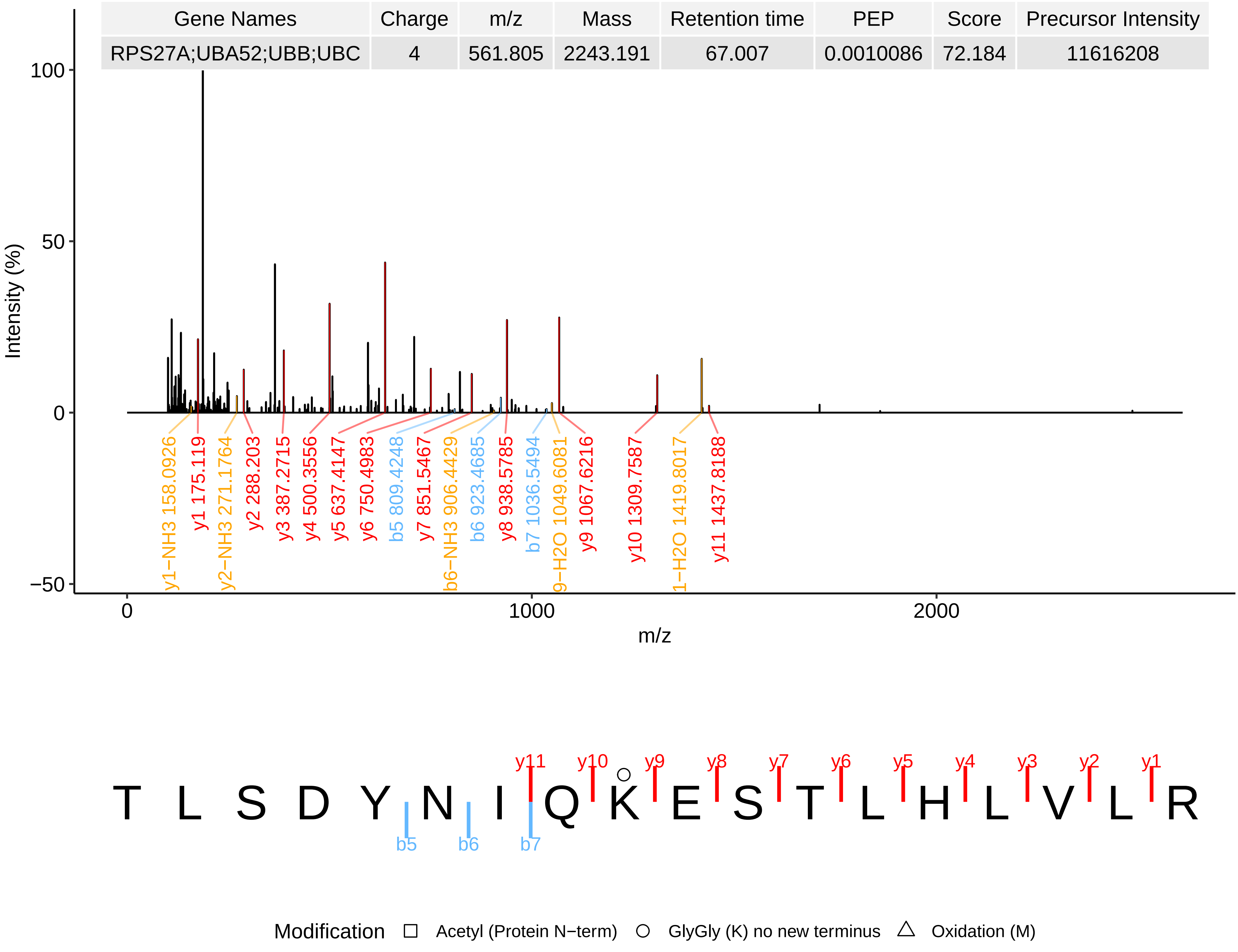

20201118_KIZ_MUb6_GlyGly_2 − Scan number: 22663

Gene Names	Charge	m/z	Mass	Retention time	PEP	Score	Precursor Intensity
RPS27A;UBA52;UBB;UBC	4	561.805	2243.191	76.168	2.6791e−17	134.85	28339850

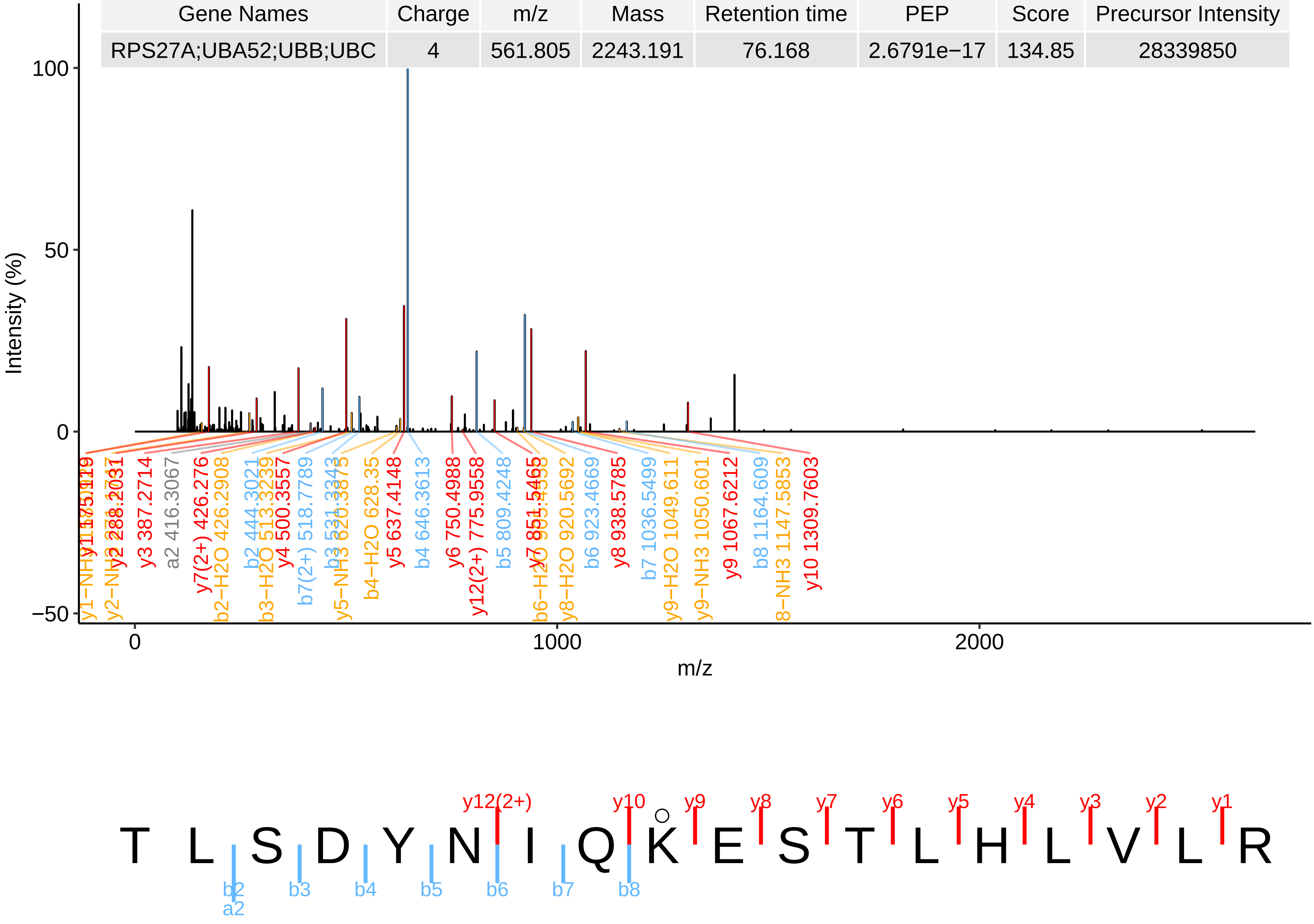

Modification □ Acetyl (Protein N−term) ○ GlyGly (K) no new terminus △ Oxidation (M)

20201118_KIZ_MUb6_GlyGly_2 − Scan number: 22815

Gene Names	Charge	m/z	Mass	Retention time	PEP	Score	Precursor Intensity
RPS27A;UBA52;UBB;UBC	4	561.805	2243.191	76.671	2.6791e−17	134.85	109525496

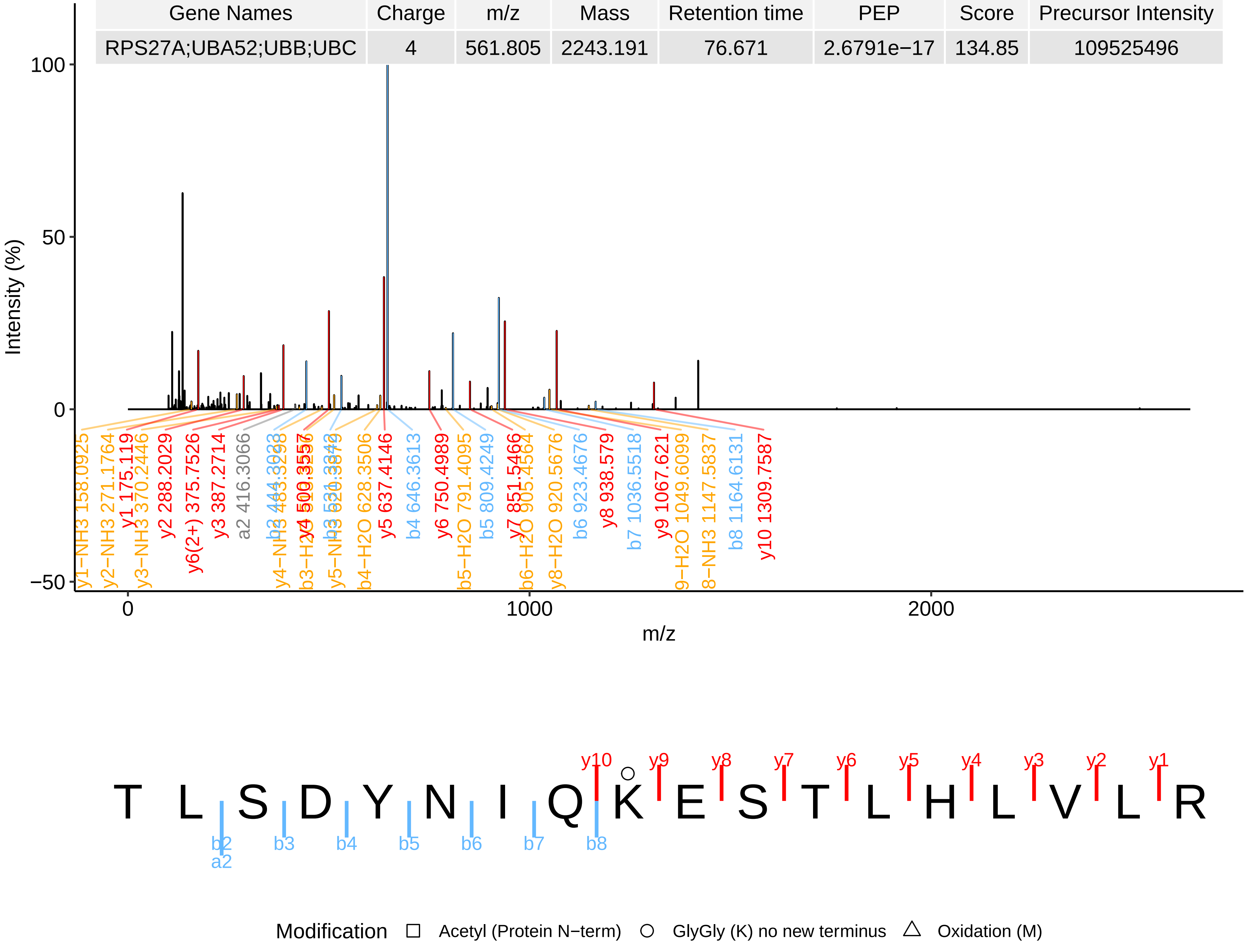

20201118_KIZ_MUb6_GlyGly_2 − Scan number: 22695

Gene Names	Charge	m/z	Mass	Retention time	PEP	Score	Precursor Intensity
RPS27A;UBA52;UBB;UBC	3	748.7376	2243.191	76.266	1.4814e−30	168.47	16912096

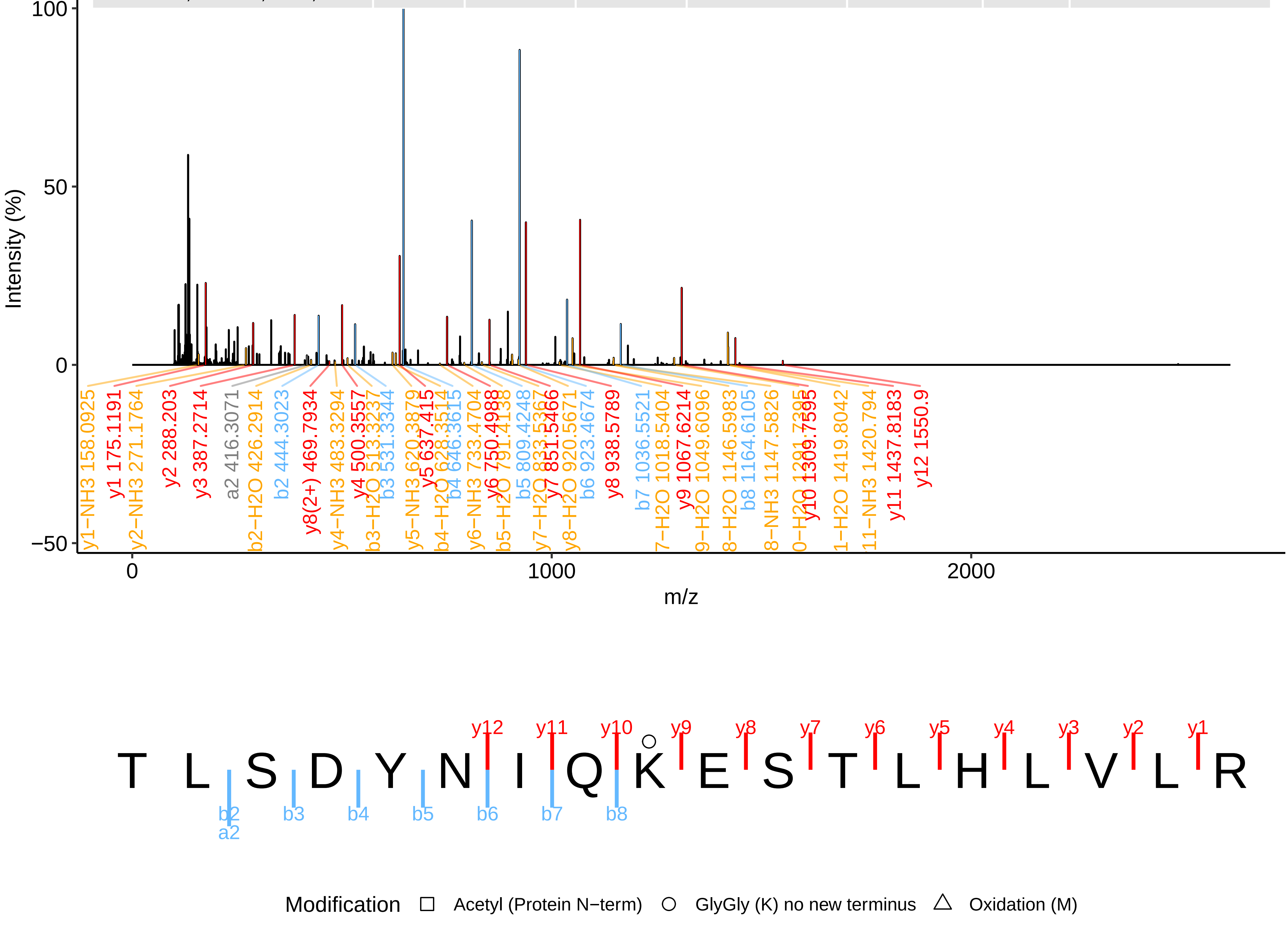

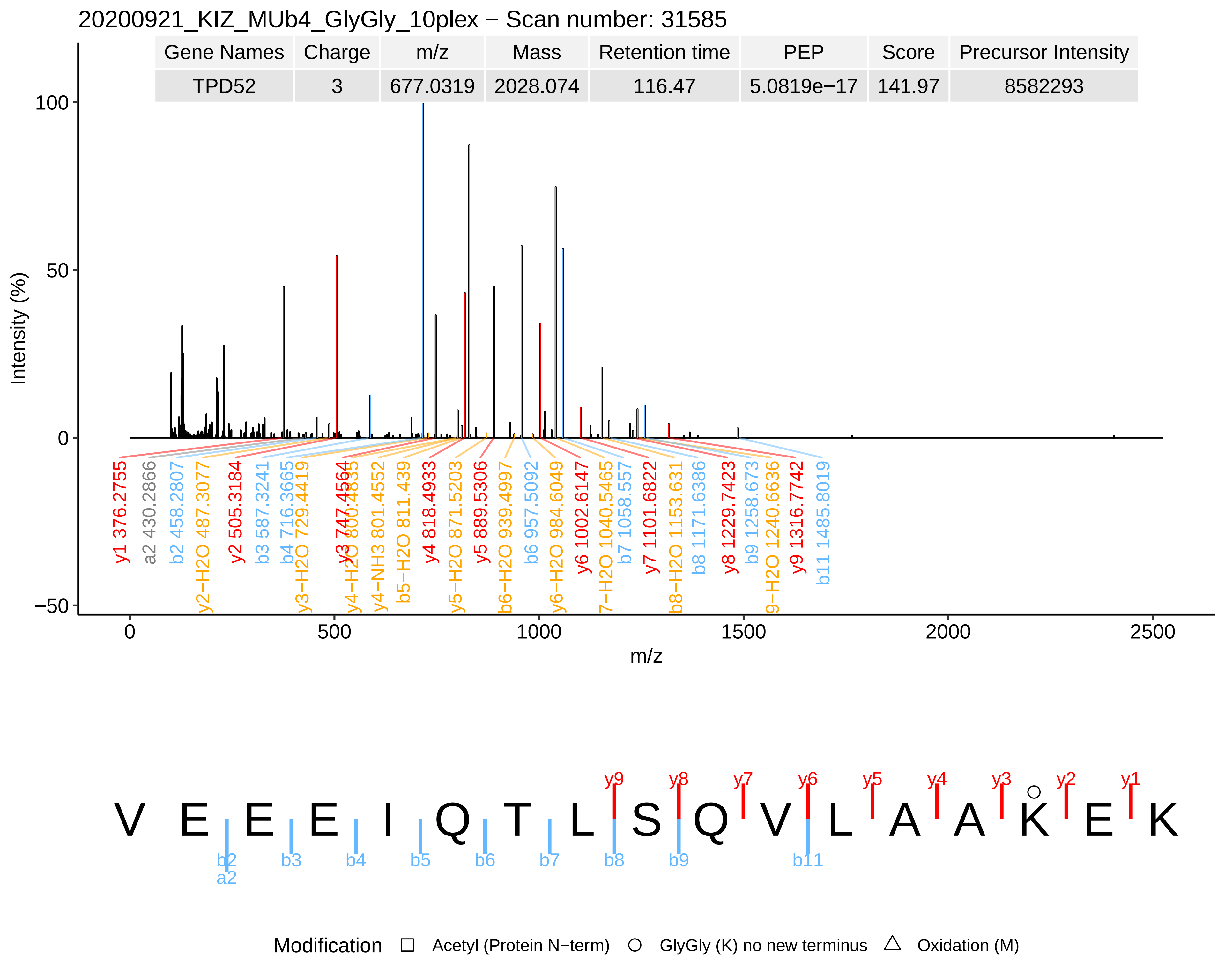
20200921_KIZ_MUb4_GlyGly_10plex − Scan number: 31585
Gene Names | Charge | m/z | Mass | Retention time | PEP | Score | Precursor Intensity
TPD52 | 3 | 677.0319 | 2028.074 | 116.47 | 5.0819e−17 | 141.97 | 8582293
Intensity (%)
100
50
0
−50
m/z
0
500
1000
1500
2000
2500
y1 376.2755
a2 430.2866
b2 458.2807
y2−H2O 487.3077
y2 505.3184
b3 587.3241
b4 716.3665
y3−H2O 729.4419
y3 747.4564
y4−H2O 800.4835
y4−NH3 801.4552
b5−H2O 811.439
y4 818.4933
y5−H2O 871.5203
y5 889.5306
b6−H2O 939.4997
b6 957.5092
y6−H2O 984.6049
y6 1002.6147
7−H2O 1040.5465
b7 1058.557
y7 1101.6822
b8−H2O 1153.631
b8 1171.6386
y8 1229.7423
b9 1258.673
9−H2O 1240.6636
y9 1316.7742
b11 1485.8019
V E E E I Q T L S Q V L A A K E K
y9 y8 y7 y6 y5 y4 y3 y2 y1
b2 a2 b3 b4 b5 b6 b7 b8 b9 b11
Modification
Acetyl (Protein N−term)
GlyGly (K) no new terminus
Oxidation (M)

20200921_KIZ_MUb4_GlyGly_10plex − Scan number: 31598

Gene Names	Charge	m/z	Mass	Retention time	PEP	Score	Precursor Intensity
TPD52	3	677.0319	2028.074	116.52	1.0421e−16	133.28	12126455

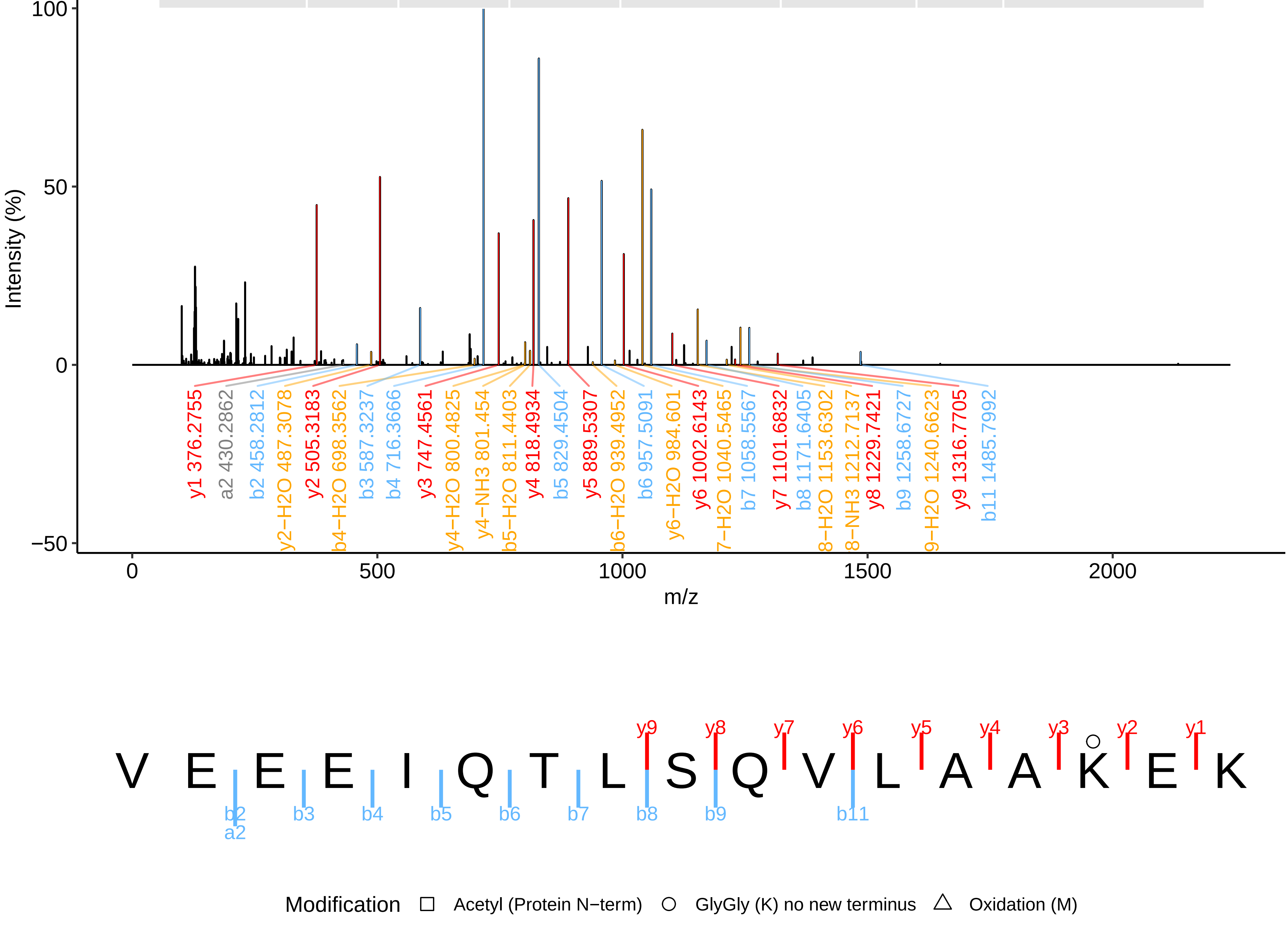

20200921_KIZ_MUb4_GlyGly_10plex_2 − Scan number: 28274

Gene Names	Charge	m/z	Mass	Retention time	PEP	Score	Precursor Intensity
TPD52	3	677.0319	2028.074	112.17	5.9525e−06	81.428	509393.8

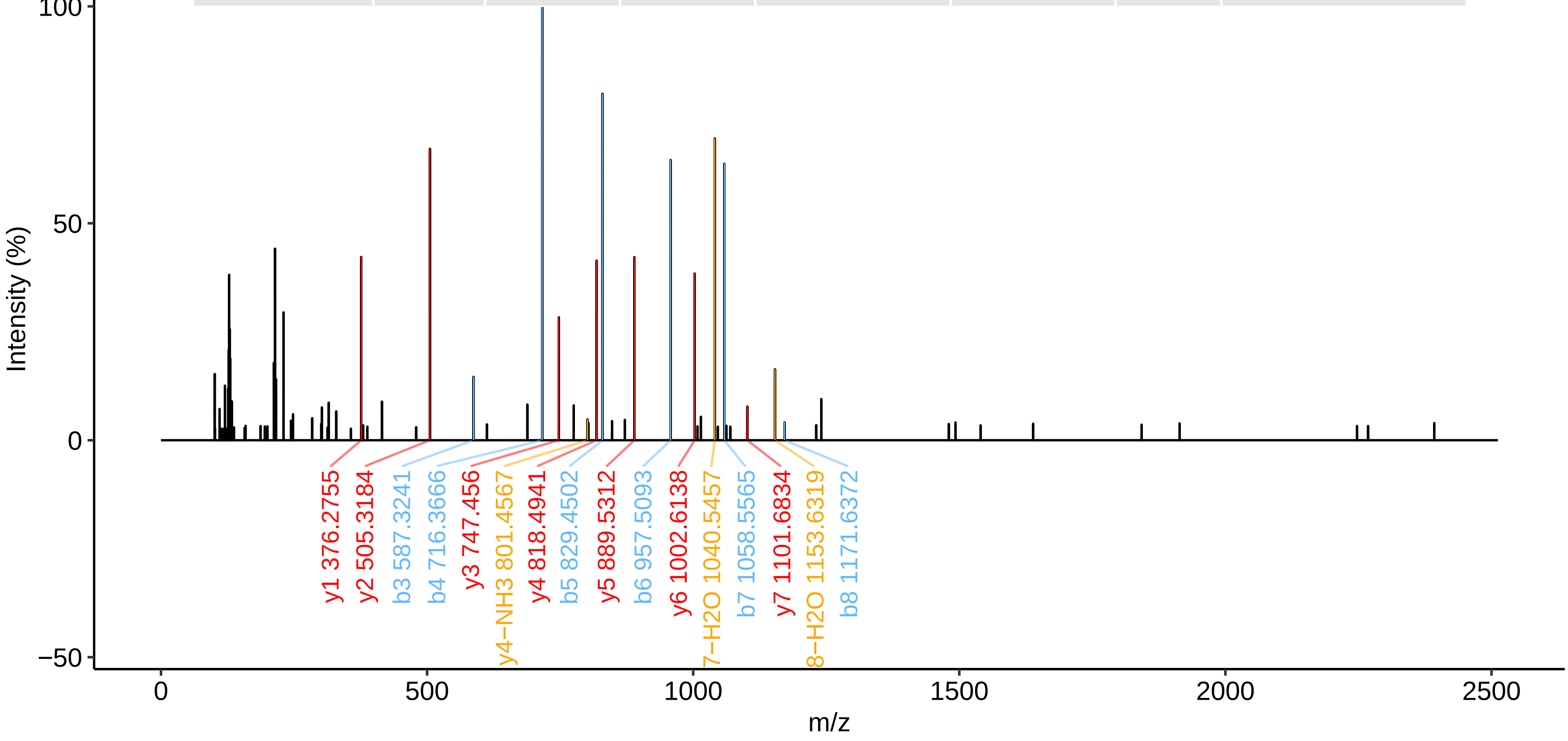

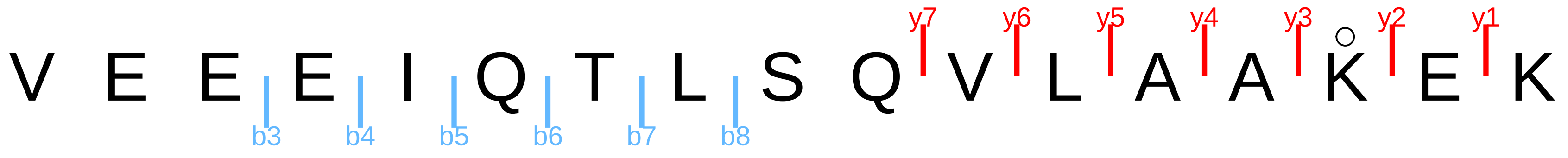

Modification □ Acetyl (Protein N−term) ○ GlyGly (K) no new terminus △ Oxidation (M)

20200921_KIZ_MUb4_GlyGly_10plex_2 − Scan number: 28290

Gene Names	Charge	m/z	Mass	Retention time	PEP	Score	Precursor Intensity
TPD52	4	508.0258	2028.074	112.23	0.00178	66.335	561467.8

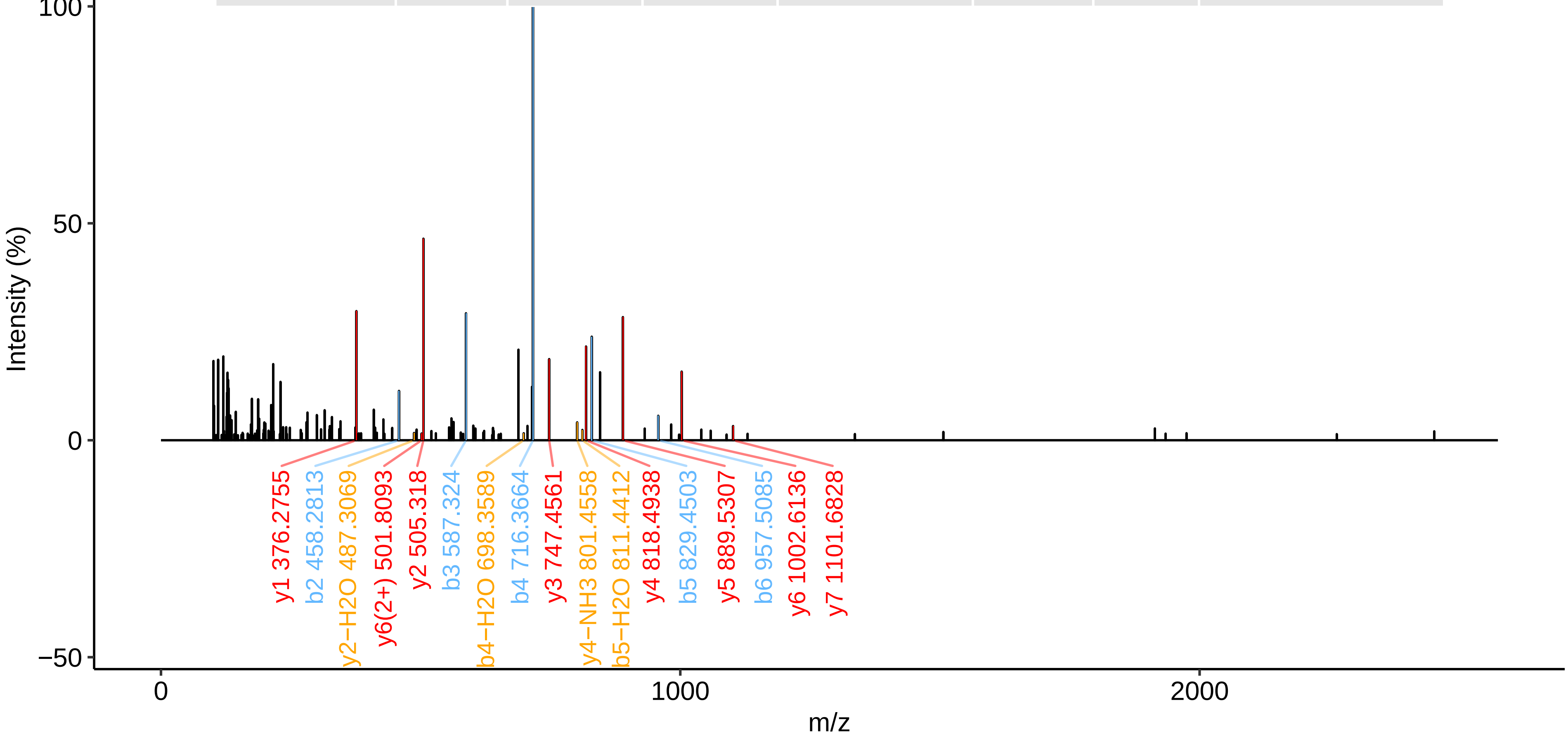

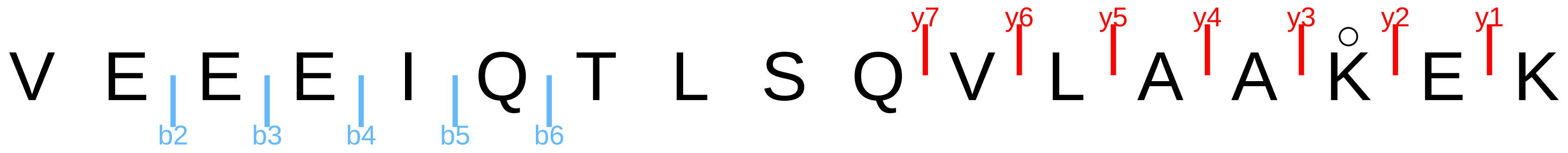

Modification □ Acetyl (Protein N−term) ○ GlyGly (K) no new terminus △ Oxidation (M)

20200924_KIZ_MUb5_GlyGly_1 − Scan number: 32037

Gene Names	Charge	m/z	Mass	Retention time	PEP	Score	Precursor Intensity
TPD52	3	677.0319	2028.074	102.19	1.5833e−22	150.28	9835481

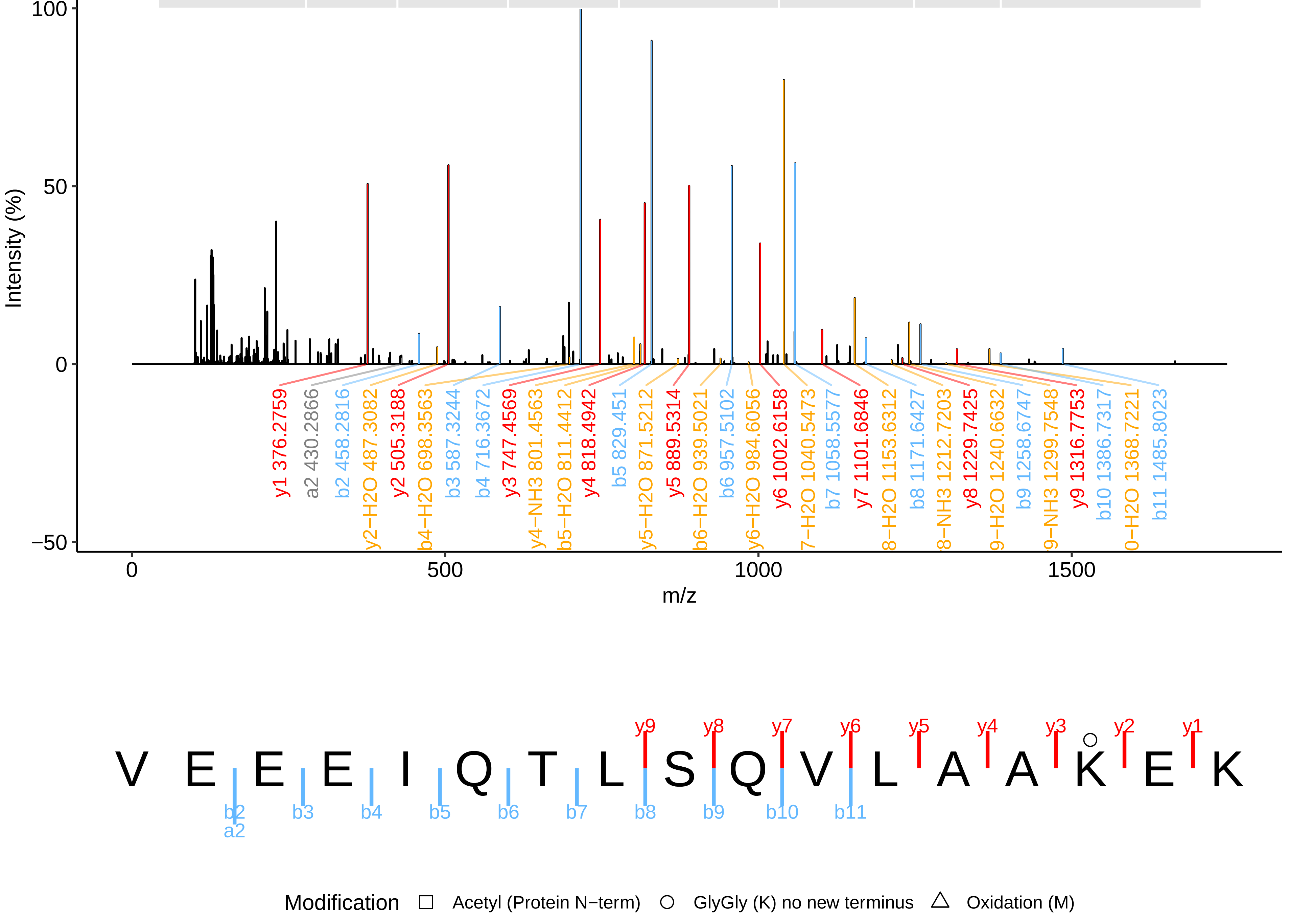

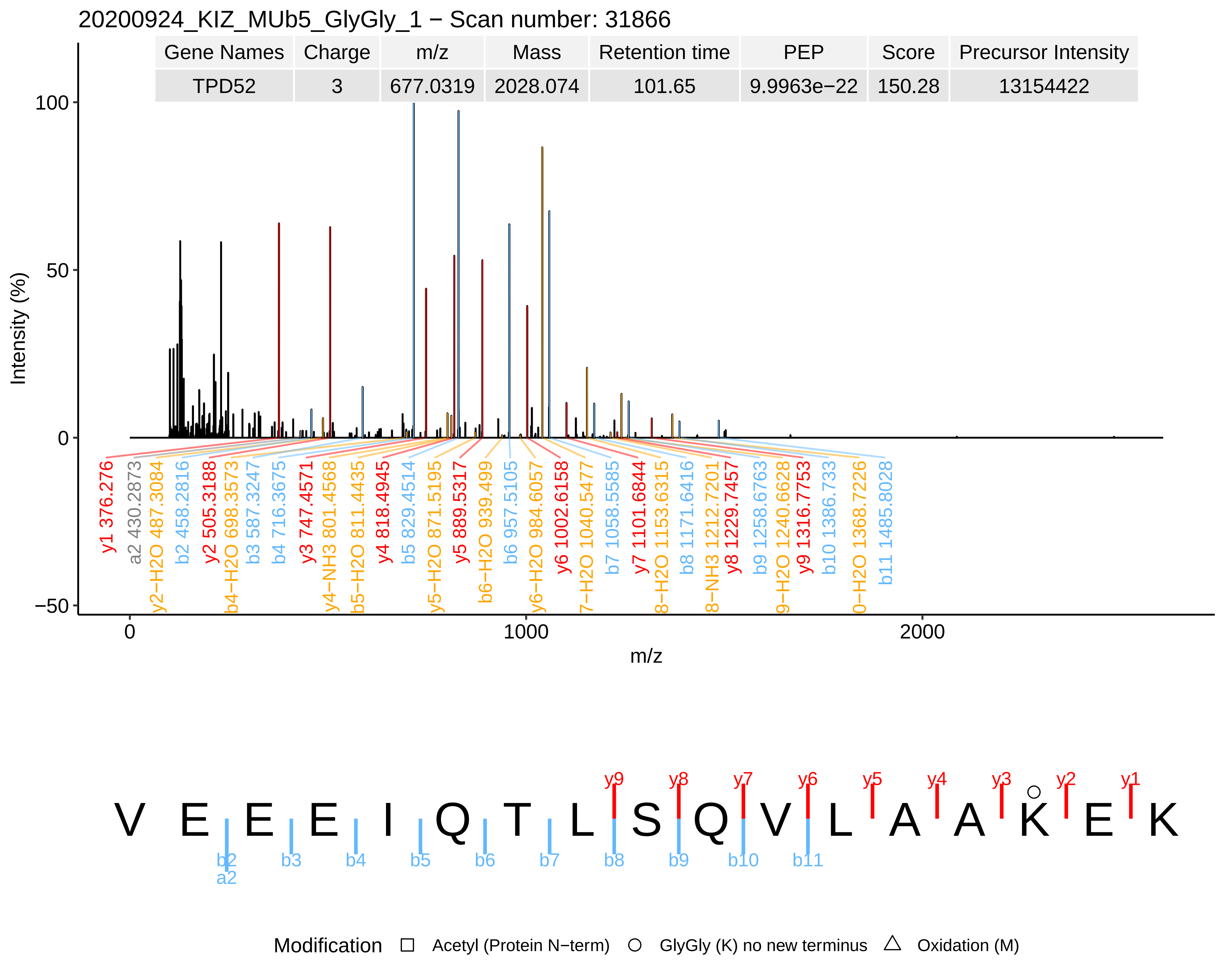
20200924_KIZ_MUb5_GlyGly_1 − Scan number: 31866
Gene Names | Charge | m/z | Mass | Retention time | PEP | Score | Precursor Intensity
TPD52 | 3 | 677.0319 | 2028.074 | 101.65 | 9.9963e−22 | 150.28 | 13154422
Intensity (%)
100
50
0
−50
0
1000
2000
m/z
y1 376.276
a2 430.2873
y2−H2O 487.3084
b2 458.2816
y2 505.3188
b4−H2O 698.3573
b3 587.3247
b4 716.3675
y3 747.4571
y4−NH3 801.4568
b5−H2O 811.4435
y4 818.4945
b5 829.4514
y5−H2O 871.5195
y5 889.5317
b6−H2O 939.499
b6 957.5105
y6−H2O 984.6057
y6 1002.6158
7−H2O 1040.5477
b7 1058.5585
y7 1101.6844
8−H2O 1153.6315
b8 1171.6416
8−NH3 1212.7201
y8 1229.7457
b9 1258.6763
9−H2O 1240.6628
y9 1316.7753
b10 1386.733
0−H2O 1368.7226
b11 1485.8028
V E E E I Q T L S Q V L A A K E K
y9 y8 y7 y6 y5 y4 y3 y2 y1
b2 a2 b3 b4 b5 b6 b7 b8 b9 b10 b11
Modification
Acetyl (Protein N−term)
GlyGly (K) no new terminus
Oxidation (M)

20201118_KIZ_MUb6_GlyGly_2 − Scan number: 35256

Gene Names	Charge	m/z	Mass	Retention time	PEP	Score	Precursor Intensity
TPD52	3	677.0319	2028.074	114.34	1.0914e−12	119.23	11349216

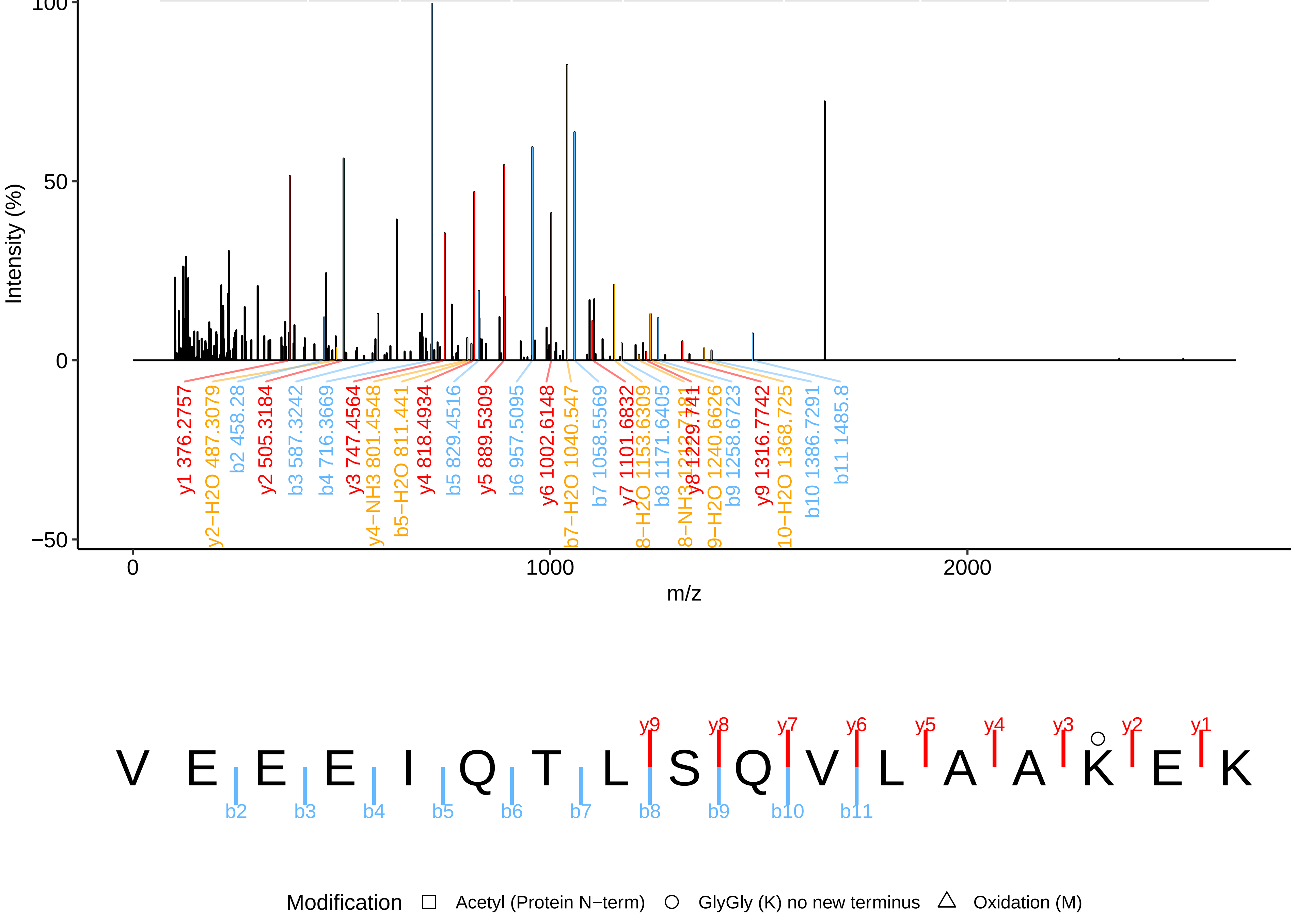

20200921_KIZ_MUb4_GlyGly_10plex − Scan number: 20106

Gene Names	Charge	m/z	Mass	Retention time	PEP	Score	Precursor Intensity
TUBA1C;TUBA1B;TUBA4A;TUBA8	3	774.757	2321.249	72.616	1.893e−21	134.52	19344584

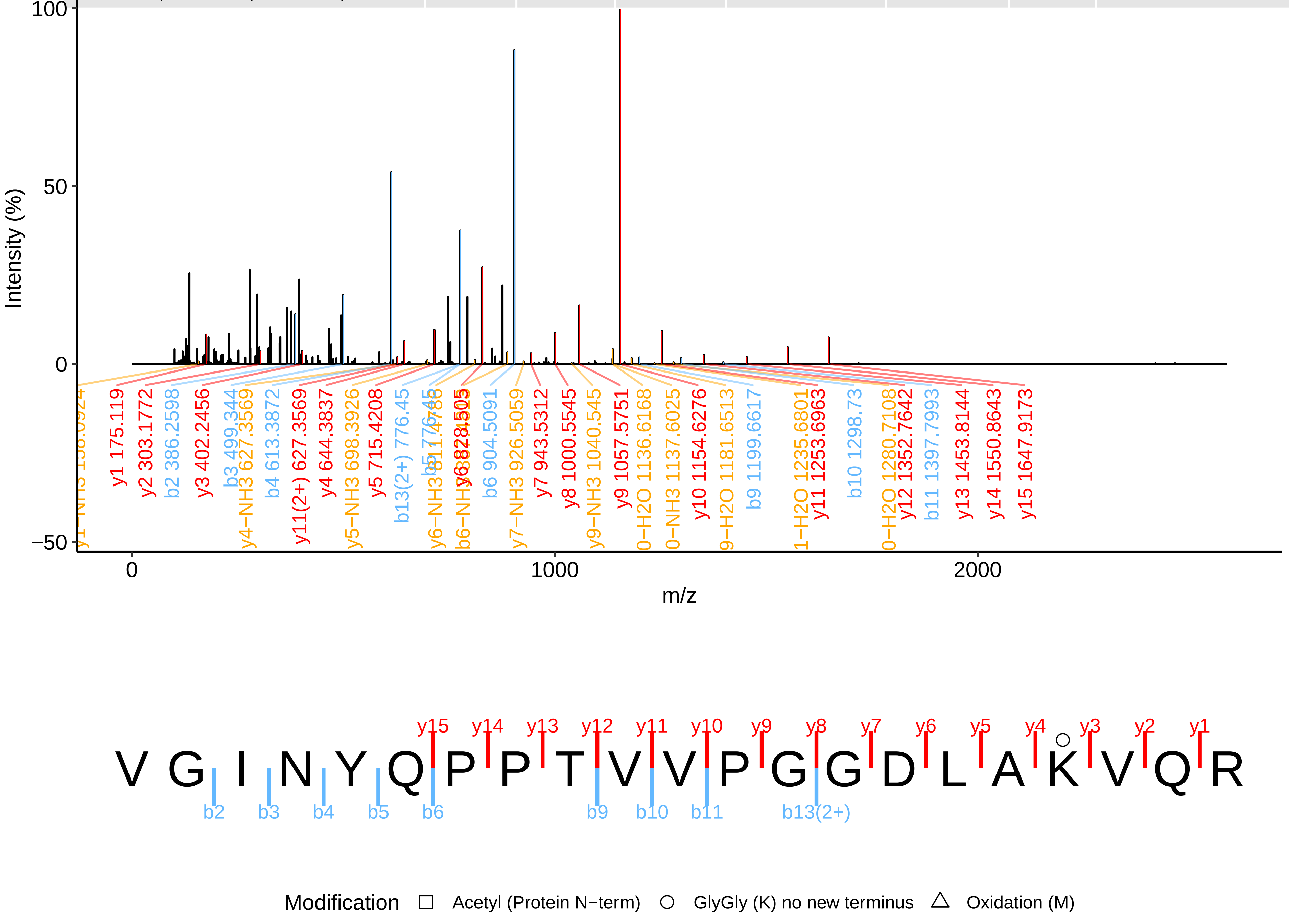

20200921_KIZ_MUb4_GlyGly_10plex − Scan number: 20238

Gene Names	Charge	m/z	Mass	Retention time	PEP	Score	Precursor Intensity
TUBA1C;TUBA1B;TUBA4A;TUBA8	3	774.757	2321.249	73.126	1.5786e−21	137.13	7576348

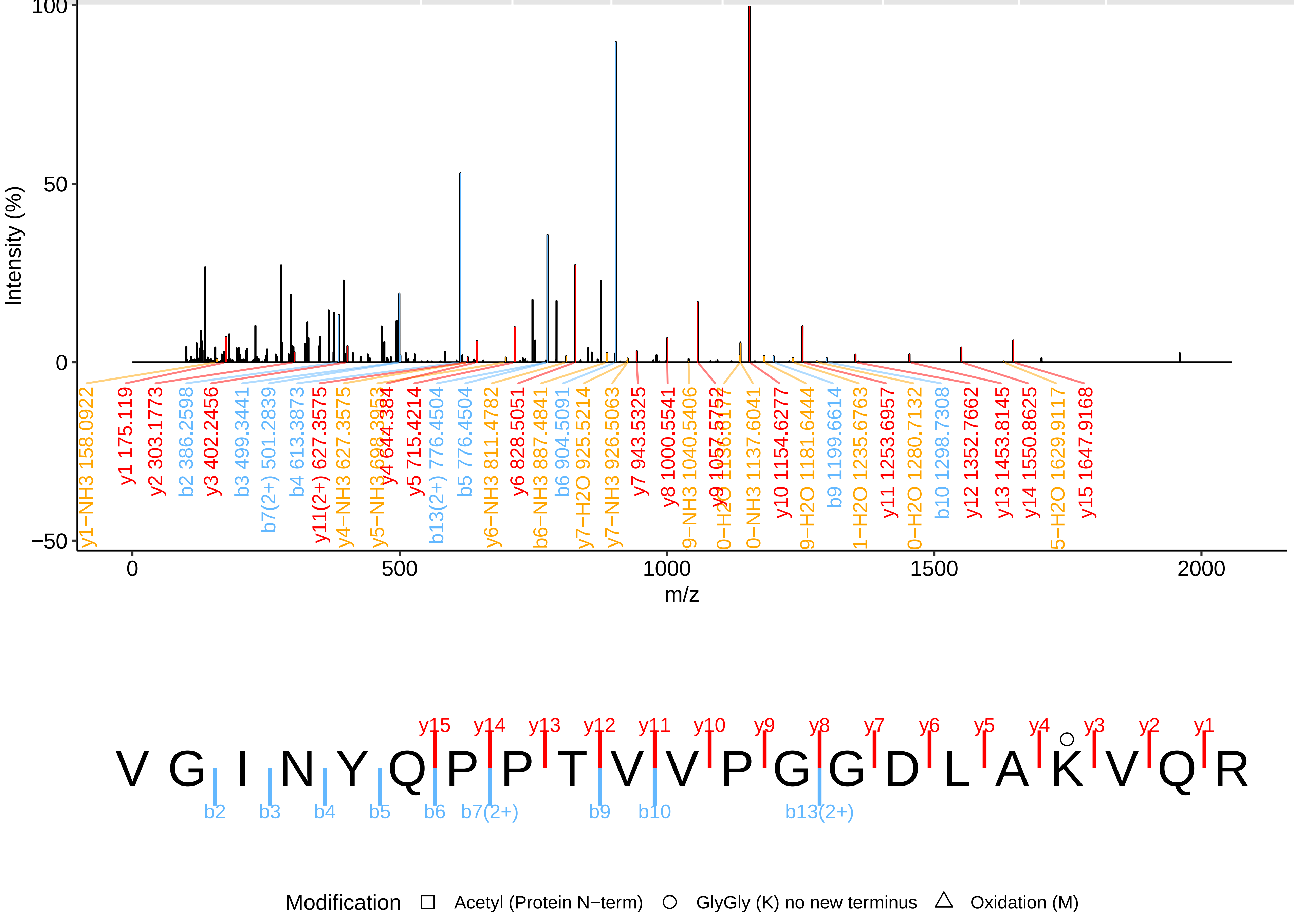

20200921_KIZ_MUb4_GlyGly_10plex_2 − Scan number: 18350

Gene Names	Charge	m/z	Mass	Retention time	PEP	Score	Precursor Intensity
TUBA1C;TUBA1B;TUBA4A;TUBA8	4	581.3196	2321.249	71.848	0.046071	44.089	1122124

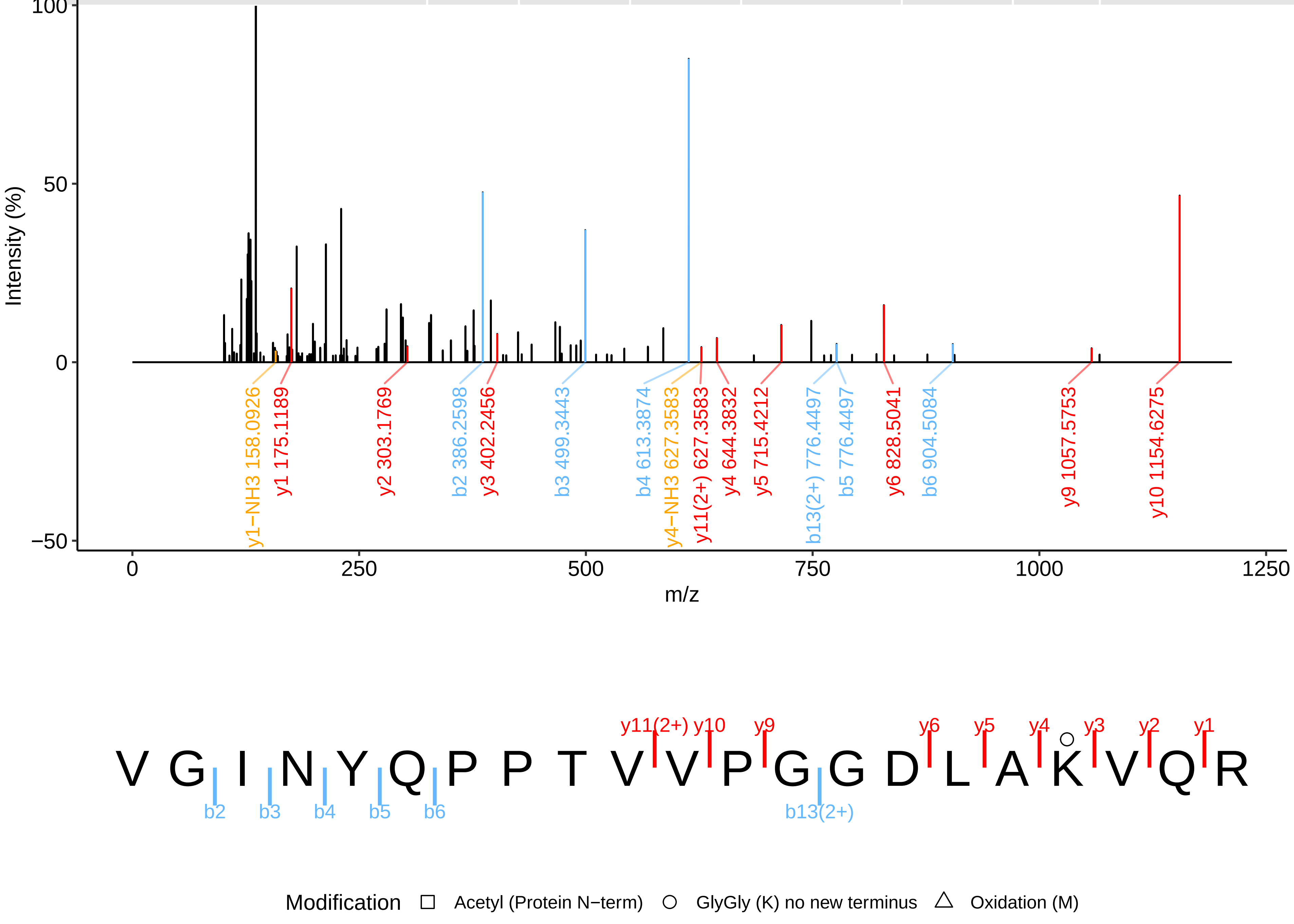

20200921_KIZ_MUb4_GlyGly_10plex_2 − Scan number: 18371

Gene Names	Charge	m/z	Mass	Retention time	PEP	Score	Precursor Intensity
TUBA1C;TUBA1B;TUBA4A;TUBA8	4	581.3196	2321.249	71.931	2.3553e−06	93.684	1086069

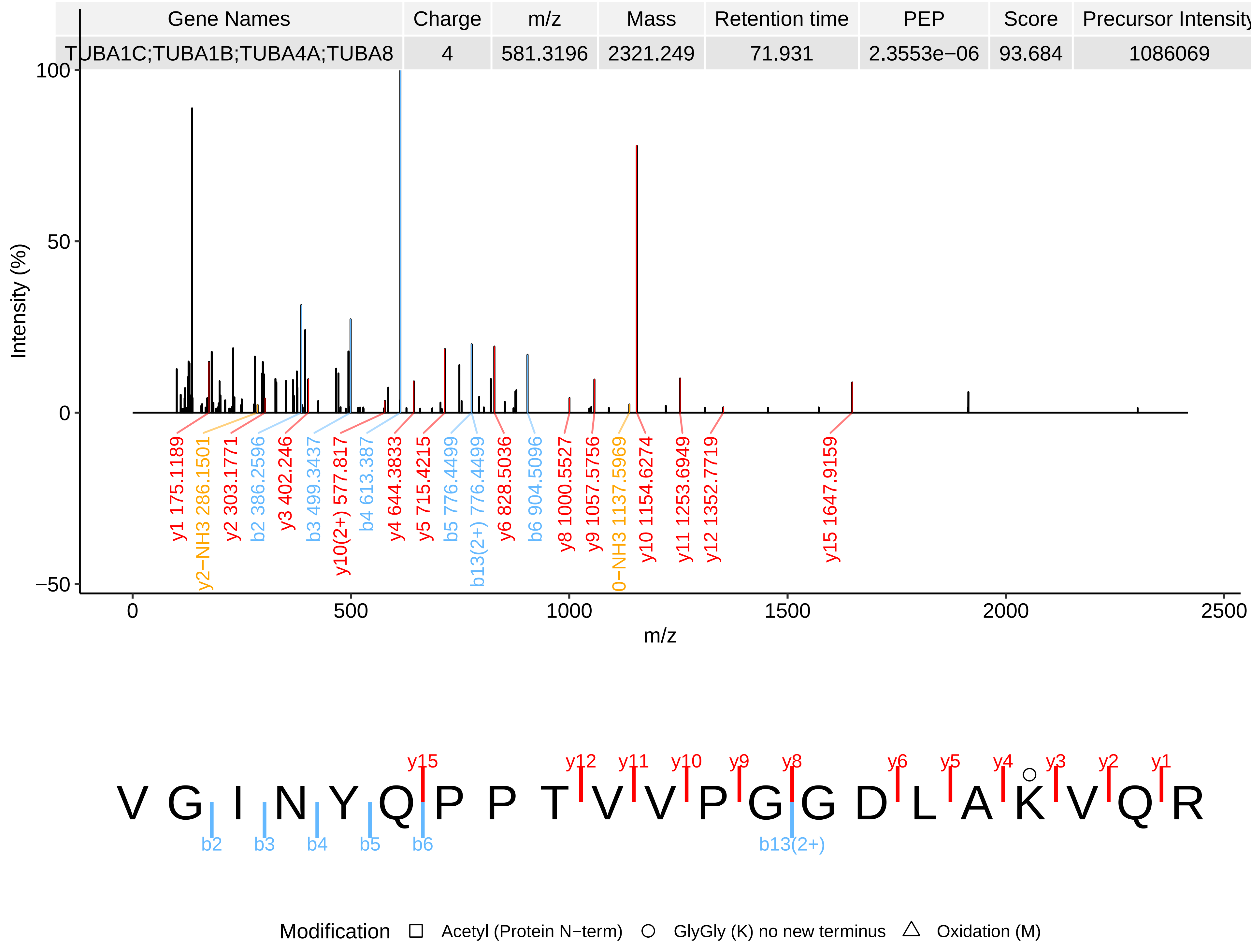

20200921_KIZ_MUb4_GlyGly_10plex_2 − Scan number: 18422

Gene Names	Charge	m/z	Mass	Retention time	PEP	Score	Precursor Intensity
TUBA1C;TUBA1B;TUBA4A;TUBA8	3	774.757	2321.249	72.136	1.0885e−16	132.74	5476124

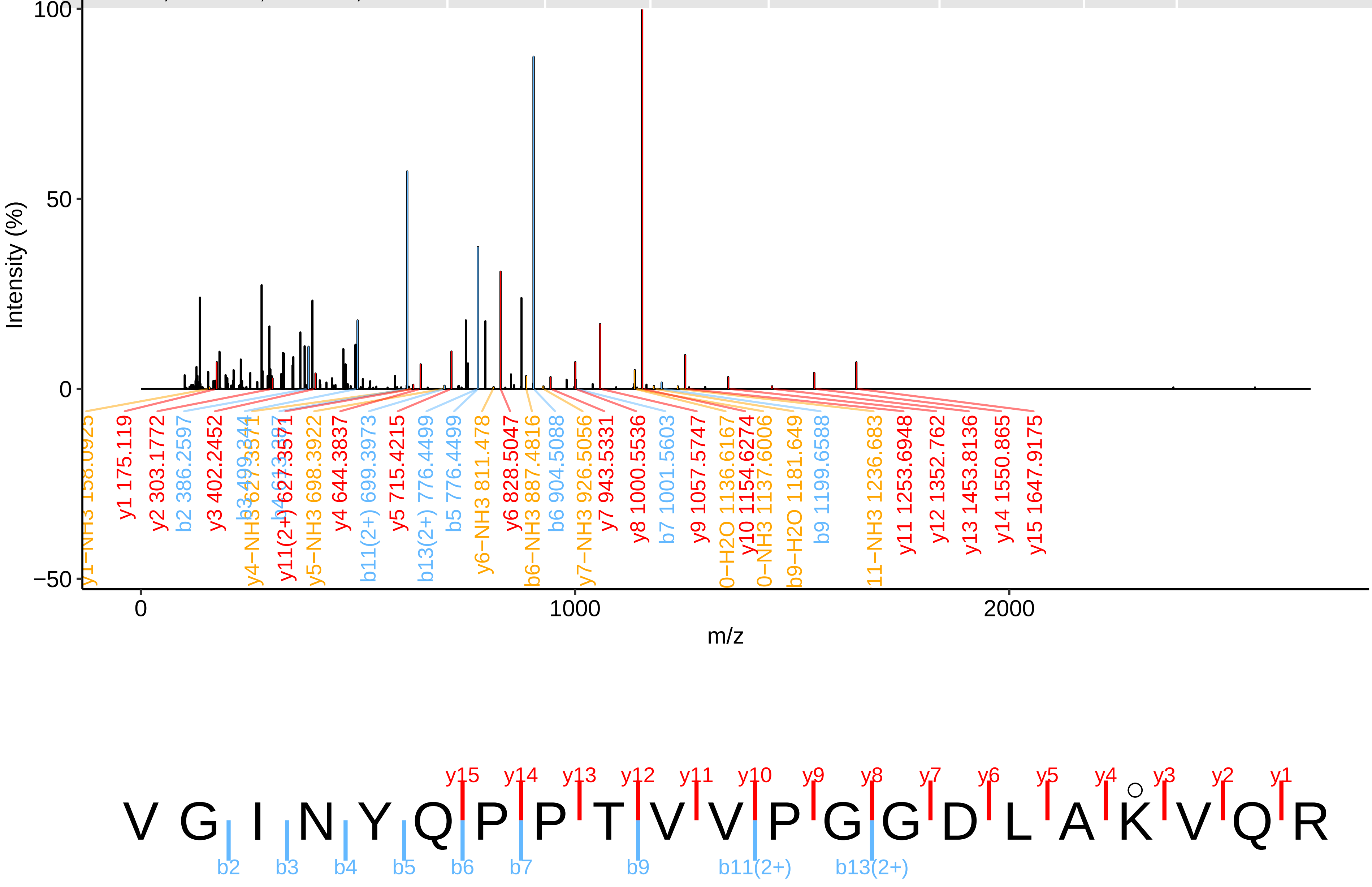

Modification □ Acetyl (Protein N−term) ○ GlyGly (K) no new terminus △ Oxidation (M)

20200924_KIZ_MUb5_GlyGly_1 − Scan number: 20411

Gene Names	Charge	m/z	Mass	Retention time	PEP	Score	Precursor Intensity
TUBA1C;TUBA1B;TUBA4A;TUBA8	3	774.757	2321.249	65.804	3.2565e−30	152.04	18848710

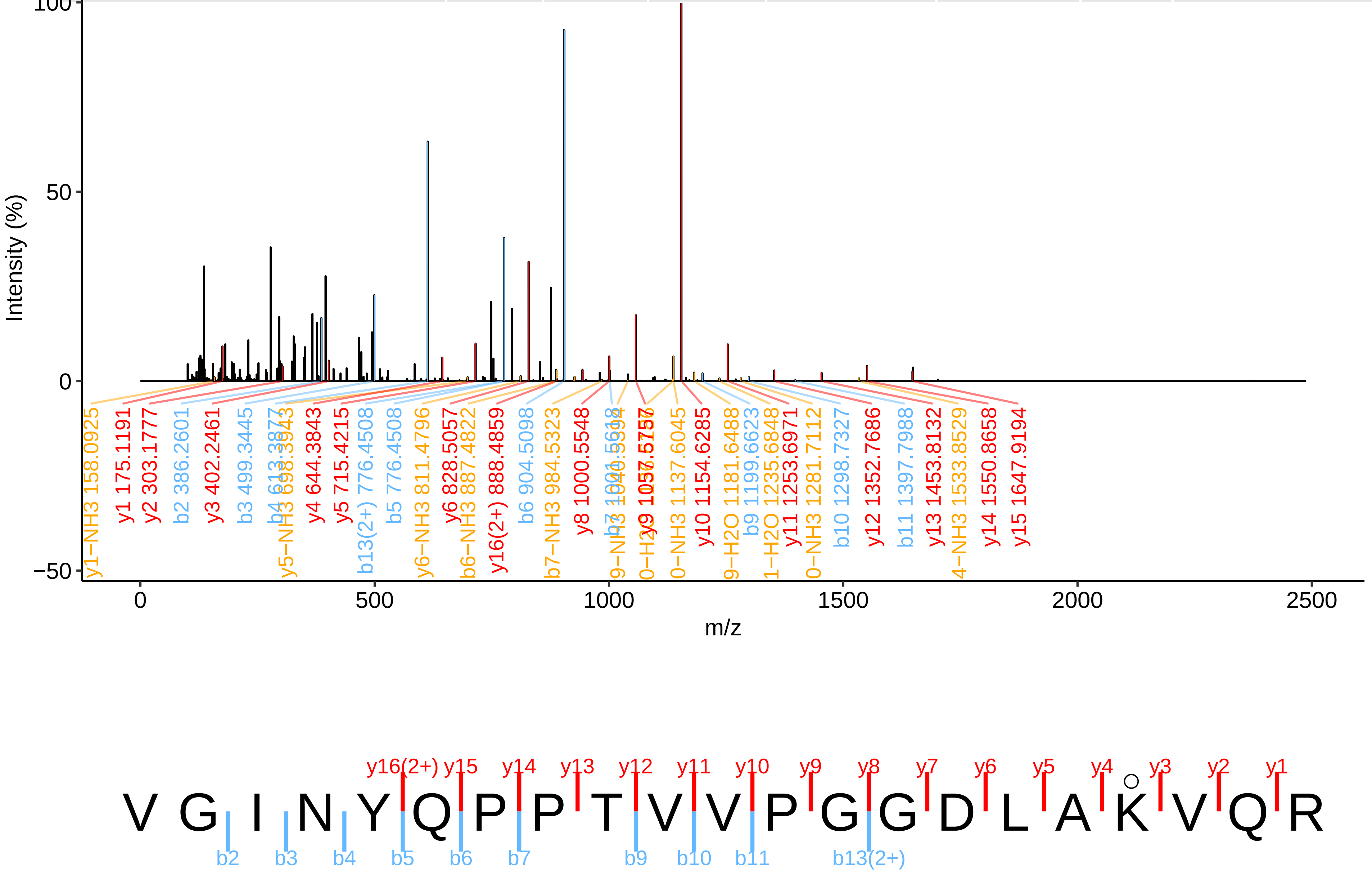

Modification □ Acetyl (Protein N−term) ○ GlyGly (K) no new terminus △ Oxidation (M)

20200924_KIZ_MUb5_GlyGly_2 − Scan number: 18373

Gene Names	Charge	m/z	Mass	Retention time	PEP	Score	Precursor Intensity
TUBA1C;TUBA1B;TUBA4A;TUBA8	3	774.757	2321.249	68.146	8.0626e−31	163.79	12334770

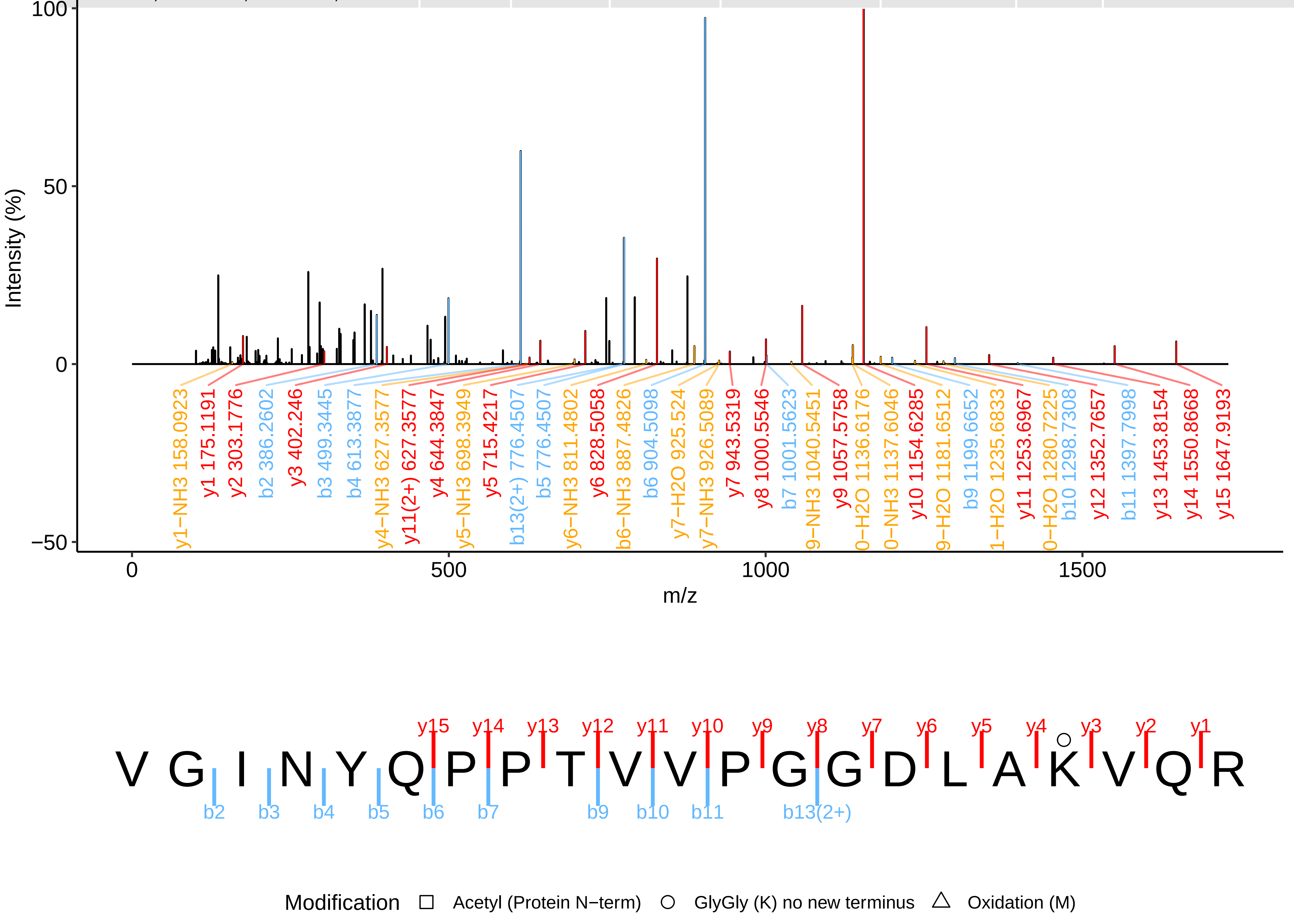

20200924_KIZ_MUb5_GlyGly_2 − Scan number: 18510

Gene Names	Charge	m/z	Mass	Retention time	PEP	Score	Precursor Intensity
TUBA1C;TUBA1B;TUBA4A;TUBA8	3	774.757	2321.249	68.66	7.1116e−22	144.33	5061315

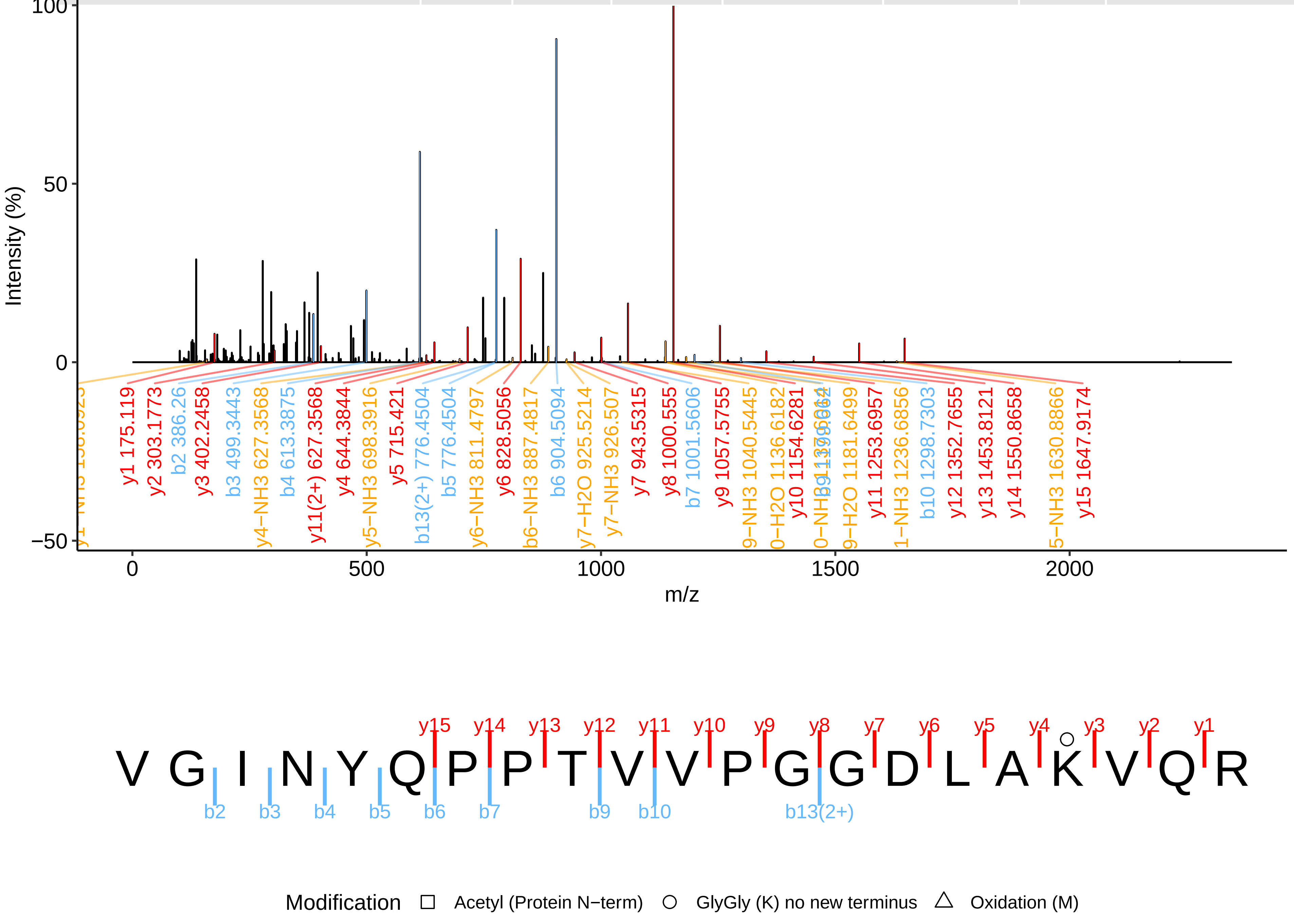

20201118_KIZ_MUb6_GlyGly_1 − Scan number: 20898

Gene Names	Charge	m/z	Mass	Retention time	PEP	Score	Precursor Intensity
TUBA1C;TUBA1B;TUBA4A;TUBA8	3	774.757	2321.249	65.311	3.7888e−10	113.49	17068324

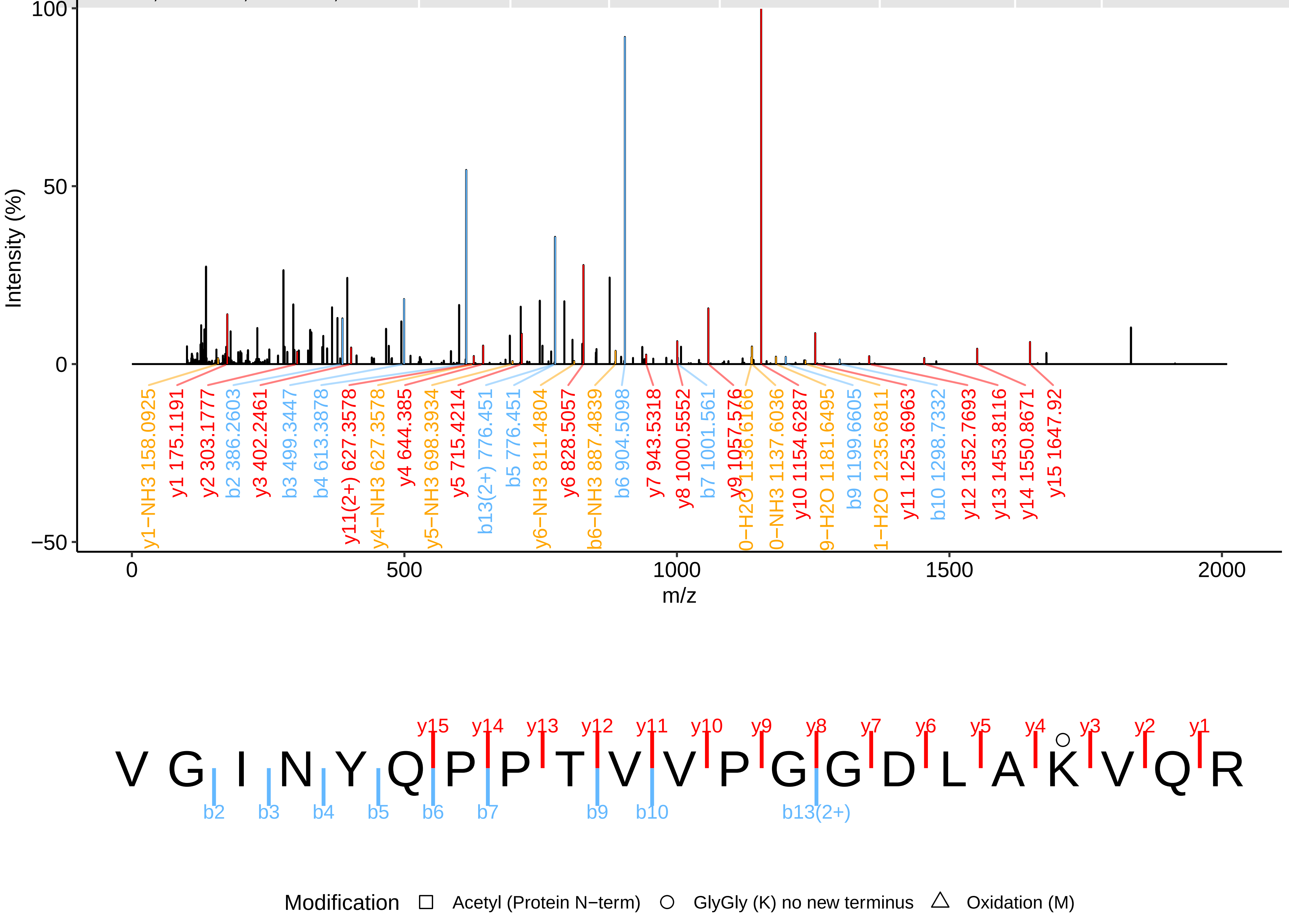

20201118_KIZ_MUb6_GlyGly_2 − Scan number: 22106

Gene Names	Charge	m/z	Mass	Retention time	PEP	Score	Precursor Intensity
TUBA1C;TUBA1B;TUBA4A;TUBA8	3	774.757	2321.249	74.314	2.3552e−22	135.43	8195336

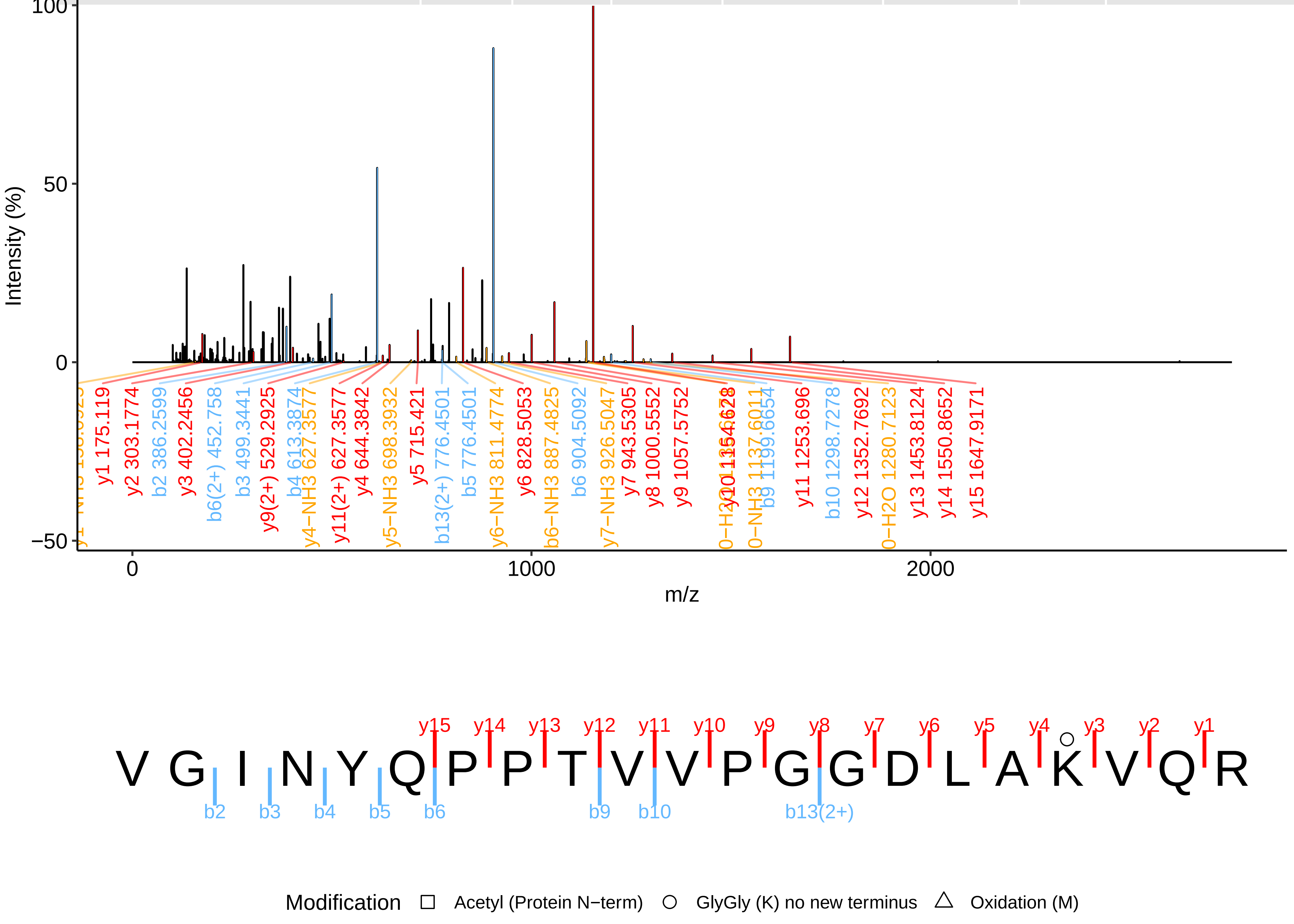

20200921_KIZ_MUb4_GlyGly_10plex_2 − Scan number: 22455

Gene Names	Charge	m/z	Mass	Retention time	PEP	Score	Precursor Intensity
SMC1A	3	764.0377	2289.091	88.524	0.015203	58.671	1717804

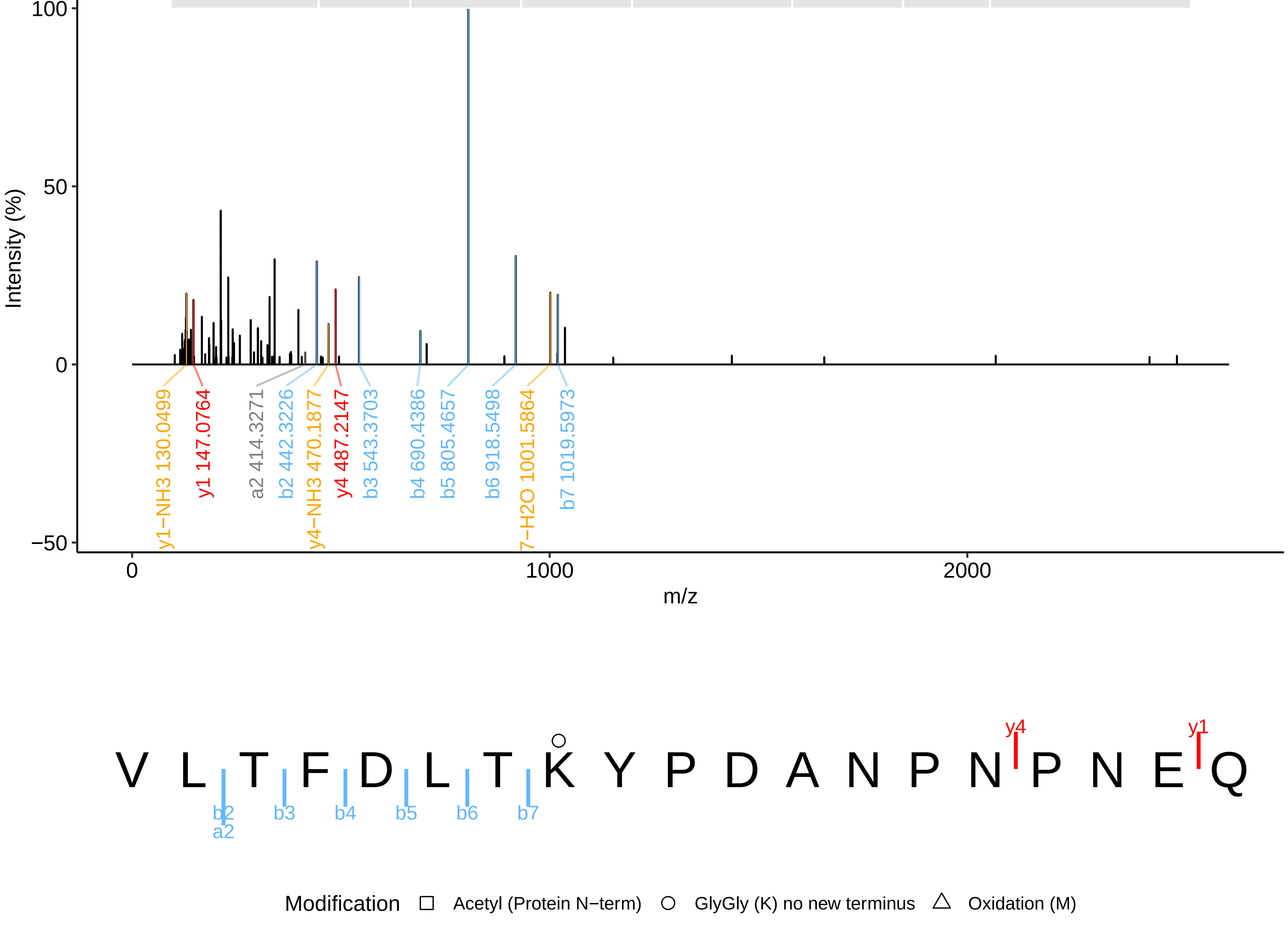

20200924_KIZ_MUb5_GlyGly_1 − Scan number: 25764

Gene Names	Charge	m/z	Mass	Retention time	PEP	Score	Precursor Intensity
SMC1A	3	764.0377	2289.091	82.577	2.8738e−06	94.882	6168653

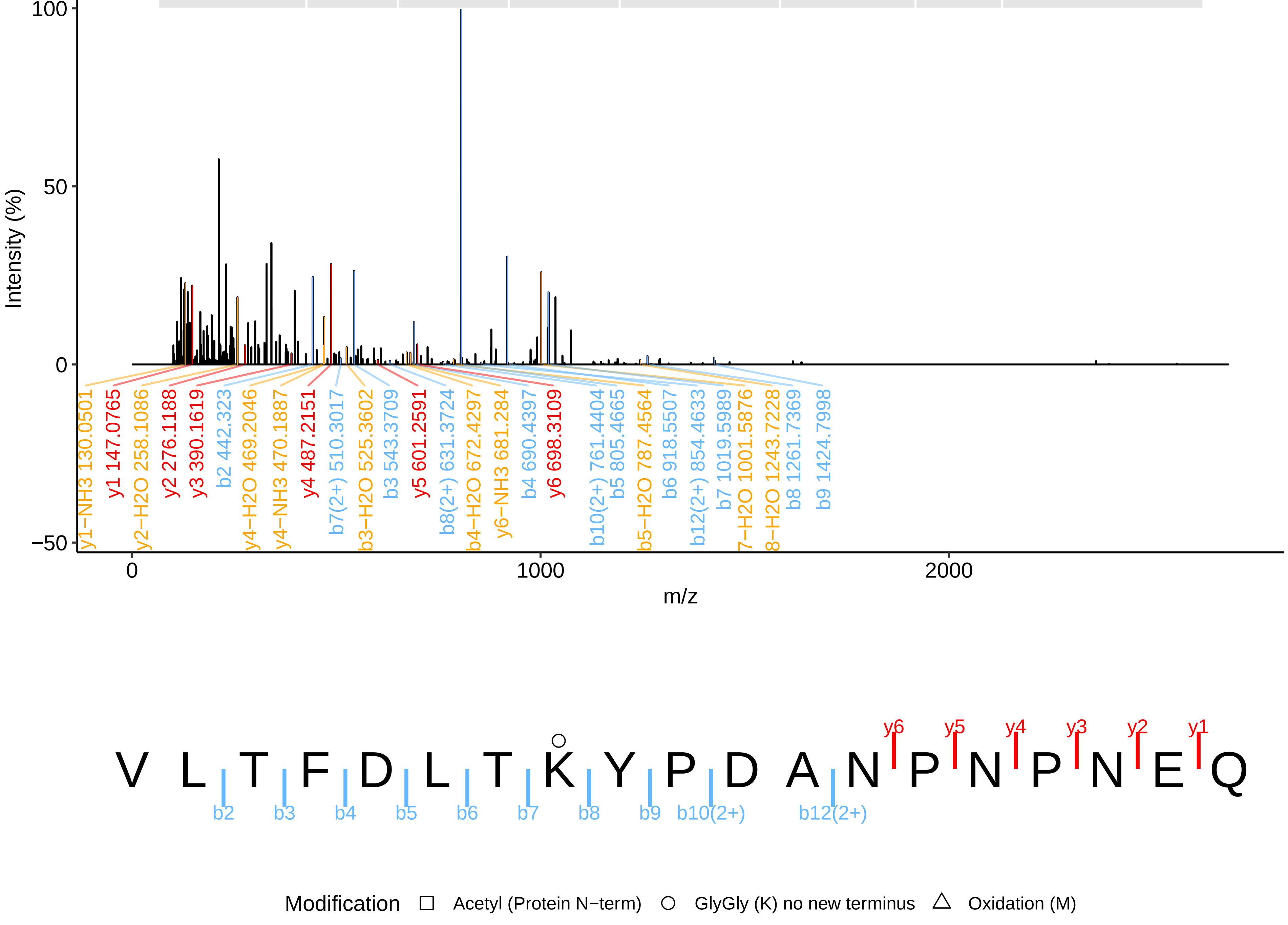

20200924_KIZ_MUb5_GlyGly_2 − Scan number: 22842

Gene Names	Charge	m/z	Mass	Retention time	PEP	Score	Precursor Intensity
SMC1A	3	764.0377	2289.091	84.607	0.0019672	65.949	4642444

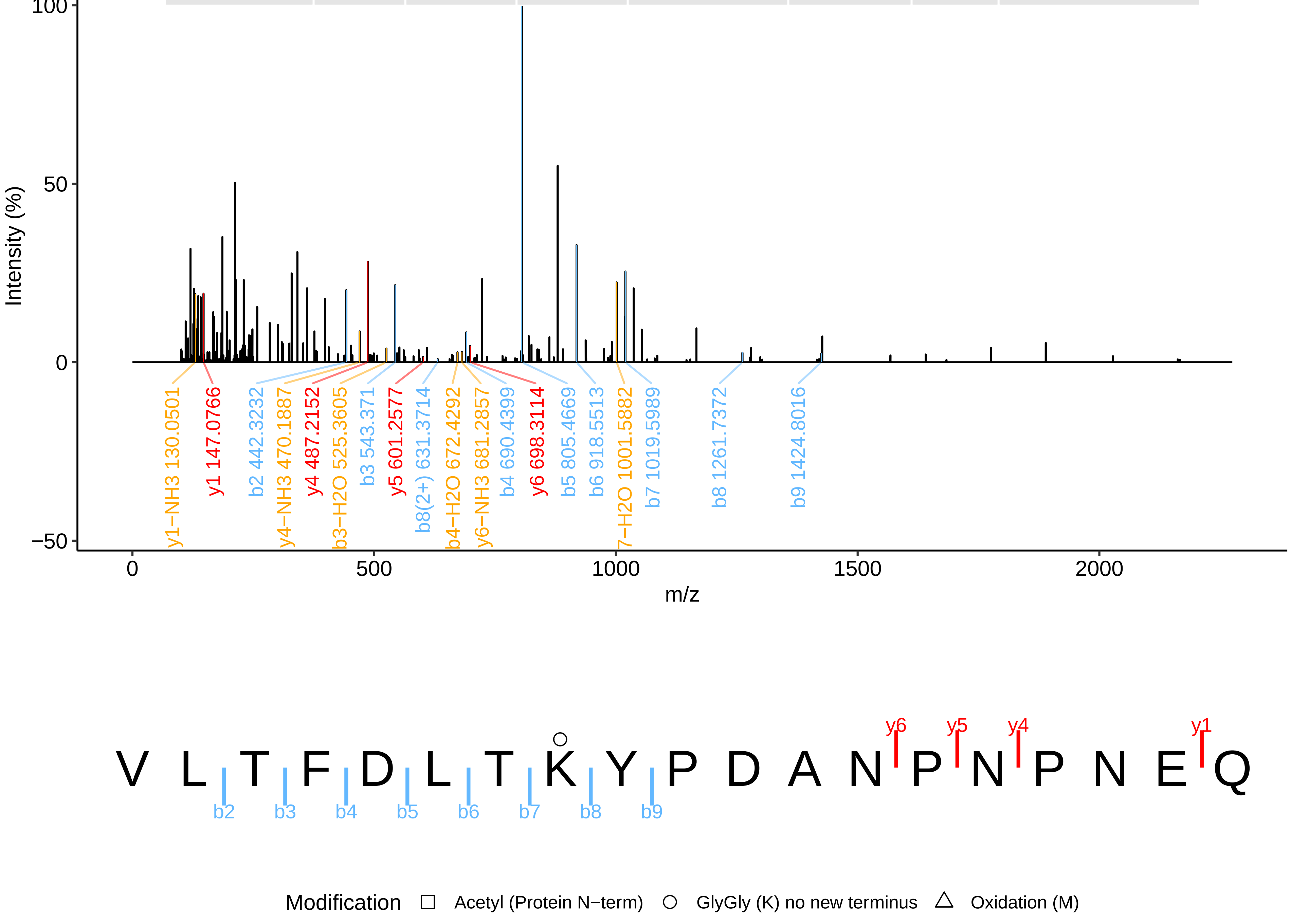

20200921_KIZ_MUb4_GlyGly_10plex_2 − Scan number: 15653

Gene Names	Charge	m/z	Mass	Retention time	PEP	Score	Precursor Intensity
CPT1A	4	504.0082	2012.004	61.261	0.0033032	64.675	983461.9

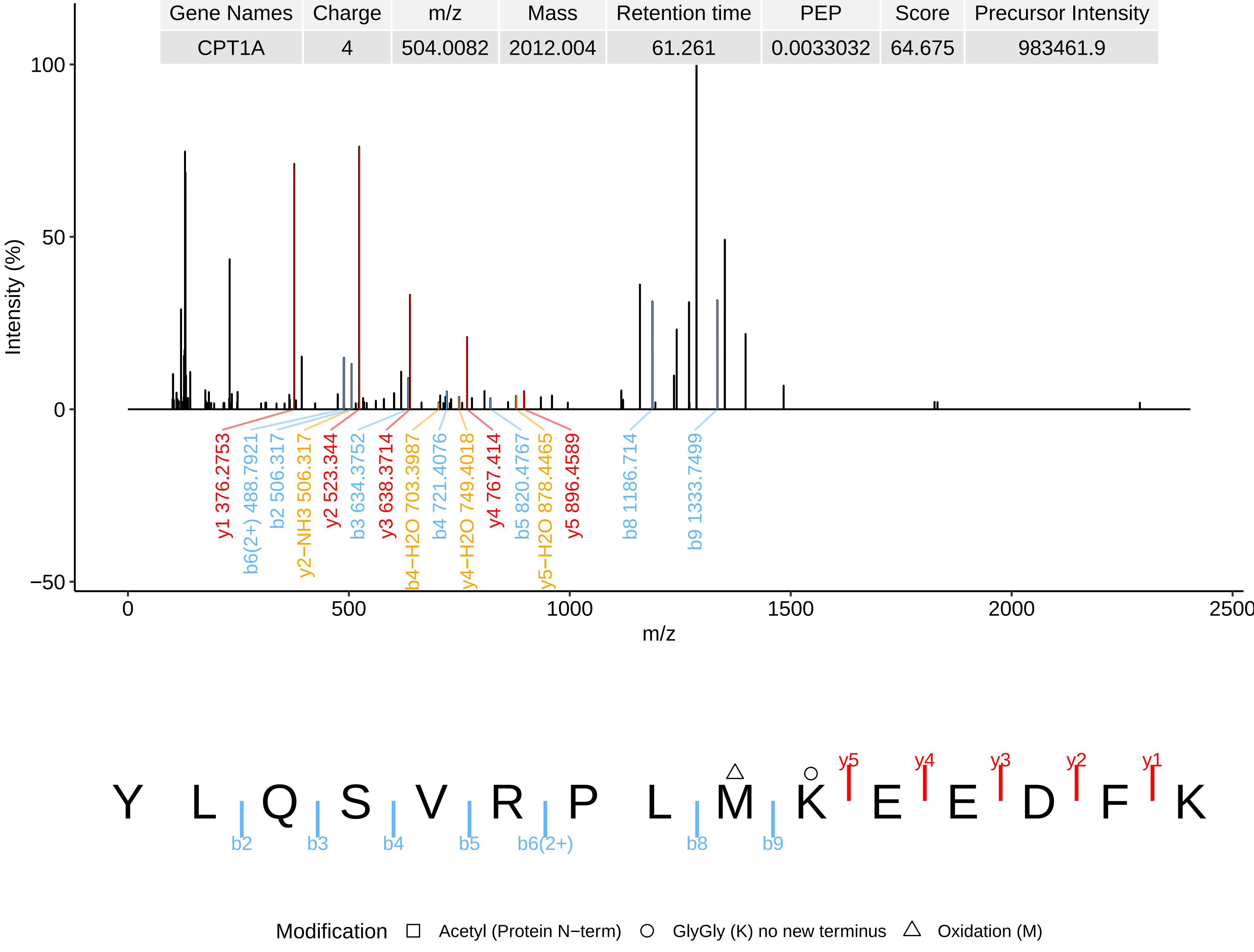

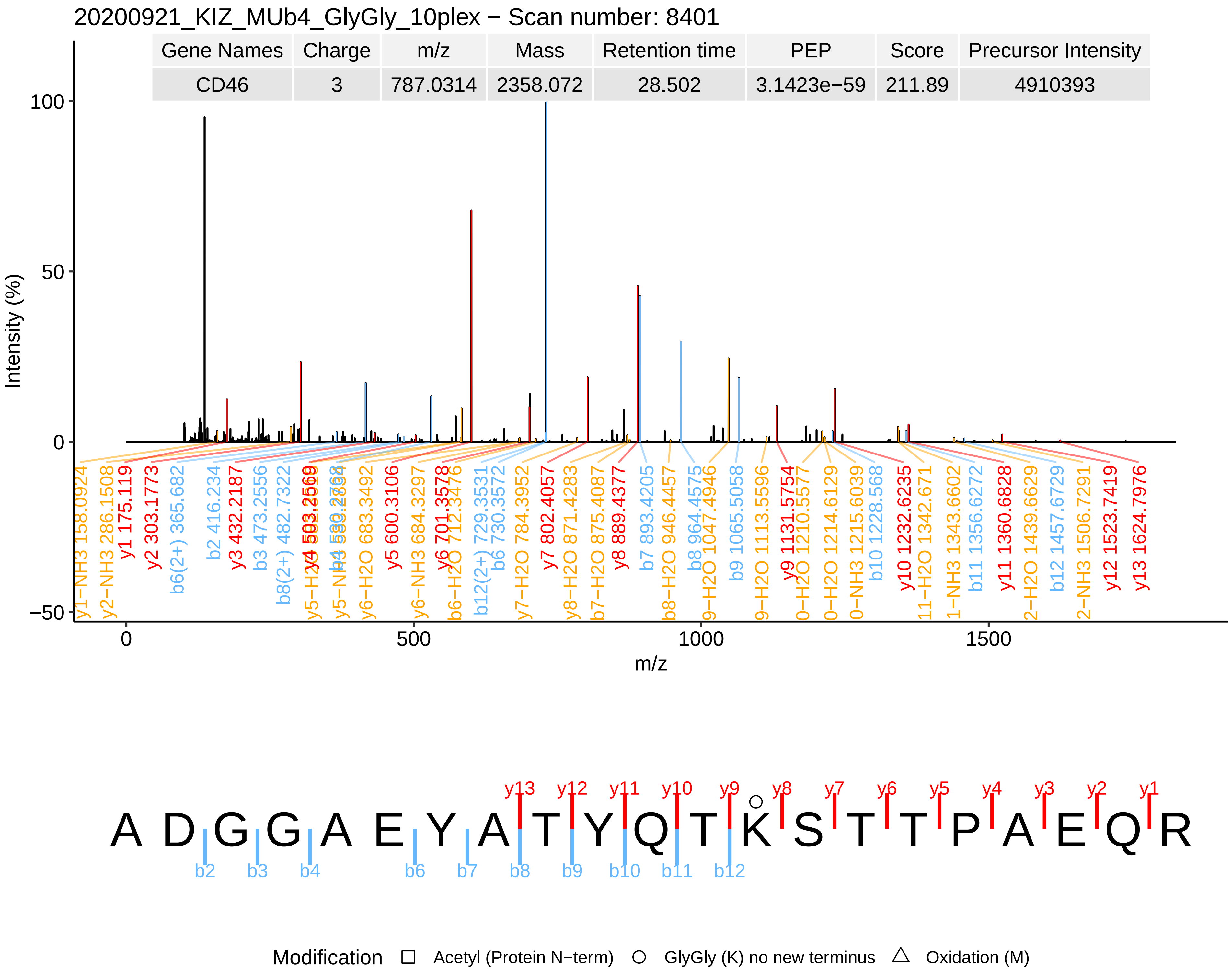

20200921_KIZ_MUb4_GlyGly_10plex − Scan number: 8401
Gene Names | Charge | m/z | Mass | Retention time | PEP | Score | Precursor Intensity
CD46 | 3 | 787.0314 | 2358.072 | 28.502 | 3.1423e−59 | 211.89 | 4910393
Intensity (%)
100
50
0
−50
m/z
0
500
1000
1500
y1−NH3 158.0924
y2−NH3 286.1508
y1 175.119
y2 303.1773
b6(2+) 365.682
b2 416.234
y3 432.2187
b3 473.2556
b8(2+) 482.7322
y5−H2O 683.3492
y5 600.3106
y6−NH3 684.3297
y6 701.3578
b6−H2O 712.3476
b12(2+) 729.3531
b6 730.3572
y7−H2O 784.3952
y7 802.4057
y8−H2O 871.4283
b7−H2O 875.4087
y8 889.4377
b7 893.4205
b8−H2O 946.4457
b8 964.4575
b9 1065.5058
y9 1131.5754
b10 1228.568
y10 1232.6235
y11 1360.6828
b11 1356.6272
b12 1457.6729
y12 1523.7419
y13 1624.7976
A D G G A E Y A T Y Q T K S T T P A E Q R
y13 y12 y11 y10 y9 y8 y7 y6 y5 y4 y3 y2 y1
b2 b3 b4 b6 b7 b8 b9 b10 b11 b12
Modification
Acetyl (Protein N−term)
GlyGly (K) no new terminus
Oxidation (M)

20200921_KIZ_MUb4_GlyGly_10plex_2 − Scan number: 7708

Gene Names	Charge	m/z	Mass	Retention time	PEP	Score	Precursor Intensity
CD46	3	787.0314	2358.072	28.851	8.9765e−22	142.78	1002339

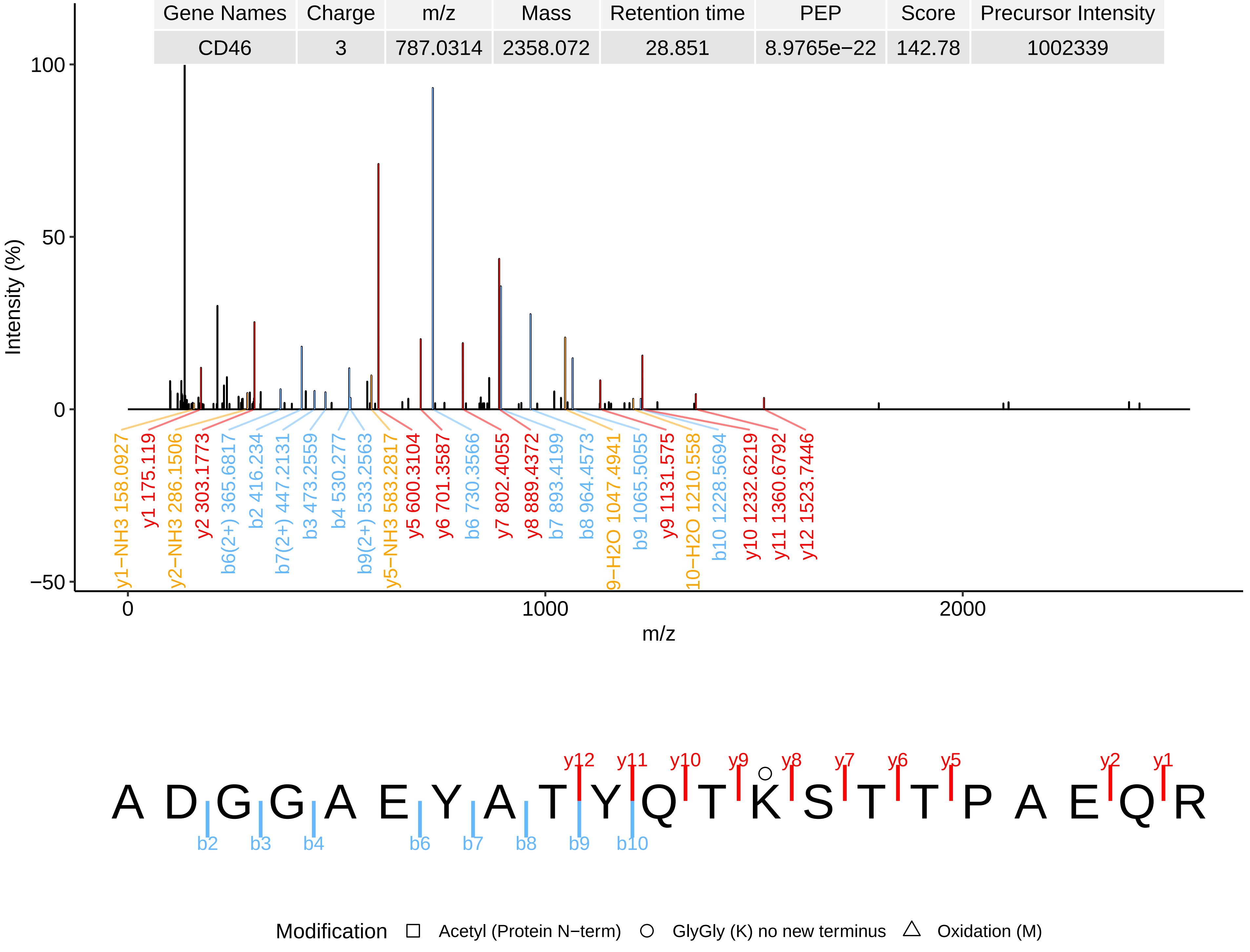

20200924_KIZ_MUb5_GlyGly_1 − Scan number: 7507

Gene Names	Charge	m/z	Mass	Retention time	PEP	Score	Precursor Intensity
CD46	3	787.0314	2358.072	23.852	2.7312e−40	181.64	6823109

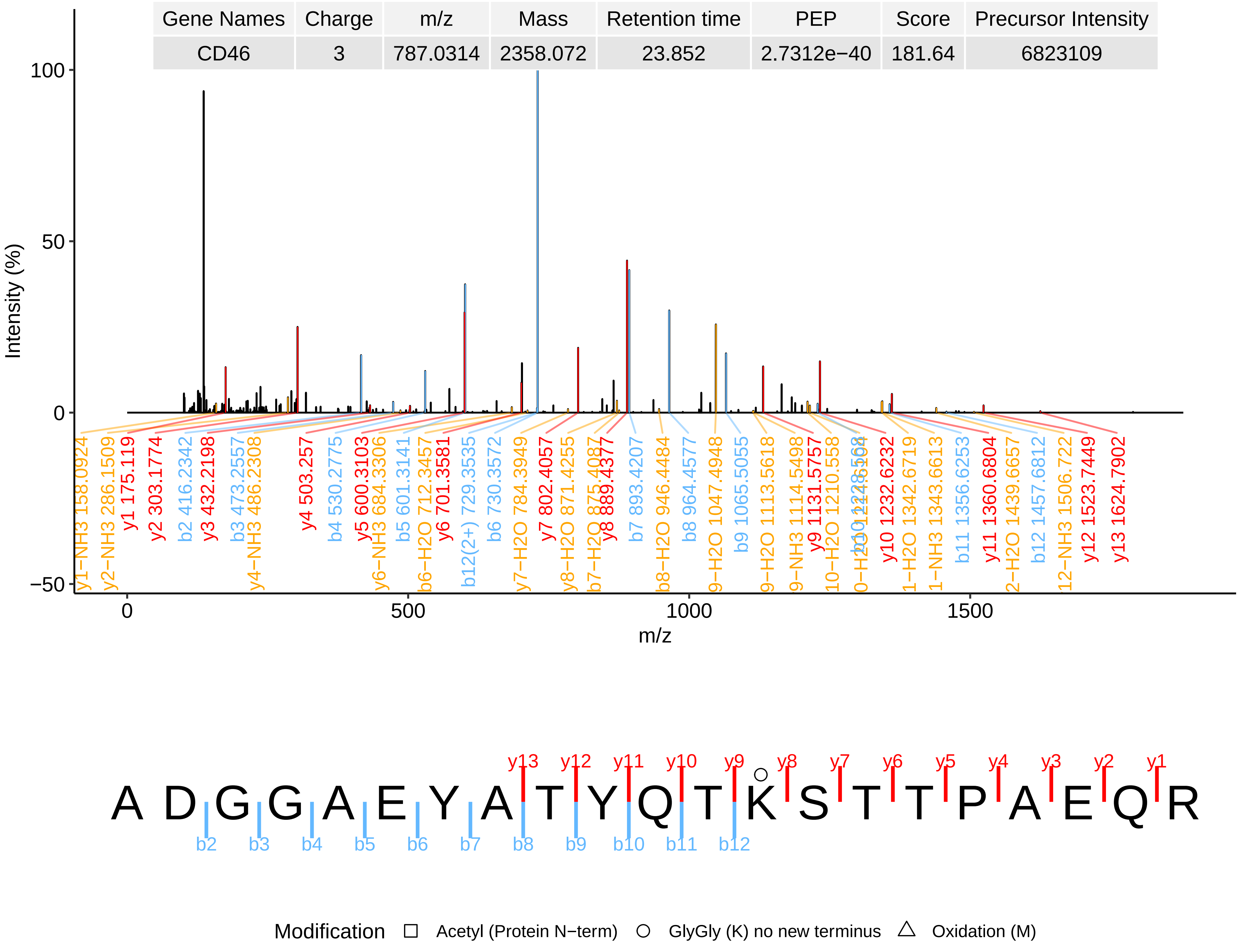

20200924_KIZ_MUb5_GlyGly_1 − Scan number: 7535

Gene Names	Charge	m/z	Mass	Retention time	PEP	Score	Precursor Intensity
CD46	3	787.0314	2358.072	23.948	1.4451e−48	190.37	12667355

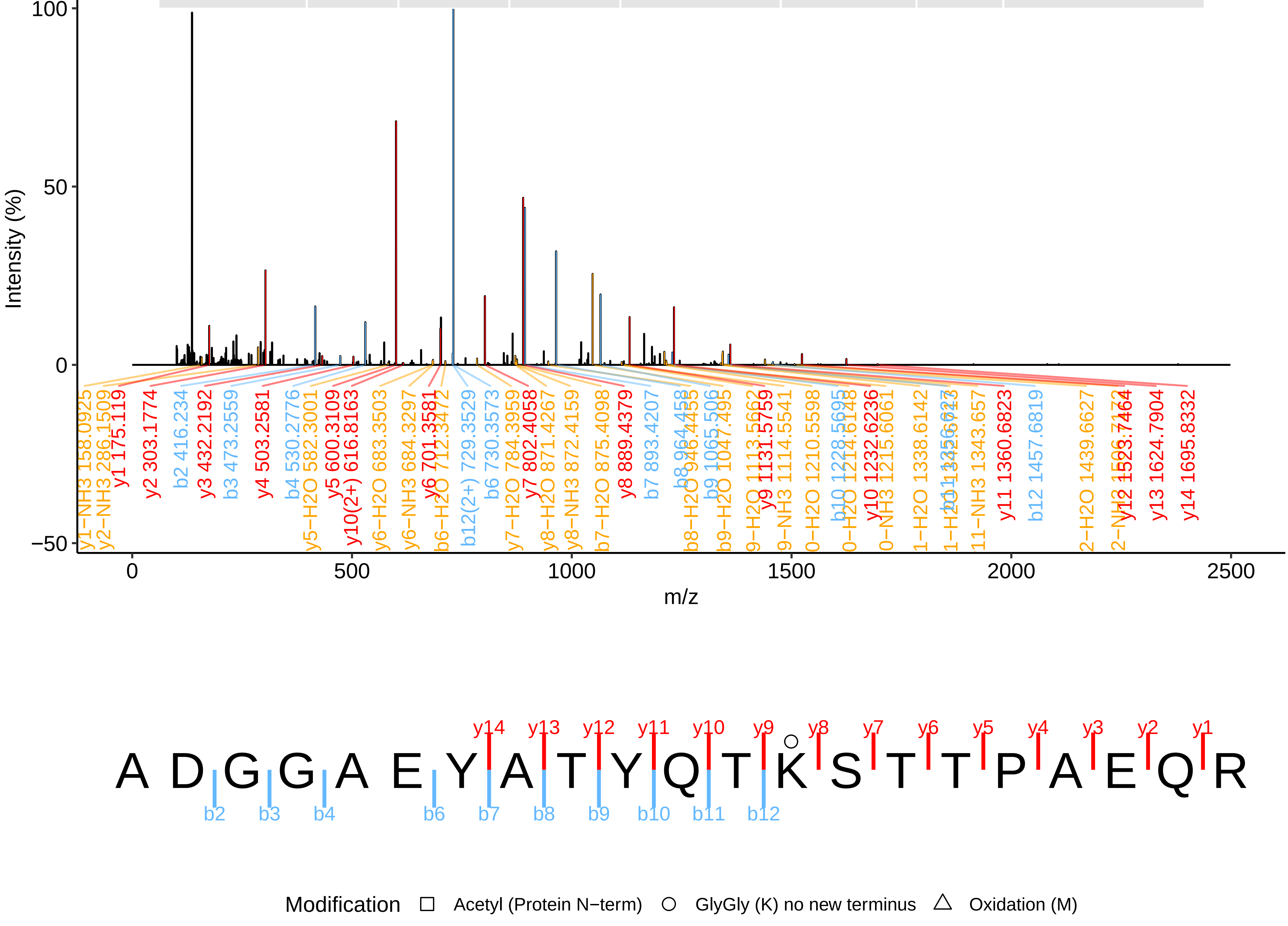

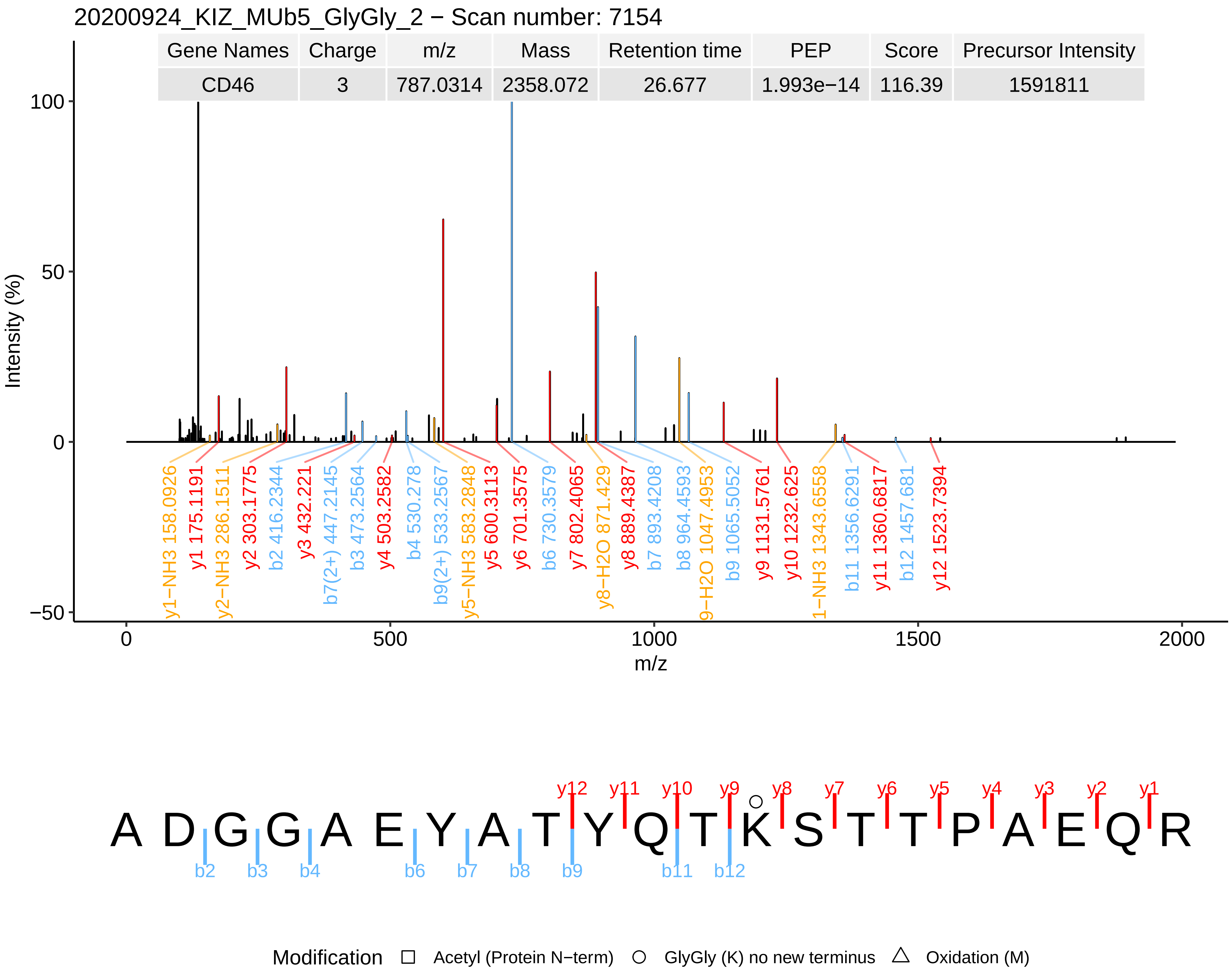

20200924_KIZ_MUb5_GlyGly_2 − Scan number: 7154
Gene Names | Charge | m/z | Mass | Retention time | PEP | Score | Precursor Intensity
CD46 | 3 | 787.0314 | 2358.072 | 26.677 | 1.993e−14 | 116.39 | 1591811
Intensity (%)
100
50
0
−50
0
500
1000
1500
2000
m/z
y1−NH3 158.0926
y1 175.1191
y2−NH3 286.1511
y2 303.1775
b2 416.2344
y3 432.221
b7(2+) 447.2145
b3 473.2564
y4 503.2582
b4 530.278
b9(2+) 533.2567
y5−NH3 583.2848
y5 600.3113
y6 701.3575
b6 730.3579
y7 802.4065
y8−H2O 871.429
y8 889.4387
b7 893.4208
b8 964.4593
9−H2O 1047.4953
b9 1065.5052
y9 1131.5761
y10 1232.625
1−NH3 1343.6558
b11 1356.6291
y11 1360.6817
b12 1457.681
y12 1523.7394
y12 y11 y10 y9 y8 y7 y6 y5 y4 y3 y2 y1
A D G G A E Y A T Y Q T K S T T P A E Q R
b2 b3 b4 b6 b7 b8 b9 b11 b12
Modification
Acetyl (Protein N−term)
GlyGly (K) no new terminus
Oxidation (M)

20200924_KIZ_MUb5_GlyGly_2 − Scan number: 7292

Gene Names	Charge	m/z	Mass	Retention time	PEP	Score	Precursor Intensity
CD46	3	787.0314	2358.072	27.212	5.9541e−06	89.011	2924934

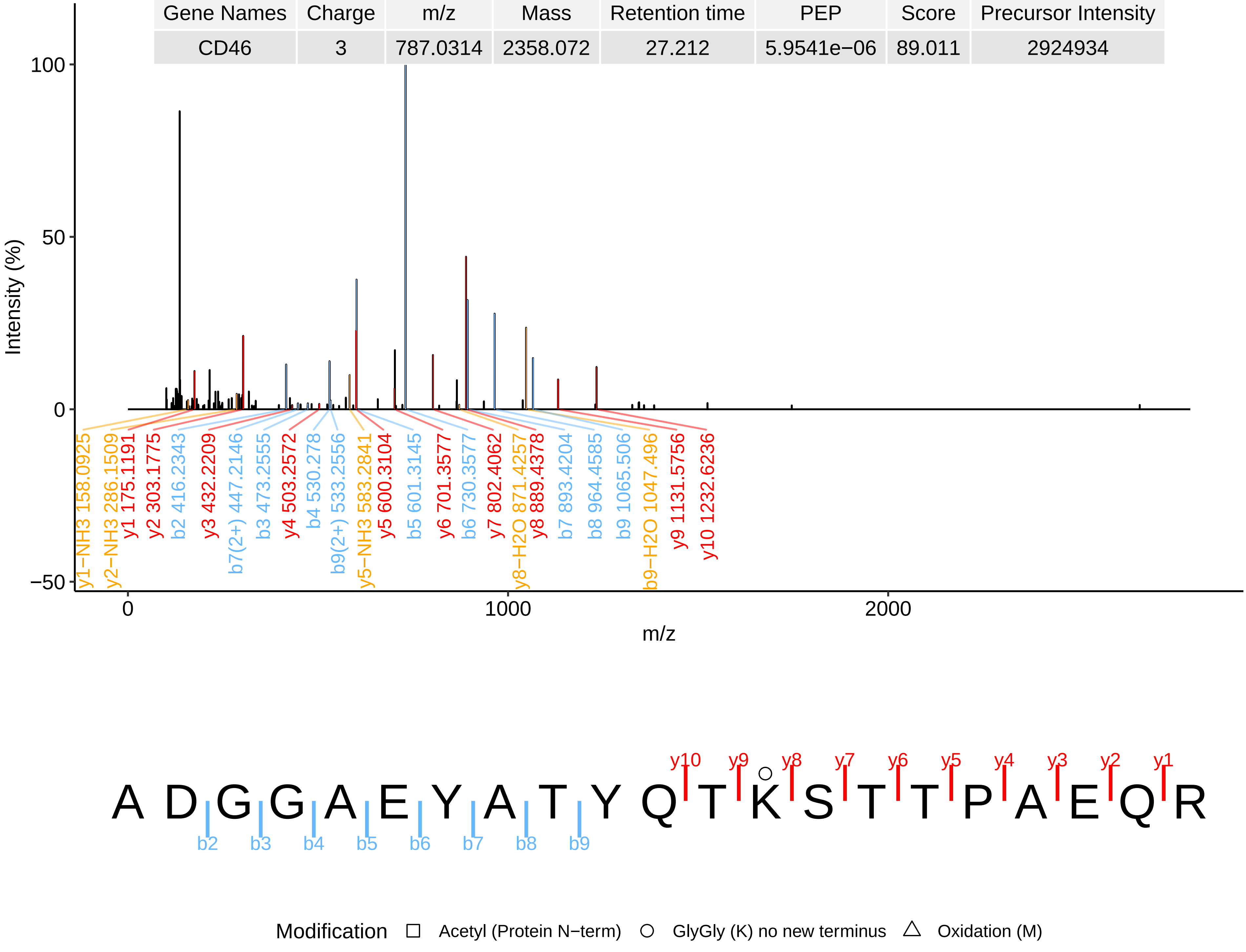

20201118_KIZ_MUb6_GlyGly_2 − Scan number: 8418

Gene Names	Charge	m/z	Mass	Retention time	PEP	Score	Precursor Intensity
CD46	3	787.0314	2358.072	28.587	1.1388e−14	123.43	5556459

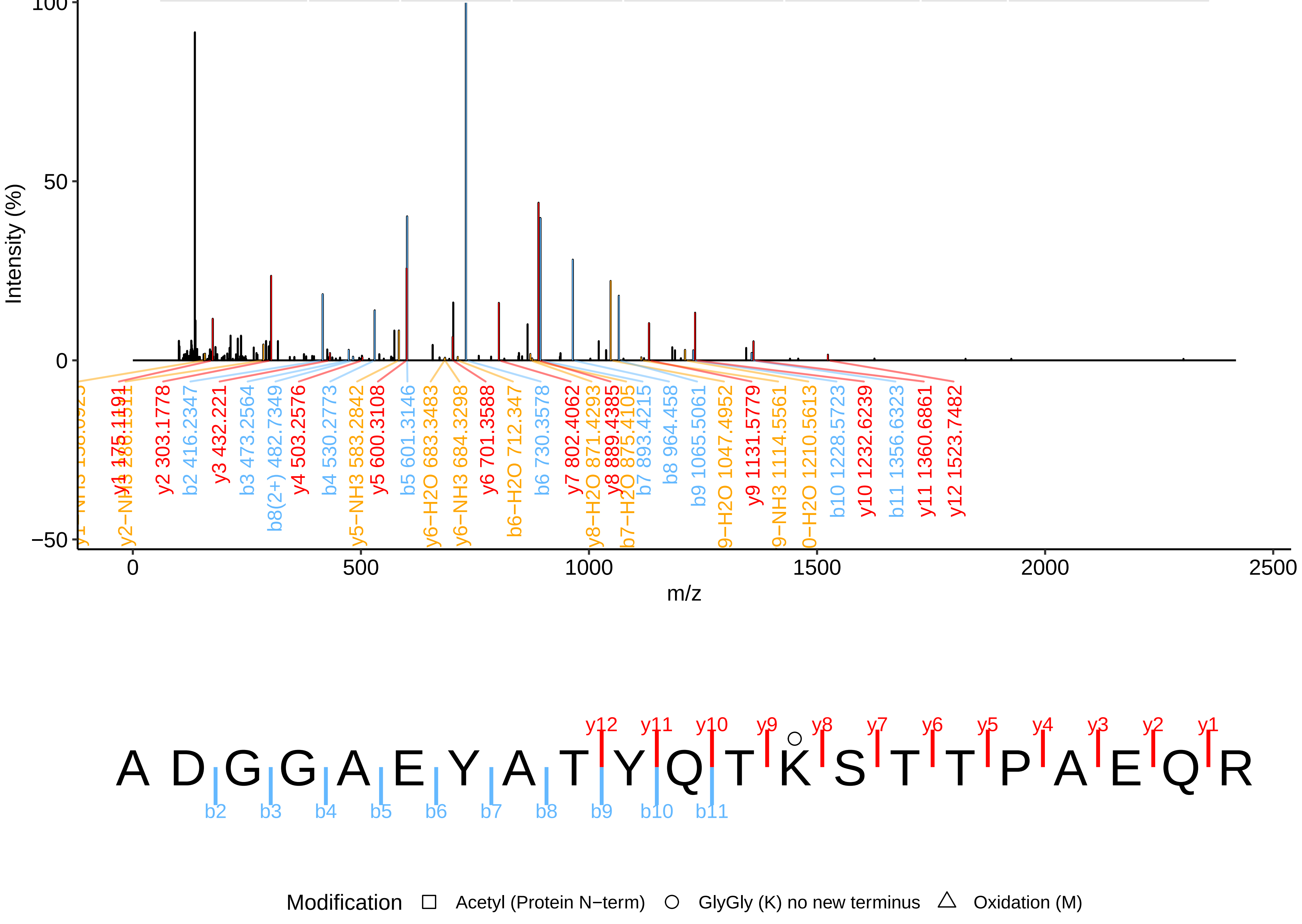

20200921_KIZ_MUb4_GlyGly_10plex − Scan number: 8380

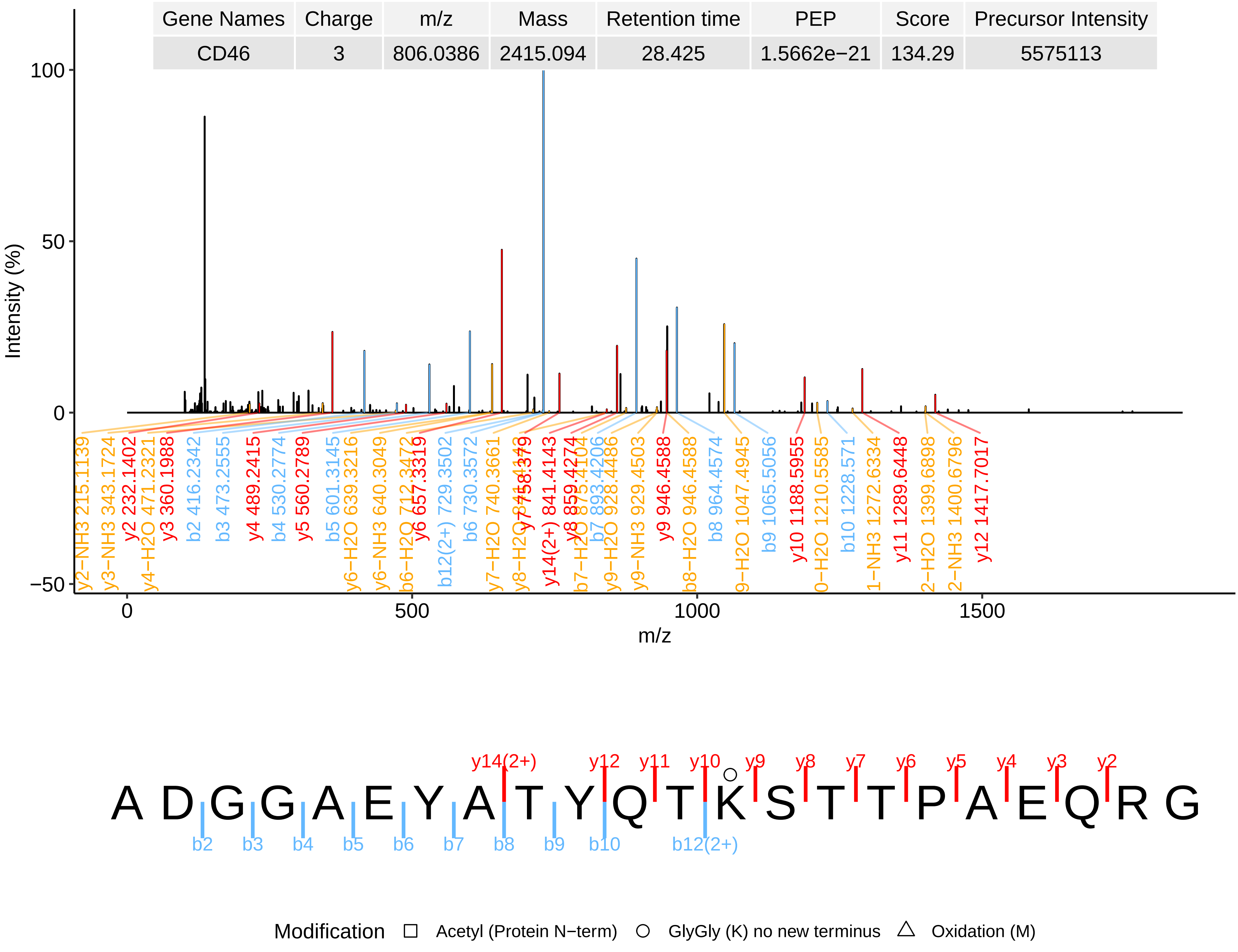

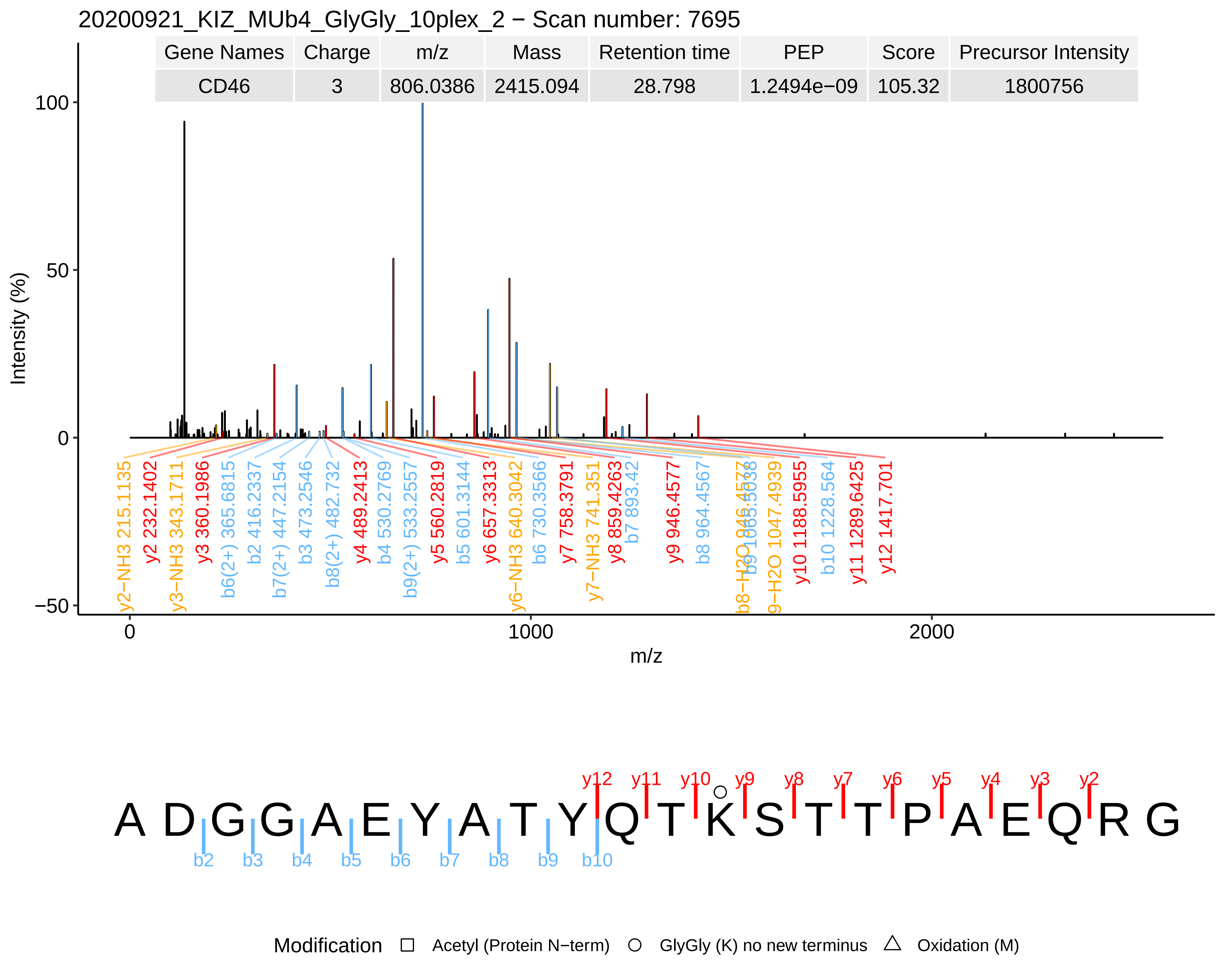

20200921_KIZ_MUb4_GlyGly_10plex_2 − Scan number: 7695
Gene Names | Charge | m/z | Mass | Retention time | PEP | Score | Precursor Intensity
CD46 | 3 | 806.0386 | 2415.094 | 28.798 | 1.2494e−09 | 105.32 | 1800756
Intensity (%)
100
50
0
−50
m/z
0
1000
2000
y2−NH3 215.1135
y2 232.1402
y3−NH3 343.1711
y3 360.1986
b6(2+) 365.6815
b2 416.2337
b7(2+) 447.2154
b3 473.2546
b8(2+) 482.732
y4 489.2413
b4 530.2769
b9(2+) 533.2557
y5 560.2819
b5 601.3144
y6 657.3313
y6−NH3 640.3042
b6 730.3566
y7 758.3791
y7−NH3 741.351
y8 859.4263
b7 893.42
y9 946.4577
b8 964.4567
y10 1188.5955
b10 1228.564
y11 1289.6425
y12 1417.701
A D G G A E Y A T Y Q T K S T T P A E Q R G
y12 y11 y10 y9 y8 y7 y6 y5 y4 y3 y2
b2 b3 b4 b5 b6 b7 b8 b9 b10
Modification
Acetyl (Protein N−term)
GlyGly (K) no new terminus
Oxidation (M)

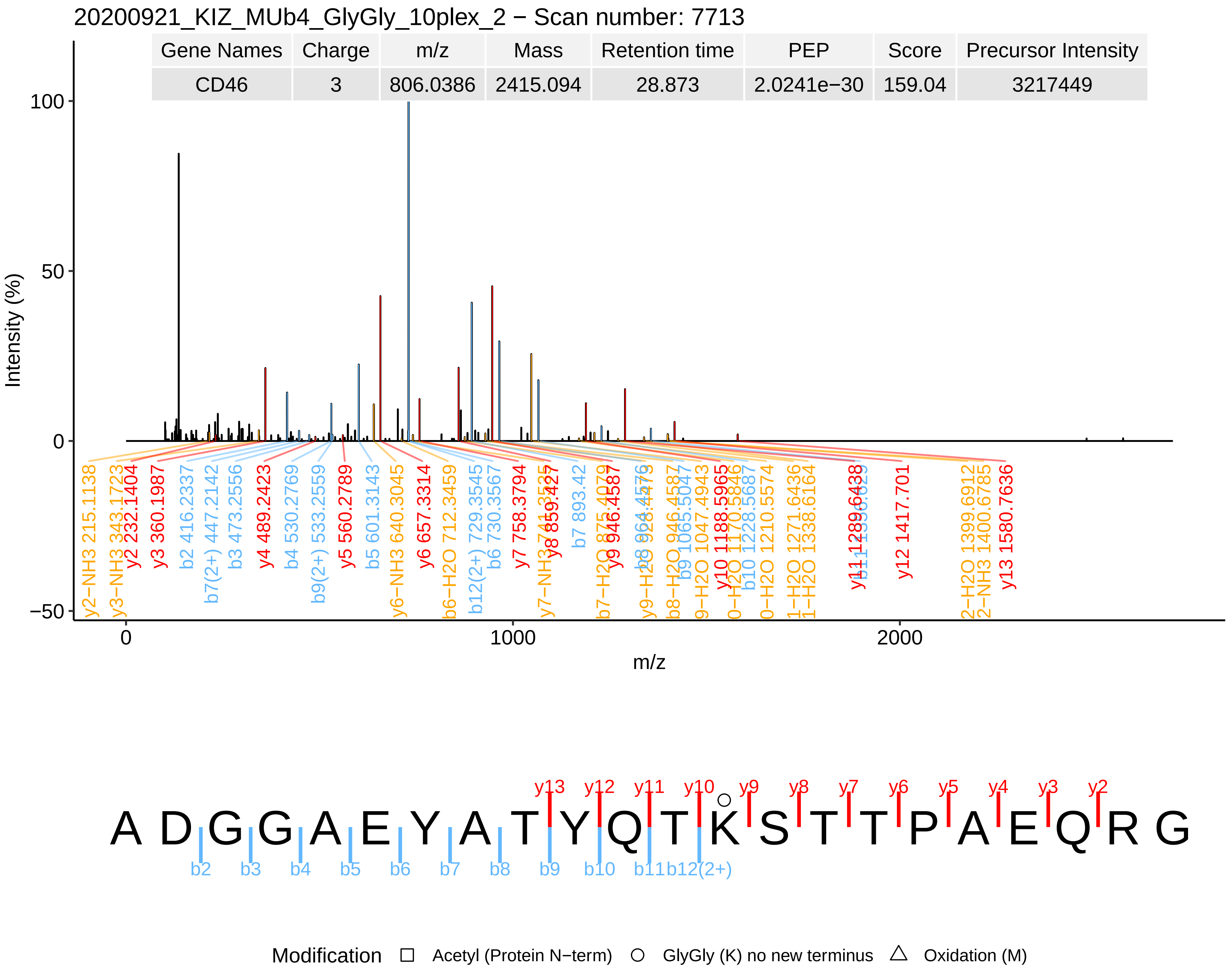
20200921_KIZ_MUb4_GlyGly_10plex_2 − Scan number: 7713
Gene Names
CD46
Charge
3
m/z
806.0386
Mass
2415.094
Retention time
28.873
PEP
2.0241e−30
Score
159.04
Precursor Intensity
3217449
Intensity (%)
100
50
0
−50
0
1000
2000
m/z
y2−NH3 215.1138
y3−NH3 343.1723
y2 232.1404
y3 360.1987
b2 416.2337
b7(2+) 447.2142
b3 473.2556
y4 489.2423
b4 530.2769
b9(2+) 533.2559
y5 560.2789
b5 601.3143
y6−NH3 640.3045
y6 657.3314
b6−H2O 712.3459
b12(2+) 729.3545
b6 730.3567
y7 758.3794
b7 893.42
y12 1417.701
y13 1580.7636
A D G G A E Y A T Y Q T K S T T P A E Q R G
y13 y12 y11 y10 y9 y8 y7 y6 y5 y4 y3 y2
b2 b3 b4 b5 b6 b7 b8 b9 b10 b11 b12(2+)
Modification
Acetyl (Protein N−term)
GlyGly (K) no new terminus
Oxidation (M)

20200924_KIZ_MUb5_GlyGly_2 − Scan number: 7210

Gene Names	Charge	m/z	Mass	Retention time	PEP	Score	Precursor Intensity
CD46	3	806.0386	2415.094	26.895	4.1908e−11	106.03	1198450

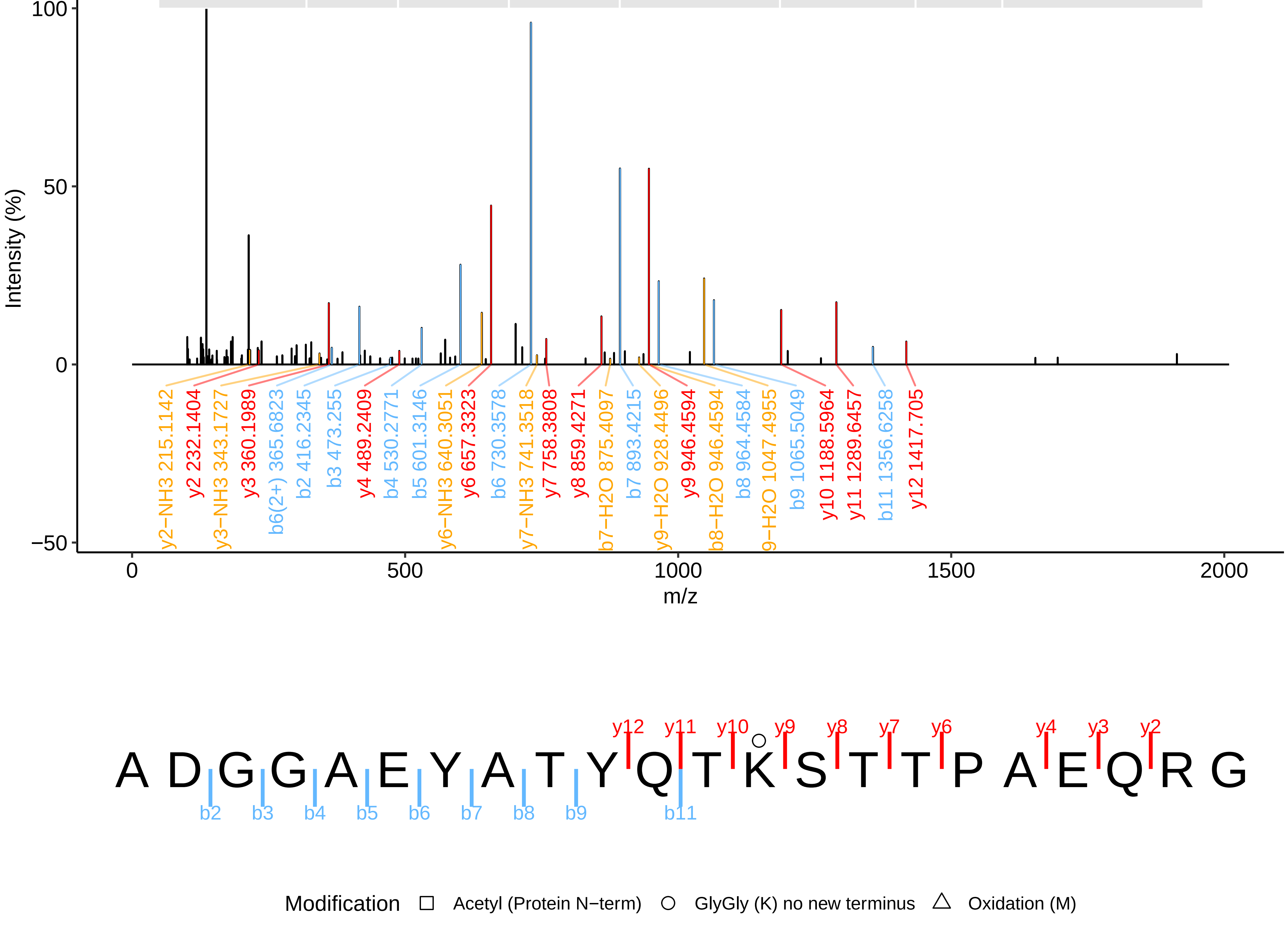

20201118_KIZ_MUb6_GlyGly_2 − Scan number: 8391

Gene Names	Charge	m/z	Mass	Retention time	PEP	Score	Precursor Intensity
CD46	3	806.0386	2415.094	28.491	2.1297e−15	130.25	4391785

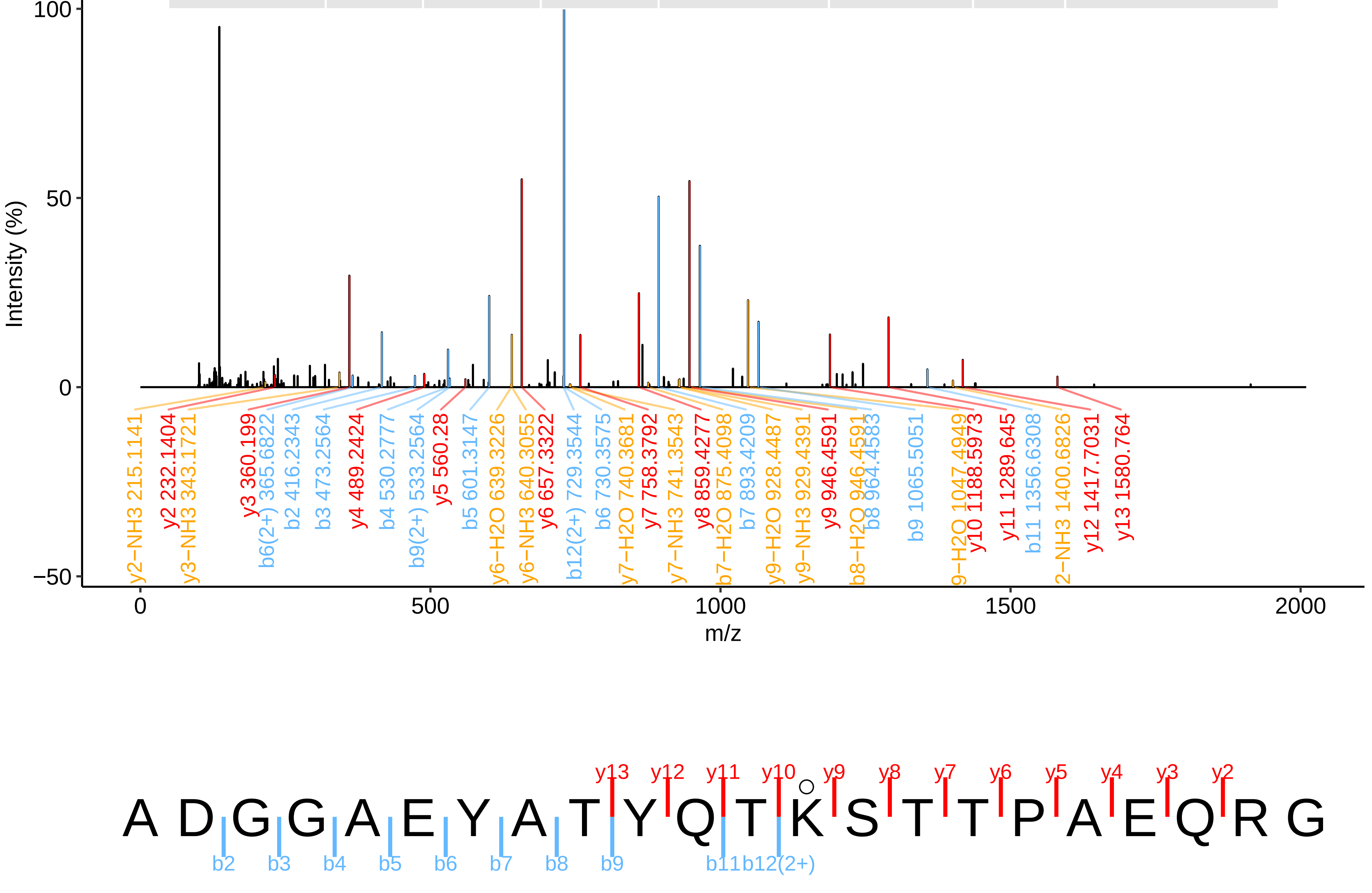

Modification □ Acetyl (Protein N−term) ○ GlyGly (K) no new terminus △ Oxidation (M)

20200921_KIZ_MUb4_GlyGly_10plex − Scan number: 5527

Gene Names	Charge	m/z	Mass	Retention time	PEP	Score	Precursor Intensity
ADRM1	4	476.4798	1901.89	18.115	3.3994e−09	120.7	3505103

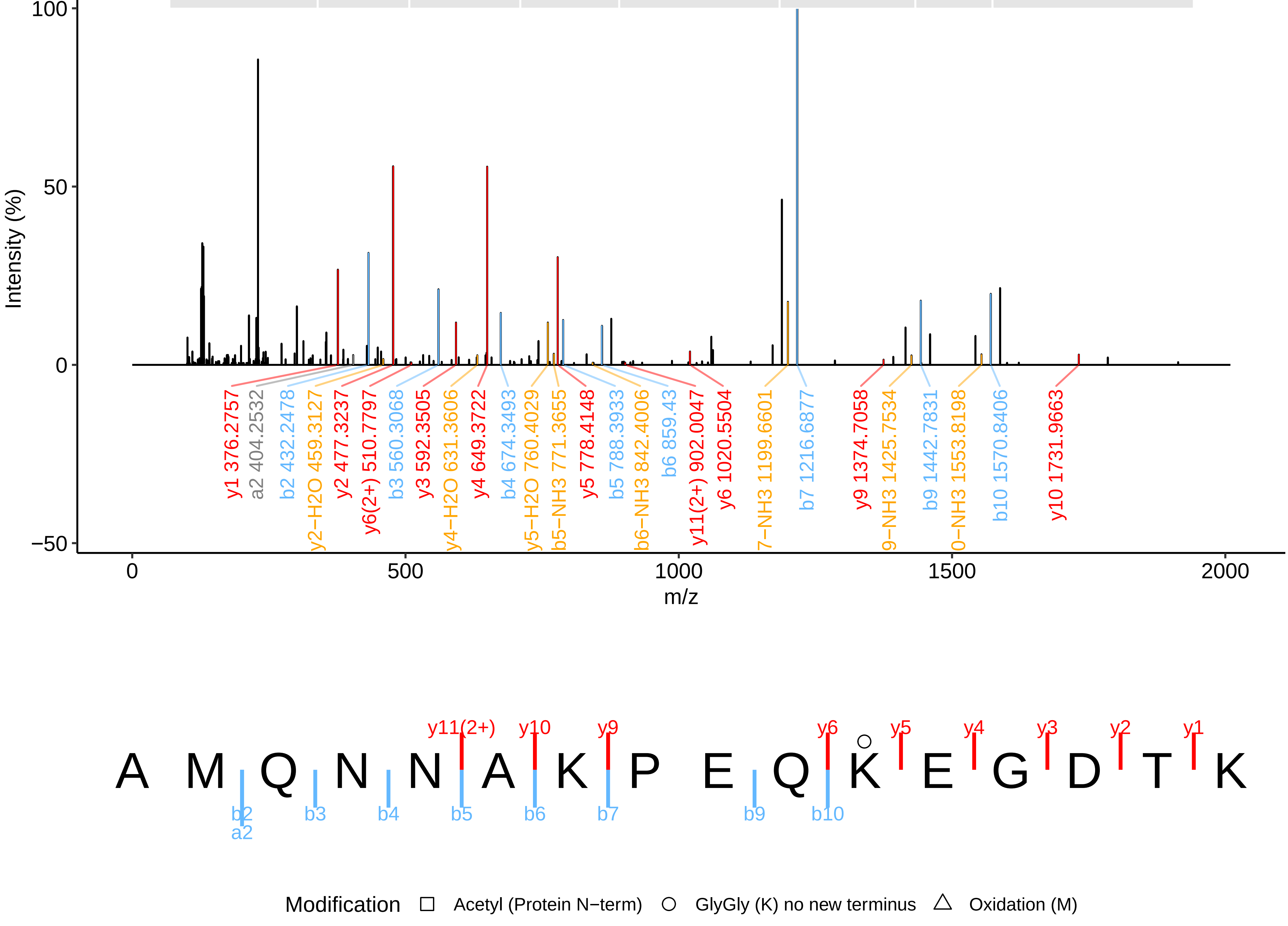

20200921_KIZ_MUb4_GlyGly_10plex_2 − Scan number: 5075

Gene Names	Charge	m/z	Mass	Retention time	PEP	Score	Precursor Intensity
ADRM1	4	476.4798	1901.89	18.081	5.2577e−05	90.367	1006947

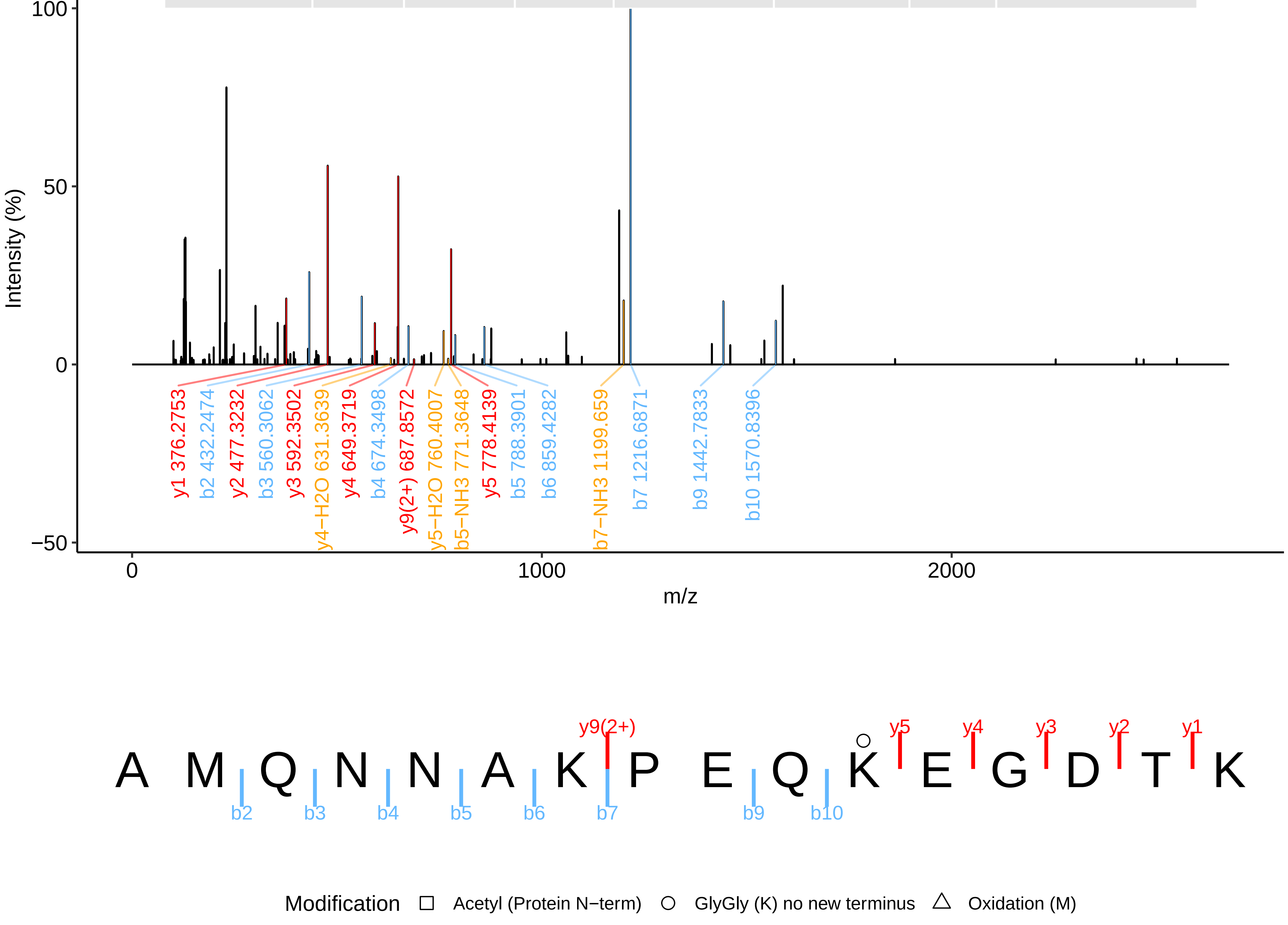

20200921_KIZ_MUb4_GlyGly_10plex_2 − Scan number: 15710

Gene Names	Charge	m/z	Mass	Retention time	PEP	Score	Precursor Intensity
ATP1A1	4	553.2835	2209.105	61.492	0.015042	58.671	NA

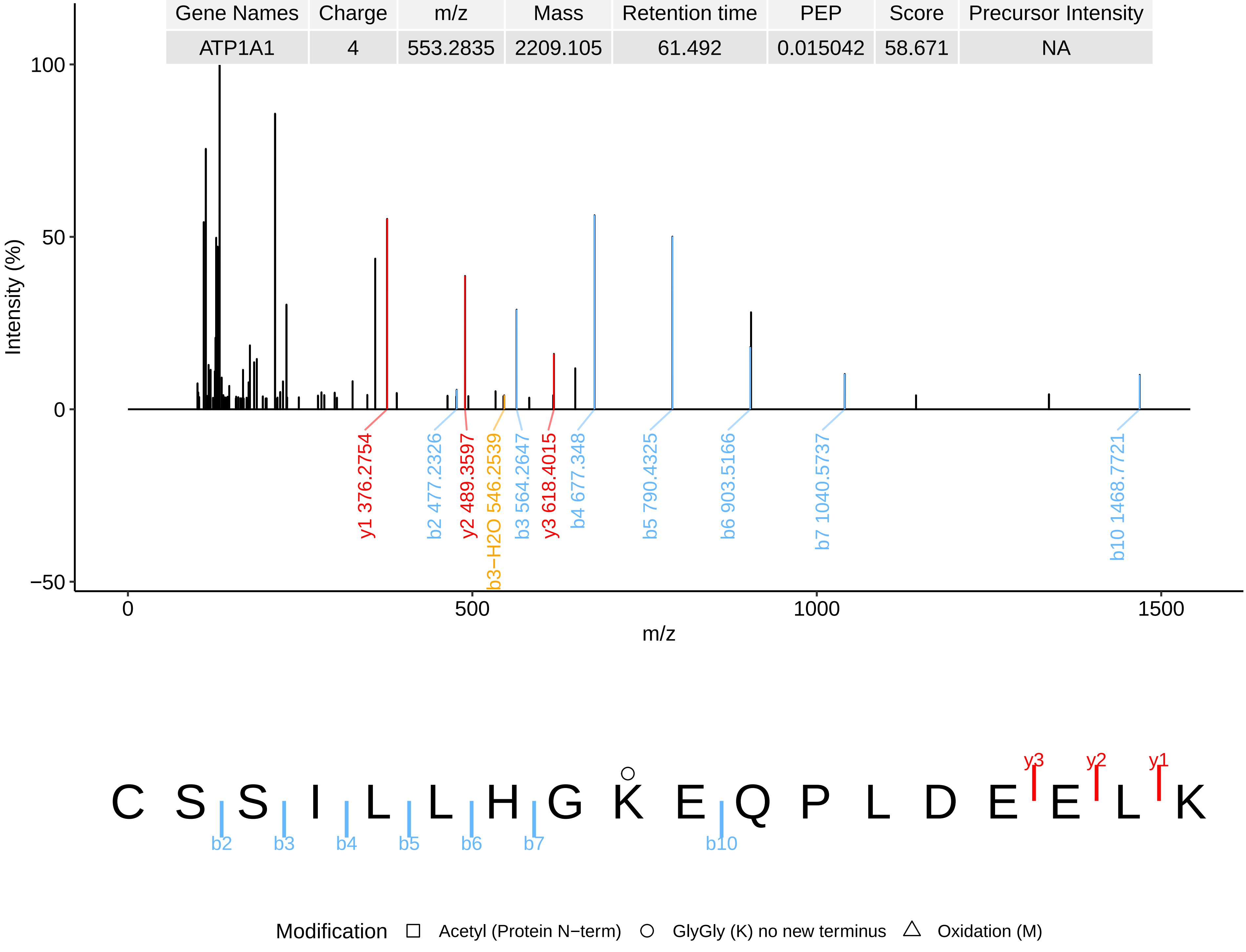

20200921_KIZ_MUb4_GlyGly_10plex_2 − Scan number: 21103

Gene Names	Charge	m/z	Mass	Retention time	PEP	Score	Precursor Intensity
EPPK1	3	736.3668	2206.079	83.043	0.014842	60.034	662192.2

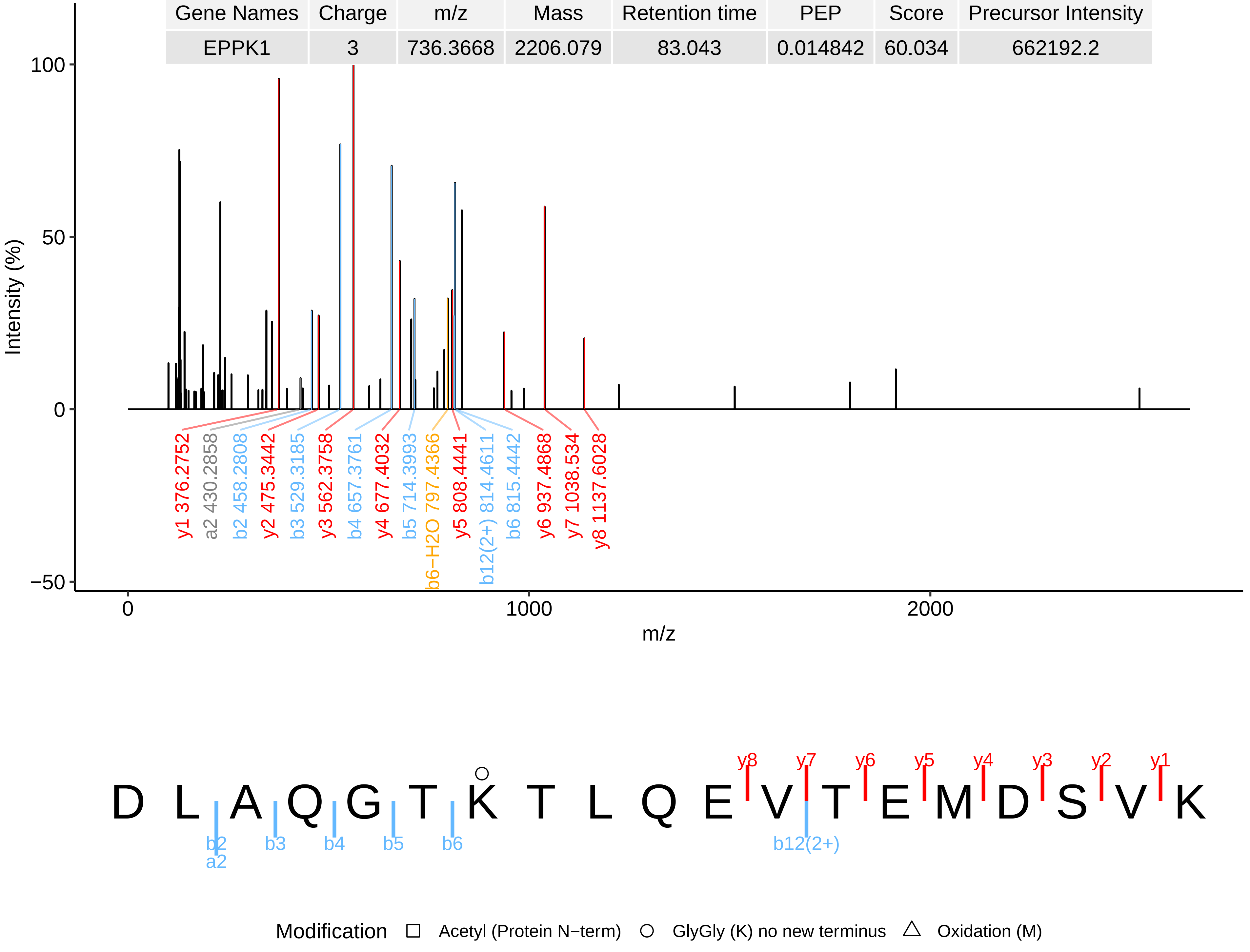

20200921_KIZ_MUb4_GlyGly_10plex_2 − Scan number: 11967

Gene Names	Charge	m/z	Mass	Retention time	PEP	Score	Precursor Intensity
SET	4	530.5039	2117.986	46.191	8.0185e−06	82.007	1092308

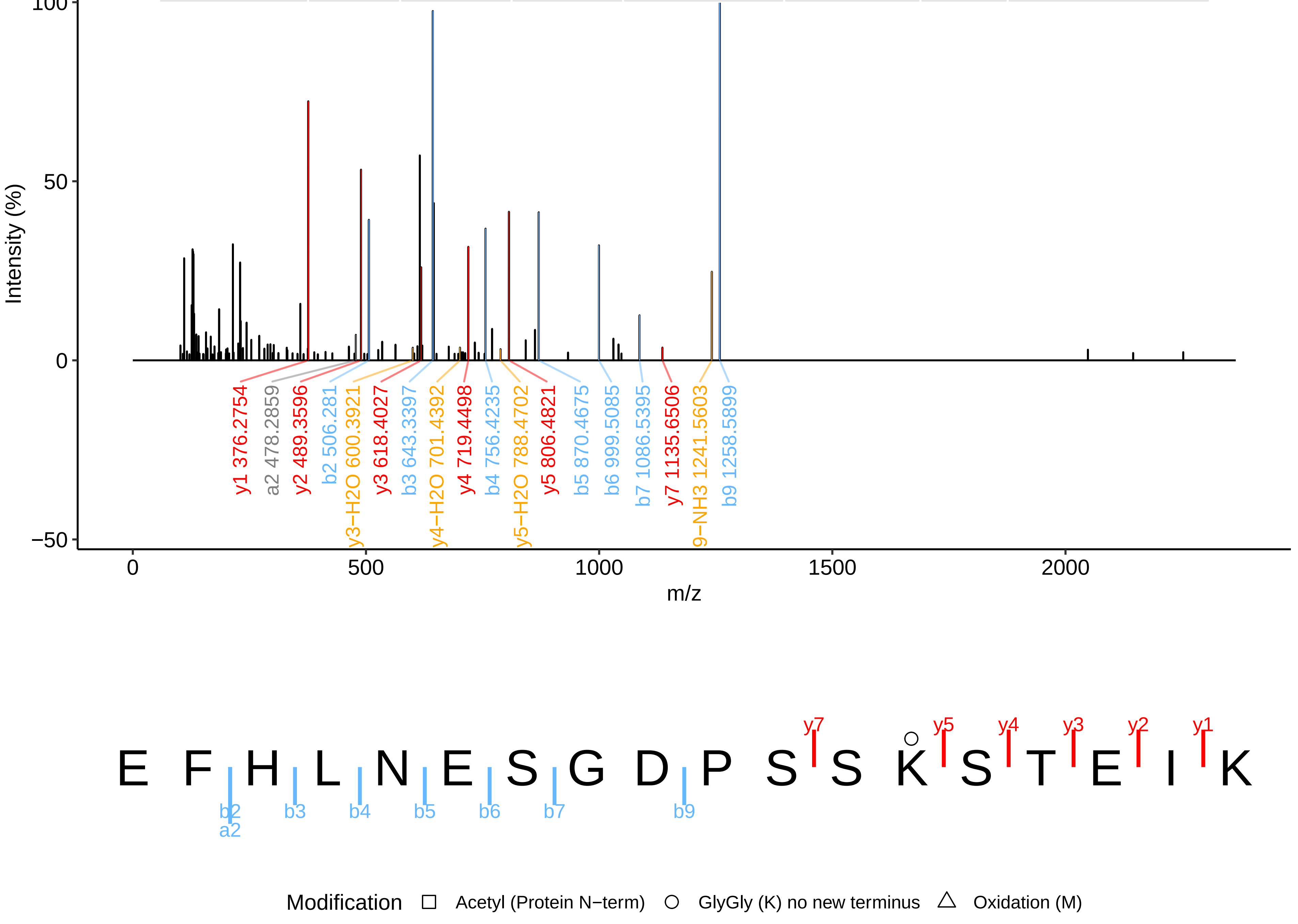

20200921_KIZ_MUb4_GlyGly_10plex_2 − Scan number: 16914

Gene Names	Charge	m/z	Mass	Retention time	PEP	Score	Precursor Intensity
TCEAL4	4	589.7638	2355.026	66.079	2.3941e−06	93.226	817203.4

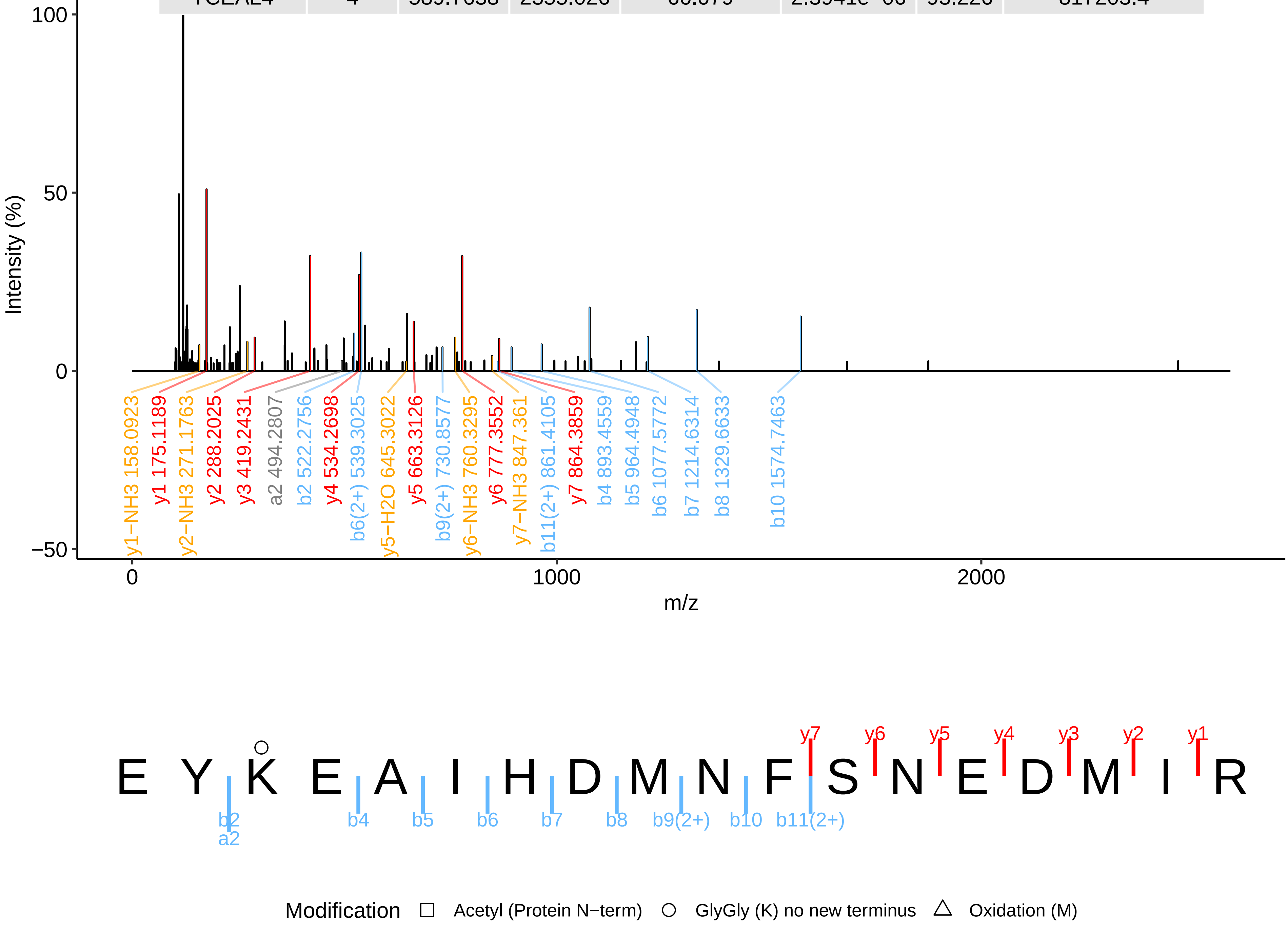

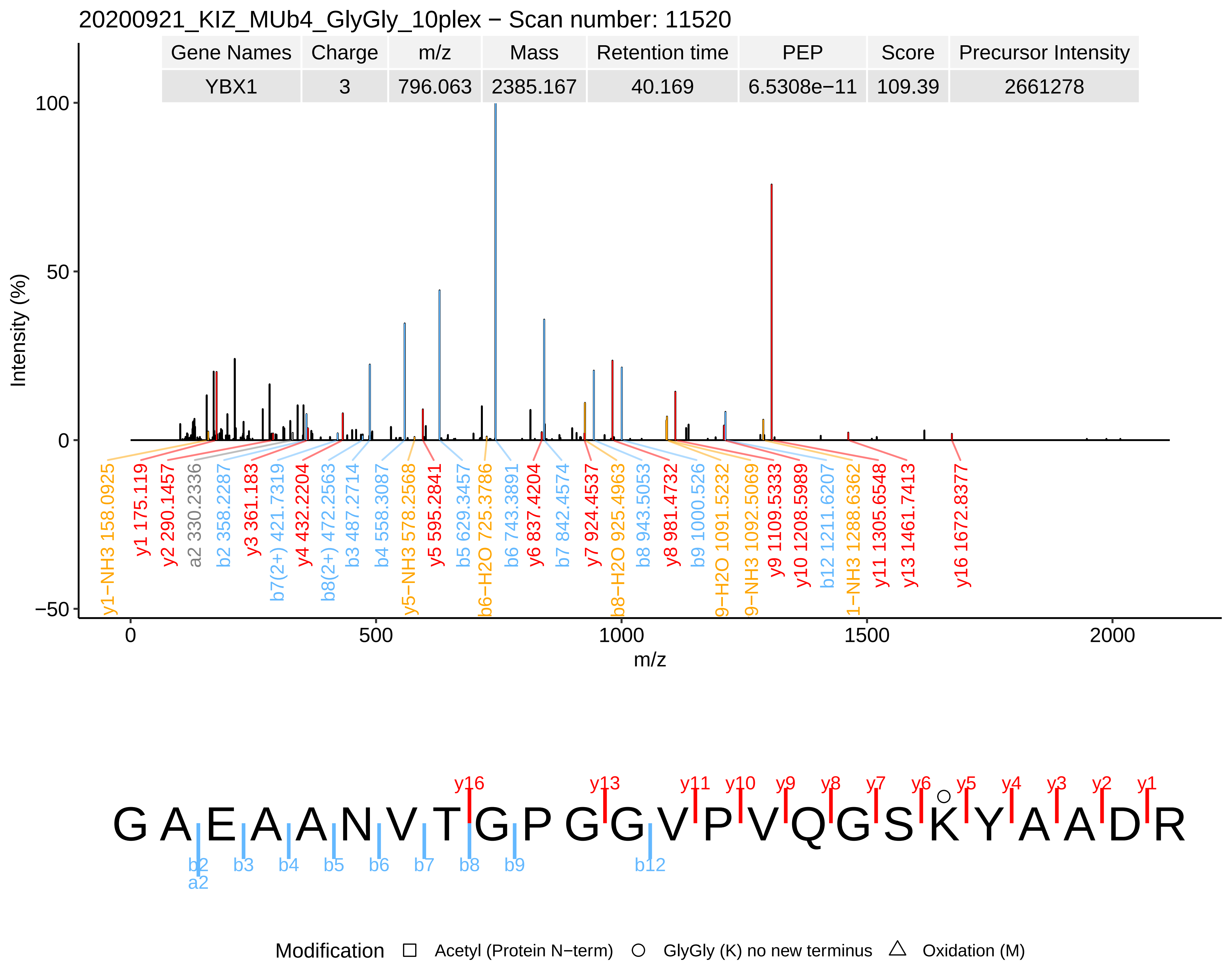

20200921_KIZ_MUb4_GlyGly_10plex − Scan number: 11520
Gene Names | Charge | m/z | Mass | Retention time | PEP | Score | Precursor Intensity
YBX1 | 3 | 796.063 | 2385.167 | 40.169 | 6.5308e−11 | 109.39 | 2661278
Intensity (%)
m/z
y1−NH3 158.0925
y1 175.119
y2 290.1457
a2 330.2336
b2 358.2287
y3 361.183
b7(2+) 421.7319
y4 432.2204
b8(2+) 472.2563
b3 487.2714
b4 558.3087
y5−NH3 578.2568
y5 595.2841
b5 629.3457
b6−H2O 725.3786
b6 743.3891
y6 837.4204
b7 842.4574
y7 924.4537
b8−H2O 925.4963
b8 943.5053
y8 981.4732
b9 1000.526
1091.5232
1092.5069
y9 1109.5333
y10 1208.5989
b12 1211.6207
1288.6362
y11 1305.6548
y13 1461.7413
y16 1672.8377
G A E A A N V T G P G G V P V Q G S K Y A A D R
Modification
Acetyl (Protein N−term)
GlyGly (K) no new terminus
Oxidation (M)

20200921_KIZ_MUb4_GlyGly_10plex_2 − Scan number: 10537

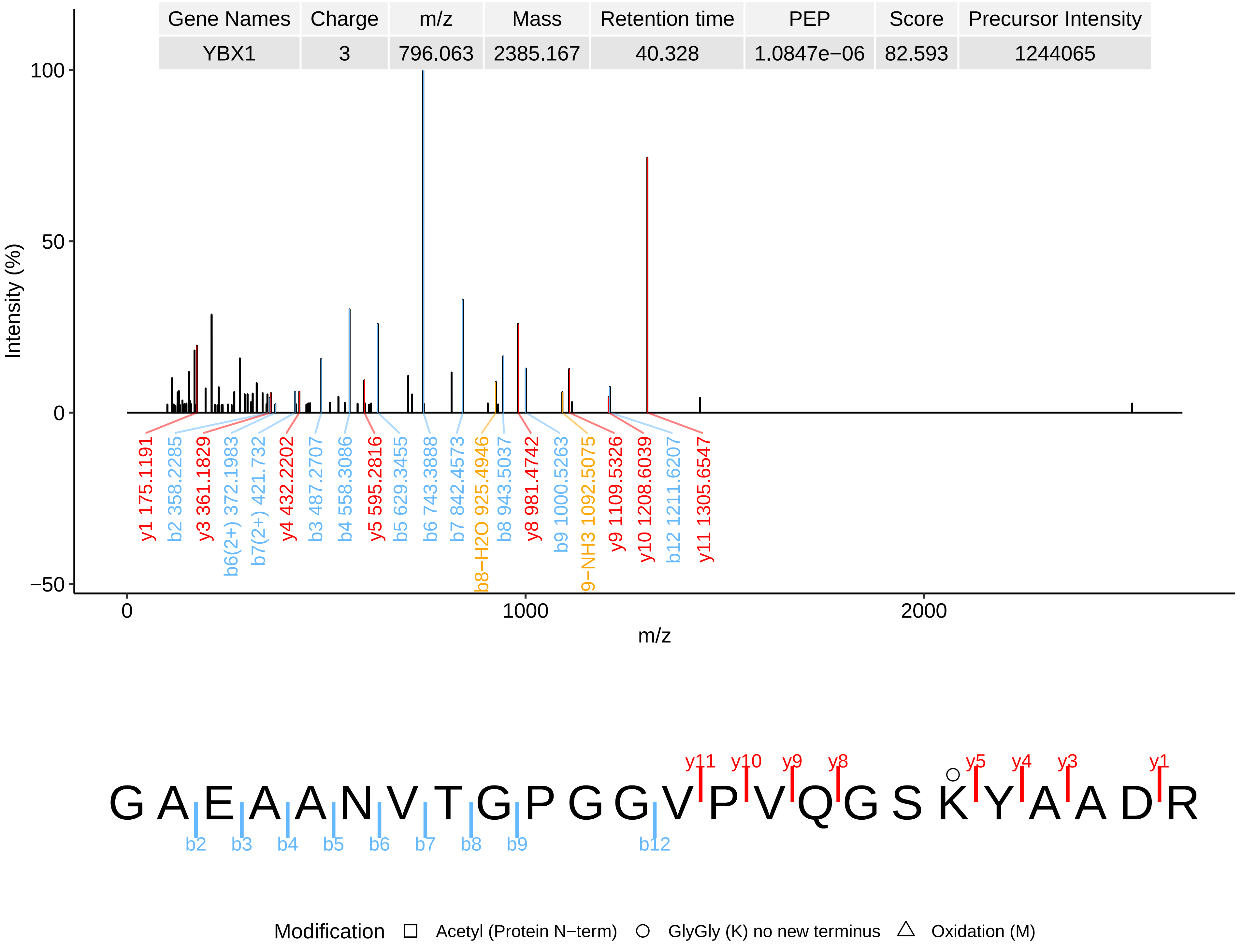

20200924_KIZ_MUb5_GlyGly_1 − Scan number: 10734

Gene Names	Charge	m/z	Mass	Retention time	PEP	Score	Precursor Intensity
YBX1	3	796.063	2385.167	34.681	5.9816e−31	154.14	NA

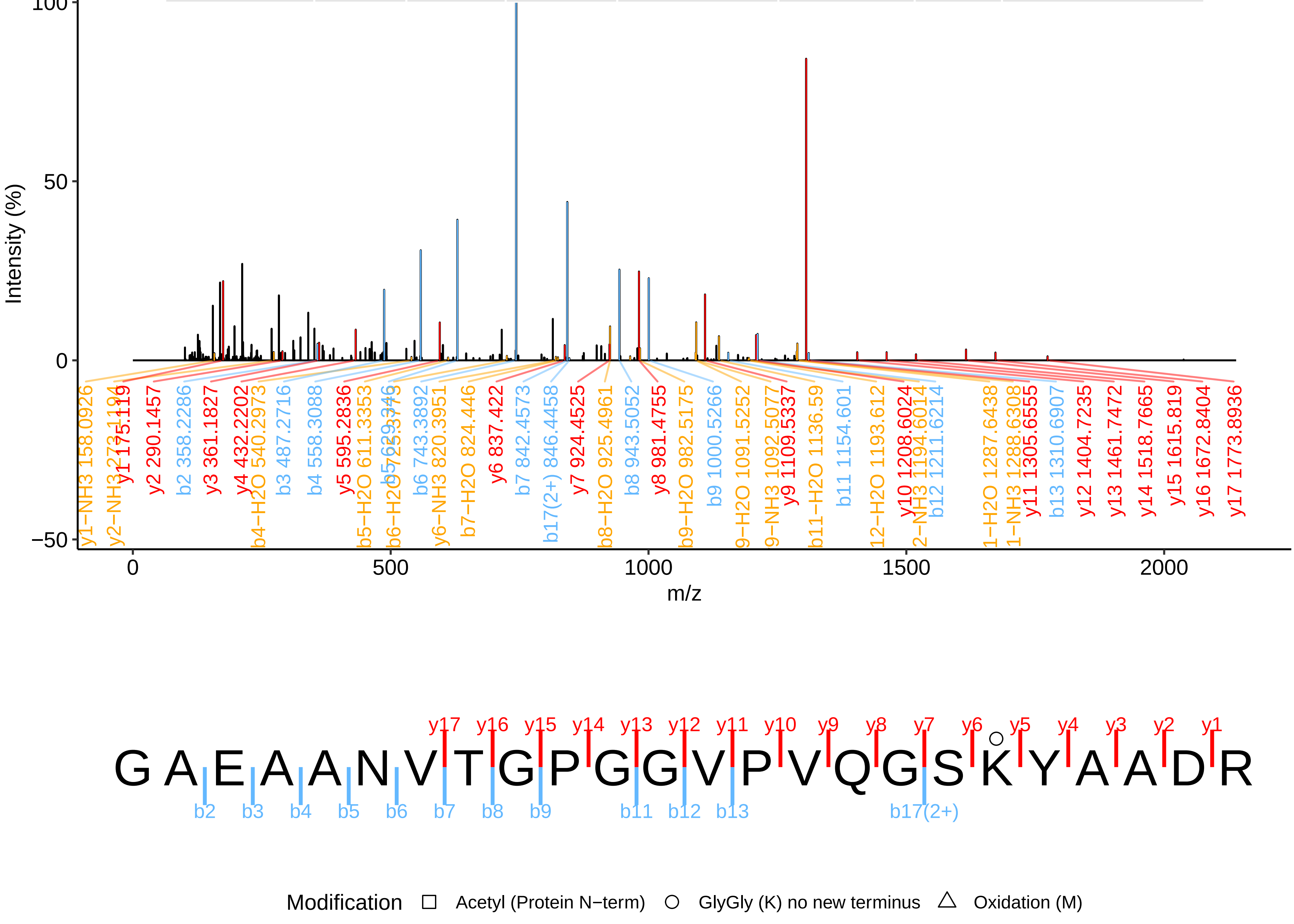

20200921_KIZ_MUb4_GlyGly_10plex − Scan number: 31275

Gene Names	Charge	m/z	Mass	Retention time	PEP	Score	Precursor Intensity
PHB2	3	720.3719	2158.094	115.25	5.3743e−06	91.725	NA

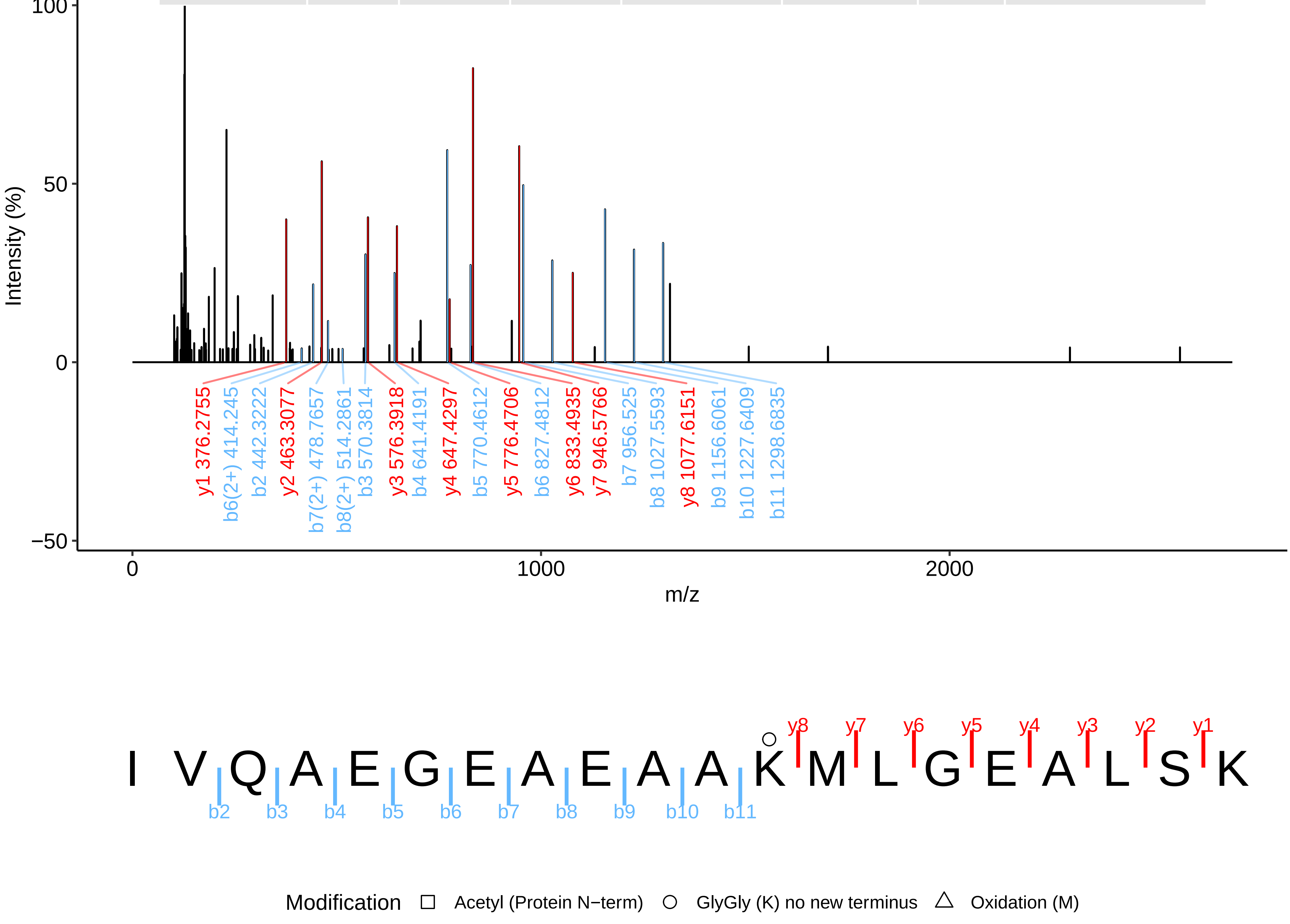

20200921_KIZ_MUb4_GlyGly_10plex − Scan number: 31282

Gene Names	Charge	m/z	Mass	Retention time	PEP	Score	Precursor Intensity
PHB2	3	720.3719	2158.094	115.28	5.3743e−06	91.725	1595602

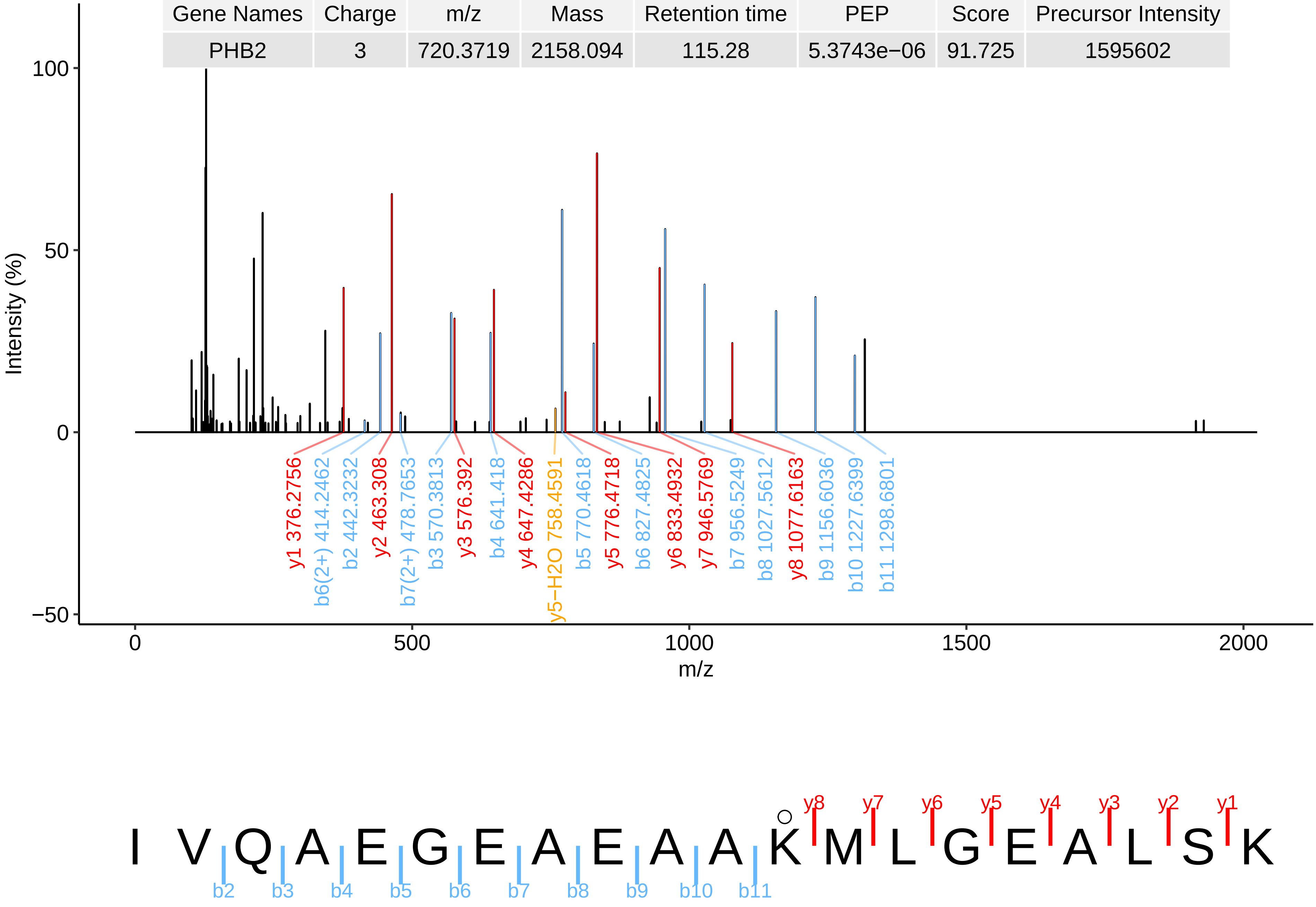

Modification □ Acetyl (Protein N−term) ○ GlyGly (K) no new terminus △ Oxidation (M)

20200921_KIZ_MUb4_GlyGly_10plex_2 − Scan number: 27912

Gene Names	Charge	m/z	Mass	Retention time	PEP	Score	Precursor Intensity
PHB2	3	720.3719	2158.094	110.72	3.3852e−09	100.67	980353.8

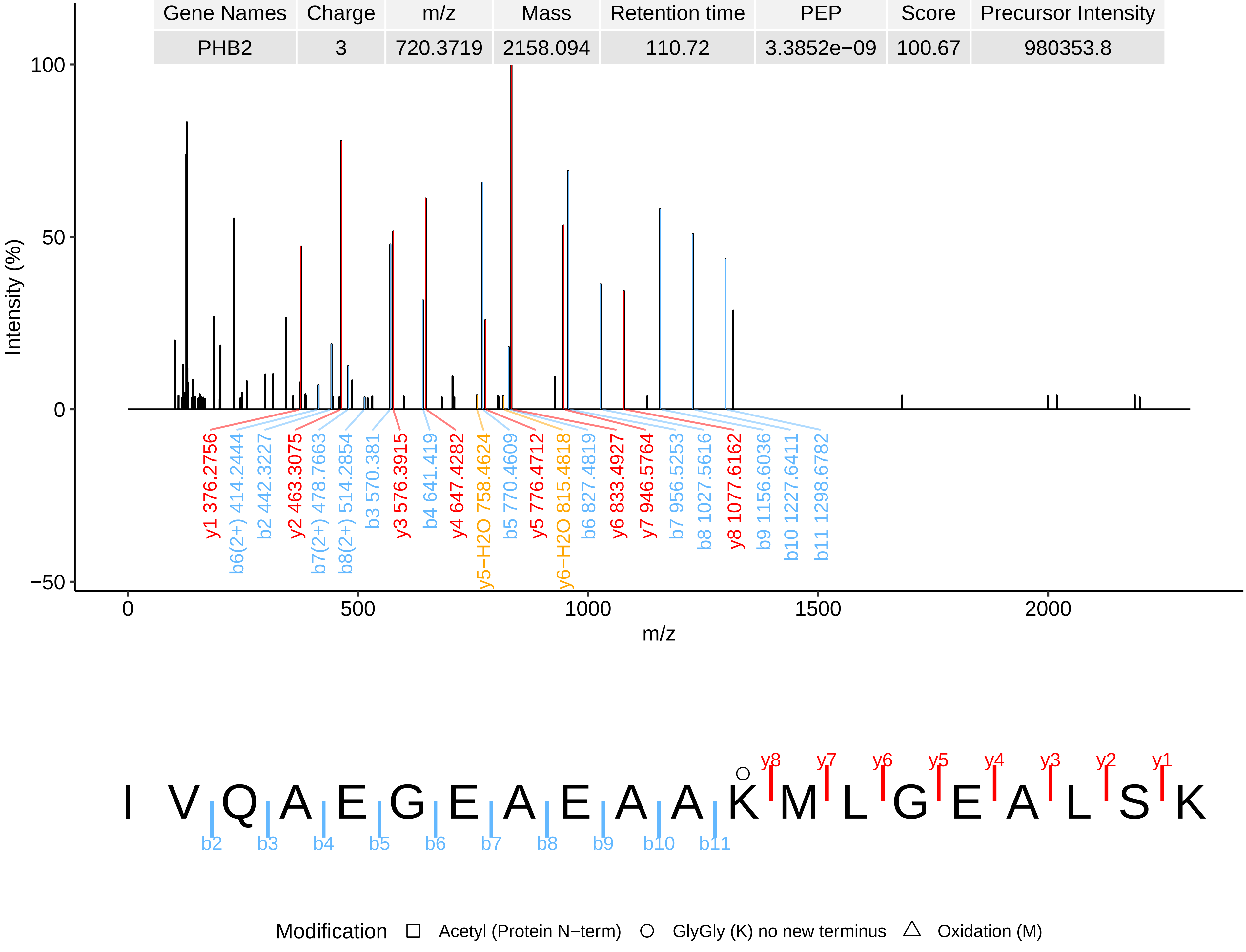

20200921_KIZ_MUb4_GlyGly_10plex_2 − Scan number: 23636

Gene Names	Charge	m/z	Mass	Retention time	PEP	Score	Precursor Intensity
PHB2	3	725.7036	2174.089	93.305	4.2931e−06	91.725	715206.9

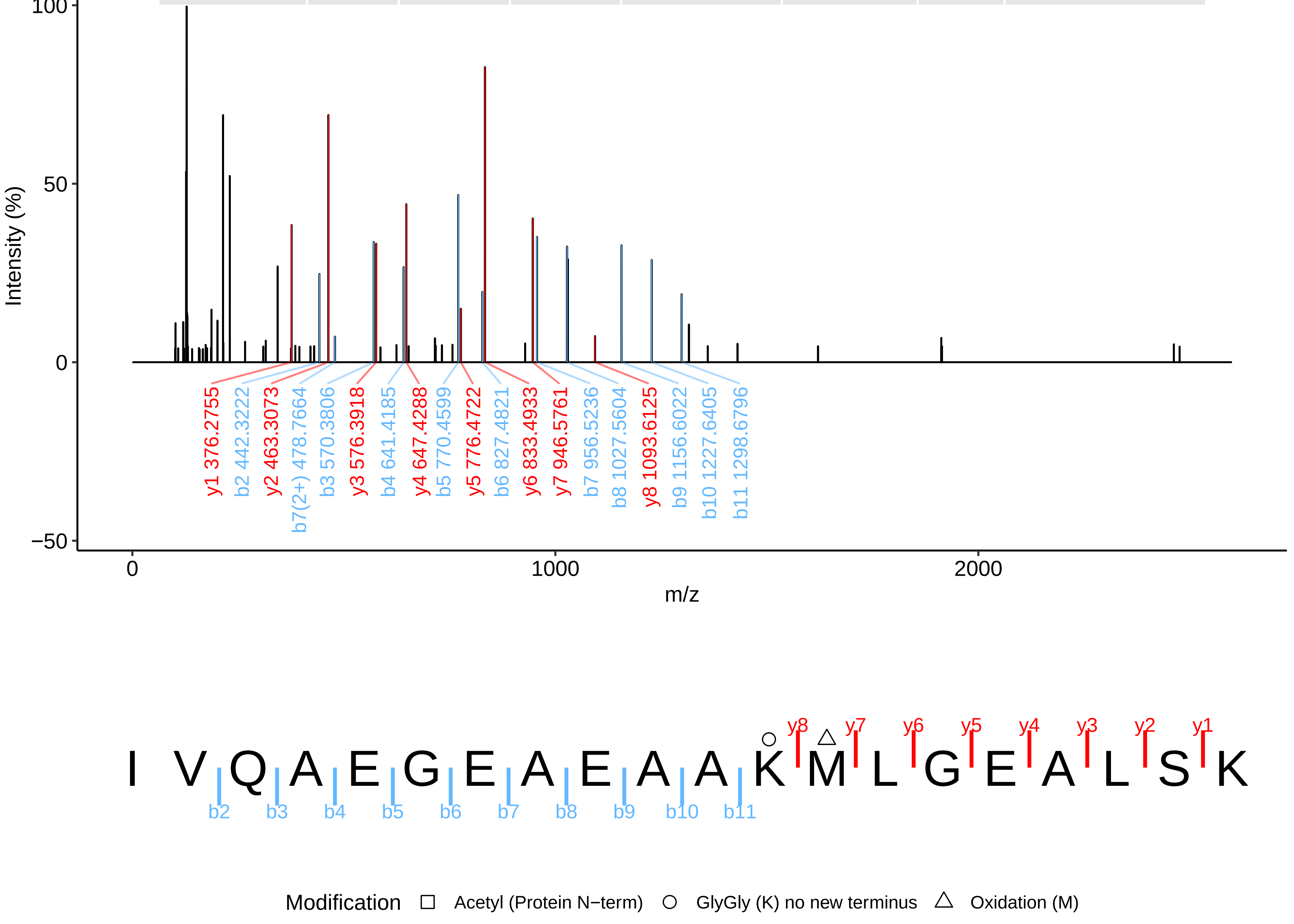

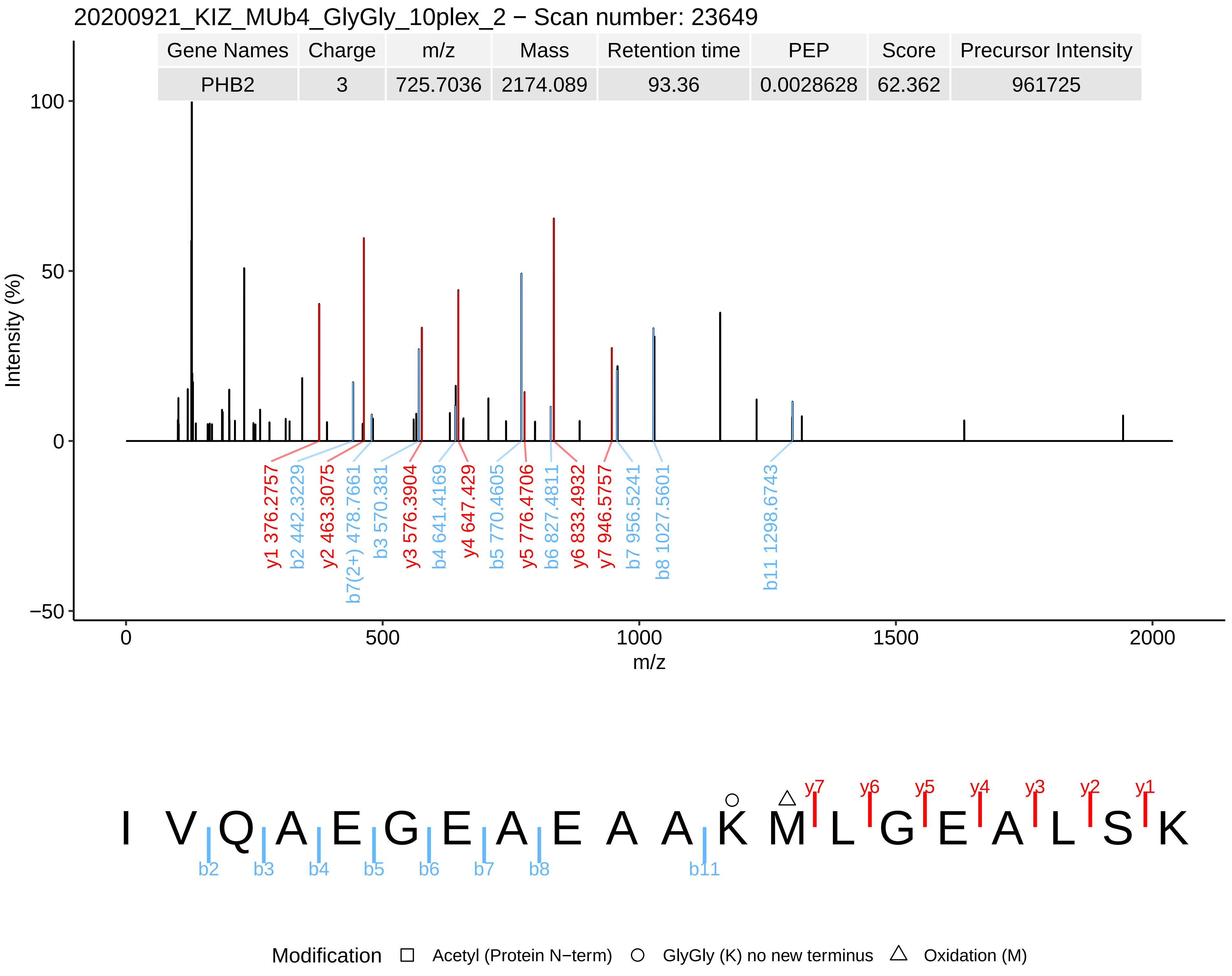
20200921_KIZ_MUb4_GlyGly_10plex_2 − Scan number: 23649
Gene Names | Charge | m/z | Mass | Retention time | PEP | Score | Precursor Intensity
PHB2 | 3 | 725.7036 | 2174.089 | 93.36 | 0.0028628 | 62.362 | 961725
Intensity (%)
100
50
0
−50
0
500
1000
1500
2000
m/z
y1 376.2757
b2 442.3229
y2 463.3075
b7(2+) 478.7661
b3 570.381
y3 576.3904
b4 641.4169
y4 647.429
b5 770.4605
y5 776.4706
b6 827.4811
y6 833.4932
y7 946.5757
b7 956.5241
b8 1027.5601
b11 1298.6743
I V Q A E G E A E A A K M L G E A L S K
y7 y6 y5 y4 y3 y2 y1
b2 b3 b4 b5 b6 b7 b8 b11
Modification
Acetyl (Protein N−term)
GlyGly (K) no new terminus
Oxidation (M)

20200921_KIZ_MUb4_GlyGly_10plex_2 − Scan number: 23776

Gene Names	Charge	m/z	Mass	Retention time	PEP	Score	Precursor Intensity
PHB2	3	725.7036	2174.089	93.875	7.9167e−06	80.236	397431

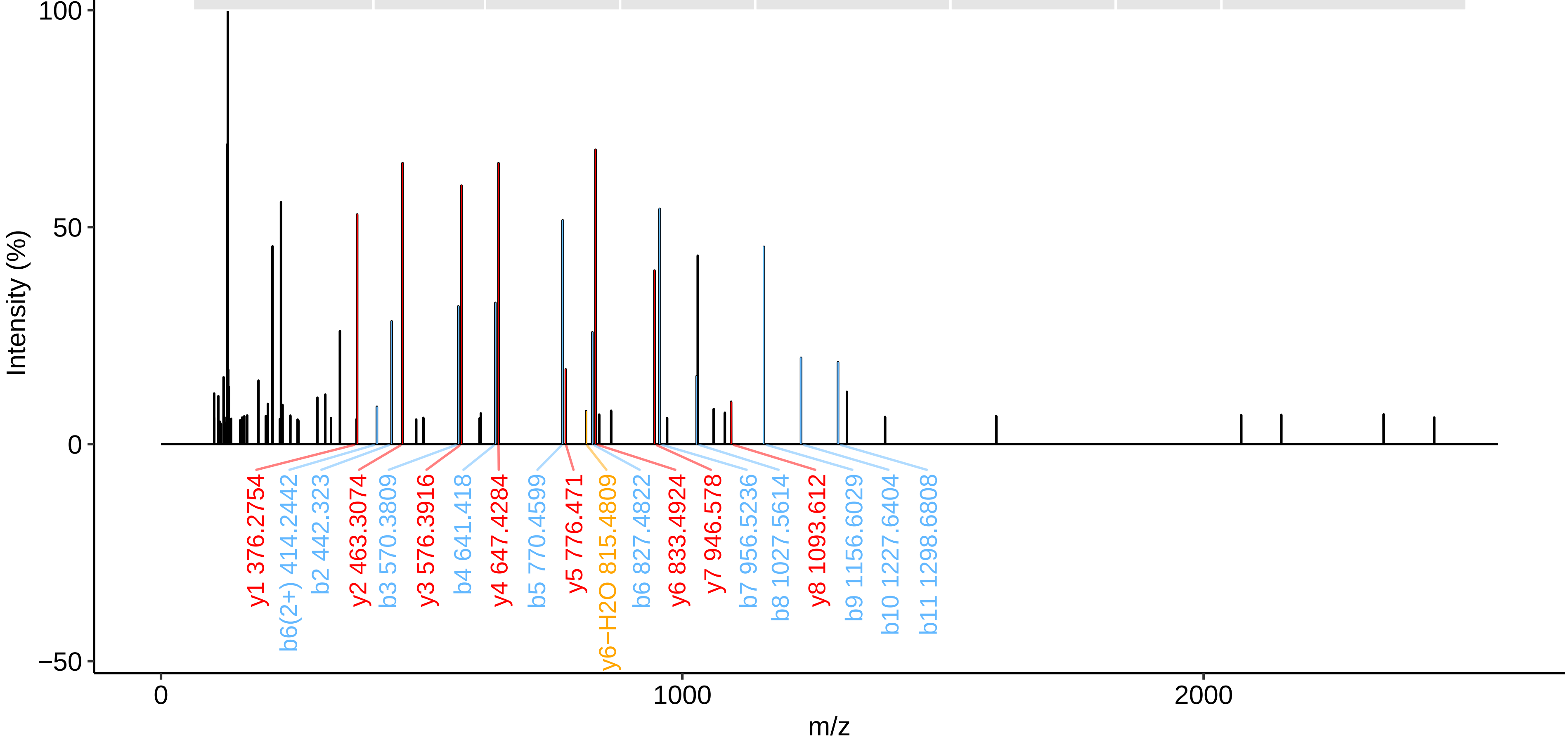

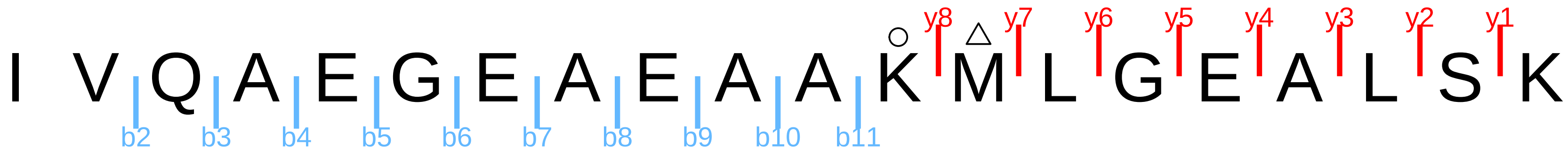

Modification ☐ Acetyl (Protein N−term) ○ GlyGly (K) no new terminus △ Oxidation (M)

20200921_KIZ_MUb4_GlyGly_10plex_2 − Scan number: 15395

Gene Names	Charge	m/z	Mass	Retention time	PEP	Score	Precursor Intensity
STAM	5	428.6266	2138.097	60.193	0.063515	51.695	766799.1

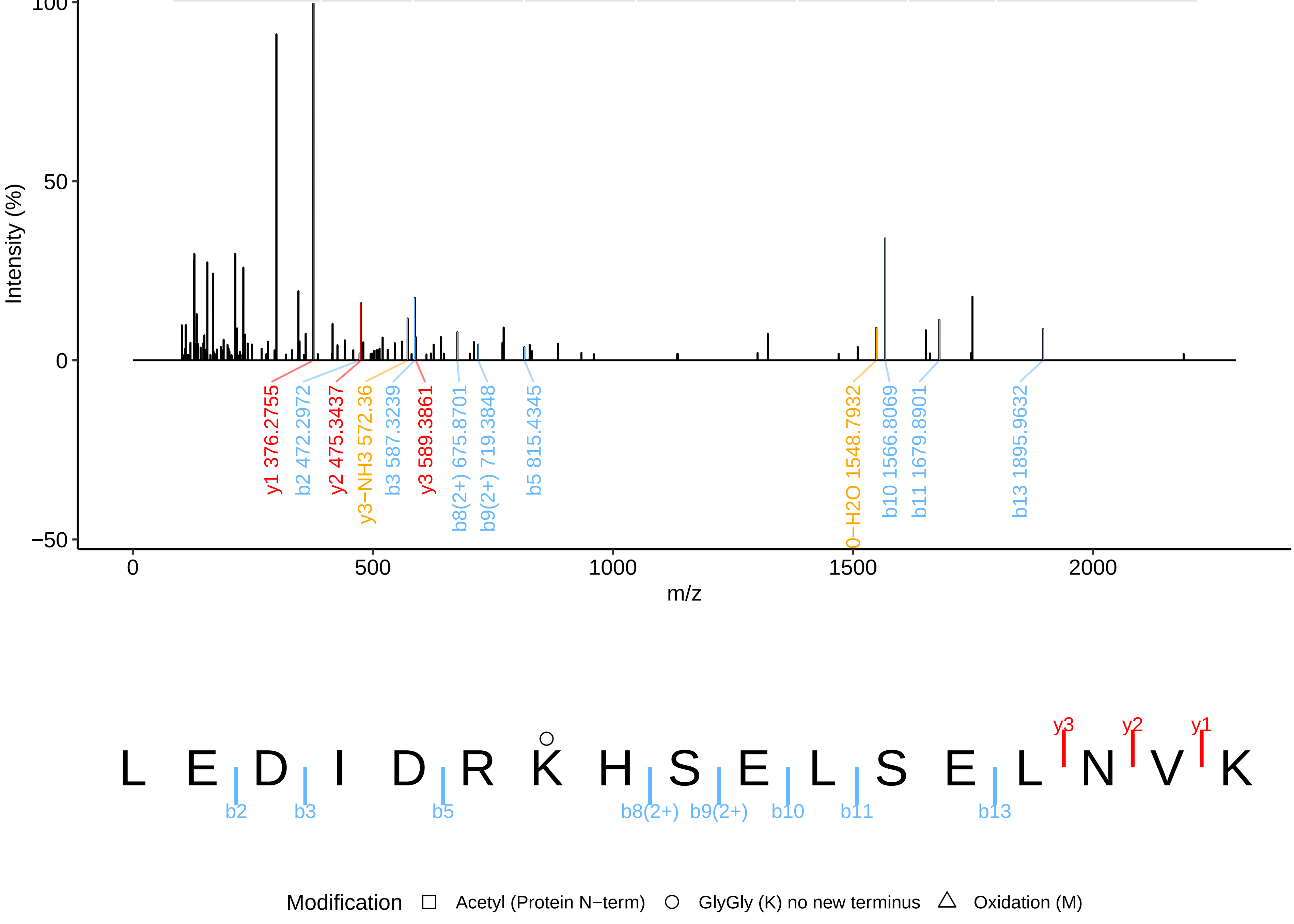

20200924_KIZ_MUb5_GlyGly_1 − Scan number: 15500

Gene Names	Charge	m/z	Mass	Retention time	PEP	Score	Precursor Intensity
STAM	5	428.6266	2138.097	50.609	0.0013912	73.208	9249472

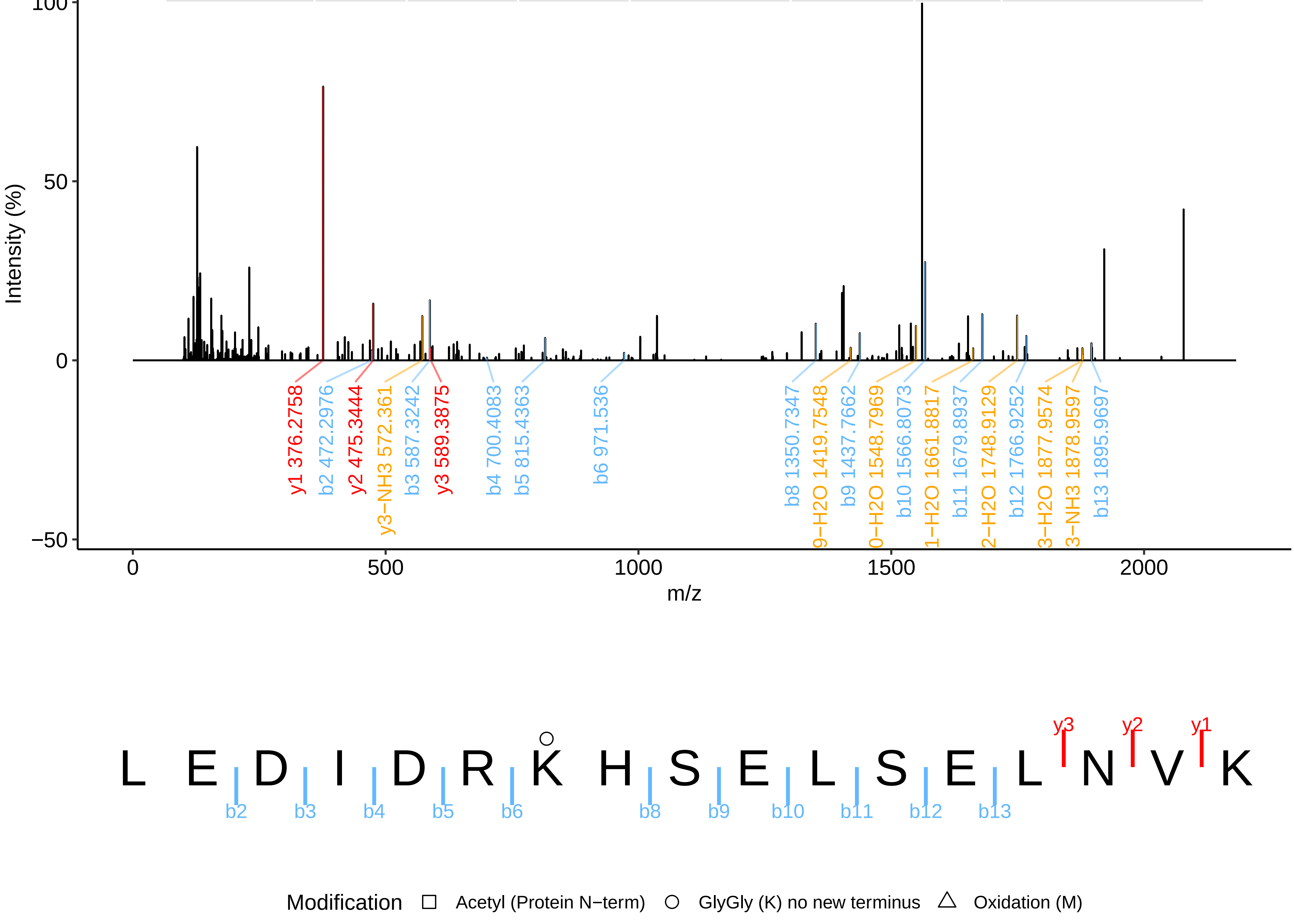

20200924_KIZ_MUb5_GlyGly_1 − Scan number: 15533

Gene Names	Charge	m/z	Mass	Retention time	PEP	Score	Precursor Intensity
STAM	4	535.5315	2138.097	50.727	6.9129e−12	119.46	3852825

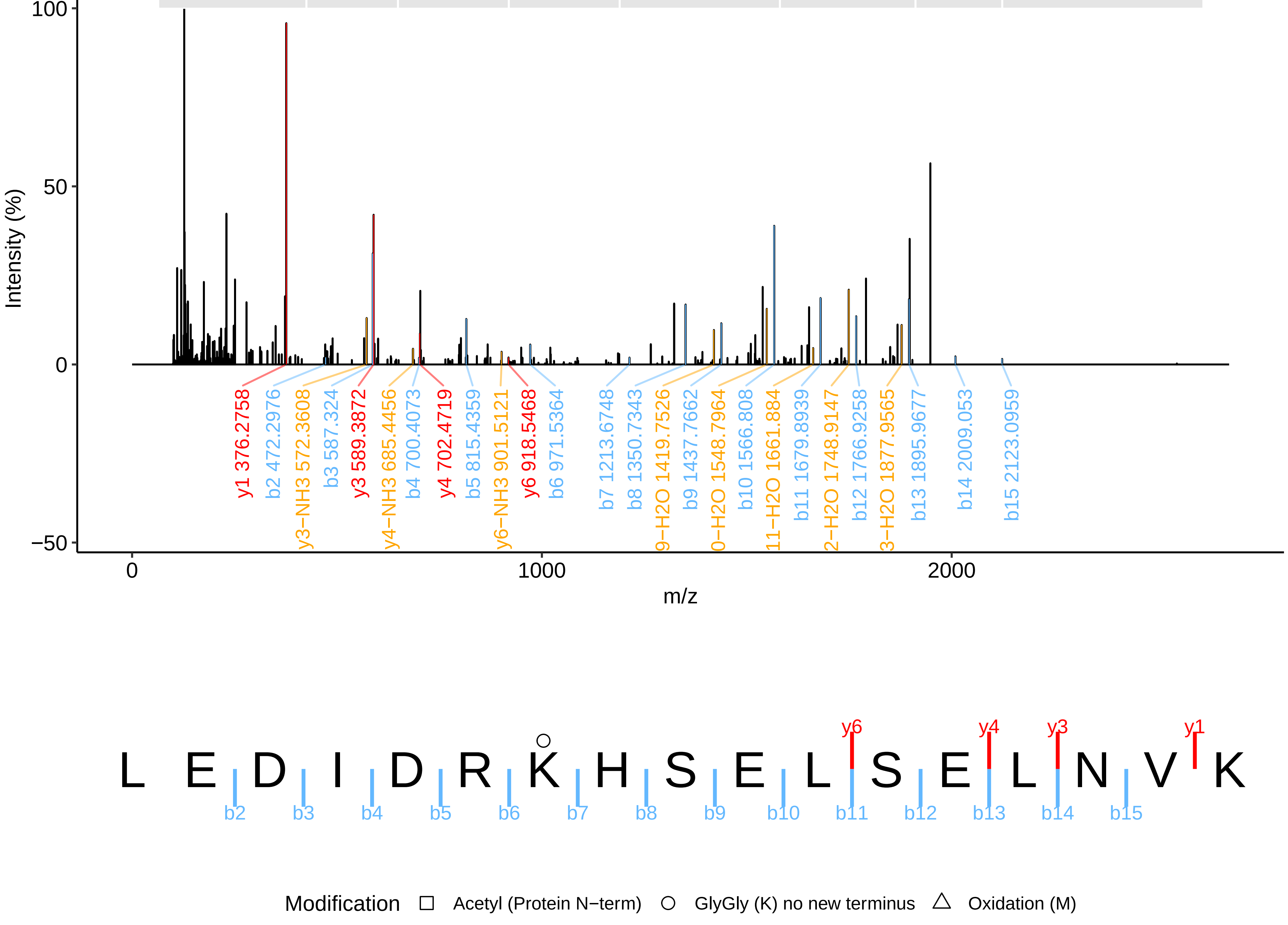

20201118_KIZ_MUb6_GlyGly_2 − Scan number: 17990

Gene Names	Charge	m/z	Mass	Retention time	PEP	Score	Precursor Intensity
STAM	5	428.6266	2138.097	60.849	3.9122e−13	128.9	10152162

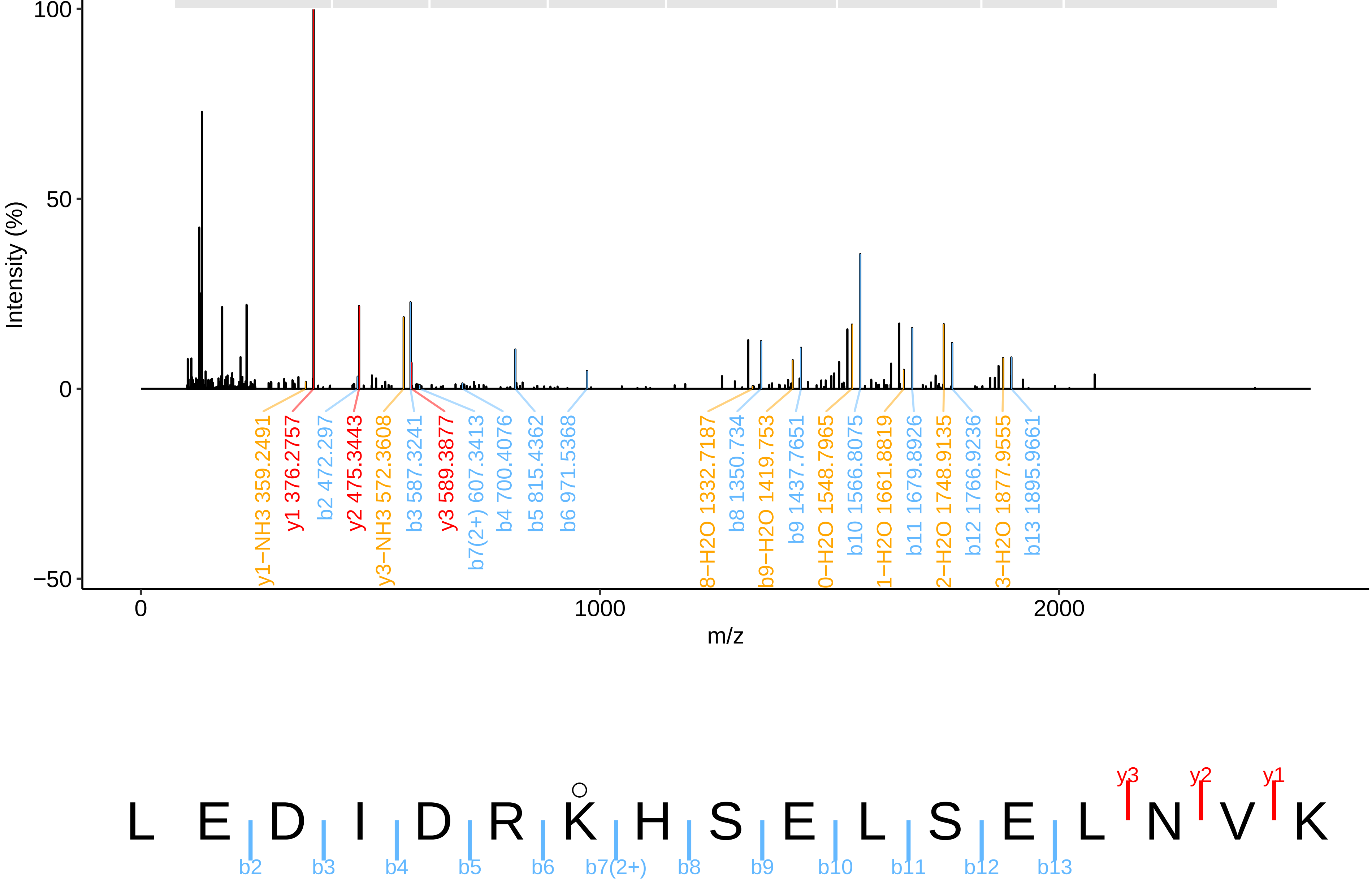

Modification □ Acetyl (Protein N−term) ○ GlyGly (K) no new terminus △ Oxidation (M)

20201118_KIZ_MUb6_GlyGly_2 − Scan number: 18016

Gene Names	Charge	m/z	Mass	Retention time	PEP	Score	Precursor Intensity
STAM	4	535.5315	2138.097	60.945	7.4239e−12	118.47	5352669

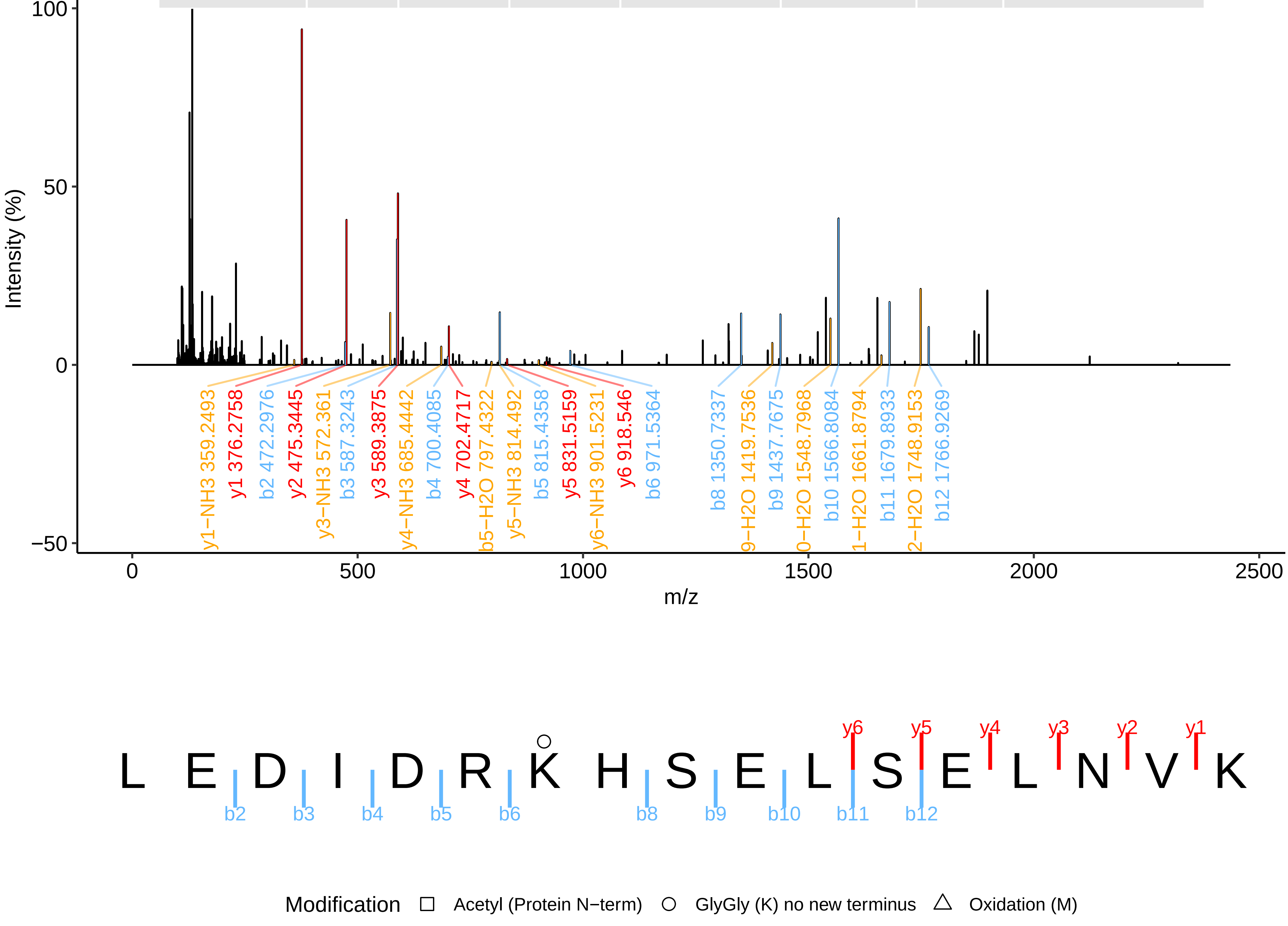

20200921_KIZ_MUb4_GlyGly_10plex − Scan number: 10844

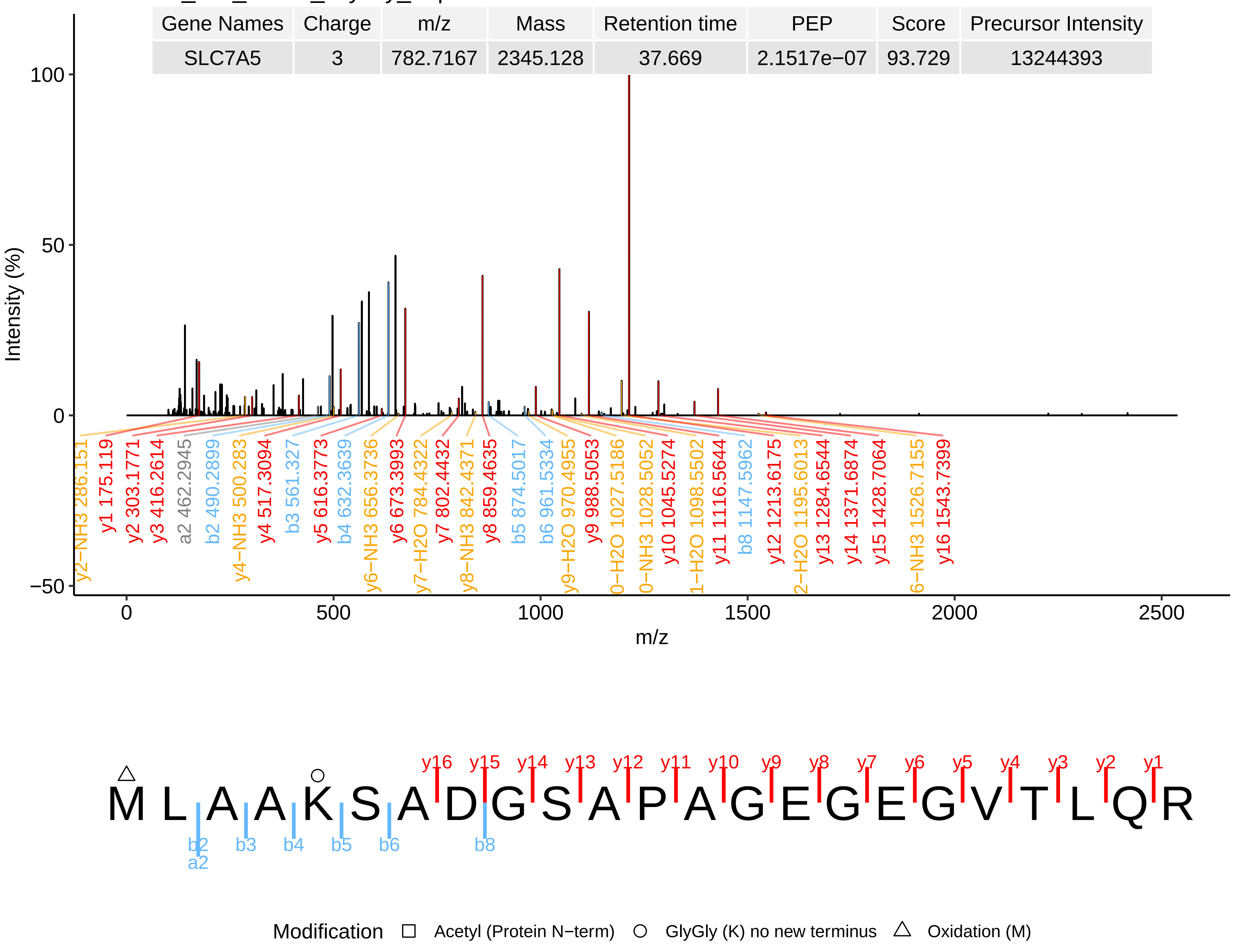

20200921_KIZ_MUb4_GlyGly_10plex − Scan number: 10984

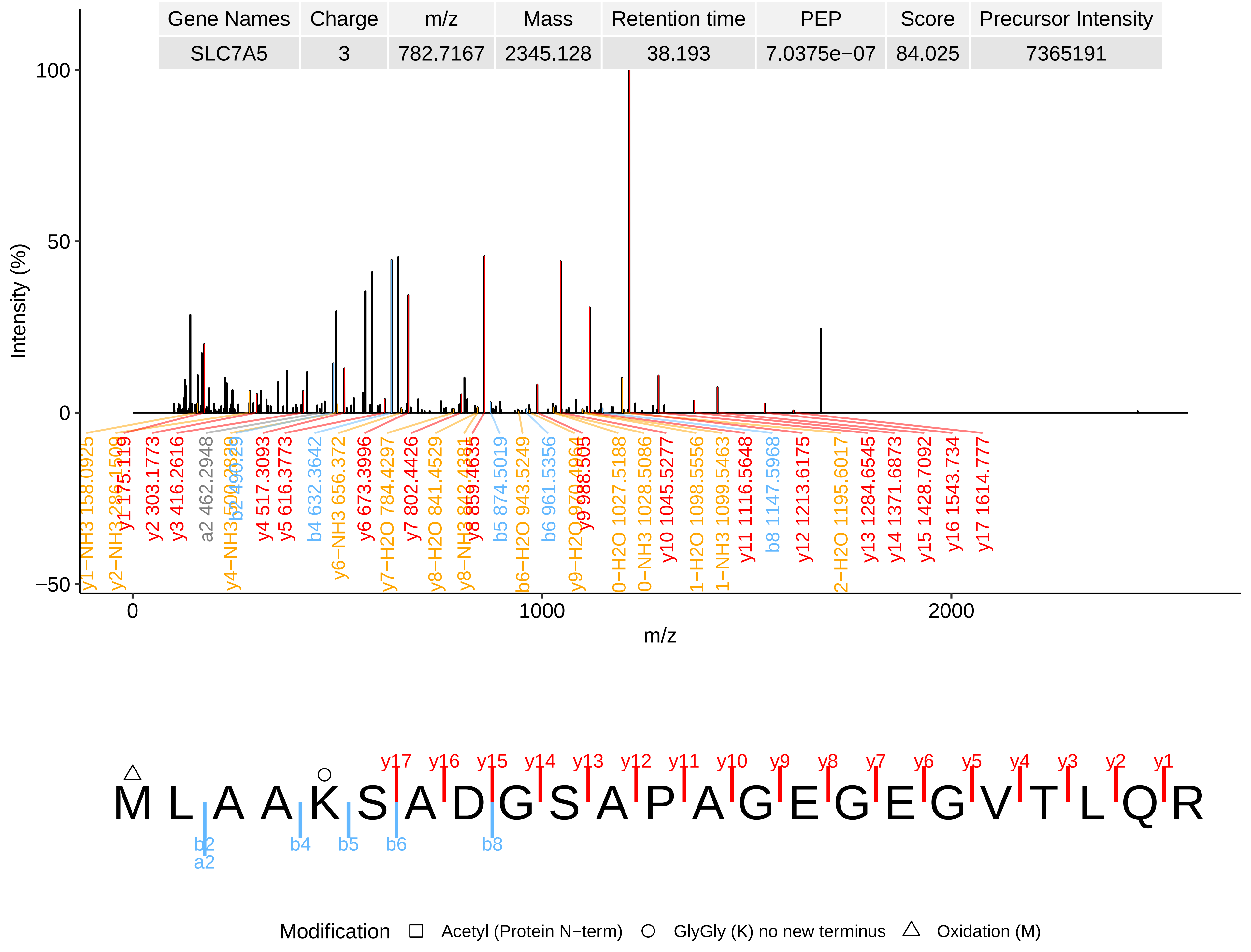

20200921_KIZ_MUb4_GlyGly_10plex_2 − Scan number: 9991

Gene Names	Charge	m/z	Mass	Retention time	PEP	Score	Precursor Intensity
SLC7A5	4	587.2893	2345.128	38.103	0.00025744	74.698	1010512

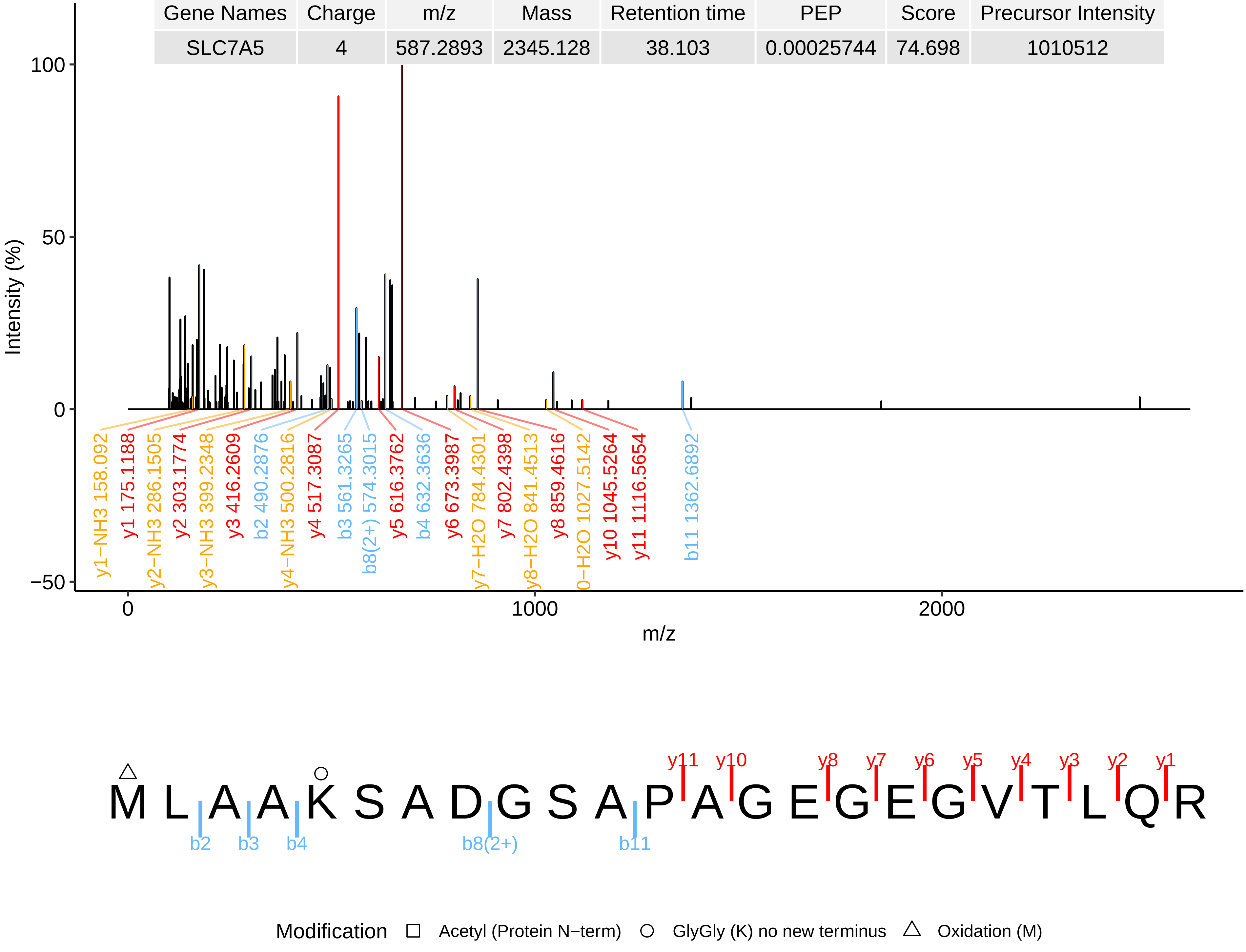

20200921_KIZ_MUb4_GlyGly_10plex_2 − Scan number: 10073

Gene Names	Charge	m/z	Mass	Retention time	PEP	Score	Precursor Intensity
SLC7A5	3	782.7167	2345.128	38.441	0.00034006	72.145	1053392

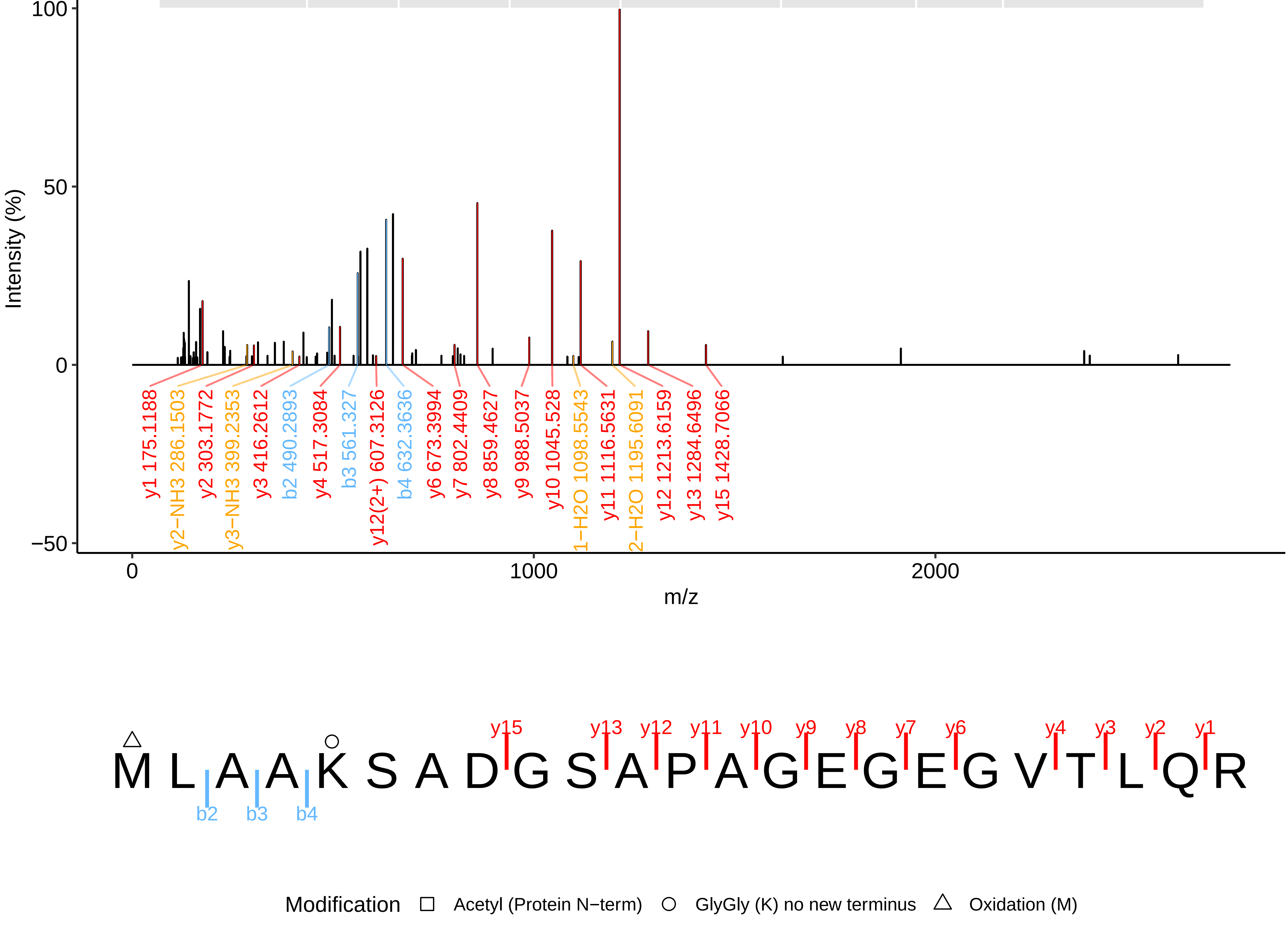

20200924_KIZ_MUb5_GlyGly_1 − Scan number: 9990

Gene Names	Charge	m/z	Mass	Retention time	PEP	Score	Precursor Intensity
SLC7A5	3	782.7167	2345.128	32.194	1.994e−10	99.93	NA

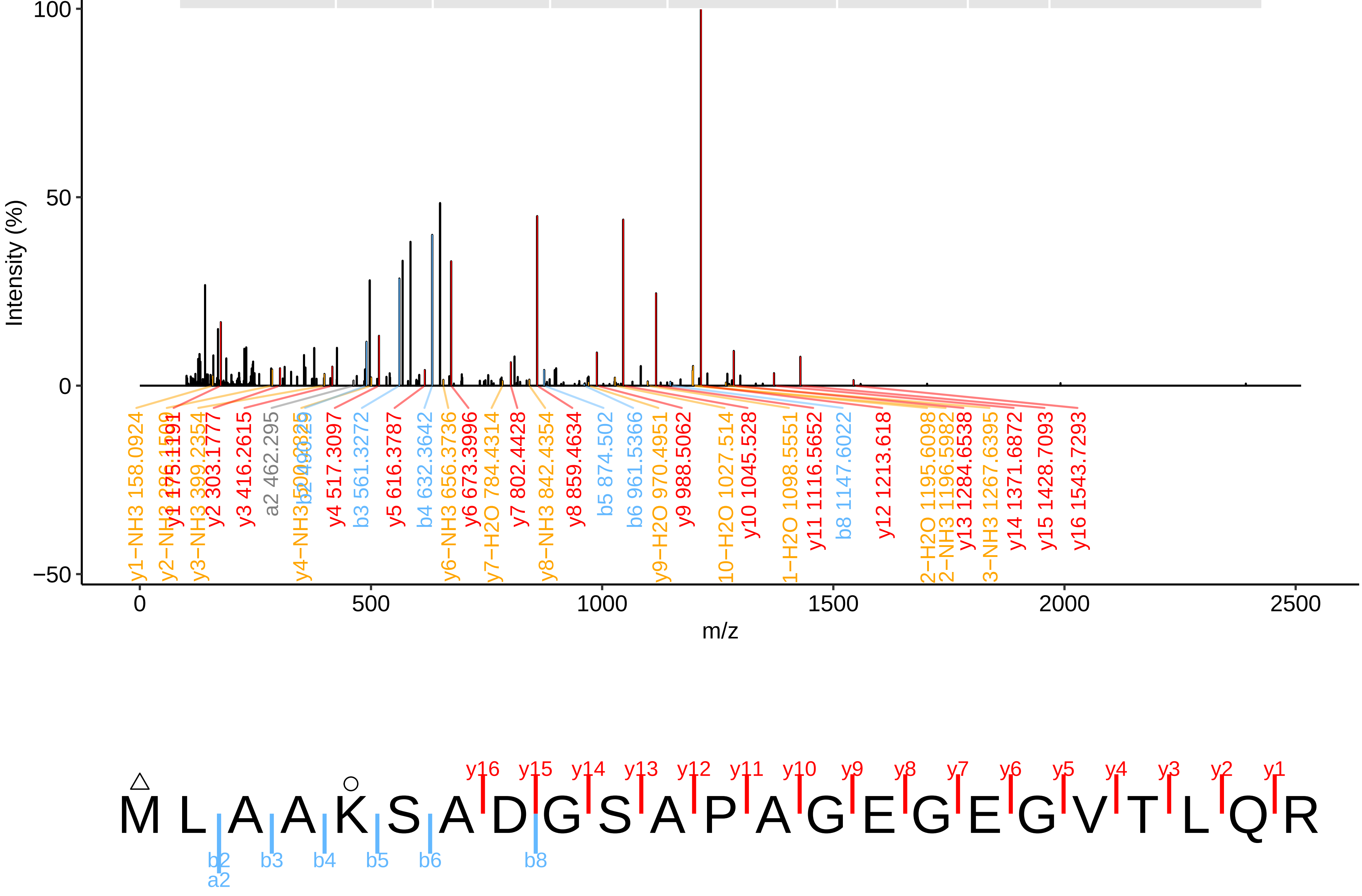

Modification □ Acetyl (Protein N−term) ○ GlyGly (K) no new terminus △ Oxidation (M)

20200924_KIZ_MUb5_GlyGly_1 − Scan number: 10030

Gene Names	Charge	m/z	Mass	Retention time	PEP	Score	Precursor Intensity
SLC7A5	3	782.7167	2345.128	32.325	3.7902e−07	90.475	10345402

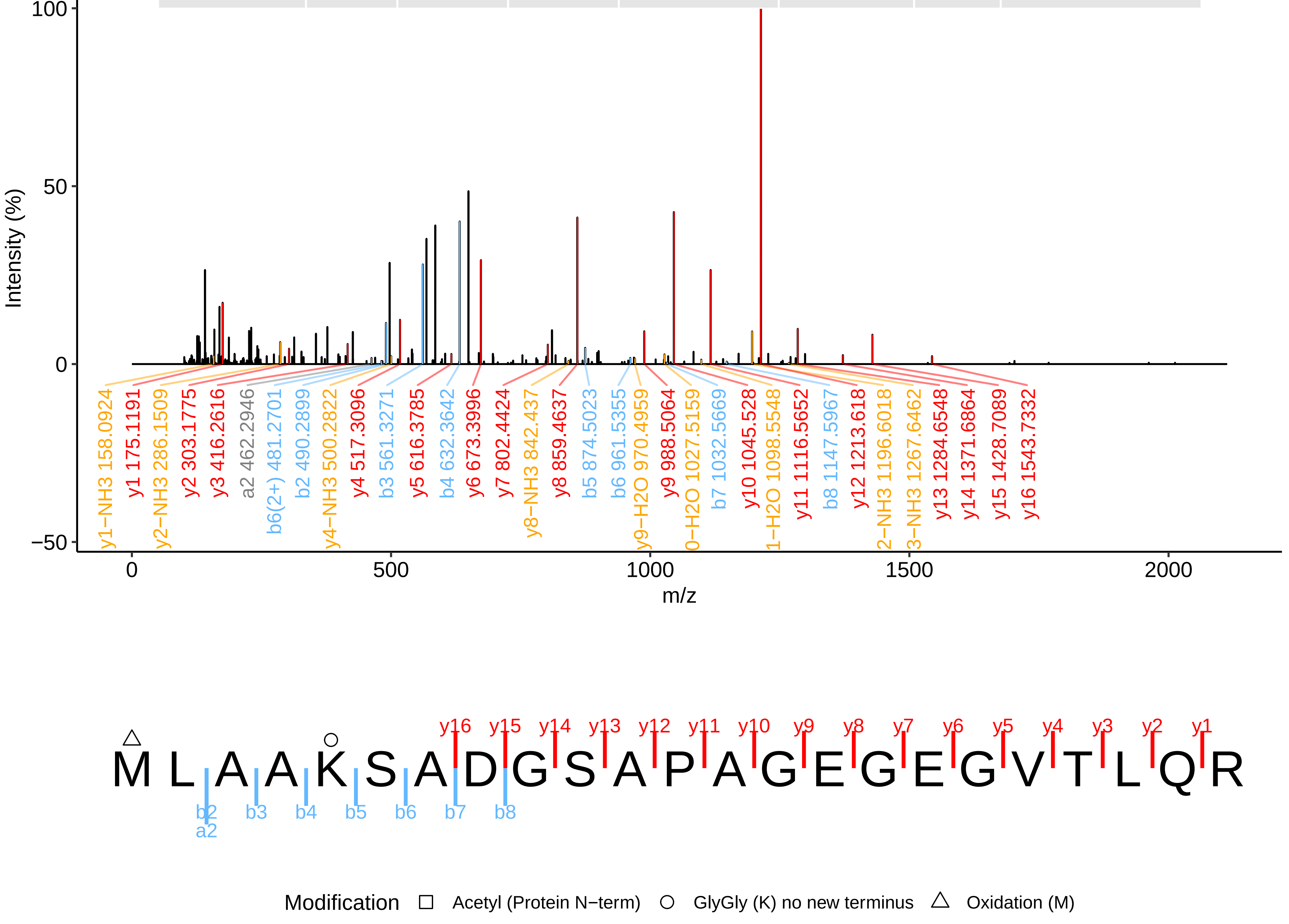

20200924_KIZ_MUb5_GlyGly_1 − Scan number: 12661

Gene Names	Charge	m/z	Mass	Retention time	PEP	Score	Precursor Intensity
SLC7A5	3	782.7167	2345.128	41.229	0.00020885	75.646	NA

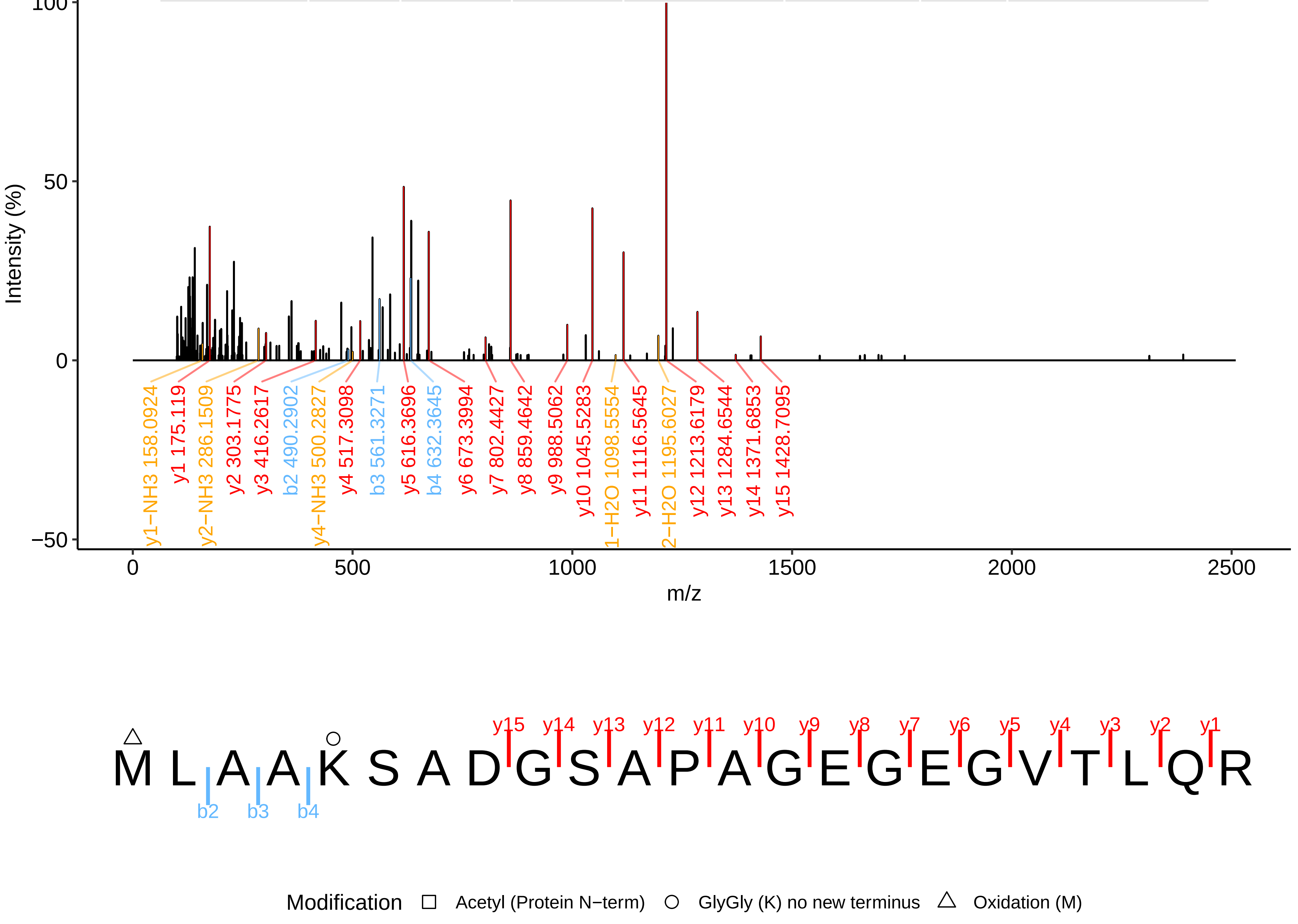

20200924_KIZ_MUb5_GlyGly_2 − Scan number: 9186

Gene Names	Charge	m/z	Mass	Retention time	PEP	Score	Precursor Intensity
SLC7A5	3	782.7167	2345.128	34.336	2.0605e−10	99.414	4098961

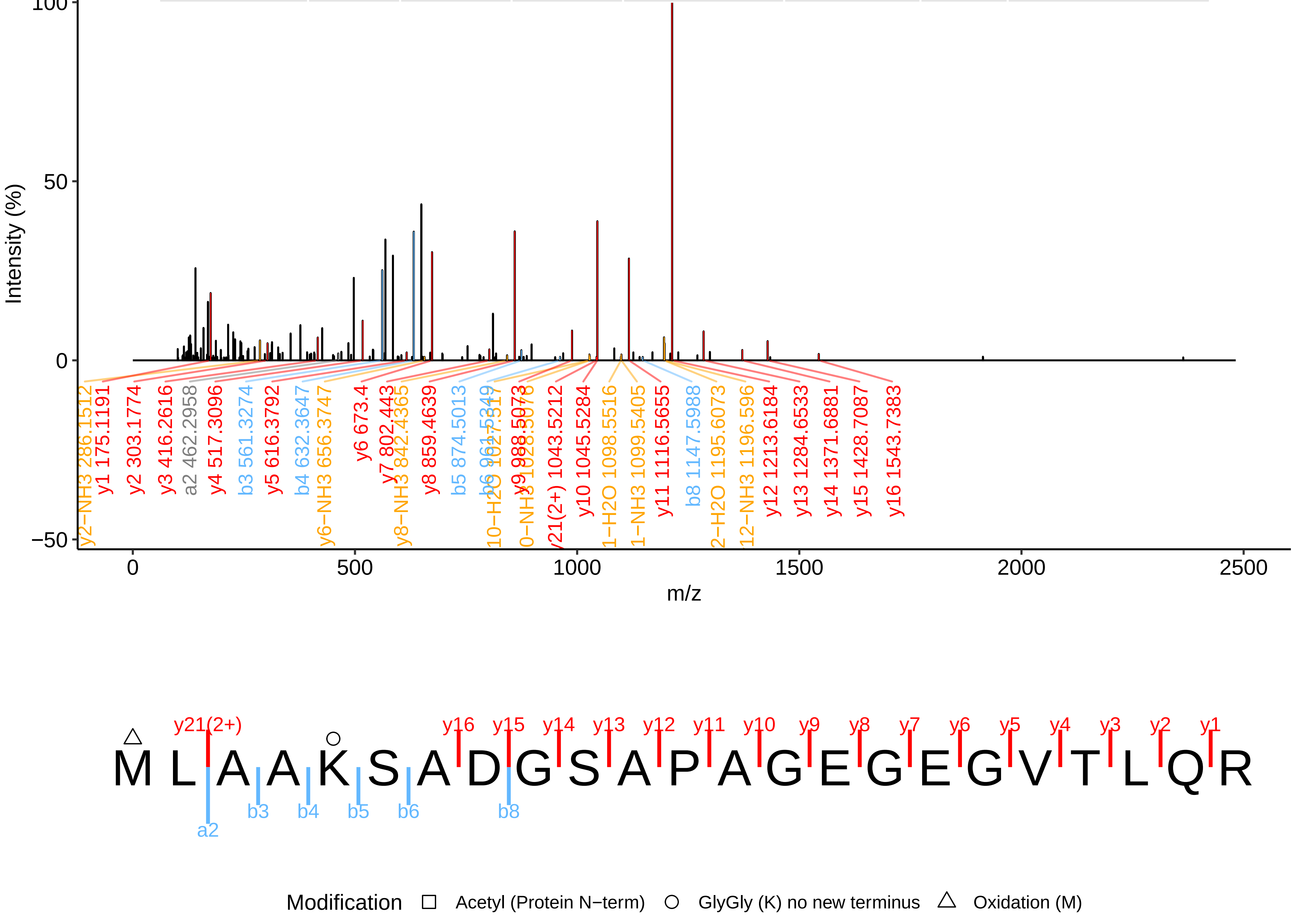

20200924_KIZ_MUb5_GlyGly_2 − Scan number: 9323

Gene Names	Charge	m/z	Mass	Retention time	PEP	Score	Precursor Intensity
SLC7A5	3	782.7167	2345.128	34.844	1.7629e−15	117.35	4726639

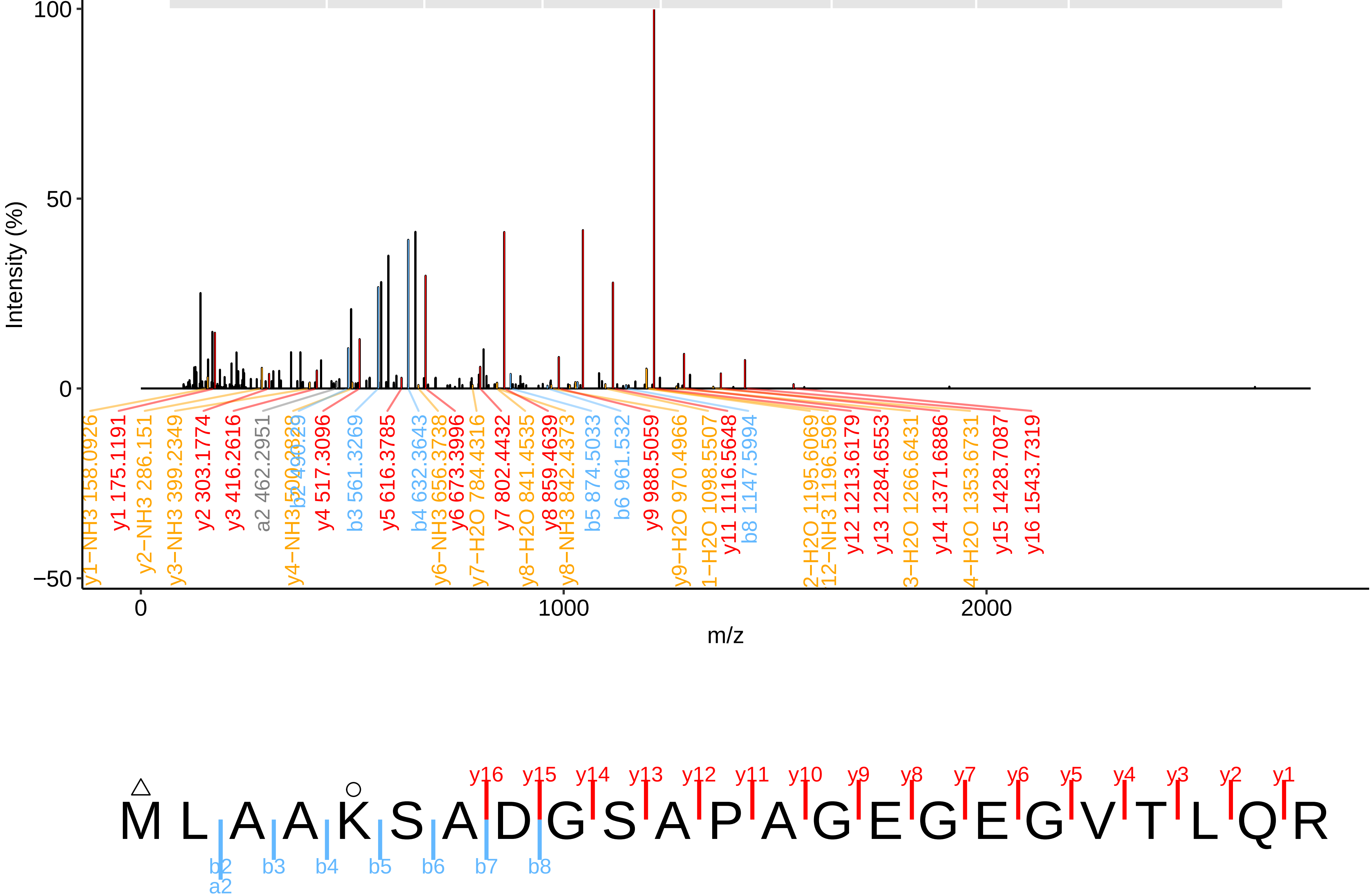

Modification □ Acetyl (Protein N−term) ○ GlyGly (K) no new terminus △ Oxidation (M)

20201118_KIZ_MUb6_GlyGly_2 − Scan number: 11356

Gene Names	Charge	m/z	Mass	Retention time	PEP	Score	Precursor Intensity
SLC7A5	3	782.7167	2345.128	38.325	1.1658e−11	99.603	4143181

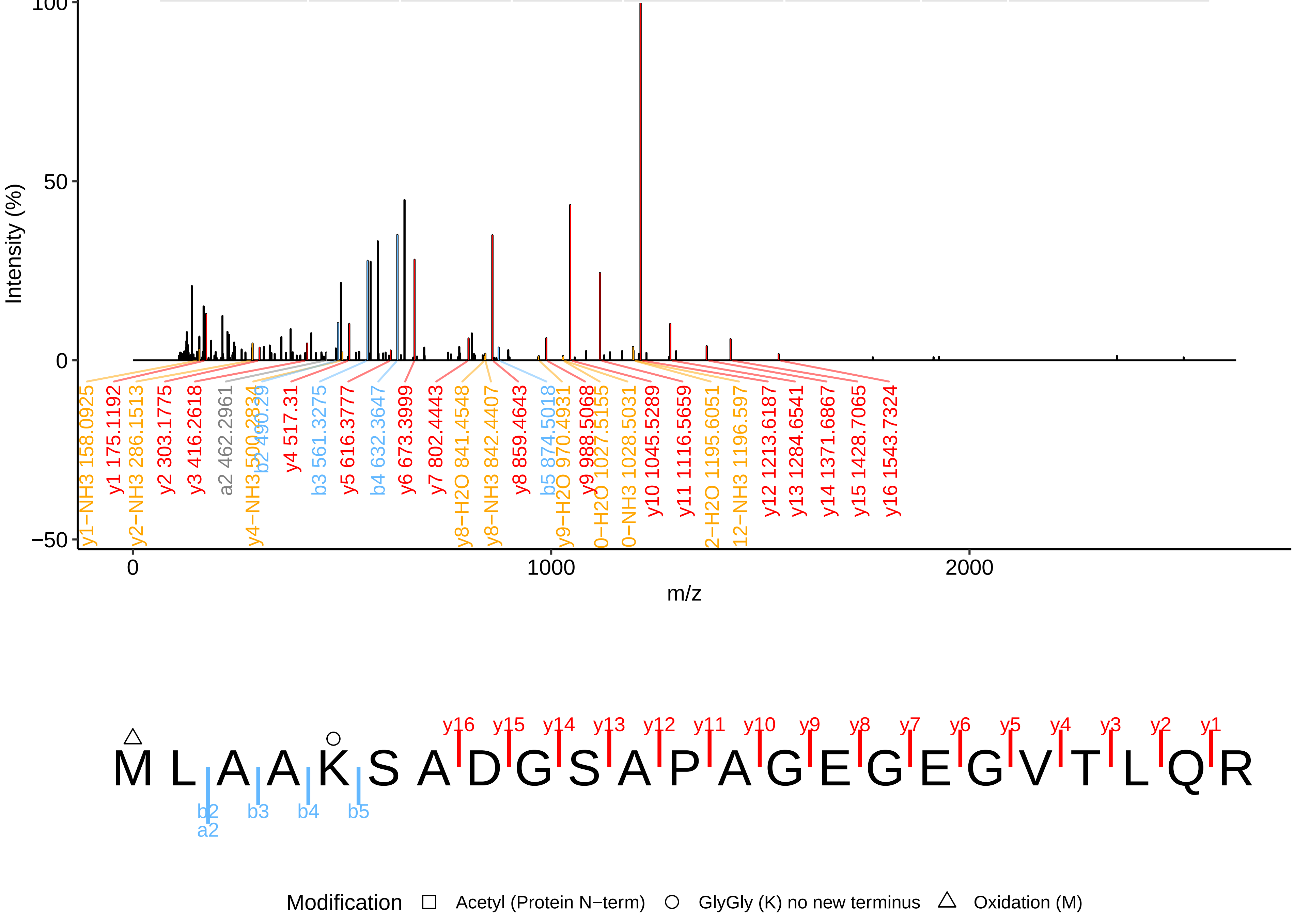

20200921_KIZ_MUb4_GlyGly_10plex − Scan number: 13093

Gene Names	Charge	m/z	Mass	Retention time	PEP	Score	Precursor Intensity
SLC1A3	4	559.7638	2235.026	46.059	7.6704e−30	171.72	20080494

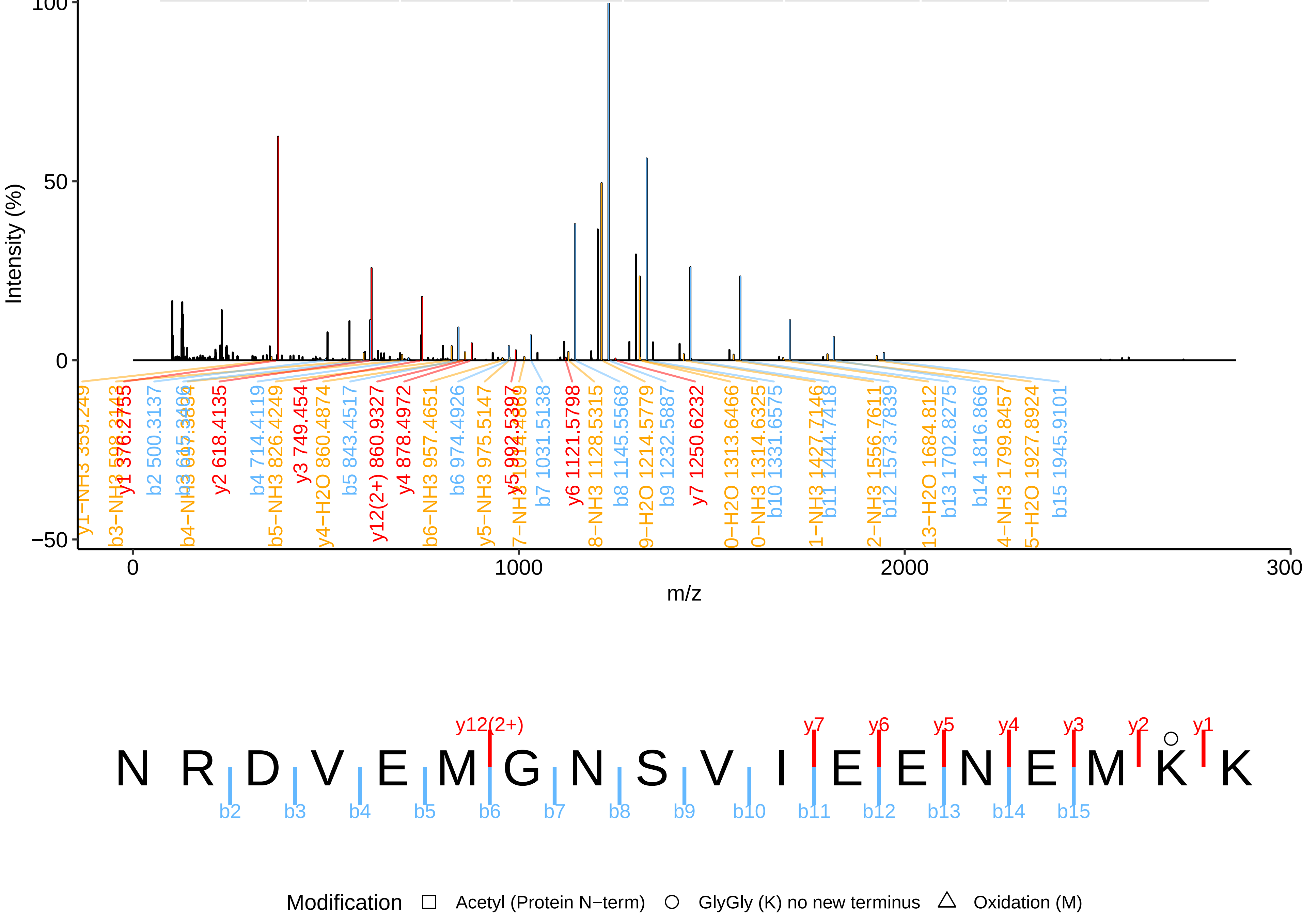

20200921_KIZ_MUb4_GlyGly_10plex_2 − Scan number: 12030

Gene Names	Charge	m/z	Mass	Retention time	PEP	Score	Precursor Intensity
SLC1A3	4	559.7638	2235.026	46.452	0.0069355	73.208	792125.4

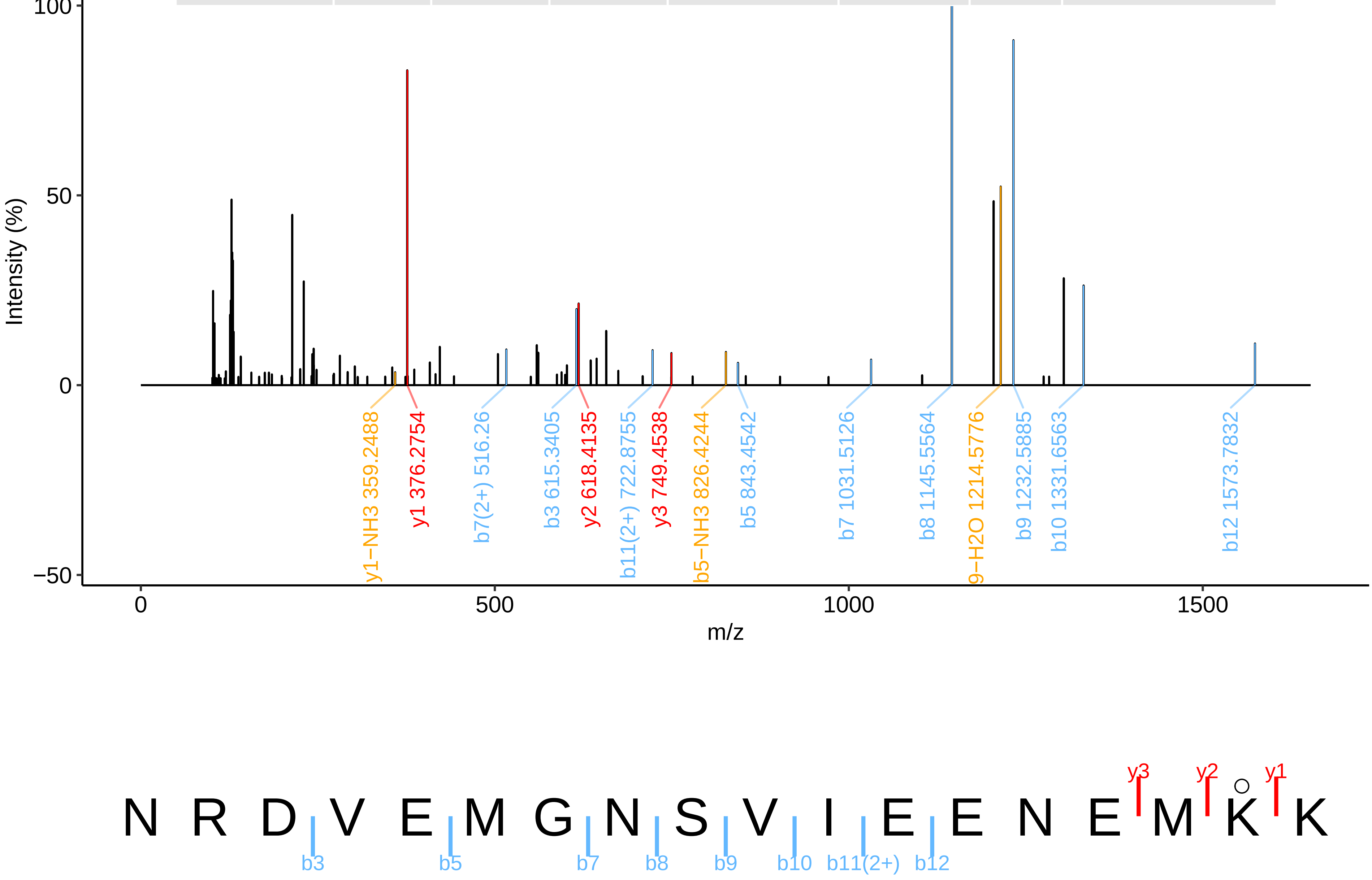

Modification □ Acetyl (Protein N−term) ○ GlyGly (K) no new terminus △ Oxidation (M)

20200921_KIZ_MUb4_GlyGly_10plex_2 − Scan number: 12032

Gene Names	Charge	m/z	Mass	Retention time	PEP	Score	Precursor Intensity
SLC1A3	4	559.7638	2235.026	46.461	6.0985e−05	82.449	1139580

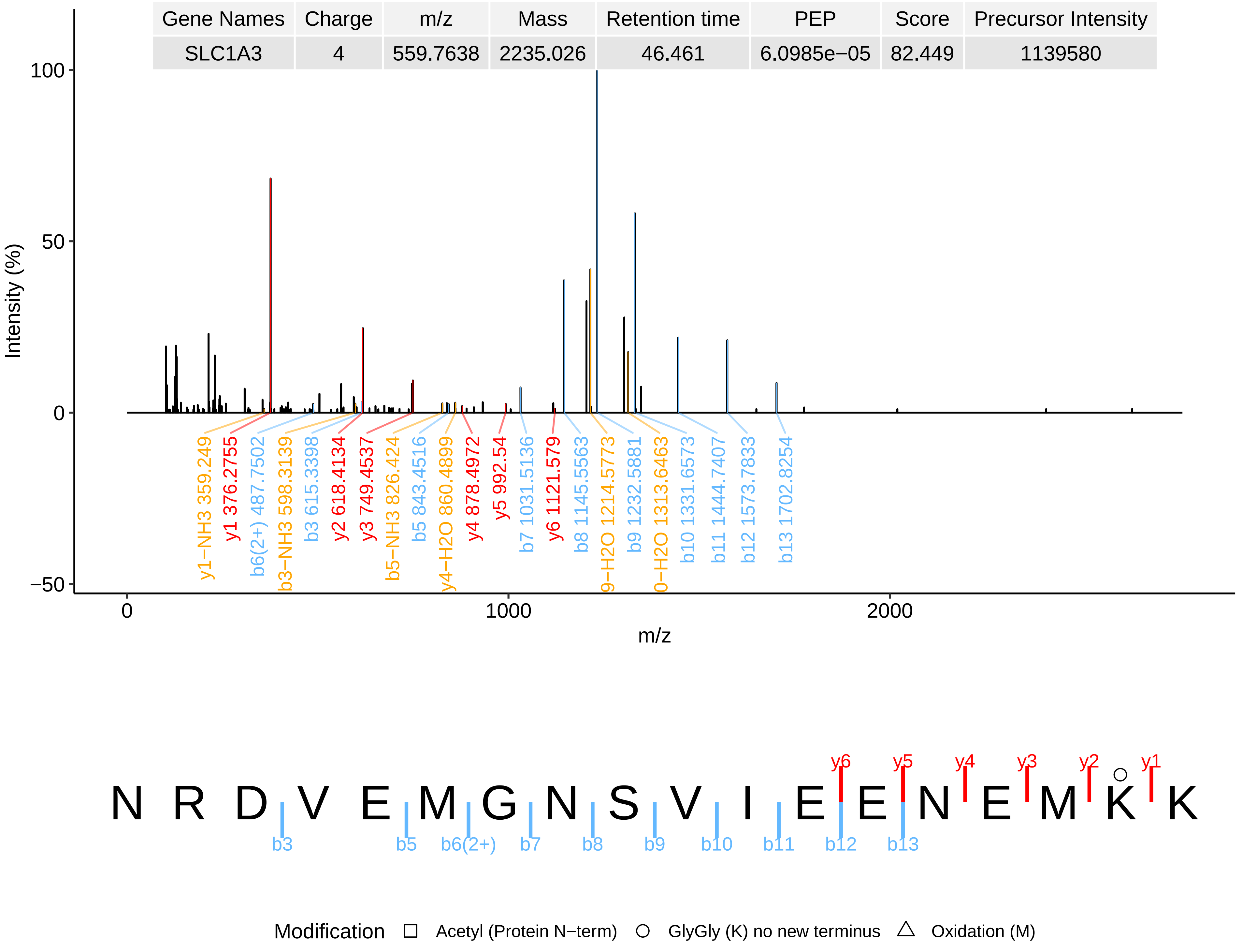

20200924_KIZ_MUb5_GlyGly_1 − Scan number: 12003

Gene Names	Charge	m/z	Mass	Retention time	PEP	Score	Precursor Intensity
SLC1A3	4	559.7638	2235.026	38.987	6.723e−30	173.37	9356172

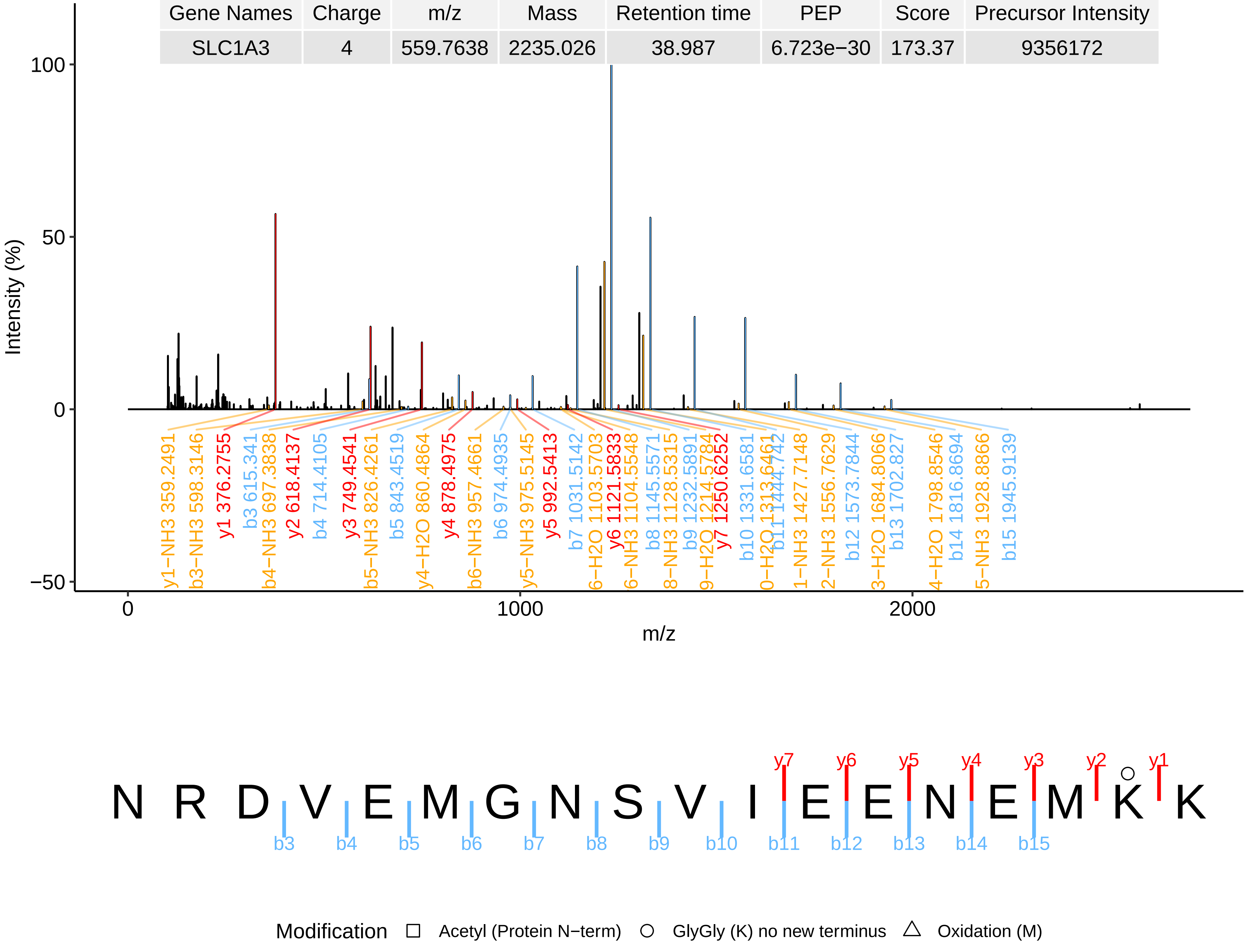

20200921_KIZ_MUb4_GlyGly_10plex − Scan number: 30686

Gene Names	Charge	m/z	Mass	Retention time	PEP	Score	Precursor Intensity
PTRF	3	739.7108	2216.111	112.84	7.6528e−21	145.88	2590476

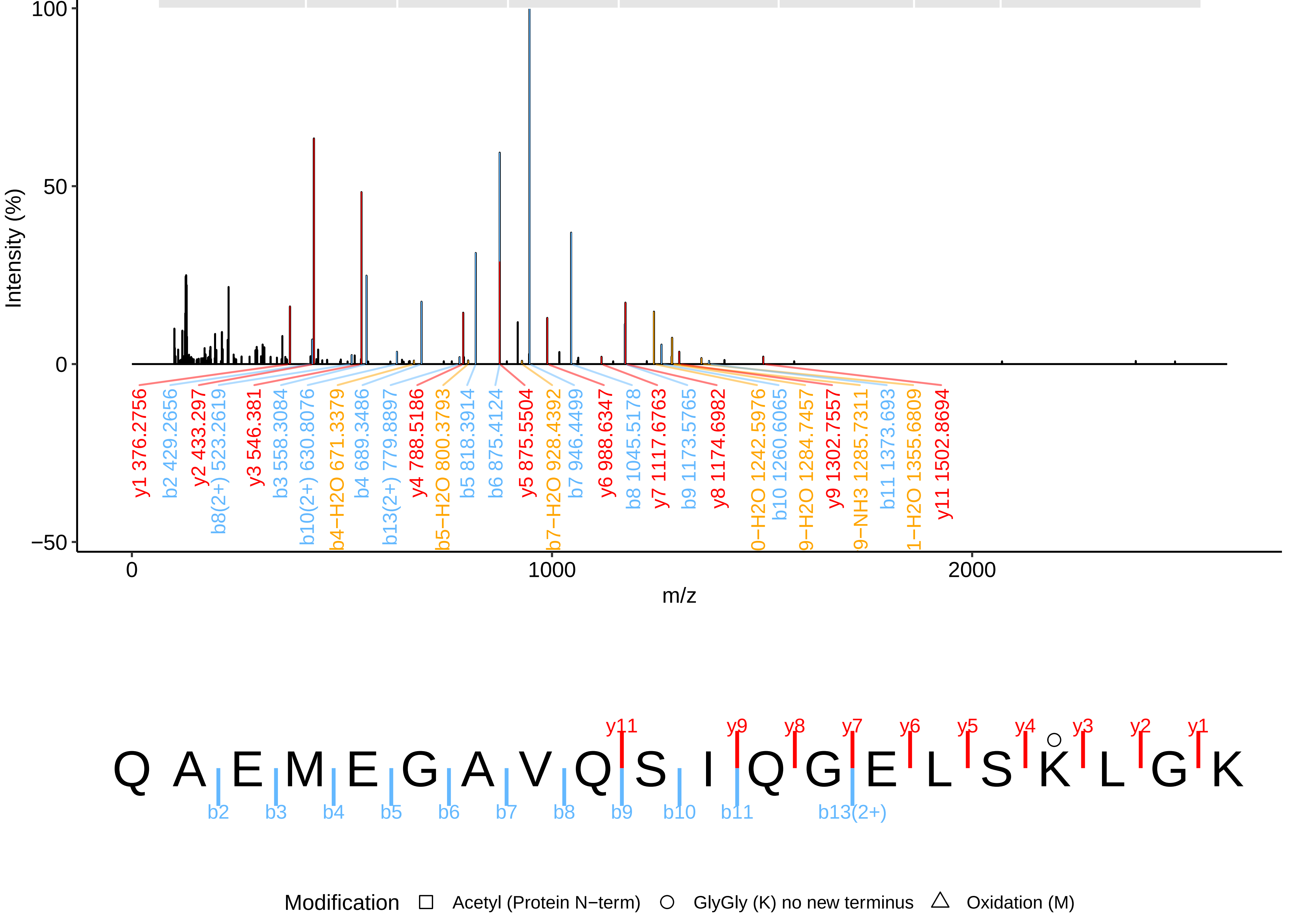

20200921_KIZ_MUb4_GlyGly_10plex_2 − Scan number: 17261

Gene Names	Charge	m/z	Mass	Retention time	PEP	Score	Precursor Intensity
INTS7	3	795.0266	2382.058	67.479	0.0015937	67.995	661619.3

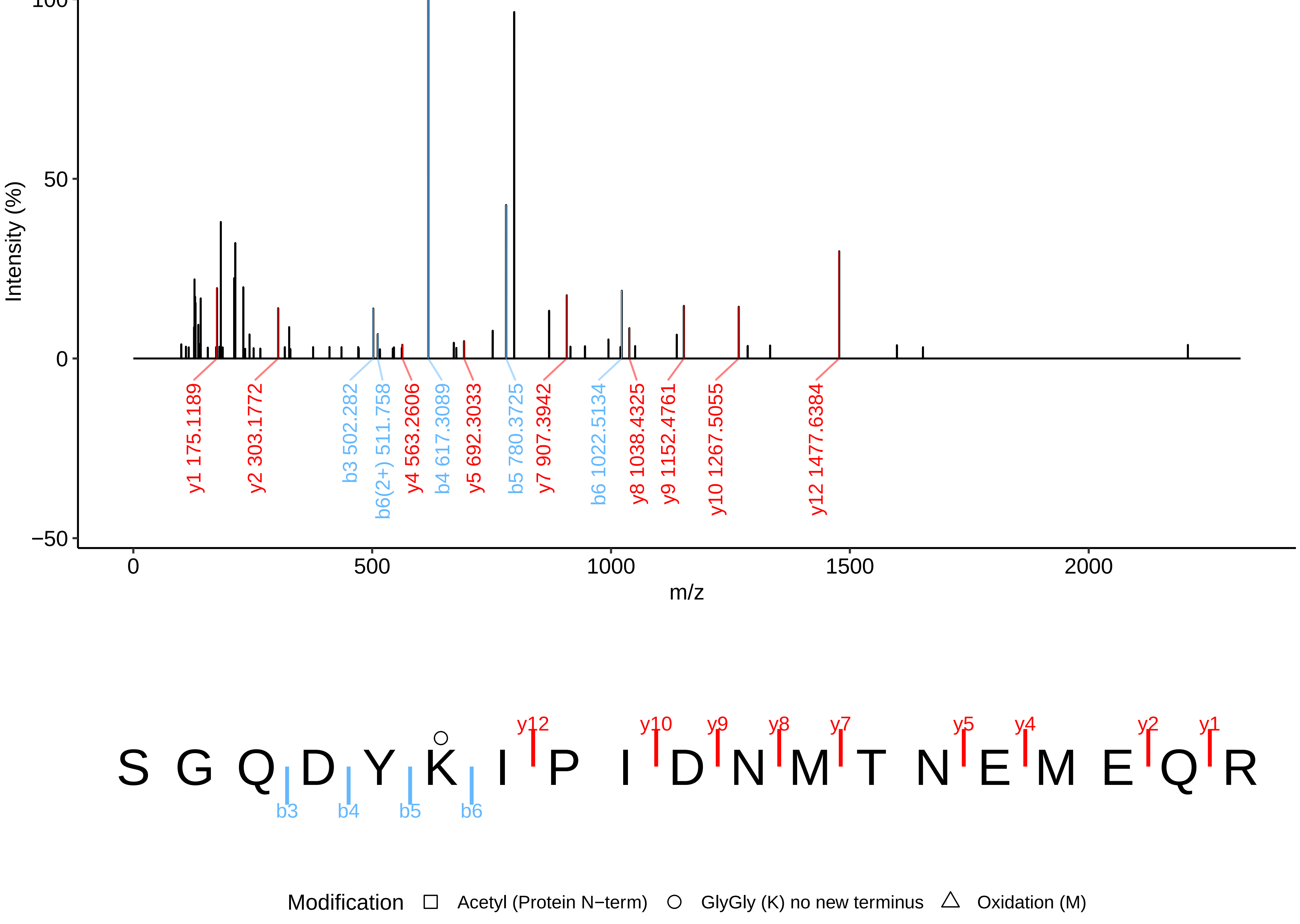

20200921_KIZ_MUb4_GlyGly_10plex − Scan number: 15590

Gene Names	Charge	m/z	Mass	Retention time	PEP	Score	Precursor Intensity
TMEM59	4	601.0462	2400.156	55.726	1.1541e−14	121.27	6090152

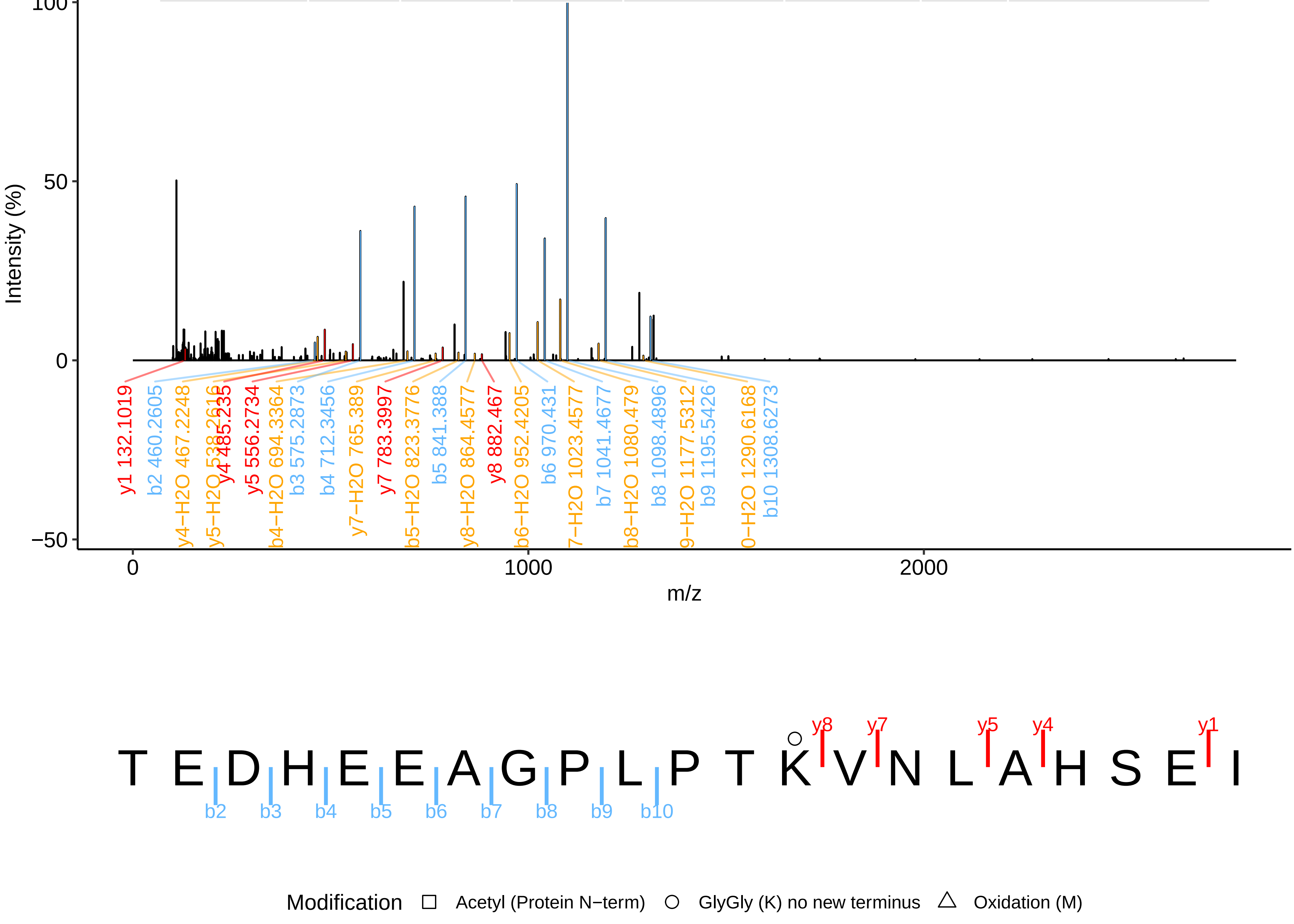

20200921_KIZ_MUb4_GlyGly_10plex − Scan number: 15721

Gene Names	Charge	m/z	Mass	Retention time	PEP	Score	Precursor Intensity
TMEM59	4	601.0462	2400.156	56.24	0.00090174	65.47	1360626

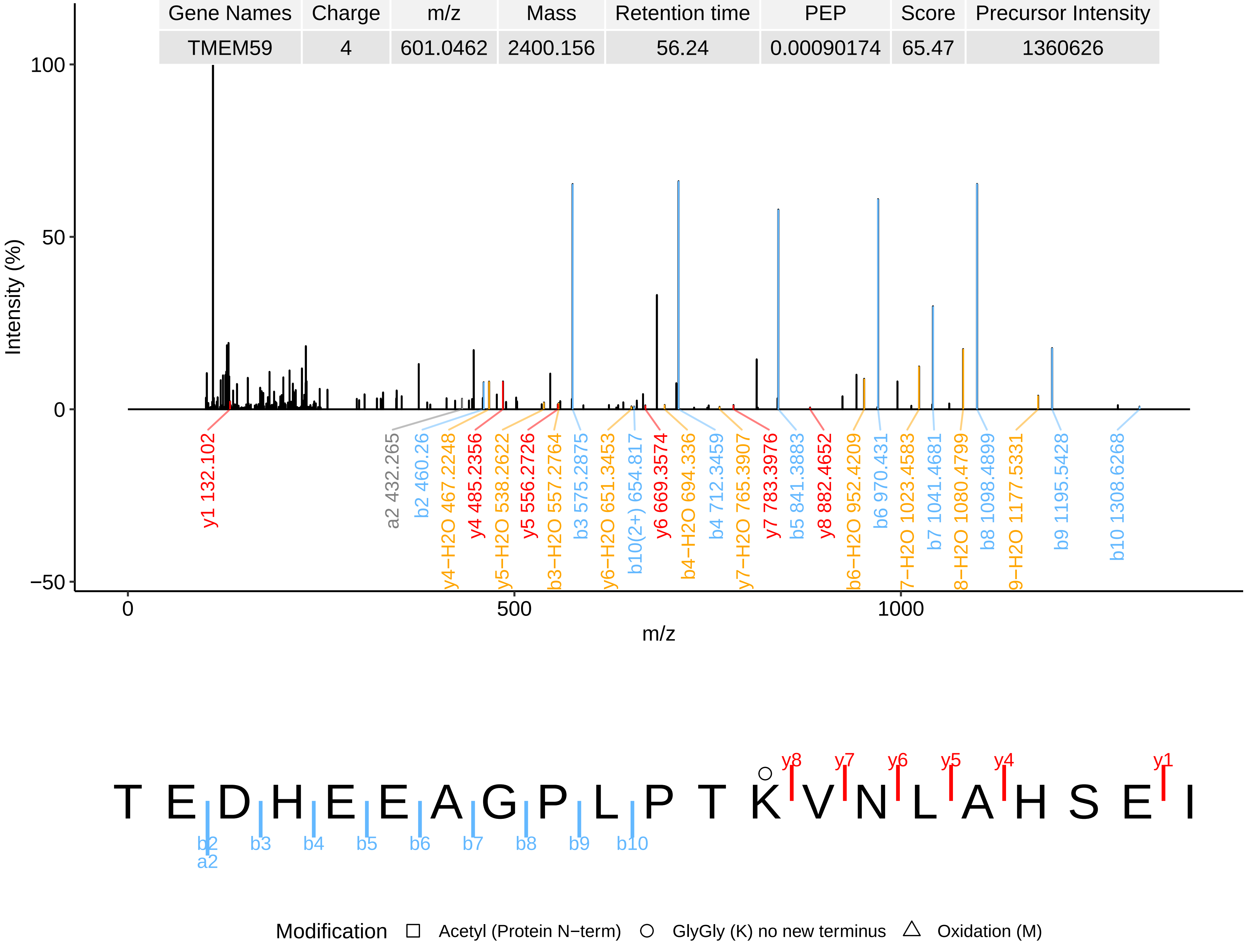

20200921_KIZ_MUb4_GlyGly_10plex − Scan number: 15725

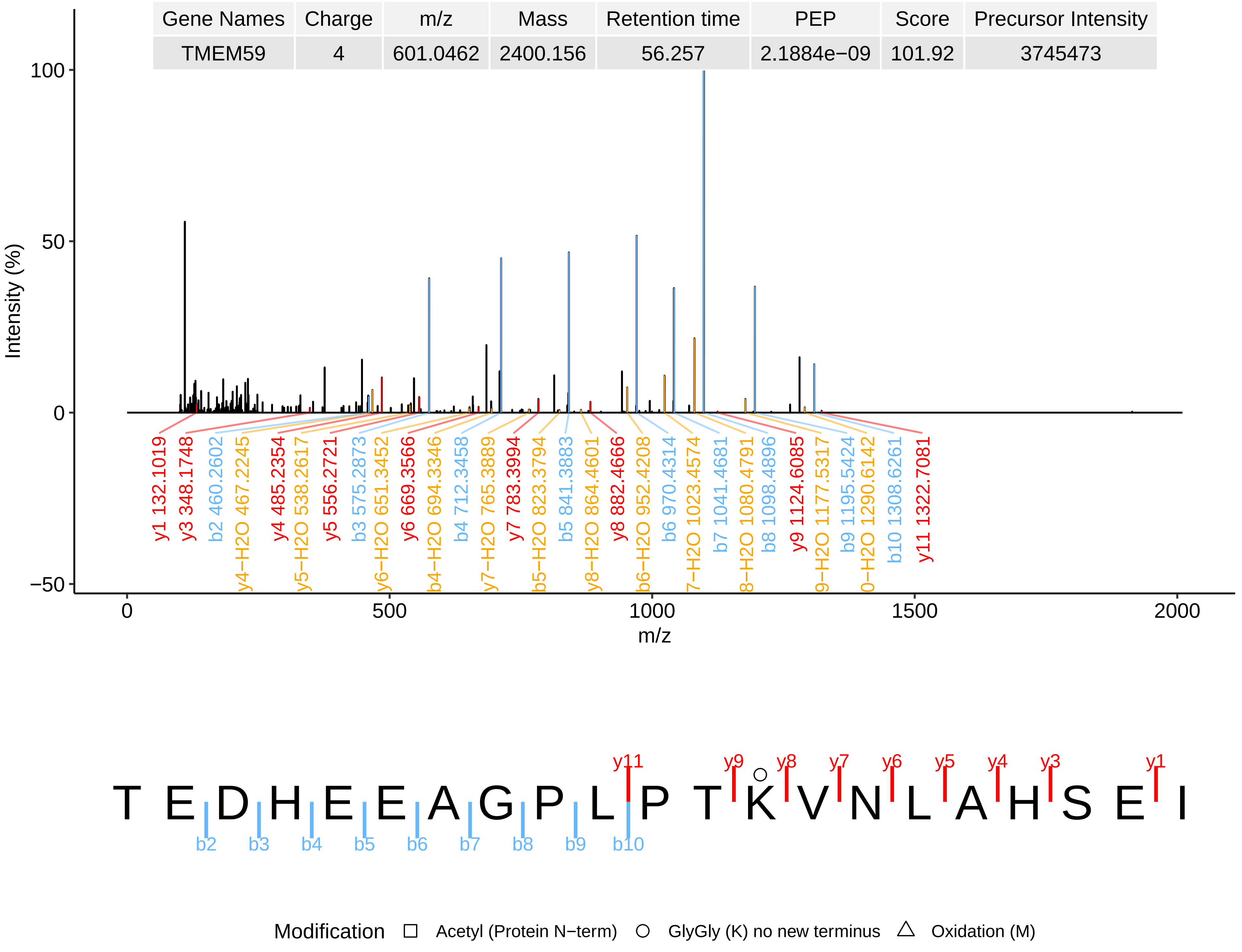

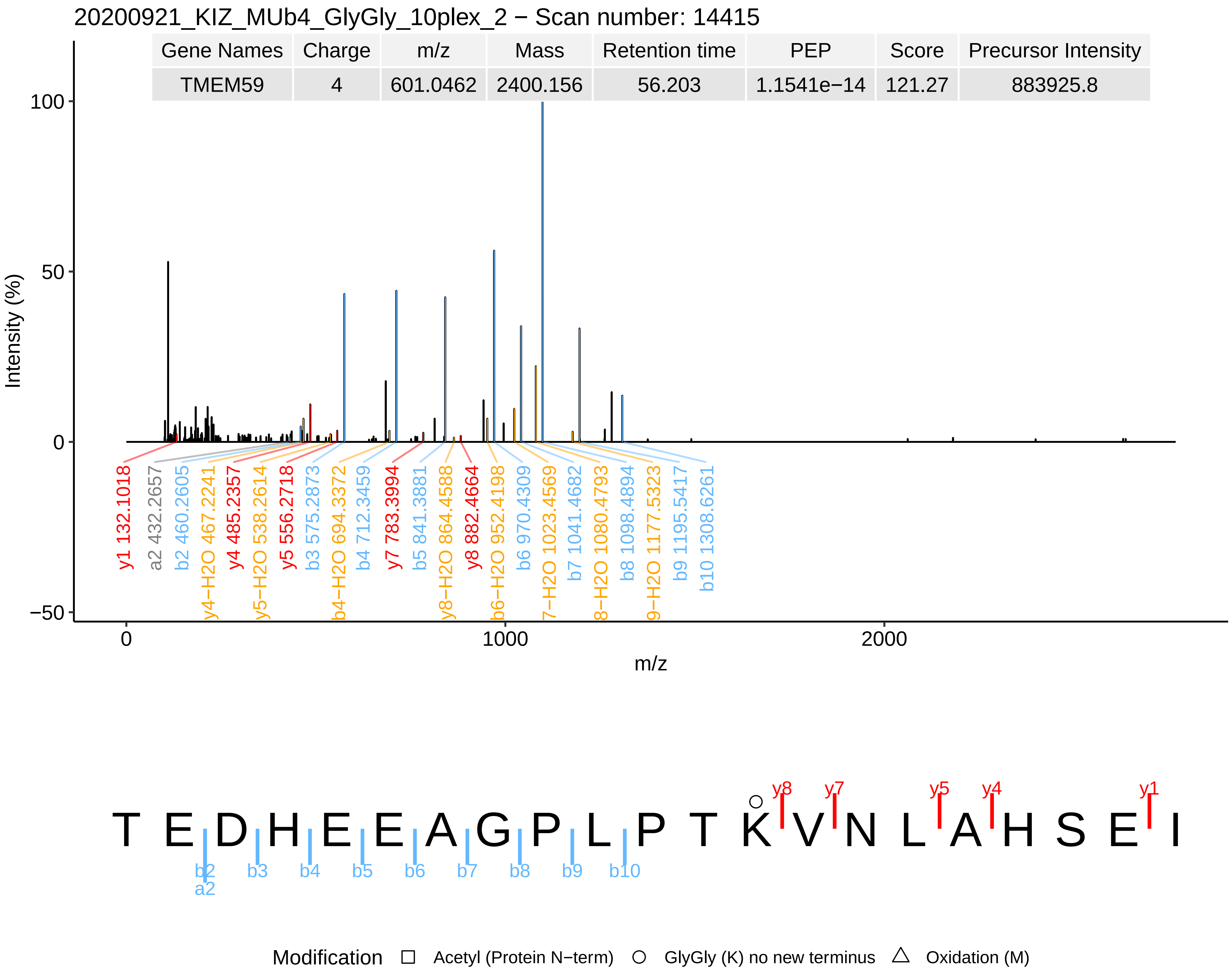
20200921_KIZ_MUb4_GlyGly_10plex_2 − Scan number: 14415
Gene Names | Charge | m/z | Mass | Retention time | PEP | Score | Precursor Intensity
TMEM59 | 4 | 601.0462 | 2400.156 | 56.203 | 1.1541e−14 | 121.27 | 883925.8
Intensity (%)
100
50
0
−50
0
1000
2000
m/z
y1 132.1018
a2 432.2657
b2 460.2605
y4−H2O 467.2241
y4 485.2357
y5−H2O 538.2614
y5 556.2718
b3 575.2873
b4−H2O 694.3372
b4 712.3459
y7 783.3994
b5 841.3881
y8−H2O 864.4588
y8 882.4664
b6−H2O 952.4198
b6 970.4309
y7−H2O 1023.4569
b7 1041.4682
y8−H2O 1080.4793
b8 1098.4894
y9−H2O 1177.5323
b9 1195.5417
b10 1308.6261
T E D H E E A G P L P T K V N L A H S E I
y8 y7 y5 y4 y1
b2 a2 b3 b4 b5 b6 b7 b8 b9 b10
Modification
Acetyl (Protein N−term)
GlyGly (K) no new terminus
Oxidation (M)

20200921_KIZ_MUb4_GlyGly_10plex_2 − Scan number: 14458

Gene Names	Charge	m/z	Mass	Retention time	PEP	Score	Precursor Intensity
TMEM59	3	801.0592	2400.156	56.375	0.00085496	68.82	1690799

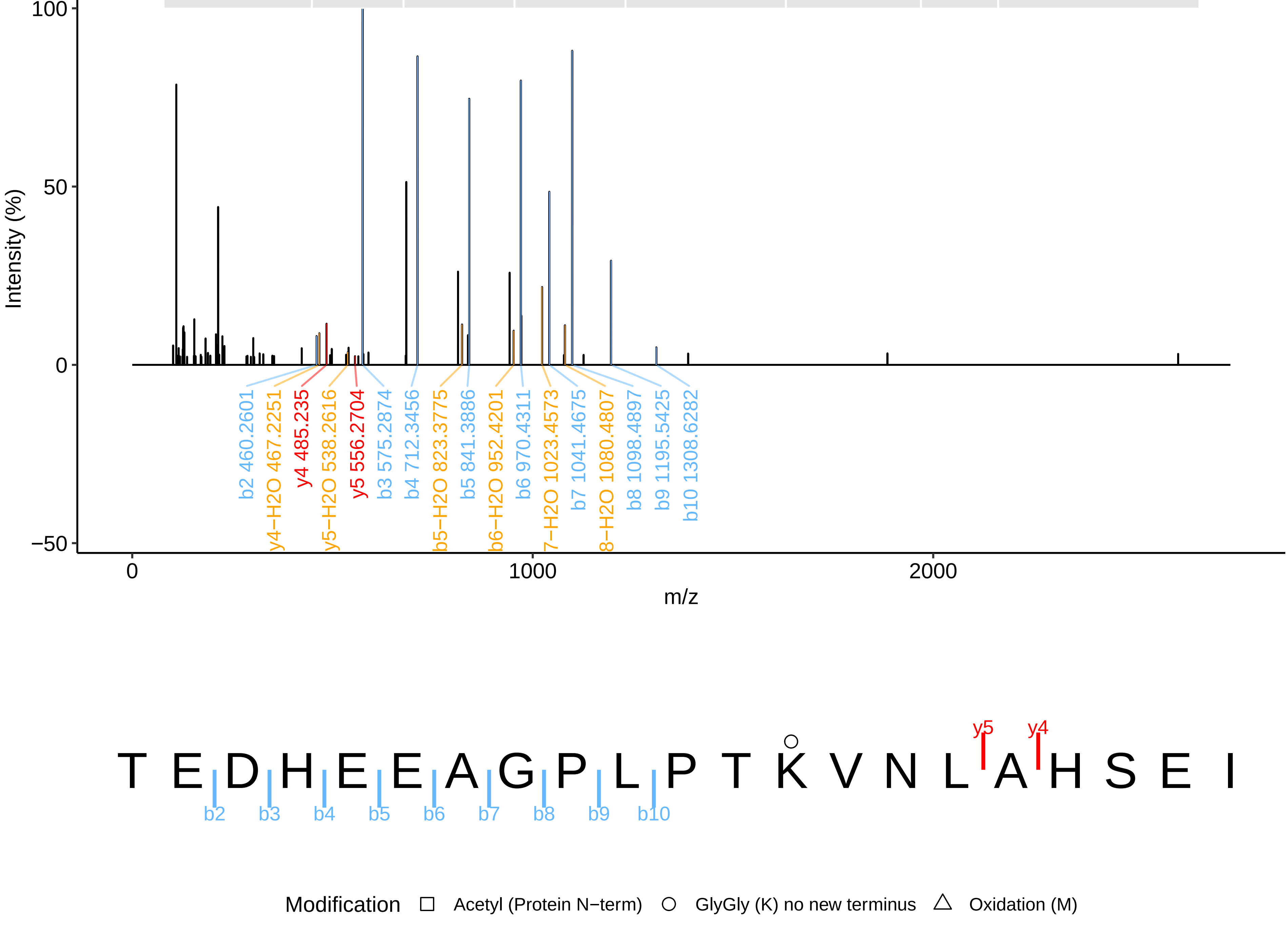

20200924_KIZ_MUb5_GlyGly_1 − Scan number: 14710

Gene Names	Charge	m/z	Mass	Retention time	PEP	Score	Precursor Intensity
TMEM59	4	601.0462	2400.156	48.052	9.8782e−16	131.84	74694264

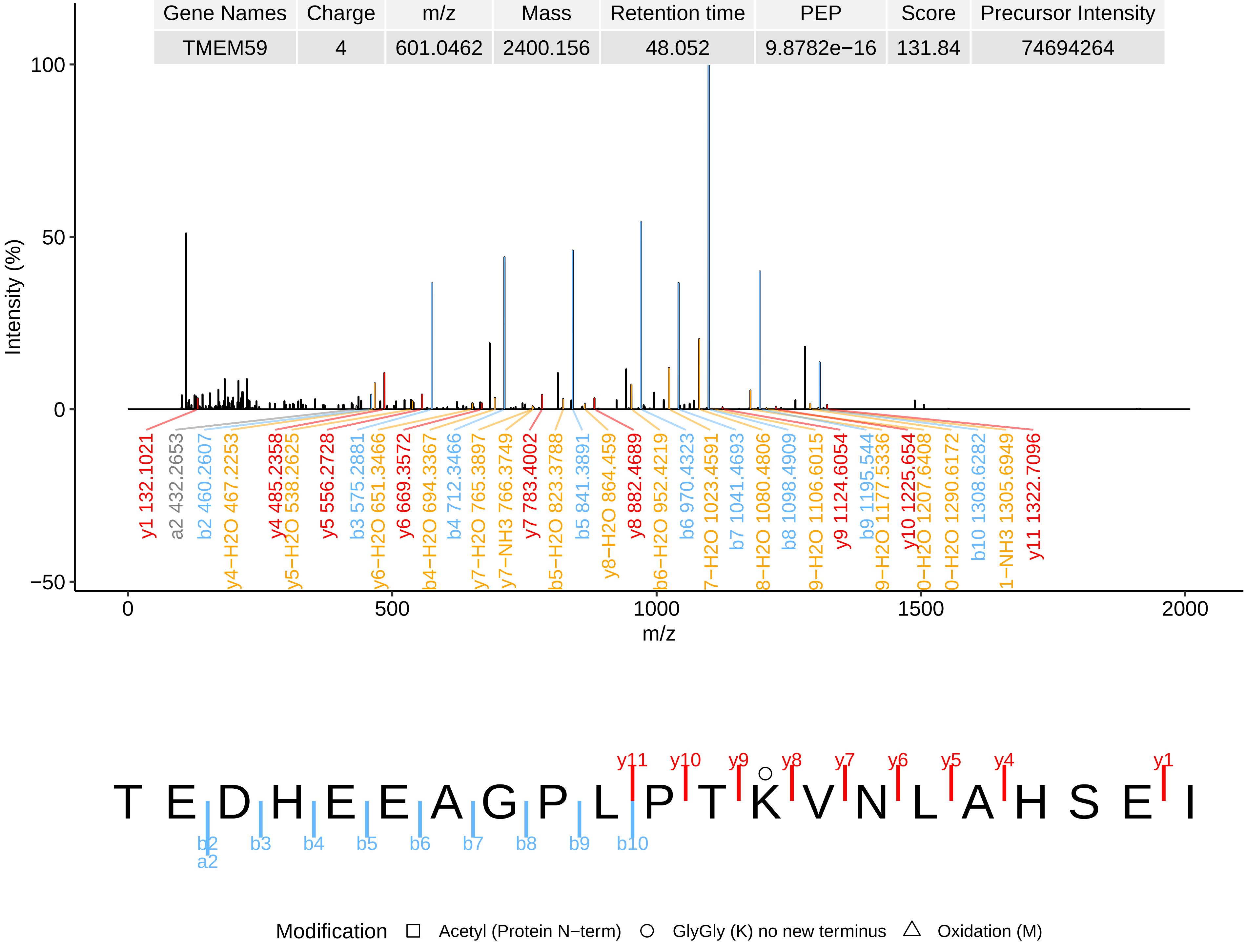

20200924_KIZ_MUb5_GlyGly_1 − Scan number: 14867

Gene Names	Charge	m/z	Mass	Retention time	PEP	Score	Precursor Intensity
TMEM59	4	601.0462	2400.156	48.555	7.9685e−15	124.84	24140612

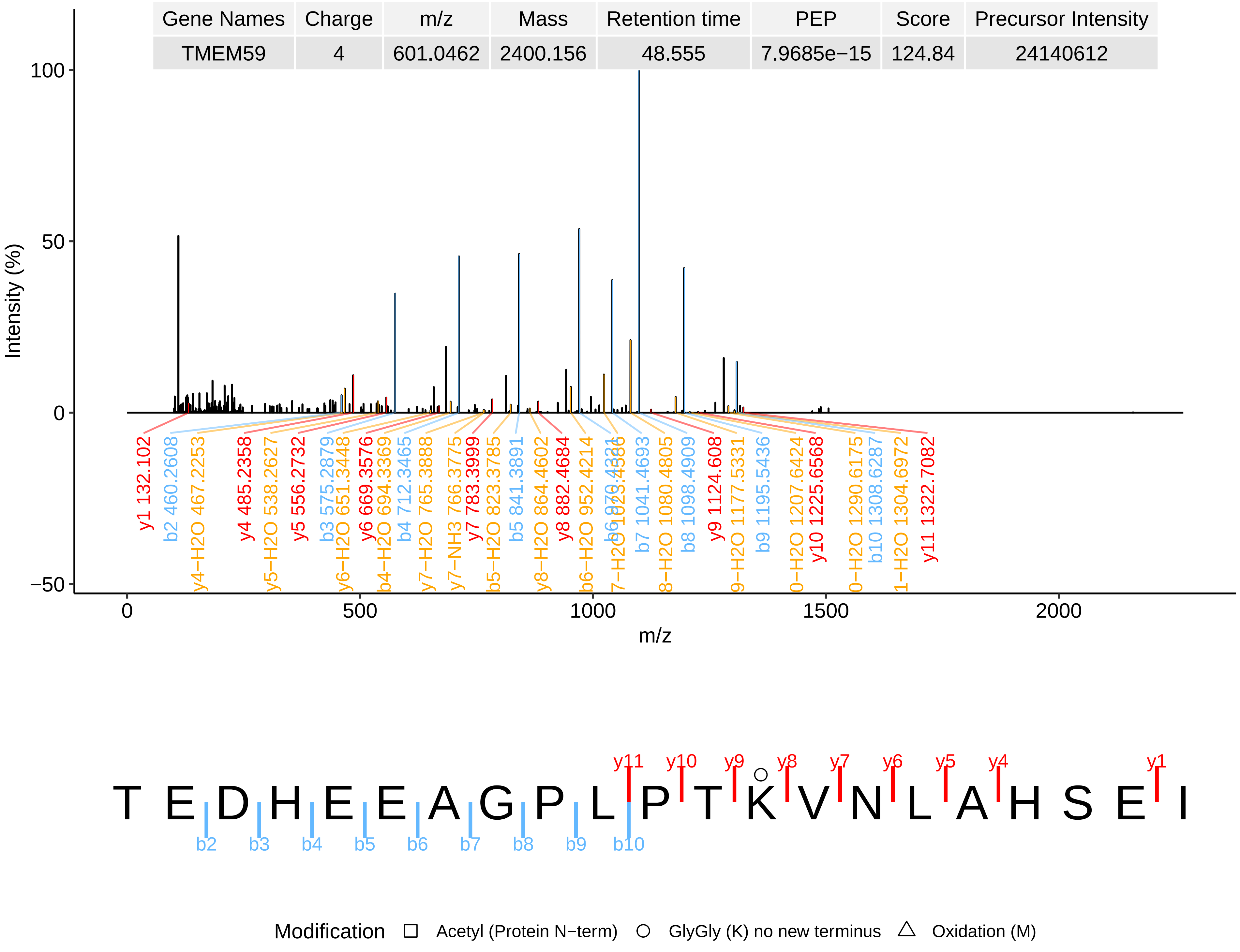

20200924_KIZ_MUb5_GlyGly_2 − Scan number: 13670

Gene Names	Charge	m/z	Mass	Retention time	PEP	Score	Precursor Intensity
TMEM59	4	601.0462	2400.156	51.106	1.6315e−21	133.78	10728392

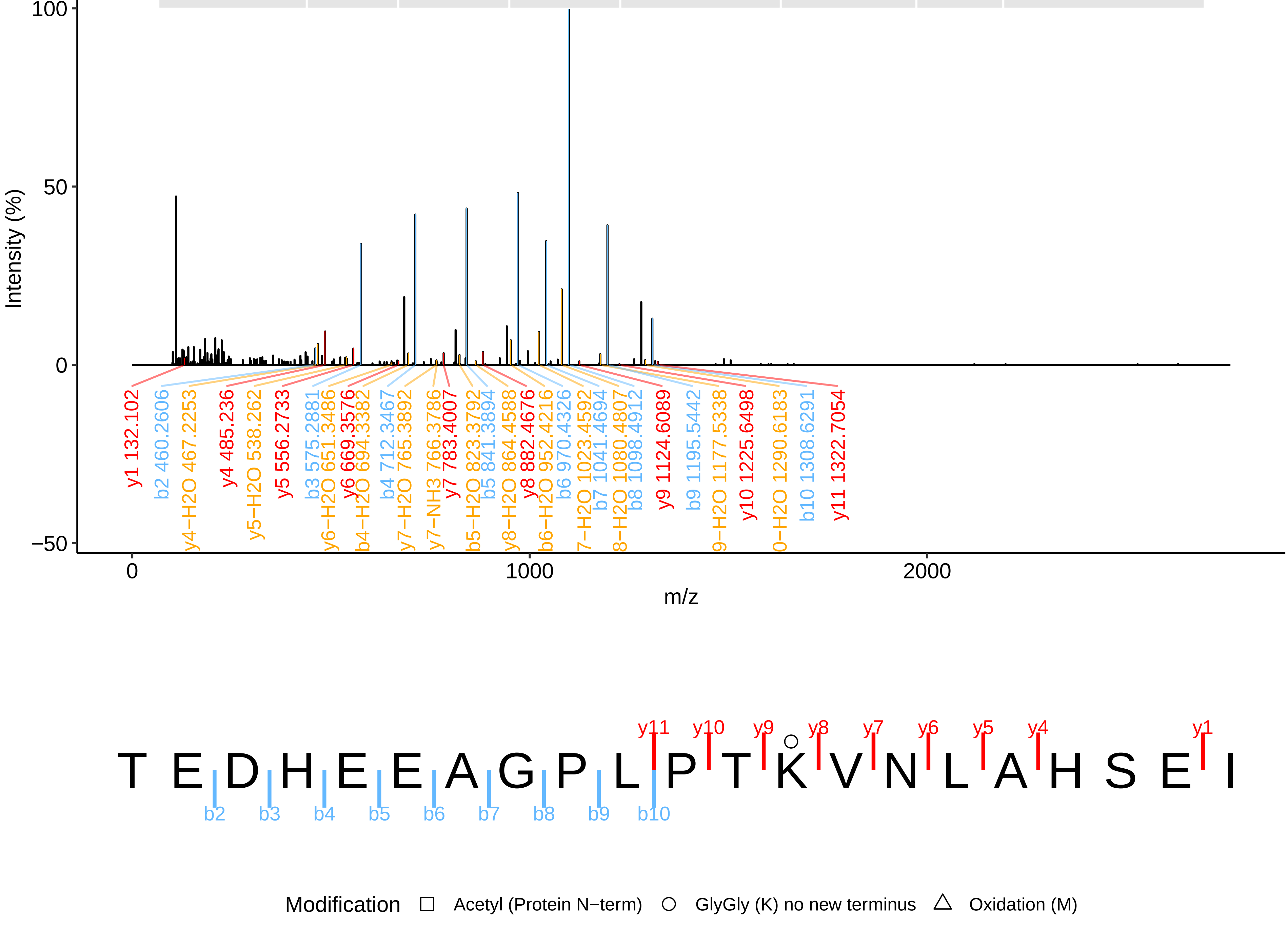

20200924_KIZ_MUb5_GlyGly_2 − Scan number: 13802

Gene Names	Charge	m/z	Mass	Retention time	PEP	Score	Precursor Intensity
TMEM59	4	601.0462	2400.156	51.618	1.2677e−21	137.45	11787471

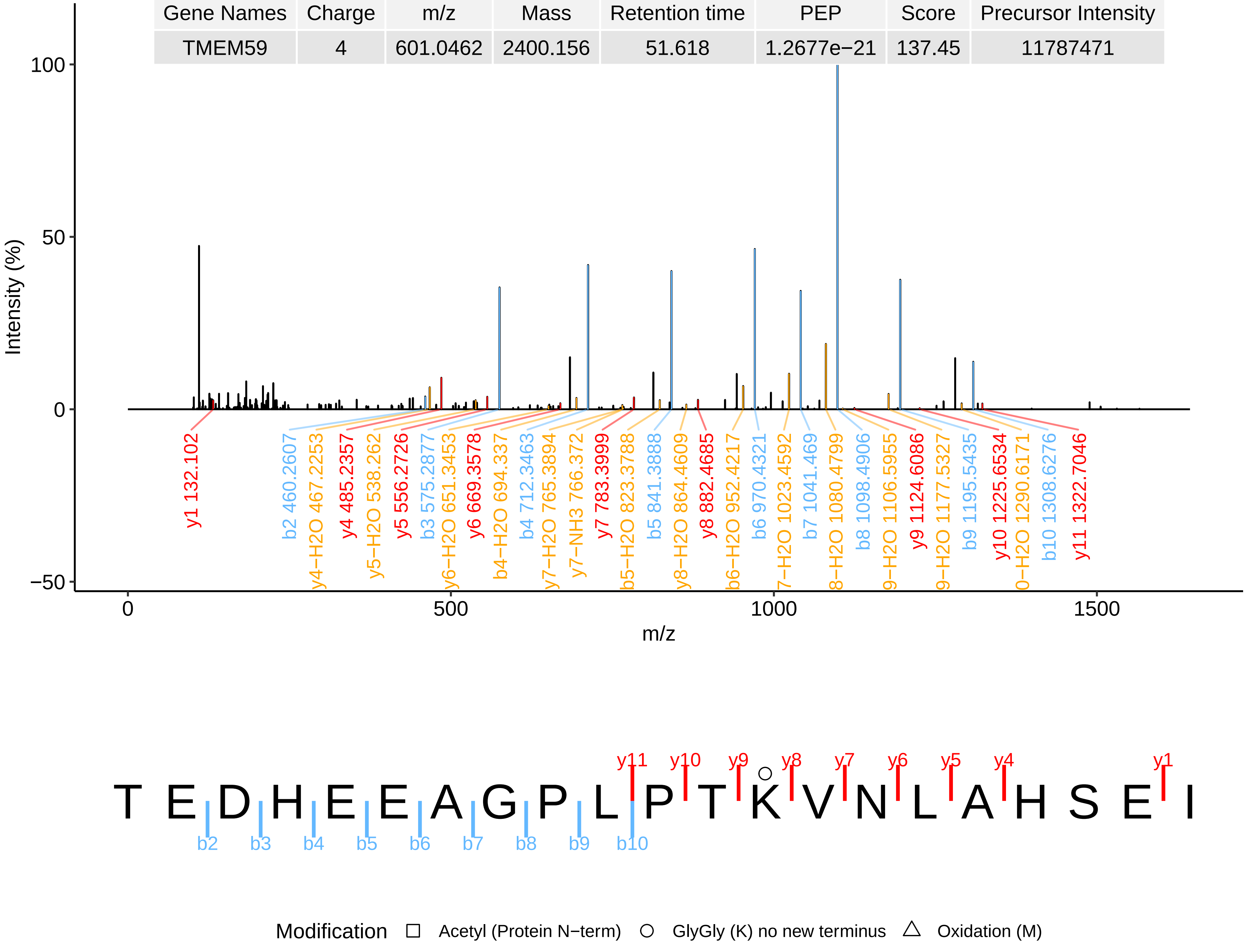

20200924_KIZ_MUb5_GlyGly_2 − Scan number: 13948

Gene Names	Charge	m/z	Mass	Retention time	PEP	Score	Precursor Intensity
TMEM59	4	601.0462	2400.156	52.167	1.4323e−14	118.48	4116668

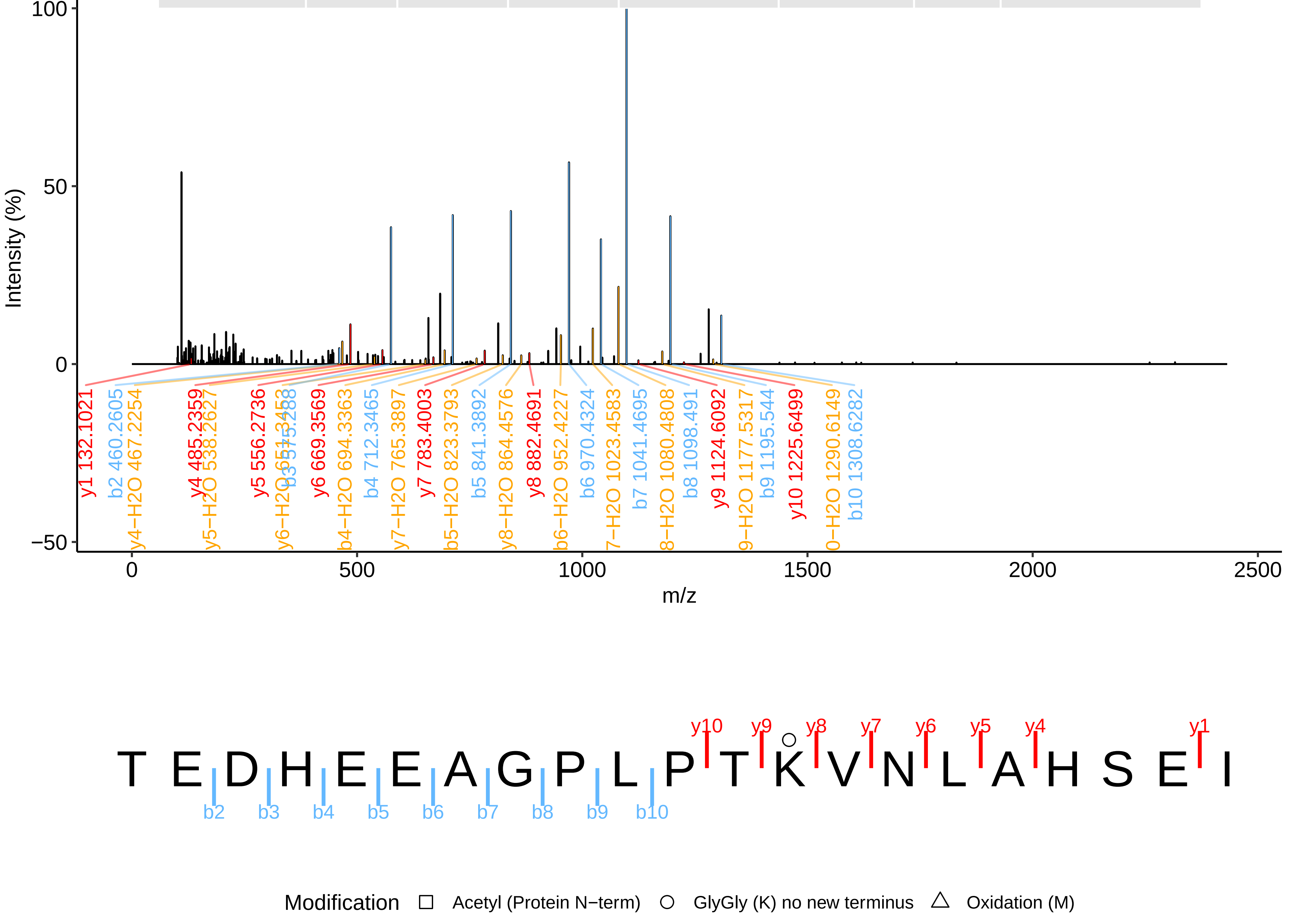

20201118_KIZ_MUb6_GlyGly_2 − Scan number: 16788

Gene Names	Charge	m/z	Mass	Retention time	PEP	Score	Precursor Intensity
TMEM59	4	601.0462	2400.156	56.744	5.495e−15	127.32	8634193

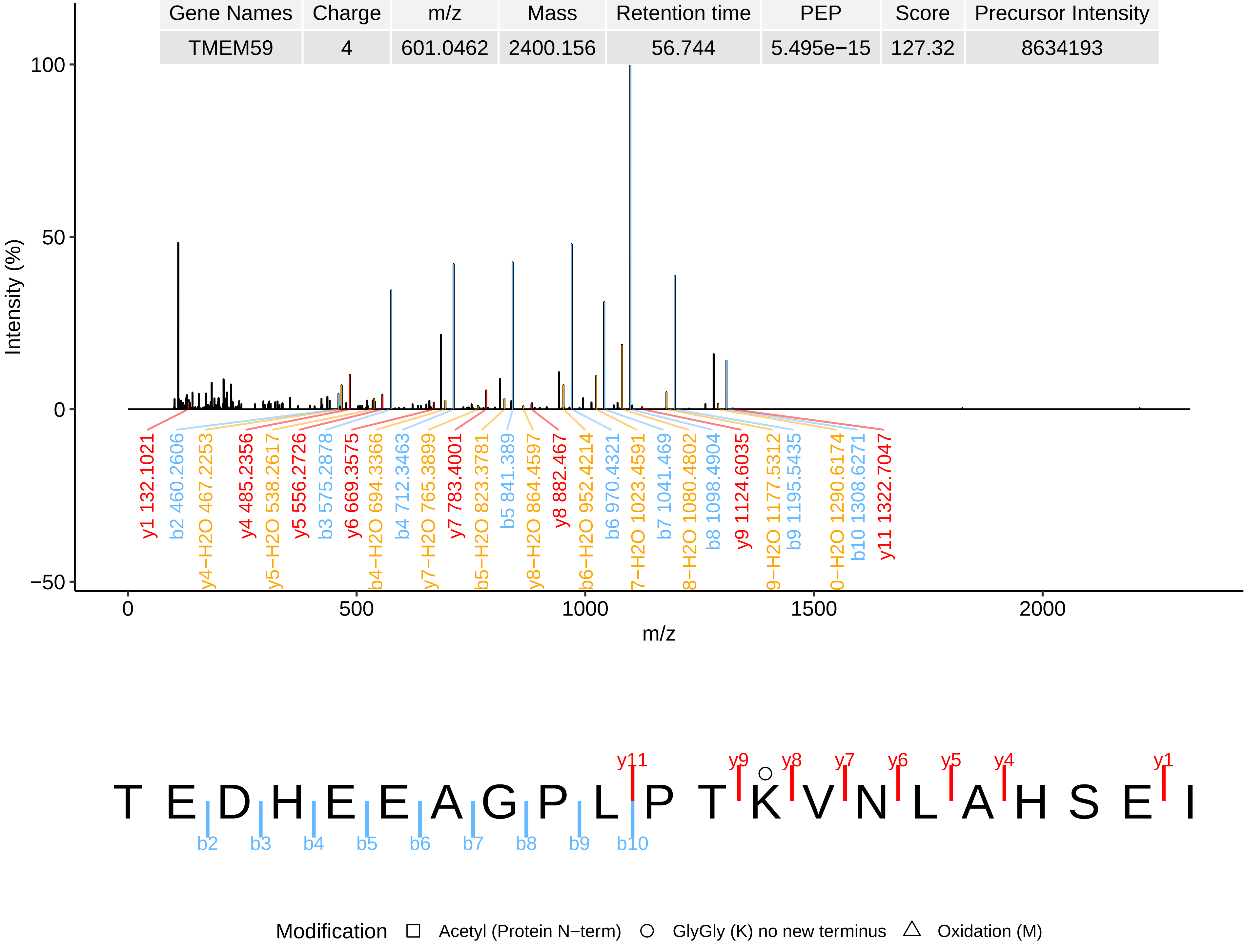

20201118_KIZ_MUb6_GlyGly_2 − Scan number: 16835

Gene Names	Charge	m/z	Mass	Retention time	PEP	Score	Precursor Intensity
TMEM59	3	801.0592	2400.156	56.918	1.1512e−07	95.954	NA

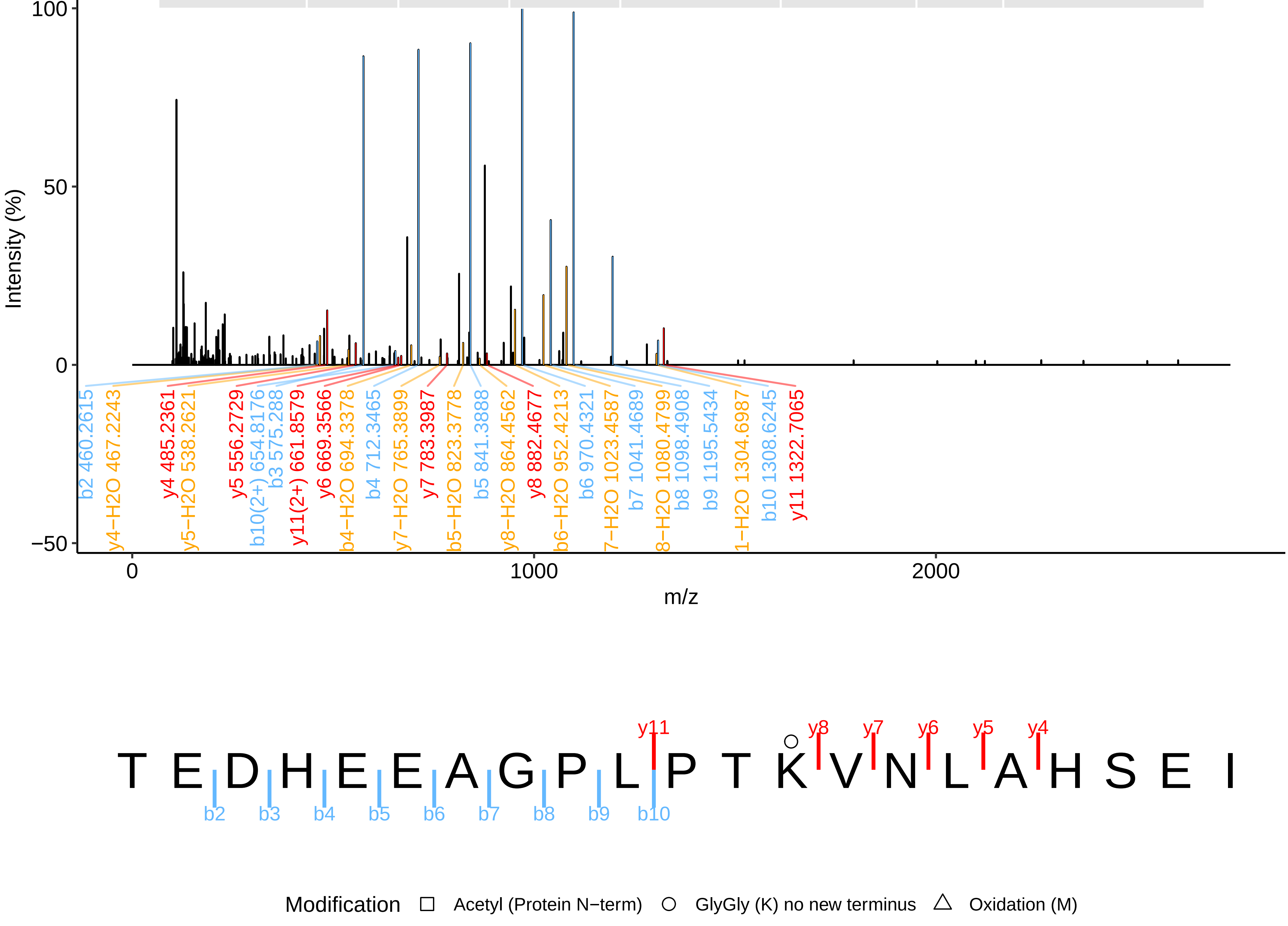

20200921_KIZ_MUb4_GlyGly_10plex − Scan number: 24627

Gene Names	Charge	m/z	Mass	Retention time	PEP	Score	Precursor Intensity
RPL9	3	814.1063	2439.297	89.572	6.8517e−15	127.17	3294140

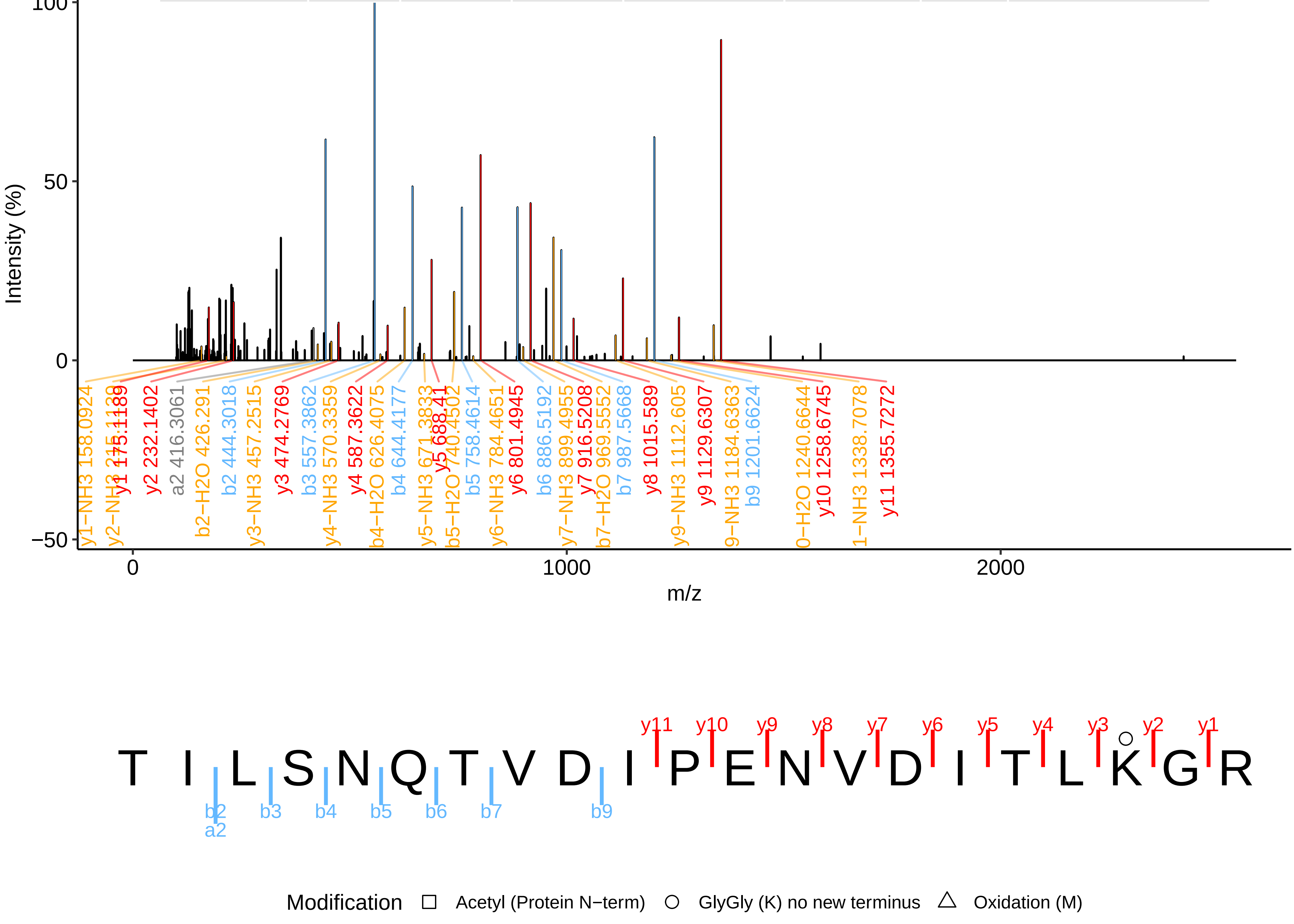

20200921_KIZ_MUb4_GlyGly_10plex − Scan number: 24632

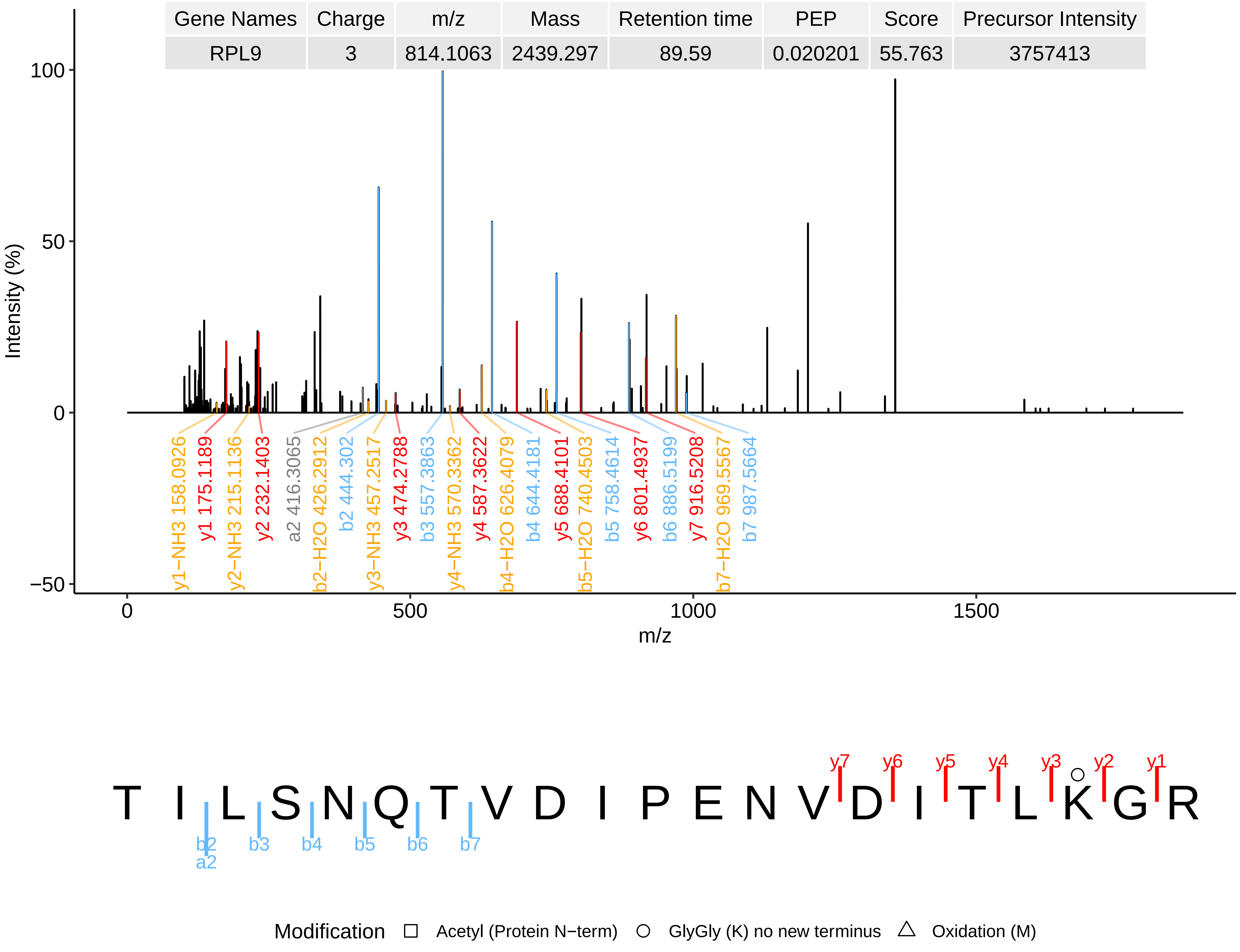

20200921_KIZ_MUb4_GlyGly_10plex − Scan number: 24782

Gene Names	Charge	m/z	Mass	Retention time	PEP	Score	Precursor Intensity
RPL9	3	814.1063	2439.297	90.157	5.8585e−10	112.44	2403440

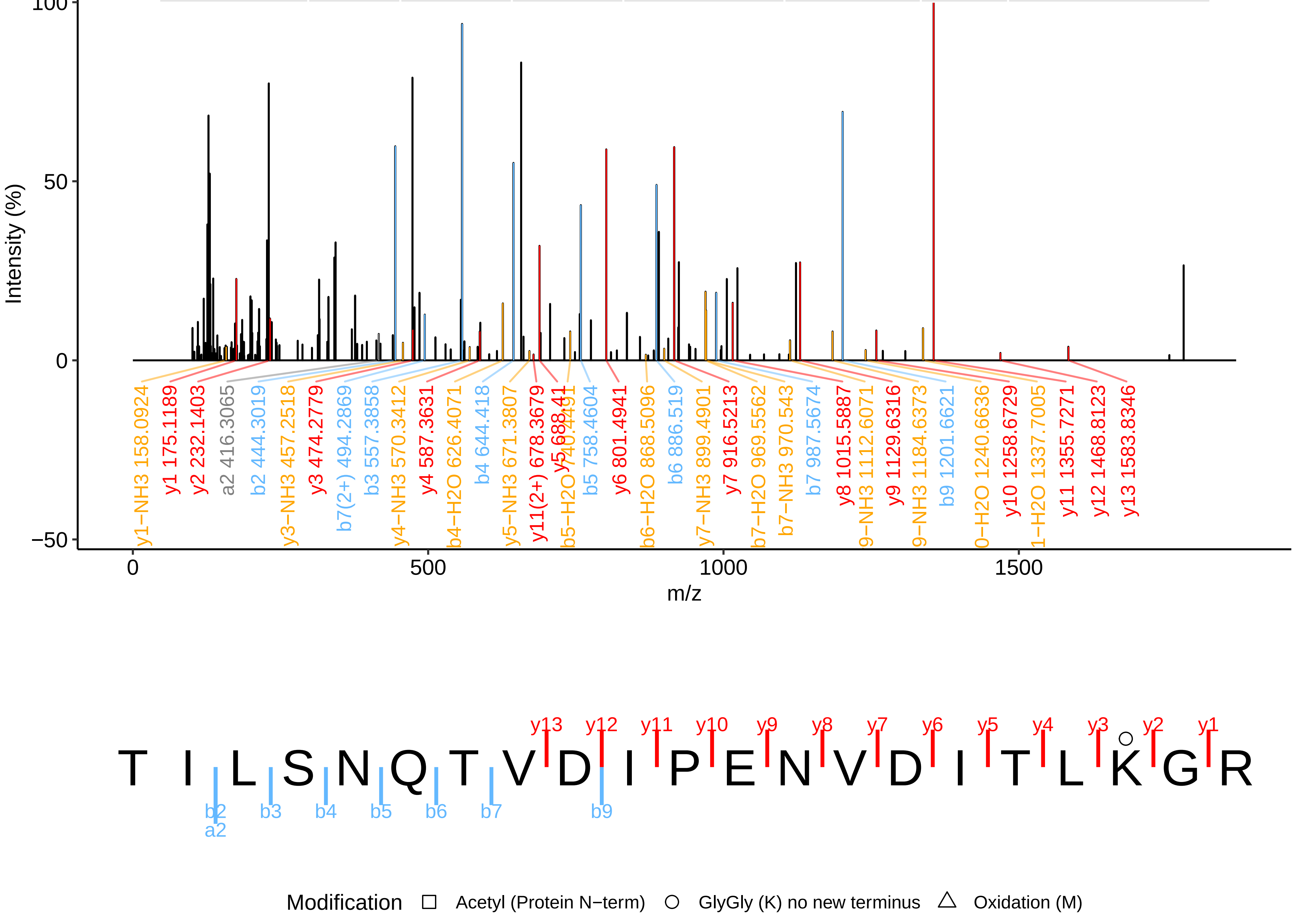

20200921_KIZ_MUb4_GlyGly_10plex_2 − Scan number: 21860

Gene Names	Charge	m/z	Mass	Retention time	PEP	Score	Precursor Intensity
RPL9	3	814.1063	2439.297	86.108	1.0749e−21	141.31	310650.9

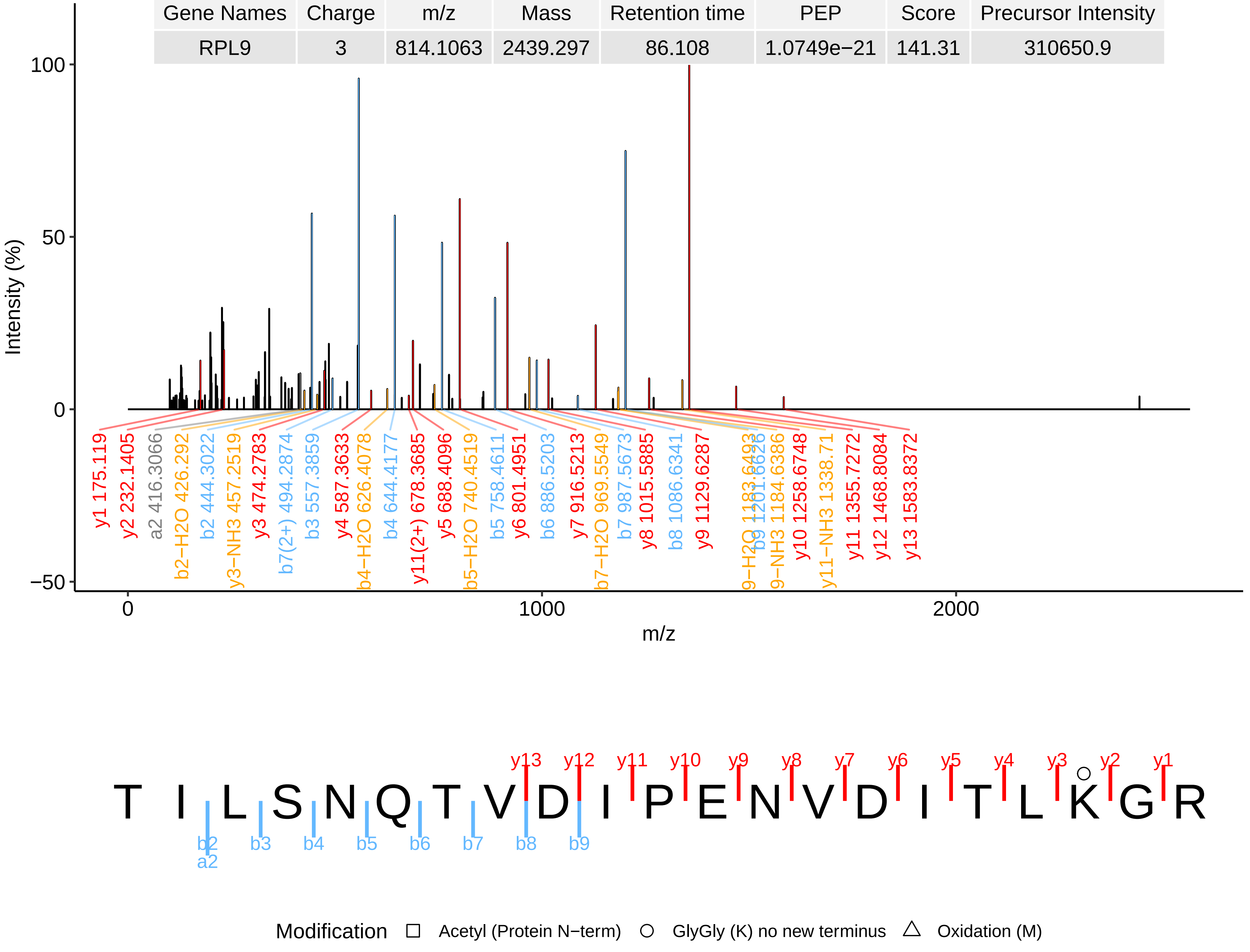

20200921_KIZ_MUb4_GlyGly_10plex_2 − Scan number: 21862

Gene Names	Charge	m/z	Mass	Retention time	PEP	Score	Precursor Intensity
RPL9	3	814.1063	2439.297	86.116	3.222e−09	99.059	801683.4

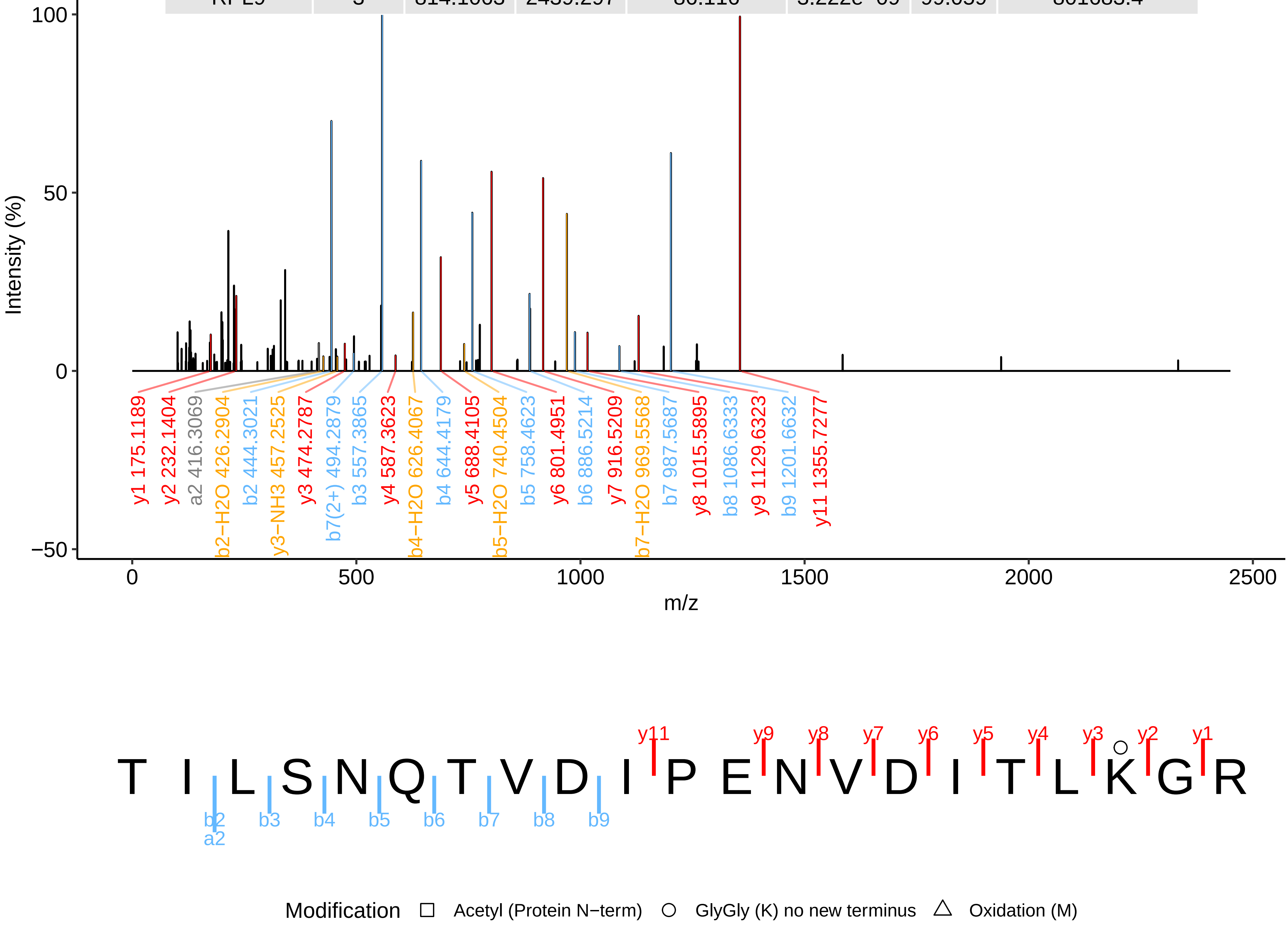

20200921_KIZ_MUb4_GlyGly_10plex_2 − Scan number: 21892

Gene Names	Charge	m/z	Mass	Retention time	PEP	Score	Precursor Intensity
RPL9	4	610.8315	2439.297	86.239	2.1126e−09	102.39	1173838

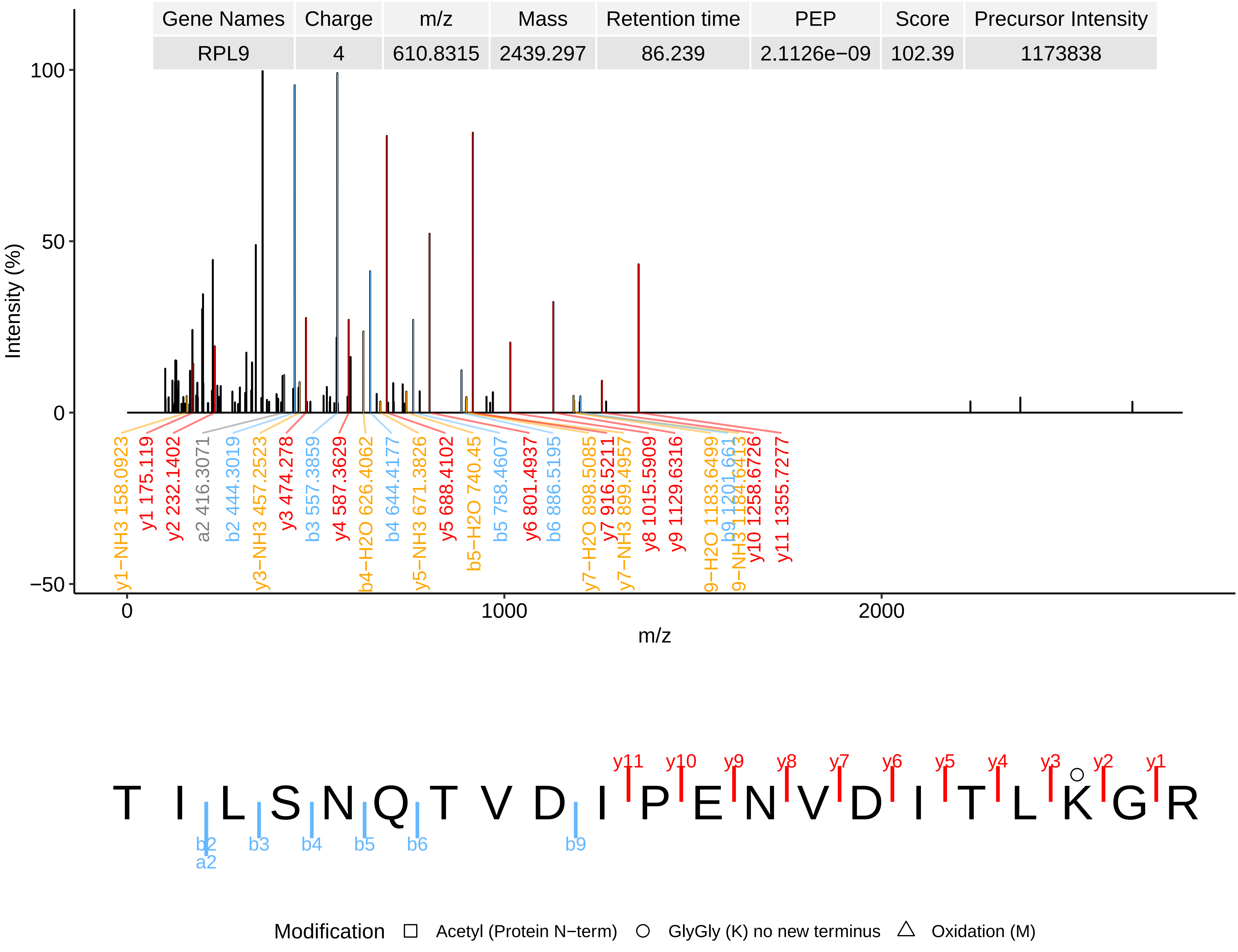

20201118_KIZ_MUb6_GlyGly_2 − Scan number: 26480

Gene Names	Charge	m/z	Mass	Retention time	PEP	Score	Precursor Intensity
RPL9	3	814.1063	2439.297	88.346	7.391e−39	179.95	4803458

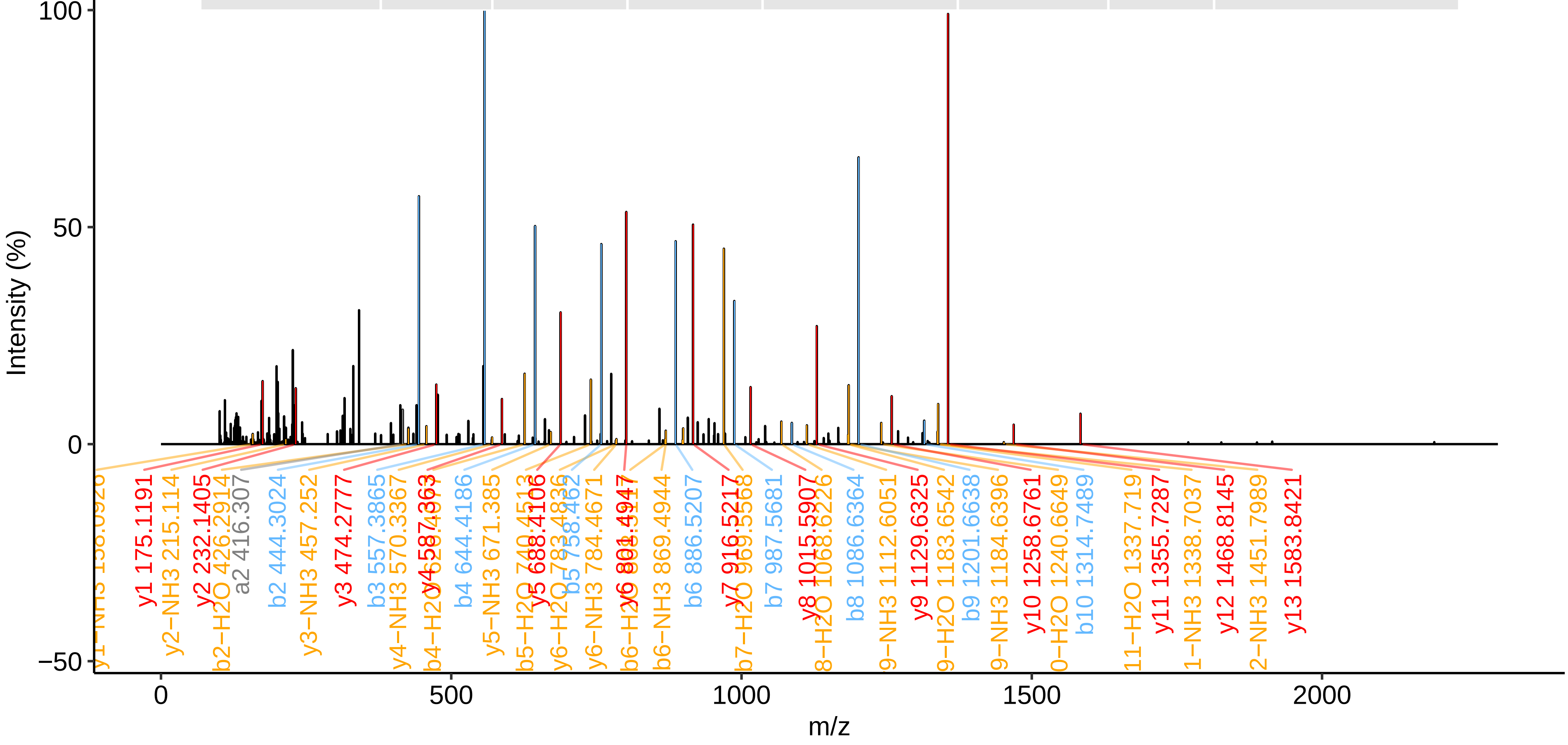

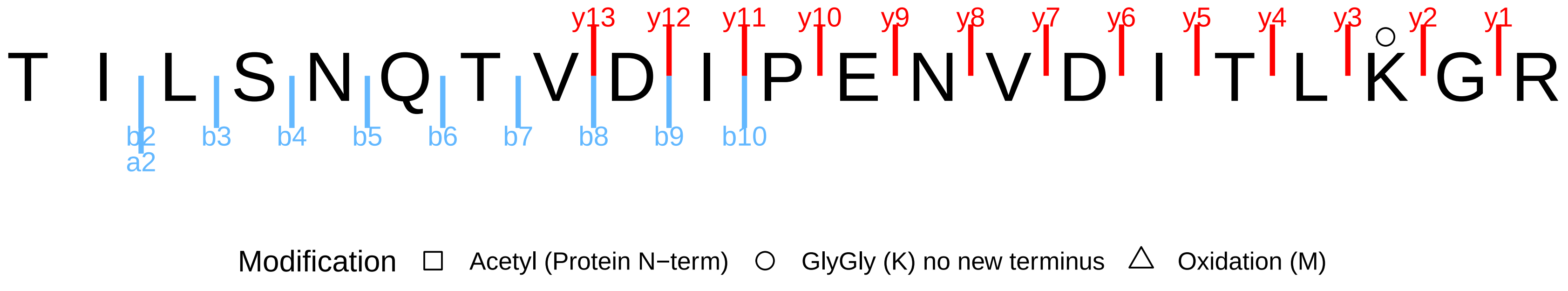

20200921_KIZ_MUb4_GlyGly_10plex_2 − Scan number: 11205

Gene Names	Charge	m/z	Mass	Retention time	PEP	Score	Precursor Intensity
EPS15L1	3	783.3702	2347.089	43.066	5.575e−07	97.284	1227962

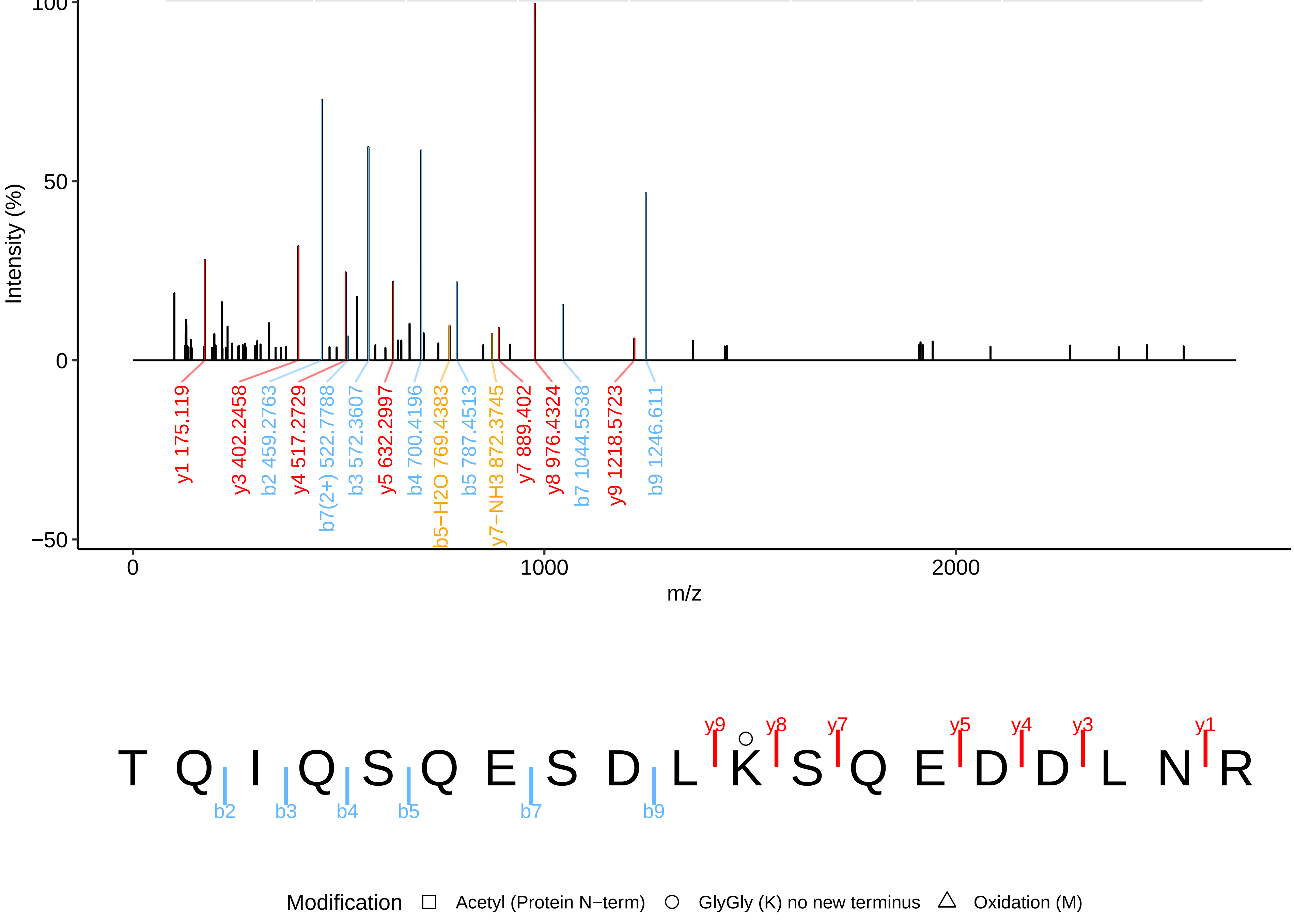

20200924_KIZ_MUb5_GlyGly_1 − Scan number: 11429

Gene Names	Charge	m/z	Mass	Retention time	PEP	Score	Precursor Intensity
EPS15L1	3	783.3702	2347.089	37.025	9.4412e−26	151.41	NA

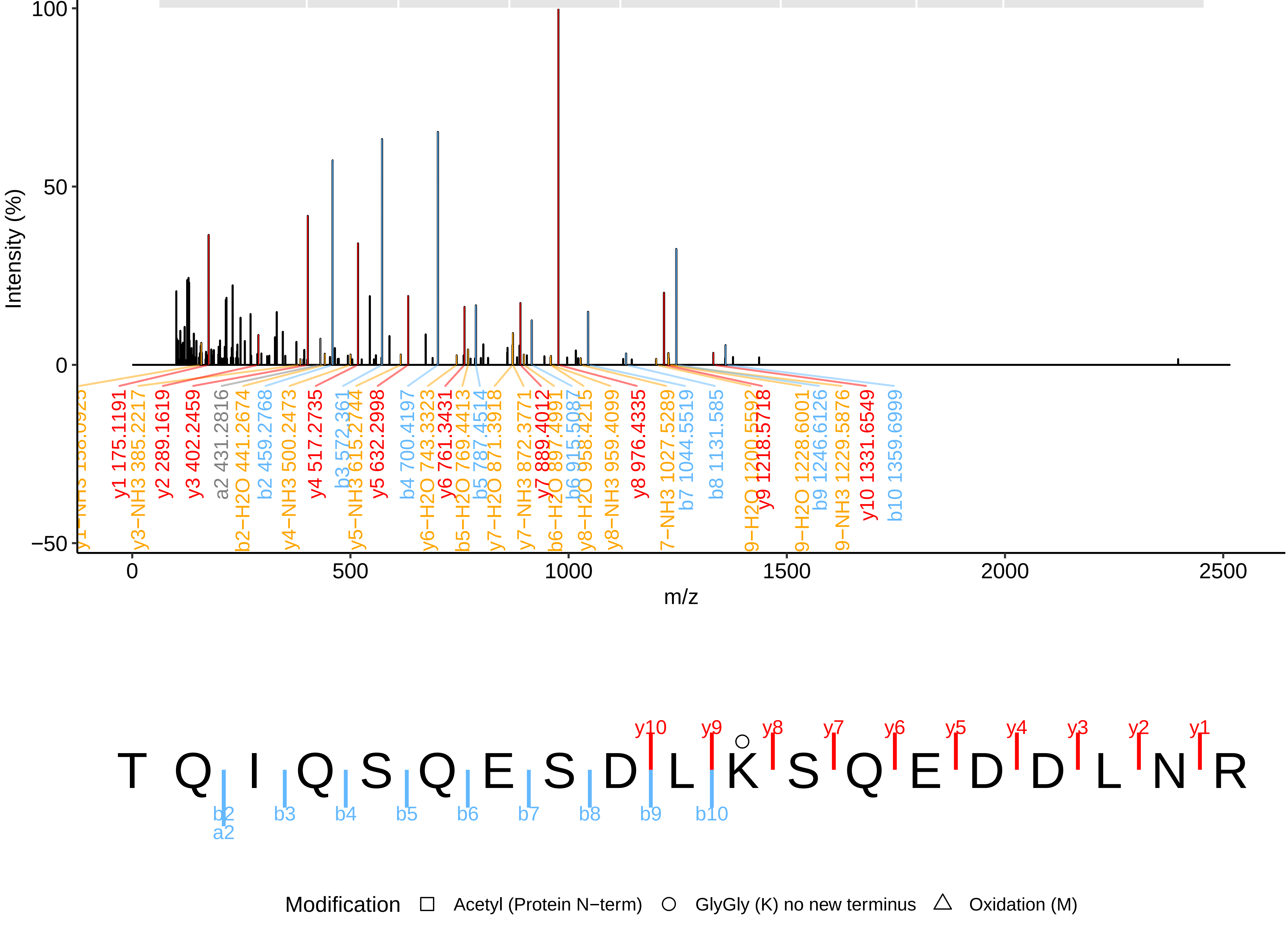

20200921_KIZ_MUb4_GlyGly_10plex − Scan number: 27568

Gene Names	Charge	m/z	Mass	Retention time	PEP	Score	Precursor Intensity
CYB5R3	3	781.7677	2342.281	100.64	9.1193e−15	115.66	4229662

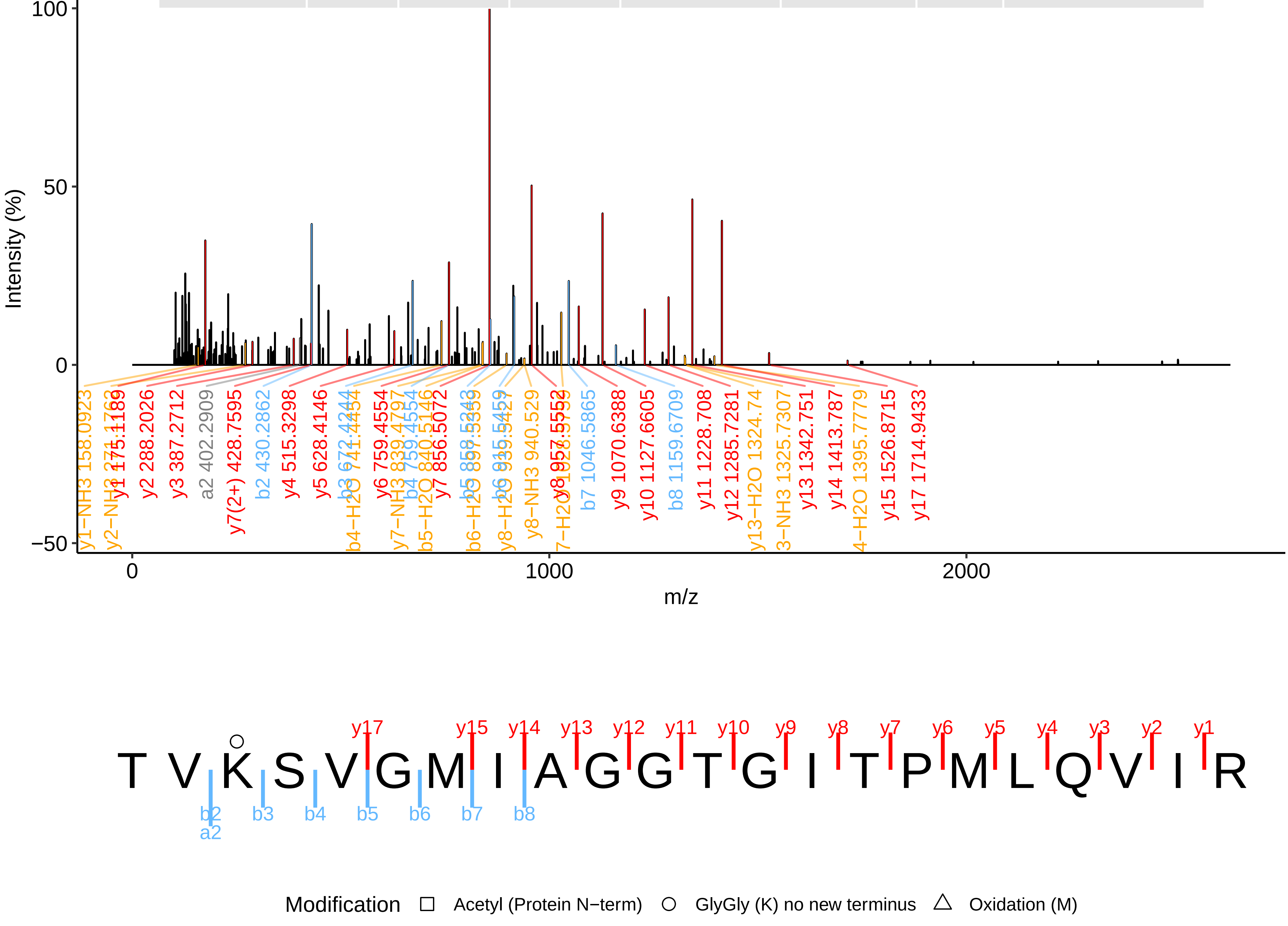

20200921_KIZ_MUb4_GlyGly_10plex_2 − Scan number: 20356

Gene Names	Charge	m/z	Mass	Retention time	PEP	Score	Precursor Intensity
ATXN10	3	804.4149	2410.223	80.004	4.1576e−06	91.725	1138015

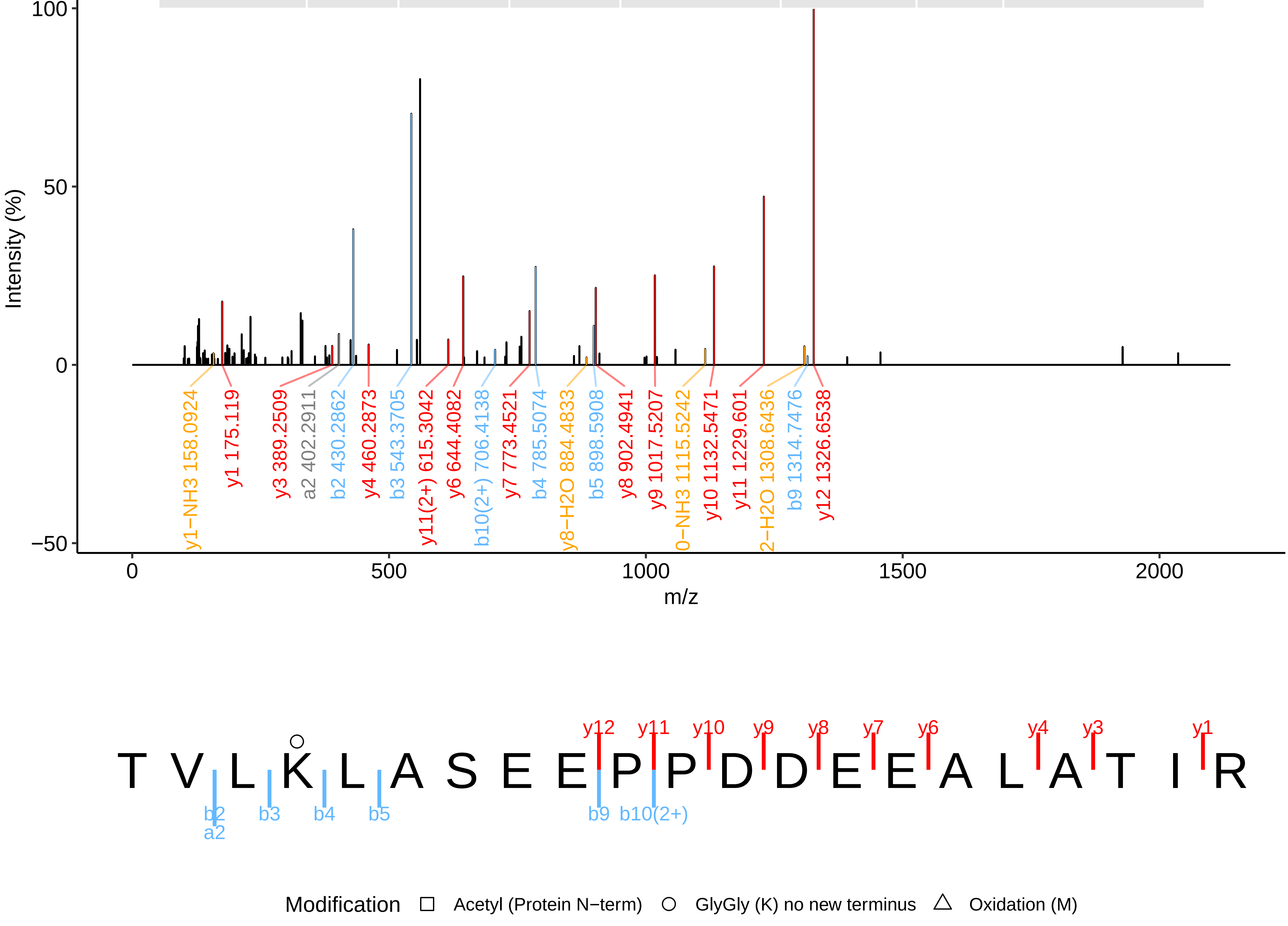

20200921_KIZ_MUb4_GlyGly_10plex − Scan number: 16537

Gene Names	Charge	m/z	Mass	Retention time	PEP	Score	Precursor Intensity
ACTB;ACTG1	4	603.0776	2408.281	59.426	5.8842e−22	144.3	5938682

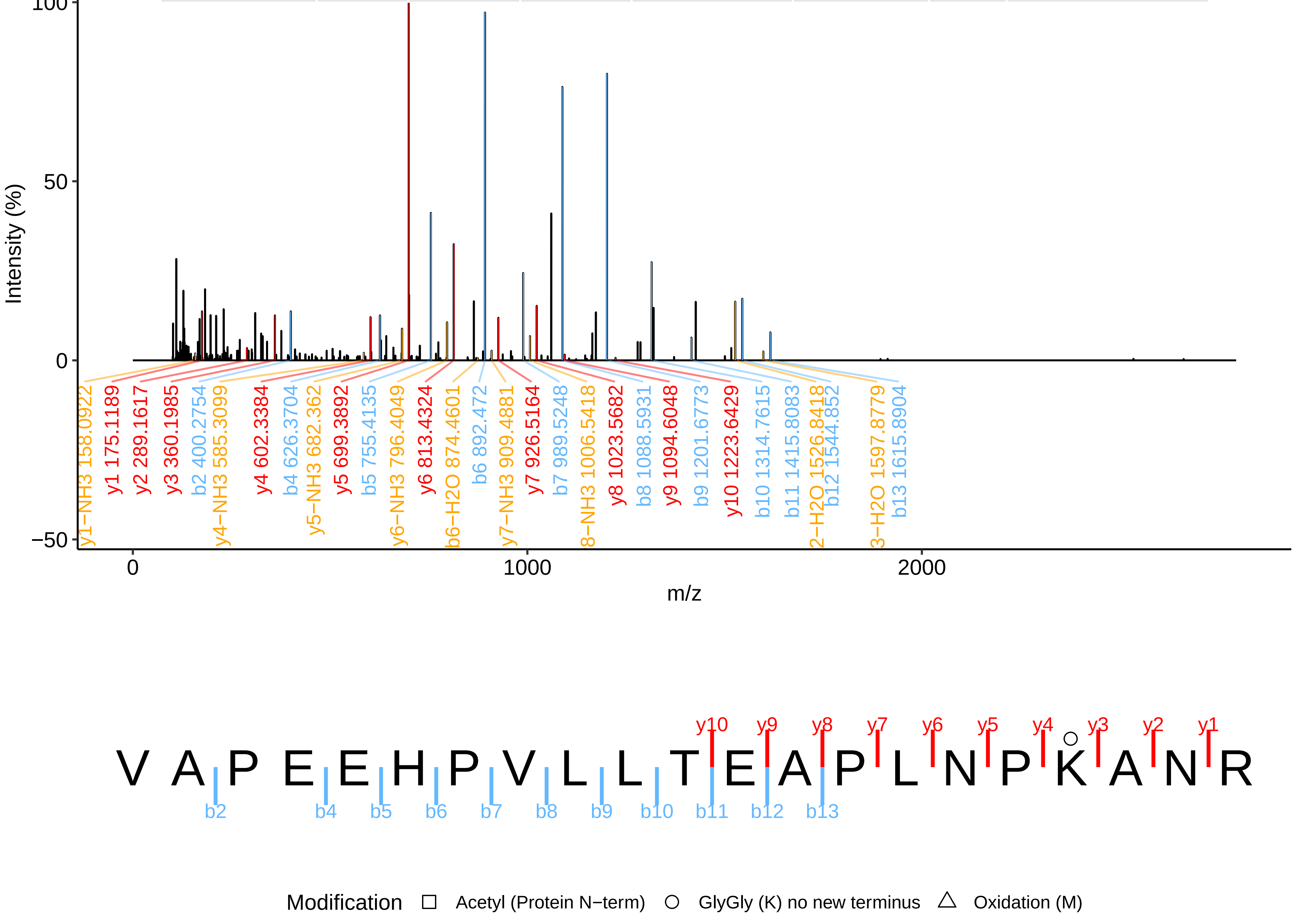

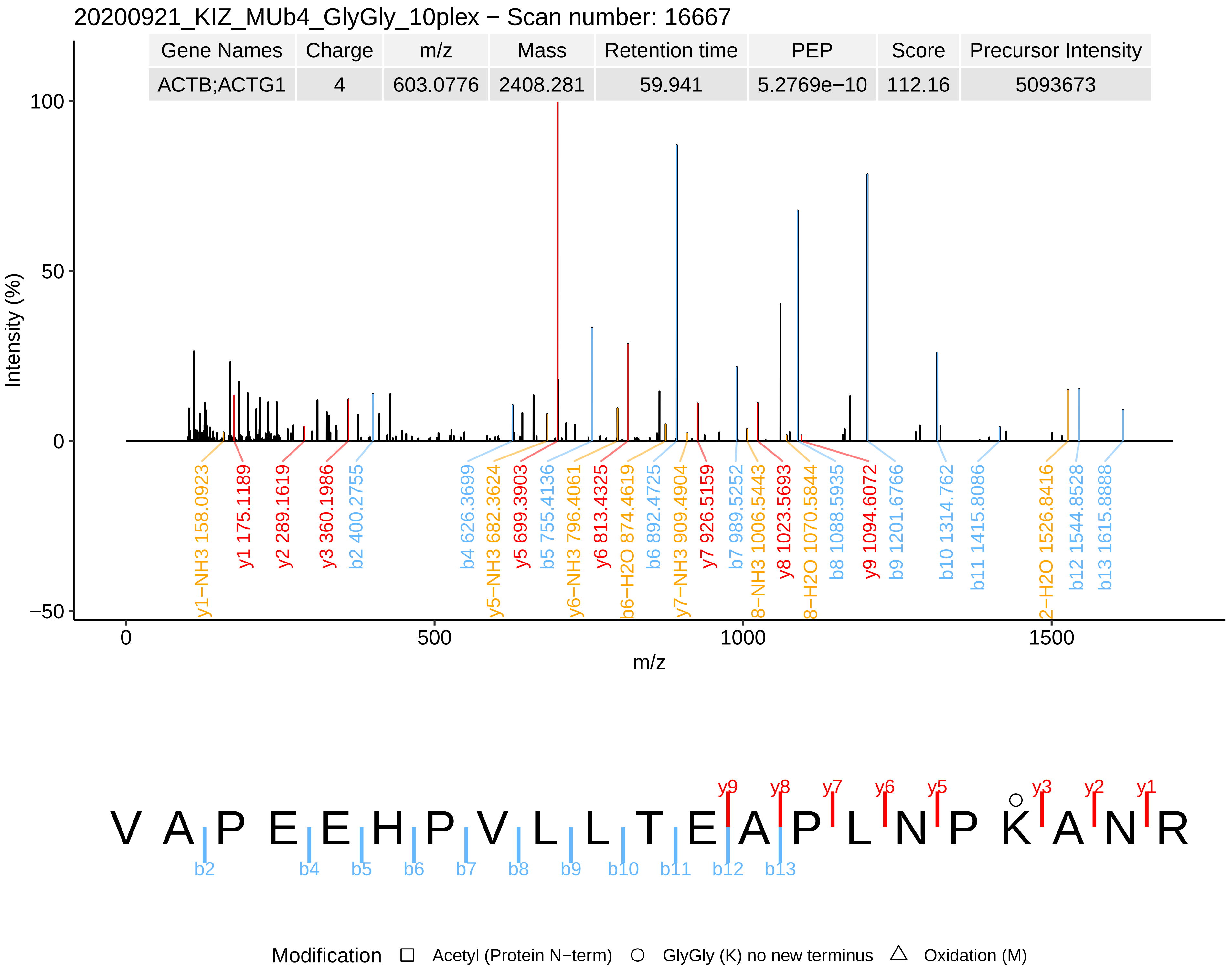
20200921_KIZ_MUb4_GlyGly_10plex − Scan number: 16667
Gene Names
Charge
m/z
Mass
Retention time
PEP
Score
Precursor Intensity
ACTB;ACTG1
4
603.0776
2408.281
59.941
5.2769e−10
112.16
5093673
Intensity (%)
100
50
0
−50
0
500
1000
1500
m/z
y1−NH3 158.0923
y1 175.1189
y2 289.1619
y3 360.1986
b2 400.2755
b4 626.3699
y5−NH3 682.3624
y5 699.3903
b5 755.4136
y6−NH3 796.4061
y6 813.4325
b6−H2O 874.4619
b6 892.4725
y7−NH3 909.4904
y7 926.5159
b7 989.5252
8−NH3 1006.5443
y8 1023.5693
8−H2O 1070.5844
b8 1088.5935
y9 1094.6072
b9 1201.6766
b10 1314.762
b11 1415.8086
2−H2O 1526.8416
b12 1544.8528
b13 1615.8888
V A P E E H P V L L T E A P L N P K A N R
y9 y8 y7 y6 y5 y3 y2 y1
b2 b4 b5 b6 b7 b8 b9 b10 b11 b12 b13
Modification
Acetyl (Protein N−term)
GlyGly (K) no new terminus
Oxidation (M)

20200921_KIZ_MUb4_GlyGly_10plex_2 − Scan number: 15329

Gene Names	Charge	m/z	Mass	Retention time	PEP	Score	Precursor Intensity
ACTB;ACTG1	4	603.0776	2408.281	59.92	1.3762e−09	106.93	725362.9

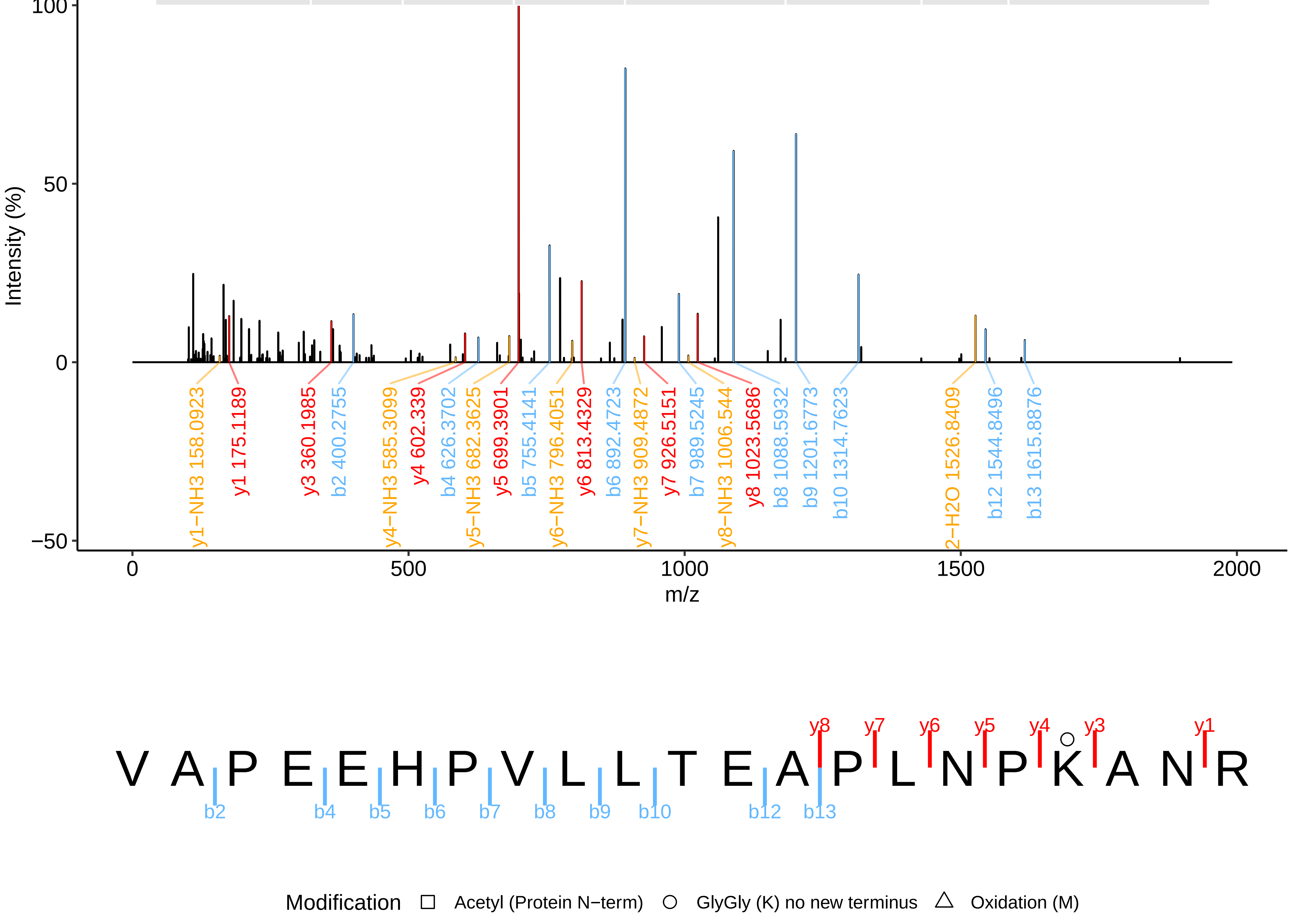

20200921_KIZ_MUb4_GlyGly_10plex_2 − Scan number: 27075

Gene Names	Charge	m/z	Mass	Retention time	PEP	Score	Precursor Intensity
FKBP8	3	712.3733	2134.098	107.31	0.0016934	69.431	495683.7

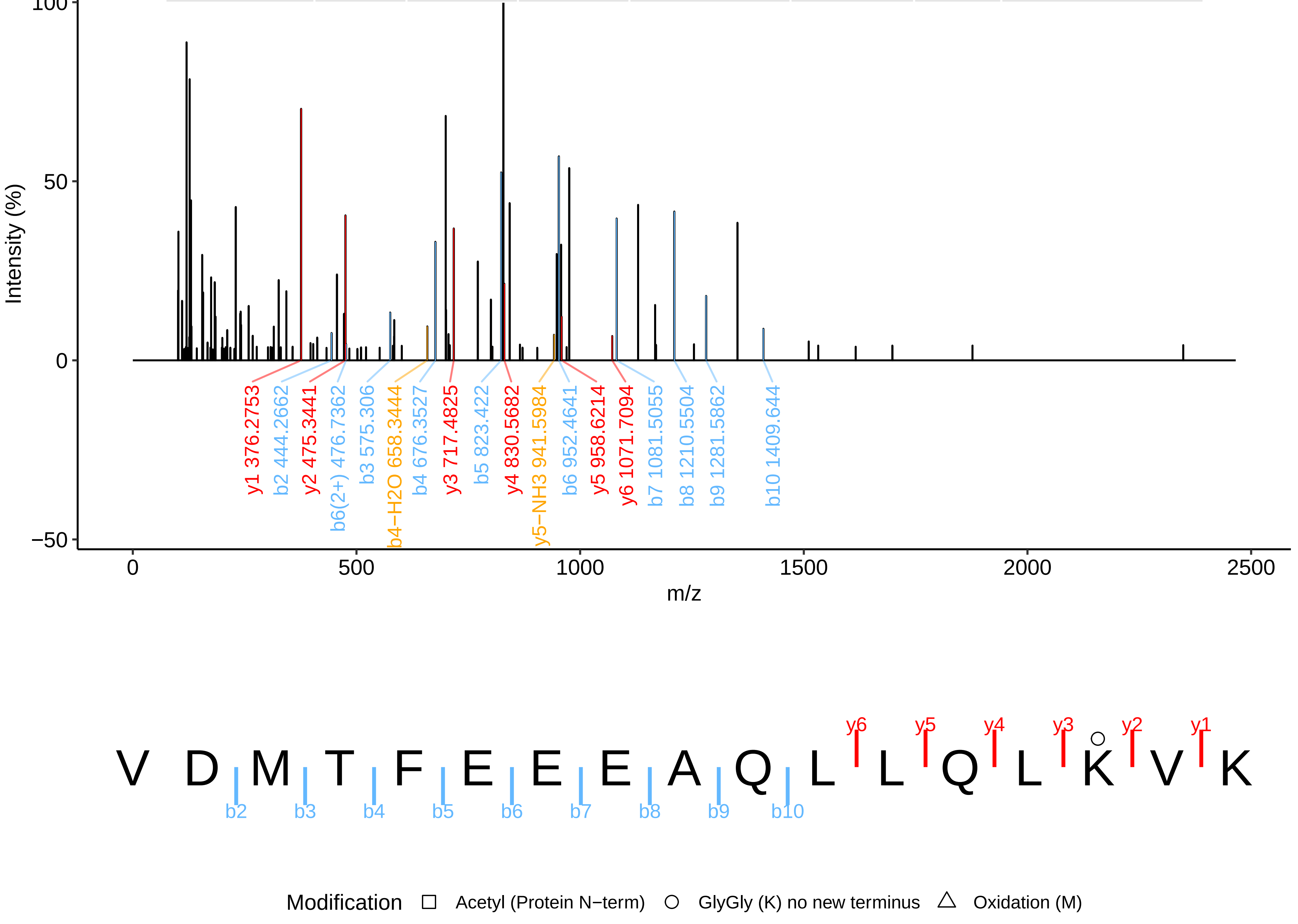

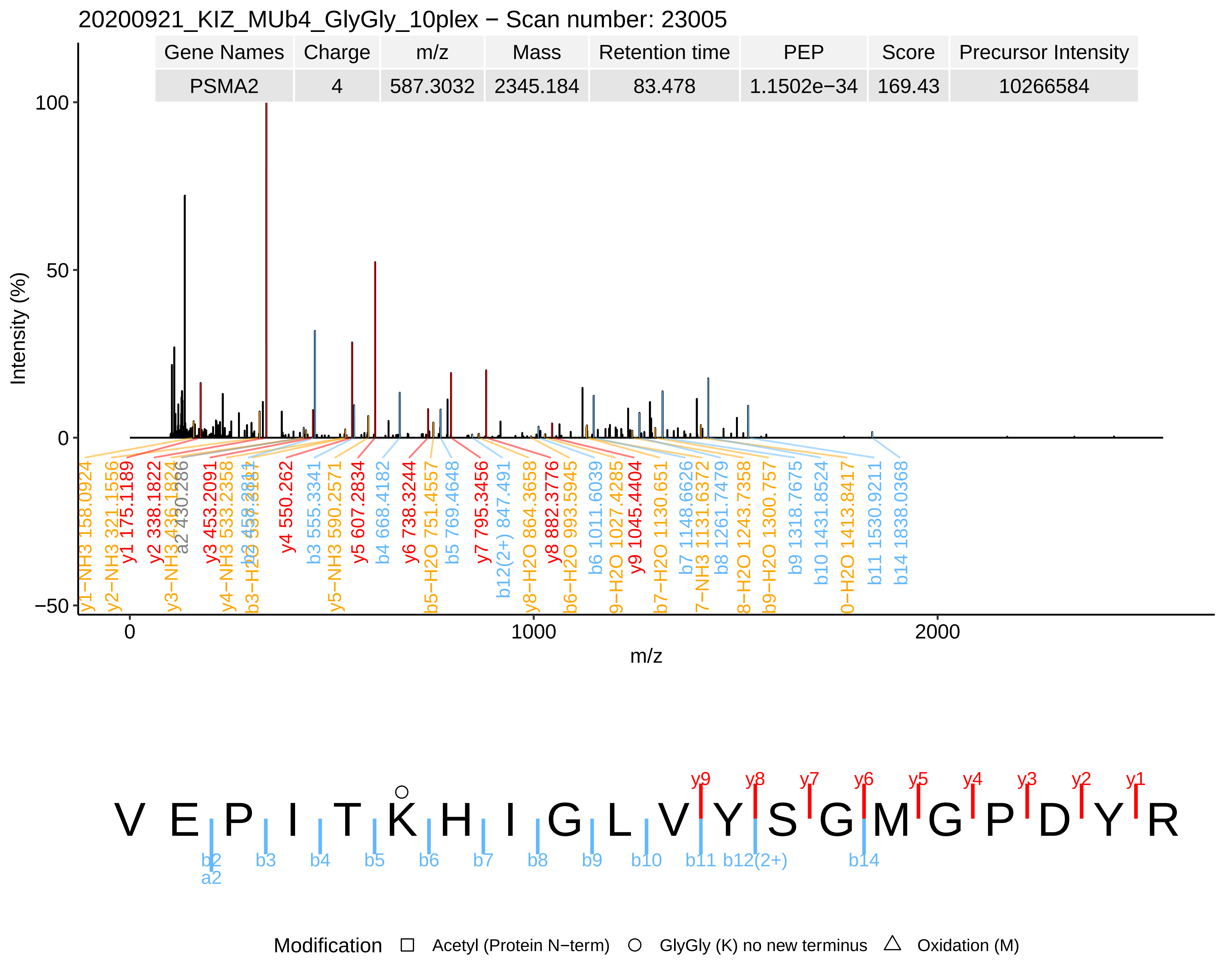

20200921_KIZ_MUb4_GlyGly_10plex − Scan number: 23005
Gene Names | Charge | m/z | Mass | Retention time | PEP | Score | Precursor Intensity
PSMA2 | 4 | 587.3032 | 2345.184 | 83.478 | 1.1502e−34 | 169.43 | 10266584
Intensity (%)
100
50
0
−50
0
1000
2000
m/z
y1−NH3 158.0924
y2−NH3 321.1556
y1 175.1189
y2 338.1822
y3−NH3 436.1824
a2 430.286
y3 453.2091
y4−NH3 533.2358
b2 458.2811
b3−H2O 537.3187
y4 550.262
b3 555.3341
y5−NH3 590.2571
y5 607.2834
b4 668.4182
y6 738.3244
b5−H2O 751.4557
b5 769.4648
y7 795.3456
b12(2+) 847.491
y8−H2O 864.3658
y8 882.3776
b6−H2O 993.5945
b6 1011.6039
y9−H2O 1027.4285
y9 1045.4404
b7−H2O 1130.651
b7 1148.6626
b7−NH3 1131.6372
b8 1261.7479
b8−H2O 1243.7358
b9−H2O 1300.757
b9 1318.7675
b10 1431.8524
b10−H2O 1413.8417
b11 1530.9211
b14 1838.0368
V E P I T K H I G L V Y S G M G P D Y R
y9 y8 y7 y6 y5 y4 y3 y2 y1
b2 b3 b4 b5 b6 b7 b8 b9 b10 b11 b12(2+) b14
a2
Modification
Acetyl (Protein N−term)
GlyGly (K) no new terminus
Oxidation (M)

20200921_KIZ_MUb4_GlyGly_10plex_2 − Scan number: 18545

Gene Names	Charge	m/z	Mass	Retention time	PEP	Score	Precursor Intensity
PSMA2	4	587.3032	2345.184	72.64	0.025697	50.938	395493.5

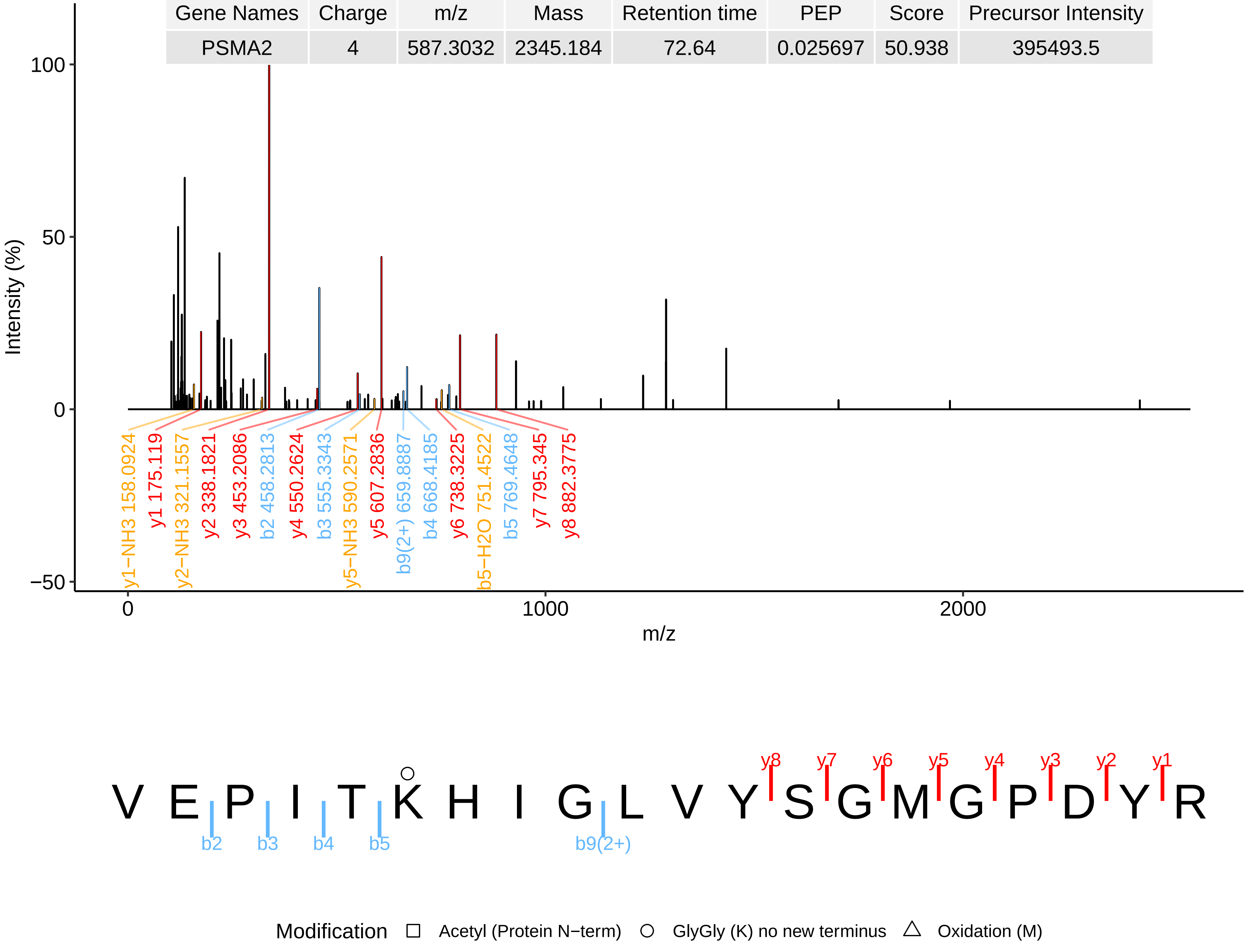

20200921_KIZ_MUb4_GlyGly_10plex_2 − Scan number: 16550

Gene Names	Charge	m/z	Mass	Retention time	PEP	Score	Precursor Intensity
PSMA2	4	591.302	2361.179	64.62	0.0003238	76.704	1060984

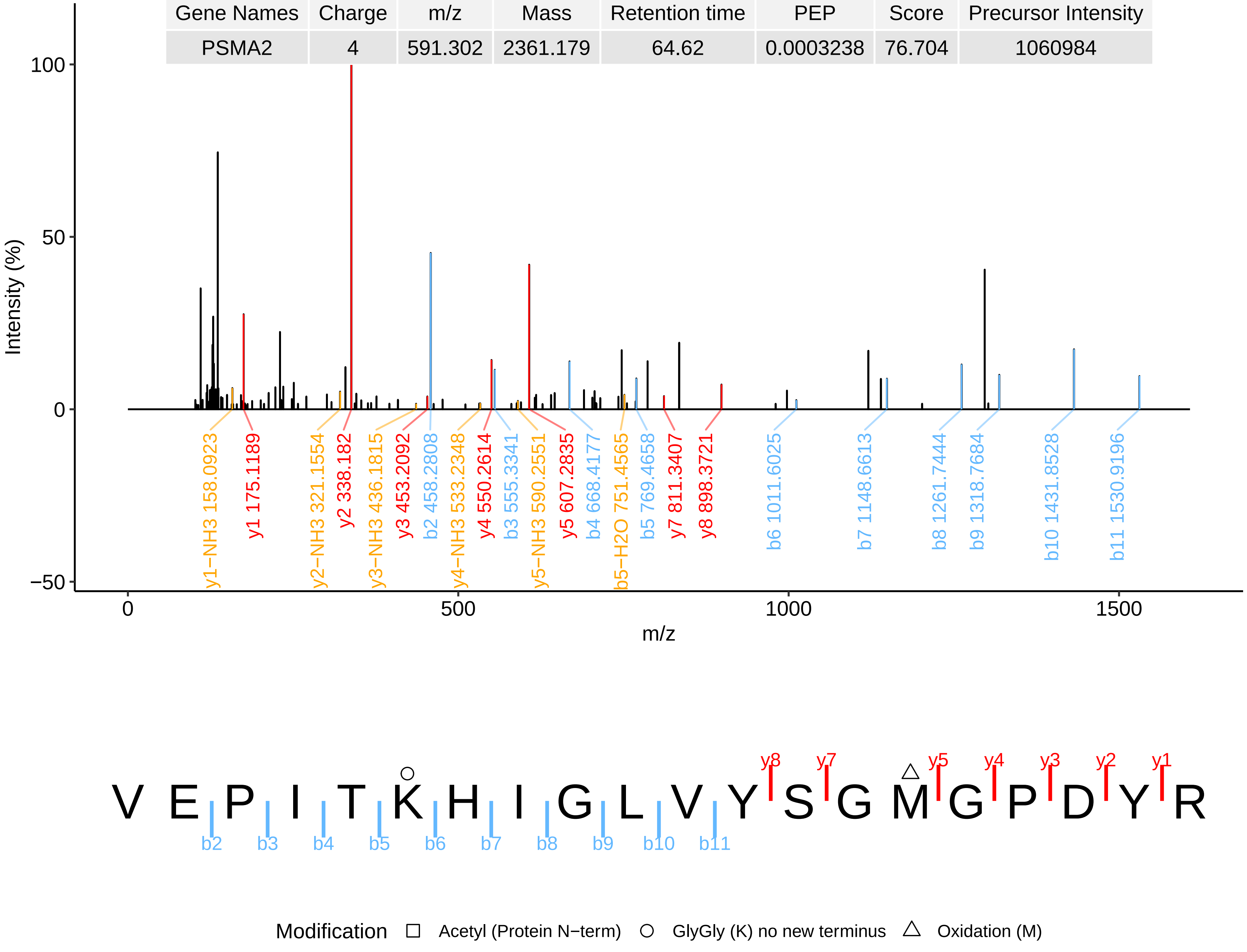

20201118_KIZ_MUb6_GlyGly_2 − Scan number: 21316

Gene Names	Charge	m/z	Mass	Retention time	PEP	Score	Precursor Intensity
TNFRSF10B	4	590.551	2358.175	71.821	0.0013277	77.871	9062602

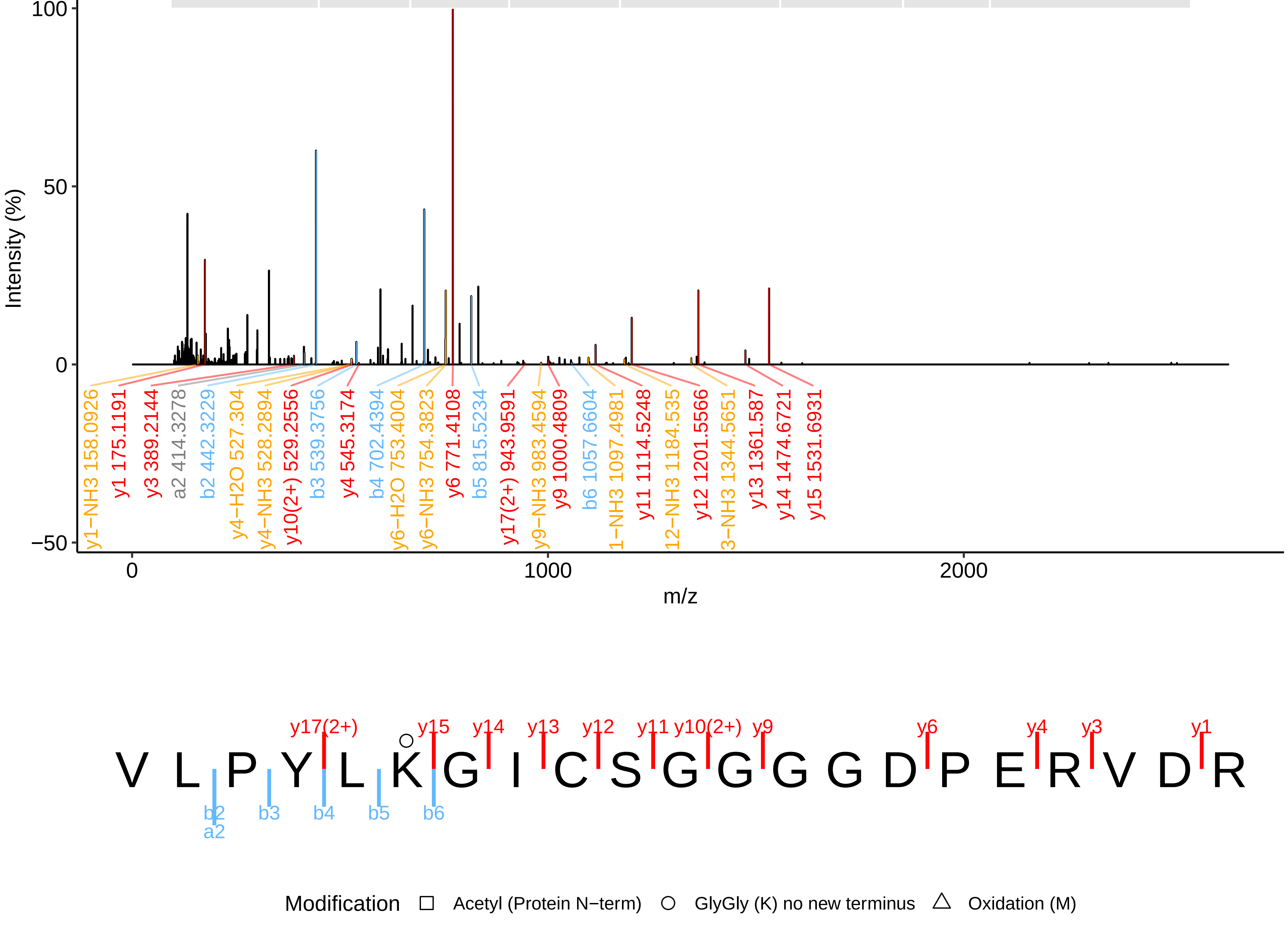

20200921_KIZ_MUb4_GlyGly_10plex − Scan number: 23288

Gene Names	Charge	m/z	Mass	Retention time	PEP	Score	Precursor Intensity
VDAC1	3	739.7461	2216.216	84.525	7.8809e−07	97.083	2501881

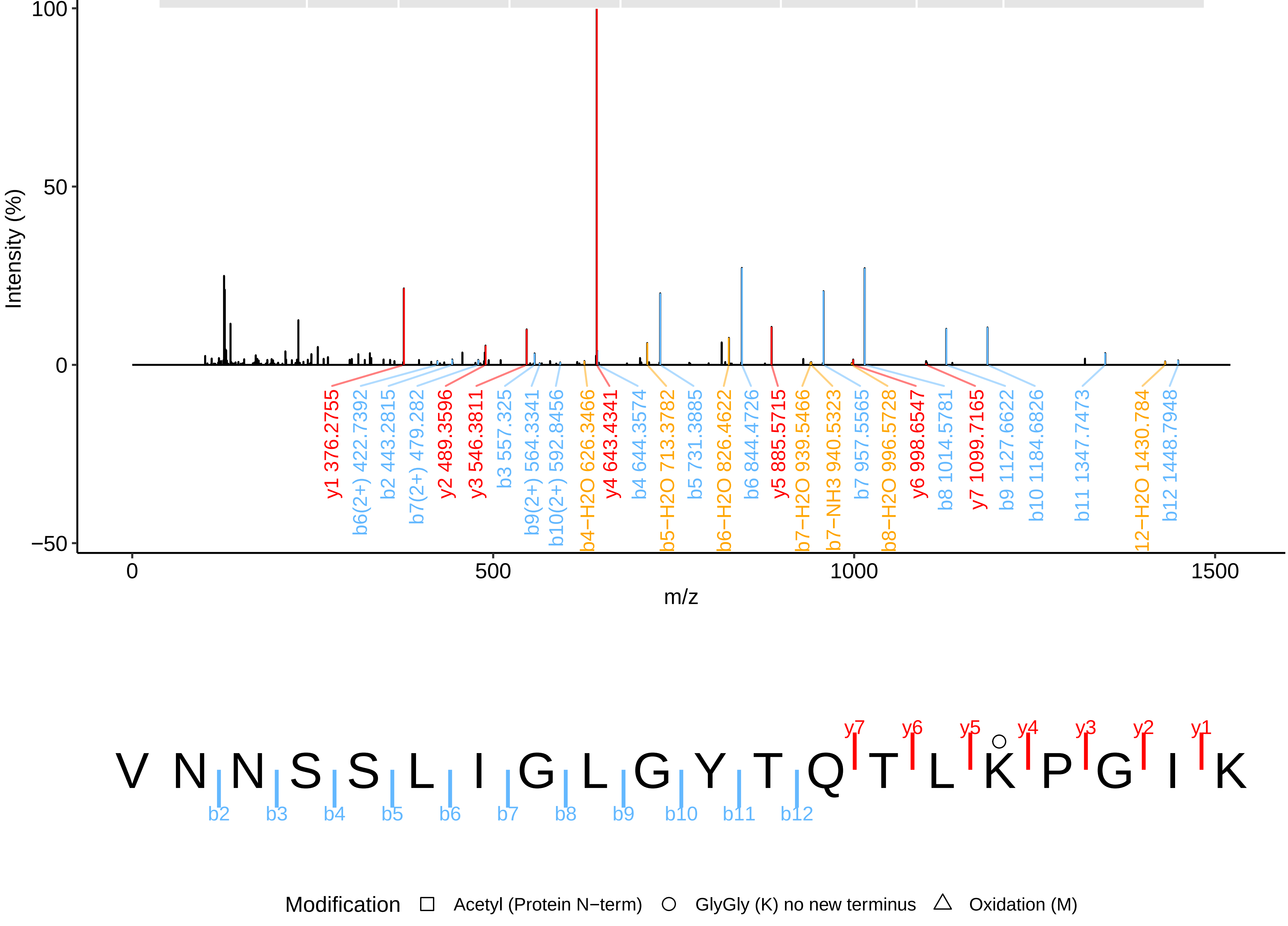

20200921_KIZ_MUb4_GlyGly_10plex_2 − Scan number: 20545

Gene Names	Charge	m/z	Mass	Retention time	PEP	Score	Precursor Intensity
VDAC1	3	739.7461	2216.216	80.764	1.0439e−05	85.808	896909.4

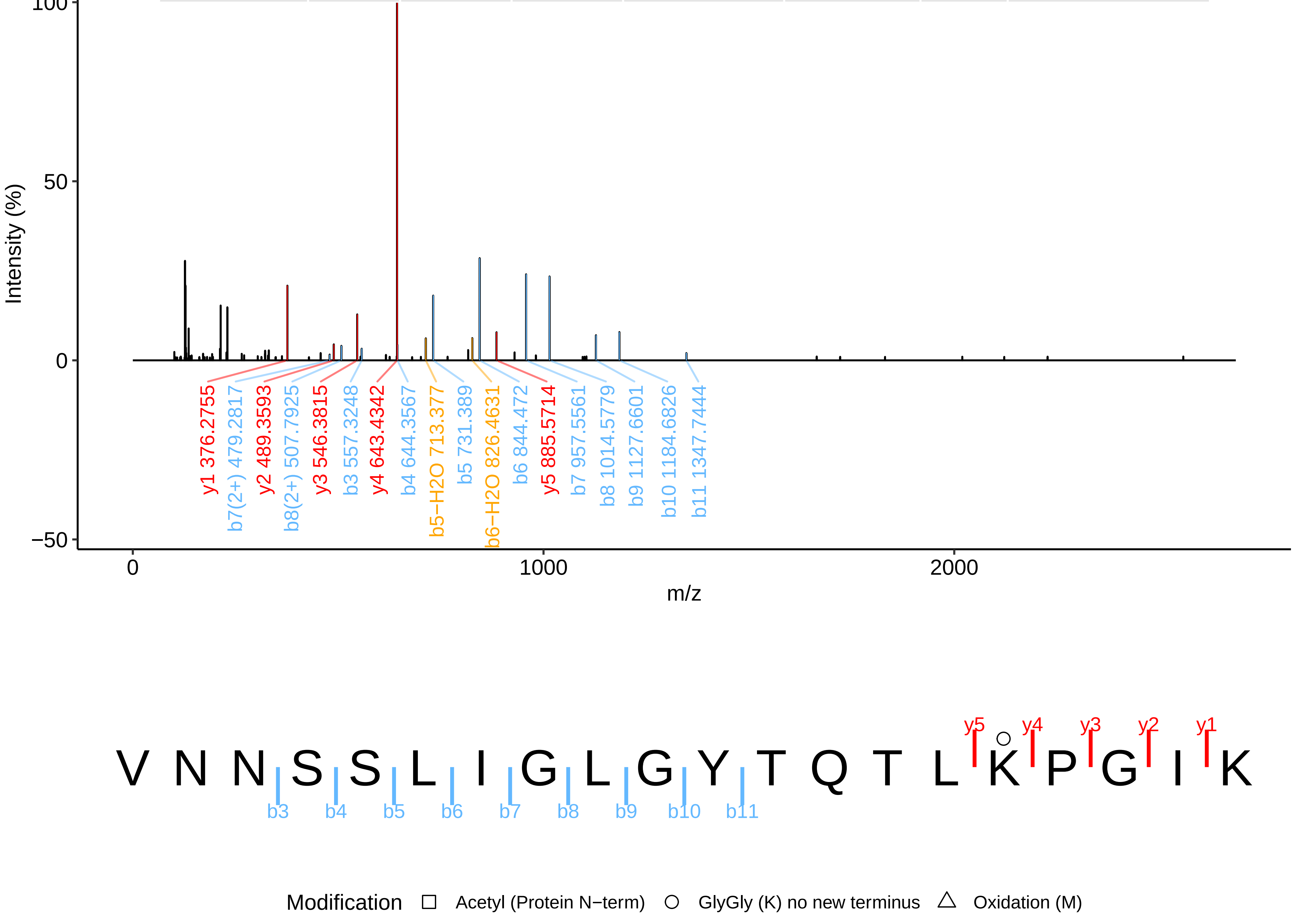

20200921_KIZ_MUb4_GlyGly_10plex_2 − Scan number: 20582

Gene Names	Charge	m/z	Mass	Retention time	PEP	Score	Precursor Intensity
VDAC1	3	739.7461	2216.216	80.919	0.012948	58.49	1573192

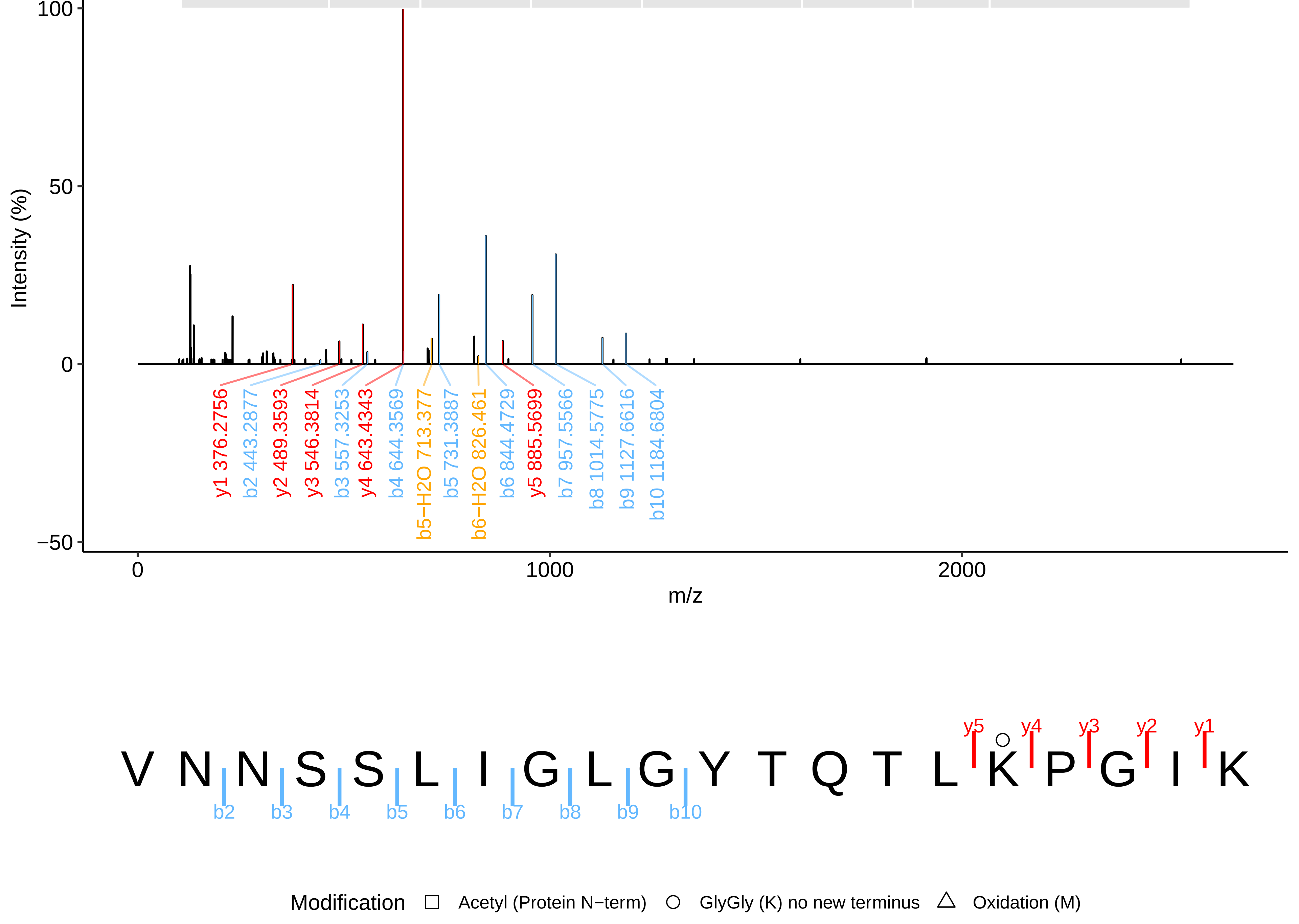

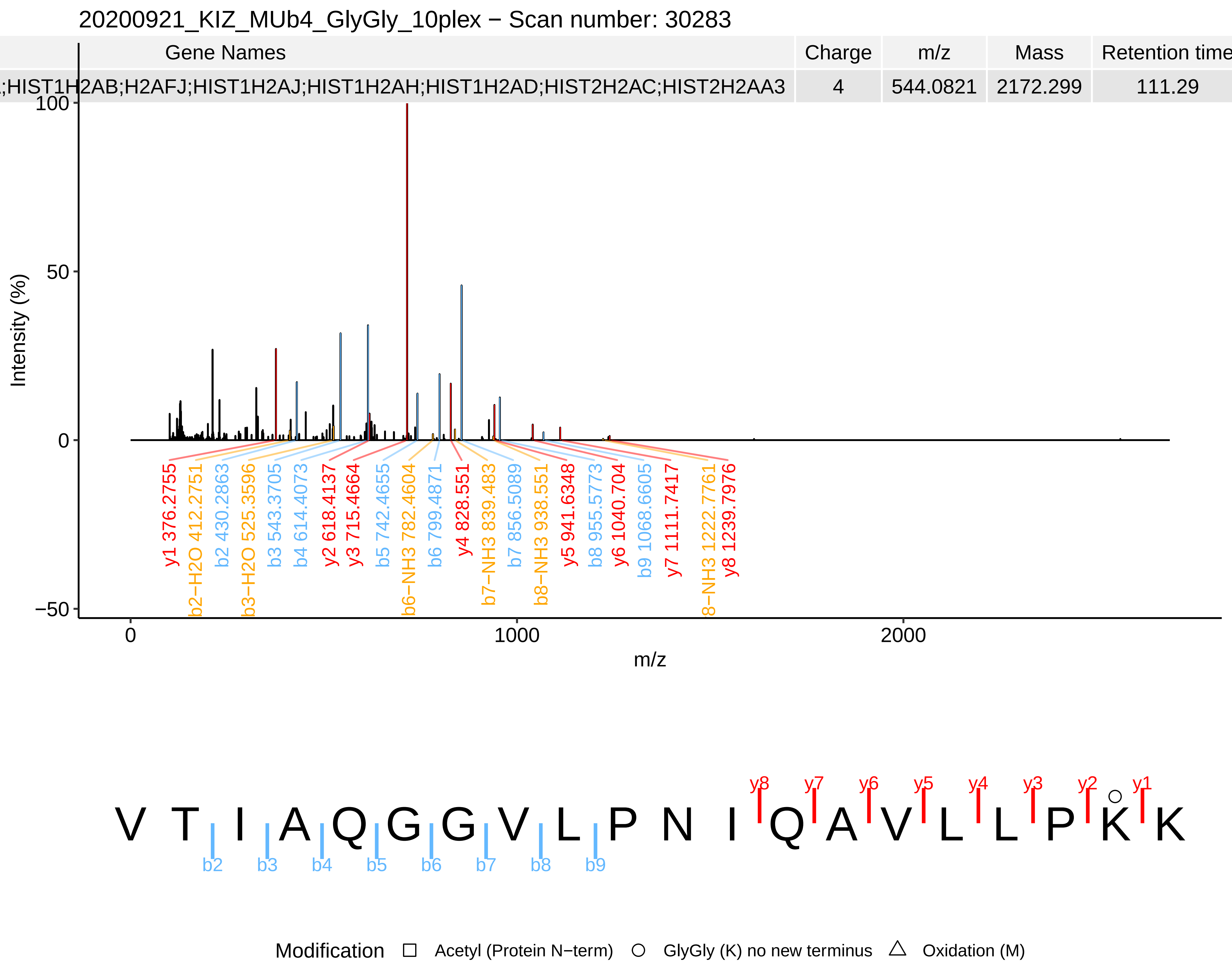
20200921_KIZ_MUb4_GlyGly_10plex − Scan number: 30283
Gene Names
Charge
m/z
Mass
Retention time
;HIST1H2AB;H2AFJ;HIST1H2AJ;HIST1H2AH;HIST1H2AD;HIST2H2AC;HIST2H2AA3
4
544.0821
2172.299
111.29
Intensity (%)
m/z
y1 376.2755
b2−H2O 412.2751
b2 430.2863
b3−H2O 525.3596
b3 543.3705
b4 614.4073
y2 618.4137
y3 715.4664
b5 742.4655
b6−NH3 782.4604
b6 799.4871
y4 828.551
b7−NH3 839.483
b7 856.5089
b8−NH3 938.551
y5 941.6348
b8 955.5773
y6 1040.704
b9 1068.6605
y7 1111.7417
8−NH3 1222.7761
y8 1239.7976
V T I A Q G G V L P N I Q A V L L P K K
b2 b3 b4 b5 b6 b7 b8 b9
y8 y7 y6 y5 y4 y3 y2 y1
Modification
Acetyl (Protein N−term)
GlyGly (K) no new terminus
Oxidation (M)

20200921_KIZ_MUb4_GlyGly_10plex − Scan number: 30406

Gene Names	Charge	m/z	Mass	Retention time
;HIST1H2AB;H2AFJ;HIST1H2AJ;HIST1H2AH;HIST1H2AD;HIST2H2AC;HIST2H2AA3	4	544.0821	2172.299	111.79

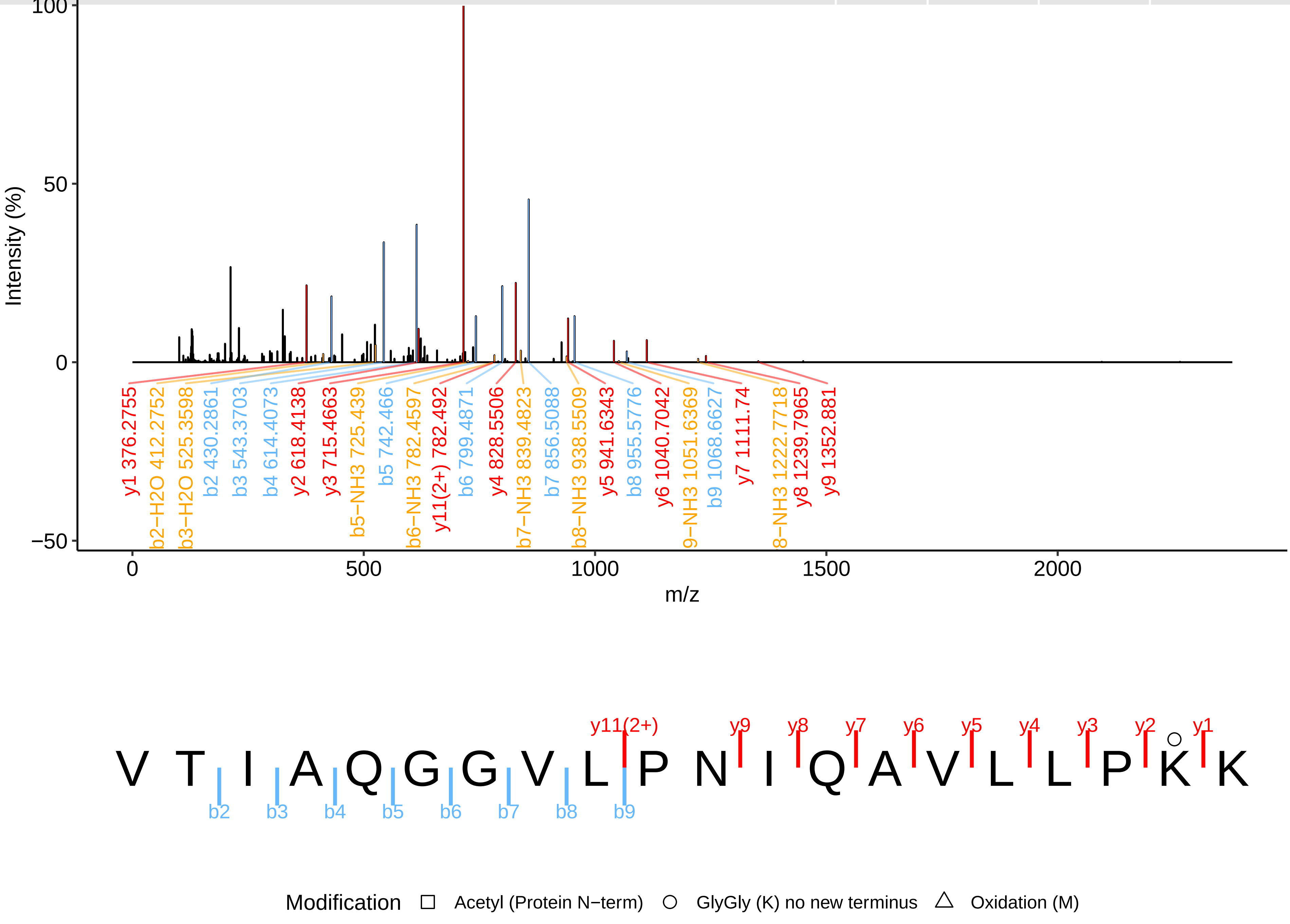

20200921_KIZ_MUb4_GlyGly_10plex − Scan number: 30422

Gene Names	Charge	m/z	Mass	Retention time
;HIST1H2AB;H2AFJ;HIST1H2AJ;HIST1H2AH;HIST1H2AD;HIST2H2AC;HIST2H2AA3	3	725.1071	2172.299	111.86

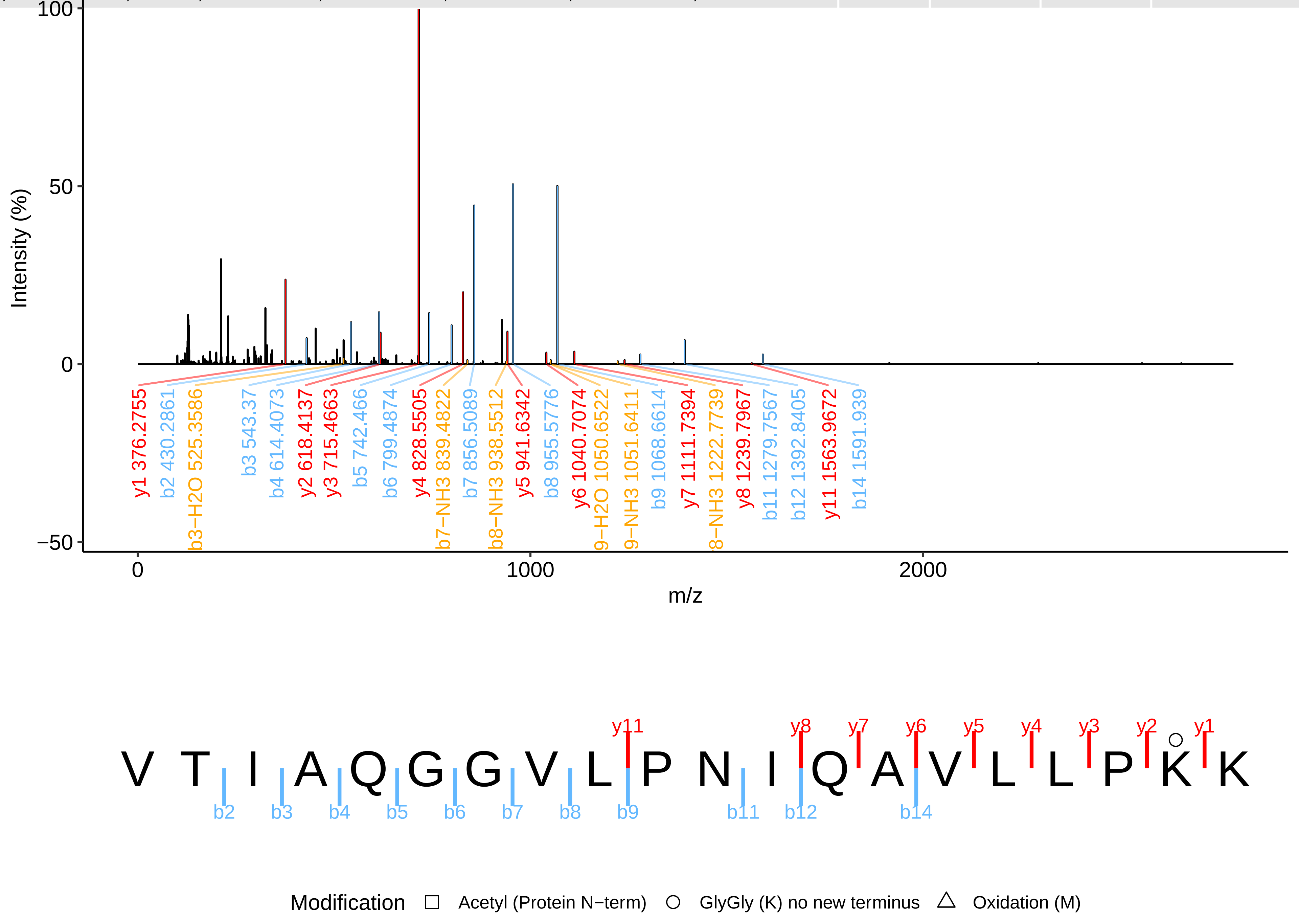

20200921_KIZ_MUb4_GlyGly_10plex_2 − Scan number: 27310

Gene Names	Charge	m/z	Mass	Retention time
A;HIST1H2AB;H2AFJ;HIST1H2AJ;HIST1H2AH;HIST1H2AD;HIST2H2AC;HIST2H2AA3	4	544.0821	2172.299	108.27

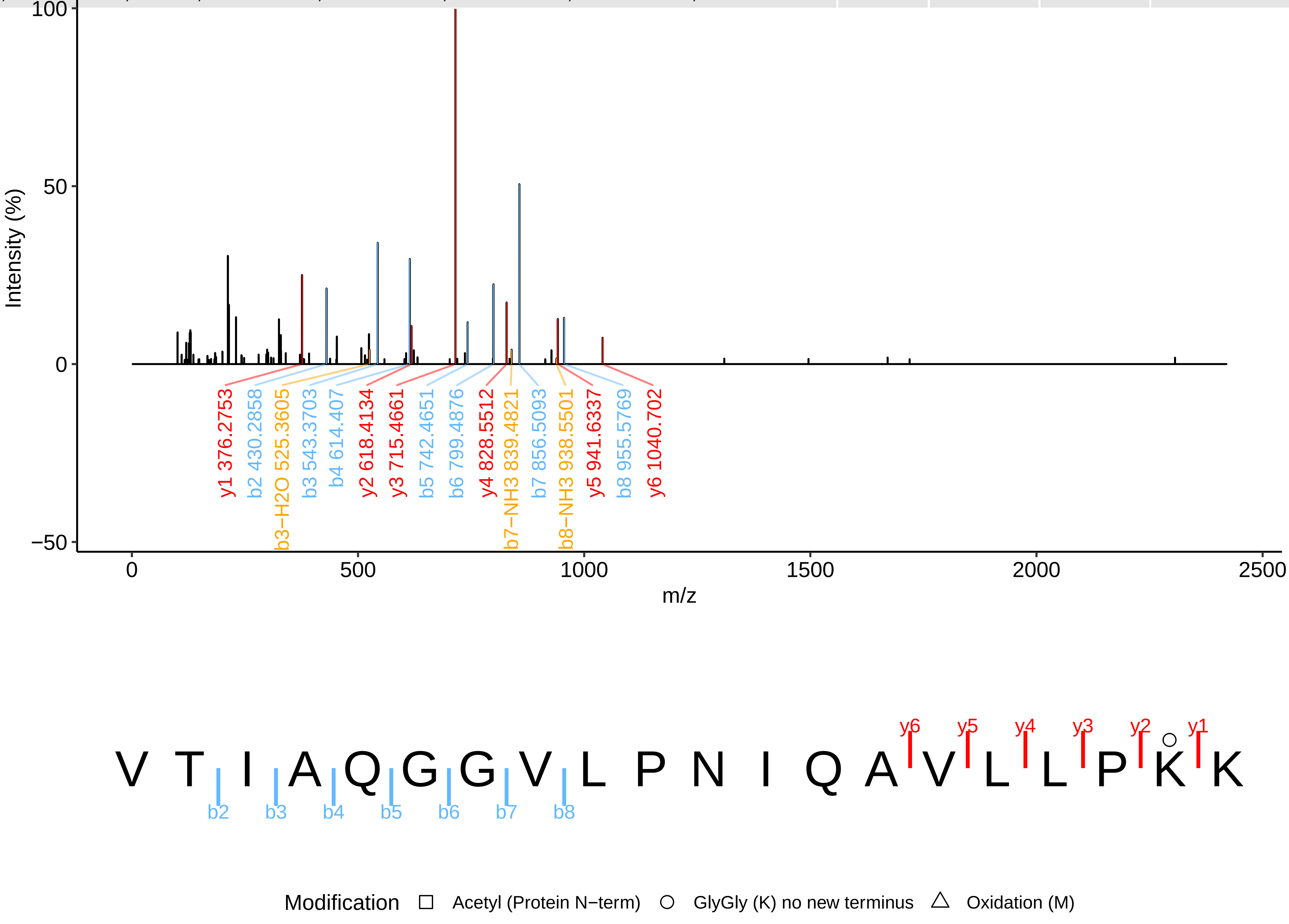

20200921_KIZ_MUb4_GlyGly_10plex_2 − Scan number: 27319

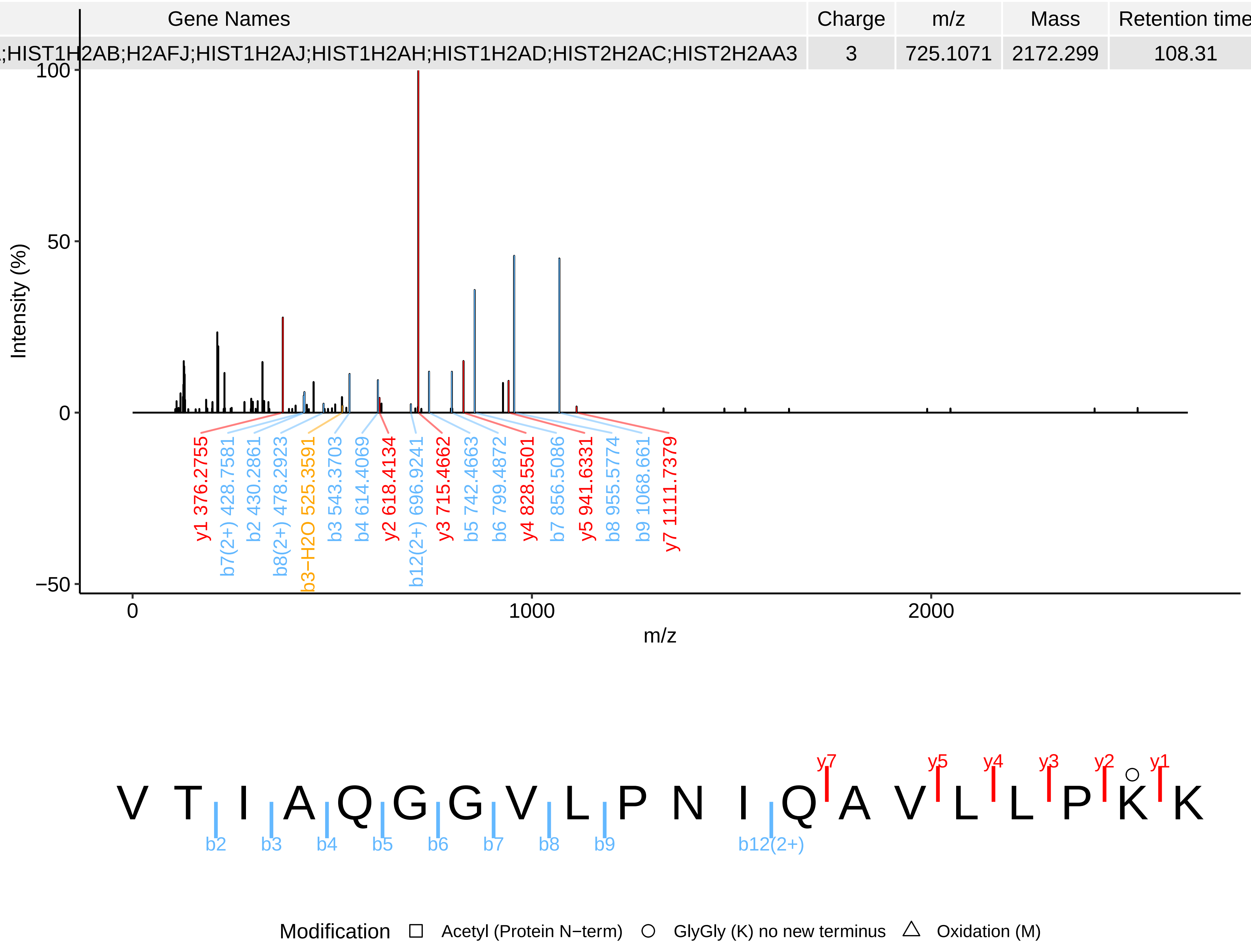

20200924_KIZ_MUb5_GlyGly_1 − Scan number: 30839

Gene Names	Charge	m/z	Mass	Retention time
;HIST1H2AB;H2AFJ;HIST1H2AJ;HIST1H2AH;HIST1H2AD;HIST2H2AC;HIST2H2AA3	4	544.0821	2172.299	98.378

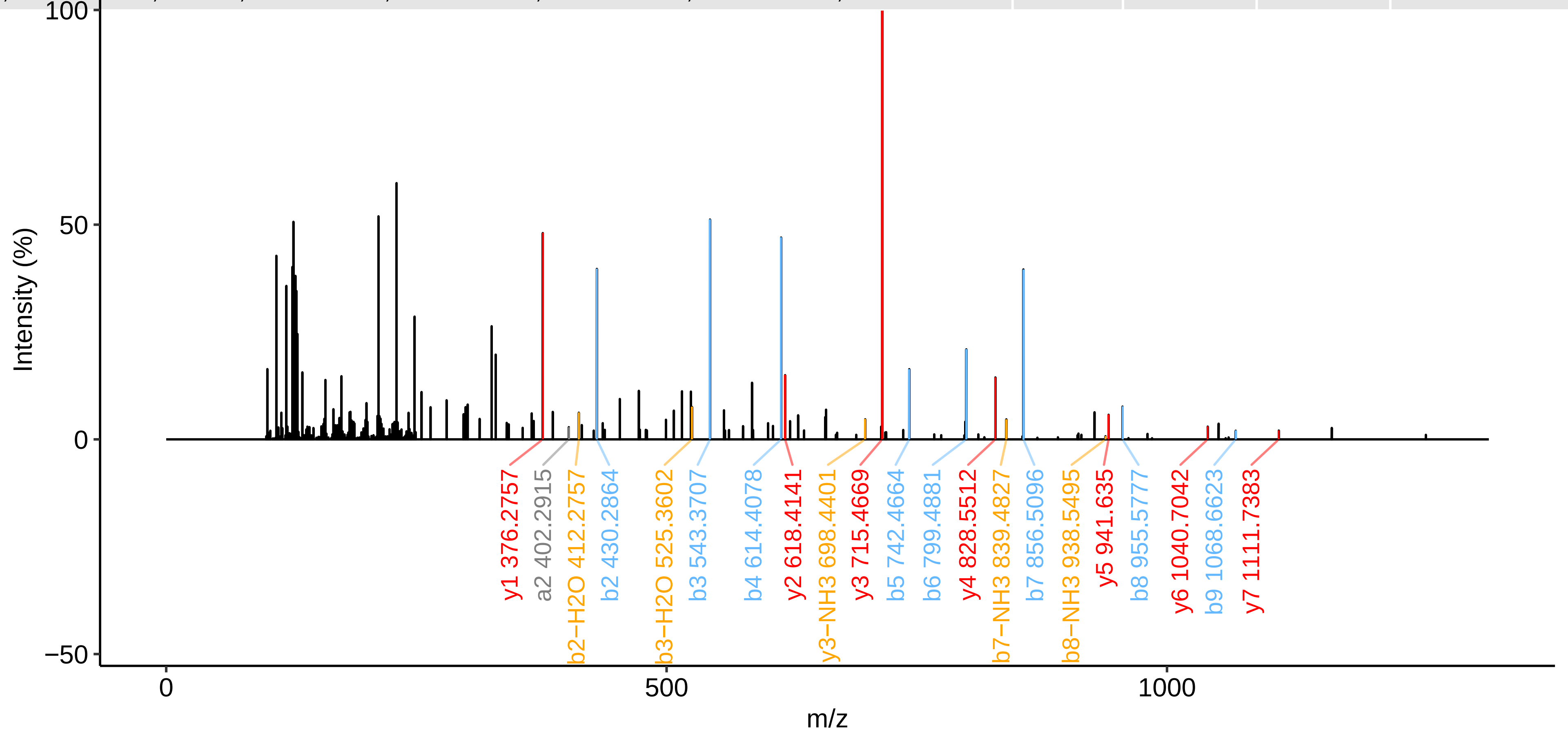

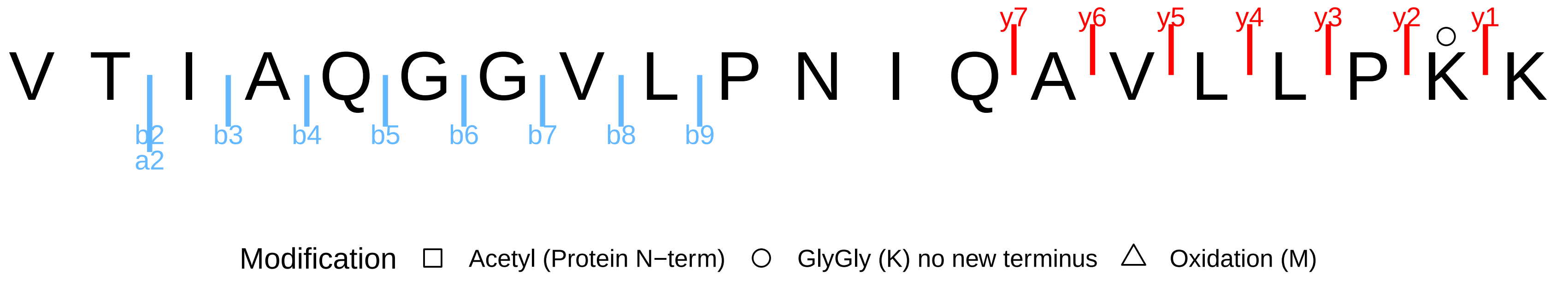

20200924_KIZ_MUb5_GlyGly_1 − Scan number: 30916

Gene Names	Charge	m/z	Mass	Retention time
;HIST1H2AB;H2AFJ;HIST1H2AJ;HIST1H2AH;HIST1H2AD;HIST2H2AC;HIST2H2AA3	4	544.0821	2172.299	98.625

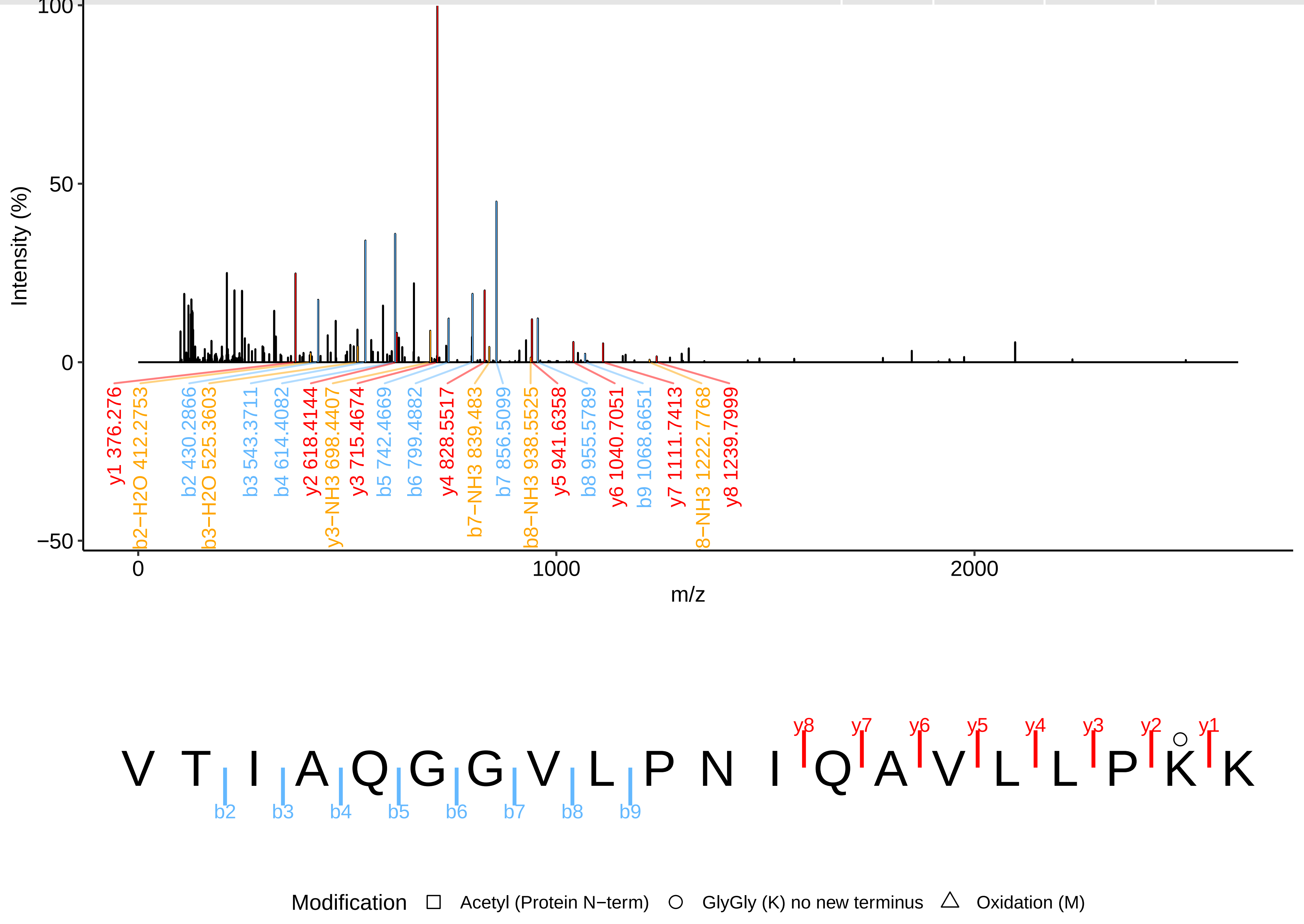

20200924_KIZ_MUb5_GlyGly_1 − Scan number: 31080

Gene Names	Charge	m/z	Mass	Retention time
A;HIST1H2AB;H2AFJ;HIST1H2AJ;HIST1H2AH;HIST1H2AD;HIST2H2AC;HIST2H2AA3	4	544.0821	2172.299	99.144

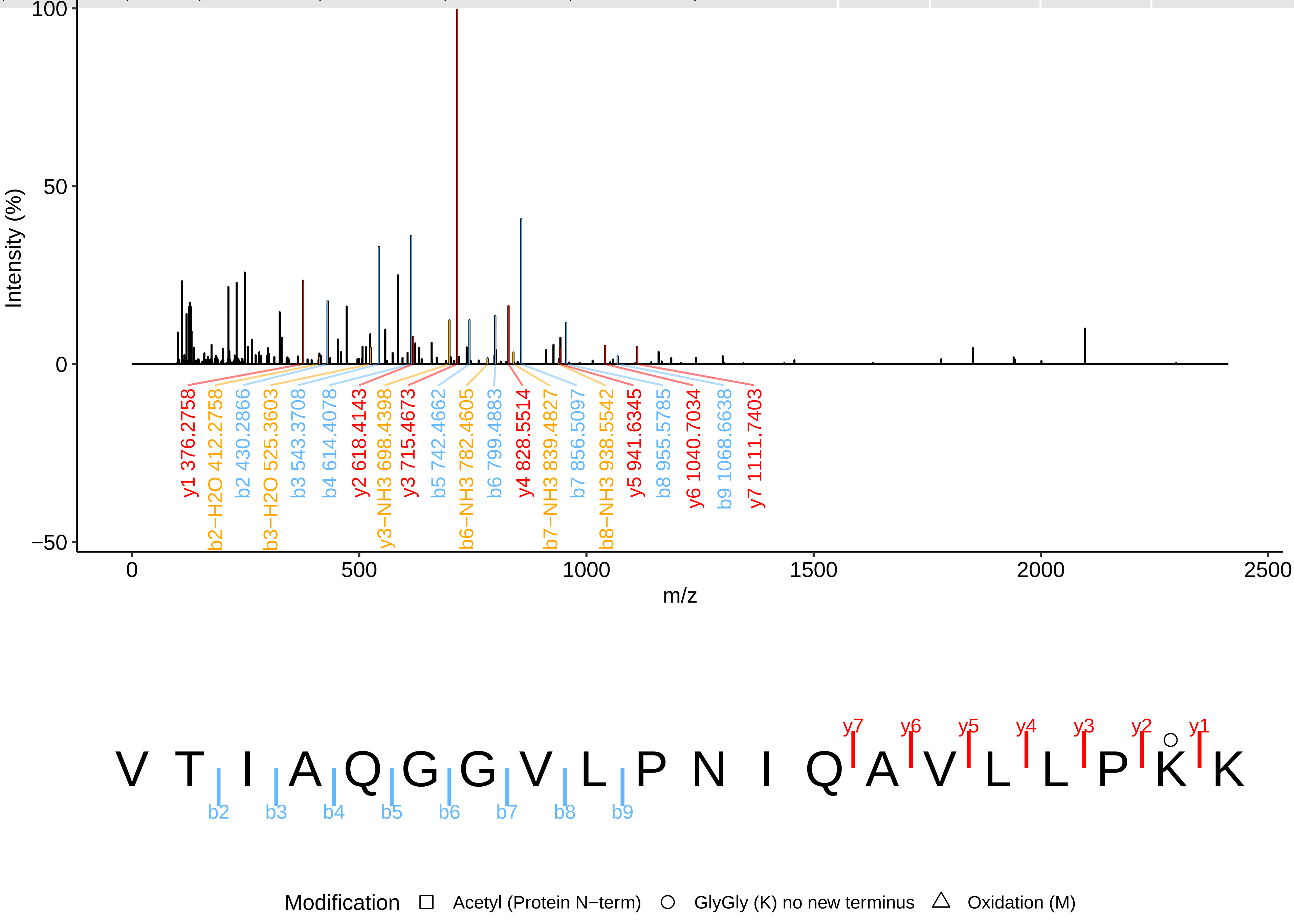

20200924_KIZ_MUb5_GlyGly_1 − Scan number: 31124

Gene Names	Charge	m/z	Mass	Retention time
;HIST1H2AB;H2AFJ;HIST1H2AJ;HIST1H2AH;HIST1H2AD;HIST2H2AC;HIST2H2AA3	3	725.1071	2172.299	99.28

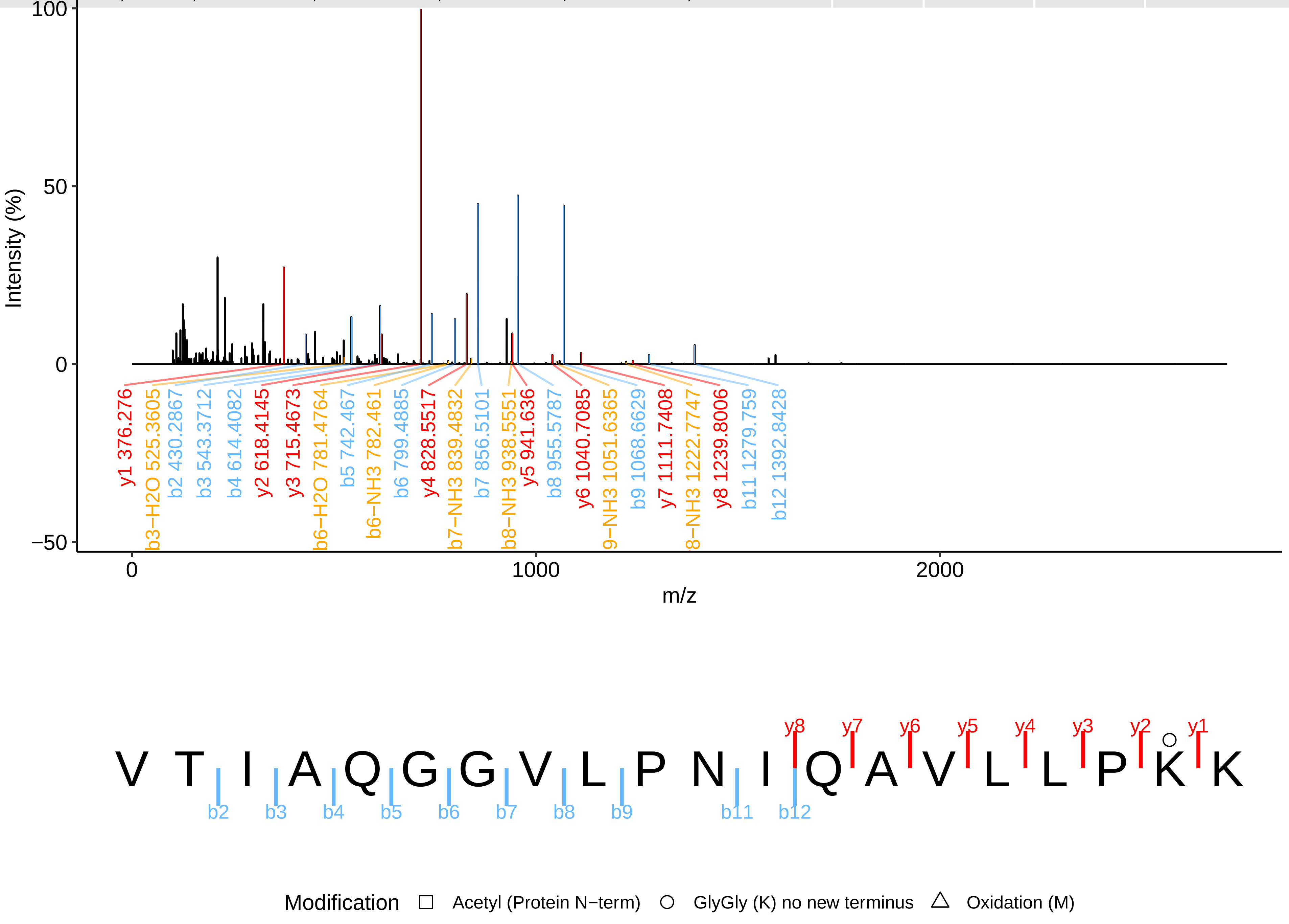

20200924_KIZ_MUb5_GlyGly_1 − Scan number: 31181

Gene Names	Charge	m/z	Mass	Retention time
;HIST1H2AB;H2AFJ;HIST1H2AJ;HIST1H2AH;HIST1H2AD;HIST2H2AC;HIST2H2AA3	3	725.1071	2172.299	99.471

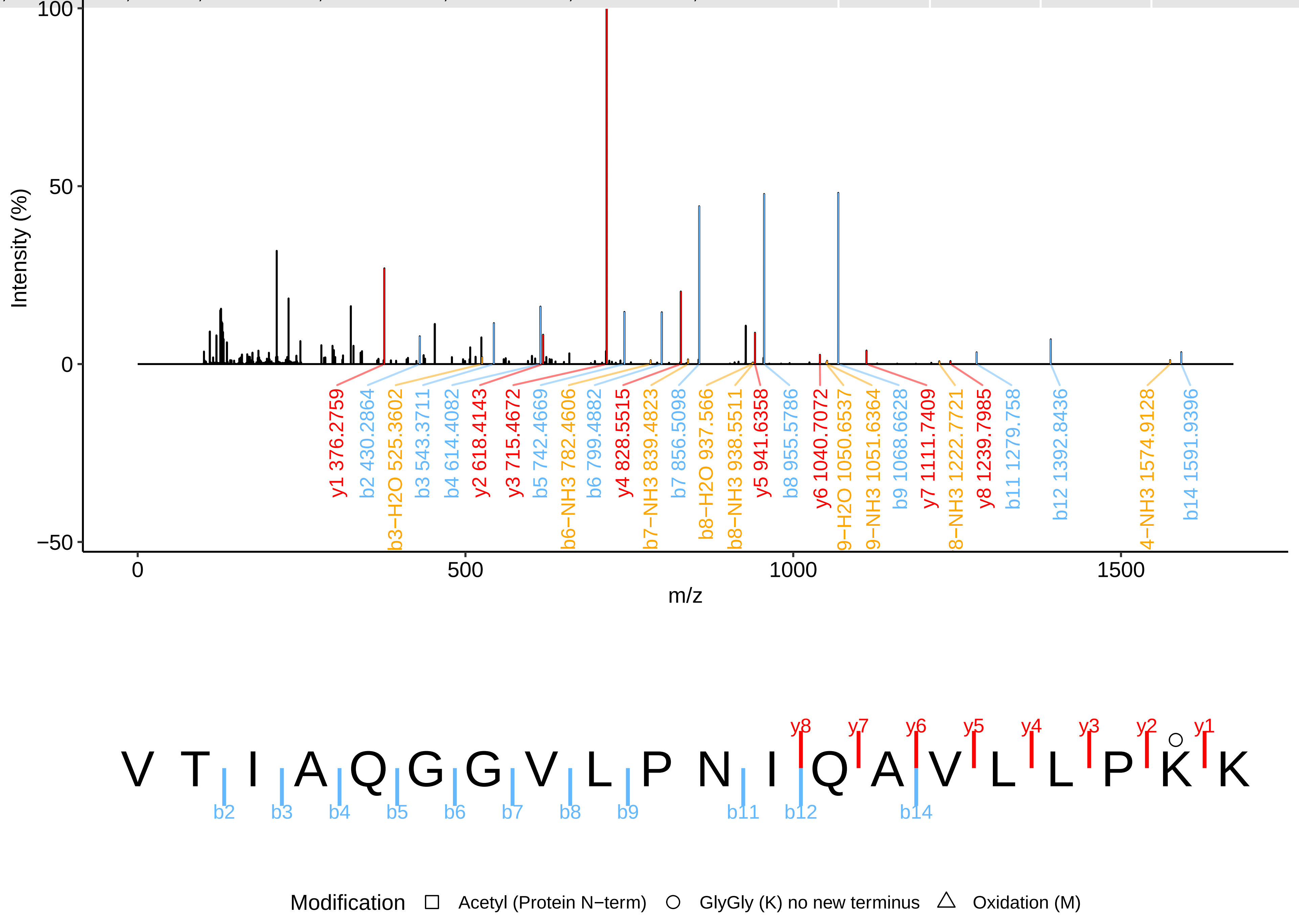

20200924_KIZ_MUb5_GlyGly_1 − Scan number: 31208

Gene Names	Charge	m/z	Mass	Retention time
;HIST1H2AB;H2AFJ;HIST1H2AJ;HIST1H2AH;HIST1H2AD;HIST2H2AC;HIST2H2AA3	3	725.1071	2172.299	99.559

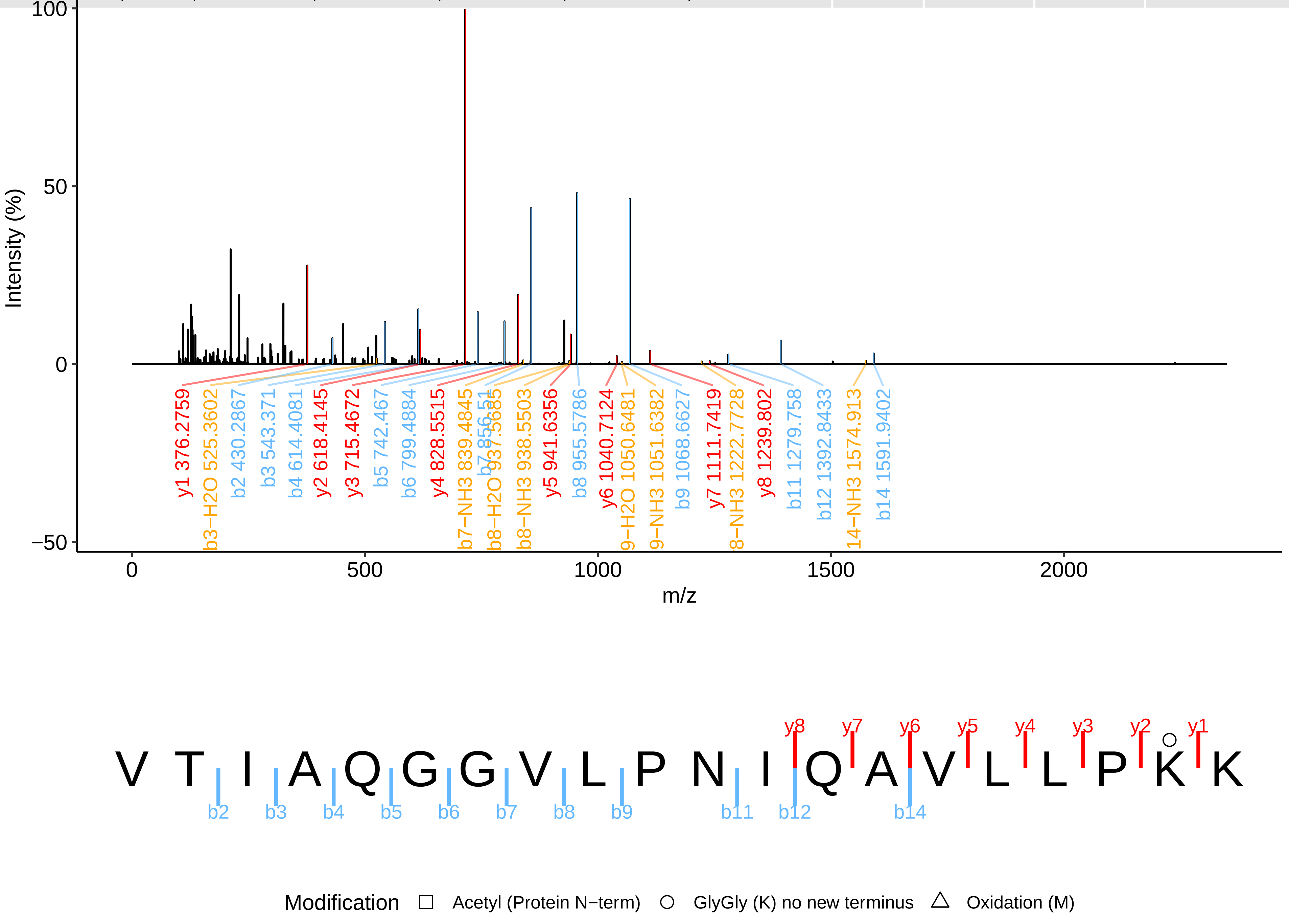

20200924_KIZ_MUb5_GlyGly_1 − Scan number: 31223

Gene Names	Charge	m/z	Mass	Retention time
;HIST1H2AB;H2AFJ;HIST1H2AJ;HIST1H2AH;HIST1H2AD;HIST2H2AC;HIST2H2AA3	3	725.1071	2172.299	99.609

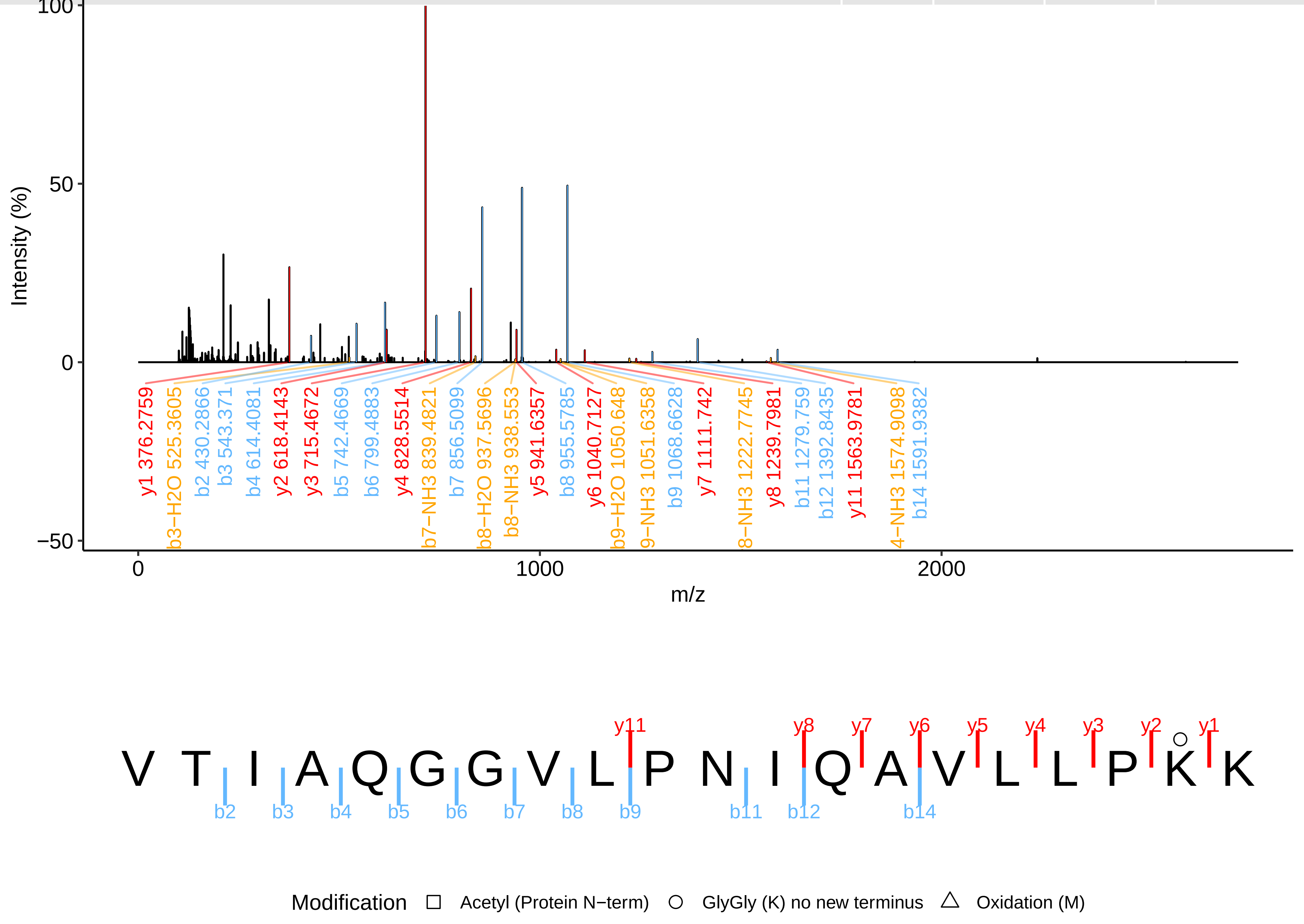

20200924_KIZ_MUb5_GlyGly_2 − Scan number: 27444

Gene Names	Charge	m/z	Mass	Retention time
;HIST1H2AB;H2AFJ;HIST1H2AJ;HIST1H2AH;HIST1H2AD;HIST2H2AC;HIST2H2AA3	3	725.1071	2172.299	101.64

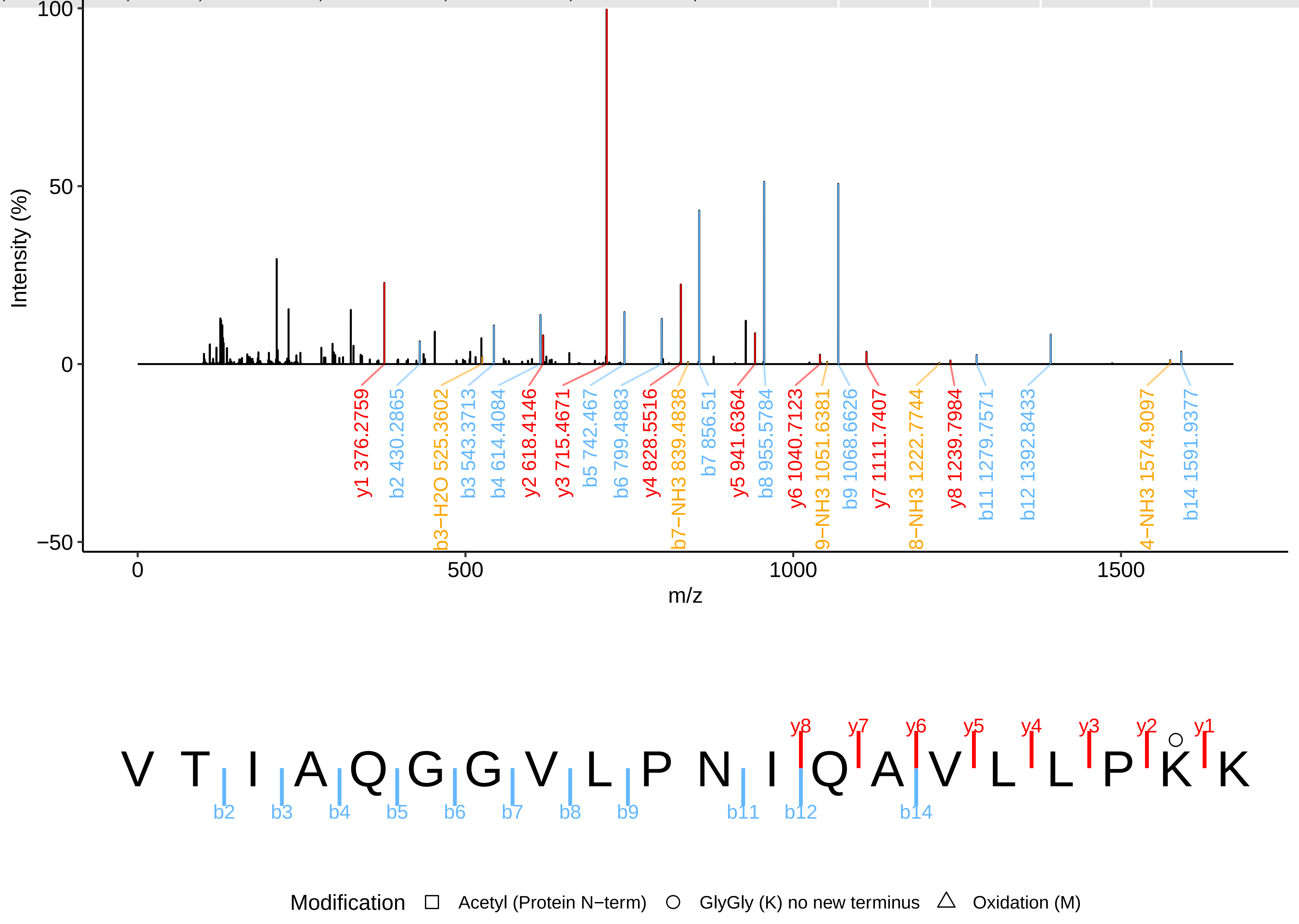

20200924_KIZ_MUb5_GlyGly_2 − Scan number: 27508

Gene Names	Charge	m/z	Mass	Retention time
;HIST1H2AB;H2AFJ;HIST1H2AJ;HIST1H2AH;HIST1H2AD;HIST2H2AC;HIST2H2AA3	4	544.0821	2172.299	101.89

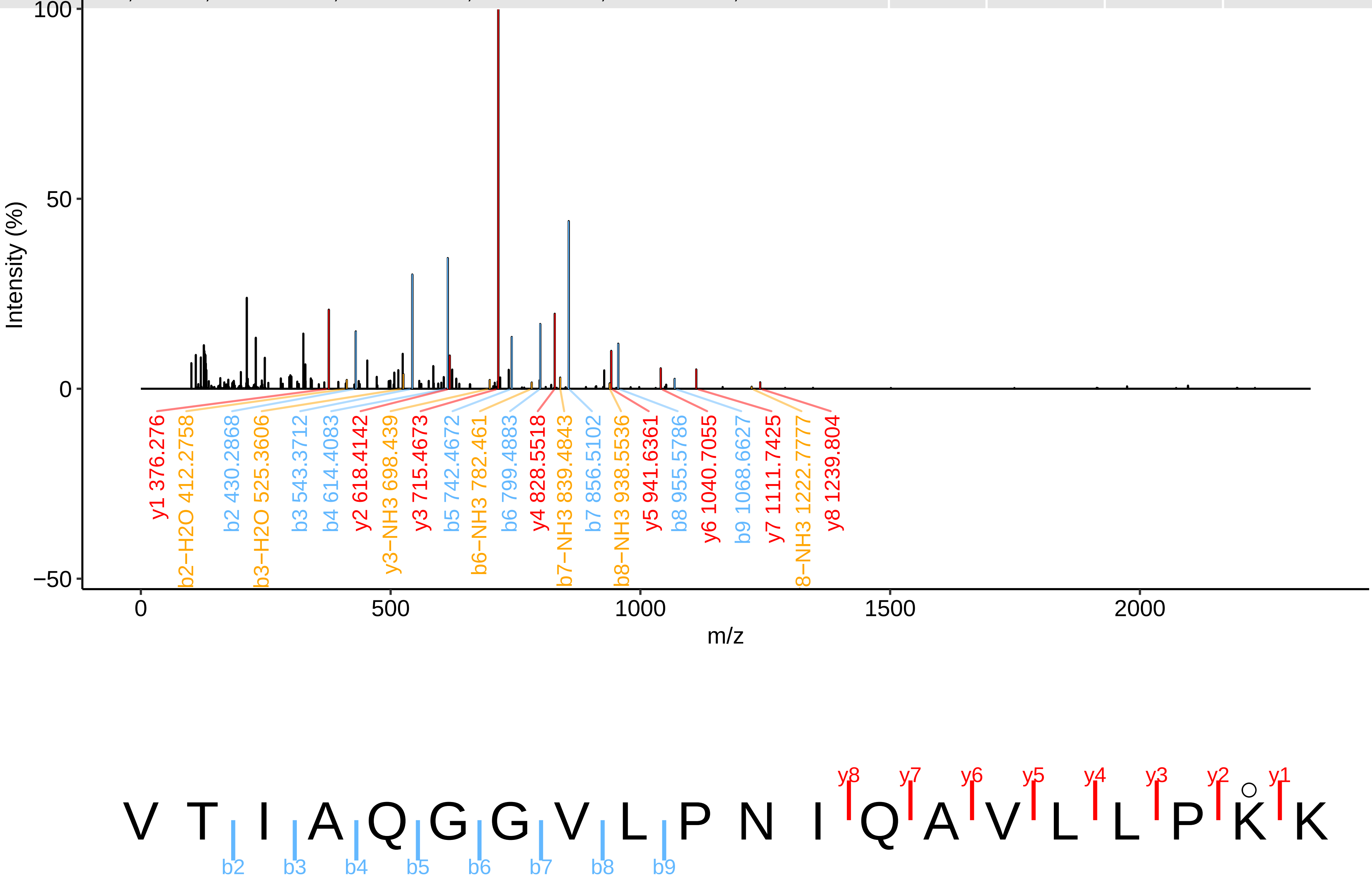

Modification □ Acetyl (Protein N−term) ○ GlyGly (K) no new terminus △ Oxidation (M)

20200924_KIZ_MUb5_GlyGly_2 − Scan number: 27654

Gene Names	Charge	m/z	Mass	Retention time
;HIST1H2AB;H2AFJ;HIST1H2AJ;HIST1H2AH;HIST1H2AD;HIST2H2AC;HIST2H2AA3	4	544.0821	2172.299	102.45

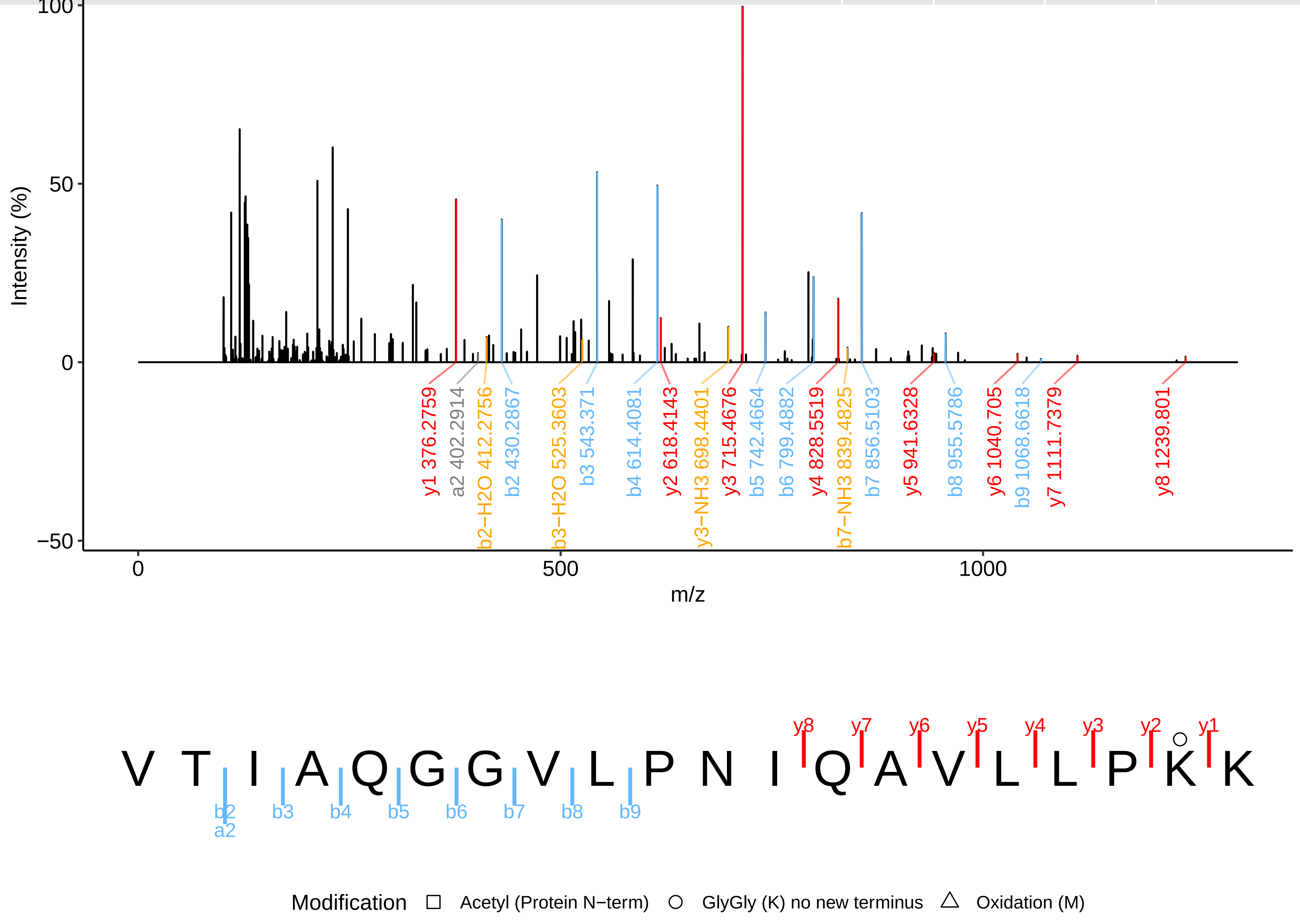

20200921_KIZ_MUb4_GlyGly_10plex − Scan number: 32347

Gene Names	Charge	m/z	Mass	Retention time	PEP	Score	Precursor Intensity
FASN	3	785.7615	2354.263	119.54	2.6093e−21	136.04	6963792

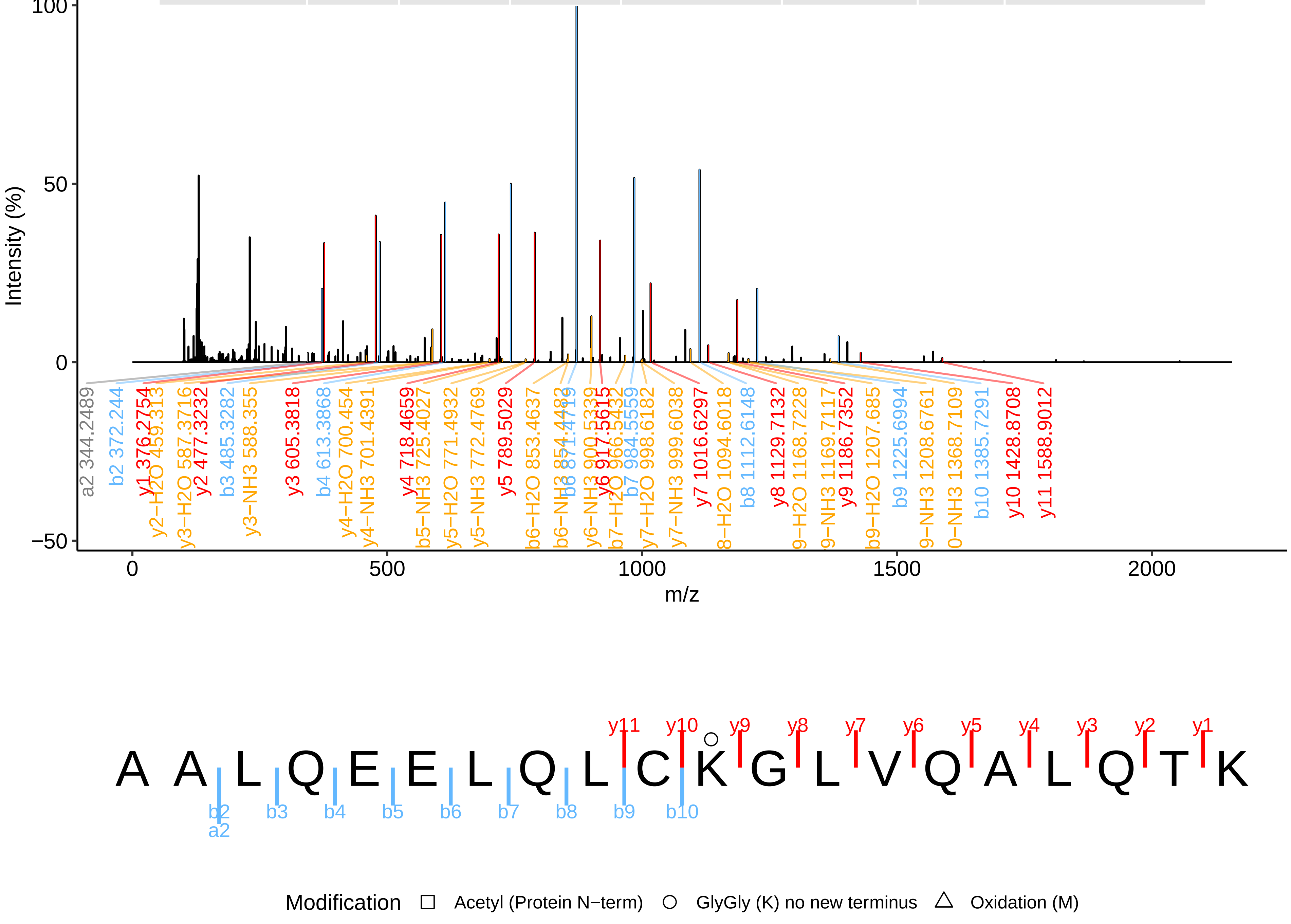

20200921_KIZ_MUb4_GlyGly_10plex_2 − Scan number: 15663

Gene Names	Charge	m/z	Mass	Retention time	PEP	Score	Precursor Intensity
ABCG2	4	514.0349	2052.11	61.302	2.8642e−05	85.619	936292.2

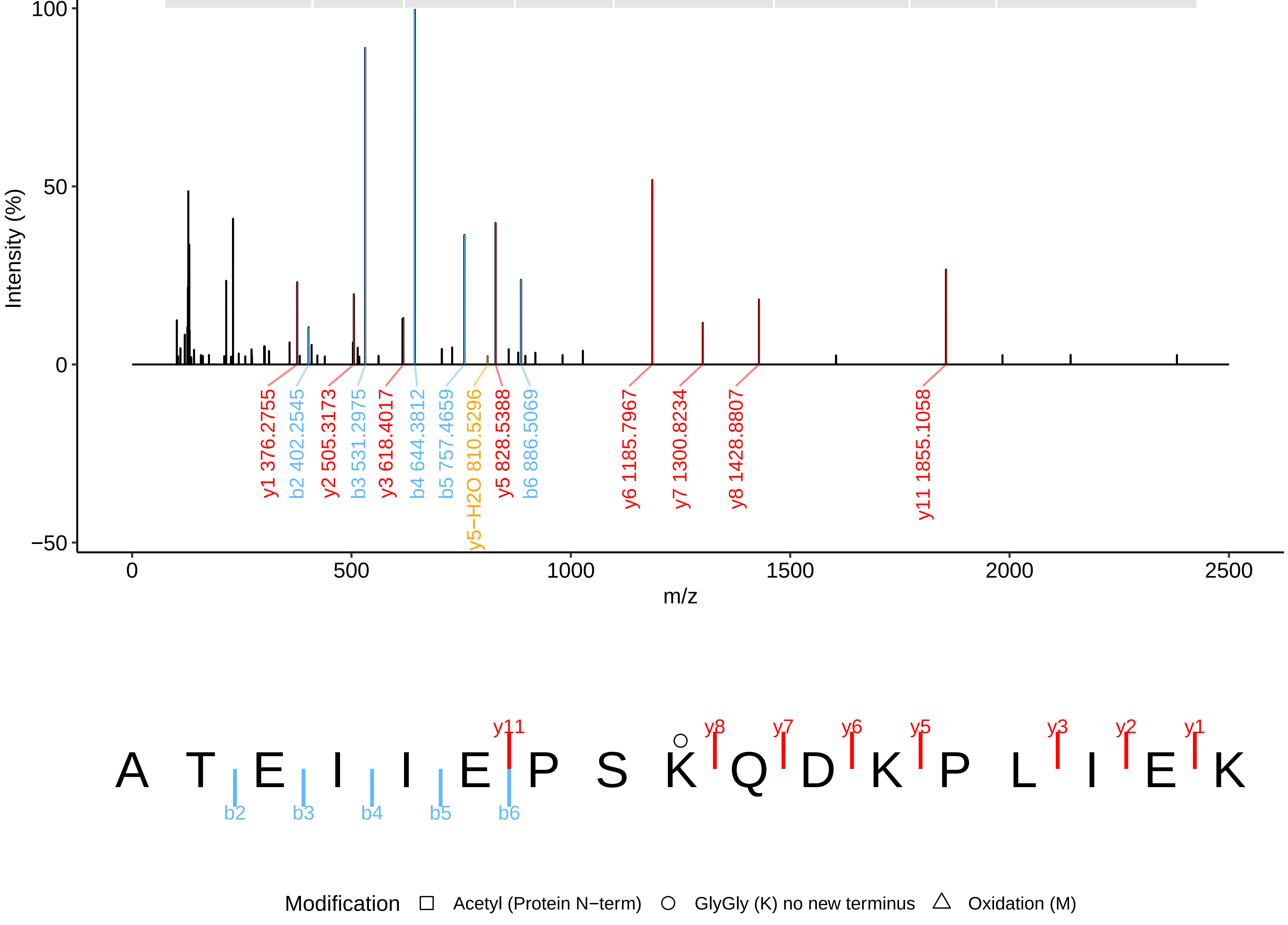

20200921_KIZ_MUb4_GlyGly_10plex_2 − Scan number: 22769

Gene Names	Charge	m/z	Mass	Retention time	PEP	Score	Precursor Intensity
SND1	3	844.0505	2529.13	89.811	7.4757e−10	106.03	1079176

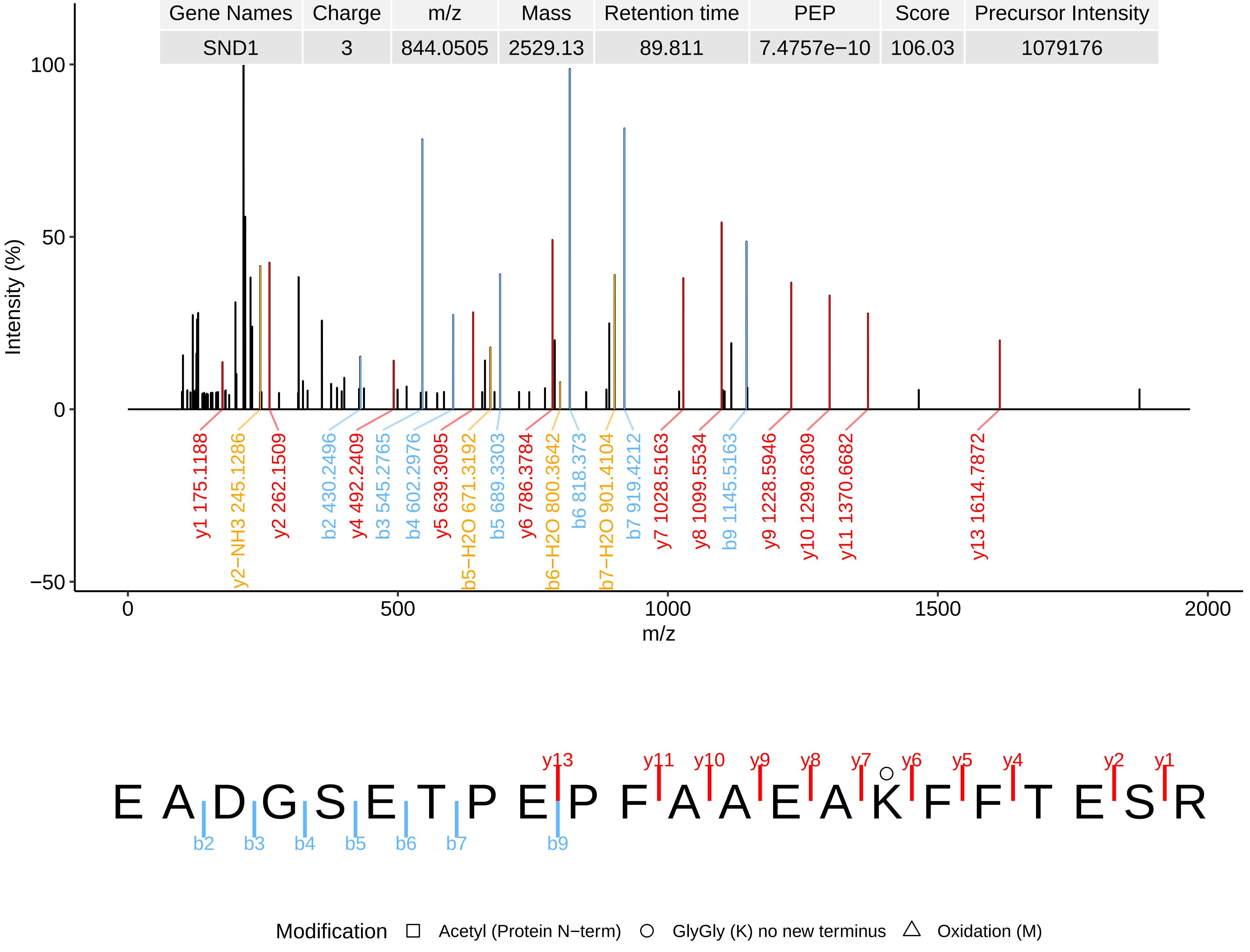

20200921_KIZ_MUb4_GlyGly_10plex − Scan number: 11679

Gene Names	Charge	m/z	Mass	Retention time	PEP	Score	Precursor Intensity
SLC43A3	4	590.7924	2359.14	40.76	1.8994e−20	138.66	4288456

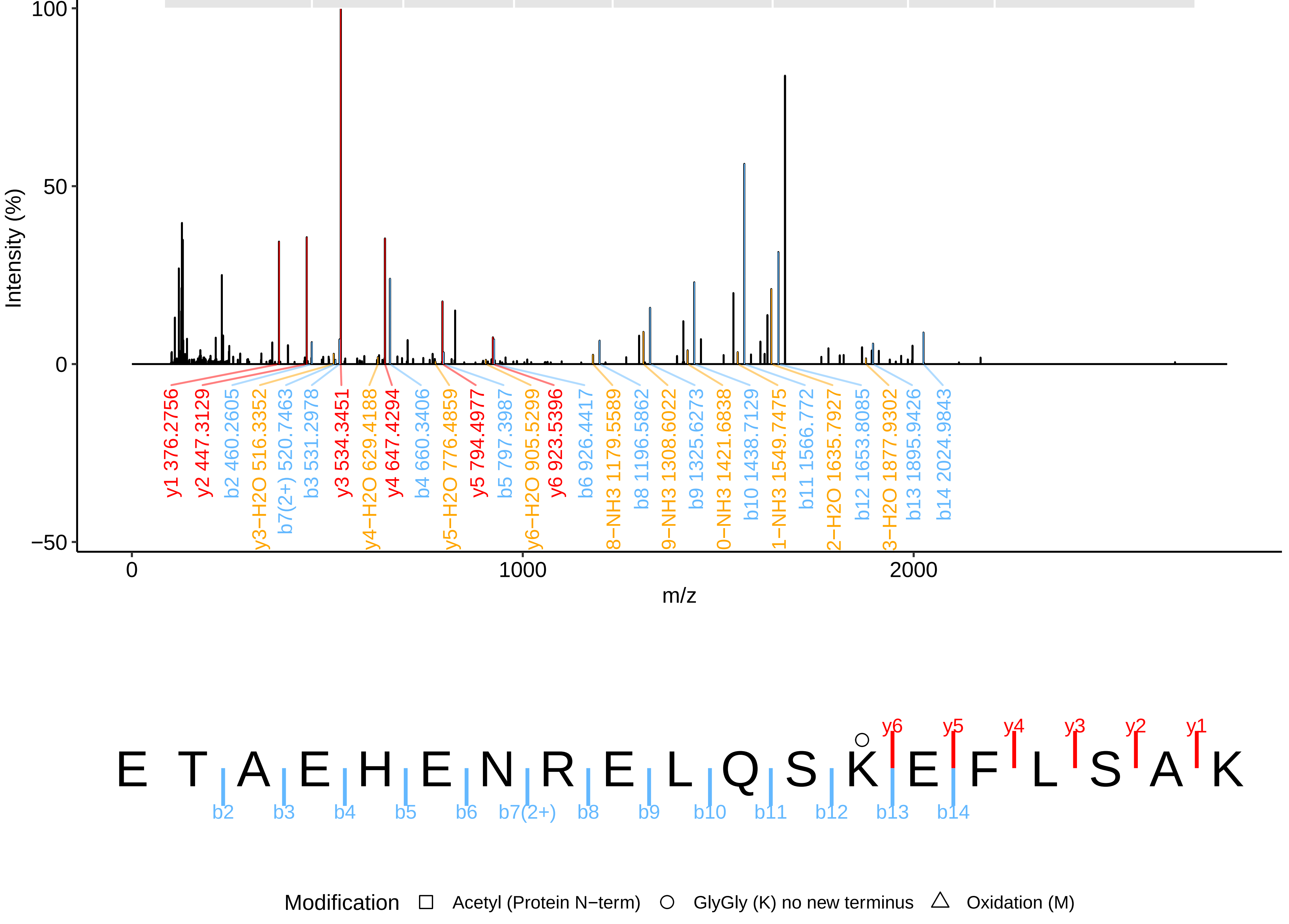

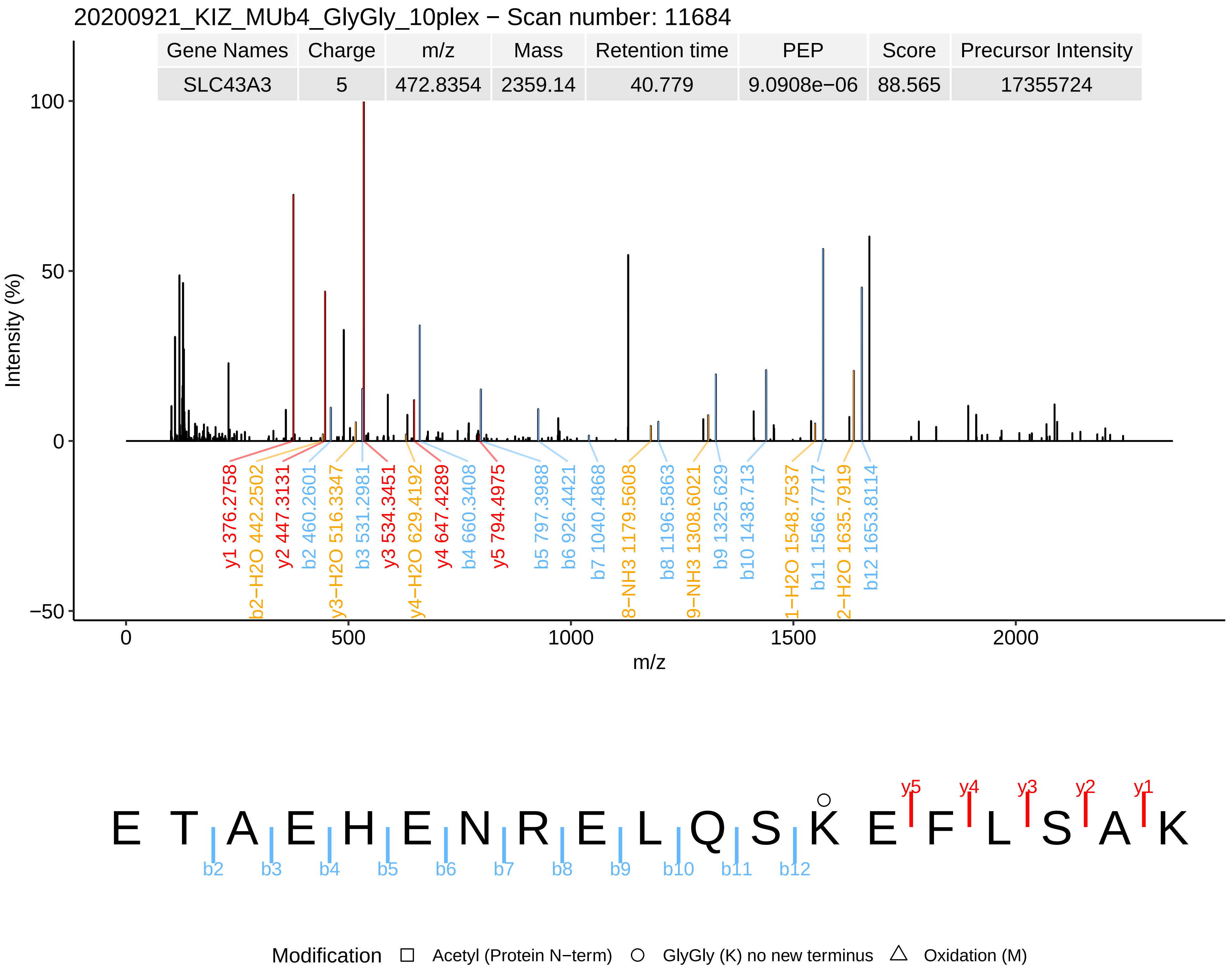

20200921_KIZ_MUb4_GlyGly_10plex − Scan number: 11684
Gene Names | Charge | m/z | Mass | Retention time | PEP | Score | Precursor Intensity
SLC43A3 | 5 | 472.8354 | 2359.14 | 40.779 | 9.0908e−06 | 88.565 | 17355724
Intensity (%)
100
50
0
−50
0
500
1000
1500
2000
m/z
y1 376.2758
b2−H2O 442.2502
y2 447.3131
b2 460.2601
y3−H2O 516.3347
b3 531.2981
y3 534.3451
y4−H2O 629.4192
y4 647.4289
b4 660.3408
y5 794.4975
b5 797.3988
b6 926.4421
b7 1040.4868
8−NH3 1179.5608
b8 1196.5863
9−NH3 1308.6021
b9 1325.629
b10 1438.713
1−H2O 1548.7537
b11 1566.7717
2−H2O 1635.7919
b12 1653.8114
E T A E H E N R E L Q S K E F L S A K
y5 y4 y3 y2 y1
b2 b3 b4 b5 b6 b7 b8 b9 b10 b11 b12
Modification
Acetyl (Protein N−term)
GlyGly (K) no new terminus
Oxidation (M)

20200921_KIZ_MUb4_GlyGly_10plex_2 − Scan number: 10783

Gene Names	Charge	m/z	Mass	Retention time	PEP	Score	Precursor Intensity
SLC43A3	5	472.8354	2359.14	41.331	0.057956	47.752	2259893

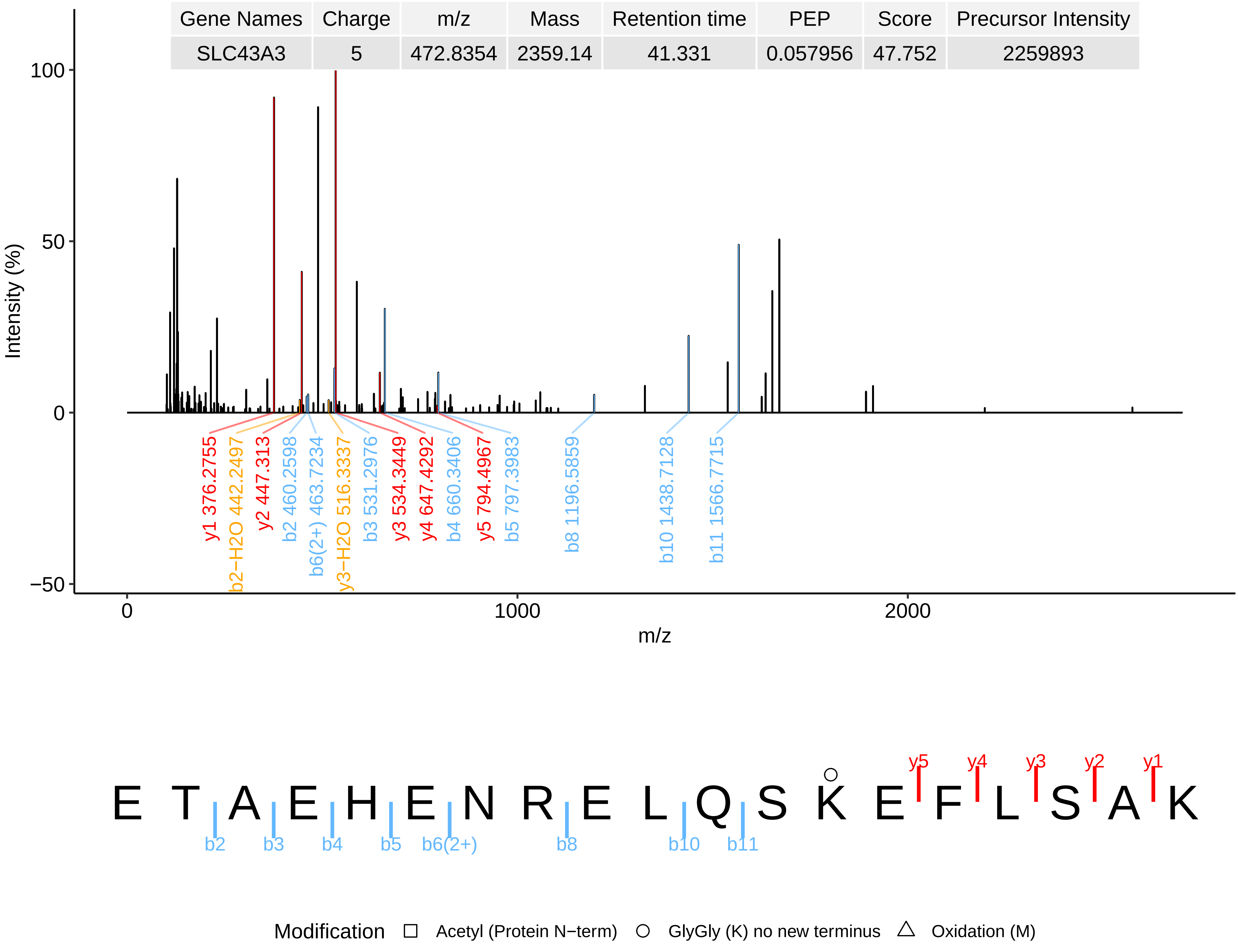

20200921_KIZ_MUb4_GlyGly_10plex_2 − Scan number: 10907

Gene Names	Charge	m/z	Mass	Retention time	PEP	Score	Precursor Intensity
SLC43A3	5	472.8354	2359.14	41.852	5.4859e−06	92.307	3690299

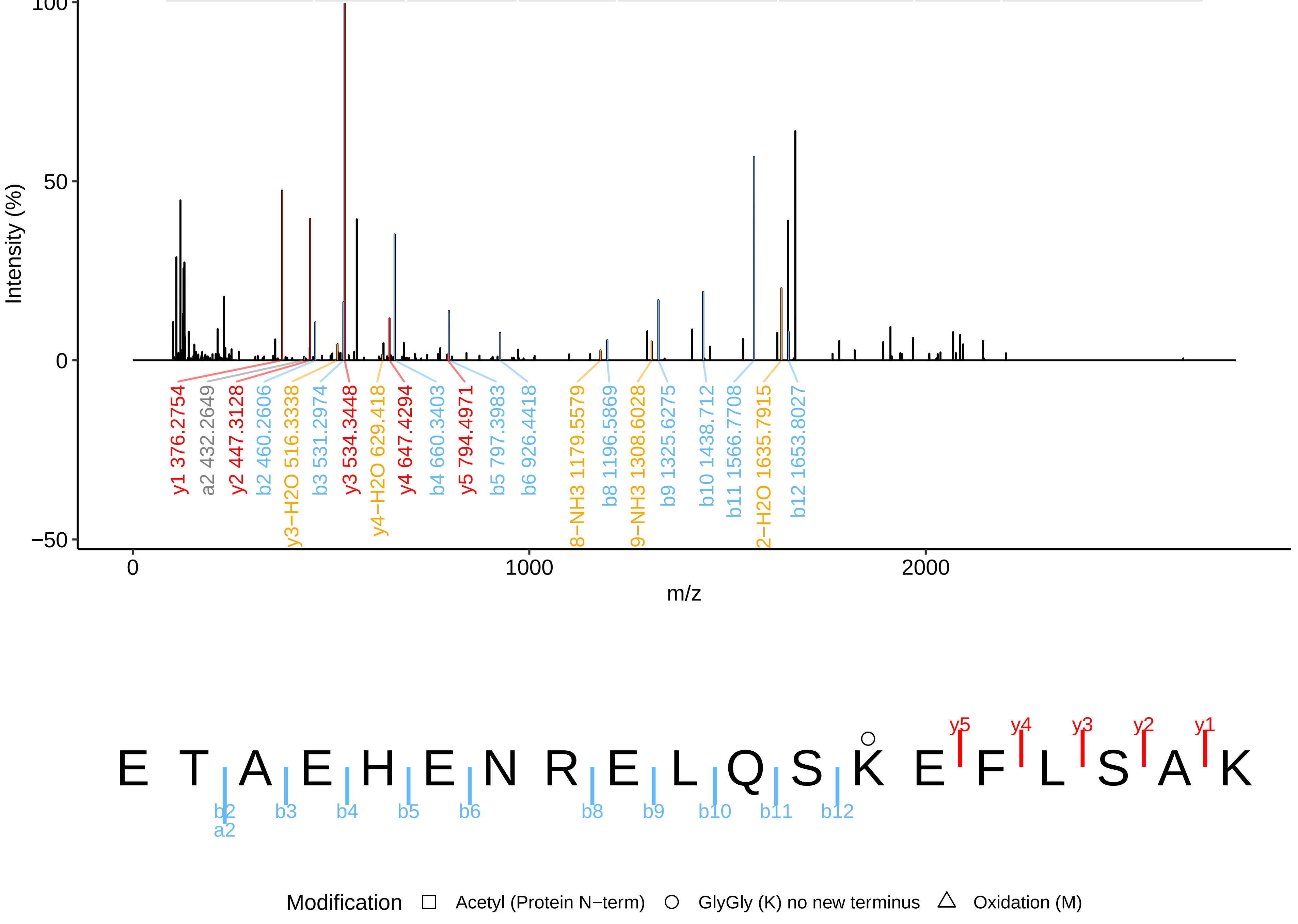

20200921_KIZ_MUb4_GlyGly_10plex_2 − Scan number: 10792

Gene Names	Charge	m/z	Mass	Retention time	PEP	Score	Precursor Intensity
SLC43A3	4	590.7924	2359.14	41.37	4.7225e−05	88.565	1422462

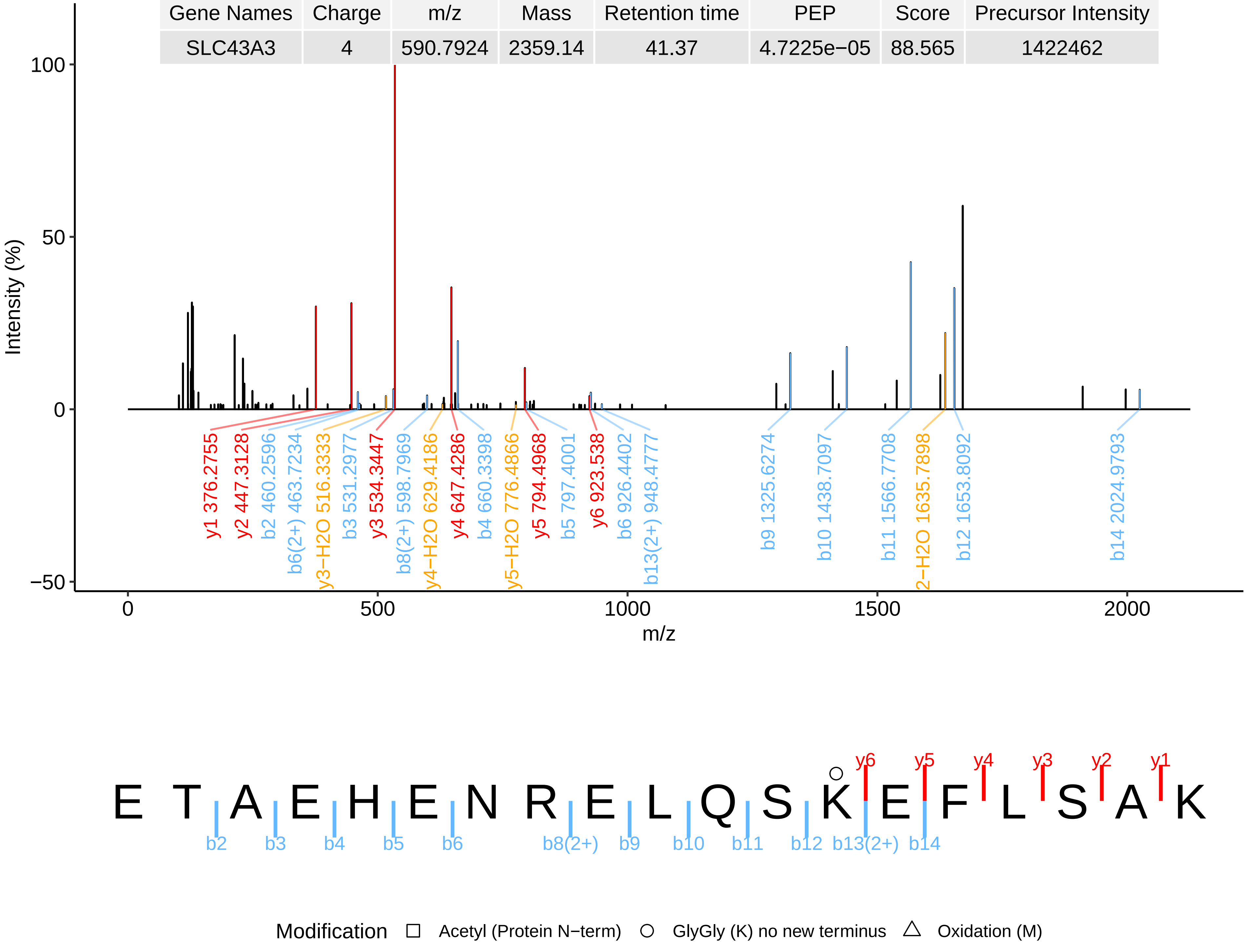

20200924_KIZ_MUb5_GlyGly_1 − Scan number: 10008

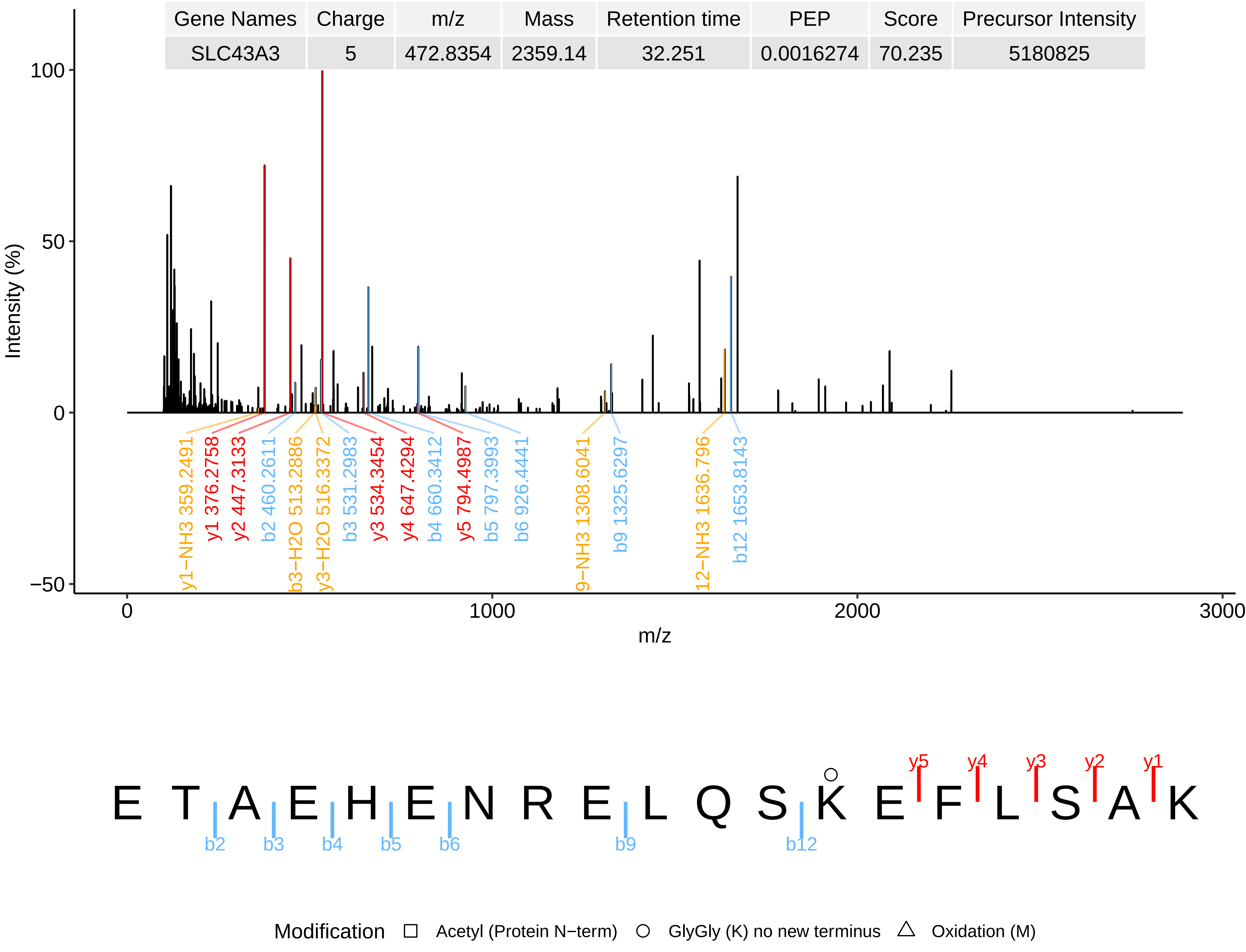

20200924_KIZ_MUb5_GlyGly_1 − Scan number: 10043

Gene Names	Charge	m/z	Mass	Retention time	PEP	Score	Precursor Intensity
SLC43A3	5	472.8354	2359.14	32.366	8.7344e−07	97.095	6994330

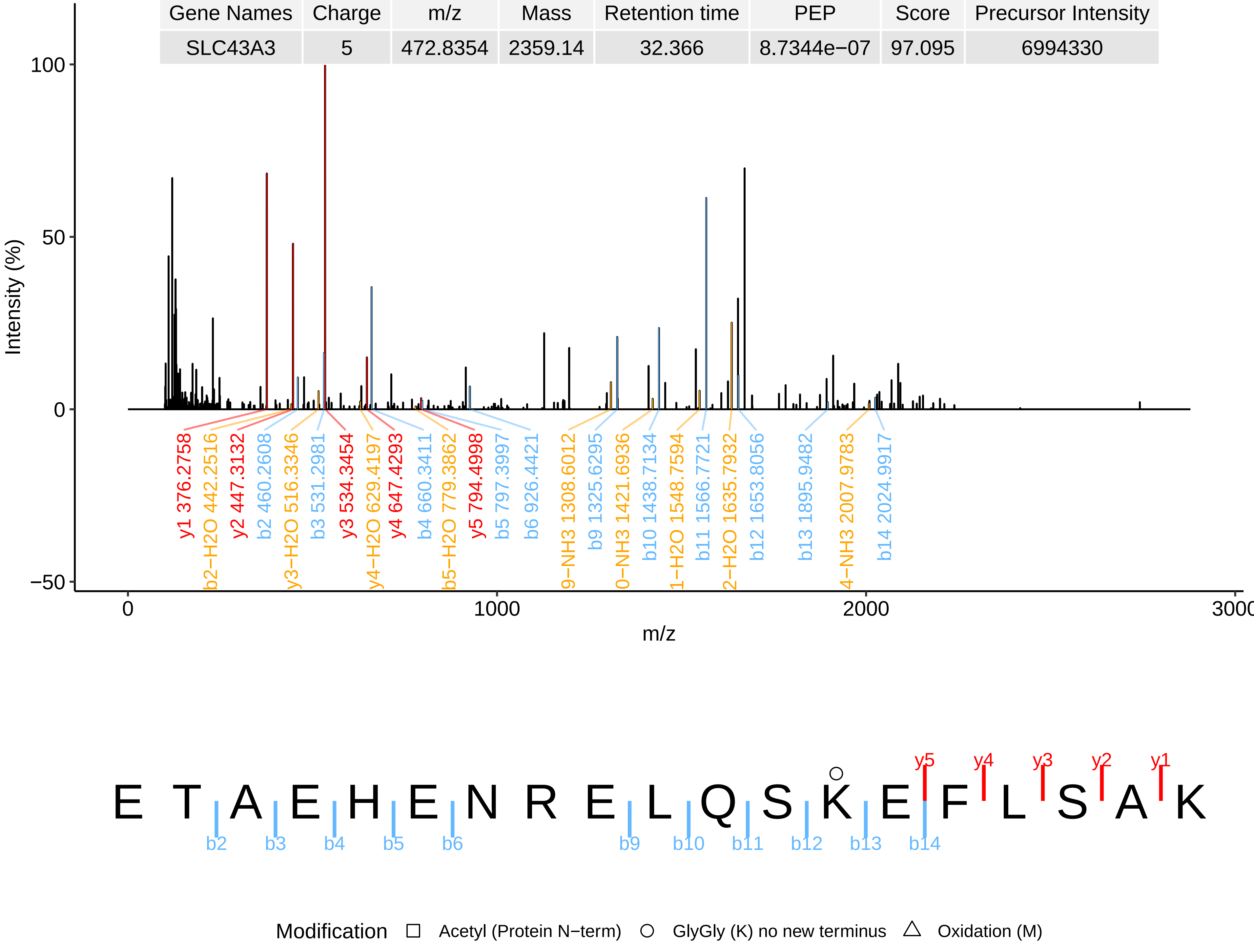

20200924_KIZ_MUb5_GlyGly_1 − Scan number: 10320

Gene Names	Charge	m/z	Mass	Retention time	PEP	Score	Precursor Intensity
SLC43A3	5	472.8354	2359.14	33.283	1.1045e−09	109.42	12014076

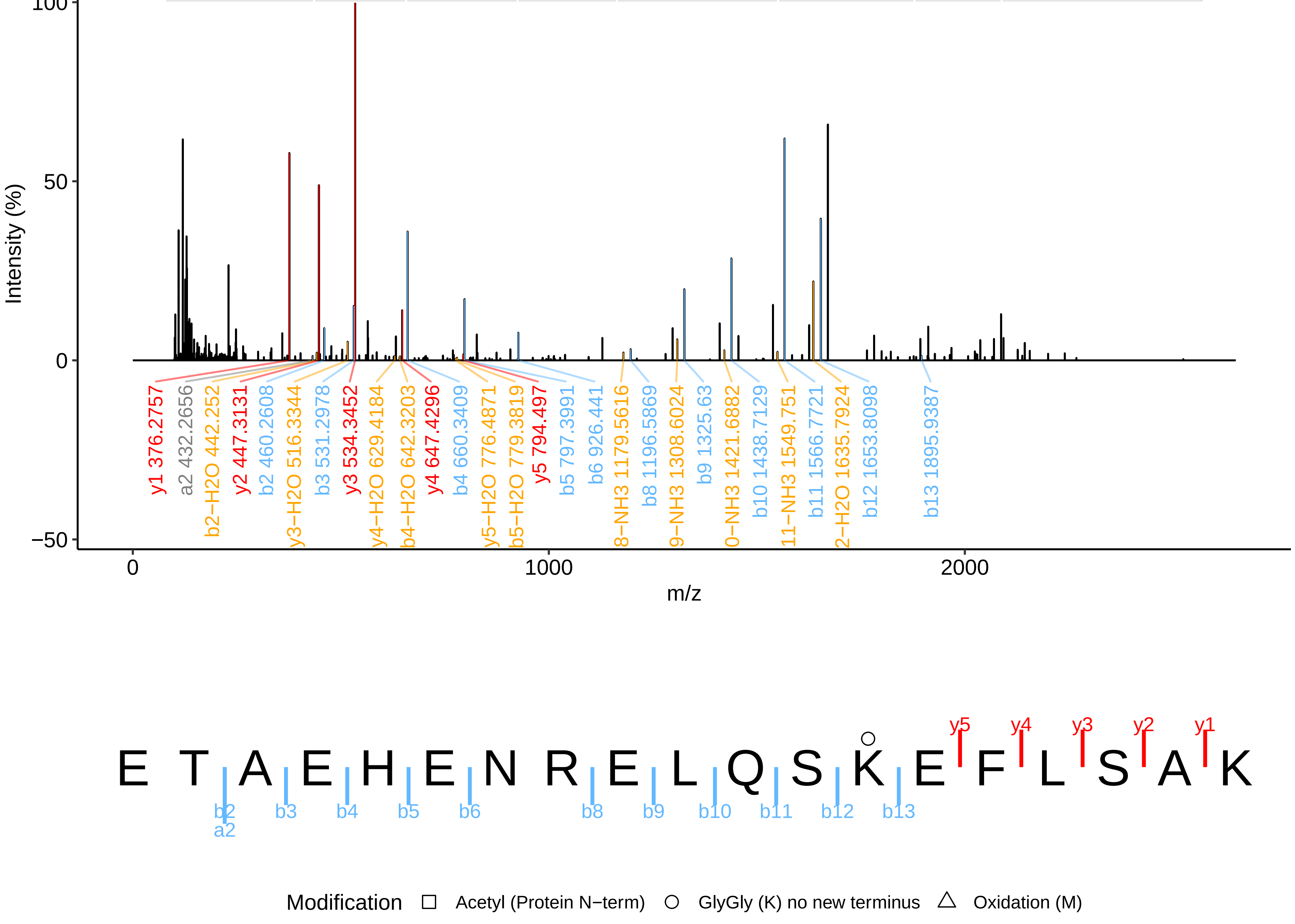

20200924_KIZ_MUb5_GlyGly_2 − Scan number: 9648

Gene Names	Charge	m/z	Mass	Retention time	PEP	Score	Precursor Intensity
SLC43A3	5	472.8354	2359.14	36.038	0.0010527	73.928	4237293

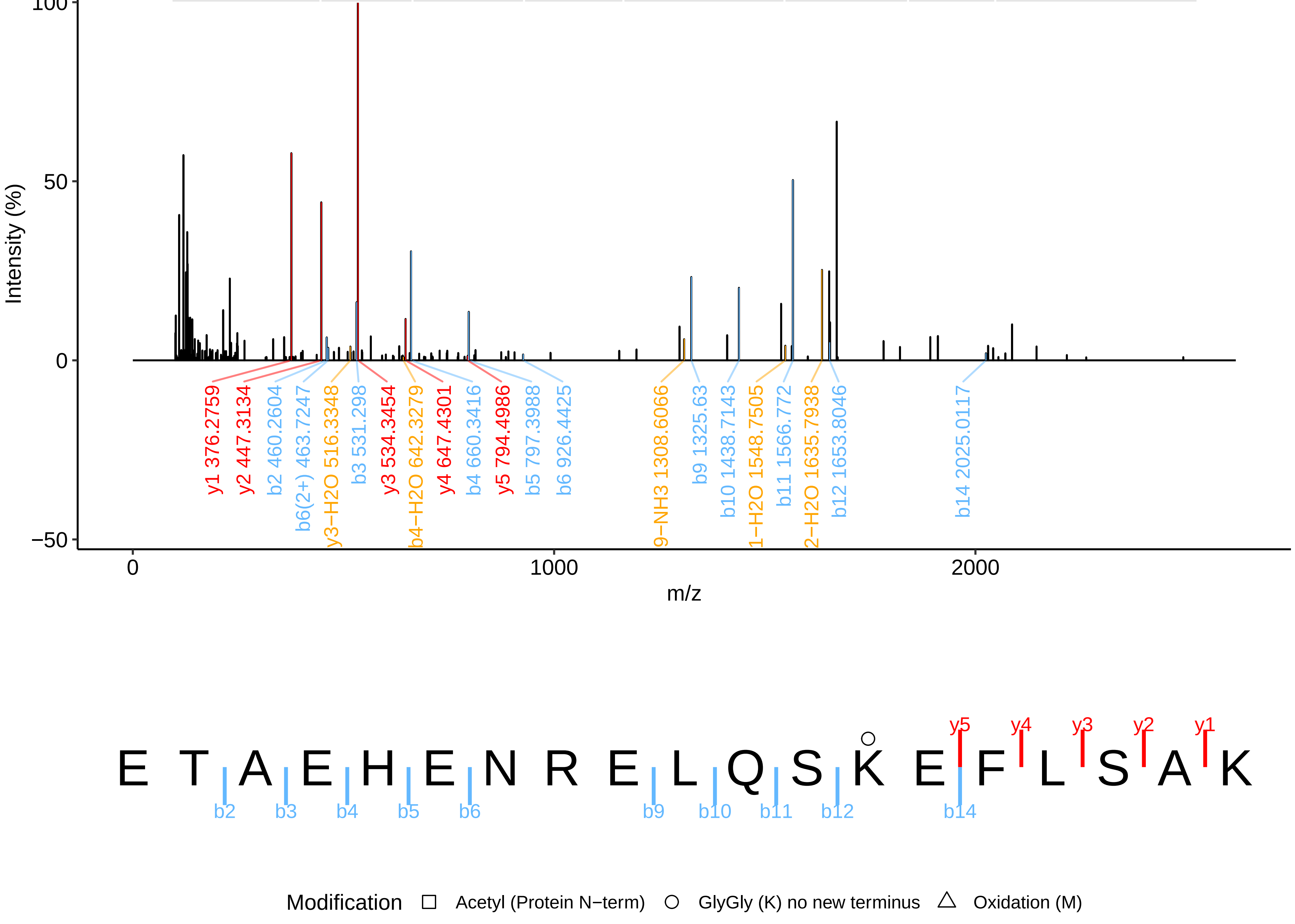

20201118_KIZ_MUb6_GlyGly_2 − Scan number: 12424

Gene Names	Charge	m/z	Mass	Retention time	PEP	Score	Precursor Intensity
SLC43A3	4	590.7924	2359.14	41.818	6.474e−14	116.29	5371991

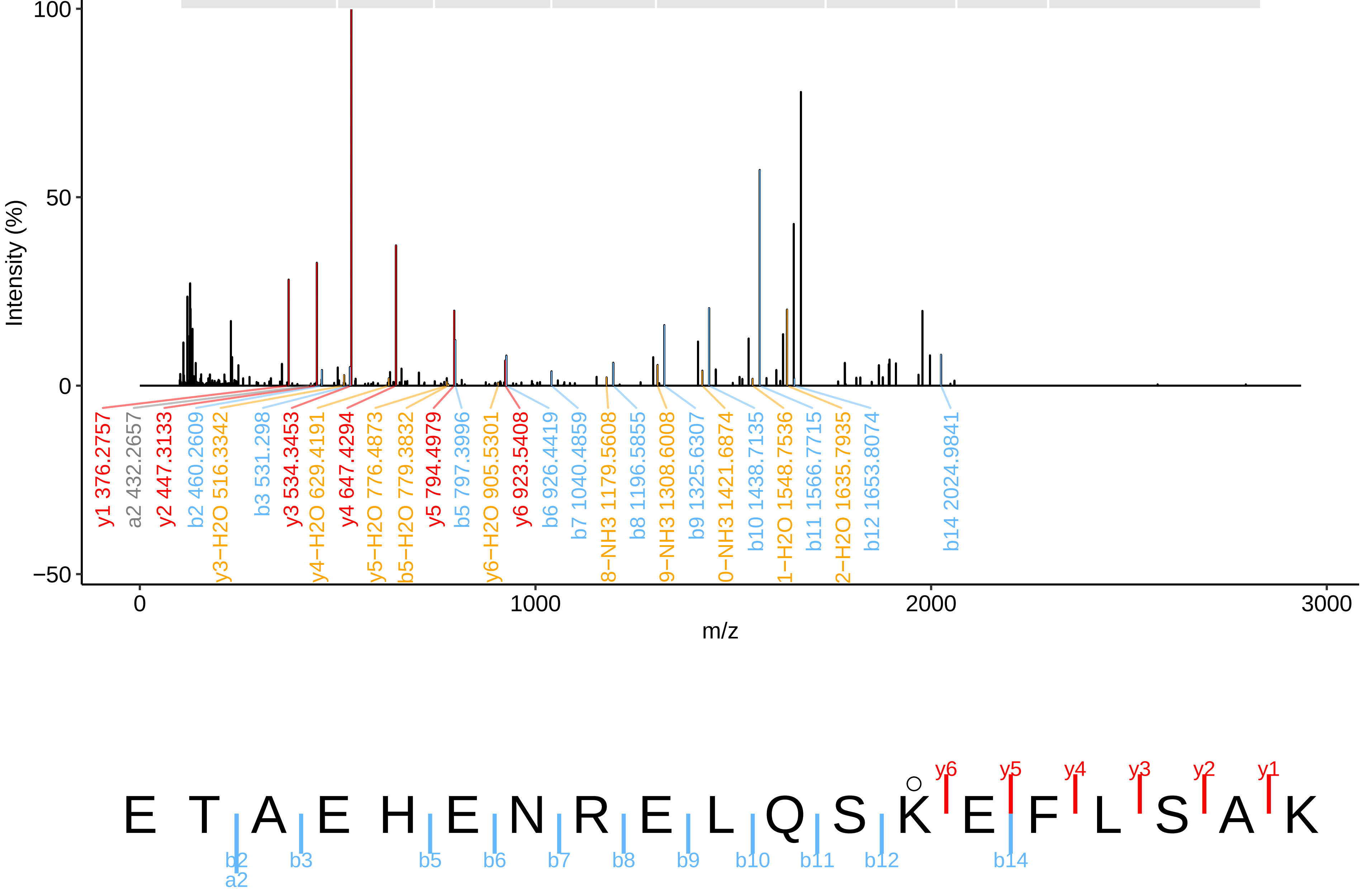

Modification □ Acetyl (Protein N−term) ○ GlyGly (K) no new terminus △ Oxidation (M)

20201118_KIZ_MUb6_GlyGly_2 − Scan number: 12437

Gene Names	Charge	m/z	Mass	Retention time	PEP	Score	Precursor Intensity
SLC43A3	5	472.8354	2359.14	41.867	7.0781e−06	90.654	21765716

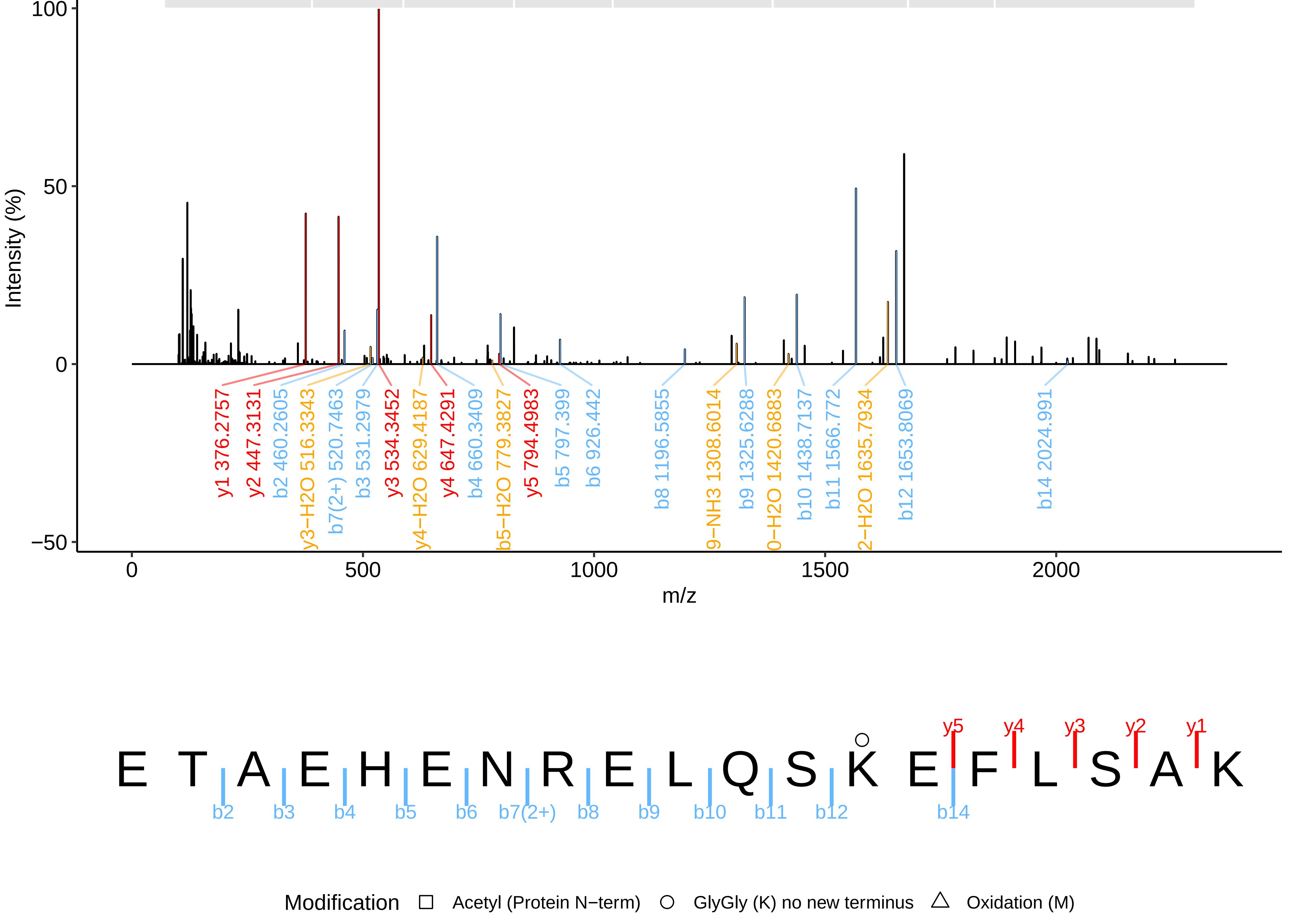

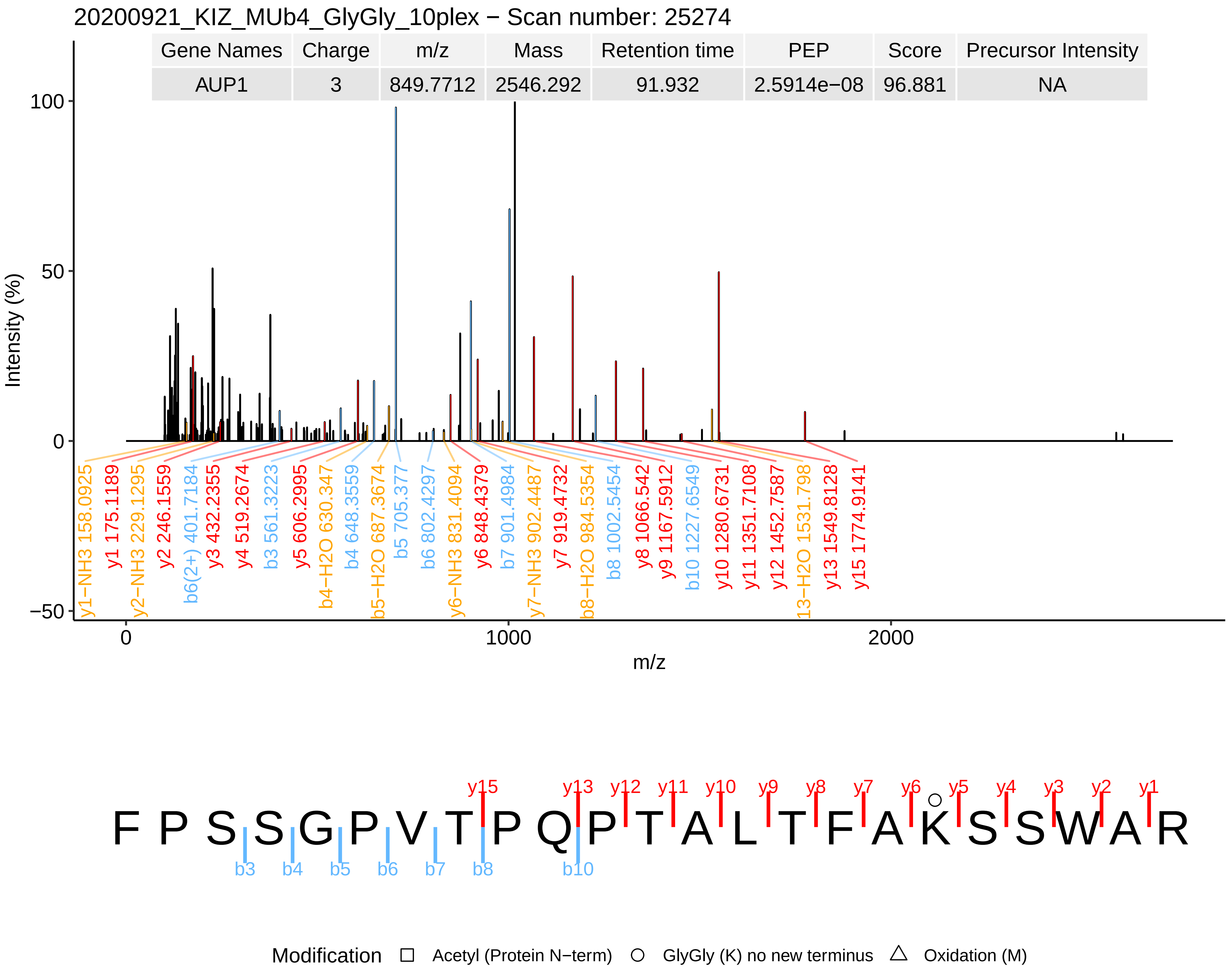
20200921_KIZ_MUb4_GlyGly_10plex − Scan number: 25274
Gene Names | Charge | m/z | Mass | Retention time | PEP | Score | Precursor Intensity
AUP1 | 3 | 849.7712 | 2546.292 | 91.932 | 2.5914e−08 | 96.881 | NA
Intensity (%)
100
50
0
−50
0
1000
2000
m/z
y1−NH3 158.0925
y1 175.1189
y2−NH3 229.1295
y2 246.1559
b6(2+) 401.7184
y3 432.2355
y4 519.2674
b3 561.3223
y5 606.2995
b4−H2O 630.347
b4 648.3559
b5−H2O 687.3674
b5 705.377
b6 802.4297
y6−NH3 831.4094
y6 848.4379
b7 901.4984
y7−NH3 902.4487
y7 919.4732
b8−H2O 984.5354
b8 1002.5454
y8 1066.542
y9 1167.5912
b10 1227.6549
y10 1280.6731
y11 1351.7108
y12 1452.7587
13−H2O 1531.798
y13 1549.8128
y15 1774.9141
y15 y13 y12 y11 y10 y9 y8 y7 y6 y5 y4 y3 y2 y1
F P S S G P V T P Q P T A L T F A K S S W A R
b3 b4 b5 b6 b7 b8 b10
Modification
Acetyl (Protein N−term)
GlyGly (K) no new terminus
Oxidation (M)

20200921_KIZ_MUb4_GlyGly_10plex_2 − Scan number: 22229

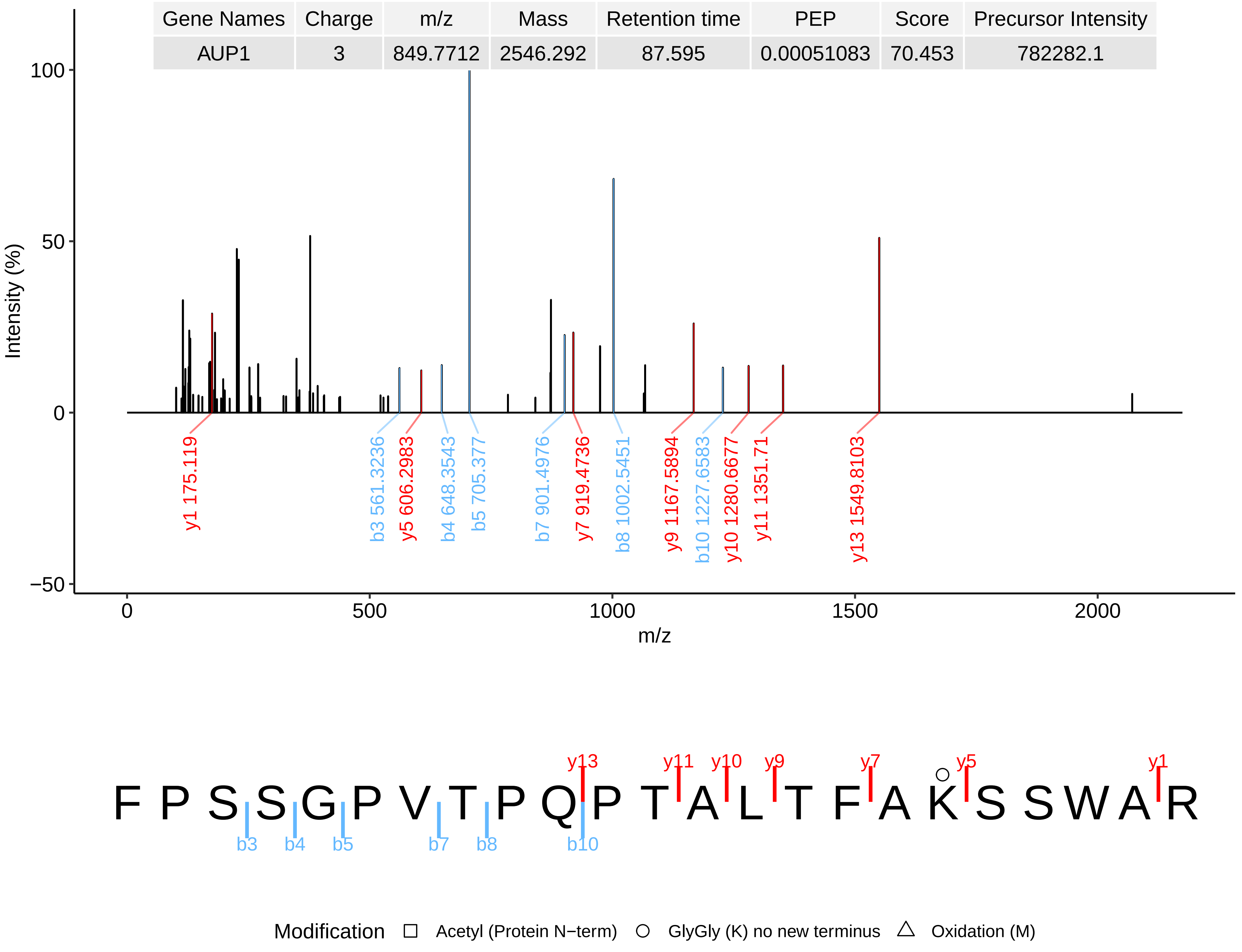

20200921_KIZ_MUb4_GlyGly_10plex_2 − Scan number: 19323

Gene Names	Charge	m/z	Mass	Retention time	PEP	Score	Precursor Intensity
GPRC5C	3	767.361	2299.061	75.834	3.3777e−06	94.057	1706139

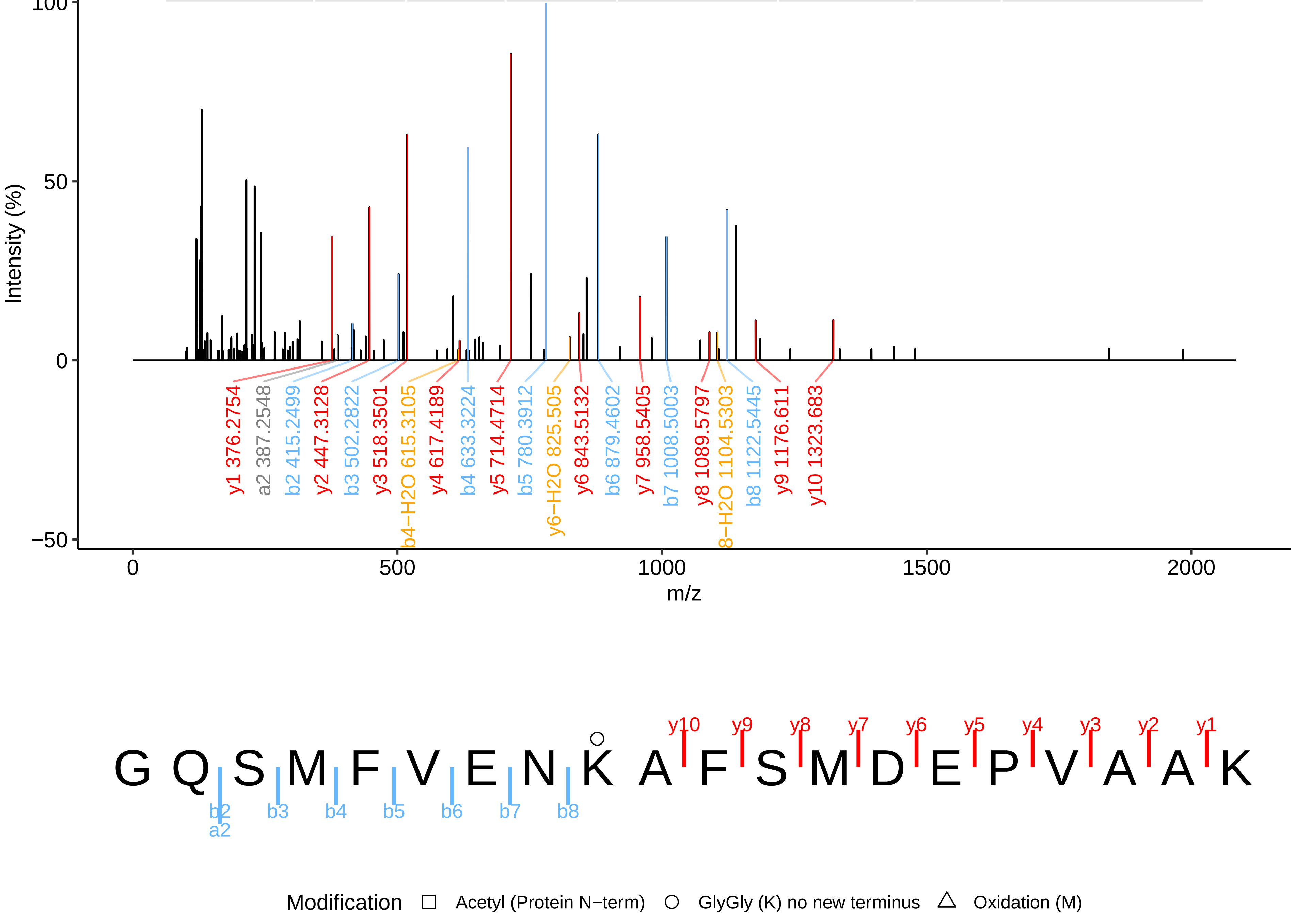

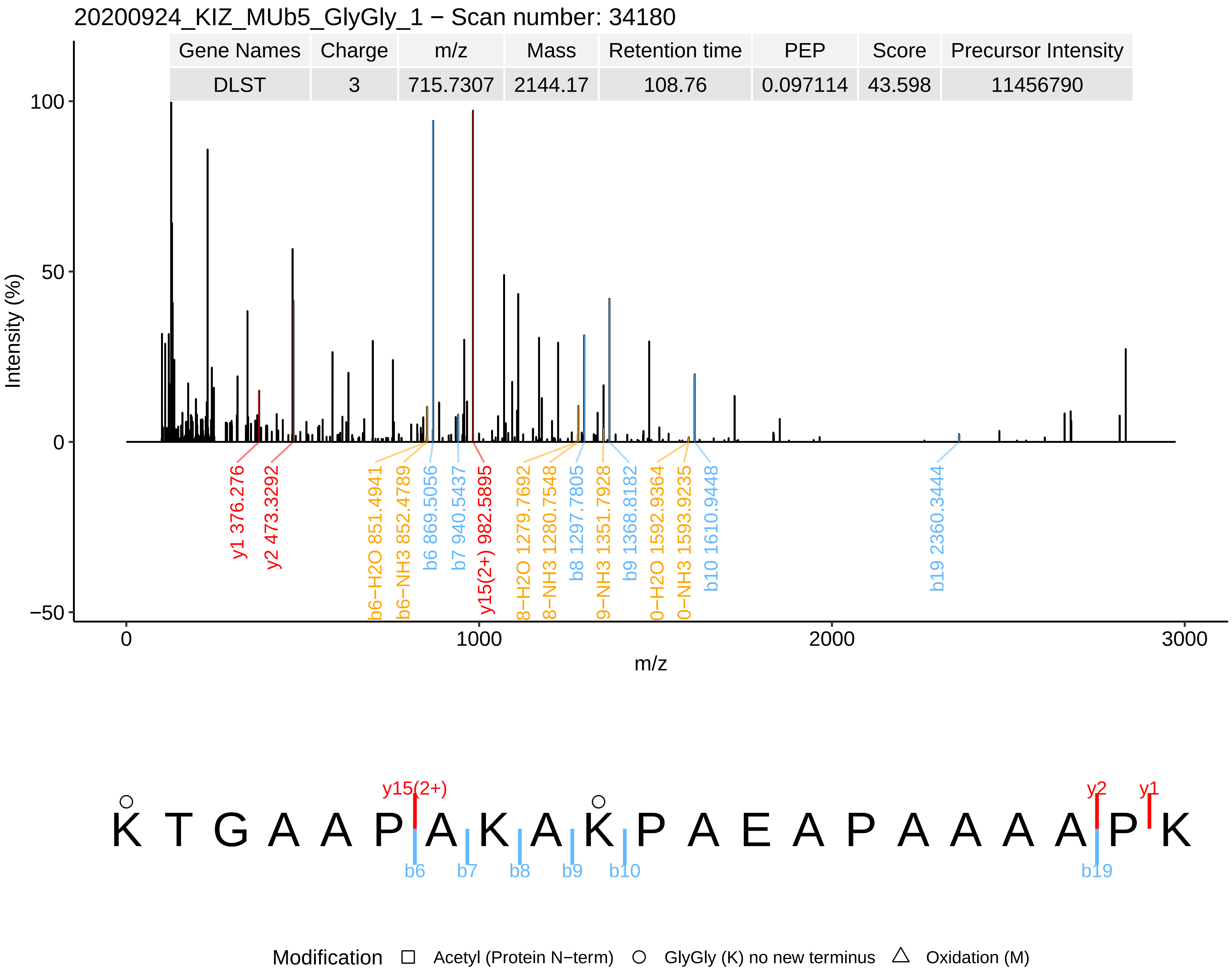
20200924_KIZ_MUb5_GlyGly_1 − Scan number: 34180
Gene Names | Charge | m/z | Mass | Retention time | PEP | Score | Precursor Intensity
DLST | 3 | 715.7307 | 2144.17 | 108.76 | 0.097114 | 43.598 | 11456790
Intensity (%)
m/z
y1 376.276
y2 473.3292
b6−H2O 851.4941
b6−NH3 852.4789
b6 869.5056
b7 940.5437
y15(2+) 982.5895
b8 1297.7805
b9 1368.8182
b10 1610.9448
b19 2360.3444
K T G A A P A K A K P A E A P A A A A P K
Modification
Acetyl (Protein N−term)
GlyGly (K) no new terminus
Oxidation (M)

20200921_KIZ_MUb4_GlyGly_10plex − Scan number: 29473

Gene Names	Charge	m/z	Mass	Retention time	PEP	Score	Precursor Intensity
HSPA1L	3	778.0424	2331.105	108.08	0.00034682	76.768	1587211

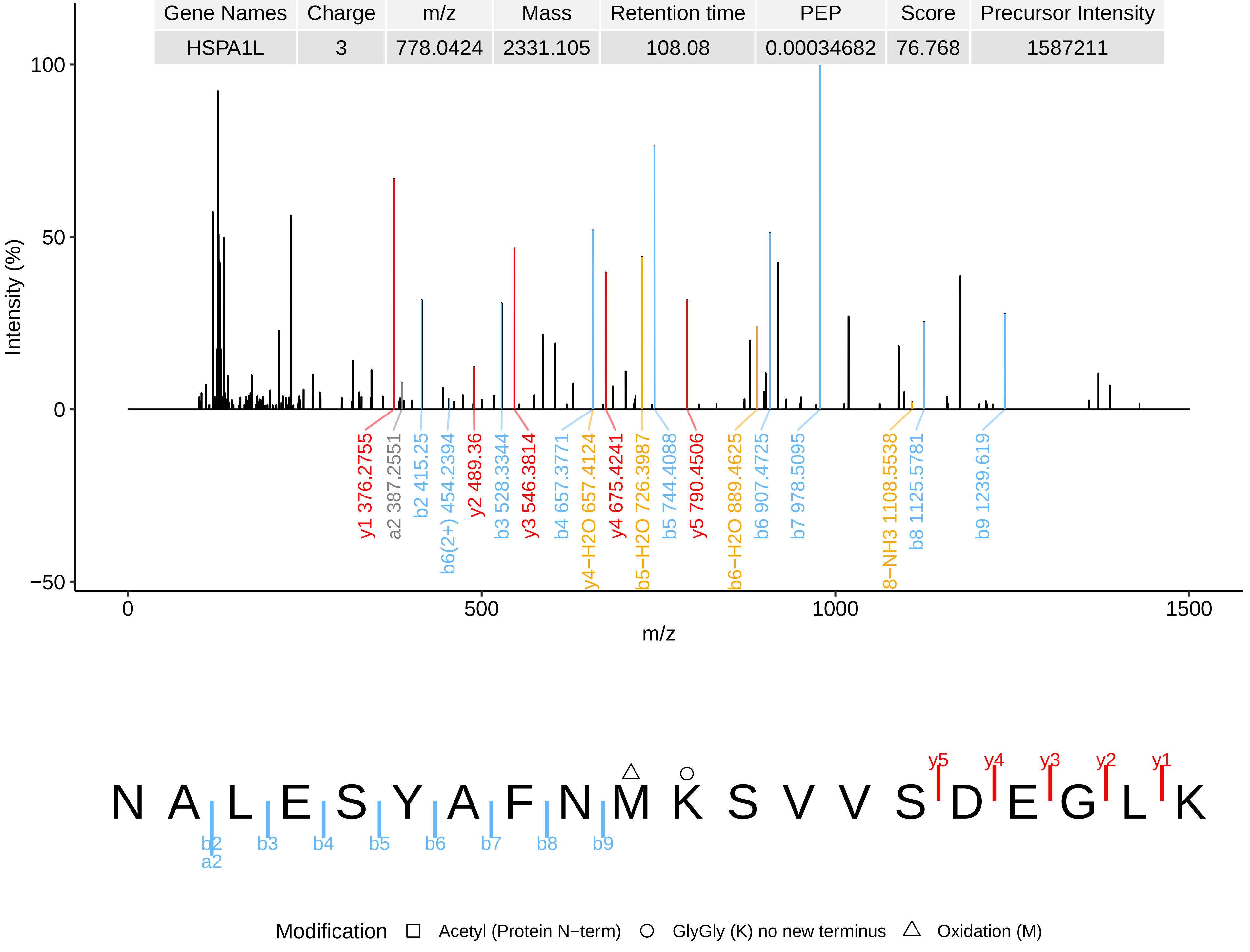

20200921_KIZ_MUb4_GlyGly_10plex − Scan number: 24619

Gene Names	Charge	m/z	Mass	Retention time	PEP	Score	Precursor Intensity
STEAP4	3	754.0486	2259.124	89.543	2.1104e−14	116.65	2816724

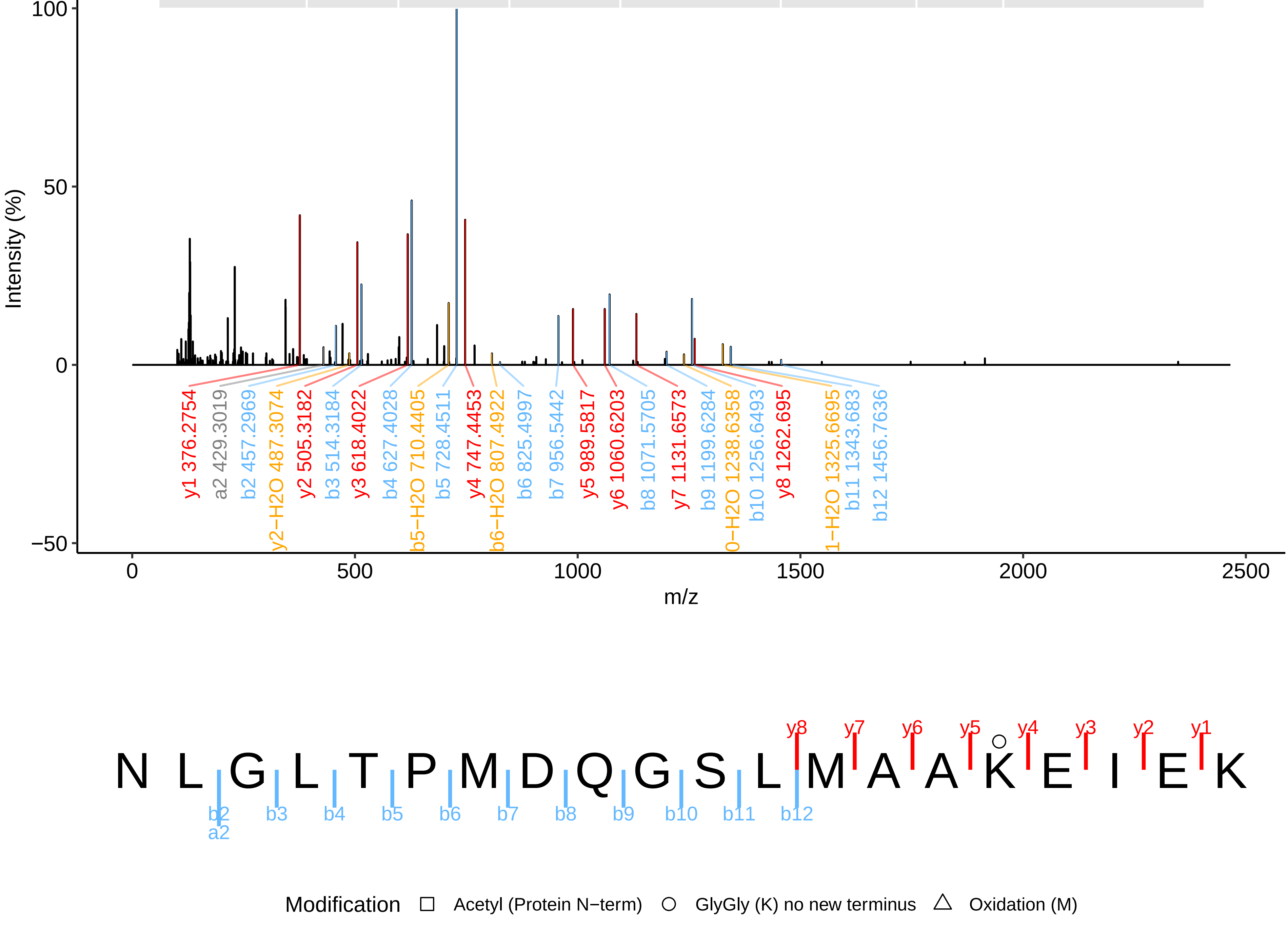

20200921_KIZ_MUb4_GlyGly_10plex_2 − Scan number: 21654

Gene Names	Charge	m/z	Mass	Retention time	PEP	Score	Precursor Intensity
STEAP4	3	754.0486	2259.124	85.277	1.0933e−05	85.231	614544.1

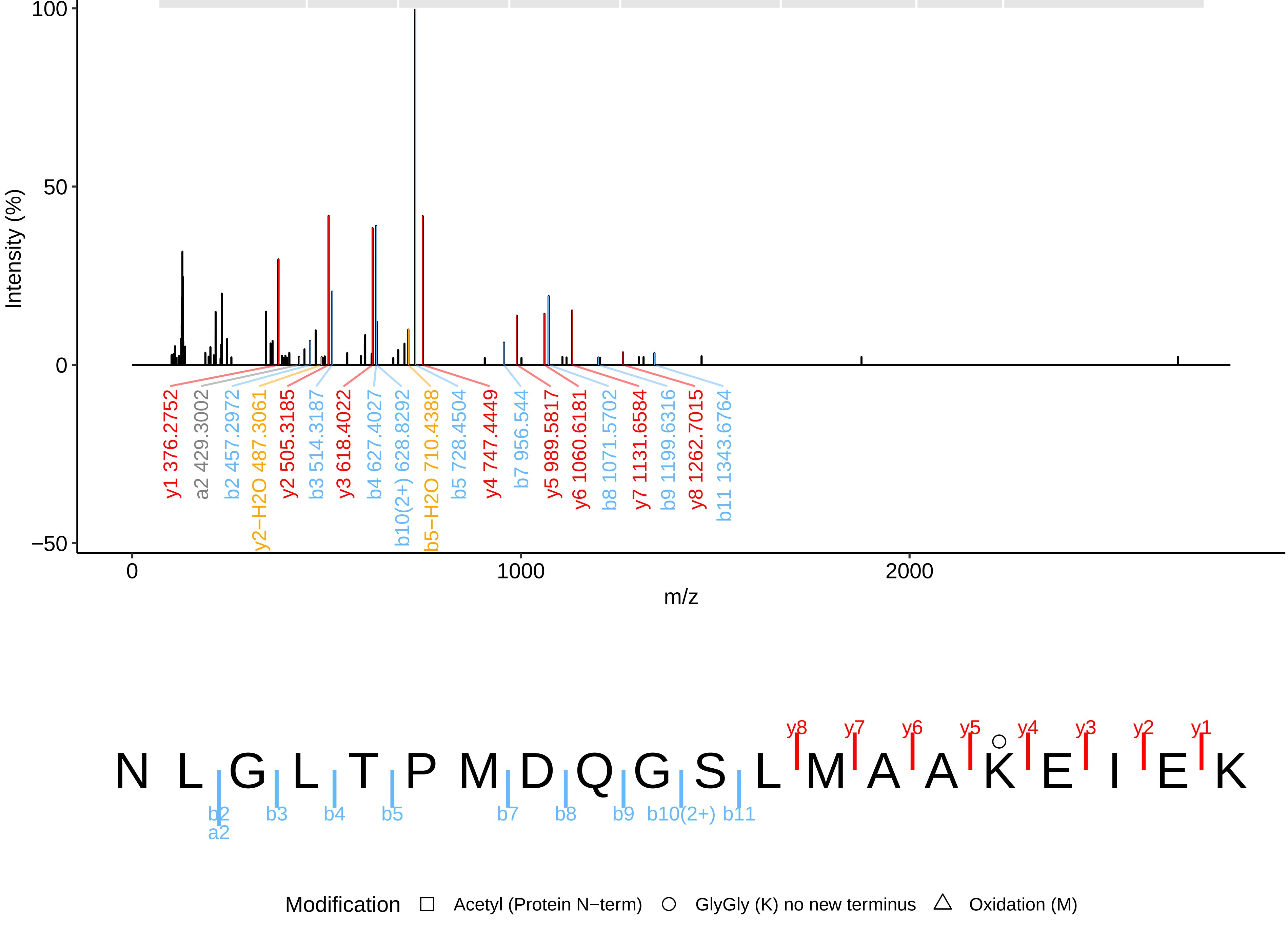

20200921_KIZ_MUb4_GlyGly_10plex_2 − Scan number: 21685

Gene Names	Charge	m/z	Mass	Retention time	PEP	Score	Precursor Intensity
STEAP4	3	754.0486	2259.124	85.401	0.00024591	78.088	2246251

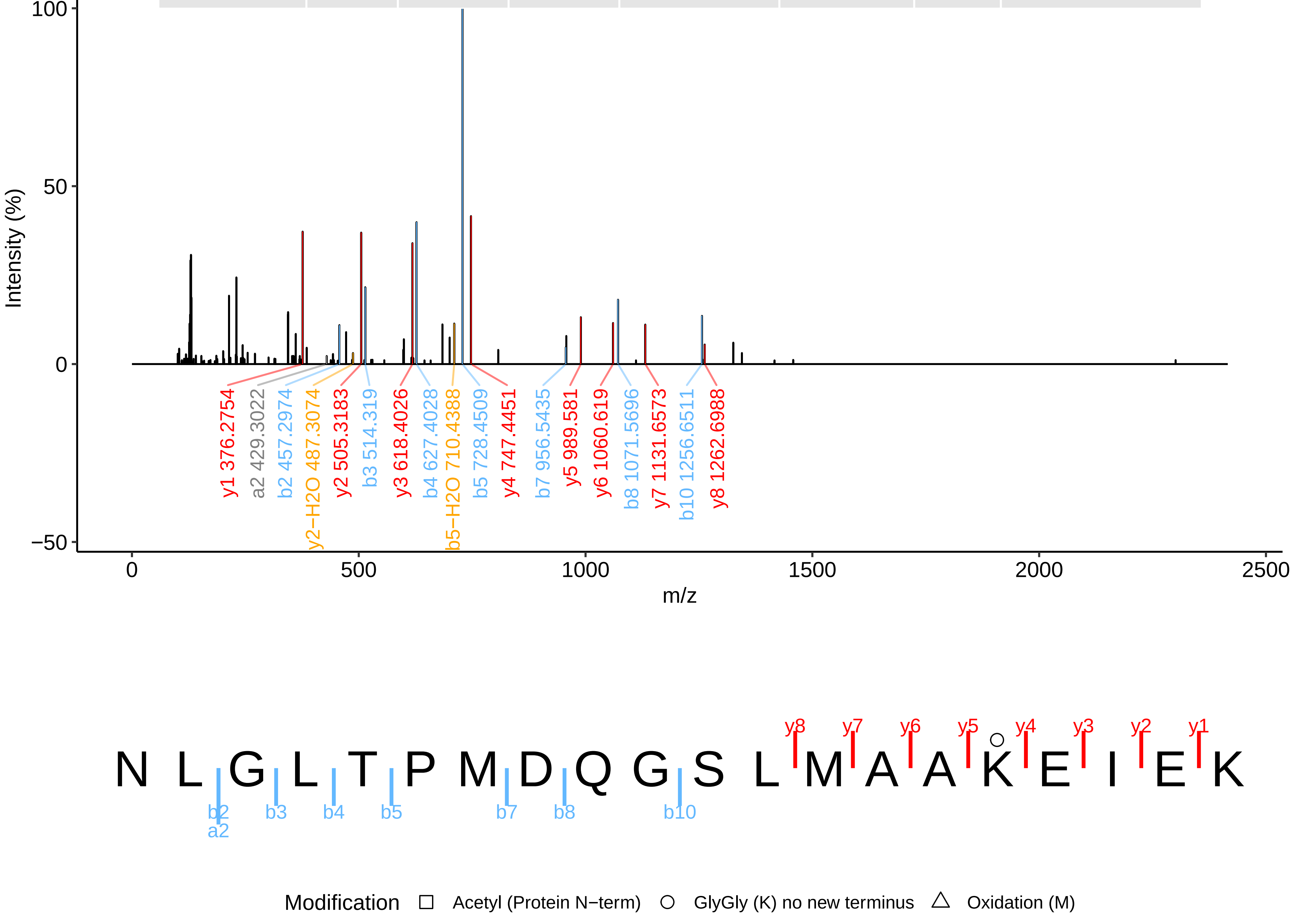

20200924_KIZ_MUb5_GlyGly_1 − Scan number: 24392

Gene Names	Charge	m/z	Mass	Retention time	PEP	Score	Precursor Intensity
STEAP4	3	754.0486	2259.124	78.376	7.6862e−35	176.46	21633240

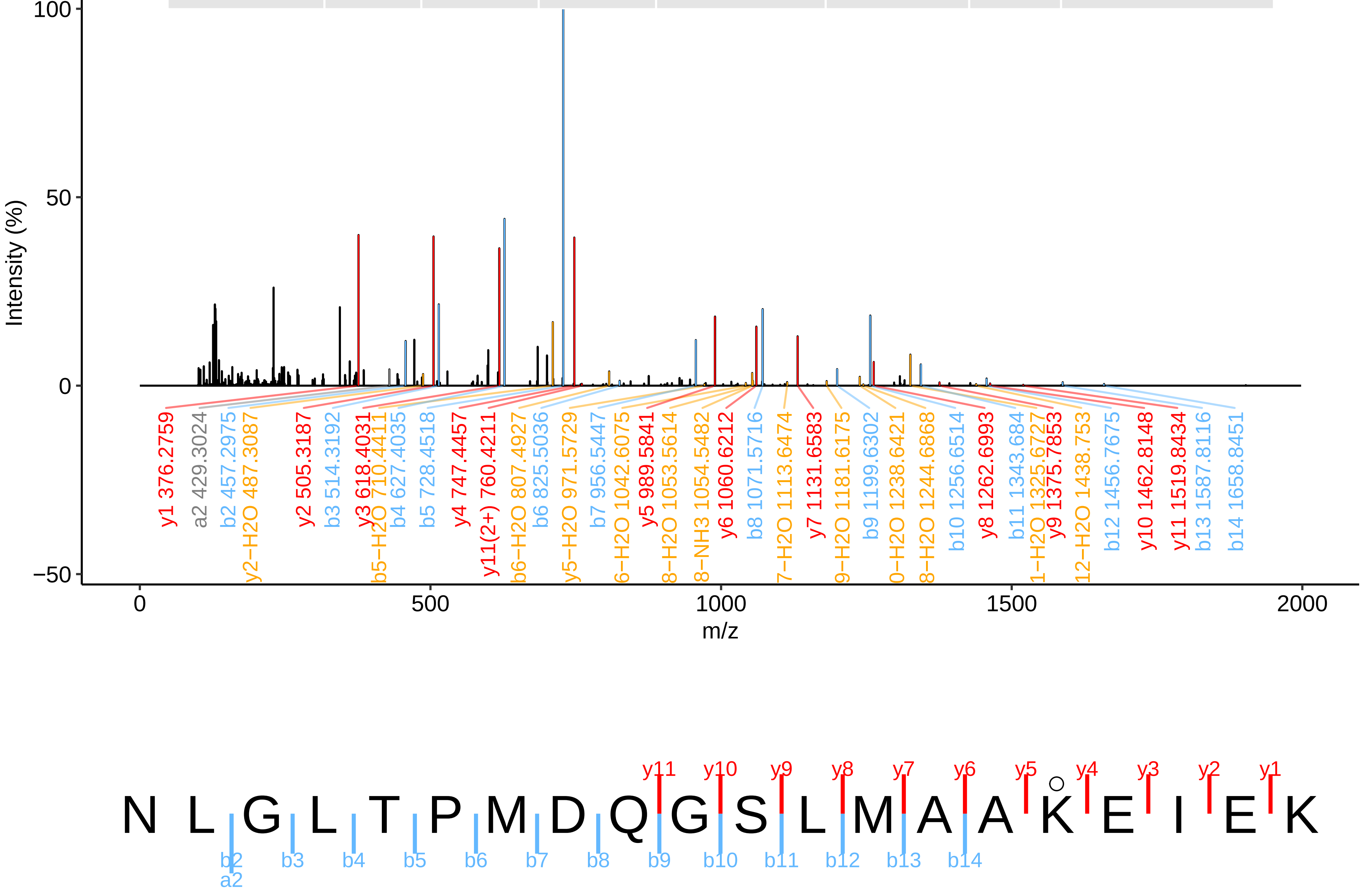

Modification □ Acetyl (Protein N−term) ○ GlyGly (K) no new terminus △ Oxidation (M)

20200924_KIZ_MUb5_GlyGly_1 − Scan number: 24227

Gene Names	Charge	m/z	Mass	Retention time	PEP	Score	Precursor Intensity
STEAP4	3	754.0486	2259.124	77.858	2.1223e−42	187.06	8019854

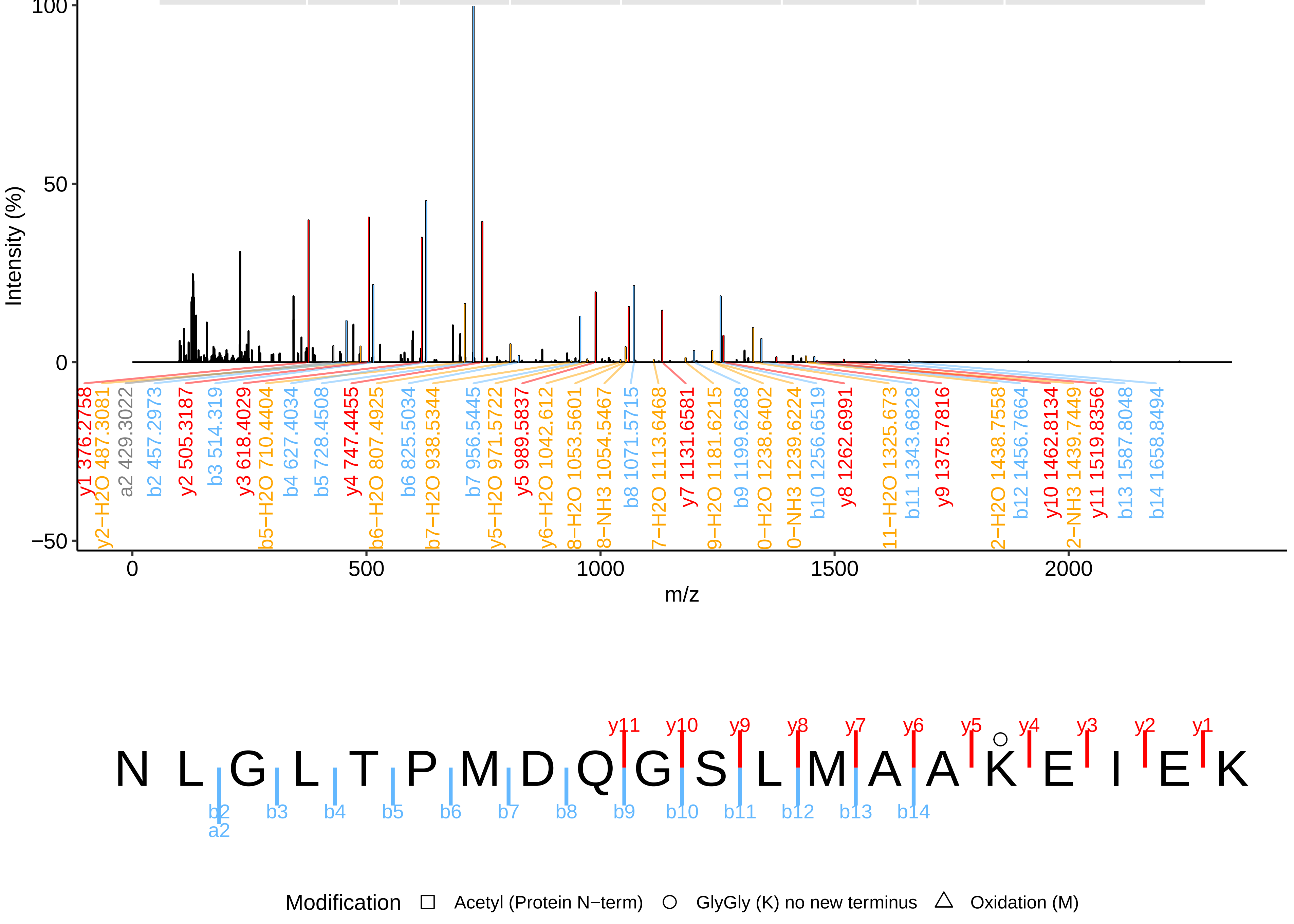

20200924_KIZ_MUb5_GlyGly_2 − Scan number: 21794

Gene Names	Charge	m/z	Mass	Retention time	PEP	Score	Precursor Intensity
STEAP4	3	754.0486	2259.124	80.708	2.0158e−14	117.37	3323706

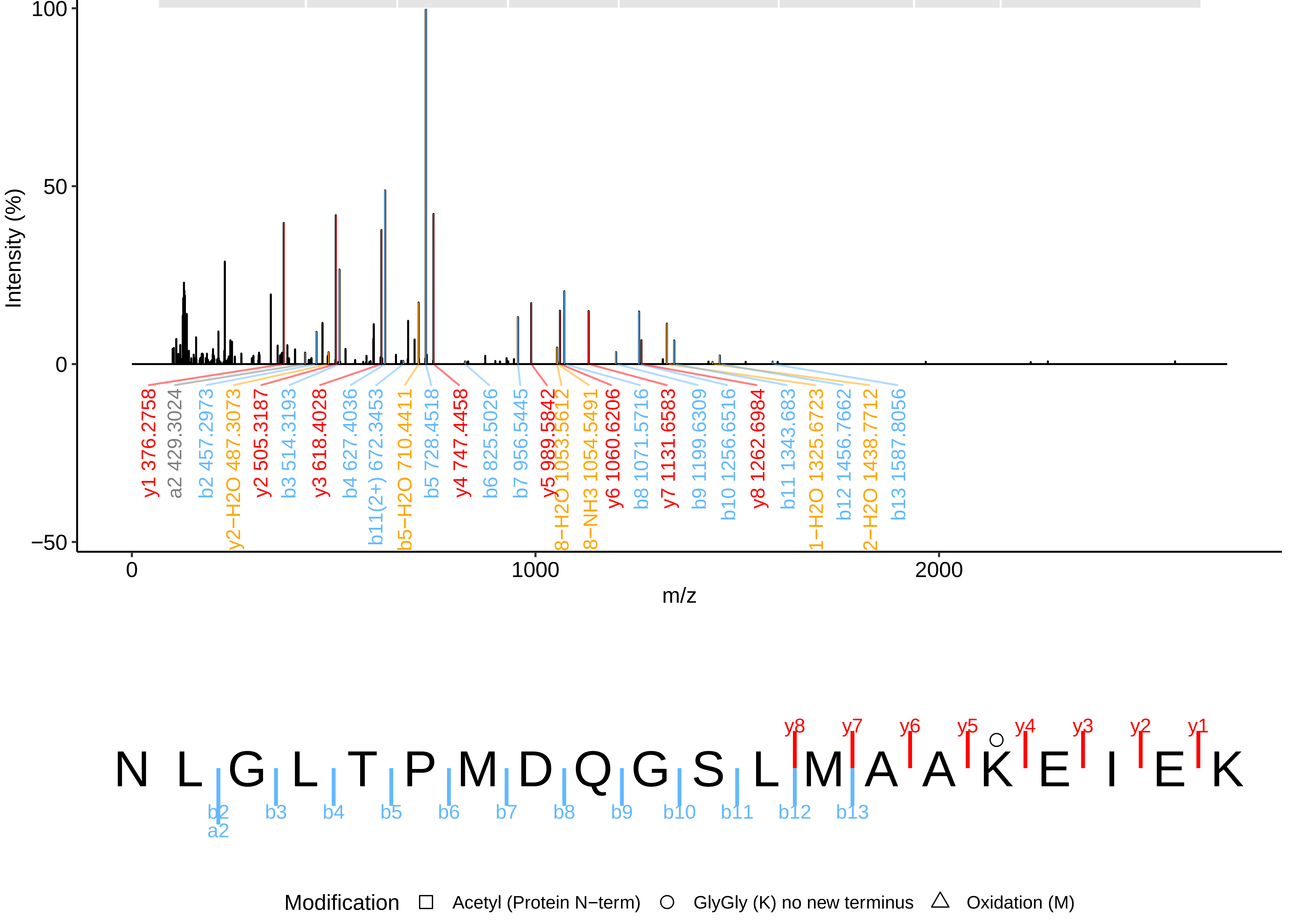

20201118_KIZ_MUb6_GlyGly_2 − Scan number: 26435

Gene Names	Charge	m/z	Mass	Retention time	PEP	Score	Precursor Intensity
STEAP4	3	754.0486	2259.124	88.198	1.909e−20	139.98	8343125

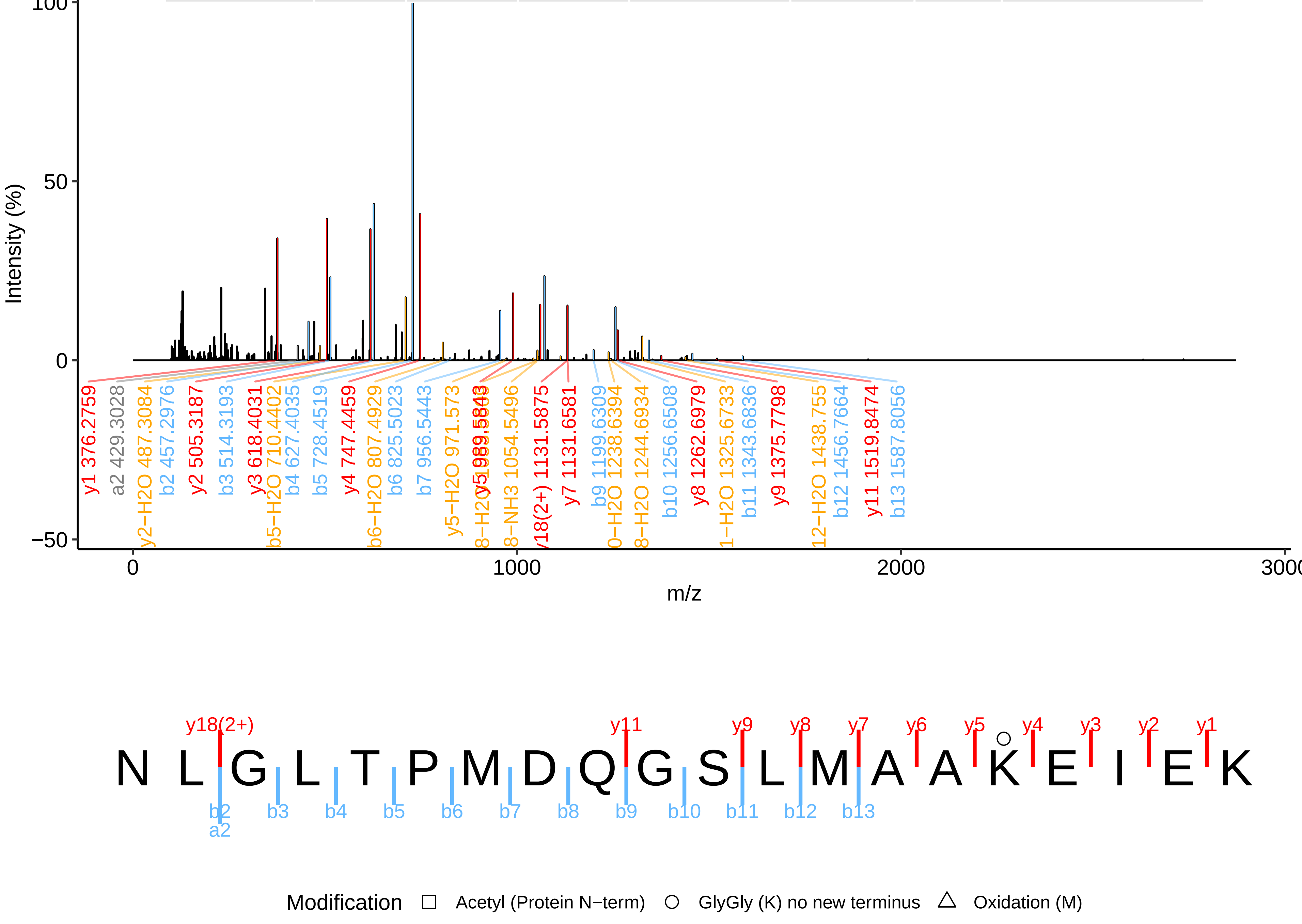

20200921_KIZ_MUb4_GlyGly_10plex_2 − Scan number: 19644

Gene Names	Charge	m/z	Mass	Retention time	PEP	Score	Precursor Intensity
STEAP4	3	759.3802	2275.119	77.124	9.2379e−06	84.493	1217630

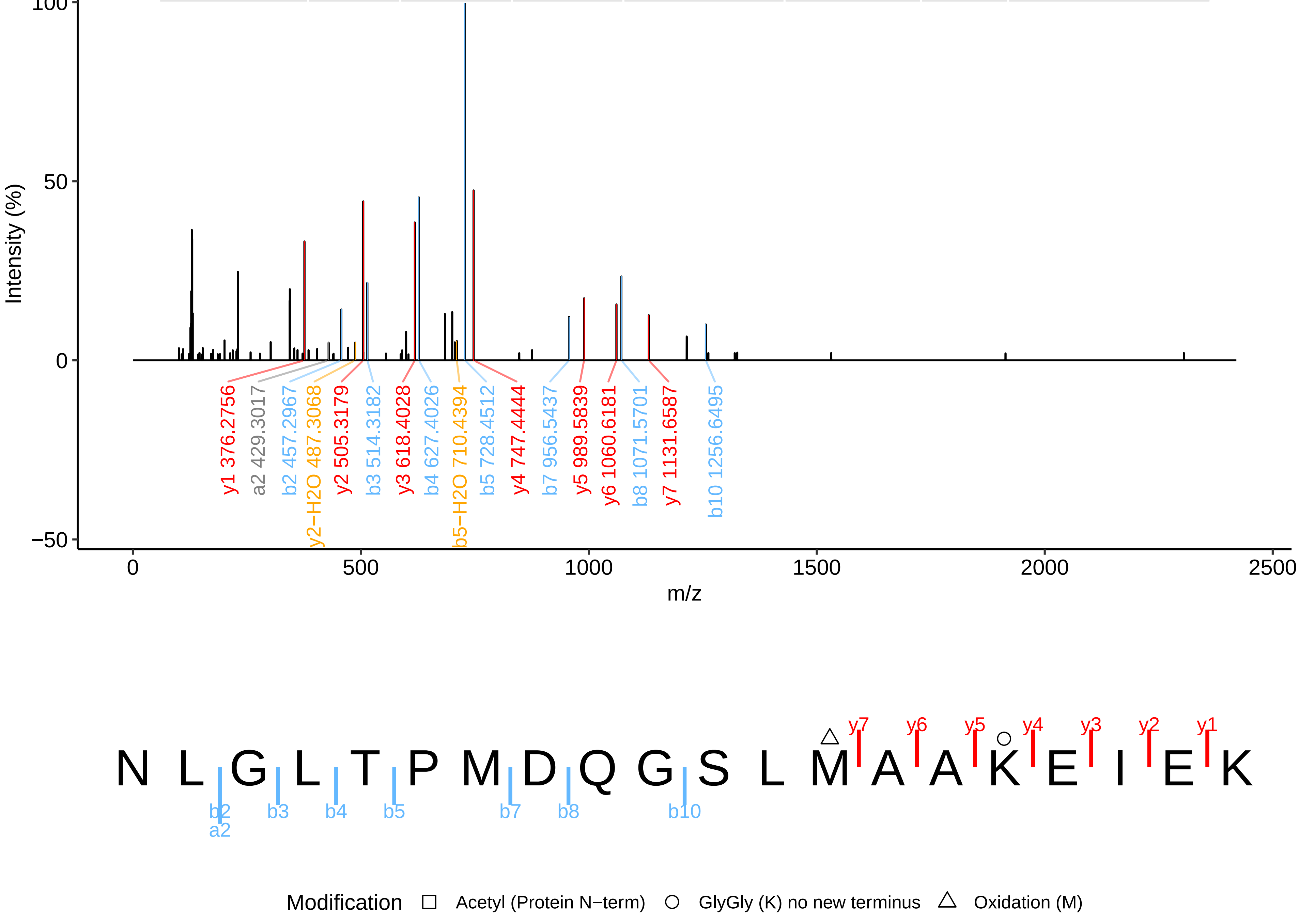

20200924_KIZ_MUb5_GlyGly_2 − Scan number: 19651

Gene Names	Charge	m/z	Mass	Retention time	PEP	Score	Precursor Intensity
STEAP4	3	759.3802	2275.119	72.867	5.6388e−06	85.231	NA

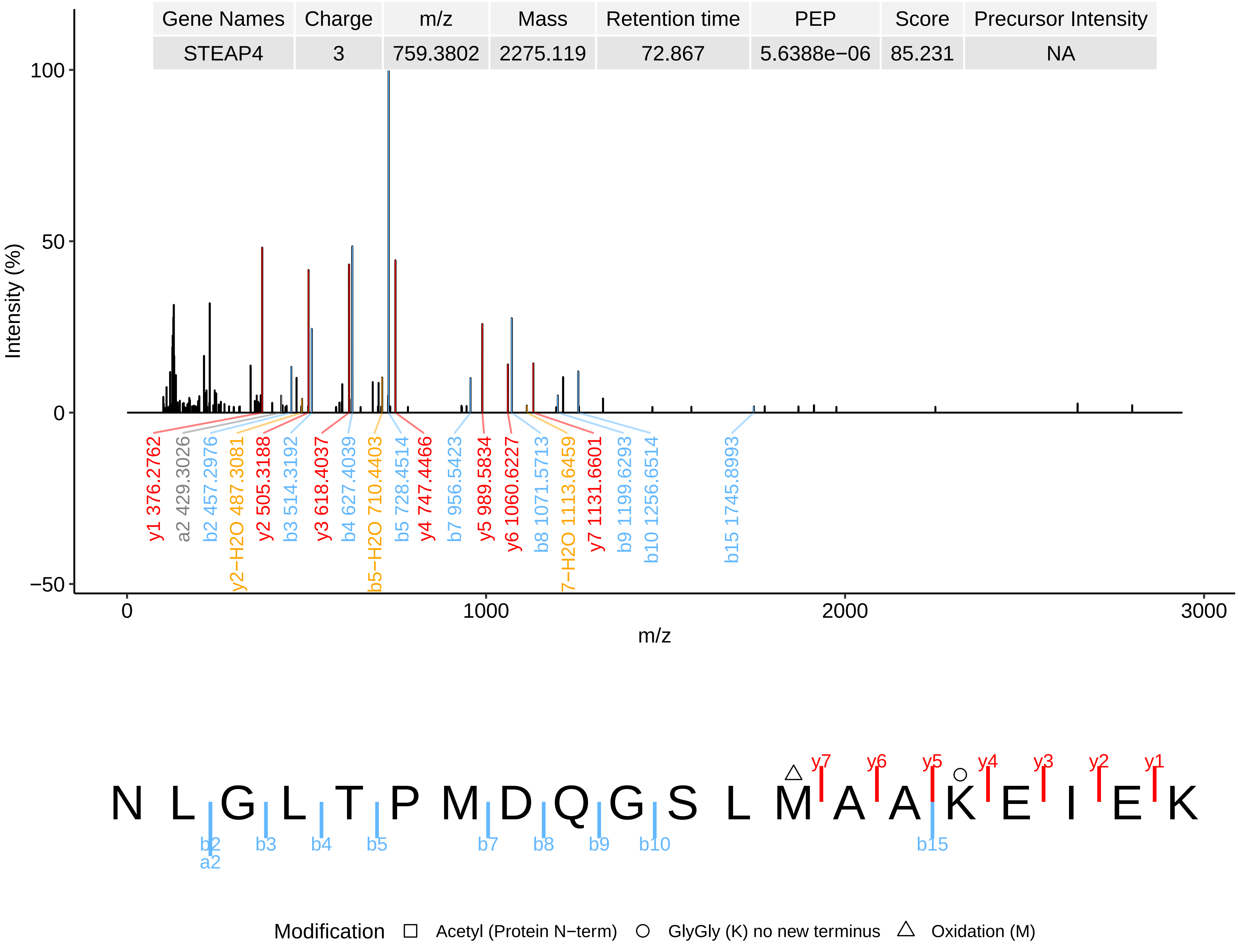

20200921_KIZ_MUb4_GlyGly_10plex_2 − Scan number: 17176

Gene Names	Charge	m/z	Mass	Retention time	PEP	Score	Precursor Intensity
STEAP4	3	764.7119	2291.114	67.128	0.010328	58.024	742880.7

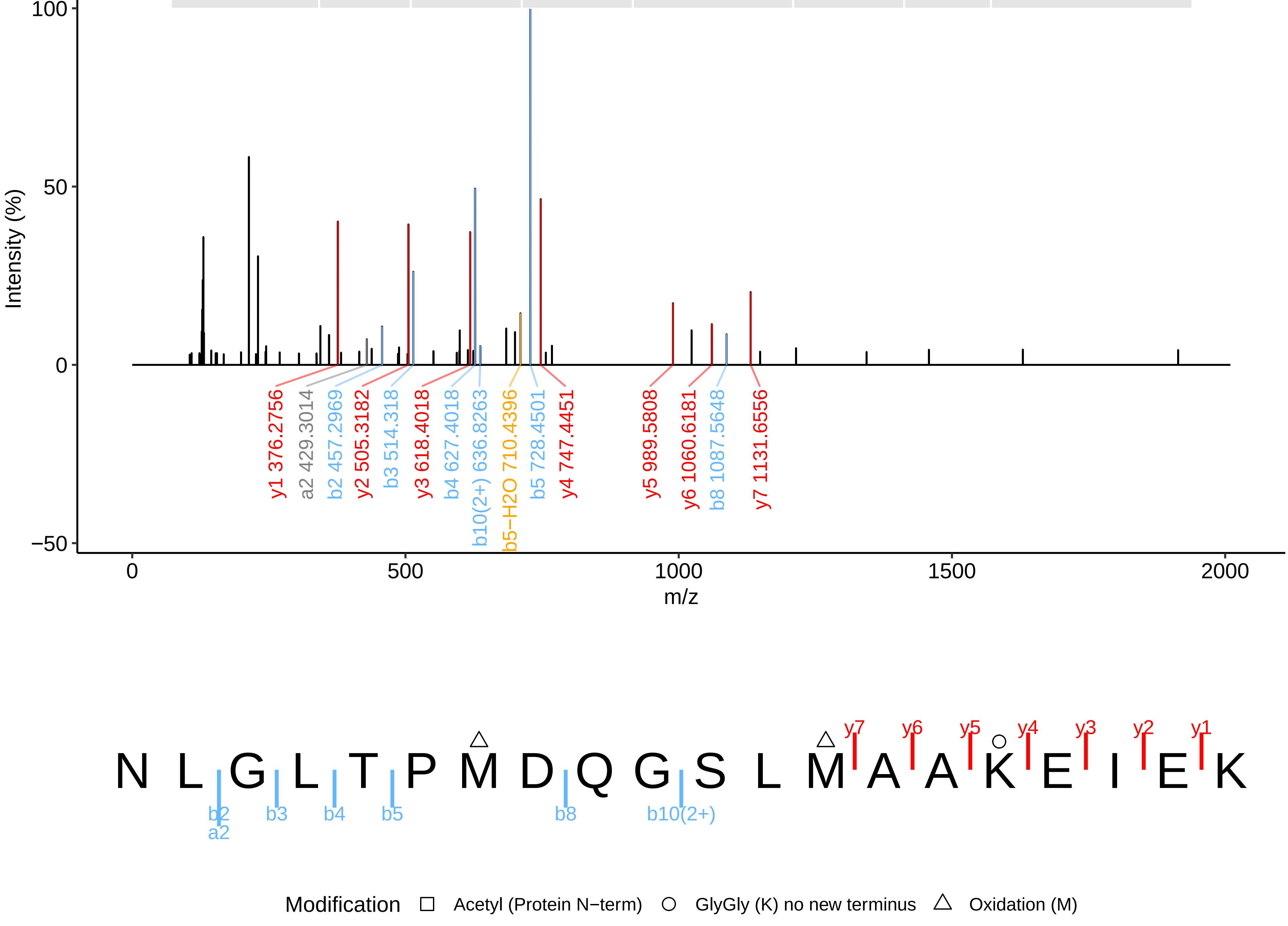

20200921_KIZ_MUb4_GlyGly_10plex − Scan number: 24453

Gene Names	Charge	m/z	Mass	Retention time	PEP	Score	Precursor Intensity
GPRC5A	3	755.6941	2264.061	88.919	4.0198e−10	111.06	2223113

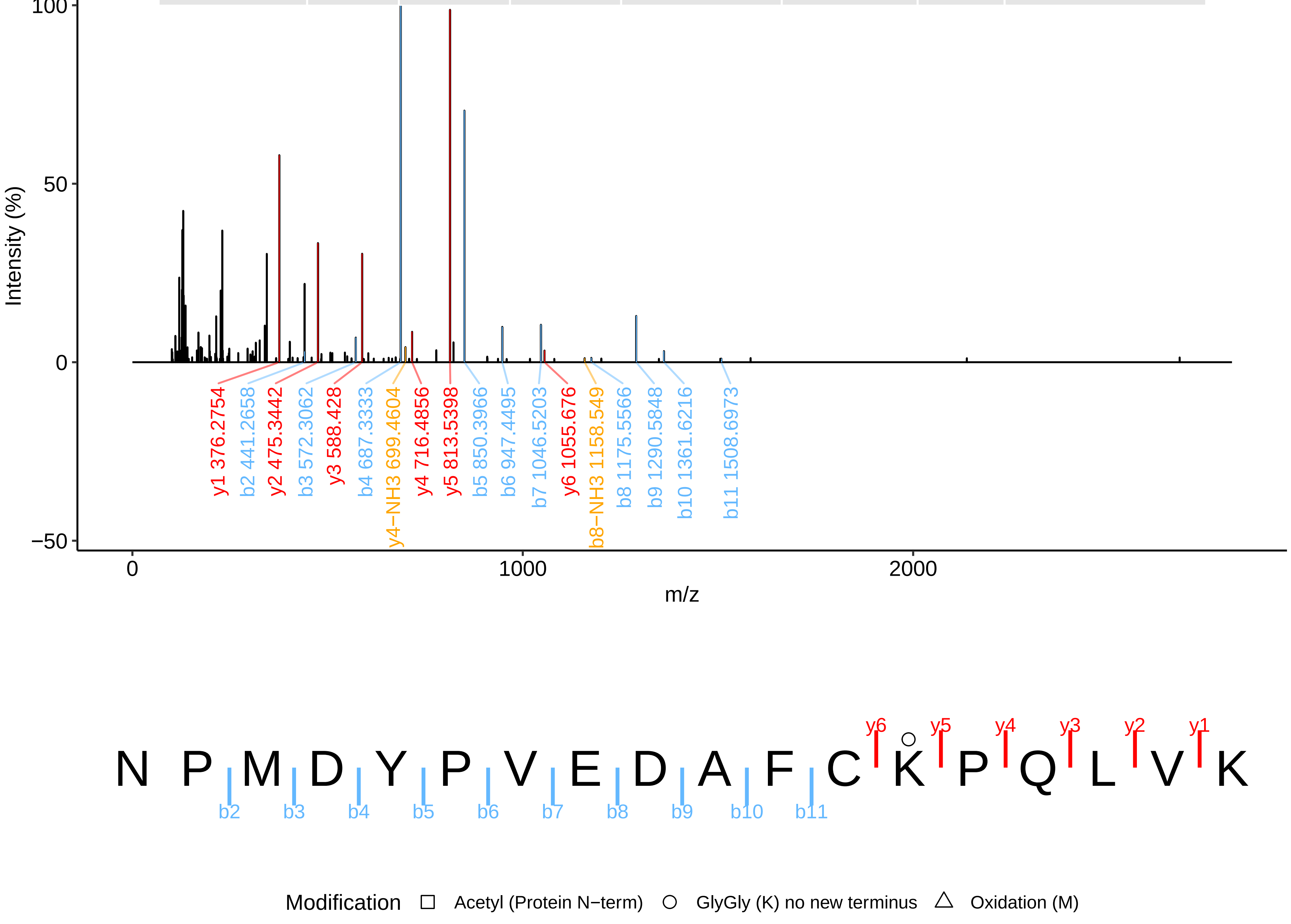

20200921_KIZ_MUb4_GlyGly_10plex − Scan number: 24457

Gene Names	Charge	m/z	Mass	Retention time	PEP	Score	Precursor Intensity
GPRC5A	3	755.6941	2264.061	88.937	6.523e−06	87.287	1507721

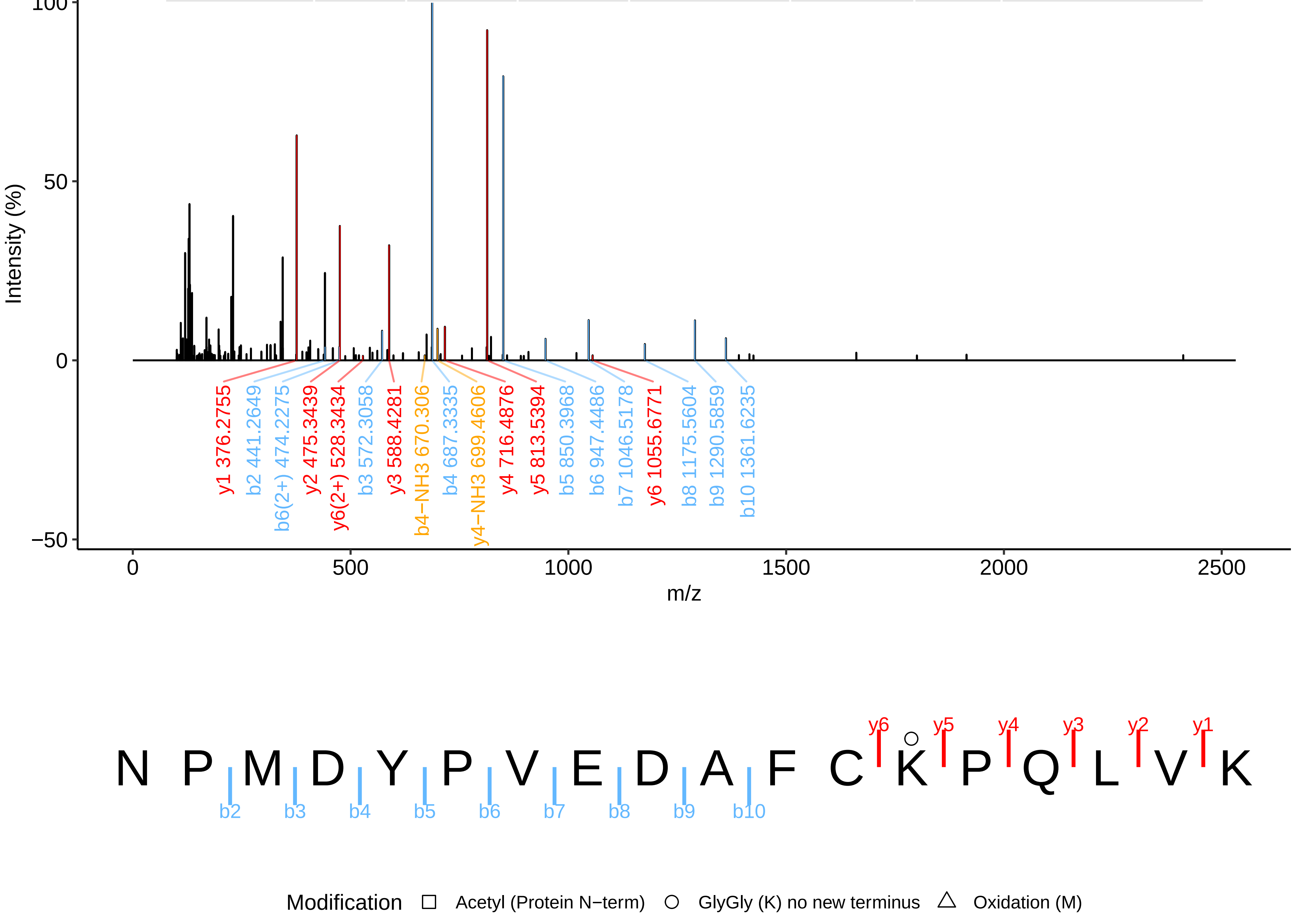

20200921_KIZ_MUb4_GlyGly_10plex − Scan number: 10518

Gene Names	Charge	m/z	Mass	Retention time	PEP	Score	Precursor Intensity
SLC1A3	4	563.7625	2251.021	36.424	7.5942e−10	105.79	9498698

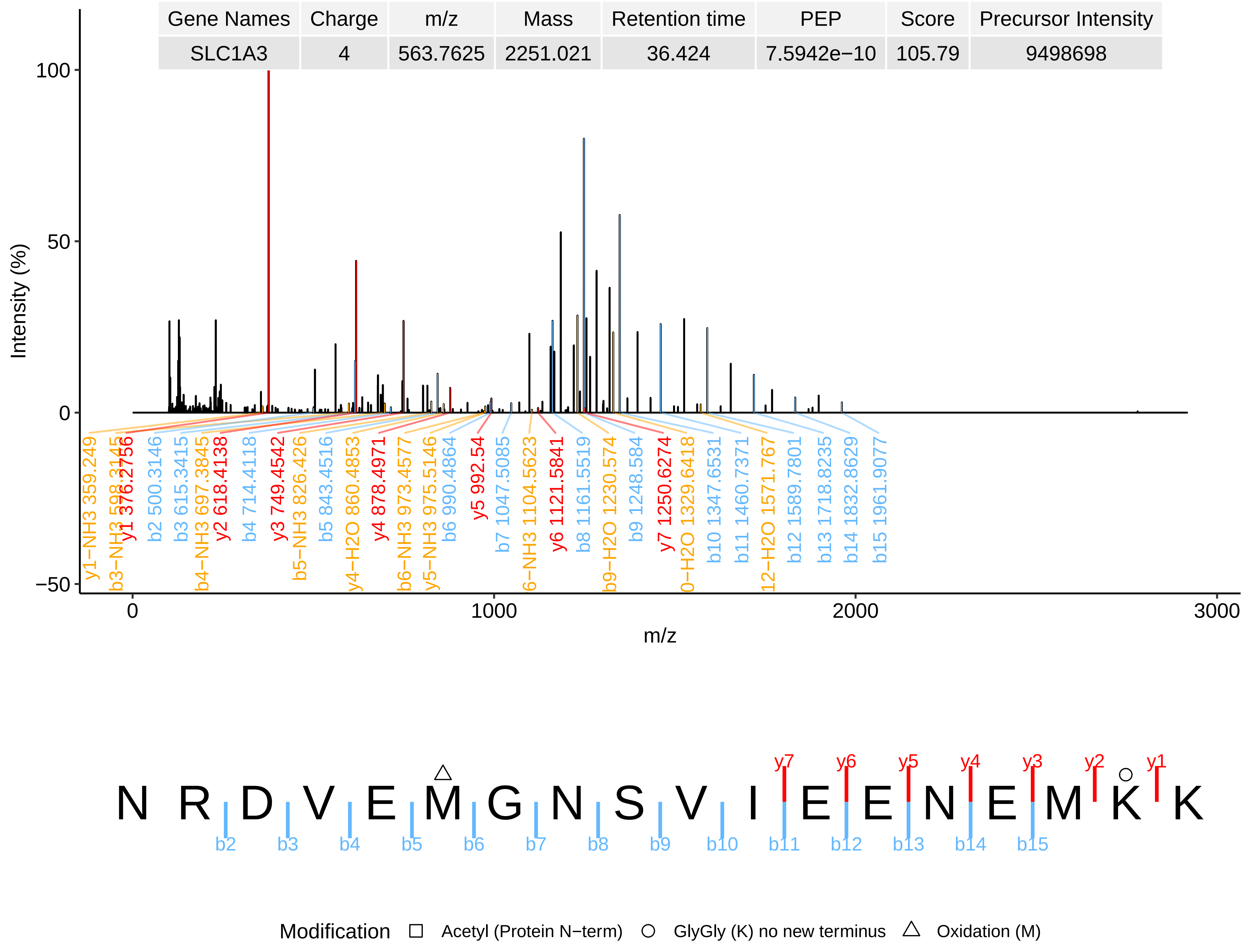

20200921_KIZ_MUb4_GlyGly_10plex − Scan number: 11306

Gene Names	Charge	m/z	Mass	Retention time	PEP	Score	Precursor Intensity
SLC1A3	4	563.7625	2251.021	39.331	2.309e−17	137.45	4427320

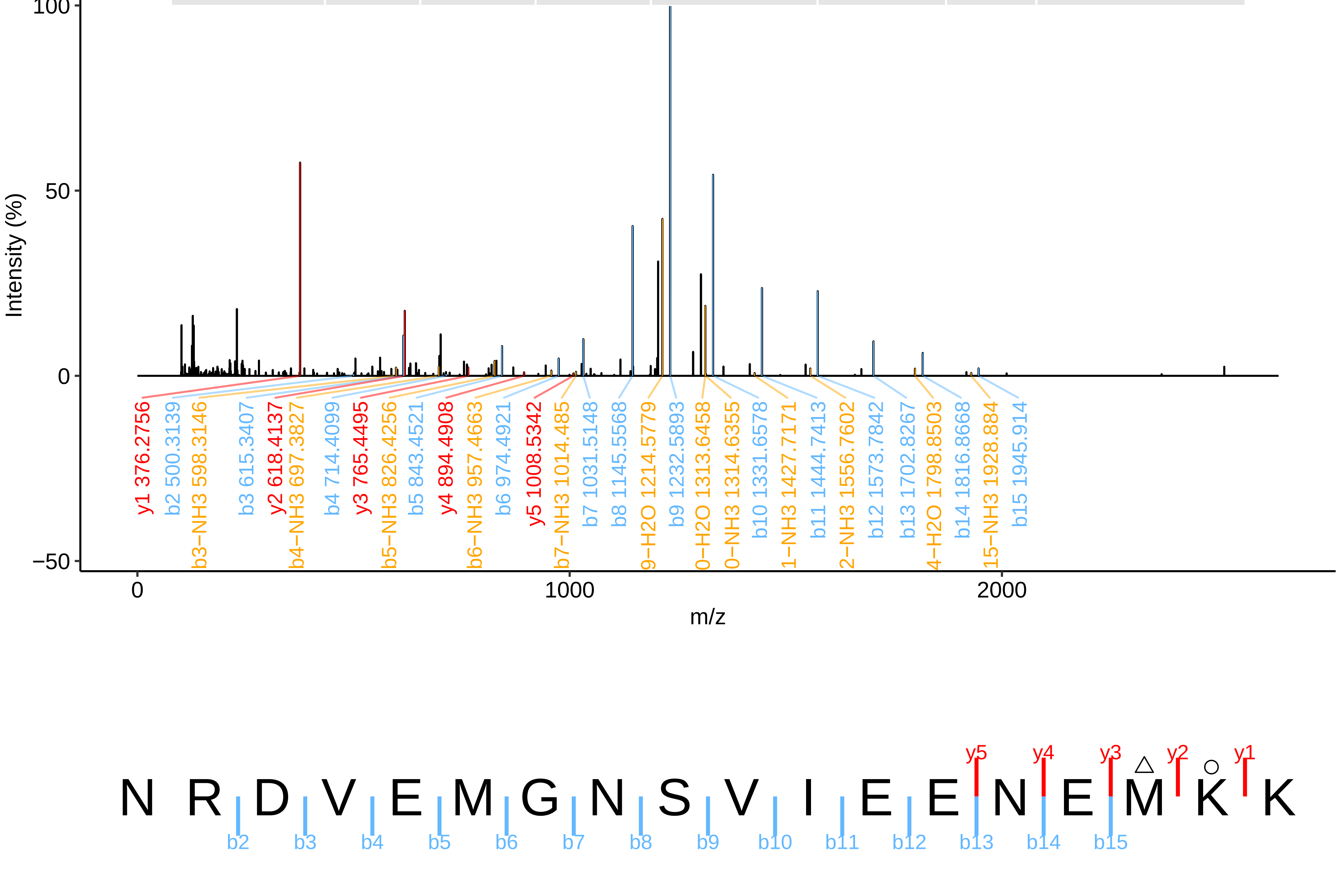

Modification □ Acetyl (Protein N−term) ○ GlyGly (K) no new terminus △ Oxidation (M)

20200921_KIZ_MUb4_GlyGly_10plex_2 − Scan number: 9705

Gene Names	Charge	m/z	Mass	Retention time	PEP	Score	Precursor Intensity
SLC1A3	4	563.7625	2251.021	36.983	2.8912e−06	92.439	839163.6

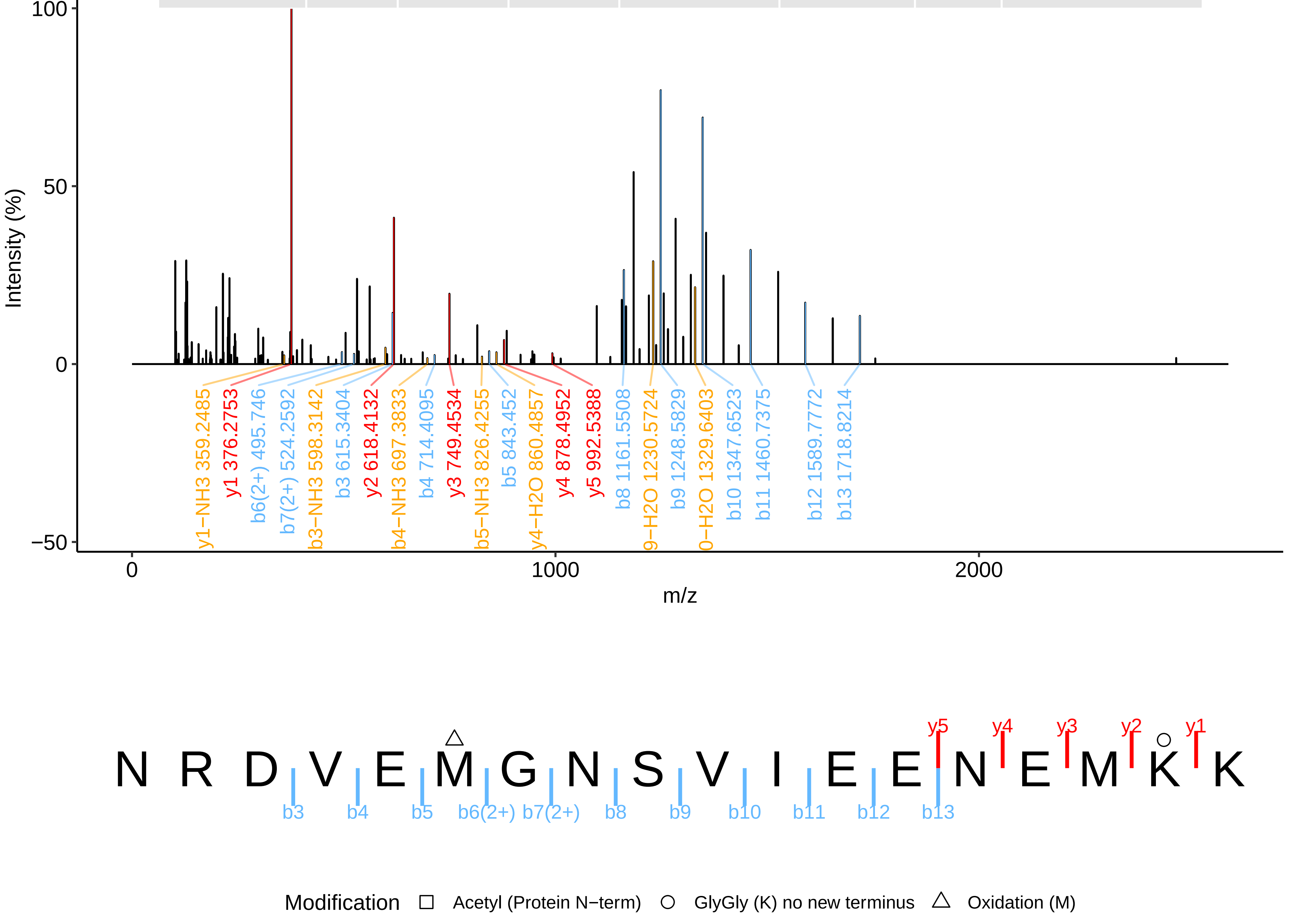

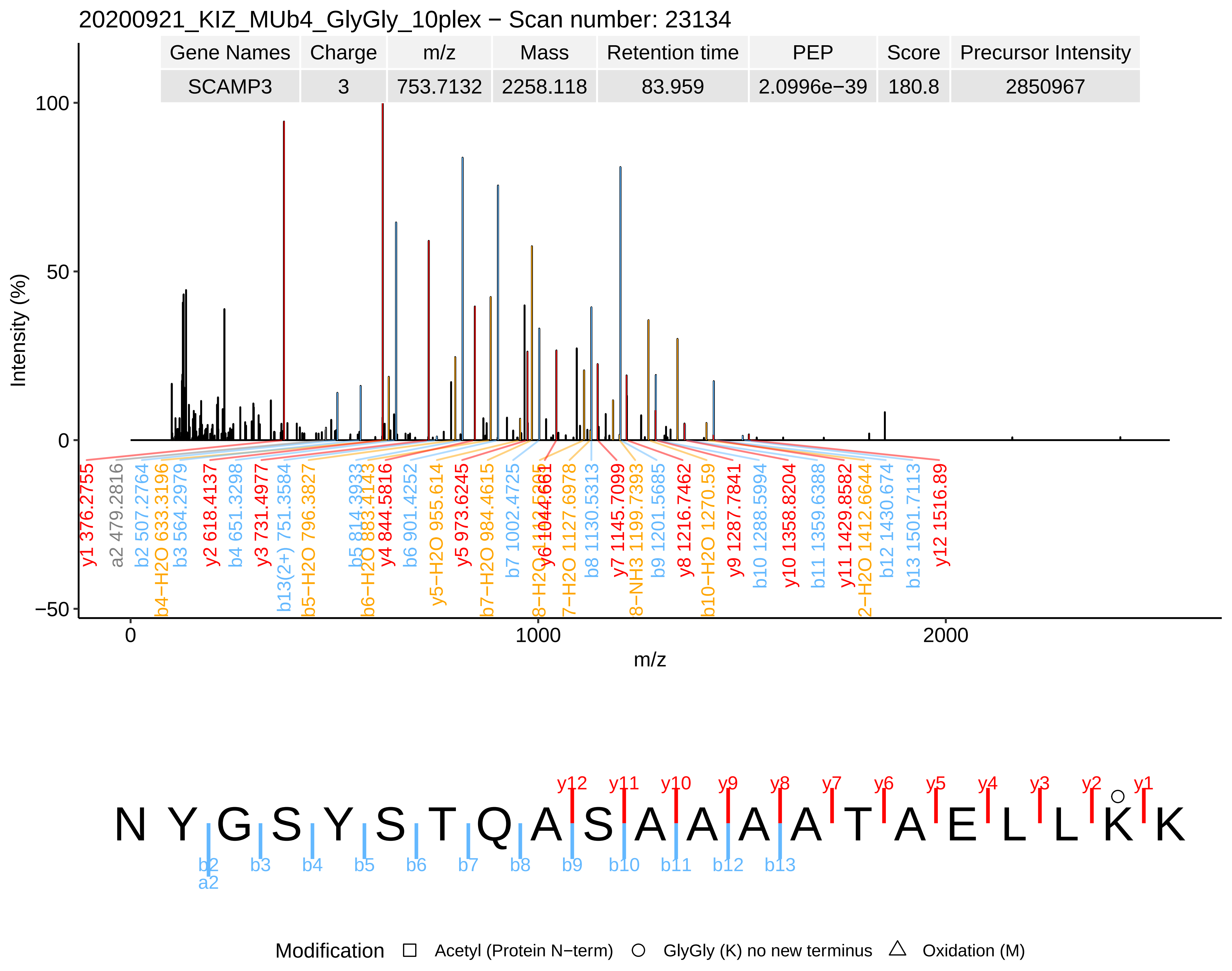
20200921_KIZ_MUb4_GlyGly_10plex − Scan number: 23134
Gene Names | Charge | m/z | Mass | Retention time | PEP | Score | Precursor Intensity
SCAMP3 | 3 | 753.7132 | 2258.118 | 83.959 | 2.0996e−39 | 180.8 | 2850967
Intensity (%)
m/z
y1 376.2755
a2 479.2816
b2 507.2764
b4−H2O 633.3196
b3 564.2979
y2 618.4137
b4 651.3298
y3 731.4977
b13(2+) 751.3584
b5−H2O 796.3827
b5 814.3933
b6−H2O 883.4143
y4 844.5816
b6 901.4252
y5−H2O 955.614
y5 973.6245
b7−H2O 984.4615
b7 1002.4725
y6 1044.661
7−H2O 1127.6978
b8 1130.5313
y7 1145.7099
8−NH3 1199.7393
b9 1201.5685
y8 1216.7462
b10−H2O 1270.59
y9 1287.7841
b10 1288.5994
y10 1358.8204
b11 1359.6388
y11 1429.8582
2−H2O 1412.6644
b12 1430.674
b13 1501.7113
y12 1516.89
N Y G S Y S T Q A S A A A A T A E L L K K
Modification
Acetyl (Protein N−term)
GlyGly (K) no new terminus
Oxidation (M)

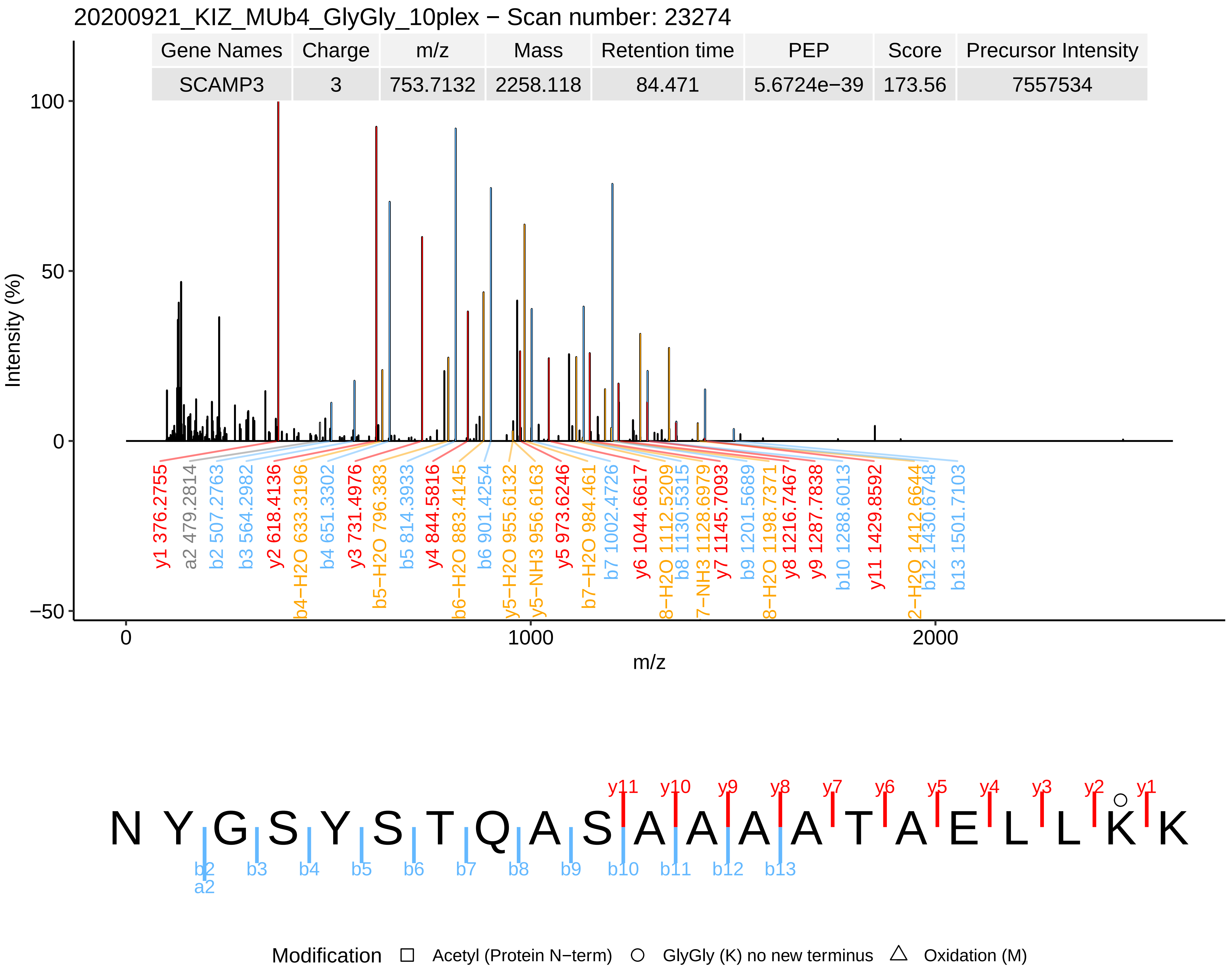
20200921_KIZ_MUb4_GlyGly_10plex − Scan number: 23274
Gene Names | Charge | m/z | Mass | Retention time | PEP | Score | Precursor Intensity
SCAMP3 | 3 | 753.7132 | 2258.118 | 84.471 | 5.6724e−39 | 173.56 | 7557534
Intensity (%)
100
50
0
−50
0
1000
2000
m/z
y1 376.2755
a2 479.2814
b2 507.2763
b3 564.2982
y2 618.4136
b4−H2O 633.3196
b4 651.3302
y3 731.4976
b5−H2O 796.383
b5 814.3933
y4 844.5816
b6−H2O 883.4145
b6 901.4254
y5−H2O 955.6132
y5−NH3 956.6163
y5 973.6246
b7−H2O 984.461
b7 1002.4726
y6 1044.6617
8−H2O 1112.5209
b8 1130.5315
7−NH3 1128.6979
y7 1145.7093
b9 1201.5689
8−H2O 1198.7371
y8 1216.7467
y9 1287.7838
b10 1288.6013
y11 1429.8592
2−H2O 1412.6644
b12 1430.6748
b13 1501.7103
N Y G S Y S T Q A S A A A A T A E L L K K
y11 y10 y9 y8 y7 y6 y5 y4 y3 y2 y1
b2 b3 b4 b5 b6 b7 b8 b9 b10 b11 b12 b13
a2
Modification
Acetyl (Protein N−term)
GlyGly (K) no new terminus
Oxidation (M)

20200921_KIZ_MUb4_GlyGly_10plex_2 − Scan number: 17753

Gene Names	Charge	m/z	Mass	Retention time	PEP	Score	Precursor Intensity
SCAMP3	4	565.5367	2258.118	69.454	6.2609e−06	84.493	2059545

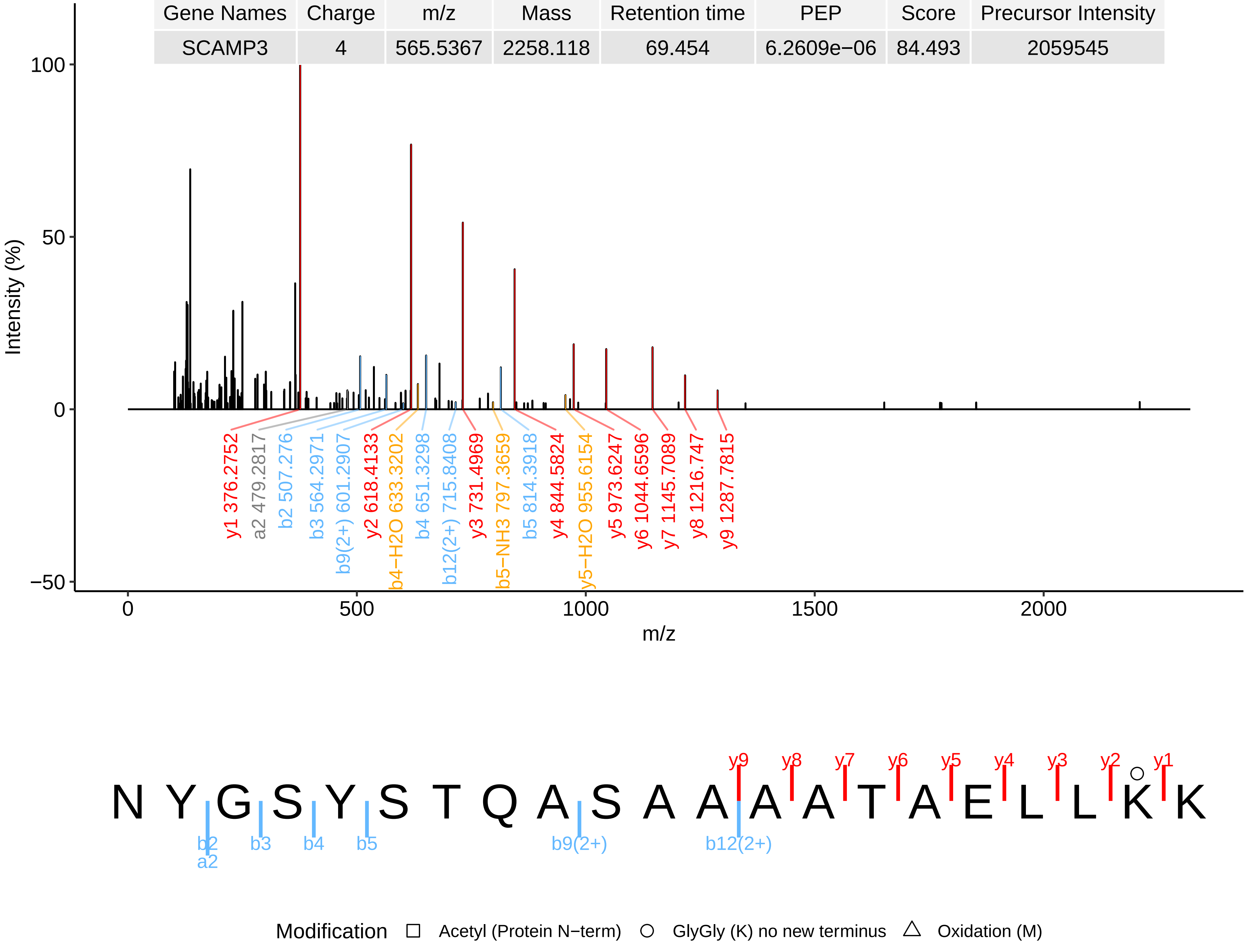

20200921_KIZ_MUb4_GlyGly_10plex_2 − Scan number: 20149

Gene Names	Charge	m/z	Mass	Retention time	PEP	Score	Precursor Intensity
SCAMP3	3	753.7132	2258.118	79.177	1.7303e−05	93.776	2020162

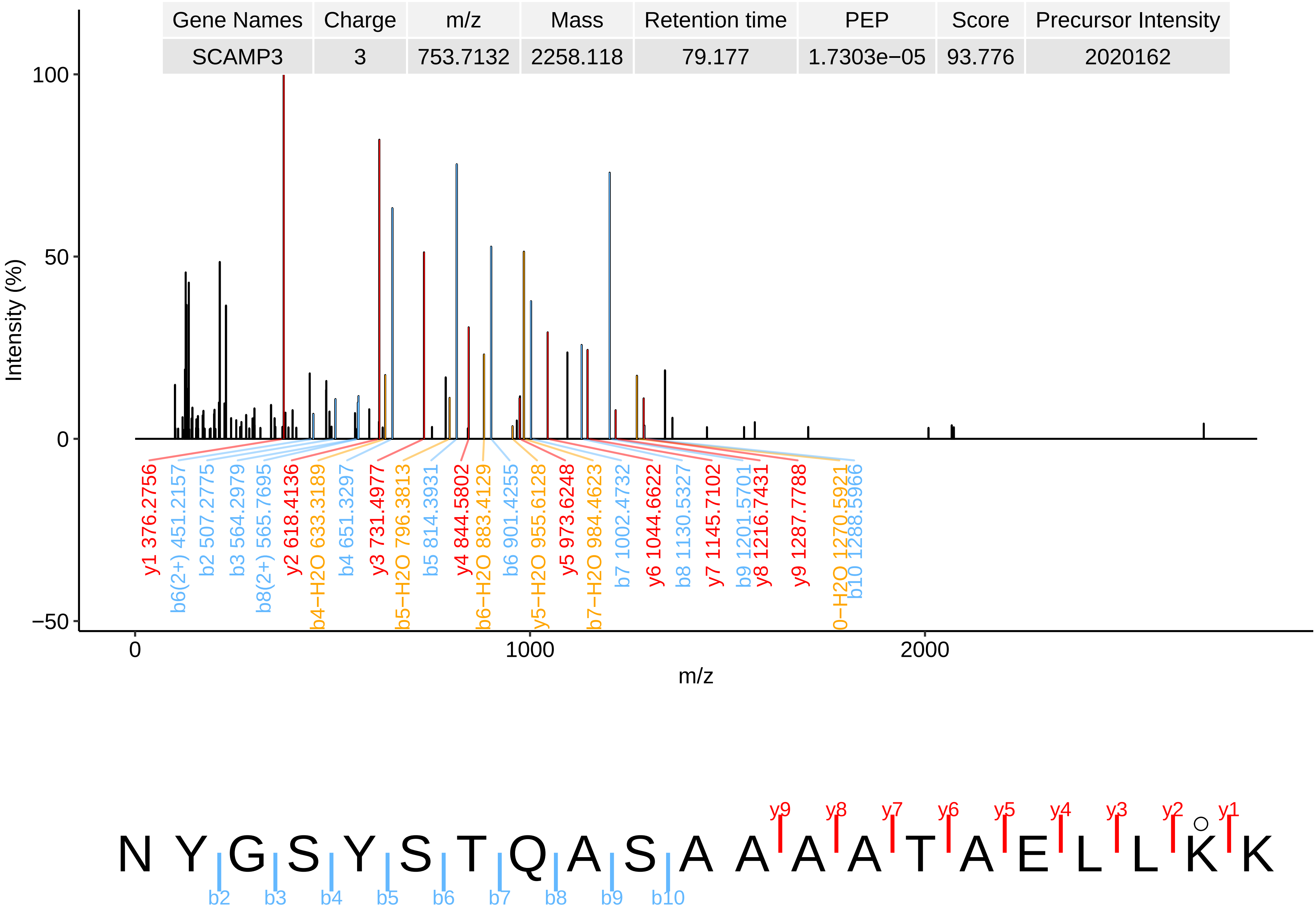

Modification □ Acetyl (Protein N−term) ○ GlyGly (K) no new terminus △ Oxidation (M)

20200921_KIZ_MUb4_GlyGly_10plex_2 − Scan number: 20169

Gene Names	Charge	m/z	Mass	Retention time	PEP	Score	Precursor Intensity
SCAMP3	3	753.7132	2258.118	79.258	1.1583e−13	117.37	5785118

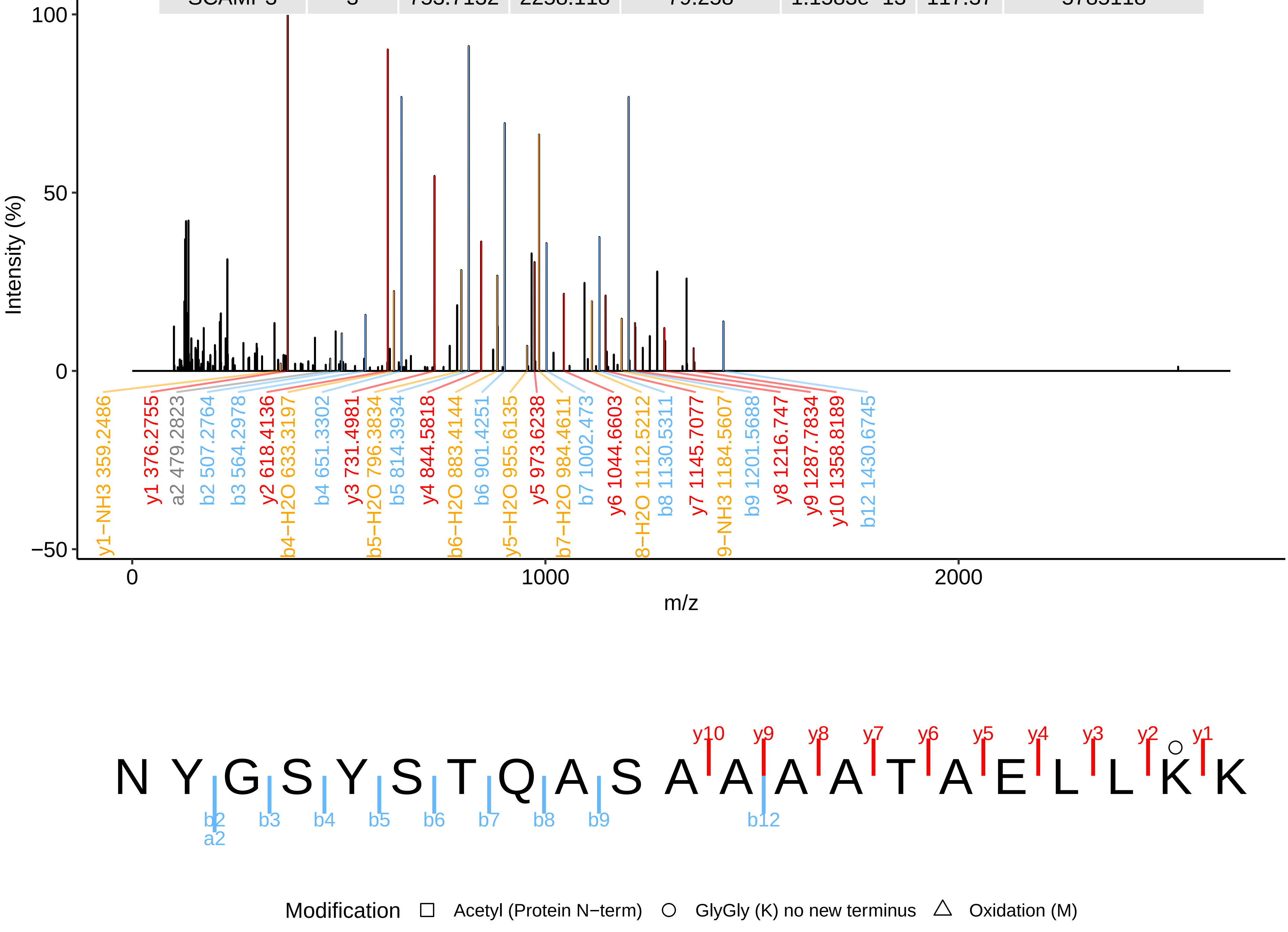

20200921_KIZ_MUb4_GlyGly_10plex_2 − Scan number: 21851

Gene Names	Charge	m/z	Mass	Retention time	PEP	Score	Precursor Intensity
EIF2S3;EIF2S3L	4	620.8393	2479.328	86.071	8.1459e−06	83.063	574785.4

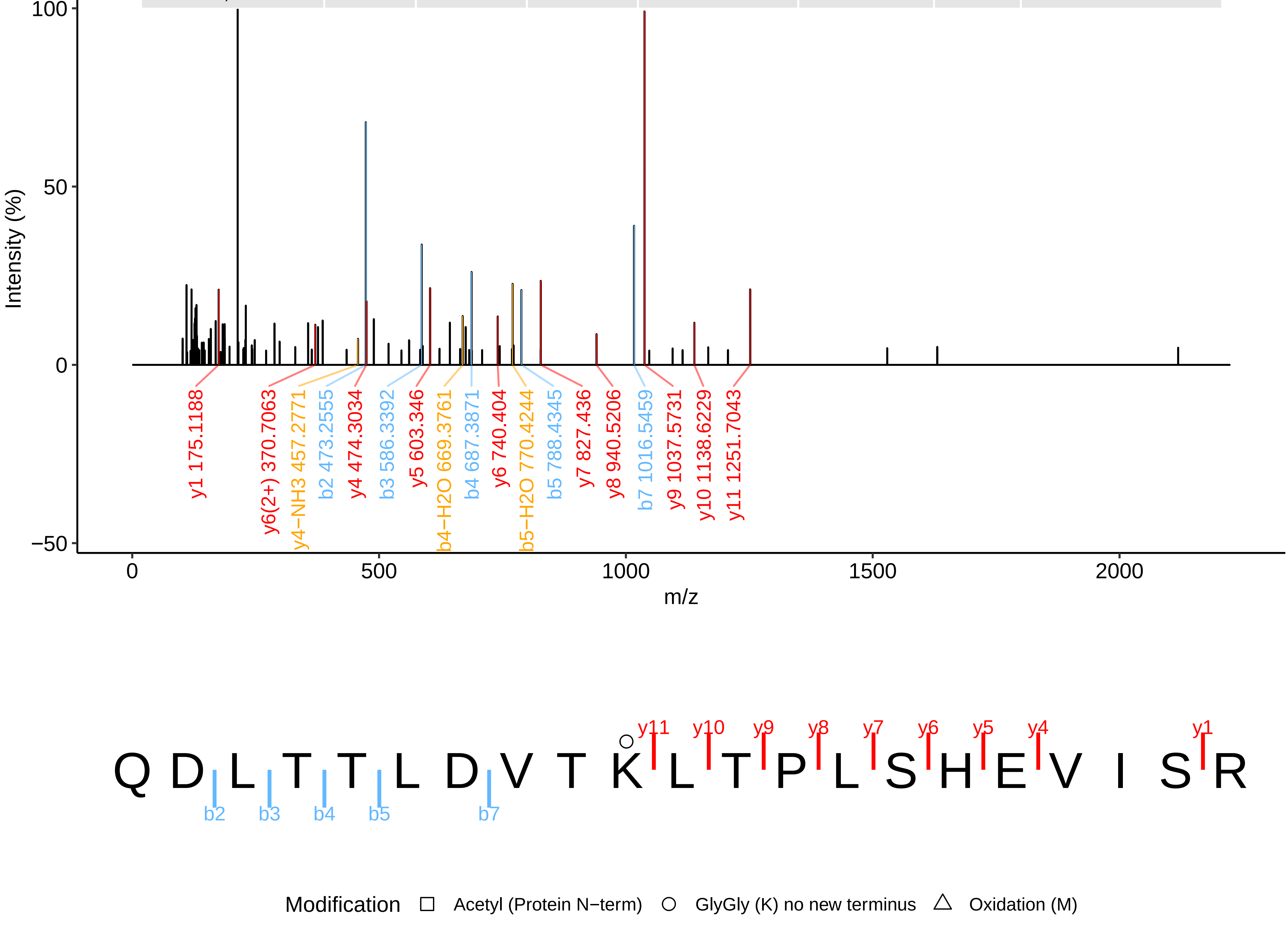

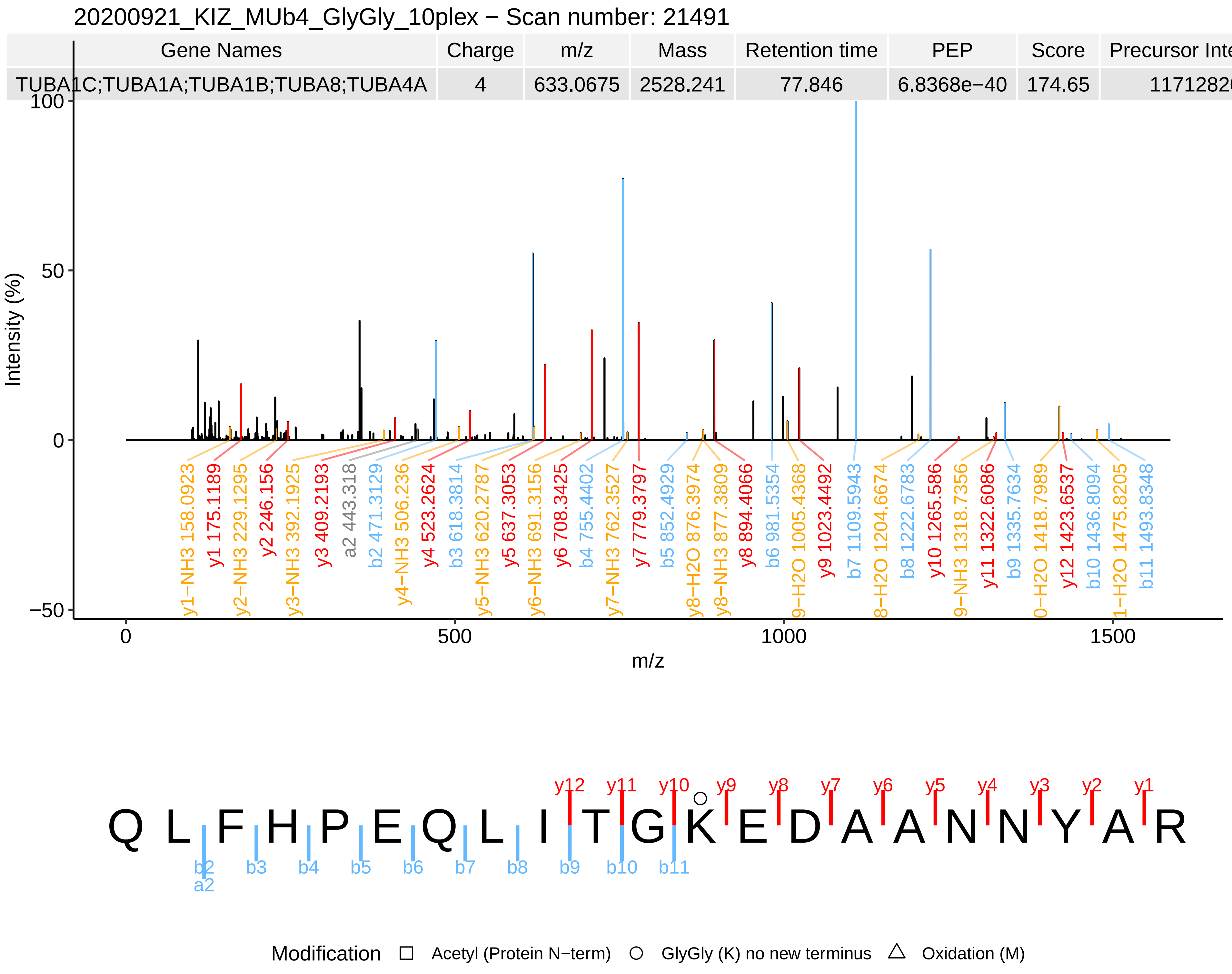

20200921_KIZ_MUb4_GlyGly_10plex − Scan number: 21491
Gene Names | Charge | m/z | Mass | Retention time | PEP | Score | Precursor Inte
TUBA1C;TUBA1A;TUBA1B;TUBA8;TUBA4A | 4 | 633.0675 | 2528.241 | 77.846 | 6.8368e−40 | 174.65 | 1171282
Intensity (%)
100
50
0
−50
m/z
0
500
1000
1500
y1−NH3 158.0923
y1 175.1189
y2−NH3 229.1295
y2 246.156
y3−NH3 392.1925
y3 409.2193
a2 443.318
b2 471.3129
y4−NH3 506.236
y4 523.2624
b3 618.3814
y5−NH3 620.2787
y5 637.3053
y6−NH3 691.3156
y6 708.3425
b4 755.4402
y7−NH3 762.3527
y7 779.3797
b5 852.4929
y8−H2O 876.3974
y8−NH3 877.3809
y8 894.4066
b6 981.5354
9−H2O 1005.4368
y9 1023.4492
b7 1109.5943
8−H2O 1204.6674
b8 1222.6783
y10 1265.586
9−NH3 1318.7356
y11 1322.6086
b9 1335.7634
0−H2O 1418.7989
y12 1423.6537
b10 1436.8094
1−H2O 1475.8205
b11 1493.8348
Q L F H P E Q L I T G K E D A A N N Y A R
y12 y11 y10 y9 y8 y7 y6 y5 y4 y3 y2 y1
b2 b3 b4 b5 b6 b7 b8 b9 b10 b11
a2
Modification
Acetyl (Protein N−term)
GlyGly (K) no new terminus
Oxidation (M)

20200921_KIZ_MUb4_GlyGly_10plex − Scan number: 21628

Gene Names	Charge	m/z	Mass	Retention time	PEP	Score	Precursor Inte
TUBA1C;TUBA1A;TUBA1B;TUBA8;TUBA4A	4	633.0675	2528.241	78.366	1.1222e−39	167.97	6560470

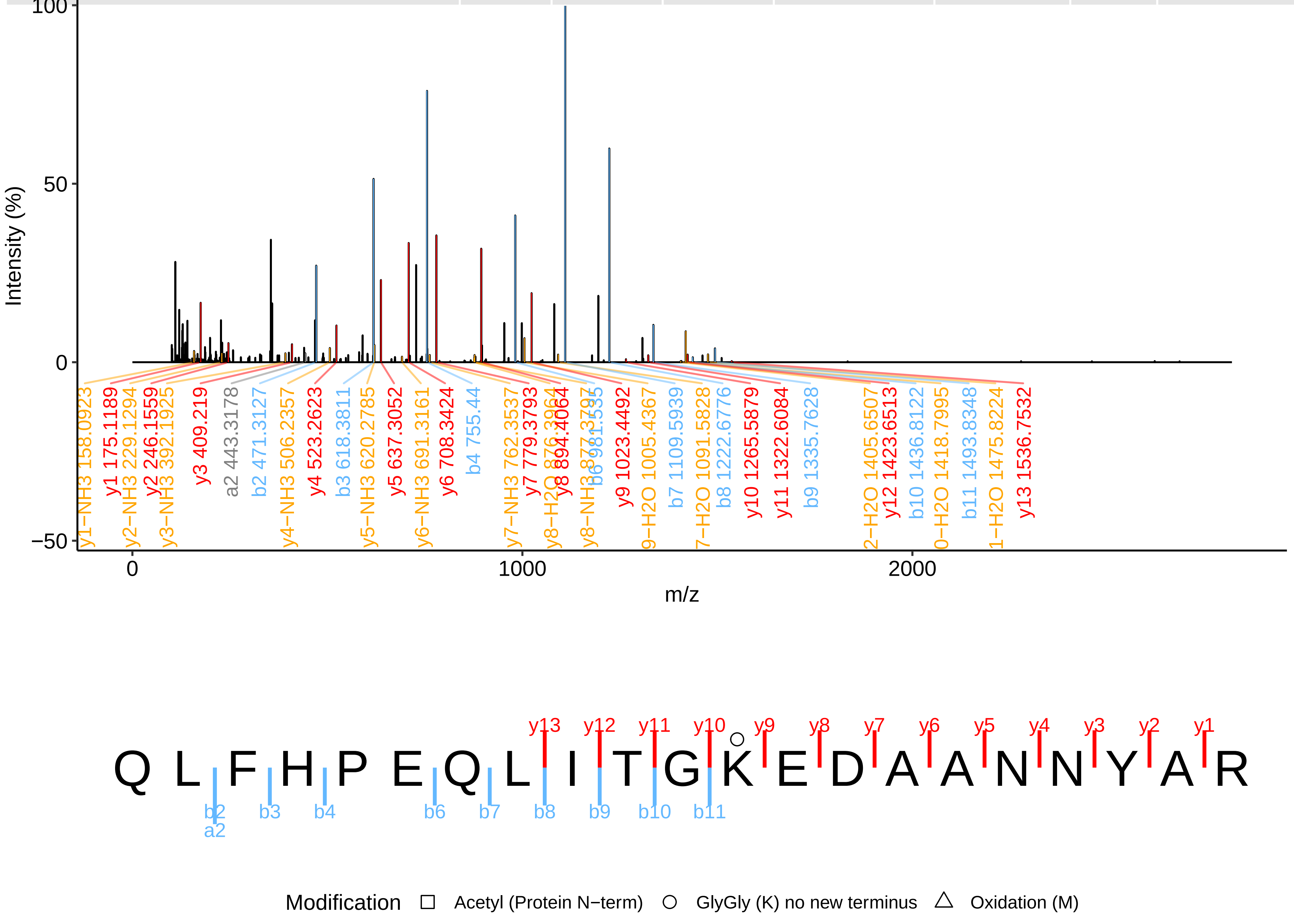

20200921_KIZ_MUb4_GlyGly_10plex_2 − Scan number: 17489

Gene Names	Charge	m/z	Mass	Retention time	PEP	Score	Precursor Inter
TUBA1C;TUBA1A;TUBA1B;TUBA8;TUBA4A	4	633.0675	2528.241	68.398	0.050711	43.164	NA

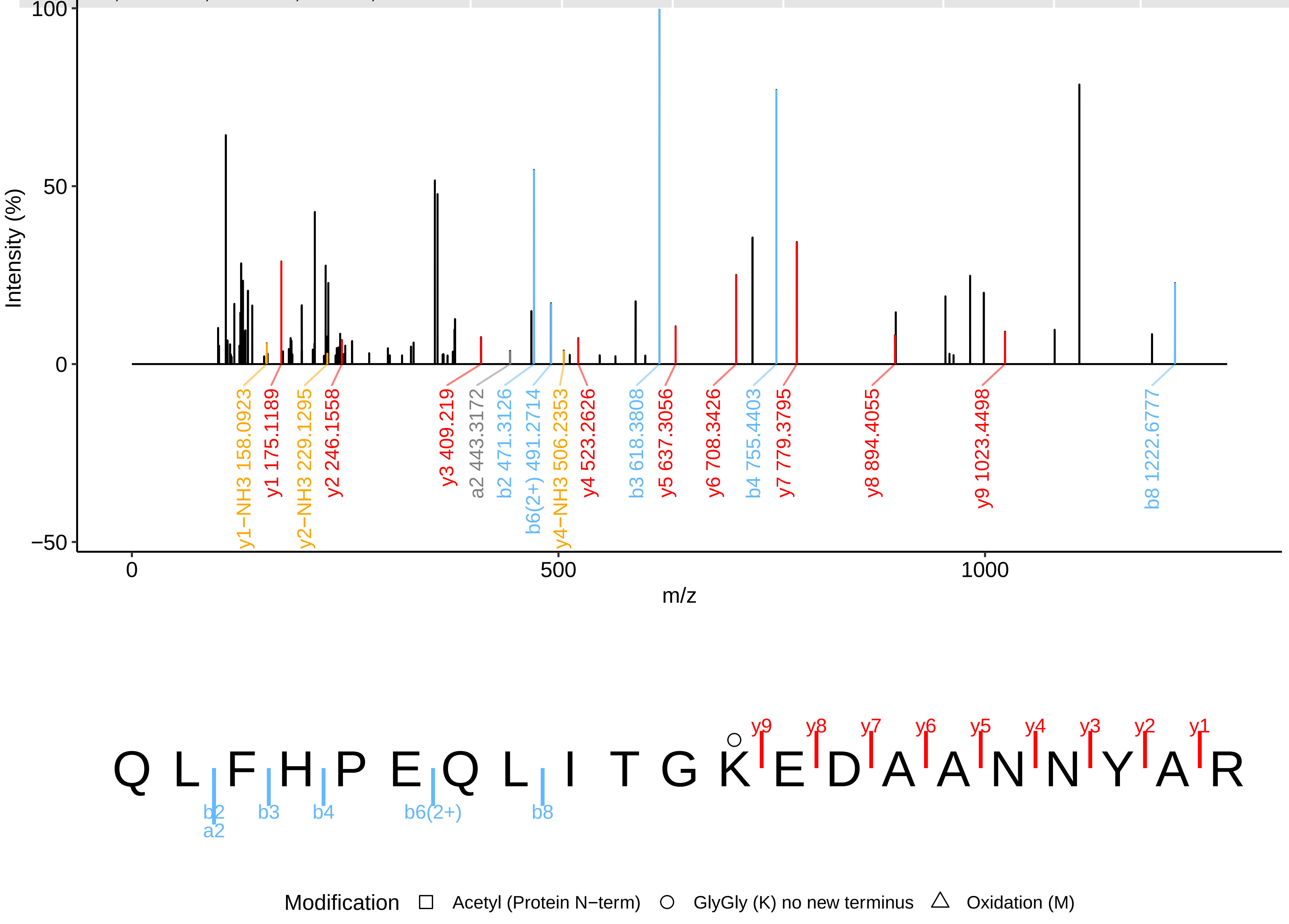

20200924_KIZ_MUb5_GlyGly_1 − Scan number: 18548

Gene Names	Charge	m/z	Mass	Retention time	PEP	Score	Precursor Inte
TUBA1C;TUBA1A;TUBA1B;TUBA8;TUBA4A	4	633.0675	2528.241	59.954	8.0864e−15	124.73	3652722[illegible]

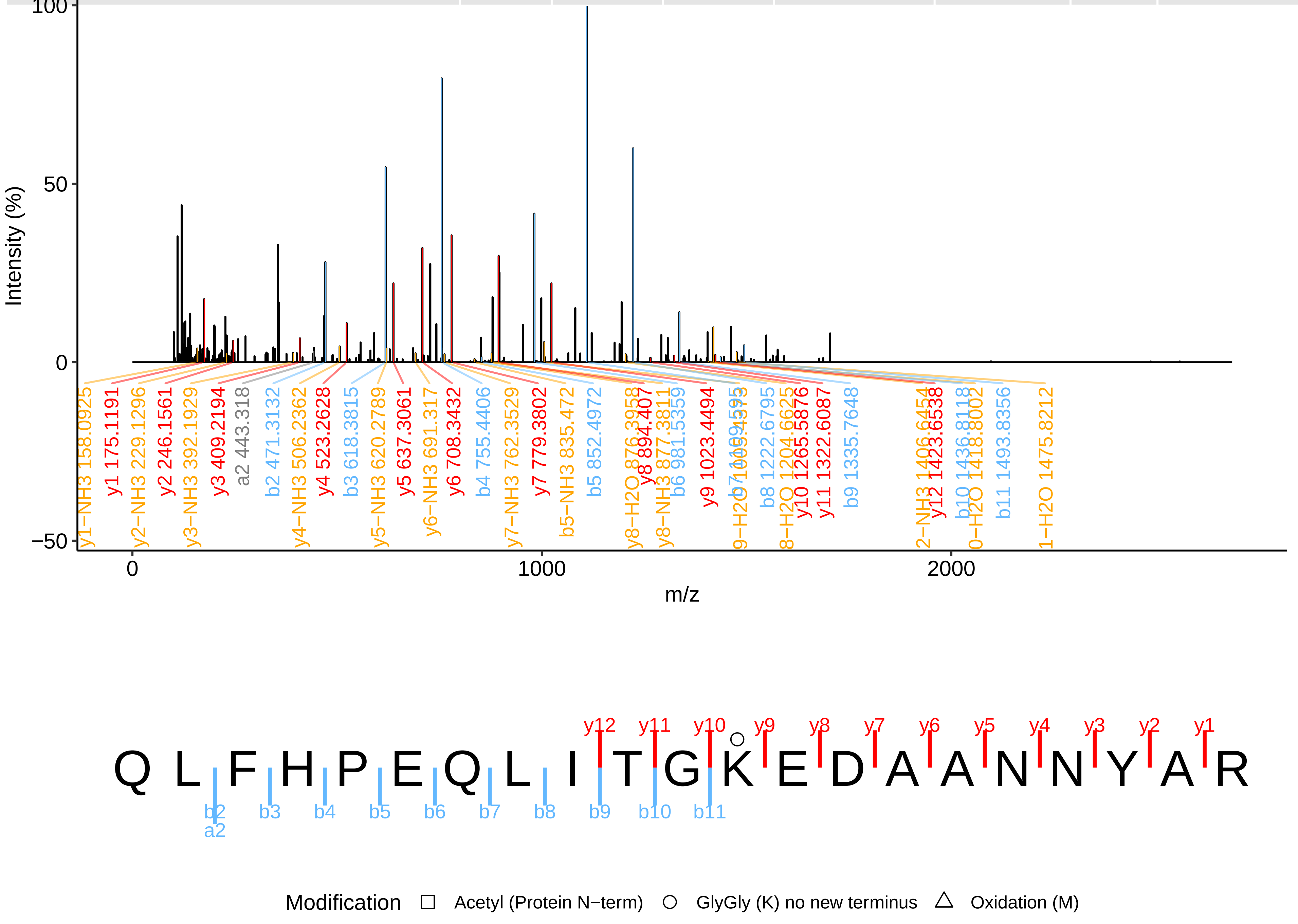

20200924_KIZ_MUb5_GlyGly_1 − Scan number: 18707

Gene Names	Charge	m/z	Mass	Retention time	PEP	Score	Precursor Inte
TUBA1C;TUBA1A;TUBA1B;TUBA8;TUBA4A	4	633.0675	2528.241	60.455	6.7762e−15	126.04	1851483

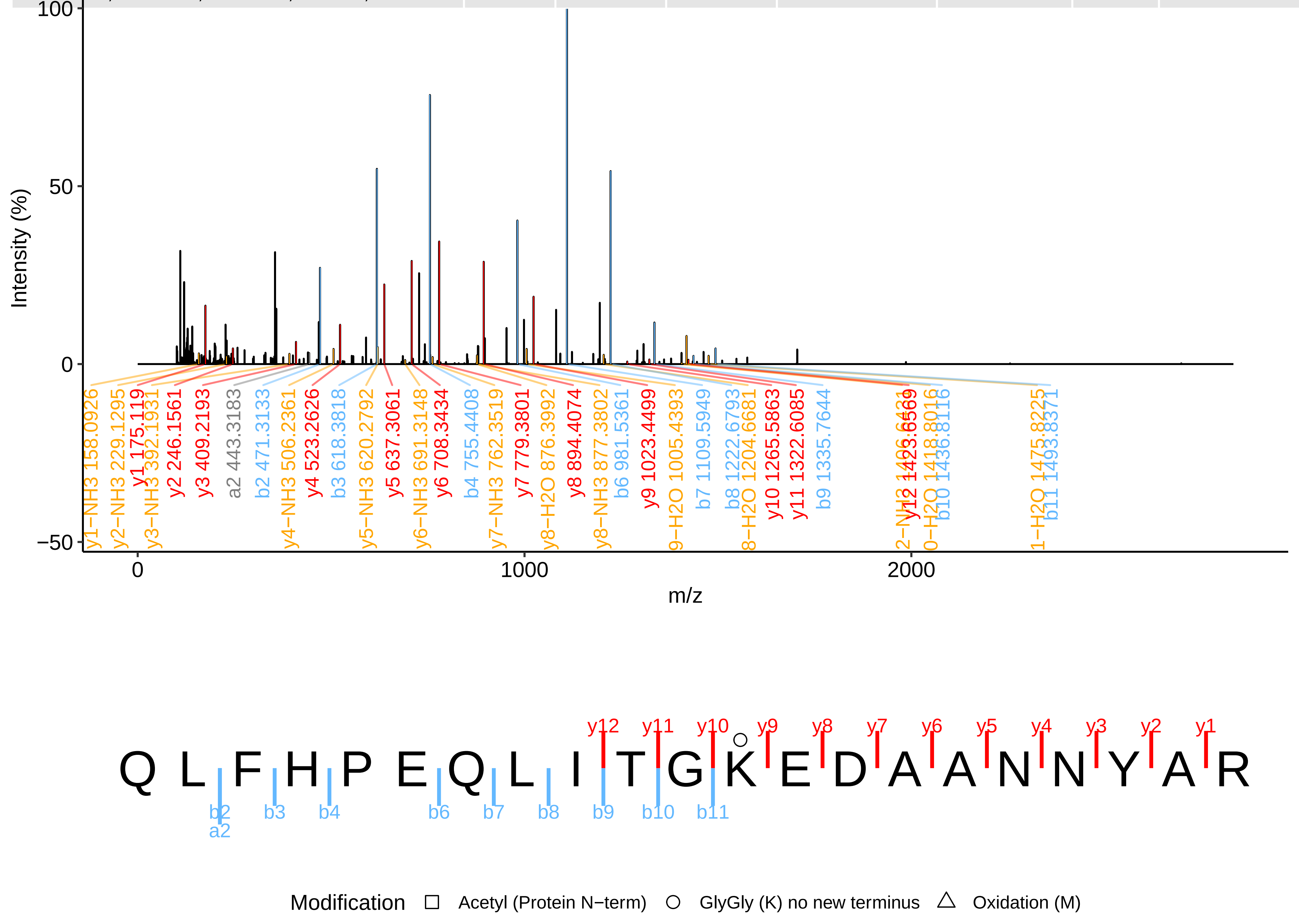

20200924_KIZ_MUb5_GlyGly_2 − Scan number: 17230

Gene Names	Charge	m/z	Mass	Retention time	PEP	Score	Precursor Inte
TUBA1C;TUBA1A;TUBA1B;TUBA8;TUBA4A	4	633.0675	2528.241	63.881	1.5587e−21	134.52	8236001

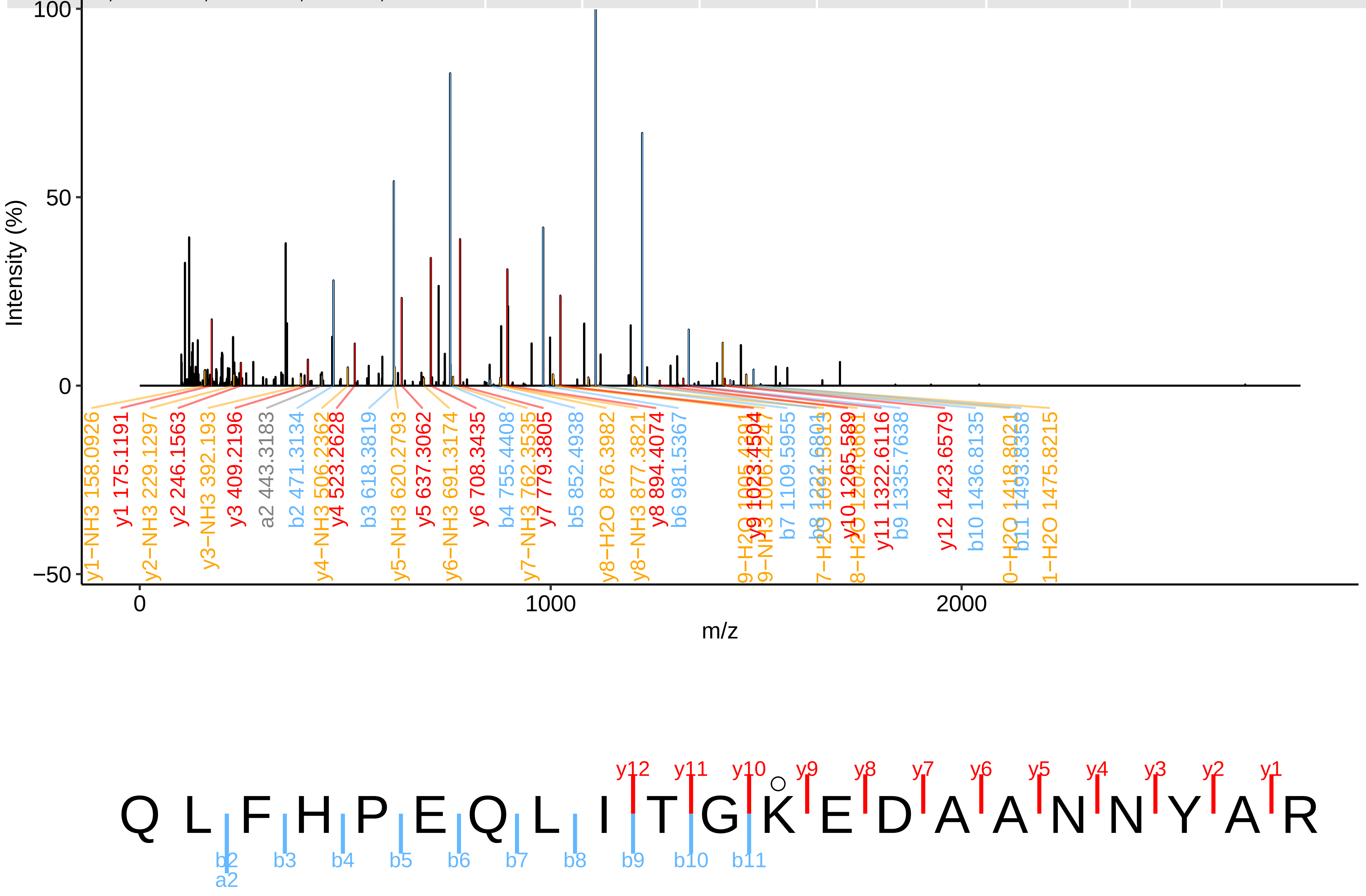

Modification □ Acetyl (Protein N−term) ○ GlyGly (K) no new terminus △ Oxidation (M)

20200924_KIZ_MUb5_GlyGly_2 − Scan number: 17428

Gene Names	Charge	m/z	Mass	Retention time	PEP	Score	Precursor Inte
TUBA1C;TUBA1A;TUBA1B;TUBA8;TUBA4A	4	633.0675	2528.241	64.591	5.3279e−15	127.49	1376233

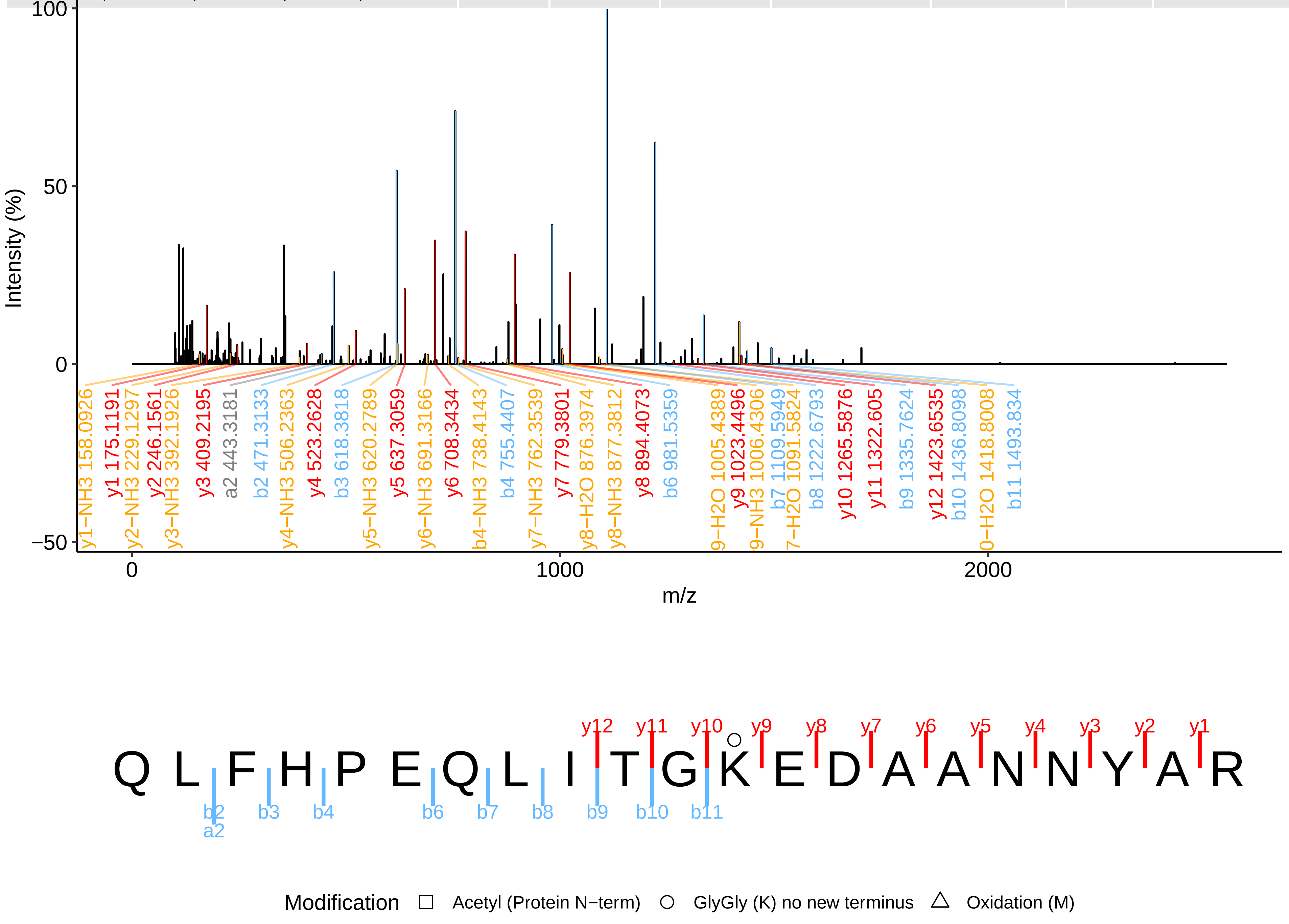

20201118_KIZ_MUb6_GlyGly_2 − Scan number: 20721

Gene Names	Charge	m/z	Mass	Retention time	PEP	Score	Precursor Inte
TUBA1C;TUBA1A;TUBA1B;TUBA8;TUBA4A	4	633.0675	2528.241	69.839	6.8368e−40	174.65	23763600

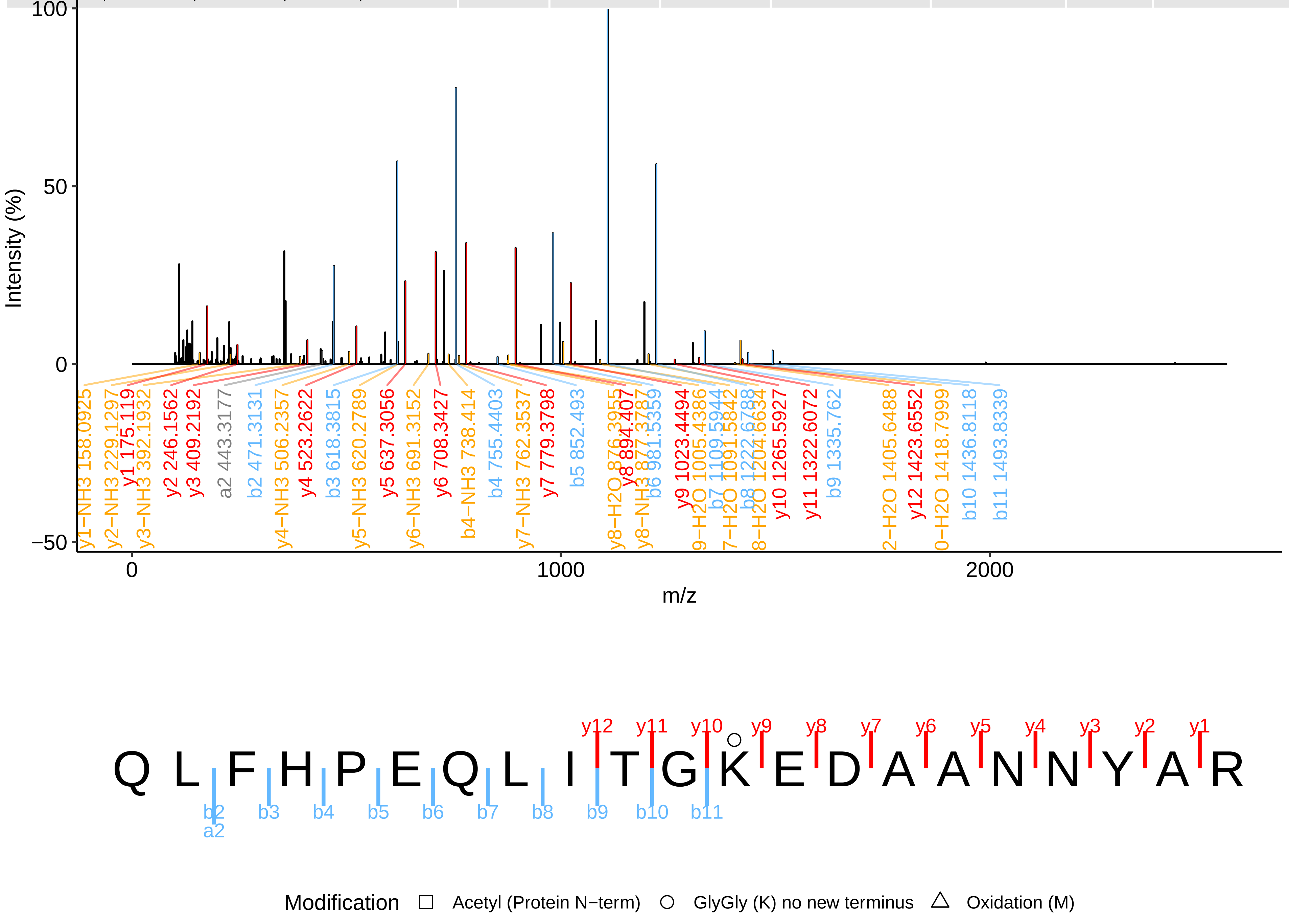

20200921_KIZ_MUb4_GlyGly_10plex − Scan number: 20641

Gene Names	Charge	m/z	Mass	Retention time	PEP	Score	Precursor Intensity
SLC3A2	3	782.7229	2345.147	74.64	8.6334e−11	108.71	1926036

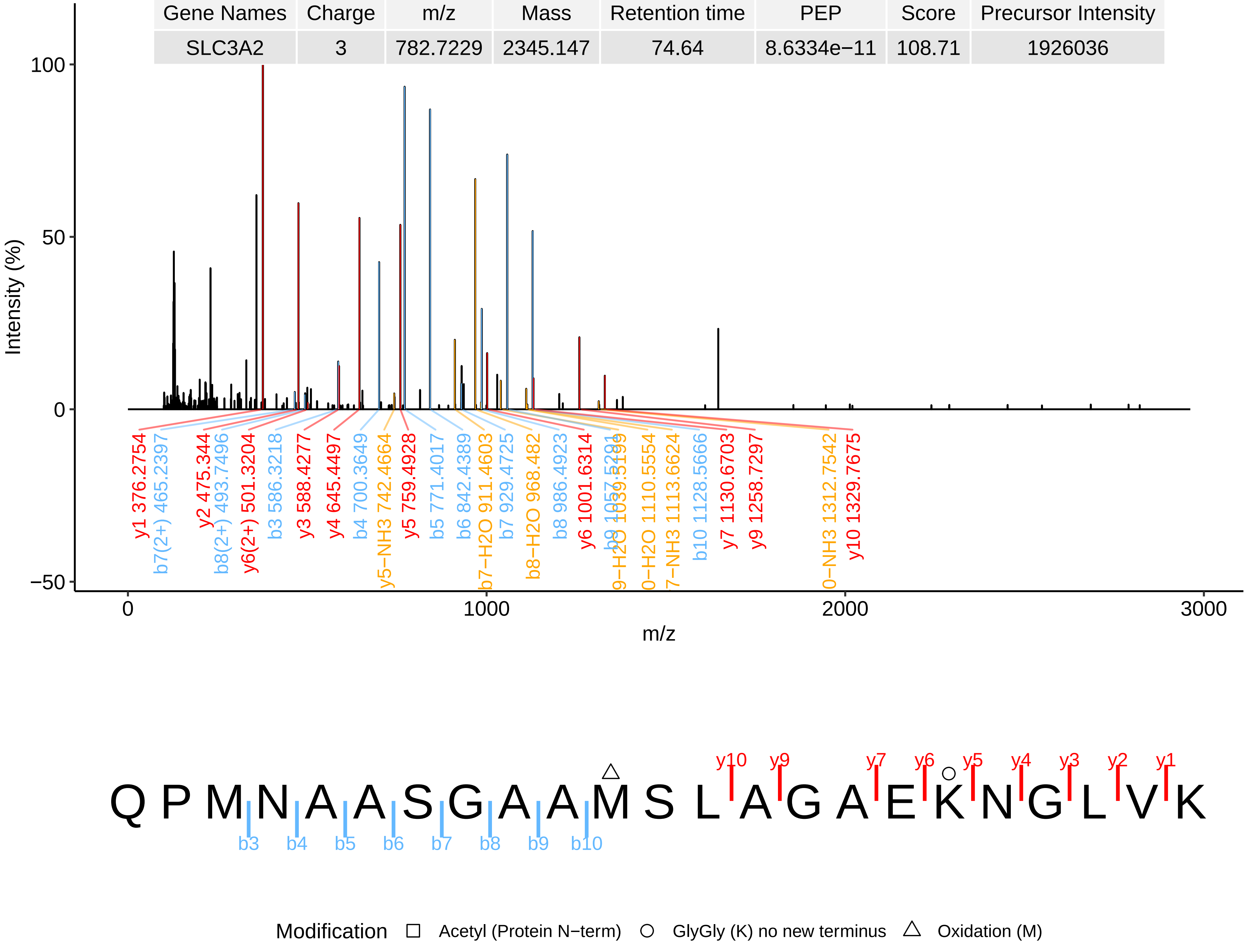

20200921_KIZ_MUb4_GlyGly_10plex_2 − Scan number: 18165

Gene Names	Charge	m/z	Mass	Retention time	PEP	Score	Precursor Intensity
SLC3A2	3	782.7229	2345.147	71.104	1.101e−10	106.86	848988.8

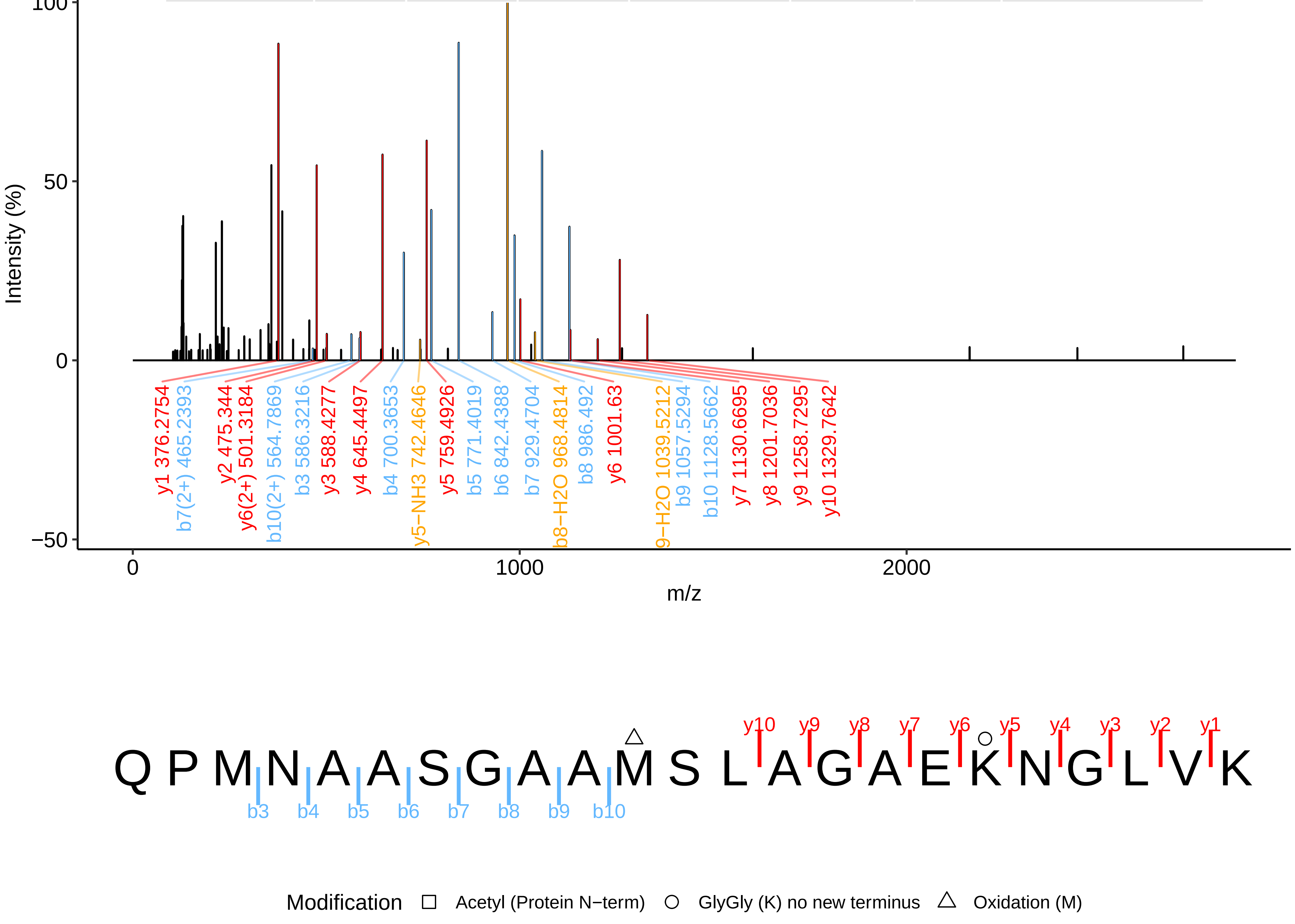

20200921_KIZ_MUb4_GlyGly_10plex_2 − Scan number: 18179

Gene Names	Charge	m/z	Mass	Retention time	PEP	Score	Precursor Intensity
SLC3A2	3	782.7229	2345.147	71.159	5.0183e−07	88.036	1466770

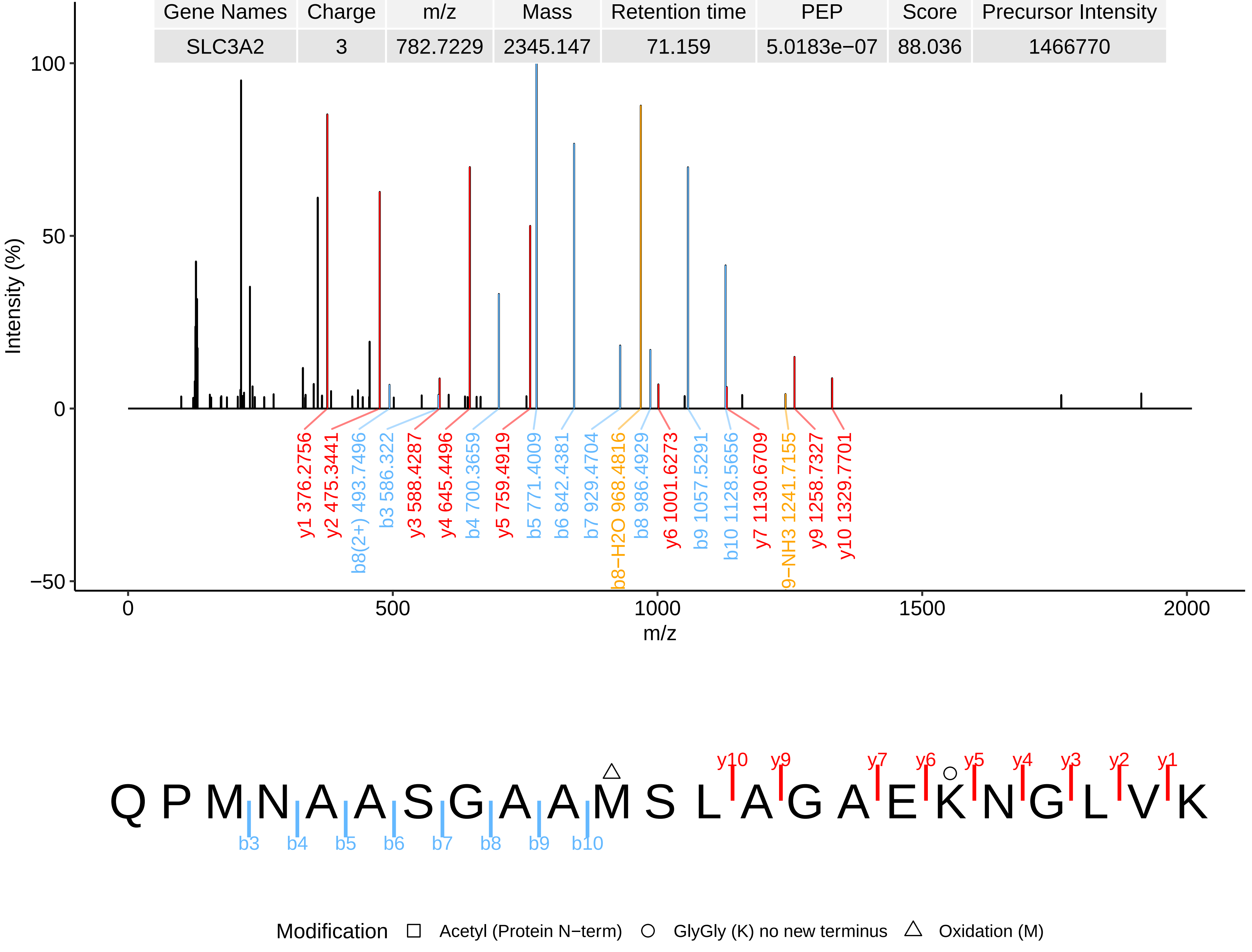

20200921_KIZ_MUb4_GlyGly_10plex_2 − Scan number: 17196

Gene Names	Charge	m/z	Mass	Retention time	PEP	Score	Precursor Intensity
SLC3A2	3	782.7229	2345.147	67.212	0.001022	62.684	740232.9

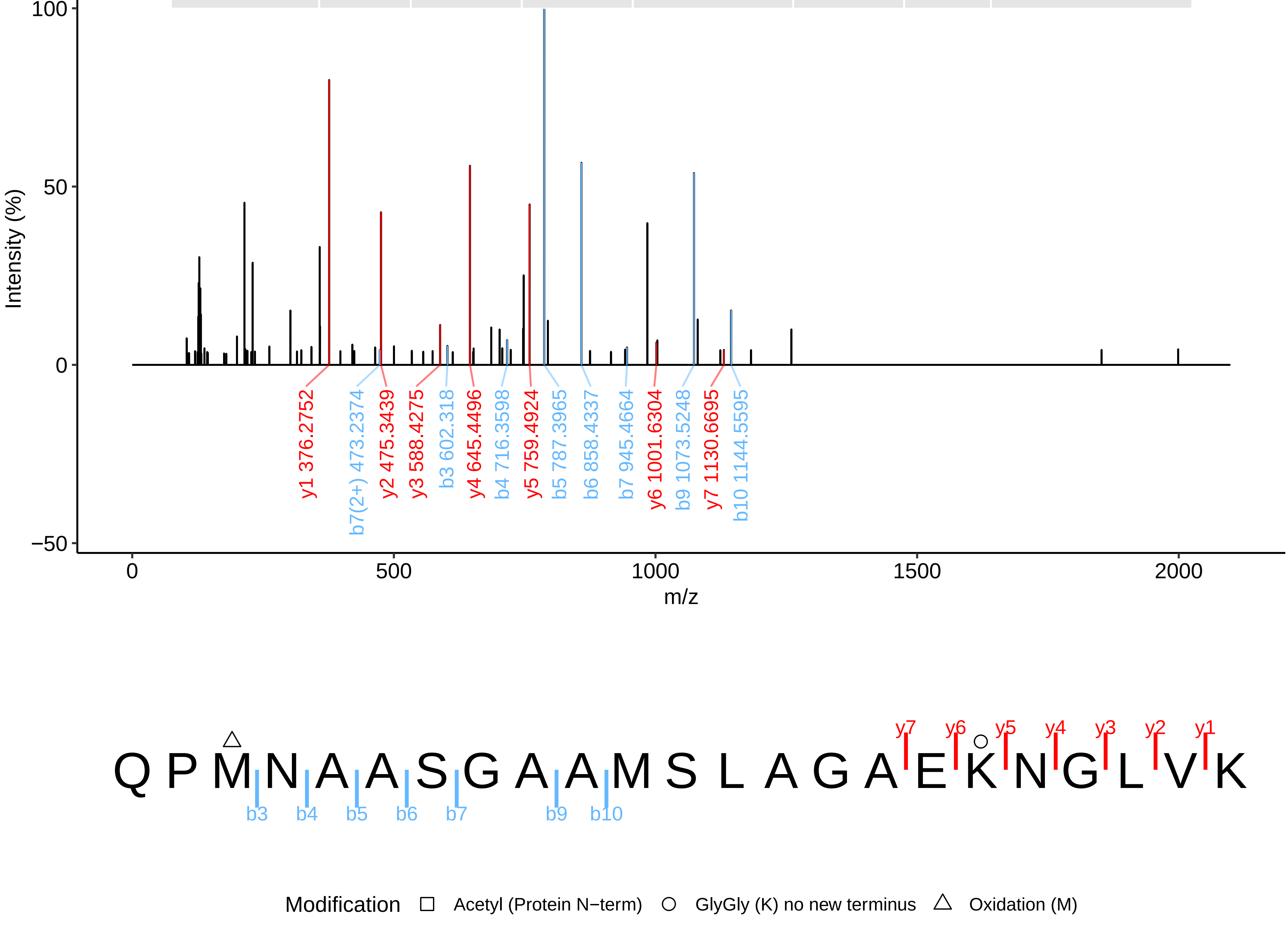

20200921_KIZ_MUb4_GlyGly_10plex − Scan number: 22996

Gene Names	Charge	m/z	Mass	Retention time	PEP	Score	Precursor Intensity
SLC3A2	3	777.3912	2329.152	83.44	1.1176e−40	168.78	13629504

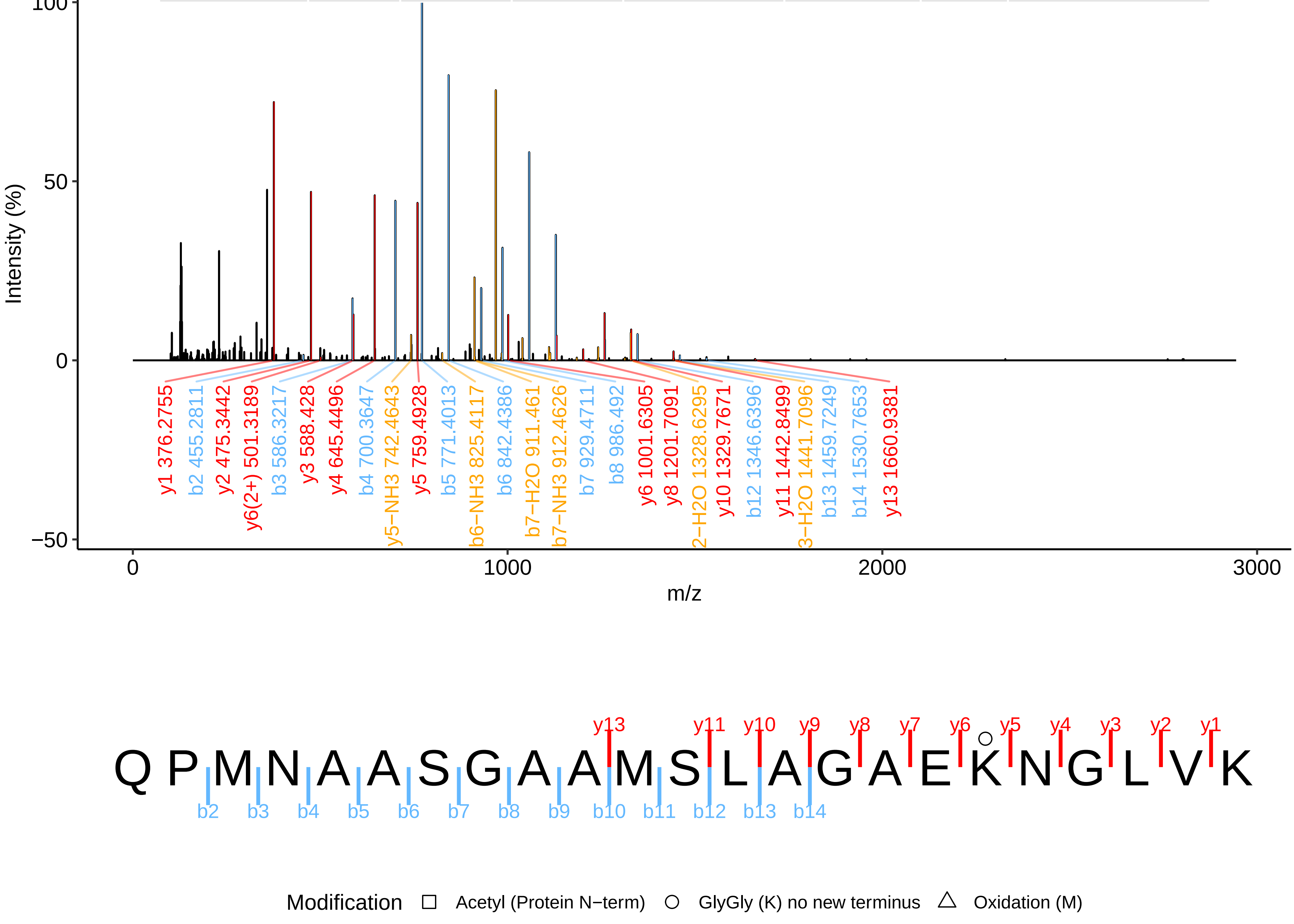

20200921_KIZ_MUb4_GlyGly_10plex − Scan number: 23026

Gene Names	Charge	m/z	Mass	Retention time	PEP	Score	Precursor Intensity
SLC3A2	4	583.2952	2329.152	83.557	1.8338e−10	101.6	NA

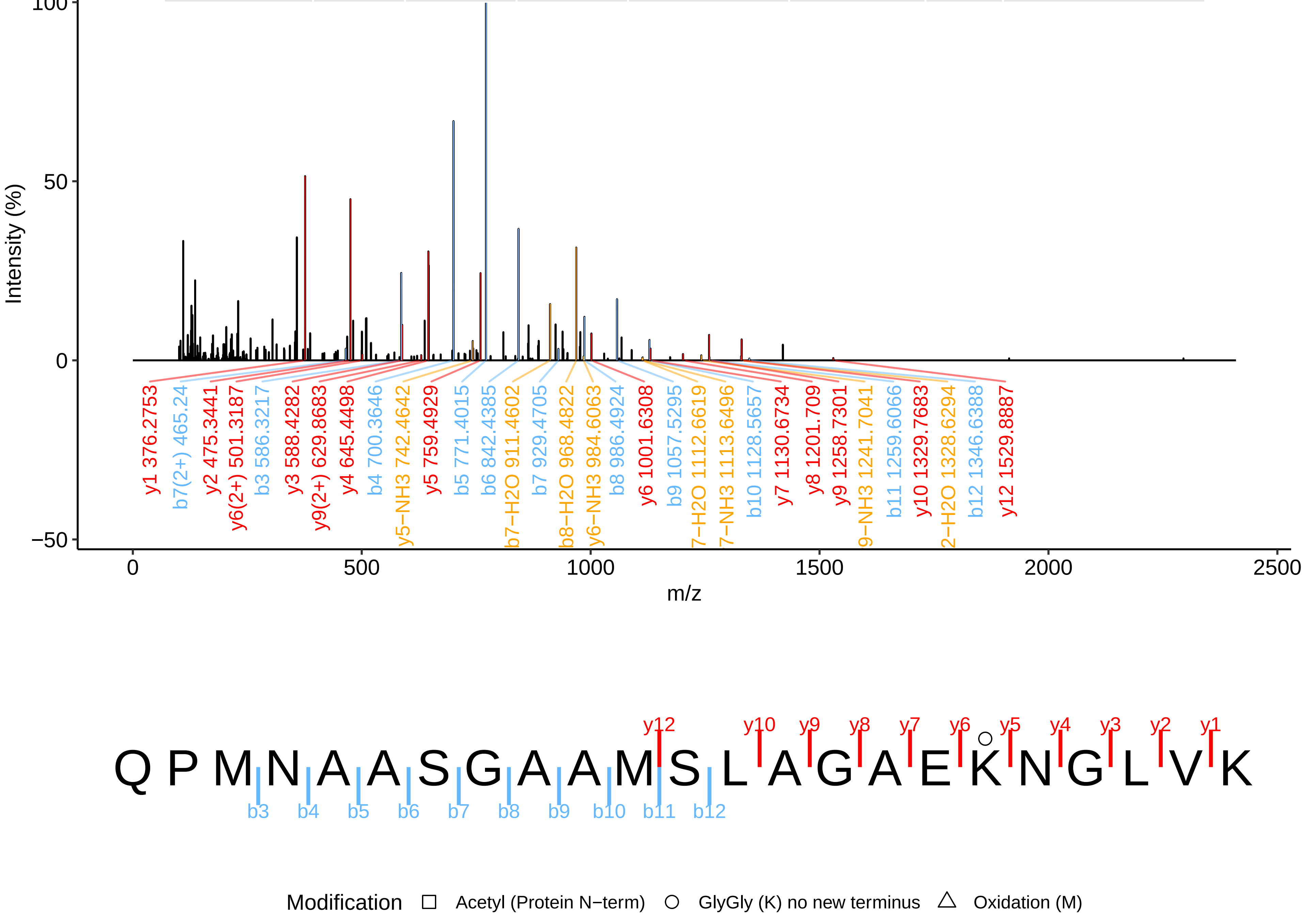

20200921_KIZ_MUb4_GlyGly_10plex − Scan number: 22853

Gene Names	Charge	m/z	Mass	Retention time	PEP	Score	Precursor Intensity
SLC3A2	3	777.3912	2329.152	82.941	1.4521e−53	191.65	6514320

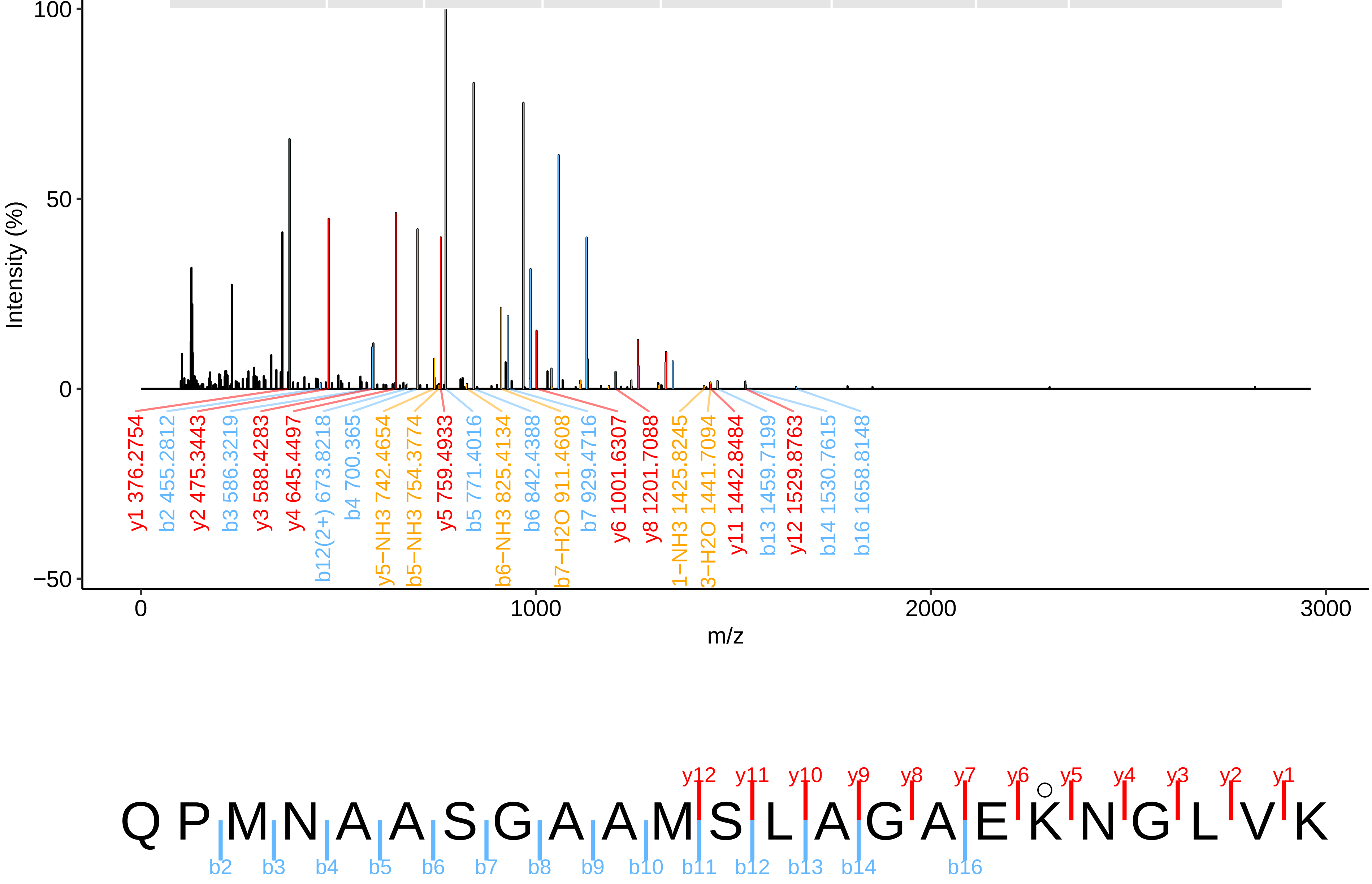

Modification □ Acetyl (Protein N−term) ○ GlyGly (K) no new terminus △ Oxidation (M)

20200921_KIZ_MUb4_GlyGly_10plex_2 − Scan number: 19888

Gene Names	Charge	m/z	Mass	Retention time	PEP	Score	Precursor Intensity
SLC3A2	3	777.3912	2329.152	78.12	0.0007326	66.045	1099275

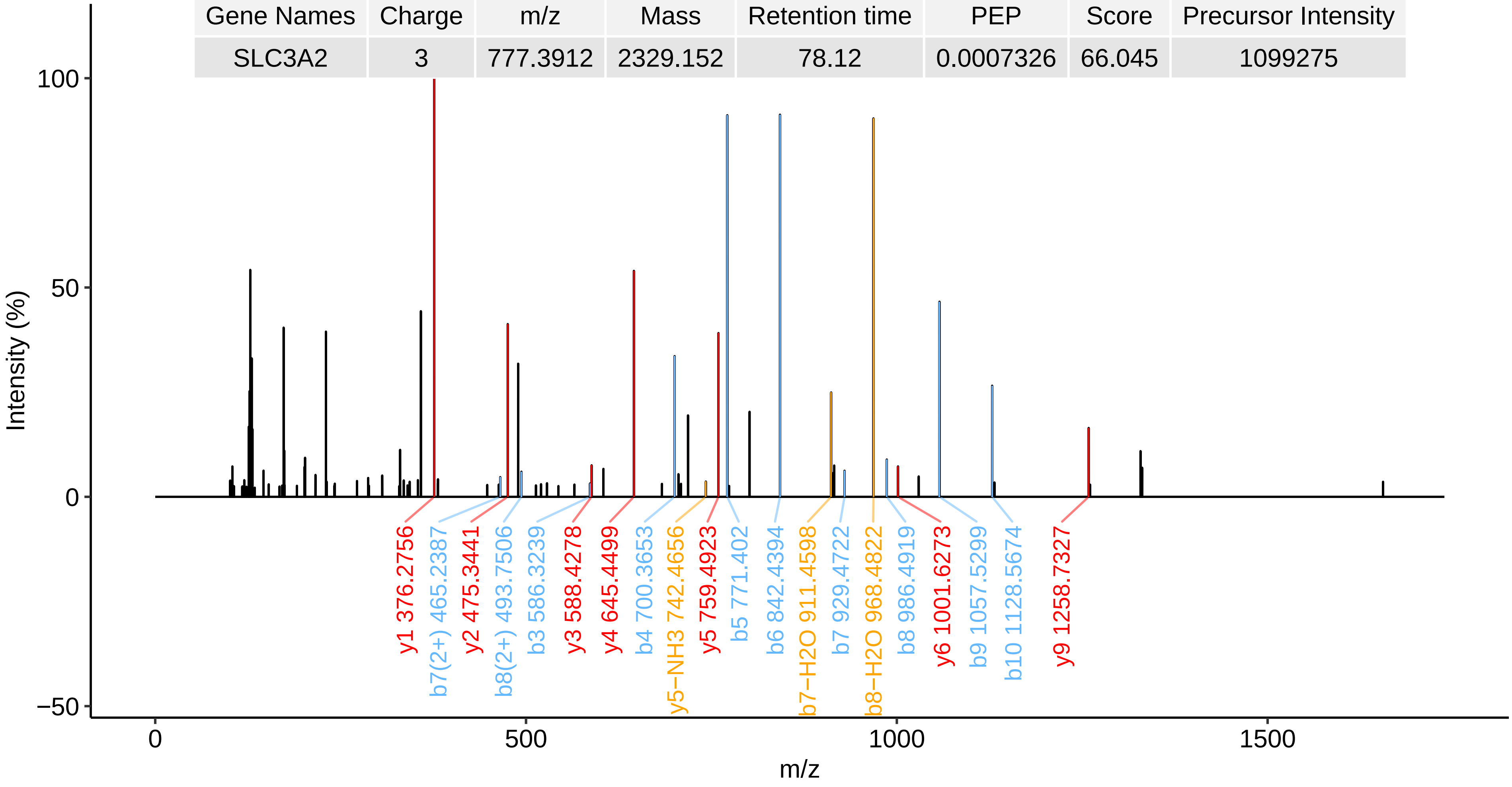

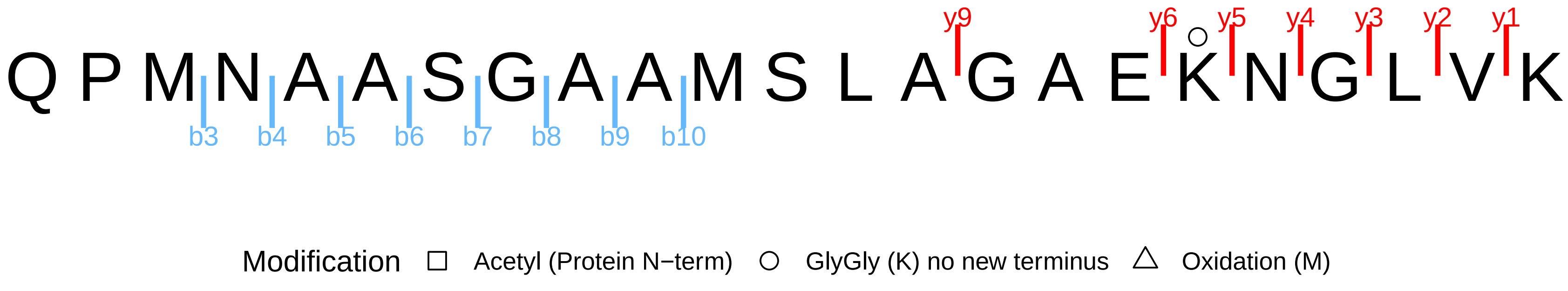

20200921_KIZ_MUb4_GlyGly_10plex_2 − Scan number: 19916

Gene Names	Charge	m/z	Mass	Retention time	PEP	Score	Precursor Intensity
SLC3A2	4	583.2952	2329.152	78.23	2.0434e−15	115.42	1570544

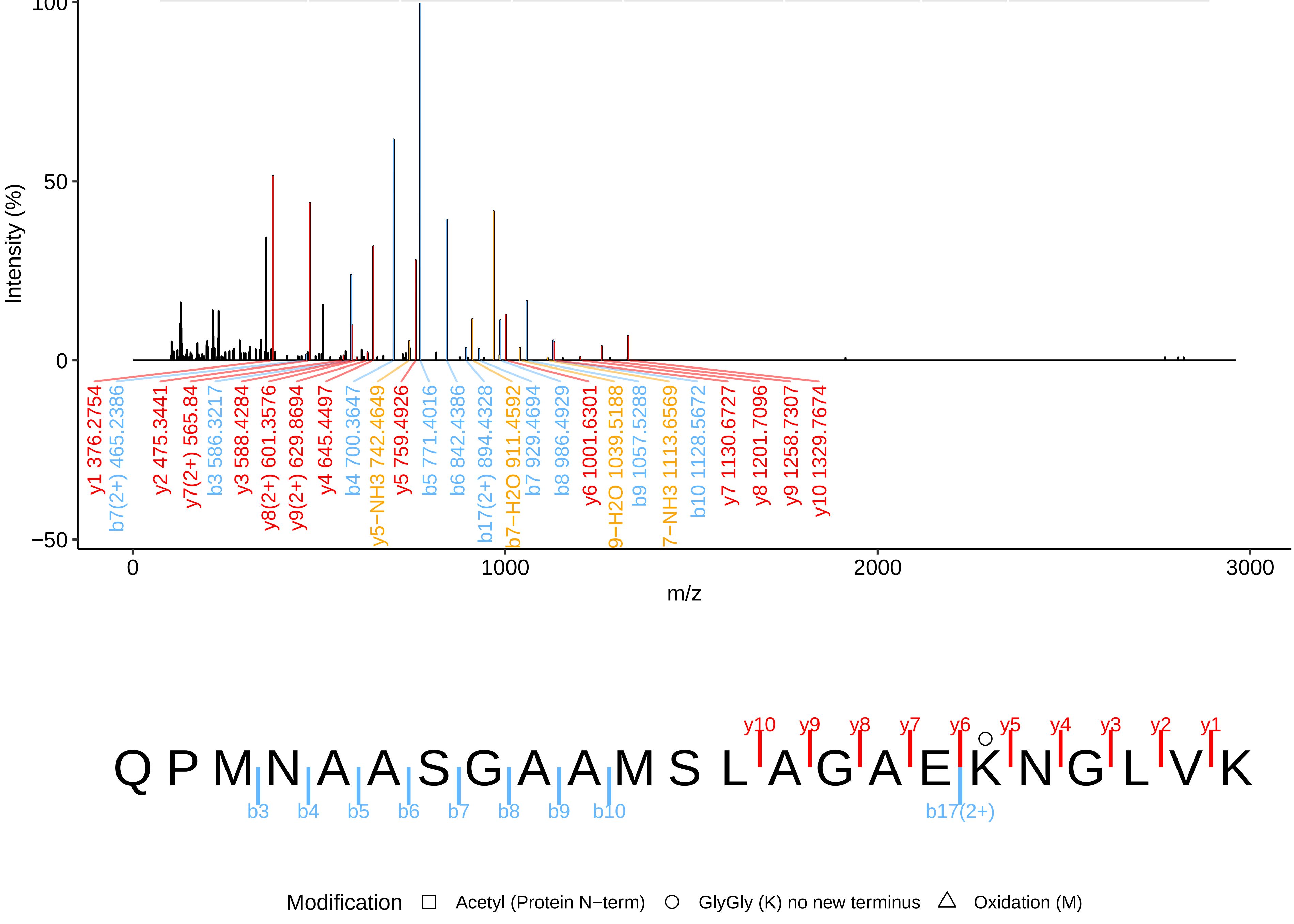

20200921_KIZ_MUb4_GlyGly_10plex_2 − Scan number: 19950

Gene Names	Charge	m/z	Mass	Retention time	PEP	Score	Precursor Intensity
SLC3A2	4	583.2952	2329.152	78.365	0.00011436	77.847	NA

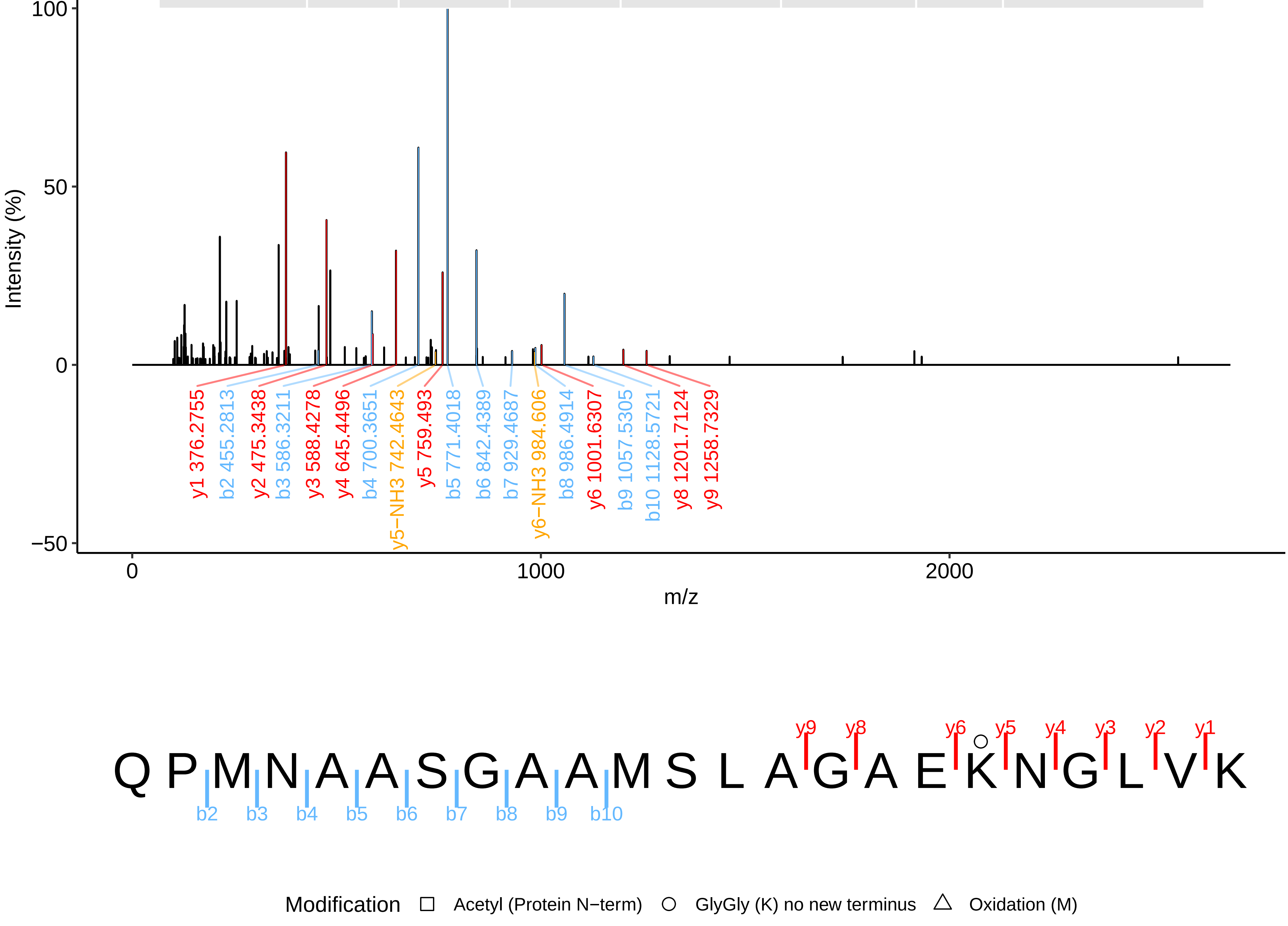

20200921_KIZ_MUb4_GlyGly_10plex_2 − Scan number: 15595

Gene Names	Charge	m/z	Mass	Retention time	PEP	Score	Precursor Intensity
SLC3A2	3	788.0545	2361.142	61.019	0.067101	40.81	754648.9

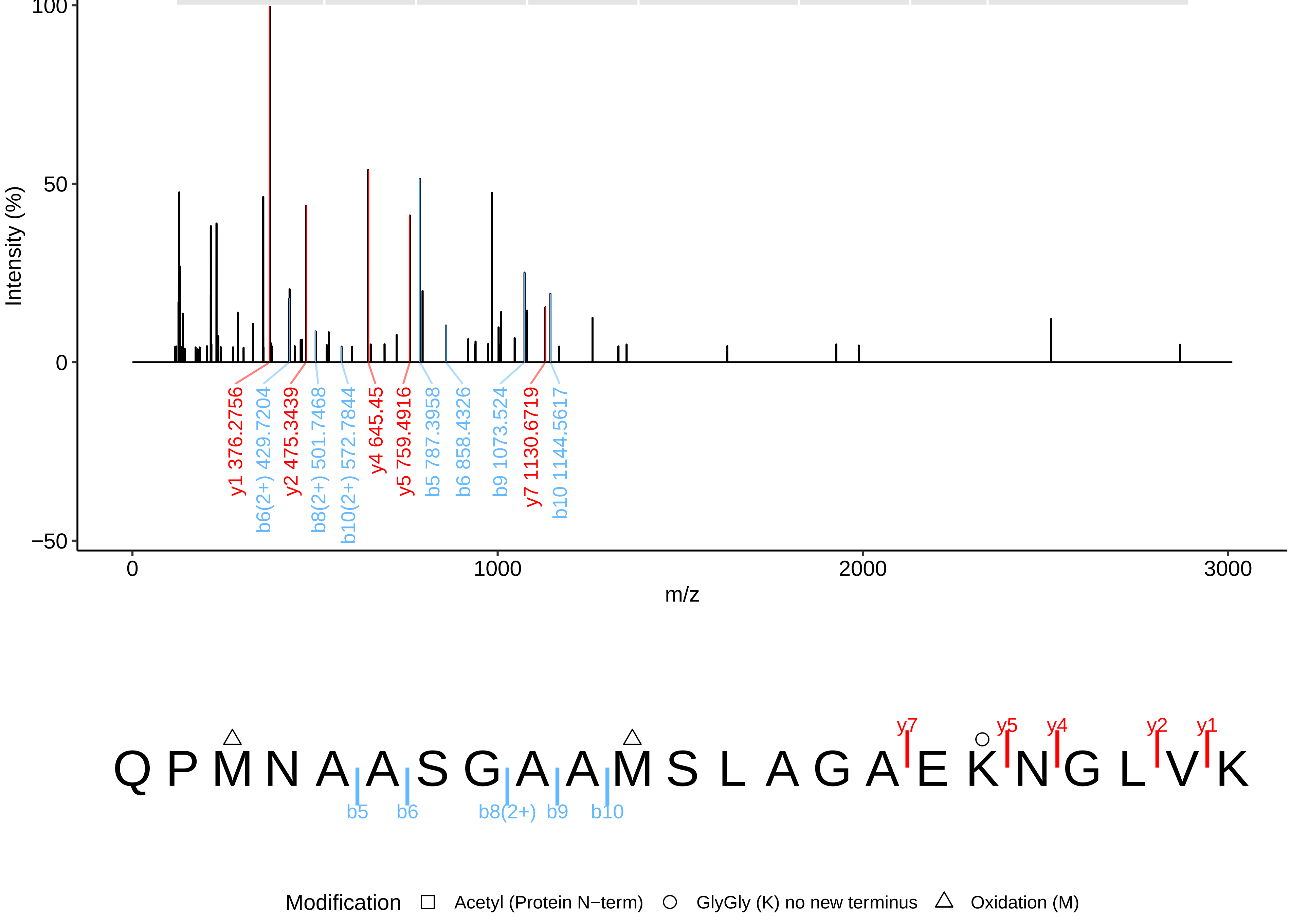

20200921_KIZ_MUb4_GlyGly_10plex − Scan number: 21746

Gene Names	Charge	m/z	Mass	Retention time	PEP	Score	Precursor Intensity
VIM	3	831.0746	2490.202	78.812	0.021046	57.142	2509969

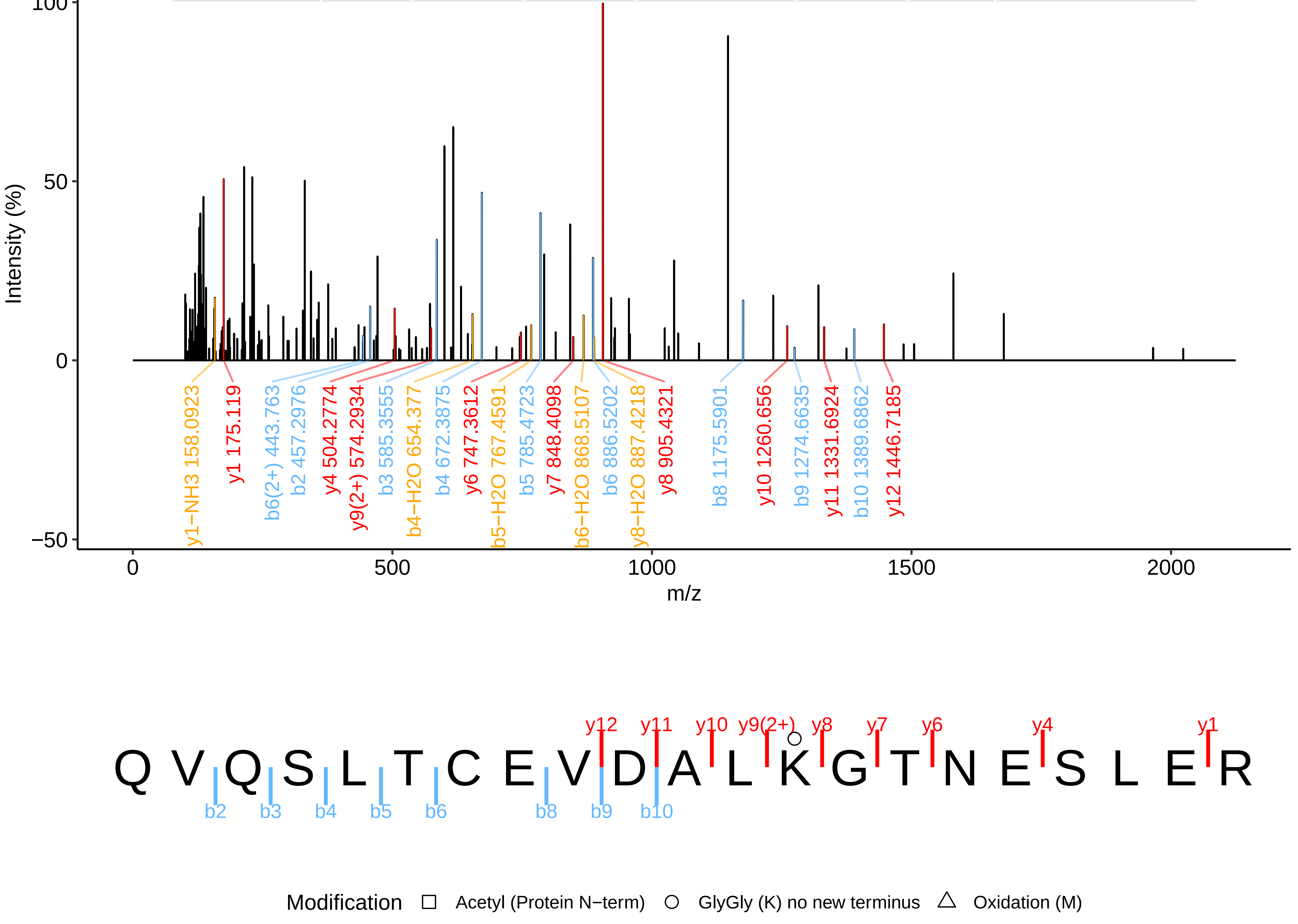

20201118_KIZ_MUb6_GlyGly_2 − Scan number: 24196

Gene Names	Charge	m/z	Mass	Retention time	PEP	Score	Precursor Intensity
SLC3A2	3	840.0549	2517.143	81.07	1.1496e−06	60.564	NA

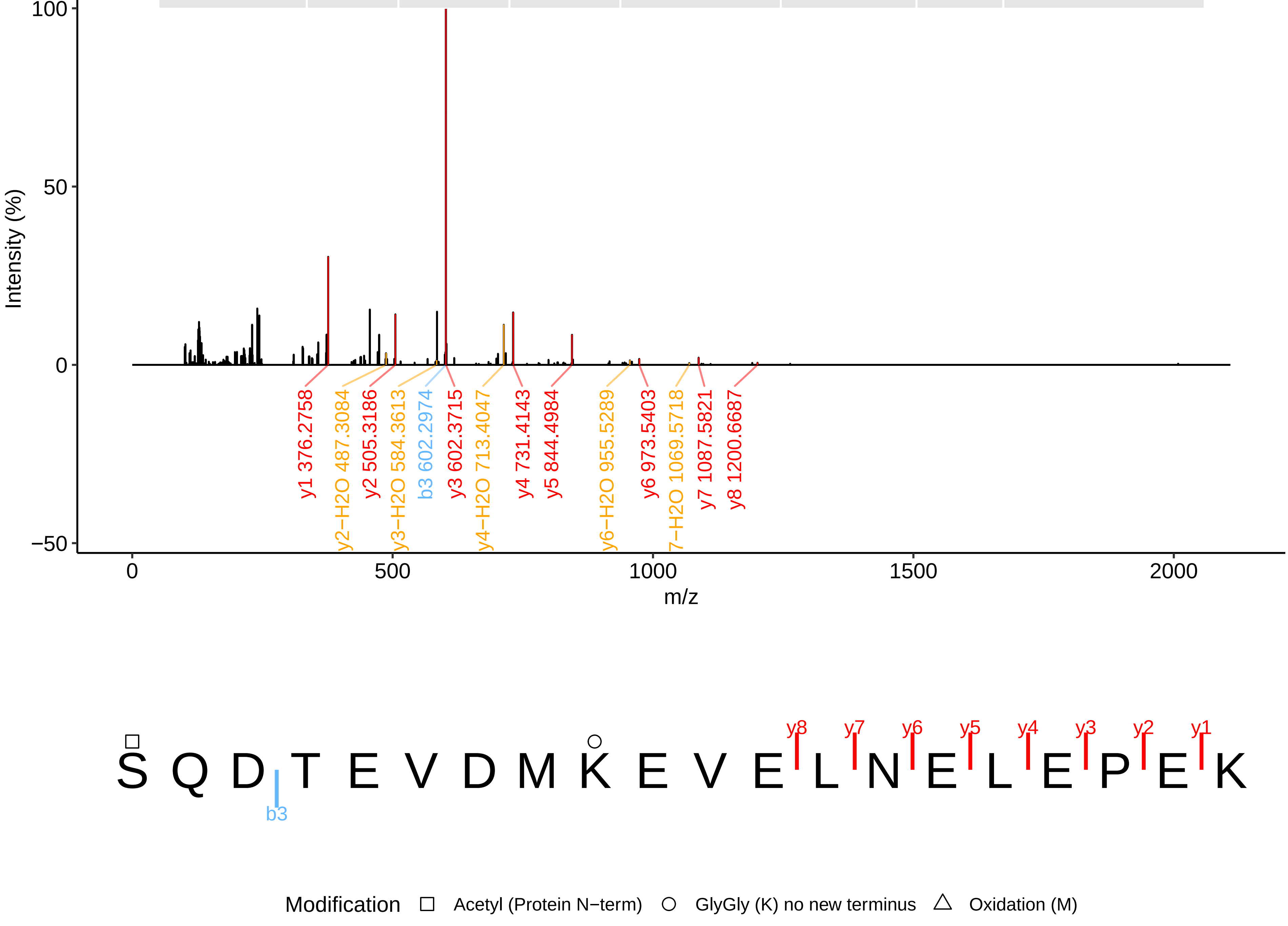

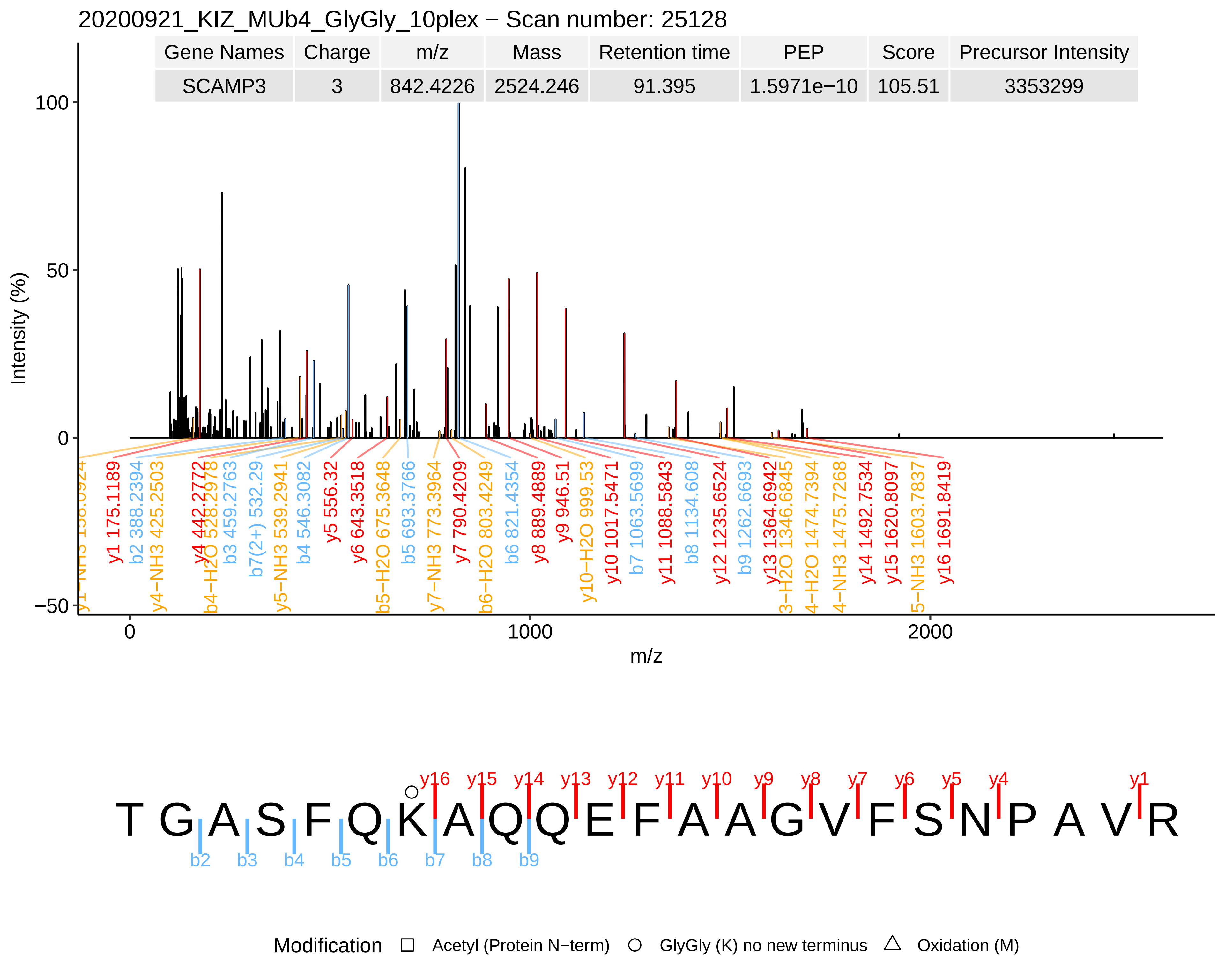

20200921_KIZ_MUb4_GlyGly_10plex − Scan number: 25128
Gene Names | Charge | m/z | Mass | Retention time | PEP | Score | Precursor Intensity
SCAMP3 | 3 | 842.4226 | 2524.246 | 91.395 | 1.5971e−10 | 105.51 | 3353299
Intensity (%)
100
50
0
−50
m/z
0
1000
2000
y1 175.1189
b2 388.2394
y4−NH3 425.2503
y4 442.2772
b4−H2O 528.2978
b3 459.2763
b7(2+) 532.29
y5−NH3 539.2941
b4 546.3082
y5 556.32
y6 643.3518
b5−H2O 675.3648
b5 693.3766
y7−NH3 773.3964
y7 790.4209
b6−H2O 803.4249
b6 821.4354
y8 889.4889
y9 946.51
y10−H2O 999.53
y10 1017.5471
b7 1063.5699
y11 1088.5843
b8 1134.608
y12 1235.6524
b9 1262.6693
y13 1364.6942
y14 1492.7534
y15 1620.8097
y16 1691.8419
T G A S F Q K A Q Q E F A A G V F S N P A V R
y16 y15 y14 y13 y12 y11 y10 y9 y8 y7 y6 y5 y4 y1
b2 b3 b4 b5 b6 b7 b8 b9
Modification
Acetyl (Protein N−term)
GlyGly (K) no new terminus
Oxidation (M)

20200921_KIZ_MUb4_GlyGly_10plex − Scan number: 25154

Gene Names	Charge	m/z	Mass	Retention time	PEP	Score	Precursor Intensity
SCAMP3	3	842.4226	2524.246	91.494	1.2013e−15	124.4	NA

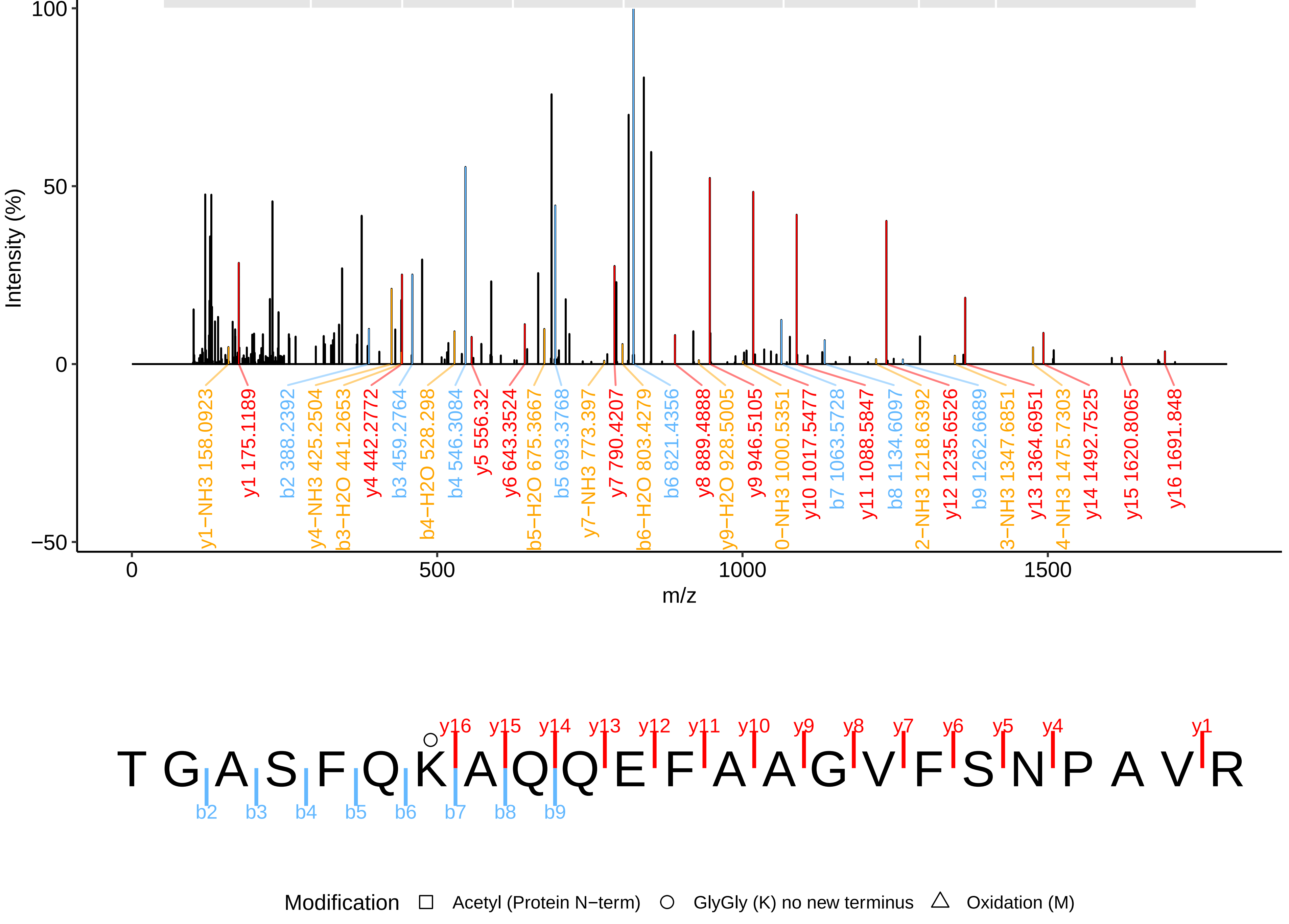

20200921_KIZ_MUb4_GlyGly_10plex_2 − Scan number: 21614

Gene Names	Charge	m/z	Mass	Retention time	PEP	Score	Precursor Intensity
SCAMP3	3	842.4226	2524.246	85.115	0.00048747	70.917	562789.6

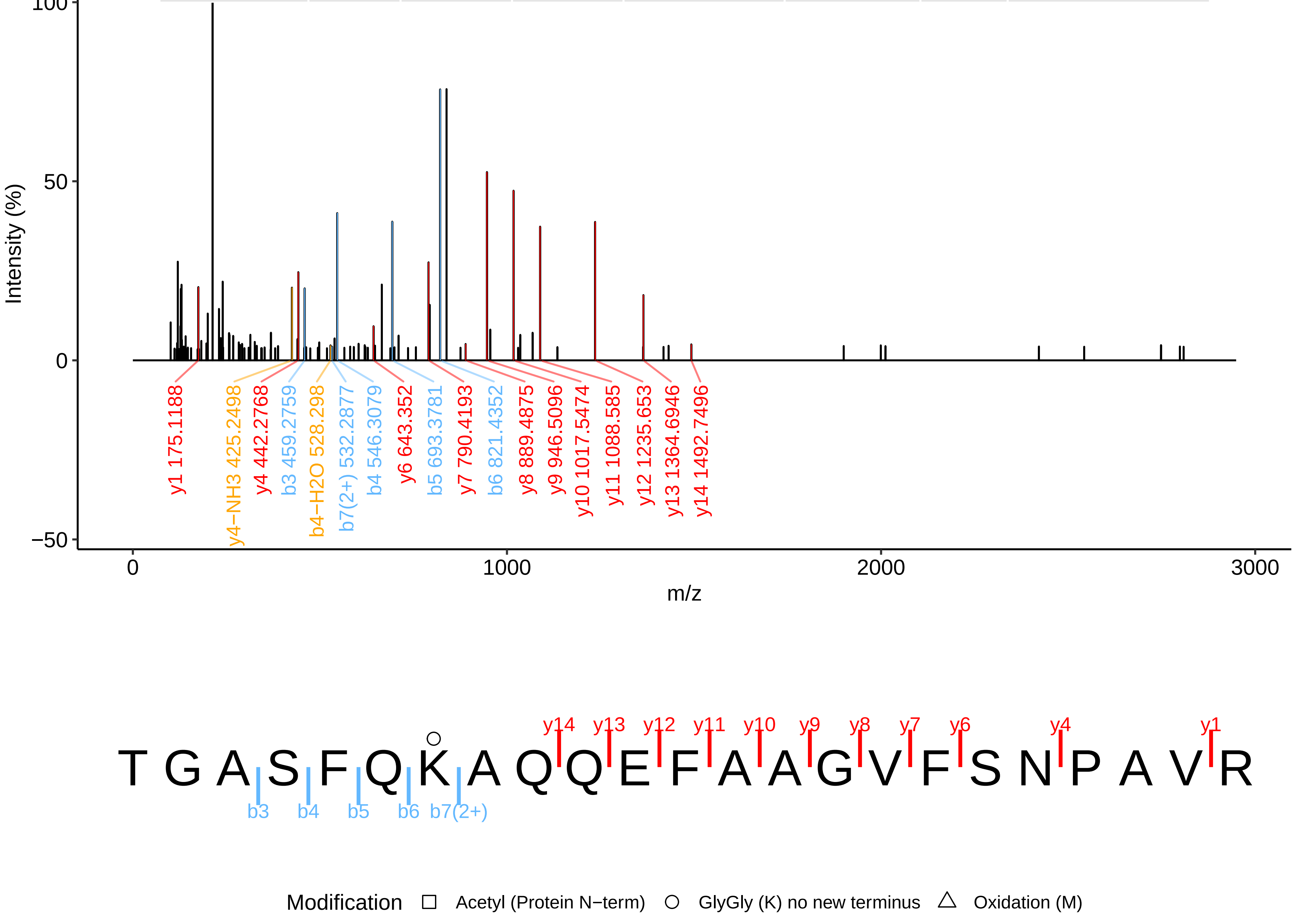

20200921_KIZ_MUb4_GlyGly_10plex − Scan number: 24528

Gene Names	Charge	m/z	Mass	Retention time	PEP	Score	Precursor Intensity
ITGB1	3	778.3972	2332.17	89.2	1.1057e−05	85.087	4348985

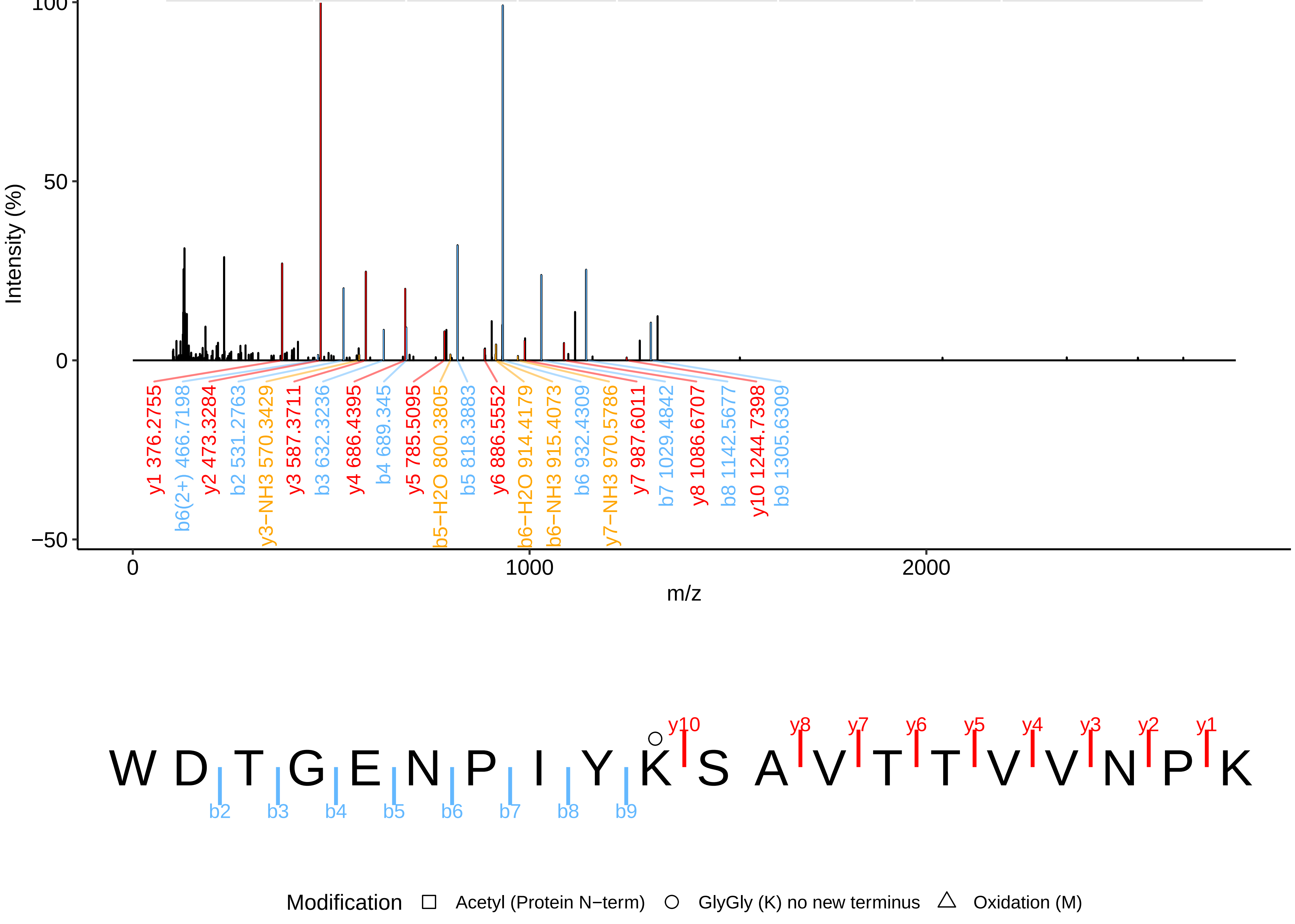

20200921_KIZ_MUb4_GlyGly_10plex_2 − Scan number: 21576

Gene Names	Charge	m/z	Mass	Retention time	PEP	Score	Precursor Intensity
ITGB1	3	778.3972	2332.17	84.96	3.0977e−09	101.92	2105537

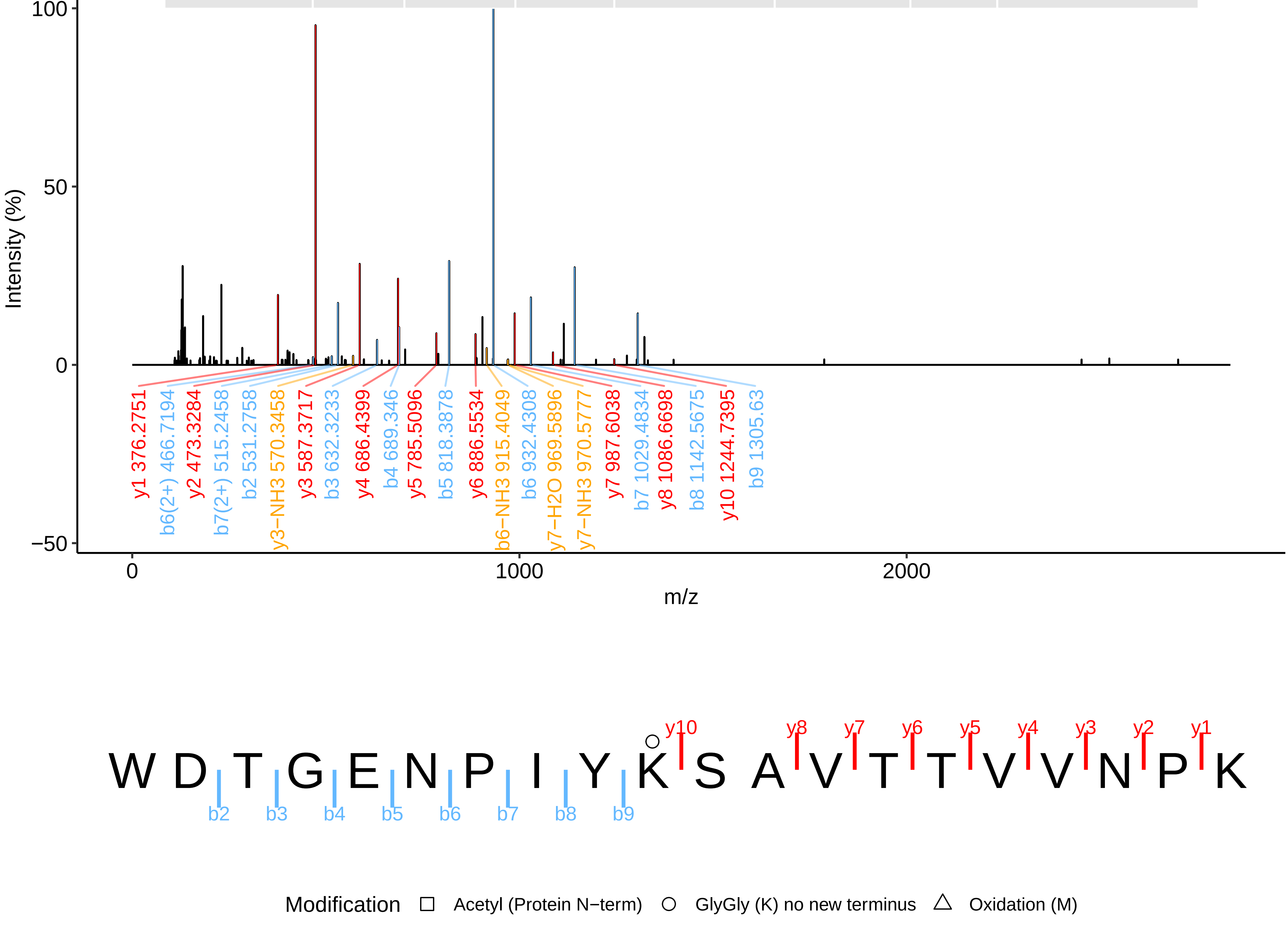

20200921_KIZ_MUb4_GlyGly_10plex_2 − Scan number: 6924

Gene Names	Charge	m/z	Mass	Retention time	PEP	Score	Precursor Intensity
SLC29A1	4	622.2993	2485.168	25.651	1.6129e−10	103.26	1319764

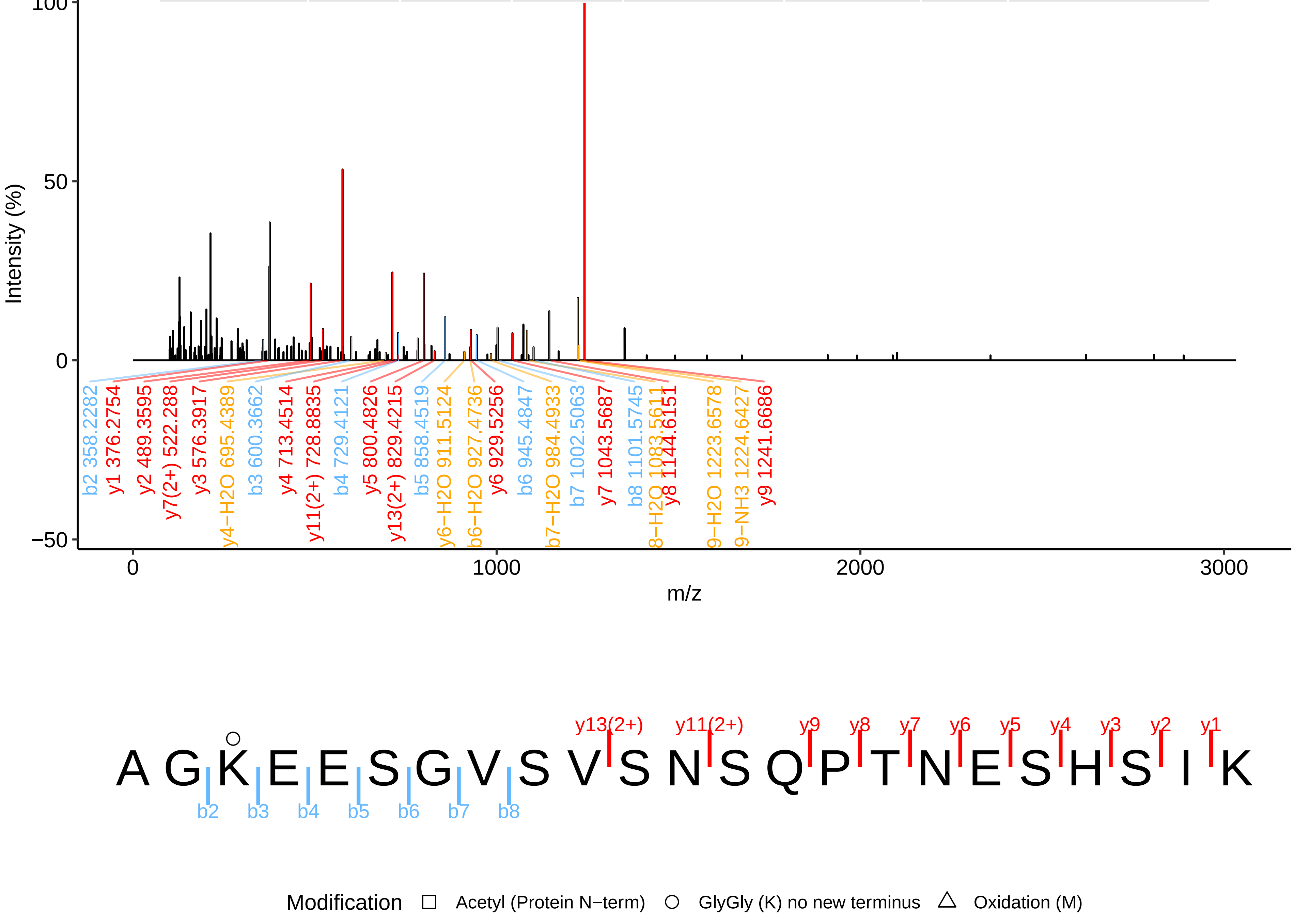

20200921_KIZ_MUb4_GlyGly_10plex − Scan number: 28268

Gene Names	Charge	m/z	Mass	Retention time	PEP	Score	Precursor Intensity
ALDOA	4	681.1098	2720.41	103.39	5.211e−31	153.36	2221951

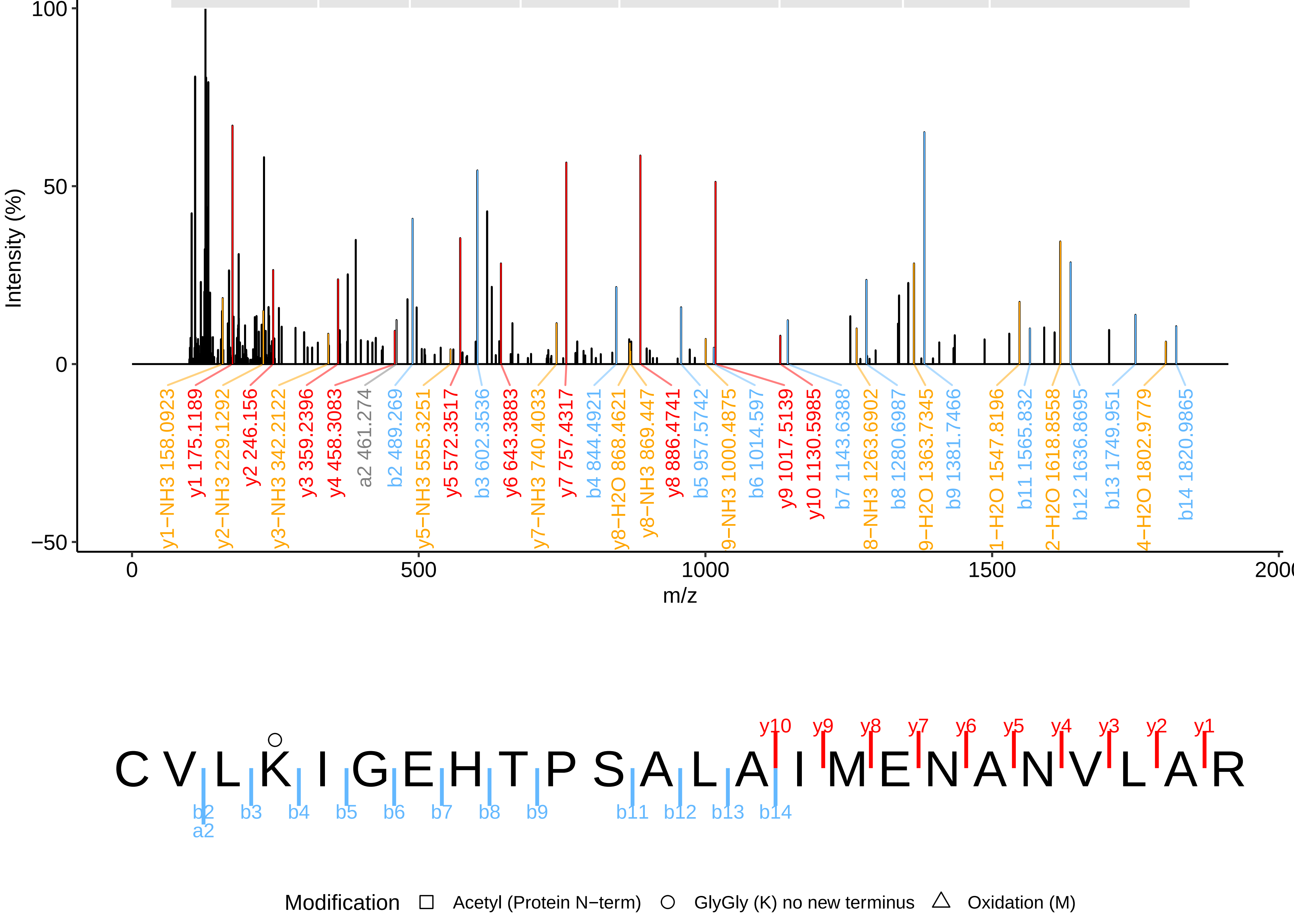

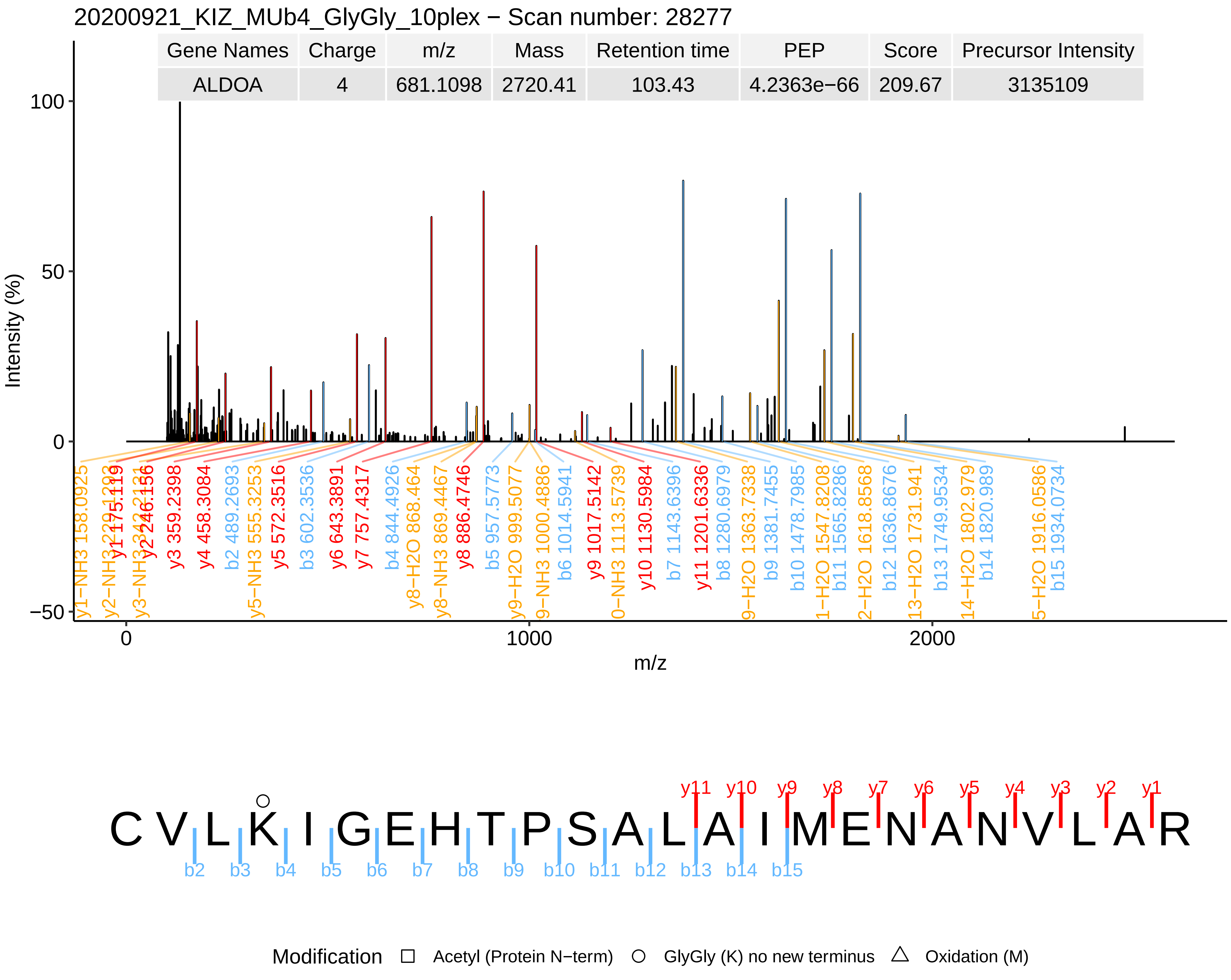

20200921_KIZ_MUb4_GlyGly_10plex − Scan number: 28277
Gene Names | Charge | m/z | Mass | Retention time | PEP | Score | Precursor Intensity
ALDOA | 4 | 681.1098 | 2720.41 | 103.43 | 4.2363e−66 | 209.67 | 3135109
Intensity (%)
100
50
0
−50
0
1000
2000
m/z
y1−NH3 158.0925
y2−NH3 229.1293
y1 175.119
y3−NH3 342.2131
y2 246.156
y3 359.2398
y4 458.3084
b2 489.2693
y5−NH3 555.3253
y5 572.3516
b3 602.3536
y6 643.3891
y7 757.4317
b4 844.4926
y8−H2O 868.464
y8−NH3 869.4467
y8 886.4746
b5 957.5773
y9−H2O 999.5077
9−NH3 1000.4886
b6 1014.5941
y9 1017.5142
0−NH3 1113.5739
y10 1130.5984
b7 1143.6396
y11 1201.6336
b8 1280.6979
9−H2O 1363.7338
b9 1381.7455
b10 1478.7985
1−H2O 1547.8208
b11 1565.8286
2−H2O 1618.8568
b12 1636.8676
13−H2O 1731.941
b13 1749.9534
14−H2O 1802.979
b14 1820.989
5−H2O 1916.0586
b15 1934.0734
C V L K I G E H T P S A L A I M E N A N V L A R
y11 y10 y9 y8 y7 y6 y5 y4 y3 y2 y1
b2 b3 b4 b5 b6 b7 b8 b9 b10 b11 b12 b13 b14 b15
Modification
Acetyl (Protein N−term)
GlyGly (K) no new terminus
Oxidation (M)

20200921_KIZ_MUb4_GlyGly_10plex_2 − Scan number: 12902

Gene Names	Charge	m/z	Mass	Retention time	PEP	Score	Precursor Intensity
SLC43A3	4	602.054	2404.187	50.029	0.033977	47.082	849631.7

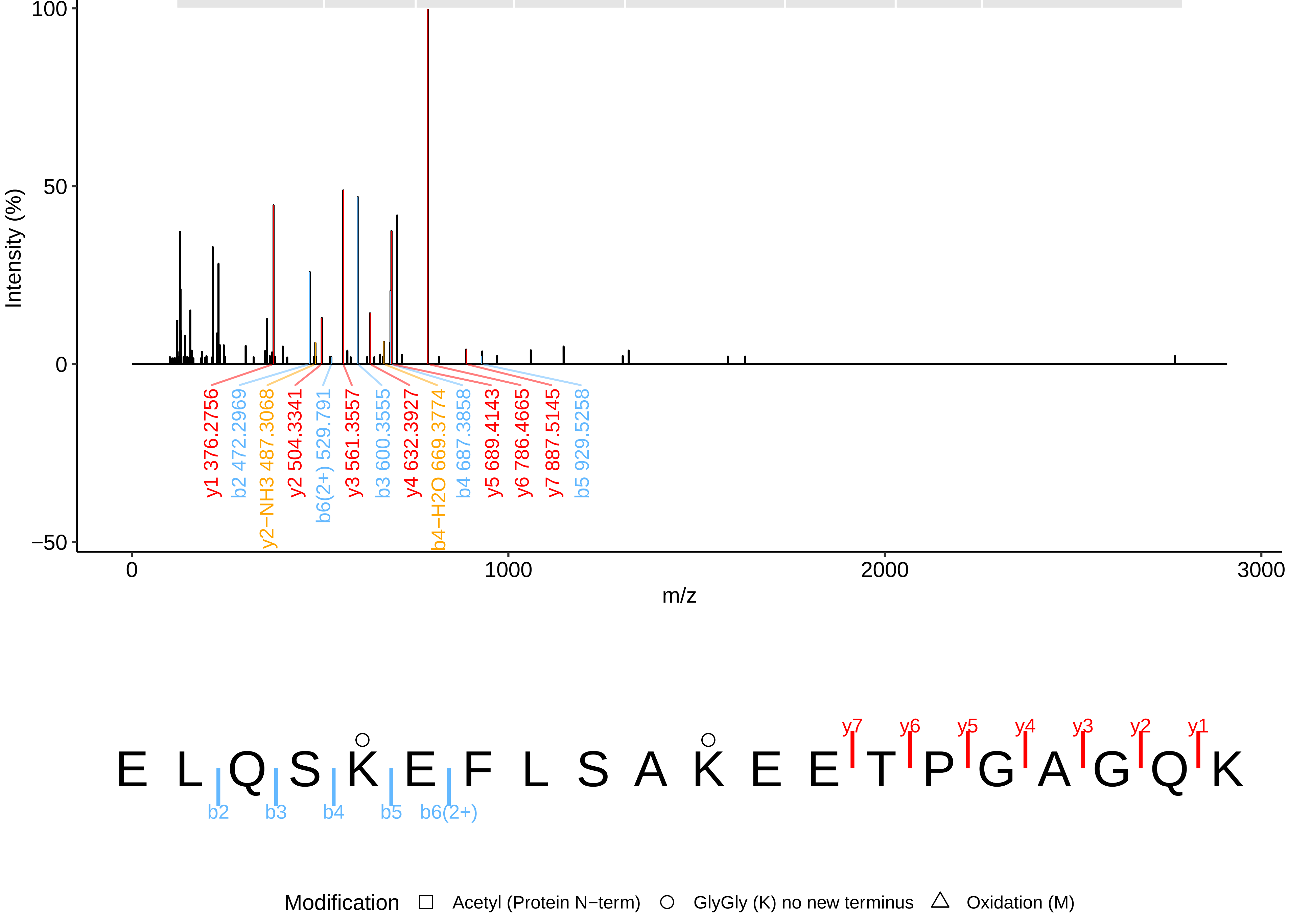

20200921_KIZ_MUb4_GlyGly_10plex_2 − Scan number: 14643

Gene Names	Charge	m/z	Mass	Retention time	PEP	Score	Precursor Intensity
SLC43A3	4	573.5433	2290.144	57.12	4.0517e−05	90.654	418801

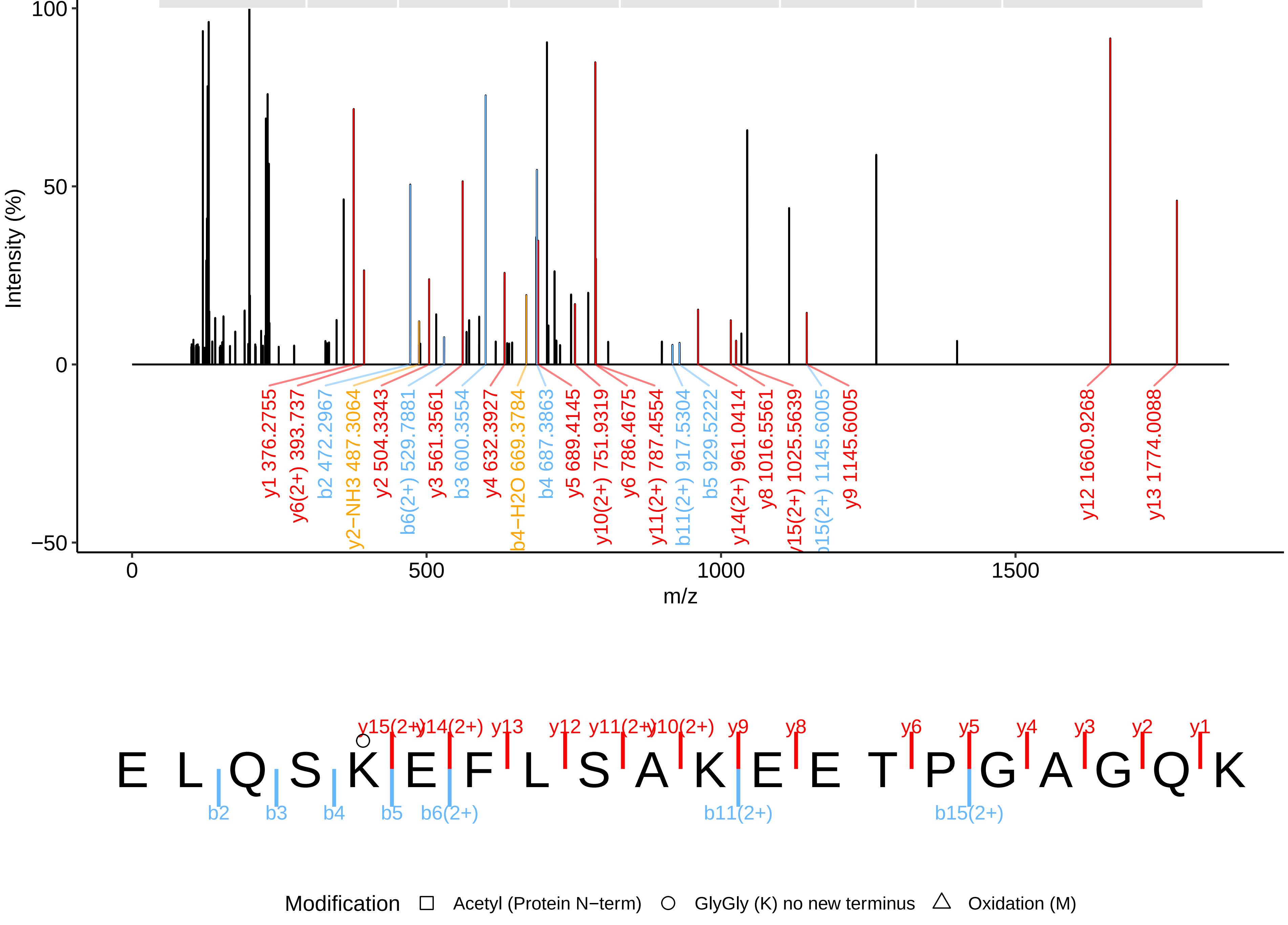

20200921_KIZ_MUb4_GlyGly_10plex_2 − Scan number: 20971

Gene Names	Charge	m/z	Mass	Retention time	PEP	Score	Precursor Intensity
EPN1	4	634.305	2533.191	82.506	0.0082644	66.874	353832.1

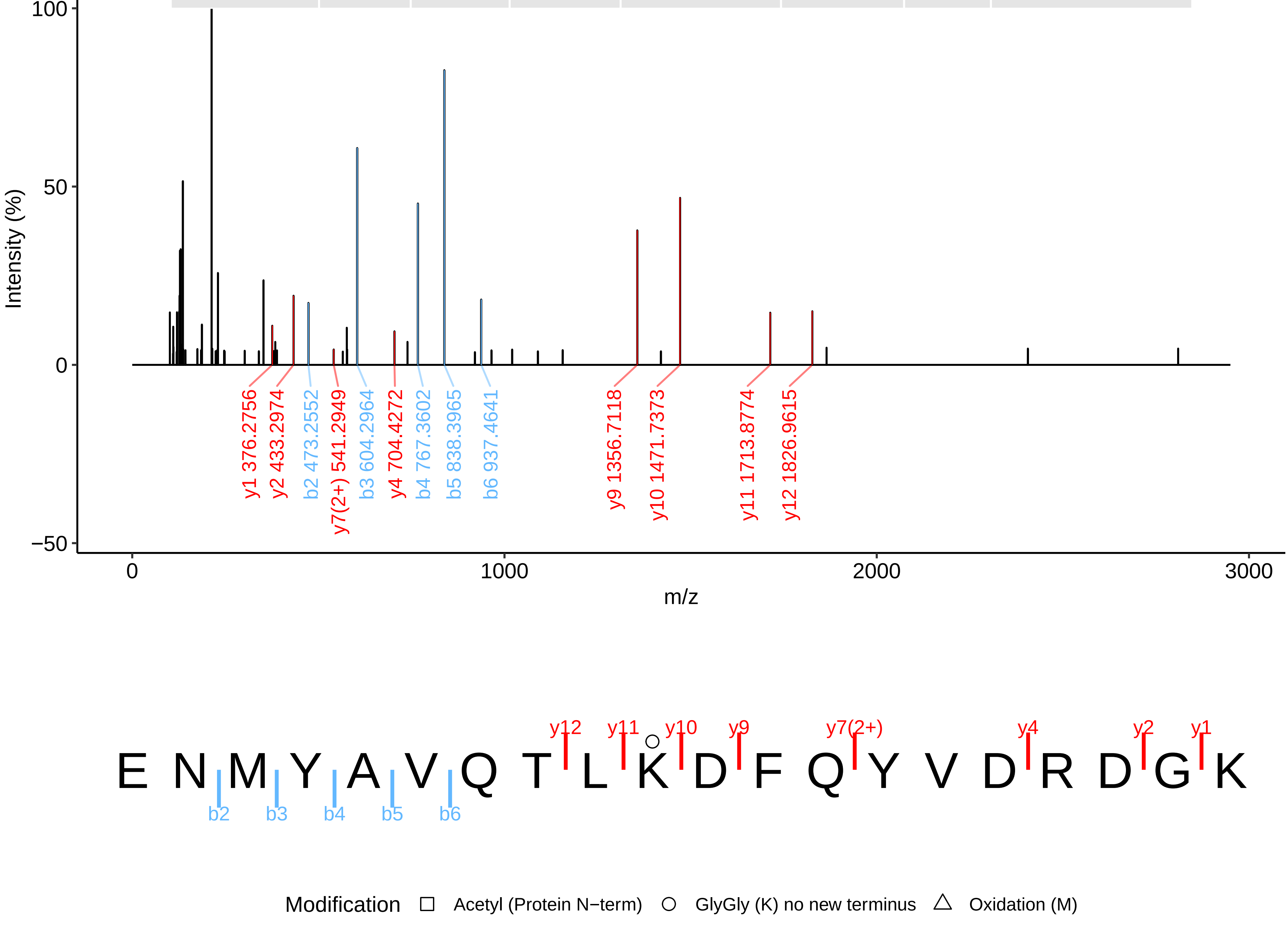

20200921_KIZ_MUb4_GlyGly_10plex − Scan number: 12379

Gene Names	Charge	m/z	Mass	Retention time	PEP	Score	Precursor Intensity
SLC16A1	4	576.7855	2303.113	43.378	6.5364e−21	144.72	6656828

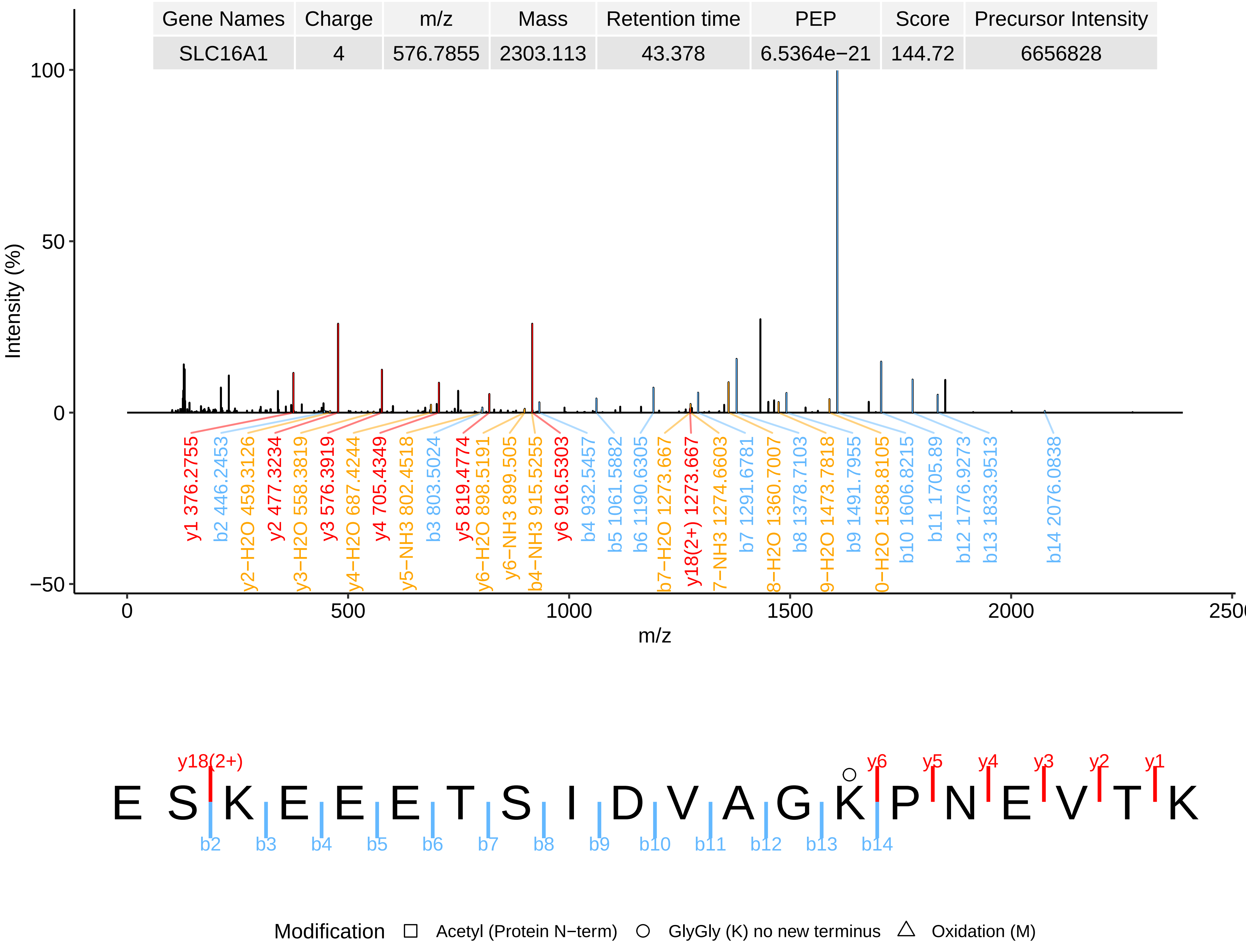

20200921_KIZ_MUb4_GlyGly_10plex − Scan number: 12666

Gene Names	Charge	m/z	Mass	Retention time	PEP	Score	Precursor Intensity
SLC16A1	4	576.7855	2303.113	44.495	1.2895e−13	117.48	4942915

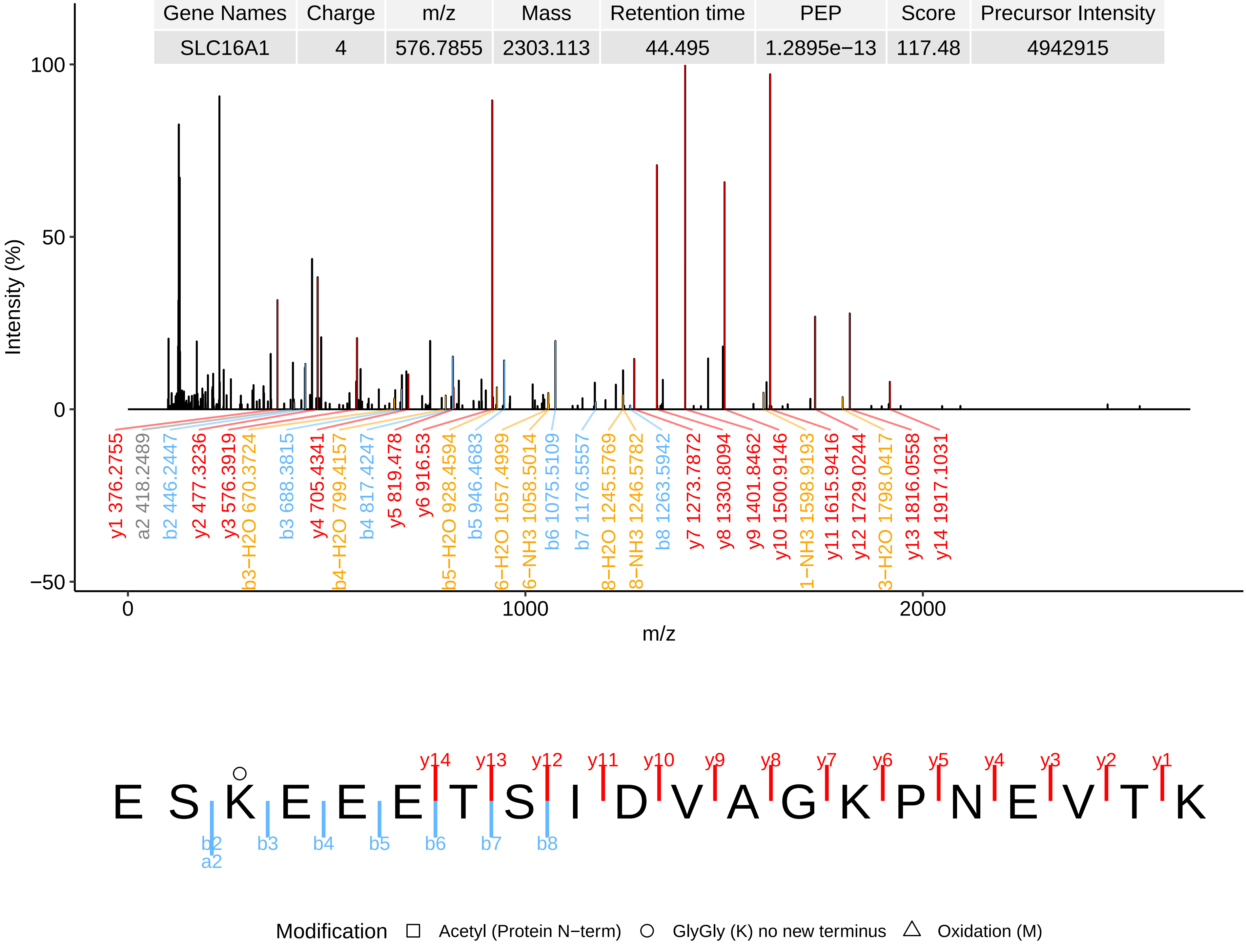

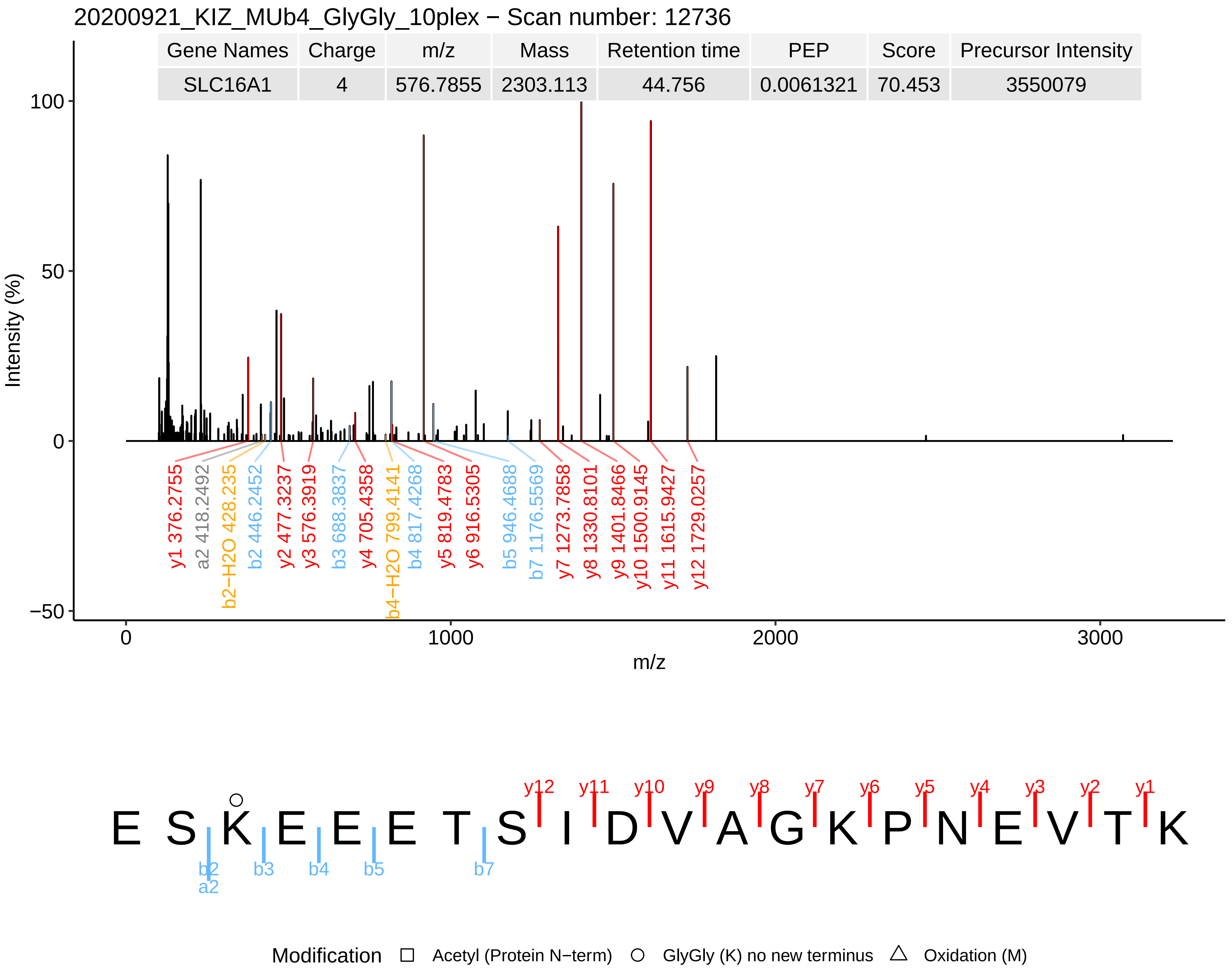

20200921_KIZ_MUb4_GlyGly_10plex − Scan number: 12736
Gene Names | Charge | m/z | Mass | Retention time | PEP | Score | Precursor Intensity
SLC16A1 | 4 | 576.7855 | 2303.113 | 44.756 | 0.0061321 | 70.453 | 3550079
Intensity (%)
m/z
y1 376.2755
a2 418.2492
b2−H2O 428.235
b2 446.2452
y2 477.3237
y3 576.3919
b3 688.3837
y4 705.4358
b4−H2O 799.4141
b4 817.4268
y5 819.4783
y6 916.5305
b5 946.4688
b7 1176.5569
y7 1273.7858
y8 1330.8101
y9 1401.8466
y10 1500.9145
y11 1615.9427
y12 1729.0257
E S K E E E T S I D V A G K P N E V T K
y12 y11 y10 y9 y8 y7 y6 y5 y4 y3 y2 y1
b2 a2 b3 b4 b5 b7
Modification
Acetyl (Protein N−term)
GlyGly (K) no new terminus
Oxidation (M)

20200921_KIZ_MUb4_GlyGly_10plex_2 − Scan number: 11362

Gene Names	Charge	m/z	Mass	Retention time	PEP	Score	Precursor Intensity
SLC16A1	4	576.7855	2303.113	43.718	9.953e−27	157.79	3472516

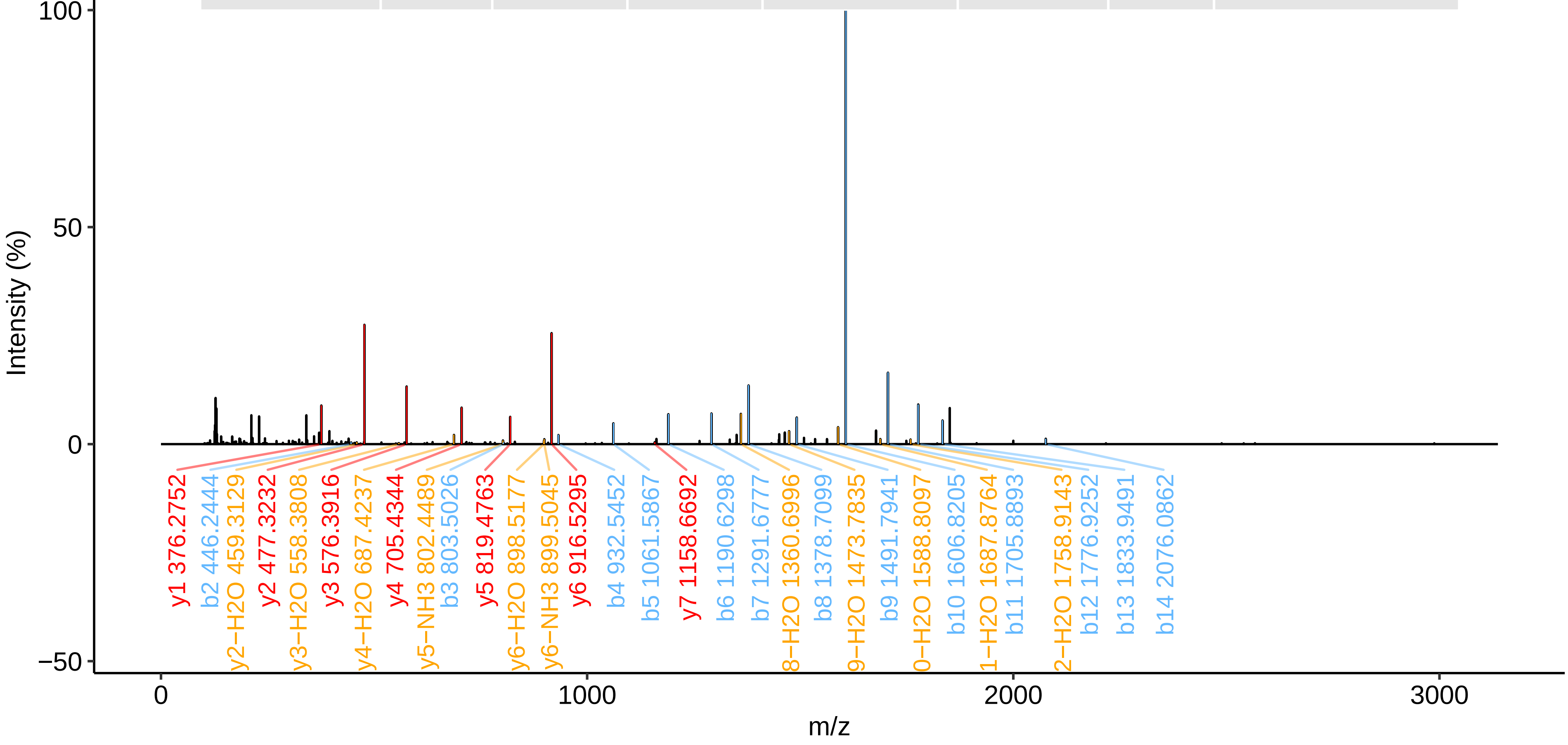

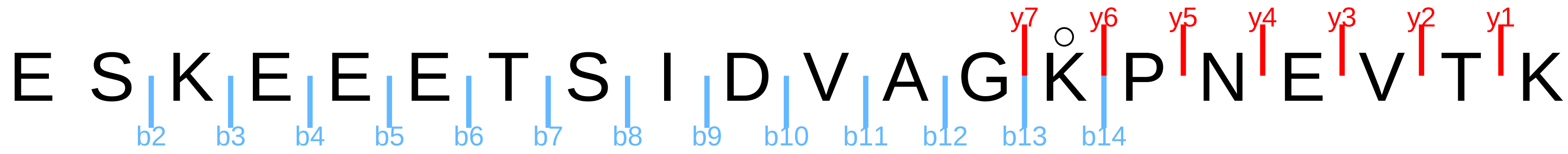

Modification □ Acetyl (Protein N−term) ○ GlyGly (K) no new terminus △ Oxidation (M)

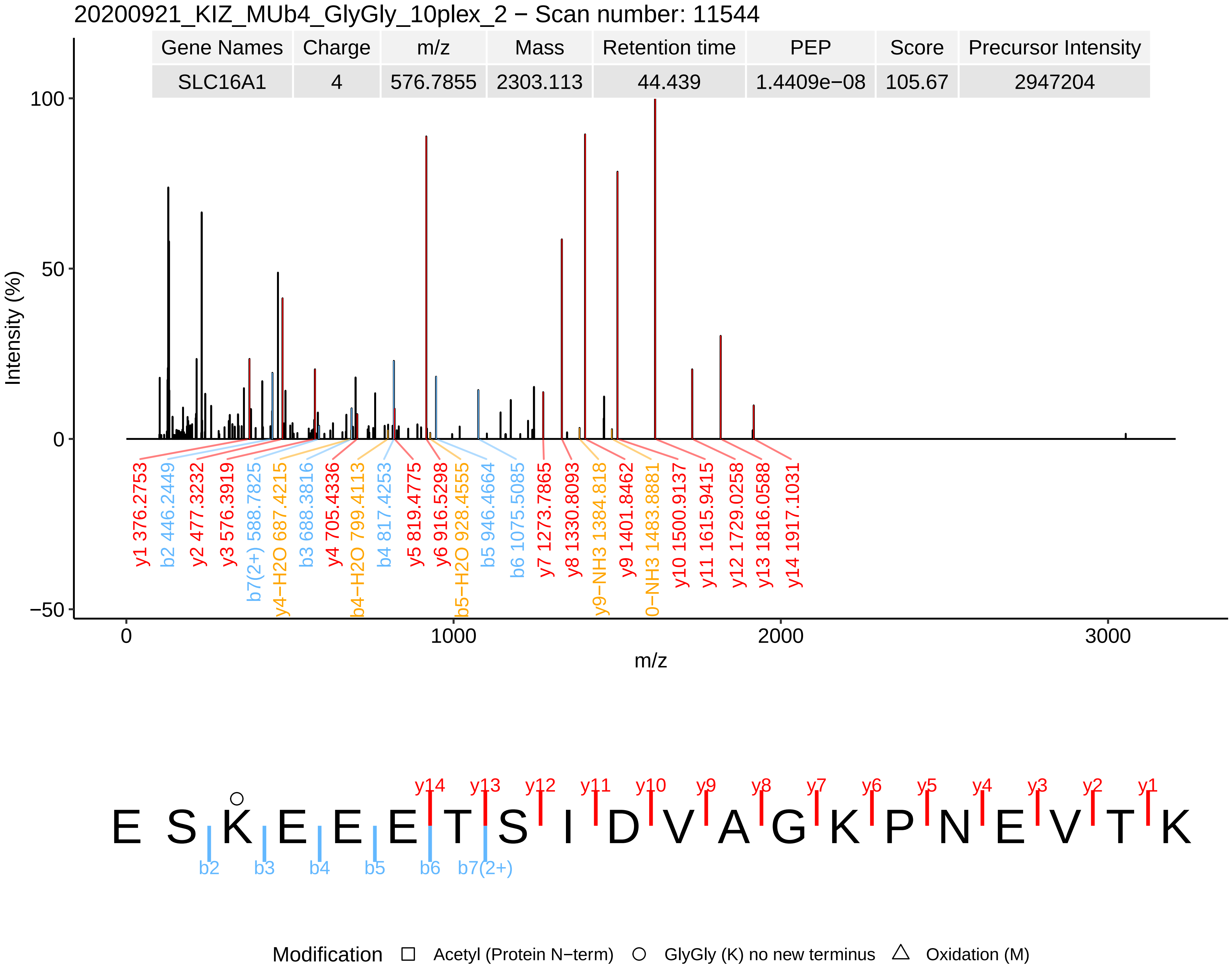

20200921_KIZ_MUb4_GlyGly_10plex_2 − Scan number: 11544
Gene Names | Charge | m/z | Mass | Retention time | PEP | Score | Precursor Intensity
SLC16A1 | 4 | 576.7855 | 2303.113 | 44.439 | 1.4409e−08 | 105.67 | 2947204
Intensity (%)
100
50
0
−50
0
1000
2000
3000
m/z
y1 376.2753
b2 446.2449
y2 477.3232
y3 576.3919
b7(2+) 588.7825
y4−H2O 687.4215
b3 688.3816
y4 705.4336
b4−H2O 799.4113
b4 817.4253
y5 819.4775
y6 916.5298
b5−H2O 928.4555
b5 946.4664
b6 1075.5085
y7 1273.7865
y8 1330.8093
y9−NH3 1384.818
y9 1401.8462
0−NH3 1483.8881
y10 1500.9137
y11 1615.9415
y12 1729.0258
y13 1816.0588
y14 1917.1031
y14 y13 y12 y11 y10 y9 y8 y7 y6 y5 y4 y3 y2 y1
E S K E E E T S I D V A G K P N E V T K
b2 b3 b4 b5 b6 b7(2+)
Modification
Acetyl (Protein N−term)
GlyGly (K) no new terminus
Oxidation (M)

20200921_KIZ_MUb4_GlyGly_10plex − Scan number: 29214

Gene Names	Charge	m/z	Mass	Retention time	PEP	Score	Precursor Intensity
ABCC4	3	798.0618	2391.163	107.04	2.5808e−08	105.31	NA

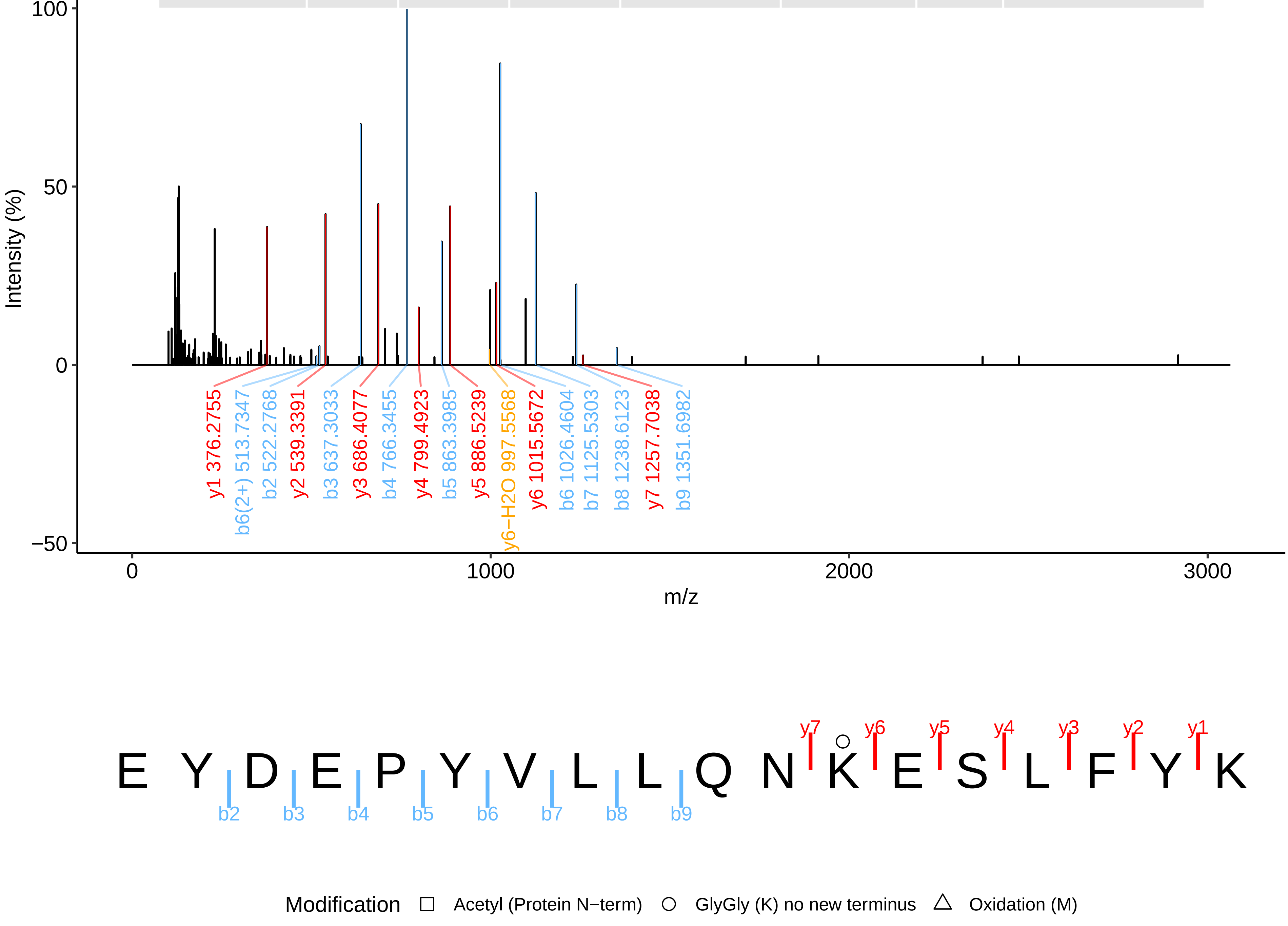

20200921_KIZ_MUb4_GlyGly_10plex − Scan number: 25612

Gene Names	Charge	m/z	Mass	Retention time	PEP	Score	Precursor Intensity
LDHB	4	554.0589	2212.206	93.194	5.3224e−14	119.23	2958853

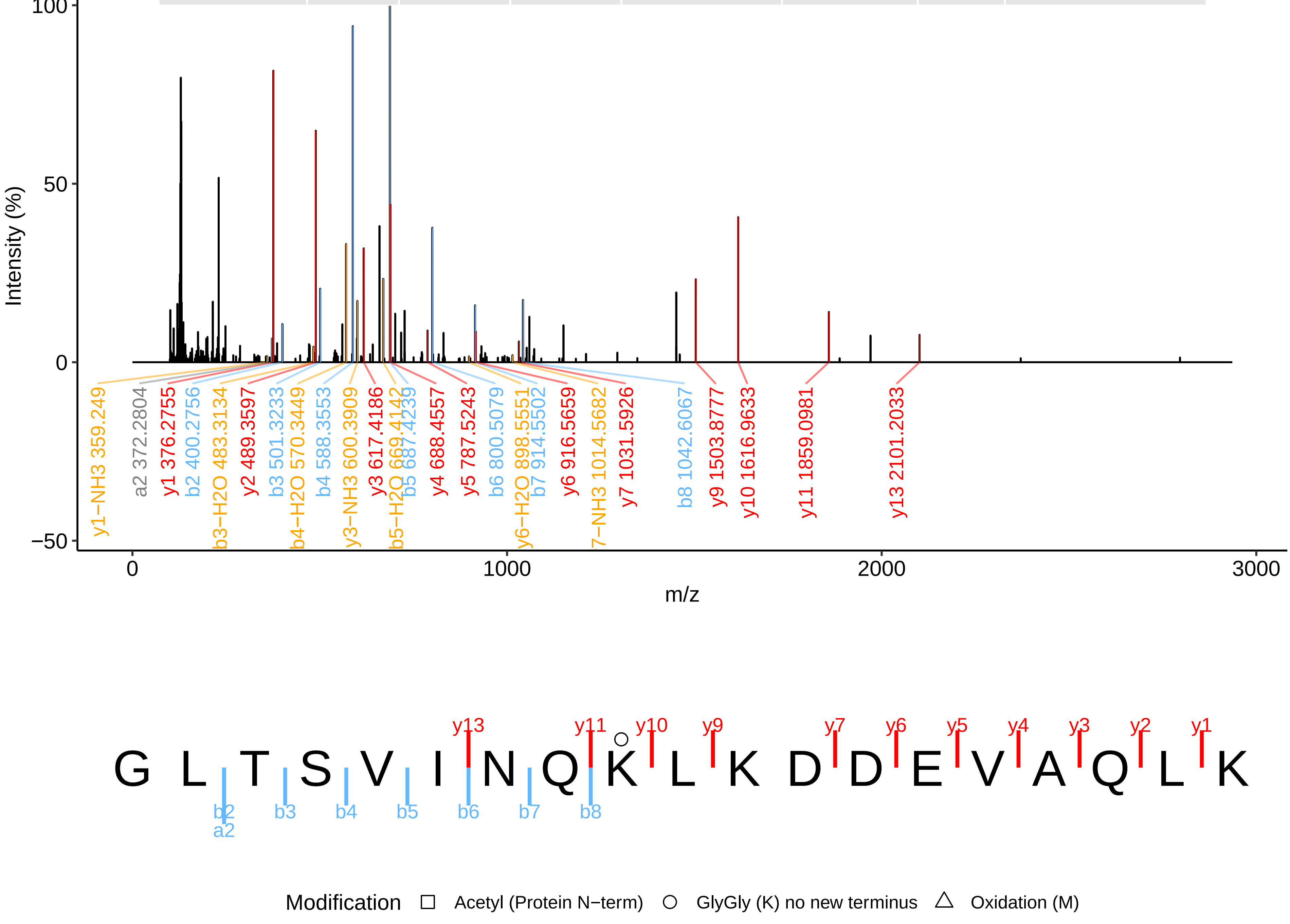

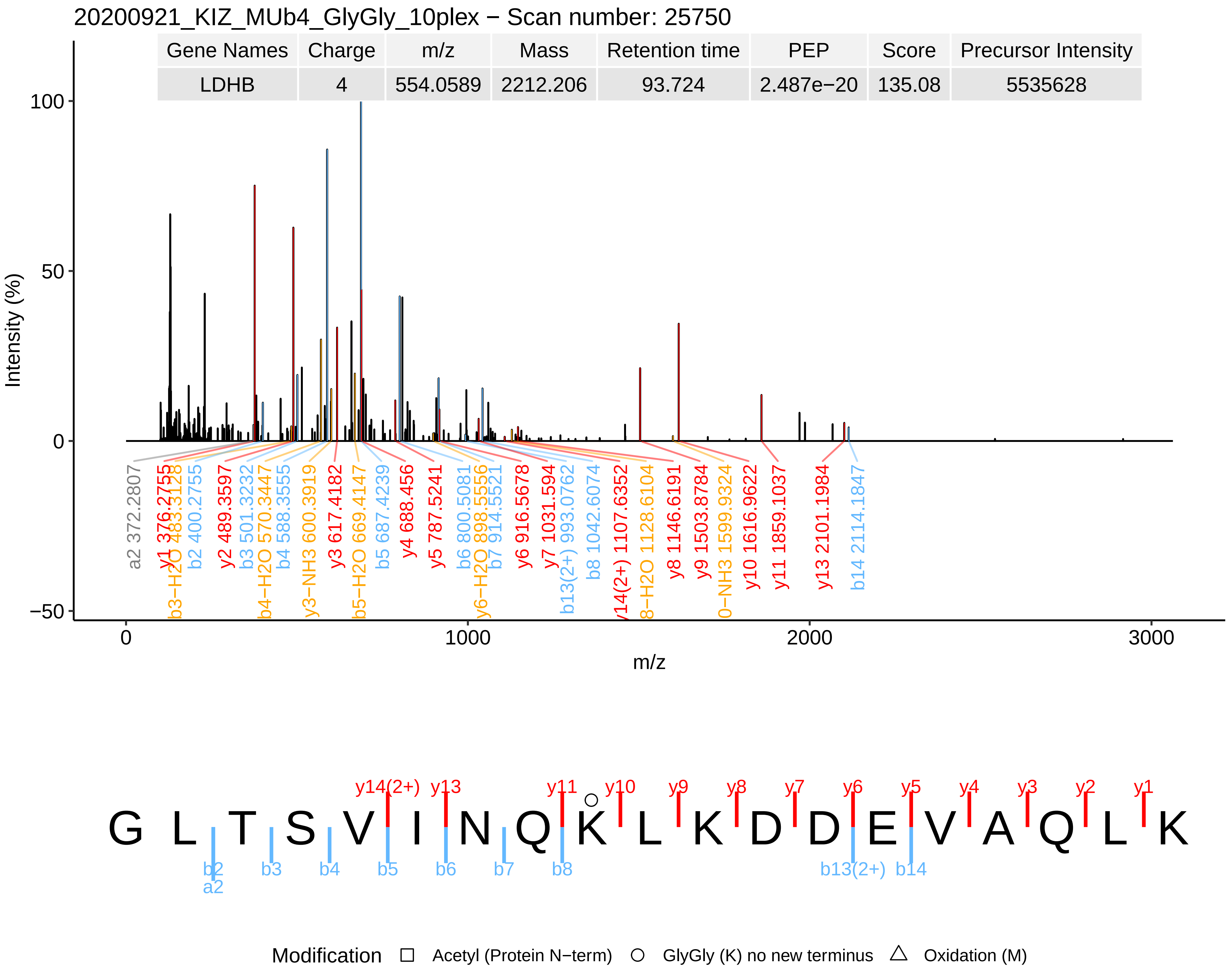

20200921_KIZ_MUb4_GlyGly_10plex − Scan number: 25750
Gene Names
Charge
m/z
Mass
Retention time
PEP
Score
Precursor Intensity
LDHB
4
554.0589
2212.206
93.724
2.487e−20
135.08
5535628
Intensity (%)
m/z
a2 372.2807
y1 376.2755
b2 400.2755
y2 489.3597
b3 501.3232
b4 588.3555
y3−NH3 600.3919
y3 617.4182
b5−H2O 669.4147
b5 687.4239
y4 688.456
y5 787.5241
b6 800.5081
b7 914.5521
y6 916.5678
y7 1031.594
b13(2+) 993.0762
b8 1042.6074
y8 1146.6191
y9 1503.8784
y10 1616.9622
y11 1859.1037
y13 2101.1984
b14 2114.1847
G L T S V I N Q K L K D D E V A Q L K
y14(2+)
y13
y11
y10
y9
y8
y7
y6
y5
y4
y3
y2
y1
b2
a2
b3
b4
b5
b6
b7
b8
b13(2+)
b14
Modification
Acetyl (Protein N−term)
GlyGly (K) no new terminus
Oxidation (M)

20200921_KIZ_MUb4_GlyGly_10plex_2 − Scan number: 21218

Gene Names	Charge	m/z	Mass	Retention time	PEP	Score	Precursor Intensity
LDHB	4	554.0589	2212.206	83.507	1.6642e−08	98.035	713071.4

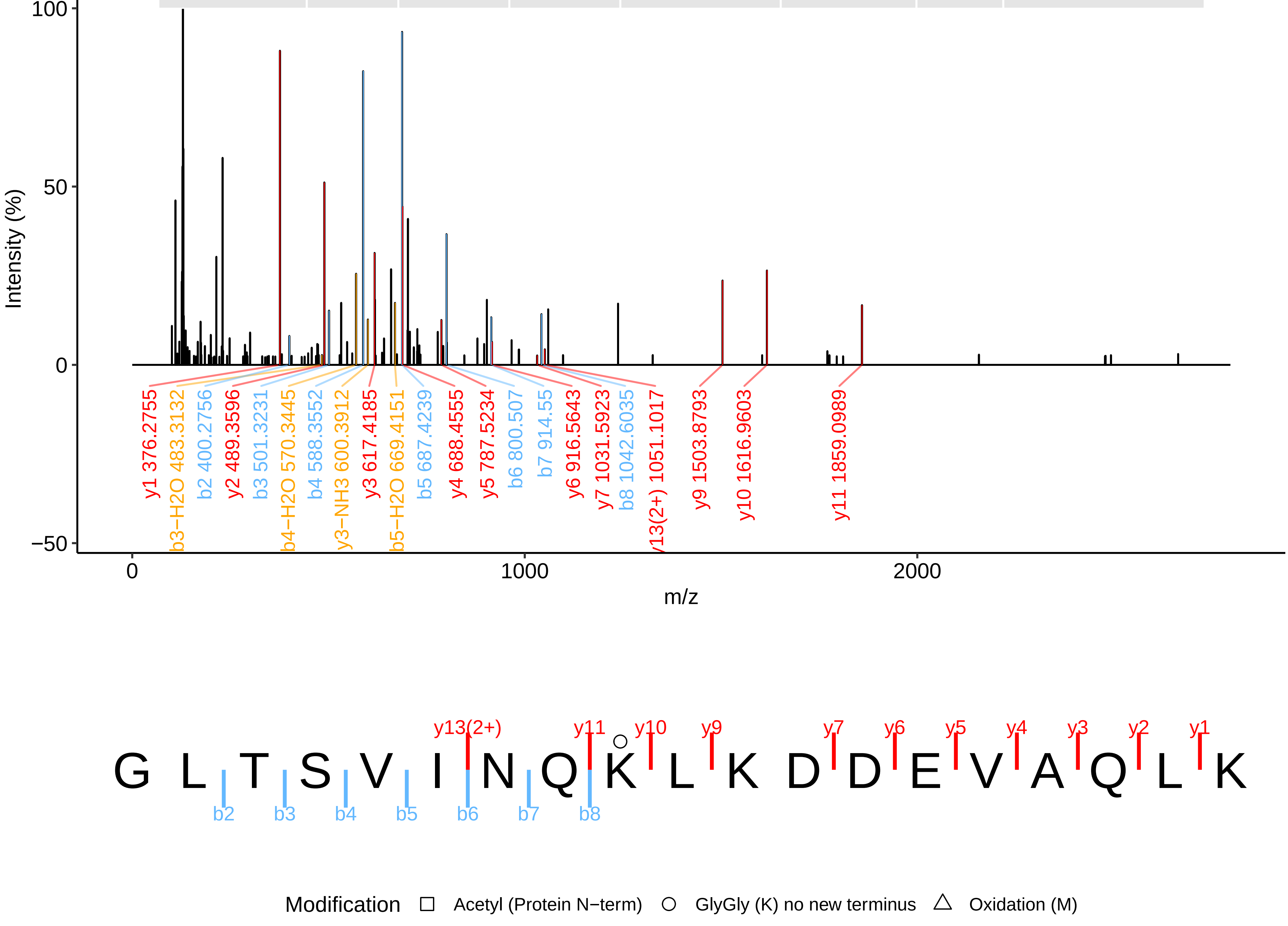

20200921_KIZ_MUb4_GlyGly_10plex_2 − Scan number: 21229

Gene Names	Charge	m/z	Mass	Retention time	PEP	Score	Precursor Intensity
LDHB	4	554.0589	2212.206	83.553	3.3439e−14	124.29	NA

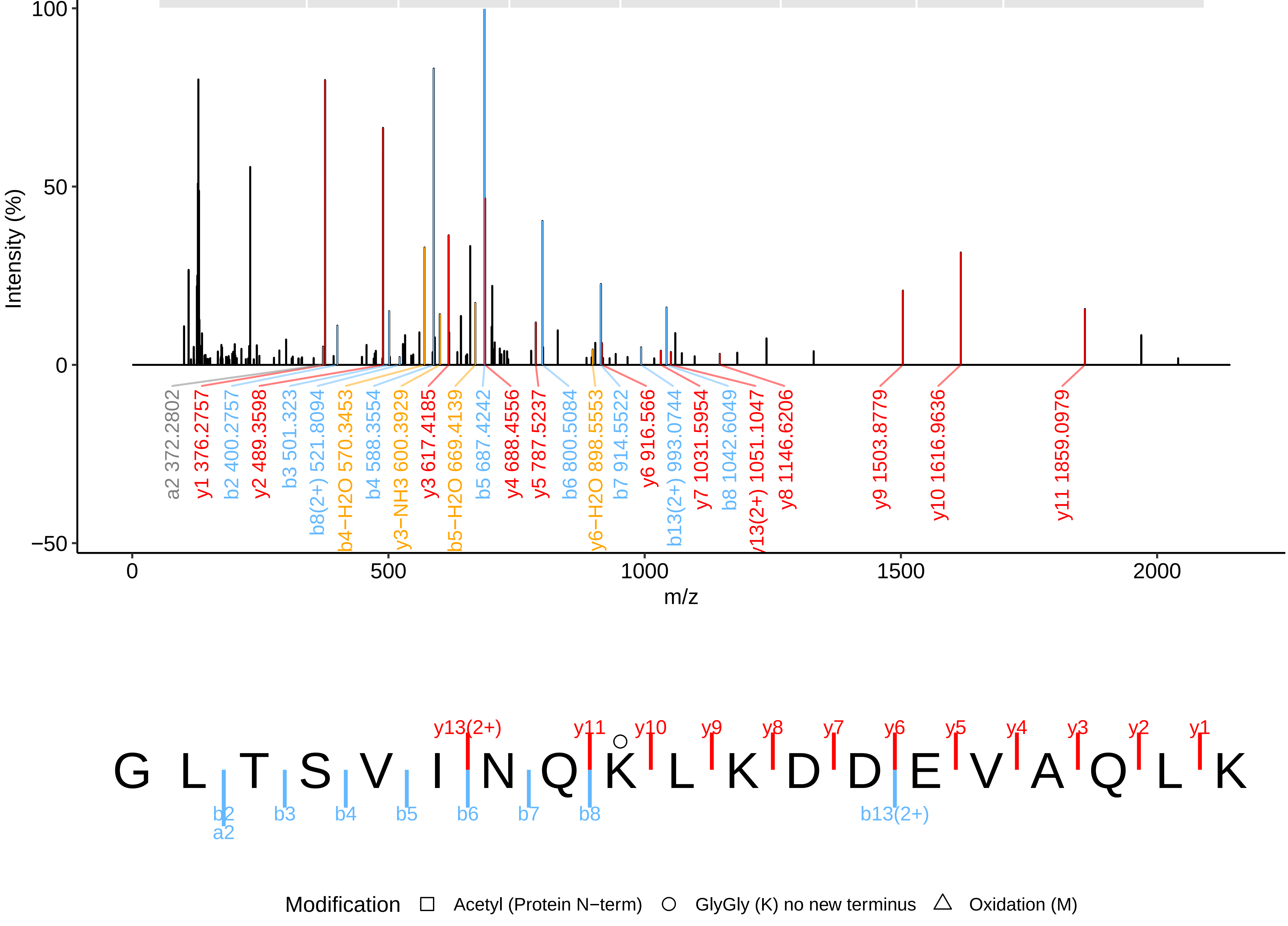

20200921_KIZ_MUb4_GlyGly_10plex − Scan number: 24684

Gene Names	Charge	m/z	Mass	Retention time	PEP	Score	Precursor Intensity
RPS27A;UBA52;UBB;UBC	4	631.5855	2522.313	89.786	7.2417e−14	123.55	5399775

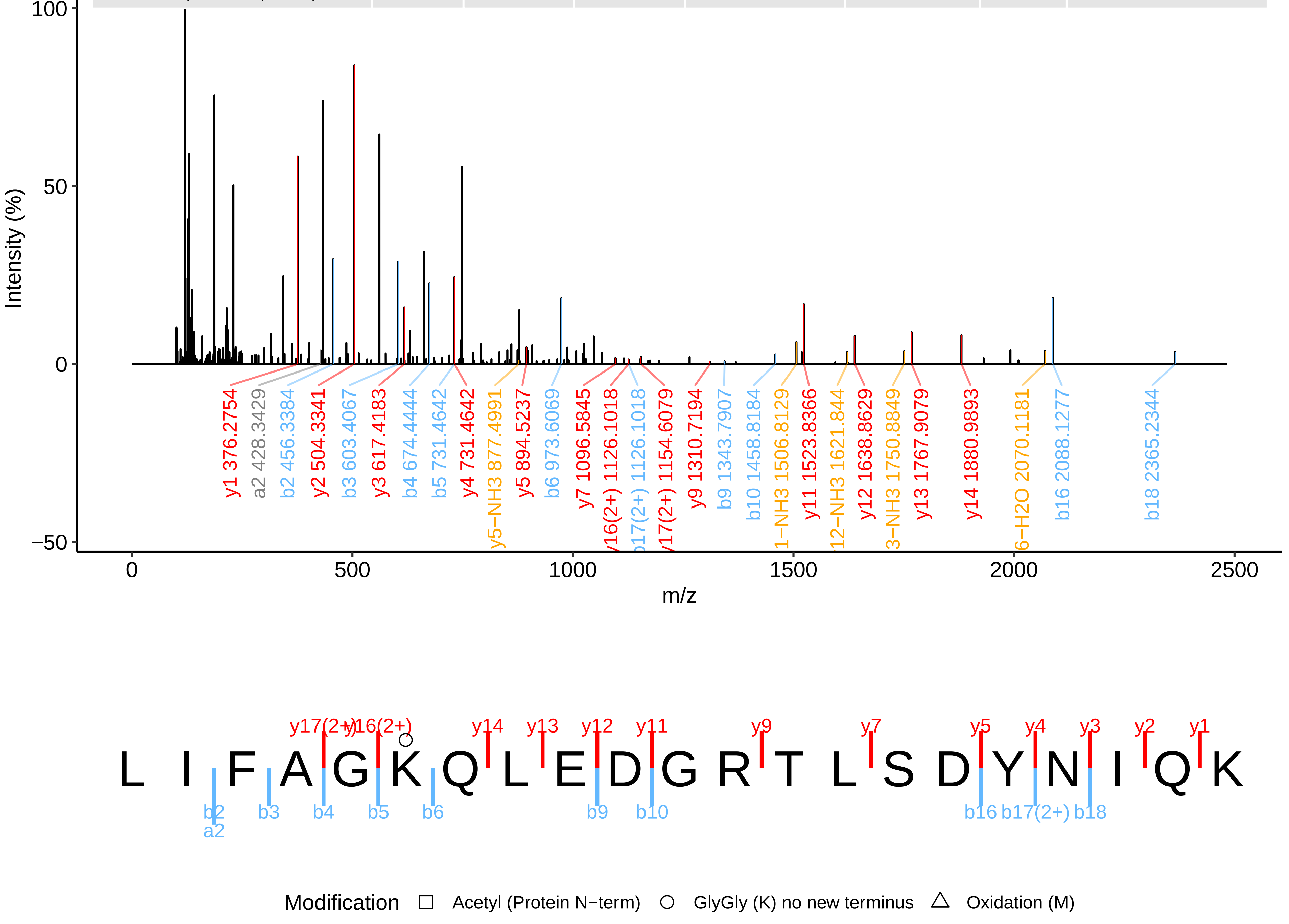

20200921_KIZ_MUb4_GlyGly_10plex − Scan number: 24767

Gene Names	Charge	m/z	Mass	Retention time	PEP	Score	Precursor Intensity
RPS27A;UBA52;UBB;UBC	3	841.7782	2522.313	90.102	9.7191e−22	145.05	2604790

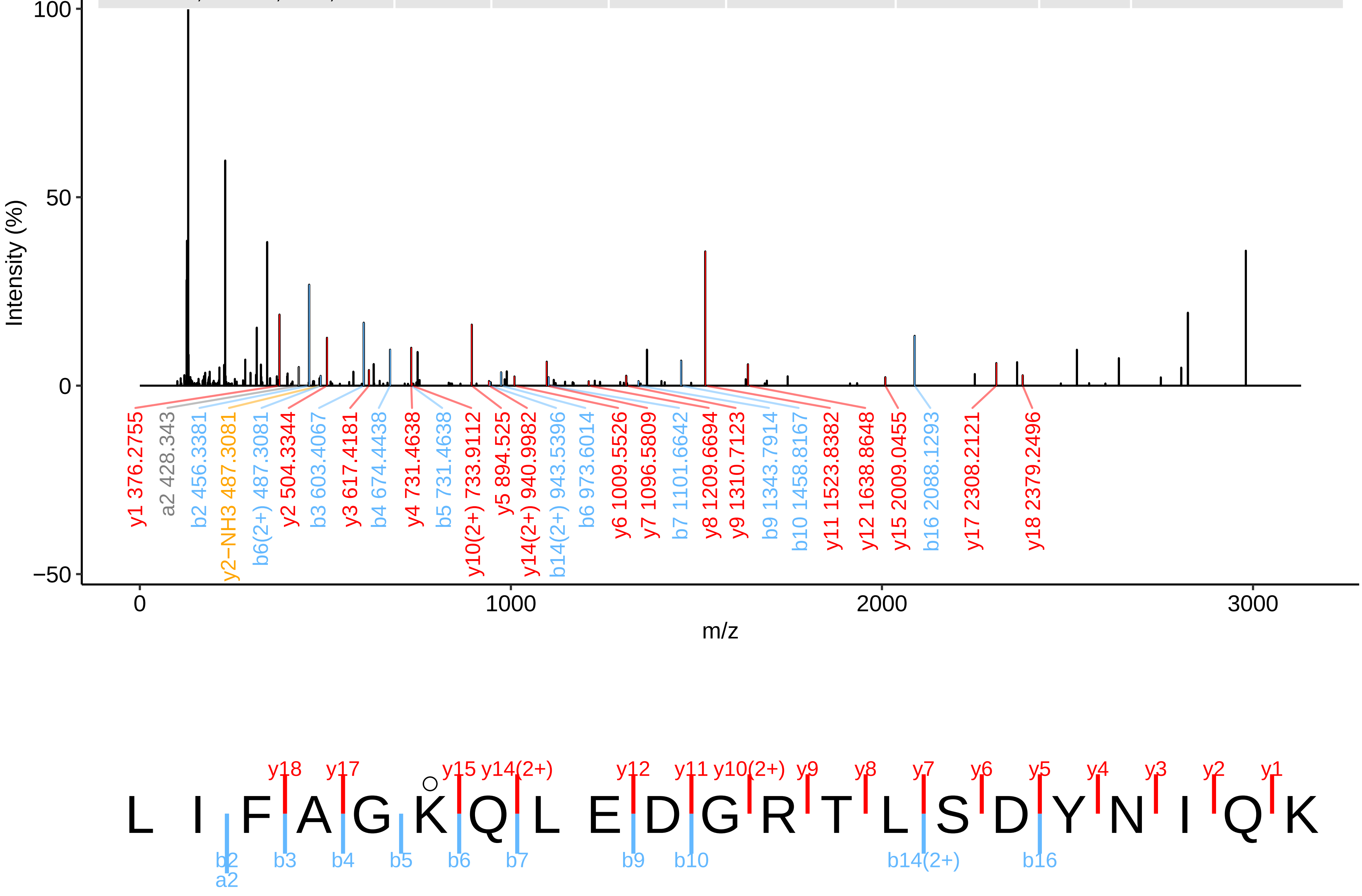

Modification □ Acetyl (Protein N−term) ○ GlyGly (K) no new terminus △ Oxidation (M)

20200921_KIZ_MUb4_GlyGly_10plex − Scan number: 24772

Gene Names	Charge	m/z	Mass	Retention time	PEP	Score	Precursor Intensity
RPS27A;UBA52;UBB;UBC	5	505.4699	2522.313	90.12	0.0011835	67.43	5744472

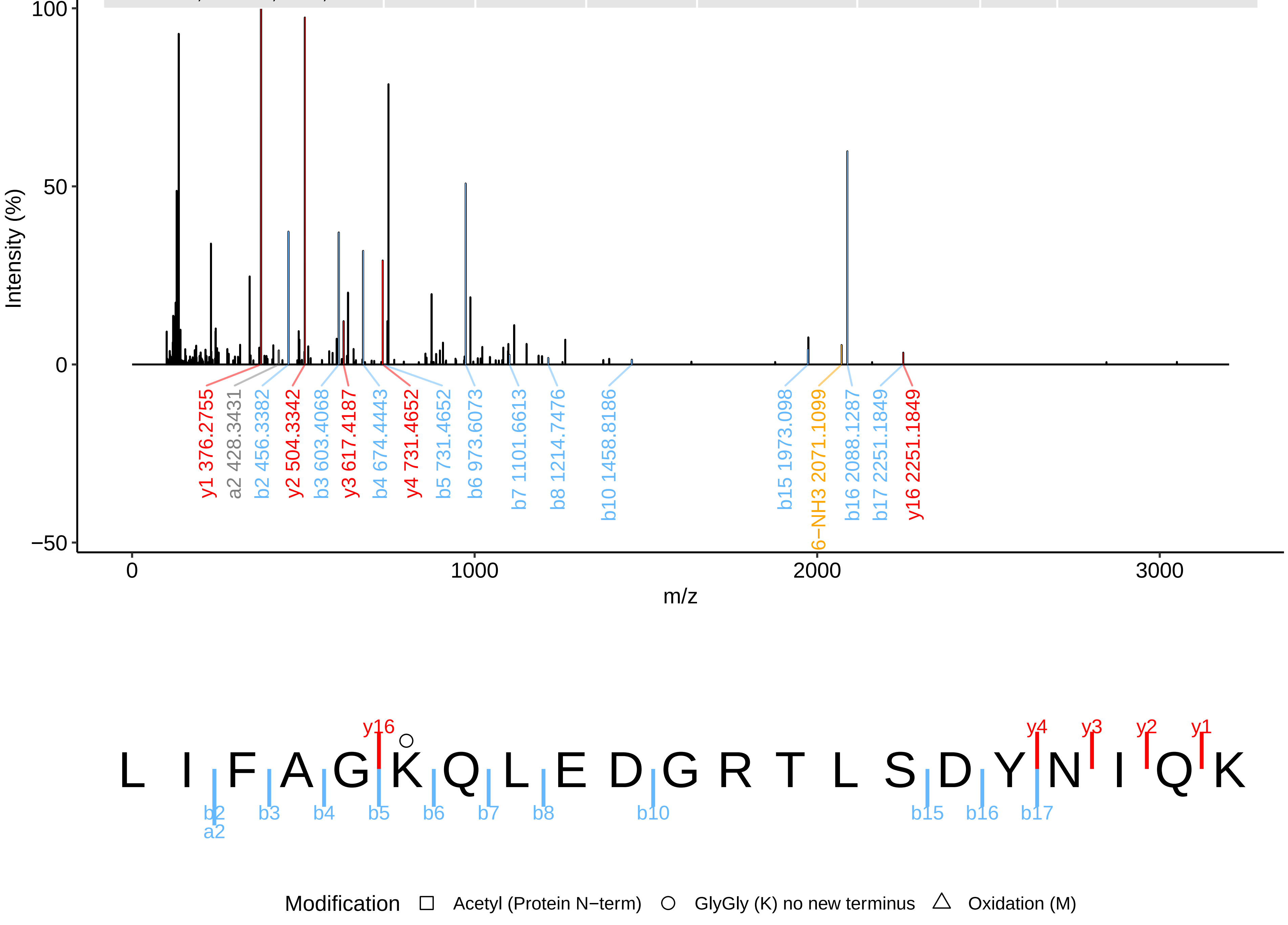

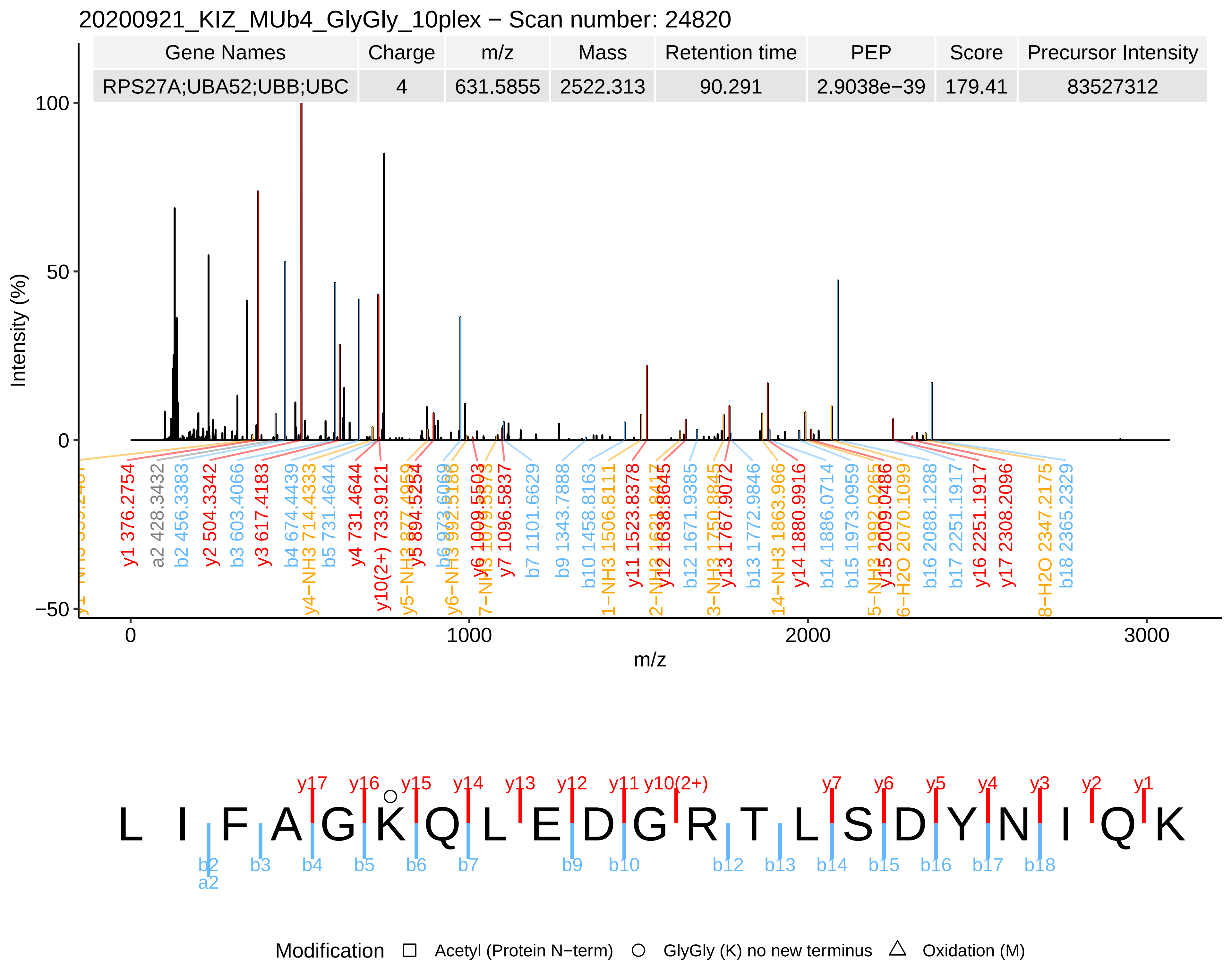
20200921_KIZ_MUb4_GlyGly_10plex − Scan number: 24820
Gene Names | Charge | m/z | Mass | Retention time | PEP | Score | Precursor Intensity
RPS27A;UBA52;UBB;UBC | 4 | 631.5855 | 2522.313 | 90.291 | 2.9038e−39 | 179.41 | 83527312
Intensity (%)
m/z
y1 376.2754
a2 428.3432
b2 456.3383
y2 504.3342
b3 603.4066
y3 617.4183
b4 674.4439
y4−NH3 714.4333
b5 731.4644
y4 731.4644
y10(2+) 733.9121
y7 1096.5837
b7 1101.6629
b9 1343.7888
b10 1458.8163
y11 1523.8378
b12 1671.9385
b13 1772.9846
y14 1880.9916
b14 1886.0714
b15 1973.0959
b16 2088.1288
b17 2251.1917
y16 2251.1917
y17 2308.2096
b18 2365.2329
L I F A G K Q L E D G R T L S D Y N I Q K
Modification
Acetyl (Protein N−term)
GlyGly (K) no new terminus
Oxidation (M)

20200921_KIZ_MUb4_GlyGly_10plex − Scan number: 24918

Gene Names	Charge	m/z	Mass	Retention time	PEP	Score	Precursor Intensity
RPS27A;UBA52;UBB;UBC	5	505.4699	2522.313	90.631	0.032922	53.278	NA

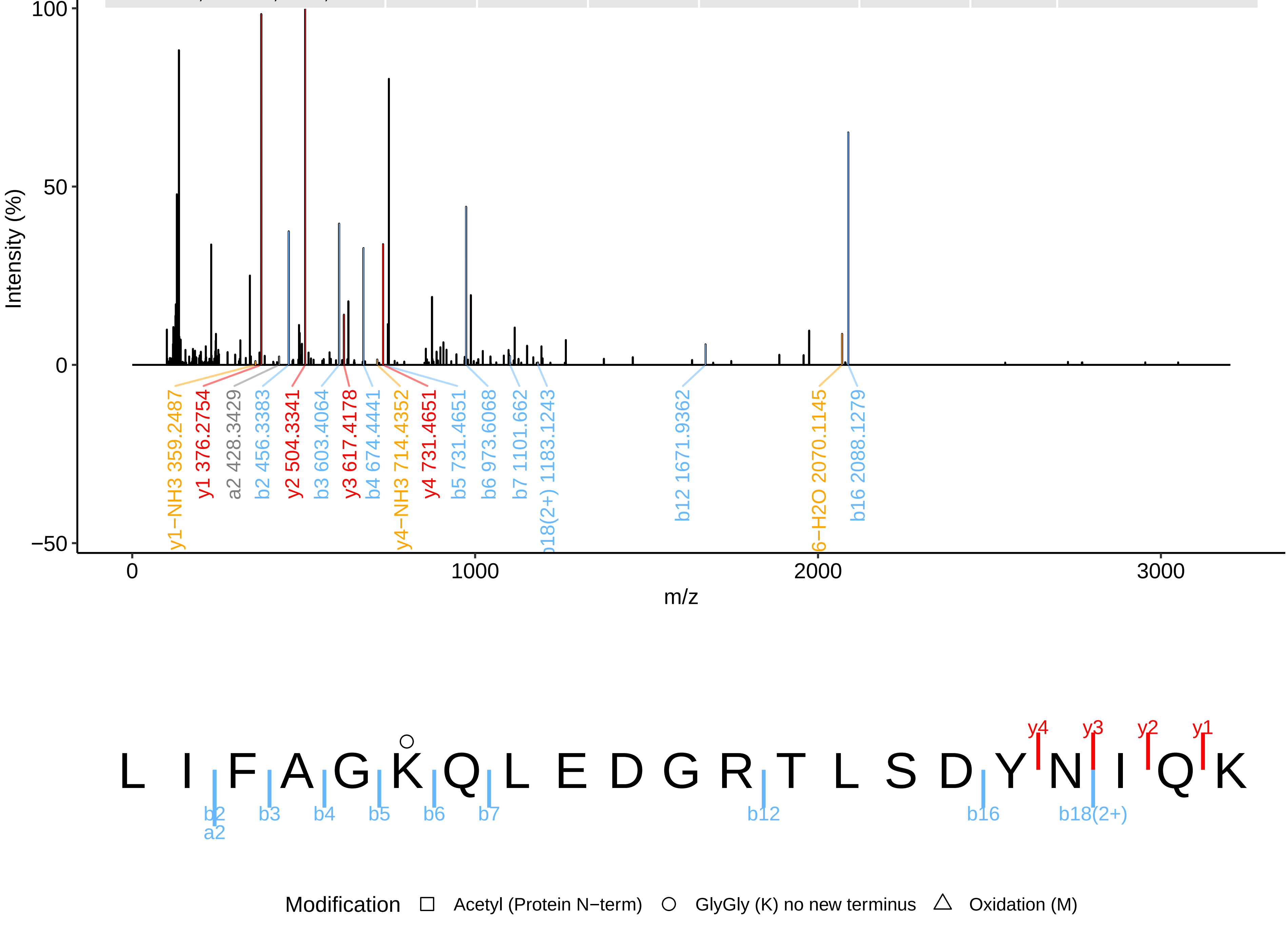

20200921_KIZ_MUb4_GlyGly_10plex − Scan number: 24965

Gene Names	Charge	m/z	Mass	Retention time	PEP	Score	Precursor Intensity
RPS27A;UBA52;UBB;UBC	4	631.5855	2522.313	90.796	4.4188e−39	176.46	504243872

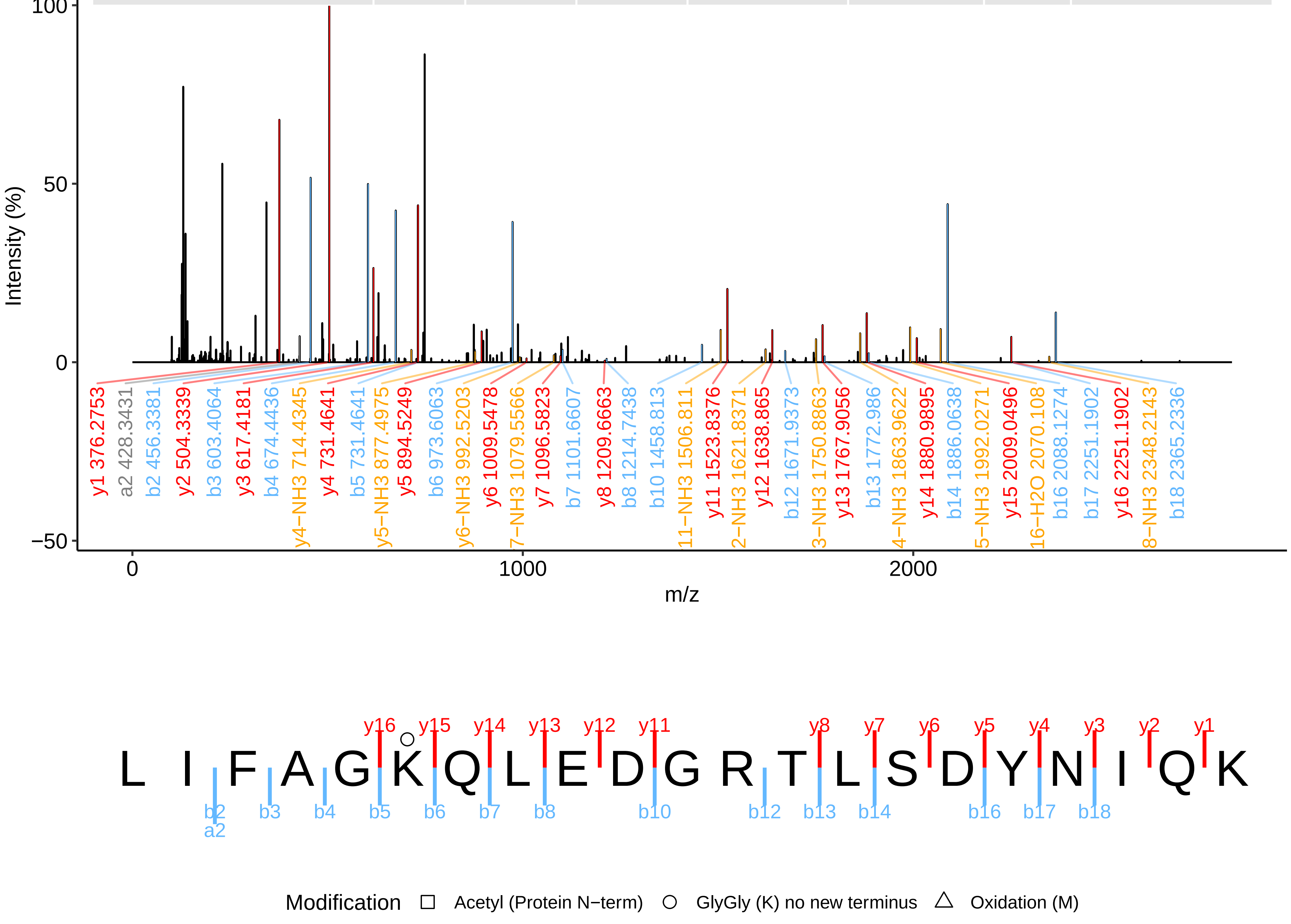

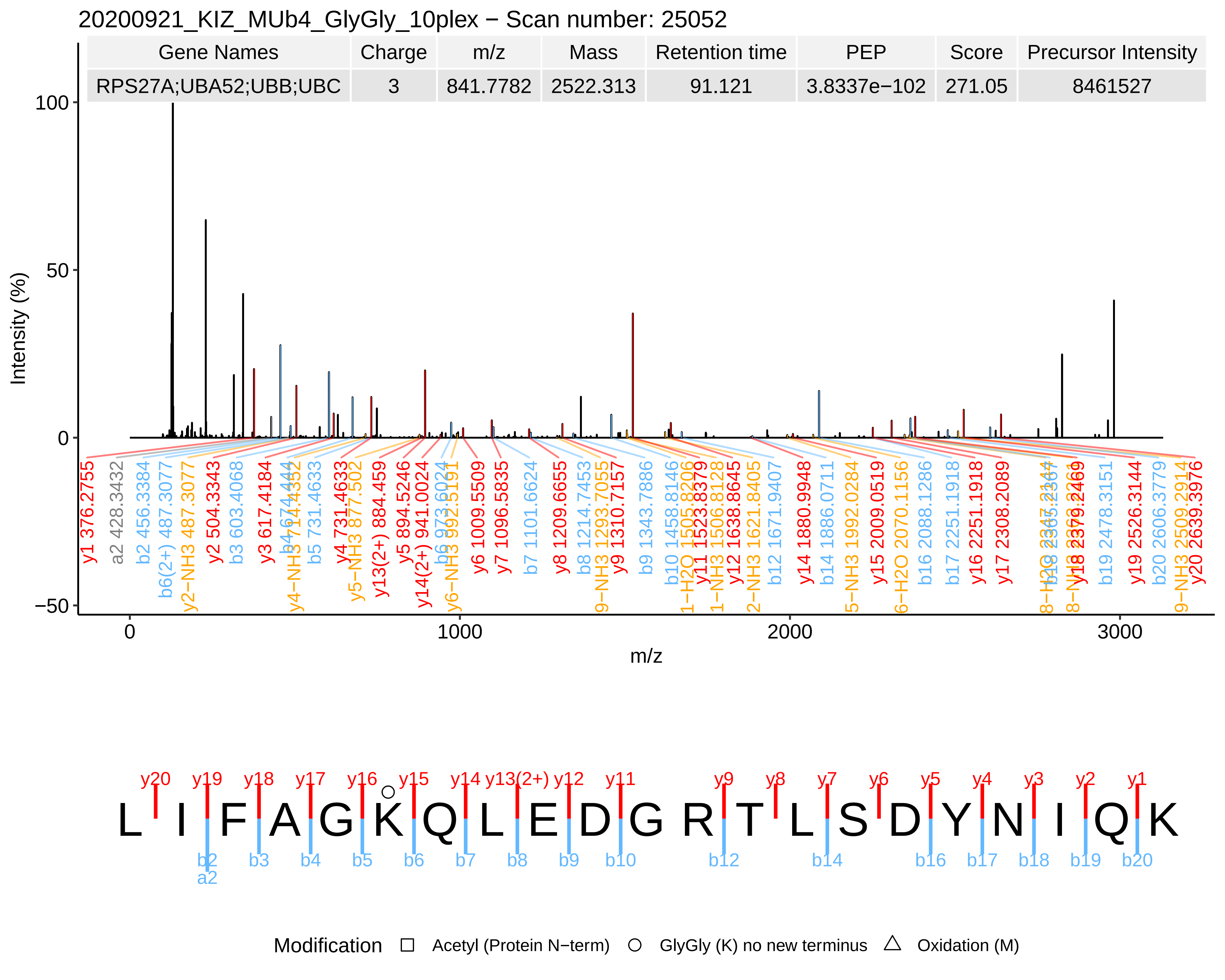

20200921_KIZ_MUb4_GlyGly_10plex − Scan number: 25052
Gene Names | Charge | m/z | Mass | Retention time | PEP | Score | Precursor Intensity
RPS27A;UBA52;UBB;UBC | 3 | 841.7782 | 2522.313 | 91.121 | 3.8337e−102 | 271.05 | 8461527
Intensity (%)
m/z
y1 376.2755
a2 428.3432
b2 456.3384
b6(2+) 487.3077
y2−NH3 487.3077
y2 504.3343
b3 603.4068
y3 617.4184
y13(2+) 884.459
y5 894.5246
y14(2+) 941.0024
y6 1009.5509
y7 1096.5835
b7 1101.6624
y8 1209.6655
b8 1214.7453
y9 1310.7157
b9 1343.7886
b10 1458.8146
y12 1638.8645
b12 1671.9407
y14 1880.9948
b14 1886.0711
y15 2009.0519
b16 2088.1286
b17 2251.1918
y16 2251.1918
y17 2308.2089
b19 2478.3151
y19 2526.3144
b20 2606.3779
y20 2639.3976
L I F A G K Q L E D G R T L S D Y N I Q K
Modification
Acetyl (Protein N−term)
GlyGly (K) no new terminus
Oxidation (M)

20200921_KIZ_MUb4_GlyGly_10plex − Scan number: 25057

Gene Names	Charge	m/z	Mass	Retention time	PEP	Score	Precursor Intensity
RPS27A;UBA52;UBB;UBC	5	505.4699	2522.313	91.139	0.0010825	68.567	19151110

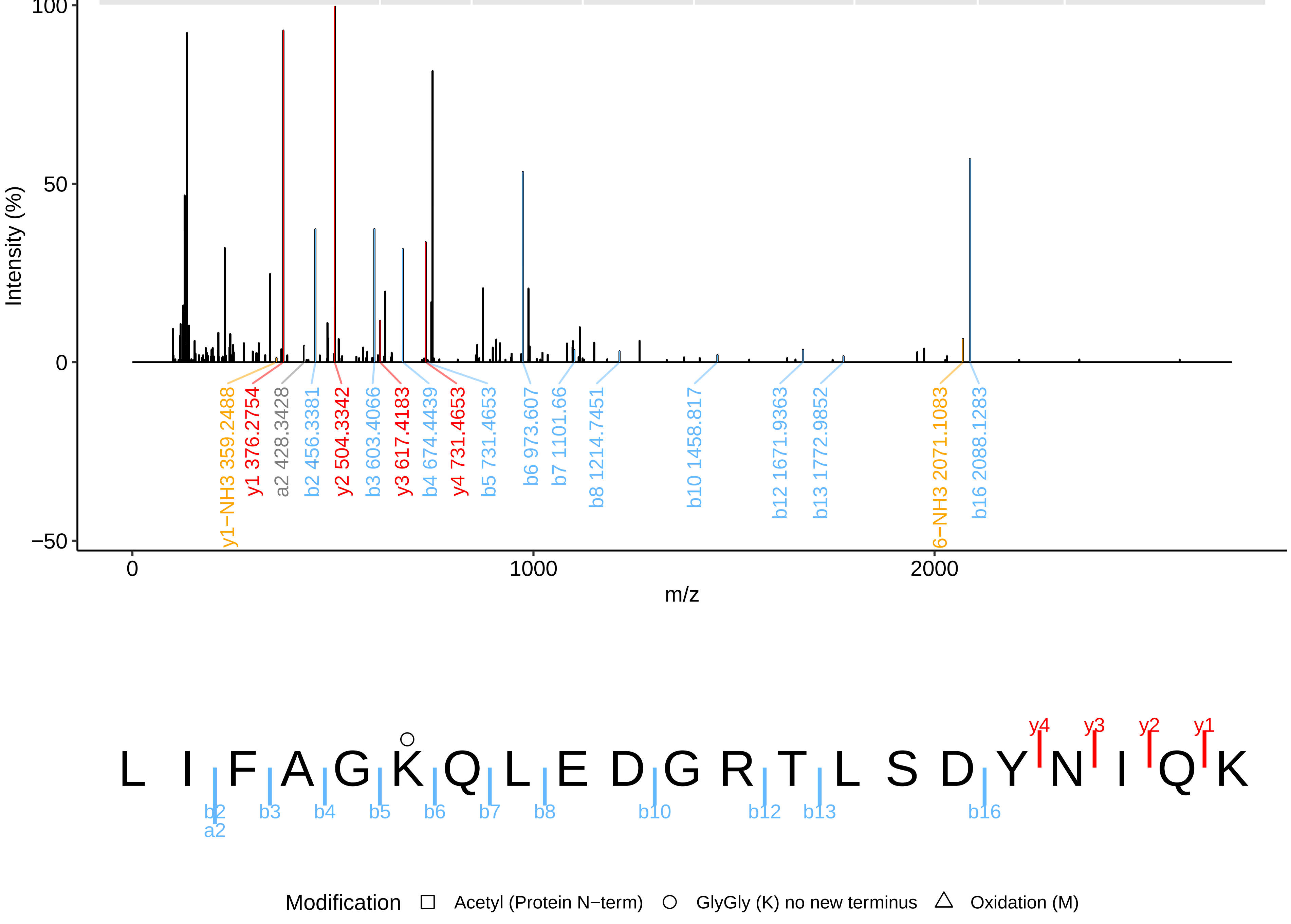

20200921_KIZ_MUb4_GlyGly_10plex − Scan number: 25106

Gene Names	Charge	m/z	Mass	Retention time	PEP	Score	Precursor Intensity
RPS27A;UBA52;UBB;UBC	4	631.5855	2522.313	91.315	1.1642e−47	187.33	33363594

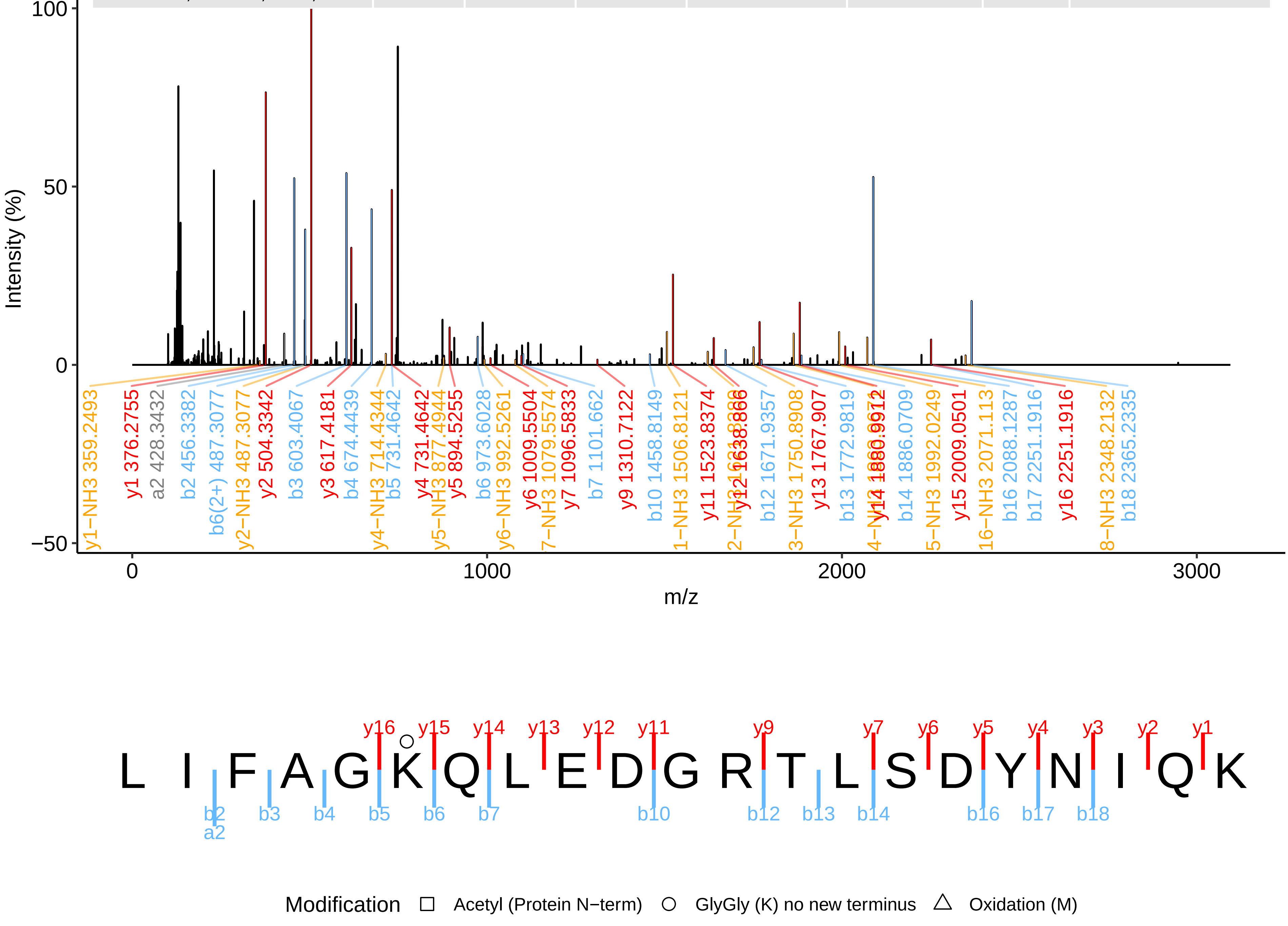

20200921_KIZ_MUb4_GlyGly_10plex − Scan number: 25246

Gene Names	Charge	m/z	Mass	Retention time	PEP	Score	Precursor Intensity
RPS27A;UBA52;UBB;UBC	4	631.5855	2522.313	91.827	5.6261e−21	142.98	5022704

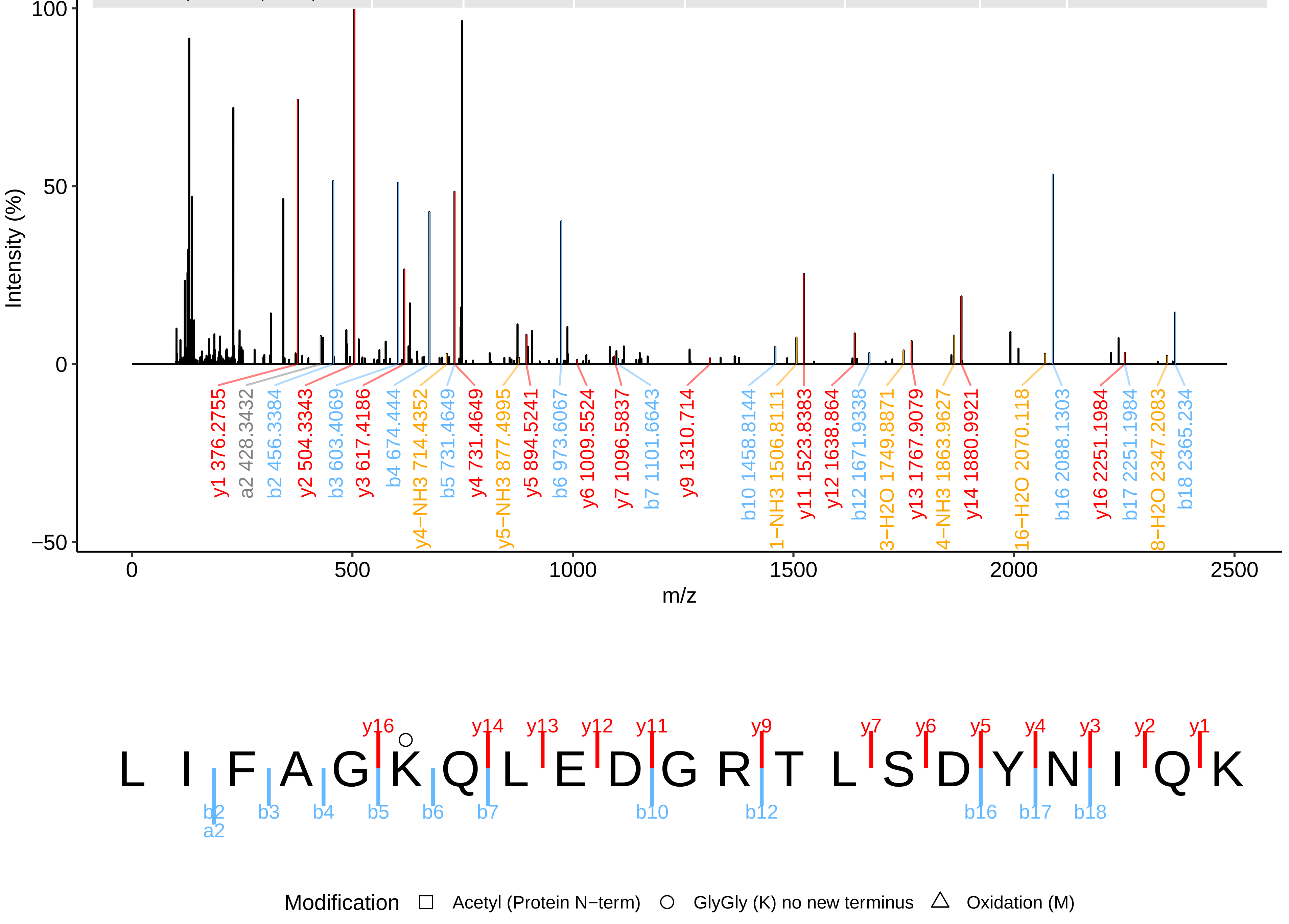

20200921_KIZ_MUb4_GlyGly_10plex − Scan number: 24911

Gene Names	Charge	m/z	Mass	Retention time	PEP	Score	Precursor Intensity
RPS27A;UBA52;UBB;UBC	3	841.7782	2522.313	90.607	1.856e−57	206.28	7912290

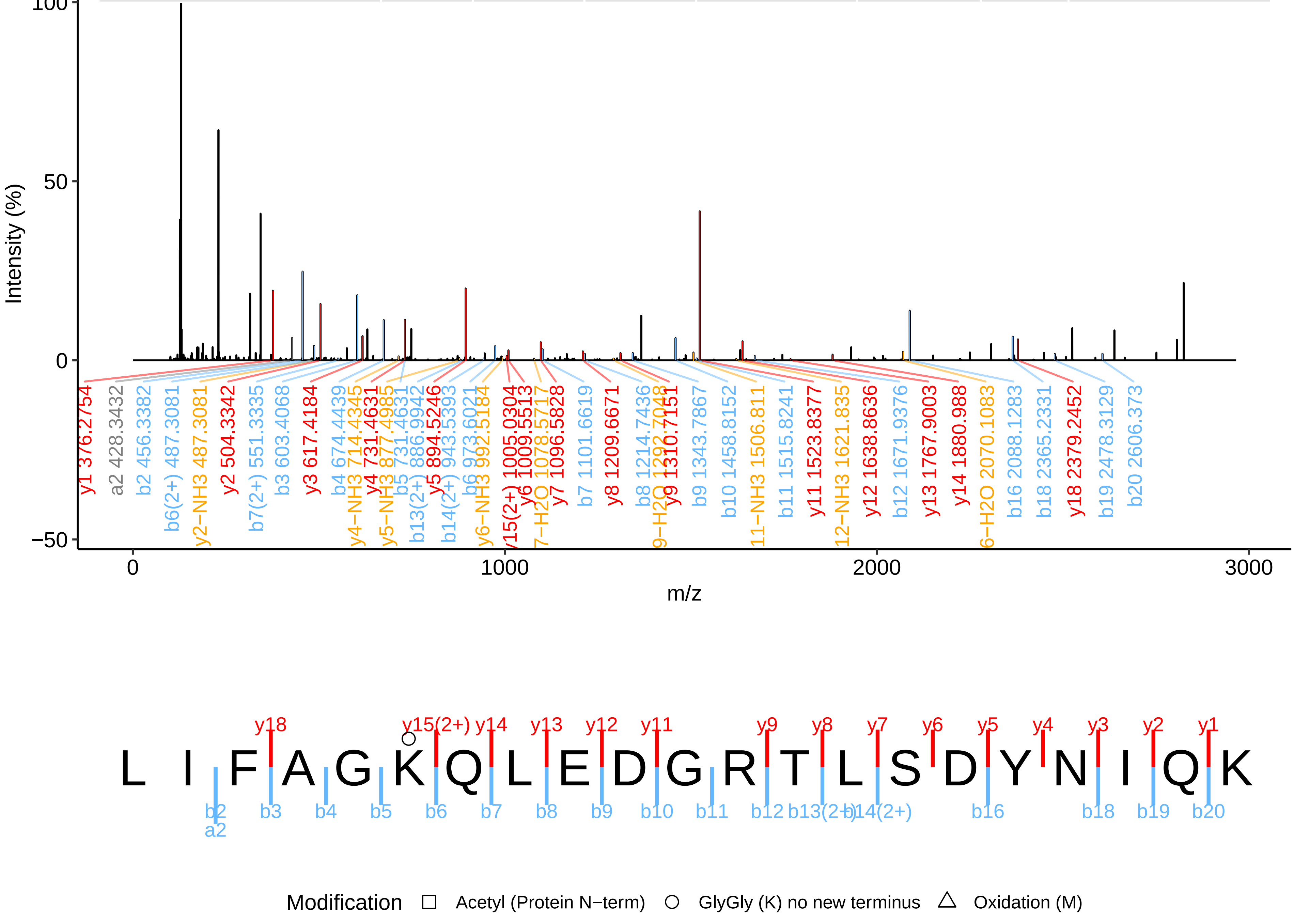

20200921_KIZ_MUb4_GlyGly_10plex − Scan number: 25049

Gene Names	Charge	m/z	Mass	Retention time	PEP	Score	Precursor Intensity
RPS27A;UBA52;UBB;UBC	3	841.7782	2522.313	91.111	5.3154e−47	191.64	6995755

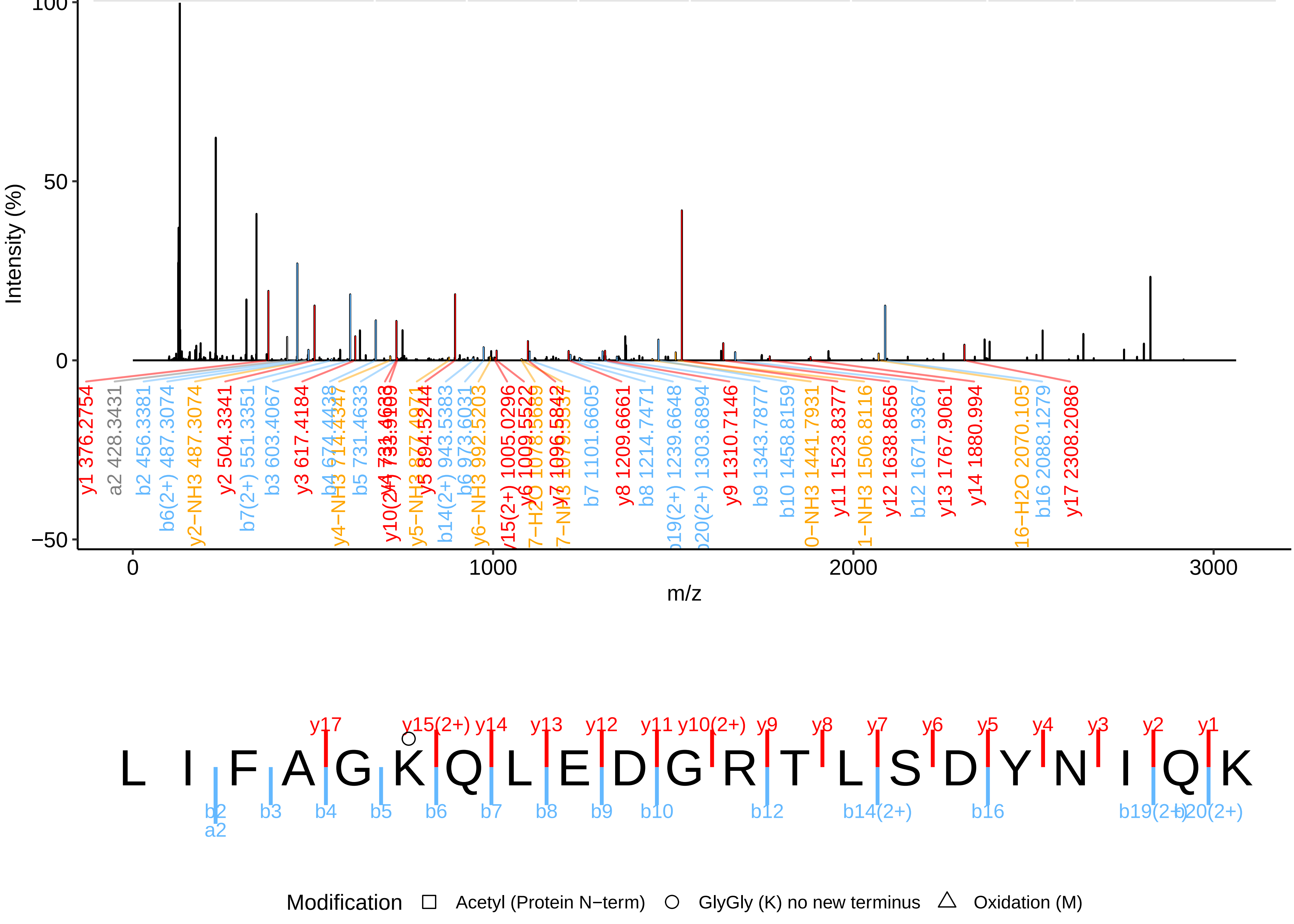

20200921_KIZ_MUb4_GlyGly_10plex_2 − Scan number: 20666

Gene Names	Charge	m/z	Mass	Retention time	PEP	Score	Precursor Intensity
RPS27A;UBA52;UBB;UBC	3	841.7782	2522.313	81.264	0.00037416	77.39	1698563

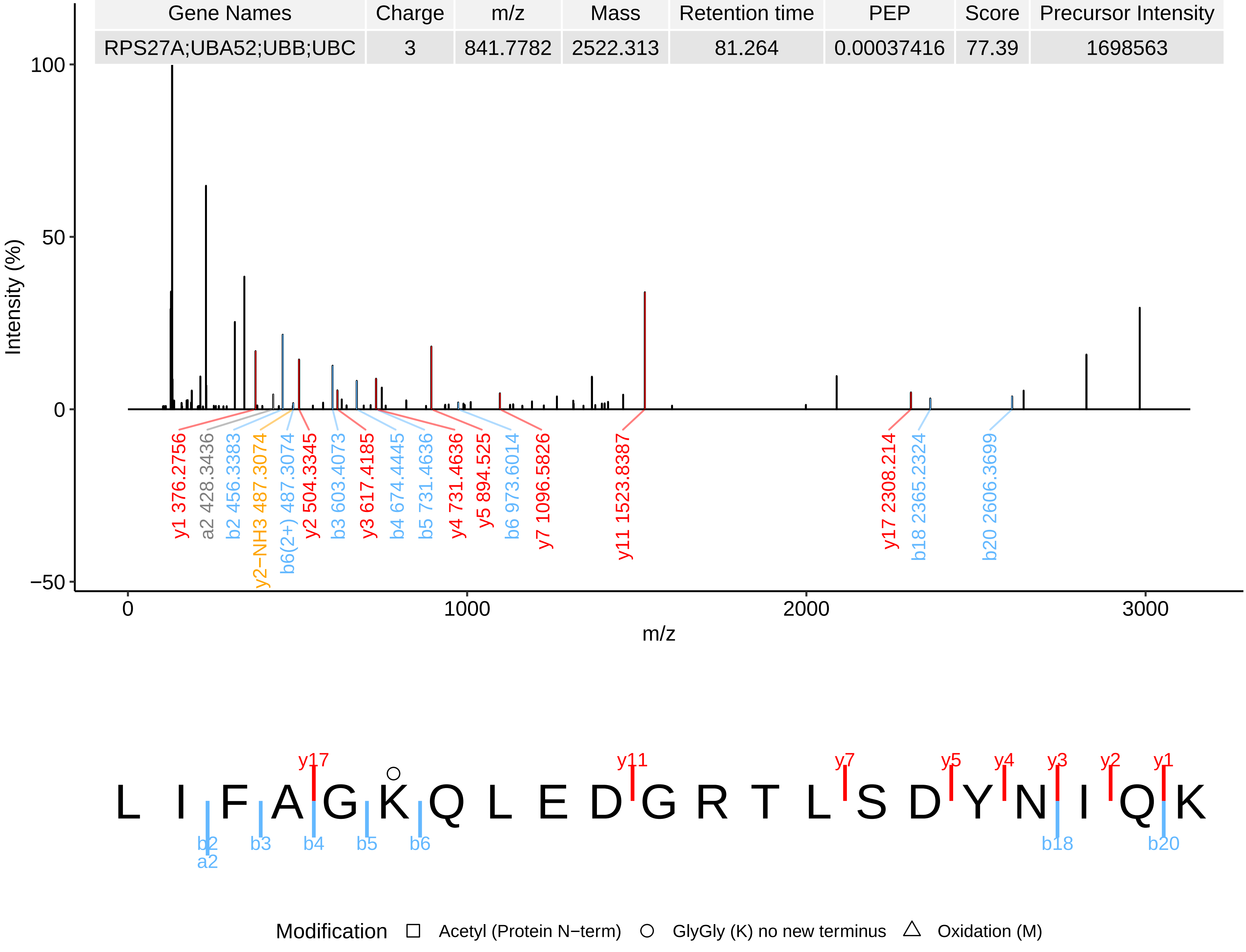

20200921_KIZ_MUb4_GlyGly_10plex_2 − Scan number: 20705

Gene Names	Charge	m/z	Mass	Retention time	PEP	Score	Precursor Intensity
RPS27A;UBA52;UBB;UBC	4	631.5855	2522.313	81.42	7.2686e−39	170.91	83628368

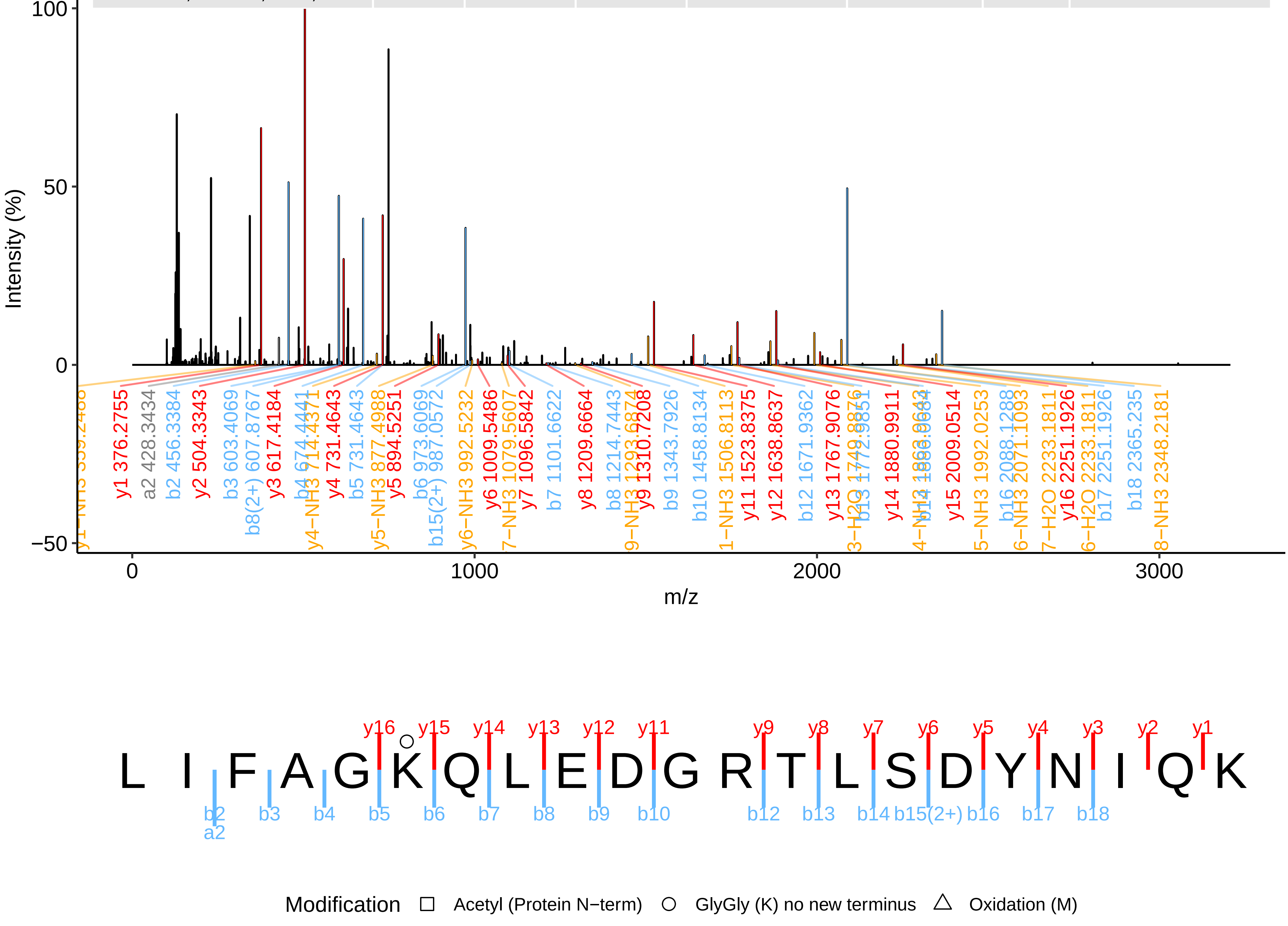

20200924_KIZ_MUb5_GlyGly_1 − Scan number: 22063

Gene Names	Charge	m/z	Mass	Retention time	PEP	Score	Precursor Intensity
RPS27A;UBA52;UBB;UBC	4	631.5855	2522.313	71.037	4.4188e−39	176.46	11460278

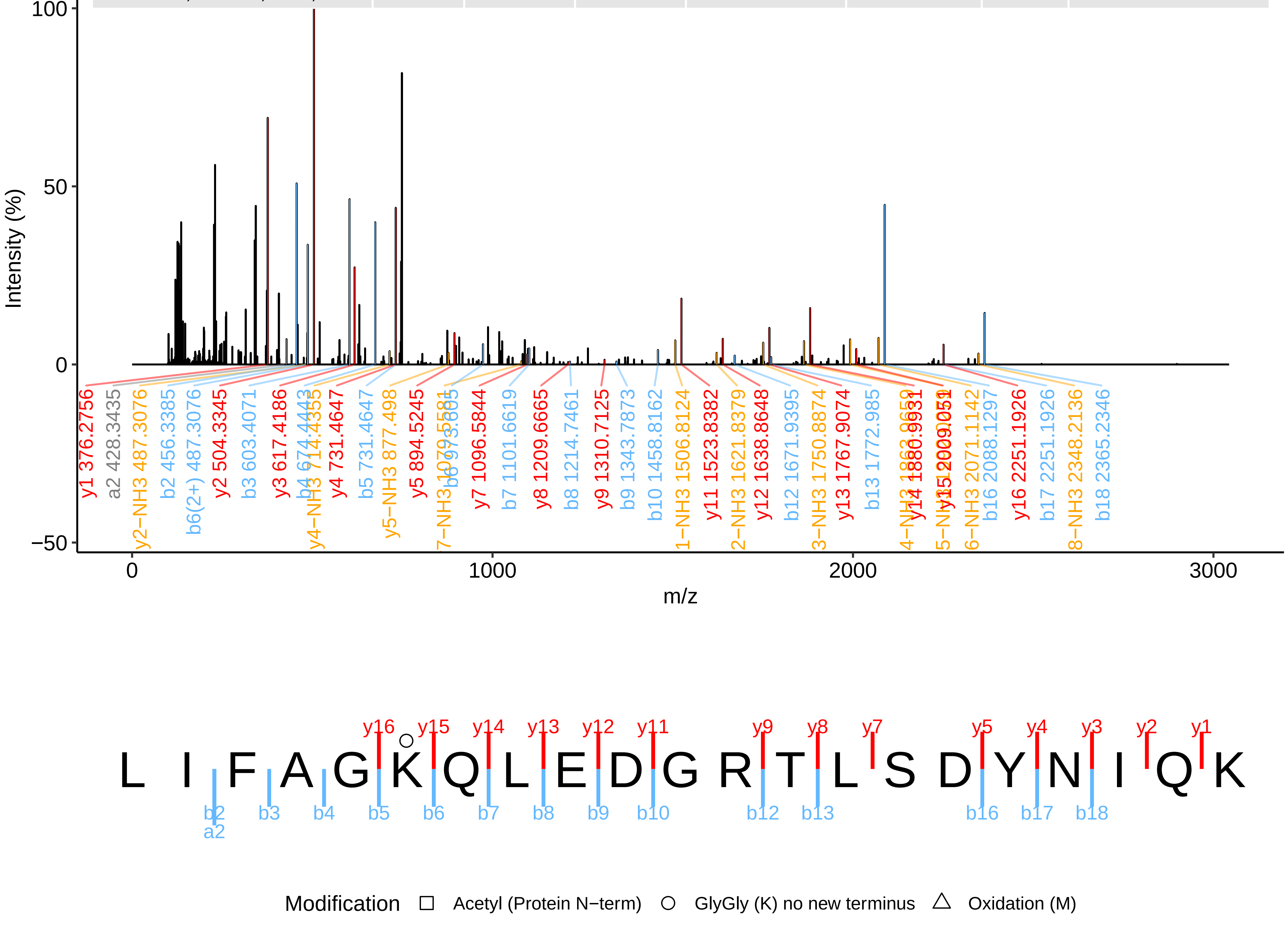

20200924_KIZ_MUb5_GlyGly_1 − Scan number: 22064

Gene Names	Charge	m/z	Mass	Retention time	PEP	Score	Precursor Intensity
RPS27A;UBA52;UBB;UBC	4	631.5855	2522.313	71.039	1.6977e−48	200.27	40181412

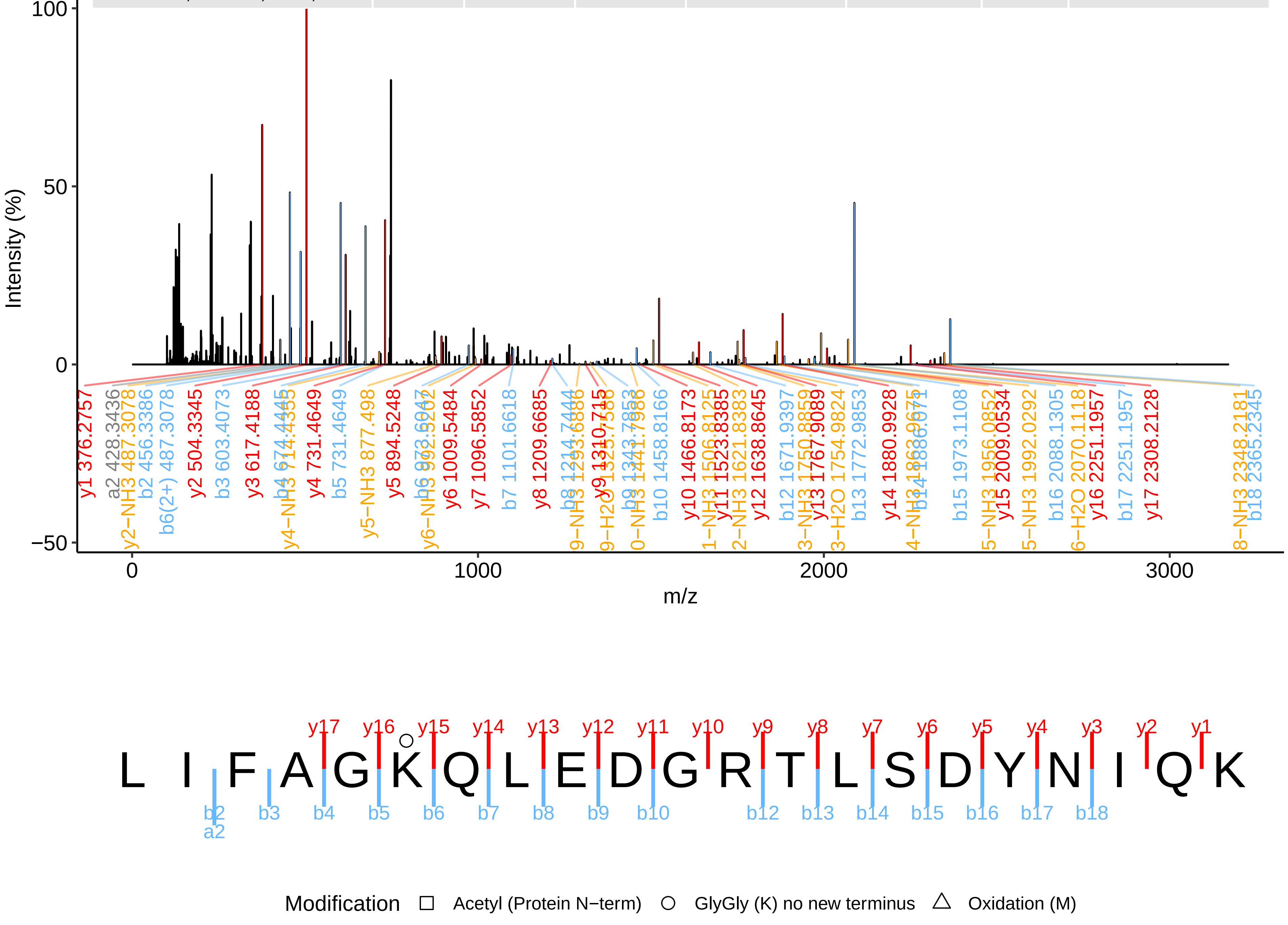

20200924_KIZ_MUb5_GlyGly_1 − Scan number: 22232

Gene Names	Charge	m/z	Mass	Retention time	PEP	Score	Precursor Intensity
RPS27A;UBA52;UBB;UBC	4	631.5855	2522.313	71.542	9.1988e−48	190.51	81156664

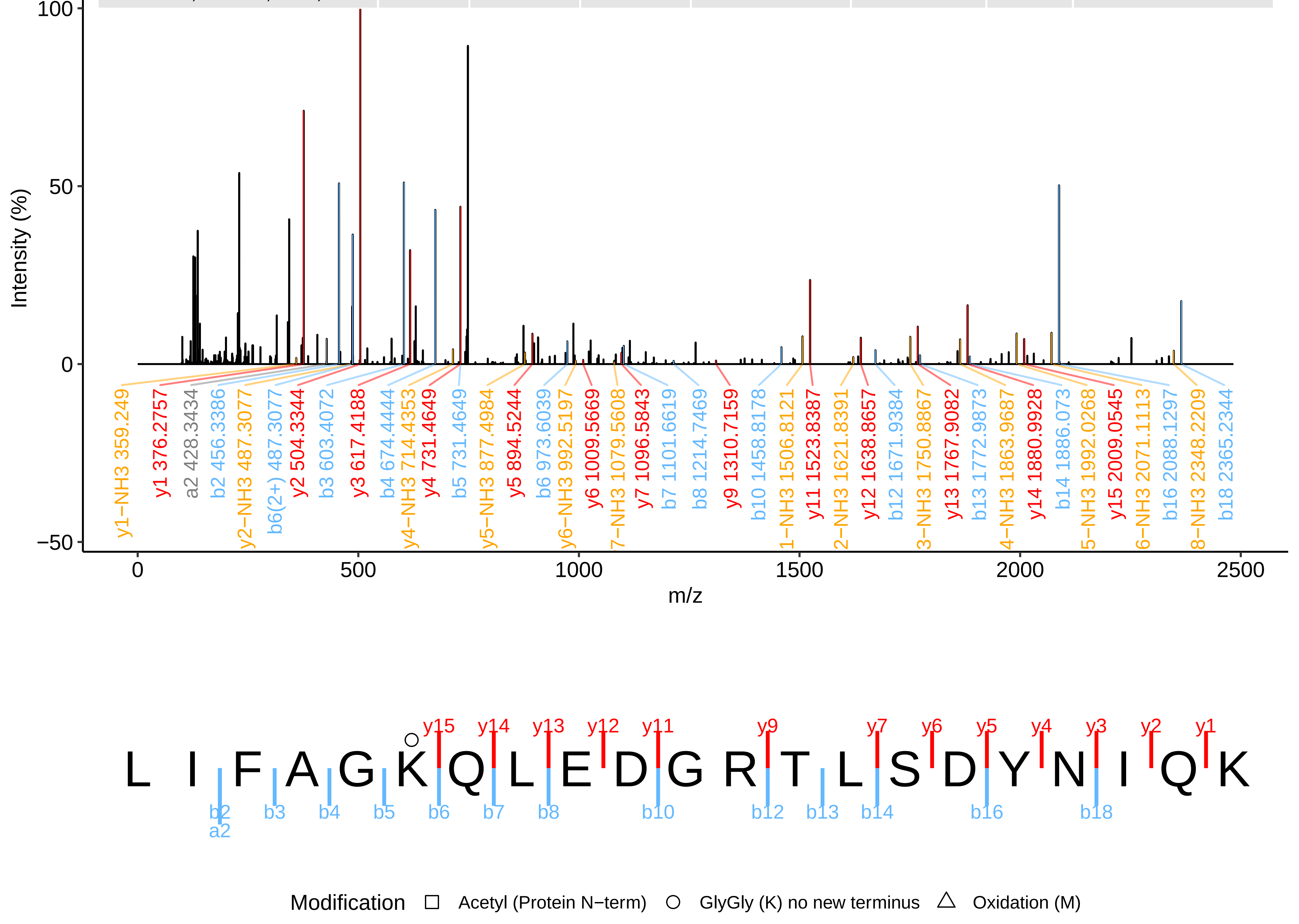

20200924_KIZ_MUb5_GlyGly_1 − Scan number: 22390

Gene Names	Charge	m/z	Mass	Retention time	PEP	Score	Precursor Intensity
RPS27A;UBA52;UBB;UBC	4	631.5855	2522.313	72.046	1.2624e−47	186.06	338972896

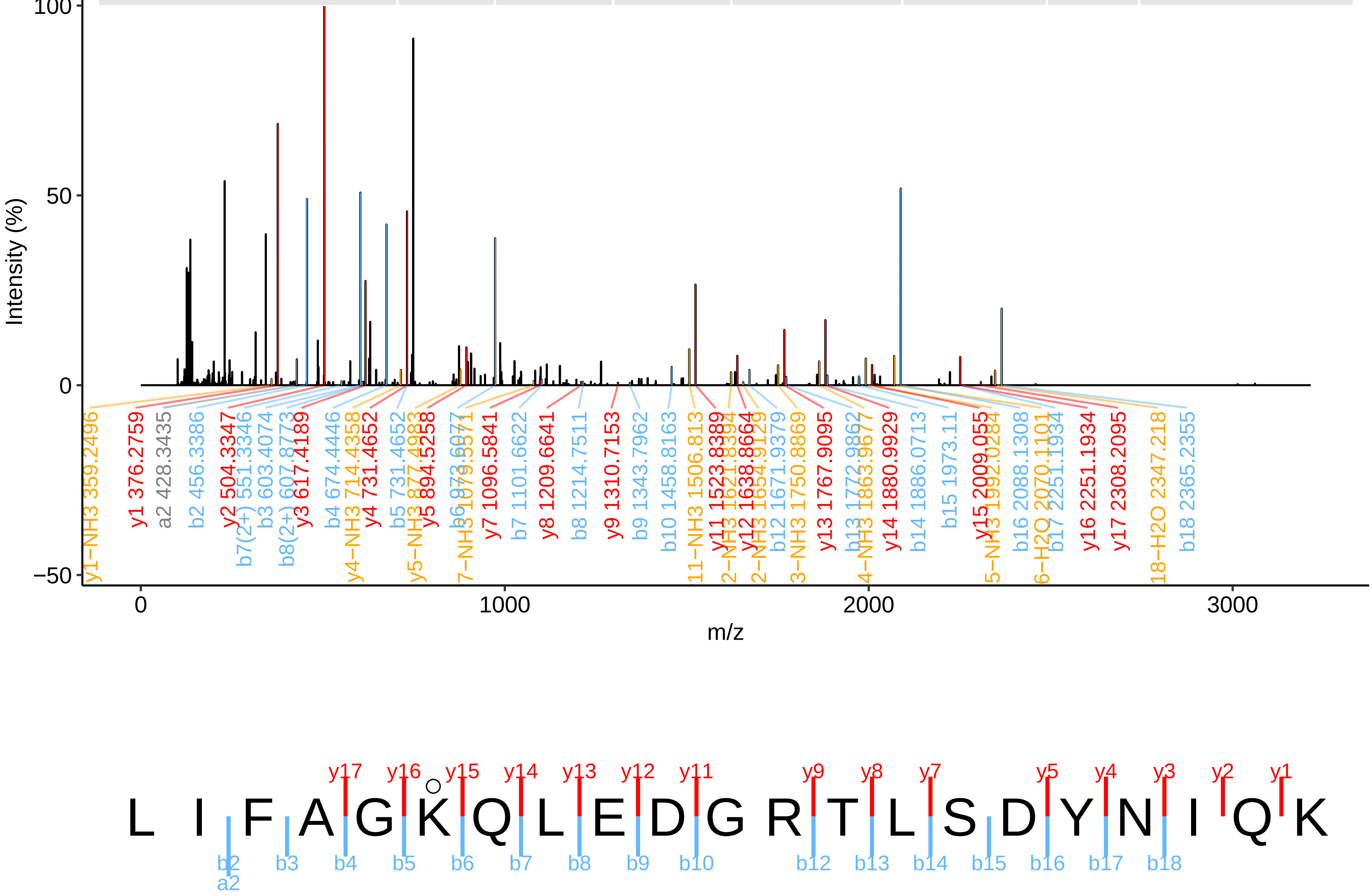

Modification □ Acetyl (Protein N−term) ○ GlyGly (K) no new terminus △ Oxidation (M)

20200924_KIZ_MUb5_GlyGly_1 − Scan number: 22552

Gene Names	Charge	m/z	Mass	Retention time	PEP	Score	Precursor Intensity
RPS27A;UBA52;UBB;UBC	4	631.5855	2522.313	72.553	9.292e−49	201.27	553580672

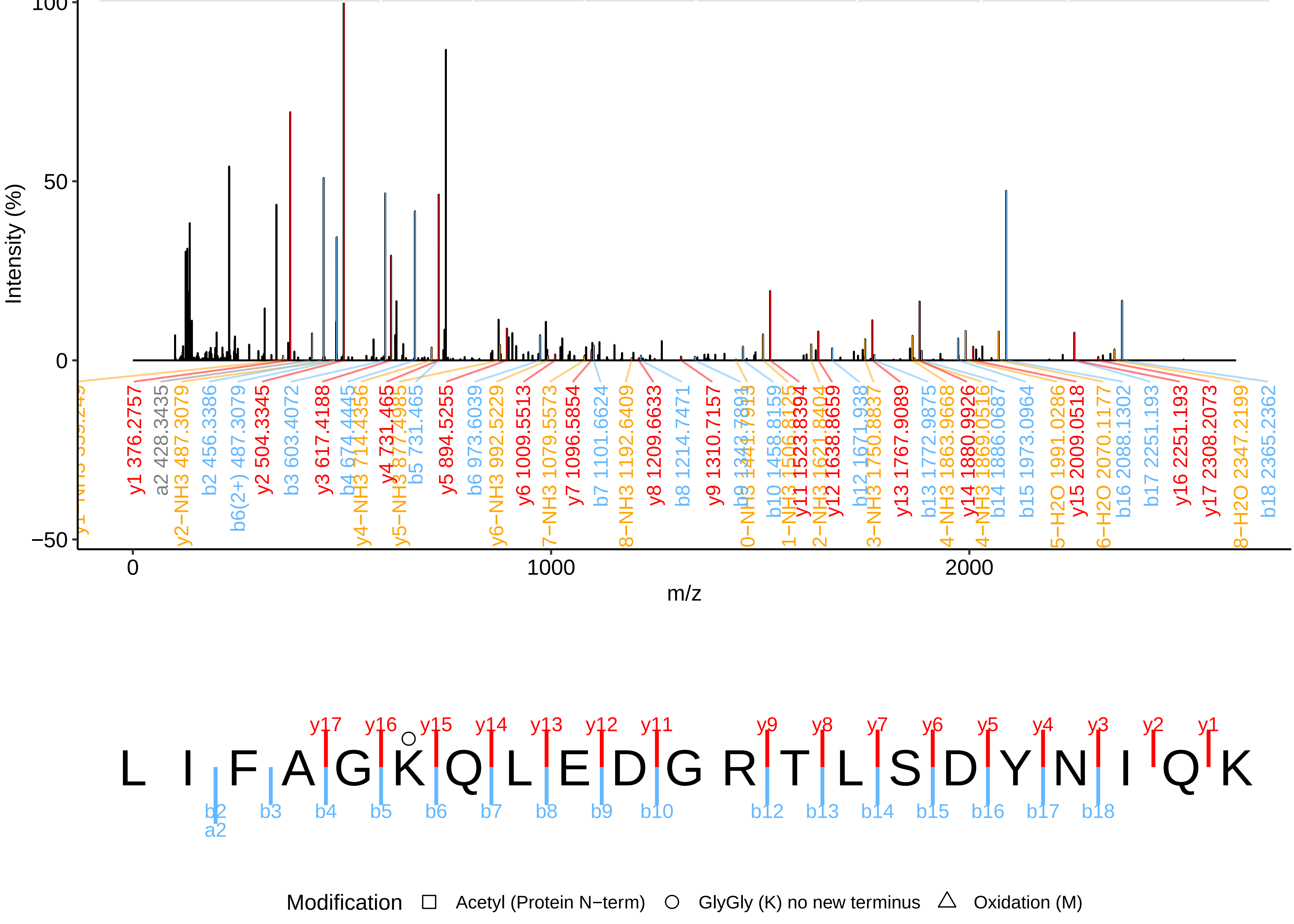

20200924_KIZ_MUb5_GlyGly_1 − Scan number: 22717

Gene Names	Charge	m/z	Mass	Retention time	PEP	Score	Precursor Intensity
RPS27A;UBA52;UBB;UBC	4	631.5855	2522.313	73.062	5.0319e−48	195.93	530959840

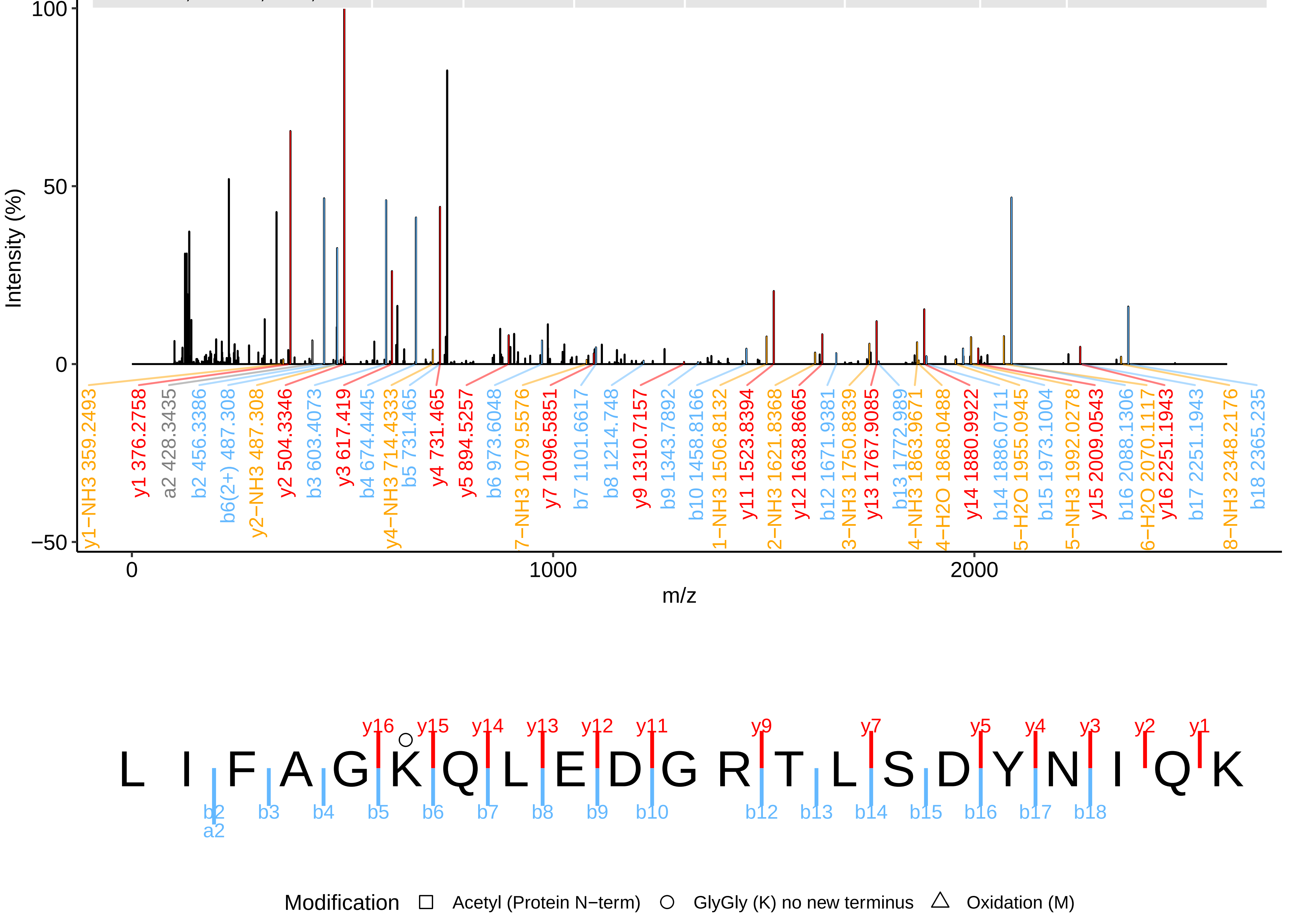

20200924_KIZ_MUb5_GlyGly_1 − Scan number: 22553

Gene Names	Charge	m/z	Mass	Retention time	PEP	Score	Precursor Intensity
RPS27A;UBA52;UBB;UBC	3	841.7782	2522.313	72.556	3.272e−15	131.09	NA

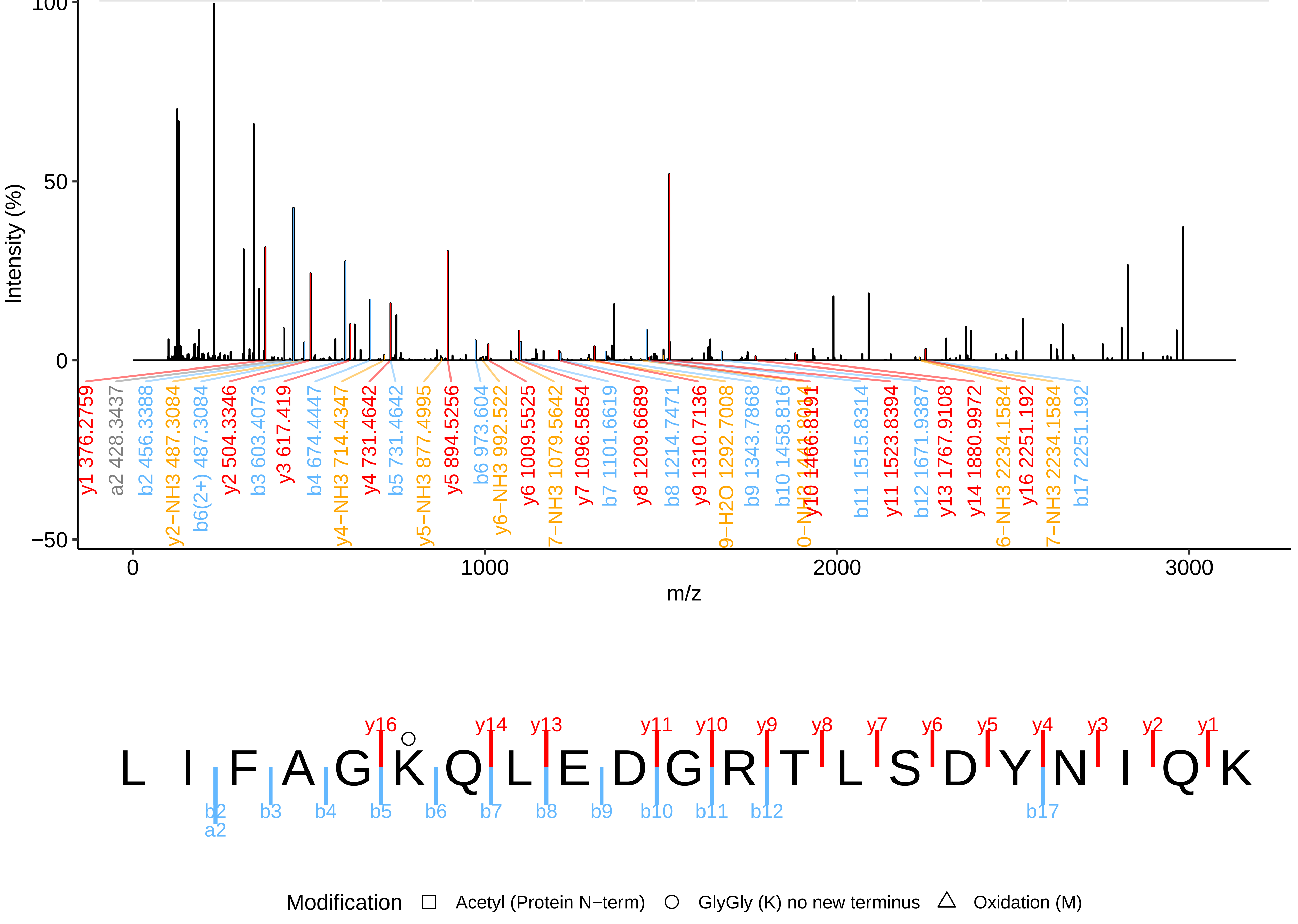

20200924_KIZ_MUb5_GlyGly_1 − Scan number: 22684

Gene Names	Charge	m/z	Mass	Retention time	PEP	Score	Precursor Intensity
RPS27A;UBA52;UBB;UBC	5	505.4699	2522.313	72.961	3.187e−09	102.37	94475552

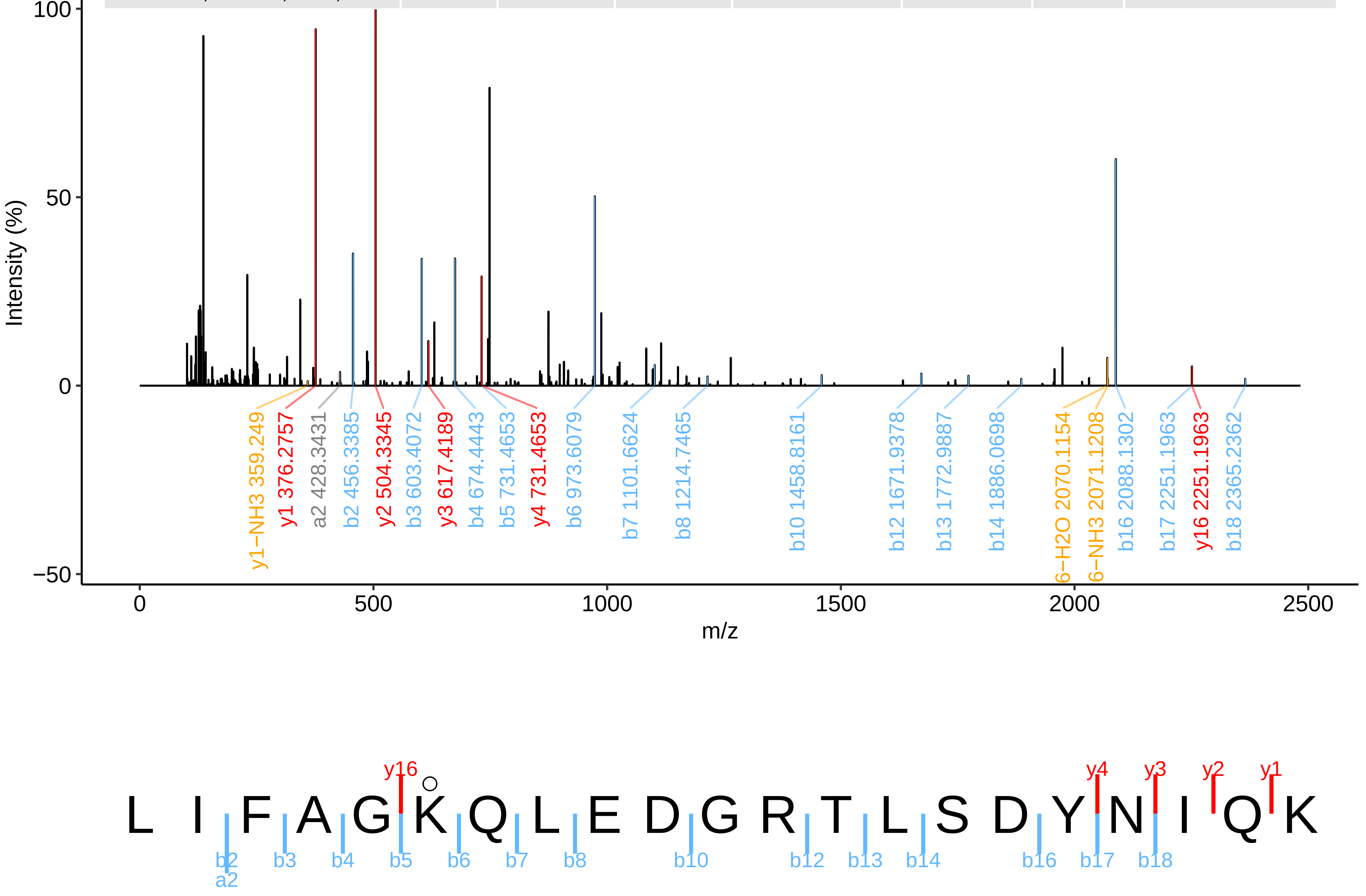

Modification □ Acetyl (Protein N−term) ○ GlyGly (K) no new terminus △ Oxidation (M)

20200924_KIZ_MUb5_GlyGly_1 − Scan number: 22846

Gene Names	Charge	m/z	Mass	Retention time	PEP	Score	Precursor Intensity
RPS27A;UBA52;UBB;UBC	5	505.4699	2522.313	73.47	3.5465e−06	93.684	63828176

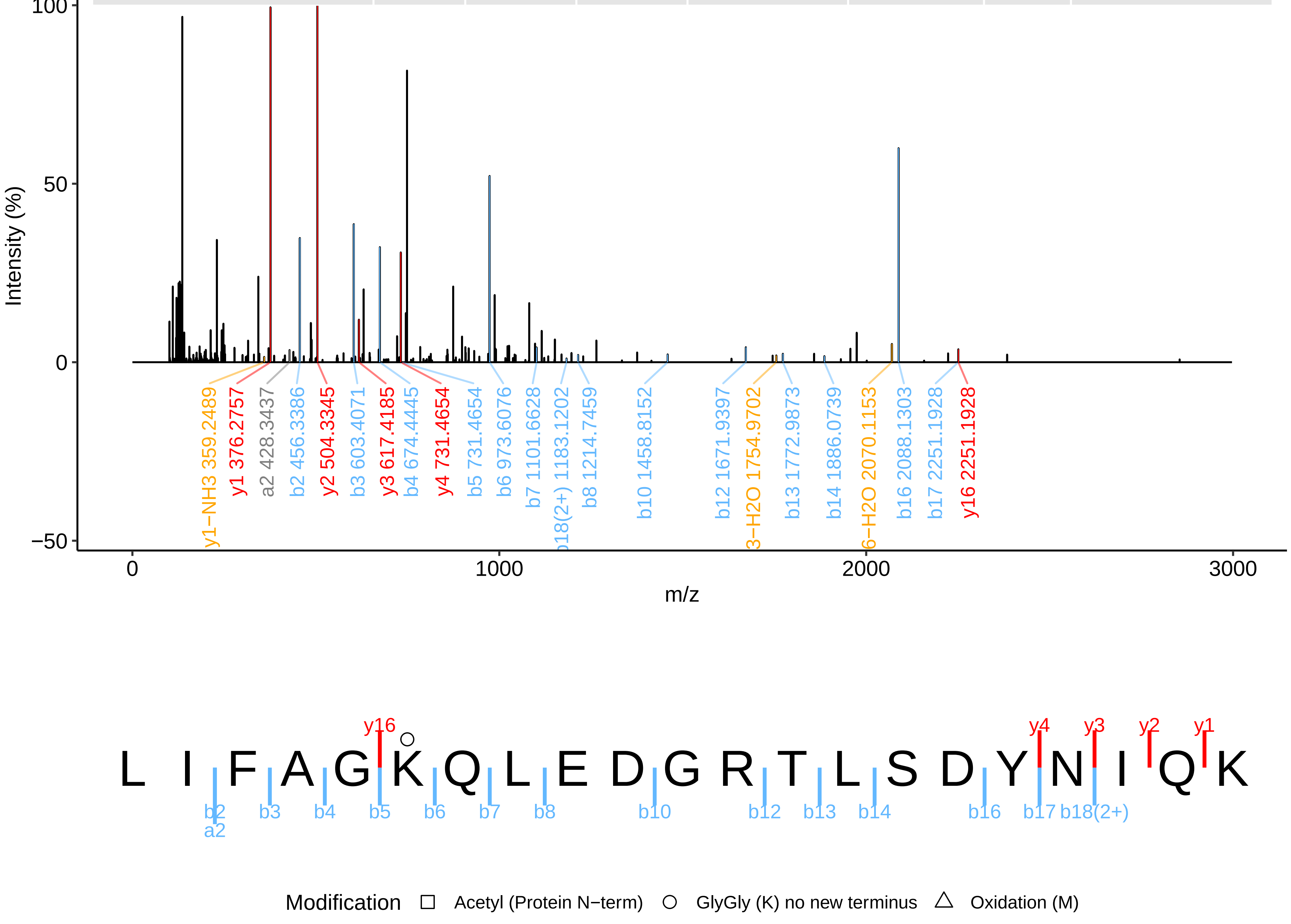

20200924_KIZ_MUb5_GlyGly_1 − Scan number: 22875

Gene Names	Charge	m/z	Mass	Retention time	PEP	Score	Precursor Intensity
RPS27A;UBA52;UBB;UBC	4	631.5855	2522.313	73.562	4.0782e−58	203.77	265953840

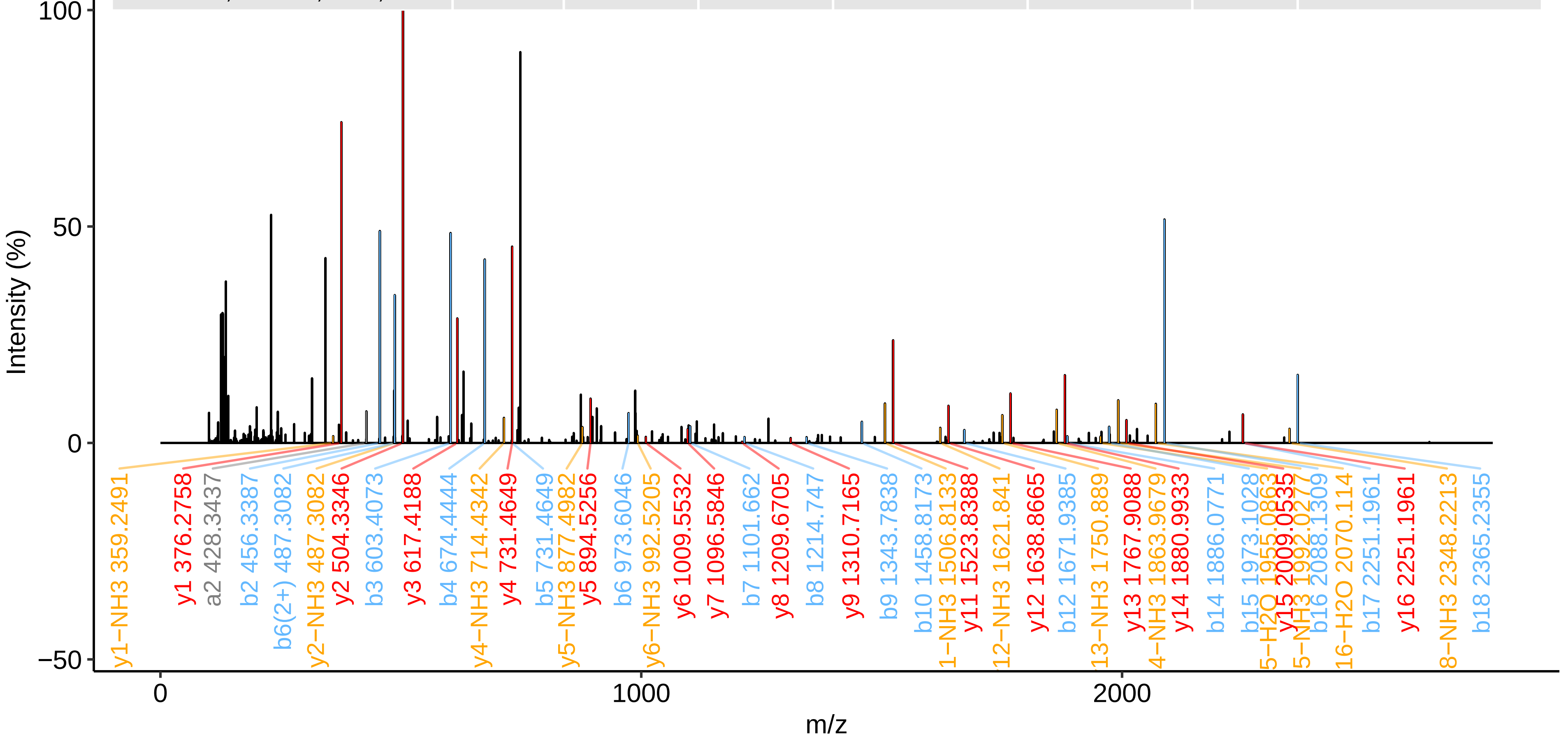

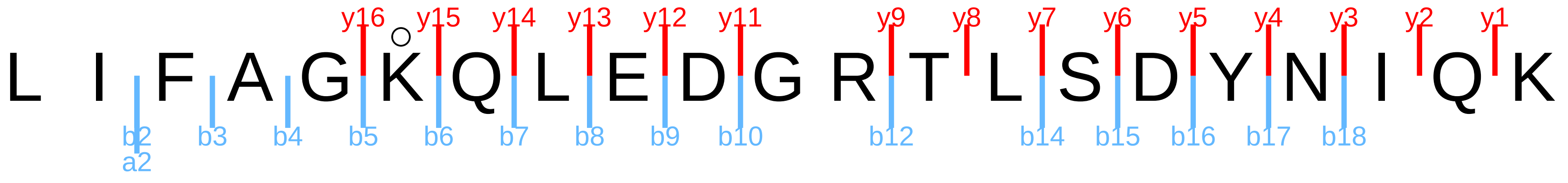

Modification □ Acetyl (Protein N−term) ○ GlyGly (K) no new terminus △ Oxidation (M)

20200924_KIZ_MUb5_GlyGly_1 − Scan number: 23036

Gene Names	Charge	m/z	Mass	Retention time	PEP	Score	Precursor Intensity
RPS27A;UBA52;UBB;UBC	4	631.5855	2522.313	74.071	2.2794e−70	219.91	296433696

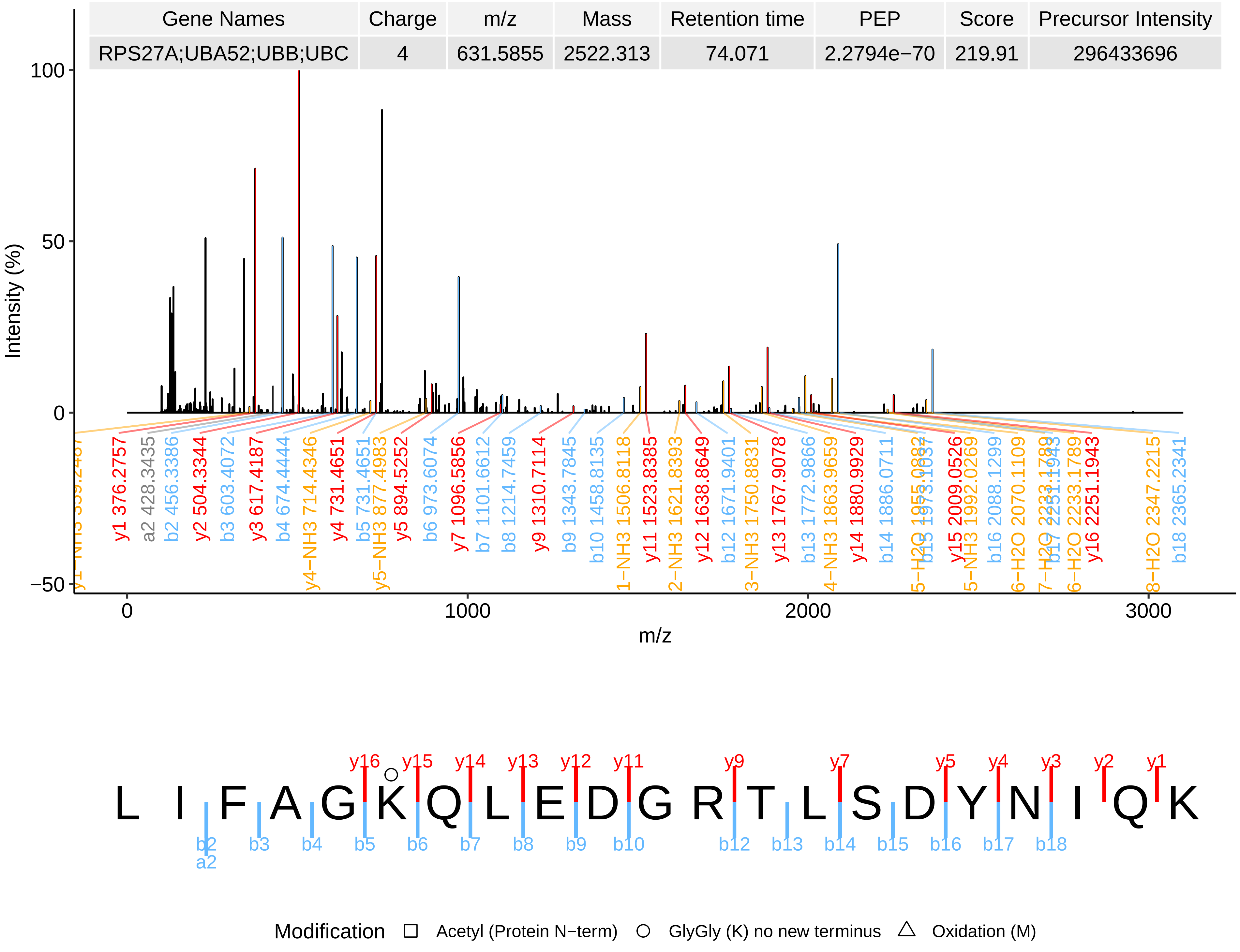

20200924_KIZ_MUb5_GlyGly_1 − Scan number: 23058

Gene Names	Charge	m/z	Mass	Retention time	PEP	Score	Precursor Intensity
RPS27A;UBA52;UBB;UBC	3	841.7782	2522.313	74.142	1.7964e−138	289.96	14714276

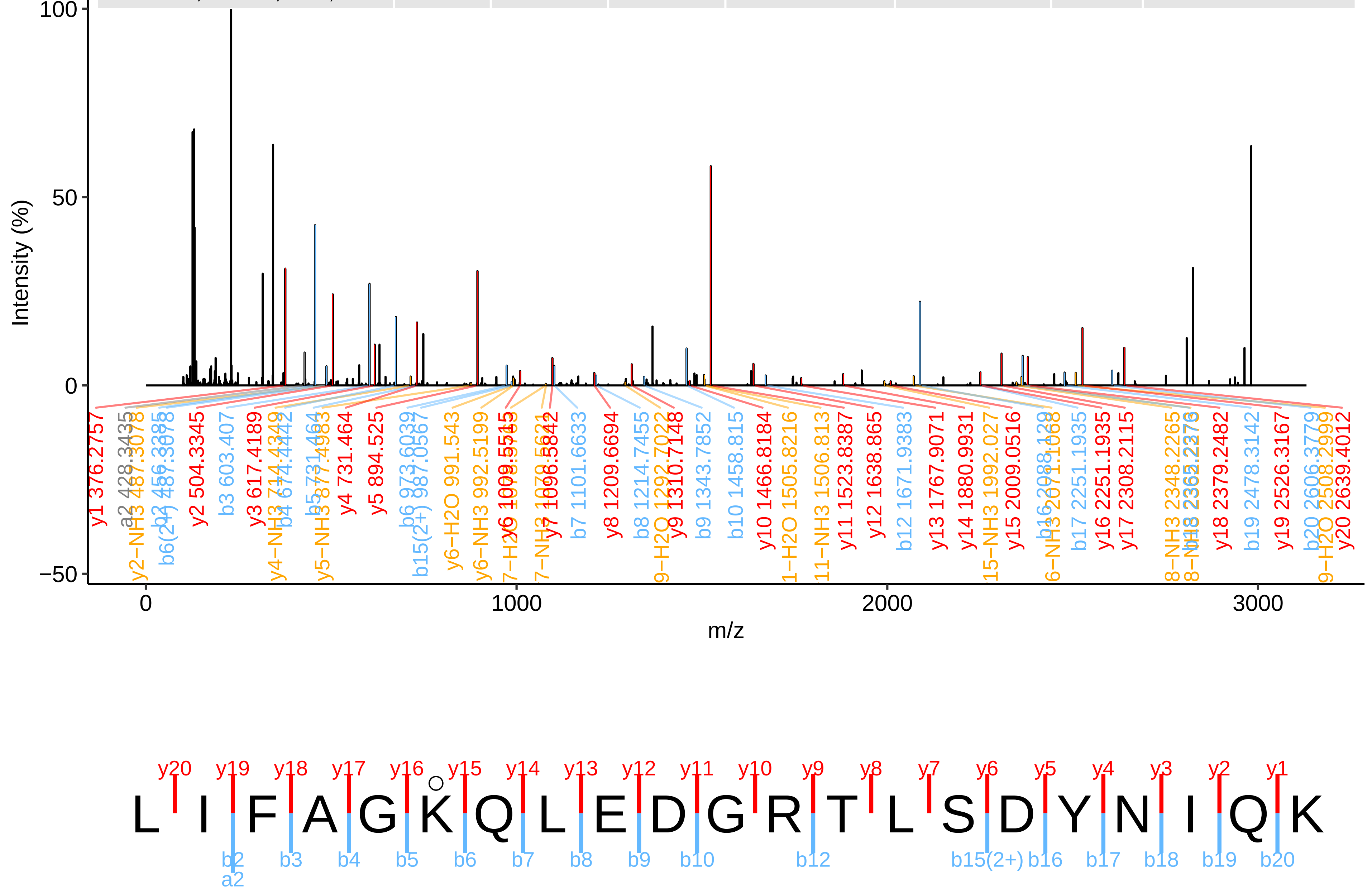

Modification □ Acetyl (Protein N−term) ○ GlyGly (K) no new terminus △ Oxidation (M)

20200924_KIZ_MUb5_GlyGly_1 − Scan number: 22494

Gene Names	Charge	m/z	Mass	Retention time	PEP	Score	Precursor Intensity
RPS27A;UBA52;UBB;UBC	3	841.7782	2522.313	72.383	2.2103e−85	253.31	7233476

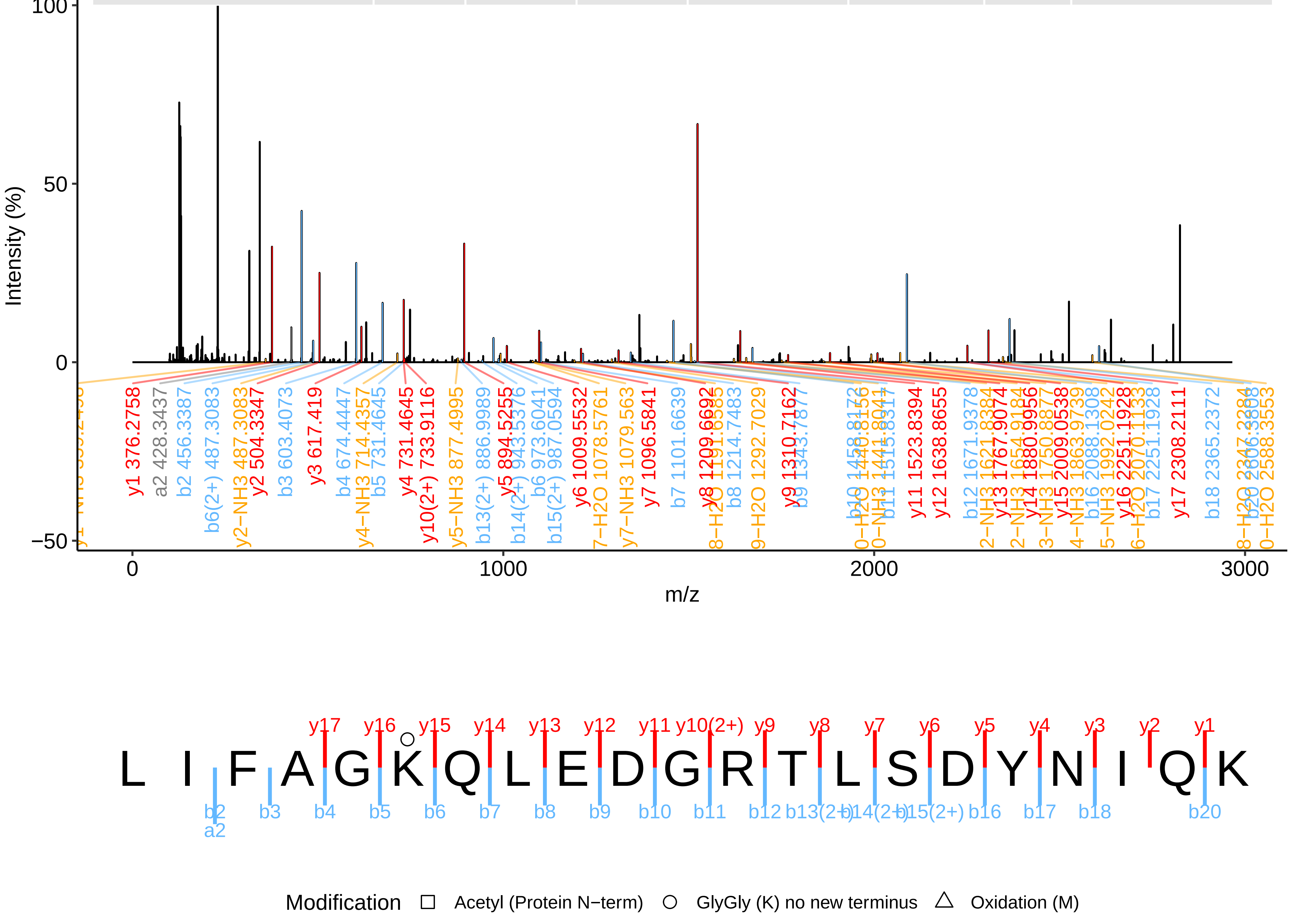

20200924_KIZ_MUb5_GlyGly_1 − Scan number: 22888

Gene Names	Charge	m/z	Mass	Retention time	PEP	Score	Precursor Intensity
RPS27A;UBA52;UBB;UBC	3	841.7782	2522.313	73.607	8.4665e−59	218.88	11119241

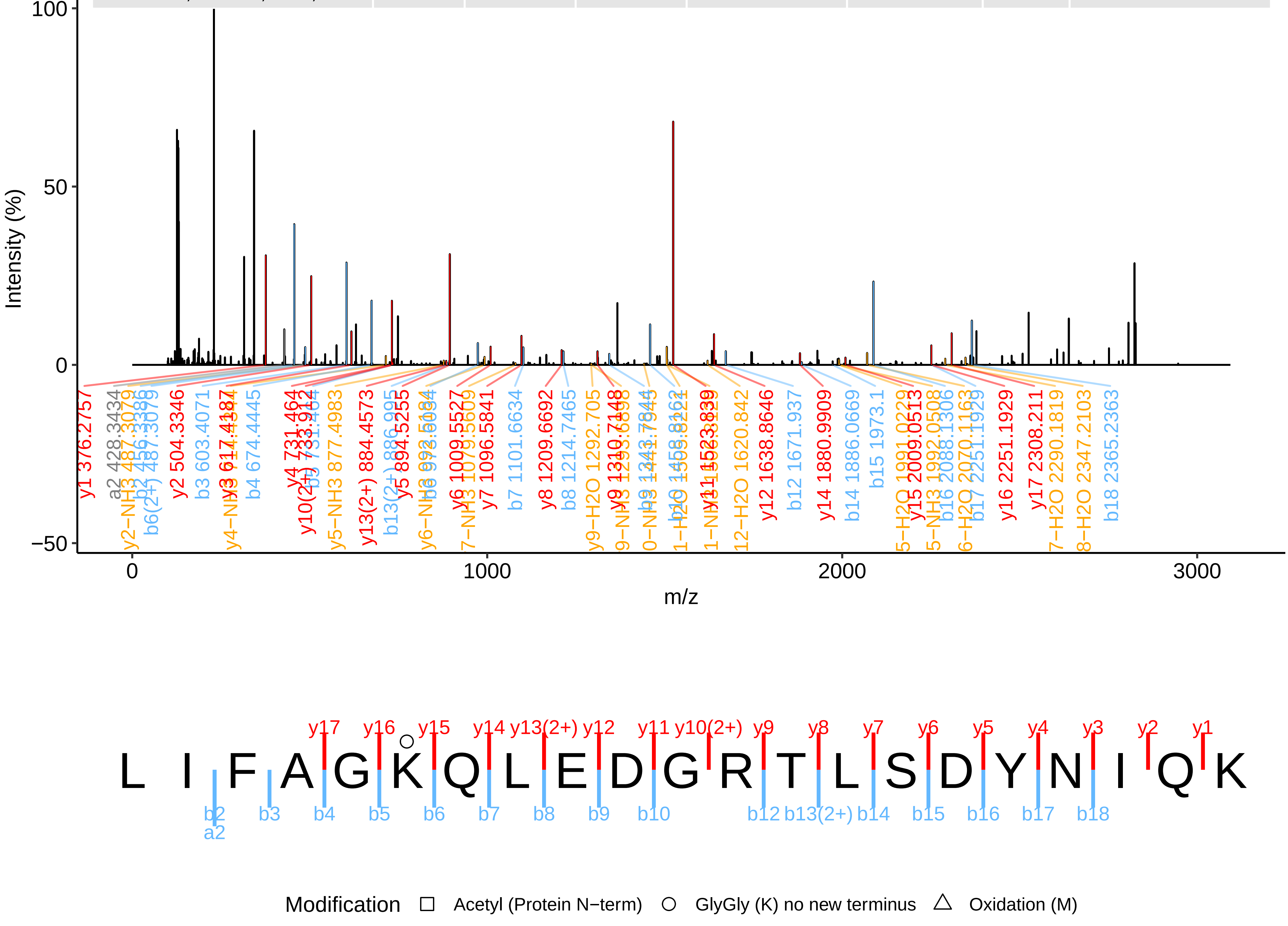

20200924_KIZ_MUb5_GlyGly_2 − Scan number: 19965

Gene Names	Charge	m/z	Mass	Retention time	PEP	Score	Precursor Intensity
RPS27A;UBA52;UBB;UBC	4	631.5855	2522.313	74.002	1.5818e−29	155.88	6487214

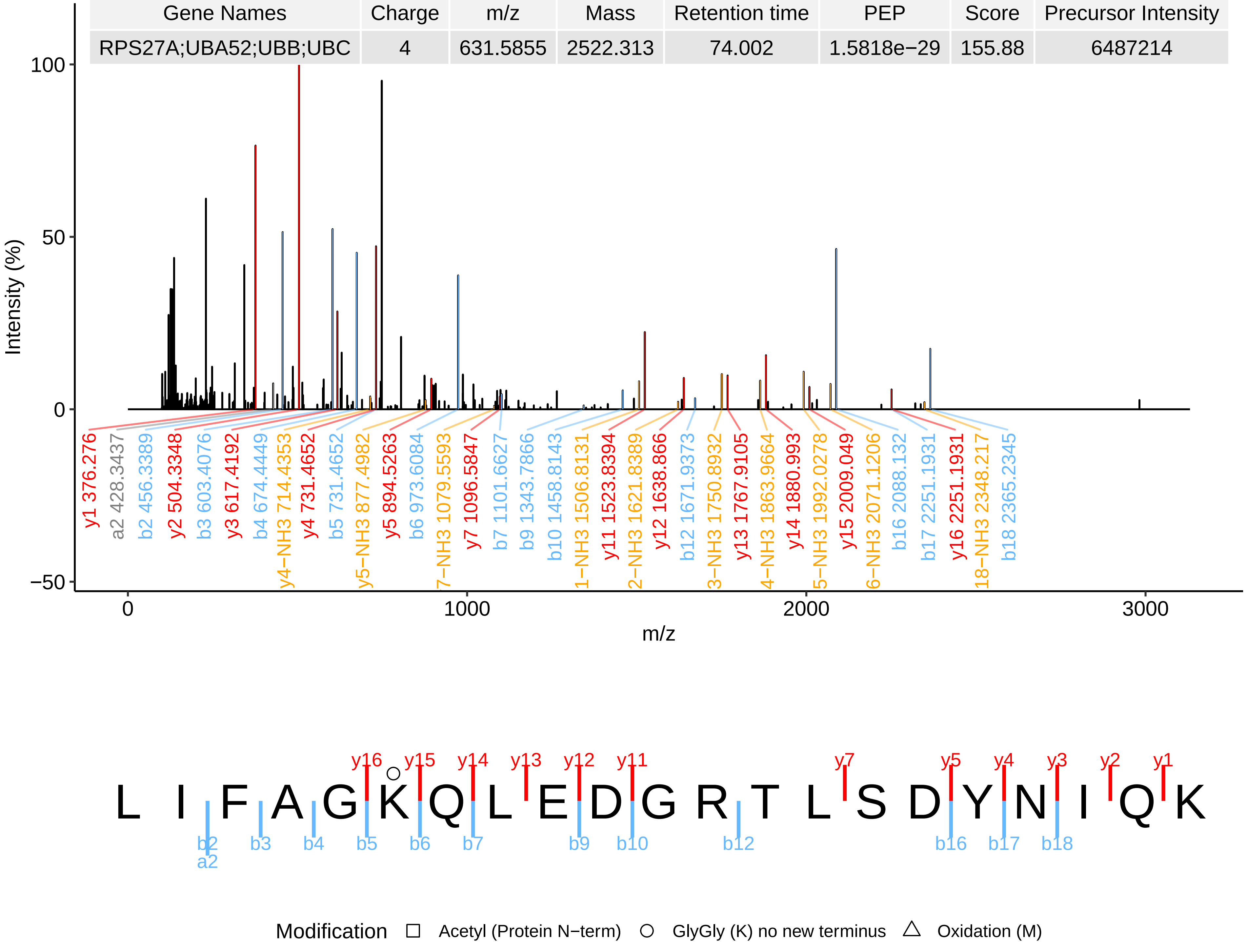

20200924_KIZ_MUb5_GlyGly_2 − Scan number: 20147

Gene Names	Charge	m/z	Mass	Retention time	PEP	Score	Precursor Intensity
RPS27A;UBA52;UBB;UBC	4	631.5855	2522.313	74.663	3.1745e−39	178.88	50848232

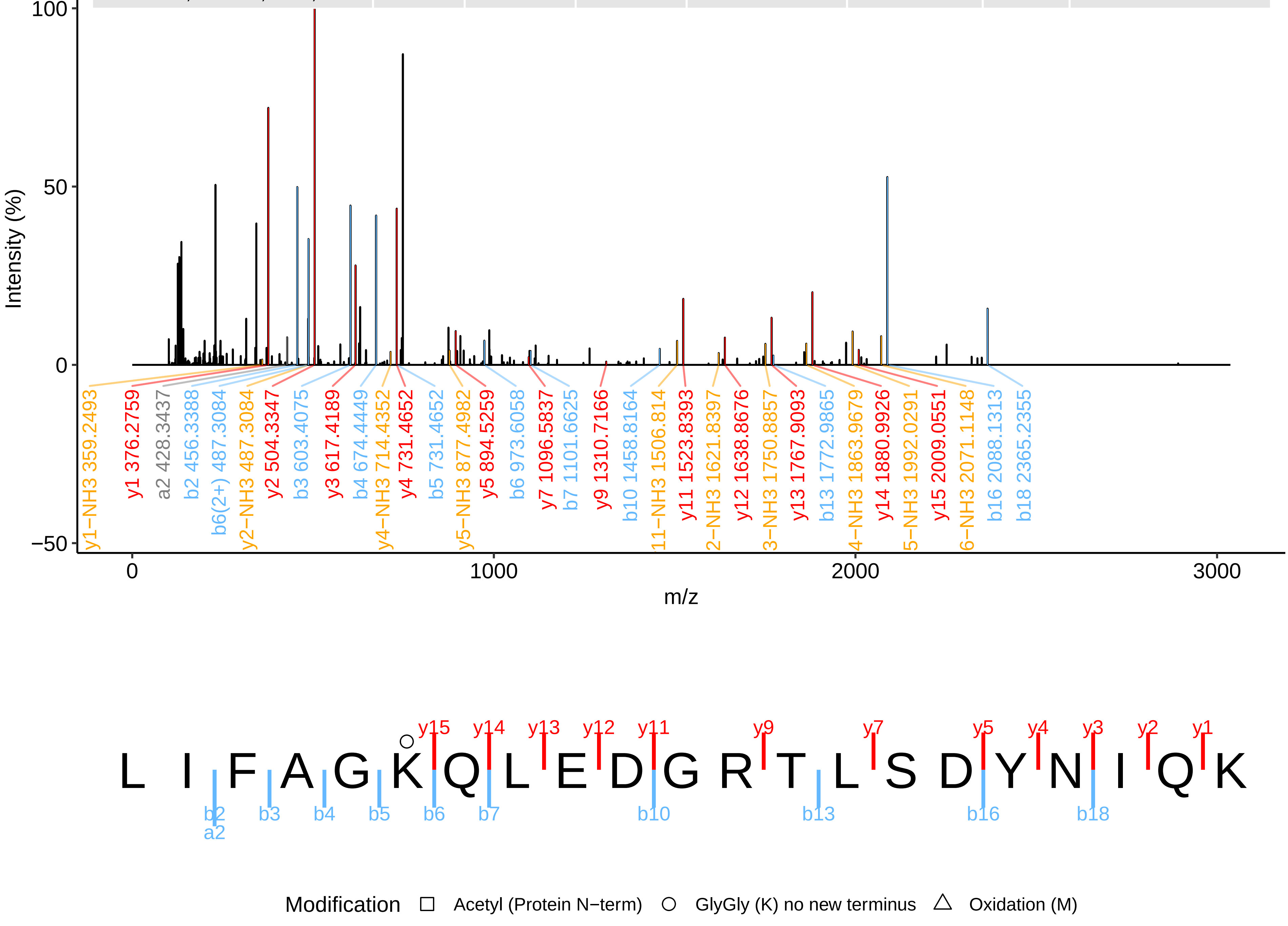

20200924_KIZ_MUb5_GlyGly_2 − Scan number: 20287

Gene Names	Charge	m/z	Mass	Retention time	PEP	Score	Precursor Intensity
RPS27A;UBA52;UBB;UBC	4	631.5855	2522.313	75.173	1.7081e−48	200.26	159238048

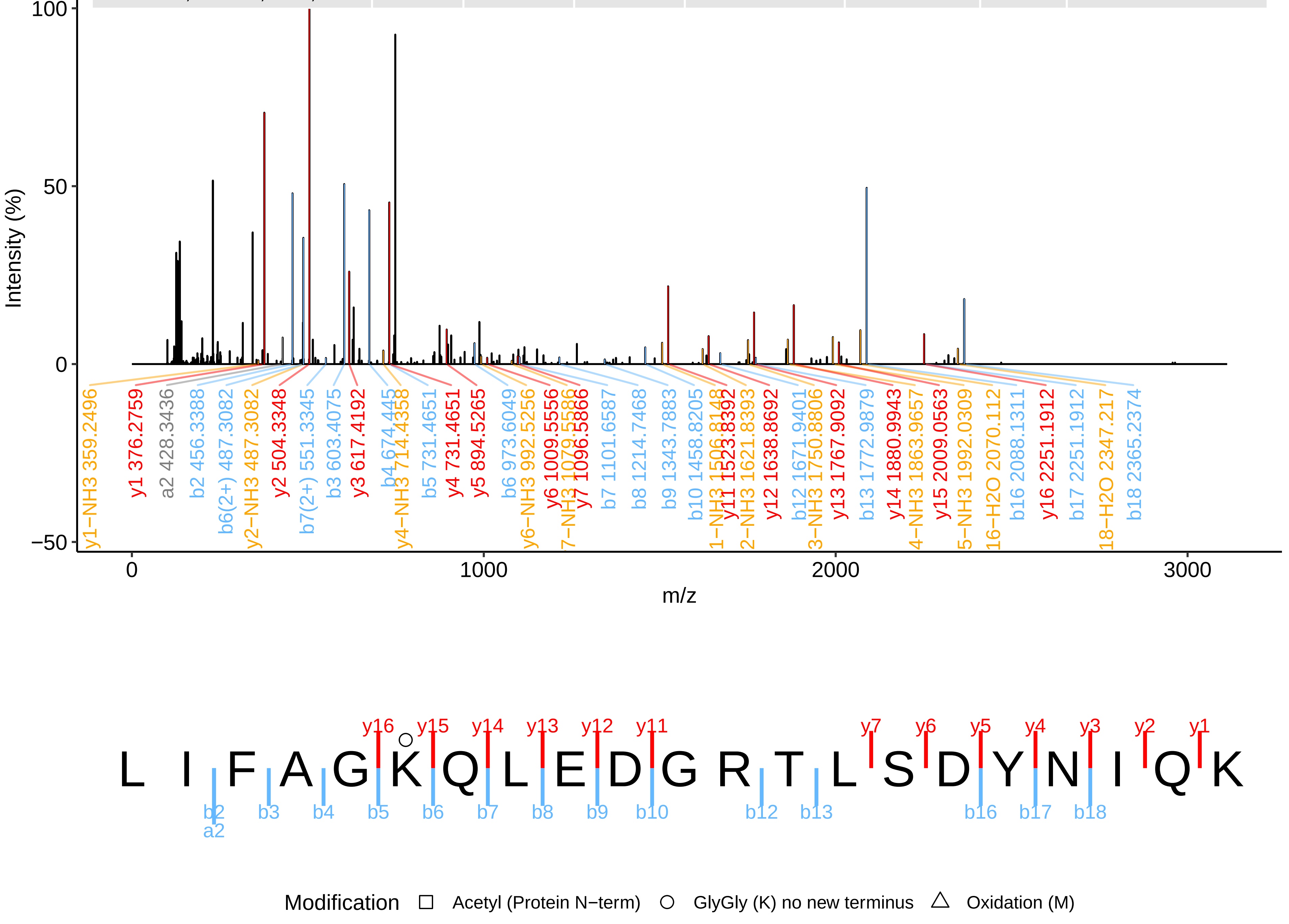

20200924_KIZ_MUb5_GlyGly_2 − Scan number: 20431

Gene Names	Charge	m/z	Mass	Retention time	PEP	Score	Precursor Intensity
RPS27A;UBA52;UBB;UBC	4	631.5855	2522.313	75.695	8.2032e−59	216.65	186536064

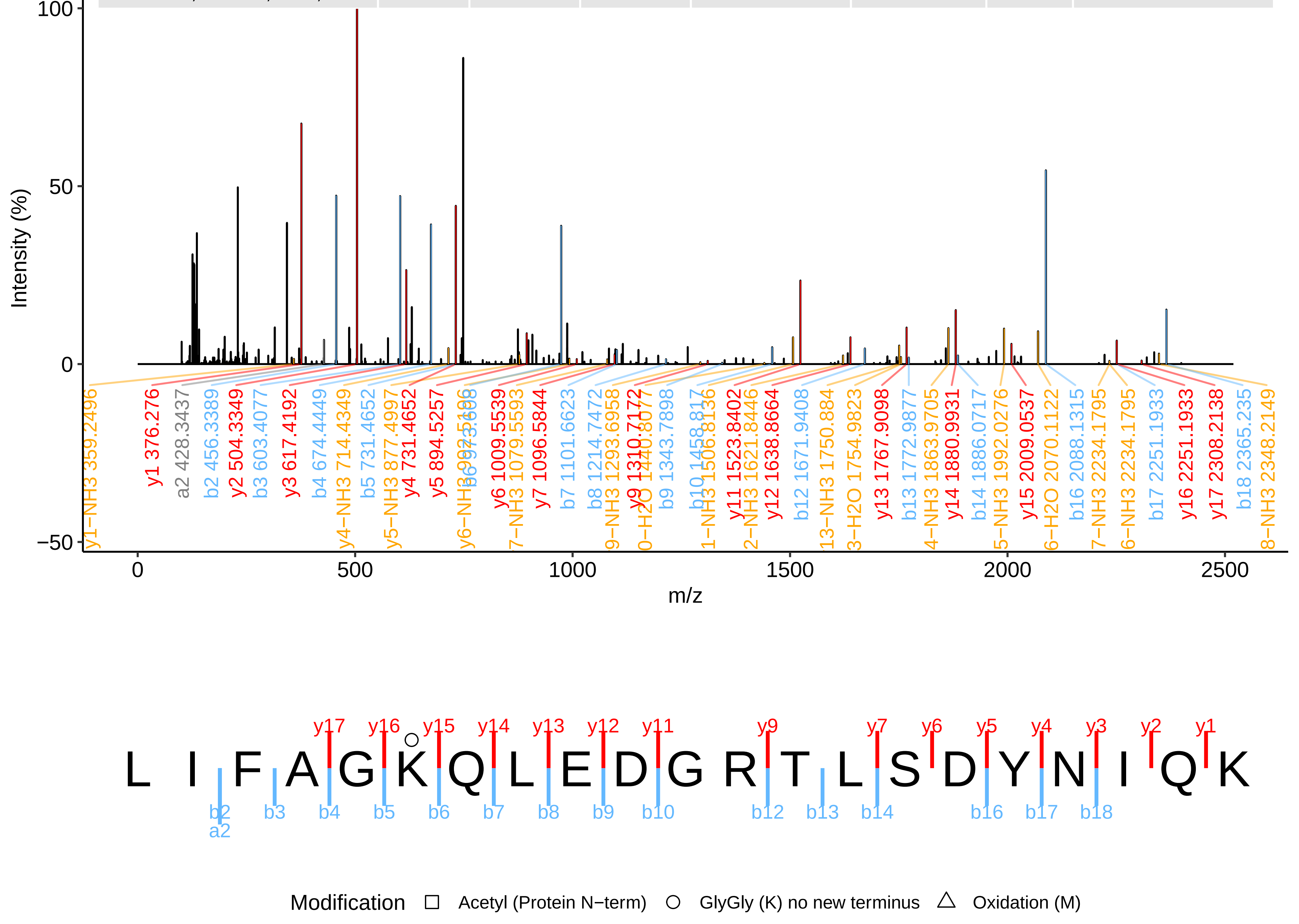

20200924_KIZ_MUb5_GlyGly_2 − Scan number: 20579

Gene Names	Charge	m/z	Mass	Retention time	PEP	Score	Precursor Intensity
RPS27A;UBA52;UBB;UBC	4	631.5855	2522.313	76.201	1.0837e−47	188.38	119272456

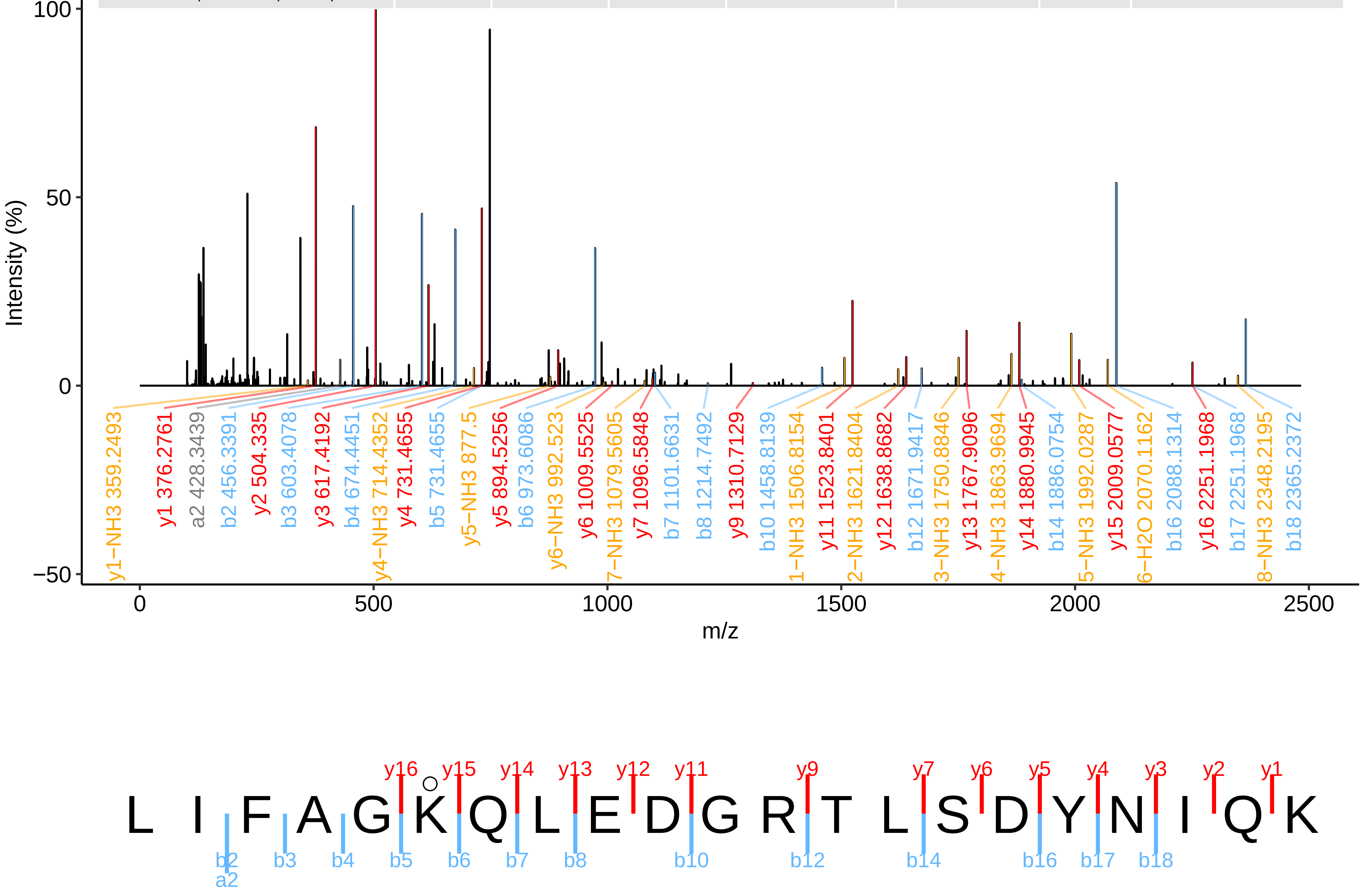

Modification □ Acetyl (Protein N−term) ○ GlyGly (K) no new terminus △ Oxidation (M)

20200924_KIZ_MUb5_GlyGly_2 − Scan number: 20273

Gene Names	Charge	m/z	Mass	Retention time	PEP	Score	Precursor Intensity
RPS27A;UBA52;UBB;UBC	5	505.4699	2522.313	75.124	0.0011334	67.995	23788616

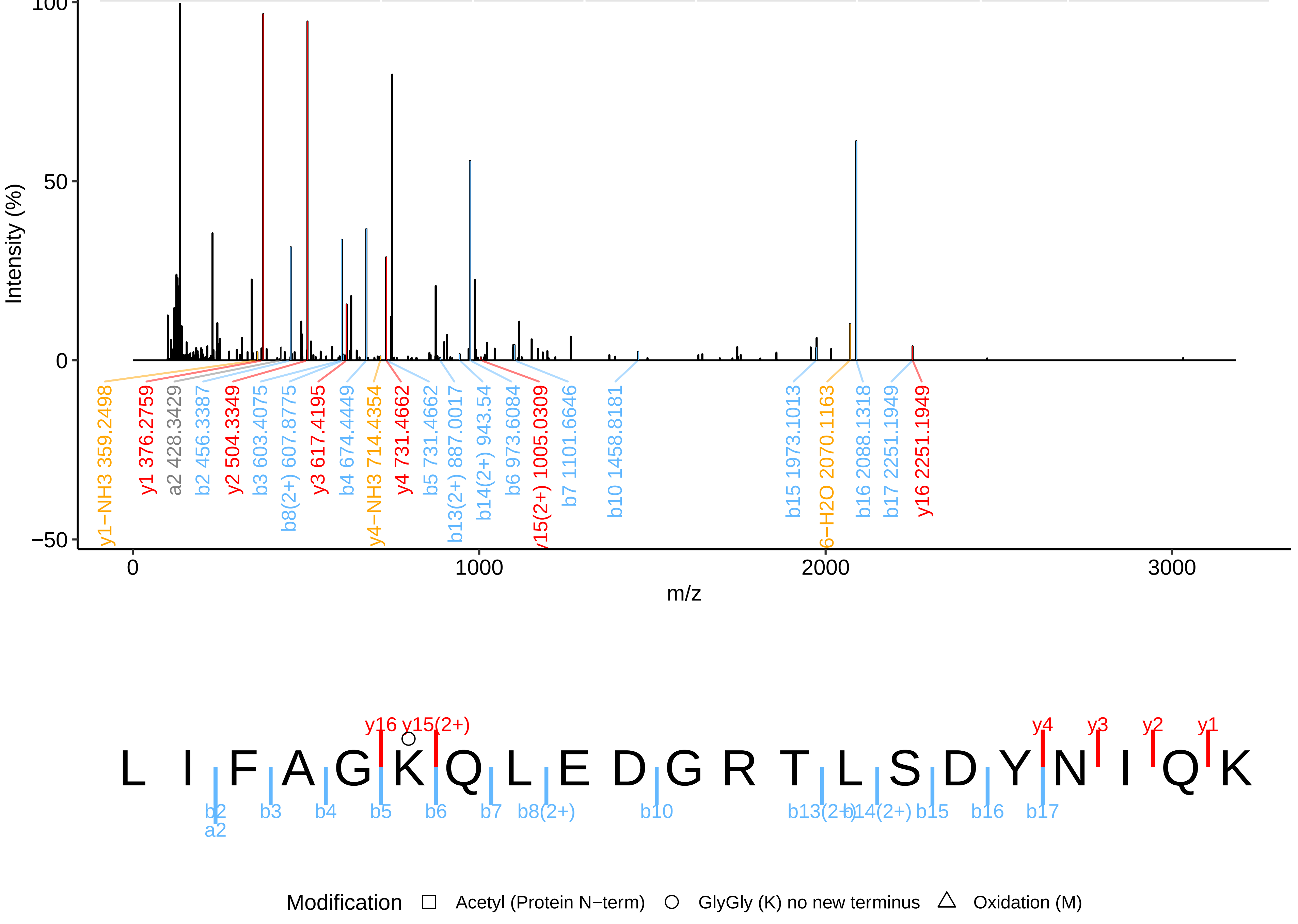

20200924_KIZ_MUb5_GlyGly_2 − Scan number: 20418

Gene Names	Charge	m/z	Mass	Retention time	PEP	Score	Precursor Intensity
RPS27A;UBA52;UBB;UBC	5	505.4699	2522.313	75.649	3.2399e−06	94.057	36355068

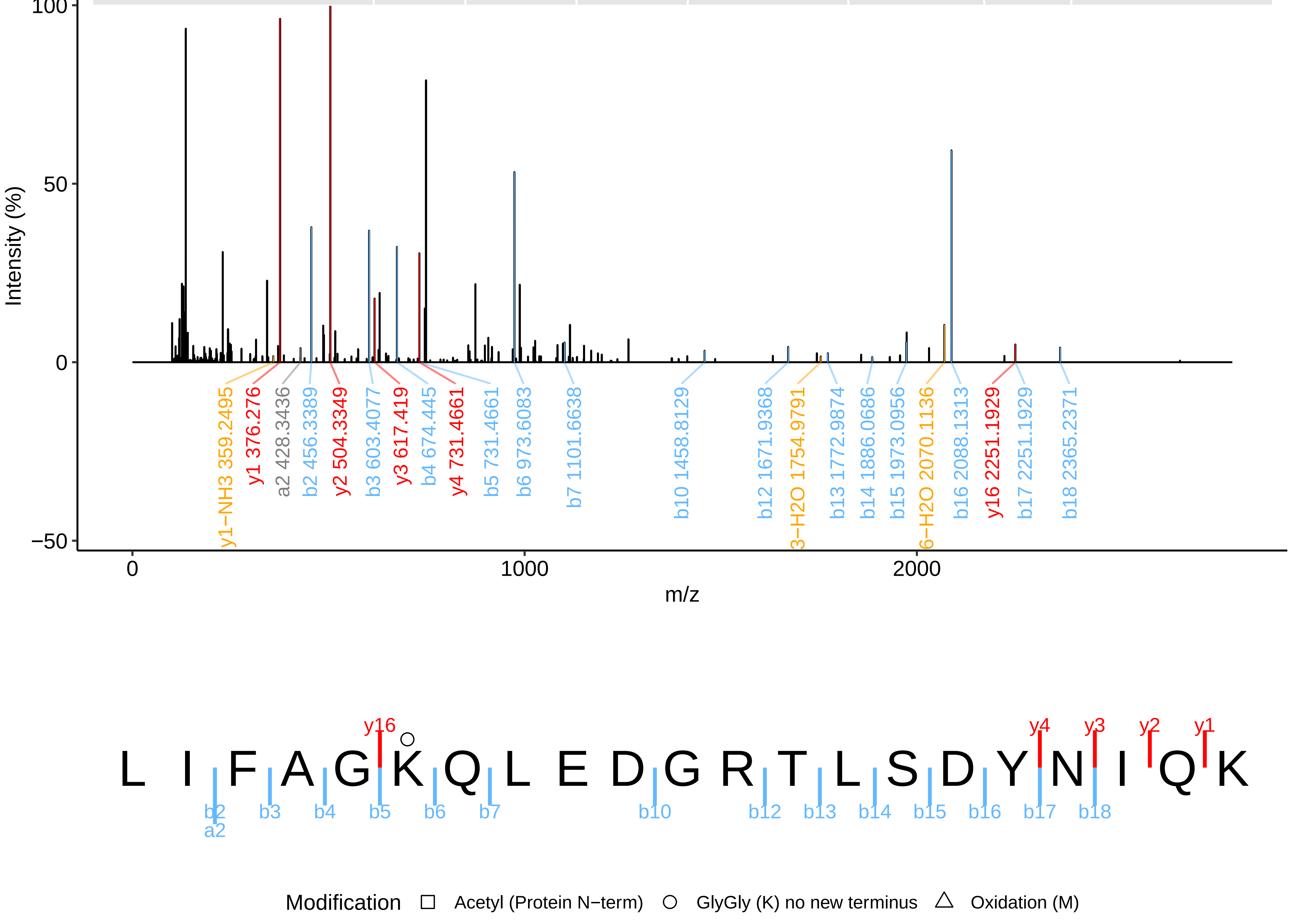

20200924_KIZ_MUb5_GlyGly_2 − Scan number: 20722

Gene Names	Charge	m/z	Mass	Retention time	PEP	Score	Precursor Intensity
RPS27A;UBA52;UBB;UBC	4	631.5855	2522.313	76.707	1.2655e−47	186.01	82304072

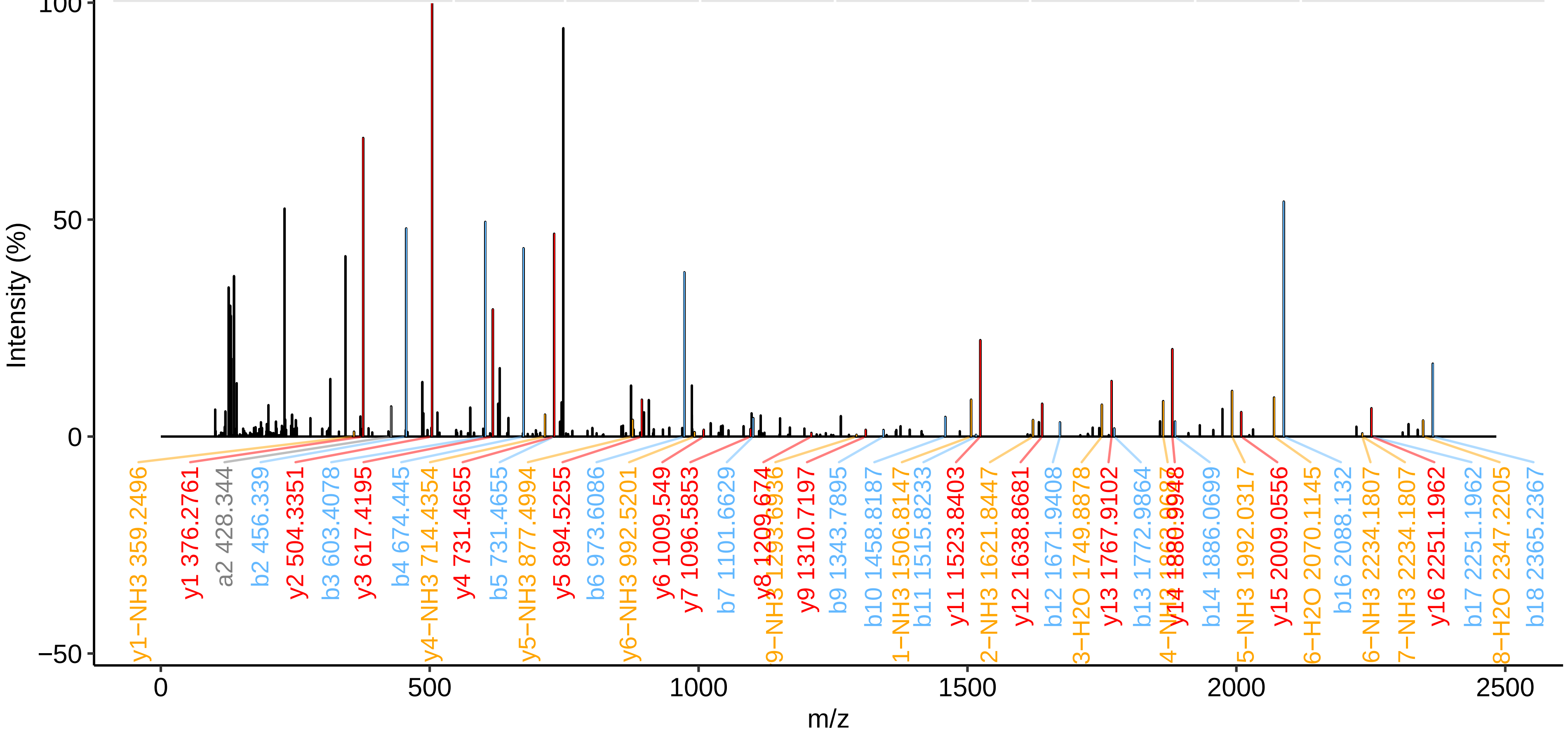

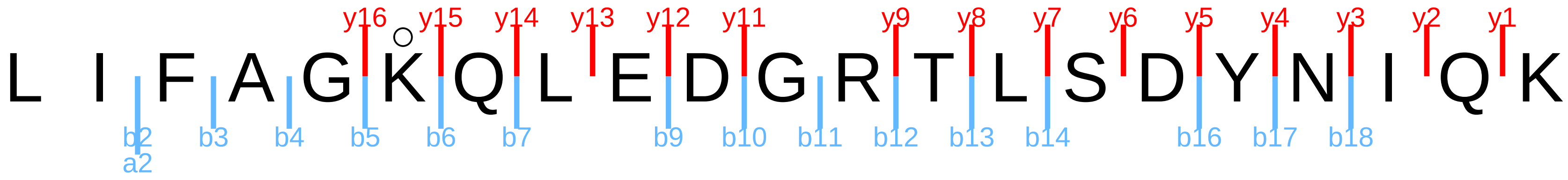

Modification □ Acetyl (Protein N−term) ○ GlyGly (K) no new terminus △ Oxidation (M)

20200924_KIZ_MUb5_GlyGly_2 − Scan number: 20862

Gene Names	Charge	m/z	Mass	Retention time	PEP	Score	Precursor Intensity
RPS27A;UBA52;UBB;UBC	4	631.5855	2522.313	77.217	8.9501e−48	190.83	40846004

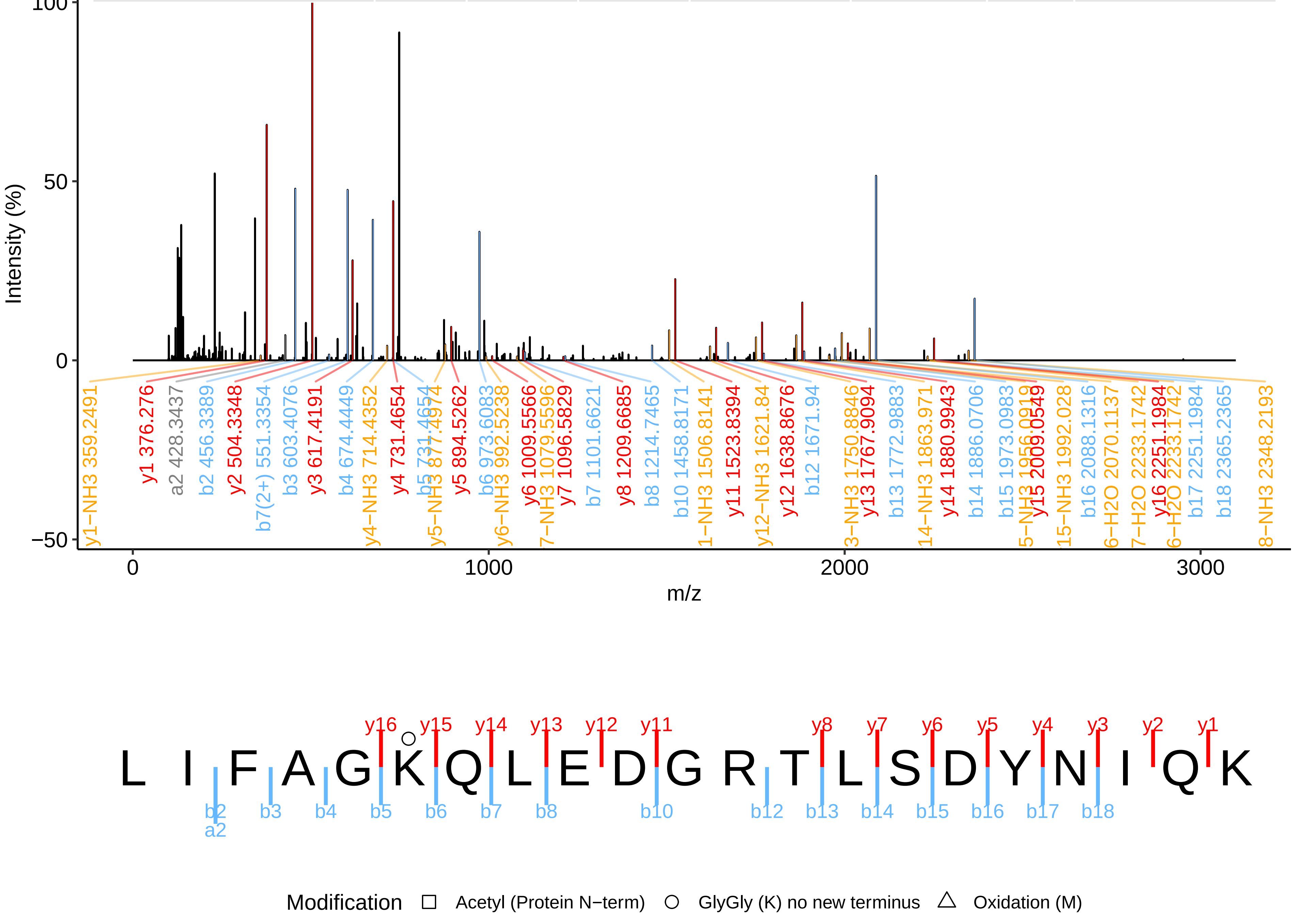

20200924_KIZ_MUb5_GlyGly_2 − Scan number: 21002

Gene Names	Charge	m/z	Mass	Retention time	PEP	Score	Precursor Intensity
RPS27A;UBA52;UBB;UBC	4	631.5855	2522.313	77.732	0.068381	59.512	9339211

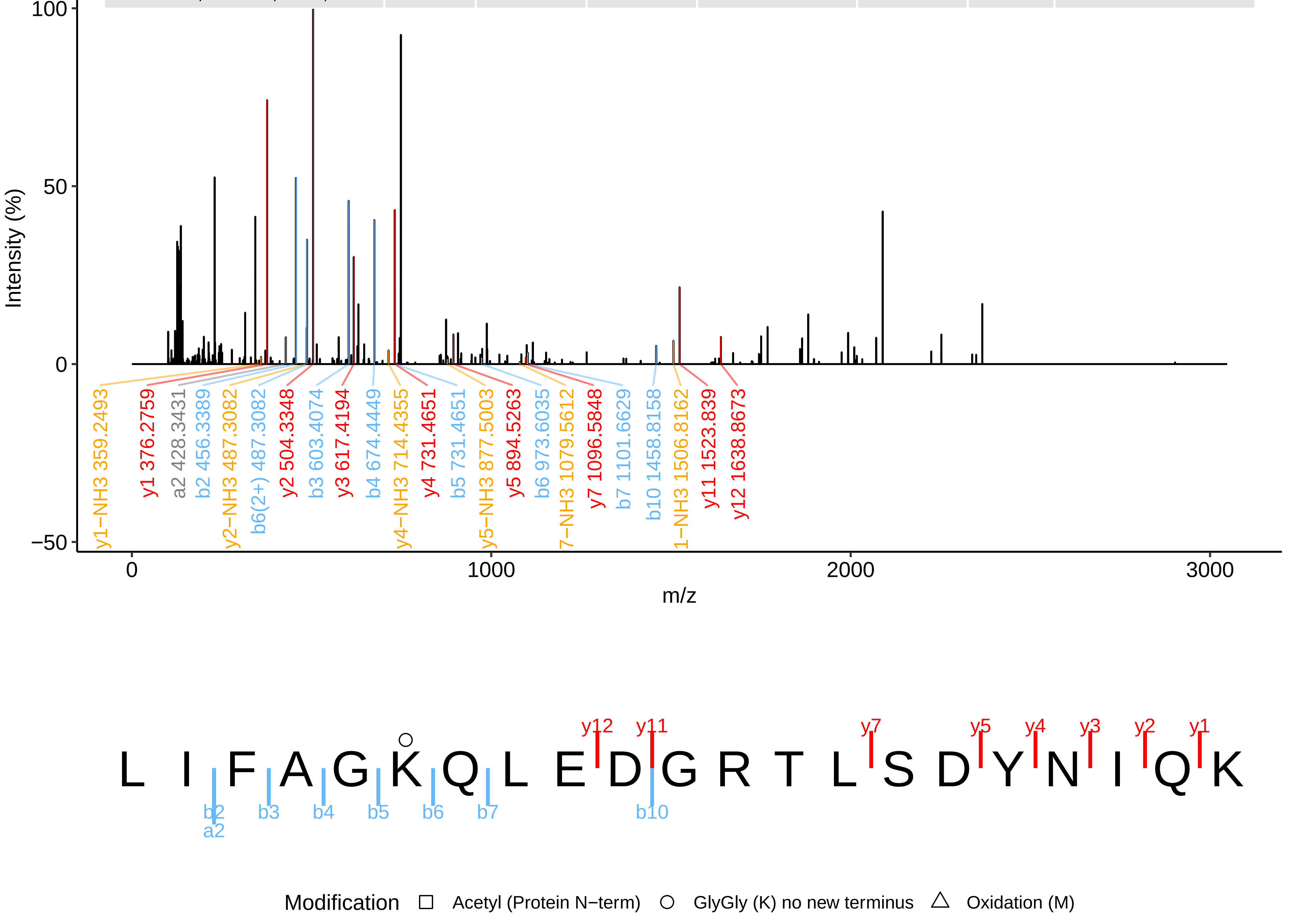

20200924_KIZ_MUb5_GlyGly_2 − Scan number: 20627

Gene Names	Charge	m/z	Mass	Retention time	PEP	Score	Precursor Intensity
RPS27A;UBA52;UBB;UBC	3	841.7782	2522.313	76.372	8.1259e−39	181.4	NA

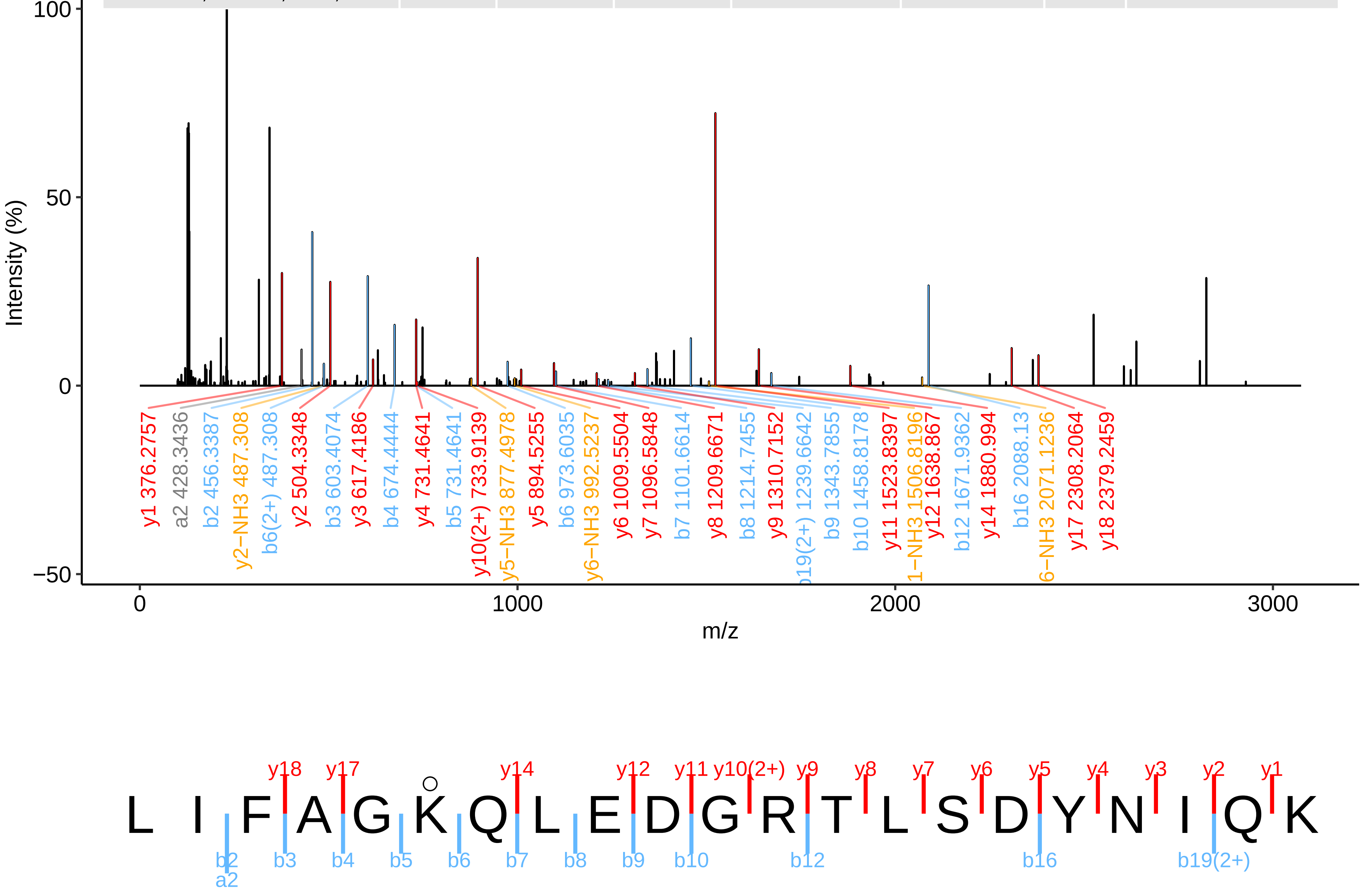

Modification □ Acetyl (Protein N−term) ○ GlyGly (K) no new terminus △ Oxidation (M)

20201118_KIZ_MUb6_GlyGly_1 − Scan number: 23364

Gene Names	Charge	m/z	Mass	Retention time	PEP	Score	Precursor Intensity
RPS27A;UBA52;UBB;UBC	4	631.5855	2522.313	72.306	7.4663e−39	170.53	35177576

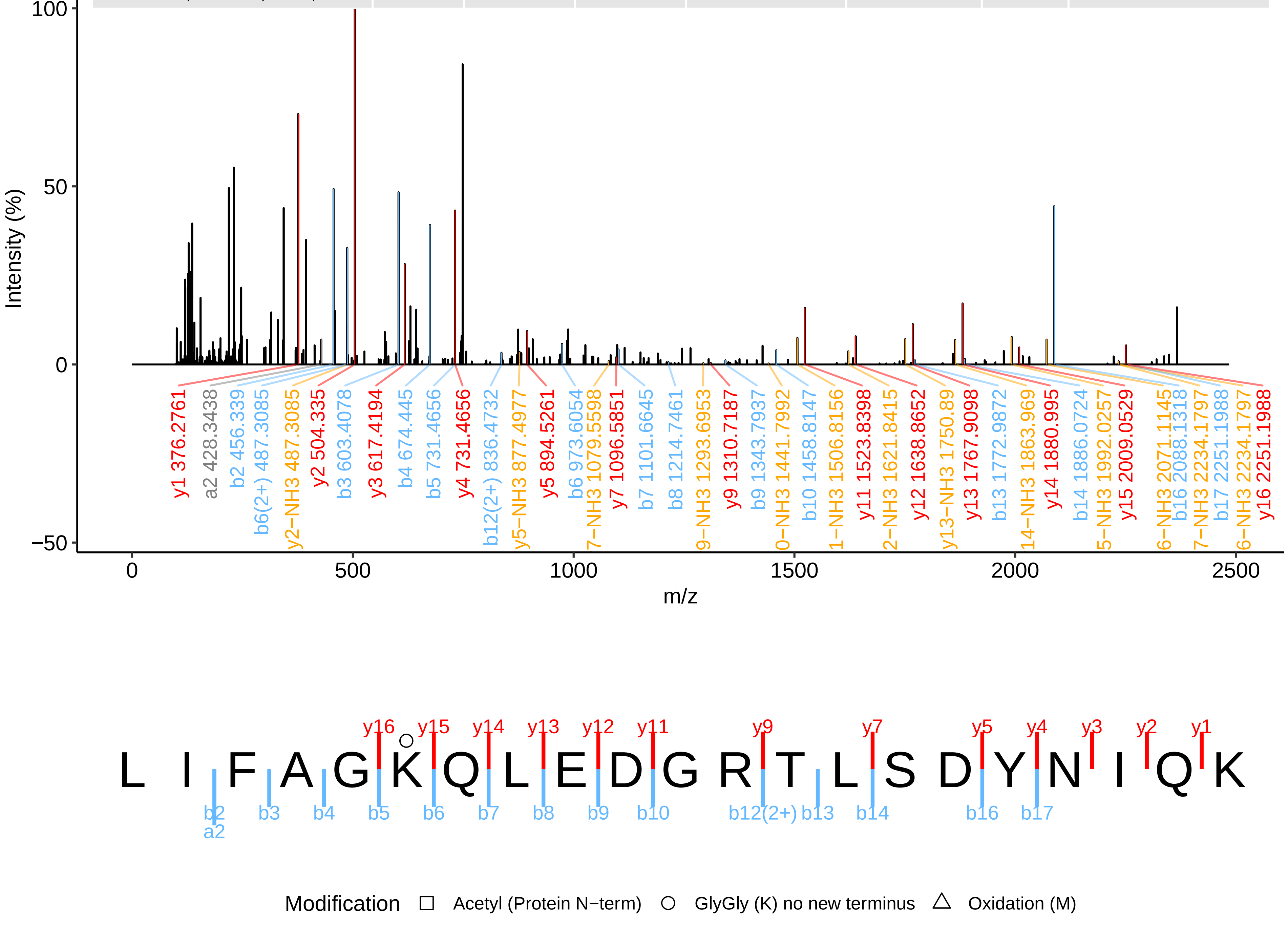

20201118_KIZ_MUb6_GlyGly_1 − Scan number: 23553

Gene Names	Charge	m/z	Mass	Retention time	PEP	Score	Precursor Intensity
RPS27A;UBA52;UBB;UBC	4	631.5855	2522.313	72.806	1.2764e−47	185.87	950023744

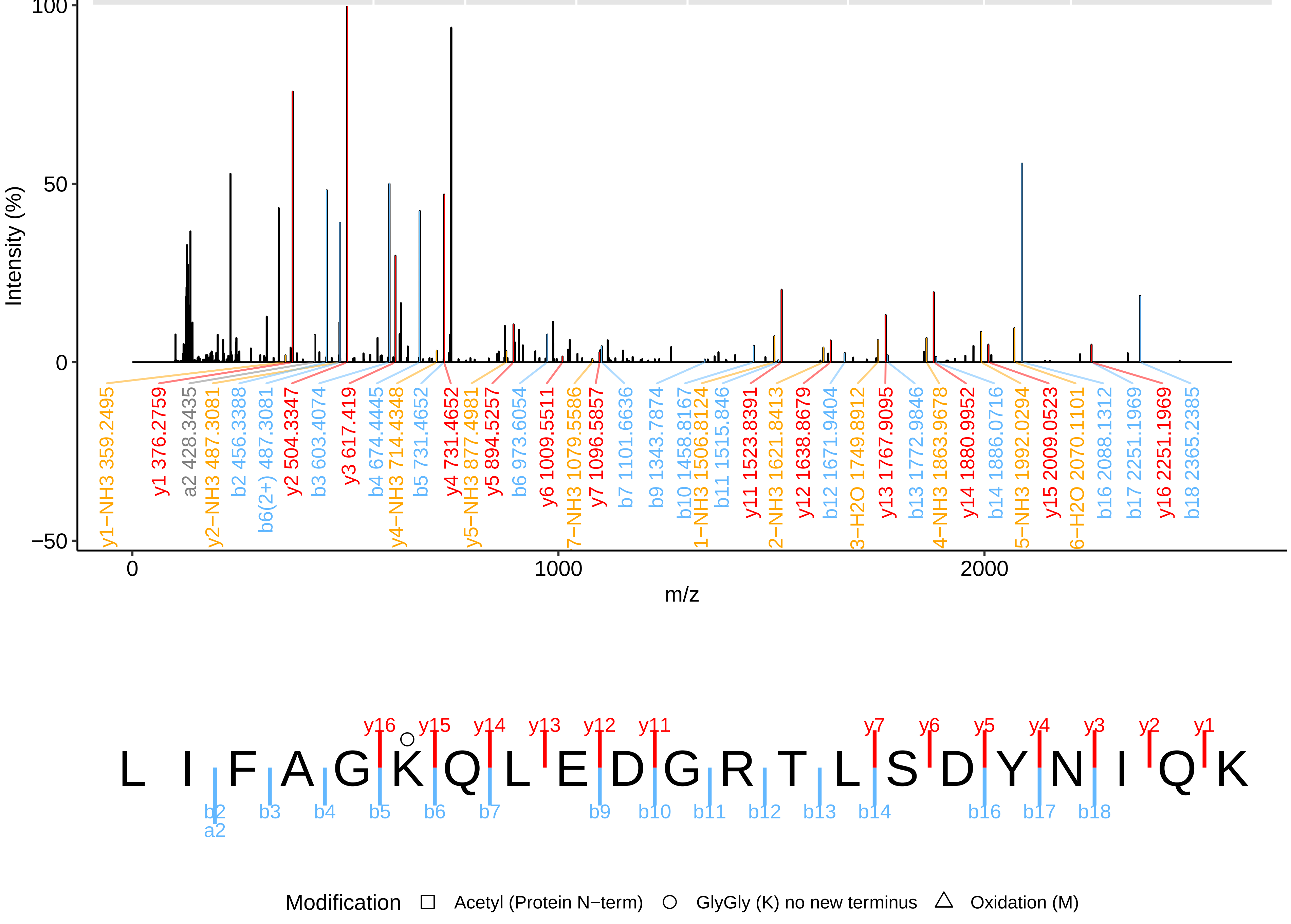

20201118_KIZ_MUb6_GlyGly_1 − Scan number: 23394

Gene Names	Charge	m/z	Mass	Retention time	PEP	Score	Precursor Intensity
RPS27A;UBA52;UBB;UBC	3	841.7782	2522.313	72.379	2.4807e−48	189.11	14376168

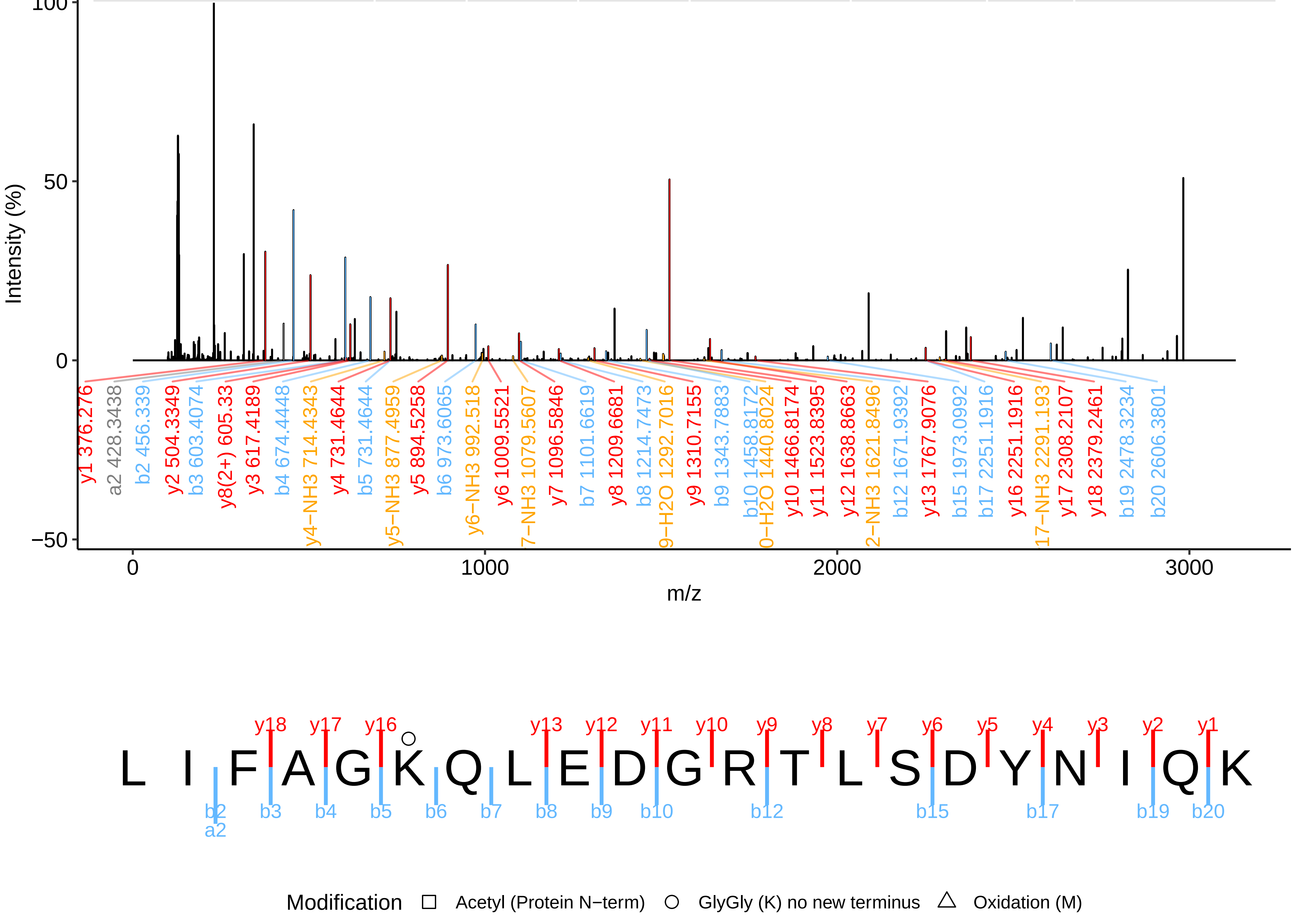

20201118_KIZ_MUb6_GlyGly_1 − Scan number: 23421

Gene Names	Charge	m/z	Mass	Retention time	PEP	Score	Precursor Intensity
RPS27A;UBA52;UBB;UBC	3	841.7782	2522.313	72.442	4.9755e−160	313.4	67894888

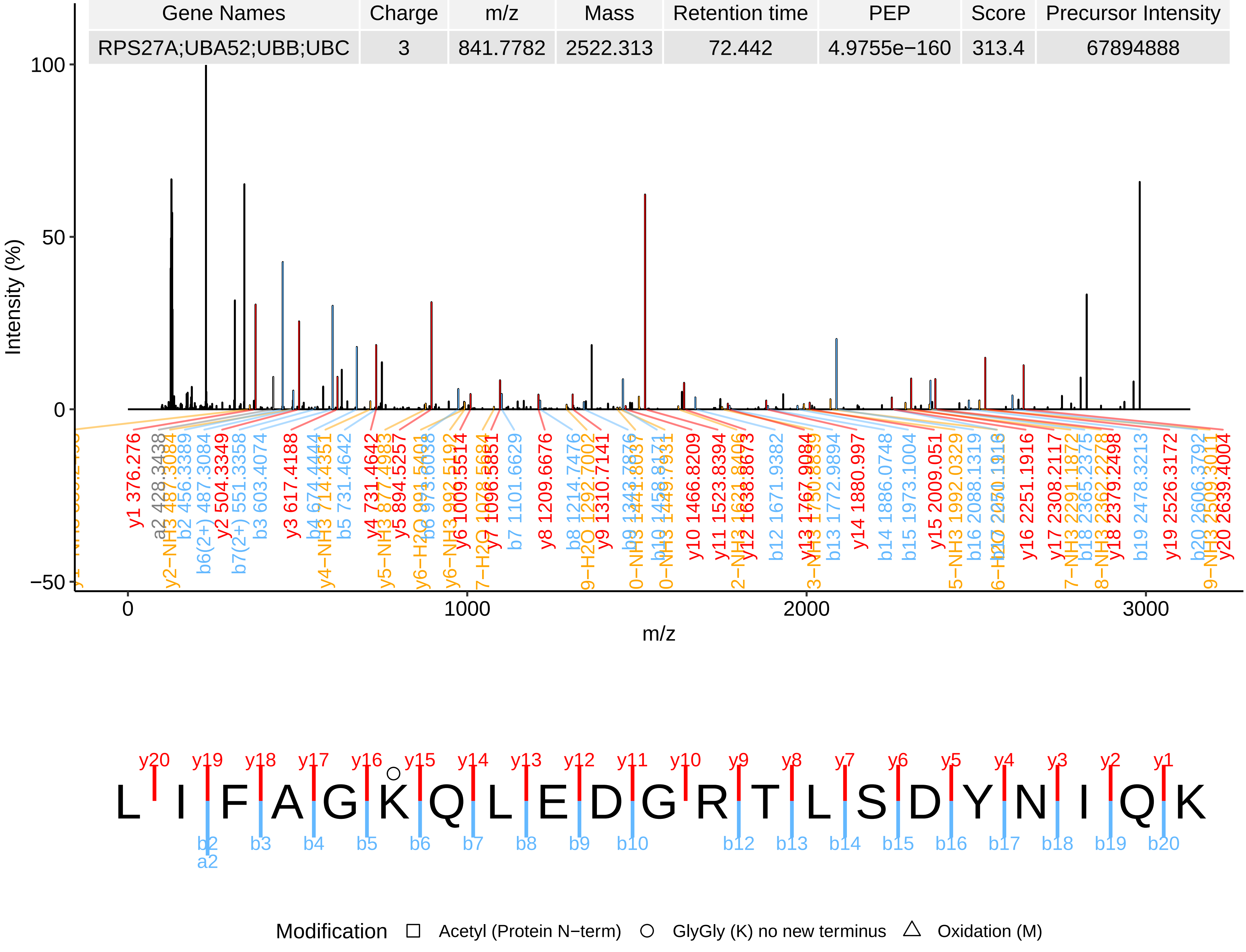

20201118_KIZ_MUb6_GlyGly_1 − Scan number: 23404

Gene Names	Charge	m/z	Mass	Retention time	PEP	Score	Precursor Intensity
RPS27A;UBA52;UBB;UBC	5	505.4699	2522.313	72.403	0.0011334	67.995	368883584

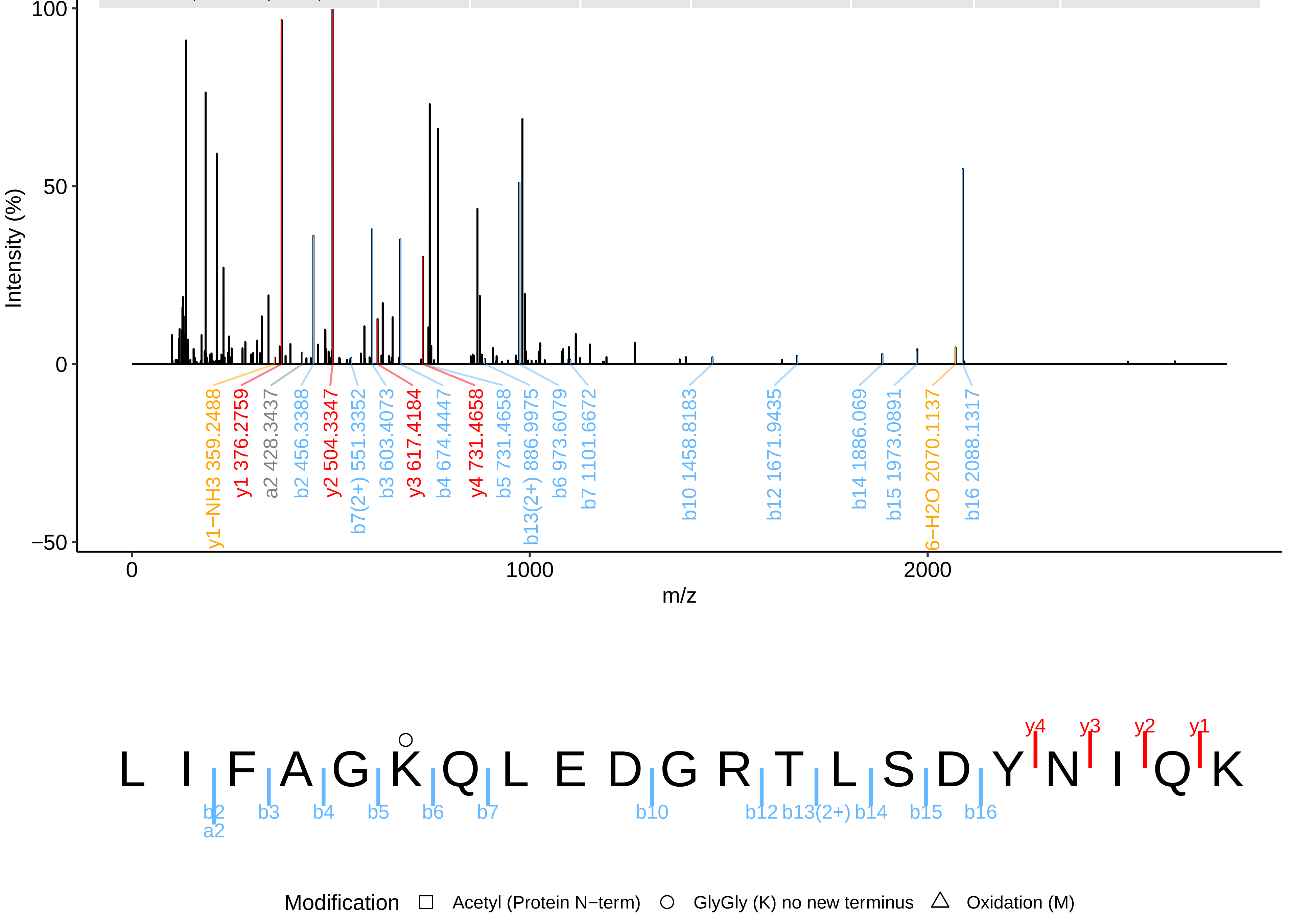

20201118_KIZ_MUb6_GlyGly_1 − Scan number: 23592

Gene Names	Charge	m/z	Mass	Retention time	PEP	Score	Precursor Intensity
RPS27A;UBA52;UBB;UBC	5	505.4699	2522.313	72.919	0.0004195	76.027	29441298

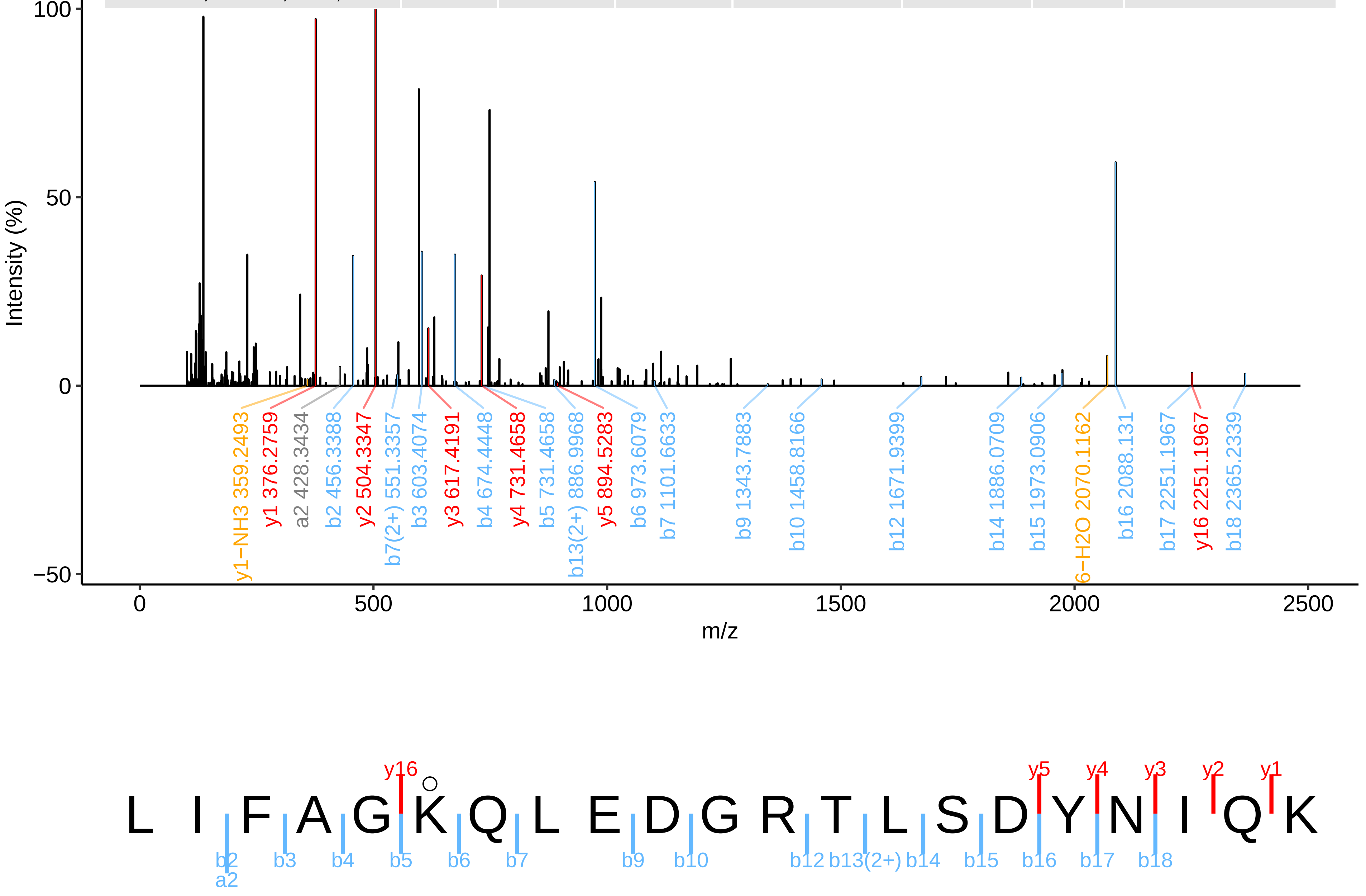

Modification □ Acetyl (Protein N−term) ○ GlyGly (K) no new terminus △ Oxidation (M)

20201118_KIZ_MUb6_GlyGly_2 − Scan number: 24941

Gene Names	Charge	m/z	Mass	Retention time	PEP	Score	Precursor Intensity
RPS27A;UBA52;UBB;UBC	4	631.5855	2522.313	83.408	1.2438e−47	186.3	28700162

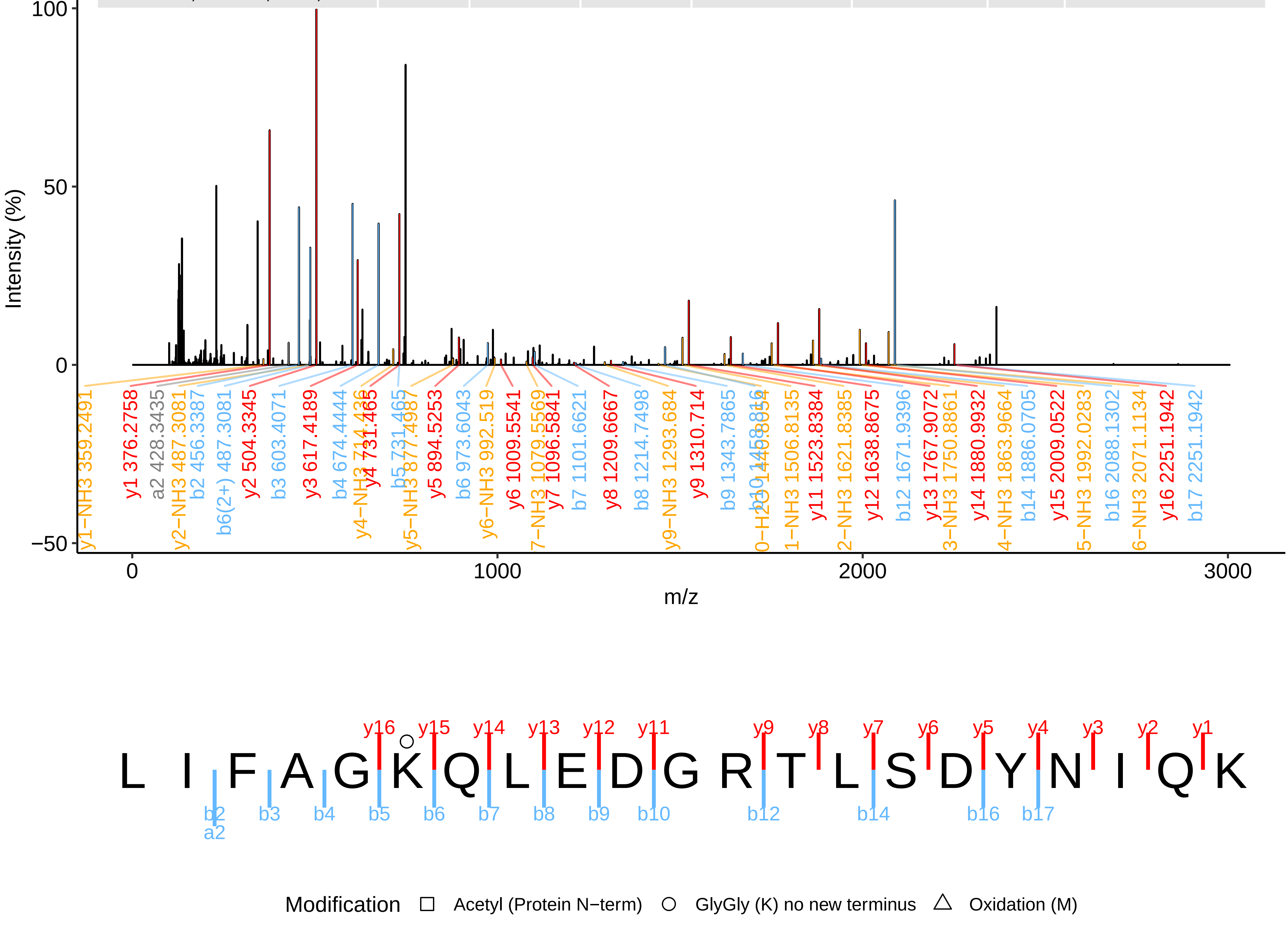

20201118_KIZ_MUb6_GlyGly_2 − Scan number: 24949

Gene Names	Charge	m/z	Mass	Retention time	PEP	Score	Precursor Intensity
RPS27A;UBA52;UBB;UBC	5	505.4699	2522.313	83.434	1.1092e−05	84.493	23418416

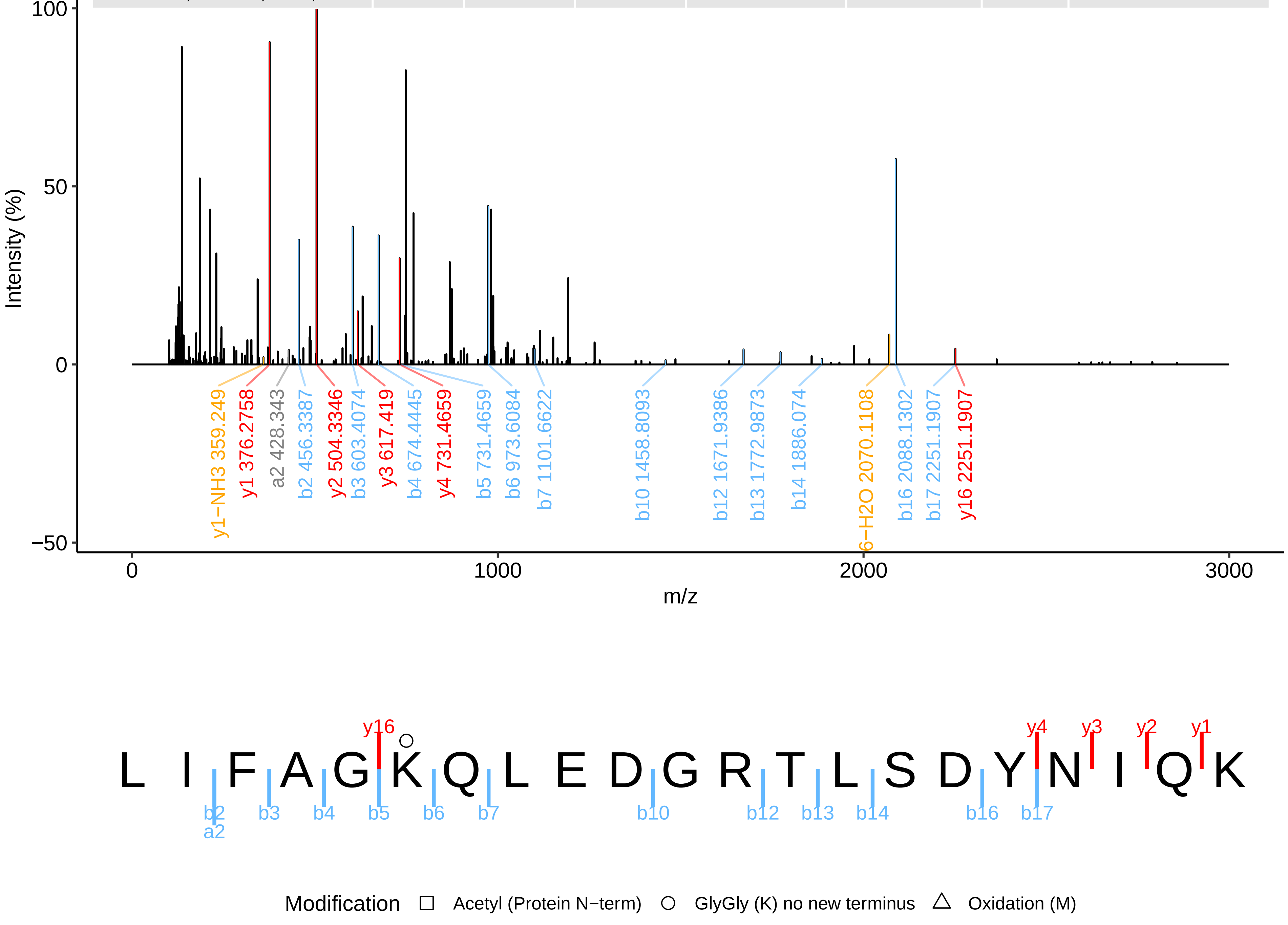

20201118_KIZ_MUb6_GlyGly_2 − Scan number: 25100

Gene Names	Charge	m/z	Mass	Retention time	PEP	Score	Precursor Intensity
RPS27A;UBA52;UBB;UBC	4	631.5855	2522.313	83.918	1.2624e−47	186.06	464361600

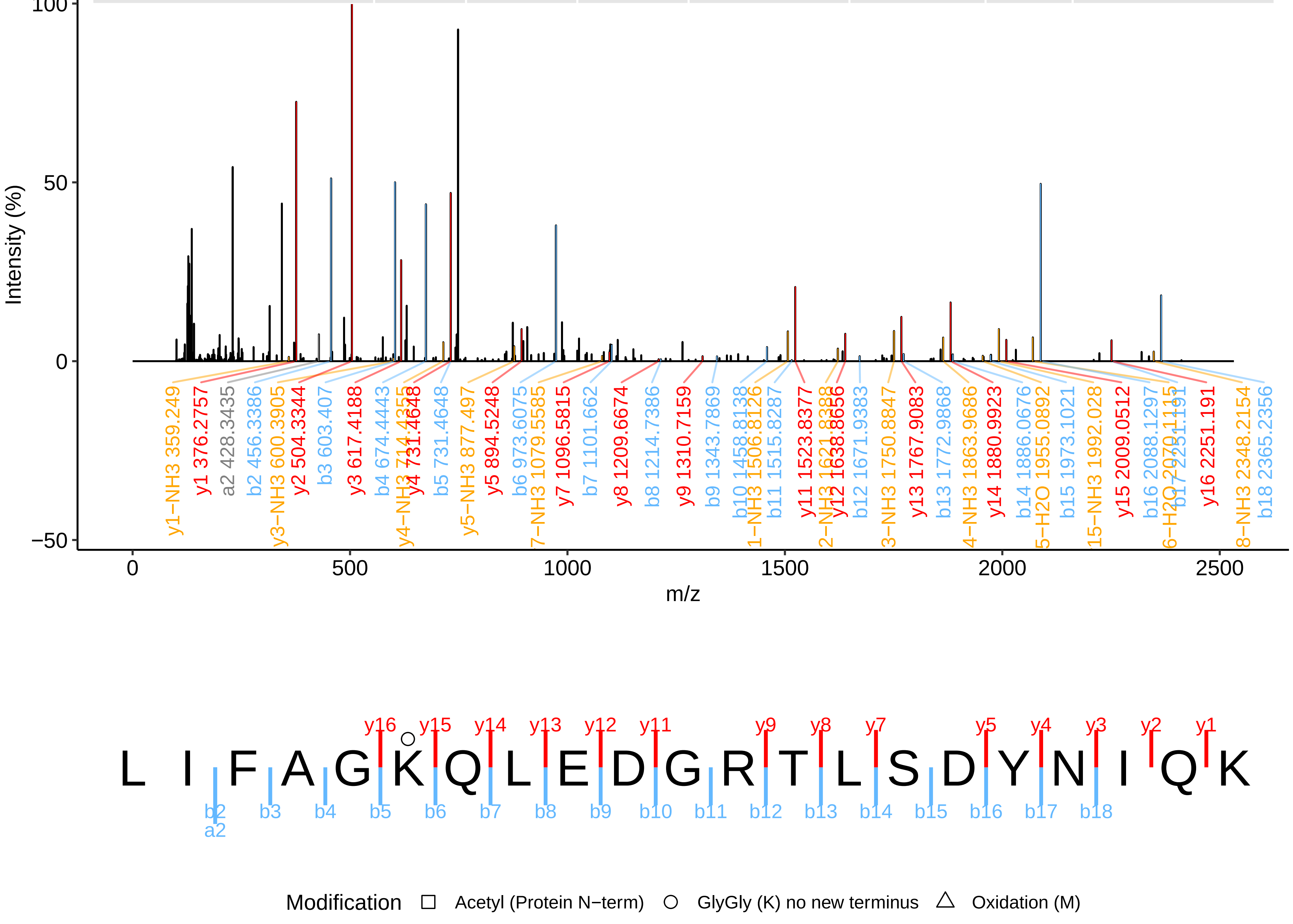

20201118_KIZ_MUb6_GlyGly_2 − Scan number: 25108

Gene Names	Charge	m/z	Mass	Retention time	PEP	Score	Precursor Intensity
RPS27A;UBA52;UBB;UBC	5	505.4699	2522.313	83.942	0.032922	53.278	47834172

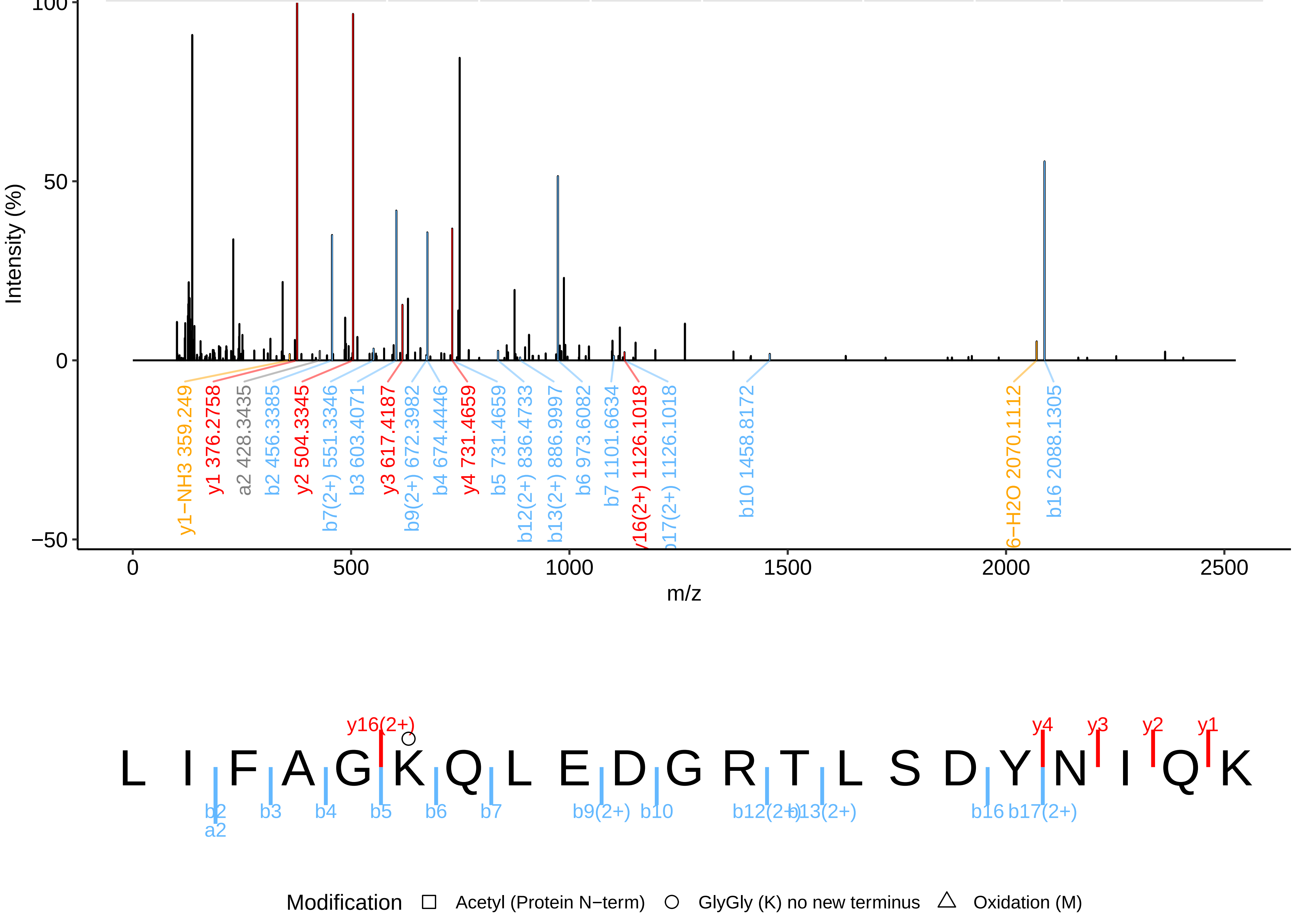

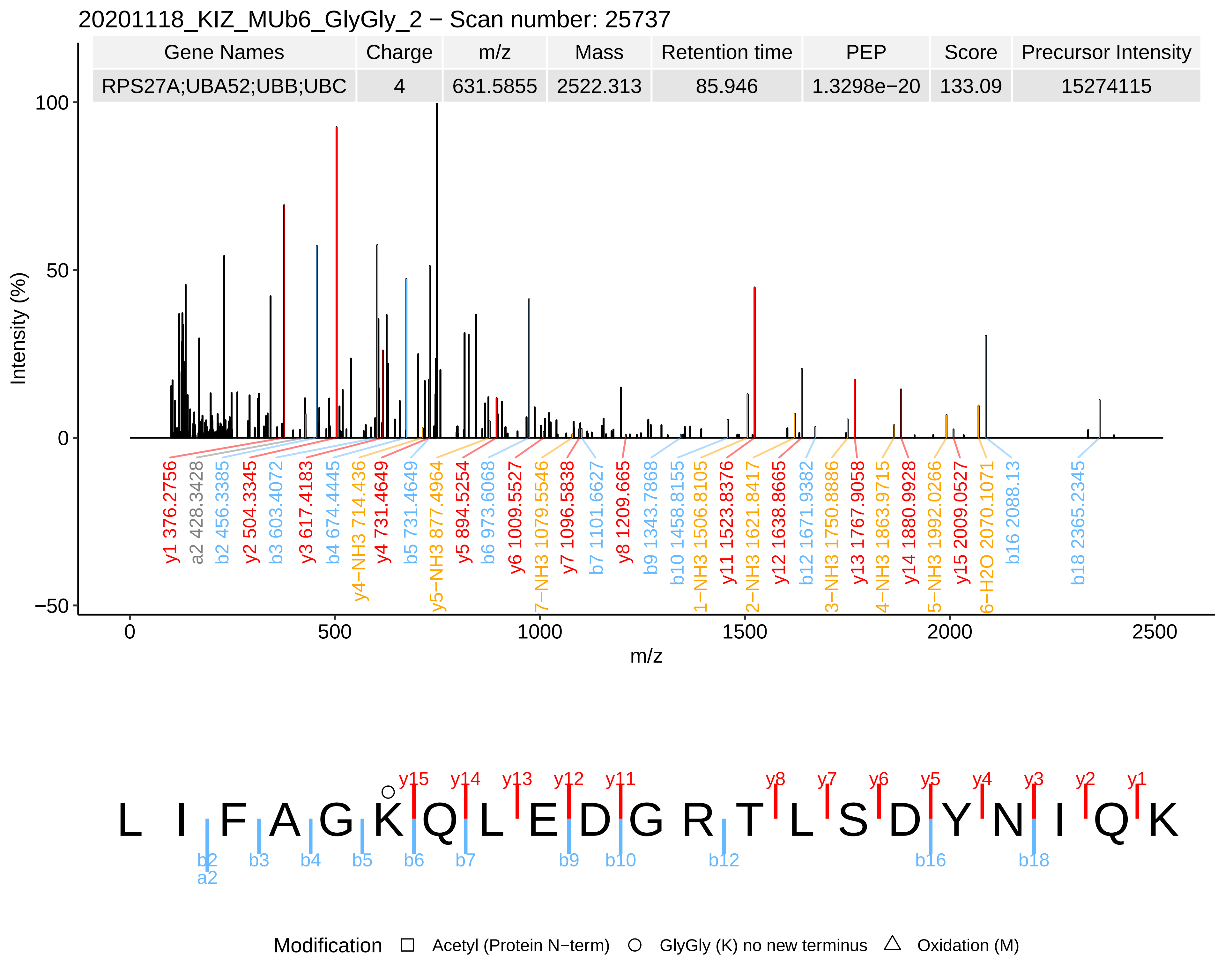
20201118_KIZ_MUb6_GlyGly_2 − Scan number: 25737
Gene Names | Charge | m/z | Mass | Retention time | PEP | Score | Precursor Intensity
RPS27A;UBA52;UBB;UBC | 4 | 631.5855 | 2522.313 | 85.946 | 1.3298e−20 | 133.09 | 15274115
Intensity (%)
m/z
y1 376.2756
a2 428.3428
b2 456.3385
y2 504.3345
b3 603.4072
y3 617.4183
b4 674.4445
y4−NH3 714.436
y4 731.4649
b5 731.4649
y5−NH3 877.4964
y5 894.5254
b6 973.6068
y6 1009.5527
y7−NH3 1079.5546
y7 1096.5838
b7 1101.6627
y8 1209.665
b9 1343.7868
b10 1458.8155
y11−NH3 1506.8105
y11 1523.8376
y12−NH3 1621.8417
y12 1638.8665
b12 1671.9382
y13−NH3 1750.8886
y13 1767.9058
y14−NH3 1863.9715
y14 1880.9928
y15−NH3 1992.0266
y15 2009.0527
y16−H2O 2070.1071
b16 2088.13
b18 2365.2345
L I F A G K Q L E D G R T L S D Y N I Q K
y15 y14 y13 y12 y11 y8 y7 y6 y5 y4 y3 y2 y1
b2 a2 b3 b4 b5 b6 b7 b9 b10 b12 b16 b18
Modification □ Acetyl (Protein N−term) ○ GlyGly (K) no new terminus △ Oxidation (M)

20200921_KIZ_MUb4_GlyGly_10plex_2 − Scan number: 20539

Gene Names	Charge	m/z	Mass	Retention time	PEP	Score	Precursor Intensity
HSPA1B;HSPA1A	3	814.431	2440.271	80.74	0.00058215	70.99	633754.9

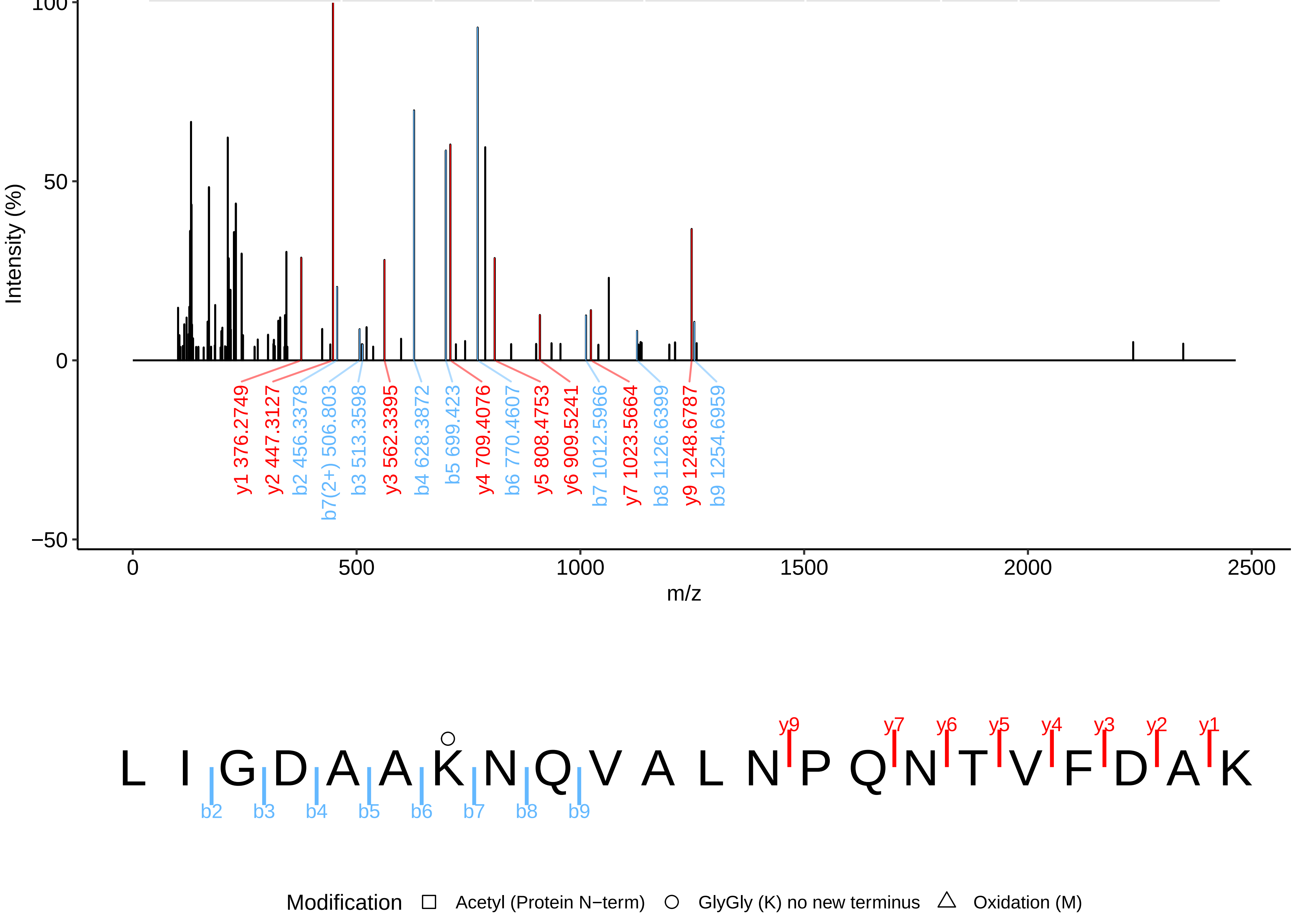

20200921_KIZ_MUb4_GlyGly_10plex_2 − Scan number: 18233

Gene Names	Charge	m/z	Mass	Retention time	PEP	Score	Precursor Intensity
HSPA8	3	816.7444	2447.211	71.375	0.00079138	64.203	1068775

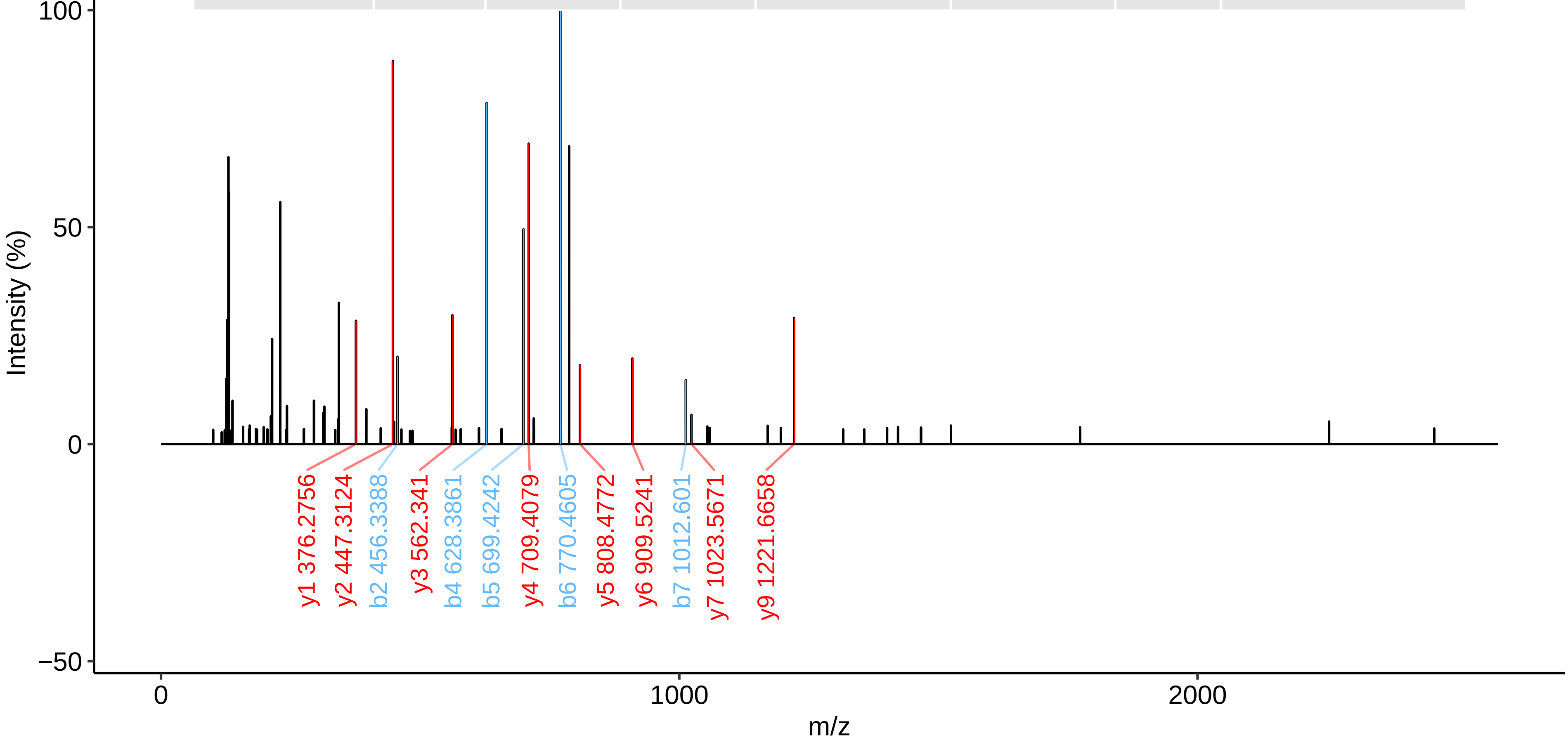

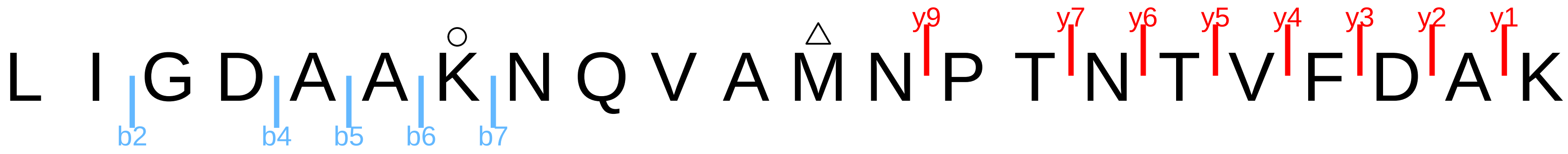

Modification □ Acetyl (Protein N−term) ○ GlyGly (K) no new terminus △ Oxidation (M)

20200921_KIZ_MUb4_GlyGly_10plex_2 − Scan number: 18250

Gene Names	Charge	m/z	Mass	Retention time	PEP	Score	Precursor Intensity
HSPA8	3	816.7444	2447.211	71.444	3.4662e−06	81.665	1732115

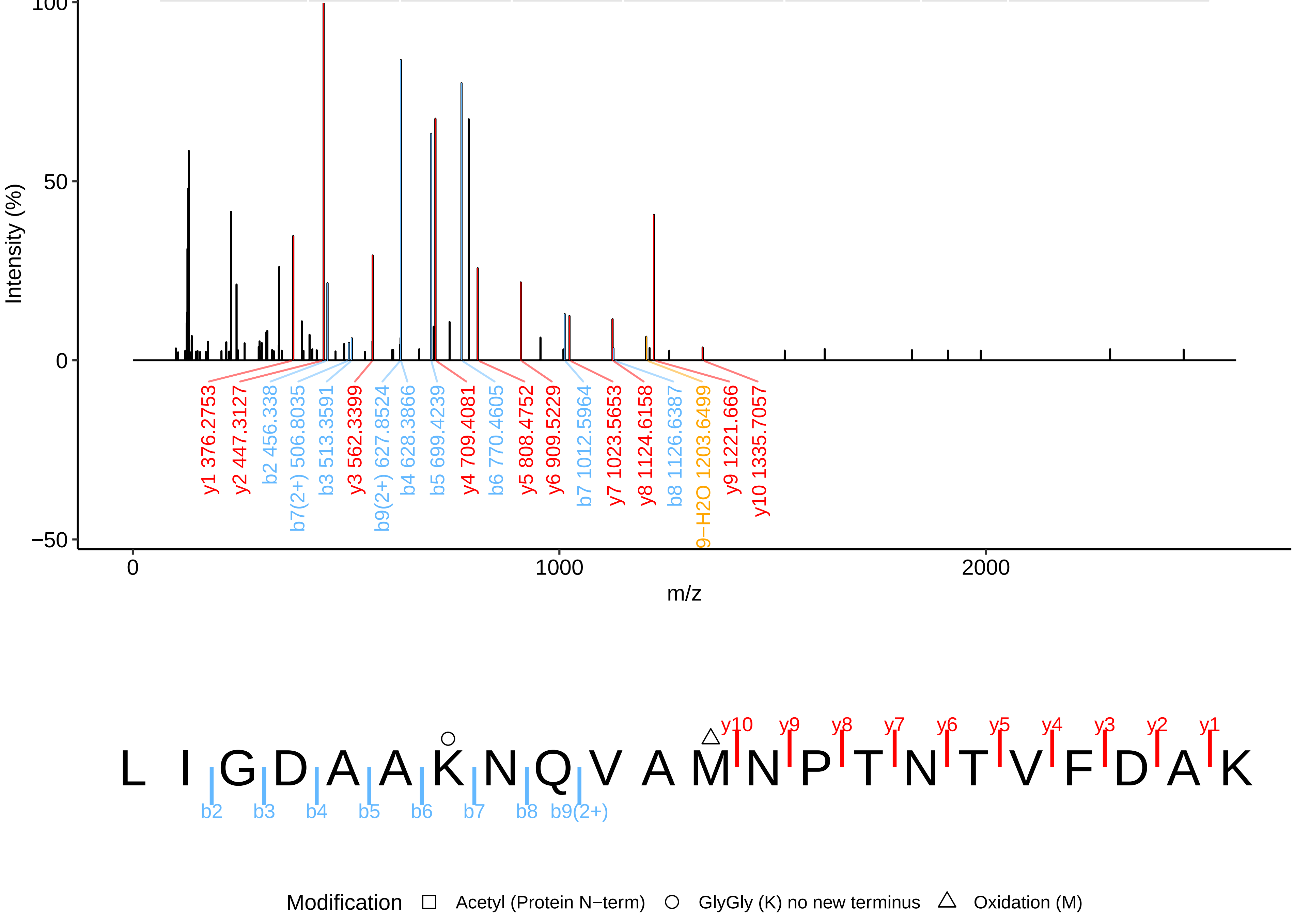

20200921_KIZ_MUb4_GlyGly_10plex_2 − Scan number: 20246

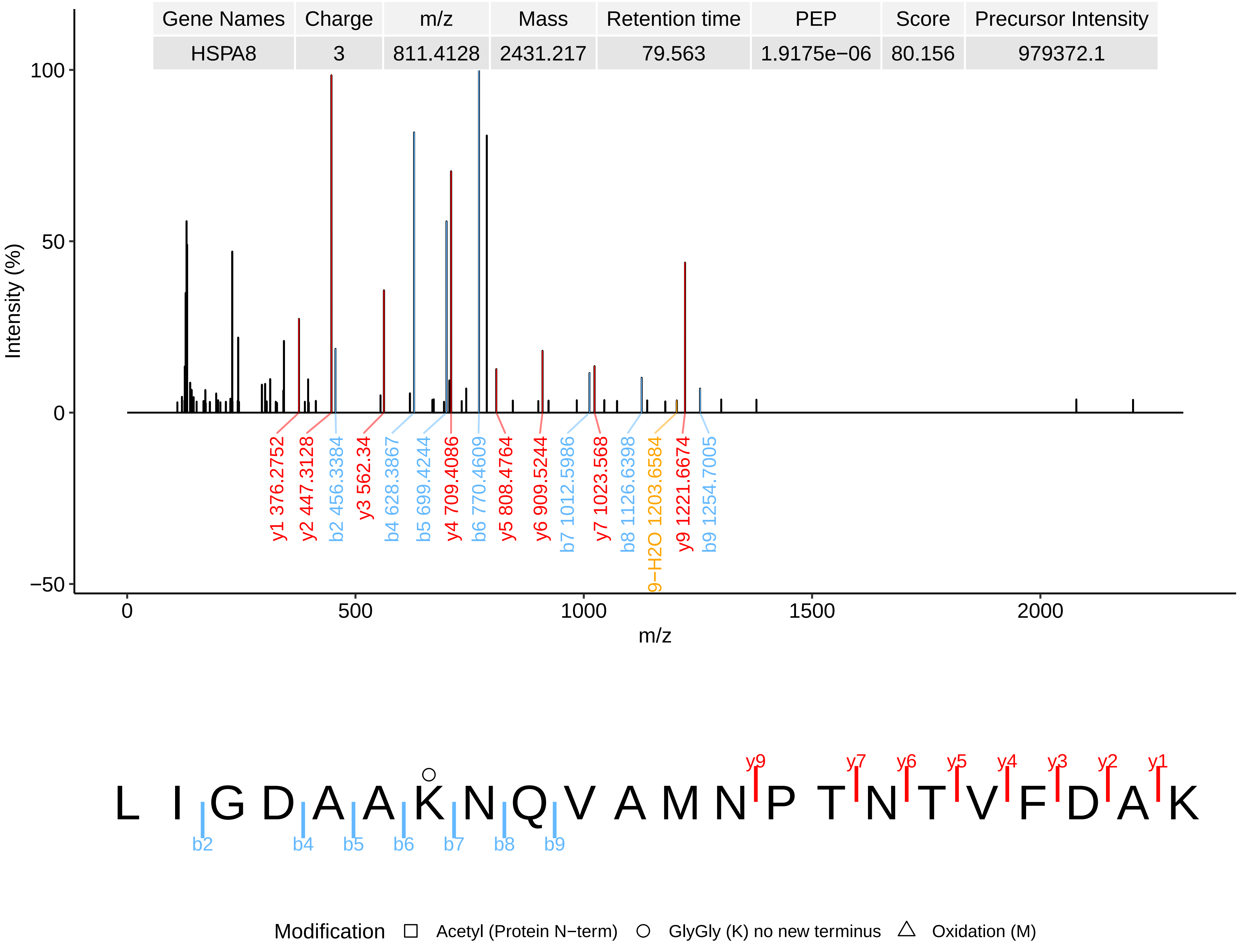

20200924_KIZ_MUb5_GlyGly_1 − Scan number: 22578

Gene Names	Charge	m/z	Mass	Retention time	PEP	Score	Precursor Intensity
HSPA8	3	811.4128	2431.217	72.631	1.177e−16	125.73	NA

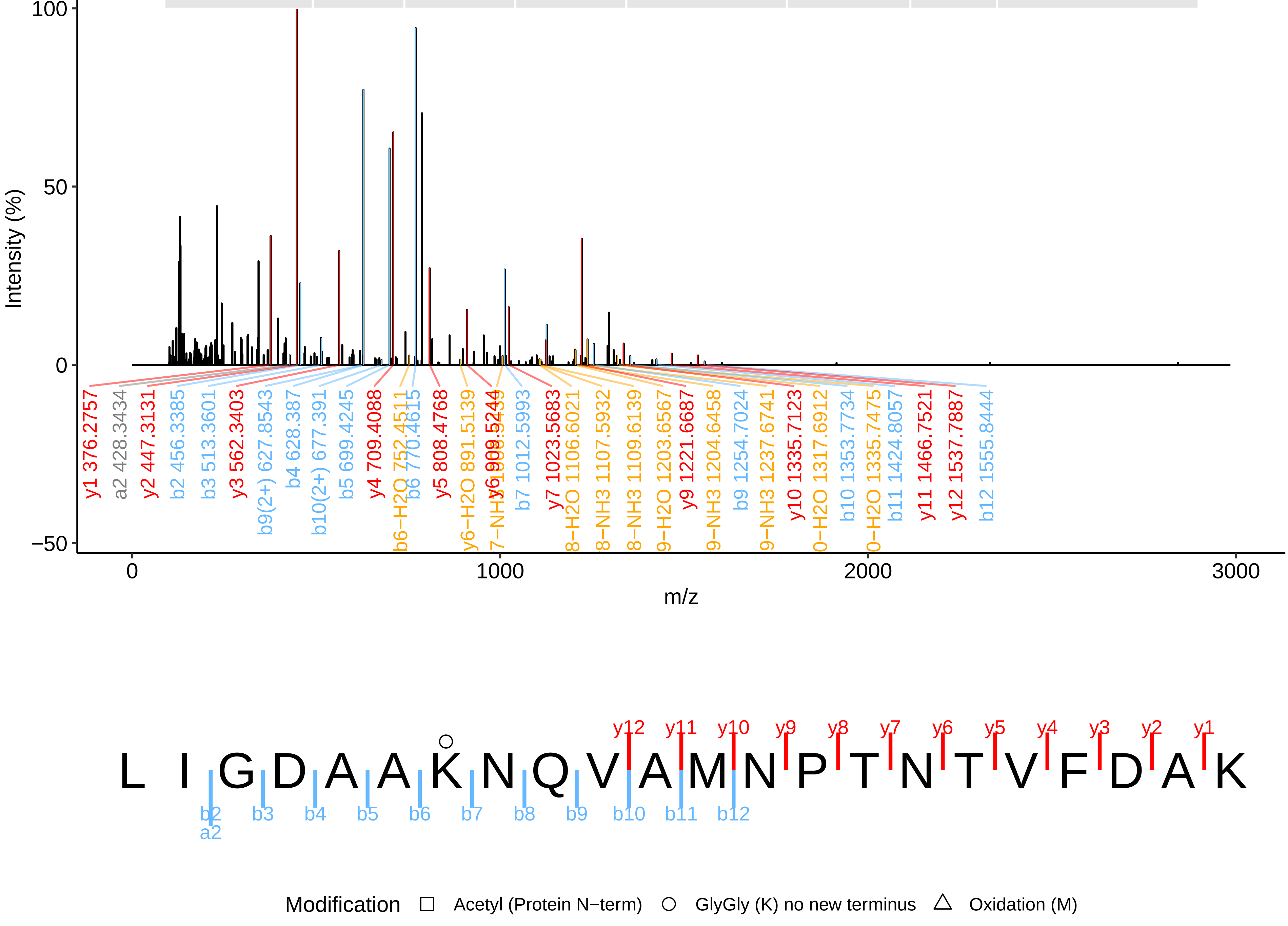

20200921_KIZ_MUb4_GlyGly_10plex − Scan number: 13414

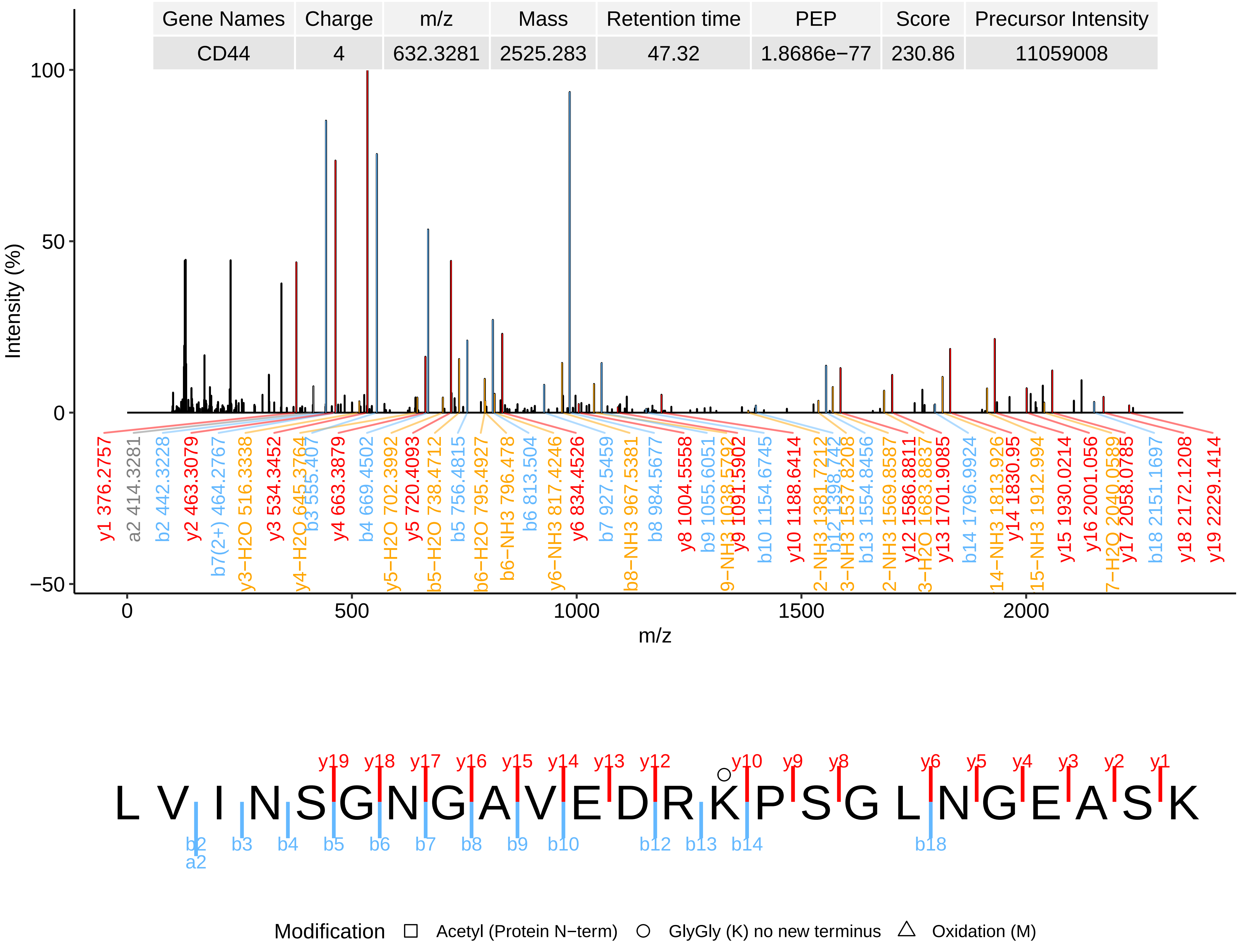

20200921_KIZ_MUb4_GlyGly_10plex_2 − Scan number: 12356

Gene Names	Charge	m/z	Mass	Retention time	PEP	Score	Precursor Intensity
CD44	4	632.3281	2525.283	47.788	2.7165e−06	92.01	973372.6

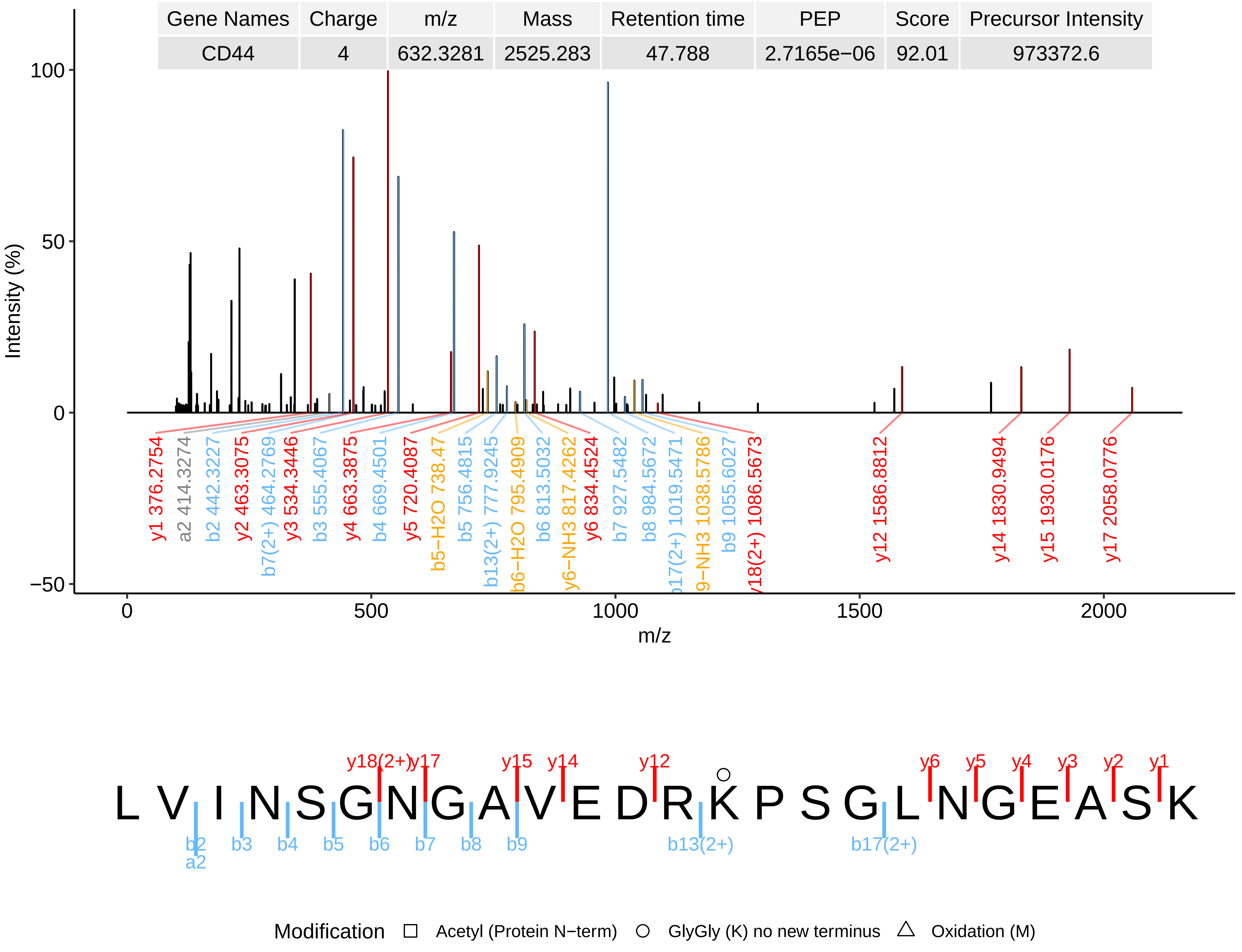

20200921_KIZ_MUb4_GlyGly_10plex_2 − Scan number: 20770

Gene Names	Charge	m/z	Mass	Retention time	PEP	Score	Precursor Intensity
PEG10	3	797.4516	2389.333	81.692	0.00041405	73.783	877817.6

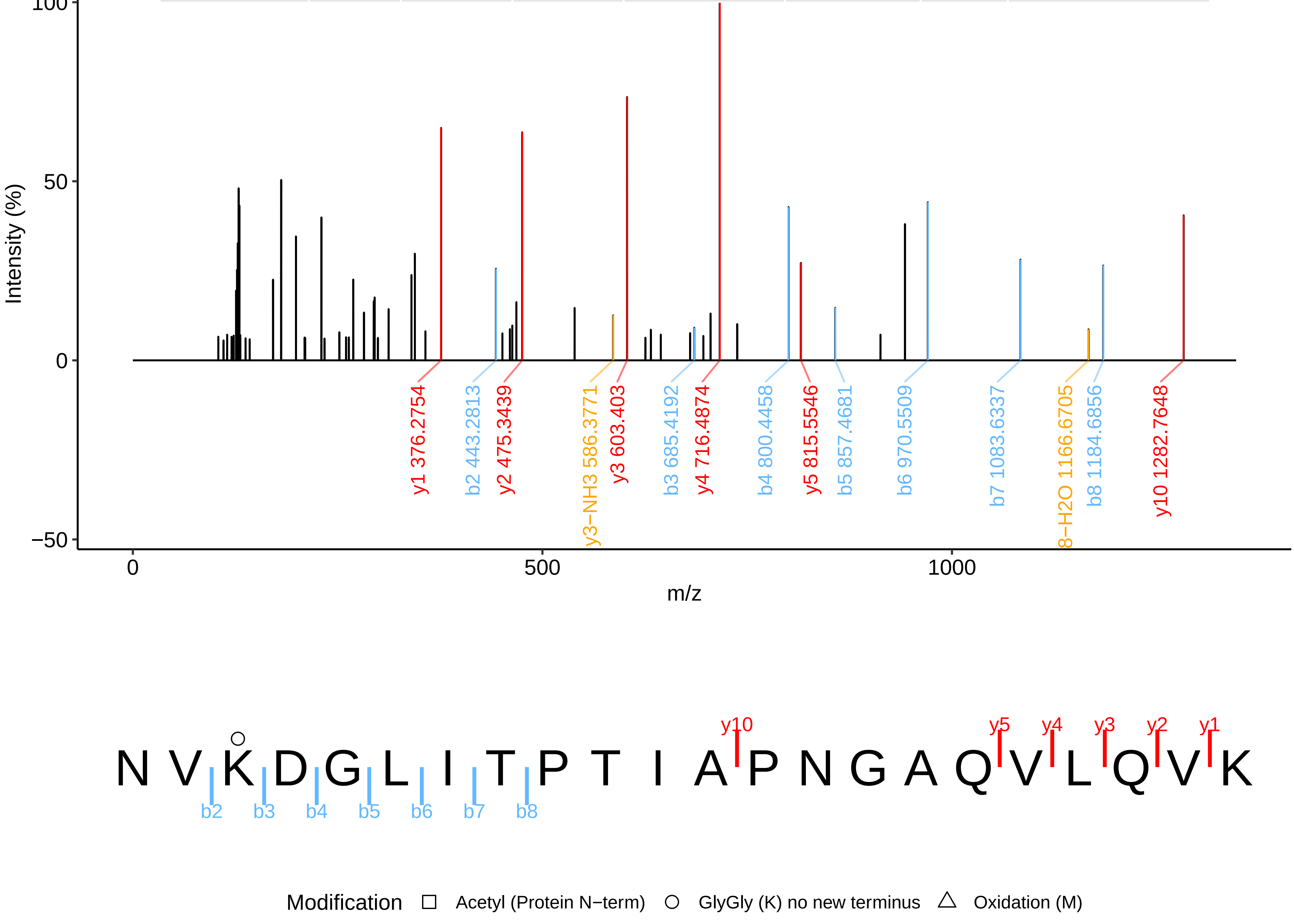

20200921_KIZ_MUb4_GlyGly_10plex_2 − Scan number: 20788

Gene Names	Charge	m/z	Mass	Retention time	PEP	Score	Precursor Intensity
PEG10	4	598.3405	2389.333	81.765	3.5071e−06	81.967	973606.4

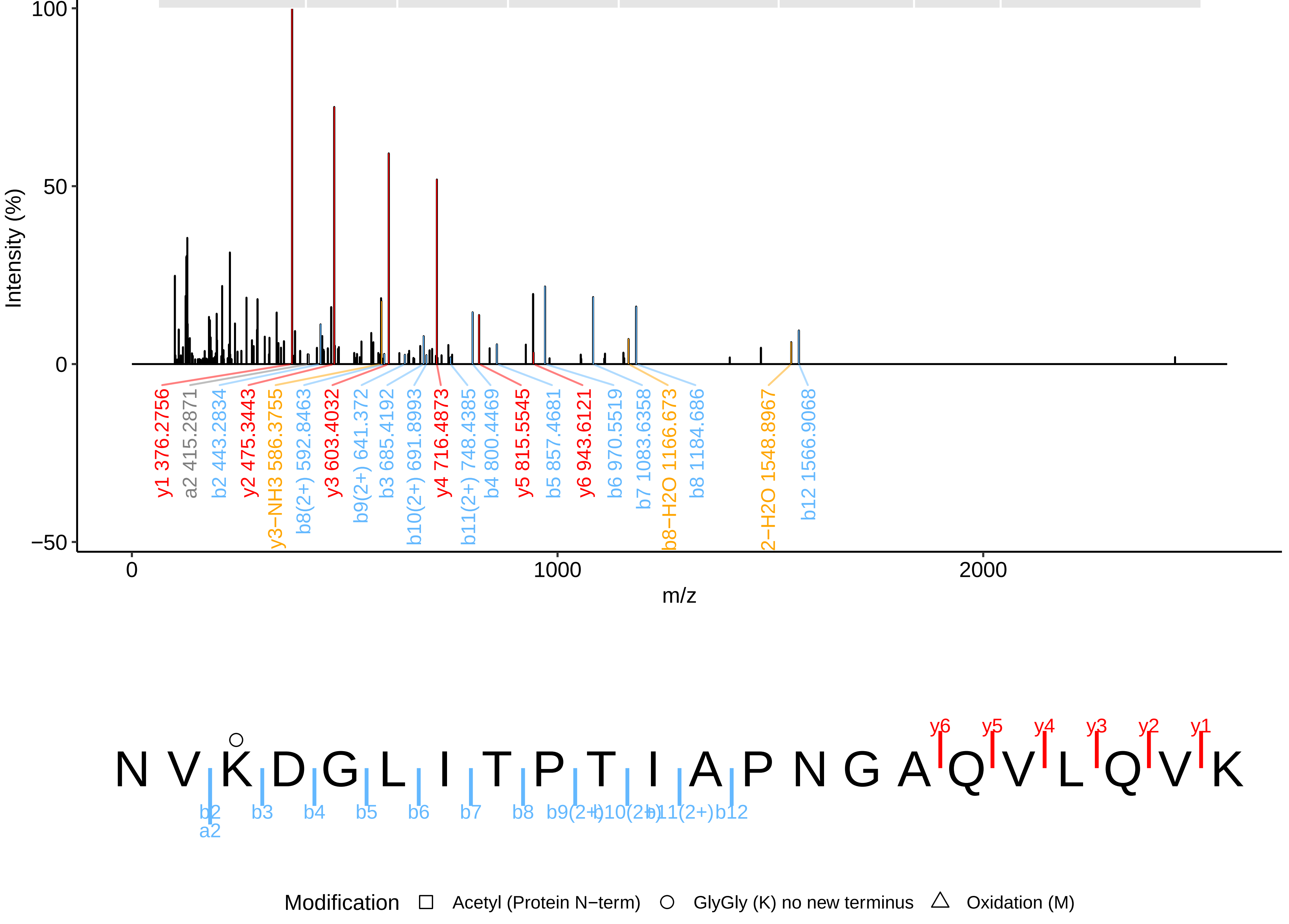

20201118_KIZ_MUb6_GlyGly_2 − Scan number: 25439

Gene Names	Charge	m/z	Mass	Retention time	PEP	Score	Precursor Intensity
PEG10	3	797.4516	2389.333	85.003	2.4452e−16	118.52	NA

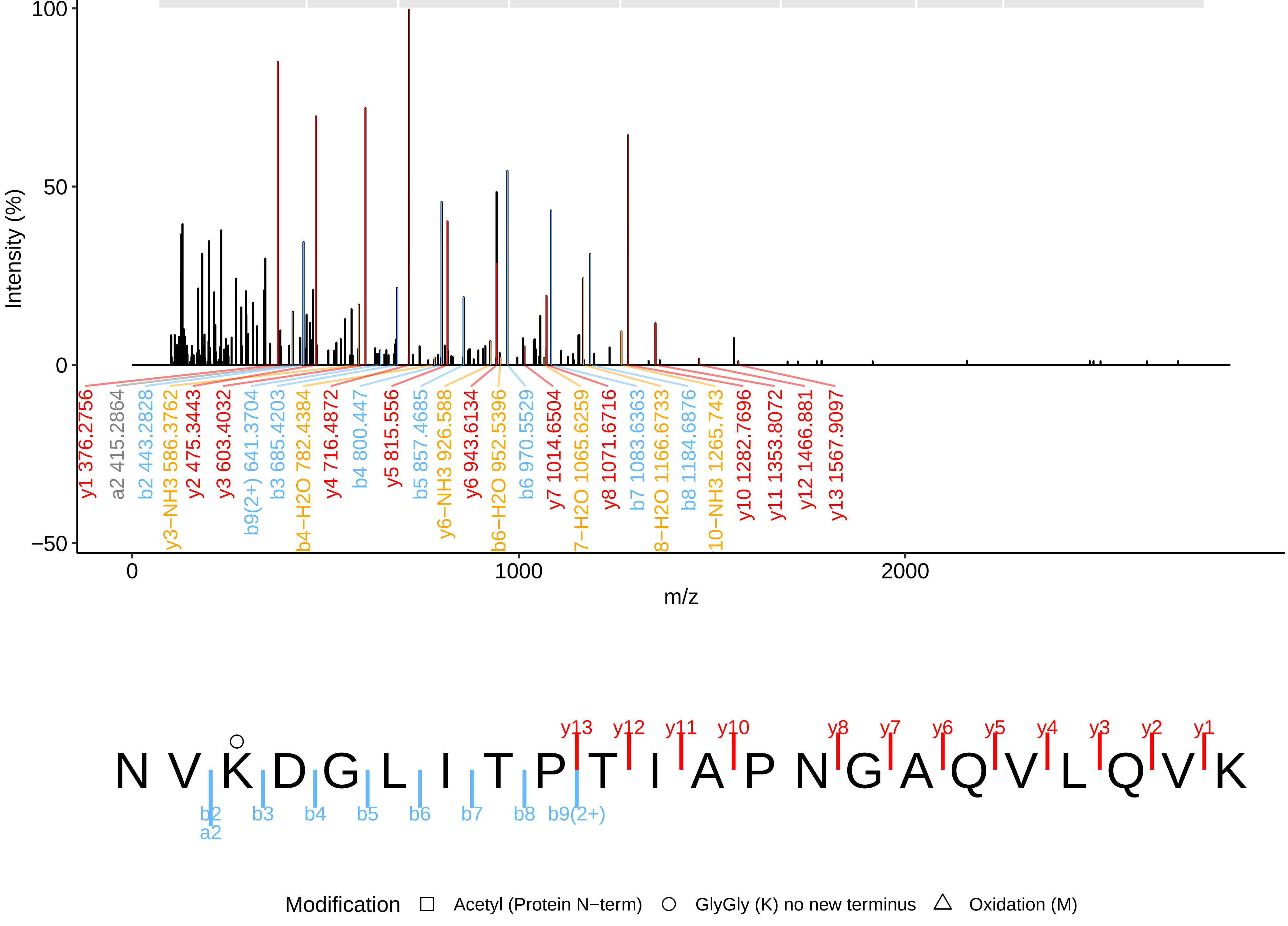

20200921_KIZ_MUb4_GlyGly_10plex − Scan number: 8136

Gene Names	Charge	m/z	Mass	Retention time	PEP	Score	Precursor Intensity
RAD23B	3	892.0861	2673.237	27.53	2.4913e−14	117.82	2826462

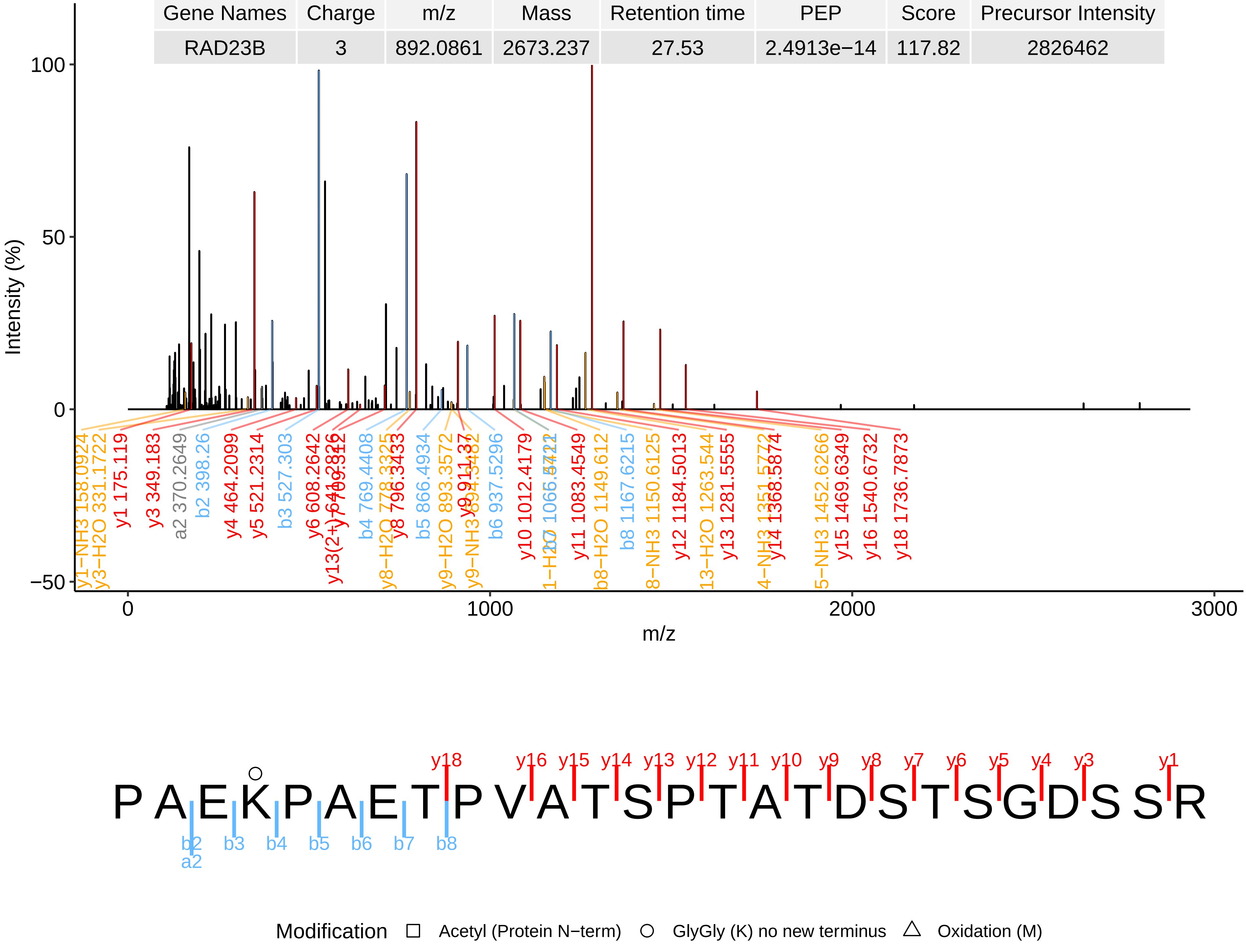

20200921_KIZ_MUb4_GlyGly_10plex_2 − Scan number: 7426

Gene Names	Charge	m/z	Mass	Retention time	PEP	Score	Precursor Intensity
RAD23B	3	892.0861	2673.237	27.707	0.0023877	67.33	778708.6

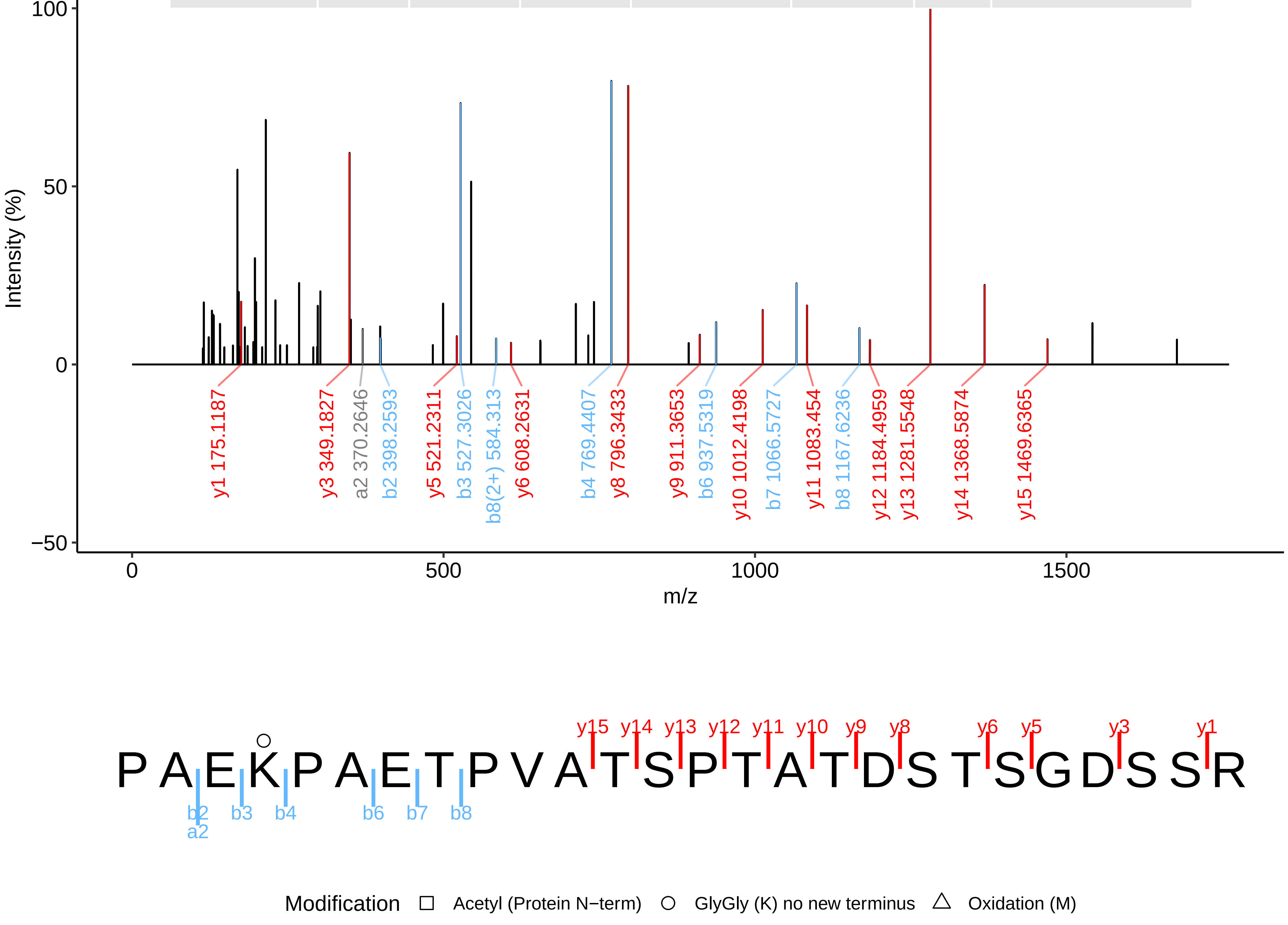

20200921_KIZ_MUb4_GlyGly_10plex_2 − Scan number: 7453

Gene Names	Charge	m/z	Mass	Retention time	PEP	Score	Precursor Intensity
RAD23B	3	892.0861	2673.237	27.814	1.7665e−05	84.404	567144.9

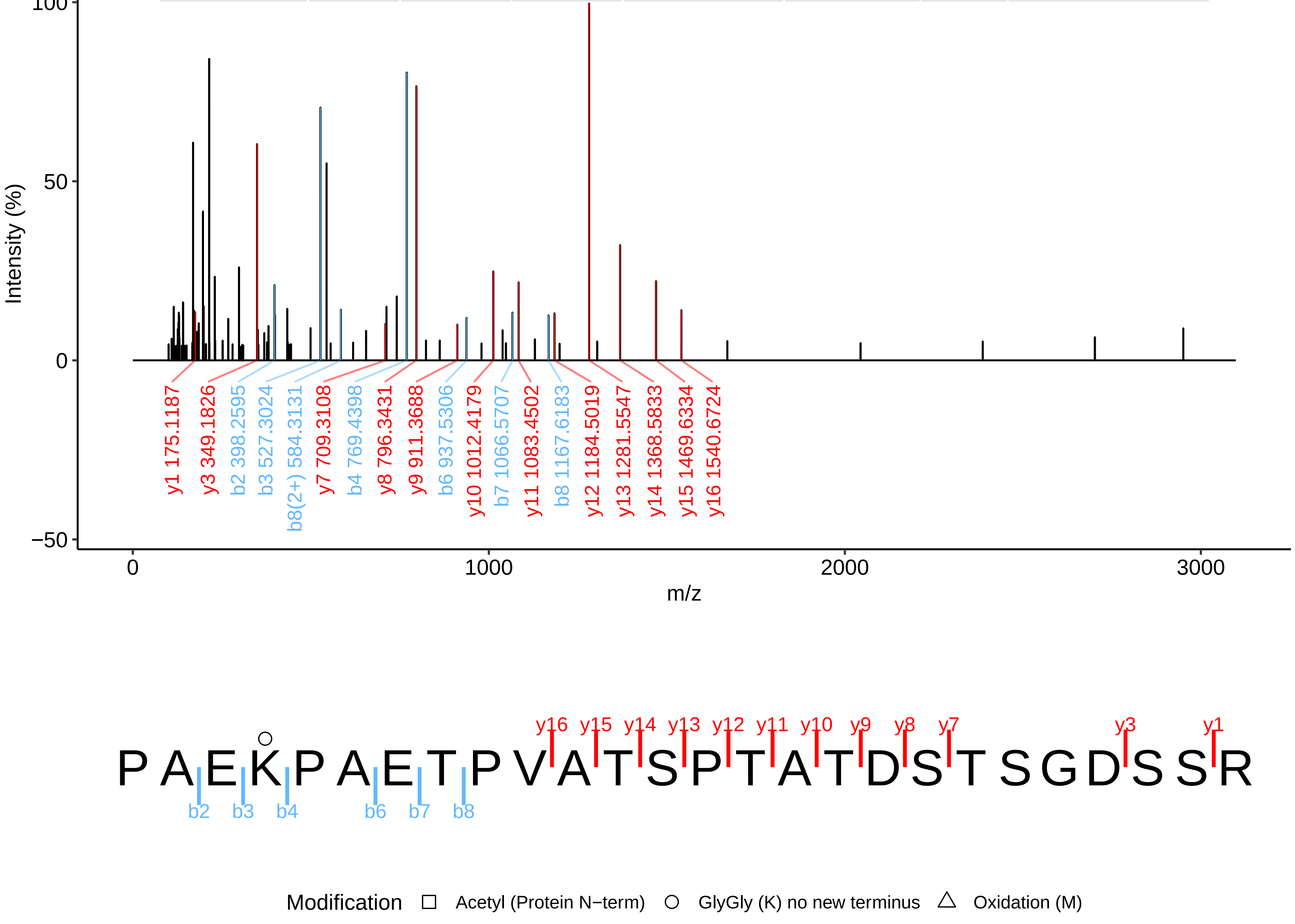

20200924_KIZ_MUb5_GlyGly_2 − Scan number: 6820

Gene Names	Charge	m/z	Mass	Retention time	PEP	Score	Precursor Intensity
RAD23B	3	892.0861	2673.237	25.44	1.4693e−12	99.135	2196709

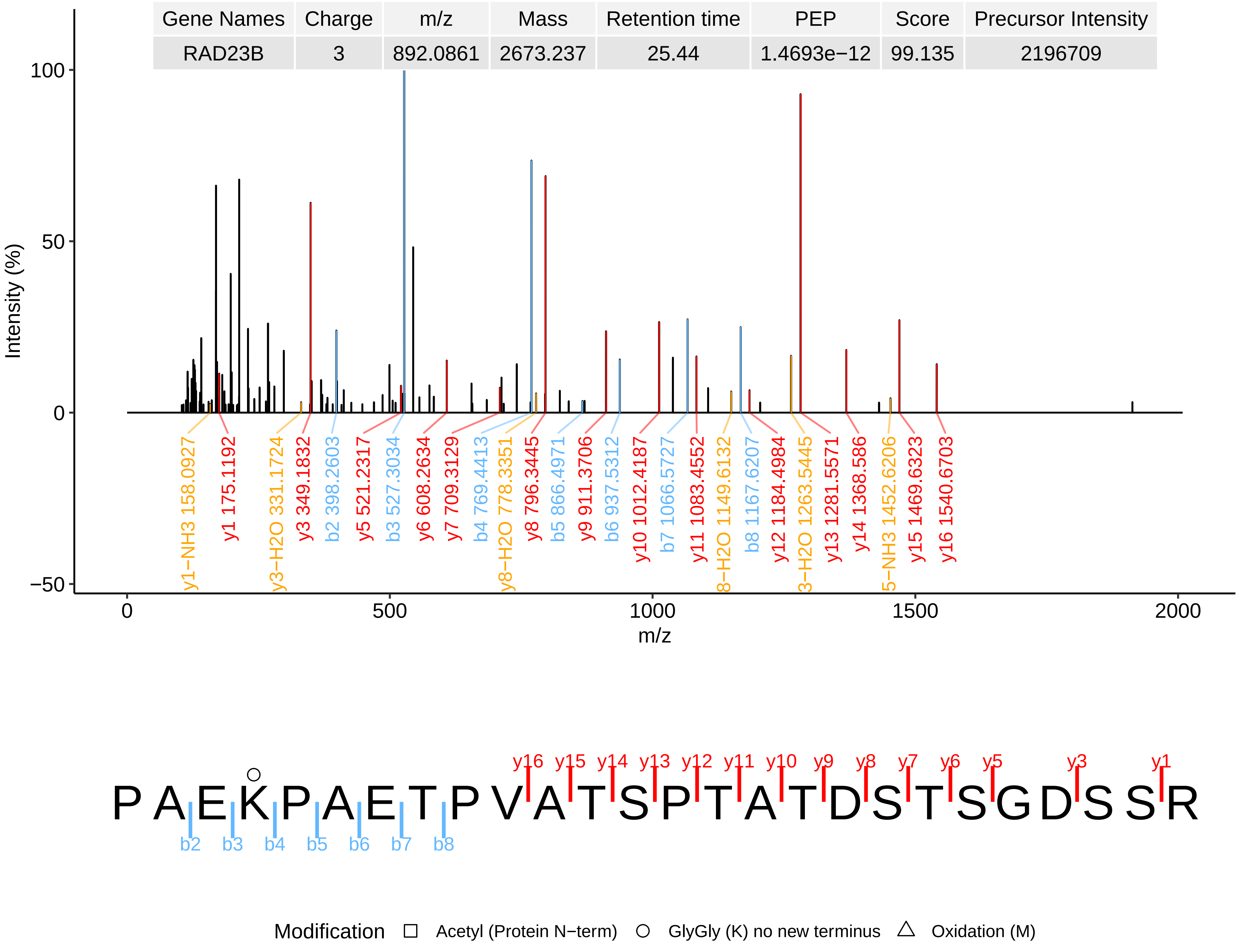

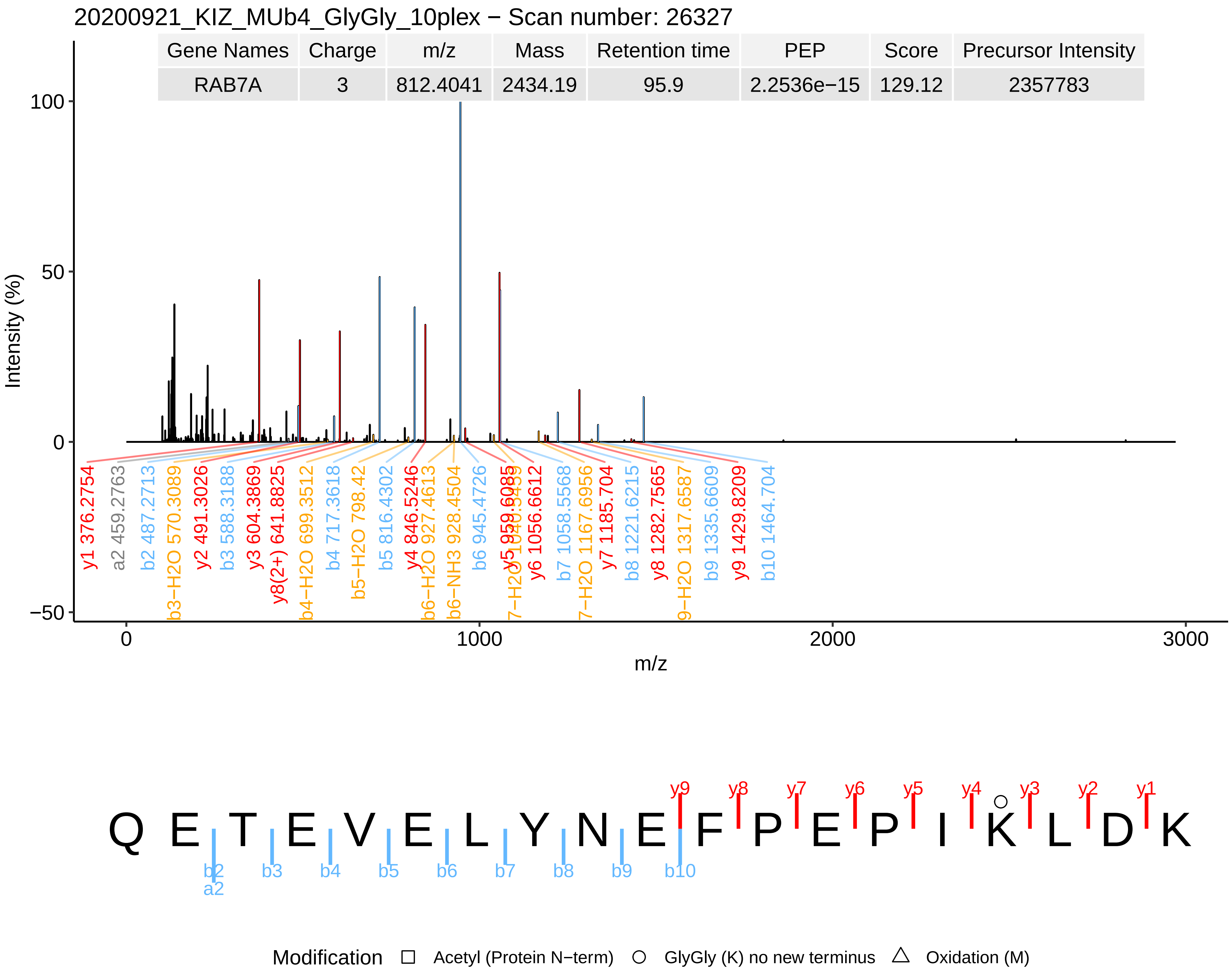
20200921_KIZ_MUb4_GlyGly_10plex − Scan number: 26327
Gene Names | Charge | m/z | Mass | Retention time | PEP | Score | Precursor Intensity
RAB7A | 3 | 812.4041 | 2434.19 | 95.9 | 2.2536e−15 | 129.12 | 2357783
Intensity (%)
100
50
0
−50
0
1000
2000
3000
m/z
y1 376.2754
a2 459.2763
b2 487.2713
b3−H2O 570.3089
y2 491.3026
b3 588.3188
y3 604.3869
y8(2+) 641.8825
b4−H2O 699.3512
b4 717.3618
b5−H2O 798.42
b5 816.4302
y4 846.5246
b6−H2O 927.4613
b6−NH3 928.4504
b6 945.4726
y5 959.6085
y6 1056.6612
b7 1058.5568
y7 1185.704
b8 1221.6215
y8 1282.7565
b9 1335.6609
y9 1429.8209
b10 1464.704
Q E T E V E L Y N E F P E P I K L D K
y9 y8 y7 y6 y5 y4 y3 y2 y1
b2 a2 b3 b4 b5 b6 b7 b8 b9 b10
Modification
Acetyl (Protein N−term)
GlyGly (K) no new terminus
Oxidation (M)

20200921_KIZ_MUb4_GlyGly_10plex − Scan number: 26459

Gene Names	Charge	m/z	Mass	Retention time	PEP	Score	Precursor Intensity
RAB7A	3	812.4041	2434.19	96.412	5.5805e−10	111.66	3648420

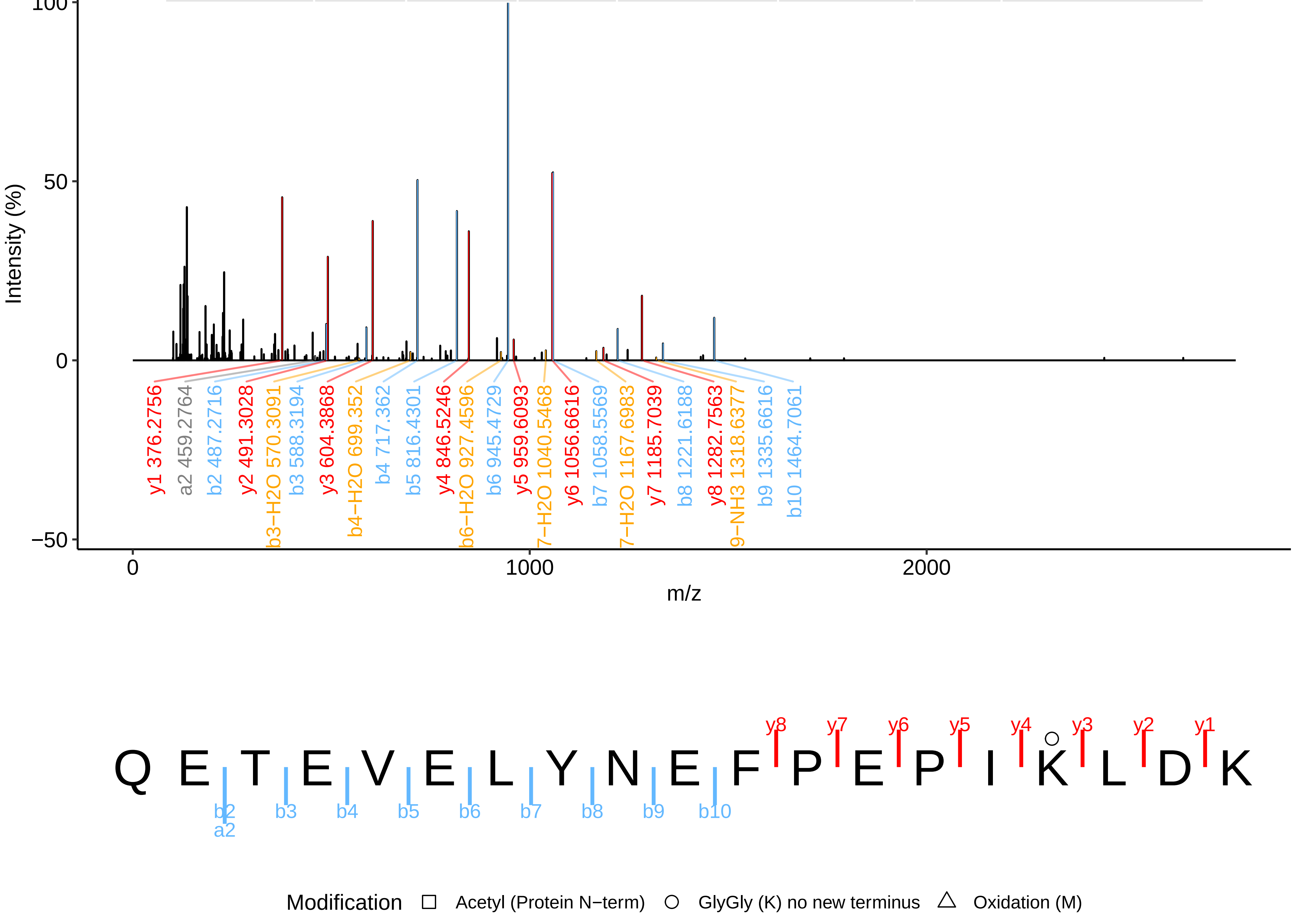

20200921_KIZ_MUb4_GlyGly_10plex_2 − Scan number: 23232

Gene Names	Charge	m/z	Mass	Retention time	PEP	Score	Precursor Intensity
RAB7A	3	812.4041	2434.19	91.674	0.00033537	78.021	NA

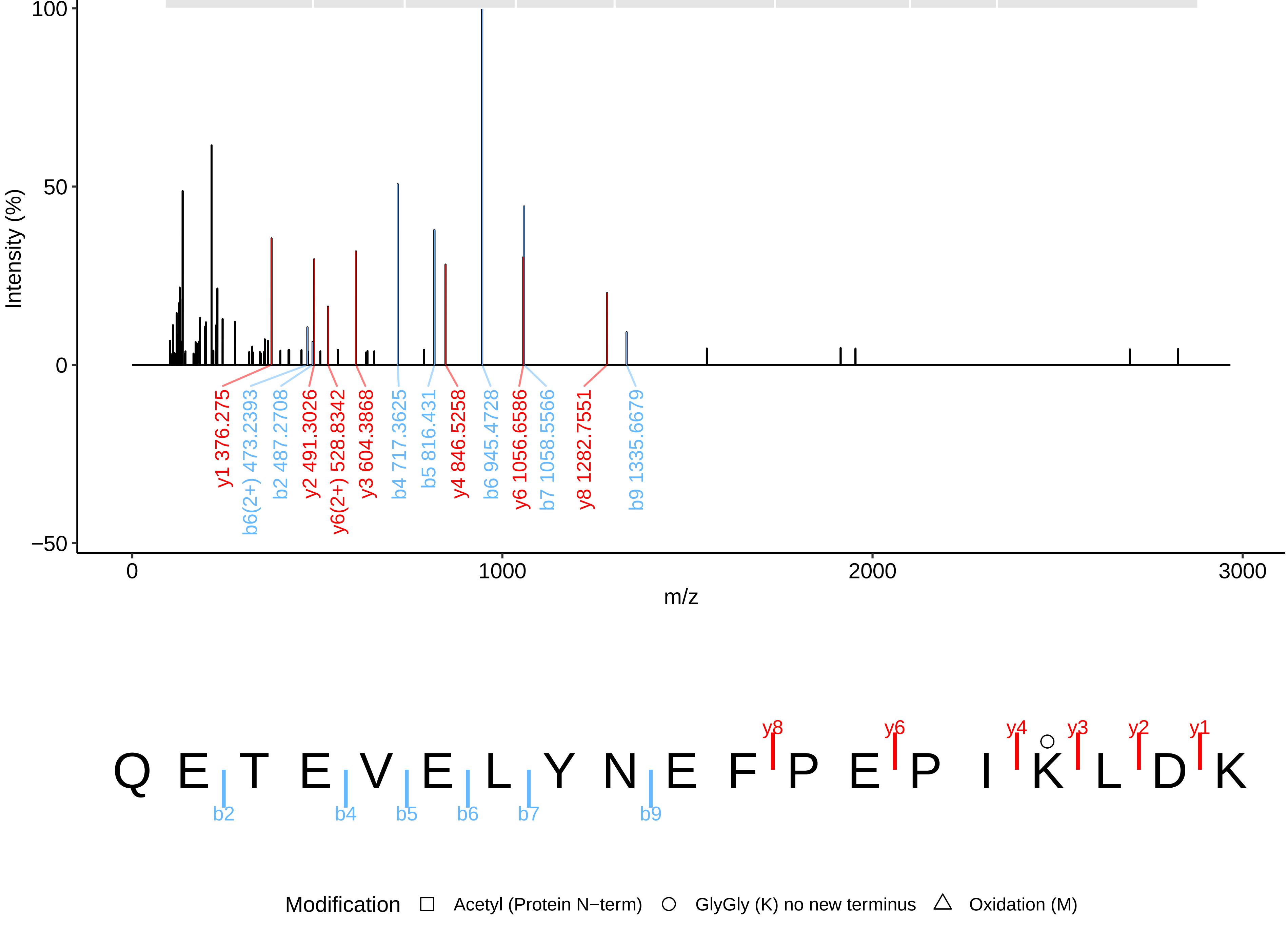

20200921_KIZ_MUb4_GlyGly_10plex_2 − Scan number: 23243

Gene Names	Charge	m/z	Mass	Retention time	PEP	Score	Precursor Intensity
RAB7A	4	609.5549	2434.19	91.72	0.016645	56.936	690392.4

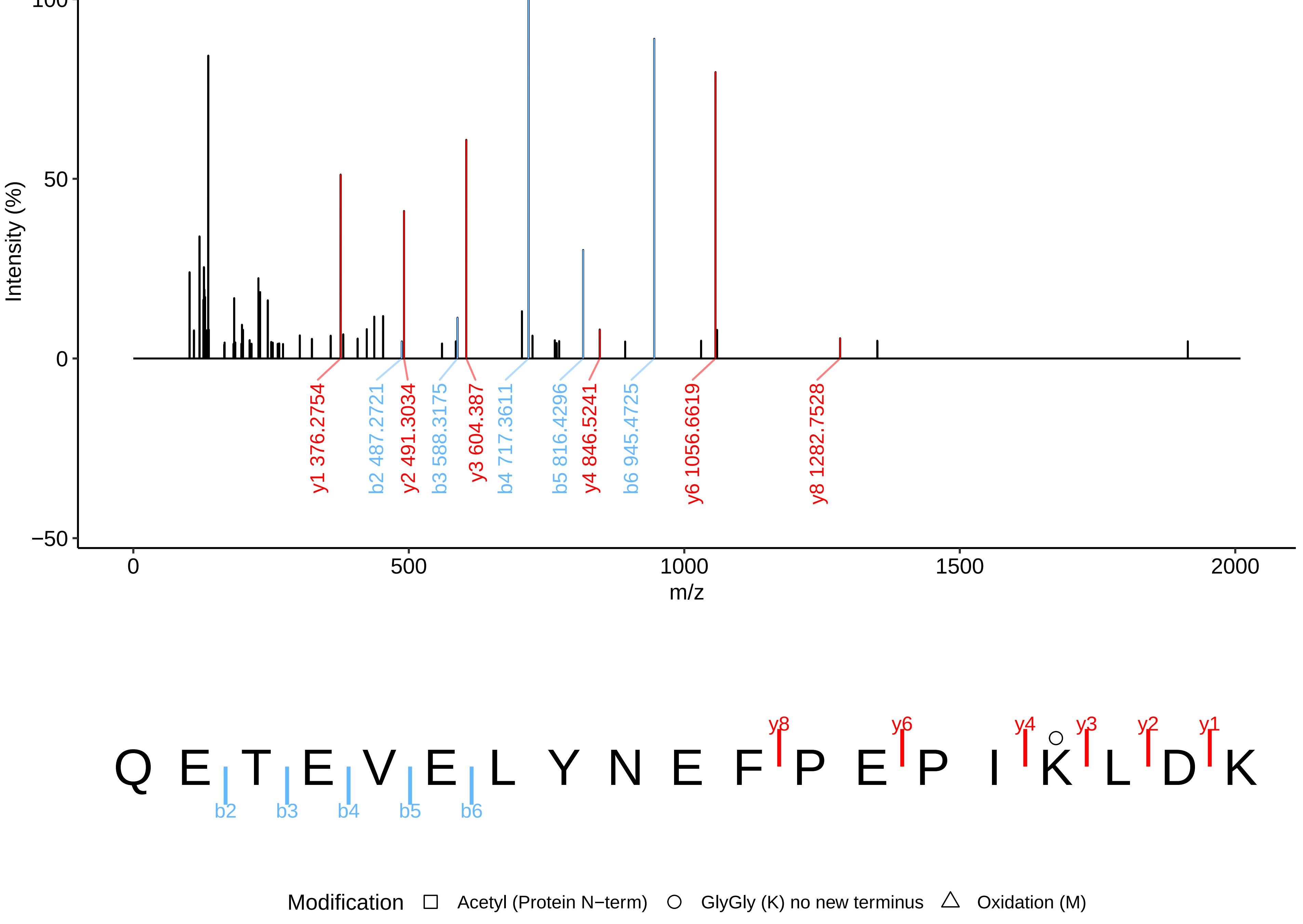

20200924_KIZ_MUb5_GlyGly_1 − Scan number: 26240

Gene Names	Charge	m/z	Mass	Retention time	PEP	Score	Precursor Intensity
RAB7A	3	812.4041	2434.19	84.063	4.7558e−20	142.88	NA

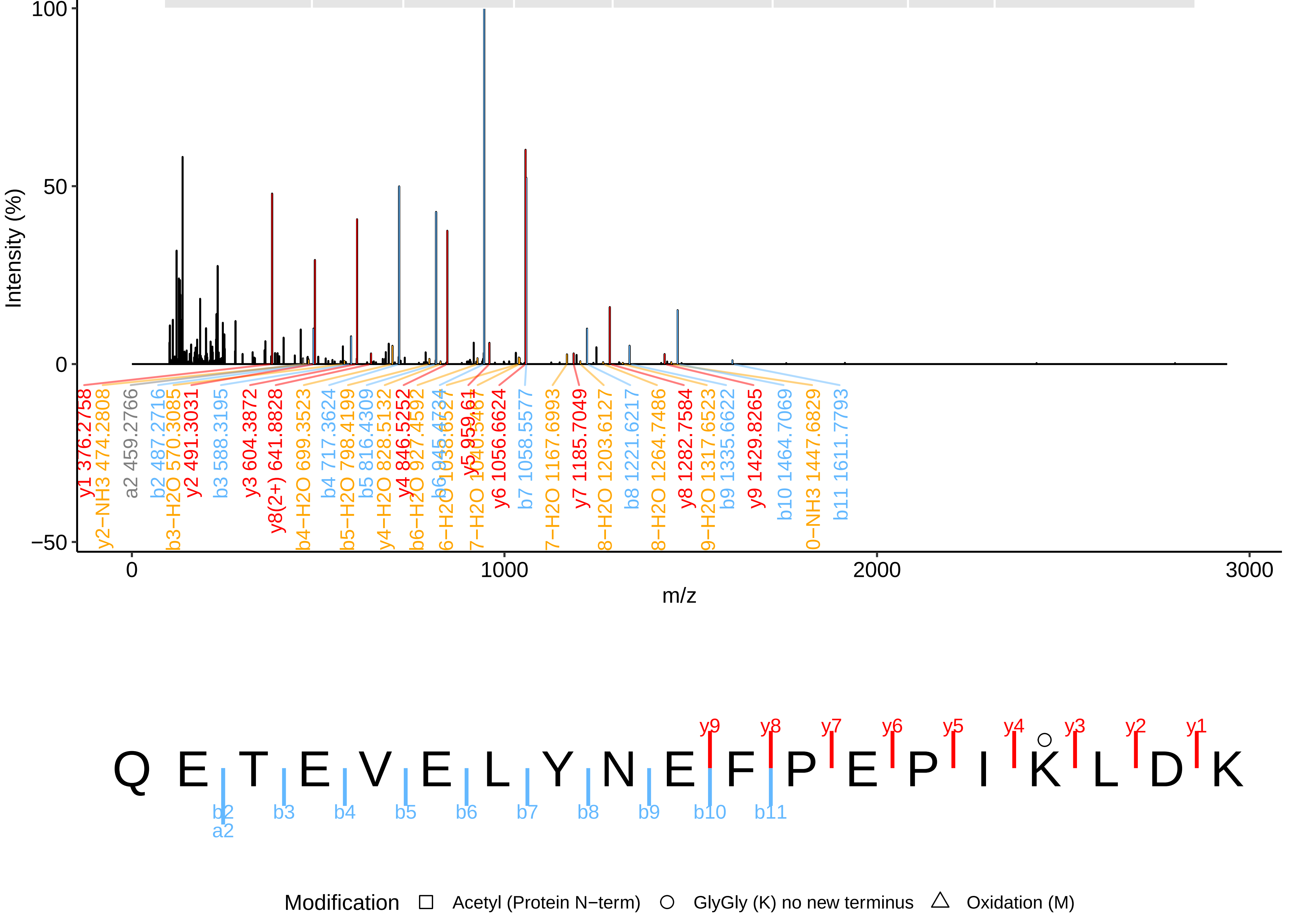

20200921_KIZ_MUb4_GlyGly_10plex_2 − Scan number: 21648

Gene Names	Charge	m/z	Mass	Retention time	PEP	Score	Precursor Intensity
PIK3C2B	3	921.7718	2762.294	85.25	0.013085	58.904	345793.9

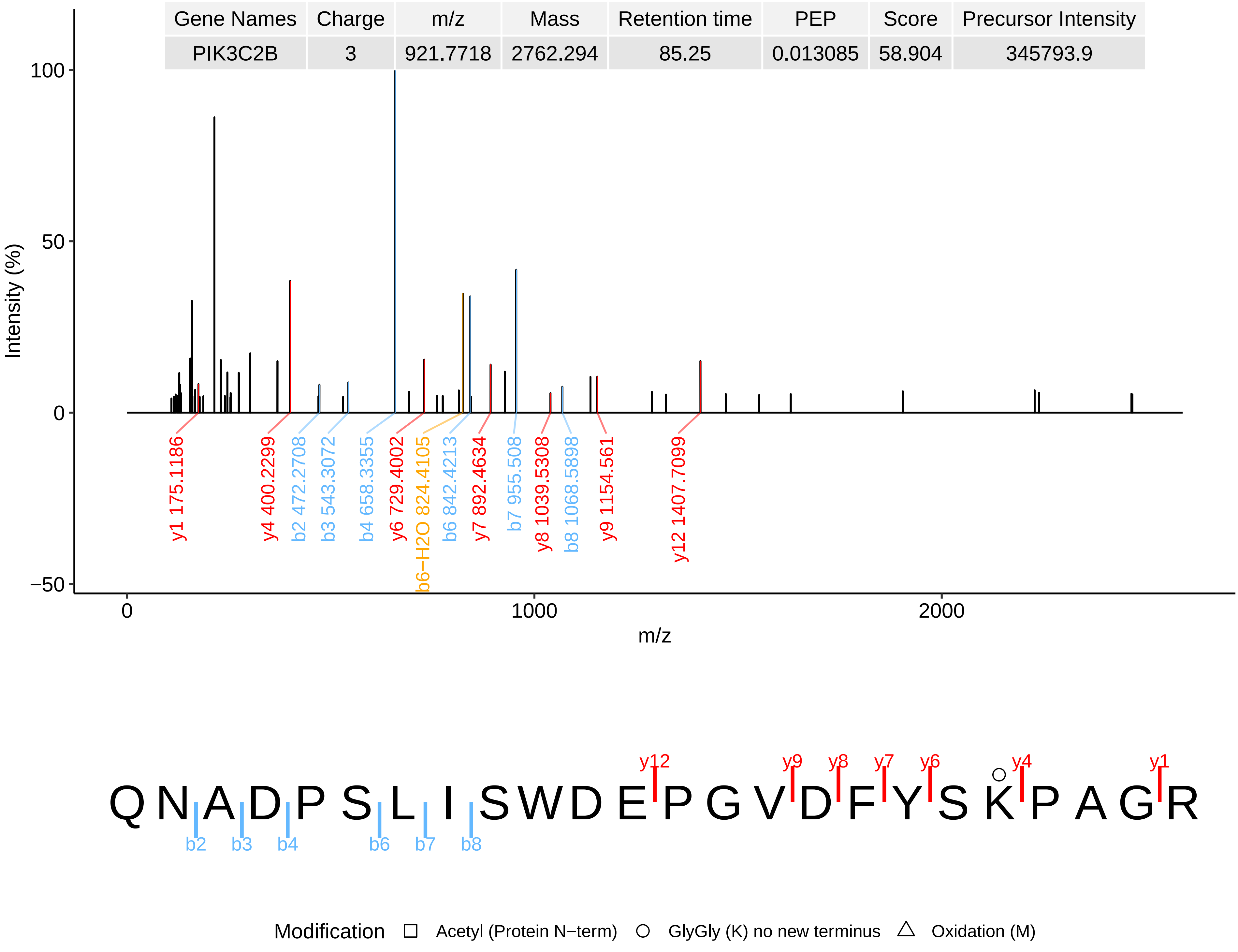

20200921_KIZ_MUb4_GlyGly_10plex_2 − Scan number: 16983

Gene Names	Charge	m/z	Mass	Retention time	PEP	Score	Precursor Intensity
EIF2S3	3	819.3727	2455.096	66.359	0.00037909	74.364	812799.7

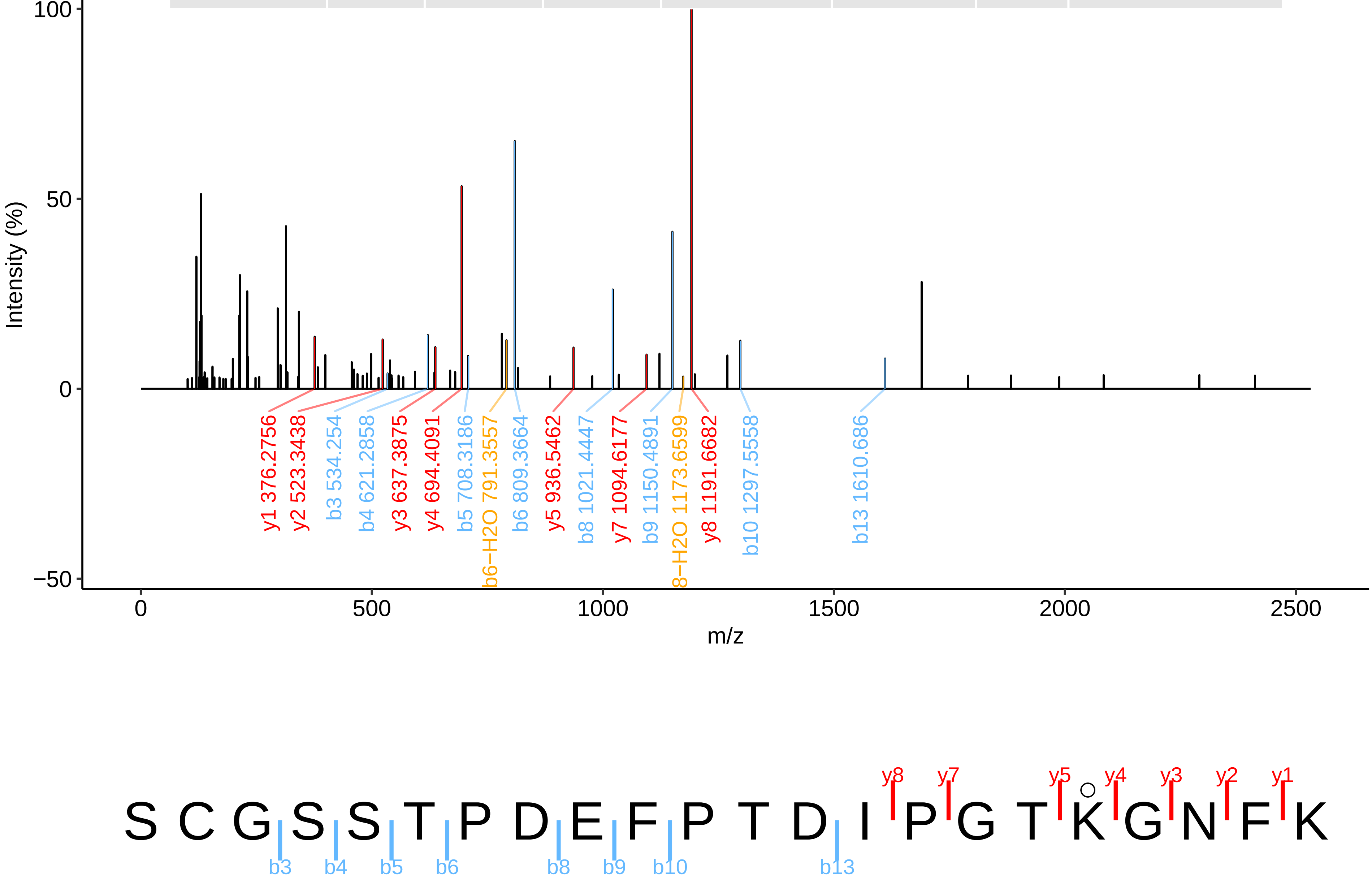

Modification □ Acetyl (Protein N−term) ○ GlyGly (K) no new terminus △ Oxidation (M)

20200921_KIZ_MUb4_GlyGly_10plex − Scan number: 26607

Gene Names	Charge	m/z	Mass	Retention time	PEP	Score	Precursor Intensity
SLC1A5	4	682.8631	2727.423	96.961	1.4691e−15	120.31	2754421

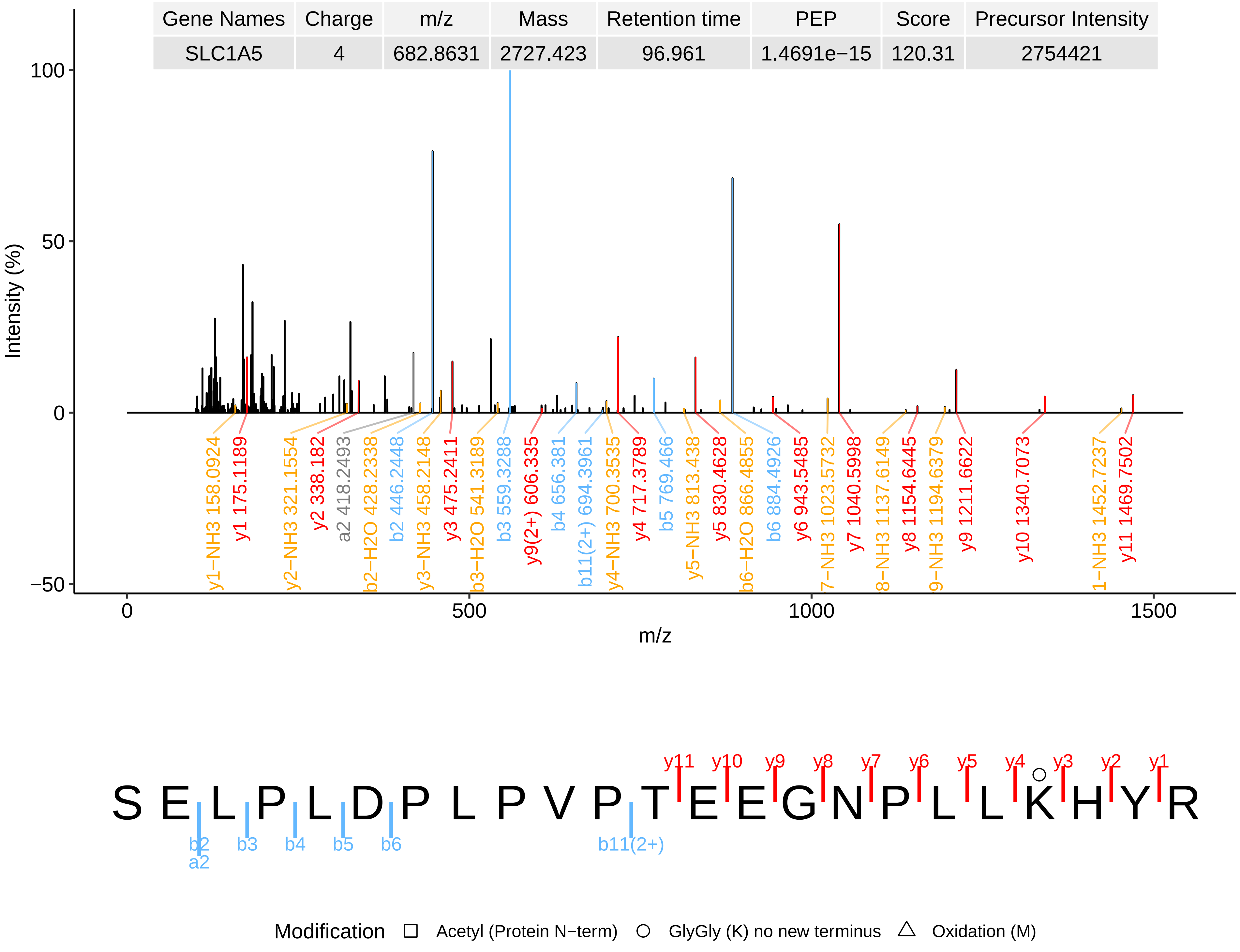

20200921_KIZ_MUb4_GlyGly_10plex − Scan number: 26625

Gene Names	Charge	m/z	Mass	Retention time	PEP	Score	Precursor Intensity
SLC1A5	4	682.8631	2727.423	97.03	2.4326e−22	139.11	4281291

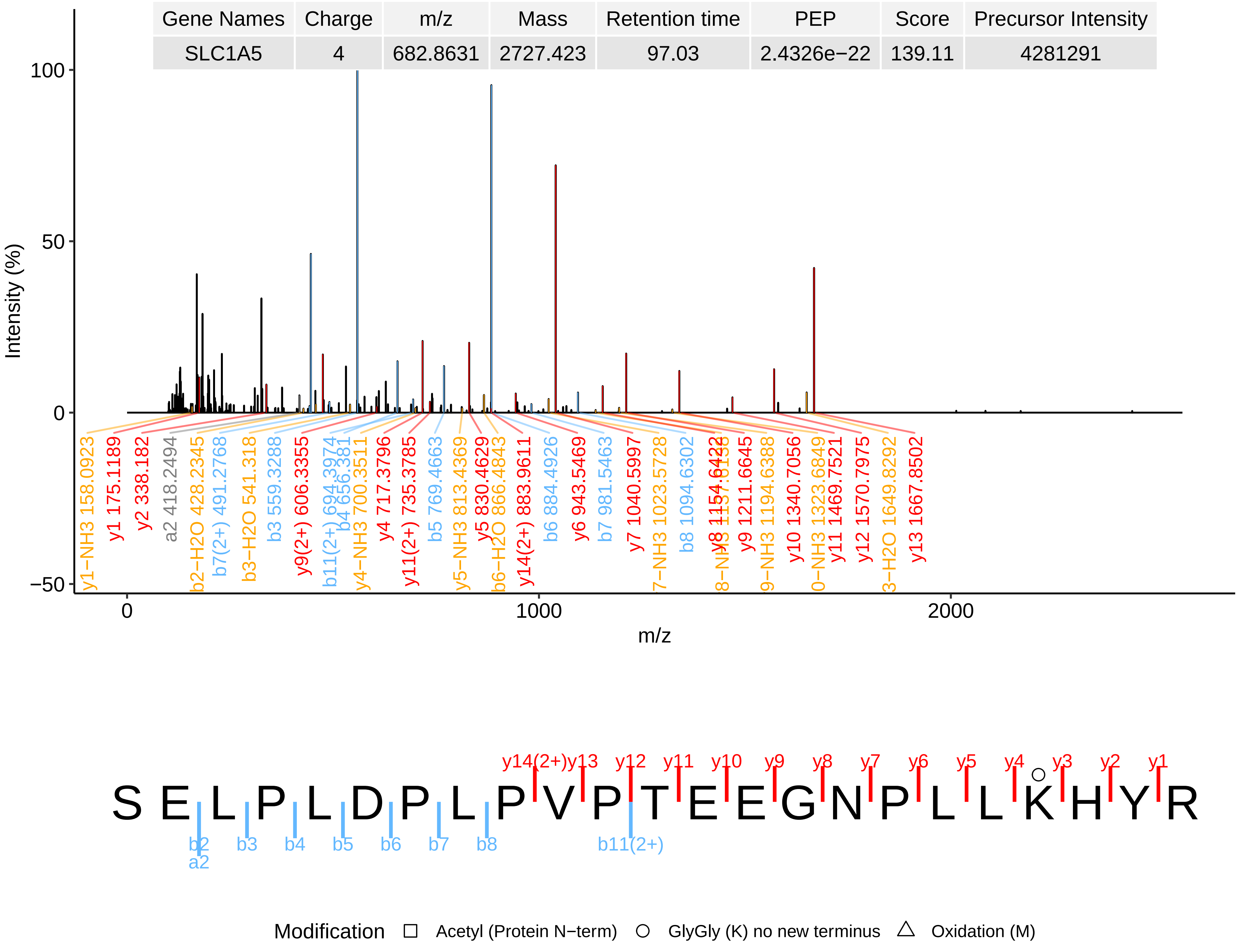

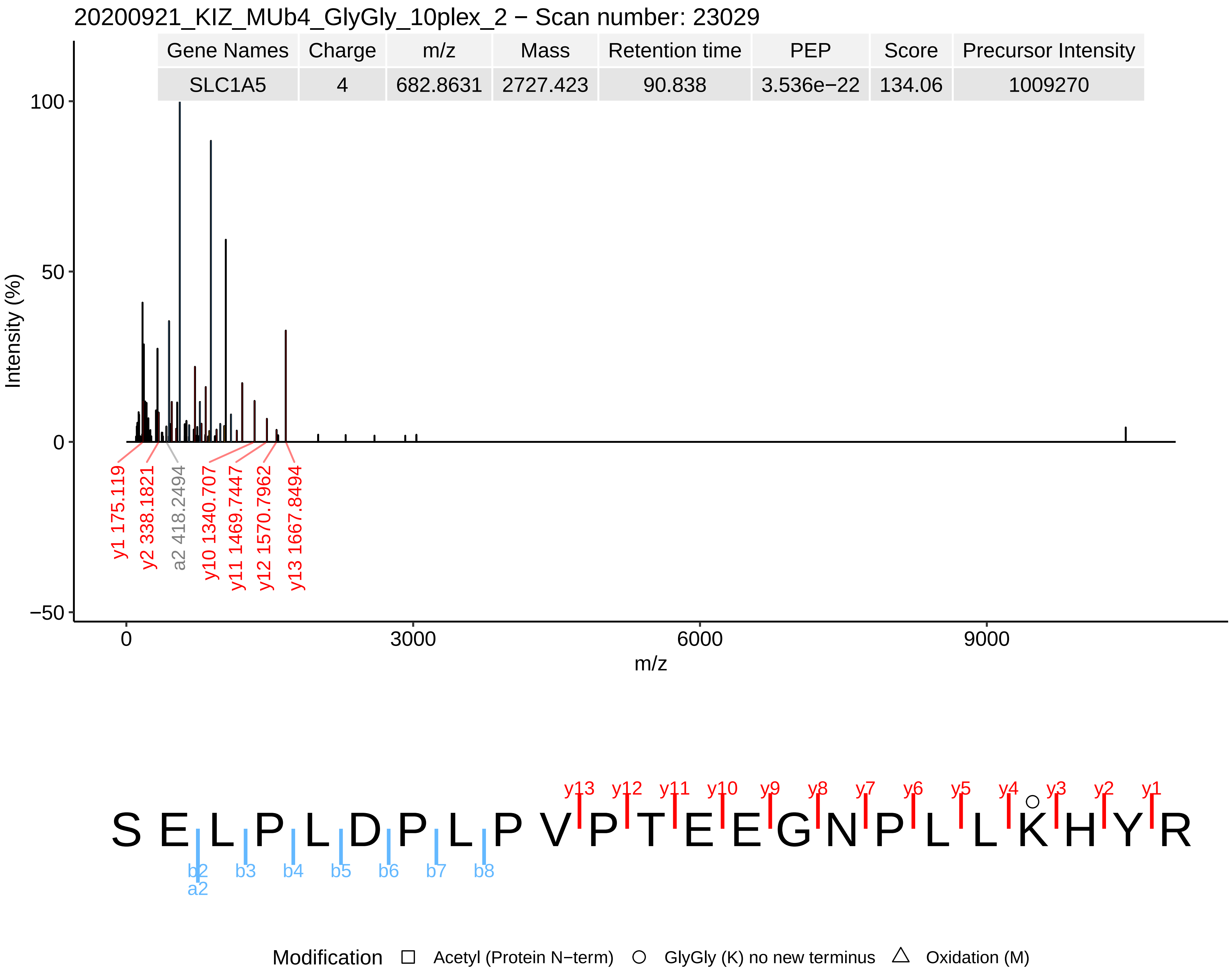
20200921_KIZ_MUb4_GlyGly_10plex_2 − Scan number: 23029
Gene Names | Charge | m/z | Mass | Retention time | PEP | Score | Precursor Intensity
SLC1A5 | 4 | 682.8631 | 2727.423 | 90.838 | 3.536e−22 | 134.06 | 1009270
Intensity (%)
100
50
0
−50
0
3000
6000
9000
m/z
y1 175.119
y2 338.1821
a2 418.2494
y10 1340.707
y11 1469.7447
y12 1570.7962
y13 1667.8494
S E L P L D P L P V P T E E G N P L L K H Y R
y13 y12 y11 y10 y9 y8 y7 y6 y5 y4 y3 y2 y1
b2 b3 b4 b5 b6 b7 b8
a2
Modification
Acetyl (Protein N−term)
GlyGly (K) no new terminus
Oxidation (M)

20200921_KIZ_MUb4_GlyGly_10plex_2 − Scan number: 23046

Gene Names	Charge	m/z	Mass	Retention time	PEP	Score	Precursor Intensity
SLC1A5	4	682.8631	2727.423	90.911	0.025145	54.117	2101026

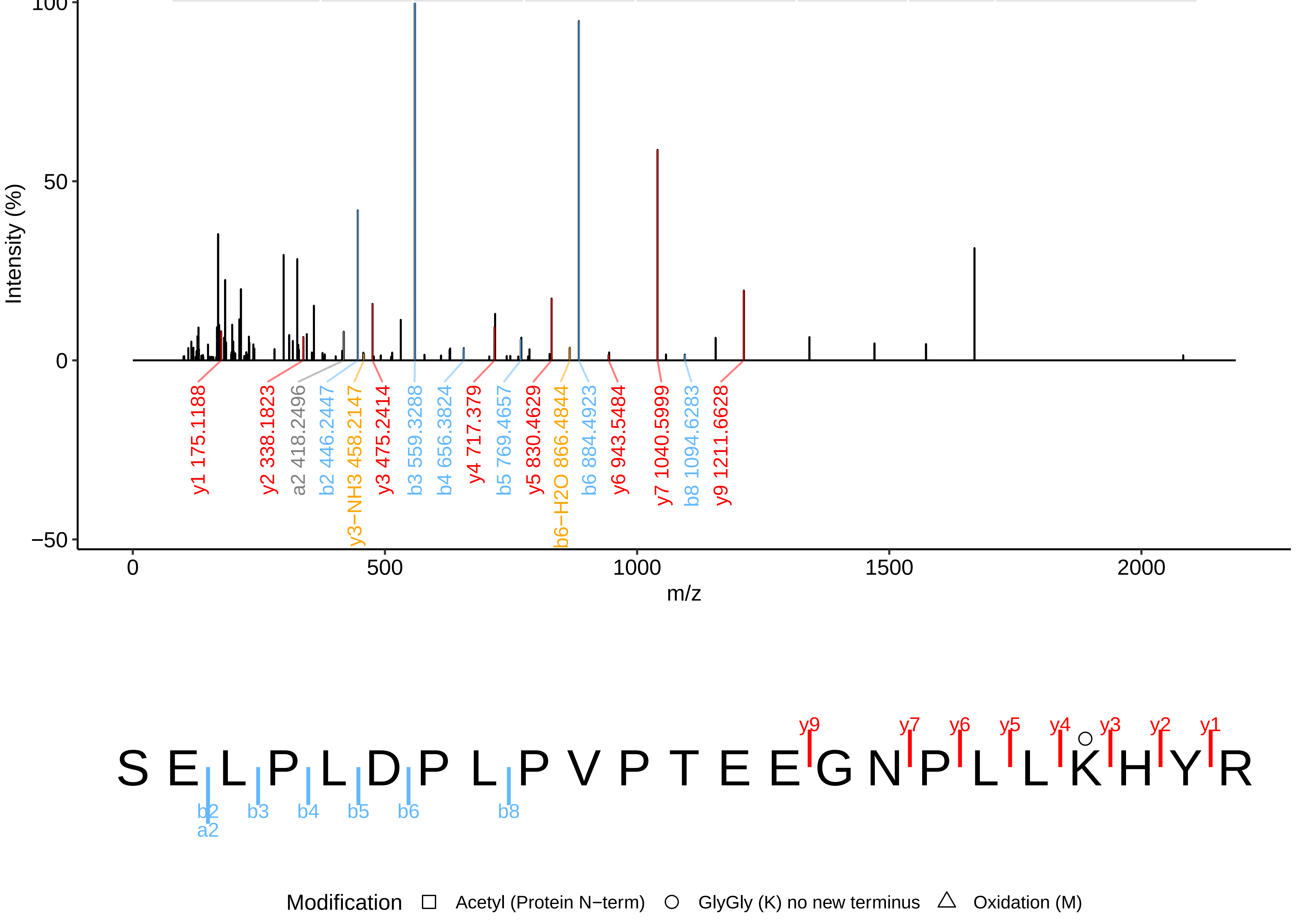

20200921_KIZ_MUb4_GlyGly_10plex − Scan number: 32324

Gene Names	Charge	m/z	Mass	Retention time	PEP	Score	Precursor Intensity
UBA1	4	681.6076	2722.401	119.45	1.7852e−15	128.74	2625725

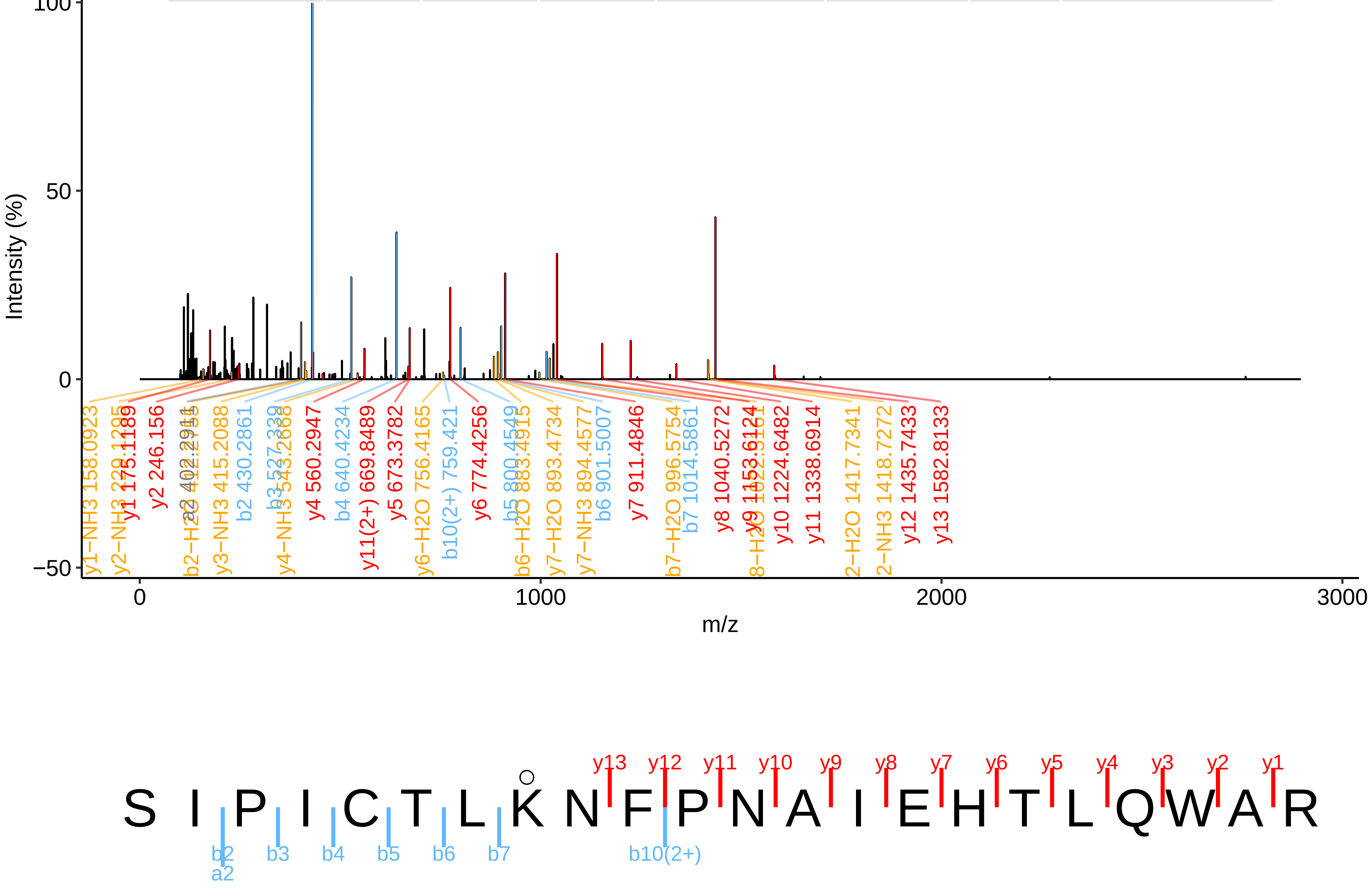

Modification □ Acetyl (Protein N−term) ○ GlyGly (K) no new terminus △ Oxidation (M)

20200921_KIZ_MUb4_GlyGly_10plex − Scan number: 27995

Gene Names	Charge	m/z	Mass	Retention time	PEP	Score	Precursor Intensity
OSMR	3	823.1037	2466.289	102.32	0.00031045	77.39	2015965

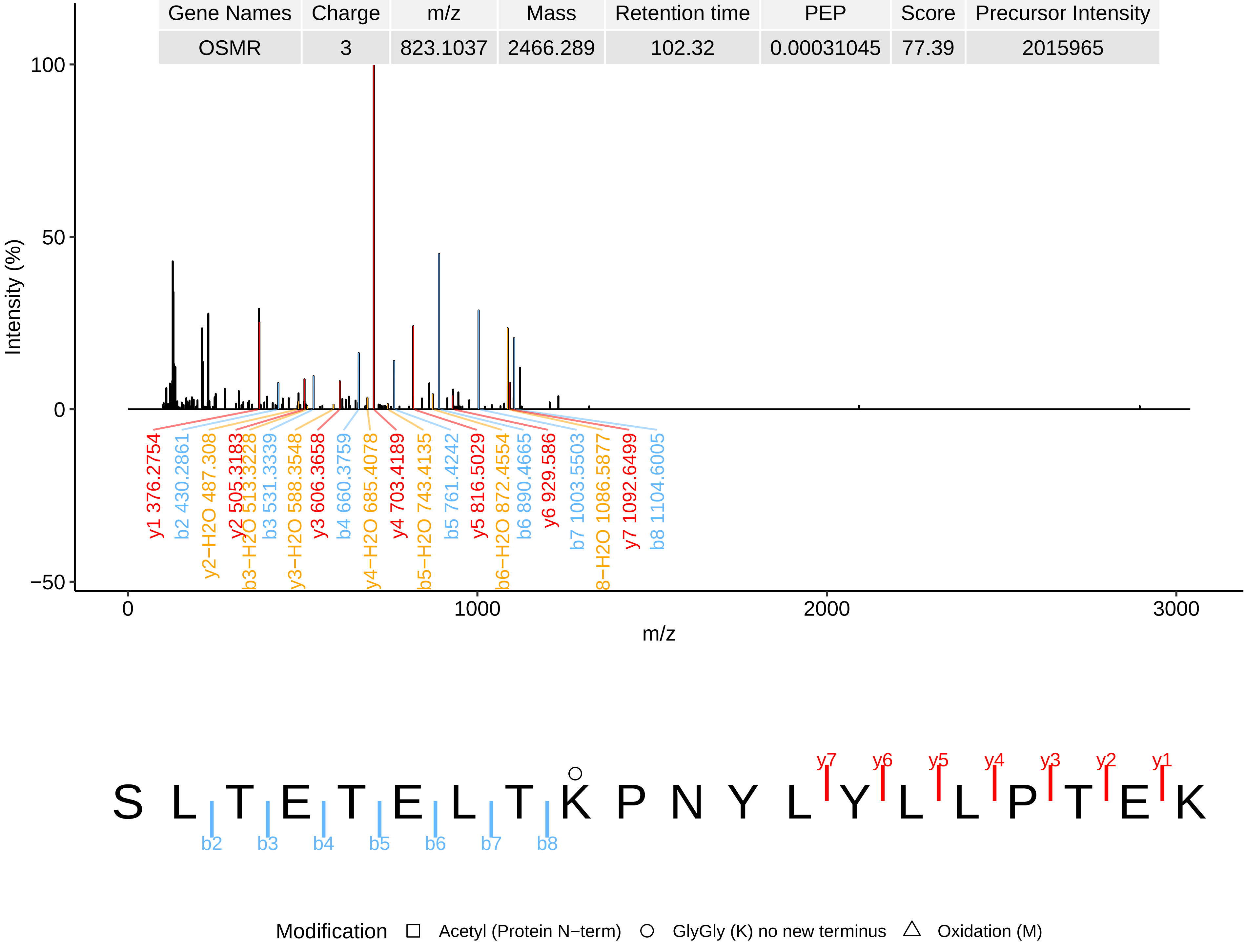

20200921_KIZ_MUb4_GlyGly_10plex_2 − Scan number: 24924

Gene Names	Charge	m/z	Mass	Retention time	PEP	Score	Precursor Intensity
OSMR	3	823.1037	2466.289	98.523	0.00019289	78.661	598475.4

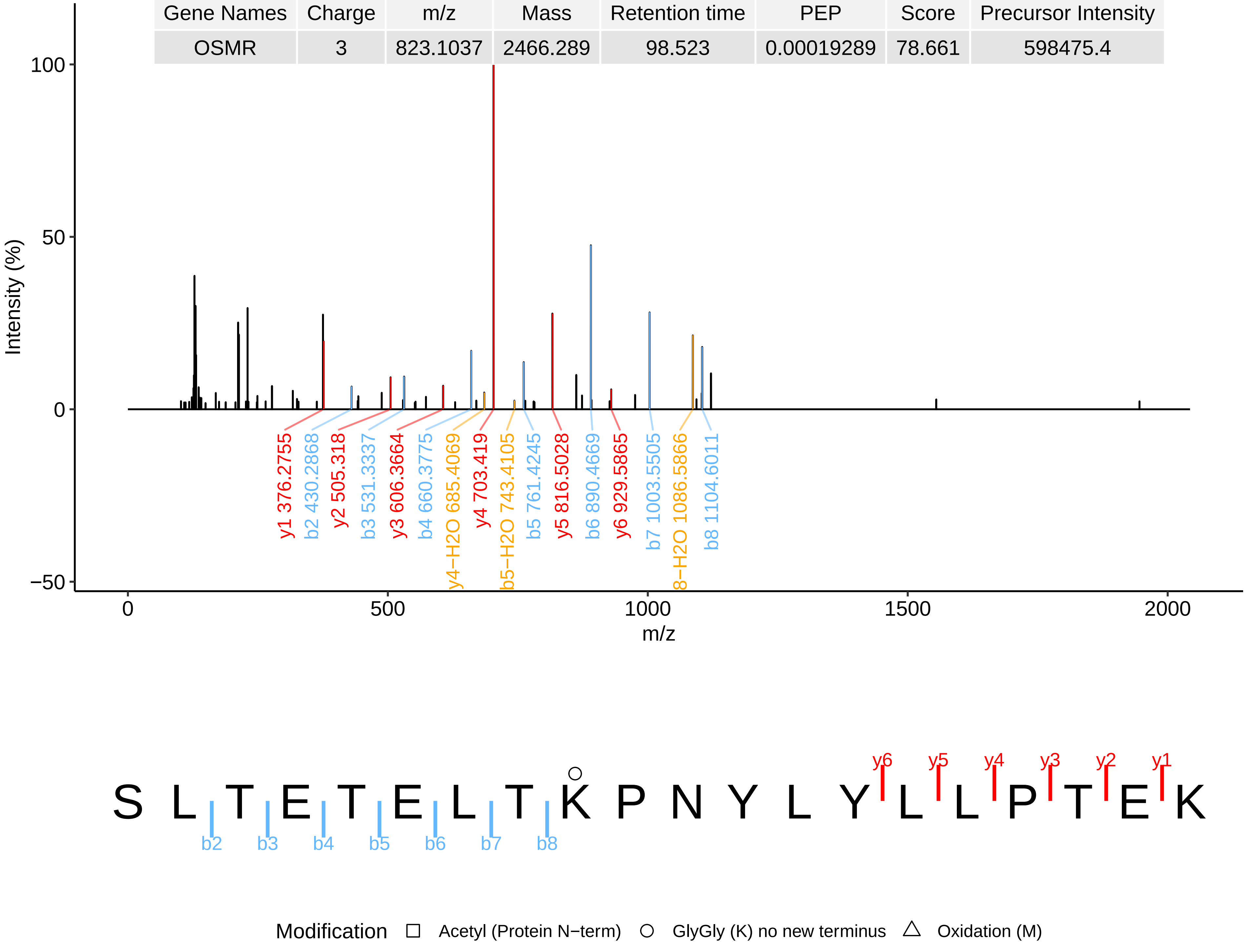

20201118_KIZ_MUb6_GlyGly_1 − Scan number: 33016

Gene Names	Charge	m/z	Mass	Retention time	PEP	Score	Precursor Intensity
THNSL1	5	505.4656	2522.292	97.454	0.036386	52.185	18573718

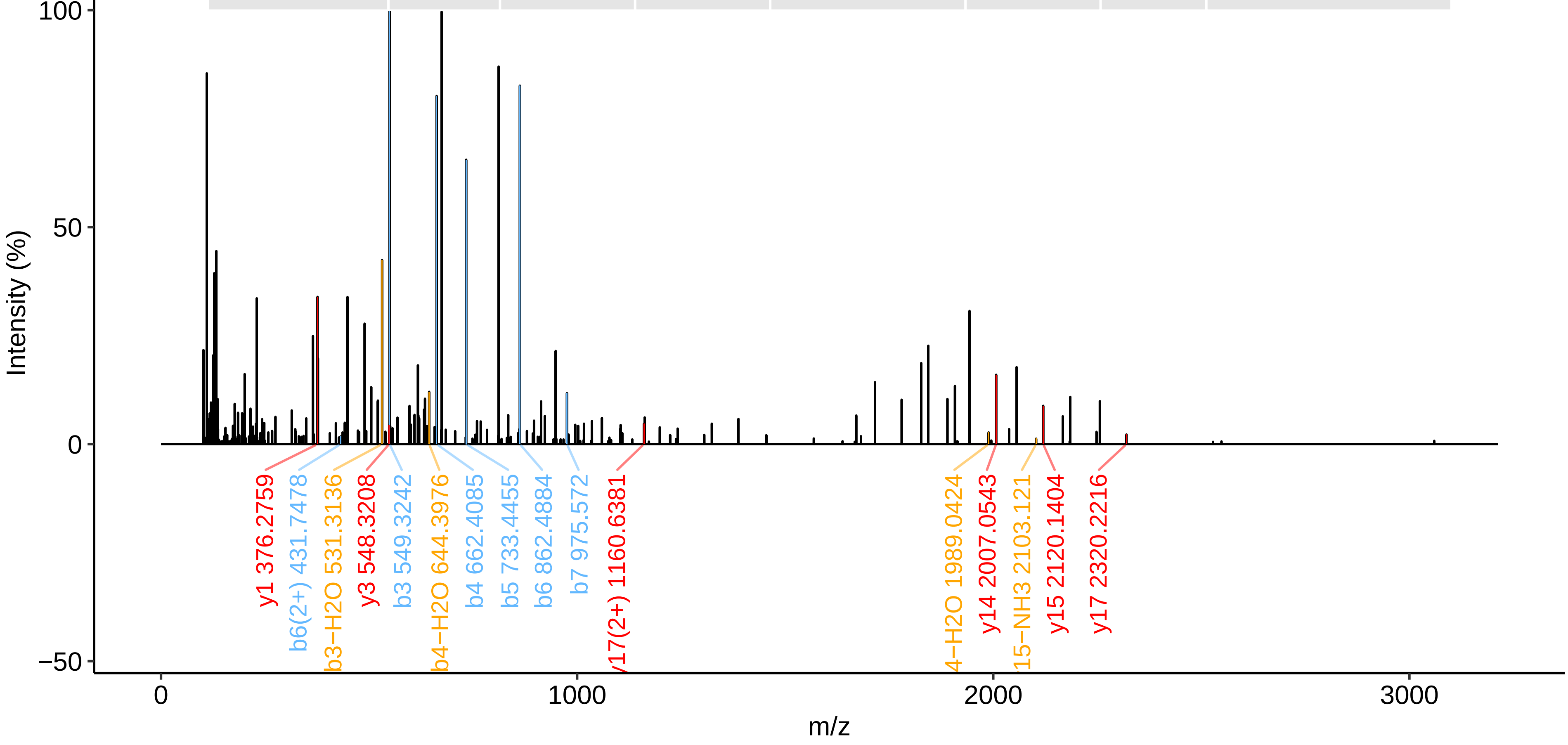

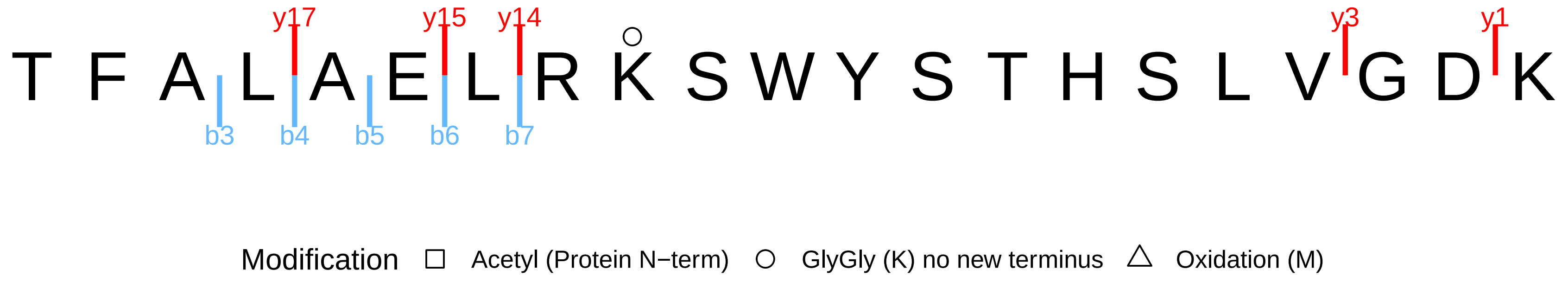

20200921_KIZ_MUb4_GlyGly_10plex_2 − Scan number: 19023

Gene Names	Charge	m/z	Mass	Retention time	PEP	Score	Precursor Intensity
TUBA1C;TUBA1A;TUBA1B	4	653.5598	2610.21	74.606	6.9964e−07	85.932	1464978

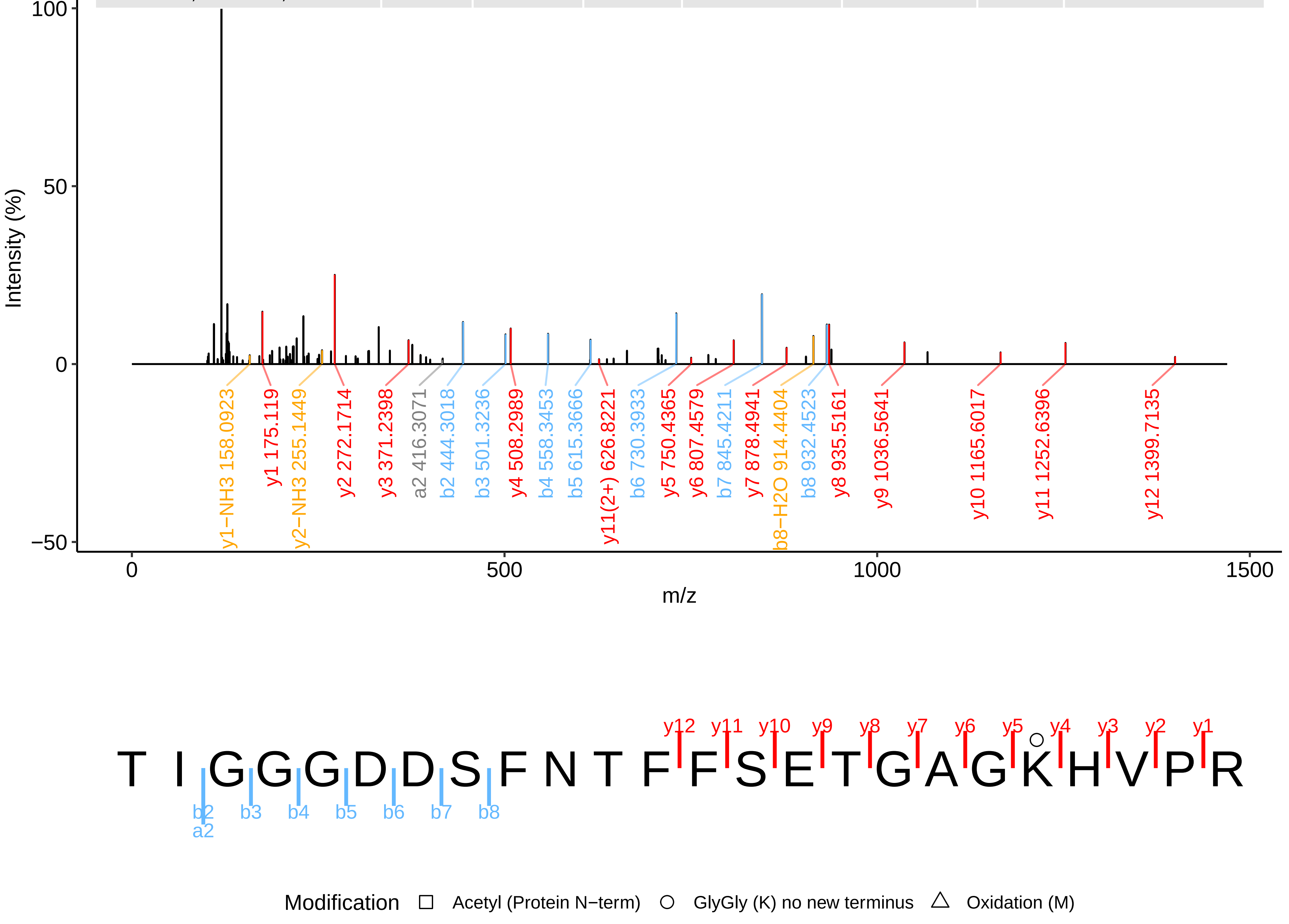

20200921_KIZ_MUb4_GlyGly_10plex_2 − Scan number: 19036

Gene Names	Charge	m/z	Mass	Retention time	PEP	Score	Precursor Intensity
TUBA1C;TUBA1A;TUBA1B	4	653.5598	2610.21	74.659	3.3647e−16	127.01	967770.8

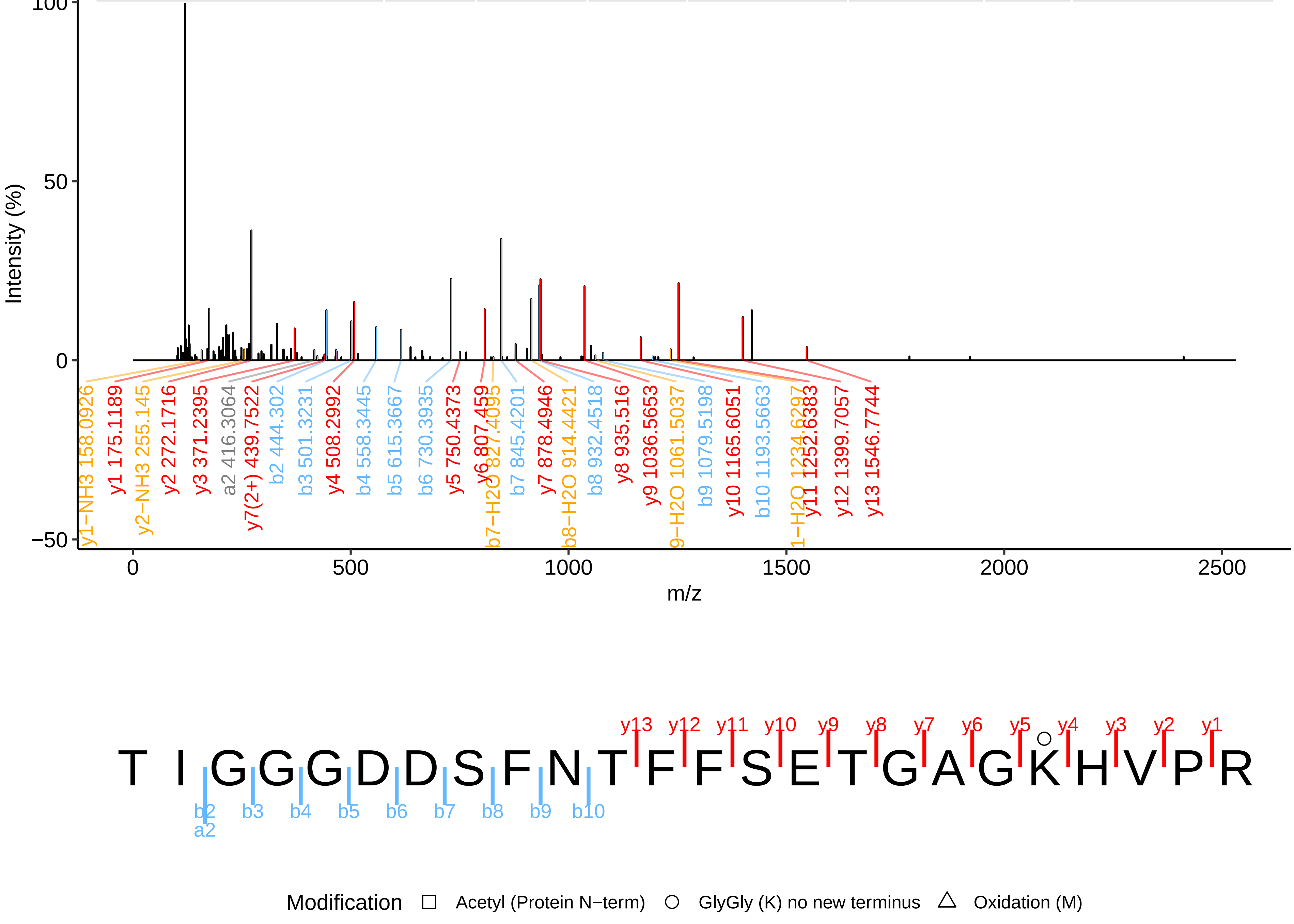

20200921_KIZ_MUb4_GlyGly_10plex − Scan number: 22799

Gene Names	Charge	m/z	Mass	Retention time	PEP	Score	Precursor Intensity
RPS27A;UBA52;UBC;UBB	3	801.4269	2401.259	82.737	4.333e−59	208.86	185002016

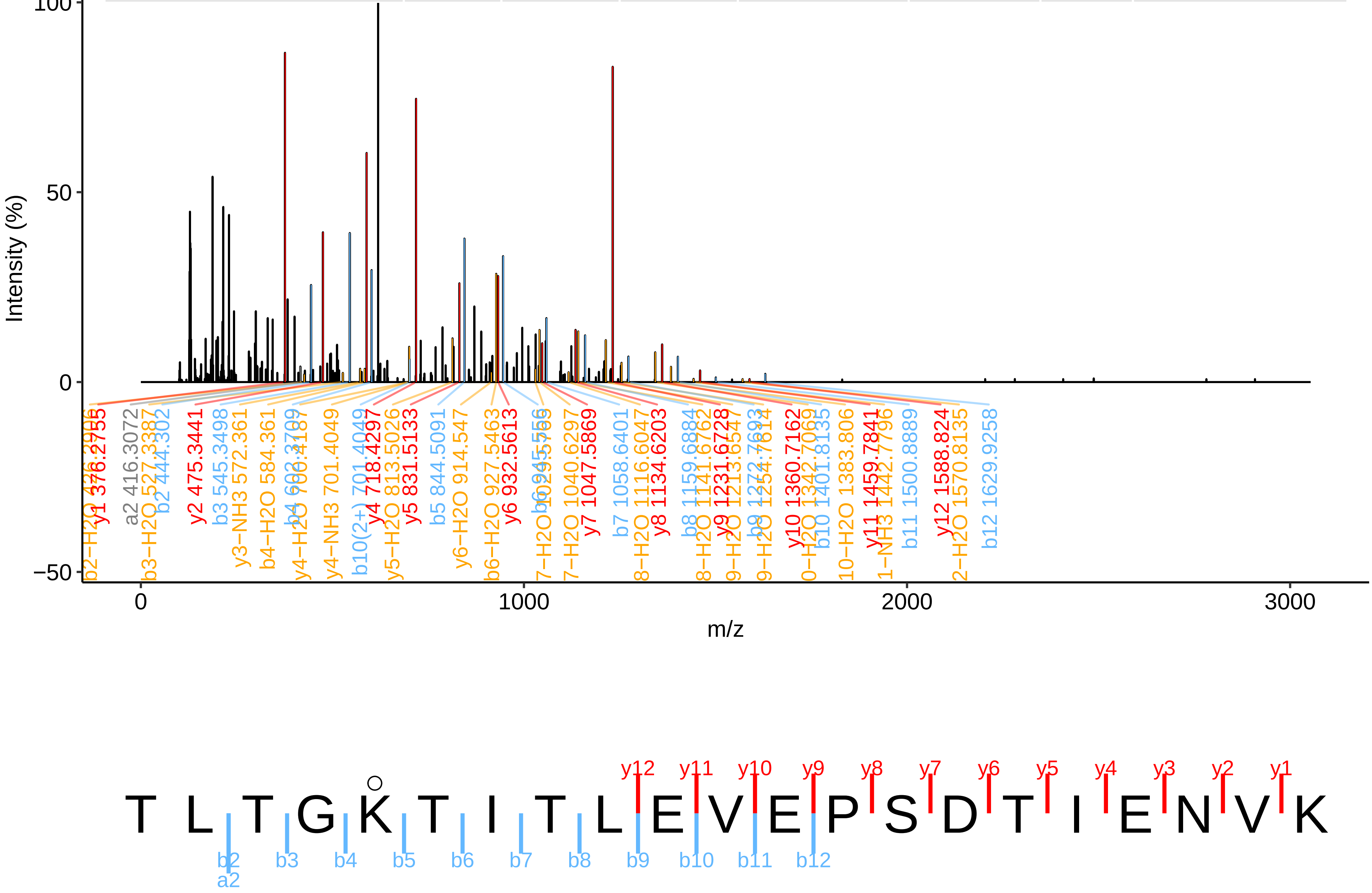

Modification □ Acetyl (Protein N−term) ○ GlyGly (K) no new terminus △ Oxidation (M)

20200921_KIZ_MUb4_GlyGly_10plex − Scan number: 22938

Gene Names	Charge	m/z	Mass	Retention time	PEP	Score	Precursor Intensity
RPS27A;UBA52;UBC;UBB	3	801.4269	2401.259	83.24	4.333e−59	208.86	219219152

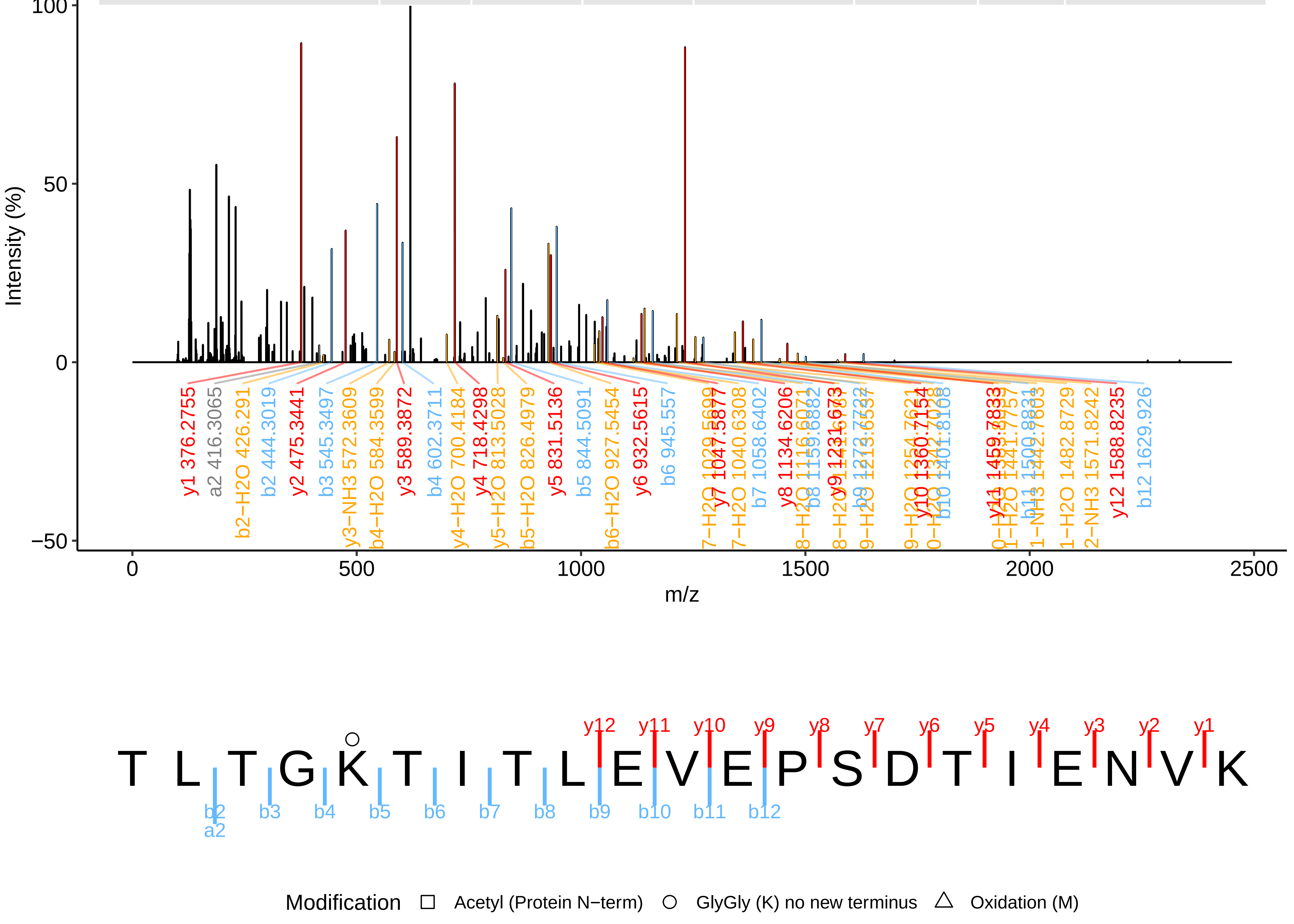

20200921_KIZ_MUb4_GlyGly_10plex − Scan number: 23080

Gene Names	Charge	m/z	Mass	Retention time	PEP	Score	Precursor Intensity
RPS27A;UBA52;UBC;UBB	3	801.4269	2401.259	83.747	1.5514e−48	189.48	45843360

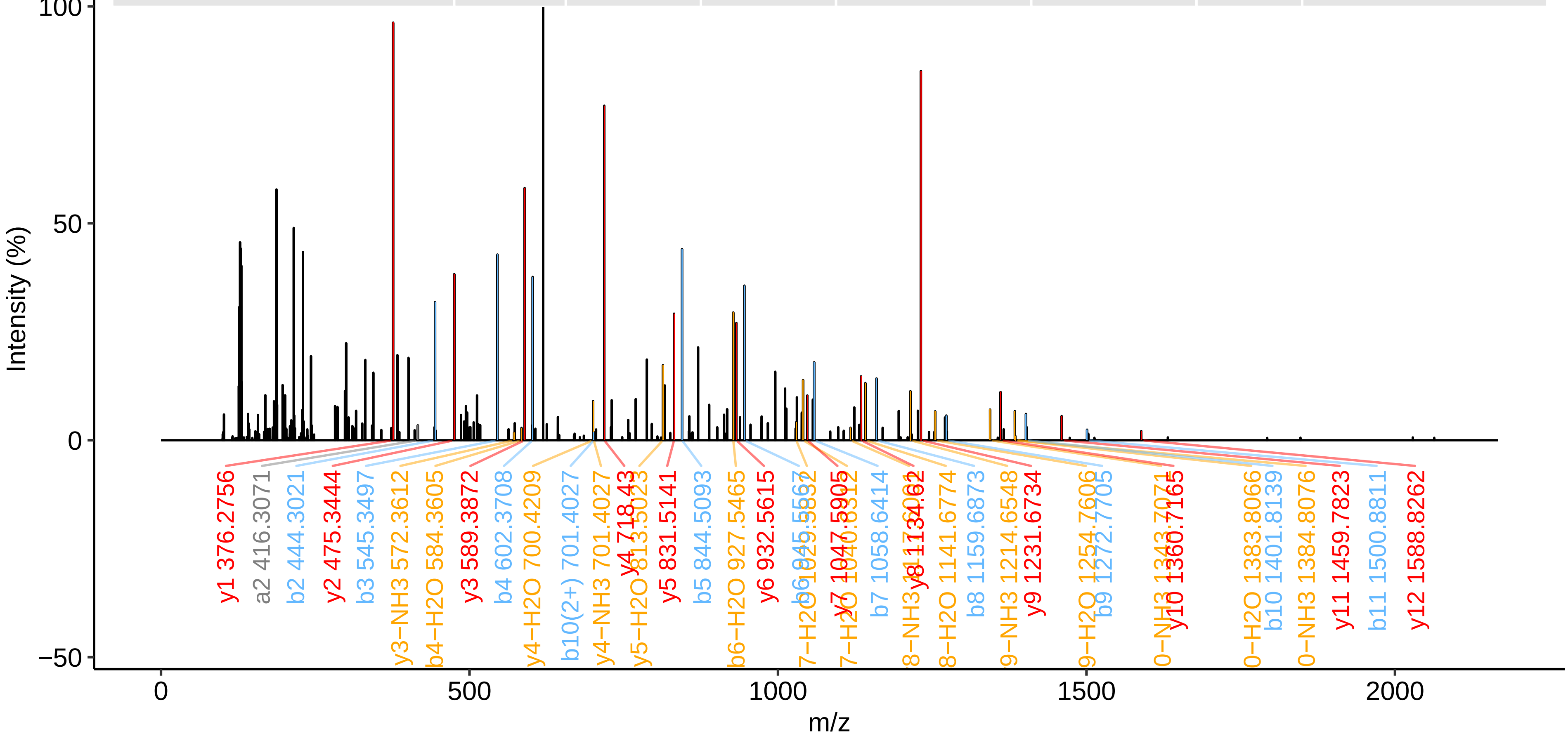

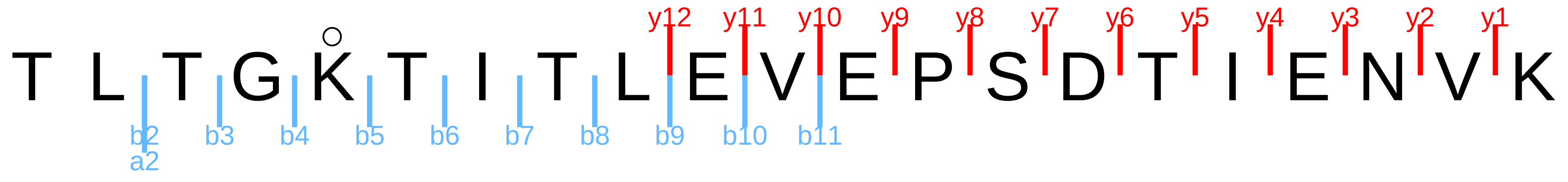

Modification □ Acetyl (Protein N−term) ○ GlyGly (K) no new terminus △ Oxidation (M)

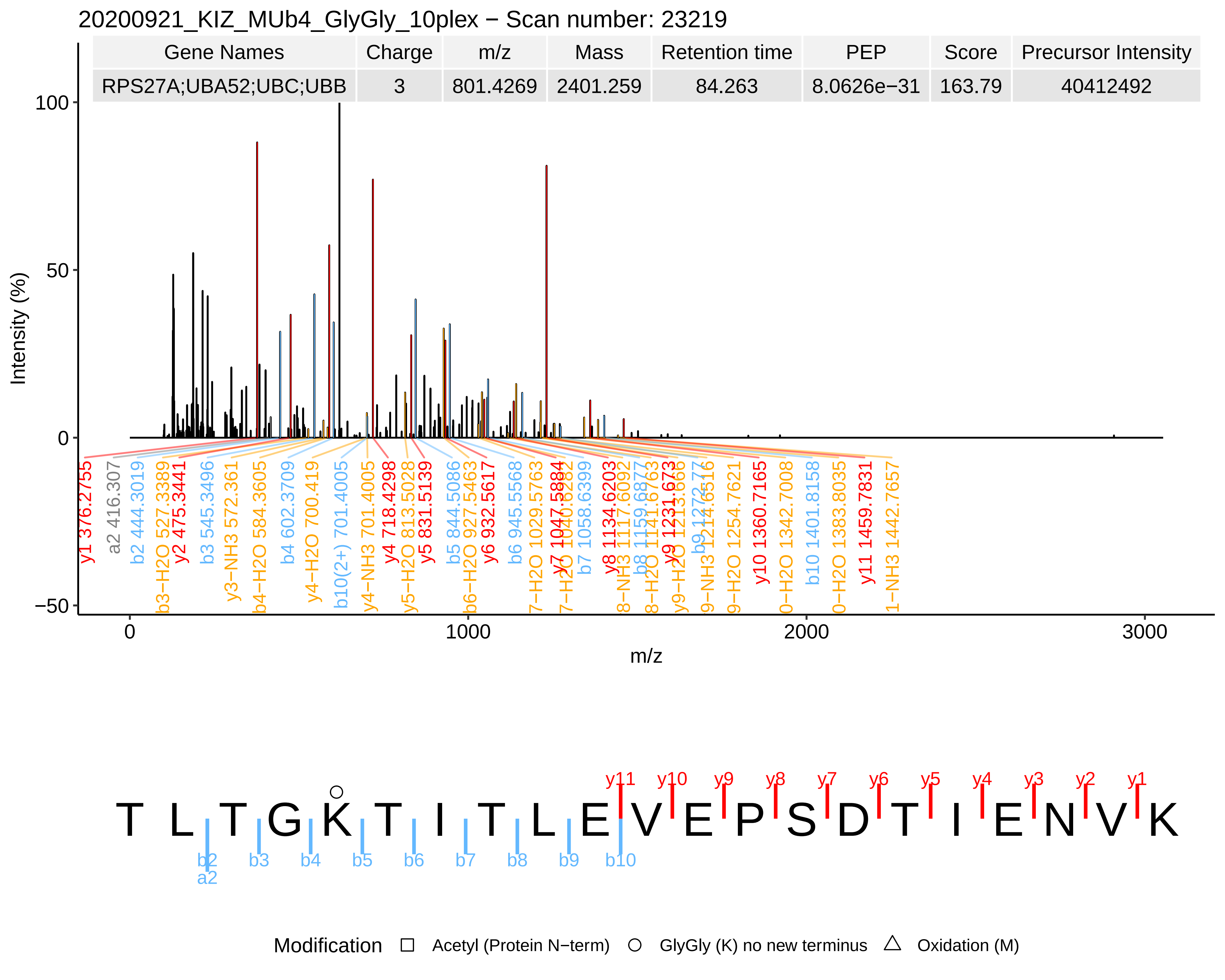

20200921_KIZ_MUb4_GlyGly_10plex − Scan number: 23219
Gene Names | Charge | m/z | Mass | Retention time | PEP | Score | Precursor Intensity
RPS27A;UBA52;UBC;UBB | 3 | 801.4269 | 2401.259 | 84.263 | 8.0626e−31 | 163.79 | 40412492
Intensity (%)
100
50
0
−50
0
1000
2000
3000
m/z
y1 376.2755
a2 416.307
b2 444.3019
b3−H2O 527.3389
y2 475.3441
b3 545.3496
y3−NH3 572.361
b4−H2O 584.3605
b4 602.3709
y4−H2O 700.419
b10(2+) 701.4005
y4−NH3 701.4005
y4 718.4298
y5−H2O 813.5028
y5 831.5139
b5 844.5086
b6−H2O 927.5463
y6 932.5617
b6 945.5568
y7 1047.5884
b7 1058.6399
y8 1134.6203
y9 1231.673
y10 1360.7165
b10 1401.8158
y11 1459.7831
T L T G K T I T L E V E P S D T I E N V K
y11 y10 y9 y8 y7 y6 y5 y4 y3 y2 y1
b2 b3 b4 b5 b6 b7 b8 b9 b10
a2
Modification
Acetyl (Protein N−term)
GlyGly (K) no new terminus
Oxidation (M)

20200921_KIZ_MUb4_GlyGly_10plex − Scan number: 23357

Gene Names	Charge	m/z	Mass	Retention time	PEP	Score	Precursor Intensity
RPS27A;UBA52;UBC;UBB	3	801.4269	2401.259	84.775	1.2814e−30	161.51	24930172

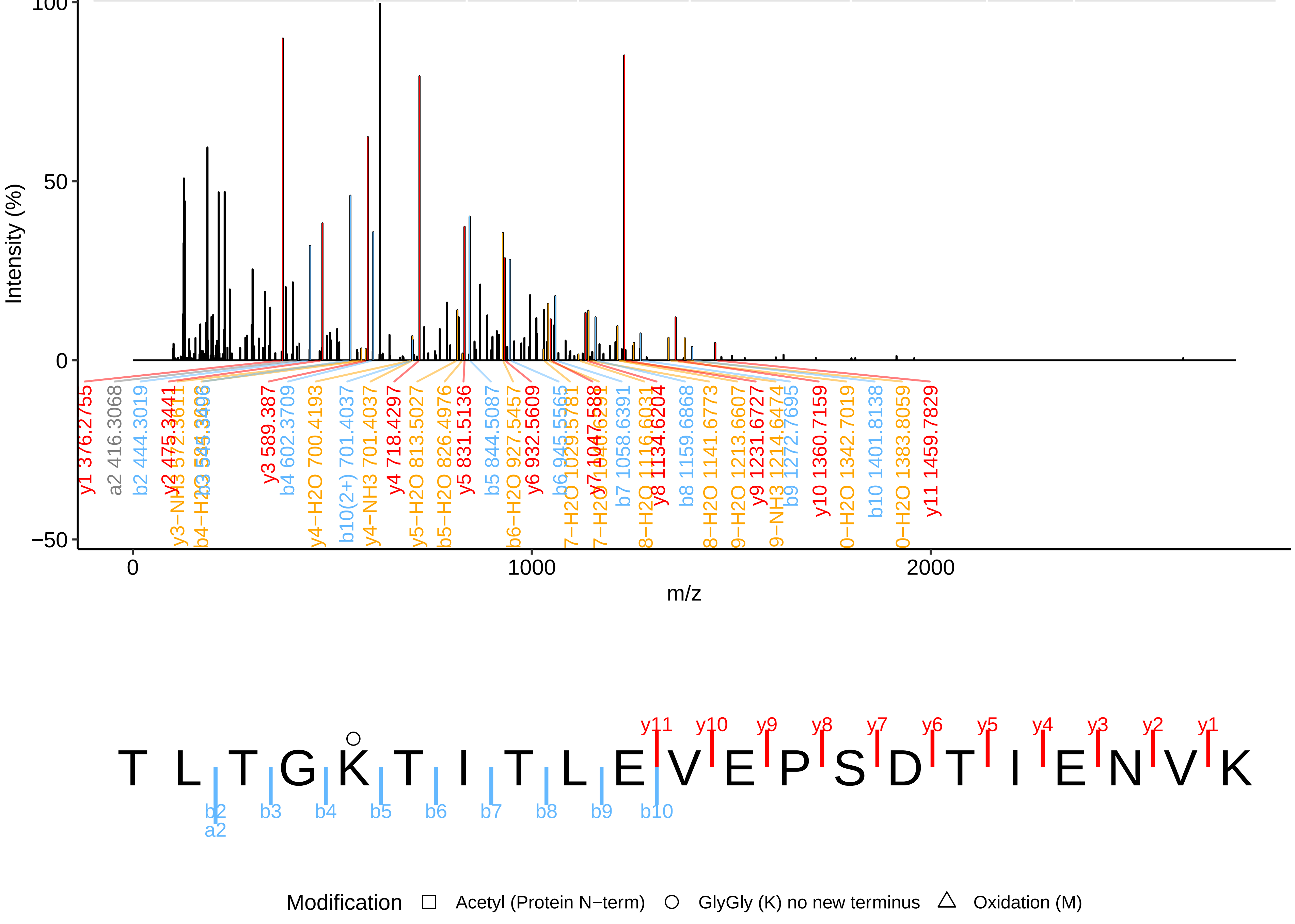

20200921_KIZ_MUb4_GlyGly_10plex − Scan number: 22829

Gene Names	Charge	m/z	Mass	Retention time	PEP	Score	Precursor Intensity
RPS27A;UBA52;UBC;UBB	4	601.322	2401.259	82.847	2.6575e−30	152.18	NA

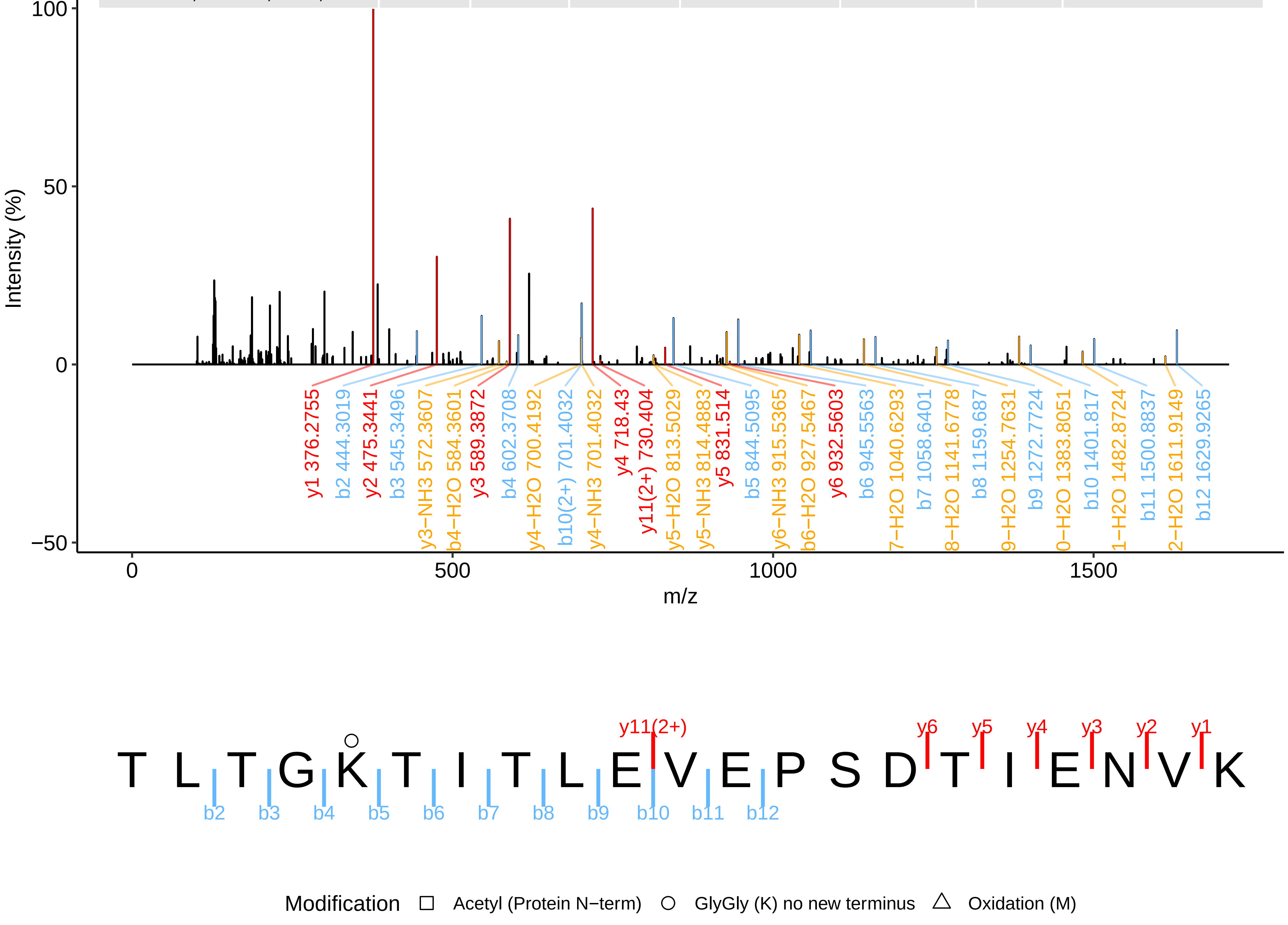

20200921_KIZ_MUb4_GlyGly_10plex − Scan number: 22973

Gene Names	Charge	m/z	Mass	Retention time	PEP	Score	Precursor Intensity
RPS27A;UBA52;UBC;UBB	4	601.322	2401.259	83.358	1.546e−30	158.65	27788146

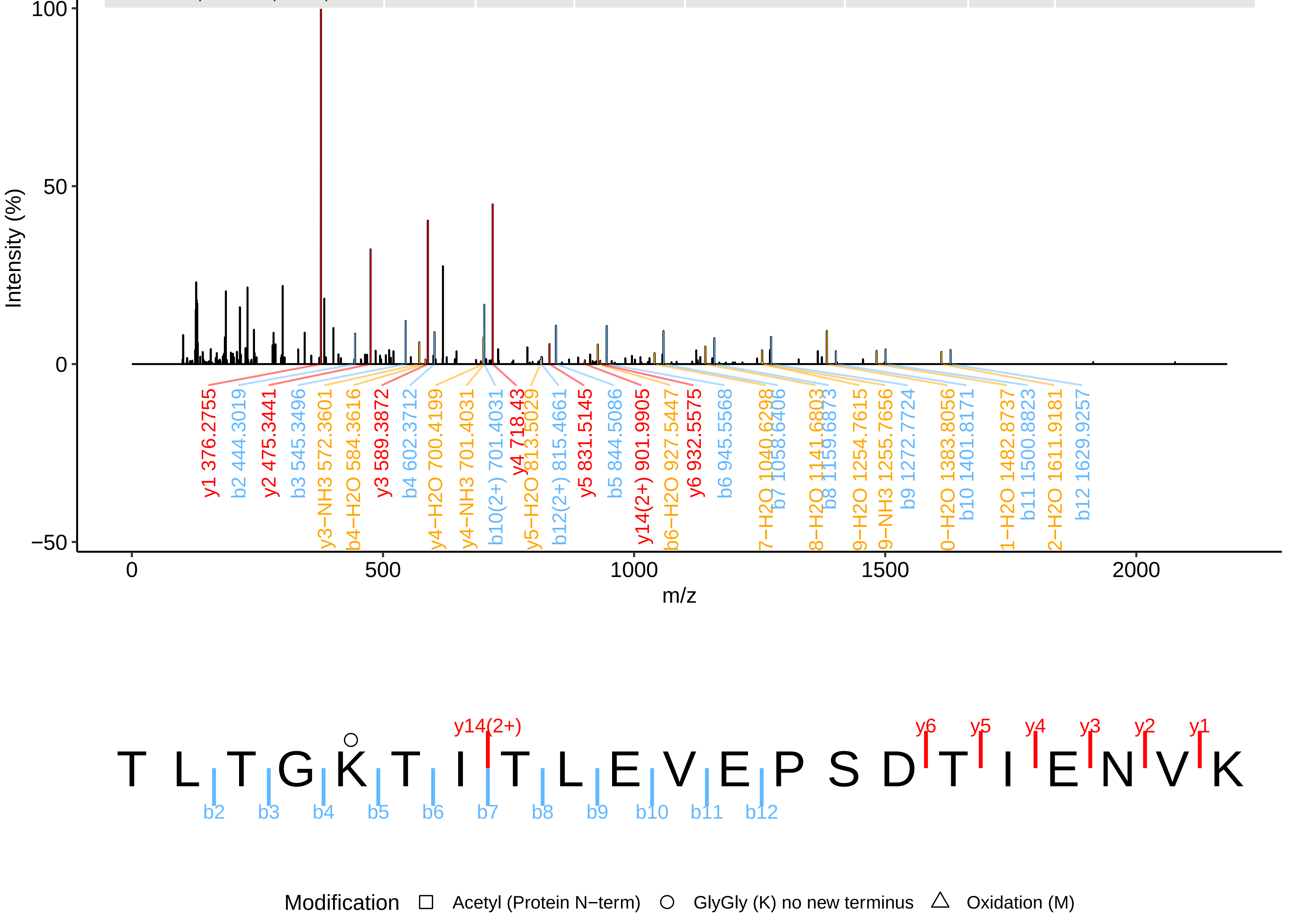

20200921_KIZ_MUb4_GlyGly_10plex − Scan number: 23110

Gene Names	Charge	m/z	Mass	Retention time	PEP	Score	Precursor Intensity
RPS27A;UBA52;UBC;UBB	4	601.322	2401.259	83.866	1.6147e−48	186.05	10026205

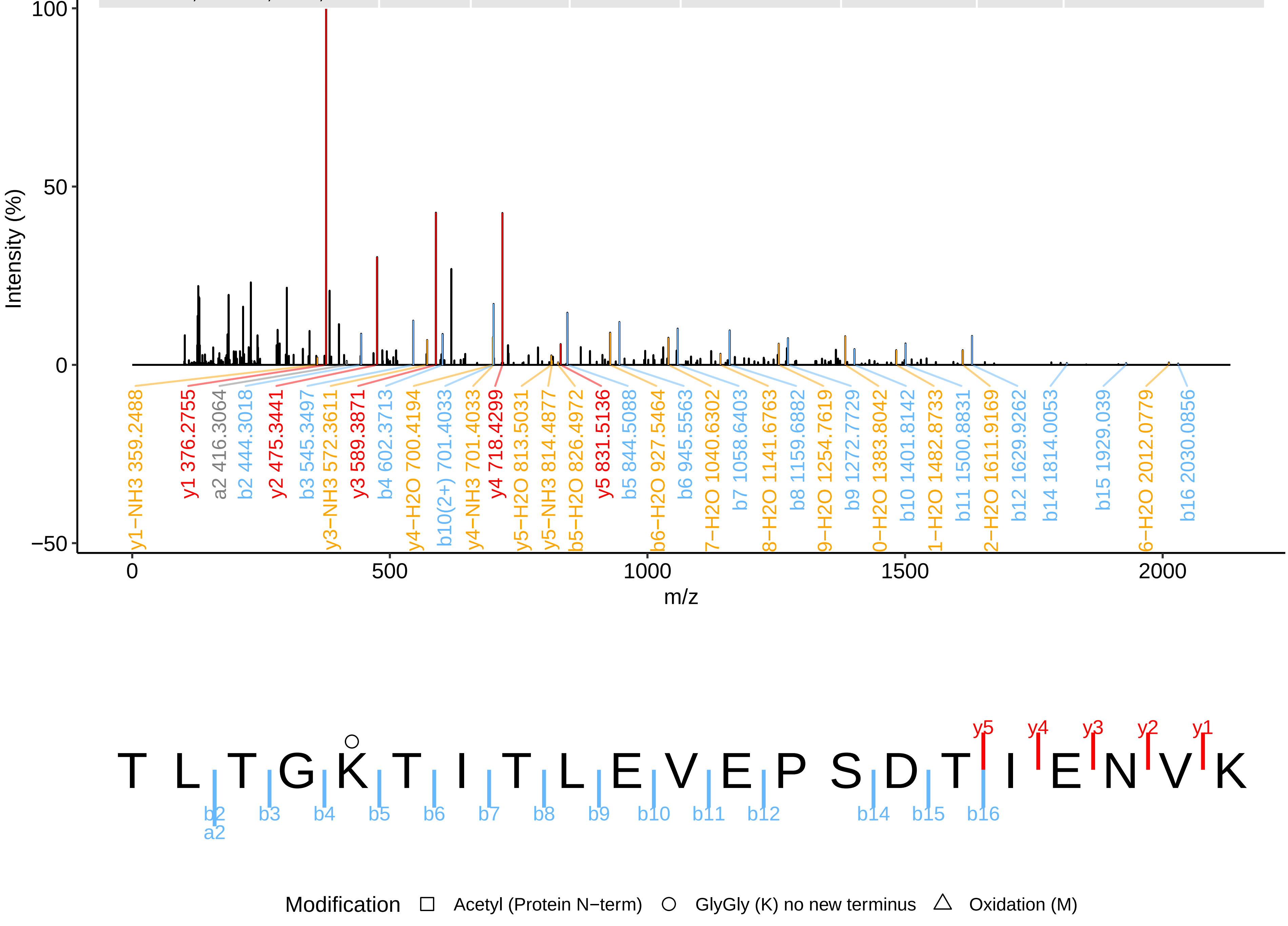

20200921_KIZ_MUb4_GlyGly_10plex − Scan number: 23248

Gene Names	Charge	m/z	Mass	Retention time	PEP	Score	Precursor Intensity
RPS27A;UBA52;UBC;UBB	4	601.322	2401.259	84.371	2.3125e−30	154.19	9218470

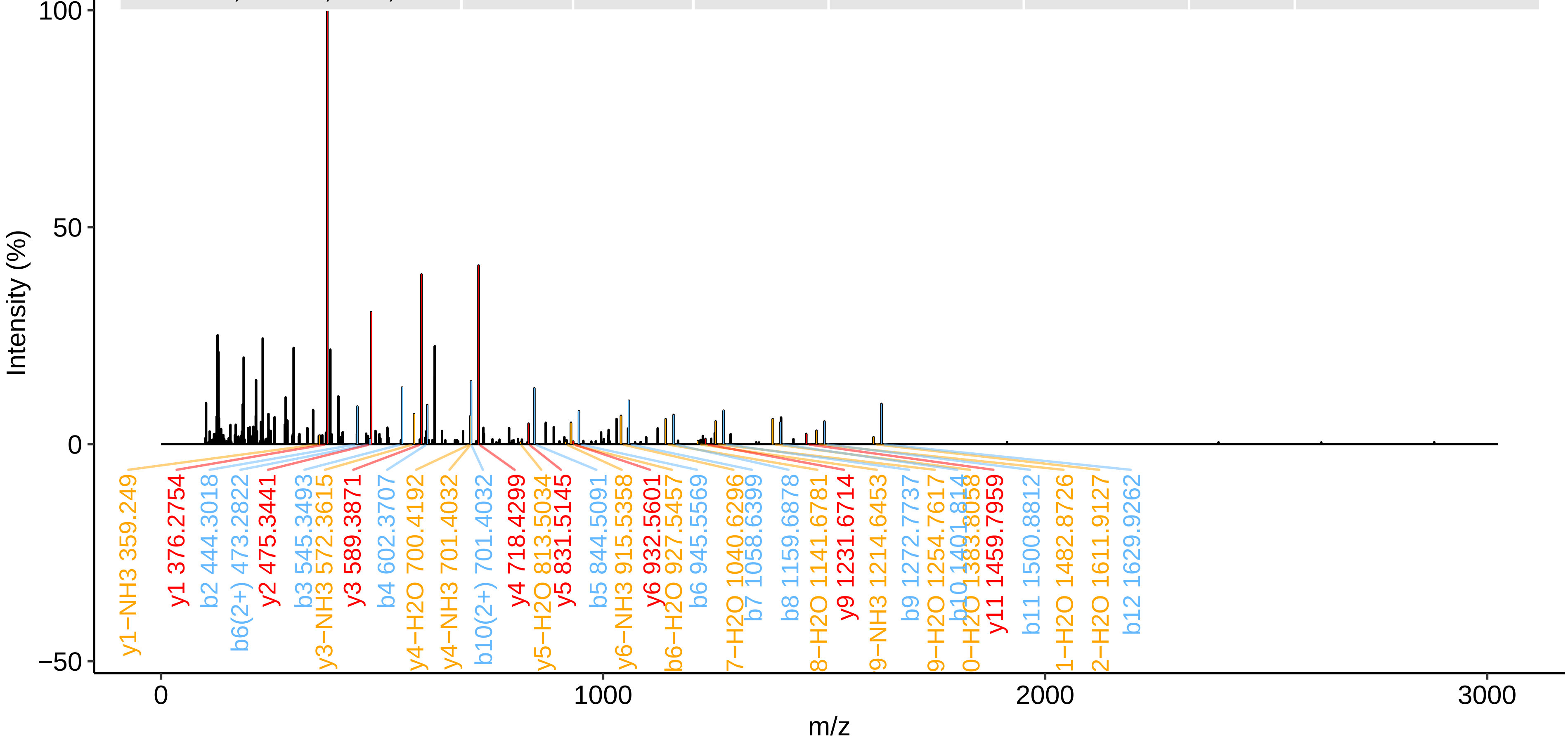

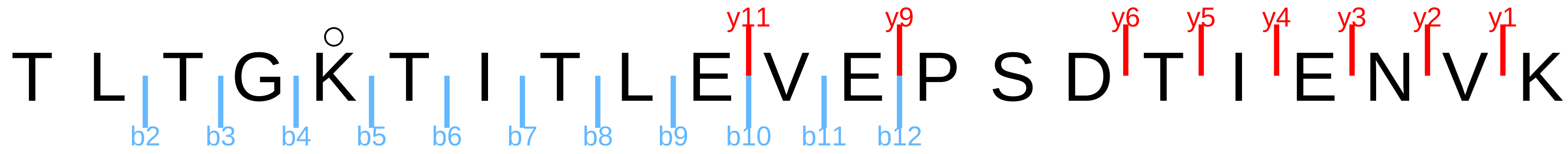

Modification □ Acetyl (Protein N−term) ○ GlyGly (K) no new terminus △ Oxidation (M)

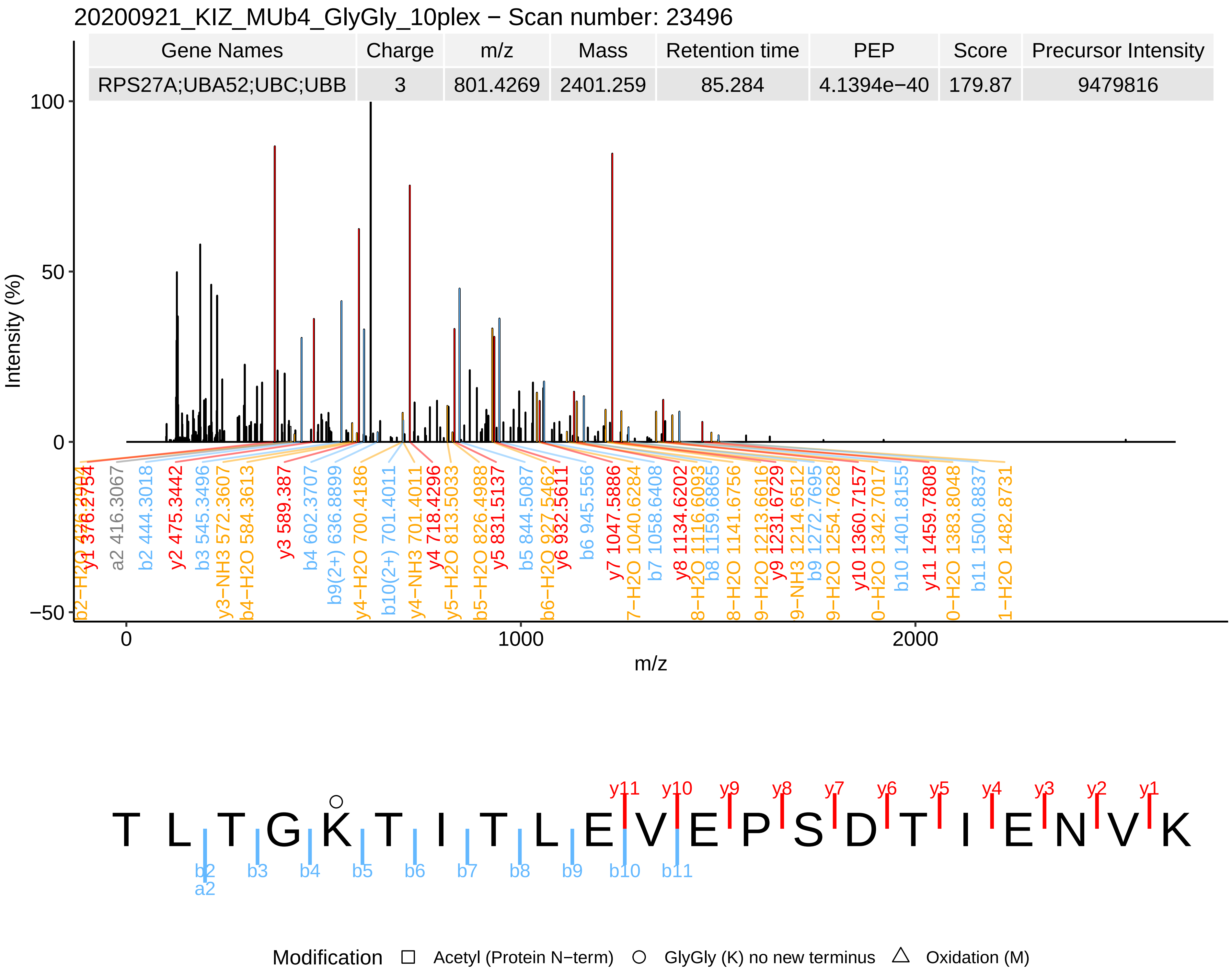
20200921_KIZ_MUb4_GlyGly_10plex − Scan number: 23496
Gene Names | Charge | m/z | Mass | Retention time | PEP | Score | Precursor Intensity
RPS27A;UBA52;UBC;UBB | 3 | 801.4269 | 2401.259 | 85.284 | 4.1394e−40 | 179.87 | 9479816
Intensity (%)
m/z
y1 376.2754
a2 416.3067
b2 444.3018
y2 475.3442
b3 545.3496
y3−NH3 572.3607
b4−H2O 584.3613
y3 589.387
b4 602.3707
b9(2+) 636.8899
y4−H2O 700.4186
b10(2+) 701.4011
y4−NH3 701.4011
y4 718.4296
y5−H2O 813.5033
b5−H2O 826.4988
y5 831.5137
b5 844.5087
b6−H2O 927.5462
y6 932.5611
b6 945.556
y7 1047.5886
y7−H2O 1040.6284
b7 1058.6408
y8 1134.6202
y8−H2O 1116.6093
b8 1159.6865
b8−H2O 1141.6756
y9−H2O 1213.6616
y9 1231.6729
y9−NH3 1214.6512
b9 1272.7695
b9−H2O 1254.7628
y10 1360.7157
y10−H2O 1342.7017
b10 1401.8155
y11 1459.7808
y11−H2O 1383.8048
b11 1500.8837
b11−H2O 1482.8731
T L T G K T I T L E V E P S D T I E N V K
Modification
Acetyl (Protein N−term)
GlyGly (K) no new terminus
Oxidation (M)

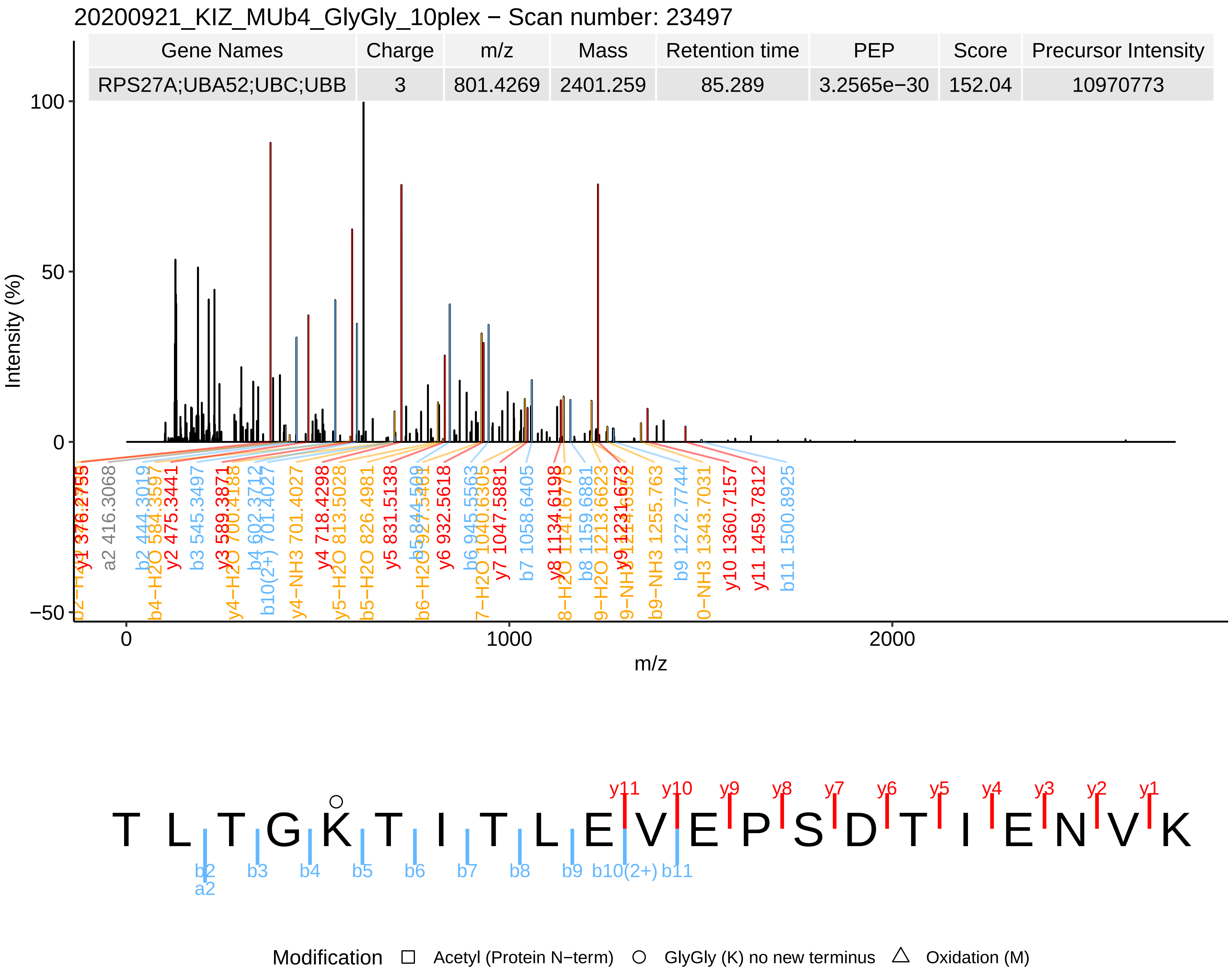
20200921_KIZ_MUb4_GlyGly_10plex − Scan number: 23497
Gene Names | Charge | m/z | Mass | Retention time | PEP | Score | Precursor Intensity
RPS27A;UBA52;UBC;UBB | 3 | 801.4269 | 2401.259 | 85.289 | 3.2565e−30 | 152.04 | 10970773
Intensity (%)
m/z
a2 416.3068
b2 444.3019
y2 475.3441
b3 545.3497
y3 589.3871
y4 718.4298
y5−H2O 813.5028
b5−H2O 826.4981
y5 831.5138
y6 932.5618
y7 1047.5881
b7 1058.6405
b8 1159.6881
b9−NH3 1255.763
b9 1272.7744
y10 1360.7157
y11 1459.7812
b11 1500.8925
T L T G K T I T L E V E P S D T I E N V K
y11 y10 y9 y8 y7 y6 y5 y4 y3 y2 y1
b2 a2 b3 b4 b5 b6 b7 b8 b9 b10(2+) b11
Modification
Acetyl (Protein N−term)
GlyGly (K) no new terminus
Oxidation (M)

20200921_KIZ_MUb4_GlyGly_10plex − Scan number: 23632

Gene Names	Charge	m/z	Mass	Retention time	PEP	Score	Precursor Intensity
RPS27A;UBA52;UBC;UBB	3	801.4269	2401.259	85.8	9.7473e−40	172.84	8945027

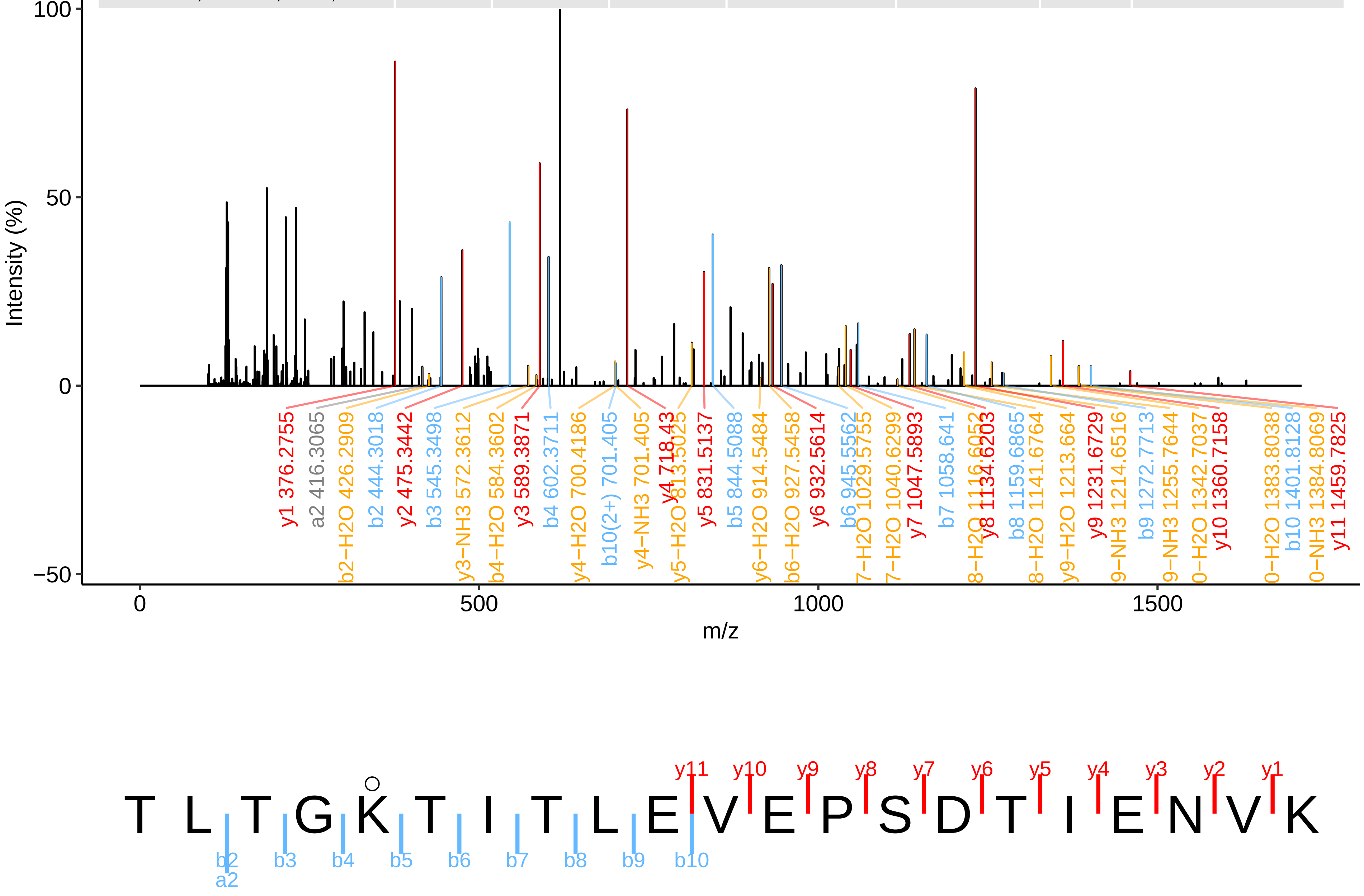

Modification □ Acetyl (Protein N−term) ○ GlyGly (K) no new terminus △ Oxidation (M)

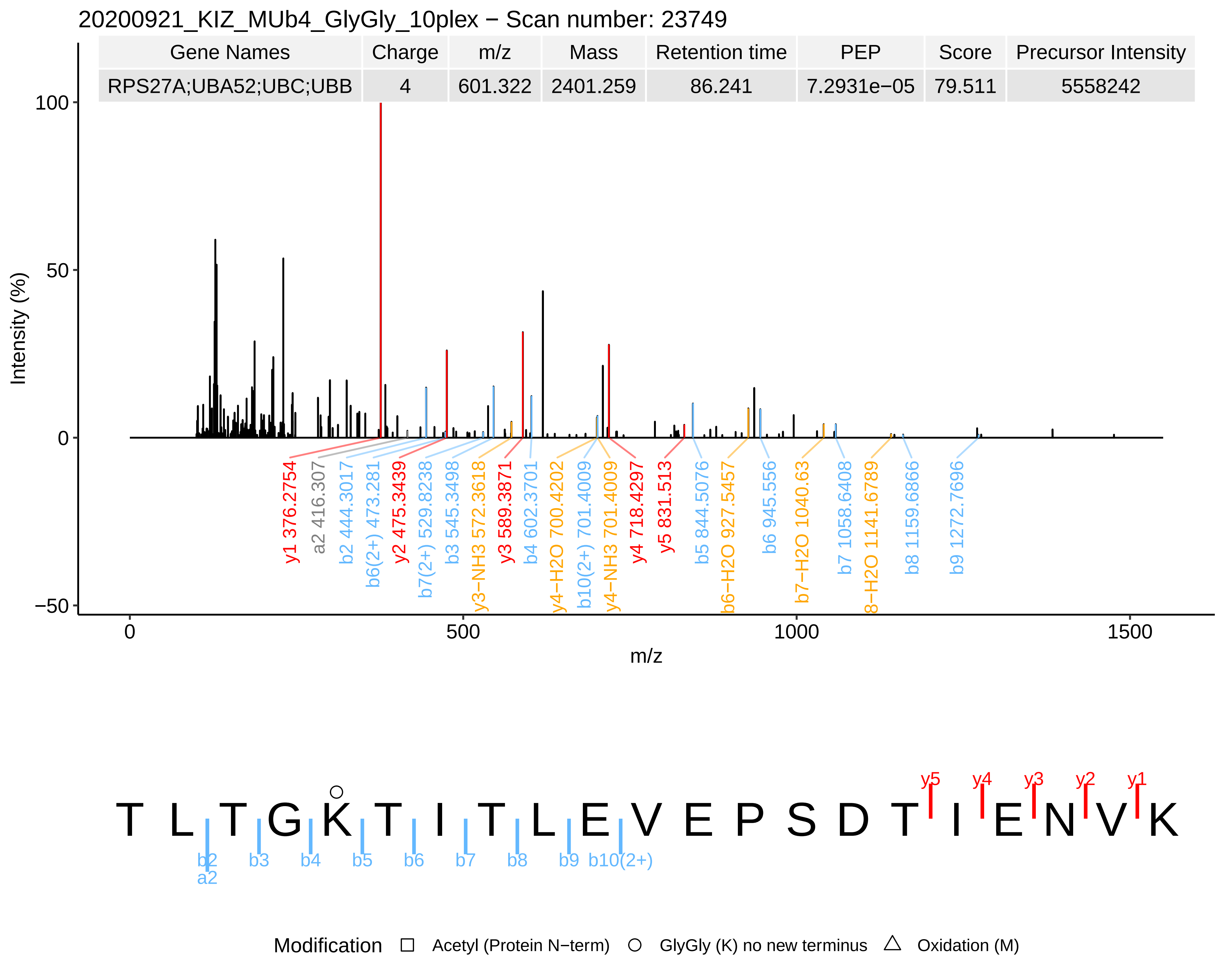

20200921_KIZ_MUb4_GlyGly_10plex − Scan number: 23749
Gene Names | Charge | m/z | Mass | Retention time | PEP | Score | Precursor Intensity
RPS27A;UBA52;UBC;UBB | 4 | 601.322 | 2401.259 | 86.241 | 7.2931e−05 | 79.511 | 5558242
Intensity (%)
m/z
y1 376.2754
a2 416.307
b2 444.3017
b6(2+) 473.281
y2 475.3439
b7(2+) 529.8238
b3 545.3498
y3−NH3 572.3618
y3 589.3871
b4 602.3701
y4−H2O 700.4202
b10(2+) 701.4009
y4−NH3 701.4009
y4 718.4297
y5 831.513
b5 844.5076
b6−H2O 927.5457
b6 945.556
b7−H2O 1040.63
b7 1058.6408
b8−H2O 1141.6789
b8 1159.6866
b9 1272.7696
T L T G K T I T L E V E P S D T I E N V K
y5 y4 y3 y2 y1
b2 a2 b3 b4 b5 b6 b7 b8 b9 b10(2+)
Modification
Acetyl (Protein N−term)
GlyGly (K) no new terminus
Oxidation (M)

20200921_KIZ_MUb4_GlyGly_10plex − Scan number: 23757

Gene Names	Charge	m/z	Mass	Retention time	PEP	Score	Precursor Intensity
RPS27A;UBA52;UBC;UBB	4	601.322	2401.259	86.276	2.9551e−30	150.45	5327048

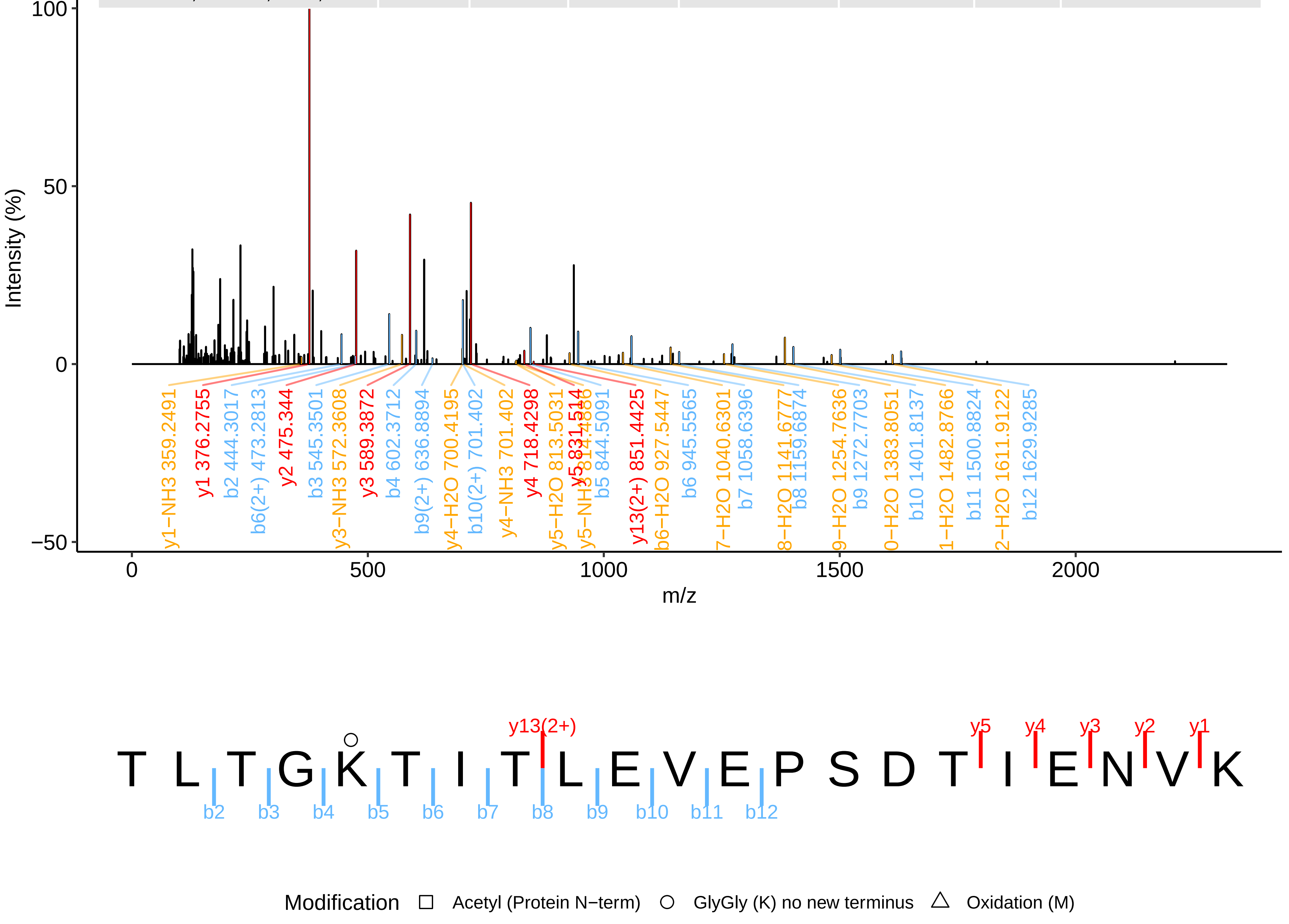

20200921_KIZ_MUb4_GlyGly_10plex − Scan number: 23762

Gene Names	Charge	m/z	Mass	Retention time	PEP	Score	Precursor Intensity
RPS27A;UBA52;UBC;UBB	4	601.322	2401.259	86.299	1.2517e−21	137.61	3421946

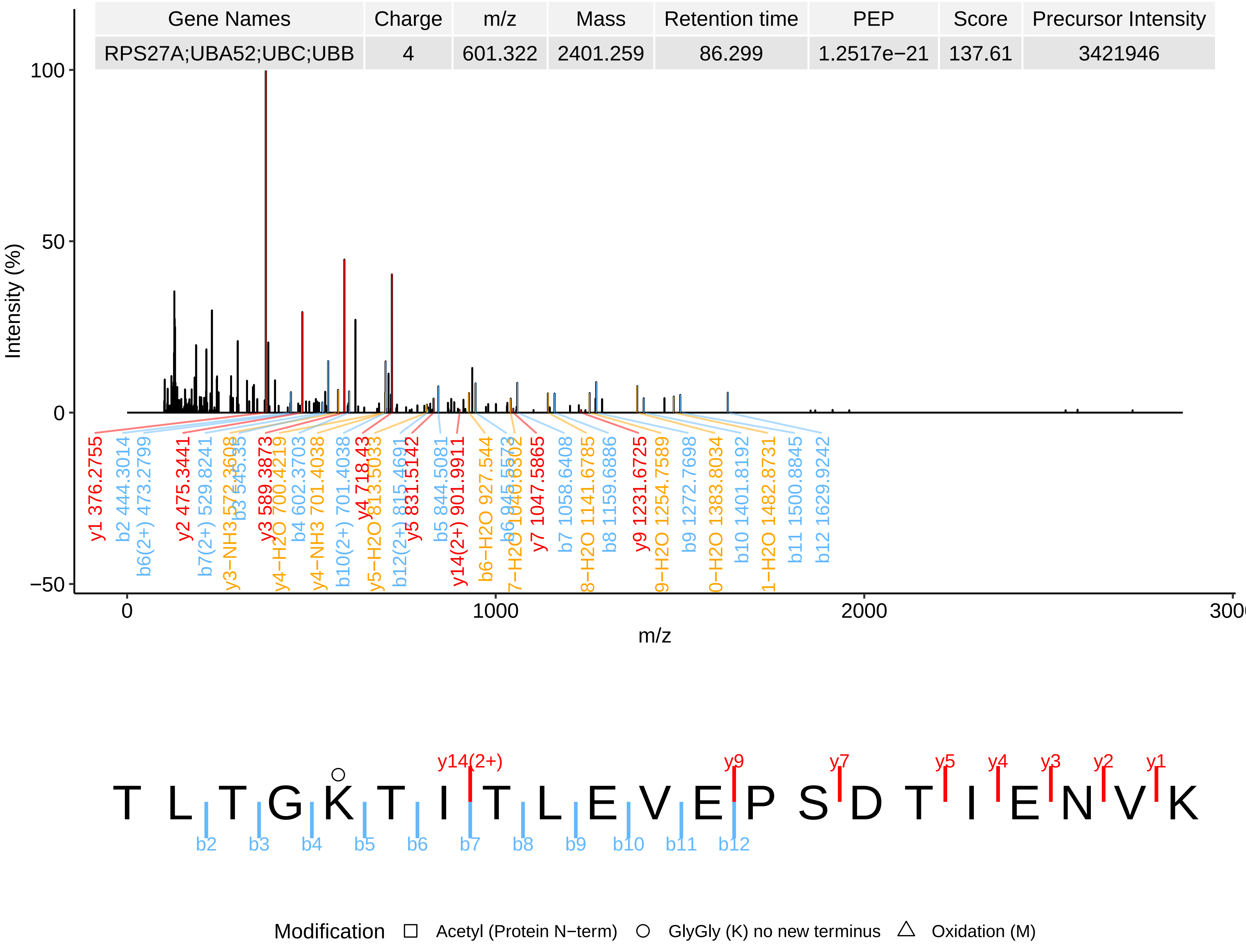

20200921_KIZ_MUb4_GlyGly_10plex − Scan number: 23764

Gene Names	Charge	m/z	Mass	Retention time	PEP	Score	Precursor Intensity
RPS27A;UBA52;UBC;UBB	3	801.4269	2401.259	86.304	5.5094e−49	197.86	7509000

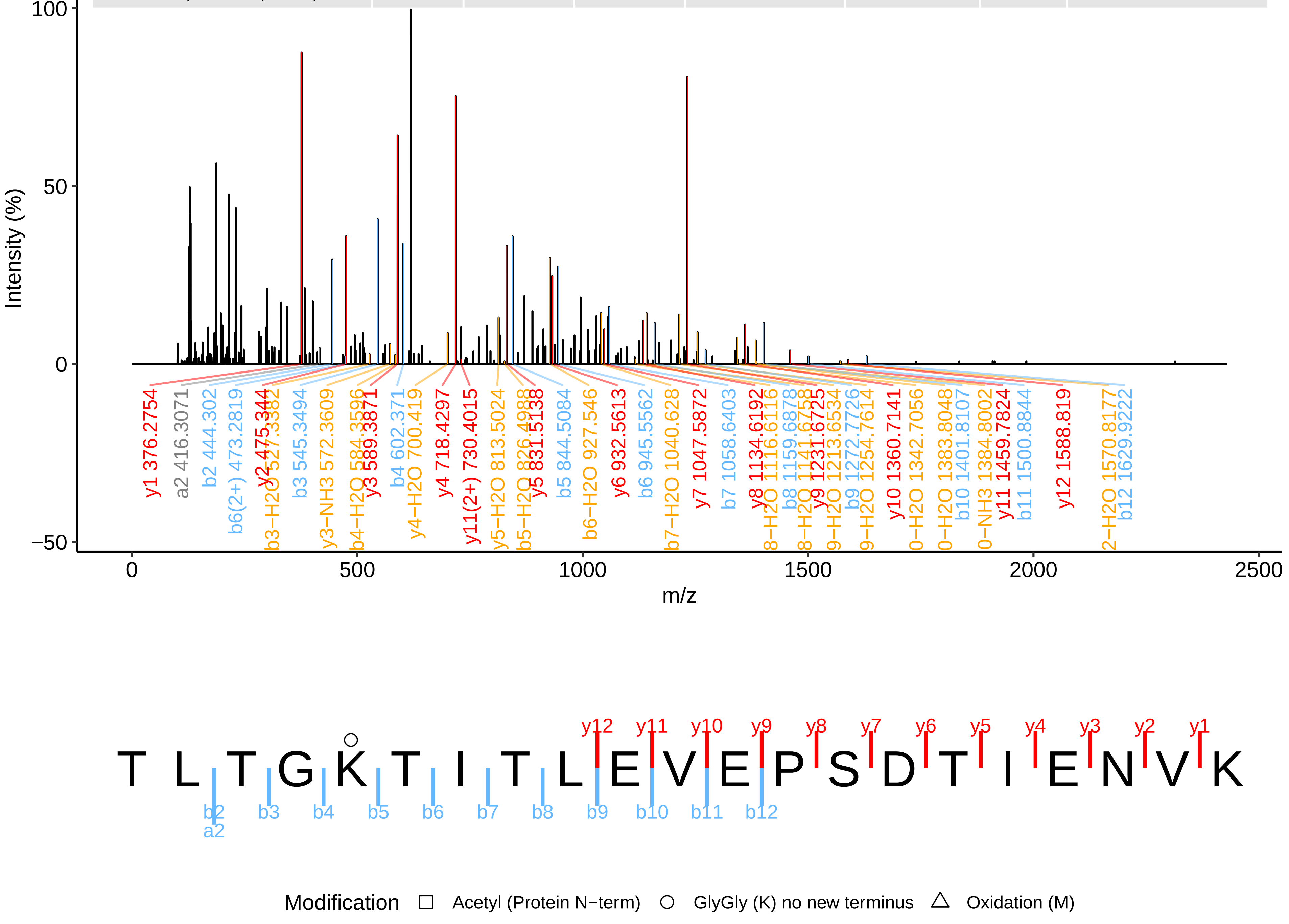

20200921_KIZ_MUb4_GlyGly_10plex − Scan number: 24082

Gene Names	Charge	m/z	Mass	Retention time	PEP	Score	Precursor Intensity
RPS27A;UBA52;UBC;UBB	3	801.4269	2401.259	87.471	1.3393e−39	168.27	5296167

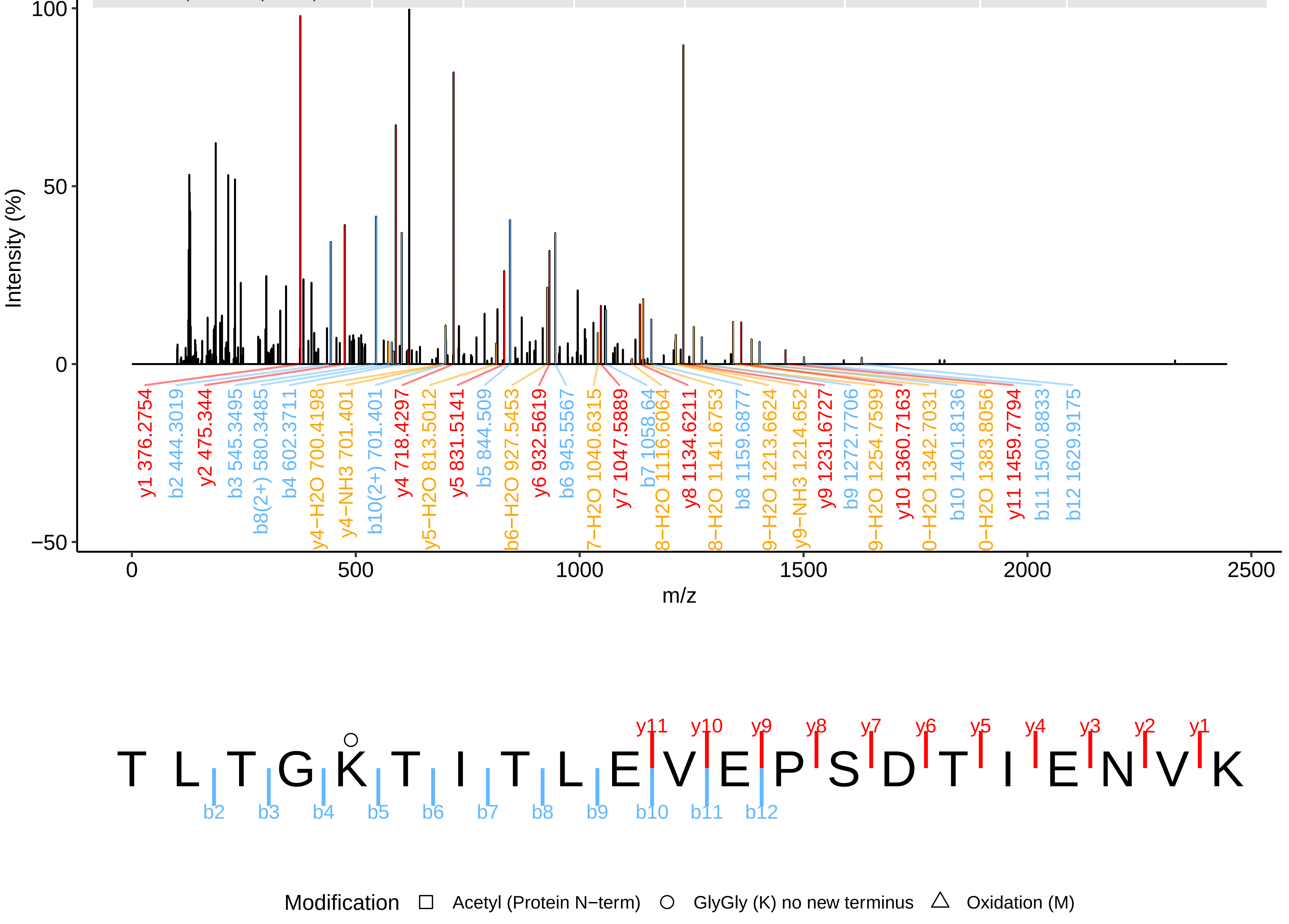

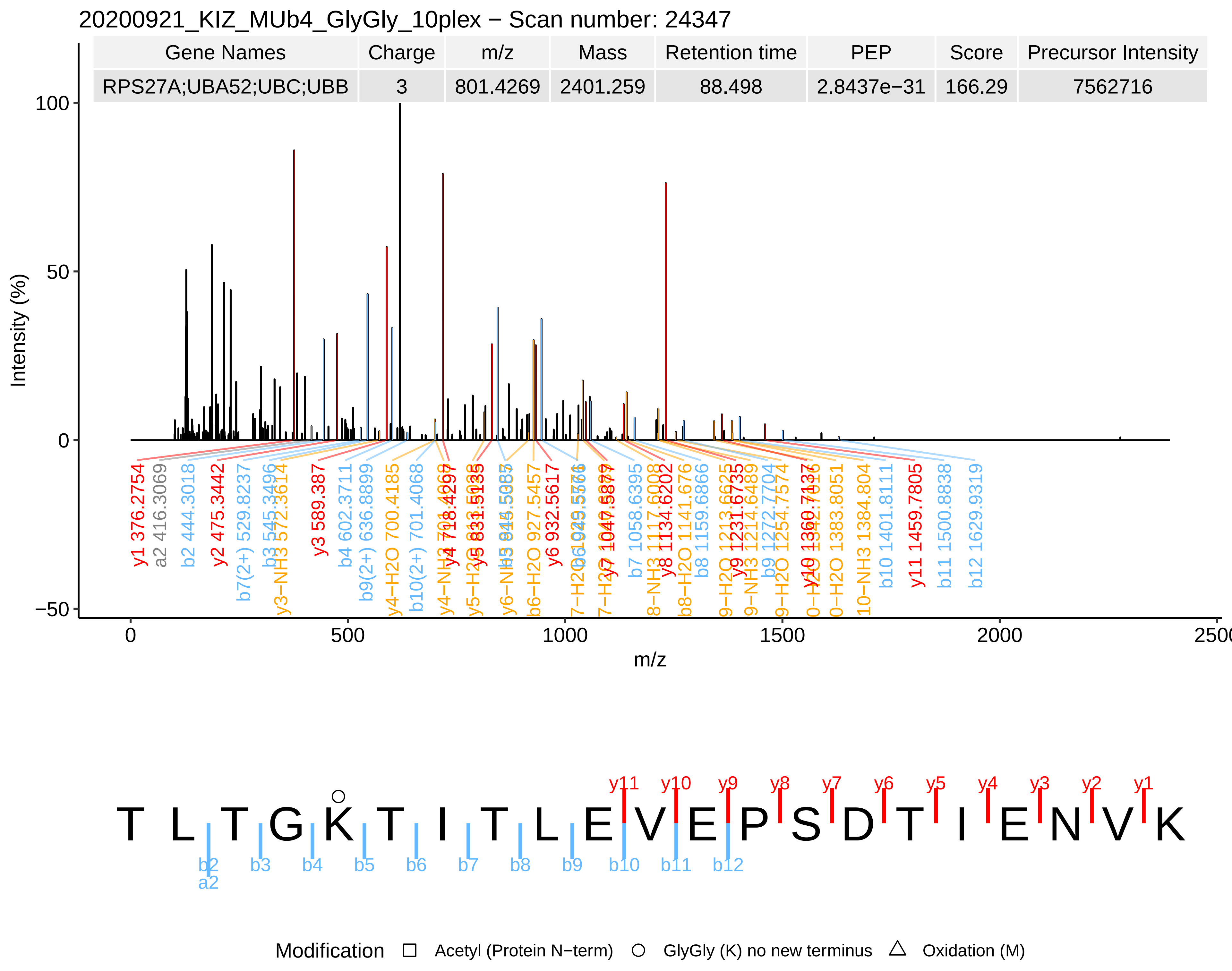

Gene Names	Charge	m/z	Mass	Retention time	PEP	Score	Precursor Intensity
RPS27A;UBA52;UBC;UBB	3	801.4269	2401.259	88.498	2.8437e−31	166.29	7562716

20200921_KIZ_MUb4_GlyGly_10plex − Scan number: 24752

Gene Names	Charge	m/z	Mass	Retention time	PEP	Score	Precursor Intensity
RPS27A;UBA52;UBC;UBB	3	801.4269	2401.259	90.044	9.3998e−22	142.43	3500227

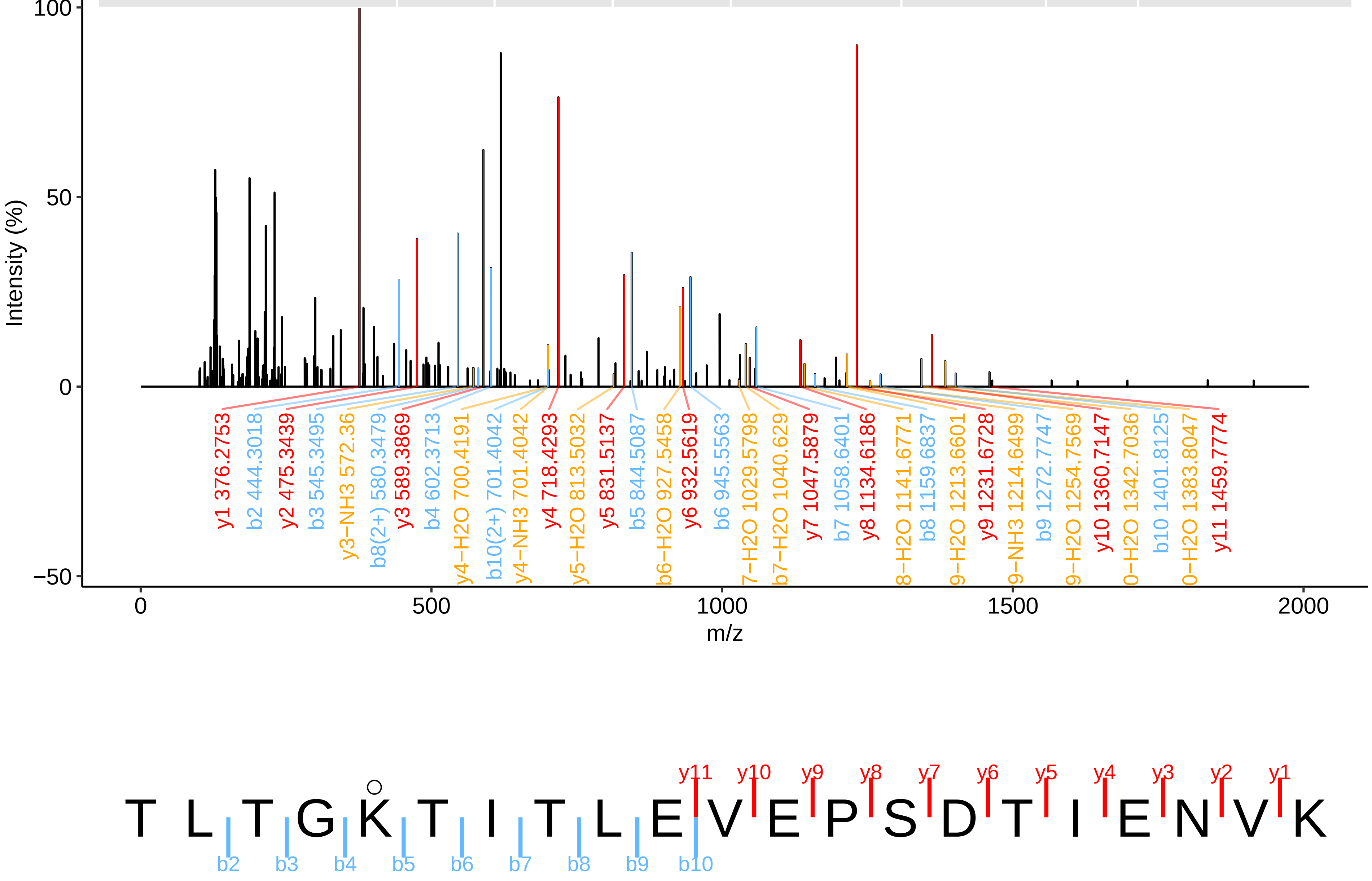

Modification □ Acetyl (Protein N−term) ○ GlyGly (K) no new terminus △ Oxidation (M)

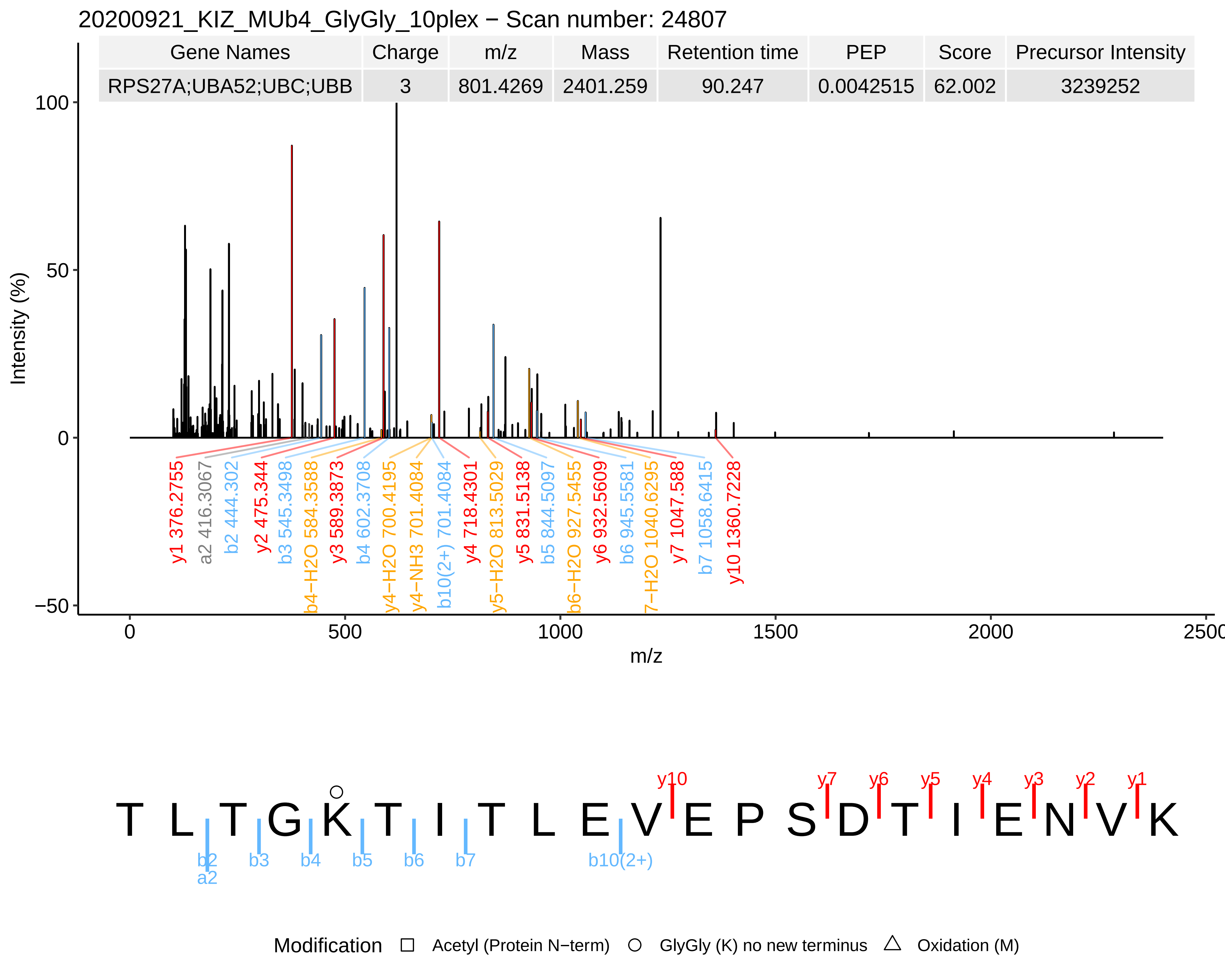
20200921_KIZ_MUb4_GlyGly_10plex − Scan number: 24807
Gene Names
Charge
m/z
Mass
Retention time
PEP
Score
Precursor Intensity
RPS27A;UBA52;UBC;UBB
3
801.4269
2401.259
90.247
0.0042515
62.002
3239252
Intensity (%)
m/z
y1 376.2755
a2 416.3067
b2 444.302
y2 475.344
b3 545.3498
b4−H2O 584.3588
y3 589.3873
b4 602.3708
y4−H2O 700.4195
y4−NH3 701.4084
b10(2+) 701.4084
y4 718.4301
y5−H2O 813.5029
y5 831.5138
b5 844.5097
b6−H2O 927.5455
y6 932.5609
b6 945.5581
7−H2O 1040.6295
y7 1047.588
b7 1058.6415
y10 1360.7228
T L T G K T I T L E V E P S D T I E N V K
y10
y7
y6
y5
y4
y3
y2
y1
b2
a2
b3
b4
b5
b6
b7
b10(2+)
Modification
Acetyl (Protein N−term)
GlyGly (K) no new terminus
Oxidation (M)

20200921_KIZ_MUb4_GlyGly_10plex − Scan number: 25657

Gene Names	Charge	m/z	Mass	Retention time	PEP	Score	Precursor Intensity
RPS27A;UBA52;UBC;UBB	3	801.4269	2401.259	93.365	4.6588e−06	90.968	3706675

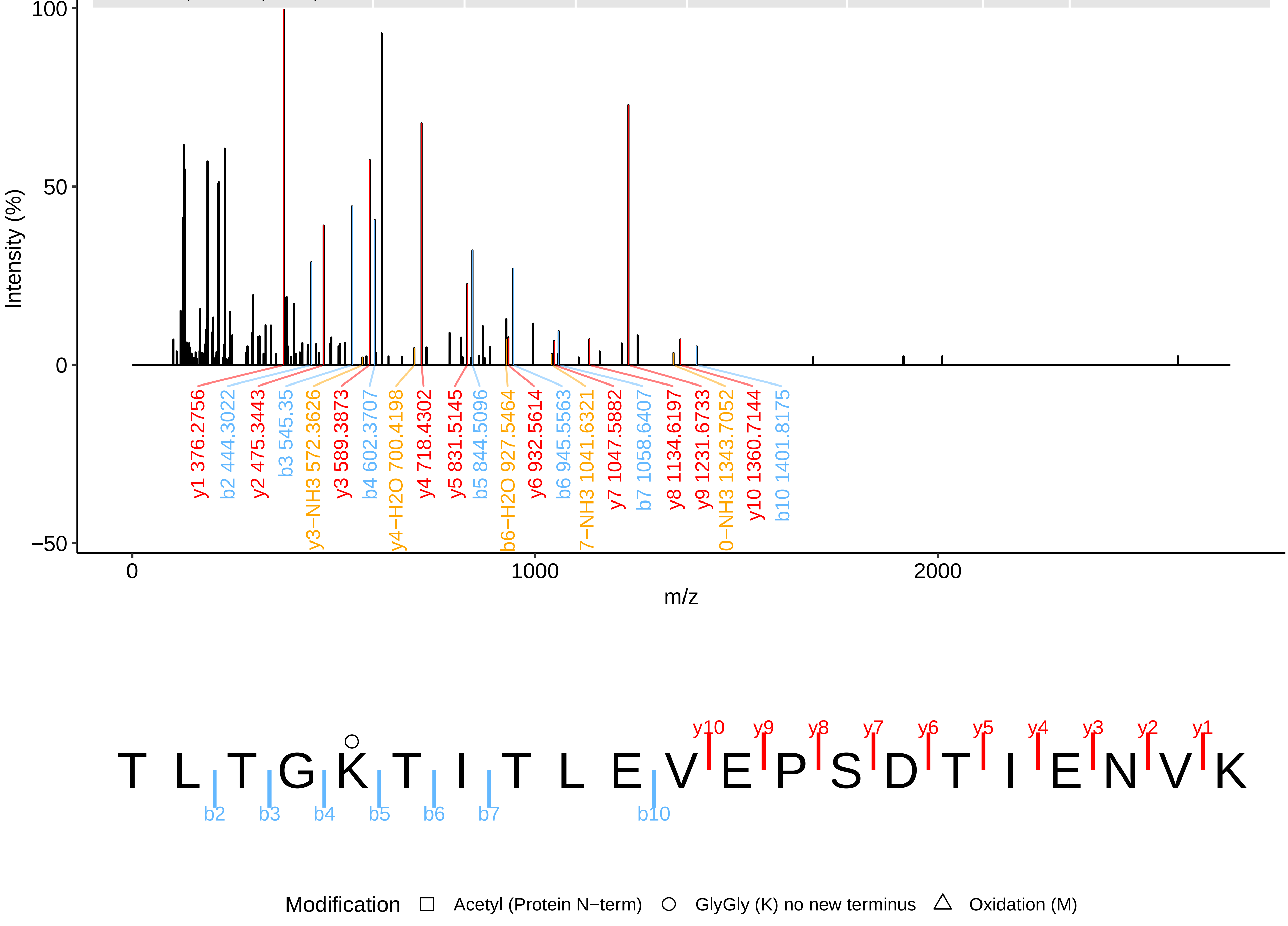

20200921_KIZ_MUb4_GlyGly_10plex − Scan number: 25789

Gene Names	Charge	m/z	Mass	Retention time	PEP	Score	Precursor Intensity
RPS27A;UBA52;UBC;UBB	3	801.4269	2401.259	93.874	2.6189e−22	148.06	2199154

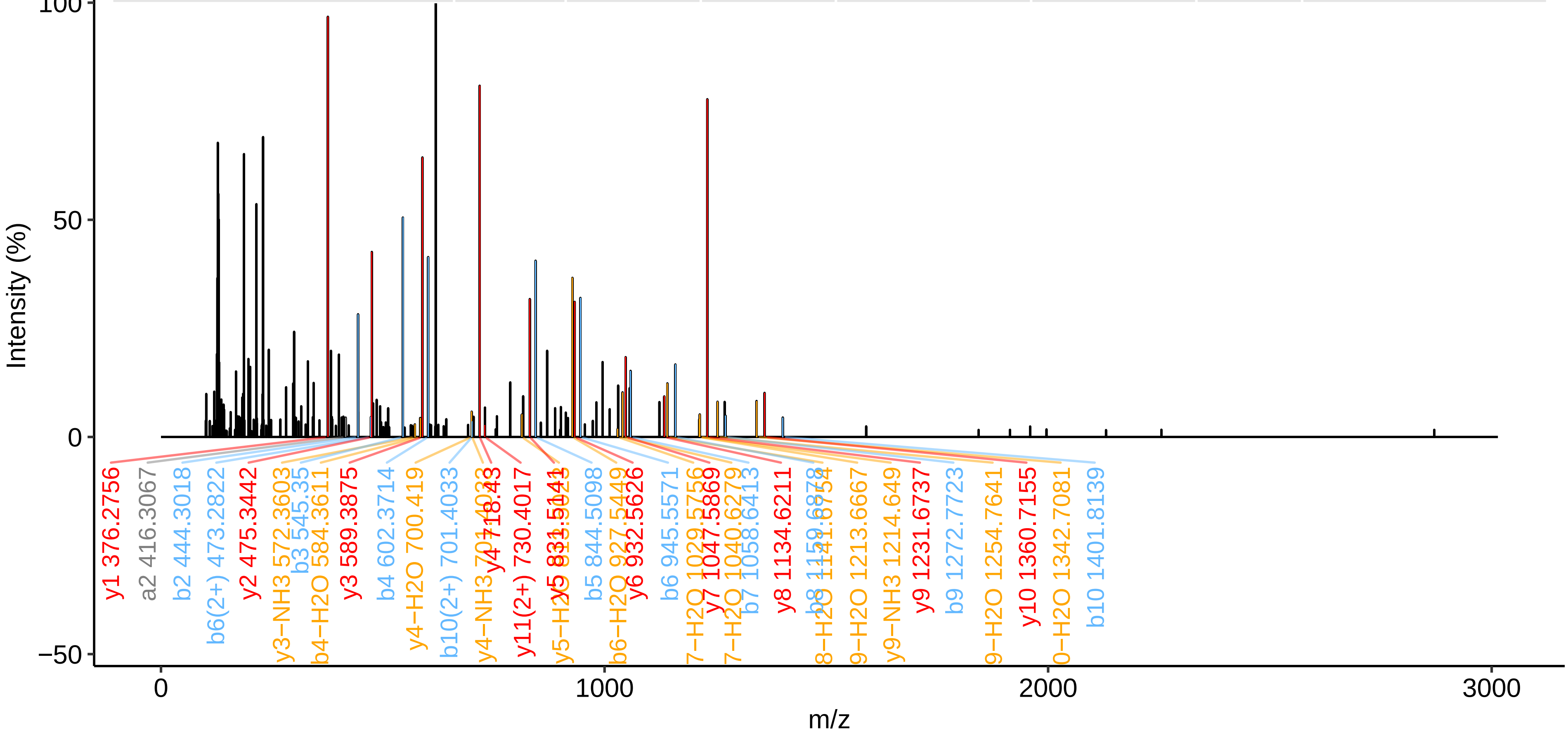

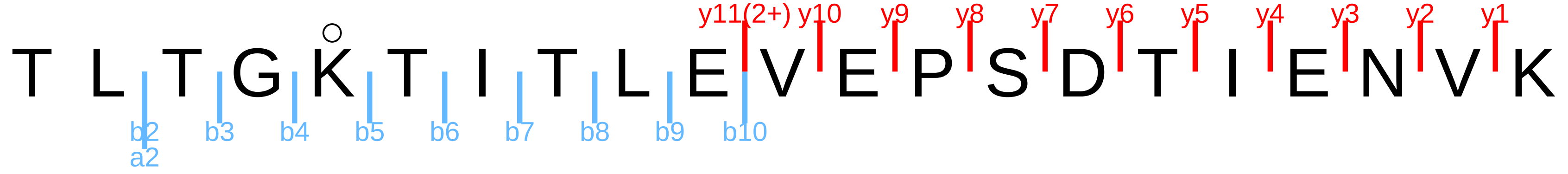

Modification □ Acetyl (Protein N−term) ○ GlyGly (K) no new terminus △ Oxidation (M)

20200921_KIZ_MUb4_GlyGly_10plex − Scan number: 26536

Gene Names	Charge	m/z	Mass	Retention time	PEP	Score	Precursor Intensity
RPS27A;UBA52;UBC;UBB	3	801.4269	2401.259	96.695	3.2565e−30	152.04	1342263

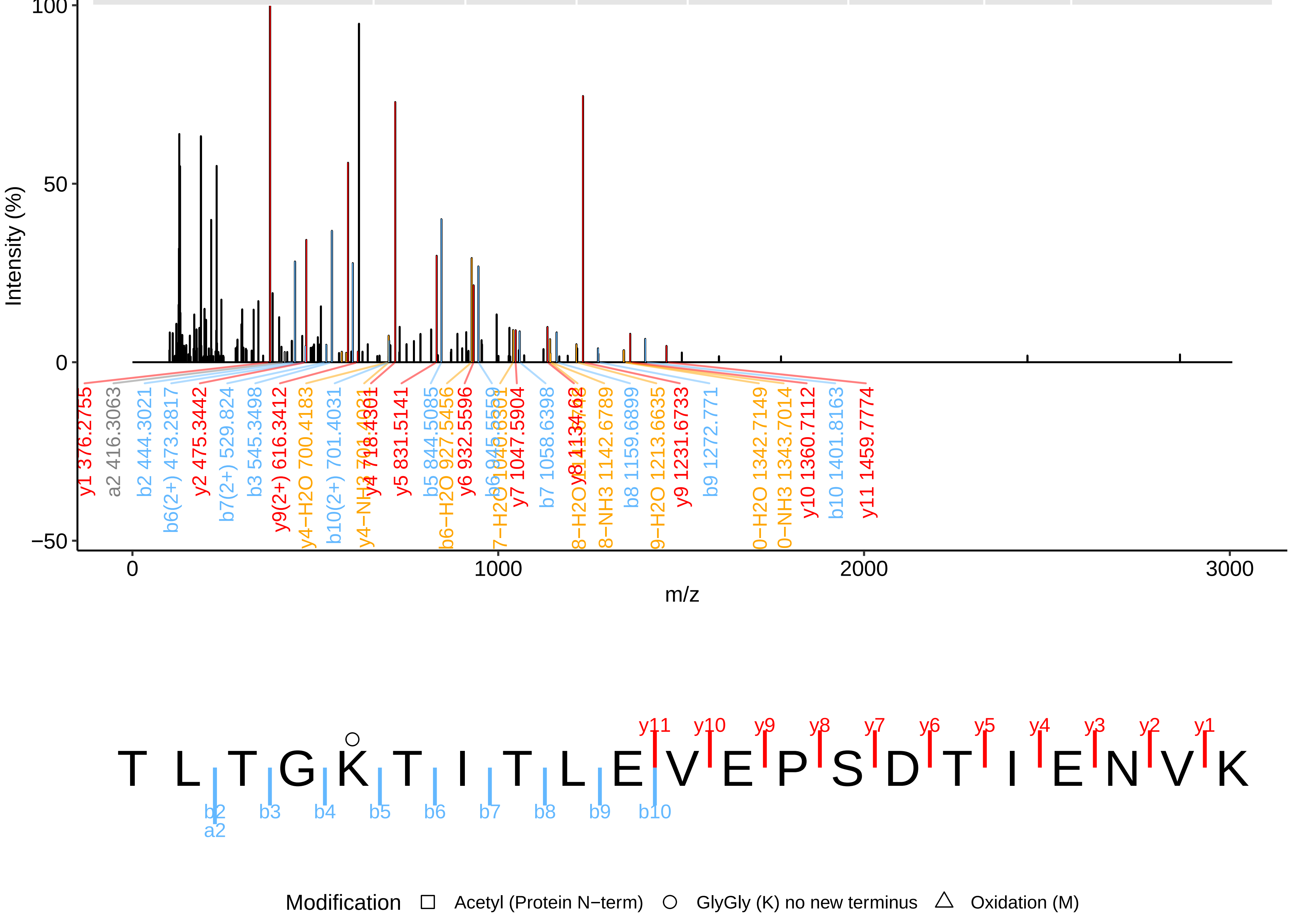

20200921_KIZ_MUb4_GlyGly_10plex − Scan number: 26538

Gene Names	Charge	m/z	Mass	Retention time	PEP	Score	Precursor Intensity
RPS27A;UBA52;UBC;UBB	3	801.4269	2401.259	96.703	9.9547e−22	141.97	1342263

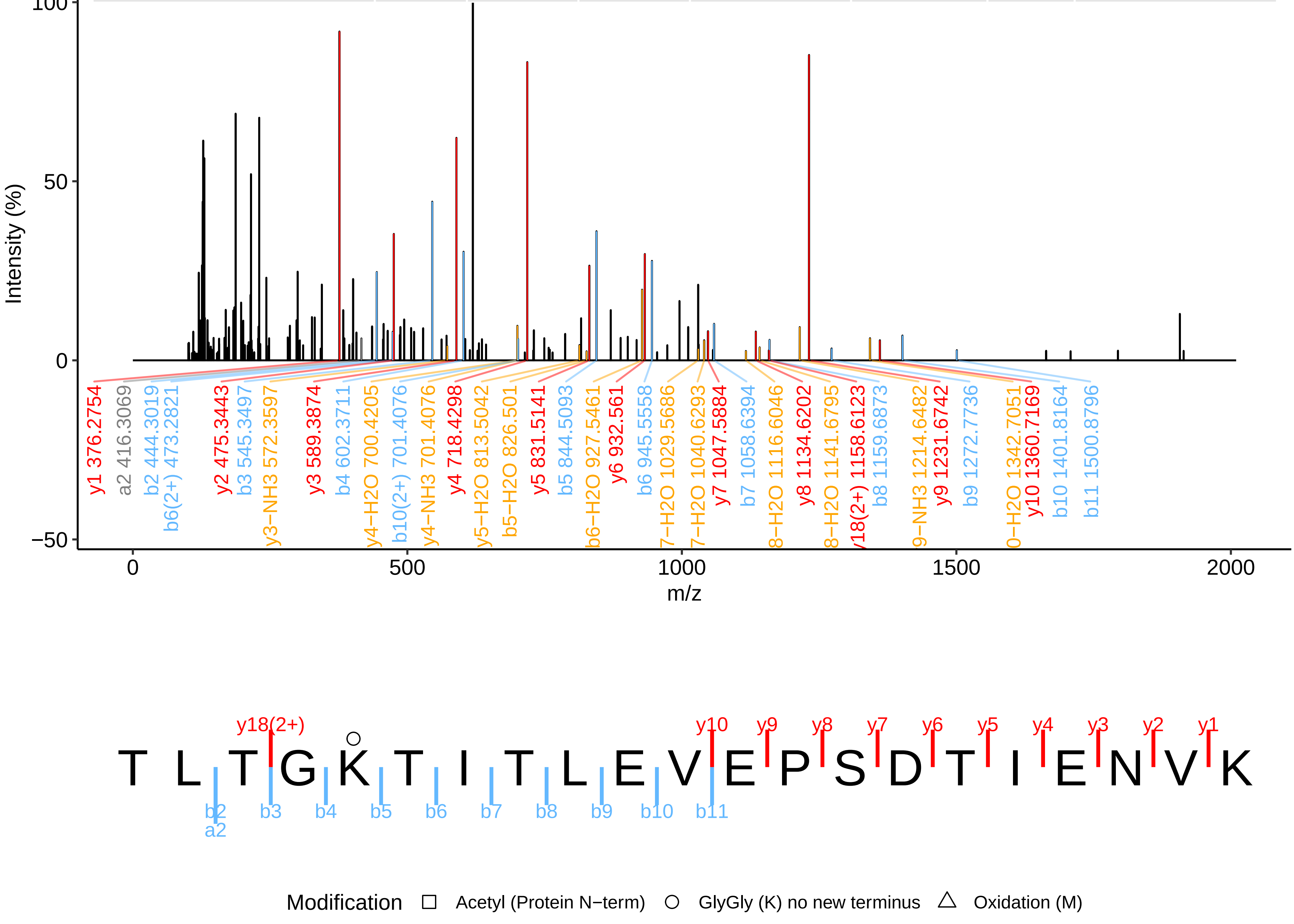

20200921_KIZ_MUb4_GlyGly_10plex − Scan number: 30326

Gene Names	Charge	m/z	Mass	Retention time	PEP	Score	Precursor Intensity
RPS27A;UBA52;UBC;UBB	3	801.4269	2401.259	111.46	1.0276e−14	124.35	1985522

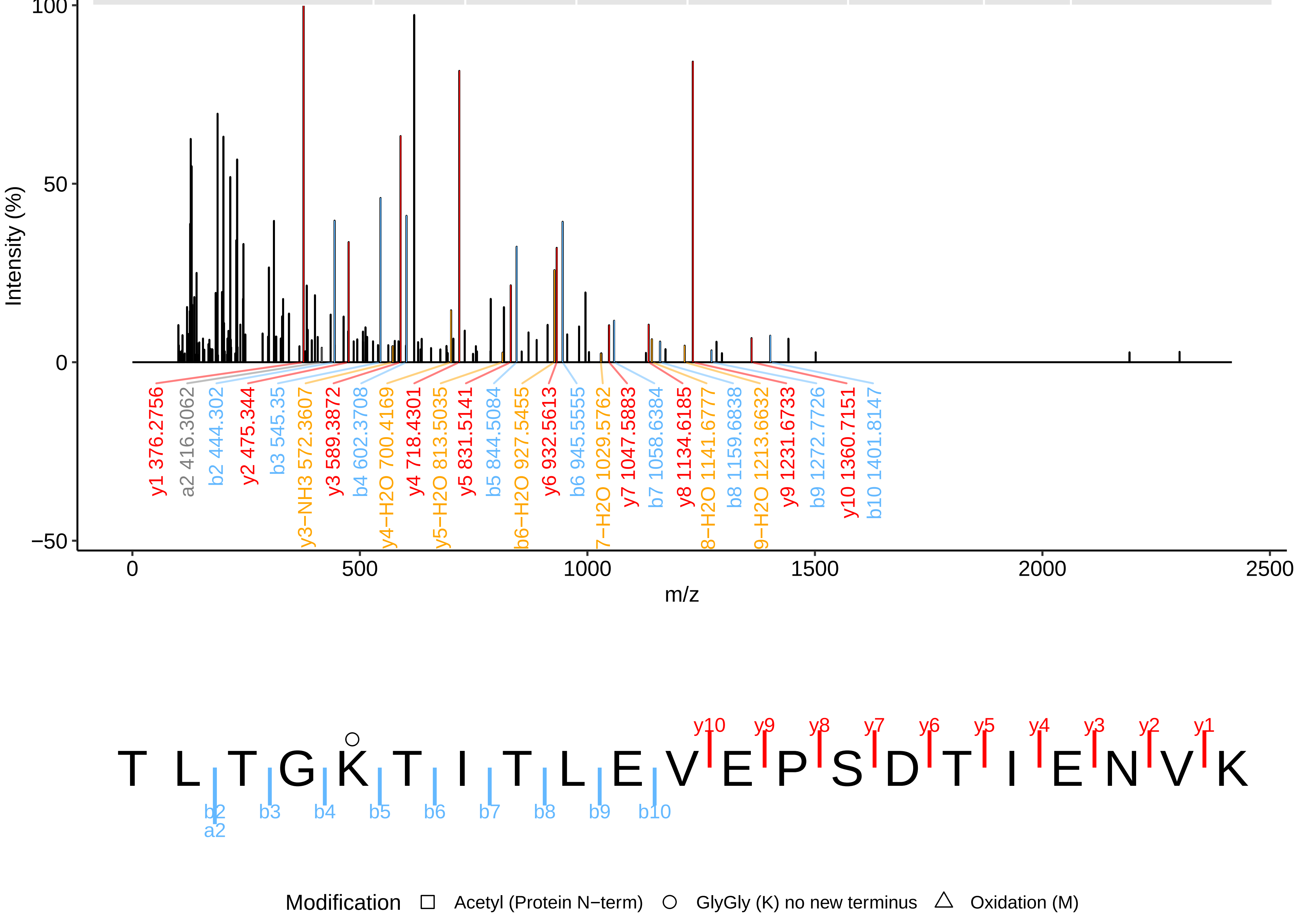

20200921_KIZ_MUb4_GlyGly_10plex − Scan number: 30346

Gene Names	Charge	m/z	Mass	Retention time	PEP	Score	Precursor Intensity
RPS27A;UBA52;UBC;UBB	3	801.4269	2401.259	111.55	6.648e−22	144.72	1688822

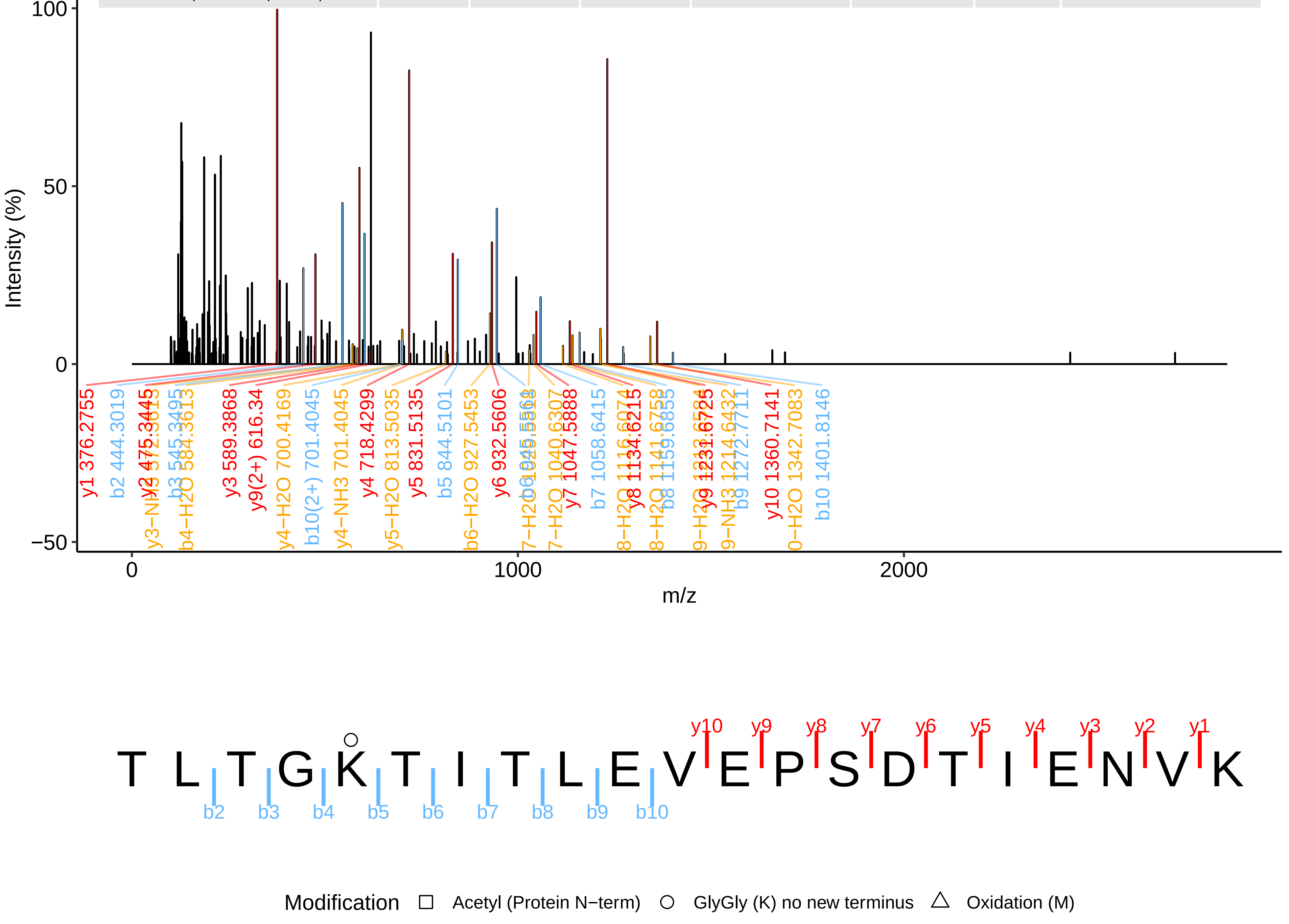

20200921_KIZ_MUb4_GlyGly_10plex − Scan number: 30475

Gene Names	Charge	m/z	Mass	Retention time	PEP	Score	Precursor Intensity
RPS27A;UBA52;UBC;UBB	3	801.4269	2401.259	112.07	3.0642e−09	99.86	2374319

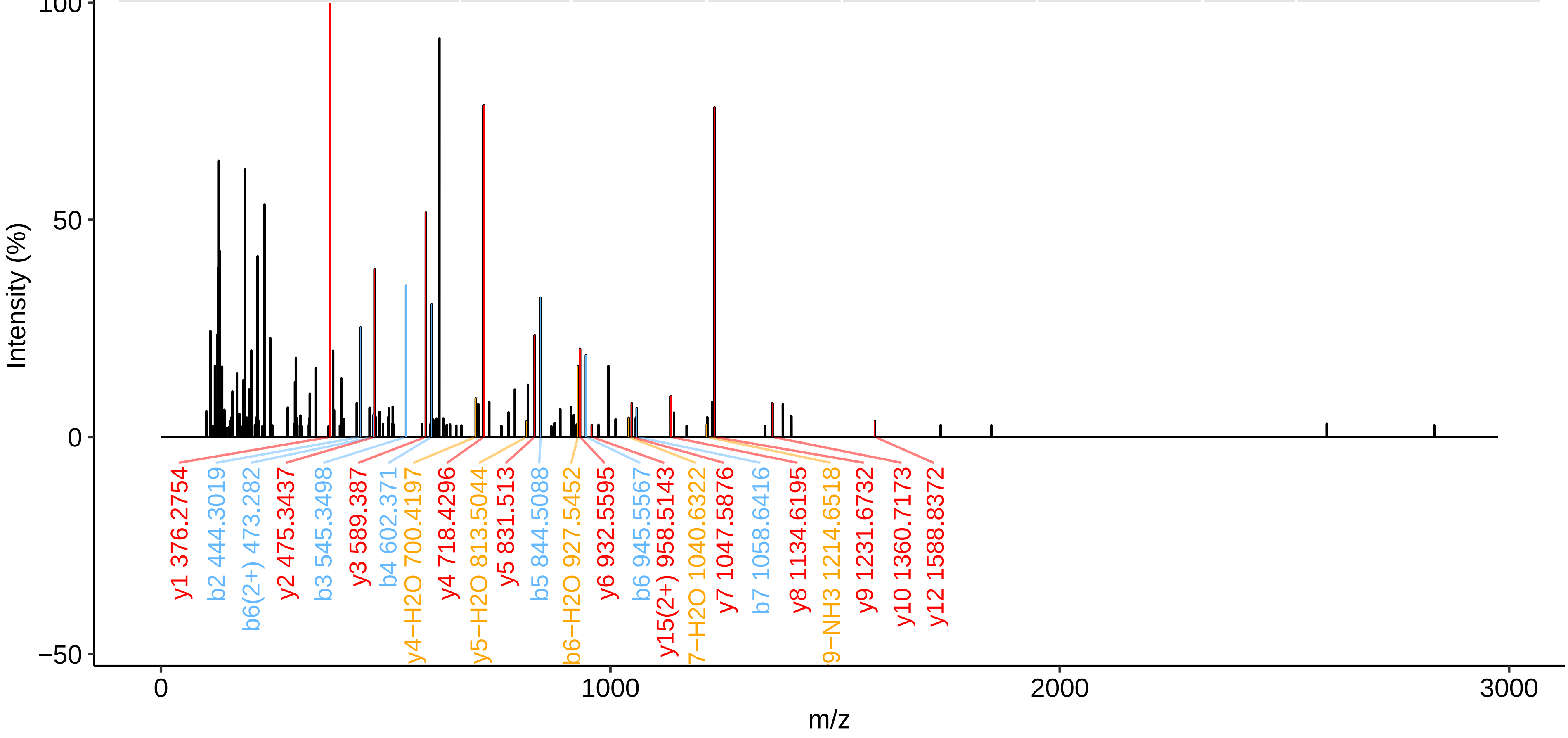

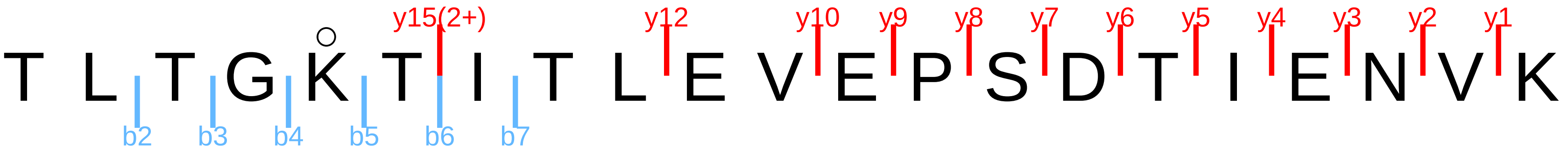

Modification □ Acetyl (Protein N−term) ○ GlyGly (K) no new terminus △ Oxidation (M)

20200921_KIZ_MUb4_GlyGly_10plex − Scan number: 34657

Gene Names	Charge	m/z	Mass	Retention time	PEP	Score	Precursor Intensity
RPS27A;UBA52;UBC;UBB	3	801.4269	2401.259	127.15	0.066795	42.309	202259.4

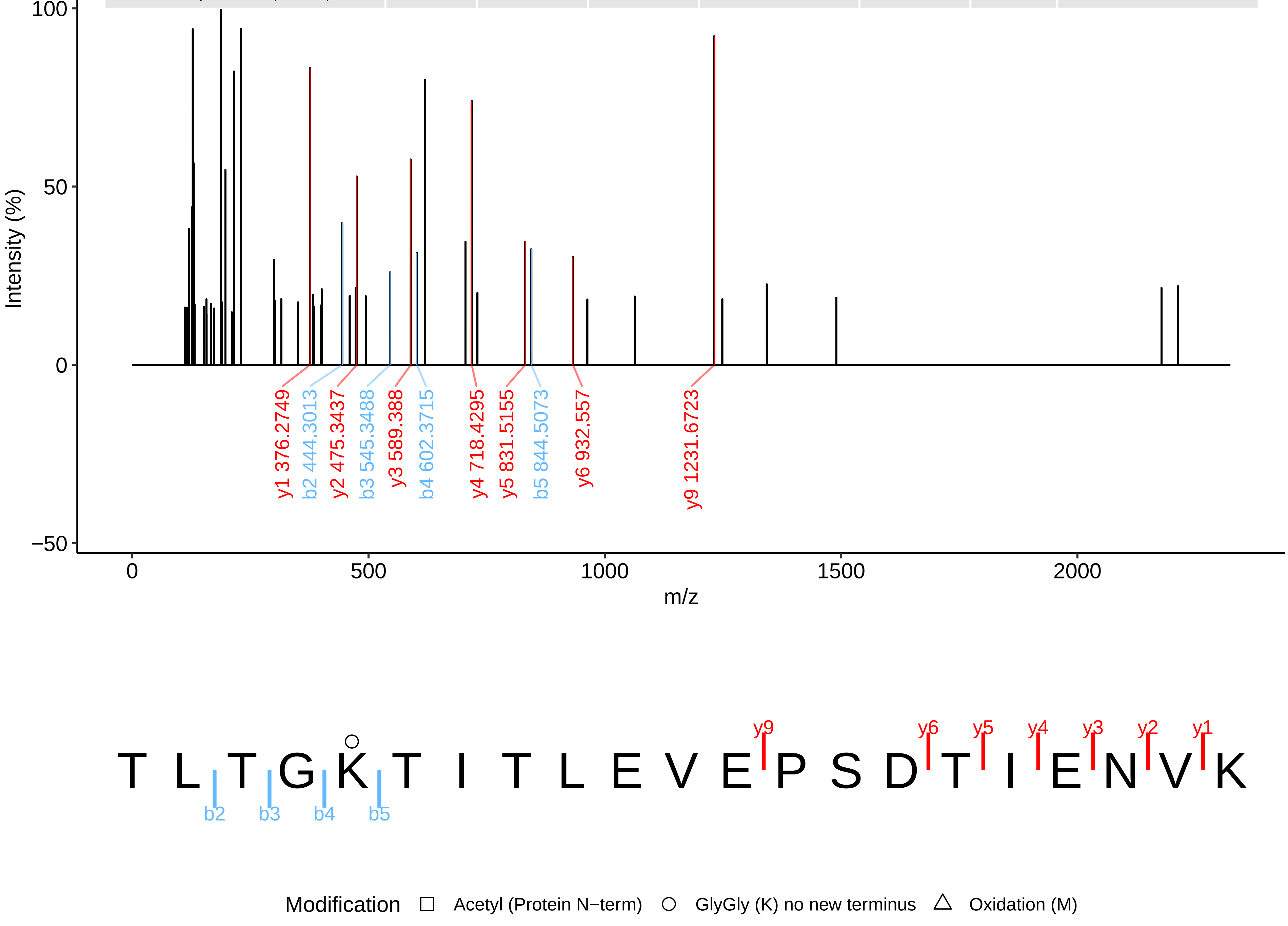

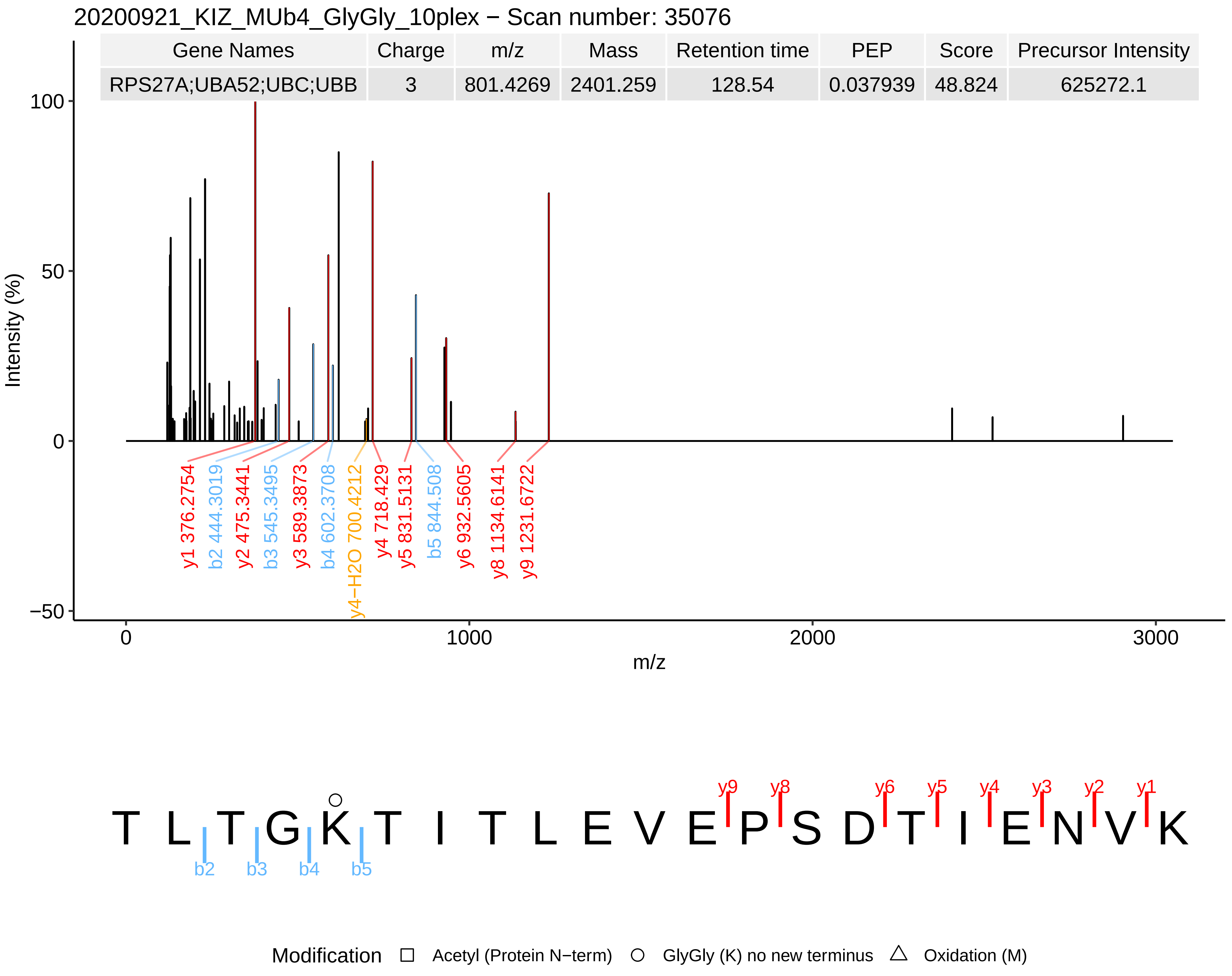
20200921_KIZ_MUb4_GlyGly_10plex − Scan number: 35076
Gene Names | Charge | m/z | Mass | Retention time | PEP | Score | Precursor Intensity
RPS27A;UBA52;UBC;UBB | 3 | 801.4269 | 2401.259 | 128.54 | 0.037939 | 48.824 | 625272.1
Intensity (%)
m/z
y1 376.2754
b2 444.3019
y2 475.3441
b3 545.3495
y3 589.3873
b4 602.3708
y4−H2O 700.4212
y4 718.429
y5 831.5131
b5 844.508
y6 932.5605
y8 1134.6141
y9 1231.6722
T L T G K T I T L E V E P S D T I E N V K
y9 y8 y6 y5 y4 y3 y2 y1
b2 b3 b4 b5
Modification
Acetyl (Protein N−term)
GlyGly (K) no new terminus
Oxidation (M)

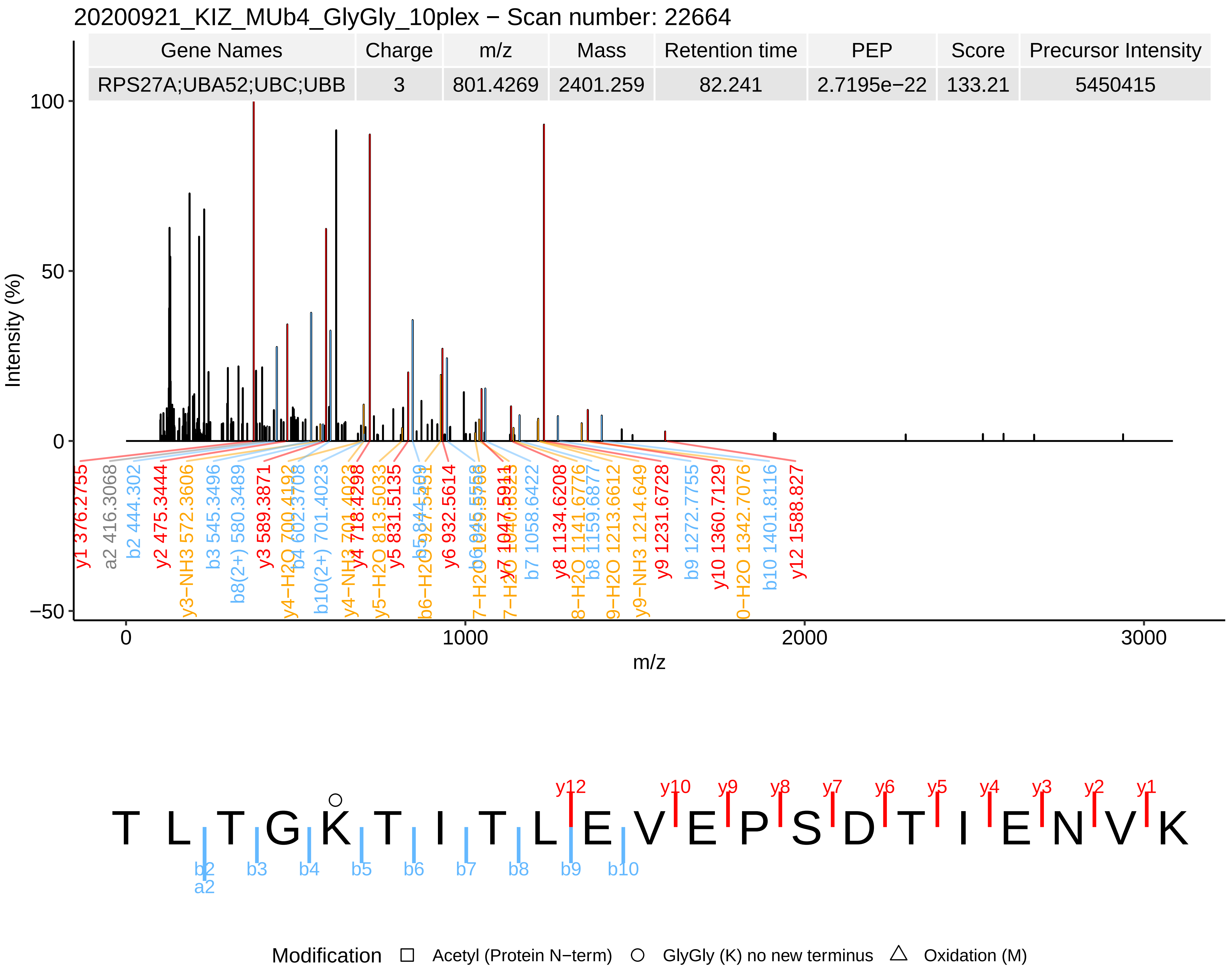
20200921_KIZ_MUb4_GlyGly_10plex − Scan number: 22664
Gene Names | Charge | m/z | Mass | Retention time | PEP | Score | Precursor Intensity
RPS27A;UBA52;UBC;UBB | 3 | 801.4269 | 2401.259 | 82.241 | 2.7195e−22 | 133.21 | 5450415
Intensity (%)
m/z
y1 376.2755
a2 416.3068
b2 444.302
y2 475.3444
y3−NH3 572.3606
b3 545.3496
b8(2+) 580.3489
y3 589.3871
b10(2+) 701.4023
y6 932.5614
b7 1058.6422
y8 1134.6208
b8 1159.6877
y9−NH3 1214.649
y9 1231.6728
b9 1272.7755
y10 1360.7129
b10 1401.8116
y12 1588.827
T L T G K T I T L E V E P S D T I E N V K
Modification
Acetyl (Protein N−term)
GlyGly (K) no new terminus
Oxidation (M)

20200921_KIZ_MUb4_GlyGly_10plex − Scan number: 24479

Gene Names	Charge	m/z	Mass	Retention time	PEP	Score	Precursor Intensity
RPS27A;UBA52;UBC;UBB	3	801.4269	2401.259	89.019	1.8902e−30	160.13	7493580

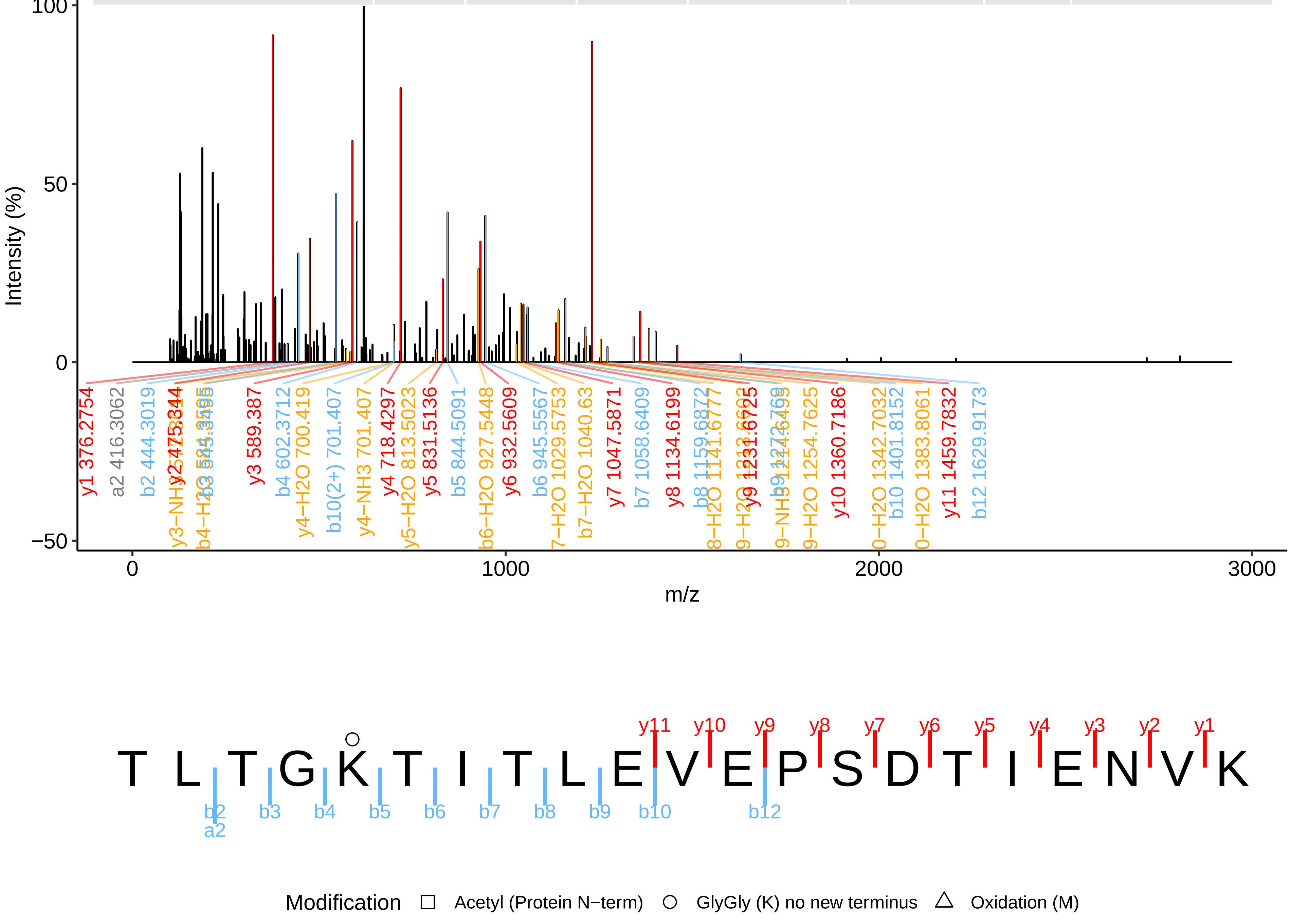

20200921_KIZ_MUb4_GlyGly_10plex − Scan number: 24962

Gene Names	Charge	m/z	Mass	Retention time	PEP	Score	Precursor Intensity
RPS27A;UBA52;UBC;UBB	3	801.4269	2401.259	90.788	2.4904e−38	168.79	5769507

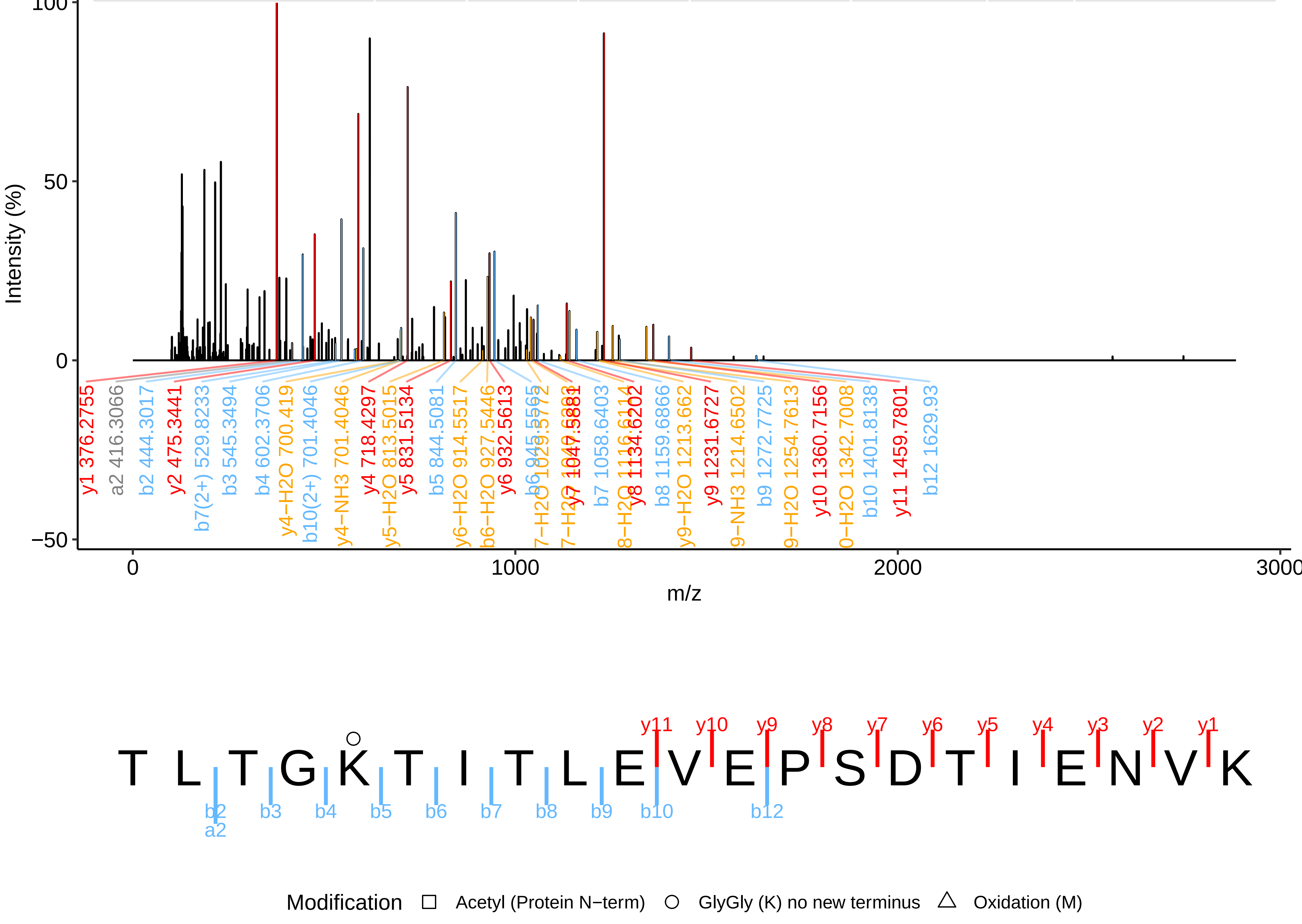

20200921_KIZ_MUb4_GlyGly_10plex − Scan number: 25528

Gene Names	Charge	m/z	Mass	Retention time	PEP	Score	Precursor Intensity
RPS27A;UBA52;UBC;UBB	3	801.4269	2401.259	92.88	7.0537e−23	145.52	NA

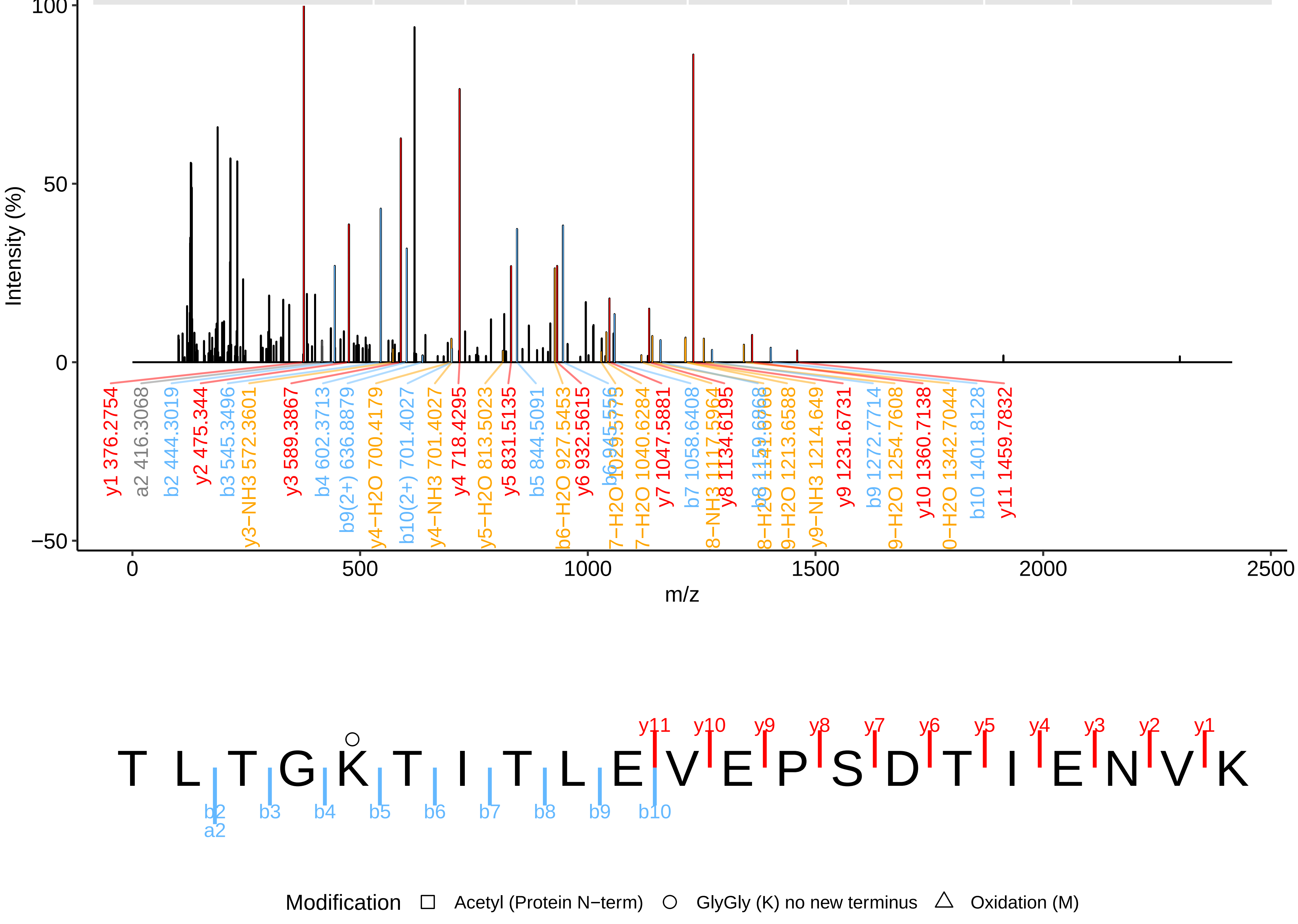

20200921_KIZ_MUb4_GlyGly_10plex − Scan number: 26246

Gene Names	Charge	m/z	Mass	Retention time	PEP	Score	Precursor Intensity
RPS27A;UBA52;UBC;UBB	3	801.4269	2401.259	95.584	2.5799e−22	134.06	3363804

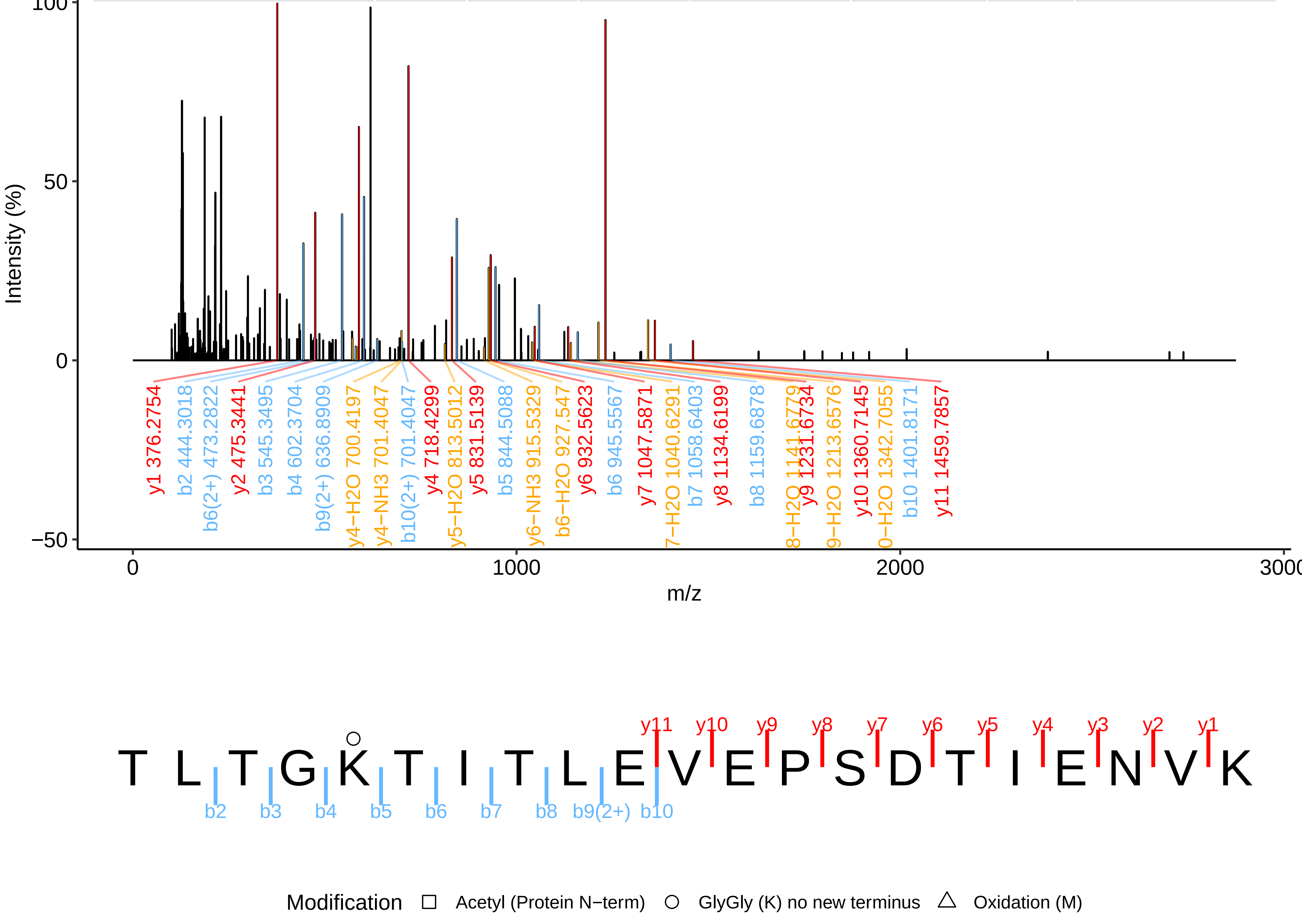

20200921_KIZ_MUb4_GlyGly_10plex − Scan number: 27285

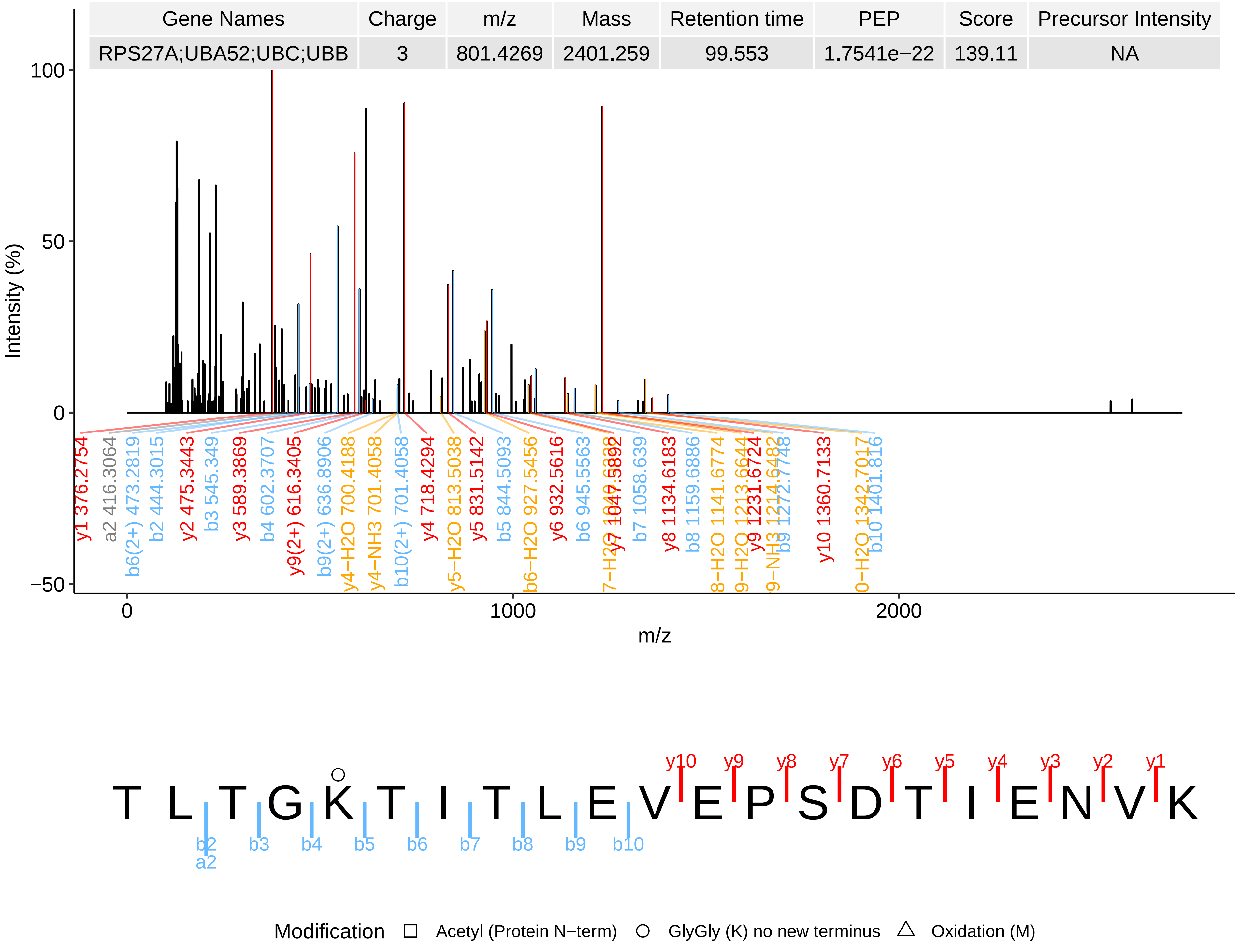

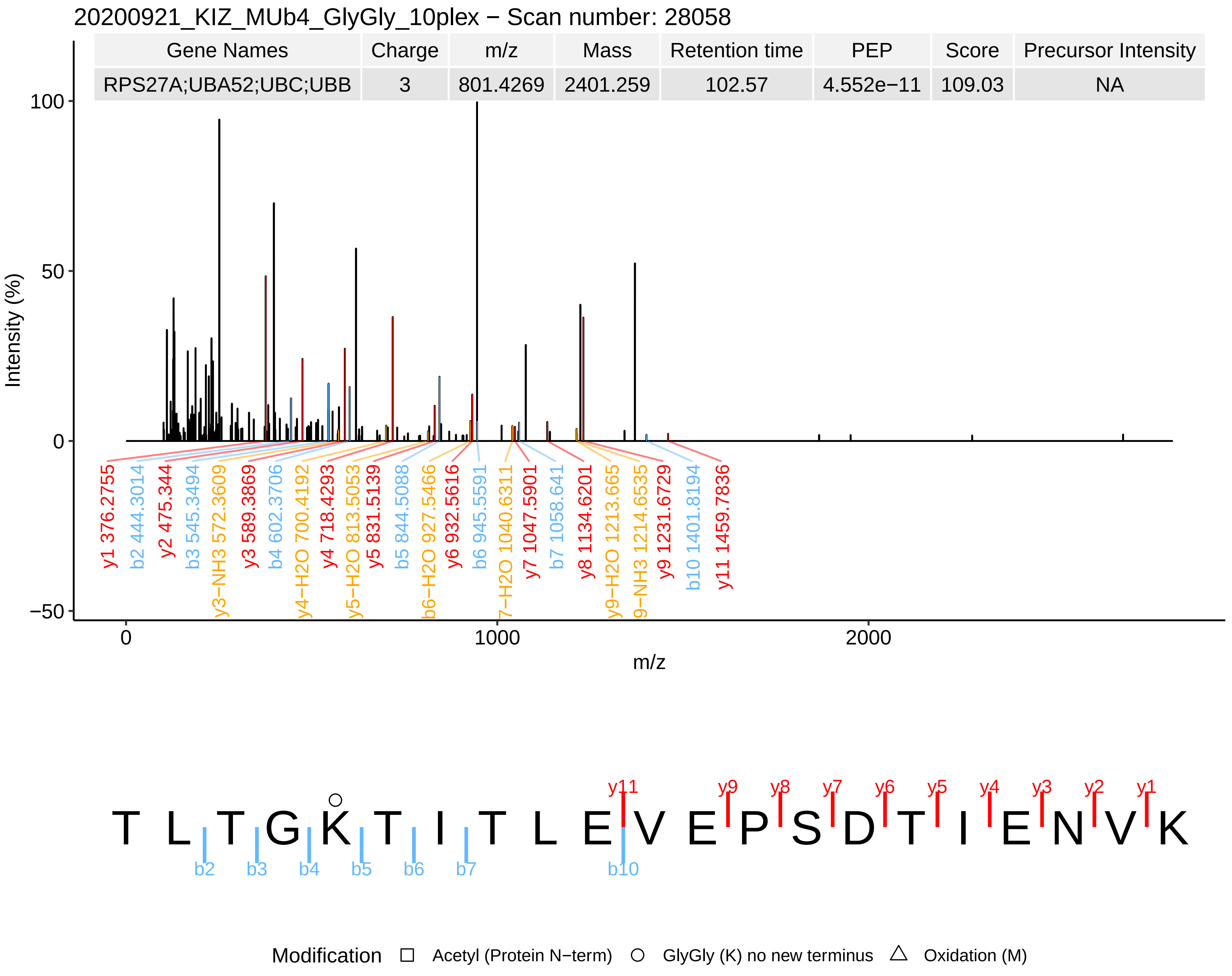

20200921_KIZ_MUb4_GlyGly_10plex − Scan number: 28058
Gene Names
Charge
m/z
Mass
Retention time
PEP
Score
Precursor Intensity
RPS27A;UBA52;UBC;UBB
3
801.4269
2401.259
102.57
4.552e−11
109.03
NA
Intensity (%)
100
50
0
−50
0
1000
2000
m/z
y1 376.2755
b2 444.3014
y2 475.344
b3 545.3494
y3−NH3 572.3609
y3 589.3869
b4 602.3706
y4−H2O 700.4192
y4 718.4293
y5−H2O 813.5053
y5 831.5139
b5 844.5088
b6−H2O 927.5466
y6 932.5616
b6 945.5591
7−H2O 1040.6311
y7 1047.5901
b7 1058.641
y8 1134.6201
y9−H2O 1213.665
9−NH3 1214.6535
y9 1231.6729
b10 1401.8194
y11 1459.7836
T L T G K T I T L E V E P S D T I E N V K
y11 y9 y8 y7 y6 y5 y4 y3 y2 y1
b2 b3 b4 b5 b6 b7 b10
Modification
Acetyl (Protein N−term)
GlyGly (K) no new terminus
Oxidation (M)

20200921_KIZ_MUb4_GlyGly_10plex − Scan number: 28222

Gene Names	Charge	m/z	Mass	Retention time	PEP	Score	Precursor Intensity
RPS27A;UBA52;UBC;UBB	3	801.4269	2401.259	103.22	3.3326e−16	126.22	NA

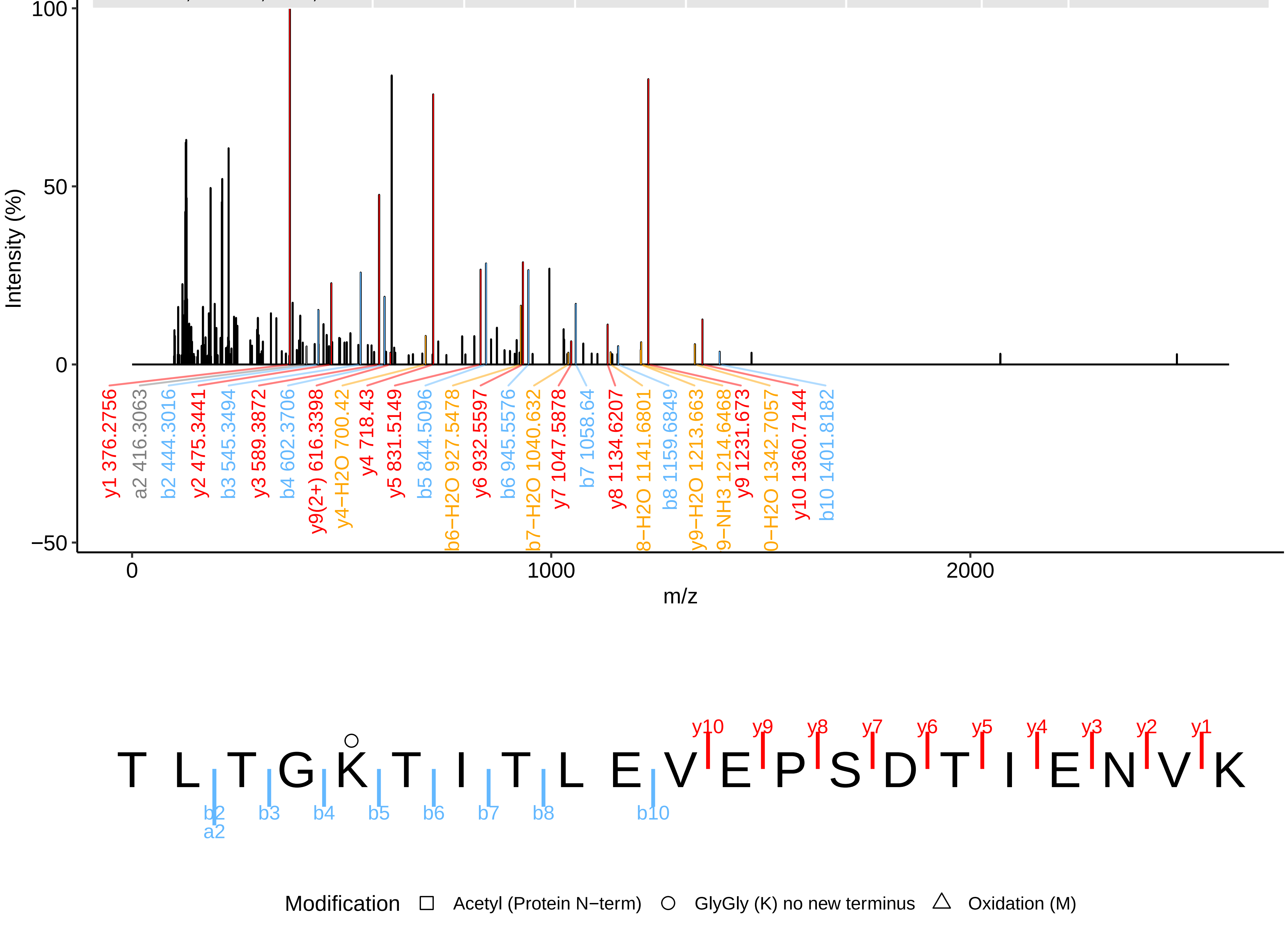

20200921_KIZ_MUb4_GlyGly_10plex − Scan number: 28764

Gene Names	Charge	m/z	Mass	Retention time	PEP	Score	Precursor Intensity
RPS27A;UBA52;UBC;UBB	3	801.4269	2401.259	105.28	1.1265e−10	100.21	NA

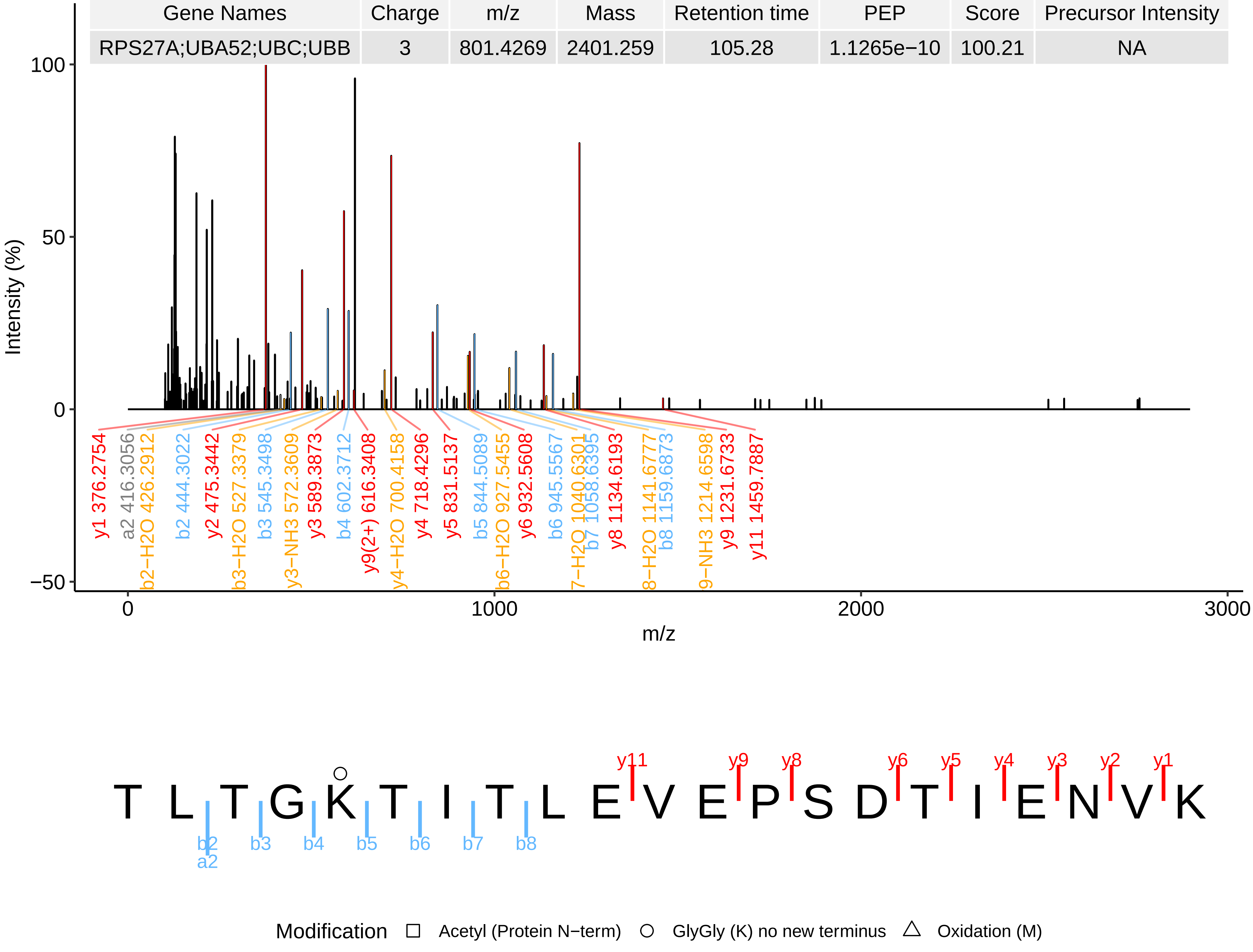

20200921_KIZ_MUb4_GlyGly_10plex − Scan number: 29587

Gene Names	Charge	m/z	Mass	Retention time	PEP	Score	Precursor Intensity
RPS27A;UBA52;UBC;UBB	3	801.4269	2401.259	108.52	2.7195e−22	133.21	NA

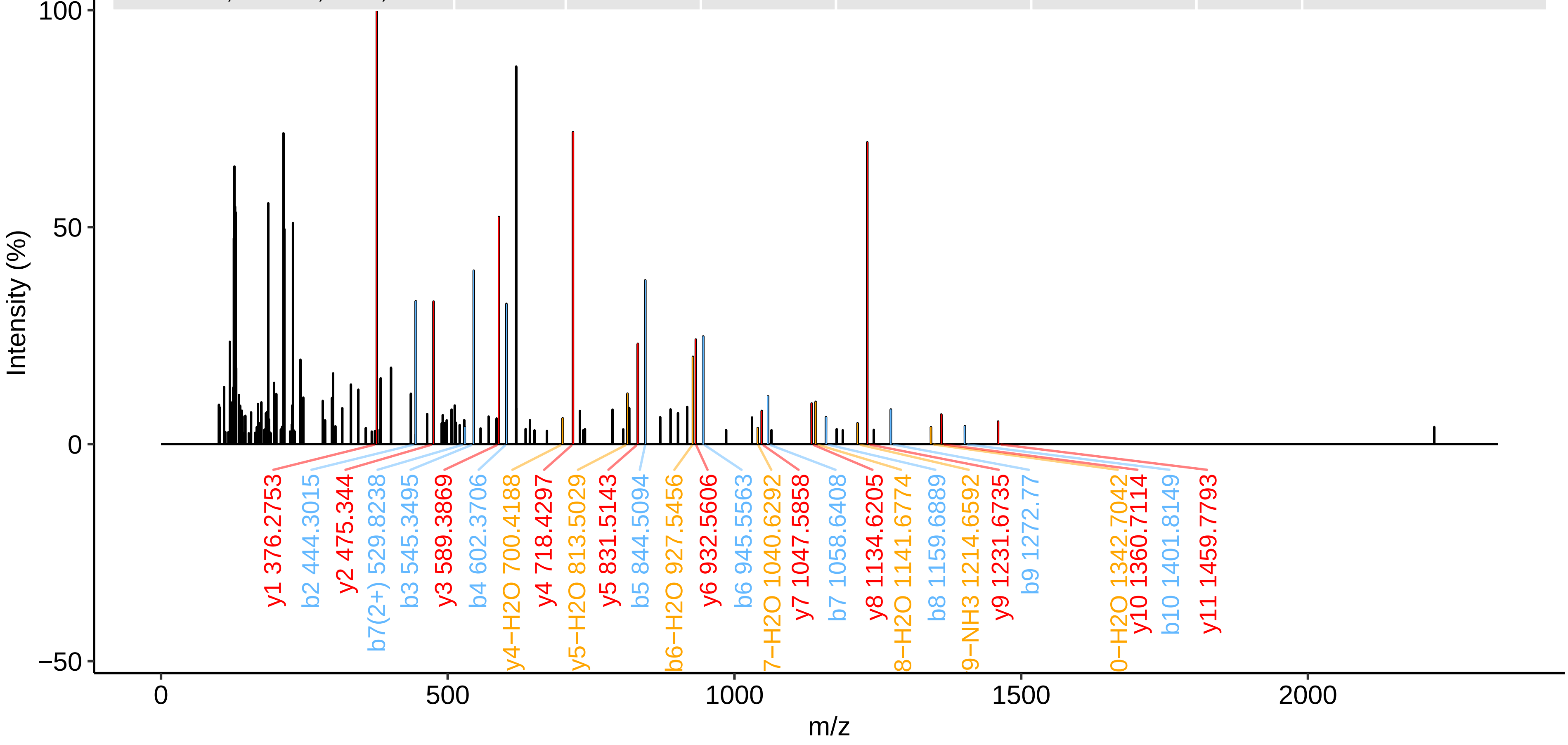

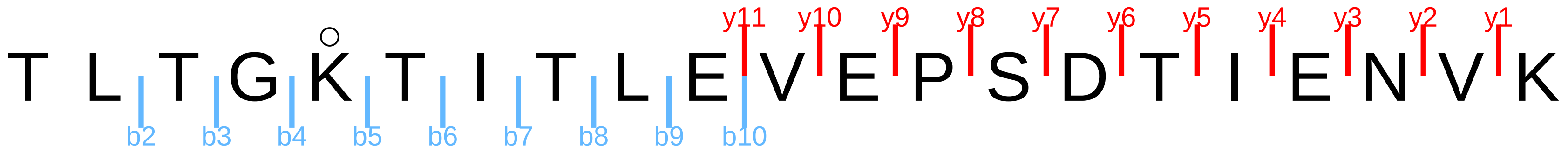

Modification □ Acetyl (Protein N−term) ○ GlyGly (K) no new terminus △ Oxidation (M)

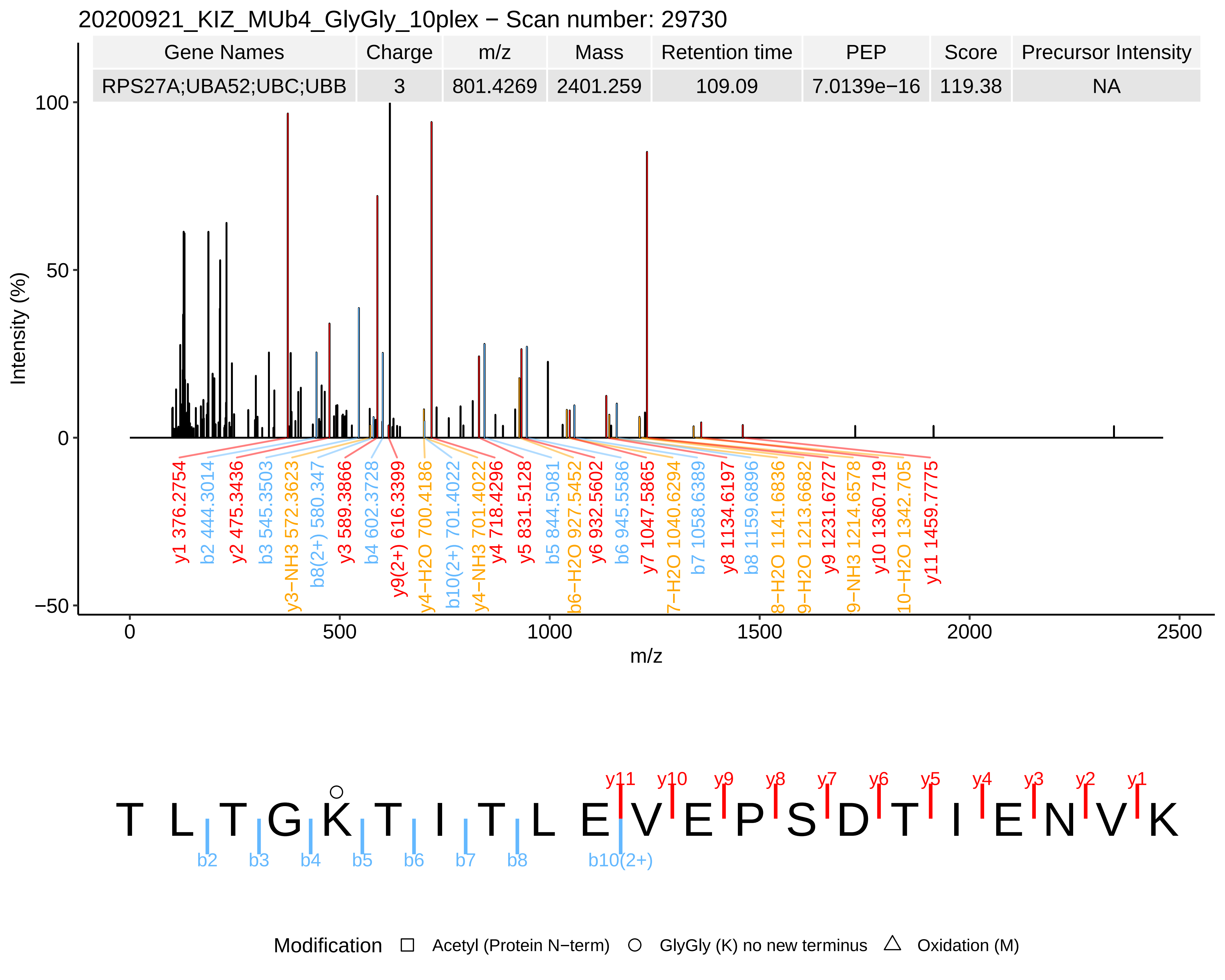
20200921_KIZ_MUb4_GlyGly_10plex − Scan number: 29730
Gene Names | Charge | m/z | Mass | Retention time | PEP | Score | Precursor Intensity
RPS27A;UBA52;UBC;UBB | 3 | 801.4269 | 2401.259 | 109.09 | 7.0139e−16 | 119.38 | NA
Intensity (%)
m/z
y1 376.2754
b2 444.3014
y2 475.3436
b3 545.3503
y3−NH3 572.3623
b8(2+) 580.347
y3 589.3866
b4 602.3728
y9(2+) 616.3399
y4−H2O 700.4186
b10(2+) 701.4022
y4−NH3 701.4022
y4 718.4296
y5 831.5128
b5 844.5081
b6−H2O 927.5452
y6 932.5602
b6 945.5586
y7 1047.5865
y7−H2O 1040.6294
b7 1058.6389
y8 1134.6197
b8 1159.6896
y8−H2O 1141.6836
y9−H2O 1213.6682
y9 1231.6727
y9−NH3 1214.6578
y10 1360.719
y10−H2O 1342.705
y11 1459.7775
T L T G K T I T L E V E P S D T I E N V K
y11 y10 y9 y8 y7 y6 y5 y4 y3 y2 y1
b2 b3 b4 b5 b6 b7 b8 b10(2+)
Modification
Acetyl (Protein N−term)
GlyGly (K) no new terminus
Oxidation (M)

20200921_KIZ_MUb4_GlyGly_10plex − Scan number: 29897

Gene Names	Charge	m/z	Mass	Retention time	PEP	Score	Precursor Intensity
RPS27A;UBA52;UBC;UBB	3	801.4269	2401.259	109.75	4.3387e−16	124.35	2714673

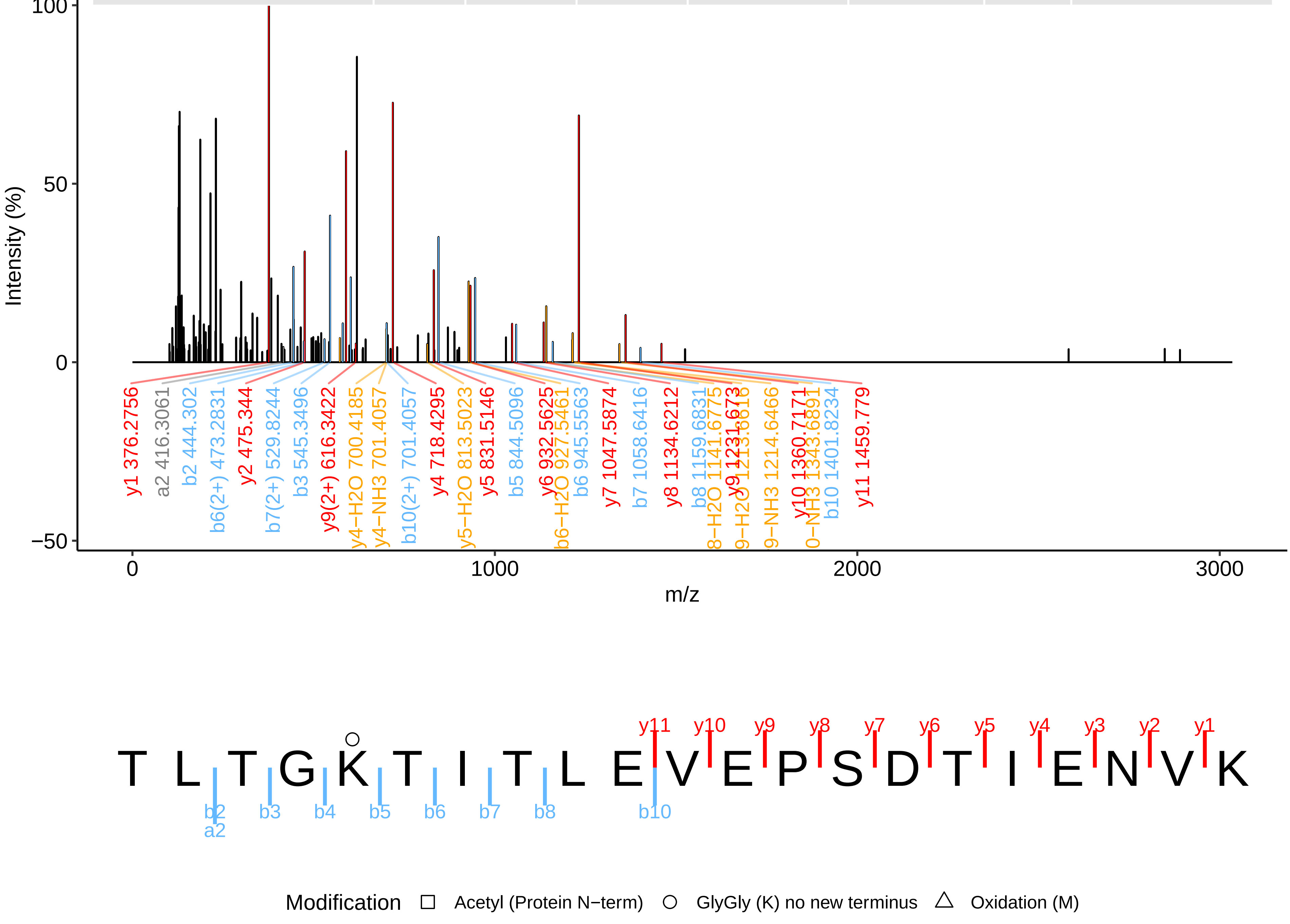

20200921_KIZ_MUb4_GlyGly_10plex − Scan number: 31475

Gene Names	Charge	m/z	Mass	Retention time	PEP	Score	Precursor Intensity
RPS27A;UBA52;UBC;UBB	3	801.4269	2401.259	116.04	0.00018002	76.704	1391403

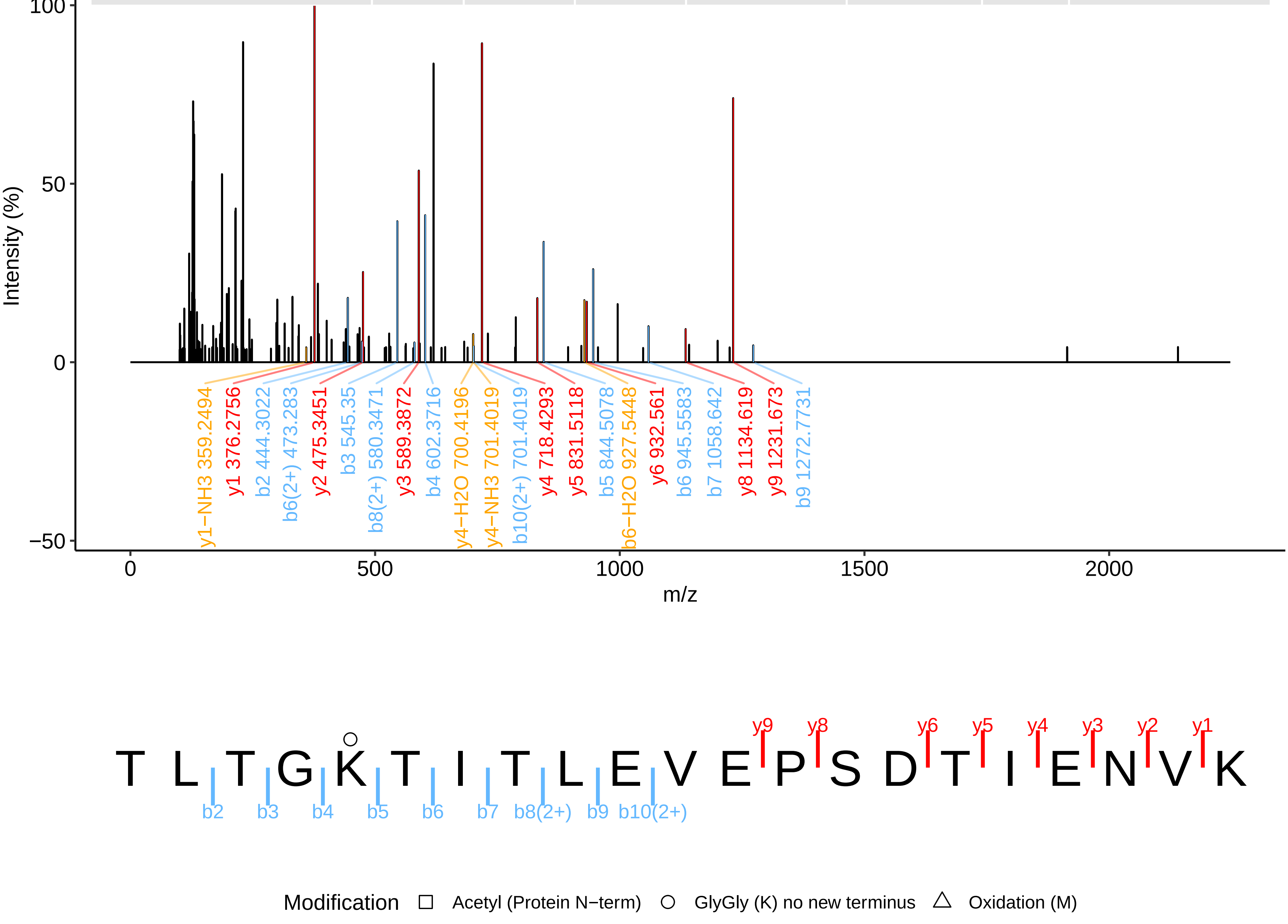

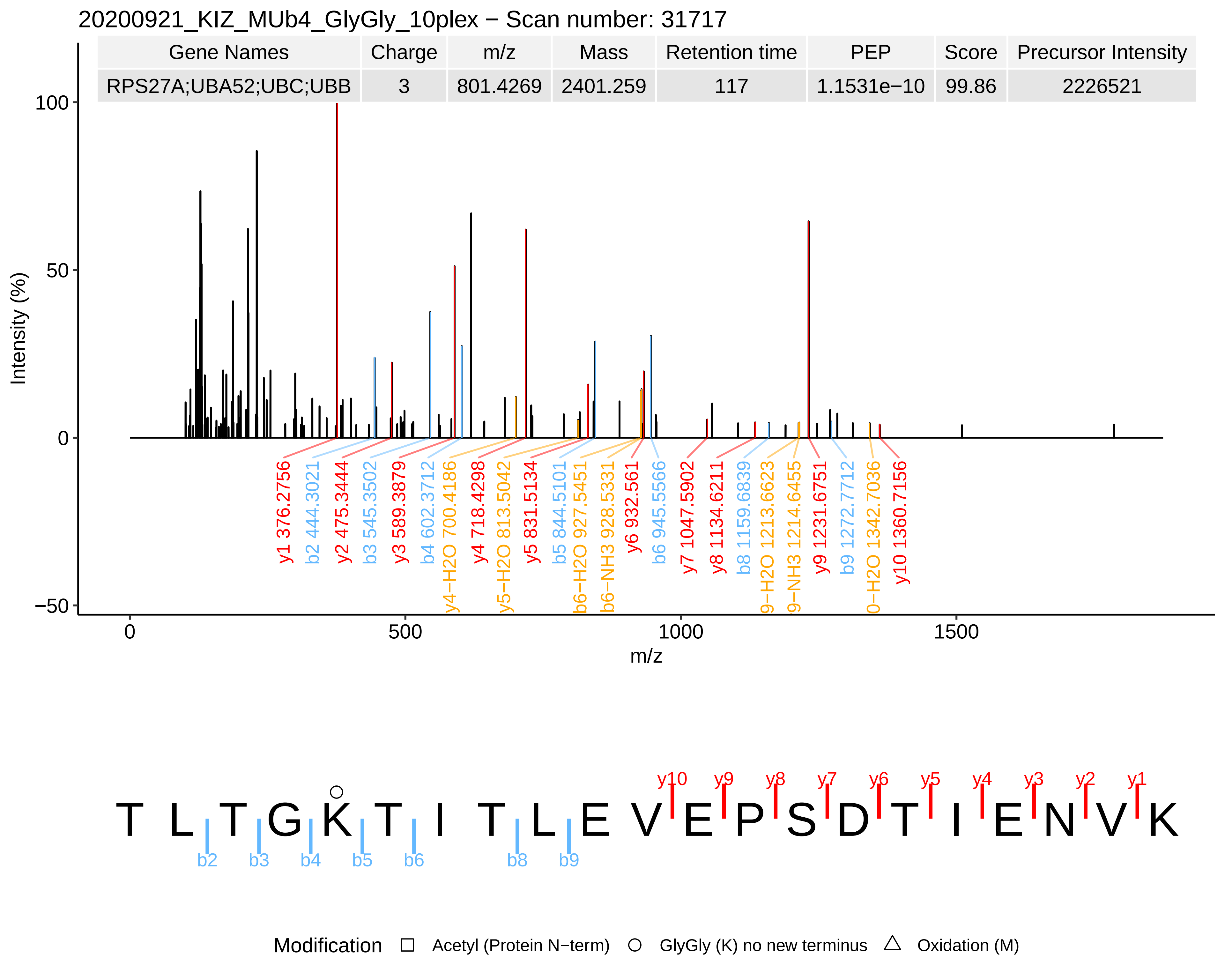

20200921_KIZ_MUb4_GlyGly_10plex − Scan number: 31717
Gene Names | Charge | m/z | Mass | Retention time | PEP | Score | Precursor Intensity
RPS27A;UBA52;UBC;UBB | 3 | 801.4269 | 2401.259 | 117 | 1.1531e−10 | 99.86 | 2226521
Intensity (%)
100
50
0
−50
0
500
1000
1500
m/z
y1 376.2756
b2 444.3021
y2 475.3444
b3 545.3502
y3 589.3879
b4 602.3712
y4−H2O 700.4186
y4 718.4298
y5−H2O 813.5042
y5 831.5134
b5 844.5101
b6−H2O 927.5451
b6−NH3 928.5331
y6 932.561
b6 945.5566
y7 1047.5902
y8 1134.6211
b8 1159.6839
9−H2O 1213.6623
9−NH3 1214.6455
y9 1231.6751
b9 1272.7712
0−H2O 1342.7036
y10 1360.7156
T L T G K T I T L E V E P S D T I E N V K
y10 y9 y8 y7 y6 y5 y4 y3 y2 y1
b2 b3 b4 b5 b6 b8 b9
Modification
Acetyl (Protein N−term)
GlyGly (K) no new terminus
Oxidation (M)

20200921_KIZ_MUb4_GlyGly_10plex − Scan number: 32137

Gene Names	Charge	m/z	Mass	Retention time	PEP	Score	Precursor Intensity
RPS27A;UBA52;UBC;UBB	3	801.4269	2401.259	118.7	3.7809e−05	79.467	NA

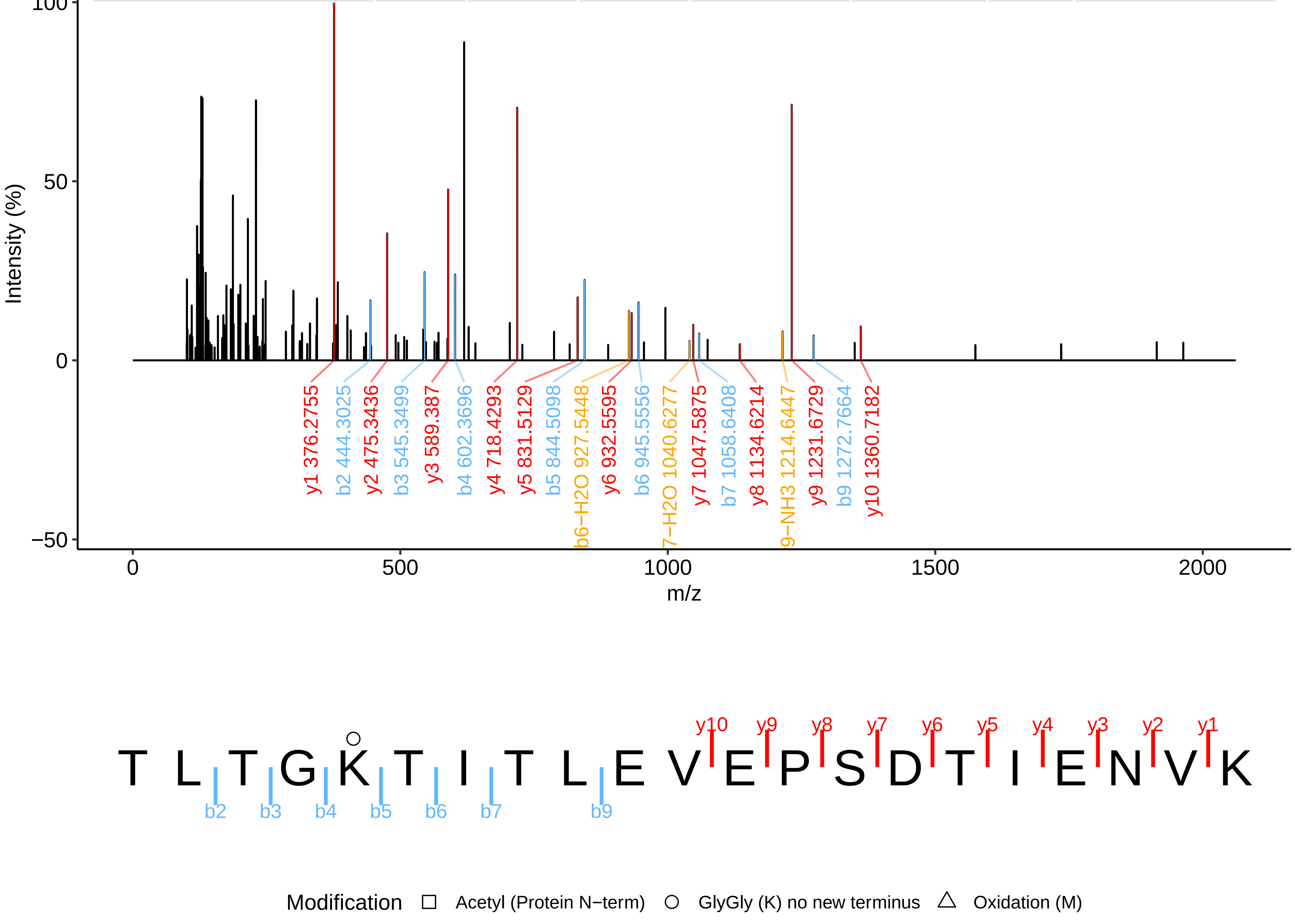

20200921_KIZ_MUb4_GlyGly_10plex − Scan number: 35366

Gene Names	Charge	m/z	Mass	Retention time	PEP	Score	Precursor Intensity
RPS27A;UBA52;UBC;UBB	3	801.4269	2401.259	129.62	1.0978e−06	81.967	456876.8

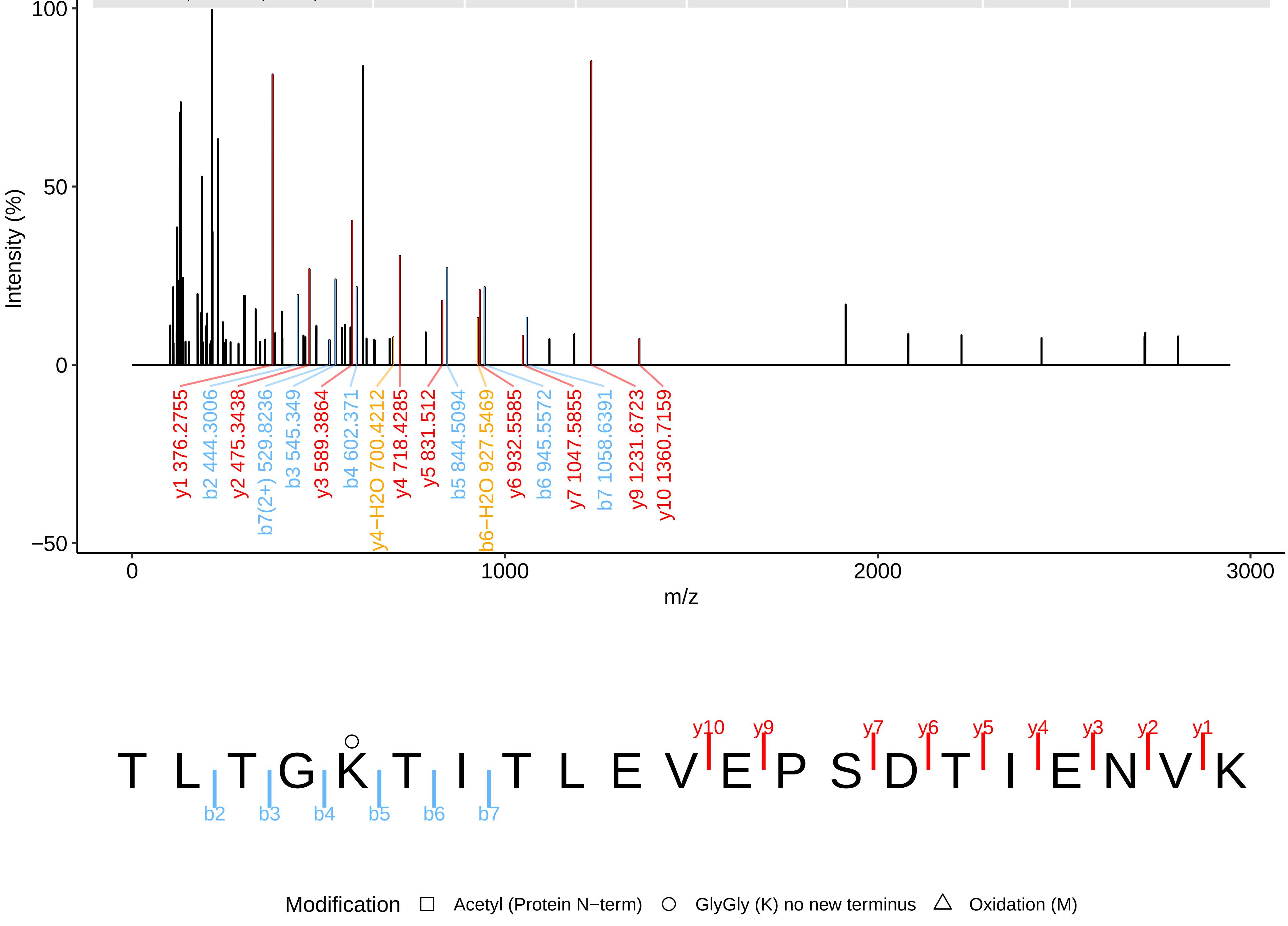

20200921_KIZ_MUb4_GlyGly_10plex_2 − Scan number: 20012

Gene Names	Charge	m/z	Mass	Retention time	PEP	Score	Precursor Intensity
RPS27A;UBA52;UBC;UBB	3	801.4269	2401.259	78.613	0.064777	42.64	938298.4

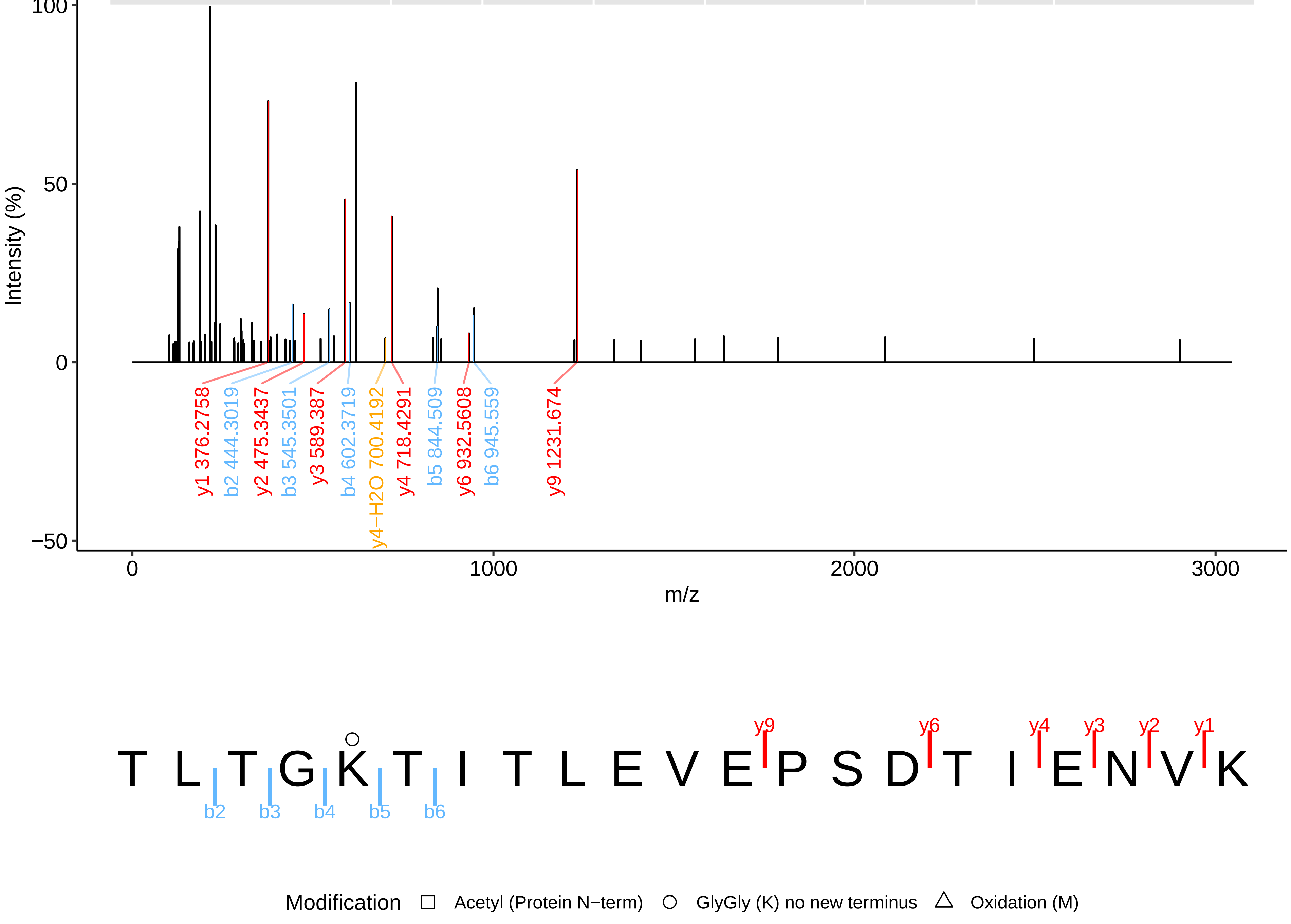

20200921_KIZ_MUb4_GlyGly_10plex_2 − Scan number: 20048

Gene Names	Charge	m/z	Mass	Retention time	PEP	Score	Precursor Intensity
RPS27A;UBA52;UBC;UBB	3	801.4269	2401.259	78.769	2.7424e−09	101.49	1394358

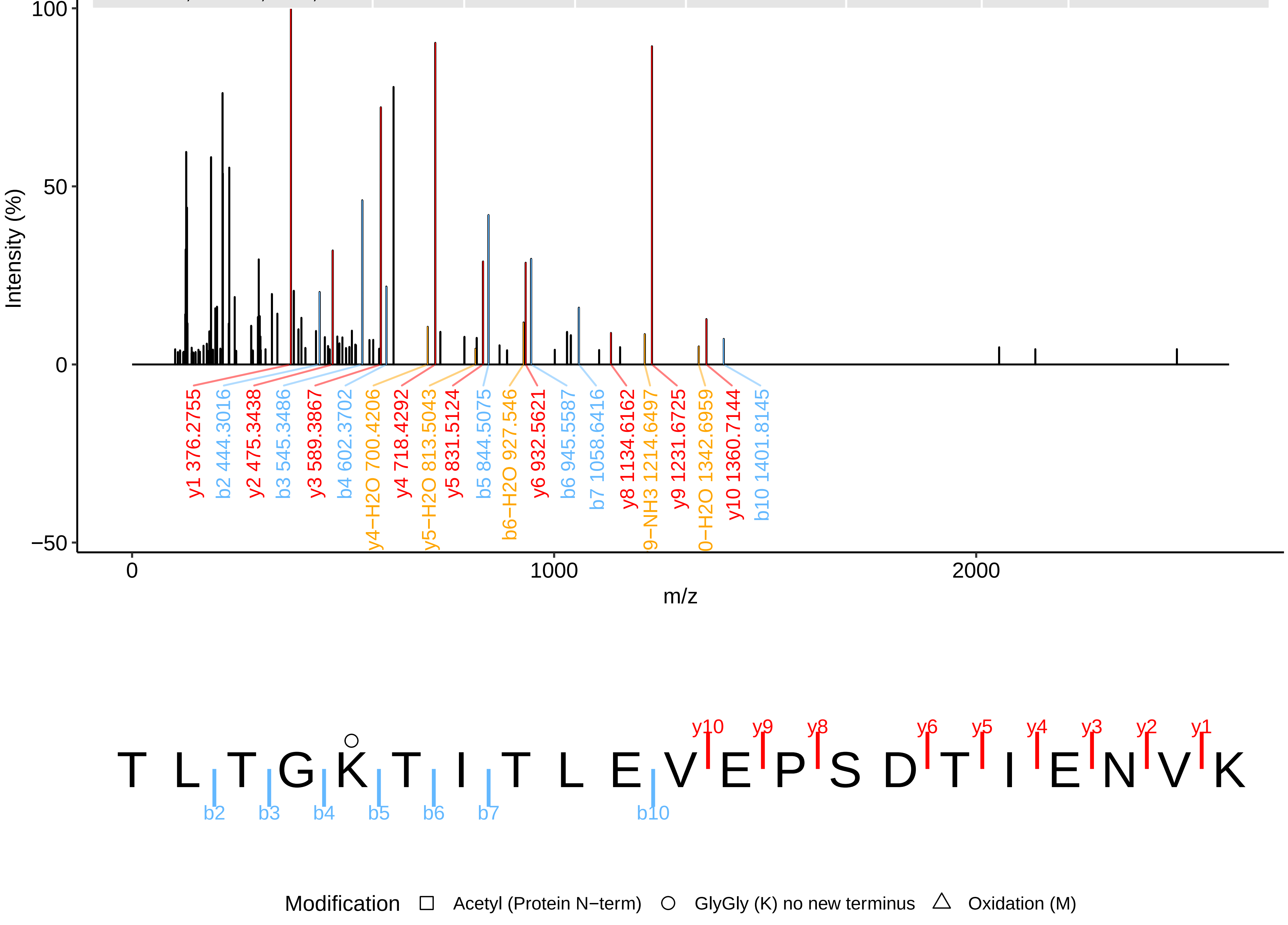

20200921_KIZ_MUb4_GlyGly_10plex_2 − Scan number: 20173

Gene Names	Charge	m/z	Mass	Retention time	PEP	Score	Precursor Intensity
RPS27A;UBA52;UBC;UBB	3	801.4269	2401.259	79.272	1.2978e−39	168.79	566326336

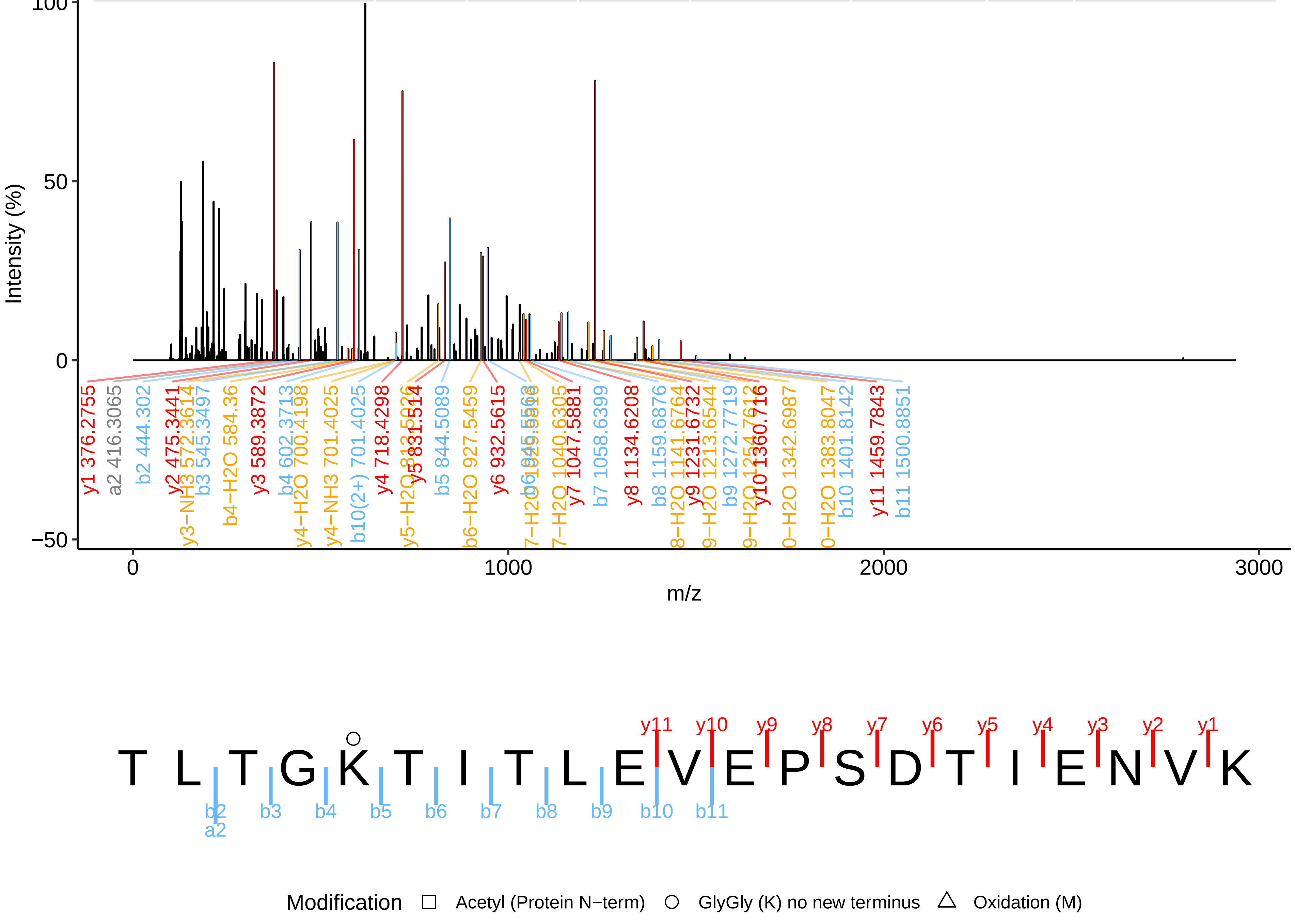

20200921_KIZ_MUb4_GlyGly_10plex_2 − Scan number: 20179

Gene Names	Charge	m/z	Mass	Retention time	PEP	Score	Precursor Intensity
RPS27A;UBA52;UBC;UBB	3	801.4269	2401.259	79.293	1.9158e−14	117.03	28216674

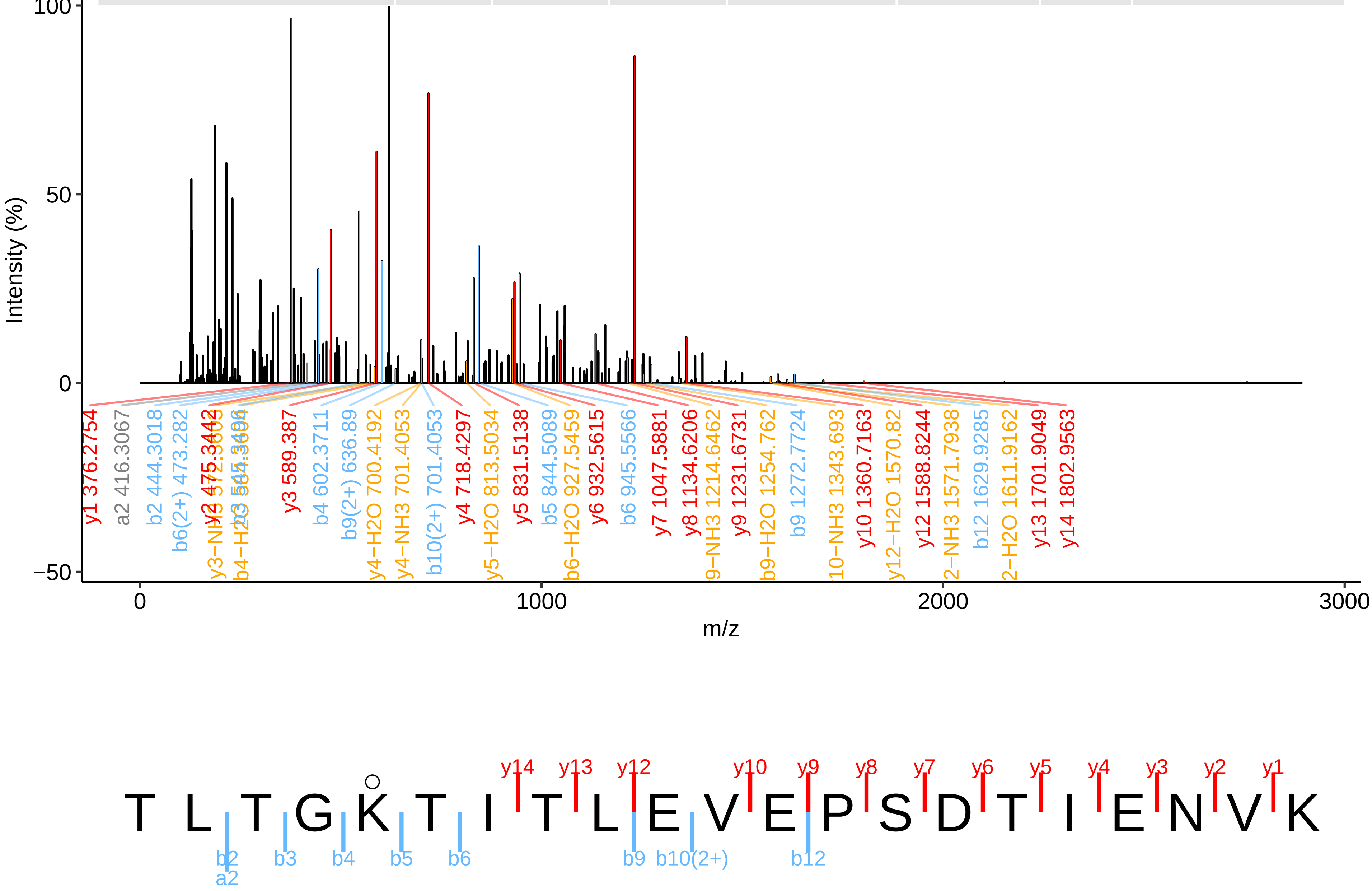

Modification □ Acetyl (Protein N−term) ○ GlyGly (K) no new terminus △ Oxidation (M)

20200921_KIZ_MUb4_GlyGly_10plex_2 − Scan number: 20305

Gene Names	Charge	m/z	Mass	Retention time	PEP	Score	Precursor Intensity
RPS27A;UBA52;UBC;UBB	3	801.4269	2401.259	79.798	1.0933e−39	171.35	10560347

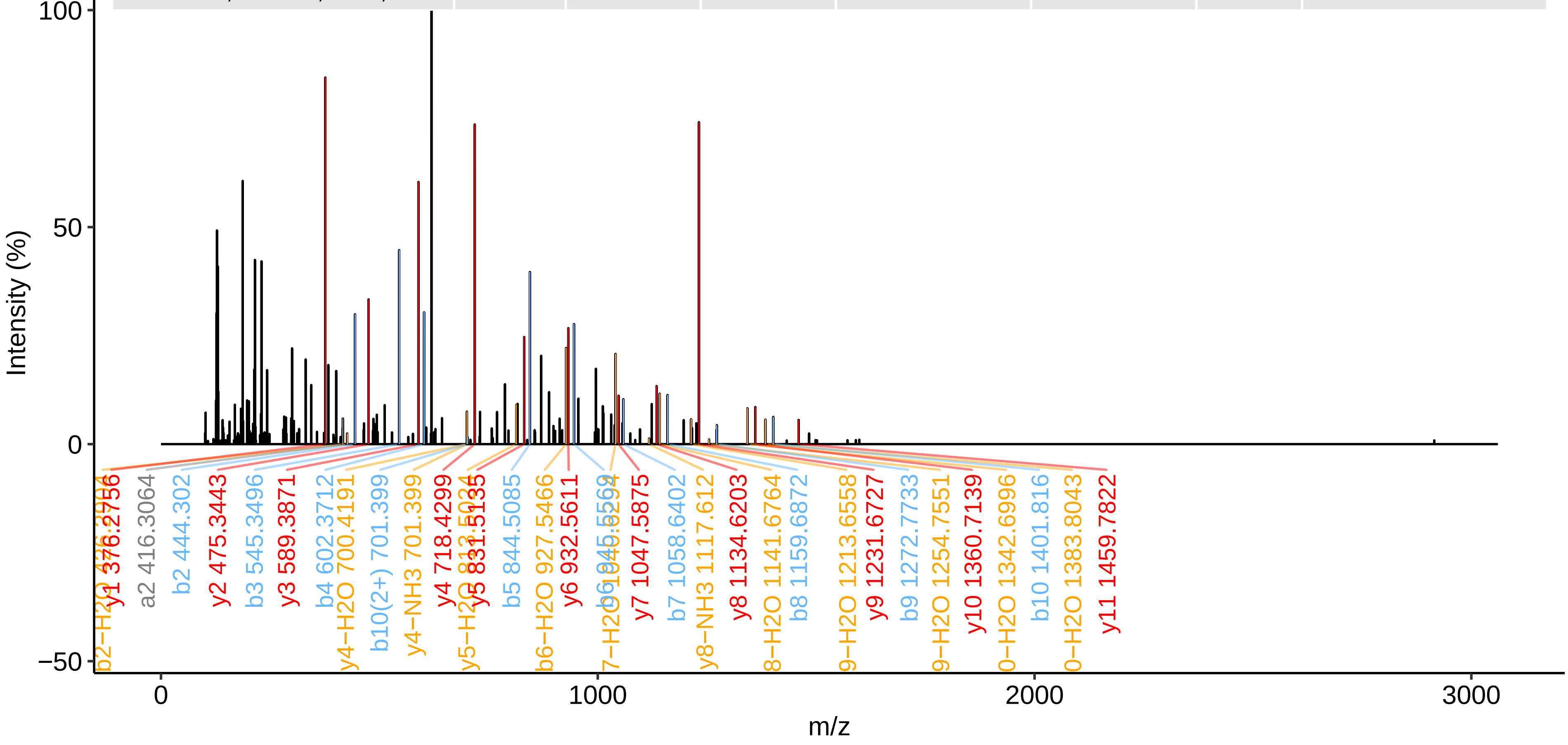

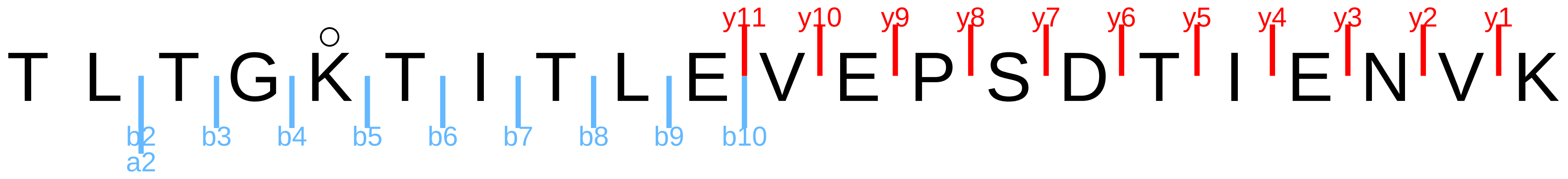

Modification □ Acetyl (Protein N−term) ○ GlyGly (K) no new terminus △ Oxidation (M)

20200921_KIZ_MUb4_GlyGly_10plex_2 − Scan number: 20435

Gene Names	Charge	m/z	Mass	Retention time	PEP	Score	Precursor Intensity
RPS27A;UBA52;UBC;UBB	3	801.4269	2401.259	80.32	1.0276e−14	124.35	2746473

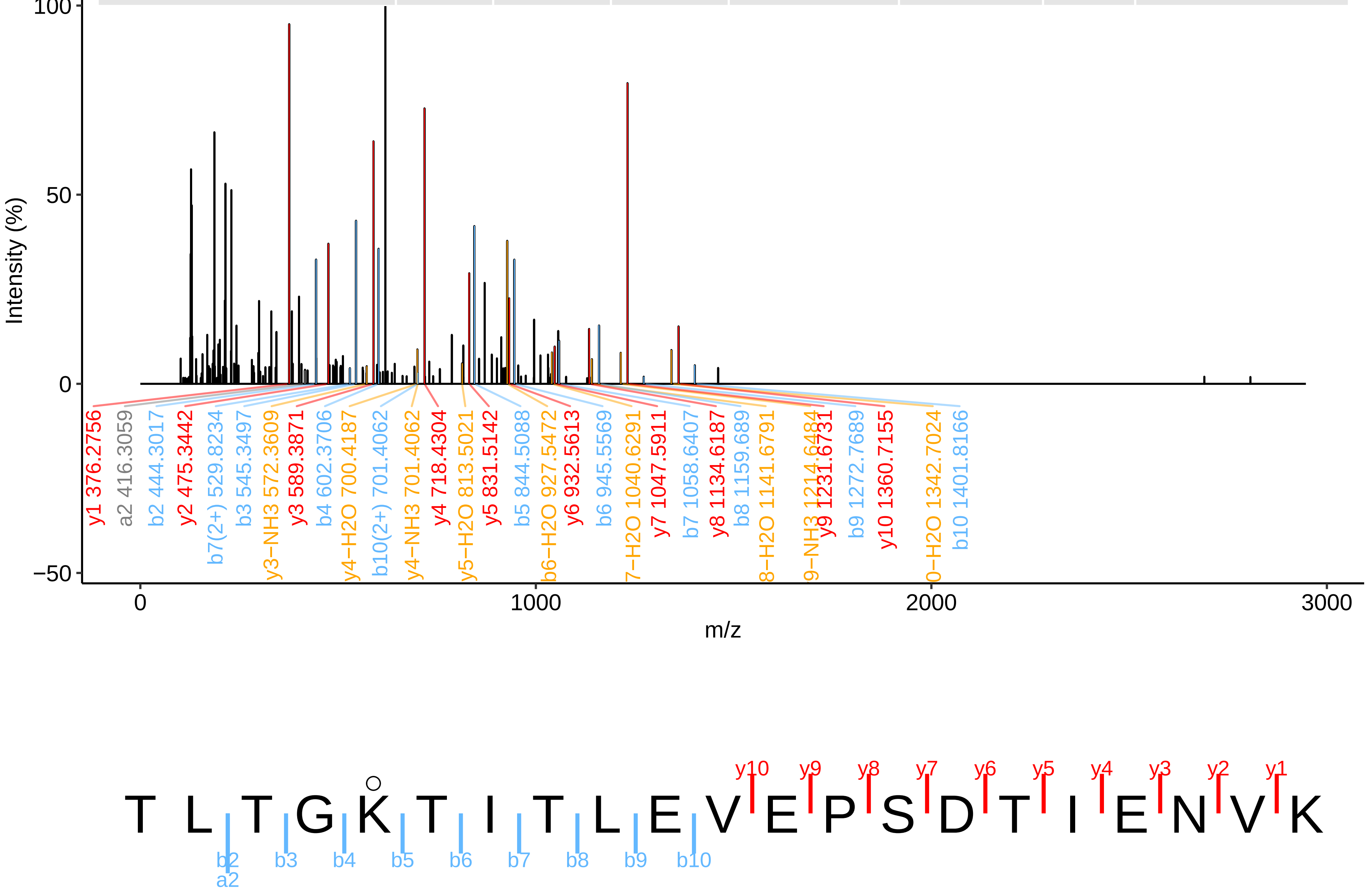

Modification □ Acetyl (Protein N−term) ○ GlyGly (K) no new terminus △ Oxidation (M)

20200921_KIZ_MUb4_GlyGly_10plex_2 − Scan number: 20563

Gene Names	Charge	m/z	Mass	Retention time	PEP	Score	Precursor Intensity
RPS27A;UBA52;UBC;UBB	3	801.4269	2401.259	80.84	9.7934e−06	83.213	1225292

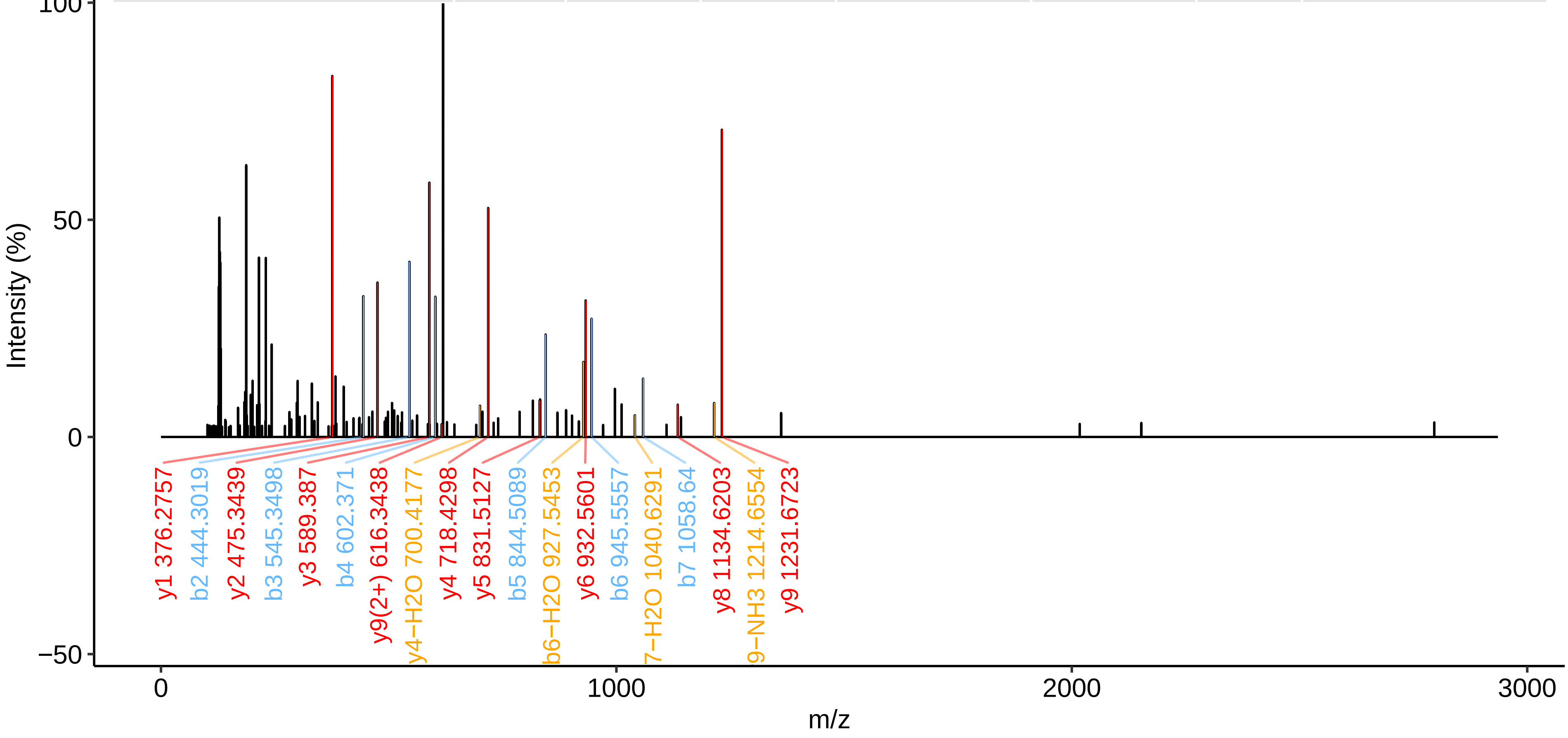

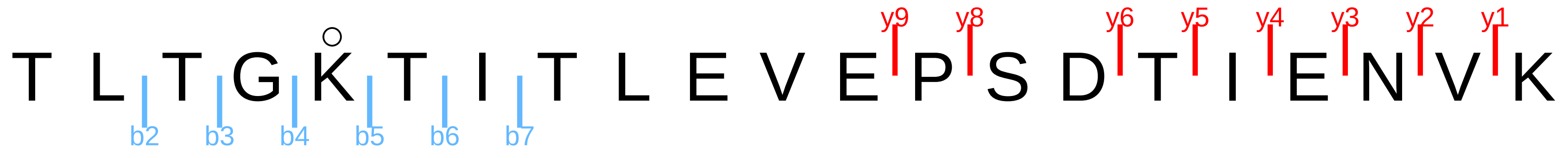

Modification □ Acetyl (Protein N−term) ○ GlyGly (K) no new terminus △ Oxidation (M)

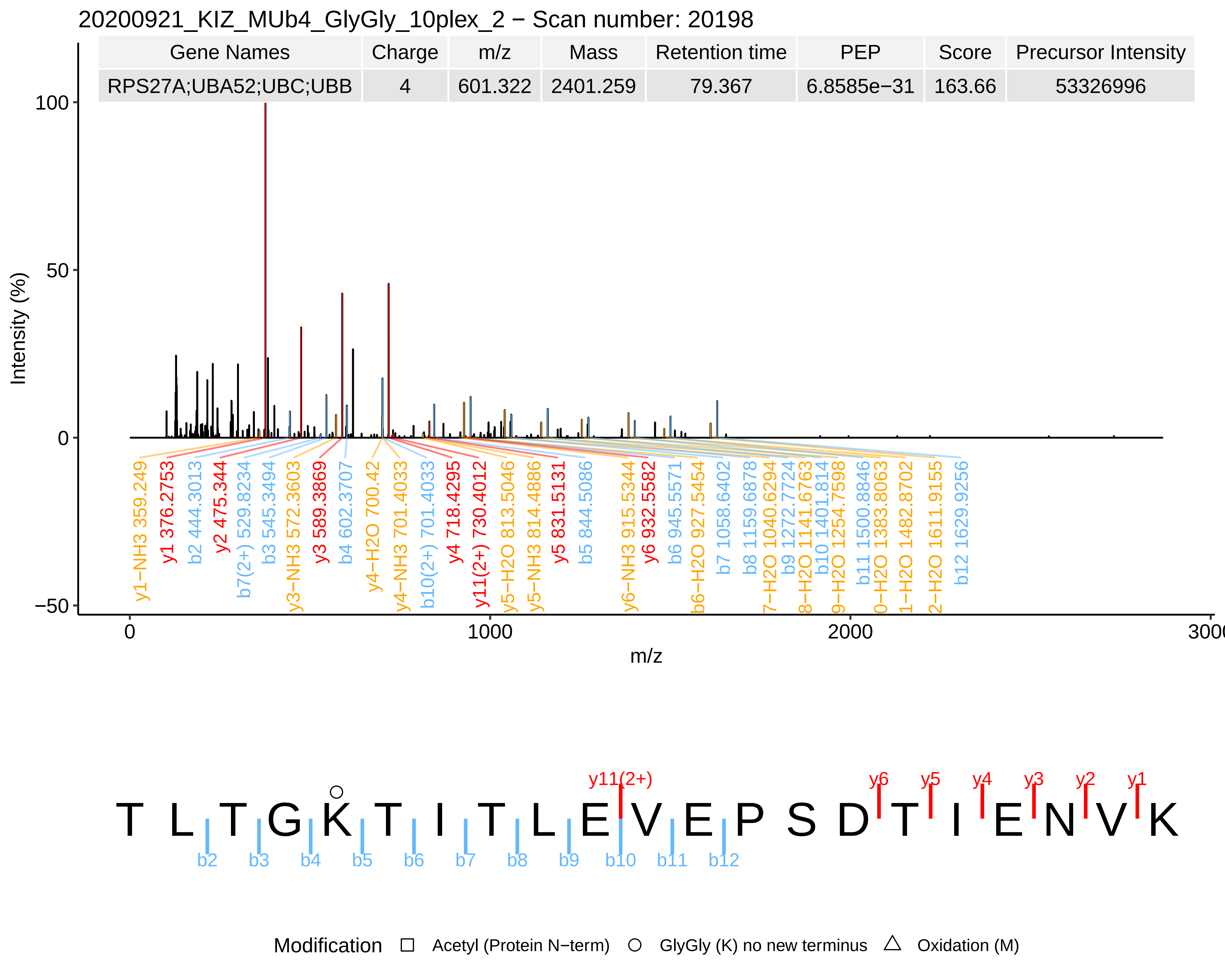

20200921_KIZ_MUb4_GlyGly_10plex_2 − Scan number: 20198
Gene Names | Charge | m/z | Mass | Retention time | PEP | Score | Precursor Intensity
RPS27A;UBA52;UBC;UBB | 4 | 601.322 | 2401.259 | 79.367 | 6.8585e−31 | 163.66 | 53326996
Intensity (%)
100
50
0
−50
0
1000
2000
m/z
y1−NH3 359.249
y1 376.2753
b2 444.3013
y2 475.344
b7(2+) 529.8234
b3 545.3494
y3−NH3 572.3603
y3 589.3869
b4 602.3707
y4−H2O 700.42
y4−NH3 701.4033
b10(2+) 701.4033
y4 718.4295
y11(2+) 730.4012
y5−H2O 813.5046
y5−NH3 814.4886
y5 831.5131
b5 844.5086
y6−NH3 915.5344
y6 932.5582
b6 945.5571
b6−H2O 927.5454
b7 1058.6402
b8 1159.6878
b9 1272.7724
b10 1401.814
b11 1500.8846
b12 1629.9256
T L T G K T I T L E V E P S D T I E N V K
y11(2+) y6 y5 y4 y3 y2 y1
b2 b3 b4 b5 b6 b7 b8 b9 b10 b11 b12
Modification
Acetyl (Protein N−term)
GlyGly (K) no new terminus
Oxidation (M)

20200921_KIZ_MUb4_GlyGly_10plex_2 − Scan number: 20324

Gene Names	Charge	m/z	Mass	Retention time	PEP	Score	Precursor Intensity
RPS27A;UBA52;UBC;UBB	4	601.322	2401.259	79.876	7.5944e−23	149.47	2790130

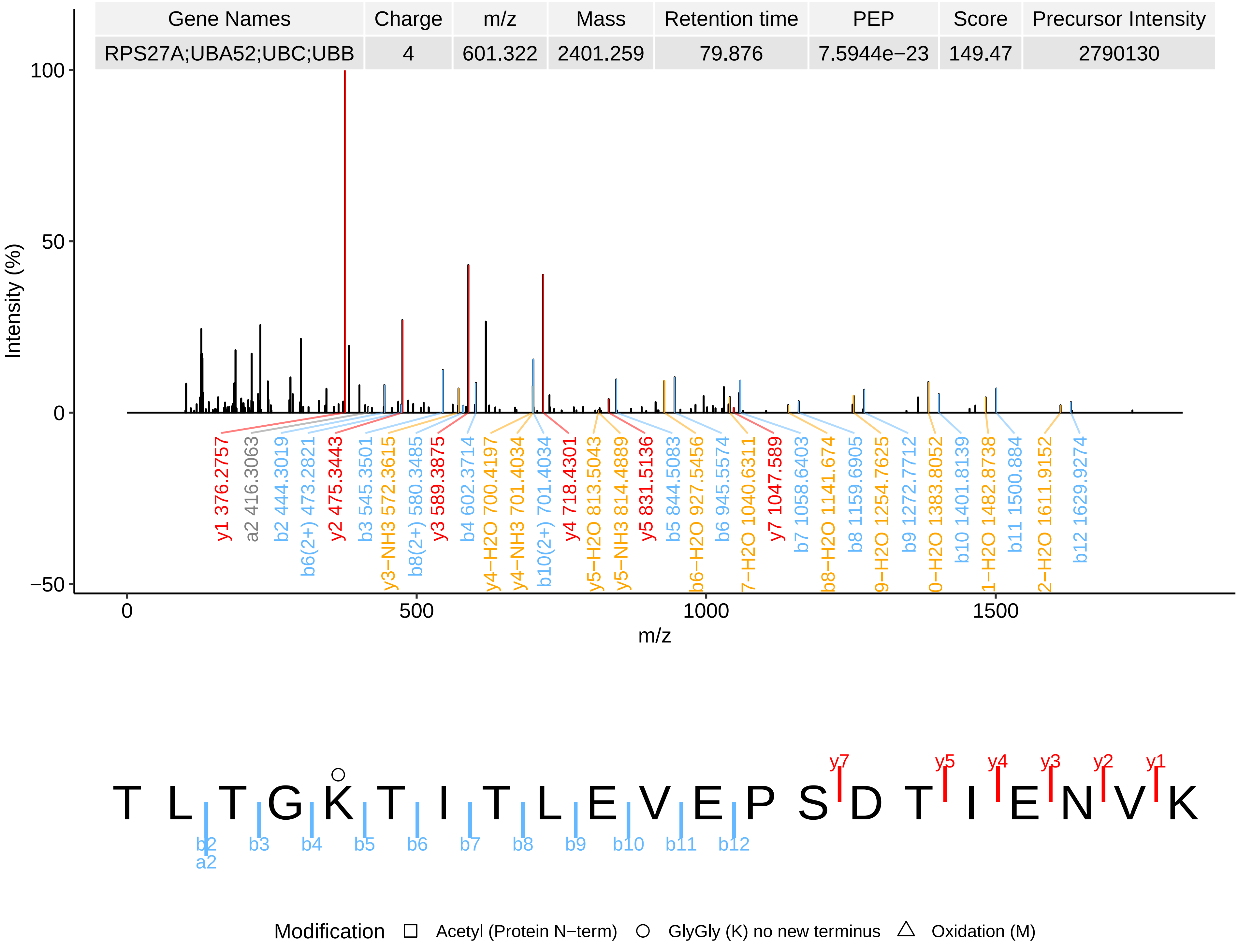

20200921_KIZ_MUb4_GlyGly_10plex_2 − Scan number: 20686

Gene Names	Charge	m/z	Mass	Retention time	PEP	Score	Precursor Intensity
RPS27A;UBA52;UBC;UBB	3	801.4269	2401.259	81.35	0.071593	41.521	896493.2

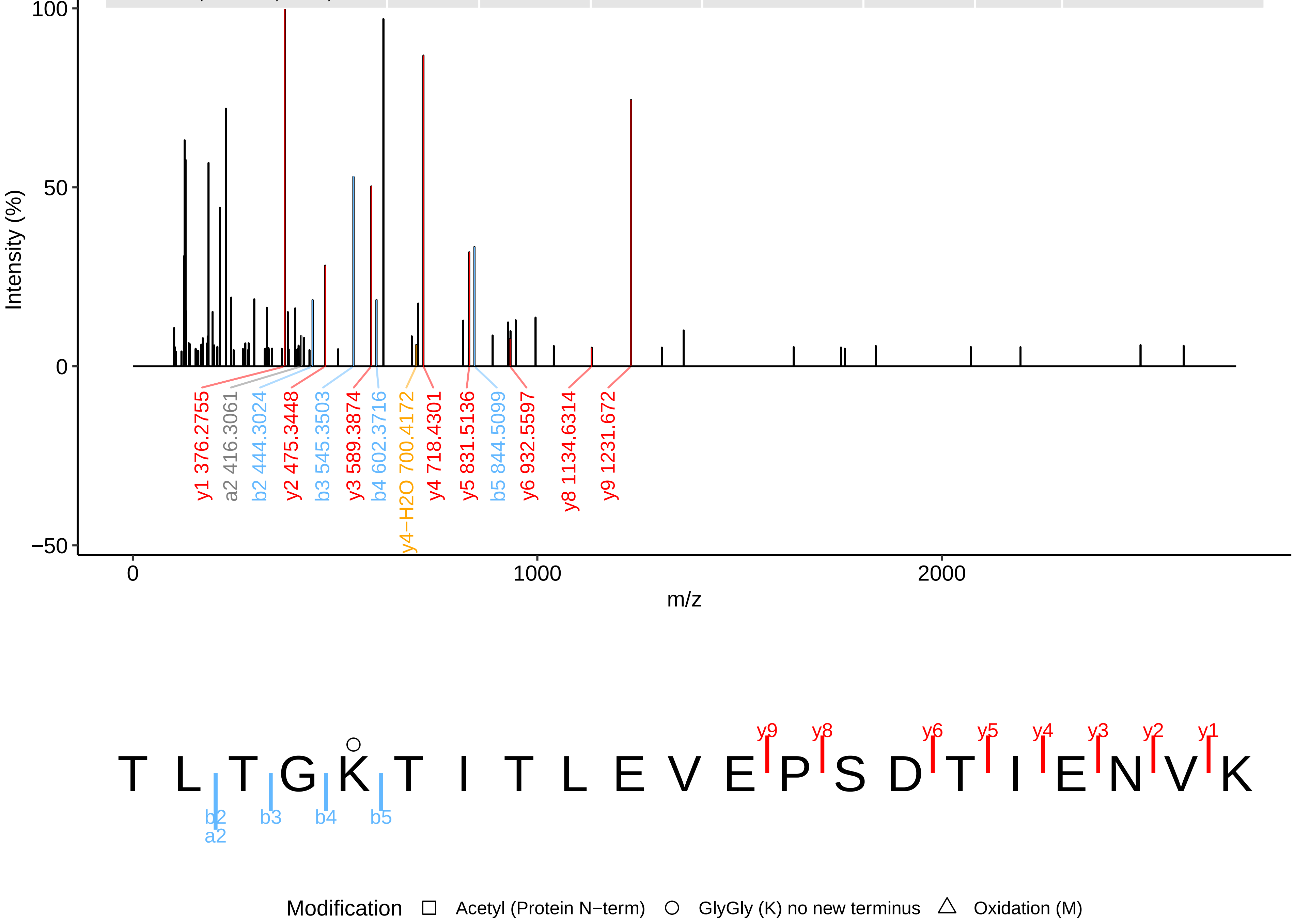

20200921_KIZ_MUb4_GlyGly_10plex_2 − Scan number: 20810

Gene Names	Charge	m/z	Mass	Retention time	PEP	Score	Precursor Intensity
RPS27A;UBA52;UBC;UBB	3	801.4269	2401.259	81.856	0.0017873	62.966	832801.8

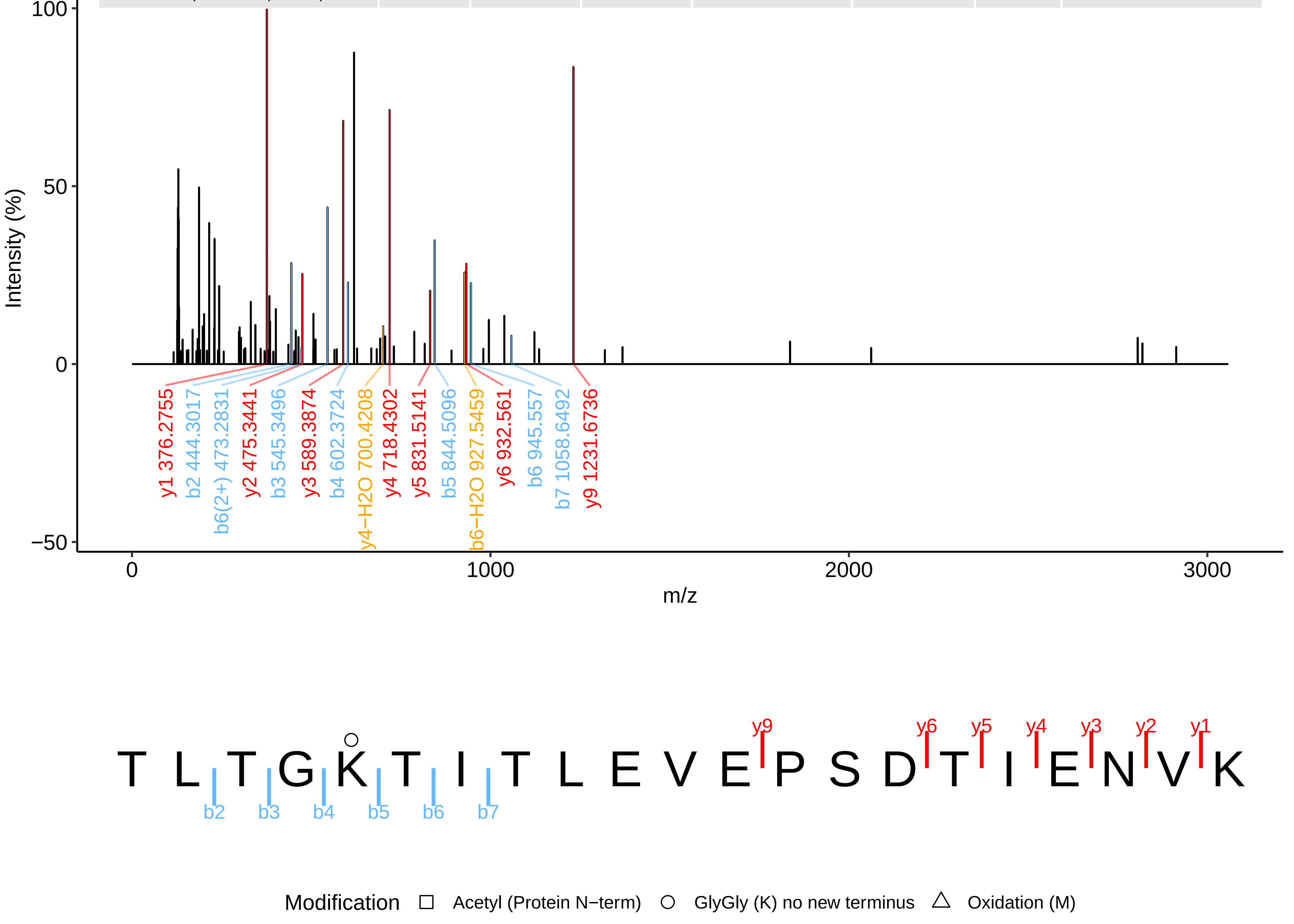

20200921_KIZ_MUb4_GlyGly_10plex_2 − Scan number: 21355

Gene Names	Charge	m/z	Mass	Retention time	PEP	Score	Precursor Intensity
RPS27A;UBA52;UBC;UBB	3	801.4269	2401.259	84.073	0.037939	48.824	556775.8

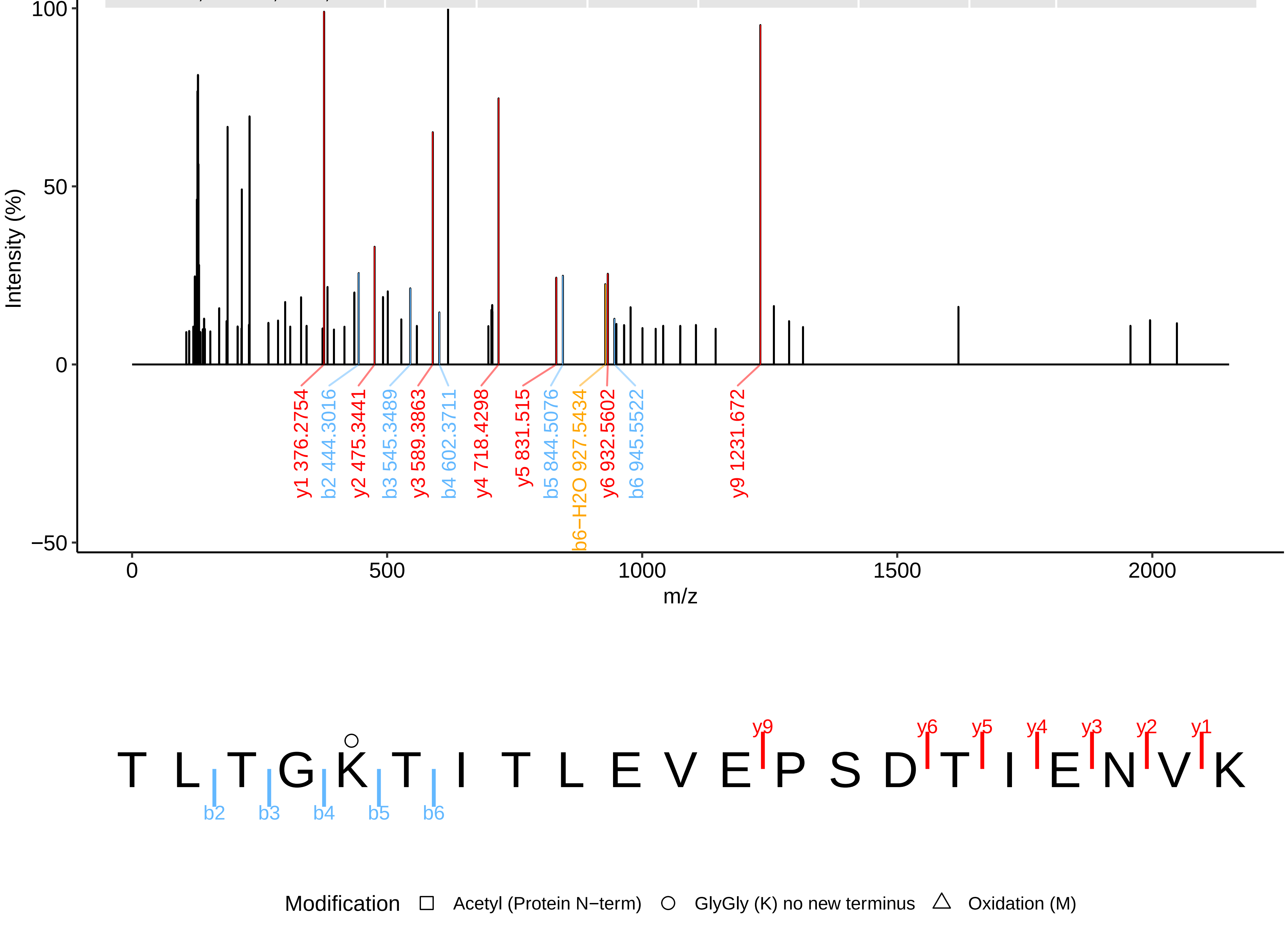

20200921_KIZ_MUb4_GlyGly_10plex_2 − Scan number: 21217

Gene Names	Charge	m/z	Mass	Retention time	PEP	Score	Precursor Intensity
RPS27A;UBA52;UBC;UBB	3	801.4269	2401.259	83.503	0.11102	42.309	NA

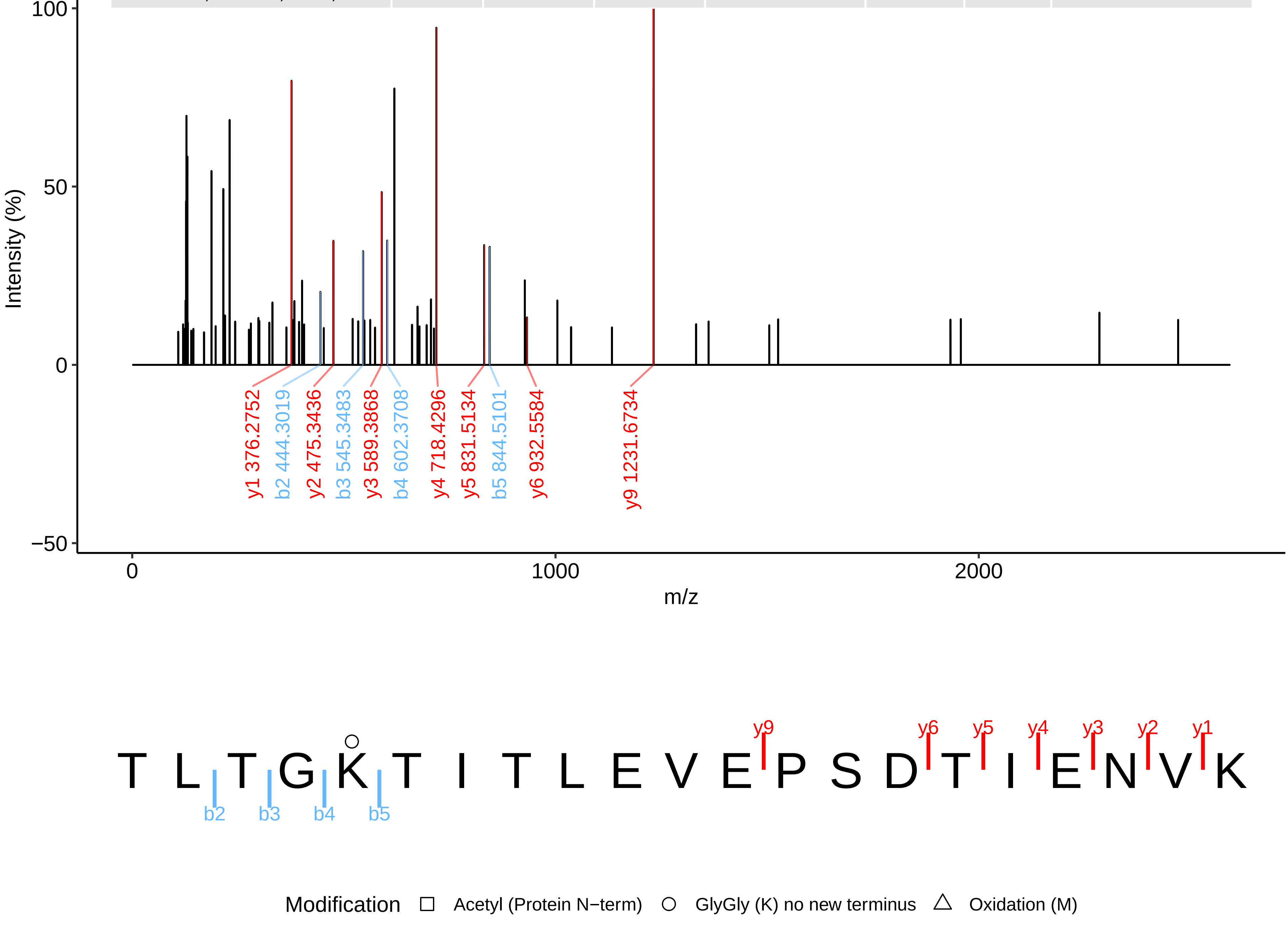

20200921_KIZ_MUb4_GlyGly_10plex_2 − Scan number: 21436

Gene Names	Charge	m/z	Mass	Retention time	PEP	Score	Precursor Intensity
RPS27A;UBA52;UBC;UBB	3	801.4269	2401.259	84.405	0.04674	49.973	NA

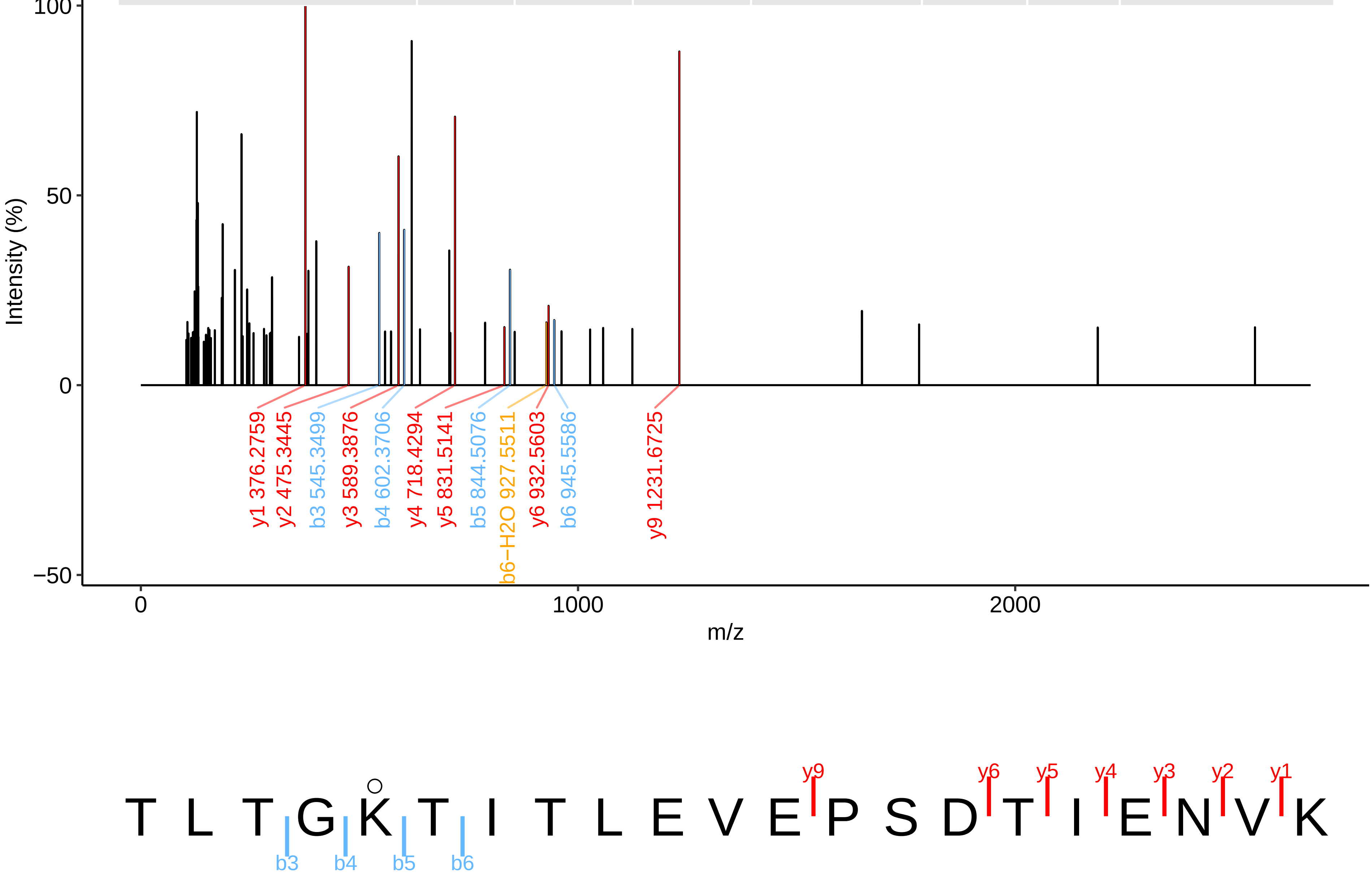

Modification □ Acetyl (Protein N−term) ○ GlyGly (K) no new terminus △ Oxidation (M)

20200924_KIZ_MUb5_GlyGly_1 − Scan number: 22341

Gene Names	Charge	m/z	Mass	Retention time	PEP	Score	Precursor Intensity
RPS27A;UBA52;UBC;UBB	4	601.322	2401.259	71.893	1.0246e−21	139.9	34431724

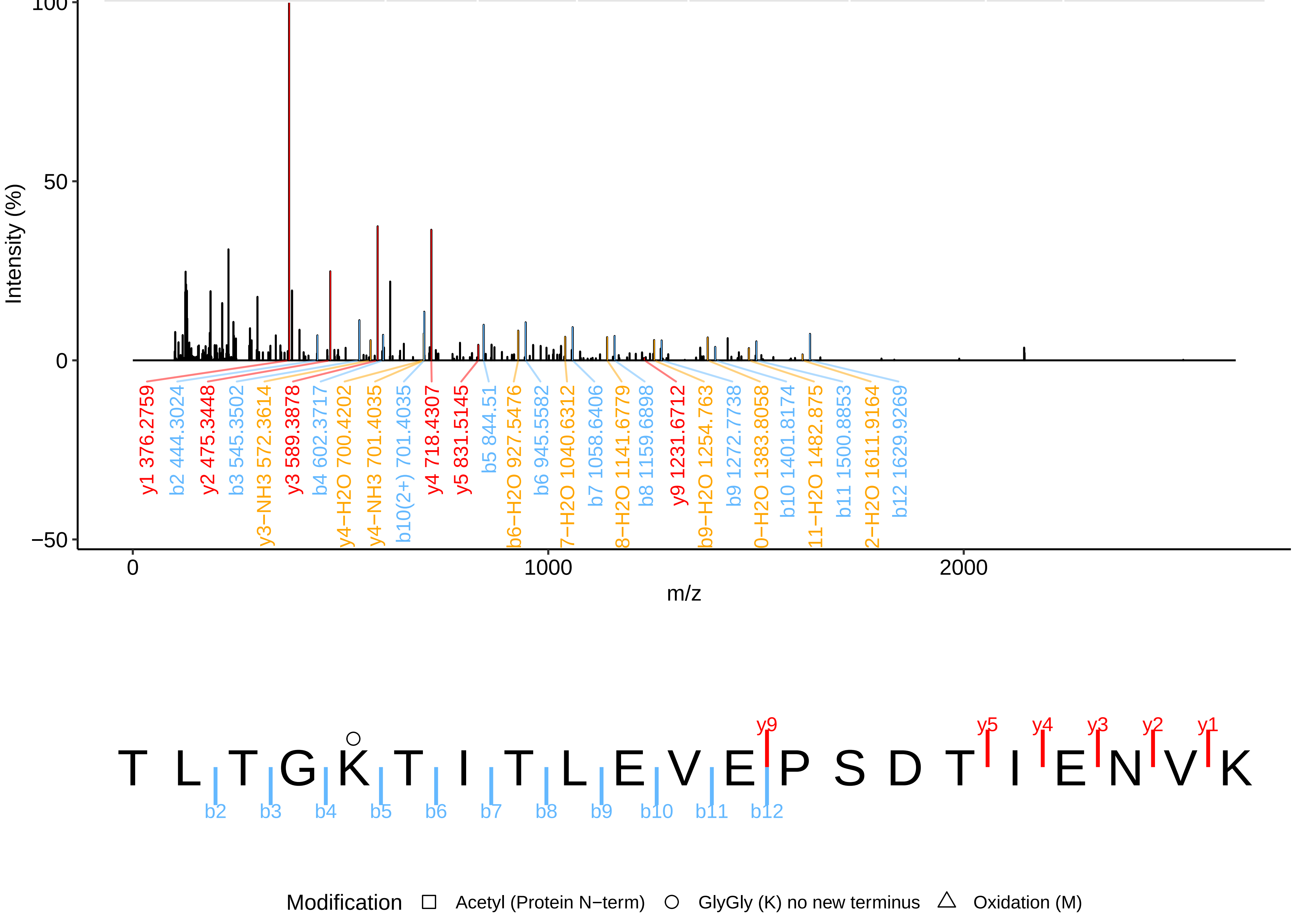

20200924_KIZ_MUb5_GlyGly_1 − Scan number: 22444

Gene Names	Charge	m/z	Mass	Retention time	PEP	Score	Precursor Intensity
RPS27A;UBA52;UBC;UBB	3	801.4269	2401.259	72.23	5.5094e−49	197.86	216474384

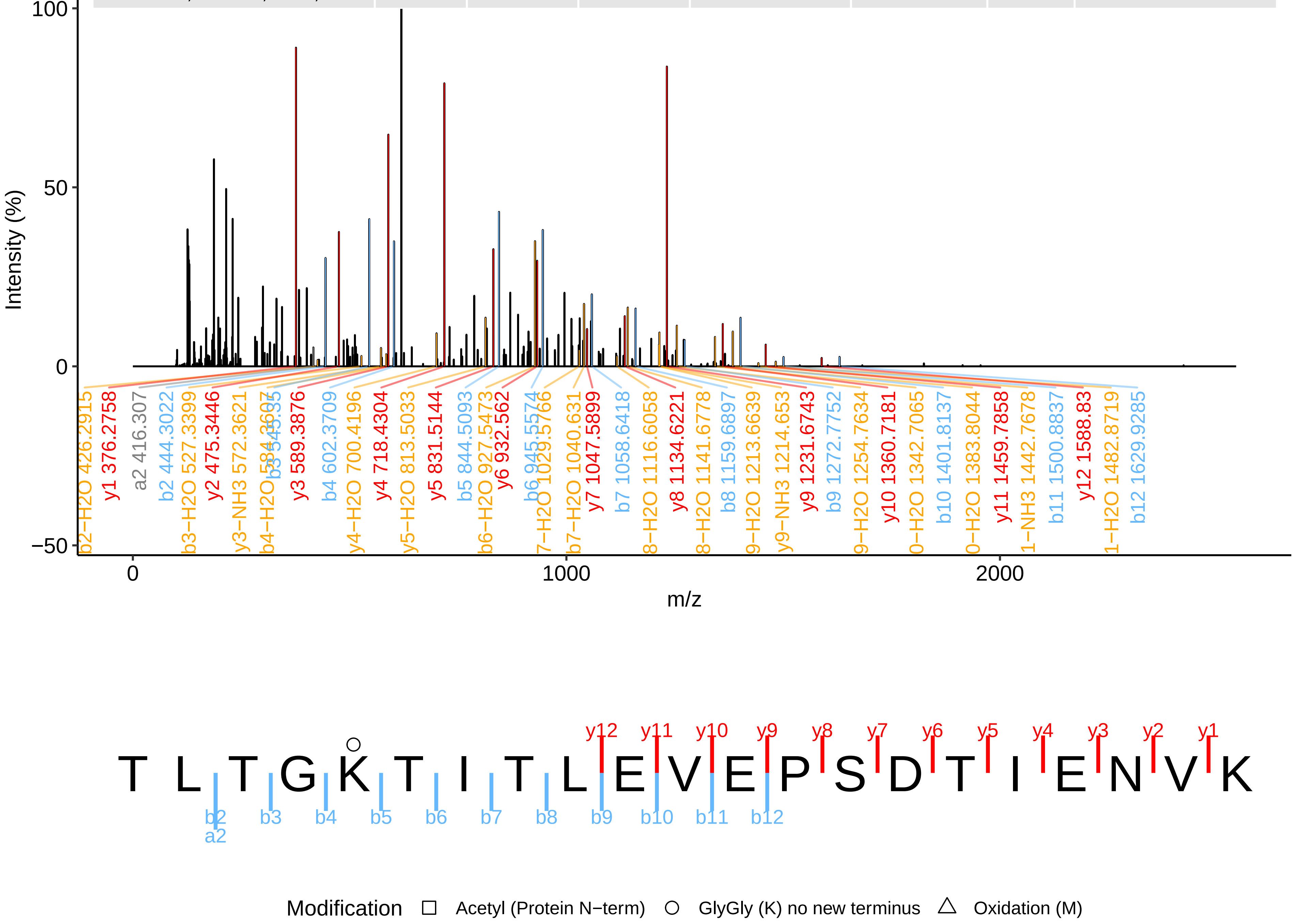

20200924_KIZ_MUb5_GlyGly_1 − Scan number: 22611

Gene Names	Charge	m/z	Mass	Retention time	PEP	Score	Precursor Intensity
RPS27A;UBA52;UBC;UBB	3	801.4269	2401.259	72.738	1.1314e−40	183.65	653567168

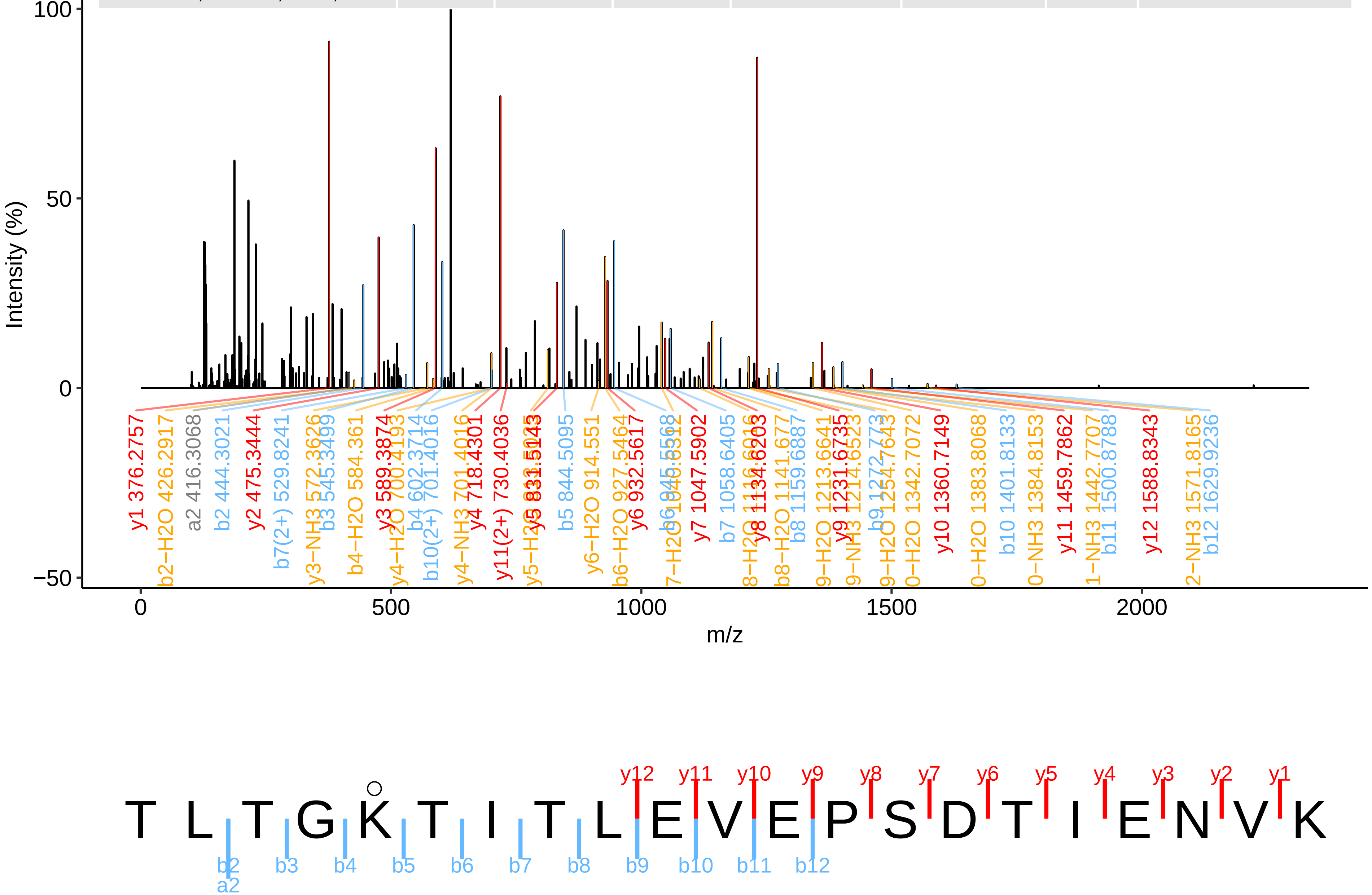

Modification □ Acetyl (Protein N−term) ○ GlyGly (K) no new terminus △ Oxidation (M)

20200924_KIZ_MUb5_GlyGly_1 − Scan number: 22774

Gene Names	Charge	m/z	Mass	Retention time	PEP	Score	Precursor Intensity
RPS27A;UBA52;UBC;UBB	3	801.4269	2401.259	73.246	5.5094e−49	197.86	186398144

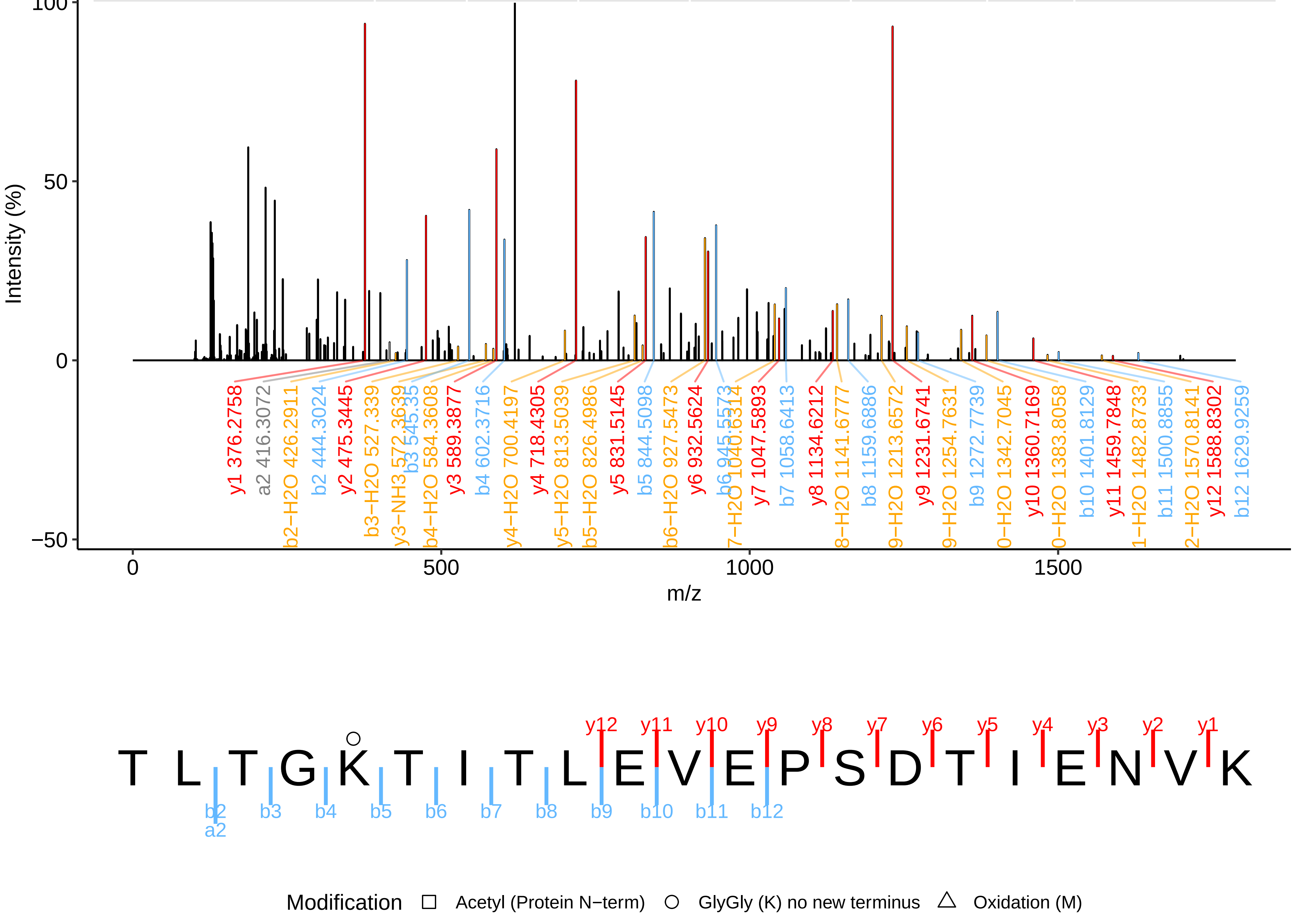

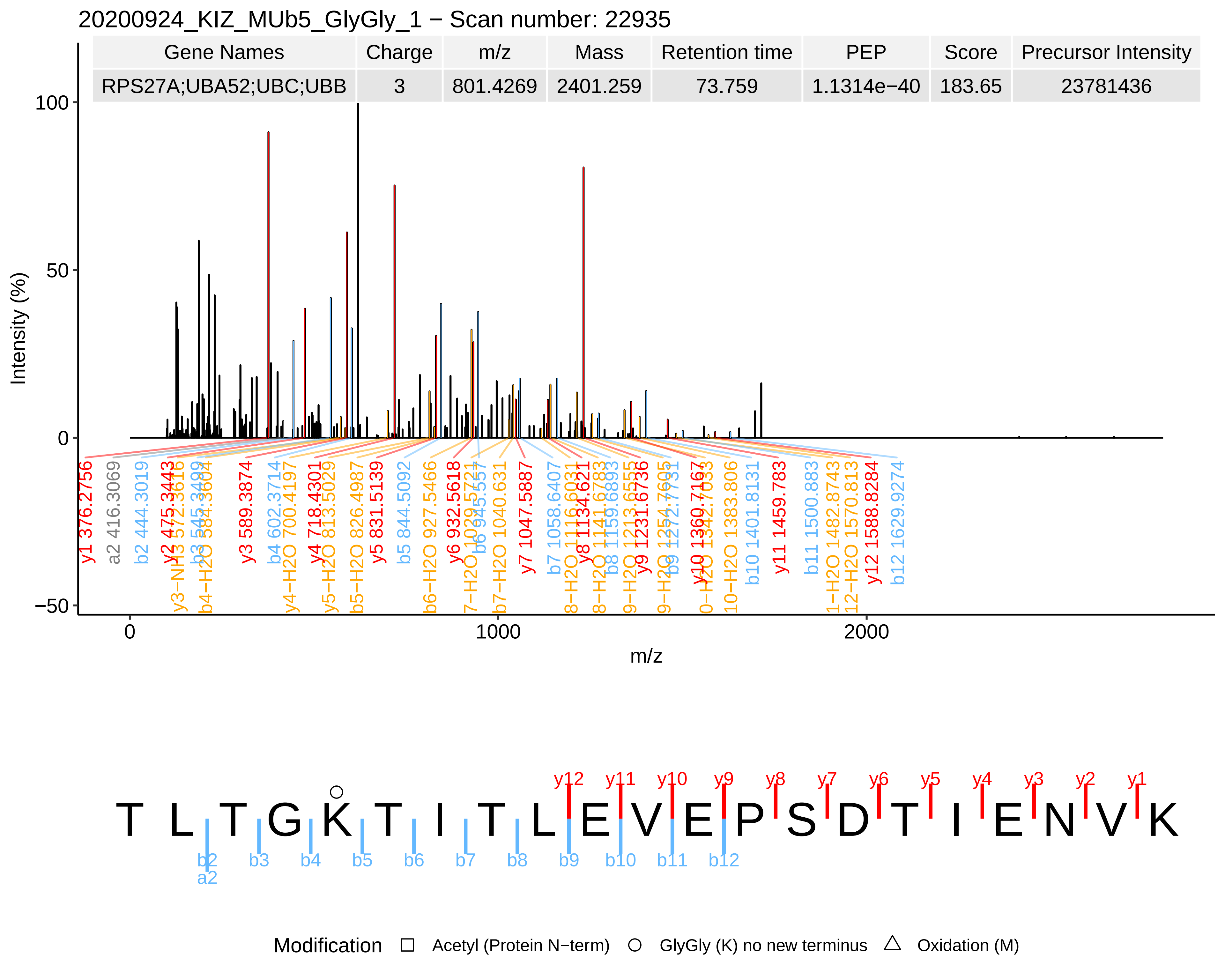
20200924_KIZ_MUb5_GlyGly_1 − Scan number: 22935
Gene Names
Charge
m/z
Mass
Retention time
PEP
Score
Precursor Intensity
RPS27A;UBA52;UBC;UBB
3
801.4269
2401.259
73.759
1.1314e−40
183.65
23781436
Intensity (%)
m/z
T L T G K T I T L E V E P S D T I E N V K
Modification
Acetyl (Protein N−term)
GlyGly (K) no new terminus
Oxidation (M)

20200924_KIZ_MUb5_GlyGly_1 − Scan number: 22516

Gene Names	Charge	m/z	Mass	Retention time	PEP	Score	Precursor Intensity
RPS27A;UBA52;UBC;UBB	4	601.322	2401.259	72.449	9.099e−40	171.21	212864016

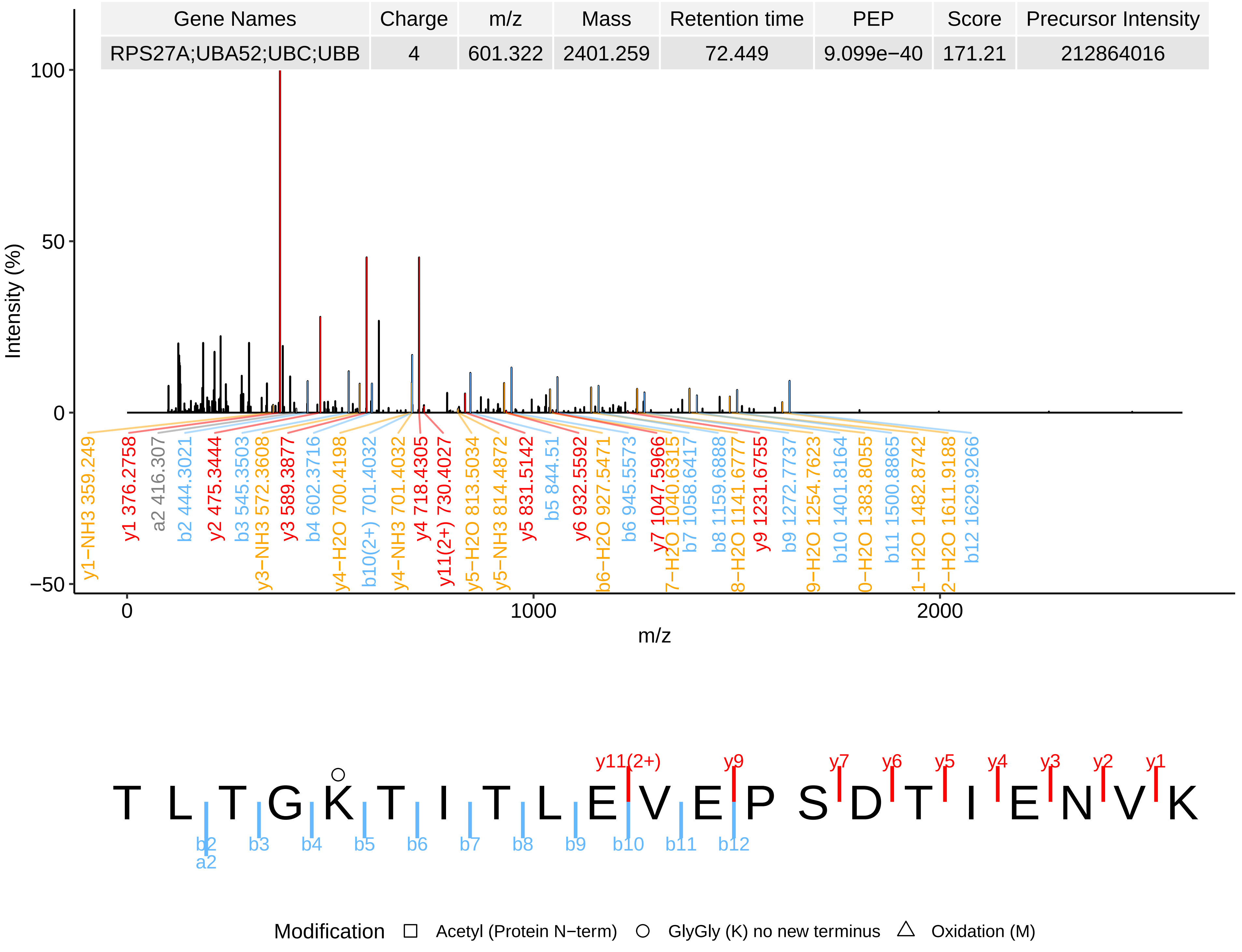

20200924_KIZ_MUb5_GlyGly_1 − Scan number: 22683

Gene Names	Charge	m/z	Mass	Retention time	PEP	Score	Precursor Intensity
RPS27A;UBA52;UBC;UBB	4	601.322	2401.259	72.959	1.8761e−30	156.73	255199408

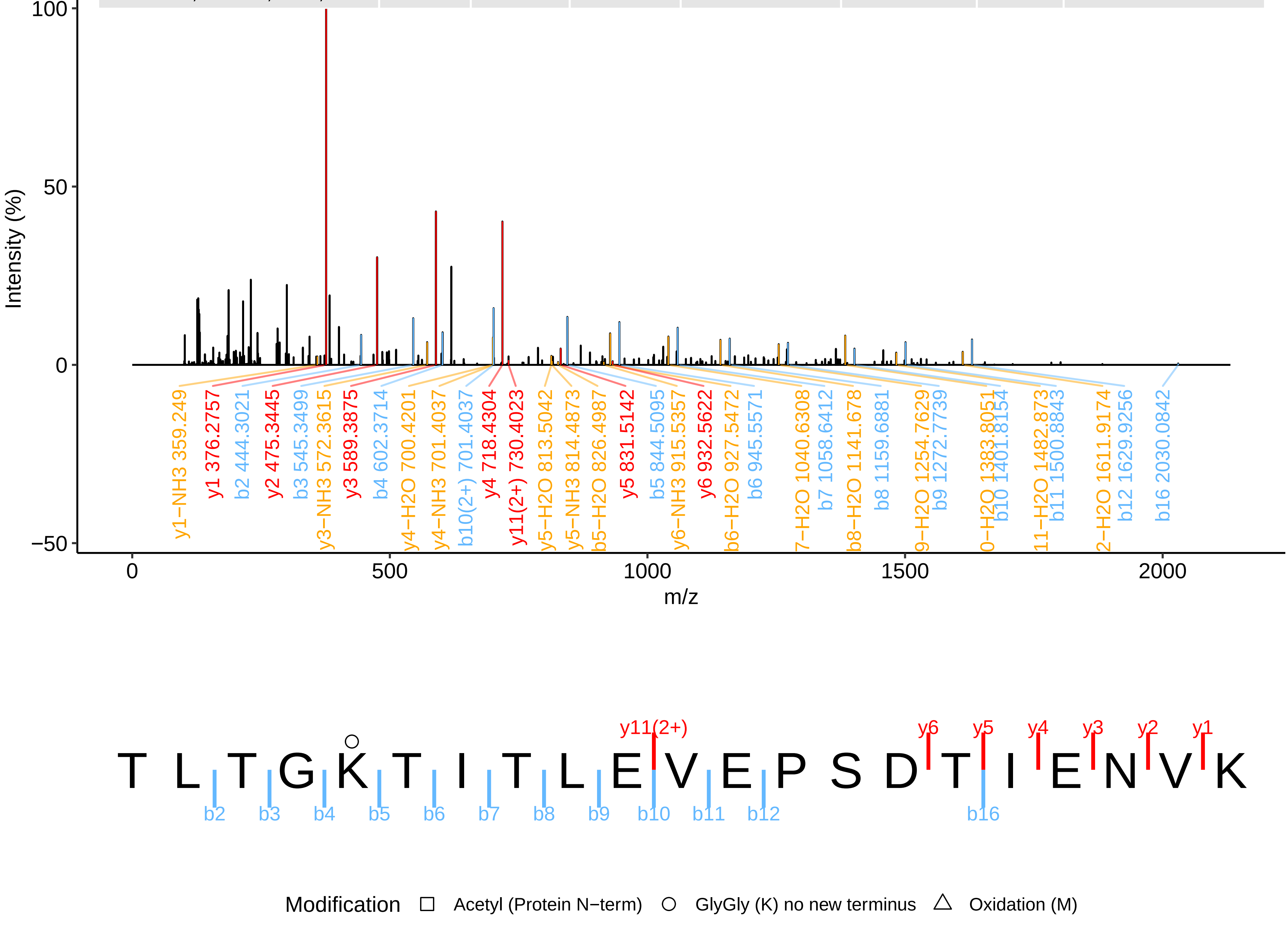

20200924_KIZ_MUb5_GlyGly_1 − Scan number: 22844

Gene Names	Charge	m/z	Mass	Retention time	PEP	Score	Precursor Intensity
RPS27A;UBA52;UBC;UBB	4	601.322	2401.259	73.465	8.9273e−23	149.34	39390376

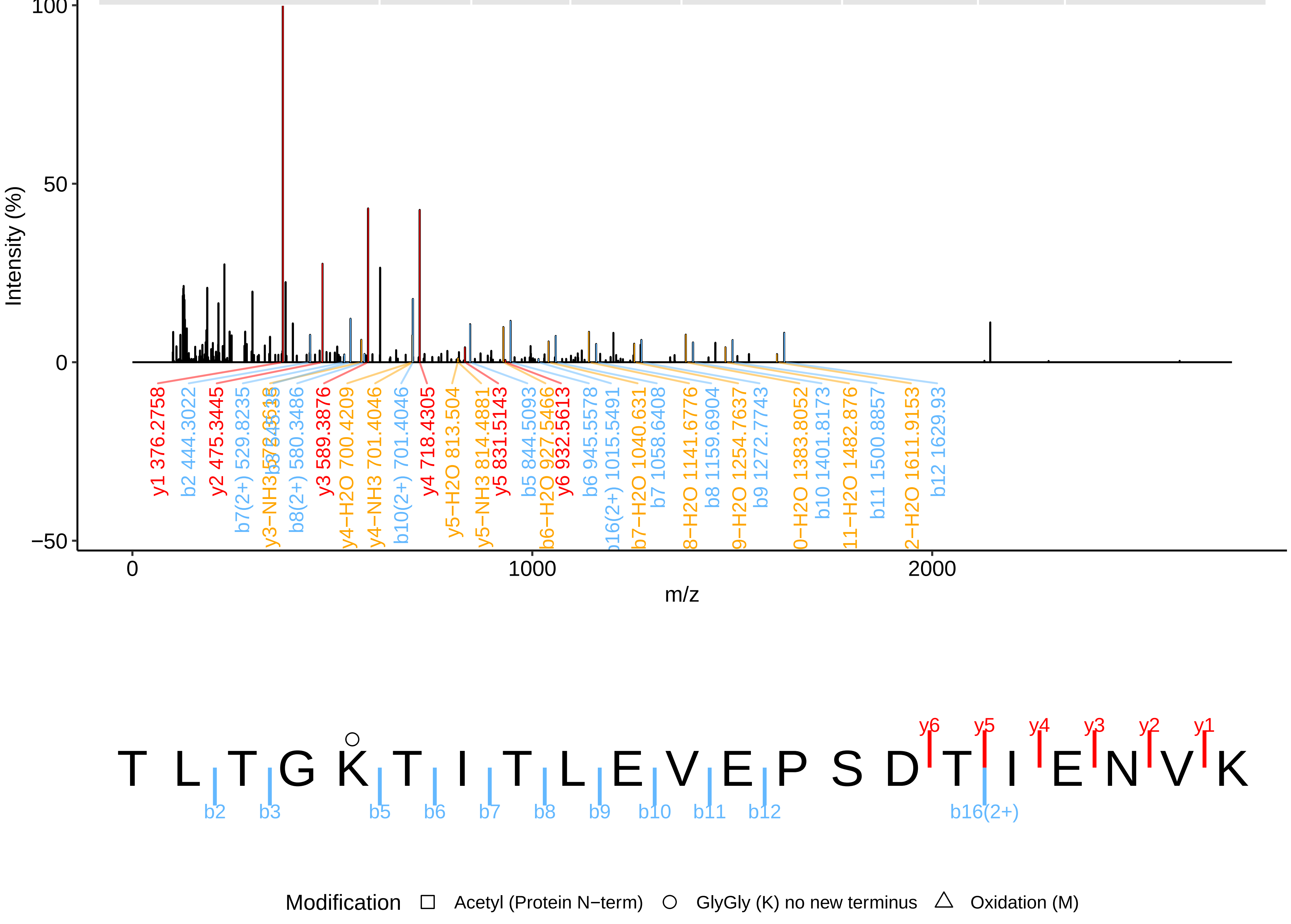

20200924_KIZ_MUb5_GlyGly_2 − Scan number: 19985

Gene Names	Charge	m/z	Mass	Retention time	PEP	Score	Precursor Intensity
RPS27A;UBA52;UBC;UBB	3	801.4269	2401.259	74.074	9.1766e−40	173.56	11899854

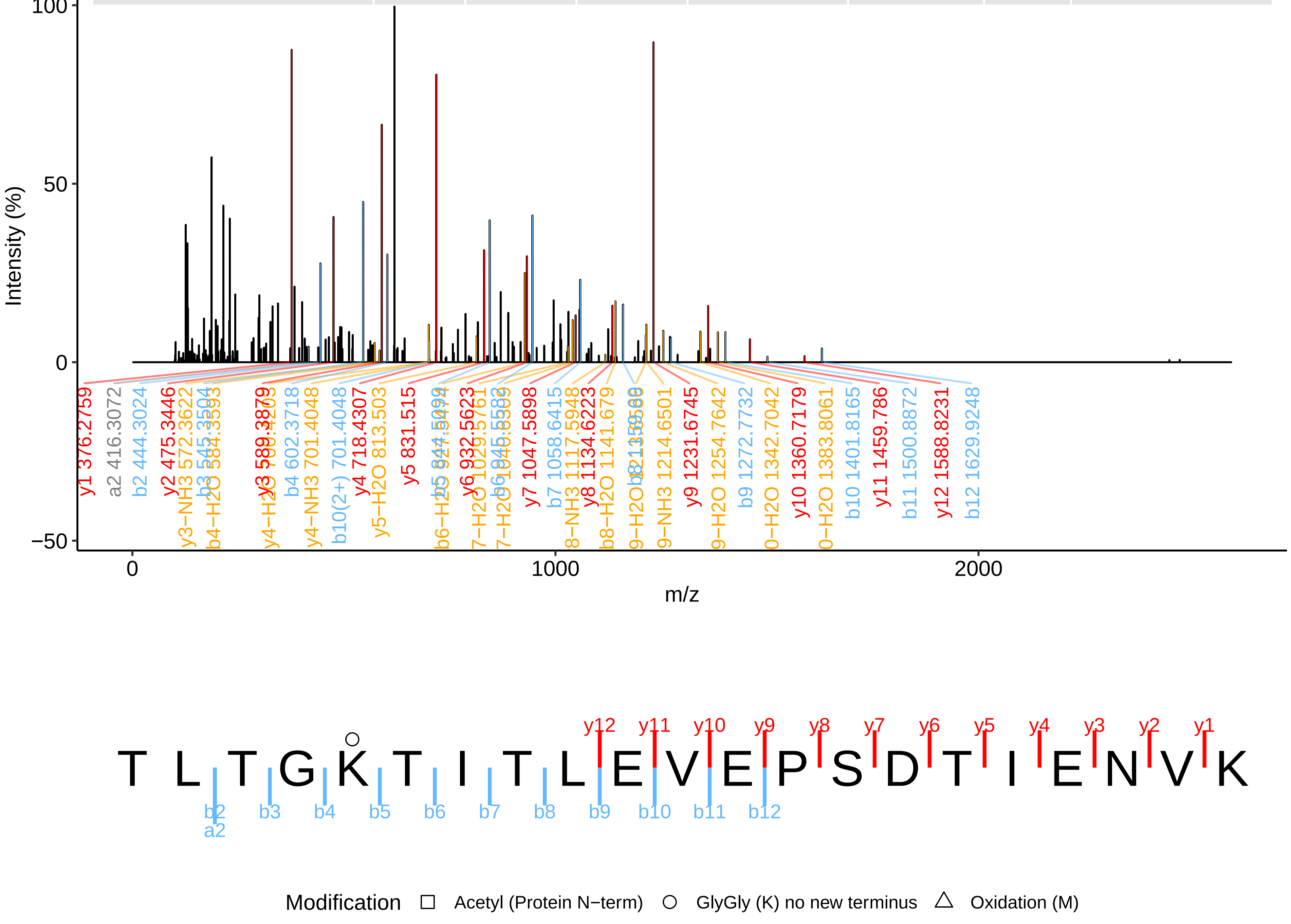

20200924_KIZ_MUb5_GlyGly_2 − Scan number: 20122

Gene Names	Charge	m/z	Mass	Retention time	PEP	Score	Precursor Intensity
RPS27A;UBA52;UBC;UBB	3	801.4269	2401.259	74.577	2.4949e−49	200.39	125380312

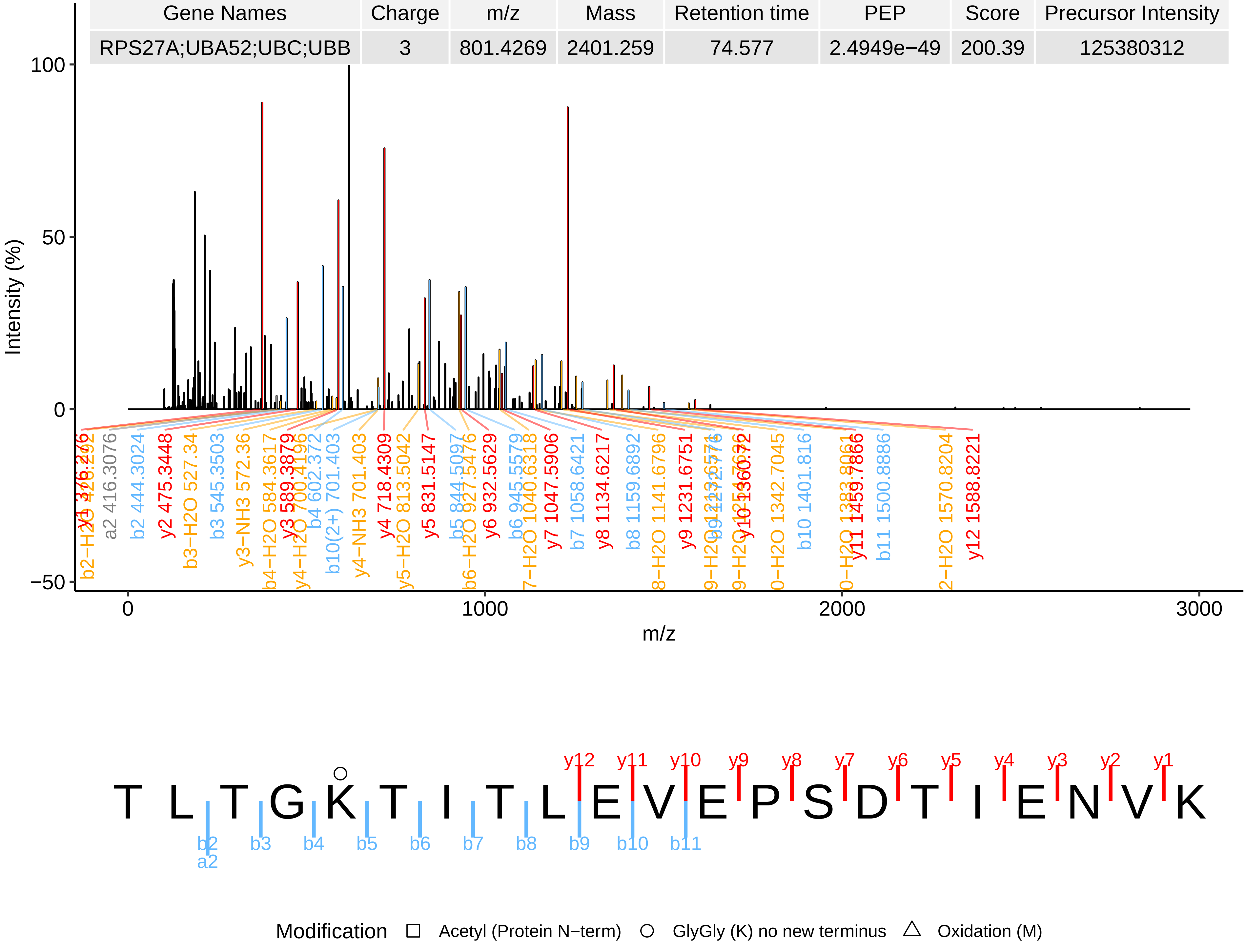

20200924_KIZ_MUb5_GlyGly_2 − Scan number: 20261

Gene Names	Charge	m/z	Mass	Retention time	PEP	Score	Precursor Intensity
RPS27A;UBA52;UBC;UBB	3	801.4269	2401.259	75.084	5.1713e−33	167.63	169469376

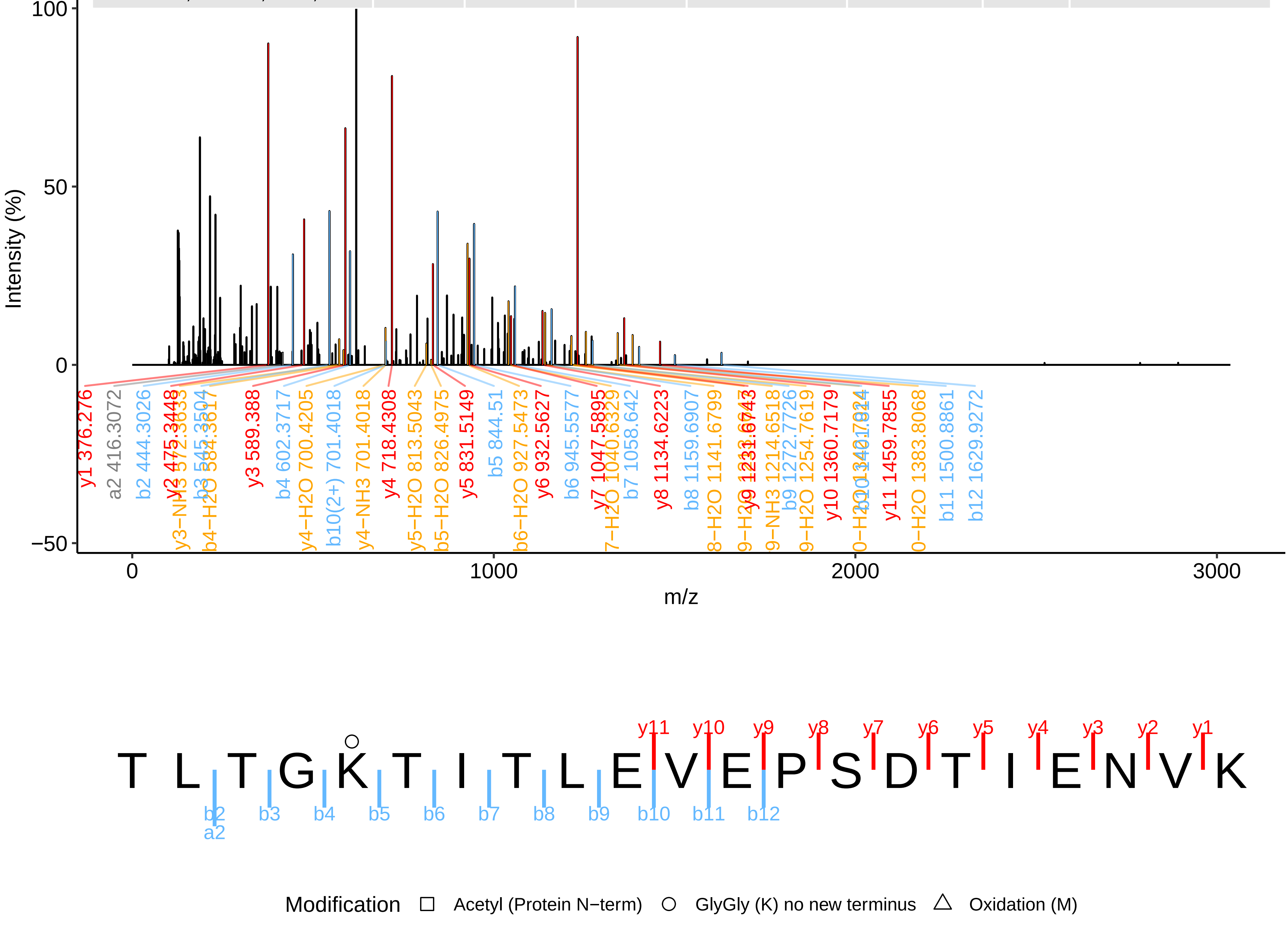

20200924_KIZ_MUb5_GlyGly_2 − Scan number: 20405

Gene Names	Charge	m/z	Mass	Retention time	PEP	Score	Precursor Intensity
RPS27A;UBA52;UBC;UBB	3	801.4269	2401.259	75.607	9.1766e−40	173.56	17823538

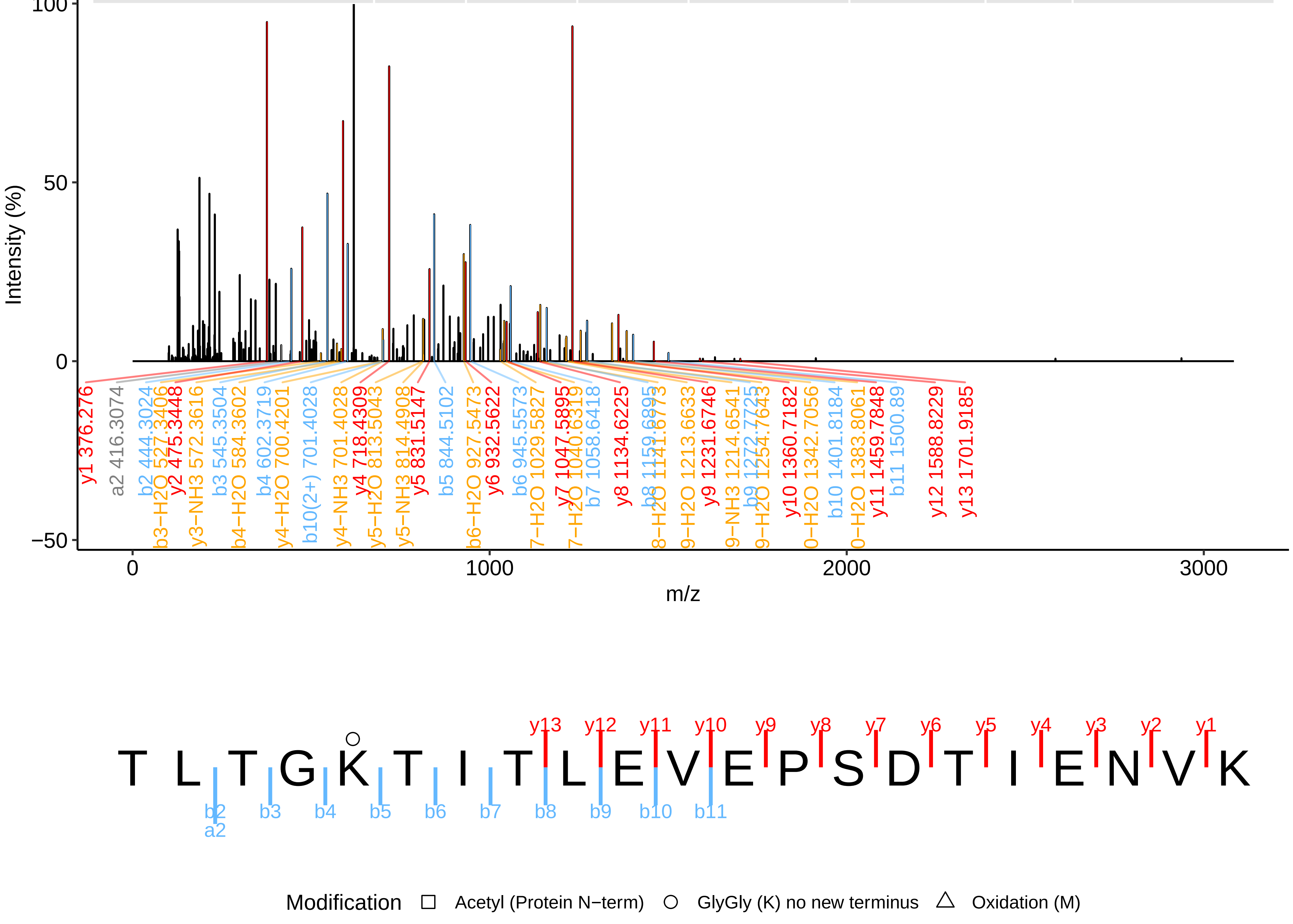

20200924_KIZ_MUb5_GlyGly_2 − Scan number: 20291

Gene Names	Charge	m/z	Mass	Retention time	PEP	Score	Precursor Intensity
RPS27A;UBA52;UBC;UBB	4	601.322	2401.259	75.187	1.0299e−30	161.65	36173636

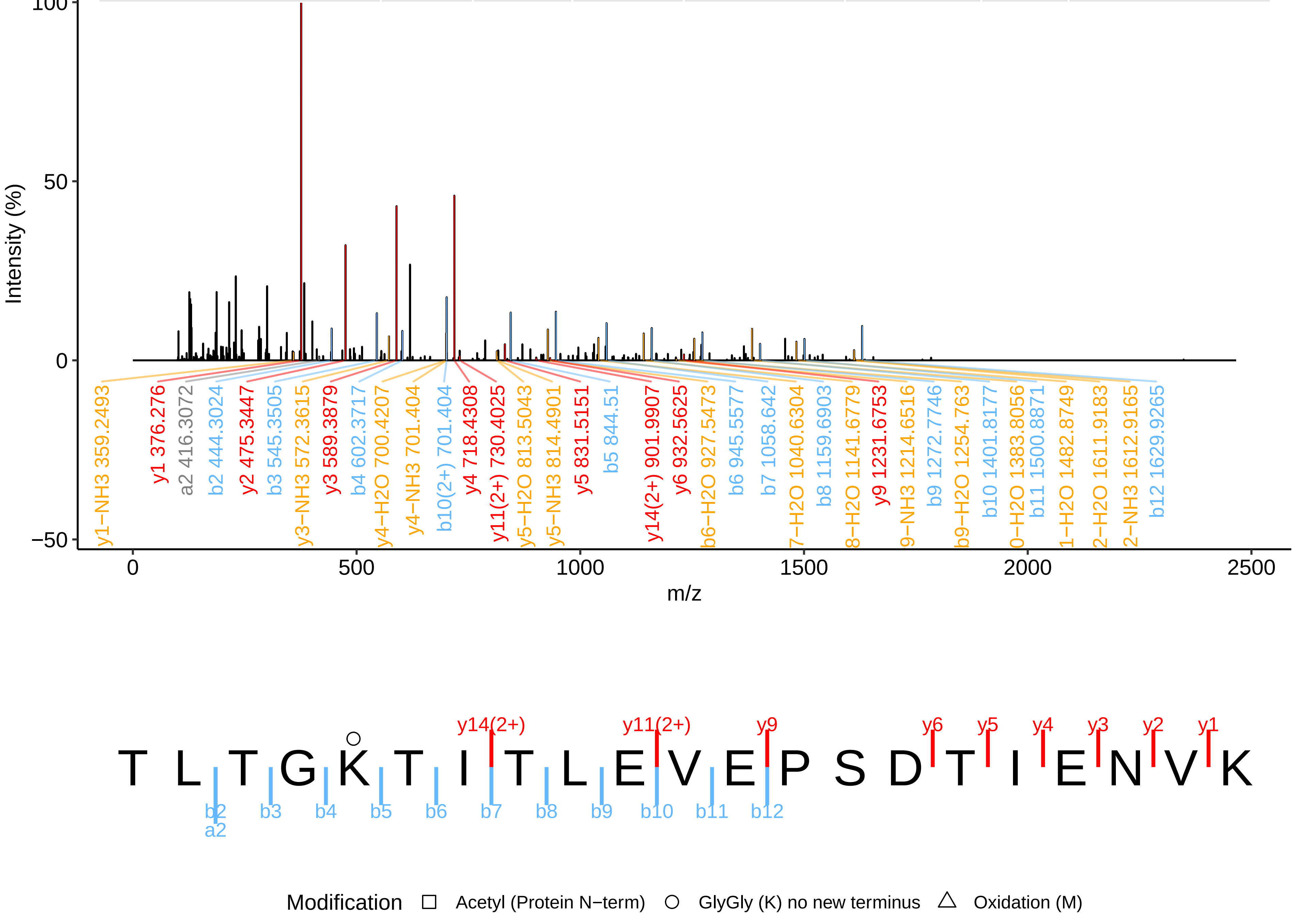

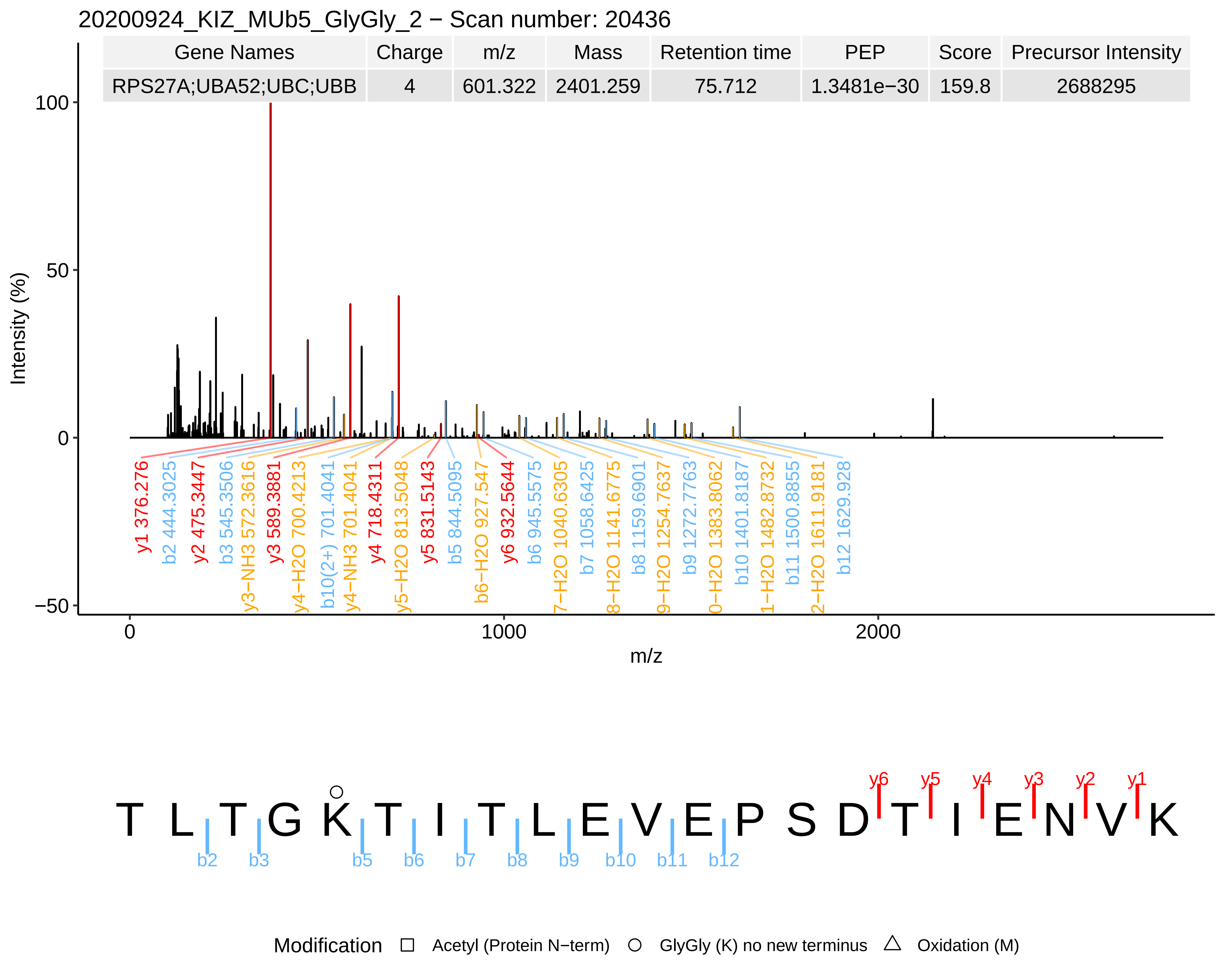
20200924_KIZ_MUb5_GlyGly_2 − Scan number: 20436
Gene Names | Charge | m/z | Mass | Retention time | PEP | Score | Precursor Intensity
RPS27A;UBA52;UBC;UBB | 4 | 601.322 | 2401.259 | 75.712 | 1.3481e−30 | 159.8 | 2688295
Intensity (%)
100
50
0
−50
0
1000
2000
m/z
y1 376.276
b2 444.3025
y2 475.3447
b3 545.3506
y3−NH3 572.3616
y3 589.3881
y4−H2O 700.4213
b10(2+) 701.4041
y4−NH3 701.4041
y4 718.4311
y5−H2O 813.5048
y5 831.5143
b5 844.5095
b6−H2O 927.547
y6 932.5644
b6 945.5575
7−H2O 1040.6305
b7 1058.6425
8−H2O 1141.6775
b8 1159.6901
9−H2O 1254.7637
b9 1272.7763
0−H2O 1383.8062
b10 1401.8187
1−H2O 1482.8732
b11 1500.8855
2−H2O 1611.9181
b12 1629.928
T L T G K T I T L E V E P S D T I E N V K
y6 y5 y4 y3 y2 y1
b2 b3 b5 b6 b7 b8 b9 b10 b11 b12
Modification
Acetyl (Protein N−term)
GlyGly (K) no new terminus
Oxidation (M)

20201118_KIZ_MUb6_GlyGly_1 − Scan number: 23226

Gene Names	Charge	m/z	Mass	Retention time	PEP	Score	Precursor Intensity
RPS27A;UBA52;UBC;UBB	3	801.4269	2401.259	71.937	3.1439e−71	221.85	34295480

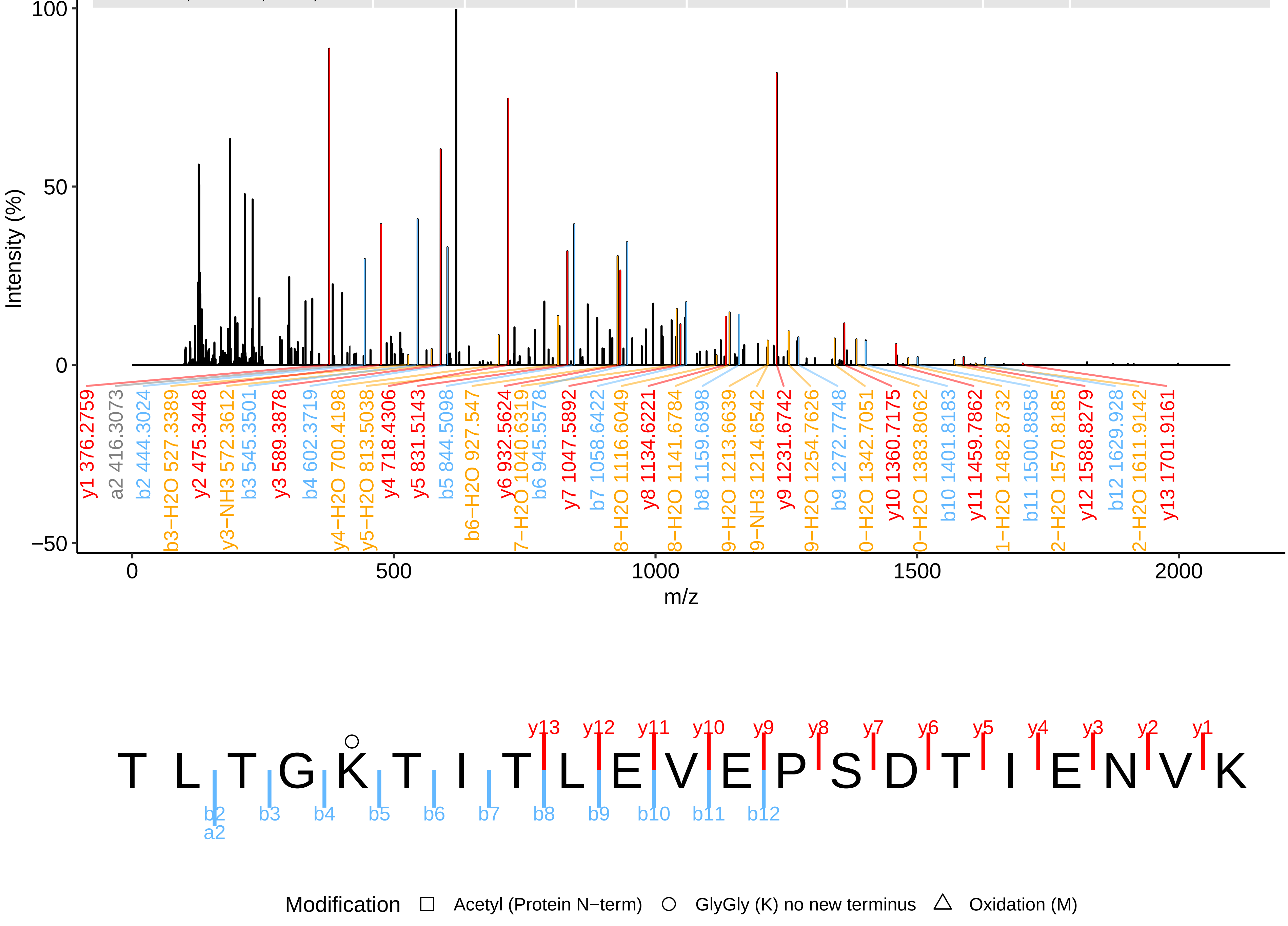

20201118_KIZ_MUb6_GlyGly_1 − Scan number: 23238

Gene Names	Charge	m/z	Mass	Retention time	PEP	Score	Precursor Intensity
RPS27A;UBA52;UBC;UBB	4	601.322	2401.259	71.968	3.1483e−22	147.06	42832368

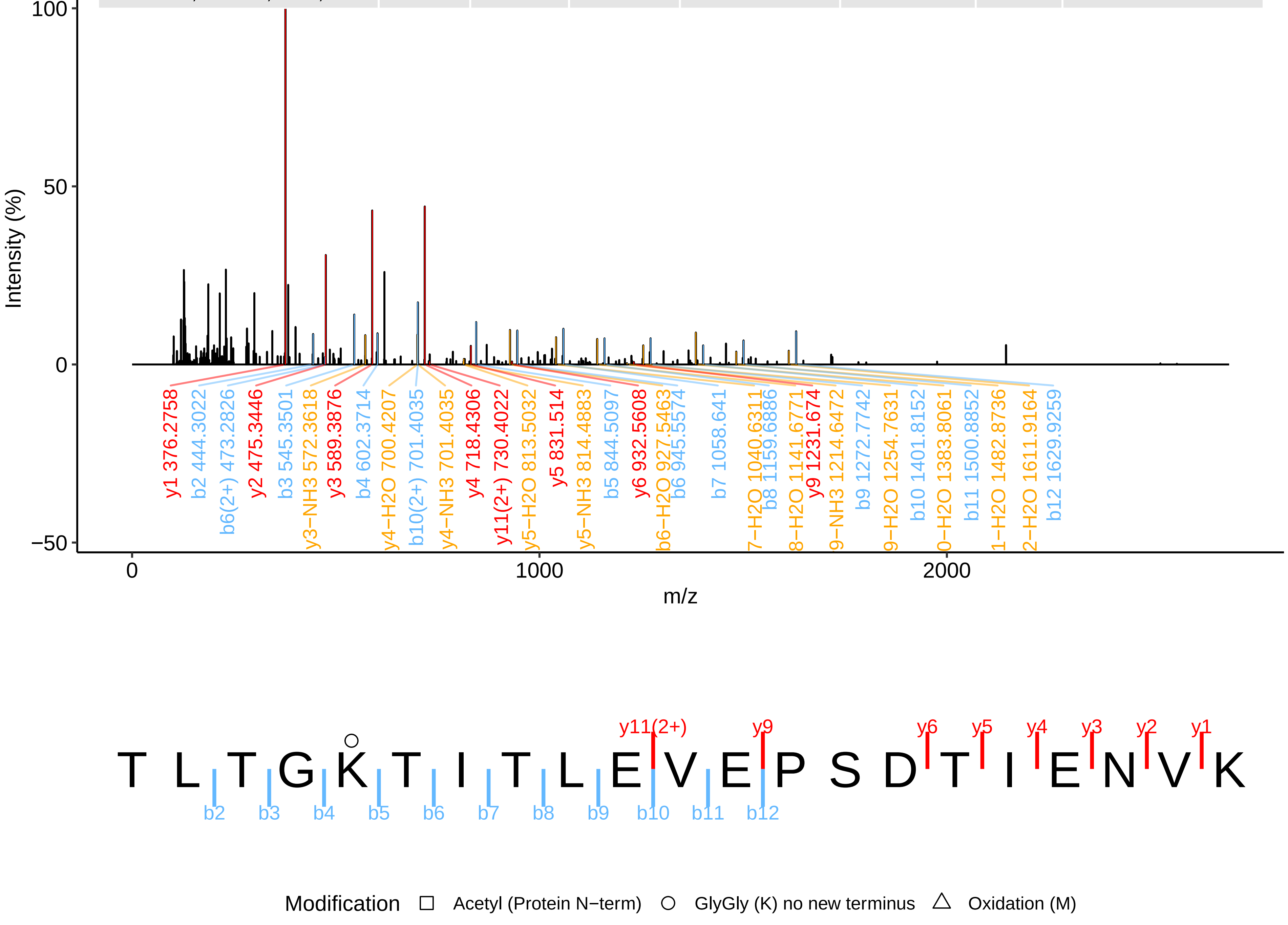

20201118_KIZ_MUb6_GlyGly_2 − Scan number: 24346

Gene Names	Charge	m/z	Mass	Retention time	PEP	Score	Precursor Intensity
RPS27A;UBA52;UBC;UBB	3	801.4269	2401.259	81.538	0.00041531	74.953	7116111

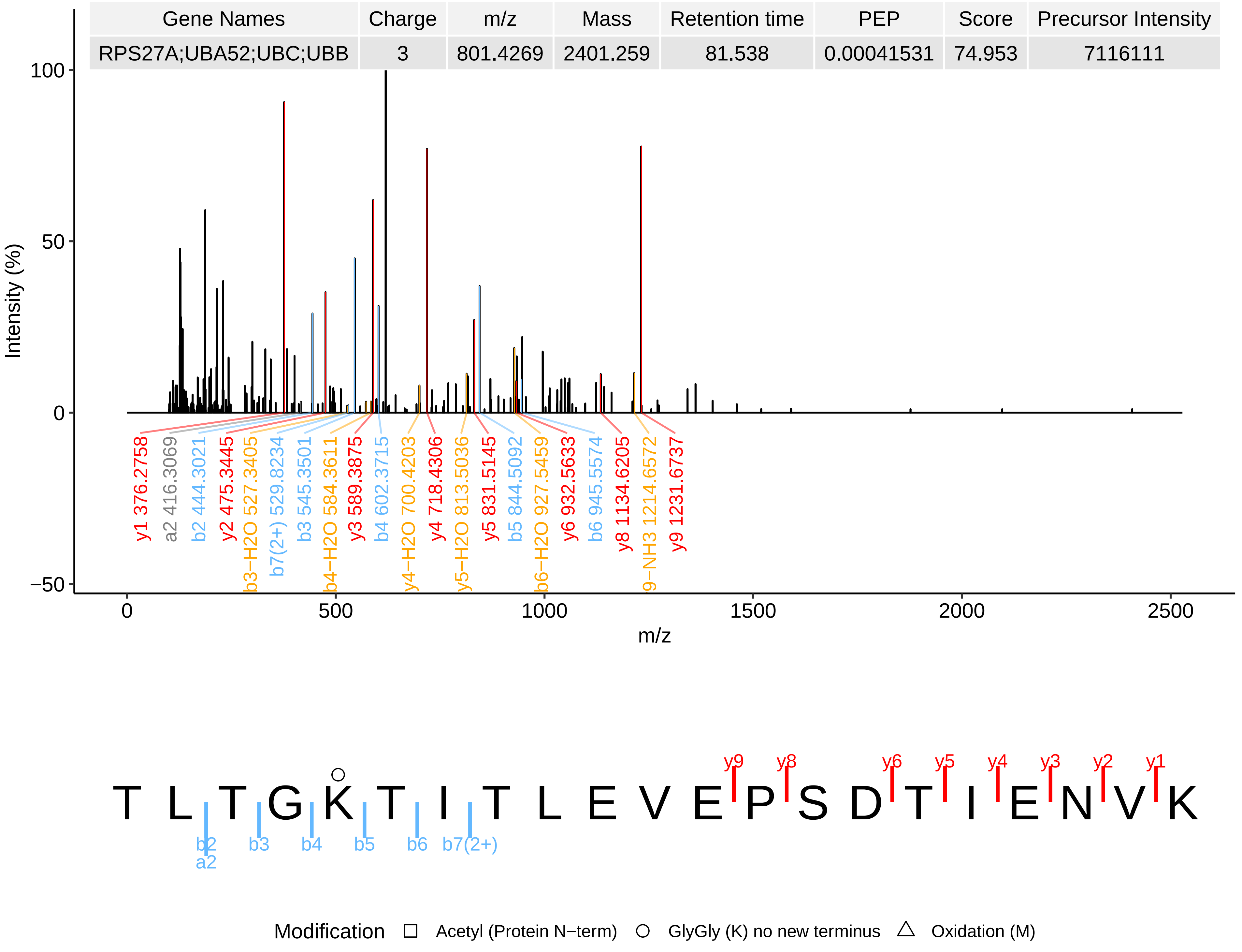

20201118_KIZ_MUb6_GlyGly_2 − Scan number: 24349

Gene Names	Charge	m/z	Mass	Retention time	PEP	Score	Precursor Intensity
RPS27A;UBA52;UBC;UBB	3	801.4269	2401.259	81.549	1.0667e−48	193.54	28231542

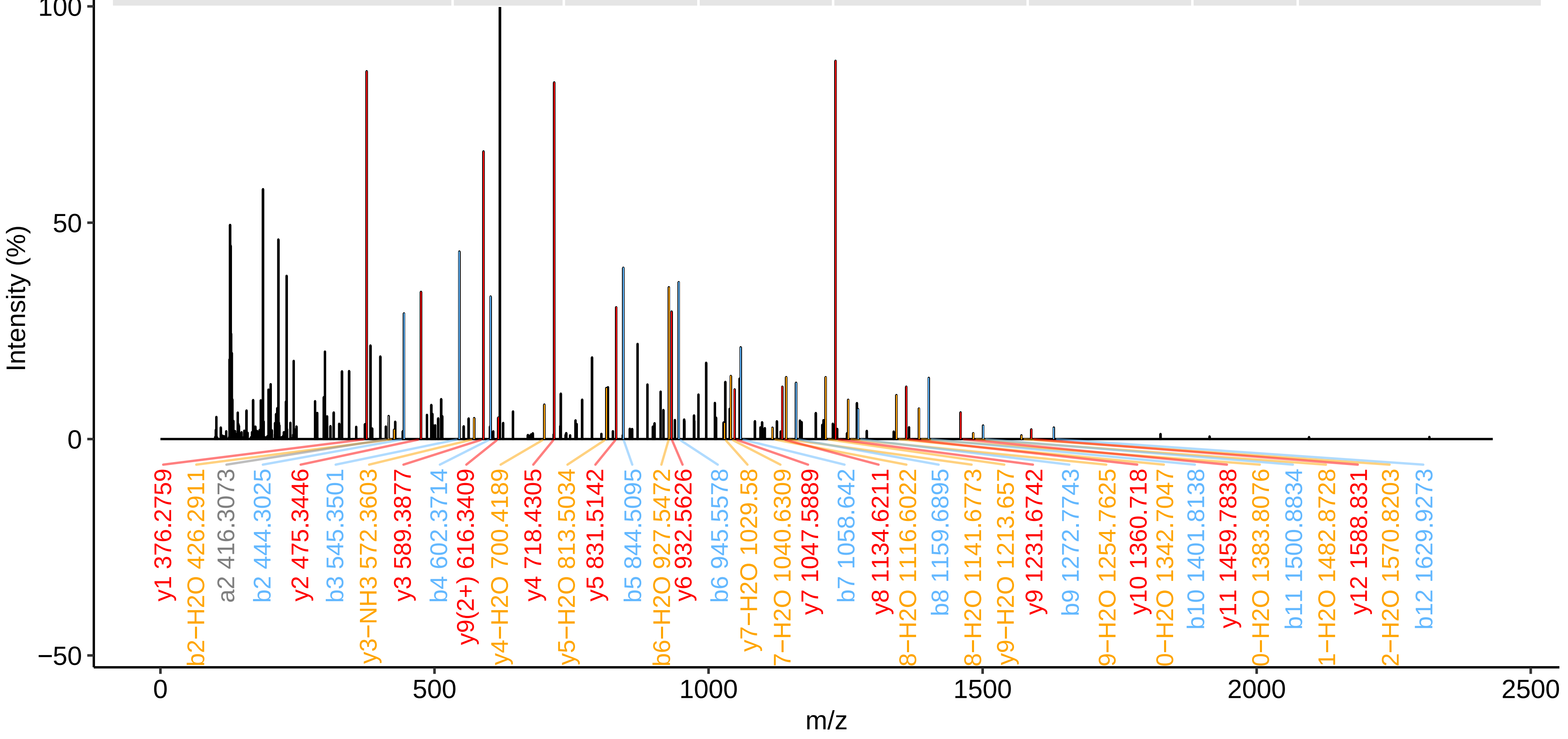

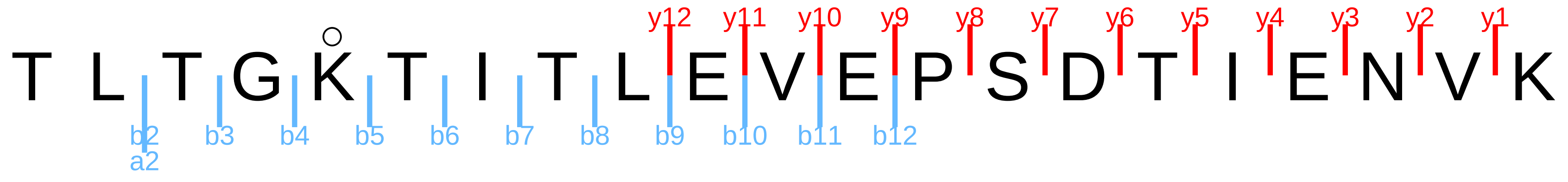

Modification □ Acetyl (Protein N−term) ○ GlyGly (K) no new terminus △ Oxidation (M)

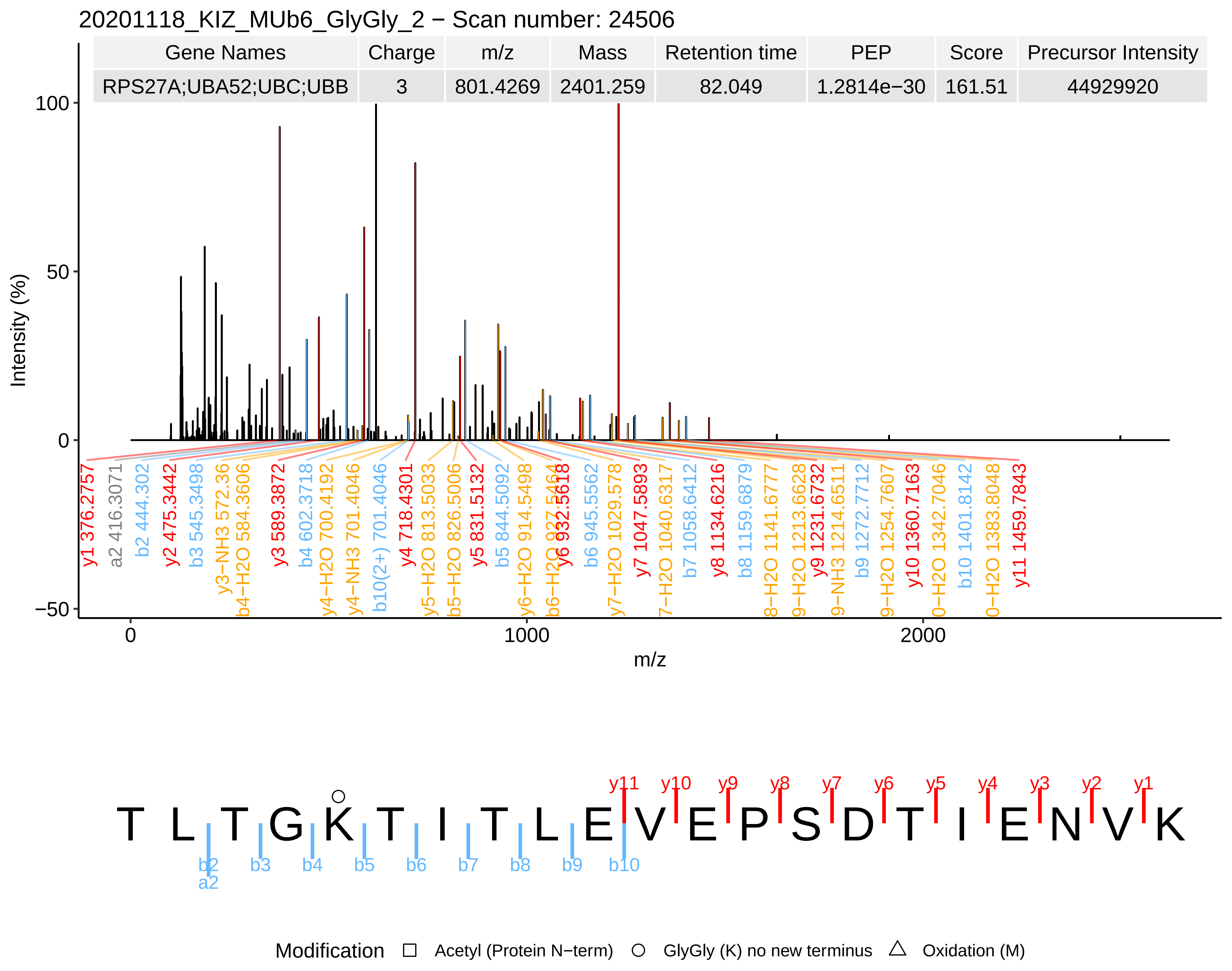
20201118_KIZ_MUb6_GlyGly_2 − Scan number: 24506
Gene Names | Charge | m/z | Mass | Retention time | PEP | Score | Precursor Intensity
RPS27A;UBA52;UBC;UBB | 3 | 801.4269 | 2401.259 | 82.049 | 1.2814e−30 | 161.51 | 44929920
Intensity (%)
100
50
0
−50
0
1000
2000
m/z
y1 376.2757
a2 416.3071
b2 444.302
y2 475.3442
b3 545.3498
y3−NH3 572.36
b4−H2O 584.3606
y3 589.3872
b4 602.3718
y4−H2O 700.4192
y4−NH3 701.4046
b10(2+) 701.4046
y4 718.4301
y5−H2O 813.5033
b5−H2O 826.5006
y5 831.5132
b5 844.5092
y6−H2O 914.5498
y6 932.5618
b6 945.5562
y7−H2O 1029.578
y7 1047.5893
b7 1058.6412
y8 1134.6216
b8 1159.6879
y9 1231.6732
b9 1272.7712
y10 1360.7163
b10 1401.8142
y11 1459.7843
T L T G K T I T L E V E P S D T I E N V K
y11 y10 y9 y8 y7 y6 y5 y4 y3 y2 y1
b2 b3 b4 b5 b6 b7 b8 b9 b10
a2
Modification
Acetyl (Protein N−term)
GlyGly (K) no new terminus
Oxidation (M)

20201118_KIZ_MUb6_GlyGly_2 − Scan number: 24353

Gene Names	Charge	m/z	Mass	Retention time	PEP	Score	Precursor Intensity
RPS27A;UBA52;UBC;UBB	4	601.322	2401.259	81.558	6.1108e−10	111.65	24378488

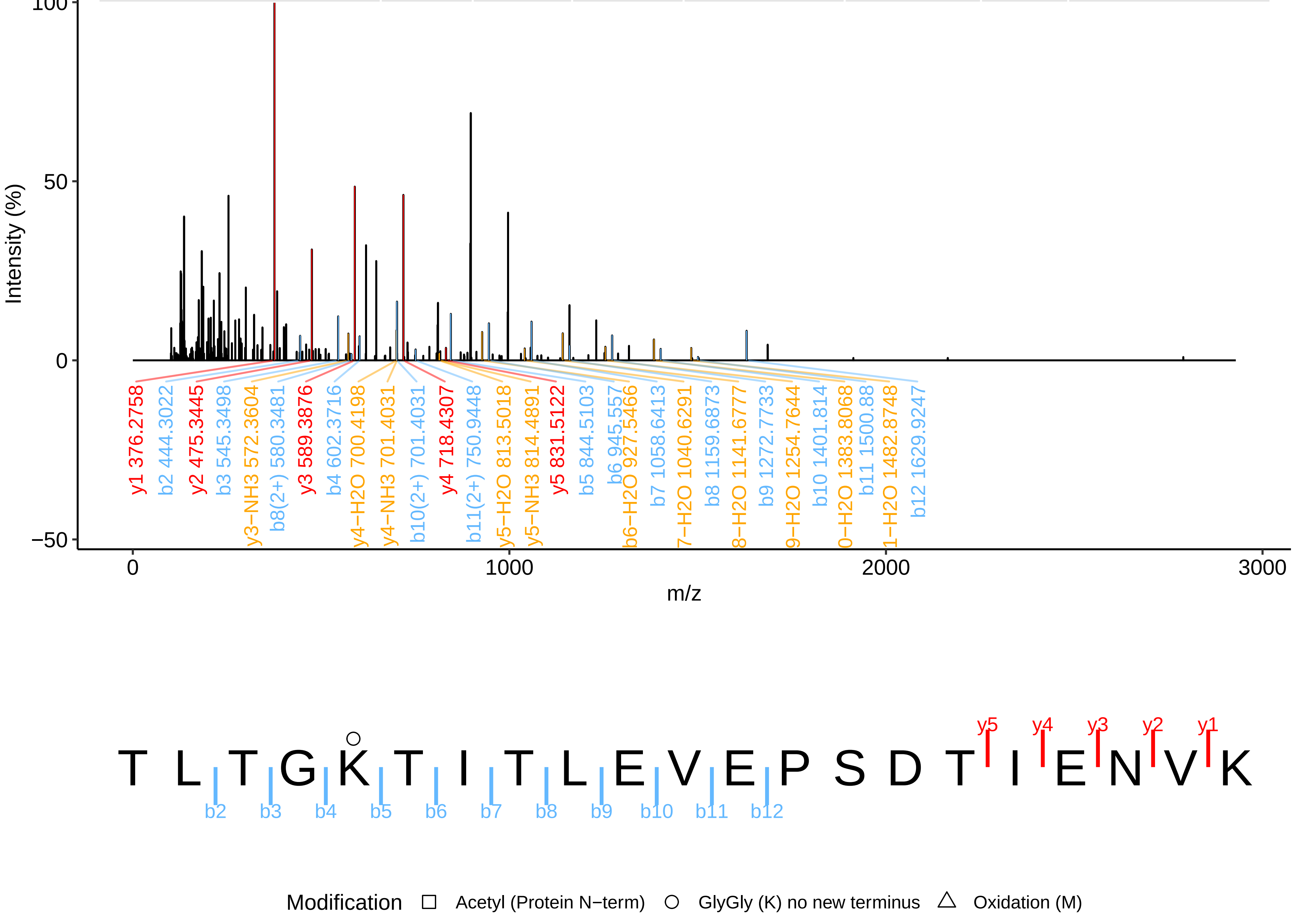

20201118_KIZ_MUb6_GlyGly_2 − Scan number: 24511

Gene Names	Charge	m/z	Mass	Retention time	PEP	Score	Precursor Intensity
RPS27A;UBA52;UBC;UBB	4	601.322	2401.259	82.065	1.0299e−30	161.65	26888988

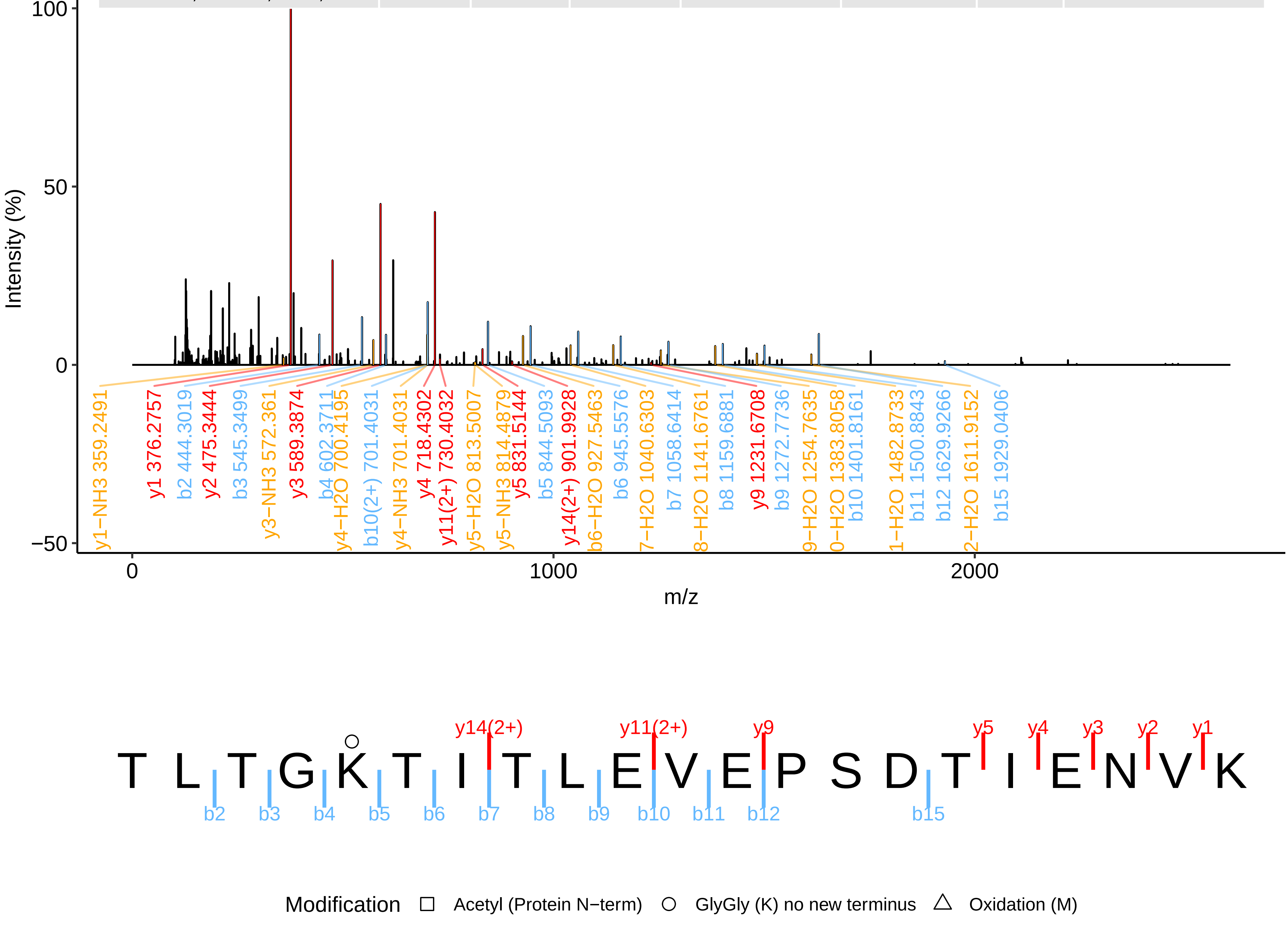

20200921_KIZ_MUb4_GlyGly_10plex − Scan number: 23097

Gene Names	Charge	m/z	Mass	Retention time	PEP	Score	Precursor Intensity
SCAMP1	3	893.4425	2677.306	83.817	0.0031912	69.981	4579250

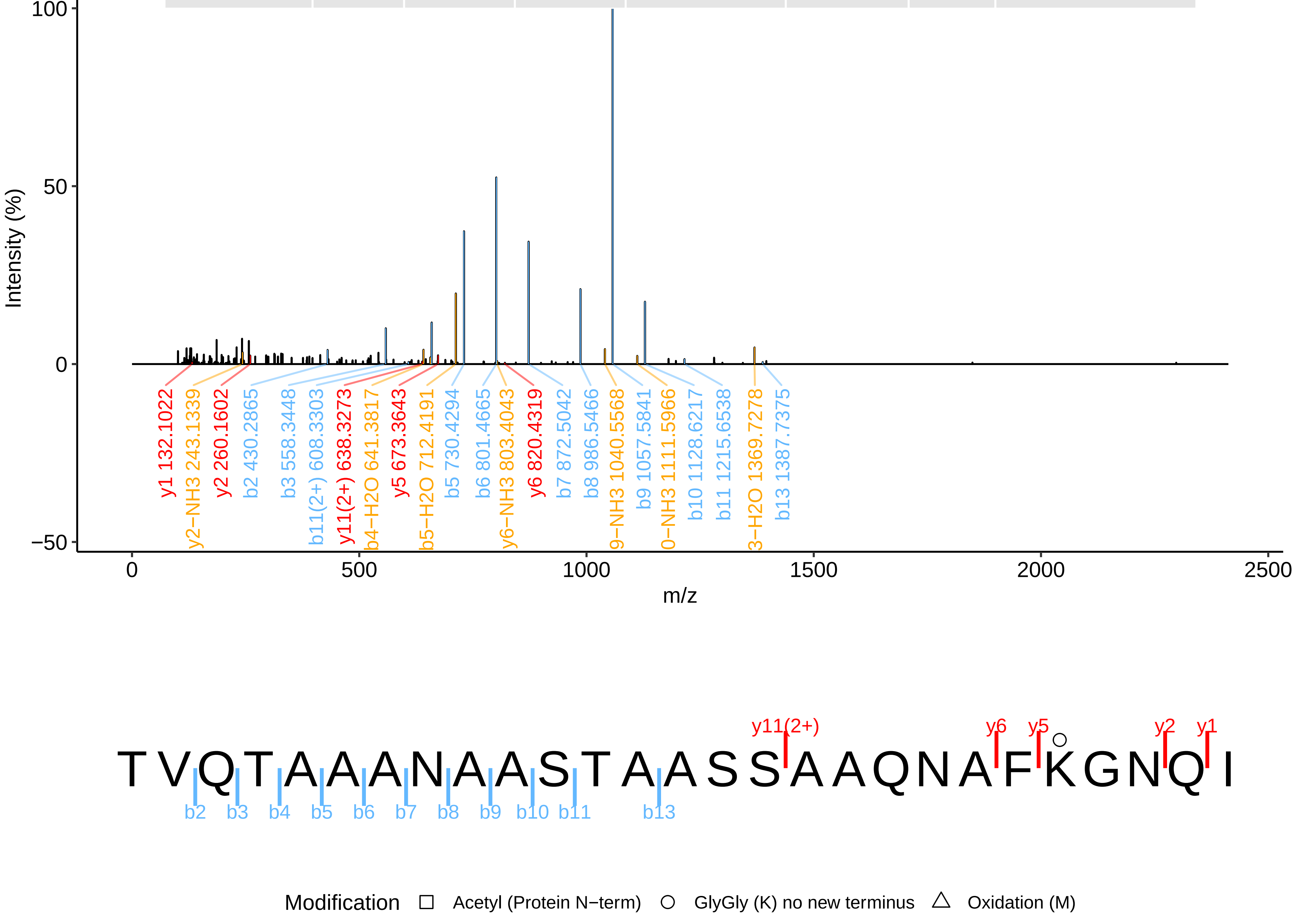

20200921_KIZ_MUb4_GlyGly_10plex − Scan number: 23166

Gene Names	Charge	m/z	Mass	Retention time	PEP	Score	Precursor Intensity
SCAMP1	3	893.4425	2677.306	84.076	8.6365e−09	102.16	19539014

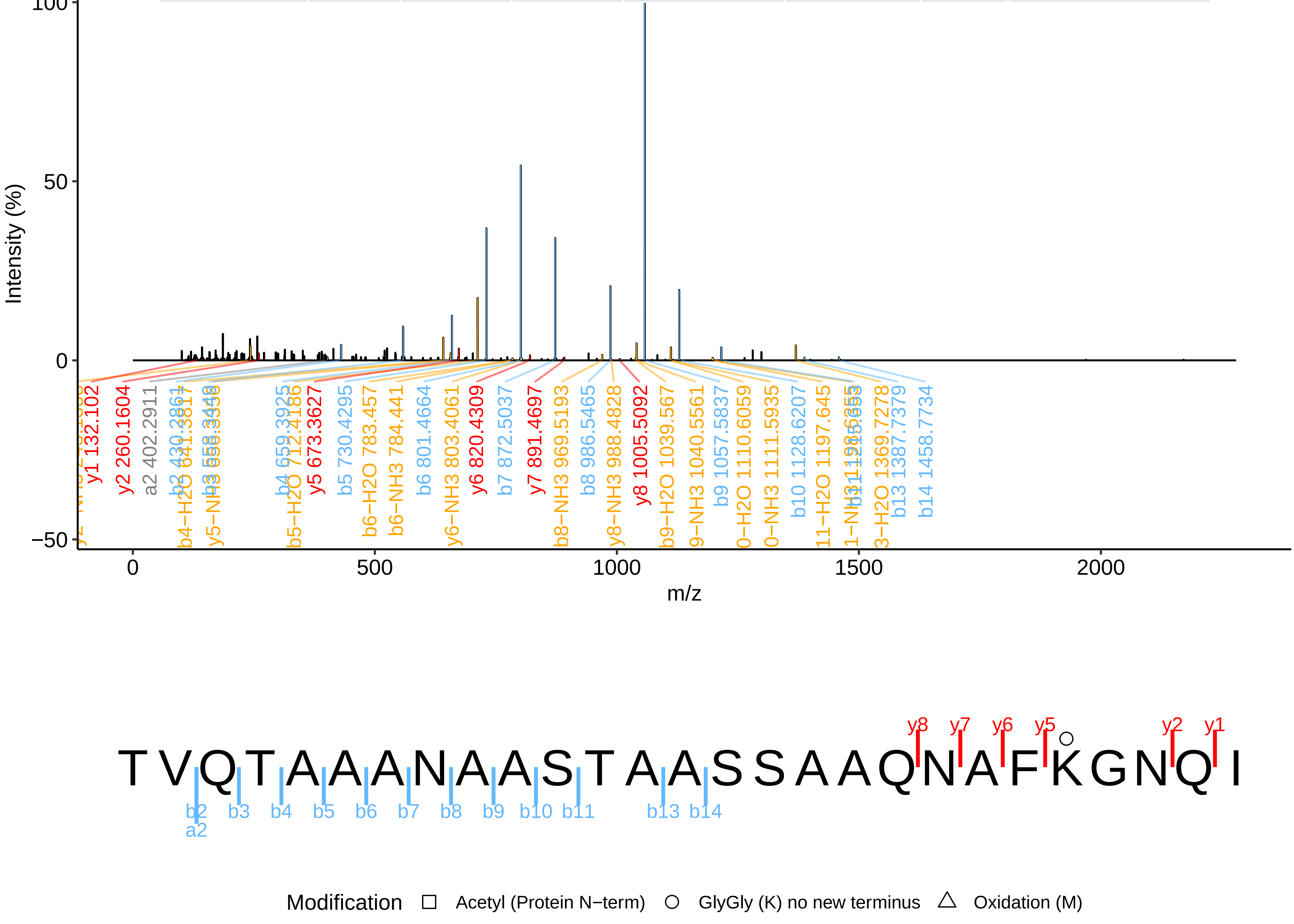

20201118_KIZ_MUb6_GlyGly_1 − Scan number: 24210

Gene Names	Charge	m/z	Mass	Retention time	PEP	Score	Precursor Intensity
SCAMP1	3	893.4425	2677.306	74.602	9.2192e−06	94.019	17994694

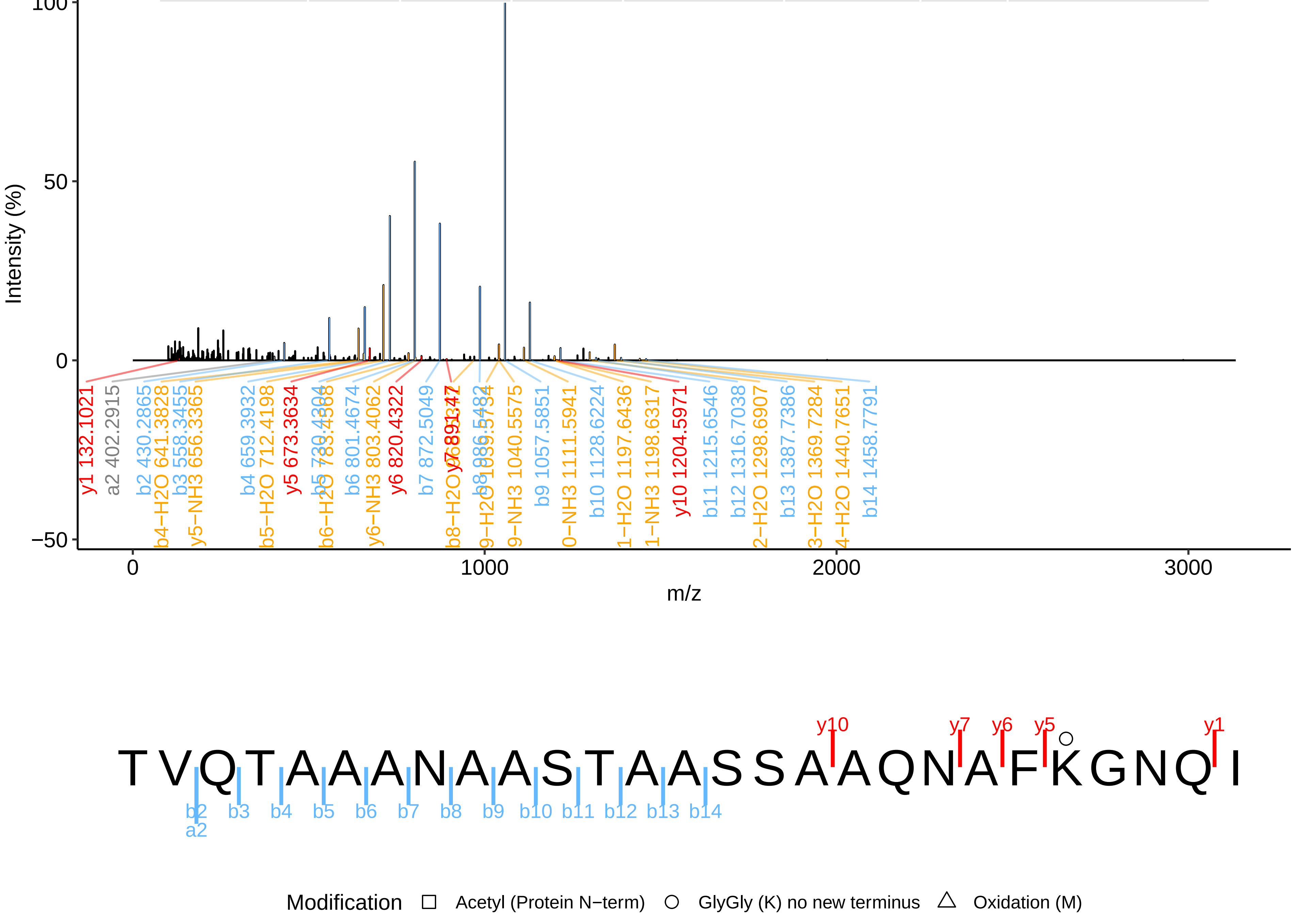

20201118_KIZ_MUb6_GlyGly_1 − Scan number: 24220

Gene Names	Charge	m/z	Mass	Retention time	PEP	Score	Precursor Intensity
SCAMP1	3	893.4425	2677.306	74.627	1.4912e−19	120.85	14037331

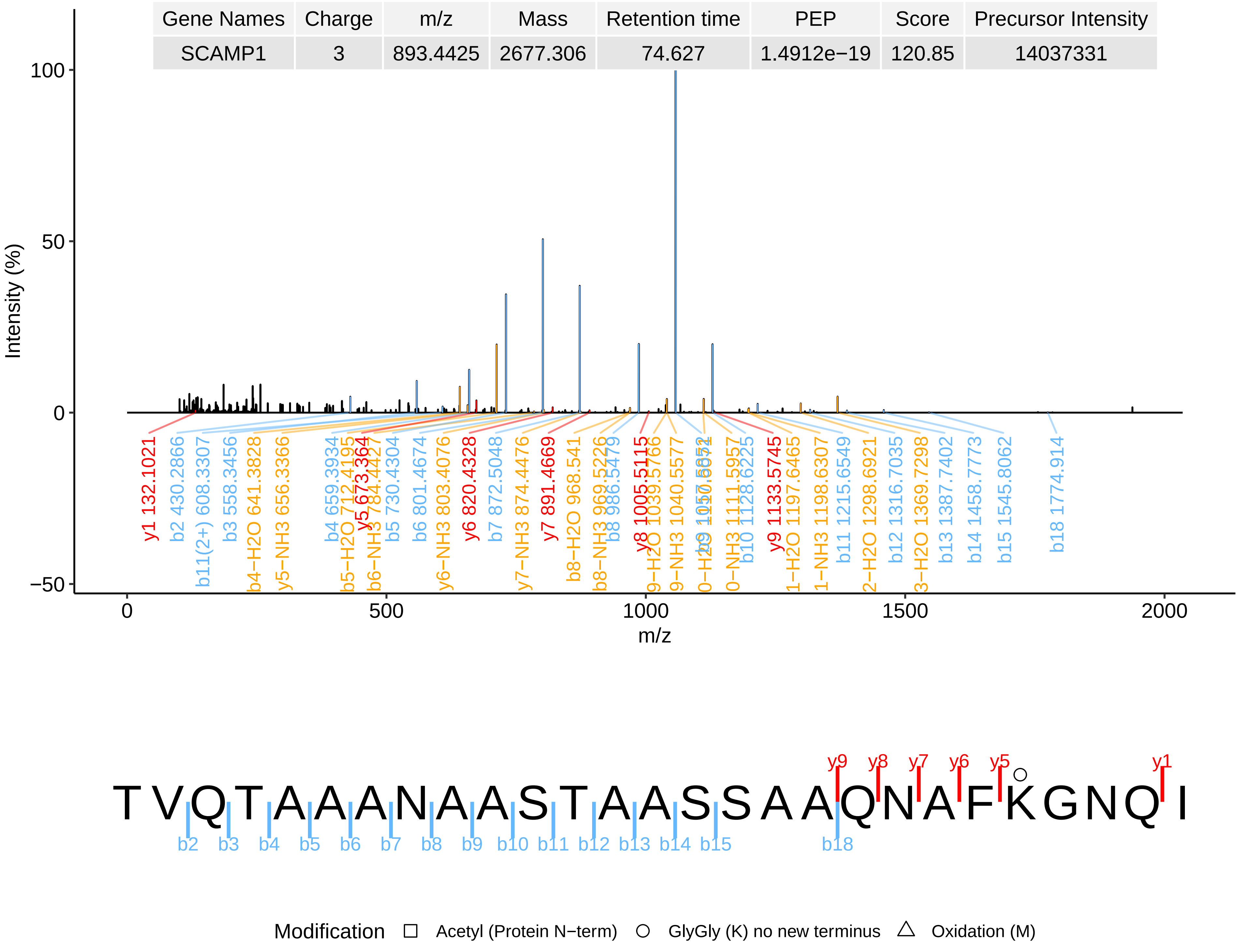

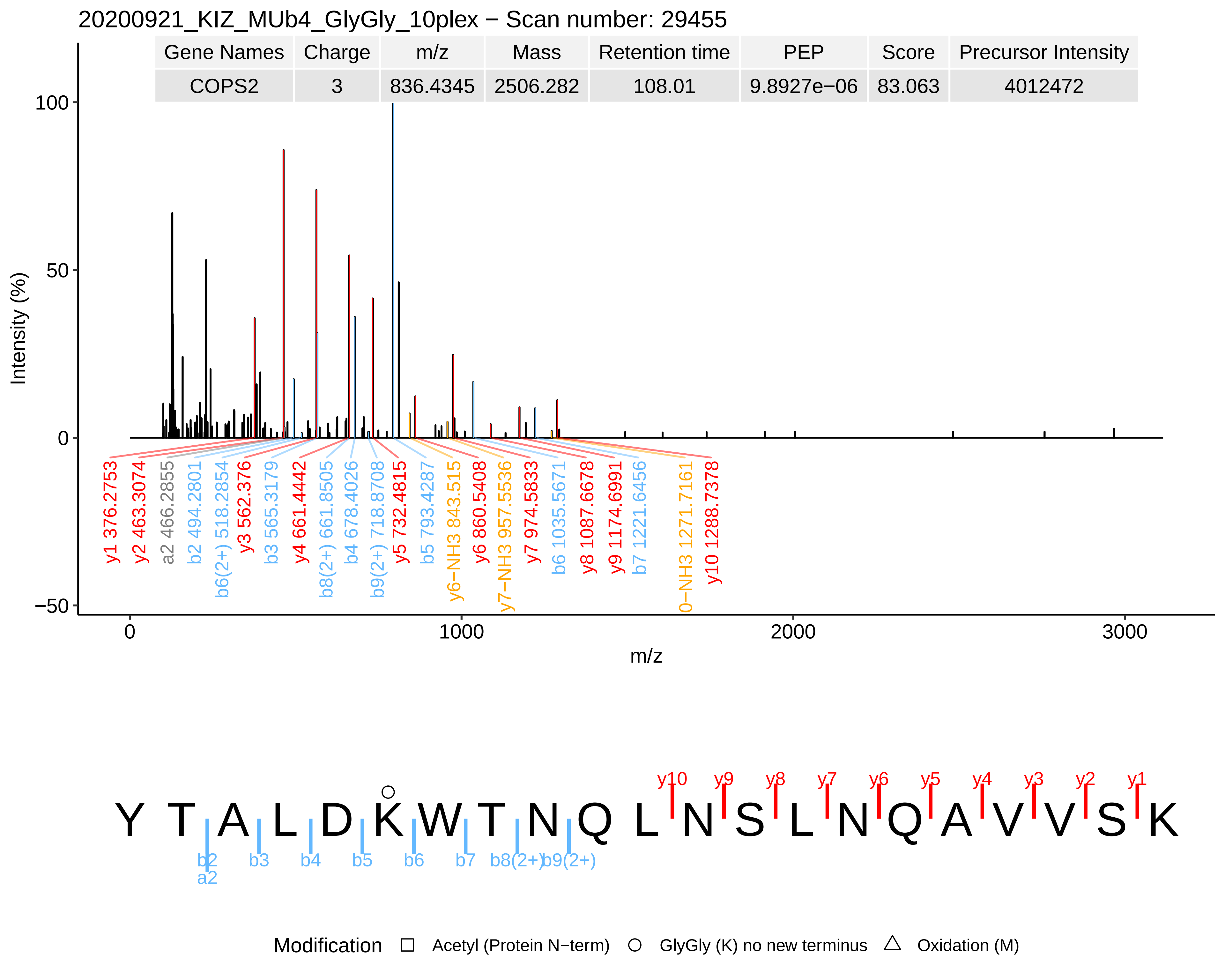
20200921_KIZ_MUb4_GlyGly_10plex − Scan number: 29455
Gene Names | Charge | m/z | Mass | Retention time | PEP | Score | Precursor Intensity
COPS2 | 3 | 836.4345 | 2506.282 | 108.01 | 9.8927e−06 | 83.063 | 4012472
Intensity (%)
100
50
0
−50
m/z
0
1000
2000
3000
y1 376.2753
y2 463.3074
a2 466.2855
b2 494.2801
b6(2+) 518.2854
y3 562.376
b3 565.3179
y4 661.4442
b8(2+) 661.8505
b4 678.4026
b9(2+) 718.8708
y5 732.4815
b5 793.4287
y6−NH3 843.515
y6 860.5408
y7−NH3 957.5536
y7 974.5833
b6 1035.5671
y8 1087.6678
y9 1174.6991
b7 1221.6456
0−NH3 1271.7161
y10 1288.7378
Y T A L D K W T N Q L N S L N Q A V V S K
y10 y9 y8 y7 y6 y5 y4 y3 y2 y1
b2 a2 b3 b4 b5 b6 b7 b8(2+) b9(2+)
Modification
Acetyl (Protein N−term)
GlyGly (K) no new terminus
Oxidation (M)

20200921_KIZ_MUb4_GlyGly_10plex − Scan number: 31699

Gene Names	Charge	m/z	Mass	Retention time	PEP	Score	Precursor Intensity
TUBB	3	940.7874	2819.34	116.92	4.4283e−19	130.09	2710434

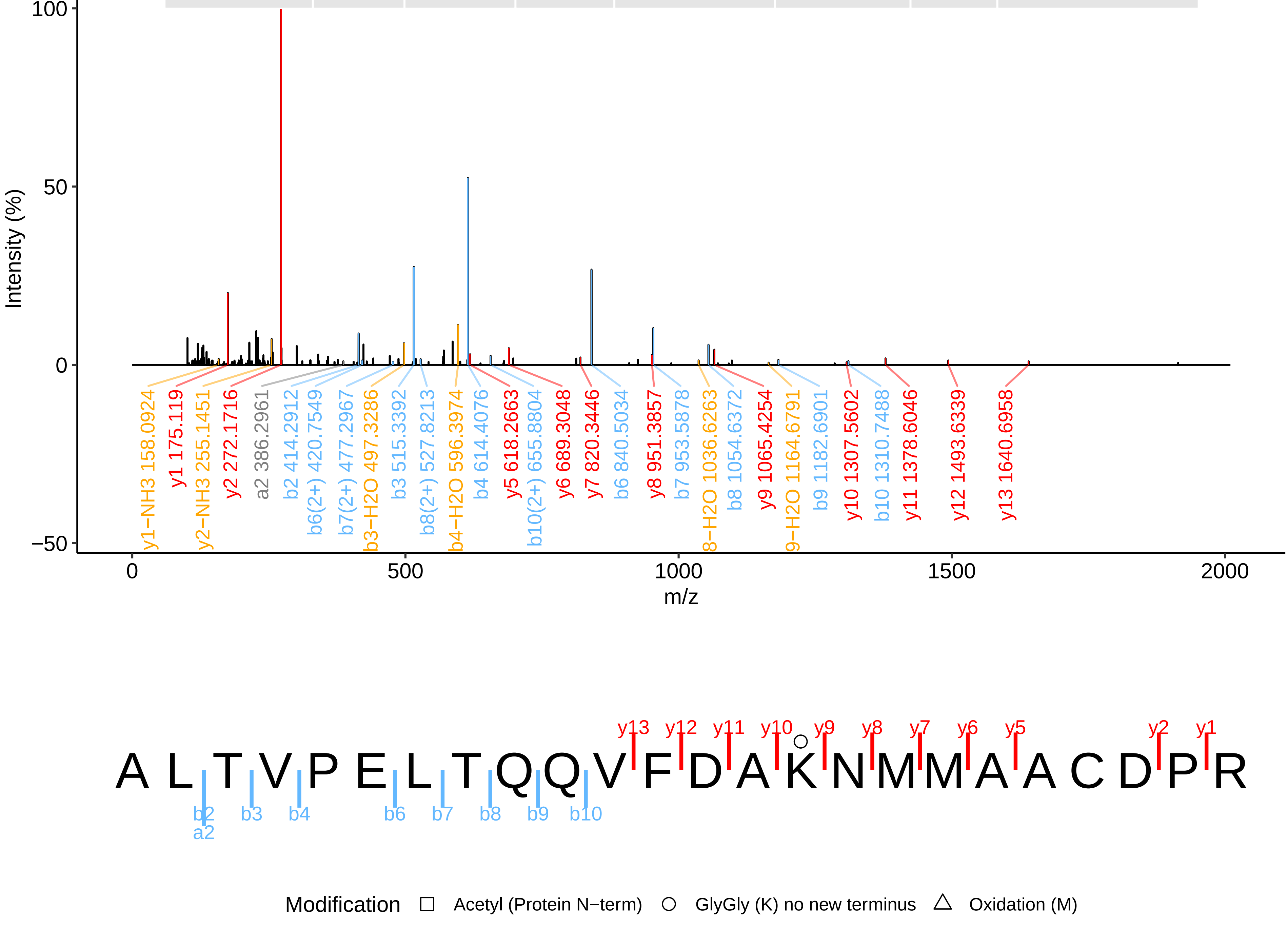

20200924_KIZ_MUb5_GlyGly_1 − Scan number: 32753

Gene Names	Charge	m/z	Mass	Retention time	PEP	Score	Precursor Intensity
TUBB	3	940.7874	2819.34	104.42	1.2028e−25	138.35	NA

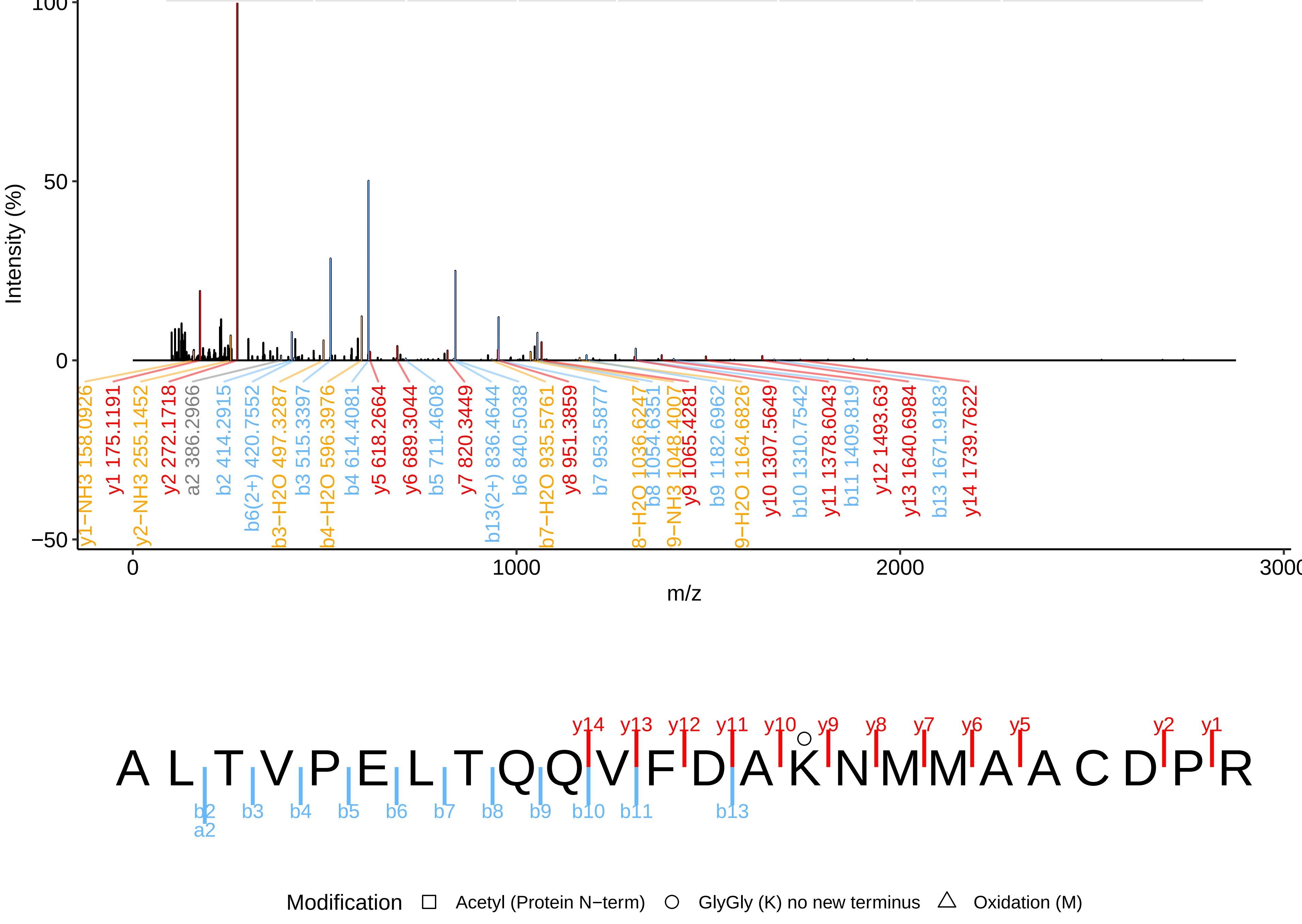

20200921_KIZ_MUb4_GlyGly_10plex − Scan number: 7513

Gene Names	Charge	m/z	Mass	Retention time	PEP	Score	Precursor Intensity
PSMD2	4	655.0796	2616.289	25.237	7.1231e−22	141.45	6441180

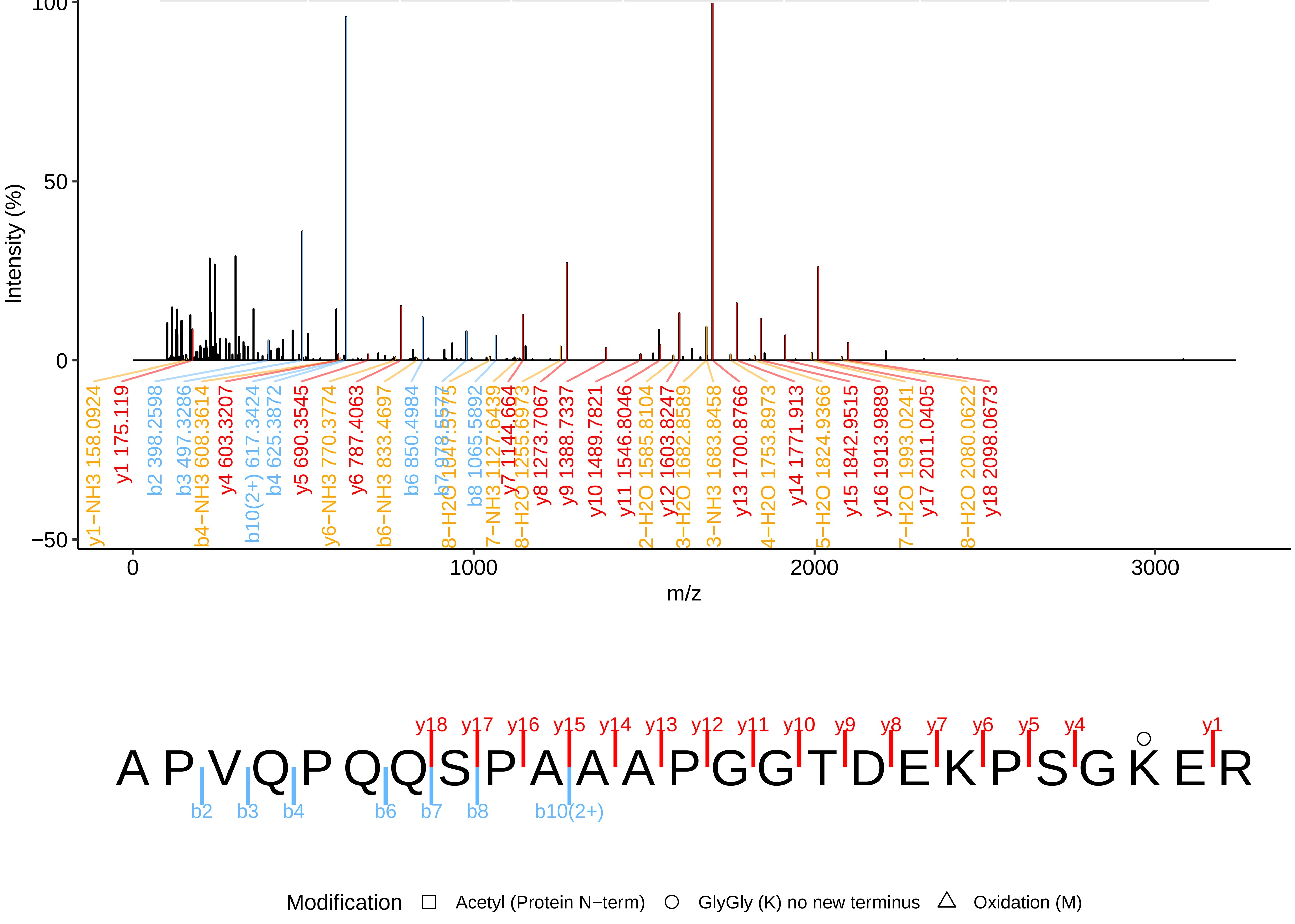

20200921_KIZ_MUb4_GlyGly_10plex_2 − Scan number: 6891

Gene Names	Charge	m/z	Mass	Retention time	PEP	Score	Precursor Intensity
PSMD2	4	655.0796	2616.289	25.521	0.00031941	79.762	1305591

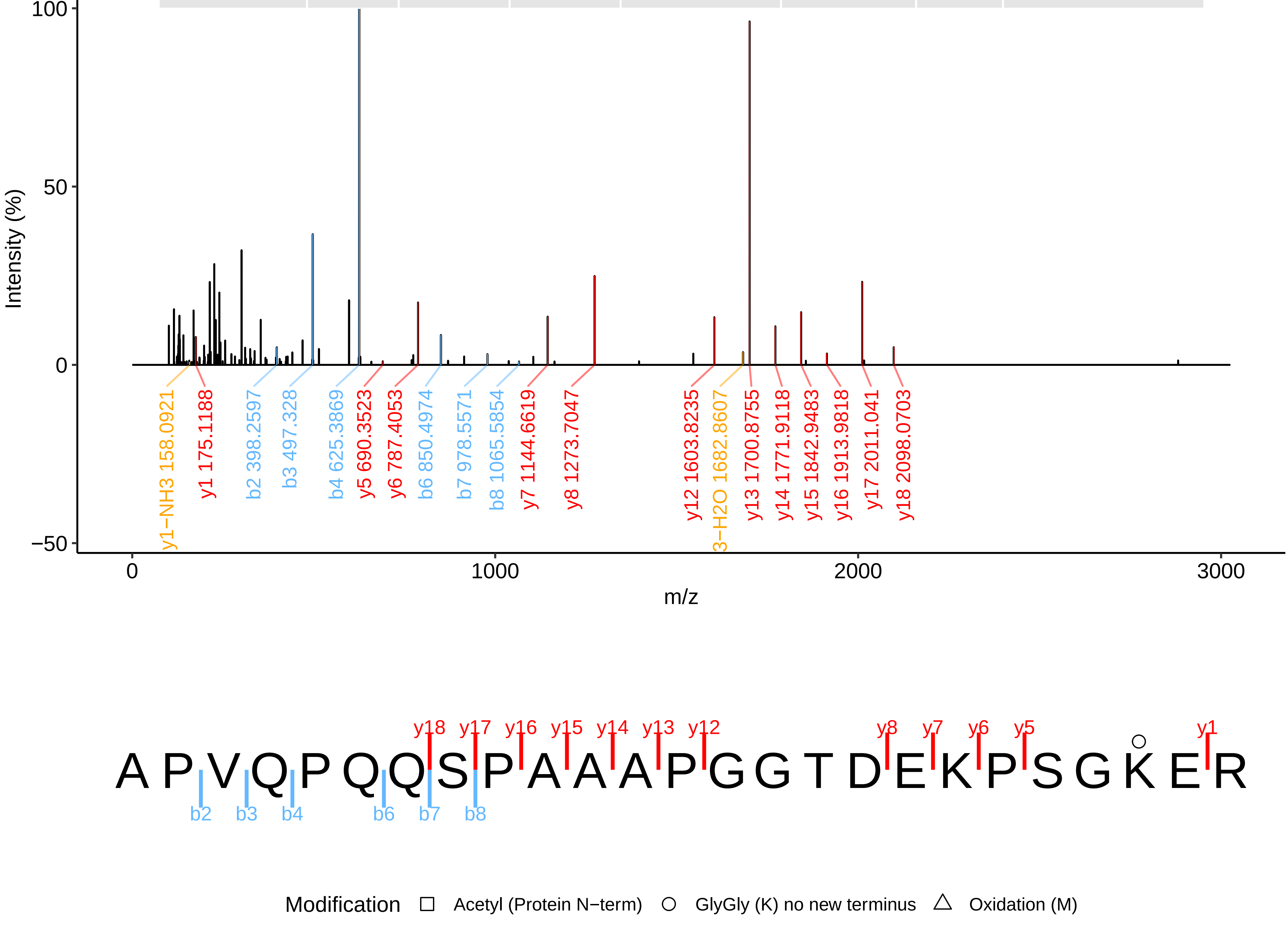

20200921_KIZ_MUb4_GlyGly_10plex − Scan number: 23726

Gene Names	Charge	m/z	Mass	Retention time	PEP	Score	Precursor Intensity
EIF3A	3	970.1648	2907.473	86.157	2.9849e−09	97.509	2741523

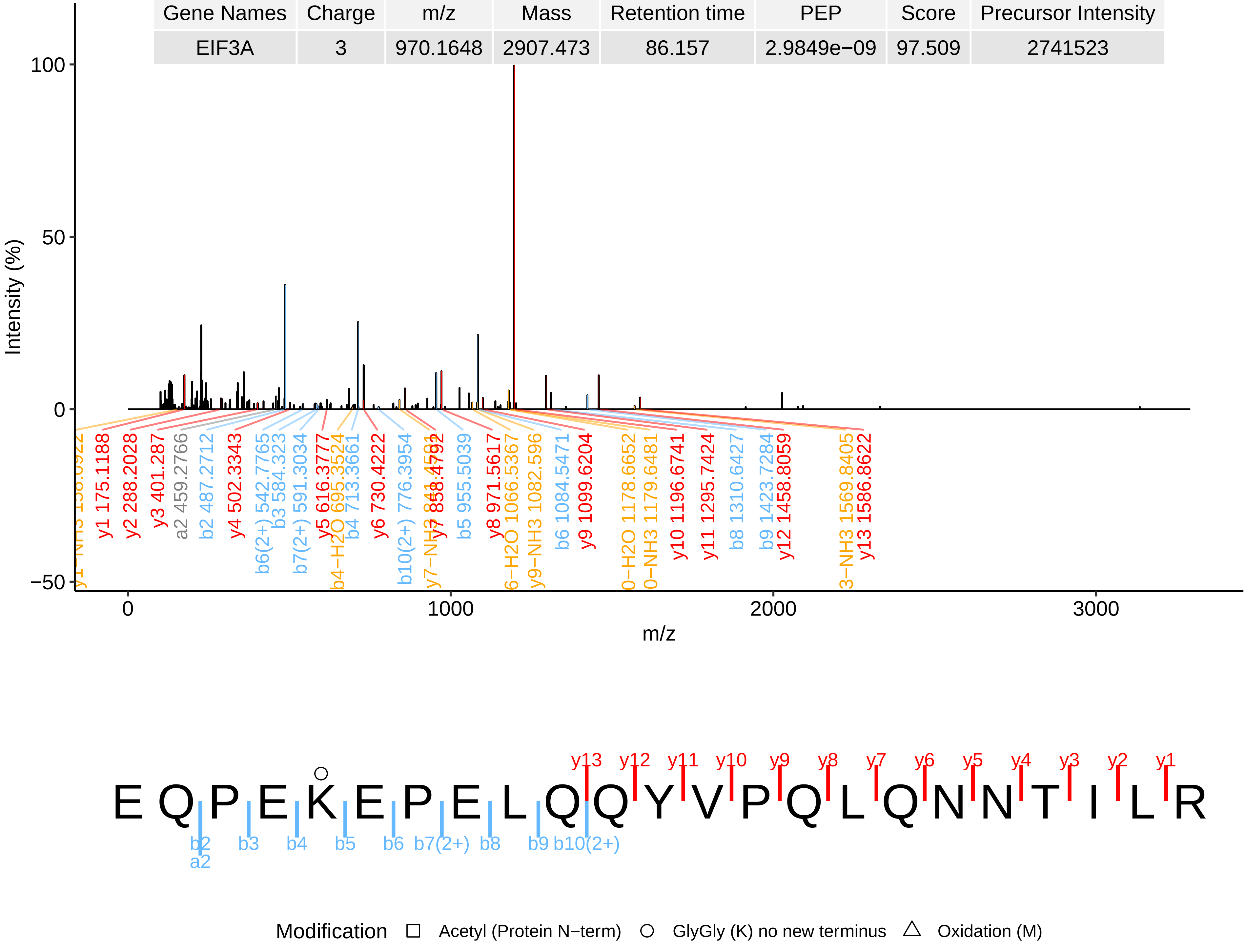

20200924_KIZ_MUb5_GlyGly_1 − Scan number: 26406

Gene Names	Charge	m/z	Mass	Retention time	PEP	Score	Precursor Intensity
USP13	3	944.1417	2829.403	84.559	0.018654	48.759	NA

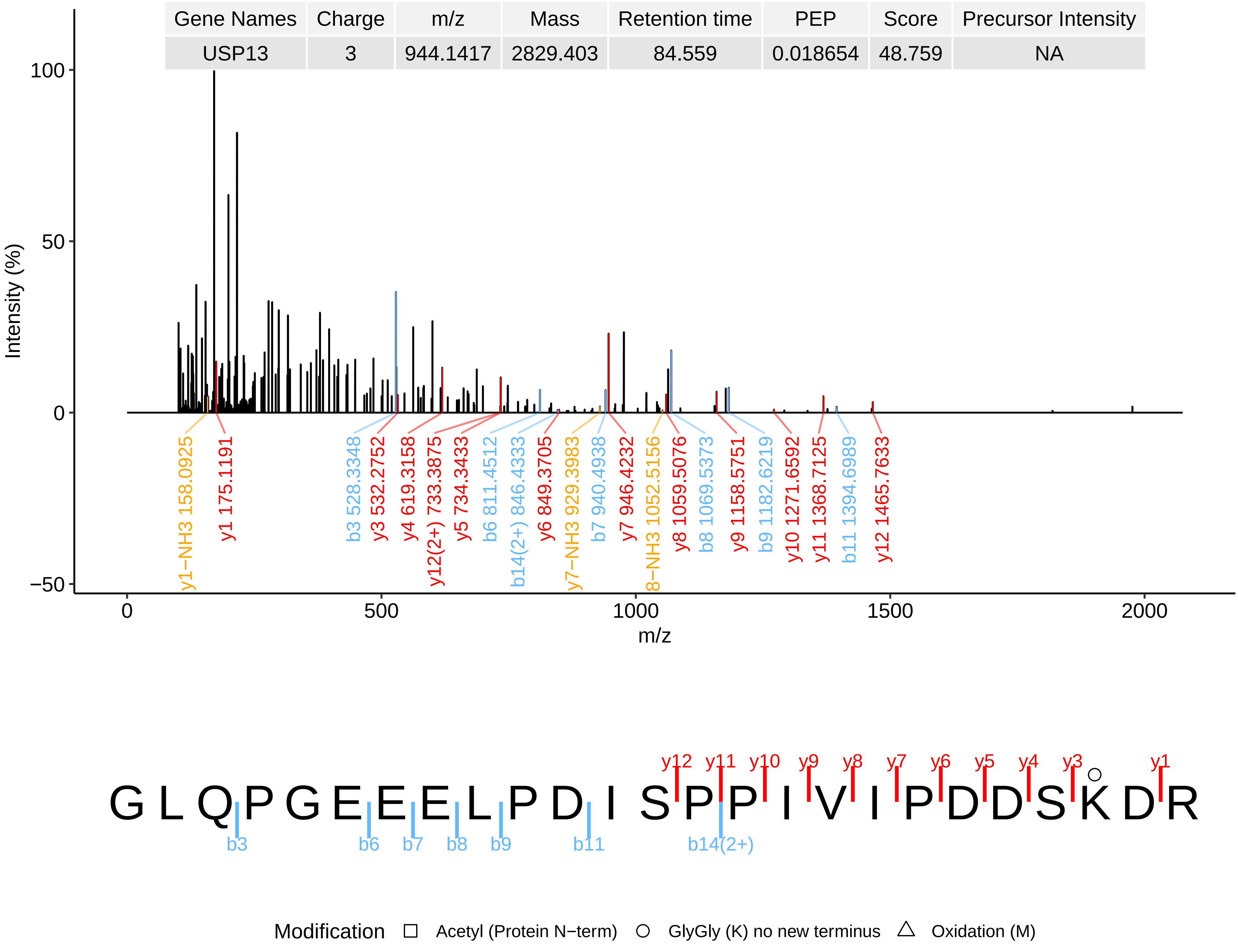

20200924_KIZ_MUb5_GlyGly_2 − Scan number: 32280

Gene Names	Charge	m/z	Mass	Retention time	PEP	Score	Precursor Intensity
RB1CC1	4	655.5958	2618.354	119.85	0.014282	62.475	59755696

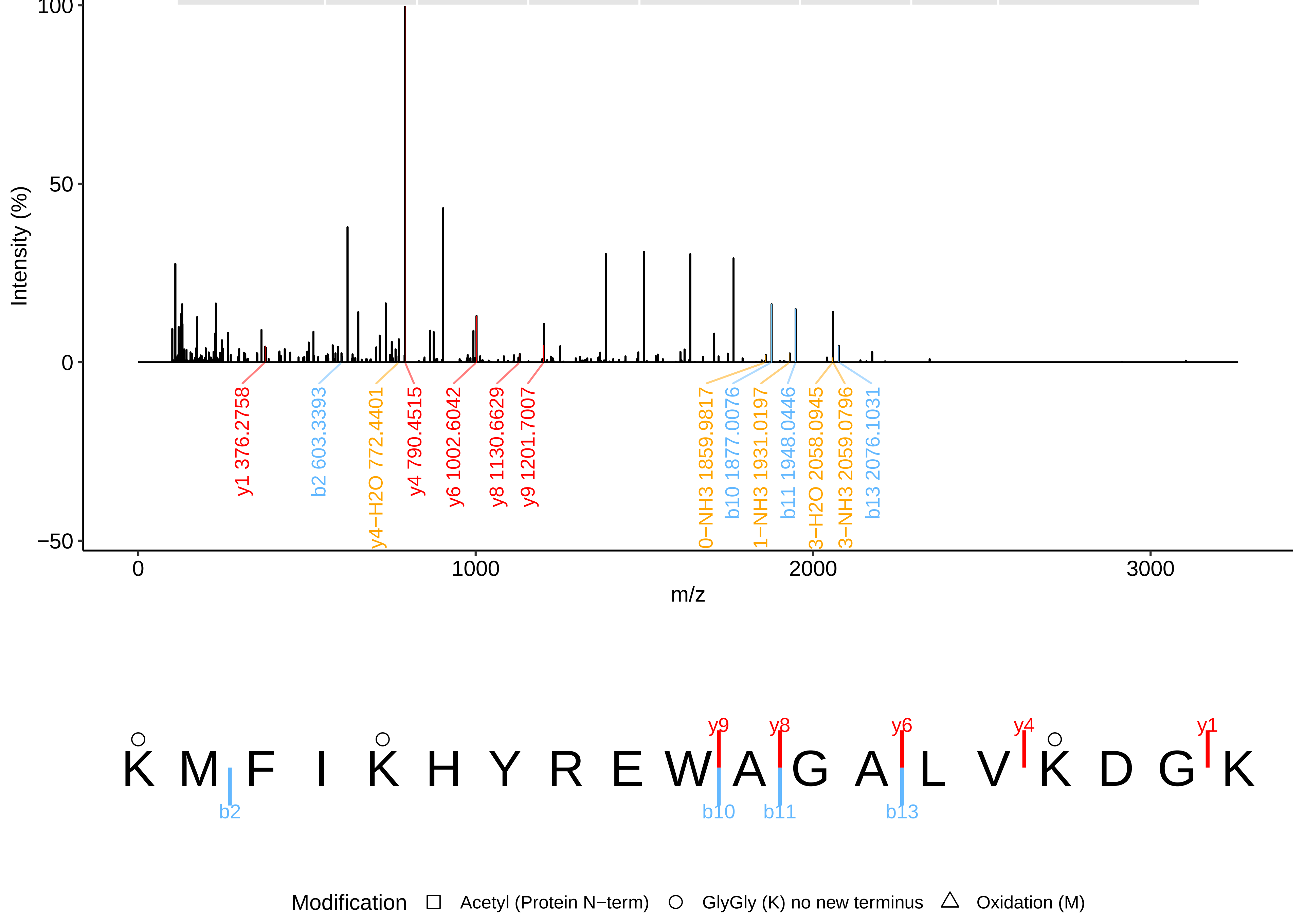

20200921_KIZ_MUb4_GlyGly_10plex − Scan number: 12141

Gene Names	Charge	m/z	Mass	Retention time	PEP	Score	Precursor Intensity
TFRC	4	604.3003	2413.172	42.494	1.6618e−29	155.28	4213569

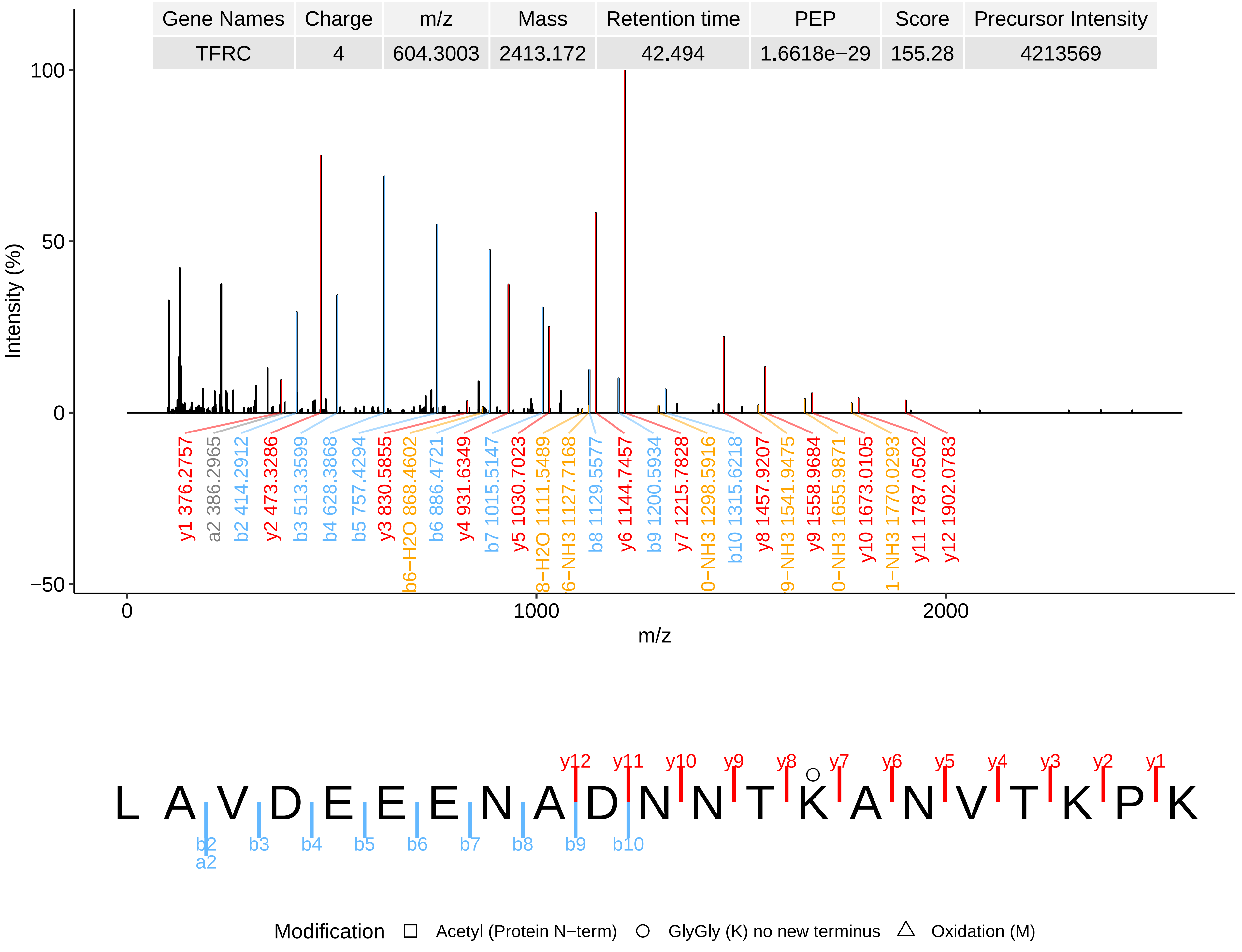

20200921_KIZ_MUb4_GlyGly_10plex_2 − Scan number: 11189

Gene Names	Charge	m/z	Mass	Retention time	PEP	Score	Precursor Intensity
TFRC	4	604.3003	2413.172	43.003	4.7628e−09	111.66	1571001

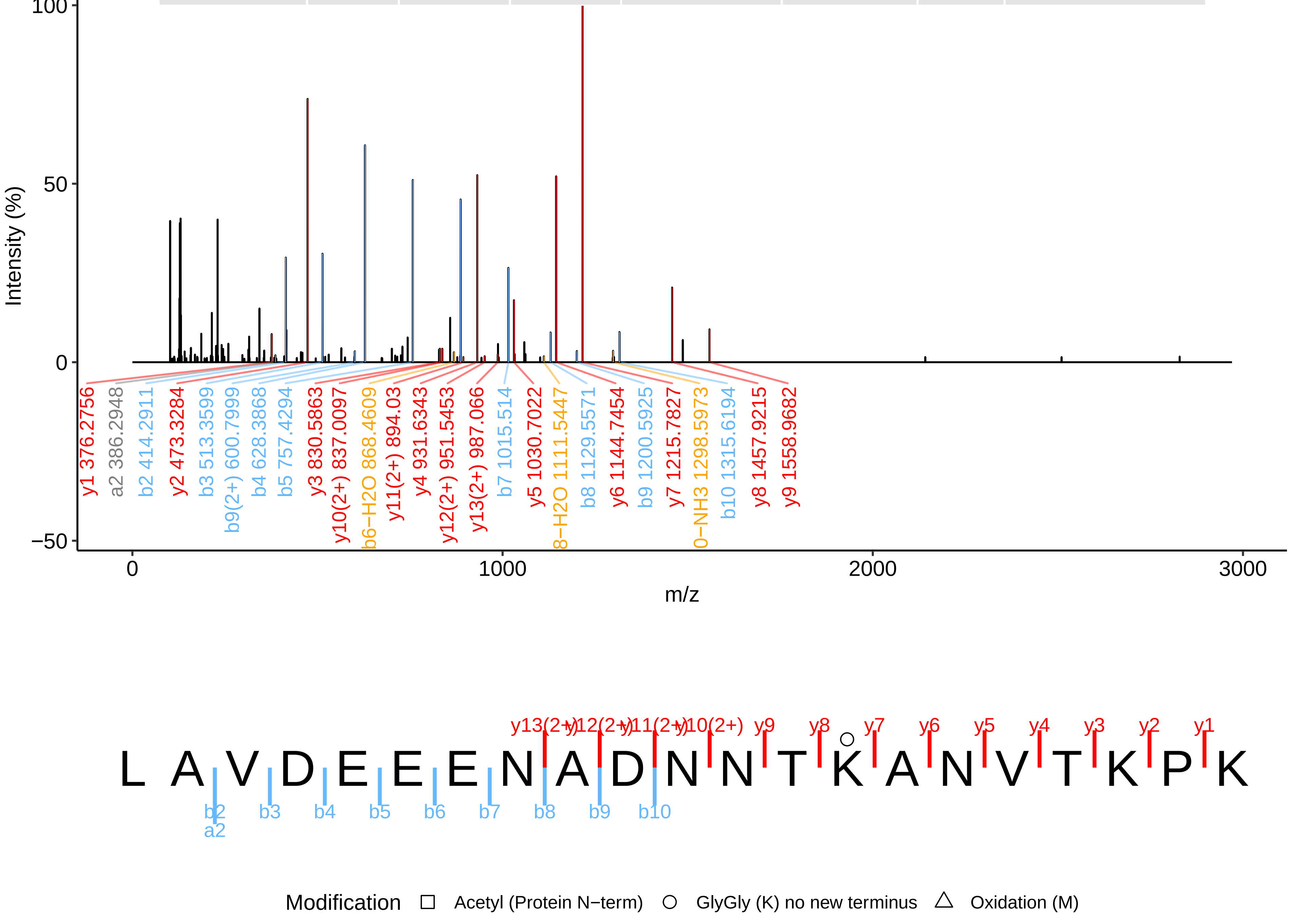

20200921_KIZ_MUb4_GlyGly_10plex − Scan number: 25934

Gene Names	Charge	m/z	Mass	Retention time	PEP	Score	Precursor Intensity
USP5	3	864.7695	2591.287	94.417	2.8358e−06	87.326	1670628

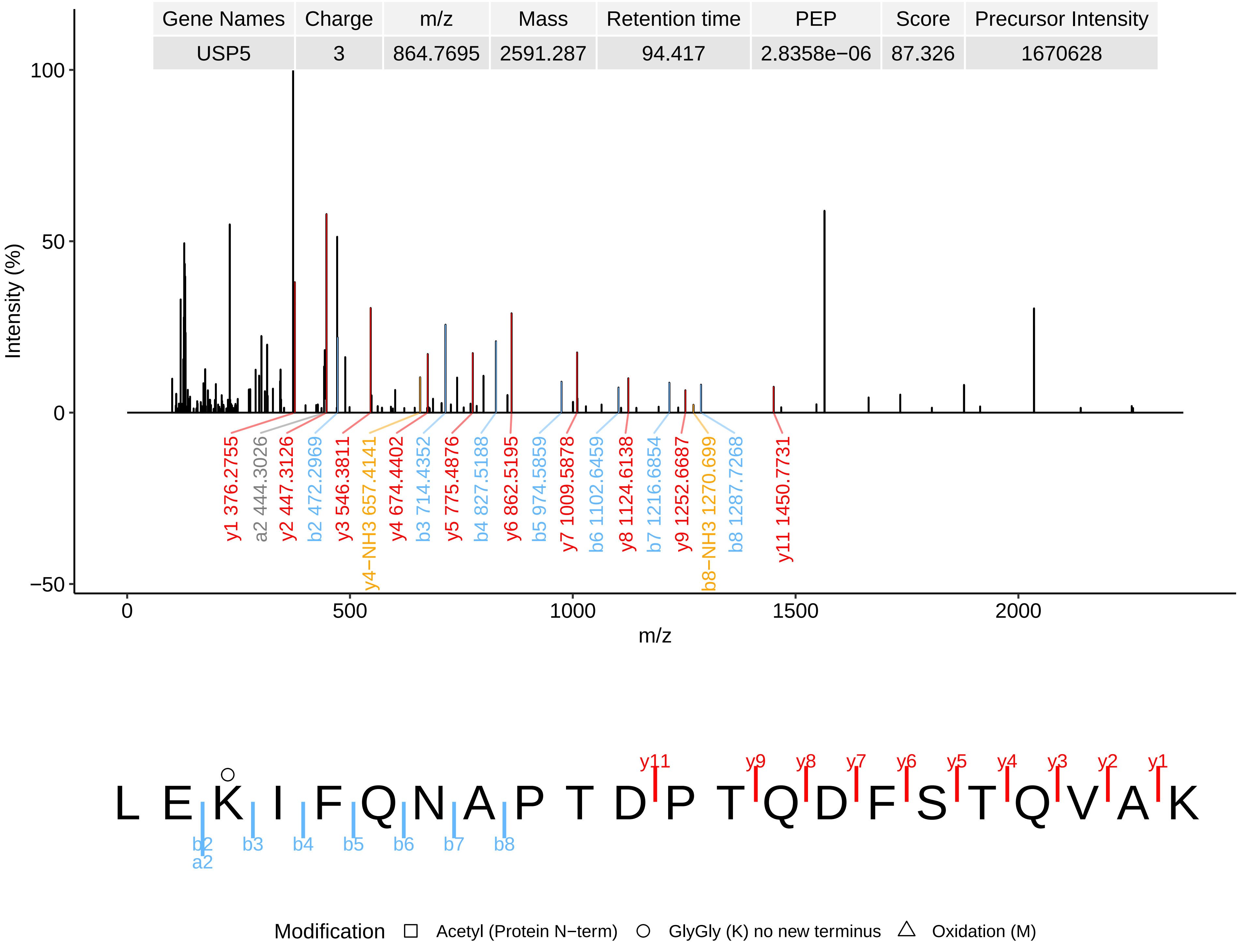

20200921_KIZ_MUb4_GlyGly_10plex − Scan number: 25963

Gene Names	Charge	m/z	Mass	Retention time	PEP	Score	Precursor Intensity
USP5	4	648.829	2591.287	94.522	1.7021e−06	90.22	3947970

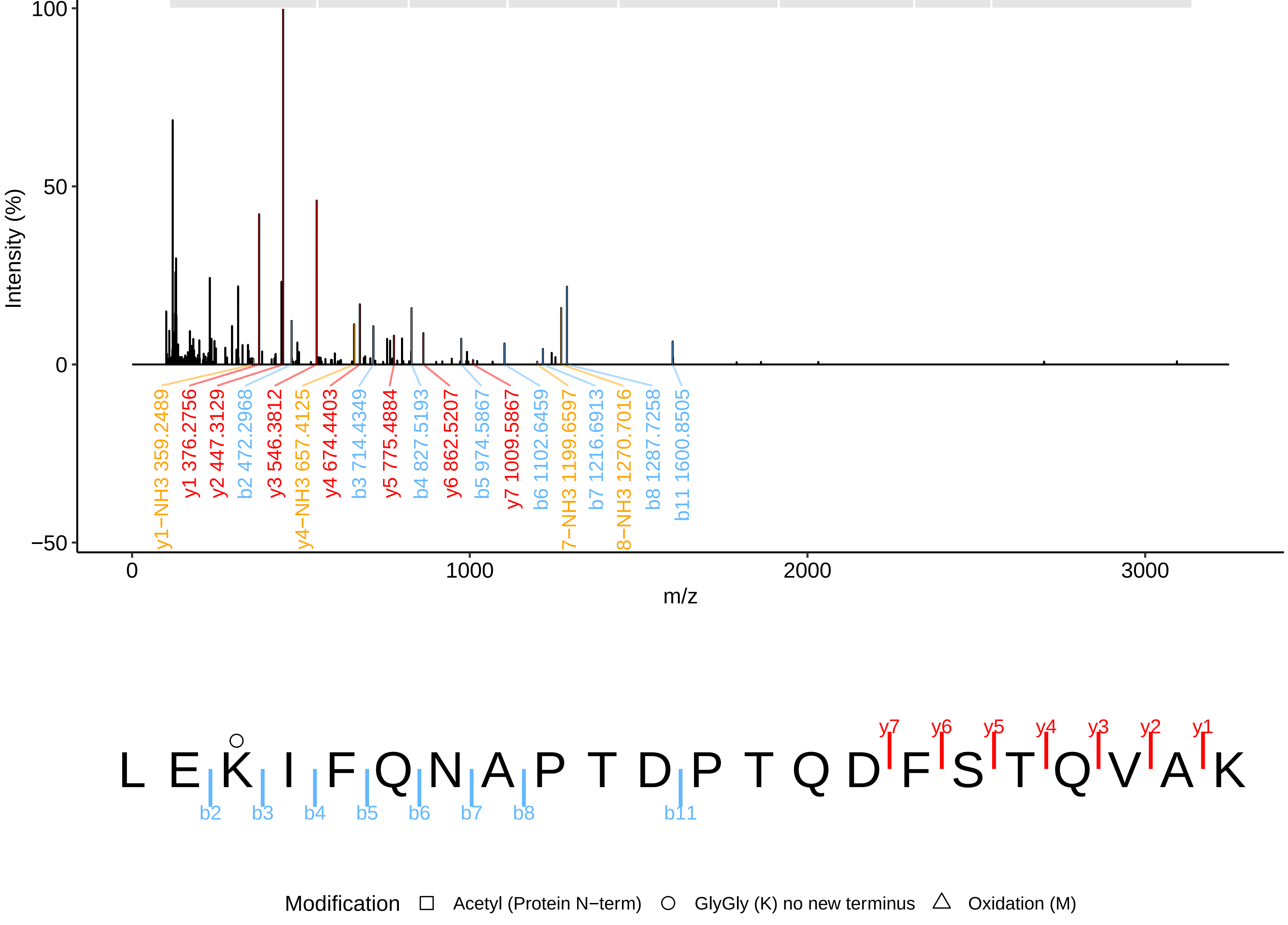

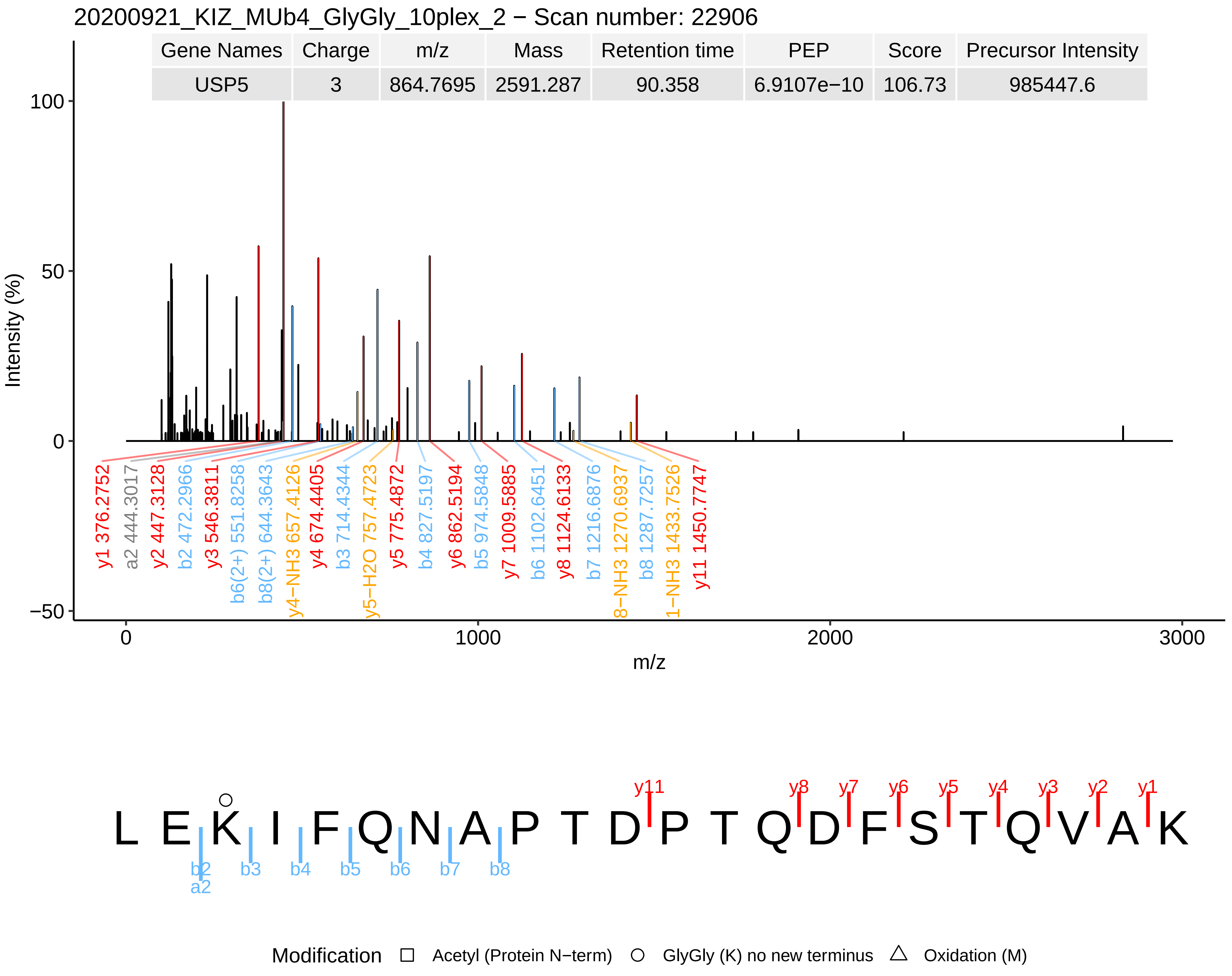
20200921_KIZ_MUb4_GlyGly_10plex_2 − Scan number: 22906
Gene Names | Charge | m/z | Mass | Retention time | PEP | Score | Precursor Intensity
USP5 | 3 | 864.7695 | 2591.287 | 90.358 | 6.9107e−10 | 106.73 | 985447.6
Intensity (%)
100
50
0
−50
m/z
0
1000
2000
3000
y1 376.2752
a2 444.3017
y2 447.3128
b2 472.2966
y3 546.3811
b6(2+) 551.8258
b8(2+) 644.3643
y4−NH3 657.4126
y4 674.4405
b3 714.4344
y5−H2O 757.4723
y5 775.4872
b4 827.5197
y6 862.5194
b5 974.5848
y7 1009.5885
b6 1102.6451
y8 1124.6133
b7 1216.6876
8−NH3 1270.6937
b8 1287.7257
1−NH3 1433.7526
y11 1450.7747
L E K I F Q N A P T D P T Q D F S T Q V A K
y11 y8 y7 y6 y5 y4 y3 y2 y1
b2 b3 b4 b5 b6 b7 b8
a2
Modification
Acetyl (Protein N−term)
GlyGly (K) no new terminus
Oxidation (M)

20200921_KIZ_MUb4_GlyGly_10plex_2 − Scan number: 22907

Gene Names	Charge	m/z	Mass	Retention time	PEP	Score	Precursor Intensity
USP5	4	648.829	2591.287	90.362	0.028438	52.541	896439.2

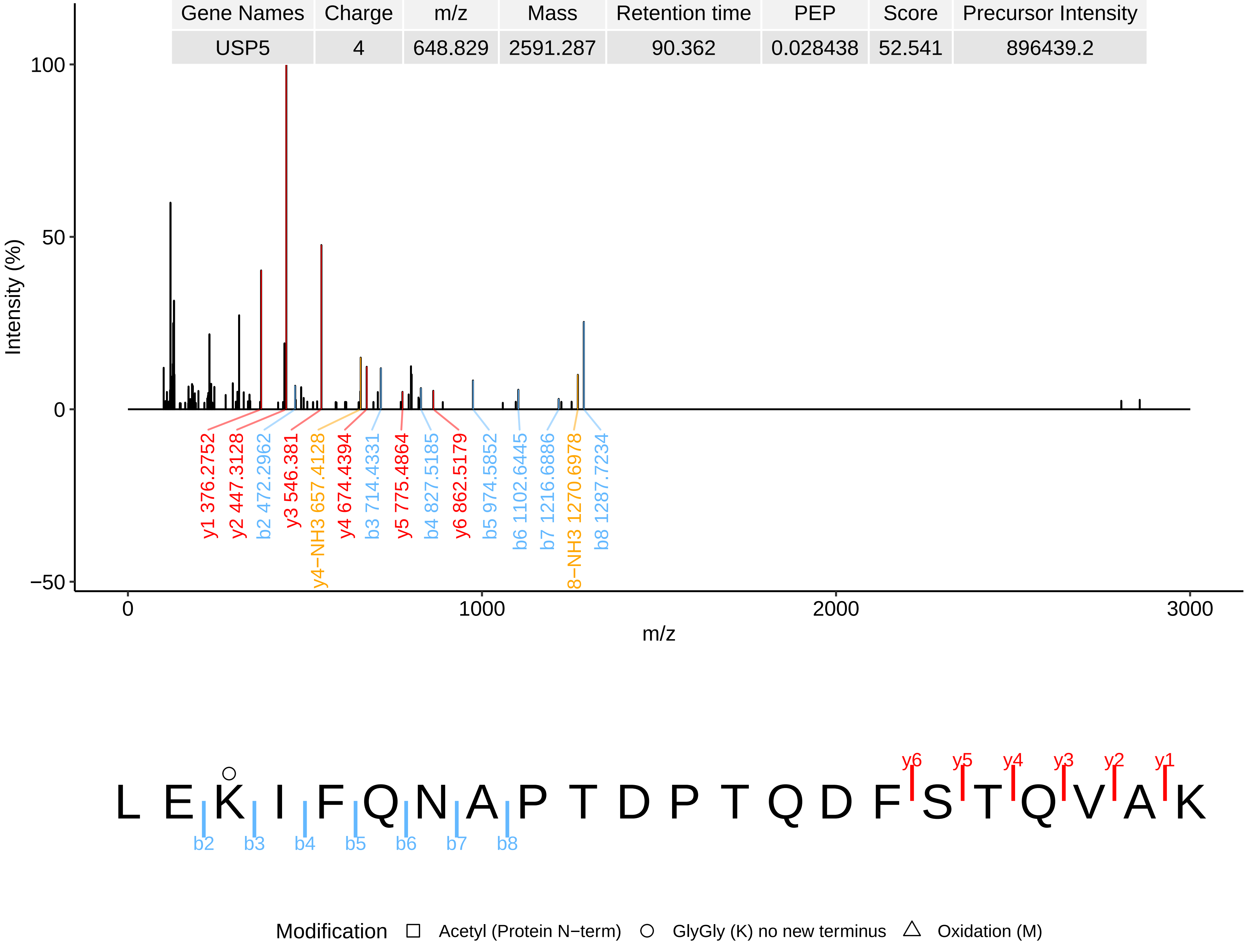

20200924_KIZ_MUb5_GlyGly_1 − Scan number: 26009

Gene Names	Charge	m/z	Mass	Retention time	PEP	Score	Precursor Intensity
USP5	4	648.829	2591.287	83.339	0.027483	52.909	NA

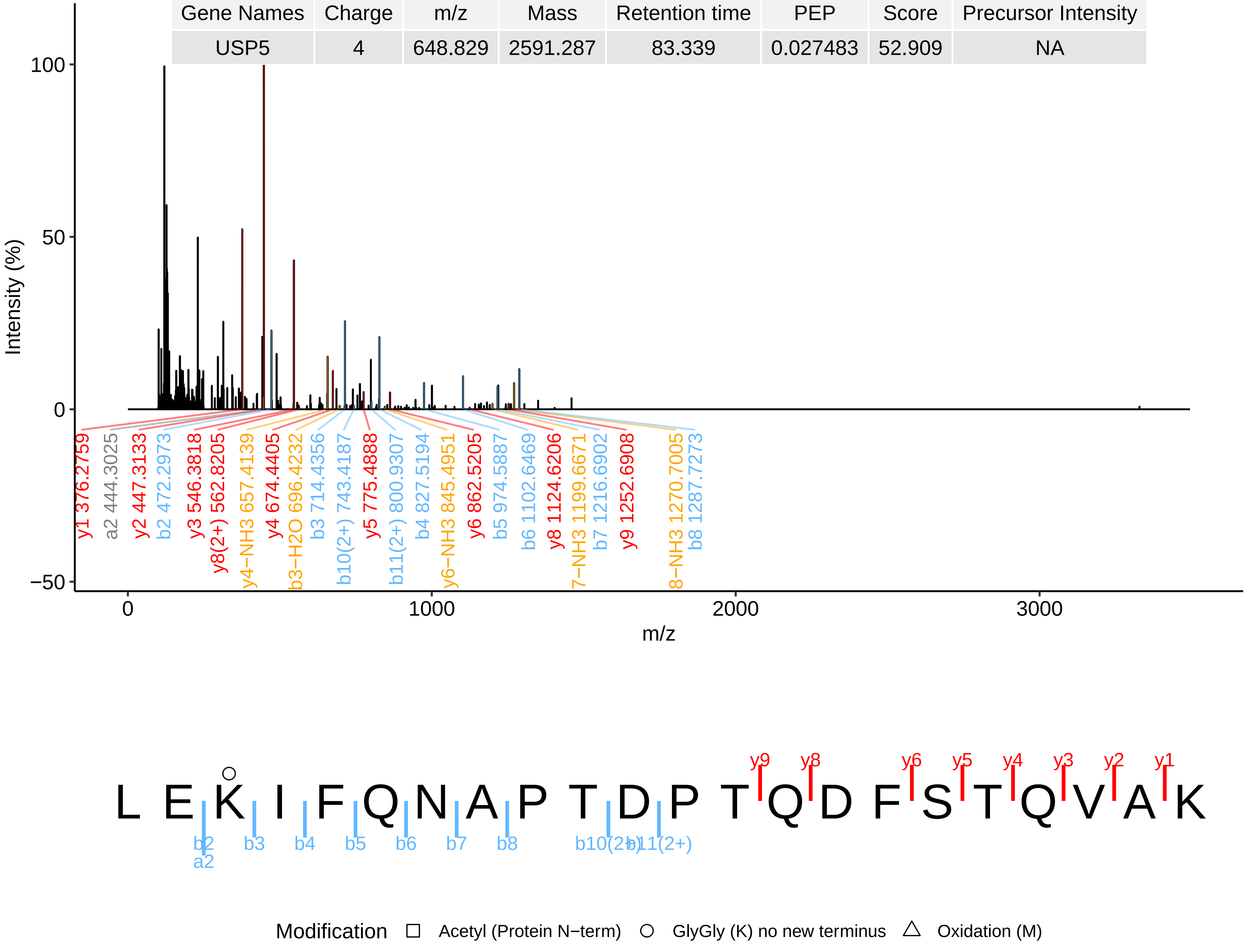

20200924_KIZ_MUb5_GlyGly_1 − Scan number: 26133

Gene Names	Charge	m/z	Mass	Retention time	PEP	Score	Precursor Intensity
USP5	4	648.829	2591.287	83.732	2.0175e−30	151.45	19721780

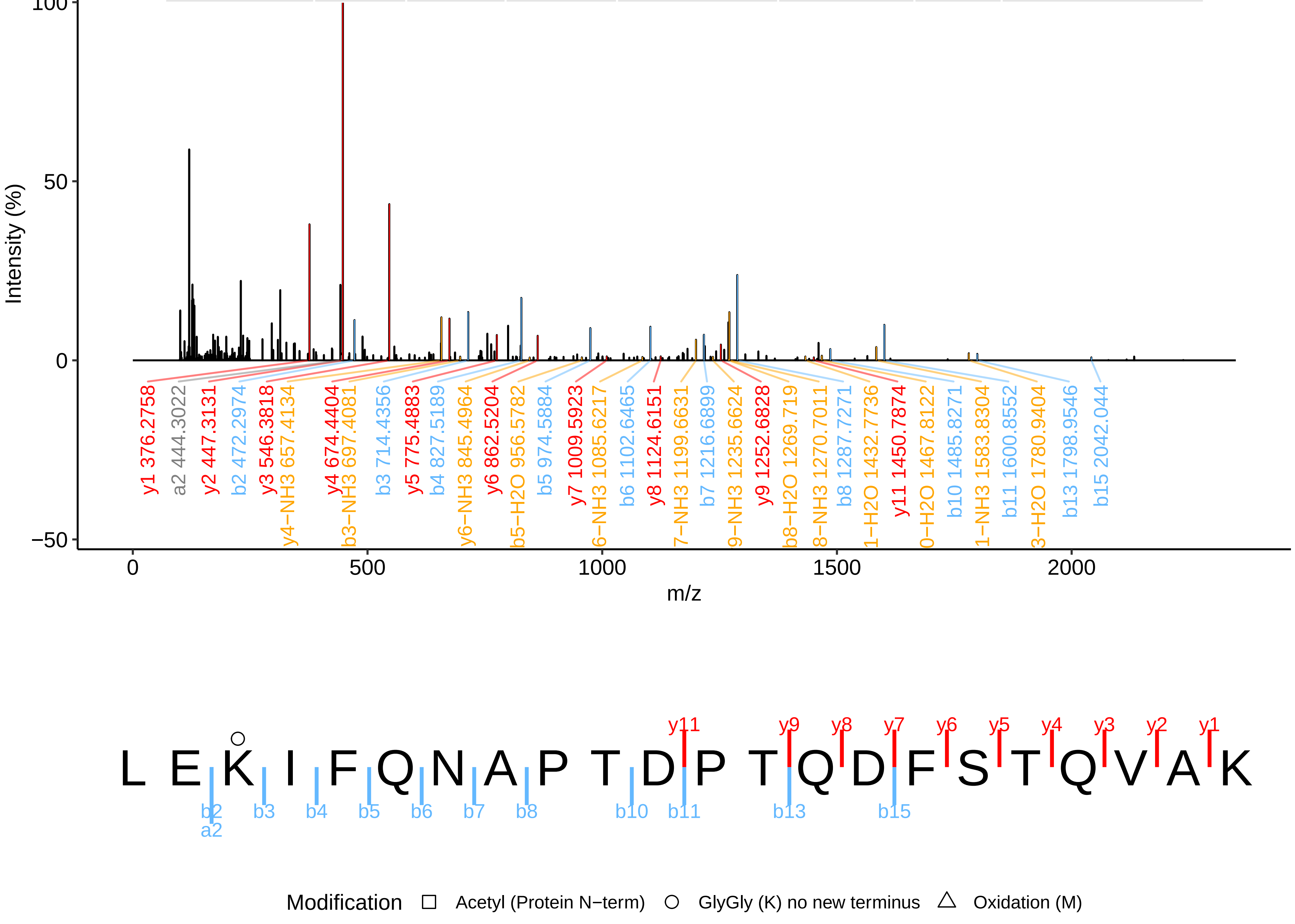

20200924_KIZ_MUb5_GlyGly_2 − Scan number: 23274

Gene Names	Charge	m/z	Mass	Retention time	PEP	Score	Precursor Intensity
USP5	4	648.829	2591.287	86.181	5.6644e−15	119.87	7337618

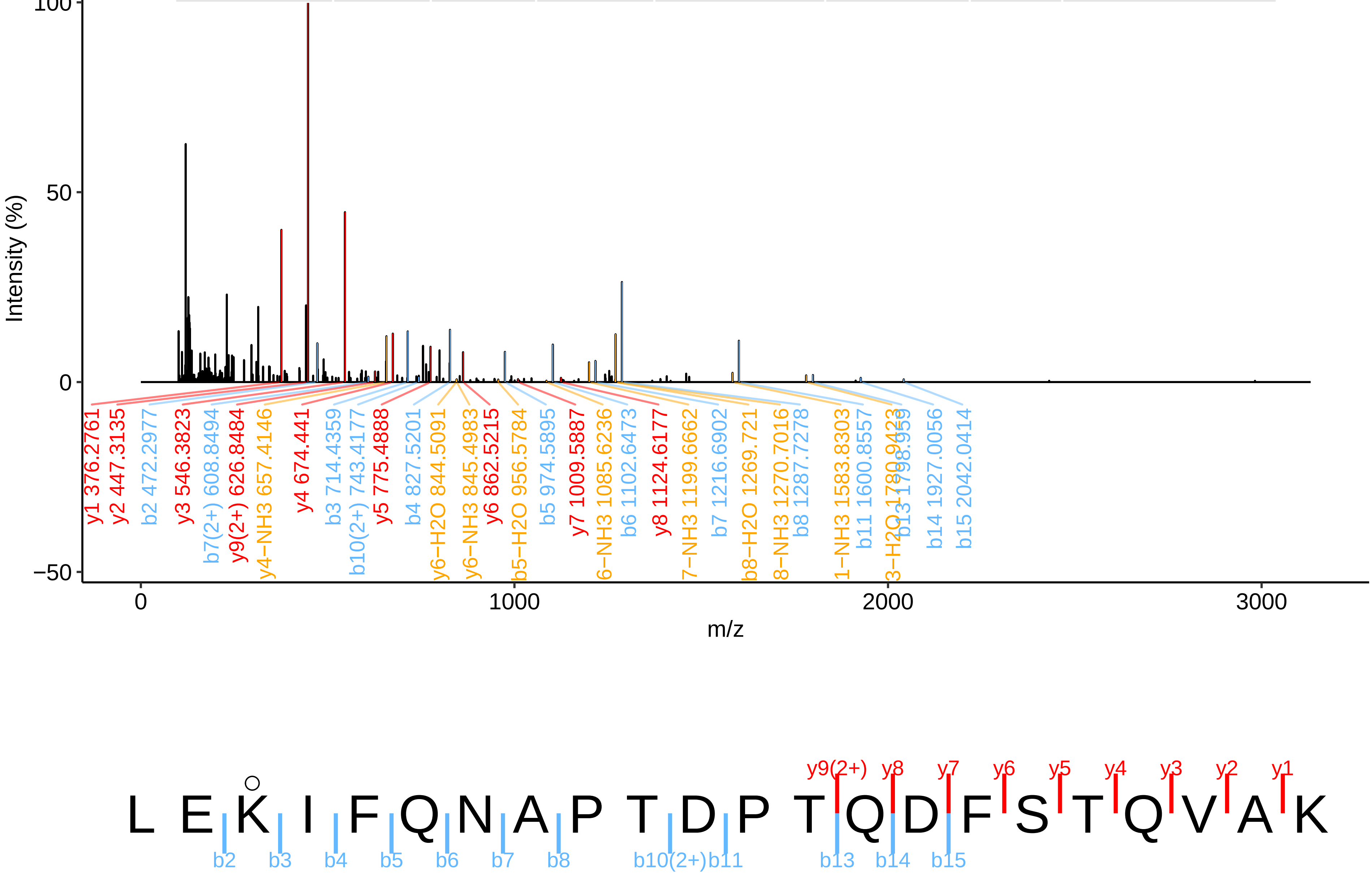

Modification □ Acetyl (Protein N−term) ○ GlyGly (K) no new terminus △ Oxidation (M)

20200921_KIZ_MUb4_GlyGly_10plex − Scan number: 30860

Gene Names	Charge	m/z	Mass	Retention time	PEP	Score	Precursor Intensity
TIMM13	3	952.7775	2855.311	113.55	0.027363	49.287	NA

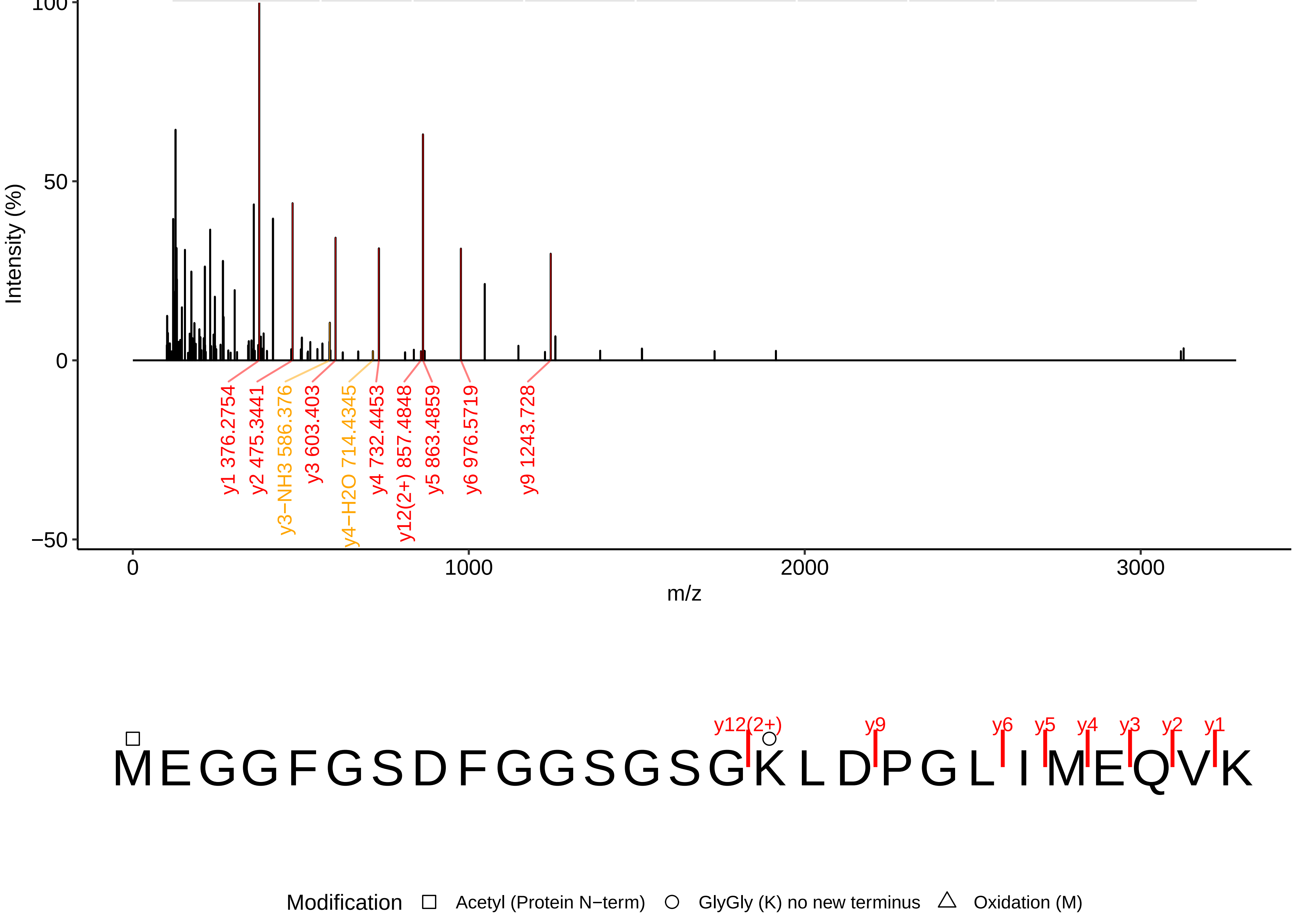

20200921_KIZ_MUb4_GlyGly_10plex − Scan number: 30884

Gene Names	Charge	m/z	Mass	Retention time	PEP	Score	Precursor Intensity
TIMM13	3	952.7775	2855.311	113.64	0.01321	48.867	2028070

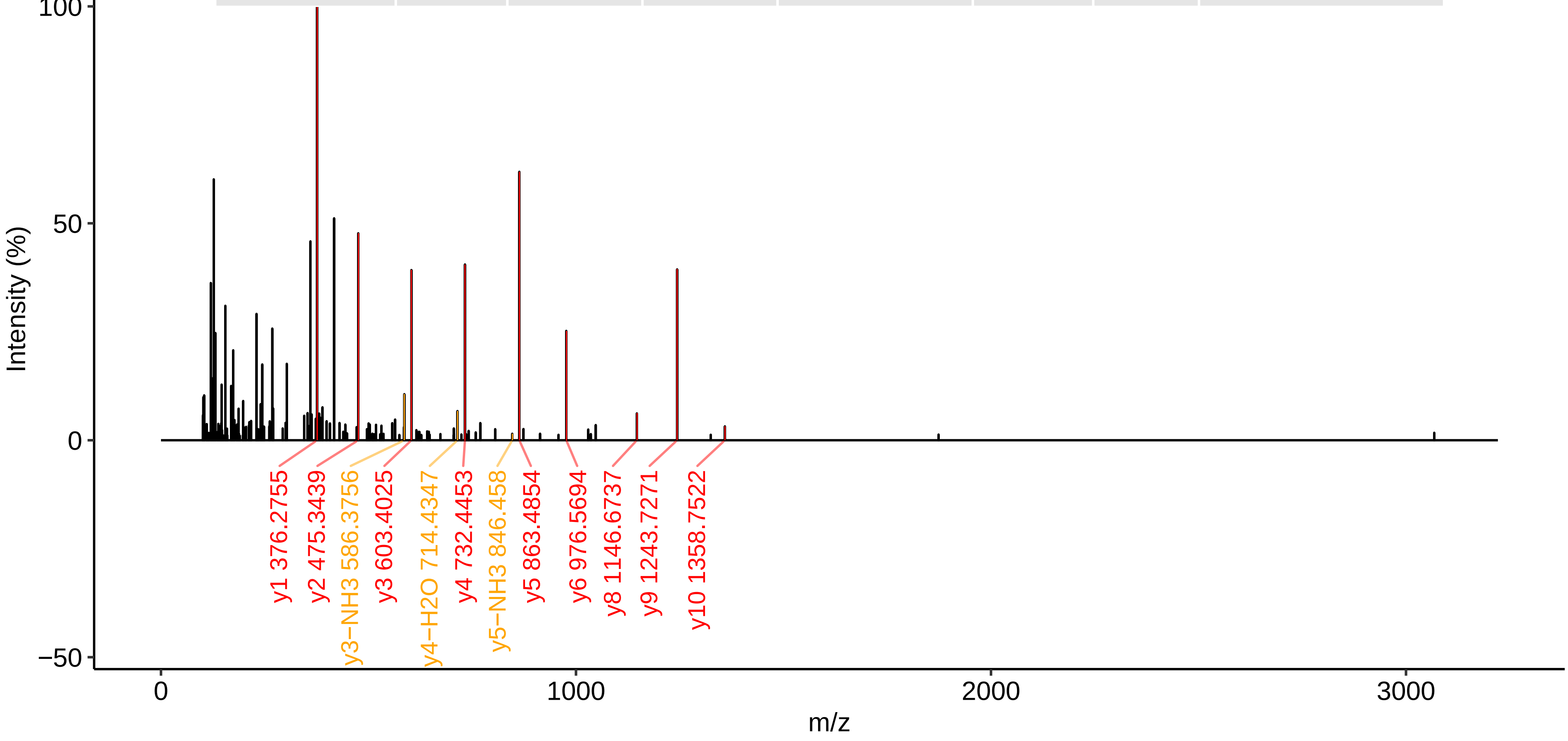

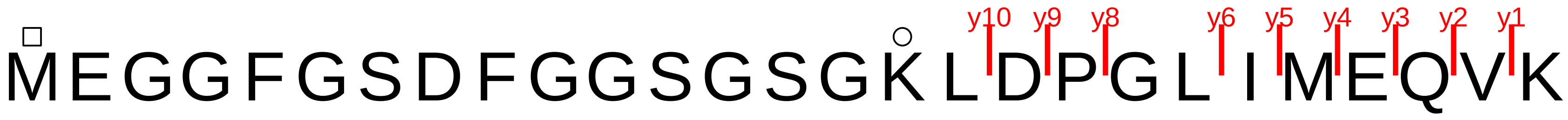

Modification □ Acetyl (Protein N−term) ○ GlyGly (K) no new terminus △ Oxidation (M)

20200921_KIZ_MUb4_GlyGly_10plex − Scan number: 30897

Gene Names	Charge	m/z	Mass	Retention time	PEP	Score	Precursor Intensity
TIMM13	3	952.7775	2855.311	113.69	1.2673e−05	56.95	2015021

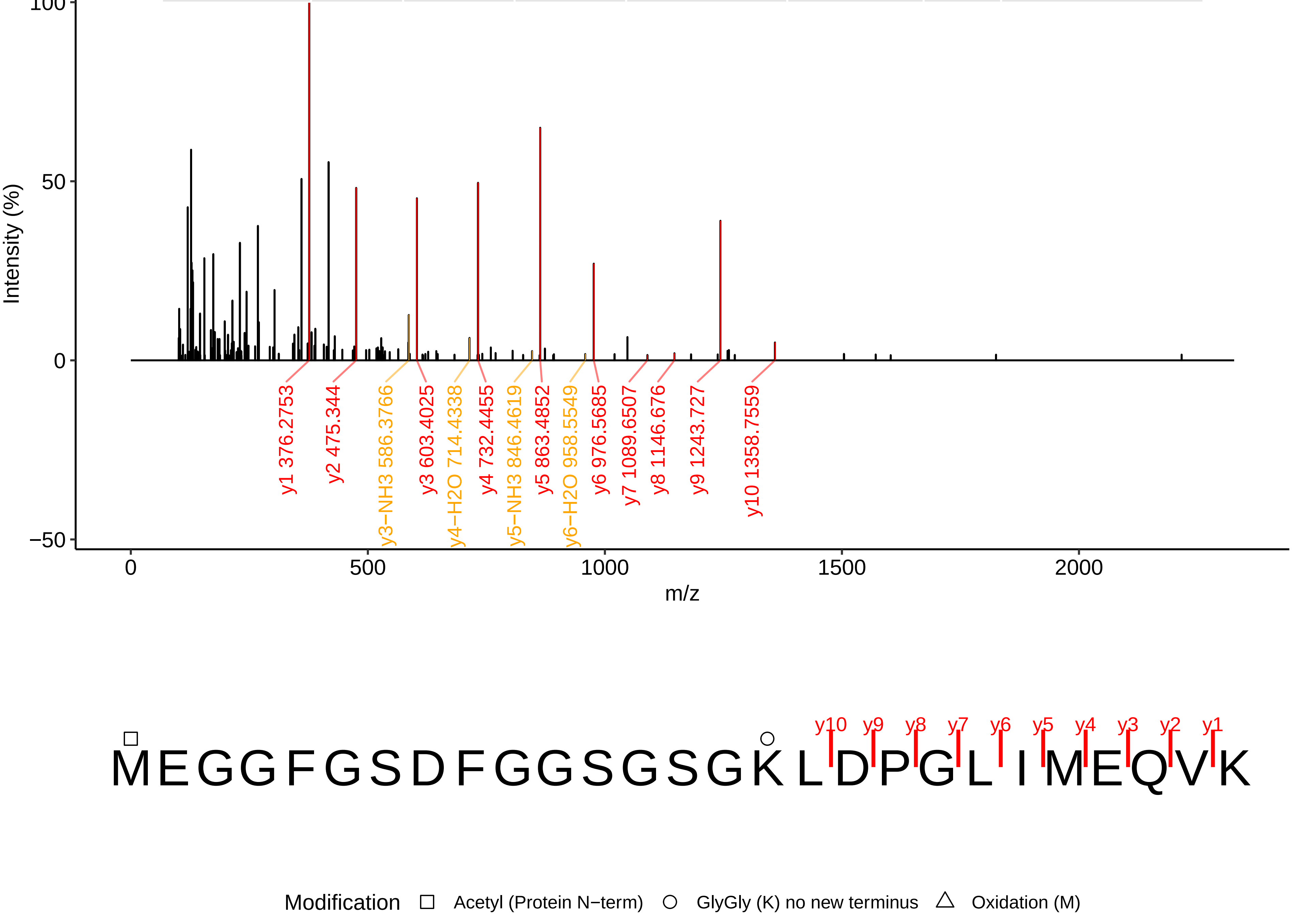

20201118_KIZ_MUb6_GlyGly_2 − Scan number: 22324

Gene Names	Charge	m/z	Mass	Retention time	PEP	Score	Precursor Intensity
RPS27A;UBA52;UBB;UBC	5	589.3124	2941.526	75.062	2.461e−22	133.95	10957687

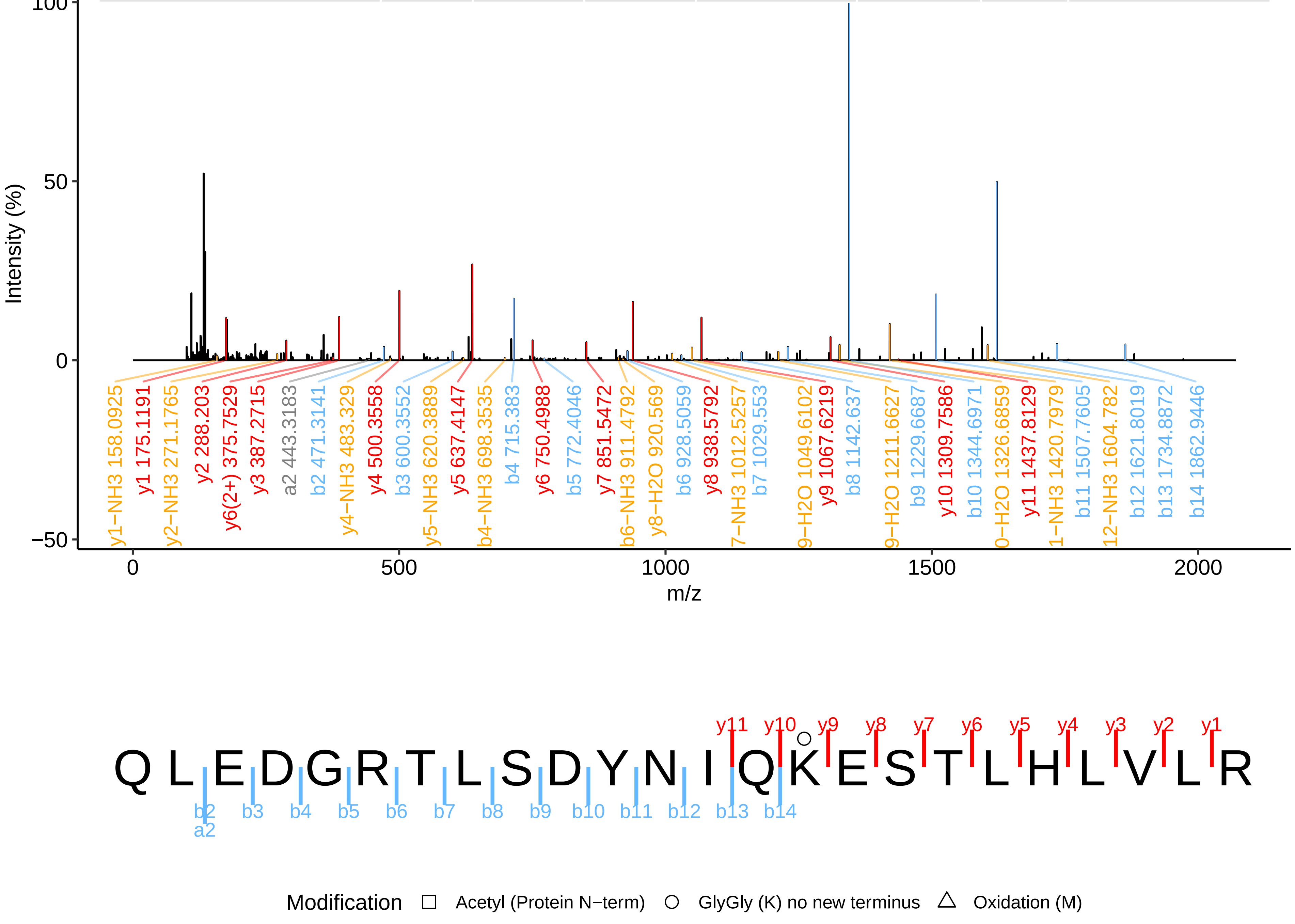

20200921_KIZ_MUb4_GlyGly_10plex_2 − Scan number: 17683

Gene Names	Charge	m/z	Mass	Retention time	PEP	Score	Precursor Intensity
SLC38A2	4	658.8214	2631.256	69.169	6.3599e−16	131.11	1678296

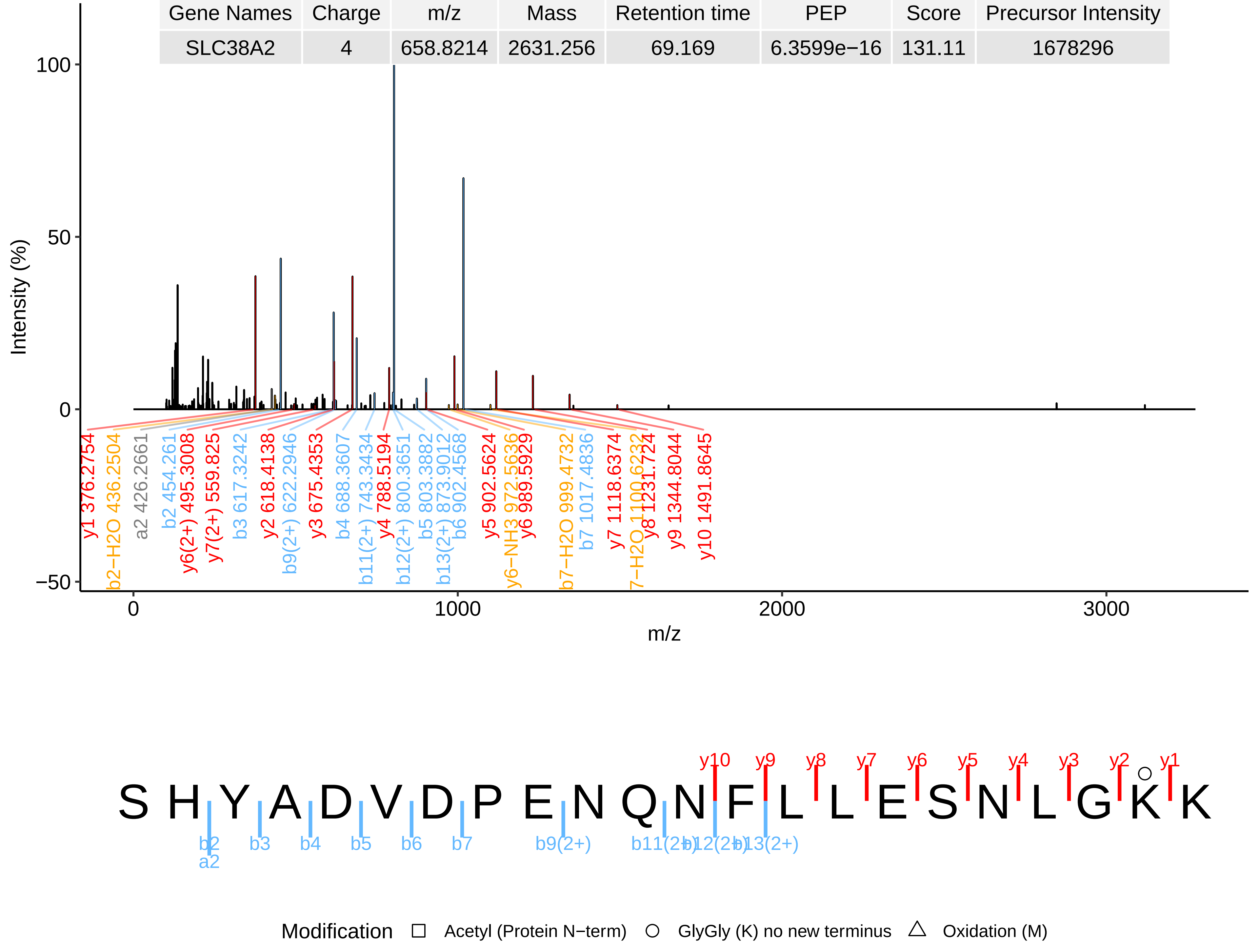

20200921_KIZ_MUb4_GlyGly_10plex_2 − Scan number: 17815

Gene Names	Charge	m/z	Mass	Retention time	PEP	Score	Precursor Intensity
SLC38A2	4	658.8214	2631.256	69.7	0.00021537	75.55	819102.8

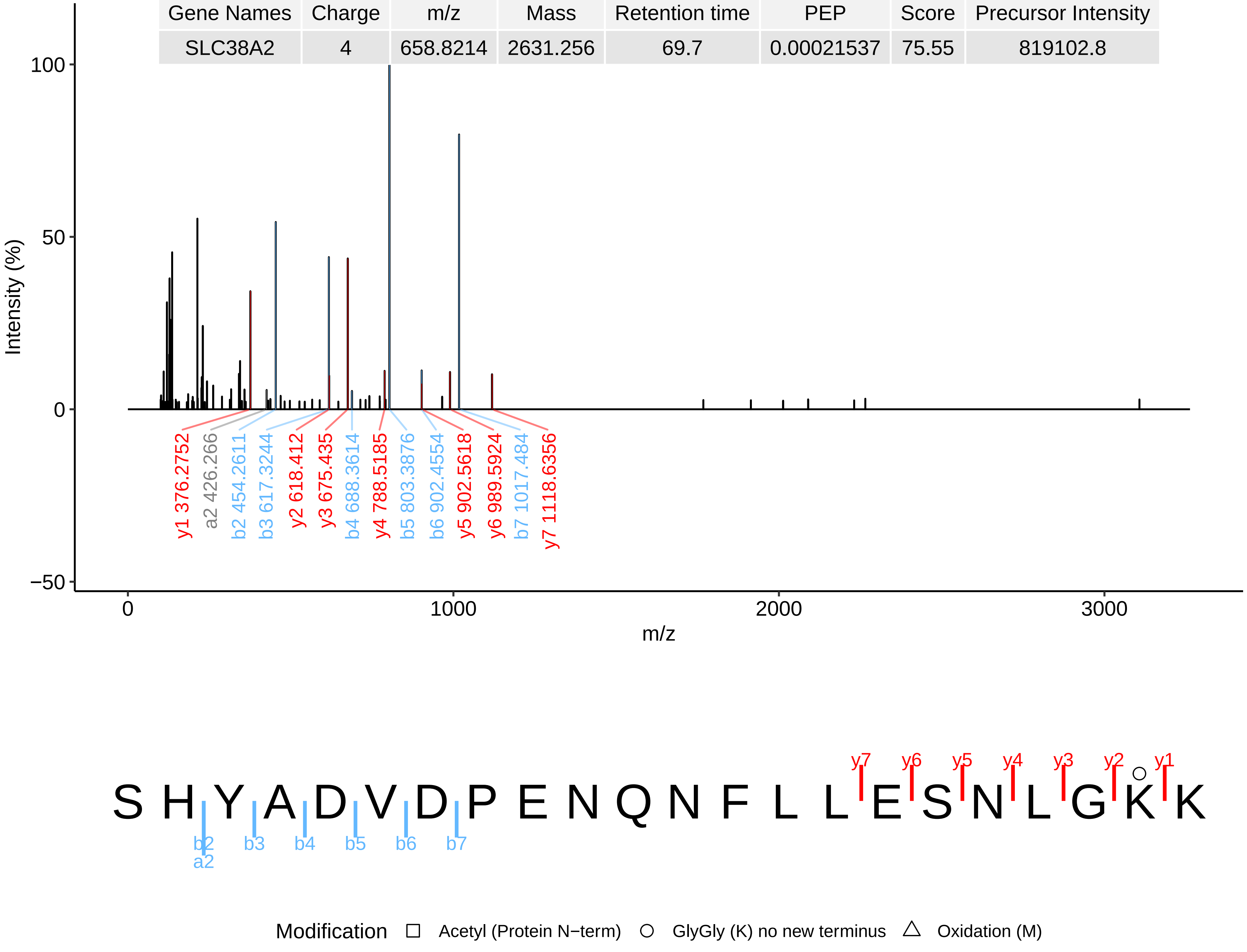

20200921_KIZ_MUb4_GlyGly_10plex_2 − Scan number: 13373

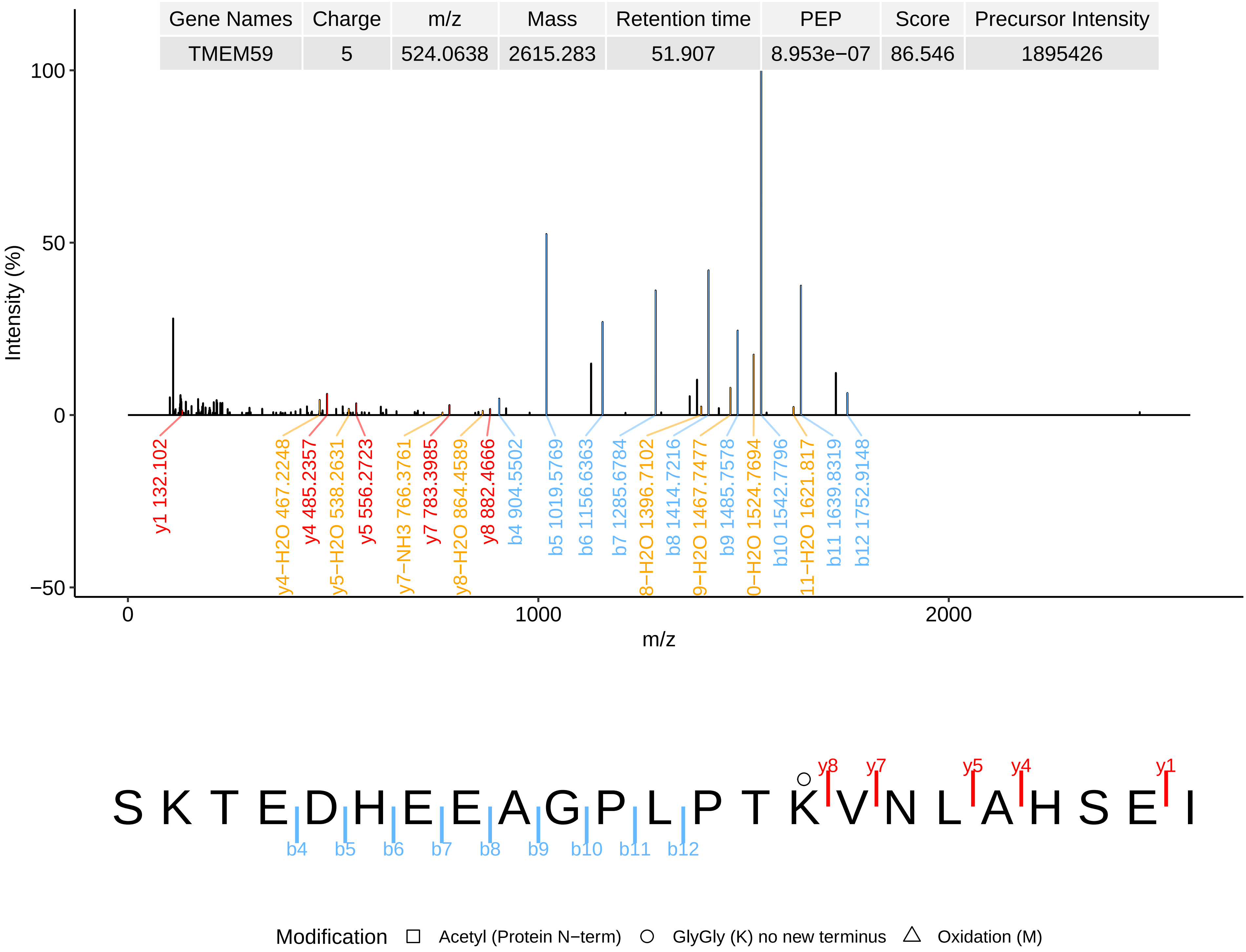

20200921_KIZ_MUb4_GlyGly_10plex_2 − Scan number: 13503

Gene Names	Charge	m/z	Mass	Retention time	PEP	Score	Precursor Intensity
TMEM59	5	524.0638	2615.283	52.442	0.00063336	70.453	726853.6

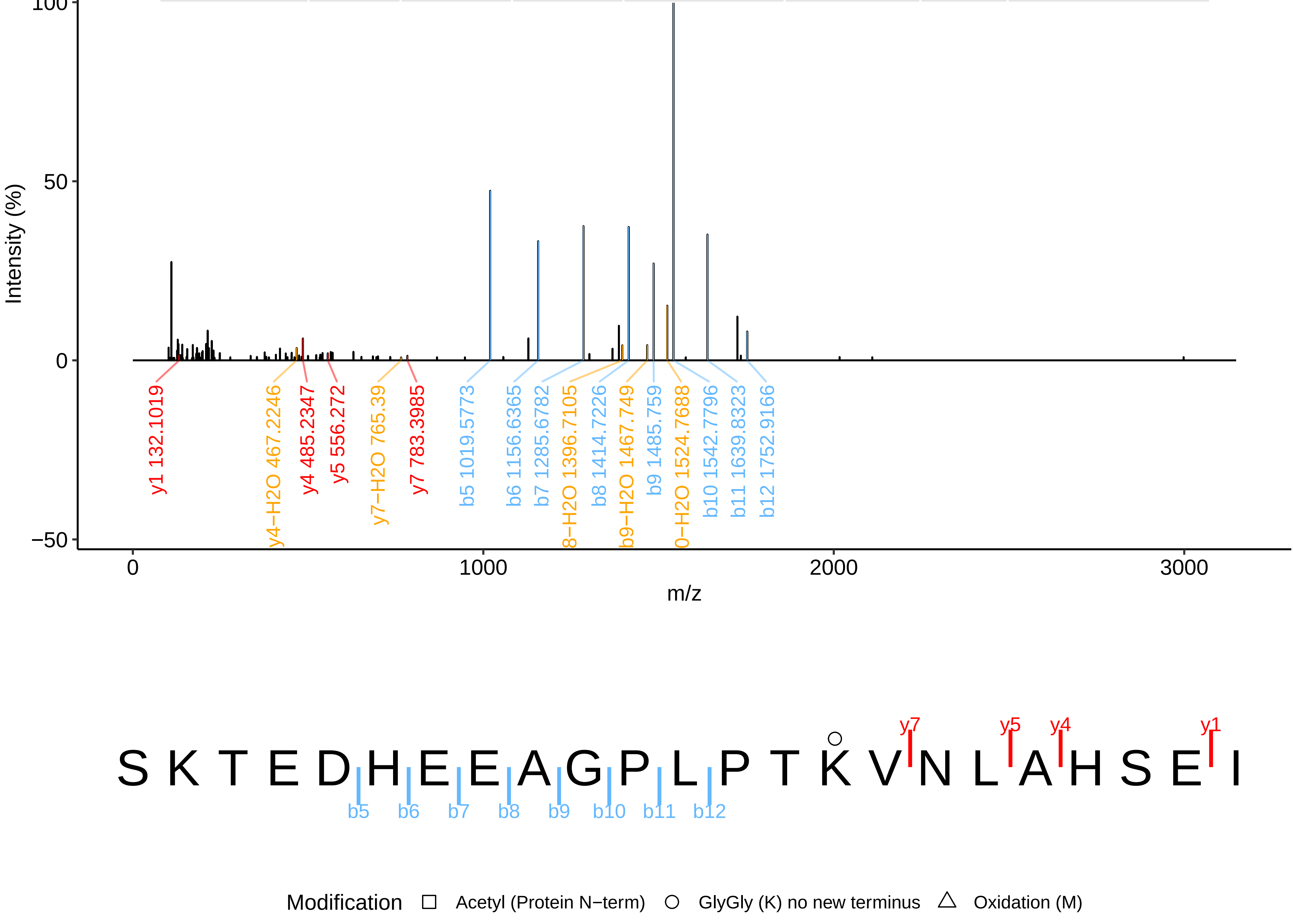

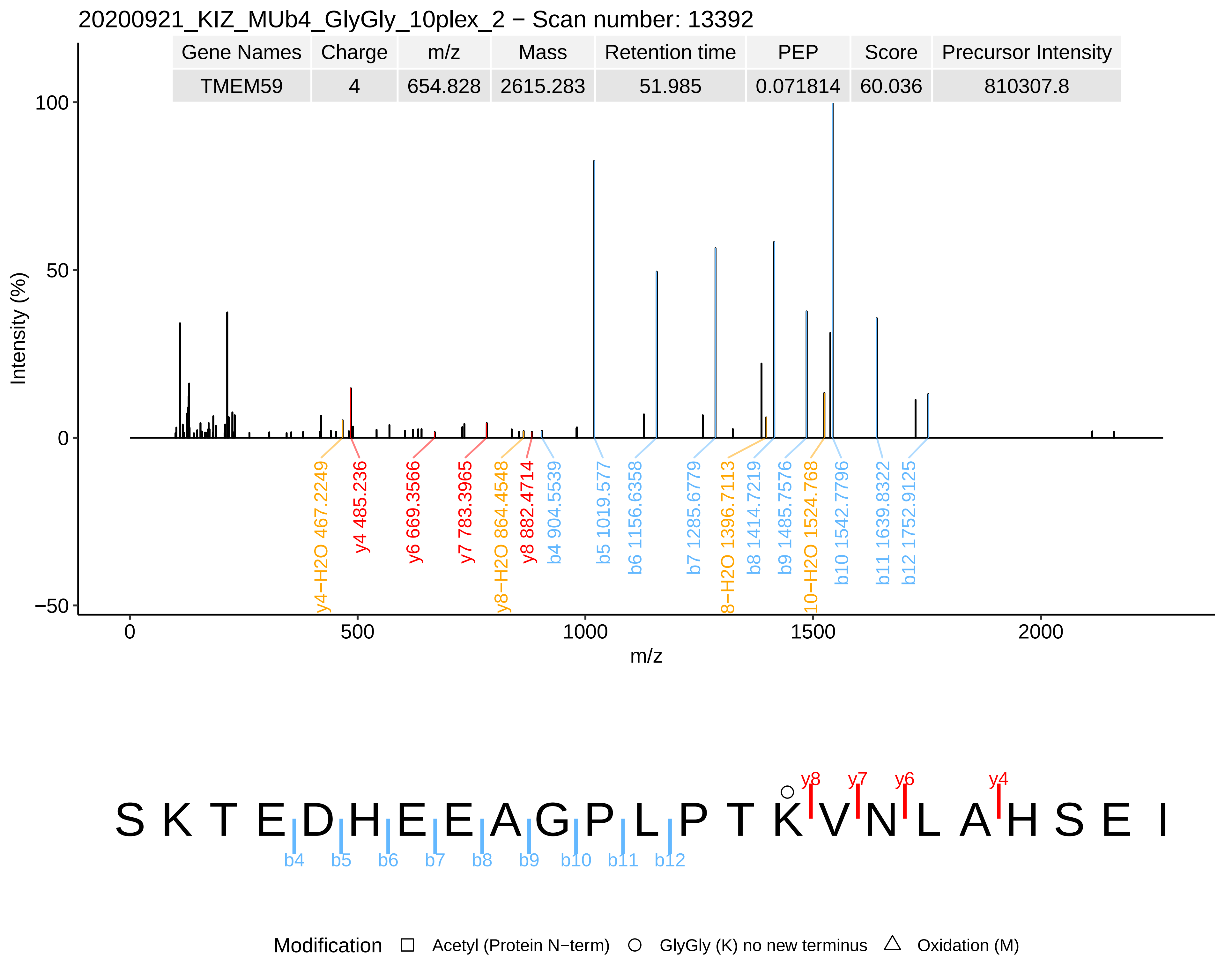
20200921_KIZ_MUb4_GlyGly_10plex_2 − Scan number: 13392
Gene Names
Charge
m/z
Mass
Retention time
PEP
Score
Precursor Intensity
TMEM59
4
654.828
2615.283
51.985
0.071814
60.036
810307.8
Intensity (%)
100
50
0
−50
m/z
0
500
1000
1500
2000
y4−H2O 467.2249
y4 485.236
y6 669.3566
y7 783.3965
y8−H2O 864.4548
y8 882.4714
b4 904.5539
b5 1019.577
b6 1156.6358
b7 1285.6779
8−H2O 1396.7113
b8 1414.7219
b9 1485.7576
10−H2O 1524.768
b10 1542.7796
b11 1639.8322
b12 1752.9125
S K T E D H E E A G P L P T K V N L A H S E I
y8
y7
y6
y4
b4
b5
b6
b7
b8
b9
b10
b11
b12
Modification
Acetyl (Protein N−term)
GlyGly (K) no new terminus
Oxidation (M)

20200924_KIZ_MUb5_GlyGly_2 − Scan number: 13376

Gene Names	Charge	m/z	Mass	Retention time	PEP	Score	Precursor Intensity
RAD18	4	670.5923	2678.34	50.037	1.12e−09	99.366	2687759

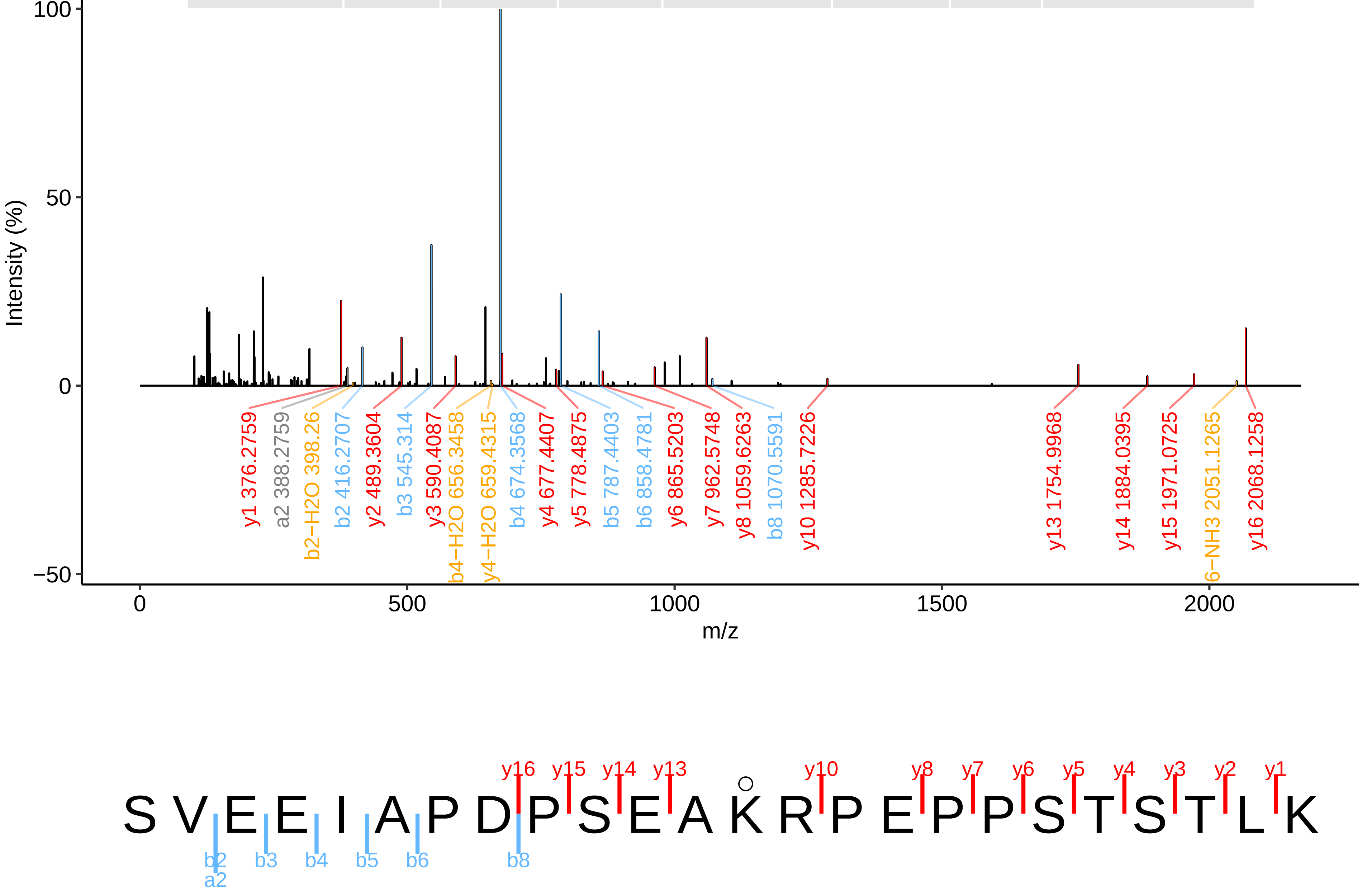

Modification □ Acetyl (Protein N−term) ○ GlyGly (K) no new terminus △ Oxidation (M)

20200921_KIZ_MUb4_GlyGly_10plex − Scan number: 28073

Gene Names	Charge	m/z	Mass	Retention time	PEP	Score	Precursor Intensity
EEF1A1;EEF1A1P5	3	877.1472	2628.42	102.63	0.00012353	77.681	2478210

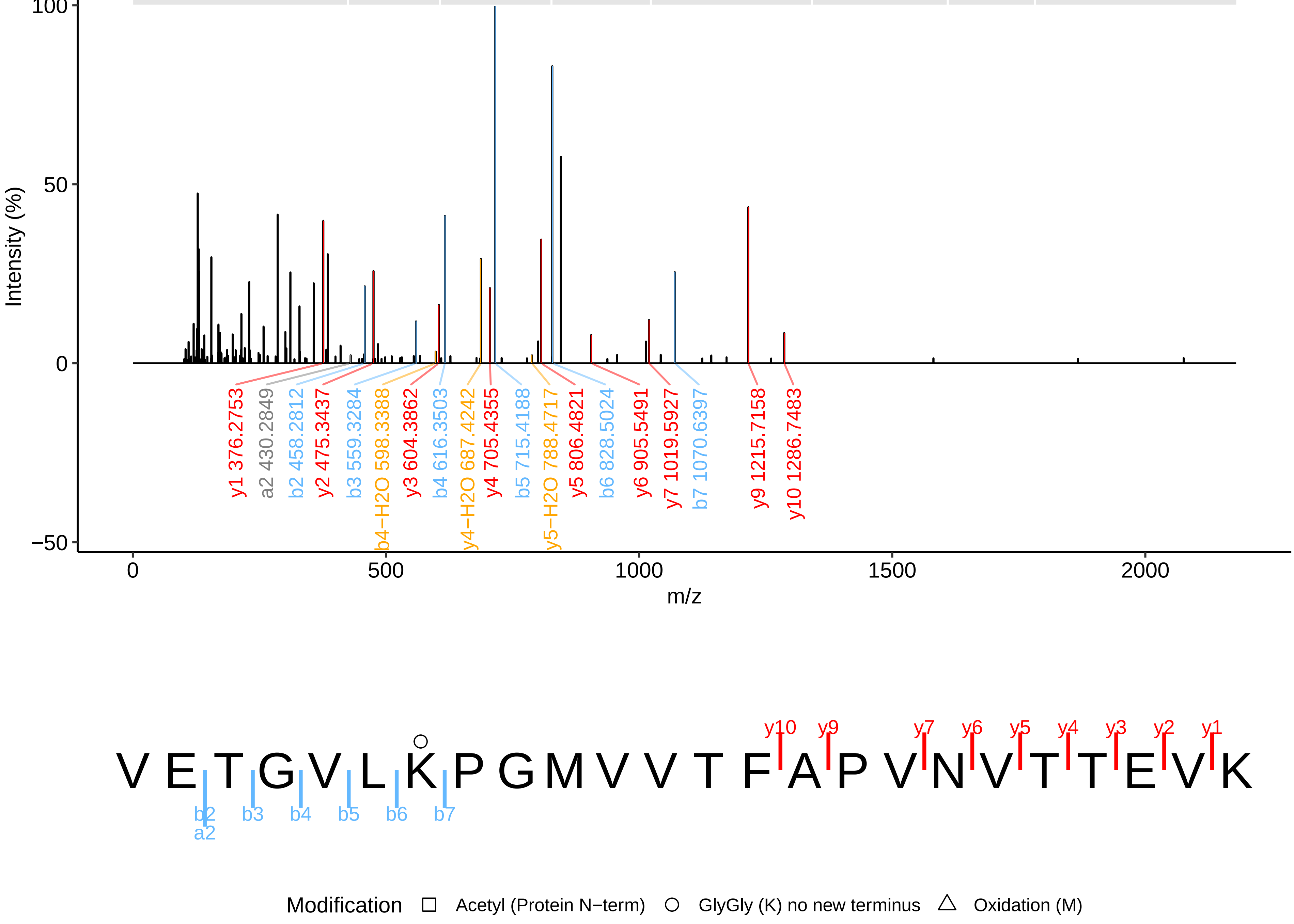

20200921_KIZ_MUb4_GlyGly_10plex − Scan number: 28087

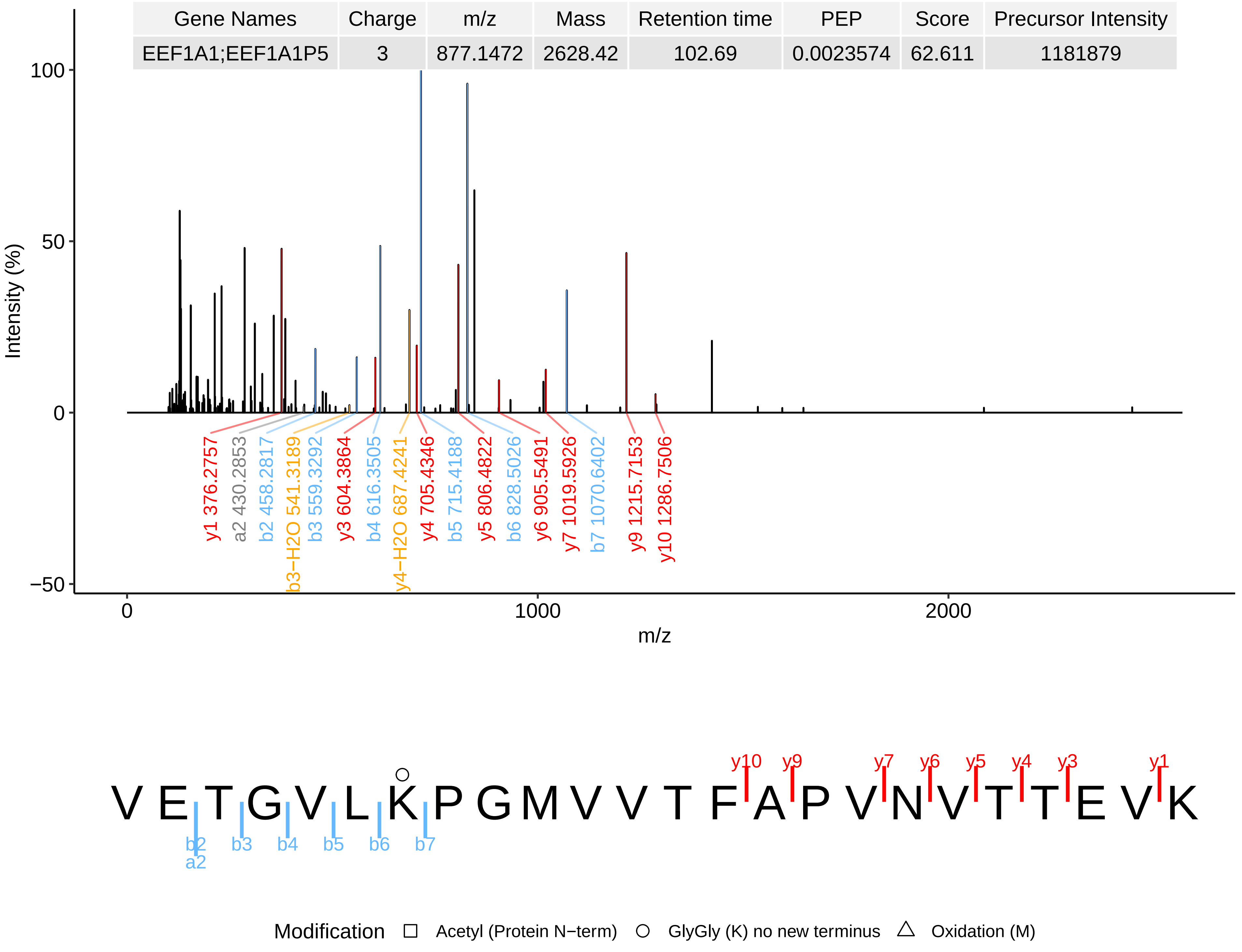

20200921_KIZ_MUb4_GlyGly_10plex − Scan number: 28086

Gene Names	Charge	m/z	Mass	Retention time	PEP	Score	Precursor Intensity
EEF1A1;EEF1A1P5	4	658.1122	2628.42	102.68	0.00041819	68.552	NA

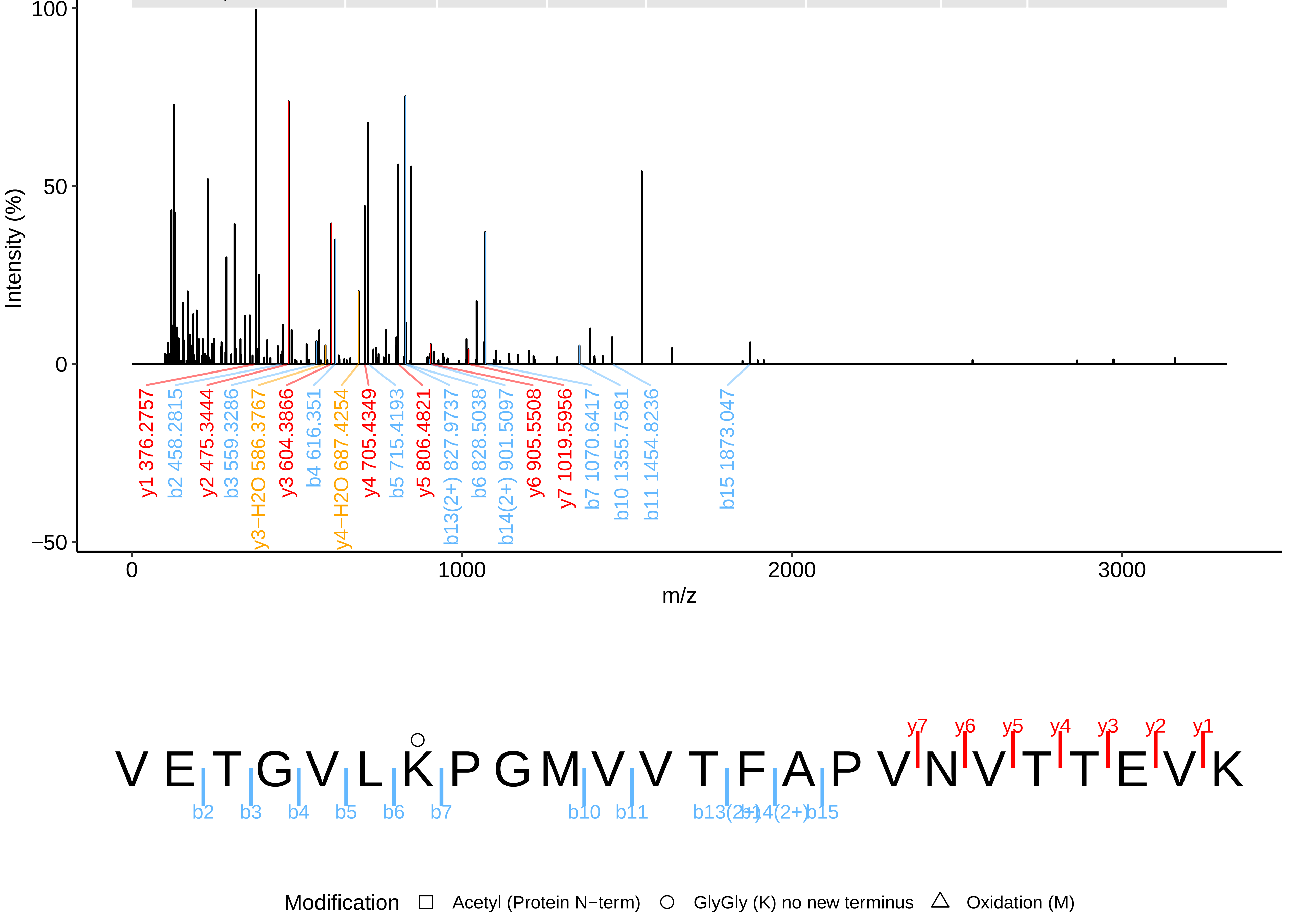

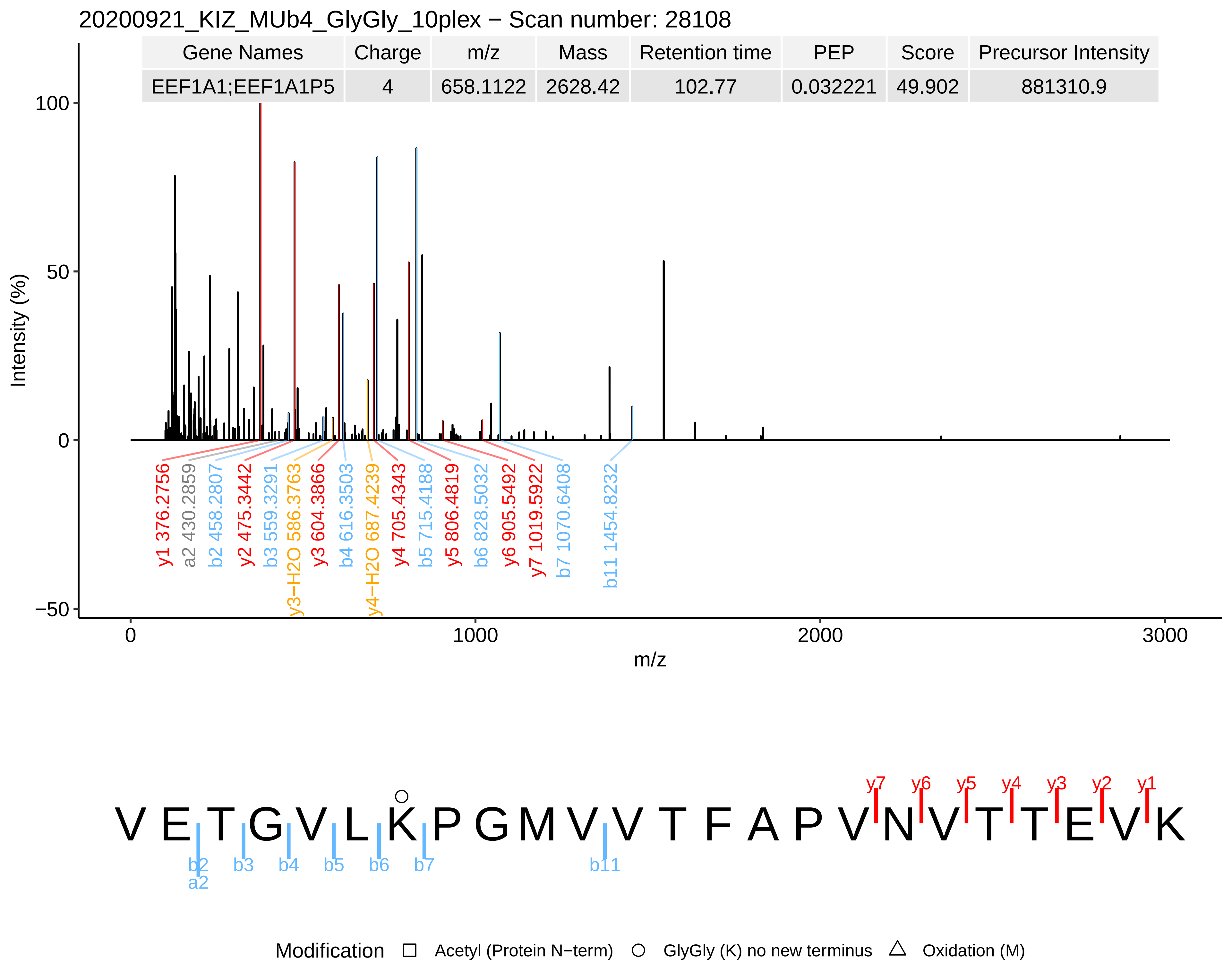
20200921_KIZ_MUb4_GlyGly_10plex − Scan number: 28108
Gene Names | Charge | m/z | Mass | Retention time | PEP | Score | Precursor Intensity
EEF1A1;EEF1A1P5 | 4 | 658.1122 | 2628.42 | 102.77 | 0.032221 | 49.902 | 881310.9
Intensity (%)
m/z
y1 376.2756
a2 430.2859
b2 458.2807
y2 475.3442
b3 559.3291
y3−H2O 586.3763
y3 604.3866
b4 616.3503
y4−H2O 687.4239
y4 705.4343
b5 715.4188
y5 806.4819
b6 828.5032
y6 905.5492
y7 1019.5922
b7 1070.6408
b11 1454.8232
V E T G V L K P G M V V T F A P V N V T T E V K
Modification: Acetyl (Protein N−term), GlyGly (K) no new terminus, Oxidation (M)

20200921_KIZ_MUb4_GlyGly_10plex_2 − Scan number: 25245

Gene Names	Charge	m/z	Mass	Retention time	PEP	Score	Precursor Intensity
EEF1A1;EEF1A1P5	3	877.1472	2628.42	99.846	0.019269	56.766	703004.4

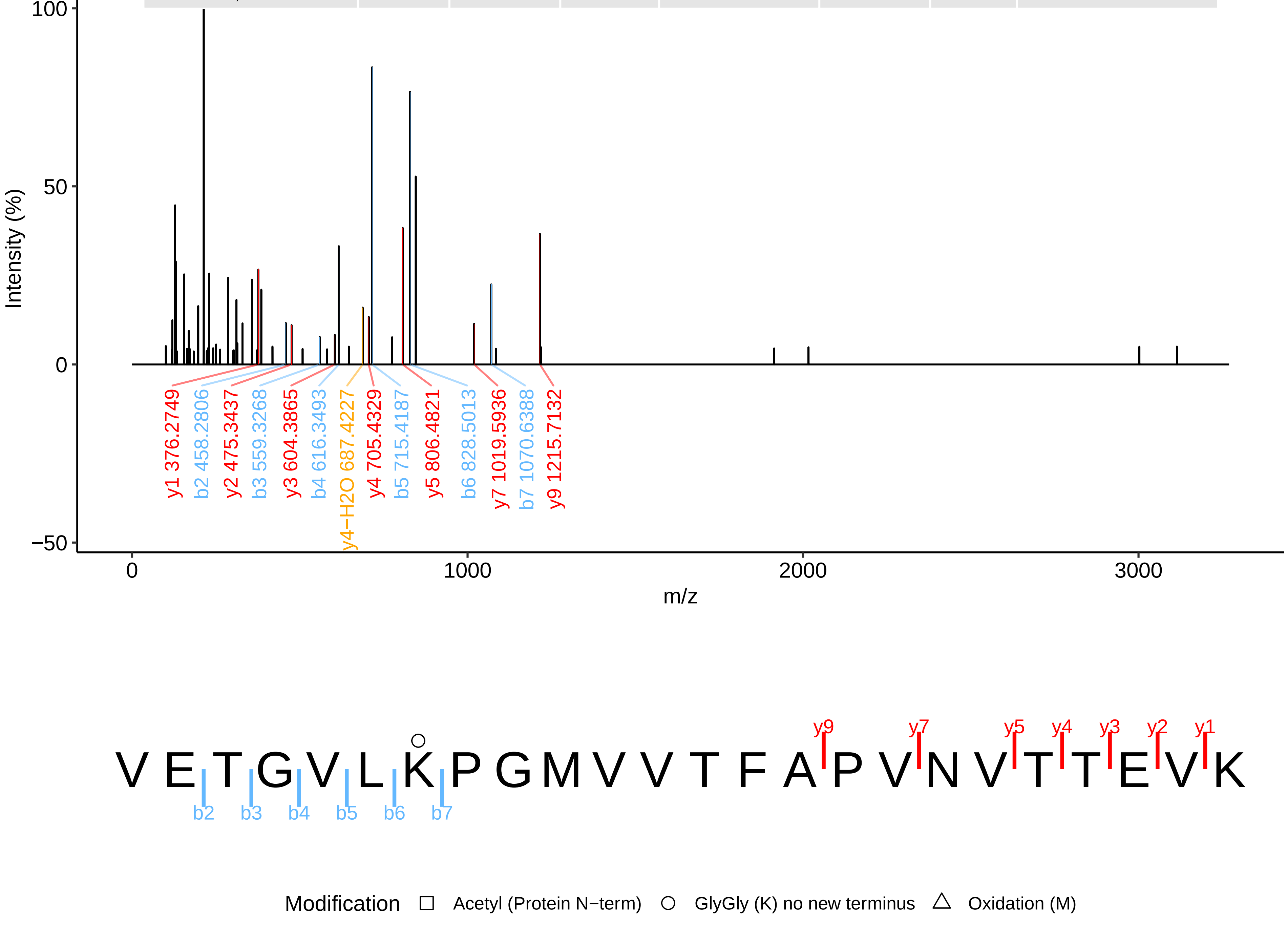

20200921_KIZ_MUb4_GlyGly_10plex_2 − Scan number: 25264

Gene Names	Charge	m/z	Mass	Retention time	PEP	Score	Precursor Intensity
EEF1A1;EEF1A1P5	3	877.1472	2628.42	99.922	0.00075451	63.165	386451.4

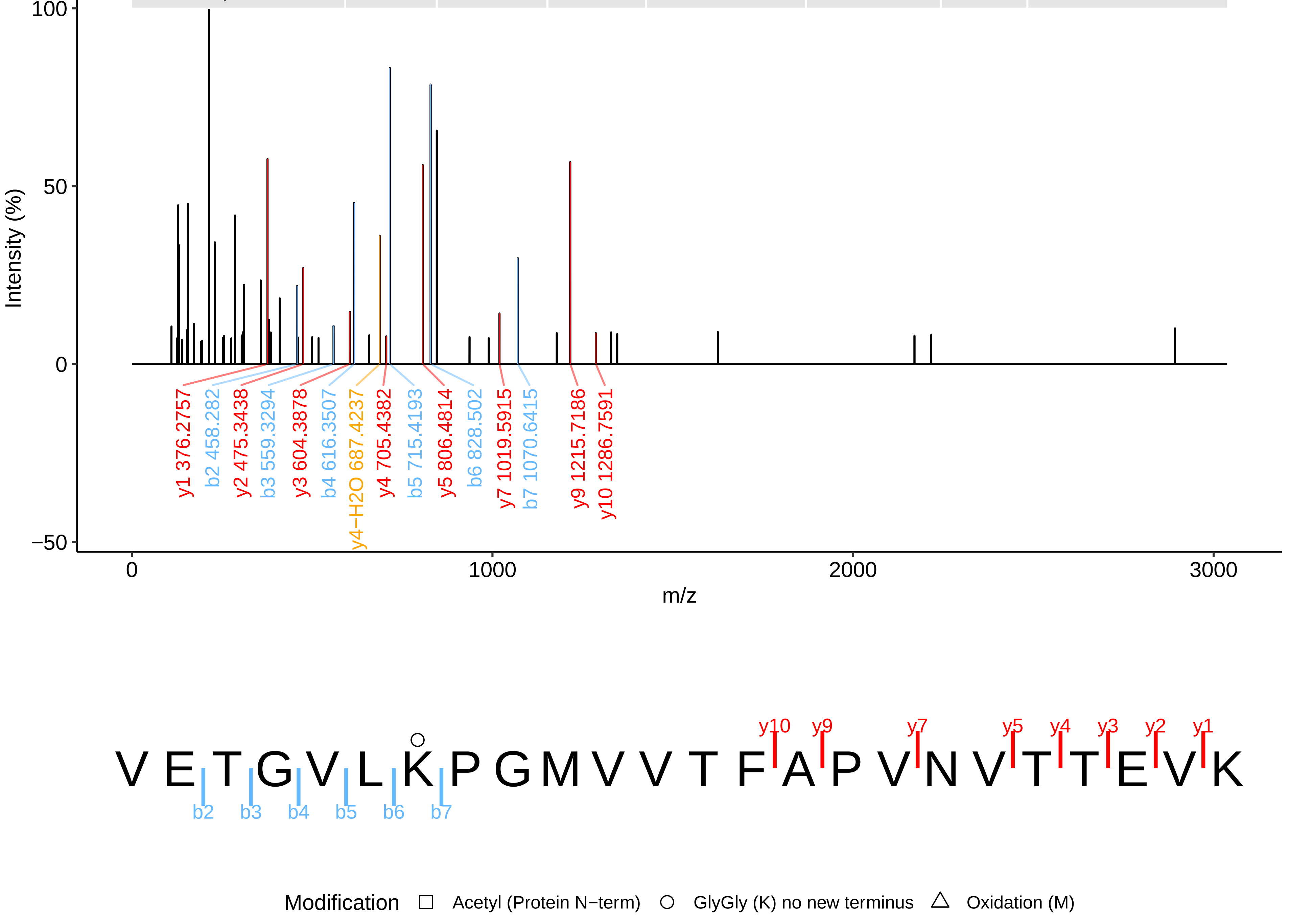

20201118_KIZ_MUb6_GlyGly_2 − Scan number: 33904

Gene Names	Charge	m/z	Mass	Retention time	PEP	Score	Precursor Intensity
MED13L	4	679.8517	2715.378	110.5	0.032976	46.159	15337111

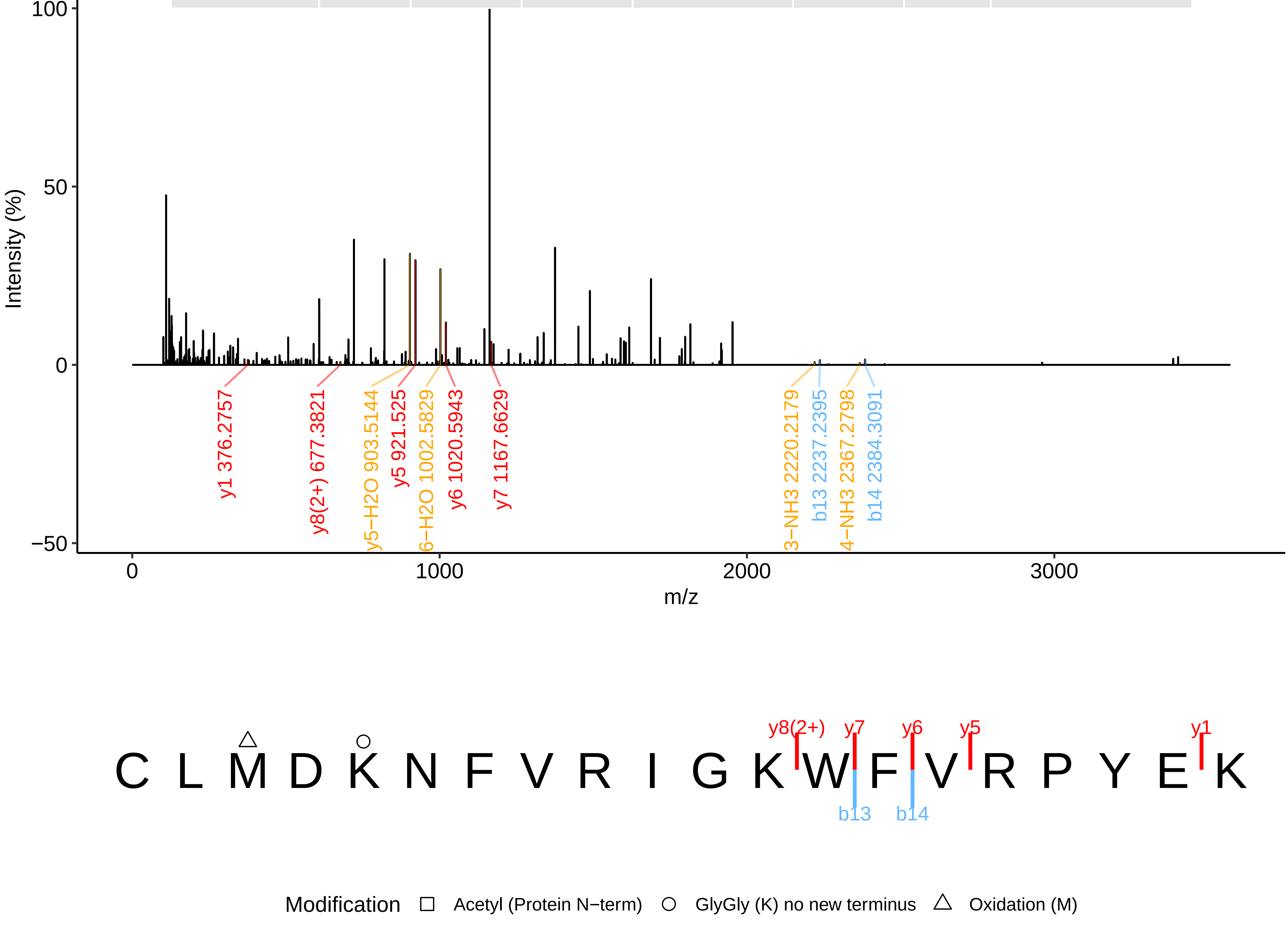

20200921_KIZ_MUb4_GlyGly_10plex_2 − Scan number: 13668

Gene Names	Charge	m/z	Mass	Retention time	PEP	Score	Precursor Intensity
SLC16A1	4	697.3386	2785.325	53.115	1.8663e−14	120.64	2301245

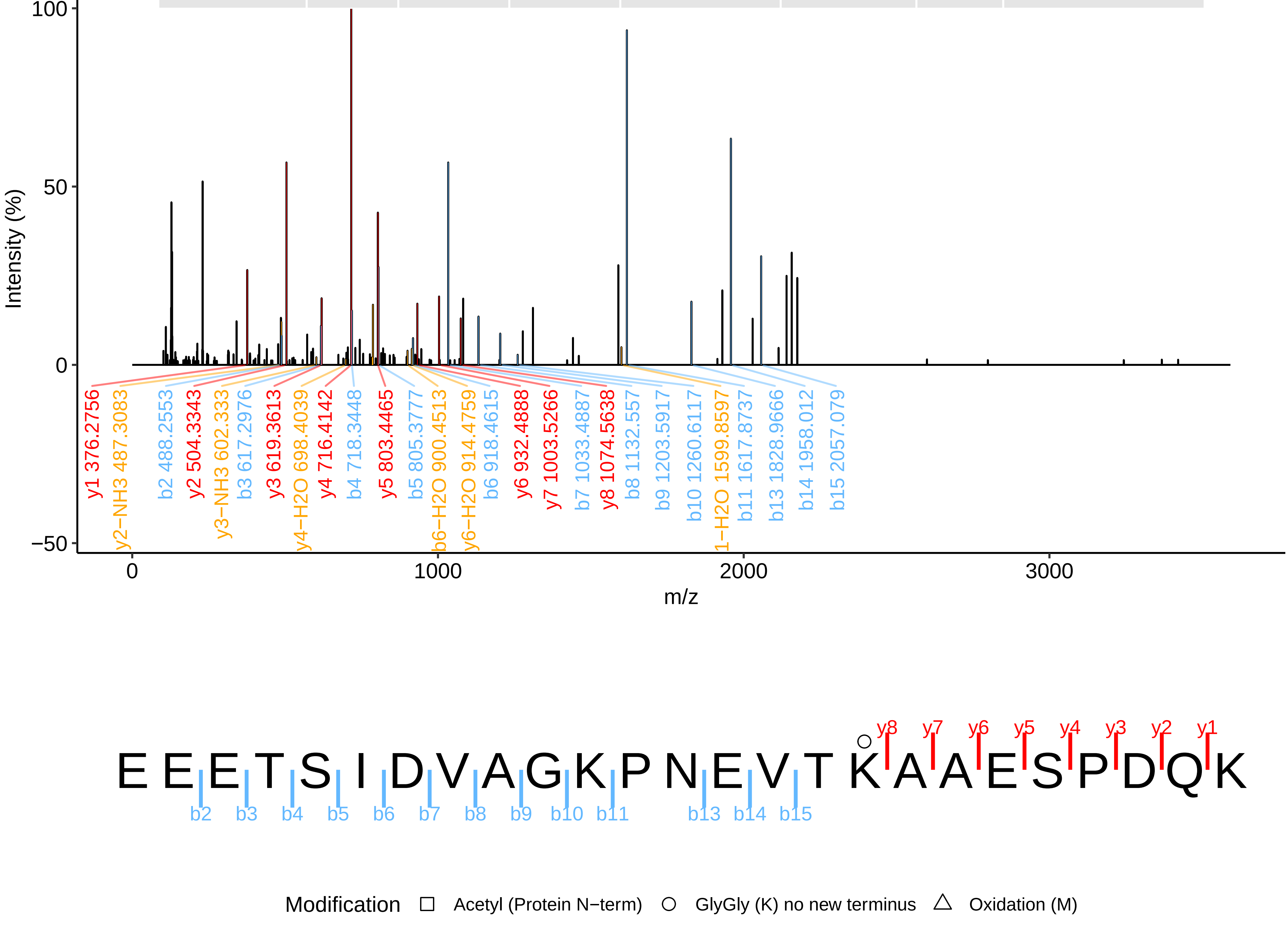

20200921_KIZ_MUb4_GlyGly_10plex_2 − Scan number: 14178

Gene Names	Charge	m/z	Mass	Retention time	PEP	Score	Precursor Intensity
SLC16A3	5	586.0966	2925.447	55.222	0.00099863	76.198	1841418

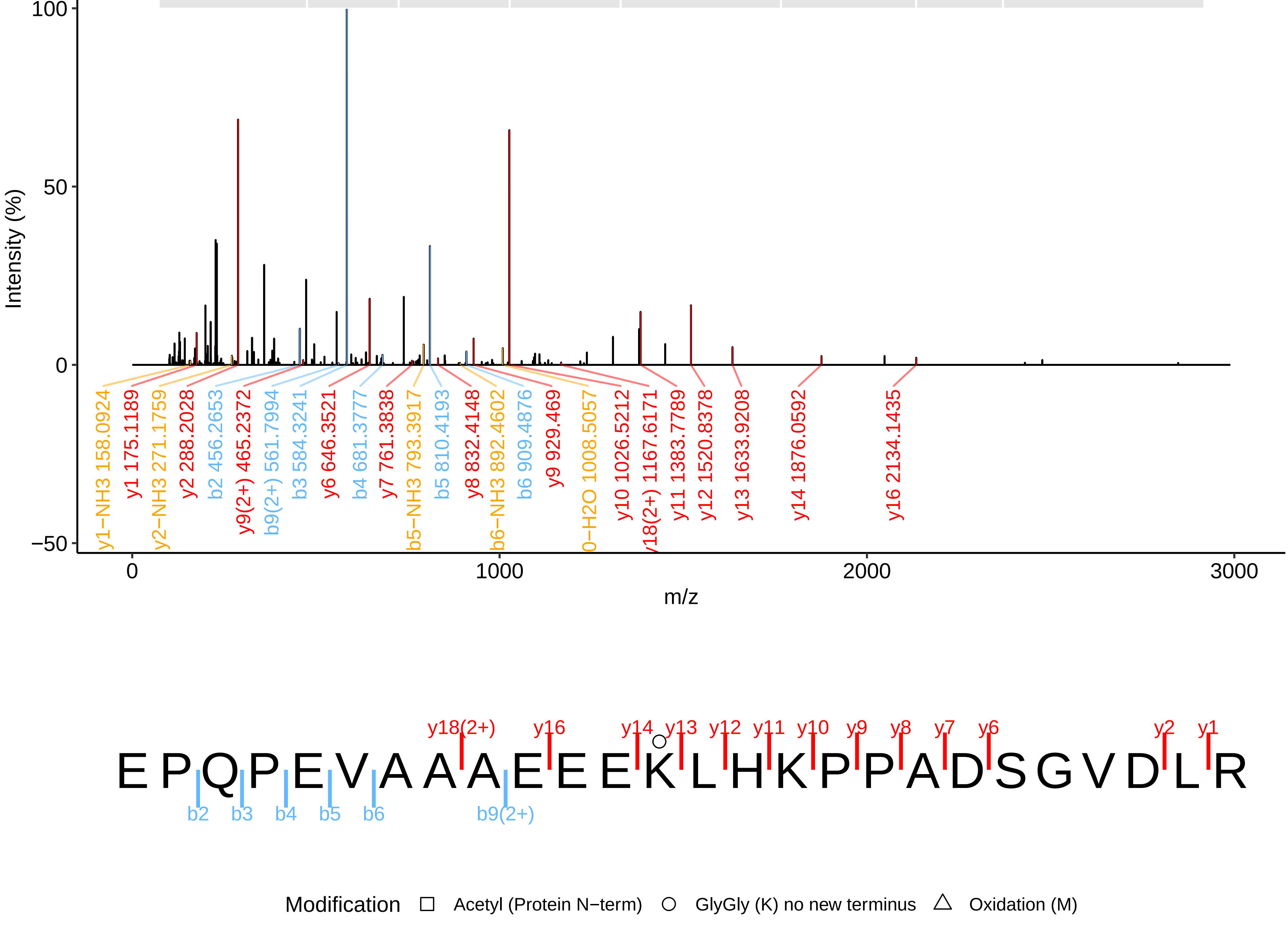

20200921_KIZ_MUb4_GlyGly_10plex_2 − Scan number: 20350

Gene Names	Charge	m/z	Mass	Retention time	PEP	Score	Precursor Intensity
TPI1	4	718.3598	2869.41	79.98	0.0088173	60.278	1309960

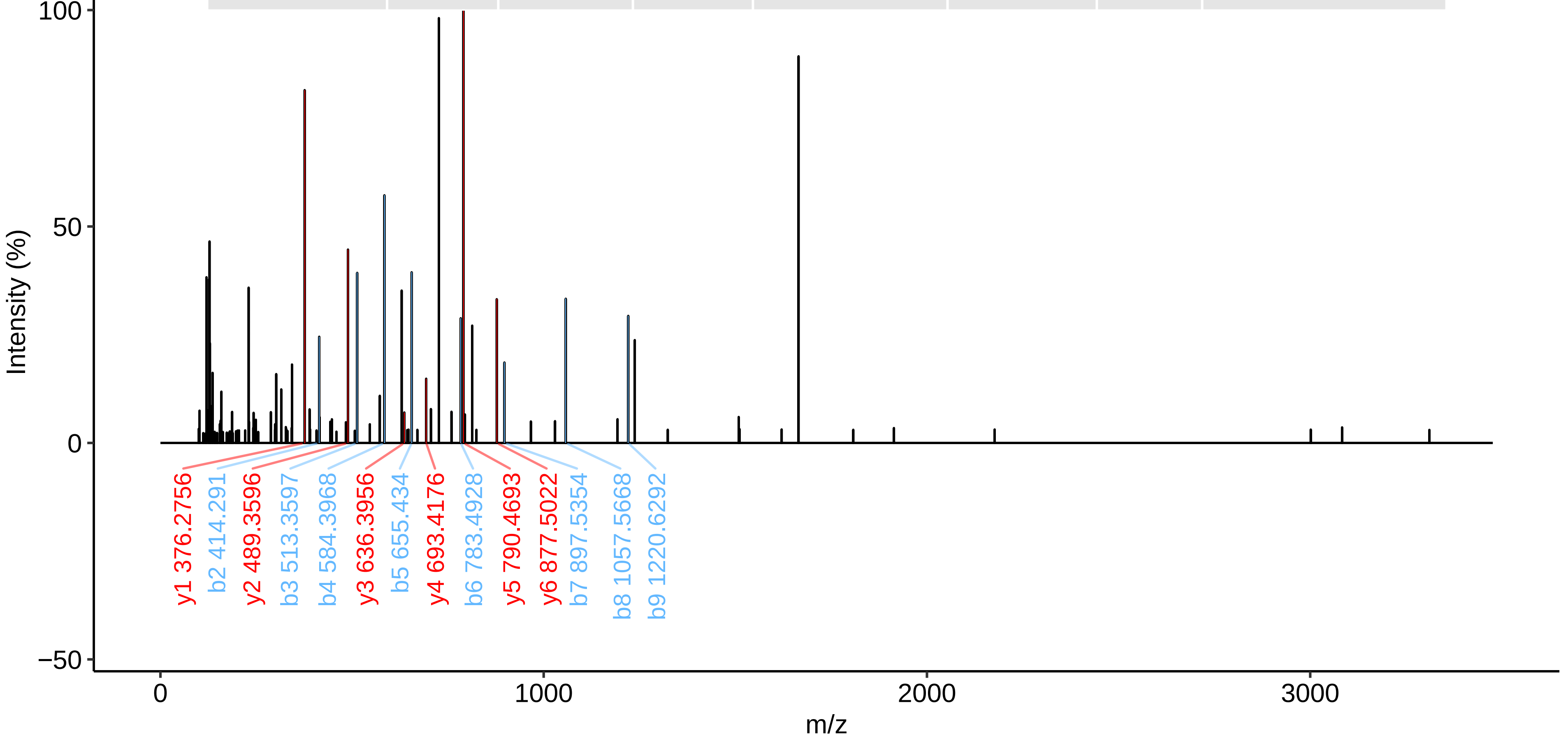

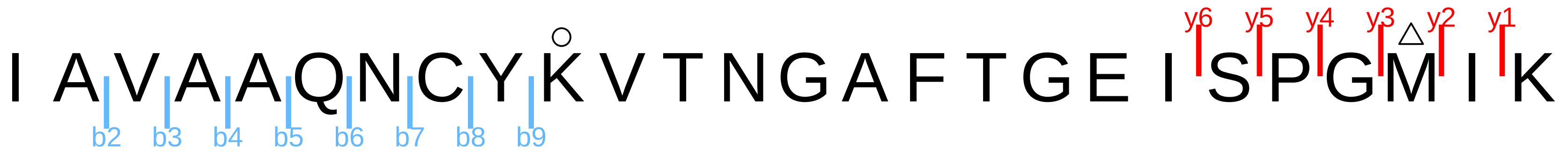

Modification □ Acetyl (Protein N−term) ○ GlyGly (K) no new terminus △ Oxidation (M)

20200921_KIZ_MUb4_GlyGly_10plex_2 − Scan number: 20385

Gene Names	Charge	m/z	Mass	Retention time	PEP	Score	Precursor Intensity
TPI1	4	718.3598	2869.41	80.121	0.048603	44.258	943271.1

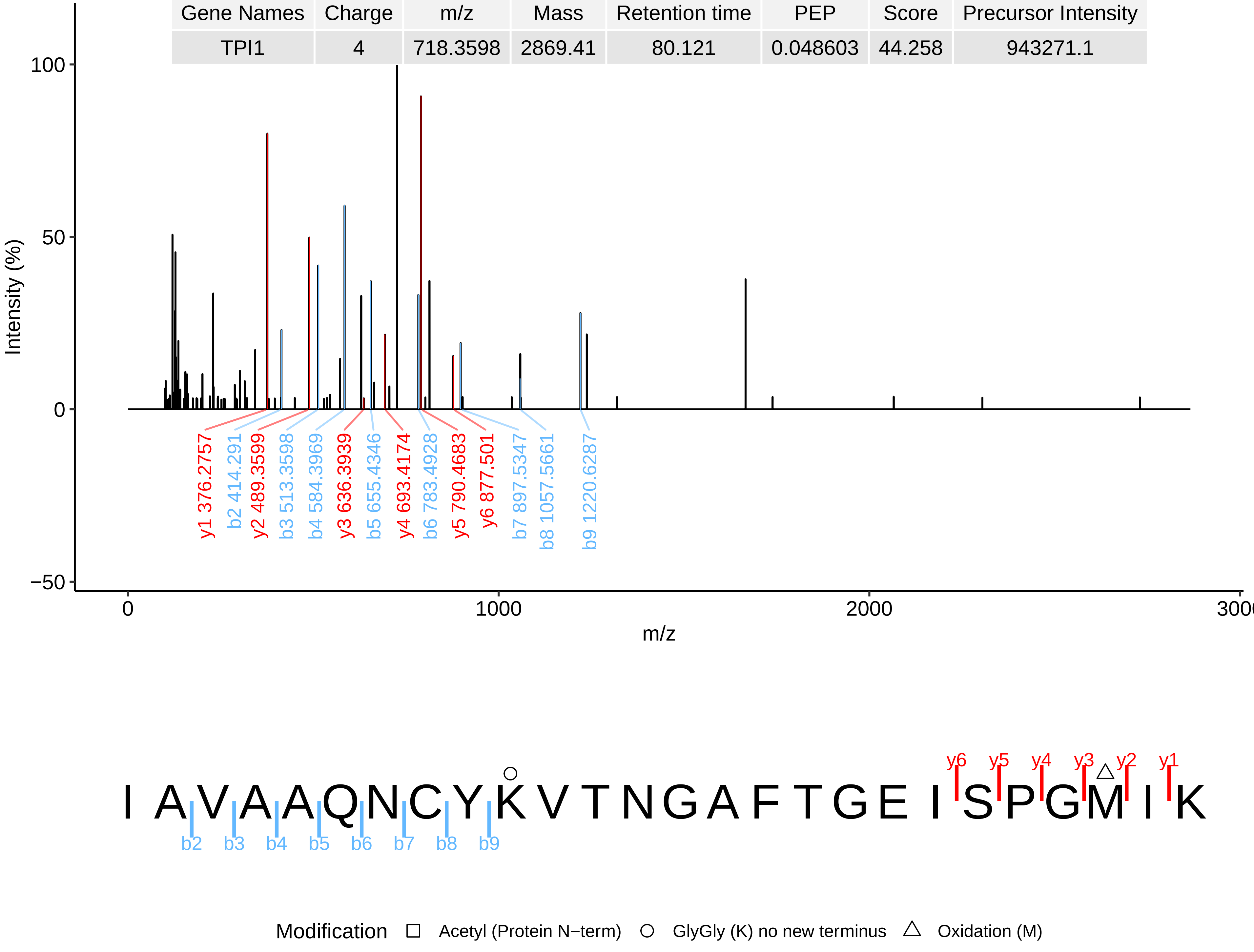

20200921_KIZ_MUb4_GlyGly_10plex_2 − Scan number: 22055

Gene Names	Charge	m/z	Mass	Retention time	PEP	Score	Precursor Intensity
TPI1	4	714.3611	2853.415	86.904	0.0083833	60.278	1541913

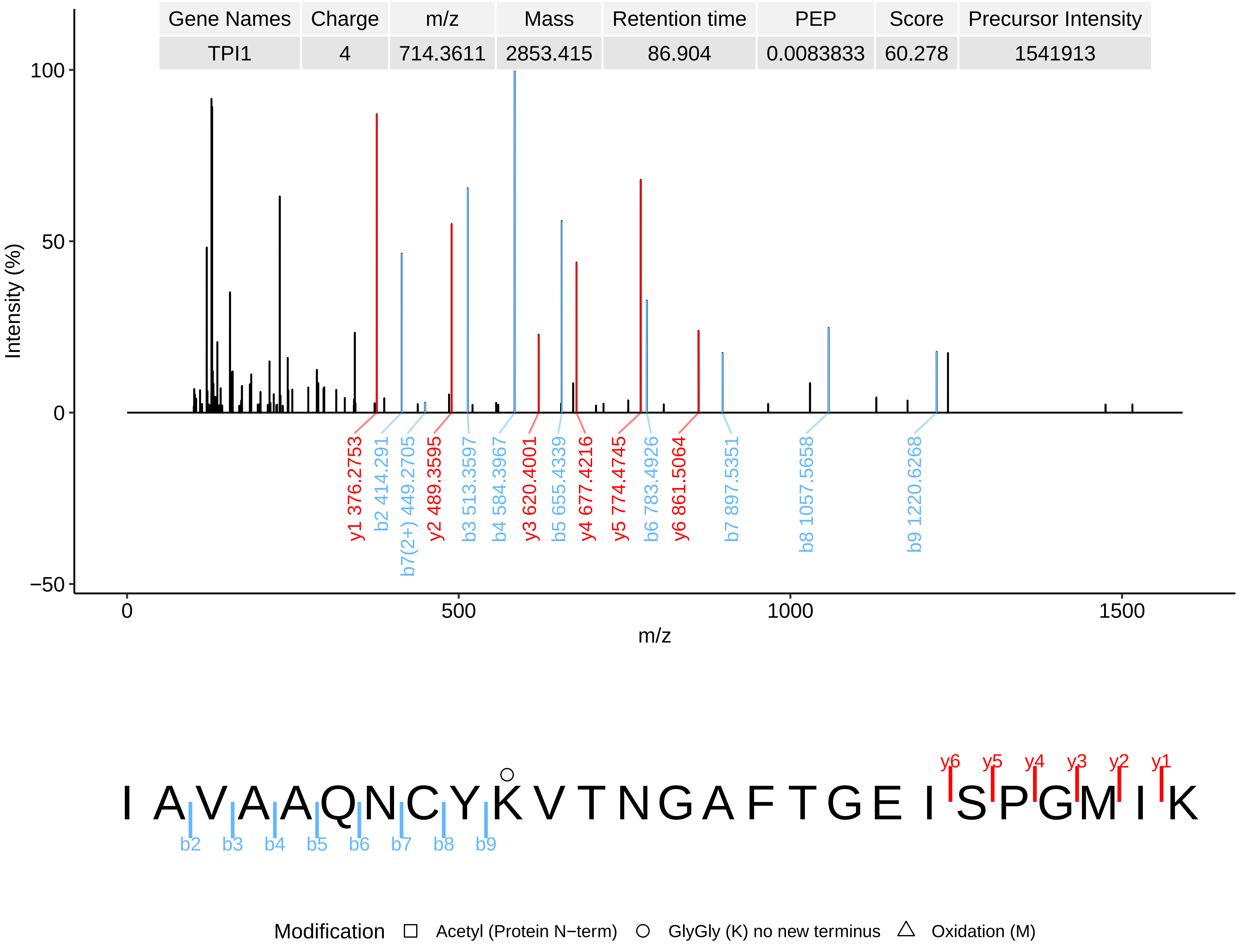

20200921_KIZ_MUb4_GlyGly_10plex_2 − Scan number: 22062

Gene Names	Charge	m/z	Mass	Retention time	PEP	Score	Precursor Intensity
TPI1	4	714.3611	2853.415	86.932	0.0017383	68.88	2281327

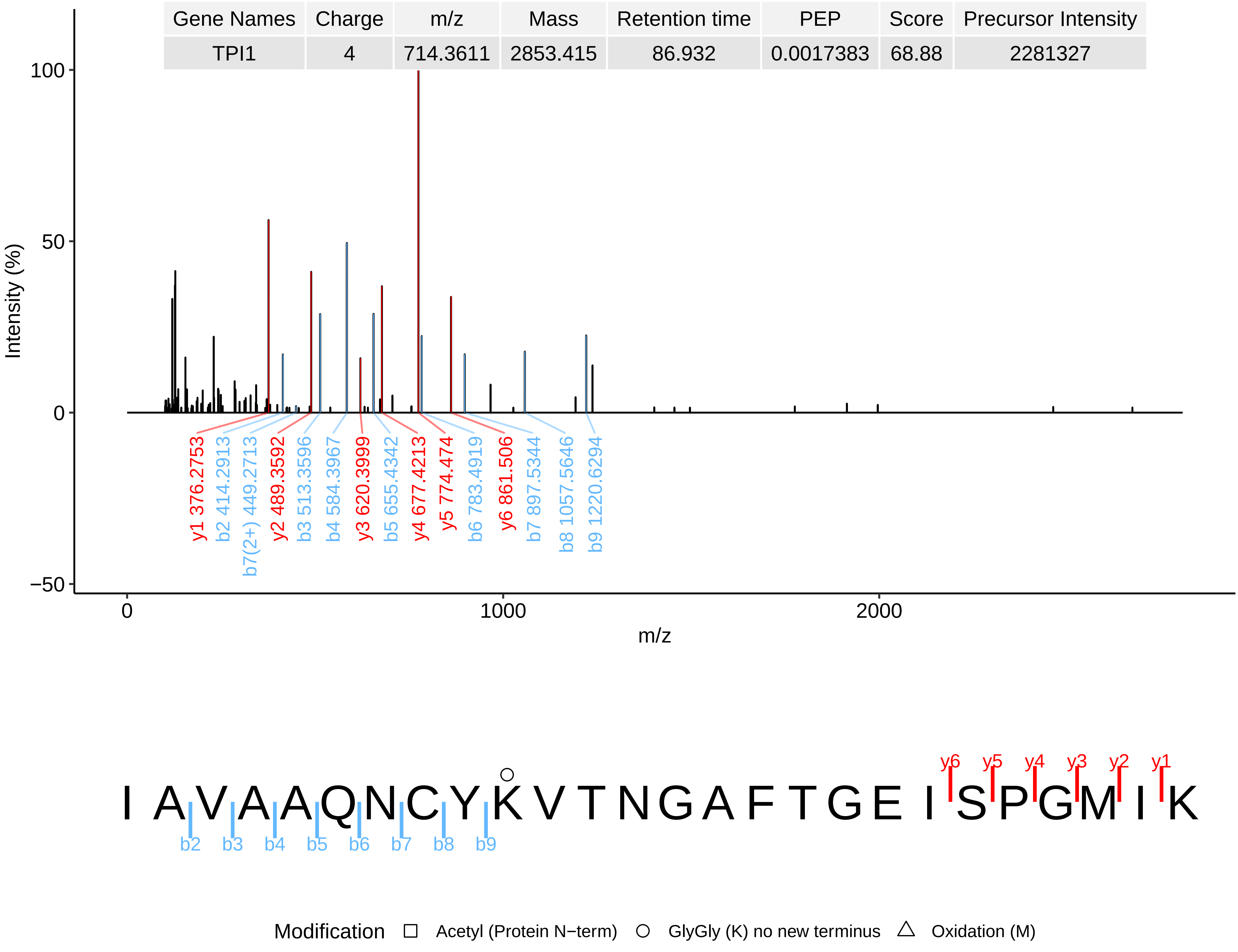

20201118_KIZ_MUb6_GlyGly_2 − Scan number: 32200

Gene Names	Charge	m/z	Mass	Retention time	PEP	Score	Precursor Intensity
SGOL2	4	792.4196	3165.649	105.59	0.033986	49.559	13816369

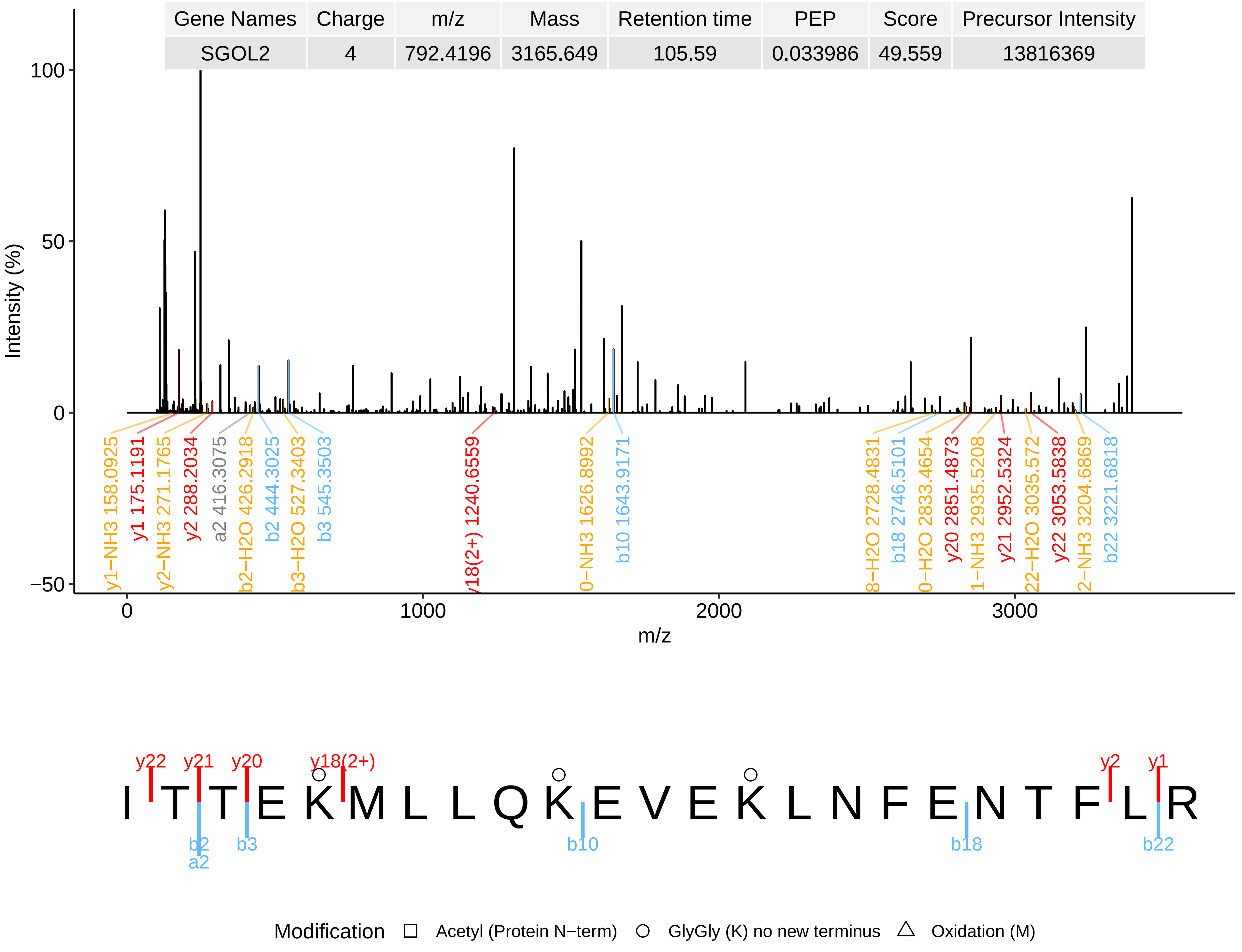

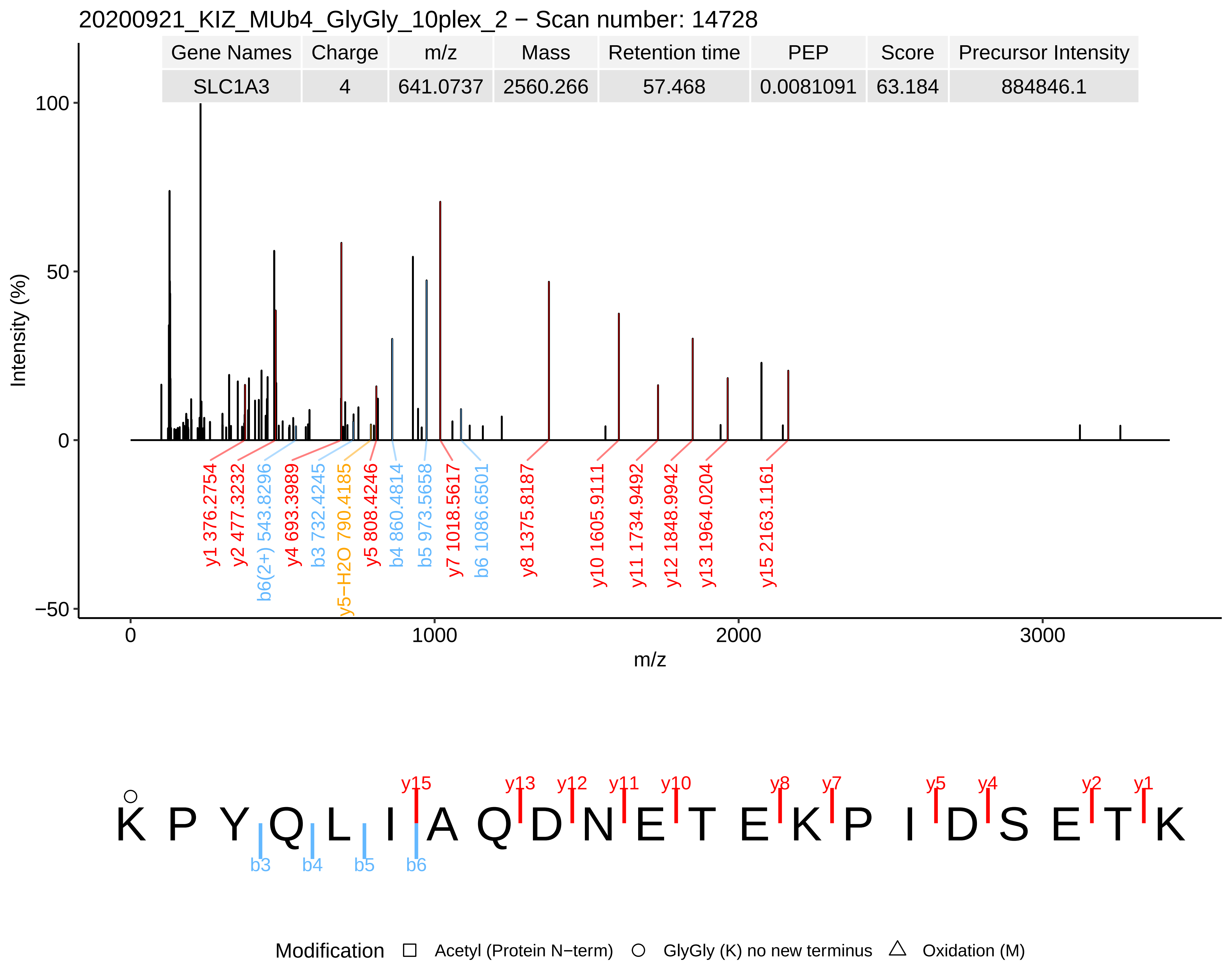
20200921_KIZ_MUb4_GlyGly_10plex_2 − Scan number: 14728
Gene Names | Charge | m/z | Mass | Retention time | PEP | Score | Precursor Intensity
SLC1A3 | 4 | 641.0737 | 2560.266 | 57.468 | 0.0081091 | 63.184 | 884846.1
Intensity (%)
100
50
0
−50
m/z
0
1000
2000
3000
y1 376.2754
y2 477.3232
b6(2+) 543.8296
y4 693.3989
b3 732.4245
y5−H2O 790.4185
y5 808.4246
b4 860.4814
b5 973.5658
y7 1018.5617
b6 1086.6501
y8 1375.8187
y10 1605.9111
y11 1734.9492
y12 1848.9942
y13 1964.0204
y15 2163.1161
K P Y Q L I A Q D N E T E K P I D S E T K
y15 y13 y12 y11 y10 y8 y7 y5 y4 y2 y1
b3 b4 b5 b6
Modification
Acetyl (Protein N−term)
GlyGly (K) no new terminus
Oxidation (M)

20201118_KIZ_MUb6_GlyGly_2 − Scan number: 17486

Gene Names	Charge	m/z	Mass	Retention time	PEP	Score	Precursor Intensity
SLC1A3	4	641.0737	2560.266	59.089	1.4987e−70	225.87	15957670

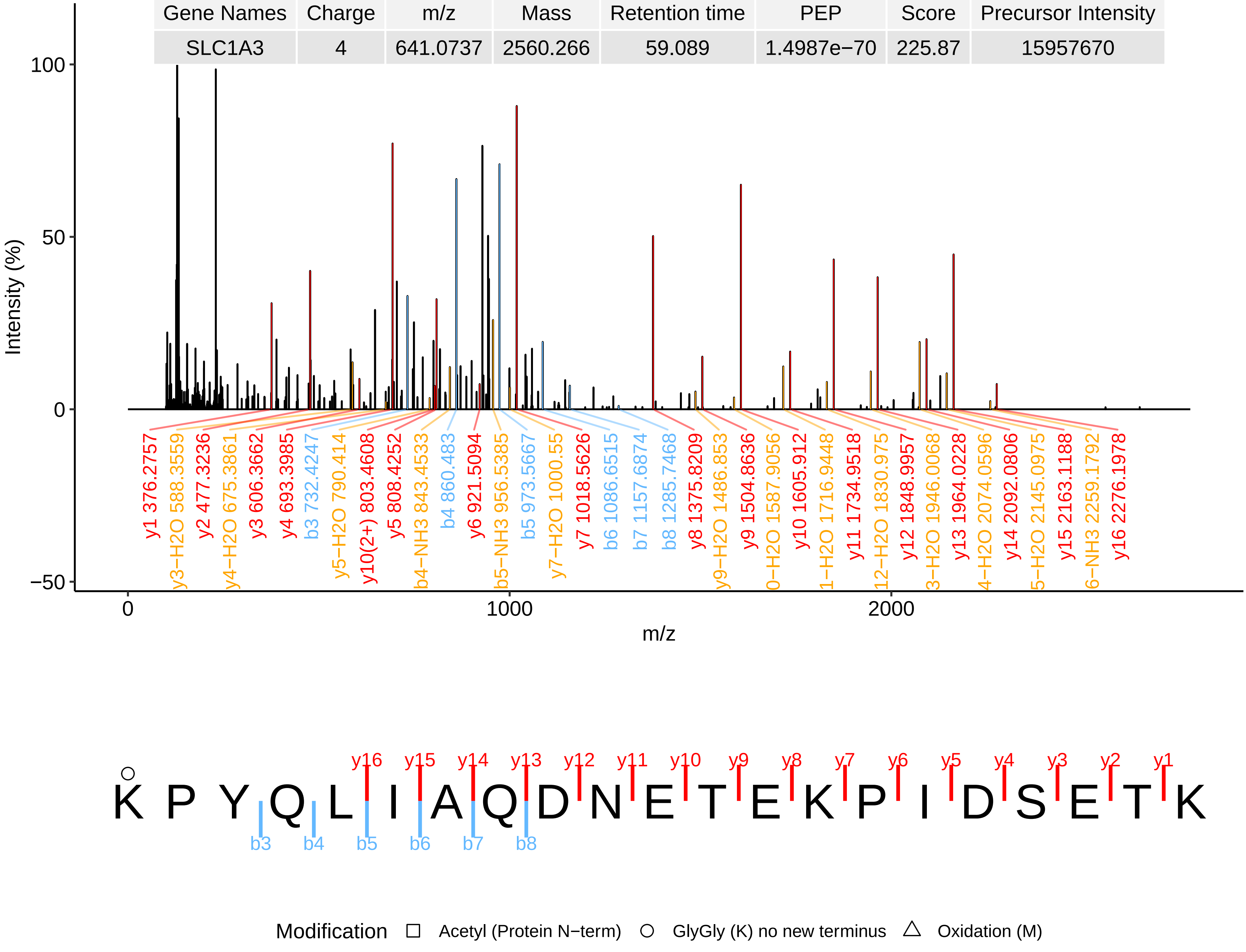

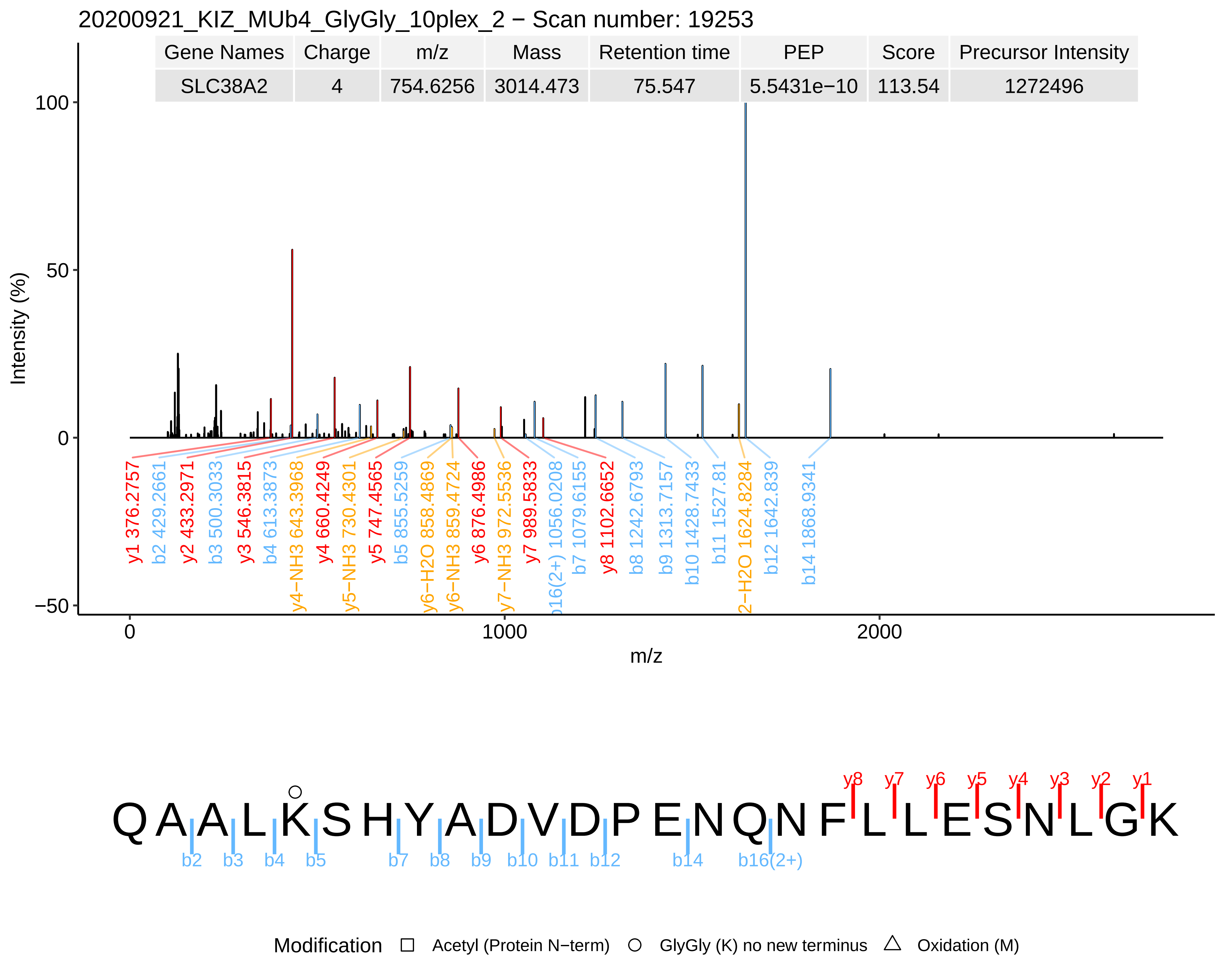

20200921_KIZ_MUb4_GlyGly_10plex_2 − Scan number: 19253
Gene Names | Charge | m/z | Mass | Retention time | PEP | Score | Precursor Intensity
SLC38A2 | 4 | 754.6256 | 3014.473 | 75.547 | 5.5431e−10 | 113.54 | 1272496
Intensity (%)
100
50
0
−50
0
1000
2000
m/z
y1 376.2757
b2 429.2661
y2 433.2971
b3 500.3033
y3 546.3815
b4 613.3873
y4−NH3 643.3968
y4 660.4249
y5−NH3 730.4301
y5 747.4565
b5 855.5259
y6−H2O 858.4869
y6−NH3 859.4724
y6 876.4986
y7−NH3 972.5536
y7 989.5833
b16(2+) 1056.0208
b7 1079.6155
y8 1102.6652
b8 1242.6793
b9 1313.7157
b10 1428.7433
b11 1527.81
2−H2O 1624.8284
b12 1642.839
b14 1868.9341
Q A A L K S H Y A D V D P E N Q N F L L E S N L G K
y8 y7 y6 y5 y4 y3 y2 y1
b2 b3 b4 b5 b7 b8 b9 b10 b11 b12 b14 b16(2+)
Modification
Acetyl (Protein N−term)
GlyGly (K) no new terminus
Oxidation (M)

20201118_KIZ_MUb6_GlyGly_2 − Scan number: 23187

Gene Names	Charge	m/z	Mass	Retention time	PEP	Score	Precursor Intensity
SLC38A2	4	754.6256	3014.473	77.841	5.4193e−68	214.49	11666137

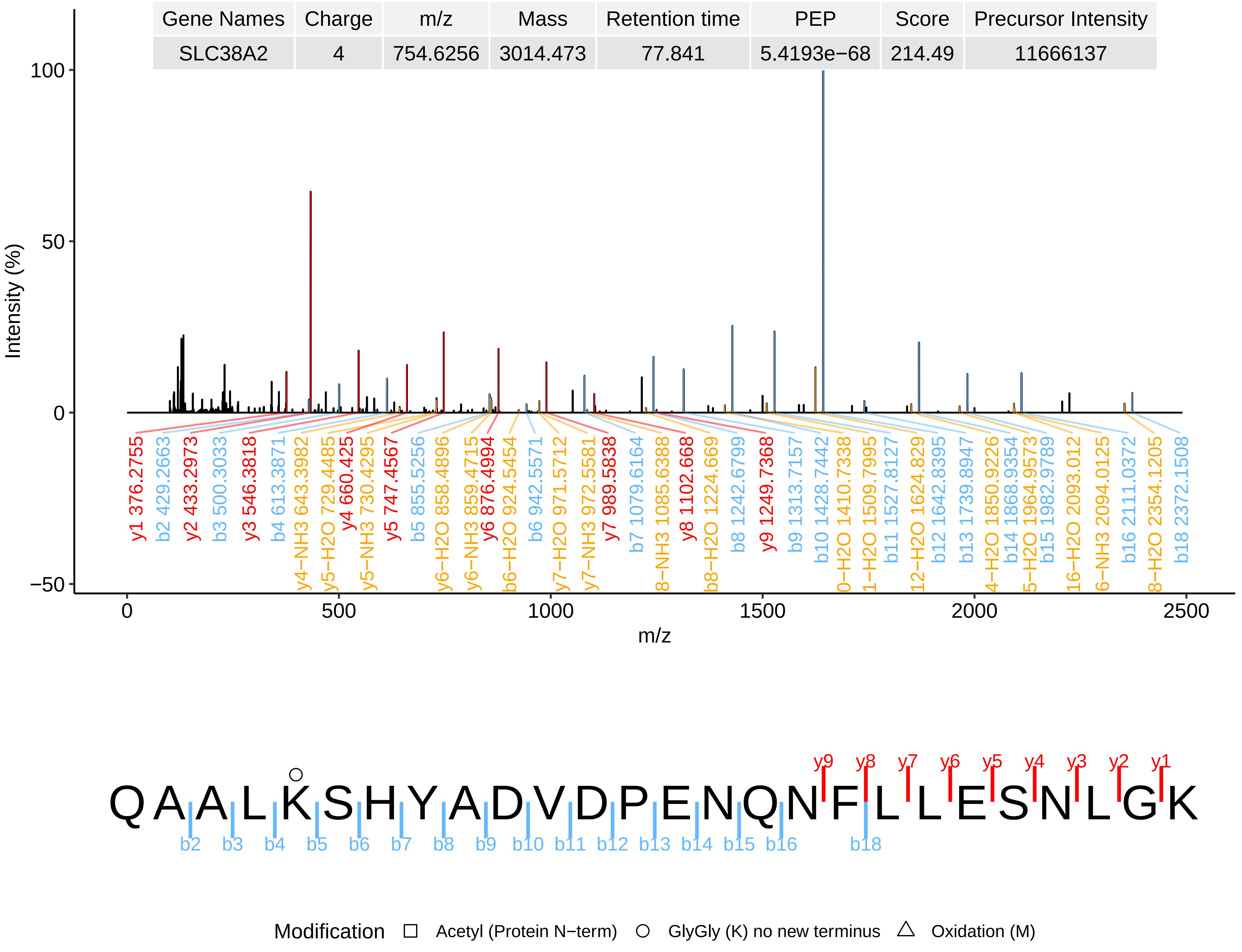

20201118_KIZ_MUb6_GlyGly_2 − Scan number: 23195

Gene Names	Charge	m/z	Mass	Retention time	PEP	Score	Precursor Intensity
SLC38A2	4	754.6256	3014.473	77.867	1.8225e−41	173.47	3375366

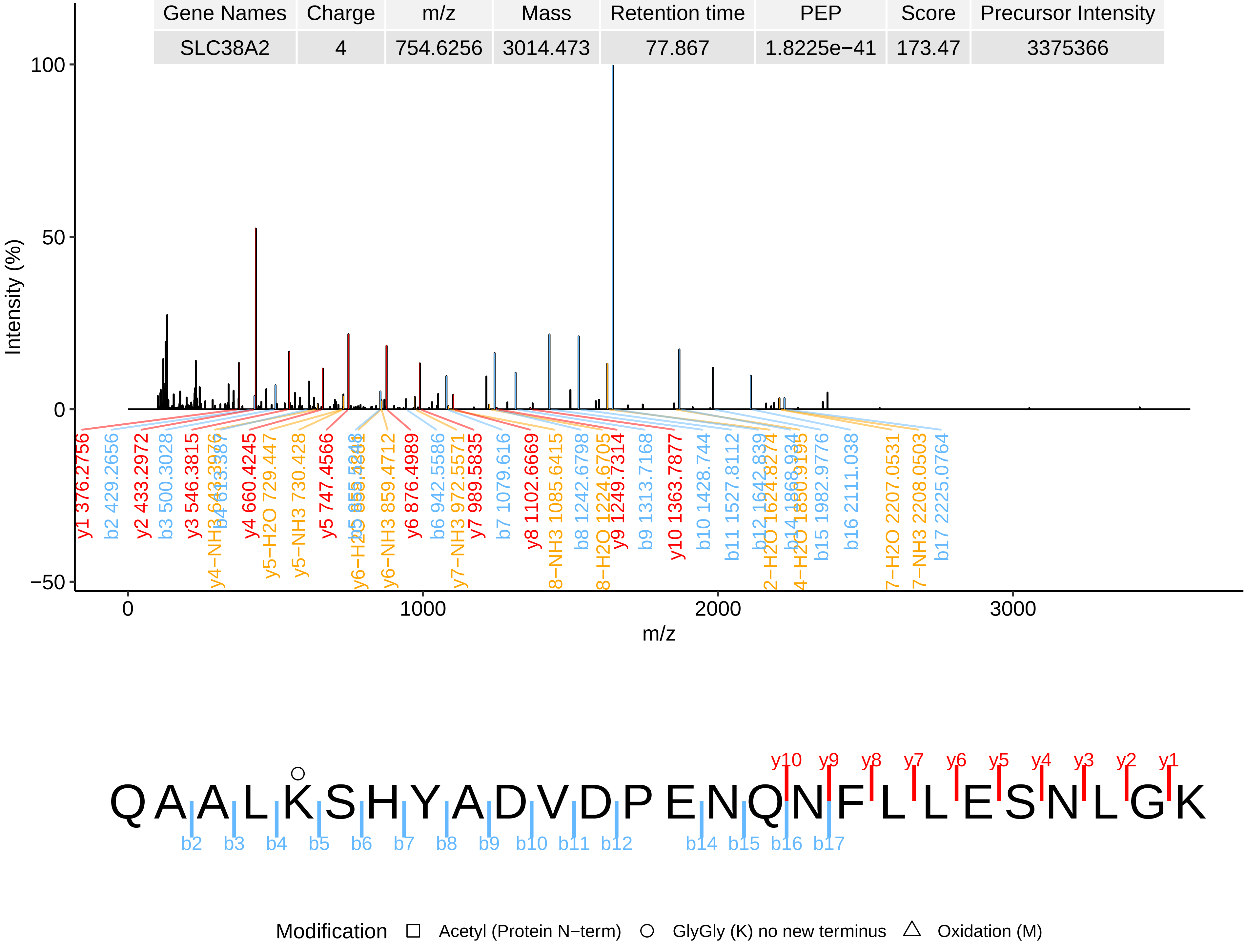

20200921_KIZ_MUb4_GlyGly_10plex_2 − Scan number: 13589

Gene Names	Charge	m/z	Mass	Retention time	PEP	Score	Precursor Intensity
TFRC	4	754.5863	3014.316	52.796	0.00059074	76.691	1598962

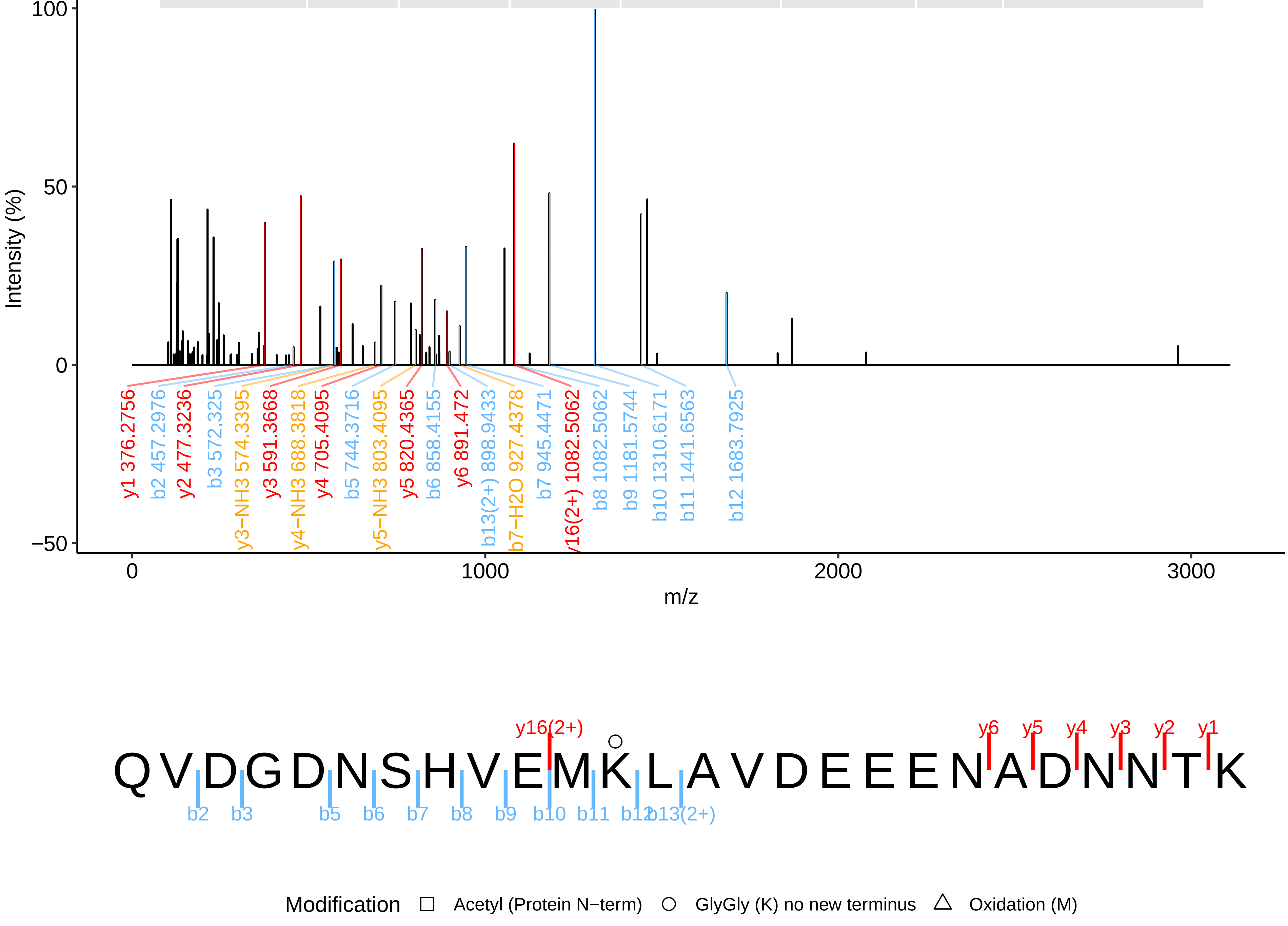

20200921_KIZ_MUb4_GlyGly_10plex_2 − Scan number: 11831

Gene Names	Charge	m/z	Mass	Retention time	PEP	Score	Precursor Intensity
VDAC3	4	727.0781	2904.283	45.635	3.2392e−06	96.303	710265.6

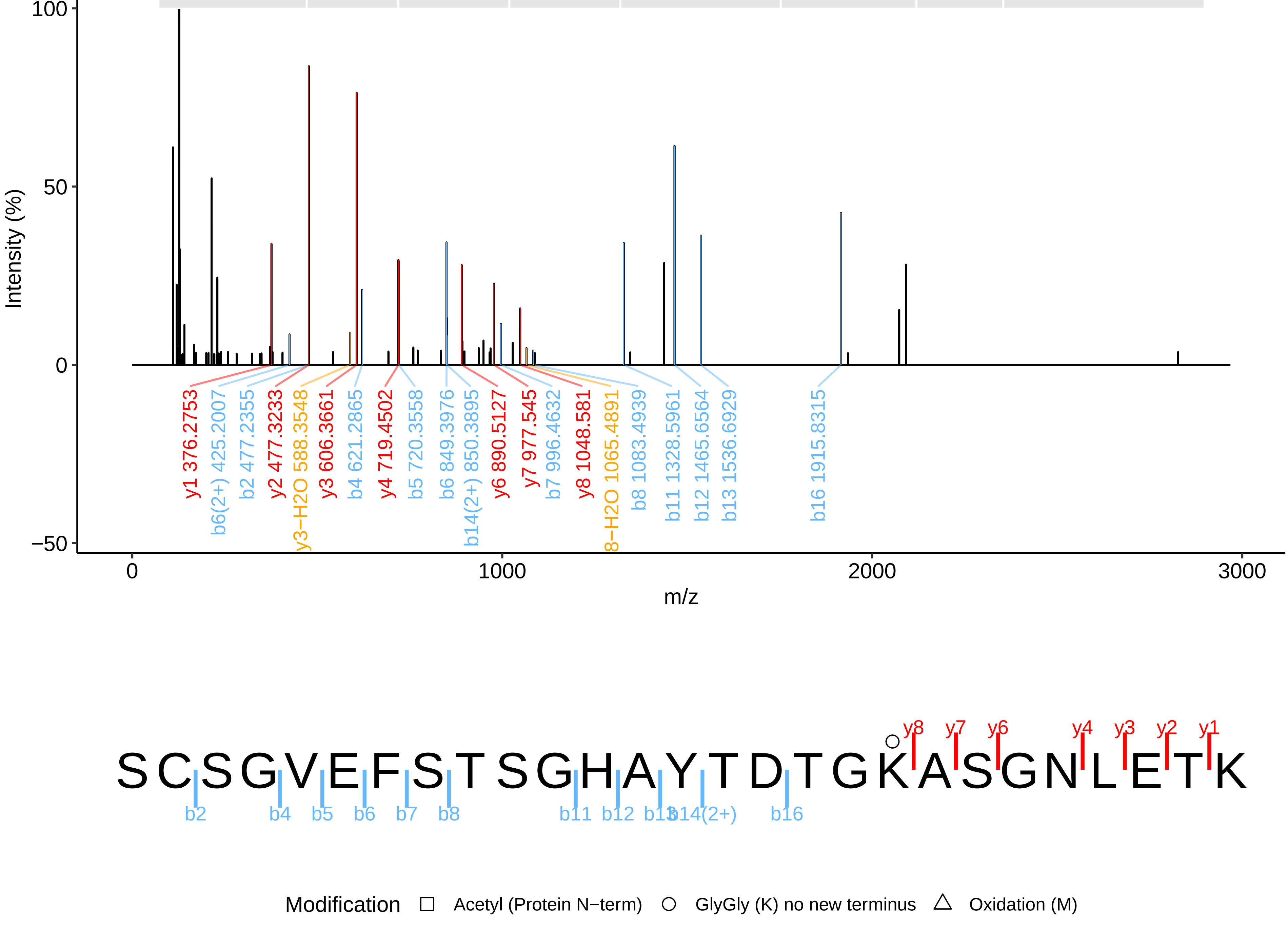

20200921_KIZ_MUb4_GlyGly_10plex − Scan number: 16199

Gene Names	Charge	m/z	Mass	Retention time	PEP	Score	Precursor Intensity
VDAC2	4	713.5779	2850.282	58.114	0.0015902	74.222	9806814

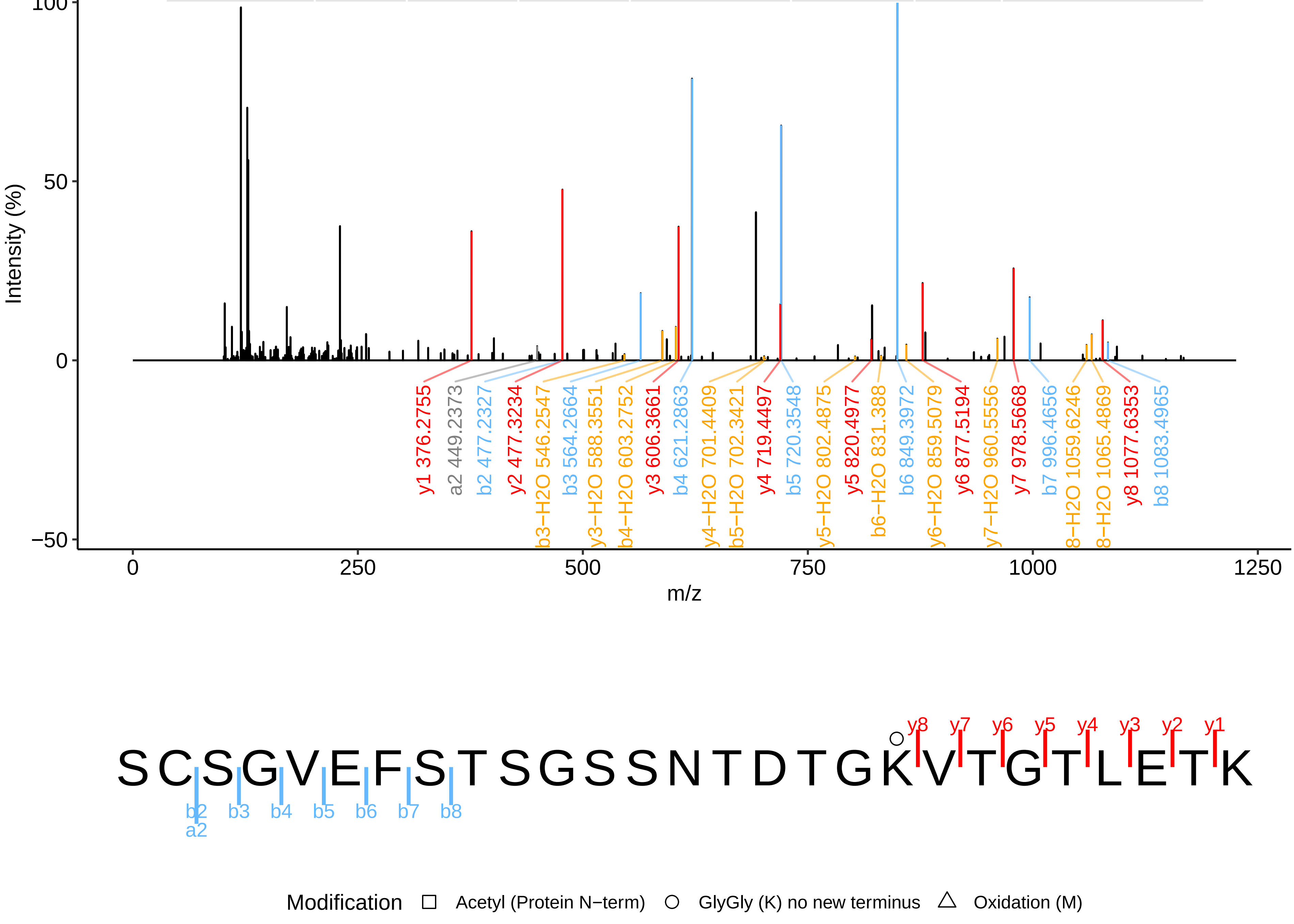

20200921_KIZ_MUb4_GlyGly_10plex − Scan number: 16225

Gene Names	Charge	m/z	Mass	Retention time	PEP	Score	Precursor Intensity
VDAC2	4	713.5779	2850.282	58.215	3.5617e−09	108.77	6029261

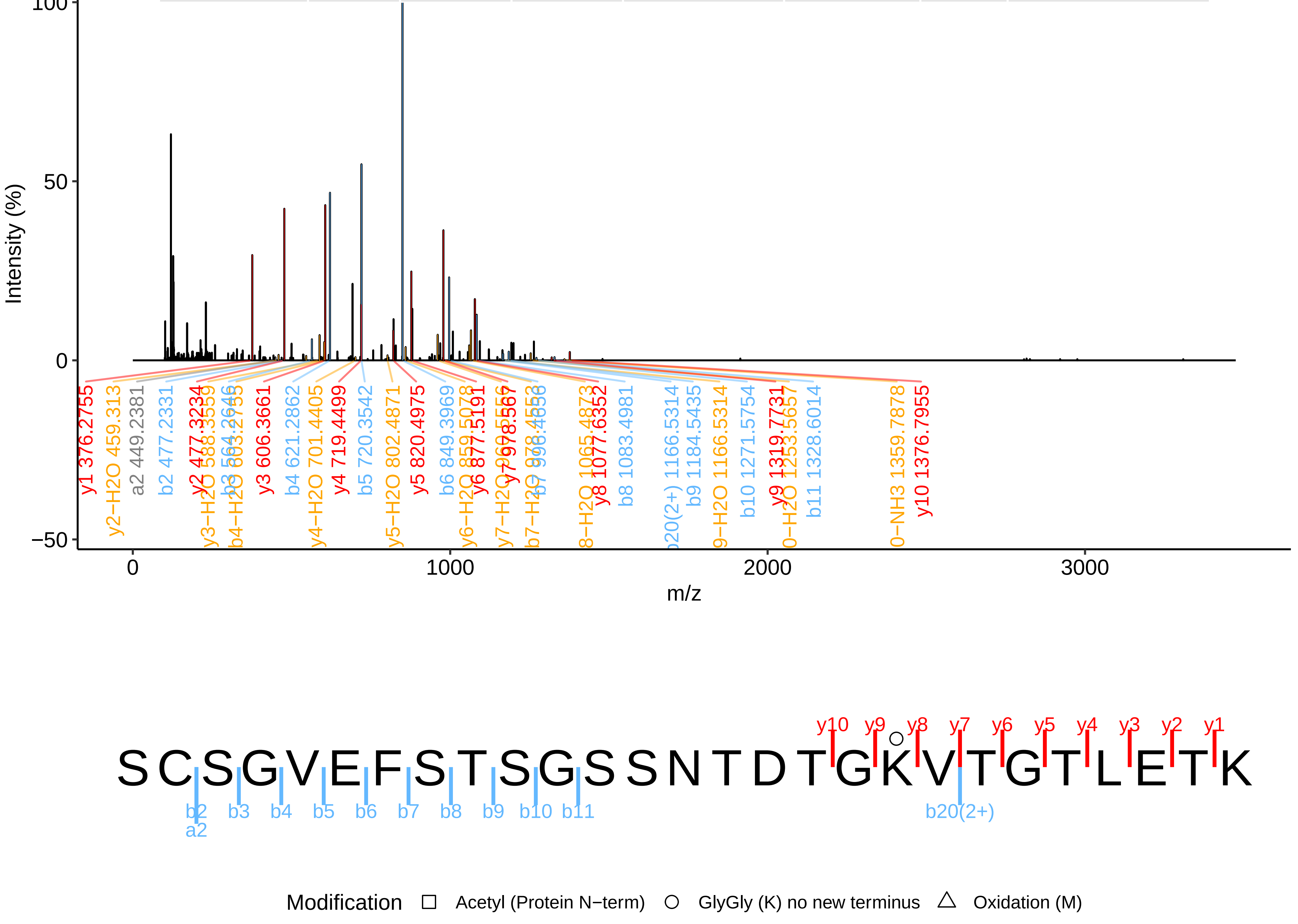

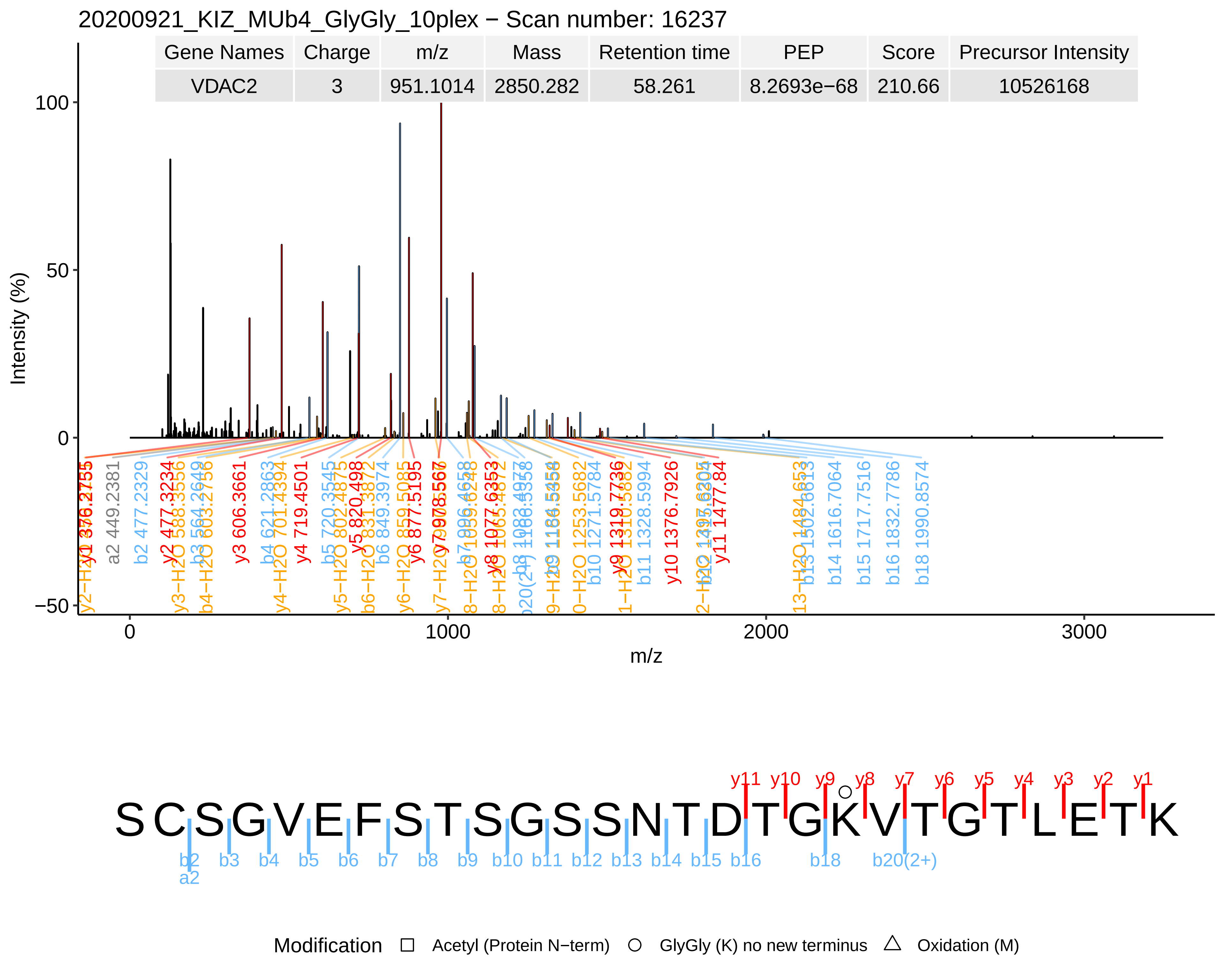

20200921_KIZ_MUb4_GlyGly_10plex − Scan number: 16237
Gene Names | Charge | m/z | Mass | Retention time | PEP | Score | Precursor Intensity
VDAC2 | 3 | 951.1014 | 2850.282 | 58.261 | 8.2693e−68 | 210.66 | 10526168
Intensity (%)
m/z
S C S G V E F S T S G S S N T D T G K V T G T L E T K
y11 y10 y9 y8 y7 y6 y5 y4 y3 y2 y1
b2 b3 b4 b5 b6 b7 b8 b9 b10 b11 b12 b13 b14 b15 b16 b18 b20(2+)
a2
Modification
Acetyl (Protein N−term)
GlyGly (K) no new terminus
Oxidation (M)

20200921_KIZ_MUb4_GlyGly_10plex − Scan number: 16371

Gene Names	Charge	m/z	Mass	Retention time	PEP	Score	Precursor Intensity
VDAC2	3	951.1014	2850.282	58.773	1.0427e−53	193.77	5247108

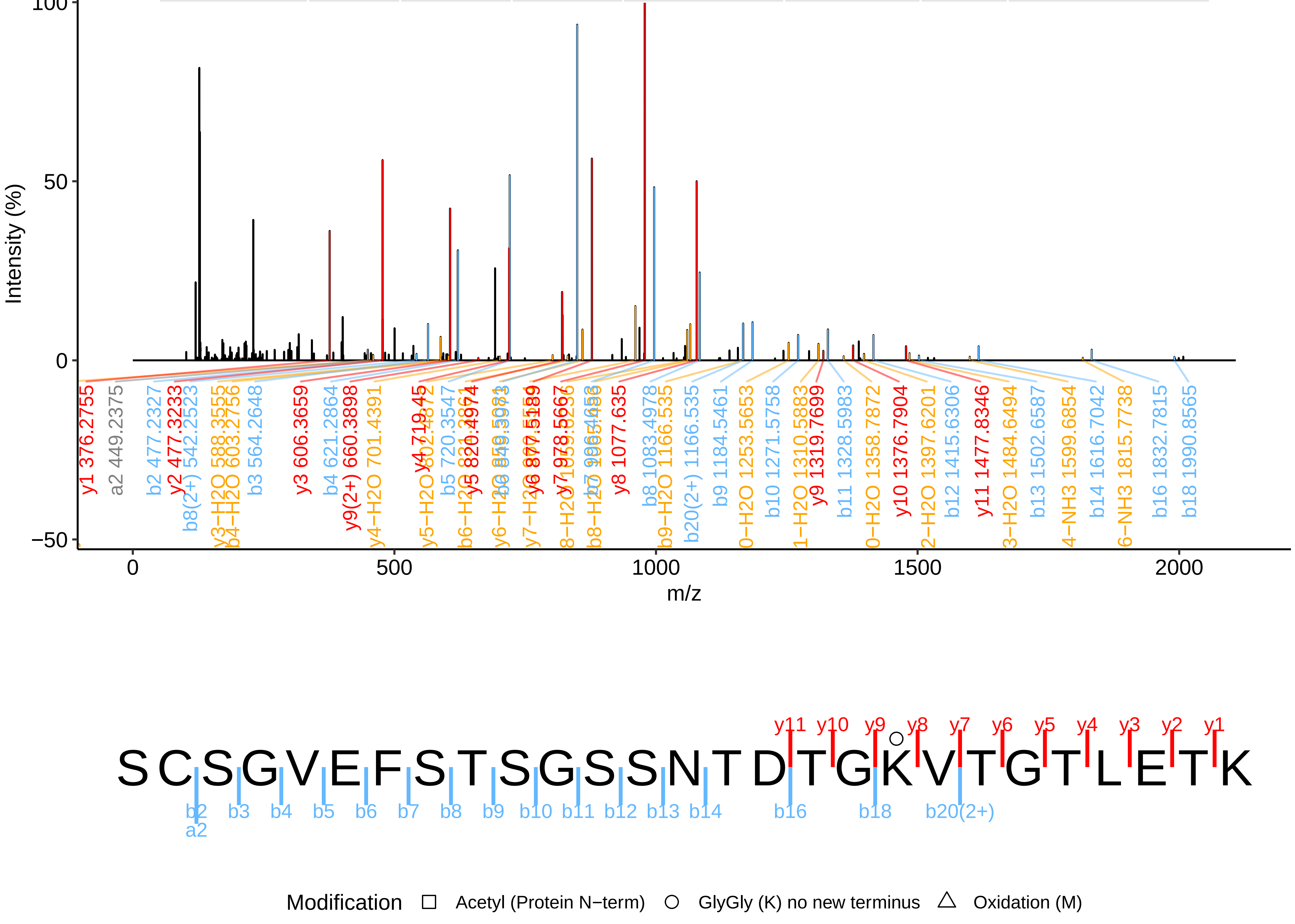

20200921_KIZ_MUb4_GlyGly_10plex − Scan number: 16358

Gene Names	Charge	m/z	Mass	Retention time	PEP	Score	Precursor Intensity
VDAC2	4	713.5779	2850.282	58.721	5.2905e−22	146.25	3961413

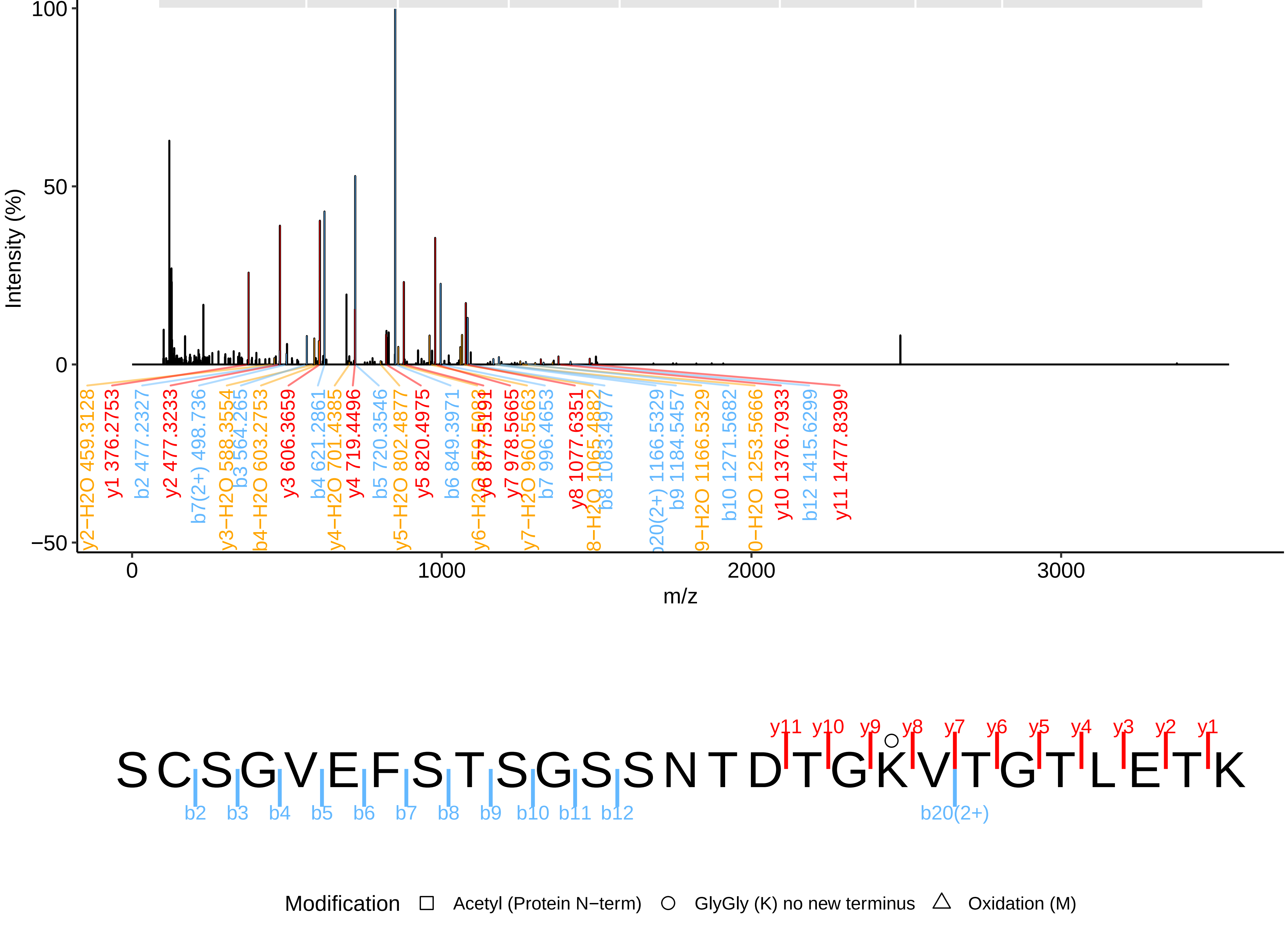

20200921_KIZ_MUb4_GlyGly_10plex − Scan number: 16501

Gene Names	Charge	m/z	Mass	Retention time	PEP	Score	Precursor Intensity
VDAC2	3	951.1014	2850.282	59.283	3.0887e−83	221.39	8899165

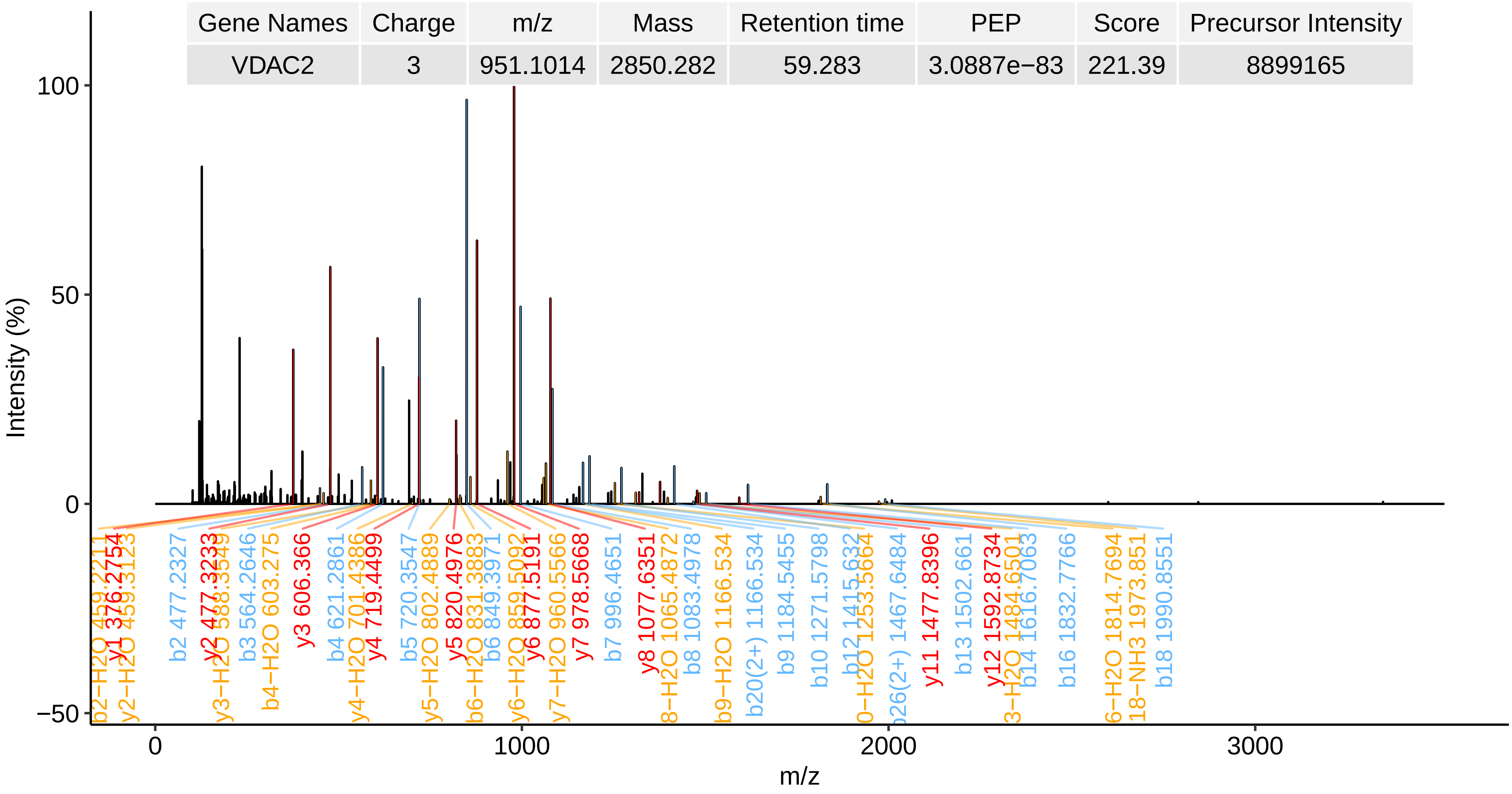

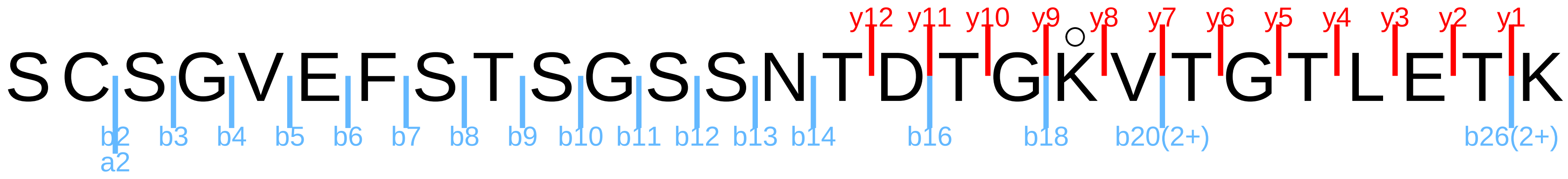

Modification □ Acetyl (Protein N−term) ○ GlyGly (K) no new terminus △ Oxidation (M)

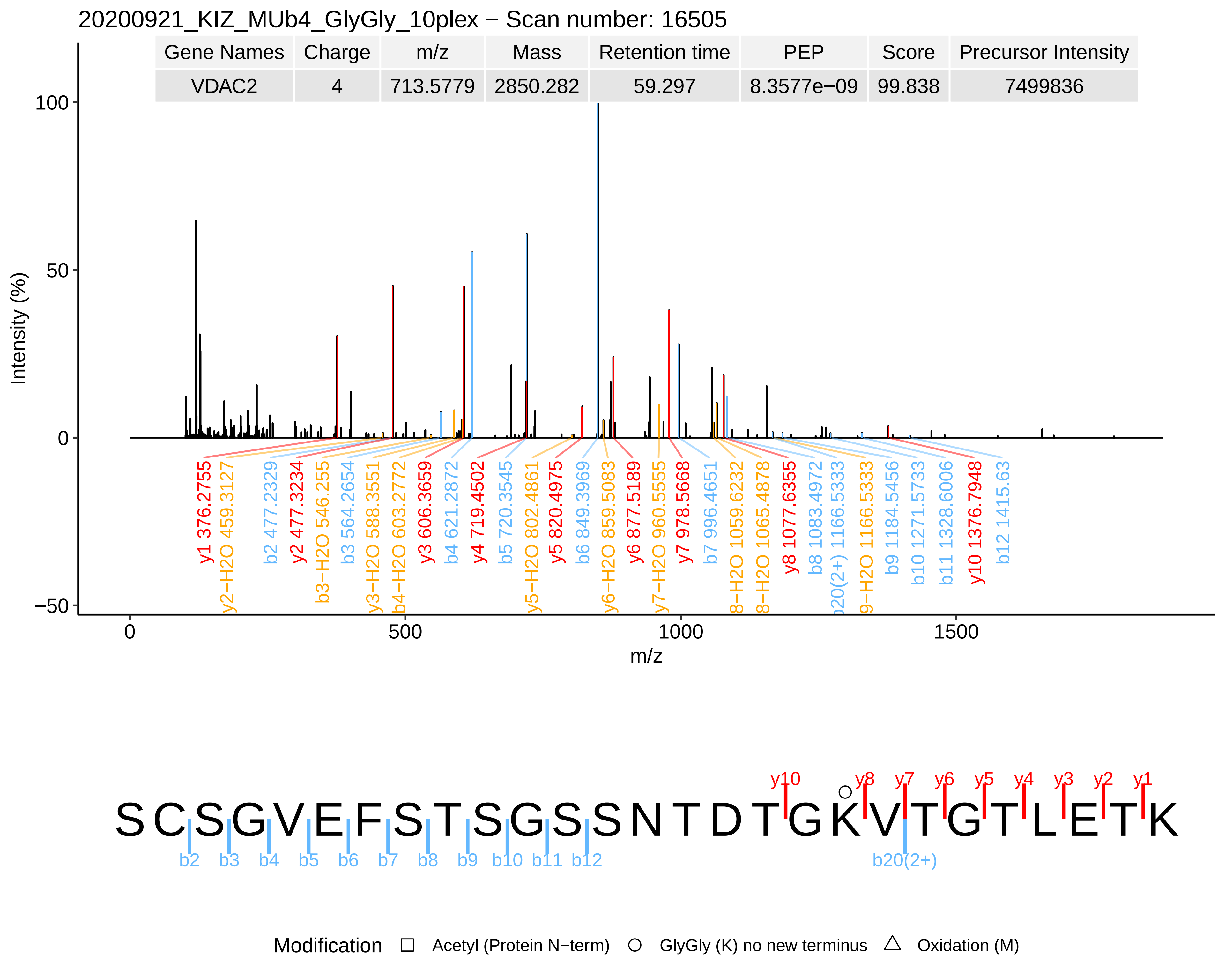

20200921_KIZ_MUb4_GlyGly_10plex − Scan number: 16505
Gene Names | Charge | m/z | Mass | Retention time | PEP | Score | Precursor Intensity
VDAC2 | 4 | 713.5779 | 2850.282 | 59.297 | 8.3577e−09 | 99.838 | 7499836
Intensity (%)
100
50
0
−50
0
500
1000
1500
m/z
y1 376.2755
y2−H2O 459.3127
b2 477.2329
y2 477.3234
b3−H2O 546.255
b3 564.2654
y3−H2O 588.3551
b4−H2O 603.2772
y3 606.3659
b4 621.2872
y4 719.4502
b5 720.3545
y5−H2O 802.4861
y5 820.4975
b6 849.3969
y6−H2O 859.5083
y6 877.5189
y7−H2O 960.5555
y7 978.5668
b7 996.4651
8−H2O 1059.6232
8−H2O 1065.4878
y8 1077.6355
b8 1083.4972
20(2+) 1166.5333
9−H2O 1166.5333
b9 1184.5456
b10 1271.5733
b11 1328.6006
y10 1376.7948
b12 1415.63
y10 y8 y7 y6 y5 y4 y3 y2 y1
S C S G V E F S T S G S S N T D T G K V T G T L E T K
b2 b3 b4 b5 b6 b7 b8 b9 b10 b11 b12 b20(2+)
Modification
Acetyl (Protein N−term)
GlyGly (K) no new terminus
Oxidation (M)

20200921_KIZ_MUb4_GlyGly_10plex − Scan number: 16631

Gene Names	Charge	m/z	Mass	Retention time	PEP	Score	Precursor Intensity
VDAC2	3	951.1014	2850.282	59.8	8.2693e−68	210.66	2633697

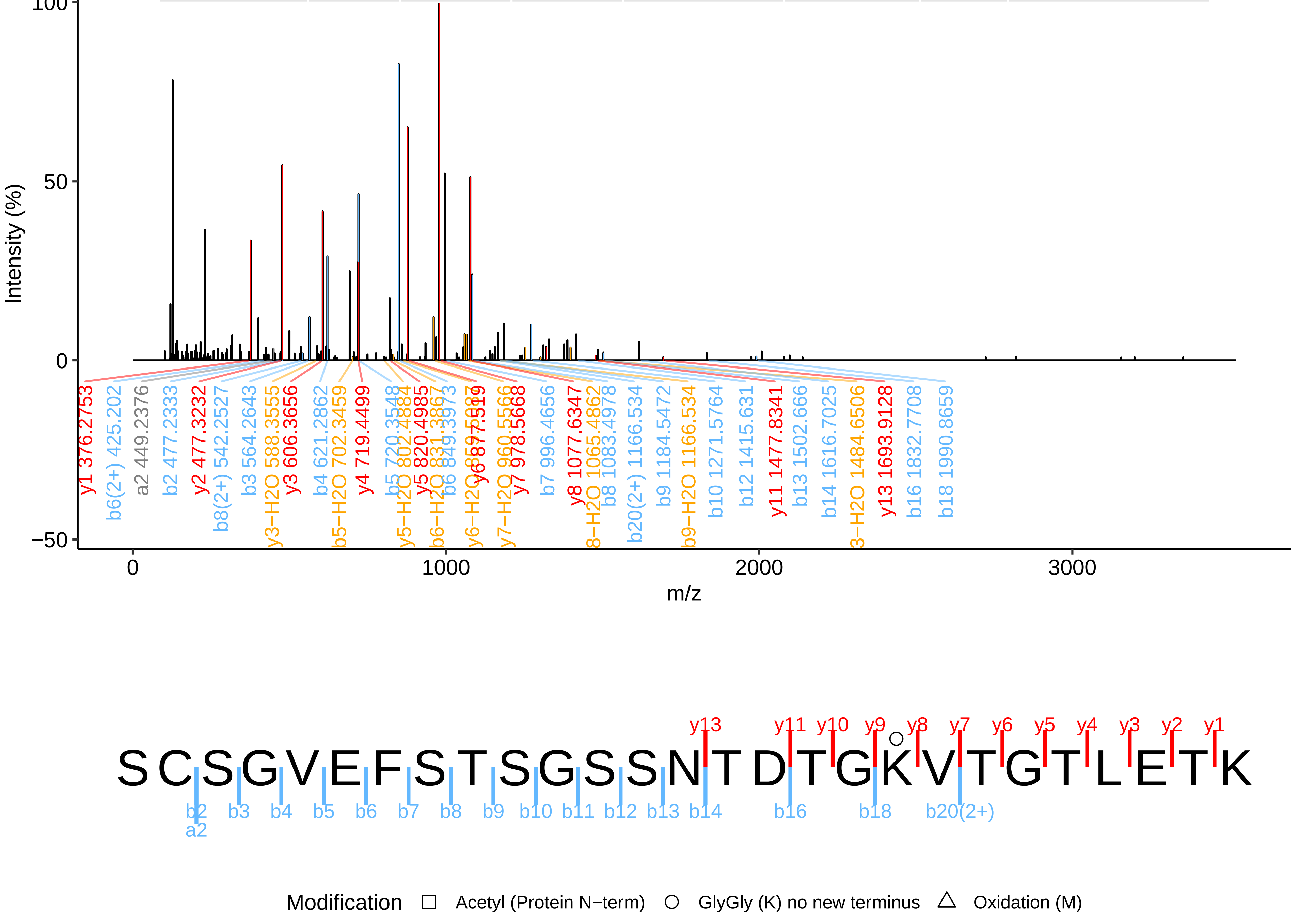

20200921_KIZ_MUb4_GlyGly_10plex − Scan number: 16761

Gene Names	Charge	m/z	Mass	Retention time	PEP	Score	Precursor Intensity
VDAC2	3	951.1014	2850.282	60.316	1.0889e−30	161.04	1637844

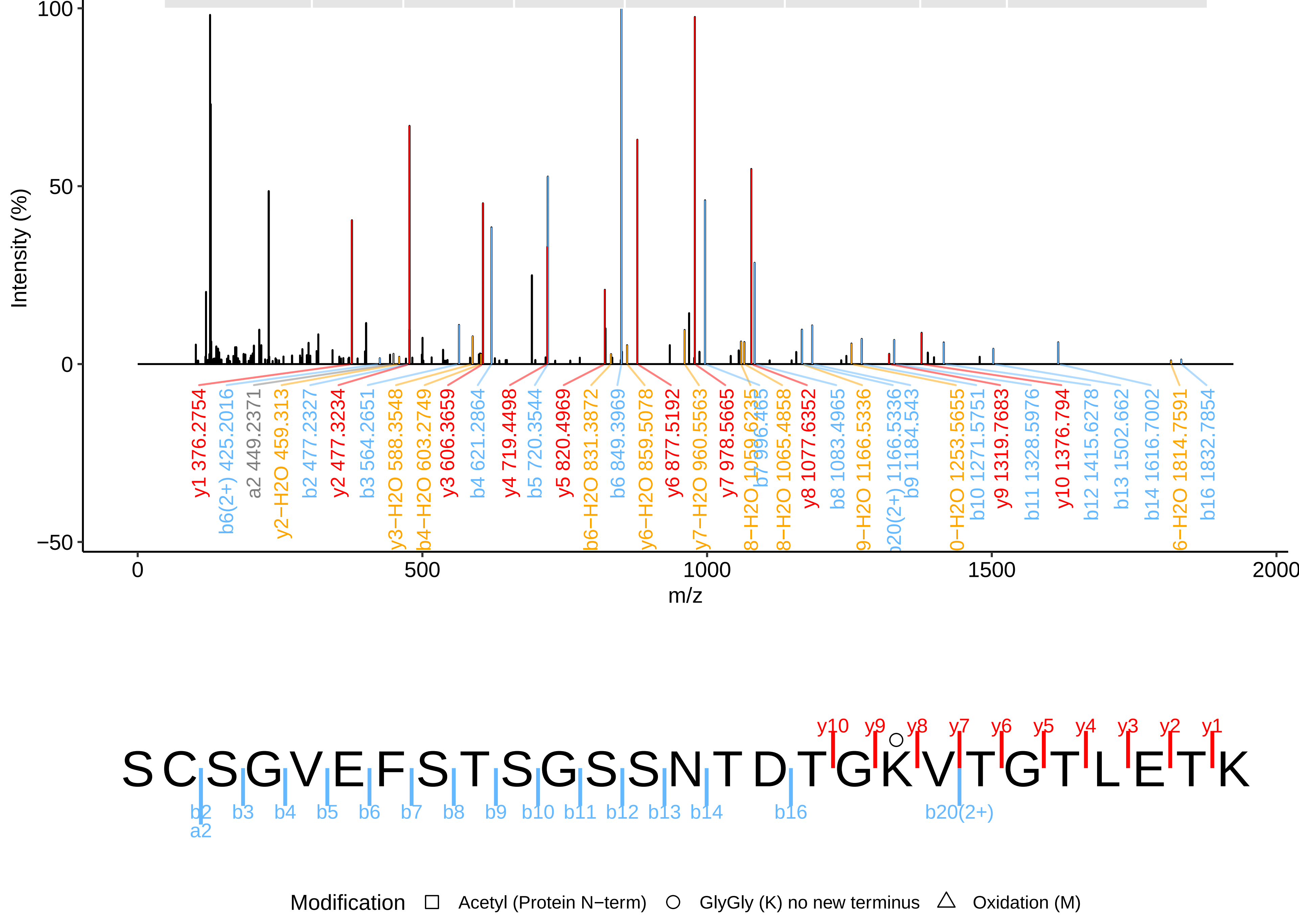

20200921_KIZ_MUb4_GlyGly_10plex − Scan number: 16919

Gene Names	Charge	m/z	Mass	Retention time	PEP	Score	Precursor Intensity
VDAC2	3	951.1014	2850.282	60.948	1.4821e−37	152.07	1733476

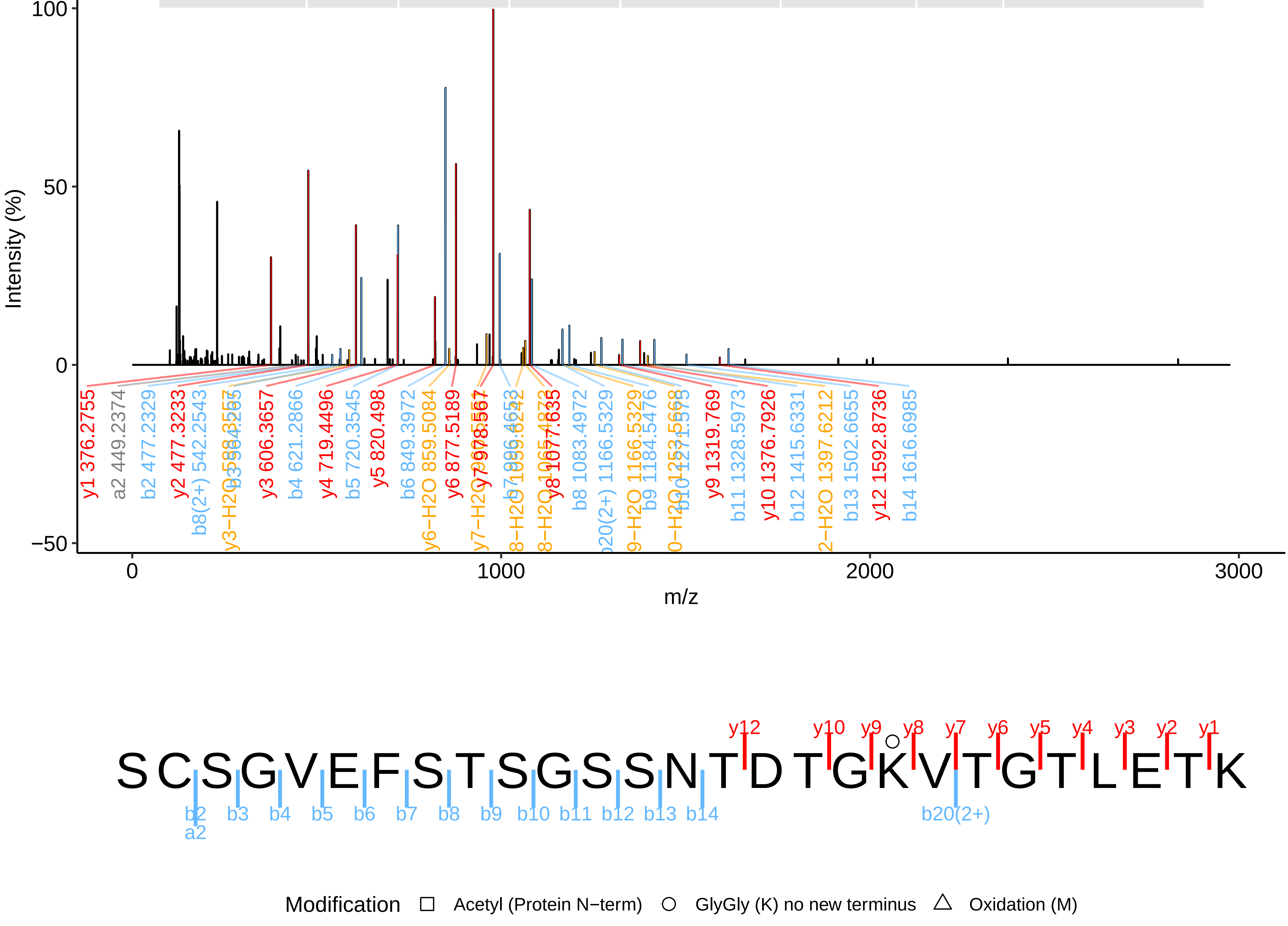

20200921_KIZ_MUb4_GlyGly_10plex_2 − Scan number: 14512

Gene Names	Charge	m/z	Mass	Retention time	PEP	Score	Precursor Intensity
VDAC2	3	951.1014	2850.282	56.593	1.5454e−21	140.66	1721158

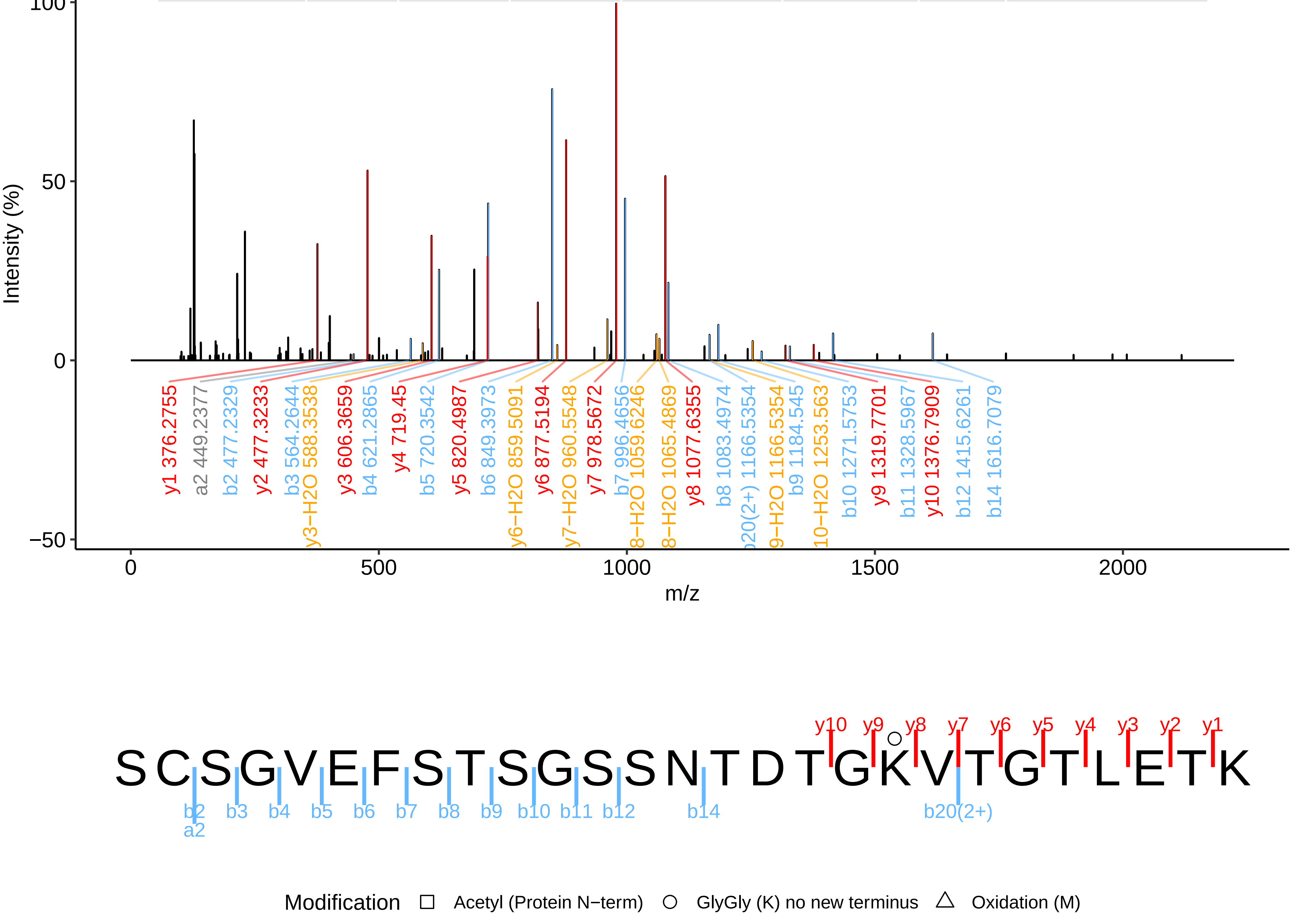

20200921_KIZ_MUb4_GlyGly_10plex_2 − Scan number: 14640

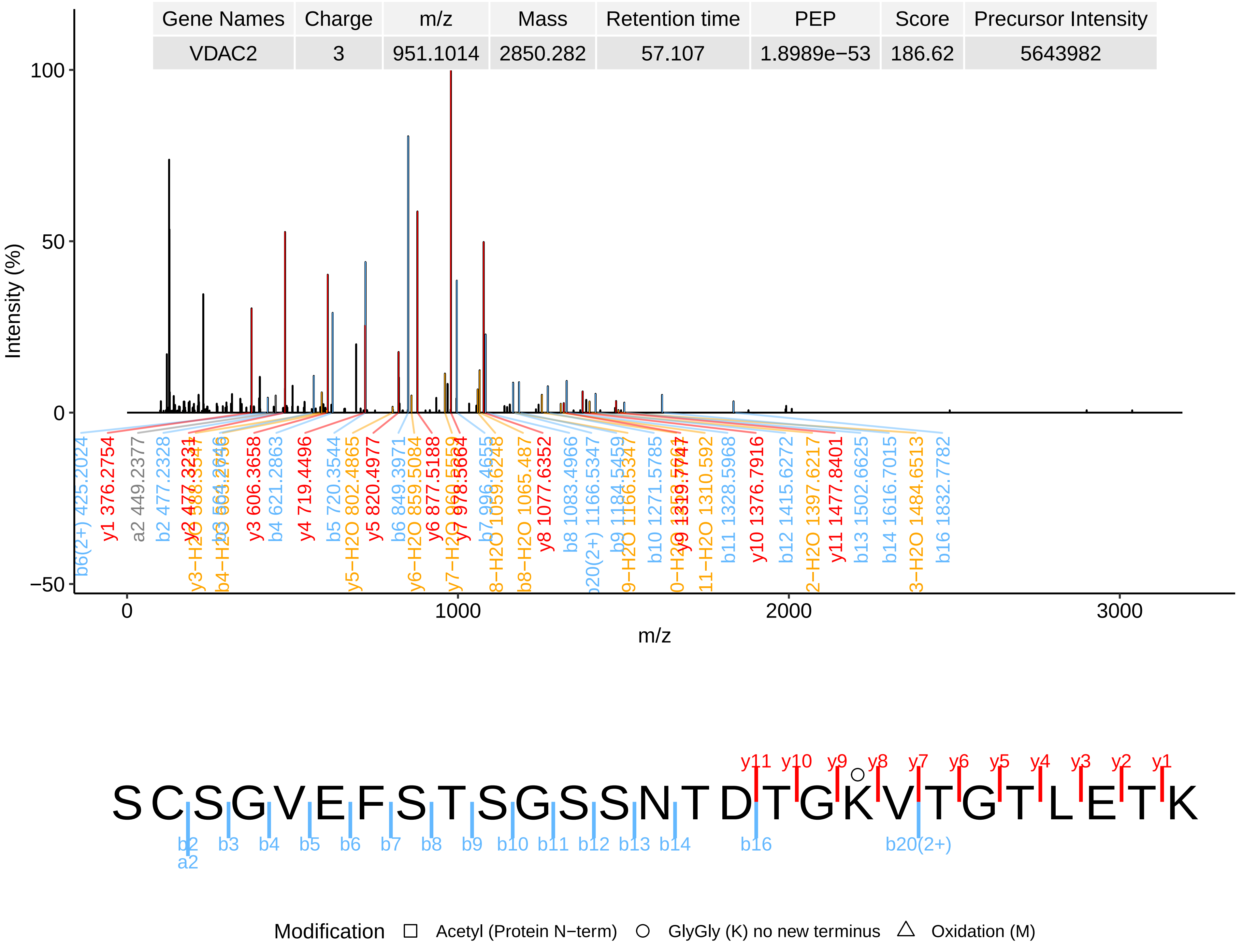

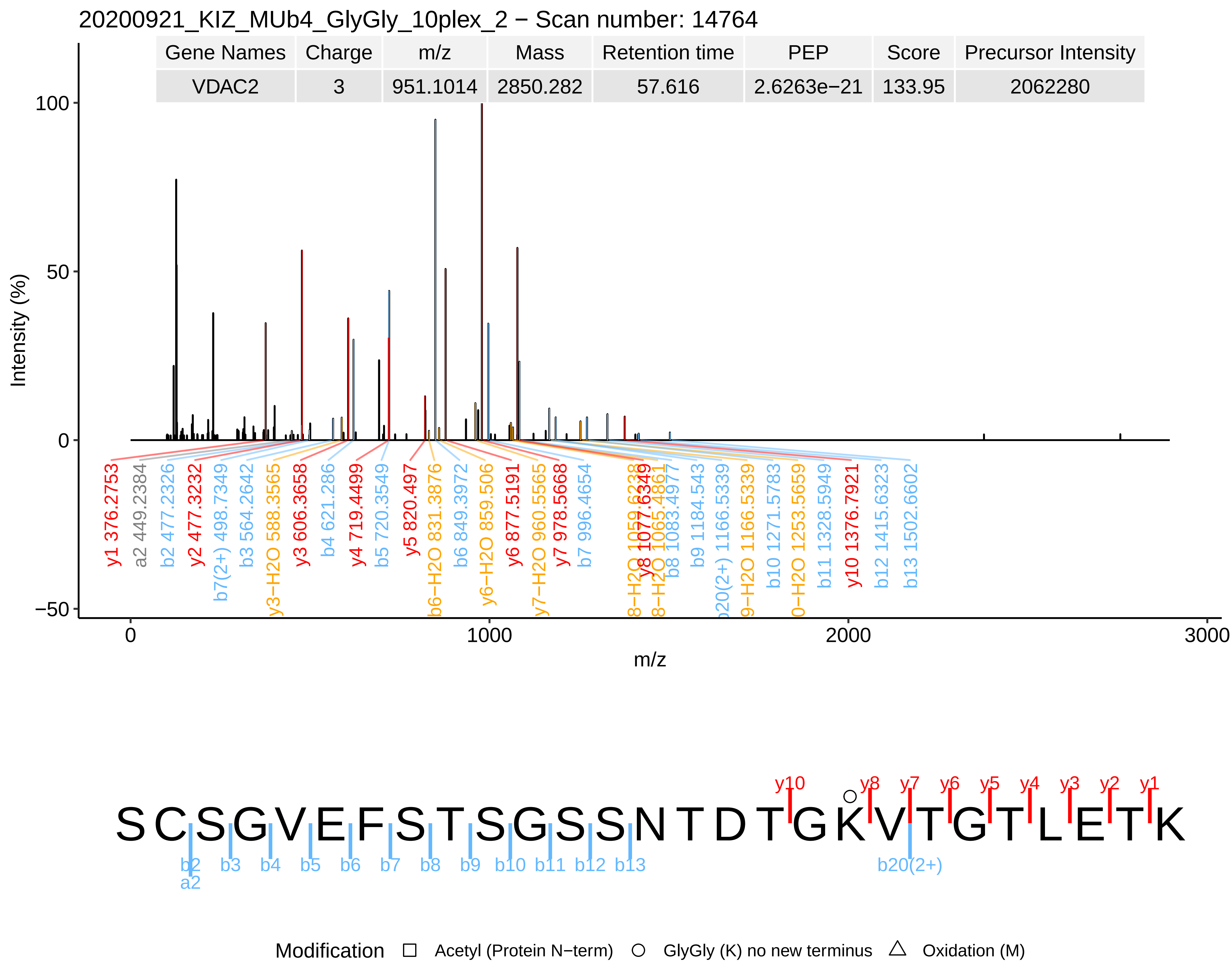
20200921_KIZ_MUb4_GlyGly_10plex_2 − Scan number: 14764
Gene Names
Charge
m/z
Mass
Retention time
PEP
Score
Precursor Intensity
VDAC2
3
951.1014
2850.282
57.616
2.6263e−21
133.95
2062280
Intensity (%)
m/z
y1 376.2753
a2 449.2384
b2 477.2326
y2 477.3232
b7(2+) 498.7349
b3 564.2642
y3−H2O 588.3565
y3 606.3658
b4 621.286
y4 719.4499
b5 720.3549
y5 820.497
b6−H2O 831.3876
b6 849.3972
y6−H2O 859.506
y6 877.5191
y7−H2O 960.5565
y7 978.5668
b7 996.4654
b8 1083.4977
b9 1184.543
b10 1271.5783
b11 1328.5949
y10 1376.7921
b12 1415.6323
b13 1502.6602
S C S G V E F S T S G S S N T D T G K V T G T L E T K
y10 y8 y7 y6 y5 y4 y3 y2 y1
b2 b3 b4 b5 b6 b7 b8 b9 b10 b11 b12 b13 b20(2+)
a2
Modification
Acetyl (Protein N−term)
GlyGly (K) no new terminus
Oxidation (M)

20200921_KIZ_MUb4_GlyGly_10plex_2 − Scan number: 15206

Gene Names	Charge	m/z	Mass	Retention time	PEP	Score	Precursor Intensity
VDAC2	3	951.1014	2850.282	59.412	0.0059733	54.658	556565.5

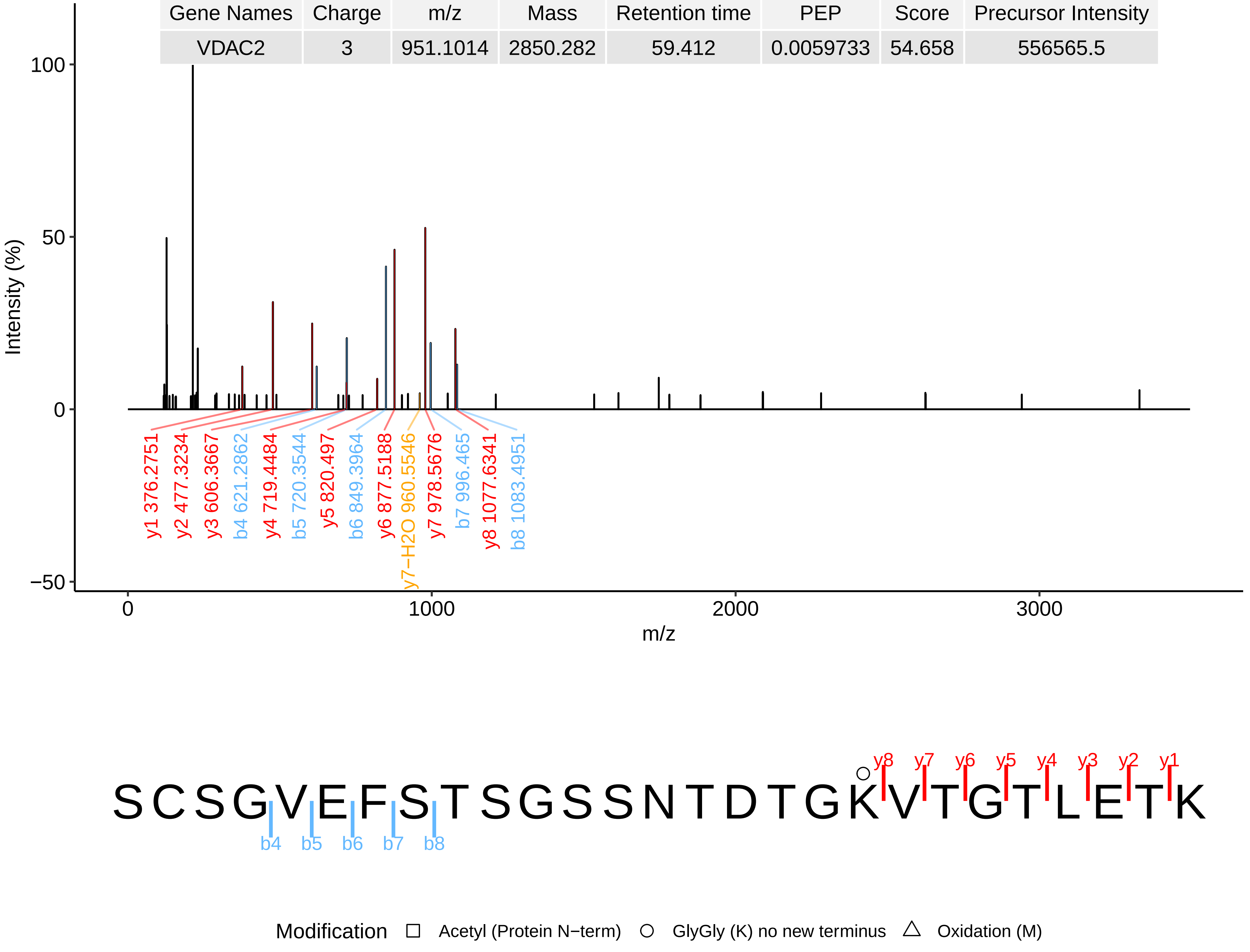

20200924_KIZ_MUb5_GlyGly_2 − Scan number: 14819

Gene Names	Charge	m/z	Mass	Retention time	PEP	Score	Precursor Intensity
VDAC2	3	951.1014	2850.282	55.368	2.2307e−19	115.11	1027333

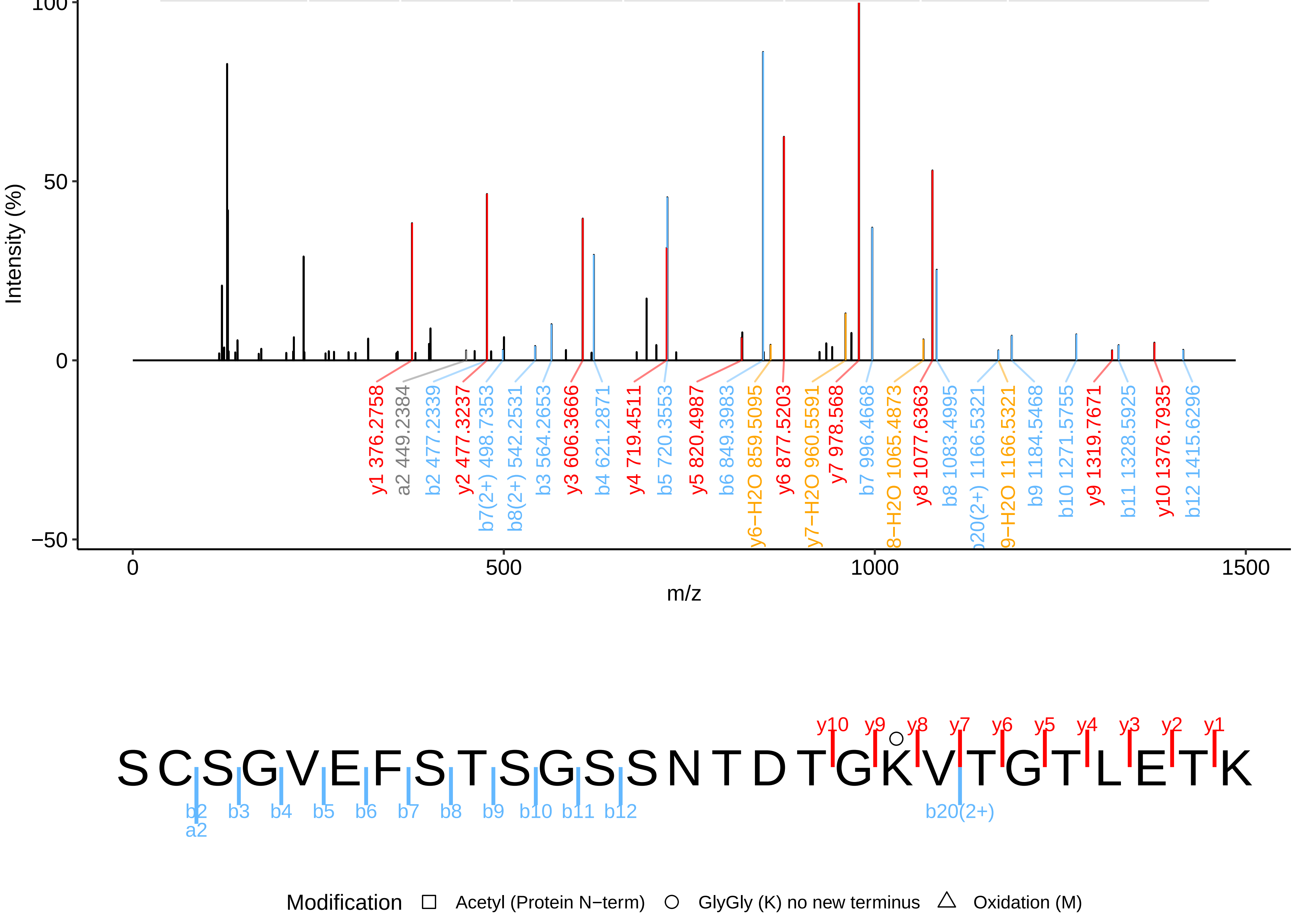

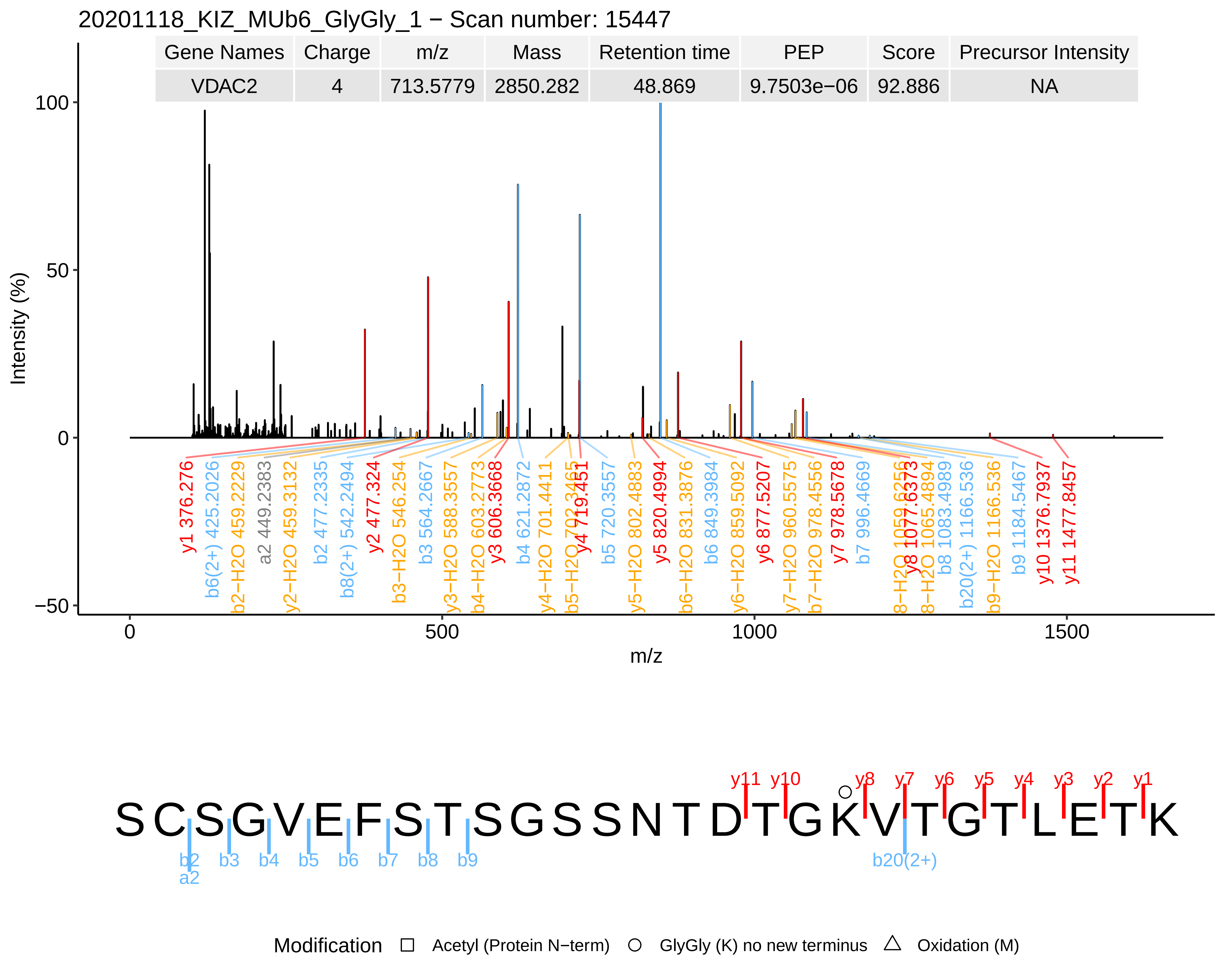

20201118_KIZ_MUb6_GlyGly_1 − Scan number: 15447
Gene Names | Charge | m/z | Mass | Retention time | PEP | Score | Precursor Intensity
VDAC2 | 4 | 713.5779 | 2850.282 | 48.869 | 9.7503e−06 | 92.886 | NA
Intensity (%)
100
50
0
−50
m/z
0
500
1000
1500
y1 376.276
b6(2+) 425.2026
b2−H2O 459.2229
a2 449.2383
y2−H2O 459.3132
b2 477.2335
b8(2+) 542.2494
y2 477.324
b3−H2O 546.254
b3 564.2667
y3−H2O 588.3557
b4−H2O 603.2773
y3 606.3668
b4 621.2872
y4−H2O 701.4411
b5−H2O 702.3465
y4 719.451
b5 720.3557
y5−H2O 802.4883
y5 820.4994
b6−H2O 831.3876
b6 849.3984
y6−H2O 859.5092
y6 877.5207
y7−H2O 960.5575
b7−H2O 978.4556
y7 978.5678
b7 996.4669
8−H2O 1059.6256
y8 1077.6373
8−H2O 1065.4894
b8 1083.4989
b20(2+) 1166.536
b9−H2O 1166.536
b9 1184.5467
y10 1376.7937
y11 1477.8457
y11 y10 y8 y7 y6 y5 y4 y3 y2 y1
S C S G V E F S T S G S S N T D T G K V T G T L E T K
b2 b3 b4 b5 b6 b7 b8 b9 b20(2+)
a2
Modification
Acetyl (Protein N−term)
GlyGly (K) no new terminus
Oxidation (M)

20201118_KIZ_MUb6_GlyGly_1 − Scan number: 15452

Gene Names	Charge	m/z	Mass	Retention time	PEP	Score	Precursor Intensity
VDAC2	4	713.5779	2850.282	48.885	1.5143e−41	179.5	36513584

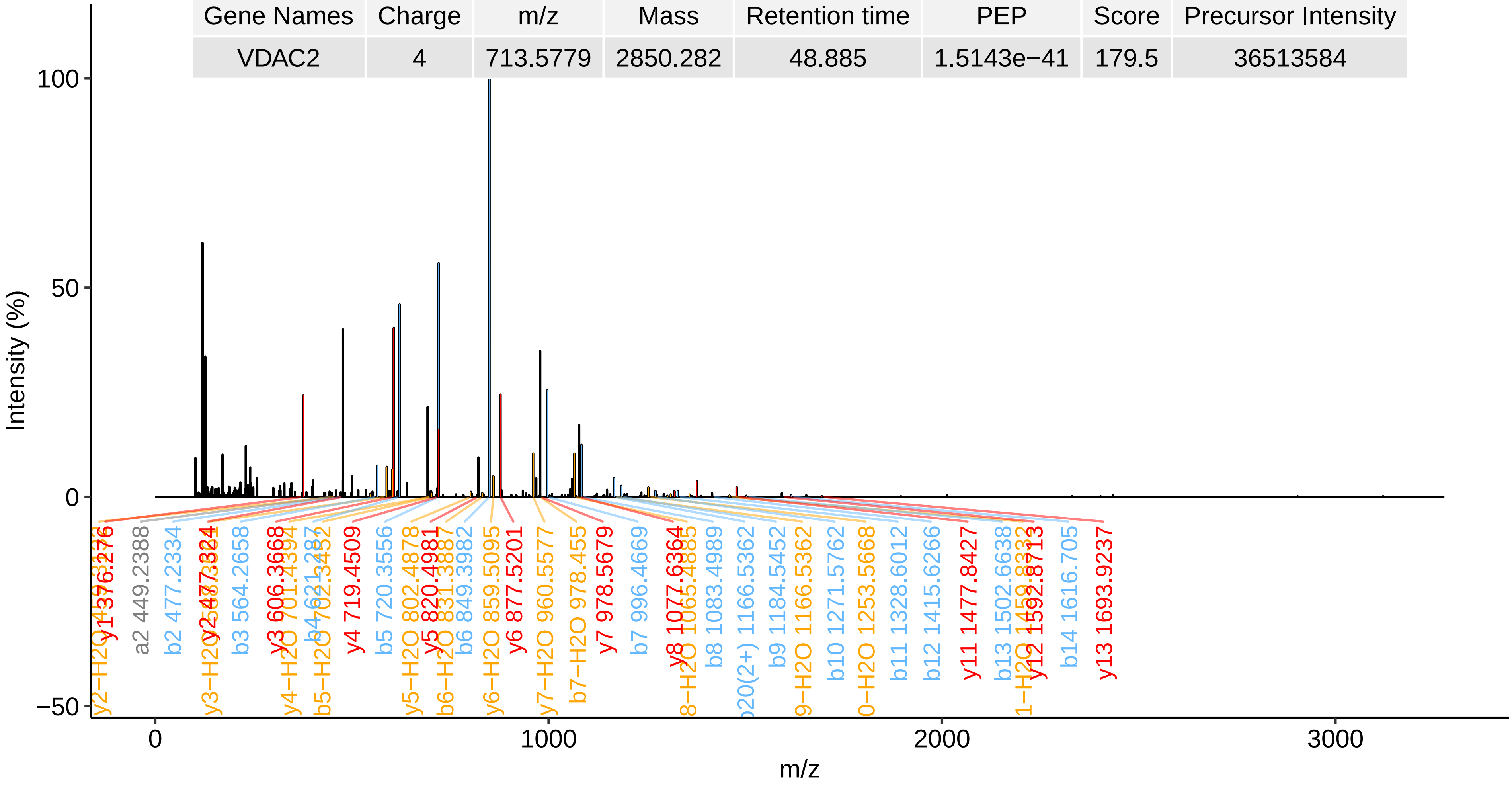

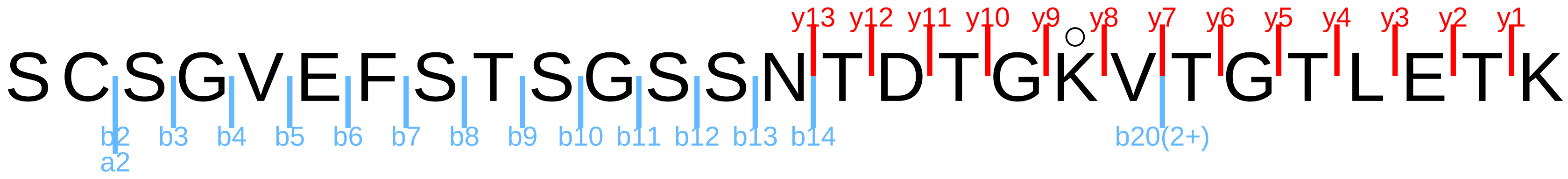

Modification □ Acetyl (Protein N−term) ○ GlyGly (K) no new terminus △ Oxidation (M)

20201118_KIZ_MUb6_GlyGly_1 − Scan number: 15448

Gene Names	Charge	m/z	Mass	Retention time	PEP	Score	Precursor Intensity
VDAC2	3	951.1014	2850.282	48.872	6.9026e−115	259.93	19556110

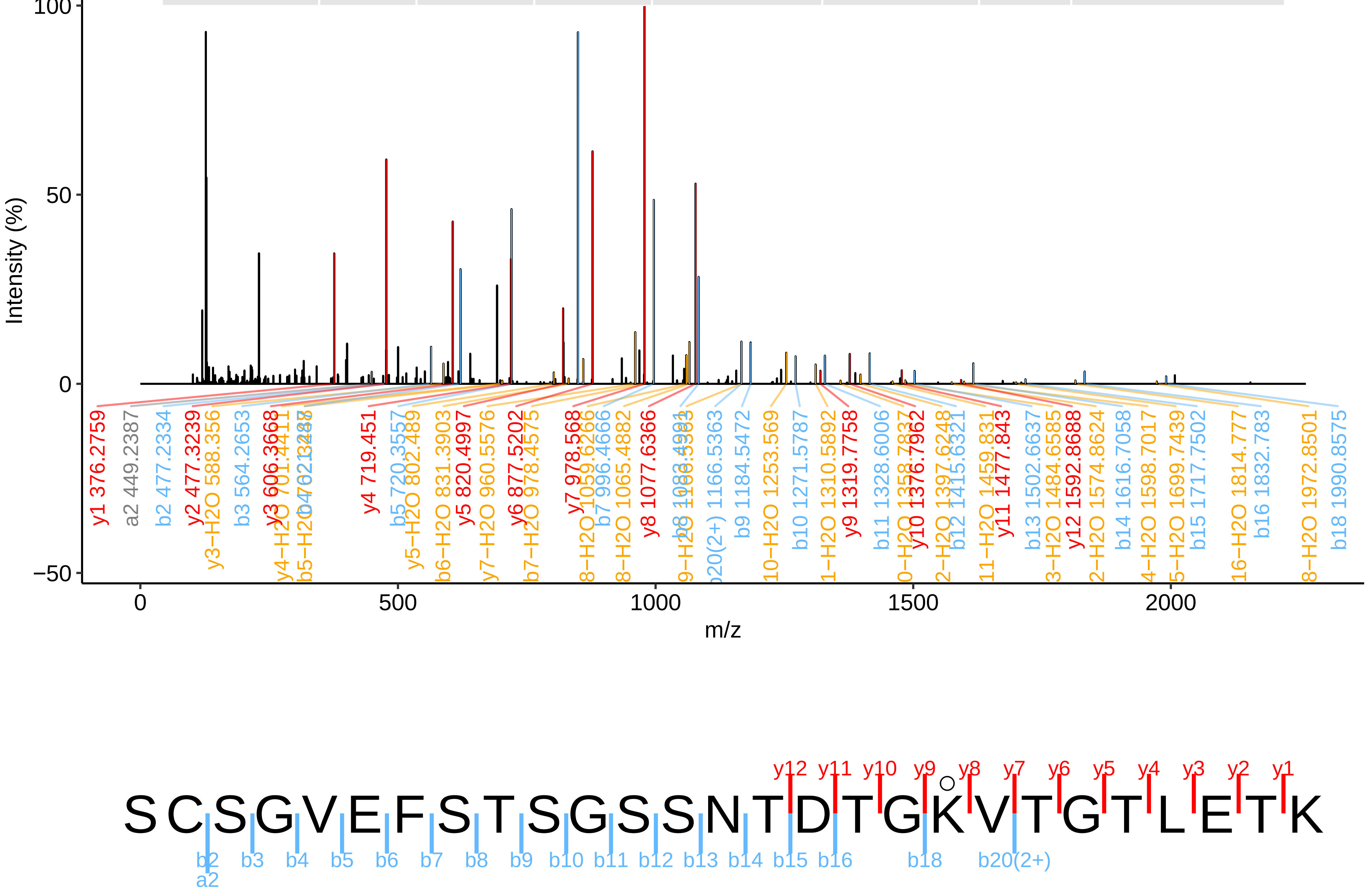

Modification □ Acetyl (Protein N−term) ○ GlyGly (K) no new terminus △ Oxidation (M)

20201118_KIZ_MUb6_GlyGly_1 − Scan number: 15456

Gene Names	Charge	m/z	Mass	Retention time	PEP	Score	Precursor Intensity
VDAC2	3	951.1014	2850.282	48.9	1.7579e−53	187.79	24835432

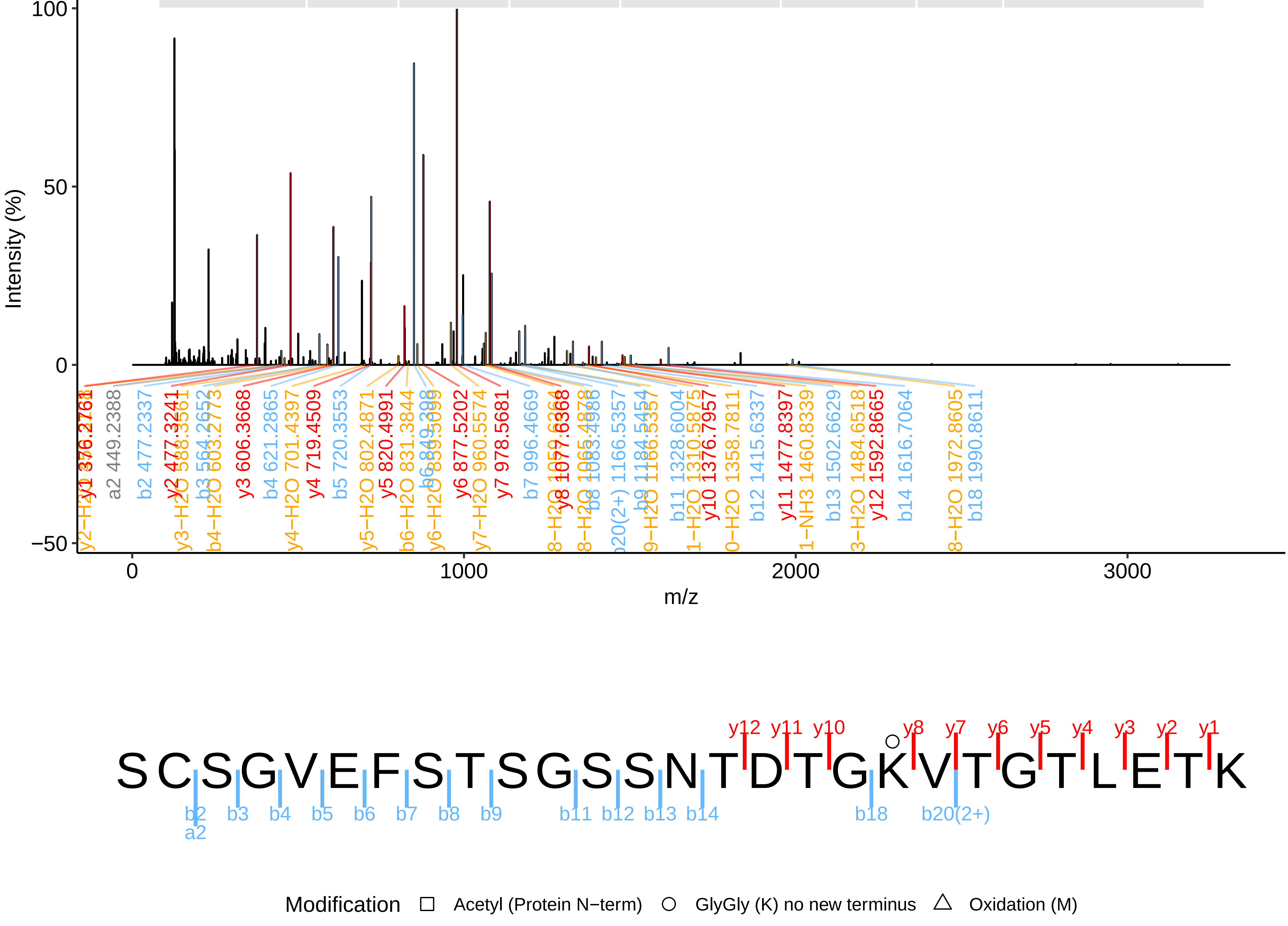

20201118_KIZ_MUb6_GlyGly_2 − Scan number: 16776

Gene Names	Charge	m/z	Mass	Retention time	PEP	Score	Precursor Intensity
VDAC2	4	713.5779	2850.282	56.698	2.7013e−05	83.826	10833723

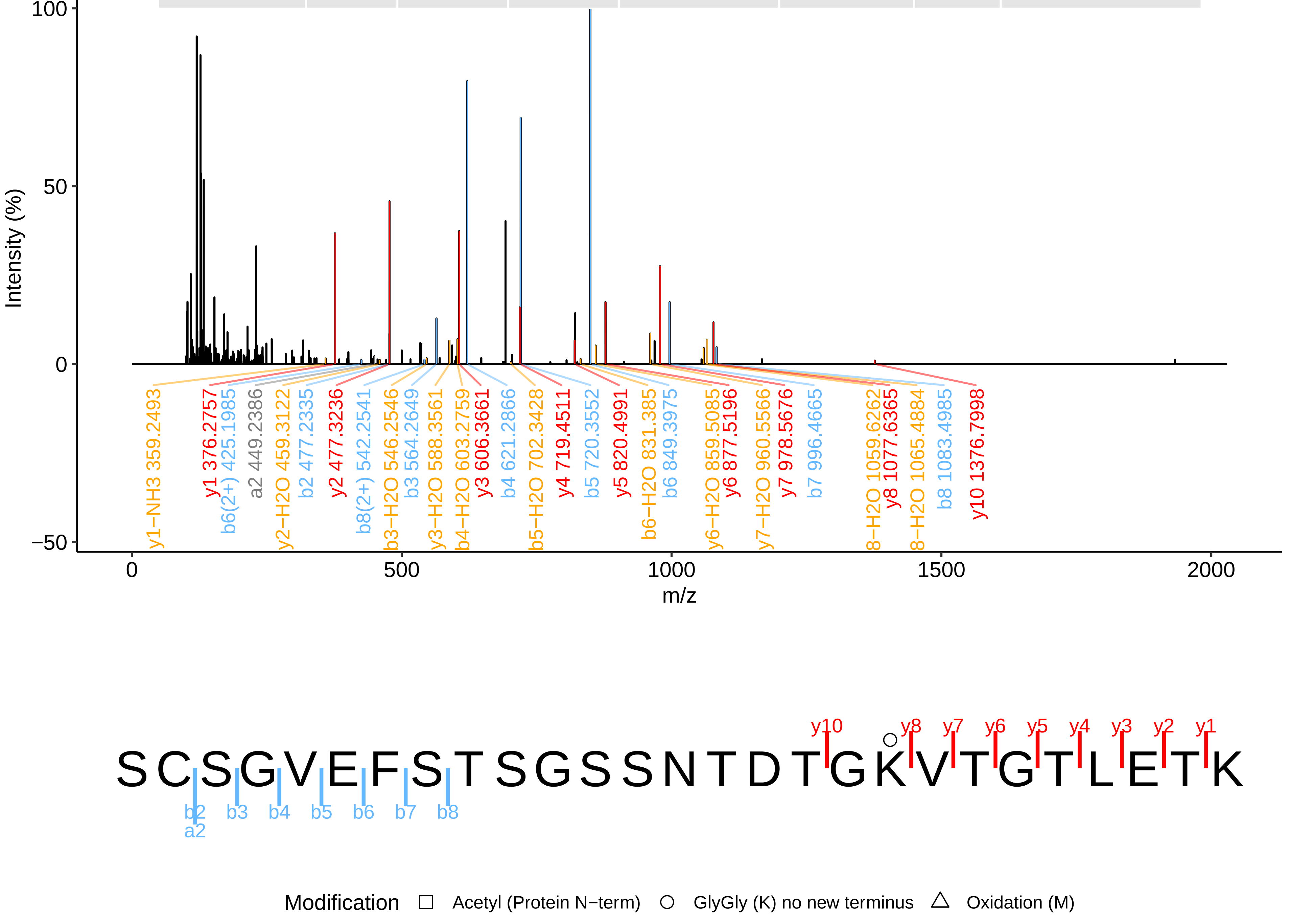

20201118_KIZ_MUb6_GlyGly_2 − Scan number: 16783

Gene Names	Charge	m/z	Mass	Retention time	PEP	Score	Precursor Intensity
VDAC2	4	713.5779	2850.282	56.722	1.4377e−31	166.59	7671571

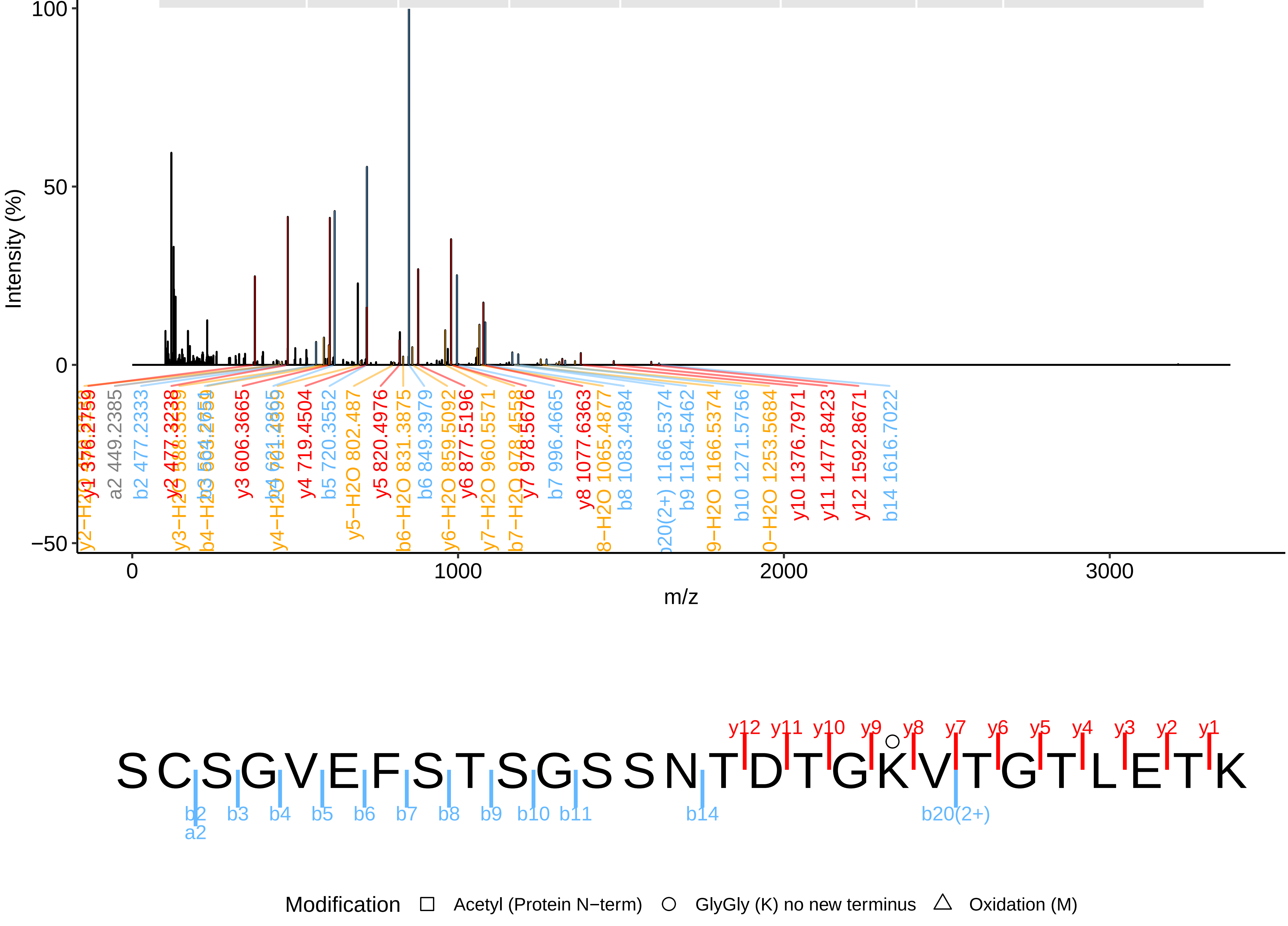

20201118_KIZ_MUb6_GlyGly_2 − Scan number: 16779

Gene Names	Charge	m/z	Mass	Retention time	PEP	Score	Precursor Intensity
VDAC2	3	951.1014	2850.282	56.707	2.7322e−30	151.06	NA

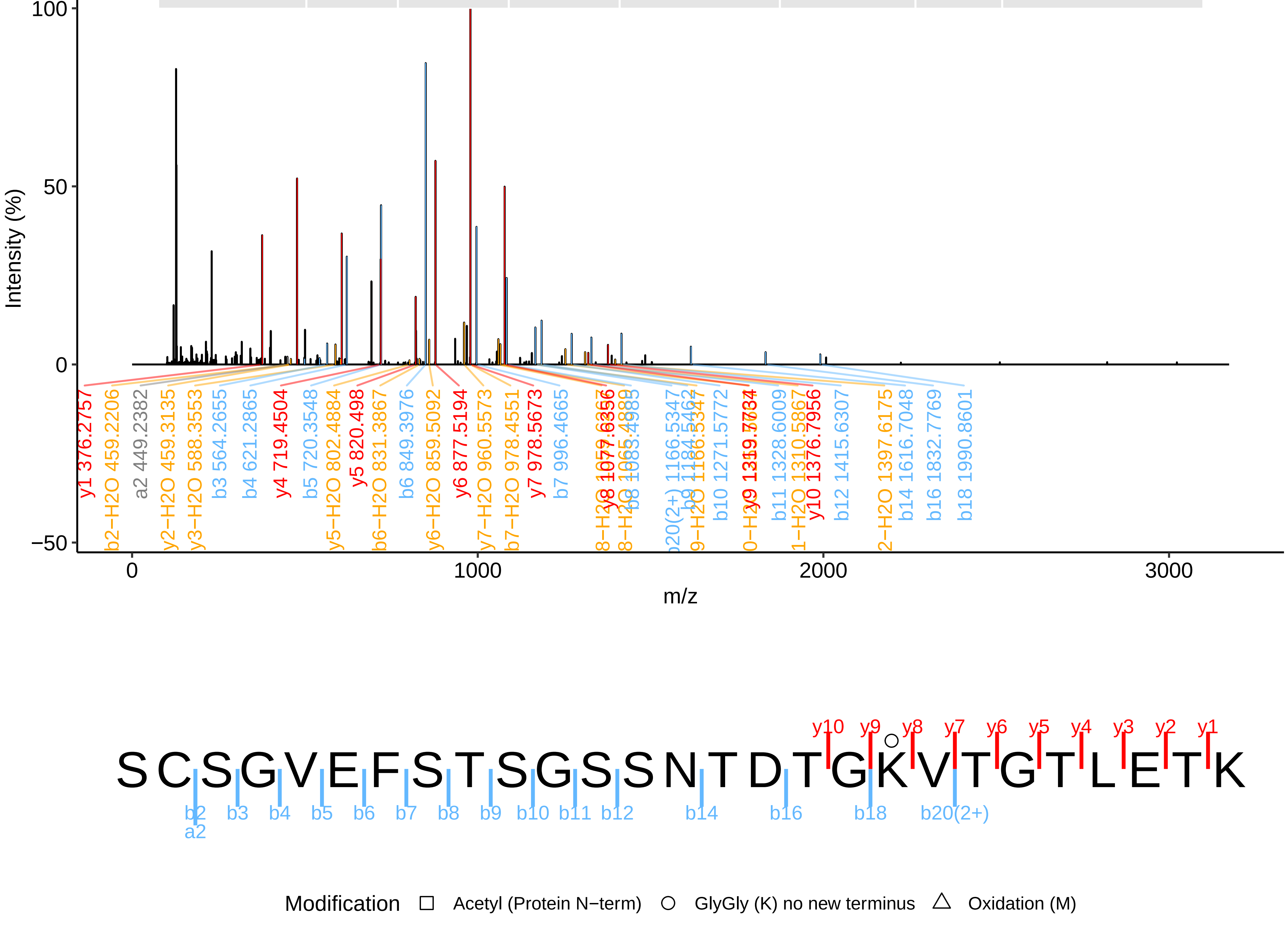

20200921_KIZ_MUb4_GlyGly_10plex − Scan number: 18732

Gene Names	Charge	m/z	Mass	Retention time	PEP	Score	Precursor Intensity
VDAC1	4	723.0954	2888.352	67.433	1.4417e−42	184.53	10189767

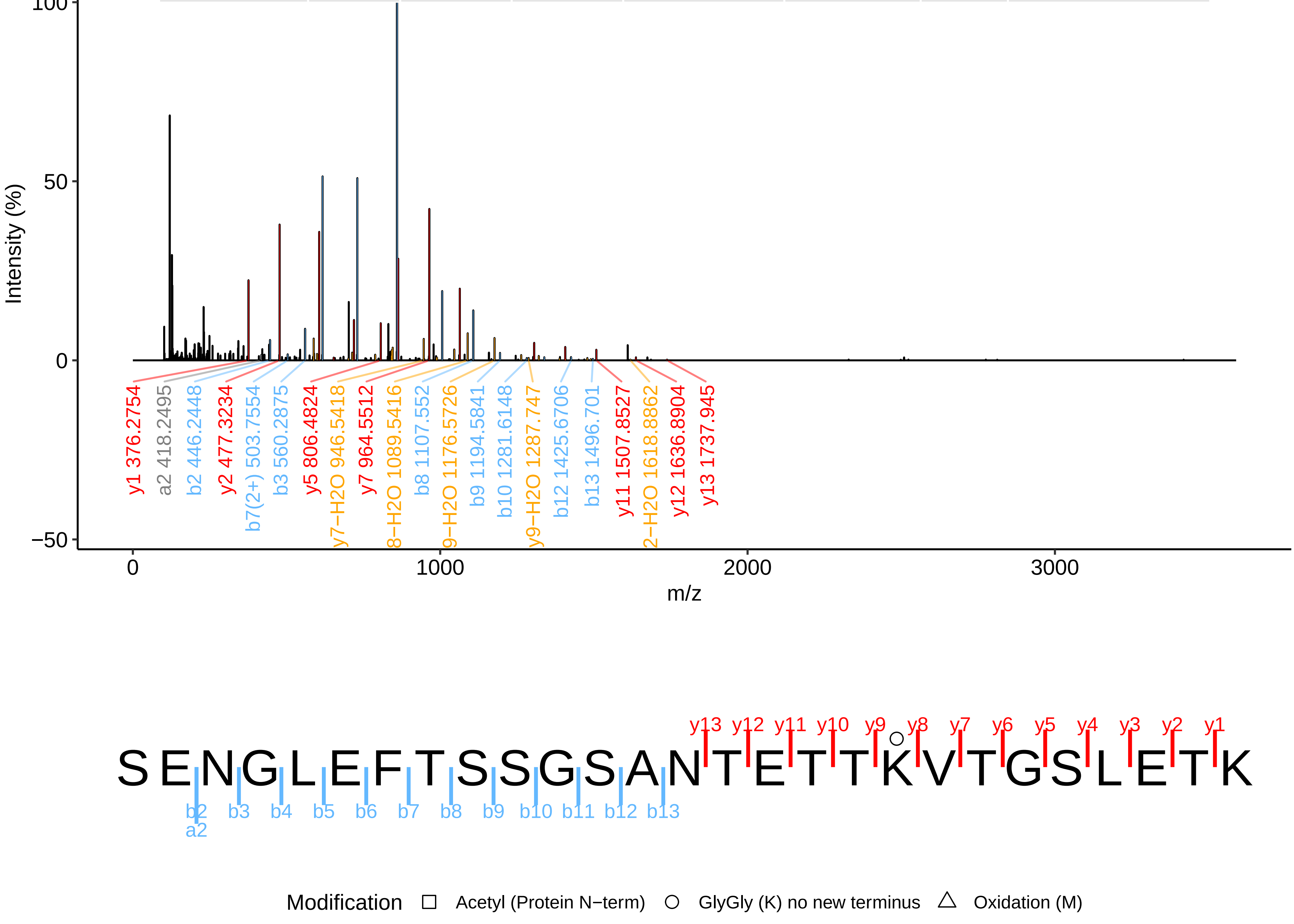

20200921_KIZ_MUb4_GlyGly_10plex − Scan number: 18819

Gene Names	Charge	m/z	Mass	Retention time	PEP	Score	Precursor Intensity
VDAC1	3	963.7914	2888.352	67.77	1.1489e−67	207.07	25807812

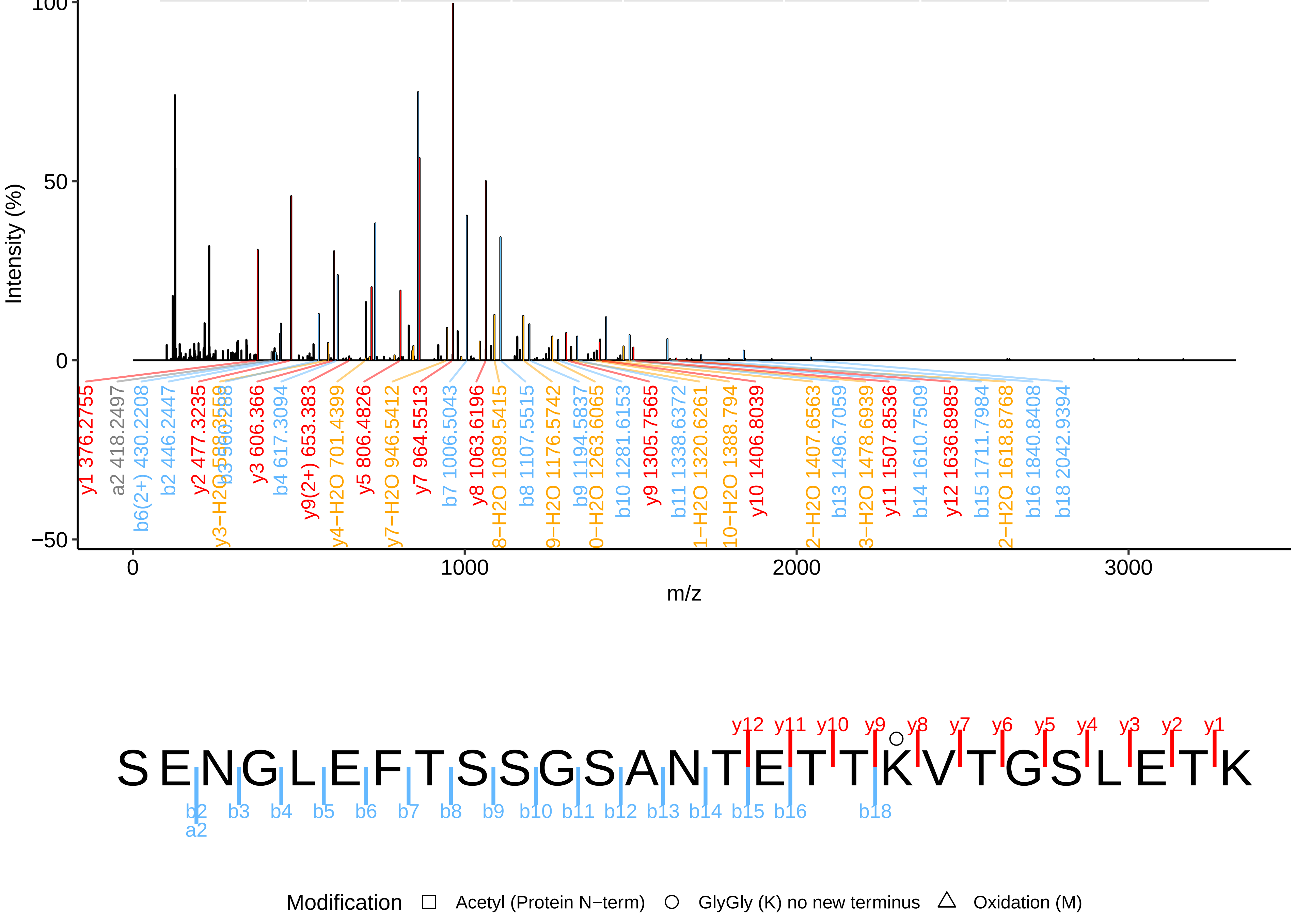

20200921_KIZ_MUb4_GlyGly_10plex − Scan number: 18863

Gene Names	Charge	m/z	Mass	Retention time	PEP	Score	Precursor Intensity
VDAC1	4	723.0954	2888.352	67.946	2.6831e−68	216.25	19930472

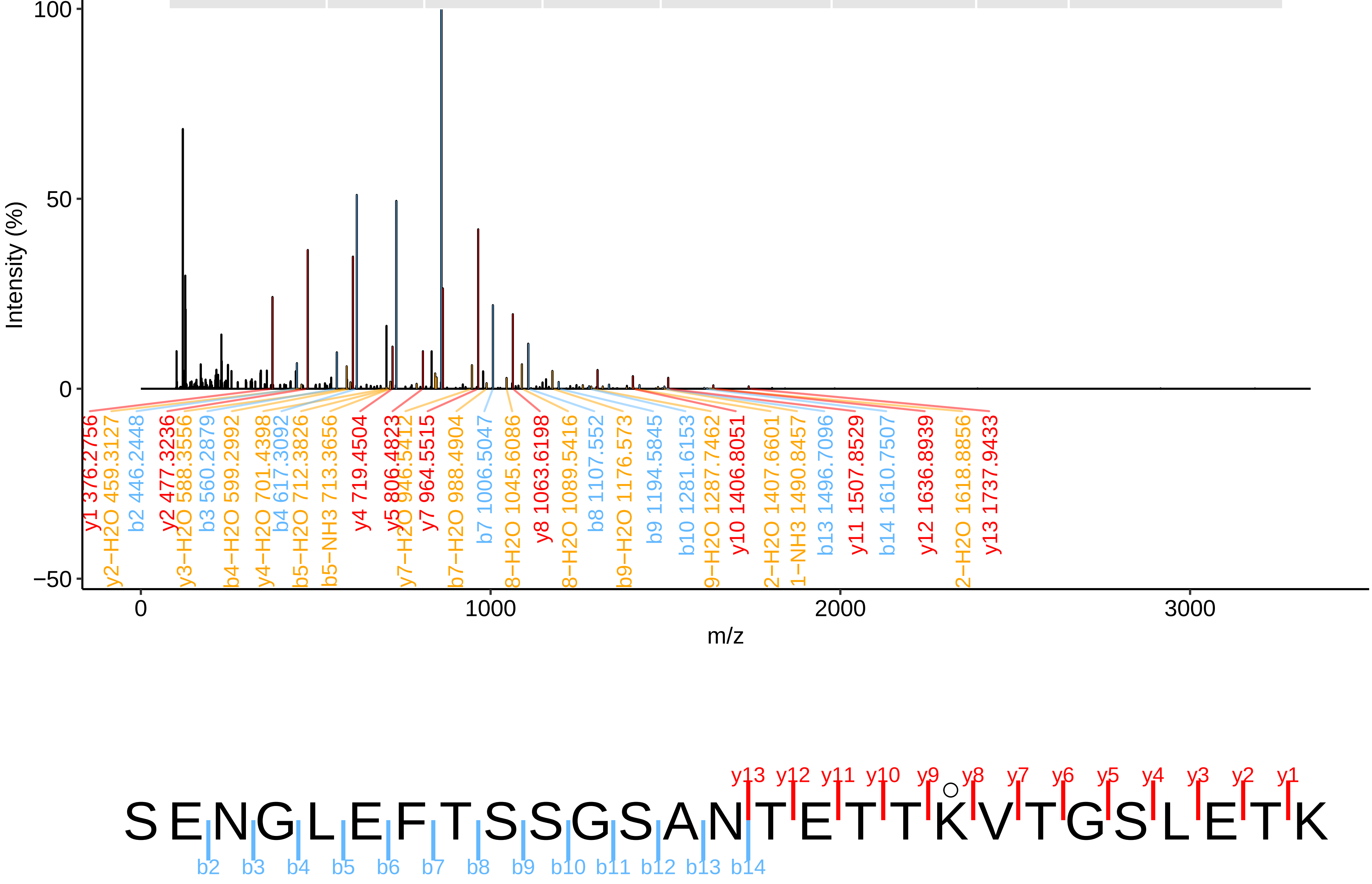

Modification □ Acetyl (Protein N−term) ○ GlyGly (K) no new terminus △ Oxidation (M)

20200921_KIZ_MUb4_GlyGly_10plex − Scan number: 18959

Gene Names	Charge	m/z	Mass	Retention time	PEP	Score	Precursor Intensity
VDAC1	3	963.7914	2888.352	68.276	2.201e−115	268.24	14340857

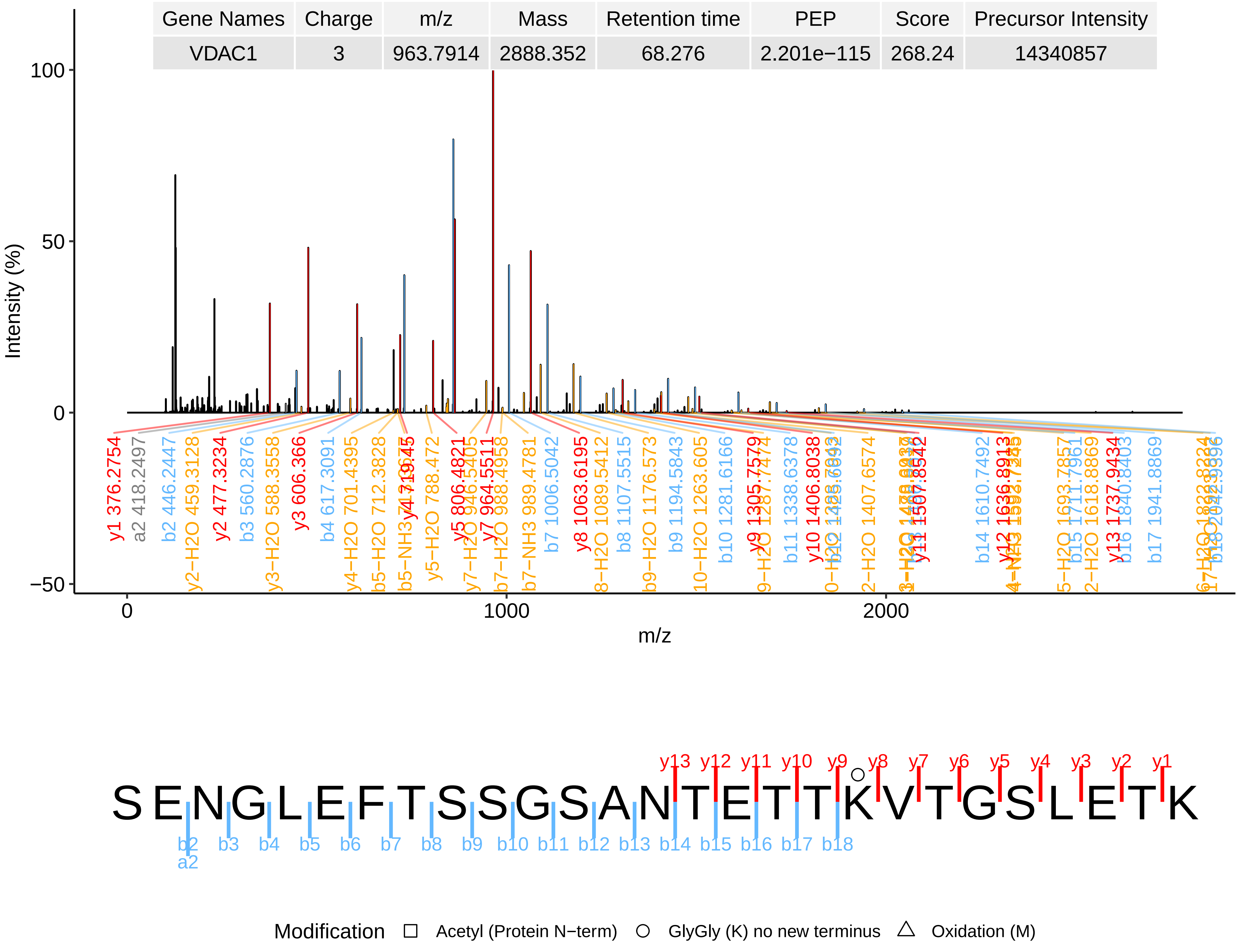

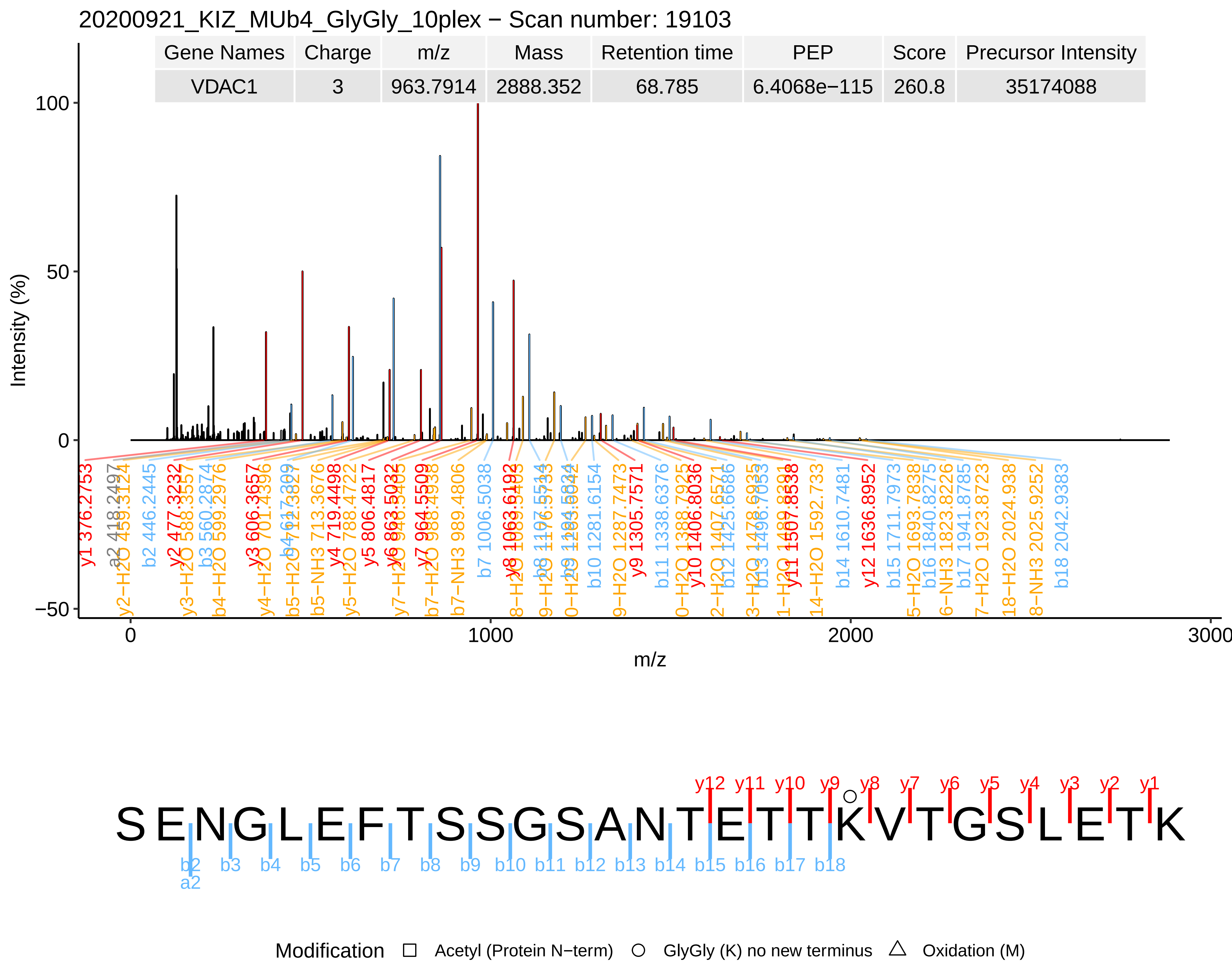
20200921_KIZ_MUb4_GlyGly_10plex − Scan number: 19103
Gene Names | Charge | m/z | Mass | Retention time | PEP | Score | Precursor Intensity
VDAC1 | 3 | 963.7914 | 2888.352 | 68.785 | 6.4068e−115 | 260.8 | 35174088
Intensity (%)
m/z
SENGLEFTSSGSANTETTKVTGSLETK
Modification
Acetyl (Protein N−term)
GlyGly (K) no new terminus
Oxidation (M)

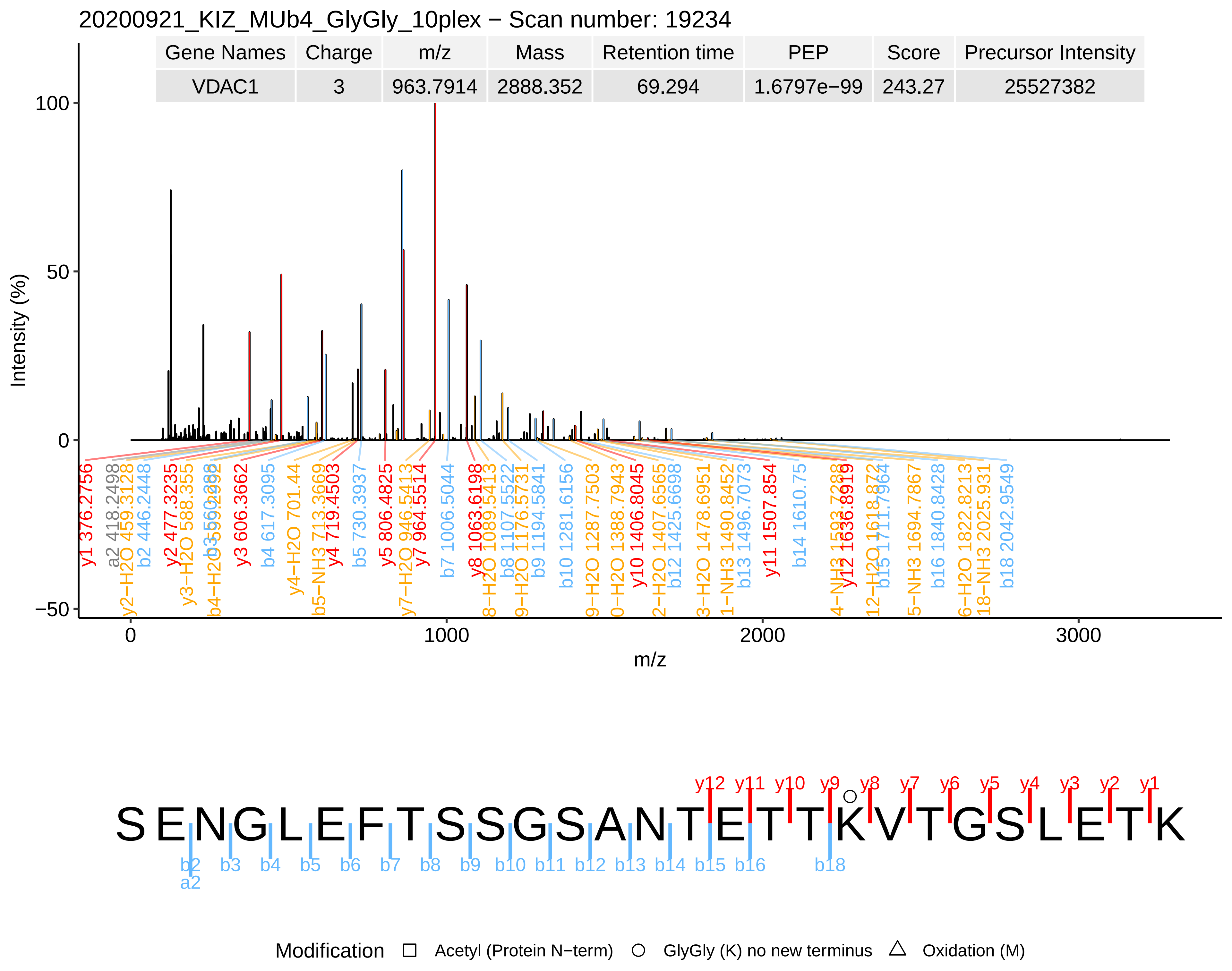
20200921_KIZ_MUb4_GlyGly_10plex − Scan number: 19234
Gene Names
Charge
m/z
Mass
Retention time
PEP
Score
Precursor Intensity
VDAC1
3
963.7914
2888.352
69.294
1.6797e−99
243.27
25527382
Intensity (%)
m/z
y1 376.2756
a2 418.2498
b2 446.2448
y2 477.3235
y3 606.3662
b4 617.3095
y4−H2O 701.44
b5−NH3 713.3669
y4 719.4503
b5 730.3937
y5 806.4825
y7−H2O 946.5413
y7 964.5514
b7 1006.5044
y8 1063.6198
b8 1107.5522
b9 1194.5841
b10 1281.6156
y10 1406.8045
b12 1425.6698
b13 1496.7073
y11 1507.854
b14 1610.75
b16 1840.8428
b18 2042.9549
S E N G L E F T S S G S A N T E T T K V T G S L E T K
y12 y11 y10 y9 y8 y7 y6 y5 y4 y3 y2 y1
b2 b3 b4 b5 b6 b7 b8 b9 b10 b11 b12 b13 b14 b15 b16 b18
a2
Modification
Acetyl (Protein N−term)
GlyGly (K) no new terminus
Oxidation (M)

20200921_KIZ_MUb4_GlyGly_10plex − Scan number: 19362

Gene Names	Charge	m/z	Mass	Retention time	PEP	Score	Precursor Intensity
VDAC1	3	963.7914	2888.352	69.799	2.4575e−99	237.97	24874746

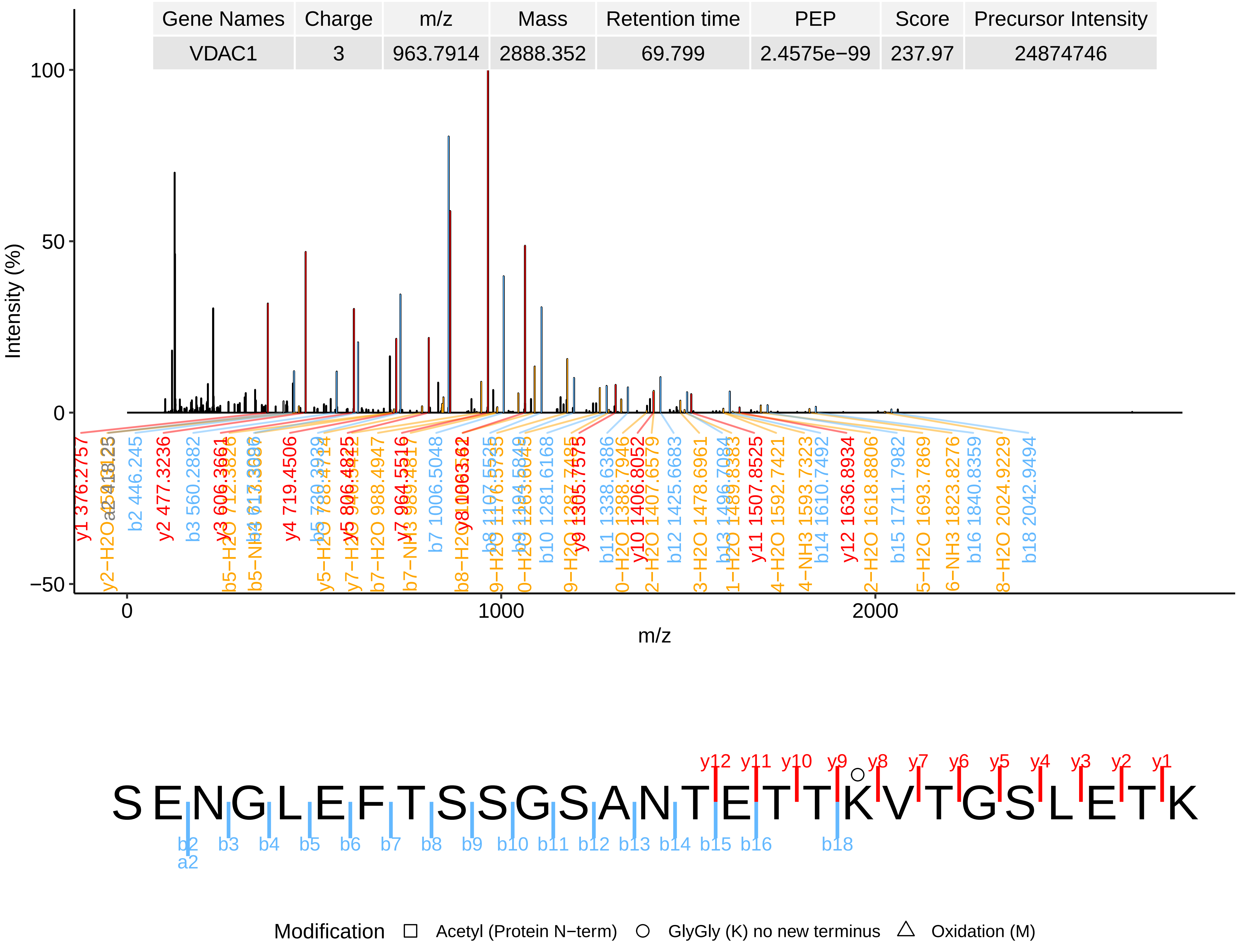

20200921_KIZ_MUb4_GlyGly_10plex − Scan number: 19494

Gene Names	Charge	m/z	Mass	Retention time	PEP	Score	Precursor Intensity
VDAC1	3	963.7914	2888.352	70.315	4.5344e−69	219.38	18846894

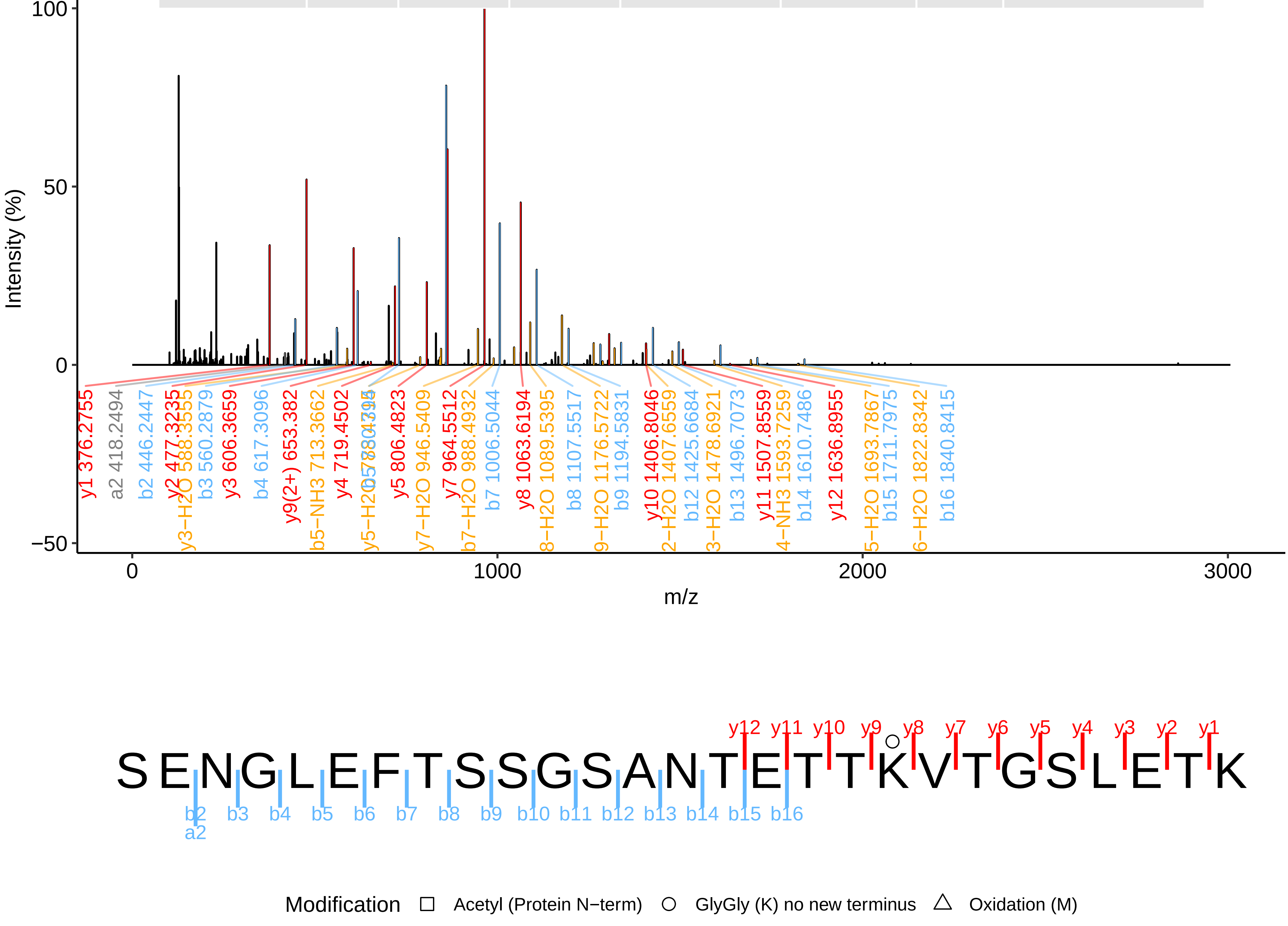

20200921_KIZ_MUb4_GlyGly_10plex − Scan number: 19007

Gene Names	Charge	m/z	Mass	Retention time	PEP	Score	Precursor Intensity
VDAC1	4	723.0954	2888.352	68.447	6.648e−31	162.75	50987272

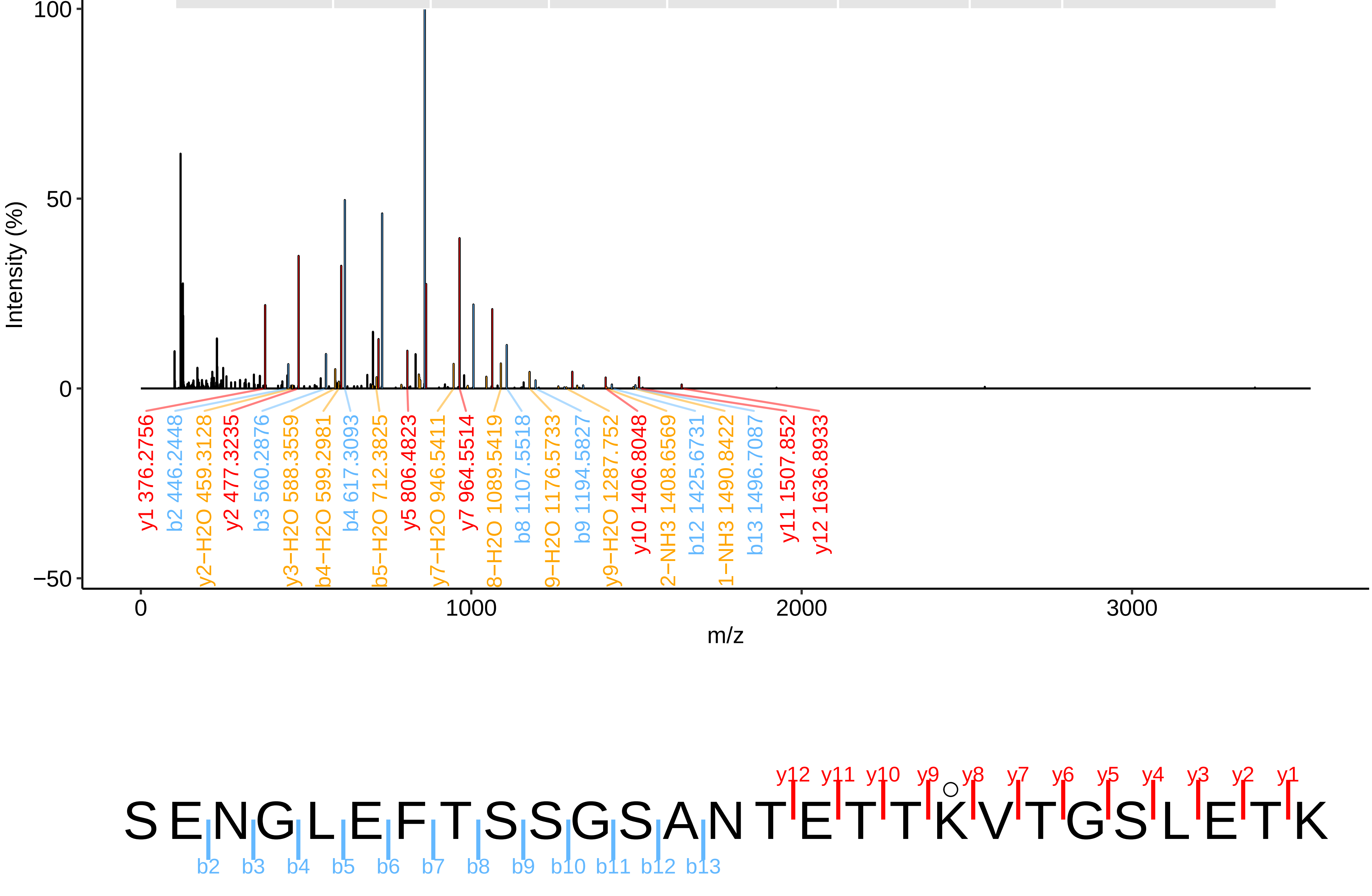

Modification □ Acetyl (Protein N−term) ○ GlyGly (K) no new terminus △ Oxidation (M)

20200921_KIZ_MUb4_GlyGly_10plex − Scan number: 19147

Gene Names	Charge	m/z	Mass	Retention time	PEP	Score	Precursor Intensity
VDAC1	4	723.0954	2888.352	68.958	1.8722e−32	167.51	34785556

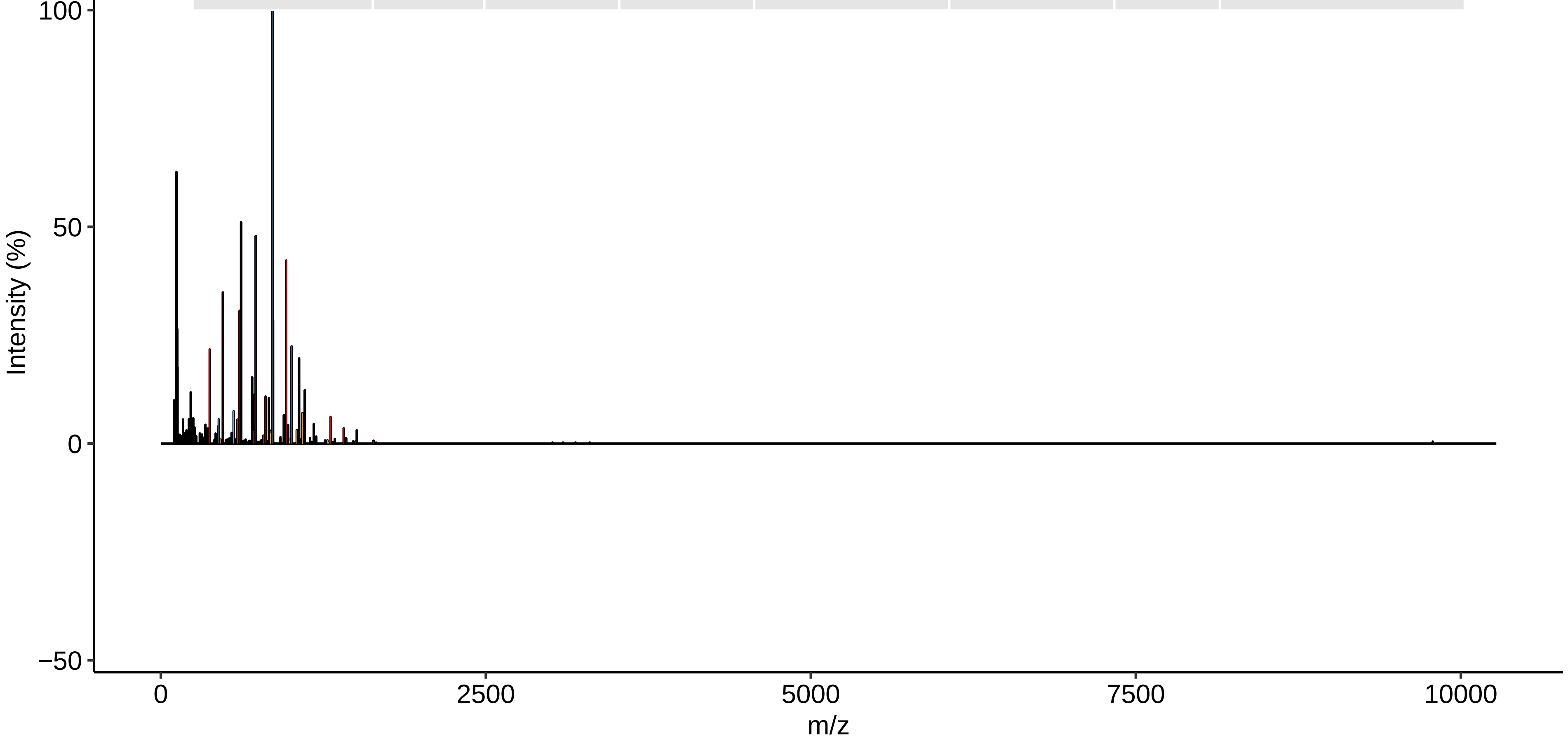

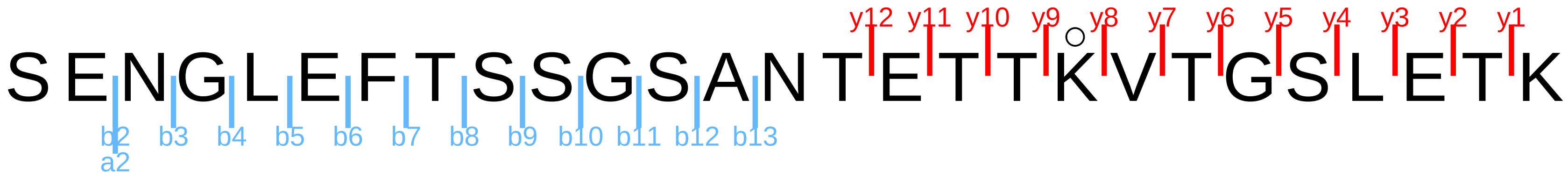

Modification □ Acetyl (Protein N−term) ○ GlyGly (K) no new terminus △ Oxidation (M)

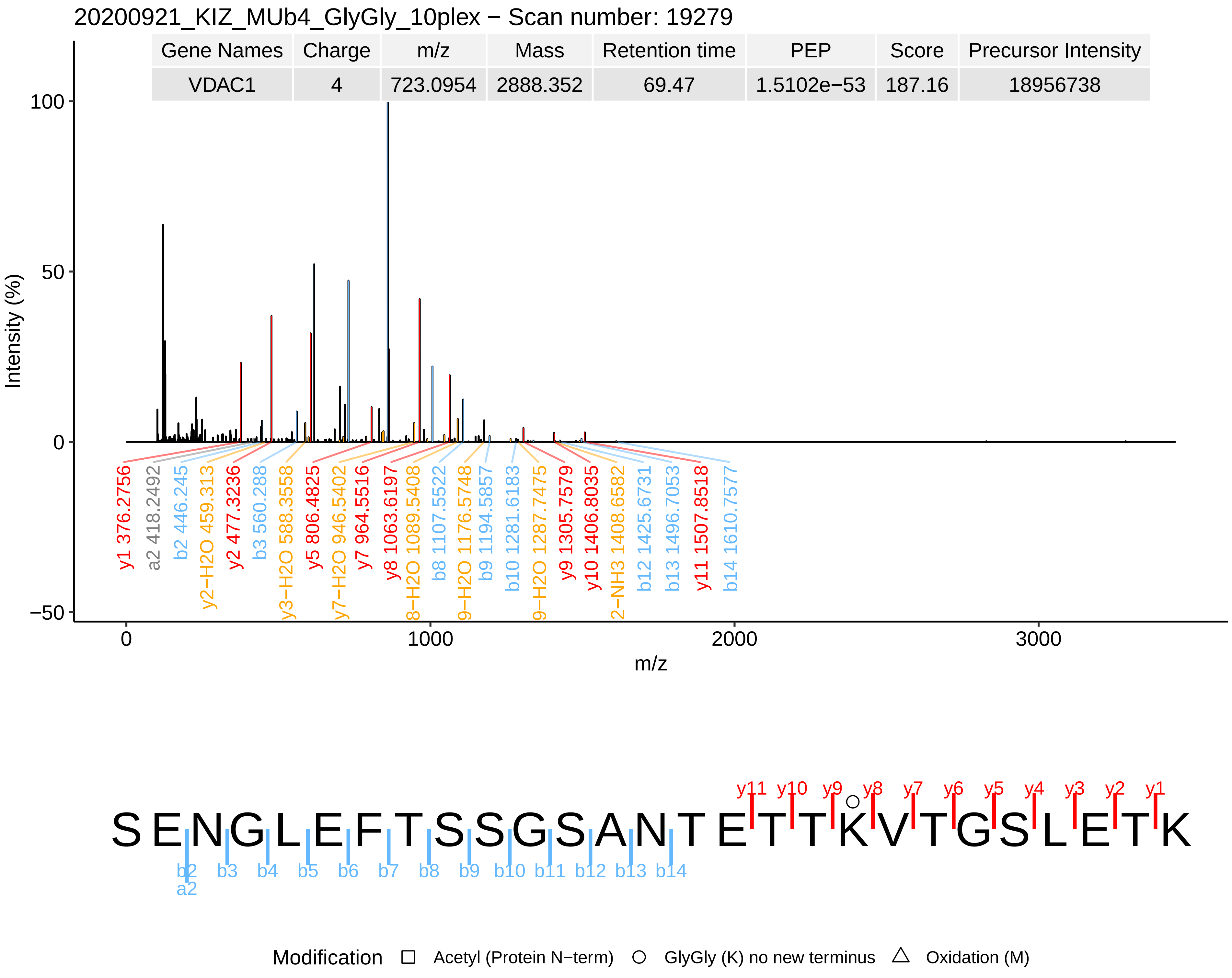

20200921_KIZ_MUb4_GlyGly_10plex − Scan number: 19279
Gene Names
Charge
m/z
Mass
Retention time
PEP
Score
Precursor Intensity
VDAC1
4
723.0954
2888.352
69.47
1.5102e−53
187.16
18956738
Intensity (%)
100
50
0
−50
0
1000
2000
3000
m/z
y1 376.2756
a2 418.2492
b2 446.245
y2−H2O 459.313
y2 477.3236
b3 560.288
y3−H2O 588.3558
y5 806.4825
y7−H2O 946.5402
y7 964.5516
y8 1063.6197
8−H2O 1089.5408
b8 1107.5522
9−H2O 1176.5748
b9 1194.5857
b10 1281.6183
9−H2O 1287.7475
y9 1305.7579
y10 1406.8035
2−NH3 1408.6582
b12 1425.6731
b13 1496.7053
y11 1507.8518
b14 1610.7577
y11 y10 y9 y8 y7 y6 y5 y4 y3 y2 y1
S E N G L E F T S S G S A N T E T T K V T G S L E T K
b2 b3 b4 b5 b6 b7 b8 b9 b10 b11 b12 b13 b14
a2
Modification
Acetyl (Protein N−term)
GlyGly (K) no new terminus
Oxidation (M)

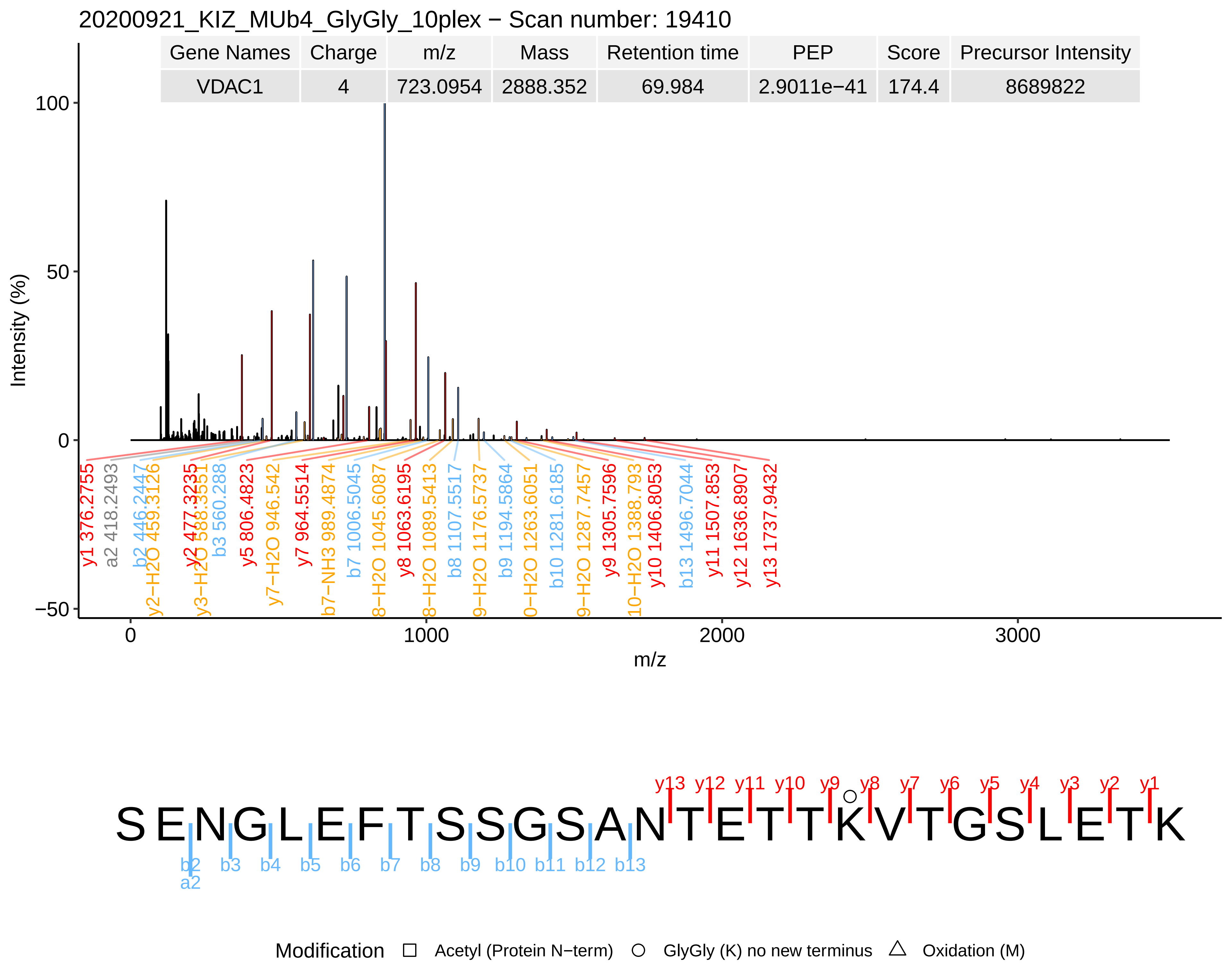
20200921_KIZ_MUb4_GlyGly_10plex − Scan number: 19410
Gene Names
Charge
m/z
Mass
Retention time
PEP
Score
Precursor Intensity
VDAC1
4
723.0954
2888.352
69.984
2.9011e−41
174.4
8689822
Intensity (%)
m/z
y1 376.2755
a2 418.2493
b2 446.2447
y2 477.3235
b3 560.288
y5 806.4823
y7−H2O 946.542
y7 964.5514
b7−NH3 989.4874
b7 1006.5045
y8 1063.6195
b8 1107.5517
b9 1194.5864
b10 1281.6185
y9 1305.7596
y10 1406.8053
b13 1496.7044
y11 1507.853
y12 1636.8907
y13 1737.9432
SENGLEFTSSGSANTETTKVTGSLETK
Modification
Acetyl (Protein N−term)
GlyGly (K) no new terminus
Oxidation (M)

20200921_KIZ_MUb4_GlyGly_10plex − Scan number: 19544

Gene Names	Charge	m/z	Mass	Retention time	PEP	Score	Precursor Intensity
VDAC1	4	723.0954	2888.352	70.503	2.1348e−30	151.91	3346135

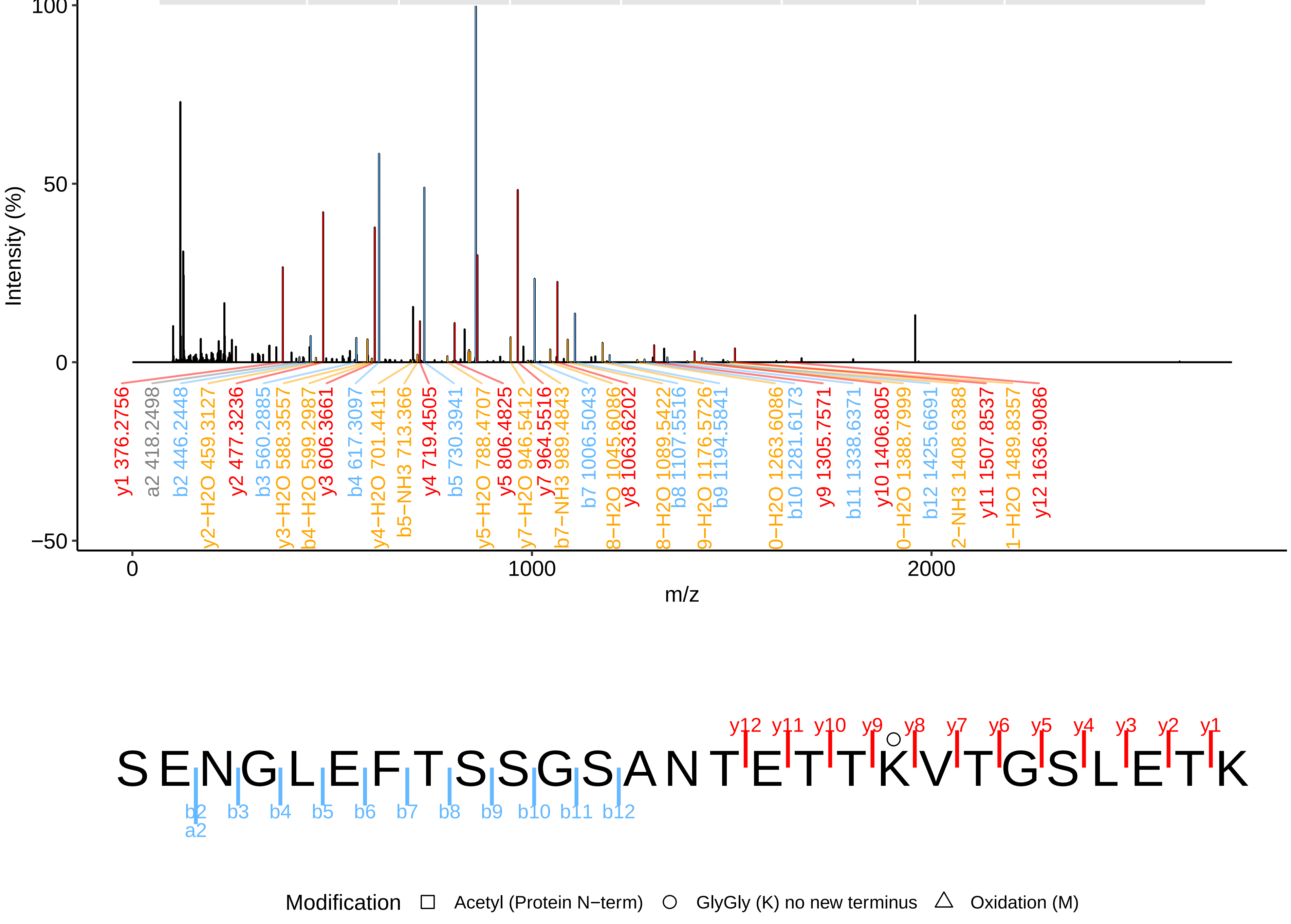

20200921_KIZ_MUb4_GlyGly_10plex − Scan number: 19815

Gene Names	Charge	m/z	Mass	Retention time	PEP	Score	Precursor Intensity
VDAC1	4	723.0954	2888.352	71.516	1.5093e−53	187.17	8480398

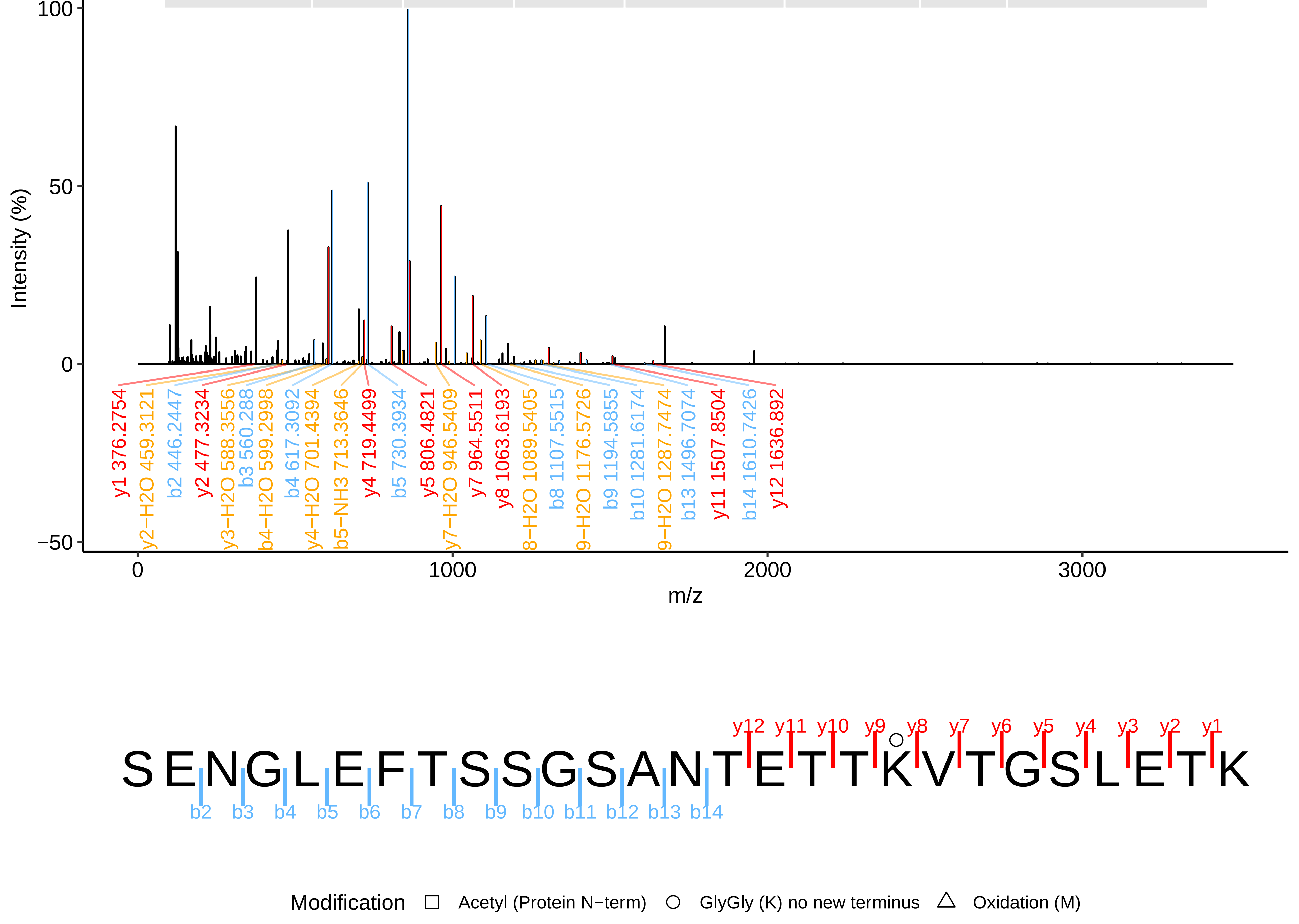

20200921_KIZ_MUb4_GlyGly_10plex − Scan number: 19898

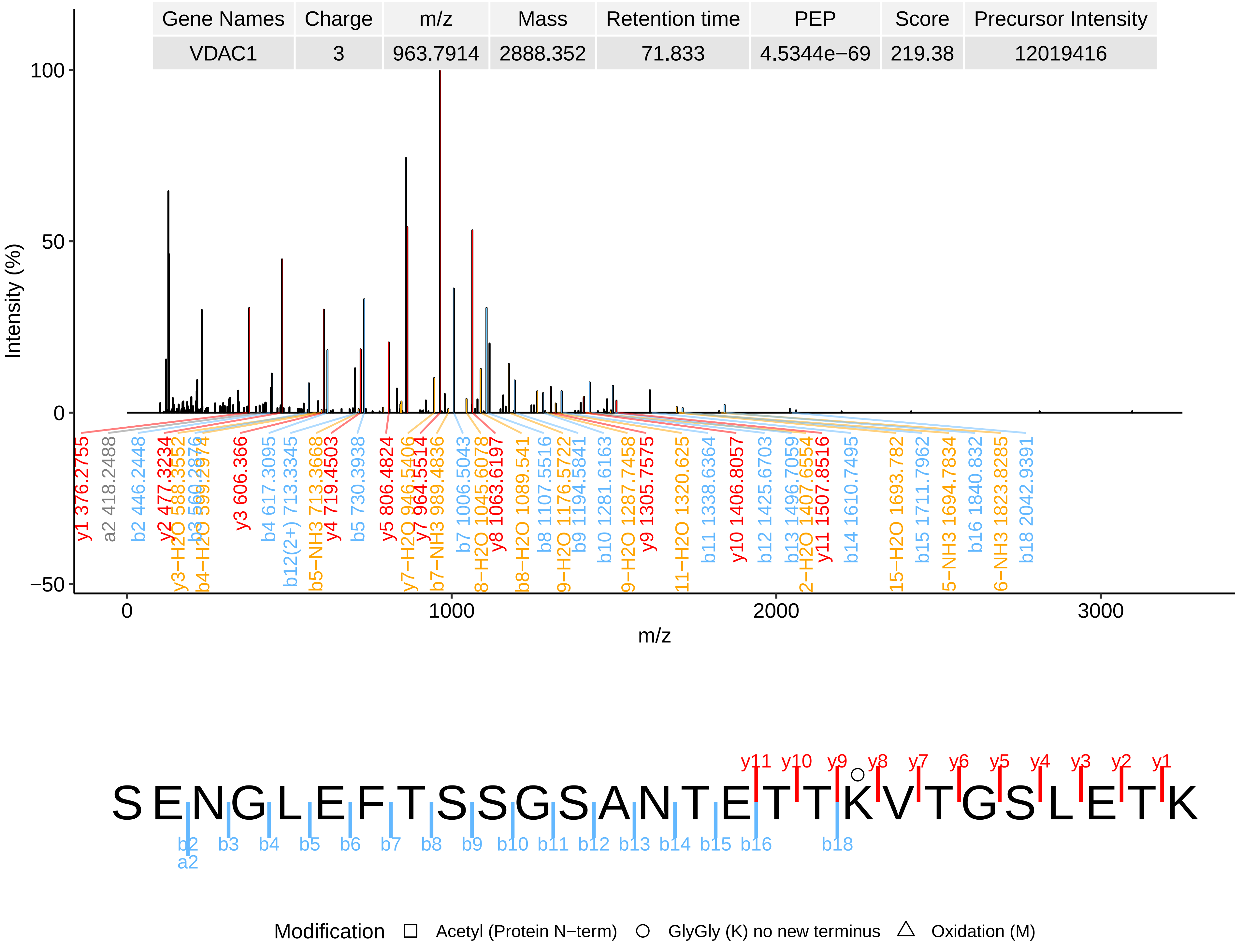

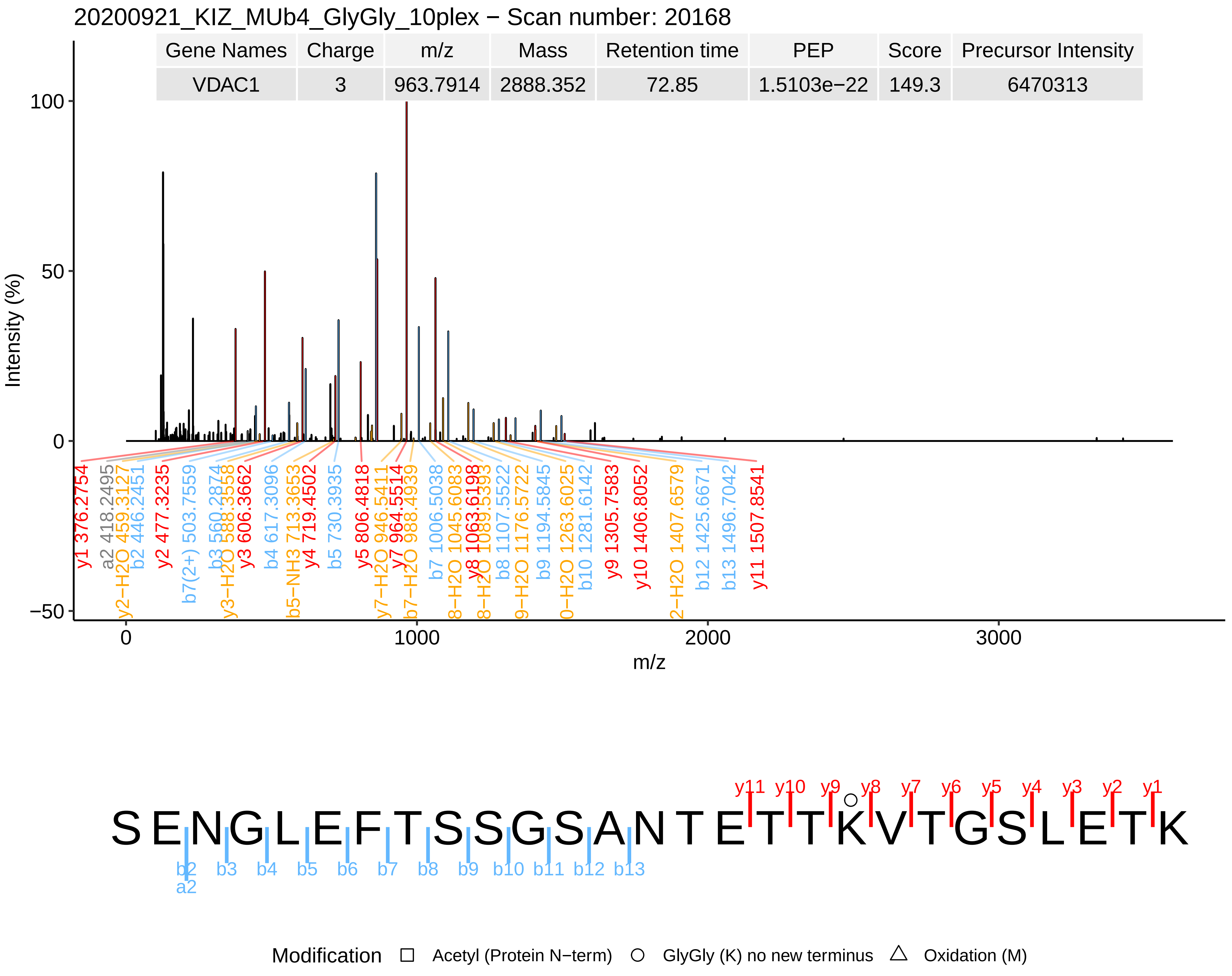
20200921_KIZ_MUb4_GlyGly_10plex − Scan number: 20168
Gene Names | Charge | m/z | Mass | Retention time | PEP | Score | Precursor Intensity
VDAC1 | 3 | 963.7914 | 2888.352 | 72.85 | 1.5103e−22 | 149.3 | 6470313
Intensity (%)
100
50
0
−50
0
1000
2000
3000
m/z
y1 376.2754
a2 418.2495
y2−H2O 459.3127
b2 446.2451
y2 477.3235
b7(2+) 503.7559
b3 560.2874
y3−H2O 588.3558
y3 606.3662
b4 617.3096
b5−NH3 713.3653
y4 719.4502
b5 730.3935
y5 806.4818
y7−H2O 946.5411
y7 964.5514
b7−H2O 988.4939
b7 1006.5038
8−H2O 1045.6083
y8 1063.6198
8−H2O 1089.5393
b8 1107.5522
9−H2O 1176.5722
b9 1194.5845
0−H2O 1263.6025
b10 1281.6142
y9 1305.7583
y10 1406.8052
2−H2O 1407.6579
b12 1425.6671
b13 1496.7042
y11 1507.8541
y11 y10 y9 y8 y7 y6 y5 y4 y3 y2 y1
S E N G L E F T S S G S A N T E T T K V T G S L E T K
b2 b3 b4 b5 b6 b7 b8 b9 b10 b11 b12 b13
a2
Modification
Acetyl (Protein N−term)
GlyGly (K) no new terminus
Oxidation (M)

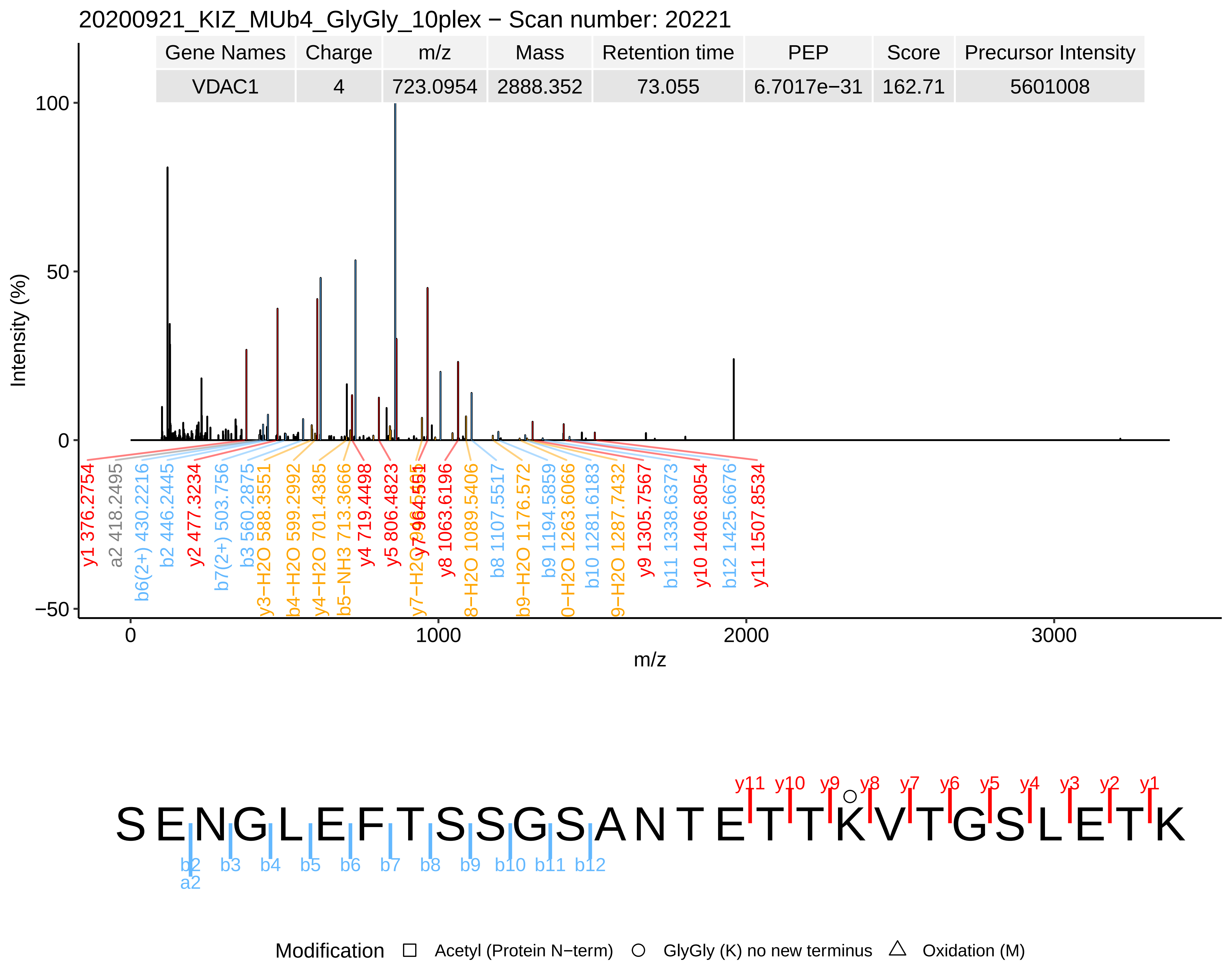

20200921_KIZ_MUb4_GlyGly_10plex − Scan number: 20221

Gene Names	Charge	m/z	Mass	Retention time	PEP	Score	Precursor Intensity
VDAC1	4	723.0954	2888.352	73.055	6.7017e−31	162.71	5601008

Intensity (%)
m/z
y1 376.2754
a2 418.2495
b6(2+) 430.2216
b2 446.2445
y2 477.3234
b7(2+) 503.756
b3 560.2875
y3−H2O 588.3551
b4−H2O 599.2992
y4−H2O 701.4385
b5−NH3 713.3666
y4 719.4498
y5 806.4823
y8 1063.6196
b8 1107.5517
b9 1194.5859
b10 1281.6183
y9 1305.7567
b11 1338.6373
y10 1406.8054
b12 1425.6676
y11 1507.8534
S E N G L E F T S S G S A N T E T T K V T G S L E T K
y11 y10 y9 y8 y7 y6 y5 y4 y3 y2 y1
b2 b3 b4 b5 b6 b7 b8 b9 b10 b11 b12
a2
Modification
Acetyl (Protein N−term)
GlyGly (K) no new terminus
Oxidation (M)

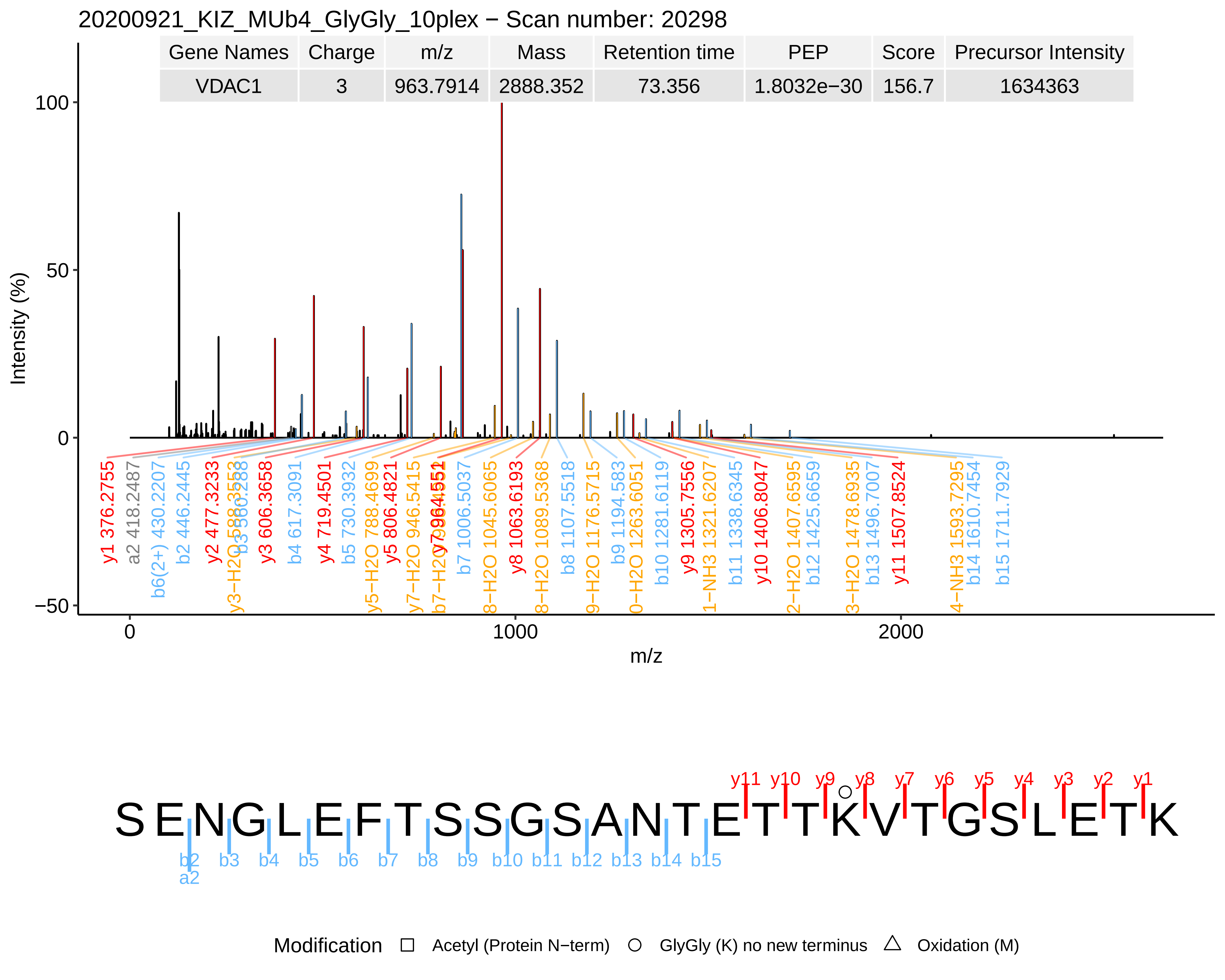
20200921_KIZ_MUb4_GlyGly_10plex − Scan number: 20298
Gene Names | Charge | m/z | Mass | Retention time | PEP | Score | Precursor Intensity
VDAC1 | 3 | 963.7914 | 2888.352 | 73.356 | 1.8032e−30 | 156.7 | 1634363
Intensity (%)
100
50
0
−50
m/z
0
1000
2000
y1 376.2755
a2 418.2487
b6(2+) 430.2207
b2 446.2445
y2 477.3233
y3 606.3658
b4 617.3091
y4 719.4501
b5 730.3932
y5−H2O 788.4699
y5 806.4821
y7−H2O 946.5415
b7 1006.5037
8−H2O 1045.6065
y8 1063.6193
8−H2O 1089.5368
b8 1107.5518
9−H2O 1176.5715
b9 1194.583
0−H2O 1263.6051
b10 1281.6119
y9 1305.7556
1−NH3 1321.6207
b11 1338.6345
y10 1406.8047
2−H2O 1407.6595
b12 1425.6659
3−H2O 1478.6935
b13 1496.7007
y11 1507.8524
4−NH3 1593.7295
b14 1610.7454
b15 1711.7929
y11 y10 y9 y8 y7 y6 y5 y4 y3 y2 y1
S E N G L E F T S S G S A N T E T T K V T G S L E T K
b2 b3 b4 b5 b6 b7 b8 b9 b10 b11 b12 b13 b14 b15
a2
Modification
Acetyl (Protein N−term)
GlyGly (K) no new terminus
Oxidation (M)

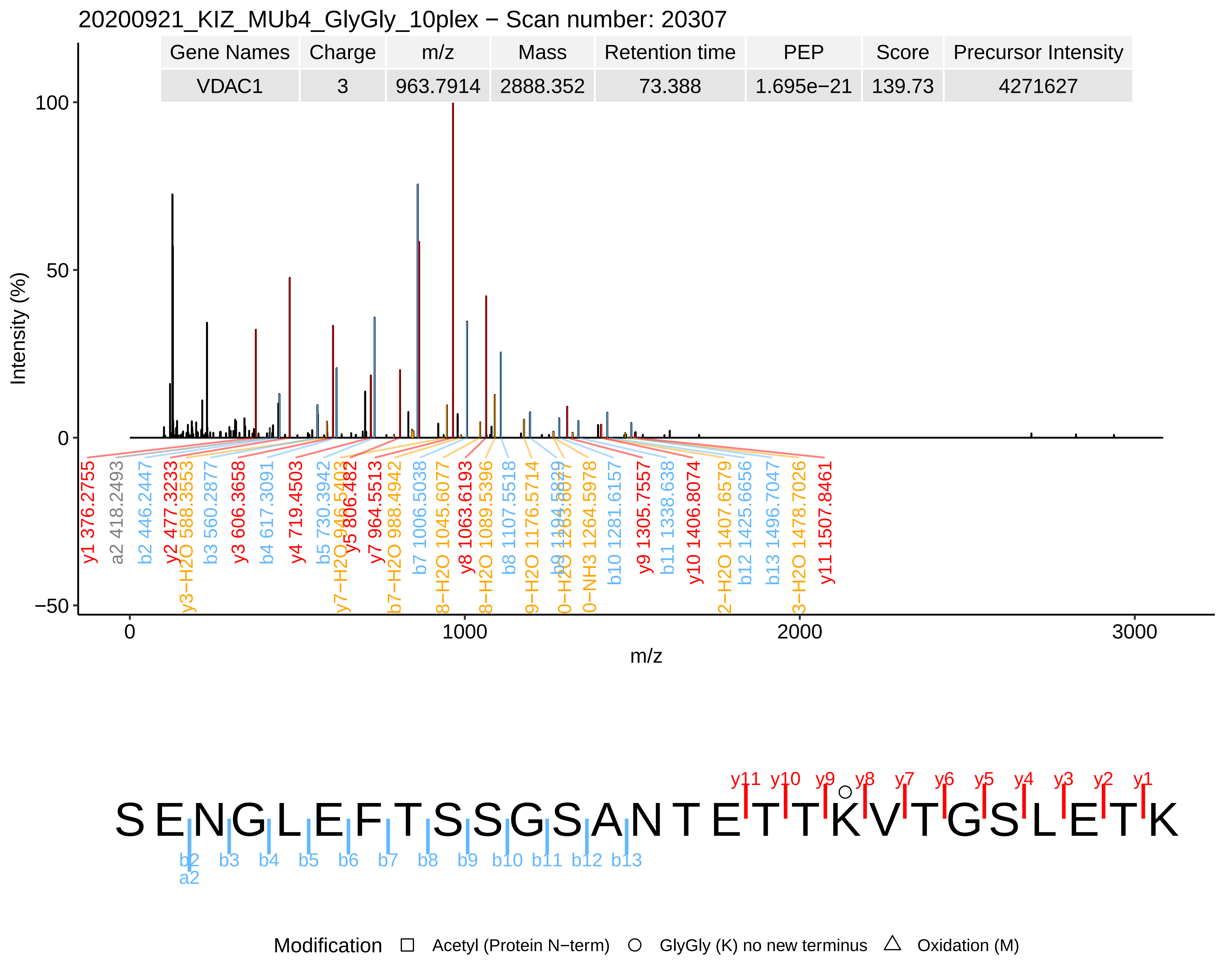

20200921_KIZ_MUb4_GlyGly_10plex − Scan number: 20307
Gene Names | Charge | m/z | Mass | Retention time | PEP | Score | Precursor Intensity
VDAC1 | 3 | 963.7914 | 2888.352 | 73.388 | 1.695e−21 | 139.73 | 4271627
Intensity (%)
m/z
y1 376.2755
a2 418.2493
b2 446.2447
y2 477.3233
y3−H2O 588.3553
b3 560.2877
y3 606.3658
b4 617.3091
y4 719.4503
b5 730.3942
y5 806.482
y7 964.5513
b7−H2O 988.4942
b7 1006.5038
y8 1063.6193
b8 1107.5518
b10 1281.6157
y9 1305.7557
b11 1338.638
y10 1406.8074
b12 1425.6656
b13 1496.7047
y11 1507.8461
S E N G L E F T S S G S A N T E T T K V T G S L E T K
Modification
Acetyl (Protein N−term)
GlyGly (K) no new terminus
Oxidation (M)

20200921_KIZ_MUb4_GlyGly_10plex − Scan number: 20368

Gene Names	Charge	m/z	Mass	Retention time	PEP	Score	Precursor Intensity
VDAC1	4	723.0954	2888.352	73.623	2.3401e−14	123.44	14330555

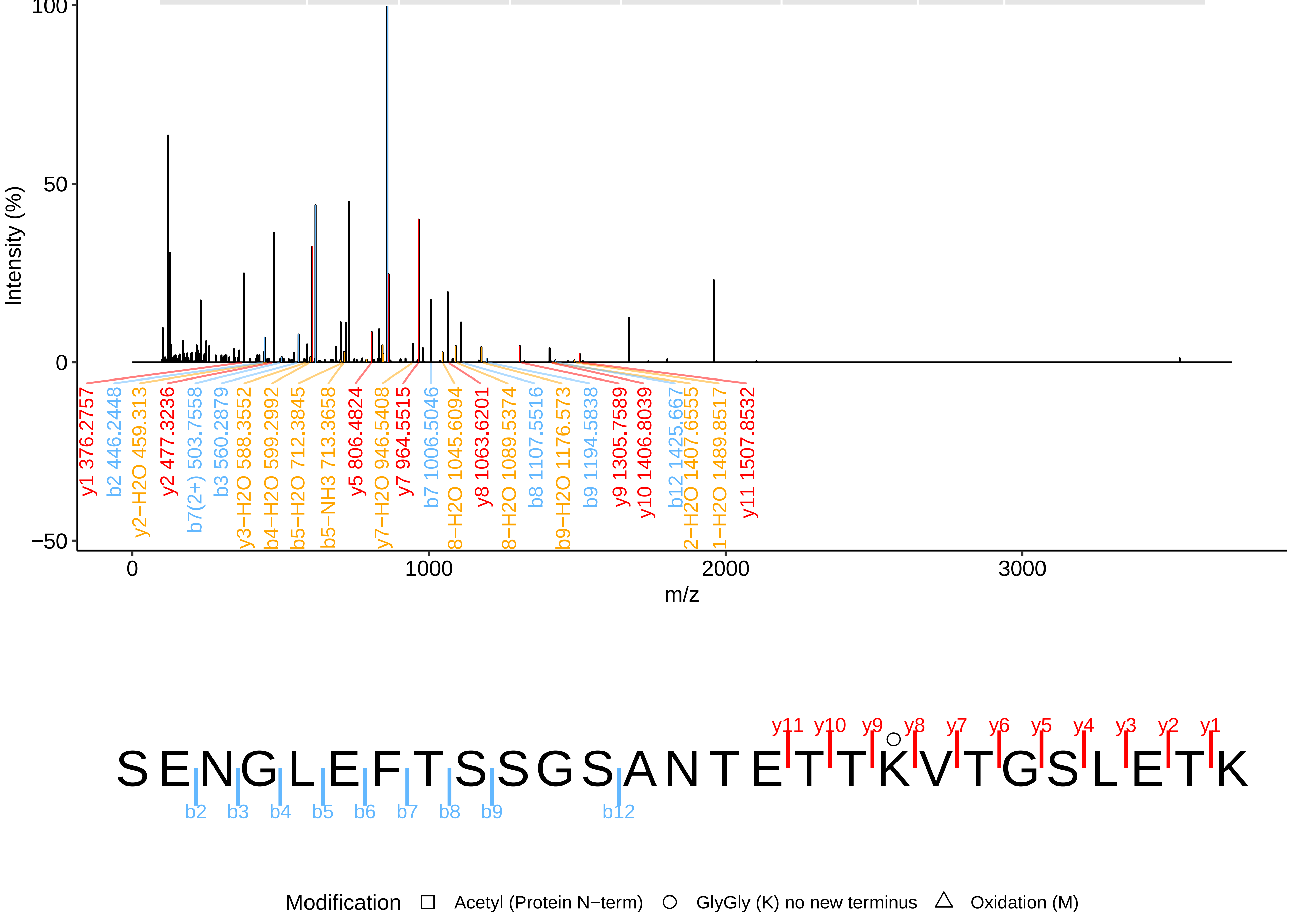

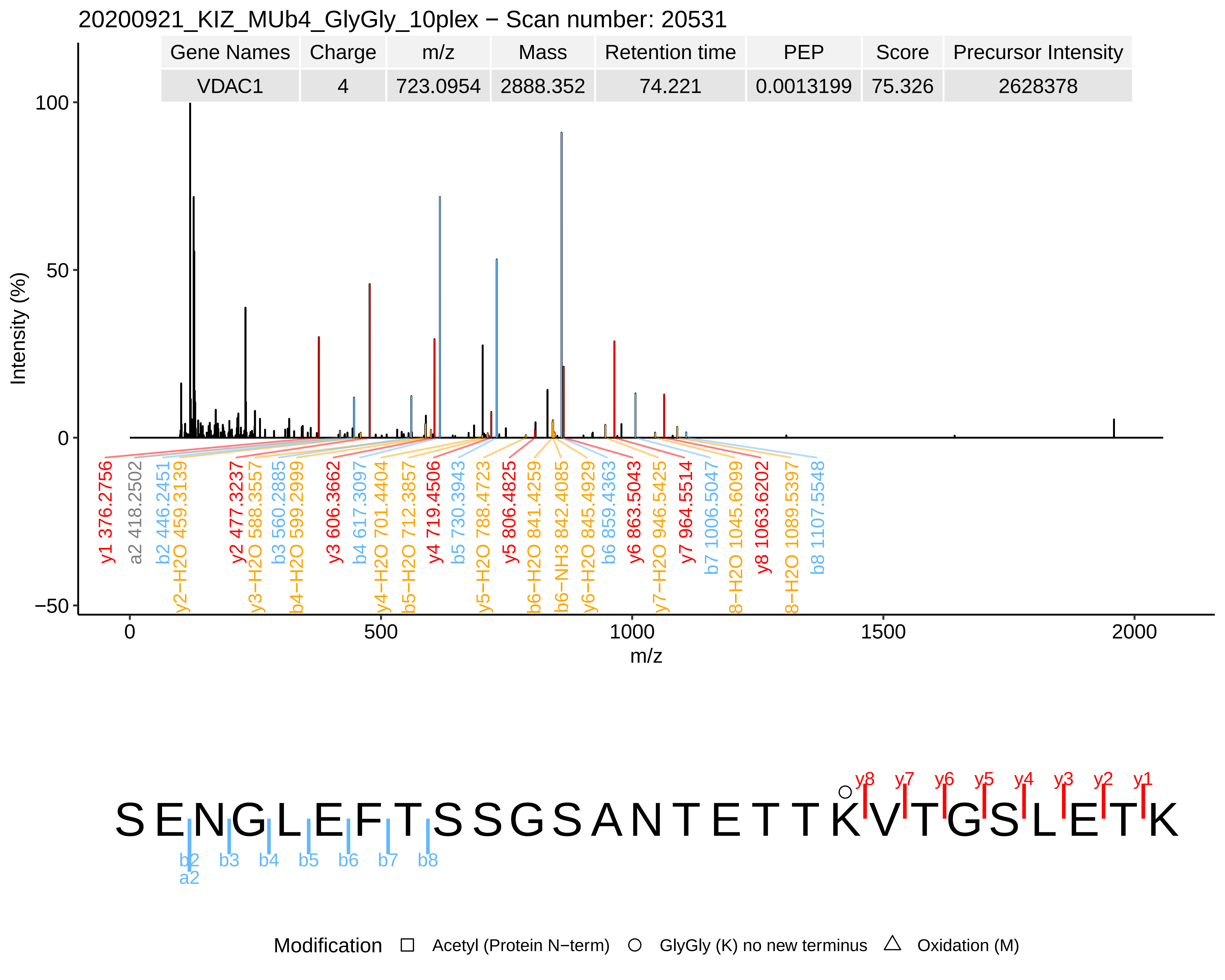

20200921_KIZ_MUb4_GlyGly_10plex − Scan number: 20531
Gene Names | Charge | m/z | Mass | Retention time | PEP | Score | Precursor Intensity
VDAC1 | 4 | 723.0954 | 2888.352 | 74.221 | 0.0013199 | 75.326 | 2628378
Intensity (%)
100
50
0
−50
0
500
1000
1500
2000
m/z
y1 376.2756
a2 418.2502
b2 446.2451
y2−H2O 459.3139
y2 477.3237
y3−H2O 588.3557
b3 560.2885
b4−H2O 599.2999
y3 606.3662
b4 617.3097
y4−H2O 701.4404
b5−H2O 712.3857
y4 719.4506
b5 730.3943
y5−H2O 788.4723
y5 806.4825
b6−H2O 841.4259
b6−NH3 842.4085
y6−H2O 845.4929
b6 859.4363
y6 863.5043
y7−H2O 946.5425
y7 964.5514
b7 1006.5047
8−H2O 1045.6099
y8 1063.6202
8−H2O 1089.5397
b8 1107.5548
y8 y7 y6 y5 y4 y3 y2 y1
S E N G L E F T S S G S A N T E T T K V T G S L E T K
b2 b3 b4 b5 b6 b7 b8
a2
Modification
Acetyl (Protein N−term)
GlyGly (K) no new terminus
Oxidation (M)

20200921_KIZ_MUb4_GlyGly_10plex − Scan number: 20532

Gene Names	Charge	m/z	Mass	Retention time	PEP	Score	Precursor Intensity
VDAC1	4	723.0954	2888.352	74.225	6.6761e−15	130.15	9742324

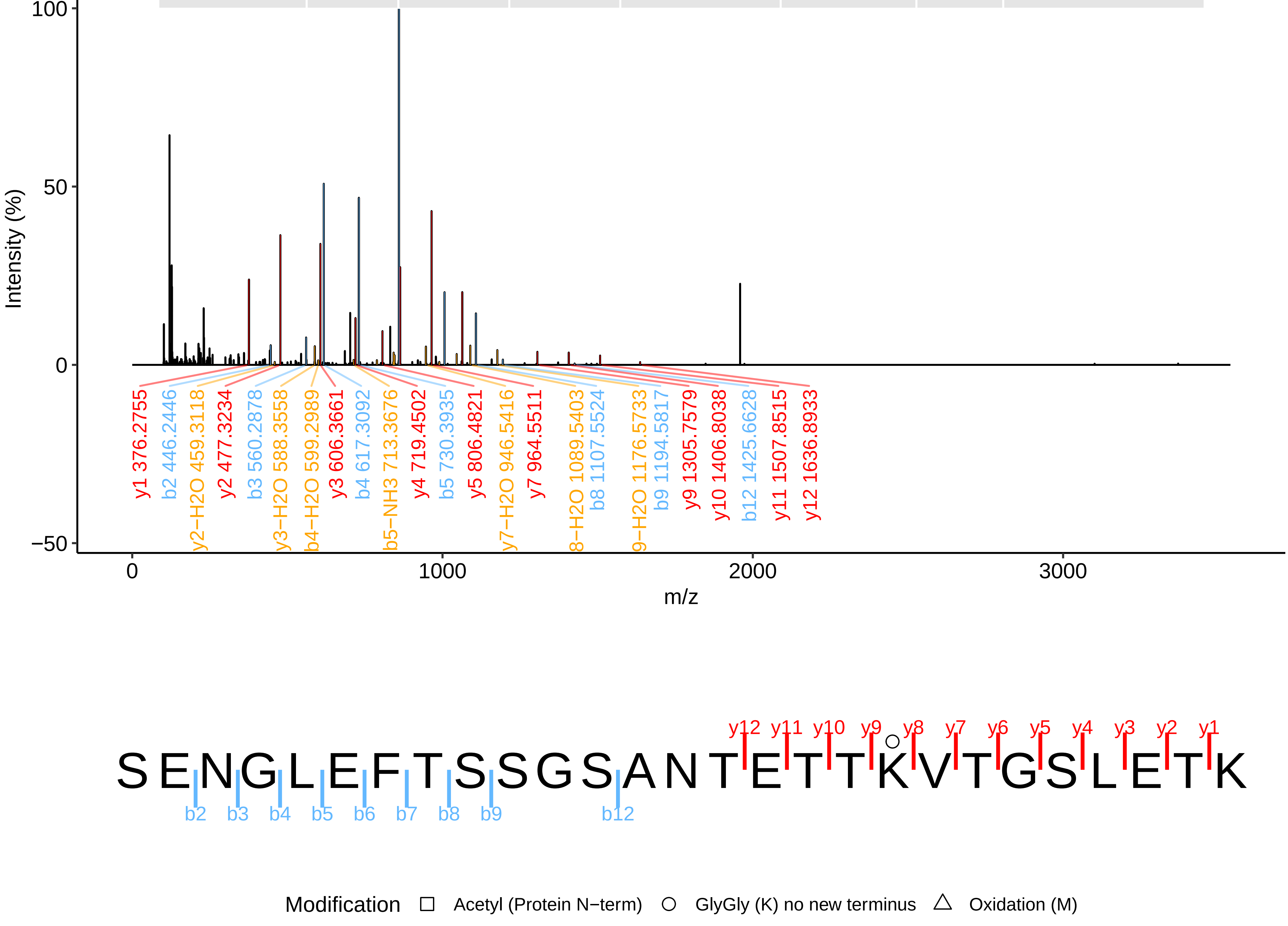

20200921_KIZ_MUb4_GlyGly_10plex − Scan number: 20584

Gene Names	Charge	m/z	Mass	Retention time	PEP	Score	Precursor Intensity
VDAC1	3	963.7914	2888.352	74.429	1.4125e−21	141.48	3752811

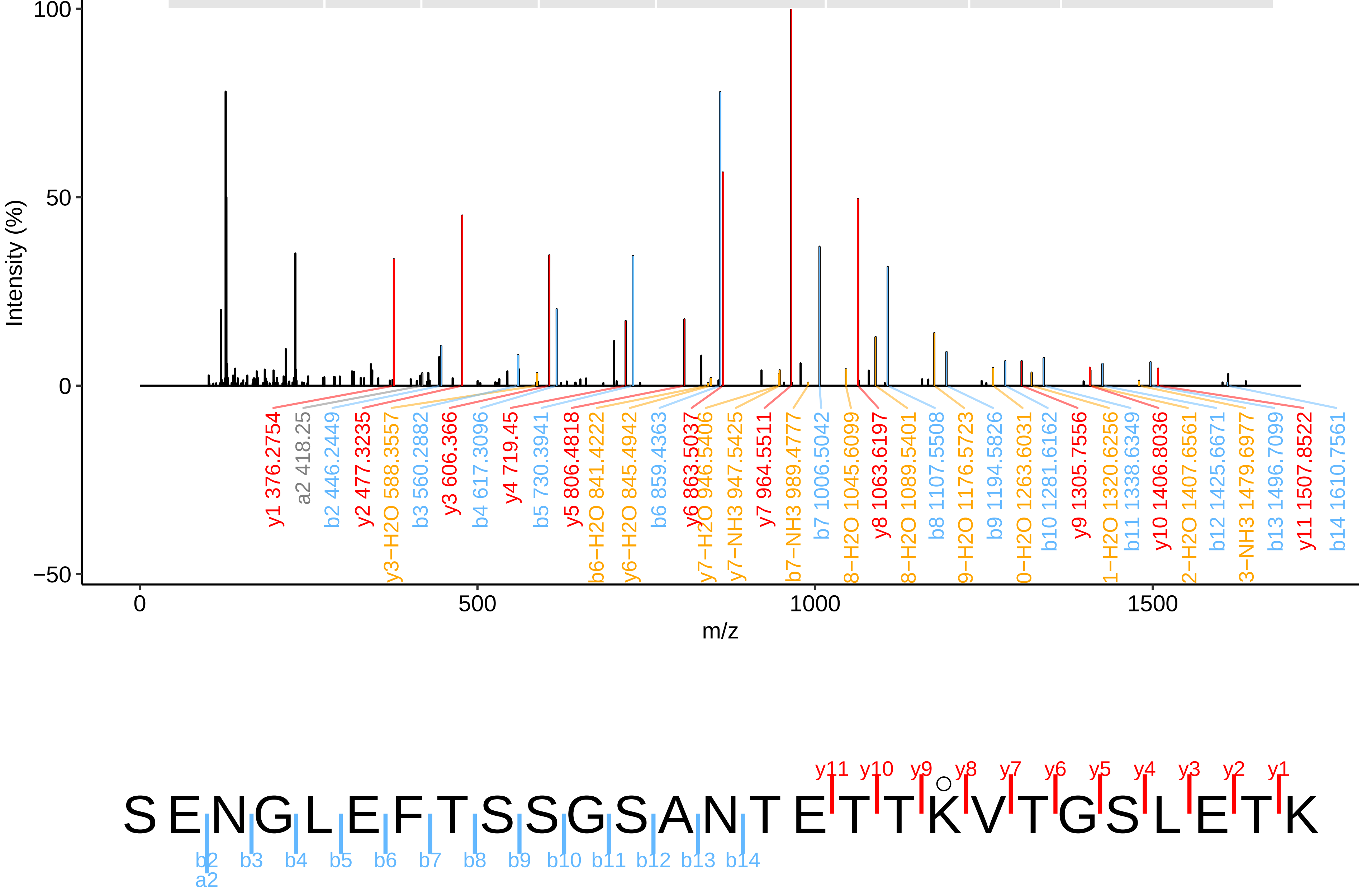

Modification □ Acetyl (Protein N−term) ○ GlyGly (K) no new terminus △ Oxidation (M)

20200921_KIZ_MUb4_GlyGly_10plex − Scan number: 21101

Gene Names	Charge	m/z	Mass	Retention time	PEP	Score	Precursor Intensity
VDAC1	3	963.7914	2888.352	76.359	2.9379e−31	165.87	2937373

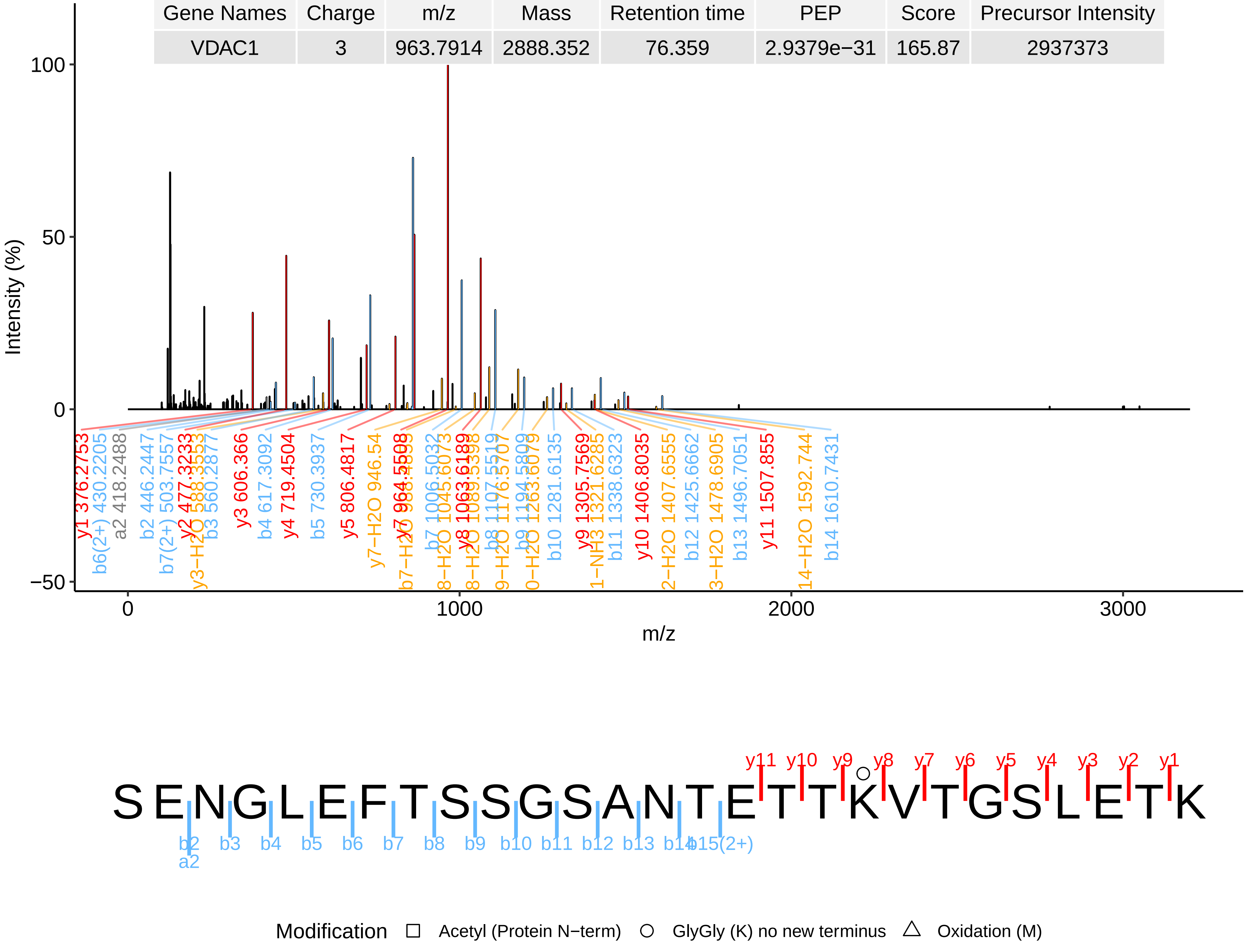

20200921_KIZ_MUb4_GlyGly_10plex − Scan number: 21336

Gene Names	Charge	m/z	Mass	Retention time	PEP	Score	Precursor Intensity
VDAC1	3	963.7914	2888.352	77.249	2.1891e−06	97.057	3802664

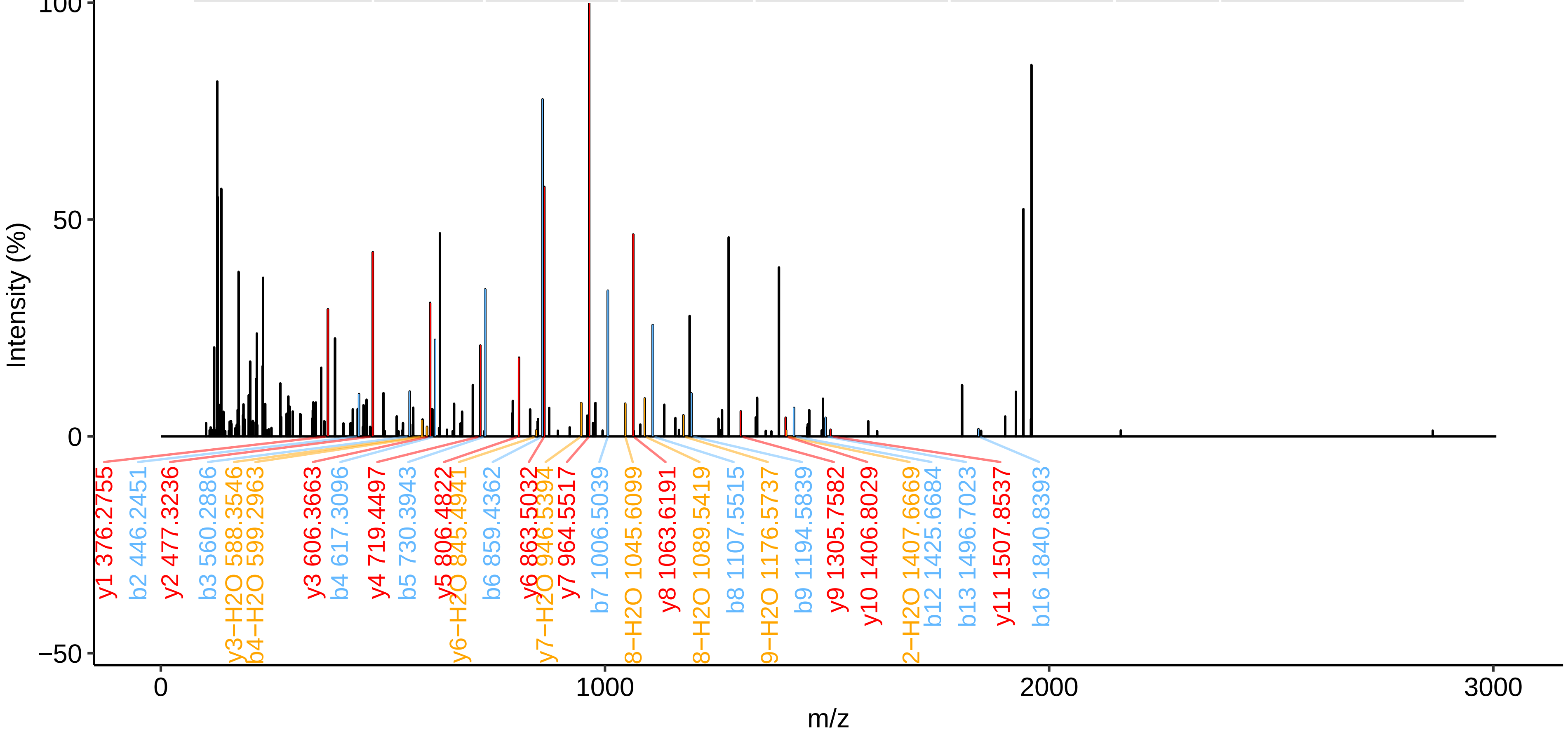

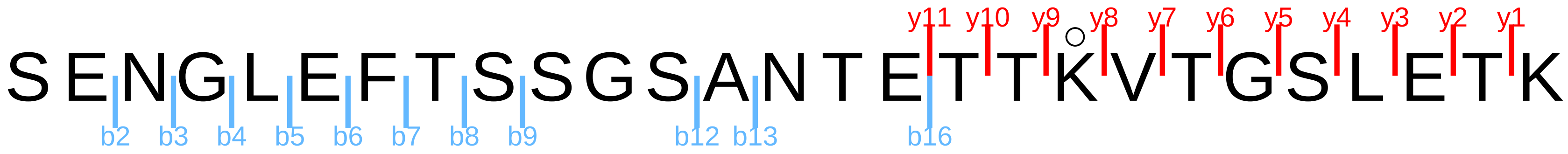

Modification □ Acetyl (Protein N−term) ○ GlyGly (K) no new terminus △ Oxidation (M)

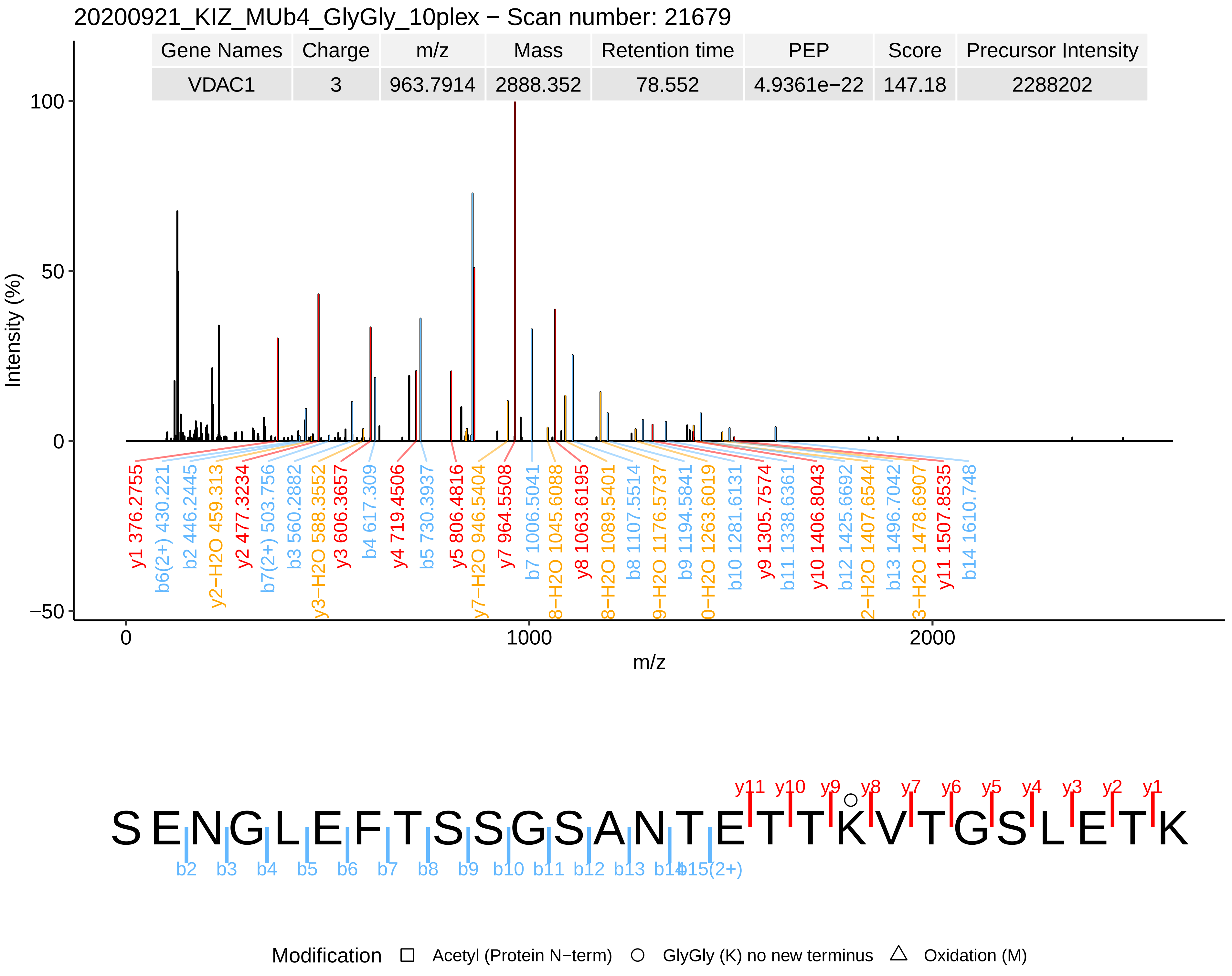
20200921_KIZ_MUb4_GlyGly_10plex − Scan number: 21679
Gene Names | Charge | m/z | Mass | Retention time | PEP | Score | Precursor Intensity
VDAC1 | 3 | 963.7914 | 2888.352 | 78.552 | 4.9361e−22 | 147.18 | 2288202
Intensity (%)
100
50
0
−50
0
1000
2000
m/z
y1 376.2755
b6(2+) 430.221
b2 446.2445
y2−H2O 459.313
y2 477.3234
b7(2+) 503.756
b3 560.2882
y3−H2O 588.3552
y3 606.3657
b4 617.309
y4 719.4506
b5 730.3937
y5 806.4816
y7−H2O 946.5404
y7 964.5508
b7 1006.5041
8−H2O 1045.6088
y8 1063.6195
8−H2O 1089.5401
b8 1107.5514
9−H2O 1176.5737
b9 1194.5841
0−H2O 1263.6019
b10 1281.6131
y9 1305.7574
b11 1338.6361
y10 1406.8043
b12 1425.6692
2−H2O 1407.6544
b13 1496.7042
3−H2O 1478.6907
y11 1507.8535
b14 1610.748
y11 y10 y9 y8 y7 y6 y5 y4 y3 y2 y1
S E N G L E F T S S G S A N T E T T K V T G S L E T K
b2 b3 b4 b5 b6 b7 b8 b9 b10 b11 b12 b13 b14 b15(2+)
Modification
Acetyl (Protein N−term)
GlyGly (K) no new terminus
Oxidation (M)

20200921_KIZ_MUb4_GlyGly_10plex − Scan number: 21683

Gene Names	Charge	m/z	Mass	Retention time	PEP	Score	Precursor Intensity
VDAC1	3	963.7914	2888.352	78.569	1.4798e−30	158.67	2288202

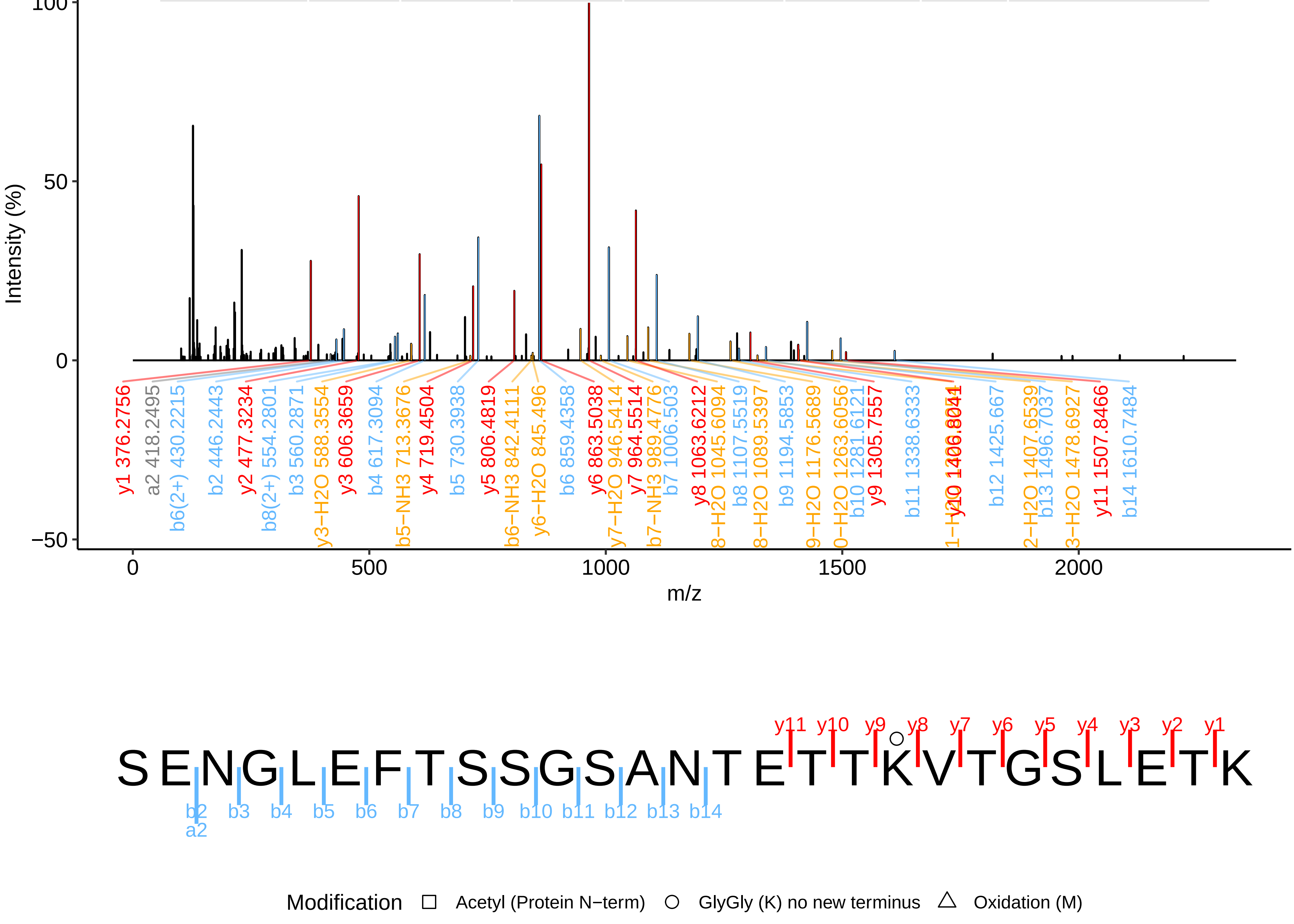

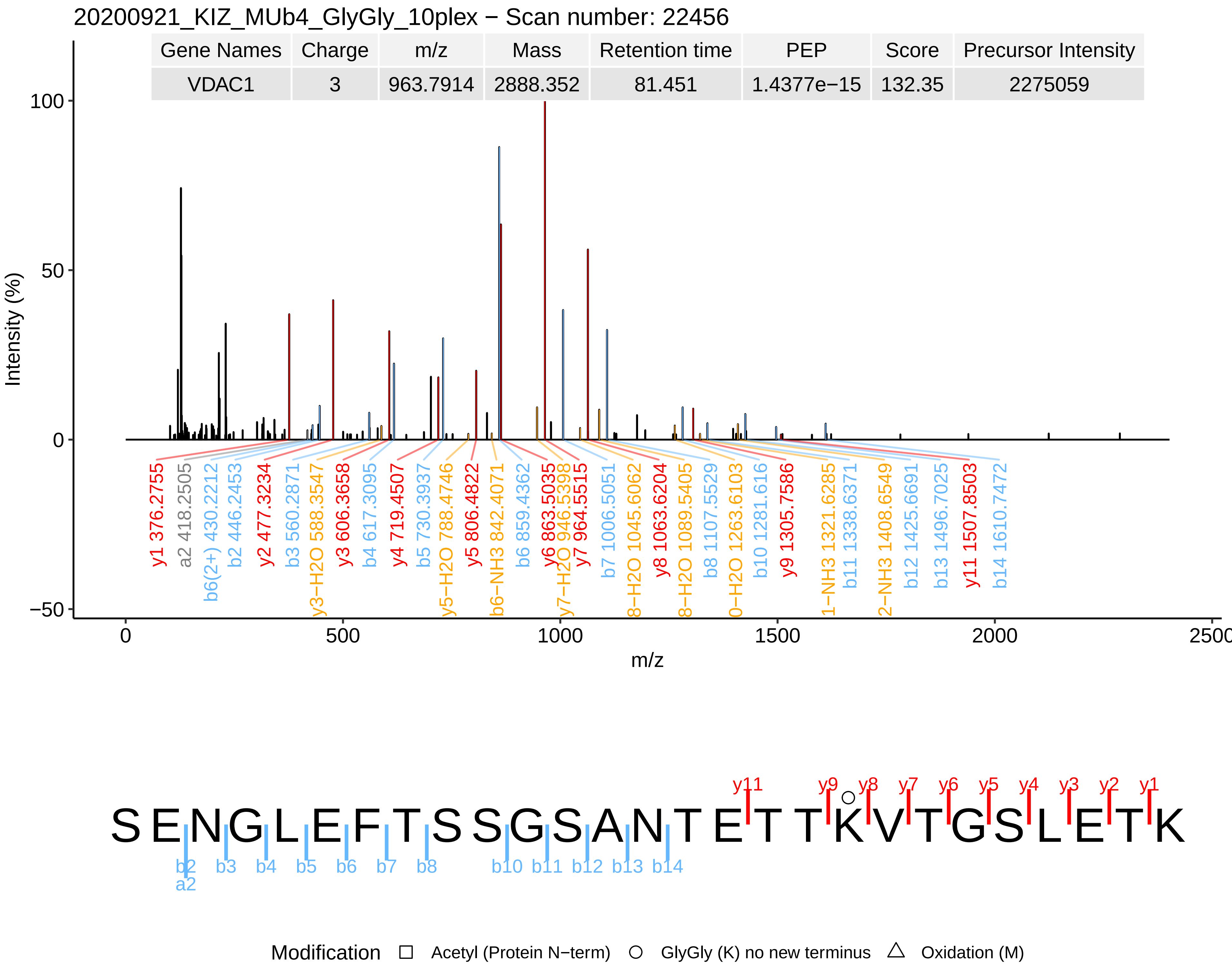

20200921_KIZ_MUb4_GlyGly_10plex − Scan number: 22456
Gene Names | Charge | m/z | Mass | Retention time | PEP | Score | Precursor Intensity
VDAC1 | 3 | 963.7914 | 2888.352 | 81.451 | 1.4377e−15 | 132.35 | 2275059
Intensity (%)
100
50
0
−50
y1 376.2755
a2 418.2505
b6(2+) 430.2212
b2 446.2453
y2 477.3234
b3 560.2871
y3−H2O 588.3547
y3 606.3658
b4 617.3095
y4 719.4507
b5 730.3937
y5−H2O 788.4746
y5 806.4822
b6−NH3 842.4071
b6 859.4362
y6 863.5035
y7−H2O 946.5398
y7 964.5515
b7 1006.5051
8−H2O 1045.6062
y8 1063.6204
8−H2O 1089.5405
b8 1107.5529
0−H2O 1263.6103
b10 1281.616
y9 1305.7586
1−NH3 1321.6285
b11 1338.6371
2−NH3 1408.6549
b12 1425.6691
b13 1496.7025
y11 1507.8503
b14 1610.7472
0
500
1000
1500
2000
2500
m/z
y11 y9 y8 y7 y6 y5 y4 y3 y2 y1
S E N G L E F T S S G S A N T E T T K V T G S L E T K
b2 b3 b4 b5 b6 b7 b8 b10 b11 b12 b13 b14
a2
Modification □ Acetyl (Protein N−term) ○ GlyGly (K) no new terminus △ Oxidation (M)

20200921_KIZ_MUb4_GlyGly_10plex − Scan number: 22466

Gene Names	Charge	m/z	Mass	Retention time	PEP	Score	Precursor Intensity
VDAC1	3	963.7914	2888.352	81.49	0.0025809	72.035	2117099

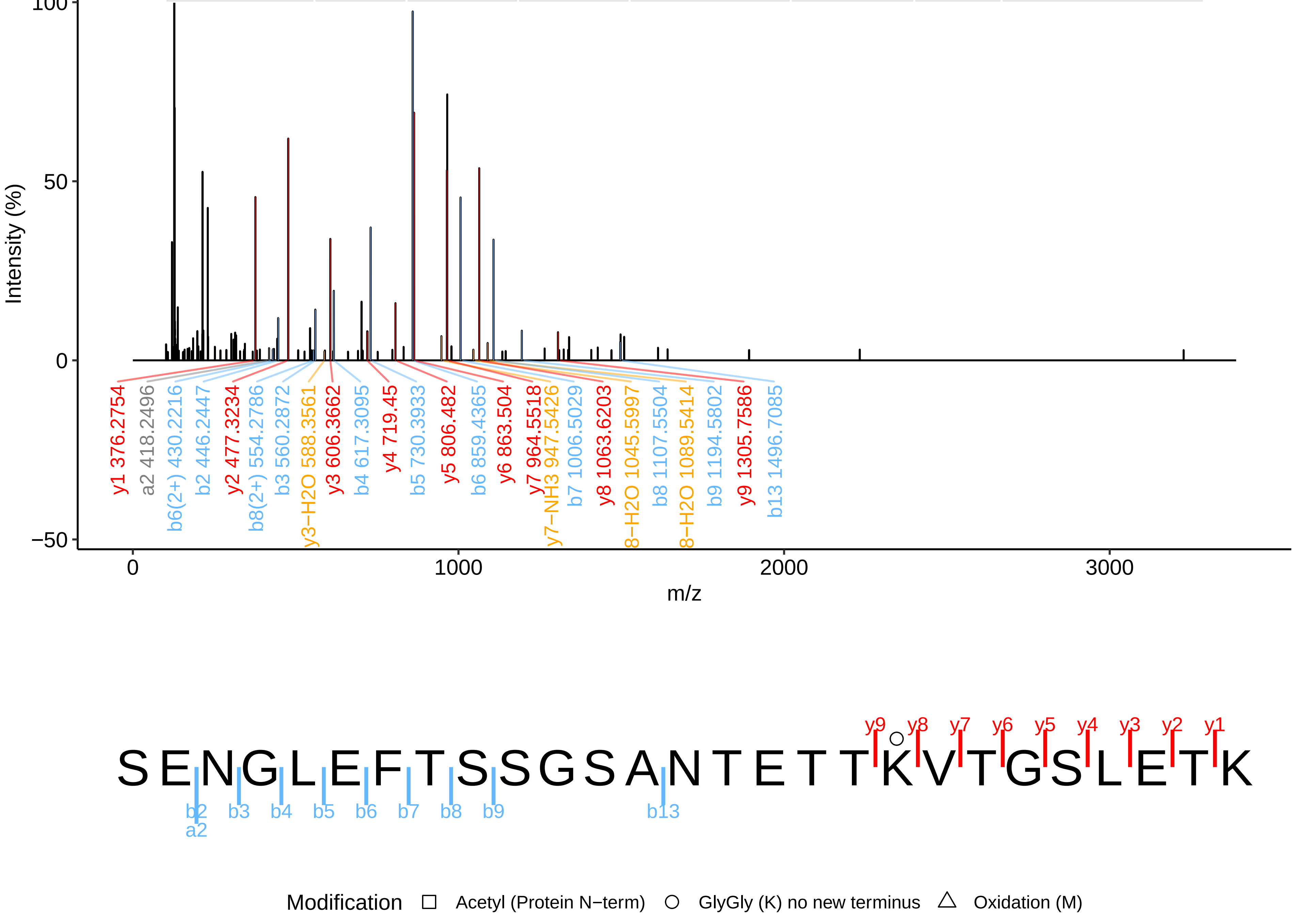

20200921_KIZ_MUb4_GlyGly_10plex − Scan number: 23582

Gene Names	Charge	m/z	Mass	Retention time	PEP	Score	Precursor Intensity
VDAC1	3	963.7914	2888.352	85.608	4.9903e−14	116.35	3366002

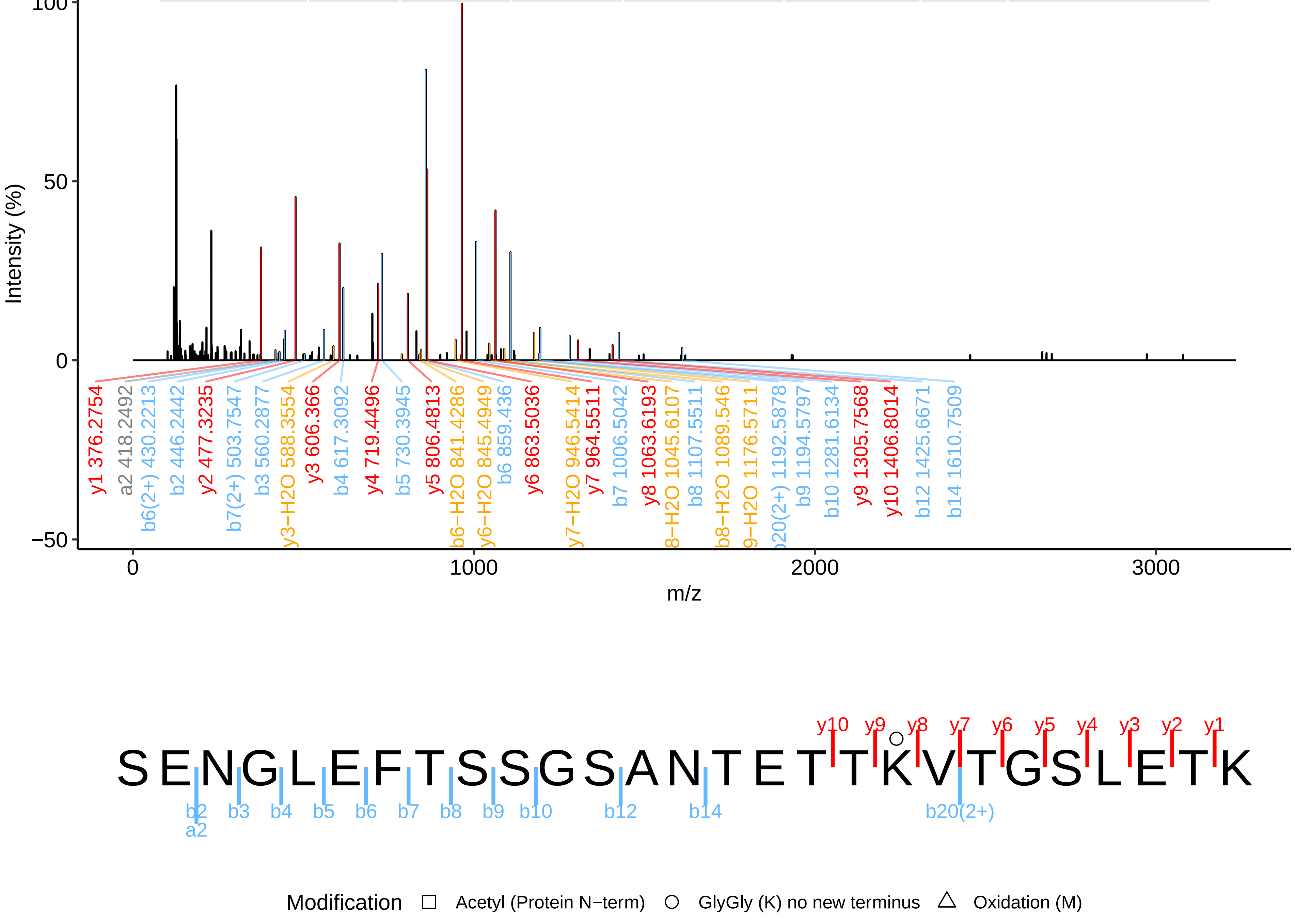

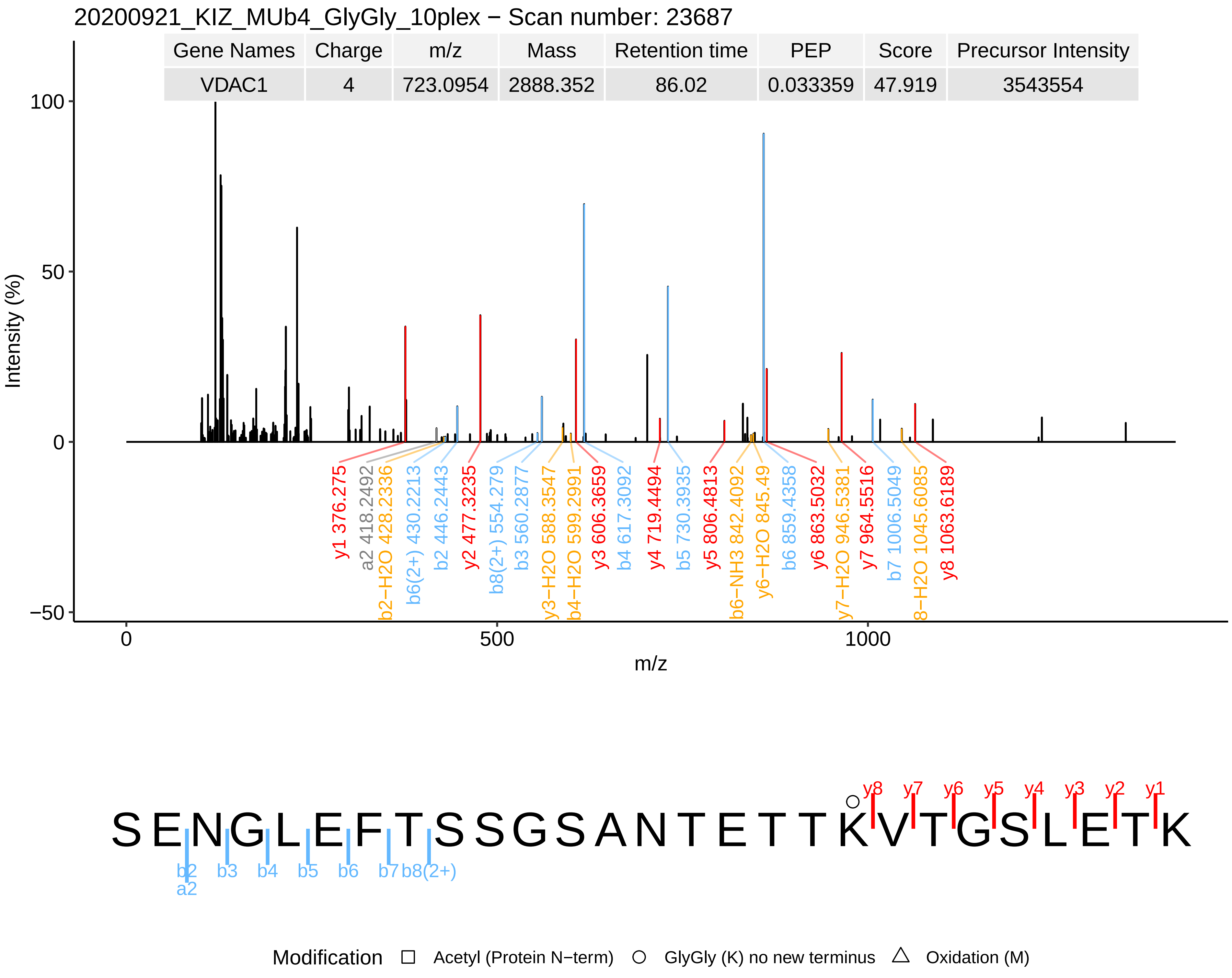
20200921_KIZ_MUb4_GlyGly_10plex − Scan number: 23687
Gene Names | Charge | m/z | Mass | Retention time | PEP | Score | Precursor Intensity
VDAC1 | 4 | 723.0954 | 2888.352 | 86.02 | 0.033359 | 47.919 | 3543554
Intensity (%)
100
50
0
−50
0
500
1000
m/z
y1 376.275
a2 418.2492
b2−H2O 428.2336
b6(2+) 430.2213
b2 446.2443
y2 477.3235
b8(2+) 554.279
b3 560.2877
y3−H2O 588.3547
b4−H2O 599.2991
y3 606.3659
b4 617.3092
y4 719.4494
b5 730.3935
y5 806.4813
b6−NH3 842.4092
y6−H2O 845.49
b6 859.4358
y6 863.5032
y7−H2O 946.5381
y7 964.5516
b7 1006.5049
8−H2O 1045.6085
y8 1063.6189
S E N G L E F T S S G S A N T E T T K V T G S L E T K
y8 y7 y6 y5 y4 y3 y2 y1
b2 b3 b4 b5 b6 b7 b8(2+)
a2
Modification
Acetyl (Protein N−term)
GlyGly (K) no new terminus
Oxidation (M)

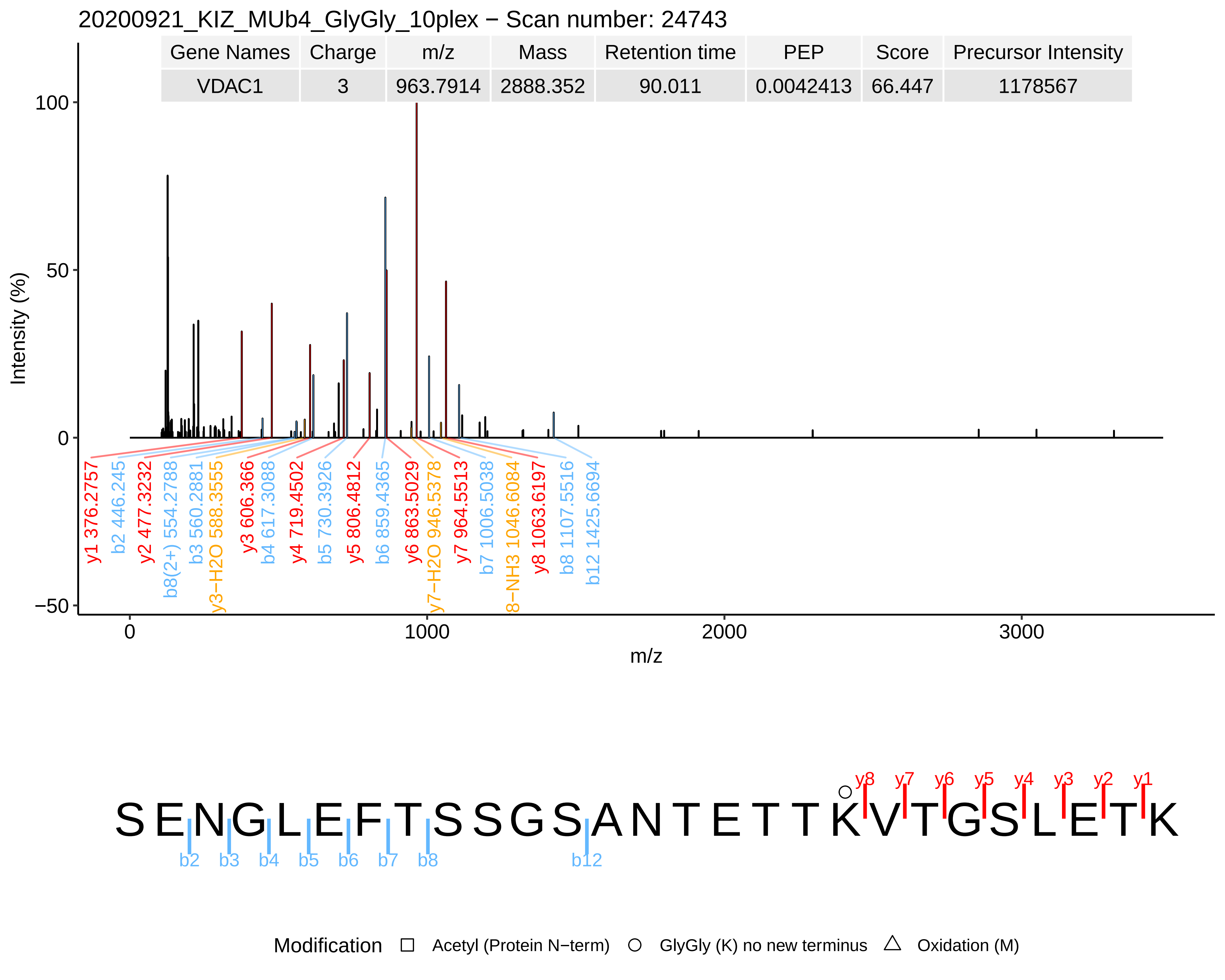
20200921_KIZ_MUb4_GlyGly_10plex − Scan number: 24743
Gene Names
Charge
m/z
Mass
Retention time
PEP
Score
Precursor Intensity
VDAC1
3
963.7914
2888.352
90.011
0.0042413
66.447
1178567
Intensity (%)
100
50
0
−50
0
1000
2000
3000
m/z
y1 376.2757
b2 446.245
y2 477.3232
b8(2+) 554.2788
b3 560.2881
y3−H2O 588.3555
y3 606.366
b4 617.3088
y4 719.4502
b5 730.3926
y5 806.4812
b6 859.4365
y6 863.5029
y7−H2O 946.5378
y7 964.5513
b7 1006.5038
8−NH3 1046.6084
y8 1063.6197
b8 1107.5516
b12 1425.6694
S E N G L E F T S S G S A N T E T T K V T G S L E T K
y8 y7 y6 y5 y4 y3 y2 y1
b2 b3 b4 b5 b6 b7 b8 b12
Modification
Acetyl (Protein N−term)
GlyGly (K) no new terminus
Oxidation (M)

20200921_KIZ_MUb4_GlyGly_10plex − Scan number: 27247

Gene Names	Charge	m/z	Mass	Retention time	PEP	Score	Precursor Intensity
VDAC1	3	963.7914	2888.352	99.412	4.0069e−09	109.26	2055425

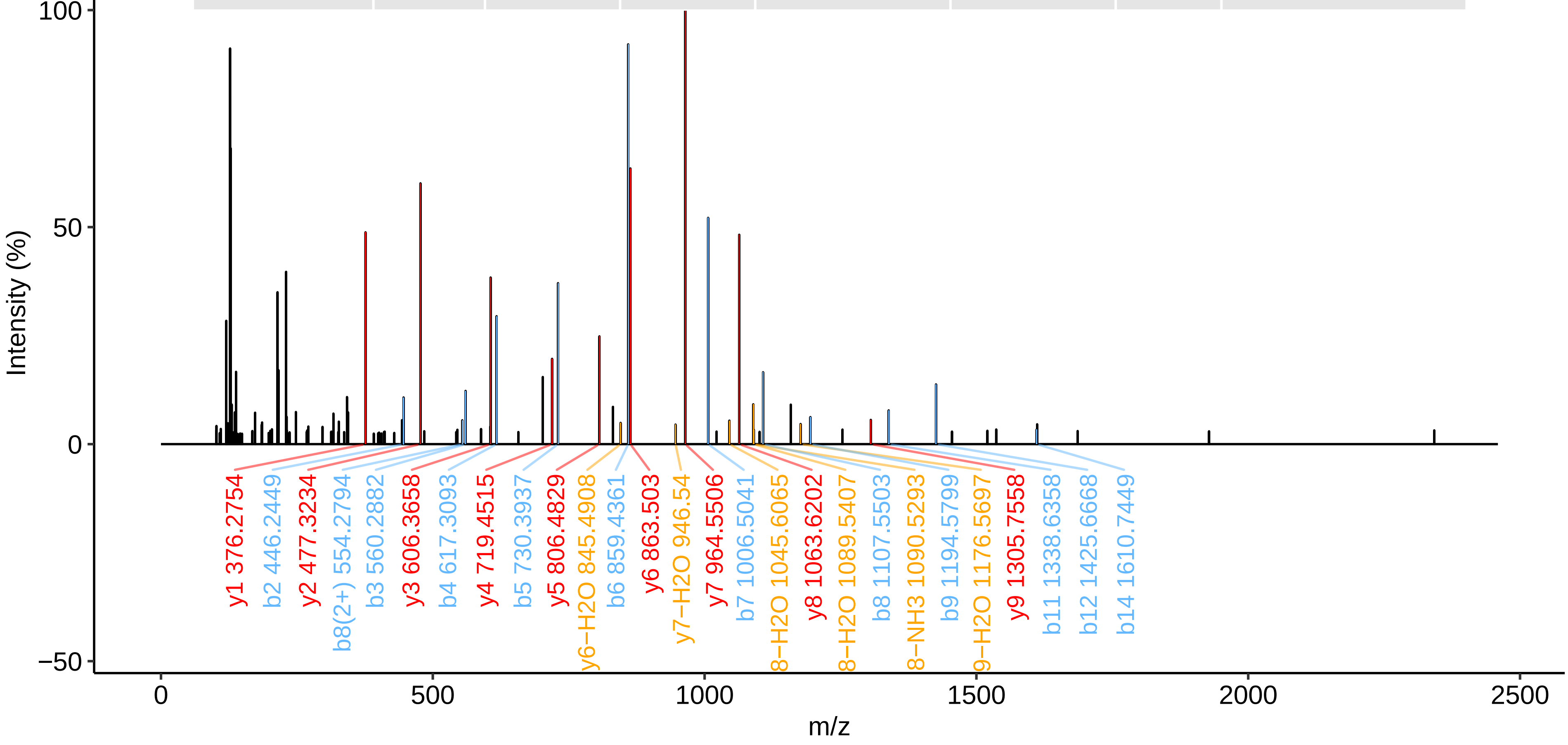

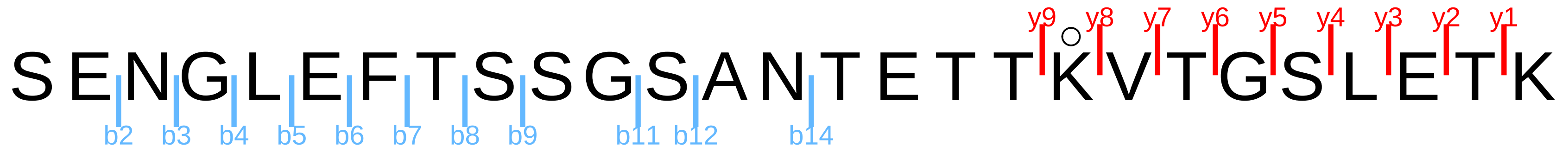

Modification □ Acetyl (Protein N−term) ○ GlyGly (K) no new terminus △ Oxidation (M)

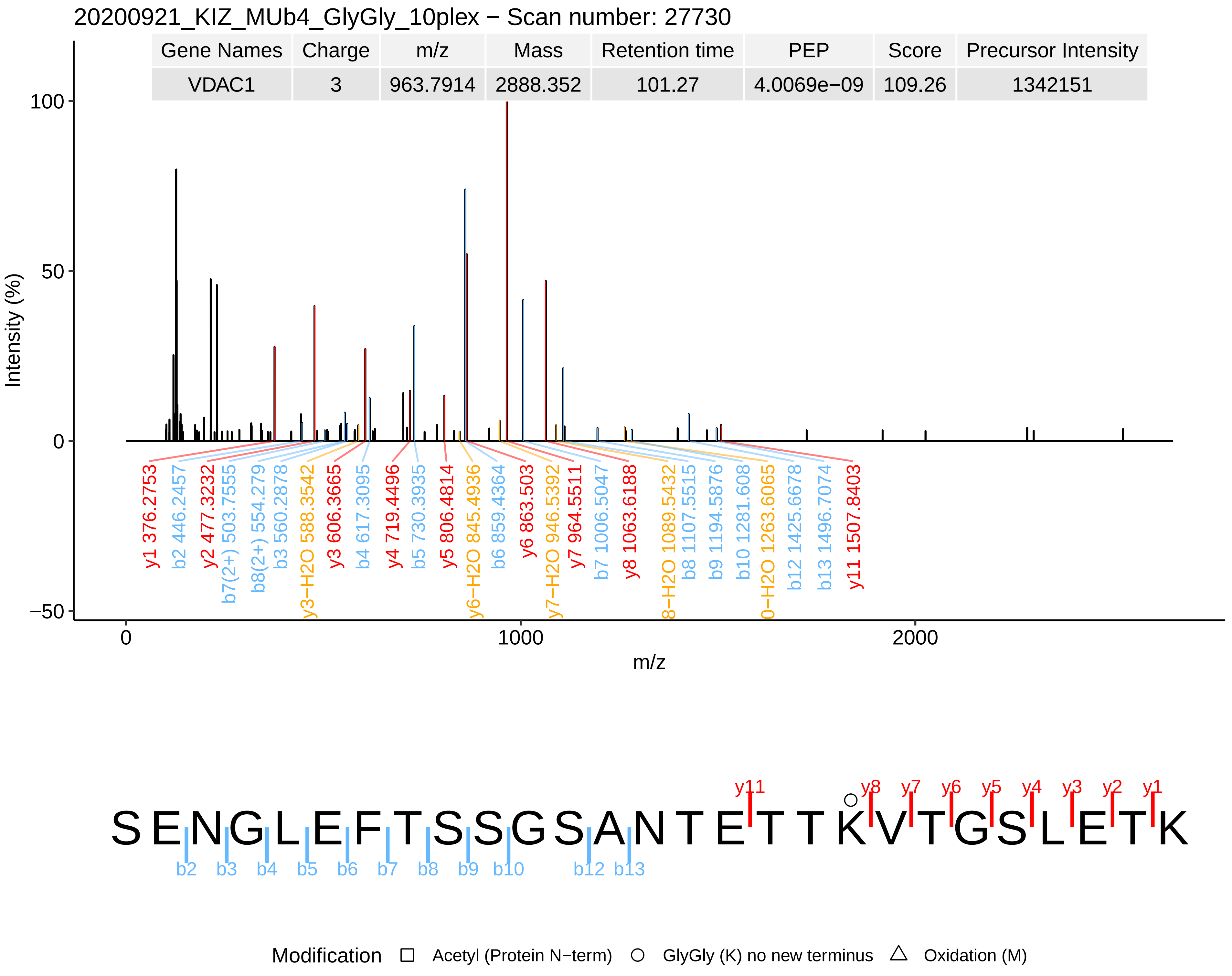

20200921_KIZ_MUb4_GlyGly_10plex − Scan number: 27730
Gene Names | Charge | m/z | Mass | Retention time | PEP | Score | Precursor Intensity
VDAC1 | 3 | 963.7914 | 2888.352 | 101.27 | 4.0069e−09 | 109.26 | 1342151
Intensity (%)
100
50
0
−50
0
1000
2000
m/z
y1 376.2753
b2 446.2457
y2 477.3232
b7(2+) 503.7555
b8(2+) 554.279
b3 560.2878
y3−H2O 588.3542
y3 606.3665
b4 617.3095
y4 719.4496
b5 730.3935
y5 806.4814
y6−H2O 845.4936
b6 859.4364
y6 863.503
y7−H2O 946.5392
y7 964.5511
b7 1006.5047
y8 1063.6188
8−H2O 1089.5432
b8 1107.5515
b9 1194.5876
b10 1281.608
0−H2O 1263.6065
b12 1425.6678
b13 1496.7074
y11 1507.8403
S E N G L E F T S S G S A N T E T T K V T G S L E T K
y11 y8 y7 y6 y5 y4 y3 y2 y1
b2 b3 b4 b5 b6 b7 b8 b9 b10 b12 b13
Modification
Acetyl (Protein N−term)
GlyGly (K) no new terminus
Oxidation (M)

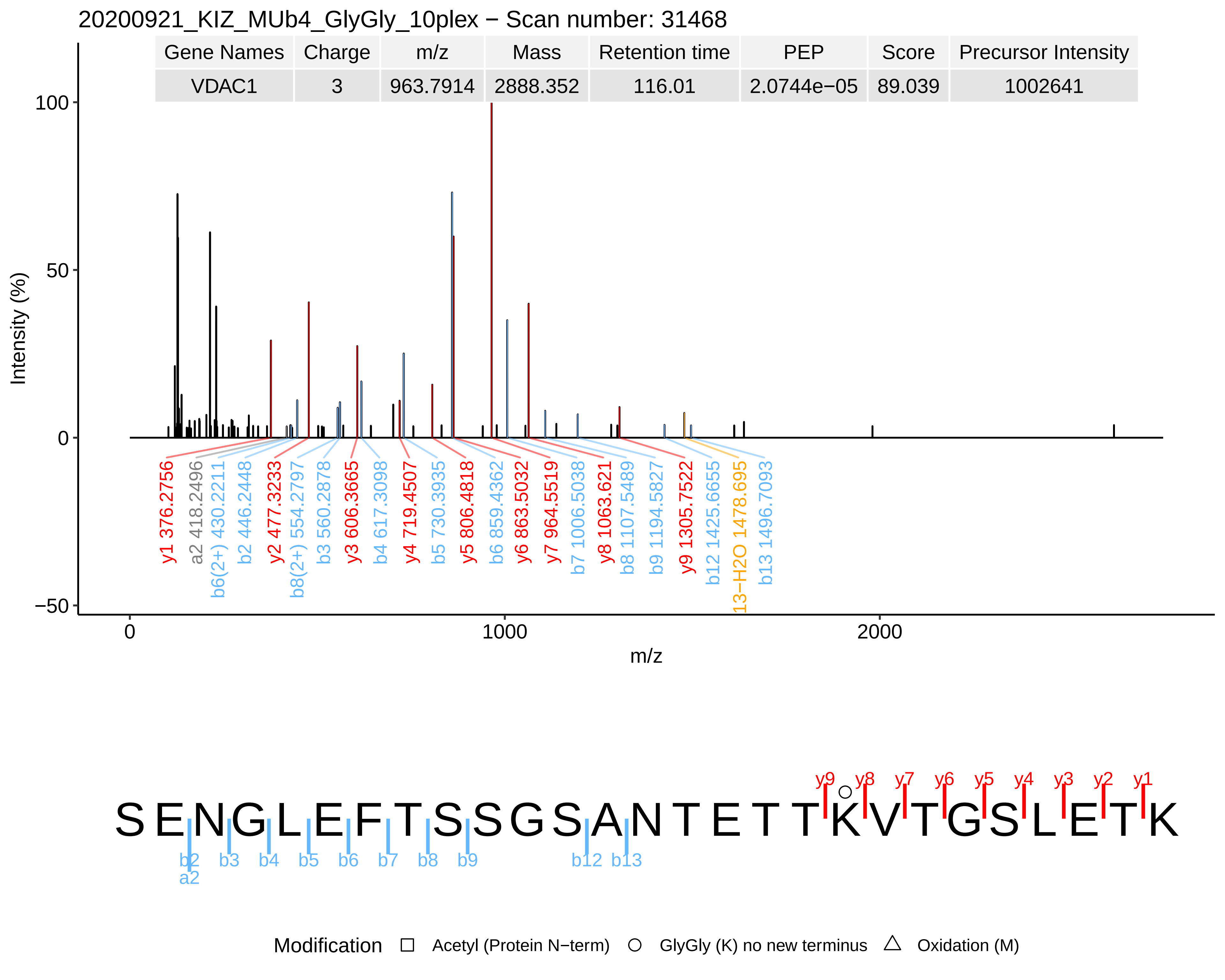

20200921_KIZ_MUb4_GlyGly_10plex − Scan number: 31468
Gene Names | Charge | m/z | Mass | Retention time | PEP | Score | Precursor Intensity
VDAC1 | 3 | 963.7914 | 2888.352 | 116.01 | 2.0744e−05 | 89.039 | 1002641
Intensity (%)
100
50
0
−50
y1 376.2756
a2 418.2496
b6(2+) 430.2211
b2 446.2448
y2 477.3233
b8(2+) 554.2797
b3 560.2878
y3 606.3665
b4 617.3098
y4 719.4507
b5 730.3935
y5 806.4818
b6 859.4362
y6 863.5032
y7 964.5519
b7 1006.5038
y8 1063.621
b8 1107.5489
b9 1194.5827
y9 1305.7522
b12 1425.6655
13−H2O 1478.695
b13 1496.7093
0
1000
2000
m/z
y9 y8 y7 y6 y5 y4 y3 y2 y1
S E N G L E F T S S G S A N T E T T K V T G S L E T K
b2 b3 b4 b5 b6 b7 b8 b9 b12 b13
a2
Modification
Acetyl (Protein N−term)
GlyGly (K) no new terminus
Oxidation (M)

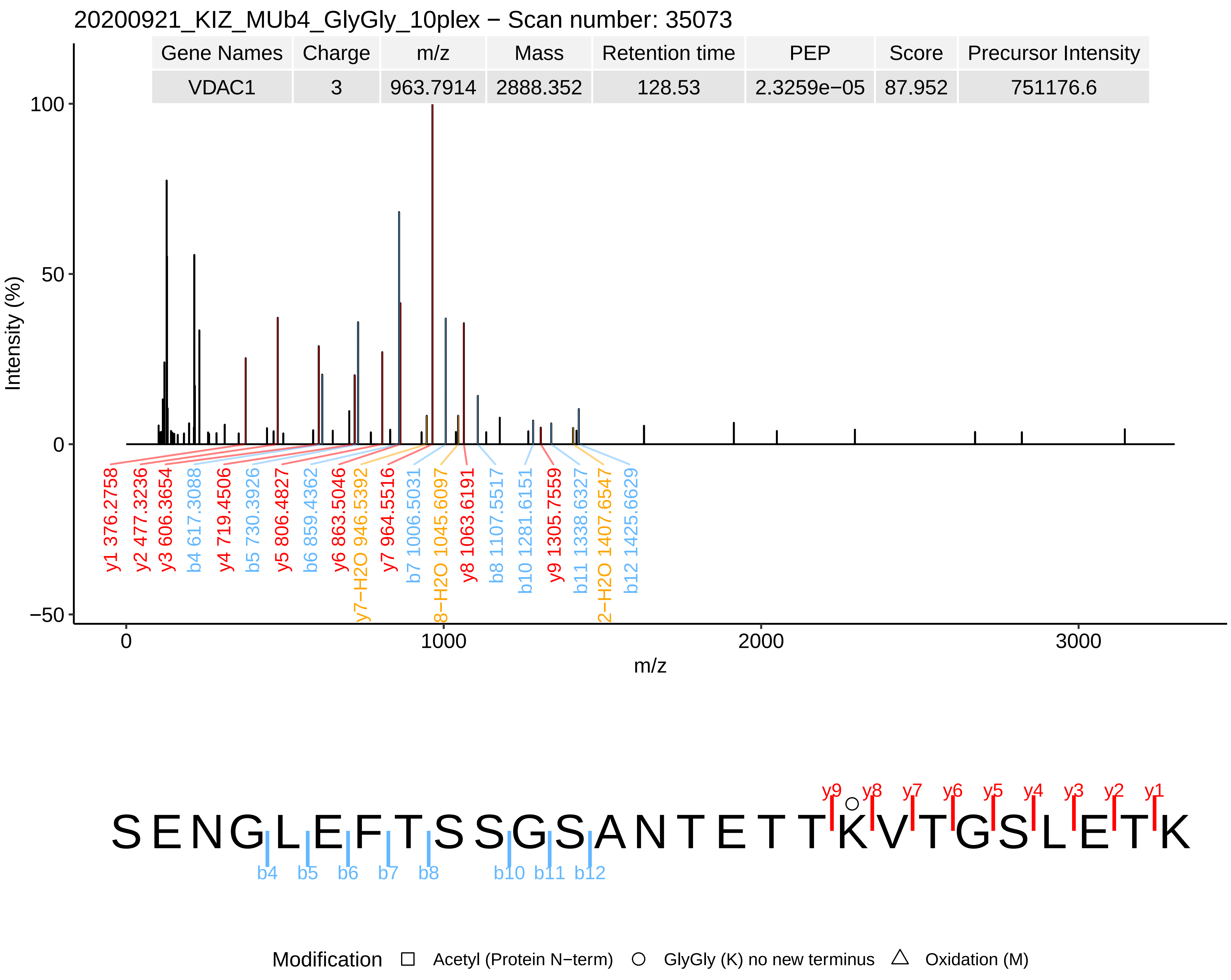

20200921_KIZ_MUb4_GlyGly_10plex − Scan number: 35073
Gene Names | Charge | m/z | Mass | Retention time | PEP | Score | Precursor Intensity
VDAC1 | 3 | 963.7914 | 2888.352 | 128.53 | 2.3259e−05 | 87.952 | 751176.6
Intensity (%)
m/z
y1 376.2758
y2 477.3236
y3 606.3654
b4 617.3088
y4 719.4506
b5 730.3926
y5 806.4827
b6 859.4362
y6 863.5046
y7−H2O 946.5392
y7 964.5516
b7 1006.5031
8−H2O 1045.6097
y8 1063.6191
b8 1107.5517
b10 1281.6151
y9 1305.7559
b11 1338.6327
2−H2O 1407.6547
b12 1425.6629
S E N G L E F T S S G S A N T E T T K V T G S L E T K
Modification: Acetyl (Protein N−term), GlyGly (K) no new terminus, Oxidation (M)

20200921_KIZ_MUb4_GlyGly_10plex − Scan number: 35204

Gene Names	Charge	m/z	Mass	Retention time	PEP	Score	Precursor Intensity
VDAC1	3	963.7914	2888.352	129.03	0.0023296	72.881	658387.8

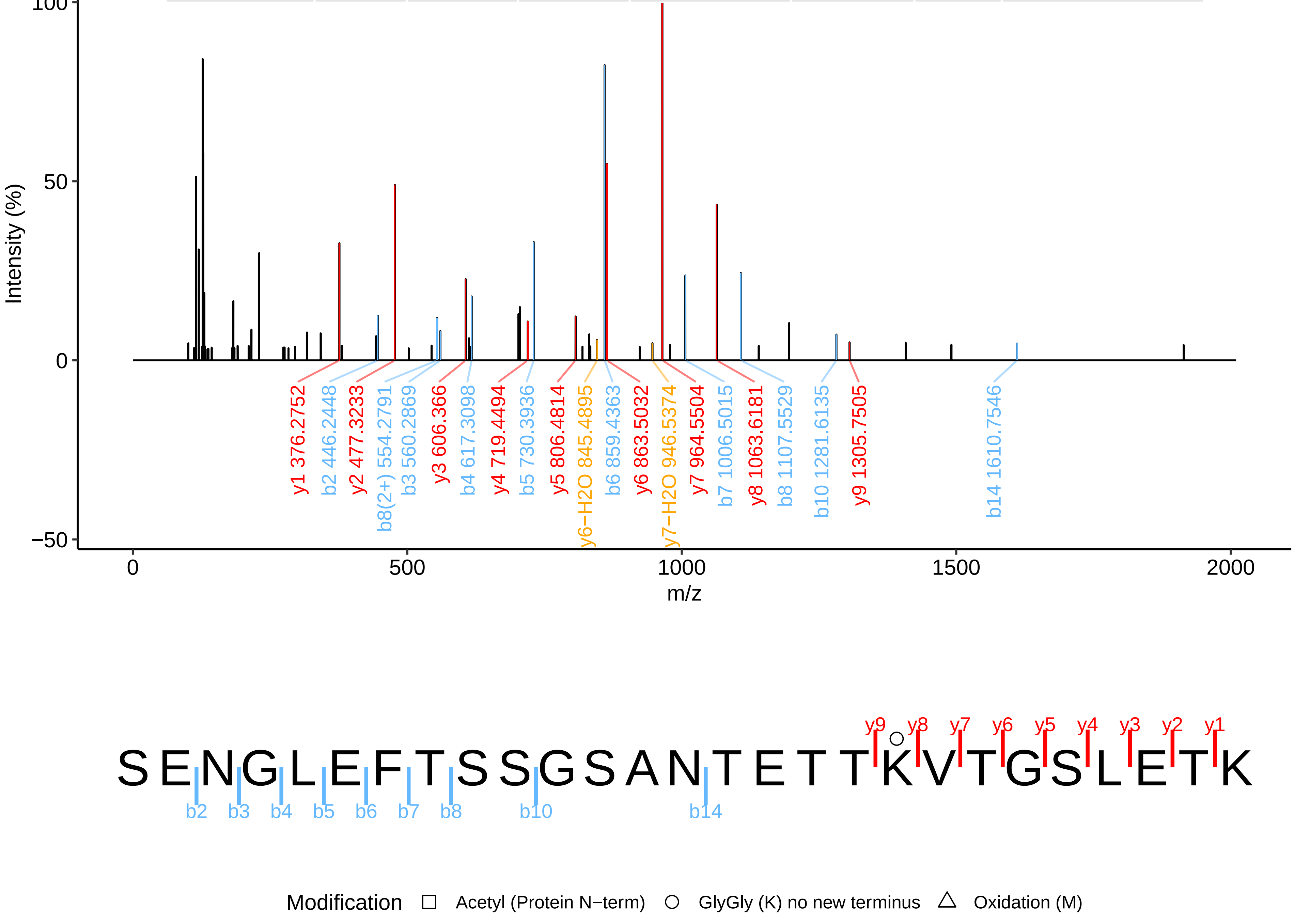

20200921_KIZ_MUb4_GlyGly_10plex − Scan number: 35383

Gene Names	Charge	m/z	Mass	Retention time	PEP	Score	Precursor Intensity
VDAC1	4	723.0954	2888.352	129.68	1.7972e−05	88.571	21772820

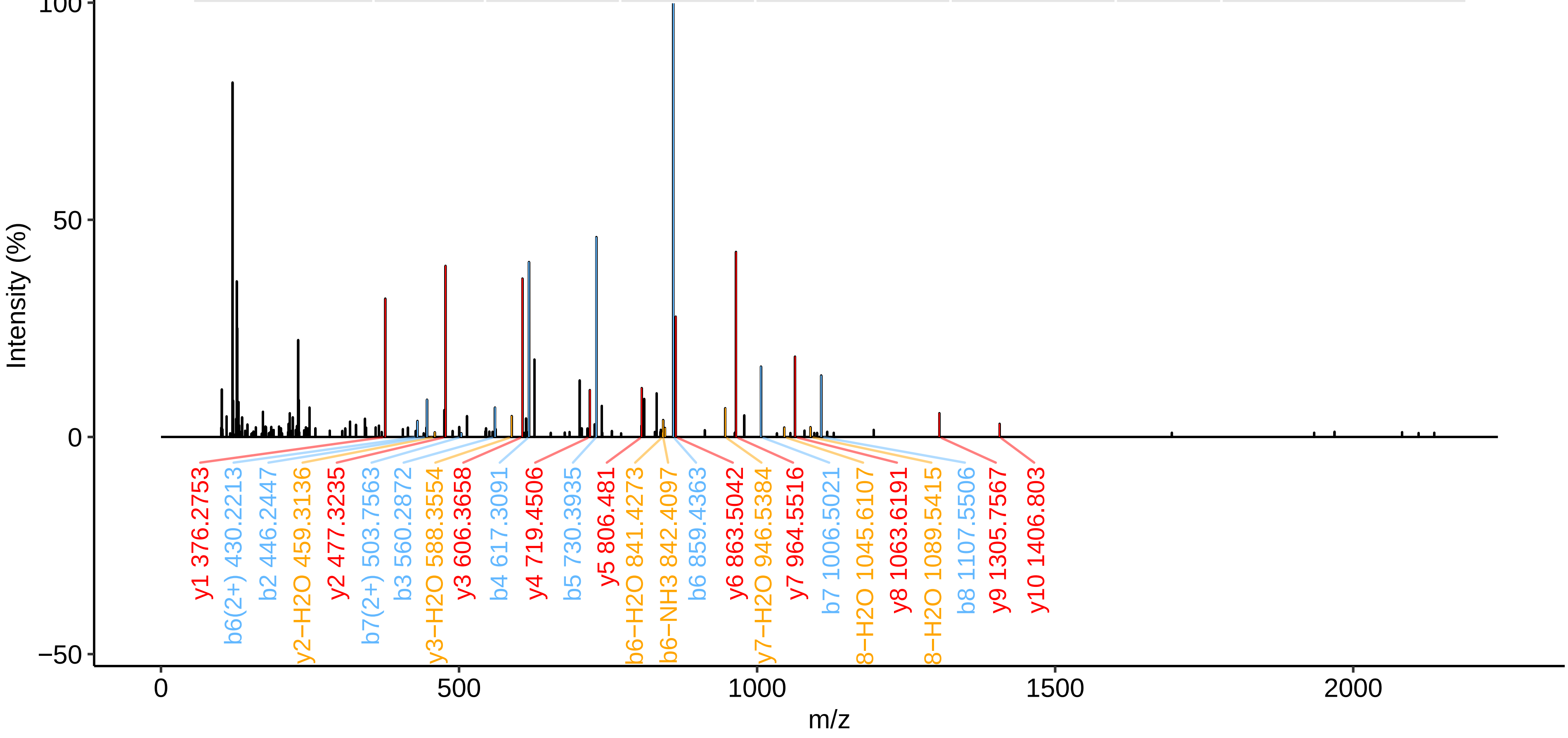

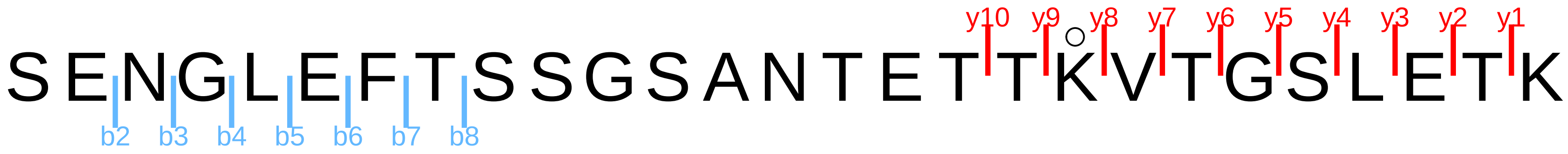

Modification □ Acetyl (Protein N−term) ○ GlyGly (K) no new terminus △ Oxidation (M)

20200921_KIZ_MUb4_GlyGly_10plex − Scan number: 20877

Gene Names	Charge	m/z	Mass	Retention time	PEP	Score	Precursor Intensity
VDAC1	3	963.7914	2888.352	75.521	7.7055e−38	159.36	NA

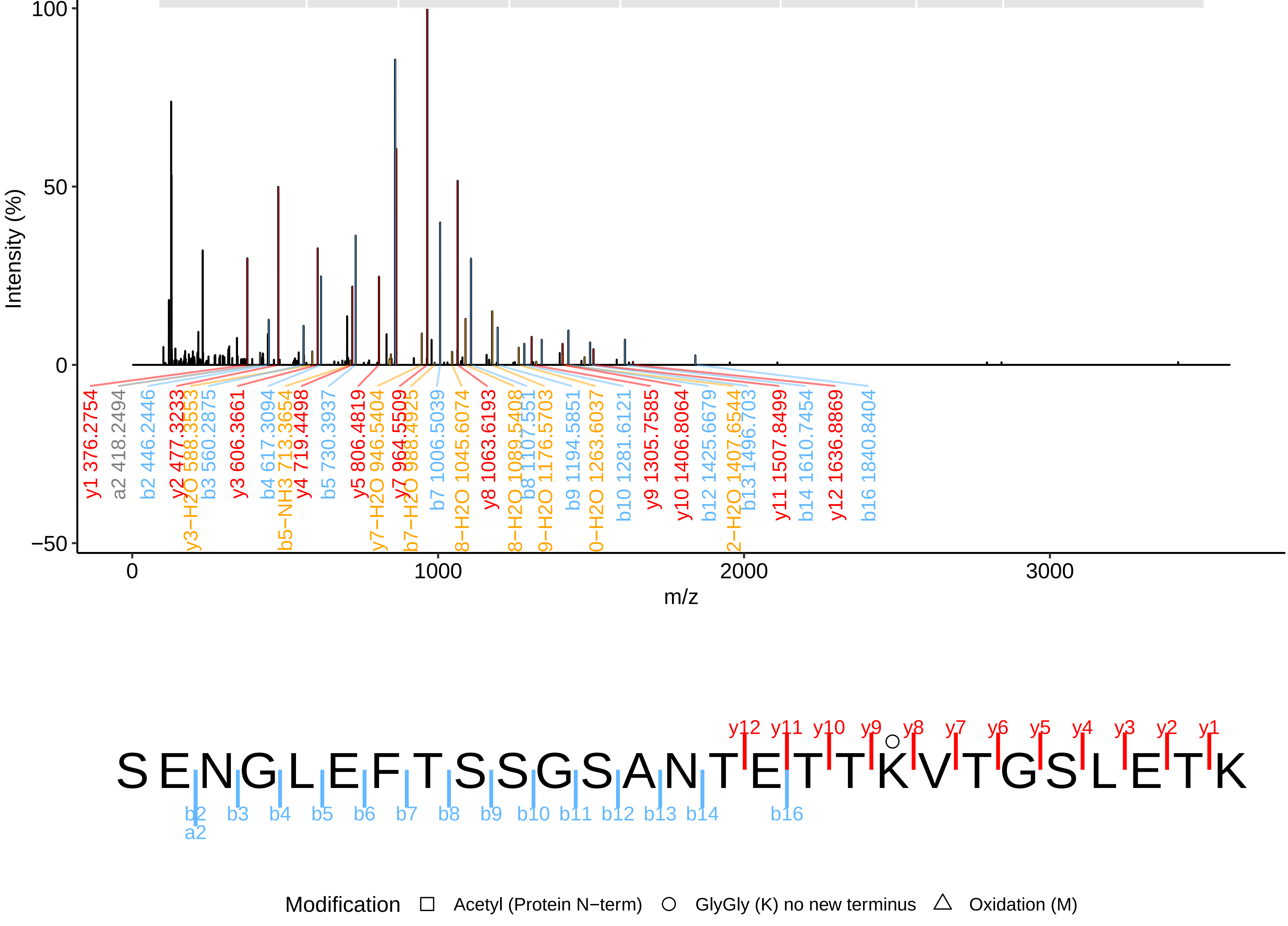

20200921_KIZ_MUb4_GlyGly_10plex − Scan number: 21410

Gene Names	Charge	m/z	Mass	Retention time	PEP	Score	Precursor Intensity
VDAC1	3	963.7914	2888.352	77.537	1.6906e−37	149.94	2756707

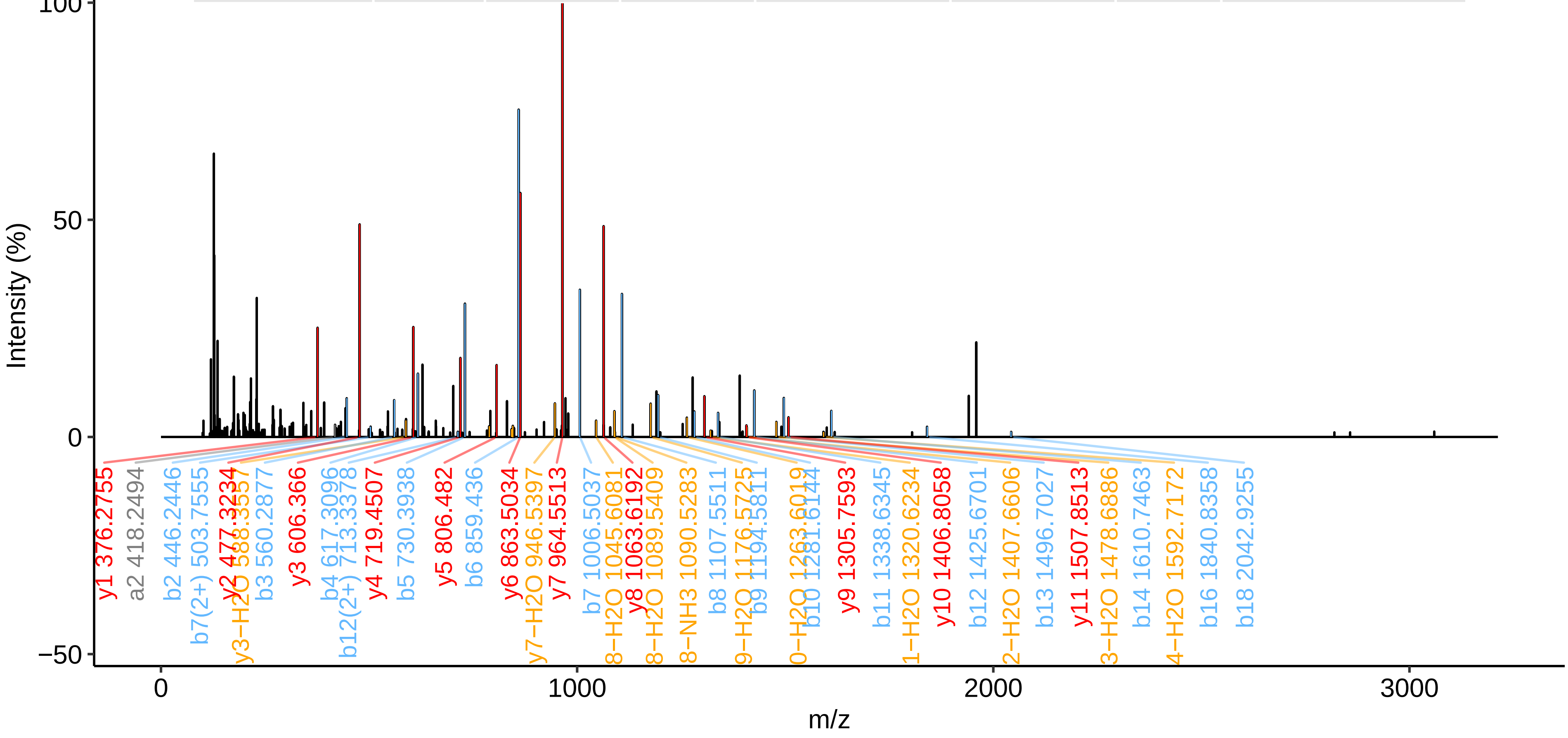

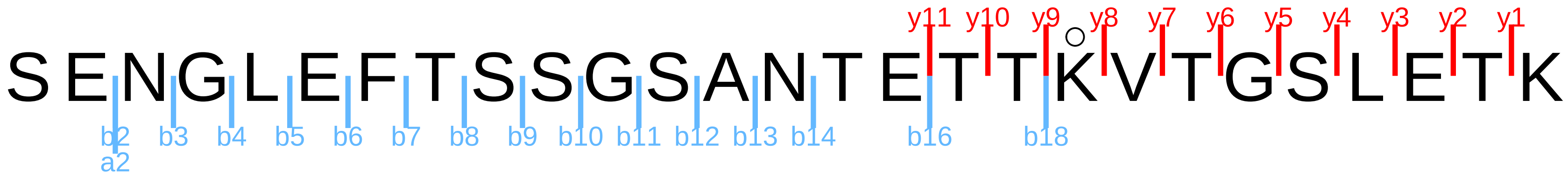

Modification □ Acetyl (Protein N−term) ○ GlyGly (K) no new terminus △ Oxidation (M)

20200921_KIZ_MUb4_GlyGly_10plex − Scan number: 21829

Gene Names	Charge	m/z	Mass	Retention time	PEP	Score	Precursor Intensity
VDAC1	3	963.7914	2888.352	79.116	2.1867e−28	147.18	4116649

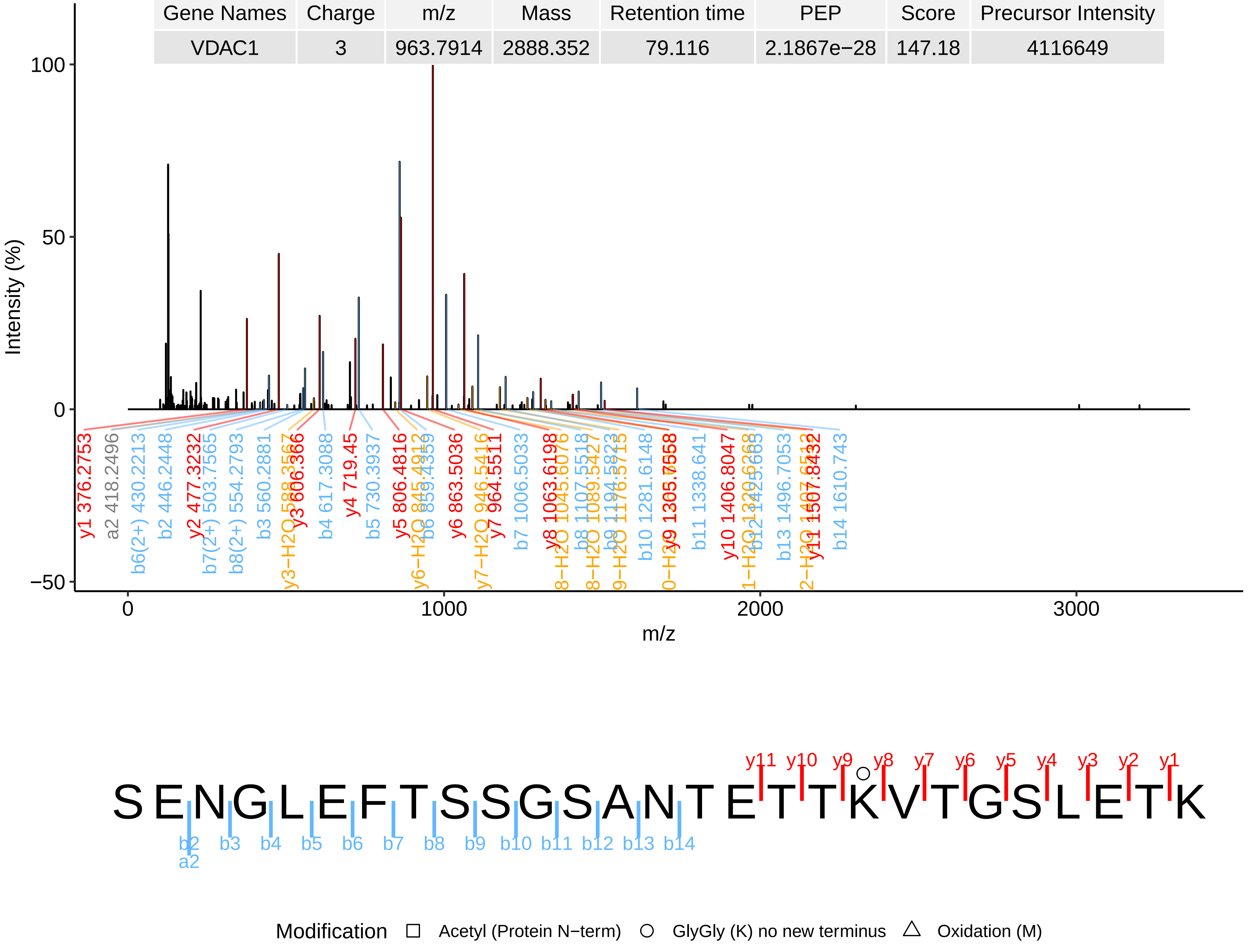

20200921_KIZ_MUb4_GlyGly_10plex − Scan number: 24750

Gene Names	Charge	m/z	Mass	Retention time	PEP	Score	Precursor Intensity
VDAC1	3	963.7914	2888.352	90.037	1.5264e−19	120.57	NA

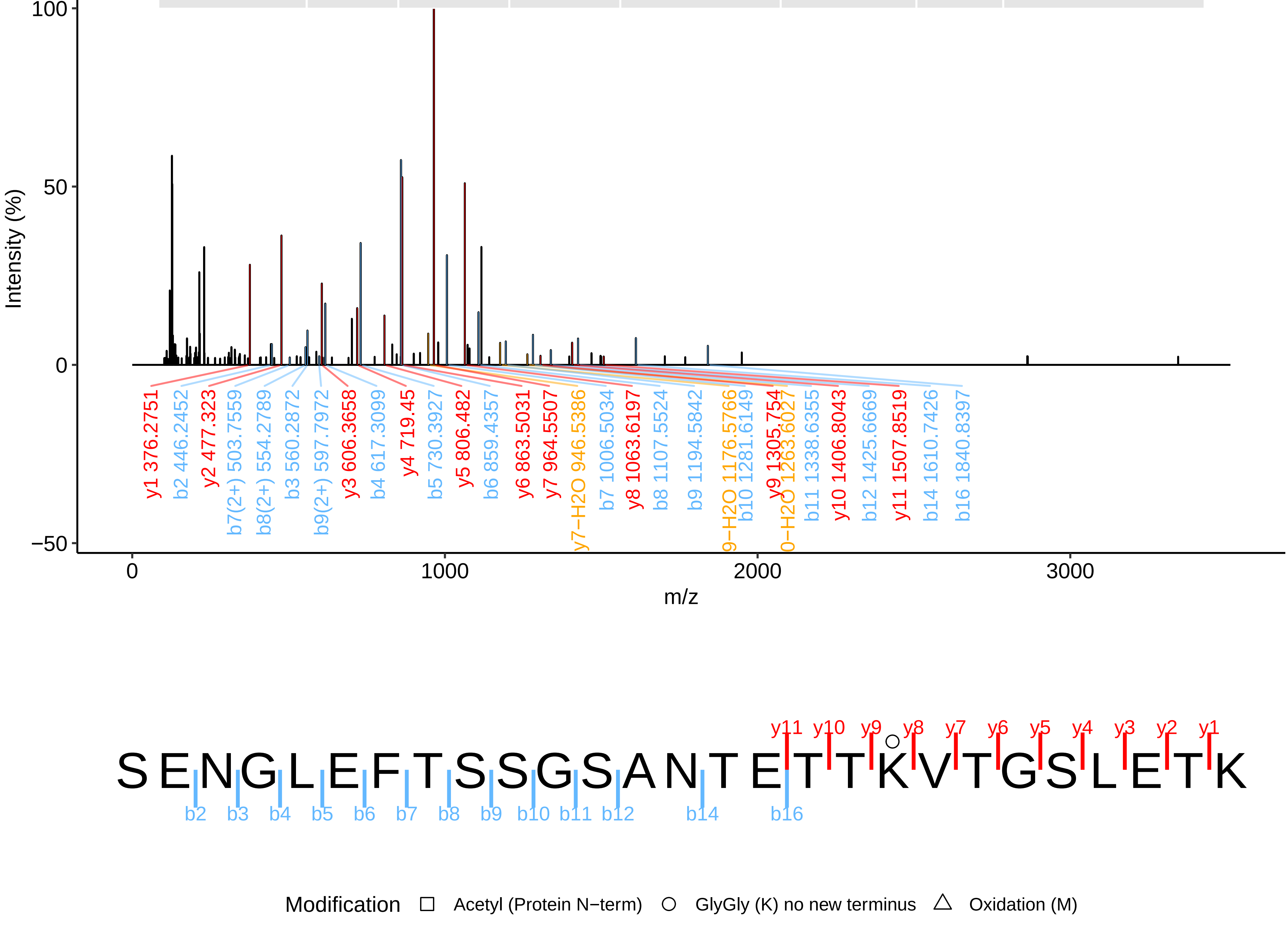

20200921_KIZ_MUb4_GlyGly_10plex − Scan number: 26379

Gene Names	Charge	m/z	Mass	Retention time	PEP	Score	Precursor Intensity
VDAC1	3	963.7914	2888.352	96.102	1.6324e−13	107.87	1131125

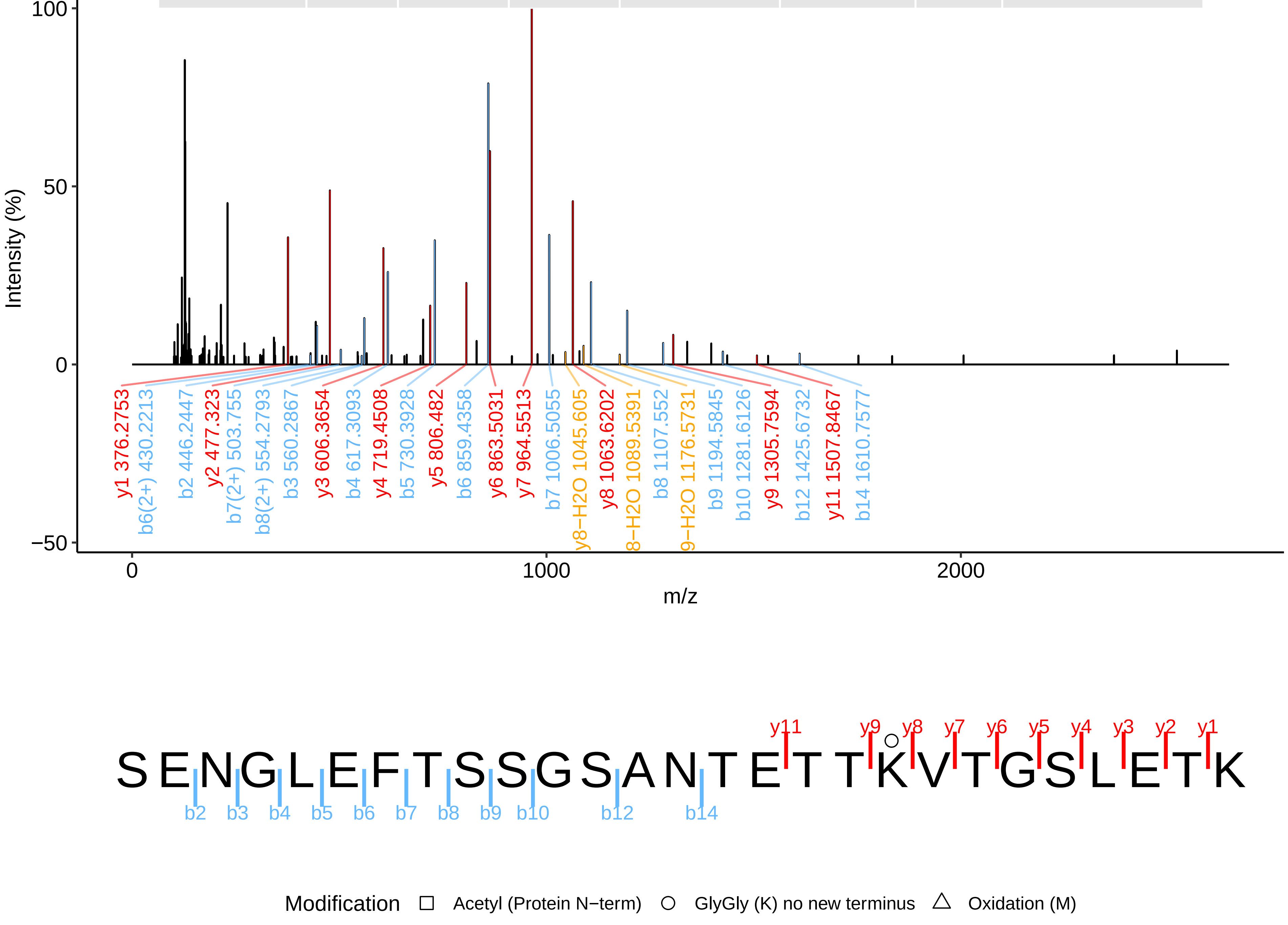

20200921_KIZ_MUb4_GlyGly_10plex − Scan number: 27879

Gene Names	Charge	m/z	Mass	Retention time	PEP	Score	Precursor Intensity
VDAC1	3	963.7914	2888.352	101.88	3.3398e−09	92.412	NA

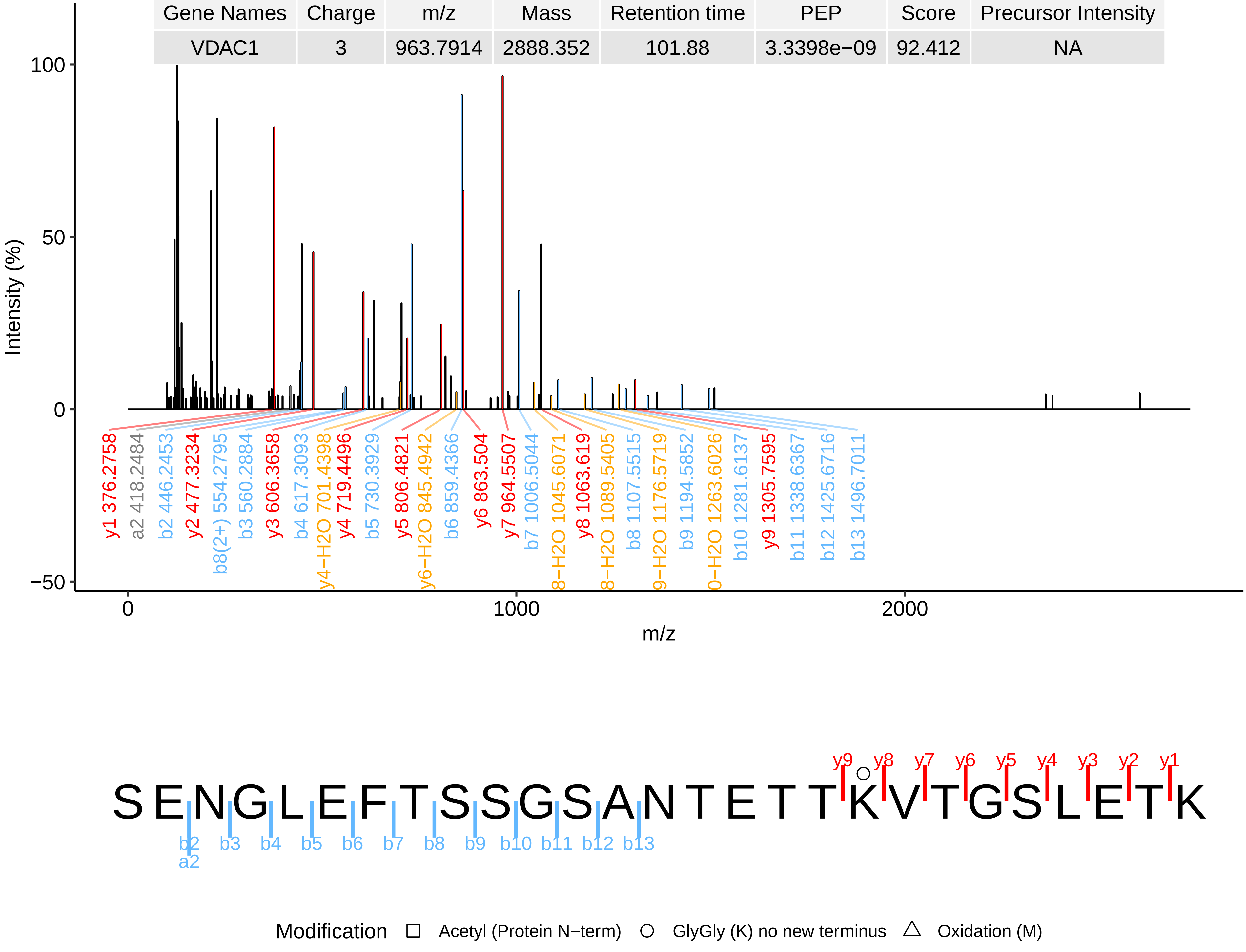

20200921_KIZ_MUb4_GlyGly_10plex − Scan number: 32142

Gene Names	Charge	m/z	Mass	Retention time	PEP	Score	Precursor Intensity
VDAC1	3	963.7914	2888.352	118.72	1.0885e−08	80.711	NA

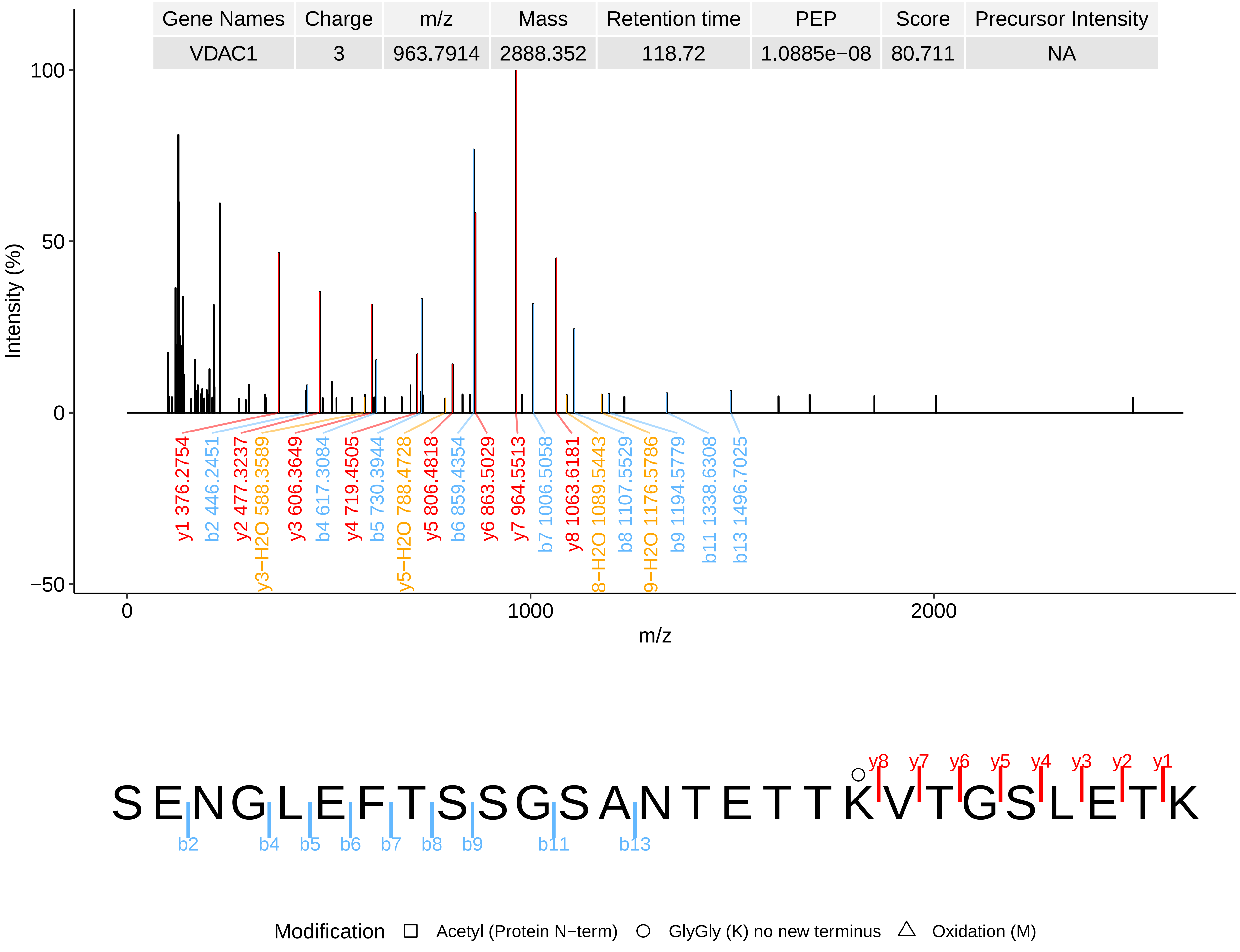

20200921_KIZ_MUb4_GlyGly_10plex_2 − Scan number: 16585

Gene Names	Charge	m/z	Mass	Retention time	PEP	Score	Precursor Intensity
VDAC1	3	963.7914	2888.352	64.759	1.1156e−21	143.32	1820652

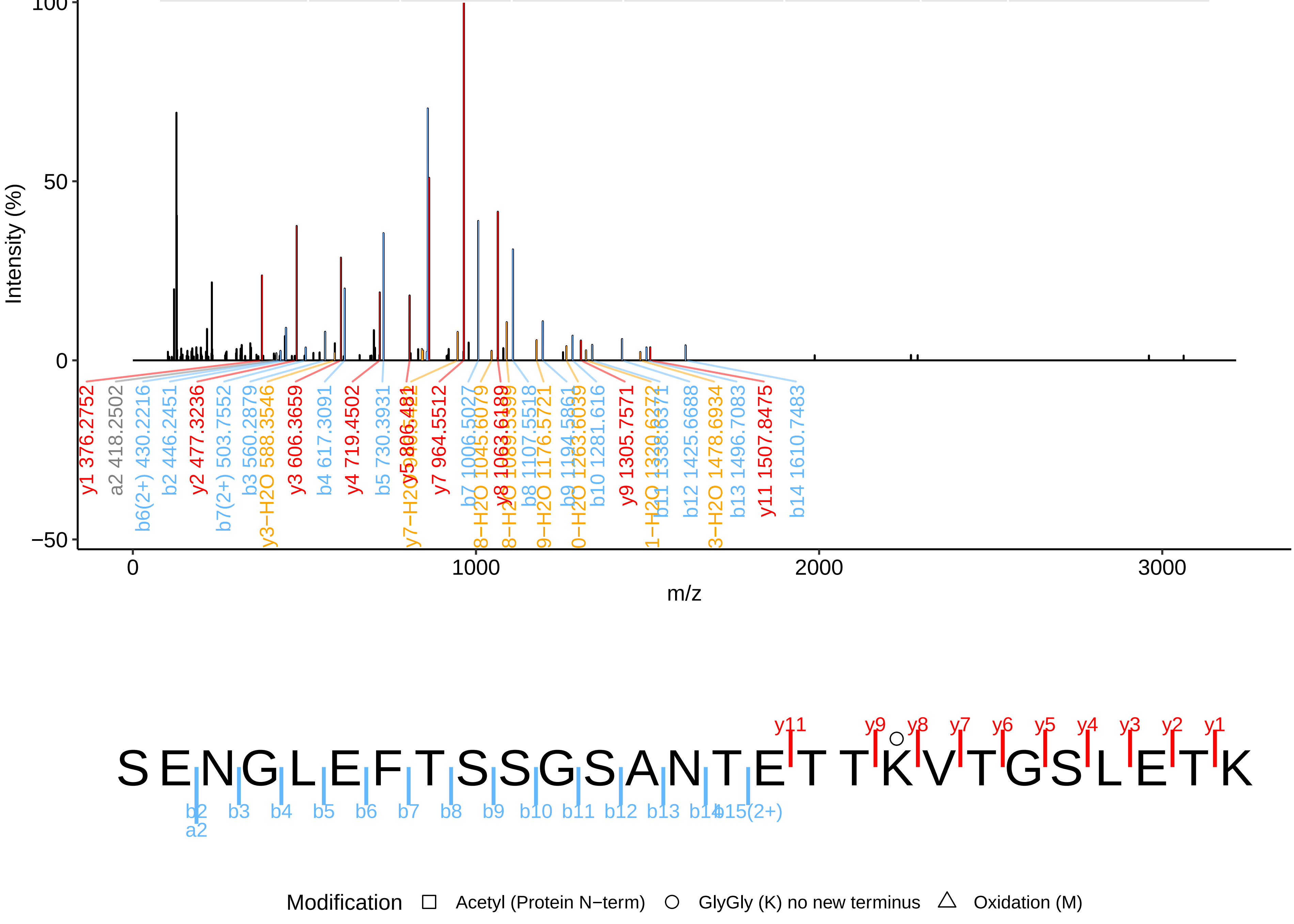

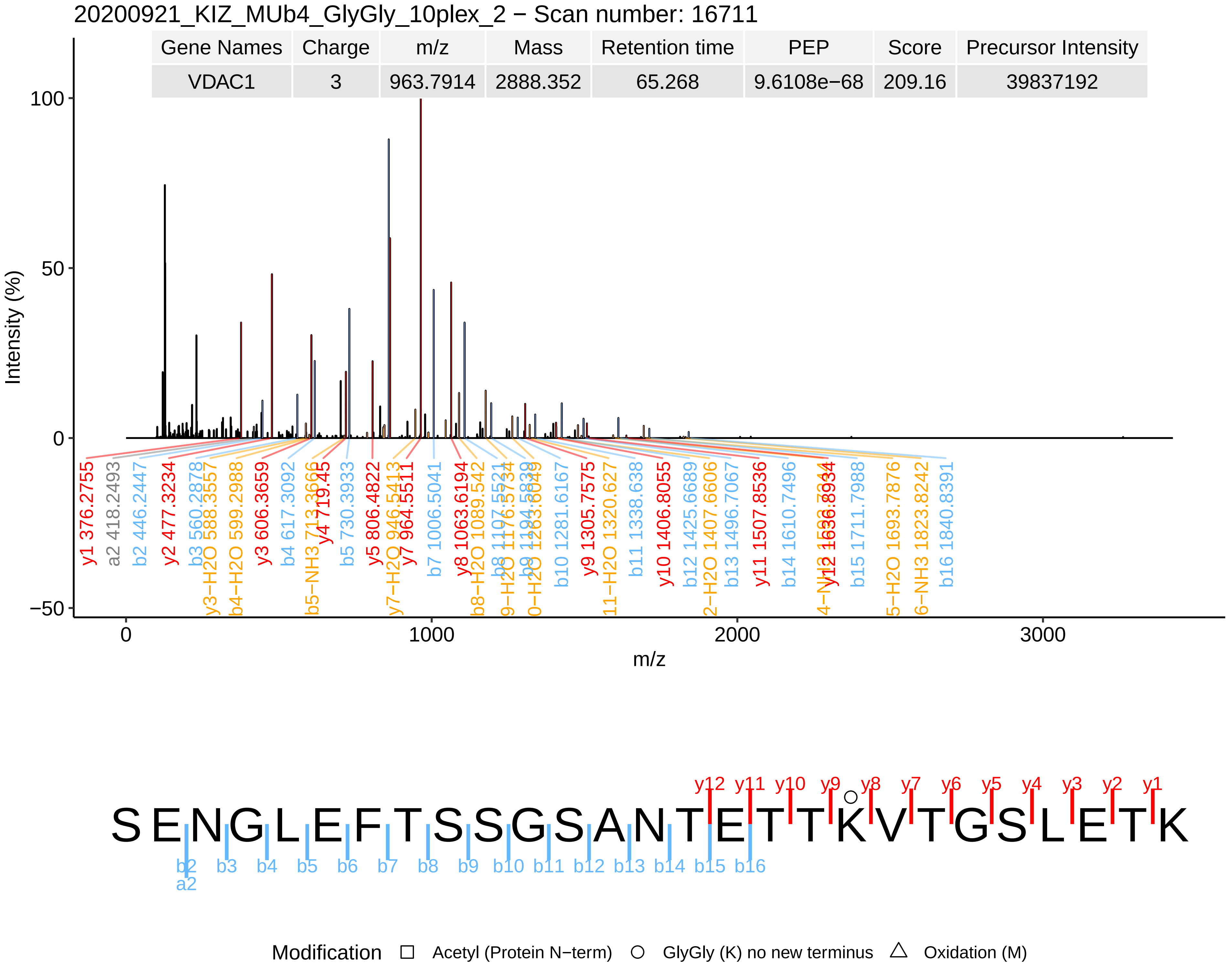
20200921_KIZ_MUb4_GlyGly_10plex_2 − Scan number: 16711
Gene Names | Charge | m/z | Mass | Retention time | PEP | Score | Precursor Intensity
VDAC1 | 3 | 963.7914 | 2888.352 | 65.268 | 9.6108e−68 | 209.16 | 39837192
Intensity (%)
m/z
S E N G L E F T S S G S A N T E T T K V T G S L E T K
Modification □ Acetyl (Protein N−term) ○ GlyGly (K) no new terminus △ Oxidation (M)

20200921_KIZ_MUb4_GlyGly_10plex_2 − Scan number: 16841

Gene Names	Charge	m/z	Mass	Retention time	PEP	Score	Precursor Intensity
VDAC1	3	963.7914	2888.352	65.791	1.9365e−41	179.2	10563181

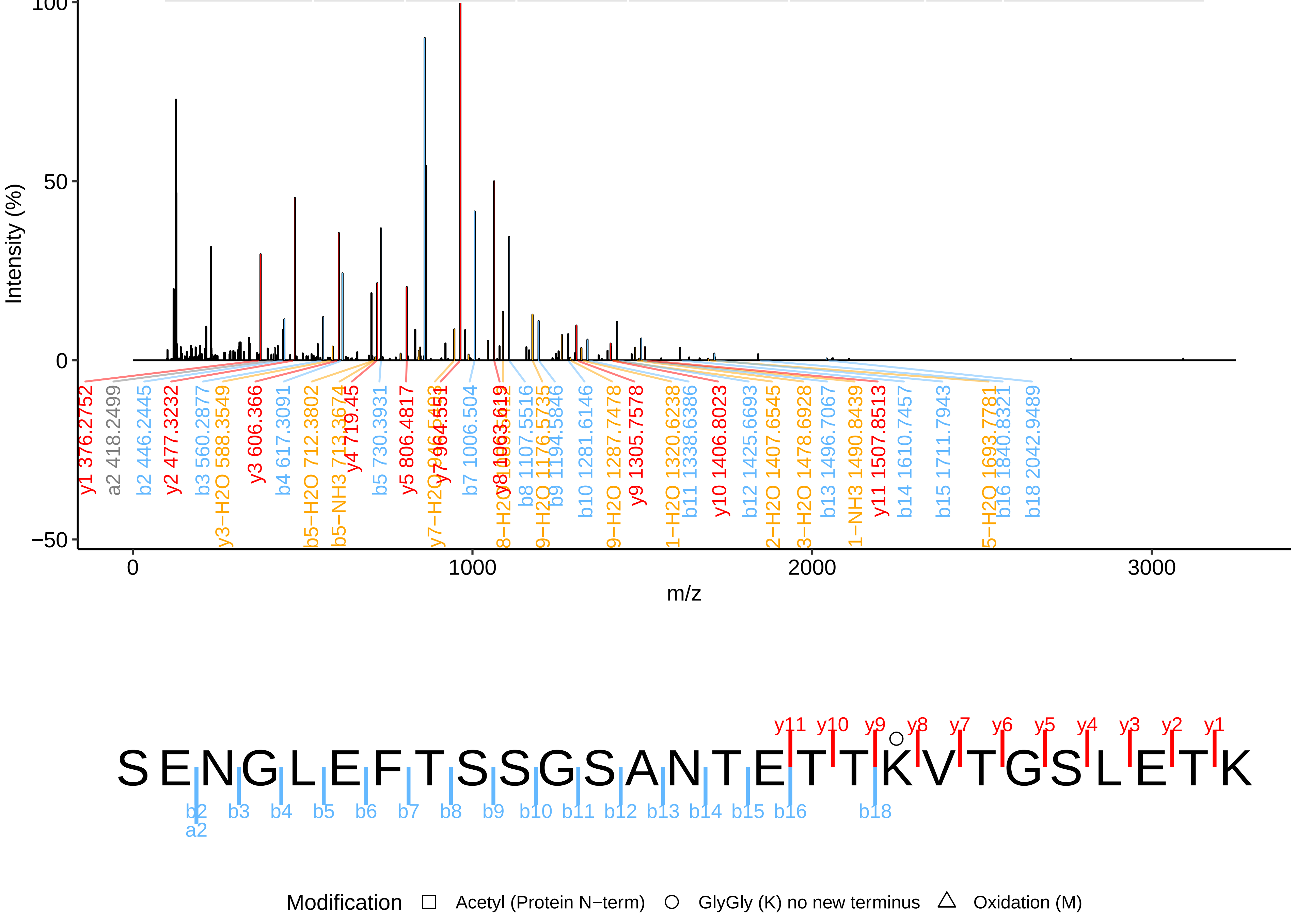

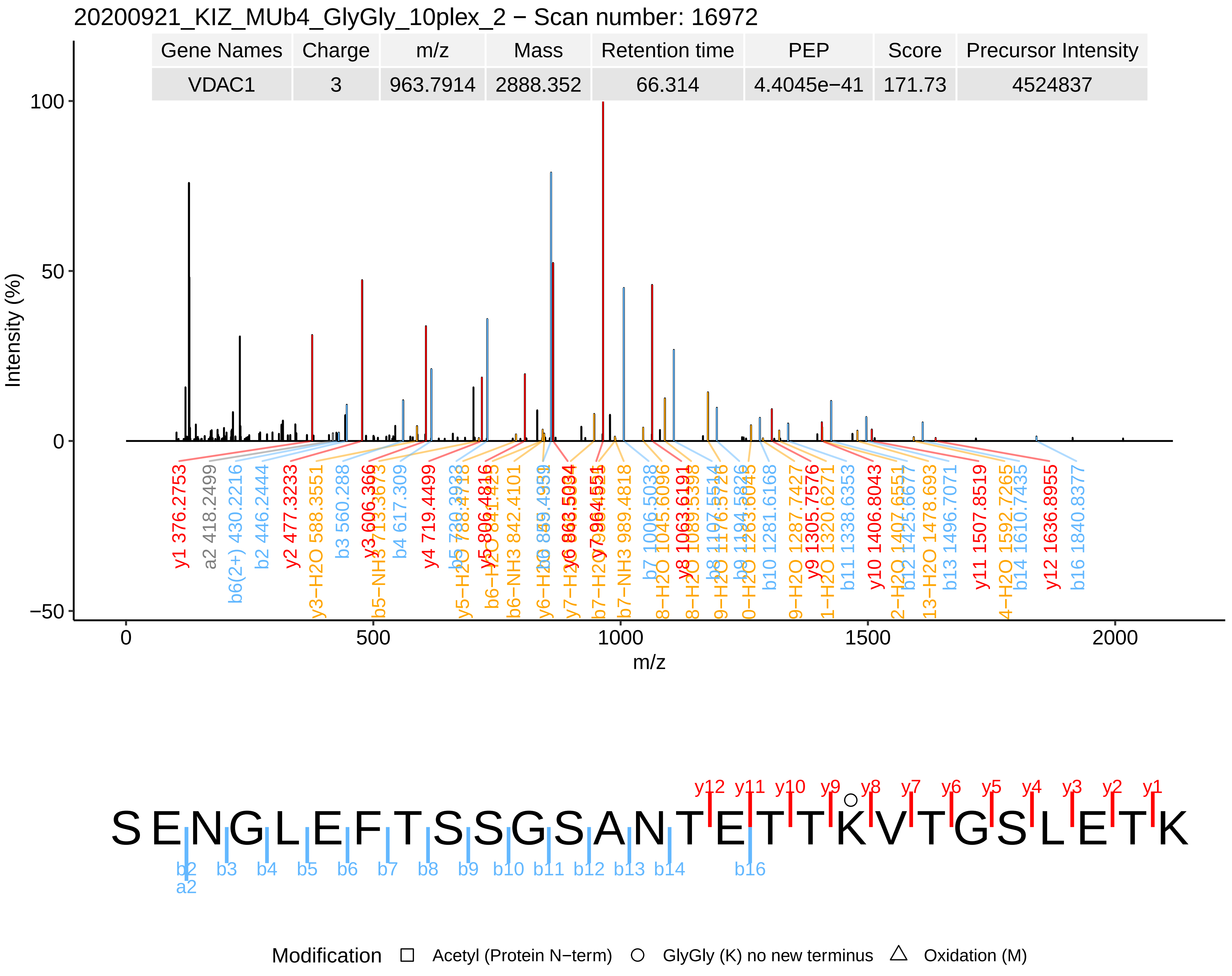
20200921_KIZ_MUb4_GlyGly_10plex_2 − Scan number: 16972
Gene Names | Charge | m/z | Mass | Retention time | PEP | Score | Precursor Intensity
VDAC1 | 3 | 963.7914 | 2888.352 | 66.314 | 4.4045e−41 | 171.73 | 4524837
Intensity (%)
m/z
S E N G L E F T S S G S A N T E T T K V T G S L E T K
y12 y11 y10 y9 y8 y7 y6 y5 y4 y3 y2 y1
b2 b3 b4 b5 b6 b7 b8 b9 b10 b11 b12 b13 b14 b16
a2
Modification
Acetyl (Protein N−term)
GlyGly (K) no new terminus
Oxidation (M)

20200921_KIZ_MUb4_GlyGly_10plex_2 − Scan number: 16587

Gene Names	Charge	m/z	Mass	Retention time	PEP	Score	Precursor Intensity
VDAC1	4	723.0954	2888.352	64.767	1.7619e−09	112.13	2049043

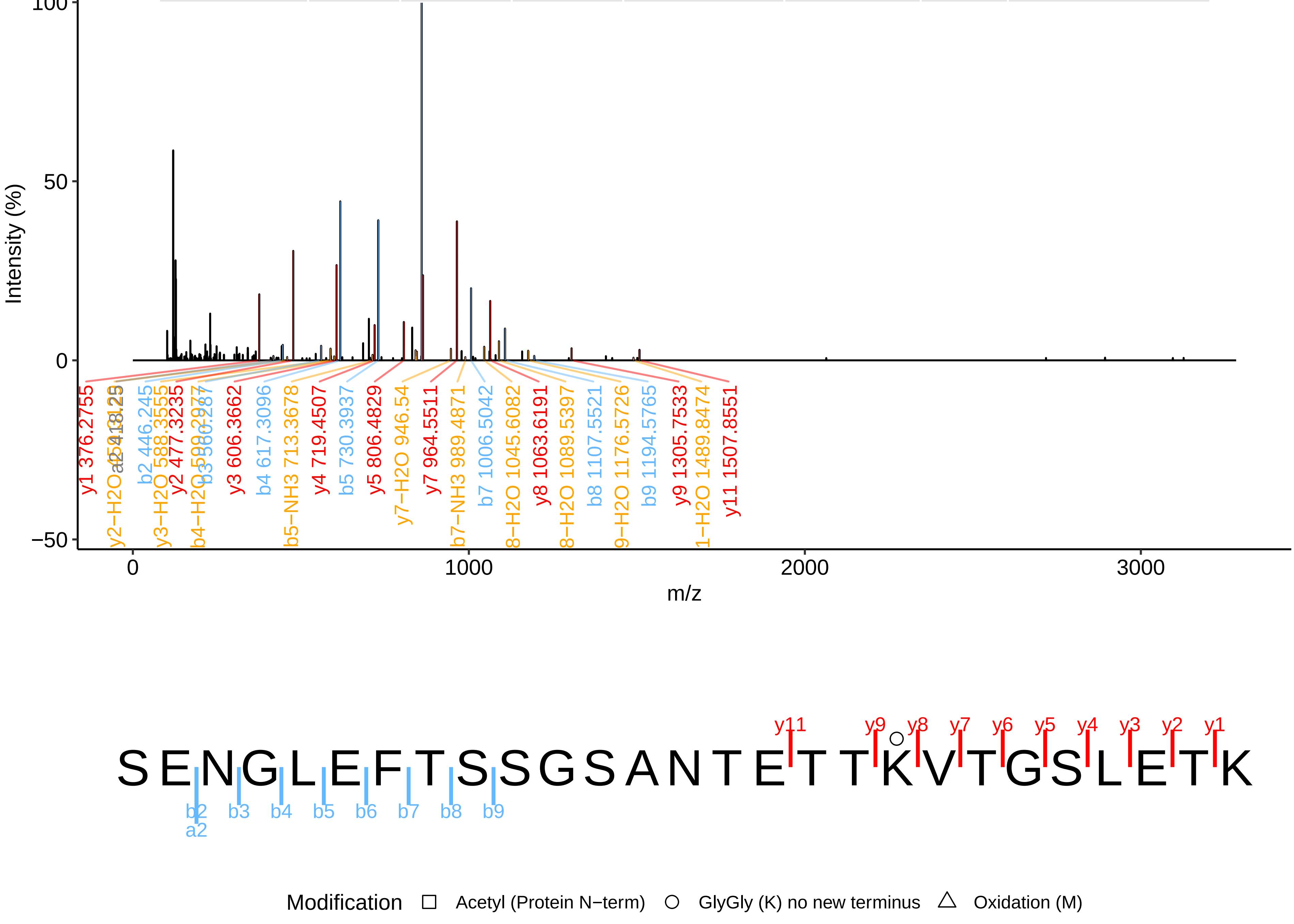

20200921_KIZ_MUb4_GlyGly_10plex_2 − Scan number: 16712

Gene Names	Charge	m/z	Mass	Retention time	PEP	Score	Precursor Intensity
VDAC1	4	723.0954	2888.352	65.27	2.1348e−30	151.91	24399546

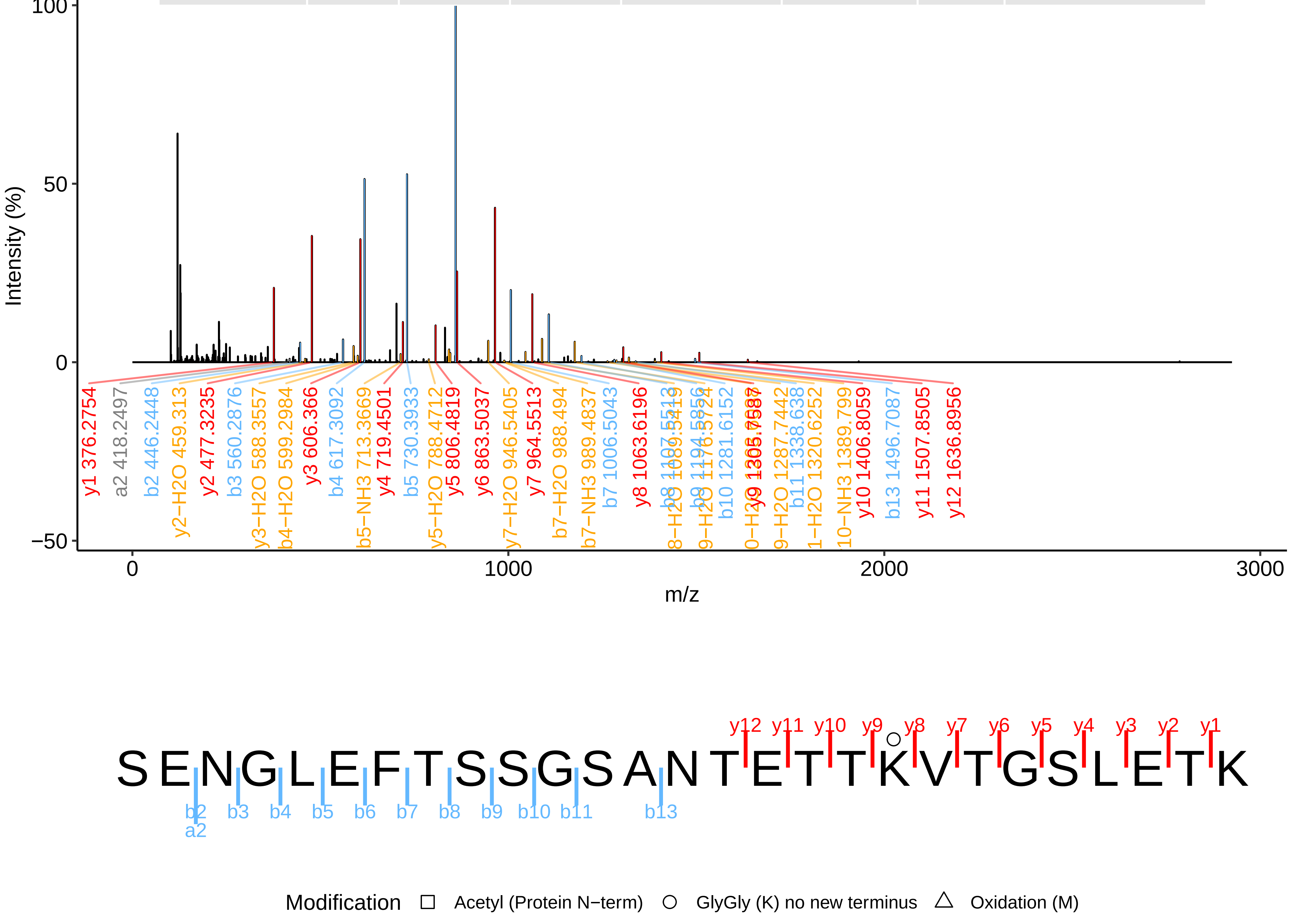

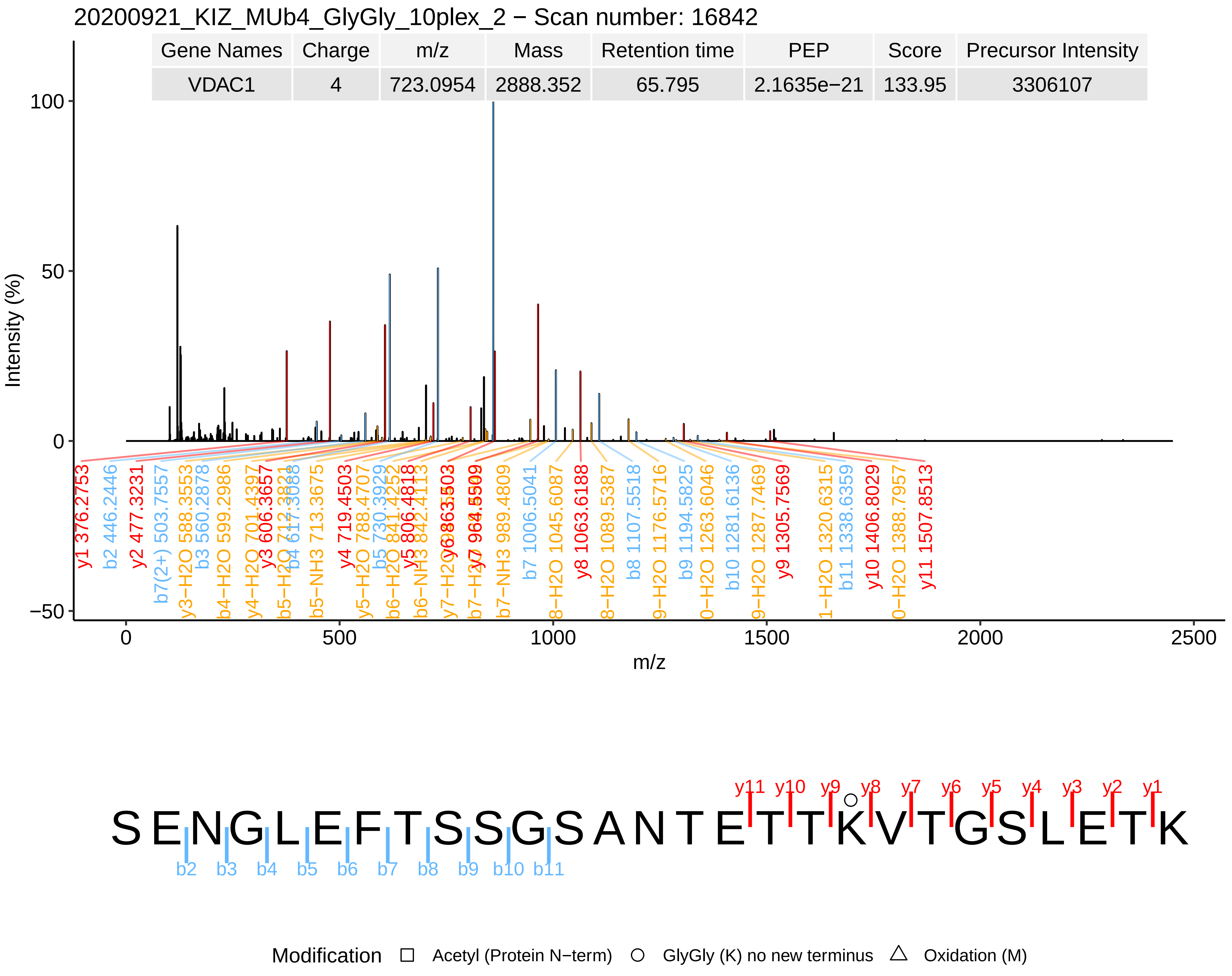

20200921_KIZ_MUb4_GlyGly_10plex_2 − Scan number: 16842
Gene Names | Charge | m/z | Mass | Retention time | PEP | Score | Precursor Intensity
VDAC1 | 4 | 723.0954 | 2888.352 | 65.795 | 2.1635e−21 | 133.95 | 3306107
Intensity (%)
m/z
y1 376.2753
b2 446.2446
y2 477.3231
b7(2+) 503.7557
b3 560.2878
y3 606.3657
b4 617.3088
y4 719.4503
b5 730.3929
y5 806.4818
y8 1063.6188
b7 1006.5041
b8 1107.5518
b9 1194.5825
b10 1281.6136
y9 1305.7569
b11 1338.6359
y10 1406.8029
y11 1507.8513
S E N G L E F T S S G S A N T E T T K V T G S L E T K
y11 y10 y9 y8 y7 y6 y5 y4 y3 y2 y1
b2 b3 b4 b5 b6 b7 b8 b9 b10 b11
Modification
Acetyl (Protein N−term)
GlyGly (K) no new terminus
Oxidation (M)

20200921_KIZ_MUb4_GlyGly_10plex_2 − Scan number: 16973

Gene Names	Charge	m/z	Mass	Retention time	PEP	Score	Precursor Intensity
VDAC1	4	723.0954	2888.352	66.318	4.1092e−14	116.35	2867889

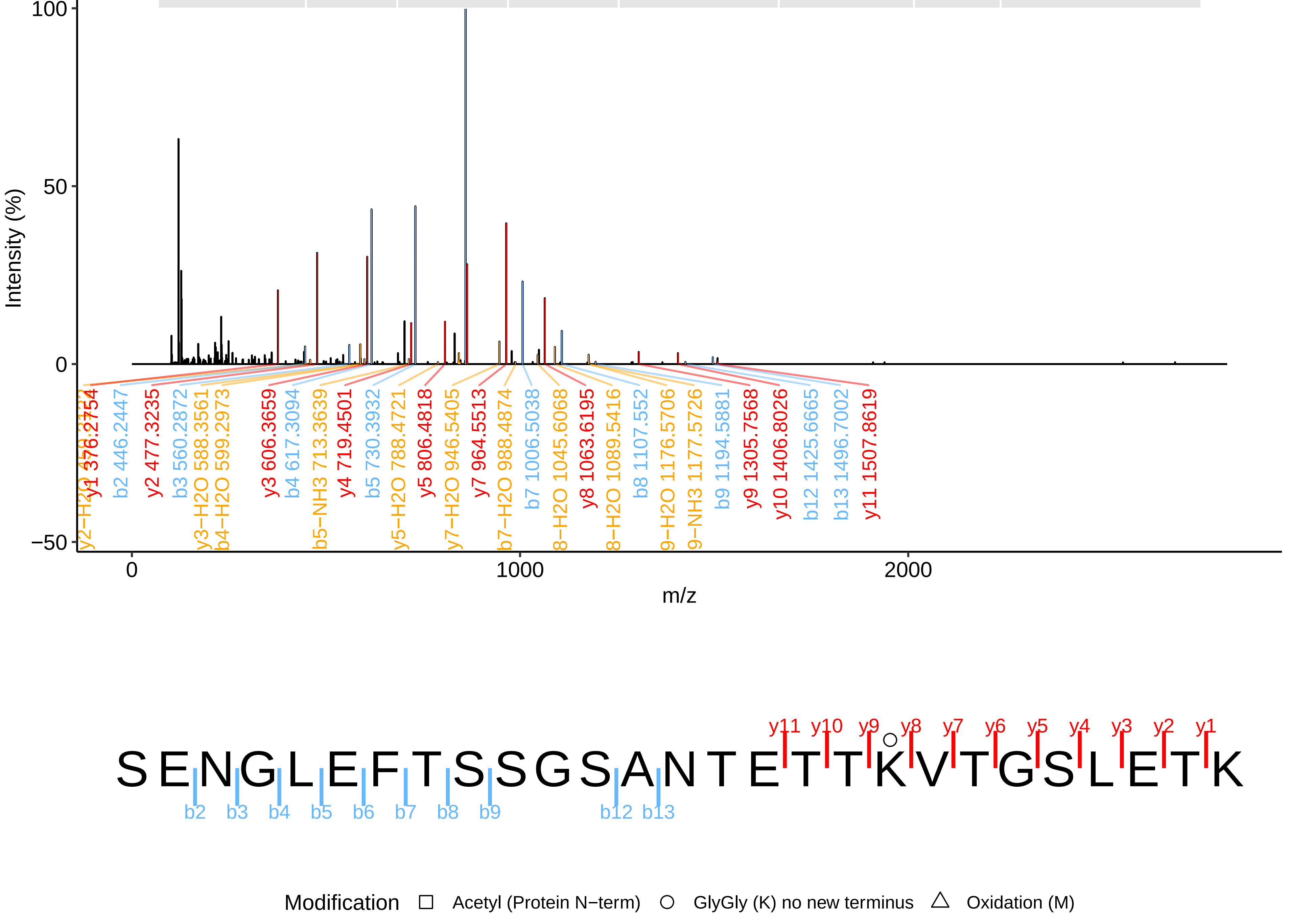

20200921_KIZ_MUb4_GlyGly_10plex_2 − Scan number: 17100

Gene Names	Charge	m/z	Mass	Retention time	PEP	Score	Precursor Intensity
VDAC1	3	963.7914	2888.352	66.819	3.4813e−41	174.53	4999645

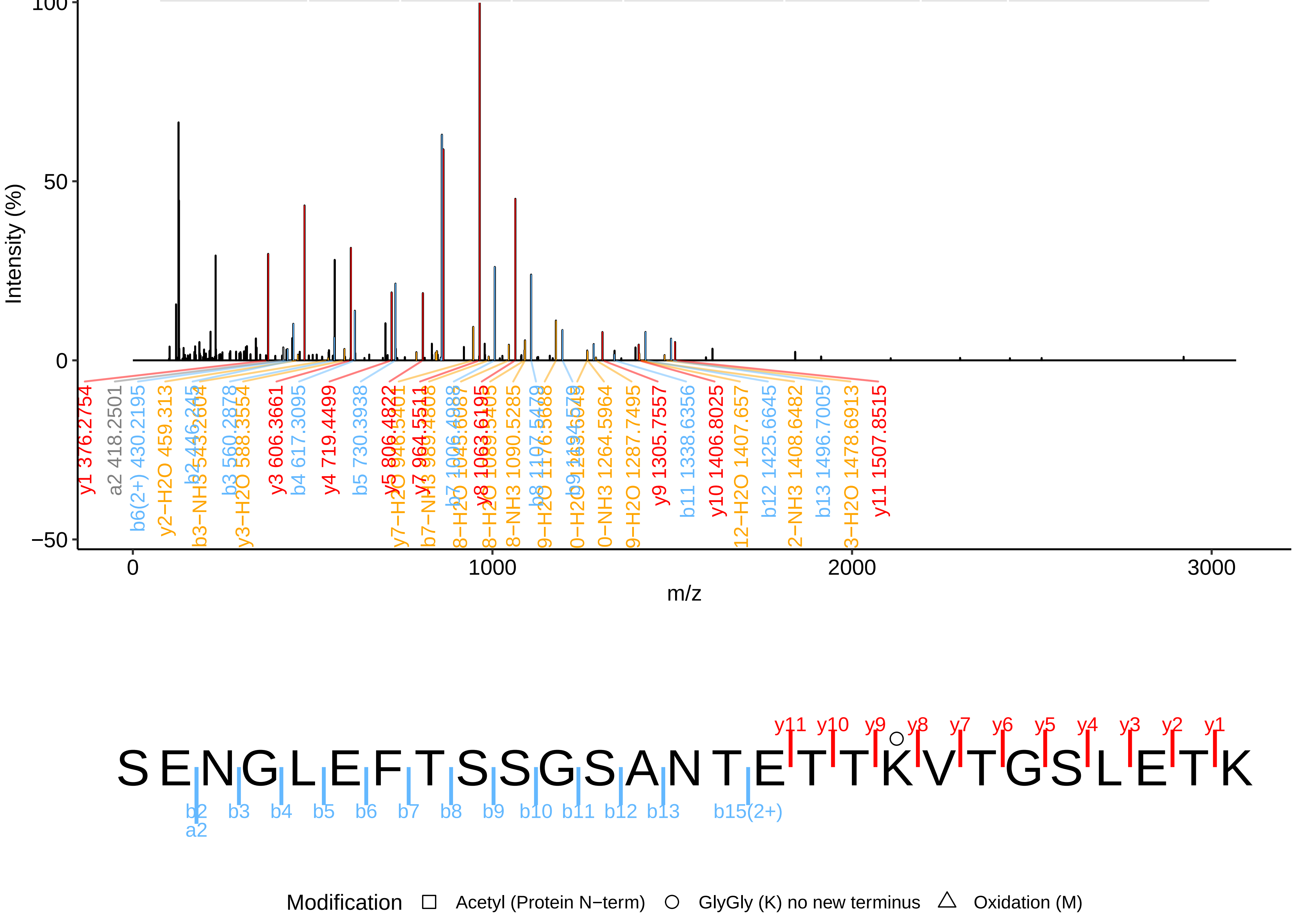

20200921_KIZ_MUb4_GlyGly_10plex_2 − Scan number: 17101

Gene Names	Charge	m/z	Mass	Retention time	PEP	Score	Precursor Intensity
VDAC1	4	723.0954	2888.352	66.823	0.0018624	73.109	2902425

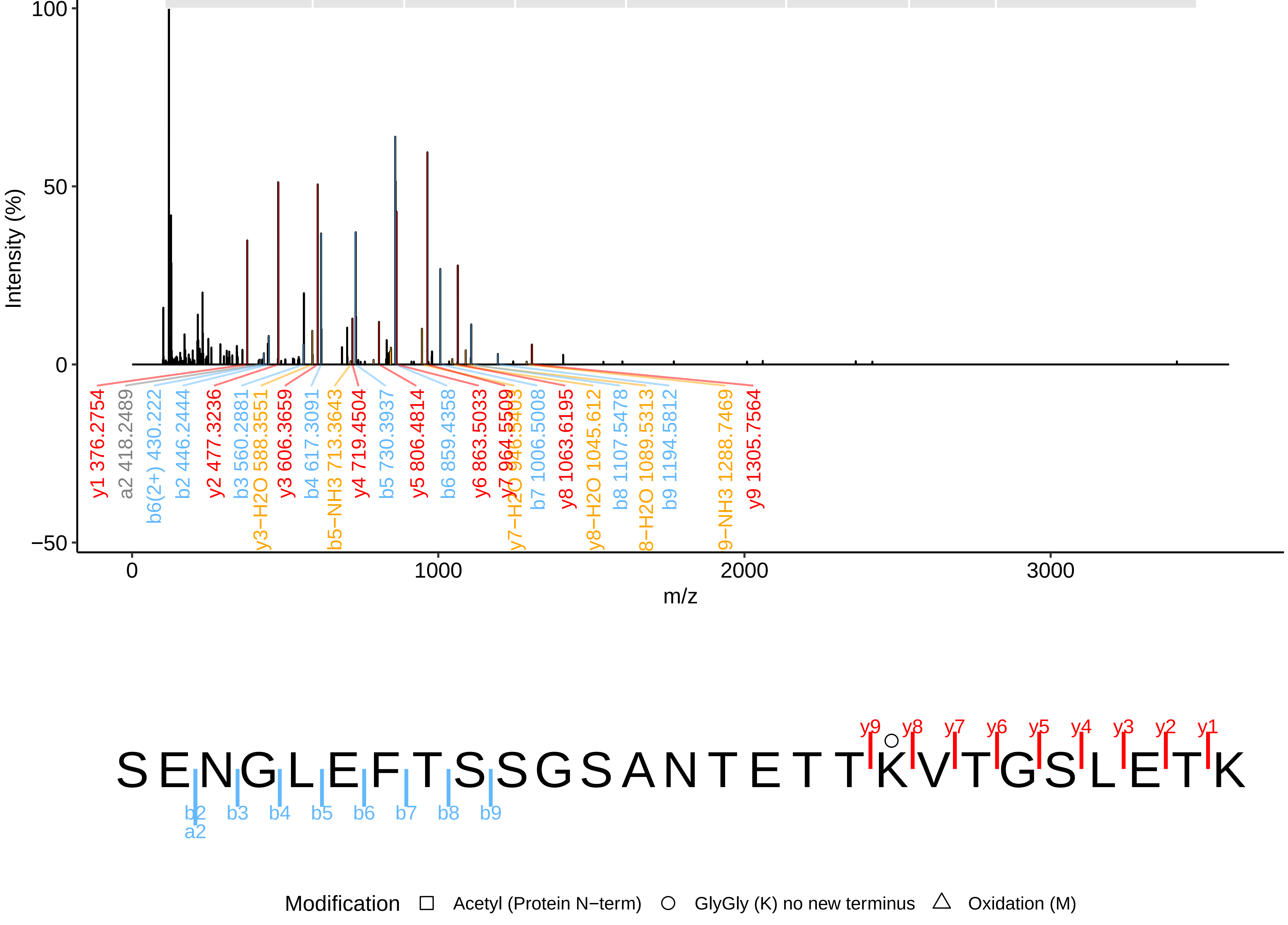

20200921_KIZ_MUb4_GlyGly_10plex_2 − Scan number: 17227

Gene Names	Charge	m/z	Mass	Retention time	PEP	Score	Precursor Intensity
VDAC1	3	963.7914	2888.352	67.342	2.0193e−30	155.39	3045172

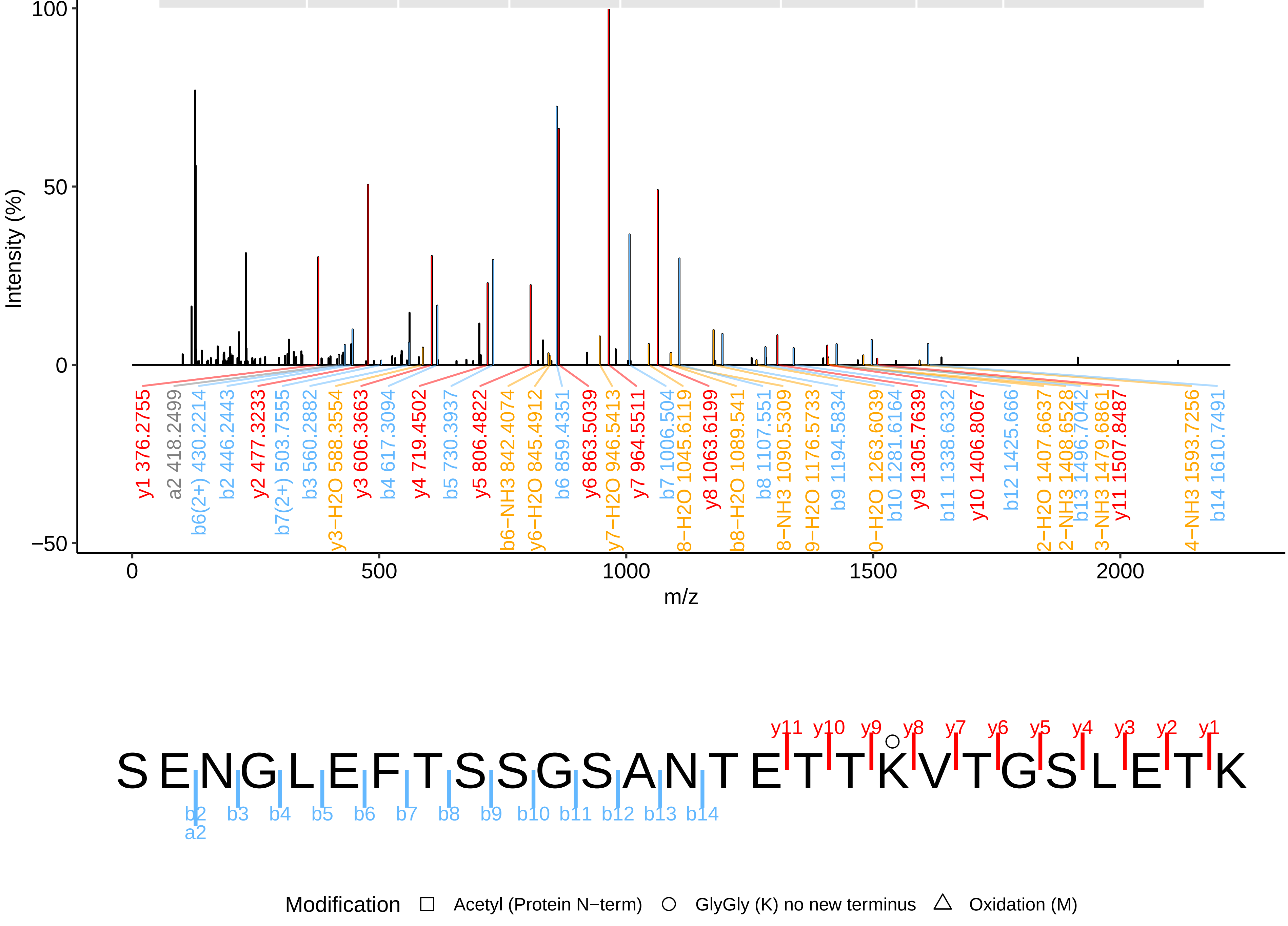

20200921_KIZ_MUb4_GlyGly_10plex_2 − Scan number: 17355

Gene Names	Charge	m/z	Mass	Retention time	PEP	Score	Precursor Intensity
VDAC1	3	963.7914	2888.352	67.865	8.5269e−15	130.01	2403633

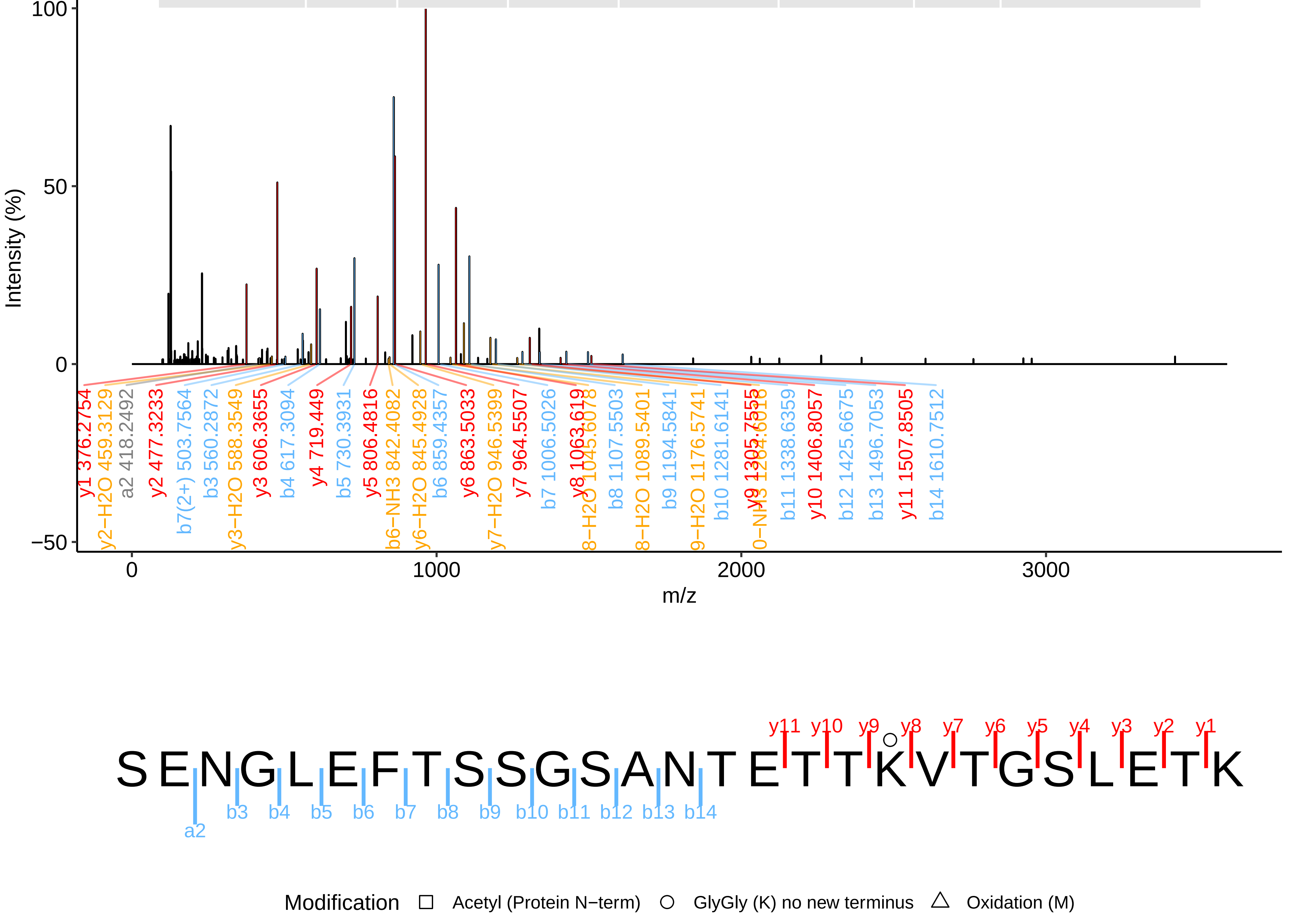

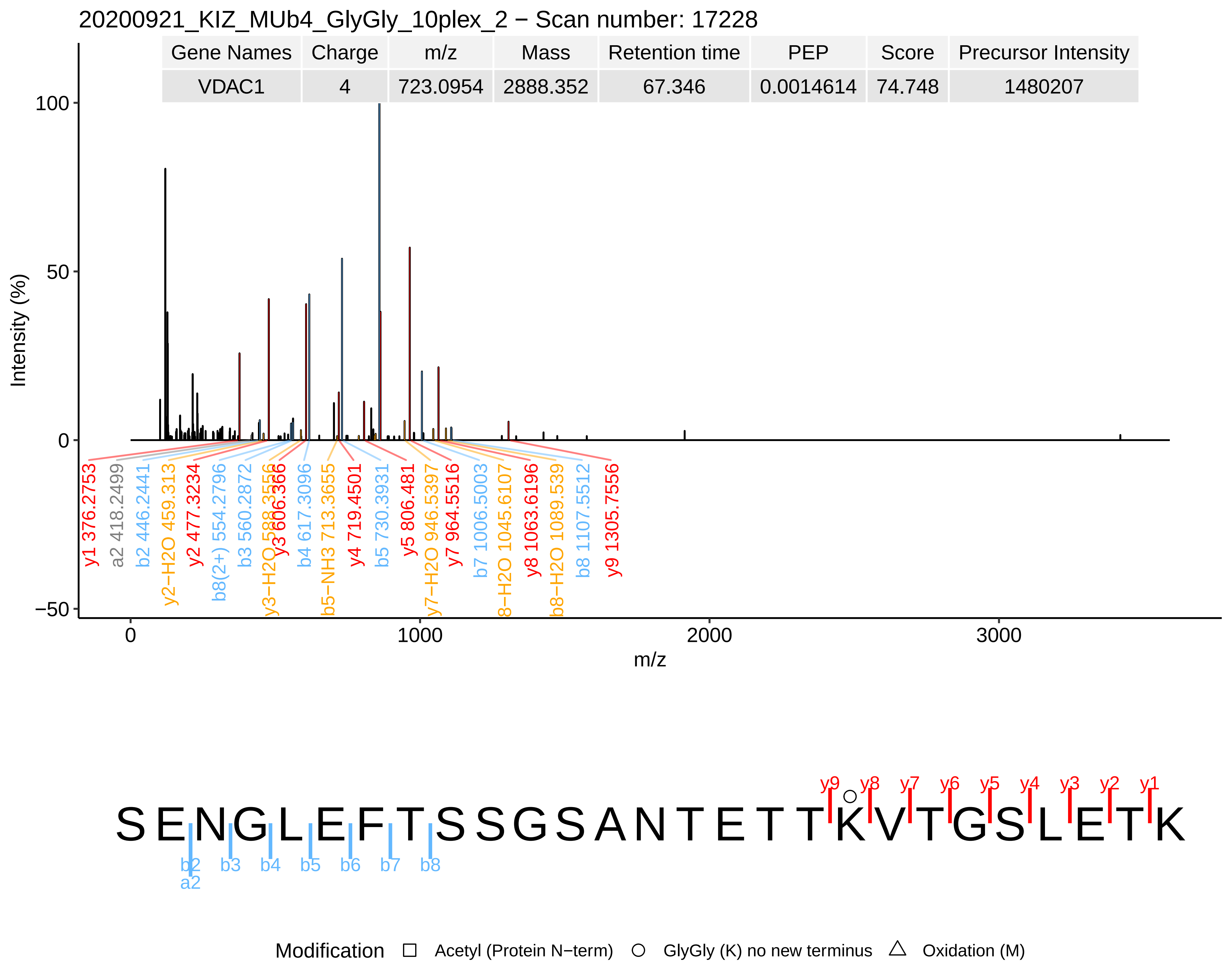
20200921_KIZ_MUb4_GlyGly_10plex_2 − Scan number: 17228
Gene Names | Charge | m/z | Mass | Retention time | PEP | Score | Precursor Intensity
VDAC1 | 4 | 723.0954 | 2888.352 | 67.346 | 0.0014614 | 74.748 | 1480207
Intensity (%)
100
50
0
−50
0
1000
2000
3000
m/z
y1 376.2753
a2 418.2499
b2 446.2441
y2−H2O 459.313
y2 477.3234
b8(2+) 554.2796
b3 560.2872
y3−H2O 588.3556
y3 606.366
b4 617.3096
b5−NH3 713.3655
y4 719.4501
b5 730.3931
y5 806.481
y7−H2O 946.5397
y7 964.5516
b7 1006.5003
8−H2O 1045.6107
y8 1063.6196
b8−H2O 1089.539
b8 1107.5512
y9 1305.7556
y9 y8 y7 y6 y5 y4 y3 y2 y1
S E N G L E F T S S G S A N T E T T K V T G S L E T K
b2 b3 b4 b5 b6 b7 b8
a2
Modification
Acetyl (Protein N−term)
GlyGly (K) no new terminus
Oxidation (M)

20200921_KIZ_MUb4_GlyGly_10plex_2 − Scan number: 17356

Gene Names	Charge	m/z	Mass	Retention time	PEP	Score	Precursor Intensity
VDAC1	4	723.0954	2888.352	67.869	0.001594	74.206	547076.8

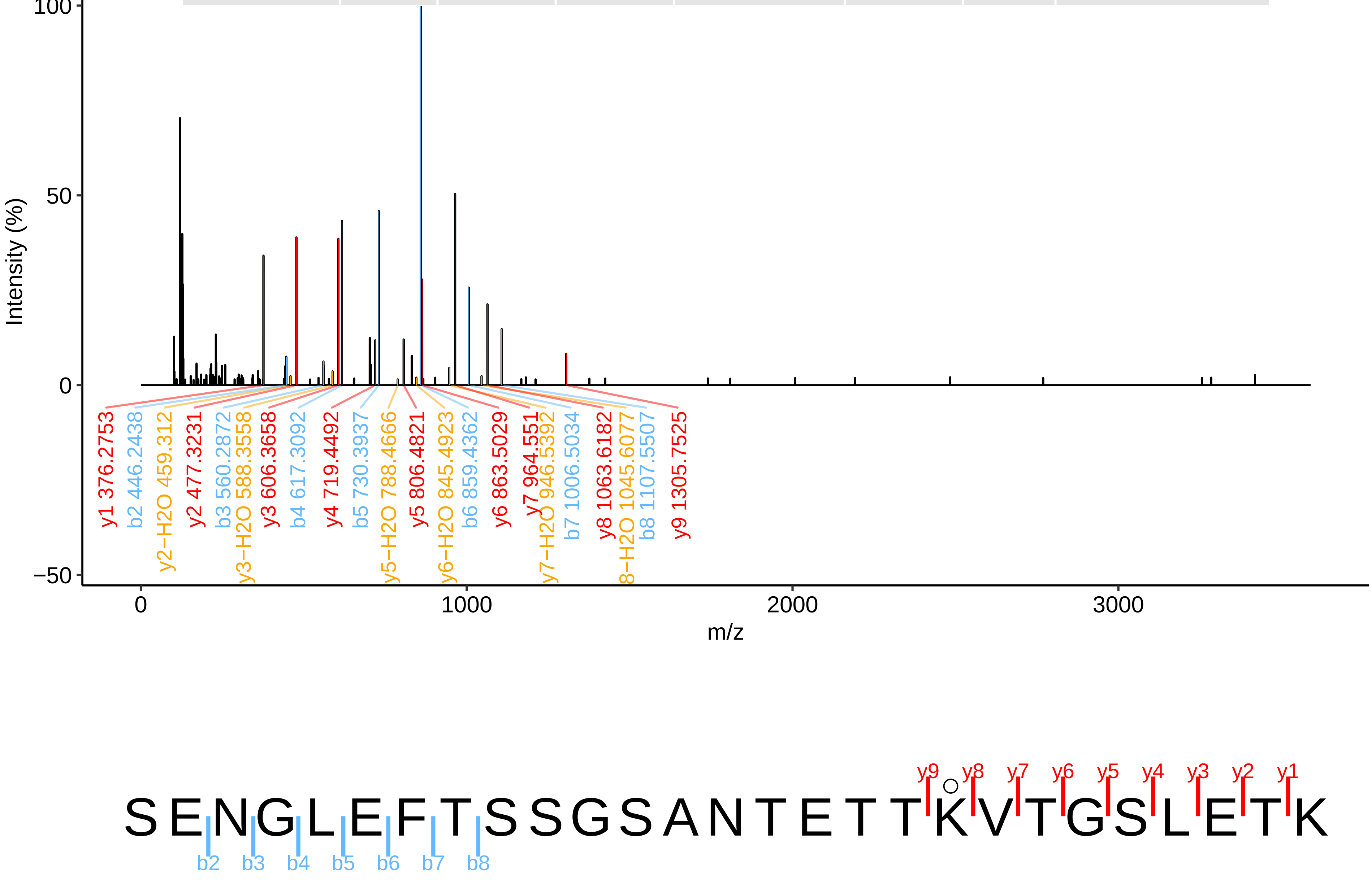

Modification □ Acetyl (Protein N−term) ○ GlyGly (K) no new terminus △ Oxidation (M)

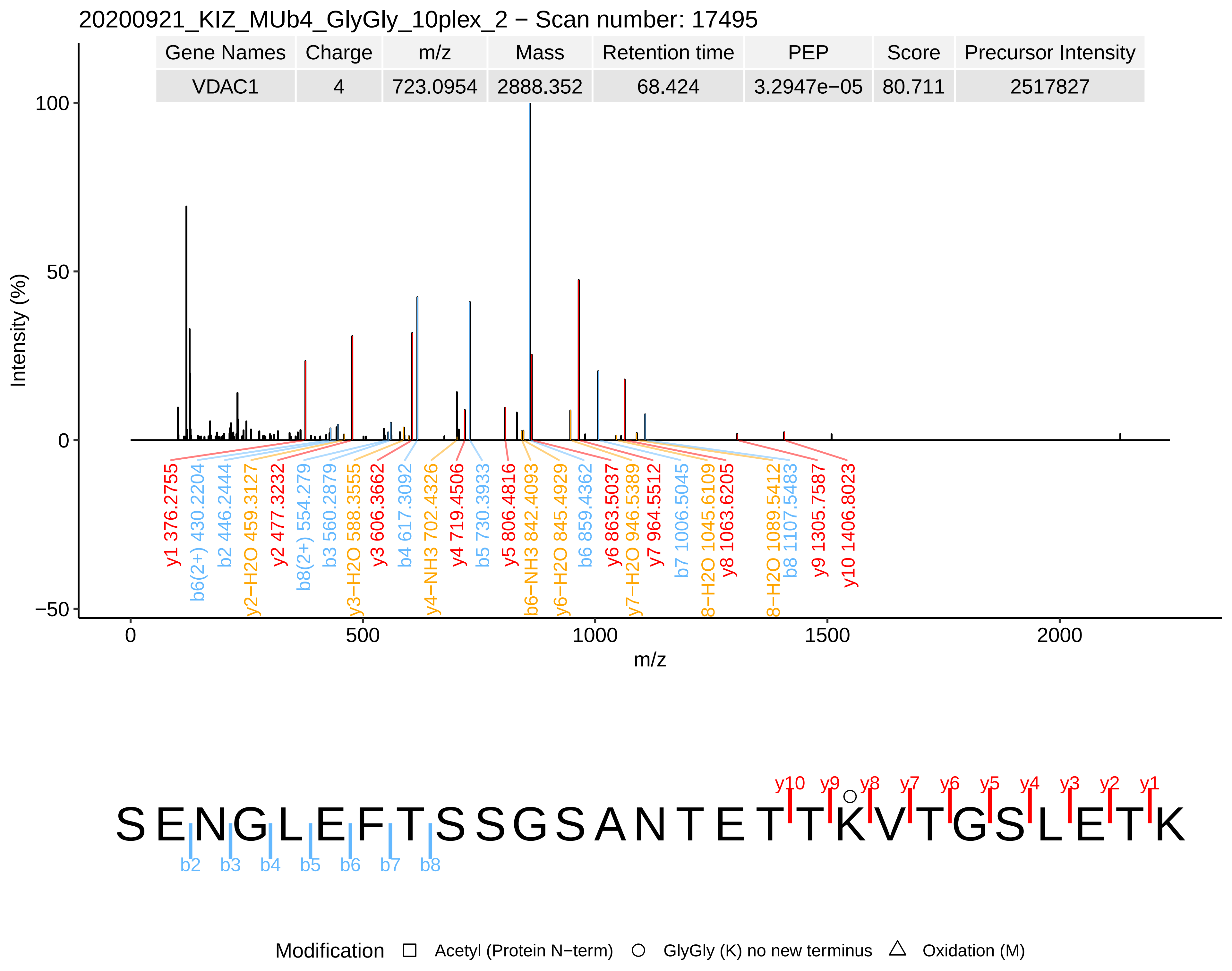
20200921_KIZ_MUb4_GlyGly_10plex_2 − Scan number: 17495
Gene Names
Charge
m/z
Mass
Retention time
PEP
Score
Precursor Intensity
VDAC1
4
723.0954
2888.352
68.424
3.2947e−05
80.711
2517827
Intensity (%)
100
50
0
−50
m/z
0
500
1000
1500
2000
y1 376.2755
b6(2+) 430.2204
b2 446.2444
y2−H2O 459.3127
y2 477.3232
b8(2+) 554.279
b3 560.2879
y3−H2O 588.3555
y3 606.3662
b4 617.3092
y4−NH3 702.4326
y4 719.4506
b5 730.3933
y5 806.4816
b6−NH3 842.4093
y6−H2O 845.4929
b6 859.4362
y6 863.5037
y7−H2O 946.5389
y7 964.5512
b7 1006.5045
8−H2O 1045.6109
y8 1063.6205
8−H2O 1089.5412
b8 1107.5483
y9 1305.7587
y10 1406.8023
SENGLEFTSSGSANTETTKVTGSLETK
y10 y9 y8 y7 y6 y5 y4 y3 y2 y1
b2 b3 b4 b5 b6 b7 b8
Modification
Acetyl (Protein N−term)
GlyGly (K) no new terminus
Oxidation (M)

20200921_KIZ_MUb4_GlyGly_10plex_2 − Scan number: 17634

Gene Names	Charge	m/z	Mass	Retention time	PEP	Score	Precursor Intensity
VDAC1	3	963.7914	2888.352	68.969	1.4377e−15	132.35	1774524

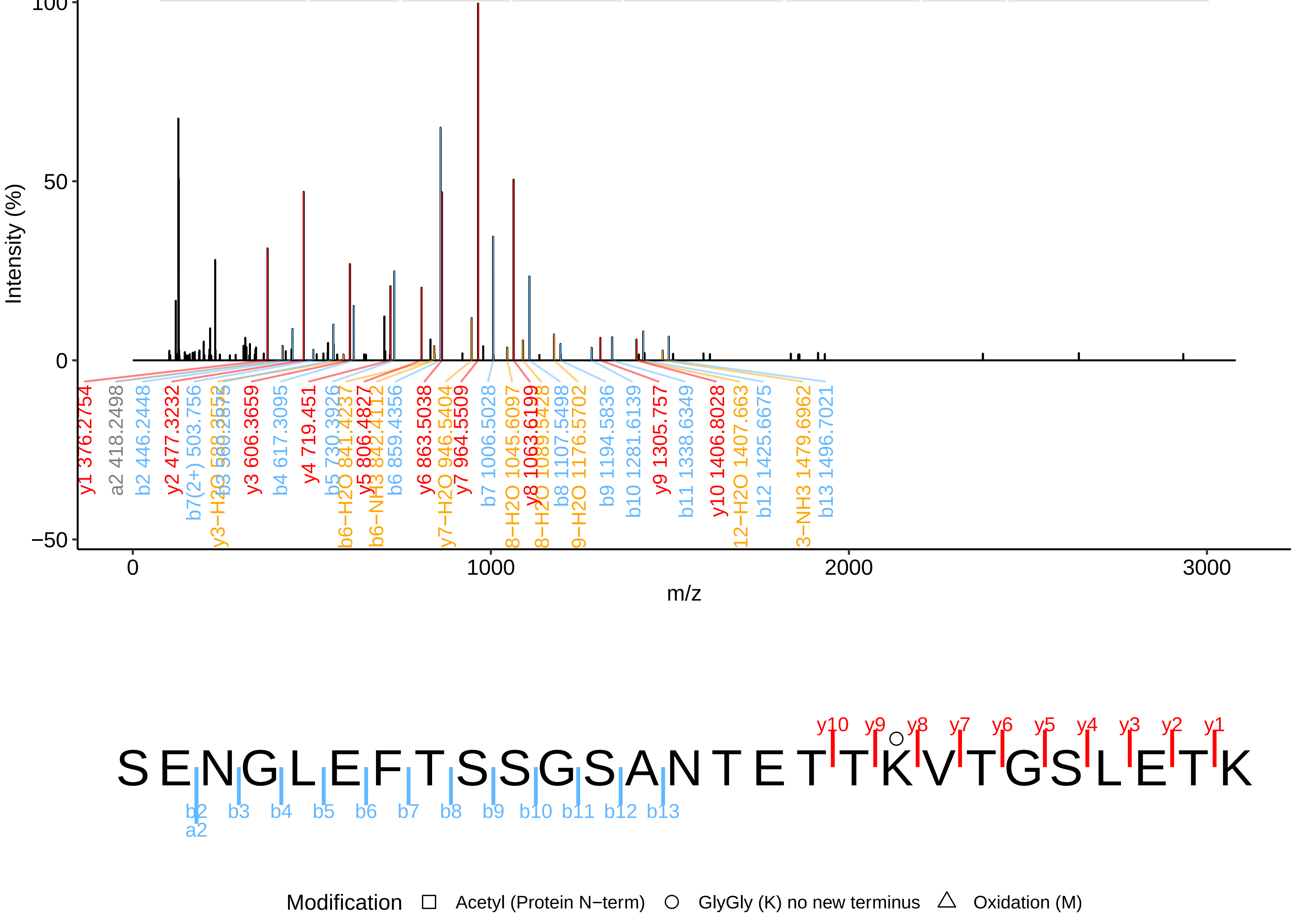

20200921_KIZ_MUb4_GlyGly_10plex_2 − Scan number: 17760

Gene Names	Charge	m/z	Mass	Retention time	PEP	Score	Precursor Intensity
VDAC1	3	963.7914	2888.352	69.48	2.4677e−05	87.339	1485904

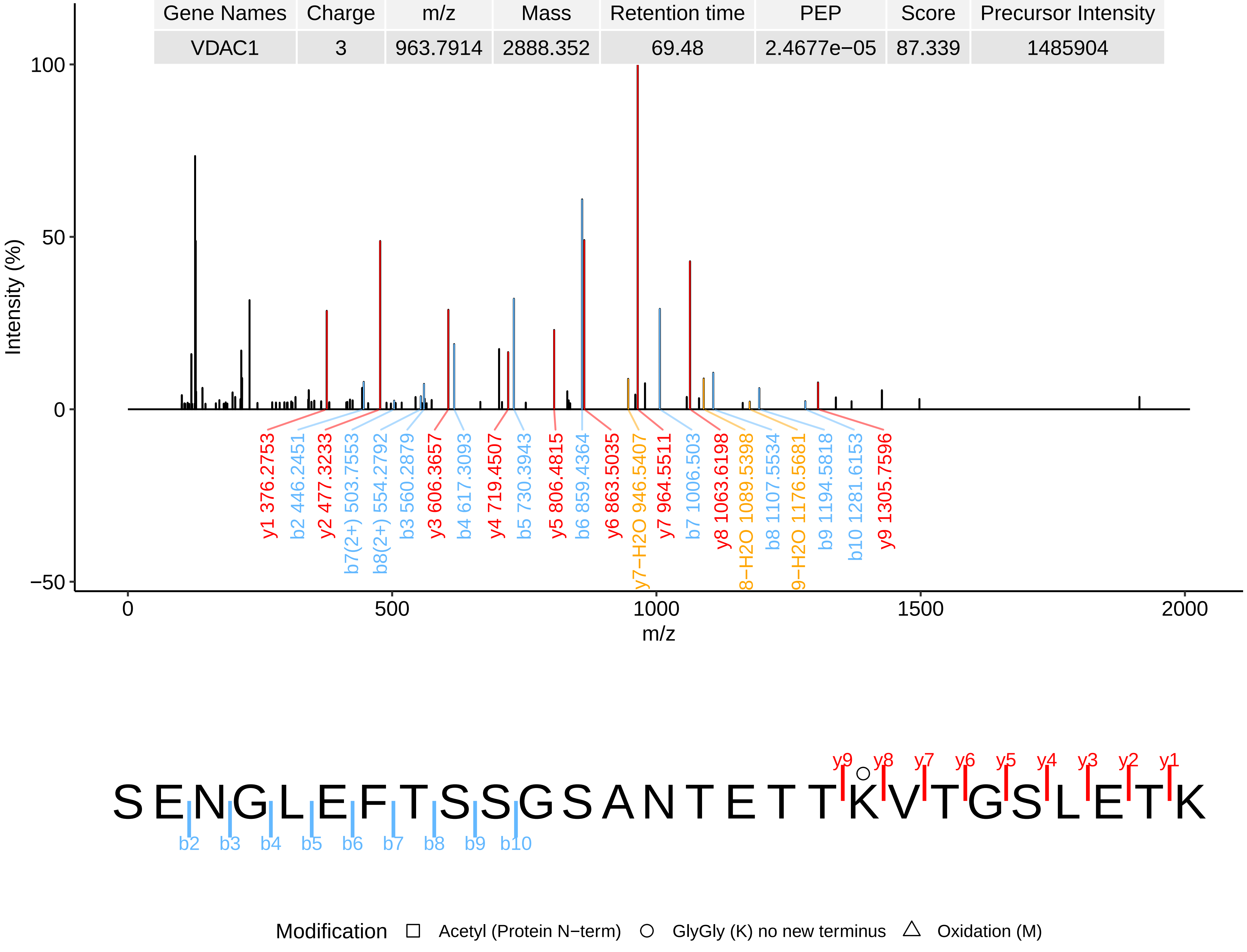

20200921_KIZ_MUb4_GlyGly_10plex_2 − Scan number: 17891

Gene Names	Charge	m/z	Mass	Retention time	PEP	Score	Precursor Intensity
VDAC1	3	963.7914	2888.352	70.008	0.0020549	73.805	1159291

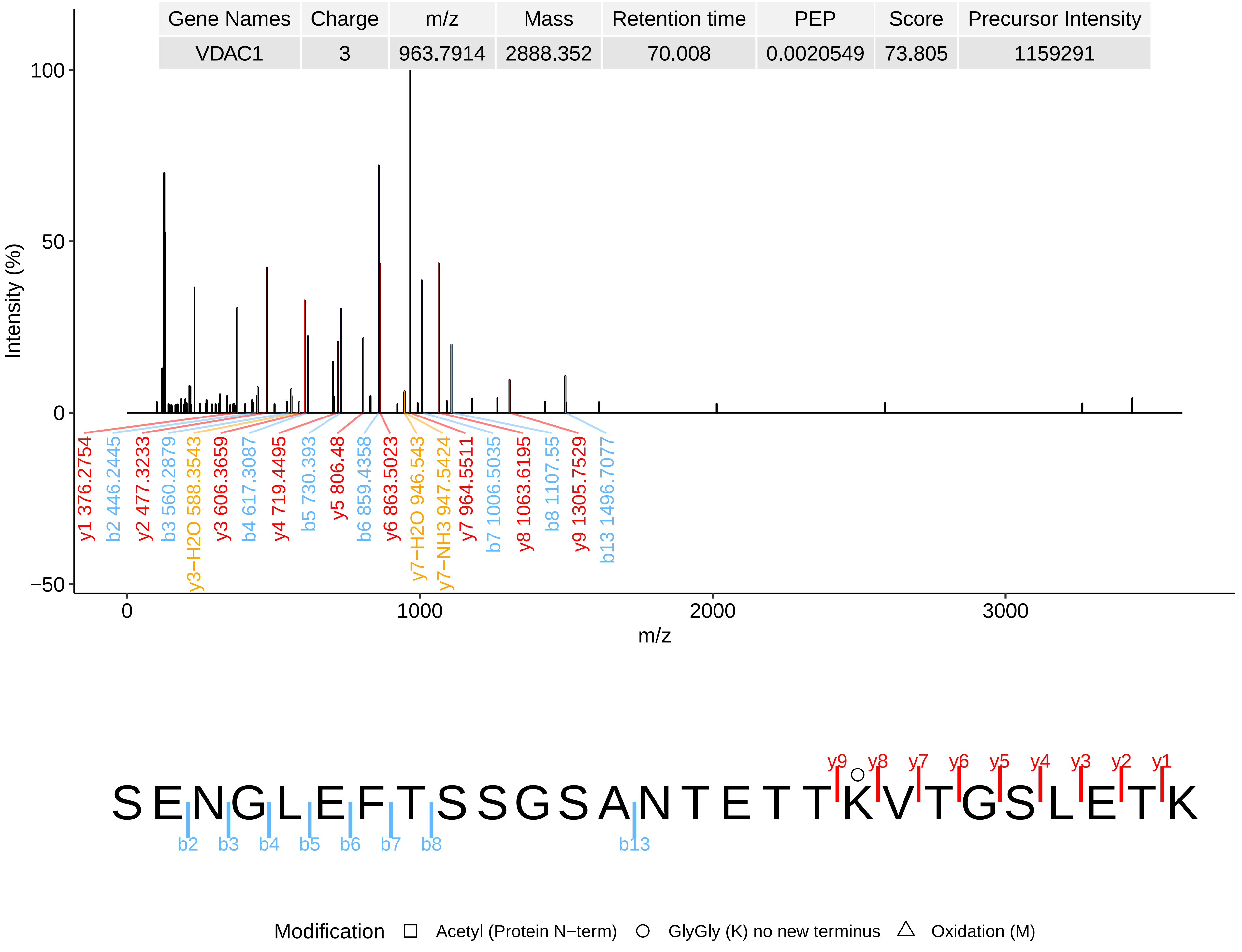

20200921_KIZ_MUb4_GlyGly_10plex_2 − Scan number: 17883

Gene Names	Charge	m/z	Mass	Retention time	PEP	Score	Precursor Intensity
VDAC1	4	723.0954	2888.352	69.976	0.015656	57.21	863843.5

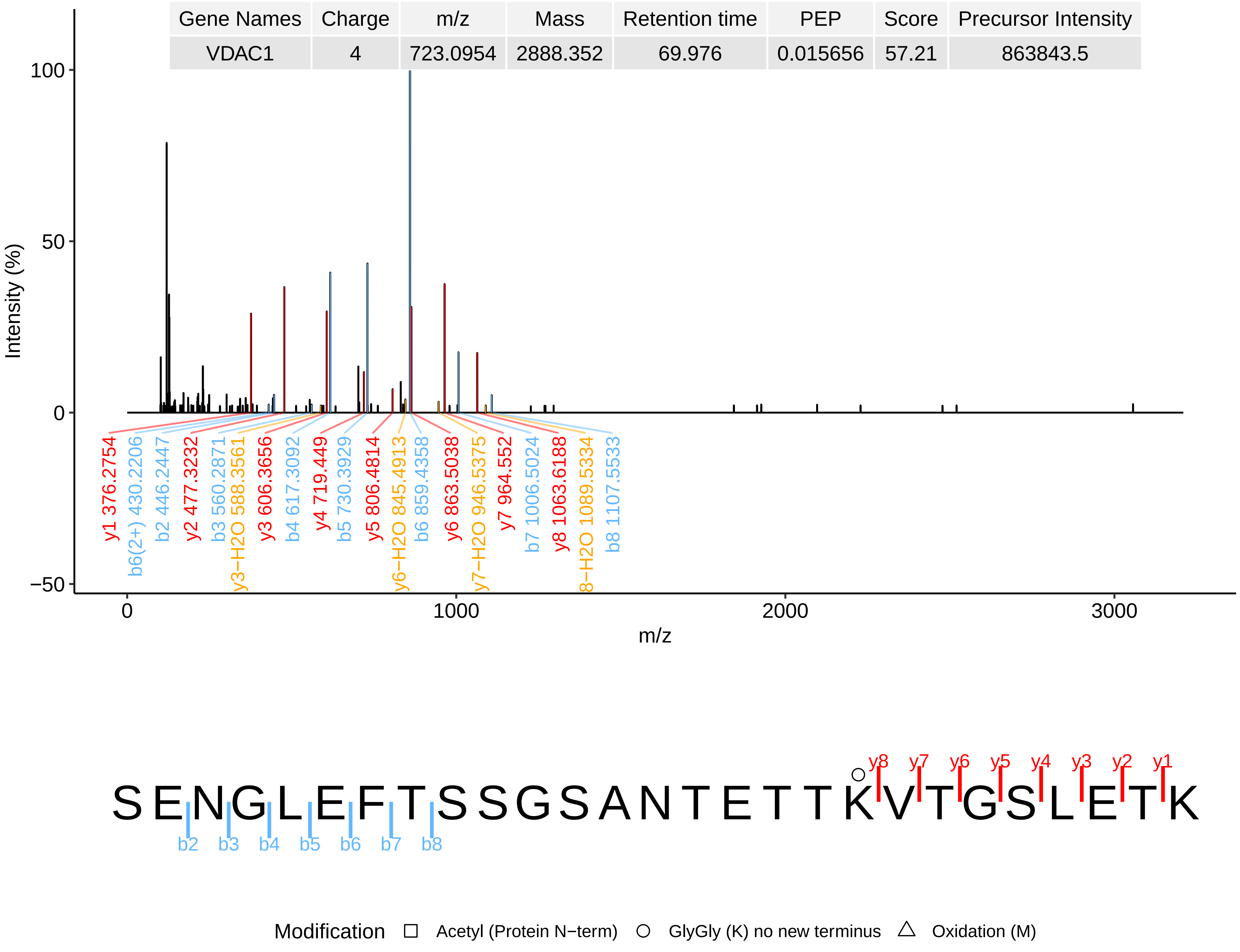

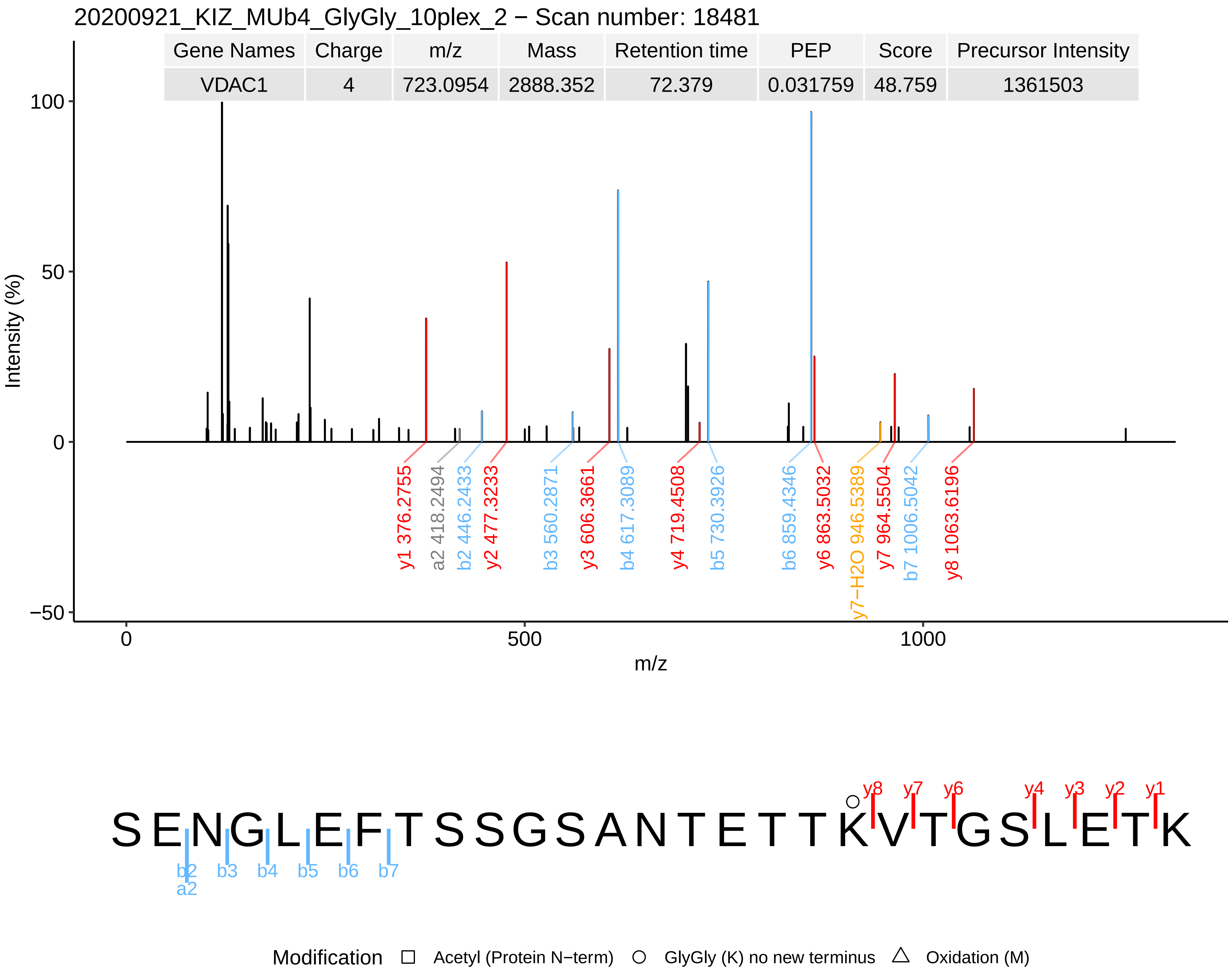
20200921_KIZ_MUb4_GlyGly_10plex_2 − Scan number: 18481
Gene Names | Charge | m/z | Mass | Retention time | PEP | Score | Precursor Intensity
VDAC1 | 4 | 723.0954 | 2888.352 | 72.379 | 0.031759 | 48.759 | 1361503
Intensity (%)
100
50
0
−50
0
500
1000
m/z
y1 376.2755
a2 418.2494
b2 446.2433
y2 477.3233
b3 560.2871
y3 606.3661
b4 617.3089
y4 719.4508
b5 730.3926
b6 859.4346
y6 863.5032
y7−H2O 946.5389
y7 964.5504
b7 1006.5042
y8 1063.6196
S E N G L E F T S S G S A N T E T T K V T G S L E T K
y8 y7 y6 y4 y3 y2 y1
b2 b3 b4 b5 b6 b7
a2
Modification
Acetyl (Protein N−term)
GlyGly (K) no new terminus
Oxidation (M)

20200921_KIZ_MUb4_GlyGly_10plex_2 − Scan number: 18509

Gene Names	Charge	m/z	Mass	Retention time	PEP	Score	Precursor Intensity
VDAC1	4	723.0954	2888.352	72.494	0.0030558	68.231	460148.2

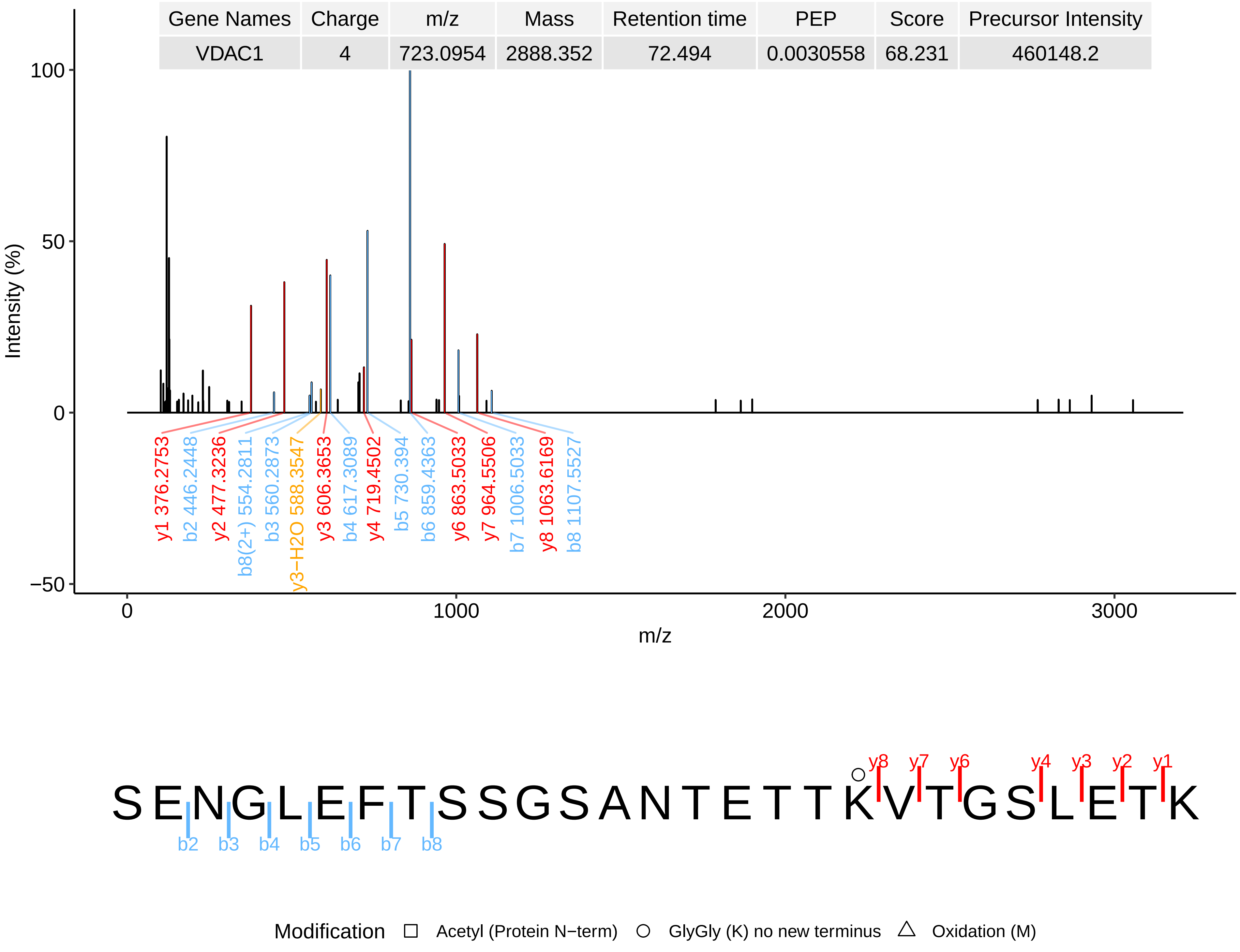

20200921_KIZ_MUb4_GlyGly_10plex_2 − Scan number: 18566

Gene Names	Charge	m/z	Mass	Retention time	PEP	Score	Precursor Intensity
VDAC1	3	963.7914	2888.352	72.73	0.0039789	67.33	591581.6

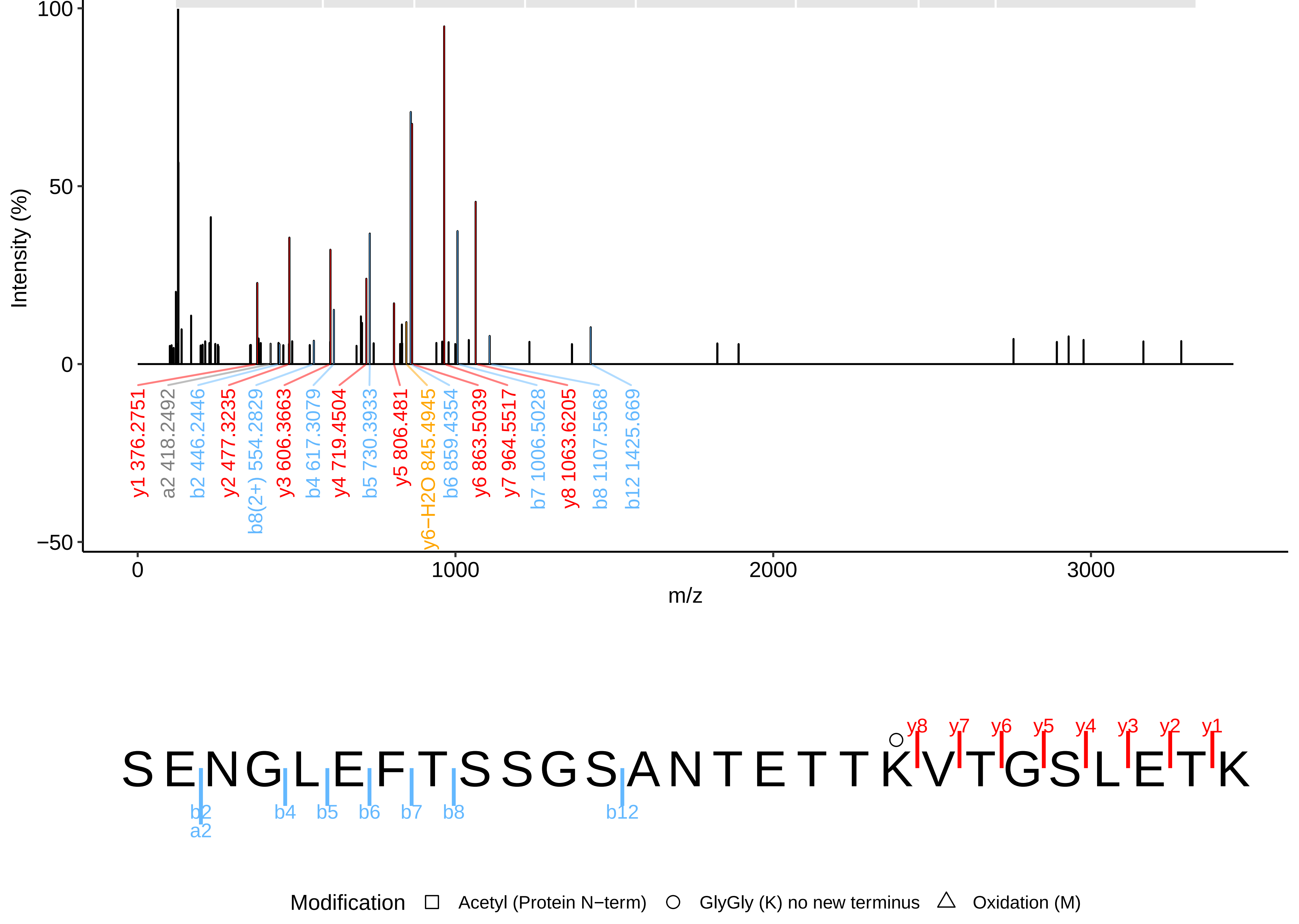

20200921_KIZ_MUb4_GlyGly_10plex_2 − Scan number: 19213

Gene Names	Charge	m/z	Mass	Retention time	PEP	Score	Precursor Intensity
VDAC1	3	963.7914	2888.352	75.384	0.012235	60.139	461353.9

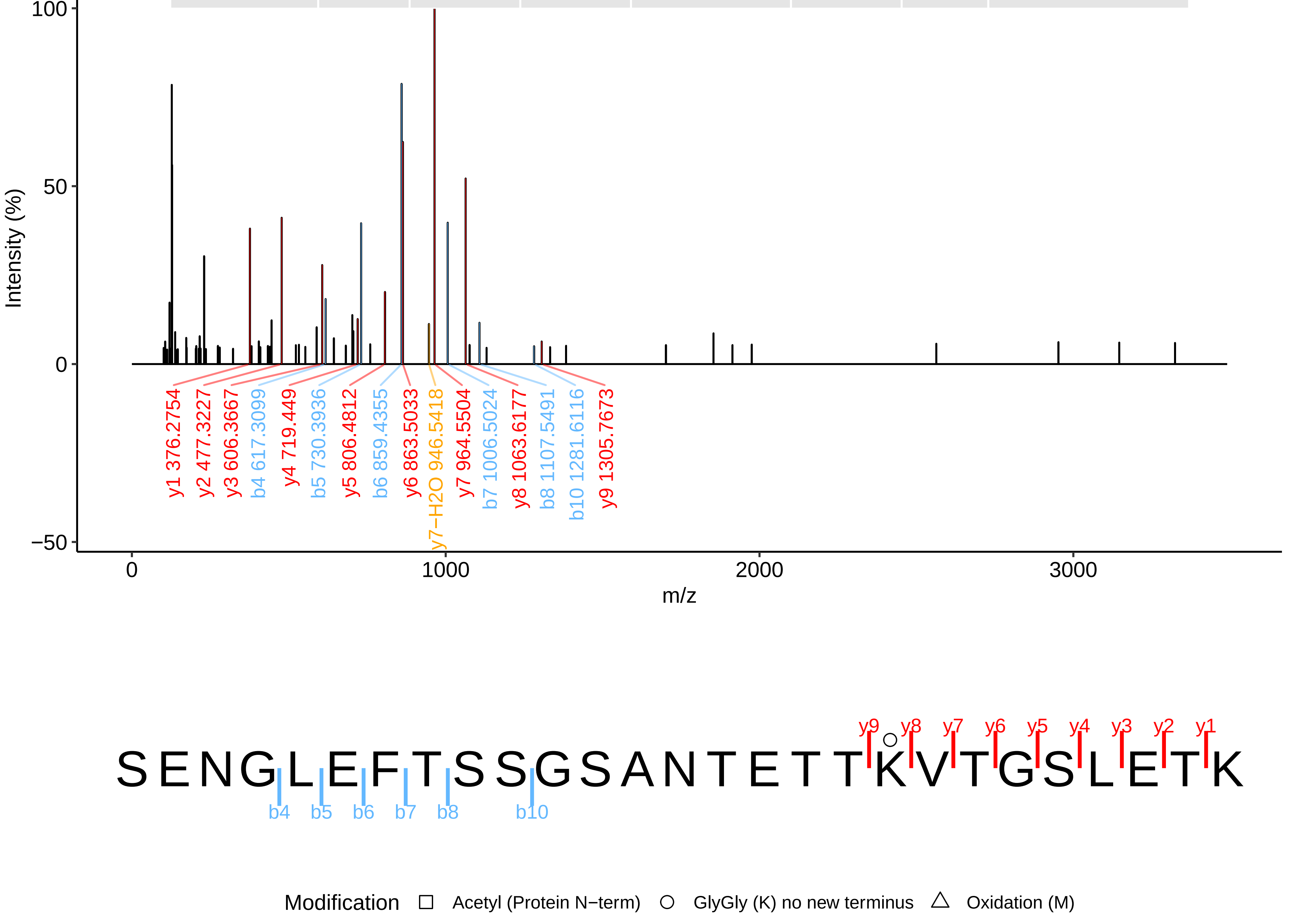

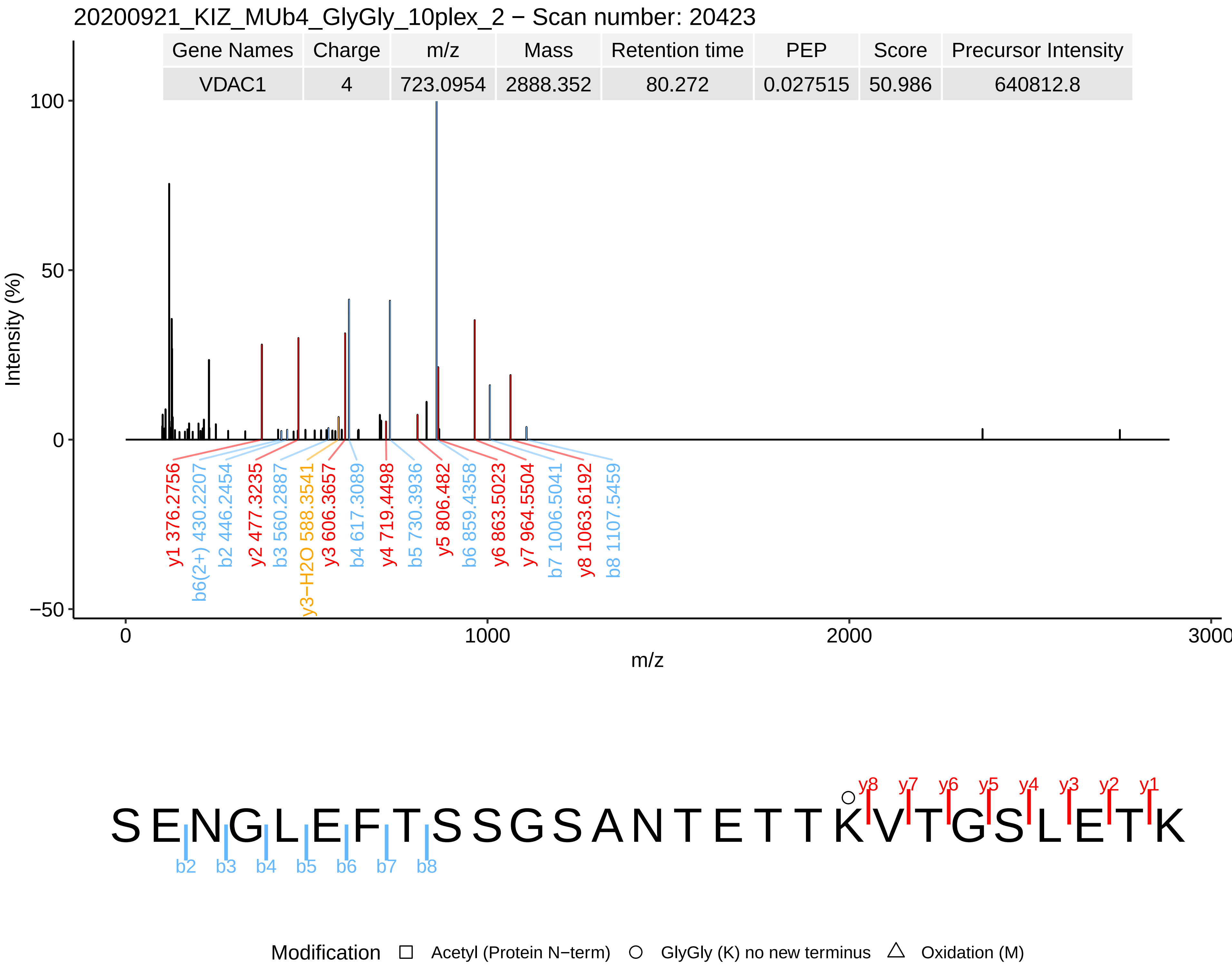
20200921_KIZ_MUb4_GlyGly_10plex_2 − Scan number: 20423
Gene Names | Charge | m/z | Mass | Retention time | PEP | Score | Precursor Intensity
VDAC1 | 4 | 723.0954 | 2888.352 | 80.272 | 0.027515 | 50.986 | 640812.8
Intensity (%)
100
50
0
−50
0
1000
2000
3000
m/z
y1 376.2756
b6(2+) 430.2207
b2 446.2454
y2 477.3235
b3 560.2887
y3−H2O 588.3541
y3 606.3657
b4 617.3089
y4 719.4498
b5 730.3936
y5 806.482
b6 859.4358
y6 863.5023
y7 964.5504
b7 1006.5041
y8 1063.6192
b8 1107.5459
y8 y7 y6 y5 y4 y3 y2 y1
S E N G L E F T S S G S A N T E T T K V T G S L E T K
b2 b3 b4 b5 b6 b7 b8
Modification
Acetyl (Protein N−term)
GlyGly (K) no new terminus
Oxidation (M)

20200921_KIZ_MUb4_GlyGly_10plex_2 − Scan number: 21378

Gene Names	Charge	m/z	Mass	Retention time	PEP	Score	Precursor Intensity
VDAC1	3	963.7914	2888.352	84.168	0.029433	52.707	799661.6

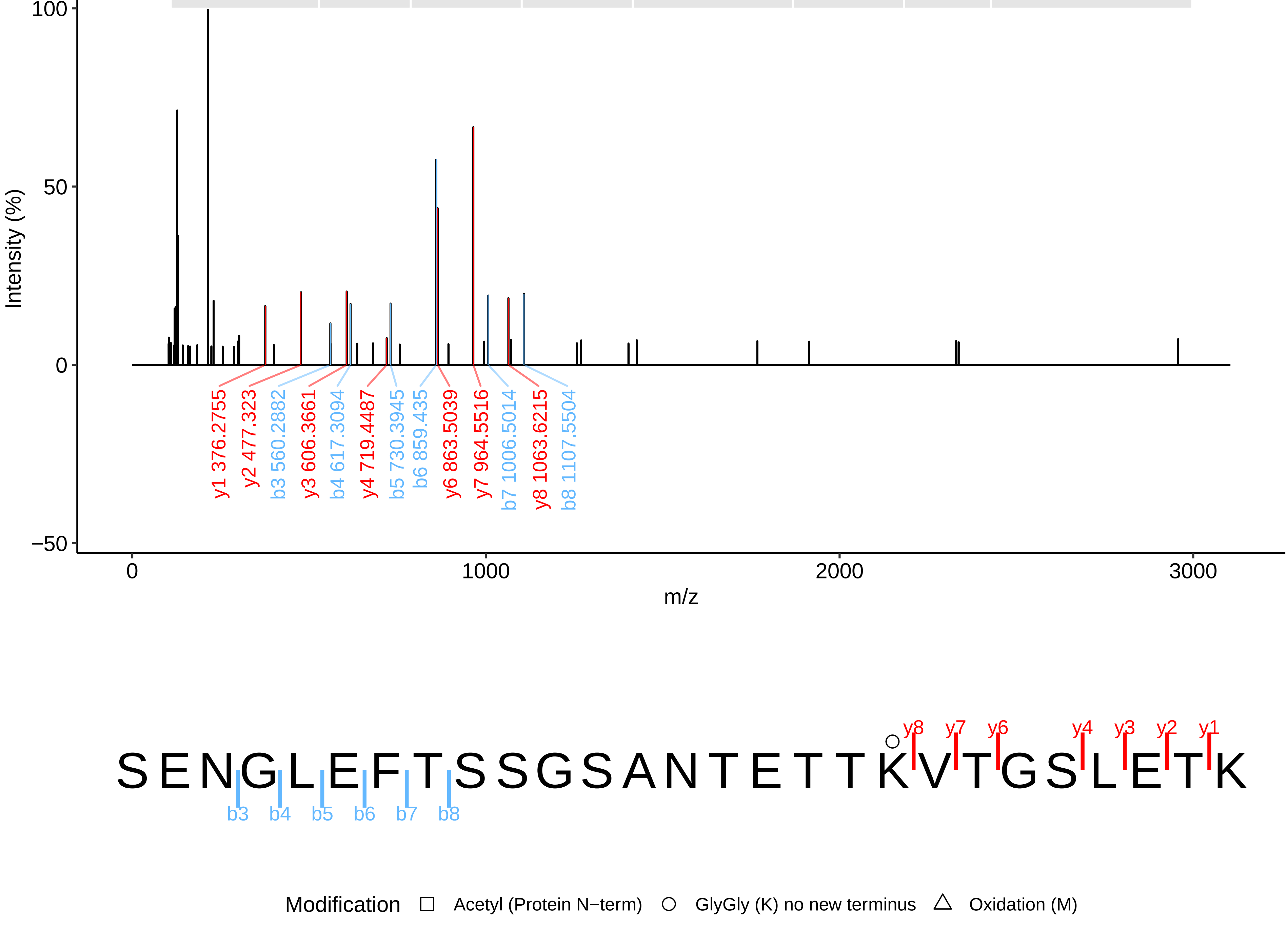

20200921_KIZ_MUb4_GlyGly_10plex_2 − Scan number: 22282

Gene Names	Charge	m/z	Mass	Retention time	PEP	Score	Precursor Intensity
VDAC1	3	963.7914	2888.352	87.817	0.030307	52.329	371106.9

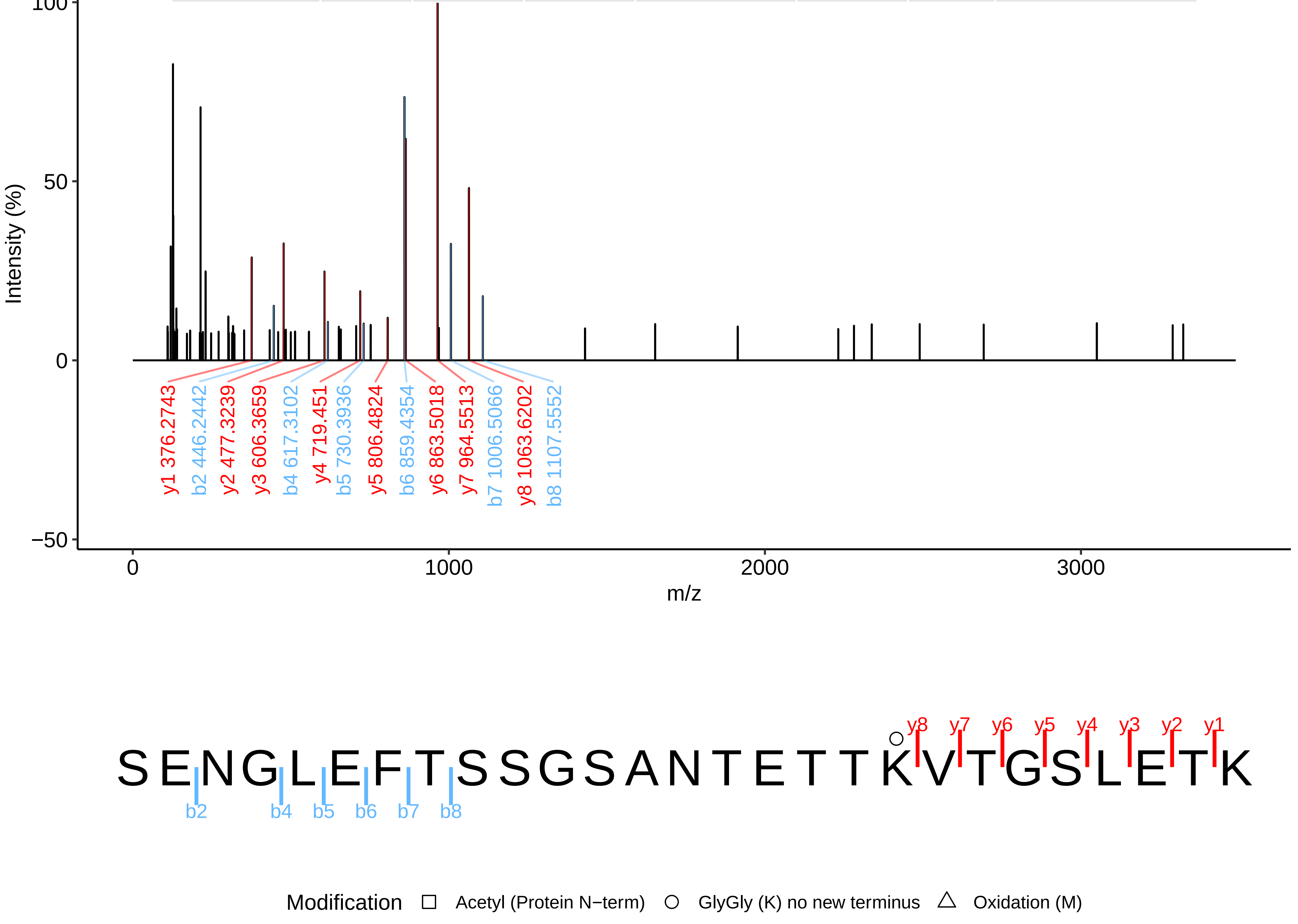

20200921_KIZ_MUb4_GlyGly_10plex_2 − Scan number: 28239

Gene Names	Charge	m/z	Mass	Retention time	PEP	Score	Precursor Intensity
VDAC1	4	723.0954	2888.352	112.02	0.033359	47.919	831450.5

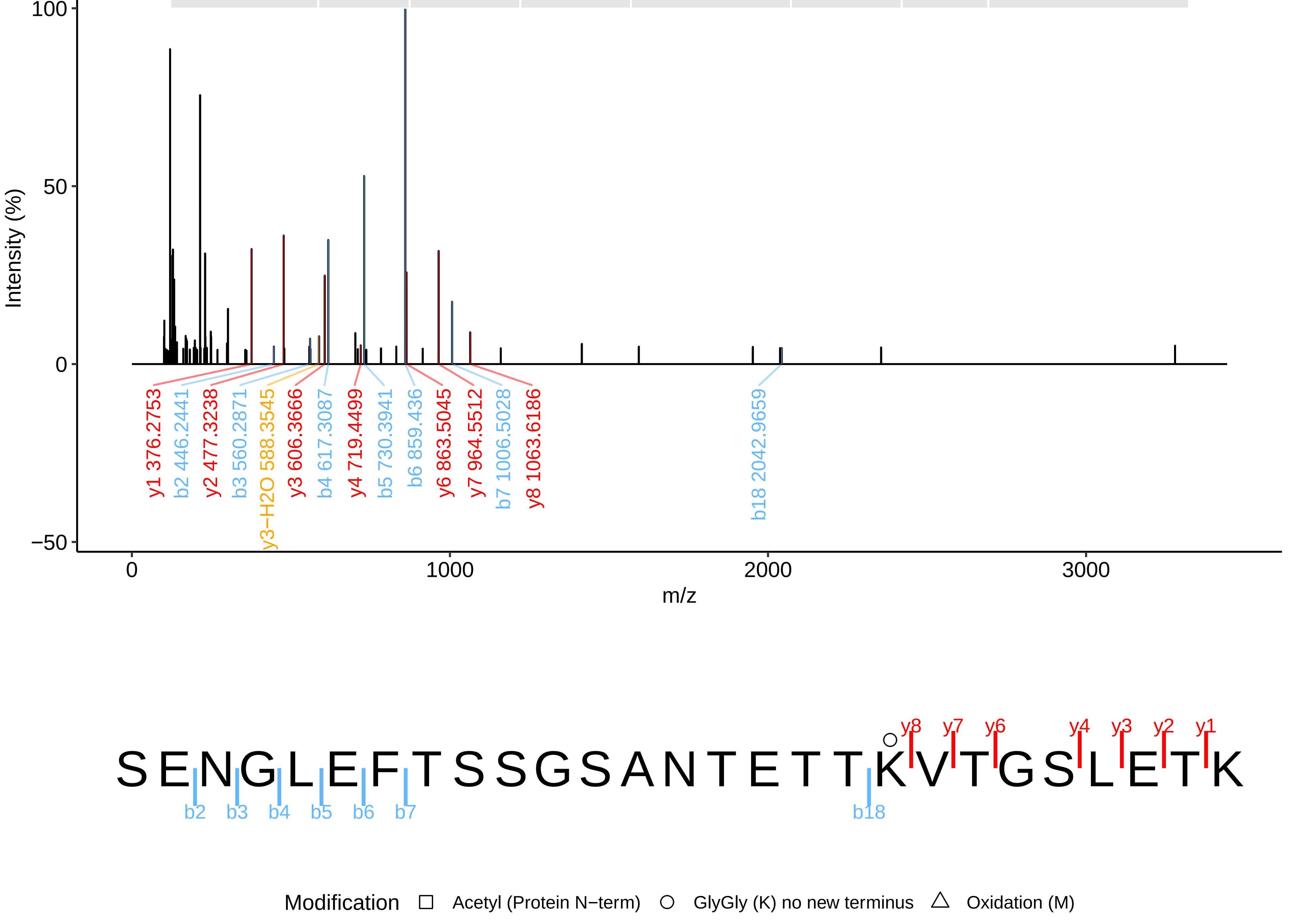

20200921_KIZ_MUb4_GlyGly_10plex_2 − Scan number: 33001

Gene Names	Charge	m/z	Mass	Retention time	PEP	Score	Precursor Intensity
VDAC1	4	723.0954	2888.352	129.71	0.0010204	76.55	8413593

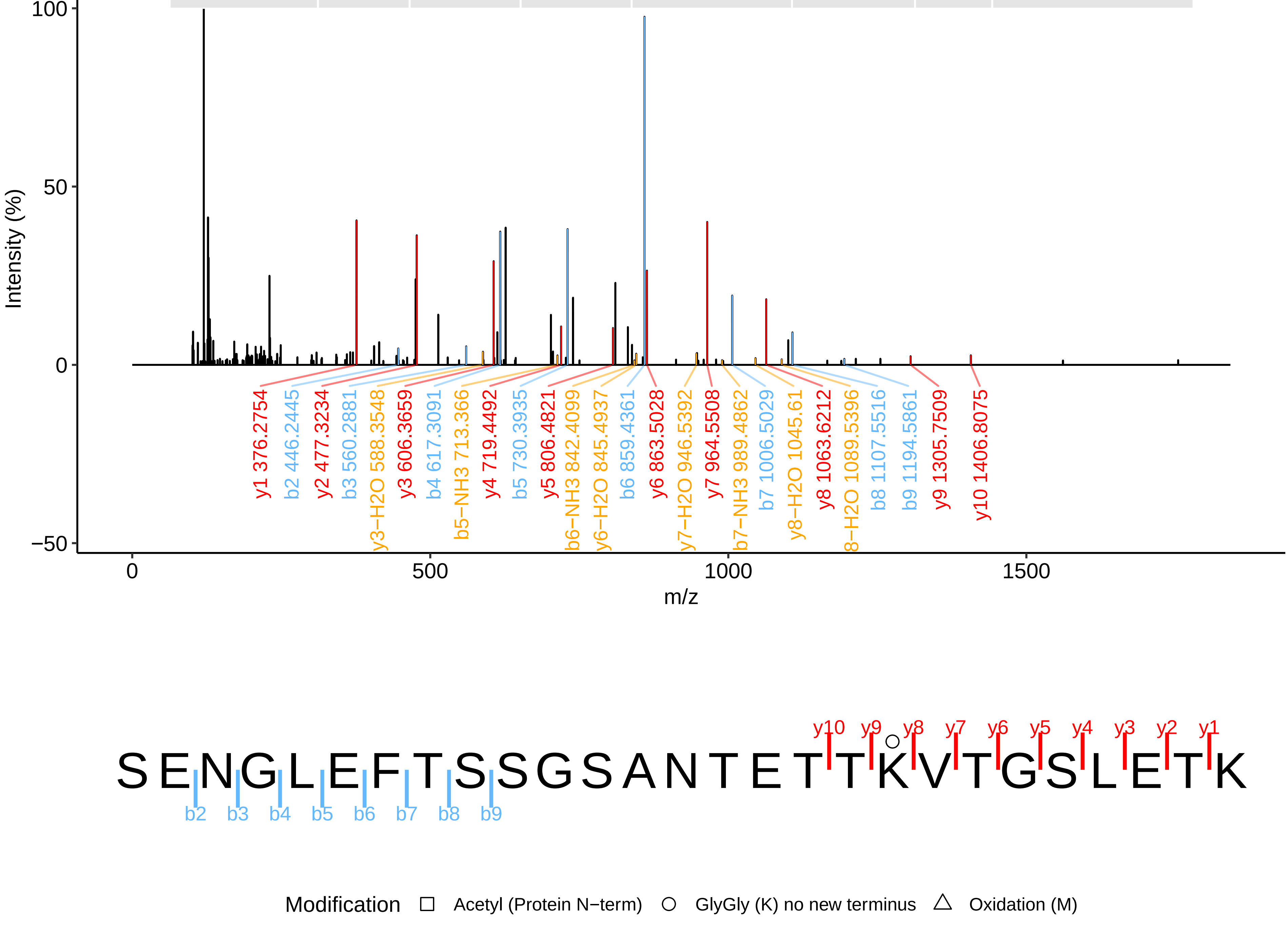

20200921_KIZ_MUb4_GlyGly_10plex_2 − Scan number: 18402

Gene Names	Charge	m/z	Mass	Retention time	PEP	Score	Precursor Intensity
VDAC1	3	963.7914	2888.352	72.056	1.0158e−08	81.839	427243.3

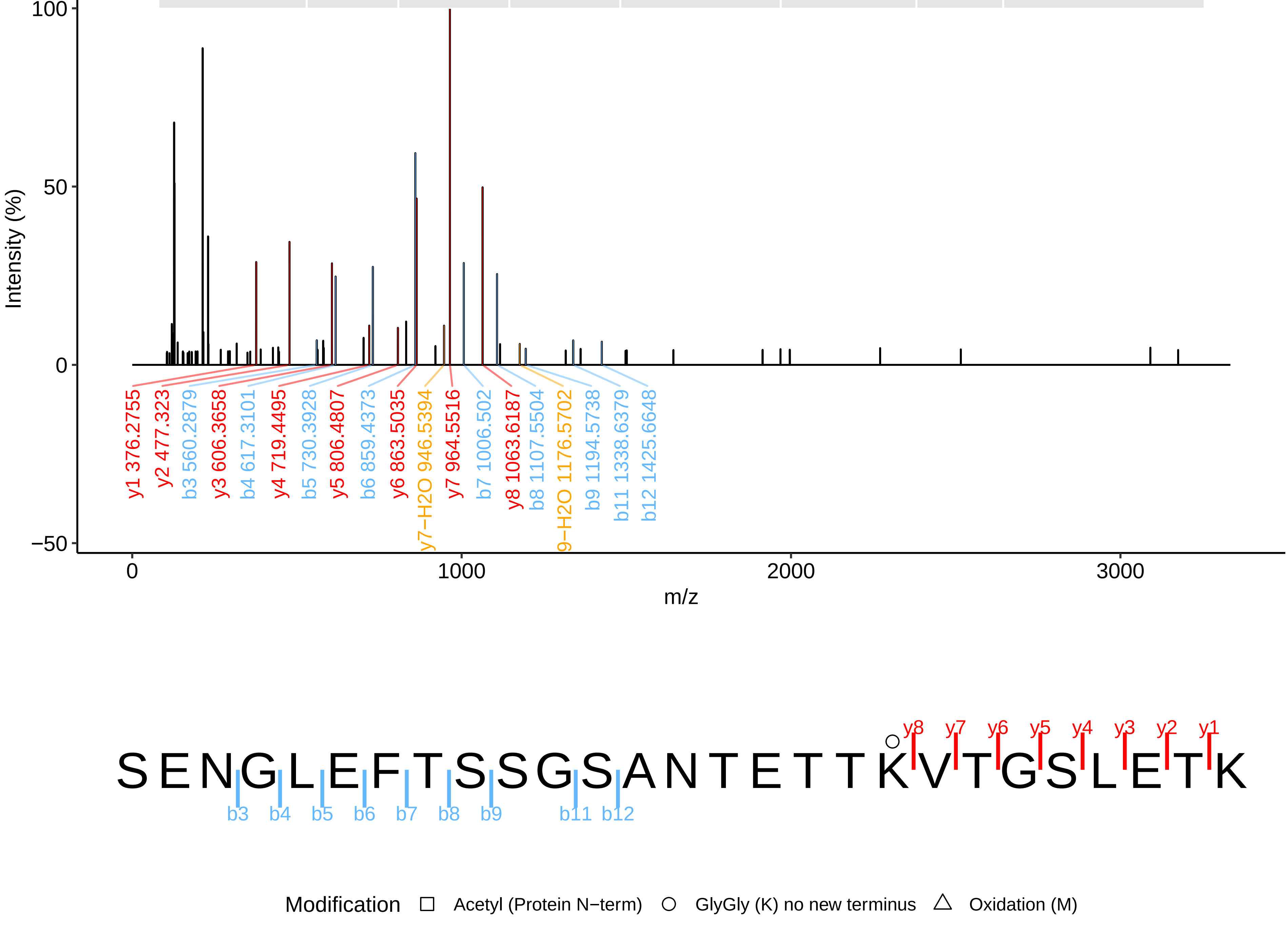

20200921_KIZ_MUb4_GlyGly_10plex_2 − Scan number: 19298

Gene Names	Charge	m/z	Mass	Retention time	PEP	Score	Precursor Intensity
VDAC1	3	963.7914	2888.352	75.732	1.1138e−05	74.206	NA

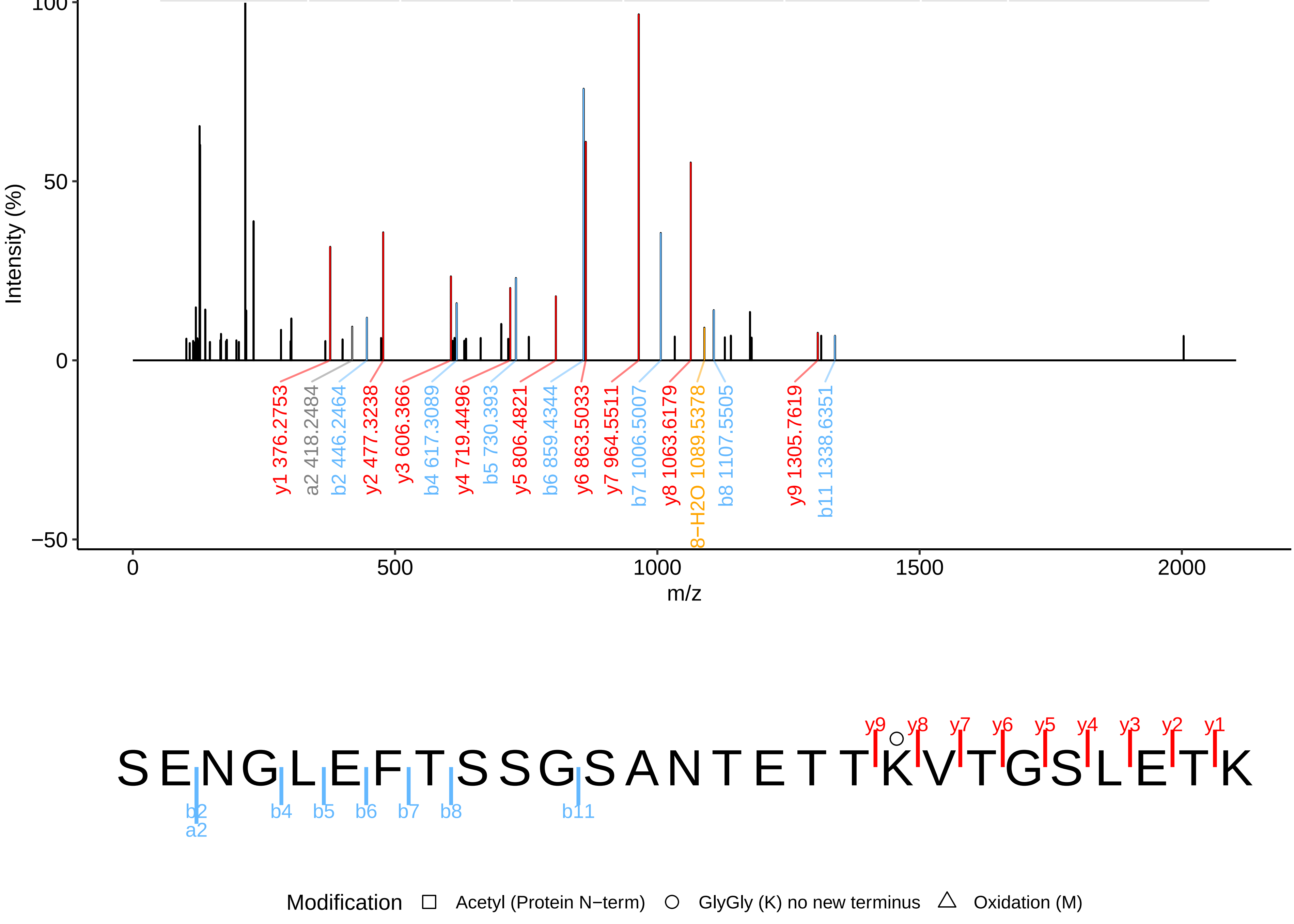

20200921_KIZ_MUb4_GlyGly_10plex_2 − Scan number: 20628

Gene Names	Charge	m/z	Mass	Retention time	PEP	Score	Precursor Intensity
VDAC1	3	963.7914	2888.352	81.105	0.010606	48.337	669550.1

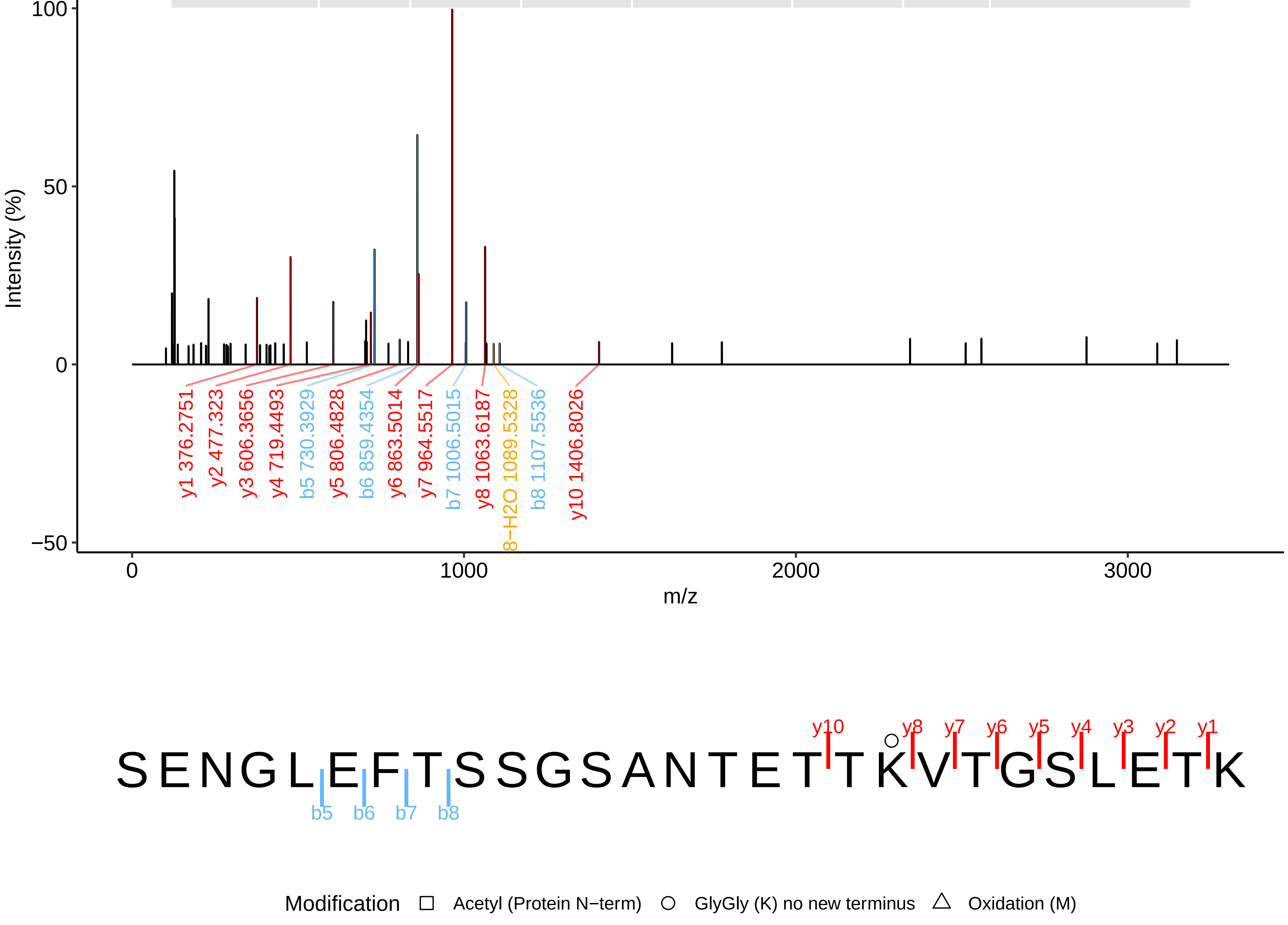

20200921_KIZ_MUb4_GlyGly_10plex_2 − Scan number: 25157

Gene Names	Charge	m/z	Mass	Retention time	PEP	Score	Precursor Intensity
VDAC1	3	963.7914	2888.352	99.482	0.013887	45.269	295324.2

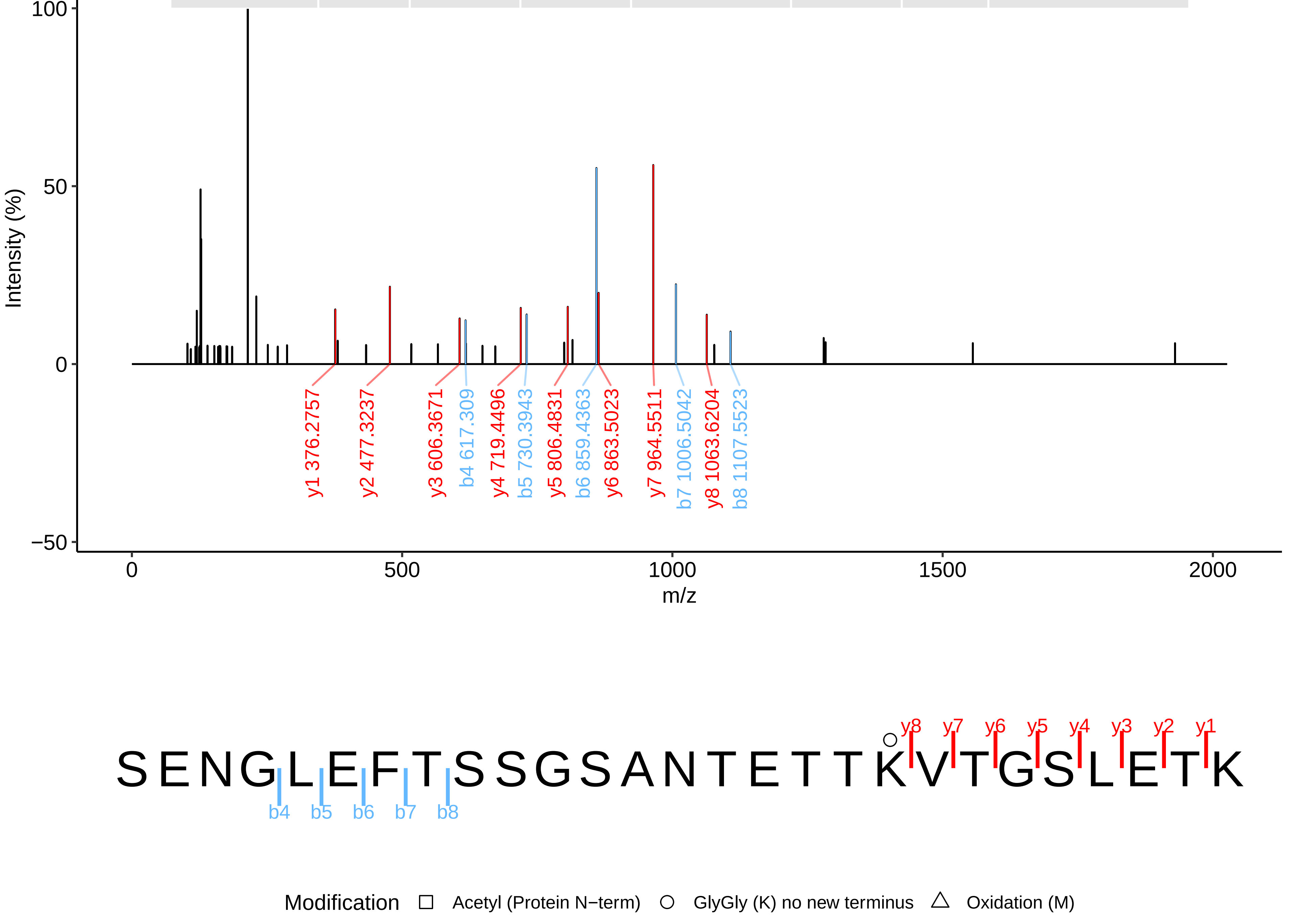

20200921_KIZ_MUb4_GlyGly_10plex_2 − Scan number: 27286

Gene Names	Charge	m/z	Mass	Retention time	PEP	Score	Precursor Intensity
VDAC1	3	963.7914	2888.352	108.18	0.0068405	53.475	NA

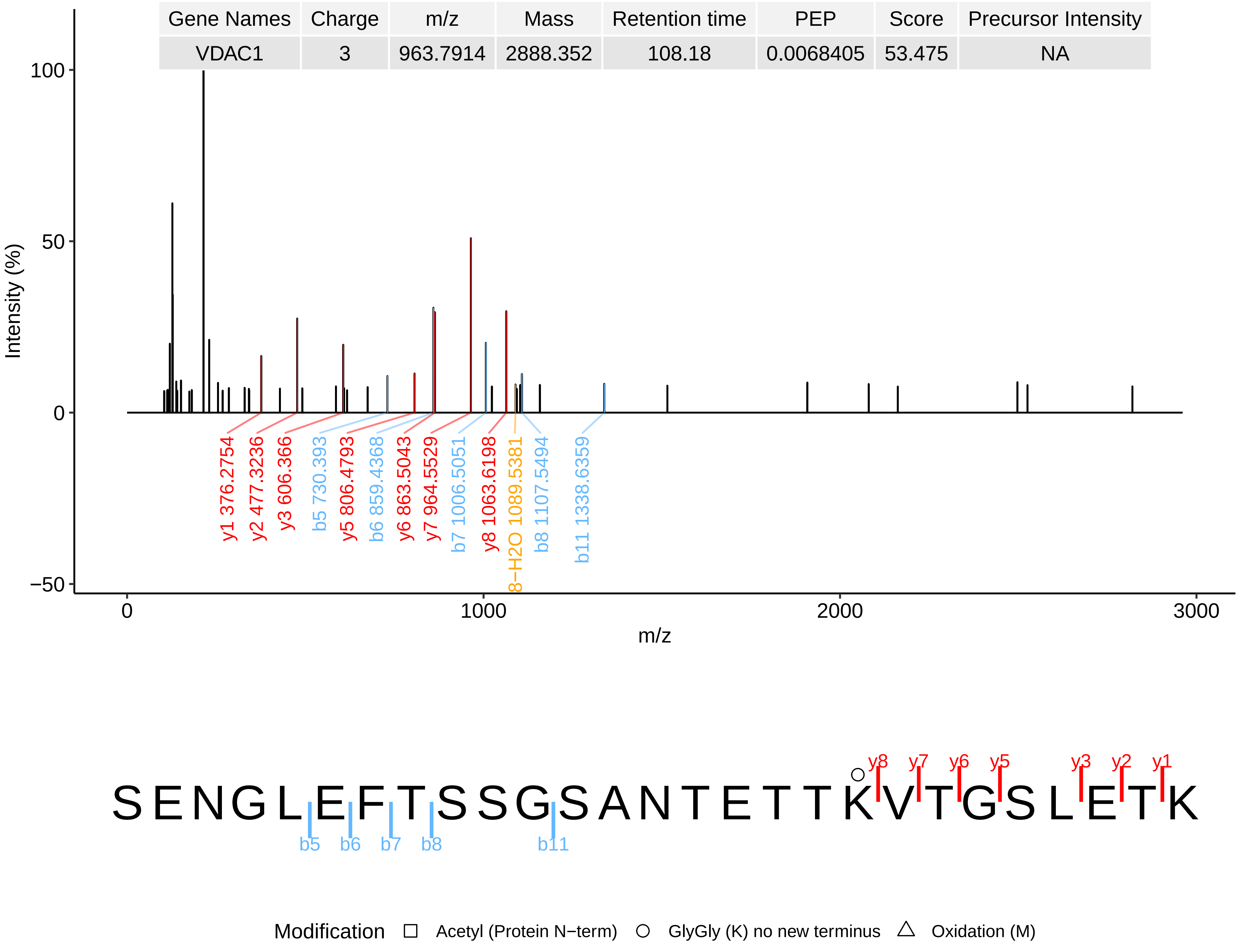

20200924_KIZ_MUb5_GlyGly_1 − Scan number: 18808

Gene Names	Charge	m/z	Mass	Retention time	PEP	Score	Precursor Intensity
VDAC1	4	723.0954	2888.352	60.778	2.1348e−30	151.91	20238294

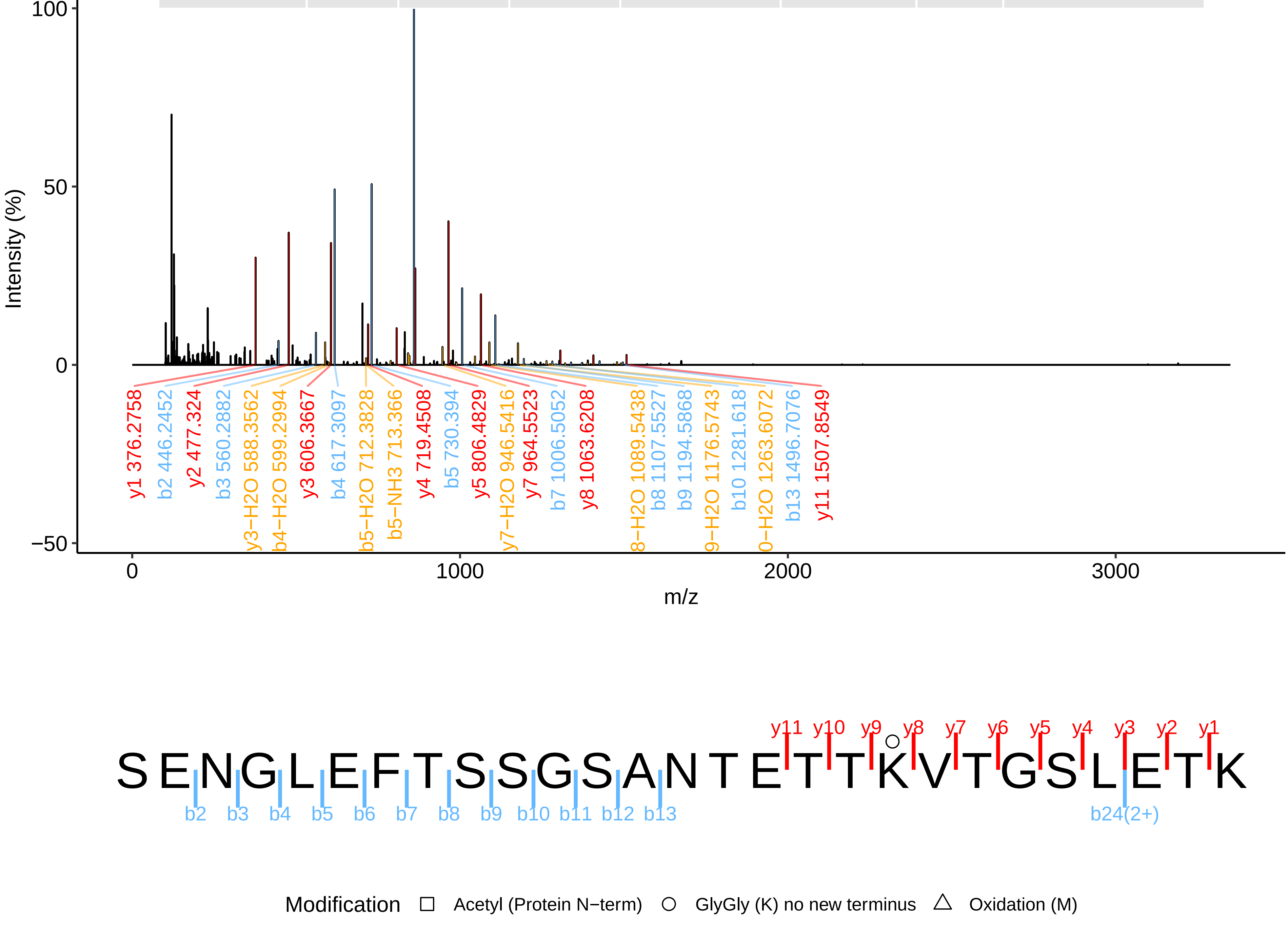

20200924_KIZ_MUb5_GlyGly_1 − Scan number: 18960

Gene Names	Charge	m/z	Mass	Retention time	PEP	Score	Precursor Intensity
VDAC1	3	963.7914	2888.352	61.269	9.6108e−68	209.16	10050890

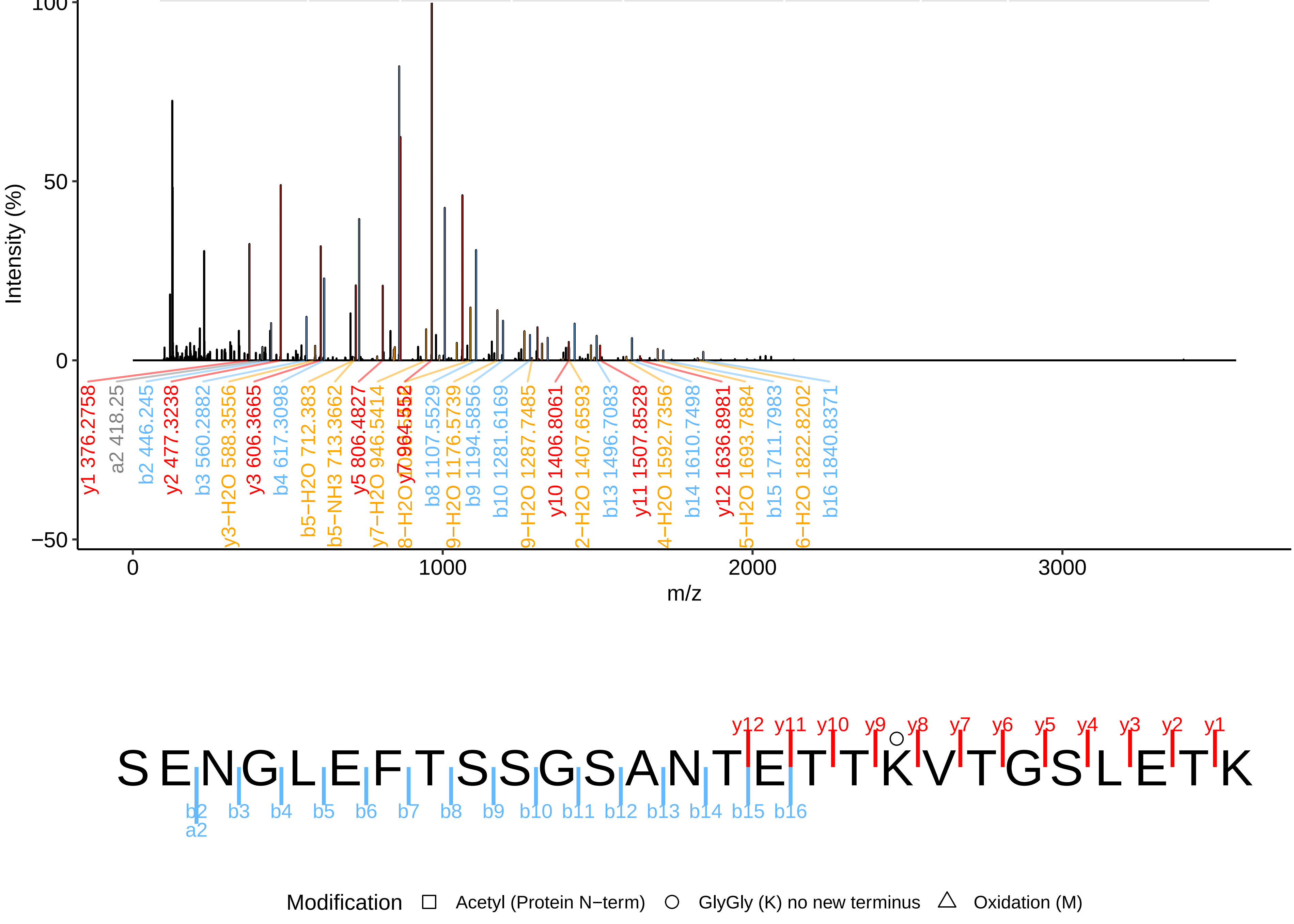

20200924_KIZ_MUb5_GlyGly_1 − Scan number: 18963

Gene Names	Charge	m/z	Mass	Retention time	PEP	Score	Precursor Intensity
VDAC1	3	963.7914	2888.352	61.279	4.5344e−69	219.38	13231479

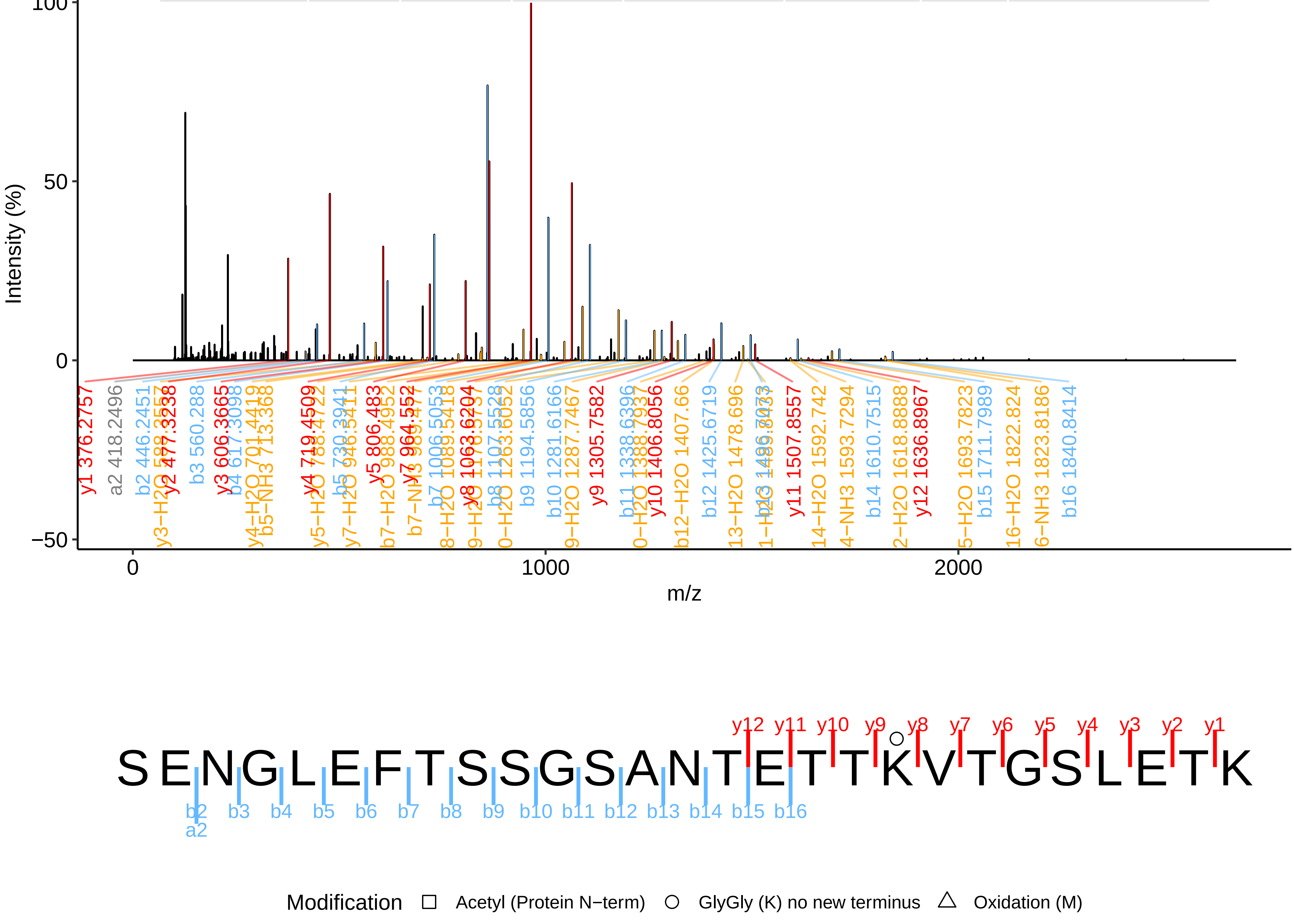

20200924_KIZ_MUb5_GlyGly_1 − Scan number: 19114

Gene Names	Charge	m/z	Mass	Retention time	PEP	Score	Precursor Intensity
VDAC1	3	963.7914	2888.352	61.789	1.1181e−98	246.21	14542877

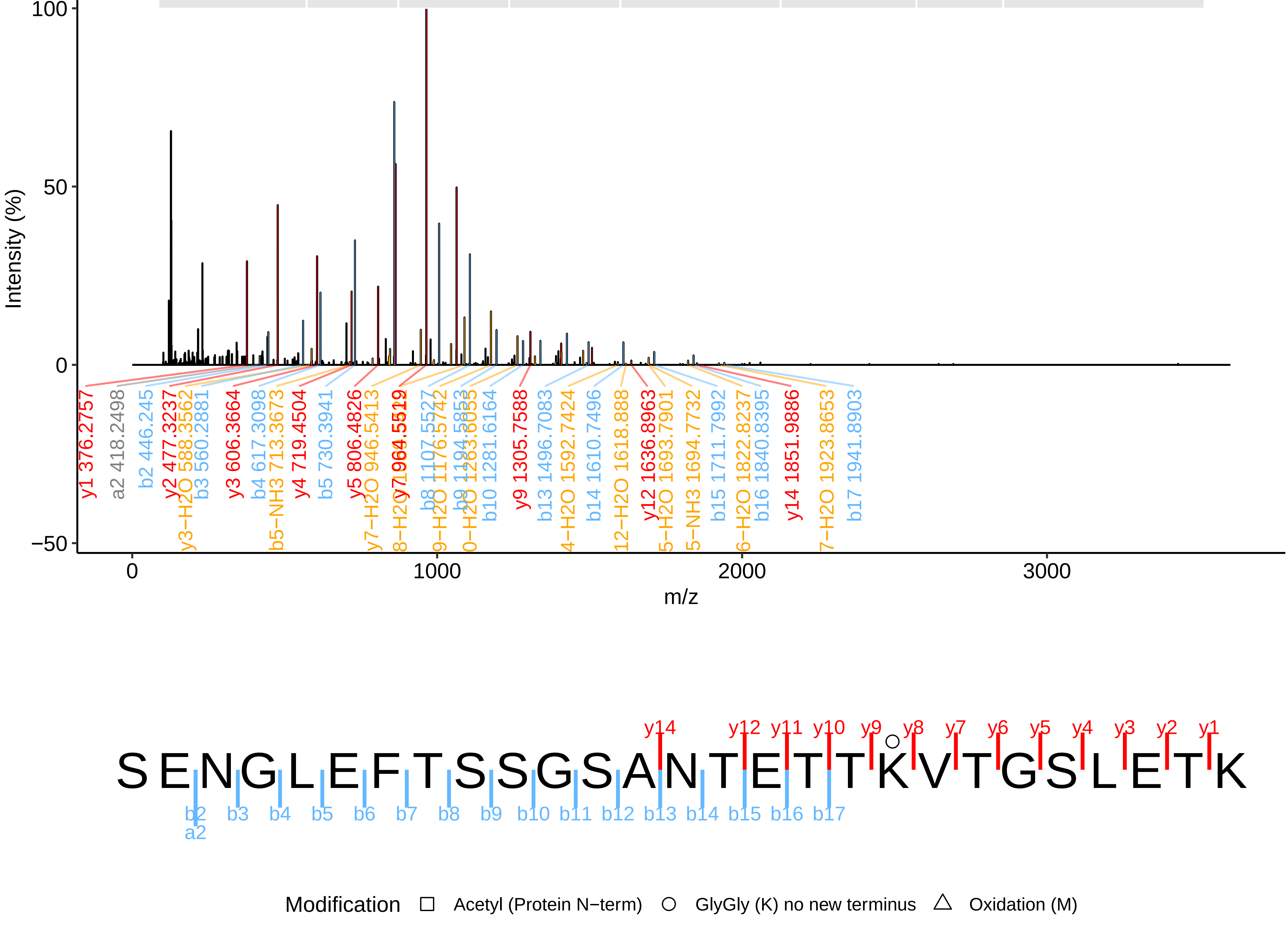

20200924_KIZ_MUb5_GlyGly_1 − Scan number: 19810

Gene Names	Charge	m/z	Mass	Retention time	PEP	Score	Precursor Intensity
VDAC1	3	963.7914	2888.352	63.991	1.6442e−84	221.26	NA

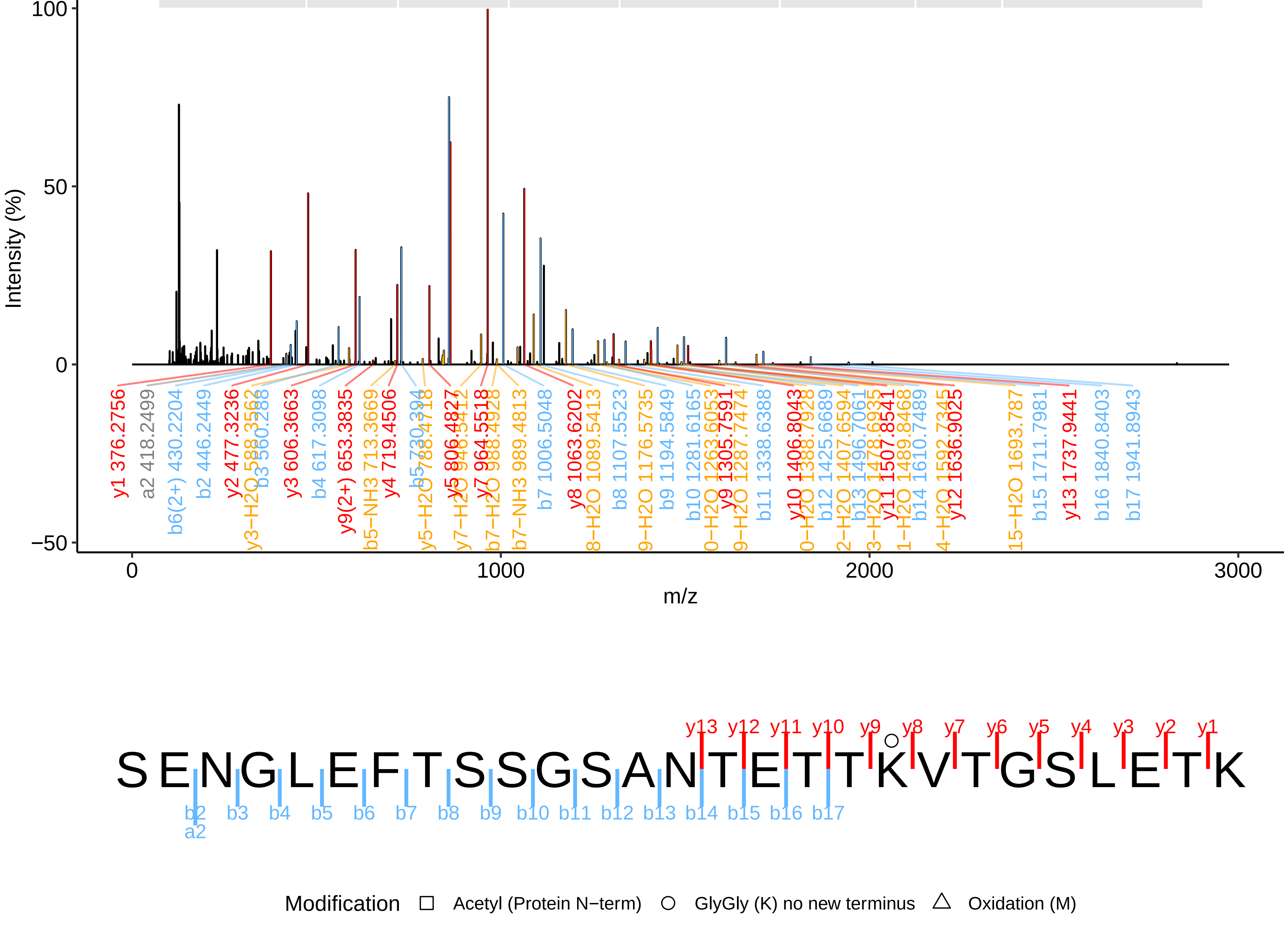

20200924_KIZ_MUb5_GlyGly_2 − Scan number: 16954

Gene Names	Charge	m/z	Mass	Retention time	PEP	Score	Precursor Intensity
VDAC1	3	963.7914	2888.352	62.91	4.2121e−41	172.32	4382393

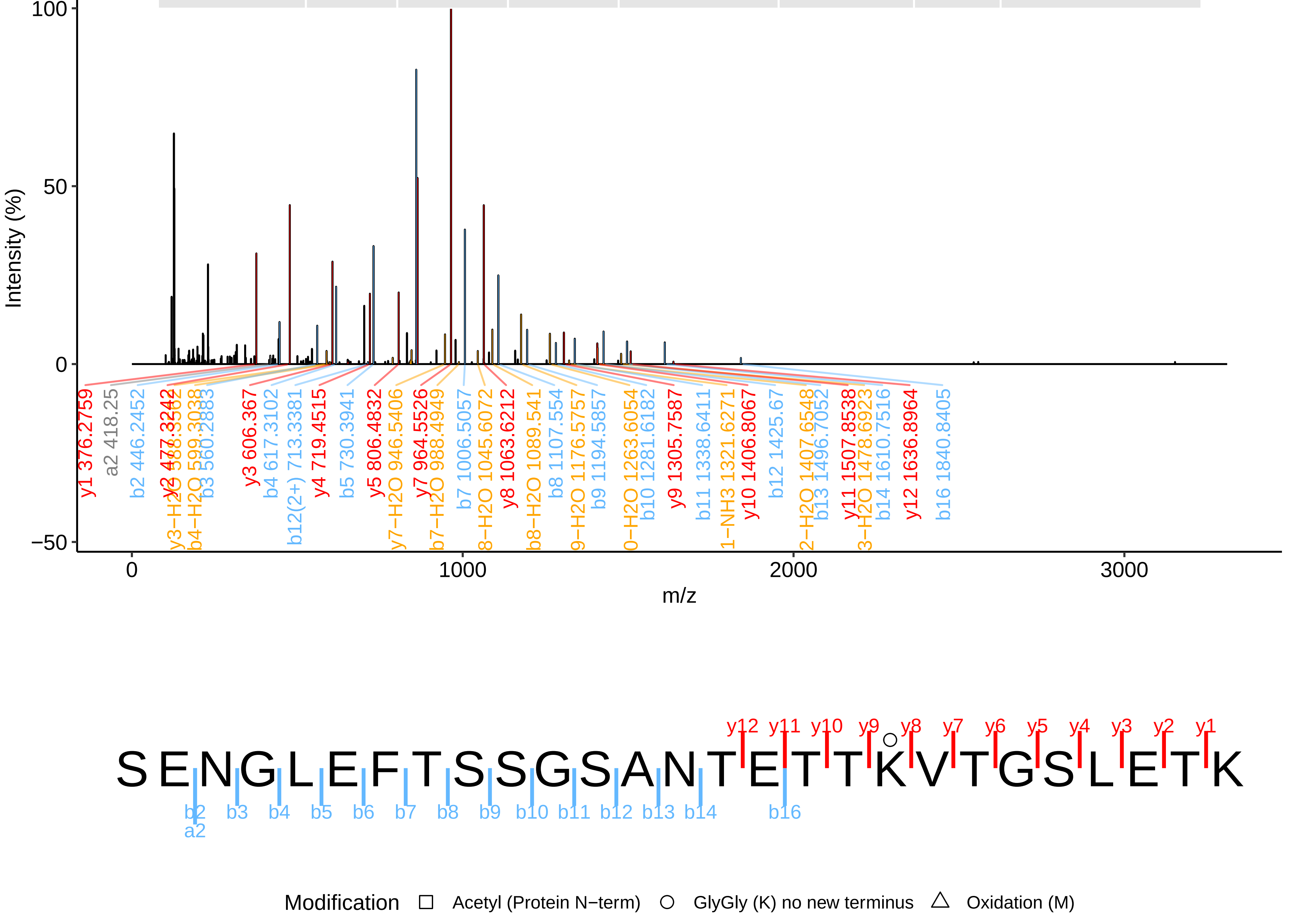

20200924_KIZ_MUb5_GlyGly_2 − Scan number: 17094

Gene Names	Charge	m/z	Mass	Retention time	PEP	Score	Precursor Intensity
VDAC1	3	963.7914	2888.352	63.413	1.4798e−30	158.67	2455562

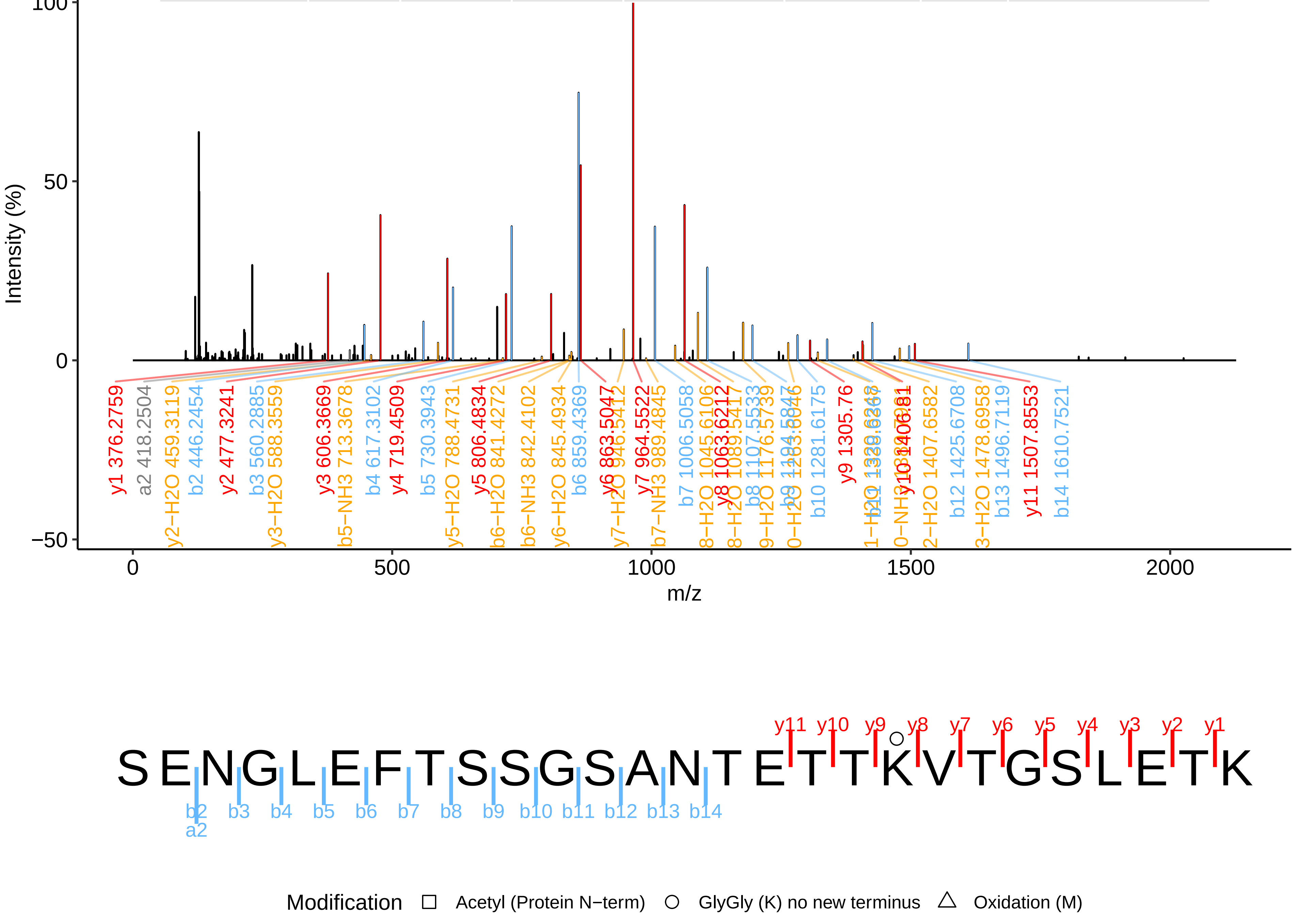

20200924_KIZ_MUb5_GlyGly_2 − Scan number: 17214

Gene Names	Charge	m/z	Mass	Retention time	PEP	Score	Precursor Intensity
VDAC1	4	723.0954	2888.352	63.821	2.0356e−21	134.91	4781162

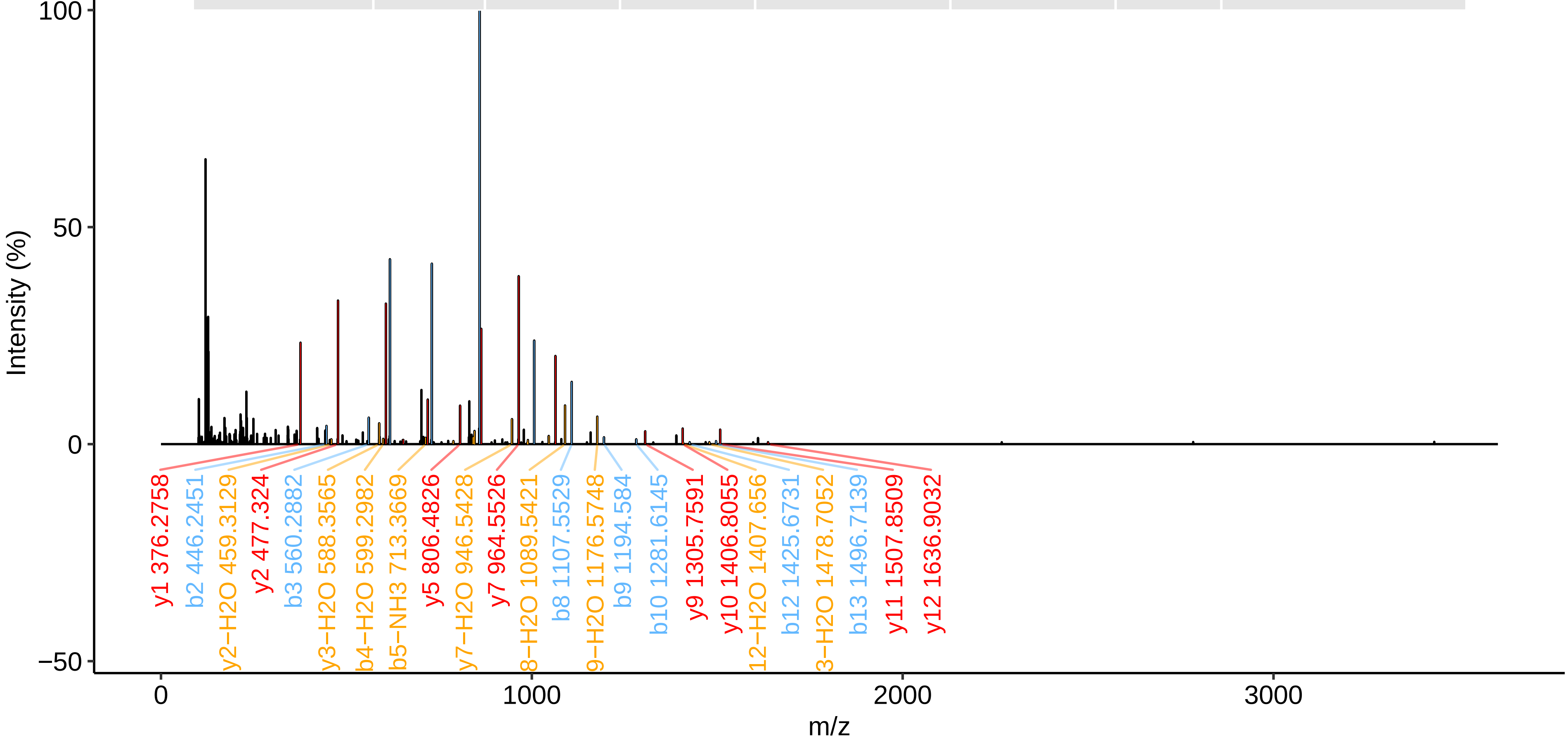

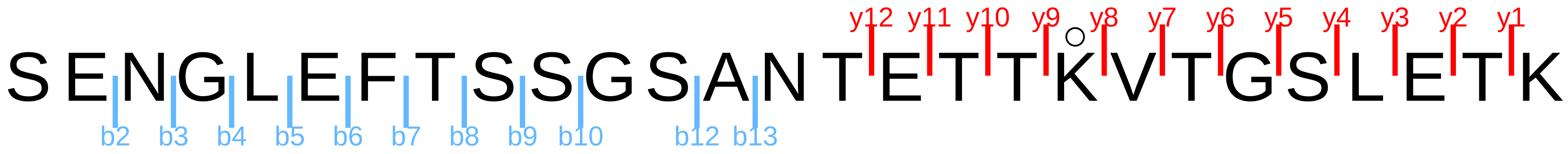

Modification □ Acetyl (Protein N−term) ○ GlyGly (K) no new terminus △ Oxidation (M)

20200924_KIZ_MUb5_GlyGly_2 − Scan number: 17254

Gene Names	Charge	m/z	Mass	Retention time	PEP	Score	Precursor Intensity
VDAC1	3	963.7914	2888.352	63.972	8.5677e−09	102.27	3623247

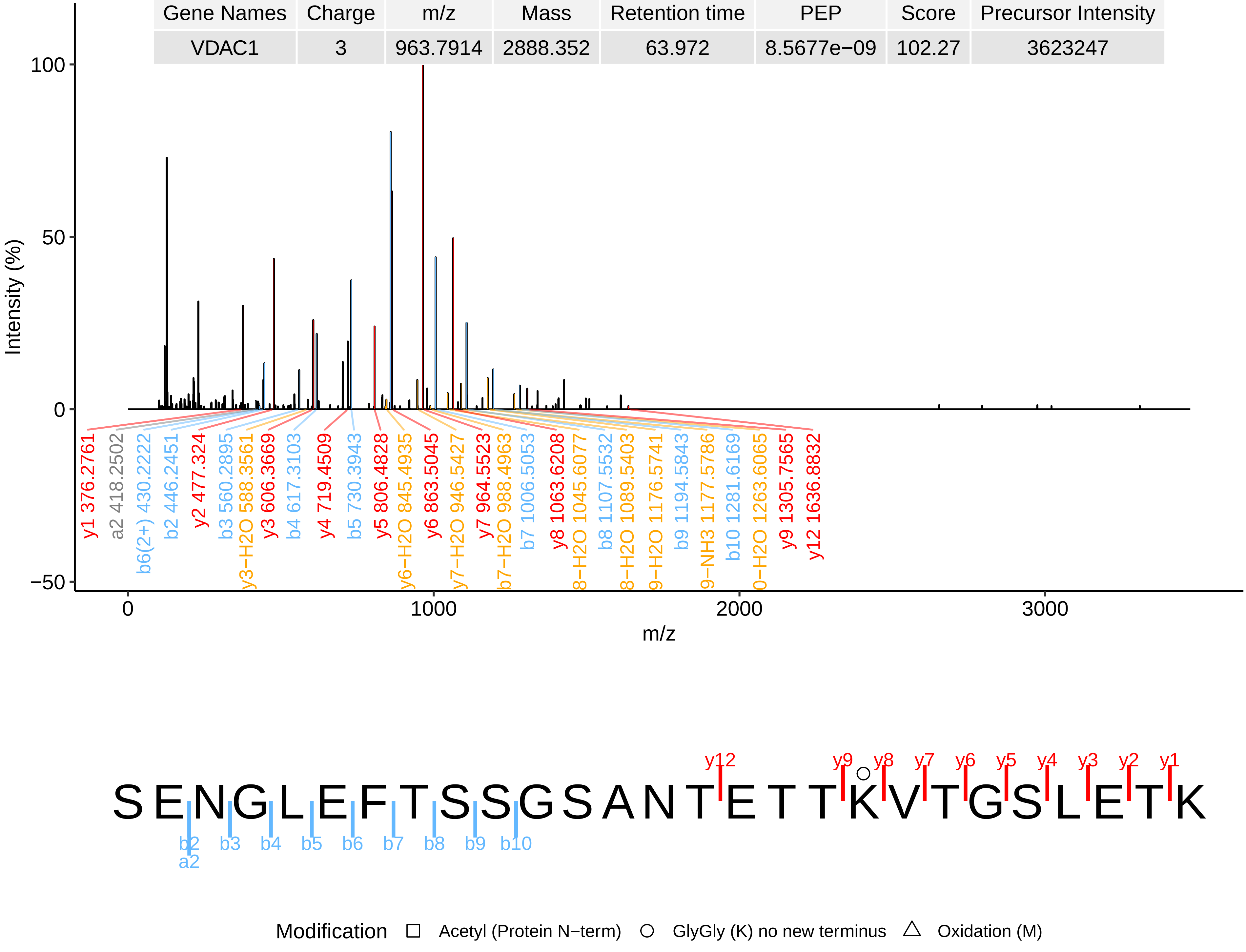

20200924_KIZ_MUb5_GlyGly_2 − Scan number: 17403

Gene Names	Charge	m/z	Mass	Retention time	PEP	Score	Precursor Intensity
VDAC1	3	963.7914	2888.352	64.505	2.3682e−30	153.27	4692119

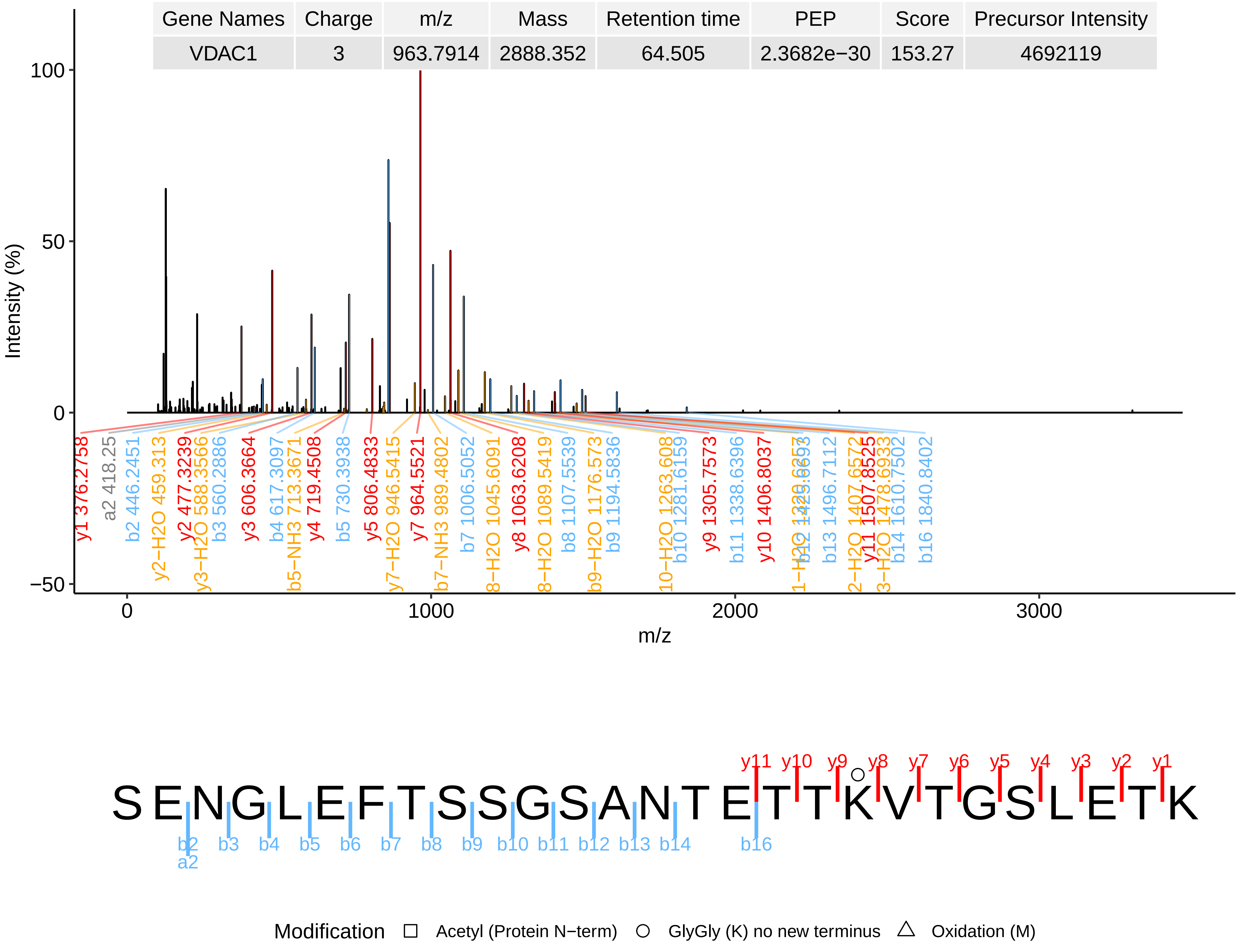

20200924_KIZ_MUb5_GlyGly_2 − Scan number: 17682

Gene Names	Charge	m/z	Mass	Retention time	PEP	Score	Precursor Intensity
VDAC1	3	963.7914	2888.352	65.533	2.6274e−21	133.95	7902535

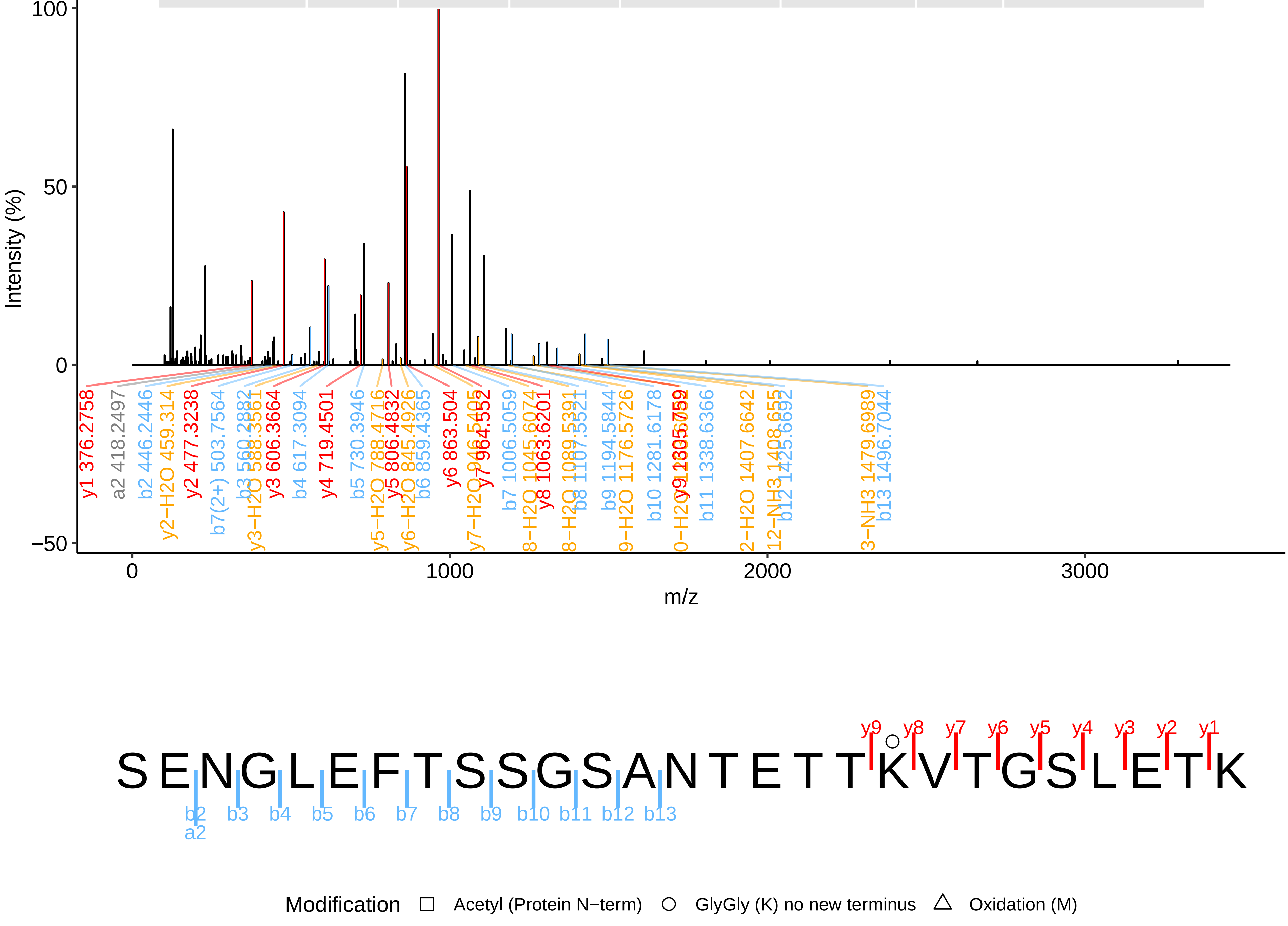

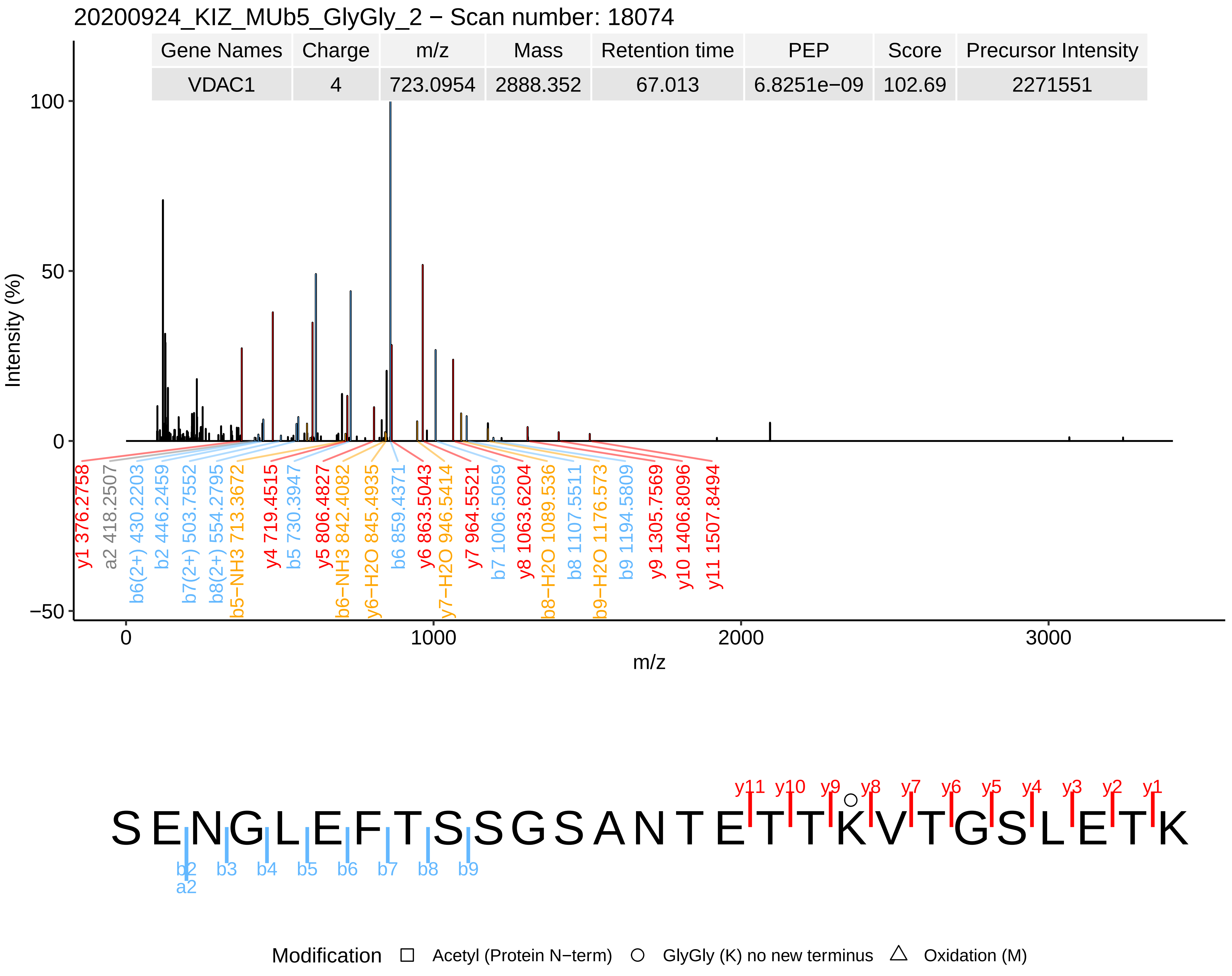
20200924_KIZ_MUb5_GlyGly_2 − Scan number: 18074
Gene Names | Charge | m/z | Mass | Retention time | PEP | Score | Precursor Intensity
VDAC1 | 4 | 723.0954 | 2888.352 | 67.013 | 6.8251e−09 | 102.69 | 2271551
Intensity (%)
100
50
0
−50
m/z
0
1000
2000
3000
y1 376.2758
a2 418.2507
b6(2+) 430.2203
b2 446.2459
b7(2+) 503.7552
b8(2+) 554.2795
b5−NH3 713.3672
y4 719.4515
b5 730.3947
y5 806.4827
b6−NH3 842.4082
y6−H2O 845.4935
b6 859.4371
y6 863.5043
y7−H2O 946.5414
y7 964.5521
b7 1006.5059
y8 1063.6204
b8−H2O 1089.536
b8 1107.5511
b9−H2O 1176.573
b9 1194.5809
y9 1305.7569
y10 1406.8096
y11 1507.8494
y11 y10 y9 y8 y7 y6 y5 y4 y3 y2 y1
S E N G L E F T S S G S A N T E T T K V T G S L E T K
b2 b3 b4 b5 b6 b7 b8 b9
a2
Modification
Acetyl (Protein N−term)
GlyGly (K) no new terminus
Oxidation (M)

20201118_KIZ_MUb6_GlyGly_1 − Scan number: 18222

Gene Names	Charge	m/z	Mass	Retention time	PEP	Score	Precursor Intensity
VDAC1	3	963.7914	2888.352	57.31	2.6416e−83	223.69	16638496

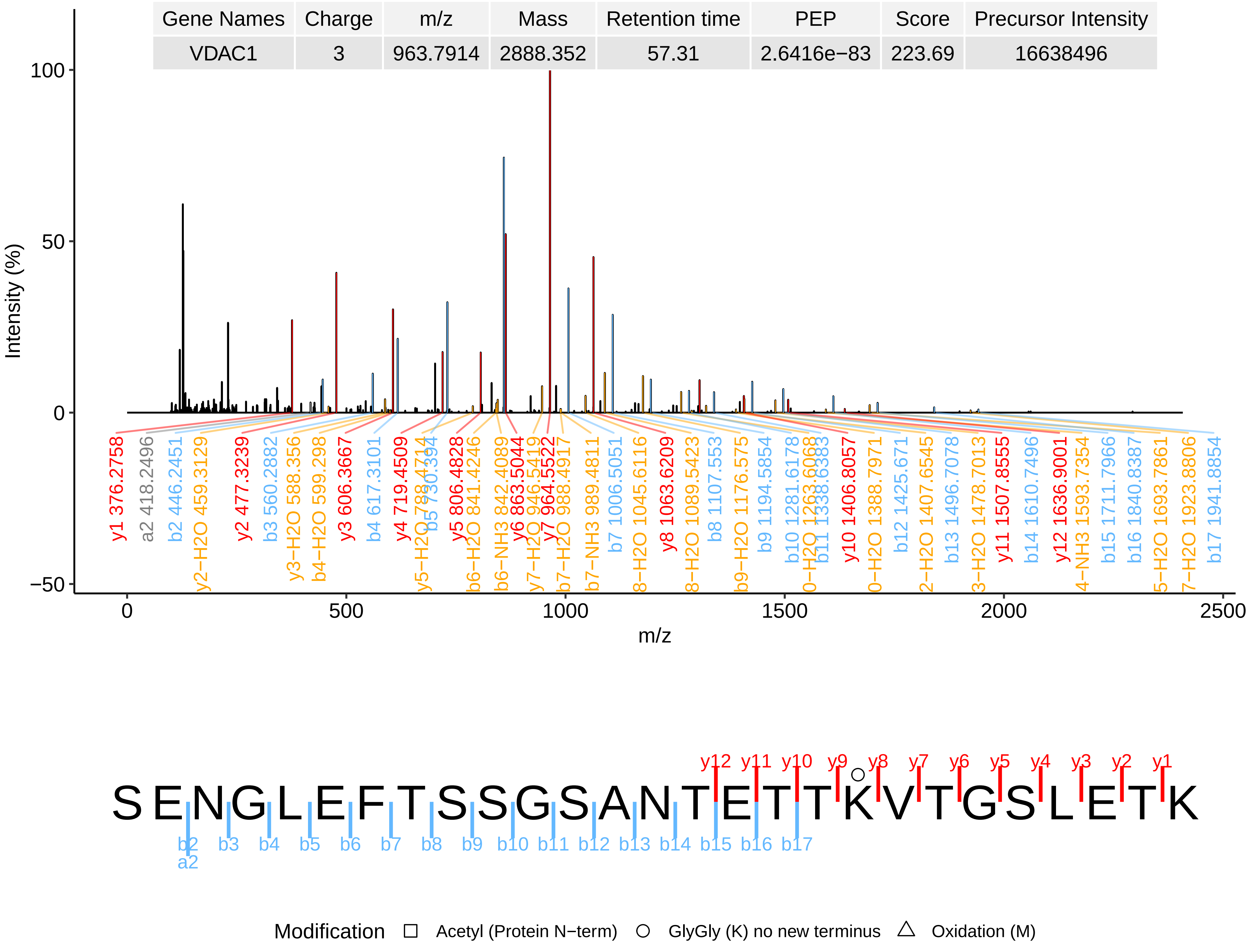

20201118_KIZ_MUb6_GlyGly_1 − Scan number: 18382

Gene Names	Charge	m/z	Mass	Retention time	PEP	Score	Precursor Intensity
VDAC1	3	963.7914	2888.352	57.81	3.4892e−101	254.48	54750800

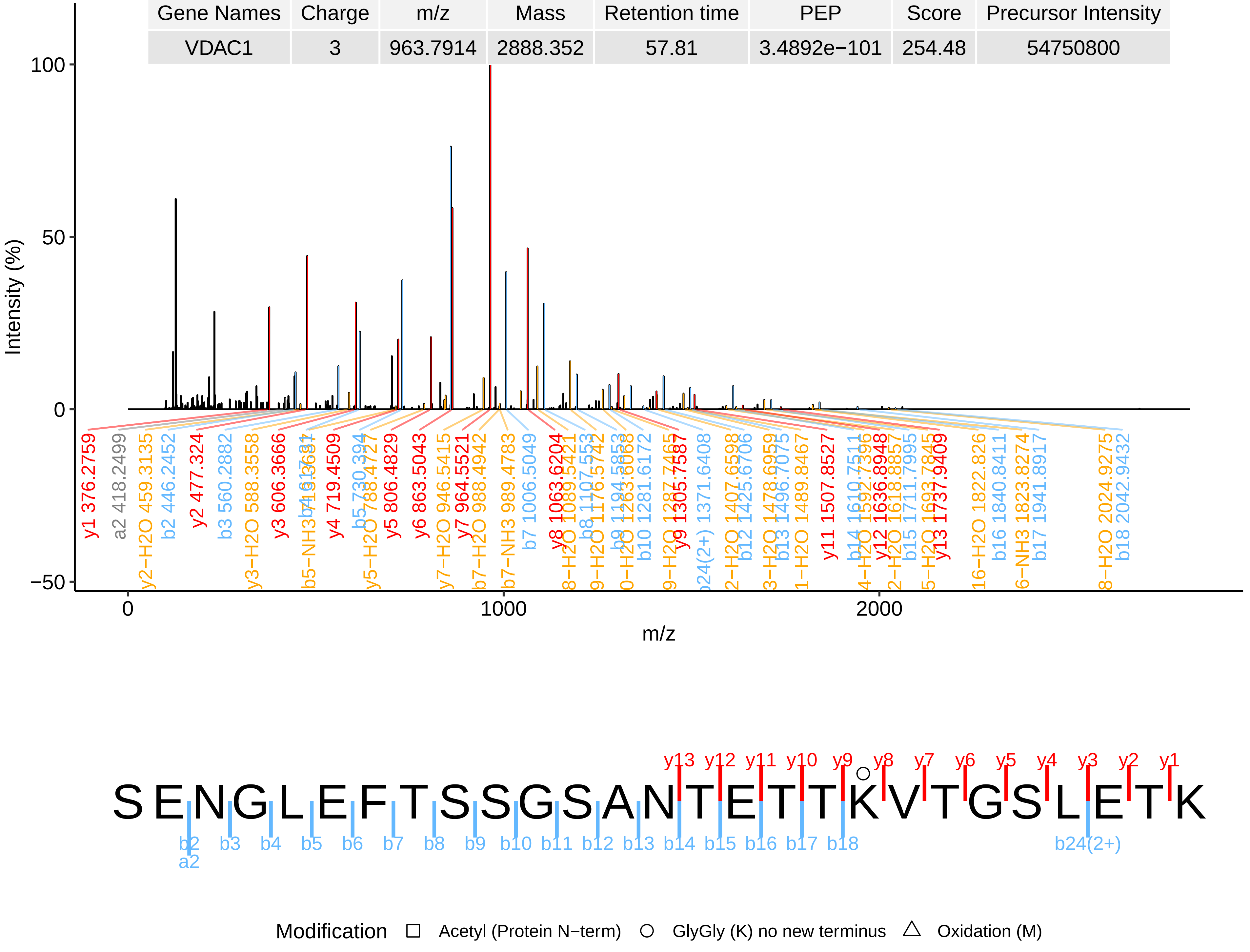

20201118_KIZ_MUb6_GlyGly_1 − Scan number: 18223

Gene Names	Charge	m/z	Mass	Retention time	PEP	Score	Precursor Intensity
VDAC1	4	723.0954	2888.352	57.314	2.802e−06	96.533	10204882

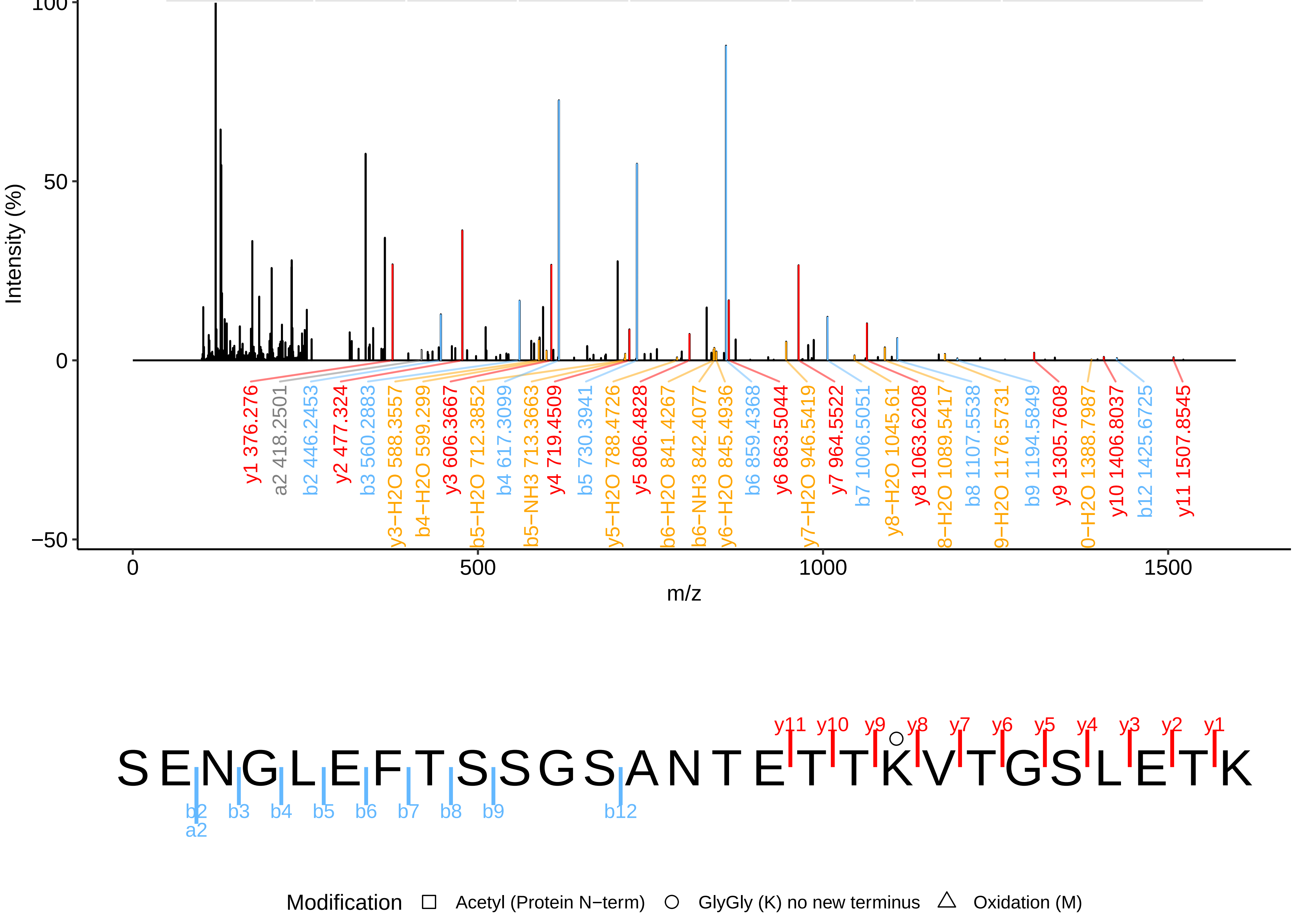

20201118_KIZ_MUb6_GlyGly_1 − Scan number: 18394

Gene Names	Charge	m/z	Mass	Retention time	PEP	Score	Precursor Intensity
VDAC1	4	723.0954	2888.352	57.847	1.7464e−99	240.26	42870936

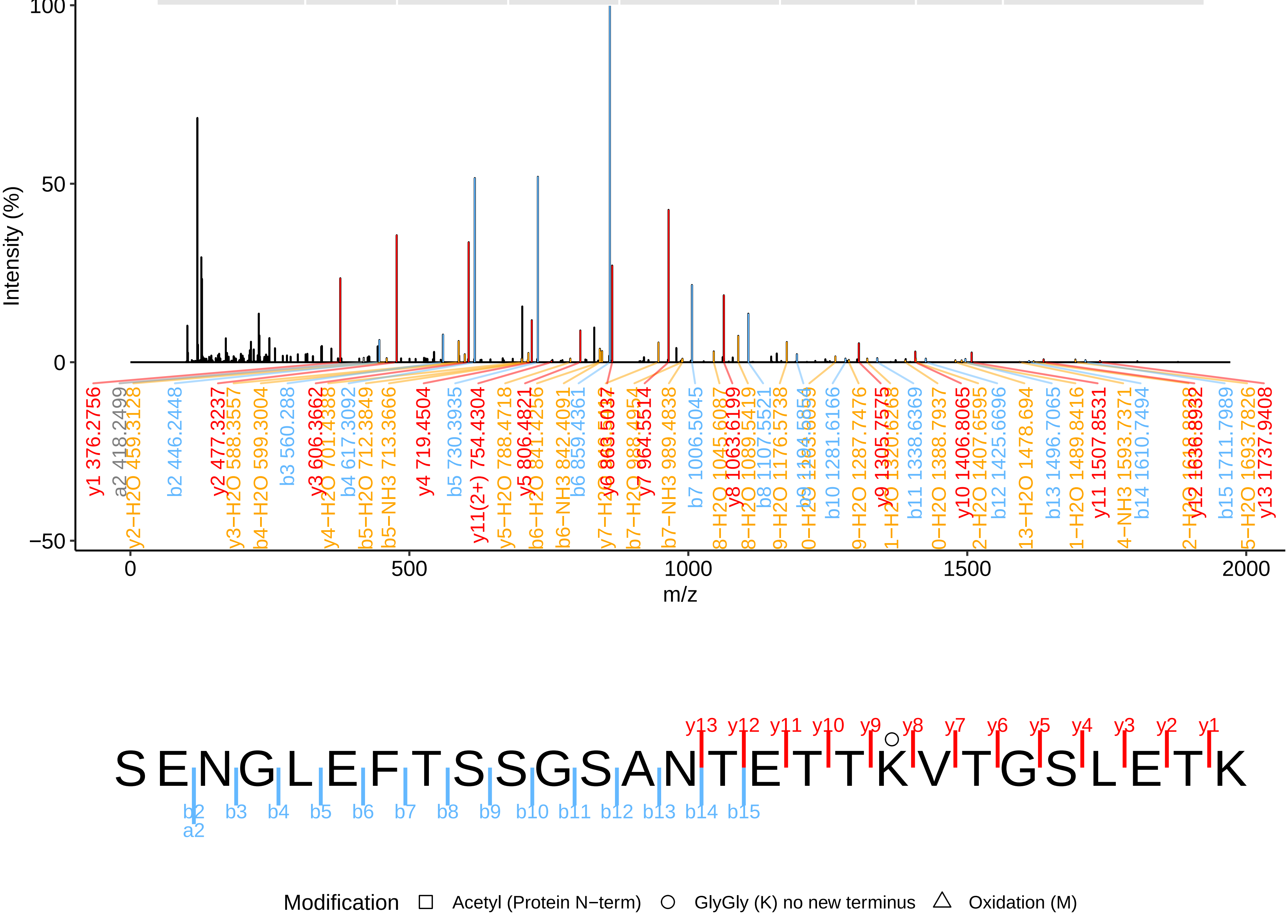

20201118_KIZ_MUb6_GlyGly_2 − Scan number: 19350

Gene Names	Charge	m/z	Mass	Retention time	PEP	Score	Precursor Intensity
VDAC1	3	963.7914	2888.352	65.297	5.9109e−54	197.54	7954715

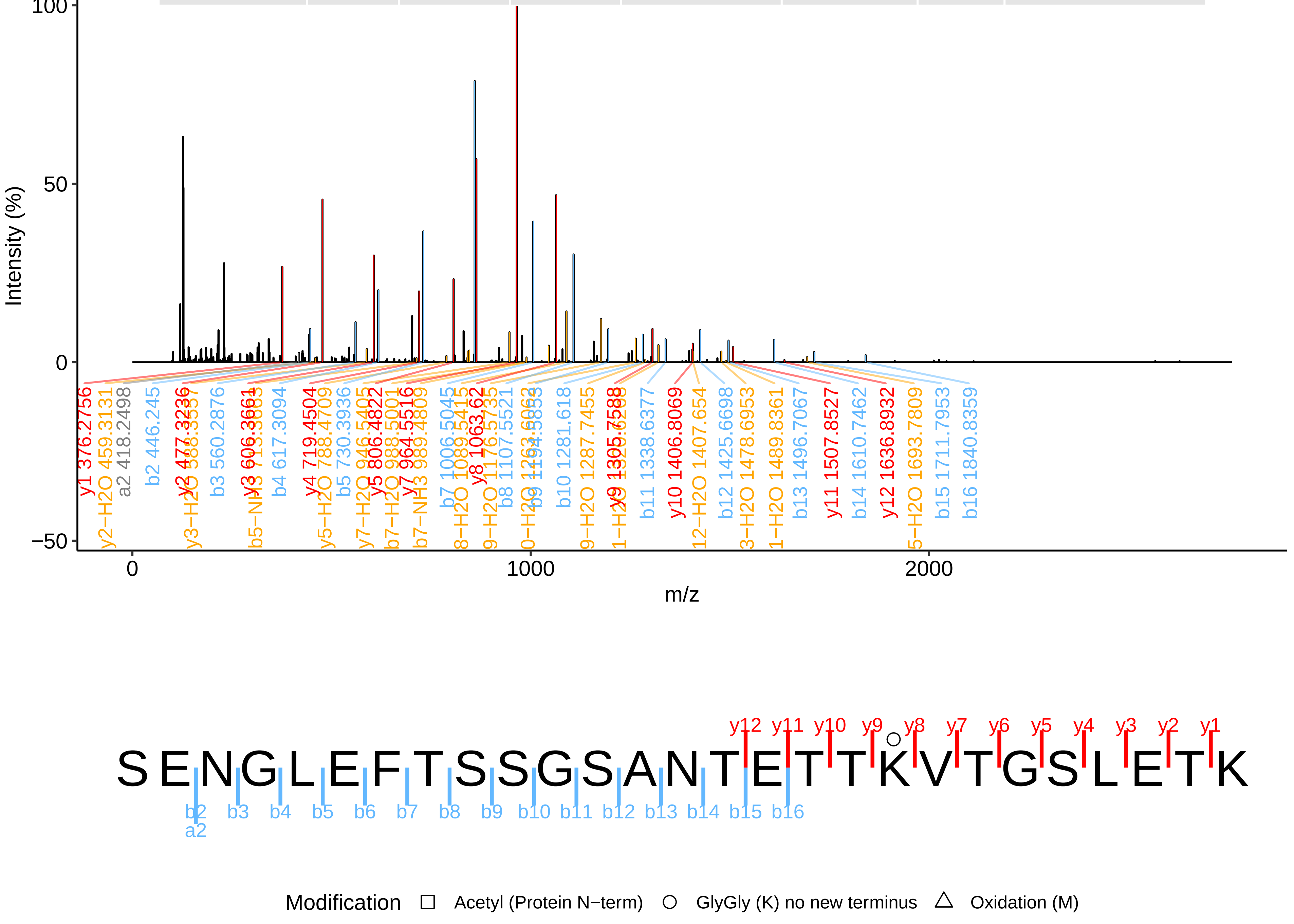

20201118_KIZ_MUb6_GlyGly_2 − Scan number: 19501

Gene Names	Charge	m/z	Mass	Retention time	PEP	Score	Precursor Intensity
VDAC1	3	963.7914	2888.352	65.806	9.856e−68	208.89	21404796

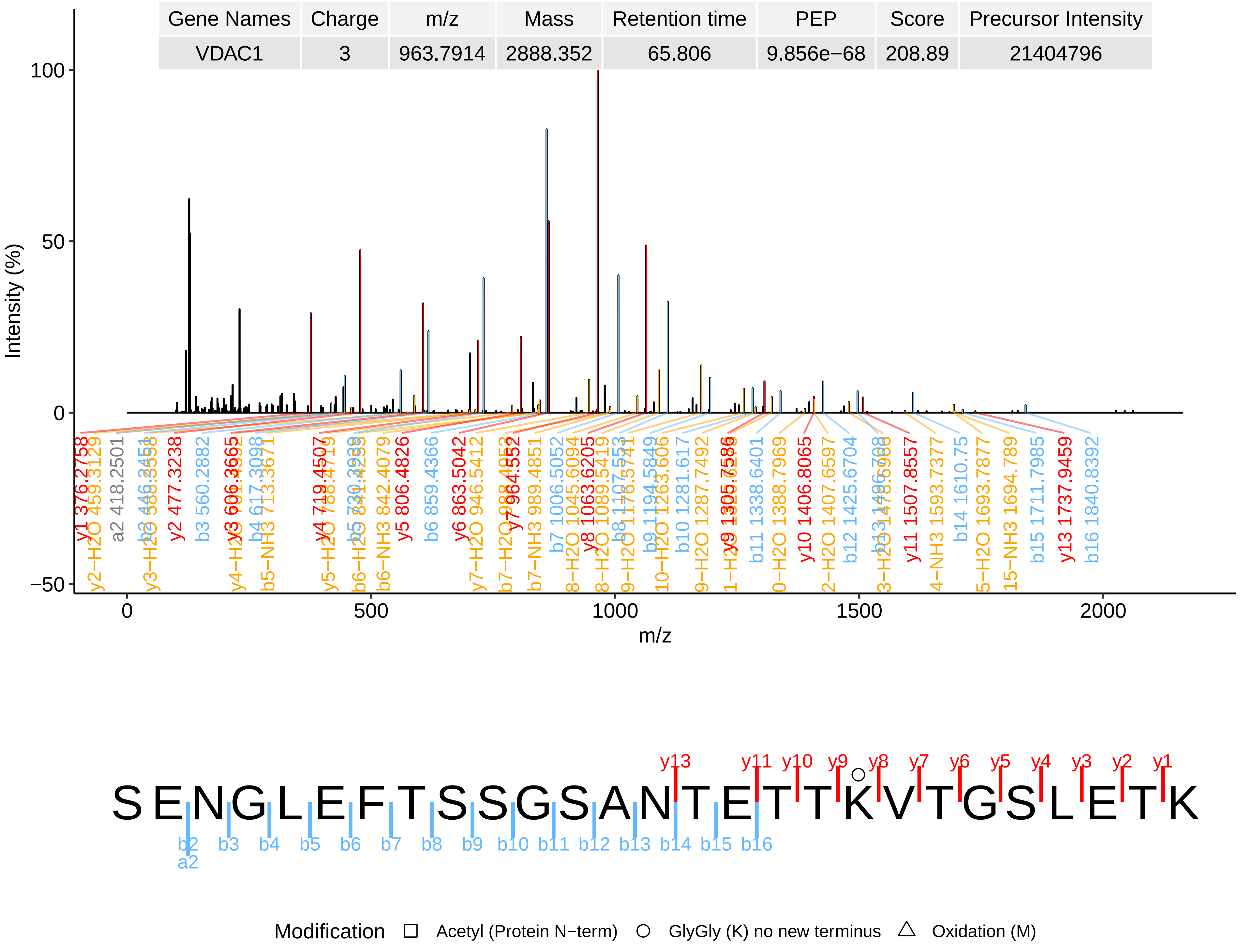

20201118_KIZ_MUb6_GlyGly_2 − Scan number: 19657

Gene Names	Charge	m/z	Mass	Retention time	PEP	Score	Precursor Intensity
VDAC1	3	963.7914	2888.352	66.317	2.4963e−84	236.02	3295331

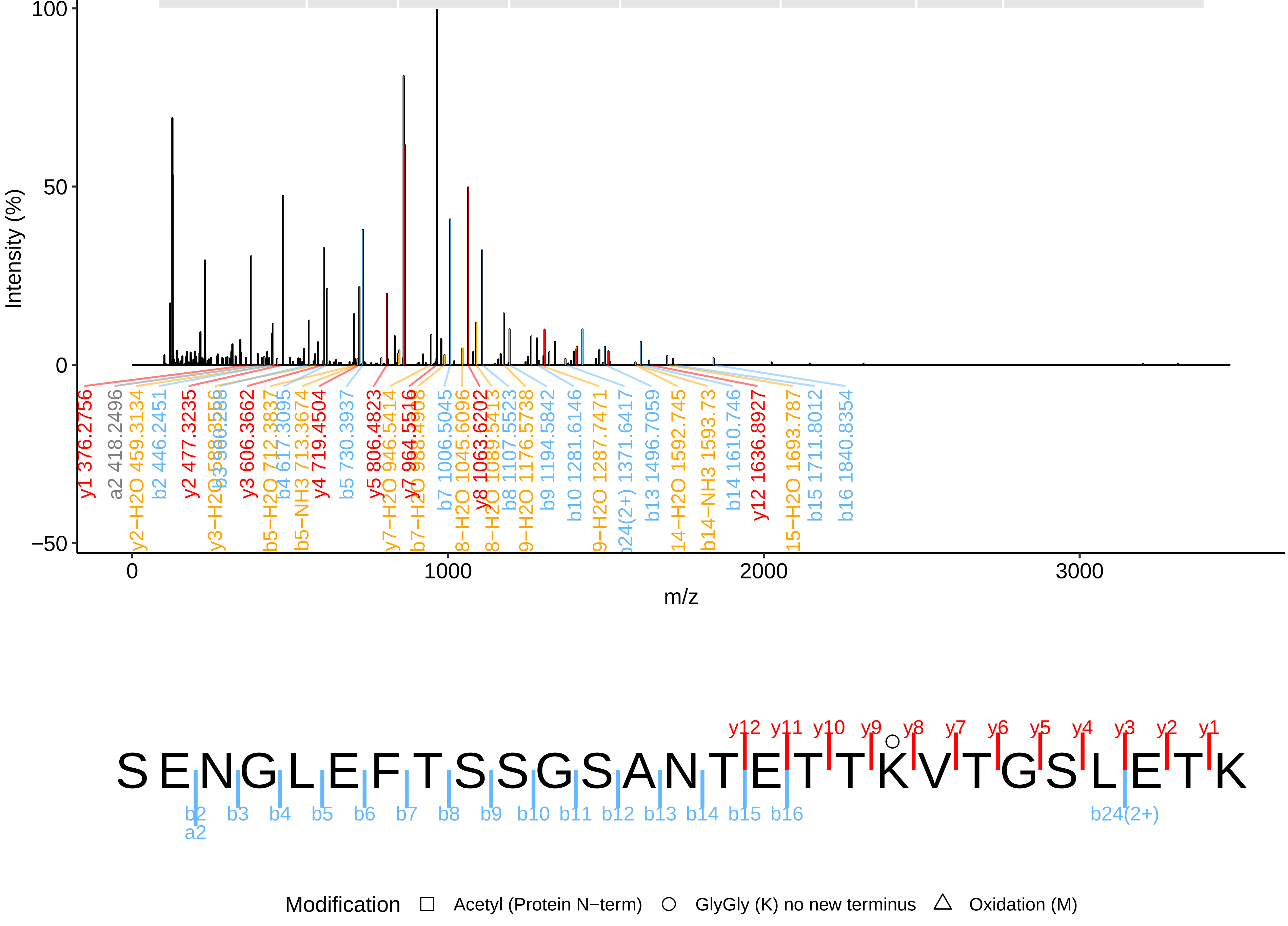

20201118_KIZ_MUb6_GlyGly_2 − Scan number: 19658

Gene Names	Charge	m/z	Mass	Retention time	PEP	Score	Precursor Intensity
VDAC1	3	963.7914	2888.352	66.321	4.3425e−56	202.44	3295331

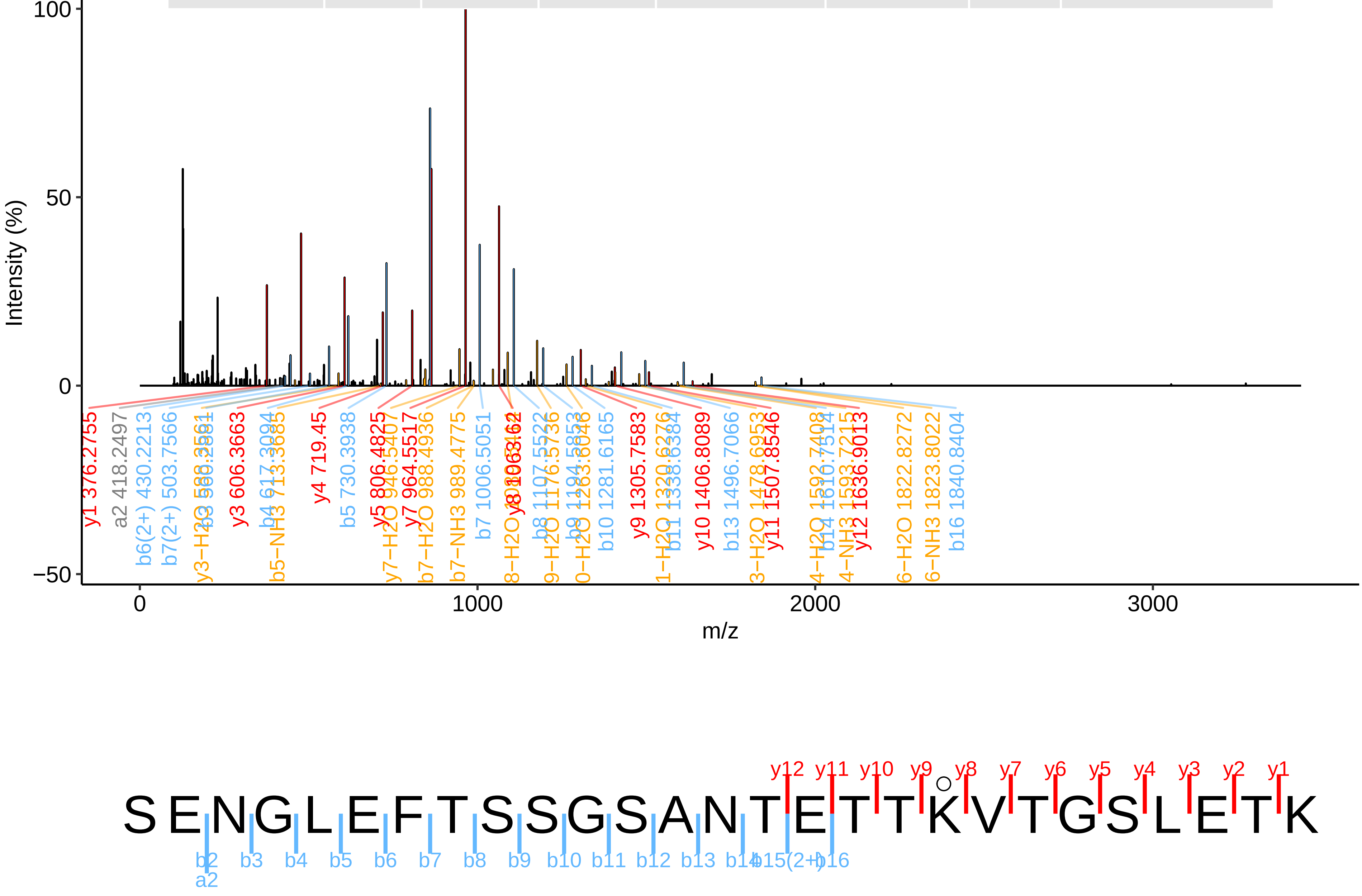

Modification □ Acetyl (Protein N−term) ○ GlyGly (K) no new terminus △ Oxidation (M)

20201118_KIZ_MUb6_GlyGly_2 − Scan number: 19353

Gene Names	Charge	m/z	Mass	Retention time	PEP	Score	Precursor Intensity
VDAC1	4	723.0954	2888.352	65.307	8.8217e−54	193.53	12942125

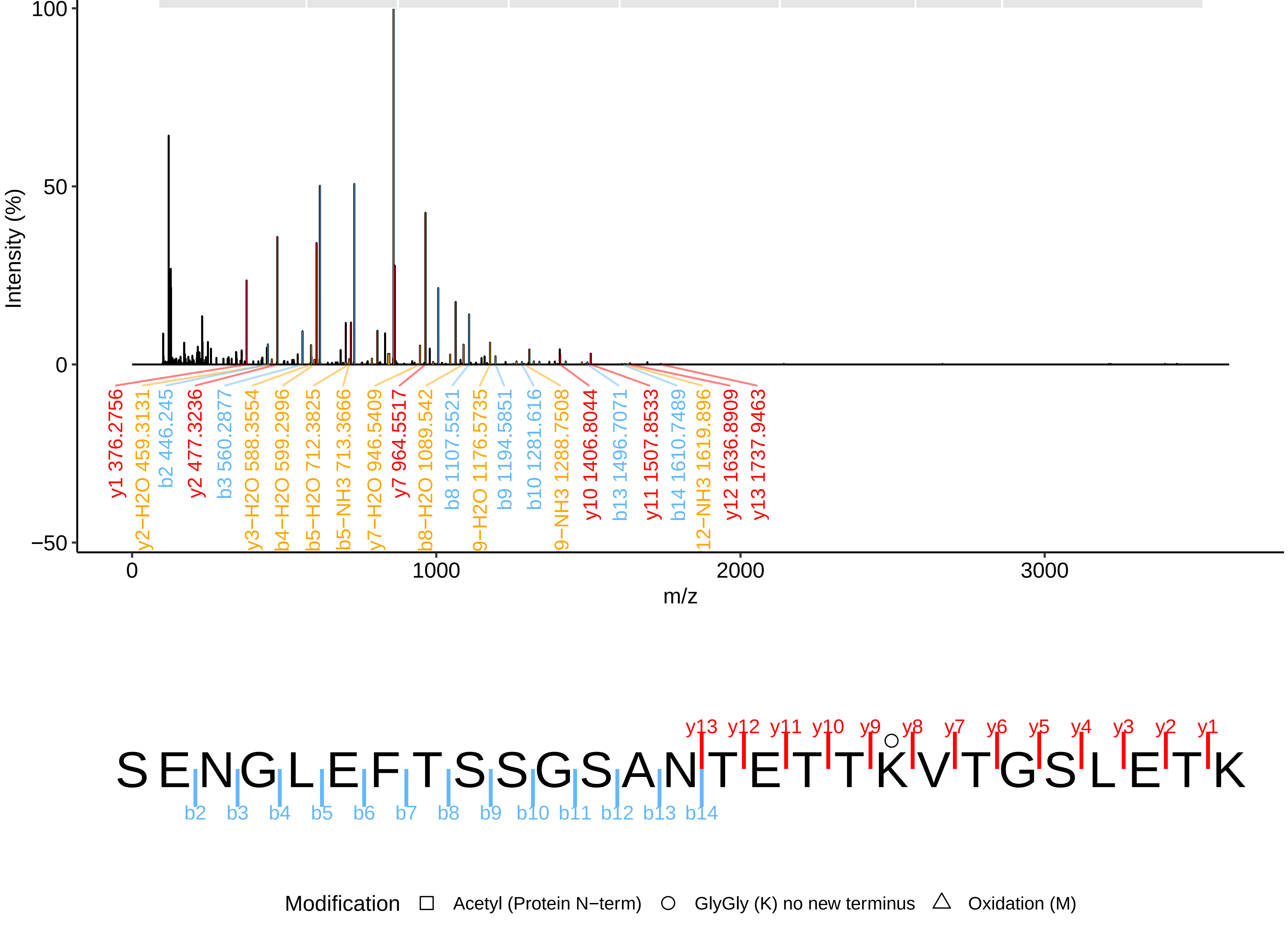

20201118_KIZ_MUb6_GlyGly_2 − Scan number: 19505

Gene Names	Charge	m/z	Mass	Retention time	PEP	Score	Precursor Intensity
VDAC1	4	723.0954	2888.352	65.818	8.8217e−54	193.53	31437404

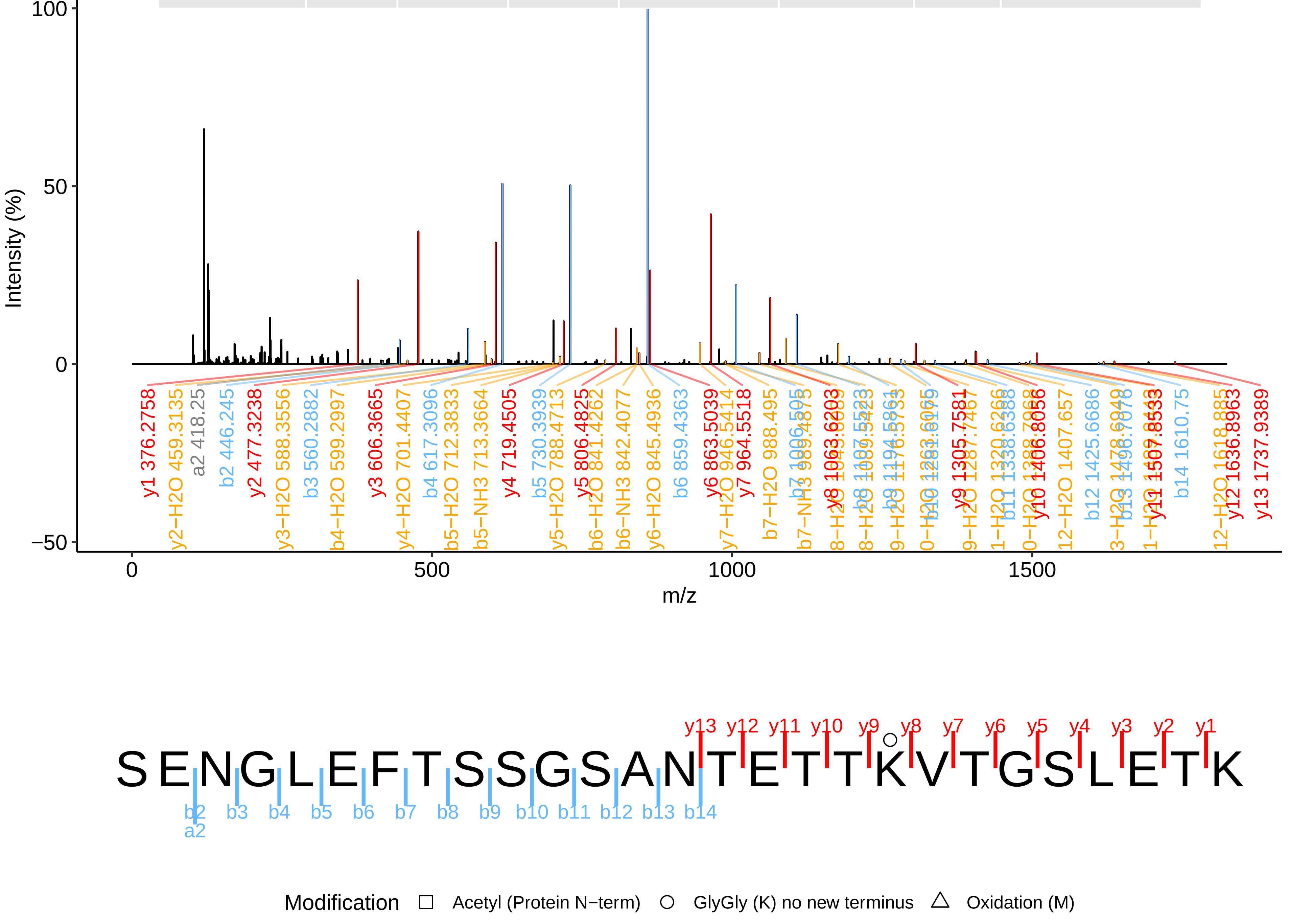

20201118_KIZ_MUb6_GlyGly_2 − Scan number: 19665

Gene Names	Charge	m/z	Mass	Retention time	PEP	Score	Precursor Intensity
VDAC1	4	723.0954	2888.352	66.344	1.7899e−30	154.45	18319004

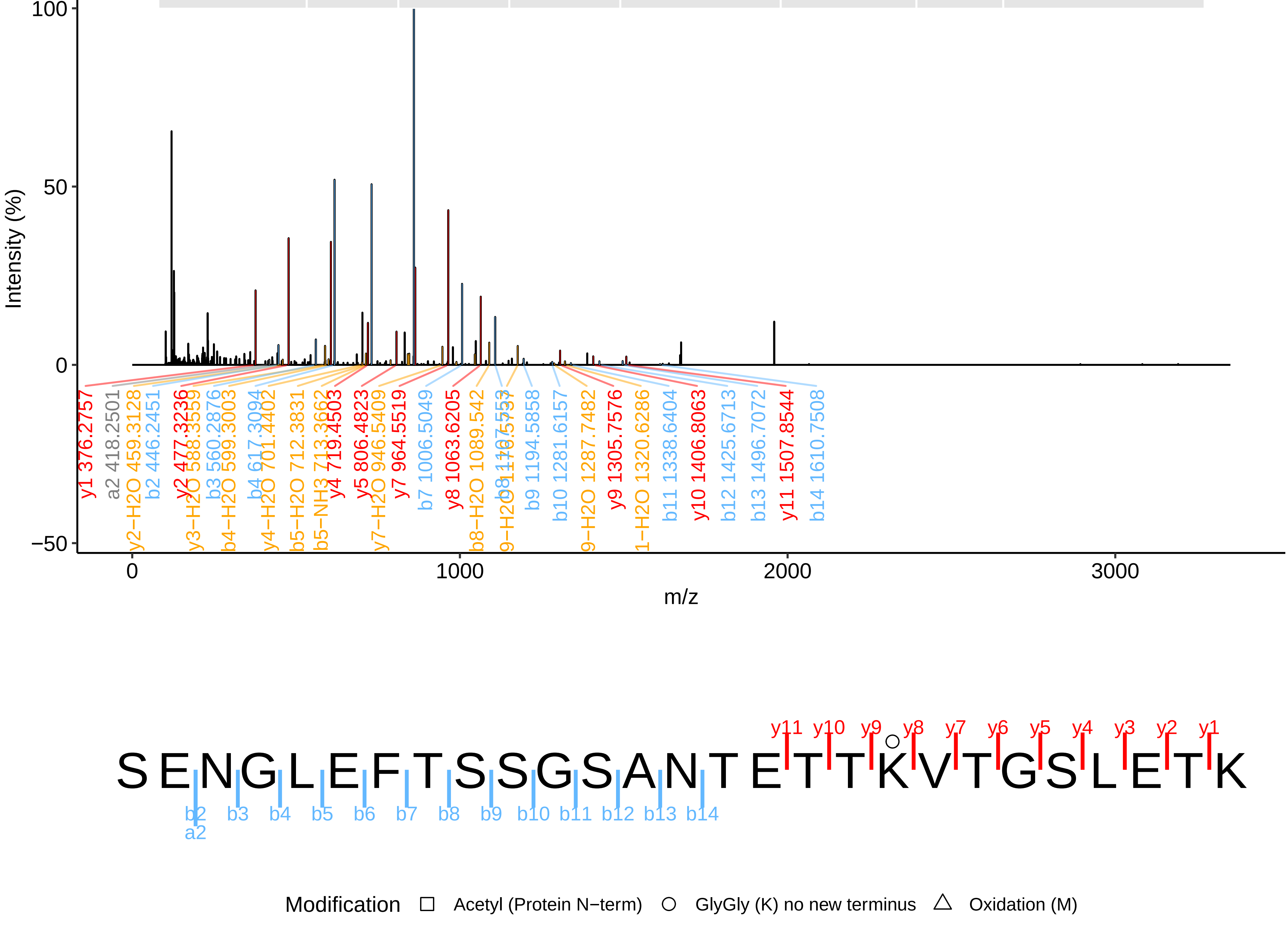

20201118_KIZ_MUb6_GlyGly_2 − Scan number: 19971

Gene Names	Charge	m/z	Mass	Retention time	PEP	Score	Precursor Intensity
VDAC1	3	963.7914	2888.352	67.359	0.0017281	74.905	9698631

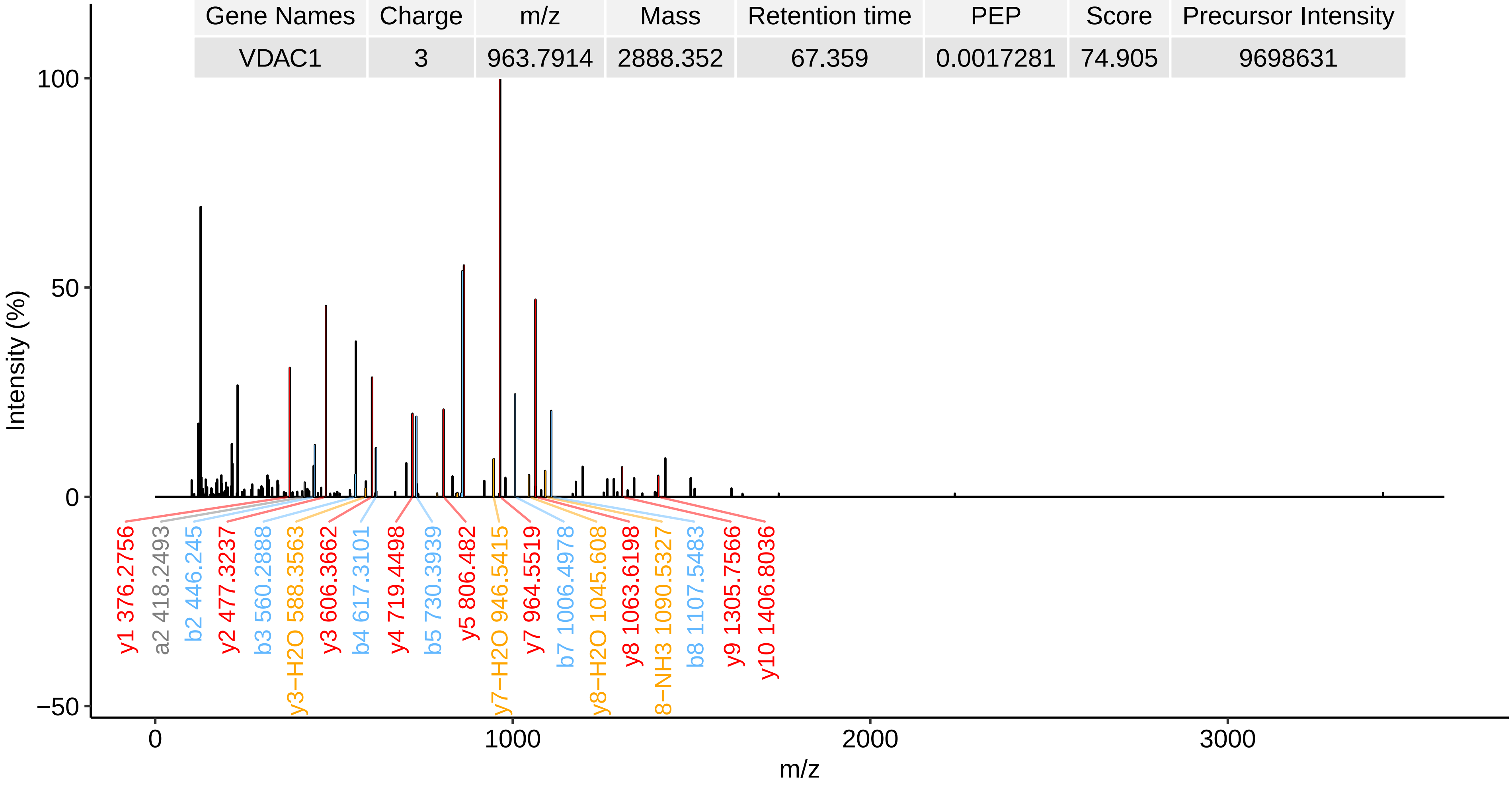

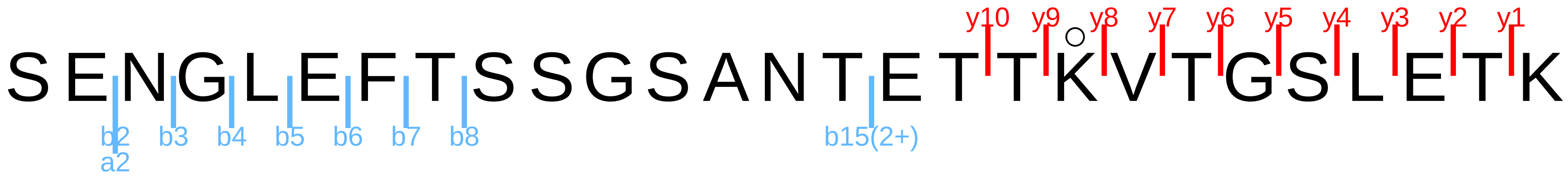

Modification □ Acetyl (Protein N−term) ○ GlyGly (K) no new terminus △ Oxidation (M)

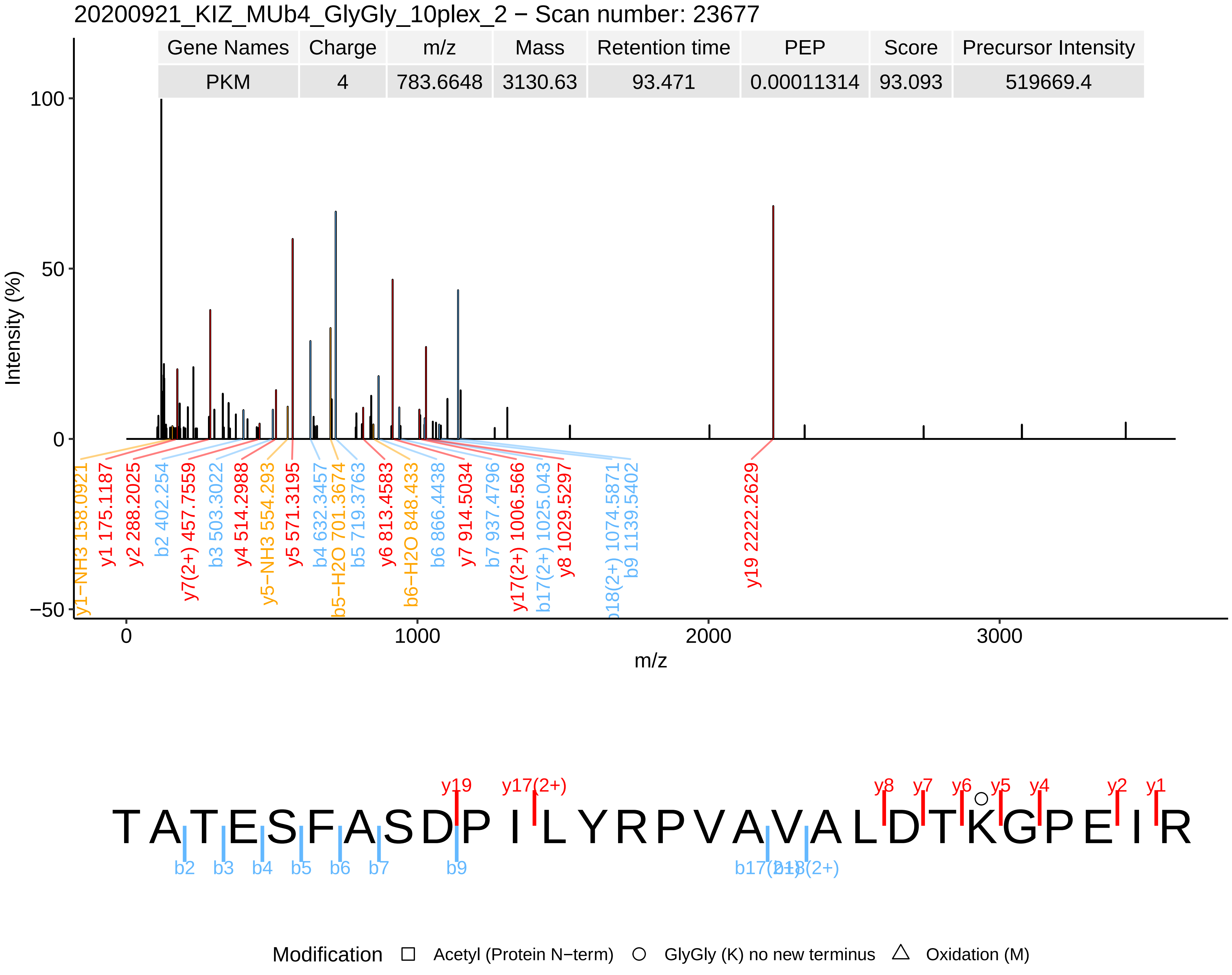
20200921_KIZ_MUb4_GlyGly_10plex_2 − Scan number: 23677
Gene Names | Charge | m/z | Mass | Retention time | PEP | Score | Precursor Intensity
PKM | 4 | 783.6648 | 3130.63 | 93.471 | 0.00011314 | 93.093 | 519669.4
Intensity (%)
m/z
y1−NH3 158.0921
y1 175.1187
y2 288.2025
b2 402.254
y7(2+) 457.7559
b3 503.3022
y4 514.2988
y5−NH3 554.293
y5 571.3195
b4 632.3457
b5−H2O 701.3674
b5 719.3763
y6 813.4583
b6−H2O 848.433
b6 866.4438
y7 914.5034
b7 937.4796
y17(2+) 1006.566
b17(2+) 1025.043
y8 1029.5297
b18(2+) 1074.5871
b9 1139.5402
y19 2222.2629
T A T E S F A S D P I L Y R P V A V A L D T K G P E I R
y19 y17(2+) y8 y7 y6 y5 y4 y2 y1
b2 b3 b4 b5 b6 b7 b9 b17(2+) b18(2+)
Modification
Acetyl (Protein N−term)
GlyGly (K) no new terminus
Oxidation (M)

20200921_KIZ_MUb4_GlyGly_10plex − Scan number: 25735

Gene Names	Charge	m/z	Mass	Retention time	PEP	Score	Precursor Intensity
SLC3A2	4	782.8773	3127.48	93.664	0.00092364	77.522	3155755

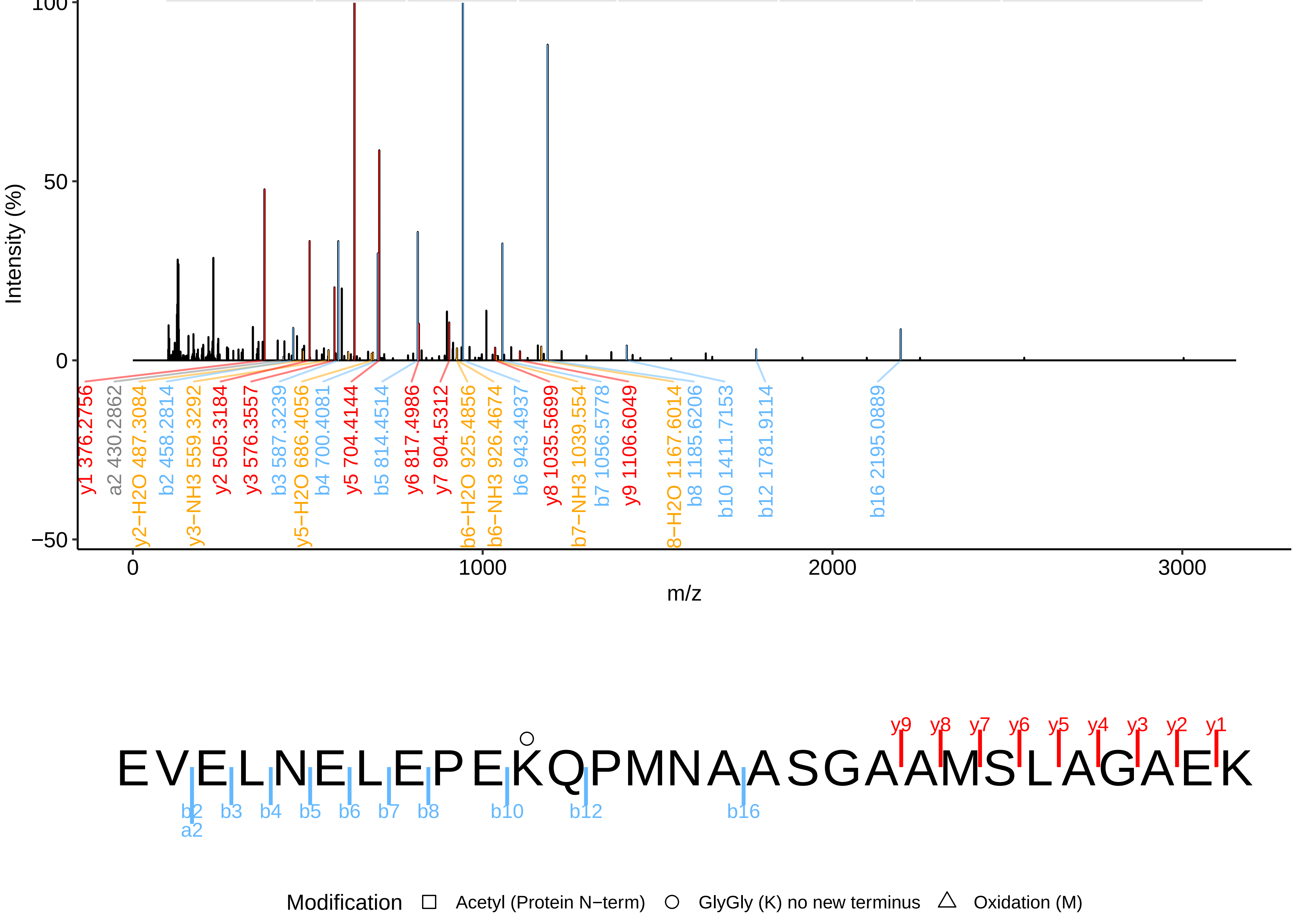

20200921_KIZ_MUb4_GlyGly_10plex − Scan number: 25748

Gene Names	Charge	m/z	Mass	Retention time	PEP	Score	Precursor Intensity
SLC3A2	3	1043.501	3127.48	93.716	4.9664e−05	88.014	NA

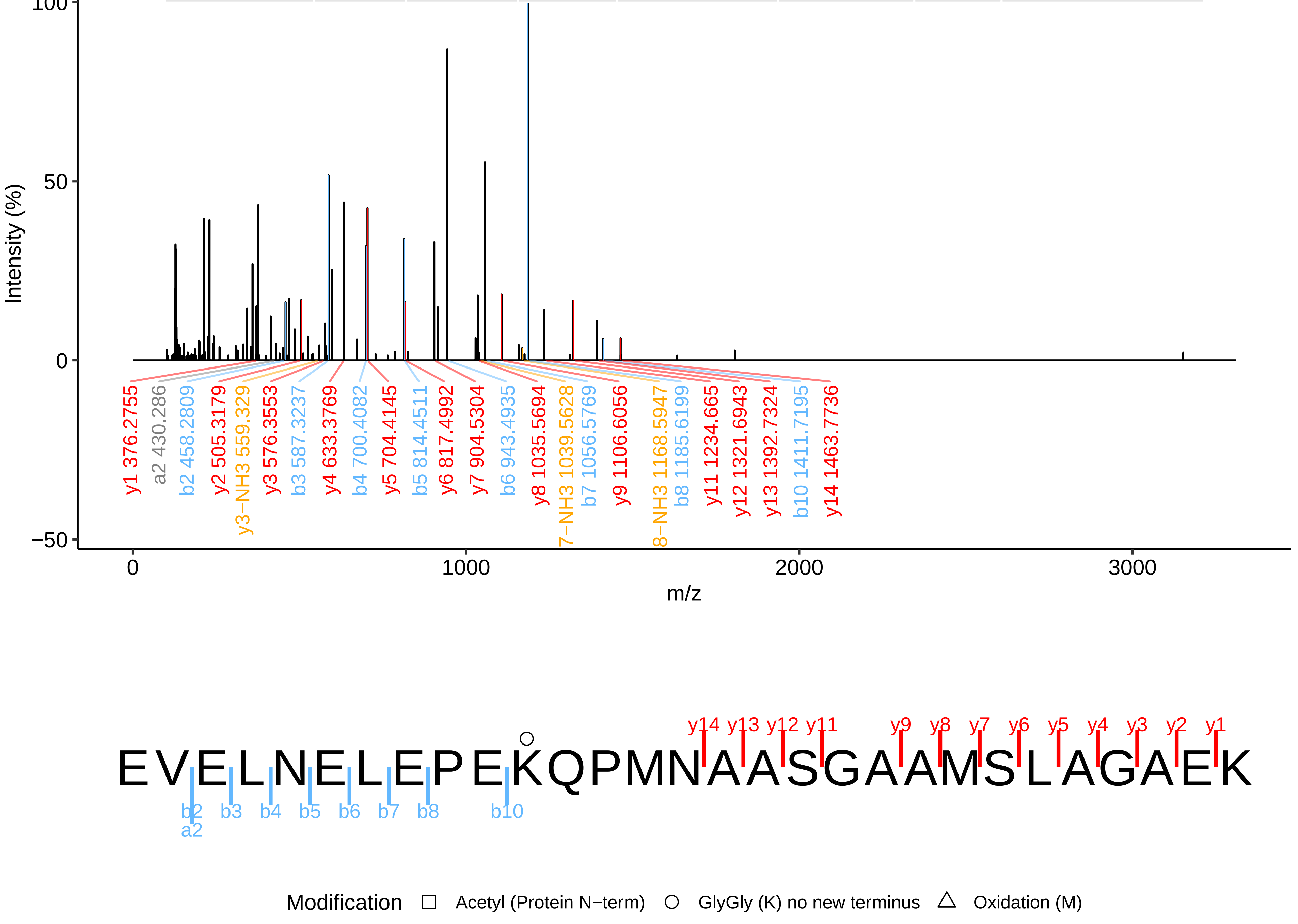

20200921_KIZ_MUb4_GlyGly_10plex_2 − Scan number: 22686

Gene Names	Charge	m/z	Mass	Retention time	PEP	Score	Precursor Intensity
SLC3A2	4	782.8773	3127.48	89.475	0.0042463	65.581	603056.7

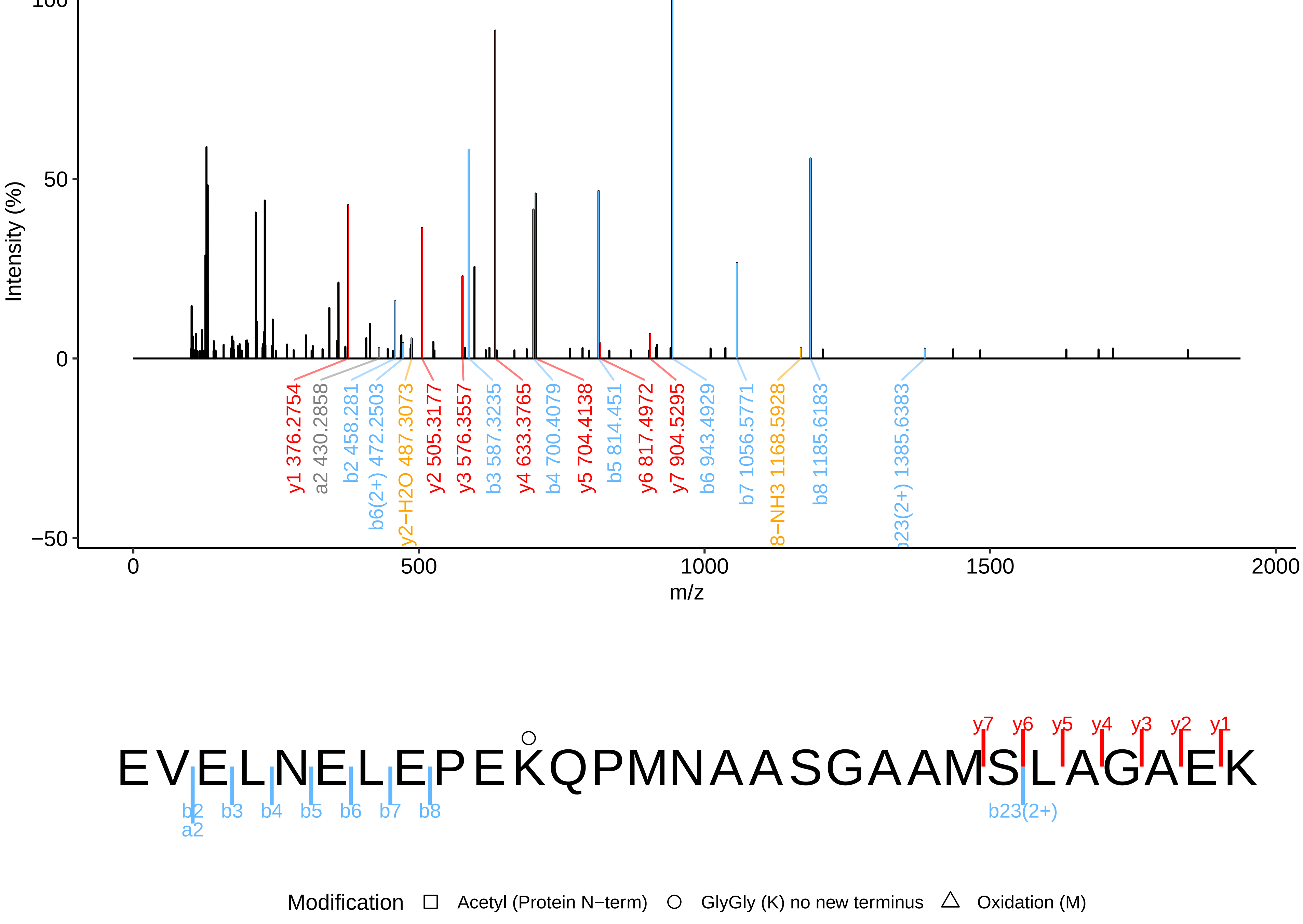

20200921_KIZ_MUb4_GlyGly_10plex_2 − Scan number: 22691

Gene Names	Charge	m/z	Mass	Retention time	PEP	Score	Precursor Intensity
SLC3A2	4	782.8773	3127.48	89.494	0.00066546	78.45	1097626

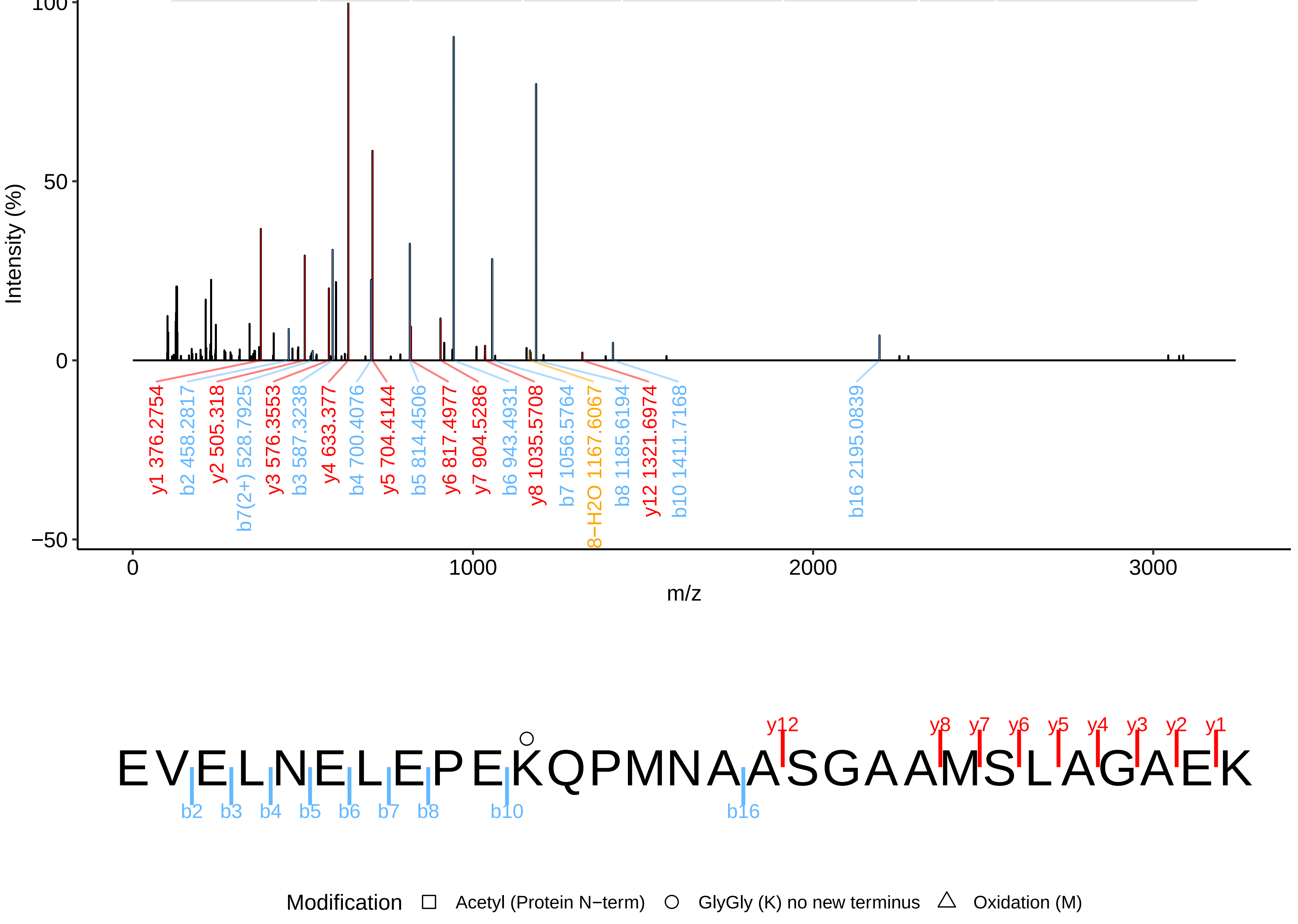

20200921_KIZ_MUb4_GlyGly_10plex_2 − Scan number: 21099

Gene Names	Charge	m/z	Mass	Retention time	PEP	Score	Precursor Intensity
SLC3A2	4	786.876	3143.475	83.028	0.025623	50.144	1975139

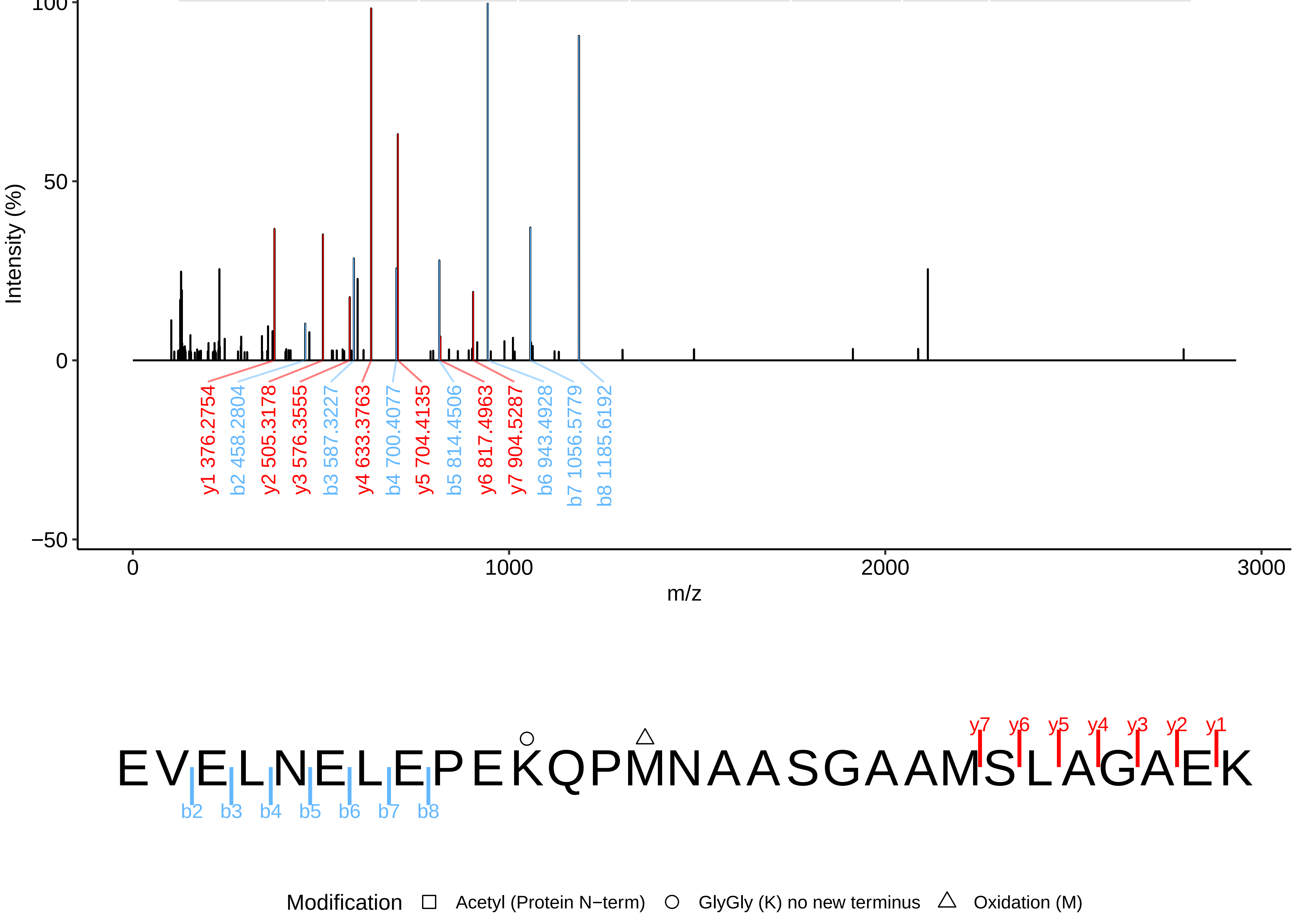

20200921_KIZ_MUb4_GlyGly_10plex − Scan number: 26980

Gene Names	Charge	m/z	Mass	Retention time	PEP	Score	Precursor Intensity
SCAMP1	4	915.1945	3656.749	98.391	7.4926e−07	81.068	2297664

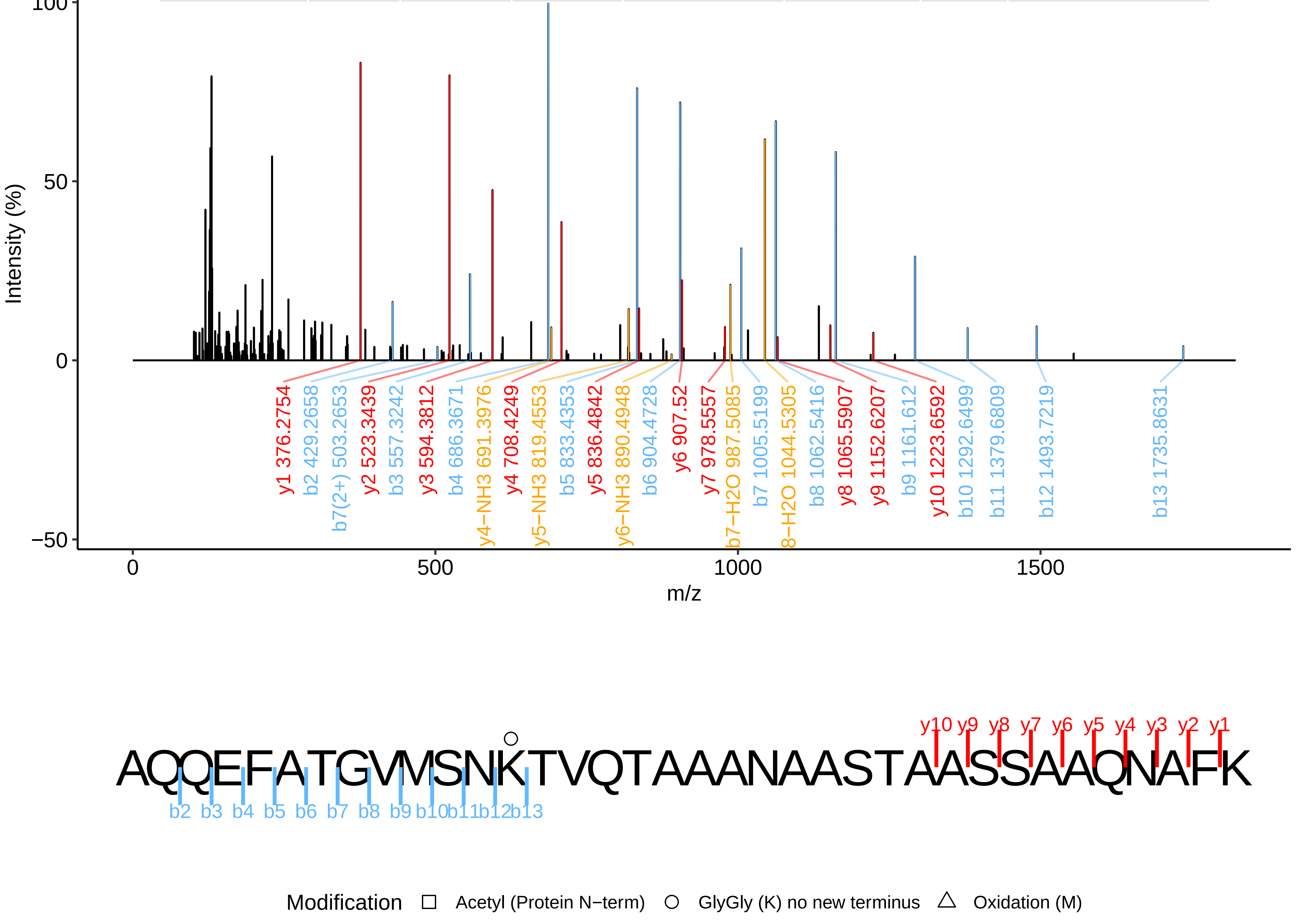

20200921_KIZ_MUb4_GlyGly_10plex − Scan number: 27010

Gene Names	Charge	m/z	Mass	Retention time	PEP	Score	Precursor Intensity
SCAMP1	4	915.1945	3656.749	98.5	1.0598e−29	156.67	4266158

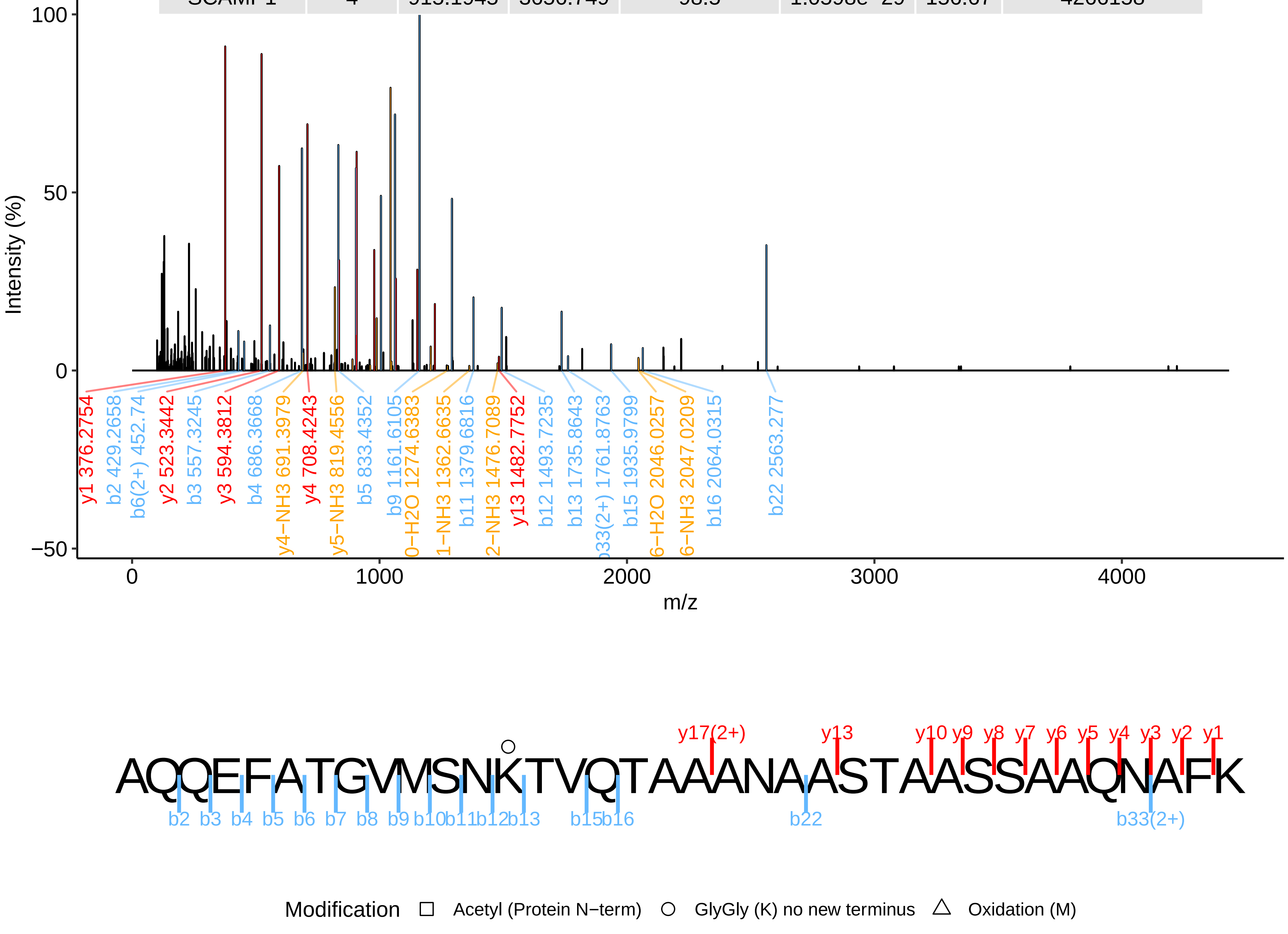

20200921_KIZ_MUb4_GlyGly_10plex − Scan number: 27021

Gene Names	Charge	m/z	Mass	Retention time	PEP	Score	Precursor Intensity
SCAMP1	4	915.1945	3656.749	98.542	3.6013e−30	163.92	3801231

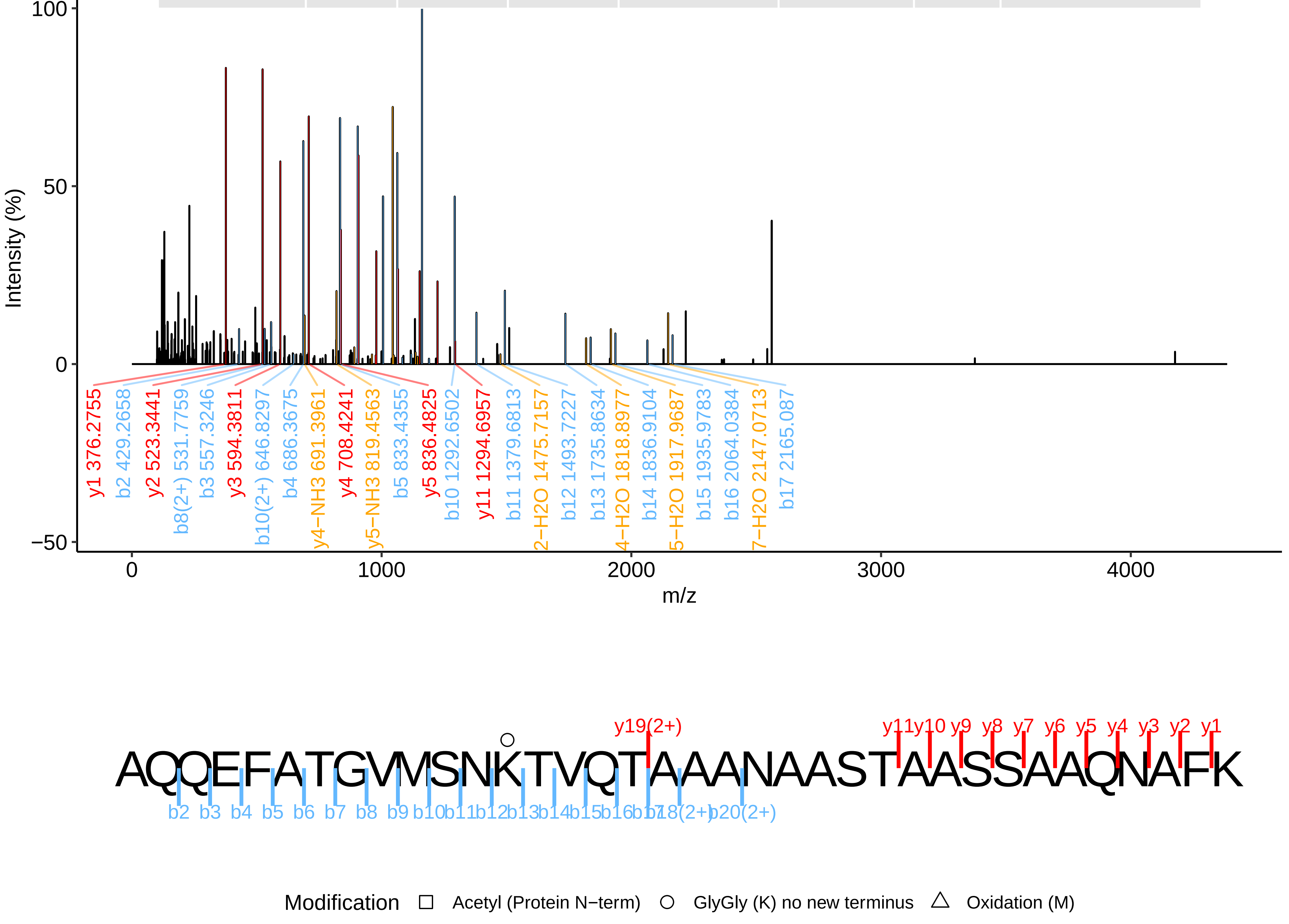

20200921_KIZ_MUb4_GlyGly_10plex_2 − Scan number: 13354

Gene Names	Charge	m/z	Mass	Retention time	PEP	Score	Precursor Intensity
SLC16A1	5	626.9063	3129.495	51.832	2.4911e−09	113.4	NA

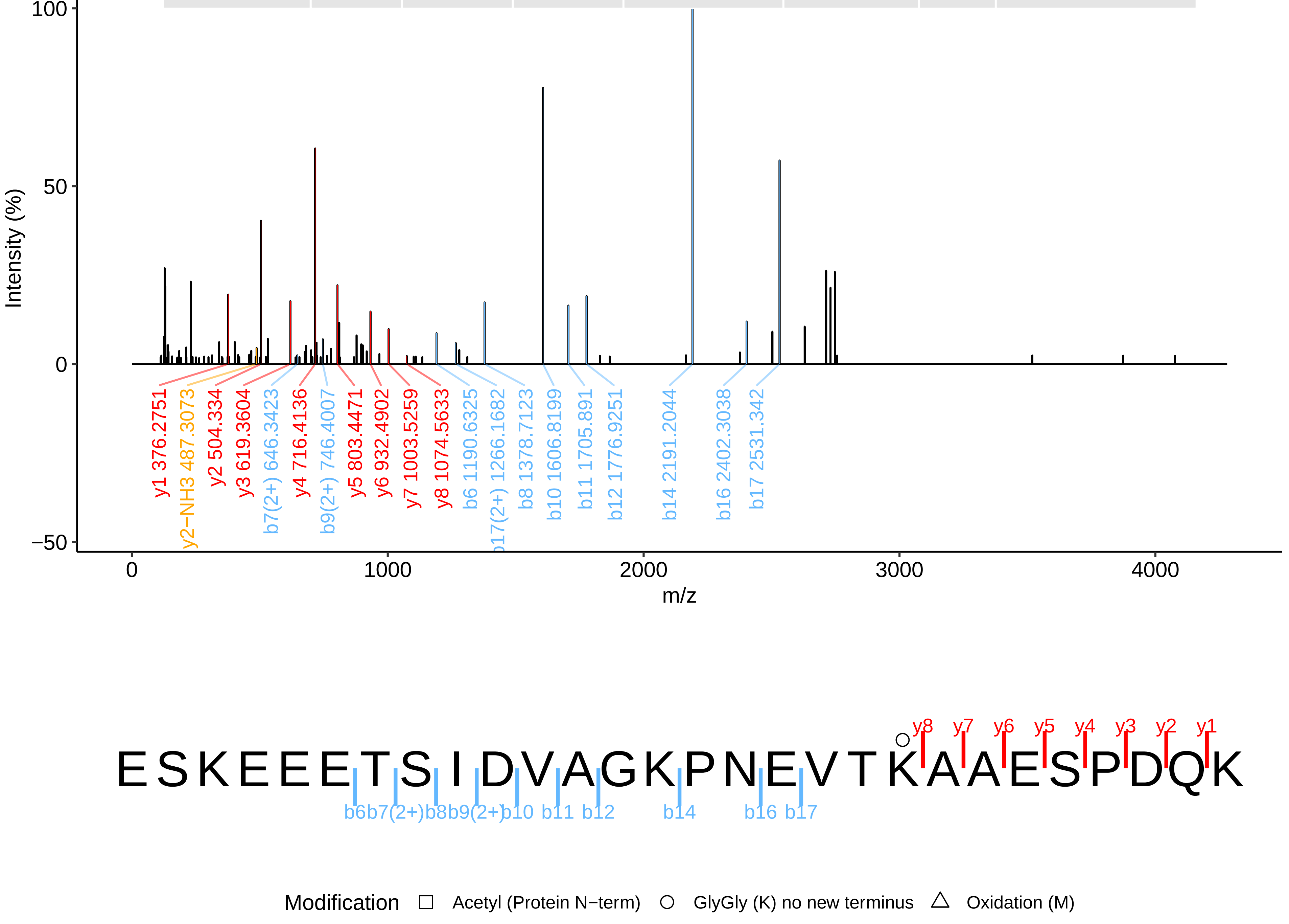

20200921_KIZ_MUb4_GlyGly_10plex_2 − Scan number: 13408

Gene Names	Charge	m/z	Mass	Retention time	PEP	Score	Precursor Intensity
SLC16A1	5	626.9063	3129.495	52.053	5.0839e−05	82.342	529695.8

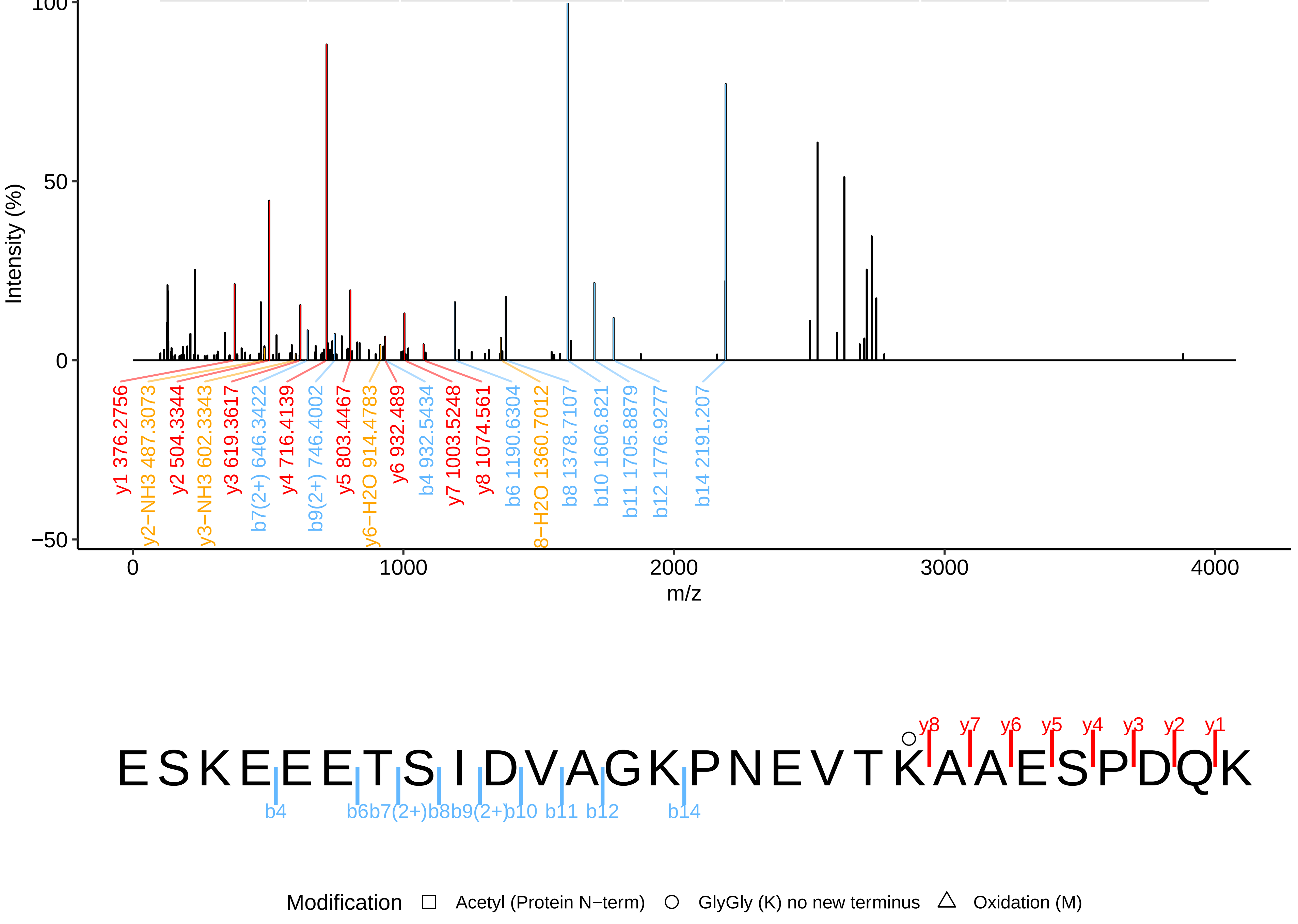

20200921_KIZ_MUb4_GlyGly_10plex_2 − Scan number: 20420

Gene Names	Charge	m/z	Mass	Retention time	PEP	Score	Precursor Intensity
SLC38A2	4	863.1377	3448.522	80.259	0.018197	52.707	382795.8

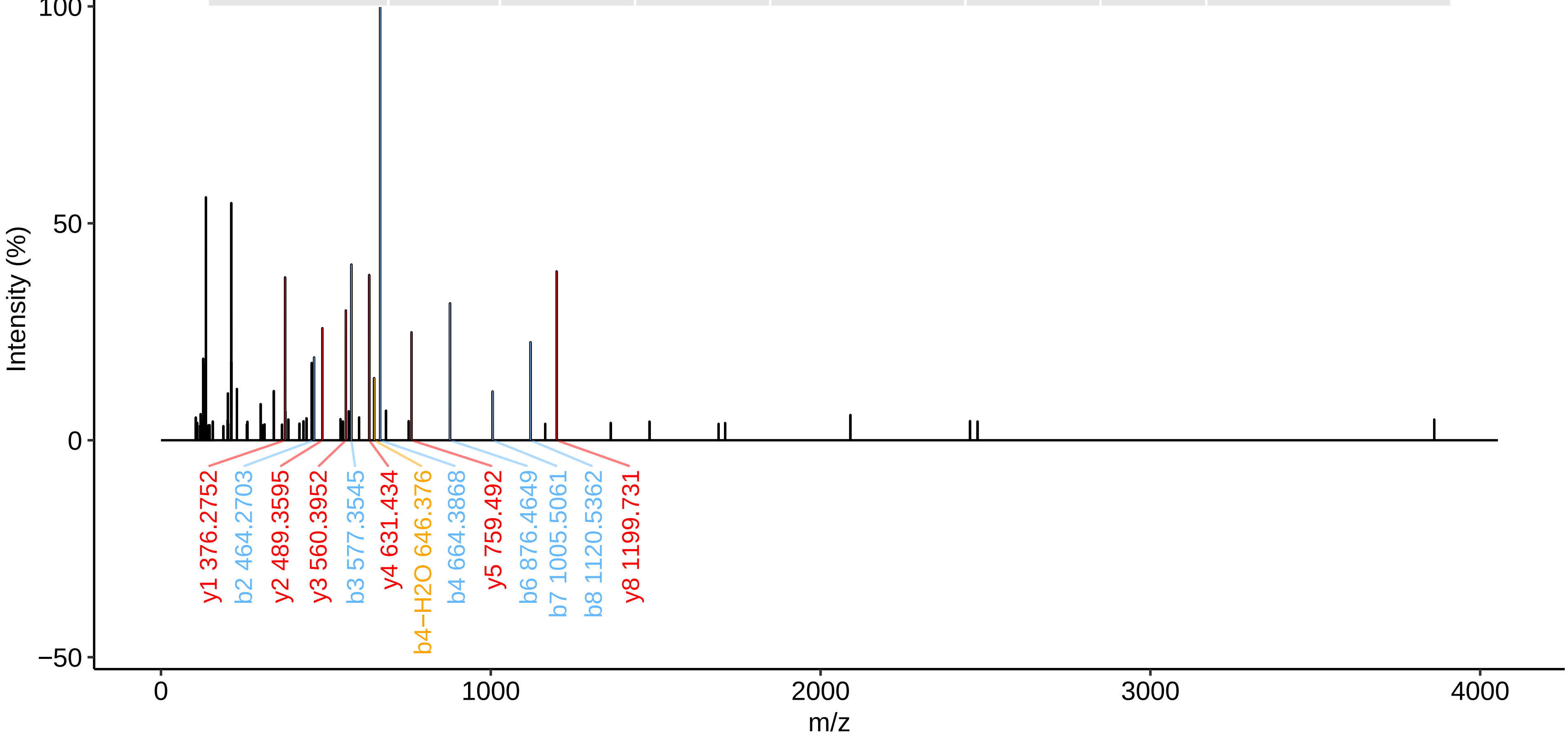

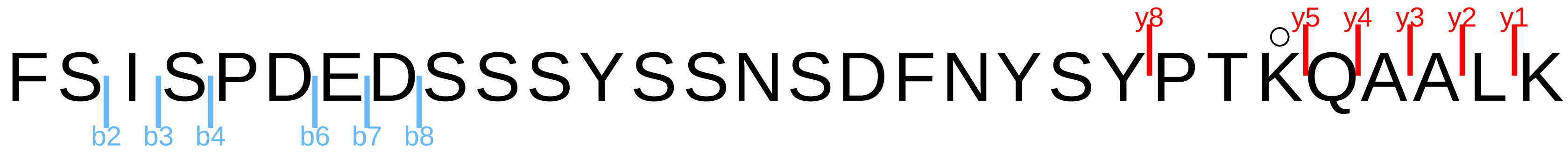

Modification □ Acetyl (Protein N−term) ○ GlyGly (K) no new terminus △ Oxidation (M)

20200921_KIZ_MUb4_GlyGly_10plex_2 − Scan number: 20427

Gene Names	Charge	m/z	Mass	Retention time	PEP	Score	Precursor Intensity
SLC38A2	3	1150.515	3448.522	80.289	0.011325	59.691	427320.8

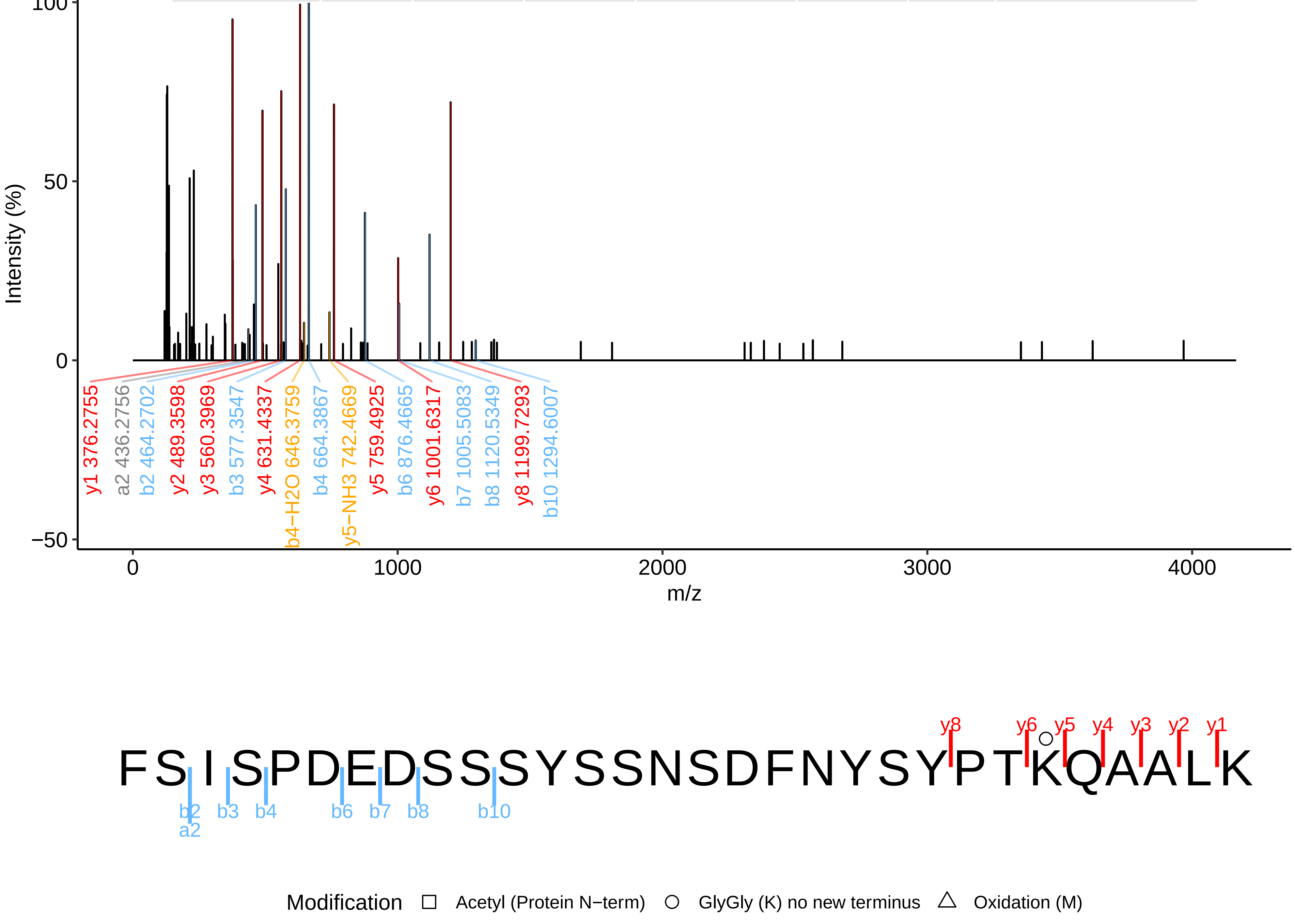

20200921_KIZ_MUb4_GlyGly_10plex_2 − Scan number: 19638

Gene Names	Charge	m/z	Mass	Retention time	PEP	Score	Precursor Intensity
RABGEF1	4	848.9024	3391.58	77.099	3.9027e−08	112.8	882721.4

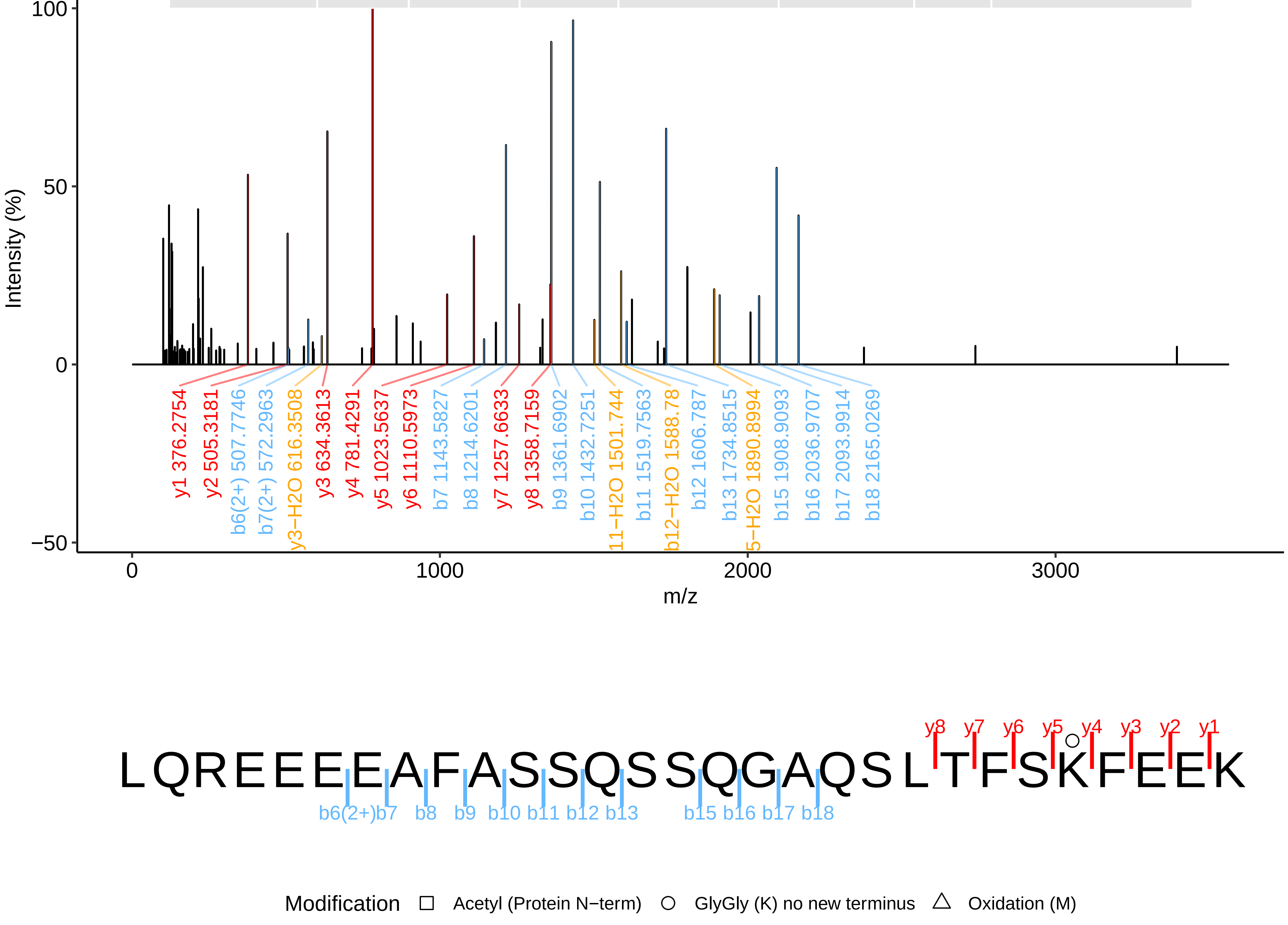

20200921_KIZ_MUb4_GlyGly_10plex − Scan number: 32364

Gene Names	Charge	m/z	Mass	Retention time	PEP	Score	Precursor Intensity
SLC1A5	4	849.9592	3395.808	119.61	1.6462e−39	175.9	48554664

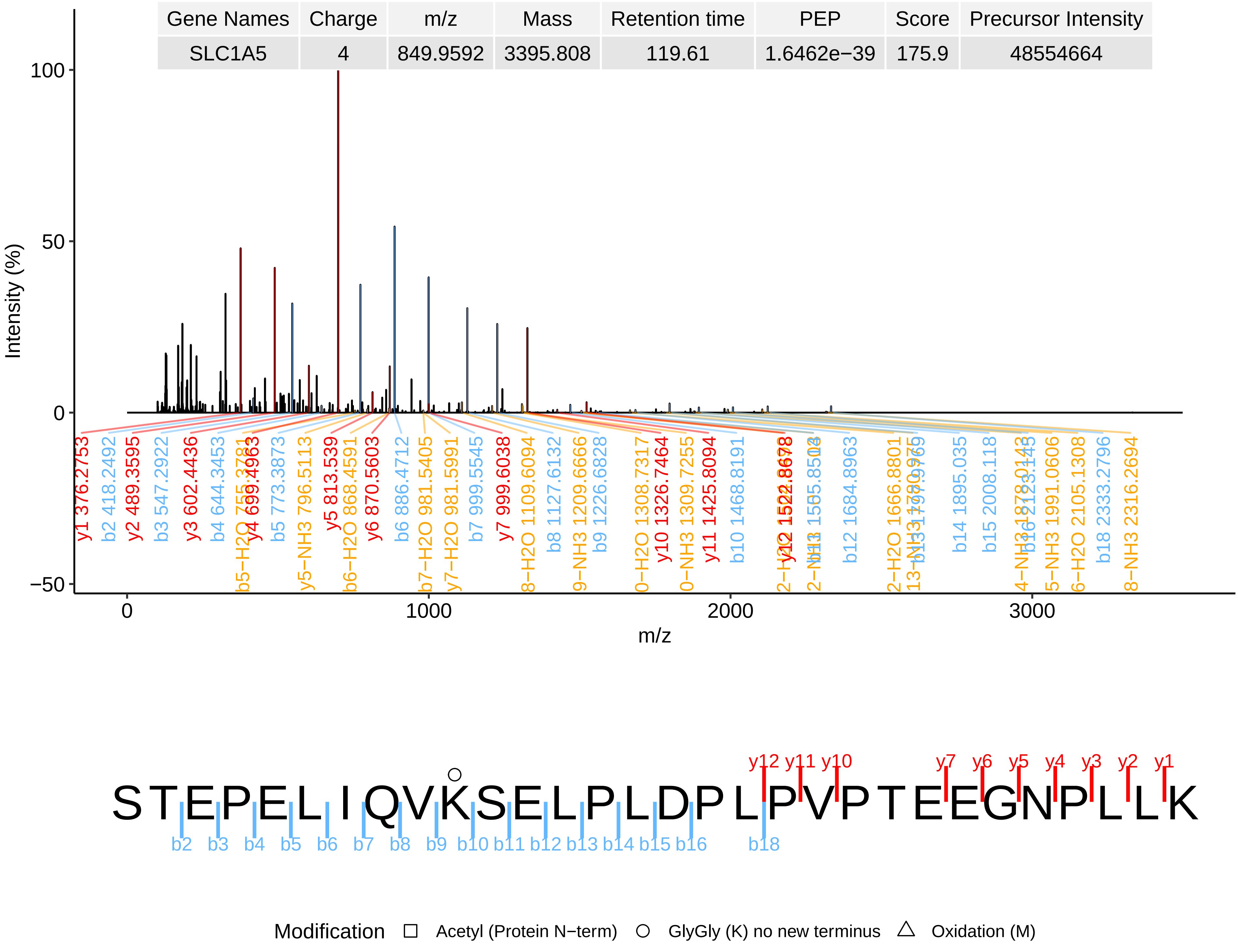

20200921_KIZ_MUb4_GlyGly_10plex − Scan number: 32365

Gene Names	Charge	m/z	Mass	Retention time	PEP	Score	Precursor Intensity
SLC1A5	5	680.1688	3395.808	119.61	9.0971e−14	128.27	23446090

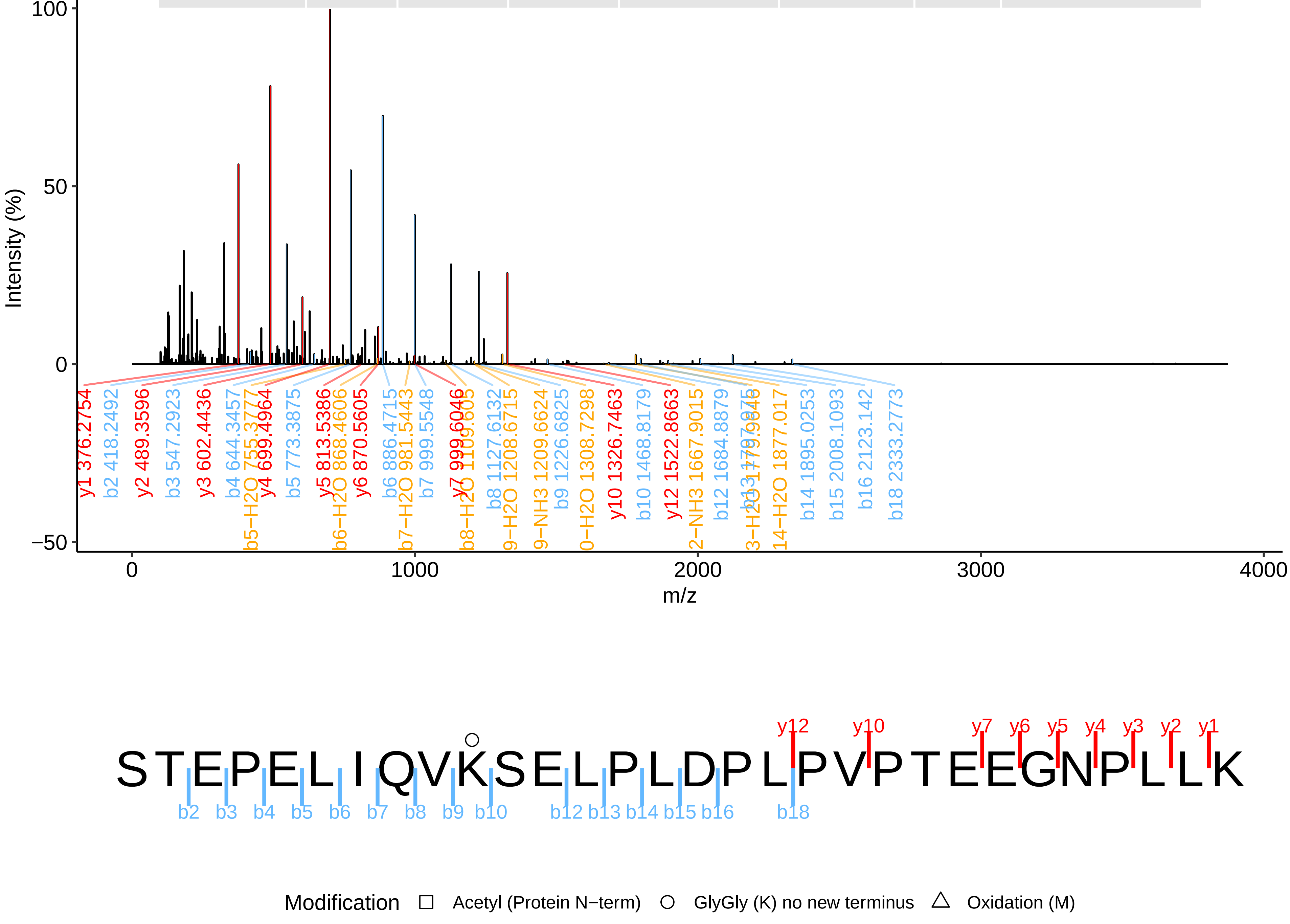

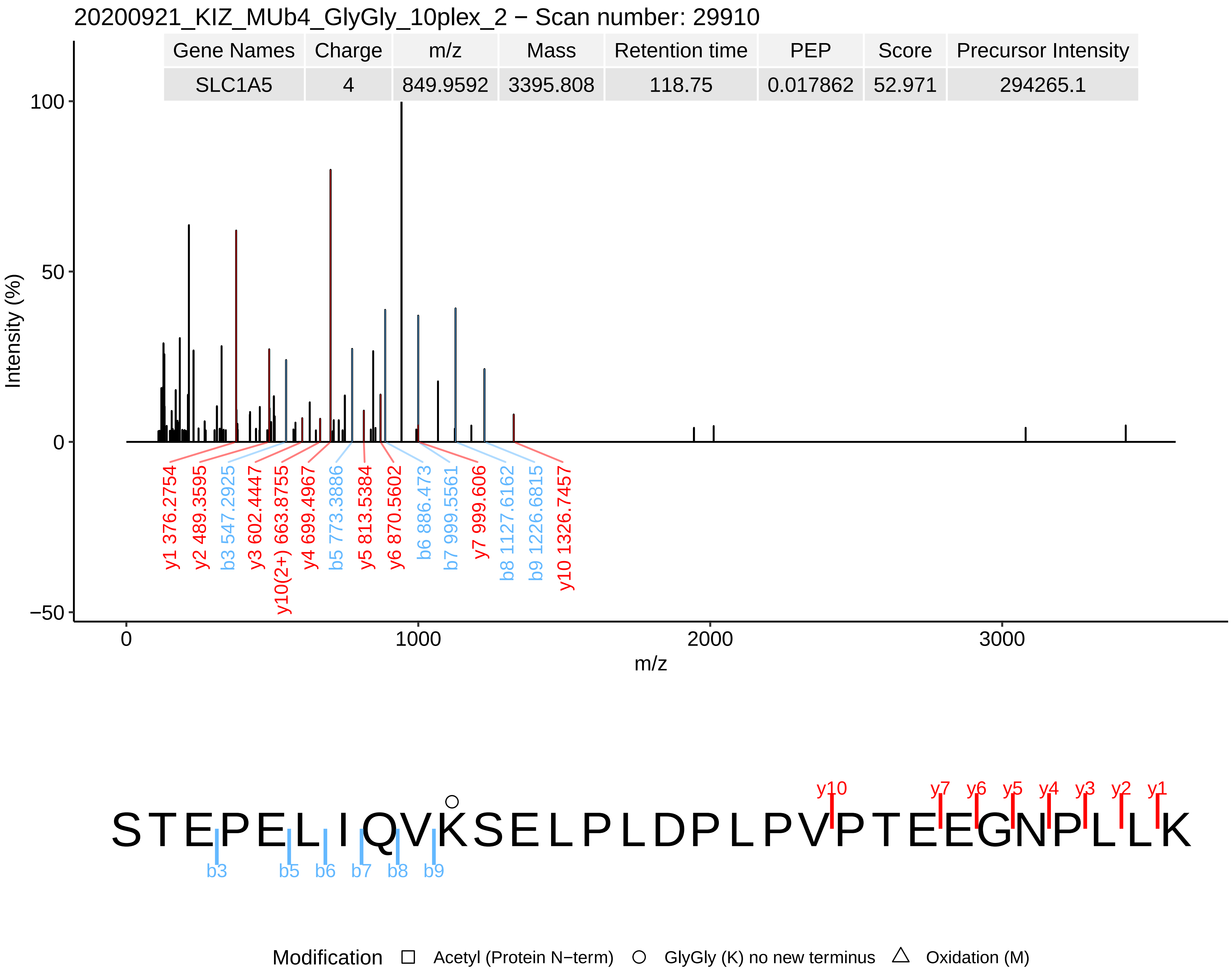
20200921_KIZ_MUb4_GlyGly_10plex_2 − Scan number: 29910
Gene Names | Charge | m/z | Mass | Retention time | PEP | Score | Precursor Intensity
SLC1A5 | 4 | 849.9592 | 3395.808 | 118.75 | 0.017862 | 52.971 | 294265.1
Intensity (%)
100
50
0
−50
0
1000
2000
3000
m/z
y1 376.2754
y2 489.3595
b3 547.2925
y3 602.4447
y10(2+) 663.8755
y4 699.4967
b5 773.3886
y5 813.5384
y6 870.5602
b6 886.473
b7 999.5561
y7 999.606
b8 1127.6162
b9 1226.6815
y10 1326.7457
y10 y7 y6 y5 y4 y3 y2 y1
S T E P E L I Q V K S E L P L D P L P V P T E E G N P L L K
b3 b5 b6 b7 b8 b9
Modification
Acetyl (Protein N−term)
GlyGly (K) no new terminus
Oxidation (M)

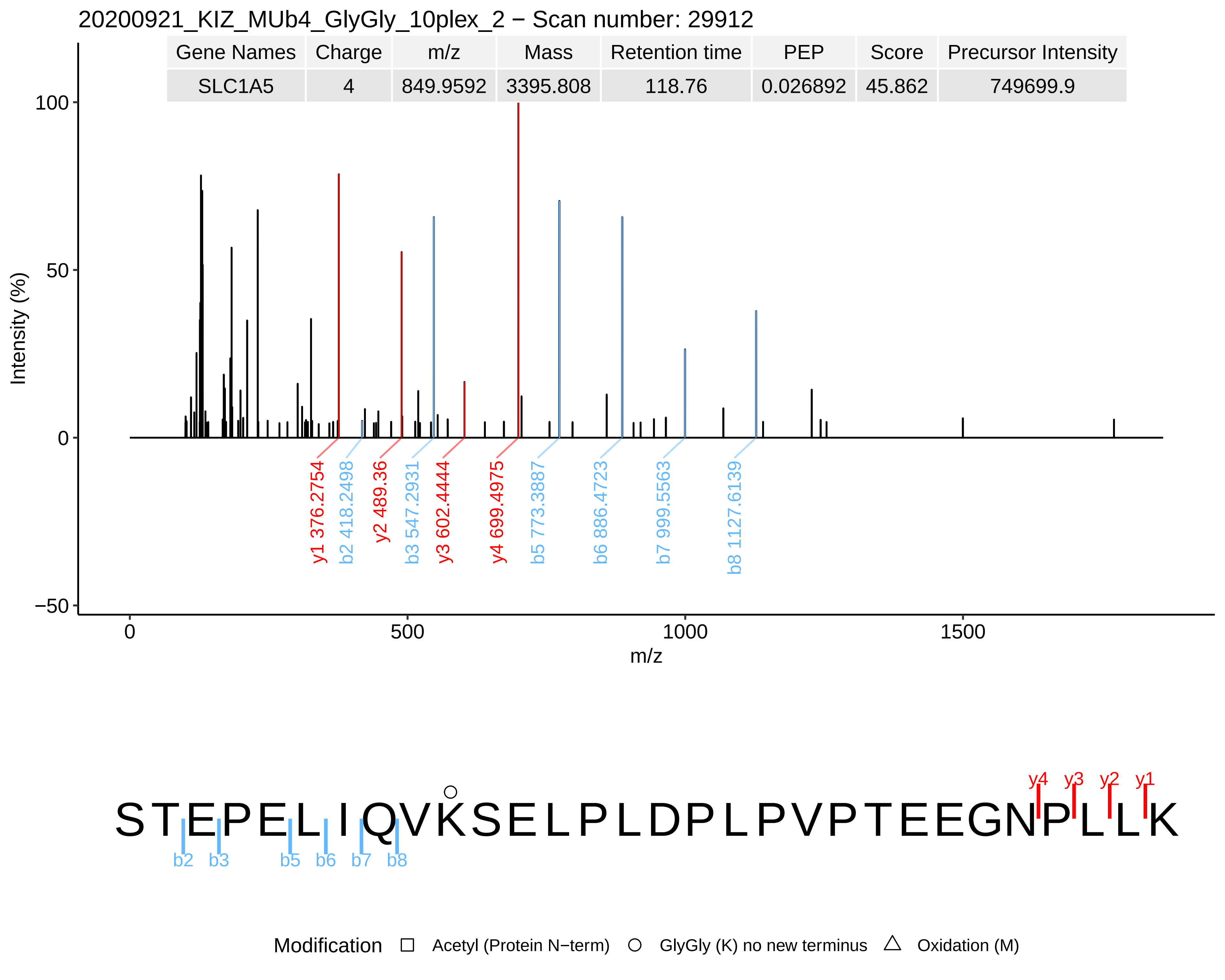
20200921_KIZ_MUb4_GlyGly_10plex_2 − Scan number: 29912
Gene Names | Charge | m/z | Mass | Retention time | PEP | Score | Precursor Intensity
SLC1A5 | 4 | 849.9592 | 3395.808 | 118.76 | 0.026892 | 45.862 | 749699.9
Intensity (%)
100
50
0
−50
0
500
1000
1500
m/z
y1 376.2754
b2 418.2498
y2 489.36
b3 547.2931
y3 602.4444
y4 699.4975
b5 773.3887
b6 886.4723
b7 999.5563
b8 1127.6139
STEPELIQVKSELPLDPLPVPTEEGNPLLK
y4 y3 y2 y1
b2 b3 b5 b6 b7 b8
Modification
Acetyl (Protein N−term)
GlyGly (K) no new terminus
Oxidation (M)

20201118_KIZ_MUb6_GlyGly_1 − Scan number: 38821

Gene Names	Charge	m/z	Mass	Retention time	PEP	Score	Precursor Intensity
SLC1A5	4	849.9592	3395.808	111.54	1.9314e−39	174.31	33405666

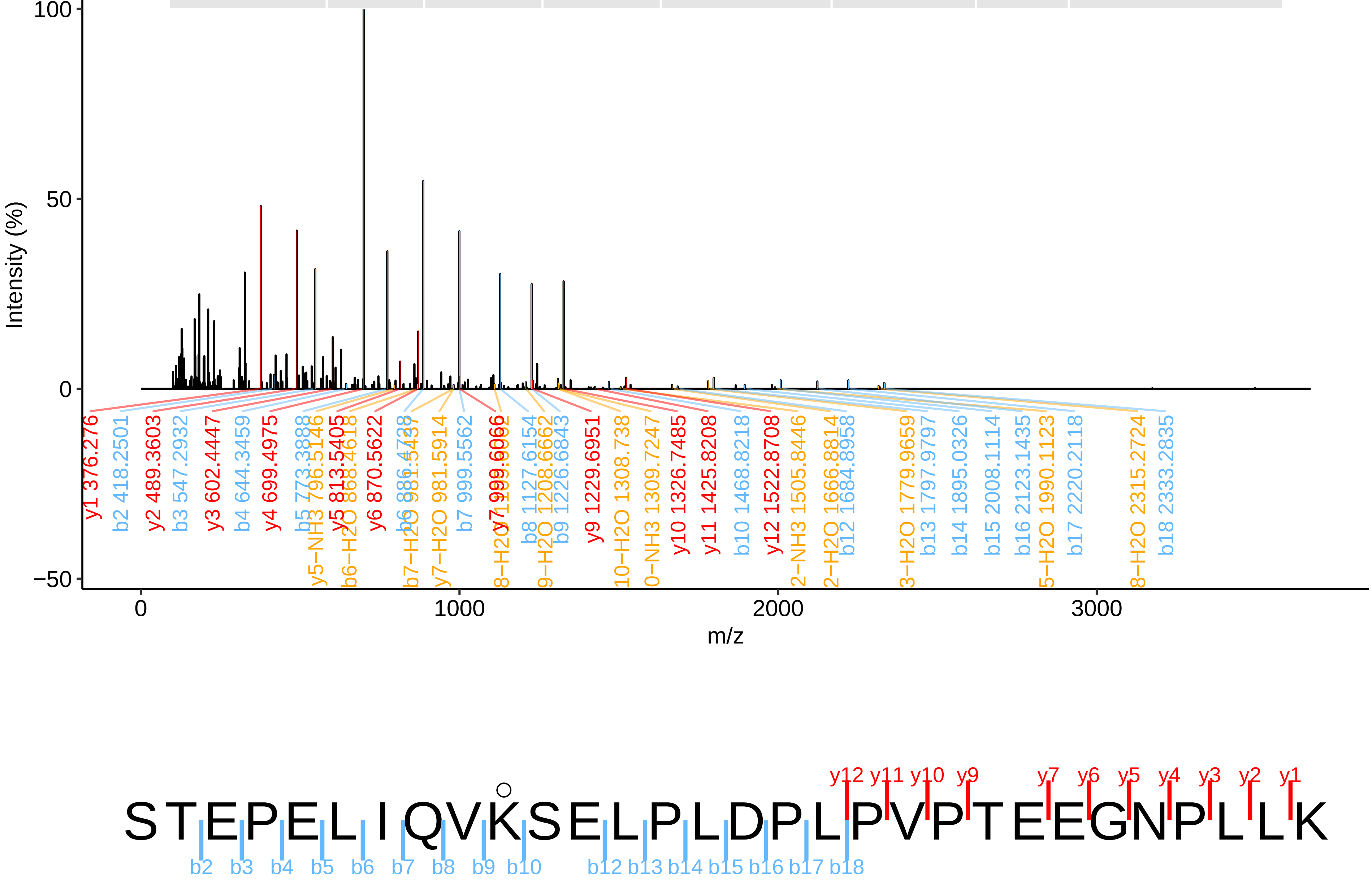

Modification ☐ Acetyl (Protein N−term) ○ GlyGly (K) no new terminus △ Oxidation (M)

20201118_KIZ_MUb6_GlyGly_1 − Scan number: 38854

Gene Names	Charge	m/z	Mass	Retention time	PEP	Score	Precursor Intensity
SLC1A5	4	849.9592	3395.808	111.62	2.6986e−13	119.97	41877600

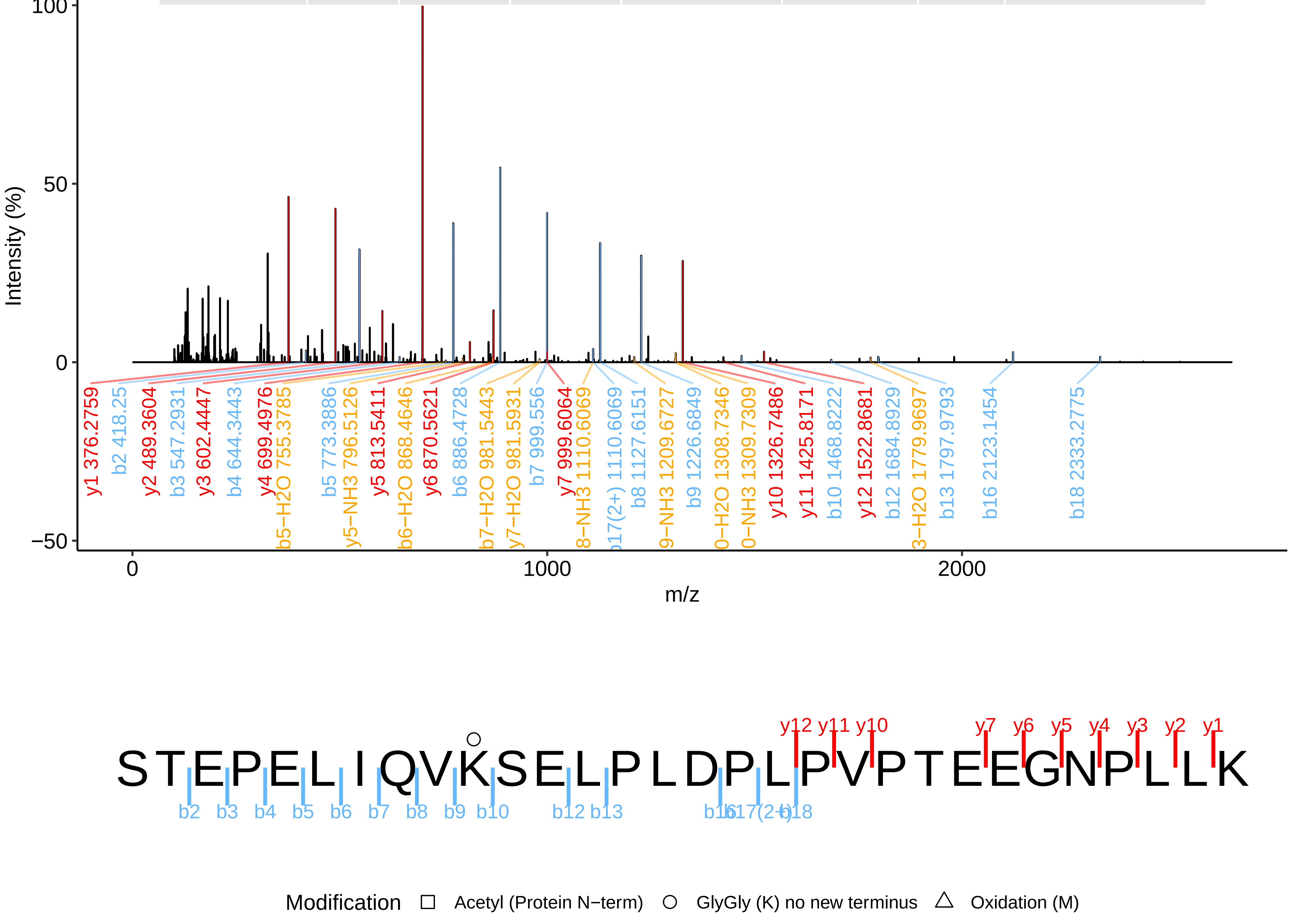

20201118_KIZ_MUb6_GlyGly_1 − Scan number: 42324

Gene Names	Charge	m/z	Mass	Retention time	PEP	Score	Precursor Intensity
MYH7B	4	947.7509	3786.975	120.06	0.014443	68.231	152072160

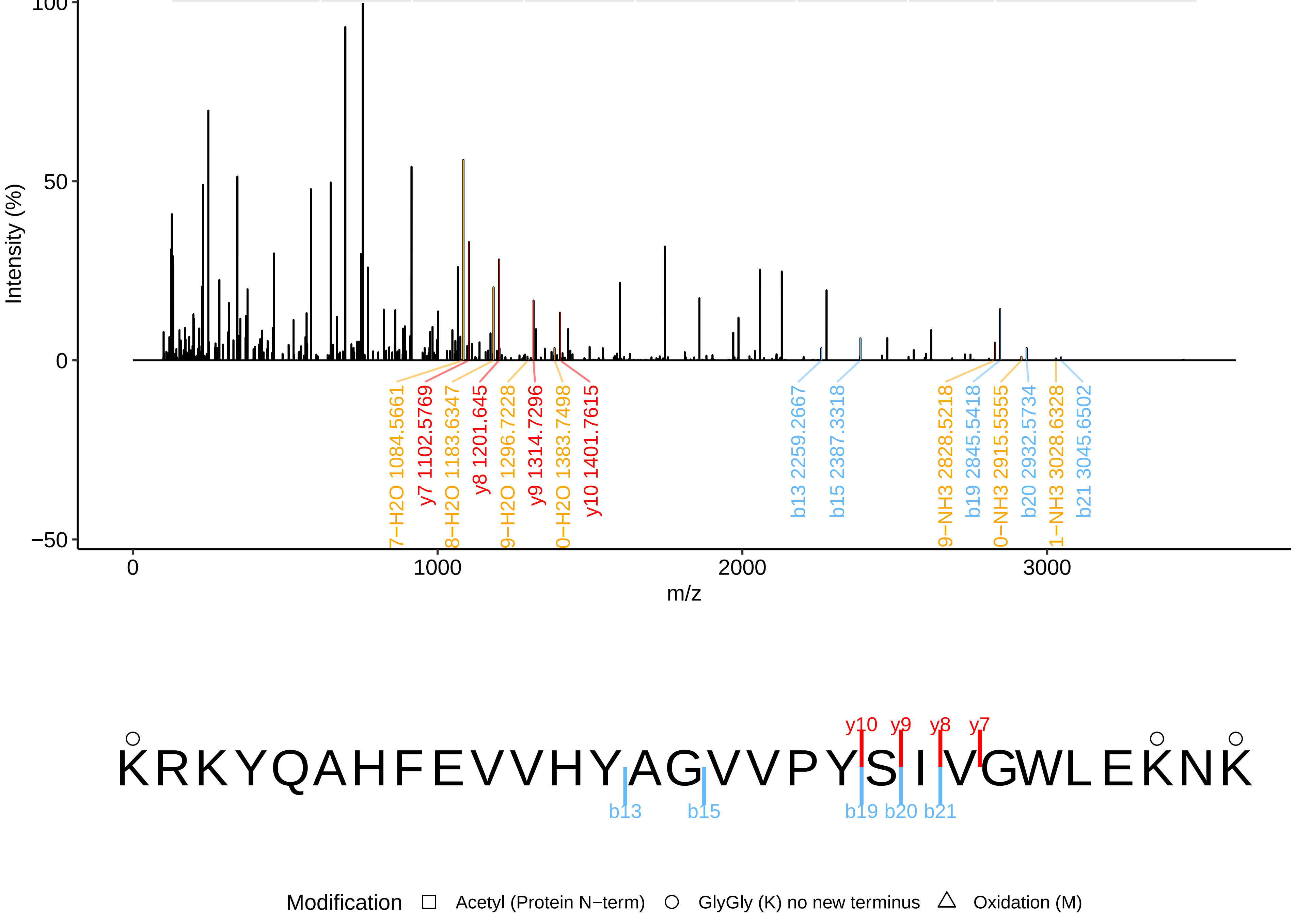

Supplement: Supplemental Data [file mmc8.pdf]
